# Telomere length de novo assembly of all 7 chromosomes and mitogenome sequencing of the model entomopathogenic fungus, *Metarhizium brunneum,* by means of a novel assembly pipeline

**DOI:** 10.1186/s12864-021-07390-y

**Published:** 2021-01-28

**Authors:** Zack Saud, Alexandra M. Kortsinoglou, Vassili N. Kouvelis, Tariq M. Butt

**Affiliations:** 1 Department of Biosciences, College of Science, Swansea University, Singleton Park, Swansea, Wales SA2 8PP UK; 2 Department of Genetics and Biotechnology, Faculty of Biology, National and Kapodistrian University of Athens, Panepistimiopolis, 15701 Athens, Greece

**Keywords:** *Metarhizium*, Fungi, Genome, Nanopore, Long-read, WGS, *Hypocreales*

## Abstract

**Background:**

More accurate and complete reference genomes have improved understanding of gene function, biology, and evolutionary mechanisms. Hybrid genome assembly approaches leverage benefits of both long, relatively error-prone reads from third-generation sequencing technologies and short, accurate reads from second-generation sequencing technologies, to produce more accurate and contiguous de novo genome assemblies in comparison to using either technology independently. In this study, we present a novel hybrid assembly pipeline that allowed for both mitogenome de novo assembly and telomere length de novo assembly of all 7 chromosomes of the model entomopathogenic fungus, *Metarhizium brunneum*.

**Results:**

The improved assembly allowed for better ab initio gene prediction and a more BUSCO complete proteome set has been generated in comparison to the eight current NCBI reference *Metarhizium* spp. genomes. Remarkably, we note that including the mitogenome in ab initio gene prediction training improved overall gene prediction. The assembly was further validated by comparing contig assembly agreement across various assemblers, assessing the assembly performance of each tool. Genomic synteny and orthologous protein clusters were compared between *Metarhizium brunneum* and three other *Hypocreales* species with complete genomes, identifying core proteins, and listing orthologous protein clusters shared uniquely between the two entomopathogenic fungal species, so as to further facilitate the understanding of molecular mechanisms underpinning fungal-insect pathogenesis.

**Conclusions:**

The novel assembly pipeline may be used for other haploid fungal species, facilitating the need to produce high-quality reference fungal genomes, leading to better understanding of fungal genomic evolution, chromosome structuring and gene regulation.

**Supplementary Information:**

The online version contains supplementary material available at 10.1186/s12864-021-07390-y.

## Background

The production of more complete and accurate genome assemblies has further improved understanding of gene function, biology, and evolutionary mechanisms [1]. High quality, accurate genome assemblies are essential for efficient genome mining, allowing for the identification of useful genes and gene clusters that drive advances in downstream applications such as metabolic engineering, synthetic biology, biotechnology-based drug development, and protein engineering [2]. The advent of second-generation sequencing technologies, such as Illumina’s sequencing by synthesis approach [3], and third generation sequencing technologies, such as Oxford Nanopore [4, 5] and Pacific Biosystems single molecule sequencing platforms [6], have reduced the cost and time of genome assembly projects in comparison to first generation Sanger (dideoxy-chain termination) sequencing [7] methods. The current state-of-the-art genome assembly approach, termed hybrid assembly, leverages benefits of both long, relatively error-prone reads from third-generation sequencing technologies, and short, accurate reads from second-generation sequencing technologies to produce more accurate and contiguous de novo genome assemblies than could be achieved using either technology independently [8]. More contiguous assemblies hold richer information about repetitive regions and chromosome structure, allowing better inferences to be made about macro-molecular genomic variations that lead to adaptation and speciation [9, 10]. Furthermore, it has been demonstrated that gene content can vary significantly between genome assemblies of differing quality made from the same read set, presumably due to the availability of new gene evidence for ab initio prediction algorithms, genome mis-assembly events and local sequence variations [11].

Fungi within the genus *Metarhizium* (Division: *Ascomycota*, Class: *Sordariomycetes*, Order: *Hypocreales*, Family: *Clavicipitaceae*) have a worldwide distribution. Besides being applied as biological control agents for pest control [12], species within the genus are frequently used as model organisms to investigate infection processes and host defence mechanisms of various arthropod hosts [13]. Research is also focused on their symbiotic relationship with plants, as they have been shown to improve plant growth and health through poorly understood mechanisms [14]. Additionally, some isolates of *Metarhizium* are capable of producing bioactive metabolites such as Swainsonine and Destruxins, compounds that have been explored as potential pharmaceuticals to treat cancer, osteoporosis, Alzheimer’s disease, and hepatitis B [15]. Given these interesting properties, there are currently only 8 species of *Metarhizium* with genomes deposited within GenBank, despite at least 50 species having been described within the genus. Different isolates (variants) of the same species have been found to vary greatly in their phenotypes [16], but due to the relatively small number of isolates sequenced, the extent of genomic variation between strains is poorly understood. Owing to their genomes having multiple chromosomes that contribute to their relatively large genome sizes (30–45 Mb) in comparison to bacterial microbes (around 5 Mb), de novo genome assemblies of *Metarhizium* spp. using first generation sequencing is very costly, and second-generation sequencing results in assemblies that are highly contiguous, falling apart around repeat rich and homologous regions of the genome. The assembled reference genomes of all 8 species currently accessible in GenBank were produced using reads from second generation sequencing technology, with some of the assemblies making use of optical mapping data to further improve assembly quality [17–22]. It is speculated that chromosome duplications and rearrangements are responsible for the differing phenotypic attributes of *Metarhizium* spp. strains [23], but as of yet, none of the *Metarhizium* genome assemblies have produced contigs or scaffolds that are chromosome length, a requirement for meaningful chromosomal macro-synteny comparisons between different strains and/or species. Karyotyping experiments carried out using pulse-field gel electrophoresis suggest the presence of 7–8 chromosomes in *Metarhizium anisopliae* (MAN), with chromosomes varying in size from an estimated 1.8 to 7.4 megabase pairs [23, 24]. A separate study provided evidence showing the smallest chromosome to be disposable in a strain of *M. brunneum* (strain V275 formerly classified as *M. anisopliae*) without having lethal effects [25].

In this study, we present a novel hybrid de novo assembly pipeline, incorporating Illumina and Nanopore sequencing reads, that allowed for telomere length assemblies of all 7 chromosomes of *M. brunneum* isolate ARSEF 4556, as well as the generation of the full circular mitochondrial genome. We benchmark this assembly against the current NCBI reference *Metarhizium* spp. genomes, providing evidence that the assembly is superior in terms of both standard assembly metrics, as well as gene content as determined by BUSCO scoring. Furthermore, we validate this assembly by comparing it against assemblies produced by various long read assemblers using the same read set, assessing fungal genome assembly performance. We perform genomic synteny and orthologous protein cluster comparisons of this assembly with three other complete genome assemblies of species within the Order *Hypocreales*, listing orthologous protein clusters shared uniquely between two of the entomopathogenic species, as well as compiling a list of core orthologous *Hypocreales* proteins shared across all four species. We present an improved genome sequence for the genus, as well as a hybrid assembly pipeline that could be used for other haploid fungal species, in order to facilitate efforts to produce high-quality genomes, ultimately leading to a better understanding of fungal genomic evolution.

## Results

### Sequencing

A total of 16,630,587 Illumina reads were produced for each pair-end read set- a theoretical coverage of around 131x of the 38 Mb sized *M. brunneum* genome. After end trimming, the theoretical coverage of the cumulative number of bases was reduced to around 105x. For the Nanopore sequencing run, a total of 1,839,242 raw long reads were produced. After length filtering, trimming and correction, the > 3000 bp long read dataset contained a total of 777,731 reads (N50 = 7156), containing 5,075,705,440 bases, a theoretical coverage of around 134x. The > 5000 bp long read dataset contained a total of 453,256 reads (N50 = 8530), containing 3,798,611,962 bases, a theoretical coverage of around 100x.

### Genome assembly

**Fig. 1:**
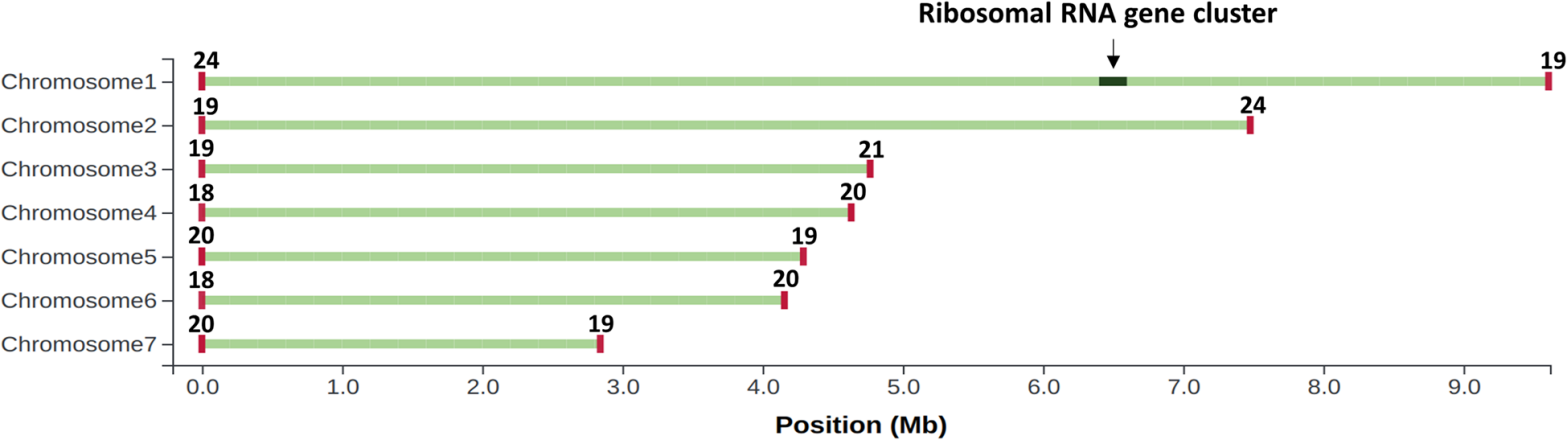
Tapestry output of complete chromosomes. Terminal telomere sequence counts (CCCTAA/ TTAGGG) are given above the terminal ends (red). The green lines depict mapped long reads to each chromosome. Read mapping depths were uniform across chromosomes, with no breaks detected, however, a pile up of reads was observed around the 18 s/28 s ribosomal RNA gene cluster in chromosome

Attempts to further reduce the number of steps in the assembly pipeline by removing individual correction steps resulted in suboptimal assemblies in comparison to using the full assembly pipeline. A tangled Flye assembly graph was produced from assembly of the FMLRC corrected long reads without the Canu trimming step (see additional file 1.A). The Flye assembly graph of the Canu trimmed long reads without the FMLRC correction step was seen to have smaller contigs, and larger contigs that failed to reach chromosome length (see additional file 1.B). The Flye assembly graph of the > 5000 bp read set with the information used to manually resolve complete chromosomes can be seen in additional file 1.C. Read assemblies of chromosomes 2, 4, 5 and 6 were found to traverse an Eulerian path, were assembled telomere to telomere, and required no further resolving. Read assemblies of chromosomes 3 and 7 were found to traverse an Eulerian path in the Flye assembly of the > 3000 bp read set (with two rounds of polishing). Chromosome 1 was deduced by subtracting chromosome 7 and using coverage depth information to deduce the correct edges between contigs, and the 5231 bp end was manually added to the end as described in the methods section. A dotplot illustrating good synteny observed between the contigs and scaffolds of the previous *M. brunneum* reference assembly and the 7 full length chromosomes produced in this study is presented in additional file 1.D. Tapestry output of terminal telomere counts, chromosome lengths, and long read mapping agreement can be found in Fig. 1.

### Validation of the assembly and comparison of long read assembly performance

The metrics for the various assemblers tested are listed in Table 1. The assemblers generally produced better results with the FMLRC/Canu trimmed reads used as input (as opposed to raw long reads), with the exceptions of Canu (produced a total assembly size that was three times as large as the other assemblers) and Shasta (produced a total assembly length of 104,717 bp). The Raven, Shasta and wtdbg2 assemblies suffered with telomere sequence loss irrespective of whether corrected or raw reads were used as input. The Canu assembly with raw reads produced a fragmented assembly. Necat and Flye produced the best assemblies in terms of N50, production of telomere length contigs, and telomere length presence, and Flye’s metrics were relatively robust irrespective of which corrected reads were used as input. The Flye assembly with the Ratatosk corrected reads contained 1 inter-chromosomal mis-assembly wherein a telomere repeat sequence was found in the central region of a chromosome. Aside from the Canu and Shasta assemblies with corrected reads used as input, the predicted genes and total lengths of the assemblies were moderately consistent. Assembly graphs showing TTAGGG_n5_ sequences detected in contigs produced by all assemblers, and colour coded blast hits of chromosomes from the final complete assembly from which mis-assemblies were inferred can be found in additional file 2.

**Table 1:** Validation of final assembly and comparison of long read assembler performance

	Raw reads							FMLRC/Canu trimmed corrected reads				Flye assemblies of corrected reads		
Assembler/read correction	Canu	Flye	Miniasm/Minipolish	NECAT	Raven	Shasta	wtdbg2	Canu	Raven	Shasta	wtdbg2	Ratatosk corrected	NECAT corrected	Canu corrected
**# contigs**	101	37	30	11	23	158	36	5184	33	29	39	12	12	20
**# telomere length contigs**	0	0	1	4	0	0	0	0	0	0	0	2	3	1
**# telomere ends**	13	10	10	14	2	1	3	63	1	0	4	12 (1 internal)	12	12
**Largest contig**	7,044,699	7,995,000	7,469,658	7,468,681	7,206,934	4,617,081	5,443,409	272,978	5,081,705	11,177	7,124,112	9,037,030	9,016,449	8,996,444
**Total length**	38,376,550	37,703,727	37,958,957	37,735,059	37,936,851	37,183,651	37,096,535	103,756,147	37,639,251	104,717	37,144,653	37,798,138	37,733,634	37,685,204
**N50**	1,881,118	3,318,636	3,131,456	4,285,476	3,481,404	1,876,944	3,054,299	24,679	2,314,652	5391	2,990,362	5,751,808	4,624,294	4,146,824
**N75**	767,395	2,040,015	2,377,107	3,139,426	2,013,237	930,220	1,521,719	14,748	1,029,583	3459	1,523,795	4,158,166	4,289,507	1,999,248
**# misassembled contigs**	0	1	0	0	2	0	0	–	2	–	2	1	0	0
**Genome fraction (%)**	99.478	99.56	99.711	99.972	99.836	97.935	98.252	99.898	99.257	0.269	98.296	99.883	99.916	99.824
**Duplication ratio**	1.021	1.002	1.008	0.999	1.005	1.005	0.998	2.744	1.004	1.03	1	1.001	1	0.999
**# predicted genes**	10,951	11,041	11,116	11,040	11,171	10,897	10,418	31,240	11,219	–	11,163	11,289	11,073	10,855
**Total aligned length**	38,336,586	37,639,589	37,919,257,	37,732,682	37,899,111	37,159,837	36,997,473	103,544,164	37,609,029	104,319	37,120,414	37,774,636	37,704,114	37,680,427

The final polished assembly was compared to assemblies produced by alternate long read assemblers. Mis-assemblies were detected by blasting the final chromosomes against each assembly in bandage and telomere presence was assessed by blasting searching for the telomere sequence TTAGGG_n5_

### Genome annotation

**Fig. 2:**
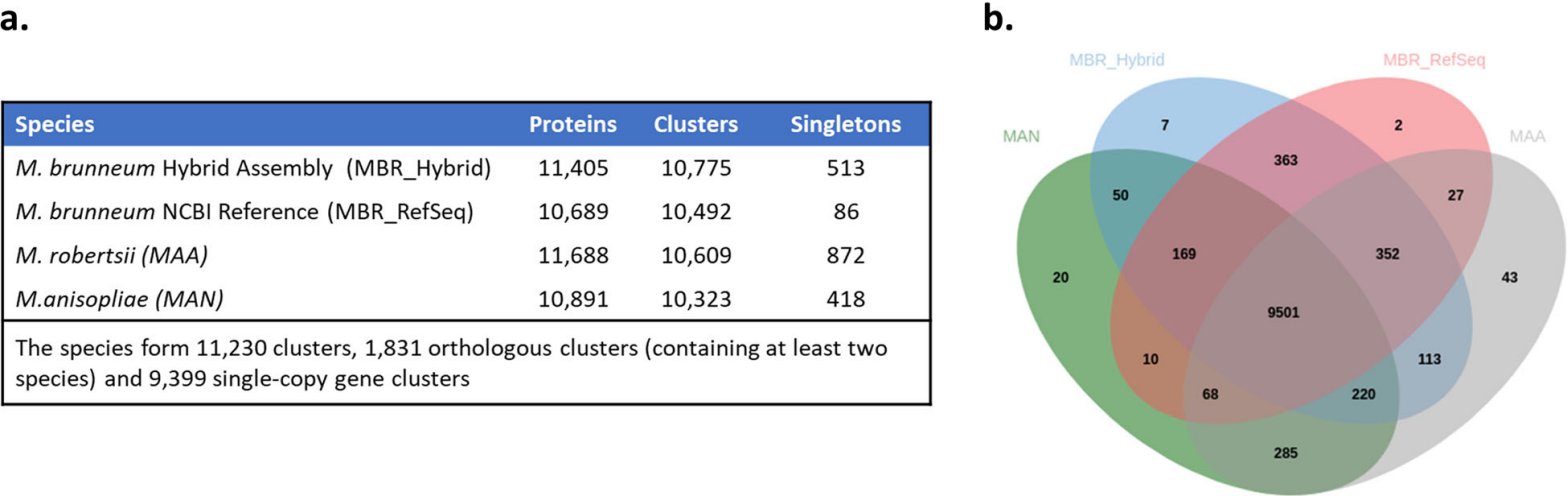
Comparison of orthologous gene clusters between *Metarhizium* protein sets. Comparison of the protein set produced in this study with the NCBI reference protein sets for *M.brunneum*, *M.robertsii* and *M.anisopliae.* **a** Number of proteins, orthologous clusters and singletons predicted for each assembly. **b** Venn diagram comparing orthologous protein cluster numbers between the four protein sets

**Fig. 3:**
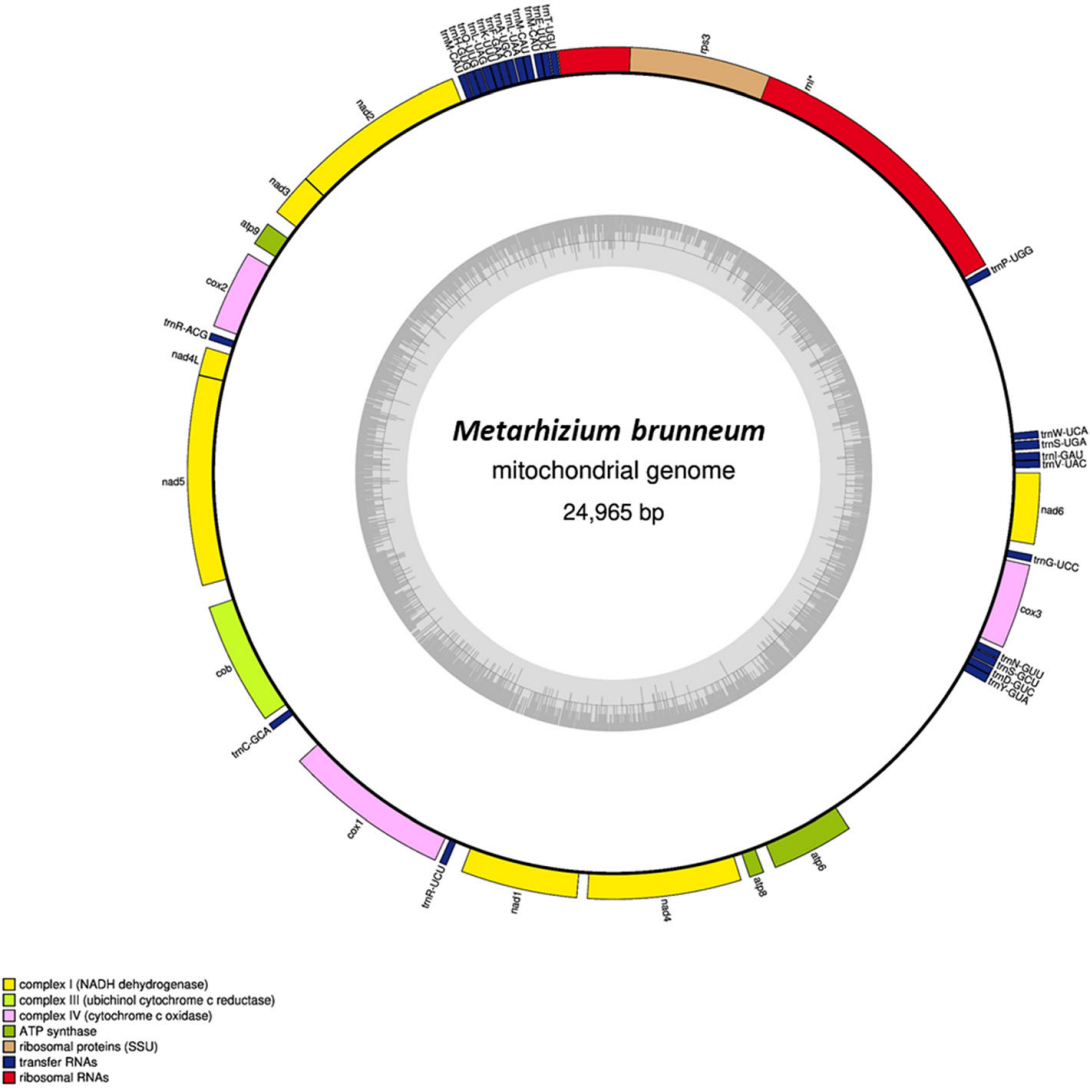
*Metarhizium brunneum* mitogenome map. Mitochondrial gene families are colour coded as per the legend. The circle inside the inner GC content graph marks the 50% threshold

A list of each chromosome’s length, GC content, tRNA genes, rRNA genes and notable genes include; specialist entomopathogenic, endophytic and mating-type genes, are detailed in Table 2. All chromosomes were numbered according to the convention of numbering chromosomes according to size, with chromosome 1 being the largest. All chromosomes were found to be oriented in the direction of the telomere sequence CCCTAA at the 5′ chromosome end and TTAGGG at the 3′ chromosome end, further validating assembly correctness. The tRNAscan-SE tool predicted a total of 124 tRNA genes in the genome assembly and RNAmmer predicted a total of 27 rRNA genes present in the genome assembly. Table 3 lists the assembly metrics, predicted proteins and protein BUSCO scores of all NCBI Reference *Metarhizium* spp. Genomes, as well as the assembly produced in this study, which was found to have the highest protein BUSCO score of 99.1% (*N* = 4494). The protein set generated in this study was found to have a total of 4455 complete BUSCOs of which 4441 were found to be complete and single copy, 14 BUSCOs were found to be complete and duplicated, 18 BUSCOs were found to be fragmented and 21 BUSCOs were found to be missing. In contrast, the current *M. brunneum* NCBI reference protein set was found to have a BUSCO score of 97.0% (*N* = 4494), and the best *Metarhizium* spp. protein BUSCO score of the NCBI reference sequences was that of *M. robertsii* with a score of 98.5% (*N* = 4494). The BUSCO scores for the four ab initio gene prediction tools used are listed in Table 4. As running a native version of the latest version of GeneMarkES with the mitogenome included proved to be best, it was this gene set that was carried forward for functional analyses. A total of 11,406 genes and 11,405 proteins were predicted using this tool, of which 1251 proteins passed the SignalP5.0 threshold for containing a signal peptide sequences. A summary of the SignalP5.0 results can be found in additional file 3 and a list of the mature proteins that were found to have a signal sequence are presented in additional file 4. Comparisons of the protein sets produced in this study with the NCBI reference protein sets for *M. brunneum*, *M. robertsii* and *M. anisopliae* are illustrated in Fig. 2. The numbers of proteins, orthologous clusters and singletons of all four protein sets are give in Fig. 2a. In comparison to the previous *M. brunneum* NCBI reference protein set, the protein set generated in this study contained more predicted proteins (11,405 vs 10,689), and contained more orthologous protein clusters (10,775 vs 10,492). A Venn diagram showing the orthologous protein clusters shared between the four protein sets is depicted in Fig. 2b. In comparison to the previous *M. brunneum* NCBI reference protein set, the protein set generated in this study was found to share more orthologous protein clusters with both *M. robertsii* (10,186 vs 9948) and *M. ansiopliae* (9940 vs 9748). The Unicycler assembly produced a circular mtDNA genome of 24,965 base pairs (Fig. 3). Identified genes included; *cox*1–3, *nad*1–6 and *nad*4L, *cob*, *atp*6, *atp*8, *atp*9, *rnl* and *rps*3. A total of 25 tRNA gene sequences were identified within the mitogenome.

**Table 2:** *Metarhizium brunneum* ARSEF 4556 chromosomal lengths, GC content, ab initio predicted tRNA, rRNA, and notable genes

Chromosome number	Length	GC %	tRNA genes	rRNA genes	Notable genes
**1**	9,606,624	51.8	34	1 × 18 s/28 s cluster (tandem repeats) 3 × 8 s	Hydrophobin 1
					Hydrophobin 2
					PR1
					Lipoxegynase
**2**	7,478,350	51	21	4 × 8 s	MAT-1-2
					MAT_Switching
					CYP6001C17
**3**	4,766,907	49.3	24	5 × 8 s	CYP52
					CYP5081A
					CYP5081B
					CYP5081C
					CYP5081D
**4**	4,632,031	49.3	11	2 × 8 s	NRPS-like antibiotic synthetase
**5**	4,290,503	51	14	1 × 8 s	MAD1
					MAD2
					Mrt
**6**	4,155,369	51.3	8	5 × 8 s	Secretory lipase
					Heterokaryon incompatibility protein
**7**	2,842,132	49.1	12	6 × 8 s	DtxS1
					DtxS2
					DtxS3
					DtxS4
					Chymotrypsin
					Bassianolide synthetase

**Table 3:** Assembly and annotation metrics for all NCBI representative genome assemblies of Metarhizium species

Species	Isolate	Assembly Accession	Total Length	Scaffolds	Scaffold N50	Full Chromosomes (plasmids)	Predicted Proteins	Protein Busco (***N*** = 4494)
*M. album*	ARSEF 1941	GCA_000804445.1	30,449,065	257	1,086,596	0 (0)	8389	96.2%
*M. acridum*	CQMa 102	GCA_000187405.1	39,422,329	241	54,747	0 (0)	9830	95.2%
*M. anisopliae*	ARSEF 549	GCA_000814975.1	38,504,274	74	2,048,875	0 (0)	10,891	97.2%
***M. brunneum***	**ARSEF 4556**	**GCA_013426205.1**	**37,796,881**			**7 (1)**	**11,405**	**99.1%**
*M. brunneum*	ARSEF 3297	GCF_000814965.1	37,066,166	92	1,825,569	0 (0)	10,689	97.0%
*M. guizhouense*	ARSEF 977	GCA_000814955.1	43,465,197	563	554,408	0 (0)	11,727	96.4%
*M. majus*	ARSEF 297	GCA_000814945.1	42,062,993	1134	364,403	0 (0)	11,394	96.8%
*M. rileyi*	RCEF 4871	GCA_001636745.1	32,013,981	389	886,790	0 (0)	8763	98.2%
*M.robertsii*	ARSEF 23	GCA_000187425.2	41,656,800	90	4,491,770	0 (0)	11,688	98.5%

The long-read assembly generated in this study is highlighted in bold text

**Table 4:** Percentage of protein Busco completion of protein sets generated from the long-read *M. brunneum* assembly predicted with various ab-initio gene prediction tools and approaches

Prediction tool	Protein BUSCO (***N*** = 4494)	Complete Busco	Single copy	Duplicated	Fragmented	Missing	Number of predicted chromosomal genes
Augustus	96.3%	4325	4313	12	58	111	10,805
GeneMarkES	99.0%	4450	4435	15	23	21	11,284
GeneMark-ES-Native no mtDNA	99.1%	4454	4439	15	19	21	11,389
**GeneMark-ES-Native with mtDNA**	**99.1%**	**4455**	**4441**	**14**	**18**	**21**	**11,406**
GlimmerM	15.6%	704	702	2	197	3593	7529

The final gene set used for functional analysis, which was subsequently deposited in the GenBank is highlighted in bold text. Note that one predicted gene in the final gene set was found to be non-protein coding. Remarkably, *ab intio* gene prediction of chromosomal genes was superior in terms of BUSCO score when the mitogenome was included for training of the prediction model

### Full genome sequence-based synteny and pan-genome analyses of *Hypocreales* fungi

**Fig. 4:**
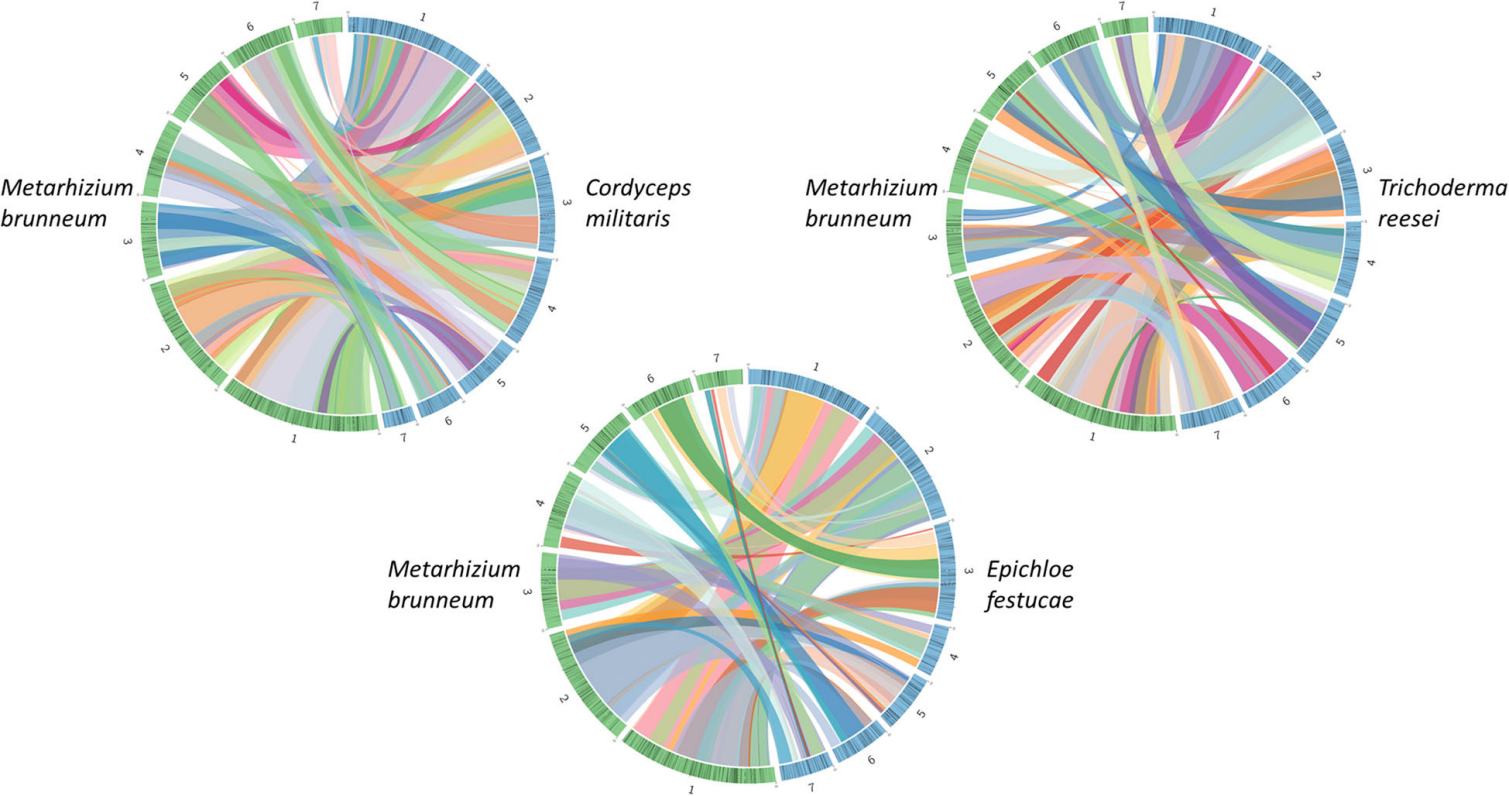
Sequence-synteny analyses between *Hypocreale* species. The Circo plots represent syntenic blocks greater than 1000 bp that were present in all 4 species analysed. Clear mesosynteny was observed between the different species of *Hypocreales* fungi, with no single chromosome showing major synteny with an individual chromosome in another species. The outer numbers indicate chromosome numbers. *M. brunneum* chromosomes are shown in green. *T. reesei*, *E. festucae* and *C. militaris* chromosomes are shown in blue

**Fig. 5:**
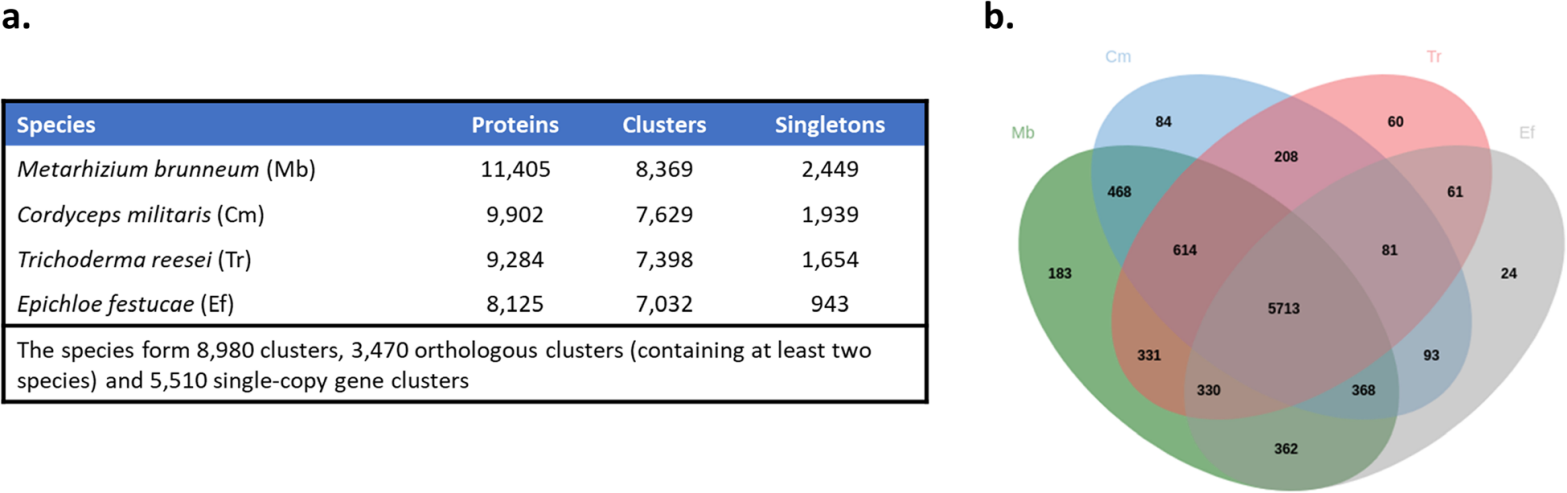
Comparison of orthologous gene clusters between the four *Hypocreales* fungi protein sets. Comparison of the protein set produced in this study with the chromosome length assemblies of the *Hypocreales* fungi; *Cordyceps militaris*, *Epichloe festucae* and *Trichoderma reesei* (a.) Number of proteins, orthologous clusters and singletons predicted for each assembly. (b.) Venn diagram comparing orthologous protein cluster numbers between the four protein sets

Abundant syntenic blocks were seen to be shared across *C. militaris*, *E. festucae, Trichoderma reesei*, and *M. brunneum* (Fig. 4). There was no discernible pattern in the sharing of these syntenic blocks amongst the chromosomes, with any individual chromosome of one species being found to share syntenic blocks with numerous other chromosomes in the other species. Assembly and annotation metrics of the *C. militaris*, *E. festucae,* and *Trichoderma reesei* genomes are stated in Table 5. A total of 9902, 9284, 8125 genes were predicted for *C. militaris*, *E. festucae,* and *Trichoderma reesei*, respectively. This is in contrast to the 11,406 genes predicted for *M. brunneum* long read assembly. Furthermore, the *M. brunneum* assembly produced in this study was found to have the highest protein BUSCO completion score of all four *Hypocreales* species. The results of comparing orthologous gene clusters between these species are presented in Fig. 5. There were 2449, 1939, 1654, and 943 singleton proteins detected with no ortholog/paralog for *M. brunneum*, *C. militaris*, *E. festucae,* and *Trichoderma reesei*, respectively. A total core set of 5713 clusters of proteins were found to be shared across all 4 species (see additional file 5). One hundred eighty-three unique orthologous clusters were formed between *M. brunneum* proteins (see additional file 6). Four hundred sixty-eight unique orthologous clusters were formed between the two entomopathogenic *Hypocreales* fungi in the comparison test- *M. brunneum* and *C. militaris* (see additional file 7). A list of the *M. brunneum* singleton proteins can be found in additional file 8. Interestingly, this number was the highest number of shared orthologous clusters between two different species in the whole comparison.

**Table 5:** Assembly and annotation metrics for complete chromosome length assemblies of fungal species within the Order *Hypocreales*

Species	Isolate	Taxonomic Family	Accession	Total Length	Number of Chromosomes	Predicted Proteins	Protein BUSCO Completion (***N*** = 4494)	Reference
*Cordyceps militaris*	ATCC 34164	*Cordycipitaceae*	GCA_008080495.1	33,618,380	7	9902	96.0%	[26]
*Epichloe festucae*	Fl1	C*lavicipitaceae*	GCA_003814445.1	35,023,690	7	9284	97.6%	[27]
*Trichoderma reesei*	QM6a	Hypocreaceae	GCA_002006585.1	34,922,528	7	8125	99.0%	[28]

## Discussion

The full genome sequence of *M. brunneum* has been assembled, producing telomere length sequences for all 7 chromosomes, a full mitogenome, and a more comprehensive protein set as determined by BUSCO analyses and analyses of orthologous protein clusters. The assembly and annotations are an improvement on the current *M. brunneum* reference assembly produced using optical mapping and mate-pair Illumina reads [18]. The seven assembled chromosomes match the number of total chromosomes predicted by pulsed-field gel electrophoresis [23, 24]. Certain genes were found to be in close proximity, as previously shown. For instance, dtx1 and dtx2 encoding Destruxins 1 and 2 were found in close proximity to dtx3 and dtx4 (which encode Destruxins 3 and 4), with the ORFs for the former being on one DNA strand and the ORFs for the latter being found on the complementary strand as previously described [29]. Furthermore, these genes were correctly placed on chromosome 7 in this assembly (the smallest chromosome), which has been shown to be dispensable, with *M. brunneum* losing its capacity to produce destruxins when this chromosome is lost [25]. Remarkably, chromosome 7, the smallest chromosome assembled, contained the greatest number of predicted 8 s rRNA genes. The mating-type genes MAT-1-2 and MAT_Switching were detected in full on chromosome 2. None of the MAT-1-1 type genes were detected in this assembly, excepting for a small 162 bp end segment (representing 15% of the full gene) of MAT-1-1-1, corroborating with previous work that has shown individual mating-type genes to be absent in some species of *Metarhizium* [20].

The circularised mtDNA matched the sequence produced by Sanger sequencing of the closely related *Metarhizium anisopliae* strain ME1 mtDNA, with 97.41% identity and 97% coverage. The current *M. brunneum* reference sequence was found to have a mitogenome of 50,066 bp, and both the mitogenome from the hybrid assembly, and the previously sequenced *M. anisopliae* ME1 mitogenome mapped this 50,066 bp sequence, if duplicated, with near 100% identity, signifying that it is most likely an incorrect concatemer that arose from a mis-assembly event. This further highlights the advantage of adopting hybrid assembly approaches for fungal genome assembly.

The majority of assemblers tested were found to produce assemblies in agreement with the complete genome, and further validate assembly correctness. Flye appears to be the most robust, producing telomere length chromosomes and good assembly N50 values regardless of the read correction strategy used, although the assembly with uncorrected reads produced no telomere length contigs. The other assembler found to produce good results with this fungal genome was NECAT. Raven, Shasta and wtdbg2 all suffered from loss of telomere sequences, a problem that would likely recur for all fungal assemblies. Canu performed better with raw reads, however the N50 value of the assembly was low. The Canu assembler was found to be the most customizable out of the assemblers tested, however, it also had the longest run time. Canu did not perform well when corrected reads were used as input. The Flye assembly using the NECAT corrected reads was the best assembly of the two self-corrected read sets, and this assembly pipeline was found to be best for assemblies with short reads. The results corroborate previous findings by Wick and Holt [30], who compared these assemblers with bacterial genomes. Their results agree with our findings, excepting their ranking of the Raven assembler, which we found to perform poorly with this fungal genome. However, the difference in performance of this assembler may be due to most bacterial genomes being circular. The differences in assemblies that result from differing read correction methods have been observed before by Fu, Wanf and Au [31], who produced an excellent comparative evaluation of long read correction tools.

In terms of cost, the hybrid assembly approach costs as little as €1500. Although this assembly vastly improves on the Illumina read only assemblies, further improvements could be made when conducting hybrid assembly by producing ultra-long nanopore reads [32], particularly for fungal species that contain genomic regions with large sections of tandem repeats. For this assembly, DNA was extracted using a spin column. Longer reads may be obtained by using gravimetric DNA extraction kits, a more traditional phenol-chloroform, or utilizing agarose plug DNA extractions. Given that longer reads are known to have a higher propensity to clog the nanopores, it may be beneficial to produce two sets of nanopore data, an initial run using the relatively shorter fragmented DNA to ensure good coverage, and, when good coverage is reached, perform an additional run with the ultra-long reads. The MinION sequencer is well suited for this task, as read output can be monitored in real-time. As Nanopore sequencing read accuracy continues to improve, through both software and hardware enhancements, it is unknown for how long one may need to produce short-read Illumina sequencing data to polish long read assemblies.

In comparing the whole genomes of 4 *Hypocreale* species, we confirmed the previous finding of the existence of mesosynteny within the *Ascomycota* Phylum [33]. No discernible pattern was observed between the syntenic blocks in the comparisons of any two species, with an individual chromosome sharing syntenic blocks with multiple other chromosomes of the other species. A protein list of core orthologous proteins shared across all four *Hypocreales* species as been compiled. This protein set may prove useful in aiding future research by narrowing the search space for molecular underpinnings of specific phenotypic functions that are unique to a *Hypocreales* species, as proteins in this list are unlikely to carry out unique functions given that they are shared across all four of these species, and it is know that orthologs are likely to carry out similar functions [34]. Likewise, the lists of orthologous proteins shared uniquely between the entomopathogenic species *C. militaris* and *M. brunneum*, the *M. brunneum* self-clusters and singletons may also aid further research into as of yet unknown molecular underpinnings related to entomopathogenesis. A list has been compiled of mature proteins resulting from removal of theoretical signal peptides, which may aid future research into *M. brunneum* protein function. The list may assist the recombinant production of proteins in non-fungal species, as well as allow for the production of active mature proteins as oppose to unknowingly cloning protein precursors that may not be functional.

## Conclusion

In this study, we present a complete genome assembly with functional annotations, of the entomopathogenic fungi *M. brunneum.* This is the first Nanopore/Illumina complete de novo hybrid assembly, to our knowledge, of a fungus in the *Sordariomycete* class. We have demonstrated that a hybrid assembly approach can be used to cheaply produce a better genome assembly, with telomere-to-telomere chromosome assemblies that can allow for chromosomal macrosynteny comparisons between strains and species. The generation of more complete fungal genomes will lead to a better understanding of fungal evolution at a finer resolution, ultimately allowing for better understanding of the genomic underpinnings of phenotypic variation. The methodology may also prove useful for quality control purposes of commercially produced fungal-based products, given the continued decline in cost of whole genome sequencing technologies.

## Methods

### Insect inoculation and DNA extraction

*M. brunneum* ARSEF strain 4556, obtained from the U.S. Department of Agriculture’s ARS Collection of Entomopathogenic Fungal Cultures (ARSEF), was cultured in SDA medium plates and incubated at 25 °C for 10 days. Sample details were deposited at the NCBI under the BioSample accession: SAMN15394350. Conidia were collected after 10 days by flooding the dish with 20 mL of 0.04% Tween 80 and scraping the surface with a scalpel. The collected conidial suspension was vortexed until complete homogenization and filtered using a sterile nylon membrane. Concentration of conidial suspension was adjusted to 1 × 10^8^ spores mL^− 1^ using a hemocytometer (Neubauer, Germany). Spore viability was verified and spores were considered to have germinated if they had formed a germ-tube that was as long as spore width.

Larvae of the greater wax moth, *Galleria mellonella*, were immersed in 10 ml of conidial suspension for 10 s and were placed on moist filter paper in petri dishes in order to encourage sporulation and fungal growth. Controls were included with insects immersed in pure 0.04% Tween 80, in order to ensure that insect death was a result of fungal infection. Plates were incubated in the dark at 25 °C and were inspected daily. After fungal growth was observed, mycelia were collected and grown on SDA media for DNA extraction.

A total of 100 mg of conidia was scraped off the plate under a laminar flow hood, and collected into a sterile 1.5 mL DNA LoBind tube (Eppendorf, Hamburg, Germany). The conidia were ground in the tube with a micro-pestle, and DNA was extracted using the PureLink® Plant Total DNA Purification Kit (Invitrogen, Carlsbad, USA), following the manufacturer’s guidelines. The DNA was checked for purity on a Nanodrop (Thermo Scientific, USA), and DNA concentrations were measured using the Qubit broad range DNA assay kit (Thermo Scientific, USA).

### Illumina sequencing

Illumina DNA library preparation and sequencing were outsourced to Eurofins Genomics GmbH, Ebersberg, Germany. Illumina paired-end reads (2 × 150 bp) were produced using the ‘INVIEW Resequencing Sequencing of Fungi 50x Coverage’ package. Illumina reads were trimmed using Trimmomatic version 0.38 [35], setting the HEADCROP configuration to 15 and the CROP configuration to 120. Read qualities were assessed with FastQC [36].

### Nanopore sequencing

A total of 1 μg of genomic DNA was used for Nanopore library preparation using a 1D Ligation Sequencing Kit (SQK-LSK109, Oxford Nanopore Technologies). Sequencing was performed on a MinION device (Oxford Nanopore Technologies), equipped with a R9.4.1 MinION flow cell. Base calling was performed offline with ONT’s Guppy software pipeline version 3.4.5, enabling the --pt_scaling flag and setting the --trim_strategy flag to DNA.

### Long read filtering and correction

Long read adapter trimming was performed with Porechop version 0.2.4 (www.github.com/rrwick/Porechop), setting the --adapter_threshold to 96, and enabling the --no_split flag. In order to retrieve any circular contig assemblies (e.g. mitochondrial DNA), adapter trimmed long reads and trimmed Illumina paired-end reads were used as input for Unicycler version 0.4.8-beta [37], using the default settings. The trimmed long reads were filtered to remove reads under 3000 bases in length using NanoFilt version 2.6.0 [38], and were subsequently converted from FASTQ to FASTA format using a custom AWK script- [‘BEGIN {*P* = 1}{if(*P*==1||*P*==2){gsub(/^[@]/,” > “);print}; if(*P*==4) *P* = 0; P++}’ in.fastq > out.fasta]. The trimmed long reads were corrected using the trimmed Illumina short reads with FMLRC version 1.0.0 [39]. These corrected reads were further trimmed with Canu version 1.9 [40], using the -trim option, setting the genome size to 38 Mb, and disabling the stop on low coverage and stop on low quality features. Two filtered read sets were generated from the Canu output using SeqKit version 0.11.0 [41], one set filtered to contain reads with > 3000 bases and the other to contain reads with > 5000 bases.

### Long read assembly

One assembly was carried out per read set using Flye version 2.7 [42] using the --nano-corr flag, setting the genome size to 38 Mb and enabling the --trestle flag. Each of the two assemblies were then used to generate an additional assembly by subjecting each output to a total of two rounds of polishing with Flye (as opposed to the default of one round). Evidence from all assemblies were used to manually resolve tangles. Mapping of reads to a short contig of 5231 bp, which contained the telomere sequence TTAGGG at its terminal end, showed the contig to overlap with an end repeat region of Chromosome 1, and they were combined manually with the aid of CAP3 [43], thus producing, in combination with the manual resolving of tangles, a FASTA file containing all 7 complete chromosomes.

### Validation of assembly and comparison of long read assembler performance

In order to validate the final complete assembly and compare long read assembler performance of a fungal genome, assemblies were carried out on both the adapter trimmed long reads (> 3000 bp) and the FMLRC corrected Canu trimmed long read (> 3000 bp) using various assemblers. Assemblers tested included; Canu version 2.0, Flye version 2.7, Miniasm/Minipolish version 0.1.3 [44] Raven version 1.1.10 [45], NECAT version 0.01 [46], wtdbg2 version 2.5 [47], and shasta version 0.5.1 [48]. All assemblers were run with default parameters (flagging raw or corrected reads depending on read input, Raven was run with the --weaken flag when corrected reads were used). Additional Flye assemblies were performed using both Canu and NECAT self-corrected read sets and an additional short-read corrected read set corrected with Ratatosk version 0.1 [49], in order to assess read correction strategy performance. The Ratatosk corrected reads were Canu trimmed using the same settings as for the FMLRC corrected read set. Assemblies were compared using Quast version 5.0.2 [50]. Bandage version 0.8.1 [51] was used to visualize assembly graphs and search for telomere sequences by using the built-in blast function to search the telomere sequence TTAGGG_n5_, as well as blast searching the complete assembly against each assembly to determine inter-chromosomal mis-assembly events.

### Assembly polishing

**Fig. 6:**
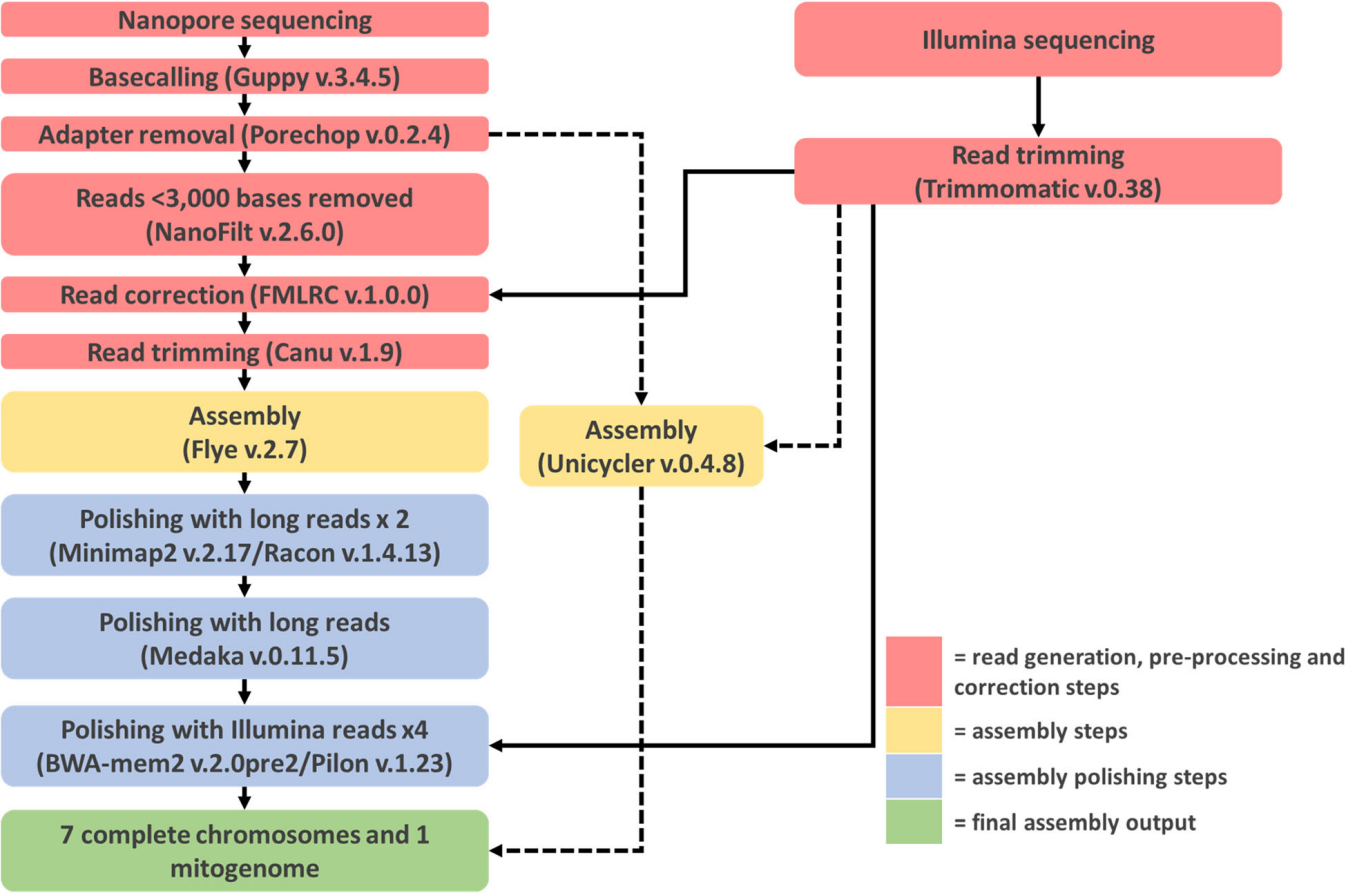
Novel assembly pipeline used to generate telomere length de novo assembly and mitogenome assembly of *Metarhizium brunneum*. An overview of the steps and tools versions used to generate the complete assembly. Arrows with dashed lines represent mitogenome assembly steps. Arrows with solid lines represent the chromosomal assembly steps

The uncorrected, adapter trimmed > 3000 bp long reads were realigned to the manually resolved assembly with minimap2 version 2.17-r941 [52] and the resulting alignment file was used to polish the assembly with Racon version v1.4.13 [53], using default parameters with the --no-trimming flag enabled. A total of two rounds of racon polishing were performed in this manner. The corrected consensus was further polished with the same long read set using Medaka version 0.11.5 (https://github.com/nanoporetech/medaka). The trimmed short-read pair-end Illumina reads were mapped to the long-read polished contigs using BWA-mem2 version 2.0pre2 [54], and the assembly was further polished with Pilon version 1.23 [55], enabling the --fix all and --changes flags. In total, four iterations of polishing with the Illumina reads were performed in this manner, and further polishing yielded no additional changes. A summary of the full assembly pipeline is shown in Fig. 6. A dotplot comparison of the scaffolds and contigs from the NCBI reference *M. brunneum* ARSEF 3297 assembly (GCF_000814965.1) against the complete assembly produced in this study was made using Mummer version 3 [56].

### Gene prediction and functional annotation

BUSCO analyses were performed with BUSCO version 4.0.2 [57], using the hypocreales_odb10 lineage gene set. Chromosomes were visualized in Tapestry version 1.0.0 (https://github.com/johnomics/tapestry) in order to determine chromosome completeness (by checking for long read mapping gaps), and setting the telomere sequence as TTAGGG- a common eukaryotic telomere repeat sequence previously shown to be present in *Metarhizium* telomeres [58]. All assembly annotations were performed in GenSAS version 6.0 [59], unless otherwise stated. Low complexity regions and repeats were detected and masked using RepeatModeler version 1.0.11 [60] and RepeatMasker version 4.0.7 [61], setting the DNA source to fungi and the speed/sensitivity parameter to slow. A masked consensus sequence was generated on which ab initio gene prediction was performed using the following tools; 1. GeneMarkES version 4.33 [62] with default parameters, 2. Augustus version 3.3.1 using *Fusarium graminearum* as the species, but otherwise keeping the default parameters, 3. GlimmerM version 2.5.1 [63] selecting *Aspergillus* as the organism. Two separate standalone ab initio gene predictions were conducted on the masked consensus sequence (one including the mitogenome sequence and the other without) using the latest version of GeneMarkES (4.48_3.60.lic), enabling the --ES and --Fungus flags. The highest BUSCO scoring ab initio predicted protein set was used for functional analyses using InterProScan version 5.25–68.0 [64], a native version of SignalP version 5.0 [65] setting the -org flag to eukaryote, and identifying ab initio predicted proteins with blastp [66] by conducting a protein vs protein search against the SwissProt protein data set to determine best matches. Ribosomal RNA genes were detected using RNAmmer version 1.2 [67]. tRNA genes were determined using tRNAscan-SE version 2.0.3 [68]. Comparison of orthologous gene clusters between the protein set generated in this study and the NCBI reference *M. brunneum, M. anisopliae* and *M. robertsii* protein sets was performed using OrthoVenn2 [69], with default parameters. The mitogenome, including previously described manual annotations [70], was visualized using the GeSeq tool in Chlorobox [71], selecting a circular mitochondrial sequence.

### Full genome sequence-based synteny and pan-genome analyses of *Hypocreales* fungi

Synteny analyses were performed by comparing the *M. brunneum* complete genome assembly to three other species within the order *Hypocreales* that had genome assemblies that are designated as complete by the NCBI (full telomere length chromosomes). These included the genomes of the entomopathogenic fungus *Cordyceps militaris* [26], the systemic endophytic fungus *Epichloe festucae* [27], and the cellulolytic, endophytic fungus *Trichoderma reesei* [28]. Genomes were aligned with progressiveMauve v2.4.0 [72], using default settings. Alignment blocks were filtered to remove syntenic blocks that were less than 1000 bp in size, and also those which were not present in all 4 species. Synteny was inferred with i-ADHoRe v3.0 [73] running default parameters, and whole genome synteny between each species were visualized with Circos plots using Circos v2.40.1 [74]. Ab-initio gene prediction was performed on the three genome assemblies of the other *Hypocreales* species using GeneMarkES (4.48_3.60.lic), enabling the --ES and --Fungus flags. In order to determine the core genes shared across the 4 species, comparison of orthologous gene clusters between the protein sets for each of the *Hypocreales* fungi were performed with OrthoVenn2 using default parameters.

## Supplementary Information

**Additional file 1. **Pipeline and assembly validation. A) a Flye assembly graph of the FMLRC corrected long reads without the Canu trimming step. B) a Flye assembly graph of the Canu trimmed long reads without the FMLRC correction step. Both assemblies failed to generate telomere length contigs. C) Manual resolving of tangles in Flye assembly (> 5000) graph. Evidence was used from both assemblies to resolve the final tangles. Chromosome 7 was telomere length in the > 3000 read length assembly, and, along with coverage data, allowed the tangle in the assembly graph between chromosome 1 and chromosome 7 to be resolved (blue). Chromosome 3 was also fully telomere length in the in the > 3000 read length assembly (pink). Mapping reads to the 5231 bp contig, which contained a telomere sequence at its terminal, showed the contig to overlap with the end repeat contig of chromosome one (purple). D) Dotplot comparison of the long read assembly *M. brunneum* reference assembly. Good synteny is observed between the 7 complete chromosomes and the contigs and scaffolds from the previous reference assembly.

**Additional file 2.** Comparison of assemblers. Assembly graphs showing TTAGGG_n5_ sequences detected in contigs produced by all assemblers, and colour coded blast hits of chromosomes from the final complete assembly from which mis-assemblies were inferred.

**Additional file 3. **Summary of SignalP results. SingalP likelihood scores, signal peptide type (if present) and signal peptide positions for all *M brunneum* proteins.

**Additional file 4. **Mature proteins. *M brunneum* mature protein sequences with signal peptides removed.

**Additional file 5. **Core *Hypocreales* protein set. A list of core proteins found to be orthologous between the four *Hypocreales* fungi; *Metarhizum brunneum*, *Cordyceps militaris*, *Epichloe festucae* and *Trichoderma reesei*.

**Additional file 6. ***Metarhizium brunneum* self-cluster. The 183 unique orthologous clusters that were formed between *M. brunneum* proteins.

**Additional file 7. **Orthologous clusters formed between the entomopathogenic fungi. The 468 unique orthologous clusters that were formed between the two entomopathogenic *Hypocreales* fungi in the comparison test- *Metarhizium brunneum* and *Cordyceps militaris*. Proteins within these clusters may be involved with the entomopathogenic process.

**Additional file 8. ***M brunneum* singletons. *Metarhizium brunneum* proteins that did not form orthologous clusters with any other proteins.

## Data Availability

All data generated in this study has been deposited at the NCBI under Bioproject PRJNA608152. Illumina sequencing read data can be accessed at the NCBI Sequence Reads Archive (SRA) using the accession number SRX7785787. Nanopore sequencing read data can be accessed at the NCBI SRA using the accession number SRX7785786. Sample information can be accessed at the NCBI BioSample repository using the accession number SAMN14166897. The genome assembly generated in this study can be accessed in NCBI’s GenBank database using the accession number GCA_013426205.1. Gene and protein names and functional annotations (GO terms, InterPro, PFAM) are included in GenBank entries. All output files have been deposited in the following GitHub repository- https://github.com/zacksaud/Metarhizium-Brunneum-ARSEF4556-Assembly-Project. The following genomes and/or information on the genome assemblies were retrieved from NCBI’s GenBank database; *Metarhizium album* ARSEF 1941 (accession number: GCA_000804445.1), *Metarhizium acridum* CQMa 102 (accession number: GCA_000187405.1), *Metarhizium anisopliae* ARSEF 549 (accession number: GCA_000814975.1), *Metarhizium brunneum* ARSEF 3297 (accession number: GCF_000814965.1), *Metarhizium guizhouense* ARSEF 977 (accession number: GCA_000814955.1), *Metarhizium majus* ARSEF 297 (accession number: GCA_000814945.1), *Metarhizium rileyi* RCEF 4871 (accession number: GCA_001636745.1), *Metarhizium robertsii* ARSEF 23 (accession number: GCA_000187425.2), *Cordyceps militaris* ATCC 34164 (accession number: GCA_008080495.1), *Epichloe festucae* Fl1 (accession number: GCA_003814445.1), and *Trichoderma reesei* QM6a (accession number: GCA_002006585.1).

## Abbreviations

ARSEF: ARS Collection of Entomopathogenic Fungal Cultures
BUSCO: Benchmarking Universal Single-Copy Orthologs
CM: *Cordyceps militaris*
DNA: Deoxyribonucleic acid
EF: *Epichloe festucae*
MAA: *Metarhizium robertsii*
MAN: *Metarhizium anisopliae*
Mb: Million base pairs
MBR: *Metarhizium brunneum*
NCBI: National center for biotechnology information
ORF: Open reading frame
rRNA: Ribosomal ribonucleic acid
SDA: Sabouraud dextrose agar
TR: *Trichoderma reesei*
tRNA: Transfer ribonucleic acid
