# Supplementary material for: Telomere length de novo assembly of all 7 chromosomes and mitogenome sequencing of the model entomopathogenic fungus, Metarhizium brunneum, by means of a novel assembly pipeline
Source: BMC Genomics. 2021 Jan 28;22:87. doi: 10.1186/s12864-021-07390-y (PMC7842015; doi:10.1186/s12864-021-07390-y)
Supplement: Supplementary file 1 — Additional file 1. Pipeline and assembly validation. A) a Flye assembly graph of the FMLRC corrected long reads without the Canu trimming step. B) a Flye assembly graph of the Canu trimmed long reads without the FMLRC correction step. Both assemblies failed to generate telomere length contigs. C) Manual resolving of tangles in Flye assembly (> 5000) graph. Evidence was used from both assemblies to resolve the final tangles. Chromosome 7 was telomere length in the > 3000 read length assembly, and, along with coverage data, allowed the tangle in the assembly graph between chromosome 1 and chromosome 7 to be resolved (blue). Chromosome 3 was also fully telomere length in the in the > 3000 read length assembly (pink). Mapping reads to the 5231 bp contig, which contained a telomere sequence at its terminal, showed the contig to overlap with the end repeat contig of chromosome one (purple). D) Dotplot comparison of the long read assembly M. brunneum reference assembly. Good synteny is observed between the 7 complete chromosomes and the contigs and scaffolds from the previous reference assembly. [file 12864_2021_7390_MOESM1_ESM.pdf]

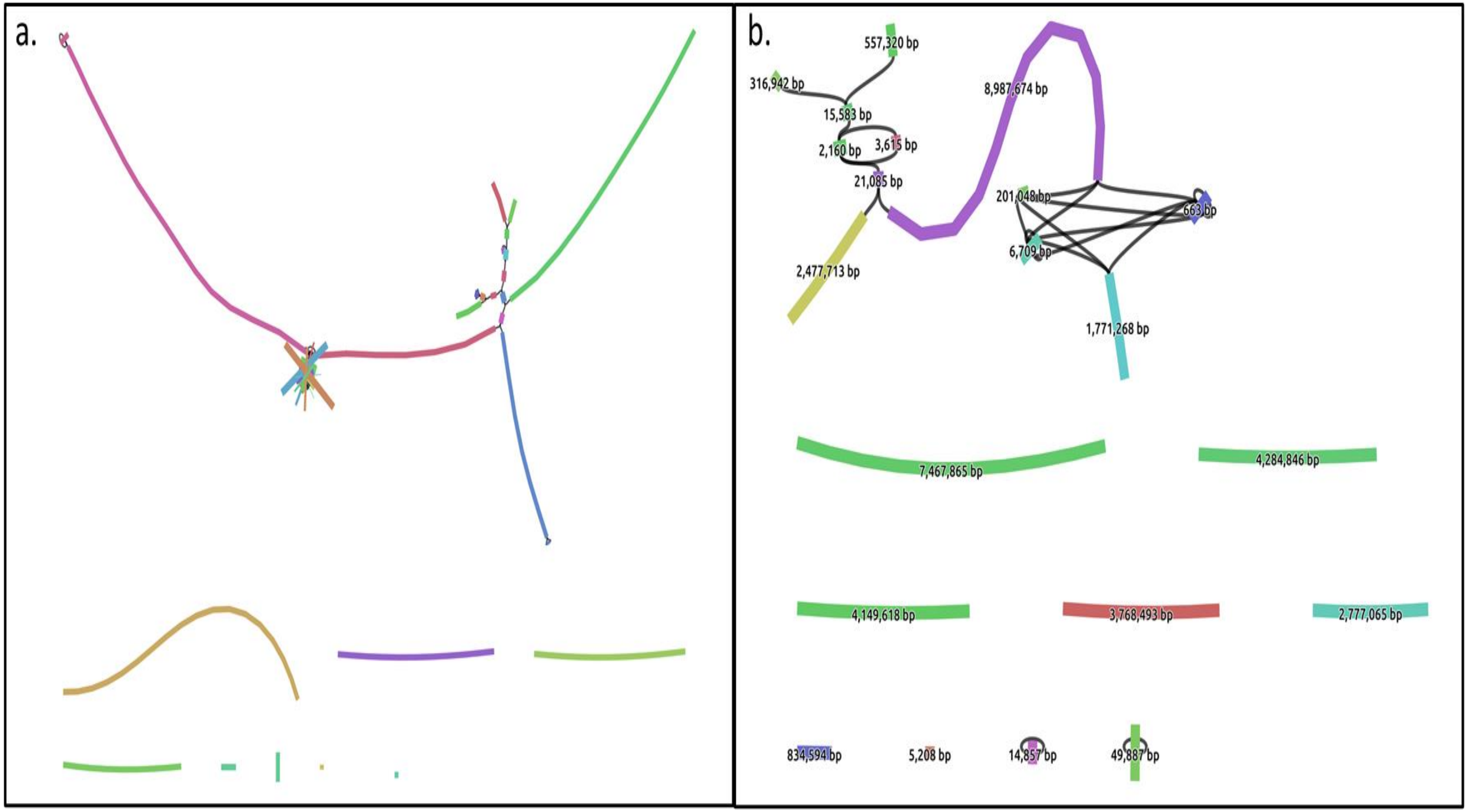

Figures S1 A) & B) Suboptimal Flye assembly graphs resulting from omitting a correction step from the assembly pipeline. A) a Flye assembly graph of the FMLRC corrected long reads without the Canu trimming step. B) a Flye assembly graph of the Canu trimmed long reads without the FMLRC correction step. Both assemblies failed to generate telomere length contigs.

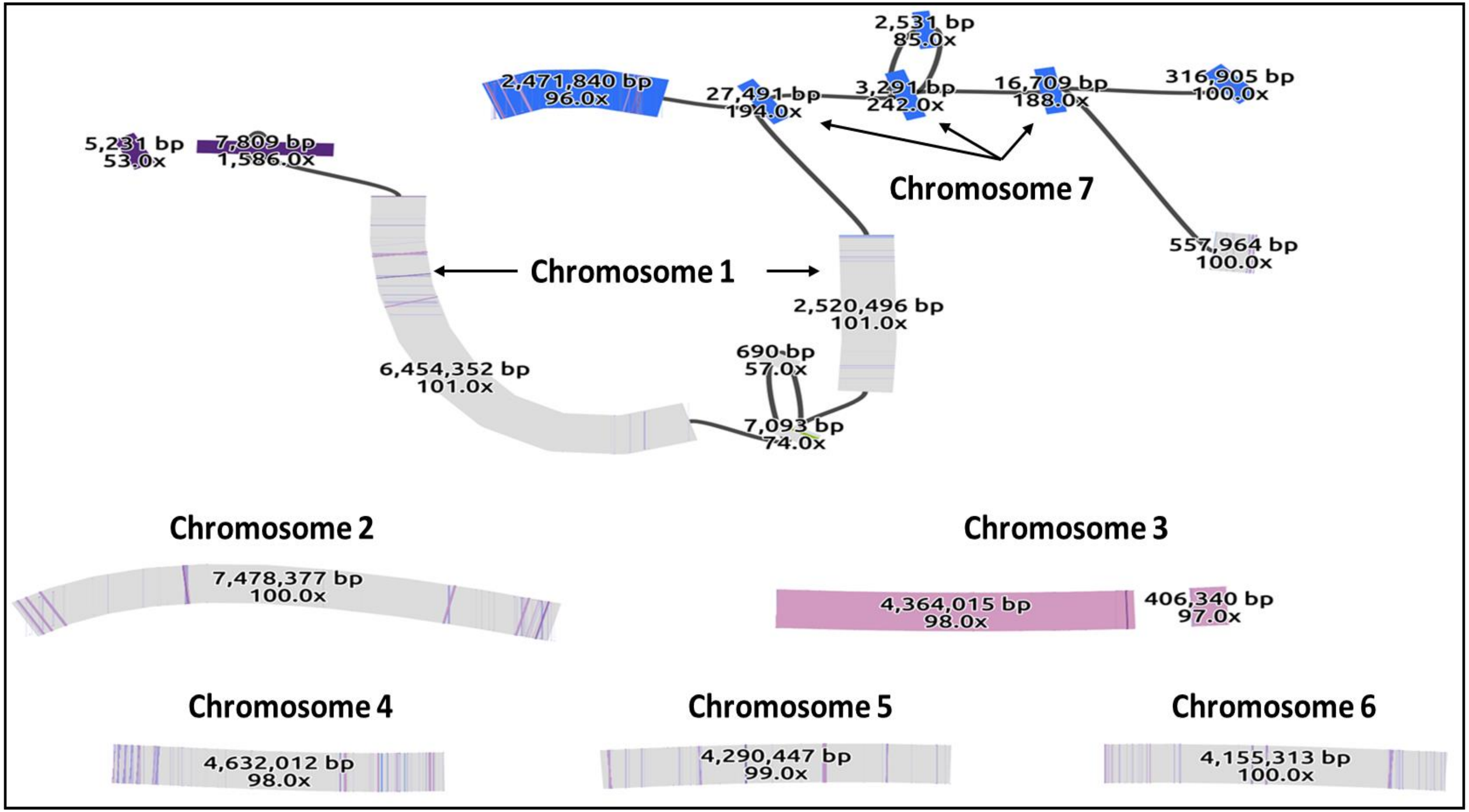

Figure S1 C) Manual resolving of tangles in Flye assembly (>5000) graph. Evidence was used from both assemblies to resolve the final tangles. Chromosome 7 was telomere length in the >3000 read length assembly, and, along with coverage data, allowed the tangle in the assembly graph between chromosome 1 and chromosome 7 to be resolved (blue). Chromosome 3 was also fully telomere length in the in the >3000 read length assembly (pink). Mapping reads to the 5,231 bp contig, which contained a telomere sequence at its terminal, showed the contig to overlap with the end repeat contig of chromosome one (purple).

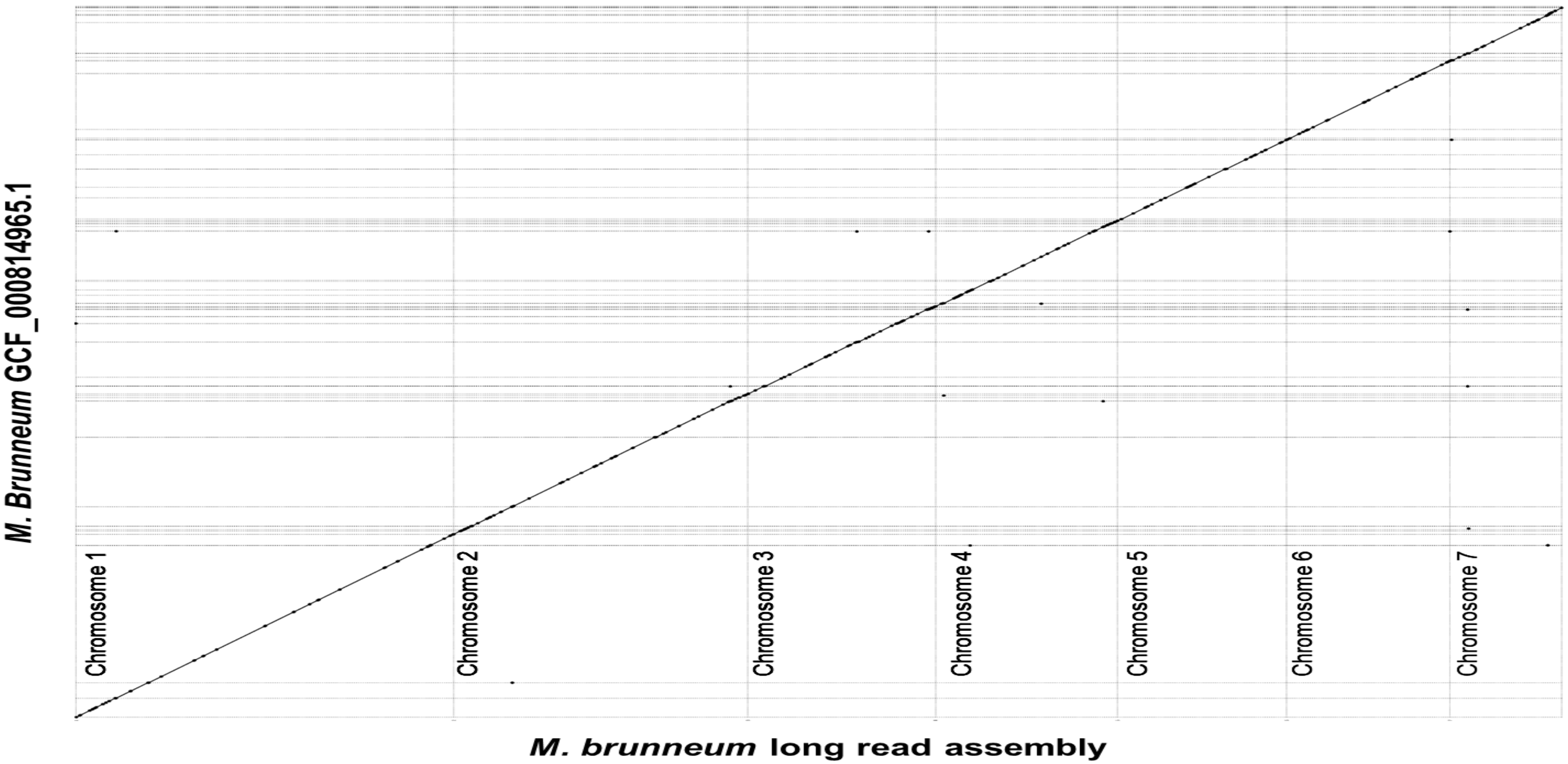

Figure S1 D) Dotplot comparison of the long read assembly M. brunneum reference assembly. Good synteny is observed between the 7 complete chromosomes and the contigs and scaffolds from the previous reference assembly.
