# Supplementary material for: Telomere length de novo assembly of all 7 chromosomes and mitogenome sequencing of the model entomopathogenic fungus, Metarhizium brunneum, by means of a novel assembly pipeline
Source: BMC Genomics. 2021 Jan 28;22:87. doi: 10.1186/s12864-021-07390-y (PMC7842015; doi:10.1186/s12864-021-07390-y)
Supplement: Supplementary file 2 — Additional file 2. Comparison of assemblers. Assembly graphs showing TTAGGGn5 sequences detected in contigs produced by all assemblers, and colour coded blast hits of chromosomes from the final complete assembly from which mis-assemblies were inferred. [file 12864_2021_7390_MOESM2_ESM.pdf]

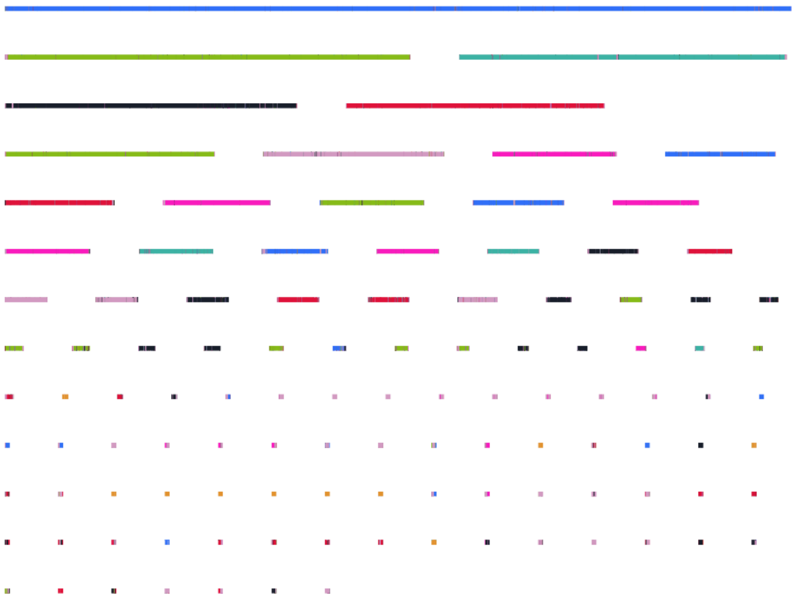

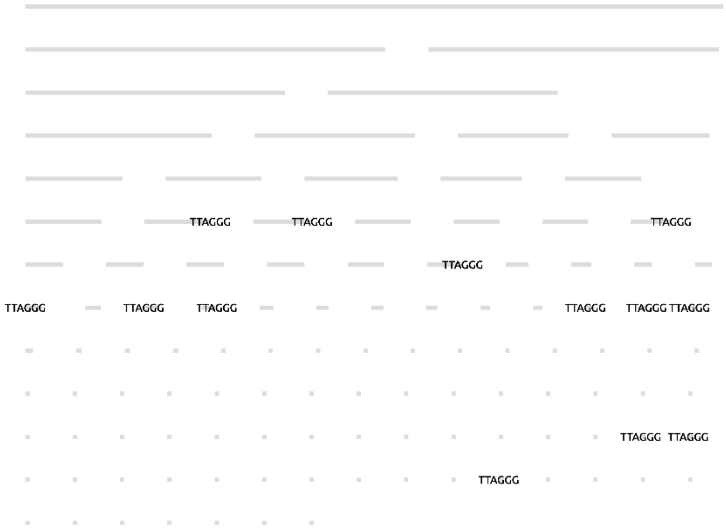

Canu uncorrected reads assembly

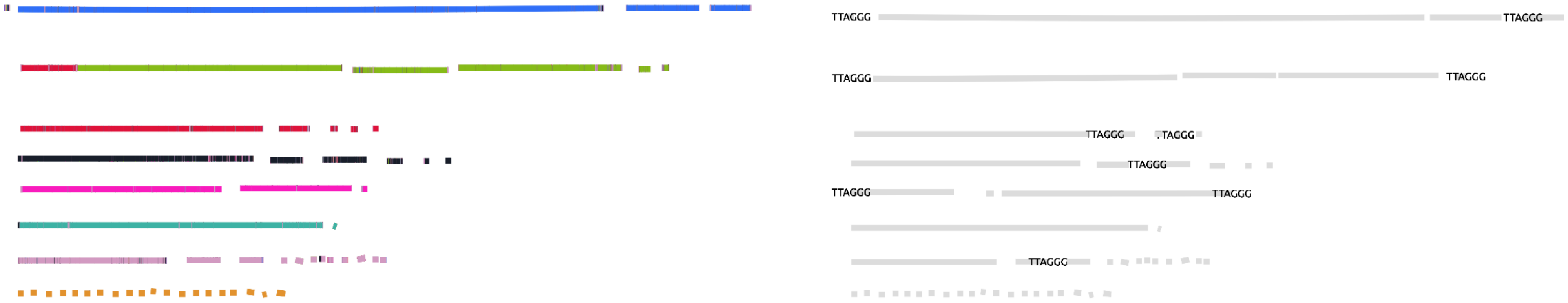

Flye uncorrected reads assembly

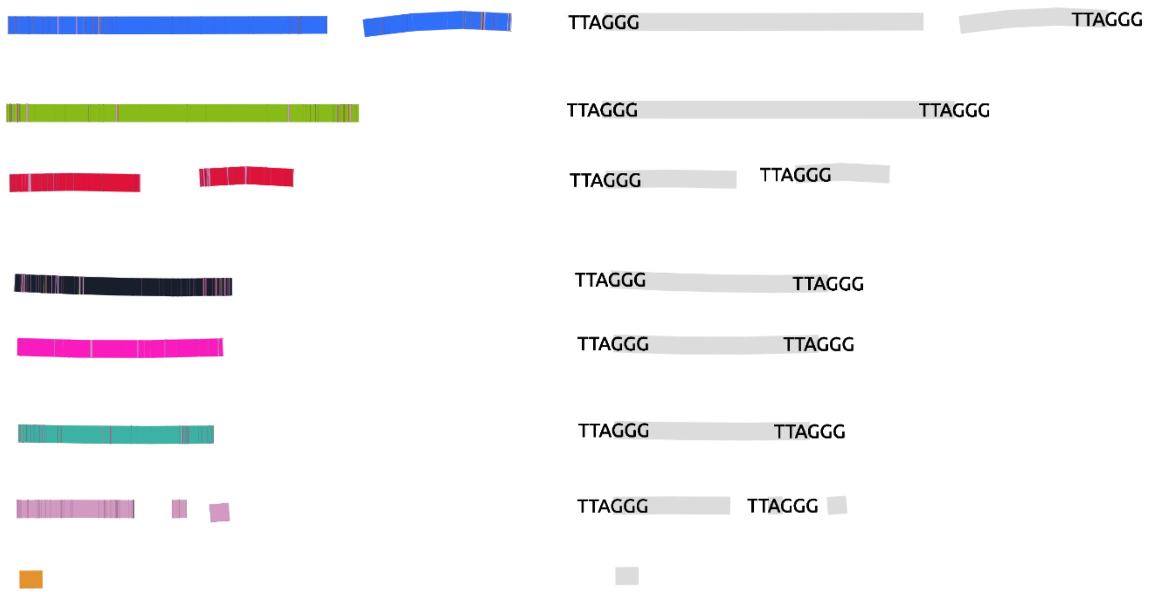

NECAT uncorrected reads assembly

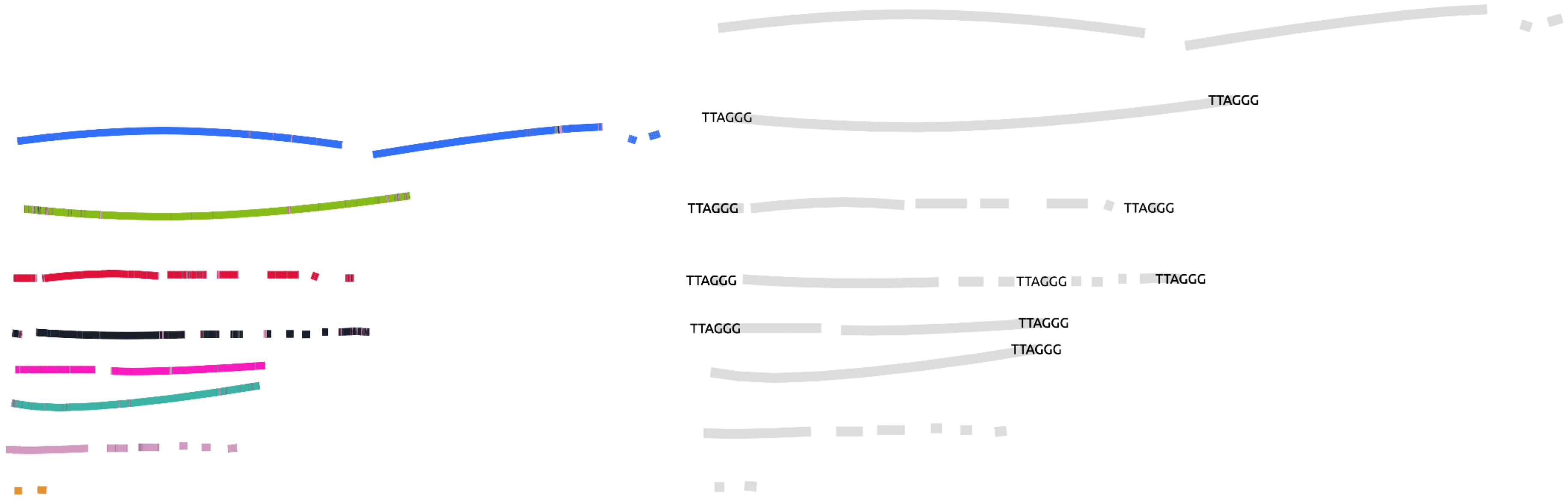

Miniasm/Minipolish uncorrected reads assembly

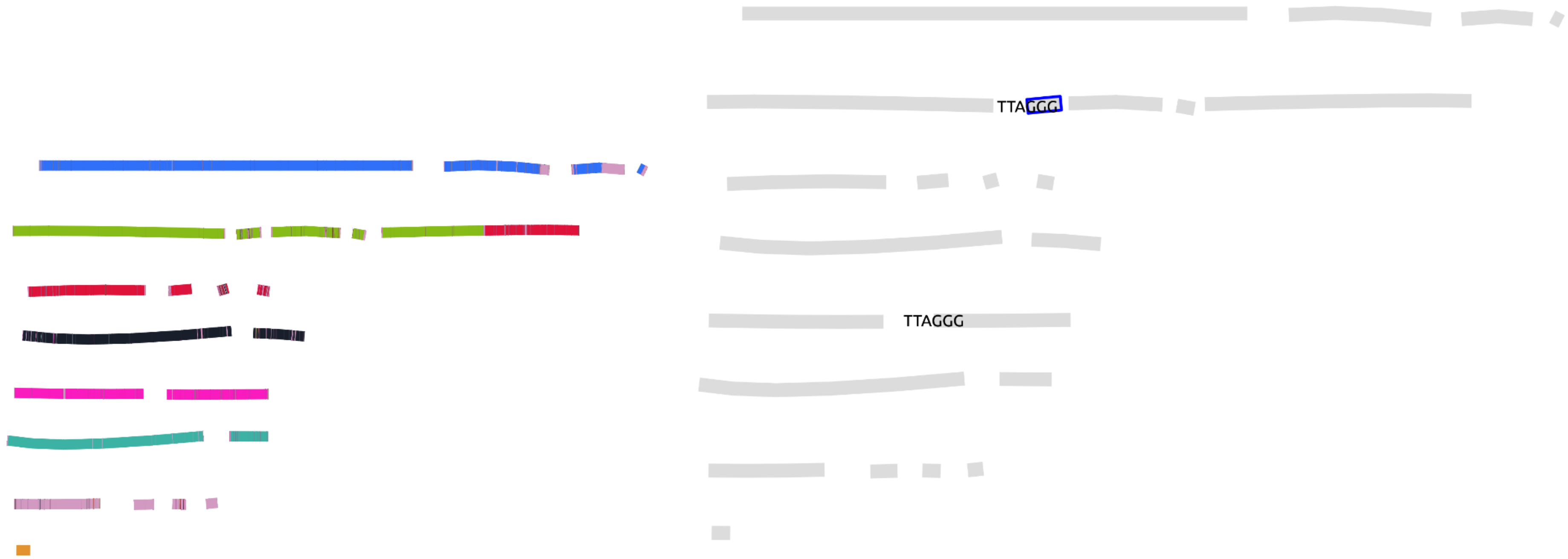

Raven uncorrected reads assembly

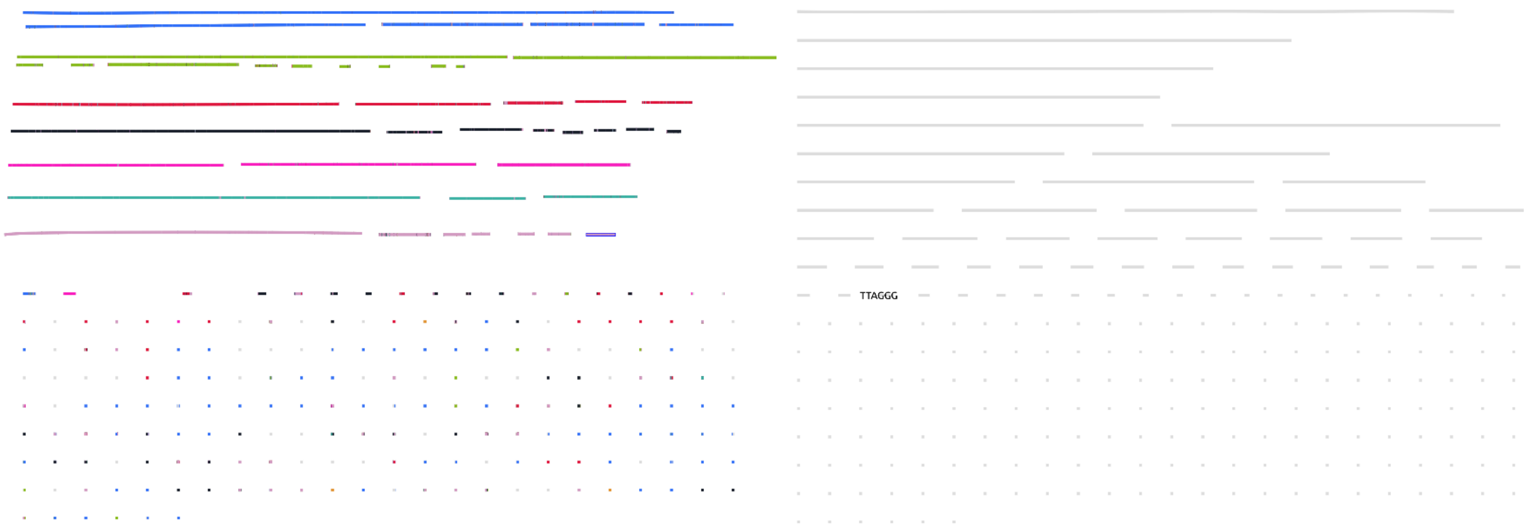

Shasta uncorrected reads assembly

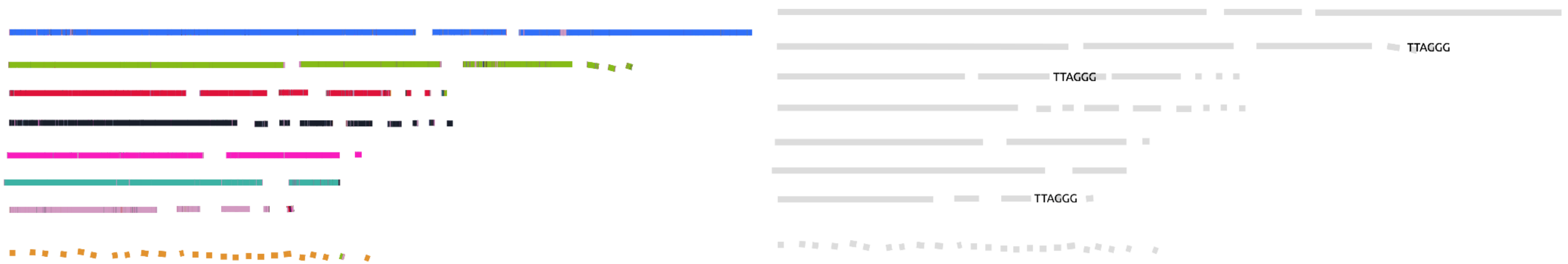

Wtdbg2 uncorrected reads assembly

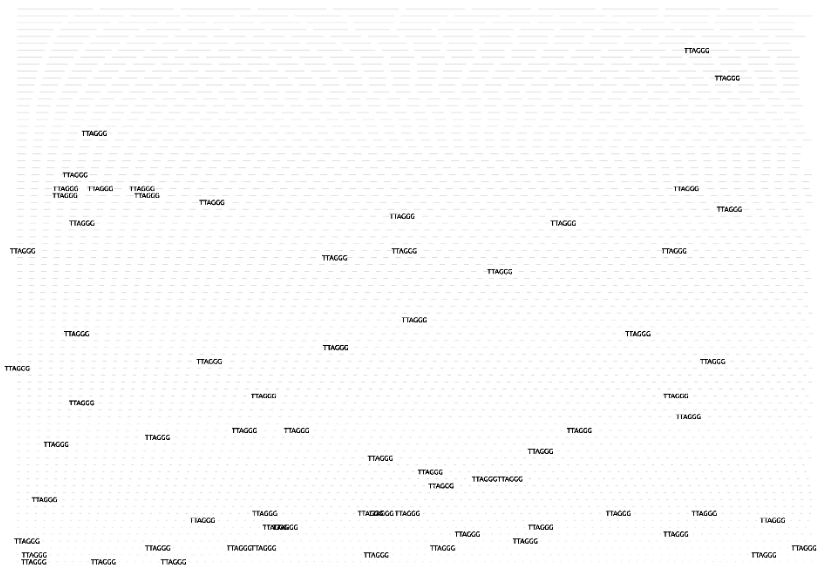

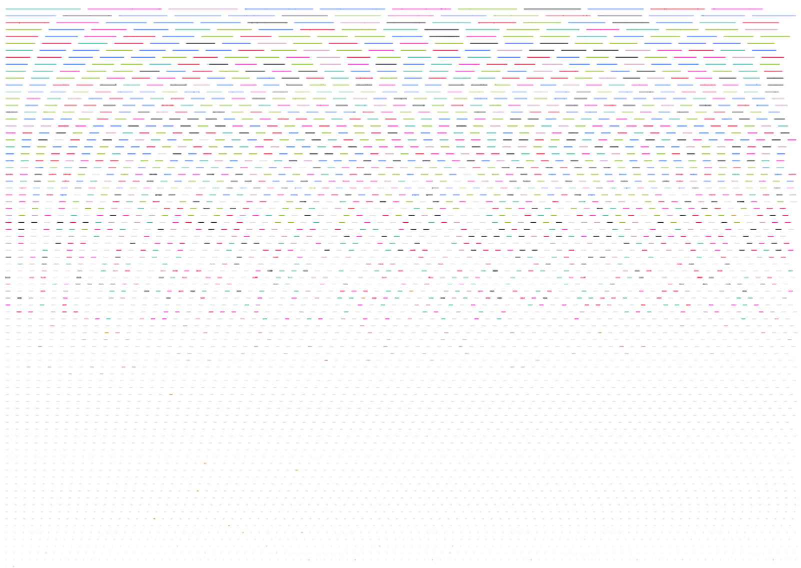

Canu assembly with FMLRC corrected reads

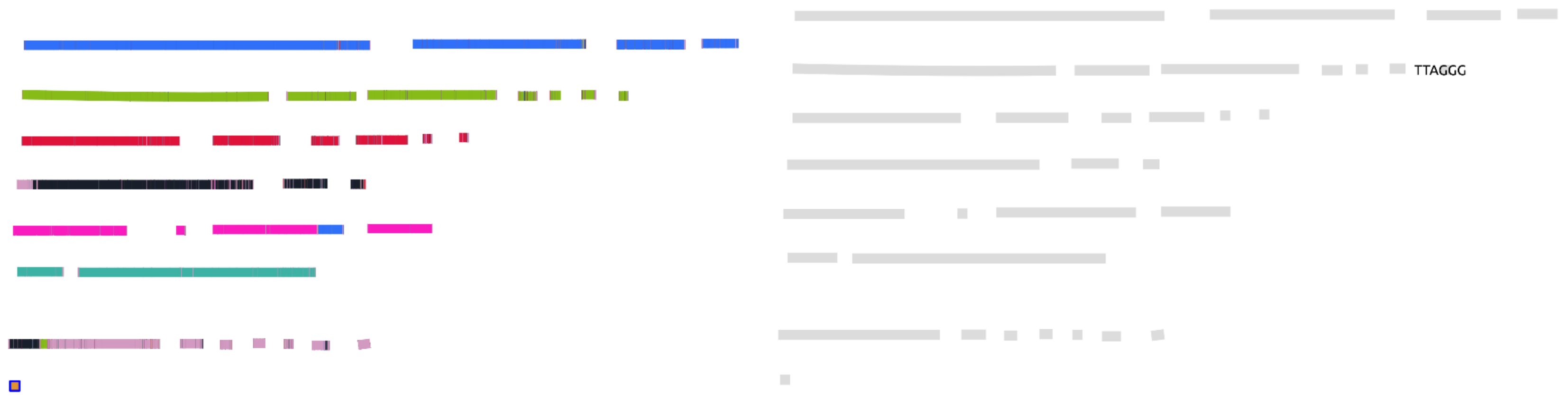

Raven assembly with FMLRC corrected reads

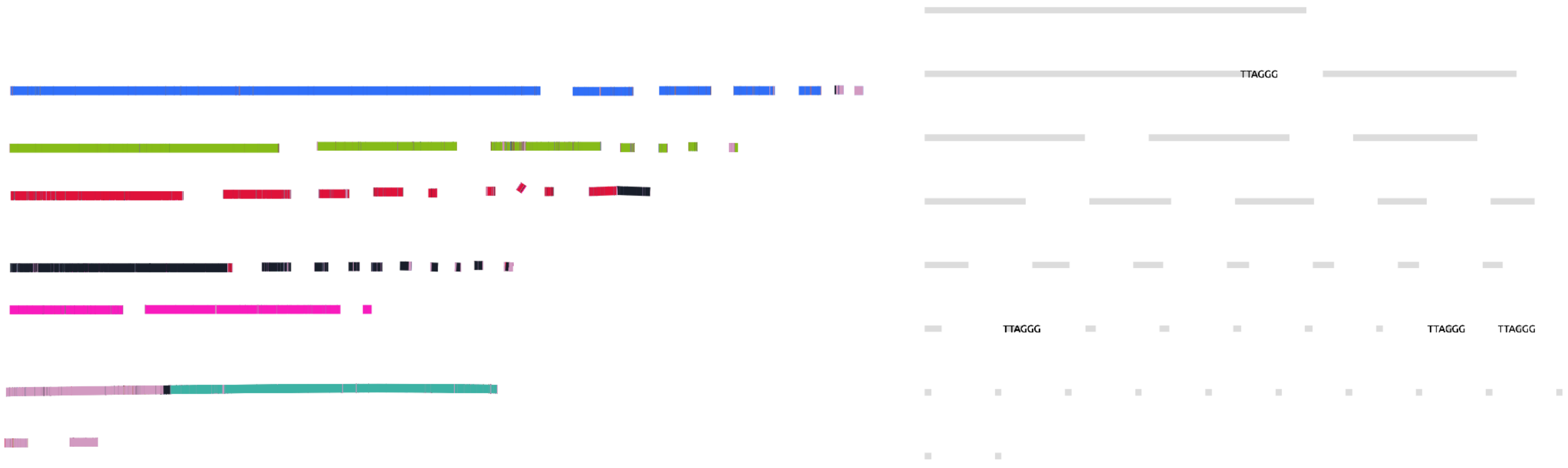

Wtdbg2 assembly with FMLRC corrected reads

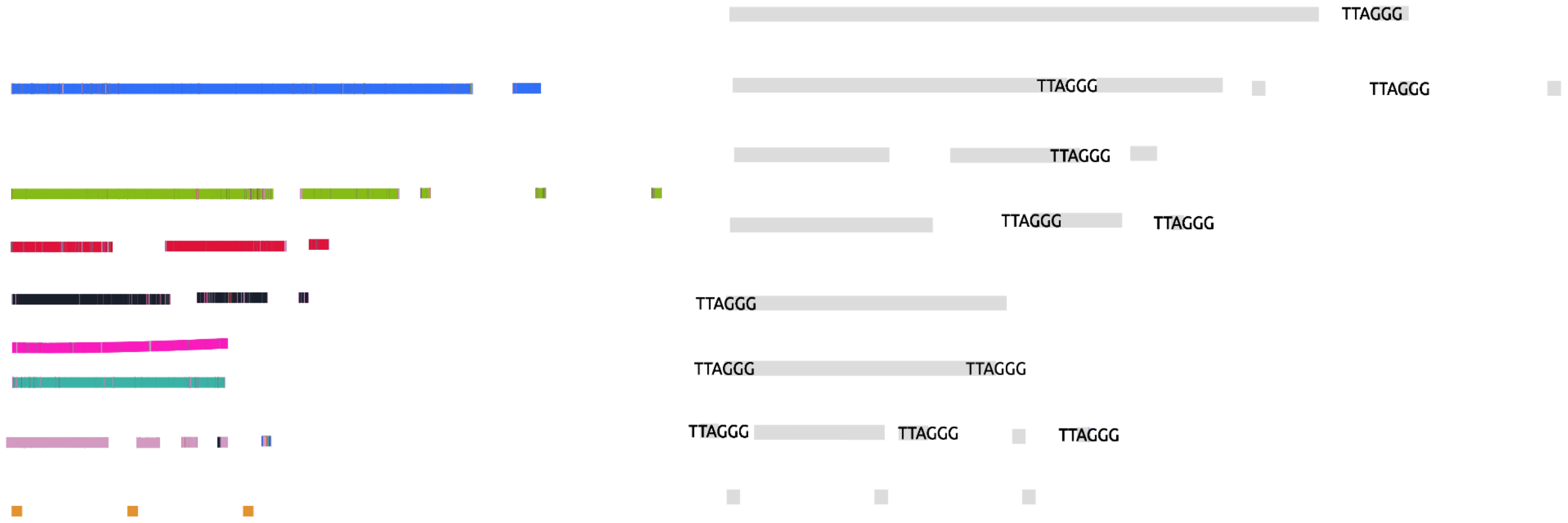

Flye assembly with Canu corrected reads

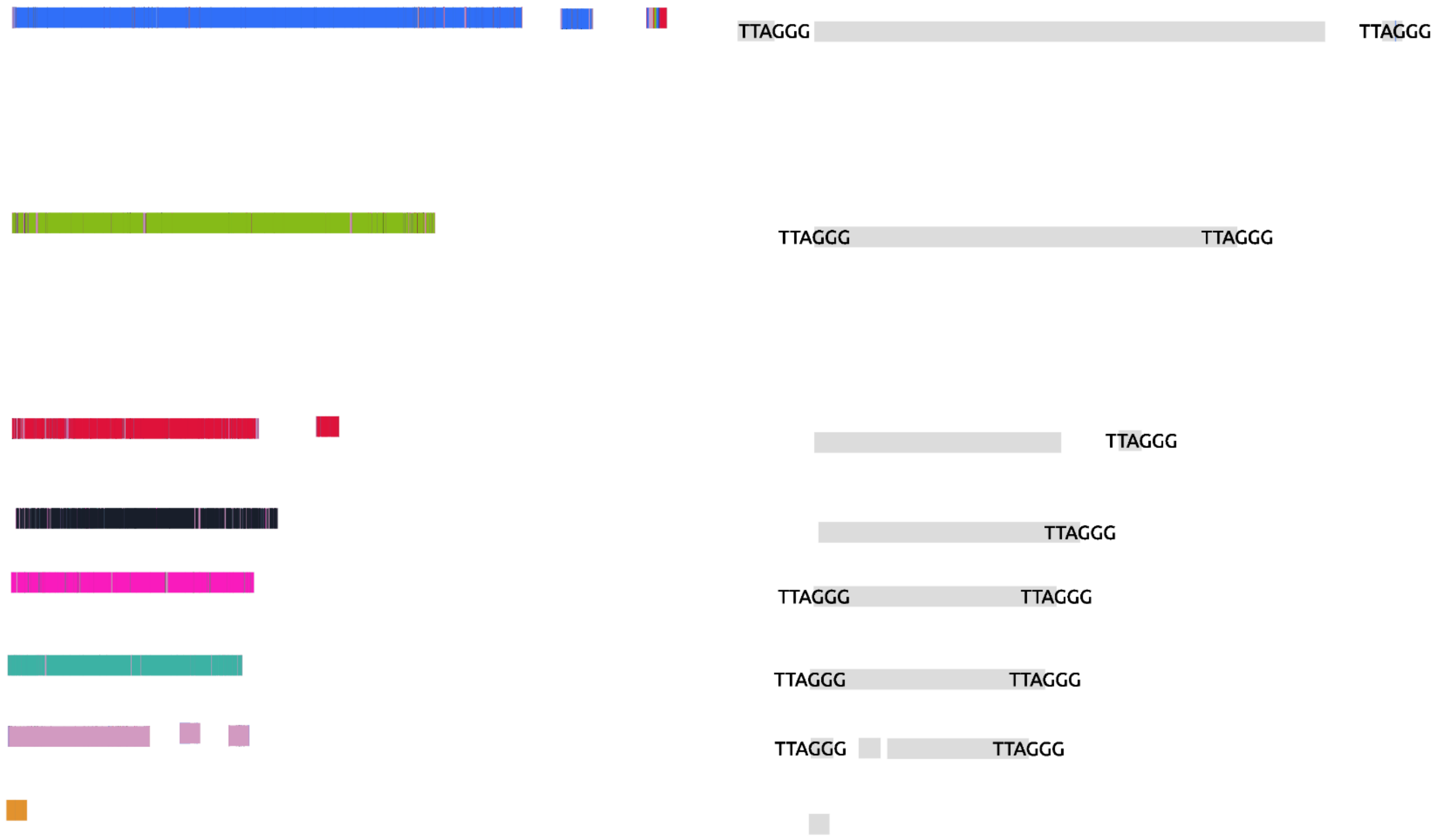

Flye assembly with NECAT corrected reads

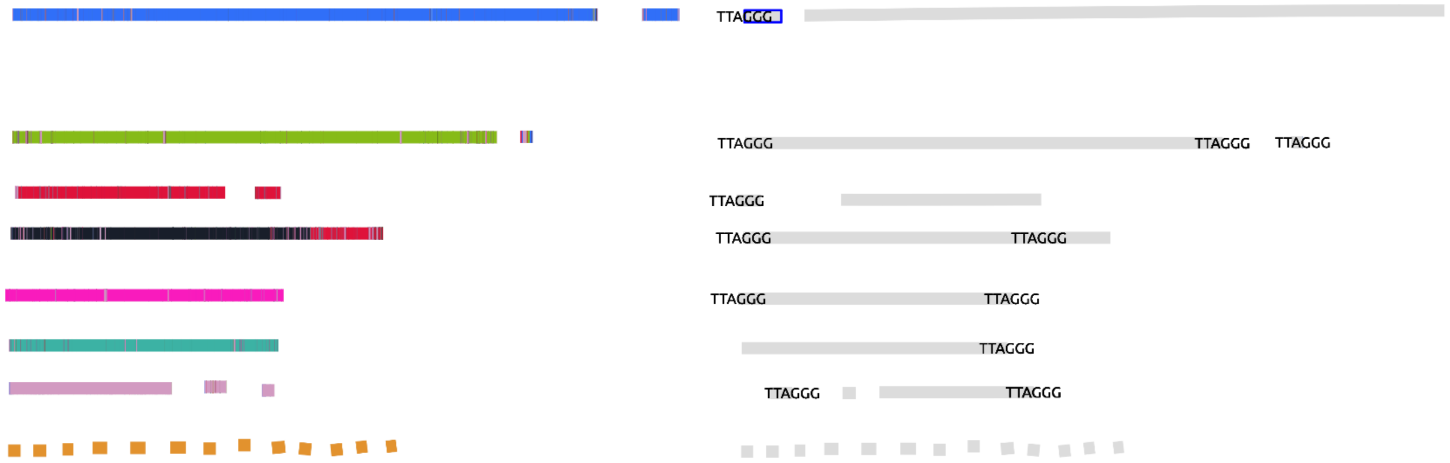

Flye assembly with Ratatosk corrected reads
