# Supplementary material for: Telomere length de novo assembly of all 7 chromosomes and mitogenome sequencing of the model entomopathogenic fungus, Metarhizium brunneum, by means of a novel assembly pipeline
Source: BMC Genomics. 2021 Jan 28;22:87. doi: 10.1186/s12864-021-07390-y (PMC7842015; doi:10.1186/s12864-021-07390-y)
Supplement: Supplementary file 3 — Additional file 3. Summary of SignalP results. SingalP likelihood scores, signal peptide type (if present) and signal peptide positions for all M brunneum proteins. [file 12864_2021_7390_MOESM3_ESM.pdf]

SignalP-5.0 Organism: euk Timestamp: 20200804202117

# ID	Prediction	SP(Sec/SPI)	OTHER	CS Position
QLI63422.1	OTHER	0.009168	0.990832	
QLI63423.1	OTHER	0.001500	0.998500	
QLI63424.1	OTHER	0.000772	0.999228	
QLI63425.1	OTHER	0.002737	0.997263	
QLI63426.1	OTHER	0.060737	0.939263	
QLI63427.1	OTHER	0.482449	0.517551	
QLI63428.1	OTHER	0.002420	0.997580	
QLI63429.1	OTHER	0.007037	0.992963	
QLI63430.1	OTHER	0.192447	0.807553	
QLI63431.1	OTHER	0.001804	0.998196	
QLI63432.1	OTHER	0.002737	0.997263	
QLI63433.1	OTHER	0.001210	0.998790	
QLI63434.1	OTHER	0.002007	0.997993	
QLI63435.1	OTHER	0.000996	0.999004	
QLI63436.1	OTHER	0.000933	0.999067	
QLI63437.1	OTHER	0.000805	0.999195	
QLI63438.1	SP(Sec/SPI)	0.975875	0.024125	CS pos: 17-18. AAA-GP. Pr: 0.7263
QLI63439.1	OTHER	0.371471	0.628529	
QLI63440.1	OTHER	0.001816	0.998184	
QLI63441.1	OTHER	0.005774	0.994226	
QLI63442.1	OTHER	0.001045	0.998955	
QLI63443.1	OTHER	0.005996	0.994004	
QLI63444.1	OTHER	0.000894	0.999106	
QLI63445.1	OTHER	0.001673	0.998327	
QLI63446.1	OTHER	0.000949	0.999051	
QLI63447.1	OTHER	0.177312	0.822688	
QLI63448.1	OTHER	0.000551	0.999449	
QLI63449.1	OTHER	0.000367	0.999633	
QLI63450.1	OTHER	0.001955	0.998045	

QLI63451.1	OTHER	0.001570	0.998430	
QLI63452.1	OTHER	0.001109	0.998891	
QLI63453.1	OTHER	0.000478	0.999522	
QLI63454.1	OTHER	0.002118	0.997882	
QLI63455.1	OTHER	0.000771	0.999229	
QLI63456.1	OTHER	0.001330	0.998670	
QLI63457.1	OTHER	0.000428	0.999572	
QLI63458.1	OTHER	0.001400	0.998600	
QLI63459.1	OTHER	0.000445	0.999555	
QLI63460.1	OTHER	0.000613	0.999387	
QLI63461.1	OTHER	0.001954	0.998046	
QLI63462.1	OTHER	0.000864	0.999136	
QLI63463.1	OTHER	0.002734	0.997266	
QLI63464.1	OTHER	0.000433	0.999567	
QLI63465.1	OTHER	0.001730	0.998270	
QLI63466.1	OTHER	0.002656	0.997344	
QLI63467.1	OTHER	0.000646	0.999354	
QLI63468.1	OTHER	0.000514	0.999486	
QLI63469.1	OTHER	0.000778	0.999222	
QLI63470.1	OTHER	0.000321	0.999679	
QLI63471.1	OTHER	0.001737	0.998263	
QLI63472.1	OTHER	0.001394	0.998606	
QLI63473.1	OTHER	0.005646	0.994354	
QLI63474.1	OTHER	0.001698	0.998302	
QLI63475.1	SP(Sec/SPI)	0.979104	0.020896	CS pos: 18-19. ALA-SP. Pr: 0.6265
QLI63476.1	OTHER	0.004267	0.995733	
QLI63477.1	OTHER	0.000357	0.999643	
QLI63478.1	OTHER	0.000750	0.999250	
QLI63479.1	OTHER	0.001066	0.998934	
QLI63480.1	OTHER	0.003538	0.996462	
QLI63481.1	SP(Sec/SPI)	0.989269	0.010731	CS pos: 20-21. ASA-VT. Pr: 0.8279

QLI63482.1	OTHER	0.003753	0.996247	
QLI63483.1	OTHER	0.001506	0.998494	
QLI63484.1	OTHER	0.000406	0.999594	
QLI63485.1	OTHER	0.002613	0.997387	
QLI63486.1	OTHER	0.001231	0.998769	
QLI63487.1	SP(Sec/SPI)	0.767121	0.232879	CS pos: 19-20. TGG-KL. Pr: 0.3842
QLI63488.1	OTHER	0.001235	0.998765	
QLI63489.1	OTHER	0.009057	0.990943	
QLI63490.1	OTHER	0.000589	0.999411	
QLI63491.1	OTHER	0.003485	0.996515	
QLI63492.1	OTHER	0.001410	0.998590	
QLI63493.1	OTHER	0.000406	0.999594	
QLI63494.1	OTHER	0.000703	0.999297	
QLI63495.1	OTHER	0.000208	0.999792	
QLI63496.1	OTHER	0.002073	0.997927	
QLI63497.1	OTHER	0.000311	0.999689	
QLI63498.1	OTHER	0.003333	0.996667	
QLI63499.1	OTHER	0.000406	0.999594	
QLI63500.1	OTHER	0.000390	0.999610	
QLI63501.1	OTHER	0.004805	0.995195	
QLI63502.1	SP(Sec/SPI)	0.938965	0.061035	CS pos: 22-23. ALA-SR. Pr: 0.8862
QLI63503.1	OTHER	0.000929	0.999071	
QLI63504.1	OTHER	0.001094	0.998906	
QLI63505.1	OTHER	0.001139	0.998861	
QLI63506.1	OTHER	0.000661	0.999339	
QLI63507.1	OTHER	0.002369	0.997631	
QLI63508.1	OTHER	0.011315	0.988685	
QLI63509.1	OTHER	0.001584	0.998416	
QLI63510.1	OTHER	0.494951	0.505049	
QLI63511.1	OTHER	0.001076	0.998924	
QLI63512.1	SP(Sec/SPI)	0.962587	0.037413	CS pos: 17-18. ATC-HQ. Pr: 0.5298

QLI63513.1	OTHER	0.002337	0.997663	
QLI63514.1	OTHER	0.003264	0.996736	
QLI63515.1	OTHER	0.000867	0.999133	
QLI63516.1	OTHER	0.002558	0.997442	
QLI63517.1	OTHER	0.000873	0.999127	
QLI63518.1	OTHER	0.000749	0.999251	
QLI63519.1	OTHER	0.000818	0.999182	
QLI63520.1	OTHER	0.004294	0.995706	
QLI63521.1	OTHER	0.004226	0.995774	
QLI63522.1	OTHER	0.000788	0.999212	
QLI63523.1	OTHER	0.001022	0.998978	
QLI63524.1	OTHER	0.000888	0.999112	
QLI63525.1	OTHER	0.001110	0.998890	
QLI63526.1	SP(Sec/SPI)	0.987593	0.012407	CS pos: 23-24. ATA-QP. Pr: 0.6060
QLI63527.1	OTHER	0.002135	0.997865	
QLI63528.1	OTHER	0.001461	0.998539	
QLI63529.1	OTHER	0.000220	0.999780	
QLI63530.1	OTHER	0.002059	0.997941	
QLI63531.1	OTHER	0.004011	0.995989	
QLI63532.1	OTHER	0.000626	0.999374	
QLI63533.1	OTHER	0.000346	0.999654	
QLI63534.1	SP(Sec/SPI)	0.971119	0.028881	CS pos: 18-19. GLA-LV. Pr: 0.6756
QLI63535.1	OTHER	0.000955	0.999045	
QLI63536.1	OTHER	0.001345	0.998655	
QLI63537.1	OTHER	0.364263	0.635737	
QLI63538.1	OTHER	0.001883	0.998117	
QLI63539.1	OTHER	0.002482	0.997518	
QLI63540.1	OTHER	0.001174	0.998826	
QLI63541.1	OTHER	0.000876	0.999124	
QLI63542.1	OTHER	0.001771	0.998229	
QLI63543.1	OTHER	0.001110	0.998890	

QLI63544.1	OTHER	0.001030	0.998970	
QLI63545.1	OTHER	0.001795	0.998205	
QLI63546.1	OTHER	0.000619	0.999381	
QLI63547.1	OTHER	0.028424	0.971576	
QLI63548.1	OTHER	0.235028	0.764972	
QLI63549.1	OTHER	0.001224	0.998776	
QLI63550.1	OTHER	0.001221	0.998779	
QLI63551.1	OTHER	0.000825	0.999175	
QLI63552.1	OTHER	0.004618	0.995382	
QLI63553.1	OTHER	0.000138	0.999862	
QLI63554.1	OTHER	0.166799	0.833201	
QLI63555.1	OTHER	0.001130	0.998870	
QLI63556.1	OTHER	0.165849	0.834151	
QLI63557.1	OTHER	0.000383	0.999617	
QLI63558.1	OTHER	0.000601	0.999399	
QLI63559.1	OTHER	0.000324	0.999676	
QLI63560.1	SP(Sec/SPI)	0.995172	0.004828	CS pos: 20-21. IHA-VE. Pr: 0.8823
QLI63561.1	OTHER	0.002287	0.997713	
QLI63562.1	OTHER	0.003640	0.996360	
QLI63563.1	OTHER	0.000482	0.999518	
QLI63564.1	OTHER	0.000597	0.999403	
QLI63565.1	OTHER	0.000802	0.999198	
QLI63566.1	OTHER	0.000652	0.999348	
QLI63567.1	OTHER	0.000366	0.999634	
QLI63568.1	OTHER	0.001742	0.998258	
QLI63569.1	OTHER	0.000321	0.999679	
QLI63570.1	OTHER	0.000337	0.999663	
QLI63571.1	OTHER	0.006113	0.993887	
QLI63572.1	OTHER	0.001517	0.998483	
QLI63573.1	OTHER	0.003683	0.996317	
QLI63574.1	OTHER	0.000811	0.999189	

QLI63575.1	OTHER	0.011914	0.988086	
QLI63576.1	OTHER	0.000999	0.999001	
QLI63577.1	OTHER	0.000502	0.999498	
QLI63578.1	OTHER	0.001310	0.998690	
QLI63579.1	OTHER	0.000829	0.999171	
QLI63580.1	OTHER	0.000696	0.999304	
QLI63581.1	SP(Sec/SPI)	0.771571	0.228429	CS pos: 23-24. VKA-TE. Pr: 0.5922
QLI63582.1	OTHER	0.002502	0.997498	
QLI63583.1	OTHER	0.002186	0.997814	
QLI63584.1	OTHER	0.000751	0.999249	
QLI63585.1	OTHER	0.002535	0.997465	
QLI63586.1	OTHER	0.002763	0.997237	
QLI63587.1	OTHER	0.000375	0.999625	
QLI63588.1	OTHER	0.000951	0.999049	
QLI63589.1	OTHER	0.000677	0.999323	
QLI63590.1	OTHER	0.001169	0.998831	
QLI63591.1	OTHER	0.000443	0.999557	
QLI63592.1	OTHER	0.000993	0.999007	
QLI63593.1	OTHER	0.001207	0.998793	
QLI63594.1	OTHER	0.001105	0.998895	
QLI63595.1	OTHER	0.001313	0.998687	
QLI63596.1	OTHER	0.002283	0.997717	
QLI63597.1	OTHER	0.025349	0.974651	
QLI63598.1	OTHER	0.000327	0.999673	
QLI63599.1	OTHER	0.005604	0.994396	
QLI63600.1	OTHER	0.001364	0.998636	
QLI63601.1	OTHER	0.002663	0.997337	
QLI63602.1	SP(Sec/SPI)	0.960444	0.039556	CS pos: 20-21. ISA-QS. Pr: 0.3472
QLI63603.1	SP(Sec/SPI)	0.996760	0.003240	CS pos: 16-17. ALG-LN. Pr: 0.4397
QLI63604.1	OTHER	0.004606	0.995394	
QLI63605.1	OTHER	0.001744	0.998256	

QLI63606.1	OTHER	0.000534	0.999466	
QLI63607.1	OTHER	0.001043	0.998957	
QLI63608.1	OTHER	0.003435	0.996565	
QLI63609.1	OTHER	0.002024	0.997976	
QLI63610.1	OTHER	0.001085	0.998915	
QLI63611.1	OTHER	0.000799	0.999201	
QLI63612.1	OTHER	0.000623	0.999377	
QLI63613.1	OTHER	0.002259	0.997741	
QLI63614.1	SP(Sec/SPI)	0.967481	0.032519	CS pos: 21-22. CQG-HG. Pr: 0.6829
QLI63615.1	OTHER	0.002748	0.997252	
QLI63616.1	OTHER	0.004873	0.995127	
QLI63617.1	SP(Sec/SPI)	0.997340	0.002660	CS pos: 20-21. AEA-SG. Pr: 0.6343
QLI63618.1	OTHER	0.016518	0.983482	
QLI63619.1	SP(Sec/SPI)	0.996131	0.003869	CS pos: 21-22. ASA-AT. Pr: 0.6136
QLI63620.1	OTHER	0.000965	0.999035	
QLI63621.1	OTHER	0.001998	0.998002	
QLI63622.1	OTHER	0.001196	0.998804	
QLI63623.1	OTHER	0.000757	0.999243	
QLI63624.1	OTHER	0.030756	0.969244	
QLI63625.1	OTHER	0.008851	0.991149	
QLI63626.1	SP(Sec/SPI)	0.997138	0.002862	CS pos: 20-21. TFA-KP. Pr: 0.9540
QLI63627.1	OTHER	0.000346	0.999654	
QLI63628.1	OTHER	0.000766	0.999234	
QLI63629.1	OTHER	0.007147	0.992853	
QLI63630.1	OTHER	0.000424	0.999576	
QLI63631.1	OTHER	0.036593	0.963407	
QLI63632.1	OTHER	0.000563	0.999437	
QLI63633.1	SP(Sec/SPI)	0.998356	0.001644	CS pos: 18-19. AEA-SP. Pr: 0.8453
QLI63634.1	SP(Sec/SPI)	0.996203	0.003797	CS pos: 18-19. VSA-AT. Pr: 0.8574
QLI63635.1	OTHER	0.000203	0.999797	
QLI63636.1	OTHER	0.002671	0.997329	

QLI63637.1	OTHER	0.002010	0.997990	
QLI63638.1	SP(Sec/SPI)	0.999140	0.000860	CS pos: 22-23. VQA-TQ. Pr: 0.9892
QLI63639.1	OTHER	0.000954	0.999046	
QLI63640.1	OTHER	0.001881	0.998119	
QLI63641.1	SP(Sec/SPI)	0.854931	0.145069	CS pos: 21-22. LQG-SN. Pr: 0.3688
QLI63642.1	OTHER	0.003934	0.996066	
QLI63643.1	OTHER	0.034457	0.965543	
QLI63644.1	OTHER	0.000954	0.999046	
QLI63645.1	OTHER	0.000474	0.999526	
QLI63646.1	OTHER	0.000916	0.999084	
QLI63647.1	OTHER	0.000797	0.999203	
QLI63648.1	SP(Sec/SPI)	0.941987	0.058013	CS pos: 19-20. TSA-FY. Pr: 0.8176
QLI63649.1	OTHER	0.001082	0.998918	
QLI63650.1	OTHER	0.003857	0.996143	
QLI63651.1	OTHER	0.001159	0.998841	
QLI63652.1	OTHER	0.001798	0.998202	
QLI63653.1	OTHER	0.001552	0.998448	
QLI63654.1	OTHER	0.000366	0.999634	
QLI63655.1	OTHER	0.000694	0.999306	
QLI63656.1	OTHER	0.018816	0.981184	
QLI63657.1	OTHER	0.002587	0.997413	
QLI63658.1	OTHER	0.000516	0.999484	
QLI63659.1	OTHER	0.005295	0.994705	
QLI63660.1	OTHER	0.001506	0.998494	
QLI63661.1	SP(Sec/SPI)	0.993758	0.006242	CS pos: 17-18. ANA-VC. Pr: 0.4680
QLI63662.1	OTHER	0.000793	0.999207	
QLI63663.1	OTHER	0.000765	0.999235	
QLI63664.1	OTHER	0.000604	0.999396	
QLI63665.1	OTHER	0.003102	0.996898	
QLI63666.1	SP(Sec/SPI)	0.993433	0.006567	CS pos: 17-18. AMA-AP. Pr: 0.7460
QLI63667.1	OTHER	0.000950	0.999050	

QLI63668.1	OTHER	0.000259	0.999741	
QLI63669.1	OTHER	0.003451	0.996549	
QLI63670.1	OTHER	0.005831	0.994169	
QLI63671.1	OTHER	0.000805	0.999195	
QLI63672.1	OTHER	0.001096	0.998904	
QLI63673.1	OTHER	0.001376	0.998624	
QLI63674.1	OTHER	0.001499	0.998501	
QLI63675.1	OTHER	0.002269	0.997731	
QLI63676.1	OTHER	0.003200	0.996800	
QLI63677.1	OTHER	0.005405	0.994595	
QLI63678.1	OTHER	0.002283	0.997717	
QLI63679.1	OTHER	0.001603	0.998397	
QLI63680.1	SP(Sec/SPI)	0.784754	0.215246	CS pos: 19-20. CGA-LP. Pr: 0.6639
QLI63681.1	OTHER	0.001295	0.998705	
QLI63682.1	OTHER	0.000230	0.999770	
QLI63683.1	OTHER	0.083997	0.916003	
QLI63684.1	OTHER	0.000599	0.999401	
QLI63685.1	OTHER	0.002390	0.997610	
QLI63686.1	OTHER	0.001714	0.998286	
QLI63687.1	OTHER	0.002407	0.997593	
QLI63688.1	OTHER	0.000217	0.999783	
QLI63689.1	OTHER	0.002465	0.997535	
QLI63690.1	OTHER	0.001093	0.998907	
QLI63691.1	SP(Sec/SPI)	0.997845	0.002155	CS pos: 15-16. AAA-QG. Pr: 0.3923
QLI63692.1	OTHER	0.015329	0.984671	
QLI63693.1	OTHER	0.003677	0.996323	
QLI63694.1	OTHER	0.000582	0.999418	
QLI63695.1	SP(Sec/SPI)	0.993220	0.006780	CS pos: 16-17. AHT-LF. Pr: 0.4359
QLI63696.1	OTHER	0.003515	0.996485	
QLI63697.1	OTHER	0.009384	0.990616	
QLI63698.1	OTHER	0.005114	0.994886	

QLI63699.1	OTHER	0.000646	0.999354	
QLI63700.1	SP(Sec/SPI)	0.999545	0.000455	CS pos: 18-19. AYA-AL. Pr: 0.9012
QLI63701.1	SP(Sec/SPI)	0.976053	0.023947	CS pos: 16-17. ALG-QQ. Pr: 0.7921
QLI63702.1	OTHER	0.001231	0.998769	
QLI63703.1	OTHER	0.001253	0.998747	
QLI63704.1	OTHER	0.016258	0.983742	
QLI63705.1	OTHER	0.002412	0.997588	
QLI63706.1	OTHER	0.001150	0.998850	
QLI63707.1	OTHER	0.005515	0.994485	
QLI63708.1	OTHER	0.000434	0.999566	
QLI63709.1	OTHER	0.002543	0.997457	
QLI63710.1	SP(Sec/SPI)	0.976204	0.023796	CS pos: 19-20. ALA-GV. Pr: 0.7479
QLI63711.1	OTHER	0.002983	0.997017	
QLI63712.1	OTHER	0.003259	0.996741	
QLI63713.1	OTHER	0.000594	0.999406	
QLI63714.1	OTHER	0.001404	0.998596	
QLI63715.1	OTHER	0.004531	0.995469	
QLI63716.1	OTHER	0.004057	0.995943	
QLI63717.1	OTHER	0.001216	0.998784	
QLI63718.1	OTHER	0.003573	0.996427	
QLI63719.1	OTHER	0.220353	0.779647	
QLI63720.1	OTHER	0.003169	0.996831	
QLI63721.1	OTHER	0.006896	0.993104	
QLI63722.1	OTHER	0.000530	0.999470	
QLI63723.1	OTHER	0.001416	0.998584	
QLI63724.1	OTHER	0.003033	0.996967	
QLI63725.1	OTHER	0.000796	0.999204	
QLI63726.1	OTHER	0.003980	0.996020	
QLI63727.1	OTHER	0.001283	0.998717	
QLI63728.1	OTHER	0.002086	0.997914	
QLI63729.1	OTHER	0.000160	0.999840	

QLI63730.1	OTHER	0.001235	0.998765	
QLI63731.1	OTHER	0.173732	0.826268	
QLI63732.1	OTHER	0.000682	0.999318	
QLI63733.1	OTHER	0.000293	0.999707	
QLI63734.1	OTHER	0.000604	0.999396	
QLI63735.1	OTHER	0.013206	0.986794	
QLI63736.1	OTHER	0.000956	0.999044	
QLI63737.1	OTHER	0.000961	0.999039	
QLI63738.1	OTHER	0.005969	0.994031	
QLI63739.1	OTHER	0.002278	0.997722	
QLI63740.1	OTHER	0.001658	0.998342	
QLI63741.1	OTHER	0.002307	0.997693	
QLI63742.1	OTHER	0.000883	0.999117	
QLI63743.1	OTHER	0.001002	0.998998	
QLI63744.1	SP(Sec/SPI)	0.997133	0.002867	CS pos: 21-22. AVA-NP. Pr: 0.5408
QLI63745.1	OTHER	0.009571	0.990429	
QLI63746.1	OTHER	0.001662	0.998338	
QLI63747.1	OTHER	0.000592	0.999408	
QLI63748.1	OTHER	0.040905	0.959095	
QLI63749.1	OTHER	0.001265	0.998735	
QLI63750.1	OTHER	0.001430	0.998570	
QLI63751.1	OTHER	0.001504	0.998496	
QLI63752.1	OTHER	0.000913	0.999087	
QLI63753.1	SP(Sec/SPI)	0.989243	0.010757	CS pos: 20-21. ALG-QS. Pr: 0.8130
QLI63754.1	OTHER	0.000927	0.999073	
QLI63755.1	OTHER	0.001450	0.998550	
QLI63756.1	OTHER	0.175419	0.824581	
QLI63757.1	OTHER	0.000834	0.999166	
QLI63758.1	SP(Sec/SPI)	0.782576	0.217424	CS pos: 18-19. AAA-FQ. Pr: 0.3543
QLI63759.1	OTHER	0.005846	0.994154	
QLI63760.1	OTHER	0.002340	0.997660	

QLI63761.1	OTHER	0.001366	0.998634	
QLI63762.1	OTHER	0.001399	0.998601	
QLI63763.1	OTHER	0.000363	0.999637	
QLI63764.1	OTHER	0.000924	0.999076	
QLI63765.1	OTHER	0.001205	0.998795	
QLI63766.1	SP(Sec/SPI)	0.996360	0.003640	CS pos: 17-18. AAG-SQ. Pr: 0.6778
QLI63767.1	OTHER	0.001962	0.998038	
QLI63768.1	OTHER	0.000931	0.999069	
QLI63769.1	OTHER	0.001093	0.998907	
QLI63770.1	OTHER	0.000176	0.999824	
QLI63771.1	OTHER	0.003309	0.996691	
QLI63772.1	OTHER	0.002373	0.997627	
QLI63773.1	OTHER	0.001850	0.998150	
QLI63774.1	OTHER	0.000835	0.999165	
QLI63775.1	OTHER	0.000333	0.999667	
QLI63776.1	OTHER	0.002714	0.997286	
QLI63777.1	OTHER	0.000342	0.999658	
QLI63778.1	OTHER	0.002309	0.997691	
QLI63779.1	OTHER	0.001149	0.998851	
QLI63780.1	OTHER	0.000422	0.999578	
QLI63781.1	OTHER	0.001684	0.998316	
QLI63782.1	OTHER	0.009499	0.990501	
QLI63783.1	OTHER	0.002995	0.997005	
QLI63784.1	OTHER	0.000512	0.999488	
QLI63785.1	OTHER	0.000914	0.999086	
QLI63786.1	OTHER	0.001913	0.998087	
QLI63787.1	OTHER	0.001119	0.998881	
QLI63788.1	OTHER	0.000147	0.999853	
QLI63789.1	OTHER	0.001042	0.998958	
QLI63790.1	OTHER	0.000901	0.999099	
QLI63791.1	OTHER	0.001230	0.998770	

QLI63792.1	OTHER	0.001680	0.998320	
QLI63793.1	OTHER	0.008892	0.991108	
QLI63794.1	OTHER	0.005097	0.994903	
QLI63795.1	OTHER	0.124074	0.875926	
QLI63796.1	OTHER	0.000600	0.999400	
QLI63797.1	OTHER	0.000258	0.999742	
QLI63798.1	SP(Sec/SPI)	0.923481	0.076519	CS pos: 18-19. AAG-VP. Pr: 0.6079
QLI63799.1	OTHER	0.001612	0.998388	
QLI63800.1	OTHER	0.000503	0.999497	
QLI63801.1	OTHER	0.021891	0.978109	
QLI63802.1	OTHER	0.002539	0.997461	
QLI63803.1	OTHER	0.000396	0.999604	
QLI63804.1	OTHER	0.000293	0.999707	
QLI63805.1	OTHER	0.002916	0.997084	
QLI63806.1	OTHER	0.000521	0.999479	
QLI63807.1	OTHER	0.001989	0.998011	
QLI63808.1	OTHER	0.000751	0.999249	
QLI63809.1	OTHER	0.001583	0.998417	
QLI63810.1	OTHER	0.245265	0.754735	
QLI63811.1	OTHER	0.000388	0.999612	
QLI63812.1	OTHER	0.000738	0.999262	
QLI63813.1	OTHER	0.043645	0.956355	
QLI63814.1	SP(Sec/SPI)	0.994955	0.005045	CS pos: 18-19. AAA-AP. Pr: 0.8402
QLI63815.1	OTHER	0.002364	0.997636	
QLI63816.1	OTHER	0.000459	0.999541	
QLI63817.1	OTHER	0.001466	0.998534	
QLI63818.1	OTHER	0.001281	0.998719	
QLI63819.1	OTHER	0.000199	0.999801	
QLI63820.1	OTHER	0.000560	0.999440	
QLI63821.1	OTHER	0.001843	0.998157	
QLI63822.1	OTHER	0.002698	0.997302	

QLI63823.1	OTHER	0.001373	0.998627	
QLI63824.1	OTHER	0.004311	0.995689	
QLI63825.1	OTHER	0.158066	0.841934	
QLI63826.1	OTHER	0.000624	0.999376	
QLI63827.1	OTHER	0.000203	0.999797	
QLI63828.1	OTHER	0.001252	0.998748	
QLI63829.1	OTHER	0.001280	0.998720	
QLI63830.1	OTHER	0.000337	0.999663	
QLI63831.1	OTHER	0.001152	0.998848	
QLI63832.1	OTHER	0.161042	0.838958	
QLI63833.1	OTHER	0.006316	0.993684	
QLI63834.1	OTHER	0.001610	0.998390	
QLI63835.1	OTHER	0.006218	0.993782	
QLI63836.1	OTHER	0.000826	0.999174	
QLI63837.1	OTHER	0.000529	0.999471	
QLI63838.1	OTHER	0.001447	0.998553	
QLI63839.1	OTHER	0.000455	0.999545	
QLI63840.1	OTHER	0.031769	0.968231	
QLI63841.1	OTHER	0.000830	0.999170	
QLI63842.1	OTHER	0.000767	0.999233	
QLI63843.1	SP(Sec/SPI)	0.702113	0.297887	CS pos: 24-25. TNA-AN. Pr: 0.6364
QLI63844.1	OTHER	0.001015	0.998985	
QLI63845.1	OTHER	0.001055	0.998945	
QLI63846.1	OTHER	0.000914	0.999086	
QLI63847.1	SP(Sec/SPI)	0.735040	0.264960	CS pos: 33-34. ARA-PA. Pr: 0.4780
QLI63848.1	OTHER	0.001894	0.998106	
QLI63849.1	OTHER	0.001070	0.998930	
QLI63850.1	OTHER	0.000767	0.999233	
QLI63851.1	OTHER	0.000354	0.999646	
QLI63852.1	OTHER	0.000503	0.999497	
QLI63853.1	OTHER	0.000715	0.999285	

QLI63854.1	OTHER	0.445916	0.554084	
QLI63855.1	OTHER	0.017439	0.982561	
QLI63856.1	OTHER	0.002639	0.997361	
QLI63857.1	OTHER	0.000941	0.999059	
QLI63858.1	OTHER	0.000691	0.999309	
QLI63859.1	OTHER	0.001310	0.998690	
QLI63860.1	OTHER	0.000548	0.999452	
QLI63861.1	OTHER	0.000385	0.999615	
QLI63862.1	OTHER	0.001554	0.998446	
QLI63863.1	OTHER	0.000584	0.999416	
QLI63864.1	OTHER	0.001233	0.998767	
QLI63865.1	OTHER	0.000745	0.999255	
QLI63866.1	OTHER	0.004930	0.995070	
QLI63867.1	OTHER	0.000508	0.999492	
QLI63868.1	OTHER	0.001378	0.998622	
QLI63869.1	OTHER	0.008252	0.991748	
QLI63870.1	OTHER	0.020247	0.979753	
QLI63871.1	OTHER	0.000895	0.999105	
QLI63872.1	OTHER	0.002365	0.997635	
QLI63873.1	OTHER	0.001253	0.998747	
QLI63874.1	SP(Sec/SPI)	0.985364	0.014636	CS pos: 19-20. LRA-AE. Pr: 0.8397
QLI63875.1	OTHER	0.006234	0.993766	
QLI63876.1	OTHER	0.007693	0.992307	
QLI63877.1	OTHER	0.001292	0.998708	
QLI63878.1	OTHER	0.002521	0.997479	
QLI63879.1	OTHER	0.000210	0.999790	
QLI63880.1	OTHER	0.108404	0.891596	
QLI63881.1	OTHER	0.000853	0.999147	
QLI63882.1	OTHER	0.000312	0.999688	
QLI63883.1	OTHER	0.004275	0.995725	
QLI63884.1	OTHER	0.001423	0.998577	

QLI63885.1	OTHER	0.017628	0.982372	
QLI63886.1	OTHER	0.002677	0.997323	
QLI63887.1	OTHER	0.003022	0.996978	
QLI63888.1	OTHER	0.000335	0.999665	
QLI63889.1	OTHER	0.001037	0.998963	
QLI63890.1	OTHER	0.001481	0.998519	
QLI63891.1	SP(Sec/SPI)	0.634509	0.365491	CS pos: 19-20. AAA-NV. Pr: 0.4048
QLI63892.1	OTHER	0.004230	0.995770	
QLI63893.1	OTHER	0.002030	0.997970	
QLI63894.1	OTHER	0.002253	0.997747	
QLI63895.1	OTHER	0.001120	0.998880	
QLI63896.1	OTHER	0.068420	0.931580	
QLI63897.1	OTHER	0.028355	0.971645	
QLI63898.1	OTHER	0.000893	0.999107	
QLI63899.1	OTHER	0.000404	0.999596	
QLI63900.1	OTHER	0.003064	0.996936	
QLI63901.1	OTHER	0.005474	0.994526	
QLI63902.1	OTHER	0.001060	0.998940	
QLI63903.1	OTHER	0.000771	0.999229	
QLI63904.1	SP(Sec/SPI)	0.857489	0.142511	CS pos: 18-19. AYA-AA. Pr: 0.3532
QLI63905.1	OTHER	0.000494	0.999506	
QLI63906.1	OTHER	0.000254	0.999746	
QLI63907.1	OTHER	0.002336	0.997664	
QLI63908.1	OTHER	0.001138	0.998862	
QLI63909.1	OTHER	0.001635	0.998365	
QLI63910.1	SP(Sec/SPI)	0.964164	0.035836	CS pos: 18-19. AVA-VP. Pr: 0.6145
QLI63911.1	OTHER	0.002609	0.997391	
QLI63912.1	OTHER	0.000762	0.999238	
QLI63913.1	OTHER	0.000707	0.999293	
QLI63914.1	OTHER	0.001106	0.998894	
QLI63915.1	OTHER	0.000297	0.999703	

QLI63916.1	OTHER	0.014134	0.985866	
QLI63917.1	OTHER	0.004733	0.995267	
QLI63918.1	OTHER	0.001300	0.998700	
QLI63919.1	OTHER	0.007804	0.992196	
QLI63920.1	OTHER	0.002353	0.997647	
QLI63921.1	OTHER	0.000941	0.999059	
QLI63922.1	SP(Sec/SPI)	0.946374	0.053626	CS pos: 20-21. AAG-GL. Pr: 0.5722
QLI63923.1	OTHER	0.011377	0.988623	
QLI63924.1	OTHER	0.003341	0.996659	
QLI63925.1	OTHER	0.345125	0.654875	
QLI63926.1	OTHER	0.000660	0.999340	
QLI63927.1	OTHER	0.002804	0.997196	
QLI63928.1	OTHER	0.036061	0.963939	
QLI63929.1	OTHER	0.000723	0.999277	
QLI63930.1	SP(Sec/SPI)	0.961371	0.038629	CS pos: 19-20. ALA-AL. Pr: 0.5059
QLI63931.1	OTHER	0.000426	0.999574	
QLI63932.1	OTHER	0.001616	0.998384	
QLI63933.1	OTHER	0.001047	0.998953	
QLI63934.1	OTHER	0.378232	0.621768	
QLI63935.1	OTHER	0.001132	0.998868	
QLI63936.1	OTHER	0.435893	0.564107	
QLI63937.1	OTHER	0.001748	0.998252	
QLI63938.1	OTHER	0.000943	0.999057	
QLI63939.1	OTHER	0.003770	0.996230	
QLI63940.1	SP(Sec/SPI)	0.989602	0.010398	CS pos: 20-21. AVA-YG. Pr: 0.8071
QLI63941.1	OTHER	0.001333	0.998667	
QLI63942.1	OTHER	0.001143	0.998857	
QLI63943.1	OTHER	0.240635	0.759365	
QLI63944.1	OTHER	0.003438	0.996562	
QLI63945.1	OTHER	0.000915	0.999085	
QLI63946.1	OTHER	0.000918	0.999082	

QLI63947.1	OTHER	0.064934	0.935066	
QLI63948.1	OTHER	0.001848	0.998152	
QLI63949.1	OTHER	0.001237	0.998763	
QLI63950.1	OTHER	0.000999	0.999001	
QLI63951.1	OTHER	0.001190	0.998810	
QLI63952.1	OTHER	0.020714	0.979286	
QLI63953.1	OTHER	0.002283	0.997717	
QLI63954.1	OTHER	0.001410	0.998590	
QLI63955.1	OTHER	0.000666	0.999334	
QLI63956.1	OTHER	0.001368	0.998632	
QLI63957.1	OTHER	0.001130	0.998870	
QLI63958.1	OTHER	0.009788	0.990212	
QLI63959.1	SP(Sec/SPI)	0.992382	0.007618	CS pos: 19-20. ATA-SF. Pr: 0.8379
QLI63960.1	OTHER	0.030162	0.969838	
QLI63961.1	OTHER	0.002268	0.997732	
QLI63962.1	OTHER	0.008618	0.991382	
QLI63963.1	OTHER	0.007641	0.992359	
QLI63964.1	SP(Sec/SPI)	0.532921	0.467079	CS pos: 26-27. AAA-SA. Pr: 0.0914
QLI63965.1	OTHER	0.001078	0.998922	
QLI63966.1	OTHER	0.002023	0.997977	
QLI63967.1	OTHER	0.000817	0.999183	
QLI63968.1	OTHER	0.001320	0.998680	
QLI63969.1	OTHER	0.001538	0.998462	
QLI63970.1	OTHER	0.000832	0.999168	
QLI63971.1	OTHER	0.000542	0.999458	
QLI63972.1	OTHER	0.000921	0.999079	
QLI63973.1	OTHER	0.001826	0.998174	
QLI63974.1	OTHER	0.000741	0.999259	
QLI63975.1	OTHER	0.000879	0.999121	
QLI63976.1	OTHER	0.001169	0.998831	
QLI63977.1	SP(Sec/SPI)	0.761076	0.238924	CS pos: 22-23. ANP-SS. Pr: 0.3081

QLI63978.1	OTHER	0.001959	0.998041	
QLI63979.1	OTHER	0.304804	0.695196	
QLI63980.1	OTHER	0.000282	0.999718	
QLI63981.1	OTHER	0.000620	0.999380	
QLI63982.1	SP(Sec/SPI)	0.982721	0.017279	CS pos: 17-18. ASA-HP. Pr: 0.5539
QLI63983.1	OTHER	0.008862	0.991138	
QLI63984.1	OTHER	0.001712	0.998288	
QLI63985.1	OTHER	0.013555	0.986445	
QLI63986.1	SP(Sec/SPI)	0.996470	0.003530	CS pos: 25-26. AQA-AR. Pr: 0.3758
QLI63987.1	OTHER	0.000260	0.999740	
QLI63988.1	OTHER	0.127160	0.872840	
QLI63989.1	OTHER	0.002169	0.997831	
QLI63990.1	OTHER	0.000578	0.999422	
QLI63991.1	OTHER	0.000943	0.999057	
QLI63992.1	OTHER	0.000661	0.999339	
QLI63993.1	OTHER	0.000133	0.999867	
QLI63994.1	OTHER	0.000819	0.999181	
QLI63995.1	OTHER	0.000928	0.999072	
QLI63996.1	OTHER	0.000359	0.999641	
QLI63997.1	OTHER	0.001755	0.998245	
QLI63998.1	SP(Sec/SPI)	0.995941	0.004059	CS pos: 19-20. AYA-VP. Pr: 0.9007
QLI63999.1	OTHER	0.000779	0.999221	
QLI64000.1	OTHER	0.001685	0.998315	
QLI64001.1	OTHER	0.010237	0.989763	
QLI64002.1	OTHER	0.001778	0.998222	
QLI64003.1	OTHER	0.105985	0.894015	
QLI64004.1	SP(Sec/SPI)	0.979196	0.020804	CS pos: 17-18. SLA-SP. Pr: 0.5096
QLI64005.1	OTHER	0.000215	0.999785	
QLI64006.1	OTHER	0.000935	0.999065	
QLI64007.1	OTHER	0.001067	0.998933	
QLI64008.1	OTHER	0.001324	0.998676	

QLI64009.1	OTHER	0.021597	0.978403	
QLI64010.1	OTHER	0.000334	0.999666	
QLI64011.1	OTHER	0.004408	0.995592	
QLI64012.1	OTHER	0.000283	0.999717	
QLI64013.1	SP(Sec/SPI)	0.991443	0.008557	CS pos: 17-18. AYA-AP. Pr: 0.8032
QLI64014.1	SP(Sec/SPI)	0.878481	0.121519	CS pos: 17-18. AAA-IP. Pr: 0.3408
QLI64015.1	OTHER	0.024673	0.975327	
QLI64016.1	SP(Sec/SPI)	0.917511	0.082489	CS pos: 19-20. AGA-TT. Pr: 0.4190
QLI64017.1	OTHER	0.000586	0.999414	
QLI64018.1	OTHER	0.001060	0.998940	
QLI64019.1	OTHER	0.001138	0.998862	
QLI64020.1	OTHER	0.094088	0.905912	
QLI64021.1	OTHER	0.000412	0.999588	
QLI64022.1	OTHER	0.001397	0.998603	
QLI64023.1	OTHER	0.000539	0.999461	
QLI64024.1	OTHER	0.002104	0.997896	
QLI64025.1	OTHER	0.000732	0.999268	
QLI64026.1	OTHER	0.003622	0.996378	
QLI64027.1	OTHER	0.000761	0.999239	
QLI64028.1	OTHER	0.000273	0.999727	
QLI64029.1	OTHER	0.001280	0.998720	
QLI64030.1	SP(Sec/SPI)	0.998008	0.001992	CS pos: 21-22. VEA-TP. Pr: 0.9712
QLI64031.1	SP(Sec/SPI)	0.990890	0.009110	CS pos: 17-18. ASA-AE. Pr: 0.6808
QLI64032.1	OTHER	0.000724	0.999276	
QLI64033.1	OTHER	0.272950	0.727050	
QLI64034.1	OTHER	0.001410	0.998590	
QLI64035.1	SP(Sec/SPI)	0.809599	0.190401	CS pos: 26-27. GLS-LS. Pr: 0.4803
QLI64036.1	OTHER	0.000620	0.999380	
QLI64037.1	OTHER	0.002144	0.997856	
QLI64038.1	OTHER	0.002811	0.997189	
QLI64039.1	OTHER	0.355006	0.644994	

QLI64040.1	OTHER	0.001556	0.998444	
QLI64041.1	SP(Sec/SPI)	0.995963	0.004037	CS pos: 20-21. AYS-EV. Pr: 0.5688
QLI64042.1	OTHER	0.000433	0.999567	
QLI64043.1	OTHER	0.001647	0.998353	
QLI64044.1	OTHER	0.001344	0.998656	
QLI64045.1	OTHER	0.000604	0.999396	
QLI64046.1	OTHER	0.001344	0.998656	
QLI64047.1	OTHER	0.001034	0.998966	
QLI64048.1	OTHER	0.023392	0.976608	
QLI64049.1	OTHER	0.006247	0.993753	
QLI64050.1	OTHER	0.000342	0.999658	
QLI64051.1	SP(Sec/SPI)	0.998151	0.001849	CS pos: 14-15. VSA-VP. Pr: 0.7487
QLI64052.1	OTHER	0.004386	0.995614	
QLI64053.1	OTHER	0.000742	0.999258	
QLI64054.1	SP(Sec/SPI)	0.916792	0.083208	CS pos: 35-36. ADA-QP. Pr: 0.8658
QLI64055.1	SP(Sec/SPI)	0.992945	0.007055	CS pos: 23-24. AVS-QS. Pr: 0.5267
QLI64056.1	OTHER	0.000906	0.999094	
QLI64057.1	OTHER	0.253983	0.746017	
QLI64058.1	OTHER	0.352167	0.647833	
QLI64059.1	OTHER	0.005476	0.994524	
QLI64060.1	OTHER	0.004224	0.995776	
QLI64061.1	SP(Sec/SPI)	0.926540	0.073460	CS pos: 21-22. VGA-EE. Pr: 0.5170
QLI64062.1	OTHER	0.001869	0.998131	
QLI64063.1	OTHER	0.001900	0.998100	
QLI64064.1	OTHER	0.002684	0.997316	
QLI64065.1	OTHER	0.002558	0.997442	
QLI64066.1	OTHER	0.005681	0.994319	
QLI64067.1	OTHER	0.004819	0.995181	
QLI64068.1	OTHER	0.004148	0.995852	
QLI64069.1	SP(Sec/SPI)	0.995061	0.004939	CS pos: 16-17. AWA-AG. Pr: 0.4185
QLI64070.1	OTHER	0.016511	0.983489	

QLI64071.1	OTHER	0.002917	0.997083
QLI64072.1	OTHER	0.003064	0.996936
QLI64073.1	OTHER	0.002159	0.997841
QLI64074.1	OTHER	0.000302	0.999698
QLI64075.1	OTHER	0.001188	0.998812
QLI64076.1	OTHER	0.001920	0.998080
QLI64077.1	OTHER	0.002268	0.997732
QLI64078.1	OTHER	0.000470	0.999530
QLI64079.1	OTHER	0.000564	0.999436
QLI64080.1	OTHER	0.001128	0.998872
QLI64081.1	OTHER	0.000910	0.999090
QLI64082.1	OTHER	0.001098	0.998902
QLI64083.1	OTHER	0.001814	0.998186
QLI64084.1	OTHER	0.001142	0.998858
QLI64085.1	OTHER	0.000646	0.999354
QLI64086.1	OTHER	0.000720	0.999280
QLI64087.1	OTHER	0.002441	0.997559
QLI64088.1	OTHER	0.000845	0.999155
QLI64089.1	OTHER	0.001520	0.998480
QLI64090.1	OTHER	0.027206	0.972794
QLI64091.1	OTHER	0.004948	0.995052
QLI64092.1	OTHER	0.001374	0.998626
QLI64093.1	OTHER	0.091937	0.908063
QLI64094.1	OTHER	0.000990	0.999010
QLI64095.1	OTHER	0.001594	0.998406
QLI64096.1	OTHER	0.002345	0.997655
QLI64097.1	OTHER	0.000955	0.999045
QLI64098.1	OTHER	0.051215	0.948785
QLI64099.1	OTHER	0.003344	0.996656
QLI64100.1	OTHER	0.000301	0.999699
QLI64101.1	OTHER	0.261496	0.738504

QLI64102.1	OTHER	0.006292	0.993708	
QLI64103.1	OTHER	0.008625	0.991375	
QLI64104.1	OTHER	0.001420	0.998580	
QLI64105.1	OTHER	0.000470	0.999530	
QLI64106.1	OTHER	0.000483	0.999517	
QLI64107.1	OTHER	0.002695	0.997305	
QLI64108.1	OTHER	0.000769	0.999231	
QLI64109.1	OTHER	0.004548	0.995452	
QLI64110.1	OTHER	0.000994	0.999006	
QLI64111.1	OTHER	0.004245	0.995755	
QLI64112.1	OTHER	0.012124	0.987876	
QLI64113.1	OTHER	0.007037	0.992963	
QLI64114.1	OTHER	0.498489	0.501511	
QLI64115.1	OTHER	0.001874	0.998126	
QLI64116.1	SP(Sec/SPI)	0.769861	0.230139	CS pos: 17-18. TYA-QI. Pr: 0.7159
QLI64117.1	OTHER	0.003309	0.996691	
QLI64118.1	OTHER	0.007691	0.992309	
QLI64119.1	OTHER	0.001953	0.998047	
QLI64120.1	OTHER	0.000809	0.999191	
QLI64121.1	SP(Sec/SPI)	0.998041	0.001959	CS pos: 18-19. ASA-YQ. Pr: 0.9034
QLI64122.1	OTHER	0.001273	0.998727	
QLI64123.1	OTHER	0.000596	0.999404	
QLI64124.1	OTHER	0.024375	0.975625	
QLI64125.1	OTHER	0.000219	0.999781	
QLI64126.1	OTHER	0.000400	0.999600	
QLI64127.1	OTHER	0.000326	0.999674	
QLI64128.1	OTHER	0.003613	0.996387	
QLI64129.1	OTHER	0.001281	0.998719	
QLI64130.1	OTHER	0.011157	0.988843	
QLI64131.1	OTHER	0.001611	0.998389	
QLI64132.1	OTHER	0.001969	0.998031	

QLI64133.1	OTHER	0.001982	0.998018	
QLI64134.1	OTHER	0.001694	0.998306	
QLI64135.1	SP(Sec/SPI)	0.981688	0.018312	CS pos: 22-23. AAA-VP. Pr: 0.6384
QLI64136.1	OTHER	0.004240	0.995760	
QLI64137.1	OTHER	0.003325	0.996675	
QLI64138.1	OTHER	0.012647	0.987353	
QLI64139.1	OTHER	0.000435	0.999565	
QLI64140.1	OTHER	0.000597	0.999403	
QLI64141.1	OTHER	0.001032	0.998968	
QLI64142.1	OTHER	0.006763	0.993237	
QLI64143.1	SP(Sec/SPI)	0.657493	0.342507	CS pos: 20-21. SRA-LE. Pr: 0.4387
QLI64144.1	OTHER	0.002665	0.997335	
QLI64145.1	OTHER	0.001491	0.998509	
QLI64146.1	OTHER	0.000531	0.999469	
QLI64147.1	OTHER	0.000906	0.999094	
QLI64148.1	OTHER	0.002368	0.997632	
QLI64149.1	OTHER	0.000418	0.999582	
QLI64150.1	SP(Sec/SPI)	0.970333	0.029667	CS pos: 20-21. TAA-LE. Pr: 0.7184
QLI64151.1	OTHER	0.001423	0.998577	
QLI64152.1	OTHER	0.000644	0.999356	
QLI64153.1	OTHER	0.001136	0.998864	
QLI64154.1	OTHER	0.000569	0.999431	
QLI64155.1	OTHER	0.000752	0.999248	
QLI64156.1	OTHER	0.015438	0.984562	
QLI64157.1	OTHER	0.001570	0.998430	
QLI64158.1	OTHER	0.002375	0.997625	
QLI64159.1	OTHER	0.000462	0.999538	
QLI64160.1	OTHER	0.041511	0.958489	
QLI64161.1	OTHER	0.000519	0.999481	
QLI64162.1	OTHER	0.000741	0.999259	
QLI64163.1	OTHER	0.005125	0.994875	

QLI64164.1	OTHER	0.000430	0.999570	
QLI64165.1	OTHER	0.000714	0.999286	
QLI64166.1	OTHER	0.001000	0.999000	
QLI64167.1	OTHER	0.000796	0.999204	
QLI64168.1	OTHER	0.000705	0.999295	
QLI64169.1	OTHER	0.008066	0.991934	
QLI64170.1	OTHER	0.001310	0.998690	
QLI64171.1	OTHER	0.000610	0.999390	
QLI64172.1	OTHER	0.000809	0.999191	
QLI64173.1	OTHER	0.001915	0.998085	
QLI64174.1	OTHER	0.001086	0.998914	
QLI64175.1	OTHER	0.000547	0.999453	
QLI64176.1	OTHER	0.000635	0.999365	
QLI64177.1	OTHER	0.000335	0.999665	
QLI64178.1	OTHER	0.001122	0.998878	
QLI64179.1	OTHER	0.001432	0.998568	
QLI64180.1	OTHER	0.001936	0.998064	
QLI64181.1	OTHER	0.000455	0.999545	
QLI64182.1	OTHER	0.000931	0.999069	
QLI64183.1	SP(Sec/SPI)	0.975209	0.024791	CS pos: 19-20. AAA-LP. Pr: 0.7028
QLI64184.1	OTHER	0.001444	0.998556	
QLI64185.1	OTHER	0.001136	0.998864	
QLI64186.1	OTHER	0.000421	0.999579	
QLI64187.1	OTHER	0.022341	0.977659	
QLI64188.1	OTHER	0.043392	0.956608	
QLI64189.1	OTHER	0.002611	0.997389	
QLI64190.1	OTHER	0.003755	0.996245	
QLI64191.1	OTHER	0.000340	0.999660	
QLI64192.1	OTHER	0.002506	0.997494	
QLI64193.1	SP(Sec/SPI)	0.972373	0.027627	CS pos: 18-19. TAA-NV. Pr: 0.6927
QLI64194.1	OTHER	0.002705	0.997295	

QLI64195.1	OTHER	0.000758	0.999242	
QLI64196.1	OTHER	0.023093	0.976907	
QLI64197.1	OTHER	0.001075	0.998925	
QLI64198.1	OTHER	0.001176	0.998824	
QLI64199.1	OTHER	0.003370	0.996630	
QLI64200.1	OTHER	0.001994	0.998006	
QLI64201.1	OTHER	0.004181	0.995819	
QLI64202.1	OTHER	0.001319	0.998681	
QLI64203.1	SP(Sec/SPI)	0.986970	0.013030	CS pos: 20-21. ALA-AP. Pr: 0.8048
QLI64204.1	OTHER	0.001704	0.998296	
QLI64205.1	OTHER	0.000879	0.999121	
QLI64206.1	SP(Sec/SPI)	0.595191	0.404809	CS pos: 19-20. CSA-AS. Pr: 0.3697
QLI64207.1	OTHER	0.000473	0.999527	
QLI64208.1	OTHER	0.002729	0.997271	
QLI64209.1	OTHER	0.001018	0.998982	
QLI64210.1	OTHER	0.001725	0.998275	
QLI64211.1	OTHER	0.002803	0.997197	
QLI64212.1	OTHER	0.006687	0.993313	
QLI64213.1	OTHER	0.000811	0.999189	
QLI64214.1	OTHER	0.000151	0.999849	
QLI64215.1	OTHER	0.001609	0.998391	
QLI64216.1	OTHER	0.001336	0.998664	
QLI64217.1	SP(Sec/SPI)	0.986323	0.013677	CS pos: 20-21. ASA-AN. Pr: 0.8657
QLI64218.1	SP(Sec/SPI)	0.999467	0.000533	CS pos: 21-22. GAA-AY. Pr: 0.5407
QLI64219.1	OTHER	0.000569	0.999431	
QLI64220.1	OTHER	0.001739	0.998261	
QLI64221.1	SP(Sec/SPI)	0.995099	0.004901	CS pos: 21-22. TQA-WN. Pr: 0.8332
QLI64222.1	SP(Sec/SPI)	0.867285	0.132715	CS pos: 21-22. SNA-QF. Pr: 0.7900
QLI64223.1	OTHER	0.006786	0.993214	
QLI64224.1	SP(Sec/SPI)	0.962625	0.037375	CS pos: 20-21. VLA-GP. Pr: 0.8212
QLI64225.1	OTHER	0.000478	0.999522	

QLI64226.1	OTHER	0.001202	0.998798	
QLI64227.1	OTHER	0.006372	0.993628	
QLI64228.1	OTHER	0.002605	0.997395	
QLI64229.1	OTHER	0.000766	0.999234	
QLI64230.1	OTHER	0.001147	0.998853	
QLI64231.1	OTHER	0.000921	0.999079	
QLI64232.1	OTHER	0.085273	0.914727	
QLI64233.1	OTHER	0.003573	0.996427	
QLI64234.1	OTHER	0.000705	0.999295	
QLI64235.1	OTHER	0.000423	0.999577	
QLI64236.1	OTHER	0.010903	0.989097	
QLI64237.1	OTHER	0.001682	0.998318	
QLI64238.1	OTHER	0.002195	0.997805	
QLI64239.1	OTHER	0.000498	0.999502	
QLI64240.1	OTHER	0.000583	0.999417	
QLI64241.1	SP(Sec/SPI)	0.995105	0.004895	CS pos: 18-19. AIA-DG. Pr: 0.4992
QLI64242.1	OTHER	0.001377	0.998623	
QLI64243.1	OTHER	0.000847	0.999153	
QLI64244.1	OTHER	0.001211	0.998789	
QLI64245.1	OTHER	0.003226	0.996774	
QLI64246.1	OTHER	0.000424	0.999576	
QLI64247.1	OTHER	0.001155	0.998845	
QLI64248.1	OTHER	0.001389	0.998611	
QLI64249.1	OTHER	0.006293	0.993707	
QLI64250.1	OTHER	0.002669	0.997331	
QLI64251.1	OTHER	0.001805	0.998195	
QLI64252.1	OTHER	0.001388	0.998612	
QLI64253.1	OTHER	0.001222	0.998778	
QLI64254.1	OTHER	0.002397	0.997603	
QLI64255.1	OTHER	0.001195	0.998805	
QLI64256.1	OTHER	0.002469	0.997531	

QLI64257.1	OTHER	0.002269	0.997731	
QLI64258.1	SP(Sec/SPI)	0.992095	0.007905	CS pos: 16-17. ASA-RQ. Pr: 0.7806
QLI64259.1	OTHER	0.002220	0.997780	
QLI64260.1	OTHER	0.265503	0.734497	
QLI64261.1	OTHER	0.000450	0.999550	
QLI64262.1	OTHER	0.003903	0.996097	
QLI64263.1	OTHER	0.000967	0.999033	
QLI64264.1	OTHER	0.000989	0.999011	
QLI64265.1	OTHER	0.009014	0.990986	
QLI64266.1	OTHER	0.001921	0.998079	
QLI64267.1	OTHER	0.000506	0.999494	
QLI64268.1	OTHER	0.007939	0.992061	
QLI64269.1	OTHER	0.000727	0.999273	
QLI64270.1	OTHER	0.134807	0.865193	
QLI64271.1	OTHER	0.000525	0.999475	
QLI64272.1	OTHER	0.002169	0.997831	
QLI64273.1	OTHER	0.000949	0.999051	
QLI64274.1	OTHER	0.025686	0.974314	
QLI64275.1	SP(Sec/SPI)	0.984439	0.015561	CS pos: 17-18. VAA-KP. Pr: 0.6460
QLI64276.1	OTHER	0.000294	0.999706	
QLI64277.1	OTHER	0.001816	0.998184	
QLI64278.1	OTHER	0.003931	0.996069	
QLI64279.1	OTHER	0.001121	0.998879	
QLI64280.1	OTHER	0.000552	0.999448	
QLI64281.1	OTHER	0.000392	0.999608	
QLI64282.1	OTHER	0.002788	0.997212	
QLI64283.1	OTHER	0.088443	0.911557	
QLI64284.1	OTHER	0.001258	0.998742	
QLI64285.1	OTHER	0.013037	0.986963	
QLI64286.1	OTHER	0.001883	0.998117	
QLI64287.1	OTHER	0.003214	0.996786	

QLI64288.1	OTHER	0.001690	0.998310	
QLI64289.1	OTHER	0.000660	0.999340	
QLI64290.1	OTHER	0.000840	0.999160	
QLI64291.1	SP(Sec/SPI)	0.947863	0.052137	CS pos: 23-24. TLA-GF. Pr: 0.7326
QLI64292.1	SP(Sec/SPI)	0.944273	0.055727	CS pos: 18-19. GFS-AK. Pr: 0.5030
QLI64293.1	OTHER	0.000322	0.999678	
QLI64294.1	OTHER	0.005338	0.994662	
QLI64295.1	OTHER	0.000474	0.999526	
QLI64296.1	OTHER	0.002257	0.997743	
QLI64297.1	OTHER	0.001610	0.998390	
QLI64298.1	SP(Sec/SPI)	0.745824	0.254176	CS pos: 23-24. VGA-AQ. Pr: 0.2945
QLI64299.1	OTHER	0.000490	0.999510	
QLI64300.1	OTHER	0.045513	0.954487	
QLI64301.1	OTHER	0.004969	0.995031	
QLI64302.1	OTHER	0.000228	0.999772	
QLI64303.1	OTHER	0.002202	0.997798	
QLI64304.1	OTHER	0.000694	0.999306	
QLI64305.1	OTHER	0.001913	0.998087	
QLI64306.1	OTHER	0.000813	0.999187	
QLI64307.1	OTHER	0.000484	0.999516	
QLI64308.1	OTHER	0.003313	0.996687	
QLI64309.1	OTHER	0.001344	0.998656	
QLI64310.1	OTHER	0.000540	0.999460	
QLI64311.1	OTHER	0.001060	0.998940	
QLI64312.1	OTHER	0.001480	0.998520	
QLI64313.1	OTHER	0.000423	0.999577	
QLI64314.1	OTHER	0.004129	0.995871	
QLI64315.1	OTHER	0.001212	0.998788	
QLI64316.1	OTHER	0.000710	0.999290	
QLI64317.1	OTHER	0.072374	0.927626	
QLI64318.1	OTHER	0.002089	0.997911	

QLI64319.1	OTHER	0.002039	0.997961	
QLI64320.1	OTHER	0.007045	0.992955	
QLI64321.1	OTHER	0.000480	0.999520	
QLI64322.1	SP(Sec/SPI)	0.951908	0.048092	CS pos: 19-20. ASS-QC. Pr: 0.6454
QLI64323.1	OTHER	0.000741	0.999259	
QLI64324.1	OTHER	0.001001	0.998999	
QLI64325.1	SP(Sec/SPI)	0.722943	0.277057	CS pos: 21-22. VIG-RP. Pr: 0.2733
QLI64326.1	OTHER	0.001253	0.998747	
QLI64327.1	OTHER	0.000372	0.999628	
QLI64328.1	OTHER	0.004701	0.995299	
QLI64329.1	OTHER	0.004037	0.995963	
QLI64330.1	SP(Sec/SPI)	0.979751	0.020249	CS pos: 19-20. AAA-AT. Pr: 0.4930
QLI64331.1	OTHER	0.001416	0.998584	
QLI64332.1	OTHER	0.001291	0.998709	
QLI64333.1	OTHER	0.000550	0.999450	
QLI64334.1	OTHER	0.000321	0.999679	
QLI64335.1	OTHER	0.000472	0.999528	
QLI64336.1	OTHER	0.002259	0.997741	
QLI64337.1	SP(Sec/SPI)	0.996182	0.003818	CS pos: 21-22. VSS-RV. Pr: 0.5878
QLI64338.1	OTHER	0.003921	0.996079	
QLI64339.1	OTHER	0.005845	0.994155	
QLI64340.1	OTHER	0.030046	0.969954	
QLI64341.1	SP(Sec/SPI)	0.959869	0.040131	CS pos: 19-20. AGA-VF. Pr: 0.6839
QLI64342.1	OTHER	0.001003	0.998997	
QLI64343.1	SP(Sec/SPI)	0.946393	0.053607	CS pos: 30-31. AKA-AP. Pr: 0.2773
QLI64344.1	OTHER	0.290038	0.709962	
QLI64345.1	OTHER	0.001557	0.998443	
QLI64346.1	OTHER	0.001947	0.998053	
QLI64347.1	OTHER	0.001459	0.998541	
QLI64348.1	OTHER	0.001619	0.998381	
QLI64349.1	OTHER	0.000229	0.999771	

QLI64350.1	OTHER	0.001217	0.998783	
QLI64351.1	OTHER	0.021026	0.978974	
QLI64352.1	OTHER	0.201374	0.798626	
QLI64353.1	OTHER	0.001334	0.998666	
QLI64354.1	OTHER	0.003876	0.996124	
QLI64355.1	OTHER	0.001108	0.998892	
QLI64356.1	OTHER	0.003897	0.996103	
QLI64357.1	OTHER	0.001346	0.998654	
QLI64358.1	OTHER	0.002833	0.997167	
QLI64359.1	SP(Sec/SPI)	0.979464	0.020536	CS pos: 20-21. AHA-LA. Pr: 0.4959
QLI64360.1	OTHER	0.000445	0.999555	
QLI64361.1	OTHER	0.001597	0.998403	
QLI64362.1	OTHER	0.000881	0.999119	
QLI64363.1	OTHER	0.001594	0.998406	
QLI64364.1	OTHER	0.000714	0.999286	
QLI64365.1	OTHER	0.001354	0.998646	
QLI64366.1	OTHER	0.137716	0.862284	
QLI64367.1	OTHER	0.000760	0.999240	
QLI64368.1	OTHER	0.003568	0.996432	
QLI64369.1	OTHER	0.001422	0.998578	
QLI64370.1	OTHER	0.001472	0.998528	
QLI64371.1	OTHER	0.001149	0.998851	
QLI64372.1	OTHER	0.000338	0.999662	
QLI64373.1	OTHER	0.000748	0.999252	
QLI64374.1	OTHER	0.003160	0.996840	
QLI64375.1	OTHER	0.004158	0.995842	
QLI64376.1	OTHER	0.000370	0.999630	
QLI64377.1	OTHER	0.003164	0.996836	
QLI64378.1	OTHER	0.000997	0.999003	
QLI64379.1	OTHER	0.001211	0.998789	
QLI64380.1	OTHER	0.006577	0.993423	

QLI64381.1	OTHER	0.001824	0.998176	
QLI64382.1	OTHER	0.023116	0.976884	
QLI64383.1	OTHER	0.008243	0.991757	
QLI64384.1	SP(Sec/SPI)	0.988794	0.011206	CS pos: 18-19. TLA-AQ. Pr: 0.5479
QLI64385.1	OTHER	0.001055	0.998945	
QLI64386.1	SP(Sec/SPI)	0.987283	0.012717	CS pos: 20-21. VHA-IP. Pr: 0.8114
QLI64387.1	OTHER	0.000933	0.999067	
QLI64388.1	OTHER	0.000612	0.999388	
QLI64389.1	OTHER	0.001184	0.998816	
QLI64390.1	OTHER	0.000579	0.999421	
QLI64391.1	OTHER	0.001119	0.998881	
QLI64392.1	OTHER	0.000776	0.999224	
QLI64393.1	OTHER	0.001558	0.998442	
QLI64394.1	OTHER	0.000333	0.999667	
QLI64395.1	OTHER	0.000746	0.999254	
QLI64396.1	OTHER	0.394418	0.605582	
QLI64397.1	SP(Sec/SPI)	0.858882	0.141118	CS pos: 24-25. AYP-RP. Pr: 0.6748
QLI64398.1	OTHER	0.000840	0.999160	
QLI64399.1	OTHER	0.000108	0.999892	
QLI64400.1	OTHER	0.000559	0.999441	
QLI64401.1	OTHER	0.001375	0.998625	
QLI64402.1	OTHER	0.001042	0.998958	
QLI64403.1	SP(Sec/SPI)	0.975974	0.024026	CS pos: 20-21. VAA-DW. Pr: 0.8891
QLI64404.1	OTHER	0.001446	0.998554	
QLI64405.1	OTHER	0.347293	0.652707	
QLI64406.1	OTHER	0.389942	0.610058	
QLI64407.1	OTHER	0.004411	0.995589	
QLI64408.1	SP(Sec/SPI)	0.885099	0.114901	CS pos: 16-17. AAA-SP. Pr: 0.5100
QLI64409.1	OTHER	0.001231	0.998769	
QLI64410.1	OTHER	0.000743	0.999257	
QLI64411.1	SP(Sec/SPI)	0.911818	0.088182	CS pos: 22-23. ISA-FP. Pr: 0.8335

QLI64412.1	OTHER	0.002001	0.997999	
QLI64413.1	OTHER	0.006650	0.993350	
QLI64414.1	OTHER	0.000625	0.999375	
QLI64415.1	OTHER	0.002646	0.997354	
QLI64416.1	OTHER	0.000813	0.999187	
QLI64417.1	OTHER	0.047854	0.952146	
QLI64418.1	OTHER	0.000986	0.999014	
QLI64419.1	OTHER	0.001838	0.998162	
QLI64420.1	OTHER	0.000408	0.999592	
QLI64421.1	OTHER	0.002768	0.997232	
QLI64422.1	OTHER	0.000642	0.999358	
QLI64423.1	OTHER	0.000701	0.999299	
QLI64424.1	OTHER	0.001030	0.998970	
QLI64425.1	OTHER	0.159845	0.840155	
QLI64426.1	OTHER	0.180409	0.819591	
QLI64427.1	OTHER	0.013559	0.986441	
QLI64428.1	OTHER	0.001951	0.998049	
QLI64429.1	OTHER	0.000602	0.999398	
QLI64430.1	OTHER	0.005223	0.994777	
QLI64431.1	OTHER	0.000396	0.999604	
QLI64432.1	OTHER	0.000291	0.999709	
QLI64433.1	OTHER	0.001267	0.998733	
QLI64434.1	OTHER	0.000906	0.999094	
QLI64435.1	OTHER	0.027653	0.972347	
QLI64436.1	OTHER	0.007664	0.992336	
QLI64437.1	SP(Sec/SPI)	0.962689	0.037311	CS pos: 15-16. VLA-AP. Pr: 0.3964
QLI64438.1	OTHER	0.000614	0.999386	
QLI64439.1	OTHER	0.000742	0.999258	
QLI64440.1	OTHER	0.002958	0.997042	
QLI64441.1	OTHER	0.000596	0.999404	
QLI64442.1	OTHER	0.000700	0.999300	

QLI64443.1	OTHER	0.004911	0.995089	
QLI64444.1	OTHER	0.000301	0.999699	
QLI64445.1	OTHER	0.003465	0.996535	
QLI64446.1	OTHER	0.001821	0.998179	
QLI64447.1	OTHER	0.000243	0.999757	
QLI64448.1	OTHER	0.029688	0.970312	
QLI64449.1	OTHER	0.079399	0.920601	
QLI64450.1	OTHER	0.000847	0.999153	
QLI64451.1	OTHER	0.002412	0.997588	
QLI64452.1	OTHER	0.001214	0.998786	
QLI64453.1	OTHER	0.000689	0.999311	
QLI64454.1	OTHER	0.001492	0.998508	
QLI64455.1	OTHER	0.002472	0.997528	
QLI64456.1	SP(Sec/SPI)	0.996464	0.003536	CS pos: 21-22. TTA-NS. Pr: 0.5237
QLI64457.1	OTHER	0.001378	0.998622	
QLI64458.1	SP(Sec/SPI)	0.964184	0.035816	CS pos: 27-28. AEA-EV. Pr: 0.2478
QLI64459.1	OTHER	0.001329	0.998671	
QLI64460.1	OTHER	0.002857	0.997143	
QLI64461.1	OTHER	0.001222	0.998778	
QLI64462.1	OTHER	0.003543	0.996457	
QLI64463.1	OTHER	0.002006	0.997994	
QLI64464.1	OTHER	0.003086	0.996914	
QLI64465.1	OTHER	0.009093	0.990907	
QLI64466.1	OTHER	0.001958	0.998042	
QLI64467.1	OTHER	0.000577	0.999423	
QLI64468.1	OTHER	0.000402	0.999598	
QLI64469.1	OTHER	0.000448	0.999552	
QLI64470.1	OTHER	0.000481	0.999519	
QLI64471.1	OTHER	0.063230	0.936770	
QLI64472.1	OTHER	0.002628	0.997372	
QLI64473.1	OTHER	0.005088	0.994912	

QLI64474.1	OTHER	0.301778	0.698222	
QLI64475.1	SP(Sec/SPI)	0.992611	0.007389	CS pos: 23-24. TCA-AG. Pr: 0.3719
QLI64476.1	SP(Sec/SPI)	0.955503	0.044497	CS pos: 18-19. AHG-LV. Pr: 0.4947
QLI64477.1	OTHER	0.002529	0.997471	
QLI64478.1	OTHER	0.001074	0.998926	
QLI64479.1	OTHER	0.003593	0.996407	
QLI64480.1	OTHER	0.000520	0.999480	
QLI64481.1	OTHER	0.000656	0.999344	
QLI64482.1	OTHER	0.011794	0.988206	
QLI64483.1	OTHER	0.000910	0.999090	
QLI64484.1	OTHER	0.001453	0.998547	
QLI64485.1	OTHER	0.001671	0.998329	
QLI64486.1	OTHER	0.000312	0.999688	
QLI64487.1	OTHER	0.000761	0.999239	
QLI64488.1	OTHER	0.001178	0.998822	
QLI64489.1	OTHER	0.000795	0.999205	
QLI64490.1	OTHER	0.000638	0.999362	
QLI64491.1	OTHER	0.004147	0.995853	
QLI64492.1	OTHER	0.000465	0.999535	
QLI64493.1	OTHER	0.000717	0.999283	
QLI64494.1	OTHER	0.000285	0.999715	
QLI64495.1	OTHER	0.005492	0.994508	
QLI64496.1	OTHER	0.000778	0.999222	
QLI64497.1	OTHER	0.003590	0.996410	
QLI64498.1	OTHER	0.001242	0.998758	
QLI64499.1	OTHER	0.000356	0.999644	
QLI64500.1	OTHER	0.000715	0.999285	
QLI64501.1	OTHER	0.002568	0.997432	
QLI64502.1	OTHER	0.000724	0.999276	
QLI64503.1	OTHER	0.001620	0.998380	
QLI64504.1	OTHER	0.000617	0.999383	

QLI64505.1	OTHER	0.000843	0.999157	
QLI64506.1	SP(Sec/SPI)	0.993715	0.006285	CS pos: 20-21. FEA-KV. Pr: 0.8362
QLI64507.1	OTHER	0.002293	0.997707	
QLI64508.1	OTHER	0.001212	0.998788	
QLI64509.1	OTHER	0.002033	0.997967	
QLI64510.1	OTHER	0.001726	0.998274	
QLI64511.1	OTHER	0.000918	0.999082	
QLI64512.1	OTHER	0.004871	0.995129	
QLI64513.1	OTHER	0.000578	0.999422	
QLI64514.1	OTHER	0.001651	0.998349	
QLI64515.1	SP(Sec/SPI)	0.998033	0.001967	CS pos: 17-18. AYA-MP. Pr: 0.7609
QLI64516.1	SP(Sec/SPI)	0.959622	0.040378	CS pos: 18-19. AVA-AR. Pr: 0.3947
QLI64517.1	OTHER	0.005454	0.994546	
QLI64518.1	OTHER	0.004913	0.995087	
QLI64519.1	OTHER	0.001420	0.998580	
QLI64520.1	OTHER	0.000526	0.999474	
QLI64521.1	OTHER	0.001435	0.998565	
QLI64522.1	SP(Sec/SPI)	0.996773	0.003227	CS pos: 17-18. AFA-AP. Pr: 0.9666
QLI64523.1	OTHER	0.000170	0.999830	
QLI64524.1	OTHER	0.006325	0.993675	
QLI64525.1	OTHER	0.000440	0.999560	
QLI64526.1	OTHER	0.018383	0.981617	
QLI64527.1	OTHER	0.005818	0.994182	
QLI64528.1	OTHER	0.001132	0.998868	
QLI64529.1	OTHER	0.001107	0.998893	
QLI64530.1	OTHER	0.000570	0.999430	
QLI64531.1	OTHER	0.001552	0.998448	
QLI64532.1	OTHER	0.002482	0.997518	
QLI64533.1	SP(Sec/SPI)	0.979156	0.020844	CS pos: 20-21. VAA-TA. Pr: 0.5710
QLI64534.1	OTHER	0.000363	0.999637	
QLI64535.1	OTHER	0.000638	0.999362	

QLI64536.1	OTHER	0.003279	0.996721
QLI64537.1	OTHER	0.000509	0.999491
QLI64538.1	OTHER	0.000609	0.999391
QLI64539.1	OTHER	0.000727	0.999273
QLI64540.1	OTHER	0.001542	0.998458
QLI64541.1	OTHER	0.001248	0.998752
QLI64542.1	OTHER	0.002307	0.997693
QLI64543.1	OTHER	0.001350	0.998650
QLI64544.1	OTHER	0.000758	0.999242
QLI64545.1	OTHER	0.000755	0.999245
QLI64546.1	OTHER	0.000211	0.999789
QLI64547.1	OTHER	0.002046	0.997954
QLI64548.1	OTHER	0.009715	0.990285
QLI64549.1	OTHER	0.002486	0.997514
QLI64550.1	OTHER	0.004609	0.995391
QLI64551.1	OTHER	0.000292	0.999708
QLI64552.1	OTHER	0.000488	0.999512
QLI64553.1	OTHER	0.002287	0.997713
QLI64554.1	OTHER	0.004553	0.995447
QLI64555.1	OTHER	0.001297	0.998703
QLI64556.1	OTHER	0.001833	0.998167
QLI64557.1	OTHER	0.000655	0.999345
QLI64558.1	OTHER	0.001441	0.998559
QLI64559.1	OTHER	0.000411	0.999589
QLI64560.1	OTHER	0.000457	0.999543
QLI64561.1	OTHER	0.004191	0.995809
QLI64562.1	OTHER	0.014305	0.985695
QLI64563.1	OTHER	0.000753	0.999247
QLI64564.1	OTHER	0.000522	0.999478
QLI64565.1	OTHER	0.000656	0.999344
QLI64566.1	OTHER	0.002425	0.997575

QLI64567.1	SP(Sec/SPI)	0.908962	0.091038	CS pos: 17-18. VSA-WL. Pr: 0.6571
QLI64568.1	OTHER	0.000703	0.999297	
QLI64569.1	OTHER	0.013954	0.986046	
QLI64570.1	SP(Sec/SPI)	0.980692	0.019308	CS pos: 18-19. AFA-NS. Pr: 0.9231
QLI64571.1	OTHER	0.005940	0.994060	
QLI64572.1	OTHER	0.001123	0.998877	
QLI64573.1	OTHER	0.001339	0.998661	
QLI64574.1	OTHER	0.002621	0.997379	
QLI64575.1	OTHER	0.000788	0.999212	
QLI64576.1	OTHER	0.000553	0.999447	
QLI64577.1	OTHER	0.001159	0.998841	
QLI64578.1	OTHER	0.002909	0.997091	
QLI64579.1	OTHER	0.001639	0.998361	
QLI64580.1	OTHER	0.000535	0.999465	
QLI64581.1	OTHER	0.001091	0.998909	
QLI64582.1	OTHER	0.000306	0.999694	
QLI64583.1	OTHER	0.001053	0.998947	
QLI64584.1	OTHER	0.000387	0.999613	
QLI64585.1	OTHER	0.005682	0.994318	
QLI64586.1	OTHER	0.000429	0.999571	
QLI64587.1	SP(Sec/SPI)	0.932609	0.067391	CS pos: 22-23. ISG-RA. Pr: 0.6180
QLI64588.1	OTHER	0.000742	0.999258	
QLI64589.1	OTHER	0.001373	0.998627	
QLI64590.1	OTHER	0.001217	0.998783	
QLI64591.1	SP(Sec/SPI)	0.818724	0.181276	CS pos: 24-25. TSA-FY. Pr: 0.7504
QLI64592.1	OTHER	0.000635	0.999365	
QLI64593.1	OTHER	0.000895	0.999105	
QLI64594.1	OTHER	0.011372	0.988628	
QLI64595.1	OTHER	0.001391	0.998609	
QLI64596.1	OTHER	0.001692	0.998308	
QLI64597.1	OTHER	0.000362	0.999638	

QLI64598.1	SP(Sec/SPI)	0.780844	0.219156	CS pos: 19-20. VVA-AN. Pr: 0.3705
QLI64599.1	OTHER	0.003135	0.996865	
QLI64600.1	OTHER	0.002825	0.997175	
QLI64601.1	OTHER	0.001227	0.998773	
QLI64602.1	OTHER	0.000801	0.999199	
QLI64603.1	OTHER	0.331761	0.668239	
QLI64604.1	OTHER	0.006096	0.993904	
QLI64605.1	OTHER	0.001723	0.998277	
QLI64606.1	OTHER	0.001736	0.998264	
QLI64607.1	OTHER	0.001171	0.998829	
QLI64608.1	OTHER	0.002526	0.997474	
QLI64609.1	OTHER	0.000785	0.999215	
QLI64610.1	OTHER	0.001379	0.998621	
QLI64611.1	OTHER	0.001540	0.998460	
QLI64612.1	OTHER	0.003490	0.996510	
QLI64613.1	OTHER	0.000379	0.999621	
QLI64614.1	OTHER	0.005155	0.994845	
QLI64615.1	OTHER	0.002362	0.997638	
QLI64616.1	SP(Sec/SPI)	0.948338	0.051662	CS pos: 18-19. AFA-SP. Pr: 0.7786
QLI64617.1	OTHER	0.000822	0.999178	
QLI64618.1	OTHER	0.001488	0.998512	
QLI64619.1	OTHER	0.001178	0.998822	
QLI64620.1	OTHER	0.001198	0.998802	
QLI64621.1	OTHER	0.002522	0.997478	
QLI64622.1	SP(Sec/SPI)	0.987436	0.012564	CS pos: 24-25. SMA-SP. Pr: 0.8163
QLI64623.1	OTHER	0.000892	0.999108	
QLI64624.1	OTHER	0.002671	0.997329	
QLI64625.1	SP(Sec/SPI)	0.933797	0.066203	CS pos: 18-19. ATA-AP. Pr: 0.7324
QLI64626.1	OTHER	0.000807	0.999193	
QLI64627.1	OTHER	0.000760	0.999240	
QLI64628.1	OTHER	0.001173	0.998827	

QLI64629.1	OTHER	0.000505	0.999495	
QLI64630.1	OTHER	0.001617	0.998383	
QLI64631.1	OTHER	0.004578	0.995422	
QLI64632.1	OTHER	0.001615	0.998385	
QLI64633.1	OTHER	0.001008	0.998992	
QLI64634.1	OTHER	0.001971	0.998029	
QLI64635.1	OTHER	0.001075	0.998925	
QLI64636.1	OTHER	0.001642	0.998358	
QLI64637.1	OTHER	0.002143	0.997857	
QLI64638.1	OTHER	0.000749	0.999251	
QLI64639.1	OTHER	0.000324	0.999676	
QLI64640.1	OTHER	0.000919	0.999081	
QLI64641.1	OTHER	0.002479	0.997521	
QLI64642.1	OTHER	0.000379	0.999621	
QLI64643.1	OTHER	0.000448	0.999552	
QLI64644.1	OTHER	0.000559	0.999441	
QLI64645.1	OTHER	0.045309	0.954691	
QLI64646.1	OTHER	0.001023	0.998977	
QLI64647.1	SP(Sec/SPI)	0.985851	0.014149	CS pos: 23-24. AHA-GY. Pr: 0.9649
QLI64648.1	OTHER	0.001718	0.998282	
QLI64649.1	OTHER	0.000200	0.999800	
QLI64650.1	OTHER	0.000458	0.999542	
QLI64651.1	OTHER	0.001156	0.998844	
QLI64652.1	OTHER	0.004135	0.995865	
QLI64653.1	OTHER	0.003065	0.996935	
QLI64654.1	OTHER	0.000399	0.999601	
QLI64655.1	OTHER	0.012787	0.987213	
QLI64656.1	OTHER	0.000707	0.999293	
QLI64657.1	OTHER	0.000409	0.999591	
QLI64658.1	OTHER	0.000349	0.999651	
QLI64659.1	OTHER	0.000448	0.999552	

QLI64660.1	OTHER	0.000323	0.999677	
QLI64661.1	OTHER	0.001127	0.998873	
QLI64662.1	SP(Sec/SPI)	0.998166	0.001834	CS pos: 17-18. ADA-AD. Pr: 0.9357
QLI64663.1	OTHER	0.001882	0.998118	
QLI64664.1	OTHER	0.001569	0.998431	
QLI64665.1	OTHER	0.003693	0.996307	
QLI64666.1	OTHER	0.001994	0.998006	
QLI64667.1	OTHER	0.001048	0.998952	
QLI64668.1	OTHER	0.001490	0.998510	
QLI64669.1	OTHER	0.409225	0.590775	
QLI64670.1	OTHER	0.000680	0.999320	
QLI64671.1	OTHER	0.001391	0.998609	
QLI64672.1	SP(Sec/SPI)	0.796295	0.203705	CS pos: 21-22. GTG-LD. Pr: 0.4057
QLI64673.1	OTHER	0.000973	0.999027	
QLI64674.1	OTHER	0.001182	0.998818	
QLI64675.1	OTHER	0.000271	0.999729	
QLI64676.1	OTHER	0.001189	0.998811	
QLI64677.1	OTHER	0.000851	0.999149	
QLI64678.1	OTHER	0.001374	0.998626	
QLI64679.1	OTHER	0.003252	0.996748	
QLI64680.1	OTHER	0.000728	0.999272	
QLI64681.1	OTHER	0.000932	0.999068	
QLI64682.1	OTHER	0.359300	0.640700	
QLI64683.1	OTHER	0.000865	0.999135	
QLI64684.1	OTHER	0.003848	0.996152	
QLI64685.1	OTHER	0.000600	0.999400	
QLI64686.1	OTHER	0.002488	0.997512	
QLI64687.1	OTHER	0.000609	0.999391	
QLI64688.1	OTHER	0.001051	0.998949	
QLI64689.1	OTHER	0.016566	0.983434	
QLI64690.1	OTHER	0.000984	0.999016	

QLI64691.1	OTHER	0.000443	0.999557	
QLI64692.1	OTHER	0.002764	0.997236	
QLI64693.1	OTHER	0.000830	0.999170	
QLI64694.1	OTHER	0.002093	0.997907	
QLI64695.1	SP(Sec/SPI)	0.660887	0.339113	CS pos: 18-19. ISA-NN. Pr: 0.3911
QLI64696.1	OTHER	0.003128	0.996872	
QLI64697.1	OTHER	0.001433	0.998567	
QLI64698.1	OTHER	0.082714	0.917286	
QLI64699.1	OTHER	0.000620	0.999380	
QLI64700.1	OTHER	0.014206	0.985794	
QLI64701.1	OTHER	0.172193	0.827807	
QLI64702.1	OTHER	0.000626	0.999374	
QLI64703.1	OTHER	0.001144	0.998856	
QLI64704.1	OTHER	0.001993	0.998007	
QLI64705.1	OTHER	0.001176	0.998824	
QLI64706.1	OTHER	0.000788	0.999212	
QLI64707.1	OTHER	0.001189	0.998811	
QLI64708.1	OTHER	0.000799	0.999201	
QLI64709.1	SP(Sec/SPI)	0.979902	0.020098	CS pos: 21-22. VEA-RQ. Pr: 0.5318
QLI64710.1	OTHER	0.001586	0.998414	
QLI64711.1	OTHER	0.000726	0.999274	
QLI64712.1	OTHER	0.001124	0.998876	
QLI64713.1	OTHER	0.022358	0.977642	
QLI64714.1	OTHER	0.000337	0.999663	
QLI64715.1	OTHER	0.000938	0.999062	
QLI64716.1	OTHER	0.001357	0.998643	
QLI64717.1	SP(Sec/SPI)	0.994735	0.005265	CS pos: 19-20. VTA-FP. Pr: 0.6686
QLI64718.1	OTHER	0.001245	0.998755	
QLI64719.1	OTHER	0.444924	0.555076	
QLI64720.1	SP(Sec/SPI)	0.994755	0.005245	CS pos: 17-18. TDA-NI. Pr: 0.9218
QLI64721.1	OTHER	0.000291	0.999709	

QLI64722.1	OTHER	0.003298	0.996702	
QLI64723.1	OTHER	0.002494	0.997506	
QLI64724.1	OTHER	0.000563	0.999437	
QLI64725.1	OTHER	0.000419	0.999581	
QLI64726.1	OTHER	0.000490	0.999510	
QLI64727.1	OTHER	0.000988	0.999012	
QLI64728.1	OTHER	0.000484	0.999516	
QLI64729.1	OTHER	0.001352	0.998648	
QLI64730.1	OTHER	0.000542	0.999458	
QLI64731.1	OTHER	0.002606	0.997394	
QLI64732.1	OTHER	0.001835	0.998165	
QLI64733.1	OTHER	0.002301	0.997699	
QLI64734.1	OTHER	0.007392	0.992608	
QLI64735.1	OTHER	0.001289	0.998711	
QLI64736.1	OTHER	0.000654	0.999346	
QLI64737.1	SP(Sec/SPI)	0.970285	0.029715	CS pos: 19-20. CQA-SN. Pr: 0.6251
QLI64738.1	SP(Sec/SPI)	0.966421	0.033579	CS pos: 17-18. AFA-AP. Pr: 0.7116
QLI64739.1	OTHER	0.001184	0.998816	
QLI64740.1	OTHER	0.019527	0.980473	
QLI64741.1	SP(Sec/SPI)	0.971095	0.028905	CS pos: 18-19. VSA-SP. Pr: 0.6041
QLI64742.1	OTHER	0.000725	0.999275	
QLI64743.1	OTHER	0.000756	0.999244	
QLI64744.1	OTHER	0.000934	0.999066	
QLI64745.1	OTHER	0.029050	0.970950	
QLI64746.1	OTHER	0.003489	0.996511	
QLI64747.1	OTHER	0.003272	0.996728	
QLI64748.1	OTHER	0.017176	0.982824	
QLI64749.1	OTHER	0.000682	0.999318	
QLI64750.1	OTHER	0.004313	0.995687	
QLI64751.1	OTHER	0.001287	0.998713	
QLI64752.1	OTHER	0.000698	0.999302	

QLI64753.1	SP(Sec/SPI)	0.964744	0.035256	CS pos: 18-19. AIA-SA. Pr: 0.4077
QLI64754.1	OTHER	0.001030	0.998970	
QLI64755.1	OTHER	0.000924	0.999076	
QLI64756.1	OTHER	0.002899	0.997101	
QLI64757.1	OTHER	0.000964	0.999036	
QLI64758.1	OTHER	0.002567	0.997433	
QLI64759.1	OTHER	0.001001	0.998999	
QLI64760.1	OTHER	0.001304	0.998696	
QLI64761.1	SP(Sec/SPI)	0.915099	0.084901	CS pos: 19-20. QTA-IP. Pr: 0.2655
QLI64762.1	OTHER	0.002155	0.997845	
QLI64763.1	OTHER	0.012698	0.987302	
QLI64764.1	OTHER	0.000657	0.999343	
QLI64765.1	SP(Sec/SPI)	0.938954	0.061046	CS pos: 18-19. VVG-QA. Pr: 0.5326
QLI64766.1	OTHER	0.080065	0.919935	
QLI64767.1	SP(Sec/SPI)	0.999344	0.000656	CS pos: 16-17. VMA-AP. Pr: 0.8194
QLI64768.1	OTHER	0.000680	0.999320	
QLI64769.1	OTHER	0.006335	0.993665	
QLI64770.1	OTHER	0.001145	0.998855	
QLI64771.1	OTHER	0.000751	0.999249	
QLI64772.1	OTHER	0.000370	0.999630	
QLI64773.1	OTHER	0.001025	0.998975	
QLI64774.1	OTHER	0.001566	0.998434	
QLI64775.1	OTHER	0.003955	0.996045	
QLI64776.1	OTHER	0.001522	0.998478	
QLI64777.1	OTHER	0.002440	0.997560	
QLI64778.1	OTHER	0.001027	0.998973	
QLI64779.1	OTHER	0.001021	0.998979	
QLI64780.1	OTHER	0.001230	0.998770	
QLI64781.1	OTHER	0.002986	0.997014	
QLI64782.1	OTHER	0.000327	0.999673	
QLI64783.1	OTHER	0.001369	0.998631	

QLI64784.1	OTHER	0.003479	0.996521	
QLI64785.1	OTHER	0.154040	0.845960	
QLI64786.1	SP(Sec/SPI)	0.995216	0.004784	CS pos: 23-24. SLA-AP. Pr: 0.9386
QLI64787.1	OTHER	0.002995	0.997005	
QLI64788.1	OTHER	0.000426	0.999574	
QLI64789.1	OTHER	0.000671	0.999329	
QLI64790.1	OTHER	0.000562	0.999438	
QLI64791.1	OTHER	0.004626	0.995374	
QLI64792.1	SP(Sec/SPI)	0.837739	0.162261	CS pos: 22-23. GHA-AI. Pr: 0.5057
QLI64793.1	OTHER	0.001365	0.998635	
QLI64794.1	OTHER	0.001291	0.998709	
QLI64795.1	OTHER	0.001184	0.998816	
QLI64796.1	OTHER	0.000626	0.999374	
QLI64797.1	OTHER	0.000800	0.999200	
QLI64798.1	OTHER	0.000821	0.999179	
QLI64799.1	OTHER	0.019978	0.980022	
QLI64800.1	OTHER	0.000807	0.999193	
QLI64801.1	OTHER	0.001919	0.998081	
QLI64802.1	OTHER	0.105493	0.894507	
QLI64803.1	OTHER	0.001090	0.998910	
QLI64804.1	SP(Sec/SPI)	0.995372	0.004628	CS pos: 19-20. ASA-AS. Pr: 0.6550
QLI64805.1	OTHER	0.000620	0.999380	
QLI64806.1	SP(Sec/SPI)	0.943792	0.056208	CS pos: 20-21. AHG-AQ. Pr: 0.8553
QLI64807.1	OTHER	0.000431	0.999569	
QLI64808.1	OTHER	0.000525	0.999475	
QLI64809.1	OTHER	0.001175	0.998825	
QLI64810.1	OTHER	0.003282	0.996718	
QLI64811.1	OTHER	0.000871	0.999129	
QLI64812.1	OTHER	0.000798	0.999202	
QLI64813.1	OTHER	0.001819	0.998181	
QLI64814.1	OTHER	0.044354	0.955646	

QLI64815.1	OTHER	0.000921	0.999079	
QLI64816.1	OTHER	0.002203	0.997797	
QLI64817.1	OTHER	0.001615	0.998385	
QLI64818.1	OTHER	0.001516	0.998484	
QLI64819.1	OTHER	0.004697	0.995303	
QLI64820.1	OTHER	0.000711	0.999289	
QLI64821.1	SP(Sec/SPI)	0.996910	0.003090	CS pos: 21-22. VLA-GS. Pr: 0.8597
QLI64822.1	OTHER	0.000998	0.999002	
QLI64823.1	OTHER	0.003590	0.996410	
QLI64824.1	OTHER	0.000298	0.999702	
QLI64825.1	OTHER	0.000855	0.999145	
QLI64826.1	OTHER	0.000899	0.999101	
QLI64827.1	OTHER	0.001324	0.998676	
QLI64828.1	OTHER	0.001544	0.998456	
QLI64829.1	OTHER	0.000900	0.999100	
QLI64830.1	SP(Sec/SPI)	0.802948	0.197052	CS pos: 15-16. VSA-TG. Pr: 0.3542
QLI64831.1	OTHER	0.000407	0.999593	
QLI64832.1	OTHER	0.000806	0.999194	
QLI64833.1	OTHER	0.001641	0.998359	
QLI64834.1	OTHER	0.000960	0.999040	
QLI64835.1	SP(Sec/SPI)	0.999190	0.000810	CS pos: 18-19. AVA-AP. Pr: 0.8760
QLI64836.1	OTHER	0.000687	0.999313	
QLI64837.1	OTHER	0.003526	0.996474	
QLI64838.1	OTHER	0.003192	0.996808	
QLI64839.1	SP(Sec/SPI)	0.735198	0.264802	CS pos: 27-28. VTA-ES. Pr: 0.5372
QLI64840.1	OTHER	0.001958	0.998042	
QLI64841.1	OTHER	0.096109	0.903891	
QLI64842.1	OTHER	0.006237	0.993763	
QLI64843.1	OTHER	0.002619	0.997381	
QLI64844.1	OTHER	0.004687	0.995313	
QLI64845.1	OTHER	0.001614	0.998386	

QLI64846.1	OTHER	0.000688	0.999312	
QLI64847.1	OTHER	0.001447	0.998553	
QLI64848.1	OTHER	0.001641	0.998359	
QLI64849.1	OTHER	0.097700	0.902300	
QLI64850.1	OTHER	0.001170	0.998830	
QLI64851.1	OTHER	0.001191	0.998809	
QLI64852.1	OTHER	0.001854	0.998146	
QLI64853.1	OTHER	0.000922	0.999078	
QLI64854.1	OTHER	0.049099	0.950901	
QLI64855.1	OTHER	0.244869	0.755131	
QLI64856.1	OTHER	0.000722	0.999278	
QLI64857.1	OTHER	0.002733	0.997267	
QLI64858.1	OTHER	0.001025	0.998975	
QLI64859.1	OTHER	0.005787	0.994213	
QLI64860.1	OTHER	0.000467	0.999533	
QLI64861.1	SP(Sec/SPI)	0.993637	0.006363	CS pos: 22-23. VHA-MY. Pr: 0.9679
QLI64862.1	OTHER	0.002655	0.997345	
QLI64863.1	OTHER	0.027014	0.972986	
QLI64864.1	OTHER	0.000699	0.999301	
QLI64865.1	OTHER	0.001174	0.998826	
QLI64866.1	OTHER	0.000730	0.999270	
QLI64867.1	OTHER	0.002481	0.997519	
QLI64868.1	OTHER	0.004763	0.995237	
QLI64869.1	OTHER	0.001030	0.998970	
QLI64870.1	OTHER	0.000377	0.999623	
QLI64871.1	OTHER	0.001790	0.998210	
QLI64872.1	OTHER	0.024783	0.975217	
QLI64873.1	OTHER	0.000542	0.999458	
QLI64874.1	OTHER	0.003451	0.996549	
QLI64875.1	SP(Sec/SPI)	0.897592	0.102408	CS pos: 22-23. VYS-EK. Pr: 0.7723
QLI64876.1	OTHER	0.001588	0.998412	

QLI64877.1	OTHER	0.000735	0.999265	
QLI64878.1	OTHER	0.000733	0.999267	
QLI64879.1	SP(Sec/SPI)	0.925650	0.074350	CS pos: 14-15. ATC-QE. Pr: 0.6531
QLI64880.1	OTHER	0.000376	0.999624	
QLI64881.1	OTHER	0.001417	0.998583	
QLI64882.1	OTHER	0.000607	0.999393	
QLI64883.1	OTHER	0.002076	0.997924	
QLI64884.1	OTHER	0.000415	0.999585	
QLI64885.1	OTHER	0.000252	0.999748	
QLI64886.1	OTHER	0.001497	0.998503	
QLI64887.1	OTHER	0.000435	0.999565	
QLI64888.1	OTHER	0.003165	0.996835	
QLI64889.1	OTHER	0.003152	0.996848	
QLI64890.1	OTHER	0.004675	0.995325	
QLI64891.1	OTHER	0.004441	0.995559	
QLI64892.1	SP(Sec/SPI)	0.987250	0.012750	CS pos: 19-20. VQA-DS. Pr: 0.9446
QLI64893.1	OTHER	0.000320	0.999680	
QLI64894.1	SP(Sec/SPI)	0.991969	0.008031	CS pos: 14-15. AAA-AP. Pr: 0.3304
QLI64895.1	OTHER	0.213495	0.786505	
QLI64896.1	OTHER	0.000805	0.999195	
QLI64897.1	SP(Sec/SPI)	0.507072	0.492928	CS pos: 17-18. AMA-RR. Pr: 0.3154
QLI64898.1	OTHER	0.000702	0.999298	
QLI64899.1	OTHER	0.000515	0.999485	
QLI64900.1	SP(Sec/SPI)	0.856456	0.143544	CS pos: 20-21. AAG-KV. Pr: 0.5131
QLI64901.1	OTHER	0.000359	0.999641	
QLI64902.1	OTHER	0.479495	0.520505	
QLI64903.1	OTHER	0.003185	0.996815	
QLI64904.1	OTHER	0.001932	0.998068	
QLI64905.1	OTHER	0.000457	0.999543	
QLI64906.1	OTHER	0.001295	0.998705	
QLI64907.1	OTHER	0.001017	0.998983	

QLI64908.1	OTHER	0.002104	0.997896	
QLI64909.1	OTHER	0.000425	0.999575	
QLI64910.1	OTHER	0.001651	0.998349	
QLI64911.1	OTHER	0.000821	0.999179	
QLI64912.1	SP(Sec/SPI)	0.993441	0.006559	CS pos: 17-18. AAA-LP. Pr: 0.2605
QLI64913.1	OTHER	0.022705	0.977295	
QLI64914.1	OTHER	0.001060	0.998940	
QLI64915.1	OTHER	0.002175	0.997825	
QLI64916.1	OTHER	0.001449	0.998551	
QLI64917.1	OTHER	0.000268	0.999732	
QLI64918.1	SP(Sec/SPI)	0.830452	0.169548	CS pos: 23-24. ATA-AE. Pr: 0.2740
QLI64919.1	OTHER	0.000566	0.999434	
QLI64920.1	OTHER	0.001559	0.998441	
QLI64921.1	OTHER	0.002824	0.997176	
QLI64922.1	OTHER	0.000673	0.999327	
QLI64923.1	OTHER	0.002395	0.997605	
QLI64924.1	OTHER	0.428201	0.571799	
QLI64925.1	OTHER	0.001669	0.998331	
QLI64926.1	OTHER	0.002301	0.997699	
QLI64927.1	OTHER	0.000545	0.999455	
QLI64928.1	OTHER	0.001500	0.998500	
QLI64929.1	OTHER	0.000668	0.999332	
QLI64930.1	SP(Sec/SPI)	0.987926	0.012074	CS pos: 21-22. ALA-IR. Pr: 0.6246
QLI64931.1	OTHER	0.000887	0.999113	
QLI64932.1	OTHER	0.000502	0.999498	
QLI64933.1	OTHER	0.008048	0.991952	
QLI64934.1	OTHER	0.001551	0.998449	
QLI64935.1	OTHER	0.008989	0.991011	
QLI64936.1	OTHER	0.001233	0.998767	
QLI64937.1	OTHER	0.000801	0.999199	
QLI64938.1	OTHER	0.001147	0.998853	

QLI64939.1	OTHER	0.003367	0.996633	
QLI64940.1	SP(Sec/SPI)	0.617029	0.382971	CS pos: 15-16. ASA-AL. Pr: 0.1805
QLI64941.1	OTHER	0.000398	0.999602	
QLI64942.1	OTHER	0.001141	0.998859	
QLI64943.1	OTHER	0.000341	0.999659	
QLI64944.1	OTHER	0.006212	0.993788	
QLI64945.1	OTHER	0.004706	0.995294	
QLI64946.1	OTHER	0.002259	0.997741	
QLI64947.1	OTHER	0.005883	0.994117	
QLI64948.1	OTHER	0.000870	0.999130	
QLI64949.1	OTHER	0.001908	0.998092	
QLI64950.1	OTHER	0.002386	0.997614	
QLI64951.1	OTHER	0.000406	0.999594	
QLI64952.1	SP(Sec/SPI)	0.995963	0.004037	CS pos: 16-17. VSA-AT. Pr: 0.5437
QLI64953.1	OTHER	0.024865	0.975135	
QLI64954.1	OTHER	0.001220	0.998780	
QLI64955.1	OTHER	0.001084	0.998916	
QLI64956.1	OTHER	0.001378	0.998622	
QLI64957.1	OTHER	0.000738	0.999262	
QLI64958.1	SP(Sec/SPI)	0.964122	0.035878	CS pos: 19-20. TWT-TP. Pr: 0.6745
QLI64959.1	OTHER	0.027665	0.972335	
QLI64960.1	OTHER	0.001004	0.998996	
QLI64961.1	OTHER	0.000521	0.999479	
QLI64962.1	OTHER	0.000540	0.999460	
QLI64963.1	OTHER	0.006923	0.993077	
QLI64964.1	OTHER	0.001224	0.998776	
QLI64965.1	OTHER	0.000437	0.999563	
QLI64966.1	SP(Sec/SPI)	0.917166	0.082834	CS pos: 16-17. AAA-AD. Pr: 0.5605
QLI64967.1	OTHER	0.000926	0.999074	
QLI64968.1	OTHER	0.002704	0.997296	
QLI64969.1	OTHER	0.000490	0.999510	

QLI64970.1	OTHER	0.000750	0.999250	
QLI64971.1	OTHER	0.001989	0.998011	
QLI64972.1	SP(Sec/SPI)	0.904726	0.095274	CS pos: 21-22. ALA-TS. Pr: 0.6524
QLI64973.1	OTHER	0.013065	0.986935	
QLI64974.1	OTHER	0.001572	0.998428	
QLI64975.1	OTHER	0.013419	0.986581	
QLI64976.1	OTHER	0.137292	0.862708	
QLI64977.1	SP(Sec/SPI)	0.756740	0.243260	CS pos: 17-18. ANA-FW. Pr: 0.6892
QLI64978.1	OTHER	0.001340	0.998660	
QLI64979.1	OTHER	0.001130	0.998870	
QLI64980.1	OTHER	0.000223	0.999777	
QLI64981.1	SP(Sec/SPI)	0.975977	0.024023	CS pos: 23-24. THA-SW. Pr: 0.8714
QLI64982.1	OTHER	0.001060	0.998940	
QLI64983.1	OTHER	0.001787	0.998213	
QLI64984.1	OTHER	0.001381	0.998619	
QLI64985.1	OTHER	0.003757	0.996243	
QLI64986.1	SP(Sec/SPI)	0.916751	0.083249	CS pos: 17-18. SAA-LS. Pr: 0.3471
QLI64987.1	OTHER	0.001412	0.998588	
QLI64988.1	OTHER	0.001102	0.998898	
QLI64989.1	OTHER	0.020991	0.979009	
QLI64990.1	OTHER	0.010322	0.989678	
QLI64991.1	OTHER	0.001151	0.998849	
QLI64992.1	OTHER	0.016880	0.983120	
QLI64993.1	SP(Sec/SPI)	0.998945	0.001055	CS pos: 19-20. AQA-TT. Pr: 0.5827
QLI64994.1	OTHER	0.004997	0.995003	
QLI64995.1	SP(Sec/SPI)	0.800741	0.199259	CS pos: 23-24. VWA-VA. Pr: 0.6995
QLI64996.1	SP(Sec/SPI)	0.945198	0.054802	CS pos: 20-21. VEA-HG. Pr: 0.6250
QLI64997.1	OTHER	0.000881	0.999119	
QLI64998.1	SP(Sec/SPI)	0.973452	0.026548	CS pos: 18-19. SVA-GP. Pr: 0.6986
QLI64999.1	OTHER	0.011572	0.988428	
QLI65000.1	OTHER	0.000506	0.999494	

QLI65001.1	OTHER	0.001363	0.998637	
QLI65002.1	OTHER	0.002965	0.997035	
QLI65003.1	OTHER	0.000471	0.999529	
QLI65004.1	OTHER	0.000753	0.999247	
QLI65005.1	OTHER	0.003417	0.996583	
QLI65006.1	OTHER	0.002199	0.997801	
QLI65007.1	OTHER	0.000279	0.999721	
QLI65008.1	OTHER	0.000801	0.999199	
QLI65009.1	OTHER	0.000488	0.999512	
QLI65010.1	OTHER	0.069591	0.930409	
QLI65011.1	OTHER	0.000347	0.999653	
QLI65012.1	OTHER	0.013067	0.986933	
QLI65013.1	SP(Sec/SPI)	0.997381	0.002619	CS pos: 19-20. AQA-DT. Pr: 0.9352
QLI65014.1	OTHER	0.001216	0.998784	
QLI65015.1	OTHER	0.000466	0.999534	
QLI65016.1	OTHER	0.001285	0.998715	
QLI65017.1	OTHER	0.001539	0.998461	
QLI65018.1	OTHER	0.000703	0.999297	
QLI65019.1	OTHER	0.001510	0.998490	
QLI65020.1	OTHER	0.001721	0.998279	
QLI65021.1	OTHER	0.001110	0.998890	
QLI65022.1	OTHER	0.001771	0.998229	
QLI65023.1	OTHER	0.002641	0.997359	
QLI65024.1	OTHER	0.001801	0.998199	
QLI65025.1	OTHER	0.001675	0.998325	
QLI65026.1	OTHER	0.019474	0.980526	
QLI65027.1	OTHER	0.002301	0.997699	
QLI65028.1	OTHER	0.000764	0.999236	
QLI65029.1	OTHER	0.000230	0.999770	
QLI65030.1	OTHER	0.001201	0.998799	
QLI65031.1	OTHER	0.056539	0.943461	

QLI65032.1	SP(Sec/SPI)	0.570826	0.429174	CS pos: 20-21. SHS-WV. Pr: 0.2531
QLI65033.1	OTHER	0.001921	0.998079	
QLI65034.1	OTHER	0.002724	0.997276	
QLI65035.1	OTHER	0.001279	0.998721	
QLI65036.1	SP(Sec/SPI)	0.998242	0.001758	CS pos: 19-20. ATA-AN. Pr: 0.7255
QLI65037.1	OTHER	0.000656	0.999344	
QLI65038.1	SP(Sec/SPI)	0.907147	0.092853	CS pos: 22-23. CNG-RA. Pr: 0.6246
QLI65039.1	OTHER	0.000885	0.999115	
QLI65040.1	OTHER	0.000397	0.999603	
QLI65041.1	OTHER	0.000648	0.999352	
QLI65042.1	OTHER	0.001175	0.998825	
QLI65043.1	OTHER	0.000400	0.999600	
QLI65044.1	OTHER	0.000787	0.999213	
QLI65045.1	OTHER	0.000868	0.999132	
QLI65046.1	OTHER	0.001373	0.998627	
QLI65047.1	OTHER	0.001585	0.998415	
QLI65048.1	OTHER	0.001338	0.998662	
QLI65049.1	OTHER	0.000835	0.999165	
QLI65050.1	OTHER	0.000765	0.999235	
QLI65051.1	OTHER	0.000941	0.999059	
QLI65052.1	OTHER	0.000968	0.999032	
QLI65053.1	OTHER	0.001399	0.998601	
QLI65054.1	OTHER	0.303599	0.696401	
QLI65055.1	OTHER	0.001712	0.998288	
QLI65056.1	OTHER	0.000854	0.999146	
QLI65057.1	SP(Sec/SPI)	0.982175	0.017825	CS pos: 20-21. AVA-AT. Pr: 0.6264
QLI65058.1	OTHER	0.000673	0.999327	
QLI65059.1	SP(Sec/SPI)	0.969140	0.030860	CS pos: 18-19. AAA-KE. Pr: 0.7676
QLI65060.1	OTHER	0.000535	0.999465	
QLI65061.1	SP(Sec/SPI)	0.984383	0.015617	CS pos: 18-19. VTA-AA. Pr: 0.6264
QLI65062.1	SP(Sec/SPI)	0.981240	0.018760	CS pos: 18-19. ASA-KV. Pr: 0.6921

QLI65063.1	OTHER	0.033045	0.966955	
QLI65064.1	OTHER	0.003260	0.996740	
QLI65065.1	OTHER	0.001214	0.998786	
QLI65066.1	OTHER	0.001093	0.998907	
QLI65067.1	OTHER	0.008762	0.991238	
QLI65068.1	SP(Sec/SPI)	0.990652	0.009348	CS pos: 20-21. TAA-WS. Pr: 0.4510
QLI65069.1	OTHER	0.000446	0.999554	
QLI65070.1	OTHER	0.047672	0.952328	
QLI65071.1	OTHER	0.000417	0.999583	
QLI65072.1	OTHER	0.001425	0.998575	
QLI65073.1	OTHER	0.001652	0.998348	
QLI65074.1	OTHER	0.002253	0.997747	
QLI65075.1	OTHER	0.000283	0.999717	
QLI65076.1	OTHER	0.001913	0.998087	
QLI65077.1	OTHER	0.002115	0.997885	
QLI65078.1	OTHER	0.001860	0.998140	
QLI65079.1	OTHER	0.001752	0.998248	
QLI65080.1	OTHER	0.000443	0.999557	
QLI65081.1	OTHER	0.001051	0.998949	
QLI65082.1	OTHER	0.000268	0.999732	
QLI65083.1	OTHER	0.004483	0.995517	
QLI65084.1	OTHER	0.005417	0.994583	
QLI65085.1	OTHER	0.001237	0.998763	
QLI65086.1	OTHER	0.001264	0.998736	
QLI65087.1	OTHER	0.005260	0.994740	
QLI65088.1	OTHER	0.000823	0.999177	
QLI65089.1	OTHER	0.000997	0.999003	
QLI65090.1	OTHER	0.001818	0.998182	
QLI65091.1	OTHER	0.001383	0.998617	
QLI65092.1	OTHER	0.005277	0.994723	
QLI65093.1	OTHER	0.000534	0.999466	

QLI65094.1	OTHER	0.005304	0.994696
QLI65095.1	OTHER	0.035749	0.964251
QLI65096.1	OTHER	0.001881	0.998119
QLI65097.1	OTHER	0.001053	0.998947
QLI65098.1	OTHER	0.000745	0.999255
QLI65099.1	OTHER	0.001498	0.998502
QLI65100.1	OTHER	0.000370	0.999630
QLI65101.1	OTHER	0.001802	0.998198
QLI65102.1	OTHER	0.003364	0.996636
QLI65103.1	OTHER	0.001691	0.998309
QLI65104.1	OTHER	0.001778	0.998222
QLI65105.1	OTHER	0.001895	0.998105
QLI65106.1	OTHER	0.000430	0.999570
QLI65107.1	OTHER	0.000374	0.999626
QLI65108.1	OTHER	0.001219	0.998781
QLI65109.1	OTHER	0.001069	0.998931
QLI65110.1	OTHER	0.000293	0.999707
QLI65111.1	OTHER	0.006192	0.993808
QLI65112.1	OTHER	0.028109	0.971891
QLI65113.1	OTHER	0.001621	0.998379
QLI65114.1	OTHER	0.000668	0.999332
QLI65115.1	OTHER	0.000422	0.999578
QLI65116.1	OTHER	0.001227	0.998773
QLI65117.1	OTHER	0.000293	0.999707
QLI65118.1	OTHER	0.001779	0.998221
QLI65119.1	OTHER	0.000691	0.999309
QLI65120.1	OTHER	0.000631	0.999369
QLI65121.1	OTHER	0.001372	0.998628
QLI65122.1	OTHER	0.002821	0.997179
QLI65123.1	OTHER	0.001148	0.998852
QLI65124.1	OTHER	0.001124	0.998876

QLI65125.1	OTHER	0.000775	0.999225	
QLI65126.1	OTHER	0.001947	0.998053	
QLI65127.1	OTHER	0.001523	0.998477	
QLI65128.1	SP(Sec/SPI)	0.821087	0.178913	CS pos: 20-21. AWA-SL. Pr: 0.3933
QLI65129.1	OTHER	0.004971	0.995029	
QLI65130.1	OTHER	0.000821	0.999179	
QLI65131.1	OTHER	0.000851	0.999149	
QLI65132.1	OTHER	0.000933	0.999067	
QLI65133.1	OTHER	0.001750	0.998250	
QLI65134.1	OTHER	0.000425	0.999575	
QLI65135.1	OTHER	0.000302	0.999698	
QLI65136.1	OTHER	0.000678	0.999322	
QLI65137.1	OTHER	0.007140	0.992860	
QLI65138.1	OTHER	0.009256	0.990744	
QLI65139.1	OTHER	0.027919	0.972081	
QLI65140.1	OTHER	0.002272	0.997728	
QLI65141.1	OTHER	0.011308	0.988692	
QLI65142.1	OTHER	0.000937	0.999063	
QLI65143.1	OTHER	0.000647	0.999353	
QLI65144.1	OTHER	0.000544	0.999456	
QLI65145.1	SP(Sec/SPI)	0.984197	0.015803	CS pos: 16-17. GSA-HF. Pr: 0.7985
QLI65146.1	SP(Sec/SPI)	0.750472	0.249528	CS pos: 23-24. CTG-LP. Pr: 0.3319
QLI65147.1	OTHER	0.000459	0.999541	
QLI65148.1	OTHER	0.026037	0.973963	
QLI65149.1	SP(Sec/SPI)	0.999214	0.000786	CS pos: 17-18. VSA-AP. Pr: 0.4660
QLI65150.1	OTHER	0.002967	0.997033	
QLI65151.1	OTHER	0.000525	0.999475	
QLI65152.1	OTHER	0.002284	0.997716	
QLI65153.1	OTHER	0.000355	0.999645	
QLI65154.1	SP(Sec/SPI)	0.929092	0.070908	CS pos: 16-17. AAA-LP. Pr: 0.5161
QLI65155.1	SP(Sec/SPI)	0.839784	0.160216	CS pos: 13-14. ALG-SE. Pr: 0.5211

QLI65156.1	OTHER	0.008163	0.991837	
QLI65157.1	OTHER	0.001412	0.998588	
QLI65158.1	OTHER	0.057605	0.942395	
QLI65159.1	OTHER	0.000708	0.999292	
QLI65160.1	OTHER	0.000169	0.999831	
QLI65161.1	OTHER	0.000759	0.999241	
QLI65162.1	OTHER	0.001068	0.998932	
QLI65163.1	OTHER	0.001988	0.998012	
QLI65164.1	OTHER	0.002068	0.997932	
QLI65165.1	OTHER	0.000853	0.999147	
QLI65166.1	OTHER	0.000544	0.999456	
QLI65167.1	SP(Sec/SPI)	0.993956	0.006044	CS pos: 19-20. AAA-SE. Pr: 0.3937
QLI65168.1	OTHER	0.002792	0.997208	
QLI65169.1	OTHER	0.001825	0.998175	
QLI65170.1	OTHER	0.001334	0.998666	
QLI65171.1	OTHER	0.000497	0.999503	
QLI65172.1	OTHER	0.003173	0.996827	
QLI65173.1	OTHER	0.001252	0.998748	
QLI65174.1	OTHER	0.001209	0.998791	
QLI65175.1	OTHER	0.001544	0.998456	
QLI65176.1	OTHER	0.002105	0.997895	
QLI65177.1	OTHER	0.001834	0.998166	
QLI65178.1	SP(Sec/SPI)	0.921091	0.078909	CS pos: 18-19. VNA-LV. Pr: 0.4491
QLI65179.1	OTHER	0.026155	0.973845	
QLI65180.1	OTHER	0.000991	0.999009	
QLI65181.1	OTHER	0.001344	0.998656	
QLI65182.1	OTHER	0.000491	0.999509	
QLI65183.1	OTHER	0.001462	0.998538	
QLI65184.1	OTHER	0.001441	0.998559	
QLI65185.1	OTHER	0.000715	0.999285	
QLI65186.1	OTHER	0.000315	0.999685	

QLI65187.1	OTHER	0.001149	0.998851	
QLI65188.1	OTHER	0.016112	0.983888	
QLI65189.1	OTHER	0.000384	0.999616	
QLI65190.1	OTHER	0.000217	0.999783	
QLI65191.1	SP(Sec/SPI)	0.890901	0.109099	CS pos: 28-29. SLT-DI. Pr: 0.3179
QLI65192.1	OTHER	0.000625	0.999375	
QLI65193.1	OTHER	0.001229	0.998771	
QLI65194.1	OTHER	0.172747	0.827253	
QLI65195.1	OTHER	0.000745	0.999255	
QLI65196.1	OTHER	0.257200	0.742800	
QLI65197.1	OTHER	0.007470	0.992530	
QLI65198.1	OTHER	0.001948	0.998052	
QLI65199.1	OTHER	0.001898	0.998102	
QLI65200.1	OTHER	0.000857	0.999143	
QLI65201.1	OTHER	0.001655	0.998345	
QLI65202.1	OTHER	0.000627	0.999373	
QLI65203.1	OTHER	0.013177	0.986823	
QLI65204.1	OTHER	0.000711	0.999289	
QLI65205.1	OTHER	0.001058	0.998942	
QLI65206.1	OTHER	0.000812	0.999188	
QLI65207.1	OTHER	0.001526	0.998474	
QLI65208.1	OTHER	0.005252	0.994748	
QLI65209.1	SP(Sec/SPI)	0.999567	0.000433	CS pos: 18-19. ASA-VT. Pr: 0.7488
QLI65210.1	OTHER	0.000430	0.999570	
QLI65211.1	OTHER	0.000822	0.999178	
QLI65212.1	OTHER	0.001119	0.998881	
QLI65213.1	OTHER	0.002332	0.997668	
QLI65214.1	OTHER	0.001048	0.998952	
QLI65215.1	OTHER	0.000495	0.999505	
QLI65216.1	OTHER	0.001755	0.998245	
QLI65217.1	OTHER	0.000412	0.999588	

QLI65218.1	OTHER	0.001015	0.998985	
QLI65219.1	OTHER	0.001458	0.998542	
QLI65220.1	OTHER	0.001054	0.998946	
QLI65221.1	OTHER	0.000948	0.999052	
QLI65222.1	OTHER	0.000508	0.999492	
QLI65223.1	OTHER	0.001686	0.998314	
QLI65224.1	OTHER	0.000469	0.999531	
QLI65225.1	OTHER	0.000621	0.999379	
QLI65226.1	OTHER	0.008139	0.991861	
QLI65227.1	SP(Sec/SPI)	0.766937	0.233063	CS pos: 18-19. AAA-QQ. Pr: 0.3586
QLI65228.1	OTHER	0.000653	0.999347	
QLI65229.1	OTHER	0.000169	0.999831	
QLI65230.1	OTHER	0.003483	0.996517	
QLI65231.1	OTHER	0.001802	0.998198	
QLI65232.1	OTHER	0.003234	0.996766	
QLI65233.1	OTHER	0.002009	0.997991	
QLI65234.1	SP(Sec/SPI)	0.935461	0.064539	CS pos: 20-21. AAA-LP. Pr: 0.5220
QLI65235.1	OTHER	0.002378	0.997622	
QLI65236.1	OTHER	0.001894	0.998106	
QLI65237.1	OTHER	0.017122	0.982878	
QLI65238.1	SP(Sec/SPI)	0.995272	0.004728	CS pos: 23-24. VQG-AP. Pr: 0.9601
QLI65239.1	OTHER	0.000175	0.999825	
QLI65240.1	OTHER	0.001264	0.998736	
QLI65241.1	SP(Sec/SPI)	0.972370	0.027630	CS pos: 18-19. ASA-LP. Pr: 0.4859
QLI65242.1	OTHER	0.000303	0.999697	
QLI65243.1	OTHER	0.001049	0.998951	
QLI65244.1	OTHER	0.001167	0.998833	
QLI65245.1	OTHER	0.000675	0.999325	
QLI65246.1	OTHER	0.118515	0.881485	
QLI65247.1	OTHER	0.001125	0.998875	
QLI65248.1	OTHER	0.000656	0.999344	

QLI65249.1	OTHER	0.271078	0.728922	
QLI65250.1	OTHER	0.002018	0.997982	
QLI65251.1	OTHER	0.028639	0.971361	
QLI65252.1	OTHER	0.012056	0.987944	
QLI65253.1	OTHER	0.001276	0.998724	
QLI65254.1	OTHER	0.001259	0.998741	
QLI65255.1	OTHER	0.000650	0.999350	
QLI65256.1	OTHER	0.000612	0.999388	
QLI65257.1	SP(Sec/SPI)	0.819942	0.180058	CS pos: 16-17. ALA-VP. Pr: 0.5012
QLI65258.1	OTHER	0.001365	0.998635	
QLI65259.1	OTHER	0.000469	0.999531	
QLI65260.1	OTHER	0.001083	0.998917	
QLI65261.1	OTHER	0.005539	0.994461	
QLI65262.1	OTHER	0.040930	0.959070	
QLI65263.1	OTHER	0.006884	0.993116	
QLI65264.1	OTHER	0.000931	0.999069	
QLI65265.1	OTHER	0.002003	0.997997	
QLI65266.1	OTHER	0.004674	0.995326	
QLI65267.1	OTHER	0.002867	0.997133	
QLI65268.1	OTHER	0.000527	0.999473	
QLI65269.1	OTHER	0.356717	0.643283	
QLI65270.1	OTHER	0.001118	0.998882	
QLI65271.1	OTHER	0.000750	0.999250	
QLI65272.1	OTHER	0.000430	0.999570	
QLI65273.1	OTHER	0.001493	0.998507	
QLI65274.1	OTHER	0.001337	0.998663	
QLI65275.1	OTHER	0.002016	0.997984	
QLI65276.1	OTHER	0.000918	0.999082	
QLI65277.1	OTHER	0.000658	0.999342	
QLI65278.1	SP(Sec/SPI)	0.993649	0.006351	CS pos: 21-22. AMA-NN. Pr: 0.9079
QLI65279.1	OTHER	0.002052	0.997948	

QLI65280.1	OTHER	0.001275	0.998725	
QLI65281.1	OTHER	0.002491	0.997509	
QLI65282.1	OTHER	0.000291	0.999709	
QLI65283.1	OTHER	0.001481	0.998519	
QLI65284.1	OTHER	0.001212	0.998788	
QLI65285.1	OTHER	0.000747	0.999253	
QLI65286.1	OTHER	0.001103	0.998897	
QLI65287.1	OTHER	0.004005	0.995995	
QLI65288.1	OTHER	0.003845	0.996155	
QLI65289.1	OTHER	0.000520	0.999480	
QLI65290.1	OTHER	0.000548	0.999452	
QLI65291.1	OTHER	0.000575	0.999425	
QLI65292.1	OTHER	0.021013	0.978987	
QLI65293.1	OTHER	0.000268	0.999732	
QLI65294.1	OTHER	0.001826	0.998174	
QLI65295.1	OTHER	0.016588	0.983412	
QLI65296.1	SP(Sec/SPI)	0.986120	0.013880	CS pos: 20-21. AGA-GP. Pr: 0.5400
QLI65297.1	OTHER	0.000204	0.999796	
QLI65298.1	OTHER	0.001194	0.998806	
QLI65299.1	SP(Sec/SPI)	0.895117	0.104883	CS pos: 29-30. CEA-AL. Pr: 0.3288
QLI65300.1	OTHER	0.004420	0.995580	
QLI65301.1	SP(Sec/SPI)	0.982106	0.017894	CS pos: 18-19. AQA-GF. Pr: 0.8699
QLI65302.1	OTHER	0.000768	0.999232	
QLI65303.1	OTHER	0.004961	0.995039	
QLI65304.1	OTHER	0.000808	0.999192	
QLI65305.1	SP(Sec/SPI)	0.998364	0.001636	CS pos: 19-20. ALA-AP. Pr: 0.9077
QLI65306.1	OTHER	0.001057	0.998943	
QLI65307.1	OTHER	0.277065	0.722935	
QLI65308.1	OTHER	0.000804	0.999196	
QLI65309.1	OTHER	0.000810	0.999190	
QLI65310.1	OTHER	0.001012	0.998988	

QLI65311.1	OTHER	0.000845	0.999155	
QLI65312.1	SP(Sec/SPI)	0.998595	0.001405	CS pos: 21-22. VSG-LD. Pr: 0.9653
QLI65313.1	OTHER	0.000377	0.999623	
QLI65314.1	OTHER	0.001555	0.998445	
QLI65315.1	OTHER	0.003723	0.996277	
QLI65316.1	OTHER	0.002824	0.997176	
QLI65317.1	OTHER	0.000843	0.999157	
QLI65318.1	OTHER	0.027338	0.972662	
QLI65319.1	OTHER	0.001395	0.998605	
QLI65320.1	SP(Sec/SPI)	0.991443	0.008557	CS pos: 20-21. AQA-DH. Pr: 0.9320
QLI65321.1	OTHER	0.000690	0.999310	
QLI65322.1	OTHER	0.002195	0.997805	
QLI65323.1	OTHER	0.001388	0.998612	
QLI65324.1	OTHER	0.005242	0.994758	
QLI65325.1	OTHER	0.001894	0.998106	
QLI65326.1	OTHER	0.001227	0.998773	
QLI65327.1	OTHER	0.236923	0.763077	
QLI65328.1	OTHER	0.000845	0.999155	
QLI65329.1	OTHER	0.029099	0.970901	
QLI65330.1	SP(Sec/SPI)	0.907620	0.092380	CS pos: 22-23. CQG-LA. Pr: 0.5360
QLI65331.1	OTHER	0.000287	0.999713	
QLI65332.1	OTHER	0.006876	0.993124	
QLI65333.1	OTHER	0.000736	0.999264	
QLI65334.1	OTHER	0.013609	0.986391	
QLI65335.1	OTHER	0.000817	0.999183	
QLI65336.1	OTHER	0.001572	0.998428	
QLI65337.1	OTHER	0.000674	0.999326	
QLI65338.1	OTHER	0.000631	0.999369	
QLI65339.1	OTHER	0.002295	0.997705	
QLI65340.1	OTHER	0.000671	0.999329	
QLI65341.1	OTHER	0.000902	0.999098	

QLI65342.1	OTHER	0.001563	0.998437	
QLI65343.1	OTHER	0.000491	0.999509	
QLI65344.1	OTHER	0.001468	0.998532	
QLI65345.1	SP(Sec/SPI)	0.991211	0.008789	CS pos: 20-21. VSA-AD. Pr: 0.5969
QLI65346.1	SP(Sec/SPI)	0.728303	0.271697	CS pos: 23-24. SYL-HP. Pr: 0.3042
QLI65347.1	OTHER	0.001519	0.998481	
QLI65348.1	OTHER	0.002904	0.997096	
QLI65349.1	OTHER	0.001332	0.998668	
QLI65350.1	OTHER	0.000505	0.999495	
QLI65351.1	OTHER	0.001169	0.998831	
QLI65352.1	OTHER	0.001655	0.998345	
QLI65353.1	OTHER	0.001455	0.998545	
QLI65354.1	OTHER	0.000864	0.999136	
QLI65355.1	OTHER	0.043197	0.956803	
QLI65356.1	OTHER	0.023627	0.976373	
QLI65357.1	OTHER	0.000207	0.999793	
QLI65358.1	SP(Sec/SPI)	0.747576	0.252424	CS pos: 25-26. VSS-RA. Pr: 0.2559
QLI65359.1	OTHER	0.000996	0.999004	
QLI65360.1	OTHER	0.000309	0.999691	
QLI65361.1	SP(Sec/SPI)	0.998856	0.001144	CS pos: 22-23. AFA-QE. Pr: 0.9078
QLI65362.1	OTHER	0.004461	0.995539	
QLI65363.1	OTHER	0.000937	0.999063	
QLI65364.1	OTHER	0.000776	0.999224	
QLI65365.1	SP(Sec/SPI)	0.940368	0.059632	CS pos: 35-36. ALG-AP. Pr: 0.6456
QLI65366.1	SP(Sec/SPI)	0.970972	0.029028	CS pos: 18-19. AHA-TY. Pr: 0.9131
QLI65367.1	OTHER	0.135098	0.864902	
QLI65368.1	OTHER	0.000615	0.999385	
QLI65369.1	OTHER	0.000299	0.999701	
QLI65370.1	OTHER	0.001374	0.998626	
QLI65371.1	OTHER	0.002358	0.997642	
QLI65372.1	OTHER	0.002033	0.997967	

QLI65373.1	OTHER	0.001210	0.998790	
QLI65374.1	OTHER	0.001191	0.998809	
QLI65375.1	OTHER	0.000953	0.999047	
QLI65376.1	OTHER	0.001603	0.998397	
QLI65377.1	OTHER	0.005508	0.994492	
QLI65378.1	OTHER	0.009307	0.990693	
QLI65379.1	OTHER	0.003720	0.996280	
QLI65380.1	OTHER	0.000565	0.999435	
QLI65381.1	OTHER	0.000902	0.999098	
QLI65382.1	SP(Sec/SPI)	0.757525	0.242475	CS pos: 20-21. VRA-GS. Pr: 0.6217
QLI65383.1	OTHER	0.007328	0.992672	
QLI65384.1	OTHER	0.006182	0.993818	
QLI65385.1	OTHER	0.000831	0.999169	
QLI65386.1	OTHER	0.002720	0.997280	
QLI65387.1	OTHER	0.001447	0.998553	
QLI65388.1	SP(Sec/SPI)	0.946149	0.053851	CS pos: 22-23. SQT-LV. Pr: 0.3806
QLI65389.1	OTHER	0.001306	0.998694	
QLI65390.1	OTHER	0.000343	0.999657	
QLI65391.1	OTHER	0.002614	0.997386	
QLI65392.1	OTHER	0.000671	0.999329	
QLI65393.1	OTHER	0.000259	0.999741	
QLI65394.1	OTHER	0.021319	0.978681	
QLI65395.1	OTHER	0.001342	0.998658	
QLI65396.1	OTHER	0.000555	0.999445	
QLI65397.1	OTHER	0.000970	0.999030	
QLI65398.1	OTHER	0.001335	0.998665	
QLI65399.1	OTHER	0.124331	0.875669	
QLI65400.1	OTHER	0.000483	0.999517	
QLI65401.1	OTHER	0.002498	0.997502	
QLI65402.1	OTHER	0.007079	0.992921	
QLI65403.1	SP(Sec/SPI)	0.601243	0.398757	CS pos: 17-18. AAA-VL. Pr: 0.2545

QLI65404.1	OTHER	0.000347	0.999653	
QLI65405.1	OTHER	0.000384	0.999616	
QLI65406.1	OTHER	0.002620	0.997380	
QLI65407.1	OTHER	0.001589	0.998411	
QLI65408.1	OTHER	0.003674	0.996326	
QLI65409.1	OTHER	0.026608	0.973392	
QLI65410.1	OTHER	0.002252	0.997748	
QLI65411.1	OTHER	0.000420	0.999580	
QLI65412.1	OTHER	0.000553	0.999447	
QLI65413.1	OTHER	0.009794	0.990206	
QLI65414.1	OTHER	0.051371	0.948629	
QLI65415.1	OTHER	0.001393	0.998607	
QLI65416.1	OTHER	0.000767	0.999233	
QLI65417.1	OTHER	0.002354	0.997646	
QLI65418.1	OTHER	0.001093	0.998907	
QLI65419.1	OTHER	0.002512	0.997488	
QLI65420.1	SP(Sec/SPI)	0.944667	0.055333	CS pos: 24-25. VNA-FY. Pr: 0.9286
QLI65421.1	OTHER	0.005067	0.994933	
QLI65422.1	OTHER	0.002699	0.997301	
QLI65423.1	OTHER	0.002754	0.997246	
QLI65424.1	SP(Sec/SPI)	0.952619	0.047381	CS pos: 19-20. ALS-NQ. Pr: 0.8161
QLI65425.1	OTHER	0.000899	0.999101	
QLI65426.1	OTHER	0.002138	0.997862	
QLI65427.1	OTHER	0.038544	0.961456	
QLI65428.1	OTHER	0.000554	0.999446	
QLI65429.1	SP(Sec/SPI)	0.994283	0.005717	CS pos: 16-17. VVA-QE. Pr: 0.8974
QLI65430.1	OTHER	0.001019	0.998981	
QLI65431.1	OTHER	0.002071	0.997929	
QLI65432.1	SP(Sec/SPI)	0.974880	0.025120	CS pos: 17-18. GLA-TP. Pr: 0.5665
QLI65433.1	OTHER	0.000947	0.999053	
QLI65434.1	OTHER	0.000581	0.999419	

QLI65435.1	SP(Sec/SPI)	0.971828	0.028172	CS pos: 17-18. ATA-HF. Pr: 0.6738
QLI65436.1	OTHER	0.000569	0.999431	
QLI65437.1	OTHER	0.001122	0.998878	
QLI65438.1	OTHER	0.000721	0.999279	
QLI65439.1	OTHER	0.001570	0.998430	
QLI65440.1	OTHER	0.002159	0.997841	
QLI65441.1	OTHER	0.000816	0.999184	
QLI65442.1	OTHER	0.001925	0.998075	
QLI65443.1	OTHER	0.004502	0.995498	
QLI65444.1	OTHER	0.001528	0.998472	
QLI65445.1	OTHER	0.001479	0.998521	
QLI65446.1	SP(Sec/SPI)	0.995125	0.004875	CS pos: 17-18. GFA-SP. Pr: 0.7101
QLI65447.1	OTHER	0.001095	0.998905	
QLI65448.1	SP(Sec/SPI)	0.922837	0.077163	CS pos: 17-18. VTA-EP. Pr: 0.7092
QLI65449.1	OTHER	0.000876	0.999124	
QLI65450.1	OTHER	0.000582	0.999418	
QLI65451.1	OTHER	0.000206	0.999794	
QLI65452.1	OTHER	0.003435	0.996565	
QLI65453.1	SP(Sec/SPI)	0.986914	0.013086	CS pos: 19-20. AAA-HM. Pr: 0.5580
QLI65454.1	OTHER	0.001195	0.998805	
QLI65455.1	OTHER	0.000325	0.999675	
QLI65456.1	OTHER	0.008648	0.991352	
QLI65457.1	OTHER	0.001058	0.998942	
QLI65458.1	OTHER	0.004431	0.995569	
QLI65459.1	OTHER	0.001487	0.998513	
QLI65460.1	OTHER	0.000811	0.999189	
QLI65461.1	OTHER	0.000828	0.999172	
QLI65462.1	OTHER	0.001988	0.998012	
QLI65463.1	OTHER	0.000414	0.999586	
QLI65464.1	OTHER	0.001117	0.998883	
QLI65465.1	OTHER	0.008742	0.991258	

QLI65466.1	OTHER	0.010889	0.989111	
QLI65467.1	OTHER	0.001100	0.998900	
QLI65468.1	OTHER	0.004193	0.995807	
QLI65469.1	OTHER	0.000767	0.999233	
QLI65470.1	SP(Sec/SPI)	0.990374	0.009626	CS pos: 22-23. ASA-DA. Pr: 0.5449
QLI65471.1	OTHER	0.000232	0.999768	
QLI65472.1	OTHER	0.002323	0.997677	
QLI65473.1	SP(Sec/SPI)	0.701871	0.298129	CS pos: 19-20. AGA-IC. Pr: 0.2846
QLI65474.1	OTHER	0.000351	0.999649	
QLI65475.1	SP(Sec/SPI)	0.819978	0.180022	CS pos: 19-20. ARS-AS. Pr: 0.3335
QLI65476.1	OTHER	0.001685	0.998315	
QLI65477.1	OTHER	0.003662	0.996338	
QLI65478.1	OTHER	0.009276	0.990724	
QLI65479.1	OTHER	0.000534	0.999466	
QLI65480.1	OTHER	0.001682	0.998318	
QLI65481.1	OTHER	0.000289	0.999711	
QLI65482.1	SP(Sec/SPI)	0.962962	0.037038	CS pos: 17-18. AWA-IN. Pr: 0.7182
QLI65483.1	OTHER	0.001239	0.998761	
QLI65484.1	SP(Sec/SPI)	0.988819	0.011181	CS pos: 21-22. AWG-AP. Pr: 0.9085
QLI65485.1	OTHER	0.003640	0.996360	
QLI65486.1	OTHER	0.000447	0.999553	
QLI65487.1	OTHER	0.091217	0.908783	
QLI65488.1	OTHER	0.000924	0.999076	
QLI65489.1	OTHER	0.002330	0.997670	
QLI65490.1	OTHER	0.000715	0.999285	
QLI65491.1	OTHER	0.000369	0.999631	
QLI65492.1	OTHER	0.108439	0.891561	
QLI65493.1	OTHER	0.000719	0.999281	
QLI65494.1	OTHER	0.001321	0.998679	
QLI65495.1	OTHER	0.016757	0.983243	
QLI65496.1	OTHER	0.000338	0.999662	

QLI65497.1	OTHER	0.005391	0.994609	
QLI65498.1	OTHER	0.000644	0.999356	
QLI65499.1	OTHER	0.000465	0.999535	
QLI65500.1	OTHER	0.001262	0.998738	
QLI65501.1	OTHER	0.007217	0.992783	
QLI65502.1	OTHER	0.054662	0.945338	
QLI65503.1	OTHER	0.000280	0.999720	
QLI65504.1	OTHER	0.000867	0.999133	
QLI65505.1	OTHER	0.007737	0.992263	
QLI65506.1	OTHER	0.000731	0.999269	
QLI65507.1	OTHER	0.000988	0.999012	
QLI65508.1	OTHER	0.000590	0.999410	
QLI65509.1	OTHER	0.000814	0.999186	
QLI65510.1	OTHER	0.004420	0.995580	
QLI65511.1	OTHER	0.001376	0.998624	
QLI65512.1	OTHER	0.001771	0.998229	
QLI65513.1	SP(Sec/SPI)	0.528746	0.471254	CS pos: 20-21. AFG-SI. Pr: 0.2659
QLI65514.1	OTHER	0.005244	0.994756	
QLI65515.1	SP(Sec/SPI)	0.995884	0.004116	CS pos: 18-19. AVA-AE. Pr: 0.6100
QLI65516.1	OTHER	0.002644	0.997356	
QLI65517.1	OTHER	0.000550	0.999450	
QLI65518.1	OTHER	0.013322	0.986678	
QLI65519.1	SP(Sec/SPI)	0.689310	0.310690	CS pos: 25-26. TLA-SL. Pr: 0.5455
QLI65520.1	OTHER	0.021425	0.978575	
QLI65521.1	OTHER	0.213162	0.786838	
QLI65522.1	OTHER	0.000337	0.999663	
QLI65523.1	OTHER	0.000539	0.999461	
QLI65524.1	OTHER	0.001115	0.998885	
QLI65525.1	OTHER	0.000980	0.999020	
QLI65526.1	OTHER	0.014529	0.985471	
QLI65527.1	SP(Sec/SPI)	0.976001	0.023999	CS pos: 18-19. VSA-VS. Pr: 0.4248

QLI65528.1	OTHER	0.001004	0.998996	
QLI65529.1	OTHER	0.000204	0.999796	
QLI65530.1	OTHER	0.001468	0.998532	
QLI65531.1	OTHER	0.014203	0.985797	
QLI65532.1	OTHER	0.137241	0.862759	
QLI65533.1	SP(Sec/SPI)	0.993041	0.006959	CS pos: 18-19. VLA-QG. Pr: 0.3838
QLI65534.1	OTHER	0.001478	0.998522	
QLI65535.1	OTHER	0.004643	0.995357	
QLI65536.1	OTHER	0.001369	0.998631	
QLI65537.1	OTHER	0.002632	0.997368	
QLI65538.1	OTHER	0.002305	0.997695	
QLI65539.1	OTHER	0.060504	0.939496	
QLI65540.1	OTHER	0.000443	0.999557	
QLI65541.1	OTHER	0.001294	0.998706	
QLI65542.1	OTHER	0.000444	0.999556	
QLI65543.1	OTHER	0.000856	0.999144	
QLI65544.1	OTHER	0.001476	0.998524	
QLI65545.1	OTHER	0.003759	0.996241	
QLI65546.1	OTHER	0.001544	0.998456	
QLI65547.1	OTHER	0.002549	0.997451	
QLI65548.1	OTHER	0.002758	0.997242	
QLI65549.1	OTHER	0.002149	0.997851	
QLI65550.1	OTHER	0.001331	0.998669	
QLI65551.1	OTHER	0.001368	0.998632	
QLI65552.1	OTHER	0.005953	0.994047	
QLI65553.1	OTHER	0.000705	0.999295	
QLI65554.1	OTHER	0.001808	0.998192	
QLI65555.1	OTHER	0.003145	0.996855	
QLI65556.1	SP(Sec/SPI)	0.915834	0.084166	CS pos: 22-23. ASG-KL. Pr: 0.4645
QLI65557.1	OTHER	0.001441	0.998559	
QLI65558.1	OTHER	0.000506	0.999494	

QLI65559.1	OTHER	0.000553	0.999447	
QLI65560.1	OTHER	0.002358	0.997642	
QLI65561.1	OTHER	0.000446	0.999554	
QLI65562.1	OTHER	0.001685	0.998315	
QLI65563.1	OTHER	0.001455	0.998545	
QLI65564.1	OTHER	0.001743	0.998257	
QLI65565.1	SP(Sec/SPI)	0.792746	0.207254	CS pos: 21-22. ILA-QD. Pr: 0.6693
QLI65566.1	OTHER	0.005789	0.994211	
QLI65567.1	OTHER	0.001051	0.998949	
QLI65568.1	SP(Sec/SPI)	0.996972	0.003028	CS pos: 17-18. ASA-QN. Pr: 0.7727
QLI65569.1	OTHER	0.000389	0.999611	
QLI65570.1	OTHER	0.006810	0.993190	
QLI65571.1	OTHER	0.000909	0.999091	
QLI65572.1	OTHER	0.002564	0.997436	
QLI65573.1	OTHER	0.000908	0.999092	
QLI65574.1	SP(Sec/SPI)	0.997367	0.002633	CS pos: 18-19. TDA-AP. Pr: 0.8842
QLI65575.1	OTHER	0.000369	0.999631	
QLI65576.1	OTHER	0.002217	0.997783	
QLI65577.1	OTHER	0.001793	0.998207	
QLI65578.1	OTHER	0.002252	0.997748	
QLI65579.1	OTHER	0.000261	0.999739	
QLI65580.1	OTHER	0.000607	0.999393	
QLI65581.1	OTHER	0.001422	0.998578	
QLI65582.1	SP(Sec/SPI)	0.986163	0.013837	CS pos: 17-18. ALA-AR. Pr: 0.7453
QLI65583.1	OTHER	0.001030	0.998970	
QLI65584.1	SP(Sec/SPI)	0.719480	0.280520	CS pos: 23-24. GSS-ST. Pr: 0.1732
QLI65585.1	OTHER	0.164333	0.835667	
QLI65586.1	OTHER	0.020677	0.979323	
QLI65587.1	SP(Sec/SPI)	0.929357	0.070643	CS pos: 17-18. AIG-AV. Pr: 0.3602
QLI65588.1	OTHER	0.001316	0.998684	
QLI65589.1	OTHER	0.001690	0.998310	

QLI65590.1	OTHER	0.000426	0.999574	
QLI65591.1	OTHER	0.003594	0.996406	
QLI65592.1	OTHER	0.001786	0.998214	
QLI65593.1	OTHER	0.001366	0.998634	
QLI65594.1	OTHER	0.001044	0.998956	
QLI65595.1	OTHER	0.001152	0.998848	
QLI65596.1	OTHER	0.002000	0.998000	
QLI65597.1	OTHER	0.000515	0.999485	
QLI65598.1	OTHER	0.001769	0.998231	
QLI65599.1	OTHER	0.000816	0.999184	
QLI65600.1	OTHER	0.011171	0.988829	
QLI65601.1	OTHER	0.001045	0.998955	
QLI65602.1	OTHER	0.000369	0.999631	
QLI65603.1	OTHER	0.000390	0.999610	
QLI65604.1	OTHER	0.001910	0.998090	
QLI65605.1	OTHER	0.000480	0.999520	
QLI65606.1	OTHER	0.000262	0.999738	
QLI65607.1	OTHER	0.000775	0.999225	
QLI65608.1	SP(Sec/SPI)	0.839211	0.160789	CS pos: 22-23. VVA-AS. Pr: 0.3918
QLI65609.1	OTHER	0.004420	0.995580	
QLI65610.1	OTHER	0.002619	0.997381	
QLI65611.1	OTHER	0.001432	0.998568	
QLI65612.1	OTHER	0.000755	0.999245	
QLI65613.1	OTHER	0.000844	0.999156	
QLI65614.1	OTHER	0.001424	0.998576	
QLI65615.1	OTHER	0.000232	0.999768	
QLI65616.1	OTHER	0.000768	0.999232	
QLI65617.1	OTHER	0.003864	0.996136	
QLI65618.1	OTHER	0.000971	0.999029	
QLI65619.1	OTHER	0.004027	0.995973	
QLI65620.1	OTHER	0.003685	0.996315	

QLI65621.1	OTHER	0.000732	0.999268	
QLI65622.1	OTHER	0.310717	0.689283	
QLI65623.1	OTHER	0.001105	0.998895	
QLI65624.1	OTHER	0.001162	0.998838	
QLI65625.1	OTHER	0.003054	0.996946	
QLI65626.1	OTHER	0.001949	0.998051	
QLI65627.1	OTHER	0.001263	0.998737	
QLI65628.1	OTHER	0.000646	0.999354	
QLI65629.1	OTHER	0.006970	0.993030	
QLI65630.1	SP(Sec/SPI)	0.998095	0.001905	CS pos: 19-20. ALC-EP. Pr: 0.8386
QLI65631.1	SP(Sec/SPI)	0.995035	0.004965	CS pos: 19-20. VNA-AP. Pr: 0.9045
QLI65632.1	OTHER	0.000222	0.999778	
QLI65633.1	OTHER	0.000988	0.999012	
QLI65634.1	OTHER	0.000795	0.999205	
QLI65635.1	OTHER	0.000489	0.999511	
QLI65636.1	OTHER	0.000782	0.999218	
QLI65637.1	OTHER	0.000403	0.999597	
QLI65638.1	OTHER	0.003026	0.996974	
QLI65639.1	OTHER	0.000982	0.999018	
QLI65640.1	SP(Sec/SPI)	0.745746	0.254254	CS pos: 34-35. VQS-TT. Pr: 0.4390
QLI65641.1	OTHER	0.001428	0.998572	
QLI65642.1	OTHER	0.000921	0.999079	
QLI65643.1	OTHER	0.000998	0.999002	
QLI65644.1	OTHER	0.001629	0.998371	
QLI65645.1	OTHER	0.001397	0.998603	
QLI65646.1	OTHER	0.001324	0.998676	
QLI65647.1	SP(Sec/SPI)	0.982344	0.017656	CS pos: 19-20. AFA-WP. Pr: 0.6514
QLI65648.1	OTHER	0.001004	0.998996	
QLI65649.1	OTHER	0.001786	0.998214	
QLI65650.1	OTHER	0.004999	0.995001	
QLI65651.1	OTHER	0.000557	0.999443	

QLI65652.1	OTHER	0.003162	0.996838	
QLI65653.1	OTHER	0.001052	0.998948	
QLI65654.1	OTHER	0.001225	0.998775	
QLI65655.1	OTHER	0.002674	0.997326	
QLI65656.1	OTHER	0.001551	0.998449	
QLI65657.1	OTHER	0.001602	0.998398	
QLI65658.1	OTHER	0.001217	0.998783	
QLI65659.1	OTHER	0.091354	0.908646	
QLI65660.1	OTHER	0.000639	0.999361	
QLI65661.1	OTHER	0.000294	0.999706	
QLI65662.1	OTHER	0.000823	0.999177	
QLI65663.1	OTHER	0.001290	0.998710	
QLI65664.1	SP(Sec/SPI)	0.996924	0.003076	CS pos: 19-20. VLG-AE. Pr: 0.8211
QLI65665.1	OTHER	0.000781	0.999219	
QLI65666.1	OTHER	0.003029	0.996971	
QLI65667.1	OTHER	0.001182	0.998818	
QLI65668.1	OTHER	0.000499	0.999501	
QLI65669.1	OTHER	0.000380	0.999620	
QLI65670.1	OTHER	0.004581	0.995419	
QLI65671.1	OTHER	0.000473	0.999527	
QLI65672.1	OTHER	0.000221	0.999779	
QLI65673.1	OTHER	0.000780	0.999220	
QLI65674.1	OTHER	0.002238	0.997762	
QLI65675.1	OTHER	0.000239	0.999761	
QLI65676.1	OTHER	0.018374	0.981626	
QLI65677.1	SP(Sec/SPI)	0.984606	0.015394	CS pos: 21-22. VSA-AD. Pr: 0.8335
QLI65678.1	OTHER	0.001896	0.998104	
QLI65679.1	OTHER	0.250557	0.749443	
QLI65680.1	OTHER	0.001031	0.998969	
QLI65681.1	OTHER	0.000414	0.999586	
QLI65682.1	OTHER	0.002431	0.997569	

QLI65683.1	SP(Sec/SPI)	0.985688	0.014312	CS pos: 17-18. AQA-DS. Pr: 0.7532
QLI65684.1	OTHER	0.010837	0.989163	
QLI65685.1	SP(Sec/SPI)	0.941326	0.058674	CS pos: 21-22. VHA-AS. Pr: 0.8480
QLI65686.1	OTHER	0.000589	0.999411	
QLI65687.1	OTHER	0.010970	0.989030	
QLI65688.1	OTHER	0.000528	0.999472	
QLI65689.1	OTHER	0.001627	0.998373	
QLI65690.1	OTHER	0.000604	0.999396	
QLI65691.1	OTHER	0.000234	0.999766	
QLI65692.1	OTHER	0.000367	0.999633	
QLI65693.1	OTHER	0.001398	0.998602	
QLI65694.1	OTHER	0.008923	0.991077	
QLI65695.1	OTHER	0.000429	0.999571	
QLI65696.1	OTHER	0.004935	0.995065	
QLI65697.1	SP(Sec/SPI)	0.986215	0.013785	CS pos: 19-20. AVA-RE. Pr: 0.7994
QLI65698.1	OTHER	0.000394	0.999606	
QLI65699.1	OTHER	0.004824	0.995176	
QLI65700.1	OTHER	0.002554	0.997446	
QLI65701.1	OTHER	0.000786	0.999214	
QLI65702.1	OTHER	0.003336	0.996664	
QLI65703.1	OTHER	0.000342	0.999658	
QLI65704.1	OTHER	0.158508	0.841492	
QLI65705.1	OTHER	0.000788	0.999212	
QLI65706.1	OTHER	0.000260	0.999740	
QLI65707.1	OTHER	0.000334	0.999666	
QLI65708.1	OTHER	0.001746	0.998254	
QLI65709.1	OTHER	0.001642	0.998358	
QLI65710.1	OTHER	0.001811	0.998189	
QLI65711.1	OTHER	0.002243	0.997757	
QLI65712.1	OTHER	0.000477	0.999523	
QLI65713.1	OTHER	0.000785	0.999215	

QLI65714.1	OTHER	0.000843	0.999157	
QLI65715.1	OTHER	0.000304	0.999696	
QLI65716.1	OTHER	0.001417	0.998583	
QLI65717.1	OTHER	0.004465	0.995535	
QLI65718.1	OTHER	0.002490	0.997510	
QLI65719.1	OTHER	0.000496	0.999504	
QLI65720.1	OTHER	0.000587	0.999413	
QLI65721.1	OTHER	0.015331	0.984669	
QLI65722.1	OTHER	0.000512	0.999488	
QLI65723.1	OTHER	0.001263	0.998737	
QLI65724.1	OTHER	0.000629	0.999371	
QLI65725.1	OTHER	0.000570	0.999430	
QLI65726.1	OTHER	0.000450	0.999550	
QLI65727.1	OTHER	0.005190	0.994810	
QLI65728.1	SP(Sec/SPI)	0.814083	0.185917	CS pos: 16-17. ASA-AF. Pr: 0.3612
QLI65729.1	OTHER	0.007332	0.992668	
QLI65730.1	OTHER	0.001218	0.998782	
QLI65731.1	OTHER	0.001200	0.998800	
QLI65732.1	OTHER	0.000217	0.999783	
QLI65733.1	OTHER	0.000776	0.999224	
QLI65734.1	OTHER	0.001219	0.998781	
QLI65735.1	OTHER	0.002660	0.997340	
QLI65736.1	OTHER	0.001034	0.998966	
QLI65737.1	OTHER	0.003980	0.996020	
QLI65738.1	OTHER	0.000692	0.999308	
QLI65739.1	SP(Sec/SPI)	0.990608	0.009392	CS pos: 20-21. AAA-VP. Pr: 0.5244
QLI65740.1	OTHER	0.001020	0.998980	
QLI65741.1	OTHER	0.000312	0.999688	
QLI65742.1	OTHER	0.001185	0.998815	
QLI65743.1	OTHER	0.003848	0.996152	
QLI65744.1	OTHER	0.000305	0.999695	

QLI65745.1	OTHER	0.001219	0.998781	
QLI65746.1	OTHER	0.000950	0.999050	
QLI65747.1	OTHER	0.000473	0.999527	
QLI65748.1	OTHER	0.000843	0.999157	
QLI65749.1	OTHER	0.001051	0.998949	
QLI65750.1	OTHER	0.001316	0.998684	
QLI65751.1	OTHER	0.379216	0.620784	
QLI65752.1	OTHER	0.002288	0.997712	
QLI65753.1	OTHER	0.001573	0.998427	
QLI65754.1	OTHER	0.000656	0.999344	
QLI65755.1	OTHER	0.001497	0.998503	
QLI65756.1	SP(Sec/SPI)	0.556016	0.443984	CS pos: 18-19. SDS-CP. Pr: 0.3779
QLI65757.1	OTHER	0.003718	0.996282	
QLI65758.1	OTHER	0.001441	0.998559	
QLI65759.1	OTHER	0.010099	0.989901	
QLI65760.1	OTHER	0.001029	0.998971	
QLI65761.1	SP(Sec/SPI)	0.991831	0.008169	CS pos: 18-19. SYA-GH. Pr: 0.8799
QLI65762.1	OTHER	0.000848	0.999152	
QLI65763.1	OTHER	0.001298	0.998702	
QLI65764.1	OTHER	0.000742	0.999258	
QLI65765.1	OTHER	0.000620	0.999380	
QLI65766.1	SP(Sec/SPI)	0.996842	0.003158	CS pos: 20-21. CNA-ND. Pr: 0.9428
QLI65767.1	OTHER	0.000745	0.999255	
QLI65768.1	OTHER	0.000555	0.999445	
QLI65769.1	OTHER	0.019117	0.980883	
QLI65770.1	OTHER	0.006780	0.993220	
QLI65771.1	OTHER	0.000825	0.999175	
QLI65772.1	OTHER	0.000759	0.999241	
QLI65773.1	SP(Sec/SPI)	0.994661	0.005339	CS pos: 20-21. TLA-LP. Pr: 0.5509
QLI65774.1	OTHER	0.001541	0.998459	
QLI65775.1	OTHER	0.000815	0.999185	

QLI65776.1	OTHER	0.002149	0.997851	
QLI65777.1	OTHER	0.000182	0.999818	
QLI65778.1	OTHER	0.000365	0.999635	
QLI65779.1	OTHER	0.000802	0.999198	
QLI65780.1	OTHER	0.001518	0.998482	
QLI65781.1	OTHER	0.000877	0.999123	
QLI65782.1	OTHER	0.001128	0.998872	
QLI65783.1	OTHER	0.000790	0.999210	
QLI65784.1	SP(Sec/SPI)	0.935469	0.064531	CS pos: 23-24. VAA-GG. Pr: 0.3795
QLI65785.1	OTHER	0.001357	0.998643	
QLI65786.1	OTHER	0.001929	0.998071	
QLI65787.1	OTHER	0.000892	0.999108	
QLI65788.1	OTHER	0.000566	0.999434	
QLI65789.1	OTHER	0.002670	0.997330	
QLI65790.1	SP(Sec/SPI)	0.993870	0.006130	CS pos: 21-22. ALG-DT. Pr: 0.5359
QLI65791.1	SP(Sec/SPI)	0.999116	0.000884	CS pos: 21-22. AQG-CT. Pr: 0.6729
QLI65792.1	OTHER	0.002419	0.997581	
QLI65793.1	OTHER	0.052907	0.947093	
QLI65794.1	OTHER	0.066589	0.933411	
QLI65795.1	OTHER	0.000648	0.999352	
QLI65796.1	OTHER	0.003580	0.996420	
QLI65797.1	OTHER	0.011105	0.988895	
QLI65798.1	OTHER	0.001521	0.998479	
QLI65799.1	OTHER	0.067093	0.932907	
QLI65800.1	SP(Sec/SPI)	0.879973	0.120027	CS pos: 18-19. GSA-LP. Pr: 0.3730
QLI65801.1	OTHER	0.000780	0.999220	
QLI65802.1	OTHER	0.001426	0.998574	
QLI65803.1	OTHER	0.001158	0.998842	
QLI65804.1	SP(Sec/SPI)	0.871178	0.128822	CS pos: 23-24. SVG-RR. Pr: 0.3425
QLI65805.1	OTHER	0.004713	0.995287	
QLI65806.1	OTHER	0.001339	0.998661	

QLI65807.1	OTHER	0.002545	0.997455	
QLI65808.1	OTHER	0.000387	0.999613	
QLI65809.1	OTHER	0.000917	0.999083	
QLI65810.1	OTHER	0.001458	0.998542	
QLI65811.1	OTHER	0.000690	0.999310	
QLI65812.1	OTHER	0.001001	0.998999	
QLI65813.1	OTHER	0.000535	0.999465	
QLI65814.1	OTHER	0.003436	0.996564	
QLI65815.1	OTHER	0.159345	0.840655	
QLI65816.1	OTHER	0.000804	0.999196	
QLI65817.1	OTHER	0.000270	0.999730	
QLI65818.1	OTHER	0.000730	0.999270	
QLI65819.1	OTHER	0.003794	0.996206	
QLI65820.1	OTHER	0.002002	0.997998	
QLI65821.1	OTHER	0.000970	0.999030	
QLI65822.1	OTHER	0.005072	0.994928	
QLI65823.1	OTHER	0.001035	0.998965	
QLI65824.1	OTHER	0.000852	0.999148	
QLI65825.1	OTHER	0.000819	0.999181	
QLI65826.1	OTHER	0.031178	0.968822	
QLI65827.1	OTHER	0.000424	0.999576	
QLI65828.1	OTHER	0.001747	0.998253	
QLI65829.1	OTHER	0.000673	0.999327	
QLI65830.1	SP(Sec/SPI)	0.745200	0.254800	CS pos: 23-24. VSA-AR. Pr: 0.6033
QLI65831.1	OTHER	0.000431	0.999569	
QLI65832.1	OTHER	0.003664	0.996336	
QLI65833.1	OTHER	0.000752	0.999248	
QLI65834.1	OTHER	0.000565	0.999435	
QLI65835.1	SP(Sec/SPI)	0.972516	0.027484	CS pos: 15-16. AIA-HT. Pr: 0.7278
QLI65836.1	OTHER	0.001598	0.998402	
QLI65837.1	OTHER	0.002179	0.997821	

QLI65838.1	OTHER	0.001259	0.998741	
QLI65839.1	OTHER	0.001674	0.998326	
QLI65840.1	OTHER	0.001047	0.998953	
QLI65841.1	OTHER	0.003309	0.996691	
QLI65842.1	OTHER	0.002187	0.997813	
QLI65843.1	OTHER	0.005445	0.994555	
QLI65844.1	OTHER	0.001581	0.998419	
QLI65845.1	OTHER	0.000717	0.999283	
QLI65846.1	OTHER	0.000560	0.999440	
QLI65847.1	OTHER	0.003809	0.996191	
QLI65848.1	OTHER	0.002741	0.997259	
QLI65849.1	OTHER	0.000606	0.999394	
QLI65850.1	OTHER	0.001230	0.998770	
QLI65851.1	OTHER	0.053113	0.946887	
QLI65852.1	OTHER	0.000815	0.999185	
QLI65853.1	OTHER	0.002221	0.997779	
QLI65854.1	OTHER	0.003008	0.996992	
QLI65855.1	OTHER	0.000838	0.999162	
QLI65856.1	OTHER	0.000868	0.999132	
QLI65857.1	OTHER	0.001151	0.998849	
QLI65858.1	OTHER	0.002639	0.997361	
QLI65859.1	OTHER	0.000525	0.999475	
QLI65860.1	OTHER	0.001914	0.998086	
QLI65861.1	OTHER	0.001978	0.998022	
QLI65862.1	OTHER	0.001698	0.998302	
QLI65863.1	OTHER	0.001870	0.998130	
QLI65864.1	OTHER	0.001248	0.998752	
QLI65865.1	OTHER	0.001243	0.998757	
QLI65866.1	SP(Sec/SPI)	0.982031	0.017969	CS pos: 18-19. LLA-FR. Pr: 0.5133
QLI65867.1	OTHER	0.003431	0.996569	
QLI65868.1	OTHER	0.002019	0.997981	

QLI65869.1	OTHER	0.003690	0.996310	
QLI65870.1	OTHER	0.149620	0.850380	
QLI65871.1	OTHER	0.001371	0.998629	
QLI65872.1	OTHER	0.000832	0.999168	
QLI65873.1	OTHER	0.000824	0.999176	
QLI65874.1	OTHER	0.001195	0.998805	
QLI65875.1	OTHER	0.000583	0.999417	
QLI65876.1	OTHER	0.003883	0.996117	
QLI65877.1	OTHER	0.001363	0.998637	
QLI65878.1	OTHER	0.019114	0.980886	
QLI65879.1	OTHER	0.018675	0.981325	
QLI65880.1	OTHER	0.007254	0.992746	
QLI65881.1	OTHER	0.005940	0.994060	
QLI65882.1	OTHER	0.002560	0.997440	
QLI65883.1	OTHER	0.002889	0.997111	
QLI65884.1	OTHER	0.000882	0.999118	
QLI65885.1	OTHER	0.001947	0.998053	
QLI65886.1	OTHER	0.077150	0.922850	
QLI65887.1	SP(Sec/SPI)	0.998262	0.001738	CS pos: 21-22. ANA-DY. Pr: 0.8979
QLI65888.1	SP(Sec/SPI)	0.754602	0.245398	CS pos: 18-19. AVG-LQ. Pr: 0.3784
QLI65889.1	OTHER	0.000864	0.999136	
QLI65890.1	OTHER	0.001172	0.998828	
QLI65891.1	OTHER	0.002770	0.997230	
QLI65892.1	OTHER	0.006523	0.993477	
QLI65893.1	OTHER	0.002372	0.997628	
QLI65894.1	OTHER	0.001471	0.998529	
QLI65895.1	OTHER	0.000331	0.999669	
QLI65896.1	OTHER	0.001283	0.998717	
QLI65897.1	SP(Sec/SPI)	0.995715	0.004285	CS pos: 18-19. ALA-AP. Pr: 0.8300
QLI65898.1	OTHER	0.010808	0.989192	
QLI65899.1	OTHER	0.006594	0.993406	

QLI65900.1	OTHER	0.002350	0.997650	
QLI65901.1	OTHER	0.003041	0.996959	
QLI65902.1	OTHER	0.000486	0.999514	
QLI65903.1	OTHER	0.144668	0.855332	
QLI65904.1	OTHER	0.001497	0.998503	
QLI65905.1	OTHER	0.000613	0.999387	
QLI65906.1	OTHER	0.000695	0.999305	
QLI65907.1	SP(Sec/SPI)	0.989275	0.010725	CS pos: 19-20. ASA-VP. Pr: 0.7518
QLI65908.1	SP(Sec/SPI)	0.926899	0.073101	CS pos: 20-21. VSA-AT. Pr: 0.8203
QLI65909.1	OTHER	0.119020	0.880980	
QLI65910.1	OTHER	0.001686	0.998314	
QLI65911.1	OTHER	0.000910	0.999090	
QLI65912.1	OTHER	0.000903	0.999097	
QLI65913.1	SP(Sec/SPI)	0.889012	0.110988	CS pos: 19-20. VSA-RA. Pr: 0.2830
QLI65914.1	OTHER	0.001623	0.998377	
QLI65915.1	OTHER	0.001735	0.998265	
QLI65916.1	OTHER	0.001893	0.998107	
QLI65917.1	OTHER	0.000670	0.999330	
QLI65918.1	OTHER	0.000800	0.999200	
QLI65919.1	OTHER	0.000350	0.999650	
QLI65920.1	OTHER	0.001005	0.998995	
QLI65921.1	OTHER	0.001406	0.998594	
QLI65922.1	OTHER	0.001304	0.998696	
QLI65923.1	OTHER	0.000208	0.999792	
QLI65924.1	OTHER	0.000473	0.999527	
QLI65925.1	OTHER	0.000933	0.999067	
QLI65926.1	OTHER	0.000628	0.999372	
QLI65927.1	SP(Sec/SPI)	0.719015	0.280985	CS pos: 20-21. GAA-AP. Pr: 0.2217
QLI65928.1	OTHER	0.000561	0.999439	
QLI65929.1	OTHER	0.000816	0.999184	
QLI65930.1	OTHER	0.000906	0.999094	

QLI65931.1	OTHER	0.001963	0.998037	
QLI65932.1	SP(Sec/SPI)	0.990990	0.009010	CS pos: 17-18. AIA-GD. Pr: 0.7879
QLI65933.1	OTHER	0.002200	0.997800	
QLI65934.1	OTHER	0.000957	0.999043	
QLI65935.1	OTHER	0.000617	0.999383	
QLI65936.1	OTHER	0.000943	0.999057	
QLI65937.1	OTHER	0.000847	0.999153	
QLI65938.1	OTHER	0.001538	0.998462	
QLI65939.1	OTHER	0.001763	0.998237	
QLI65940.1	OTHER	0.001000	0.999000	
QLI65941.1	OTHER	0.002260	0.997740	
QLI65942.1	OTHER	0.001361	0.998639	
QLI65943.1	OTHER	0.002801	0.997199	
QLI65944.1	SP(Sec/SPI)	0.938944	0.061056	CS pos: 23-24. GRA-QT. Pr: 0.7938
QLI65945.1	OTHER	0.006018	0.993982	
QLI65946.1	OTHER	0.001997	0.998003	
QLI65947.1	OTHER	0.000313	0.999687	
QLI65948.1	OTHER	0.008080	0.991920	
QLI65949.1	OTHER	0.000753	0.999247	
QLI65950.1	OTHER	0.005026	0.994974	
QLI65951.1	OTHER	0.000197	0.999803	
QLI65952.1	OTHER	0.091633	0.908367	
QLI65953.1	OTHER	0.000885	0.999115	
QLI65954.1	OTHER	0.002373	0.997627	
QLI65955.1	SP(Sec/SPI)	0.964019	0.035981	CS pos: 16-17. ANA-FP. Pr: 0.8320
QLI65956.1	OTHER	0.001264	0.998736	
QLI65957.1	OTHER	0.001368	0.998632	
QLI65958.1	OTHER	0.001420	0.998580	
QLI65959.1	OTHER	0.002958	0.997042	
QLI65960.1	OTHER	0.005062	0.994938	
QLI65961.1	OTHER	0.000836	0.999164	

QLI65962.1	OTHER	0.000583	0.999417	
QLI65963.1	OTHER	0.010043	0.989957	
QLI65964.1	SP(Sec/SPI)	0.920213	0.079787	CS pos: 21-22. IAA-FP. Pr: 0.6774
QLI65965.1	OTHER	0.000448	0.999552	
QLI65966.1	OTHER	0.000604	0.999396	
QLI65967.1	OTHER	0.001645	0.998355	
QLI65968.1	OTHER	0.001676	0.998324	
QLI65969.1	OTHER	0.001772	0.998228	
QLI65970.1	OTHER	0.000772	0.999228	
QLI65971.1	OTHER	0.002204	0.997796	
QLI65972.1	OTHER	0.235055	0.764945	
QLI65973.1	SP(Sec/SPI)	0.980629	0.019371	CS pos: 19-20. ALG-KP. Pr: 0.9367
QLI65974.1	OTHER	0.001852	0.998148	
QLI65975.1	OTHER	0.000988	0.999012	
QLI65976.1	SP(Sec/SPI)	0.968644	0.031356	CS pos: 20-21. ATA-AA. Pr: 0.3768
QLI65977.1	OTHER	0.037265	0.962735	
QLI65978.1	OTHER	0.000345	0.999655	
QLI65979.1	OTHER	0.001165	0.998835	
QLI65980.1	OTHER	0.000626	0.999374	
QLI65981.1	OTHER	0.000452	0.999548	
QLI65982.1	OTHER	0.001241	0.998759	
QLI65983.1	OTHER	0.002201	0.997799	
QLI65984.1	OTHER	0.003050	0.996950	
QLI65985.1	OTHER	0.002115	0.997885	
QLI65986.1	OTHER	0.000263	0.999737	
QLI65987.1	OTHER	0.000745	0.999255	
QLI65988.1	OTHER	0.000359	0.999641	
QLI65989.1	OTHER	0.001144	0.998856	
QLI65990.1	SP(Sec/SPI)	0.856767	0.143233	CS pos: 21-22. VLS-AQ. Pr: 0.4538
QLI65991.1	OTHER	0.002135	0.997865	
QLI65992.1	OTHER	0.000703	0.999297	

QLI65993.1	OTHER	0.003329	0.996671	
QLI65994.1	OTHER	0.002907	0.997093	
QLI65995.1	OTHER	0.001104	0.998896	
QLI65996.1	OTHER	0.000324	0.999676	
QLI65997.1	OTHER	0.000662	0.999338	
QLI65998.1	OTHER	0.005485	0.994515	
QLI65999.1	SP(Sec/SPI)	0.992436	0.007564	CS pos: 21-22. AVA-RS. Pr: 0.8786
QLI66000.1	OTHER	0.002072	0.997928	
QLI66001.1	OTHER	0.000684	0.999316	
QLI66002.1	OTHER	0.001068	0.998932	
QLI66003.1	OTHER	0.000751	0.999249	
QLI66004.1	OTHER	0.004854	0.995146	
QLI66005.1	OTHER	0.001687	0.998313	
QLI66006.1	OTHER	0.001754	0.998246	
QLI66007.1	OTHER	0.000736	0.999264	
QLI66008.1	OTHER	0.002950	0.997050	
QLI66009.1	OTHER	0.001977	0.998023	
QLI66010.1	OTHER	0.000887	0.999113	
QLI66011.1	OTHER	0.003179	0.996821	
QLI66012.1	OTHER	0.000528	0.999472	
QLI66013.1	OTHER	0.000834	0.999166	
QLI66014.1	OTHER	0.000514	0.999486	
QLI66015.1	OTHER	0.000770	0.999230	
QLI66016.1	OTHER	0.000693	0.999307	
QLI66017.1	SP(Sec/SPI)	0.979074	0.020926	CS pos: 16-17. ATA-TP. Pr: 0.5303
QLI66018.1	OTHER	0.137296	0.862704	
QLI66019.1	OTHER	0.001037	0.998963	
QLI66020.1	OTHER	0.002691	0.997309	
QLI66021.1	OTHER	0.000537	0.999463	
QLI66022.1	OTHER	0.002141	0.997859	
QLI66023.1	OTHER	0.004293	0.995707	

QLI66024.1	OTHER	0.002970	0.997030	
QLI66025.1	OTHER	0.000344	0.999656	
QLI66026.1	OTHER	0.001378	0.998622	
QLI66027.1	OTHER	0.000666	0.999334	
QLI66028.1	OTHER	0.000322	0.999678	
QLI66029.1	OTHER	0.321603	0.678397	
QLI66030.1	OTHER	0.001721	0.998279	
QLI66031.1	OTHER	0.001701	0.998299	
QLI66032.1	OTHER	0.000200	0.999800	
QLI66033.1	OTHER	0.000843	0.999157	
QLI66034.1	OTHER	0.004827	0.995173	
QLI66035.1	OTHER	0.001930	0.998070	
QLI66036.1	OTHER	0.000678	0.999322	
QLI66037.1	OTHER	0.001253	0.998747	
QLI66038.1	OTHER	0.002177	0.997823	
QLI66039.1	OTHER	0.020981	0.979019	
QLI66040.1	OTHER	0.471898	0.528102	
QLI66041.1	OTHER	0.022509	0.977491	
QLI66042.1	OTHER	0.002727	0.997273	
QLI66043.1	OTHER	0.007624	0.992376	
QLI66044.1	SP(Sec/SPI)	0.969022	0.030978	CS pos: 15-16. VSA-LP. Pr: 0.6330
QLI66045.1	SP(Sec/SPI)	0.855087	0.144913	CS pos: 21-22. AAA-TP. Pr: 0.5845
QLI66046.1	OTHER	0.017038	0.982962	
QLI66047.1	OTHER	0.001708	0.998292	
QLI66048.1	OTHER	0.001433	0.998567	
QLI66049.1	OTHER	0.001032	0.998968	
QLI66050.1	OTHER	0.135105	0.864895	
QLI66051.1	OTHER	0.000623	0.999377	
QLI66052.1	OTHER	0.000588	0.999412	
QLI66053.1	OTHER	0.002592	0.997408	
QLI66054.1	OTHER	0.000791	0.999209	

QLI66055.1	OTHER	0.014716	0.985284	
QLI66056.1	OTHER	0.001466	0.998534	
QLI66057.1	OTHER	0.064335	0.935665	
QLI66058.1	OTHER	0.000548	0.999452	
QLI66059.1	OTHER	0.010609	0.989391	
QLI66060.1	OTHER	0.003382	0.996618	
QLI66061.1	OTHER	0.000635	0.999365	
QLI66062.1	OTHER	0.000650	0.999350	
QLI66063.1	OTHER	0.000566	0.999434	
QLI66064.1	OTHER	0.000395	0.999605	
QLI66065.1	SP(Sec/SPI)	0.781426	0.218574	CS pos: 22-23. TEG-MA. Pr: 0.3031
QLI66066.1	OTHER	0.000756	0.999244	
QLI66067.1	OTHER	0.001361	0.998639	
QLI66068.1	OTHER	0.003698	0.996302	
QLI66069.1	OTHER	0.000978	0.999022	
QLI66070.1	OTHER	0.001028	0.998972	
QLI66071.1	OTHER	0.005928	0.994072	
QLI66072.1	OTHER	0.000372	0.999628	
QLI66073.1	OTHER	0.000664	0.999336	
QLI66074.1	OTHER	0.000627	0.999373	
QLI66075.1	OTHER	0.000773	0.999227	
QLI66076.1	OTHER	0.001298	0.998702	
QLI66077.1	OTHER	0.003617	0.996383	
QLI66078.1	OTHER	0.001670	0.998330	
QLI66079.1	SP(Sec/SPI)	0.982798	0.017202	CS pos: 23-24. ASA-SR. Pr: 0.6647
QLI66080.1	OTHER	0.001051	0.998949	
QLI66081.1	OTHER	0.000759	0.999241	
QLI66082.1	OTHER	0.000839	0.999161	
QLI66083.1	OTHER	0.001787	0.998213	
QLI66084.1	OTHER	0.006418	0.993582	
QLI66085.1	OTHER	0.000895	0.999105	

QLI66086.1	SP(Sec/SPI)	0.945541	0.054459	CS pos: 19-20. SIA-IG. Pr: 0.5817
QLI66087.1	OTHER	0.000476	0.999524	
QLI66088.1	OTHER	0.001651	0.998349	
QLI66089.1	OTHER	0.000737	0.999263	
QLI66090.1	OTHER	0.003626	0.996374	
QLI66091.1	SP(Sec/SPI)	0.890681	0.109319	CS pos: 18-19. SAA-DE. Pr: 0.5899
QLI66092.1	OTHER	0.001244	0.998756	
QLI66093.1	OTHER	0.000400	0.999600	
QLI66094.1	OTHER	0.000463	0.999537	
QLI66095.1	SP(Sec/SPI)	0.961518	0.038482	CS pos: 21-22. AAA-AT. Pr: 0.3934
QLI66096.1	OTHER	0.000587	0.999413	
QLI66097.1	OTHER	0.000964	0.999036	
QLI66098.1	OTHER	0.004493	0.995507	
QLI66099.1	OTHER	0.000825	0.999175	
QLI66100.1	OTHER	0.000921	0.999079	
QLI66101.1	OTHER	0.009109	0.990891	
QLI66102.1	SP(Sec/SPI)	0.862547	0.137453	CS pos: 18-19. ATT-IG. Pr: 0.3063
QLI66103.1	SP(Sec/SPI)	0.997241	0.002759	CS pos: 19-20. CDT-FS. Pr: 0.2757
QLI66104.1	OTHER	0.000991	0.999009	
QLI66105.1	OTHER	0.000880	0.999120	
QLI66106.1	OTHER	0.000500	0.999500	
QLI66107.1	OTHER	0.000628	0.999372	
QLI66108.1	OTHER	0.001044	0.998956	
QLI66109.1	OTHER	0.000899	0.999101	
QLI66110.1	OTHER	0.001667	0.998333	
QLI66111.1	OTHER	0.000753	0.999247	
QLI66112.1	OTHER	0.000689	0.999311	
QLI66113.1	OTHER	0.002366	0.997634	
QLI66114.1	OTHER	0.001444	0.998556	
QLI66115.1	OTHER	0.000677	0.999323	
QLI66116.1	OTHER	0.003285	0.996715	

QLI66117.1	OTHER	0.001296	0.998704	
QLI66118.1	OTHER	0.001223	0.998777	
QLI66119.1	OTHER	0.002091	0.997909	
QLI66120.1	OTHER	0.002017	0.997983	
QLI66121.1	SP(Sec/SPI)	0.978562	0.021438	CS pos: 23-24. AAA-AN. Pr: 0.6700
QLI66122.1	SP(Sec/SPI)	0.966002	0.033998	CS pos: 18-19. SLG-FP. Pr: 0.8037
QLI66123.1	OTHER	0.000604	0.999396	
QLI66124.1	OTHER	0.001436	0.998564	
QLI66125.1	SP(Sec/SPI)	0.915316	0.084684	CS pos: 22-23. CDA-TS. Pr: 0.6313
QLI66126.1	OTHER	0.001116	0.998884	
QLI66127.1	OTHER	0.000253	0.999747	
QLI66128.1	OTHER	0.005601	0.994399	
QLI66129.1	SP(Sec/SPI)	0.588136	0.411864	CS pos: 18-19. AVT-AP. Pr: 0.3043
QLI66130.1	OTHER	0.002148	0.997852	
QLI66131.1	OTHER	0.000606	0.999394	
QLI66132.1	OTHER	0.002176	0.997824	
QLI66133.1	OTHER	0.002409	0.997591	
QLI66134.1	OTHER	0.001064	0.998936	
QLI66135.1	OTHER	0.004286	0.995714	
QLI66136.1	OTHER	0.003077	0.996923	
QLI66137.1	OTHER	0.002249	0.997751	
QLI66138.1	SP(Sec/SPI)	0.936870	0.063130	CS pos: 22-23. VRG-FA. Pr: 0.3648
QLI66139.1	OTHER	0.003870	0.996130	
QLI66140.1	SP(Sec/SPI)	0.785910	0.214090	CS pos: 22-23. ALF-RD. Pr: 0.3111
QLI66141.1	OTHER	0.001024	0.998976	
QLI66142.1	OTHER	0.000916	0.999084	
QLI66143.1	OTHER	0.001125	0.998875	
QLI66144.1	OTHER	0.000866	0.999134	
QLI66145.1	OTHER	0.040373	0.959627	
QLI66146.1	OTHER	0.001887	0.998113	
QLI66147.1	OTHER	0.001106	0.998894	

QLI66148.1	OTHER	0.004338	0.995662	
QLI66149.1	OTHER	0.006980	0.993020	
QLI66150.1	OTHER	0.000476	0.999524	
QLI66151.1	OTHER	0.001735	0.998265	
QLI66152.1	SP(Sec/SPI)	0.988097	0.011903	CS pos: 18-19. VNG-LP. Pr: 0.7930
QLI66153.1	OTHER	0.005264	0.994736	
QLI66154.1	OTHER	0.015187	0.984813	
QLI66155.1	OTHER	0.002097	0.997903	
QLI66156.1	SP(Sec/SPI)	0.996396	0.003604	CS pos: 19-20. TTS-LS. Pr: 0.4733
QLI66157.1	OTHER	0.001047	0.998953	
QLI66158.1	OTHER	0.000315	0.999685	
QLI66159.1	OTHER	0.003586	0.996414	
QLI66160.1	OTHER	0.000543	0.999457	
QLI66161.1	SP(Sec/SPI)	0.962168	0.037832	CS pos: 25-26. VNG-QT. Pr: 0.8360
QLI66162.1	OTHER	0.000848	0.999152	
QLI66163.1	SP(Sec/SPI)	0.816192	0.183808	CS pos: 28-29. VYG-TY. Pr: 0.4889
QLI66164.1	OTHER	0.004206	0.995794	
QLI66165.1	OTHER	0.001380	0.998620	
QLI66166.1	OTHER	0.001952	0.998048	
QLI66167.1	OTHER	0.001216	0.998784	
QLI66168.1	OTHER	0.000692	0.999308	
QLI66169.1	OTHER	0.001484	0.998516	
QLI66170.1	OTHER	0.000704	0.999296	
QLI66171.1	OTHER	0.000913	0.999087	
QLI66172.1	OTHER	0.002183	0.997817	
QLI66173.1	OTHER	0.002535	0.997465	
QLI66174.1	OTHER	0.001794	0.998206	
QLI66175.1	OTHER	0.001483	0.998517	
QLI66176.1	OTHER	0.000411	0.999589	
QLI66177.1	OTHER	0.007229	0.992771	
QLI66178.1	OTHER	0.002973	0.997027	

QLI66179.1	OTHER	0.000127	0.999873	
QLI66180.1	OTHER	0.009390	0.990610	
QLI66181.1	OTHER	0.000910	0.999090	
QLI66182.1	OTHER	0.001030	0.998970	
QLI66183.1	OTHER	0.000790	0.999210	
QLI66184.1	OTHER	0.205847	0.794153	
QLI66185.1	OTHER	0.001783	0.998217	
QLI66186.1	OTHER	0.030772	0.969228	
QLI66187.1	OTHER	0.001436	0.998564	
QLI66188.1	OTHER	0.000516	0.999484	
QLI66189.1	SP(Sec/SPI)	0.978987	0.021013	CS pos: 20-21. ALA-WG. Pr: 0.5422
QLI66190.1	OTHER	0.001392	0.998608	
QLI66191.1	OTHER	0.000446	0.999554	
QLI66192.1	OTHER	0.003009	0.996991	
QLI66193.1	OTHER	0.001693	0.998307	
QLI66194.1	SP(Sec/SPI)	0.994359	0.005641	CS pos: 20-21. GLA-AP. Pr: 0.8635
QLI66195.1	OTHER	0.000769	0.999231	
QLI66196.1	OTHER	0.001895	0.998105	
QLI66197.1	OTHER	0.001501	0.998499	
QLI66198.1	OTHER	0.000919	0.999081	
QLI66199.1	OTHER	0.283480	0.716520	
QLI66200.1	OTHER	0.000394	0.999606	
QLI66201.1	OTHER	0.001831	0.998169	
QLI66202.1	OTHER	0.003609	0.996391	
QLI66203.1	OTHER	0.000642	0.999358	
QLI66204.1	OTHER	0.023273	0.976727	
QLI66205.1	OTHER	0.000581	0.999419	
QLI66206.1	OTHER	0.000578	0.999422	
QLI66207.1	OTHER	0.001506	0.998494	
QLI66208.1	OTHER	0.001291	0.998709	
QLI66209.1	OTHER	0.000776	0.999224	

QLI66210.1	OTHER	0.034109	0.965891
QLI66211.1	OTHER	0.000537	0.999463
QLI66212.1	OTHER	0.001381	0.998619
QLI66213.1	OTHER	0.000932	0.999068
QLI66214.1	OTHER	0.003211	0.996789
QLI66215.1	OTHER	0.000176	0.999824
QLI66216.1	OTHER	0.005553	0.994447
QLI66217.1	OTHER	0.001593	0.998407
QLI66218.1	OTHER	0.001301	0.998699
QLI66219.1	OTHER	0.001017	0.998983
QLI66220.1	OTHER	0.003155	0.996845
QLI66221.1	OTHER	0.002272	0.997728
QLI66222.1	OTHER	0.000352	0.999648
QLI66223.1	OTHER	0.000621	0.999379
QLI66224.1	OTHER	0.002064	0.997936
QLI66225.1	OTHER	0.000534	0.999466
QLI66226.1	OTHER	0.000811	0.999189
QLI66227.1	OTHER	0.001966	0.998034
QLI66228.1	OTHER	0.000655	0.999345
QLI66229.1	OTHER	0.001650	0.998350
QLI66230.1	OTHER	0.000880	0.999120
QLI66231.1	OTHER	0.000477	0.999523
QLI66232.1	OTHER	0.015624	0.984376
QLI66233.1	OTHER	0.003122	0.996878
QLI66234.1	OTHER	0.001066	0.998934
QLI66235.1	OTHER	0.000908	0.999092
QLI66236.1	OTHER	0.001249	0.998751
QLI66237.1	OTHER	0.001731	0.998269
QLI66238.1	OTHER	0.000571	0.999429
QLI66239.1	OTHER	0.001482	0.998518
QLI66240.1	OTHER	0.016191	0.983809

QLI66241.1	OTHER	0.000442	0.999558	
QLI66242.1	SP(Sec/SPI)	0.993074	0.006926	CS pos: 19-20. VLA-TP. Pr: 0.9208
QLI66243.1	OTHER	0.004359	0.995641	
QLI66244.1	OTHER	0.000438	0.999562	
QLI66245.1	OTHER	0.021831	0.978169	
QLI66246.1	SP(Sec/SPI)	0.616435	0.383565	CS pos: 21-22. LFS-RR. Pr: 0.2702
QLI66247.1	OTHER	0.016567	0.983433	
QLI66248.1	OTHER	0.001623	0.998377	
QLI66249.1	OTHER	0.000797	0.999203	
QLI66250.1	OTHER	0.001602	0.998398	
QLI66251.1	OTHER	0.020199	0.979801	
QLI66252.1	OTHER	0.003933	0.996067	
QLI66253.1	OTHER	0.003422	0.996578	
QLI66254.1	OTHER	0.000514	0.999486	
QLI66255.1	OTHER	0.000856	0.999144	
QLI66256.1	OTHER	0.000814	0.999186	
QLI66257.1	OTHER	0.000796	0.999204	
QLI66258.1	OTHER	0.001095	0.998905	
QLI66259.1	OTHER	0.000637	0.999363	
QLI66260.1	SP(Sec/SPI)	0.970574	0.029426	CS pos: 23-24. AGA-GP. Pr: 0.6880
QLI66261.1	OTHER	0.000681	0.999319	
QLI66262.1	OTHER	0.000861	0.999139	
QLI66263.1	OTHER	0.031690	0.968310	
QLI66264.1	OTHER	0.001528	0.998472	
QLI66265.1	OTHER	0.001191	0.998809	
QLI66266.1	OTHER	0.000633	0.999367	
QLI66267.1	SP(Sec/SPI)	0.988640	0.011360	CS pos: 21-22. CQG-HL. Pr: 0.8376
QLI66268.1	OTHER	0.002613	0.997387	
QLI66269.1	SP(Sec/SPI)	0.998066	0.001934	CS pos: 19-20. VLS-GG. Pr: 0.8275
QLI66270.1	OTHER	0.002207	0.997793	
QLI66271.1	OTHER	0.002001	0.997999	

QLI66272.1	SP(Sec/SPI)	0.997985	0.002015	CS pos: 17-18. VFA-AP. Pr: 0.6756
QLI66273.1	OTHER	0.001901	0.998099	
QLI66274.1	OTHER	0.000479	0.999521	
QLI66275.1	OTHER	0.007416	0.992584	
QLI66276.1	OTHER	0.000961	0.999039	
QLI66277.1	OTHER	0.000754	0.999246	
QLI66278.1	OTHER	0.001356	0.998644	
QLI66279.1	OTHER	0.000511	0.999489	
QLI66280.1	OTHER	0.004815	0.995185	
QLI66281.1	OTHER	0.000339	0.999661	
QLI66282.1	OTHER	0.005965	0.994035	
QLI66283.1	OTHER	0.001861	0.998139	
QLI66284.1	SP(Sec/SPI)	0.673830	0.326170	CS pos: 32-33. VLA-EG. Pr: 0.4964
QLI66285.1	OTHER	0.000415	0.999585	
QLI66286.1	OTHER	0.001654	0.998346	
QLI66287.1	OTHER	0.011128	0.988872	
QLI66288.1	OTHER	0.000771	0.999229	
QLI66289.1	OTHER	0.000502	0.999498	
QLI66290.1	OTHER	0.000237	0.999763	
QLI66291.1	OTHER	0.000838	0.999162	
QLI66292.1	OTHER	0.002240	0.997760	
QLI66293.1	OTHER	0.072059	0.927941	
QLI66294.1	OTHER	0.000901	0.999099	
QLI66295.1	OTHER	0.000465	0.999535	
QLI66296.1	OTHER	0.001813	0.998187	
QLI66297.1	OTHER	0.000751	0.999249	
QLI66298.1	OTHER	0.001080	0.998920	
QLI66299.1	OTHER	0.001673	0.998327	
QLI66300.1	SP(Sec/SPI)	0.987326	0.012674	CS pos: 18-19. AAA-QV. Pr: 0.4768
QLI66301.1	OTHER	0.001079	0.998921	
QLI66302.1	SP(Sec/SPI)	0.997643	0.002357	CS pos: 19-20. AVA-GL. Pr: 0.7059

QLI66303.1	OTHER	0.001297	0.998703	
QLI66304.1	OTHER	0.027920	0.972080	
QLI66305.1	OTHER	0.001320	0.998680	
QLI66306.1	OTHER	0.000380	0.999620	
QLI66307.1	OTHER	0.001219	0.998781	
QLI66308.1	OTHER	0.003891	0.996109	
QLI66309.1	OTHER	0.000691	0.999309	
QLI66310.1	OTHER	0.001530	0.998470	
QLI66311.1	OTHER	0.000264	0.999736	
QLI66312.1	OTHER	0.001752	0.998248	
QLI66313.1	OTHER	0.000533	0.999467	
QLI66314.1	OTHER	0.000619	0.999381	
QLI66315.1	OTHER	0.006175	0.993825	
QLI66316.1	SP(Sec/SPI)	0.971672	0.028328	CS pos: 30-31. SAS-VK. Pr: 0.4873
QLI66317.1	OTHER	0.004699	0.995301	
QLI66318.1	SP(Sec/SPI)	0.996414	0.003586	CS pos: 19-20. ALG-AP. Pr: 0.8574
QLI66319.1	OTHER	0.001695	0.998305	
QLI66320.1	OTHER	0.001245	0.998755	
QLI66321.1	OTHER	0.001100	0.998900	
QLI66322.1	OTHER	0.000717	0.999283	
QLI66323.1	SP(Sec/SPI)	0.858004	0.141996	CS pos: 18-19. VSA-AT. Pr: 0.6774
QLI66324.1	OTHER	0.000952	0.999048	
QLI66325.1	OTHER	0.000806	0.999194	
QLI66326.1	OTHER	0.000530	0.999470	
QLI66327.1	OTHER	0.004317	0.995683	
QLI66328.1	OTHER	0.001409	0.998591	
QLI66329.1	OTHER	0.001389	0.998611	
QLI66330.1	OTHER	0.021064	0.978936	
QLI66331.1	OTHER	0.000318	0.999682	
QLI66332.1	OTHER	0.000387	0.999613	
QLI66333.1	OTHER	0.001129	0.998871	

QLI66334.1	OTHER	0.003568	0.996432
QLI66335.1	OTHER	0.001589	0.998411
QLI66336.1	OTHER	0.000919	0.999081
QLI66337.1	OTHER	0.000443	0.999557
QLI66338.1	OTHER	0.000843	0.999157
QLI66339.1	OTHER	0.001052	0.998948
QLI66340.1	OTHER	0.000322	0.999678
QLI66341.1	OTHER	0.000731	0.999269
QLI66342.1	OTHER	0.003033	0.996967
QLI66343.1	OTHER	0.000504	0.999496
QLI66344.1	OTHER	0.000580	0.999420
QLI66345.1	OTHER	0.000728	0.999272
QLI66346.1	OTHER	0.002624	0.997376
QLI66347.1	OTHER	0.413243	0.586757
QLI66348.1	OTHER	0.003736	0.996264
QLI66349.1	OTHER	0.004041	0.995959
QLI66350.1	OTHER	0.000472	0.999528
QLI66351.1	OTHER	0.000278	0.999722
QLI66352.1	OTHER	0.000481	0.999519
QLI66353.1	OTHER	0.004274	0.995726
QLI66354.1	OTHER	0.001631	0.998369
QLI66355.1	OTHER	0.000526	0.999474
QLI66356.1	OTHER	0.000679	0.999321
QLI66357.1	OTHER	0.000832	0.999168
QLI66358.1	OTHER	0.013002	0.986998
QLI66359.1	OTHER	0.000756	0.999244
QLI66360.1	OTHER	0.005022	0.994978
QLI66361.1	OTHER	0.005521	0.994479
QLI66362.1	OTHER	0.000600	0.999400
QLI66363.1	OTHER	0.000878	0.999122
QLI66364.1	OTHER	0.007476	0.992524

QLI66365.1	OTHER	0.004841	0.995159	
QLI66366.1	OTHER	0.005971	0.994029	
QLI66367.1	OTHER	0.001169	0.998831	
QLI66368.1	OTHER	0.003980	0.996020	
QLI66369.1	OTHER	0.140106	0.859894	
QLI66370.1	OTHER	0.004766	0.995234	
QLI66371.1	OTHER	0.129022	0.870978	
QLI66372.1	OTHER	0.001453	0.998547	
QLI66373.1	OTHER	0.000699	0.999301	
QLI66374.1	OTHER	0.000429	0.999571	
QLI66375.1	OTHER	0.000542	0.999458	
QLI66376.1	OTHER	0.000580	0.999420	
QLI66377.1	OTHER	0.002112	0.997888	
QLI66378.1	OTHER	0.000890	0.999110	
QLI66379.1	OTHER	0.008670	0.991330	
QLI66380.1	OTHER	0.000382	0.999618	
QLI66381.1	OTHER	0.000413	0.999587	
QLI66382.1	OTHER	0.001204	0.998796	
QLI66383.1	OTHER	0.001571	0.998429	
QLI66384.1	OTHER	0.001916	0.998084	
QLI66385.1	OTHER	0.000837	0.999163	
QLI66386.1	OTHER	0.002484	0.997516	
QLI66387.1	OTHER	0.004648	0.995352	
QLI66388.1	OTHER	0.001239	0.998761	
QLI66389.1	OTHER	0.004831	0.995169	
QLI66390.1	OTHER	0.001063	0.998937	
QLI66391.1	OTHER	0.002793	0.997207	
QLI66392.1	OTHER	0.000973	0.999027	
QLI66393.1	OTHER	0.000586	0.999414	
QLI66394.1	SP(Sec/SPI)	0.688166	0.311834	CS pos: 29-30. VAG-NV. Pr: 0.5090
QLI66395.1	SP(Sec/SPI)	0.833959	0.166041	CS pos: 26-27. SHS-SP. Pr: 0.6074

QLI66396.1	OTHER	0.000653	0.999347	
QLI66397.1	OTHER	0.000357	0.999643	
QLI66398.1	OTHER	0.000507	0.999493	
QLI66399.1	OTHER	0.000412	0.999588	
QLI66400.1	OTHER	0.001781	0.998219	
QLI66401.1	OTHER	0.000291	0.999709	
QLI66402.1	OTHER	0.013247	0.986753	
QLI66403.1	OTHER	0.001670	0.998330	
QLI66404.1	SP(Sec/SPI)	0.999629	0.000371	CS pos: 19-20. AAA-QP. Pr: 0.8087
QLI66405.1	OTHER	0.002018	0.997982	
QLI66406.1	OTHER	0.002197	0.997803	
QLI66407.1	OTHER	0.001299	0.998701	
QLI66408.1	OTHER	0.003181	0.996819	
QLI66409.1	OTHER	0.001454	0.998546	
QLI66410.1	OTHER	0.001118	0.998882	
QLI66411.1	OTHER	0.021420	0.978580	
QLI66412.1	OTHER	0.000901	0.999099	
QLI66413.1	OTHER	0.000760	0.999240	
QLI66414.1	OTHER	0.002047	0.997953	
QLI66415.1	OTHER	0.001180	0.998820	
QLI66416.1	OTHER	0.001512	0.998488	
QLI66417.1	OTHER	0.000427	0.999573	
QLI66418.1	SP(Sec/SPI)	0.990893	0.009107	CS pos: 25-26. TAA-QN. Pr: 0.6629
QLI66419.1	OTHER	0.001066	0.998934	
QLI66420.1	OTHER	0.002450	0.997550	
QLI66421.1	OTHER	0.009662	0.990338	
QLI66422.1	OTHER	0.136973	0.863027	
QLI66423.1	OTHER	0.004954	0.995046	
QLI66424.1	OTHER	0.002380	0.997620	
QLI66425.1	OTHER	0.001081	0.998919	
QLI66426.1	OTHER	0.003803	0.996197	

QLI66427.1	OTHER	0.003857	0.996143	
QLI66428.1	OTHER	0.000529	0.999471	
QLI66429.1	OTHER	0.001492	0.998508	
QLI66430.1	OTHER	0.021339	0.978661	
QLI66431.1	OTHER	0.000708	0.999292	
QLI66432.1	OTHER	0.001381	0.998619	
QLI66433.1	SP(Sec/SPI)	0.998652	0.001348	CS pos: 16-17. VLA-TP. Pr: 0.5973
QLI66434.1	OTHER	0.003936	0.996064	
QLI66435.1	OTHER	0.001238	0.998762	
QLI66436.1	OTHER	0.002211	0.997789	
QLI66437.1	OTHER	0.001432	0.998568	
QLI66438.1	SP(Sec/SPI)	0.990633	0.009367	CS pos: 20-21. ASA-IQ. Pr: 0.4535
QLI66439.1	OTHER	0.000349	0.999651	
QLI66440.1	OTHER	0.000383	0.999617	
QLI66441.1	OTHER	0.000747	0.999253	
QLI66442.1	OTHER	0.001331	0.998669	
QLI66443.1	SP(Sec/SPI)	0.808296	0.191704	CS pos: 18-19. ALA-AT. Pr: 0.5730
QLI66444.1	OTHER	0.000772	0.999228	
QLI66445.1	OTHER	0.001524	0.998476	
QLI66446.1	OTHER	0.000706	0.999294	
QLI66447.1	OTHER	0.001187	0.998813	
QLI66448.1	OTHER	0.001547	0.998453	
QLI66449.1	OTHER	0.008307	0.991693	
QLI66450.1	OTHER	0.000325	0.999675	
QLI66451.1	OTHER	0.001859	0.998141	
QLI66452.1	OTHER	0.000816	0.999184	
QLI66453.1	OTHER	0.001265	0.998735	
QLI66454.1	OTHER	0.422166	0.577834	
QLI66455.1	OTHER	0.033297	0.966703	
QLI66456.1	OTHER	0.000803	0.999197	
QLI66457.1	OTHER	0.001006	0.998994	

QLI66458.1	OTHER	0.000828	0.999172	
QLI66459.1	OTHER	0.001251	0.998749	
QLI66460.1	OTHER	0.000265	0.999735	
QLI66461.1	OTHER	0.000921	0.999079	
QLI66462.1	OTHER	0.001420	0.998580	
QLI66463.1	OTHER	0.004297	0.995703	
QLI66464.1	OTHER	0.012977	0.987023	
QLI66465.1	OTHER	0.000584	0.999416	
QLI66466.1	OTHER	0.008712	0.991288	
QLI66467.1	OTHER	0.001838	0.998162	
QLI66468.1	OTHER	0.000322	0.999678	
QLI66469.1	OTHER	0.003093	0.996907	
QLI66470.1	OTHER	0.002096	0.997904	
QLI66471.1	OTHER	0.000966	0.999034	
QLI66472.1	OTHER	0.001924	0.998076	
QLI66473.1	OTHER	0.062757	0.937243	
QLI66474.1	OTHER	0.000778	0.999222	
QLI66475.1	OTHER	0.000809	0.999191	
QLI66476.1	OTHER	0.000492	0.999508	
QLI66477.1	OTHER	0.309952	0.690048	
QLI66478.1	OTHER	0.006529	0.993471	
QLI66479.1	OTHER	0.001712	0.998288	
QLI66480.1	OTHER	0.000342	0.999658	
QLI66481.1	OTHER	0.010774	0.989226	
QLI66482.1	OTHER	0.001274	0.998726	
QLI66483.1	OTHER	0.001516	0.998484	
QLI66484.1	OTHER	0.003607	0.996393	
QLI66485.1	OTHER	0.001014	0.998986	
QLI66486.1	OTHER	0.001248	0.998752	
QLI66487.1	OTHER	0.001521	0.998479	
QLI66488.1	SP(Sec/SPI)	0.816336	0.183664	CS pos: 22-23. ILG-VP. Pr: 0.5085

QLI66489.1	OTHER	0.017152	0.982848	
QLI66490.1	OTHER	0.001354	0.998646	
QLI66491.1	SP(Sec/SPI)	0.968841	0.031159	CS pos: 17-18. AAP-GP. Pr: 0.3395
QLI66492.1	OTHER	0.001987	0.998013	
QLI66493.1	OTHER	0.002251	0.997749	
QLI66494.1	OTHER	0.010766	0.989234	
QLI66495.1	OTHER	0.000856	0.999144	
QLI66496.1	OTHER	0.056583	0.943417	
QLI66497.1	OTHER	0.001045	0.998955	
QLI66498.1	OTHER	0.088550	0.911450	
QLI66499.1	OTHER	0.001089	0.998911	
QLI66500.1	OTHER	0.002799	0.997201	
QLI66501.1	OTHER	0.002237	0.997763	
QLI66502.1	OTHER	0.002955	0.997045	
QLI66503.1	OTHER	0.002179	0.997821	
QLI66504.1	OTHER	0.000968	0.999032	
QLI66505.1	OTHER	0.004634	0.995366	
QLI66506.1	OTHER	0.000895	0.999105	
QLI66507.1	OTHER	0.000503	0.999497	
QLI66508.1	OTHER	0.000806	0.999194	
QLI66509.1	OTHER	0.010751	0.989249	
QLI66510.1	OTHER	0.000550	0.999450	
QLI66511.1	OTHER	0.001335	0.998665	
QLI66512.1	OTHER	0.000254	0.999746	
QLI66513.1	OTHER	0.000590	0.999410	
QLI66514.1	OTHER	0.000670	0.999330	
QLI66515.1	OTHER	0.002466	0.997534	
QLI66516.1	OTHER	0.001405	0.998595	
QLI66517.1	OTHER	0.000725	0.999275	
QLI66518.1	OTHER	0.001015	0.998985	
QLI66519.1	OTHER	0.004442	0.995558	

QLI66520.1	OTHER	0.000287	0.999713
QLI66521.1	OTHER	0.006909	0.993091
QLI66522.1	OTHER	0.000575	0.999425
QLI66523.1	OTHER	0.000964	0.999036
QLI66524.1	OTHER	0.001089	0.998911
QLI66525.1	OTHER	0.002587	0.997413
QLI66526.1	OTHER	0.001889	0.998111
QLI66527.1	OTHER	0.001779	0.998221
QLI66528.1	OTHER	0.005162	0.994838
QLI66529.1	OTHER	0.000929	0.999071
QLI66530.1	OTHER	0.001846	0.998154
QLI66531.1	OTHER	0.000186	0.999814
QLI66532.1	OTHER	0.000885	0.999115
QLI66533.1	OTHER	0.009102	0.990898
QLI66534.1	OTHER	0.000377	0.999623
QLI66535.1	OTHER	0.000718	0.999282
QLI66536.1	OTHER	0.002909	0.997091
QLI66537.1	OTHER	0.006586	0.993414
QLI66538.1	OTHER	0.000939	0.999061
QLI66539.1	OTHER	0.119008	0.880992
QLI66540.1	OTHER	0.001540	0.998460
QLI66541.1	OTHER	0.003799	0.996201
QLI66542.1	OTHER	0.000925	0.999075
QLI66543.1	OTHER	0.002650	0.997350
QLI66544.1	OTHER	0.000321	0.999679
QLI66545.1	OTHER	0.001178	0.998822
QLI66546.1	OTHER	0.000619	0.999381
QLI66547.1	OTHER	0.000899	0.999101
QLI66548.1	OTHER	0.064968	0.935032
QLI66549.1	OTHER	0.003741	0.996259
QLI66550.1	OTHER	0.001328	0.998672

QLI66551.1	OTHER	0.001080	0.998920	
QLI66552.1	OTHER	0.001867	0.998133	
QLI66553.1	OTHER	0.000425	0.999575	
QLI66554.1	OTHER	0.001038	0.998962	
QLI66555.1	OTHER	0.001520	0.998480	
QLI66556.1	OTHER	0.000444	0.999556	
QLI66557.1	OTHER	0.000925	0.999075	
QLI66558.1	OTHER	0.017039	0.982961	
QLI66559.1	OTHER	0.049390	0.950610	
QLI66560.1	OTHER	0.000974	0.999026	
QLI66561.1	OTHER	0.015960	0.984040	
QLI66562.1	OTHER	0.001043	0.998957	
QLI66563.1	OTHER	0.002218	0.997782	
QLI66564.1	OTHER	0.000537	0.999463	
QLI66565.1	OTHER	0.000761	0.999239	
QLI66566.1	SP(Sec/SPI)	0.976422	0.023578	CS pos: 18-19. ALA-CT. Pr: 0.5185
QLI66567.1	OTHER	0.002138	0.997862	
QLI66568.1	SP(Sec/SPI)	0.901235	0.098765	CS pos: 25-26. AHS-HD. Pr: 0.3449
QLI66569.1	OTHER	0.000879	0.999121	
QLI66570.1	OTHER	0.003068	0.996932	
QLI66571.1	OTHER	0.001290	0.998710	
QLI66572.1	OTHER	0.000848	0.999152	
QLI66573.1	OTHER	0.001992	0.998008	
QLI66574.1	OTHER	0.000519	0.999481	
QLI66575.1	SP(Sec/SPI)	0.915182	0.084818	CS pos: 19-20. AAA-RD. Pr: 0.3416
QLI66576.1	OTHER	0.001018	0.998982	
QLI66577.1	SP(Sec/SPI)	0.977616	0.022384	CS pos: 22-23. AHA-HV. Pr: 0.8435
QLI66578.1	OTHER	0.000475	0.999525	
QLI66579.1	OTHER	0.001850	0.998150	
QLI66580.1	OTHER	0.001951	0.998049	
QLI66581.1	OTHER	0.000892	0.999108	

QLI66582.1	OTHER	0.005476	0.994524
QLI66583.1	OTHER	0.000261	0.999739
QLI66584.1	OTHER	0.022907	0.977093
QLI66585.1	OTHER	0.003159	0.996841
QLI66586.1	OTHER	0.001448	0.998552
QLI66587.1	OTHER	0.002552	0.997448
QLI66588.1	OTHER	0.004088	0.995912
QLI66589.1	OTHER	0.006726	0.993274
QLI66590.1	OTHER	0.001701	0.998299
QLI66591.1	OTHER	0.000661	0.999339
QLI66592.1	OTHER	0.001854	0.998146
QLI66593.1	OTHER	0.001163	0.998837
QLI66594.1	OTHER	0.000605	0.999395
QLI66595.1	OTHER	0.001752	0.998248
QLI66596.1	OTHER	0.000917	0.999083
QLI66597.1	OTHER	0.001516	0.998484
QLI66598.1	OTHER	0.022308	0.977692
QLI66599.1	OTHER	0.000693	0.999307
QLI66600.1	OTHER	0.000697	0.999303
QLI66601.1	OTHER	0.001718	0.998282
QLI66602.1	OTHER	0.001624	0.998376
QLI66603.1	OTHER	0.001125	0.998875
QLI66604.1	OTHER	0.001275	0.998725
QLI66605.1	OTHER	0.001070	0.998930
QLI66606.1	OTHER	0.000615	0.999385
QLI66607.1	OTHER	0.002924	0.997076
QLI66608.1	OTHER	0.002076	0.997924
QLI66609.1	OTHER	0.002570	0.997430
QLI66610.1	OTHER	0.001657	0.998343
QLI66611.1	OTHER	0.001659	0.998341
QLI66612.1	OTHER	0.000853	0.999147

QLI66613.1	OTHER	0.005424	0.994576	
QLI66614.1	OTHER	0.000451	0.999549	
QLI66615.1	OTHER	0.000786	0.999214	
QLI66616.1	OTHER	0.000371	0.999629	
QLI66617.1	OTHER	0.000315	0.999685	
QLI66618.1	OTHER	0.001028	0.998972	
QLI66619.1	OTHER	0.000733	0.999267	
QLI66620.1	OTHER	0.001453	0.998547	
QLI66621.1	OTHER	0.003017	0.996983	
QLI66622.1	OTHER	0.003445	0.996555	
QLI66623.1	OTHER	0.001005	0.998995	
QLI66624.1	OTHER	0.004228	0.995772	
QLI66625.1	OTHER	0.000561	0.999439	
QLI66626.1	OTHER	0.002038	0.997962	
QLI66627.1	OTHER	0.011564	0.988436	
QLI66628.1	OTHER	0.000846	0.999154	
QLI66629.1	OTHER	0.000466	0.999534	
QLI66630.1	SP(Sec/SPI)	0.989746	0.010254	CS pos: 17-18. SIA-AP. Pr: 0.3436
QLI66631.1	OTHER	0.000423	0.999577	
QLI66632.1	OTHER	0.001338	0.998662	
QLI66633.1	OTHER	0.001554	0.998446	
QLI66634.1	OTHER	0.002180	0.997820	
QLI66635.1	OTHER	0.000524	0.999476	
QLI66636.1	OTHER	0.019451	0.980549	
QLI66637.1	OTHER	0.001395	0.998605	
QLI66638.1	OTHER	0.011639	0.988361	
QLI66639.1	OTHER	0.001976	0.998024	
QLI66640.1	OTHER	0.156832	0.843168	
QLI66641.1	OTHER	0.002012	0.997988	
QLI66642.1	OTHER	0.001054	0.998946	
QLI66643.1	OTHER	0.003009	0.996991	

QLI66644.1	OTHER	0.001214	0.998786	
QLI66645.1	OTHER	0.001984	0.998016	
QLI66646.1	SP(Sec/SPI)	0.969552	0.030448	CS pos: 17-18. AAG-NP. Pr: 0.6487
QLI66647.1	OTHER	0.000397	0.999603	
QLI66648.1	SP(Sec/SPI)	0.882315	0.117685	CS pos: 20-21. AVS-QH. Pr: 0.4673
QLI66649.1	SP(Sec/SPI)	0.844053	0.155947	CS pos: 16-17. CNG-YF. Pr: 0.7360
QLI66650.1	OTHER	0.000319	0.999681	
QLI66651.1	OTHER	0.001560	0.998440	
QLI66652.1	SP(Sec/SPI)	0.993869	0.006131	CS pos: 18-19. SYG-DK. Pr: 0.9297
QLI66653.1	OTHER	0.004424	0.995576	
QLI66654.1	OTHER	0.000997	0.999003	
QLI66655.1	OTHER	0.000928	0.999072	
QLI66656.1	OTHER	0.001216	0.998784	
QLI66657.1	OTHER	0.002994	0.997006	
QLI66658.1	SP(Sec/SPI)	0.991593	0.008407	CS pos: 17-18. AGA-IE. Pr: 0.6461
QLI66659.1	OTHER	0.002187	0.997813	
QLI66660.1	SP(Sec/SPI)	0.997258	0.002742	CS pos: 18-19. THA-NP. Pr: 0.9207
QLI66661.1	OTHER	0.007586	0.992414	
QLI66662.1	OTHER	0.000421	0.999579	
QLI66663.1	OTHER	0.001229	0.998771	
QLI66664.1	OTHER	0.011904	0.988096	
QLI66665.1	OTHER	0.001516	0.998484	
QLI66666.1	OTHER	0.345629	0.654371	
QLI66667.1	OTHER	0.001735	0.998265	
QLI66668.1	OTHER	0.000465	0.999535	
QLI66669.1	OTHER	0.000429	0.999571	
QLI66670.1	OTHER	0.000465	0.999535	
QLI66671.1	SP(Sec/SPI)	0.992245	0.007755	CS pos: 19-20. AFA-RT. Pr: 0.6049
QLI66672.1	OTHER	0.000325	0.999675	
QLI66673.1	OTHER	0.001085	0.998915	
QLI66674.1	OTHER	0.002271	0.997729	

QLI66675.1	OTHER	0.002250	0.997750	
QLI66676.1	SP(Sec/SPI)	0.984205	0.015795	CS pos: 22-23. AQA-GP. Pr: 0.9514
QLI66677.1	OTHER	0.000667	0.999333	
QLI66678.1	SP(Sec/SPI)	0.957459	0.042541	CS pos: 16-17. AVA-AN. Pr: 0.4550
QLI66679.1	SP(Sec/SPI)	0.996387	0.003613	CS pos: 20-21. VAG-ES. Pr: 0.6812
QLI66680.1	OTHER	0.000901	0.999099	
QLI66681.1	OTHER	0.003121	0.996879	
QLI66682.1	OTHER	0.000415	0.999585	
QLI66683.1	SP(Sec/SPI)	0.805372	0.194628	CS pos: 19-20. VAA-LY. Pr: 0.6719
QLI66684.1	OTHER	0.001434	0.998566	
QLI66685.1	OTHER	0.002585	0.997415	
QLI66686.1	OTHER	0.034826	0.965174	
QLI66687.1	SP(Sec/SPI)	0.865012	0.134988	CS pos: 20-21. LSA-QQ. Pr: 0.3802
QLI66688.1	OTHER	0.002798	0.997202	
QLI66689.1	SP(Sec/SPI)	0.925271	0.074729	CS pos: 22-23. AVA-VP. Pr: 0.6501
QLI66690.1	OTHER	0.001440	0.998560	
QLI66691.1	OTHER	0.003425	0.996575	
QLI66692.1	OTHER	0.005857	0.994143	
QLI66693.1	OTHER	0.002400	0.997600	
QLI66694.1	SP(Sec/SPI)	0.992462	0.007538	CS pos: 20-21. AFA-AP. Pr: 0.9155
QLI66695.1	OTHER	0.003936	0.996064	
QLI66696.1	OTHER	0.001017	0.998983	
QLI66697.1	OTHER	0.000424	0.999576	
QLI66698.1	OTHER	0.000796	0.999204	
QLI66699.1	OTHER	0.000507	0.999493	
QLI66700.1	OTHER	0.001500	0.998500	
QLI66701.1	OTHER	0.107788	0.892212	
QLI66702.1	OTHER	0.001600	0.998400	
QLI66703.1	OTHER	0.002942	0.997058	
QLI66704.1	OTHER	0.001488	0.998512	
QLI66705.1	OTHER	0.001048	0.998952	

QLI66706.1	OTHER	0.002083	0.997917	
QLI66707.1	OTHER	0.000739	0.999261	
QLI66708.1	OTHER	0.000975	0.999025	
QLI66709.1	OTHER	0.001339	0.998661	
QLI66710.1	OTHER	0.001506	0.998494	
QLI66711.1	OTHER	0.011036	0.988964	
QLI66712.1	OTHER	0.262316	0.737684	
QLI66713.1	OTHER	0.003872	0.996128	
QLI66714.1	OTHER	0.002186	0.997814	
QLI66715.1	OTHER	0.014647	0.985353	
QLI66716.1	OTHER	0.000780	0.999220	
QLI66717.1	OTHER	0.001646	0.998354	
QLI66718.1	OTHER	0.002382	0.997618	
QLI66719.1	OTHER	0.000846	0.999154	
QLI66720.1	OTHER	0.016535	0.983465	
QLI66721.1	SP(Sec/SPI)	0.992606	0.007394	CS pos: 17-18. ASA-TS. Pr: 0.4269
QLI66722.1	OTHER	0.003069	0.996931	
QLI66723.1	OTHER	0.001219	0.998781	
QLI66724.1	OTHER	0.001428	0.998572	
QLI66725.1	OTHER	0.004436	0.995564	
QLI66726.1	OTHER	0.000688	0.999312	
QLI66727.1	OTHER	0.000977	0.999023	
QLI66728.1	SP(Sec/SPI)	0.997199	0.002801	CS pos: 18-19. ASA-AR. Pr: 0.6500
QLI66729.1	OTHER	0.001587	0.998413	
QLI66730.1	OTHER	0.005274	0.994726	
QLI66731.1	OTHER	0.019185	0.980815	
QLI66732.1	OTHER	0.000523	0.999477	
QLI66733.1	OTHER	0.002320	0.997680	
QLI66734.1	OTHER	0.002414	0.997586	
QLI66735.1	OTHER	0.009553	0.990447	
QLI66736.1	OTHER	0.001062	0.998938	

QLI66737.1	OTHER	0.002253	0.997747	
QLI66738.1	OTHER	0.000275	0.999725	
QLI66739.1	OTHER	0.003991	0.996009	
QLI66740.1	OTHER	0.010844	0.989156	
QLI66741.1	OTHER	0.001236	0.998764	
QLI66742.1	OTHER	0.000596	0.999404	
QLI66743.1	OTHER	0.001851	0.998149	
QLI66744.1	OTHER	0.002936	0.997064	
QLI66745.1	OTHER	0.000803	0.999197	
QLI66746.1	OTHER	0.001923	0.998077	
QLI66747.1	OTHER	0.000981	0.999019	
QLI66748.1	OTHER	0.001915	0.998085	
QLI66749.1	OTHER	0.001313	0.998687	
QLI66750.1	OTHER	0.000141	0.999859	
QLI66751.1	OTHER	0.000587	0.999413	
QLI66752.1	OTHER	0.000827	0.999173	
QLI66753.1	OTHER	0.000760	0.999240	
QLI66754.1	SP(Sec/SPI)	0.989072	0.010928	CS pos: 20-21. GNA-TP. Pr: 0.7846
QLI66755.1	OTHER	0.332131	0.667869	
QLI66756.1	OTHER	0.000419	0.999581	
QLI66757.1	OTHER	0.001940	0.998060	
QLI66758.1	OTHER	0.002704	0.997296	
QLI66759.1	SP(Sec/SPI)	0.982650	0.017350	CS pos: 17-18. AAS-LK. Pr: 0.2713
QLI66760.1	OTHER	0.001528	0.998472	
QLI66761.1	OTHER	0.000816	0.999184	
QLI66762.1	OTHER	0.001278	0.998722	
QLI66763.1	SP(Sec/SPI)	0.993054	0.006946	CS pos: 16-17. VSA-AP. Pr: 0.7578
QLI66764.1	OTHER	0.001400	0.998600	
QLI66765.1	OTHER	0.000414	0.999586	
QLI66766.1	OTHER	0.000874	0.999126	
QLI66767.1	OTHER	0.000243	0.999757	

QLI66768.1	OTHER	0.004685	0.995315	
QLI66769.1	OTHER	0.002797	0.997203	
QLI66770.1	OTHER	0.004505	0.995495	
QLI66771.1	OTHER	0.000495	0.999505	
QLI66772.1	OTHER	0.000896	0.999104	
QLI66773.1	OTHER	0.000699	0.999301	
QLI66774.1	OTHER	0.000862	0.999138	
QLI66775.1	OTHER	0.001682	0.998318	
QLI66776.1	OTHER	0.011537	0.988463	
QLI66777.1	OTHER	0.000358	0.999642	
QLI66778.1	OTHER	0.001372	0.998628	
QLI66779.1	SP(Sec/SPI)	0.920247	0.079753	CS pos: 18-19. ATA-NN. Pr: 0.6469
QLI66780.1	SP(Sec/SPI)	0.936972	0.063028	CS pos: 22-23. ASA-VA. Pr: 0.4849
QLI66781.1	SP(Sec/SPI)	0.984286	0.015714	CS pos: 22-23. SLA-TQ. Pr: 0.7943
QLI66782.1	OTHER	0.000850	0.999150	
QLI66783.1	OTHER	0.001111	0.998889	
QLI66784.1	OTHER	0.093245	0.906755	
QLI66785.1	OTHER	0.005010	0.994990	
QLI66786.1	SP(Sec/SPI)	0.997839	0.002161	CS pos: 15-16. AAA-DV. Pr: 0.5881
QLI66787.1	OTHER	0.001396	0.998604	
QLI66788.1	OTHER	0.000482	0.999518	
QLI66789.1	OTHER	0.003105	0.996895	
QLI66790.1	OTHER	0.001401	0.998599	
QLI66791.1	OTHER	0.000383	0.999617	
QLI66792.1	OTHER	0.002487	0.997513	
QLI66793.1	OTHER	0.002078	0.997922	
QLI66794.1	OTHER	0.000404	0.999596	
QLI66795.1	OTHER	0.001578	0.998422	
QLI66796.1	SP(Sec/SPI)	0.803214	0.196786	CS pos: 19-20. ALA-MP. Pr: 0.2155
QLI66797.1	OTHER	0.001032	0.998968	
QLI66798.1	OTHER	0.001339	0.998661	

QLI66799.1	OTHER	0.000399	0.999601	
QLI66800.1	OTHER	0.001518	0.998482	
QLI66801.1	OTHER	0.001249	0.998751	
QLI66802.1	OTHER	0.001152	0.998848	
QLI66803.1	OTHER	0.241852	0.758148	
QLI66804.1	OTHER	0.001635	0.998365	
QLI66805.1	OTHER	0.000617	0.999383	
QLI66806.1	SP(Sec/SPI)	0.879732	0.120268	CS pos: 23-24. SLA-HS. Pr: 0.6267
QLI66807.1	OTHER	0.001041	0.998959	
QLI66808.1	OTHER	0.000976	0.999024	
QLI66809.1	OTHER	0.004233	0.995767	
QLI66810.1	OTHER	0.000544	0.999456	
QLI66811.1	OTHER	0.003163	0.996837	
QLI66812.1	OTHER	0.004401	0.995599	
QLI66813.1	OTHER	0.001079	0.998921	
QLI66814.1	OTHER	0.000324	0.999676	
QLI66815.1	OTHER	0.001143	0.998857	
QLI66816.1	OTHER	0.003018	0.996982	
QLI66817.1	OTHER	0.002087	0.997913	
QLI66818.1	SP(Sec/SPI)	0.998291	0.001709	CS pos: 16-17. AIA-AP. Pr: 0.5364
QLI66819.1	OTHER	0.003632	0.996368	
QLI66820.1	OTHER	0.001365	0.998635	
QLI66821.1	OTHER	0.001408	0.998592	
QLI66822.1	OTHER	0.001229	0.998771	
QLI66823.1	OTHER	0.000537	0.999463	
QLI66824.1	OTHER	0.001370	0.998630	
QLI66825.1	OTHER	0.001037	0.998963	
QLI66826.1	OTHER	0.015078	0.984922	
QLI66827.1	OTHER	0.009073	0.990927	
QLI66828.1	OTHER	0.000935	0.999065	
QLI66829.1	OTHER	0.000449	0.999551	

QLI66830.1	OTHER	0.003801	0.996199	
QLI66831.1	OTHER	0.000959	0.999041	
QLI66832.1	OTHER	0.000883	0.999117	
QLI66833.1	OTHER	0.000590	0.999410	
QLI66834.1	SP(Sec/SPI)	0.955740	0.044260	CS pos: 21-22. IAA-DD. Pr: 0.5109
QLI66835.1	OTHER	0.000671	0.999329	
QLI66836.1	OTHER	0.000494	0.999506	
QLI66837.1	SP(Sec/SPI)	0.552636	0.447364	CS pos: 22-23. SIA-QL. Pr: 0.3792
QLI66838.1	SP(Sec/SPI)	0.991475	0.008525	CS pos: 27-28. AFA-QN. Pr: 0.9227
QLI66839.1	OTHER	0.000541	0.999459	
QLI66840.1	OTHER	0.002991	0.997009	
QLI66841.1	OTHER	0.000592	0.999408	
QLI66842.1	SP(Sec/SPI)	0.992051	0.007949	CS pos: 17-18. AST-AP. Pr: 0.4679
QLI66843.1	OTHER	0.001548	0.998452	
QLI66844.1	SP(Sec/SPI)	0.623437	0.376563	CS pos: 22-23. AQS-KL. Pr: 0.5205
QLI66845.1	OTHER	0.006399	0.993601	
QLI66846.1	OTHER	0.001441	0.998559	
QLI66847.1	OTHER	0.000556	0.999444	
QLI66848.1	OTHER	0.001364	0.998636	
QLI66849.1	OTHER	0.000128	0.999872	
QLI66850.1	OTHER	0.000908	0.999092	
QLI66851.1	OTHER	0.001959	0.998041	
QLI66852.1	OTHER	0.000606	0.999394	
QLI66853.1	OTHER	0.005136	0.994864	
QLI66854.1	OTHER	0.002792	0.997208	
QLI66855.1	SP(Sec/SPI)	0.996579	0.003421	CS pos: 17-18. TVA-AP. Pr: 0.4730
QLI66856.1	OTHER	0.000863	0.999137	
QLI66857.1	OTHER	0.000698	0.999302	
QLI66858.1	OTHER	0.019828	0.980172	
QLI66859.1	OTHER	0.000433	0.999567	
QLI66860.1	OTHER	0.019738	0.980262	

QLI66861.1	SP(Sec/SPI)	0.998505	0.001495	CS pos: 17-18. VSA-QS. Pr: 0.6429
QLI66862.1	OTHER	0.001098	0.998902	
QLI66863.1	OTHER	0.000688	0.999312	
QLI66864.1	OTHER	0.000252	0.999748	
QLI66865.1	OTHER	0.041152	0.958848	
QLI66866.1	OTHER	0.001466	0.998534	
QLI66867.1	SP(Sec/SPI)	0.988428	0.011572	CS pos: 25-26. ASA-QG. Pr: 0.3904
QLI66868.1	OTHER	0.009022	0.990978	
QLI66869.1	OTHER	0.002515	0.997485	
QLI66870.1	OTHER	0.000806	0.999194	
QLI66871.1	SP(Sec/SPI)	0.980278	0.019722	CS pos: 20-21. ASA-AT. Pr: 0.8575
QLI66872.1	OTHER	0.001234	0.998766	
QLI66873.1	OTHER	0.001359	0.998641	
QLI66874.1	SP(Sec/SPI)	0.962766	0.037234	CS pos: 19-20. TQA-MS. Pr: 0.4336
QLI66875.1	OTHER	0.297599	0.702401	
QLI66876.1	OTHER	0.000852	0.999148	
QLI66877.1	OTHER	0.000572	0.999428	
QLI66878.1	OTHER	0.138880	0.861120	
QLI66879.1	OTHER	0.007741	0.992259	
QLI66880.1	OTHER	0.002683	0.997317	
QLI66881.1	OTHER	0.001177	0.998823	
QLI66882.1	OTHER	0.001228	0.998772	
QLI66883.1	OTHER	0.000639	0.999361	
QLI66884.1	OTHER	0.000999	0.999001	
QLI66885.1	SP(Sec/SPI)	0.913184	0.086816	CS pos: 20-21. VEA-RN. Pr: 0.8744
QLI66886.1	OTHER	0.000845	0.999155	
QLI66887.1	OTHER	0.000374	0.999626	
QLI66888.1	SP(Sec/SPI)	0.909057	0.090943	CS pos: 20-21. AYA-WG. Pr: 0.7134
QLI66889.1	OTHER	0.000658	0.999342	
QLI66890.1	OTHER	0.002004	0.997996	
QLI66891.1	OTHER	0.037919	0.962081	

QLI66892.1	OTHER	0.003476	0.996524	
QLI66893.1	OTHER	0.012069	0.987931	
QLI66894.1	OTHER	0.000716	0.999284	
QLI66895.1	OTHER	0.002103	0.997897	
QLI66896.1	OTHER	0.000335	0.999665	
QLI66897.1	OTHER	0.000608	0.999392	
QLI66898.1	OTHER	0.000357	0.999643	
QLI66899.1	OTHER	0.003367	0.996633	
QLI66900.1	OTHER	0.000751	0.999249	
QLI66901.1	OTHER	0.000948	0.999052	
QLI66902.1	OTHER	0.003754	0.996246	
QLI66903.1	OTHER	0.001675	0.998325	
QLI66904.1	OTHER	0.018195	0.981805	
QLI66905.1	OTHER	0.000766	0.999234	
QLI66906.1	OTHER	0.010029	0.989971	
QLI66907.1	OTHER	0.005260	0.994740	
QLI66908.1	OTHER	0.008710	0.991290	
QLI66909.1	OTHER	0.000470	0.999530	
QLI66910.1	OTHER	0.000517	0.999483	
QLI66911.1	OTHER	0.002302	0.997698	
QLI66912.1	OTHER	0.003586	0.996414	
QLI66913.1	OTHER	0.002172	0.997828	
QLI66914.1	OTHER	0.000412	0.999588	
QLI66915.1	OTHER	0.000424	0.999576	
QLI66916.1	OTHER	0.000462	0.999538	
QLI66917.1	OTHER	0.003969	0.996031	
QLI66918.1	OTHER	0.002050	0.997950	
QLI66919.1	OTHER	0.000821	0.999179	
QLI66920.1	OTHER	0.000867	0.999133	
QLI66921.1	OTHER	0.001428	0.998572	
QLI66922.1	SP(Sec/SPI)	0.784240	0.215760	CS pos: 18-19. AVA-DP. Pr: 0.7238

QLI66923.1	OTHER	0.000291	0.999709	
QLI66924.1	OTHER	0.000835	0.999165	
QLI66925.1	OTHER	0.003342	0.996658	
QLI66926.1	OTHER	0.023138	0.976862	
QLI66927.1	SP(Sec/SPI)	0.924787	0.075213	CS pos: 21-22. IDS-AV. Pr: 0.4386
QLI66928.1	OTHER	0.001086	0.998914	
QLI66929.1	SP(Sec/SPI)	0.990266	0.009734	CS pos: 21-22. ACA-TT. Pr: 0.7898
QLI66930.1	OTHER	0.000918	0.999082	
QLI66931.1	OTHER	0.000435	0.999565	
QLI66932.1	OTHER	0.001871	0.998129	
QLI66933.1	OTHER	0.002915	0.997085	
QLI66934.1	SP(Sec/SPI)	0.967871	0.032129	CS pos: 19-20. ASG-KP. Pr: 0.8760
QLI66935.1	OTHER	0.000348	0.999652	
QLI66936.1	OTHER	0.002751	0.997249	
QLI66937.1	OTHER	0.000426	0.999574	
QLI66938.1	OTHER	0.001561	0.998439	
QLI66939.1	OTHER	0.005073	0.994927	
QLI66940.1	OTHER	0.000878	0.999122	
QLI66941.1	OTHER	0.000280	0.999720	
QLI66942.1	SP(Sec/SPI)	0.935702	0.064298	CS pos: 26-27. AHS-GP. Pr: 0.8623
QLI66943.1	OTHER	0.001194	0.998806	
QLI66944.1	OTHER	0.000689	0.999311	
QLI66945.1	SP(Sec/SPI)	0.926211	0.073789	CS pos: 16-17. AMA-MF. Pr: 0.6426
QLI66946.1	OTHER	0.037827	0.962173	
QLI66947.1	OTHER	0.007386	0.992614	
QLI66948.1	SP(Sec/SPI)	0.649655	0.350345	CS pos: 21-22. ASA-LS. Pr: 0.5763
QLI66949.1	OTHER	0.004442	0.995558	
QLI66950.1	SP(Sec/SPI)	0.755107	0.244893	CS pos: 20-21. SHG-LR. Pr: 0.4765
QLI66951.1	OTHER	0.075073	0.924927	
QLI66952.1	OTHER	0.000817	0.999183	
QLI66953.1	SP(Sec/SPI)	0.989983	0.010017	CS pos: 21-22. ALS-VP. Pr: 0.6476

QLI66954.1	OTHER	0.002648	0.997352	
QLI66955.1	OTHER	0.001372	0.998628	
QLI66956.1	OTHER	0.001327	0.998673	
QLI66957.1	SP(Sec/SPI)	0.999577	0.000423	CS pos: 17-18. AAA-WP. Pr: 0.5055
QLI66958.1	OTHER	0.000797	0.999203	
QLI66959.1	OTHER	0.000491	0.999509	
QLI66960.1	OTHER	0.001162	0.998838	
QLI66961.1	OTHER	0.001698	0.998302	
QLI66962.1	OTHER	0.000229	0.999771	
QLI66963.1	SP(Sec/SPI)	0.913783	0.086217	CS pos: 16-17. ASA-LP. Pr: 0.6404
QLI66964.1	OTHER	0.000586	0.999414	
QLI66965.1	OTHER	0.001177	0.998823	
QLI66966.1	OTHER	0.001345	0.998655	
QLI66967.1	OTHER	0.000508	0.999492	
QLI66968.1	OTHER	0.002365	0.997635	
QLI66969.1	OTHER	0.001397	0.998603	
QLI66970.1	OTHER	0.000439	0.999561	
QLI66971.1	OTHER	0.000396	0.999604	
QLI66972.1	SP(Sec/SPI)	0.970454	0.029546	CS pos: 18-19. IAA-TP. Pr: 0.6805
QLI66973.1	OTHER	0.001073	0.998927	
QLI66974.1	OTHER	0.007356	0.992644	
QLI66975.1	OTHER	0.000733	0.999267	
QLI66976.1	OTHER	0.003544	0.996456	
QLI66977.1	OTHER	0.000843	0.999157	
QLI66978.1	OTHER	0.003248	0.996752	
QLI66979.1	OTHER	0.000713	0.999287	
QLI66980.1	OTHER	0.001845	0.998155	
QLI66981.1	OTHER	0.001604	0.998396	
QLI66982.1	OTHER	0.000856	0.999144	
QLI66983.1	OTHER	0.000806	0.999194	
QLI66984.1	OTHER	0.001503	0.998497	

QLI66985.1	OTHER	0.001081	0.998919	
QLI66986.1	OTHER	0.001284	0.998716	
QLI66987.1	OTHER	0.000485	0.999515	
QLI66988.1	OTHER	0.000760	0.999240	
QLI66989.1	OTHER	0.006297	0.993703	
QLI66990.1	OTHER	0.001296	0.998704	
QLI66991.1	OTHER	0.003414	0.996586	
QLI66992.1	OTHER	0.003288	0.996712	
QLI66993.1	OTHER	0.000413	0.999587	
QLI66994.1	OTHER	0.000477	0.999523	
QLI66995.1	OTHER	0.001211	0.998789	
QLI66996.1	OTHER	0.001288	0.998712	
QLI66997.1	OTHER	0.000379	0.999621	
QLI66998.1	OTHER	0.071500	0.928500	
QLI66999.1	OTHER	0.000309	0.999691	
QLI67000.1	OTHER	0.330559	0.669441	
QLI67001.1	OTHER	0.000663	0.999337	
QLI67002.1	OTHER	0.000750	0.999250	
QLI67003.1	SP(Sec/SPI)	0.933253	0.066747	CS pos: 19-20. SRA-AP. Pr: 0.7665
QLI67004.1	OTHER	0.020492	0.979508	
QLI67005.1	OTHER	0.002830	0.997170	
QLI67006.1	OTHER	0.000827	0.999173	
QLI67007.1	OTHER	0.001090	0.998910	
QLI67008.1	OTHER	0.001344	0.998656	
QLI67009.1	OTHER	0.001014	0.998986	
QLI67010.1	OTHER	0.001185	0.998815	
QLI67011.1	OTHER	0.000943	0.999057	
QLI67012.1	OTHER	0.000416	0.999584	
QLI67013.1	OTHER	0.002162	0.997838	
QLI67014.1	OTHER	0.008268	0.991732	
QLI67015.1	OTHER	0.003900	0.996100	

QLI67016.1	OTHER	0.001080	0.998920	
QLI67017.1	SP(Sec/SPI)	0.972430	0.027570	CS pos: 24-25. VAA-AV. Pr: 0.6176
QLI67018.1	OTHER	0.009799	0.990201	
QLI67019.1	OTHER	0.000967	0.999033	
QLI67020.1	OTHER	0.000901	0.999099	
QLI67021.1	OTHER	0.000876	0.999124	
QLI67022.1	OTHER	0.000659	0.999341	
QLI67023.1	OTHER	0.005566	0.994434	
QLI67024.1	OTHER	0.003485	0.996515	
QLI67025.1	OTHER	0.000905	0.999095	
QLI67026.1	OTHER	0.000515	0.999485	
QLI67027.1	OTHER	0.002140	0.997860	
QLI67028.1	OTHER	0.011063	0.988937	
QLI67029.1	OTHER	0.001545	0.998455	
QLI67030.1	SP(Sec/SPI)	0.997881	0.002119	CS pos: 17-18. VLA-AP. Pr: 0.8122
QLI67031.1	SP(Sec/SPI)	0.994179	0.005821	CS pos: 17-18. ALA-MP. Pr: 0.8596
QLI67032.1	OTHER	0.001334	0.998666	
QLI67033.1	OTHER	0.002125	0.997875	
QLI67034.1	OTHER	0.000875	0.999125	
QLI67035.1	OTHER	0.000409	0.999591	
QLI67036.1	OTHER	0.001332	0.998668	
QLI67037.1	OTHER	0.000930	0.999070	
QLI67038.1	OTHER	0.002416	0.997584	
QLI67039.1	OTHER	0.001436	0.998564	
QLI67040.1	OTHER	0.000629	0.999371	
QLI67041.1	OTHER	0.023367	0.976633	
QLI67042.1	OTHER	0.009663	0.990337	
QLI67043.1	OTHER	0.001465	0.998535	
QLI67044.1	OTHER	0.002428	0.997572	
QLI67045.1	OTHER	0.002118	0.997882	
QLI67046.1	OTHER	0.007677	0.992323	

QLI67047.1	OTHER	0.000469	0.999531	
QLI67048.1	OTHER	0.001572	0.998428	
QLI67049.1	OTHER	0.000631	0.999369	
QLI67050.1	OTHER	0.000253	0.999747	
QLI67051.1	OTHER	0.010354	0.989646	
QLI67052.1	OTHER	0.003659	0.996341	
QLI67053.1	OTHER	0.001877	0.998123	
QLI67054.1	OTHER	0.004250	0.995750	
QLI67055.1	OTHER	0.000269	0.999731	
QLI67056.1	OTHER	0.001214	0.998786	
QLI67057.1	OTHER	0.000629	0.999371	
QLI67058.1	OTHER	0.005072	0.994928	
QLI67059.1	OTHER	0.001573	0.998427	
QLI67060.1	OTHER	0.002029	0.997971	
QLI67061.1	OTHER	0.008573	0.991427	
QLI67062.1	OTHER	0.008165	0.991835	
QLI67063.1	OTHER	0.002086	0.997914	
QLI67064.1	OTHER	0.001041	0.998959	
QLI67065.1	OTHER	0.001361	0.998639	
QLI67066.1	OTHER	0.000541	0.999459	
QLI67067.1	OTHER	0.001285	0.998715	
QLI67068.1	OTHER	0.033035	0.966965	
QLI67069.1	OTHER	0.000633	0.999367	
QLI67070.1	SP(Sec/SPI)	0.784781	0.215219	CS pos: 17-18. ALT-AS. Pr: 0.2448
QLI67071.1	OTHER	0.005619	0.994381	
QLI67072.1	OTHER	0.003323	0.996677	
QLI67073.1	OTHER	0.004968	0.995032	
QLI67074.1	OTHER	0.000462	0.999538	
QLI67075.1	SP(Sec/SPI)	0.995560	0.004440	CS pos: 17-18. VAA-AP. Pr: 0.7176
QLI67076.1	OTHER	0.000641	0.999359	
QLI67077.1	SP(Sec/SPI)	0.961839	0.038161	CS pos: 21-22. AHG-NQ. Pr: 0.3317

QLI67078.1	OTHER	0.001300	0.998700	
QLI67079.1	OTHER	0.000684	0.999316	
QLI67080.1	OTHER	0.002267	0.997733	
QLI67081.1	OTHER	0.000514	0.999486	
QLI67082.1	OTHER	0.000266	0.999734	
QLI67083.1	OTHER	0.012996	0.987004	
QLI67084.1	SP(Sec/SPI)	0.998783	0.001217	CS pos: 17-18. VMA-AP. Pr: 0.9265
QLI67085.1	OTHER	0.002892	0.997108	
QLI67086.1	OTHER	0.010356	0.989644	
QLI67087.1	OTHER	0.002509	0.997491	
QLI67088.1	OTHER	0.004202	0.995798	
QLI67089.1	OTHER	0.003760	0.996240	
QLI67090.1	OTHER	0.015280	0.984720	
QLI67091.1	OTHER	0.032895	0.967105	
QLI67092.1	OTHER	0.001021	0.998979	
QLI67093.1	OTHER	0.000373	0.999627	
QLI67094.1	OTHER	0.001226	0.998774	
QLI67095.1	OTHER	0.005876	0.994124	
QLI67096.1	OTHER	0.000811	0.999189	
QLI67097.1	OTHER	0.000500	0.999500	
QLI67098.1	OTHER	0.002241	0.997759	
QLI67099.1	OTHER	0.001899	0.998101	
QLI67100.1	OTHER	0.001441	0.998559	
QLI67101.1	OTHER	0.001188	0.998812	
QLI67102.1	OTHER	0.000785	0.999215	
QLI67103.1	OTHER	0.002019	0.997981	
QLI67104.1	OTHER	0.000754	0.999246	
QLI67105.1	OTHER	0.001819	0.998181	
QLI67106.1	OTHER	0.000861	0.999139	
QLI67107.1	OTHER	0.003404	0.996596	
QLI67108.1	OTHER	0.001626	0.998374	

QLI67109.1	OTHER	0.000954	0.999046	
QLI67110.1	OTHER	0.003177	0.996823	
QLI67111.1	OTHER	0.002616	0.997384	
QLI67112.1	SP(Sec/SPI)	0.990681	0.009319	CS pos: 17-18. ALA-AP. Pr: 0.6821
QLI67113.1	OTHER	0.000338	0.999662	
QLI67114.1	OTHER	0.000870	0.999130	
QLI67115.1	OTHER	0.000236	0.999764	
QLI67116.1	OTHER	0.000839	0.999161	
QLI67117.1	OTHER	0.002291	0.997709	
QLI67118.1	OTHER	0.059430	0.940570	
QLI67119.1	OTHER	0.305002	0.694998	
QLI67120.1	OTHER	0.001628	0.998372	
QLI67121.1	OTHER	0.002928	0.997072	
QLI67122.1	OTHER	0.000837	0.999163	
QLI67123.1	OTHER	0.026054	0.973946	
QLI67124.1	OTHER	0.001065	0.998935	
QLI67125.1	OTHER	0.000860	0.999140	
QLI67126.1	SP(Sec/SPI)	0.972334	0.027666	CS pos: 19-20. SWA-AK. Pr: 0.7201
QLI67127.1	OTHER	0.000428	0.999572	
QLI67128.1	OTHER	0.001789	0.998211	
QLI67129.1	OTHER	0.003839	0.996161	
QLI67130.1	OTHER	0.002675	0.997325	
QLI67131.1	SP(Sec/SPI)	0.973408	0.026592	CS pos: 16-17. IIA-NN. Pr: 0.6540
QLI67132.1	OTHER	0.002011	0.997989	
QLI67133.1	OTHER	0.000591	0.999409	
QLI67134.1	OTHER	0.001062	0.998938	
QLI67135.1	OTHER	0.005009	0.994991	
QLI67136.1	OTHER	0.002188	0.997812	
QLI67137.1	OTHER	0.001496	0.998504	
QLI67138.1	SP(Sec/SPI)	0.996461	0.003539	CS pos: 23-24. ALA-KC. Pr: 0.3327
QLI67139.1	OTHER	0.003398	0.996602	

QLI67140.1	OTHER	0.001328	0.998672	
QLI67141.1	OTHER	0.000798	0.999202	
QLI67142.1	OTHER	0.000732	0.999268	
QLI67143.1	OTHER	0.001709	0.998291	
QLI67144.1	OTHER	0.002967	0.997033	
QLI67145.1	OTHER	0.000160	0.999840	
QLI67146.1	OTHER	0.000390	0.999610	
QLI67147.1	OTHER	0.000769	0.999231	
QLI67148.1	OTHER	0.000519	0.999481	
QLI67149.1	OTHER	0.001260	0.998740	
QLI67150.1	OTHER	0.001485	0.998515	
QLI67151.1	OTHER	0.000323	0.999677	
QLI67152.1	OTHER	0.001358	0.998642	
QLI67153.1	OTHER	0.002162	0.997838	
QLI67154.1	SP(Sec/SPI)	0.784542	0.215458	CS pos: 23-24. ACA-SS. Pr: 0.4621
QLI67155.1	SP(Sec/SPI)	0.976707	0.023293	CS pos: 18-19. VLG-AP. Pr: 0.8664
QLI67156.1	OTHER	0.001118	0.998882	
QLI67157.1	OTHER	0.000387	0.999613	
QLI67158.1	OTHER	0.001404	0.998596	
QLI67159.1	OTHER	0.000862	0.999138	
QLI67160.1	OTHER	0.000502	0.999498	
QLI67161.1	OTHER	0.000399	0.999601	
QLI67162.1	OTHER	0.000725	0.999275	
QLI67163.1	SP(Sec/SPI)	0.987097	0.012903	CS pos: 21-22. VSS-VD. Pr: 0.5363
QLI67164.1	OTHER	0.213579	0.786421	
QLI67165.1	OTHER	0.001200	0.998800	
QLI67166.1	OTHER	0.000628	0.999372	
QLI67167.1	OTHER	0.000527	0.999473	
QLI67168.1	OTHER	0.000694	0.999306	
QLI67169.1	OTHER	0.000747	0.999253	
QLI67170.1	OTHER	0.004248	0.995752	

QLI67171.1	OTHER	0.000783	0.999217	
QLI67172.1	OTHER	0.000472	0.999528	
QLI67173.1	OTHER	0.030087	0.969913	
QLI67174.1	SP(Sec/SPI)	0.993488	0.006512	CS pos: 16-17. VAA-DK. Pr: 0.8804
QLI67175.1	OTHER	0.002329	0.997671	
QLI67176.1	OTHER	0.023906	0.976094	
QLI67177.1	OTHER	0.001664	0.998336	
QLI67178.1	OTHER	0.001059	0.998941	
QLI67179.1	OTHER	0.023181	0.976819	
QLI67180.1	OTHER	0.001373	0.998627	
QLI67181.1	SP(Sec/SPI)	0.852820	0.147180	CS pos: 20-21. GSA-HM. Pr: 0.4898
QLI67182.1	OTHER	0.001451	0.998549	
QLI67183.1	OTHER	0.000185	0.999815	
QLI67184.1	OTHER	0.000617	0.999383	
QLI67185.1	SP(Sec/SPI)	0.615927	0.384073	CS pos: 28-29. TSA-MG. Pr: 0.3948
QLI67186.1	OTHER	0.011980	0.988020	
QLI67187.1	OTHER	0.001093	0.998907	
QLI67188.1	OTHER	0.000449	0.999551	
QLI67189.1	OTHER	0.000452	0.999548	
QLI67190.1	OTHER	0.001411	0.998589	
QLI67191.1	OTHER	0.001063	0.998937	
QLI67192.1	OTHER	0.001346	0.998654	
QLI67193.1	OTHER	0.001387	0.998613	
QLI67194.1	OTHER	0.149495	0.850505	
QLI67195.1	SP(Sec/SPI)	0.978879	0.021121	CS pos: 18-19. AVA-AV. Pr: 0.4029
QLI67196.1	OTHER	0.001553	0.998447	
QLI67197.1	SP(Sec/SPI)	0.994591	0.005409	CS pos: 19-20. AMA-LP. Pr: 0.7765
QLI67198.1	OTHER	0.001161	0.998839	
QLI67199.1	SP(Sec/SPI)	0.949247	0.050753	CS pos: 17-18. VAA-AP. Pr: 0.6568
QLI67200.1	OTHER	0.001693	0.998307	
QLI67201.1	OTHER	0.020364	0.979636	

QLI67202.1	OTHER	0.000611	0.999389	
QLI67203.1	OTHER	0.000927	0.999073	
QLI67204.1	OTHER	0.002664	0.997336	
QLI67205.1	SP(Sec/SPI)	0.755106	0.244894	CS pos: 19-20. VHG-WP. Pr: 0.6907
QLI67206.1	OTHER	0.000793	0.999207	
QLI67207.1	OTHER	0.002902	0.997098	
QLI67208.1	SP(Sec/SPI)	0.995131	0.004869	CS pos: 16-17. AAA-HT. Pr: 0.5688
QLI67209.1	OTHER	0.000911	0.999089	
QLI67210.1	OTHER	0.000316	0.999684	
QLI67211.1	SP(Sec/SPI)	0.993142	0.006858	CS pos: 19-20. VGA-SL. Pr: 0.5985
QLI67212.1	OTHER	0.000325	0.999675	
QLI67213.1	OTHER	0.001622	0.998378	
QLI67214.1	SP(Sec/SPI)	0.625023	0.374977	CS pos: 19-20. STA-ER. Pr: 0.4550
QLI67215.1	OTHER	0.000498	0.999502	
QLI67216.1	OTHER	0.000515	0.999485	
QLI67217.1	OTHER	0.002638	0.997362	
QLI67218.1	SP(Sec/SPI)	0.977192	0.022808	CS pos: 19-20. VAG-TK. Pr: 0.3182
QLI67219.1	OTHER	0.001587	0.998413	
QLI67220.1	SP(Sec/SPI)	0.998832	0.001168	CS pos: 22-23. CVA-DA. Pr: 0.5706
QLI67221.1	OTHER	0.000626	0.999374	
QLI67222.1	OTHER	0.006945	0.993055	
QLI67223.1	OTHER	0.000516	0.999484	
QLI67224.1	OTHER	0.000836	0.999164	
QLI67225.1	SP(Sec/SPI)	0.931749	0.068251	CS pos: 16-17. AAA-IP. Pr: 0.7713
QLI67226.1	SP(Sec/SPI)	0.912530	0.087470	CS pos: 20-21. VQA-VP. Pr: 0.8929
QLI67227.1	OTHER	0.000542	0.999458	
QLI67228.1	OTHER	0.001108	0.998892	
QLI67229.1	OTHER	0.000418	0.999582	
QLI67230.1	OTHER	0.002121	0.997879	
QLI67231.1	OTHER	0.000235	0.999765	
QLI67232.1	OTHER	0.005549	0.994451	

QLI67233.1	OTHER	0.006954	0.993046	
QLI67234.1	OTHER	0.000275	0.999725	
QLI67235.1	OTHER	0.008170	0.991830	
QLI67236.1	OTHER	0.002554	0.997446	
QLI67237.1	OTHER	0.003029	0.996971	
QLI67238.1	OTHER	0.000977	0.999023	
QLI67239.1	OTHER	0.001155	0.998845	
QLI67240.1	OTHER	0.000300	0.999700	
QLI67241.1	OTHER	0.006287	0.993713	
QLI67242.1	OTHER	0.000299	0.999701	
QLI67243.1	OTHER	0.001827	0.998173	
QLI67244.1	OTHER	0.001888	0.998112	
QLI67245.1	OTHER	0.000263	0.999737	
QLI67246.1	OTHER	0.108140	0.891860	
QLI67247.1	OTHER	0.001605	0.998395	
QLI67248.1	OTHER	0.002227	0.997773	
QLI67249.1	OTHER	0.001126	0.998874	
QLI67250.1	OTHER	0.000943	0.999057	
QLI67251.1	OTHER	0.005031	0.994969	
QLI67252.1	OTHER	0.000770	0.999230	
QLI67253.1	OTHER	0.000247	0.999753	
QLI67254.1	OTHER	0.001432	0.998568	
QLI67255.1	OTHER	0.001691	0.998309	
QLI67256.1	OTHER	0.009329	0.990671	
QLI67257.1	OTHER	0.001499	0.998501	
QLI67258.1	OTHER	0.001544	0.998456	
QLI67259.1	SP(Sec/SPI)	0.876744	0.123256	CS pos: 17-18. VIS-AP. Pr: 0.2938
QLI67260.1	OTHER	0.182660	0.817340	
QLI67261.1	OTHER	0.000938	0.999062	
QLI67262.1	OTHER	0.002292	0.997708	
QLI67263.1	OTHER	0.008679	0.991321	

QLI67264.1	OTHER	0.014594	0.985406	
QLI67265.1	SP(Sec/SPI)	0.992933	0.007067	CS pos: 18-19. ALA-GT. Pr: 0.4782
QLI67266.1	OTHER	0.001467	0.998533	
QLI67267.1	OTHER	0.056454	0.943546	
QLI67268.1	OTHER	0.000512	0.999488	
QLI67269.1	OTHER	0.000348	0.999652	
QLI67270.1	OTHER	0.007464	0.992536	
QLI67271.1	SP(Sec/SPI)	0.801118	0.198882	CS pos: 18-19. VLA-HP. Pr: 0.6158
QLI67272.1	OTHER	0.001783	0.998217	
QLI67273.1	OTHER	0.000813	0.999187	
QLI67274.1	OTHER	0.002498	0.997502	
QLI67275.1	OTHER	0.000828	0.999172	
QLI67276.1	OTHER	0.001168	0.998832	
QLI67277.1	OTHER	0.000384	0.999616	
QLI67278.1	OTHER	0.000733	0.999267	
QLI67279.1	OTHER	0.002661	0.997339	
QLI67280.1	OTHER	0.002531	0.997469	
QLI67281.1	OTHER	0.004206	0.995794	
QLI67282.1	OTHER	0.002921	0.997079	
QLI67283.1	OTHER	0.006272	0.993728	
QLI67284.1	OTHER	0.002080	0.997920	
QLI67285.1	OTHER	0.000564	0.999436	
QLI67286.1	OTHER	0.001765	0.998235	
QLI67287.1	SP(Sec/SPI)	0.985994	0.014006	CS pos: 21-22. CNA-RA. Pr: 0.7387
QLI67288.1	OTHER	0.000454	0.999546	
QLI67289.1	OTHER	0.004709	0.995291	
QLI67290.1	OTHER	0.002434	0.997566	
QLI67291.1	SP(Sec/SPI)	0.977643	0.022357	CS pos: 23-24. AAA-AS. Pr: 0.2404
QLI67292.1	OTHER	0.000614	0.999386	
QLI67293.1	OTHER	0.001816	0.998184	
QLI67294.1	OTHER	0.001427	0.998573	

QLI67295.1	OTHER	0.000595	0.999405	
QLI67296.1	OTHER	0.000716	0.999284	
QLI67297.1	OTHER	0.000783	0.999217	
QLI67298.1	OTHER	0.002956	0.997044	
QLI67299.1	SP(Sec/SPI)	0.884731	0.115269	CS pos: 21-22. VNA-LR. Pr: 0.5374
QLI67300.1	OTHER	0.000367	0.999633	
QLI67301.1	OTHER	0.000681	0.999319	
QLI67302.1	SP(Sec/SPI)	0.999912	0.000088	CS pos: 18-19. VQA-DD. Pr: 0.9901
QLI67303.1	OTHER	0.000535	0.999465	
QLI67304.1	OTHER	0.001011	0.998989	
QLI67305.1	OTHER	0.001405	0.998595	
QLI67306.1	OTHER	0.002991	0.997009	
QLI67307.1	OTHER	0.001266	0.998734	
QLI67308.1	OTHER	0.002645	0.997355	
QLI67309.1	OTHER	0.000423	0.999577	
QLI67310.1	OTHER	0.002236	0.997764	
QLI67311.1	OTHER	0.022793	0.977207	
QLI67312.1	OTHER	0.006194	0.993806	
QLI67313.1	OTHER	0.005896	0.994104	
QLI67314.1	OTHER	0.004835	0.995165	
QLI67315.1	OTHER	0.000740	0.999260	
QLI67316.1	OTHER	0.000583	0.999417	
QLI67317.1	OTHER	0.430099	0.569901	
QLI67318.1	OTHER	0.000895	0.999105	
QLI67319.1	SP(Sec/SPI)	0.997113	0.002887	CS pos: 24-25. VTG-QK. Pr: 0.5360
QLI67320.1	OTHER	0.000677	0.999323	
QLI67321.1	OTHER	0.000212	0.999788	
QLI67322.1	OTHER	0.002874	0.997126	
QLI67323.1	OTHER	0.000625	0.999375	
QLI67324.1	OTHER	0.001019	0.998981	
QLI67325.1	OTHER	0.003229	0.996771	

QLI67326.1	OTHER	0.001361	0.998639	
QLI67327.1	OTHER	0.000287	0.999713	
QLI67328.1	OTHER	0.000811	0.999189	
QLI67329.1	OTHER	0.000697	0.999303	
QLI67330.1	OTHER	0.110012	0.889988	
QLI67331.1	OTHER	0.014556	0.985444	
QLI67332.1	OTHER	0.003097	0.996903	
QLI67333.1	OTHER	0.000714	0.999286	
QLI67334.1	OTHER	0.000634	0.999366	
QLI67335.1	OTHER	0.000178	0.999822	
QLI67336.1	OTHER	0.001427	0.998573	
QLI67337.1	OTHER	0.001426	0.998574	
QLI67338.1	OTHER	0.000229	0.999771	
QLI67339.1	OTHER	0.001590	0.998410	
QLI67340.1	SP(Sec/SPI)	0.966748	0.033252	CS pos: 19-20. GQA-AF. Pr: 0.6313
QLI67341.1	SP(Sec/SPI)	0.801006	0.198994	CS pos: 19-20. CLS-AV. Pr: 0.4846
QLI67342.1	OTHER	0.003573	0.996427	
QLI67343.1	OTHER	0.000841	0.999159	
QLI67344.1	OTHER	0.001471	0.998529	
QLI67345.1	OTHER	0.001576	0.998424	
QLI67346.1	SP(Sec/SPI)	0.996900	0.003100	CS pos: 19-20. VQG-NF. Pr: 0.5701
QLI67347.1	OTHER	0.000482	0.999518	
QLI67348.1	OTHER	0.000735	0.999265	
QLI67349.1	OTHER	0.009413	0.990587	
QLI67350.1	SP(Sec/SPI)	0.992382	0.007618	CS pos: 17-18. ATA-QQ. Pr: 0.3519
QLI67351.1	OTHER	0.000804	0.999196	
QLI67352.1	OTHER	0.006354	0.993646	
QLI67353.1	OTHER	0.000918	0.999082	
QLI67354.1	OTHER	0.001667	0.998333	
QLI67355.1	OTHER	0.000958	0.999042	
QLI67356.1	SP(Sec/SPI)	0.859272	0.140728	CS pos: 25-26. ANA-EN. Pr: 0.3094

QLI67357.1	OTHER	0.000768	0.999232	
QLI67358.1	OTHER	0.002574	0.997426	
QLI67359.1	OTHER	0.001274	0.998726	
QLI67360.1	OTHER	0.000680	0.999320	
QLI67361.1	OTHER	0.001820	0.998180	
QLI67362.1	OTHER	0.497717	0.502283	
QLI67363.1	OTHER	0.001725	0.998275	
QLI67364.1	OTHER	0.002462	0.997538	
QLI67365.1	OTHER	0.002041	0.997959	
QLI67366.1	OTHER	0.001414	0.998586	
QLI67367.1	OTHER	0.001117	0.998883	
QLI67368.1	OTHER	0.000545	0.999455	
QLI67369.1	OTHER	0.007502	0.992498	
QLI67370.1	OTHER	0.001797	0.998203	
QLI67371.1	OTHER	0.004131	0.995869	
QLI67372.1	OTHER	0.000786	0.999214	
QLI67373.1	OTHER	0.000661	0.999339	
QLI67374.1	OTHER	0.002778	0.997222	
QLI67375.1	OTHER	0.001610	0.998390	
QLI67376.1	OTHER	0.001229	0.998771	
QLI67377.1	OTHER	0.001344	0.998656	
QLI67378.1	OTHER	0.001660	0.998340	
QLI67379.1	OTHER	0.000712	0.999288	
QLI67380.1	OTHER	0.000518	0.999482	
QLI67381.1	OTHER	0.000861	0.999139	
QLI67382.1	OTHER	0.000817	0.999183	
QLI67383.1	OTHER	0.003619	0.996381	
QLI67384.1	OTHER	0.000526	0.999474	
QLI67385.1	SP(Sec/SPI)	0.955831	0.044169	CS pos: 16-17. TLA-CP. Pr: 0.6949
QLI67386.1	OTHER	0.001816	0.998184	
QLI67387.1	OTHER	0.000380	0.999620	

QLI67388.1	OTHER	0.360546	0.639454	
QLI67389.1	OTHER	0.002012	0.997988	
QLI67390.1	OTHER	0.001442	0.998558	
QLI67391.1	OTHER	0.003475	0.996525	
QLI67392.1	OTHER	0.000401	0.999599	
QLI67393.1	OTHER	0.000444	0.999556	
QLI67394.1	OTHER	0.001355	0.998645	
QLI67395.1	OTHER	0.000453	0.999547	
QLI67396.1	OTHER	0.001649	0.998351	
QLI67397.1	OTHER	0.000954	0.999046	
QLI67398.1	OTHER	0.001312	0.998688	
QLI67399.1	SP(Sec/SPI)	0.952455	0.047545	CS pos: 17-18. VSG-HG. Pr: 0.7498
QLI67400.1	OTHER	0.004208	0.995792	
QLI67401.1	OTHER	0.000814	0.999186	
QLI67402.1	OTHER	0.004159	0.995841	
QLI67403.1	OTHER	0.000995	0.999005	
QLI67404.1	OTHER	0.001898	0.998102	
QLI67405.1	OTHER	0.000806	0.999194	
QLI67406.1	OTHER	0.000900	0.999100	
QLI67407.1	OTHER	0.001286	0.998714	
QLI67408.1	OTHER	0.000391	0.999609	
QLI67409.1	OTHER	0.000376	0.999624	
QLI67410.1	OTHER	0.002050	0.997950	
QLI67411.1	OTHER	0.004541	0.995459	
QLI67412.1	OTHER	0.000521	0.999479	
QLI67413.1	SP(Sec/SPI)	0.990807	0.009193	CS pos: 20-21. VIA-AP. Pr: 0.8180
QLI67414.1	OTHER	0.001972	0.998028	
QLI67415.1	OTHER	0.001306	0.998694	
QLI67416.1	OTHER	0.002554	0.997446	
QLI67417.1	OTHER	0.001176	0.998824	
QLI67418.1	OTHER	0.001758	0.998242	

QLI67419.1	OTHER	0.001573	0.998427	
QLI67420.1	OTHER	0.000931	0.999069	
QLI67421.1	OTHER	0.000899	0.999101	
QLI67422.1	OTHER	0.000392	0.999608	
QLI67423.1	OTHER	0.000103	0.999897	
QLI67424.1	OTHER	0.000761	0.999239	
QLI67425.1	OTHER	0.000654	0.999346	
QLI67426.1	OTHER	0.000869	0.999131	
QLI67427.1	OTHER	0.000499	0.999501	
QLI67428.1	OTHER	0.024360	0.975640	
QLI67429.1	OTHER	0.000393	0.999607	
QLI67430.1	SP(Sec/SPI)	0.854817	0.145183	CS pos: 17-18. AHA-GV. Pr: 0.8226
QLI67431.1	OTHER	0.459947	0.540053	
QLI67432.1	OTHER	0.001013	0.998987	
QLI67433.1	OTHER	0.000314	0.999686	
QLI67434.1	OTHER	0.000810	0.999190	
QLI67435.1	OTHER	0.001068	0.998932	
QLI67436.1	OTHER	0.000213	0.999787	
QLI67437.1	OTHER	0.001536	0.998464	
QLI67438.1	OTHER	0.013607	0.986393	
QLI67439.1	OTHER	0.000597	0.999403	
QLI67440.1	OTHER	0.000447	0.999553	
QLI67441.1	OTHER	0.000515	0.999485	
QLI67442.1	OTHER	0.000419	0.999581	
QLI67443.1	OTHER	0.003016	0.996984	
QLI67444.1	OTHER	0.001341	0.998659	
QLI67445.1	SP(Sec/SPI)	0.995458	0.004542	CS pos: 18-19. VFT-SP. Pr: 0.7044
QLI67446.1	OTHER	0.000647	0.999353	
QLI67447.1	OTHER	0.000228	0.999772	
QLI67448.1	OTHER	0.000799	0.999201	
QLI67449.1	OTHER	0.001393	0.998607	

QLI67450.1	OTHER	0.000726	0.999274	
QLI67451.1	OTHER	0.000283	0.999717	
QLI67452.1	OTHER	0.008193	0.991807	
QLI67453.1	OTHER	0.000321	0.999679	
QLI67454.1	OTHER	0.004477	0.995523	
QLI67455.1	OTHER	0.001301	0.998699	
QLI67456.1	OTHER	0.000206	0.999794	
QLI67457.1	OTHER	0.000919	0.999081	
QLI67458.1	OTHER	0.000508	0.999492	
QLI67459.1	OTHER	0.000746	0.999254	
QLI67460.1	OTHER	0.004579	0.995421	
QLI67461.1	OTHER	0.001110	0.998890	
QLI67462.1	OTHER	0.000600	0.999400	
QLI67463.1	OTHER	0.001627	0.998373	
QLI67464.1	OTHER	0.001716	0.998284	
QLI67465.1	OTHER	0.031093	0.968907	
QLI67466.1	OTHER	0.096867	0.903133	
QLI67467.1	OTHER	0.008081	0.991919	
QLI67468.1	OTHER	0.001260	0.998740	
QLI67469.1	OTHER	0.000492	0.999508	
QLI67470.1	OTHER	0.001945	0.998055	
QLI67471.1	OTHER	0.000565	0.999435	
QLI67472.1	OTHER	0.001382	0.998618	
QLI67473.1	OTHER	0.001308	0.998692	
QLI67474.1	OTHER	0.298409	0.701591	
QLI67475.1	OTHER	0.001060	0.998940	
QLI67476.1	OTHER	0.000424	0.999576	
QLI67477.1	SP(Sec/SPI)	0.934875	0.065125	CS pos: 18-19. VVA-DL. Pr: 0.8345
QLI67478.1	OTHER	0.001885	0.998115	
QLI67479.1	OTHER	0.005913	0.994087	
QLI67480.1	OTHER	0.000298	0.999702	

QLI67481.1	SP(Sec/SPI)	0.914152	0.085848	CS pos: 27-28. VAG-YP. Pr: 0.5731
QLI67482.1	OTHER	0.001745	0.998255	
QLI67483.1	OTHER	0.000717	0.999283	
QLI67484.1	OTHER	0.004558	0.995442	
QLI67485.1	OTHER	0.000763	0.999237	
QLI67486.1	SP(Sec/SPI)	0.935988	0.064012	CS pos: 33-34. VQA-DV. Pr: 0.5957
QLI67487.1	OTHER	0.001665	0.998335	
QLI67488.1	OTHER	0.000295	0.999705	
QLI67489.1	OTHER	0.003838	0.996162	
QLI67490.1	OTHER	0.000369	0.999631	
QLI67491.1	OTHER	0.000413	0.999587	
QLI67492.1	OTHER	0.000656	0.999344	
QLI67493.1	OTHER	0.001631	0.998369	
QLI67494.1	OTHER	0.003323	0.996677	
QLI67495.1	OTHER	0.003973	0.996027	
QLI67496.1	OTHER	0.000661	0.999339	
QLI67497.1	OTHER	0.004102	0.995898	
QLI67498.1	OTHER	0.001538	0.998462	
QLI67499.1	OTHER	0.002939	0.997061	
QLI67500.1	OTHER	0.021280	0.978720	
QLI67501.1	OTHER	0.458049	0.541951	
QLI67502.1	OTHER	0.000988	0.999012	
QLI67503.1	OTHER	0.000381	0.999619	
QLI67504.1	OTHER	0.122786	0.877214	
QLI67505.1	OTHER	0.001739	0.998261	
QLI67506.1	SP(Sec/SPI)	0.982528	0.017472	CS pos: 19-20. VAA-TK. Pr: 0.5400
QLI67507.1	OTHER	0.001160	0.998840	
QLI67508.1	OTHER	0.003528	0.996472	
QLI67509.1	OTHER	0.000489	0.999511	
QLI67510.1	OTHER	0.000479	0.999521	
QLI67511.1	SP(Sec/SPI)	0.981388	0.018612	CS pos: 17-18. ASA-GR. Pr: 0.8049

QLI67512.1	OTHER	0.002619	0.997381	
QLI67513.1	OTHER	0.000748	0.999252	
QLI67514.1	OTHER	0.002575	0.997425	
QLI67515.1	OTHER	0.001211	0.998789	
QLI67516.1	OTHER	0.000796	0.999204	
QLI67517.1	OTHER	0.005178	0.994822	
QLI67518.1	OTHER	0.001291	0.998709	
QLI67519.1	OTHER	0.000409	0.999591	
QLI67520.1	OTHER	0.003211	0.996789	
QLI67521.1	OTHER	0.020307	0.979693	
QLI67522.1	OTHER	0.053031	0.946969	
QLI67523.1	OTHER	0.000397	0.999603	
QLI67524.1	OTHER	0.000527	0.999473	
QLI67525.1	OTHER	0.000552	0.999448	
QLI67526.1	OTHER	0.001453	0.998547	
QLI67527.1	OTHER	0.000573	0.999427	
QLI67528.1	OTHER	0.001326	0.998674	
QLI67529.1	OTHER	0.303332	0.696668	
QLI67530.1	OTHER	0.000995	0.999005	
QLI67531.1	OTHER	0.002577	0.997423	
QLI67532.1	OTHER	0.002934	0.997066	
QLI67533.1	OTHER	0.000458	0.999542	
QLI67534.1	SP(Sec/SPI)	0.816184	0.183816	CS pos: 24-25. AKS-DS. Pr: 0.5722
QLI67535.1	OTHER	0.002247	0.997753	
QLI67536.1	OTHER	0.000242	0.999758	
QLI67537.1	OTHER	0.001469	0.998531	
QLI67538.1	OTHER	0.000948	0.999052	
QLI67539.1	OTHER	0.004614	0.995386	
QLI67540.1	OTHER	0.001561	0.998439	
QLI67541.1	OTHER	0.000331	0.999669	
QLI67542.1	SP(Sec/SPI)	0.997738	0.002262	CS pos: 19-20. VIA-EE. Pr: 0.9256

QLI67543.1	OTHER	0.001633	0.998367	
QLI67544.1	OTHER	0.002159	0.997841	
QLI67545.1	OTHER	0.000417	0.999583	
QLI67546.1	OTHER	0.000587	0.999413	
QLI67547.1	OTHER	0.078645	0.921355	
QLI67548.1	SP(Sec/SPI)	0.999827	0.000173	CS pos: 16-17. ALA-AP. Pr: 0.7591
QLI67549.1	OTHER	0.010998	0.989002	
QLI67550.1	OTHER	0.000477	0.999523	
QLI67551.1	OTHER	0.001454	0.998546	
QLI67552.1	SP(Sec/SPI)	0.993683	0.006317	CS pos: 22-23. VGS-SK. Pr: 0.3745
QLI67553.1	OTHER	0.001210	0.998790	
QLI67554.1	OTHER	0.001152	0.998848	
QLI67555.1	SP(Sec/SPI)	0.998672	0.001328	CS pos: 18-19. TMA-GY. Pr: 0.6700
QLI67556.1	OTHER	0.000298	0.999702	
QLI67557.1	OTHER	0.000821	0.999179	
QLI67558.1	OTHER	0.001026	0.998974	
QLI67559.1	OTHER	0.000779	0.999221	
QLI67560.1	OTHER	0.001338	0.998662	
QLI67561.1	OTHER	0.002559	0.997441	
QLI67562.1	OTHER	0.002120	0.997880	
QLI67563.1	SP(Sec/SPI)	0.993917	0.006083	CS pos: 17-18. AAA-LP. Pr: 0.4422
QLI67564.1	OTHER	0.002282	0.997718	
QLI67565.1	OTHER	0.000375	0.999625	
QLI67566.1	OTHER	0.000856	0.999144	
QLI67567.1	OTHER	0.254757	0.745243	
QLI67568.1	OTHER	0.000721	0.999279	
QLI67569.1	OTHER	0.001153	0.998847	
QLI67570.1	OTHER	0.000718	0.999282	
QLI67571.1	OTHER	0.000131	0.999869	
QLI67572.1	OTHER	0.000591	0.999409	
QLI67573.1	OTHER	0.000452	0.999548	

QLI67574.1	OTHER	0.002145	0.997855	
QLI67575.1	OTHER	0.000750	0.999250	
QLI67576.1	SP(Sec/SPI)	0.994451	0.005549	CS pos: 18-19. SLA-EN. Pr: 0.4996
QLI67577.1	OTHER	0.228433	0.771567	
QLI67578.1	OTHER	0.002230	0.997770	
QLI67579.1	OTHER	0.003230	0.996770	
QLI67580.1	OTHER	0.006750	0.993250	
QLI67581.1	OTHER	0.002675	0.997325	
QLI67582.1	OTHER	0.001746	0.998254	
QLI67583.1	OTHER	0.001728	0.998272	
QLI67584.1	OTHER	0.005516	0.994484	
QLI67585.1	OTHER	0.000416	0.999584	
QLI67586.1	OTHER	0.001198	0.998802	
QLI67587.1	OTHER	0.001034	0.998966	
QLI67588.1	OTHER	0.000672	0.999328	
QLI67589.1	OTHER	0.000426	0.999574	
QLI67590.1	OTHER	0.002218	0.997782	
QLI67591.1	OTHER	0.000955	0.999045	
QLI67592.1	OTHER	0.001265	0.998735	
QLI67593.1	OTHER	0.002327	0.997673	
QLI67594.1	OTHER	0.001099	0.998901	
QLI67595.1	OTHER	0.001393	0.998607	
QLI67596.1	OTHER	0.000378	0.999622	
QLI67597.1	OTHER	0.000224	0.999776	
QLI67598.1	OTHER	0.000944	0.999056	
QLI67599.1	OTHER	0.002479	0.997521	
QLI67600.1	OTHER	0.129141	0.870859	
QLI67601.1	OTHER	0.001680	0.998320	
QLI67602.1	OTHER	0.001299	0.998701	
QLI67603.1	OTHER	0.001139	0.998861	
QLI67604.1	OTHER	0.000787	0.999213	

QLI67605.1	OTHER	0.000394	0.999606
QLI67606.1	OTHER	0.000532	0.999468
QLI67607.1	OTHER	0.000751	0.999249
QLI67608.1	OTHER	0.004046	0.995954
QLI67609.1	OTHER	0.003875	0.996125
QLI67610.1	OTHER	0.003086	0.996914
QLI67611.1	OTHER	0.004930	0.995070
QLI67612.1	OTHER	0.000277	0.999723
QLI67613.1	OTHER	0.002473	0.997527
QLI67614.1	OTHER	0.000672	0.999328
QLI67615.1	OTHER	0.000759	0.999241
QLI67616.1	OTHER	0.000892	0.999108
QLI67617.1	OTHER	0.001627	0.998373
QLI67618.1	OTHER	0.000429	0.999571
QLI67619.1	OTHER	0.190417	0.809583
QLI67620.1	OTHER	0.001057	0.998943
QLI67621.1	OTHER	0.015304	0.984696
QLI67622.1	OTHER	0.006368	0.993632
QLI67623.1	OTHER	0.004600	0.995400
QLI67624.1	OTHER	0.000815	0.999185
QLI67625.1	OTHER	0.000347	0.999653
QLI67626.1	OTHER	0.000743	0.999257
QLI67627.1	OTHER	0.001633	0.998367
QLI67628.1	OTHER	0.361234	0.638766
QLI67629.1	OTHER	0.001674	0.998326
QLI67630.1	OTHER	0.001264	0.998736
QLI67631.1	OTHER	0.003721	0.996279
QLI67632.1	OTHER	0.008881	0.991119
QLI67633.1	OTHER	0.006777	0.993223
QLI67634.1	OTHER	0.000424	0.999576
QLI67635.1	OTHER	0.000247	0.999753

QLI67636.1	SP(Sec/SPI)	0.965795	0.034205	CS pos: 21-22. VRA-VL. Pr: 0.6970
QLI67637.1	SP(Sec/SPI)	0.967058	0.032942	CS pos: 19-20. AWA-LP. Pr: 0.8396
QLI67638.1	OTHER	0.003576	0.996424	
QLI67639.1	OTHER	0.000637	0.999363	
QLI67640.1	SP(Sec/SPI)	0.727874	0.272126	CS pos: 24-25. ATG-YD. Pr: 0.5275
QLI67641.1	OTHER	0.001924	0.998076	
QLI67642.1	OTHER	0.000762	0.999238	
QLI67643.1	OTHER	0.010521	0.989479	
QLI67644.1	OTHER	0.001577	0.998423	
QLI67645.1	OTHER	0.000824	0.999176	
QLI67646.1	OTHER	0.001486	0.998514	
QLI67647.1	OTHER	0.001137	0.998863	
QLI67648.1	OTHER	0.000433	0.999567	
QLI67649.1	OTHER	0.002543	0.997457	
QLI67650.1	OTHER	0.000373	0.999627	
QLI67651.1	OTHER	0.001143	0.998857	
QLI67652.1	OTHER	0.000546	0.999454	
QLI67653.1	OTHER	0.000559	0.999441	
QLI67654.1	OTHER	0.000130	0.999870	
QLI67655.1	OTHER	0.000575	0.999425	
QLI67656.1	OTHER	0.001395	0.998605	
QLI67657.1	OTHER	0.001981	0.998019	
QLI67658.1	OTHER	0.001307	0.998693	
QLI67659.1	OTHER	0.002968	0.997032	
QLI67660.1	OTHER	0.005717	0.994283	
QLI67661.1	OTHER	0.001488	0.998512	
QLI67662.1	OTHER	0.005137	0.994863	
QLI67663.1	OTHER	0.000386	0.999614	
QLI67664.1	OTHER	0.001052	0.998948	
QLI67665.1	OTHER	0.219307	0.780693	
QLI67666.1	OTHER	0.000884	0.999116	

QLI67667.1	OTHER	0.001846	0.998154	
QLI67668.1	OTHER	0.001371	0.998629	
QLI67669.1	OTHER	0.002708	0.997292	
QLI67670.1	OTHER	0.001942	0.998058	
QLI67671.1	OTHER	0.001522	0.998478	
QLI67672.1	OTHER	0.000443	0.999557	
QLI67673.1	OTHER	0.001083	0.998917	
QLI67674.1	OTHER	0.000523	0.999477	
QLI67675.1	OTHER	0.002979	0.997021	
QLI67676.1	SP(Sec/SPI)	0.994669	0.005331	CS pos: 19-20. GIA-AP. Pr: 0.7820
QLI67677.1	OTHER	0.000972	0.999028	
QLI67678.1	OTHER	0.000629	0.999371	
QLI67679.1	OTHER	0.001000	0.999000	
QLI67680.1	OTHER	0.003409	0.996591	
QLI67681.1	OTHER	0.002414	0.997586	
QLI67682.1	OTHER	0.000229	0.999771	
QLI67683.1	OTHER	0.000393	0.999607	
QLI67684.1	OTHER	0.000517	0.999483	
QLI67685.1	SP(Sec/SPI)	0.986221	0.013779	CS pos: 16-17. AIA-VP. Pr: 0.6954
QLI67686.1	OTHER	0.006108	0.993892	
QLI67687.1	OTHER	0.000428	0.999572	
QLI67688.1	OTHER	0.001982	0.998018	
QLI67689.1	OTHER	0.001277	0.998723	
QLI67690.1	OTHER	0.000705	0.999295	
QLI67691.1	OTHER	0.001324	0.998676	
QLI67692.1	OTHER	0.001995	0.998005	
QLI67693.1	SP(Sec/SPI)	0.790954	0.209046	CS pos: 15-16. AAA-QL. Pr: 0.3125
QLI67694.1	OTHER	0.000705	0.999295	
QLI67695.1	OTHER	0.001560	0.998440	
QLI67696.1	OTHER	0.034081	0.965919	
QLI67697.1	OTHER	0.002011	0.997989	

QLI67698.1	OTHER	0.001009	0.998991	
QLI67699.1	OTHER	0.000631	0.999369	
QLI67700.1	OTHER	0.000150	0.999850	
QLI67701.1	OTHER	0.000914	0.999086	
QLI67702.1	OTHER	0.001438	0.998562	
QLI67703.1	OTHER	0.049289	0.950711	
QLI67704.1	OTHER	0.012727	0.987273	
QLI67705.1	OTHER	0.001218	0.998782	
QLI67706.1	OTHER	0.000740	0.999260	
QLI67707.1	OTHER	0.000586	0.999414	
QLI67708.1	OTHER	0.002034	0.997966	
QLI67709.1	OTHER	0.000961	0.999039	
QLI67710.1	SP(Sec/SPI)	0.961521	0.038479	CS pos: 15-16. ASA-IP. Pr: 0.6434
QLI67711.1	OTHER	0.000435	0.999565	
QLI67712.1	OTHER	0.001176	0.998824	
QLI67713.1	OTHER	0.000700	0.999300	
QLI67714.1	OTHER	0.002229	0.997771	
QLI67715.1	OTHER	0.001253	0.998747	
QLI67716.1	OTHER	0.018499	0.981501	
QLI67717.1	OTHER	0.000732	0.999268	
QLI67718.1	OTHER	0.002357	0.997643	
QLI67719.1	OTHER	0.005611	0.994389	
QLI67720.1	OTHER	0.057146	0.942854	
QLI67721.1	OTHER	0.001176	0.998824	
QLI67722.1	OTHER	0.000807	0.999193	
QLI67723.1	OTHER	0.000442	0.999558	
QLI67724.1	OTHER	0.003102	0.996898	
QLI67725.1	OTHER	0.007852	0.992148	
QLI67726.1	OTHER	0.009243	0.990757	
QLI67727.1	OTHER	0.002087	0.997913	
QLI67728.1	OTHER	0.002105	0.997895	

QLI67729.1	OTHER	0.001964	0.998036	
QLI67730.1	OTHER	0.002552	0.997448	
QLI67731.1	OTHER	0.001795	0.998205	
QLI67732.1	OTHER	0.016101	0.983899	
QLI67733.1	OTHER	0.001625	0.998375	
QLI67734.1	OTHER	0.000883	0.999117	
QLI67735.1	OTHER	0.001929	0.998071	
QLI67736.1	OTHER	0.000822	0.999178	
QLI67737.1	OTHER	0.009629	0.990371	
QLI67738.1	OTHER	0.000595	0.999405	
QLI67739.1	OTHER	0.000520	0.999480	
QLI67740.1	OTHER	0.001450	0.998550	
QLI67741.1	OTHER	0.001706	0.998294	
QLI67742.1	OTHER	0.002291	0.997709	
QLI67743.1	OTHER	0.000959	0.999041	
QLI67744.1	OTHER	0.000442	0.999558	
QLI67745.1	OTHER	0.000955	0.999045	
QLI67746.1	OTHER	0.000543	0.999457	
QLI67747.1	OTHER	0.000653	0.999347	
QLI67748.1	OTHER	0.028197	0.971803	
QLI67749.1	OTHER	0.000219	0.999781	
QLI67750.1	OTHER	0.000842	0.999158	
QLI67751.1	OTHER	0.010873	0.989127	
QLI67752.1	OTHER	0.000358	0.999642	
QLI67753.1	SP(Sec/SPI)	0.997423	0.002577	CS pos: 20-21. SSG-LP. Pr: 0.7602
QLI67754.1	OTHER	0.001327	0.998673	
QLI67755.1	OTHER	0.000218	0.999782	
QLI67756.1	OTHER	0.002761	0.997239	
QLI67757.1	OTHER	0.001041	0.998959	
QLI67758.1	OTHER	0.002616	0.997384	
QLI67759.1	OTHER	0.002476	0.997524	

QLI67760.1	OTHER	0.397690	0.602310	
QLI67761.1	OTHER	0.000821	0.999179	
QLI67762.1	OTHER	0.001094	0.998906	
QLI67763.1	OTHER	0.001324	0.998676	
QLI67764.1	SP(Sec/SPI)	0.993479	0.006521	CS pos: 19-20. ARA-KN. Pr: 0.8670
QLI67765.1	SP(Sec/SPI)	0.994793	0.005207	CS pos: 17-18. VFA-SG. Pr: 0.6166
QLI67766.1	OTHER	0.000309	0.999691	
QLI67767.1	OTHER	0.002441	0.997559	
QLI67768.1	OTHER	0.002709	0.997291	
QLI67769.1	OTHER	0.001127	0.998873	
QLI67770.1	OTHER	0.002557	0.997443	
QLI67771.1	OTHER	0.011757	0.988243	
QLI67772.1	OTHER	0.000869	0.999131	
QLI67773.1	OTHER	0.000433	0.999567	
QLI67774.1	OTHER	0.030911	0.969089	
QLI67775.1	OTHER	0.002247	0.997753	
QLI67776.1	OTHER	0.002140	0.997860	
QLI67777.1	OTHER	0.001259	0.998741	
QLI67778.1	OTHER	0.010597	0.989403	
QLI67779.1	OTHER	0.000354	0.999646	
QLI67780.1	OTHER	0.001051	0.998949	
QLI67781.1	OTHER	0.002926	0.997074	
QLI67782.1	OTHER	0.001485	0.998515	
QLI67783.1	OTHER	0.001966	0.998034	
QLI67784.1	OTHER	0.001192	0.998808	
QLI67785.1	OTHER	0.010338	0.989662	
QLI67786.1	OTHER	0.196124	0.803876	
QLI67787.1	OTHER	0.001083	0.998917	
QLI67788.1	OTHER	0.001508	0.998492	
QLI67789.1	OTHER	0.001907	0.998093	
QLI67790.1	OTHER	0.000962	0.999038	

QLI67791.1	OTHER	0.000822	0.999178	
QLI67792.1	OTHER	0.043154	0.956846	
QLI67793.1	OTHER	0.002210	0.997790	
QLI67794.1	OTHER	0.000815	0.999185	
QLI67795.1	OTHER	0.001259	0.998741	
QLI67796.1	OTHER	0.003303	0.996697	
QLI67797.1	OTHER	0.001309	0.998691	
QLI67798.1	OTHER	0.001022	0.998978	
QLI67799.1	OTHER	0.000780	0.999220	
QLI67800.1	OTHER	0.000728	0.999272	
QLI67801.1	OTHER	0.001217	0.998783	
QLI67802.1	OTHER	0.001641	0.998359	
QLI67803.1	OTHER	0.000829	0.999171	
QLI67804.1	OTHER	0.045990	0.954010	
QLI67805.1	OTHER	0.000637	0.999363	
QLI67806.1	OTHER	0.001012	0.998988	
QLI67807.1	OTHER	0.002893	0.997107	
QLI67808.1	OTHER	0.001072	0.998928	
QLI67809.1	OTHER	0.000451	0.999549	
QLI67810.1	SP(Sec/SPI)	0.994838	0.005162	CS pos: 25-26. VAA-LP. Pr: 0.8015
QLI67811.1	OTHER	0.000825	0.999175	
QLI67812.1	OTHER	0.000686	0.999314	
QLI67813.1	OTHER	0.000370	0.999630	
QLI67814.1	OTHER	0.000442	0.999558	
QLI67815.1	OTHER	0.001037	0.998963	
QLI67816.1	OTHER	0.058085	0.941915	
QLI67817.1	OTHER	0.000816	0.999184	
QLI67818.1	OTHER	0.002176	0.997824	
QLI67819.1	OTHER	0.000634	0.999366	
QLI67820.1	OTHER	0.002700	0.997300	
QLI67821.1	OTHER	0.000178	0.999822	

QLI67822.1	OTHER	0.001139	0.998861	
QLI67823.1	OTHER	0.004632	0.995368	
QLI67824.1	OTHER	0.001725	0.998275	
QLI67825.1	OTHER	0.000709	0.999291	
QLI67826.1	SP(Sec/SPI)	0.698963	0.301037	CS pos: 24-25. SSA-RT. Pr: 0.2845
QLI67827.1	OTHER	0.002379	0.997621	
QLI67828.1	OTHER	0.000962	0.999038	
QLI67829.1	OTHER	0.000900	0.999100	
QLI67830.1	OTHER	0.000982	0.999018	
QLI67831.1	SP(Sec/SPI)	0.869650	0.130350	CS pos: 19-20. VAA-QT. Pr: 0.5057
QLI67832.1	OTHER	0.035074	0.964926	
QLI67833.1	OTHER	0.004410	0.995590	
QLI67834.1	OTHER	0.000196	0.999804	
QLI67835.1	OTHER	0.001271	0.998729	
QLI67836.1	OTHER	0.002741	0.997259	
QLI67837.1	OTHER	0.001907	0.998093	
QLI67838.1	OTHER	0.004447	0.995553	
QLI67839.1	SP(Sec/SPI)	0.993370	0.006630	CS pos: 23-24. SHA-AA. Pr: 0.8723
QLI67840.1	OTHER	0.001462	0.998538	
QLI67841.1	OTHER	0.000326	0.999674	
QLI67842.1	OTHER	0.001921	0.998079	
QLI67843.1	OTHER	0.001455	0.998545	
QLI67844.1	OTHER	0.002255	0.997745	
QLI67845.1	OTHER	0.000421	0.999579	
QLI67846.1	OTHER	0.001372	0.998628	
QLI67847.1	SP(Sec/SPI)	0.993753	0.006247	CS pos: 18-19. TAA-TE. Pr: 0.5073
QLI67848.1	OTHER	0.001495	0.998505	
QLI67849.1	OTHER	0.000755	0.999245	
QLI67850.1	SP(Sec/SPI)	0.902960	0.097040	CS pos: 19-20. AVA-HP. Pr: 0.7469
QLI67851.1	SP(Sec/SPI)	0.960804	0.039196	CS pos: 25-26. AVA-ES. Pr: 0.6782
QLI67852.1	OTHER	0.001253	0.998747	

QLI67853.1	OTHER	0.000934	0.999066	
QLI67854.1	OTHER	0.001130	0.998870	
QLI67855.1	OTHER	0.001441	0.998559	
QLI67856.1	OTHER	0.003191	0.996809	
QLI67857.1	OTHER	0.000932	0.999068	
QLI67858.1	OTHER	0.000408	0.999592	
QLI67859.1	OTHER	0.071432	0.928568	
QLI67860.1	OTHER	0.000584	0.999416	
QLI67861.1	OTHER	0.000776	0.999224	
QLI67862.1	OTHER	0.000434	0.999566	
QLI67863.1	OTHER	0.003545	0.996455	
QLI67864.1	OTHER	0.026860	0.973140	
QLI67865.1	SP(Sec/SPI)	0.582148	0.417852	CS pos: 18-19. VYG-HP. Pr: 0.4134
QLI67866.1	OTHER	0.000183	0.999817	
QLI67867.1	OTHER	0.002748	0.997252	
QLI67868.1	OTHER	0.000467	0.999533	
QLI67869.1	OTHER	0.004077	0.995923	
QLI67870.1	OTHER	0.004802	0.995198	
QLI67871.1	OTHER	0.000722	0.999278	
QLI67872.1	OTHER	0.001034	0.998966	
QLI67873.1	OTHER	0.009781	0.990219	
QLI67874.1	OTHER	0.000506	0.999494	
QLI67875.1	OTHER	0.001006	0.998994	
QLI67876.1	OTHER	0.004392	0.995608	
QLI67877.1	OTHER	0.004675	0.995325	
QLI67878.1	OTHER	0.011644	0.988356	
QLI67879.1	OTHER	0.000564	0.999436	
QLI67880.1	OTHER	0.008832	0.991168	
QLI67881.1	OTHER	0.000741	0.999259	
QLI67882.1	OTHER	0.000553	0.999447	
QLI67883.1	OTHER	0.005186	0.994814	

QLI67884.1	OTHER	0.000537	0.999463	
QLI67885.1	OTHER	0.138095	0.861905	
QLI67886.1	OTHER	0.003192	0.996808	
QLI67887.1	OTHER	0.000449	0.999551	
QLI67888.1	OTHER	0.000496	0.999504	
QLI67889.1	OTHER	0.004963	0.995037	
QLI67890.1	OTHER	0.000494	0.999506	
QLI67891.1	OTHER	0.066883	0.933117	
QLI67892.1	OTHER	0.001652	0.998348	
QLI67893.1	OTHER	0.002387	0.997613	
QLI67894.1	SP(Sec/SPI)	0.994602	0.005398	CS pos: 19-20. AAA-RT. Pr: 0.6033
QLI67895.1	SP(Sec/SPI)	0.613372	0.386628	CS pos: 22-23. TSA-YV. Pr: 0.3818
QLI67896.1	OTHER	0.001163	0.998837	
QLI67897.1	OTHER	0.002379	0.997621	
QLI67898.1	OTHER	0.000626	0.999374	
QLI67899.1	OTHER	0.000746	0.999254	
QLI67900.1	OTHER	0.001125	0.998875	
QLI67901.1	OTHER	0.002268	0.997732	
QLI67902.1	OTHER	0.000557	0.999443	
QLI67903.1	OTHER	0.003242	0.996758	
QLI67904.1	OTHER	0.002005	0.997995	
QLI67905.1	OTHER	0.001126	0.998874	
QLI67906.1	SP(Sec/SPI)	0.990141	0.009859	CS pos: 28-29. ADA-ID. Pr: 0.8449
QLI67907.1	OTHER	0.001130	0.998870	
QLI67908.1	OTHER	0.002610	0.997390	
QLI67909.1	OTHER	0.046309	0.953691	
QLI67910.1	OTHER	0.003049	0.996951	
QLI67911.1	OTHER	0.000852	0.999148	
QLI67912.1	OTHER	0.012438	0.987562	
QLI67913.1	OTHER	0.007582	0.992418	
QLI67914.1	OTHER	0.000121	0.999879	

QLI67915.1	OTHER	0.001159	0.998841	
QLI67916.1	OTHER	0.001178	0.998822	
QLI67917.1	OTHER	0.003656	0.996344	
QLI67918.1	OTHER	0.001272	0.998728	
QLI67919.1	OTHER	0.002461	0.997539	
QLI67920.1	OTHER	0.000274	0.999726	
QLI67921.1	SP(Sec/SPI)	0.974541	0.025459	CS pos: 24-25. CRA-AT. Pr: 0.8486
QLI67922.1	OTHER	0.002239	0.997761	
QLI67923.1	SP(Sec/SPI)	0.962333	0.037667	CS pos: 20-21. ALA-AP. Pr: 0.7551
QLI67924.1	OTHER	0.001133	0.998867	
QLI67925.1	OTHER	0.000549	0.999451	
QLI67926.1	OTHER	0.000503	0.999497	
QLI67927.1	OTHER	0.000855	0.999145	
QLI67928.1	OTHER	0.001758	0.998242	
QLI67929.1	OTHER	0.001478	0.998522	
QLI67930.1	OTHER	0.015594	0.984406	
QLI67931.1	OTHER	0.000468	0.999532	
QLI67932.1	OTHER	0.001436	0.998564	
QLI67933.1	OTHER	0.003282	0.996718	
QLI67934.1	OTHER	0.001410	0.998590	
QLI67935.1	OTHER	0.000519	0.999481	
QLI67936.1	OTHER	0.000529	0.999471	
QLI67937.1	OTHER	0.000371	0.999629	
QLI67938.1	OTHER	0.001833	0.998167	
QLI67939.1	OTHER	0.001775	0.998225	
QLI67940.1	OTHER	0.000964	0.999036	
QLI67941.1	OTHER	0.001456	0.998544	
QLI67942.1	OTHER	0.455198	0.544802	
QLI67943.1	OTHER	0.000325	0.999675	
QLI67944.1	OTHER	0.001348	0.998652	
QLI67945.1	OTHER	0.001056	0.998944	

QLI67946.1	OTHER	0.005087	0.994913	
QLI67947.1	OTHER	0.014389	0.985611	
QLI67948.1	SP(Sec/SPI)	0.949260	0.050740	CS pos: 20-21. AAA-AI. Pr: 0.5202
QLI67949.1	SP(Sec/SPI)	0.989107	0.010893	CS pos: 17-18. ASA-AA. Pr: 0.4487
QLI67950.1	OTHER	0.008801	0.991199	
QLI67951.1	OTHER	0.000966	0.999034	
QLI67952.1	OTHER	0.002468	0.997532	
QLI67953.1	OTHER	0.000571	0.999429	
QLI67954.1	OTHER	0.022820	0.977180	
QLI67955.1	OTHER	0.000919	0.999081	
QLI67956.1	OTHER	0.000588	0.999412	
QLI67957.1	OTHER	0.001025	0.998975	
QLI67958.1	OTHER	0.001449	0.998551	
QLI67959.1	OTHER	0.002436	0.997564	
QLI67960.1	OTHER	0.001345	0.998655	
QLI67961.1	OTHER	0.001354	0.998646	
QLI67962.1	OTHER	0.038456	0.961544	
QLI67963.1	OTHER	0.007884	0.992116	
QLI67964.1	OTHER	0.001193	0.998807	
QLI67965.1	OTHER	0.000971	0.999029	
QLI67966.1	OTHER	0.000772	0.999228	
QLI67967.1	OTHER	0.000735	0.999265	
QLI67968.1	OTHER	0.011657	0.988343	
QLI67969.1	OTHER	0.003902	0.996098	
QLI67970.1	OTHER	0.000545	0.999455	
QLI67971.1	OTHER	0.003436	0.996564	
QLI67972.1	OTHER	0.010661	0.989339	
QLI67973.1	SP(Sec/SPI)	0.997337	0.002663	CS pos: 20-21. AGA-SP. Pr: 0.8077
QLI67974.1	OTHER	0.133089	0.866911	
QLI67975.1	OTHER	0.001002	0.998998	
QLI67976.1	OTHER	0.000602	0.999398	

QLI67977.1	OTHER	0.001012	0.998988	
QLI67978.1	OTHER	0.006826	0.993174	
QLI67979.1	OTHER	0.001414	0.998586	
QLI67980.1	OTHER	0.001713	0.998287	
QLI67981.1	OTHER	0.000739	0.999261	
QLI67982.1	OTHER	0.004205	0.995795	
QLI67983.1	SP(Sec/SPI)	0.996019	0.003981	CS pos: 27-28. SLS-FD. Pr: 0.8778
QLI67984.1	OTHER	0.001246	0.998754	
QLI67985.1	OTHER	0.000362	0.999638	
QLI67986.1	OTHER	0.001776	0.998224	
QLI67987.1	OTHER	0.004553	0.995447	
QLI67988.1	OTHER	0.050727	0.949273	
QLI67989.1	OTHER	0.000440	0.999560	
QLI67990.1	OTHER	0.001914	0.998086	
QLI67991.1	OTHER	0.058408	0.941592	
QLI67992.1	OTHER	0.000728	0.999272	
QLI67993.1	OTHER	0.000525	0.999475	
QLI67994.1	OTHER	0.000639	0.999361	
QLI67995.1	OTHER	0.002130	0.997870	
QLI67996.1	OTHER	0.003433	0.996567	
QLI67997.1	OTHER	0.010210	0.989790	
QLI67998.1	OTHER	0.001424	0.998576	
QLI67999.1	OTHER	0.001011	0.998989	
QLI68000.1	SP(Sec/SPI)	0.923974	0.076026	CS pos: 27-28. TLA-AQ. Pr: 0.7060
QLI68001.1	OTHER	0.000813	0.999187	
QLI68002.1	OTHER	0.100758	0.899242	
QLI68003.1	OTHER	0.000365	0.999635	
QLI68004.1	OTHER	0.000380	0.999620	
QLI68005.1	OTHER	0.001509	0.998491	
QLI68006.1	OTHER	0.000310	0.999690	
QLI68007.1	SP(Sec/SPI)	0.984269	0.015731	CS pos: 18-19. SVA-QF. Pr: 0.6956

QLI68008.1	OTHER	0.001195	0.998805	
QLI68009.1	OTHER	0.000627	0.999373	
QLI68010.1	OTHER	0.000901	0.999099	
QLI68011.1	OTHER	0.000868	0.999132	
QLI68012.1	OTHER	0.000559	0.999441	
QLI68013.1	OTHER	0.000721	0.999279	
QLI68014.1	OTHER	0.001330	0.998670	
QLI68015.1	OTHER	0.001097	0.998903	
QLI68016.1	SP(Sec/SPI)	0.975437	0.024563	CS pos: 19-20. VLG-VP. Pr: 0.4965
QLI68017.1	SP(Sec/SPI)	0.853292	0.146708	CS pos: 21-22. GYA-LD. Pr: 0.7588
QLI68018.1	OTHER	0.000864	0.999136	
QLI68019.1	OTHER	0.011263	0.988737	
QLI68020.1	OTHER	0.000588	0.999412	
QLI68021.1	OTHER	0.065178	0.934822	
QLI68022.1	OTHER	0.008863	0.991137	
QLI68023.1	OTHER	0.004575	0.995425	
QLI68024.1	OTHER	0.001837	0.998163	
QLI68025.1	SP(Sec/SPI)	0.963704	0.036296	CS pos: 21-22. VTA-GP. Pr: 0.7372
QLI68026.1	OTHER	0.001856	0.998144	
QLI68027.1	OTHER	0.001133	0.998867	
QLI68028.1	SP(Sec/SPI)	0.974791	0.025209	CS pos: 17-18. AVA-VP. Pr: 0.8967
QLI68029.1	OTHER	0.000480	0.999520	
QLI68030.1	OTHER	0.003501	0.996499	
QLI68031.1	OTHER	0.000497	0.999503	
QLI68032.1	OTHER	0.003222	0.996778	
QLI68033.1	OTHER	0.004871	0.995129	
QLI68034.1	OTHER	0.000503	0.999497	
QLI68035.1	OTHER	0.001713	0.998287	
QLI68036.1	OTHER	0.002855	0.997145	
QLI68037.1	OTHER	0.000533	0.999467	
QLI68038.1	OTHER	0.002497	0.997503	

QLI68039.1	OTHER	0.000729	0.999271	
QLI68040.1	OTHER	0.000650	0.999350	
QLI68041.1	OTHER	0.001651	0.998349	
QLI68042.1	OTHER	0.000660	0.999340	
QLI68043.1	OTHER	0.001975	0.998025	
QLI68044.1	OTHER	0.000759	0.999241	
QLI68045.1	OTHER	0.006178	0.993822	
QLI68046.1	OTHER	0.001626	0.998374	
QLI68047.1	OTHER	0.012505	0.987495	
QLI68048.1	OTHER	0.000372	0.999628	
QLI68049.1	OTHER	0.000351	0.999649	
QLI68050.1	OTHER	0.001753	0.998247	
QLI68051.1	OTHER	0.001522	0.998478	
QLI68052.1	OTHER	0.001443	0.998557	
QLI68053.1	OTHER	0.002275	0.997725	
QLI68054.1	OTHER	0.000921	0.999079	
QLI68055.1	OTHER	0.001104	0.998896	
QLI68056.1	OTHER	0.000818	0.999182	
QLI68057.1	OTHER	0.001380	0.998620	
QLI68058.1	OTHER	0.000366	0.999634	
QLI68059.1	OTHER	0.000949	0.999051	
QLI68060.1	OTHER	0.001428	0.998572	
QLI68061.1	OTHER	0.000744	0.999256	
QLI68062.1	OTHER	0.000267	0.999733	
QLI68063.1	OTHER	0.001485	0.998515	
QLI68064.1	OTHER	0.173838	0.826162	
QLI68065.1	OTHER	0.000757	0.999243	
QLI68066.1	OTHER	0.017820	0.982180	
QLI68067.1	OTHER	0.002777	0.997223	
QLI68068.1	SP(Sec/SPI)	0.941621	0.058379	CS pos: 19-20. SRA-IS. Pr: 0.5870
QLI68069.1	OTHER	0.000417	0.999583	

QLI68070.1	OTHER	0.000426	0.999574	
QLI68071.1	OTHER	0.002332	0.997668	
QLI68072.1	OTHER	0.000733	0.999267	
QLI68073.1	OTHER	0.007758	0.992242	
QLI68074.1	OTHER	0.005170	0.994830	
QLI68075.1	OTHER	0.004666	0.995334	
QLI68076.1	OTHER	0.000583	0.999417	
QLI68077.1	OTHER	0.000771	0.999229	
QLI68078.1	OTHER	0.001236	0.998764	
QLI68079.1	OTHER	0.002783	0.997217	
QLI68080.1	OTHER	0.000731	0.999269	
QLI68081.1	OTHER	0.001726	0.998274	
QLI68082.1	OTHER	0.000727	0.999273	
QLI68083.1	OTHER	0.002209	0.997791	
QLI68084.1	OTHER	0.031186	0.968814	
QLI68085.1	OTHER	0.004463	0.995537	
QLI68086.1	OTHER	0.031689	0.968311	
QLI68087.1	OTHER	0.001288	0.998712	
QLI68088.1	OTHER	0.001735	0.998265	
QLI68089.1	OTHER	0.001649	0.998351	
QLI68090.1	OTHER	0.001375	0.998625	
QLI68091.1	SP(Sec/SPI)	0.989640	0.010360	CS pos: 28-29. AAA-FG. Pr: 0.6957
QLI68092.1	OTHER	0.148580	0.851420	
QLI68093.1	OTHER	0.001657	0.998343	
QLI68094.1	OTHER	0.277140	0.722860	
QLI68095.1	OTHER	0.000770	0.999230	
QLI68096.1	OTHER	0.006900	0.993100	
QLI68097.1	SP(Sec/SPI)	0.938161	0.061839	CS pos: 17-18. VAG-QY. Pr: 0.7591
QLI68098.1	OTHER	0.000697	0.999303	
QLI68099.1	OTHER	0.000728	0.999272	
QLI68100.1	SP(Sec/SPI)	0.506585	0.493415	CS pos: 31-32. IHS-KD. Pr: 0.1768

QLI68101.1	OTHER	0.000564	0.999436	
QLI68102.1	OTHER	0.000849	0.999151	
QLI68103.1	OTHER	0.000735	0.999265	
QLI68104.1	SP(Sec/SPI)	0.911318	0.088682	CS pos: 17-18. ATA-QF. Pr: 0.4785
QLI68105.1	SP(Sec/SPI)	0.893847	0.106153	CS pos: 18-19. AVA-FD. Pr: 0.7999
QLI68106.1	OTHER	0.001533	0.998467	
QLI68107.1	OTHER	0.000948	0.999052	
QLI68108.1	OTHER	0.008252	0.991748	
QLI68109.1	OTHER	0.002972	0.997028	
QLI68110.1	OTHER	0.000898	0.999102	
QLI68111.1	OTHER	0.001994	0.998006	
QLI68112.1	OTHER	0.001440	0.998560	
QLI68113.1	OTHER	0.000632	0.999368	
QLI68114.1	OTHER	0.000548	0.999452	
QLI68115.1	OTHER	0.000942	0.999058	
QLI68116.1	OTHER	0.002062	0.997938	
QLI68117.1	OTHER	0.001000	0.999000	
QLI68118.1	OTHER	0.001774	0.998226	
QLI68119.1	OTHER	0.001098	0.998902	
QLI68120.1	OTHER	0.000363	0.999637	
QLI68121.1	OTHER	0.015181	0.984819	
QLI68122.1	OTHER	0.001674	0.998326	
QLI68123.1	OTHER	0.000532	0.999468	
QLI68124.1	OTHER	0.000362	0.999638	
QLI68125.1	OTHER	0.000951	0.999049	
QLI68126.1	OTHER	0.000957	0.999043	
QLI68127.1	OTHER	0.000847	0.999153	
QLI68128.1	OTHER	0.000191	0.999809	
QLI68129.1	OTHER	0.000758	0.999242	
QLI68130.1	OTHER	0.000433	0.999567	
QLI68131.1	OTHER	0.001197	0.998803	

QLI68132.1	OTHER	0.006152	0.993848	
QLI68133.1	SP(Sec/SPI)	0.520396	0.479604	CS pos: 32-33. GAA-VA. Pr: 0.1556
QLI68134.1	OTHER	0.002490	0.997510	
QLI68135.1	OTHER	0.001171	0.998829	
QLI68136.1	OTHER	0.290415	0.709585	
QLI68137.1	OTHER	0.152499	0.847501	
QLI68138.1	OTHER	0.001005	0.998995	
QLI68139.1	OTHER	0.001256	0.998744	
QLI68140.1	OTHER	0.000321	0.999679	
QLI68141.1	OTHER	0.000245	0.999755	
QLI68142.1	OTHER	0.029668	0.970332	
QLI68143.1	OTHER	0.000888	0.999112	
QLI68144.1	OTHER	0.001920	0.998080	
QLI68145.1	OTHER	0.000276	0.999724	
QLI68146.1	OTHER	0.000502	0.999498	
QLI68147.1	OTHER	0.001619	0.998381	
QLI68148.1	OTHER	0.002208	0.997792	
QLI68149.1	OTHER	0.024304	0.975696	
QLI68150.1	OTHER	0.000645	0.999355	
QLI68151.1	SP(Sec/SPI)	0.971348	0.028652	CS pos: 22-23. VNG-QS. Pr: 0.8520
QLI68152.1	OTHER	0.001001	0.998999	
QLI68153.1	OTHER	0.010616	0.989384	
QLI68154.1	OTHER	0.000963	0.999037	
QLI68155.1	OTHER	0.002053	0.997947	
QLI68156.1	OTHER	0.000406	0.999594	
QLI68157.1	OTHER	0.000627	0.999373	
QLI68158.1	OTHER	0.001884	0.998116	
QLI68159.1	OTHER	0.007063	0.992937	
QLI68160.1	OTHER	0.000640	0.999360	
QLI68161.1	OTHER	0.000775	0.999225	
QLI68162.1	OTHER	0.003049	0.996951	

QLI68163.1	OTHER	0.004181	0.995819	
QLI68164.1	SP(Sec/SPI)	0.996026	0.003974	CS pos: 18-19. VYS-LV. Pr: 0.6247
QLI68165.1	OTHER	0.020485	0.979515	
QLI68166.1	OTHER	0.001228	0.998772	
QLI68167.1	OTHER	0.000942	0.999058	
QLI68168.1	OTHER	0.000745	0.999255	
QLI68169.1	OTHER	0.002818	0.997182	
QLI68170.1	OTHER	0.001529	0.998471	
QLI68171.1	OTHER	0.000556	0.999444	
QLI68172.1	OTHER	0.001130	0.998870	
QLI68173.1	OTHER	0.000395	0.999605	
QLI68174.1	OTHER	0.000506	0.999494	
QLI68175.1	SP(Sec/SPI)	0.864264	0.135736	CS pos: 22-23. CNA-AA. Pr: 0.6421
QLI68176.1	SP(Sec/SPI)	0.730121	0.269879	CS pos: 26-27. ADG-HD. Pr: 0.4923
QLI68177.1	OTHER	0.000653	0.999347	
QLI68178.1	OTHER	0.018729	0.981271	
QLI68179.1	OTHER	0.000937	0.999063	
QLI68180.1	OTHER	0.000672	0.999328	
QLI68181.1	OTHER	0.000469	0.999531	
QLI68182.1	OTHER	0.007629	0.992371	
QLI68183.1	OTHER	0.000404	0.999596	
QLI68184.1	OTHER	0.000376	0.999624	
QLI68185.1	OTHER	0.013345	0.986655	
QLI68186.1	OTHER	0.000981	0.999019	
QLI68187.1	OTHER	0.001231	0.998769	
QLI68188.1	OTHER	0.001309	0.998691	
QLI68189.1	OTHER	0.002456	0.997544	
QLI68190.1	OTHER	0.020718	0.979282	
QLI68191.1	OTHER	0.002001	0.997999	
QLI68192.1	SP(Sec/SPI)	0.887650	0.112350	CS pos: 28-29. AAA-AA. Pr: 0.2570
QLI68193.1	OTHER	0.002240	0.997760	

QLI68194.1	OTHER	0.000743	0.999257	
QLI68195.1	OTHER	0.002175	0.997825	
QLI68196.1	OTHER	0.000845	0.999155	
QLI68197.1	OTHER	0.220627	0.779373	
QLI68198.1	OTHER	0.003836	0.996164	
QLI68199.1	OTHER	0.002872	0.997128	
QLI68200.1	OTHER	0.001023	0.998977	
QLI68201.1	OTHER	0.001113	0.998887	
QLI68202.1	OTHER	0.000707	0.999293	
QLI68203.1	OTHER	0.001326	0.998674	
QLI68204.1	OTHER	0.000533	0.999467	
QLI68205.1	OTHER	0.014023	0.985977	
QLI68206.1	OTHER	0.003026	0.996974	
QLI68207.1	OTHER	0.000515	0.999485	
QLI68208.1	OTHER	0.000484	0.999516	
QLI68209.1	OTHER	0.000340	0.999660	
QLI68210.1	OTHER	0.001297	0.998703	
QLI68211.1	OTHER	0.001264	0.998736	
QLI68212.1	SP(Sec/SPI)	0.995700	0.004300	CS pos: 18-19. AQA-GI. Pr: 0.9114
QLI68213.1	OTHER	0.002194	0.997806	
QLI68214.1	OTHER	0.000586	0.999414	
QLI68215.1	OTHER	0.001087	0.998913	
QLI68216.1	OTHER	0.001796	0.998204	
QLI68217.1	OTHER	0.003935	0.996065	
QLI68218.1	OTHER	0.000918	0.999082	
QLI68219.1	OTHER	0.002231	0.997769	
QLI68220.1	OTHER	0.002926	0.997074	
QLI68221.1	OTHER	0.001278	0.998722	
QLI68222.1	OTHER	0.000570	0.999430	
QLI68223.1	OTHER	0.000831	0.999169	
QLI68224.1	OTHER	0.001152	0.998848	

QLI68225.1	OTHER	0.000634	0.999366	
QLI68226.1	OTHER	0.002559	0.997441	
QLI68227.1	OTHER	0.001226	0.998774	
QLI68228.1	OTHER	0.007834	0.992166	
QLI68229.1	OTHER	0.000342	0.999658	
QLI68230.1	OTHER	0.001427	0.998573	
QLI68231.1	OTHER	0.000528	0.999472	
QLI68232.1	OTHER	0.001821	0.998179	
QLI68233.1	OTHER	0.004467	0.995533	
QLI68234.1	SP(Sec/SPI)	0.575897	0.424103	CS pos: 25-26. LRK-ES. Pr: 0.0865
QLI68235.1	OTHER	0.000985	0.999015	
QLI68236.1	OTHER	0.000584	0.999416	
QLI68237.1	OTHER	0.000924	0.999076	
QLI68238.1	OTHER	0.000622	0.999378	
QLI68239.1	OTHER	0.001280	0.998720	
QLI68240.1	OTHER	0.000833	0.999167	
QLI68241.1	SP(Sec/SPI)	0.990445	0.009555	CS pos: 17-18. AAA-LK. Pr: 0.7682
QLI68242.1	OTHER	0.071974	0.928026	
QLI68243.1	OTHER	0.000729	0.999271	
QLI68244.1	OTHER	0.001807	0.998193	
QLI68245.1	OTHER	0.001910	0.998090	
QLI68246.1	OTHER	0.001187	0.998813	
QLI68247.1	OTHER	0.001103	0.998897	
QLI68248.1	SP(Sec/SPI)	0.995219	0.004781	CS pos: 16-17. VAA-AP. Pr: 0.4547
QLI68249.1	OTHER	0.001024	0.998976	
QLI68250.1	SP(Sec/SPI)	0.997511	0.002489	CS pos: 17-18. ATS-MP. Pr: 0.3518
QLI68251.1	OTHER	0.002538	0.997462	
QLI68252.1	OTHER	0.000609	0.999391	
QLI68253.1	OTHER	0.001194	0.998806	
QLI68254.1	OTHER	0.003057	0.996943	
QLI68255.1	OTHER	0.010509	0.989491	

QLI68256.1	OTHER	0.000420	0.999580	
QLI68257.1	OTHER	0.059438	0.940562	
QLI68258.1	OTHER	0.000617	0.999383	
QLI68259.1	OTHER	0.006725	0.993275	
QLI68260.1	OTHER	0.001827	0.998173	
QLI68261.1	OTHER	0.001602	0.998398	
QLI68262.1	OTHER	0.000753	0.999247	
QLI68263.1	OTHER	0.005350	0.994650	
QLI68264.1	OTHER	0.019620	0.980380	
QLI68265.1	OTHER	0.001817	0.998183	
QLI68266.1	OTHER	0.000726	0.999274	
QLI68267.1	OTHER	0.000803	0.999197	
QLI68268.1	OTHER	0.000414	0.999586	
QLI68269.1	OTHER	0.001538	0.998462	
QLI68270.1	OTHER	0.003714	0.996286	
QLI68271.1	OTHER	0.000464	0.999536	
QLI68272.1	OTHER	0.363529	0.636471	
QLI68273.1	OTHER	0.014178	0.985822	
QLI68274.1	SP(Sec/SPI)	0.996577	0.003423	CS pos: 20-21. TSA-AL. Pr: 0.5050
QLI68275.1	OTHER	0.001723	0.998277	
QLI68276.1	OTHER	0.020957	0.979043	
QLI68277.1	OTHER	0.001223	0.998777	
QLI68278.1	OTHER	0.003251	0.996749	
QLI68279.1	OTHER	0.012581	0.987419	
QLI68280.1	OTHER	0.000363	0.999637	
QLI68281.1	OTHER	0.000863	0.999137	
QLI68282.1	SP(Sec/SPI)	0.895753	0.104247	CS pos: 16-17. TFA-KN. Pr: 0.6896
QLI68283.1	OTHER	0.001419	0.998581	
QLI68284.1	OTHER	0.003453	0.996547	
QLI68285.1	OTHER	0.001676	0.998324	
QLI68286.1	OTHER	0.009519	0.990481	

QLI68287.1	OTHER	0.000774	0.999226	
QLI68288.1	OTHER	0.001646	0.998354	
QLI68289.1	SP(Sec/SPI)	0.999826	0.000174	CS pos: 18-19. ATA-DD. Pr: 0.8334
QLI68290.1	OTHER	0.001067	0.998933	
QLI68291.1	OTHER	0.002680	0.997320	
QLI68292.1	OTHER	0.000627	0.999373	
QLI68293.1	OTHER	0.002059	0.997941	
QLI68294.1	OTHER	0.001483	0.998517	
QLI68295.1	OTHER	0.002111	0.997889	
QLI68296.1	OTHER	0.000726	0.999274	
QLI68297.1	SP(Sec/SPI)	0.962330	0.037670	CS pos: 17-18. AIS-GP. Pr: 0.6678
QLI68298.1	OTHER	0.006813	0.993187	
QLI68299.1	OTHER	0.000933	0.999067	
QLI68300.1	OTHER	0.001210	0.998790	
QLI68301.1	OTHER	0.000815	0.999185	
QLI68302.1	OTHER	0.000312	0.999688	
QLI68303.1	OTHER	0.001247	0.998753	
QLI68304.1	OTHER	0.000712	0.999288	
QLI68305.1	SP(Sec/SPI)	0.992931	0.007069	CS pos: 17-18. AAA-EQ. Pr: 0.8773
QLI68306.1	SP(Sec/SPI)	0.988038	0.011962	CS pos: 20-21. VQS-HF. Pr: 0.7570
QLI68307.1	OTHER	0.000954	0.999046	
QLI68308.1	OTHER	0.003526	0.996474	
QLI68309.1	OTHER	0.001071	0.998929	
QLI68310.1	OTHER	0.001003	0.998997	
QLI68311.1	OTHER	0.004072	0.995928	
QLI68312.1	OTHER	0.000703	0.999297	
QLI68313.1	OTHER	0.001369	0.998631	
QLI68314.1	OTHER	0.000707	0.999293	
QLI68315.1	OTHER	0.001256	0.998744	
QLI68316.1	OTHER	0.003108	0.996892	
QLI68317.1	OTHER	0.002196	0.997804	

QLI68318.1	OTHER	0.000841	0.999159	
QLI68319.1	OTHER	0.000737	0.999263	
QLI68320.1	OTHER	0.000531	0.999469	
QLI68321.1	OTHER	0.000809	0.999191	
QLI68322.1	OTHER	0.001944	0.998056	
QLI68323.1	OTHER	0.004066	0.995934	
QLI68324.1	OTHER	0.000305	0.999695	
QLI68325.1	OTHER	0.000812	0.999188	
QLI68326.1	OTHER	0.002150	0.997850	
QLI68327.1	OTHER	0.067004	0.932996	
QLI68328.1	OTHER	0.000844	0.999156	
QLI68329.1	OTHER	0.001835	0.998165	
QLI68330.1	OTHER	0.002057	0.997943	
QLI68331.1	SP(Sec/SPI)	0.998581	0.001419	CS pos: 18-19. ALA-DD. Pr: 0.9826
QLI68332.1	OTHER	0.000796	0.999204	
QLI68333.1	OTHER	0.000750	0.999250	
QLI68334.1	OTHER	0.003671	0.996329	
QLI68335.1	OTHER	0.001193	0.998807	
QLI68336.1	OTHER	0.001514	0.998486	
QLI68337.1	OTHER	0.001471	0.998529	
QLI68338.1	OTHER	0.000989	0.999011	
QLI68339.1	OTHER	0.002456	0.997544	
QLI68340.1	OTHER	0.001542	0.998458	
QLI68341.1	OTHER	0.001740	0.998260	
QLI68342.1	OTHER	0.000139	0.999861	
QLI68343.1	OTHER	0.001861	0.998139	
QLI68344.1	OTHER	0.001894	0.998106	
QLI68345.1	OTHER	0.005495	0.994505	
QLI68346.1	SP(Sec/SPI)	0.998700	0.001300	CS pos: 20-21. ALA-HN. Pr: 0.9131
QLI68347.1	OTHER	0.000378	0.999622	
QLI68348.1	OTHER	0.001984	0.998016	

QLI68349.1	OTHER	0.000661	0.999339	
QLI68350.1	OTHER	0.000701	0.999299	
QLI68351.1	OTHER	0.000846	0.999154	
QLI68352.1	OTHER	0.001180	0.998820	
QLI68353.1	OTHER	0.002331	0.997669	
QLI68354.1	OTHER	0.065112	0.934888	
QLI68355.1	OTHER	0.000461	0.999539	
QLI68356.1	OTHER	0.000454	0.999546	
QLI68357.1	OTHER	0.001695	0.998305	
QLI68358.1	OTHER	0.001022	0.998978	
QLI68359.1	SP(Sec/SPI)	0.928865	0.071135	CS pos: 13-14. ANA-AV. Pr: 0.5288
QLI68360.1	OTHER	0.000637	0.999363	
QLI68361.1	OTHER	0.002391	0.997609	
QLI68362.1	OTHER	0.001033	0.998967	
QLI68363.1	OTHER	0.013258	0.986742	
QLI68364.1	OTHER	0.000392	0.999608	
QLI68365.1	OTHER	0.000578	0.999422	
QLI68366.1	OTHER	0.000768	0.999232	
QLI68367.1	OTHER	0.002164	0.997836	
QLI68368.1	OTHER	0.013993	0.986007	
QLI68369.1	OTHER	0.000511	0.999489	
QLI68370.1	OTHER	0.002556	0.997444	
QLI68371.1	OTHER	0.000473	0.999527	
QLI68372.1	OTHER	0.001418	0.998582	
QLI68373.1	OTHER	0.002193	0.997807	
QLI68374.1	OTHER	0.003290	0.996710	
QLI68375.1	OTHER	0.001268	0.998732	
QLI68376.1	OTHER	0.000394	0.999606	
QLI68377.1	OTHER	0.000294	0.999706	
QLI68378.1	OTHER	0.002568	0.997432	
QLI68379.1	OTHER	0.019245	0.980755	

QLI68380.1	OTHER	0.002203	0.997797	
QLI68381.1	OTHER	0.381220	0.618780	
QLI68382.1	SP(Sec/SPI)	0.971295	0.028705	CS pos: 20-21. ASA-LP. Pr: 0.7006
QLI68383.1	OTHER	0.002175	0.997825	
QLI68384.1	OTHER	0.001730	0.998270	
QLI68385.1	OTHER	0.001351	0.998649	
QLI68386.1	SP(Sec/SPI)	0.973814	0.026186	CS pos: 20-21. AYA-AA. Pr: 0.5418
QLI68387.1	OTHER	0.001626	0.998374	
QLI68388.1	SP(Sec/SPI)	0.991278	0.008722	CS pos: 17-18. VSA-AP. Pr: 0.5545
QLI68389.1	OTHER	0.007669	0.992331	
QLI68390.1	OTHER	0.000559	0.999441	
QLI68391.1	OTHER	0.001042	0.998958	
QLI68392.1	OTHER	0.001230	0.998770	
QLI68393.1	OTHER	0.000336	0.999664	
QLI68394.1	OTHER	0.000418	0.999582	
QLI68395.1	SP(Sec/SPI)	0.697511	0.302489	CS pos: 23-24. LVA-QD. Pr: 0.4111
QLI68396.1	OTHER	0.001544	0.998456	
QLI68397.1	OTHER	0.000402	0.999598	
QLI68398.1	OTHER	0.003274	0.996726	
QLI68399.1	OTHER	0.000751	0.999249	
QLI68400.1	OTHER	0.000812	0.999188	
QLI68401.1	OTHER	0.002627	0.997373	
QLI68402.1	OTHER	0.000598	0.999402	
QLI68403.1	OTHER	0.000139	0.999861	
QLI68404.1	OTHER	0.001185	0.998815	
QLI68405.1	OTHER	0.002135	0.997865	
QLI68406.1	OTHER	0.005481	0.994519	
QLI68407.1	OTHER	0.094959	0.905041	
QLI68408.1	OTHER	0.001194	0.998806	
QLI68409.1	OTHER	0.001461	0.998539	
QLI68410.1	OTHER	0.000342	0.999658	

QLI68411.1	SP(Sec/SPI)	0.993847	0.006153	CS pos: 19-20. SAG-QT. Pr: 0.4387
QLI68412.1	OTHER	0.001056	0.998944	
QLI68413.1	SP(Sec/SPI)	0.996022	0.003978	CS pos: 17-18. ASA-HG. Pr: 0.5253
QLI68414.1	OTHER	0.007784	0.992216	
QLI68415.1	OTHER	0.000857	0.999143	
QLI68416.1	OTHER	0.094821	0.905179	
QLI68417.1	SP(Sec/SPI)	0.912415	0.087585	CS pos: 21-22. GLA-QD. Pr: 0.8530
QLI68418.1	OTHER	0.006537	0.993463	
QLI68419.1	OTHER	0.018098	0.981902	
QLI68420.1	OTHER	0.001122	0.998878	
QLI68421.1	OTHER	0.072047	0.927953	
QLI68422.1	OTHER	0.001060	0.998940	
QLI68423.1	OTHER	0.004205	0.995795	
QLI68424.1	OTHER	0.002220	0.997780	
QLI68425.1	OTHER	0.000449	0.999551	
QLI68426.1	OTHER	0.001444	0.998556	
QLI68427.1	OTHER	0.002852	0.997148	
QLI68428.1	OTHER	0.001493	0.998507	
QLI68429.1	OTHER	0.001193	0.998807	
QLI68430.1	OTHER	0.021649	0.978351	
QLI68431.1	OTHER	0.002946	0.997054	
QLI68432.1	OTHER	0.000712	0.999288	
QLI68433.1	OTHER	0.001094	0.998906	
QLI68434.1	OTHER	0.001116	0.998884	
QLI68435.1	SP(Sec/SPI)	0.924772	0.075228	CS pos: 19-20. AAA-AA. Pr: 0.2997
QLI68436.1	OTHER	0.001185	0.998815	
QLI68437.1	OTHER	0.003189	0.996811	
QLI68438.1	OTHER	0.001880	0.998120	
QLI68439.1	OTHER	0.001909	0.998091	
QLI68440.1	OTHER	0.001275	0.998725	
QLI68441.1	OTHER	0.001653	0.998347	

QLI68442.1	OTHER	0.001655	0.998345	
QLI68443.1	OTHER	0.001020	0.998980	
QLI68444.1	SP(Sec/SPI)	0.999663	0.000337	CS pos: 16-17. ALG-SP. Pr: 0.3333
QLI68445.1	OTHER	0.000659	0.999341	
QLI68446.1	OTHER	0.002170	0.997830	
QLI68447.1	OTHER	0.000333	0.999667	
QLI68448.1	OTHER	0.018099	0.981901	
QLI68449.1	OTHER	0.002726	0.997274	
QLI68450.1	OTHER	0.001903	0.998097	
QLI68451.1	OTHER	0.004525	0.995475	
QLI68452.1	OTHER	0.001649	0.998351	
QLI68453.1	OTHER	0.011169	0.988831	
QLI68454.1	OTHER	0.001271	0.998729	
QLI68455.1	OTHER	0.003969	0.996031	
QLI68456.1	OTHER	0.031281	0.968719	
QLI68457.1	OTHER	0.001503	0.998497	
QLI68458.1	OTHER	0.006580	0.993420	
QLI68459.1	OTHER	0.000583	0.999417	
QLI68460.1	OTHER	0.000281	0.999719	
QLI68461.1	OTHER	0.008026	0.991974	
QLI68462.1	OTHER	0.001494	0.998506	
QLI68463.1	SP(Sec/SPI)	0.967736	0.032264	CS pos: 20-21. AQS-HL. Pr: 0.5497
QLI68464.1	OTHER	0.002314	0.997686	
QLI68465.1	OTHER	0.003560	0.996440	
QLI68466.1	SP(Sec/SPI)	0.959636	0.040364	CS pos: 22-23. VSA-AS. Pr: 0.7734
QLI68467.1	OTHER	0.002489	0.997511	
QLI68468.1	OTHER	0.003381	0.996619	
QLI68469.1	OTHER	0.001163	0.998837	
QLI68470.1	OTHER	0.002408	0.997592	
QLI68471.1	OTHER	0.001455	0.998545	
QLI68472.1	OTHER	0.001142	0.998858	

QLI68473.1	OTHER	0.000337	0.999663	
QLI68474.1	OTHER	0.001502	0.998498	
QLI68475.1	OTHER	0.002873	0.997127	
QLI68476.1	OTHER	0.000807	0.999193	
QLI68477.1	OTHER	0.003158	0.996842	
QLI68478.1	OTHER	0.001095	0.998905	
QLI68479.1	SP(Sec/SPI)	0.907973	0.092027	CS pos: 18-19. VSA-TL. Pr: 0.4768
QLI68480.1	SP(Sec/SPI)	0.998209	0.001791	CS pos: 20-21. ASA-SA. Pr: 0.3769
QLI68481.1	OTHER	0.002355	0.997645	
QLI68482.1	OTHER	0.009124	0.990876	
QLI68483.1	SP(Sec/SPI)	0.989691	0.010309	CS pos: 19-20. ALA-AA. Pr: 0.4987
QLI68484.1	SP(Sec/SPI)	0.958956	0.041044	CS pos: 15-16. TAA-AP. Pr: 0.3892
QLI68485.1	OTHER	0.005301	0.994699	
QLI68486.1	OTHER	0.000310	0.999690	
QLI68487.1	OTHER	0.007392	0.992608	
QLI68488.1	OTHER	0.006266	0.993734	
QLI68489.1	OTHER	0.000602	0.999398	
QLI68490.1	OTHER	0.003119	0.996881	
QLI68491.1	OTHER	0.005308	0.994692	
QLI68492.1	OTHER	0.000707	0.999293	
QLI68493.1	OTHER	0.029988	0.970012	
QLI68494.1	OTHER	0.004169	0.995831	
QLI68495.1	OTHER	0.006147	0.993853	
QLI68496.1	OTHER	0.005073	0.994927	
QLI68497.1	OTHER	0.001716	0.998284	
QLI68498.1	OTHER	0.015897	0.984103	
QLI68499.1	OTHER	0.023031	0.976969	
QLI68500.1	OTHER	0.000754	0.999246	
QLI68501.1	OTHER	0.001654	0.998346	
QLI68502.1	OTHER	0.000474	0.999526	
QLI68503.1	OTHER	0.002147	0.997853	

QLI68504.1	OTHER	0.000607	0.999393	
QLI68505.1	OTHER	0.001154	0.998846	
QLI68506.1	OTHER	0.000717	0.999283	
QLI68507.1	OTHER	0.000492	0.999508	
QLI68508.1	OTHER	0.000522	0.999478	
QLI68509.1	OTHER	0.001827	0.998173	
QLI68510.1	SP(Sec/SPI)	0.998494	0.001506	CS pos: 22-23. ASS-AP. Pr: 0.6212
QLI68511.1	OTHER	0.018581	0.981419	
QLI68512.1	OTHER	0.001766	0.998234	
QLI68513.1	OTHER	0.001989	0.998011	
QLI68514.1	OTHER	0.000822	0.999178	
QLI68515.1	SP(Sec/SPI)	0.682534	0.317466	CS pos: 35-36. TLA-AC. Pr: 0.2333
QLI68516.1	SP(Sec/SPI)	0.982947	0.017053	CS pos: 16-17. ALA-VP. Pr: 0.6414
QLI68517.1	OTHER	0.001740	0.998260	
QLI68518.1	OTHER	0.000345	0.999655	
QLI68519.1	OTHER	0.020548	0.979452	
QLI68520.1	OTHER	0.004085	0.995915	
QLI68521.1	OTHER	0.000855	0.999145	
QLI68522.1	OTHER	0.001478	0.998522	
QLI68523.1	OTHER	0.327396	0.672604	
QLI68524.1	OTHER	0.000900	0.999100	
QLI68525.1	OTHER	0.000899	0.999101	
QLI68526.1	OTHER	0.001581	0.998419	
QLI68527.1	OTHER	0.005359	0.994641	
QLI68528.1	OTHER	0.001802	0.998198	
QLI68529.1	OTHER	0.000628	0.999372	
QLI68530.1	OTHER	0.001371	0.998629	
QLI68531.1	OTHER	0.000595	0.999405	
QLI68532.1	OTHER	0.001991	0.998009	
QLI68533.1	OTHER	0.000657	0.999343	
QLI68534.1	OTHER	0.001096	0.998904	

QLI68535.1	OTHER	0.001871	0.998129	
QLI68536.1	OTHER	0.000501	0.999499	
QLI68537.1	OTHER	0.001136	0.998864	
QLI68538.1	OTHER	0.001912	0.998088	
QLI68539.1	OTHER	0.000854	0.999146	
QLI68540.1	OTHER	0.000759	0.999241	
QLI68541.1	OTHER	0.000658	0.999342	
QLI68542.1	OTHER	0.002092	0.997908	
QLI68543.1	OTHER	0.002190	0.997810	
QLI68544.1	OTHER	0.003225	0.996775	
QLI68545.1	OTHER	0.051405	0.948595	
QLI68546.1	OTHER	0.000720	0.999280	
QLI68547.1	OTHER	0.000960	0.999040	
QLI68548.1	OTHER	0.001023	0.998977	
QLI68549.1	OTHER	0.000884	0.999116	
QLI68550.1	OTHER	0.000774	0.999226	
QLI68551.1	SP(Sec/SPI)	0.821969	0.178031	CS pos: 19-20. AQS-KP. Pr: 0.3475
QLI68552.1	OTHER	0.000377	0.999623	
QLI68553.1	OTHER	0.000205	0.999795	
QLI68554.1	OTHER	0.001807	0.998193	
QLI68555.1	SP(Sec/SPI)	0.996044	0.003956	CS pos: 25-26. GEA-AP. Pr: 0.7898
QLI68556.1	OTHER	0.000899	0.999101	
QLI68557.1	OTHER	0.001121	0.998879	
QLI68558.1	OTHER	0.001621	0.998379	
QLI68559.1	OTHER	0.002455	0.997545	
QLI68560.1	OTHER	0.000273	0.999727	
QLI68561.1	OTHER	0.002450	0.997550	
QLI68562.1	OTHER	0.001884	0.998116	
QLI68563.1	OTHER	0.001284	0.998716	
QLI68564.1	OTHER	0.001021	0.998979	
QLI68565.1	OTHER	0.000256	0.999744	

QLI68566.1	OTHER	0.001329	0.998671	
QLI68567.1	OTHER	0.000462	0.999538	
QLI68568.1	SP(Sec/SPI)	0.981010	0.018990	CS pos: 18-19. TMA-TV. Pr: 0.6045
QLI68569.1	OTHER	0.000687	0.999313	
QLI68570.1	OTHER	0.003654	0.996346	
QLI68571.1	OTHER	0.001509	0.998491	
QLI68572.1	OTHER	0.000693	0.999307	
QLI68573.1	OTHER	0.000285	0.999715	
QLI68574.1	OTHER	0.000551	0.999449	
QLI68575.1	OTHER	0.000977	0.999023	
QLI68576.1	OTHER	0.001656	0.998344	
QLI68577.1	OTHER	0.001596	0.998404	
QLI68578.1	OTHER	0.147618	0.852382	
QLI68579.1	OTHER	0.000564	0.999436	
QLI68580.1	OTHER	0.010364	0.989636	
QLI68581.1	OTHER	0.175996	0.824004	
QLI68582.1	OTHER	0.119944	0.880056	
QLI68583.1	OTHER	0.000600	0.999400	
QLI68584.1	SP(Sec/SPI)	0.993303	0.006697	CS pos: 17-18. ALA-AP. Pr: 0.6863
QLI68585.1	OTHER	0.000796	0.999204	
QLI68586.1	OTHER	0.000463	0.999537	
QLI68587.1	OTHER	0.001304	0.998696	
QLI68588.1	OTHER	0.019318	0.980682	
QLI68589.1	OTHER	0.000898	0.999102	
QLI68590.1	OTHER	0.000138	0.999862	
QLI68591.1	OTHER	0.002705	0.997295	
QLI68592.1	OTHER	0.000612	0.999388	
QLI68593.1	OTHER	0.001483	0.998517	
QLI68594.1	OTHER	0.000682	0.999318	
QLI68595.1	OTHER	0.002880	0.997120	
QLI68596.1	OTHER	0.000680	0.999320	

QLI68597.1	OTHER	0.000806	0.999194	
QLI68598.1	OTHER	0.003016	0.996984	
QLI68599.1	OTHER	0.002111	0.997889	
QLI68600.1	OTHER	0.000446	0.999554	
QLI68601.1	OTHER	0.000255	0.999745	
QLI68602.1	SP(Sec/SPI)	0.997118	0.002882	CS pos: 18-19. VFA-RN. Pr: 0.9278
QLI68603.1	OTHER	0.003836	0.996164	
QLI68604.1	OTHER	0.001105	0.998895	
QLI68605.1	OTHER	0.001423	0.998577	
QLI68606.1	OTHER	0.002831	0.997169	
QLI68607.1	OTHER	0.023230	0.976770	
QLI68608.1	OTHER	0.002985	0.997015	
QLI68609.1	OTHER	0.000308	0.999692	
QLI68610.1	SP(Sec/SPI)	0.996649	0.003351	CS pos: 19-20. ASA-QD. Pr: 0.8822
QLI68611.1	OTHER	0.003954	0.996046	
QLI68612.1	OTHER	0.001006	0.998994	
QLI68613.1	OTHER	0.001959	0.998041	
QLI68614.1	OTHER	0.001833	0.998167	
QLI68615.1	OTHER	0.001398	0.998602	
QLI68616.1	OTHER	0.001189	0.998811	
QLI68617.1	OTHER	0.000841	0.999159	
QLI68618.1	OTHER	0.008430	0.991570	
QLI68619.1	OTHER	0.000755	0.999245	
QLI68620.1	OTHER	0.006080	0.993920	
QLI68621.1	OTHER	0.001567	0.998433	
QLI68622.1	OTHER	0.012573	0.987427	
QLI68623.1	OTHER	0.005328	0.994672	
QLI68624.1	SP(Sec/SPI)	0.967354	0.032646	CS pos: 21-22. ASA-QF. Pr: 0.7357
QLI68625.1	OTHER	0.000666	0.999334	
QLI68626.1	OTHER	0.001560	0.998440	
QLI68627.1	OTHER	0.030069	0.969931	

QLI68628.1	OTHER	0.000864	0.999136
QLI68629.1	OTHER	0.001923	0.998077
QLI68630.1	OTHER	0.002082	0.997918
QLI68631.1	OTHER	0.006233	0.993767
QLI68632.1	OTHER	0.008455	0.991545
QLI68633.1	OTHER	0.001393	0.998607
QLI68634.1	OTHER	0.001023	0.998977
QLI68635.1	OTHER	0.001165	0.998835
QLI68636.1	OTHER	0.002228	0.997772
QLI68637.1	OTHER	0.001912	0.998088
QLI68638.1	OTHER	0.001375	0.998625
QLI68639.1	OTHER	0.000808	0.999192
QLI68640.1	OTHER	0.000728	0.999272
QLI68641.1	OTHER	0.010598	0.989402
QLI68642.1	OTHER	0.000589	0.999411
QLI68643.1	OTHER	0.000427	0.999573
QLI68644.1	OTHER	0.001032	0.998968
QLI68645.1	OTHER	0.017435	0.982565
QLI68646.1	OTHER	0.002021	0.997979
QLI68647.1	OTHER	0.000901	0.999099
QLI68648.1	OTHER	0.000467	0.999533
QLI68649.1	OTHER	0.000833	0.999167
QLI68650.1	OTHER	0.002448	0.997552
QLI68651.1	OTHER	0.232586	0.767414
QLI68652.1	OTHER	0.005158	0.994842
QLI68653.1	OTHER	0.000446	0.999554
QLI68654.1	OTHER	0.000542	0.999458
QLI68655.1	OTHER	0.005640	0.994360
QLI68656.1	OTHER	0.000806	0.999194
QLI68657.1	OTHER	0.000543	0.999457
QLI68658.1	OTHER	0.000814	0.999186

QLI68659.1	SP(Sec/SPI)	0.939712	0.060288	CS pos: 19-20. ISS-IP. Pr: 0.5774
QLI68660.1	OTHER	0.000631	0.999369	
QLI68661.1	OTHER	0.001392	0.998608	
QLI68662.1	OTHER	0.003062	0.996938	
QLI68663.1	OTHER	0.001182	0.998818	
QLI68664.1	SP(Sec/SPI)	0.971607	0.028393	CS pos: 17-18. ALG-IN. Pr: 0.3105
QLI68665.1	OTHER	0.000945	0.999055	
QLI68666.1	OTHER	0.001832	0.998168	
QLI68667.1	OTHER	0.335312	0.664688	
QLI68668.1	OTHER	0.003595	0.996405	
QLI68669.1	OTHER	0.000360	0.999640	
QLI68670.1	OTHER	0.000743	0.999257	
QLI68671.1	OTHER	0.001969	0.998031	
QLI68672.1	OTHER	0.000267	0.999733	
QLI68673.1	OTHER	0.000406	0.999594	
QLI68674.1	OTHER	0.001318	0.998682	
QLI68675.1	OTHER	0.004336	0.995664	
QLI68676.1	OTHER	0.001977	0.998023	
QLI68677.1	OTHER	0.001302	0.998698	
QLI68678.1	OTHER	0.001467	0.998533	
QLI68679.1	OTHER	0.001113	0.998887	
QLI68680.1	SP(Sec/SPI)	0.610568	0.389432	CS pos: 19-20. GFA-LD. Pr: 0.5165
QLI68681.1	OTHER	0.003008	0.996992	
QLI68682.1	OTHER	0.003085	0.996915	
QLI68683.1	OTHER	0.000529	0.999471	
QLI68684.1	OTHER	0.001070	0.998930	
QLI68685.1	OTHER	0.000788	0.999212	
QLI68686.1	OTHER	0.001891	0.998109	
QLI68687.1	OTHER	0.002414	0.997586	
QLI68688.1	OTHER	0.000875	0.999125	
QLI68689.1	OTHER	0.001177	0.998823	

QLI68690.1	OTHER	0.001208	0.998792	
QLI68691.1	OTHER	0.226093	0.773907	
QLI68692.1	OTHER	0.000748	0.999252	
QLI68693.1	OTHER	0.001576	0.998424	
QLI68694.1	OTHER	0.001526	0.998474	
QLI68695.1	OTHER	0.002721	0.997279	
QLI68696.1	OTHER	0.001473	0.998527	
QLI68697.1	OTHER	0.007501	0.992499	
QLI68698.1	OTHER	0.002497	0.997503	
QLI68699.1	SP(Sec/SPI)	0.999162	0.000838	CS pos: 19-20. VSA-YP. Pr: 0.9673
QLI68700.1	OTHER	0.084972	0.915028	
QLI68701.1	OTHER	0.000484	0.999516	
QLI68702.1	OTHER	0.000649	0.999351	
QLI68703.1	OTHER	0.000617	0.999383	
QLI68704.1	OTHER	0.157662	0.842338	
QLI68705.1	OTHER	0.001649	0.998351	
QLI68706.1	OTHER	0.001626	0.998374	
QLI68707.1	OTHER	0.065980	0.934020	
QLI68708.1	OTHER	0.000721	0.999279	
QLI68709.1	OTHER	0.000901	0.999099	
QLI68710.1	OTHER	0.002550	0.997450	
QLI68711.1	OTHER	0.202321	0.797679	
QLI68712.1	OTHER	0.037889	0.962111	
QLI68713.1	OTHER	0.000829	0.999171	
QLI68714.1	OTHER	0.000580	0.999420	
QLI68715.1	OTHER	0.000505	0.999495	
QLI68716.1	SP(Sec/SPI)	0.982708	0.017292	CS pos: 19-20. IQA-AD. Pr: 0.8237
QLI68717.1	OTHER	0.000481	0.999519	
QLI68718.1	OTHER	0.002384	0.997616	
QLI68719.1	OTHER	0.002743	0.997257	
QLI68720.1	OTHER	0.019600	0.980400	

QLI68721.1	SP(Sec/SPI)	0.919426	0.080574	CS pos: 20-21. VIA-QP. Pr: 0.7926
QLI68722.1	SP(Sec/SPI)	0.995149	0.004851	CS pos: 21-22. AWA-AT. Pr: 0.8773
QLI68723.1	OTHER	0.002270	0.997730	
QLI68724.1	OTHER	0.076840	0.923160	
QLI68725.1	OTHER	0.000176	0.999824	
QLI68726.1	OTHER	0.001933	0.998067	
QLI68727.1	SP(Sec/SPI)	0.984421	0.015579	CS pos: 22-23. TAA-LQ. Pr: 0.4888
QLI68728.1	OTHER	0.000556	0.999444	
QLI68729.1	OTHER	0.002483	0.997517	
QLI68730.1	OTHER	0.001613	0.998387	
QLI68731.1	OTHER	0.000590	0.999410	
QLI68732.1	OTHER	0.003097	0.996903	
QLI68733.1	OTHER	0.001184	0.998816	
QLI68734.1	OTHER	0.003480	0.996520	
QLI68735.1	OTHER	0.106175	0.893825	
QLI68736.1	OTHER	0.034597	0.965403	
QLI68737.1	SP(Sec/SPI)	0.997758	0.002242	CS pos: 17-18. VSA-TD. Pr: 0.7741
QLI68738.1	OTHER	0.000961	0.999039	
QLI68739.1	OTHER	0.001460	0.998540	
QLI68740.1	OTHER	0.000590	0.999410	
QLI68741.1	OTHER	0.002217	0.997783	
QLI68742.1	OTHER	0.000805	0.999195	
QLI68743.1	OTHER	0.000880	0.999120	
QLI68744.1	SP(Sec/SPI)	0.826236	0.173764	CS pos: 18-19. ANT-AS. Pr: 0.2173
QLI68745.1	OTHER	0.000275	0.999725	
QLI68746.1	OTHER	0.000696	0.999304	
QLI68747.1	OTHER	0.184420	0.815580	
QLI68748.1	OTHER	0.001003	0.998997	
QLI68749.1	OTHER	0.000602	0.999398	
QLI68750.1	OTHER	0.001771	0.998229	
QLI68751.1	OTHER	0.001158	0.998842	

QLI68752.1	OTHER	0.001527	0.998473	
QLI68753.1	OTHER	0.000940	0.999060	
QLI68754.1	OTHER	0.000245	0.999755	
QLI68755.1	OTHER	0.000696	0.999304	
QLI68756.1	OTHER	0.001818	0.998182	
QLI68757.1	SP(Sec/SPI)	0.948541	0.051459	CS pos: 16-17. IYG-QS. Pr: 0.4699
QLI68758.1	OTHER	0.000779	0.999221	
QLI68759.1	OTHER	0.021320	0.978680	
QLI68760.1	OTHER	0.002032	0.997968	
QLI68761.1	OTHER	0.000544	0.999456	
QLI68762.1	OTHER	0.002071	0.997929	
QLI68763.1	OTHER	0.002236	0.997764	
QLI68764.1	OTHER	0.009068	0.990932	
QLI68765.1	OTHER	0.000977	0.999023	
QLI68766.1	OTHER	0.001926	0.998074	
QLI68767.1	OTHER	0.002421	0.997579	
QLI68768.1	OTHER	0.000474	0.999526	
QLI68769.1	OTHER	0.000581	0.999419	
QLI68770.1	OTHER	0.005338	0.994662	
QLI68771.1	SP(Sec/SPI)	0.997963	0.002037	CS pos: 21-22. CRS-HP. Pr: 0.9326
QLI68772.1	OTHER	0.001611	0.998389	
QLI68773.1	OTHER	0.000564	0.999436	
QLI68774.1	OTHER	0.001645	0.998355	
QLI68775.1	OTHER	0.229107	0.770893	
QLI68776.1	OTHER	0.001597	0.998403	
QLI68777.1	OTHER	0.132725	0.867275	
QLI68778.1	OTHER	0.001769	0.998231	
QLI68779.1	OTHER	0.001599	0.998401	
QLI68780.1	OTHER	0.001978	0.998022	
QLI68781.1	OTHER	0.001601	0.998399	
QLI68782.1	OTHER	0.001913	0.998087	

QLI68783.1	OTHER	0.000428	0.999572
QLI68784.1	OTHER	0.002144	0.997856
QLI68785.1	OTHER	0.000536	0.999464
QLI68786.1	OTHER	0.001392	0.998608
QLI68787.1	OTHER	0.000911	0.999089
QLI68788.1	OTHER	0.000643	0.999357
QLI68789.1	OTHER	0.008660	0.991340
QLI68790.1	OTHER	0.001154	0.998846
QLI68791.1	OTHER	0.001221	0.998779
QLI68792.1	OTHER	0.000386	0.999614
QLI68793.1	OTHER	0.001293	0.998707
QLI68794.1	OTHER	0.002116	0.997884
QLI68795.1	OTHER	0.001059	0.998941
QLI68796.1	OTHER	0.000640	0.999360
QLI68797.1	OTHER	0.000518	0.999482
QLI68798.1	OTHER	0.001483	0.998517
QLI68799.1	OTHER	0.001862	0.998138
QLI68800.1	OTHER	0.000825	0.999175
QLI68801.1	OTHER	0.001028	0.998972
QLI68802.1	OTHER	0.001633	0.998367
QLI68803.1	OTHER	0.002124	0.997876
QLI68804.1	OTHER	0.001180	0.998820
QLI68805.1	OTHER	0.001120	0.998880
QLI68806.1	OTHER	0.234413	0.765587
QLI68807.1	OTHER	0.001327	0.998673
QLI68808.1	OTHER	0.001414	0.998586
QLI68809.1	OTHER	0.032598	0.967402
QLI68810.1	OTHER	0.004217	0.995783
QLI68811.1	OTHER	0.000914	0.999086
QLI68812.1	OTHER	0.000378	0.999622
QLI68813.1	OTHER	0.005346	0.994654

QLI68814.1	OTHER	0.001044	0.998956	
QLI68815.1	OTHER	0.452928	0.547072	
QLI68816.1	OTHER	0.018494	0.981506	
QLI68817.1	OTHER	0.000625	0.999375	
QLI68818.1	OTHER	0.000415	0.999585	
QLI68819.1	OTHER	0.000964	0.999036	
QLI68820.1	OTHER	0.001398	0.998602	
QLI68821.1	OTHER	0.041836	0.958164	
QLI68822.1	OTHER	0.002934	0.997066	
QLI68823.1	OTHER	0.000333	0.999667	
QLI68824.1	OTHER	0.001991	0.998009	
QLI68825.1	OTHER	0.000834	0.999166	
QLI68826.1	OTHER	0.001845	0.998155	
QLI68827.1	OTHER	0.000859	0.999141	
QLI68828.1	OTHER	0.000982	0.999018	
QLI68829.1	SP(Sec/SPI)	0.899161	0.100839	CS pos: 22-23. GLA-IN. Pr: 0.6662
QLI68830.1	OTHER	0.014260	0.985740	
QLI68831.1	OTHER	0.000625	0.999375	
QLI68832.1	SP(Sec/SPI)	0.996373	0.003627	CS pos: 16-17. ATA-HM. Pr: 0.7139
QLI68833.1	OTHER	0.010336	0.989664	
QLI68834.1	OTHER	0.003076	0.996924	
QLI68835.1	OTHER	0.022742	0.977258	
QLI68836.1	OTHER	0.000328	0.999672	
QLI68837.1	OTHER	0.000811	0.999189	
QLI68838.1	OTHER	0.021004	0.978996	
QLI68839.1	SP(Sec/SPI)	0.656553	0.343447	CS pos: 29-30. VSA-AY. Pr: 0.5132
QLI68840.1	OTHER	0.019530	0.980470	
QLI68841.1	OTHER	0.000572	0.999428	
QLI68842.1	SP(Sec/SPI)	0.987088	0.012912	CS pos: 19-20. IFA-AA. Pr: 0.7738
QLI68843.1	OTHER	0.000944	0.999056	
QLI68844.1	OTHER	0.002619	0.997381	

QLI68845.1	OTHER	0.000835	0.999165	
QLI68846.1	OTHER	0.000856	0.999144	
QLI68847.1	OTHER	0.002913	0.997087	
QLI68848.1	OTHER	0.000917	0.999083	
QLI68849.1	OTHER	0.000935	0.999065	
QLI68850.1	OTHER	0.000738	0.999262	
QLI68851.1	OTHER	0.001109	0.998891	
QLI68852.1	SP(Sec/SPI)	0.585428	0.414572	CS pos: 17-18. AAA-AV. Pr: 0.1802
QLI68853.1	OTHER	0.000418	0.999582	
QLI68854.1	OTHER	0.001123	0.998877	
QLI68855.1	OTHER	0.001074	0.998926	
QLI68856.1	OTHER	0.001596	0.998404	
QLI68857.1	OTHER	0.000535	0.999465	
QLI68858.1	OTHER	0.001430	0.998570	
QLI68859.1	OTHER	0.088082	0.911918	
QLI68860.1	OTHER	0.011761	0.988239	
QLI68861.1	OTHER	0.002082	0.997918	
QLI68862.1	OTHER	0.439196	0.560804	
QLI68863.1	OTHER	0.000752	0.999248	
QLI68864.1	OTHER	0.083644	0.916356	
QLI68865.1	OTHER	0.001879	0.998121	
QLI68866.1	OTHER	0.010383	0.989617	
QLI68867.1	SP(Sec/SPI)	0.992492	0.007508	CS pos: 16-17. ATA-QF. Pr: 0.8814
QLI68868.1	OTHER	0.134355	0.865645	
QLI68869.1	OTHER	0.001038	0.998962	
QLI68870.1	OTHER	0.000338	0.999662	
QLI68871.1	OTHER	0.004366	0.995634	
QLI68872.1	OTHER	0.001210	0.998790	
QLI68873.1	OTHER	0.419328	0.580672	
QLI68874.1	OTHER	0.000727	0.999273	
QLI68875.1	OTHER	0.003000	0.997000	

QLI68876.1	OTHER	0.001148	0.998852	
QLI68877.1	OTHER	0.000508	0.999492	
QLI68878.1	OTHER	0.001026	0.998974	
QLI68879.1	OTHER	0.000301	0.999699	
QLI68880.1	OTHER	0.009157	0.990843	
QLI68881.1	SP(Sec/SPI)	0.997615	0.002385	CS pos: 21-22. CQS-QL. Pr: 0.7599
QLI68882.1	OTHER	0.000356	0.999644	
QLI68883.1	SP(Sec/SPI)	0.987605	0.012395	CS pos: 19-20. ASA-AP. Pr: 0.8445
QLI68884.1	OTHER	0.000335	0.999665	
QLI68885.1	OTHER	0.012453	0.987547	
QLI68886.1	OTHER	0.001045	0.998955	
QLI68887.1	OTHER	0.003188	0.996812	
QLI68888.1	OTHER	0.002313	0.997687	
QLI68889.1	OTHER	0.001257	0.998743	
QLI68890.1	OTHER	0.001560	0.998440	
QLI68891.1	SP(Sec/SPI)	0.997751	0.002249	CS pos: 17-18. CLA-GP. Pr: 0.8797
QLI68892.1	OTHER	0.000602	0.999398	
QLI68893.1	OTHER	0.001968	0.998032	
QLI68894.1	SP(Sec/SPI)	0.515211	0.484789	CS pos: 29-30. ASA-FS. Pr: 0.2562
QLI68895.1	OTHER	0.001005	0.998995	
QLI68896.1	OTHER	0.000658	0.999342	
QLI68897.1	OTHER	0.002341	0.997659	
QLI68898.1	OTHER	0.000930	0.999070	
QLI68899.1	OTHER	0.003838	0.996162	
QLI68900.1	OTHER	0.001808	0.998192	
QLI68901.1	OTHER	0.000826	0.999174	
QLI68902.1	OTHER	0.000717	0.999283	
QLI68903.1	OTHER	0.003013	0.996987	
QLI68904.1	OTHER	0.002627	0.997373	
QLI68905.1	OTHER	0.001985	0.998015	
QLI68906.1	OTHER	0.001536	0.998464	

QLI68907.1	OTHER	0.067369	0.932631	
QLI68908.1	SP(Sec/SPI)	0.988587	0.011413	CS pos: 26-27. VLA-LP. Pr: 0.9268
QLI68909.1	OTHER	0.000605	0.999395	
QLI68910.1	OTHER	0.000561	0.999439	
QLI68911.1	OTHER	0.003988	0.996012	
QLI68912.1	OTHER	0.001322	0.998678	
QLI68913.1	SP(Sec/SPI)	0.920305	0.079695	CS pos: 18-19. TAA-KP. Pr: 0.5582
QLI68914.1	SP(Sec/SPI)	0.999060	0.000940	CS pos: 19-20. ALA-GP. Pr: 0.9193
QLI68915.1	OTHER	0.001065	0.998935	
QLI68916.1	OTHER	0.000478	0.999522	
QLI68917.1	SP(Sec/SPI)	0.993059	0.006941	CS pos: 15-16. VAA-LP. Pr: 0.3489
QLI68918.1	OTHER	0.001038	0.998962	
QLI68919.1	OTHER	0.000735	0.999265	
QLI68920.1	OTHER	0.001234	0.998766	
QLI68921.1	OTHER	0.000831	0.999169	
QLI68922.1	OTHER	0.003653	0.996347	
QLI68923.1	SP(Sec/SPI)	0.922897	0.077103	CS pos: 20-21. ASA-SV. Pr: 0.7719
QLI68924.1	OTHER	0.000509	0.999491	
QLI68925.1	OTHER	0.000954	0.999046	
QLI68926.1	OTHER	0.000515	0.999485	
QLI68927.1	OTHER	0.001139	0.998861	
QLI68928.1	OTHER	0.021636	0.978364	
QLI68929.1	OTHER	0.002337	0.997663	
QLI68930.1	OTHER	0.099239	0.900761	
QLI68931.1	OTHER	0.002889	0.997111	
QLI68932.1	OTHER	0.000150	0.999850	
QLI68933.1	OTHER	0.002132	0.997868	
QLI68934.1	OTHER	0.009812	0.990188	
QLI68935.1	OTHER	0.196582	0.803418	
QLI68936.1	OTHER	0.004886	0.995114	
QLI68937.1	OTHER	0.002341	0.997659	

QLI68938.1	OTHER	0.002527	0.997473	
QLI68939.1	OTHER	0.000801	0.999199	
QLI68940.1	OTHER	0.001045	0.998955	
QLI68941.1	OTHER	0.000224	0.999776	
QLI68942.1	OTHER	0.001111	0.998889	
QLI68943.1	SP(Sec/SPI)	0.960857	0.039143	CS pos: 19-20. ASA-SS. Pr: 0.6939
QLI68944.1	OTHER	0.002127	0.997873	
QLI68945.1	OTHER	0.000701	0.999299	
QLI68946.1	OTHER	0.001534	0.998466	
QLI68947.1	OTHER	0.016797	0.983203	
QLI68948.1	OTHER	0.000655	0.999345	
QLI68949.1	OTHER	0.001042	0.998958	
QLI68950.1	OTHER	0.000580	0.999420	
QLI68951.1	OTHER	0.000950	0.999050	
QLI68952.1	OTHER	0.002029	0.997971	
QLI68953.1	SP(Sec/SPI)	0.910619	0.089381	CS pos: 19-20. AAA-EK. Pr: 0.4316
QLI68954.1	OTHER	0.012919	0.987081	
QLI68955.1	OTHER	0.000682	0.999318	
QLI68956.1	OTHER	0.002366	0.997634	
QLI68957.1	OTHER	0.000518	0.999482	
QLI68958.1	OTHER	0.011798	0.988202	
QLI68959.1	OTHER	0.001089	0.998911	
QLI68960.1	OTHER	0.000489	0.999511	
QLI68961.1	SP(Sec/SPI)	0.898700	0.101300	CS pos: 21-22. GLS-FP. Pr: 0.6683
QLI68962.1	OTHER	0.001214	0.998786	
QLI68963.1	OTHER	0.000707	0.999293	
QLI68964.1	OTHER	0.172677	0.827323	
QLI68965.1	OTHER	0.000628	0.999372	
QLI68966.1	OTHER	0.001513	0.998487	
QLI68967.1	OTHER	0.001092	0.998908	
QLI68968.1	OTHER	0.492665	0.507335	

QLI68969.1	OTHER	0.005591	0.994409	
QLI68970.1	OTHER	0.004160	0.995840	
QLI68971.1	OTHER	0.108360	0.891640	
QLI68972.1	OTHER	0.000991	0.999009	
QLI68973.1	OTHER	0.001722	0.998278	
QLI68974.1	SP(Sec/SPI)	0.914322	0.085678	CS pos: 17-18. AAA-QF. Pr: 0.6289
QLI68975.1	OTHER	0.006448	0.993552	
QLI68976.1	OTHER	0.002657	0.997343	
QLI68977.1	OTHER	0.003071	0.996929	
QLI68978.1	OTHER	0.002034	0.997966	
QLI68979.1	OTHER	0.001690	0.998310	
QLI68980.1	OTHER	0.023590	0.976410	
QLI68981.1	SP(Sec/SPI)	0.932416	0.067584	CS pos: 21-22. TSA-TG. Pr: 0.6172
QLI68982.1	OTHER	0.001657	0.998343	
QLI68983.1	OTHER	0.001573	0.998427	
QLI68984.1	OTHER	0.001398	0.998602	
QLI68985.1	SP(Sec/SPI)	0.996640	0.003360	CS pos: 19-20. ADA-AP. Pr: 0.8988
QLI68986.1	OTHER	0.043091	0.956909	
QLI68987.1	OTHER	0.002581	0.997419	
QLI68988.1	OTHER	0.000514	0.999486	
QLI68989.1	OTHER	0.001243	0.998757	
QLI68990.1	OTHER	0.001124	0.998876	
QLI68991.1	OTHER	0.013302	0.986698	
QLI68992.1	OTHER	0.001945	0.998055	
QLI68993.1	OTHER	0.000645	0.999355	
QLI68994.1	OTHER	0.000702	0.999298	
QLI68995.1	OTHER	0.031927	0.968073	
QLI68996.1	OTHER	0.000507	0.999493	
QLI68997.1	SP(Sec/SPI)	0.982912	0.017088	CS pos: 20-21. CSG-TG. Pr: 0.3920
QLI68998.1	OTHER	0.001681	0.998319	
QLI68999.1	OTHER	0.000848	0.999152	

QLI69000.1	OTHER	0.001218	0.998782	
QLI69001.1	OTHER	0.001388	0.998612	
QLI69002.1	OTHER	0.001343	0.998657	
QLI69003.1	OTHER	0.000915	0.999085	
QLI69004.1	OTHER	0.004243	0.995757	
QLI69005.1	SP(Sec/SPI)	0.987451	0.012549	CS pos: 21-22. ATA-VP. Pr: 0.6854
QLI69006.1	OTHER	0.000569	0.999431	
QLI69007.1	OTHER	0.000580	0.999420	
QLI69008.1	OTHER	0.006090	0.993910	
QLI69009.1	SP(Sec/SPI)	0.782293	0.217707	CS pos: 21-22. VSS-TG. Pr: 0.2719
QLI69010.1	OTHER	0.002372	0.997628	
QLI69011.1	OTHER	0.004486	0.995514	
QLI69012.1	OTHER	0.001071	0.998929	
QLI69013.1	OTHER	0.015823	0.984177	
QLI69014.1	OTHER	0.004649	0.995351	
QLI69015.1	OTHER	0.002314	0.997686	
QLI69016.1	SP(Sec/SPI)	0.993059	0.006941	CS pos: 20-21. SLA-AE. Pr: 0.5271
QLI69017.1	SP(Sec/SPI)	0.990406	0.009594	CS pos: 17-18. ATA-LI. Pr: 0.5086
QLI69018.1	SP(Sec/SPI)	0.985471	0.014529	CS pos: 23-24. LLA-RS. Pr: 0.6432
QLI69019.1	OTHER	0.001365	0.998635	
QLI69020.1	OTHER	0.000780	0.999220	
QLI69021.1	OTHER	0.001546	0.998454	
QLI69022.1	OTHER	0.230099	0.769901	
QLI69023.1	OTHER	0.037028	0.962972	
QLI69024.1	OTHER	0.013030	0.986970	
QLI69025.1	OTHER	0.000240	0.999760	
QLI69026.1	OTHER	0.000251	0.999749	
QLI69027.1	SP(Sec/SPI)	0.976313	0.023687	CS pos: 20-21. ATA-LP. Pr: 0.7509
QLI69028.1	OTHER	0.005859	0.994141	
QLI69029.1	SP(Sec/SPI)	0.700481	0.299519	CS pos: 19-20. LHG-YH. Pr: 0.6355
QLI69030.1	OTHER	0.000839	0.999161	

QLI69031.1	OTHER	0.001731	0.998269	
QLI69032.1	OTHER	0.001767	0.998233	
QLI69033.1	OTHER	0.000446	0.999554	
QLI69034.1	SP(Sec/SPI)	0.906105	0.093895	CS pos: 27-28. VSS-TR. Pr: 0.2253
QLI69035.1	OTHER	0.002378	0.997622	
QLI69036.1	SP(Sec/SPI)	0.962176	0.037824	CS pos: 25-26. ALS-AP. Pr: 0.5004
QLI69037.1	OTHER	0.003793	0.996207	
QLI69038.1	OTHER	0.003596	0.996404	
QLI69039.1	SP(Sec/SPI)	0.995369	0.004631	CS pos: 20-21. ALA-AP. Pr: 0.9603
QLI69040.1	OTHER	0.000583	0.999417	
QLI69041.1	OTHER	0.020050	0.979950	
QLI69042.1	OTHER	0.001808	0.998192	
QLI69043.1	OTHER	0.000374	0.999626	
QLI69044.1	OTHER	0.005855	0.994145	
QLI69045.1	SP(Sec/SPI)	0.996262	0.003738	CS pos: 22-23. TDT-SI. Pr: 0.3702
QLI69046.1	OTHER	0.006243	0.993757	
QLI69047.1	OTHER	0.114159	0.885841	
QLI69048.1	SP(Sec/SPI)	0.691552	0.308448	CS pos: 17-18. CHA-LS. Pr: 0.2912
QLI69049.1	SP(Sec/SPI)	0.975513	0.024487	CS pos: 19-20. AMA-GL. Pr: 0.6845
QLI69050.1	OTHER	0.000773	0.999227	
QLI69051.1	OTHER	0.000876	0.999124	
QLI69052.1	OTHER	0.000608	0.999392	
QLI69053.1	OTHER	0.001356	0.998644	
QLI69054.1	OTHER	0.000861	0.999139	
QLI69055.1	OTHER	0.001777	0.998223	
QLI69056.1	OTHER	0.001838	0.998162	
QLI69057.1	OTHER	0.008091	0.991909	
QLI69058.1	OTHER	0.002898	0.997102	
QLI69059.1	OTHER	0.204638	0.795362	
QLI69060.1	SP(Sec/SPI)	0.999339	0.000661	CS pos: 19-20. ASA-YA. Pr: 0.5300
QLI69061.1	OTHER	0.019260	0.980740	

QLI69062.1	OTHER	0.256814	0.743186	
QLI69063.1	SP(Sec/SPI)	0.996108	0.003892	CS pos: 21-22. CQA-HN. Pr: 0.9788
QLI69064.1	SP(Sec/SPI)	0.969835	0.030165	CS pos: 17-18. AIG-DA. Pr: 0.4855
QLI69065.1	OTHER	0.003328	0.996672	
QLI69066.1	OTHER	0.008357	0.991643	
QLI69067.1	OTHER	0.000375	0.999625	
QLI69068.1	OTHER	0.002295	0.997705	
QLI69069.1	OTHER	0.001720	0.998280	
QLI69070.1	OTHER	0.000663	0.999337	
QLI69071.1	OTHER	0.000876	0.999124	
QLI69072.1	OTHER	0.001092	0.998908	
QLI69073.1	OTHER	0.002401	0.997599	
QLI69074.1	OTHER	0.005786	0.994214	
QLI69075.1	OTHER	0.000998	0.999002	
QLI69076.1	OTHER	0.000598	0.999402	
QLI69077.1	OTHER	0.000732	0.999268	
QLI69078.1	OTHER	0.001833	0.998167	
QLI69079.1	OTHER	0.001280	0.998720	
QLI69080.1	OTHER	0.142056	0.857944	
QLI69081.1	OTHER	0.000653	0.999347	
QLI69082.1	SP(Sec/SPI)	0.899444	0.100556	CS pos: 18-19. AAG-QT. Pr: 0.4751
QLI69083.1	SP(Sec/SPI)	0.994037	0.005963	CS pos: 20-21. GAA-DG. Pr: 0.4557
QLI69084.1	OTHER	0.002062	0.997938	
QLI69085.1	OTHER	0.008305	0.991695	
QLI69086.1	OTHER	0.003115	0.996885	
QLI69087.1	OTHER	0.000969	0.999031	
QLI69088.1	OTHER	0.001204	0.998796	
QLI69089.1	OTHER	0.002816	0.997184	
QLI69090.1	OTHER	0.001161	0.998839	
QLI69091.1	OTHER	0.002441	0.997559	
QLI69092.1	OTHER	0.001277	0.998723	

QLI69093.1	OTHER	0.002819	0.997181	
QLI69094.1	SP(Sec/SPI)	0.993746	0.006254	CS pos: 25-26. LQA-AN. Pr: 0.4965
QLI69095.1	OTHER	0.002384	0.997616	
QLI69096.1	OTHER	0.003251	0.996749	
QLI69097.1	OTHER	0.003896	0.996104	
QLI69098.1	OTHER	0.000541	0.999459	
QLI69099.1	OTHER	0.001358	0.998642	
QLI69100.1	OTHER	0.001344	0.998656	
QLI69101.1	OTHER	0.000417	0.999583	
QLI69102.1	SP(Sec/SPI)	0.862301	0.137699	CS pos: 28-29. VSS-QD. Pr: 0.7823
QLI69103.1	OTHER	0.002642	0.997358	
QLI69104.1	SP(Sec/SPI)	0.854615	0.145385	CS pos: 22-23. AQS-TW. Pr: 0.5995
QLI69105.1	OTHER	0.000447	0.999553	
QLI69106.1	OTHER	0.000529	0.999471	
QLI69107.1	OTHER	0.000485	0.999515	
QLI69108.1	OTHER	0.002280	0.997720	
QLI69109.1	OTHER	0.003009	0.996991	
QLI69110.1	OTHER	0.000360	0.999640	
QLI69111.1	OTHER	0.000813	0.999187	
QLI69112.1	OTHER	0.010340	0.989660	
QLI69113.1	OTHER	0.000960	0.999040	
QLI69114.1	SP(Sec/SPI)	0.793853	0.206147	CS pos: 23-24. AYT-HL. Pr: 0.6257
QLI69115.1	OTHER	0.001483	0.998517	
QLI69116.1	OTHER	0.004318	0.995682	
QLI69117.1	OTHER	0.003132	0.996868	
QLI69118.1	OTHER	0.002347	0.997653	
QLI69119.1	OTHER	0.001793	0.998207	
QLI69120.1	OTHER	0.000663	0.999337	
QLI69121.1	SP(Sec/SPI)	0.990199	0.009801	CS pos: 19-20. ALG-AP. Pr: 0.7232
QLI69122.1	OTHER	0.006206	0.993794	
QLI69123.1	OTHER	0.037927	0.962073	

QLI69124.1	OTHER	0.000760	0.999240	
QLI69125.1	OTHER	0.001712	0.998288	
QLI69126.1	OTHER	0.002564	0.997436	
QLI69127.1	OTHER	0.001785	0.998215	
QLI69128.1	OTHER	0.022362	0.977638	
QLI69129.1	SP(Sec/SPI)	0.862224	0.137776	CS pos: 25-26. AHA-AA. Pr: 0.5503
QLI69130.1	OTHER	0.000272	0.999728	
QLI69131.1	SP(Sec/SPI)	0.988512	0.011488	CS pos: 22-23. AGA-IE. Pr: 0.7885
QLI69132.1	OTHER	0.000930	0.999070	
QLI69133.1	OTHER	0.002016	0.997984	
QLI69134.1	OTHER	0.000329	0.999671	
QLI69135.1	OTHER	0.000991	0.999009	
QLI69136.1	OTHER	0.001289	0.998711	
QLI69137.1	SP(Sec/SPI)	0.547008	0.452992	CS pos: 28-29. GLP-DL. Pr: 0.1690
QLI69138.1	SP(Sec/SPI)	0.946699	0.053301	CS pos: 22-23. VSG-TT. Pr: 0.2834
QLI69139.1	OTHER	0.001195	0.998805	
QLI69140.1	OTHER	0.002927	0.997073	
QLI69141.1	SP(Sec/SPI)	0.910997	0.089003	CS pos: 19-20. SAA-AP. Pr: 0.5271
QLI69142.1	OTHER	0.000608	0.999392	
QLI69143.1	SP(Sec/SPI)	0.967443	0.032557	CS pos: 19-20. VSA-KV. Pr: 0.8534
QLI69144.1	OTHER	0.000551	0.999449	
QLI69145.1	OTHER	0.003295	0.996705	
QLI69146.1	OTHER	0.000759	0.999241	
QLI69147.1	SP(Sec/SPI)	0.862668	0.137332	CS pos: 27-28. VAA-HR. Pr: 0.5406
QLI69148.1	OTHER	0.000728	0.999272	
QLI69149.1	OTHER	0.000794	0.999206	
QLI69150.1	OTHER	0.000849	0.999151	
QLI69151.1	OTHER	0.001069	0.998931	
QLI69152.1	SP(Sec/SPI)	0.944215	0.055785	CS pos: 22-23. AQA-DT. Pr: 0.7044
QLI69153.1	OTHER	0.000652	0.999348	
QLI69154.1	OTHER	0.052446	0.947554	

QLI69155.1	OTHER	0.000750	0.999250	
QLI69156.1	OTHER	0.000712	0.999288	
QLI69157.1	OTHER	0.000718	0.999282	
QLI69158.1	SP(Sec/SPI)	0.878112	0.121888	CS pos: 26-27. TLG-AT. Pr: 0.7323
QLI69159.1	OTHER	0.001360	0.998640	
QLI69160.1	OTHER	0.002424	0.997576	
QLI69161.1	OTHER	0.000967	0.999033	
QLI69162.1	OTHER	0.008725	0.991275	
QLI69163.1	OTHER	0.000843	0.999157	
QLI69164.1	OTHER	0.001868	0.998132	
QLI69165.1	OTHER	0.000553	0.999447	
QLI69166.1	OTHER	0.000645	0.999355	
QLI69167.1	OTHER	0.001490	0.998510	
QLI69168.1	OTHER	0.001193	0.998807	
QLI69169.1	OTHER	0.000507	0.999493	
QLI69170.1	OTHER	0.000662	0.999338	
QLI69171.1	OTHER	0.000853	0.999147	
QLI69172.1	OTHER	0.001327	0.998673	
QLI69173.1	OTHER	0.000658	0.999342	
QLI69174.1	OTHER	0.002596	0.997404	
QLI69175.1	OTHER	0.000911	0.999089	
QLI69176.1	OTHER	0.001924	0.998076	
QLI69177.1	OTHER	0.000800	0.999200	
QLI69178.1	SP(Sec/SPI)	0.993213	0.006787	CS pos: 17-18. AQA-MR. Pr: 0.8130
QLI69179.1	SP(Sec/SPI)	0.997675	0.002325	CS pos: 17-18. TLA-QD. Pr: 0.9378
QLI69180.1	OTHER	0.001068	0.998932	
QLI69181.1	OTHER	0.000235	0.999765	
QLI69182.1	OTHER	0.000791	0.999209	
QLI69183.1	OTHER	0.000509	0.999491	
QLI69184.1	OTHER	0.000983	0.999017	
QLI69185.1	OTHER	0.002465	0.997535	

QLI69186.1	OTHER	0.000461	0.999539	
QLI69187.1	OTHER	0.004217	0.995783	
QLI69188.1	OTHER	0.002200	0.997800	
QLI69189.1	SP(Sec/SPI)	0.984220	0.015780	CS pos: 17-18. CKA-VY. Pr: 0.8375
QLI69190.1	OTHER	0.008837	0.991163	
QLI69191.1	OTHER	0.001479	0.998521	
QLI69192.1	SP(Sec/SPI)	0.960470	0.039530	CS pos: 23-24. AQA-TY. Pr: 0.4357
QLI69193.1	OTHER	0.000479	0.999521	
QLI69194.1	OTHER	0.030419	0.969581	
QLI69195.1	OTHER	0.009275	0.990725	
QLI69196.1	OTHER	0.000264	0.999736	
QLI69197.1	OTHER	0.000789	0.999211	
QLI69198.1	OTHER	0.001822	0.998178	
QLI69199.1	OTHER	0.028727	0.971273	
QLI69200.1	OTHER	0.008997	0.991003	
QLI69201.1	OTHER	0.005864	0.994136	
QLI69202.1	OTHER	0.000527	0.999473	
QLI69203.1	OTHER	0.000359	0.999641	
QLI69204.1	SP(Sec/SPI)	0.999317	0.000683	CS pos: 17-18. ALA-AP. Pr: 0.9154
QLI69205.1	OTHER	0.001793	0.998207	
QLI69206.1	OTHER	0.487199	0.512801	
QLI69207.1	OTHER	0.001565	0.998435	
QLI69208.1	OTHER	0.033503	0.966497	
QLI69209.1	OTHER	0.003176	0.996824	
QLI69210.1	OTHER	0.000889	0.999111	
QLI69211.1	OTHER	0.000490	0.999510	
QLI69212.1	OTHER	0.004448	0.995552	
QLI69213.1	OTHER	0.000472	0.999528	
QLI69214.1	SP(Sec/SPI)	0.954861	0.045139	CS pos: 18-19. VQA-AA. Pr: 0.5455
QLI69215.1	OTHER	0.002387	0.997613	
QLI69216.1	OTHER	0.004754	0.995246	

QLI69217.1	OTHER	0.001508	0.998492	
QLI69218.1	OTHER	0.000801	0.999199	
QLI69219.1	OTHER	0.000982	0.999018	
QLI69220.1	OTHER	0.004324	0.995676	
QLI69221.1	OTHER	0.001332	0.998668	
QLI69222.1	OTHER	0.001000	0.999000	
QLI69223.1	OTHER	0.000678	0.999322	
QLI69224.1	OTHER	0.001856	0.998144	
QLI69225.1	OTHER	0.000870	0.999130	
QLI69226.1	OTHER	0.004543	0.995457	
QLI69227.1	OTHER	0.000770	0.999230	
QLI69228.1	OTHER	0.034062	0.965938	
QLI69229.1	OTHER	0.003572	0.996428	
QLI69230.1	OTHER	0.006814	0.993186	
QLI69231.1	OTHER	0.003366	0.996634	
QLI69232.1	OTHER	0.000710	0.999290	
QLI69233.1	OTHER	0.000725	0.999275	
QLI69234.1	OTHER	0.008046	0.991954	
QLI69235.1	SP(Sec/SPI)	0.933179	0.066821	CS pos: 19-20. ALS-IP. Pr: 0.3983
QLI69236.1	OTHER	0.001376	0.998624	
QLI69237.1	OTHER	0.001957	0.998043	
QLI69238.1	OTHER	0.001533	0.998467	
QLI69239.1	OTHER	0.000665	0.999335	
QLI69240.1	OTHER	0.001984	0.998016	
QLI69241.1	OTHER	0.000900	0.999100	
QLI69242.1	OTHER	0.000502	0.999498	
QLI69243.1	OTHER	0.001891	0.998109	
QLI69244.1	OTHER	0.000915	0.999085	
QLI69245.1	OTHER	0.002226	0.997774	
QLI69246.1	OTHER	0.000745	0.999255	
QLI69247.1	SP(Sec/SPI)	0.971871	0.028129	CS pos: 18-19. AVA-DD. Pr: 0.7159

QLI69248.1	SP(Sec/SPI)	0.541185	0.458815	CS pos: 15-16. ANC-FD. Pr: 0.3383
QLI69249.1	OTHER	0.001123	0.998877	
QLI69250.1	OTHER	0.001200	0.998800	
QLI69251.1	OTHER	0.000187	0.999813	
QLI69252.1	OTHER	0.000520	0.999480	
QLI69253.1	SP(Sec/SPI)	0.701241	0.298759	CS pos: 22-23. GSA-RS. Pr: 0.2244
QLI69254.1	OTHER	0.001032	0.998968	
QLI69255.1	OTHER	0.000552	0.999448	
QLI69256.1	SP(Sec/SPI)	0.979418	0.020582	CS pos: 17-18. ANA-RI. Pr: 0.7092
QLI69257.1	OTHER	0.000275	0.999725	
QLI69258.1	OTHER	0.003802	0.996198	
QLI69259.1	OTHER	0.001676	0.998324	
QLI69260.1	OTHER	0.001368	0.998632	
QLI69261.1	OTHER	0.000565	0.999435	
QLI69262.1	OTHER	0.004460	0.995540	
QLI69263.1	SP(Sec/SPI)	0.804468	0.195532	CS pos: 18-19. GAA-FT. Pr: 0.5679
QLI69264.1	OTHER	0.000413	0.999587	
QLI69265.1	OTHER	0.000853	0.999147	
QLI69266.1	OTHER	0.267077	0.732923	
QLI69267.1	OTHER	0.001347	0.998653	
QLI69268.1	OTHER	0.000655	0.999345	
QLI69269.1	OTHER	0.000599	0.999401	
QLI69270.1	OTHER	0.001389	0.998611	
QLI69271.1	SP(Sec/SPI)	0.995546	0.004454	CS pos: 21-22. TSA-AP. Pr: 0.5330
QLI69272.1	OTHER	0.001165	0.998835	
QLI69273.1	OTHER	0.000773	0.999227	
QLI69274.1	OTHER	0.000792	0.999208	
QLI69275.1	OTHER	0.000732	0.999268	
QLI69276.1	OTHER	0.001624	0.998376	
QLI69277.1	SP(Sec/SPI)	0.556092	0.443908	CS pos: 18-19. VLC-RF. Pr: 0.3293
QLI69278.1	OTHER	0.000158	0.999842	

QLI69279.1	OTHER	0.001413	0.998587
QLI69280.1	OTHER	0.001090	0.998910
QLI69281.1	OTHER	0.402787	0.597213
QLI69282.1	OTHER	0.001395	0.998605
QLI69283.1	OTHER	0.002713	0.997287
QLI69284.1	OTHER	0.000980	0.999020
QLI69285.1	OTHER	0.001329	0.998671
QLI69286.1	OTHER	0.151226	0.848774
QLI69287.1	OTHER	0.002577	0.997423
QLI69288.1	OTHER	0.001121	0.998879
QLI69289.1	OTHER	0.199873	0.800127
QLI69290.1	OTHER	0.000754	0.999246
QLI69291.1	OTHER	0.006496	0.993504
QLI69292.1	OTHER	0.001127	0.998873
QLI69293.1	OTHER	0.003494	0.996506
QLI69294.1	OTHER	0.003957	0.996043
QLI69295.1	OTHER	0.394898	0.605102
QLI69296.1	OTHER	0.002758	0.997242
QLI69297.1	OTHER	0.003816	0.996184
QLI69298.1	OTHER	0.000760	0.999240
QLI69299.1	OTHER	0.001835	0.998165
QLI69300.1	OTHER	0.002102	0.997898
QLI69301.1	OTHER	0.001477	0.998523
QLI69302.1	OTHER	0.002807	0.997193
QLI69303.1	OTHER	0.322894	0.677106
QLI69304.1	OTHER	0.000817	0.999183
QLI69305.1	OTHER	0.000703	0.999297
QLI69306.1	OTHER	0.001399	0.998601
QLI69307.1	OTHER	0.003175	0.996825
QLI69308.1	OTHER	0.000616	0.999384
QLI69309.1	OTHER	0.002249	0.997751

QLI69310.1	OTHER	0.000974	0.999026	
QLI69311.1	OTHER	0.001674	0.998326	
QLI69312.1	OTHER	0.001101	0.998899	
QLI69313.1	OTHER	0.000336	0.999664	
QLI69314.1	OTHER	0.002252	0.997748	
QLI69315.1	OTHER	0.043607	0.956393	
QLI69316.1	OTHER	0.000484	0.999516	
QLI69317.1	OTHER	0.004900	0.995100	
QLI69318.1	OTHER	0.001598	0.998402	
QLI69319.1	OTHER	0.001245	0.998755	
QLI69320.1	OTHER	0.001256	0.998744	
QLI69321.1	OTHER	0.001027	0.998973	
QLI69322.1	OTHER	0.023832	0.976168	
QLI69323.1	OTHER	0.160643	0.839357	
QLI69324.1	OTHER	0.007873	0.992127	
QLI69325.1	OTHER	0.001573	0.998427	
QLI69326.1	OTHER	0.000909	0.999091	
QLI69327.1	OTHER	0.062463	0.937537	
QLI69328.1	OTHER	0.004344	0.995656	
QLI69329.1	OTHER	0.008858	0.991142	
QLI69330.1	OTHER	0.001913	0.998087	
QLI69331.1	OTHER	0.001375	0.998625	
QLI69332.1	OTHER	0.001509	0.998491	
QLI69333.1	OTHER	0.000931	0.999069	
QLI69334.1	OTHER	0.000564	0.999436	
QLI69335.1	OTHER	0.000346	0.999654	
QLI69336.1	OTHER	0.001291	0.998709	
QLI69337.1	OTHER	0.002141	0.997859	
QLI69338.1	OTHER	0.000585	0.999415	
QLI69339.1	OTHER	0.000929	0.999071	
QLI69340.1	SP(Sec/SPI)	0.703294	0.296706	CS pos: 21-22. AVA-VP. Pr: 0.4639

QLI69341.1	OTHER	0.000629	0.999371	
QLI69342.1	OTHER	0.000982	0.999018	
QLI69343.1	OTHER	0.003207	0.996793	
QLI69344.1	OTHER	0.000660	0.999340	
QLI69345.1	OTHER	0.001100	0.998900	
QLI69346.1	OTHER	0.000321	0.999679	
QLI69347.1	OTHER	0.000557	0.999443	
QLI69348.1	OTHER	0.001879	0.998121	
QLI69349.1	OTHER	0.003231	0.996769	
QLI69350.1	OTHER	0.002559	0.997441	
QLI69351.1	OTHER	0.000256	0.999744	
QLI69352.1	OTHER	0.000462	0.999538	
QLI69353.1	OTHER	0.000420	0.999580	
QLI69354.1	OTHER	0.001100	0.998900	
QLI69355.1	SP(Sec/SPI)	0.976256	0.023744	CS pos: 20-21. ALG-IN. Pr: 0.5524
QLI69356.1	OTHER	0.000317	0.999683	
QLI69357.1	OTHER	0.000719	0.999281	
QLI69358.1	OTHER	0.000903	0.999097	
QLI69359.1	OTHER	0.000576	0.999424	
QLI69360.1	OTHER	0.002853	0.997147	
QLI69361.1	OTHER	0.001872	0.998128	
QLI69362.1	OTHER	0.001309	0.998691	
QLI69363.1	OTHER	0.006484	0.993516	
QLI69364.1	OTHER	0.001235	0.998765	
QLI69365.1	OTHER	0.000752	0.999248	
QLI69366.1	SP(Sec/SPI)	0.772667	0.227333	CS pos: 29-30. SNA-SV. Pr: 0.5235
QLI69367.1	OTHER	0.001308	0.998692	
QLI69368.1	OTHER	0.000515	0.999485	
QLI69369.1	OTHER	0.004150	0.995850	
QLI69370.1	OTHER	0.001821	0.998179	
QLI69371.1	OTHER	0.000592	0.999408	

QLI69372.1	OTHER	0.374253	0.625747	
QLI69373.1	SP(Sec/SPI)	0.981298	0.018702	CS pos: 20-21. GSA-VL. Pr: 0.3130
QLI69374.1	OTHER	0.000601	0.999399	
QLI69375.1	SP(Sec/SPI)	0.997876	0.002124	CS pos: 18-19. ATG-LP. Pr: 0.8342
QLI69376.1	OTHER	0.001885	0.998115	
QLI69377.1	OTHER	0.004967	0.995033	
QLI69378.1	OTHER	0.001336	0.998664	
QLI69379.1	OTHER	0.026143	0.973857	
QLI69380.1	OTHER	0.001030	0.998970	
QLI69381.1	SP(Sec/SPI)	0.955414	0.044586	CS pos: 19-20. SLA-TK. Pr: 0.6751
QLI69382.1	OTHER	0.002751	0.997249	
QLI69383.1	OTHER	0.011384	0.988616	
QLI69384.1	SP(Sec/SPI)	0.944234	0.055766	CS pos: 28-29. AIA-GD. Pr: 0.7411
QLI69385.1	OTHER	0.001300	0.998700	
QLI69386.1	OTHER	0.000522	0.999478	
QLI69387.1	OTHER	0.022737	0.977263	
QLI69388.1	OTHER	0.172584	0.827416	
QLI69389.1	OTHER	0.001192	0.998808	
QLI69390.1	SP(Sec/SPI)	0.963994	0.036006	CS pos: 22-23. CHG-AW. Pr: 0.8994
QLI69391.1	OTHER	0.001029	0.998971	
QLI69392.1	OTHER	0.002499	0.997501	
QLI69393.1	OTHER	0.000805	0.999195	
QLI69394.1	OTHER	0.000469	0.999531	
QLI69395.1	OTHER	0.000412	0.999588	
QLI69396.1	OTHER	0.000682	0.999318	
QLI69397.1	OTHER	0.001340	0.998660	
QLI69398.1	OTHER	0.001135	0.998865	
QLI69399.1	OTHER	0.002654	0.997346	
QLI69400.1	OTHER	0.001859	0.998141	
QLI69401.1	OTHER	0.000971	0.999029	
QLI69402.1	OTHER	0.000947	0.999053	

QLI69403.1	OTHER	0.001329	0.998671	
QLI69404.1	OTHER	0.000938	0.999062	
QLI69405.1	OTHER	0.000865	0.999135	
QLI69406.1	OTHER	0.000504	0.999496	
QLI69407.1	OTHER	0.000558	0.999442	
QLI69408.1	OTHER	0.005389	0.994611	
QLI69409.1	SP(Sec/SPI)	0.992832	0.007168	CS pos: 25-26. AQG-KQ. Pr: 0.9185
QLI69410.1	OTHER	0.004056	0.995944	
QLI69411.1	OTHER	0.004098	0.995902	
QLI69412.1	OTHER	0.000742	0.999258	
QLI69413.1	OTHER	0.002447	0.997553	
QLI69414.1	OTHER	0.000208	0.999792	
QLI69415.1	OTHER	0.001320	0.998680	
QLI69416.1	OTHER	0.000525	0.999475	
QLI69417.1	OTHER	0.001250	0.998750	
QLI69418.1	OTHER	0.003070	0.996930	
QLI69419.1	OTHER	0.005681	0.994319	
QLI69420.1	SP(Sec/SPI)	0.963688	0.036312	CS pos: 20-21. AQG-GS. Pr: 0.5700
QLI69421.1	OTHER	0.000562	0.999438	
QLI69422.1	OTHER	0.003336	0.996664	
QLI69423.1	OTHER	0.001852	0.998148	
QLI69424.1	OTHER	0.005930	0.994070	
QLI69425.1	OTHER	0.000639	0.999361	
QLI69426.1	OTHER	0.000758	0.999242	
QLI69427.1	OTHER	0.002908	0.997092	
QLI69428.1	OTHER	0.008437	0.991563	
QLI69429.1	OTHER	0.003715	0.996285	
QLI69430.1	OTHER	0.026144	0.973856	
QLI69431.1	SP(Sec/SPI)	0.899617	0.100383	CS pos: 20-21. AAG-AP. Pr: 0.5953
QLI69432.1	OTHER	0.002442	0.997558	
QLI69433.1	OTHER	0.199848	0.800152	

QLI69434.1	OTHER	0.002027	0.997973	
QLI69435.1	OTHER	0.000973	0.999027	
QLI69436.1	SP(Sec/SPI)	0.978568	0.021432	CS pos: 21-22. ATA-AP. Pr: 0.7928
QLI69437.1	OTHER	0.000945	0.999055	
QLI69438.1	OTHER	0.000791	0.999209	
QLI69439.1	SP(Sec/SPI)	0.995900	0.004100	CS pos: 18-19. ASA-EN. Pr: 0.9026
QLI69440.1	OTHER	0.010494	0.989506	
QLI69441.1	OTHER	0.000706	0.999294	
QLI69442.1	OTHER	0.006795	0.993205	
QLI69443.1	OTHER	0.000737	0.999263	
QLI69444.1	OTHER	0.002214	0.997786	
QLI69445.1	OTHER	0.000287	0.999713	
QLI69446.1	OTHER	0.001146	0.998854	
QLI69447.1	OTHER	0.000325	0.999675	
QLI69448.1	SP(Sec/SPI)	0.993644	0.006356	CS pos: 17-18. TSA-AI. Pr: 0.4099
QLI69449.1	OTHER	0.005255	0.994745	
QLI69450.1	OTHER	0.006821	0.993179	
QLI69451.1	OTHER	0.000239	0.999761	
QLI69452.1	OTHER	0.004677	0.995323	
QLI69453.1	OTHER	0.000491	0.999509	
QLI69454.1	OTHER	0.000424	0.999576	
QLI69455.1	OTHER	0.001715	0.998285	
QLI69456.1	SP(Sec/SPI)	0.580995	0.419005	CS pos: 16-17. SLA-IS. Pr: 0.3066
QLI69457.1	OTHER	0.002896	0.997104	
QLI69458.1	OTHER	0.002163	0.997837	
QLI69459.1	SP(Sec/SPI)	0.966457	0.033543	CS pos: 17-18. SLG-IP. Pr: 0.7229
QLI69460.1	OTHER	0.000103	0.999897	
QLI69461.1	OTHER	0.001101	0.998899	
QLI69462.1	OTHER	0.015017	0.984983	
QLI69463.1	OTHER	0.000894	0.999106	
QLI69464.1	OTHER	0.008420	0.991580	

QLI69465.1	OTHER	0.000321	0.999679	
QLI69466.1	OTHER	0.374918	0.625082	
QLI69467.1	OTHER	0.030363	0.969637	
QLI69468.1	OTHER	0.002862	0.997138	
QLI69469.1	OTHER	0.001257	0.998743	
QLI69470.1	OTHER	0.000262	0.999738	
QLI69471.1	OTHER	0.001391	0.998609	
QLI69472.1	OTHER	0.000511	0.999489	
QLI69473.1	OTHER	0.001362	0.998638	
QLI69474.1	OTHER	0.000699	0.999301	
QLI69475.1	OTHER	0.003738	0.996262	
QLI69476.1	OTHER	0.000344	0.999656	
QLI69477.1	OTHER	0.003746	0.996254	
QLI69478.1	OTHER	0.000423	0.999577	
QLI69479.1	OTHER	0.000607	0.999393	
QLI69480.1	SP(Sec/SPI)	0.823367	0.176633	CS pos: 20-21. AIA-GV. Pr: 0.3618
QLI69481.1	OTHER	0.001663	0.998337	
QLI69482.1	SP(Sec/SPI)	0.926822	0.073178	CS pos: 19-20. ISA-SD. Pr: 0.5918
QLI69483.1	OTHER	0.002918	0.997082	
QLI69484.1	OTHER	0.000587	0.999413	
QLI69485.1	OTHER	0.002103	0.997897	
QLI69486.1	OTHER	0.000319	0.999681	
QLI69487.1	OTHER	0.000549	0.999451	
QLI69488.1	OTHER	0.000173	0.999827	
QLI69489.1	OTHER	0.000404	0.999596	
QLI69490.1	SP(Sec/SPI)	0.985701	0.014299	CS pos: 18-19. ASA-RP. Pr: 0.4258
QLI69491.1	OTHER	0.059212	0.940788	
QLI69492.1	OTHER	0.001140	0.998860	
QLI69493.1	OTHER	0.001767	0.998233	
QLI69494.1	OTHER	0.015467	0.984533	
QLI69495.1	OTHER	0.001601	0.998399	

QLI69496.1	OTHER	0.003115	0.996885	
QLI69497.1	OTHER	0.002428	0.997572	
QLI69498.1	OTHER	0.001070	0.998930	
QLI69499.1	OTHER	0.004947	0.995053	
QLI69500.1	OTHER	0.002221	0.997779	
QLI69501.1	OTHER	0.143272	0.856728	
QLI69502.1	OTHER	0.003131	0.996869	
QLI69503.1	OTHER	0.000537	0.999463	
QLI69504.1	OTHER	0.000839	0.999161	
QLI69505.1	OTHER	0.028205	0.971795	
QLI69506.1	OTHER	0.038101	0.961899	
QLI69507.1	OTHER	0.000328	0.999672	
QLI69508.1	OTHER	0.071625	0.928375	
QLI69509.1	SP(Sec/SPI)	0.990799	0.009201	CS pos: 18-19. AVA-QY. Pr: 0.7950
QLI69510.1	OTHER	0.001689	0.998311	
QLI69511.1	OTHER	0.000554	0.999446	
QLI69512.1	OTHER	0.000975	0.999025	
QLI69513.1	OTHER	0.005812	0.994188	
QLI69514.1	OTHER	0.000421	0.999579	
QLI69515.1	OTHER	0.000553	0.999447	
QLI69516.1	OTHER	0.000624	0.999376	
QLI69517.1	OTHER	0.010574	0.989426	
QLI69518.1	OTHER	0.000520	0.999480	
QLI69519.1	OTHER	0.001407	0.998593	
QLI69520.1	OTHER	0.001217	0.998783	
QLI69521.1	OTHER	0.028759	0.971241	
QLI69522.1	OTHER	0.000877	0.999123	
QLI69523.1	SP(Sec/SPI)	0.965522	0.034478	CS pos: 18-19. AES-TC. Pr: 0.3775
QLI69524.1	OTHER	0.000651	0.999349	
QLI69525.1	OTHER	0.000701	0.999299	
QLI69526.1	OTHER	0.000522	0.999478	

QLI69527.1	OTHER	0.001113	0.998887	
QLI69528.1	OTHER	0.001304	0.998696	
QLI69529.1	OTHER	0.000530	0.999470	
QLI69530.1	SP(Sec/SPI)	0.995287	0.004713	CS pos: 16-17. CSA-AP. Pr: 0.4935
QLI69531.1	OTHER	0.001145	0.998855	
QLI69532.1	OTHER	0.002916	0.997084	
QLI69533.1	OTHER	0.000934	0.999066	
QLI69534.1	OTHER	0.002977	0.997023	
QLI69535.1	SP(Sec/SPI)	0.910852	0.089148	CS pos: 28-29. AHS-WI. Pr: 0.6329
QLI69536.1	OTHER	0.033422	0.966578	
QLI69537.1	OTHER	0.000609	0.999391	
QLI69538.1	OTHER	0.001307	0.998693	
QLI69539.1	OTHER	0.000439	0.999561	
QLI69540.1	OTHER	0.000355	0.999645	
QLI69541.1	OTHER	0.000726	0.999274	
QLI69542.1	OTHER	0.002108	0.997892	
QLI69543.1	OTHER	0.001490	0.998510	
QLI69544.1	OTHER	0.002024	0.997976	
QLI69545.1	OTHER	0.004461	0.995539	
QLI69546.1	SP(Sec/SPI)	0.838092	0.161908	CS pos: 22-23. VFS-AA. Pr: 0.2998
QLI69547.1	OTHER	0.000426	0.999574	
QLI69548.1	OTHER	0.001576	0.998424	
QLI69549.1	OTHER	0.116782	0.883218	
QLI69550.1	OTHER	0.000295	0.999705	
QLI69551.1	OTHER	0.000384	0.999616	
QLI69552.1	OTHER	0.005772	0.994228	
QLI69553.1	OTHER	0.001234	0.998766	
QLI69554.1	OTHER	0.000247	0.999753	
QLI69555.1	OTHER	0.001826	0.998174	
QLI69556.1	OTHER	0.000544	0.999456	
QLI69557.1	OTHER	0.001010	0.998990	

QLI69558.1	OTHER	0.002017	0.997983	
QLI69559.1	OTHER	0.418145	0.581855	
QLI69560.1	OTHER	0.007714	0.992286	
QLI69561.1	OTHER	0.000644	0.999356	
QLI69562.1	SP(Sec/SPI)	0.997097	0.002903	CS pos: 17-18. AAA-KI. Pr: 0.7875
QLI69563.1	OTHER	0.001555	0.998445	
QLI69564.1	OTHER	0.000313	0.999687	
QLI69565.1	OTHER	0.001353	0.998647	
QLI69566.1	OTHER	0.004521	0.995479	
QLI69567.1	OTHER	0.002770	0.997230	
QLI69568.1	OTHER	0.205961	0.794039	
QLI69569.1	OTHER	0.010217	0.989783	
QLI69570.1	OTHER	0.016993	0.983007	
QLI69571.1	SP(Sec/SPI)	0.870415	0.129585	CS pos: 37-38. TAA-SH. Pr: 0.6158
QLI69572.1	OTHER	0.002035	0.997965	
QLI69573.1	SP(Sec/SPI)	0.971235	0.028765	CS pos: 20-21. TLA-AM. Pr: 0.6629
QLI69574.1	OTHER	0.080364	0.919636	
QLI69575.1	OTHER	0.000434	0.999566	
QLI69576.1	SP(Sec/SPI)	0.994509	0.005491	CS pos: 17-18. TAG-SP. Pr: 0.6440
QLI69577.1	SP(Sec/SPI)	0.990090	0.009910	CS pos: 18-19. AFA-AP. Pr: 0.7379
QLI69578.1	OTHER	0.005514	0.994486	
QLI69579.1	SP(Sec/SPI)	0.998726	0.001274	CS pos: 17-18. VSA-FP. Pr: 0.7540
QLI69580.1	OTHER	0.010951	0.989049	
QLI69581.1	OTHER	0.417331	0.582669	
QLI69582.1	OTHER	0.001459	0.998541	
QLI69583.1	OTHER	0.000520	0.999480	
QLI69584.1	OTHER	0.001293	0.998707	
QLI69585.1	OTHER	0.007453	0.992547	
QLI69586.1	OTHER	0.000708	0.999292	
QLI69587.1	OTHER	0.003762	0.996238	
QLI69588.1	OTHER	0.000528	0.999472	

QLI69589.1	OTHER	0.000468	0.999532	
QLI69590.1	SP(Sec/SPI)	0.890384	0.109616	CS pos: 23-24. VFC-IQ. Pr: 0.7532
QLI69591.1	OTHER	0.195038	0.804962	
QLI69592.1	OTHER	0.026586	0.973414	
QLI69593.1	OTHER	0.006980	0.993020	
QLI69594.1	OTHER	0.002472	0.997528	
QLI69595.1	SP(Sec/SPI)	0.963563	0.036437	CS pos: 20-21. AVA-LT. Pr: 0.6187
QLI69596.1	SP(Sec/SPI)	0.981769	0.018231	CS pos: 23-24. ASA-KD. Pr: 0.8992
QLI69597.1	OTHER	0.000353	0.999647	
QLI69598.1	OTHER	0.003604	0.996396	
QLI69599.1	OTHER	0.001991	0.998009	
QLI69600.1	OTHER	0.003459	0.996541	
QLI69601.1	OTHER	0.001812	0.998188	
QLI69602.1	SP(Sec/SPI)	0.991570	0.008430	CS pos: 19-20. AQA-RY. Pr: 0.5480
QLI69603.1	SP(Sec/SPI)	0.985442	0.014558	CS pos: 21-22. AWA-QP. Pr: 0.8714
QLI69604.1	OTHER	0.002384	0.997616	
QLI69605.1	SP(Sec/SPI)	0.990718	0.009282	CS pos: 26-27. SRA-LP. Pr: 0.3524
QLI69606.1	OTHER	0.000323	0.999677	
QLI69607.1	OTHER	0.445452	0.554548	
QLI69608.1	OTHER	0.001431	0.998569	
QLI69609.1	OTHER	0.002348	0.997652	
QLI69610.1	OTHER	0.001457	0.998543	
QLI69611.1	OTHER	0.001507	0.998493	
QLI69612.1	OTHER	0.001939	0.998061	
QLI69613.1	OTHER	0.002018	0.997982	
QLI69614.1	SP(Sec/SPI)	0.898074	0.101926	CS pos: 19-20. VSA-GT. Pr: 0.5155
QLI69615.1	OTHER	0.002250	0.997750	
QLI69616.1	OTHER	0.000948	0.999052	
QLI69617.1	OTHER	0.000422	0.999578	
QLI69618.1	OTHER	0.001614	0.998386	
QLI69619.1	OTHER	0.002529	0.997471	

QLI69620.1	OTHER	0.000775	0.999225	
QLI69621.1	OTHER	0.000589	0.999411	
QLI69622.1	OTHER	0.112720	0.887280	
QLI69623.1	OTHER	0.000381	0.999619	
QLI69624.1	OTHER	0.000429	0.999571	
QLI69625.1	OTHER	0.003254	0.996746	
QLI69626.1	OTHER	0.002531	0.997469	
QLI69627.1	OTHER	0.003225	0.996775	
QLI69628.1	OTHER	0.001330	0.998670	
QLI69629.1	SP(Sec/SPI)	0.997059	0.002941	CS pos: 21-22. VSS-HP. Pr: 0.8822
QLI69630.1	SP(Sec/SPI)	0.986420	0.013580	CS pos: 18-19. VQA-AP. Pr: 0.9327
QLI69631.1	OTHER	0.001082	0.998918	
QLI69632.1	OTHER	0.000523	0.999477	
QLI69633.1	OTHER	0.001510	0.998490	
QLI69634.1	OTHER	0.002360	0.997640	
QLI69635.1	SP(Sec/SPI)	0.995002	0.004998	CS pos: 18-19. SNA-VL. Pr: 0.6636
QLI69636.1	OTHER	0.001077	0.998923	
QLI69637.1	OTHER	0.000415	0.999585	
QLI69638.1	OTHER	0.000492	0.999508	
QLI69639.1	OTHER	0.000831	0.999169	
QLI69640.1	OTHER	0.001240	0.998760	
QLI69641.1	OTHER	0.000385	0.999615	
QLI69642.1	SP(Sec/SPI)	0.728086	0.271914	CS pos: 16-17. SSA-HI. Pr: 0.4572
QLI69643.1	OTHER	0.002210	0.997790	
QLI69644.1	SP(Sec/SPI)	0.989299	0.010701	CS pos: 22-23. ADS-YT. Pr: 0.6420
QLI69645.1	OTHER	0.000793	0.999207	
QLI69646.1	OTHER	0.000786	0.999214	
QLI69647.1	OTHER	0.001569	0.998431	
QLI69648.1	OTHER	0.000472	0.999528	
QLI69649.1	SP(Sec/SPI)	0.997367	0.002633	CS pos: 17-18. VFA-AD. Pr: 0.7521
QLI69650.1	OTHER	0.000855	0.999145	

QLI69651.1	OTHER	0.002560	0.997440	
QLI69652.1	OTHER	0.001119	0.998881	
QLI69653.1	OTHER	0.003164	0.996836	
QLI69654.1	OTHER	0.010457	0.989543	
QLI69655.1	OTHER	0.001064	0.998936	
QLI69656.1	OTHER	0.002560	0.997440	
QLI69657.1	OTHER	0.000854	0.999146	
QLI69658.1	OTHER	0.020349	0.979651	
QLI69659.1	OTHER	0.006873	0.993127	
QLI69660.1	OTHER	0.000153	0.999847	
QLI69661.1	OTHER	0.029023	0.970977	
QLI69662.1	OTHER	0.003138	0.996862	
QLI69663.1	OTHER	0.000794	0.999206	
QLI69664.1	OTHER	0.000433	0.999567	
QLI69665.1	OTHER	0.000265	0.999735	
QLI69666.1	OTHER	0.002694	0.997306	
QLI69667.1	OTHER	0.000449	0.999551	
QLI69668.1	OTHER	0.005117	0.994883	
QLI69669.1	SP(Sec/SPI)	0.930330	0.069670	CS pos: 20-21. SRS-AL. Pr: 0.4648
QLI69670.1	OTHER	0.000538	0.999462	
QLI69671.1	OTHER	0.001090	0.998910	
QLI69672.1	OTHER	0.000412	0.999588	
QLI69673.1	OTHER	0.001846	0.998154	
QLI69674.1	OTHER	0.000474	0.999526	
QLI69675.1	OTHER	0.000447	0.999553	
QLI69676.1	OTHER	0.001245	0.998755	
QLI69677.1	OTHER	0.001948	0.998052	
QLI69678.1	OTHER	0.001525	0.998475	
QLI69679.1	OTHER	0.001228	0.998772	
QLI69680.1	OTHER	0.084105	0.915895	
QLI69681.1	SP(Sec/SPI)	0.984509	0.015491	CS pos: 21-22. SQA-QV. Pr: 0.9296

QLI69682.1	OTHER	0.001085	0.998915	
QLI69683.1	OTHER	0.001601	0.998399	
QLI69684.1	OTHER	0.000705	0.999295	
QLI69685.1	OTHER	0.000593	0.999407	
QLI69686.1	SP(Sec/SPI)	0.822523	0.177477	CS pos: 18-19. VAG-LP. Pr: 0.1827
QLI69687.1	OTHER	0.000826	0.999174	
QLI69688.1	OTHER	0.000618	0.999382	
QLI69689.1	OTHER	0.001070	0.998930	
QLI69690.1	OTHER	0.000479	0.999521	
QLI69691.1	OTHER	0.000203	0.999797	
QLI69692.1	OTHER	0.000375	0.999625	
QLI69693.1	SP(Sec/SPI)	0.924672	0.075328	CS pos: 20-21. SSG-RG. Pr: 0.2257
QLI69694.1	OTHER	0.001118	0.998882	
QLI69695.1	OTHER	0.015674	0.984326	
QLI69696.1	OTHER	0.000587	0.999413	
QLI69697.1	OTHER	0.092935	0.907065	
QLI69698.1	OTHER	0.001279	0.998721	
QLI69699.1	OTHER	0.000907	0.999093	
QLI69700.1	OTHER	0.003329	0.996671	
QLI69701.1	OTHER	0.001763	0.998237	
QLI69702.1	OTHER	0.007585	0.992415	
QLI69703.1	OTHER	0.001247	0.998753	
QLI69704.1	OTHER	0.000663	0.999337	
QLI69705.1	OTHER	0.000963	0.999037	
QLI69706.1	OTHER	0.001267	0.998733	
QLI69707.1	OTHER	0.000806	0.999194	
QLI69708.1	OTHER	0.003141	0.996859	
QLI69709.1	OTHER	0.002061	0.997939	
QLI69710.1	OTHER	0.001031	0.998969	
QLI69711.1	OTHER	0.001524	0.998476	
QLI69712.1	SP(Sec/SPI)	0.997914	0.002086	CS pos: 19-20. VIA-QN. Pr: 0.8805

QLI69713.1	SP(Sec/SPI)	0.982909	0.017091	CS pos: 19-20. VFS-AA. Pr: 0.4632
QLI69714.1	OTHER	0.001229	0.998771	
QLI69715.1	OTHER	0.061610	0.938390	
QLI69716.1	OTHER	0.007183	0.992817	
QLI69717.1	OTHER	0.002216	0.997784	
QLI69718.1	SP(Sec/SPI)	0.908305	0.091695	CS pos: 21-22. AVA-SN. Pr: 0.5856
QLI69719.1	OTHER	0.000728	0.999272	
QLI69720.1	OTHER	0.000628	0.999372	
QLI69721.1	OTHER	0.001052	0.998948	
QLI69722.1	OTHER	0.001677	0.998323	
QLI69723.1	SP(Sec/SPI)	0.998882	0.001118	CS pos: 16-17. AAA-QN. Pr: 0.8554
QLI69724.1	OTHER	0.067886	0.932114	
QLI69725.1	OTHER	0.006418	0.993582	
QLI69726.1	OTHER	0.000993	0.999007	
QLI69727.1	SP(Sec/SPI)	0.863333	0.136667	CS pos: 21-22. VSA-KV. Pr: 0.7190
QLI69728.1	OTHER	0.000488	0.999512	
QLI69729.1	OTHER	0.002494	0.997506	
QLI69730.1	SP(Sec/SPI)	0.982225	0.017775	CS pos: 17-18. AIA-AP. Pr: 0.6254
QLI69731.1	OTHER	0.000859	0.999141	
QLI69732.1	OTHER	0.000463	0.999537	
QLI69733.1	OTHER	0.000922	0.999078	
QLI69734.1	SP(Sec/SPI)	0.900104	0.099896	CS pos: 25-26. VLG-HP. Pr: 0.7851
QLI69735.1	OTHER	0.001032	0.998968	
QLI69736.1	OTHER	0.001582	0.998418	
QLI69737.1	OTHER	0.000549	0.999451	
QLI69738.1	OTHER	0.001672	0.998328	
QLI69739.1	OTHER	0.011096	0.988904	
QLI69740.1	OTHER	0.002856	0.997144	
QLI69741.1	OTHER	0.001743	0.998257	
QLI69742.1	OTHER	0.000603	0.999397	
QLI69743.1	OTHER	0.001663	0.998337	

QLI69744.1	OTHER	0.000634	0.999366	
QLI69745.1	OTHER	0.000411	0.999589	
QLI69746.1	SP(Sec/SPI)	0.993900	0.006100	CS pos: 18-19. SLG-SS. Pr: 0.7276
QLI69747.1	OTHER	0.001316	0.998684	
QLI69748.1	OTHER	0.000950	0.999050	
QLI69749.1	OTHER	0.023027	0.976973	
QLI69750.1	SP(Sec/SPI)	0.969192	0.030808	CS pos: 18-19. AIA-AP. Pr: 0.7812
QLI69751.1	OTHER	0.000578	0.999422	
QLI69752.1	OTHER	0.000882	0.999118	
QLI69753.1	SP(Sec/SPI)	0.997425	0.002575	CS pos: 21-22. VSA-IP. Pr: 0.7712
QLI69754.1	SP(Sec/SPI)	0.980362	0.019638	CS pos: 26-27. VHA-VA. Pr: 0.7993
QLI69755.1	SP(Sec/SPI)	0.677483	0.322517	CS pos: 22-23. VAA-AT. Pr: 0.3342
QLI69756.1	OTHER	0.000968	0.999032	
QLI69757.1	OTHER	0.001002	0.998998	
QLI69758.1	OTHER	0.002427	0.997573	
QLI69759.1	OTHER	0.000332	0.999668	
QLI69760.1	OTHER	0.019652	0.980348	
QLI69761.1	OTHER	0.000920	0.999080	
QLI69762.1	OTHER	0.000669	0.999331	
QLI69763.1	OTHER	0.018920	0.981080	
QLI69764.1	OTHER	0.000985	0.999015	
QLI69765.1	OTHER	0.002721	0.997279	
QLI69766.1	OTHER	0.033161	0.966839	
QLI69767.1	OTHER	0.000879	0.999121	
QLI69768.1	SP(Sec/SPI)	0.939180	0.060820	CS pos: 20-21. ATA-TP. Pr: 0.7063
QLI69769.1	OTHER	0.000858	0.999142	
QLI69770.1	OTHER	0.001785	0.998215	
QLI69771.1	OTHER	0.002115	0.997885	
QLI69772.1	OTHER	0.001073	0.998927	
QLI69773.1	OTHER	0.000549	0.999451	
QLI69774.1	OTHER	0.002304	0.997696	

QLI69775.1	OTHER	0.029728	0.970272	
QLI69776.1	OTHER	0.001970	0.998030	
QLI69777.1	SP(Sec/SPI)	0.995322	0.004678	CS pos: 18-19. ARA-ST. Pr: 0.8153
QLI69778.1	OTHER	0.000695	0.999305	
QLI69779.1	SP(Sec/SPI)	0.964742	0.035258	CS pos: 24-25. SVA-DP. Pr: 0.5127
QLI69780.1	OTHER	0.003794	0.996206	
QLI69781.1	OTHER	0.001047	0.998953	
QLI69782.1	OTHER	0.000390	0.999610	
QLI69783.1	OTHER	0.179920	0.820080	
QLI69784.1	OTHER	0.004391	0.995609	
QLI69785.1	OTHER	0.003751	0.996249	
QLI69786.1	OTHER	0.000981	0.999019	
QLI69787.1	OTHER	0.000174	0.999826	
QLI69788.1	SP(Sec/SPI)	0.985836	0.014164	CS pos: 16-17. VNA-IL. Pr: 0.5696
QLI69789.1	OTHER	0.003597	0.996403	
QLI69790.1	OTHER	0.000798	0.999202	
QLI69791.1	OTHER	0.000388	0.999612	
QLI69792.1	OTHER	0.000412	0.999588	
QLI69793.1	SP(Sec/SPI)	0.942457	0.057543	CS pos: 17-18. SLA-LP. Pr: 0.6052
QLI69794.1	SP(Sec/SPI)	0.991791	0.008209	CS pos: 19-20. VSG-QD. Pr: 0.9535
QLI69795.1	OTHER	0.001623	0.998377	
QLI69796.1	OTHER	0.001083	0.998917	
QLI69797.1	OTHER	0.011894	0.988106	
QLI69798.1	OTHER	0.000443	0.999557	
QLI69799.1	SP(Sec/SPI)	0.986041	0.013959	CS pos: 17-18. ALA-HP. Pr: 0.4904
QLI69800.1	OTHER	0.001274	0.998726	
QLI69801.1	OTHER	0.000395	0.999605	
QLI69802.1	OTHER	0.004032	0.995968	
QLI69803.1	OTHER	0.000228	0.999772	
QLI69804.1	OTHER	0.014188	0.985812	
QLI69805.1	OTHER	0.000617	0.999383	

QLI69806.1	OTHER	0.002592	0.997408	
QLI69807.1	OTHER	0.003465	0.996535	
QLI69808.1	OTHER	0.011772	0.988228	
QLI69809.1	SP(Sec/SPI)	0.986400	0.013600	CS pos: 15-16. VAA-GV. Pr: 0.6265
QLI69810.1	OTHER	0.001632	0.998368	
QLI69811.1	OTHER	0.002935	0.997065	
QLI69812.1	SP(Sec/SPI)	0.837864	0.162136	CS pos: 24-25. AQA-AL. Pr: 0.4416
QLI69813.1	OTHER	0.004394	0.995606	
QLI69814.1	SP(Sec/SPI)	0.645716	0.354284	CS pos: 27-28. VLG-IR. Pr: 0.3955
QLI69815.1	SP(Sec/SPI)	0.999512	0.000488	CS pos: 19-20. AIA-HS. Pr: 0.5905
QLI69816.1	OTHER	0.036920	0.963080	
QLI69817.1	SP(Sec/SPI)	0.988527	0.011473	CS pos: 22-23. VAG-QW. Pr: 0.7687
QLI69818.1	OTHER	0.000557	0.999443	
QLI69819.1	OTHER	0.000806	0.999194	
QLI69820.1	OTHER	0.172428	0.827572	
QLI69821.1	OTHER	0.001301	0.998699	
QLI69822.1	OTHER	0.000738	0.999262	
QLI69823.1	OTHER	0.004373	0.995627	
QLI69824.1	OTHER	0.000496	0.999504	
QLI69825.1	OTHER	0.010114	0.989886	
QLI69826.1	OTHER	0.014132	0.985868	
QLI69827.1	OTHER	0.000987	0.999013	
QLI69828.1	OTHER	0.002752	0.997248	
QLI69829.1	SP(Sec/SPI)	0.987146	0.012854	CS pos: 16-17. ACA-AP. Pr: 0.4842
QLI69830.1	OTHER	0.002734	0.997266	
QLI69831.1	OTHER	0.001138	0.998862	
QLI69832.1	OTHER	0.000535	0.999465	
QLI69833.1	OTHER	0.000565	0.999435	
QLI69834.1	OTHER	0.000588	0.999412	
QLI69835.1	OTHER	0.002438	0.997562	
QLI69836.1	OTHER	0.012203	0.987797	

QLI69837.1	OTHER	0.009262	0.990738	
QLI69838.1	SP(Sec/SPI)	0.962380	0.037620	CS pos: 18-19. ALA-AA. Pr: 0.5069
QLI69839.1	OTHER	0.001923	0.998077	
QLI69840.1	OTHER	0.001747	0.998253	
QLI69841.1	OTHER	0.002508	0.997492	
QLI69842.1	OTHER	0.006127	0.993873	
QLI69843.1	OTHER	0.000858	0.999142	
QLI69844.1	OTHER	0.000592	0.999408	
QLI69845.1	SP(Sec/SPI)	0.991134	0.008866	CS pos: 23-24. VSG-QL. Pr: 0.5535
QLI69846.1	SP(Sec/SPI)	0.992974	0.007026	CS pos: 17-18. AAA-TD. Pr: 0.4927
QLI69847.1	OTHER	0.001596	0.998404	
QLI69848.1	OTHER	0.006433	0.993567	
QLI69849.1	OTHER	0.001468	0.998532	
QLI69850.1	OTHER	0.005927	0.994073	
QLI69851.1	OTHER	0.000959	0.999041	
QLI69852.1	OTHER	0.000615	0.999385	
QLI69853.1	OTHER	0.000590	0.999410	
QLI69854.1	OTHER	0.001292	0.998708	
QLI69855.1	OTHER	0.012700	0.987300	
QLI69856.1	OTHER	0.003172	0.996828	
QLI69857.1	SP(Sec/SPI)	0.998394	0.001606	CS pos: 19-20. VSA-QV. Pr: 0.5921
QLI69858.1	OTHER	0.000977	0.999023	
QLI69859.1	OTHER	0.000358	0.999642	
QLI69860.1	OTHER	0.000806	0.999194	
QLI69861.1	OTHER	0.002222	0.997778	
QLI69862.1	OTHER	0.000423	0.999577	
QLI69863.1	OTHER	0.003698	0.996302	
QLI69864.1	SP(Sec/SPI)	0.636806	0.363194	CS pos: 18-19. CKA-AL. Pr: 0.4824
QLI69865.1	SP(Sec/SPI)	0.979523	0.020477	CS pos: 21-22. STA-DK. Pr: 0.8149
QLI69866.1	OTHER	0.000295	0.999705	
QLI69867.1	OTHER	0.003900	0.996100	

QLI69868.1	OTHER	0.000908	0.999092	
QLI69869.1	OTHER	0.001405	0.998595	
QLI69870.1	OTHER	0.004526	0.995474	
QLI69871.1	OTHER	0.003880	0.996120	
QLI69872.1	OTHER	0.002627	0.997373	
QLI69873.1	OTHER	0.063283	0.936717	
QLI69874.1	OTHER	0.000430	0.999570	
QLI69875.1	OTHER	0.001851	0.998149	
QLI69876.1	OTHER	0.000691	0.999309	
QLI69877.1	OTHER	0.005567	0.994433	
QLI69878.1	OTHER	0.003067	0.996933	
QLI69879.1	OTHER	0.002765	0.997235	
QLI69880.1	OTHER	0.001200	0.998800	
QLI69881.1	OTHER	0.000561	0.999439	
QLI69882.1	OTHER	0.000584	0.999416	
QLI69883.1	OTHER	0.004728	0.995272	
QLI69884.1	OTHER	0.001214	0.998786	
QLI69885.1	OTHER	0.003355	0.996645	
QLI69886.1	OTHER	0.004734	0.995266	
QLI69887.1	OTHER	0.000699	0.999301	
QLI69888.1	OTHER	0.001855	0.998145	
QLI69889.1	OTHER	0.001464	0.998536	
QLI69890.1	OTHER	0.000311	0.999689	
QLI69891.1	OTHER	0.004131	0.995869	
QLI69892.1	OTHER	0.000444	0.999556	
QLI69893.1	OTHER	0.000397	0.999603	
QLI69894.1	SP(Sec/SPI)	0.928603	0.071397	CS pos: 18-19. SSA-AT. Pr: 0.4521
QLI69895.1	OTHER	0.000266	0.999734	
QLI69896.1	OTHER	0.001657	0.998343	
QLI69897.1	OTHER	0.004864	0.995136	
QLI69898.1	OTHER	0.000652	0.999348	

QLI69899.1	OTHER	0.206412	0.793588	
QLI69900.1	OTHER	0.001080	0.998920	
QLI69901.1	OTHER	0.007391	0.992609	
QLI69902.1	OTHER	0.002420	0.997580	
QLI69903.1	SP(Sec/SPI)	0.950588	0.049412	CS pos: 15-16. AMA-AP. Pr: 0.3485
QLI69904.1	OTHER	0.002769	0.997231	
QLI69905.1	SP(Sec/SPI)	0.998638	0.001362	CS pos: 15-16. AAA-IP. Pr: 0.5743
QLI69906.1	OTHER	0.000547	0.999453	
QLI69907.1	OTHER	0.449598	0.550402	
QLI69908.1	OTHER	0.001515	0.998485	
QLI69909.1	SP(Sec/SPI)	0.995735	0.004265	CS pos: 17-18. AHA-TH. Pr: 0.8694
QLI69910.1	OTHER	0.000909	0.999091	
QLI69911.1	OTHER	0.000907	0.999093	
QLI69912.1	SP(Sec/SPI)	0.999858	0.000142	CS pos: 18-19. ALA-AP. Pr: 0.9530
QLI69913.1	SP(Sec/SPI)	0.798716	0.201284	CS pos: 20-21. TSA-TN. Pr: 0.4421
QLI69914.1	OTHER	0.002088	0.997912	
QLI69915.1	OTHER	0.031687	0.968313	
QLI69916.1	OTHER	0.294471	0.705529	
QLI69917.1	SP(Sec/SPI)	0.996703	0.003297	CS pos: 18-19. ALA-AP. Pr: 0.8620
QLI69918.1	OTHER	0.000351	0.999649	
QLI69919.1	OTHER	0.029322	0.970678	
QLI69920.1	OTHER	0.003021	0.996979	
QLI69921.1	OTHER	0.002790	0.997210	
QLI69922.1	OTHER	0.001945	0.998055	
QLI69923.1	OTHER	0.396762	0.603238	
QLI69924.1	OTHER	0.000402	0.999598	
QLI69925.1	OTHER	0.001666	0.998334	
QLI69926.1	OTHER	0.005451	0.994549	
QLI69927.1	OTHER	0.002230	0.997770	
QLI69928.1	OTHER	0.001024	0.998976	
QLI69929.1	OTHER	0.000398	0.999602	

QLI69930.1	OTHER	0.000520	0.999480	
QLI69931.1	OTHER	0.002364	0.997636	
QLI69932.1	OTHER	0.000804	0.999196	
QLI69933.1	OTHER	0.000875	0.999125	
QLI69934.1	OTHER	0.001039	0.998961	
QLI69935.1	OTHER	0.001426	0.998574	
QLI69936.1	SP(Sec/SPI)	0.934643	0.065357	CS pos: 17-18. SIC-QS. Pr: 0.4533
QLI69937.1	OTHER	0.001270	0.998730	
QLI69938.1	OTHER	0.005880	0.994120	
QLI69939.1	OTHER	0.006358	0.993642	
QLI69940.1	OTHER	0.001119	0.998881	
QLI69941.1	SP(Sec/SPI)	0.999760	0.000240	CS pos: 19-20. IMA-QD. Pr: 0.9266
QLI69942.1	OTHER	0.013043	0.986957	
QLI69943.1	OTHER	0.000696	0.999304	
QLI69944.1	OTHER	0.001994	0.998006	
QLI69945.1	OTHER	0.001319	0.998681	
QLI69946.1	OTHER	0.011504	0.988496	
QLI69947.1	OTHER	0.000743	0.999257	
QLI69948.1	OTHER	0.003519	0.996481	
QLI69949.1	OTHER	0.001982	0.998018	
QLI69950.1	OTHER	0.005047	0.994953	
QLI69951.1	OTHER	0.002319	0.997681	
QLI69952.1	OTHER	0.001648	0.998352	
QLI69953.1	OTHER	0.001630	0.998370	
QLI69954.1	OTHER	0.001694	0.998306	
QLI69955.1	OTHER	0.003445	0.996555	
QLI69956.1	OTHER	0.000872	0.999128	
QLI69957.1	OTHER	0.003601	0.996399	
QLI69958.1	OTHER	0.001369	0.998631	
QLI69959.1	OTHER	0.000424	0.999576	
QLI69960.1	OTHER	0.000494	0.999506	

QLI69961.1	OTHER	0.001833	0.998167	
QLI69962.1	OTHER	0.000894	0.999106	
QLI69963.1	OTHER	0.232850	0.767150	
QLI69964.1	OTHER	0.000343	0.999657	
QLI69965.1	OTHER	0.047110	0.952890	
QLI69966.1	OTHER	0.004362	0.995638	
QLI69967.1	OTHER	0.001536	0.998464	
QLI69968.1	SP(Sec/SPI)	0.994317	0.005683	CS pos: 24-25. AMA-CR. Pr: 0.8887
QLI69969.1	OTHER	0.001512	0.998488	
QLI69970.1	OTHER	0.000630	0.999370	
QLI69971.1	OTHER	0.001231	0.998769	
QLI69972.1	SP(Sec/SPI)	0.923846	0.076154	CS pos: 18-19. SLG-LP. Pr: 0.6327
QLI69973.1	OTHER	0.002555	0.997445	
QLI69974.1	OTHER	0.001120	0.998880	
QLI69975.1	OTHER	0.000461	0.999539	
QLI69976.1	OTHER	0.001612	0.998388	
QLI69977.1	OTHER	0.000804	0.999196	
QLI69978.1	SP(Sec/SPI)	0.964762	0.035238	CS pos: 18-19. ASA-YY. Pr: 0.8188
QLI69979.1	OTHER	0.039568	0.960432	
QLI69980.1	OTHER	0.104928	0.895072	
QLI69981.1	OTHER	0.000656	0.999344	
QLI69982.1	OTHER	0.001706	0.998294	
QLI69983.1	OTHER	0.001967	0.998033	
QLI69984.1	OTHER	0.001859	0.998141	
QLI69985.1	OTHER	0.000578	0.999422	
QLI69986.1	SP(Sec/SPI)	0.992944	0.007056	CS pos: 18-19. ALA-QV. Pr: 0.8544
QLI69987.1	OTHER	0.000511	0.999489	
QLI69988.1	OTHER	0.005431	0.994569	
QLI69989.1	OTHER	0.000296	0.999704	
QLI69990.1	OTHER	0.001772	0.998228	
QLI69991.1	OTHER	0.003833	0.996167	

QLI69992.1	OTHER	0.000290	0.999710	
QLI69993.1	OTHER	0.000480	0.999520	
QLI69994.1	OTHER	0.001119	0.998881	
QLI69995.1	OTHER	0.354258	0.645742	
QLI69996.1	OTHER	0.002851	0.997149	
QLI69997.1	OTHER	0.001791	0.998209	
QLI69998.1	OTHER	0.000853	0.999147	
QLI69999.1	SP(Sec/SPI)	0.980671	0.019329	CS pos: 16-17. AQG-AP. Pr: 0.6470
QLI70000.1	OTHER	0.009034	0.990966	
QLI70001.1	SP(Sec/SPI)	0.967437	0.032563	CS pos: 18-19. VLS-QF. Pr: 0.8941
QLI70002.1	OTHER	0.350236	0.649764	
QLI70003.1	OTHER	0.001278	0.998722	
QLI70004.1	OTHER	0.006866	0.993134	
QLI70005.1	OTHER	0.000937	0.999063	
QLI70006.1	OTHER	0.000806	0.999194	
QLI70007.1	OTHER	0.001559	0.998441	
QLI70008.1	SP(Sec/SPI)	0.973355	0.026645	CS pos: 23-24. ASG-EK. Pr: 0.6963
QLI70009.1	OTHER	0.000926	0.999074	
QLI70010.1	OTHER	0.003031	0.996969	
QLI70011.1	OTHER	0.000824	0.999176	
QLI70012.1	OTHER	0.000388	0.999612	
QLI70013.1	OTHER	0.000529	0.999471	
QLI70014.1	OTHER	0.002028	0.997972	
QLI70015.1	OTHER	0.000285	0.999715	
QLI70016.1	OTHER	0.001473	0.998527	
QLI70017.1	OTHER	0.007288	0.992712	
QLI70018.1	OTHER	0.001494	0.998506	
QLI70019.1	OTHER	0.000605	0.999395	
QLI70020.1	SP(Sec/SPI)	0.614104	0.385896	CS pos: 14-15. ASA-AI. Pr: 0.3338
QLI70021.1	OTHER	0.001961	0.998039	
QLI70022.1	OTHER	0.002679	0.997321	

QLI70023.1	SP(Sec/SPI)	0.967866	0.032134	CS pos: 22-23. TNA-QY. Pr: 0.6820
QLI70024.1	OTHER	0.001671	0.998329	
QLI70025.1	OTHER	0.001149	0.998851	
QLI70026.1	SP(Sec/SPI)	0.722097	0.277903	CS pos: 17-18. TQA-LL. Pr: 0.5458
QLI70027.1	OTHER	0.001095	0.998905	
QLI70028.1	OTHER	0.000789	0.999211	
QLI70029.1	OTHER	0.000917	0.999083	
QLI70030.1	OTHER	0.000690	0.999310	
QLI70031.1	OTHER	0.002398	0.997602	
QLI70032.1	OTHER	0.003049	0.996951	
QLI70033.1	OTHER	0.000705	0.999295	
QLI70034.1	OTHER	0.005439	0.994561	
QLI70035.1	OTHER	0.001041	0.998959	
QLI70036.1	OTHER	0.001506	0.998494	
QLI70037.1	OTHER	0.000315	0.999685	
QLI70038.1	OTHER	0.000809	0.999191	
QLI70039.1	OTHER	0.000846	0.999154	
QLI70040.1	OTHER	0.001865	0.998135	
QLI70041.1	OTHER	0.002800	0.997200	
QLI70042.1	SP(Sec/SPI)	0.999203	0.000797	CS pos: 19-20. INA-DY. Pr: 0.9490
QLI70043.1	OTHER	0.003774	0.996226	
QLI70044.1	OTHER	0.017796	0.982204	
QLI70045.1	OTHER	0.022818	0.977182	
QLI70046.1	OTHER	0.000988	0.999012	
QLI70047.1	OTHER	0.003506	0.996494	
QLI70048.1	OTHER	0.003696	0.996304	
QLI70049.1	OTHER	0.000660	0.999340	
QLI70050.1	OTHER	0.001126	0.998874	
QLI70051.1	OTHER	0.001401	0.998599	
QLI70052.1	OTHER	0.015933	0.984067	
QLI70053.1	OTHER	0.003111	0.996889	

QLI70054.1	OTHER	0.000986	0.999014	
QLI70055.1	OTHER	0.005893	0.994107	
QLI70056.1	OTHER	0.000559	0.999441	
QLI70057.1	OTHER	0.000378	0.999622	
QLI70058.1	OTHER	0.001604	0.998396	
QLI70059.1	OTHER	0.002100	0.997900	
QLI70060.1	OTHER	0.004791	0.995209	
QLI70061.1	OTHER	0.001428	0.998572	
QLI70062.1	OTHER	0.004702	0.995298	
QLI70063.1	OTHER	0.001097	0.998903	
QLI70064.1	OTHER	0.000736	0.999264	
QLI70065.1	SP(Sec/SPI)	0.904544	0.095456	CS pos: 18-19. ALG-IN. Pr: 0.3274
QLI70066.1	OTHER	0.002886	0.997114	
QLI70067.1	OTHER	0.000562	0.999438	
QLI70068.1	SP(Sec/SPI)	0.994771	0.005229	CS pos: 23-24. AHA-QE. Pr: 0.9873
QLI70069.1	OTHER	0.007804	0.992196	
QLI70070.1	OTHER	0.000789	0.999211	
QLI70071.1	OTHER	0.000534	0.999466	
QLI70072.1	OTHER	0.000852	0.999148	
QLI70073.1	OTHER	0.105828	0.894172	
QLI70074.1	OTHER	0.001286	0.998714	
QLI70075.1	OTHER	0.001000	0.999000	
QLI70076.1	OTHER	0.002298	0.997702	
QLI70077.1	OTHER	0.035089	0.964911	
QLI70078.1	OTHER	0.000626	0.999374	
QLI70079.1	OTHER	0.305098	0.694902	
QLI70080.1	OTHER	0.001366	0.998634	
QLI70081.1	OTHER	0.002402	0.997598	
QLI70082.1	OTHER	0.000258	0.999742	
QLI70083.1	OTHER	0.014619	0.985381	
QLI70084.1	OTHER	0.002905	0.997095	

QLI70085.1	OTHER	0.000791	0.999209	
QLI70086.1	OTHER	0.000846	0.999154	
QLI70087.1	OTHER	0.001538	0.998462	
QLI70088.1	OTHER	0.001220	0.998780	
QLI70089.1	OTHER	0.002431	0.997569	
QLI70090.1	OTHER	0.000858	0.999142	
QLI70091.1	SP(Sec/SPI)	0.999003	0.000997	CS pos: 18-19. TDA-TP. Pr: 0.9681
QLI70092.1	OTHER	0.000800	0.999200	
QLI70093.1	OTHER	0.004575	0.995425	
QLI70094.1	SP(Sec/SPI)	0.995765	0.004235	CS pos: 16-17. AAA-VP. Pr: 0.7061
QLI70095.1	OTHER	0.005677	0.994323	
QLI70096.1	OTHER	0.000453	0.999547	
QLI70097.1	OTHER	0.402900	0.597100	
QLI70098.1	OTHER	0.000604	0.999396	
QLI70099.1	SP(Sec/SPI)	0.980483	0.019517	CS pos: 20-21. ASA-SP. Pr: 0.8266
QLI70100.1	OTHER	0.000331	0.999669	
QLI70101.1	OTHER	0.000672	0.999328	
QLI70102.1	OTHER	0.001435	0.998565	
QLI70103.1	SP(Sec/SPI)	0.980941	0.019059	CS pos: 15-16. VKA-ML. Pr: 0.6960
QLI70104.1	OTHER	0.005313	0.994687	
QLI70105.1	OTHER	0.003113	0.996887	
QLI70106.1	OTHER	0.157090	0.842910	
QLI70107.1	OTHER	0.001439	0.998561	
QLI70108.1	OTHER	0.002461	0.997539	
QLI70109.1	OTHER	0.000411	0.999589	
QLI70110.1	OTHER	0.000397	0.999603	
QLI70111.1	OTHER	0.004009	0.995991	
QLI70112.1	OTHER	0.000381	0.999619	
QLI70113.1	SP(Sec/SPI)	0.970238	0.029762	CS pos: 16-17. AAA-NP. Pr: 0.7599
QLI70114.1	SP(Sec/SPI)	0.999186	0.000814	CS pos: 23-24. ADA-AS. Pr: 0.8928
QLI70115.1	OTHER	0.001000	0.999000	

QLI70116.1	OTHER	0.000245	0.999755	
QLI70117.1	OTHER	0.000928	0.999072	
QLI70118.1	OTHER	0.002080	0.997920	
QLI70119.1	OTHER	0.000440	0.999560	
QLI70120.1	OTHER	0.000250	0.999750	
QLI70121.1	OTHER	0.000443	0.999557	
QLI70122.1	OTHER	0.347978	0.652022	
QLI70123.1	SP(Sec/SPI)	0.980517	0.019483	CS pos: 26-27. ALA-IP. Pr: 0.4253
QLI70124.1	OTHER	0.000988	0.999012	
QLI70125.1	OTHER	0.006708	0.993292	
QLI70126.1	OTHER	0.043373	0.956627	
QLI70127.1	OTHER	0.000431	0.999569	
QLI70128.1	OTHER	0.002457	0.997543	
QLI70129.1	OTHER	0.003119	0.996881	
QLI70130.1	OTHER	0.002393	0.997607	
QLI70131.1	SP(Sec/SPI)	0.927459	0.072541	CS pos: 24-25. AHS-KI. Pr: 0.6494
QLI70132.1	OTHER	0.000937	0.999063	
QLI70133.1	OTHER	0.184000	0.816000	
QLI70134.1	OTHER	0.001209	0.998791	
QLI70135.1	OTHER	0.009449	0.990551	
QLI70136.1	OTHER	0.000283	0.999717	
QLI70137.1	OTHER	0.001556	0.998444	
QLI70138.1	SP(Sec/SPI)	0.790457	0.209543	CS pos: 18-19. VSA-GV. Pr: 0.4373
QLI70139.1	OTHER	0.000613	0.999387	
QLI70140.1	OTHER	0.000613	0.999387	
QLI70141.1	OTHER	0.001677	0.998323	
QLI70142.1	OTHER	0.000600	0.999400	
QLI70143.1	OTHER	0.000790	0.999210	
QLI70144.1	OTHER	0.001001	0.998999	
QLI70145.1	OTHER	0.011351	0.988649	
QLI70146.1	OTHER	0.000696	0.999304	

QLI70147.1	OTHER	0.000679	0.999321	
QLI70148.1	OTHER	0.000943	0.999057	
QLI70149.1	OTHER	0.000840	0.999160	
QLI70150.1	OTHER	0.010674	0.989326	
QLI70151.1	SP(Sec/SPI)	0.994126	0.005874	CS pos: 17-18. AAA-DP. Pr: 0.8664
QLI70152.1	OTHER	0.002851	0.997149	
QLI70153.1	OTHER	0.000953	0.999047	
QLI70154.1	OTHER	0.001093	0.998907	
QLI70155.1	SP(Sec/SPI)	0.998737	0.001263	CS pos: 18-19. ALA-IP. Pr: 0.8819
QLI70156.1	OTHER	0.000208	0.999792	
QLI70157.1	SP(Sec/SPI)	0.956715	0.043285	CS pos: 22-23. AVS-QT. Pr: 0.7683
QLI70158.1	OTHER	0.001276	0.998724	
QLI70159.1	OTHER	0.000844	0.999156	
QLI70160.1	OTHER	0.000418	0.999582	
QLI70161.1	OTHER	0.000758	0.999242	
QLI70162.1	OTHER	0.000754	0.999246	
QLI70163.1	OTHER	0.000973	0.999027	
QLI70164.1	OTHER	0.019397	0.980603	
QLI70165.1	OTHER	0.004232	0.995768	
QLI70166.1	OTHER	0.000687	0.999313	
QLI70167.1	OTHER	0.009896	0.990104	
QLI70168.1	OTHER	0.001508	0.998492	
QLI70169.1	OTHER	0.001125	0.998875	
QLI70170.1	OTHER	0.001681	0.998319	
QLI70171.1	OTHER	0.002029	0.997971	
QLI70172.1	OTHER	0.003106	0.996894	
QLI70173.1	SP(Sec/SPI)	0.982027	0.017973	CS pos: 19-20. VSA-DL. Pr: 0.8870
QLI70174.1	OTHER	0.003268	0.996732	
QLI70175.1	OTHER	0.007736	0.992264	
QLI70176.1	OTHER	0.001758	0.998242	
QLI70177.1	OTHER	0.000640	0.999360	

QLI70178.1	OTHER	0.003964	0.996036	
QLI70179.1	OTHER	0.000880	0.999120	
QLI70180.1	OTHER	0.000288	0.999712	
QLI70181.1	OTHER	0.001226	0.998774	
QLI70182.1	OTHER	0.001429	0.998571	
QLI70183.1	OTHER	0.000670	0.999330	
QLI70184.1	OTHER	0.000162	0.999838	
QLI70185.1	OTHER	0.000834	0.999166	
QLI70186.1	OTHER	0.023971	0.976029	
QLI70187.1	OTHER	0.213214	0.786786	
QLI70188.1	OTHER	0.021223	0.978777	
QLI70189.1	SP(Sec/SPI)	0.981412	0.018588	CS pos: 19-20. ALA-VP. Pr: 0.8092
QLI70190.1	OTHER	0.263500	0.736500	
QLI70191.1	OTHER	0.000453	0.999547	
QLI70192.1	OTHER	0.006651	0.993349	
QLI70193.1	SP(Sec/SPI)	0.964065	0.035935	CS pos: 16-17. GWA-VP. Pr: 0.6648
QLI70194.1	OTHER	0.000900	0.999100	
QLI70195.1	SP(Sec/SPI)	0.949081	0.050919	CS pos: 23-24. STA-IN. Pr: 0.7048
QLI70196.1	OTHER	0.000969	0.999031	
QLI70197.1	OTHER	0.001614	0.998386	
QLI70198.1	OTHER	0.000465	0.999535	
QLI70199.1	OTHER	0.002314	0.997686	
QLI70200.1	OTHER	0.000189	0.999811	
QLI70201.1	OTHER	0.000766	0.999234	
QLI70202.1	OTHER	0.001658	0.998342	
QLI70203.1	SP(Sec/SPI)	0.873029	0.126971	CS pos: 19-20. GHA-AT. Pr: 0.7822
QLI70204.1	OTHER	0.000951	0.999049	
QLI70205.1	OTHER	0.000390	0.999610	
QLI70206.1	OTHER	0.001845	0.998155	
QLI70207.1	OTHER	0.001825	0.998175	
QLI70208.1	SP(Sec/SPI)	0.965882	0.034118	CS pos: 23-24. AQA-QT. Pr: 0.5811

QLI70209.1	OTHER	0.000806	0.999194	
QLI70210.1	OTHER	0.000590	0.999410	
QLI70211.1	OTHER	0.002325	0.997675	
QLI70212.1	OTHER	0.008968	0.991032	
QLI70213.1	OTHER	0.055414	0.944586	
QLI70214.1	OTHER	0.001988	0.998012	
QLI70215.1	OTHER	0.004713	0.995287	
QLI70216.1	OTHER	0.000795	0.999205	
QLI70217.1	SP(Sec/SPI)	0.894682	0.105318	CS pos: 27-28. ASA-AV. Pr: 0.4321
QLI70218.1	OTHER	0.001965	0.998035	
QLI70219.1	OTHER	0.008637	0.991363	
QLI70220.1	SP(Sec/SPI)	0.897579	0.102421	CS pos: 24-25. GLA-AP. Pr: 0.7387
QLI70221.1	OTHER	0.001858	0.998142	
QLI70222.1	OTHER	0.008163	0.991837	
QLI70223.1	OTHER	0.003590	0.996410	
QLI70224.1	OTHER	0.014710	0.985290	
QLI70225.1	OTHER	0.000893	0.999107	
QLI70226.1	OTHER	0.000417	0.999583	
QLI70227.1	OTHER	0.003675	0.996325	
QLI70228.1	OTHER	0.002535	0.997465	
QLI70229.1	OTHER	0.001099	0.998901	
QLI70230.1	SP(Sec/SPI)	0.988410	0.011590	CS pos: 16-17. AQA-LY. Pr: 0.9051
QLI70231.1	OTHER	0.009603	0.990397	
QLI70232.1	OTHER	0.044813	0.955187	
QLI70233.1	OTHER	0.001850	0.998150	
QLI70234.1	OTHER	0.000995	0.999005	
QLI70235.1	OTHER	0.001139	0.998861	
QLI70236.1	OTHER	0.049547	0.950453	
QLI70237.1	OTHER	0.000928	0.999072	
QLI70238.1	OTHER	0.003052	0.996948	
QLI70239.1	OTHER	0.001074	0.998926	

QLI70240.1	OTHER	0.000213	0.999787	
QLI70241.1	OTHER	0.000456	0.999544	
QLI70242.1	OTHER	0.000745	0.999255	
QLI70243.1	OTHER	0.000186	0.999814	
QLI70244.1	OTHER	0.001895	0.998105	
QLI70245.1	OTHER	0.002314	0.997686	
QLI70246.1	OTHER	0.001301	0.998699	
QLI70247.1	OTHER	0.000442	0.999558	
QLI70248.1	OTHER	0.001789	0.998211	
QLI70249.1	OTHER	0.000994	0.999006	
QLI70250.1	SP(Sec/SPI)	0.976795	0.023205	CS pos: 18-19. AMS-RD. Pr: 0.5995
QLI70251.1	OTHER	0.000208	0.999792	
QLI70252.1	OTHER	0.000729	0.999271	
QLI70253.1	OTHER	0.000538	0.999462	
QLI70254.1	OTHER	0.001509	0.998491	
QLI70255.1	OTHER	0.000446	0.999554	
QLI70256.1	OTHER	0.000985	0.999015	
QLI70257.1	OTHER	0.001102	0.998898	
QLI70258.1	SP(Sec/SPI)	0.953351	0.046649	CS pos: 19-20. SLA-TP. Pr: 0.6080
QLI70259.1	OTHER	0.002908	0.997092	
QLI70260.1	SP(Sec/SPI)	0.998185	0.001815	CS pos: 22-23. TAA-AP. Pr: 0.3182
QLI70261.1	OTHER	0.001577	0.998423	
QLI70262.1	OTHER	0.489018	0.510982	
QLI70263.1	OTHER	0.001129	0.998871	
QLI70264.1	OTHER	0.001133	0.998867	
QLI70265.1	OTHER	0.000468	0.999532	
QLI70266.1	OTHER	0.001256	0.998744	
QLI70267.1	OTHER	0.001120	0.998880	
QLI70268.1	OTHER	0.000885	0.999115	
QLI70269.1	OTHER	0.000516	0.999484	
QLI70270.1	OTHER	0.000541	0.999459	

QLI70271.1	OTHER	0.000597	0.999403	
QLI70272.1	OTHER	0.008701	0.991299	
QLI70273.1	OTHER	0.000338	0.999662	
QLI70274.1	OTHER	0.004226	0.995774	
QLI70275.1	OTHER	0.000632	0.999368	
QLI70276.1	SP(Sec/SPI)	0.864755	0.135245	CS pos: 18-19. SLA-AT. Pr: 0.4601
QLI70277.1	OTHER	0.088591	0.911409	
QLI70278.1	OTHER	0.001374	0.998626	
QLI70279.1	OTHER	0.002123	0.997877	
QLI70280.1	OTHER	0.002528	0.997472	
QLI70281.1	OTHER	0.001071	0.998929	
QLI70282.1	OTHER	0.000470	0.999530	
QLI70283.1	OTHER	0.006641	0.993359	
QLI70284.1	OTHER	0.001375	0.998625	
QLI70285.1	OTHER	0.000884	0.999116	
QLI70286.1	OTHER	0.001874	0.998126	
QLI70287.1	OTHER	0.000320	0.999680	
QLI70288.1	OTHER	0.001660	0.998340	
QLI70289.1	OTHER	0.255675	0.744325	
QLI70290.1	OTHER	0.001470	0.998530	
QLI70291.1	OTHER	0.000310	0.999690	
QLI70292.1	OTHER	0.001192	0.998808	
QLI70293.1	OTHER	0.000361	0.999639	
QLI70294.1	OTHER	0.000174	0.999826	
QLI70295.1	OTHER	0.001014	0.998986	
QLI70296.1	SP(Sec/SPI)	0.620971	0.379029	CS pos: 32-33. VFA-QQ. Pr: 0.3460
QLI70297.1	OTHER	0.006147	0.993853	
QLI70298.1	OTHER	0.001134	0.998866	
QLI70299.1	SP(Sec/SPI)	0.972483	0.027517	CS pos: 23-24. VSG-MV. Pr: 0.7881
QLI70300.1	OTHER	0.002473	0.997527	
QLI70301.1	OTHER	0.000343	0.999657	

QLI70302.1	OTHER	0.003048	0.996952	
QLI70303.1	OTHER	0.001369	0.998631	
QLI70304.1	OTHER	0.000553	0.999447	
QLI70305.1	OTHER	0.144310	0.855690	
QLI70306.1	SP(Sec/SPI)	0.978558	0.021442	CS pos: 20-21. ATA-AS. Pr: 0.6502
QLI70307.1	OTHER	0.001041	0.998959	
QLI70308.1	OTHER	0.001256	0.998744	
QLI70309.1	OTHER	0.255542	0.744458	
QLI70310.1	OTHER	0.000311	0.999689	
QLI70311.1	SP(Sec/SPI)	0.999124	0.000876	CS pos: 17-18. ASA-AV. Pr: 0.4037
QLI70312.1	OTHER	0.000383	0.999617	
QLI70313.1	SP(Sec/SPI)	0.979465	0.020535	CS pos: 16-17. ALA-AP. Pr: 0.7512
QLI70314.1	SP(Sec/SPI)	0.985455	0.014545	CS pos: 19-20. VLG-SP. Pr: 0.8833
QLI70315.1	OTHER	0.232235	0.767765	
QLI70316.1	OTHER	0.254778	0.745222	
QLI70317.1	OTHER	0.000390	0.999610	
QLI70318.1	OTHER	0.001362	0.998638	
QLI70319.1	OTHER	0.001583	0.998417	
QLI70320.1	OTHER	0.000688	0.999312	
QLI70321.1	OTHER	0.000414	0.999586	
QLI70322.1	OTHER	0.000437	0.999563	
QLI70323.1	OTHER	0.003958	0.996042	
QLI70324.1	OTHER	0.000756	0.999244	
QLI70325.1	OTHER	0.002413	0.997587	
QLI70326.1	OTHER	0.001666	0.998334	
QLI70327.1	OTHER	0.001508	0.998492	
QLI70328.1	OTHER	0.001226	0.998774	
QLI70329.1	OTHER	0.001344	0.998656	
QLI70330.1	OTHER	0.000876	0.999124	
QLI70331.1	OTHER	0.001128	0.998872	
QLI70332.1	SP(Sec/SPI)	0.994166	0.005834	CS pos: 15-16. IWA-SP. Pr: 0.7935

QLI70333.1	OTHER	0.000552	0.999448	
QLI70334.1	SP(Sec/SPI)	0.882293	0.117707	CS pos: 16-17. ASA-HI. Pr: 0.6534
QLI70335.1	SP(Sec/SPI)	0.986451	0.013549	CS pos: 16-17. AAA-ES. Pr: 0.5460
QLI70336.1	OTHER	0.001236	0.998764	
QLI70337.1	OTHER	0.021037	0.978963	
QLI70338.1	OTHER	0.000878	0.999122	
QLI70339.1	OTHER	0.001969	0.998031	
QLI70340.1	OTHER	0.002449	0.997551	
QLI70341.1	OTHER	0.000445	0.999555	
QLI70342.1	SP(Sec/SPI)	0.990406	0.009594	CS pos: 17-18. AAA-AP. Pr: 0.7991
QLI70343.1	OTHER	0.000471	0.999529	
QLI70344.1	OTHER	0.000780	0.999220	
QLI70345.1	SP(Sec/SPI)	0.996204	0.003796	CS pos: 17-18. ASA-AP. Pr: 0.8841
QLI70346.1	OTHER	0.001277	0.998723	
QLI70347.1	OTHER	0.001285	0.998715	
QLI70348.1	OTHER	0.000702	0.999298	
QLI70349.1	OTHER	0.005964	0.994036	
QLI70350.1	OTHER	0.001433	0.998567	
QLI70351.1	OTHER	0.001146	0.998854	
QLI70352.1	OTHER	0.000132	0.999868	
QLI70353.1	OTHER	0.000789	0.999211	
QLI70354.1	OTHER	0.014180	0.985820	
QLI70355.1	OTHER	0.020813	0.979187	
QLI70356.1	OTHER	0.000972	0.999028	
QLI70357.1	OTHER	0.000285	0.999715	
QLI70358.1	OTHER	0.000376	0.999624	
QLI70359.1	OTHER	0.001014	0.998986	
QLI70360.1	OTHER	0.001129	0.998871	
QLI70361.1	OTHER	0.000542	0.999458	
QLI70362.1	SP(Sec/SPI)	0.981812	0.018188	CS pos: 21-22. AAA-DG. Pr: 0.6619
QLI70363.1	OTHER	0.003040	0.996960	

QLI70364.1	OTHER	0.000846	0.999154	
QLI70365.1	SP(Sec/SPI)	0.998614	0.001386	CS pos: 19-20. AQA-QD. Pr: 0.9032
QLI70366.1	OTHER	0.051581	0.948419	
QLI70367.1	OTHER	0.001803	0.998197	
QLI70368.1	OTHER	0.013321	0.986679	
QLI70369.1	OTHER	0.000323	0.999677	
QLI70370.1	OTHER	0.001244	0.998756	
QLI70371.1	OTHER	0.008263	0.991737	
QLI70372.1	OTHER	0.000204	0.999796	
QLI70373.1	OTHER	0.004135	0.995865	
QLI70374.1	SP(Sec/SPI)	0.982103	0.017897	CS pos: 19-20. ALA-SG. Pr: 0.3487
QLI70375.1	OTHER	0.003327	0.996673	
QLI70376.1	OTHER	0.001210	0.998790	
QLI70377.1	OTHER	0.001144	0.998856	
QLI70378.1	OTHER	0.001638	0.998362	
QLI70379.1	OTHER	0.007471	0.992529	
QLI70380.1	OTHER	0.001588	0.998412	
QLI70381.1	OTHER	0.000815	0.999185	
QLI70382.1	OTHER	0.000757	0.999243	
QLI70383.1	SP(Sec/SPI)	0.989724	0.010276	CS pos: 23-24. ANA-LK. Pr: 0.9657
QLI70384.1	OTHER	0.000842	0.999158	
QLI70385.1	OTHER	0.000950	0.999050	
QLI70386.1	OTHER	0.001878	0.998122	
QLI70387.1	OTHER	0.002757	0.997243	
QLI70388.1	OTHER	0.000815	0.999185	
QLI70389.1	OTHER	0.001959	0.998041	
QLI70390.1	OTHER	0.262886	0.737114	
QLI70391.1	OTHER	0.011918	0.988082	
QLI70392.1	OTHER	0.001200	0.998800	
QLI70393.1	OTHER	0.000366	0.999634	
QLI70394.1	OTHER	0.000328	0.999672	

QLI70395.1	OTHER	0.000812	0.999188	
QLI70396.1	OTHER	0.001481	0.998519	
QLI70397.1	OTHER	0.001191	0.998809	
QLI70398.1	OTHER	0.000854	0.999146	
QLI70399.1	SP(Sec/SPI)	0.985653	0.014347	CS pos: 21-22. AAA-SD. Pr: 0.4456
QLI70400.1	OTHER	0.001316	0.998684	
QLI70401.1	OTHER	0.419787	0.580213	
QLI70402.1	OTHER	0.001549	0.998451	
QLI70403.1	SP(Sec/SPI)	0.971079	0.028921	CS pos: 20-21. ALA-AS. Pr: 0.4408
QLI70404.1	OTHER	0.001391	0.998609	
QLI70405.1	OTHER	0.087833	0.912167	
QLI70406.1	OTHER	0.000194	0.999806	
QLI70407.1	SP(Sec/SPI)	0.941193	0.058807	CS pos: 27-28. STA-ID. Pr: 0.6700
QLI70408.1	SP(Sec/SPI)	0.992315	0.007685	CS pos: 20-21. VSA-RP. Pr: 0.7871
QLI70409.1	OTHER	0.003301	0.996699	
QLI70410.1	OTHER	0.000911	0.999089	
QLI70411.1	SP(Sec/SPI)	0.996977	0.003023	CS pos: 20-21. PQA-KP. Pr: 0.4682
QLI70412.1	OTHER	0.000916	0.999084	
QLI70413.1	OTHER	0.004436	0.995564	
QLI70414.1	OTHER	0.001809	0.998191	
QLI70415.1	OTHER	0.000706	0.999294	
QLI70416.1	OTHER	0.000773	0.999227	
QLI70417.1	OTHER	0.015927	0.984073	
QLI70418.1	OTHER	0.002374	0.997626	
QLI70419.1	OTHER	0.001385	0.998615	
QLI70420.1	OTHER	0.000330	0.999670	
QLI70421.1	OTHER	0.001218	0.998782	
QLI70422.1	OTHER	0.000407	0.999593	
QLI70423.1	OTHER	0.000180	0.999820	
QLI70424.1	OTHER	0.001140	0.998860	
QLI70425.1	OTHER	0.001880	0.998120	

QLI70426.1	OTHER	0.000796	0.999204	
QLI70427.1	SP(Sec/SPI)	0.909582	0.090418	CS pos: 20-21. TAA-LD. Pr: 0.7433
QLI70428.1	OTHER	0.001104	0.998896	
QLI70429.1	OTHER	0.000614	0.999386	
QLI70430.1	OTHER	0.002381	0.997619	
QLI70431.1	OTHER	0.001113	0.998887	
QLI70432.1	OTHER	0.003127	0.996873	
QLI70433.1	OTHER	0.001013	0.998987	
QLI70434.1	OTHER	0.000949	0.999051	
QLI70435.1	OTHER	0.097495	0.902505	
QLI70436.1	OTHER	0.000359	0.999641	
QLI70437.1	SP(Sec/SPI)	0.949580	0.050420	CS pos: 21-22. VDG-AS. Pr: 0.2998
QLI70438.1	SP(Sec/SPI)	0.995989	0.004011	CS pos: 17-18. AQA-VQ. Pr: 0.9105
QLI70439.1	OTHER	0.002937	0.997063	
QLI70440.1	OTHER	0.001042	0.998958	
QLI70441.1	OTHER	0.001081	0.998919	
QLI70442.1	OTHER	0.000889	0.999111	
QLI70443.1	OTHER	0.001353	0.998647	
QLI70444.1	OTHER	0.000936	0.999064	
QLI70445.1	SP(Sec/SPI)	0.578541	0.421459	CS pos: 20-21. AMG-IP. Pr: 0.2426
QLI70446.1	OTHER	0.000506	0.999494	
QLI70447.1	OTHER	0.001656	0.998344	
QLI70448.1	OTHER	0.001360	0.998640	
QLI70449.1	OTHER	0.099497	0.900503	
QLI70450.1	SP(Sec/SPI)	0.885893	0.114107	CS pos: 21-22. AQA-LD. Pr: 0.4851
QLI70451.1	OTHER	0.006190	0.993810	
QLI70452.1	OTHER	0.001190	0.998810	
QLI70453.1	OTHER	0.000949	0.999051	
QLI70454.1	OTHER	0.004814	0.995186	
QLI70455.1	OTHER	0.000592	0.999408	
QLI70456.1	OTHER	0.002854	0.997146	

QLI70457.1	OTHER	0.041892	0.958108	
QLI70458.1	OTHER	0.001112	0.998888	
QLI70459.1	OTHER	0.001981	0.998019	
QLI70460.1	OTHER	0.014889	0.985111	
QLI70461.1	SP(Sec/SPI)	0.989883	0.010117	CS pos: 24-25. CQA-HN. Pr: 0.9692
QLI70462.1	OTHER	0.001205	0.998795	
QLI70463.1	OTHER	0.000723	0.999277	
QLI70464.1	OTHER	0.000972	0.999028	
QLI70465.1	OTHER	0.001149	0.998851	
QLI70466.1	OTHER	0.008355	0.991645	
QLI70467.1	OTHER	0.002160	0.997840	
QLI70468.1	OTHER	0.000427	0.999573	
QLI70469.1	OTHER	0.001535	0.998465	
QLI70470.1	OTHER	0.001071	0.998929	
QLI70471.1	SP(Sec/SPI)	0.914795	0.085205	CS pos: 17-18. SLA-RE. Pr: 0.7734
QLI70472.1	OTHER	0.000345	0.999655	
QLI70473.1	OTHER	0.000463	0.999537	
QLI70474.1	OTHER	0.001759	0.998241	
QLI70475.1	OTHER	0.000771	0.999229	
QLI70476.1	SP(Sec/SPI)	0.984876	0.015124	CS pos: 17-18. VSA-IP. Pr: 0.6745
QLI70477.1	SP(Sec/SPI)	0.927346	0.072654	CS pos: 17-18. CLA-AR. Pr: 0.5539
QLI70478.1	OTHER	0.136066	0.863934	
QLI70479.1	OTHER	0.002545	0.997455	
QLI70480.1	OTHER	0.004037	0.995963	
QLI70481.1	OTHER	0.015843	0.984157	
QLI70482.1	OTHER	0.003873	0.996127	
QLI70483.1	OTHER	0.001155	0.998845	
QLI70484.1	OTHER	0.001990	0.998010	
QLI70485.1	OTHER	0.001003	0.998997	
QLI70486.1	OTHER	0.000970	0.999030	
QLI70487.1	OTHER	0.000561	0.999439	

QLI70488.1	SP(Sec/SPI)	0.609702	0.390298	CS pos: 20-21. AIA-NK. Pr: 0.5057
QLI70489.1	OTHER	0.000292	0.999708	
QLI70490.1	OTHER	0.002164	0.997836	
QLI70491.1	SP(Sec/SPI)	0.996597	0.003403	CS pos: 23-24. AAP-AS. Pr: 0.2696
QLI70492.1	OTHER	0.008271	0.991729	
QLI70493.1	OTHER	0.001201	0.998799	
QLI70494.1	OTHER	0.023618	0.976382	
QLI70495.1	OTHER	0.000617	0.999383	
QLI70496.1	OTHER	0.002094	0.997906	
QLI70497.1	SP(Sec/SPI)	0.994858	0.005142	CS pos: 21-22. ASA-VA. Pr: 0.5411
QLI70498.1	OTHER	0.001109	0.998891	
QLI70499.1	SP(Sec/SPI)	0.908548	0.091452	CS pos: 20-21. AAA-RD. Pr: 0.5588
QLI70500.1	OTHER	0.000416	0.999584	
QLI70501.1	OTHER	0.000600	0.999400	
QLI70502.1	OTHER	0.002520	0.997480	
QLI70503.1	OTHER	0.000579	0.999421	
QLI70504.1	SP(Sec/SPI)	0.996110	0.003890	CS pos: 24-25. AAA-AA. Pr: 0.3753
QLI70505.1	OTHER	0.001186	0.998814	
QLI70506.1	OTHER	0.000206	0.999794	
QLI70507.1	SP(Sec/SPI)	0.996756	0.003244	CS pos: 16-17. ALA-AP. Pr: 0.8521
QLI70508.1	OTHER	0.003240	0.996760	
QLI70509.1	OTHER	0.004286	0.995714	
QLI70510.1	OTHER	0.000462	0.999538	
QLI70511.1	OTHER	0.003319	0.996681	
QLI70512.1	OTHER	0.000740	0.999260	
QLI70513.1	OTHER	0.144884	0.855116	
QLI70514.1	OTHER	0.000354	0.999646	
QLI70515.1	OTHER	0.000733	0.999267	
QLI70516.1	OTHER	0.000526	0.999474	
QLI70517.1	SP(Sec/SPI)	0.998801	0.001199	CS pos: 19-20. SVA-DK. Pr: 0.8232
QLI70518.1	SP(Sec/SPI)	0.996756	0.003244	CS pos: 18-19. AGA-KR. Pr: 0.5479

QLI70519.1	OTHER	0.000607	0.999393	
QLI70520.1	OTHER	0.000388	0.999612	
QLI70521.1	OTHER	0.000632	0.999368	
QLI70522.1	SP(Sec/SPI)	0.989715	0.010285	CS pos: 17-18. IAA-SP. Pr: 0.5060
QLI70523.1	OTHER	0.001236	0.998764	
QLI70524.1	OTHER	0.000609	0.999391	
QLI70525.1	OTHER	0.000572	0.999428	
QLI70526.1	OTHER	0.001113	0.998887	
QLI70527.1	OTHER	0.000614	0.999386	
QLI70528.1	OTHER	0.001546	0.998454	
QLI70529.1	SP(Sec/SPI)	0.971169	0.028831	CS pos: 20-21. AIC-AE. Pr: 0.5459
QLI70530.1	OTHER	0.001371	0.998629	
QLI70531.1	OTHER	0.002317	0.997683	
QLI70532.1	OTHER	0.004133	0.995867	
QLI70533.1	OTHER	0.003752	0.996248	
QLI70534.1	OTHER	0.002116	0.997884	
QLI70535.1	OTHER	0.000360	0.999640	
QLI70536.1	SP(Sec/SPI)	0.995383	0.004617	CS pos: 18-19. ASA-AP. Pr: 0.9461
QLI70537.1	OTHER	0.001795	0.998205	
QLI70538.1	OTHER	0.000273	0.999727	
QLI70539.1	OTHER	0.000391	0.999609	
QLI70540.1	OTHER	0.001159	0.998841	
QLI70541.1	OTHER	0.000844	0.999156	
QLI70542.1	SP(Sec/SPI)	0.883540	0.116460	CS pos: 19-20. AVS-NG. Pr: 0.2428
QLI70543.1	OTHER	0.000816	0.999184	
QLI70544.1	OTHER	0.001269	0.998731	
QLI70545.1	OTHER	0.105008	0.894992	
QLI70546.1	OTHER	0.053312	0.946688	
QLI70547.1	OTHER	0.000811	0.999189	
QLI70548.1	OTHER	0.002003	0.997997	
QLI70549.1	OTHER	0.000435	0.999565	

QLI70550.1	SP(Sec/SPI)	0.537520	0.462480	CS pos: 26-27. TFA-HK. Pr: 0.3923
QLI70551.1	SP(Sec/SPI)	0.999078	0.000922	CS pos: 16-17. ASA-SP. Pr: 0.7482
QLI70552.1	OTHER	0.000777	0.999223	
QLI70553.1	OTHER	0.001807	0.998193	
QLI70554.1	OTHER	0.001294	0.998706	
QLI70555.1	OTHER	0.001504	0.998496	
QLI70556.1	OTHER	0.000517	0.999483	
QLI70557.1	OTHER	0.002100	0.997900	
QLI70558.1	OTHER	0.001476	0.998524	
QLI70559.1	SP(Sec/SPI)	0.997195	0.002805	CS pos: 17-18. ANA-VG. Pr: 0.4070
QLI70560.1	OTHER	0.159262	0.840738	
QLI70561.1	SP(Sec/SPI)	0.975882	0.024118	CS pos: 22-23. ARA-NL. Pr: 0.7623
QLI70562.1	OTHER	0.000521	0.999479	
QLI70563.1	OTHER	0.001173	0.998827	
QLI70564.1	SP(Sec/SPI)	0.785836	0.214164	CS pos: 25-26. SAA-SP. Pr: 0.6141
QLI70565.1	OTHER	0.005097	0.994903	
QLI70566.1	OTHER	0.000572	0.999428	
QLI70567.1	OTHER	0.001024	0.998976	
QLI70568.1	OTHER	0.000890	0.999110	
QLI70569.1	OTHER	0.000225	0.999775	
QLI70570.1	OTHER	0.000312	0.999688	
QLI70571.1	OTHER	0.001083	0.998917	
QLI70572.1	OTHER	0.000665	0.999335	
QLI70573.1	OTHER	0.000856	0.999144	
QLI70574.1	OTHER	0.000575	0.999425	
QLI70575.1	OTHER	0.002042	0.997958	
QLI70576.1	OTHER	0.000993	0.999007	
QLI70577.1	OTHER	0.000668	0.999332	
QLI70578.1	OTHER	0.001794	0.998206	
QLI70579.1	OTHER	0.000675	0.999325	
QLI70580.1	OTHER	0.000390	0.999610	

QLI70581.1	OTHER	0.001562	0.998438	
QLI70582.1	OTHER	0.024243	0.975757	
QLI70583.1	OTHER	0.001171	0.998829	
QLI70584.1	OTHER	0.001219	0.998781	
QLI70585.1	OTHER	0.000565	0.999435	
QLI70586.1	SP(Sec/SPI)	0.990105	0.009895	CS pos: 16-17. VAA-EP. Pr: 0.6885
QLI70587.1	OTHER	0.001544	0.998456	
QLI70588.1	OTHER	0.000244	0.999756	
QLI70589.1	OTHER	0.002816	0.997184	
QLI70590.1	OTHER	0.003213	0.996787	
QLI70591.1	OTHER	0.000520	0.999480	
QLI70592.1	OTHER	0.000787	0.999213	
QLI70593.1	OTHER	0.000247	0.999753	
QLI70594.1	SP(Sec/SPI)	0.573642	0.426358	CS pos: 21-22. TMA-EG. Pr: 0.4201
QLI70595.1	OTHER	0.001757	0.998243	
QLI70596.1	OTHER	0.000475	0.999525	
QLI70597.1	OTHER	0.015960	0.984040	
QLI70598.1	SP(Sec/SPI)	0.570429	0.429571	CS pos: 29-30. ITA-PV. Pr: 0.2038
QLI70599.1	SP(Sec/SPI)	0.962644	0.037356	CS pos: 17-18. GLA-NK. Pr: 0.4423
QLI70600.1	OTHER	0.001205	0.998795	
QLI70601.1	OTHER	0.000334	0.999666	
QLI70602.1	OTHER	0.000922	0.999078	
QLI70603.1	SP(Sec/SPI)	0.975888	0.024112	CS pos: 18-19. VAA-AP. Pr: 0.6848
QLI70604.1	OTHER	0.003763	0.996237	
QLI70605.1	OTHER	0.001568	0.998432	
QLI70606.1	OTHER	0.001187	0.998813	
QLI70607.1	OTHER	0.004423	0.995577	
QLI70608.1	OTHER	0.001558	0.998442	
QLI70609.1	OTHER	0.128133	0.871867	
QLI70610.1	OTHER	0.003955	0.996045	
QLI70611.1	OTHER	0.009066	0.990934	

QLI70612.1	OTHER	0.000872	0.999128	
QLI70613.1	OTHER	0.000976	0.999024	
QLI70614.1	OTHER	0.001143	0.998857	
QLI70615.1	OTHER	0.002643	0.997357	
QLI70616.1	OTHER	0.002000	0.998000	
QLI70617.1	OTHER	0.003284	0.996716	
QLI70618.1	SP(Sec/SPI)	0.962804	0.037196	CS pos: 23-24. ALA-DN. Pr: 0.8248
QLI70619.1	OTHER	0.000333	0.999667	
QLI70620.1	OTHER	0.335044	0.664956	
QLI70621.1	OTHER	0.393045	0.606955	
QLI70622.1	OTHER	0.002347	0.997653	
QLI70623.1	OTHER	0.000633	0.999367	
QLI70624.1	OTHER	0.002434	0.997566	
QLI70625.1	OTHER	0.000868	0.999132	
QLI70626.1	OTHER	0.000822	0.999178	
QLI70627.1	OTHER	0.001002	0.998998	
QLI70628.1	OTHER	0.000475	0.999525	
QLI70629.1	OTHER	0.001365	0.998635	
QLI70630.1	OTHER	0.002485	0.997515	
QLI70631.1	OTHER	0.004630	0.995370	
QLI70632.1	OTHER	0.000456	0.999544	
QLI70633.1	OTHER	0.000641	0.999359	
QLI70634.1	OTHER	0.002724	0.997276	
QLI70635.1	OTHER	0.001846	0.998154	
QLI70636.1	OTHER	0.004451	0.995549	
QLI70637.1	OTHER	0.000880	0.999120	
QLI70638.1	OTHER	0.000515	0.999485	
QLI70639.1	OTHER	0.024093	0.975907	
QLI70640.1	OTHER	0.000694	0.999306	
QLI70641.1	OTHER	0.000317	0.999683	
QLI70642.1	SP(Sec/SPI)	0.976230	0.023770	CS pos: 24-25. VLG-EC. Pr: 0.8467

QLI70643.1	OTHER	0.000425	0.999575	
QLI70644.1	OTHER	0.000308	0.999692	
QLI70645.1	OTHER	0.002357	0.997643	
QLI70646.1	OTHER	0.000729	0.999271	
QLI70647.1	OTHER	0.002369	0.997631	
QLI70648.1	OTHER	0.000410	0.999590	
QLI70649.1	OTHER	0.215953	0.784047	
QLI70650.1	OTHER	0.000749	0.999251	
QLI70651.1	OTHER	0.000650	0.999350	
QLI70652.1	SP(Sec/SPI)	0.924796	0.075204	CS pos: 20-21. ILA-VR. Pr: 0.7289
QLI70653.1	OTHER	0.015352	0.984648	
QLI70654.1	OTHER	0.003047	0.996953	
QLI70655.1	OTHER	0.000526	0.999474	
QLI70656.1	SP(Sec/SPI)	0.900192	0.099808	CS pos: 19-20. ALA-AP. Pr: 0.7211
QLI70657.1	OTHER	0.000646	0.999354	
QLI70658.1	OTHER	0.000874	0.999126	
QLI70659.1	OTHER	0.078904	0.921096	
QLI70660.1	OTHER	0.056065	0.943935	
QLI70661.1	OTHER	0.001851	0.998149	
QLI70662.1	OTHER	0.003109	0.996891	
QLI70663.1	OTHER	0.000458	0.999542	
QLI70664.1	OTHER	0.001821	0.998179	
QLI70665.1	OTHER	0.001223	0.998777	
QLI70666.1	SP(Sec/SPI)	0.984577	0.015423	CS pos: 18-19. ASA-QE. Pr: 0.9203
QLI70667.1	OTHER	0.002017	0.997983	
QLI70668.1	OTHER	0.076784	0.923216	
QLI70669.1	OTHER	0.000534	0.999466	
QLI70670.1	OTHER	0.002096	0.997904	
QLI70671.1	OTHER	0.000588	0.999412	
QLI70672.1	SP(Sec/SPI)	0.994132	0.005868	CS pos: 17-18. TAA-FP. Pr: 0.5643
QLI70673.1	OTHER	0.002773	0.997227	

QLI70674.1	OTHER	0.001193	0.998807	
QLI70675.1	OTHER	0.011677	0.988323	
QLI70676.1	OTHER	0.000714	0.999286	
QLI70677.1	OTHER	0.075201	0.924799	
QLI70678.1	SP(Sec/SPI)	0.983021	0.016979	CS pos: 23-24. IYA-KP. Pr: 0.9414
QLI70679.1	OTHER	0.000521	0.999479	
QLI70680.1	OTHER	0.011383	0.988617	
QLI70681.1	OTHER	0.001063	0.998937	
QLI70682.1	SP(Sec/SPI)	0.942929	0.057071	CS pos: 23-24. VLA-CV. Pr: 0.6128
QLI70683.1	OTHER	0.002533	0.997467	
QLI70684.1	OTHER	0.004857	0.995143	
QLI70685.1	OTHER	0.000804	0.999196	
QLI70686.1	OTHER	0.000734	0.999266	
QLI70687.1	OTHER	0.000848	0.999152	
QLI70688.1	OTHER	0.001772	0.998228	
QLI70689.1	OTHER	0.000646	0.999354	
QLI70690.1	OTHER	0.000677	0.999323	
QLI70691.1	OTHER	0.000799	0.999201	
QLI70692.1	OTHER	0.000797	0.999203	
QLI70693.1	SP(Sec/SPI)	0.992265	0.007735	CS pos: 20-21. ASA-TL. Pr: 0.4586
QLI70694.1	OTHER	0.001883	0.998117	
QLI70695.1	OTHER	0.005551	0.994449	
QLI70696.1	OTHER	0.000749	0.999251	
QLI70697.1	OTHER	0.000444	0.999556	
QLI70698.1	OTHER	0.012386	0.987614	
QLI70699.1	OTHER	0.000472	0.999528	
QLI70700.1	OTHER	0.000300	0.999700	
QLI70701.1	OTHER	0.001465	0.998535	
QLI70702.1	SP(Sec/SPI)	0.948349	0.051651	CS pos: 18-19. VAA-HP. Pr: 0.6401
QLI70703.1	OTHER	0.002886	0.997114	
QLI70704.1	OTHER	0.001031	0.998969	

QLI70705.1	OTHER	0.001672	0.998328	
QLI70706.1	OTHER	0.000699	0.999301	
QLI70707.1	OTHER	0.006742	0.993258	
QLI70708.1	OTHER	0.000438	0.999562	
QLI70709.1	OTHER	0.000672	0.999328	
QLI70710.1	OTHER	0.000297	0.999703	
QLI70711.1	SP(Sec/SPI)	0.991319	0.008681	CS pos: 16-17. ALA-AP. Pr: 0.6104
QLI70712.1	SP(Sec/SPI)	0.994165	0.005835	CS pos: 19-20. ASA-AT. Pr: 0.7929
QLI70713.1	SP(Sec/SPI)	0.971636	0.028364	CS pos: 18-19. VQG-LN. Pr: 0.8637
QLI70714.1	OTHER	0.000576	0.999424	
QLI70715.1	OTHER	0.397743	0.602257	
QLI70716.1	OTHER	0.000455	0.999545	
QLI70717.1	OTHER	0.005939	0.994061	
QLI70718.1	OTHER	0.000991	0.999009	
QLI70719.1	OTHER	0.000295	0.999705	
QLI70720.1	OTHER	0.000544	0.999456	
QLI70721.1	OTHER	0.003952	0.996048	
QLI70722.1	OTHER	0.001556	0.998444	
QLI70723.1	OTHER	0.001134	0.998866	
QLI70724.1	OTHER	0.005278	0.994722	
QLI70725.1	OTHER	0.001829	0.998171	
QLI70726.1	OTHER	0.000495	0.999505	
QLI70727.1	SP(Sec/SPI)	0.993776	0.006224	CS pos: 21-22. AQA-SS. Pr: 0.6922
QLI70728.1	OTHER	0.001567	0.998433	
QLI70729.1	OTHER	0.001120	0.998880	
QLI70730.1	OTHER	0.000894	0.999106	
QLI70731.1	SP(Sec/SPI)	0.995093	0.004907	CS pos: 16-17. AQA-GP. Pr: 0.8835
QLI70732.1	OTHER	0.002578	0.997422	
QLI70733.1	OTHER	0.000569	0.999431	
QLI70734.1	OTHER	0.000191	0.999809	
QLI70735.1	OTHER	0.003926	0.996074	

QLI70736.1	SP(Sec/SPI)	0.848782	0.151218	CS pos: 15-16. VMA-DS. Pr: 0.5515
QLI70737.1	OTHER	0.001022	0.998978	
QLI70738.1	OTHER	0.000405	0.999595	
QLI70739.1	OTHER	0.000922	0.999078	
QLI70740.1	OTHER	0.021706	0.978294	
QLI70741.1	OTHER	0.001184	0.998816	
QLI70742.1	OTHER	0.000199	0.999801	
QLI70743.1	OTHER	0.049001	0.950999	
QLI70744.1	OTHER	0.001490	0.998510	
QLI70745.1	OTHER	0.001012	0.998988	
QLI70746.1	OTHER	0.001431	0.998569	
QLI70747.1	OTHER	0.000664	0.999336	
QLI70748.1	OTHER	0.000976	0.999024	
QLI70749.1	OTHER	0.008266	0.991734	
QLI70750.1	OTHER	0.000665	0.999335	
QLI70751.1	OTHER	0.000325	0.999675	
QLI70752.1	OTHER	0.000791	0.999209	
QLI70753.1	OTHER	0.003621	0.996379	
QLI70754.1	OTHER	0.005146	0.994854	
QLI70755.1	SP(Sec/SPI)	0.999348	0.000652	CS pos: 22-23. VSA-QT. Pr: 0.8240
QLI70756.1	OTHER	0.001601	0.998399	
QLI70757.1	OTHER	0.002166	0.997834	
QLI70758.1	OTHER	0.002548	0.997452	
QLI70759.1	OTHER	0.001065	0.998935	
QLI70760.1	OTHER	0.000606	0.999394	
QLI70761.1	OTHER	0.003265	0.996735	
QLI70762.1	OTHER	0.005976	0.994024	
QLI70763.1	OTHER	0.000315	0.999685	
QLI70764.1	OTHER	0.001151	0.998849	
QLI70765.1	OTHER	0.005896	0.994104	
QLI70766.1	OTHER	0.001321	0.998679	

QLI70767.1	OTHER	0.001216	0.998784	
QLI70768.1	OTHER	0.003692	0.996308	
QLI70769.1	OTHER	0.000760	0.999240	
QLI70770.1	OTHER	0.001053	0.998947	
QLI70771.1	OTHER	0.002681	0.997319	
QLI70772.1	OTHER	0.000553	0.999447	
QLI70773.1	OTHER	0.002200	0.997800	
QLI70774.1	OTHER	0.001204	0.998796	
QLI70775.1	SP(Sec/SPI)	0.937821	0.062179	CS pos: 28-29. TLA-GD. Pr: 0.7187
QLI70776.1	OTHER	0.000359	0.999641	
QLI70777.1	OTHER	0.012589	0.987411	
QLI70778.1	OTHER	0.000307	0.999693	
QLI70779.1	OTHER	0.001005	0.998995	
QLI70780.1	OTHER	0.001260	0.998740	
QLI70781.1	OTHER	0.001325	0.998675	
QLI70782.1	OTHER	0.000184	0.999816	
QLI70783.1	OTHER	0.002193	0.997807	
QLI70784.1	OTHER	0.011120	0.988880	
QLI70785.1	OTHER	0.070223	0.929777	
QLI70786.1	OTHER	0.002197	0.997803	
QLI70787.1	OTHER	0.150089	0.849911	
QLI70788.1	OTHER	0.001213	0.998787	
QLI70789.1	OTHER	0.001194	0.998806	
QLI70790.1	OTHER	0.104844	0.895156	
QLI70791.1	OTHER	0.000866	0.999134	
QLI70792.1	OTHER	0.001743	0.998257	
QLI70793.1	SP(Sec/SPI)	0.659507	0.340493	CS pos: 18-19. VAA-SR. Pr: 0.3430
QLI70794.1	OTHER	0.001443	0.998557	
QLI70795.1	OTHER	0.000425	0.999575	
QLI70796.1	OTHER	0.005183	0.994817	
QLI70797.1	OTHER	0.004056	0.995944	

QLI70798.1	OTHER	0.000507	0.999493	
QLI70799.1	OTHER	0.005541	0.994459	
QLI70800.1	SP(Sec/SPI)	0.994608	0.005392	CS pos: 21-22. THG-ET. Pr: 0.5298
QLI70801.1	SP(Sec/SPI)	0.940098	0.059902	CS pos: 19-20. ASS-LV. Pr: 0.1952
QLI70802.1	OTHER	0.007412	0.992588	
QLI70803.1	OTHER	0.000893	0.999107	
QLI70804.1	OTHER	0.000421	0.999579	
QLI70805.1	OTHER	0.000826	0.999174	
QLI70806.1	OTHER	0.000549	0.999451	
QLI70807.1	OTHER	0.006847	0.993153	
QLI70808.1	OTHER	0.016670	0.983330	
QLI70809.1	SP(Sec/SPI)	0.982184	0.017816	CS pos: 15-16. SIA-AP. Pr: 0.3843
QLI70810.1	OTHER	0.003139	0.996861	
QLI70811.1	OTHER	0.015098	0.984902	
QLI70812.1	OTHER	0.001511	0.998489	
QLI70813.1	OTHER	0.000658	0.999342	
QLI70814.1	OTHER	0.002712	0.997288	
QLI70815.1	OTHER	0.017646	0.982354	
QLI70816.1	OTHER	0.001700	0.998300	
QLI70817.1	OTHER	0.001667	0.998333	
QLI70818.1	OTHER	0.002593	0.997407	
QLI70819.1	OTHER	0.001281	0.998719	
QLI70820.1	OTHER	0.001087	0.998913	
QLI70821.1	OTHER	0.004955	0.995045	
QLI70822.1	OTHER	0.000805	0.999195	
QLI70823.1	OTHER	0.007013	0.992987	
QLI70824.1	OTHER	0.001556	0.998444	
QLI70825.1	OTHER	0.005323	0.994677	
QLI70826.1	OTHER	0.000706	0.999294	
QLI70827.1	OTHER	0.002426	0.997574	
QLI70828.1	OTHER	0.003317	0.996683	

QLI70829.1	OTHER	0.000478	0.999522
QLI70830.1	OTHER	0.001050	0.998950
QLI70831.1	OTHER	0.002400	0.997600
QLI70832.1	OTHER	0.000737	0.999263
QLI70833.1	OTHER	0.000560	0.999440
QLI70834.1	OTHER	0.039433	0.960567
QLI70835.1	OTHER	0.006552	0.993448
QLI70836.1	OTHER	0.001304	0.998696
QLI70837.1	OTHER	0.000584	0.999416
QLI70838.1	OTHER	0.453288	0.546712
QLI70839.1	OTHER	0.000282	0.999718
QLI70840.1	OTHER	0.004923	0.995077
QLI70841.1	OTHER	0.001130	0.998870
QLI70842.1	OTHER	0.001487	0.998513
QLI70843.1	OTHER	0.018251	0.981749
QLI70844.1	OTHER	0.000629	0.999371
QLI70845.1	OTHER	0.000729	0.999271
QLI70846.1	OTHER	0.000941	0.999059
QLI70847.1	OTHER	0.001696	0.998304
QLI70848.1	OTHER	0.005465	0.994535
QLI70849.1	OTHER	0.000911	0.999089
QLI70850.1	OTHER	0.001457	0.998543
QLI70851.1	OTHER	0.000604	0.999396
QLI70852.1	OTHER	0.001053	0.998947
QLI70853.1	OTHER	0.003997	0.996003
QLI70854.1	OTHER	0.001711	0.998289
QLI70855.1	OTHER	0.001180	0.998820
QLI70856.1	OTHER	0.002918	0.997082
QLI70857.1	OTHER	0.001942	0.998058
QLI70858.1	OTHER	0.001417	0.998583
QLI70859.1	OTHER	0.002533	0.997467

QLI70860.1	OTHER	0.002098	0.997902	
QLI70861.1	SP(Sec/SPI)	0.923177	0.076823	CS pos: 16-17. AFA-AN. Pr: 0.7524
QLI70862.1	OTHER	0.005327	0.994673	
QLI70863.1	SP(Sec/SPI)	0.990447	0.009553	CS pos: 18-19. GGA-TD. Pr: 0.5459
QLI70864.1	OTHER	0.013476	0.986524	
QLI70865.1	SP(Sec/SPI)	0.973489	0.026511	CS pos: 21-22. ASA-KP. Pr: 0.8964
QLI70866.1	OTHER	0.000794	0.999206	
QLI70867.1	OTHER	0.001581	0.998419	
QLI70868.1	OTHER	0.000908	0.999092	
QLI70869.1	OTHER	0.000322	0.999678	
QLI70870.1	OTHER	0.002073	0.997927	
QLI70871.1	OTHER	0.134450	0.865550	
QLI70872.1	OTHER	0.000295	0.999705	
QLI70873.1	OTHER	0.271466	0.728534	
QLI70874.1	OTHER	0.001482	0.998518	
QLI70875.1	OTHER	0.000792	0.999208	
QLI70876.1	SP(Sec/SPI)	0.990318	0.009682	CS pos: 16-17. AWA-QQ. Pr: 0.9063
QLI70877.1	OTHER	0.069114	0.930886	
QLI70878.1	OTHER	0.010118	0.989882	
QLI70879.1	SP(Sec/SPI)	0.999639	0.000361	CS pos: 17-18. AIA-AP. Pr: 0.7969
QLI70880.1	SP(Sec/SPI)	0.985873	0.014127	CS pos: 19-20. TAC-LN. Pr: 0.4862
QLI70881.1	OTHER	0.002989	0.997011	
QLI70882.1	OTHER	0.021559	0.978441	
QLI70883.1	OTHER	0.001399	0.998601	
QLI70884.1	OTHER	0.000836	0.999164	
QLI70885.1	OTHER	0.000872	0.999128	
QLI70886.1	OTHER	0.018918	0.981082	
QLI70887.1	OTHER	0.055997	0.944003	
QLI70888.1	OTHER	0.002848	0.997152	
QLI70889.1	OTHER	0.000419	0.999581	
QLI70890.1	OTHER	0.000832	0.999168	

QLI70891.1	OTHER	0.000391	0.999609	
QLI70892.1	OTHER	0.000733	0.999267	
QLI70893.1	OTHER	0.001188	0.998812	
QLI70894.1	OTHER	0.000212	0.999788	
QLI70895.1	OTHER	0.003149	0.996851	
QLI70896.1	OTHER	0.002160	0.997840	
QLI70897.1	OTHER	0.000909	0.999091	
QLI70898.1	OTHER	0.000656	0.999344	
QLI70899.1	SP(Sec/SPI)	0.888095	0.111905	CS pos: 16-17. ATA-ER. Pr: 0.4786
QLI70900.1	OTHER	0.002072	0.997928	
QLI70901.1	OTHER	0.008676	0.991324	
QLI70902.1	OTHER	0.003948	0.996052	
QLI70903.1	OTHER	0.000748	0.999252	
QLI70904.1	OTHER	0.002293	0.997707	
QLI70905.1	OTHER	0.001491	0.998509	
QLI70906.1	SP(Sec/SPI)	0.992533	0.007467	CS pos: 19-20. CAA-VP. Pr: 0.8419
QLI70907.1	OTHER	0.001148	0.998852	
QLI70908.1	OTHER	0.001000	0.999000	
QLI70909.1	OTHER	0.002478	0.997522	
QLI70910.1	OTHER	0.027998	0.972002	
QLI70911.1	OTHER	0.000740	0.999260	
QLI70912.1	OTHER	0.005634	0.994366	
QLI70913.1	SP(Sec/SPI)	0.999459	0.000541	CS pos: 20-21. CLA-QT. Pr: 0.8526
QLI70914.1	OTHER	0.000974	0.999026	
QLI70915.1	OTHER	0.000793	0.999207	
QLI70916.1	SP(Sec/SPI)	0.972839	0.027161	CS pos: 18-19. SLG-SP. Pr: 0.3963
QLI70917.1	SP(Sec/SPI)	0.995505	0.004495	CS pos: 21-22. GAS-VL. Pr: 0.6706
QLI70918.1	OTHER	0.001256	0.998744	
QLI70919.1	OTHER	0.001986	0.998014	
QLI70920.1	OTHER	0.000851	0.999149	
QLI70921.1	SP(Sec/SPI)	0.747255	0.252745	CS pos: 42-43. VGA-EP. Pr: 0.3597

QLI70922.1	OTHER	0.292157	0.707843	
QLI70923.1	OTHER	0.002005	0.997995	
QLI70924.1	SP(Sec/SPI)	0.981461	0.018539	CS pos: 17-18. VSG-HG. Pr: 0.6737
QLI70925.1	OTHER	0.000743	0.999257	
QLI70926.1	OTHER	0.001135	0.998865	
QLI70927.1	OTHER	0.001394	0.998606	
QLI70928.1	OTHER	0.000987	0.999013	
QLI70929.1	OTHER	0.002365	0.997635	
QLI70930.1	OTHER	0.002308	0.997692	
QLI70931.1	OTHER	0.000336	0.999664	
QLI70932.1	OTHER	0.001305	0.998695	
QLI70933.1	OTHER	0.001162	0.998838	
QLI70934.1	OTHER	0.001308	0.998692	
QLI70935.1	OTHER	0.000852	0.999148	
QLI70936.1	OTHER	0.002205	0.997795	
QLI70937.1	OTHER	0.005415	0.994585	
QLI70938.1	OTHER	0.009334	0.990666	
QLI70939.1	OTHER	0.001365	0.998635	
QLI70940.1	OTHER	0.000621	0.999379	
QLI70941.1	OTHER	0.000459	0.999541	
QLI70942.1	OTHER	0.003671	0.996329	
QLI70943.1	OTHER	0.001296	0.998704	
QLI70944.1	OTHER	0.001694	0.998306	
QLI70945.1	OTHER	0.000862	0.999138	
QLI70946.1	OTHER	0.001232	0.998768	
QLI70947.1	OTHER	0.000256	0.999744	
QLI70948.1	SP(Sec/SPI)	0.993211	0.006789	CS pos: 19-20. VLG-SP. Pr: 0.8904
QLI70949.1	SP(Sec/SPI)	0.999339	0.000661	CS pos: 19-20. AVA-DC. Pr: 0.8003
QLI70950.1	OTHER	0.000583	0.999417	
QLI70951.1	OTHER	0.001035	0.998965	
QLI70952.1	SP(Sec/SPI)	0.996189	0.003811	CS pos: 17-18. ASA-HP. Pr: 0.7628

QLI70953.1	OTHER	0.000481	0.999519	
QLI70954.1	OTHER	0.001235	0.998765	
QLI70955.1	OTHER	0.001577	0.998423	
QLI70956.1	OTHER	0.002025	0.997975	
QLI70957.1	OTHER	0.314852	0.685148	
QLI70958.1	OTHER	0.000198	0.999802	
QLI70959.1	OTHER	0.081787	0.918213	
QLI70960.1	OTHER	0.001906	0.998094	
QLI70961.1	OTHER	0.000874	0.999126	
QLI70962.1	OTHER	0.000379	0.999621	
QLI70963.1	OTHER	0.013007	0.986993	
QLI70964.1	OTHER	0.001097	0.998903	
QLI70965.1	OTHER	0.000762	0.999238	
QLI70966.1	OTHER	0.000545	0.999455	
QLI70967.1	OTHER	0.001404	0.998596	
QLI70968.1	OTHER	0.000560	0.999440	
QLI70969.1	OTHER	0.014276	0.985724	
QLI70970.1	OTHER	0.464617	0.535383	
QLI70971.1	OTHER	0.000390	0.999610	
QLI70972.1	OTHER	0.000550	0.999450	
QLI70973.1	SP(Sec/SPI)	0.977902	0.022098	CS pos: 25-26. AEA-QY. Pr: 0.9038
QLI70974.1	OTHER	0.002663	0.997337	
QLI70975.1	OTHER	0.005759	0.994241	
QLI70976.1	OTHER	0.000958	0.999042	
QLI70977.1	OTHER	0.003255	0.996745	
QLI70978.1	OTHER	0.016824	0.983176	
QLI70979.1	OTHER	0.001921	0.998079	
QLI70980.1	SP(Sec/SPI)	0.995825	0.004175	CS pos: 32-33. AHA-ER. Pr: 0.9549
QLI70981.1	OTHER	0.002819	0.997181	
QLI70982.1	SP(Sec/SPI)	0.976601	0.023399	CS pos: 18-19. AAG-HP. Pr: 0.8426
QLI70983.1	OTHER	0.035562	0.964438	

QLI70984.1	OTHER	0.000756	0.999244	
QLI70985.1	OTHER	0.000567	0.999433	
QLI70986.1	OTHER	0.001390	0.998610	
QLI70987.1	OTHER	0.000919	0.999081	
QLI70988.1	OTHER	0.000989	0.999011	
QLI70989.1	OTHER	0.000534	0.999466	
QLI70990.1	OTHER	0.000489	0.999511	
QLI70991.1	OTHER	0.001748	0.998252	
QLI70992.1	OTHER	0.001067	0.998933	
QLI70993.1	OTHER	0.002394	0.997606	
QLI70994.1	OTHER	0.001935	0.998065	
QLI70995.1	OTHER	0.000790	0.999210	
QLI70996.1	OTHER	0.007577	0.992423	
QLI70997.1	OTHER	0.003014	0.996986	
QLI70998.1	OTHER	0.000688	0.999312	
QLI70999.1	OTHER	0.002479	0.997521	
QLI71000.1	OTHER	0.000864	0.999136	
QLI71001.1	OTHER	0.003851	0.996149	
QLI71002.1	OTHER	0.000439	0.999561	
QLI71003.1	OTHER	0.001566	0.998434	
QLI71004.1	OTHER	0.000140	0.999860	
QLI71005.1	OTHER	0.006369	0.993631	
QLI71006.1	SP(Sec/SPI)	0.760625	0.239375	CS pos: 22-23. SSG-EP. Pr: 0.2524
QLI71007.1	OTHER	0.000490	0.999510	
QLI71008.1	OTHER	0.000608	0.999392	
QLI71009.1	OTHER	0.001242	0.998758	
QLI71010.1	OTHER	0.002314	0.997686	
QLI71011.1	OTHER	0.001072	0.998928	
QLI71012.1	OTHER	0.003651	0.996349	
QLI71013.1	OTHER	0.002132	0.997868	
QLI71014.1	SP(Sec/SPI)	0.994915	0.005085	CS pos: 19-20. VLA-MN. Pr: 0.8972

QLI71015.1	OTHER	0.000922	0.999078	
QLI71016.1	SP(Sec/SPI)	0.989058	0.010942	CS pos: 18-19. TDA-RR. Pr: 0.7955
QLI71017.1	OTHER	0.001804	0.998196	
QLI71018.1	SP(Sec/SPI)	0.856040	0.143960	CS pos: 24-25. SAG-SV. Pr: 0.2964
QLI71019.1	OTHER	0.000587	0.999413	
QLI71020.1	OTHER	0.000576	0.999424	
QLI71021.1	SP(Sec/SPI)	0.962605	0.037395	CS pos: 18-19. VKS-SA. Pr: 0.4068
QLI71022.1	SP(Sec/SPI)	0.972886	0.027114	CS pos: 16-17. VLA-MT. Pr: 0.3896
QLI71023.1	OTHER	0.003804	0.996196	
QLI71024.1	OTHER	0.000621	0.999379	
QLI71025.1	OTHER	0.000536	0.999464	
QLI71026.1	OTHER	0.002042	0.997958	
QLI71027.1	OTHER	0.000361	0.999639	
QLI71028.1	OTHER	0.000649	0.999351	
QLI71029.1	OTHER	0.001519	0.998481	
QLI71030.1	OTHER	0.000438	0.999562	
QLI71031.1	OTHER	0.156917	0.843083	
QLI71032.1	OTHER	0.001197	0.998803	
QLI71033.1	OTHER	0.000913	0.999087	
QLI71034.1	OTHER	0.001486	0.998514	
QLI71035.1	OTHER	0.002358	0.997642	
QLI71036.1	OTHER	0.000594	0.999406	
QLI71037.1	OTHER	0.000536	0.999464	
QLI71038.1	OTHER	0.001792	0.998208	
QLI71039.1	OTHER	0.000297	0.999703	
QLI71040.1	OTHER	0.000341	0.999659	
QLI71041.1	OTHER	0.006182	0.993818	
QLI71042.1	OTHER	0.000620	0.999380	
QLI71043.1	OTHER	0.004981	0.995019	
QLI71044.1	OTHER	0.000675	0.999325	
QLI71045.1	OTHER	0.001077	0.998923	

QLI71046.1	OTHER	0.001008	0.998992	
QLI71047.1	OTHER	0.002192	0.997808	
QLI71048.1	OTHER	0.000828	0.999172	
QLI71049.1	OTHER	0.000525	0.999475	
QLI71050.1	SP(Sec/SPI)	0.993824	0.006176	CS pos: 16-17. VWA-SP. Pr: 0.9423
QLI71051.1	OTHER	0.000358	0.999642	
QLI71052.1	OTHER	0.001342	0.998658	
QLI71053.1	OTHER	0.000308	0.999692	
QLI71054.1	OTHER	0.002550	0.997450	
QLI71055.1	OTHER	0.022170	0.977830	
QLI71056.1	OTHER	0.002591	0.997409	
QLI71057.1	OTHER	0.001850	0.998150	
QLI71058.1	OTHER	0.001139	0.998861	
QLI71059.1	OTHER	0.000725	0.999275	
QLI71060.1	SP(Sec/SPI)	0.979524	0.020476	CS pos: 17-18. ALA-AE. Pr: 0.7202
QLI71061.1	OTHER	0.000720	0.999280	
QLI71062.1	OTHER	0.000950	0.999050	
QLI71063.1	SP(Sec/SPI)	0.985941	0.014059	CS pos: 18-19. VRG-MV. Pr: 0.6897
QLI71064.1	SP(Sec/SPI)	0.997185	0.002815	CS pos: 21-22. IAA-DP. Pr: 0.9264
QLI71065.1	OTHER	0.000536	0.999464	
QLI71066.1	SP(Sec/SPI)	0.970863	0.029137	CS pos: 19-20. IAA-AP. Pr: 0.6136
QLI71067.1	OTHER	0.000589	0.999411	
QLI71068.1	OTHER	0.001338	0.998662	
QLI71069.1	OTHER	0.002889	0.997111	
QLI71070.1	OTHER	0.006508	0.993492	
QLI71071.1	OTHER	0.000736	0.999264	
QLI71072.1	OTHER	0.001530	0.998470	
QLI71073.1	OTHER	0.001327	0.998673	
QLI71074.1	SP(Sec/SPI)	0.973855	0.026145	CS pos: 17-18. VAA-QP. Pr: 0.6541
QLI71075.1	OTHER	0.001316	0.998684	
QLI71076.1	OTHER	0.001060	0.998940	

QLI71077.1	OTHER	0.001626	0.998374	
QLI71078.1	OTHER	0.001309	0.998691	
QLI71079.1	OTHER	0.001160	0.998840	
QLI71080.1	OTHER	0.001064	0.998936	
QLI71081.1	OTHER	0.000443	0.999557	
QLI71082.1	OTHER	0.000353	0.999647	
QLI71083.1	OTHER	0.000479	0.999521	
QLI71084.1	OTHER	0.003653	0.996347	
QLI71085.1	OTHER	0.002623	0.997377	
QLI71086.1	OTHER	0.005076	0.994924	
QLI71087.1	SP(Sec/SPI)	0.969765	0.030235	CS pos: 18-19. AQG-SS. Pr: 0.5337
QLI71088.1	SP(Sec/SPI)	0.936945	0.063055	CS pos: 19-20. AVA-VP. Pr: 0.7497
QLI71089.1	OTHER	0.014050	0.985950	
QLI71090.1	SP(Sec/SPI)	0.952851	0.047149	CS pos: 19-20. AHA-GH. Pr: 0.7138
QLI71091.1	OTHER	0.005858	0.994142	
QLI71092.1	OTHER	0.001459	0.998541	
QLI71093.1	OTHER	0.003666	0.996334	
QLI71094.1	OTHER	0.000848	0.999152	
QLI71095.1	SP(Sec/SPI)	0.873928	0.126072	CS pos: 21-22. GAA-VQ. Pr: 0.4838
QLI71096.1	SP(Sec/SPI)	0.998271	0.001729	CS pos: 18-19. ALA-AI. Pr: 0.7574
QLI71097.1	OTHER	0.000965	0.999035	
QLI71098.1	OTHER	0.005275	0.994725	
QLI71099.1	OTHER	0.001078	0.998922	
QLI71100.1	OTHER	0.001023	0.998977	
QLI71101.1	OTHER	0.001320	0.998680	
QLI71102.1	OTHER	0.000902	0.999098	
QLI71103.1	SP(Sec/SPI)	0.986262	0.013738	CS pos: 17-18. ASA-YT. Pr: 0.8868
QLI71104.1	OTHER	0.001235	0.998765	
QLI71105.1	OTHER	0.000357	0.999643	
QLI71106.1	OTHER	0.000392	0.999608	
QLI71107.1	OTHER	0.003184	0.996816	

QLI71108.1	OTHER	0.000402	0.999598	
QLI71109.1	OTHER	0.002078	0.997922	
QLI71110.1	OTHER	0.000219	0.999781	
QLI71111.1	OTHER	0.002210	0.997790	
QLI71112.1	OTHER	0.006578	0.993422	
QLI71113.1	SP(Sec/SPI)	0.999573	0.000427	CS pos: 16-17. AMA-YE. Pr: 0.9194
QLI71114.1	OTHER	0.001955	0.998045	
QLI71115.1	SP(Sec/SPI)	0.994091	0.005909	CS pos: 19-20. VAA-QV. Pr: 0.6171
QLI71116.1	OTHER	0.001307	0.998693	
QLI71117.1	OTHER	0.001388	0.998612	
QLI71118.1	OTHER	0.001467	0.998533	
QLI71119.1	OTHER	0.000728	0.999272	
QLI71120.1	OTHER	0.000619	0.999381	
QLI71121.1	OTHER	0.001976	0.998024	
QLI71122.1	OTHER	0.000535	0.999465	
QLI71123.1	OTHER	0.001425	0.998575	
QLI71124.1	OTHER	0.006190	0.993810	
QLI71125.1	OTHER	0.001649	0.998351	
QLI71126.1	OTHER	0.002251	0.997749	
QLI71127.1	OTHER	0.001184	0.998816	
QLI71128.1	OTHER	0.000854	0.999146	
QLI71129.1	OTHER	0.000473	0.999527	
QLI71130.1	OTHER	0.001623	0.998377	
QLI71131.1	SP(Sec/SPI)	0.991260	0.008740	CS pos: 19-20. VSC-EP. Pr: 0.8193
QLI71132.1	OTHER	0.000646	0.999354	
QLI71133.1	OTHER	0.001282	0.998718	
QLI71134.1	OTHER	0.000885	0.999115	
QLI71135.1	SP(Sec/SPI)	0.977626	0.022374	CS pos: 18-19. ASA-VR. Pr: 0.8372
QLI71136.1	OTHER	0.002884	0.997116	
QLI71137.1	OTHER	0.001083	0.998917	
QLI71138.1	SP(Sec/SPI)	0.518188	0.481812	CS pos: 18-19. AAG-RT. Pr: 0.2074

QLI71139.1	OTHER	0.001384	0.998616	
QLI71140.1	OTHER	0.000468	0.999532	
QLI71141.1	OTHER	0.000795	0.999205	
QLI71142.1	OTHER	0.000941	0.999059	
QLI71143.1	OTHER	0.002435	0.997565	
QLI71144.1	SP(Sec/SPI)	0.977530	0.022470	CS pos: 20-21. ADA-AA. Pr: 0.5527
QLI71145.1	OTHER	0.302599	0.697401	
QLI71146.1	OTHER	0.000657	0.999343	
QLI71147.1	OTHER	0.005081	0.994919	
QLI71148.1	OTHER	0.009326	0.990674	
QLI71149.1	SP(Sec/SPI)	0.864658	0.135342	CS pos: 16-17. VAG-DS. Pr: 0.6118
QLI71150.1	SP(Sec/SPI)	0.506214	0.493786	CS pos: 25-26. THA-SA. Pr: 0.2409
QLI71151.1	OTHER	0.004024	0.995976	
QLI71152.1	OTHER	0.000639	0.999361	
QLI71153.1	OTHER	0.001018	0.998982	
QLI71154.1	OTHER	0.007616	0.992384	
QLI71155.1	OTHER	0.002517	0.997483	
QLI71156.1	OTHER	0.000174	0.999826	
QLI71157.1	OTHER	0.000955	0.999045	
QLI71158.1	OTHER	0.002262	0.997738	
QLI71159.1	OTHER	0.000648	0.999352	
QLI71160.1	OTHER	0.001489	0.998511	
QLI71161.1	OTHER	0.000629	0.999371	
QLI71162.1	OTHER	0.000520	0.999480	
QLI71163.1	OTHER	0.000965	0.999035	
QLI71164.1	SP(Sec/SPI)	0.995940	0.004060	CS pos: 21-22. ISG-DN. Pr: 0.8627
QLI71165.1	OTHER	0.002622	0.997378	
QLI71166.1	OTHER	0.006846	0.993154	
QLI71167.1	OTHER	0.001249	0.998751	
QLI71168.1	OTHER	0.000544	0.999456	
QLI71169.1	OTHER	0.002246	0.997754	

QLI71170.1	OTHER	0.000821	0.999179	
QLI71171.1	SP(Sec/SPI)	0.999254	0.000746	CS pos: 20-21. SYA-WD. Pr: 0.9518
QLI71172.1	OTHER	0.003078	0.996922	
QLI71173.1	OTHER	0.000945	0.999055	
QLI71174.1	SP(Sec/SPI)	0.985437	0.014563	CS pos: 18-19. VTA-SP. Pr: 0.7534
QLI71175.1	OTHER	0.000279	0.999721	
QLI71176.1	OTHER	0.000270	0.999730	
QLI71177.1	OTHER	0.002225	0.997775	
QLI71178.1	SP(Sec/SPI)	0.910694	0.089306	CS pos: 19-20. AAG-HM. Pr: 0.4794
QLI71179.1	OTHER	0.003576	0.996424	
QLI71180.1	OTHER	0.003009	0.996991	
QLI71181.1	OTHER	0.001248	0.998752	
QLI71182.1	OTHER	0.001667	0.998333	
QLI71183.1	OTHER	0.000401	0.999599	
QLI71184.1	OTHER	0.004978	0.995022	
QLI71185.1	SP(Sec/SPI)	0.964709	0.035291	CS pos: 17-18. VAA-VP. Pr: 0.7763
QLI71186.1	OTHER	0.000524	0.999476	
QLI71187.1	OTHER	0.000446	0.999554	
QLI71188.1	SP(Sec/SPI)	0.985405	0.014595	CS pos: 20-21. ATA-TA. Pr: 0.4077
QLI71189.1	SP(Sec/SPI)	0.759935	0.240065	CS pos: 20-21. ASA-SC. Pr: 0.4850
QLI71190.1	OTHER	0.000870	0.999130	
QLI71191.1	OTHER	0.005027	0.994973	
QLI71192.1	OTHER	0.000694	0.999306	
QLI71193.1	SP(Sec/SPI)	0.996349	0.003651	CS pos: 19-20. ALA-AP. Pr: 0.9695
QLI71194.1	OTHER	0.000907	0.999093	
QLI71195.1	SP(Sec/SPI)	0.874563	0.125437	CS pos: 18-19. AYA-IN. Pr: 0.7361
QLI71196.1	OTHER	0.001248	0.998752	
QLI71197.1	OTHER	0.002913	0.997087	
QLI71198.1	SP(Sec/SPI)	0.962573	0.037427	CS pos: 20-21. ATC-QF. Pr: 0.6463
QLI71199.1	OTHER	0.003116	0.996884	
QLI71200.1	OTHER	0.000572	0.999428	

QLI71201.1	OTHER	0.000744	0.999256	
QLI71202.1	OTHER	0.003514	0.996486	
QLI71203.1	SP(Sec/SPI)	0.996075	0.003925	CS pos: 19-20. CLA-LP. Pr: 0.8916
QLI71204.1	OTHER	0.001639	0.998361	
QLI71205.1	OTHER	0.000430	0.999570	
QLI71206.1	SP(Sec/SPI)	0.744463	0.255537	CS pos: 17-18. AGA-MR. Pr: 0.3662
QLI71207.1	OTHER	0.000735	0.999265	
QLI71208.1	OTHER	0.001231	0.998769	
QLI71209.1	OTHER	0.002445	0.997555	
QLI71210.1	SP(Sec/SPI)	0.989147	0.010853	CS pos: 23-24. ALA-GF. Pr: 0.8956
QLI71211.1	OTHER	0.000425	0.999575	
QLI71212.1	OTHER	0.000359	0.999641	
QLI71213.1	OTHER	0.001937	0.998063	
QLI71214.1	SP(Sec/SPI)	0.996836	0.003164	CS pos: 20-21. VWG-GP. Pr: 0.9786
QLI71215.1	OTHER	0.000603	0.999397	
QLI71216.1	OTHER	0.001072	0.998928	
QLI71217.1	OTHER	0.057549	0.942451	
QLI71218.1	OTHER	0.000803	0.999197	
QLI71219.1	OTHER	0.002591	0.997409	
QLI71220.1	OTHER	0.000910	0.999090	
QLI71221.1	OTHER	0.000399	0.999601	
QLI71222.1	OTHER	0.000980	0.999020	
QLI71223.1	OTHER	0.000537	0.999463	
QLI71224.1	OTHER	0.001448	0.998552	
QLI71225.1	OTHER	0.002023	0.997977	
QLI71226.1	SP(Sec/SPI)	0.989818	0.010182	CS pos: 16-17. AAA-AP. Pr: 0.8218
QLI71227.1	OTHER	0.003166	0.996834	
QLI71228.1	OTHER	0.002975	0.997025	
QLI71229.1	OTHER	0.001111	0.998889	
QLI71230.1	OTHER	0.143563	0.856437	
QLI71231.1	OTHER	0.000946	0.999054	

QLI71232.1	OTHER	0.001130	0.998870	
QLI71233.1	OTHER	0.003589	0.996411	
QLI71234.1	OTHER	0.006076	0.993924	
QLI71235.1	OTHER	0.002950	0.997050	
QLI71236.1	OTHER	0.000746	0.999254	
QLI71237.1	OTHER	0.001027	0.998973	
QLI71238.1	OTHER	0.002225	0.997775	
QLI71239.1	OTHER	0.000188	0.999812	
QLI71240.1	OTHER	0.000470	0.999530	
QLI71241.1	OTHER	0.000201	0.999799	
QLI71242.1	OTHER	0.002686	0.997314	
QLI71243.1	OTHER	0.001485	0.998515	
QLI71244.1	OTHER	0.295903	0.704097	
QLI71245.1	OTHER	0.000767	0.999233	
QLI71246.1	OTHER	0.014385	0.985615	
QLI71247.1	OTHER	0.002295	0.997705	
QLI71248.1	SP(Sec/SPI)	0.865733	0.134267	CS pos: 31-32. VGA-NP. Pr: 0.8127
QLI71249.1	OTHER	0.001579	0.998421	
QLI71250.1	OTHER	0.001160	0.998840	
QLI71251.1	OTHER	0.001007	0.998993	
QLI71252.1	OTHER	0.002623	0.997377	
QLI71253.1	OTHER	0.000884	0.999116	
QLI71254.1	OTHER	0.000747	0.999253	
QLI71255.1	OTHER	0.037243	0.962757	
QLI71256.1	OTHER	0.000736	0.999264	
QLI71257.1	OTHER	0.030415	0.969585	
QLI71258.1	OTHER	0.082539	0.917461	
QLI71259.1	OTHER	0.001230	0.998770	
QLI71260.1	OTHER	0.000751	0.999249	
QLI71261.1	OTHER	0.001033	0.998967	
QLI71262.1	OTHER	0.000505	0.999495	

QLI71263.1	OTHER	0.000457	0.999543	
QLI71264.1	OTHER	0.000729	0.999271	
QLI71265.1	OTHER	0.001423	0.998577	
QLI71266.1	OTHER	0.083957	0.916043	
QLI71267.1	OTHER	0.000886	0.999114	
QLI71268.1	OTHER	0.206257	0.793743	
QLI71269.1	OTHER	0.000437	0.999563	
QLI71270.1	OTHER	0.013556	0.986444	
QLI71271.1	SP(Sec/SPI)	0.998638	0.001362	CS pos: 18-19. AWA-KN. Pr: 0.8426
QLI71272.1	SP(Sec/SPI)	0.989843	0.010157	CS pos: 23-24. SSC-AN. Pr: 0.2921
QLI71273.1	OTHER	0.000356	0.999644	
QLI71274.1	OTHER	0.001438	0.998562	
QLI71275.1	OTHER	0.001873	0.998127	
QLI71276.1	OTHER	0.000248	0.999752	
QLI71277.1	OTHER	0.002094	0.997906	
QLI71278.1	OTHER	0.001945	0.998055	
QLI71279.1	OTHER	0.228527	0.771473	
QLI71280.1	OTHER	0.000288	0.999712	
QLI71281.1	SP(Sec/SPI)	0.983495	0.016505	CS pos: 23-24. SHA-DA. Pr: 0.5291
QLI71282.1	OTHER	0.001105	0.998895	
QLI71283.1	OTHER	0.000953	0.999047	
QLI71284.1	OTHER	0.001072	0.998928	
QLI71285.1	OTHER	0.000812	0.999188	
QLI71286.1	OTHER	0.000370	0.999630	
QLI71287.1	OTHER	0.000336	0.999664	
QLI71288.1	OTHER	0.007154	0.992846	
QLI71289.1	SP(Sec/SPI)	0.997827	0.002173	CS pos: 22-23. AWA-AD. Pr: 0.9599
QLI71290.1	SP(Sec/SPI)	0.994139	0.005861	CS pos: 19-20. ASC-FG. Pr: 0.5766
QLI71291.1	OTHER	0.000276	0.999724	
QLI71292.1	OTHER	0.001047	0.998953	
QLI71293.1	OTHER	0.005822	0.994178	

QLI71294.1	OTHER	0.000818	0.999182	
QLI71295.1	OTHER	0.001379	0.998621	
QLI71296.1	OTHER	0.000089	0.999911	
QLI71297.1	OTHER	0.001643	0.998357	
QLI71298.1	OTHER	0.001989	0.998011	
QLI71299.1	OTHER	0.001541	0.998459	
QLI71300.1	OTHER	0.005309	0.994691	
QLI71301.1	OTHER	0.000326	0.999674	
QLI71302.1	SP(Sec/SPI)	0.997048	0.002952	CS pos: 20-21. ATA-SR. Pr: 0.6868
QLI71303.1	OTHER	0.027738	0.972262	
QLI71304.1	OTHER	0.001564	0.998436	
QLI71305.1	OTHER	0.000770	0.999230	
QLI71306.1	OTHER	0.001047	0.998953	
QLI71307.1	OTHER	0.001745	0.998255	
QLI71308.1	OTHER	0.001777	0.998223	
QLI71309.1	OTHER	0.134025	0.865975	
QLI71310.1	OTHER	0.001065	0.998935	
QLI71311.1	OTHER	0.000488	0.999512	
QLI71312.1	OTHER	0.011506	0.988494	
QLI71313.1	OTHER	0.001769	0.998231	
QLI71314.1	SP(Sec/SPI)	0.994452	0.005548	CS pos: 19-20. ARA-LP. Pr: 0.8475
QLI71315.1	OTHER	0.000804	0.999196	
QLI71316.1	OTHER	0.000645	0.999355	
QLI71317.1	OTHER	0.001372	0.998628	
QLI71318.1	OTHER	0.399908	0.600092	
QLI71319.1	OTHER	0.001404	0.998596	
QLI71320.1	OTHER	0.000459	0.999541	
QLI71321.1	OTHER	0.002427	0.997573	
QLI71322.1	OTHER	0.002250	0.997750	
QLI71323.1	OTHER	0.000832	0.999168	
QLI71324.1	OTHER	0.000893	0.999107	

QLI71325.1	OTHER	0.002239	0.997761	
QLI71326.1	OTHER	0.001902	0.998098	
QLI71327.1	OTHER	0.007411	0.992589	
QLI71328.1	OTHER	0.003480	0.996520	
QLI71329.1	OTHER	0.002540	0.997460	
QLI71330.1	OTHER	0.000830	0.999170	
QLI71331.1	OTHER	0.000800	0.999200	
QLI71332.1	OTHER	0.002139	0.997861	
QLI71333.1	OTHER	0.000740	0.999260	
QLI71334.1	OTHER	0.000581	0.999419	
QLI71335.1	OTHER	0.000363	0.999637	
QLI71336.1	SP(Sec/SPI)	0.886232	0.113768	CS pos: 17-18. AWA-LP. Pr: 0.8186
QLI71337.1	OTHER	0.001679	0.998321	
QLI71338.1	OTHER	0.000584	0.999416	
QLI71339.1	OTHER	0.000880	0.999120	
QLI71340.1	OTHER	0.002768	0.997232	
QLI71341.1	OTHER	0.001069	0.998931	
QLI71342.1	OTHER	0.002590	0.997410	
QLI71343.1	OTHER	0.000394	0.999606	
QLI71344.1	OTHER	0.001033	0.998967	
QLI71345.1	OTHER	0.000579	0.999421	
QLI71346.1	OTHER	0.011631	0.988369	
QLI71347.1	OTHER	0.001606	0.998394	
QLI71348.1	OTHER	0.010972	0.989028	
QLI71349.1	OTHER	0.000859	0.999141	
QLI71350.1	OTHER	0.011171	0.988829	
QLI71351.1	OTHER	0.001528	0.998472	
QLI71352.1	OTHER	0.000613	0.999387	
QLI71353.1	OTHER	0.001732	0.998268	
QLI71354.1	OTHER	0.001342	0.998658	
QLI71355.1	OTHER	0.001192	0.998808	

QLI71356.1	OTHER	0.000697	0.999303	
QLI71357.1	OTHER	0.001309	0.998691	
QLI71358.1	SP(Sec/SPI)	0.980943	0.019057	CS pos: 20-21. VAA-VE. Pr: 0.7858
QLI71359.1	SP(Sec/SPI)	0.998344	0.001656	CS pos: 24-25. VAG-ED. Pr: 0.9318
QLI71360.1	SP(Sec/SPI)	0.993870	0.006130	CS pos: 18-19. VLG-QI. Pr: 0.8971
QLI71361.1	OTHER	0.000533	0.999467	
QLI71362.1	SP(Sec/SPI)	0.994693	0.005307	CS pos: 17-18. TTA-QQ. Pr: 0.9147
QLI71363.1	OTHER	0.002099	0.997901	
QLI71364.1	OTHER	0.000778	0.999222	
QLI71365.1	OTHER	0.001259	0.998741	
QLI71366.1	OTHER	0.011511	0.988489	
QLI71367.1	SP(Sec/SPI)	0.835291	0.164709	CS pos: 24-25. CLG-QN. Pr: 0.7228
QLI71368.1	SP(Sec/SPI)	0.997187	0.002813	CS pos: 16-17. ILA-AT. Pr: 0.4938
QLI71369.1	OTHER	0.000334	0.999666	
QLI71370.1	OTHER	0.000546	0.999454	
QLI71371.1	OTHER	0.001969	0.998031	
QLI71372.1	OTHER	0.001683	0.998317	
QLI71373.1	OTHER	0.000412	0.999588	
QLI71374.1	SP(Sec/SPI)	0.902506	0.097494	CS pos: 20-21. VAA-SV. Pr: 0.3927
QLI71375.1	OTHER	0.000968	0.999032	
QLI71376.1	OTHER	0.000982	0.999018	
QLI71377.1	OTHER	0.001624	0.998376	
QLI71378.1	OTHER	0.004092	0.995908	
QLI71379.1	OTHER	0.004133	0.995867	
QLI71380.1	OTHER	0.001025	0.998975	
QLI71381.1	OTHER	0.001305	0.998695	
QLI71382.1	SP(Sec/SPI)	0.996345	0.003655	CS pos: 18-19. VSA-LP. Pr: 0.3244
QLI71383.1	OTHER	0.001274	0.998726	
QLI71384.1	SP(Sec/SPI)	0.956175	0.043825	CS pos: 22-23. GLA-TP. Pr: 0.5545
QLI71385.1	OTHER	0.001786	0.998214	
QLI71386.1	OTHER	0.003896	0.996104	

QLI71387.1	OTHER	0.001745	0.998255	
QLI71388.1	OTHER	0.000278	0.999722	
QLI71389.1	OTHER	0.000819	0.999181	
QLI71390.1	OTHER	0.000196	0.999804	
QLI71391.1	OTHER	0.000542	0.999458	
QLI71392.1	OTHER	0.000997	0.999003	
QLI71393.1	OTHER	0.001124	0.998876	
QLI71394.1	OTHER	0.000837	0.999163	
QLI71395.1	SP(Sec/SPI)	0.954354	0.045646	CS pos: 25-26. VWP-DG. Pr: 0.2610
QLI71396.1	OTHER	0.003363	0.996637	
QLI71397.1	OTHER	0.001442	0.998558	
QLI71398.1	SP(Sec/SPI)	0.992979	0.007021	CS pos: 19-20. VLA-LD. Pr: 0.8964
QLI71399.1	OTHER	0.000621	0.999379	
QLI71400.1	OTHER	0.001244	0.998756	
QLI71401.1	OTHER	0.007412	0.992588	
QLI71402.1	OTHER	0.002961	0.997039	
QLI71403.1	OTHER	0.001332	0.998668	
QLI71404.1	OTHER	0.000466	0.999534	
QLI71405.1	OTHER	0.001624	0.998376	
QLI71406.1	OTHER	0.003567	0.996433	
QLI71407.1	OTHER	0.000446	0.999554	
QLI71408.1	OTHER	0.001335	0.998665	
QLI71409.1	OTHER	0.003104	0.996896	
QLI71410.1	OTHER	0.001848	0.998152	
QLI71411.1	OTHER	0.002863	0.997137	
QLI71412.1	OTHER	0.001263	0.998737	
QLI71413.1	OTHER	0.026124	0.973876	
QLI71414.1	OTHER	0.000717	0.999283	
QLI71415.1	OTHER	0.001746	0.998254	
QLI71416.1	SP(Sec/SPI)	0.984730	0.015270	CS pos: 25-26. VNA-AG. Pr: 0.7960
QLI71417.1	OTHER	0.003632	0.996368	

QLI71418.1	SP(Sec/SPI)	0.987802	0.012198	CS pos: 19-20. ALG-AD. Pr: 0.8773
QLI71419.1	OTHER	0.002321	0.997679	
QLI71420.1	OTHER	0.001432	0.998568	
QLI71421.1	OTHER	0.005173	0.994827	
QLI71422.1	OTHER	0.021179	0.978821	
QLI71423.1	OTHER	0.003424	0.996576	
QLI71424.1	OTHER	0.001074	0.998926	
QLI71425.1	OTHER	0.000586	0.999414	
QLI71426.1	OTHER	0.001020	0.998980	
QLI71427.1	OTHER	0.000592	0.999408	
QLI71428.1	OTHER	0.000702	0.999298	
QLI71429.1	OTHER	0.069190	0.930810	
QLI71430.1	OTHER	0.000324	0.999676	
QLI71431.1	OTHER	0.000882	0.999118	
QLI71432.1	OTHER	0.011783	0.988217	
QLI71433.1	OTHER	0.024402	0.975598	
QLI71434.1	OTHER	0.003397	0.996603	
QLI71435.1	OTHER	0.001209	0.998791	
QLI71436.1	OTHER	0.020314	0.979686	
QLI71437.1	OTHER	0.001012	0.998988	
QLI71438.1	OTHER	0.000483	0.999517	
QLI71439.1	SP(Sec/SPI)	0.981869	0.018131	CS pos: 17-18. TLA-SP. Pr: 0.7339
QLI71440.1	OTHER	0.008673	0.991327	
QLI71441.1	OTHER	0.037444	0.962556	
QLI71442.1	OTHER	0.004734	0.995266	
QLI71443.1	OTHER	0.003793	0.996207	
QLI71444.1	OTHER	0.001231	0.998769	
QLI71445.1	OTHER	0.000376	0.999624	
QLI71446.1	OTHER	0.000355	0.999645	
QLI71447.1	OTHER	0.001338	0.998662	
QLI71448.1	OTHER	0.001385	0.998615	

QLI71449.1	OTHER	0.002548	0.997452	
QLI71450.1	OTHER	0.002804	0.997196	
QLI71451.1	OTHER	0.001474	0.998526	
QLI71452.1	OTHER	0.003065	0.996935	
QLI71453.1	OTHER	0.001913	0.998087	
QLI71454.1	OTHER	0.000345	0.999655	
QLI71455.1	OTHER	0.002905	0.997095	
QLI71456.1	OTHER	0.001414	0.998586	
QLI71457.1	OTHER	0.001683	0.998317	
QLI71458.1	OTHER	0.009508	0.990492	
QLI71459.1	OTHER	0.002756	0.997244	
QLI71460.1	OTHER	0.006419	0.993581	
QLI71461.1	OTHER	0.004115	0.995885	
QLI71462.1	OTHER	0.000356	0.999644	
QLI71463.1	OTHER	0.000762	0.999238	
QLI71464.1	OTHER	0.006490	0.993510	
QLI71465.1	OTHER	0.000586	0.999414	
QLI71466.1	OTHER	0.000665	0.999335	
QLI71467.1	OTHER	0.001188	0.998812	
QLI71468.1	OTHER	0.000641	0.999359	
QLI71469.1	SP(Sec/SPI)	0.991578	0.008422	CS pos: 19-20. AFA-TE. Pr: 0.7047
QLI71470.1	OTHER	0.059205	0.940795	
QLI71471.1	OTHER	0.000608	0.999392	
QLI71472.1	OTHER	0.000892	0.999108	
QLI71473.1	OTHER	0.000782	0.999218	
QLI71474.1	OTHER	0.001927	0.998073	
QLI71475.1	OTHER	0.001833	0.998167	
QLI71476.1	SP(Sec/SPI)	0.619266	0.380734	CS pos: 31-32. PRA-KV. Pr: 0.1752
QLI71477.1	OTHER	0.001002	0.998998	
QLI71478.1	OTHER	0.001828	0.998172	
QLI71479.1	OTHER	0.265190	0.734810	

QLI71480.1	OTHER	0.000416	0.999584	
QLI71481.1	SP(Sec/SPI)	0.995100	0.004900	CS pos: 19-20. ALA-SP. Pr: 0.6000
QLI71482.1	OTHER	0.000380	0.999620	
QLI71483.1	OTHER	0.348520	0.651480	
QLI71484.1	OTHER	0.000841	0.999159	
QLI71485.1	OTHER	0.000912	0.999088	
QLI71486.1	SP(Sec/SPI)	0.969540	0.030460	CS pos: 17-18. VTA-SS. Pr: 0.4366
QLI71487.1	OTHER	0.001572	0.998428	
QLI71488.1	OTHER	0.000705	0.999295	
QLI71489.1	OTHER	0.000998	0.999002	
QLI71490.1	OTHER	0.000529	0.999471	
QLI71491.1	OTHER	0.005554	0.994446	
QLI71492.1	OTHER	0.002562	0.997438	
QLI71493.1	OTHER	0.003901	0.996099	
QLI71494.1	OTHER	0.000379	0.999621	
QLI71495.1	OTHER	0.010343	0.989657	
QLI71496.1	OTHER	0.000790	0.999210	
QLI71497.1	OTHER	0.000752	0.999248	
QLI71498.1	SP(Sec/SPI)	0.992718	0.007282	CS pos: 20-21. AAA-AE. Pr: 0.5718
QLI71499.1	OTHER	0.001221	0.998779	
QLI71500.1	OTHER	0.003537	0.996463	
QLI71501.1	OTHER	0.003309	0.996691	
QLI71502.1	OTHER	0.003223	0.996777	
QLI71503.1	SP(Sec/SPI)	0.968008	0.031992	CS pos: 19-20. ASA-AN. Pr: 0.8902
QLI71504.1	OTHER	0.000635	0.999365	
QLI71505.1	OTHER	0.010040	0.989960	
QLI71506.1	SP(Sec/SPI)	0.908168	0.091832	CS pos: 25-26. AAA-FG. Pr: 0.5730
QLI71507.1	OTHER	0.000531	0.999469	
QLI71508.1	OTHER	0.004468	0.995532	
QLI71509.1	OTHER	0.003422	0.996578	
QLI71510.1	OTHER	0.125312	0.874688	

QLI71511.1	OTHER	0.000965	0.999035	
QLI71512.1	OTHER	0.001446	0.998554	
QLI71513.1	OTHER	0.000473	0.999527	
QLI71514.1	OTHER	0.007101	0.992899	
QLI71515.1	OTHER	0.001181	0.998819	
QLI71516.1	OTHER	0.002466	0.997534	
QLI71517.1	OTHER	0.031872	0.968128	
QLI71518.1	OTHER	0.115784	0.884216	
QLI71519.1	OTHER	0.001393	0.998607	
QLI71520.1	OTHER	0.004285	0.995715	
QLI71521.1	OTHER	0.001269	0.998731	
QLI71522.1	OTHER	0.000888	0.999112	
QLI71523.1	OTHER	0.001375	0.998625	
QLI71524.1	SP(Sec/SPI)	0.991198	0.008802	CS pos: 18-19. AAA-AT. Pr: 0.3318
QLI71525.1	OTHER	0.000169	0.999831	
QLI71526.1	OTHER	0.015045	0.984955	
QLI71527.1	OTHER	0.001417	0.998583	
QLI71528.1	SP(Sec/SPI)	0.815508	0.184492	CS pos: 16-17. FGA-QK. Pr: 0.2728
QLI71529.1	OTHER	0.003509	0.996491	
QLI71530.1	OTHER	0.001795	0.998205	
QLI71531.1	OTHER	0.000940	0.999060	
QLI71532.1	OTHER	0.000806	0.999194	
QLI71533.1	OTHER	0.001913	0.998087	
QLI71534.1	OTHER	0.001365	0.998635	
QLI71535.1	OTHER	0.000499	0.999501	
QLI71536.1	OTHER	0.000576	0.999424	
QLI71537.1	OTHER	0.001125	0.998875	
QLI71538.1	OTHER	0.022414	0.977586	
QLI71539.1	OTHER	0.000585	0.999415	
QLI71540.1	OTHER	0.000366	0.999634	
QLI71541.1	OTHER	0.002036	0.997964	

QLI71542.1	OTHER	0.002066	0.997934	
QLI71543.1	OTHER	0.000934	0.999066	
QLI71544.1	OTHER	0.000883	0.999117	
QLI71545.1	OTHER	0.001997	0.998003	
QLI71546.1	OTHER	0.001095	0.998905	
QLI71547.1	OTHER	0.017076	0.982924	
QLI71548.1	OTHER	0.002031	0.997969	
QLI71549.1	OTHER	0.000651	0.999349	
QLI71550.1	SP(Sec/SPI)	0.977108	0.022892	CS pos: 17-18. ASA-YQ. Pr: 0.8534
QLI71551.1	OTHER	0.007577	0.992423	
QLI71552.1	SP(Sec/SPI)	0.975698	0.024302	CS pos: 18-19. AIA-QY. Pr: 0.8981
QLI71553.1	OTHER	0.002244	0.997756	
QLI71554.1	OTHER	0.000597	0.999403	
QLI71555.1	OTHER	0.000605	0.999395	
QLI71556.1	SP(Sec/SPI)	0.998208	0.001792	CS pos: 16-17. AAA-VP. Pr: 0.8084
QLI71557.1	SP(Sec/SPI)	0.962516	0.037484	CS pos: 19-20. SAA-TP. Pr: 0.3624
QLI71558.1	OTHER	0.001140	0.998860	
QLI71559.1	OTHER	0.000605	0.999395	
QLI71560.1	SP(Sec/SPI)	0.955095	0.044905	CS pos: 24-25. ANA-IN. Pr: 0.9059
QLI71561.1	OTHER	0.000813	0.999187	
QLI71562.1	OTHER	0.001161	0.998839	
QLI71563.1	OTHER	0.003550	0.996450	
QLI71564.1	OTHER	0.004127	0.995873	
QLI71565.1	OTHER	0.002246	0.997754	
QLI71566.1	OTHER	0.001591	0.998409	
QLI71567.1	OTHER	0.200112	0.799888	
QLI71568.1	OTHER	0.001996	0.998004	
QLI71569.1	OTHER	0.000541	0.999459	
QLI71570.1	OTHER	0.000823	0.999177	
QLI71571.1	SP(Sec/SPI)	0.920783	0.079217	CS pos: 22-23. MRA-DY. Pr: 0.4802
QLI71572.1	OTHER	0.001103	0.998897	

QLI71573.1	OTHER	0.001220	0.998780	
QLI71574.1	OTHER	0.001448	0.998552	
QLI71575.1	SP(Sec/SPI)	0.957488	0.042512	CS pos: 21-22. SRA-LN. Pr: 0.8312
QLI71576.1	OTHER	0.243189	0.756811	
QLI71577.1	OTHER	0.001493	0.998507	
QLI71578.1	OTHER	0.001177	0.998823	
QLI71579.1	OTHER	0.000598	0.999402	
QLI71580.1	OTHER	0.000474	0.999526	
QLI71581.1	OTHER	0.001551	0.998449	
QLI71582.1	OTHER	0.000687	0.999313	
QLI71583.1	OTHER	0.002226	0.997774	
QLI71584.1	OTHER	0.001146	0.998854	
QLI71585.1	OTHER	0.001957	0.998043	
QLI71586.1	OTHER	0.000902	0.999098	
QLI71587.1	SP(Sec/SPI)	0.947810	0.052190	CS pos: 23-24. ASA-LE. Pr: 0.6981
QLI71588.1	OTHER	0.006035	0.993965	
QLI71589.1	OTHER	0.000440	0.999560	
QLI71590.1	OTHER	0.002929	0.997071	
QLI71591.1	OTHER	0.001406	0.998594	
QLI71592.1	OTHER	0.002402	0.997598	
QLI71593.1	OTHER	0.047738	0.952262	
QLI71594.1	OTHER	0.000463	0.999537	
QLI71595.1	OTHER	0.004254	0.995746	
QLI71596.1	OTHER	0.001594	0.998406	
QLI71597.1	OTHER	0.000797	0.999203	
QLI71598.1	OTHER	0.002081	0.997919	
QLI71599.1	SP(Sec/SPI)	0.888854	0.111146	CS pos: 20-21. VDA-IW. Pr: 0.8457
QLI71600.1	OTHER	0.000880	0.999120	
QLI71601.1	OTHER	0.005290	0.994710	
QLI71602.1	OTHER	0.001963	0.998037	
QLI71603.1	OTHER	0.000891	0.999109	

QLI71604.1	OTHER	0.008548	0.991452	
QLI71605.1	OTHER	0.003092	0.996908	
QLI71606.1	OTHER	0.001346	0.998654	
QLI71607.1	SP(Sec/SPI)	0.996757	0.003243	CS pos: 23-24. VYA-QP. Pr: 0.9828
QLI71608.1	OTHER	0.001041	0.998959	
QLI71609.1	OTHER	0.000287	0.999713	
QLI71610.1	OTHER	0.005451	0.994549	
QLI71611.1	OTHER	0.000807	0.999193	
QLI71612.1	SP(Sec/SPI)	0.998790	0.001210	CS pos: 20-21. ANA-RE. Pr: 0.6605
QLI71613.1	OTHER	0.002132	0.997868	
QLI71614.1	OTHER	0.001852	0.998148	
QLI71615.1	OTHER	0.002331	0.997669	
QLI71616.1	OTHER	0.000784	0.999216	
QLI71617.1	OTHER	0.000284	0.999716	
QLI71618.1	OTHER	0.000469	0.999531	
QLI71619.1	OTHER	0.022144	0.977856	
QLI71620.1	OTHER	0.000812	0.999188	
QLI71621.1	OTHER	0.000955	0.999045	
QLI71622.1	OTHER	0.004063	0.995937	
QLI71623.1	OTHER	0.003974	0.996026	
QLI71624.1	OTHER	0.000205	0.999795	
QLI71625.1	SP(Sec/SPI)	0.940970	0.059030	CS pos: 23-24. CEA-TE. Pr: 0.8415
QLI71626.1	OTHER	0.004021	0.995979	
QLI71627.1	OTHER	0.001536	0.998464	
QLI71628.1	OTHER	0.015871	0.984129	
QLI71629.1	OTHER	0.001013	0.998987	
QLI71630.1	OTHER	0.012964	0.987036	
QLI71631.1	OTHER	0.001268	0.998732	
QLI71632.1	OTHER	0.002944	0.997056	
QLI71633.1	OTHER	0.000474	0.999526	
QLI71634.1	OTHER	0.000898	0.999102	

QLI71635.1	OTHER	0.001046	0.998954	
QLI71636.1	OTHER	0.001627	0.998373	
QLI71637.1	OTHER	0.000624	0.999376	
QLI71638.1	OTHER	0.007659	0.992341	
QLI71639.1	OTHER	0.000355	0.999645	
QLI71640.1	OTHER	0.001101	0.998899	
QLI71641.1	OTHER	0.001032	0.998968	
QLI71642.1	OTHER	0.000757	0.999243	
QLI71643.1	OTHER	0.000810	0.999190	
QLI71644.1	OTHER	0.001681	0.998319	
QLI71645.1	OTHER	0.001366	0.998634	
QLI71646.1	SP(Sec/SPI)	0.993165	0.006835	CS pos: 19-20. ATA-AP. Pr: 0.7842
QLI71647.1	OTHER	0.001632	0.998368	
QLI71648.1	SP(Sec/SPI)	0.954244	0.045756	CS pos: 24-25. GLA-AP. Pr: 0.7253
QLI71649.1	OTHER	0.000682	0.999318	
QLI71650.1	OTHER	0.006733	0.993267	
QLI71651.1	OTHER	0.001023	0.998977	
QLI71652.1	OTHER	0.000486	0.999514	
QLI71653.1	OTHER	0.000736	0.999264	
QLI71654.1	OTHER	0.000892	0.999108	
QLI71655.1	SP(Sec/SPI)	0.999013	0.000987	CS pos: 22-23. ANG-DG. Pr: 0.8068
QLI71656.1	OTHER	0.000760	0.999240	
QLI71657.1	OTHER	0.001119	0.998881	
QLI71658.1	OTHER	0.001239	0.998761	
QLI71659.1	OTHER	0.000281	0.999719	
QLI71660.1	OTHER	0.000646	0.999354	
QLI71661.1	OTHER	0.001606	0.998394	
QLI71662.1	OTHER	0.001076	0.998924	
QLI71663.1	OTHER	0.000417	0.999583	
QLI71664.1	OTHER	0.003937	0.996063	
QLI71665.1	OTHER	0.000775	0.999225	

QLI71666.1	OTHER	0.001074	0.998926	
QLI71667.1	OTHER	0.323934	0.676066	
QLI71668.1	OTHER	0.000847	0.999153	
QLI71669.1	OTHER	0.005312	0.994688	
QLI71670.1	OTHER	0.001300	0.998700	
QLI71671.1	OTHER	0.002218	0.997782	
QLI71672.1	OTHER	0.002399	0.997601	
QLI71673.1	OTHER	0.002366	0.997634	
QLI71674.1	OTHER	0.006308	0.993692	
QLI71675.1	OTHER	0.000490	0.999510	
QLI71676.1	OTHER	0.001049	0.998951	
QLI71677.1	OTHER	0.001840	0.998160	
QLI71678.1	OTHER	0.000332	0.999668	
QLI71679.1	OTHER	0.087275	0.912725	
QLI71680.1	OTHER	0.010603	0.989397	
QLI71681.1	OTHER	0.001479	0.998521	
QLI71682.1	OTHER	0.168481	0.831519	
QLI71683.1	OTHER	0.140691	0.859309	
QLI71684.1	OTHER	0.069710	0.930290	
QLI71685.1	OTHER	0.000616	0.999384	
QLI71686.1	OTHER	0.000345	0.999655	
QLI71687.1	OTHER	0.000712	0.999288	
QLI71688.1	OTHER	0.000495	0.999505	
QLI71689.1	OTHER	0.001348	0.998652	
QLI71690.1	OTHER	0.000531	0.999469	
QLI71691.1	OTHER	0.000538	0.999462	
QLI71692.1	OTHER	0.000702	0.999298	
QLI71693.1	OTHER	0.001504	0.998496	
QLI71694.1	SP(Sec/SPI)	0.855398	0.144602	CS pos: 20-21. TVG-WG. Pr: 0.3013
QLI71695.1	OTHER	0.000528	0.999472	
QLI71696.1	OTHER	0.005799	0.994201	

QLI71697.1	OTHER	0.001773	0.998227	
QLI71698.1	SP(Sec/SPI)	0.899342	0.100658	CS pos: 19-20. SYA-FY. Pr: 0.4916
QLI71699.1	OTHER	0.000736	0.999264	
QLI71700.1	OTHER	0.000505	0.999495	
QLI71701.1	OTHER	0.009951	0.990049	
QLI71702.1	OTHER	0.001433	0.998567	
QLI71703.1	OTHER	0.000604	0.999396	
QLI71704.1	SP(Sec/SPI)	0.982552	0.017448	CS pos: 18-19. ILG-GE. Pr: 0.2861
QLI71705.1	OTHER	0.002867	0.997133	
QLI71706.1	OTHER	0.000522	0.999478	
QLI71707.1	OTHER	0.001389	0.998611	
QLI71708.1	OTHER	0.002827	0.997173	
QLI71709.1	OTHER	0.001986	0.998014	
QLI71710.1	SP(Sec/SPI)	0.989752	0.010248	CS pos: 16-17. CLA-AR. Pr: 0.6670
QLI71711.1	OTHER	0.052607	0.947393	
QLI71712.1	OTHER	0.001575	0.998425	
QLI71713.1	OTHER	0.001614	0.998386	
QLI71714.1	SP(Sec/SPI)	0.704073	0.295927	CS pos: 25-26. VIS-IP. Pr: 0.5297
QLI71715.1	OTHER	0.001138	0.998862	
QLI71716.1	OTHER	0.020755	0.979245	
QLI71717.1	OTHER	0.001512	0.998488	
QLI71718.1	OTHER	0.001096	0.998904	
QLI71719.1	OTHER	0.000606	0.999394	
QLI71720.1	SP(Sec/SPI)	0.702585	0.297415	CS pos: 16-17. GLA-RP. Pr: 0.1956
QLI71721.1	OTHER	0.000727	0.999273	
QLI71722.1	OTHER	0.004673	0.995327	
QLI71723.1	OTHER	0.001153	0.998847	
QLI71724.1	OTHER	0.001471	0.998529	
QLI71725.1	OTHER	0.002099	0.997901	
QLI71726.1	OTHER	0.000851	0.999149	
QLI71727.1	OTHER	0.000504	0.999496	

QLI71728.1	OTHER	0.002700	0.997300	
QLI71729.1	OTHER	0.002291	0.997709	
QLI71730.1	OTHER	0.002399	0.997601	
QLI71731.1	SP(Sec/SPI)	0.972057	0.027943	CS pos: 18-19. TTA-AP. Pr: 0.6336
QLI71732.1	OTHER	0.007364	0.992636	
QLI71733.1	OTHER	0.001067	0.998933	
QLI71734.1	OTHER	0.002589	0.997411	
QLI71735.1	OTHER	0.000761	0.999239	
QLI71736.1	OTHER	0.000185	0.999815	
QLI71737.1	OTHER	0.001474	0.998526	
QLI71738.1	OTHER	0.001769	0.998231	
QLI71739.1	OTHER	0.001862	0.998138	
QLI71740.1	OTHER	0.001235	0.998765	
QLI71741.1	OTHER	0.001985	0.998015	
QLI71742.1	OTHER	0.041024	0.958976	
QLI71743.1	OTHER	0.027784	0.972216	
QLI71744.1	OTHER	0.001024	0.998976	
QLI71745.1	OTHER	0.002702	0.997298	
QLI71746.1	OTHER	0.000541	0.999459	
QLI71747.1	OTHER	0.001430	0.998570	
QLI71748.1	OTHER	0.000924	0.999076	
QLI71749.1	OTHER	0.010049	0.989951	
QLI71750.1	SP(Sec/SPI)	0.992081	0.007919	CS pos: 16-17. ASA-HE. Pr: 0.7453
QLI71751.1	OTHER	0.000664	0.999336	
QLI71752.1	OTHER	0.316197	0.683803	
QLI71753.1	OTHER	0.004437	0.995563	
QLI71754.1	OTHER	0.001473	0.998527	
QLI71755.1	OTHER	0.004137	0.995863	
QLI71756.1	OTHER	0.000458	0.999542	
QLI71757.1	OTHER	0.004368	0.995632	
QLI71758.1	OTHER	0.000996	0.999004	

QLI71759.1	OTHER	0.001603	0.998397	
QLI71760.1	OTHER	0.001469	0.998531	
QLI71761.1	OTHER	0.001344	0.998656	
QLI71762.1	OTHER	0.012336	0.987664	
QLI71763.1	OTHER	0.000803	0.999197	
QLI71764.1	SP(Sec/SPI)	0.997350	0.002650	CS pos: 19-20. AAA-AP. Pr: 0.8444
QLI71765.1	SP(Sec/SPI)	0.738325	0.261675	CS pos: 20-21. ASA-SA. Pr: 0.3592
QLI71766.1	OTHER	0.001366	0.998634	
QLI71767.1	OTHER	0.001849	0.998151	
QLI71768.1	OTHER	0.000266	0.999734	
QLI71769.1	SP(Sec/SPI)	0.900425	0.099575	CS pos: 22-23. LLA-WD. Pr: 0.7826
QLI71770.1	OTHER	0.000314	0.999686	
QLI71771.1	OTHER	0.000370	0.999630	
QLI71772.1	OTHER	0.000707	0.999293	
QLI71773.1	OTHER	0.000822	0.999178	
QLI71774.1	OTHER	0.001366	0.998634	
QLI71775.1	OTHER	0.000673	0.999327	
QLI71776.1	OTHER	0.000928	0.999072	
QLI71777.1	SP(Sec/SPI)	0.962500	0.037500	CS pos: 18-19. ALA-VD. Pr: 0.8841
QLI71778.1	OTHER	0.000937	0.999063	
QLI71779.1	OTHER	0.000565	0.999435	
QLI71780.1	OTHER	0.000152	0.999848	
QLI71781.1	OTHER	0.007315	0.992685	
QLI71782.1	OTHER	0.001477	0.998523	
QLI71783.1	OTHER	0.000896	0.999104	
QLI71784.1	OTHER	0.001314	0.998686	
QLI71785.1	OTHER	0.000408	0.999592	
QLI71786.1	OTHER	0.003109	0.996891	
QLI71787.1	OTHER	0.002016	0.997984	
QLI71788.1	OTHER	0.350361	0.649639	
QLI71789.1	OTHER	0.000711	0.999289	

QLI71790.1	OTHER	0.000968	0.999032	
QLI71791.1	SP(Sec/SPI)	0.996094	0.003906	CS pos: 17-18. ALA-AP. Pr: 0.7768
QLI71792.1	OTHER	0.001599	0.998401	
QLI71793.1	OTHER	0.006592	0.993408	
QLI71794.1	OTHER	0.001460	0.998540	
QLI71795.1	OTHER	0.000948	0.999052	
QLI71796.1	OTHER	0.000280	0.999720	
QLI71797.1	OTHER	0.000358	0.999642	
QLI71798.1	OTHER	0.001743	0.998257	
QLI71799.1	OTHER	0.001952	0.998048	
QLI71800.1	OTHER	0.001909	0.998091	
QLI71801.1	OTHER	0.002256	0.997744	
QLI71802.1	OTHER	0.003713	0.996287	
QLI71803.1	OTHER	0.001206	0.998794	
QLI71804.1	SP(Sec/SPI)	0.996609	0.003391	CS pos: 15-16. ILA-IE. Pr: 0.7390
QLI71805.1	OTHER	0.000686	0.999314	
QLI71806.1	OTHER	0.001283	0.998717	
QLI71807.1	OTHER	0.001696	0.998304	
QLI71808.1	SP(Sec/SPI)	0.992644	0.007356	CS pos: 19-20. VLA-HV. Pr: 0.8138
QLI71809.1	OTHER	0.000331	0.999669	
QLI71810.1	OTHER	0.000700	0.999300	
QLI71811.1	OTHER	0.000593	0.999407	
QLI71812.1	OTHER	0.000375	0.999625	
QLI71813.1	OTHER	0.000298	0.999702	
QLI71814.1	OTHER	0.001659	0.998341	
QLI71815.1	OTHER	0.000891	0.999109	
QLI71816.1	OTHER	0.002977	0.997023	
QLI71817.1	OTHER	0.001072	0.998928	
QLI71818.1	OTHER	0.000909	0.999091	
QLI71819.1	OTHER	0.000735	0.999265	
QLI71820.1	SP(Sec/SPI)	0.538983	0.461017	CS pos: 23-24. CFC-QK. Pr: 0.3128

QLI71821.1	SP(Sec/SPI)	0.984703	0.015297	CS pos: 17-18. GAG-AP. Pr: 0.4682
QLI71822.1	OTHER	0.001006	0.998994	
QLI71823.1	OTHER	0.000378	0.999622	
QLI71824.1	OTHER	0.000735	0.999265	
QLI71825.1	OTHER	0.000949	0.999051	
QLI71826.1	OTHER	0.000586	0.999414	
QLI71827.1	SP(Sec/SPI)	0.946254	0.053746	CS pos: 20-21. ASA-TL. Pr: 0.7936
QLI71828.1	OTHER	0.001981	0.998019	
QLI71829.1	OTHER	0.001467	0.998533	
QLI71830.1	SP(Sec/SPI)	0.926806	0.073194	CS pos: 17-18. VLA-IG. Pr: 0.4023
QLI71831.1	OTHER	0.000709	0.999291	
QLI71832.1	OTHER	0.002117	0.997883	
QLI71833.1	OTHER	0.003570	0.996430	
QLI71834.1	OTHER	0.001280	0.998720	
QLI71835.1	OTHER	0.002672	0.997328	
QLI71836.1	OTHER	0.001075	0.998925	
QLI71837.1	OTHER	0.002819	0.997181	
QLI71838.1	SP(Sec/SPI)	0.964744	0.035256	CS pos: 18-19. TAA-ET. Pr: 0.8159
QLI71839.1	OTHER	0.002158	0.997842	
QLI71840.1	OTHER	0.000792	0.999208	
QLI71841.1	OTHER	0.000515	0.999485	
QLI71842.1	OTHER	0.039763	0.960237	
QLI71843.1	OTHER	0.000845	0.999155	
QLI71844.1	SP(Sec/SPI)	0.956985	0.043015	CS pos: 25-26. AVG-AQ. Pr: 0.2400
QLI71845.1	OTHER	0.000871	0.999129	
QLI71846.1	OTHER	0.001008	0.998992	
QLI71847.1	OTHER	0.179489	0.820511	
QLI71848.1	SP(Sec/SPI)	0.989756	0.010244	CS pos: 18-19. AHA-AD. Pr: 0.8834
QLI71849.1	SP(Sec/SPI)	0.979647	0.020353	CS pos: 20-21. ALA-CG. Pr: 0.7916
QLI71850.1	OTHER	0.002416	0.997584	
QLI71851.1	OTHER	0.000630	0.999370	

QLI71852.1	OTHER	0.002696	0.997304	
QLI71853.1	OTHER	0.000686	0.999314	
QLI71854.1	OTHER	0.000438	0.999562	
QLI71855.1	SP(Sec/SPI)	0.792369	0.207631	CS pos: 19-20. ALG-NP. Pr: 0.6722
QLI71856.1	OTHER	0.000721	0.999279	
QLI71857.1	OTHER	0.000956	0.999044	
QLI71858.1	OTHER	0.004602	0.995398	
QLI71859.1	OTHER	0.001254	0.998746	
QLI71860.1	OTHER	0.002782	0.997218	
QLI71861.1	OTHER	0.004130	0.995870	
QLI71862.1	OTHER	0.000283	0.999717	
QLI71863.1	SP(Sec/SPI)	0.978763	0.021237	CS pos: 20-21. THG-WP. Pr: 0.7235
QLI71864.1	OTHER	0.000965	0.999035	
QLI71865.1	OTHER	0.176539	0.823461	
QLI71866.1	OTHER	0.001378	0.998622	
QLI71867.1	OTHER	0.006036	0.993964	
QLI71868.1	OTHER	0.001456	0.998544	
QLI71869.1	OTHER	0.000391	0.999609	
QLI71870.1	OTHER	0.002537	0.997463	
QLI71871.1	OTHER	0.000322	0.999678	
QLI71872.1	SP(Sec/SPI)	0.961531	0.038469	CS pos: 28-29. AVA-AA. Pr: 0.2535
QLI71873.1	OTHER	0.001103	0.998897	
QLI71874.1	OTHER	0.010520	0.989480	
QLI71875.1	OTHER	0.002106	0.997894	
QLI71876.1	OTHER	0.001031	0.998969	
QLI71877.1	OTHER	0.002063	0.997937	
QLI71878.1	OTHER	0.013120	0.986880	
QLI71879.1	SP(Sec/SPI)	0.962468	0.037532	CS pos: 18-19. AIA-QD. Pr: 0.8634
QLI71880.1	OTHER	0.000415	0.999585	
QLI71881.1	OTHER	0.001259	0.998741	
QLI71882.1	OTHER	0.000408	0.999592	

QLI71883.1	OTHER	0.000521	0.999479	
QLI71884.1	OTHER	0.009205	0.990795	
QLI71885.1	OTHER	0.006699	0.993301	
QLI71886.1	SP(Sec/SPI)	0.998921	0.001079	CS pos: 17-18. ALA-RD. Pr: 0.9372
QLI71887.1	OTHER	0.001317	0.998683	
QLI71888.1	OTHER	0.000296	0.999704	
QLI71889.1	OTHER	0.005339	0.994661	
QLI71890.1	OTHER	0.001474	0.998526	
QLI71891.1	OTHER	0.001274	0.998726	
QLI71892.1	SP(Sec/SPI)	0.992496	0.007504	CS pos: 23-24. AHA-NG. Pr: 0.6495
QLI71893.1	OTHER	0.000491	0.999509	
QLI71894.1	OTHER	0.000768	0.999232	
QLI71895.1	OTHER	0.002500	0.997500	
QLI71896.1	OTHER	0.001028	0.998972	
QLI71897.1	OTHER	0.002524	0.997476	
QLI71898.1	OTHER	0.006955	0.993045	
QLI71899.1	OTHER	0.000312	0.999688	
QLI71900.1	OTHER	0.001062	0.998938	
QLI71901.1	SP(Sec/SPI)	0.955884	0.044116	CS pos: 21-22. AHA-PV. Pr: 0.2856
QLI71902.1	OTHER	0.002365	0.997635	
QLI71903.1	OTHER	0.001183	0.998817	
QLI71904.1	OTHER	0.000532	0.999468	
QLI71905.1	OTHER	0.188695	0.811305	
QLI71906.1	SP(Sec/SPI)	0.985515	0.014485	CS pos: 18-19. VNA-SA. Pr: 0.6473
QLI71907.1	OTHER	0.005616	0.994384	
QLI71908.1	OTHER	0.007914	0.992086	
QLI71909.1	OTHER	0.002699	0.997301	
QLI71910.1	SP(Sec/SPI)	0.790102	0.209898	CS pos: 26-27. CLA-SP. Pr: 0.5382
QLI71911.1	OTHER	0.000470	0.999530	
QLI71912.1	OTHER	0.029937	0.970063	
QLI71913.1	OTHER	0.001561	0.998439	

QLI71914.1	OTHER	0.002624	0.997376	
QLI71915.1	OTHER	0.017328	0.982672	
QLI71916.1	OTHER	0.000766	0.999234	
QLI71917.1	OTHER	0.001615	0.998385	
QLI71918.1	OTHER	0.001344	0.998656	
QLI71919.1	SP(Sec/SPI)	0.959359	0.040641	CS pos: 18-19. VAG-LP. Pr: 0.4115
QLI71920.1	OTHER	0.003098	0.996902	
QLI71921.1	OTHER	0.000637	0.999363	
QLI71922.1	OTHER	0.002223	0.997777	
QLI71923.1	OTHER	0.000457	0.999543	
QLI71924.1	SP(Sec/SPI)	0.914254	0.085746	CS pos: 21-22. AAA-RP. Pr: 0.5693
QLI71925.1	OTHER	0.000390	0.999610	
QLI71926.1	OTHER	0.007620	0.992380	
QLI71927.1	OTHER	0.000933	0.999067	
QLI71928.1	OTHER	0.013009	0.986991	
QLI71929.1	OTHER	0.003736	0.996264	
QLI71930.1	OTHER	0.055234	0.944766	
QLI71931.1	OTHER	0.449441	0.550559	
QLI71932.1	OTHER	0.001031	0.998969	
QLI71933.1	OTHER	0.001897	0.998103	
QLI71934.1	OTHER	0.001379	0.998621	
QLI71935.1	OTHER	0.000417	0.999583	
QLI71936.1	OTHER	0.002632	0.997368	
QLI71937.1	OTHER	0.000806	0.999194	
QLI71938.1	OTHER	0.001884	0.998116	
QLI71939.1	OTHER	0.000360	0.999640	
QLI71940.1	OTHER	0.008470	0.991530	
QLI71941.1	OTHER	0.011768	0.988232	
QLI71942.1	OTHER	0.022580	0.977420	
QLI71943.1	SP(Sec/SPI)	0.990576	0.009424	CS pos: 17-18. SLA-GP. Pr: 0.5837
QLI71944.1	OTHER	0.012825	0.987175	

QLI71945.1	SP(Sec/SPI)	0.996166	0.003834	CS pos: 15-16. VQA-AK. Pr: 0.8256
QLI71946.1	OTHER	0.000549	0.999451	
QLI71947.1	OTHER	0.000461	0.999539	
QLI71948.1	OTHER	0.113250	0.886750	
QLI71949.1	OTHER	0.001113	0.998887	
QLI71950.1	OTHER	0.001081	0.998919	
QLI71951.1	OTHER	0.001197	0.998803	
QLI71952.1	OTHER	0.000756	0.999244	
QLI71953.1	OTHER	0.006193	0.993807	
QLI71954.1	OTHER	0.002717	0.997283	
QLI71955.1	OTHER	0.000787	0.999213	
QLI71956.1	OTHER	0.000757	0.999243	
QLI71957.1	OTHER	0.000803	0.999197	
QLI71958.1	OTHER	0.000584	0.999416	
QLI71959.1	SP(Sec/SPI)	0.987330	0.012670	CS pos: 19-20. ASA-VN. Pr: 0.4653
QLI71960.1	OTHER	0.000745	0.999255	
QLI71961.1	OTHER	0.005738	0.994262	
QLI71962.1	SP(Sec/SPI)	0.999079	0.000921	CS pos: 16-17. ASA-HS. Pr: 0.7019
QLI71963.1	OTHER	0.000521	0.999479	
QLI71964.1	OTHER	0.009487	0.990513	
QLI71965.1	OTHER	0.116501	0.883499	
QLI71966.1	OTHER	0.001118	0.998882	
QLI71967.1	OTHER	0.000545	0.999455	
QLI71968.1	OTHER	0.000410	0.999590	
QLI71969.1	OTHER	0.001063	0.998937	
QLI71970.1	OTHER	0.001396	0.998604	
QLI71971.1	OTHER	0.003592	0.996408	
QLI71972.1	OTHER	0.012208	0.987792	
QLI71973.1	OTHER	0.000817	0.999183	
QLI71974.1	OTHER	0.001983	0.998017	
QLI71975.1	OTHER	0.002085	0.997915	

QLI71976.1	OTHER	0.000732	0.999268	
QLI71977.1	OTHER	0.001774	0.998226	
QLI71978.1	OTHER	0.036686	0.963314	
QLI71979.1	OTHER	0.001695	0.998305	
QLI71980.1	OTHER	0.019594	0.980406	
QLI71981.1	OTHER	0.001011	0.998989	
QLI71982.1	OTHER	0.000615	0.999385	
QLI71983.1	OTHER	0.000823	0.999177	
QLI71984.1	OTHER	0.002884	0.997116	
QLI71985.1	OTHER	0.019901	0.980099	
QLI71986.1	OTHER	0.000925	0.999075	
QLI71987.1	OTHER	0.000643	0.999357	
QLI71988.1	OTHER	0.004247	0.995753	
QLI71989.1	OTHER	0.002538	0.997462	
QLI71990.1	OTHER	0.002220	0.997780	
QLI71991.1	SP(Sec/SPI)	0.979784	0.020216	CS pos: 17-18. SLA-AE. Pr: 0.7656
QLI71992.1	OTHER	0.000561	0.999439	
QLI71993.1	OTHER	0.042079	0.957921	
QLI71994.1	OTHER	0.005168	0.994832	
QLI71995.1	OTHER	0.214669	0.785331	
QLI71996.1	OTHER	0.000620	0.999380	
QLI71997.1	OTHER	0.000505	0.999495	
QLI71998.1	OTHER	0.087176	0.912824	
QLI71999.1	OTHER	0.007907	0.992093	
QLI72000.1	OTHER	0.001399	0.998601	
QLI72001.1	OTHER	0.001130	0.998870	
QLI72002.1	OTHER	0.002618	0.997382	
QLI72003.1	OTHER	0.042977	0.957023	
QLI72004.1	OTHER	0.128165	0.871835	
QLI72005.1	OTHER	0.002199	0.997801	
QLI72006.1	OTHER	0.039590	0.960410	

QLI72007.1	OTHER	0.010713	0.989287	
QLI72008.1	OTHER	0.003182	0.996818	
QLI72009.1	OTHER	0.000942	0.999058	
QLI72010.1	OTHER	0.001743	0.998257	
QLI72011.1	OTHER	0.000681	0.999319	
QLI72012.1	SP(Sec/SPI)	0.947397	0.052603	CS pos: 24-25. ASA-EE. Pr: 0.4965
QLI72013.1	OTHER	0.000576	0.999424	
QLI72014.1	OTHER	0.000843	0.999157	
QLI72015.1	OTHER	0.002280	0.997720	
QLI72016.1	OTHER	0.005321	0.994679	
QLI72017.1	OTHER	0.001072	0.998928	
QLI72018.1	OTHER	0.003222	0.996778	
QLI72019.1	OTHER	0.000647	0.999353	
QLI72020.1	OTHER	0.001119	0.998881	
QLI72021.1	OTHER	0.000583	0.999417	
QLI72022.1	OTHER	0.001706	0.998294	
QLI72023.1	OTHER	0.002757	0.997243	
QLI72024.1	OTHER	0.001025	0.998975	
QLI72025.1	OTHER	0.001620	0.998380	
QLI72026.1	OTHER	0.000901	0.999099	
QLI72027.1	SP(Sec/SPI)	0.987273	0.012727	CS pos: 18-19. CHA-LQ. Pr: 0.6420
QLI72028.1	OTHER	0.003598	0.996402	
QLI72029.1	OTHER	0.000820	0.999180	
QLI72030.1	OTHER	0.002192	0.997808	
QLI72031.1	OTHER	0.001985	0.998015	
QLI72032.1	OTHER	0.007642	0.992358	
QLI72033.1	OTHER	0.000907	0.999093	
QLI72034.1	OTHER	0.000471	0.999529	
QLI72035.1	OTHER	0.010154	0.989846	
QLI72036.1	SP(Sec/SPI)	0.996160	0.003840	CS pos: 19-20. ANA-AK. Pr: 0.9146
QLI72037.1	OTHER	0.001574	0.998426	

QLI72038.1	OTHER	0.000808	0.999192	
QLI72039.1	OTHER	0.001847	0.998153	
QLI72040.1	OTHER	0.237931	0.762069	
QLI72041.1	OTHER	0.007830	0.992170	
QLI72042.1	SP(Sec/SPI)	0.995765	0.004235	CS pos: 16-17. ALA-AP. Pr: 0.6454
QLI72043.1	OTHER	0.003285	0.996715	
QLI72044.1	OTHER	0.004836	0.995164	
QLI72045.1	OTHER	0.002916	0.997084	
QLI72046.1	OTHER	0.006977	0.993023	
QLI72047.1	SP(Sec/SPI)	0.977666	0.022334	CS pos: 19-20. AVA-SP. Pr: 0.6460
QLI72048.1	OTHER	0.000887	0.999113	
QLI72049.1	OTHER	0.000727	0.999273	
QLI72050.1	SP(Sec/SPI)	0.902696	0.097304	CS pos: 15-16. VHG-LS. Pr: 0.7208
QLI72051.1	OTHER	0.000390	0.999610	
QLI72052.1	OTHER	0.001989	0.998011	
QLI72053.1	OTHER	0.000726	0.999274	
QLI72054.1	OTHER	0.000718	0.999282	
QLI72055.1	OTHER	0.000652	0.999348	
QLI72056.1	OTHER	0.000579	0.999421	
QLI72057.1	OTHER	0.003109	0.996891	
QLI72058.1	OTHER	0.182466	0.817534	
QLI72059.1	OTHER	0.014248	0.985752	
QLI72060.1	OTHER	0.002181	0.997819	
QLI72061.1	OTHER	0.001192	0.998808	
QLI72062.1	OTHER	0.004391	0.995609	
QLI72063.1	OTHER	0.010013	0.989987	
QLI72064.1	OTHER	0.000621	0.999379	
QLI72065.1	OTHER	0.003714	0.996286	
QLI72066.1	OTHER	0.001145	0.998855	
QLI72067.1	OTHER	0.001649	0.998351	
QLI72068.1	OTHER	0.000236	0.999764	

QLI72069.1	SP(Sec/SPI)	0.990891	0.009109	CS pos: 19-20. ALA-AD. Pr: 0.6710
QLI72070.1	OTHER	0.001031	0.998969	
QLI72071.1	OTHER	0.021452	0.978548	
QLI72072.1	OTHER	0.000673	0.999327	
QLI72073.1	OTHER	0.001590	0.998410	
QLI72074.1	OTHER	0.000298	0.999702	
QLI72075.1	OTHER	0.002136	0.997864	
QLI72076.1	OTHER	0.000602	0.999398	
QLI72077.1	OTHER	0.010856	0.989144	
QLI72078.1	OTHER	0.005476	0.994524	
QLI72079.1	OTHER	0.003143	0.996857	
QLI72080.1	OTHER	0.041905	0.958095	
QLI72081.1	OTHER	0.038892	0.961108	
QLI72082.1	OTHER	0.000311	0.999689	
QLI72083.1	OTHER	0.003630	0.996370	
QLI72084.1	OTHER	0.003732	0.996268	
QLI72085.1	OTHER	0.000713	0.999287	
QLI72086.1	OTHER	0.000589	0.999411	
QLI72087.1	OTHER	0.007608	0.992392	
QLI72088.1	OTHER	0.037293	0.962707	
QLI72089.1	SP(Sec/SPI)	0.688197	0.311803	CS pos: 21-22. SSA-AP. Pr: 0.4186
QLI72090.1	OTHER	0.041440	0.958560	
QLI72091.1	OTHER	0.015428	0.984572	
QLI72092.1	OTHER	0.001612	0.998388	
QLI72093.1	SP(Sec/SPI)	0.949505	0.050495	CS pos: 23-24. SHQ-KC. Pr: 0.6931
QLI72094.1	OTHER	0.002050	0.997950	
QLI72095.1	OTHER	0.000888	0.999112	
QLI72096.1	OTHER	0.000651	0.999349	
QLI72097.1	OTHER	0.003220	0.996780	
QLI72098.1	OTHER	0.001785	0.998215	
QLI72099.1	OTHER	0.002658	0.997342	

QLI72100.1	OTHER	0.000916	0.999084	
QLI72101.1	SP(Sec/SPI)	0.992436	0.007564	CS pos: 17-18. AEA-CA. Pr: 0.5976
QLI72102.1	OTHER	0.000575	0.999425	
QLI72103.1	OTHER	0.001534	0.998466	
QLI72104.1	SP(Sec/SPI)	0.888339	0.111661	CS pos: 15-16. AAA-AP. Pr: 0.4025
QLI72105.1	SP(Sec/SPI)	0.539766	0.460234	CS pos: 17-18. VYC-TT. Pr: 0.2741
QLI72106.1	OTHER	0.000693	0.999307	
QLI72107.1	OTHER	0.000751	0.999249	
QLI72108.1	OTHER	0.000884	0.999116	
QLI72109.1	OTHER	0.002022	0.997978	
QLI72110.1	OTHER	0.002560	0.997440	
QLI72111.1	OTHER	0.001090	0.998910	
QLI72112.1	OTHER	0.001414	0.998586	
QLI72113.1	OTHER	0.001238	0.998762	
QLI72114.1	OTHER	0.000742	0.999258	
QLI72115.1	OTHER	0.000307	0.999693	
QLI72116.1	OTHER	0.000573	0.999427	
QLI72117.1	OTHER	0.001108	0.998892	
QLI72118.1	OTHER	0.000807	0.999193	
QLI72119.1	OTHER	0.040699	0.959301	
QLI72120.1	OTHER	0.000688	0.999312	
QLI72121.1	OTHER	0.002010	0.997990	
QLI72122.1	OTHER	0.001051	0.998949	
QLI72123.1	OTHER	0.000558	0.999442	
QLI72124.1	OTHER	0.001340	0.998660	
QLI72125.1	OTHER	0.000350	0.999650	
QLI72126.1	OTHER	0.003509	0.996491	
QLI72127.1	OTHER	0.009933	0.990067	
QLI72128.1	OTHER	0.001903	0.998097	
QLI72129.1	OTHER	0.000713	0.999287	
QLI72130.1	OTHER	0.001933	0.998067	

QLI72131.1	OTHER	0.000410	0.999590	
QLI72132.1	OTHER	0.000715	0.999285	
QLI72133.1	OTHER	0.001735	0.998265	
QLI72134.1	OTHER	0.000578	0.999422	
QLI72135.1	OTHER	0.000197	0.999803	
QLI72136.1	OTHER	0.000920	0.999080	
QLI72137.1	OTHER	0.000371	0.999629	
QLI72138.1	OTHER	0.001344	0.998656	
QLI72139.1	OTHER	0.000670	0.999330	
QLI72140.1	OTHER	0.001386	0.998614	
QLI72141.1	OTHER	0.148372	0.851628	
QLI72142.1	OTHER	0.001195	0.998805	
QLI72143.1	SP(Sec/SPI)	0.705443	0.294557	CS pos: 22-23. VLS-AD. Pr: 0.2248
QLI72144.1	OTHER	0.001982	0.998018	
QLI72145.1	OTHER	0.001186	0.998814	
QLI72146.1	OTHER	0.001134	0.998866	
QLI72147.1	OTHER	0.048743	0.951257	
QLI72148.1	OTHER	0.001270	0.998730	
QLI72149.1	OTHER	0.005658	0.994342	
QLI72150.1	OTHER	0.004105	0.995895	
QLI72151.1	OTHER	0.002202	0.997798	
QLI72152.1	OTHER	0.001647	0.998353	
QLI72153.1	OTHER	0.002248	0.997752	
QLI72154.1	OTHER	0.000433	0.999567	
QLI72155.1	OTHER	0.000666	0.999334	
QLI72156.1	OTHER	0.000910	0.999090	
QLI72157.1	OTHER	0.001147	0.998853	
QLI72158.1	OTHER	0.000312	0.999688	
QLI72159.1	OTHER	0.000334	0.999666	
QLI72160.1	SP(Sec/SPI)	0.719392	0.280608	CS pos: 19-20. VTA-LV. Pr: 0.4081
QLI72161.1	OTHER	0.001244	0.998756	

QLI72162.1	OTHER	0.001058	0.998942	
QLI72163.1	OTHER	0.001869	0.998131	
QLI72164.1	SP(Sec/SPI)	0.942744	0.057256	CS pos: 17-18. AIA-VP. Pr: 0.7406
QLI72165.1	OTHER	0.001167	0.998833	
QLI72166.1	SP(Sec/SPI)	0.906514	0.093486	CS pos: 27-28. VHA-QE. Pr: 0.4375
QLI72167.1	SP(Sec/SPI)	0.908693	0.091307	CS pos: 18-19. VYA-QI. Pr: 0.7859
QLI72168.1	OTHER	0.001207	0.998793	
QLI72169.1	OTHER	0.000558	0.999442	
QLI72170.1	OTHER	0.005031	0.994969	
QLI72171.1	OTHER	0.001533	0.998467	
QLI72172.1	SP(Sec/SPI)	0.951486	0.048514	CS pos: 17-18. VAA-SP. Pr: 0.5573
QLI72173.1	SP(Sec/SPI)	0.998719	0.001281	CS pos: 18-19. VNA-VI. Pr: 0.9675
QLI72174.1	OTHER	0.100552	0.899448	
QLI72175.1	OTHER	0.001287	0.998713	
QLI72176.1	OTHER	0.000830	0.999170	
QLI72177.1	OTHER	0.000712	0.999288	
QLI72178.1	OTHER	0.000858	0.999142	
QLI72179.1	OTHER	0.004020	0.995980	
QLI72180.1	OTHER	0.002090	0.997910	
QLI72181.1	SP(Sec/SPI)	0.989094	0.010906	CS pos: 22-23. CHG-SQ. Pr: 0.9072
QLI72182.1	OTHER	0.001226	0.998774	
QLI72183.1	SP(Sec/SPI)	0.949344	0.050656	CS pos: 24-25. SEA-DE. Pr: 0.8499
QLI72184.1	OTHER	0.001449	0.998551	
QLI72185.1	OTHER	0.000798	0.999202	
QLI72186.1	OTHER	0.001751	0.998249	
QLI72187.1	OTHER	0.001075	0.998925	
QLI72188.1	OTHER	0.002025	0.997975	
QLI72189.1	OTHER	0.000326	0.999674	
QLI72190.1	OTHER	0.275315	0.724685	
QLI72191.1	OTHER	0.002819	0.997181	
QLI72192.1	OTHER	0.013704	0.986296	

QLI72193.1	OTHER	0.000639	0.999361	
QLI72194.1	OTHER	0.001708	0.998292	
QLI72195.1	OTHER	0.001175	0.998825	
QLI72196.1	OTHER	0.001032	0.998968	
QLI72197.1	OTHER	0.023973	0.976027	
QLI72198.1	OTHER	0.004171	0.995829	
QLI72199.1	OTHER	0.010401	0.989599	
QLI72200.1	OTHER	0.000968	0.999032	
QLI72201.1	OTHER	0.000728	0.999272	
QLI72202.1	OTHER	0.001168	0.998832	
QLI72203.1	OTHER	0.001263	0.998737	
QLI72204.1	OTHER	0.001392	0.998608	
QLI72205.1	OTHER	0.000743	0.999257	
QLI72206.1	OTHER	0.001918	0.998082	
QLI72207.1	OTHER	0.001695	0.998305	
QLI72208.1	OTHER	0.000141	0.999859	
QLI72209.1	OTHER	0.000337	0.999663	
QLI72210.1	OTHER	0.001261	0.998739	
QLI72211.1	SP(Sec/SPI)	0.995853	0.004147	CS pos: 22-23. AFG-NP. Pr: 0.6624
QLI72212.1	SP(Sec/SPI)	0.969002	0.030998	CS pos: 21-22. VAA-QP. Pr: 0.5958
QLI72213.1	OTHER	0.004183	0.995817	
QLI72214.1	OTHER	0.004925	0.995075	
QLI72215.1	OTHER	0.000575	0.999425	
QLI72216.1	OTHER	0.000548	0.999452	
QLI72217.1	OTHER	0.000953	0.999047	
QLI72218.1	OTHER	0.001318	0.998682	
QLI72219.1	OTHER	0.001160	0.998840	
QLI72220.1	SP(Sec/SPI)	0.997348	0.002652	CS pos: 19-20. IHA-GV. Pr: 0.8006
QLI72221.1	OTHER	0.000284	0.999716	
QLI72222.1	OTHER	0.000670	0.999330	
QLI72223.1	OTHER	0.001540	0.998460	

QLI72224.1	OTHER	0.001032	0.998968	
QLI72225.1	OTHER	0.001349	0.998651	
QLI72226.1	OTHER	0.000802	0.999198	
QLI72227.1	OTHER	0.000129	0.999871	
QLI72228.1	OTHER	0.000989	0.999011	
QLI72229.1	OTHER	0.000396	0.999604	
QLI72230.1	OTHER	0.002180	0.997820	
QLI72231.1	OTHER	0.000637	0.999363	
QLI72232.1	OTHER	0.000709	0.999291	
QLI72233.1	OTHER	0.000260	0.999740	
QLI72234.1	OTHER	0.000675	0.999325	
QLI72235.1	OTHER	0.001377	0.998623	
QLI72236.1	OTHER	0.003046	0.996954	
QLI72237.1	OTHER	0.002591	0.997409	
QLI72238.1	OTHER	0.000192	0.999808	
QLI72239.1	SP(Sec/SPI)	0.805769	0.194231	CS pos: 15-16. ADS-LK. Pr: 0.3440
QLI72240.1	OTHER	0.043150	0.956850	
QLI72241.1	OTHER	0.007063	0.992937	
QLI72242.1	OTHER	0.000264	0.999736	
QLI72243.1	SP(Sec/SPI)	0.959189	0.040811	CS pos: 19-20. IAG-QE. Pr: 0.7224
QLI72244.1	OTHER	0.000408	0.999592	
QLI72245.1	OTHER	0.000532	0.999468	
QLI72246.1	OTHER	0.000482	0.999518	
QLI72247.1	OTHER	0.000460	0.999540	
QLI72248.1	OTHER	0.001379	0.998621	
QLI72249.1	OTHER	0.003126	0.996874	
QLI72250.1	OTHER	0.001474	0.998526	
QLI72251.1	OTHER	0.000615	0.999385	
QLI72252.1	OTHER	0.001340	0.998660	
QLI72253.1	OTHER	0.000428	0.999572	
QLI72254.1	OTHER	0.000875	0.999125	

QLI72255.1	OTHER	0.001620	0.998380	
QLI72256.1	OTHER	0.003347	0.996653	
QLI72257.1	OTHER	0.000947	0.999053	
QLI72258.1	OTHER	0.000523	0.999477	
QLI72259.1	OTHER	0.000732	0.999268	
QLI72260.1	OTHER	0.000496	0.999504	
QLI72261.1	SP(Sec/SPI)	0.996342	0.003658	CS pos: 19-20. VAA-TE. Pr: 0.5194
QLI72262.1	SP(Sec/SPI)	0.749586	0.250414	CS pos: 23-24. RDA-AE. Pr: 0.2414
QLI72263.1	OTHER	0.001991	0.998009	
QLI72264.1	OTHER	0.001494	0.998506	
QLI72265.1	OTHER	0.002458	0.997542	
QLI72266.1	OTHER	0.000385	0.999615	
QLI72267.1	OTHER	0.048572	0.951428	
QLI72268.1	OTHER	0.002592	0.997408	
QLI72269.1	OTHER	0.000177	0.999823	
QLI72270.1	OTHER	0.005252	0.994748	
QLI72271.1	OTHER	0.000236	0.999764	
QLI72272.1	OTHER	0.001082	0.998918	
QLI72273.1	SP(Sec/SPI)	0.933328	0.066672	CS pos: 16-17. AAA-VR. Pr: 0.3625
QLI72274.1	SP(Sec/SPI)	0.999707	0.000293	CS pos: 19-20. SFA-QD. Pr: 0.8214
QLI72275.1	OTHER	0.000494	0.999506	
QLI72276.1	OTHER	0.000417	0.999583	
QLI72277.1	OTHER	0.441869	0.558131	
QLI72278.1	OTHER	0.000959	0.999041	
QLI72279.1	OTHER	0.001582	0.998418	
QLI72280.1	OTHER	0.001175	0.998825	
QLI72281.1	OTHER	0.022770	0.977230	
QLI72282.1	OTHER	0.003638	0.996362	
QLI72283.1	OTHER	0.001553	0.998447	
QLI72284.1	SP(Sec/SPI)	0.922991	0.077009	CS pos: 19-20. ASA-AT. Pr: 0.4064
QLI72285.1	OTHER	0.003115	0.996885	

QLI72286.1	OTHER	0.008673	0.991327	
QLI72287.1	OTHER	0.000788	0.999212	
QLI72288.1	SP(Sec/SPI)	0.999304	0.000696	CS pos: 21-22. VSA-GS. Pr: 0.9148
QLI72289.1	OTHER	0.001353	0.998647	
QLI72290.1	SP(Sec/SPI)	0.969383	0.030617	CS pos: 16-17. ALA-AS. Pr: 0.4903
QLI72291.1	OTHER	0.002067	0.997933	
QLI72292.1	OTHER	0.000604	0.999396	
QLI72293.1	OTHER	0.001672	0.998328	
QLI72294.1	OTHER	0.000362	0.999638	
QLI72295.1	OTHER	0.000822	0.999178	
QLI72296.1	OTHER	0.006996	0.993004	
QLI72297.1	OTHER	0.009748	0.990252	
QLI72298.1	OTHER	0.002326	0.997674	
QLI72299.1	OTHER	0.000512	0.999488	
QLI72300.1	OTHER	0.001330	0.998670	
QLI72301.1	OTHER	0.001435	0.998565	
QLI72302.1	OTHER	0.123030	0.876970	
QLI72303.1	OTHER	0.000845	0.999155	
QLI72304.1	OTHER	0.000554	0.999446	
QLI72305.1	OTHER	0.000937	0.999063	
QLI72306.1	OTHER	0.013428	0.986572	
QLI72307.1	OTHER	0.000723	0.999277	
QLI72308.1	OTHER	0.001938	0.998062	
QLI72309.1	OTHER	0.001665	0.998335	
QLI72310.1	OTHER	0.005485	0.994515	
QLI72311.1	OTHER	0.001973	0.998027	
QLI72312.1	OTHER	0.001442	0.998558	
QLI72313.1	OTHER	0.001009	0.998991	
QLI72314.1	OTHER	0.001740	0.998260	
QLI72315.1	OTHER	0.004370	0.995630	
QLI72316.1	OTHER	0.040659	0.959341	

QLI72317.1	OTHER	0.000846	0.999154	
QLI72318.1	OTHER	0.001550	0.998450	
QLI72319.1	OTHER	0.000554	0.999446	
QLI72320.1	OTHER	0.001261	0.998739	
QLI72321.1	SP(Sec/SPI)	0.981673	0.018327	CS pos: 19-20. VEG-AE. Pr: 0.7543
QLI72322.1	OTHER	0.001449	0.998551	
QLI72323.1	OTHER	0.001821	0.998179	
QLI72324.1	OTHER	0.000645	0.999355	
QLI72325.1	OTHER	0.002111	0.997889	
QLI72326.1	OTHER	0.007800	0.992200	
QLI72327.1	OTHER	0.000601	0.999399	
QLI72328.1	OTHER	0.002447	0.997553	
QLI72329.1	OTHER	0.000948	0.999052	
QLI72330.1	OTHER	0.000712	0.999288	
QLI72331.1	OTHER	0.002419	0.997581	
QLI72332.1	OTHER	0.000489	0.999511	
QLI72333.1	OTHER	0.000348	0.999652	
QLI72334.1	OTHER	0.001839	0.998161	
QLI72335.1	OTHER	0.001024	0.998976	
QLI72336.1	OTHER	0.001561	0.998439	
QLI72337.1	OTHER	0.002426	0.997574	
QLI72338.1	OTHER	0.000543	0.999457	
QLI72339.1	OTHER	0.000737	0.999263	
QLI72340.1	OTHER	0.001008	0.998992	
QLI72341.1	OTHER	0.000187	0.999813	
QLI72342.1	OTHER	0.001321	0.998679	
QLI72343.1	OTHER	0.003089	0.996911	
QLI72344.1	OTHER	0.000437	0.999563	
QLI72345.1	OTHER	0.006348	0.993652	
QLI72346.1	OTHER	0.001365	0.998635	
QLI72347.1	OTHER	0.000548	0.999452	

QLI72348.1	OTHER	0.003766	0.996234	
QLI72349.1	OTHER	0.002824	0.997176	
QLI72350.1	OTHER	0.000658	0.999342	
QLI72351.1	OTHER	0.000421	0.999579	
QLI72352.1	OTHER	0.002350	0.997650	
QLI72353.1	OTHER	0.001106	0.998894	
QLI72354.1	OTHER	0.001941	0.998059	
QLI72355.1	OTHER	0.023680	0.976320	
QLI72356.1	OTHER	0.001121	0.998879	
QLI72357.1	OTHER	0.112883	0.887117	
QLI72358.1	OTHER	0.321891	0.678109	
QLI72359.1	OTHER	0.001707	0.998293	
QLI72360.1	OTHER	0.000633	0.999367	
QLI72361.1	OTHER	0.000533	0.999467	
QLI72362.1	OTHER	0.000744	0.999256	
QLI72363.1	OTHER	0.001175	0.998825	
QLI72364.1	OTHER	0.001351	0.998649	
QLI72365.1	OTHER	0.000743	0.999257	
QLI72366.1	SP(Sec/SPI)	0.986810	0.013190	CS pos: 18-19. VTA-GI. Pr: 0.6819
QLI72367.1	OTHER	0.003715	0.996285	
QLI72368.1	OTHER	0.007453	0.992547	
QLI72369.1	OTHER	0.000689	0.999311	
QLI72370.1	OTHER	0.000780	0.999220	
QLI72371.1	OTHER	0.144132	0.855868	
QLI72372.1	OTHER	0.001547	0.998453	
QLI72373.1	OTHER	0.004522	0.995478	
QLI72374.1	OTHER	0.001028	0.998972	
QLI72375.1	OTHER	0.001711	0.998289	
QLI72376.1	OTHER	0.000616	0.999384	
QLI72377.1	OTHER	0.001614	0.998386	
QLI72378.1	OTHER	0.009454	0.990546	

QLI72379.1	OTHER	0.000850	0.999150	
QLI72380.1	OTHER	0.002137	0.997863	
QLI72381.1	OTHER	0.000697	0.999303	
QLI72382.1	OTHER	0.005977	0.994023	
QLI72383.1	OTHER	0.003024	0.996976	
QLI72384.1	OTHER	0.000889	0.999111	
QLI72385.1	SP(Sec/SPI)	0.995517	0.004483	CS pos: 17-18. VQA-AP. Pr: 0.9055
QLI72386.1	OTHER	0.181817	0.818183	
QLI72387.1	OTHER	0.001117	0.998883	
QLI72388.1	OTHER	0.001663	0.998337	
QLI72389.1	OTHER	0.004604	0.995396	
QLI72390.1	OTHER	0.008600	0.991400	
QLI72391.1	SP(Sec/SPI)	0.532026	0.467974	CS pos: 23-24. LNA-TD. Pr: 0.4966
QLI72392.1	SP(Sec/SPI)	0.994324	0.005676	CS pos: 20-21. TDA-GR. Pr: 0.9028
QLI72393.1	OTHER	0.001406	0.998594	
QLI72394.1	OTHER	0.002146	0.997854	
QLI72395.1	OTHER	0.002121	0.997879	
QLI72396.1	OTHER	0.001459	0.998541	
QLI72397.1	OTHER	0.000685	0.999315	
QLI72398.1	OTHER	0.000263	0.999737	
QLI72399.1	OTHER	0.000789	0.999211	
QLI72400.1	OTHER	0.004347	0.995653	
QLI72401.1	OTHER	0.005057	0.994943	
QLI72402.1	OTHER	0.001321	0.998679	
QLI72403.1	OTHER	0.001547	0.998453	
QLI72404.1	OTHER	0.000467	0.999533	
QLI72405.1	OTHER	0.012002	0.987998	
QLI72406.1	OTHER	0.000744	0.999256	
QLI72407.1	OTHER	0.000570	0.999430	
QLI72408.1	OTHER	0.001127	0.998873	
QLI72409.1	OTHER	0.001270	0.998730	

QLI72410.1	OTHER	0.000893	0.999107	
QLI72411.1	SP(Sec/SPI)	0.636012	0.363988	CS pos: 25-26. ALT-AT. Pr: 0.2688
QLI72412.1	SP(Sec/SPI)	0.777266	0.222734	CS pos: 20-21. SFS-WP. Pr: 0.3199
QLI72413.1	OTHER	0.002066	0.997934	
QLI72414.1	OTHER	0.000188	0.999812	
QLI72415.1	OTHER	0.000358	0.999642	
QLI72416.1	OTHER	0.000874	0.999126	
QLI72417.1	OTHER	0.002540	0.997460	
QLI72418.1	OTHER	0.000228	0.999772	
QLI72419.1	OTHER	0.003428	0.996572	
QLI72420.1	OTHER	0.007135	0.992865	
QLI72421.1	OTHER	0.009734	0.990266	
QLI72422.1	OTHER	0.089863	0.910137	
QLI72423.1	OTHER	0.000336	0.999664	
QLI72424.1	OTHER	0.001279	0.998721	
QLI72425.1	OTHER	0.001987	0.998013	
QLI72426.1	OTHER	0.001276	0.998724	
QLI72427.1	OTHER	0.000349	0.999651	
QLI72428.1	OTHER	0.001215	0.998785	
QLI72429.1	OTHER	0.000838	0.999162	
QLI72430.1	OTHER	0.000682	0.999318	
QLI72431.1	SP(Sec/SPI)	0.518938	0.481062	CS pos: 23-24. AGA-LP. Pr: 0.3021
QLI72432.1	OTHER	0.003294	0.996706	
QLI72433.1	OTHER	0.001419	0.998581	
QLI72434.1	OTHER	0.001084	0.998916	
QLI72435.1	OTHER	0.001650	0.998350	
QLI72436.1	OTHER	0.001095	0.998905	
QLI72437.1	OTHER	0.000182	0.999818	
QLI72438.1	OTHER	0.000696	0.999304	
QLI72439.1	OTHER	0.036657	0.963343	
QLI72440.1	OTHER	0.000354	0.999646	

QLI72441.1	OTHER	0.002071	0.997929	
QLI72442.1	OTHER	0.000910	0.999090	
QLI72443.1	OTHER	0.003139	0.996861	
QLI72444.1	OTHER	0.000710	0.999290	
QLI72445.1	OTHER	0.001591	0.998409	
QLI72446.1	OTHER	0.000812	0.999188	
QLI72447.1	OTHER	0.001457	0.998543	
QLI72448.1	OTHER	0.001844	0.998156	
QLI72449.1	OTHER	0.001235	0.998765	
QLI72450.1	OTHER	0.001228	0.998772	
QLI72451.1	OTHER	0.009559	0.990441	
QLI72452.1	OTHER	0.000567	0.999433	
QLI72453.1	OTHER	0.002133	0.997867	
QLI72454.1	OTHER	0.003281	0.996719	
QLI72455.1	OTHER	0.000848	0.999152	
QLI72456.1	OTHER	0.001679	0.998321	
QLI72457.1	SP(Sec/SPI)	0.992344	0.007656	CS pos: 17-18. ALA-VP. Pr: 0.9136
QLI72458.1	SP(Sec/SPI)	0.866661	0.133339	CS pos: 20-21. SSA-LG. Pr: 0.3735
QLI72459.1	SP(Sec/SPI)	0.983875	0.016125	CS pos: 21-22. CLG-AS. Pr: 0.6049
QLI72460.1	OTHER	0.002120	0.997880	
QLI72461.1	SP(Sec/SPI)	0.992796	0.007204	CS pos: 16-17. GAT-AP. Pr: 0.3946
QLI72462.1	OTHER	0.065800	0.934200	
QLI72463.1	OTHER	0.000691	0.999309	
QLI72464.1	OTHER	0.002247	0.997753	
QLI72465.1	OTHER	0.062455	0.937545	
QLI72466.1	OTHER	0.003179	0.996821	
QLI72467.1	OTHER	0.479296	0.520704	
QLI72468.1	SP(Sec/SPI)	0.934588	0.065412	CS pos: 21-22. SRA-AD. Pr: 0.6442
QLI72469.1	OTHER	0.013188	0.986812	
QLI72470.1	OTHER	0.000533	0.999467	
QLI72471.1	OTHER	0.005177	0.994823	

QLI72472.1	SP(Sec/SPI)	0.859220	0.140780	CS pos: 20-21. ASA-KT. Pr: 0.6792
QLI72473.1	OTHER	0.005974	0.994026	
QLI72474.1	OTHER	0.000850	0.999150	
QLI72475.1	OTHER	0.008804	0.991196	
QLI72476.1	OTHER	0.001927	0.998073	
QLI72477.1	OTHER	0.005165	0.994835	
QLI72478.1	SP(Sec/SPI)	0.943755	0.056245	CS pos: 19-20. SSA-AG. Pr: 0.3890
QLI72479.1	SP(Sec/SPI)	0.980931	0.019069	CS pos: 19-20. GMA-QT. Pr: 0.6512
QLI72480.1	OTHER	0.005366	0.994634	
QLI72481.1	OTHER	0.001017	0.998983	
QLI72482.1	OTHER	0.000586	0.999414	
QLI72483.1	OTHER	0.000769	0.999231	
QLI72484.1	OTHER	0.002917	0.997083	
QLI72485.1	OTHER	0.005783	0.994217	
QLI72486.1	OTHER	0.001071	0.998929	
QLI72487.1	OTHER	0.000771	0.999229	
QLI72488.1	SP(Sec/SPI)	0.513027	0.486973	CS pos: 20-21. PSA-KD. Pr: 0.2141
QLI72489.1	OTHER	0.001200	0.998800	
QLI72490.1	OTHER	0.001743	0.998257	
QLI72491.1	OTHER	0.004696	0.995304	
QLI72492.1	OTHER	0.001123	0.998877	
QLI72493.1	OTHER	0.277890	0.722110	
QLI72494.1	OTHER	0.012584	0.987416	
QLI72495.1	OTHER	0.000477	0.999523	
QLI72496.1	OTHER	0.000241	0.999759	
QLI72497.1	OTHER	0.001689	0.998311	
QLI72498.1	OTHER	0.119056	0.880944	
QLI72499.1	OTHER	0.000740	0.999260	
QLI72500.1	OTHER	0.331057	0.668943	
QLI72501.1	SP(Sec/SPI)	0.996066	0.003934	CS pos: 20-21. SSA-AP. Pr: 0.7889
QLI72502.1	OTHER	0.001315	0.998685	

QLI72503.1	OTHER	0.009087	0.990913	
QLI72504.1	OTHER	0.002037	0.997963	
QLI72505.1	OTHER	0.003166	0.996834	
QLI72506.1	OTHER	0.000403	0.999597	
QLI72507.1	SP(Sec/SPI)	0.938724	0.061276	CS pos: 20-21. VSA-VG. Pr: 0.6527
QLI72508.1	OTHER	0.000278	0.999722	
QLI72509.1	OTHER	0.003510	0.996490	
QLI72510.1	OTHER	0.000775	0.999225	
QLI72511.1	OTHER	0.000871	0.999129	
QLI72512.1	OTHER	0.000886	0.999114	
QLI72513.1	OTHER	0.002087	0.997913	
QLI72514.1	OTHER	0.004030	0.995970	
QLI72515.1	OTHER	0.001945	0.998055	
QLI72516.1	OTHER	0.000501	0.999499	
QLI72517.1	OTHER	0.002448	0.997552	
QLI72518.1	SP(Sec/SPI)	0.929145	0.070855	CS pos: 21-22. SIA-VD. Pr: 0.6381
QLI72519.1	OTHER	0.000469	0.999531	
QLI72520.1	OTHER	0.002537	0.997463	
QLI72521.1	OTHER	0.001616	0.998384	
QLI72522.1	SP(Sec/SPI)	0.976611	0.023389	CS pos: 19-20. VSA-AT. Pr: 0.8165
QLI72523.1	OTHER	0.223001	0.776999	
QLI72524.1	OTHER	0.001614	0.998386	
QLI72525.1	OTHER	0.001617	0.998383	
QLI72526.1	OTHER	0.000944	0.999056	
QLI72527.1	OTHER	0.000709	0.999291	
QLI72528.1	OTHER	0.000741	0.999259	
QLI72529.1	OTHER	0.000205	0.999795	
QLI72530.1	OTHER	0.003222	0.996778	
QLI72531.1	OTHER	0.001826	0.998174	
QLI72532.1	OTHER	0.002934	0.997066	
QLI72533.1	OTHER	0.000719	0.999281	

QLI72534.1	OTHER	0.038334	0.961666	
QLI72535.1	SP(Sec/SPI)	0.987169	0.012831	CS pos: 21-22. ASA-VE. Pr: 0.3920
QLI72536.1	SP(Sec/SPI)	0.994317	0.005683	CS pos: 20-21. LGA-RQ. Pr: 0.3214
QLI72537.1	OTHER	0.019154	0.980846	
QLI72538.1	SP(Sec/SPI)	0.546977	0.453023	CS pos: 27-28. GFS-LV. Pr: 0.2113
QLI72539.1	SP(Sec/SPI)	0.991994	0.008006	CS pos: 26-27. GEA-AP. Pr: 0.9573
QLI72540.1	OTHER	0.000951	0.999049	
QLI72541.1	OTHER	0.001540	0.998460	
QLI72542.1	OTHER	0.003119	0.996881	
QLI72543.1	SP(Sec/SPI)	0.604188	0.395812	CS pos: 26-27. VQS-AT. Pr: 0.3280
QLI72544.1	OTHER	0.001705	0.998295	
QLI72545.1	OTHER	0.001103	0.998897	
QLI72546.1	OTHER	0.000999	0.999001	
QLI72547.1	OTHER	0.000927	0.999073	
QLI72548.1	OTHER	0.000248	0.999752	
QLI72549.1	OTHER	0.002027	0.997973	
QLI72550.1	OTHER	0.001785	0.998215	
QLI72551.1	SP(Sec/SPI)	0.908081	0.091919	CS pos: 25-26. GFA-AG. Pr: 0.4758
QLI72552.1	OTHER	0.001257	0.998743	
QLI72553.1	OTHER	0.000459	0.999541	
QLI72554.1	OTHER	0.001130	0.998870	
QLI72555.1	OTHER	0.002255	0.997745	
QLI72556.1	OTHER	0.000884	0.999116	
QLI72557.1	OTHER	0.000167	0.999833	
QLI72558.1	OTHER	0.000218	0.999782	
QLI72559.1	OTHER	0.002095	0.997905	
QLI72560.1	OTHER	0.001915	0.998085	
QLI72561.1	OTHER	0.001365	0.998635	
QLI72562.1	SP(Sec/SPI)	0.999418	0.000582	CS pos: 17-18. VAA-SP. Pr: 0.5738
QLI72563.1	OTHER	0.326545	0.673455	
QLI72564.1	OTHER	0.046122	0.953878	

QLI72565.1	OTHER	0.014032	0.985968	
QLI72566.1	OTHER	0.001435	0.998565	
QLI72567.1	OTHER	0.000672	0.999328	
QLI72568.1	OTHER	0.001320	0.998680	
QLI72569.1	OTHER	0.001093	0.998907	
QLI72570.1	OTHER	0.002990	0.997010	
QLI72571.1	OTHER	0.001349	0.998651	
QLI72572.1	SP(Sec/SPI)	0.971372	0.028628	CS pos: 21-22. AVA-QN. Pr: 0.5940
QLI72573.1	OTHER	0.001814	0.998186	
QLI72574.1	OTHER	0.003861	0.996139	
QLI72575.1	OTHER	0.000903	0.999097	
QLI72576.1	OTHER	0.021983	0.978017	
QLI72577.1	OTHER	0.000592	0.999408	
QLI72578.1	OTHER	0.002070	0.997930	
QLI72579.1	OTHER	0.174644	0.825356	
QLI72580.1	OTHER	0.020169	0.979831	
QLI72581.1	OTHER	0.000378	0.999622	
QLI72582.1	OTHER	0.001346	0.998654	
QLI72583.1	OTHER	0.000840	0.999160	
QLI72584.1	SP(Sec/SPI)	0.975810	0.024190	CS pos: 21-22. CDG-RE. Pr: 0.5013
QLI72585.1	OTHER	0.001817	0.998183	
QLI72586.1	OTHER	0.000789	0.999211	
QLI72587.1	OTHER	0.000219	0.999781	
QLI72588.1	OTHER	0.000597	0.999403	
QLI72589.1	OTHER	0.000290	0.999710	
QLI72590.1	OTHER	0.001226	0.998774	
QLI72591.1	OTHER	0.002354	0.997646	
QLI72592.1	SP(Sec/SPI)	0.999065	0.000935	CS pos: 19-20. TLA-HE. Pr: 0.9758
QLI72593.1	OTHER	0.001017	0.998983	
QLI72594.1	OTHER	0.010702	0.989298	
QLI72595.1	OTHER	0.000816	0.999184	

QLI72596.1	OTHER	0.004791	0.995209	
QLI72597.1	OTHER	0.000513	0.999487	
QLI72598.1	OTHER	0.001414	0.998586	
QLI72599.1	OTHER	0.027439	0.972561	
QLI72600.1	OTHER	0.000602	0.999398	
QLI72601.1	OTHER	0.001314	0.998686	
QLI72602.1	OTHER	0.000885	0.999115	
QLI72603.1	SP(Sec/SPI)	0.993180	0.006820	CS pos: 19-20. ASA-KP. Pr: 0.8132
QLI72604.1	SP(Sec/SPI)	0.745471	0.254529	CS pos: 22-23. CNT-LL. Pr: 0.3609
QLI72605.1	OTHER	0.000719	0.999281	
QLI72606.1	OTHER	0.078871	0.921129	
QLI72607.1	OTHER	0.003497	0.996503	
QLI72608.1	OTHER	0.001186	0.998814	
QLI72609.1	OTHER	0.003033	0.996967	
QLI72610.1	OTHER	0.000754	0.999246	
QLI72611.1	OTHER	0.002822	0.997178	
QLI72612.1	OTHER	0.000555	0.999445	
QLI72613.1	OTHER	0.001299	0.998701	
QLI72614.1	OTHER	0.001425	0.998575	
QLI72615.1	OTHER	0.001135	0.998865	
QLI72616.1	OTHER	0.058440	0.941560	
QLI72617.1	OTHER	0.000469	0.999531	
QLI72618.1	OTHER	0.001077	0.998923	
QLI72619.1	OTHER	0.001185	0.998815	
QLI72620.1	OTHER	0.002913	0.997087	
QLI72621.1	OTHER	0.002529	0.997471	
QLI72622.1	OTHER	0.000667	0.999333	
QLI72623.1	OTHER	0.002910	0.997090	
QLI72624.1	OTHER	0.000994	0.999006	
QLI72625.1	OTHER	0.000600	0.999400	
QLI72626.1	OTHER	0.000541	0.999459	

QLI72627.1	SP(Sec/SPI)	0.991054	0.008946	CS pos: 17-18. ASA-HT. Pr: 0.4802
QLI72628.1	OTHER	0.001021	0.998979	
QLI72629.1	OTHER	0.000996	0.999004	
QLI72630.1	OTHER	0.000750	0.999250	
QLI72631.1	OTHER	0.003642	0.996358	
QLI72632.1	OTHER	0.000887	0.999113	
QLI72633.1	OTHER	0.007519	0.992481	
QLI72634.1	OTHER	0.000369	0.999631	
QLI72635.1	SP(Sec/SPI)	0.954255	0.045745	CS pos: 19-20. VLA-VP. Pr: 0.8355
QLI72636.1	OTHER	0.001081	0.998919	
QLI72637.1	OTHER	0.001309	0.998691	
QLI72638.1	OTHER	0.000399	0.999601	
QLI72639.1	OTHER	0.030907	0.969093	
QLI72640.1	OTHER	0.003418	0.996582	
QLI72641.1	OTHER	0.002605	0.997395	
QLI72642.1	OTHER	0.001769	0.998231	
QLI72643.1	OTHER	0.000699	0.999301	
QLI72644.1	OTHER	0.003589	0.996411	
QLI72645.1	OTHER	0.000941	0.999059	
QLI72646.1	OTHER	0.000900	0.999100	
QLI72647.1	OTHER	0.001347	0.998653	
QLI72648.1	OTHER	0.001302	0.998698	
QLI72649.1	OTHER	0.364158	0.635842	
QLI72650.1	OTHER	0.000441	0.999559	
QLI72651.1	OTHER	0.000594	0.999406	
QLI72652.1	OTHER	0.000924	0.999076	
QLI72653.1	OTHER	0.001457	0.998543	
QLI72654.1	OTHER	0.012386	0.987614	
QLI72655.1	OTHER	0.000467	0.999533	
QLI72656.1	OTHER	0.000623	0.999377	
QLI72657.1	OTHER	0.008534	0.991466	

QLI72658.1	OTHER	0.000942	0.999058	
QLI72659.1	OTHER	0.000557	0.999443	
QLI72660.1	OTHER	0.000986	0.999014	
QLI72661.1	OTHER	0.001178	0.998822	
QLI72662.1	OTHER	0.001334	0.998666	
QLI72663.1	OTHER	0.000710	0.999290	
QLI72664.1	OTHER	0.000462	0.999538	
QLI72665.1	OTHER	0.002808	0.997192	
QLI72666.1	OTHER	0.000589	0.999411	
QLI72667.1	OTHER	0.000684	0.999316	
QLI72668.1	OTHER	0.001085	0.998915	
QLI72669.1	OTHER	0.002370	0.997630	
QLI72670.1	OTHER	0.001647	0.998353	
QLI72671.1	OTHER	0.002031	0.997969	
QLI72672.1	OTHER	0.001154	0.998846	
QLI72673.1	OTHER	0.003042	0.996958	
QLI72674.1	OTHER	0.001146	0.998854	
QLI72675.1	OTHER	0.000748	0.999252	
QLI72676.1	OTHER	0.000527	0.999473	
QLI72677.1	SP(Sec/SPI)	0.929945	0.070055	CS pos: 19-20. AAA-TF. Pr: 0.5903
QLI72678.1	OTHER	0.001425	0.998575	
QLI72679.1	OTHER	0.001299	0.998701	
QLI72680.1	SP(Sec/SPI)	0.997462	0.002538	CS pos: 18-19. SIA-AP. Pr: 0.6079
QLI72681.1	OTHER	0.002577	0.997423	
QLI72682.1	OTHER	0.000690	0.999310	
QLI72683.1	OTHER	0.000776	0.999224	
QLI72684.1	OTHER	0.000310	0.999690	
QLI72685.1	OTHER	0.005617	0.994383	
QLI72686.1	OTHER	0.004445	0.995555	
QLI72687.1	OTHER	0.000732	0.999268	
QLI72688.1	OTHER	0.004150	0.995850	

QLI72689.1	OTHER	0.004508	0.995492	
QLI72690.1	OTHER	0.000948	0.999052	
QLI72691.1	OTHER	0.001094	0.998906	
QLI72692.1	OTHER	0.001323	0.998677	
QLI72693.1	OTHER	0.001311	0.998689	
QLI72694.1	OTHER	0.002108	0.997892	
QLI72695.1	OTHER	0.000482	0.999518	
QLI72696.1	OTHER	0.001906	0.998094	
QLI72697.1	OTHER	0.001282	0.998718	
QLI72698.1	OTHER	0.001030	0.998970	
QLI72699.1	OTHER	0.018210	0.981790	
QLI72700.1	OTHER	0.000277	0.999723	
QLI72701.1	SP(Sec/SPI)	0.996873	0.003127	CS pos: 17-18. ASA-AP. Pr: 0.8589
QLI72702.1	OTHER	0.001648	0.998352	
QLI72703.1	OTHER	0.000548	0.999452	
QLI72704.1	OTHER	0.000974	0.999026	
QLI72705.1	OTHER	0.000482	0.999518	
QLI72706.1	OTHER	0.002753	0.997247	
QLI72707.1	SP(Sec/SPI)	0.933868	0.066132	CS pos: 24-25. FAA-GP. Pr: 0.1897
QLI72708.1	OTHER	0.000758	0.999242	
QLI72709.1	SP(Sec/SPI)	0.585071	0.414929	CS pos: 31-32. GDD-AP. Pr: 0.1959
QLI72710.1	OTHER	0.000605	0.999395	
QLI72711.1	OTHER	0.005609	0.994391	
QLI72712.1	OTHER	0.003508	0.996492	
QLI72713.1	OTHER	0.000985	0.999015	
QLI72714.1	OTHER	0.002829	0.997171	
QLI72715.1	OTHER	0.000636	0.999364	
QLI72716.1	OTHER	0.000566	0.999434	
QLI72717.1	SP(Sec/SPI)	0.998560	0.001440	CS pos: 16-17. GSA-GT. Pr: 0.4779
QLI72718.1	OTHER	0.000970	0.999030	
QLI72719.1	OTHER	0.008373	0.991627	

QLI72720.1	SP(Sec/SPI)	0.919557	0.080443	CS pos: 21-22. AAA-CP. Pr: 0.4281
QLI72721.1	OTHER	0.000278	0.999722	
QLI72722.1	OTHER	0.000542	0.999458	
QLI72723.1	OTHER	0.000603	0.999397	
QLI72724.1	OTHER	0.003777	0.996223	
QLI72725.1	OTHER	0.000933	0.999067	
QLI72726.1	SP(Sec/SPI)	0.659039	0.340961	CS pos: 28-29. LKS-SV. Pr: 0.1620
QLI72727.1	OTHER	0.000891	0.999109	
QLI72728.1	OTHER	0.002252	0.997748	
QLI72729.1	OTHER	0.001143	0.998857	
QLI72730.1	OTHER	0.002095	0.997905	
QLI72731.1	OTHER	0.000355	0.999645	
QLI72732.1	OTHER	0.001503	0.998497	
QLI72733.1	OTHER	0.001180	0.998820	
QLI72734.1	OTHER	0.004362	0.995638	
QLI72735.1	OTHER	0.000610	0.999390	
QLI72736.1	OTHER	0.000755	0.999245	
QLI72737.1	OTHER	0.001191	0.998809	
QLI72738.1	OTHER	0.003732	0.996268	
QLI72739.1	OTHER	0.002869	0.997131	
QLI72740.1	OTHER	0.016932	0.983068	
QLI72741.1	SP(Sec/SPI)	0.990288	0.009712	CS pos: 15-16. VSA-AP. Pr: 0.7553
QLI72742.1	OTHER	0.000618	0.999382	
QLI72743.1	OTHER	0.000241	0.999759	
QLI72744.1	OTHER	0.003000	0.997000	
QLI72745.1	OTHER	0.000962	0.999038	
QLI72746.1	OTHER	0.001018	0.998982	
QLI72747.1	OTHER	0.005462	0.994538	
QLI72748.1	OTHER	0.001018	0.998982	
QLI72749.1	OTHER	0.000829	0.999171	
QLI72750.1	OTHER	0.001212	0.998788	

QLI72751.1	SP(Sec/SPI)	0.994707	0.005293	CS pos: 19-20. AAA-VA. Pr: 0.3454
QLI72752.1	OTHER	0.001795	0.998205	
QLI72753.1	OTHER	0.000320	0.999680	
QLI72754.1	OTHER	0.004032	0.995968	
QLI72755.1	OTHER	0.026732	0.973268	
QLI72756.1	OTHER	0.001930	0.998070	
QLI72757.1	OTHER	0.000800	0.999200	
QLI72758.1	OTHER	0.000810	0.999190	
QLI72759.1	OTHER	0.011398	0.988602	
QLI72760.1	OTHER	0.000197	0.999803	
QLI72761.1	OTHER	0.000461	0.999539	
QLI72762.1	OTHER	0.001163	0.998837	
QLI72763.1	OTHER	0.009573	0.990427	
QLI72764.1	OTHER	0.005061	0.994939	
QLI72765.1	OTHER	0.000577	0.999423	
QLI72766.1	OTHER	0.000657	0.999343	
QLI72767.1	OTHER	0.001317	0.998683	
QLI72768.1	OTHER	0.001766	0.998234	
QLI72769.1	OTHER	0.001605	0.998395	
QLI72770.1	OTHER	0.020049	0.979951	
QLI72771.1	OTHER	0.000297	0.999703	
QLI72772.1	OTHER	0.001460	0.998540	
QLI72773.1	OTHER	0.001229	0.998771	
QLI72774.1	OTHER	0.002144	0.997856	
QLI72775.1	OTHER	0.000898	0.999102	
QLI72776.1	OTHER	0.000513	0.999487	
QLI72777.1	OTHER	0.118758	0.881242	
QLI72778.1	OTHER	0.000973	0.999027	
QLI72779.1	OTHER	0.028635	0.971365	
QLI72780.1	OTHER	0.001133	0.998867	
QLI72781.1	OTHER	0.001900	0.998100	

QLI72782.1	OTHER	0.001655	0.998345	
QLI72783.1	SP(Sec/SPI)	0.987960	0.012040	CS pos: 19-20. TSA-AE. Pr: 0.5671
QLI72784.1	OTHER	0.000315	0.999685	
QLI72785.1	OTHER	0.000629	0.999371	
QLI72786.1	OTHER	0.002815	0.997185	
QLI72787.1	OTHER	0.004377	0.995623	
QLI72788.1	OTHER	0.000333	0.999667	
QLI72789.1	OTHER	0.000606	0.999394	
QLI72790.1	OTHER	0.000637	0.999363	
QLI72791.1	OTHER	0.025157	0.974843	
QLI72792.1	OTHER	0.000885	0.999115	
QLI72793.1	OTHER	0.000505	0.999495	
QLI72794.1	OTHER	0.000519	0.999481	
QLI72795.1	OTHER	0.005522	0.994478	
QLI72796.1	OTHER	0.000865	0.999135	
QLI72797.1	OTHER	0.000482	0.999518	
QLI72798.1	OTHER	0.001188	0.998812	
QLI72799.1	OTHER	0.001844	0.998156	
QLI72800.1	OTHER	0.000360	0.999640	
QLI72801.1	SP(Sec/SPI)	0.866378	0.133622	CS pos: 19-20. TVA-IP. Pr: 0.6013
QLI72802.1	SP(Sec/SPI)	0.998894	0.001106	CS pos: 16-17. ATA-AE. Pr: 0.6850
QLI72803.1	OTHER	0.000651	0.999349	
QLI72804.1	OTHER	0.000526	0.999474	
QLI72805.1	OTHER	0.000857	0.999143	
QLI72806.1	OTHER	0.002637	0.997363	
QLI72807.1	OTHER	0.186779	0.813221	
QLI72808.1	OTHER	0.014710	0.985290	
QLI72809.1	OTHER	0.001235	0.998765	
QLI72810.1	OTHER	0.000684	0.999316	
QLI72811.1	OTHER	0.000209	0.999791	
QLI72812.1	OTHER	0.001183	0.998817	

QLI72813.1	OTHER	0.003929	0.996071	
QLI72814.1	OTHER	0.008137	0.991863	
QLI72815.1	OTHER	0.000479	0.999521	
QLI72816.1	OTHER	0.000878	0.999122	
QLI72817.1	OTHER	0.000411	0.999589	
QLI72818.1	OTHER	0.000463	0.999537	
QLI72819.1	OTHER	0.000986	0.999014	
QLI72820.1	OTHER	0.000485	0.999515	
QLI72821.1	OTHER	0.000291	0.999709	
QLI72822.1	OTHER	0.055138	0.944862	
QLI72823.1	OTHER	0.001114	0.998886	
QLI72824.1	OTHER	0.151435	0.848565	
QLI72825.1	OTHER	0.000327	0.999673	
QLI72826.1	OTHER	0.000604	0.999396	
QLI72827.1	OTHER	0.000799	0.999201	
QLI72828.1	OTHER	0.000936	0.999064	
QLI72829.1	OTHER	0.215187	0.784813	
QLI72830.1	OTHER	0.000906	0.999094	
QLI72831.1	OTHER	0.002993	0.997007	
QLI72832.1	OTHER	0.001292	0.998708	
QLI72833.1	SP(Sec/SPI)	0.952631	0.047369	CS pos: 18-19. ALA-VP. Pr: 0.6155
QLI72834.1	OTHER	0.000533	0.999467	
QLI72835.1	OTHER	0.001218	0.998782	
QLI72836.1	OTHER	0.001698	0.998302	
QLI72837.1	OTHER	0.003352	0.996648	
QLI72838.1	OTHER	0.000689	0.999311	
QLI72839.1	OTHER	0.417570	0.582430	
QLI72840.1	OTHER	0.000642	0.999358	
QLI72841.1	OTHER	0.044722	0.955278	
QLI72842.1	SP(Sec/SPI)	0.980010	0.019990	CS pos: 23-24. AFA-LK. Pr: 0.9192
QLI72843.1	OTHER	0.001627	0.998373	

QLI72844.1	OTHER	0.000488	0.999512	
QLI72845.1	OTHER	0.000703	0.999297	
QLI72846.1	OTHER	0.000957	0.999043	
QLI72847.1	OTHER	0.000869	0.999131	
QLI72848.1	OTHER	0.000665	0.999335	
QLI72849.1	OTHER	0.002668	0.997332	
QLI72850.1	OTHER	0.000847	0.999153	
QLI72851.1	OTHER	0.001217	0.998783	
QLI72852.1	OTHER	0.001319	0.998681	
QLI72853.1	OTHER	0.002734	0.997266	
QLI72854.1	OTHER	0.000914	0.999086	
QLI72855.1	OTHER	0.001294	0.998706	
QLI72856.1	OTHER	0.000806	0.999194	
QLI72857.1	OTHER	0.003422	0.996578	
QLI72858.1	OTHER	0.001962	0.998038	
QLI72859.1	SP(Sec/SPI)	0.952772	0.047228	CS pos: 17-18. VLA-AP. Pr: 0.6079
QLI72860.1	OTHER	0.000476	0.999524	
QLI72861.1	OTHER	0.000347	0.999653	
QLI72862.1	OTHER	0.187165	0.812835	
QLI72863.1	OTHER	0.000766	0.999234	
QLI72864.1	SP(Sec/SPI)	0.999054	0.000946	CS pos: 20-21. AAA-FG. Pr: 0.7033
QLI72865.1	OTHER	0.001411	0.998589	
QLI72866.1	SP(Sec/SPI)	0.914769	0.085231	CS pos: 27-28. VMG-GQ. Pr: 0.8268
QLI72867.1	OTHER	0.001129	0.998871	
QLI72868.1	OTHER	0.092673	0.907327	
QLI72869.1	OTHER	0.001962	0.998038	
QLI72870.1	OTHER	0.002150	0.997850	
QLI72871.1	OTHER	0.001601	0.998399	
QLI72872.1	OTHER	0.001779	0.998221	
QLI72873.1	OTHER	0.000610	0.999390	
QLI72874.1	SP(Sec/SPI)	0.754806	0.245194	CS pos: 35-36. SEA-IH. Pr: 0.4784

QLI72875.1	OTHER	0.002384	0.997616	
QLI72876.1	OTHER	0.001170	0.998830	
QLI72877.1	OTHER	0.003148	0.996852	
QLI72878.1	SP(Sec/SPI)	0.989884	0.010116	CS pos: 25-26. VLA-KD. Pr: 0.9414
QLI72879.1	OTHER	0.000990	0.999010	
QLI72880.1	OTHER	0.007819	0.992181	
QLI72881.1	OTHER	0.000706	0.999294	
QLI72882.1	OTHER	0.002878	0.997122	
QLI72883.1	OTHER	0.000154	0.999846	
QLI72884.1	OTHER	0.219144	0.780856	
QLI72885.1	OTHER	0.001605	0.998395	
QLI72886.1	OTHER	0.000896	0.999104	
QLI72887.1	OTHER	0.000675	0.999325	
QLI72888.1	OTHER	0.000976	0.999024	
QLI72889.1	OTHER	0.002951	0.997049	
QLI72890.1	OTHER	0.001309	0.998691	
QLI72891.1	OTHER	0.002570	0.997430	
QLI72892.1	OTHER	0.184429	0.815571	
QLI72893.1	OTHER	0.000499	0.999501	
QLI72894.1	OTHER	0.001909	0.998091	
QLI72895.1	OTHER	0.000674	0.999326	
QLI72896.1	OTHER	0.001969	0.998031	
QLI72897.1	OTHER	0.000513	0.999487	
QLI72898.1	OTHER	0.011732	0.988268	
QLI72899.1	SP(Sec/SPI)	0.997126	0.002874	CS pos: 20-21. VSA-AP. Pr: 0.9582
QLI72900.1	OTHER	0.002214	0.997786	
QLI72901.1	OTHER	0.001026	0.998974	
QLI72902.1	SP(Sec/SPI)	0.991677	0.008323	CS pos: 19-20. VNA-AP. Pr: 0.9408
QLI72903.1	OTHER	0.000793	0.999207	
QLI72904.1	OTHER	0.002005	0.997995	
QLI72905.1	OTHER	0.000890	0.999110	

QLI72906.1	OTHER	0.000855	0.999145	
QLI72907.1	OTHER	0.004044	0.995956	
QLI72908.1	OTHER	0.000680	0.999320	
QLI72909.1	OTHER	0.001265	0.998735	
QLI72910.1	OTHER	0.000641	0.999359	
QLI72911.1	OTHER	0.000865	0.999135	
QLI72912.1	OTHER	0.001515	0.998485	
QLI72913.1	OTHER	0.001918	0.998082	
QLI72914.1	SP(Sec/SPI)	0.994315	0.005685	CS pos: 20-21. ATA-AT. Pr: 0.7610
QLI72915.1	OTHER	0.128860	0.871140	
QLI72916.1	SP(Sec/SPI)	0.804938	0.195062	CS pos: 19-20. SLA-TT. Pr: 0.5652
QLI72917.1	OTHER	0.001275	0.998725	
QLI72918.1	OTHER	0.000580	0.999420	
QLI72919.1	OTHER	0.005423	0.994577	
QLI72920.1	OTHER	0.001302	0.998698	
QLI72921.1	OTHER	0.000249	0.999751	
QLI72922.1	OTHER	0.000558	0.999442	
QLI72923.1	SP(Sec/SPI)	0.938637	0.061363	CS pos: 19-20. TIA-QS. Pr: 0.4958
QLI72924.1	OTHER	0.000749	0.999251	
QLI72925.1	OTHER	0.001069	0.998931	
QLI72926.1	OTHER	0.002007	0.997993	
QLI72927.1	OTHER	0.002846	0.997154	
QLI72928.1	OTHER	0.001084	0.998916	
QLI72929.1	OTHER	0.000359	0.999641	
QLI72930.1	OTHER	0.001178	0.998822	
QLI72931.1	OTHER	0.003446	0.996554	
QLI72932.1	OTHER	0.000365	0.999635	
QLI72933.1	OTHER	0.002967	0.997033	
QLI72934.1	OTHER	0.001228	0.998772	
QLI72935.1	OTHER	0.000243	0.999757	
QLI72936.1	OTHER	0.007385	0.992615	

QLI72937.1	OTHER	0.000608	0.999392	
QLI72938.1	OTHER	0.001642	0.998358	
QLI72939.1	OTHER	0.002324	0.997676	
QLI72940.1	OTHER	0.001037	0.998963	
QLI72941.1	SP(Sec/SPI)	0.979029	0.020971	CS pos: 15-16. ALA-IP. Pr: 0.4756
QLI72942.1	OTHER	0.005042	0.994958	
QLI72943.1	OTHER	0.003501	0.996499	
QLI72944.1	OTHER	0.001830	0.998170	
QLI72945.1	OTHER	0.002122	0.997878	
QLI72946.1	OTHER	0.001179	0.998821	
QLI72947.1	OTHER	0.003661	0.996339	
QLI72948.1	OTHER	0.002366	0.997634	
QLI72949.1	OTHER	0.001255	0.998745	
QLI72950.1	SP(Sec/SPI)	0.994861	0.005139	CS pos: 23-24. AQA-NG. Pr: 0.4931
QLI72951.1	OTHER	0.001109	0.998891	
QLI72952.1	OTHER	0.002000	0.998000	
QLI72953.1	OTHER	0.000490	0.999510	
QLI72954.1	OTHER	0.000694	0.999306	
QLI72955.1	OTHER	0.002640	0.997360	
QLI72956.1	SP(Sec/SPI)	0.999321	0.000679	CS pos: 19-20. ALA-DP. Pr: 0.9857
QLI72957.1	OTHER	0.001184	0.998816	
QLI72958.1	OTHER	0.003711	0.996289	
QLI72959.1	SP(Sec/SPI)	0.996554	0.003446	CS pos: 16-17. VGA-LP. Pr: 0.4112
QLI72960.1	SP(Sec/SPI)	0.995428	0.004572	CS pos: 20-21. TSA-VT. Pr: 0.8847
QLI72961.1	OTHER	0.003658	0.996342	
QLI72962.1	OTHER	0.000709	0.999291	
QLI72963.1	OTHER	0.000530	0.999470	
QLI72964.1	OTHER	0.000704	0.999296	
QLI72965.1	OTHER	0.000593	0.999407	
QLI72966.1	OTHER	0.000193	0.999807	
QLI72967.1	OTHER	0.000467	0.999533	

QLI72968.1	OTHER	0.000465	0.999535	
QLI72969.1	SP(Sec/SPI)	0.989643	0.010357	CS pos: 19-20. SFA-AP. Pr: 0.8728
QLI72970.1	OTHER	0.002318	0.997682	
QLI72971.1	OTHER	0.000580	0.999420	
QLI72972.1	SP(Sec/SPI)	0.997042	0.002958	CS pos: 20-21. ASA-HP. Pr: 0.9324
QLI72973.1	OTHER	0.000824	0.999176	
QLI72974.1	OTHER	0.013939	0.986061	
QLI72975.1	OTHER	0.000838	0.999162	
QLI72976.1	SP(Sec/SPI)	0.786475	0.213525	CS pos: 29-30. SFA-AS. Pr: 0.5378
QLI72977.1	OTHER	0.002107	0.997893	
QLI72978.1	OTHER	0.000673	0.999327	
QLI72979.1	OTHER	0.000717	0.999283	
QLI72980.1	OTHER	0.001598	0.998402	
QLI72981.1	OTHER	0.000189	0.999811	
QLI72982.1	OTHER	0.000637	0.999363	
QLI72983.1	OTHER	0.011564	0.988436	
QLI72984.1	OTHER	0.001351	0.998649	
QLI72985.1	OTHER	0.001299	0.998701	
QLI72986.1	OTHER	0.003437	0.996563	
QLI72987.1	OTHER	0.384562	0.615438	
QLI72988.1	OTHER	0.000814	0.999186	
QLI72989.1	OTHER	0.002837	0.997163	
QLI72990.1	OTHER	0.000492	0.999508	
QLI72991.1	OTHER	0.001422	0.998578	
QLI72992.1	SP(Sec/SPI)	0.990518	0.009482	CS pos: 17-18. ALA-AP. Pr: 0.8548
QLI72993.1	OTHER	0.002081	0.997919	
QLI72994.1	OTHER	0.001604	0.998396	
QLI72995.1	OTHER	0.001688	0.998312	
QLI72996.1	OTHER	0.017049	0.982951	
QLI72997.1	OTHER	0.423555	0.576445	
QLI72998.1	SP(Sec/SPI)	0.982937	0.017063	CS pos: 22-23. ALS-AR. Pr: 0.4710

QLI72999.1	OTHER	0.001928	0.998072	
QLI73000.1	OTHER	0.000610	0.999390	
QLI73001.1	OTHER	0.000421	0.999579	
QLI73002.1	SP(Sec/SPI)	0.907737	0.092263	CS pos: 18-19. VAG-AP. Pr: 0.7410
QLI73003.1	OTHER	0.000504	0.999496	
QLI73004.1	OTHER	0.001880	0.998120	
QLI73005.1	SP(Sec/SPI)	0.922819	0.077181	CS pos: 20-21. ARA-AP. Pr: 0.8298
QLI73006.1	OTHER	0.001267	0.998733	
QLI73007.1	OTHER	0.002072	0.997928	
QLI73008.1	OTHER	0.000329	0.999671	
QLI73009.1	OTHER	0.000453	0.999547	
QLI73010.1	OTHER	0.001308	0.998692	
QLI73011.1	OTHER	0.001203	0.998797	
QLI73012.1	OTHER	0.001676	0.998324	
QLI73013.1	OTHER	0.117798	0.882202	
QLI73014.1	SP(Sec/SPI)	0.952851	0.047149	CS pos: 19-20. ALG-KP. Pr: 0.8876
QLI73015.1	OTHER	0.028134	0.971866	
QLI73016.1	OTHER	0.000182	0.999818	
QLI73017.1	OTHER	0.000683	0.999317	
QLI73018.1	OTHER	0.001208	0.998792	
QLI73019.1	OTHER	0.001549	0.998451	
QLI73020.1	SP(Sec/SPI)	0.990092	0.009908	CS pos: 16-17. ALA-TP. Pr: 0.4449
QLI73021.1	OTHER	0.000405	0.999595	
QLI73022.1	OTHER	0.003362	0.996638	
QLI73023.1	OTHER	0.000970	0.999030	
QLI73024.1	OTHER	0.000811	0.999189	
QLI73025.1	OTHER	0.000706	0.999294	
QLI73026.1	OTHER	0.000614	0.999386	
QLI73027.1	OTHER	0.003798	0.996202	
QLI73028.1	OTHER	0.002867	0.997133	
QLI73029.1	OTHER	0.000767	0.999233	

QLI73030.1	OTHER	0.001823	0.998177	
QLI73031.1	OTHER	0.063203	0.936797	
QLI73032.1	OTHER	0.004655	0.995345	
QLI73033.1	OTHER	0.003280	0.996720	
QLI73034.1	OTHER	0.010261	0.989739	
QLI73035.1	OTHER	0.001676	0.998324	
QLI73036.1	OTHER	0.001287	0.998713	
QLI73037.1	SP(Sec/SPI)	0.936104	0.063896	CS pos: 18-19. TAA-TE. Pr: 0.3548
QLI73038.1	OTHER	0.294249	0.705751	
QLI73039.1	OTHER	0.002810	0.997190	
QLI73040.1	OTHER	0.000628	0.999372	
QLI73041.1	OTHER	0.014771	0.985229	
QLI73042.1	OTHER	0.003562	0.996438	
QLI73043.1	OTHER	0.000979	0.999021	
QLI73044.1	OTHER	0.000921	0.999079	
QLI73045.1	OTHER	0.001798	0.998202	
QLI73046.1	SP(Sec/SPI)	0.714732	0.285268	CS pos: 27-28. AAA-IT. Pr: 0.5532
QLI73047.1	OTHER	0.000852	0.999148	
QLI73048.1	OTHER	0.012041	0.987959	
QLI73049.1	OTHER	0.001806	0.998194	
QLI73050.1	OTHER	0.000361	0.999639	
QLI73051.1	OTHER	0.000292	0.999708	
QLI73052.1	OTHER	0.000244	0.999756	
QLI73053.1	OTHER	0.000847	0.999153	
QLI73054.1	OTHER	0.002190	0.997810	
QLI73055.1	OTHER	0.009327	0.990673	
QLI73056.1	OTHER	0.006432	0.993568	
QLI73057.1	OTHER	0.003496	0.996504	
QLI73058.1	OTHER	0.002623	0.997377	
QLI73059.1	SP(Sec/SPI)	0.997653	0.002347	CS pos: 19-20. SIA-DH. Pr: 0.8882
QLI73060.1	OTHER	0.003258	0.996742	

QLI73061.1	OTHER	0.000722	0.999278	
QLI73062.1	SP(Sec/SPI)	0.641883	0.358117	CS pos: 33-34. VAA-GP. Pr: 0.3949
QLI73063.1	OTHER	0.003352	0.996648	
QLI73064.1	OTHER	0.003227	0.996773	
QLI73065.1	OTHER	0.000188	0.999812	
QLI73066.1	OTHER	0.012400	0.987600	
QLI73067.1	OTHER	0.003942	0.996058	
QLI73068.1	OTHER	0.067713	0.932287	
QLI73069.1	SP(Sec/SPI)	0.589029	0.410971	CS pos: 16-17. AAA-IN. Pr: 0.2538
QLI73070.1	OTHER	0.002345	0.997655	
QLI73071.1	OTHER	0.000632	0.999368	
QLI73072.1	OTHER	0.000580	0.999420	
QLI73073.1	OTHER	0.001349	0.998651	
QLI73074.1	OTHER	0.007042	0.992958	
QLI73075.1	SP(Sec/SPI)	0.991247	0.008753	CS pos: 18-19. AVA-QS. Pr: 0.3763
QLI73076.1	OTHER	0.001083	0.998917	
QLI73077.1	OTHER	0.000892	0.999108	
QLI73078.1	OTHER	0.001629	0.998371	
QLI73079.1	OTHER	0.001457	0.998543	
QLI73080.1	SP(Sec/SPI)	0.953294	0.046706	CS pos: 22-23. VNT-DT. Pr: 0.5938
QLI73081.1	OTHER	0.001422	0.998578	
QLI73082.1	SP(Sec/SPI)	0.996888	0.003112	CS pos: 20-21. ASA-DD. Pr: 0.9543
QLI73083.1	OTHER	0.001790	0.998210	
QLI73084.1	OTHER	0.000496	0.999504	
QLI73085.1	OTHER	0.000253	0.999747	
QLI73086.1	OTHER	0.011163	0.988837	
QLI73087.1	OTHER	0.001440	0.998560	
QLI73088.1	OTHER	0.000533	0.999467	
QLI73089.1	OTHER	0.000699	0.999301	
QLI73090.1	OTHER	0.030489	0.969511	
QLI73091.1	OTHER	0.002052	0.997948	

QLI73092.1	OTHER	0.001106	0.998894	
QLI73093.1	OTHER	0.001511	0.998489	
QLI73094.1	OTHER	0.000759	0.999241	
QLI73095.1	OTHER	0.001479	0.998521	
QLI73096.1	OTHER	0.000776	0.999224	
QLI73097.1	OTHER	0.037633	0.962367	
QLI73098.1	OTHER	0.001958	0.998042	
QLI73099.1	OTHER	0.000913	0.999087	
QLI73100.1	OTHER	0.002231	0.997769	
QLI73101.1	OTHER	0.001053	0.998947	
QLI73102.1	OTHER	0.000715	0.999285	
QLI73103.1	OTHER	0.002290	0.997710	
QLI73104.1	OTHER	0.000720	0.999280	
QLI73105.1	OTHER	0.000779	0.999221	
QLI73106.1	SP(Sec/SPI)	0.996203	0.003797	CS pos: 20-21. ILA-DR. Pr: 0.9712
QLI73107.1	OTHER	0.002576	0.997424	
QLI73108.1	OTHER	0.001626	0.998374	
QLI73109.1	OTHER	0.001086	0.998914	
QLI73110.1	OTHER	0.000720	0.999280	
QLI73111.1	OTHER	0.002068	0.997932	
QLI73112.1	OTHER	0.002837	0.997163	
QLI73113.1	OTHER	0.102874	0.897126	
QLI73114.1	SP(Sec/SPI)	0.998239	0.001761	CS pos: 17-18. AHA-QT. Pr: 0.9215
QLI73115.1	OTHER	0.061641	0.938359	
QLI73116.1	OTHER	0.005586	0.994414	
QLI73117.1	OTHER	0.001242	0.998758	
QLI73118.1	OTHER	0.032023	0.967977	
QLI73119.1	OTHER	0.000349	0.999651	
QLI73120.1	OTHER	0.018357	0.981643	
QLI73121.1	OTHER	0.002351	0.997649	
QLI73122.1	OTHER	0.019251	0.980749	

QLI73123.1	OTHER	0.002762	0.997238
QLI73124.1	OTHER	0.000671	0.999329
QLI73125.1	OTHER	0.003200	0.996800
QLI73126.1	OTHER	0.000763	0.999237
QLI73127.1	OTHER	0.000810	0.999190
QLI73128.1	OTHER	0.000793	0.999207
QLI73129.1	OTHER	0.000457	0.999543
QLI73130.1	OTHER	0.001918	0.998082
QLI73131.1	OTHER	0.002909	0.997091
QLI73132.1	OTHER	0.000833	0.999167
QLI73133.1	OTHER	0.000161	0.999839
QLI73134.1	OTHER	0.001391	0.998609
QLI73135.1	OTHER	0.001417	0.998583
QLI73136.1	OTHER	0.000394	0.999606
QLI73137.1	OTHER	0.001922	0.998078
QLI73138.1	OTHER	0.000860	0.999140
QLI73139.1	OTHER	0.000671	0.999329
QLI73140.1	OTHER	0.003815	0.996185
QLI73141.1	OTHER	0.001642	0.998358
QLI73142.1	OTHER	0.000986	0.999014
QLI73143.1	OTHER	0.000502	0.999498
QLI73144.1	OTHER	0.001103	0.998897
QLI73145.1	OTHER	0.000839	0.999161
QLI73146.1	OTHER	0.000512	0.999488
QLI73147.1	OTHER	0.001832	0.998168
QLI73148.1	OTHER	0.001792	0.998208
QLI73149.1	OTHER	0.002234	0.997766
QLI73150.1	OTHER	0.007646	0.992354
QLI73151.1	OTHER	0.001611	0.998389
QLI73152.1	OTHER	0.000322	0.999678
QLI73153.1	OTHER	0.000526	0.999474

QLI73154.1	SP(Sec/SPI)	0.988687	0.011313	CS pos: 22-23. AKA-DK. Pr: 0.6556
QLI73155.1	OTHER	0.000189	0.999811	
QLI73156.1	SP(Sec/SPI)	0.991558	0.008442	CS pos: 15-16. AQA-SP. Pr: 0.5936
QLI73157.1	OTHER	0.001127	0.998873	
QLI73158.1	OTHER	0.006320	0.993680	
QLI73159.1	OTHER	0.011647	0.988353	
QLI73160.1	OTHER	0.004713	0.995287	
QLI73161.1	OTHER	0.001137	0.998863	
QLI73162.1	OTHER	0.000843	0.999157	
QLI73163.1	OTHER	0.003271	0.996729	
QLI73164.1	OTHER	0.001824	0.998176	
QLI73165.1	OTHER	0.449576	0.550424	
QLI73166.1	OTHER	0.254142	0.745858	
QLI73167.1	OTHER	0.004386	0.995614	
QLI73168.1	OTHER	0.017850	0.982150	
QLI73169.1	OTHER	0.001871	0.998129	
QLI73170.1	OTHER	0.001550	0.998450	
QLI73171.1	OTHER	0.004928	0.995072	
QLI73172.1	OTHER	0.001794	0.998206	
QLI73173.1	OTHER	0.000553	0.999447	
QLI73174.1	OTHER	0.001626	0.998374	
QLI73175.1	OTHER	0.003665	0.996335	
QLI73176.1	OTHER	0.001601	0.998399	
QLI73177.1	OTHER	0.003672	0.996328	
QLI73178.1	OTHER	0.001186	0.998814	
QLI73179.1	OTHER	0.002511	0.997489	
QLI73180.1	OTHER	0.000473	0.999527	
QLI73181.1	OTHER	0.001425	0.998575	
QLI73182.1	OTHER	0.012567	0.987433	
QLI73183.1	OTHER	0.002167	0.997833	
QLI73184.1	OTHER	0.003268	0.996732	

QLI73185.1	OTHER	0.000769	0.999231	
QLI73186.1	OTHER	0.001962	0.998038	
QLI73187.1	OTHER	0.001549	0.998451	
QLI73188.1	OTHER	0.002427	0.997573	
QLI73189.1	SP(Sec/SPI)	0.980924	0.019076	CS pos: 21-22. ATA-TL. Pr: 0.5570
QLI73190.1	OTHER	0.033760	0.966240	
QLI73191.1	OTHER	0.001520	0.998480	
QLI73192.1	OTHER	0.000669	0.999331	
QLI73193.1	OTHER	0.011626	0.988374	
QLI73194.1	OTHER	0.006329	0.993671	
QLI73195.1	OTHER	0.003082	0.996918	
QLI73196.1	OTHER	0.039379	0.960621	
QLI73197.1	OTHER	0.002716	0.997284	
QLI73198.1	SP(Sec/SPI)	0.999399	0.000601	CS pos: 17-18. AAA-QQ. Pr: 0.7275
QLI73199.1	OTHER	0.003259	0.996741	
QLI73200.1	OTHER	0.001957	0.998043	
QLI73201.1	OTHER	0.003617	0.996383	
QLI73202.1	OTHER	0.002136	0.997864	
QLI73203.1	OTHER	0.005276	0.994724	
QLI73204.1	OTHER	0.000738	0.999262	
QLI73205.1	OTHER	0.001096	0.998904	
QLI73206.1	OTHER	0.001694	0.998306	
QLI73207.1	SP(Sec/SPI)	0.703287	0.296713	CS pos: 17-18. AGG-MH. Pr: 0.4982
QLI73208.1	OTHER	0.000589	0.999411	
QLI73209.1	OTHER	0.000944	0.999056	
QLI73210.1	OTHER	0.000536	0.999464	
QLI73211.1	OTHER	0.000474	0.999526	
QLI73212.1	OTHER	0.000363	0.999637	
QLI73213.1	OTHER	0.001913	0.998087	
QLI73214.1	OTHER	0.004161	0.995839	
QLI73215.1	OTHER	0.000848	0.999152	

QLI73216.1	OTHER	0.001105	0.998895	
QLI73217.1	OTHER	0.000884	0.999116	
QLI73218.1	OTHER	0.001522	0.998478	
QLI73219.1	OTHER	0.000847	0.999153	
QLI73220.1	OTHER	0.000681	0.999319	
QLI73221.1	OTHER	0.001044	0.998956	
QLI73222.1	OTHER	0.002453	0.997547	
QLI73223.1	SP(Sec/SPI)	0.897028	0.102972	CS pos: 19-20. AHS-SQ. Pr: 0.8163
QLI73224.1	OTHER	0.000910	0.999090	
QLI73225.1	OTHER	0.002308	0.997692	
QLI73226.1	OTHER	0.000861	0.999139	
QLI73227.1	OTHER	0.002179	0.997821	
QLI73228.1	SP(Sec/SPI)	0.995904	0.004096	CS pos: 20-21. AMA-AP. Pr: 0.7773
QLI73229.1	OTHER	0.000247	0.999753	
QLI73230.1	OTHER	0.000670	0.999330	
QLI73231.1	OTHER	0.001203	0.998797	
QLI73232.1	OTHER	0.000658	0.999342	
QLI73233.1	OTHER	0.000879	0.999121	
QLI73234.1	SP(Sec/SPI)	0.914807	0.085193	CS pos: 23-24. VSA-HN. Pr: 0.8142
QLI73235.1	OTHER	0.000911	0.999089	
QLI73236.1	OTHER	0.001818	0.998182	
QLI73237.1	SP(Sec/SPI)	0.846051	0.153949	CS pos: 22-23. VLA-VV. Pr: 0.5978
QLI73238.1	OTHER	0.000344	0.999656	
QLI73239.1	OTHER	0.002021	0.997979	
QLI73240.1	OTHER	0.001108	0.998892	
QLI73241.1	OTHER	0.001422	0.998578	
QLI73242.1	OTHER	0.001342	0.998658	
QLI73243.1	OTHER	0.000829	0.999171	
QLI73244.1	OTHER	0.369602	0.630398	
QLI73245.1	OTHER	0.001596	0.998404	
QLI73246.1	OTHER	0.001928	0.998072	

QLI73247.1	SP(Sec/SPI)	0.960837	0.039163	CS pos: 21-22. AAA-AT. Pr: 0.3548
QLI73248.1	OTHER	0.001795	0.998205	
QLI73249.1	OTHER	0.000552	0.999448	
QLI73250.1	OTHER	0.005328	0.994672	
QLI73251.1	OTHER	0.000283	0.999717	
QLI73252.1	OTHER	0.037257	0.962743	
QLI73253.1	OTHER	0.000862	0.999138	
QLI73254.1	OTHER	0.000649	0.999351	
QLI73255.1	OTHER	0.001447	0.998553	
QLI73256.1	OTHER	0.001614	0.998386	
QLI73257.1	OTHER	0.010687	0.989313	
QLI73258.1	OTHER	0.001987	0.998013	
QLI73259.1	OTHER	0.001072	0.998928	
QLI73260.1	SP(Sec/SPI)	0.719643	0.280357	CS pos: 19-20. AVA-IL. Pr: 0.2579
QLI73261.1	OTHER	0.001331	0.998669	
QLI73262.1	OTHER	0.018847	0.981153	
QLI73263.1	OTHER	0.002959	0.997041	
QLI73264.1	OTHER	0.000407	0.999593	
QLI73265.1	OTHER	0.004670	0.995330	
QLI73266.1	OTHER	0.055465	0.944535	
QLI73267.1	OTHER	0.001457	0.998543	
QLI73268.1	OTHER	0.246983	0.753017	
QLI73269.1	OTHER	0.001695	0.998305	
QLI73270.1	OTHER	0.000875	0.999125	
QLI73271.1	OTHER	0.010894	0.989106	
QLI73272.1	OTHER	0.000767	0.999233	
QLI73273.1	OTHER	0.004430	0.995570	
QLI73274.1	OTHER	0.001116	0.998884	
QLI73275.1	OTHER	0.001679	0.998321	
QLI73276.1	OTHER	0.001334	0.998666	
QLI73277.1	OTHER	0.000905	0.999095	

QLI73278.1	OTHER	0.005641	0.994359	
QLI73279.1	OTHER	0.002345	0.997655	
QLI73280.1	OTHER	0.000412	0.999588	
QLI73281.1	OTHER	0.000491	0.999509	
QLI73282.1	OTHER	0.000551	0.999449	
QLI73283.1	OTHER	0.001023	0.998977	
QLI73284.1	SP(Sec/SPI)	0.974293	0.025707	CS pos: 22-23. ATA-AS. Pr: 0.3916
QLI73285.1	OTHER	0.001845	0.998155	
QLI73286.1	SP(Sec/SPI)	0.997929	0.002071	CS pos: 19-20. GQA-AD. Pr: 0.7176
QLI73287.1	OTHER	0.000360	0.999640	
QLI73288.1	OTHER	0.000746	0.999254	
QLI73289.1	OTHER	0.001536	0.998464	
QLI73290.1	OTHER	0.000346	0.999654	
QLI73291.1	OTHER	0.000342	0.999658	
QLI73292.1	OTHER	0.001955	0.998045	
QLI73293.1	OTHER	0.000597	0.999403	
QLI73294.1	OTHER	0.000320	0.999680	
QLI73295.1	OTHER	0.000953	0.999047	
QLI73296.1	OTHER	0.001052	0.998948	
QLI73297.1	OTHER	0.001626	0.998374	
QLI73298.1	OTHER	0.001086	0.998914	
QLI73299.1	OTHER	0.003118	0.996882	
QLI73300.1	OTHER	0.001143	0.998857	
QLI73301.1	OTHER	0.000908	0.999092	
QLI73302.1	OTHER	0.001138	0.998862	
QLI73303.1	OTHER	0.001108	0.998892	
QLI73304.1	OTHER	0.006716	0.993284	
QLI73305.1	OTHER	0.001031	0.998969	
QLI73306.1	OTHER	0.000337	0.999663	
QLI73307.1	OTHER	0.001077	0.998923	
QLI73308.1	OTHER	0.001507	0.998493	

QLI73309.1	OTHER	0.002909	0.997091	
QLI73310.1	OTHER	0.002288	0.997712	
QLI73311.1	OTHER	0.003979	0.996021	
QLI73312.1	OTHER	0.000581	0.999419	
QLI73313.1	OTHER	0.005124	0.994876	
QLI73314.1	OTHER	0.017052	0.982948	
QLI73315.1	OTHER	0.001062	0.998938	
QLI73316.1	OTHER	0.002027	0.997973	
QLI73317.1	OTHER	0.000262	0.999738	
QLI73318.1	SP(Sec/SPI)	0.608038	0.391962	CS pos: 28-29. VVS-RE. Pr: 0.2519
QLI73319.1	OTHER	0.068812	0.931188	
QLI73320.1	OTHER	0.000690	0.999310	
QLI73321.1	OTHER	0.002076	0.997924	
QLI73322.1	SP(Sec/SPI)	0.987908	0.012092	CS pos: 16-17. VSA-AP. Pr: 0.6379
QLI73323.1	OTHER	0.001686	0.998314	
QLI73324.1	OTHER	0.003006	0.996994	
QLI73325.1	OTHER	0.000529	0.999471	
QLI73326.1	OTHER	0.001588	0.998412	
QLI73327.1	OTHER	0.000838	0.999162	
QLI73328.1	OTHER	0.000849	0.999151	
QLI73329.1	OTHER	0.001076	0.998924	
QLI73330.1	SP(Sec/SPI)	0.979042	0.020958	CS pos: 27-28. AEA-AK. Pr: 0.9528
QLI73331.1	OTHER	0.003473	0.996527	
QLI73332.1	OTHER	0.006573	0.993427	
QLI73333.1	OTHER	0.000448	0.999552	
QLI73334.1	OTHER	0.003638	0.996362	
QLI73335.1	OTHER	0.017806	0.982194	
QLI73336.1	OTHER	0.000843	0.999157	
QLI73337.1	OTHER	0.010517	0.989483	
QLI73338.1	OTHER	0.404958	0.595042	
QLI73339.1	OTHER	0.000845	0.999155	

QLI73340.1	OTHER	0.002499	0.997501	
QLI73341.1	OTHER	0.001773	0.998227	
QLI73342.1	OTHER	0.000713	0.999287	
QLI73343.1	OTHER	0.000768	0.999232	
QLI73344.1	OTHER	0.006639	0.993361	
QLI73345.1	OTHER	0.001147	0.998853	
QLI73346.1	OTHER	0.001180	0.998820	
QLI73347.1	OTHER	0.003789	0.996211	
QLI73348.1	OTHER	0.001194	0.998806	
QLI73349.1	OTHER	0.251851	0.748149	
QLI73350.1	OTHER	0.004080	0.995920	
QLI73351.1	OTHER	0.001281	0.998719	
QLI73352.1	OTHER	0.005131	0.994869	
QLI73353.1	OTHER	0.006382	0.993618	
QLI73354.1	OTHER	0.003557	0.996443	
QLI73355.1	OTHER	0.008481	0.991519	
QLI73356.1	SP(Sec/SPI)	0.975566	0.024434	CS pos: 24-25. AQS-SS. Pr: 0.3759
QLI73357.1	OTHER	0.003113	0.996887	
QLI73358.1	OTHER	0.002430	0.997570	
QLI73359.1	OTHER	0.004875	0.995125	
QLI73360.1	OTHER	0.000208	0.999792	
QLI73361.1	OTHER	0.000684	0.999316	
QLI73362.1	OTHER	0.001264	0.998736	
QLI73363.1	OTHER	0.000863	0.999137	
QLI73364.1	OTHER	0.000625	0.999375	
QLI73365.1	OTHER	0.065403	0.934597	
QLI73366.1	OTHER	0.005445	0.994555	
QLI73367.1	SP(Sec/SPI)	0.970386	0.029614	CS pos: 18-19. LNG-HV. Pr: 0.7668
QLI73368.1	OTHER	0.000512	0.999488	
QLI73369.1	OTHER	0.015168	0.984832	
QLI73370.1	OTHER	0.001183	0.998817	

QLI73371.1	OTHER	0.003303	0.996697	
QLI73372.1	OTHER	0.000678	0.999322	
QLI73373.1	OTHER	0.001050	0.998950	
QLI73374.1	OTHER	0.000907	0.999093	
QLI73375.1	OTHER	0.000938	0.999062	
QLI73376.1	OTHER	0.017489	0.982511	
QLI73377.1	OTHER	0.001173	0.998827	
QLI73378.1	OTHER	0.001383	0.998617	
QLI73379.1	OTHER	0.001853	0.998147	
QLI73380.1	OTHER	0.000280	0.999720	
QLI73381.1	SP(Sec/SPI)	0.997403	0.002597	CS pos: 19-20. AAA-QQ. Pr: 0.6394
QLI73382.1	OTHER	0.000388	0.999612	
QLI73383.1	OTHER	0.001085	0.998915	
QLI73384.1	OTHER	0.007446	0.992554	
QLI73385.1	OTHER	0.000403	0.999597	
QLI73386.1	OTHER	0.000711	0.999289	
QLI73387.1	OTHER	0.005978	0.994022	
QLI73388.1	OTHER	0.003999	0.996001	
QLI73389.1	OTHER	0.001024	0.998976	
QLI73390.1	OTHER	0.434448	0.565552	
QLI73391.1	OTHER	0.002044	0.997956	
QLI73392.1	OTHER	0.000347	0.999653	
QLI73393.1	OTHER	0.000420	0.999580	
QLI73394.1	OTHER	0.001829	0.998171	
QLI73395.1	OTHER	0.000512	0.999488	
QLI73396.1	OTHER	0.015191	0.984809	
QLI73397.1	OTHER	0.000542	0.999458	
QLI73398.1	OTHER	0.024604	0.975396	
QLI73399.1	OTHER	0.001400	0.998600	
QLI73400.1	OTHER	0.002878	0.997122	
QLI73401.1	OTHER	0.000627	0.999373	

QLI73402.1	OTHER	0.001766	0.998234	
QLI73403.1	OTHER	0.001173	0.998827	
QLI73404.1	OTHER	0.002201	0.997799	
QLI73405.1	OTHER	0.001274	0.998726	
QLI73406.1	OTHER	0.000682	0.999318	
QLI73407.1	OTHER	0.007669	0.992331	
QLI73408.1	SP(Sec/SPI)	0.906259	0.093741	CS pos: 20-21. AVA-AT. Pr: 0.5989
QLI73409.1	OTHER	0.001273	0.998727	
QLI73410.1	OTHER	0.003585	0.996415	
QLI73411.1	OTHER	0.004235	0.995765	
QLI73412.1	OTHER	0.000922	0.999078	
QLI73413.1	OTHER	0.001810	0.998190	
QLI73414.1	OTHER	0.001383	0.998617	
QLI73415.1	OTHER	0.000800	0.999200	
QLI73416.1	OTHER	0.001563	0.998437	
QLI73417.1	SP(Sec/SPI)	0.747757	0.252243	CS pos: 25-26. VSA-TV. Pr: 0.2932
QLI73418.1	OTHER	0.000239	0.999761	
QLI73419.1	OTHER	0.000458	0.999542	
QLI73420.1	OTHER	0.000203	0.999797	
QLI73421.1	OTHER	0.006432	0.993568	
QLI73422.1	SP(Sec/SPI)	0.982622	0.017378	CS pos: 22-23. VAT-LS. Pr: 0.4925
QLI73423.1	SP(Sec/SPI)	0.752244	0.247756	CS pos: 17-18. ALT-AT. Pr: 0.2713
QLI73424.1	OTHER	0.159927	0.840073	
QLI73425.1	OTHER	0.001089	0.998911	
QLI73426.1	OTHER	0.022366	0.977634	
QLI73427.1	OTHER	0.000491	0.999509	
QLI73428.1	OTHER	0.002794	0.997206	
QLI73429.1	OTHER	0.001507	0.998493	
QLI73430.1	OTHER	0.015329	0.984671	
QLI73431.1	OTHER	0.001185	0.998815	
QLI73432.1	OTHER	0.000625	0.999375	

QLI73433.1	OTHER	0.000565	0.999435
QLI73434.1	OTHER	0.001014	0.998986
QLI73435.1	OTHER	0.001174	0.998826
QLI73436.1	OTHER	0.290935	0.709065
QLI73437.1	OTHER	0.000967	0.999033
QLI73438.1	OTHER	0.001179	0.998821
QLI73439.1	OTHER	0.000090	0.999910
QLI73440.1	OTHER	0.006862	0.993138
QLI73441.1	OTHER	0.000133	0.999867
QLI73442.1	OTHER	0.001980	0.998020
QLI73443.1	OTHER	0.001058	0.998942
QLI73444.1	OTHER	0.000910	0.999090
QLI73445.1	OTHER	0.000615	0.999385
QLI73446.1	OTHER	0.001303	0.998697
QLI73447.1	OTHER	0.001325	0.998675
QLI73448.1	OTHER	0.046296	0.953704
QLI73449.1	OTHER	0.001050	0.998950
QLI73450.1	OTHER	0.002318	0.997682
QLI73451.1	OTHER	0.006371	0.993629
QLI73452.1	OTHER	0.000646	0.999354
QLI73453.1	OTHER	0.001228	0.998772
QLI73454.1	OTHER	0.000493	0.999507
QLI73455.1	OTHER	0.002547	0.997453
QLI73456.1	OTHER	0.000786	0.999214
QLI73457.1	OTHER	0.001240	0.998760
QLI73458.1	OTHER	0.009504	0.990496
QLI73459.1	OTHER	0.000387	0.999613
QLI73460.1	OTHER	0.001437	0.998563
QLI73461.1	OTHER	0.000237	0.999763
QLI73462.1	OTHER	0.001463	0.998537
QLI73463.1	OTHER	0.001482	0.998518

QLI73464.1	OTHER	0.002102	0.997898	
QLI73465.1	OTHER	0.002818	0.997182	
QLI73466.1	SP(Sec/SPI)	0.837103	0.162897	CS pos: 17-18. ILS-VP. Pr: 0.7525
QLI73467.1	OTHER	0.003137	0.996863	
QLI73468.1	OTHER	0.001876	0.998124	
QLI73469.1	OTHER	0.000713	0.999287	
QLI73470.1	OTHER	0.001294	0.998706	
QLI73471.1	OTHER	0.002569	0.997431	
QLI73472.1	OTHER	0.002042	0.997958	
QLI73473.1	OTHER	0.000475	0.999525	
QLI73474.1	OTHER	0.000227	0.999773	
QLI73475.1	OTHER	0.001821	0.998179	
QLI73476.1	OTHER	0.001341	0.998659	
QLI73477.1	OTHER	0.002090	0.997910	
QLI73478.1	OTHER	0.000594	0.999406	
QLI73479.1	OTHER	0.001287	0.998713	
QLI73480.1	OTHER	0.000773	0.999227	
QLI73481.1	SP(Sec/SPI)	0.960670	0.039330	CS pos: 16-17. TCA-QG. Pr: 0.5698
QLI73482.1	OTHER	0.000846	0.999154	
QLI73483.1	OTHER	0.000311	0.999689	
QLI73484.1	OTHER	0.000308	0.999692	
QLI73485.1	OTHER	0.003145	0.996855	
QLI73486.1	OTHER	0.001079	0.998921	
QLI73487.1	OTHER	0.000788	0.999212	
QLI73488.1	SP(Sec/SPI)	0.968857	0.031143	CS pos: 25-26. ALA-AT. Pr: 0.4224
QLI73489.1	OTHER	0.001016	0.998984	
QLI73490.1	OTHER	0.002646	0.997354	
QLI73491.1	OTHER	0.000583	0.999417	
QLI73492.1	OTHER	0.000453	0.999547	
QLI73493.1	OTHER	0.001775	0.998225	
QLI73494.1	SP(Sec/SPI)	0.962900	0.037100	CS pos: 31-32. ASA-VQ. Pr: 0.8832

QLI73495.1	OTHER	0.001205	0.998795	
QLI73496.1	OTHER	0.211570	0.788430	
QLI73497.1	OTHER	0.001466	0.998534	
QLI73498.1	OTHER	0.018837	0.981163	
QLI73499.1	OTHER	0.000546	0.999454	
QLI73500.1	OTHER	0.000737	0.999263	
QLI73501.1	OTHER	0.004475	0.995525	
QLI73502.1	SP(Sec/SPI)	0.996090	0.003910	CS pos: 17-18. AAA-QD. Pr: 0.6802
QLI73503.1	OTHER	0.000779	0.999221	
QLI73504.1	SP(Sec/SPI)	0.998296	0.001704	CS pos: 17-18. CLA-QS. Pr: 0.9446
QLI73505.1	OTHER	0.000844	0.999156	
QLI73506.1	OTHER	0.000197	0.999803	
QLI73507.1	OTHER	0.000597	0.999403	
QLI73508.1	OTHER	0.001893	0.998107	
QLI73509.1	OTHER	0.000678	0.999322	
QLI73510.1	OTHER	0.000393	0.999607	
QLI73511.1	OTHER	0.001691	0.998309	
QLI73512.1	OTHER	0.000713	0.999287	
QLI73513.1	OTHER	0.083045	0.916955	
QLI73514.1	OTHER	0.004121	0.995879	
QLI73515.1	OTHER	0.001639	0.998361	
QLI73516.1	OTHER	0.004187	0.995813	
QLI73517.1	OTHER	0.005128	0.994872	
QLI73518.1	OTHER	0.000322	0.999678	
QLI73519.1	SP(Sec/SPI)	0.976410	0.023590	CS pos: 21-22. ISA-EQ. Pr: 0.8256
QLI73520.1	SP(Sec/SPI)	0.955966	0.044034	CS pos: 19-20. AVG-GT. Pr: 0.3983
QLI73521.1	OTHER	0.000988	0.999012	
QLI73522.1	OTHER	0.002044	0.997956	
QLI73523.1	OTHER	0.001158	0.998842	
QLI73524.1	OTHER	0.000840	0.999160	
QLI73525.1	OTHER	0.001134	0.998866	

QLI73526.1	OTHER	0.002317	0.997683	
QLI73527.1	OTHER	0.002959	0.997041	
QLI73528.1	OTHER	0.000469	0.999531	
QLI73529.1	SP(Sec/SPI)	0.963850	0.036150	CS pos: 21-22. VYS-SN. Pr: 0.8318
QLI73530.1	OTHER	0.001233	0.998767	
QLI73531.1	OTHER	0.003276	0.996724	
QLI73532.1	OTHER	0.178152	0.821848	
QLI73533.1	OTHER	0.001680	0.998320	
QLI73534.1	OTHER	0.001164	0.998836	
QLI73535.1	OTHER	0.001520	0.998480	
QLI73536.1	OTHER	0.002247	0.997753	
QLI73537.1	OTHER	0.003351	0.996649	
QLI73538.1	SP(Sec/SPI)	0.811986	0.188014	CS pos: 25-26. LQG-GP. Pr: 0.4162
QLI73539.1	OTHER	0.005188	0.994812	
QLI73540.1	OTHER	0.000968	0.999032	
QLI73541.1	OTHER	0.000946	0.999054	
QLI73542.1	OTHER	0.001341	0.998659	
QLI73543.1	OTHER	0.005922	0.994078	
QLI73544.1	OTHER	0.001863	0.998137	
QLI73545.1	OTHER	0.000719	0.999281	
QLI73546.1	OTHER	0.000507	0.999493	
QLI73547.1	OTHER	0.001189	0.998811	
QLI73548.1	OTHER	0.007078	0.992922	
QLI73549.1	OTHER	0.001122	0.998878	
QLI73550.1	OTHER	0.006527	0.993473	
QLI73551.1	OTHER	0.001246	0.998754	
QLI73552.1	OTHER	0.028154	0.971846	
QLI73553.1	OTHER	0.000226	0.999774	
QLI73554.1	OTHER	0.000876	0.999124	
QLI73555.1	OTHER	0.000938	0.999062	
QLI73556.1	OTHER	0.001389	0.998611	

QLI73557.1	OTHER	0.015248	0.984752	
QLI73558.1	OTHER	0.001721	0.998279	
QLI73559.1	OTHER	0.002438	0.997562	
QLI73560.1	OTHER	0.007915	0.992085	
QLI73561.1	OTHER	0.000519	0.999481	
QLI73562.1	OTHER	0.005351	0.994649	
QLI73563.1	OTHER	0.002274	0.997726	
QLI73564.1	OTHER	0.001689	0.998311	
QLI73565.1	OTHER	0.019253	0.980747	
QLI73566.1	OTHER	0.002411	0.997589	
QLI73567.1	OTHER	0.013693	0.986307	
QLI73568.1	OTHER	0.001931	0.998069	
QLI73569.1	OTHER	0.001166	0.998834	
QLI73570.1	OTHER	0.051173	0.948827	
QLI73571.1	OTHER	0.000321	0.999679	
QLI73572.1	OTHER	0.000710	0.999290	
QLI73573.1	OTHER	0.000142	0.999858	
QLI73574.1	OTHER	0.001268	0.998732	
QLI73575.1	OTHER	0.022846	0.977154	
QLI73576.1	OTHER	0.000399	0.999601	
QLI73577.1	OTHER	0.031935	0.968065	
QLI73578.1	OTHER	0.001399	0.998601	
QLI73579.1	SP(Sec/SPI)	0.978446	0.021554	CS pos: 19-20. AAA-AD. Pr: 0.3013
QLI73580.1	OTHER	0.004955	0.995045	
QLI73581.1	OTHER	0.359362	0.640638	
QLI73582.1	OTHER	0.000377	0.999623	
QLI73583.1	OTHER	0.002469	0.997531	
QLI73584.1	OTHER	0.000824	0.999176	
QLI73585.1	OTHER	0.183728	0.816272	
QLI73586.1	OTHER	0.003682	0.996318	
QLI73587.1	OTHER	0.000282	0.999718	

QLI73588.1	OTHER	0.001471	0.998529	
QLI73589.1	OTHER	0.003369	0.996631	
QLI73590.1	SP(Sec/SPI)	0.988696	0.011304	CS pos: 20-21. GSA-LP. Pr: 0.6316
QLI73591.1	OTHER	0.000479	0.999521	
QLI73592.1	OTHER	0.047637	0.952363	
QLI73593.1	OTHER	0.001061	0.998939	
QLI73594.1	OTHER	0.003391	0.996609	
QLI73595.1	OTHER	0.001772	0.998228	
QLI73596.1	SP(Sec/SPI)	0.990379	0.009621	CS pos: 23-24. VQA-SN. Pr: 0.8247
QLI73597.1	OTHER	0.003068	0.996932	
QLI73598.1	OTHER	0.000758	0.999242	
QLI73599.1	OTHER	0.006640	0.993360	
QLI73600.1	OTHER	0.000337	0.999663	
QLI73601.1	OTHER	0.034338	0.965662	
QLI73602.1	OTHER	0.001722	0.998278	
QLI73603.1	OTHER	0.002615	0.997385	
QLI73604.1	OTHER	0.003081	0.996919	
QLI73605.1	OTHER	0.001864	0.998136	
QLI73606.1	OTHER	0.002774	0.997226	
QLI73607.1	OTHER	0.001240	0.998760	
QLI73608.1	SP(Sec/SPI)	0.992560	0.007440	CS pos: 16-17. ASA-TV. Pr: 0.3485
QLI73609.1	OTHER	0.018736	0.981264	
QLI73610.1	OTHER	0.001144	0.998856	
QLI73611.1	OTHER	0.002659	0.997341	
QLI73612.1	OTHER	0.001127	0.998873	
QLI73613.1	OTHER	0.000814	0.999186	
QLI73614.1	OTHER	0.003749	0.996251	
QLI73615.1	OTHER	0.000947	0.999053	
QLI73616.1	OTHER	0.000978	0.999022	
QLI73617.1	OTHER	0.001287	0.998713	
QLI73618.1	OTHER	0.000285	0.999715	

QLI73619.1	OTHER	0.001124	0.998876	
QLI73620.1	OTHER	0.001231	0.998769	
QLI73621.1	OTHER	0.000868	0.999132	
QLI73622.1	SP(Sec/SPI)	0.899151	0.100849	CS pos: 19-20. SRQ-SP. Pr: 0.5151
QLI73623.1	OTHER	0.001421	0.998579	
QLI73624.1	SP(Sec/SPI)	0.961354	0.038646	CS pos: 20-21. VSA-QT. Pr: 0.8102
QLI73625.1	SP(Sec/SPI)	0.850268	0.149732	CS pos: 21-22. VAA-ST. Pr: 0.5128
QLI73626.1	SP(Sec/SPI)	0.726632	0.273368	CS pos: 35-36. ALA-AE. Pr: 0.6933
QLI73627.1	OTHER	0.000518	0.999482	
QLI73628.1	OTHER	0.001297	0.998703	
QLI73629.1	OTHER	0.005292	0.994708	
QLI73630.1	OTHER	0.248668	0.751332	
QLI73631.1	SP(Sec/SPI)	0.985297	0.014703	CS pos: 17-18. AMA-NP. Pr: 0.4263
QLI73632.1	OTHER	0.000462	0.999538	
QLI73633.1	OTHER	0.000460	0.999540	
QLI73634.1	OTHER	0.017855	0.982145	
QLI73635.1	OTHER	0.001695	0.998305	
QLI73636.1	OTHER	0.000670	0.999330	
QLI73637.1	OTHER	0.001274	0.998726	
QLI73638.1	OTHER	0.000719	0.999281	
QLI73639.1	OTHER	0.001366	0.998634	
QLI73640.1	OTHER	0.000888	0.999112	
QLI73641.1	OTHER	0.007304	0.992696	
QLI73642.1	OTHER	0.004280	0.995720	
QLI73643.1	OTHER	0.002211	0.997789	
QLI73644.1	OTHER	0.011741	0.988259	
QLI73645.1	OTHER	0.000471	0.999529	
QLI73646.1	SP(Sec/SPI)	0.995806	0.004194	CS pos: 17-18. ALA-AP. Pr: 0.8393
QLI73647.1	OTHER	0.000969	0.999031	
QLI73648.1	OTHER	0.001011	0.998989	
QLI73649.1	OTHER	0.001733	0.998267	

QLI73650.1	OTHER	0.001658	0.998342	
QLI73651.1	OTHER	0.001008	0.998992	
QLI73652.1	OTHER	0.001504	0.998496	
QLI73653.1	OTHER	0.004261	0.995739	
QLI73654.1	OTHER	0.001941	0.998059	
QLI73655.1	OTHER	0.074815	0.925185	
QLI73656.1	SP(Sec/SPI)	0.628508	0.371492	CS pos: 27-28. SSG-VD. Pr: 0.3815
QLI73657.1	OTHER	0.000322	0.999678	
QLI73658.1	OTHER	0.000924	0.999076	
QLI73659.1	OTHER	0.000412	0.999588	
QLI73660.1	OTHER	0.001353	0.998647	
QLI73661.1	SP(Sec/SPI)	0.954078	0.045922	CS pos: 15-16. VTA-QF. Pr: 0.5836
QLI73662.1	OTHER	0.000619	0.999381	
QLI73663.1	OTHER	0.000587	0.999413	
QLI73664.1	OTHER	0.001833	0.998167	
QLI73665.1	SP(Sec/SPI)	0.846469	0.153531	CS pos: 18-19. IEG-AC. Pr: 0.3516
QLI73666.1	OTHER	0.035213	0.964787	
QLI73667.1	OTHER	0.001861	0.998139	
QLI73668.1	OTHER	0.001076	0.998924	
QLI73669.1	OTHER	0.000780	0.999220	
QLI73670.1	OTHER	0.001292	0.998708	
QLI73671.1	OTHER	0.001049	0.998951	
QLI73672.1	OTHER	0.001757	0.998243	
QLI73673.1	OTHER	0.093852	0.906148	
QLI73674.1	OTHER	0.000869	0.999131	
QLI73675.1	OTHER	0.000622	0.999378	
QLI73676.1	OTHER	0.001085	0.998915	
QLI73677.1	OTHER	0.000453	0.999547	
QLI73678.1	OTHER	0.004502	0.995498	
QLI73679.1	OTHER	0.000595	0.999405	
QLI73680.1	OTHER	0.001517	0.998483	

QLI73681.1	SP(Sec/SPI)	0.980463	0.019537	CS pos: 16-17. VMA-FD. Pr: 0.8631
QLI73682.1	OTHER	0.003151	0.996849	
QLI73683.1	SP(Sec/SPI)	0.997969	0.002031	CS pos: 18-19. AAA-LT. Pr: 0.4916
QLI73684.1	OTHER	0.000664	0.999336	
QLI73685.1	SP(Sec/SPI)	0.789222	0.210778	CS pos: 22-23. VIS-SP. Pr: 0.4763
QLI73686.1	OTHER	0.000630	0.999370	
QLI73687.1	OTHER	0.001005	0.998995	
QLI73688.1	OTHER	0.000735	0.999265	
QLI73689.1	OTHER	0.001076	0.998924	
QLI73690.1	OTHER	0.004100	0.995900	
QLI73691.1	OTHER	0.000595	0.999405	
QLI73692.1	SP(Sec/SPI)	0.989802	0.010198	CS pos: 20-21. TSA-EI. Pr: 0.4202
QLI73693.1	SP(Sec/SPI)	0.826567	0.173433	CS pos: 18-19. VLA-SP. Pr: 0.4714
QLI73694.1	OTHER	0.002505	0.997495	
QLI73695.1	OTHER	0.000454	0.999546	
QLI73696.1	OTHER	0.000654	0.999346	
QLI73697.1	OTHER	0.002309	0.997691	
QLI73698.1	OTHER	0.001357	0.998643	
QLI73699.1	OTHER	0.118790	0.881210	
QLI73700.1	OTHER	0.001302	0.998698	
QLI73701.1	OTHER	0.004947	0.995053	
QLI73702.1	OTHER	0.004807	0.995193	
QLI73703.1	OTHER	0.001947	0.998053	
QLI73704.1	OTHER	0.000987	0.999013	
QLI73705.1	OTHER	0.001177	0.998823	
QLI73706.1	SP(Sec/SPI)	0.990889	0.009111	CS pos: 21-22. AHA-DK. Pr: 0.9585
QLI73707.1	OTHER	0.000336	0.999664	
QLI73708.1	OTHER	0.021167	0.978833	
QLI73709.1	OTHER	0.000299	0.999701	
QLI73710.1	OTHER	0.134217	0.865783	
QLI73711.1	OTHER	0.002246	0.997754	

QLI73712.1	OTHER	0.005568	0.994432	
QLI73713.1	OTHER	0.003480	0.996520	
QLI73714.1	OTHER	0.000555	0.999445	
QLI73715.1	OTHER	0.003168	0.996832	
QLI73716.1	OTHER	0.000967	0.999033	
QLI73717.1	OTHER	0.002748	0.997252	
QLI73718.1	SP(Sec/SPI)	0.983899	0.016101	CS pos: 17-18. ALA-LL. Pr: 0.4010
QLI73719.1	OTHER	0.001114	0.998886	
QLI73720.1	OTHER	0.001269	0.998731	
QLI73721.1	OTHER	0.000534	0.999466	
QLI73722.1	OTHER	0.000537	0.999463	
QLI73723.1	OTHER	0.000687	0.999313	
QLI73724.1	OTHER	0.001067	0.998933	
QLI73725.1	OTHER	0.000357	0.999643	
QLI73726.1	OTHER	0.005156	0.994844	
QLI73727.1	OTHER	0.015884	0.984116	
QLI73728.1	OTHER	0.001401	0.998599	
QLI73729.1	OTHER	0.001103	0.998897	
QLI73730.1	OTHER	0.003873	0.996127	
QLI73731.1	OTHER	0.000994	0.999006	
QLI73732.1	OTHER	0.002767	0.997233	
QLI73733.1	OTHER	0.002644	0.997356	
QLI73734.1	OTHER	0.000848	0.999152	
QLI73735.1	OTHER	0.012731	0.987269	
QLI73736.1	OTHER	0.001839	0.998161	
QLI73737.1	OTHER	0.001298	0.998702	
QLI73738.1	OTHER	0.001089	0.998911	
QLI73739.1	OTHER	0.001387	0.998613	
QLI73740.1	OTHER	0.001544	0.998456	
QLI73741.1	OTHER	0.000753	0.999247	
QLI73742.1	OTHER	0.002428	0.997572	

QLI73743.1	OTHER	0.001402	0.998598	
QLI73744.1	OTHER	0.001747	0.998253	
QLI73745.1	OTHER	0.000601	0.999399	
QLI73746.1	OTHER	0.001373	0.998627	
QLI73747.1	OTHER	0.002292	0.997708	
QLI73748.1	OTHER	0.000568	0.999432	
QLI73749.1	OTHER	0.000415	0.999585	
QLI73750.1	OTHER	0.001263	0.998737	
QLI73751.1	OTHER	0.000144	0.999856	
QLI73752.1	OTHER	0.002874	0.997126	
QLI73753.1	OTHER	0.000820	0.999180	
QLI73754.1	OTHER	0.009062	0.990938	
QLI73755.1	OTHER	0.006886	0.993114	
QLI73756.1	OTHER	0.001236	0.998764	
QLI73757.1	OTHER	0.002337	0.997663	
QLI73758.1	OTHER	0.001365	0.998635	
QLI73759.1	OTHER	0.000476	0.999524	
QLI73760.1	OTHER	0.000691	0.999309	
QLI73761.1	SP(Sec/SPI)	0.955286	0.044714	CS pos: 20-21. VSA-AE. Pr: 0.7296
QLI73762.1	OTHER	0.001891	0.998109	
QLI73763.1	OTHER	0.000895	0.999105	
QLI73764.1	OTHER	0.000157	0.999843	
QLI73765.1	OTHER	0.000679	0.999321	
QLI73766.1	OTHER	0.000467	0.999533	
QLI73767.1	OTHER	0.000661	0.999339	
QLI73768.1	OTHER	0.001728	0.998272	
QLI73769.1	OTHER	0.001622	0.998378	
QLI73770.1	OTHER	0.006040	0.993960	
QLI73771.1	OTHER	0.000563	0.999437	
QLI73772.1	OTHER	0.001431	0.998569	
QLI73773.1	OTHER	0.002320	0.997680	

QLI73774.1	OTHER	0.000717	0.999283	
QLI73775.1	OTHER	0.002735	0.997265	
QLI73776.1	OTHER	0.382641	0.617359	
QLI73777.1	SP(Sec/SPI)	0.998321	0.001679	CS pos: 17-18. AAA-AP. Pr: 0.8405
QLI73778.1	OTHER	0.002116	0.997884	
QLI73779.1	OTHER	0.001840	0.998160	
QLI73780.1	OTHER	0.001127	0.998873	
QLI73781.1	OTHER	0.000819	0.999181	
QLI73782.1	OTHER	0.000623	0.999377	
QLI73783.1	OTHER	0.002426	0.997574	
QLI73784.1	OTHER	0.001673	0.998327	
QLI73785.1	OTHER	0.001845	0.998155	
QLI73786.1	OTHER	0.000793	0.999207	
QLI73787.1	OTHER	0.000412	0.999588	
QLI73788.1	OTHER	0.007991	0.992009	
QLI73789.1	OTHER	0.024310	0.975690	
QLI73790.1	OTHER	0.000604	0.999396	
QLI73791.1	OTHER	0.001046	0.998954	
QLI73792.1	OTHER	0.000673	0.999327	
QLI73793.1	OTHER	0.000298	0.999702	
QLI73794.1	OTHER	0.000421	0.999579	
QLI73795.1	OTHER	0.006386	0.993614	
QLI73796.1	OTHER	0.000226	0.999774	
QLI73797.1	OTHER	0.001806	0.998194	
QLI73798.1	SP(Sec/SPI)	0.993218	0.006782	CS pos: 18-19. AAA-YP. Pr: 0.6928
QLI73799.1	OTHER	0.000277	0.999723	
QLI73800.1	OTHER	0.001997	0.998003	
QLI73801.1	OTHER	0.001286	0.998714	
QLI73802.1	OTHER	0.001137	0.998863	
QLI73803.1	SP(Sec/SPI)	0.940104	0.059896	CS pos: 15-16. ALG-AV. Pr: 0.5878
QLI73804.1	OTHER	0.000662	0.999338	

QLI73805.1	OTHER	0.002247	0.997753	
QLI73806.1	OTHER	0.001069	0.998931	
QLI73807.1	SP(Sec/SPI)	0.958236	0.041764	CS pos: 18-19. VSA-TP. Pr: 0.7373
QLI73808.1	OTHER	0.002612	0.997388	
QLI73809.1	OTHER	0.001199	0.998801	
QLI73810.1	OTHER	0.000864	0.999136	
QLI73811.1	OTHER	0.000602	0.999398	
QLI73812.1	OTHER	0.000643	0.999357	
QLI73813.1	OTHER	0.003816	0.996184	
QLI73814.1	OTHER	0.003563	0.996437	
QLI73815.1	SP(Sec/SPI)	0.997651	0.002349	CS pos: 21-22. AQA-AV. Pr: 0.6212
QLI73816.1	SP(Sec/SPI)	0.973547	0.026453	CS pos: 20-21. GDA-GV. Pr: 0.5196
QLI73817.1	OTHER	0.057998	0.942002	
QLI73818.1	OTHER	0.000774	0.999226	
QLI73819.1	OTHER	0.489012	0.510988	
QLI73820.1	OTHER	0.000642	0.999358	
QLI73821.1	OTHER	0.028546	0.971454	
QLI73822.1	OTHER	0.000173	0.999827	
QLI73823.1	OTHER	0.002701	0.997299	
QLI73824.1	OTHER	0.000578	0.999422	
QLI73825.1	OTHER	0.000732	0.999268	
QLI73826.1	OTHER	0.000467	0.999533	
QLI73827.1	OTHER	0.003285	0.996715	
QLI73828.1	OTHER	0.000594	0.999406	
QLI73829.1	OTHER	0.000633	0.999367	
QLI73830.1	OTHER	0.000457	0.999543	
QLI73831.1	OTHER	0.004431	0.995569	
QLI73832.1	OTHER	0.000617	0.999383	
QLI73833.1	OTHER	0.006953	0.993047	
QLI73834.1	SP(Sec/SPI)	0.941180	0.058820	CS pos: 19-20. TLA-GG. Pr: 0.5253
QLI73835.1	OTHER	0.001800	0.998200	

QLI73836.1	OTHER	0.005127	0.994873	
QLI73837.1	OTHER	0.000571	0.999429	
QLI73838.1	OTHER	0.000546	0.999454	
QLI73839.1	OTHER	0.000319	0.999681	
QLI73840.1	OTHER	0.000460	0.999540	
QLI73841.1	OTHER	0.000332	0.999668	
QLI73842.1	SP(Sec/SPI)	0.984200	0.015800	CS pos: 19-20. AGA-IP. Pr: 0.4732
QLI73843.1	OTHER	0.003892	0.996108	
QLI73844.1	OTHER	0.000373	0.999627	
QLI73845.1	OTHER	0.000306	0.999694	
QLI73846.1	OTHER	0.000450	0.999550	
QLI73847.1	OTHER	0.013163	0.986837	
QLI73848.1	OTHER	0.002093	0.997907	
QLI73849.1	OTHER	0.002203	0.997797	
QLI73850.1	OTHER	0.001322	0.998678	
QLI73851.1	OTHER	0.000344	0.999656	
QLI73852.1	SP(Sec/SPI)	0.998054	0.001946	CS pos: 18-19. AAA-DC. Pr: 0.5381
QLI73853.1	OTHER	0.000943	0.999057	
QLI73854.1	OTHER	0.002452	0.997548	
QLI73855.1	OTHER	0.002009	0.997991	
QLI73856.1	OTHER	0.001901	0.998099	
QLI73857.1	OTHER	0.005314	0.994686	
QLI73858.1	OTHER	0.081266	0.918734	
QLI73859.1	OTHER	0.008151	0.991849	
QLI73860.1	SP(Sec/SPI)	0.978945	0.021055	CS pos: 19-20. AAA-DT. Pr: 0.8110
QLI73861.1	SP(Sec/SPI)	0.657260	0.342740	CS pos: 18-19. GLA-LV. Pr: 0.3400
QLI73862.1	OTHER	0.241265	0.758735	
QLI73863.1	OTHER	0.004219	0.995781	
QLI73864.1	SP(Sec/SPI)	0.986066	0.013934	CS pos: 16-17. TFA-NP. Pr: 0.8997
QLI73865.1	OTHER	0.000879	0.999121	
QLI73866.1	OTHER	0.003188	0.996812	

QLI73867.1	OTHER	0.008709	0.991291	
QLI73868.1	OTHER	0.002059	0.997941	
QLI73869.1	OTHER	0.000805	0.999195	
QLI73870.1	OTHER	0.001393	0.998607	
QLI73871.1	OTHER	0.001531	0.998469	
QLI73872.1	OTHER	0.000268	0.999732	
QLI73873.1	OTHER	0.001679	0.998321	
QLI73874.1	OTHER	0.000788	0.999212	
QLI73875.1	OTHER	0.000576	0.999424	
QLI73876.1	OTHER	0.000996	0.999004	
QLI73877.1	OTHER	0.288503	0.711497	
QLI73878.1	SP(Sec/SPI)	0.982130	0.017870	CS pos: 19-20. SLA-AP. Pr: 0.7501
QLI73879.1	OTHER	0.001427	0.998573	
QLI73880.1	OTHER	0.002443	0.997557	
QLI73881.1	OTHER	0.001325	0.998675	
QLI73882.1	OTHER	0.069536	0.930464	
QLI73883.1	OTHER	0.035869	0.964131	
QLI73884.1	OTHER	0.001270	0.998730	
QLI73885.1	OTHER	0.002075	0.997925	
QLI73886.1	OTHER	0.000796	0.999204	
QLI73887.1	OTHER	0.000456	0.999544	
QLI73888.1	OTHER	0.002841	0.997159	
QLI73889.1	OTHER	0.001681	0.998319	
QLI73890.1	OTHER	0.000514	0.999486	
QLI73891.1	OTHER	0.000547	0.999453	
QLI73892.1	OTHER	0.002545	0.997455	
QLI73893.1	OTHER	0.000299	0.999701	
QLI73894.1	OTHER	0.000834	0.999166	
QLI73895.1	OTHER	0.000240	0.999760	
QLI73896.1	OTHER	0.000406	0.999594	
QLI73897.1	OTHER	0.001844	0.998156	

QLI73898.1	OTHER	0.002267	0.997733	
QLI73899.1	OTHER	0.018139	0.981861	
QLI73900.1	OTHER	0.000545	0.999455	
QLI73901.1	SP(Sec/SPI)	0.993512	0.006488	CS pos: 18-19. GHA-LT. Pr: 0.5622
QLI73902.1	OTHER	0.000639	0.999361	
QLI73903.1	OTHER	0.000259	0.999741	
QLI73904.1	OTHER	0.003184	0.996816	
QLI73905.1	SP(Sec/SPI)	0.960846	0.039154	CS pos: 29-30. VSA-SP. Pr: 0.7634
QLI73906.1	OTHER	0.093931	0.906069	
QLI73907.1	SP(Sec/SPI)	0.999032	0.000968	CS pos: 18-19. VVA-QN. Pr: 0.9199
QLI73908.1	OTHER	0.000330	0.999670	
QLI73909.1	SP(Sec/SPI)	0.910034	0.089966	CS pos: 15-16. AAA-SP. Pr: 0.4392
QLI73910.1	OTHER	0.000725	0.999275	
QLI73911.1	OTHER	0.002297	0.997703	
QLI73912.1	OTHER	0.001213	0.998787	
QLI73913.1	OTHER	0.000549	0.999451	
QLI73914.1	OTHER	0.001258	0.998742	
QLI73915.1	OTHER	0.001430	0.998570	
QLI73916.1	SP(Sec/SPI)	0.999032	0.000968	CS pos: 23-24. SYA-ST. Pr: 0.8936
QLI73917.1	OTHER	0.000926	0.999074	
QLI73918.1	OTHER	0.000355	0.999645	
QLI73919.1	OTHER	0.001213	0.998787	
QLI73920.1	OTHER	0.006819	0.993181	
QLI73921.1	OTHER	0.000448	0.999552	
QLI73922.1	OTHER	0.000971	0.999029	
QLI73923.1	SP(Sec/SPI)	0.993733	0.006267	CS pos: 20-21. SFC-SP. Pr: 0.5841
QLI73924.1	OTHER	0.002440	0.997560	
QLI73925.1	OTHER	0.136129	0.863871	
QLI73926.1	OTHER	0.000803	0.999197	
QLI73927.1	OTHER	0.005905	0.994095	
QLI73928.1	OTHER	0.000784	0.999216	

QLI73929.1	OTHER	0.000865	0.999135	
QLI73930.1	SP(Sec/SPI)	0.999287	0.000713	CS pos: 17-18. VSA-QS. Pr: 0.5324
QLI73931.1	OTHER	0.000961	0.999039	
QLI73932.1	OTHER	0.001112	0.998888	
QLI73933.1	OTHER	0.012852	0.987148	
QLI73934.1	OTHER	0.006182	0.993818	
QLI73935.1	SP(Sec/SPI)	0.907533	0.092467	CS pos: 19-20. VSA-AH. Pr: 0.7265
QLI73936.1	OTHER	0.015579	0.984421	
QLI73937.1	OTHER	0.000316	0.999684	
QLI73938.1	OTHER	0.000182	0.999818	
QLI73939.1	OTHER	0.000794	0.999206	
QLI73940.1	OTHER	0.007432	0.992568	
QLI73941.1	OTHER	0.000874	0.999126	
QLI73942.1	OTHER	0.001982	0.998018	
QLI73943.1	OTHER	0.000483	0.999517	
QLI73944.1	OTHER	0.001236	0.998764	
QLI73945.1	OTHER	0.001516	0.998484	
QLI73946.1	OTHER	0.003173	0.996827	
QLI73947.1	OTHER	0.016557	0.983443	
QLI73948.1	OTHER	0.000271	0.999729	
QLI73949.1	OTHER	0.016480	0.983520	
QLI73950.1	OTHER	0.001260	0.998740	
QLI73951.1	OTHER	0.000900	0.999100	
QLI73952.1	OTHER	0.002754	0.997246	
QLI73953.1	OTHER	0.005482	0.994518	
QLI73954.1	OTHER	0.000196	0.999804	
QLI73955.1	OTHER	0.000331	0.999669	
QLI73956.1	OTHER	0.000922	0.999078	
QLI73957.1	OTHER	0.005086	0.994914	
QLI73958.1	SP(Sec/SPI)	0.613629	0.386371	CS pos: 26-27. VQG-TA. Pr: 0.1939
QLI73959.1	OTHER	0.000551	0.999449	

QLI73960.1	OTHER	0.000662	0.999338	
QLI73961.1	OTHER	0.000632	0.999368	
QLI73962.1	OTHER	0.000866	0.999134	
QLI73963.1	OTHER	0.002386	0.997614	
QLI73964.1	OTHER	0.001540	0.998460	
QLI73965.1	OTHER	0.002210	0.997790	
QLI73966.1	OTHER	0.001684	0.998316	
QLI73967.1	OTHER	0.007062	0.992938	
QLI73968.1	OTHER	0.001440	0.998560	
QLI73969.1	OTHER	0.002362	0.997638	
QLI73970.1	OTHER	0.000196	0.999804	
QLI73971.1	SP(Sec/SPI)	0.985754	0.014246	CS pos: 27-28. AQA-QS. Pr: 0.6644
QLI73972.1	OTHER	0.000682	0.999318	
QLI73973.1	OTHER	0.000472	0.999528	
QLI73974.1	OTHER	0.001480	0.998520	
QLI73975.1	OTHER	0.000468	0.999532	
QLI73976.1	SP(Sec/SPI)	0.916814	0.083186	CS pos: 15-16. VAG-DV. Pr: 0.3500
QLI73977.1	OTHER	0.001661	0.998339	
QLI73978.1	OTHER	0.000537	0.999463	
QLI73979.1	OTHER	0.000359	0.999641	
QLI73980.1	OTHER	0.000148	0.999852	
QLI73981.1	OTHER	0.000361	0.999639	
QLI73982.1	OTHER	0.007094	0.992906	
QLI73983.1	OTHER	0.052285	0.947715	
QLI73984.1	OTHER	0.004128	0.995872	
QLI73985.1	SP(Sec/SPI)	0.999716	0.000284	CS pos: 21-22. SLA-DL. Pr: 0.5314
QLI73986.1	OTHER	0.003715	0.996285	
QLI73987.1	OTHER	0.004683	0.995317	
QLI73988.1	OTHER	0.002596	0.997404	
QLI73989.1	OTHER	0.002496	0.997504	
QLI73990.1	OTHER	0.002026	0.997974	

QLI73991.1	OTHER	0.002754	0.997246	
QLI73992.1	OTHER	0.000858	0.999142	
QLI73993.1	OTHER	0.000698	0.999302	
QLI73994.1	OTHER	0.018469	0.981531	
QLI73995.1	SP(Sec/SPI)	0.979090	0.020910	CS pos: 17-18. ALA-VS. Pr: 0.6264
QLI73996.1	OTHER	0.000852	0.999148	
QLI73997.1	OTHER	0.001679	0.998321	
QLI73998.1	OTHER	0.003686	0.996314	
QLI73999.1	OTHER	0.001146	0.998854	
QLI74000.1	OTHER	0.000981	0.999019	
QLI74001.1	OTHER	0.000526	0.999474	
QLI74002.1	SP(Sec/SPI)	0.956337	0.043663	CS pos: 19-20. VLA-AD. Pr: 0.8451
QLI74003.1	OTHER	0.001334	0.998666	
QLI74004.1	OTHER	0.000446	0.999554	
QLI74005.1	OTHER	0.001514	0.998486	
QLI74006.1	OTHER	0.001337	0.998663	
QLI74007.1	OTHER	0.001835	0.998165	
QLI74008.1	OTHER	0.001205	0.998795	
QLI74009.1	OTHER	0.000465	0.999535	
QLI74010.1	OTHER	0.004980	0.995020	
QLI74011.1	OTHER	0.007459	0.992541	
QLI74012.1	OTHER	0.001445	0.998555	
QLI74013.1	OTHER	0.006620	0.993380	
QLI74014.1	OTHER	0.000612	0.999388	
QLI74015.1	OTHER	0.000397	0.999603	
QLI74016.1	SP(Sec/SPI)	0.775863	0.224137	CS pos: 19-20. VIS-VQ. Pr: 0.4667
QLI74017.1	OTHER	0.001342	0.998658	
QLI74018.1	OTHER	0.003878	0.996122	
QLI74019.1	OTHER	0.000804	0.999196	
QLI74020.1	SP(Sec/SPI)	0.878088	0.121912	CS pos: 24-25. TGA-QY. Pr: 0.6229
QLI74021.1	OTHER	0.000806	0.999194	

QLI74022.1	OTHER	0.001496	0.998504	
QLI74023.1	OTHER	0.018407	0.981593	
QLI74024.1	OTHER	0.001944	0.998056	
QLI74025.1	OTHER	0.002766	0.997234	
QLI74026.1	OTHER	0.001114	0.998886	
QLI74027.1	OTHER	0.002774	0.997226	
QLI74028.1	OTHER	0.005166	0.994834	
QLI74029.1	OTHER	0.000787	0.999213	
QLI74030.1	OTHER	0.000442	0.999558	
QLI74031.1	OTHER	0.000358	0.999642	
QLI74032.1	OTHER	0.000176	0.999824	
QLI74033.1	OTHER	0.001527	0.998473	
QLI74034.1	OTHER	0.001398	0.998602	
QLI74035.1	OTHER	0.001989	0.998011	
QLI74036.1	SP(Sec/SPI)	0.937558	0.062442	CS pos: 25-26. VLT-AQ. Pr: 0.3934
QLI74037.1	OTHER	0.001391	0.998609	
QLI74038.1	OTHER	0.007346	0.992654	
QLI74039.1	OTHER	0.000804	0.999196	
QLI74040.1	OTHER	0.000508	0.999492	
QLI74041.1	SP(Sec/SPI)	0.991260	0.008740	CS pos: 18-19. ATA-AF. Pr: 0.6272
QLI74042.1	OTHER	0.001294	0.998706	
QLI74043.1	OTHER	0.001348	0.998652	
QLI74044.1	OTHER	0.096138	0.903862	
QLI74045.1	OTHER	0.000852	0.999148	
QLI74046.1	OTHER	0.000425	0.999575	
QLI74047.1	OTHER	0.001165	0.998835	
QLI74048.1	OTHER	0.001578	0.998422	
QLI74049.1	OTHER	0.001019	0.998981	
QLI74050.1	OTHER	0.000711	0.999289	
QLI74051.1	OTHER	0.003864	0.996136	
QLI74052.1	OTHER	0.000454	0.999546	

QLI74053.1	OTHER	0.050547	0.949453	
QLI74054.1	OTHER	0.002337	0.997663	
QLI74055.1	OTHER	0.002828	0.997172	
QLI74056.1	OTHER	0.000529	0.999471	
QLI74057.1	OTHER	0.000639	0.999361	
QLI74058.1	OTHER	0.001916	0.998084	
QLI74059.1	OTHER	0.000745	0.999255	
QLI74060.1	OTHER	0.001402	0.998598	
QLI74061.1	SP(Sec/SPI)	0.952923	0.047077	CS pos: 21-22. TVG-LL. Pr: 0.4703
QLI74062.1	OTHER	0.005780	0.994220	
QLI74063.1	OTHER	0.002276	0.997724	
QLI74064.1	SP(Sec/SPI)	0.896481	0.103519	CS pos: 21-22. VNS-DT. Pr: 0.6509
QLI74065.1	OTHER	0.000393	0.999607	
QLI74066.1	OTHER	0.000674	0.999326	
QLI74067.1	OTHER	0.033291	0.966709	
QLI74068.1	OTHER	0.000912	0.999088	
QLI74069.1	OTHER	0.026227	0.973773	
QLI74070.1	OTHER	0.001455	0.998545	
QLI74071.1	OTHER	0.000504	0.999496	
QLI74072.1	OTHER	0.000613	0.999387	
QLI74073.1	SP(Sec/SPI)	0.999854	0.000146	CS pos: 17-18. AFA-LP. Pr: 0.9420
QLI74074.1	OTHER	0.001191	0.998809	
QLI74075.1	OTHER	0.001439	0.998561	
QLI74076.1	OTHER	0.002876	0.997124	
QLI74077.1	OTHER	0.002509	0.997491	
QLI74078.1	OTHER	0.002099	0.997901	
QLI74079.1	OTHER	0.002831	0.997169	
QLI74080.1	OTHER	0.029523	0.970477	
QLI74081.1	OTHER	0.000789	0.999211	
QLI74082.1	OTHER	0.001043	0.998957	
QLI74083.1	OTHER	0.003815	0.996185	

QLI74084.1	OTHER	0.001217	0.998783	
QLI74085.1	OTHER	0.000419	0.999581	
QLI74086.1	OTHER	0.002208	0.997792	
QLI74087.1	OTHER	0.000979	0.999021	
QLI74088.1	OTHER	0.001017	0.998983	
QLI74089.1	OTHER	0.000941	0.999059	
QLI74090.1	OTHER	0.003541	0.996459	
QLI74091.1	OTHER	0.036604	0.963396	
QLI74092.1	OTHER	0.000921	0.999079	
QLI74093.1	OTHER	0.000800	0.999200	
QLI74094.1	OTHER	0.002621	0.997379	
QLI74095.1	OTHER	0.007284	0.992716	
QLI74096.1	OTHER	0.000927	0.999073	
QLI74097.1	OTHER	0.000396	0.999604	
QLI74098.1	OTHER	0.000620	0.999380	
QLI74099.1	OTHER	0.001798	0.998202	
QLI74100.1	SP(Sec/SPI)	0.993493	0.006507	CS pos: 19-20. AAA-AP. Pr: 0.6698
QLI74101.1	OTHER	0.002208	0.997792	
QLI74102.1	OTHER	0.002019	0.997981	
QLI74103.1	SP(Sec/SPI)	0.660521	0.339479	CS pos: 21-22. SGA-RI. Pr: 0.4457
QLI74104.1	OTHER	0.000251	0.999749	
QLI74105.1	OTHER	0.000549	0.999451	
QLI74106.1	OTHER	0.000567	0.999433	
QLI74107.1	OTHER	0.003444	0.996556	
QLI74108.1	SP(Sec/SPI)	0.957394	0.042606	CS pos: 18-19. AFA-AG. Pr: 0.6094
QLI74109.1	OTHER	0.010528	0.989472	
QLI74110.1	SP(Sec/SPI)	0.946863	0.053137	CS pos: 18-19. TCA-MV. Pr: 0.6411
QLI74111.1	OTHER	0.001399	0.998601	
QLI74112.1	SP(Sec/SPI)	0.982729	0.017271	CS pos: 18-19. ALA-QN. Pr: 0.8116
QLI74113.1	SP(Sec/SPI)	0.563045	0.436955	CS pos: 20-21. SHS-WV. Pr: 0.2502
QLI74114.1	OTHER	0.001588	0.998412	

QLI74115.1	OTHER	0.021658	0.978342	
QLI74116.1	OTHER	0.000499	0.999501	
QLI74117.1	OTHER	0.001528	0.998472	
QLI74118.1	OTHER	0.001601	0.998399	
QLI74119.1	SP(Sec/SPI)	0.936069	0.063931	CS pos: 22-23. AQA-TT. Pr: 0.4154
QLI74120.1	OTHER	0.000697	0.999303	
QLI74121.1	SP(Sec/SPI)	0.982610	0.017390	CS pos: 19-20. AYG-AP. Pr: 0.8086
QLI74122.1	OTHER	0.001376	0.998624	
QLI74123.1	OTHER	0.001361	0.998639	
QLI74124.1	OTHER	0.001252	0.998748	
QLI74125.1	SP(Sec/SPI)	0.754662	0.245338	CS pos: 20-21. GYG-FK. Pr: 0.4028
QLI74126.1	OTHER	0.000789	0.999211	
QLI74127.1	OTHER	0.002976	0.997024	
QLI74128.1	OTHER	0.002149	0.997851	
QLI74129.1	OTHER	0.002589	0.997411	
QLI74130.1	OTHER	0.000834	0.999166	
QLI74131.1	OTHER	0.000687	0.999313	
QLI74132.1	OTHER	0.019281	0.980719	
QLI74133.1	SP(Sec/SPI)	0.977235	0.022765	CS pos: 19-20. VWG-LA. Pr: 0.5895
QLI74134.1	OTHER	0.000590	0.999410	
QLI74135.1	OTHER	0.001676	0.998324	
QLI74136.1	SP(Sec/SPI)	0.992193	0.007807	CS pos: 17-18. ACC-QE. Pr: 0.8087
QLI74137.1	OTHER	0.000830	0.999170	
QLI74138.1	OTHER	0.001027	0.998973	
QLI74139.1	OTHER	0.000646	0.999354	
QLI74140.1	OTHER	0.003769	0.996231	
QLI74141.1	SP(Sec/SPI)	0.926261	0.073739	CS pos: 23-24. IHA-AS. Pr: 0.7048
QLI74142.1	OTHER	0.002792	0.997208	
QLI74143.1	OTHER	0.000446	0.999554	
QLI74144.1	OTHER	0.001213	0.998787	
QLI74145.1	OTHER	0.013023	0.986977	

QLI74146.1	OTHER	0.004058	0.995942	
QLI74147.1	OTHER	0.001425	0.998575	
QLI74148.1	OTHER	0.005564	0.994436	
QLI74149.1	OTHER	0.000994	0.999006	
QLI74150.1	OTHER	0.000838	0.999162	
QLI74151.1	OTHER	0.001149	0.998851	
QLI74152.1	SP(Sec/SPI)	0.996282	0.003718	CS pos: 17-18. ALA-DA. Pr: 0.5940
QLI74153.1	OTHER	0.011081	0.988919	
QLI74154.1	SP(Sec/SPI)	0.621773	0.378227	CS pos: 19-20. VSG-AL. Pr: 0.2470
QLI74155.1	OTHER	0.005101	0.994899	
QLI74156.1	OTHER	0.003909	0.996091	
QLI74157.1	OTHER	0.001702	0.998298	
QLI74158.1	OTHER	0.000242	0.999758	
QLI74159.1	SP(Sec/SPI)	0.929185	0.070815	CS pos: 20-21. AAA-AT. Pr: 0.5738
QLI74160.1	OTHER	0.000823	0.999177	
QLI74161.1	OTHER	0.003234	0.996766	
QLI74162.1	SP(Sec/SPI)	0.947670	0.052330	CS pos: 15-16. ARA-TI. Pr: 0.5159
QLI74163.1	OTHER	0.001129	0.998871	
QLI74164.1	OTHER	0.005839	0.994161	
QLI74165.1	OTHER	0.007050	0.992950	
QLI74166.1	OTHER	0.001229	0.998771	
QLI74167.1	OTHER	0.000497	0.999503	
QLI74168.1	OTHER	0.010811	0.989189	
QLI74169.1	OTHER	0.003078	0.996922	
QLI74170.1	OTHER	0.001700	0.998300	
QLI74171.1	OTHER	0.000351	0.999649	
QLI74172.1	OTHER	0.015020	0.984980	
QLI74173.1	OTHER	0.001298	0.998702	
QLI74174.1	OTHER	0.000842	0.999158	
QLI74175.1	OTHER	0.001324	0.998676	
QLI74176.1	OTHER	0.000900	0.999100	

QLI74177.1	OTHER	0.000920	0.999080	
QLI74178.1	OTHER	0.000782	0.999218	
QLI74179.1	OTHER	0.000901	0.999099	
QLI74180.1	OTHER	0.002910	0.997090	
QLI74181.1	OTHER	0.000857	0.999143	
QLI74182.1	OTHER	0.002879	0.997121	
QLI74183.1	OTHER	0.000722	0.999278	
QLI74184.1	OTHER	0.001055	0.998945	
QLI74185.1	OTHER	0.001408	0.998592	
QLI74186.1	OTHER	0.002900	0.997100	
QLI74187.1	OTHER	0.001399	0.998601	
QLI74188.1	OTHER	0.000743	0.999257	
QLI74189.1	OTHER	0.001116	0.998884	
QLI74190.1	OTHER	0.000459	0.999541	
QLI74191.1	OTHER	0.003575	0.996425	
QLI74192.1	OTHER	0.000806	0.999194	
QLI74193.1	OTHER	0.001786	0.998214	
QLI74194.1	SP(Sec/SPI)	0.947379	0.052621	CS pos: 16-17. SLA-SP. Pr: 0.4708
QLI74195.1	OTHER	0.000929	0.999071	
QLI74196.1	OTHER	0.001505	0.998495	
QLI74197.1	OTHER	0.001831	0.998169	
QLI74198.1	OTHER	0.001781	0.998219	
QLI74199.1	OTHER	0.000643	0.999357	
QLI74200.1	SP(Sec/SPI)	0.879122	0.120878	CS pos: 18-19. SLA-CV. Pr: 0.2487
QLI74201.1	OTHER	0.001078	0.998922	
QLI74202.1	OTHER	0.000268	0.999732	
QLI74203.1	OTHER	0.001189	0.998811	
QLI74204.1	SP(Sec/SPI)	0.823284	0.176716	CS pos: 20-21. SWG-IK. Pr: 0.3421
QLI74205.1	OTHER	0.001347	0.998653	
QLI74206.1	OTHER	0.067132	0.932868	
QLI74207.1	SP(Sec/SPI)	0.960356	0.039644	CS pos: 19-20. VFG-AP. Pr: 0.7122

QLI74208.1	OTHER	0.000283	0.999717	
QLI74209.1	OTHER	0.000512	0.999488	
QLI74210.1	OTHER	0.006937	0.993063	
QLI74211.1	OTHER	0.002021	0.997979	
QLI74212.1	OTHER	0.004494	0.995506	
QLI74213.1	OTHER	0.001124	0.998876	
QLI74214.1	OTHER	0.003609	0.996391	
QLI74215.1	OTHER	0.001309	0.998691	
QLI74216.1	OTHER	0.033562	0.966438	
QLI74217.1	OTHER	0.000365	0.999635	
QLI74218.1	SP(Sec/SPI)	0.692893	0.307107	CS pos: 16-17. VVA-DW. Pr: 0.3777
QLI74219.1	OTHER	0.001150	0.998850	
QLI74220.1	OTHER	0.003694	0.996306	
QLI74221.1	OTHER	0.001926	0.998074	
QLI74222.1	OTHER	0.002681	0.997319	
QLI74223.1	SP(Sec/SPI)	0.948102	0.051898	CS pos: 17-18. VNA-YN. Pr: 0.9379
QLI74224.1	OTHER	0.000880	0.999120	
QLI74225.1	OTHER	0.005172	0.994828	
QLI74226.1	SP(Sec/SPI)	0.998370	0.001630	CS pos: 19-20. AHA-HG. Pr: 0.7552
QLI74227.1	OTHER	0.001942	0.998058	
QLI74228.1	OTHER	0.000645	0.999355	
QLI74229.1	OTHER	0.000393	0.999607	
QLI74230.1	OTHER	0.004056	0.995944	
QLI74231.1	OTHER	0.000538	0.999462	
QLI74232.1	OTHER	0.000663	0.999337	
QLI74233.1	OTHER	0.000929	0.999071	
QLI74234.1	OTHER	0.004104	0.995896	
QLI74235.1	OTHER	0.001965	0.998035	
QLI74236.1	SP(Sec/SPI)	0.994086	0.005914	CS pos: 20-21. GAA-RL. Pr: 0.4893
QLI74237.1	OTHER	0.004974	0.995026	
QLI74238.1	SP(Sec/SPI)	0.992020	0.007980	CS pos: 20-21. AYS-TP. Pr: 0.5094

QLI74239.1	OTHER	0.000428	0.999572	
QLI74240.1	OTHER	0.003436	0.996564	
QLI74241.1	OTHER	0.000414	0.999586	
QLI74242.1	OTHER	0.000301	0.999699	
QLI74243.1	OTHER	0.005461	0.994539	
QLI74244.1	OTHER	0.000987	0.999013	
QLI74245.1	OTHER	0.001065	0.998935	
QLI74246.1	SP(Sec/SPI)	0.968230	0.031770	CS pos: 19-20. ASA-QS. Pr: 0.7648
QLI74247.1	OTHER	0.001355	0.998645	
QLI74248.1	OTHER	0.000404	0.999596	
QLI74249.1	OTHER	0.001189	0.998811	
QLI74250.1	SP(Sec/SPI)	0.571223	0.428777	CS pos: 22-23. PSA-KQ. Pr: 0.2252
QLI74251.1	OTHER	0.001421	0.998579	
QLI74252.1	OTHER	0.006323	0.993677	
QLI74253.1	OTHER	0.002088	0.997912	
QLI74254.1	OTHER	0.000427	0.999573	
QLI74255.1	OTHER	0.000499	0.999501	
QLI74256.1	OTHER	0.000609	0.999391	
QLI74257.1	SP(Sec/SPI)	0.978548	0.021452	CS pos: 17-18. AIA-GQ. Pr: 0.4649
QLI74258.1	OTHER	0.001454	0.998546	
QLI74259.1	SP(Sec/SPI)	0.964470	0.035530	CS pos: 17-18. ASA-LS. Pr: 0.2745
QLI74260.1	OTHER	0.003739	0.996261	
QLI74261.1	SP(Sec/SPI)	0.585533	0.414467	CS pos: 17-18. ALA-NV. Pr: 0.4201
QLI74262.1	OTHER	0.002129	0.997871	
QLI74263.1	OTHER	0.002158	0.997842	
QLI74264.1	OTHER	0.003437	0.996563	
QLI74265.1	OTHER	0.002467	0.997533	
QLI74266.1	OTHER	0.001237	0.998763	
QLI74267.1	OTHER	0.001351	0.998649	
QLI74268.1	OTHER	0.002174	0.997826	
QLI74269.1	OTHER	0.001708	0.998292	

QLI74270.1	SP(Sec/SPI)	0.736668	0.263332	CS pos: 18-19. AMA-AP. Pr: 0.5351
QLI74271.1	OTHER	0.001373	0.998627	
QLI74272.1	OTHER	0.000799	0.999201	
QLI74273.1	OTHER	0.000548	0.999452	
QLI74274.1	OTHER	0.004684	0.995316	
QLI74275.1	OTHER	0.001056	0.998944	
QLI74276.1	OTHER	0.001682	0.998318	
QLI74277.1	OTHER	0.000534	0.999466	
QLI74278.1	OTHER	0.002319	0.997681	
QLI74279.1	OTHER	0.001184	0.998816	
QLI74280.1	OTHER	0.000365	0.999635	
QLI74281.1	SP(Sec/SPI)	0.977789	0.022211	CS pos: 22-23. AVA-QS. Pr: 0.6791
QLI74282.1	SP(Sec/SPI)	0.973225	0.026775	CS pos: 24-25. ASA-VD. Pr: 0.8515
QLI74283.1	OTHER	0.008294	0.991706	
QLI74284.1	SP(Sec/SPI)	0.916939	0.083061	CS pos: 17-18. ATA-LP. Pr: 0.7063
QLI74285.1	OTHER	0.001097	0.998903	
QLI74286.1	OTHER	0.002293	0.997707	
QLI74287.1	OTHER	0.001374	0.998626	
QLI74288.1	SP(Sec/SPI)	0.957355	0.042645	CS pos: 16-17. AKA-GP. Pr: 0.5649
QLI74289.1	OTHER	0.001032	0.998968	
QLI74290.1	SP(Sec/SPI)	0.910258	0.089742	CS pos: 21-22. AVG-GA. Pr: 0.2938
QLI74291.1	OTHER	0.001660	0.998340	
QLI74292.1	OTHER	0.000623	0.999377	
QLI74293.1	OTHER	0.037045	0.962955	
QLI74294.1	OTHER	0.001008	0.998992	
QLI74295.1	OTHER	0.001240	0.998760	
QLI74296.1	OTHER	0.038468	0.961532	
QLI74297.1	SP(Sec/SPI)	0.999358	0.000642	CS pos: 20-21. VLA-QT. Pr: 0.9608
QLI74298.1	OTHER	0.001047	0.998953	
QLI74299.1	OTHER	0.000865	0.999135	
QLI74300.1	OTHER	0.001033	0.998967	

QLI74301.1	SP(Sec/SPI)	0.830958	0.169042	CS pos: 22-23. VYG-SA. Pr: 0.3063
QLI74302.1	SP(Sec/SPI)	0.822949	0.177051	CS pos: 21-22. THG-IR. Pr: 0.7920
QLI74303.1	OTHER	0.001902	0.998098	
QLI74304.1	OTHER	0.000656	0.999344	
QLI74305.1	OTHER	0.002685	0.997315	
QLI74306.1	OTHER	0.000430	0.999570	
QLI74307.1	OTHER	0.000776	0.999224	
QLI74308.1	OTHER	0.000410	0.999590	
QLI74309.1	OTHER	0.001012	0.998988	
QLI74310.1	OTHER	0.001858	0.998142	
QLI74311.1	OTHER	0.000481	0.999519	
QLI74312.1	OTHER	0.339501	0.660499	
QLI74313.1	OTHER	0.000449	0.999551	
QLI74314.1	SP(Sec/SPI)	0.895999	0.104001	CS pos: 20-21. QQA-TI. Pr: 0.2436
QLI74315.1	OTHER	0.002989	0.997011	
QLI74316.1	SP(Sec/SPI)	0.986200	0.013800	CS pos: 20-21. AAA-LQ. Pr: 0.3728
QLI74317.1	OTHER	0.003606	0.996394	
QLI74318.1	SP(Sec/SPI)	0.979493	0.020507	CS pos: 19-20. TLA-ST. Pr: 0.6457
QLI74319.1	SP(Sec/SPI)	0.970342	0.029658	CS pos: 20-21. TAA-DS. Pr: 0.6107
QLI74320.1	OTHER	0.004427	0.995573	
QLI74321.1	OTHER	0.404812	0.595188	
QLI74322.1	OTHER	0.001188	0.998812	
QLI74323.1	OTHER	0.004659	0.995341	
QLI74324.1	SP(Sec/SPI)	0.948217	0.051783	CS pos: 20-21. AAA-AR. Pr: 0.4220
QLI74325.1	SP(Sec/SPI)	0.998245	0.001755	CS pos: 17-18. TLA-MP. Pr: 0.8951
QLI74326.1	OTHER	0.001573	0.998427	
QLI74327.1	OTHER	0.280521	0.719479	
QLI74328.1	OTHER	0.000345	0.999655	
QLI74329.1	OTHER	0.003672	0.996328	
QLI74330.1	OTHER	0.002719	0.997281	
QLI74331.1	OTHER	0.000429	0.999571	

QLI74332.1	OTHER	0.000706	0.999294	
QLI74333.1	OTHER	0.001537	0.998463	
QLI74334.1	OTHER	0.003949	0.996051	
QLI74335.1	OTHER	0.000698	0.999302	
QLI74336.1	OTHER	0.000933	0.999067	
QLI74337.1	OTHER	0.001126	0.998874	
QLI74338.1	OTHER	0.004642	0.995358	
QLI74339.1	OTHER	0.000754	0.999246	
QLI74340.1	OTHER	0.002420	0.997580	
QLI74341.1	OTHER	0.002349	0.997651	
QLI74342.1	OTHER	0.001628	0.998372	
QLI74343.1	OTHER	0.005077	0.994923	
QLI74344.1	OTHER	0.003432	0.996568	
QLI74345.1	SP(Sec/SPI)	0.936490	0.063510	CS pos: 17-18. ALA-AP. Pr: 0.6958
QLI74346.1	OTHER	0.001518	0.998482	
QLI74347.1	SP(Sec/SPI)	0.996968	0.003032	CS pos: 20-21. AQS-IL. Pr: 0.8156
QLI74348.1	OTHER	0.001435	0.998565	
QLI74349.1	OTHER	0.008407	0.991593	
QLI74350.1	SP(Sec/SPI)	0.995269	0.004731	CS pos: 16-17. TSA-QV. Pr: 0.4435
QLI74351.1	OTHER	0.000339	0.999661	
QLI74352.1	OTHER	0.001900	0.998100	
QLI74353.1	OTHER	0.001842	0.998158	
QLI74354.1	OTHER	0.000539	0.999461	
QLI74355.1	OTHER	0.000831	0.999169	
QLI74356.1	OTHER	0.004164	0.995836	
QLI74357.1	OTHER	0.000806	0.999194	
QLI74358.1	OTHER	0.001586	0.998414	
QLI74359.1	OTHER	0.002639	0.997361	
QLI74360.1	OTHER	0.000414	0.999586	
QLI74361.1	OTHER	0.000995	0.999005	
QLI74362.1	SP(Sec/SPI)	0.984846	0.015154	CS pos: 17-18. AGA-LV. Pr: 0.4226

QLI74363.1	OTHER	0.425038	0.574962	
QLI74364.1	OTHER	0.001882	0.998118	
QLI74365.1	OTHER	0.000487	0.999513	
QLI74366.1	OTHER	0.001838	0.998162	
QLI74367.1	OTHER	0.005276	0.994724	
QLI74368.1	OTHER	0.001399	0.998601	
QLI74369.1	OTHER	0.000674	0.999326	
QLI74370.1	SP(Sec/SPI)	0.984127	0.015873	CS pos: 19-20. ASS-GL. Pr: 0.4003
QLI74371.1	OTHER	0.006926	0.993074	
QLI74372.1	OTHER	0.002076	0.997924	
QLI74373.1	OTHER	0.000608	0.999392	
QLI74374.1	OTHER	0.004797	0.995203	
QLI74375.1	OTHER	0.001147	0.998853	
QLI74376.1	OTHER	0.001337	0.998663	
QLI74377.1	OTHER	0.001383	0.998617	
QLI74378.1	SP(Sec/SPI)	0.983368	0.016632	CS pos: 16-17. ASA-HL. Pr: 0.4875
QLI74379.1	OTHER	0.042535	0.957465	
QLI74380.1	OTHER	0.000392	0.999608	
QLI74381.1	OTHER	0.000634	0.999366	
QLI74382.1	OTHER	0.028189	0.971811	
QLI74383.1	OTHER	0.002058	0.997942	
QLI74384.1	OTHER	0.005603	0.994397	
QLI74385.1	SP(Sec/SPI)	0.981827	0.018173	CS pos: 18-19. STA-LP. Pr: 0.4748
QLI74386.1	SP(Sec/SPI)	0.976265	0.023735	CS pos: 18-19. AIA-QY. Pr: 0.9031
QLI74387.1	OTHER	0.006622	0.993378	
QLI74388.1	OTHER	0.000356	0.999644	
QLI74389.1	SP(Sec/SPI)	0.999561	0.000439	CS pos: 18-19. CSA-QN. Pr: 0.6671
QLI74390.1	OTHER	0.001364	0.998636	
QLI74391.1	OTHER	0.000364	0.999636	
QLI74392.1	OTHER	0.001772	0.998228	
QLI74393.1	OTHER	0.000256	0.999744	

QLI74394.1	SP(Sec/SPI)	0.998035	0.001965	CS pos: 17-18. AVA-AP. Pr: 0.5804
QLI74395.1	OTHER	0.004007	0.995993	
QLI74396.1	OTHER	0.001499	0.998501	
QLI74397.1	OTHER	0.000966	0.999034	
QLI74398.1	OTHER	0.004580	0.995420	
QLI74399.1	OTHER	0.001380	0.998620	
QLI74400.1	SP(Sec/SPI)	0.994173	0.005827	CS pos: 19-20. ATA-TV. Pr: 0.5246
QLI74401.1	OTHER	0.008703	0.991297	
QLI74402.1	OTHER	0.000726	0.999274	
QLI74403.1	SP(Sec/SPI)	0.959999	0.040001	CS pos: 19-20. AHG-VR. Pr: 0.7372
QLI74404.1	SP(Sec/SPI)	0.990549	0.009451	CS pos: 17-18. ALA-AP. Pr: 0.7647
QLI74405.1	OTHER	0.023223	0.976777	
QLI74406.1	OTHER	0.002037	0.997963	
QLI74407.1	OTHER	0.001332	0.998668	
QLI74408.1	OTHER	0.063996	0.936004	
QLI74409.1	OTHER	0.056384	0.943616	
QLI74410.1	OTHER	0.047408	0.952592	
QLI74411.1	OTHER	0.000894	0.999106	
QLI74412.1	OTHER	0.000699	0.999301	
QLI74413.1	OTHER	0.000349	0.999651	
QLI74414.1	OTHER	0.003555	0.996445	
QLI74415.1	OTHER	0.024238	0.975762	
QLI74416.1	OTHER	0.000529	0.999471	
QLI74417.1	OTHER	0.000623	0.999377	
QLI74418.1	OTHER	0.000466	0.999534	
QLI74419.1	OTHER	0.003580	0.996420	
QLI74420.1	SP(Sec/SPI)	0.969821	0.030179	CS pos: 21-22. ALS-TR. Pr: 0.3293
QLI74421.1	OTHER	0.000713	0.999287	
QLI74422.1	OTHER	0.000476	0.999524	
QLI74423.1	OTHER	0.000632	0.999368	
QLI74424.1	OTHER	0.001088	0.998912	

QLI74425.1	OTHER	0.000643	0.999357	
QLI74426.1	OTHER	0.000511	0.999489	
QLI74427.1	OTHER	0.002020	0.997980	
QLI74428.1	OTHER	0.000542	0.999458	
QLI74429.1	OTHER	0.000540	0.999460	
QLI74430.1	OTHER	0.002054	0.997946	
QLI74431.1	OTHER	0.000577	0.999423	
QLI74432.1	OTHER	0.001740	0.998260	
QLI74433.1	OTHER	0.000203	0.999797	
QLI74434.1	OTHER	0.000658	0.999342	
QLI74435.1	SP(Sec/SPI)	0.994581	0.005419	CS pos: 15-16. VGA-TP. Pr: 0.6407
QLI74436.1	OTHER	0.000423	0.999577	
QLI74437.1	OTHER	0.001167	0.998833	
QLI74438.1	OTHER	0.000483	0.999517	
QLI74439.1	OTHER	0.000849	0.999151	
QLI74440.1	OTHER	0.000421	0.999579	
QLI74441.1	OTHER	0.002815	0.997185	
QLI74442.1	OTHER	0.000446	0.999554	
QLI74443.1	OTHER	0.001065	0.998935	
QLI74444.1	OTHER	0.000516	0.999484	
QLI74445.1	OTHER	0.000660	0.999340	
QLI74446.1	OTHER	0.001336	0.998664	
QLI74447.1	OTHER	0.003304	0.996696	
QLI74448.1	OTHER	0.001251	0.998749	
QLI74449.1	SP(Sec/SPI)	0.968196	0.031804	CS pos: 18-19. AVA-ID. Pr: 0.4488
QLI74450.1	OTHER	0.000406	0.999594	
QLI74451.1	SP(Sec/SPI)	0.917427	0.082573	CS pos: 16-17. TSA-GV. Pr: 0.4364
QLI74452.1	SP(Sec/SPI)	0.964030	0.035970	CS pos: 25-26. TAA-AP. Pr: 0.5912
QLI74453.1	OTHER	0.001843	0.998157	
QLI74454.1	OTHER	0.000490	0.999510	
QLI74455.1	SP(Sec/SPI)	0.988410	0.011590	CS pos: 20-21. SQA-IN. Pr: 0.7953

QLI74456.1	OTHER	0.001499	0.998501	
QLI74457.1	OTHER	0.005009	0.994991	
QLI74458.1	OTHER	0.002391	0.997609	
QLI74459.1	OTHER	0.000844	0.999156	
QLI74460.1	OTHER	0.001054	0.998946	
QLI74461.1	OTHER	0.000608	0.999392	
QLI74462.1	SP(Sec/SPI)	0.968012	0.031988	CS pos: 19-20. AHG-AV. Pr: 0.7710
QLI74463.1	OTHER	0.000620	0.999380	
QLI74464.1	OTHER	0.001125	0.998875	
QLI74465.1	OTHER	0.005520	0.994480	
QLI74466.1	OTHER	0.000291	0.999709	
QLI74467.1	OTHER	0.001225	0.998775	
QLI74468.1	SP(Sec/SPI)	0.597597	0.402403	CS pos: 22-23. SLA-AD. Pr: 0.3227
QLI74469.1	OTHER	0.002543	0.997457	
QLI74470.1	OTHER	0.003395	0.996605	
QLI74471.1	SP(Sec/SPI)	0.989761	0.010239	CS pos: 20-21. VHA-RP. Pr: 0.8712
QLI74472.1	OTHER	0.001266	0.998734	
QLI74473.1	OTHER	0.001196	0.998804	
QLI74474.1	OTHER	0.000550	0.999450	
QLI74475.1	OTHER	0.001998	0.998002	
QLI74476.1	OTHER	0.000816	0.999184	
QLI74477.1	SP(Sec/SPI)	0.963714	0.036286	CS pos: 22-23. VSA-RP. Pr: 0.6852
QLI74478.1	OTHER	0.001252	0.998748	
QLI74479.1	OTHER	0.002844	0.997156	
QLI74480.1	OTHER	0.001026	0.998974	
QLI74481.1	OTHER	0.001392	0.998608	
QLI74482.1	OTHER	0.000797	0.999203	
QLI74483.1	SP(Sec/SPI)	0.577138	0.422862	CS pos: 22-23. SLT-LK. Pr: 0.4621
QLI74484.1	OTHER	0.001230	0.998770	
QLI74485.1	OTHER	0.373815	0.626185	
QLI74486.1	OTHER	0.001109	0.998891	

QLI74487.1	OTHER	0.002388	0.997612	
QLI74488.1	OTHER	0.000431	0.999569	
QLI74489.1	OTHER	0.000487	0.999513	
QLI74490.1	OTHER	0.001243	0.998757	
QLI74491.1	OTHER	0.000607	0.999393	
QLI74492.1	OTHER	0.001345	0.998655	
QLI74493.1	SP(Sec/SPI)	0.606640	0.393360	CS pos: 18-19. ATA-FA. Pr: 0.2474
QLI74494.1	OTHER	0.000673	0.999327	
QLI74495.1	OTHER	0.001295	0.998705	
QLI74496.1	OTHER	0.055968	0.944032	
QLI74497.1	OTHER	0.001032	0.998968	
QLI74498.1	OTHER	0.009772	0.990228	
QLI74499.1	OTHER	0.001613	0.998387	
QLI74500.1	OTHER	0.008419	0.991581	
QLI74501.1	OTHER	0.001021	0.998979	
QLI74502.1	OTHER	0.102825	0.897175	
QLI74503.1	OTHER	0.001710	0.998290	
QLI74504.1	SP(Sec/SPI)	0.998002	0.001998	CS pos: 17-18. AAA-LP. Pr: 0.7575
QLI74505.1	OTHER	0.000549	0.999451	
QLI74506.1	OTHER	0.011150	0.988850	
QLI74507.1	OTHER	0.418619	0.581381	
QLI74508.1	OTHER	0.000427	0.999573	
QLI74509.1	OTHER	0.000628	0.999372	
QLI74510.1	SP(Sec/SPI)	0.990901	0.009099	CS pos: 18-19. VVA-EG. Pr: 0.5486
QLI74511.1	OTHER	0.000585	0.999415	
QLI74512.1	SP(Sec/SPI)	0.890406	0.109594	CS pos: 25-26. AIG-QK. Pr: 0.7428
QLI74513.1	OTHER	0.001449	0.998551	
QLI74514.1	SP(Sec/SPI)	0.987371	0.012629	CS pos: 19-20. VAA-AP. Pr: 0.6804
QLI74515.1	OTHER	0.001241	0.998759	
QLI74516.1	OTHER	0.001120	0.998880	
QLI74517.1	OTHER	0.032109	0.967891	

QLI74518.1	OTHER	0.000522	0.999478	
QLI74519.1	OTHER	0.000679	0.999321	
QLI74520.1	OTHER	0.008816	0.991184	
QLI74521.1	OTHER	0.001107	0.998893	
QLI74522.1	OTHER	0.003322	0.996678	
QLI74523.1	SP(Sec/SPI)	0.994678	0.005322	CS pos: 20-21. AKA-QT. Pr: 0.9128
QLI74524.1	OTHER	0.000195	0.999805	
QLI74525.1	OTHER	0.000501	0.999499	
QLI74526.1	OTHER	0.001046	0.998954	
QLI74527.1	OTHER	0.000705	0.999295	
QLI74528.1	OTHER	0.014644	0.985356	
QLI74529.1	OTHER	0.003235	0.996765	
QLI74530.1	OTHER	0.000578	0.999422	
QLI74531.1	OTHER	0.002134	0.997866	
QLI74532.1	OTHER	0.002271	0.997729	
QLI74533.1	OTHER	0.000553	0.999447	
QLI74534.1	OTHER	0.000927	0.999073	
QLI74535.1	OTHER	0.002962	0.997038	
QLI74536.1	OTHER	0.001062	0.998938	
QLI74537.1	OTHER	0.002755	0.997245	
QLI74538.1	OTHER	0.001837	0.998163	
QLI74539.1	OTHER	0.000834	0.999166	
QLI74540.1	SP(Sec/SPI)	0.986499	0.013501	CS pos: 20-21. AAA-EP. Pr: 0.7932
QLI74541.1	OTHER	0.002494	0.997506	
QLI74542.1	OTHER	0.001139	0.998861	
QLI74543.1	OTHER	0.003212	0.996788	
QLI74544.1	OTHER	0.001783	0.998217	
QLI74545.1	SP(Sec/SPI)	0.999175	0.000825	CS pos: 18-19. ASA-QQ. Pr: 0.9551
QLI74546.1	OTHER	0.006183	0.993817	
QLI74547.1	OTHER	0.000447	0.999553	
QLI74548.1	OTHER	0.003676	0.996324	

QLI74549.1	OTHER	0.001400	0.998600	
QLI74550.1	OTHER	0.002135	0.997865	
QLI74551.1	OTHER	0.000402	0.999598	
QLI74552.1	OTHER	0.000459	0.999541	
QLI74553.1	SP(Sec/SPI)	0.966834	0.033166	CS pos: 16-17. ASA-HL. Pr: 0.5008
QLI74554.1	OTHER	0.004253	0.995747	
QLI74555.1	SP(Sec/SPI)	0.987046	0.012954	CS pos: 21-22. SAA-DS. Pr: 0.4453
QLI74556.1	OTHER	0.001857	0.998143	
QLI74557.1	OTHER	0.003589	0.996411	
QLI74558.1	OTHER	0.001509	0.998491	
QLI74559.1	OTHER	0.002127	0.997873	
QLI74560.1	OTHER	0.001155	0.998845	
QLI74561.1	OTHER	0.002359	0.997641	
QLI74562.1	OTHER	0.010709	0.989291	
QLI74563.1	SP(Sec/SPI)	0.932576	0.067424	CS pos: 20-21. GLG-AD. Pr: 0.3862
QLI74564.1	SP(Sec/SPI)	0.998683	0.001317	CS pos: 19-20. VLA-VP. Pr: 0.9446
QLI74565.1	OTHER	0.000167	0.999833	
QLI74566.1	OTHER	0.000874	0.999126	
QLI74567.1	SP(Sec/SPI)	0.998672	0.001328	CS pos: 20-21. ALA-QS. Pr: 0.5085
QLI74568.1	SP(Sec/SPI)	0.900198	0.099802	CS pos: 19-20. VAA-NS. Pr: 0.6793
QLI74569.1	OTHER	0.001110	0.998890	
QLI74570.1	OTHER	0.000894	0.999106	
QLI74571.1	OTHER	0.000946	0.999054	
QLI74572.1	OTHER	0.002367	0.997633	
QLI74573.1	OTHER	0.000934	0.999066	
QLI74574.1	OTHER	0.000302	0.999698	
QLI74575.1	OTHER	0.000497	0.999503	
QLI74576.1	OTHER	0.001677	0.998323	
QLI74577.1	OTHER	0.006030	0.993970	
QLI74578.1	OTHER	0.003623	0.996377	
QLI74579.1	OTHER	0.120749	0.879251	

QLI74580.1	OTHER	0.003540	0.996460	
QLI74581.1	OTHER	0.001806	0.998194	
QLI74582.1	OTHER	0.015969	0.984031	
QLI74583.1	OTHER	0.000964	0.999036	
QLI74584.1	OTHER	0.001501	0.998499	
QLI74585.1	OTHER	0.351374	0.648626	
QLI74586.1	OTHER	0.002355	0.997645	
QLI74587.1	OTHER	0.000563	0.999437	
QLI74588.1	OTHER	0.001162	0.998838	
QLI74589.1	OTHER	0.002719	0.997281	
QLI74590.1	SP(Sec/SPI)	0.974386	0.025614	CS pos: 16-17. SLA-AP. Pr: 0.5717
QLI74591.1	OTHER	0.000517	0.999483	
QLI74592.1	OTHER	0.000474	0.999526	
QLI74593.1	OTHER	0.000475	0.999525	
QLI74594.1	OTHER	0.001915	0.998085	
QLI74595.1	OTHER	0.020751	0.979249	
QLI74596.1	SP(Sec/SPI)	0.670054	0.329946	CS pos: 24-25. VMG-MM. Pr: 0.4560
QLI74597.1	OTHER	0.000243	0.999757	
QLI74598.1	OTHER	0.001640	0.998360	
QLI74599.1	OTHER	0.000724	0.999276	
QLI74600.1	OTHER	0.000929	0.999071	
QLI74601.1	OTHER	0.399041	0.600959	
QLI74602.1	OTHER	0.001595	0.998405	
QLI74603.1	OTHER	0.002068	0.997932	
QLI74604.1	OTHER	0.001074	0.998926	
QLI74605.1	OTHER	0.019599	0.980401	
QLI74606.1	OTHER	0.000512	0.999488	
QLI74607.1	SP(Sec/SPI)	0.989935	0.010065	CS pos: 16-17. TAA-FP. Pr: 0.7561
QLI74608.1	SP(Sec/SPI)	0.944375	0.055625	CS pos: 17-18. AAA-QL. Pr: 0.6573
QLI74609.1	OTHER	0.346590	0.653410	
QLI74610.1	OTHER	0.000905	0.999095	

QLI74611.1	OTHER	0.001268	0.998732	
QLI74612.1	OTHER	0.000939	0.999061	
QLI74613.1	OTHER	0.000456	0.999544	
QLI74614.1	OTHER	0.000570	0.999430	
QLI74615.1	OTHER	0.003152	0.996848	
QLI74616.1	SP(Sec/SPI)	0.994625	0.005375	CS pos: 22-23. AQA-TP. Pr: 0.7466
QLI74617.1	OTHER	0.064619	0.935381	
QLI74618.1	OTHER	0.000545	0.999455	
QLI74619.1	OTHER	0.136366	0.863634	
QLI74620.1	OTHER	0.008659	0.991341	
QLI74621.1	SP(Sec/SPI)	0.986883	0.013117	CS pos: 22-23. TTA-AT. Pr: 0.4968
QLI74622.1	OTHER	0.301754	0.698246	
QLI74623.1	OTHER	0.001339	0.998661	
QLI74624.1	OTHER	0.004644	0.995356	
QLI74625.1	OTHER	0.001617	0.998383	
QLI74626.1	OTHER	0.025593	0.974407	
QLI74627.1	OTHER	0.000888	0.999112	
QLI74628.1	OTHER	0.000360	0.999640	
QLI74629.1	OTHER	0.002872	0.997128	
QLI74630.1	OTHER	0.000556	0.999444	
QLI74631.1	OTHER	0.001807	0.998193	
QLI74632.1	OTHER	0.001585	0.998415	
QLI74633.1	OTHER	0.001472	0.998528	
QLI74634.1	OTHER	0.002701	0.997299	
QLI74635.1	OTHER	0.001215	0.998785	
QLI74636.1	OTHER	0.004482	0.995518	
QLI74637.1	OTHER	0.001359	0.998641	
QLI74638.1	OTHER	0.002087	0.997913	
QLI74639.1	OTHER	0.005506	0.994494	
QLI74640.1	SP(Sec/SPI)	0.909635	0.090365	CS pos: 22-23. ASA-NA. Pr: 0.5256
QLI74641.1	OTHER	0.001840	0.998160	

QLI74642.1	OTHER	0.000751	0.999249	
QLI74643.1	OTHER	0.005293	0.994707	
QLI74644.1	OTHER	0.000547	0.999453	
QLI74645.1	OTHER	0.001466	0.998534	
QLI74646.1	OTHER	0.000989	0.999011	
QLI74647.1	OTHER	0.000728	0.999272	
QLI74648.1	OTHER	0.000418	0.999582	
QLI74649.1	OTHER	0.012683	0.987317	
QLI74650.1	OTHER	0.005474	0.994526	
QLI74651.1	OTHER	0.000451	0.999549	
QLI74652.1	OTHER	0.010689	0.989311	
QLI74653.1	OTHER	0.004435	0.995565	
QLI74654.1	OTHER	0.000951	0.999049	
QLI74655.1	OTHER	0.000348	0.999652	
QLI74656.1	OTHER	0.001178	0.998822	
QLI74657.1	OTHER	0.000517	0.999483	
QLI74658.1	OTHER	0.000195	0.999805	
QLI74659.1	OTHER	0.002234	0.997766	
QLI74660.1	OTHER	0.000627	0.999373	
QLI74661.1	SP(Sec/SPI)	0.835062	0.164938	CS pos: 28-29. AKA-ED. Pr: 0.5073
QLI74662.1	OTHER	0.000904	0.999096	
QLI74663.1	OTHER	0.001439	0.998561	
QLI74664.1	OTHER	0.002419	0.997581	
QLI74665.1	OTHER	0.002102	0.997898	
QLI74666.1	OTHER	0.001296	0.998704	
QLI74667.1	OTHER	0.001171	0.998829	
QLI74668.1	SP(Sec/SPI)	0.912803	0.087197	CS pos: 26-27. IQA-RS. Pr: 0.7581
QLI74669.1	OTHER	0.000486	0.999514	
QLI74670.1	OTHER	0.002565	0.997435	
QLI74671.1	OTHER	0.468355	0.531645	
QLI74672.1	OTHER	0.006411	0.993589	

QLI74673.1	OTHER	0.001185	0.998815	
QLI74674.1	OTHER	0.000968	0.999032	
QLI74675.1	OTHER	0.001124	0.998876	
QLI74676.1	OTHER	0.000628	0.999372	
QLI74677.1	OTHER	0.000894	0.999106	
QLI74678.1	SP(Sec/SPI)	0.838613	0.161387	CS pos: 32-33. SQS-LA. Pr: 0.2126
QLI74679.1	OTHER	0.003377	0.996623	
QLI74680.1	OTHER	0.022194	0.977806	
QLI74681.1	OTHER	0.020706	0.979294	
QLI74682.1	OTHER	0.000914	0.999086	
QLI74683.1	OTHER	0.002305	0.997695	
QLI74684.1	OTHER	0.001170	0.998830	
QLI74685.1	OTHER	0.001438	0.998562	
QLI74686.1	OTHER	0.006561	0.993439	
QLI74687.1	OTHER	0.002613	0.997387	
QLI74688.1	OTHER	0.006246	0.993754	
QLI74689.1	OTHER	0.001679	0.998321	
QLI74690.1	OTHER	0.005048	0.994952	
QLI74691.1	OTHER	0.010169	0.989831	
QLI74692.1	SP(Sec/SPI)	0.519972	0.480028	CS pos: 18-19. VGA-MQ. Pr: 0.2276
QLI74693.1	OTHER	0.002992	0.997008	
QLI74694.1	OTHER	0.045622	0.954378	
QLI74695.1	OTHER	0.002613	0.997387	
QLI74696.1	SP(Sec/SPI)	0.911002	0.088998	CS pos: 22-23. ASA-NG. Pr: 0.4326
QLI74697.1	OTHER	0.002825	0.997175	
QLI74698.1	OTHER	0.001788	0.998212	
QLI74699.1	OTHER	0.005151	0.994849	
QLI74700.1	OTHER	0.000471	0.999529	
QLI74701.1	OTHER	0.001960	0.998040	
QLI74702.1	OTHER	0.002279	0.997721	
QLI74703.1	OTHER	0.000504	0.999496	

QLI74704.1	OTHER	0.000559	0.999441	
QLI74705.1	OTHER	0.000572	0.999428	
QLI74706.1	SP(Sec/SPI)	0.874012	0.125988	CS pos: 20-21. ATA-RP. Pr: 0.4768
QLI74707.1	OTHER	0.003502	0.996498	
QLI74708.1	OTHER	0.000495	0.999505	
QLI74709.1	OTHER	0.004335	0.995665	
QLI74710.1	OTHER	0.002244	0.997756	
QLI74711.1	OTHER	0.140328	0.859672	
QLI74712.1	OTHER	0.001468	0.998532	
QLI74713.1	OTHER	0.001493	0.998507	
QLI74714.1	OTHER	0.000579	0.999421	
QLI74715.1	OTHER	0.000829	0.999171	
QLI74716.1	OTHER	0.001049	0.998951	
QLI74717.1	OTHER	0.000735	0.999265	
QLI74718.1	OTHER	0.001101	0.998899	
QLI74719.1	OTHER	0.002162	0.997838	
QLI74720.1	SP(Sec/SPI)	0.722280	0.277720	CS pos: 20-21. ASA-TP. Pr: 0.6050
QLI74721.1	OTHER	0.002164	0.997836	
QLI74722.1	SP(Sec/SPI)	0.975586	0.024414	CS pos: 20-21. TTA-AS. Pr: 0.4285
QLI74723.1	SP(Sec/SPI)	0.927326	0.072674	CS pos: 21-22. VSA-ES. Pr: 0.7330
QLI74724.1	OTHER	0.222469	0.777531	
QLI74725.1	OTHER	0.000125	0.999875	
QLI74726.1	OTHER	0.113270	0.886730	
QLI74727.1	OTHER	0.001903	0.998097	
QLI74728.1	OTHER	0.000424	0.999576	
QLI74729.1	SP(Sec/SPI)	0.990016	0.009984	CS pos: 17-18. AIA-HP. Pr: 0.3806
QLI74730.1	OTHER	0.002948	0.997052	
QLI74731.1	OTHER	0.004469	0.995531	
QLI74732.1	OTHER	0.000625	0.999375	
QLI74733.1	OTHER	0.000467	0.999533	
QLI74734.1	OTHER	0.013096	0.986904	

QLI74735.1	SP(Sec/SPI)	0.937020	0.062980	CS pos: 24-25. ALA-QD. Pr: 0.5883
QLI74736.1	OTHER	0.001193	0.998807	
QLI74737.1	OTHER	0.000450	0.999550	
QLI74738.1	OTHER	0.000517	0.999483	
QLI74739.1	OTHER	0.005538	0.994462	
QLI74740.1	SP(Sec/SPI)	0.930300	0.069700	CS pos: 24-25. GWA-AV. Pr: 0.4624
QLI74741.1	OTHER	0.005720	0.994280	
QLI74742.1	OTHER	0.000203	0.999797	
QLI74743.1	OTHER	0.000956	0.999044	
QLI74744.1	OTHER	0.003020	0.996980	
QLI74745.1	SP(Sec/SPI)	0.994808	0.005192	CS pos: 17-18. GSA-AP. Pr: 0.8670
QLI74746.1	OTHER	0.000284	0.999716	
QLI74747.1	OTHER	0.002268	0.997732	
QLI74748.1	OTHER	0.006144	0.993856	
QLI74749.1	OTHER	0.002423	0.997577	
QLI74750.1	OTHER	0.002019	0.997981	
QLI74751.1	OTHER	0.001216	0.998784	
QLI74752.1	OTHER	0.001518	0.998482	
QLI74753.1	OTHER	0.002775	0.997225	
QLI74754.1	SP(Sec/SPI)	0.978783	0.021217	CS pos: 17-18. ALA-AP. Pr: 0.7377
QLI74755.1	OTHER	0.000588	0.999412	
QLI74756.1	OTHER	0.001400	0.998600	
QLI74757.1	SP(Sec/SPI)	0.918930	0.081070	CS pos: 26-27. LEA-QA. Pr: 0.6052
QLI74758.1	SP(Sec/SPI)	0.997193	0.002807	CS pos: 17-18. ARA-CP. Pr: 0.8069
QLI74759.1	OTHER	0.001054	0.998946	
QLI74760.1	SP(Sec/SPI)	0.988595	0.011405	CS pos: 19-20. AAA-AP. Pr: 0.7309
QLI74761.1	OTHER	0.000646	0.999354	
QLI74762.1	OTHER	0.002416	0.997584	
QLI74763.1	OTHER	0.024375	0.975625	
QLI74764.1	OTHER	0.000628	0.999372	
QLI74765.1	SP(Sec/SPI)	0.975067	0.024933	CS pos: 18-19. AQA-AS. Pr: 0.8529

QLI74766.1	SP(Sec/SPI)	0.998417	0.001583	CS pos: 19-20. TTA-LE. Pr: 0.9005
QLI74767.1	OTHER	0.001256	0.998744	
QLI74768.1	OTHER	0.000988	0.999012	
QLI74769.1	SP(Sec/SPI)	0.998716	0.001284	CS pos: 18-19. AAA-KG. Pr: 0.5841
QLI74770.1	OTHER	0.001414	0.998586	
QLI74771.1	SP(Sec/SPI)	0.998349	0.001651	CS pos: 20-21. AWA-KT. Pr: 0.9744
QLI74772.1	OTHER	0.001278	0.998722	
QLI74773.1	OTHER	0.009748	0.990252	
QLI74774.1	OTHER	0.001224	0.998776	
QLI74775.1	SP(Sec/SPI)	0.999393	0.000607	CS pos: 16-17. ATA-AP. Pr: 0.4573
QLI74776.1	SP(Sec/SPI)	0.985081	0.014919	CS pos: 20-21. SVA-RR. Pr: 0.7742
QLI74777.1	SP(Sec/SPI)	0.870812	0.129188	CS pos: 19-20. AAA-SS. Pr: 0.5453
QLI74778.1	OTHER	0.002522	0.997478	
QLI74779.1	OTHER	0.000626	0.999374	
QLI74780.1	OTHER	0.000788	0.999212	
QLI74781.1	OTHER	0.001419	0.998581	
QLI74782.1	OTHER	0.002020	0.997980	
QLI74783.1	OTHER	0.001690	0.998310	
QLI74784.1	OTHER	0.001433	0.998567	
QLI74785.1	OTHER	0.001233	0.998767	
QLI74786.1	SP(Sec/SPI)	0.987956	0.012044	CS pos: 20-21. ANA-AP. Pr: 0.8744
QLI74787.1	OTHER	0.000639	0.999361	
QLI74788.1	OTHER	0.010901	0.989099	
QLI74789.1	OTHER	0.003063	0.996937	
QLI74790.1	OTHER	0.001945	0.998055	
QLI74791.1	OTHER	0.001854	0.998146	
QLI74792.1	OTHER	0.000733	0.999267	
QLI74793.1	OTHER	0.000225	0.999775	
QLI74794.1	OTHER	0.000910	0.999090	
QLI74795.1	OTHER	0.001522	0.998478	
QLI74796.1	OTHER	0.000618	0.999382	

QLI74797.1	OTHER	0.006542	0.993458	
QLI74798.1	OTHER	0.001850	0.998150	
QLI74799.1	OTHER	0.002684	0.997316	
QLI74800.1	OTHER	0.000594	0.999406	
QLI74801.1	OTHER	0.000630	0.999370	
QLI74802.1	OTHER	0.000655	0.999345	
QLI74803.1	OTHER	0.001260	0.998740	
QLI74804.1	OTHER	0.001611	0.998389	
QLI74805.1	SP(Sec/SPI)	0.823392	0.176608	CS pos: 21-22. AIA-GT. Pr: 0.6645
QLI74806.1	OTHER	0.000848	0.999152	
QLI74807.1	OTHER	0.007246	0.992754	
QLI74808.1	OTHER	0.001693	0.998307	
QLI74809.1	OTHER	0.005791	0.994209	
QLI74810.1	OTHER	0.000803	0.999197	
QLI74811.1	OTHER	0.000904	0.999096	
QLI74812.1	SP(Sec/SPI)	0.973975	0.026025	CS pos: 20-21. ARS-AV. Pr: 0.5144
QLI74813.1	OTHER	0.001737	0.998263	
QLI74814.1	OTHER	0.000434	0.999566	
QLI74815.1	SP(Sec/SPI)	0.992807	0.007193	CS pos: 22-23. SGA-AD. Pr: 0.4164
QLI74816.1	OTHER	0.010430	0.989570	
QLI74817.1	OTHER	0.000578	0.999422	
QLI74818.1	OTHER	0.001112	0.998888	
QLI74819.1	OTHER	0.000165	0.999835	
QLI74820.1	OTHER	0.000975	0.999025	
QLI74821.1	OTHER	0.001129	0.998871	
QLI74822.1	OTHER	0.000986	0.999014	
QLI74823.1	OTHER	0.001524	0.998476	
QLI74824.1	OTHER	0.000706	0.999294	
QLI74825.1	OTHER	0.001033	0.998967	
QLI74826.1	OTHER	0.000754	0.999246	
