# Supplementary material for: Telomere length de novo assembly of all 7 chromosomes and mitogenome sequencing of the model entomopathogenic fungus, Metarhizium brunneum, by means of a novel assembly pipeline
Source: BMC Genomics. 2021 Jan 28;22:87. doi: 10.1186/s12864-021-07390-y (PMC7842015; doi:10.1186/s12864-021-07390-y)
Supplement: Supplementary file 4 — Additional file 4. Mature proteins. M brunneum mature protein sequences with signal peptides removed. [file 12864_2021_7390_MOESM4_ESM.pdf]

>QLI63438.1

GPLKKRALTTEWVTEFVTVTVTADDSEPTPEIPAVFVETEVVTLTPSAPPKVKPTPVFPDSPSVEIKPSTSTFSPPPKPT
VIQPKPEVSTPAAPPPPATTSAAPPPSPSSGNSGSYADKMVQIHNECRSKHSSPDLIWDPNLAITAEKIAKSCKFAH
QMGVDGGSYGQNIAAASSDKNSNPTDSGVNAVGDMWYGEVKNYTPAFYRSGPSQGTPLGDYGHFTQVVWKG
STKVGCATVYCDAGTIFQMPSQFTVCNYKTQGNVGGQYPENVLPPKTG

>QLI63475.1

SPTGTPPAGDPERIATRTATAVVPAGTILGSARFGVEAFNGIPYAEPPLGPLRMKPPVRLTESLGVFDATGLARPCH
QQVVSTDSKNFLLDTLGSLANLPFVQTVTGQTEDCLTISVARPEGTTAGDNLPVLFWIFGGGFELGWTSMYDGTG
LLNHAKSINQPFVFVAVQYRVAGYGFMPGKEIMGEGSGNAGLLDQRMGLEWVADNIAAFGGDPDKVTIWGESA
GAISVLDQMIMYEGDHTYKGKPLFRGAIMNSGSVVPTNPLDHPKGQAVFDKVAETGGCGGAADKLACLRALSSN
DFLNAVTSAPGIVSYDALALSYLPRPDGKVITVSPDILALSGKYAPVPMIIGDQEDEGTFFGLFQPNLTTTRGFADYLQ
SYYFPGATTDQLVQLVETYGTGLSAITNGSPFGTGLLNEITPGFKRRAAVLGDLVFTLSRRVFLAGATATHQNVPSW
SYLASYGRGLPILGTFHGSDIVQTFYGVPDNYAANSIRTYYSNFVHNLDPNVGVQGEFPNWPRWSEGNNLAHFFR
DRSTLLKDDFRQTSYEWISNHIAELRF

>QLI63481.1

VTPISDSDMNQMLNEGGVSLAMKAQPMFFFGQAMKQPPCIPTFATTPDGKQVPSSKLCAFPDVGCECRNPGVG
IGNPSPSFPIYYSYQKCSDTSVRVAYNFDWERVIVVFTKAADGNWRPDQLFLSQHSGYGRLQWAKIQNTINREDA
AEPRGGKDGRQYLDHPKVYVEWSKHAHRDTRNTGWNDPLSQLTDNAFRSNDWWYFPKRDDYIRADRSTEIGRL
LGSFDWGDASSNPPSVHDGLCTA

>QLI63487.1

KLQTACANISSSYQSWVARGGTGTFAGIPGSTAQGCLRSMPFHPQLALQFLKEYEKYLQFHSTIHTLKHAPPSYMS
AHVDLVAHLENIRQKVVNGLYSSQFDFDWDLGRSISKANDGHLTLSLCSQRIMHFEHPPPLVSLSTDGLELPSIFTFQ
DAKLKLAGFPEVSPLVEINGVEAAYFLEANYAIKLGYQDPDARYNHLFASPSANFSGKYSGGAWTSLLGLWPGASN
RLLFSNGTTVTVKTTASWPHSNGPMNYPDGKSLFEAACVPHPDSGSAFGSYGGTYGISPQLEAPPSGASVFPDPIV
GEQRDRARGYYLDGAGCEDVAVLQVSDFRVGQNATRFASVVGRFVMQAAADGKRKLILDMSGNLGGDIAAGFN
LFRVLFPDKPIYPATRFRATELIDFMGRIFSETHKQENVTLDLPFVAPLAVGIDGTTKMGSWGDVYGPHEIMGDQM
SSMSGVFDFNLGSTSSDPISGYGDIPLFPEVQLFPANNIILMTDGQCASTCSVVAHLLKEQGVRSIVFGGRPRHGLM
QAVGGVRGAQYWALDTISEYVERARHLALAALQTDSPILSRAEMQRFNELAPLPLRDLPLRLDTYEQSGINICNAYS
PGDDSIPLQFMYEPADCRLFFTAENYMDPATAWAAAAAAMFLNGSCVDATPAGRQGLSISPAKTMAESVSLHSS
RGGLRQKFGAERSPSGKALPLPRWAA

>QLI63502.1

SRTFYNSGTLNGWDYVRKEHKGTVDQVTNVVYKGSSALKMTQTYDSNYHNRYHAEVDHNYGYKRGDSSFYGFA
FRLSDTWDFQDQSYNIAQFIANRPGAGCGGDDWMPSTMVWVQGNQLFSRIVTGHYRQPNCGRNIDTLRNLGQ
ISAGQWHKVIMQVKWASDGSGFFKIWLDGNKVVERYNTATTIDDDSVFQFRVGLYANSWHDDGYMKGNQGFR
QIWYDEIAMGTEFMDVDPDQQ

>QLI63512.1

HQQPLKDAKHRVPTSHHTVTTAAPSYRQELLSLHRQLISIPSISGDENAVGNFLVDYLTARGYHADLQPVAARDGTP
PNKQRFNVLAWKGERIPSAKVVVSSHIDVVPPHIPYSISPGEITPETMIKGRGSVDAKGSVASMIVALEQLHASKEIA
NRGDVMLLFVVGEEVAGDGMATFSDSLSRMEEPPKFDAVIFGEPTENKLACGHKGGLFCDITARGVPGHSGYPW
LGKSANELMVRALAKILDADLGSSELFGNTTFNIGRFDGGVAANVIPEKAVVKFAGRVAIGPEDKGHEIVTSKIQRIL
DEVDDEAFDMDCTHGYGSVECNCEVDGFEKITVNYGTDIPNLAGDHTRYLYGPGNILVAHGARENLTVADLEEAV
EGYQKLIVHALKQ

>QLI63526.1

QPGGNLEVSEIQKITDASQGGFRPFIEFSVAGQVMRGLLDTGSSDLNIPKTGSEFCRGEGEQCDGRTTGFKAGSLD
VNKNKTDIRDLNIPLNATFTGGAAFVGTFIESSLQVTPGGKEVPVRMGLVESGGVPPGQVSFPVMGIGPIQGESAK
DPYPNVPARFKQDDLSKSNAYGLYLGDFRSPNNGSLVWGGFDAAKFEGELKAAPLLKPDGDSLPAFVVDFSSVGL
APSRDTDQGNNSQGRPNRQKSAVSESDDSIPWSGEFKTSAFTSRRLDNARVPSYAHTSQSLSTRDLLQRQQRGNL
LSNDAPPVAWLDTGVPEIILSGGTLDALGRRLGARPGPQGEFLADCNRIRGMDLTLGMNQDNVQVRVPLDSIIVA
PDPGSGAGEPGNAGRGNSRQPQRGRNQRGPNKGQNNGKGRGGNRQGGNQQGGNQQGGNQQGGNQQGG
NQQGGNTPNGNGQGGNDNIGSGTSPGAGAGTCQLAIAPVQGNGPTDIPVLGAPALQSMYVVFDMDSKALMIA
QAKMNETRTDIREYIPGGGS

>QLI63534.1

LVGNGWSFSGKPAGGLKDVTFPFNMAGAAHTSGYYFAQQFKFNGIPDVGYCGIQNRPNKNGKSIVHGVFSSFQK
GTTTSDPNCHTGADGGPGVSCAVDFEGDYSATYNIVVENESGTKWKGTAVNAATGKSVHIGSWTLPSGARGIESS
QVGFVEYYPWNSGTHKCSDLPKTSVTMYKPTSKTAGAGAGAIGKPYEYGDCVGKVNFSTVGVPDGWKITVGF

>QLI63560.1

VEVVSDSSCDCFLTNGTQATYFTNHLFFDFRSLKDHASVPSVISDPEDTPNAPPTSDYFKSEAWTKTWALQTWANS
KRNGNGFSGDATVLMVNSPNNVFIEPNKDASASSATYLTMRTARLSNFQTAAEFQTQNSDYHYLSLRMLARTVG
PPGAVSAVFTYRDASKLADIQEADMEIVTRGPGNKVQYTNQPGYTDNGDGIKANATRNATLPRGLQWSDWAVY
RLDWTPTRSVWYVDGEEVANIAYQVPRDPAAINLNAWGDGGSWSGNMSVGAESNLQLQWIEMVYNTTEDSQ
RSRRKRRGAAAQLLTRGGKATLCGAVCSIDEGSQAGAVAKLWGVKSSSARTAASSCSLLAVAWFCLALGVFA

>QLI63581.1

TEPQPVAFYPTAWLGVDGNWSTFGFLVGSNNVNVLFSTALSEFWAVGPGGCNRNDPHCSSNRGGIYYPSDSKH
WSGLGTWELGLPDLGTGGNGQYGFDTIAGLNPMTSVGYQMSNVLISAINSTDYFLGFFGVGMRSGNFGDIVATP
PMRQAVASFGWVSSYSYGYTAGASYRNTVGSATLGGYDAARLAMHDKTFTLNQTEGVPRPLIRGIEVSSSNGSS
WKPATRILLDYKSAFTAVIDTTTPYLWLPPALCDNFAKALNLTYNETFQLYTLTNEQYRQYSSNSSLAFVFSFSSMDN
RDNFGDPLDVTGVVNITVPITAFVSLLSYPFMKGAIGYTDPAVPYFTLRKAPANSTAIIGNAFMQEAYLMTKYDSGV
FSIHQARFPSDPTGDAKLTKINRPNNSPYPPPPDPNGGNGLSTGEMVGIAVGAVAFFSTLVFAFFCYRRHKRQQRE
RSGDDFDDGKDSASTLTPDSPRSPVYRMLSRIVGRRRSRRAGTTMTAGGDPAEAPDHQIYELPAPVAPAELDAGG
SDDNSILGEADLGTDSTQHLSAYEIARRKLDRQLQGPVPEYTPPVDGTVVQLEKASVPELRPTIQTDMIDQPSPVSP
TRSRGADSTSNTFLVSEPSPVSPRGDWNSAELPSPLTASMPPRSFSSGTRSGGQSTASRSLSSNSNTPVSQVTDARP
PPIPAAFQRTPIDPSKVVCLGPLPENVRLSGHTVGTGSRIVNSDGRDIPASMFIPGSRTSEGSLGSNFTEEEVRMAEE
KPRCTNHSSMGLSHGHHGTLPSQWETDRQSLPSQPREHGSETGSPKPVHSDESSEEGRIDPGRDLIHVPQMADK
RYSWEEYRS

>QLI63602.1

QSEPQPSNPSTASVTDNYMGPAAFMWPPDRVWSGDMDNQAPCGSRAAAGNRTKFPITGGAVALVAQDDYYN
SKISISYKEDPQTNTDFSVLLETIDISDLNPGHTCVSVPDAPSRVTAGTNATLQIIYKADWDAPHNQTFYACADITYV
NQVDFKLRIPCFNATEPGEDDKKFAAEKDKPAGDSKTPTASSGGSGLKGGALAGVIVGSILGACAIAAAGLFLYRRR
EQKLRQLRLAKMEENAKKSDYRDDRSVELI

>QLI63603.1

LNSFTVDSDCDSLLAQALDGGLPQPVHWAKAGCQQALVCKSDPTKDCCISRIKNGLPLPLRYDDAVPYYEKCAGVS
VPKPRKEDDIVLPSLSCELIRDFRCGLGFASEFCDIVYAELDGCKKREQCSKQTEKDCCGAYPAKNPEAVRRYYSKCV
GTCNVVTEKVVEQEDCLAVGE

>QLI63614.1

HGLSSNTTIDRRQTGGESTYGERAVMHVYRGEIRRTPEQVKQDGGFYSRGVQRILSGNPPSVEELELGSSLYRHAA
GDTAEFTRYVSTSTDPGVSLTFAVDDERPTGKGYIYKIHASQRLVDLNRSLGKYSPYPGQHEYVAMEFIPFEQIEGW
WTVTYQDDFSDPAIGRRTRMQLREGNLRGFHQNPDFDNSFKYARTGGMAPQLAGFPRLSAAWEDRSWQEYKTI
PVSKSLDDMIGAACAGKASCRLERITPKVASWRFNKLTTSKALASNGPIDPTKQRKPFSGFRVYGKAATLVGIQALA
PHMRQVLHWLREWDHPIGHAVRWIDDHINELQQMIGGPPRSDISGNDNQAALINFFKYIFWLLQGSDRKPEQL
DLLSYGEKTRRRLVSVNDILRTCDRVDEQPPEDALLRDNLHESCSEMRDKAMEVEGLTEEEVVVGRNLCSSCQTCT
WEPEHGLCRDKAGTIVWPREPLPEGDQYAPTLQTGENPCKASRKRARGAAEA

>QLI63617.1

SGASETEKDAVQSTIFNNQTVPPLVELTPSNWADEVKKSKWLLVKHFSPWCGHCMAFAPTFQTLYEFYHTSKPKD
DSTFESYYDFRFGVIDCTVYADLCSEHNVAAFPTTVLFENGAAFDRVQGAKGITVVHNLIERGLEKEHPGSRPKTIDL
PEPGATESPKKPTATTEDDVKKDEEPKKDEEPKKNEEPKKNEEPKKNEDAKKDEDGKPSKKPLGNDWKVPTAGELE
KERKPKKPTVTPNPEGVSIPFTAESFQKLVTQTQDAWFIKFYAPWCPHCKAMGPTWEQLAKTMRGRLNIGEVNC
DKESRLCKDVHATAYPTVIFFKGGERAEYSGLRGLGDFVQFADKAVDLASGIPDVNATSFKAMEETEDVIFMYFYD
HATTSEDFRALEMIPLSLIGHAKLVKTNDPTLYERFKITTWPRLLVSREGRPTYYPPITPNEMRDKNEVLNWMRSV
WLPLVPELTPSNARQIFNGKLVVLGILSHVDKDSFSSSLREMKSAANEWMDRQIQEFQLERKKLRDSKQMRIEEAE
DRGDQRALRAAKGIRVDMNNSGRKEVTFAWVDGVYWQRWIRQTYGIDVKDGEKIIINDEDNRRYWDQTVTGN
YIMVSRTSIMETLDKIVYGPHVIKYKLTVSTIEKIFFDIKVAFVEHPYLSMGCVLGMAFGTLSWLRGRSRRARGGHFR
LDDAMGINQLKEGLLGMNGNSNQKAD

>QLI63619.1

ATPQCNTSADCLPGYICGPSDFAFSGTSSNVCVKTGTCNNKPDPQFPQDGPKCGDSIFCNVGGSCGEGYYQSGGE
TVLTQVCVNQATGQQCAASS

>QLI63626.1

KPTNDAPKNDIVDLGYAQHIPTYHNTTISGHKVKIYKNIRFANAPTGNLRFRAPDTKLPKVHGVQDGKVPWASTAC
IASAPGYIPYPEINGTTWGREDCLFLDVYVPEGVNPGDDVPVLHNFFGSAYAFGSKDIMFSPMGLFDRMFRDHAD
KFIFVSNNYRLGMPGWTYAEGEDMDGNVGMLDCLAAAEWTSKYIKDFGGDGKRITTIGQSAGAGMIYYLMVAY
AGRRKLPFQQSKAYLSSPAAPPKRNVTERQKELFNLVLKTANCTTLRCLRSVPEETMIGINDQLINQMPSQSGGGQI
GPLHGFGPAPDGKIIPDLPLAMLRRGEFYKGLNALILGSMALEGMGTSHDTGLPGYFPIMVRQQLPYASSETIKML
QDEYHDASKVSKMAWDWTTDIVFACNANNLANSLPSLARRYIMSTPPAVHGQDLDYFFNNEDIAPVNNTELVN
GFQRRLLNFVNGQKMDWPVWGSVKEMYNITDEFEGTTLPRKLMKRCDLLNQLILDPANGA

>QLI63633.1

SPVSRGESPVDSAVIIFAILRDTPIHHGLFAANGSAIWIGKDTASYCPPVVEERGECPKGKDTSFWVNDRCGMNVIV
PGGQQAYVAPDGTISYTAPHSAYIPPGSIRTGFHLIPTPFDGFWEFVIDSHEFWACPGTPGQGPWQVIIADENSSV
PGRDVTDCLKFKAYAKPVKDPVWEYLSTLGPEPIVVQEYL

>QLI63634.1

ATFTLSEVQAQIEAYQEQNGTHVAPFDPQFGCTLACGFLAFSRPGKLSYPNSLAYEFEESRYWSQQQAFTRPACRF
TPGAAVDVSLAVLTSRVTQCKFAVKSGGHAAFAGASNIEGGLTLDLRNLNRVVVSADKKQTSVGAGNIWYDVYTK
LQPMGLTVVGGRVSAIGVGGLTLGGGISFFSNRYGWSCDNVNNFQVVFADGSIRHVNPRAYPDLYWALRGGGN
NFGIVTRFDLATYPQGDMWAGSQAFLLTNETATAINNAFYHLAINSPQDPYAQIIIAYAYVQSQGVYVIASDLQYG
KPTPNPAILNNFTSIKGPVADTLRVTNLTGLTLEFNSSNPGGFRQTYWALTTGNDAGLMSEMVAIYQDEVEKIKDT
PGIVPSVIFQPISTDMTKLMSKNGGNPLGLAGQGPLNLINIDISWSNEADDMRIITAAQNMATRFAAAAEAKGLD
HPYLYQNYAALQQDVFLSYGEVNLARLKSIQAKYDPTKAWQKLQPGYFKLG

>QLI63638.1

TQIHNNPVACAVGCLNEFKGEFTSQHQDPYVGGCMGPFPNDIACCILKTCPEDQWPMFCGKDNYMNVPDKQK
CFA

>QLI63641.1

SNFNITPEFAVQNGCDEKCQATLAKVNAIDLGIFGQNFDFDWFATASNFSGSKPGDVLKMQAMDPKMLGEQVK
QGTTVYRIQYASVDLDNSTVPATGFIALPFAPASLKSKASNSSSSHQFPLVAYAHGTSGVYRGCAPSNGPALYDYDS
WQLLLQRGYAVVATDYAGLGNNQTEHKYCSFPVQVNDIVYSVVAARKAMGNIFTQEWMAVGHSEGGGAVWK
LAESRFAKDDSKYLGTVSLAPALKIADMLRNHTDFILKSGYLTLVAKALQRFSPSYNFTMLGQVQRQRMAIADASQ
PCLIAQGAISVGLTREQVLDEKGFETDLQLLQSWQDKMAPASGGRTSAPLLLVQGLNDTAVVAQVAHESWQQAC
QDQSEVHLREYTELDHSGVIVAAASEWLAWVDARFNGEATSGNCTRLQRVPFDAAHVIAPPEKPEDADA

>QLI63648.1

FYPYTPQWLKEKEDLTPLREAKRSPAVGNARDGVTFRIEKKRTQENQSPALLAAQQAAWLRHKFKHTRSGVPVNS
VEKRASQYSIMDATESNEAMAAGINQDGTDYSYFVKAGLGSKGKQVYLLVDTGAGSTWVMGSGCTDTSCSTHTT
FGPGDSSTFTDAGKSFSVSYGTGTVQGKLVTDTINVAGISLKYTFGQASKTSDVFSHFAFDGILGMSMGQGDSDNF
LKTLVEAKKLDKNLFGIHLNRASDGSNNGEIKFGSTNPDKHIGDISYTPIGSKNGDWAIQIDDMAYDGKKADAGGV
LSYIDTGTSFIFGPPSLVRKIHNTVPGSSSSDGLTYTVPCSSNKTLSFTFSGIDYKISSKDWISPKDSSGNCTSNLYGREV
VDGAWLLGDTFLKNVYAVFDADQKRIGLAAPADSKGGSSSSSSSGSSPPTQTTVGLTTVTATALTTGVTPQPDFST
QSKGPPLGLSGHETGSTRSSSATAKPTESTKSAAPDVRARQTAKLAVAMLFAALAALVA

>QLI63661.1

VCKTNFTQPPANGPNANYQDNPVYQQGQKIDVQWKSDLDTMDLFVFQQYPAAGKGVQFLKKLREGTRSTSLIW
TVSLDGLSRNVPRGENAILYFAVLRARSPDRDGTSHYFNVSVPDAASTTAPATQTSDVPTMSTTTPAATDVPESSG
ESGPGLSRGGVAGVAVGSTVGGLLLLGGMGLLAWRKMRRGRPAEDAAVSDAKPPELAGPAIHEAPAEHRPRSTP
VYEAP

>QLI63666.1

APAPAPIRDQLSAPRINQRDVAVPKTNDKQVAALYDNGYAPPHW

>QLI63680.1

LPHYDSPQQRLAKGLSLTGKYAKDNPYTPDYSDPHDHAIDAVGKGLDPRPWRNGNGATVLGPYNRDRSRQSPD
MIRPPSTDHGNIPNMRWSYTDSHVRIEEGGWTRQTTVRELPTSIELAGVNMRLDTGVIRELHWHKEAEWAYVLE
GEVRVTALDYEGGNFMEDLKKGDLWYFPSGVPHSLQGLGENGTEFLLIFDDGRFSEESTFILTDWLAHTPKSVIAKN
FRLDPQVFAHLPAGEKYIFQGSQPGAIDQERPSGKHVKKSRYQFTHRMLDQEPKQTSGGLVRITDSTNFPIAQTVA
AAHVIIEPGALREMHWHPTADEWSFFIRGRARVTVFAAEGNARTFDYVPGDVGIVPKNMGHFVENIGDEPVEML
EVFRADKFRDFSLFQWMGETPRRMVVDHLFAGDEDNGRKFWDAVRDAEKDEVKQPGDVDDDAAEE

>QLI63691.1

QGVVSATVYSQPNFEGLLRNVIGFGCVNLDDVGFKIESIELRHQTFCYAYGKVNCAGTSVYMSRSVGDLWRYLTTE
SAHCFDAS

>QLI63695.1

LFTTLYVNGENQGDGTCVRMPHDSSTANGPIYPITGDDMSCGRDGNKAVAYTCPAPHNATLTFKFREWTDGTRP
GVIAPEHKGPCSVYLKKMDDMYADNAAAGPGWFKVWEDGYDAGSGQWCVDKLIEKEGLLSVRLPEGLPAGYYL
VRPEILALHNAPQGDPQFYLGCAQIYVQEGPDGPLRIPDEANVSIPGHVSADTPGLTYNIYKKSQGEYPIPGPKVFIP

EAKSFSPSPGNQTEGAIPKDCVVKNANWCAKPLPGFSNEDGCWASVENCWSQDQACWNSVTPSGDVNCKTW
QEYCTQSETRCNSGDYAKGPVEFKGEDTMPDLPGKALGMWNDVFTATG

>QLI63700.1

ALDPSELKNVGQGNGKQFITGKCLSNADCSTNCCAGKNGGGVCSAVAVANADGKTGCGFGGGNAGNNGANAG
NNGANAGNNGANAGNNGANAGNNGANAGNNGANAGNNGANAGNGGNQGVPTGDGAENFANGAGKQFI
TGVCASDADCASTCCAKTTKACAAIGAVGAENCGFVA

>QLI63701.1

QQDLSLKSSCDDLINAGPLSPSLPSTVPWKLQGCLAARECTSQPENCRTPLAIQENRIRAGKPLPHKTDDADAFWG
QFGYGNVEMRPYKGVVLEKYNCSLLKGFVCGLGFDGQYCKLMFAQKAGCESYDQYNSSPEHSSKCKEISEKFEDNL
EPEAYHLASQCIGLVEVPMSPLGAKGQDADHLSQKSPSTSHQETASTPDKTSNEDHGREWHRVPSRASPSAQAPT
DDIRLSTTVSSRTMDTFWFPQLSLGYDDTPRKIRRGFNSDKEMVFLACCRQYPKAVGWTLLLFLTVVMEAYDKSLV
SGFLAFPAFQRKYGQPESTTPGTSVGDGSGAYQIPAPWQIALHNAAFTCEIIGLLTHGYITYNIGYRKVMMGSLVW
LCLSVFPAFFASNITTLLVSQALCGIPWGVIQTLAATYAAEVVPSGLRPYVLSNINMCWVVGQLLGTGVLRALVHSD
SQWSYRLPFALQWAWAVPLLIGVYFAPESPWWFIRHERAADARRSLGRLCNRSGSHIDDSIALMEHINRVEKELN
YGGATYSDLFKGVNRRRTEISCVVWMCQALSGSVLTAVAAYFFEQAGFDPSNSFSLSTGMYGMALIAGIISWGLLF
KIGRRKLYMFGLASAVAFLTAGGIVSVVSAGSNGADWALGALIILMTFTYDLTLGPVCYVVVAEIPSTRLRVKTVALA
RVAYNIAMIVNTSLVPKMLNPTAWNIAGKSCFVYVGTAFCCLVWCYFRLPETKGLSYLELDILFEKKAPARKFGQVQ
DRLAKSAYLTASHAERLTDAWHGWLAYS

>QLI63710.1

GVVQPISVPAVHIRNGTVVGNYIESHNQDAFLGIPFAQPPIGDLRFNAPQSISEGWKSPLNATAYGAHCINYLLGLP
LDPADLATRYPQSEDCLTINVVRPAGTKPNARLPVLTYIYGGGFQEGGSADARYNTTALVDKSVQIGQPSIVVTMN
YRLQGWGFLAGDEARKQGLLNLGIQDQRLALRWIQENIEAFGGDHRRVTIQGESAGALSVGFHLLANGGRDDGL
FNAAICQSGGPYNALSFPSDAQSQKTYESVLKAINCTDASDTLKCLRAAPFDTLNTAFASLSFLPVIDGTLVPEYASTA
LASGRFVKVPLLIGANTDEGKVFAGMGVNTTEEFAGFIEKYPYVHTTTNATIRDLLEAYPEPGTNSTHGQSDDTLPV
SAPYGAQFLRAARYTGDVMFIAGRRYTCETWAHYGVPCYSYRFNTIPGATDPLYLGATHFEEVAFVFDNVLGLGM
PSNAFDVEPAERKQSYKQLGDTMSRMWMSFSATHSPNNHRVKSMRTLWPAYNLKNPQNMVFDGNITSFVEKD
DWRIDALKLIIERSSDFSR

>QLI63744.1

NPDFSFPDIVPMAKRQNSGPAYQCHASCGYAIQNSTKDGYCQDQSWVKLLDDCLDCALKYKIWQWYGDKVSAA
AGKCGLEATPKPVDDSGSSQVGSGTAAPSTTPITTAAQQTSAPGQSTHAESRSVSGPVAPTSAVQTTAASDVAPG
HNSTASHAPTVTAGASQNMLSGRVVAGVAVLIAANMF

>QLI63753.1

QSFSRCNPQLRDGCPPNSAFGNQRASCDLVQGTCGVFTPSAGTTTITHGVNGAAFKIEEQTDAPTIETPKYIFFGKF
EVDIQAAPGAGIVTSAVLQSDDLDEIDWEIIGSDNARVQTNYFSKGNTSTYDRGKFHNVSNPTGSAHRYTIEWTKT
QLVWSIDGEPVRTLLAKDCKAGTSSGFPQTPMQIKLGTWVAGKPGSAQGTIDWAGGYADWSKKPFVAYYKSVTI
TDYAGGDGPGRAATQYVYGDKTGTWESIRVQ

>QLI63758.1

FQGFNYGSTFTDGRPKAQTDFENEFKTAAGLEGTNGGFTSARLYTMVQAGTGNDVISAIPAAISTKTSLLLGLWAS
AGQASFDNELAALKKTIDQYCSQLDGLIAGISVGSEDLYRISPTGQAASPDPGASPTQLVNYIKEVRTTIKGSCLETAP
IGHVDTWTAYVNETNKPVIDAIDWVGMDAYPYYENTKPNGIENAAKLFQAAIDNTQSASGGKPIWITETGWPVS
GPKEASAVPGVEEAQTFWREVGCPRFGNVNVWWFTLQDGAPTIPSPAFGVIGSTLTTKPLYDLSCDKKQPTSSKA

AQSSQTSAQSQAPTATGPAGTSVQGSTVQQTTVPAAGTTQQGGQSNTVVPPPASTLTTGNGGNGGNGSNGGN
GGNGSNGSNGGNGSNGGNGSNGSNGSNGGNGSNGGNGSNGGNGSNGGNGNNGSNGSNGSNGGNGINGG
GAVNGSTTYIVPGQGTATGTGGLPGVTVPPNAGSKLSAFAGVAVGAVMAVAAL

>QLI63766.1

SQWQGRDLRTCVAEVLGEGADKRVVGPEQSTYTDARMGESIQFDQMPALIAYASHASQVAPLIRCARRSRVKAV
PRAGGHHFMAYSALGGALVIDITHIDFVDVSADKTTARVGAGIRLGALYTALNLHGRDWPGGICPTVGLSGFLGAG
GFNMQMRTLGLGVDHVVAAEVVLANGSLVNASPAENSDLFWAVRGGGGGSYGIVVEWTLKVSQFPRSSMVQI
KWHEPDSRVDLATRFFDWAPRTDPAFTSSVHVYKNRTEIQGWCLGCTLDHAKALMNSSGLLAIGKPEVHISGGCN
SINARMFGFIVSECIPDTEVSKYAPPAMNVLQQPFTQVDKYPQFTWNETRQDPNSPQAQPWPRFRRMSKSFFM
QKSKKLGREVVQSLVDRLTELPDEAAGWGEWHAWNISRKGEDEAAFAWRGEAYAHLEFILTGSEDAEKHKRLAE
WKEDLEGYLRPLTGPASYAGYMDASISTDPLPSYYGHNADRLKLIKAKYDHADFFDNPLGISPGR

>QLI63798.1

VPLTDKTSVKPRQAPGAQNVVYWGQNGGGTIENNDLAAYCQPNSGIDVLVLAFLYQFGNGGNIPSGTIGQSCYIS
TSGQGQNCEALTAAIHTCQSAGVKIILSLGGATSSYSLQTQAQAEQIGQYLWDSYGNSGNKTVQRPFGSNFVNGF
DLDIEVNGGSSQYYQYMIAKLRSNFASDKSNTYLITGAPQCPIPEPNMGVIISNAVFDHLYVQFYNNNNYTVPCALG
INGNAPFNYNNWTSFIADTPSAGAKIFIGVPASPLASTGTPSGAQYYAAPEQLAAIVGEYRSDAHFGGIMMWSAG
FSDANVNDGCTYAQQAKSILVSGAPCASSGPPSSTPATAPAPTATTMPSSTSVSSPTASPTGGTVPQWGQCGGEG
YSGPTQCVPPYQCVKQGDWWSSCR

>QLI63814.1

APTPGKTVEDYFIGADKRDKTVEDYFIGADKRDKTVEDYFIGADKRDKTVEDYFIGAEKRDKTVEDYFIGADKRDKT
VEDYFIGAD

>QLI63843.1

ANLKPPVNNQCLYSGKDFILMAVGGPNTRSDYHVNQTEEWFYQLKGDMLLKIVENGSQFRDVHIREGEMFLLPG
NTPHSPIRYKDTVGLVMERTRPKESIDQLQWYCPNKKAHSEEPVIIRHEQFYCEDIETQLKLVIDDWMTNEETRKCR
ECGQTAPPY

>QLI63847.1

PAIAITIATVTVKATAMVMMRVVTVDDLIVLHRQTECRYAHVANLLEETSRSAANGRRMTRPPKKKNVPDGPATIP
NIADRGAINDHEGSSRGAAALSIGLLSHVPYSVTKASNVFGIGSEHPFANYWTCEGGLPEVISVLPDKAQADSLVAR
YFECVDPVYPMIHRQTFYADYEHFWRMKTDERNKMDPSFIALIFVILALGTQFVSSTTPKDRKQTAEFYASASNQA
LRMFSYLSSASIRSIQAMVLITYFLINDNHASDGWAFAGILIRQAYAMGLHRDPNIVTPNANPFEKQQRRKVWQA
VLLQDTFLTVLLSLPPSATHTDVSVDDLLDDSSSIASSDPTDTAYIRGSWMLANLVQETICSPRSLDVPICTTVRHKSK
LVADFRAVYRSFPDIFRSWDTDSLTAVASTNKRIVRQTLFLTSNYFHNLMLVHASESPDVPVNVRGTLEAAHDAISA
FFLMFSLLESEARVWWVFNHRAFLEALCIANVLRETAKDAAGKDMLARDPLFVRARTDITRMIQIMELIGSNNDVA
RTRVQILSEFLEDTDTA

>QLI63874.1

AELDHEKLQRSLEARTWARETRDLTSLAGQLKTCNGCKRVLSVLKALVKTGDAALVILGKKLCKINSSYDEEFCTGVV
EREAPSIASIIRTMKVGSDSCIHFCMSFLGVCDAPKIDEWNIGFTTQKCCHGAKDKILKDEDRDIIQIVHFSDIHVDPL
YEKGSNTKCGKPTCCRSYTENDKPGKTRNPAGPFGDHACDSPIALEKSMYEFIKREFPQAAFSLFTGDIVDHGLWN
TSKSYNEDLIQHSYEMMTENLNIVYGTTGNHEVHPPNIFEPVSRGNETQWVYDSLSRAWSRWIGNSSMVEARAV
GAYSTRYPKGNLRIISLNTNMYYRLNFILYQEVLEKDPNGQFEWLIKELDAAEVIGENVYIIGHMPMGDADALPNGS
NYFDQIVNRYSKTIKAMFFGHTHLDHFEISYSNYTERTHNNAVAISYICPSLTPTAGMPSFRVYDVDAETFEVIDAKT

YIADMDDESFQTSGPTWKQYYSAKEAYSEIVEPRLTEPKAELSPAFWHNVTVAFENNQTAFDLYMTRKSRGWQA
DKQCTEMCRQTEVCALRAGRAQDNCWVPTAGVHFSKRDESAHNHNKHDECGSSVTKSLLSSLMRREDMLELLQ
ESFLDVDATIEPALVW

>QLI63891.1

NVLYVSSYSGKVTTLDLNSARSPSCRVADNVPVLKAVSSTDGCAVSPSWLTLDHAHSTLFCIDEALPSDKGSLSSFKT
NSNGSLTTLNKAVTLGGPVSGAGAGALTTWDVSNPSNLTLVQSELYHMDKPGPIADRQNAPHPHQTLLDPTGKFI
LVPDLGADKIYVYAVAANSLRLTVLPPIVVKAGSGPRHVAFAVRDDKTFMYLVTELANTIIGYEVTYPDGGIEFKELF
NIDSHGAGKPVSEAVYAAEILVSPDAKFLIVSSRGDNTFSIPNFDRKNSTNITSDPLINFSIDGETGSLTPIQEAPCGGR
YPRQFSINKAGTLVAVGQQKDARVVLIKRDPESGKLGDFVGYADVAGEITSVIFNE

>QLI63904.1

AAPPAVIPTLAKFDGLALGRMVEHVQKAKDEIALVKNFIDSQPDPYIDLPSKMFPSWGCLSDRVQFNVDEVTIAISA
HEDASDNGPVTMDIAESVATVDTSTAGWSMEDSTTFGIDSSVNIGFAGIGVSGGVHHSNTNSNGHSGENSNQKE
YRKEVGRTRECPKNTHCSVQTWTYMATLKGNCPLIPMVDPVCWKPRVGKEWFPATFPAYDVLRDNKLLRGNLSL
PAVRGQDTTWDTIGDMYFTMTNKDGSPTGAVLPKEFAENVWWADEAKYSINYKFTTPCTISAPITDSQGNPRRT
QVNLKVEHGKFVRDEVQEPTGYNFTVIDVLESAFD

>QLI63910.1

VPVQLAERAALQPLPGLTAVEEDLEKRGLSFVELMARQEAAQGERPSSAAPAAASTTAAAEAPAETQNNQDQNK
DQNKDQNKDQNKDQNKDQNKDQNKDQNKDQNQNQNQDQNKDQNKDNKGQNNNNNNNNKDNNNNNN
NNNKDNNNNNNNDQNNNNNNNNNNNNNGNRNDDINQIIQQGLQGNLFQSLLTPSITAIINQMGIGQNFNFG
AIQNLGFADQIFLFNQLQLAQGFVQFGVVPRQQVITVLNNGFVSNGINVGIFKRDVEE

>QLI63922.1

GLVPSPLTGKQHYHENSTFSKLFAAPPIATGELDRAGWKVTCDSFEPGNECIKAIDGNNDTFWHTKFEGSNVPHQI
VVDFGSMHNINGISALPRQDGNNHGYIAQHDVAVSTDGSNWETVAAGTWYGGDKLLKYANFETRTARYVRLRA
TSEVSGAPWTSVAELKAYAAKSGPAAYGGVGKWGATIDFPTVPVAAAVDPVSGKVLVWSSYTYDNYLGSTQDRV
FTSLWDPATGAVTPKLVDDTDHDMFCPGISIDGTGQMVVTGGNSASKTTLYDFASGAWLPGPDMTVARGYQAS
ATLSDGRVFTIGGCWSGGWFDKNGEVYDPRARAWSGLPGALVRPMLTADAQGIFRADNHAWLFGWRNGSVF
QAGPSTAMHWYYTAGNGSVAPAGDRRSDRGTDPDAMNGNAVMFDARAGRILSFGGSPSYQNSQASAAAHLIT
IGDPGKPADVRFASNGLWSPRAFHTSAVLPDGTVFITGGQSYAVPFSDETPQLTPELYDPVADAFYKQQPNSIVRV
YHSVALLLPDATVLSAGGGLCGDCNTNHFDGQVFTPQYLLTKDGQPAVRPVIRSATLSGRTVTIETDSSVASASLIRF
GTATHTVNTDQRRVPLTLVRAGDNRYTAEVPADTGVVLPGYYMLFVMNDKGVPSVSKTLNFLV

>QLI63930.1

ALTDDDLRSIPSPGDDFDIHKGKLLAPILIPRVPGTEGNYKTQKHFVDFFKSSLPSWELAWQNSTSKTPLSGTKEVPF
SNLIFSRDPPGSHPGDVSRLTLVAHFDTLVKPEGFVGAIDSAAPCAMLMHVARSIDAALTKKWEHDDGGSGGLEV
RQGVQILFLDGEEAFKQWSATDSLYGARSLAQEWDETFHPALSTFRTPLESISLFVLLDLLGSPDPSIPSYFLPTHWTY
QSMAALEGRMRALGLLESRPAKPFLYNADKQPHQFYSGYIEDDHVPFMQRGVDVLHIIPGSFPSVWHKIDDDGD
HLDLPTTRDWARIVTAFVVEFMELGGLLDKTAATSAEKTSATQSVHSKRTEL

>QLI63940.1

YGPHFPLRNSTTNATETQAAPVVVWTTVTQIVTQTADAGCPANYALNRKPTATESPTATFVNSAPAATLSPNVHW
SYDTKAIENVIPVEPKKGCELYYGASEPSKSGYYAFVTYFFKSPAVNIDHTDHLAAEYHEKNGMTVSFNNKEAFKHAL
DTWTMANGLLLIAYIPGCGDYAKGERCYFNVTAIEYKHDQLVIVAKGDSMHPDQVTNLGETEWGWWNANKDK
AHGSASASGEAFAWGTSTASASPSSPTGASSGASKPLGQLECTAPADDAHGLPTACLGSNFDQLLDKQLGYTELSP

EAKQFLDSLSSGSNVTGPANSTLSARALRMRRRSLAQRGFWSGVWNFFKTAFTTVYNAVASVISIGGAIDRDFSFK
APDPESSNTWAKTLLGDLTQTQSPWGEAILLKSLGSPNAVGDDGVLKYMNVYCVGCGASGHAQVAGRAQWSPL
AGLQEGRLELQANMQFVLKLGIDAEFSLRQNLEYELFNYGLPALSFGVVTIGPYVSVGARVGLEAAARGKVLVGAE
MGMQDSLVVIDLVNPGNNKNSGWDPYFKPVFEASGEVALSAELGLPVGLKCGLKISSWEKAVGVVDEPSIKGTAK
VSGSVNLTDAGSIVGAFGDSDGCDGILTQISWRNRLWAGLVEPGDAPLLDTQDRALFRKCIGKDNKPARRELLDG
PRPMRITATDKTARSSTGSKSMTYNVQGVPTELYNQTLNHRLLPLVNPTRSTRIVSCANGNLYAVQNDDQDNEYC
FGLWDTTGQSDIVYDGVHRSLHYYSETMSRLGVSRLRASDAAQVPRTAVTVALVPFRNPDGDDFYVVVDHAEQV
LFPIVCDFAGETASKLFVVSDPVAGPETLLRKDVLYSVTGGDVSKCHPLALKP

>QLI63959.1

SFDGNVNYDSPSRRHESLGIDVGLVERRSWKRGNVAHEPSQLHFTHGIASGDPWPESVILWTRVAPRNESDKSEA
TVNGTAPLWSHETDKYIKADANPICVEWKVFQSKRANSTSQKVVASGKAYTTSDIDYTVKVEAKGLKPLTTYFYQF
NVCASSNKSPVGRTKTAPARDDAVSKLSFAVFSCSNFPNGYFNAYGNAARKDKHDYAIHLGDYLYETGRGGERAT
KPSGTIWTLGDYRTRHGLYRSDADLQLLSKNSPWITTWDDHEFADNGYRDGFSGLNNTEDSFLKSGTKVTVDTRK
VHAVRAYFEWMPIRQTDVDDGLRVWRSFQMGKLMDLIILDTRNYDRSITDLYWNKHYISEIADDPSRSLMGARQ
ENWFYRSLSQSKERGATWRIIGNQIVFSRILQNDNRGLNGDAWDGYIANRNRTLRHMYDNEIDNNIFLAGDSHQ
NWASDLAWLGNKPYDKATGNGAIGVELAGTAVSSSGQDGPIEPIAGDSARSLVKRNEELGWQEGYYRGYFELTIT
PDKATAQYYGCPTVATRNGWDIPLANFSIVAGGNHLERPIAGGKVESGALRDGQVKHSNLTLNTNTGKWEVIGFE
KMYL

>QLI63964.1

SAPGDQAAEELVYRCGNTHPSKELVDAHEELQKKEQQHKTRSIDANETINIDTYLHVVMSHQDNQTLASDTMLN
NQMDVLNKAFEESKFHFNLKKIRRVNNATWAVKNDSLAMRSQLRQGDQKTLNAYILQGLEGFVVSEVHKGYNLG
EALTPDQWGLHGLEADLVNIIVGTIPGGSNTKFNGGITLVHEVGHWLGLLHPHEYGCDGGDFIDDTPAMARPDYK
CTRGLDSCRGPKYKGEDPIHNYMGYGPASCLYEFTKDQRKRMRDLWERFRLFKNVPEISWKKDCQAALLPLYTDC
KADKSKKNETCATELVENAYLPCTPLSPTTKRECLDRFARYKEKCNSESCKEDFVNSYERYCAADRRVFSDASNDDQ
KPKLSQTEAGKQWRLVCRQATMLVQEQCTKSECQKEMVCGSLFTRCAKAPQDSKQCQDVLDPLVKLCETDKCRR
GLEALKSKECVAGKDPVAEYNGGIVTDWDIHGDMAPITRDEWRKRCNEVPKAMVRSCEADKACIDNVKSQNLSS
FCQKDRPQFQVDCKAEFAAATVWACASEKCRNEVLDLENKACAGRSSI

>QLI63977.1

SSSSCKKWGASPTPVPMHHLCESRQPAVSAPHPNIWGDLTDEEAAGVIELLHRQSTGLNLTTEDAAGSWDNKILL
VELLAPNKSDTLPFLNNKTTTPPPRCARATLMFGATPNPYLQDYVIGPLPVANNTQVQPLQFLYNNAGKGKVAVD
FADRSAVDHYVKDVGMSVADITKRLWDGTLNDTLGLLPIFPPWKENGSLVLWSGFVNNPTSAFDSETILPLGLYFG
VDVMGRDPSKWSVIGWYYDGKHYPTTNAFRAASASSNFTLLGANSDGPWAQIERQGSPMNFDHLPPPTAVMP
GSNRFSVDAKENYVEWMDFSFFLSYYRDNGLRLFNVQYKGRRILYELGLQESLALYAGNDPVLSGTTYFDSKPGIGP
SVVSLVDGYDCPTYSTYLNATYRQGEKSYTRFNAICLFEFDQGYPIQRHSTANYTSVTKNIAFTVRSISSIGNYDYMFS
YNFFLDGSIEVSVRASGYIHGGFSANNEEYGWKIHDNLSGSMHDHVLTYKADIDVLGEKNSLQKVEFVPATIEYPW
SNGAKRNTMKLQKSFVQNENEAKINWAPNGAAMYAIVNKEAKNKFGEYPGYRFTPATSNVIFLTTSNSSNIMNA
VNFADHHFYVTKQKDTEAQGTHPYNVLNPADPLIDFAKFFDGESLDQEDLVLWFNLGMHHAPHTGDLPNTVSTT
AHSALIIEPLNYLEMDASRATSQQVRLNYKDGKVQSIKTFGSQNMTCHVDASQLMPKLWRDEGTVSVLTFVPGQS
LIHPILDPPRQVTTMRTRKIPRVSSEYKVANVT

>QLI63982.1

HPTVSTRADEQDYNATYLGQILLDRLEKIAINEQSPPRFVSPQAWQHTCPDVLLSFQGEQVQFWHEASIHPNDTIS
KRAFYIPHVETGDINPGPGDLKLSIAKSTMNSKMESKGWTVGARVAGTFGAKDGPSANVELSASYSDTTTTTKMQ
TVTTSHDASCKPGYECRLETWTFHIAVRARTRLLPYYQTWTAGGGHGAKTETCQMKQSVATCEQFRQRLREWCD

YSPEVNNHAVAWDAWHGGVWPPAPLDRTEDIELKLPIAEANGNQIMSRIVLVSEPIMRKKSGDAPEAVKEGTQQTIKDAMERGTRFQVLDSSSSSS

>QLI63986.1

ARTPDTGFTVFVDEGCAGFRGNPKCKEYAEKCYEDSKNLATKQQVIDCTQAKLKADALGPGGAPSKEQFCAGYMKDPKCQEYAKACEREEFAKLRKAAESDSRDWNGDAEPARQLPTGQQLIYCTQEKLVGPSLLDKLCVRFGWENRQGCIAKITDCNTKGEVLADCMLPYDEYGI

>QLI63998.1

VPAAVPPNGVDEARWGPGGRWSDRPDSWNPRGGQYDLPLDWDPLGFASRIGVGSPRFDYKVFVDWTWVSNIVTTPKCYGQWNPSLCLHPQQPYWDPRNSTTFKNLTSEYADRSWRPNHFFMQDPMSIEYGSDLLHIGPVTGEAVLQLTDLQFNVSAKYGTPFPFTGIFGMSPVFRGDDVDYQSAYYQQWKNGHWRTAHTGFVYCHEESRKPVCNGHDGIQTMGGIRRDLIKNQKIWWYDVRLYPDVNTLDFVYNPPVYNYWGIELAGLKIGTEVQKIEPTSNSSGKGAIFDHAAYGRGTPLTPNAYARLAQITGGKPVELKEPPNNGEQKFFSVDCSKLKSFPSIKYKFTGSSREWAVTPEMYVEKMKDGSCVLNVRTLASGDKFIGNFGETFAKEKYIILDFEKNRVGIADMQW

>QLI64004.1

SPAYFSGEPEKPGDITINNRLNTNVNLDILQPNPIDVDEVGVYNRRVIIKGLSSYTIPEKEVPPSADLRLRVLSPMPGQGPVDVIYRSLHRGPDSPFTYDIRRNDPHFVFPGVVDIQPRPRTPQPQCRRLTSFPQGHHIPVLPKCDAGTKLLITLCEQNHPDCPPRPHPPPTEYEAEWL

>QLI64013.1

APAPAPEASPWCWRPGQPCWKVKRVAEAFSESIKSSGALKERTPEAEYSNSPGGAAYKIKRSLIELAHVASLTAREPAEYYRDLSLETRFAADEGLDKRGEVAEDKRQWCSSPGEPCWKDKRDAVAEDKRQWCSAPGEPCWKDKRWCGRPGQPCWKAKRAAEAVINEIRDMSKRDEGGDAPPSHQGGHFPAVCNGPNILCLKTKREASPEANPEANPQWCWRPGQPCWKAKRDLHALDLAARNVVESLE

>QLI64014.1

IPEDGSGAASPAPSGPAPSETALSVPAPPKTALSVPASSETALSVPASSETALSVPAPSVPATSETAPSETAPSGPAPSGPATSEPAPSAACEPLLPWVAATANSPFCKSYRGSEAMCGTKAFCESYGSEETRPDSKYDSVAKCLARHEPEPVRAGCKKSKASSPRPALNGNRPVTQGLQD

>QLI64016.1

TTVTDNAAFASNKTFDFVIVGAGLSGITVANKLSGQGHSVLLVEAGPDGSWNPAIWDVESSVYPAVFCNWRYPVYDDAGNKLNSTIDAGACIGGSTSINGMVWYRPTKAEIDKLETLGNPGWNWDNLEPYMKAIERNHPPTADQVAQGAGYDPEVHGYHGQVNTSFPTPMRIPKAVQLYKKGLPLVFTGLEVGNDLSSRTSVVSASTSWTIWNDPVTGKHVRSSAADALLWAPDQQRTTLTVLANHTVAKVTFDKDLQATGVLFASSLTRDAGKLYTVKARKSVILSAGTLATPAILERSGVGGASVLSAANVAQMIDLPGVGVNLNDQPGSTTYAHMSENHQNDTSLVDGGKLFGPEISLVNIDEIWATDAHGVAESLTSPANLASRAEELVRAGGAVSIAGAESILNATIQLTVEARLPVAEFIAETYPAVFGAPFWPLTPLSRGHVHIRSADPFEVPVITPRFLTDQFDQQVAVAVARLSRALWASTAFEGFVDDAYYDPEIGPNGTDAEYLAWLKGTAGPASHWIGSTAMMPRHLGGVVDSRLRVYGARNLRVVDAGILPFQLTSHTMSTLYAVAQKAAQLILEDCN

>QLI64030.1

TPTTTPRPLPTFPVSMADHGEVFLVIPEGPKALGTPGYFRSQGGISEGSLPAPDAETAAEWQRLRNIRPDDSEDSIDFEDLSLVLMSLVPDEAAARRQAWMMLDQRRQPAQAERVWIFRIATGPNVWLGEWQDVLGNPMSMSRALGGLPWSQIINFTELDGTNADVRNLFETLTERDPVVPPVVATMELSWMNNDDYDAAWGNYGSVRGIPHRVFDAPEG

MTRMEMARDLMALVTSSRVVPDAQRRQELRELLDWDIDAEPGRDFPLLRHGQPTTLEAAALSRIQWGQVQIPP
NIQLALASGLVTIGQCGTALRQIRDAVNKGPKGKRGVVW

>QLI64031.1

AEQFRLNQTYNASNFFKSFDFRTTGGIPTQNDDNWSWVKYQNLTNAQKKGLAKIKDGEVYLGIDHTRVLNKTSAE
GRDSLRVVSKKAFKYGWTLGVGEWPKAGEIDIYEGWNLDIHNKPVFHVGPASEFGSCTIEQGDQYSGIISSNCDNT
FADNKTQWLNQGCQSRETENTIWADPKGGIQALEWTTEYIKLFTWPHGQEPANLSDEELDTSSWGQPSVQLRAS
NCDINRIFQEQQLMFTLPVCGIPPGSDQFWKELDGGSGRTCDKVTREPTCIDYVSKNPQAFKNFYFQIKDIRYLTQK
VIKPTNHTTIATSTSTRSMNRTSTATSLTTTVSLNHTTIASSLTATVSKNQTRITTATGSTIQDGQTGIQEAVTATSTAS
KASVSCKHRHGARHI

>QLI64035.1

LSDLPSLKLMQDIACKKHFGLVTQELLPEEKCRDAAVQRILNRINIGISVSVTIGSALVAFPLGILADRVGRVPVLAASL
LSLFLSQGYAMLVCWRWEAMPLEAMWAMGVFFLLGGGQRMAEAIVFTMVADVAPRSQRASWFQWVVGAVL
AAELAGPFLSARLIESSIWLLLYVSLALVAVGGILLALLSPETLCRKTDGVKEDLDGGSPVTTKRTIFAIFSRPAVFLVPG
AVLSLPMAATLSGILFRFMPVQFHWQLSKSALLVSLRSMITLVTLLLLLPGAAYICNKKTTYSHRYRDGIFLRASALCFL
AGSAFFVLVLSQGFIIAGVVLSALGSGIPTLCRSMMVSALGEKSTGLLFGILALGEIIGFLCCTVAMGALFDVALTSWI
GYPFVLGVVLAGAIFITSWMAGTSKEKATPEKADVVLEPLKN

>QLI64041.1

EVGIYERFYFYYAYKLDAMTVGEKKIATGCGANCNFDEFINFVEENKGANLRKVSTEHFPDIERTAELYANSGAATQ
VREGRVFNGVGNYGELFEKVRTRLEGLQHKTNYEYDKLSGEELATKVQEVKTKLMESLKQVWQGRVRASLSSFKPY
EGFKIVETADGKAVDFKATLEAKENKGAITEEHLKKLWDEHVENFKGADAVKEFKPLKDWHPAIVEENGIISMRSSL
EGAVESGWIPKDSKWLQRNWKKSYDIGHSRNVDSVGDTYKALETKLCPGAPSRRDFGLLRKRQALLCIRAAEIEKT
MDIKAGDVDNGGKPPGEKPPNPGEEGKPPGEKNIPASEEQVKRANSVSENSFEKSAEKRLGKVPKSFEEKYGTNSY
KELRTKALEYKPLEPNSPKIRSGGTGLSKVGKALGAVGGVLYVKGIIDAFRDHVSPLHFFAAVTSIVPFLGCAVETGAS
VEDKVPAGFIVAEGVLCNVADALLLTPFWPVGIALHLVNAVVKFFRPPPTPPTVLELAEKRDAVWAHALEDIYRYTY
SAYETQQKSSFAFKTENALFFDSLNVLSESADTIGSLNATGSAELFDPKLFPDQSAVPDAAELETRSKEAITNLEEEEW
GAVLRRQRNTLLRTAIATANGSLFELDKAAVDVNDKFIKYIESPEFKETYPNSGWQGEKILAFFKNWATSIRKHPELG
VAGPAGLLLAIPAGEDKDFEYPDIHTFINKRVEETTEYVRSKPPVLPRALDVSFVLGQSKALLNLPKDTLSPLSFLEVEV
SSASEKPLSPLYLQTLVVRHTREIVDVMYGRMKESDLKDTDFAVQDPELLKNFRLLIAMKLGAVHDADRVAEIQRR
HPSGYDPELLNHRGLVRAITPYIPVVLENPTDPVPVSHYLTMVSGLEKTLVDEALQARVAKYVETQDEATKSFWEEQ
KKNFNSLKAIAEKITGVLARPPSMPGDFLAKHKPTCVSKGQDETMCQYLVEACYFAENMGDEFVNQCLDAAVSLE
KTREHKVDLCAKFPSYNSCELAIHDCDKTYRNSMYTVLFECAARAAPDREAEYAKMAAE

>QLI64051.1

VPIPSPASLIDINANNNDLKFPINVLGLQGNDVNALNGNLAGNSASNGQTNNNAANGGKESAPKSDNTRTNNGG
TTTTNNVNTNHASNSLIGVNANGNKLDFPINVLGAQANGVTALNGNLADNSASNGQTNNGAKPAEPKEAVSSP
NTVTNNINTNGDAENTNTNINTNANGNTVSTGTGGANTANTNTNANSNTNANTNTNTNTNTNTNTNTNTNTV
NANATPNAIVNNTKNTNTTSPSVTNTNYAGTNATNITATKTDKTTTIKTSDAPDAETDASPETDSDTQTETSAPGN
TGSQPPPVTSVPNSQENNAAPTKVITYTVSPIPVSNVVSAAQPGSAQPGSAQPGSVDVSSVVNAVNGLTAAITLLT
GAVGQGCAKQ

>QLI64054.1

QPRQAAHSIISPHVAPAAGVAVDVDGSYKDRKKPSREHHPAAFAAAAAAAARNDPAHWGATPISAATIETPSGG
DHKRSALQKQSDHQKEEQHQLQAGHGSENTPHKPRLDDNIHNHNKKQTRNDASAFATTLAPDNSVRAPSPLRR
QPSGLTSPQYARSLEDWEVEDFVLLATVDGDLYAADRKTGKQLWSLLVDQPMVETIHYRGNSSELDDDYSPVDHY

VWAIEPNQDGGVYVWIPEPNARPRWTGFTMKELVEVLSPFAGNEPEPAVVYVGDKRTTMVTLDAATGRVIKWF
GTGGSHVNQAESCLKPNALYDRDAEECSTTGTITLGRTEYTVGIERRDGKPIATLKYAEWTPNNRDKDLFHQYRVS
KDNRYISTQHDGKIYSFDYARSDSNSVPARLFSQKFEAPVARVFDVCRPWDATPDSNPDLILLSQPIMPSPDDHTSN
SIFLNRTTTGGWYALSGRAYPLIDAAPVAPASRPDGWEKMASWDSMTETKLSRLIGTHSLGSQQKVQQFPTLPDR
PATGNDREDPDNQSVPQHVPVEIEEPTIVDKVKSIPQSAATSVYDFFSNPVLIIFLIGLLVYNRKTFARAYQLWLNGS
SPKDVFSYLLPGATPDDKLATQGTDVSEPNGKQSPKPATAEDNSSEANPPELDAKAEDRSPRDDDIAKPDNESTTD
TDSTPKSASPSKPGAGKDKLADGPDNGSVAGGTGPEVKKKRAHRGRRGGVKHRKGRPRDSSQSRGDEPPNATVE
EAVSNAKKLGDRPIWEPDVLTVANDMQAVSGPVVRMGNIEVNLEEQLGTGSNGTLVFAGKFDGRDVAVKRMLI
QFYDIASQETRLLRESDDHPNVIRYYSQQTQGSFLFIALERCAASLAEIVERPHAFHELANAGKVDLPGVLYQITNGIS
HLHNLRIVHRDLKPQNILVNMGKNGKPRLLVSDFGLCKKLESEQSSFGATTGRAAGTSGWRAPELLLDDDGRDLNL
MEASTHSGSGSVLVQDGTMPHHRRATRAIDIFSLGLVFFYVLTNGSHPFDCGDRYMREVNIRKNNYSLQLLDVLG
DFAFEAKDLIMSMLNANPKQRPTATEIMCHPFFWSPKKRLSFLCDVSDHFEKEPRDPPSVALEELERHAPEITRGDF
LRSLPREFVDSLGKQRKYTGSRLLDLLRALRNKRNHYEDMSDSLKRQVGPLPDGYLSFWTTRFPQLLLVCWNVVYN
VQWEESDRFREYYEPAGL

>QLI64055.1

QSFDVVKRDDNPTPTTIPTSSSSAPKGTNSARIDNDGKDPKERGGESLSSTLSGTITDAPTSTHRPTSTLPLTIATDGP
LDNATFFNATIPNGQLPLPPRLTPGWGVAGVIMLVTGVIYTLVGIKNRWIHTFFSTAYMTALGVSVLIVYVMNVPV
SNALQGGYVVAVVMSGCAIGAAAMFFKELTEGFGCALGGFCISMWLLCLVPGGLLHAVASKAIFIAVFTVAGFAFY
FSRWTRDWALIFMISFGGATITILGIDCFSRAGLKEFWAYVWDINDNLFPLGADTYPVTKGIRVETAAVIIIFLVGIISQ
IKLWRIVREKREKRAAERAEDQRNLENEEENVGRHIEDLNARERRQWERAYGDGMSHSITDSRATDYGDVGSEKK
LRPSYVESKQRTSGEVIEMADMSESDTSRNGMDGLMANEDTDEGKITVRVAADDVPESTTDIEAERLDEKAPSSV
EANVLTDGKWYSKETTAGKRRPQAHSMTEVPEVIPLPFTVPEAADSASEGDRSSIATFADDVEATAAEPLKHRSLV
KRLSQGSVTLLRSFSHRSGRTGGVGGDVASDAGESTEELVLEKTRRPENEGGSLAATIDDESVSGGNLHSITGDDEL
RNIHEGDETNRRSIEIDAQLGGKGDTIRYSVKSTKPKTKSSPTSTATSGPVVGISEPNEDTTTELRDTLPTSEDKAMQL
GGEPYADLTPPEASERAKSVATASSTPASLTKDRLPRPLSKAAMSYRTNEWAKHLSNAECPEPDALQVTLGRAKKE
VKNSTEKAAPVNVAELQKTAKDGTPAPAVRRSISQASDMGNNTHLAKRSSKQDLSPPTRLVTPSSSTGLETSGEQR
TPLSPVIAPAALLRKSSGPRHSTSNFMPIAEENSLQAVDSPIVRDEASEKQQPASNNSSAIILDGSPATVLGRNPTSG
VVSYSSPQTLIGQREMFLRSKSQGSLLSSTPEPIFAHQGPGSDGGSLFNYPTYAAAFNVADPDDVPLSQRKEIMRQS
SLMSLSRSPSQSIKAPSRPTSGFESSESIVFNSHQPRRVSTLPTQAVRESQLASFRQSVQQELRSGTPIITPSGRETPFG
PSTLLGGGREAEVQRNIEMQRSVLMGQKEAEAQRRESQKREREHADRAFDERMRNGDMLDAHREAMRKMQK
GAR

>QLI64061.1

EEAANAQKYTNYAVSGIKSLNDHWYNVKTGIWDDAWWNSGNALTTLADFAALRLAEANKLNVGGYMRNTFVQ
AQKVNVKTAKFVNKAGMVSSVYCLDGSSGCMAKREFLGKRGFDDFINDFYDDEGWWALGWIRAYDVSGDDDY
LGAAIDLFNDMQTGLGGPCDGGIYWSKDRQYVNAIANELYLSVAASLARRVPQNGTYKAIAVQQWDWFERSGM
INAQNLINDGLDASCKNNGLQTWTYNQGVVLGGLAELFRATGDLKYAEKAVSIAQAAMKALSVDGILTETGGCDA
AGDCGRDGAQFKGVFIRNLRYLNDVAPHQEFKDFIIQNAKSIWAKDRTSDNQFGVAWAGPYYPAFGATQSSALD
ALVAAVAVS

>QLI64069.1

AGGADELDARWNEPTRTTTIVTYTTVTVCPYTTTVTHGGTTVVVTKSTLSTVTLTSCRGCEGATVTVPGPTVTQGTT
TLVDITYTTVCPVTETITAPGSTYLTTKTVTSSVVTKVLTTIYNTVVQPGVTKTATDVVLSTITSLCPVTQVTTIGGKEQT
VTFTTTSLITTLVPVTEHKTVVQPDRTKTATDVVLSTITSLCPVTQVTTIGGKEQTVTFTTTSLITTLVPVTEQKTVVQP
DRTKTEVHGVTSIITSLCPVTQVTTIGGKEQTITFTTTSLIETVVPTTLQQTVTQPGQTITATDVVKSTITSVHPVTLVTT
INGKQETITTTATSLIETQVPVTVTKTIHGEATTETLTNVAYQTVTNLVPVTEVVTVGGEKVTVVNTRTEVVKVAHSS

VIFQTVTAPGQTLSSVHVLTVTETLGGGLATQTVTVPGGPGTPVVIQSTNTVHQSAPSSTLVVTQTSAPVVTAAAQ
AHAPAVGYLFAGVMGAMALL

>QLI64116.1

QITVIQGVLTSVGGGIDGLNSAASGFNGDVDAVKSKADALVSTIKSGKSKVDSSSDLQLTDALGLTDPVQALTKKGQ
SLADNFKAKRSDVEKAGACGTVRTELKDINDNSQALIKSVVAKVPKDAQTIAQSLAAGLTKVLDQAQDDFSESNCK
DSGSPGGSSSKTGGSSQTSAAAPTTAAATGGHSSASASASAPATTLAPTSAAPSGNGTTGGNPPPVTAGASFLAPA
GALVMAVAAALL

>QLI64121.1

YQLPANLKKIYDQHKAGTCSKKLSGTFSGGAAYCGDLPNAIFLKGSSGNYDNMDIDCDGANNSAGGCANDPSGQ
GQTSFKDTVKTYGIPDLDANIHPYVVFGNEGASPSFNPQSKGMKPLSIMAVVCNNQLFYGVWGDTNGFTSTGEA
SLALGKLCFPNEGLSGDNGHDPKDVLYIGFTGSDTVPGKSGANWKAKKTTEFEDSIKALGDKLVASL

>QLI64135.1

VPTTQDTRATCGSGNLDLSKWKLQLPSGTKGHPTEISTSDLCNGYSSEFFSMQGDALVMKVPGGPSSGKCVTTPN
SKHCRTELREVKPASWDPRAKTNRLVGELKVTQNDGEICIGQIHIDDAVSTKPVAELYYNAGGDLAVGVNTCRTCG
QKRTPIGNVPKGQRLSYEIRYEGNRLSVSINGQAFKTLSTYDLDAPSSYFKAGNYNQGEGPTTVQFYKIQTSHS

>QLI64143.1

LEAVILADTNRDGQLDVTSDAQGKQAWTEERGALFMANIADTDGRCAEKITDTTPFADLDKCNDASDNVLRNPK
FLAPLQTLPNSGLTDAAQGRITVSGDLAKDKVRIFHKTGNDWAYVDDSRVFQAAELRAGLELGIDARDVRRPGGW
DGRVRVHFNLTDNGESAADNVQLRVAPVLIHHHLQKLTQVYIASIKDHSQGTRQFIENVEKYASDAGAPVAKFDT
GRDTWAQDVFEVGYTSIPGPDGPVVLRVLIRSSQTNQRDVSGRNVYKQLRSASVAAVQHLPPVVDGPLWGIDSL
GNLETVPPHSHNGKHYPNGRAVMGSKLGTKPHMVAFLEAQEVQPPIEIDTDWLRVGHTDEFMQFLPANNERG
WVMVVDDPKLALDLFKKAQQEGHGATQAMSRPHMLHDIEFLTFSTQFSYLPSDTIDGVLRRWKLEERNLLAAARI
EANIEIMKRETGITDADIIRIPALYYVHGKGWDDNRRQWGKEKGHKRSEREGLMPAFGEEDGEARLDRRQAEKPE
NLLVAFHPAVINGVVFNNGQYLAPNPWGPVIDGSDILAAAANKEYAKYGFNITYIDDWFDHHANAGEVHCATNV
ARDASKKWW

>QLI64150.1

LEPQQPLNAVDHEENAVHGNSDAADDSPAPALEAERVEFLGYVNASQVSVADCPQWPTHNQAVKVGEIWWQ
DNGWYAQPLKMWNHRHAIEWGYCIETRFGWDSDSGQIFYAQKVSESEQFSEWALKAYKIWSAALTEGVVGWN
ADKARWLPCTNVPGCGGGARFAMDKNLCFRARLKSGRVITACETFTNQGYRICGEYSRKHHGRNRCGTGRNSHY
WFGTETGCWEN

>QLI64183.1

LPADISSDLPPRMFSNLSGLQSKYARGIMAQAKKEHLGSQGCRAGIATALVESTLIMHANTAVPASLAYDFDRLGQ
DADSIGLFQQRASIYTNIKCSMDVACSAHQFFKEMKNVPQWKYMDVGNLCQEVQRSENPERYHEFMDQATEIC
QAAGF

>QLI64193.1

NVIEPRHAVRIEDVTQGDDLFKRKGGGGGGGRGGSSGGSSGGSSGGSSGGSRGGSSGSSGSSGSGSRGSGSGGG
SRGNTAPGSNAGGTSRGGSGPPRAFGGGSYYGGGAARPYRSGGISPLGGAAIGLAAGALLFWPGRWLYGAYLYP
YHNHYVFHNATTNKEESLPVVCACGTYEVCGCDENNSTMTELVGNGTYDALNKSIINVGDYKGQKTLLVNGTLPN
GTTVDGPDEGAGVGVGMKSLVEAAGLWPAVVCVIAAICLA

>QLI64203.1

APGNTPDERDNDADGSIVFPGGNGYSGWAICGDQVSKQRFPRLQVPAGAGGGCVRYYRGIDMTGVVTELHFFF
RDGFRTACDCAAKCISQPASCNNWVWKHTFMPGDGGRRSCTLYSSPNLPGNVTLDYDEAKSSGFRPLSDANNPQ
VGGDSPLTFLDDAGTKPDPDGVSGFTAIDQNGGLYC

>QLI64206.1

ASSKRGLVFTPNAEHAGDNKLWVQSGSDLTWYYNYQSSPSPAYSSLSQDQFEFIPMMWGIGSDPNDTKFLTDVK
KLIDDGTKIKHVLGFNEPDTSSAYGGSNIKPADAALAWVANFEPLGKMGVKLGLPACTGGWDSLPWLKQFLGNCS
ELVSTGKDKKNCTWDFLPVHWYDNFAGLASHIGEREATWPNKSIWITEYAFAHQDLKPTQEFYNQTIDYFDKLDSI
GRYSYFGAFRSTNSNVGANAAFLDKSGKLTDIGSWYLGFAATGVTPESSKAALLEPTVGSLILGVIAGILVGSL

>QLI64217.1

ANINKRIIGGKDAEYGEFPFIVSILSNRDHPFCGGSLLDSTTVLTAAHCITGAYSVKAGTLNSYTGGVDAKVASAKIHP
DYTDGESPWHDIGILKLLDPIDPSAIISYAKLPVNGSDPAINSTATAAGWTRGTQTAPKYHLAGIGADRLSKVVIPIRA
RQHCSNLNPRAGVDTIVCAGGDGKNVCKGDSGGPLIDRETGQLIGVASFVIKDATEKHFYYCNLSPSVYTRVSRYIT
FINENLGESGYTIPEPLSPEAQRIAKAKEQVELHCTRFMNDADACMAAAPPCIAAAEPYKTTDELLRCVDRMQVCA
SQDEPGKLNQCIENAKACVGKAEVTFWDLDALAQCAKKDL

>QLI64218.1

AYCNTSQLIHMDVIGTCDCSPEPKGCSTPCPGCGVTPTKNIRNCNGGCEDSESDCAACGIWFHTLCNCLQHPLNC
QNSGTIQKYGAPVWVLLETPPGDHNLVTTTQFLPGIRQMNAGHDEAWLFAQQKFDKTSEALAMNPVVVRTME
QVHIHVCPRNTTTASMLGKITAFSSSKLVQLADDKEMYCLGIDHSVDVKGFAGLVADFINSPPPNVCKDMVGAAII
EDDKSRRWACATTNRQGPLGKVCFH

>QLI64221.1

WNIELPPCIEPFKPFVYSGCFQDGNPNALVFRSSLDQQDMTVEKCIADCKGNGFRYAGLEYYGVCFCGATVNGAQ
LDESKCSFPCSGNKTETCGGDNTLSVWQDPTFPKSPSDVTVDDYKSLGCYTDDSPKGRTLSYPIDLDASTFTTKKCLA
ACEKQGFPFAGVEFGKECWCGVVLANDTAKADTSQCNMPCQGDSSDTCGGRGRLNLWVAKDLESLEPCGHKP
GTTTTSLPPSNTTTTKPVDTTTTKSETTTTTKPETTSTTQPETTTTTKPETTTTTKPQTTTTKPETTTTTPQTTTTTKPQ
TTTTTKPQTTTKPTTTTKDIPHTTTNLCTATVTLPPKCEWQCGSWCAPPLPHWGDKNGCIIAQKTCHKQVSSCFKN
AGWPGSIECFKFKAWCGFIDAYCASWCPSDSCGKWDCWDKHHGGGGKPPSNPPTTTTSVYPCPTTSKPATTTTK
PATSCPPEPTNICTQPTNQKYGYGPGKPVGGIPLPVVGCNDCKDEFDEKPFKYYTEAESRNCPGFPWPRWPSVCS
QACEEQYTHCVATYNKGCESLGWRDSWRKRAGESDEEFAKRWSGPDNGGSGTCAAAGSVSTGAWSARGSDSL
QCWGWGGNNPAWALERCKAQYQDCLRVNQWVDPKDKCKRWPGC

>QLI64222.1

QFSNWLSDQINTSICTWSQPRAALIRDTVYLDGGDIWWNSGLASGKFGPVLDQGNFQGYVLSYNFSESFSKDTNV
TGILVNKRMSKAIGGEQSGISSEPGYDDGGMLANDAEFFLYGGAVTANTLASNPEPAANAVLAYRRYAYGPEKPL
WQSGFNSGHLDDGVTRYIAYGGPVNAPSENKAWYFSGLTSPSRGPISVLTYENKTTLAMIPSNTLIELDMTNQLFE
KWTNITLPDNIKPRANPEVVWVPVGKQGILVVLGGVVYPDWASGDLESANETESKKLKSPEFMRTIDIYDVANKK
WYQQPTEGGPGARTRGCAVVAPASDKSSFNIYYYGGYDGIHPKNDFSDEVWVLSLPSFSWTLLNKGVPVHGRSG
HKCFKPYPDQMMVFGGYTPTIGSSFTCLDQGPVVVFNLTTGEWLDGYSPTKYGNYGVHEKIIAAVGGTASGGATV
SSPVPSGWASKELGAVFSVAYDTKKVKQYWPYDSASSPPPPPGATTSPPEQAVFPHDSHLLQIILPAVLVPIIISVGL
GIALWYCCARRKKSSAASSTDSTADESGLNIMTWARGPGAAKRSMMIQSLQKPETGTSPDLEENSTFEPIATDKTD
TTPHEMEDTQVSELCDTSSPAELHDTGFTPMSTFQKQFGPYGFETTTSQYNIQPGLGSPGADSHTHDGEREWTES
PLLGTWSQPRPRLEIPNFSSVSESSKNQDPPMVSPEERSRPDIPISPTSTSERFGGDYISATAVMSPLDRTMSGESED
GRFPSEC

>QLI64224.1

GPVDGSRTAAKRDPLGLSLPPLIPAIPGVTEPLASNAPPLPILQVPTPPVDSPPFDVSNIQPKKIGHFWTGAGDNTHK
DFLVTVSLDDDTFGTIIHISDVPTSGNSPHHLGTSLDGKTLVGGGLLSLLKTQDTAFYWDSSDPYHPKFAHSNRALLS
SITDEIRAKPEGGFLITYMGSAVGSSPGRLVETDAKGNIIHEWPEDVEGTLNILGEQFTPHGLSVDFKNNVILTSDFV
VPLSILKPVSALGIQKANTLRLWELDSRKIISTIQIPHGGGIQDVKFIPGNKESAALATAVNPGQVWIIYPFRKDANGK
QGVAELFYDLGDKAKDSVAIYSDISDDGKLAYFTFTLGNHVAALDISDLNNVKRLDNPDETQPIIGPHYVKISPDKKN
LLVTGYFVQAGDISVLNTPGDYKGHWIDILPDGSLNFNRTIDFENIFTKTRGGARPHSVVIFDLTDPTNPKYY

>QLI64241.1

DGKAITDAMASIKTKTTDLGATVSNWKGDLLGALPITVKSTQLLKEIKTATKTASKSAVLTTEEALAVAGATTDLSKA
VTASLQIIVDTKPKFDRLIILSPVVLLNLEMLKDATDDFSSEVVSKVPEALQGIAQSLIKPIEDAFAAAIKKYQLF

>QLI64258.1

RQCQNITVPVSISSRNGVFDMAPPSTDIDVTNLFLRMAWPGNNGTANLLKGYKTVSGNYTLAATYCEPDNGPGH
ALQILTHGVGFDRSYWDFSFANYNYSYVNRALDAGYSTLTWDRLGIGHSSHGDDPVNEIQIFLEIAALKALTDTARG
GQLRGVAHKFNKFVHLGHSFGSAMTYALTALHPDITDAIVLTGFSQVPQFLAYFVLGANFAPVADNHLLAGHYAA
GYVAPRDSIGVHINFFGPGDFDPEVLRVATQTGQPAAVGELLTVGSVPKSSEFPGPVMIITGDRDIPFCGGNCMNT
MAINGSAPNLVEYSRDSFKKASAFQATVVPNAGHGLNYNYNAPDVYRAITDFIKASV

>QLI64275.1

KPIATDDYGNTLETRGGNVQVSETDLTNMKFYSQHSAAAYCNYNTAVGKPIVCKDTACPLVMQNKPVVIASMVG
RLTGIGAYVAIDSIRQEVIFSIRGSNNIRNYITDVIFAWRSCDLAHQCKLHTGFAEAWDEIKDAASAAIKSAREKNPGY
KVVITGHSLGAAVAIISTAYLRRDGIPIDLYTYGAPRVGNDKFANWFSSQQGRHWRVTHENDPVPRLPPIFTGYRH
VTPEYWLSGGDAFQTVYDLSEIRVCKGIANIKCNASRFITDILAHLYYLGYTGGCSASPLRLRDGQEEDPLPQDLKDR
LTAWSQKDQQFVEENDI

>QLI64291.1

GFNSGSGKNVAVYWGQNSYNQGSGPLAQQRLGYYCSNTDIDIIPVAFMNGISPPITNFANAGDNCTAFPSNSNLL
SCPQIEADIKSCQATNGKTIILSLGGATYGQGGWSSVSAAQAAAQNVWDMFGPVPSGKAIDRPFGSAVVDGFDF
DFEASANNLPAFGQKLRSLMDAAGGKKFYLTAAPQCVFPDAAVGAALNAVSFDFIMIQFYNNWCGVSNFQEGST
TQNAFNFDVWDNWAKTTSPNKNIKLLIGVPAAPGAGGGYTSGSKLKAAINWSQKYSNFGGAMMWDMSQLYS
NSAFLGEIVSDISNGPTTTLPPGGTTTTTTTTATTTTTTTTSSTTSPAPTGTLVPQWGQCGGEGYTGPTQCQPPYKC
VYGGQWWSSCQ

>QLI64292.1

AKNERTFAVLRFTNKQLTKGRMDPIVSPGQVSTHVHSIFGGSNFGLGSTGKDLMDSKCTTAMIKGDNSNYWVPSL
YFKDPKTGKLEDVELFYANAYYFFEPTNDDIKAFPVGLSIMSGDQTIRTPPKDGATSNLDPSKGTVNPIKWTCPRSN
FDSPSWPAGSNGLAAGIQDPNNKGEGVGFPDVNCDGYASPLRADVHFPSCYNPAAGLTNYKENMAFPTDDGKG
KQDCPKGWIHVPHLFLEVYWNTPLFKDRWEQGKGQQPFVLSNGDATGYSNHADFMAAWDEKLLQHIIDTCNA
GSQGMDKCPGLNGLNKDDCTIKSQVDETVDGVLDALPGDNPITGWSYGGNGTEGGGNEPKPSQSQSTGGDNT
AKPSSTNKAGGDNTSTAKPTNSQPAQPTNSQPAQPTNSQPTQPTNSKPTGGDNTSKVSSAAPTSDNKPTSTKNP
DNATTKTQPTSEAGSSKPTLPSQPSACTPKIHTVYETITVTAQLPGATDRPVSNSTRTAGDFKYAGCFKDSRDRVLK
DDVRPNLGAISNAKCVAHCKAKGYNLAGTEYGGQCYCGNELVRSEKLDDSACNIACEDDKSDTCGGGWALTVFSK
DGEASLKDVKSRRHAHEHLQRHRSPHY

>QLI64298.1

AQINNLIWTLINYLPVSTSKAAAEQRTLQAEYLKVRRDLNATSSQDEFARWAKLRRQHDKLLEKLDASKKAMEASR
ARFDNYLTAIRMLITKVPQYFIPFWYGKEPMFWLPYGWFPYYAEWIISFPRAPMGSVSAPSWQLACSVVITLLAEIL
MFASRQISAQPKTKVAQPGEEAKKPGKQTAEKSEEKKEL

>QLI64322.1

QCPYADPGRLAARTEGRPDSREHLKGYEVDDGDVFLTSDVGGPIEDQNSLKAGERGPTLLEDFVFRQKITHFDHER
VPERAVHARGAGAHGTFTSYGDFSNITAASFLGAKDKQTPVFVRFSTVAGSRGSADTARDVHGFAVRLYTDEGNF
DIVGNNVPVFFIQDAIQFPDLVHSVKPRSDNEIPQAATAHDTAWDFFSQETSTLHTLFWAMAGYGIPRSFRHMDG
HGVHTFRLVNENGDTKLVKWHWKTKQGKASMVWDEAQHVAGKNADFHRQDLFDAIASGNFPEWELNIQMIN
EDQALAFGFDVLDPTKIIPEELAPLRPLGILRLDANPANYFAETEQIMYQPGHIVRGVDFTDDPLLQGRIFSYLDTQL
NRHGGPNFEQLPINRPVVPIHNNNRDGAAQNLIHKNTAPYIPNSMNKGFPKQANRTQGKGFFTAPGRQVSGALE
RRRSSTFQDHWSQPRLFFNSITPIEQQFLVDAIRFETSGLTREVQQNVMVQLNRISHDIAVRVGKTLGLEAPAPDDT
YYHDNTTQGLSIFGQELPTIATLRVGVLVSTESSDSLTQANSLKEQFSASKVTVVTVGESRVDGIDQTYTSAEAVGFD
GIIVTDGAEKLFDPKNKSTLFPPGRPLQIVVDGYNWGKPVGFLGKAKDVVGAAGASEGAGVFIKDGPADIVEEFKK
GLATFKFTDRFAQDGQ

>QLI64325.1

RPGSGASPANKASPEEFLHSRNLPDGYYVPSYYPAPYGGWISDWQESYRKAREFVASMTLAEKTNITAGTGIFMGS
APRVGFPQLCFNDGHNGIRQADNRAVAIGKEARGKGVNVWLGPTVGPIGRKPKGGRNWEGFGADPSLQAIGAR
ETIKGVQEQGVIATIKHFIGNEQEMYRMYNPFQYAYSANIDDRTLHEVYMWPFAEGIHAGVGSVMMAYNAVNG
TACSQHPYLINALLKDELGFQGFVQSDWLAHMSGVASAIAGLDMDMPGDTQVPLFGFSYWMYDLTRSALNGSV
PMDRLNDMATRVVASWYKMGQDNGFPETNFHTLTRDSVGMLYPAAFPDTPSVVVNNFVPVQDDHNEIARQV
AQDAITLLKNNGNLLPLRTSQSLKIFGTGAQTNPDGPNACADRNCNKGTLGQGWGSGTVDYMYLDDPISGIKTRA
QDVQFYDTDSFPDQIDQPKDTDVALVFITSDSGENTYTVENNHGDRDASGLYAWHHGDQLVKDVASKYKNVIVV
AHTVGPMILEKWIDLPAVKSVLVAHLPGQEAGQSLADVLFGDVSPNGHLPYSITKKEEDLPESVTKLISAPNIHQHQ
DTYTEGLYIDYRWLNKNNIKPRYAFGHGLSYTNFTYSNATITKVGQMTKTPPTRSPKEGILDYTQPIPEPSEAVMPD
GFHKHWRYIYSWLQASEAKNAAADVKKKYPYPSGYSTAQKPGPRAGGGQGGNPALWDVAYKLSVVVTNAGLDH
SGKASVQAYLQFPKNITYETPIIQLRDFEKTEVLRSGESTTVELQLTRKDLSVWDVVLQDWVIPVVDGGYTVWLGG
ASDDLRVSCHVDGMKCQSAATSE

>QLI64330.1

ATIRVDVGEGGAFTFSPDTIKASTGDTLDFHFYPLNHSVVMGDFSSPCAPAKTGGFFSGFMPVSSGEATDSFQVNV
NSTDPIFFYCAQTVLEHCKNGMSGVVNPSSSQTLSAYKNAAKSVSTASHPANSFGGTLVSSGQTSTSASASASPTSS
GGGTYGGGAGSGVRAPAEAVVAFAVGMAAFLVG

>QLI64337.1

RVAARDDAAPASCCASSNGASNLVARADGDVDDCQELEMYDAGTDREELQRVESGNFDGFTAITCSIIVAFLTRN
NNPWGVTSLGRRADTDETDLSECHRAQDVLKKMKIPEPCRKIKEIEFGFTLSNDWWSGTNDDIGATISGPAGKAE
FVITEQPSRGESKWVPVNMQESFKSDSIDITGINNLTLTAETFFLRGTAEDQFKVQDIRLRAKCADPGFEARNNQYV
GINAWYGRPQKGWWIFGNFYPSNFDRQTVATFQVVPGDWSFAPPCNIIKDLTYEFTLGGTSLNGDGTGDTLTLVL
GEGKINLGSSFDAGSSKKDNIDLKAAFNKDTVDIRDLKAVSINDDDSTAGTFDKGWKFQGLTLSATCADVPKKVQ
MRKFAAENKDVSHKDGEPAWSGTISPADWLEVA

>QLI64341.1

VFKDEVGDIDFHYSLVGLPQVETTFFHRPRKEDKASLLYTLGDVGVIGAINPSNGELIWRHQISHGIANGGGHLRAP
EGENWVTSAYGPKVQAWNALTGRNIWDMEFKGVVKDLEILELTESPRKDVLALFDEDGVTVLRRLHGALGTVVW
EFRETSKDLPVQVSTNIANIYVLSLHGSPGSYNLKVTSLDMATGARVDHWSVGNKGDIHGAEDVMFVGANSAAPI

AAWASRGLSKLSVNVLGTKTKQEFTLPDGTETLRIHAPHLAQSQPHFLLHIGTKTGHKAIVYHTDLKSGQVSKAYEL
PHLSGPGAFSTSSDAANVYFTRITANDISIVSSESHGILASWPYLPKDGHAAVHAVSEVIKKAGGKEFAIRSAAVTDA
DDWVLIKNGQVDWTRHEGLSGAVAAVWAEIPEAERLAEVLAEEAHANPVAAYIHRVNRHIADLEHLPAYLARLPQ
RIIDGISGADLTGHKDGLHRDTFGFSKIVVVATRRGRFYGLDTGRQGKILWSQAVFNVDPGESLAIRGIVAKDAESE
VTVVGSNGERATIASATGRILEAHQAGSFTKVASTAVIGDESSQWLLSLGANGLPVPNMPIGTIPNDTIVLRDGSLG
VKGVKFTAKDGEVIKDDIWQFQVGKGQKIVDIVSPPSHDPISSIGRVLGDRKVAYKYFNPNTVVVAAIEEATSTLSV
HLLDIISGQALASQVYNGVDSTKGLSCAMAENWYACTFFGDYTVNDGTDRTIKGFQVAVSDLYESPEPNDRGPLG
DAAEFSSLNPVDTPTGVPLPHVVSQAYVFSQQLKSLSVTQTLQGITSRQLLAYLPDSNSILAVSRHIIDPRRPVDRDPT
PAEVEAEALMRYTPQFEIDGRGIVSHELDVLGVQEILATPAVIESTSLLFAYGVDIFGTRAAPSGVFDILGKGFNKVTL
VGTVAALFVGVLFLAPVVRRKQIDRRWEAFL

>QLI64343.1

APNTPAGFSNCIFYDDFDGDEDSLPDASRWTIDLGTRYDGGPAQWGTGEIETYTDDWQNIRITADGTLKITPVRG
HGNSWTSSRIETTADWDFACARGERLRVEARIKLGDNPEGRSLGIWPAFWTLGSEYRGNYQNWPAVGEIDILESV
NGLRKIWHVAHCGTNPGGVCNEPNGLGHITEPFERGVWHTIAWEVDRSGDDEESMRWYVDEHLQWTLWEDD
VRDAGAWHAMAANSKMILLNVAVGGGFPNGVSGITTPTSDTLDGDGASMEVDYVAVYLEGGW

>QLI64359.1

LASSADLPVITLPWGKWQAEVDKTDPLLYVFRNVRFGAEPQRFGPSDFPTWANDSIQRPGSISCISVNIEGLSKPPG
GRVPLKDPESFEVPQDEDCLFLDIYVPVRAFEPNARKLSVIVWIYGGAFALGSKNDGEILYTGRPIIKASNYNTIFITGN
YRTGAFGFLAGDYMQKAGLPNAGLYDQALLFKWVSKYVEQVGGDKEKVSAWGESAGAGSILHHLIRQDGKIDPG
FQTYYAMSPAYEMSWDNAPDGRLDTYYRMYSKFADCGDKYDIDCLRRADRTKLVKANQELFDTVRQTGLFPVGP
AVDGEWIRTIPSILLSEGKSWPGIESGIISHCSNESANFNPKGVNNEGDFDKFLETFLPGSPLAPQRLEIKDFYDCPRT
HGGNFSACLETVIRDAVFTCNTRYMFEAYPDKSYMMEYAFASRESGFHGVDLIPLFTKNVAEAKEFLEKMNIAPFW
AGIYADALHKTISPKFTKYLASFAVSGDPNGAGLRPLWPMARDTEQGVLDDVMQVREILALQKFRLGKDDQNKKD
SCDLWLKIAHEVRHKPEGALGQEEL

>QLI64384.1

AQCQSIQPKSDPQTAPGVTFKVLANDLSKPRGVIADPKGNLLVVEAGAKGVRRIELDDGKGLDTCVTKSSSLVDDE
TLNHGIELSSDGKTLFVSSSTGVYAYDYDAEKGTVGKAKNIITGMDQGGHATRTLLVPKHNSNLLLVSRGSDGNIDK
DTVEIGSAKSQIRIFKIDHLLKIDSPVRYSDGEVLGWGLRNSVGVAEDPVTGYVWSVENSIDDMQRNGVDVHNSN
PGEELNFHGLPNDTTSDVYGKNFGYPSCVAIFDTSNVNGYPGGAKIGLQMVGDQMPNNYTDRWCQDDTVSPYI
TFGSHLAPLDIKFNFQGSAALISFHGSWNRKPPNGYRLSRVAFSEGYPKADKSSPTAEQELMWNKDNSYLKMLLTN
IFFASLATGELILNYDGSLEGRVEEFEVEEMVIDAVGGPSKLGALKNPLRQRDSDDPMFGSAALSADGVLHVLNKR
QSCEAGYWYCSGPCKLREQCCGNDRCIPSSAECCKNGRYCEAGNHCYILSSQGNLPVCCTDSRCTARVENGRTTYA
TSRTTTRTYTTTRKQYYYWTMTWYYYYYYWTYSTVIEASVVTSARTSTTSVLSVSTTAAAAASSYFSSISSTLSFSTPA
SATSLASLTGSTRKGAEGTDGPTATDTPNSGGGSENTADNGGGNEKSSPTTAATGATGTRRPDSPGAAASLWSS
MDILTTMSVALGVSTGALAVML

>QLI64386.1

IPQAAPTTAAVPSPSSTSSSGQACNNSPTLCGRQYNAVTHMGAHNSAFLRDSSTGNSLAGNQLKNATAALNAGLR
LLQAQVHKPNSTLELCHTSCDLLDAGALESWLKDINAWVTKNPNDVVTLLLVNSDNAPASDYGAVFESSGLAKVG
YKPQSNLLTSTWPTLQSMISANARVVTFVTNMDYSASTPYLLPEFDHVFETPFEVTAIGGFNCTVDRPSKANPASSS
LSSGFMSLVNHFKYQSLIGSIQVPDVDAISTVNSAGTSETGNLGKHLQQCKTEWNKAPNFVLVDFWDKGDPIAAL
DSMNGVTDATGRSTSQSSGQSAGSNFAKDRKFGIGALVAFVSAALLLV

>QLI64397.1

RPYAGIPYSKQAAKRLLGDVPDFVKFIQSGQDAAHFGIAQCRKLNSPLIQVFLRPFSRPFIFLDDDREVEDILATRTKE
FDRAPSTVGAFKPFFKHSSILKQTTSAWRAQRRLWADTMSTDFLRRVAAPKMHKYALELVELLRTKTVIARGRPFS
VEEDFDLATFDIIWASVLGSDLNGVLNEHAGIQSGLDSVAQPESTDSPAVMPRVQRGQMFLAAEFFNMTMEQSL
TSPLPTLQHWIWRQLPTYKRHWAVKEQIVNDLIKNARHRFAHSSETQGSGDQDTCAMDLVLRRESLAIQKSTTSST
LTPPTTAEVHDELFMFLIAGHETTATILTWSTKFLTNNPTAQNTLRAALQDAFPESSPLHLPPVEALIAAKIPYMDAT
LEECVRLANITPRLVRVATVDTHVLGYHIPKGSQIMCSPYVGEAPFDIPESLRTRKCRAAKDNVDRGWGPDMSRFC
PERWLDDAGNFNPRAFRRLAFSTGPRACFGKKLAMQELRVVMALLVLSFRFEAIPDALNGYQGRTRALHAPRQGY
VRLTPL

>QLI64403.1

DWQFRSRPDLAPPRLNITIPATNAVDKGYLFLAPFAGFPDTPTEQHGPRQAGPYIFRDNGDLVWSGYSIFSIWSTN
FQAGRWKGKDVLFSFEGDHNAGYGHGHGHITFVDQRYETIRELRAGNHKLVDKHEFHIVNEETGLIQIYQPVPRDL
TKWGASPEQQWIVNAIIQELDISTGKLLFEWSSLDHVTPDEAILPINPGQAGSGYNSSDAWDYFHINSVDKDAEGN
YLISARDACALHKINGTDGSIIWRLNGNRSDFKMAKGTKFCFQHHARWLSQEDGIETISLYDNSAHGTEHSGGSEV
HTAPTSSAKILKLNTKSWTADLVRAYFPPDHLLSKSQGSTQVLPGGNVLVGWGSEGAVTEFTSDGTPIFHAYMDSG
PLGDGVQNYRAFRYNWTGLPSEDPAIVALESNHGTAVYVSWNGDTETAVWRFYAETDEFGSRRFLGEVERRSFET
KLLVPNQKLTSVSAEAIDASGKLLISTAVVKTEQELLPPRTEATSHKVKLVEEVEEQKAFEKGGLGKEYGLLKFVRLRIP
LGDDL

>QLI64408.1

SPLGERQVVPNYPSKSTSKGFHLVVNVTDLSKDFESPVHNTYASSIHVGAGLALVGQSASTGYGRIFYQNGTVDEQ
RYSQSNILSDSGTPPFPSGLTLVKDPNSQTVSTAHLNGGAGQSGIGITRFPEPYAFLDPETWVACNESQPYYGGKYF
VILKQAETTIGDDGSINRNIPDGCAPVRLVPECTKLNDLPAGSTSSHEYALDSECYPDVKSLDWTQYGP

>QLI64411.1

FPNGKRDVVGRAPKQPAKEVFRGDFRSPRDVAKDGGFRPQGNYANNNQAFSIYRHMVGEHGREDDSDLSDEDS
DEEWTSAFVSVTTSVDTAASYGRWVYRIHATPNMIDPTEGEENQPEIFALGGVPWSQVMGWYELRFENNESVN
AEGITANDFTPNPAYQPRYDGYTLTTVEPRGPWRSHDRNYWTQFMNGPNVAPAVAFTGQFPLNFGTYSLDGLP
GPSQETQIERPPPTQPEAAALSAEDIVMAAEFLHEHNAPCDVSGVDPQTQYLILEADAGFARSLARLHRERPNDTL
PTPFDEADQQIQAIIERLRRGQEPNTCQLVGACSGLKLSQKKRATTPGAVSKLCKKIQKPKRGKNRLSPATQNSEKC
VLRSPLKIRVQLSDDTSAGSWDRLFLEMGKNARYERGQPAYLLKESPSAGDVMYLDVDLADAYPDKAVTVEDIKKV
RLVSYLDNGKEGSNELELQDITLTGKCAASPRAAEARIKARQWFTSWSSALKLKDWVWKRDCEKFTSLKFEWSLG
NKVRAGTWDDLKLGSHRDRRVRAHDVQFATNPSAGDSGGEEIKLKDMYGSASVHVDRVDFFHVYSTTRWPSSG
EDEWEFGGIKFRAQCEGFDKAVVLDKFGSDYNWHSRSNGIDLPIAAGDWRWEDENFQKQHPFPREKDEL

>QLI64437.1

APATIGRRAEPAPLFTPQAESIIADKYIVKFKDDIARIATDDTVSALTSKADFVYEHAFHGFAGSLTKEELKMLREHPG
VDFIEKDAVMRISGITEQSGAPWGLGRISHRQKGSTTYRYDDSAGEGTCVYIIDTGIEASHPEFEGRATFLKSFISGQT
SDGHGHGTHCAGTIGSKSYGVAKKAKLYGVKVLDNQGSGSYSGIISGMDYVAQDSKTRGCPKGAIASMSLGGGYS
ASVNQGAAALVNSGVFLAVAAGNDNRDAQNTSPASEPSACTVGATDSNDNRSSFSNYGKVVDIFAPGTGVLSTW
IGGSTNTISGTSMATPHIAGLAAYLSALQGKTTPAALCKKIQDTATKNALKGVPSGTVNYLAYNGNGA

>QLI64456.1

NSLPKGDVDYCSVYNRAPTPGCQPVPEGFLRSRGDSLSTPETDPRVLEDAFSALSVLQNVYFEPVNGTWPTSIDWT
GAVVETVIAGMLTTLTQSLDSTERGRDWKEKENLISSIFAQVTHSFFGQNAVAILDQAYDDVLWVVLGWLEAIKFV
RLHSSLHYPGTEQTCLNVPGRLSDALGTISFHGYNWYCTFAERARAFWDFATRGWDTSLCHGGMVWNPRLLPYK
NAVTNELWISASISMYEYFPNDEFDDAWTASQGFPTNDPLYLAAAIVGYKWLKDVNMTNSQGLYVDGYHVDMS

KPNNVECDQRDEMVYTYNQGVILTGQRGLFTVTGSPSYLEDGHTLIQNVIQATGWDLGENAAIDNVNERPRGKL
PPWRGIGRGGILEEQCDADGTCSQDSQTFKGIFFHHLTAFCAPISPVDIPGDANVNQTEFSKVQSAHDNACRGYLH
WVKYNMDAALATRDKAGRFGMWWGADIFDAFDASRQTDGIDHTTPNATDYRNKGTPMDVIWGLNMTWHP
GSKTSTGLCKNLLLRPDPKLRIGVSPELSVDSQIVLGRQNSHGDPNDRGRGRTVETQAGGLALIRAYWELSQS

>QLI64458.1

EVDAPVALPVFERGVFDAKRVNNLNFRQQDLSYLFALNQGSFNFGLLQQLALQNNFNIFAFQDLFNVGTFNINALL
QFQQLQTLLAIAQTGVFNQFDLAALNLGALNLGLINGIGAFDIASIIDVGVVPQIQSVVSTVTPTVII

>QLI64475.1

AGSESNCAISPKSIVQDACASYSTLENLNDRVKPALDDLTRTTDFFSYYRLNLFNKKCPFWDDSNGFCGNIGCAVET
LDNEEDIPEVWRAKELSKLEGPLAKHPGKQERKQHPQRPLQGELGENVGESCVVEYDDECDERDYCVIEDESASSK
GDYVSLLRNPERFTGYGGEGSKQVWDAVYRENCFQKSSFSHSANLGMSYNYNPAALGFKQVMDAAGRQAQLEA
QRLQHPNIPFVSSTGYEVDDECLEKRVFYRVMSGMHASISAHLCWDFLNQTTGEWQPNVQCYKDRLHGFSDRIS
NLYFNYALVTRAVAKLGPELAGPDYTFCTGDPTEDLVTRVKVSEVAKQAASIPQIFDESLMFVNGEGPSLKEDFRNR
FRNVSRLMDCVGCDKCRLWGKIQTNGYGTALKILFESDNHSNKIPVLKRTELVALFNTYARLSSSMRAVGKFRQMV
DAESGHKPAAATESAAVEDEKVNEEGAEEDDFDPSYLWRELEDMKRRGPKSSSFRDQWAHETALIRQSVKVVLH
GWLHGSILIYRIAVTEAHRLWLYFLGLQPGPGLVDYRWAPKDKKTEL

>QLI64476.1

LVTRIQGANGVMMPGLSVADGTPRDCSSNGCGSQADTAIIRQRDIRSGRASPLGRTQGNGPIDASVMVSSFMGG
ANTAKAPTNNGASGAAGQEDDLSALQQQRQQRREEEKRQLGQLLGGGKANGGGGLLGGLLGGGAGAGKQTN
AAPESMVADTAGQGSSQGLPTADENGEVTMVFRQINQDGAGPLSASIDATSGGTKAESFQNAEVTQDVPGLGF
GGLSTATKTDFPLKVKMPAGMTCEAKVGGAENVCIVRVQNTAAAGPFGGSAAFTQTKTARARAIAYRLKKRMEIN
RD

>QLI64506.1

KVYKKNVPTYDVGPDAKIDGKSITYRDPDCEEGLDCTITRSCSSAGTSPVLSADKKYFACCLPGTHLLGSPQTAFDCC
ADGHDLVGSPAVGYHCCPTGFEFDGTQCKEVCKNGKVLVGGKCVCPEGMVEGPDGNCKPKPKCTSGLETGKCYI
FTAENGNRLGLHNDNVYYAAPDSMIQRYGKFQLCADEKCTPGQAVNPSNEIYIRDTYGDLATGANRGQWLNNA
ANGNHIGRTPAFASAGHFSISKWPCGKYCLGGFTQGIGPACPAEIPAMTFYSQDPQMCVAFQFTEVPCDLKADVN
NCIWANGDQCCNRVDCSGKKGE

>QLI64515.1

MPTTTEASESGLERRTGPGIVAAADKMKGKPYVWGGGNIHGPTNGGFDCSGLTQYSVYQAEKKEIPRTAQAQYA
SKLGKHIPRAQAKAGDLLFWGKGGDCKTGVVHVGIFTKPGWMVNAAHTGVPVREQKIWTSYGGESICPDAVRF
W

>QLI64516.1

ARPKNAILLSQVQSLTLRGNGAKTTNRRVSAIPQLKCTSSRELCSLYSIDTMRCTNQGSSYGGEDIEWSCTATLPEEL
KLGSTDVICEGYASADDPYVLKGSCGVEYSLLLTSKGQERYPHIANPYGGYFSDGRGGTDLSAWLFTVVFVAVLGWI
VYSACVAGNGGRQARGNTGRRWGGGGGGGGGGGFGGGGWGPGNDPPPPYPGTKPSSSGSAWGPGFWSGL
AGGAAAGYLAGNRSRNDERRGYDRGWGSNTWGSGPASPRASPAASWGSSSSGGSSRHESTGFGSTRRR

>QLI64522.1

APYKRSVLGEVTNVLSGVTAKTGASQLESQLQGVFGGSFAQVEKALGGVPLANKLLDLIHGGLKPAAAQAIGQALA
MVQSGLPIDAVNAYLNKATGGAVANLENTLGASDLVGGLTGVTGLVSGLVGSV

>QLI64533.1

TALTYKLDANEKACFYTETKKDNEKIAFYFAVQSGGSFDVDYIVEGPNAKMILQGEKERQGDFVFTAQHAGEYSFCF
NNEMSTFAEKYVDFEISVENEARTAQLPSKQGTSPEQTSVLEDSIFKISSQLSTISRNQKYFRTRENRNFSTVNSTEKR
IVNFSVIQIGLIICMGALQVFVVRFFFQGARKGYV

>QLI64567.1

WLPQDRDLQAFNQTARFEKLGKRFKPSLPNGVTKIRGVNFGGWLICEPWMMQNEWRNVLGCGGSASEFDCM
NDHYKGGNREAGNQKFEKHWRDWINPDTVQSVHDVGLNTIRIPIGYWSYTDIVDKASEPFADGDRMLPYLDAVV
QKAADLGIYVVIDLHGAPGGQQQDAFTGQNNQPAGFFNDYNFGRAEKWLAWMTNRIHTNPAYASVGTIEVLNE
PVSRHDAAGRYPAPGEDPGLVQKYYPAALKAVRDAEAALGIADAKRLHVQFMSSKWDAGDARAAAAVARDPAT
AFDDHNYIGFALGSSNGDQRRLMHSACTDSRVVNGQAFAVTGEWSMTSGVDWKDAGFFKRFFTAQQQLYEKP
GMDGWIYWTWKTELNDPRWTYSYATYLNYVPTDAAALEKNVYQDVCSGFR

>QLI64570.1

NSPITFQNTSNTILRVDTGSYGPAIEEAHYFYQDWPVGFAVSSKGRIFVSYYPGNVSFTLGEVVNSTAEKAYLPQYN
VPSANSTQTIDGTLFGSQNSTGFISVQALYVTPKTANRSETLWVLDTGRPNDASQMAYGLPGGAKLVAVNLDNDT
IVRTYLFPSTVSYPDSSVNDMRFDFRENITTSGQGVAYLSDESPEGRNGIIVLDLGTGKSWRHLDRHPAGLSGYGVV
PSYQGMPFYQETPSGPFTHLPQGLDGIQLDLTGSTLFFSSMTSDYLFSIETKYLLDHTSPACIQAANSAVRNLGQRG
GNGNGFEGDSNGFIYQAMPEQNAVYAYDPTTLRVAPFIRDPRIIWPDGLSVSEDGYIYIIINQLPYQPMWNNGTDL
RQKPGALLRAKLPNNGAKVKTLF

>QLI64587.1

RAPQGPEPPSKQILDAIKDGMKDWGPAGSLKPPKDGPKAPIKNMIGGLPELHNPKPGSSDWREDTLKGPRKKPG
KTYPNSNPCSKRDVACDYGPSSSNSPSDKGKVKGKGKVGASVLNIQGGKFIDFFSELDPEAFGPLIKAIEDGEITVADI
EMALKRAISETMKQRWADFESAEKAAETLKNIAFDIFATARYATPPGFWHDVVKNLPPIAKEIHNAKTPEEKLEIAN
KNINEVVTIWSYTPVGAFNENIMKEVAKGTPTTTAVAVSVNKLWSYTPLGWLINQIAPIDSWIRGDDDQVAEPKP
KKRPDPPKKNLKPPVATWGKCKCILSRPPARGDKCANTCRAVRALGGWK

>QLI64591.1

FYLPGVAPTSYKPDDQVPLYVNKISPVAAMQDYRLHSVVSYDYYHPAFQFCQPNGGPKYVSESLGSILFGDRIMTSP
FDLRMLRNETCKPLCKVSYPEKMREFINDRIYQGYSLNWLVDGLPAGQKIQDELTGTTFYSPGFSIGDYQFTENEEE
DEKLSFNNHYEIWIEYHEVNGNPNQLRVVGVVVQPSSLAYTGEPDCADNHPKLIFEEGDKNQDVYFSYSVYWRKS
DTAWATRWDKYLHVFDPKIHWFWLIDTAIIVIILVLTVMSILVRTLKKDISRYNRLDAISLDDLNGTSAIEDGVQEDS
GWKLVHGDVFRTPSHPLLLSVLLGSGAQLFVMTGCTIAFALLGFLSPSNRGSLGTIMIILYTLLGFVGGYTSARTYKA
MQGEQWKVNIALTPVLVPAIVFAAFFLLDLFLWAKQSSGAVPFTTMLVIVGIWFVISIPLSVAGSWLGFRSPQIEAP
VRVNQIPRQIPPVTTYLRPIPSMLIVGLLPFGAIFVELYFIMSSIWFSRIYYMFGFLFLCYGLMVVVCAAVTILMTYFLL
CAENYHWQWRSFLAAGMSGGYIFLNCLLYLITKVKLGGLAGIVLYMGYSALISFLFFILAGSIGYFASWWFVTRIYSSI
KID

>QLI64598.1

ANPNNLGRDSGLKSTSYHDGAGVLKYVNPLIGTRGIIPDSNGGMIPSISTPFGMTRWTPQTRENYISQVPYSDLDR
RVHGFQATHQPAIWMGEQGQMTITPGLLVSGDSIRTQFQKRGLEFRKSDEKSTPYVYEVLLDADSIGDFNWNLTE
QWAHEEYGDGCPPCPGGAGTVPESVEEGSNGRVRKRGYTDRFDAFEERDLDESSSNSSDANLGGYEHSIKAAMS
ATSHVGHIRFDFQTNSGSKSQIRPYVVVQATRLNWTGHVEIDPQRREISGSNTQRQDYAIGPDHPKNFSAFFVSRF
SAPFESYGTSQGGKTERGHGSLHGKDVGAYATFDTAHDRIEVRTGVSFISVEQARKNLDIEIPDSHSFESTVEQTKKA
WLDQLGRITIEGVNKTDAEHDQRAIFYTGLFHSLQYPNDYSEPTGSEGVSKRRFYSGYTDSVHEEHDSYYQSWSIW
DTYRAEHALLTLFAPERVNSMMRSLLRIFDWSGRLPIWANMVETNIMIATNADAVIANALVRGFNDFDIQKAWK

AVYADAYIPPERDTLLLYFDREPKTPHEARAGLTSYLEHGWVDNDRWAEAASRTLDYSLNDFAAAVVATYAGDHA
SAKQLMTRSRNYMKLWNNETQFMQTRNSNGTFAREDWGWTEGDKWVYTFDVMHDAGGLASLFKGGKADM
KAKLDQHFSGGHNMHSNEPSHHVPYLYSAIGYPMSSAEKVRDIAWTEYNNTAAGLSGNEDLGQMSAWYVFSAL
GFYPLNPASDEYVVGTPFFDRVEIRLPSMSGADGHTLVISAPGAGTQGKAYIKSLKVDGKPVDKPLLKHEDIVNARKI
EFHMSSEPTGWGRKGTV

>QLI64616.1

SPHIRVRGGARVELARRTPAGGDPAAKQQAQIVRNYAKQLEQYAEQLENGGGTETGNGQPTPNNPSGNEDETG
NGRPTPDIPSGNGTETGNKPPTPSIPSGNGTETGNKPPTPSRPGSSPLFPGAEDDATGAGDDPISAGDDPISAGDD
PISAGDDPISAGDDPISAGDNPISAGDDSTSAGDGTETGNKRPNPSRPGSSPLSPGAGDDLIGAGDGTETGNKLPT
PSRPGSSPLSPGAGDGLISAGDGTETGNKRPNPSRPGSSPLSPGAGDDSTGDGIIASKE

>QLI64622.1

SPPILHRQDNTASNPSPRGQGPEIQFGHCYTIKNKNGEPLGHQPSAWNYLRFGAGTKPARFKVCQNLGQCKSPNT
DYQVLLNRARFWLFDVEGNYYSPRGDFVAANSPPFGSNRNLYPAGGDYRYYVDFWGENDCSRPDARPLGQCPV
KLRIDNLRDDKGLVIQDGYLKAAASGDDVVVVTFEDAKCPDE

>QLI64625.1

APLIERTGLLPPSQDPWYQAPKDLDALAPGSILKSRKPPAPIAAFGIESVNLQAAHQIMYKTTNSFNGSTATVTTVLI
PHNADLTKVLSYQSIVDAASIDCATSYALQLGSKSGVVFGTFTTQAELFLIAALLDKGWVVVLPDYLGPHSAFLANRL
AAHAVLDGIRAVTKSTAFTGVQHNATVALWGYSGGSVATGWAAELHASYAPELNIVGAALGGTVPSIPPVVKAV
NKGYAAGLLPSGFLGLAAEYPELKGIIHEQVVPQHRPAFEKVKKQCLVADLEEFFYEDIVSMLEEPSILTSGSLATILGE
VAMGKSAPKMPLYVYKPVHDEISPVANTDDLVEFYCANGGSVQYERDWVSLHGTLLATGAPKALSWLIGLVDGK
PQPTGCSTSNVFSSFLDLKALEIFPKAILDDLWALLKEPIGPRRHWYSL

>QLI64647.1

GYLLDTTYDSTNFFNSFDFFTLPDPTGGFVQYVDGQTANQEGLAVYAKGGIYMGVDSKTKNPPNGRKSVRVSSKK
SFTRGLFIADIAHMPGSICGTWPAFWLLGPGWPSSGEIDILEGVNAQTKNSITLHTANGCTMSNQGALPSTQFSSA
DCGALGASKGCQQATVDASNYGDGFNAIGGGVYATEWTSDHIAVWFFPRSNIPQDITSQNPNPASWGAPTARF
VGGNTCNINNFFKDHQIIFDTTFCGQWAGDVWKNDAKCSTLAPTCQEYVSGNPEQFANAFWVVNSVKVYQQG
GSPGPVQHFAAVNPSSGSVGPQDQNHPQPQTQVQPQSTQPATQPQPTNGQVWNGQPQPNSDTNSQPPPPK
QDNSWNGQVWNGGGWNSKRRNRAVEKRFSA

>QLI64662.1

ADLAKYVLTDTGSTGGGNTFPGVSWPFGMVKLGPDLYTGTDSYSGYQPTGNFTGFSMLHESGTGGAPKYGVVSQ
MPVVGAVTNPLSDAMNDTRAEKDFTEVGYYKATLGSGTVVGLYASRKAGLYRYEFPNTAAAKNVLVDVSHVLSSY
RGQGLEQHFVAGNISIVPTKKGNTDLKYIGWGTYDNGWNRAAPWTVYFCGHFNRPFTYKTFLGKDKTTDHLVEFS
NKSASYQSDTARLGVLFTFNHTNVVSRVGVSFISTDQACGNVDEEISQESDGWDIRNKTRDEWNINVLGRITTPDT
NVTKLTQLYTAMYFMHLLPTRKTGENPGWNSSEPYYDDIFTLWDTHRCTTALFHVLQEEYYAEFIRSLIDTYRHTG
WLPDGRSSFSNGAVQGGTNADNVLADAYVKGVAGASPNGGINWEDAYEALLKNANTMPPNNHDPRDPTGSTA
EGKGALPDYSQLGYITPSSMNDFSLAVVAAGLGKDTFATYFNRSRNWRNHWNTNMTALNFTGFLGPRNTSGFVP
QDPLSCGGCYWRDAYYQALPWEYSFNAHHDLRTVAEYMGGPERFVQRLEKTFEPGAYPGNKPFGNTIFNPGNEP
SFTTPYLYHYAGRQDLAVRRSRHVAESYYAPKPDGLPGNSDAGAMESWLLWNMIGLYPMTGTPLFLIGSPWFAD
LTIHLGGSGSAARKLRITSTGGGEDAFYVRGVTLNRAPHNQSWVDWGQVFRSGGTLEFELGPEPLNWTTGDAPP
TMGERDPEDARLALRRWVGGQA

>QLI64672.1

LDVNRALQARHDTCPETKTVTETQTITERNTVTVTTAGACTTATTPTTTTTTSKSTPTPTGCPAPPACDNLGFDWAY
YNNSAHNGDKTYSNFHPDTYKRVKPLYVGTTRYVGGLYGEKGGKDPAGRIYGSTKDLKLDYFALNHHAYFYACEAG
TYSINIPYANDAVFVWTGAKAYKGWTDDNADAKARYNQPDHIAGQASSKVNVPADTYVPIRFVYGQAQYGGGF
YFTVTTPSGQVIVSDKAHNSPYVVRHSCDGVKAPKYMPFGKEQ

>QLI64695.1

NNFRVFDSTAYTNTSIGYGTSNINWIPNYVCSPLVANNAIPSAETWQSIVLEWNIYPGYPLVLDCENIYLTSPSTADH
NLEVMKTLQTWAAQVLPSDQIIGWYGLSGNTAASLYGHYRSLIANHSAHAFFPSAYTFSSSLTTWNASLNSVLAKIK
AIDASLPVYPYTWPQYHGSPYAFYPVGLWKSQLDVLTGNQDINGFVIWGGKNHQVCGDACQASAGKNPWLEAT
RTYLKDLYGIYGGASQKQGGQLFTGV

>QLI64709.1

RQPYRLAVMALPGQSLMRRDTSGYKPDQKQCKAGNTCAESCGADYQQCAGGNPDVAHCFNPKAGESCCTDDS
GNSCQDGYYCTHDTKAQTWCCPKDMDLTACAAAYTISGLVAATSKPPPSTTSQAPTTTSIPPTTTSSSIASSTTSSTP
VITTSENTNTTTSISTASIGKSTAWTAVNSTISTHVPTQPSAGIPSVTGAPPPAPTNVNAATASGVSALLLVAAGLLAL
L

>QLI64717.1

FPVTADSLNCRAEPNTSSAVKKTYKKTDDVKISCQTEGPSINGNSIWDKTQDGCYVADYYIKTGSSGYVTGKCGGG
GGGGSNPPPSGGFCKTLNKAGTDLITRWEGFVDRPKPDPIGLPTVGYGHLCQKKGCAEVKYTFPLTKATALQLLND
DLPSYTKCLGRALDAGKVKLNENQWAALTSWVFNVGCGAAQSSSLVKRLNRGENANTVASEELPKWKMGGGRV
LPGLVKRRADEVGLFKIASSRSAFPQCQ

>QLI64720.1

NIGQDNLAPLFKSKDSVPDSYIVKFKDGISSTGFDSTLASFTDNSHHDYDAVFKGFSATLDSVAIRNLRRHPDVEFIE
QDAPFTINGFVEQKNAPWNLARISHRQRGSISYIYDDSAGEGTCSYIIDTGIDATHPQFGGRAQNIKSFVNTATDGN
GHGTHLAGIIGSAIYGVAKKTKLYGVKCLDDRGSGTTSNVIAAMDFVAKDAKTRGCPKGAMANMSLGGGYSAAV
NKAAASLVASGVFVSVAAGGSGTDAKNTSPASEPTVCTVGGSTEKDERASYSNYGPVVDIFAPGVSILSTWLNGAS
NTLSGSSMSAAHITGLGAYIAALEGFPGGEKLCKRLQELATKGVLTNVPSGTLNLLAFNGNPSG

>QLI64737.1

SNSTSRTSTITAPPTARPTTSVGTTSRTTSRTSTTVVAAGPNPVAPGLGLAALMALGALV

>QLI64738.1

APQRVLTAEEWVGDRVITQQRVLEEFRGRNIICDDEEENNRINCEIGFVSIGIEEKSHDGLSPLCEKKGGCKYCRIESG
FRNAQHFMCAYKSSNKDEPIFFPVE

>QLI64741.1

SPVEKRAIGGVLLCTGANSTGTCKYSVYELNKCHQLTEPFYQNTNTFAVDGENFYCFPKLVNCTDICTSPTGCTFGA
VDYSYEHKYNLSAISWDKYITSFQCGMKQDPQTNNNGSSSKRFLS

>QLI64753.1

SAVSQAKGLLAEPKQLEERTEDKNPYGYGNPPPVQHTPSTTTQQVQPTYGAPAYLTPSLETHFSKPTLTFQAPVYSI
LTTLQTSAKPVTSKSSTTQAATTDAAHDTAPGYGAPPAVTKSSTTQPAPTYGSYSVL

>QLI64761.1

IPCTTNAKLPSLKFRDDGKFQIAVFSDFHLAESAATPRGPKQDNKTIQVMADVLDKDRPDLVVLNGDLITGEVTLKD
NSTDYIDPLVAPLVERNLTWASTYGNHDHTFSLSAENIFNREHEYVGARTQRMVRTAEAGVSNYFLPVYARSCRDT
TACDPELLLWFFDSRGGAYYQRPTAAGAPTPQPNWVDTTVVEWFQGTNAAFVQRAGRVIPSLAFVHIPPNATSH
AQRRIRPNRNPGIDLEQVSQQSQGWCANGTQDWDNPRCRYGGFDVPFMKALATTPGLMGLFYGHDHANTWC
YRWDGEVPGTGIVARGINLCYGQHTGYGGYGDFIRGGREIVLDEGRLKRFEVDTYMRLEDGRTVGAVSLNATFNR
DWYPATPNDQTKLE

>QLI64765.1

QAPAPASVELGAGSSKAAAAHSWEAPAPTAKPDHDWEHSSAASHEWHQASSAEAQPSWGSSADRHQNATVVT
KTQVVSRYTTYCPEPTTVEIGTKTYTVTKPTTLTITECPCTVTETHKKPHPSKEAPPPHTKEIPPPKPSVQTPAVPQPPP
PAATTGVVVAGADGLTAAYGFAMAAAGLVGGVFAL

>QLI64767.1

APYDNPPPPKPNHTPGPAPGPAPGPAPGAGDKCNNDQKQVCCDKVIGLVCNLGLVNNCGGGKTYCCAANAPTT
QNGGIHILDLNVGSCNRVF

>QLI64786.1

APSPGFDVKFTNFTNVATNGSWWMADIKRQGNAPFANGTYSVFRNVKDGEFGAKGDGITDDTDAINKAISDGG
RCGDGCDSRTTTPALVYFPPGTYLVSKPIIQYYYTQLVGDAASVPTLKASANFEGIAVIDSNPYKSDGSNWHTNQN
NFFRQIRNFVIDLTDLRQETGTGIHWQVAQATSLQNIVFNMVQSKSSQNKQQGIFMENGSGGFMSDLTFNGGAL
GAFFGNQQFTTRNLKFNNCGTAIFMNWNWVWTLHGISIDNCDVGINMANGGSVQTVGSVLLTDSTISNTRVGII
TAYKPSQSGTNGTLVLDNVDTTKGVPVAVQNDRNQTILAGNSLIQSWVQGRSYVGATGKAGQDARSPVPKPAAL
TDSTGKIVTKSKPQYETVPASKFISVKSQGAKGDGKTDDTAAFQQVFDNIRPGEIVYIDHGVYVIKDTVRVPKDVKIV
GESWPLILAGGCKNFADESKPKPVFQVGQPGETGNIEMQDFILATKGPQPGAILMQVNVAGESKGSVGLWDVHF
RVAGATGTKLQSDKCSKDPKTEAPPNPRCVGSFMLVHVTRDASAYFENTWFWVADHELDLADHNQINIYNGRG
VLIESTKGLWLWGTASEHNQLYNYQLTNAENVYMSLIQTETAYMQGNPDARVPFKVNSQFSDPDFSTCTGPRCA
RTWGLRAQNSSNVFVYGGGLYSFYDNYDQECVAANNCQDEMISIENSELHMFGISTKASVSMVTLNGQAAIQDS
ENRNNFCAAIAAFASS

>QLI64792.1

AITNAVGNAGGVGRALGIDSSTPRDGTRRNPFQQDATRFRGASAKTVGETVGAGSNDVESGTQAIMSEMGGQL
PQVTPGGNLQMTLHQVNGDGAGPYTCMINSDGTGQNWKNVQVTENVEGNQRGRNNAGSASDHPLTAAIPA
GQECTGQVAGQENVCLVRCQNPARAGPFGGVVPVQMVQEAGANNGTAPNAAAPNAASPNAASPNAAAPNA
AAGTNAGTNAGTNTGKTGNAGNAAGAGNAAKKGAAKGNKNNNNNNNNAAADDDDEEENDKRSIDTGAFVK
RIDEKTRAVEFTA

>QLI64804.1

ASVTFWTLDDLVRTIYFTPNPGFPEVAPVTCNNQQKTVVNFPDQWIGNYYAVQKGQKNAPGMLGEVNFGAWG
GMTYFDVSAIVDPNDQNNVKQMYPASGKSPMSGCPVFPCNNAYYLPDDVQTKVTHESDLVTTLGEGFTGLNFS

>QLI64806.1

AQPGAVQPVAAPMRDLAWGQLNFLHTTDTHGWLSGHLLDADGARPQYSADWGDYISFSQHMKKRADDTGSD
LLLIDSGDRVEGNGLYDASTPKGLFYYDAWAEHHVDLICVGNHELYQASTADYEANTTVPKFGDKYVASNVDYVD
RATGQRKPLAQRYRKFKTKNKGLEILAFGFLFDFTGNANNTVVQPVAETIKEKWFQDAIREKPDLFVVVGHVGLR
MTEFRTIFTALRKQNWHIPIVFFGGHAHVRDALSYDSQAFAMASGRYFETVGFMSIDGIQKQSADDVSAAASLKFS
RKYIDTNLLGMYYHTGLNQTTFPTDDGKRVSKQIARARKTLNLDHRYGCAPKDLWASRVQYPNNASIYSWIQDEV
FPDVVVNTKRKDKPRLAILNTGGIRFDIFKGPFTRDSTYIVSPFTSGFNYVPDVPYNIASKVLTLLNGADKILSTHGAET

KFMAIPEVMFPSGHGQSPADAHQAEDERLELRDAKPDLIRGYTTKDDIGTDGDDTVHEAIPFYNTPNCIQSEINFPE
KGDPETVDLVYIDFIQPWIIPALKFAGGDYTADDAKVYMDGTLTYKLSEWIGKNWQGDC

>QLI64821.1

GSPDEGCSSTITKTLAETTSIIMAPTSTCNQIGTAKPTESLGSQGNHGHGNNQDKVPASSGSGGHDGGNGNGNQ
NGNGSGNGQNSSGNTPISGPDSNLGSSNELDNGSGSGSNGEAGSNLGSNSGPGNGVSSSTGSLPDEASSPTHGP
GNSNRPESGAENESGCDLGPSPGNSDGVSSGTGSEPDDGSAPGNDSGSGDMENESGSSLGSNSNSNSAPGSGIG
SGSGPKPSYESATGNGHDSVSGSDSGTGNGIYNSPGYESAKDFQPGSGSGSDSDSDSDNRGGNSSSGSESKNGASI
QPDNPGSHDSGTVNYKDNKSGKGNGSGNPTGTDAANGSGNGSGDDGFGSGSGENGSGSGKGNNAGHSHTDG
SSSIESGHGSGGPSGGVSGSDSGSGSSAHGSGSTYGNASTATPPVVTAGASPSRSSARFASSLVGLAGFLCLF

>QLI64830.1

TGLILPLYIYPAATSDDGAANWAPVFNAAASNRDLSWLTVVNPHNGPGDTHLPGNNDINYIQGVTKLNTHPNIKPI
GYVRTNYAQFSLDQVKQDVAAWKGWDTYSASNISVQGIFFDESAPNAPYMSALVGYTRAAFGRPITVTCNFGKA
VADEFYDICDVVVAFESCLNCPGLPQYKDAATIQANIPANRMGKAAVILNYFTGTAFDGTFADATLVHRYFQTARN
MGLAWAYFCSQDYNNLLAWPATIWEDVKALS

>QLI64835.1

APPPPNGSVWWHTCGNCKCGHSGAYENFKGDTGCLRFDPNQDIRAAGLTRHGRLQTTCSLFTSDNCSGKVVQSI
GVAGGTYACTAFNQNTKSIRCYYDV

>QLI64839.1

ESPNISQLRADLTSLANTLRWNITTQDTYAPVQQAALSAYSTLTSVARKHHKAIPQADQFSETLERLIAESRYLVDLS
APADYEPSVLDSVMFLTGSVDEWRFTIDTISTKLELPSSYQAPRLRQSTAATGINRETAPGTEFRDADTGPVMVVIP
TGTFTAGSTPEEHERWQVPQNRRDFELPQRRVSIATPLAIGKTEVTVDEFDTFVRETSYQPRGGARWWNPDNSTA
MVFNQDLNYLNPGFAQTPDSPVVAITRQDAVAYADWLSAITGATYRLPTEDEWEWAARGGTQDTFFWGNQLD
DVVSYANSFDITSKRVNGFRWANTPVDDGFAWTAPVASFQPNAFGLYDVTANAREFCADTWVRDLSDAAADGS
VHVGAAPFPVVRGGAWNYQPQNLRINYRSAYFSSEVATNMFGFRLVREL

>QLI64861.1

MYTSNSPVLQVNAKTYHTLIAKSNHTSIVEFYAPWCGHCKNLKPAYENAAKKLDGLAKVAAIDCDNEMNKQFCGG
MGVQGFPTLKIVRPGKKPGSKPVVEDYQGARTAKAIVEAVVSKINNHVTKVTDKDLDAFLKPQGPKAILFTDKGTT
SALLRSLAIDFLDVISVAQVRNKEAKIVEKFGITKFPTFVLVPGGDKEPIIYDGDLNKKDTVAFLKQVGQPNPDPAPAK
PKVKGDKTKAPKSEKAQSKKTKASSSASPDTEDSATTPEASTQTTATEPAASTPDVISITTVTTKDTLVEKCLSPKSHT
CVLAFIPVDASENSAKVTDSLSQLNTKYVHGHRQLFPFLAIPSNIEGLDSIRQALGLEKDVELIAINARRSWWRHYDG
NFDVESVEAWLDAIRMGEGSKKKLPTEIIAVEEVVEEDSDKKTDAEEQAPADEVKHEEL

>QLI64875.1

EKSSKGDLILCDCGIGDDKEHPDWSTSRQMNWYKDIKWPASAANYPNAPDMAVQVPYKDGIYPWIPQGVTAT
MPNGEVWTAYIEDGTPDGFKAGSAVSSKDGKNMLNCWAYRGRPVSAAINKTVSHDAICWTAFVCNHDNEPPSR
PDDMDHPSTMTSSTATPATTYYTKPPKTVTATVTTTSGQPVPTYNPNKGTLSIYAGVNPRFINWQDTWQSFINHF
VWDKNSGRCIGEPARGIGYNITIDCAGIQIDEDTHMTLLLIKALRDVGLNSLWFNQNPVIPGGNGPNNTSPNWVV
MPESFTLEATDLGTNNVVGRLAYKTHYDNFLTPPCSICEIGRFDKNFFNPIIESMKGSYPEFNNFTIKGQCDPWIACV

>QLI64879.1

QEIIGNISNGKISKLKSHIIAKRPDLVKTELDAAKDFADRYAQAKAEKPLPNITSIKAICGCPANEMFSLAYVFRAAGDK
PKIDFIPVQDTEPTYGDVQRQTTTIATDTFSWKWSHEVTLSVTVEVSFGLQELGQLFRFSVTRSTTDKTEHGITTTRT

TQFSDGRTYKCRNDHRCQLQTWTFTAEYEGDYYEMPVADFNCLSKSVKWKYKSGASRQLANSSKVSFASLLGKED SWDRLSQRYFTTRDRETGEDRAEALDQWSNVLGVDFPPDSVEIVPKNKSIARGPATFPILNDNGEPYHVTVLFDYS AVNRSTVNGSSTGKQKRDVLDKDLASDDVELEIFVVDTNIPHHDVDTVLPNGTVIRAK

>QLI64892.1

DSLKDIKHIILFMQENRSFDHYFGTMAGVRNFGDPNVQVNDGTPVWRQSMRQPKAGVDYLSPWHINYLGGDW REATQCMGGGINSWEAMHGSYNNGRGDGWAIHDTDYSMGYYKREDVPTHWDIAEGWTVMDNSYQSILGLTD PNRVMWMSGTVNTAGSYTNPDGQGGNILSNRASPGCDSPGNNCFPFVWKTMPEYLEDAGISWQVWQDFDN FEDNMLAYFKQYQDAPEGDALRENGISFPGLQAFYDACAEGSLPQVSWVIGPQELSEHTPNMPIDGAWLQKKVV EAVTNSPAYKESALIISYDEQGGWADHVIPPVAPQDAPGEWITDPFNANNGQVPIGPGPRIPRFIVSPWTRGGHV FAETGDHTSDLLFLEAWAAANGYDVFNQGISDWRRQHMTDMVNAFDFDNPDYSLPQLTEVRDPEPRTDNNWS GNLSLGSLTGAWVGPARCRNGYEHGNYPPVPYGEANAEQNMDDLVEDGFKQVRGFITEGRYITIESNGLGLGHA ENGHVVALESAEEHDDVRQRWVLHAVDGNRFGNTFHLQSTSEKSYVSKNGSLTTKKSAARAFIFEYEAQGSTYTLR AKGAKSGKYVSLGKSSKHDPEYRENAGKITWEGHGSRFKIYAVNYQS

>QLI64894.1

APAGVFESDKLLTIDKDGKDSSYDVNKRSHFVADPLLQISEDDSASSYQRSNFKADPLLEISDDPNASSYN

>QLI64897.1

RRVANVFPPPAGIVQRNAETESSESGIQVSRGKQVEGITANSRAEVIVIWLNPGNGASKTSINEQVTVTKTVTAGA GGEATQAPGGSATASAPGTGATHTITVGGANGLAFSPQELRVPMGDMLLFKFGSQNHTVTQAEFATPCKPMAG GMDSGFLANPNNTVSPPPEVAMQVMTDKPLWFFCAQKGHCGKGMVLSINPTAEKTHAMFQGMAIAQNGAGS ATPITGGEQGSTPPPPSAPGGVGGNSTGGAGGAGGSGSSGGNSGVTPGKATMGADGSYHCVVTCNSGSFPDAS QGVGAFGGMSGALPV

>QLI64900.1

KVIYALFLSPYRNVPGPRLCKITRYWAVYHDMWLRRIEKIYEWHREYGDVVMVAPGEVSFSNAALTREIYGSTGRH PKSKYFDNFLMYGERPIFCTLDVRDHRQMLKRTFTFYQPTSIYKPASLQPLWTNVRRFVDQLKRDIAVQPTVDVLLY CNFYSFDNITNLVYGPELCARTIGDAECEERAILKGWKEVEVWNNLSYNFPLVHKMIRAVVSYARKDPAFLSAEERL TDWNMDKIESARRNPDKMVAGSLLHQLSNMKTPDGEAFPTSWIAAEMLDNIHAAQTTVALSLTYTLWNLARHP EWQDRIRAELLALPVKEDGLPNFDDVMAAPILDACFRESSRLCPLSSGRAERVVPVTKAYNGVVLPAGTIVSTSTLSI HHRPDVFPDAHMYRPDRWLEADEATLRAMESCYMPFGYGARLCLGKAFAVAEIKLLIAGIVTEFRLWDDPQSPTT ERSMEQLGTQNAMPRGQRCDLGFRQLAEWERERGNGTQGAP

>QLI64912.1

LPAPADGCESDSVPAVEQTKPVGENNGKLPWLKPGQYASLCGSSKFTDEDCGTDMYCKAFDDIKNVTDRKFTSSK QCFAAHEPEPLPWREPGQFASLCGSSKFTDEDCGTDMYCRAFDDIKNVTDRKFTSSKQCFAAHEPNPNPKLPWKE PGQFASLCGSSKYTDDDCGTETYCKAFDDIKNVTDRKFNSSKQCFDAHDPKPKA

>QLI64918.1

AEAFDDEALYKKTLPEFTPDGLKIVDLRFKNDLEKRGVCGSVPGVANGECVRYYSSTGCADQDHIKGYKPTCAGNC YVDKFYSVKAIGDGTYSTNCDLFSDTSCQNKIASVGGKTGGGHCANANGWSMKCYYRC

>QLI64930.1

IRNKHVGDFRLFSDEGCSAGNLGVWTVIDDDVRDHPCQRIPDGEGVRSVFVADVNSECRFSLYEDADCQAGEQN TTQGNCYNSAAGWKAWKMTC

>QLI64940.1

ALQLPLQRPINDIAPHSAFHARPLVSSGTLQDSIDKDRLWERAEELYALARKSEDEFNHPTRVIGSAGHQATLEYIKS
ELASLGSYYNVSEQPFDAYAGRVREFRLVIHDKVPASTTAFSLTPPTKNNEPVFGQLILVRGTGCKASDYPHKVKGNI
ALMQRGTCPFGDKSQLAGRAGAIAAIIYNTVDEELHGTLGKPSPDHIATFGVSSKDAEDWVENMPRGEIFRGSAYI
DATVQTIKTNNIIAQTTEGDADNCVMLGGHSDSVEEGPGINDDGSGSLSILEVASQLAAFRIQNCVRFAWWSAEE
EGLLGSEHYVKSLSDKENAKIRLFMDYDMMASPNFAYQVYNATDAENPLGSERLRDLYTDWYTSSGLNYTMIEFD
GRSDYDPFVRAGIPAGGIATGAEGVKTPLEAGFFGGEEGEWYDKNYHQIGDDLNNLNMTAWEVNTKLIAHSVAT
YAASFEGFPERTREVGVASFRKPKCNALSPQFCHS

>QLI64952.1

ATPVGWGRAPQNSCLHEKLSPGSQVLNLTTKSIGGQNLGELQASICESEKRDKGILQSPEAVSKDPKNMLTGTRLQ
PNLIVQRSKTLPELAAEALLKLGKLSAYKQTRSLVTLDIILPLSQSSFDRLKQWDHPIGRAAKAFDDAITSMQEAIGGE
QVPEIFGNKLKLRIICYLRGEQRYKSAVDYACERLHGSNEQQALEQENEMRDKLVQLFEDCGDATKTDEQNDLLQV
CDELSRKTLDLEQATEELIAFRKQLQQKFGKAASVCPEVRIGLQKWLHGIWDL

>QLI64958.1

TPPPAAFGHNGTDFLLRGKPFVIIGGQMDPHRVPFDYWRRRIILAKALGINTIFSSIFWNEMEPEKGKWASKQPAN
NLTHFLDIAKEQGLYVVLQPGPYVGGAREWGGLPYWLSRIPGLKVRSYNEPFLNATKSYFTRLAKDLAPWQITKGG
NVIMVQIENEYGSYGADVRYKEALRNMAASLFDVPLYTADGRDKLLLEDGQTSRVLSVVKGGPQGFPLRDEAVTD
KSSLGPHLDGEAHISKPLQWGPEAKHYAMDTDQPSFVESYIRNLGMTLSANNSINMYMFHGGTNFGLSAGSLYT
GNRTTPWANSYHGESLLDEAGRTTPLYHIIRAEILKYLPDASAIPPPPNNLPLLQINEFKLEPYASILHTVTYTKTRKMP
VYMETLKQGQGLILYEHKVLAAANGTLEAGDRPRDRIIVYVNGLRKGIIDSFHACPATVKLSLQAGDILQLLVENAGR
TSHWRKGSREPNLMDDPFKGINGSVTVGSSVLRGWKIRQIPCKEPPILKKPIGGSIQKGILPVYYKGRFKLPHLGPGE
FYAGMDTYLTIEGGTRGIVWVNGFNLGRYWVSGPQQSLYVPGALLNPGHSNKIHVLELTPWNMTLTARGETTKT
WENRPDPEAPQS

>QLI64966.1

ADDDLSDFTNNLAQDLGPLLALFGEAVTKQYLSESTTFLDYFTFAMGPLGIITAVVSVIRVCGHPSLRAFIGRSQEGE
AAVEAELCASTSRDVCELFHKGGIARVLGRQPDLLELVAVSPAGGRGRGKRLEIFQDHIRNADETMYWKRSRGSVF
SPDDKSGRCQMSPTPNLSLNIGIKRPKLWVVSLVALNGLVLQAGIIVLAGFGAWRLGWNKYHPDNATARDYAPR
MFIAGSLLLSLGMWGCAVLIGQATQEVRFRRDEQAAGHKTRLIWLQPGPQLVGEQSFDPSSFFESEKQPLEYWVS
SRKRKGSERLFEVLTFLAVLASVVGYILQFIGLRGMNAWLSLAQLGVTLYMSMLRGALRIQRLQNHENKLSKIPDLV
AGHELDWLAYELAFDDSPMQATGQPQRGANWHIVSGKMPSDDKPMTVCNGGSFELSGASVSSRRNSCDKTSLR
EFIGSQGCSDLYYELFDLRVQLSGLTGNQGMASLATGSGGGKPWKSDMVAVRKSAIKLAEALGEIATALFSGENRA
KRSDLTVPVEALAHTDSEPCCSSTLTQVVGVEMKAPEPTQPKWRVNAAHIEAILGLWTWSLVSSQDVVGDSESPN
TSASSKDRASVSHQILAAGLDNEYWNSEVDIEVELNFWFGPNTHLLSRESLRLGKGMKCDNFTTLCRQLTGSGGW
EKLPRSTESSSSLLSCSKTPIFQLRLFGWNILCESLAISGTDPVGASDVVKLQSSQDDSHVSIGVQYLRSECSIIDLCTQ
QLLITLVKNLISLAALPIPESTVSEKEGFIQLQNPLVATFEKAFVDSEIGSRTDAITTIIPQLRRQLLPSDENLISSLVQSAN
TFRQKGEWARAAVLLKWGCRHFSPEHMRNRHADRALQWQFFERILRVAAEFYRYSATLKSDKMGISYAQDGLRE
MRDMCHFMLPSSEEDSVEPCQQNITSMLACYADVFDKLAAETSRPRKANSVHPFVHAIDDGNRVDALYELSFLHS
GDFGSKSLKPALPLAIRNQWTEVVDALFEMRVHPDSEDEAGRTGASHCAELGHVSLLRELAARGAFLDQSDNMA
RTPLHWAALSGQDEIVAVLLETGNVDISRRDQDGVVPFWHAIEKNHDSIVRALLDRGFTVDARRAKLDRSPISWTA
EHGQAEIVQHLVQSGAEVNKAEKGLHAGFHPLHHACEGSHEDVVRVLLEAGADPNVRSENGRTPLHLAAAAGST
AIVQILLSLVPDVDETCNSGNTALHYAASEGRVHLLKMLIDAGADIERGDDEDRRPIHAATKSNYFWSHPDAVFELL
KLGAVIDPVDKIGHTPLCFAADNGQAGIIEELLSRGANVNHADKHGQTPLYNAAQKERFAIVKRLLEAGADVHCQD
KNGNTALIAATDSLWGGSDLVQLLLEHGAVDVPRKDGATALSIARSNRFWSTEEVLLQSGNYPNTYREGQSL

>QLI64972.1

TSMQRVHNDASTAFVTLNIPKNKGEAEPLTLRLDVGESEAACGPGNLTVNGQELIQDGNGNGQGTLAAHHGSNF
TASWAFSCDARSPKMLLKMQVTQLDDLEIAPFNFSASFTQILPRQIWIMDGAAVVNRVHRLPQESNLQKGLAETT
LQDQIRDLDILRIKARQLENAILAQESKIAQKLGQKKHSPPTNIQDCTSLECVLKALGGKFRDVSEQFSGDVEDLKDFI
TDSEDGSLPVPADDYEYWYGEEEELDDNRPETHDLKDHRVKWTWVDQLPLVVSDTDGPSIEYVRPRRQPPLAMA
IALSVIALCLLSTLLILRLHRRTAGLPVAIDNEKVLFSERPRRHSKRESWAERRARRKARKHAIKEYVRNIFMRWCHKT
VSPPSPVYEESTSIEEEFASFREALSVVDSLVAVEEGRNREVPRYADVQGSAAGYEAFADDESLPPYESEDDSTPVVD
GFRYGGSTGDDRLGYDK

>QLI64977.1

FWRMECPGRVGLARMDPIINPNEASPHAHTIHGSNGFSESATTADLLDGNCTSCRVTQDKSAYWTPTIYFQDAKT
EKFEIVPQVGGMLAYYLLYGDNITAFPPGFQMISGDNDRRTYTLGDARQPDPDRSQWAGLGQTTQEALAQRAIG
FNCLNYAKNPEQTLYRHYLPPKAELDQDCTDGVRFEIMFPSCWKGGNAIDSEDHKSHMAFPDQVMTGTCPEGYP
VRTPSILYETIWNTYAFKNRDGRFVVSNGDTTGYGYHGDFMTGWDPEFLQRAINKCTDLSGLIQDCHIFDVVDKEK
ATSCKIKKPLLKALFAEDVVGPMANLPGGIKVGQVGSGDHGKKPPAASKPAASSSASSSAQSPVSKPSAAVSPLPGL
AFKENAPSSSSVPSPTTTSVTPPPPPPTTPSVQPSYYSTEYITNGNQVTQILWVEKWVTVTDYGNSPAPTPARAHD
HRRRHMRHGHVHGKF

>QLI64981.1

SWQDYEAQARTPASCPDYTDYSQKPHAPYSQGPLKLPFMRPSEECRTFTSPAVEKVIRDMKARIRDADLARLFENT
FPSTLDTTVKYFDPKVNLAFIVTGDITAQWLRDTGNQFAHLYKLLPQDDNLKALVKAIINTEARYIYQYPYCGSFQPP
PESGLKPTVNDYAAKVNVNPPVDNQTVFECKYELDSLAAFLKISRSFYQNTKDASFINDNWEKAMNKILQTINEQS
QSSWSDDWEFVSYYNWTGTAGSLSPPVPNSGNGEPKLANGLVACSHRPSDDLCVFNFITSDNAMLSVELNHIAD
MLDASRKLNQVSRQARQHARTIRNAVLHHTKTPNGIFAYETNGYGGQYIMDDANVPSLVSLPYLGFLPRSDSTYRK
TKDAMFSRSNPYYAAGKKFQGIGGPHVNATYPWPMSQVSGIYGTDDDDEIKGRLALILENTSGLGLIHESVHIYNT
SDFTRPWFAWANSYFAEMMLDLAERKPGLIFKDDTPYIVGGDDEK

>QLI64986.1

LSLFNSESQRAITSNDDLKIPGESPLEHCSDRKAEGYVEIKSVDLAPNPPSAYVLSPSRLGEMVVGYPVANDYADWF
VLFDSSGQDLVIKAEGTVKKTIKEGAYIDLTVKYGLIRLLKTKADLCEQMGEVDLKCPVEPGDRVITKTVQLPKEIPPG
TYNVEADVFAANGERITCLTATVKFNMPGMDFLGGEL

>QLI64993.1

TTESQPSSSQTLPPSPTGSICHPHGDHWHCEPTSTAAVVSTQSAAGTCTPHNDHWHCPSGVSKPATPPAQTQSSA
SASSTGGHDHDDHDHGDGKECTPHNDHWHCPSGVSSPTYPPTAVSTRSTTRAPSGTASGSAASATSTTATAGAG
RANAVLGAASLVGAVLVAAIMA

>QLI64995.1

VAGCGMDLFINVCLTILGYFPGHIHAFYLEYIYYDRREQAHQGRFPTSRAPGVYSDNVQTGGQGYGTIVQPTR

>QLI64996.1

HGKISVATGDAGGNTTALGIMGGVVPGQGPNRQTEPDTTIFRSRNAASDGLGRTKGNGANTLEAMSRVVAMSG
STLPQVSSNGGYISATYHIVTTDGAGPVRAIIDPSGTGQFSQGTEAEVMTQVPGRNGNIAPGPKSNNRPQNGQG
GGGGGLIGNLLGKRASNVDTDHPLKVAIPAGTTCQGSMGGMSNVCLLKVANPSGAGPFGGVIAFQMAGSGANS
TATDGEIAGGSGNASNSNGGNANIGNSNNSNNGGNADVGNSNNSNNGAANNGAADNGENASSGNDITSTNE
AKKGNSFSSKGNNREKRAVMFQS

>QLI64998.1

GPMDMRAVGHHCATRSPLEERGLFARQRPQQSPLPDDGQEINLGFVLHFCCGGGSQCPSDSVAEKAVEDMNGL
FANGKIKFNLQNVSRIADPLCISGVRDNKAMDQLKSRVHQGNTTTLNIVYVPTNSGAGTKGMCIVPSPDTNISKGI
GDVDGCVVAMDTLPEGNSSNGVNSGNGGNGRLQGNNGSGGRLGGLFGRLFSRQDGGVGGVGEVGGGGSAHI
SGALATSVHETGHWLGELHANGGGDVAGTENVMVPWSTFERQYSFSADQFQRMRQTALARVKDKDTPVANKT
DTGPVDGGVNTPPHPPFGNGDDVVNTGGQGNPVRPTNPGFDPIGNGDNIVNTHPQPHPPFGNGNGGDNTGG
QGNPVRPTNPGFNPFGNGNGGDNTGGQGNPVGPINPDFHPGSGRPATGNYADFWAYVKQILGDRRVSGFEVS
RIGARAEPGAEADASGLVHIARREGAEAEADASGLVHIARRDDAGEPDGSGLVHIARK

>QLI65013.1

DTIKITARNDNTFNPNSVTAKKGDILEFHFQPKNHSVVAGDYKYPCSPLPIGSGFFSGYMDVESGEGDKVFRVTWN
NTDPMAFYSSQGDECPKGMVGMVNPNGTETLDDYKKRAGGLARGVTPGNSPYGGDVTGNTSPSSSGSKNGGK
DKGDESGAGALRASLLALATAWGAIILKSSMMRGGVASSRGAARQLRAGLHHSRNQRFTGQYSSCIGRIGFRPTAI
KHFPTCQTTLPAPTALPPYTIGLRTAATIVESQLHGEGPLAEYDRRVENGLLRDDDHQRGIIESLQNLYNELRNYHAP
EVKHPSLDLLKPARKSVFSSLFGSNGKAQSAISDIPDNLPRGLYLYGDVGSGKTMLMDLFYDTLPSSVKTKTRIHFHN
FMQDVHKRLHKLKMQHGTDVDAVPFVAADIAEHGNVLCFDEFQCTDVADAMILRRLLECLMSHGVVLVTTSNR
HPDDLYKNGIQRESFIPAIKLLKNRLHVINLDSPTDYRKIPRPPSGVYHTPLDAHANSHAEKWFRFLGDSSDNPPHSE
TQKVWGREIYVPRVSGRCAWFTFDELIRQPKSAADYLELVRAYDSFIVTEVPGMTIRERDLARRFITFIDAVYEGNAK
LVLTTEKPLTELFISKDEIAESLLKNNPQSTEQGEKAVSTVKELMEDVDRQAEELKNSNLFAGEEEAFAFMRALSRLK
HMESKEWVERGMGLESQGGKEDKDNWSKTRSRQMEDSM

>QLI65032.1

WVERLMVIGTNGTMIGNPGYIRGAVSRLDPNFNDFKMQHLLPTIPDGLLTDKLCKNTQRNRTYTTDLPALQAAPG
AFIALQYQENGHVTLPGLTPQKKNSGTVYVYGTLYPRDDELLSSIHNVWNTDGTGGDGRGRLLAVRNFDDGQCY
QINTGPLSMQRQAKFHKAAMNPQGADLWCQNDIRLPVSIPFSWYTLYWVWDWPSSPSDHLPGGESEIYTSCMD
IEIQPRVQLDEMNFVDGQDLNMAGIKEQLE

>QLI65036.1

ANPTTSDDMGPAAFMWPEDRVWSAAADNTAPCGSVARVGNRTNFPLTNGQVALVDQKEAHSVQLSISYDDDP
KEDNDFTTLIKPEAMAELDKGHTCVNVQNPPPFIKAGANATFQIKYVASFDKPDKETFYACADITYVEFANFKEKVP
CFNATVPEDSKKDTPTPTSDASKNSGSASSGLSGGGTAGVVIGVVGGVALIAVAALLIYRRRQQRLRMLRQKFSPR
NIKPDGQHRDSPSVRSESA

>QLI65038.1

RAVEGEKRAAAALTWKALGGSIVGHPGIISWAPDRTDIFVRGSDNAVYHKWQLGNPGTAWGPSDTGFQNLGGSI
ISDVTVVSRGYDRLDAFVVGADNACYHGWWNGSYWSTWGTLGGAFSGDISAVAWGNGRLDLFGRGNDNAV
WHRAWDGAAWGAWESLGGAVIGSPKVVSWGANRLDVFARGTNNEVYQIAWNGSSWSGWYNHGGTVLDDIT
PVSTAPNRLDLFVRGGNNALYQKNWNGAAWSGWISLGGVIMSRPSATTWGGKYITVAAQGANNAVYLLEFNG
NSWGDWRSIGGVVTEAPVINPLGSVNAAIFARGNNAGLFVYE

>QLI65057.1

ATIDKRIKFGEAAKQGEFPSIIRIHYNTTSVLCGGSLLDNTTVLTAAHCWFEGLRTHHSEIVSVRAGSLNKNTGGEVA
KVKSIKVHPEYKPHWNRNDIAILKLSTPIQESGTIKYANLPATVLDPVAASVVVAAGWMTRAASTVVAAGWGMTE
NNILPDKLLKVVLFILDPTACLEDGEDDPEYTNYLDTKVCAGYAGKDTSVGDSGGPLFDYNTTELIGVTSFGGLSPLG
GFYTKISRYMTWINENLGDVKSLPSGVAG

>QLI65059.1

KEAPSSTTHAPACTATASSGTGGFFDLRPDTAHPSEKGKQHKTALAKDYHARGYDYGKNFTLNICGAVVDPVTEVV
GISKSQWTNVSAYYMSHGSIYSIGSESMDLVSRGRKLVLQYTGGSPCGTTKSNKSTRTPSSPSANYNYANKESALTN
QPHQVETLKDDKEEKSSRRKSTTISFLCDRDPSSSQASISFVGVDEDECSYFFEGRSIHACAQAEPHKPGSVGPGSVF
GIILVVAFLVYVLGGVFYNRTISNARGWRQLPNYSLWAGIWSFFSDMFVIAFSSCARCLPGRRGYSHLSSSPRNRNS
DAENRLIDQLDEEWDD

>QLI65061.1

AACAGNSAGDRMQWCDYSVATDYSTTVVDTSVTREYWLELTDVVVAPDGFSRPAIAVNGSIPGPTLFADWGDT
VVVHVSNSLTTSLNGTSIHFHGIRQNYTNENDGVVSITQCPLAVNQSTTYTWKATQYGTSWYHSHIGLQAWEGVF
GGIIINGPASSNYDEDLGMVFLNDWDHQTVDELYSVAQTAGPPTLGNGLINGTNVYGSDDSSSQTGTRFNVSFTS
GRSYRLRLLNAAIDTHWKFSIDNHTMTVIAADLVPIQPFTATVLNIGIAQRYDVIVTADQASIADNFWMRAIPQAA
CSDNESSDNIRGIVYYGDSPATPTTTGYNYTDGCDDETANLAPYVSKSVSDANWNDLEQATVGTNSAGLFKWYLN
STTMLVDWANPTLQSVLNGTTSYETDDAVIQLSDADQWVYLVIETAIGVPHPIHLHGHDFFVLAQGSGTYSSDTV
ALNTDNPARRDTAMLPASGYLVLAWQTDNPGVWLMHCHIGWHTSEGFALQFIERQGDIGAVTNATYVDNVCA
NWNAYQRIASIEQEDSGV

>QLI65062.1

KVMRRTACNVTEPPKNWAVALYDGLDVIDVFGPIDVLYILSLSQQLNLYFLADTMAPVWMRPPLPELNKAGSNFT
ASFNPTHTFDNPPDDIEILLVPGGLGMRRGNSSEPVVDFVRKTYPKVKYLLTTCTGAGVAARAGVLDGKRATTNKA
AWNEIVKMGPNVKWVSPARWTVDGNIWTSSGVTSGFDLVFEFVDTVYNKNTSHTIQGAVEYVRAKDPCDDPFA
KWHNIPPSGDCRANK

>QLI65068.1

WSKEDREIFRIRDEISAHEPNPAATFYDILGVPPSASFDDINRAHRKKTIALHPDKVAQRLRAERAKKAGGKGKKSAI
KPPSSAEVKAAVKQASERQTRLSLIANILKGPSRDRYDHFLAHGFPLWKGTDYYYDRYRPGLGTVMVGLFIVGGGAI
HYLILYMSWKRQREFVGRYVKFARDTAWGGNLGIPNIEPRSSTPATASDEEAPPPVPQNRKERRMQEKEAKKEGG
RGRARKVTKKTPSASRDASSGPTGVRKRVVAENGKILVVDSLGDVYLEEQDEEGNVNEYLLDPNELLQPTFRDTAV
VRLPMHIFNLTVGRFTGSRDAVSDSETVDAEEVVDVSQQTPTSESAGDDFELLDKSTDSLGKAKSSGMEKSGKSSK
RKNKKR

>QLI65128.1

SLGAPGPSIRQVKGQIGQLGDVTNKPILDGDKASTFFVGDFRDPTVVFREGLQPGFDTGLDDAAGATTPEDVFVSP
SHEASIDMSFFQQESEAQTGFVYQVTLHGLPQGHWLHDTNRNDAETADLFVVPGAIPPKNIQGVYIYRKDGSGLA
VKTKWIPNQNPPDAPRPRKRQTKKPCVPIDPNDVDPEDDDTPVVDEDGTVQEPKDPRCFTNYHGSVKDRERDKK
KRKKPGSSWRRRNCLDESEV

>QLI65145.1

HFLLNYPKSIGFDDSNEGNAPCGGFTPDLSKDLVDFHVGGDAISVKLTHPQGNWLFRVTTDEKAESGWEQIFPIVQ
QSGLGDFCEPQVTVPSKYAGKKGVLSVVSSATDGLLYQCAVVNFVDGKGDKPSACVNASSVKASFTDDSKLTALV
GSGSSSTPTSGSGSSTASKTSASSTATHSGAASSLHAWSLTSAGWGGDCENKLSG

>QLI65146.1

LPAVRDLVPGLCPRDIDEAQALQSSLSPGAKVYFPGSPEFDDASTRWSTLSPPTVNVVVVAATAKDVSETVKYANR
KKLPFLAYNSAHGAITTLGRMKSGVEIYLDQLNTIEVAEDGQTATIGGGALSKHVTDTLWDAGKQTVTGTCECVSL
MGPALGGGHGWLQGHHGLVADQFVSMDVVLADGELQTVDPESDLWWALNGAGHNFGIVTSVTTKVYDIEHR
DWAIETIVFSGDKVGAVYDAANKHLLQNGTQAADVINWSYWLHDAAADPNNPIILFYIIQEGVKAVDARYTQPFH
DLGPVAVTPMSGTYKDLAGWTGIALSSPPCQKAGLANPRFPIYLQSYNVEAQEEAYEAFADATRGSSAFNGSLFM

FEGYPVQGVQAVDGESSAFAFREAHLLAAPLITYKPDGADLDKEAGRLGNRLRDILHKKSGLCNVRAYVNYAFGNE
NPKVWYGSAKWRQERLKELKKQYDPLGKFSFFGPIA

>QLI65149.1

APANLQARDATNCGGVQYTAGAVQAAADAACNYVQSGSHAGSSTYPHRYNNYEGFDFPVAGPYNEFPILKSGQI
YSGGSPGPDRVVISNSCNLAGVITHTGASGNNFVGCSGTS

>QLI65154.1

LPGRPLKNNRGSTKRALGHQSVPNQRNTTHPQYSGNWAGAVQSGQSFTHVEGVITVPQVSGADGAAASAWVG
IDGDACQQALLQTGVSFYANGAFDAWYEWIPDASSDFDDFQLSVGDQIKMVIDATGVTTGVATLENLTTGQKVT
HTYGDSPSSLCQNSAEWIVEAFMEGDSQVVLADFGQVTFTDASATDSAGTVDGDGATIVNMQDQTGNIVTDCA
ASGSRVSCTYTGN

>QLI65155.1

SEMSLLKKQIIQIQPELVKHYTSIVKPLVPPRMWASQAGFYEPLVGAAVKPVCGCTVEEMFQLHDVFFSNITSEVDV
DFYPHADSQPLGGDIWFESSTATTDTSTFKWEHNGEFSLEWSFQVAGAGPKVGAKLGYKVGKDNTETKTTTFTEK
KRLTCPQGHLCQVQTWSFSASIPGNFFKVPAVDFTCLPACLRWKHKETGLYHELNTTSRVALAGLAGQSATWDAL
GQKYFELRDEHDKPVSSCPPGGETIVGIQFPPEGVRTVYSQARPHTPSVPIMDDSKKPYQIQIMLDFSRKSFPGETR
RDGVVANLTQEELAALKPEDAEVKAYVVGTNIPGLDADTNIGK

>QLI65167.1

SEAPCLSLTTNFPTCVLSCAQEAASKAGCTNTADLECQCNPKSSSAIQGLALGCAAACSATELPAGISAGRALCECVA
SHAAATTSGTETGTETGTETTGTETTGTETTATGTPHTGALTSEITRTQTSTGTKTGSESETTTTTGTQTETSTTTEAT
TSGATTTSPTTATQTTTTAGAGIFQVSFAVVAGAILVAAVVL

>QLI65178.1

LVLEKASAAHHLERMTGRFPNSQQATVYSEERCKGKAYTIKPDGKCTLLPGDLTGKINGAMVPEGVVCDFYIDKKC
NEPLWIGMEDPGTCNFSELEIGNQAKSVHCYDDTERGEV

>QLI65191.1

DIAAKTAAPFRQPSSSSPSSKNTSSSPAAPMTKPPVYFFSHGGPTVQYDTQHAAYPVLQQIGREITQKVKPKAVVVF
SAHWQGARDEIYVNEDEHADLIYDFYGFPDHYYQAKYPNKGDAQLASRIMVMLSEAGIASRGVSRGLDHGVWS
GFTVAFDPETNPLNVPLVQVSLFKSESPSAHYALGRAVSALRDEGVVIIGAGMSVHNLRDLHSVFDGDGAPLPYTV
SFDNALREAVEADPAQREDRMAAVCARPDARQAHPYMDHVMPVFVAAGAAYEDRGKQTWTFHEGSMGWA
QYRFGQLPE

>QLI65209.1

VTPGTKPRPSHTDIPGCPAPTTTPTTANPPHTTPGLSECGVTTTHTWYGNKTMGCQYDPLGGCIADAILTLPCGCT
KATNVVQQTTTVCATVPSAVNCVTGFMFTTTATNC

>QLI65227.1

QQSAQVFIVPAGDASSPPAITPSLARLLLLQRLAPSGRGLSLHDIPNGVGPEHAVSLISRFGKNIPPLFSDGQATEPSQ
LVLMLEGMDDKQMKDLQKALSMSPSFTIADPPSDKAHRDLMELDFYRAGVADGSKCSIQQLVNPLENCWAGKR
SAAAKFSIKKNPELLDEVPKQIKQLIRLAKSGELQTTIVALPSTKSSSNWLDEQQQELRRRQAEQVISSFDRPAANAE
PTSTPKPDPIFDSSERIKACYETKEACMASTRNCSSHGECQDKYAKVDGSKNTAACFSCHCQRTRSTSGSLTTWAG
PTCAKKDVSVAFWLFAGFTLALVGILYLAISILFNVGEEQLPGVIGAGVSRSK

>QLI65234.1

LPAAGQTQTLSINAVHNKNFKPNGPLALAKAYNKYNVPLPTELSSLVSRIEEDLGLQKRQDGTGSDPAKPPPGVGD
LEYLAEVDIGTPPQKLYLDFDTGSSDLWVFSSETQQSQVNGQKIYDIQSSSSAKPLEGASWSIQYGDGSTCQGDVYL
DQVTVGGLTVKQQAVESAKTVSAQFTRGPSQNSGLLGLAFDEINTVKPTQQKTWFSNIKDSLKAPLFTANLKHAA
DGTYNFGYIDEQEHDGEISYTELDSSRGFWGFTASGYAVGNGDLQPAQIQGIADTGTTLLLIDDGIVEAYYAQVQG
AQNNRQMGGYVFDCGATLPDFRFGVGRATITIPGSIINYAPAQESSSTCFGGIQSSGQLGQNIFGDIALKAALVVFD
AGNNRLGWAPKKA

>QLI65238.1

APPPATPGGIRARAPGKPPFVWRGETGKTRDGEPGRSPADVQRAGGMWARGFQIIDQLSPEQLEAGSGLWTHT
TNPGRTRDVTQYVSTSTSLLGAITFAVRPSPRHAQEVGYLYRIRTNWAMVDVNLSLENISPYPEQQEQAVVQGIP
WNQIMGWYEVRAADLNRILGAEDPVSQLGRFNRNADFRPDSTDPSGAQPQLAGLENPVEGRHGAWEAYIGQS
VASNLRQFIREYGPRTEAADQTTLSSLDWSAVEIPAHLRTALGQGAASAAQCAMALAAVVVSWHHDELKRSIPW
HKVDSRDTTSNLGSNACGRLATAVKDKAENKEATVKICDEPRSGGECLTIQTPPQSCVGVPLEWNDRASTILLSASA
GICQFFHDYNCAGASFQSSFPGNDHLDEFDEGRFNNMISSIRCDNTSPETQIERPPVIEVAKPDACIAKPRPLAEILW
VMINNIDGENPGDLFGSIHATDDSGRQVIYSVEKDDSVSVEPRDQITLRTSRPLAADGNFVIDLDLWDRDRDASPH
DEVSRGQIAWKVYDPANEYDTPIQTEVDGEYGKATVDYVVMSSAAQAVVQVIMLDGDGEEPADVFGRIRTSSRF
VQRDLFNRESGDSMEIYPGTAIPLSRAIMAVPMLDTLKIHVDLWDHDADWSPNDQIAYGIAEFRPRLSGSEKKVV
DGEYGKVEVSVTWGGGRPQGC

>QLI65241.1

LPATDGANGANGNTAAGTDVIINVPQLYPESADFNKKNGLTYFSKLYNAAVAVVDVSKDSVVDTITFDRTDVAEFH
ASGVEIDQKTNQLSVTINAGIAFDTAGANVSGDNFLYKVDLNGDKPKELWHANLTAVTNGAYGGCQDMVHDTC
GNTFVVCTYPGAIIKVNADGTKAIPWYLSNYTKPGTSPILPGLTGIVASGDLLIANDAQAKQLVSFNKRDSLGKRVVI
PVTGLTGESGDDGVYLPEKYGGKVLLTQLSTEGTNVFVSKDGWKSAEFVTKIASAYTPQDKGGFAVATVQIQDKIY
GVIEYFLDDKNGGLAGNRTKFELEDLTTKIDQAVQGKI

>QLI65257.1

VPFTTTAFDIATRETAPTPSPTSGPLTKTESVTLYSVRNSVLTIPSQATPMVAVPPCVQTAVPDAKGQIAPGECGAIW
SYYPIFWAALAVACLFGFLVAVHVLQAAVFKKVYVVLGHYHGHYLGDDGHSIPYVKLEEPAE

>QLI65278.1

NNAIPGNAFTNNNGLAAAEAAAPMWYMPSGTCMPSAAEDGQGHQTNGVDTDNCNINKLSHNCPPQPQWQG
TNTFYGNVAGEPFSTIPTYWSAKKCANTWKIIYYVYFKKDTGHKSDWEGIVVSFRDTGGDNWVRDTVKMEQDSN
HKQINWGDVNDTFDGTDDWQAFRQRNRDHGKFYFGKFHHSVHQDWHTTSFKDTCPPNSGDDFRNGDYQFW
ARNNLRHVSVLNPAWNWGKASSPANIDLCTY

>QLI65296.1

GPVVHDADKDVTYHGLCRNGIDVFLNIPYGQDTSGENRFRPPRPHSSRRGSAIDATAYGPACPQALGPWVPPISLT
NMTKVSEDCLNLNIARPAGESVGGEKLPVMVYIHGGSFWAGSNEEITIRPDGMILESVKNGLPVIHVAMNYRLGFF
GFARSDALRKEGSENAGLKDQRLALEWVQDNIAQFGGDPNRVTIFGQSSGGLSIGMHIMAYGGSKPAPFQQAIA
ESQALEPGITGNFTTNAMQALVDHVGCNKTPLGSASTIACLRSFPTDTVLNASLATYQSDTAHNIGDIWLPSVDGD
FLPAPPSQLIREHRFASVTAMMGWCDDDTTFFTDANITTASDTHAFLRAYAPAVSPANIRRLEALYPASDFPADAA
ANASSEFYRSARIFRDIVMACQPLGYAEQLAAAGNPVYLYDWNQTVLDAFLTQVARRPGMGPIHTSEFAYIFGNLS
HYDTGDYDFRPSPADYALRDRGSRSWSTFASTGRPGLEGHDTLQGFAPAFTPDGRVNVFVVGGPSEGLSPIDGPG
ANSALERQKLRARCAFINSPEMIEQLGY

>QLI65299.1

ALASVCPSDSVCFQWGVPESSSGSESGAMYLQLQASLSYQWIGLGTGSRMRGSNMLVVYQNGHGNVTLSTRQS
TGHSMPTYAQRPDVQLLEGSGVLNGTLRANIRCGECSDVGVGGSSNWITAWKQGPPLHSSDLSEAIAYHDGYSSF
SVDLAKAAIASDENPFLSANSSVDTHSGPSSAVSGLNTVDQTSETAALLCAHGIVMSVVFLIGFPAGSFIMLLVGRW
KIHAGWQVLSFIGLCCGFAIGVIISPRYN

>QLI65301.1

GFSIETIHKESAPILTHVDAEVVPDSYIIKFKDHVNEAAVNDHHSWIQSIHKDGEEERLELRKRSLGSSPVEAFAGLKH
TYNIASGFKGYSGHFHESVIEKVRNHGDVEFIEKDTIARILRPVTPDESVQETCTPETEKQAPWGLARVSHRKGLSFG
TYNKYLYAADGGEGVDAYVIDTGTNTDHVDFEGRAKWGKTIPAGDADEDGNGHGTHCSGTIAGKKYGVAKKAN
VYAVKVLRSNGSGTMADVVKGVEFAATRHVEQVLLAKDGKRKGFKGSVANMSLGGGKTQALDAAVNAAVKAGI
HFAVAAGNDNADACNYSPAAAELPVTVGASAFDDSRAYFSNYGKCTDIFAPGLNILSTWIGSRTAVNTISGTSMAS
PHICGLLAYYLSLQPAGDSEFSVASITPKQLKETLIDISTQGVLTDIPNDTPNKLAWNGGGCSNYSKIVAAGGYIGRPR
TSEPKPVGNAVKEEMKVLSHKVSQGVKDLGDKTEKFVEKIHDVVDKEIEHFISEISV

>QLI65305.1

APQNEVSKHALESLSLDDLDHELQTCPIVQELSAIKHAHHAAAPSSLTTRLFAVLFPGSPAVNALLATLYISGPPNFLL
ALCPTNIDPSSLSVMVAFAVGGLLGDTLFHLLPEIFLGEDEPDRARFVLVEPNRNLVLGLAILVGFMTFVAMDKGLRI
ATGGAGHDHHSHAHAHEADAQTTTAAASGADTGTGNGQVKSRRKGKGKKEHADEDHHKEVNPSVKLGGYLNLI
ADFTHNITDGLAMSASFYASPTIGATTTVAVFFHEIPHEVGDFALLVQSGFSKRAAMASQFVTAIGALMGTLIGIAIQ
EFGGGNTDGEASMPRNAGLWGTSLTWGDMLLPFTAGTFLYVGTVAVIPELLETGPNKMQELRKTLVQFSAVAVG
AGIMLYISWHD

>QLI65312.1

LDVQIQDEASIKSTAGTIAYGLMKYYTGNNTGDTPGNLPDPYYWWEAGAMFGTIIDYWHLTGDSSYNDATMQAL
LHQAGDTKDFMPKNQTRTEGNDDQGFWAMAAMSAAENKFPDPPSDQAQWLALAQAVFNQYVLRWDPADC
KGGMRWQIFQFNNGWNYKNSISNGCFFNIASRLHRYTGNNTYGEWATKIFEWQQSINLITSDYGVHDGISIDPD
GTCSRIDMLEWSYNAGIFLHGAAAMYNATSDDKWKKAVDGILKHASEKFFKDGVVYEQFCEFNKVCNNDQQSFK
GYLLRWLAATSQLVPYTYDTIKPLLEKAGTVAASTCTGSTAPPNFKGQPGTACGFSWIPQGRFDGMVGVGEQMN
ALDAVMYNLVRKSTPPVTQNNGGTSKGNPSAGSGGTEDPTVLKPLTTADKAGAGVITMLFIVGALGGTAFMLLEF
R

>QLI65320.1

DHASDLKSISLRTHSIQQPYLDSDMQSRWFDFGGDTIVRTDSYIRLTSDLPSQSGWLFSRVPLTATNWEVEVEFKIS
GKHQLYGDGFAMWITKQRGQMGPVFGSVDRFEGLGIFVDTYKNNRPGVVFPYVMAMYGDGQRSYNKNDDGK
ETELAGCSARGIRHASVPTKMRLTYIQDKQLKLDLQYREEDEWTSCFEVDNPPQIPNIAYLGFSAETGELSDNHDIIS
VSTKNLYNSPGSTTDRAGANKGRPIQSNVVKEGSSWTWFFTKIILFFVAVGGMYVGYTAYRAKSKSHRF

>QLI65330.1

LANKCMAAPDLTATQRESFDWLKGGLPAYPSSRLTSPALDGGSVRPWALSLTPSIGLWLKSYGSAAGVLHMQAGE
SWPKASQDKDQSHHSLVSCILILPWLKDYGLS

>QLI65345.1

ADSTTTVSMLQPLADDGEGLTLRGSVVGVDGDKTTYGFGCEAGASADSCLPEGTSMTVVQGPSTLVQSIQATMS
DWDGKVGGDEAEDAEDLLHQSAVVSLEHSCKLNPDQNRAECGVMAAVAVGSATFVSQGSDVLTAYKDMVFPV
VITAGVDKLSPAPGATQTGNVPAATGSAGASRTTSLATISGSKAPASTASASTAGATTTNAAGAVVPRRLGLAGVA
AGIGAALLLL

>QLI65346.1

HPDENFQGPEIIAGQIFSYPVRRTWEFTSEHPIRSVFPLWPVYGLPMLLLRWLWIGNGHDGEIPPIAVFWTLRVLM
FLISFVLEDWALHELIPSPRHRRVAVLLVASSYVTWTYQTHTFSNSVETLVVAWSLVLIQRIVDTRQQSSLLSSTVLGI
VAVFGVFNRITFPAFLLIPGLRLIPHFLNKPFSFVALASAALITTVIAIALDTAFYSSEPVTWANLISRPTITPLNFFLYNS
DTANLAQHGIHPWYQHVAANLPQLLGPAAALVFAKPHLSLRLYSAISGLFVLSIFPHQEARFLLPTVPLILSSVELPKN
KILLRTWAGAWIIFNLFLGVLMGIYHQGGVVPGQVFMSKQPDATQAIWWKTYTPPIWLLNGKNEVLQTRDVMG
LKGDALLTQLTELATCDTPADRRNQEYLKEKNGTYLIAPASATWLDPYLSNKGLHGLRFREVWRYRKHLNLDDLDF
GDDGIWNTLSRVIGRRGLIAWRVTKSCQKLSNSS

>QLI65358.1

RALNQPRALPPSIDSFYKPPAGFESKAPGTILRQRLVISSFFGLIPDPVETWQLLYRTTAIDGSPIATVTTIFKPLFPKKD
RFVSFHTAYDSSASICNPSYNYQLGALQVDLISSAEFLLLQTYLLSGYIVASPDYEGPDAAFSPGRLEGMGVLDGMRA
VVNFKNTLHFSTSTPMIVGVGYSGGAIATGWAASLQPTYAPELAMKGWVQGGTPANLTGILTFIDNTLFSGFLPAA
IDGLTKPSAYGAQLGPLVESIVTPRGRDILKFANTNCAVANLIAFPEQSVLSTSIQSLGPGLLYHPDVVAVLQRNTMG
VYKNETPTAPVFVYHASNDEVVPYANASTLVDAWCGYGASVKFTTFANGGHVTTEVVAILDALQFVKDAFAGTVP
GGCSRNTELSSTLNPIALGVALEPLLIGLIEVLAIAGKNDANILKDIKTLGKH

>QLI65361.1

QEADTLKNKIGWTEEELLRDLTPIPTGVPQNDPWALPLINGCPRNCVEAGPDSANWTQLYDQNHLSLCKYPLLFAF
NVHNGPSRYSTIRVCALNGGDGKRSTSAVVVPEKDGDAAPPATGLPTASFSECGAGESTTEATVKAGFDVVSENTR
PDAMSAANFLISHLSSNTACGNVILFAKFGVALVGLYAGADLKKDRAAELLHKYW

>QLI65365.1

APVSDLSSLELYPRNKHIPDKNEVVELIRDGKDFHKYAKEGHPHKDKAAFFSGQGRQTIAKIRDWATAKKQGLTTV
RDIWKSDNFYQQGQYKDIDAETFRDFQKAFSKYYAQQAKGKAYLIFPHDQTPKRDGIFWSVELDEIISHGKVDVII
WLDQNKIGEKEYHQDNEERIYWKKGERKPDGA

>QLI65366.1

TYWTFSDASVSVGSKTSEKTVETFSDTDRVRRTVSFGHQDTLKVALTTKEGSKAKRPHQAFLVLREASGLETPFALT
VKESGKGVVQISHKDLPAQLLTATAPLEASLVLGSTGSTKGSVTPAFDIAVKLDPGHPTPSPDAPLRYGKLAEIHHIFR
ADPKNPPRIVSLVFSLAVLATVPALFIGWIGLGGNFGHASKAIGNAPLSHALFFGSIIAMEGVFFLYYSAWNLFQTLP
VMGVVAVVAFLSGTKALGEVQARRLAGER

>QLI65382.1

GSLRDVKHIVLFMQENRAFDHYFGTMAGVRGFGDPNVQVNRDGRSTFEQPISPQNGVDILKPWHINHLGGEWL
EATQCMGAGDNGWATMHAAYNGGLGNNWAQADGPYSLGYFTRADIPTHFDIAEGWTVADMASQSILAATDP
NRITWMSGSVNIPGSPTNPDGTGGMIIDNSASPGCEAPGLNCFPFVWKTFPEYLEAAGISWQVWQDMDNFEDN
MLAYFEQYQTAKNGSALRTRGNSYPGLDAFYDAAAKGTLPQVSWIVGPQEQSEHAPNLPKDGAWLQKKVVDAIT
QSPAYNETVLVISYDEQGGWADHVVPVVAPKGTPGEWLKDPYNQFGDVPLGPGWRTPRFIISPWTRGGNVFTEF
TDHTSDIQFVEAWAAAHGYKDVRTKALTQWRRDHMSNLVNAFDFDHPDLSTPRITAVDTPESLPDDGSHWSGN
LSLGSLTGPWVGAAKCLQDHRSTKPPIPYGKENAGQDMAQLVEDGFKRVRGQLTEGRYLTFETLGLALASYAGIVV
SFAPATRNHSDPRQRWIVHAVGTGTEFTIQSAADKKYIAGTPVGLLTSDVGSAQAFRITYNPRGAKYRLSLANKPAS
SFVAPNQKSRRRAANGTVSAAAAVDWEARFGEFSIYSVSYRS

>QLI65388.1

LVLESNNYHTSCRCRPHEKCWPSKHQWSTLNSTIQGNLQAVRPVAAPCFNPSSPLATSCAVAKQNANNSVWRSA
QPGAVQWENWEAWPEKDQSCYFDQPKNIPCGQGRISLYSAVVERAEHIQAAVRFAAKYNLRLAIKNSGHCFLGR

STAPESLQISTNKIKSMAFTDRFVPEGAAGSTSESFGSAVTIGAGVQLKELYVAATKHNVTVVAGLARTIGAAGGYIQ
GGGHSPLGNWKGMASDNALEFKVVNAKGELVTANSYKNQDLFWALRGGGGGTFGVVISVTIRTFPDVPSGFVSF
GFGTLAANSSAYWDMVRAFHAHLPSVSGAGGAGYYAISFVPSDINGTKVLELTGGFGFLNVTSEEAIRKAVAPMM
AEVNRYAGRVSGYNISINPRVSDYILGLLRGEADDTGGIAVYGSRMISRDFLLSRDGAARLTDALRDIAEISPTGFIGIV
VAGGAAADPTIDSALNPAWRKTLTHIAFGAGWNSTTPVKEIRAIQEELTNIKMEKLRVLEPHMGAYLNEADPNEK
DFQKSFWGANYARLYKVKQAVDPDNLFIARKGVGSENWDDAGLCRRASKMGVDMPIESLLTKRAWMPKDSPSA
DD

>QLI65403.1

VLTLLRRRSPISLPGYKGEGSLAFLHDAKAFATKPIALIQKATRQCGDVFSIQVLSVYNVWLRGNQLNRVYLETREDV
WSFVGGMGLFLNKIIDSGYWDHYRVLLSSLSRYVSSGPAQRYAATVSVEETQKAAAEWSLWDGFELFDSVSYLVH
KIIVRSLMGEDFYEHNAAELFNLLHSMEADIGSLLSFILPDWVPHPPARRLLKARNRFKEIFMERLRERDRQERSDVR
PLQDYVAFTMEDGATVPLKYLMPSHHTMLMFAAHTSTAASISWNIIALLRHPNVMKQVTSELRSMPEGQESLLFQ
ACIKETSRYYCGMKLLRLARENVCIPGSNVNIPKGAVVSISPYLTHLDPENYSEPEVWNPQRWINNKREIVQIDNKT
DGVKYLPFGGGSHKCVGEKMAMIMVVRTVATLLREYDFAWETEDIPDKTDFSNLNFDKVGTPWLRGGVRVRMR
KATR

>QLI65420.1

FYIPGWSIKSYKEDQQIPLMVNKVYSDNTQLQYAYYDLPFVCPPTGQHKSAAGLLSGQSIPLNLGEVLRGDRIMAS
DMELAMKKDTSCNVLCTREISRSDLRRAKELVHDGYVTEWIVDNLPGATSFVTVDKTKKYYAAGFKLGYTEFSPSTG
KARYYLHNHHTIVIRYRQAAGRAGARGEKIVVGFEVYPKSIGNGNRRDTKGCPVDLQNINQPLELYMAPNKTLDAA
APIYTDSSYQPAATEVDDDSAGGTISIPYTYSVYFREDDTIEWSHRWDLYFVNQEEGSRIHWLAIVNSLIICGLLTGIV
MIILAKTIRTDIQGYKDAKAEDGKLRSKRKSRSGNRTPKEKPGLLDQGDDAENDADISSDDEALEEVTGWKLLHAD
VFRTPRSGNLLAPLVGSGMQLMFMAMGLVLLSAIGVLNPSFRGGFVSVGVGLFIFAGLFSGYFSARVFKSFDGRDY
RANALVTAFLFPGLAFALVFVLNLFVWAQASSTAIPFGTLIAIVFLWLCVQVPLVLAGSYYGYLKAGAWAHPTRTTAI
PRQVPRQAWYIKSLQSILLAGLIPFAVIFIELLFVFQSLWQDKSGYYYVFGFLAVVSMILVVTIAEVTIVTIYIQLCSENY
HWWWQSFFVGGGSALWVFAYSIWYYFFKLHITGLVSSLLFFSYSFMACCVYGLLTGTIGFLSAYAFVRRIYSAIKVD

>QLI65424.1

NQDEHLRPSAELARLRAPQIFNAVHNALRQWGSSVHHNGMSLFLATVPPGVLFHHGNLRPQSPSEPDWLAYEVE
HAEGFTRSFGPPPKQNPGSSSAHRQNVLGGGAERGWLHVYRTTRPLRFLYVDGMSGGKTDMGTLDTQDYLLRSR
YSHEDKRQGNTPIRERERAEFLCRLCEEWDLQGIIRMEAGFEIIKCDFTDGLEQVQAFRRSSRELRSETRQFEYLRGIA
ERYFDIGSSRTVIDYSSMVSAFFFSVNLTNPDAGRPDLPRLSEATGAELAAINKYFNATVEKRRDETPPSIDWQDVS
DIIVSRYADRLEYMVQHVPSVDVLALEVAFLLDVYIDYPDEDEPSLENAAQHCRDFYLQAISPASESDHLIHAAFRQV
TTEICTALFAVRELLESEQLTGEQTLAAARKRLQSLVDYLDWARFKRCSGCGVDEVCVIPMWPFGNEEDYYHPRCR
NSSDTRGGKSYWGRFTP

>QLI65429.1

QETGNHPEWQRWCGKAYQPQHPSFEPGGQTVEPDKLPGGPALDIQFKPRYSIYLESEKQAEFVVNAGISKWHGQ
PWPSLSTPGAAPRVVFTINLVSNNDLLVSDSVNVSTTGNVFAFNLSNLQPSLDPYEVVLFGATEDGASNVTVTSEL
MFLPEKTKGSVAKLDNLNGGFLFRSPATGNNFEPFLPYGYYASCDRFLCDKDYIQKVKAYKDLGLNSMVSLTTVQN
SRATYEYMDTLDLRYMYDLRGSYKNLTAVEEQVSVIRDFEGLYSYWGADEPDGHQDPFDLLPEARRLIRQLDPYHP
VSVTLNCQNFYYKEYTAGADFVMEDAYPIAINSTFSKWGTPCNTTYGDCGCDNCQGNVQDVPNRLDDLLQYEH
WLGLWPKTKAHNPQAFHGEDYWFRDPTNEEEVAMNALGFNHDAKVIASWVWPFSDSLGQIMGKFGSAVANQ
PARDLIVTGKAKRVHVEGHRVVDAAYWAGETQVLVSVVNGGYEAIDGEVSIPLPKLFALQTKDAIVWGNGTWAL
GDGEVRLSKQSAMATNMIILGVGKGKSKR

>QLI65432.1

TPILDEPREVGPLSNISPLFPRAGTGDDKTDPIKAKVTVTKGSKKDPTGTGGNQLTFDVDCWAILCKDAPKVLQRVV
KKRVTQNRKDSKACPSPYTRKKDPVTAPARNNKWAQSDFNSAEEYPFASSLQGGTGAYLVPVNGASQSTQGAQL
ANLYRANKILQFDPESTAAGASKGTWFELEFTGELGPYCDALARGDTSVCDDKHDASGPWGFDVAKFVSQWNAA
TGKYDYAGKN

>QLI65435.1

HFGLTYPPWRADTLAAENEAKYSQWSYPCAGVDYKAGNVTDWPIGGGLLALDLHHPWTYVFVNLGLGENATNF
NVSLTPQFWNVTGKGTLCVDKLPVPVDVQDGALASLQVVTSGASGSALYNCADIRFKKDAKTPGNCTTSKGITVQ
NVTGQSSAGNASGNGTSDGKQSAGVAAGANVVALVTVAALASGFAMGL

>QLI65446.1

SPVPEEEPTRVKRLQFCNQYGGCKPQPSAST

>QLI65448.1

EPVPRRSGLKPLVRRDETDSMTKETLLAAMKDASGSMALREEPFTLIVQGGAVNVILGNRQTTGDIDYIATTYSANE
PEKYGEEVLDKLKPGWKWAHQSSAKRCKPIPSKWTDNTISAFFYKKAALWEKYKSEAEAQDTVLSAEGMDDDGT
GIKFIAAPWDWQFVGKMVSSGADGKPRKPYDFDDAKFYMQKWLEKSQRQSVSYSDMKSWFSAWGKEAPSTLG
QLAREVNQKAGTDLITGISDE

>QLI65453.1

HMEMKDPPPIRSGFNPYTGWDIDYDMISPLNSDGSDFPCKGYARYLGTPKGRPVATWTAGGTYSMTISGRIPHD
GGSCQASVSYDRGNSWKVIRSWVGNCPVLGDSSYSFTLPSDLPAGDMLFGWSWFNNEGNREMYMNCAAITVK
AGNKWAQRGAADSYQSRPAMFVANVGNGVCTYEGTDVEFPQPGPDPVRTSRRPHAPGHNGCGNWGGDY

>QLI65470.1

DAPQHGTVQHLEQRQEDAKQKEELSQLFLEYAKTADDIKNLMNKLAGK

>QLI65473.1

ICLEKATTIPDGWQMLDEIPDPSTPMRFSIALRQPEMHGLASKILANGLLGMGDTRSLRTPAQDDVDHVMEWLSS
NGVSEAKVDTDWIRVATTVGEANKLLDMQLRRYSYDGKKPILRAPEYNIPDSLDTAIDFIHPIANFMTPEHEVSVAP
PLAEDAGLHRRDVACSPATTPDCINKLYGINYTTPDGKSCIRFGIAGFLEQWANYDDTRRSFEHSRPDLVKAGYNFT
VELINGGENQQDLGTAGSEANLDIQFGMAVGYPTNVVYYSTGGRGAKIGDNGEPLTGELDDNEPYLDFFHHLSSK
KNEELPHVLSISYADDELSVPKAYAIRVCNEIGMLTSRGVSVLSGSGDGGAKGGRNATCRSNDGSNKDMTISTFPAS
CPWVTSVGAVNNGQEPPNGSVFSSGGFSAYFERPDWQDAAVSEYVDALDGHLEGYYNSSMRAVPDISAIGTRFQ
TVINGSPFMLDGTSASTPVLAGMIALVNDARVRQGKPVLGWLNERLYSDEVREVLQDITAGQSFSCKFSGGKEPG
GWPATEGYDAITGLGVPNDFNKFMDVLVNMD

>QLI65475.1

ASFSIHDDLLAYPQFEVVFASDYISEKDAQALVNRNNQQHPTYSAEFSKTTGNHAHATTTTDAAAPSSNESDEIASY
SYEILNLSPHRYLCSIPVIEPPGPVNETENALAKVEEEREHQRAAVKGWELVSKLEGSCLYYVSGWWSYSFCNNREIV
QYHAISVSSNGQIPRRDPNGQEYILGRVPTLPATTGDRKKKRQQRGFDDPPRPPAELQVKGDQRYLVQKLEGGTIC
DLTGKDRKIEVQYQCVPGIKTDKIGWIKEVVTCSYVMMINTPRLCSDVAFQPPVEKIANPISCKLISESDASTLLLDQ
QPRAAQPDNDGTKENIRQKETNDEVNEKKDAAQVTVGGVLVGGKRALSTGDDDGKPLKLQGLGHLFAPQPKILQ
IIAEAASKENGGKVKGLTEEELEKLNIDPKAVQEMREKLKKLAGEKGWKMQLFQMNEDDEKELFGYVDDTEEEEG
EGKPGDGKVDSGKTKSDGDAGGEKGPNHKEPGKQRMKSREKKKDEGKGSEEKFFNRDEL

>QLI65482.1

INSSVNHTVIDDNRAVELPGVLRYPIKGVQGSSGLSKRQDGQSLHSQRHGLIYTIKLDIGTPKSTVDVQFDTGSSQL
WVNPICSHAARPADCTKLGRLRYSSTLKTLGKTHKLTYGRGYAYVKYVRDFVQVGSAKLPNQTFGVAIDSGFVSAGI
LGVGPSKRGMKNSDFVVNSLHKQGFTKSIAFGVDLQGIASARGSVVFGGVDLKKFRGTLHATPILDRAAAPDHAP
RYWINLDSISQTAGGETVTLVDQRFPVFLDTGATLSYFPPSIVRKLLQGFPGAKRDKYANYIVNCSLRRQNASVDFQ
FGSTVIRAAYKDFIYPFDDNACALGFTEATDKAYILGDSFLRSAYVVFDQTNRQLALAQAADCGTRLVAIGPGPAGP
LVGECGRPPVTSNTTFHTFTKRTSNKTSSSTKMTTTASPTYTSTLTTTKVYTITTEIVTSLTTSHPGENPKPTYRMHSRL
HRGSRLPPHPKSTAGCPNGGDCPPPAVVIPTETGVIPRPGRPLKAPVTAGASSMGAGAFILVITAIVMLYAGH

>QLI65484.1

APGYGGFTLRWQDSFPGAAGTSPNTGNWNLVDGYLNVNNELQRYSPSTRNLQISGGQTVQLVPWRDGSTRMG
WTSGRMESRYVFTPDAGRVTRVEAALRFGGAPQPAKQGIWPAFWLLGDSIRHGVQWPTGGELDIMERVNGLW
TGYGTVHCGYSASGGPCNEPNGFGGAVGIPDDGWHVWRLEFDRRSGDWAQQTITWFMDGRQFHQISGARIG
DYNLWVALCQRPQFFILNLAVGGNWPGYPNGNTQDGYGSMMEVAYVAHYST

>QLI65513.1

SIPKGYDEGGFASASGLRSFLTDFGLTRGRWTGDPSQLATRRAVVSSLGVLGAAIGAGMAIYVTDVIGRLRTWQLF
TLLWMTGFFTVTFASGNVGVLLFARIWGGIGAGGLTVVAPLYLSEIAKAKNRGMIVSVYMVVLLTTLMLGFFISYAA
RRTMASNREQYRVVLAVPQIPVGIALFCSLFLHDTPRWLASKNRHDEALIVLARLRNKSMEDPEIQSEYREMQQEV
VSKNLILADTSTWTIIKEVATVPSYRNRFLLGLAMQTFAQWSGGNGITYYIPEIFRLAGITTNRALINAGGYGATKLVF
TMVFTWGLIDYFGRRRCFMTGLAMQCATHIYMAIYMAIWRRSDNQSASTAAIASVFVYAIGWSIGLCTVQYLYGT
EILPTRVRGVCYSVNMMVHWLFQFAVVRTTPPMFRNLDVWGAYVFWALVCFIGLIVLGIWAPETKGVPLERMGE
LFEGPWYKTWKAKVNMNGDGESLHLQTSRDSVVHQHENRNKAPVD

>QLI65515.1

AETTTVKILVLGDESPFVGSVVNVDSSATTFAVQCPSGTPSDECGLPPGGATITQGESTWQWSLGMSNSEMGVLT
QKANCKLDPSKDVAACKIEFTEGSQVTSSEVTESGYKTSMFPVTITAGADKLSASPGASATNGGSNSASETATAKTT
QASKTSSTGEAGQPGEAAATSTTGKNAAPAVTQNAVLAGVAAIVGGVLAM

>QLI65519.1

SLNLPITDMFTHGAVNIDIGLPDTQYTLLFDPGSSNTWIGAQKAYVITSSSQETSDSVAVTYGSGSFSGLEYNDILTIS
GADFNQSIGVANTTTGVTEVDGLLGLGPTDLTLGTLTPDKSQTIPTVPDNLFSQGHVESATVTIVKQAITFGSTSLTN
VTYAPITEVAVAKTFWGFDASVAYGDSSLLSAKAGIIDHGSTATLLSTSSFNHLLNLTGATVDDNTGLPVLSSCSALD
PMILTLAGVNITIPVDIYRWPADNNTAIGGDKNKCYLAISNIGTSFENLSQRPNLQFRKSSFSGNQADEDNVSFILGY
TTLKHFGVVLDKENSLIGMTSQVMEAR

>QLI65527.1

VSTAGNRLLVVLDDVAEKENYKQFFGDLTERGYHITYETPKSEHVKLFHLGERTYDHLVFLPAKVKALGPNLTPNILV
DFVNANGNILVALSSTTPASSSLTSLLAQIDIGLPAERTGTVVDHFNYDALSAPESHDVLVLDAPTNVRPGLKNYFEV
PGVLSFPHGAGHTLGPGALLTPVIRAPSTAYSYNPKEQAEAVDPEDLFAAGKQLTLVSVFQARNSARVAVVGSAE
MLQDKWLDAKVSRPEGSKVKTENREFAKRLSGWAFQEIGVLRVNNIEHQLKGDNETNPEIYRIKNDVSYSISMSEY
SWNKWEPYTLPATDALQLEFSMLSPFHRLDLQPLSVSDSATVYGTSFTLPDQHGIFNFKINYKRPFLTYIEEKNTVSV
RHIAHDEWPRSYVISGAWPWISGIGATVGGFVGFCAIWMYSKPVGGKPKTK

>QLI65533.1

QGNHAIDSFAYQGCSSVDMSCFTPPVLLSAQPITPEMCQWACLGHQFAALFPEDCRCGDDPNAIKPLDERACNTP
CLGDPNHGMCGSICPAEGPAIANVYTKTEAASQQPQIETIHTTSIFALPITLISSEDCTTSDQGPVTEQPAGGLITPVG
SAPGIPTTEQPAGGIITPVGSAPGIPTTFTFVLSSSPATVTTPSEMPPAPARLTTSCPDEQSSATEEPTVAPPITTCDED

SSIPAPSSGVPVPYGTTTVSLPVYTSGAATPEATTSEATTSVGTTSEATTSEATTSEAKPATLPVPSPQSYSDPYSSQKE
QDPEATADSVPGPSQDHPDRPSISTLWSRPSDVADPTGQPPVPAQVPGSDSTHSMVPPLATIGGLALIAAIIM

>QLI65556.1

KLLKPQTNTFNSNFTLTDEQIAPLNLSSSAANNINVAAQFERSNWATGSVFGDPFYTHLPPNATSAAAGSVLKAQE
FTNTSSYTLAPALALSRIVYQSKTLNGTLVPVSAYILWPYMPRGGGLVAPLVSWGHGTTGIYAECAPSHIRSLSFHFS
GPYSLALAGYAVVGTDYAGLGVPFYPDGKNITHEYGASPAAGNDLLYAAQAAHAAFPHRISHDFVVMGHSQGGA
AAWAAAQQQVDAKVPGFMGSIAAAPVTNSFEFFTTQSNIGLVQAARAIRLVFPEIPISSILTDVGEKFVDLMGMIQ
SCNSVFATAAVELVGTDFSHPKVNLTRDEFVNSHYSKLWADLTVAGGKDFQGPLLVLQGTADHILPETLTAKYVNK
TCEKYPQNELQYVKVNSAGHVSVVYATQQIWLEWLDQRFSKRGRFDRKGTCSQLEVGSTAPLPLEEYQGEPNYFL
EYSLDSYQVA

>QLI65565.1

QDSNLESLLPRHKSPLLGDIRKSLRTACDVTEFPASIEKLCDEYCESEIAASDLKGRLCKNFRKYIESLGKCAKDTRSTV
NKQCKDKCRCSKDDDCDINDVCKDHACNPKDMICSNDHCMPKPPTCGQPGFDWAEWRGPLSWNSVKSPPFTE
YDPTVFKSQRPEHGGRTNTLLIDNPEDLYGQAIDINLASVIHQGFLLAPETGNYTFLFGQADDIVLVWLGENAFRG
WTRANADIERTYIPPPGDETRTTRHLEQGTYYPIRVAWGDKGGNTAMSVRIMAPNGTELTGQNGGYFRTEACDG
SYDKFPPYGRS

>QLI65568.1

QNAVVMNTCPTAIYVQSFPYDGSAAGPLTTVQPGKSFSEPFRASGSTVKIAKTKTLDKPLFFGYSFSSNPDYAYYEFS
TEWGNPFAASQNTLTPGDGCEVFDCQANDAGCYSTPAHKKVYGCPQPVNLTARVCK

>QLI65574.1

APVVQTEAAVPDVPHRDPADYYKVVAAQPTETASIPPHKKYRGVPQTTEGITVQNSKRWLIFGDPTLNRNKTEPV
RPQLPKTSASTNRFPRPMLPLIPCCGPCETFFEKNPAGLGVFVCPSMPSRKPSPTTTEGQGPKETGKPSSTVTEGRG
TRVTEMPSSTITLEQGPKITKRPSPTKAHGHEENKMSQL

>QLI65582.1

ARPFLNEPDTGIEDILKCSSNGTLPDIKAIVGLPDFEYVARKYLPTRNFTYYRNGAAGEYSYRNNLEVYRRYRLRPRVL
VDISNIESTLSTTILGHNFSAPFFISPCARADYAHAEAEINFVKGAAAGNILYMPALFAQRSIEDIAAAKKPGQVLFQQ
LYLESNETFNKDLFERTEKAGAKAIIFTVDSAADGNRHRAARFGVGSADSSYSAFSWTFYEQLRNQTKLPIILKGVMT
VEDAQEAVKRKVPAIILSNHGGRQLDGSPSALEVALEIYEKDPELFKKIEVLADGGIRYGVDAIMLLSLGVKAVGLGR
PFMYSNIYGQAGVEKVIQIMKHEIAIDAGNMGIADLKKVSPDFVEWKKPNGWAM

>QLI65584.1

STVAASTTAGNGTITNDAYFYGQSPPIYPSPEMTGGSQWAAAYQKAKALVSQMTLEEKVNVTGGVDLRTGCSGSI
YPNKRLNFPGMCVSDAGNGLRNTDFVNAYPAGIHVGTSWNKDLARRRGAAMGGEFRRKGVNVLLGPMVGPA
WRVVRGGRNWEGFSADPYLSGSLVAQTIQGVQSRGVQTSLKHYIANEQELNRNPSGEVEAVSSNVDDKTMHEV
YLWPFQDGVRAGTGNIMCSYQRINNSYGCANSKTLNGLLKTELGFQGFVMSDWGAQHAGVATALAGMDMVM
PSGDGFWGDHLVKAVKNGSVPESRVTDMATRILTTWYQFGQDAIFDKPGIGMPLDVRQPHETIEGRDHNDRPVL
LDGAIEGHVLVKNTKNTLPLKSPRMLSLFGYSARSADSLSPGPGDLLINLWRFGLTSVSIDDVLSSQLGGQQAKYPNI
AVNGTIMGGGGSGAGTPAVFVAPYDAISMRAAQDDTAIFHDFGMPEPVVVPSTDTCIVFGNAWASEGYDRPSL
HDNFTDSLVKTVADQCNKTVVVLHNAGPRIVESFVDHPNVTAIIFAHLPGRDSGTSLVRLLYGEAGFSGKLSYTVAR
KESDYSHLLSPDEPAGKFQKFPQSNFTEGVYLDYKYFEMHNITPRYEFGFGLSYTTFSLANLVIQRVNGGNSGEWAE
GAIIPGGQADLFDNIATVTVDVSNTGNMPGAEVPQLYVGIPGGPAKQLRGFEKRFLAPGTTVPVEFPLTRRDLSVW
DTTAQKWRLQRGQYNIYVGTSSRDVPLKSSLTIN

>QLI65587.1

AVIPNNNGFPTPNDQQKLQIAQEAGGLLPGSPPPDSLGAGSITAFQLIAFNELFETAYFSSLLHNITSGAPGYEAENT
DELAKIFSTVLAQEEQHALAAVSTLKAANAFAPSACQYQFPVSNLKDAVNLAETFTAVVLGALQGANVIFSQDKQT
GAVQTVSSVIGQEGEQNGFYRVFLDKVPSESPFLTTVPAPFAWSALQAFVVPDSCPFPLSNINLPIFPALEVNGGAIA
SVEPRDQTLSFSADVSGSEAAKAYIGRDNLFATYTTGQQLPISVAITNVEWSGGRISFQAPFPFSKFVMQGFSHVAL
TTGDTFDSADAVVQSALAAPGLIQVNNRL

>QLI65608.1

ASRRDNQTICDYYAEKVFGESNVATQLRLMQGIIAYAYAGGDTLPDPNPNSTGIFNKGKFNGYDVFLRPLFDGSKA
TTNFRDQPVPINWLDGGGLDPLLAFLNGSTVFAKIEPDSNQEILFSHWYVTFGKVYQCSNASVFVAKEYAPFTPAY
VHKFMNLNSTEVAYFIDQLMLASKYYNFSDSDAQQLAATLNTKYNNKCSPPDDNGQLNSVCYADSCPEAVPKKDC
EAYKNLEPYGFKASTPTGTATGVIPTMSSSPNSSATSSGEVAGDSSSRLSAGAIAGIVIGGSVVILMAVGMWLYFRR
QQQNKPAETPAAAQNQMSEAASPAPAYSSQVPYSHPHDSYYSRGPHDSYTVSTIGSPVSTAATWEQSKLPQLQE
MAAESPSPPPGTLSPNNQGDRCTRLRRWKVLIHRLPGAKTQNHNGVSNRIDYLSQ

>QLI65630.1

EPATSIMSEGVSYLAGGGGCYKKNQYLGHFVKDIVISGKCLDLAHTDAINFGTVDGWCWGYKSRKCEGLKKEMNF
TLGCNKLSRHGTPMSILCRLPVY

>QLI65631.1

APATMNSVSSRQAGDQGTFTISQKAPFQDTSLLPALNGASEVDLNMAGYEMGYHPNSDQYKCHGSCGIVLRISRL
PASEKCTHPYAQPFVNFCFGTCKNLAGFSPYPPALVFKNQCKLNAQEARSTTVEQAASTSAEAPASTSSSQPASTTG
SPVSRPTSSAAAPSGSSSQAPPASSSAASSPSRTSAPHTLPTGTTSPRPSHPSGVASASGTAPKATPTNAGPVTAGA
ASTSFSVIAAITVATLAMIIA

>QLI65640.1

TTGGYDGSMLNGLNILPSYTDYFKLTPATQGLNTASVFIGGFFGPMVGGVMSDKLGRRPALFWASIITMAGIVLQS
AAQNVAMFVVARVVLGFGGGISNVAAPVYLSETFPSRWRAWGVGLLNNFYYVGALMAAGVTLGTGKWDSTWA
WRCPSLLQGVFSLICILILPFVPESPRWLIRQDRYEDARLVVAQTNADGNLTDPVAMTVYQEIVDTLEWEKKQGRT
MSPLEIVQNPVARKRVLIGGSAGPFSCIAGNIIASYYLGNELSTAGVTSSDDQLKANVVLNVWCLACCLAGTQLAAS
WGRKSTALLSQGLLIACLFIIGGLSKIYADNPGGASKGLIYGDVAVMFLFQGFYSIAWTPLLTLYPPEVMDYPTRANG
VAFSQFTLNGLAMLLVFVMPIGLDNIGWRLYMINGSWDVITFLLIAVFWVETKGKTLEEIDAIFERERHSSAPGVED
VRKRRITVDEVQMEKQLQHQDVRVRETLGQ

>QLI65647.1

WPTTGGDVTIKSFQLYPENADYYAKLDRVYISVLYNSSVAVWDPTNNVIESIIEFPETNKTEYHASGIEVEAKTGLLSA
IINPGAAFDTSGADISGDSFLYKIDMKTKKWLWRKNVSKPTEGRYGGCQEAIHDRHHNTFVLCTYPGAIIKVSADGK
TATSWYESNYMKPGETPQRPGLTGGVSKDDFLLAVDDESHKLLRFDMREEKGNPVVVPIGKNNEDVGLGLDGAY
LPPKYDGQVLLVTSESKEINVIISNDNWKTAEKAGVIKNTYFDNPENNRGFSVSTVQIGEGDDAPIYSINEYFLDAQD
ASKPTVPGTLAKDRSEFPLQDITKAVDDIVWS

>QLI65664.1

AEAPIPGYGVEVLQWDIEVAVGRTEVLNGTVQEVYAQALQINPKFTLTIAAEARDVHQKQKRSSVQCGNWPLADK
GRIQQGINYLRGAPAAPRNGPGPGNCGRVSCSYNSAIWWCNDNTTPKTLDSWNWVADSAQHIVNTCAPAAGS
VSGQNFESGNWNTIVRGDSC

>QLI65677.1

ADIQKRSFSVSRVANPNFTGRNGPRALAKVYRKFLVPLPKGLEETMHAQDKRQVAAAQARKRSPEAFRPMRLRQ
PGRRGLLDDLLQGLGLEDGNQNGNKGEQNGNQGENGNKNGNKNGNQEGNQNGNKNGNQNGNQEGNQNG
NQNGNQEGNQNGNQNGNQEGNQNGNQNGNQNGNQNENGNNNGNKNGTTGAPPQGTAPGTNQTGTVEA
KPEANDAEFISPVKIGGQTVNLDFDTGSSDLWVFNTQLGTGSGAGHRVFDPQRSQTFRLMPGASFNISYGDGSGA
VGNVGTDVVDVGGASFPNQTVQLATAVSQSFVQDQSNDGLMGLAFSKINTVKPVKQATFFDNVKDSLAAPVFT
ADLRKAASGSYTFGTIDESKFKGPMAWIPVNTTQGFWQFTSERFAVNGGQPKTSTPGGQAIADTGTSLMIADSV
MVQAYYDQVQGARVNAQDGSVVFPCNAKLPDLDVDIGGVYMARVAGSDMVFADLGNGECFGGLQVSPIRNLA
IYGDIFFKSQFVAFNAGNTSLGMAPHA

>QLI65683.1

DSSHALFYKVVDRETAQRIASIKGLAAAGNDTPVFNDQGFWFSHFTVGASPDLEILIDTGSSDAILNPGVYKPSPGS
VNANRRFRISYATTNPDGSGTLTASGNVYQDVITQLSANLTVANQTLGDIQDPASPPTFPRDGLIGYASQQGSALR
GSPFINSLCDQGALSTCRFGLALRPNKTGELYYGTLATDTFTEPLTTVPLTQGEWAVQGDVTVDGAAVQHGASIITD
SGTTVIFGPTHRVADVFARAGVQAVPTSTGLAGYYNCSAPPTIGLSFAGANFDILPEALAFASDGDNCTAAVHGSD
AFGDNWLVGQAFFQGRYVDHNVADGTMGFADLK

>QLI65685.1

ASRDDELALISKRRIADLAQFPDPTWFSQISAWLDTQKSDGTWPDVNYMSGCAAQRANWPIQVHWNRIITFAAA
WSGANPAVAQNWTRNDNLMEAISRGLEYWFSNDYTPADCMGNGGKATGGCPCGTPGLWNTNWYGQAILIP
QLCSTACLLLKDANLTKDITSGCERIPRRSYDLRDGVYGSGGRMTGANAVLVMQNSASLALYSNNATILQDAYARA
MSVMTYADKTMEDGIHRDGTFLQHNGILYNGNYGKDLFNAFIQLEGEAIGTSFAAGNATRDAIAAQVRGNEWMI
FVDQQTKQEHWDFNAIGRFVAFPTQDLQANADINFNTTKLAGAVADFAGANNVSDTIRRLKSNGTEKLVGNKGF
WASDYMVHRTKSFVLGNKMLSTRSRNTESVNSANPYGYHLGQGTLFSYVEGNEYKDIMGAWDWNLIPGTTTLL
NHTSLSAGASANVGKKDFVGVVSNGRVGAAVQEYADPLDGSISYRKAWFYQDDFVLITTQDIKKTVPDASVVTVL
DNRASAHGNIWVDGRNVQADGGRTARGKTLFYGGNGYLSYGKAFDLTLFEGKRTGNWSEISTSPAGVTTVSIFSA
YATIADTCSAYAVFPASSRGRLAKELEKPSASPIIQSGISGAVGSGRLSLVFWPGGDGSIKLSLREIGWAESGSVNITS
AQPGSYLFSGTGKRGKGMTLVVTLSDPTQKAASASFSLNFDGARAKLGAVAADEGRSGNETEVKYTVDLPTGGM
AGSSTSRKMYVEFR

>QLI65697.1

RERVDTAPQAPAPRTNGRSFTLNQVQNKDFQGHDGPMSFLRAHFKYAKKLPDYLSKLVEIDPNFRIKFGLGSQAD
GQIGTVLADPTPLVDTQYAIEIGIGTPPQHIRLNLDTGSSDFWVFSTDTNPSMVDRQVLYDADKSMTSHFLAGESW
KIKYGDNASASGYVYTDRVQIGETFVDTQAVQVAVNLTKDLSSDNFVSGILGMANSAANTVRPTPQRTYIHNIKDN
LAEPLFTANLQRQAPGSYNFGYIDDSQYKGSITYTPVEPSFPLWMVSTTGYRIAKTSHNEQIKSIVDTGTSLLLLPETV
VSSYYAKVKGSSVHPQLGMRLVPCNADLPDFYLTIGSYRGRVPGSYMNYGRISGNSCFGGMQSSGDLPFSVLGDV
FLKAQFVVFDYGNARVGFANKQLYE

>QLI65728.1

AFNPLAQQQPIMPGGDRLPAQPDNKSYAQETPPPPAIKFKTGSSPPPPPPKDQDAHENKYEPQAQPQVSLSDTIG
PLRSISSFSSFTRLCESTSSLLADLSARTTVLAPLNSAVDSLPRKPWESPADYRALGSQAYDGTGGQDRANKNLLRFV
EAHLVAQSPWPRGEKAKTLAGREIWWEEESDGQRVLMPDRVAVDRVASRVSNGELWIIRGVINYT

>QLI65739.1

VPSKPASTISPTSPTSPKTDPFYAVPENIEHLPPGTLLRSRKFPFTDSPFGLTDVNFNISQQILYRTTDSHGRATATVLS
VLIASPGNNSKVLSFQPAQNAPALQCAPSHTFDAHPPSRQKPESTITRPELLSIQSALHQGWIVLIPDYQGPKAAYM
AGRLAGHAVLDGIRVAVQTPALTRIRKNPKIAMWGYSSGAAATAWAAELHQTYAPELEIVGAAVGGVPADIAAV
VDRINGRKHAGLIAGGAVGLANEYPDVAAAIYPRIRNKYQPLFNKIKTRCGDDSTKAFNNKNVITMFNTTEVRNLP

AVVDVMNQTSLGKMAPRIPIYLYHSFHDQISPVGTVNKLYDFYCANGASVLYKRDFFSTHGSAAVTGMPRALVFLI
DMMDGKGLSACSQATILSGLLESPTWGRIPEGLINALLFLLRRTA

>QLI65756.1

CPWADVNPSNRSRNRHDSIYYCQPTRQILVEPFDLGPVFRPKELAPITQPLQNPVVPAGSRNTIKLLECPIEAFLAPE
LLRRLVESSRDETTEIGEAGLS

>QLI65761.1

GHAPRDYKDNDYYVIQIDSSVPPEEIASRLHLRHEGTVGALSDHHVFRSRKTEHDVVRRELLESKRKKRTLEEYDVLD
NIRFSAKQELRRHLHKRVIPPPPPGAHIPRAVADPALSAVKKQQTIMDTLSIKDPIFTAQWHLFNSVEVGNDVNVT
GVWMEGITGKNATVAIVDDGLDMHSEDLRENYFAEGSYDFNDHDPEPAPVLSDDHHGTRCAGEVAAVRNDVC
GIGVAYESKVAGIRILSAVISDEDEAEALMYKNDKNQIYSCSWGPSDDGRTMEAPSVLIRRAMLKSIQEGRNKLGSIF
VFASGNGAKSGDNCNFDGYTNSIFSITVGAVSRDNQQTYYSEPCSAQLAVTYSSGGSTSDGFIHTTDVGSNRCTDR
HGGTSAAAPLAAGIFALVLEVDPELSWRDMQYLVMDTAKPFSAPGVVWNQTGIGKQFSHAFGYGKIDTYDLVQK
AKTWKKVKPQAWFFSPRLEVNGAIPEGPTGISANFTVTKDMLKEANLERLEHVTVFMNVNHTRRGDISVDLISPSN
MVSQIATTRSGDEHYAGYVNWTFMSVAHWGESGVGKWTLVVRDTEQNKHNGSFVDWRLKLWGESIDAKKAT
ELGMPTEDEYVDLPVTQSPTPTASPTPTATASPTTTATATSTVPPSWFPGFGSKTAMLWVIGAAVLIVLFCIGLCIYL
FIARKRRNTPRDDYEFELLDEEEADGLNSNEKGGEGRRTRGGELYDAFAGASDDEEEFDEYRDPSTERLAGNGYEE
DQYVVGEESDDEGSSGAAETMPLGGGSRS

>QLI65766.1

NDRGSTPTPTVTTAPIFIPYYSEEQWSAVRGSIVSMNQTATQTTYTIFCPTATQVACDLSLEFPFIIVEGPSTLKFHGT
LTSTYIADLECNMKGTTAATCSGYSSYCSGYTNGKYTGPTEVSWTSTLTGSNIHWGTLTLADKPSETDDSLEVTATNI
AVPTVPSSQTYNSTPTQTGGGDNLHSSRSWAPTTALLSIFASVVIM

>QLI65773.1

LPATSSAQPEASSEQWQEAFMSIWSCHCTHRGGQDYKKHVKKDMQCMIENTATTVGNFETGEKDSLVEMDKEV
EKCKAAAWQRARHSECEPGQYETECYFPIWKVYSTCVGNSPSTSKRGSDCPKNPNSKPEKVKGDKAKPQGANSEE
ADECPEDPSSKPEEAKSQEANPEEADECPEDPSSKPEEAKSLGDNSKEADECPEDPSSKPEEAKSQGAKTQGATSEE
VEPEKAKTQESKPEKAAKTQEAKQA

>QLI65784.1

GGAEGDAGRTTIGFRSVGQEEKDLLTKAGSLIRSRNTAATHIGDGVYLGNSPIKRDGESLFIVTADEAAFKAASKVW
VPQEYFTLPVERENDILGEREAAISRDQLEYYIEDQGLHSSRTVKLAGIHGSVSDDIQQMLIPNEMISHDQNGVPIET
ESKLDTKIEEPSGPMFWQVDYEEWEGIQGSRVYTEDTLRARSASLAEKATKAVTEAEKLVESDSPAAAEAIAEAVAT
AKRCAEGVGHFHRKRPDWVDFDDFNTVYKQYSNARKAEAKLRKLKLTKAVEAQKSKLTEAAEKVTDDATRASGIA
EAEQAAAESNKLLQTAADEAEGVKKAHEKLESVRKQLDGGEQEEFIELLAKNPRAVWATLQESFTSMNGKPAASL
DAAIIDEADITQEEYSIFQEGVDEAKAMATEIGEGVRALQMTVGHPEILCKRGDIDCILVPSDKEKLPTAEKPAKIEPA
NEGELIAIARQRSKESFDDLLTDFKYKSVVKHDQLYKELNERLPEFSPVSRTERIVSLSTKFGEGALAVAGLALYGKAV
ADVFSSDTSSVLDKAAVVTSILPGVGCAVQLADSVEHGNVDAGHTALCFAEDALLVSGFWEIALVMQLTESLASWI
EAGNEQDKLFDTEVLRSKGLAGWEANVDRMLKHIASDEFAANTTTRFATYQILVLYQASQLTGDLHASHKAVSGK
PGNQTDEIVPHIEPEFRRQICAAMAQSKFQLRQKLEAVALEHAKKFSEEYKDKFLKDYREAATKPIPFLGIPISFGAG
NLDEVLEDARRWPLPLHEGRIKRAIREVVERLETPAQCKCLQGSKKAPCEYADCSTPKPPRGRKDAAGRVYVTNVQ
SEDHAKQMRLWDECVSLYTTCPYPGLTGEVGRQLWCTPAS

>QLI65790.1

DTDTITWGGDNSRAGYQTNHNMDPSIVGSSQFGQLFRTKLPGVWVGQPEQIFSQPLVYTPSGGDKQFVYFATTQ
NNVYKLDAKTGQIVASRNLHIPFLTADLDGCVDINPTIGITATGVIDPATDTLYLTAKTYVDQNSGEKAQGRPAGRY
YIHALDVNDLSERPNFPVDLEGIIARNTAERMFQGGIHHQRPGLLHTGDFIYAGFASHCVQYNFTGWIIGWHKTTG
KIVEKWATEGEGVPNTIKGGGIWMSGGGIASDDKGSIFLATGNGYASQLADVPVNGFNPPTALEEAALHMTINAD
GTLRLVDFMMPHEKRELDGADKDLGTSPLEILPSEFSCGAIKRIGVVTGKSGKTYFLNLDNLGGYKNGKDGLDDVIQ
VYENLNSVYAGAGVYPLEGGYIYINVIQHPSIVFKFSCNNGSPSFSKVAETPTSNAYILGVSHGTTTSLNGQPGTGLL
WITDVQGLNLRVYDAVPQGQTMNLIQSFNIPAVTKFTRPVFGNGIVYVGTTQGYVYGFGSPVKVPLNCTSPVDFG
GVDMKNSSIAKPVTCKALIGVTVTGIELNDAKNKDFSLSGLPDLPLELAVDQTFTVNAQFSPLKVGLQSNDVVVNTT
NSVTGFSTGTHARLTGTGHSAGALLDLTPVILTFKNAVTGEDPNGISESIIASNLGNGILTVQTVLYSTTGTDGPFQA
WNGQGDLVIGKFRFQKLPPTIAPNSAATVNIQFDTSESGTYSCYVKFVSDGGNRTISVAASSGPPPKALLEFQTPDE
TGWVKYKPGTPFSFGNITENNSRSLKFRVTNSAARGAAQLSLTVSKPPFGVAGLIRALNQVDLAEGTSLGAGESAN
ATLICNVPYAQWNTAPYNGTVQWTMNTNDATLGKQFIQFFCNAVAEQAPPLLPNGLGQFKYVGCYRDNTPGRQ
LPNQILASDTMTSADCIKACHEKGYTFCATQYHRECWGGNTIPLQKVDDANCNYYCMGALKQICGGNGNNNDG
TYMSTFGDTLKWDGNTTQPPSASGPVVNPGVSGYTSIGCYTEATDGRALTNGVGTEKKTVKDCVDGCKAVNYIYA
GVEYGGECWCGNEFTAGAVPAPDKDCSMTCNDNSTEYCGGPSRLNVYKLDAGKTASATSALPSVTVKTTASVPAS
ASSTTTLAGSSTVATSSASTTTSIAEISTVTSTSSTPTPTGPVKKAKVGNWNFQGCWTEATHGRALTSRAYADDNM
TLESCAKFCHDLAYFGVEYGRECYCGDKLQAGSVRADNQDDCNFLCPGDKTEYCGAGVRLELYKYGASSSLLPTSTS
ASNPLSPGSSTKTQTPVTSVPTTRITTTKLAAATSSLGSSTSADTAVSSLSTTSLDSTSIVSSSTETSTTSATPTPTGPVIS
EGNANFTYYACVSEPSGGRLLKSQVLNTDNMTIGMCLGKCNKYQYAGVEYGQECWCGDKLNLGGDGNGSPGK
NVTDKECSFLCPGNSTEYCGAGLRLSLYSRKQITKRWNWSIL

>QLI65791.1

CTPGKQQCASVNDQHGNHLCSSCAIAIGDAGGRCITNNYCQQRDNSCTTINGSPYCV

>QLI65800.1

LPAARRGNLVVRQAQNGTAPTTEVSETELNDFDILQIALTLEHLEEAYYREGFAKFPDSDFAALGLKPEQIEDLKRIG
QTEEEHVSFLQSTLAQAGVQPVQPCQYEFNVTDAKGMATLGALFENVGVSGYLGLAKRIKDPAILSAAATIVTIESR
HQSSLRVLLGQTAVPQAFDAPLSLKSVFSIAAPFIKSCPQGSNLAVTPFPALTMEAAQSGEASAMTVGSTVRVAAA
SGAAAATHCAFTSGGVVPGGTAFTPFSESAGCEIPQGVAGVTYLSLASSAPLDGALTDDITVAGPMILAL

>QLI65804.1

RRGSKLPPGPPTLPIIGNLHQIPTTFTHLKFAEWAKQYGEIFSLKMGPATCVVLTSPRLVKQLVDKKSNLYSHRPVSHI
GYDIISQGDHILLMQYSGKWRACRRLIHQFFMESMVVNNHLALVDAEAVQMVRDFVEEPENYMQHPKRFSNSTI
MSLVFGTRTPDINATHMKRLYRLMEAWSNLQEFGSTPPVDIFPLIKYLPERLFGNWRTRAQHVSKQMNSLHQEW
LNKVVERRKSQGPRDCFLDRALDQEDSGKLPLDRHALYFLAGTLMEGGSDTTGSVLVTFLHAMTKWPNVMKKAQ
DEIDSVVGEDRSPTWGDYAQLPYVAACVKETLRWRPVIPLGFPHVVGEDDAIDGMAIPKGSQIIINAWGMQHDP
ERFADPDTFNPERYVGVPQLASELANGDWEKRDHYAYGSGRRLCPGIHLAERSLFIAMAKLLWALNIEAGVDAEG
RVMDADVSAEGAYASGLLVCPKTFPCRITPRSQARKDTIMREFGVATAEVFAKYSDDKEW

>QLI65830.1

ARLPPMAGSNNGDVLGKRQLSAAGLPDLSDEQQAELLKVYKAYSSAKDIKSFLDGVSNLPKGEAFKTLAKALGGGA
TAGNVRSALDKTPGKPQTVIITYVLGIGKCLVEGKSVTDAVDCGKKAVIGLGDLAVIKVPETCNNRLGQLELFGTGW
PAPCHDPKAIEHPQGSAAHRRLKWGFCRLFEIMTPERLRKTDQQTGGSCDSRFPTDQQIKDDLCRKYFGGAPCGA
SFPLETEEDFKKAKADEAKYKKWLESFSPQLQFEAVVGVQGEMPSMFKGKNAVPLDKETCRGGKCGETCSGDKCL
PLREVCEPACVSDEAEGGKCNCGWDQFGMKCNSCHVDKCDECVLKPITEKDGKFFAGEQPEQLHVECRDNGECY
FVPTRPSTAPAAKA

>QLI65835.1

HTPDFKTSEAGNPFVDGFYADPDNEFYNNQYWVYPTSSYEYDKQTYLDAFSSPDLVQWTKHANILVASDFTWAR
RAVWAPAPISRNGKYYLYFGANDIQTNSELGGIGVGISDQPEGPYKDALGKPLIGQYYNGAQPIDQDVFIDDDGQA
YIYYGGHGHANVAKLNEDMVTIGTFDDGTSYREITPENYVEGPQMMKRNGTYYLFWSEGGWGGPDYAVSYAMS
TSPTGPFKRLEKILQQDPAVASGSGHNGVIHVPNTDIYYIVYHRHPLGENIDANDRHLAYDRLYFNADGTIQPVRML
VHDNFNDGNMIGWTTYGGSWDAKTNALRAGASAGGKALLNTNFGDVDFSADVKMPSANGGDAGLVFRASTP
ALGTDAYDGYYAGIGTSGKLVLGRAEGGSWTQLASVKADVVPGKTYRVRVTAKKNAISVYAGDSTSAQLHVLEGT
FGSGMTGVRVYQTDAVFDNVRVEHL

>QLI65866.1

FRFNRELVANEKPMCGPGEGSCEMGSCCSEIGFCGTTAEYCSGSQCQLDYSHTCDTLVPPDGSDTSEIPRPRVGKV
PYGPMITSCKNPGMVALTFDDGPYIYTTELLDLLAAHEVKATFFITGDNRAKGHIDDPATAWPSILRRMYNAGHQV
ASHTWTHRDLTEVNETVRRAEIIHNEMALRNILGRIPTYIRPPFLECSAGSGCEETLGDLAYHSISANLDTKDYMYDD
PALIQRSKDRYSSTLSPDSKENSYIVLAHDVHEQTVHNLTEYMISLARERDYKLVTVGECLGDPEENWYRSAETRTV
GKRNLCSGPAKPQVTSVSPPLNTTTKISPNQRCGGSTGYICPGSGFGDCCSHWGYCGSTPEFCGTGCDNDFGDCD
PSPQGVLDTTNGLCGARYSATCLHFAGKTCCSKYGYWYVPFLVAKFIGVLTIQVAGVRLSIVVRGARTSTVIVTERRY
FGFKTLARATPRAPGSQTPRSIESATSVDSQFHTSNVSLWGDVRQEEAKLRWDAEEELKRVSKELLRLQKWALITGL
VVLNSALITISLLLPQAVYLFLALLSCNTILQAGMVVCIVANVIWIKIICGCRKKKNTAPATPERMVLLLPCYNETKEEIA
RSLDSLVSQKGIDEHARLVFIVVDGNVRGPGMEKTTQDYFLEDILEPGTSKYFENGYRARDGLFMPVKTRVGRYKGI
PFVFVGKRYNQGKRDSLCFARSFLYHFKKRSVNAVTIFNKELFEYLGNCLLENGLPNVDYLVGMDADTVFDDFCVV
EMVNTIRKNPKLVGVCGHVCVDFDGNNFGLWSLYQSVEYSQTQGLRRMFQSRITGKVNCLPGCCQLIKVDEATF
GDTVLRERFGYCPKPNDVMTQQIMGNYSEDSIHASIIFSLFPKKQTAQALRSKAFTIVPQTWKVFLSQRKRWALGSI
SNEFVMIFRPGIILVERLQSIVAVMTWAITPFIISAVAQLIALFAKKGAKVFEDEIFVGLISVLWVRYLYSFCIGFWLPR
NNLQRLQYFVGFIIHFLSSPFINIIILIYSLLYADDFKWGDEALFV

>QLI65887.1

DYTIDPDSVPIGTRKAWCDQEMTTCPLICQQVEPRTTIDNTCDPESLTYGCLCGNNLQPNVSQYSLSLPYFVCTQW
VIQCKDACPTDACKSDCQQKHPCGAQDPVRPNATKTAGASATSTGGSNTIYTNAPGGGSDNGKKGAGAALEVG
RTYGLAFVLTSLFAGFALL

>QLI65888.1

LQDQKVLSNPKSTLGFDFNKLGNVDFDVWTKPLREAFGEATSEAKALWQEVSMLAPEAVDAFKQHVINAKPKKH
SRIPNSKWDHVVHGADVQNIWVKNEKGESHRKVGGKLDNFSLRAKKVDPAKLGVDKVKQYSGYLDDNEQDKHL
FYWFFESRNDPATDPVVLWLNGGPGCSSLTGLFMELGPASINKKVEVVHNPYSWNANASVIFLDQPVNVGYSYG
SGTVSNTVAAGKDIYALLTLFFHQFPEYAKQDFHIAGESYAGHYIPVFTSEILSHKDRNINLKSVLIGNGLTDGYTQYE
YYRPMACGEGGYPAVLDESQCLSLDNALPRCQSLIKSCYDSESAWTCVPASIYCNNAMIGPYQRTGRNPYDVRRD
CKGGNLCYDELGYISDWLNKADVMEALGAEVDSYDSCNFDINRNFLLQGDWMKPYFKLVPKILDEIPVLIYAGDAD
FICNWLGNQAWTNKLEWSGHKGFSEAKSKGVKVSSGNGAQEYGKLKSHGNLSFLQIYKAGHMTPFDQPEASLDF
LNRWVAGRLDA

>QLI65897.1

APHEHKKPHPSYGDANAQCGNHQKLSCCNRGDSGGVLDGLLGGNCQPINILALLPIQNQCTNQVACCTGNSNGL
INLPCTNVNL

>QLI65907.1

VPPTTAFPLKTPSRPLLQWTLPPVIPQMRPAFQAKIDWDLDNNHTIQTPSGLRASWWSREGYLYDTTGRQFGEILR
ANDDGVVTSGTDPGTKYLELLISYVVKLDDGHWAYVKHTGGAIVRQYQNGIVRVETDSKKYTWLNRVDFIAPGTF

NGTEVMTVNHYFPNFEPSVDIPLEPSLVTVVCNRKDVPLNKGWLRVYLDGLDKCDVFDRKLFLAGIIITPPVTGRPF
HQIVEMT

>QLI65908.1

ATIHKRLIGGEYAKGGEFPFIVSIRQSGSHICGGSLLDSTTVLTAAHCIKCLDFVKAGTLDSRQGGVEVEVVSAALHPR
YSRIRHHDDIAILKLATPIEESDTIRYATLPASGSDPELNTTAVVAGWGLDGRNTGPADKLSKVNITIHERGECSEKLK
LGAVEDIICAGDDGKDACEGDSGGPPVDPVTGHVIGLVTWGQCRDPPTAYTRVSSYIDFINGFVGGSDSSPLFNLG
RKCRDLVQSVGNATGPGDAGIPSTPTGEEEEEEEEEEEEQGPTAFDVLIAKIRELAKQCGLKENEKMTPEIQKCIARL
HEEGVTRR

>QLI65913.1

RAGGQAPRNPNGRNTNVPPRARAHPGVPFDKWTRDGPVANKFDNADTQKYRVNGTAIPDVDFDIGEAYAGQ
MSISQDVNGPDKFYFWFQPSPNPDADKEIVIWLNGGIHYTQPGCSSLEGFIQENGPFLWQYGTYRPVANPWGW
HHLTNVLWVEQPINTGFSTGTVTAQDEEDVARQFMGFFKNFIDTFSMQGYKVYITGESYAGMYCPYIANAMINA
NDTKHFNMKGMLIYDPSITDDRVTELTLVPFVDYHHSLFPFNDSFTKQIHDIDSKCGYADARSKHLSYPPPGHLPNP
LPGIDPNTGKPRPECDDYLLANTIQQAITDLNPCFDIYQVATTCPLLWDVLGFPGSFDYLPKGASIYFDRQDVKKAIN
APLDRKWASCGGPVFVDHDKSPPSSTTVLGNVIDKTKNVIIGHGALDFILLANGTLMAIQNMTFGGQLGFQKPPV
EPFYVPYHTTGELGTIAGAGVFGTTHTERGLTYVGVSLSGHMVPQYAPSAAFRHLEFLLGRVDSLSSKKPFTTDPKFP
QPDGPLGNGTAPPGYN

>QLI65927.1

APEFDWDSISPSTDLRYHDCYAAFKCARLKLPLDWKNSTDPRTVAIAVIKLPAKIPDHDPAFGGPIFTNPGGPGGSG
VSFVVDNGHFLRDYVDSPGKKHYDIVSFDPRGTGNSRPLANCFPVAALARDAWKLETRGNGGPDKSLAAISYGLAL
YDGYGRRCEKADAEGLNGGEIFKYLGTPSVARDMVAMVDSIDELRKTEAARDGPVELLKRNEGDVPRLQYMGFSY
GTILGNYFASLFPGRIGRIVLDGVSNADDYATGPGWLTNSVDSDAIVDKFFDGCFHAGPQVCRLARPQDKSASDLR
TRFWPWVKQIDEAPVAGVGPLGSSVVLTGTDIRLVLVGAAYTPLKSFIQTAEVLDNAMVHGTYDTILAMIESGLGA
PLQDACPVANQTAKPDLGTDPLTAVLCGDGEDIRGKKPSWWARYVDRQLAQSAVFGGLMSTVRFPCSGWRFKP
NWSFRGPFTSPEPARDGEQPQRGQPAAPIMFLTNRLDPVTPLASARAMARKHPHARVVVQEAMGHCAAISAYS
ECTRGIVARYFDTGKLPDGEAVCEAECGPWDAGCARTMPKSSGEDAGEASLARRFPLGV

>QLI65932.1

GDVSTDYTTQLVTITACPESVTDCPARSTQVQTSVVPLTTSTVYSTKVHTITSCAPQVTNCPAHSTIVSTETVAVSTTV
CPVGPTSVPVPVVPVPTVPGHGNNSTGVVVPPPASSHVVPPPASSGPATQGPPPAVSTNSPVTQAPAPPACPAPS
VTAITKSYTTVLTSVEYSTIEVPCPPTGTVPQGTGIPPVPTGPANPPSGGNPPAGGNGTTPGGNPPPVTAGAASFAG
SAVFAAVAGIAAFVLA

>QLI65944.1

QTWSRCNPVQGGQCPPDTALGMAVNVDFSKGAVNSFAASGTPSYGRDGVSFTVSRSGDAPQLASLFYIMFGRV
EITMKAAPGAGIVSSLVLESDALDEIDIEWLGTNPDEIQSNYFGKGQTTTYNRGQFHSVSGTQAGFITYTIDWTQDR
IIWMAGGKVVRELKASEAQANQYPQTPMQVKFGAWAGGDPSTNAAGTVEWARGPTDYSKGPFSMLVQRVVIS
DYSTGKQYRYKDSSGSWQSIEAVDGSVNGNFGKGNSLTVTATAVGTAPTTTGIVPIPVGGIAKDGSPATATQTGW
PWVASASPSGGTVPSGWYINSNGKIMRSNAAALQKSASFSVAILGPLTLGVVVFFYRLW

>QLI65955.1

FPGMSELRHVSRAQDEDNTKKLPGDLEGQDESTMSETGKLIKNILQGNENPQDLTTAYSSVPDQNSAECKADKCCI
WKHIADEMKSKMTGDAGRCNNLARQCIRMGFHDAATWSLNTGKDGGADGSLVLARECFDRKVNNGVTDGCN
QMQAWFDTYKSFGVSMADLIQMGANVATVVCPLGPRVRSFVGRKDNANPSPDGLLPSPSDPADQLISLFANKTI

SANQLVALVGAHTTSQQFFVDTARAGDPQDSTPGVWDTNFYGETTDANSPKQVFKFQSDVSLSQDSRTKGAWT
AFTGTQGQRPWNGAYAAAYVRMSMLGVYNTNDLTECTKALPLPITSFTFPDAAALADFANGGAPDASRNASNG
DIIVV

>QLI65964.1

FPTPIGGDGGLLSLLGLGSSRNGDKNASPTPAKSNVKAKSRIVPNSYIVVYKNTTSAADVKAMTASVSSQLKKRNLN
KRGFEGQPLSTNVRSFQINNWHAMNFEAEESMALEVGEYDGVDYVENNTWFSTQELVEQTNAPVGLQRLSEAA
PVGEQAKKGSYVYDSSAGNGTTAYVVDSGCRTTHRDFQGRATTIANFVKGERATDANGHGTHVACTIAGARFGV
AKLATVKCVKVMNAKGQGTNADIIAGLQSVVEDVNKTKPQAATMNMSLGGGRSKALDMAINNVFKAGVLPVVA
AGNENQNAKNVSPAAAPNAVTVGAVNATTDQKAGFSNFGPSVDINAPGVDVQSCGIESDSDVSTKSGTSMASP
HVAGLANYLMRLENVSDPAKVTALLKNLSKDTDATVEGGRRDTTPLIANNGNQKDKNTFLDENGPVKGGTQGNG
AASN

>QLI65973.1

KPSLRRRQMDGSKNEIGAQRVTATEIAIDYLAPNPPCGGYTPWVGVWPADACNPYTADFVAWAYAERTQGRDIR
TVTINLAELDAGEYKAAFVCEDGKRKPWLVSQTFKLEDEPPAKKKQGEQCVKSDDCEGGFFCQHQCGGAMEEYG
PGSVECLIAGNPTCKPRPKKEGEGCSKHGDCAGGLYCAHQCGGRVDDYRPALCLIGGGPRCAKERQW

>QLI65976.1

AAMDKRIVNGEEAKPGDFPFIVSIGSTPSGKSHFCAGSLLDNITVLTAGHCVASAYYVRAGTQDLGEAADVAEVAY
AKSHPDFNLTTKDANYYAVNDIGIVKLSTPINRSDKIEYATLPEDGWHPAVKSTGRTAGWGPTISGKGSPKAVDHL
RVLDIPVRPLEECLQYSLVPDNKDTKICAGGDGKITTRYDGGGPFIDQDTGYLSGVVSQAMSDAKYPGTFTNVGSY
MSFIEEYRGSNGRPDPNAPSRAKLLQEAAKKFISEDEVFEHCDRTGQPRLDCYEAKKPCDTQRRKKPNQTHEEYFQ
CIDEEVIKWQKFLEEYDNQGNKKVQG

>QLI65990.1

AQISVSSPLSAQTNKSPFTGDFNSHVEVLMDEWKLAGMAVAVIDGDDVFTEAYGYSNLPDTKATPETLWFTGSTT
KAQLAASLAHLIDAKHHPALKDGWSTPISSIIRDDFVLRDRHATALLTLDDAVSHRTGVPSHDFALATYPDSNASTP
VAHAVRNLRNLPAQLEPRVEWHYCNPMWQVLSHVVETVTGASLRKTLESVIWAPLGMKSTFLDVDSARASGLDL
STGYWYDDKKGEHVAMPFMATAEVSGAGAVISSVVDYARWVKALLKKDAFLSEATHEDIVRPRFVSMAEPSKGA
DVTTYALGWFRTVLHGERTIWHSGSTGTHGALVYWLPERQYGLVVFANYPNGVLEAVTYRLLEDRLGVPQDRRID
INGLQKEALRRRKQDLANATALLFPRLPSPRLPPSVDPSLLAGTYHNDGYGRLALSEQTGDDGRTLLVADRPEFIWA
QRVTLEHASGDYWTARWRFRDESPETGQFFKARFVVGSDGEVEGLEVTYRAGVDGFLDGVVFYARTR

>QLI65999.1

RSRISCRDDLNAFITKQNHISLDGVLANIGSDGSRAQGAAAGAVVASPSKSDPDYWYTWSRDSALTFKVLIELFIGG
NKSLQPKIEQYMTAQAHLQGVSNPSGGPDTGGLGEPKFHVNLTAFTGPWGRPQRDGPPLRATALTIYANWLIAN
GGQAEAANTVWPIIAKDLAYTVQYWNRTGFDLWEEINGSSFFTLSASFRALVEGATLAKALGKQCPDCETNAPRIL
CFLQSFWASGYIDSNINVNDGRTGKDVNSIISSIHTFDPAAACTDATFQPCSSRALANHKAVVDSFRTIYAVNKGRT
PGRAAAVGRYSEDVYYNGNPWYLATMAAAEQMYAAVYQWRKIGSITVDATSLPFFIDLMPNIAAGTYAKDSDTF
TSIIKAVTAYGDDFINVVKQYTPADGSLAEQFDRETGSPKSAVHLTWSYASFVGAVERRSGVVPPSWGEPNANTVP
KICEAPPSCDSTMTFNVKVTTVPGENIYVVGSITELKNWSPADAIPLDASQYTPSNPLWSAKVTMPAGTNFEYKYIK
KTSEGSVVWESDPNRSATSSTGCRSTGTLNDEWR

>QLI66017.1

TPLSSRQEAGIAADTNTIPTLGASFCIGFTRPECKGAVGKCSDEDKVIADCIRDEHPACITDQNSPCPSGTMSPEQVV
ACKEASKGYGVATFVNNCKEENEVDESDACKTVETTFGEAWDANNCEAAFGPDEGGDQVELADEGEQ

>QLI66044.1

LPRALLDKRAADTVGPNRGRAADVSWNGQNDYSSSRGRDRHPSWQGKDRDPSWQGKDRDPSWQGKDNDPS
WQGKDNDPSWQGKDNDPSWNGPGWSEDPYLPDFPQENDRPEDNNQPEVNDQPEDNNQPEVNDQPEVND
QPEVNDQPEVNDQPEDNDKSEDNDLSEDDCPLENDGLSEDNDQQEDNDQQEDDCPDEDNTPPKPSATTTSAPD
KPVATQTEPVVSATPSPTEVPSGTNKDLYMPIVDKWLRALSLRPLKYDAELAKTALQCSELSNGALKHRGSNAQIM
APGNETAFEHVFVGGWLGERSDLFPDQEVWRNFSKAWNYRGQTGHADYLADQGTYDDGTPLKLTKIGCGWAG
GSNGGQQWNMWTCDLA

>QLI66045.1

TPIPDAEMEYLLNTAGIELAMKAQPMFLMGQAVGRAPCMPSWAIVNGTQAAPSKLCAWPDSGCDCRNPGVPL
GSPMPSFPVYFSYSRCGNAAVRIAYNLFYTKDGFIPNKIFGHPFDWERVVVIWNKNQSNGMWAPAQLYLSQHTG
YQRIEWAQIKNTFNAADASKPRGGPDGQKNLDHPKCYISSAKHDMHQEKSTAWIDVLSQLTNNAFRSDSWWYF
PTKEDYILADESTDVGKLIASFDWGDADSTPPLVAKGLCNA

>QLI66065.1

MAKMLLEHKPDLLYQSCRGETALYCASQAGDLSMADLFKSPGFYCRLVPNQTPVIFEDGQLWNMPYLEDIMPLQ
RRFKPNRQIILMMDLLVLSDMIGRKLILFAIQERKYSLFILGVPKEGTIEQCFTWRTSAQMAAITLAKNLPWSFISPY

>QLI66079.1

SRDTPRDSQVRLTGPIRHHQVDPVILDALKKHSDPVDALVSLHPEAADQLAQPRLLRVSGEPTARWMTEGDKLRL
KRKGHKFVDITDHEDFYKQNVNALAGEAHLPNLSHQHIVKPLLPQVSTKRMRHVLEHMTSYYTRYFGSVTGEESA
QWLHDHIAEIIKESPFHTHISLEVFTHSFPQPSIIARFEPKVRNFSLPLTILGAHQDSMNYLFPLLPAPGADDDCSGTV
SILEAFRVLATSGFIPINGPVEFHWYAAEEGGLLGSQAIARYKKESGARIGAMMEFDMTAFVAKNATESIGFIETEAD
APLTKWAADLASEYVSIPAKVYKLPSGAGSDYMSFTKLGYPSAFASEGNPSAGAFPGEFDPYVHTAKDTMDVDDD
TGIFSLEHMARFSELAIAFVVEQAGWDNKWR

>QLI66086.1

IGFDDFYDFADTQILLQLDARAELATSPPHYPSPWMNPQAPGWEDAYKQARDFVSRMTLAEKVNLTTGTGWMS
DNCVGNSGAVPRLGLRALCLQDGPLGIRLSDYNSAFPAGITAGATWSEHLWRDRGKALGAESRDKGIDIALGPAS
GPLGRAPTGGRNAEGFGSDPYLQGKGLANTVIGIQESGVIACAKHFIANEQEHFRQAGESVPRGFNISESLSSNVD
DKTLHEDYAWPFADAIRAGVGSIMCSYNQINNSYACQNSKLLNGILKDEMGFQGFVMSDWQAQHGGAATAVA
GLDMSMPGDTEFNTGYSFWGGNLTLAVINGTVPAYRIDDMAMRIMASFFKVGMKPGKQVPTSFSSWTRDTFGY
RQAAAKENWEQVNFQVDVRGNHAAHIRESAAKGTVILKNTGSLPLNKPKFLAVVGEDAGQNSRGPNGCDDRGC
DDGTLAMLWGSGTSQFPYLITPDSALQRQAIQDGSRYESVLSNYQWAATQRVVAQPNVTAVVFVNADSGEGYIE
VDGNAGDRKNLTLWKNGDDLIKNVSSICHNTIVVIHSVGPVLVTDWYQNPNISAIVWAGLPGQESGNSITDILYGK
TSPGRSPFTWGPTRESYGTDVLYKPNNGNDAPQQDFTEGSFIDYRHFDKVAPGKNSTGAPIYEFGYGLSWSTFEYS
NLKVEKRDVRPMSPPNGKTIAAPTFGNFSTNLKDYAFPPSIRYVYQFIYPWLNTTASGKEASGDPHYGQTAEQFLP
PGATDGSPQPRLASSGEPGGNRQLWDVVYTVSATITNTGKRVSDEIPQLYISLGGKNEPVRVLRGFNRIEGIAPGQS
VTFRHEITRREISNWDTASQNWVITKAPKKVFVGSSSRHLPLSADLS

>QLI66091.1

DEQQPSQAVNAAAESWIWKPVSKPHEPSKPPESTRPHETTRPHESSRPHVSTRPHESSKKRESTKPHESSRPHVST
RPHESSKRRESTRPHESSRPHVSTRTHESSKRRESTKTHETSKPHRTSKTTEHETTTEHKTTETEHKTKTKHETRTKEH
KTTETEHKTKTERETKTKEHKATETETERKTRTERKTKTERETETERRIRTERETETERQTKTERETKTREHKATETETE
RKTKTERETRTKEHQATETETKHKTREHKATETETEHETRTKEHKTIETERRTHTKEHKTREHTKVIPTAFTTFCPEPT
TITINREKFTVVTPTTLTITNCPCTVTEAVTTGIETEAPPPPISTAAADKANAGIGALVAAGFAAMLL

>QLI66095.1

ATIDKRILGGQPAEKGDVPFIVRFDNNCGGSLLDKTTVLTAAHCVSDEAGKLSVRAGELQRAGEGGVVAEVASIKR
HPEYIRGRLPNGIFHPNDIAIVKLSSPIEKSDTIGYARLPANGSDPVVKSMATVAGWGAQGHSKNDGNLHKVDIPV
HKRDDCSDIDKGAVRDTIVCAGAYGKTACDGDSGGPLIDRWGQLIGVVSGGGSACHEQGRETLIYTRVGSYIPFICE
NLDAPCPDTLYEPAAQPPAEPIPESEDPFWREVNSQAQQVCNDRGLTGEQAHLCEEYKQACVFDNLATDDANVI
VDCVKNKA

>QLI66102.1

IGVLLGMQAYNHAKGRPPPFSGGDDGNGLPGIGTPKGKDTVEIKCDQAFGFDPKSKGQNFILNPNPWGWKKGEE
GALCMLVNFNGNRTYATEFSAPVFNTTWQYPRVTGGGNNVHAFPNAKVDSKNFPVQIGSVSKFEFDVEWSLSLK
NDTHEEVTDADVTANQINANVAIDMFMDKDSTKAEDSEKAGFEVMVWFADFGTDAWPLGKKNTPDKGLVKSQ
TLEGVEFELYSGLNSQNQMVLSWVATKPTTTFKGDLKPLIDTIFAMNNANYPQKTDYLGYLAFGQEAYSSTANVTF
SVPSLAVDIETST

>QLI66103.1

FSCGDTVYSSKKLTTATGEACSLLKKGRSVGRNKYPHEYRNLEKIKLTGSGPYYEFPVFANGEVYDGGSPGPDRVIITK
DCKQAGVITHKGASGNSFVTCSPTSVGTLNSQSVFALCFAMMLHAVFA

>QLI66121.1

ANLTGYEYVVVGSGAGGGPLAARLALAGHKTLLIEAGDDQGNNPNYTVPAYSARSSEDEKLAWNFYVRHYADDA
RQARDYKTSYRTADGTLYTGLNPPPGAEMLGTLYPRTGTLGGCTAHNALVAIYPHRSDFEYVASLTGDASWAPDN
MRKYFVRMEKNRYLLPLLAKGHGFDGWLDTERAPITLPLEDPKLLSLIAGGSFALGNYSRLLPNLLDVALGDANEDS
ESRDRDGGYFQIPLSAGDGRRYGSREFVVATRDAKNPDGSKKYPLDVRMNCHVTRVVFDESGGGEPRATGVEFLD
GAYLYRASPRSGSAAKGVPGSATASREVIIAGGAYNSPQLLKLSGVGPASELAQFGIPLVKDLPGVGTNLQDHYEVA
VQGRLPSDWRFLSGCTFSDDNDPCLDRWRRPGLLTGRGAYVSNGFAAAMLMKSSVASEYDAFLFGGPVNFRGYF
PGYSYNITADHDVFTWAILKGHPRNTAGHVRLRSADPLDVPDVLYNYFDTGSGDSAADLTAIREAIGVARDAFSRQ
LVKVDEVLPGAGVRSAAEVEAYARDTAWGHHASSTCPIGADGDPMAVLDSSFRVRGVKGLRVVDASVYPRIPGTF
TALSTYMVGEKAADVILSQNK

>QLI66122.1

FPEFYWGSRSSPGRISITNNMNTPVQLDKVTGKSLANGIKVSRDQETVIPAHETLQIQAIDQSADLKLRVKGATHN
QVEVSYTSGNQGTYNYAIKPIEGGGFPGVVRVEPDRLQCRPERWLPGHPATPQVTCRDGVELRVYLEGPYPRVFE
AEYDDEYYDGWY

>QLI66125.1

TSHHHRMHQAYQRLAHPHSHVHSRAESNVLVKRGTCAFPTDDSNLVAVTPDQKNAGWAMSPDQECKPGSYCPI
ACKPGMVMNQWDPDSTYTYPASMNGGLHCDENGNVKKPFPNKPNCVEGTGSVKAINMCKKHMSWCQTVLP
GNEAMLIPTLVKSEAVIAVPGSSYWQSTAAHFYINPPGTGTDGCIWGTEDKAIGNWSPYVAGANTDSNGQTFVKI
GWNPIWESSGLKGTKPGFGVKIECPDGGCSGLPCQIDPSAAEGSVASKDAAVGAGGSSFCVVTVAKGSSAHIVAF
DGSGGNKEPPKQSSSAPPPPPPPSTSQQPTTTQIPTTSSTPPKPTTTSVAPTTTSQPTTTLAPTTSSSTLTTSSSSSLSS
SSSSSSSTTSYAPPTLIPGIFHENGTSLSNSTTTLASSTAPTTSIAETTVPNVPIPTEKKGEAGRQQGSAAVAGLVVALV
AAACLF

>QLI66129.1

APNVPVQPPSTSEWDLSKPLNGFTFQLAKARSAAPTIDKPALLYSRITSIKGSSSNQNAVSSLVQILQTPPSHGQHTY
QNISTTGNLSTQYAIQCGWDGAPVWLLLDTGSADTWAVQTGFECHDSHGTKHNQAACAFGNPHVKKFQNGPI
DGLHFYLKYGSGEKVFGPMGYADISCGGVSVPRQQAGLANYTYWHGNNLTVGILGLAYPSITSAFYGEIGQEAAW

NAIQYVPFLTNAIMQGSIDPVFSVALAKNSSDGIIAWGGLPRVQRASDVYAETDLIIANLVGSAETSWRYSFYTIIPD
GIIWGQRMDKSKFPYIVDTGTTMNYLPPPLAEAVANAFLPRAVYLYQWGSYFAPCDAVPPRFAVVISGIEFWLNP
ADMIYRDLVDPMTGYCAVAITSGGSGPYILGDVFLQNVVAVFDIGGAQMRFYRRA

>QLI66138.1

FADGLADQKAVSHDDQSLRVSKPDSSETHHDTKFAYDSRYMKQLLNKTAHRDAARTLIQSYLITCRELQVQTWLM
NGSLLGWWWGKKMLPWDYPSNVQITEADLHFLAAYHNASVHFHRRKGMPKGKRFLLEISPDFTNRAQTGATDV
IDARWTDLGSGLYLNITAVRYSPDHPSGEGVLYTKDGQQFHDTLLHPLRDTTFEGEPVKIPYRYKTMLAERYGEEAL
KKTHYNGHKFDTPEMQWVMQG

>QLI66140.1

RDRSPIPETSGNVSKISELPELESLLSSTTYVVVDFYADWCPPCRAIAPQFSKLADEYASKGQLAFAKVNVDHCKGAA
KHHNVTAMPTFLFFKNGQQTSVVVQSSKHGTSAKMPKDGVVEKIQGADVMLLRSTVQALAENAQR

>QLI66152.1

LPSPPLAELDAFNVIGGVTWTGEVLPGKNVTFSGTMQEVRAKILAENPDYFSAYDNEAGIEHGSHLQKRWELKQP
PDCNYGNFIRRINAIKFINELNAKGGGNAMCGAPPRRGPGLGGCSRVSCSWGSQMWLCNDNDYHLDLPCTEVA
SALFELSNVCYTVQQGGDTETQGQLFTTDNWNVIIDYYGDCHSSPTVGR

>QLI66156.1

LSITPGDDASVKSAASTIAYGLMKYYTGNNTGDTPGNLPSPYYWWEAGAMFGTMIDYWYMTGDASYNAATSQA
MIHQASPTRDFMPINQTRTEGNDDQGFWAMASMSAAENKYPDPPPDKPGWLELTQAVFNHYVSRWDEDNCG
GGLRWQVFTFNAGYNYKNSISNGCFFNIAARLARYTGNSTYADWATAIFEWEQKAGLITKEFAVLDGIHVDHGGG
GTCVNRTDTQWSYNAGIFLHGAAVMYNLTGGEVWKARTDGLVRHSLGEFARDGVIFEPACEPVRGCDYNGLSFK
GYYIRWLAATIKMAPHTYEAVYPVMRKTAEAAARACSGSDASFRGVPGTACGFAWTDGGKFDGLVGVGQQMC
ALDAVMYTLVDGVGAAVTHDTGGTSRGNPAAGGESDDSHLPLPKAITAGDRAGAAIITIILVGIGIGGSAFMLWDE
DL

>QLI66161.1

QTQCRSRDGPKLGAVASENSICSRIGTRLLEDGGNAVDALVGTVFCVGVTSMYHSGIGGGGFLLLRSPNGTYEFVD
FRETAPAAAFEDMYKGNVNASMYGGLASGVPGELRGLEYIHRKYGALKWADVVAPSIKLARFGFPVTEDLVRYMD
STSPNAFLTQDPSWAMDFAPRGFRVRLGETMTRKRYANTLETIASEGADAFYTGAIADATIRALTAANGTMTLEDL
GNYTVALREPVQLQYRGYKLTSTNAPSSGVVALSALNILSGYDAFLDPSRSNLSTHLMDEAMKFAYGQRTKLGDPS
FVAGLDKYTKDMISPQAGAEVRSKITDQKTFDVSYYDPEGLESLETPGTSHIVAADAAGMSVSMTTTVNLLFGSGL
MVPETGVIMNNEMNDFSIPGESNAFGYIPSAANFARPGKRPLSSISPIIAETADGKLYFSIGSAGGSRIITANIQNAIH
LLDGNMTVEEALGQPRLHDQLVPSRVSFEYTYNNRTVAYMKSLGYNATWVAPGQSTAQGLRLLPNGTFEAAGEP
RQKNSGGFAV

>QLI66163.1

TYQDAHSAIKYTDIDYLVFTDASRYVWHAQSPYARDTYRYTPLLAWLLLPTVSFFSFGKVVFALADLLAGWLILRILR
RRGIPPEQAGAFAALWLWNPMVATISTRGSAEGLLGVLATALVWAAEGHRVNLAAALLGLGVHFKIYPCIWAPAI
AWWMDDDRPRRKPARRRARFLSRDRIQLAAVSLATFTGLNVLMYAVYVSPPFSSPRRAAVTQTDFRSYGRPFLVH
TFFHHLTRIDHRHNFSPYNVLLYLASSRPGIPLHAESLAFLPQLSLSCVLIPLVLAKKDLATTMMAQTFAFVTFNKVCT
SQYFLWYMIFLPLYLPTSTFLRKPRLGMTALVLWLVSQGAWLHQGYELEFLGRSTFFPGLWASSVAFFLVNCWILG
VIISDGGAHTAPKARTE

>QLI66189.1

WGALGHDTTAHLASHFVSSPTRDYLKRLLRDQGDDYLAKVATWADQIRGLEVWKYTGNFHFIDAHDDPAGGSC
QVDYARDCKEGGCIISALANYTDRARDRALPRIERERAFKFLVHFIGDLHQPLHNEDVARGGTQIRVRWQKRQYNL
HAVWDTSILEEITQHLGKDPMSTAIRWADELAREISSGKYAADKKGWLENFHPGSLNVTAMAWSNEANHYVCTH
VFPPGLGPKQITQKNLFSNGYYQQAAPIVERQIARAGFRMAAWLDDVVKSIQAEEGSNETVDDEL

>QLI66194.1

APPGLTRPADLRLIKTSESDPGQWVTEQEEFDRFISQNIGFIDITDVEDDEVLSILSADPSGQDAQRQAVTYPNGAL
HLDQGNKLLANVTTDGPKSWLKTYSEFHTRYYQSGTGLQAANWLFNQAKQLAAPNPAITVQRFNHTRFQQPSIIA
RLPGNSSSLVVVGAHLDSIGSSPTGRSPGADDDGSGTVVVLEALRVLAASGLRPRNTIEFHWYAAEEVGLLGSQDV
WASYKAAGRSVVGYLNQDMAGYSPTGVPAVFQDYVDAGLTRYVTALIGDYLHVKPGTSKCGYGCSDHASARAN
GFPAAFVAEDTFENSNKYIHSANDTYDKIMWPTILLHAKMVVSYLLEASYI

>QLI66242.1

TPIDVRETTDLELLTTDVTPSSPLLRRDSCQTLDSVLDRIGTSSEVVVYASTGGLTALYVCRRSHGHNCDELAAAIAGA
IASVFLILKRTGAISARDGQSAESLVDFLRREFGEDGATFDSIEDATPHILARYESGERRPVEVASIQGLTSGNNTLNM
DVYDFGNGDGHIYLPHDVLTKRSGGDDDLASRFEKRAGAPGFKVSYTTRIKSKLTRAHQISMSQTLAHWWANRA
NCCNMHDMIGFVETGHAANFYYRIIPETVNFGLNYETVDSCGQMARFL

>QLI66246.1

RRSPYFHDRGAPPAGAWADGPLKLVDTPWTLHQKNDLFTTAATHMALLHNAIVRGYNSIYLQAPHVRGDDVPDF
AAYALTWHKFLVSHHDDEEDKLFPDMAGILRDDGIWGNMTQEHESFLPGLAAFQALLNTTDATAYDAGALRGV
MDSFAADLSAHLHNEVAVMAAMAAHENAPARESLRGALAGDMLKAWGKNTVTKAGYVDVLPFFLLNTDRTFE
GGVWRNWPRMPEAVRWSMVNLVGMRHGPRWRFSSCDAAGRPRELFALKALREEKEREKERSEL

>QLI66260.1

GPVPDMDSCPDGVRTLETQHTQILTITPVLVSTHCPYDMDLVIDHANTIPCTNAPTDISTIVTITNTGSPAMTSLEN
GTPAKPTETGKLPPPNTVTTQAESASTPGQSSQMSDIGNSNSRPTGSGASGSGYSKTSHAASHTDESSASSRPGSS
NSKPSISLTSNSASGPYSSPVSLMPEASSSGNYTWSPAFASSTANSSSGTASSEPTSSRSAASPGESSAVEGSQTTSSP
SESSAESSSQMGTLPTETSRTGILSSGSSRTATSQSINTQTETSADTTRTETSRSEVAQSMSSNMEASPTGTSQAGTS
QTGTSQTGTSQTSEAAISSSAEISPSESLSASLSSPMTDVTSSATETTITKASSTSSADASSSTSSTSSTPASSSTSASSST
SSPSAGSSPSSPATSSSTSTTSTTSTTSLTSSASAGSSTSISSSPSASSSNPASSSNPATSTTSTSSTSSNSSTSPTSSTCSP
AAPTKVCATGLPRPCKDLANLGSINLNILQDAAGCTSALGAFAKDVSQCFVLGPLDLLSPWKKVVNCLQDKIGEMC
LPQLPDACTGLAGQHGVSLANNVAACTVQLGPFAVGKTYECLKGGFFAGGFFNGDDIVACLRGTTGLGLSTCAPA
GPACVSALPPDCNLLGGLNGLDISLSKLEGCAKAIGSYGGGVAAKCLNPAAIVQTTLGGGVVACLRDALKDVCIPTL
PEACFNLITVGGERLTTQLPQCIDALGPFKSGAVLDCVTKPTSGPGVVLCLNKAIFGS

>QLI66267.1

HLKPGHSFEPTLERRGVDTESGGFDDEFPRHVYRGEIQRTPKEVQKDGGFYSRGMQRILSGDKLSWEELEDGSSLF
RHAAGDTAPFTRYVSTSADPATSLTFAVNDEDPVEKGYIYKIHADKRMVDVNRSLGKYSPYPAQTEHAAIGFIPFEQ
IEGWWEVTYKHDFADPKVGKKTQDKLRRGQLKHFHRNPHFSPNFQKLRGNGVAPQLAGFPRLSTAWDEDTWK
QFKTLPVSKSLDDMIEAVCTGNGNDNCIKQLGQPQSKPTKPKTSPSGPIDPAKPKSLATSFRVYAKAGTLVGFNVLA
PYLRNVLNQLRQWDHPIGWAVKELDDSINGFQEYIGGPRRNDISGNDNQAALINFFKRVFWILEGPRRRPQDLQL
LSYGKRNKMRLISVNDVLRTCERVDETPPDEQQLKTNLKDACTKVRKKAMELEMPTAAELEIGRAACGVCGLAWS
PQDGQCKDKTGAILWPREPPAESTCTHHDSTKCGGEQTAAQLQTGQAVCGICGSNWYPDEGKCRDAAGVLLWP
FKTCSAGAGRIQCEGGQTTAQRETGQAVCKACGKYWDPEGSKCRDEAGMLIWPPQHLPSIKPLHPSEKCRGGND
MTCQRRQIAAEAERGRIVCGTCRFSWDLRARKCRDNSGAVIWPPKE

>QLI66269.1

GGTNQPKYAYDPSTTKYCTFWYDNDDGSVACQDIPNQWFITDEQWIRWNPVIAASCGNFVIGKSYCIEASQEPIS
APPATLKSTTSGSSSSTSLSSSTATKPNNGISTPTPTQPGIVENCNKFYWVSLGERCQDIASKNGIPLTDFLNWNPNA
GQKCGSLWADTYACVSVMGYKPQPTPSKPDTSVKTPSPTQPGIVDNCDRFYLVQAGDSCVTIAANVGISVADLLK
WNPQAGSQCTGLWANAYACTGIIPAFRLRTQYHNDCTGAVYNDLSVNDGTCIRTGCSVASLDISPEGYCPDGQIQ
ISYWEKPDCTAIGFDLGQSYGTAVAHFSNGTVLQLAKVEGSQRYQAFLQSELQKQQEAWWYPPHAEIQREDLSRL
LKQYMGIGGPDGAVILAEMLIALRTSSEAVLGAPLPATVVITAPYIIAWSYEETLQISYIKRAQKLAGLRTVKMESMT
PVYLGEANTILAANRRMLCPDLFCNGPEWTNENFHKYDVVYLVSLTNHSLYTSFQISTCFFWPARSAQLGTINPRF
GLNQLEQASDQEIFWRELQDHLKSRVREYVKQPDNYRESFLVVVSGEAADNPKVVEAIRGIITDMEKDPAFRVVET
GKAPRIELLISEDPTYAAAKGVAFGQRINMDSRYCDDWFEREKAMGGGRDEDSRDEL

>QLI66272.1

APAATTGPSGKTSVHNKRADFYWGGAVQEGPPGTGWNYVQGSVVVPSFGGGDNQHSANMWVGIDGDDCTS
AILQTGLVAYGDGTFWLWTEWWKHPMQSYEASLAFSPNDTLRFTVHATSTTSGTTTVENLSSGHAVSHTFTSESA
YPLCETDAEWILEDWQYDGQPVALENWGTIKIFDTVAKSPGKQVTAAGSGIVNININGRTLTSSSVDSDGTVSVTYI
GPN

>QLI66284.1

EGPVDKKKPIYDDLDAPSANDAPAPAAPIKALQFSKPPAEEEPKKEKKPRGPSPTDRLAVHIGKARMALYRFAVCTE
NKVNETMDSAFNLEQSFTNTIASLAPSRESGEKLMPGTIYVLVAAMAGSILTRNRNILLRSTVPLAFGVGAGWTVLP
VTMRNISDLSWKYEQRFPAVAETHVNIREGLRRGISFAKVHSQVGVQYVDDKVTDAREAVEGWVKQGK

>QLI66300.1

QVHTDCNPMERDCPANPAFGTDHNFNFNSTPSSDLWETTAGTVSYDAKTGAAFTINKQGDSPTLRTKFYFFFGRT
EIHLKVAPGRGIVSSMMWLSDDLDEVDWEFLGSNKTFATTNYFGKGRQDFKNGGSHPMTGMQDDYHNYTTV
WTKDSIEWFIDGNHVRTLNSKDANNTHNYPQTPMRMSVGIWAGGDPSLPEGTRLWAGGDTDYANGPYTMYLK
SAQVTDYSSGKEYTYGDRSGSWESIKIESGNSTAVDALNKQPDKSTSEKWNDLPTGAKAGVYAGGGAVGALALGA
LLWYYIRQRRIGAAEAKAAALRDEEDHRENARFLKDGINPDGFTDHGQEYNARELRAGGMASENSYHVPESSPFD
GPFDEKSRLGSSASNVSTLVGAAGAGAVAGGAMGAAGAMRGQPGRKASPASSNHGFDFGVPPSPGHPSRSPSP
GMPPGSPRQHQMRSGSAPDPYSRMGSPGPHQQGYGLQRMQSPGAMGPQRSFTDNQQPGYGYRGPASSGG
NNGW

>QLI66302.1

GLYPGITPDNHTCALVEPVLSCSCKAKLDKVEDTCCVETFGGLLVATQLWSTFTGLEDKGQIYPKNDWSIHGLWPD
FCNGSYTQYCDLSRQYDPKPSPNTTNGQPGGIPVPAYKGEPIETWFGPYGKLDLLAYMNKYWVSQLDPNWVFW
AHEFSKHATCFSTFQKECYGPKAAEHDDLFDFFETVVTWQRRLPSFRWLSDAGIRPSNRTSYTLSDIRHALTKEFGQ
QPFIGCGGPKYNETEAGKGSKDNGRTEINELWYFYHVNGTPQRGDAEKLDAGKAGGRLTTCAQAKGAIKYYERTK
GSEE

>QLI66316.1

VKEHDFKKCDQSGFCKRNRAYADNANTHSSTWSSPYEVLPETAKFKDGQWQAVILKTINNGEKAALPITVSLLKSG
VARISIDEEKRQKKEIELRHNSKARKERYNEAEDWVIVGGLELDKQAQVAFQDKSQANIKYGGDDKFEVVIKFSPFE
VDFKRDDESHIKFNDRGLFNMEHWRPKIEKEKTEGDDSKAEENAKTDDGEDESTWWDESFGGNTDSKPRGPESV
AMDISFVGYEQVFGIPEHTGSISLKQTRGGDGNHHEPYRLYNTDVFEYILDSPMTLYGAIPFMQAHRKDSSVGVFW
LNAAETWIDIVKGKDHKNPLSLGKGARTSTHTHWISEAGILDVFVFLGPTPRDLTKKYGELTGYTAMPQEFSVGYH
QCRWNYISDDDVKDVDRRMDKYKIPYDVIWLDIEYTEEKKYFTWDPHSFADPVGMGQQLDTHGRKLVVIIDPHIK
KVDNYAVSSDMLSKDFAVHNKDDETYEGWCWPGSSNWVDCFNPKANEWWKTLFKYDSFKGTLENTFLWNDM
NEPSVFNGPETTMPKDNIHFGNWEHRDIHNLNGLTFHNASWEAMASRKKGEMRRPFILTRSFYSGSQRLGAMW

TGDNQANWEHLAASIPMVLNQGISGFPFAGADVGGFFGNPDKDLMARWYQAGIFYPFMRAHAHIDSRRREPY
MLGEPYTEILTKALRLRYALLPSWYTAFFHANRDGSPIVRPMVWTHPSEESGFAIDDQLFLGTTGLLAKPIVEKDKFS
TDIWIPDDEIYFDYTTYQILKTQKNKRVTVDAAIDSVPLLMRGGHIFPRRDIPRRSSAAMRFDDYTLVVTVSKDGSAE
GDLYADDGDTFDHEKGQYIYRKFSLADGTLSSVDAEGRSAKSVKAGGWLKAMSQVHVDRIIIVGAPASLNVKEVQI
ASEGKTWAVKVDYHSAEKGRAAFAVLGRVGAKISEDWSIKI

>QLI66318.1

APSPEGKDGSKDSSSIPPGAESQPAIHVGKNSALAASKCELYDPAFDGVCRLMIRHVQIAITVPAICAALNIAAAILTQ
FAGTLLSAAATLGRAST

>QLI66323.1

ATNNILGFNSGATFADRSAKFKKDFEAEMKGAQGLKGAPGTFNTVRLYTNIQAYSQDDPIQAFEAAMETKTQLLL
GIWASGTDNIDKEISALQKAVKTYGKEFTDLVVGISVGSEDLYRDSQTGSKNKAGVGNSPDSILKFIGDYKKAFKDSA
LSKVPVGHVDTWDAWTNSSVKPVIDAVDWVGVDEYPFYENGKGNSIENAPYLFEKAYGATVNAINGKPIWVTET
GWPTTGPDWDEAKASVENAKYYWDEVGCRSLFNKVPTFWYTLRDSNPDNKVKFAIAKDLTTPLFDLTCPKTFKTK
PKSSSVVSSATGTATGTATGTATATGAVNGTATATGASRPTSTGGSGNPGSDGSNGSNGSGNNGGSNNGGSNT
GGGSGTTSGATASSTVVKGASSTVEKFSGAAYFVLAVLAGSLTLL

>QLI66394.1

NVLPQETKTVEAHAFNVRPWPLATLAPASPLELLRRESNTICGYIGGDPNLPATCLAGSHCAADVENGVIGCCPDK
GPCNAGIFTGCVDRNSGPQTVADPYIYTCRGKNVCYKNMFEGGYFQYGCGSASALATTVVLTASGRLPLDLTTISV
KLTATVTPLSSPATLSSEPSRTSDSSTVLESPVASGKPPEPSETHGGAAPSSNGSSSTNTGAIIGGAVGGVAAVFAIAT
LAFLLWRRKSSNVRRGPGNEKDSRYIRSMAPAAHREFEPLPSSHDASEARFHAGYGNSTPGNSTPGNRTPGISTLA
ASTSDSVSAVSGRSGSPRVQPYSHDTIDAVATRSGHTNYDSDRVPLTRELDDFSHGFNSALEAIESDSDVEANRNH
MATYPGPRRGGGGGGVLWQQNRRRSRNLAWM

>QLI66395.1

SPAQSRGSAIRAHAASSGNAHDDSMRAVPQKPMIDMSRMSTYDKLAYAYPYDIETKFPNYIWQTWKTTPADSNF
AFREQEASWTLQHPTFVHEVITDSVAESLLQLFYAAVPEVLETYNALPLPVLKADFFRYLILFARGGIYSDIDTYAIKSAI
EWVPSHIPRETIGLVVGIEADPDRPDWAEWYSRRIQFCQWTIQSKPGHPVLRDIITRITNSTLALKRDGKLSSFQGK
NVVNLTGPAVWTDTIMDYLNDGHFFDMRKSQGKIDWHNFTGMETSKRVGDVIVLPITSFSPGVEQMGSKDYDD
PMAFVKHDFEGTWKPESERHIGEQNQDS

>QLI66404.1

QPPRDSPADACCCCDISSNVISCDRSILKKDCVCAAVACAANVPTVYTDSQAPPTAAVKKPETRPAQQTRAPSVAR
TGVPDTADACCCCDIRRSVISCSRSIGKDECFCAAVMCPQGVPVVWADDDKSVSDEDAVRPPPSLPVENLPPPPA
GSVAKQPTPVATTEAPHAIPSTKGKPPGLNDRPKRFVTVAKPAQPKPMPLGCLAPSSASARRFNCCCCNPGKNQV
VCQLREQQDCVCAAVACPIDAETVHVQPPVCTNI

>QLI66418.1

QNLVQVQLTSGTKITGRLNSGVETYNGIPYADPPIGPLRLRPPQKISRHLDSVDGTGIAAACPQLLLSPADKNIISSIGS
QILDLPFFKDVKGQEDCLTVSVQRPANIKPGDKLPVLFWMYGGGFALGSTNTYDGASLVREGVANKQPFIFVAVN
YRVGGFGFMPGIEMLNEGSANAGLLDQRMGLEWVADNIDQFGGNPDRVTLWGESAGSISVFYQMALYGGNSS
YKGKPLFRGGIMNSGTTIPAEPMDGFKGQATYNQVLKEAGCAGAVNNTLSCLRELDYDTFYNAVTQTFSGIFSFSG
TKISFPPRPDGKVLPLSPDELAATGQYYAVPIIAGDQEDEGTIFALYQQSVKTNDDLVNYFSQFVFPNATKEQLSELV
EAYTKDDEGSPFRTGPINSLYPMYKRIAAIIGDMTFTLSRRSFLEATAKANPNMPAWSFLSSYAHPLPILGTFHASDLI

QIFYGIPPTHATSSTRQYYFNFLYNQDPNKGNKGQLNWPVWKENNTLMWFKTQLDNGYLTDSFRNNQYIVLAKL
AKAKVLRQ

>QLI66433.1

TPTNCDPKVPSVTVPACSRGVGTVKYDKTVPDLKPFPRTQVDLCYTDTHLDIKFTARDEVNFYFNSSQGTNADIWE
YEVMEAFIYKGTDNPQTYFEFEVNPNNVTYQAFVYNPSKVRADGAPFDHFFVSDPPADGFSAVTELDKPNQLWVS
DVKIPLGLFNVDKGQAKGTQWRMNFFRTVVSPQTYPDQELGAWSVPNKASFHITPYFGHVKFV

>QLI66438.1

IQQRQEEEVPQCDEAPPSVEFVEYSKTLLQNSSSLETRQAQKRYNFRVYAHVVYSEKNSKGGYVSEENVKKDIDLLN
KRYSRAGISFTYAETDYLQNSAWAHSNDPAMNKKLRRGGYADLNLYYVAKIPGKLISGGPIGGHCTYPIVPRGNSLT
VPQATMERDGCVMIATSVPGGSSWSDLSPLTTHEVGHWLGLRHTFEGGCKDGDTIDDTFPQEKPTGRCENQPKL
PDGRPGAYACGGLHPSNSLNFMDYSSEFTLGQEQRMRQWADSRQKLGGSSTDQKPPENPKPKPKPNPPTPKPPS
NSGESKVWGQCGGRDWKGPTACSKGLVCKHFGVWYSQCVPAA

>QLI66443.1

ATTHNERRTADDVSEFDLASRSAPLMHFAKRQDQRVQDAFKEMKAAAKAAKAAIPEAEKTKVDEAKKNLKAAKE
AAQAKIPEEEKSKVEEAKKGVQAAKDAADAAIPADLKKELDDKKKAFKDAKGQEASQGAPAPPAPTEASQGAPAP
AASTEAPKL

>QLI66488.1

VPLETSTIVDYDKGDLARHAVDVGGLDEHLADLSLPIDARHDSRLVERQVQTPPTRKDMKVYLVNLEKQPAYSGCT
QKLVFWSGVTEGQARSFATKNKRFTGDVYGYFTERAVEQKDLKECQVVWCRIEKSIVVQSRKGKQVTSVAKYVLY
KDRSTKHGGLIKTIAA

>QLI66491.1

GPLPPAPPGHGLPIVKTTTTPGGQTLDWVPISSQGDIAQPPPPSLLKRAPTRQDAAVVAAIRPSDAGPAGTVPILRS
HGPTRPMKRLPRPGDNAAAAAVSARSQQGTHWYASTAQNASNHGGTATYSIFKAFVQRPSDFSLLQVAVIRNDA
AHAGTPPKSQTVEAGWINYPDQVAAPHLFSFYTTNNYESYGDGVCGWNRDVAGWVQYDGEIYPGVAFAPLATV
GGDRYEADIGFYYHQGNWWLHTLGRFVGYYPGSLFSKGVDPADTLDHHSDQINFYGEIYNSEDEMTTTDMGSGE
FPDKGFGYAAYLRKIAYYDTKDTFQNYNGSRGVVISDQSRYNLSAVWNSGLDWGSYFFIGGPGAGGVVGA

>QLI66566.1

CTPPPGSGFAKAAPPISLFIYNVGTGAITPSATALVNKFPADGGQDRTTLMTFNDPSGGTSNNCMIFFDPGDPSLTV
AGSGKLDLFTSNAPAPPGGSPGWGPPGNQRNNQLVRWTVAPGSVSTDATYGSLTVACNSLNNAGFEIVGVYDT
DTVSYKTFGVCVAYN

>QLI66568.1

HDDSFTPDIVLSVTRQNISIGGMYRETTLVNNSLPGPTLRIPEQKTIWIRVYNDIENDNVTIHWHGLAQAAYPFSDG
APLASQWPIPPKHFFDYELKAESGTAGTYYYHSHVGFQAGTATGALIVEDPAGAPYTTDGERLVFLQEYWSQSDTQ
IKNGLESIPLRWPGEPKGWLINGKSISNYRAVDPSTRALSVIDVEPGKTYRFRFVGATALSLALLGFEKHGNLEIIEAD
GEYTQPYPVDLLQIGSGQRYSALFHAKSCQDLREDGQLDYYIQLESRDRNRVVTNYAVLRYENACNMTSQPVATN
KNPETPPLNLPPTIDGYLDYALQPLEPNNFPTAEEVTRRVMLNVQVVENRYFIWTINNYTWSEDADALPATVPQTP
YLVSLYQNQSAYLPNYEDAVANGGIDPKTGTYPAKIGEVLEIVLQQFGGKSVSGGGGGMLDTHPWHGHGIHHYD
VGGGRGAWDPDTVEEKLEGKQPVLRDTTMLYRYERFVKMDEKMGWRVWRIRVEQPGVWMVHCHTLQHMIQ
GMQTVWVNGDAEDILKVGKPDVEGYLLYGGNVYGNESHTPQVVHFDEMD

>QLI66575.1

RDATNHPRRVKEVKNVAIIGAGAAGTSAAYHLQKYAEEEGLAVNITVFEKTDHIGGRTVTVNAFNDPGQPVELGAS
IFIKDNHIMYNATRDFDLSLTGLTEPQPADYTAIWDGKTILFRSEAGASKWWDAAKLFLKYGLAPYRAVQLVKSAV
GTFLRLYETPYFPFRSLTQRAFELGLLRLTGVTGEQYLRDNNINPDFVRDLMQSATRVNYASNMAYIHGLEAMVSFS
TEGAVAVAGGNWQIFEKLLQHSGAVYYPNTSVATLAFQKGADKPGSAPKYLISTKSSGSTSKAAKLPTAFDNVIIAS
PWQFSGIEAAKGVIKHRIDTIPYMKLHVTLFTSPFLLQPSFFGLEAGTKAPSNVYTTLGKDEEAHEGPEGVGRTGFYS
ISTLRTVTNPKTQGKEFLYKIFSAEPVNSTFLSDILGTPVPSCFICNETTGEVEPISWYYPHWFYSYPIESPRVTFQDPIV
GRGLYYTSGVESFISTMETSALMGMNVARLMADDFAGVTRSRGGNKDTASQDTSPRPPREDFWDSMDSEMGA
SMNFPGADEL

>QLI66577.1

HVAKSAPKDATCRKTSVAILGGGMAGITAAQALSNNSITDFVIIEYNDRVGGRATQTNFGKKEDGSPYVVELGPN
WIQGLGRPGGPENPIWTLAKKYNLKNTFSDYTSMLTYDETGYTDYSDILDEYDEAWTKASVRAGRMLAENAQDET
TRAGLAMAGWNPKHTDMKRQAVEWWNWDWDAALTPEESSLIFGAASDNLTFHQFSDHNNLVIDPRGYRHIIE
EESNTFLNKNDNRLLLKTQITNVTYSDDGVTIHNSDGSCISAAYAICTFSLGVLQNNAVAFEPQLPEWKRVAIQKFS
MGTYTKIFMQFNETFWPTDSQYFLYASPTTRGYYPVWQSLSTEGFMPGSNIIFATVTEEGSYRVEQQTDEQTKDE
ALEVLRQMFPNVTVPEPLAFMYPRWTKAPWCFGSYSNWPIGTTLEMHQNLRANTGRLWFAGEATSAENFGFLH
GAWFEGMEAGSQVAALLKGECAHVYNGAECGGRVHYETLRGTSPLENYNVLNGWASSSF

>QLI66630.1

APAAGPPASTQGKEDVTKEVGKLAEEACPRDQLVYSSRGGLLLRQEDCKRYADKCLQGGIIEPAKLKECINDARIKSK
KPAQCPKEPAKEA

>QLI66646.1

NPAMLRKRQADQGAKKALETYCGNAGFEAGDVLEADEKLQGTCRKKDGAKKEGPSVAAATIFCDSVGREFNGAG
ASYMKADYVIFCGSKVANKPDAAKNSGLALFNQIIADAGDDEKLLNKTLGQLVSEAPSKLVDMYNIIRAPPSTAKLG
LGGPARFAASAASFLLTAIPDNAKPGGLLGPDTALGRWLRINPIYGKAGTQPSPFIDTDPSGVTLLPQTQKCIAFKDE
SRWYWSKKVKTCESWHDVDPCDTEYAYDVAKNEGKTRETRCKEEANPPKEKTPAELEKEKEDKLREEEKNCFAPR
MTCSNANGKFLYCMDERDSEECKRNQWHPGQGHPRKE

>QLI66648.1

QHHVFSTTCAGKTYTYNELAGYGFVRSDALDKFGDSISLGSSIAITSWSKNGPKYQGRLYGLPDRGWNTQGTINFQ
PRVHEFLVTLTPNTSASAAHPSPPNVAFVYQNSTLLTGPDGTPMTGLDADQTGGLQYPGFPLLPAATSAGDGFGG
QGPGWKRVALDPEALVLAKDGGFWIGDEYGPYIYKFDKNGRMTAAVAPPNALLPLRNGTVRSVFRLEGAVFGLT
RRQCGSFSSNNPPAYNLTKKPIPSNPTQGRQNNQGFEGMTASPDGQTLWALLQSAAVQDGGSSSKTRQYTRLLQ
YSTEDKQAVVYQGEYVVPLPTFKDASGKTAVASQSDMHFVSDTQFFFLPRDSNNGGNLSSWESIYRHVDIFDISNA
TNVKGPAHDAFNASIASTAGVLKSDITPATVCPLIDFNVKAQLSRFGLHNGGPRDGGLLNEKWEGIALVPVENEDG
KHSAEEYFLFASSDNDFITQHGFINFGKTPYADKSGLNLDHQMLVFKITLPKGSRPLVG

>QLI66649.1

YFLGPVFNTLRPGRHEQPRIAVSPLELEQFARTEKRADSVGKKLTRIPPEDLEETGKILAKALKNDPQVKKYLALLEPLI
AAASKTTKCYENYKNSIDAVIKVCNDKYAKVVDLKNRILSLASMPGFRTSLLFWQKLPNVTHSRESLQGDATKARDF
IDAISGVNKVLDEANKWKPIAEQLHRYHEHLKSLRKAISDAEDCYEAFITKNETERLESPEATSEVLKPCADQYAIVKD
RRHELEEFQNGTLKVSGGKAFADLELANLPALEEKTAALETDRARTAELVWEGSDEVRQLNATIFDEINLAFNNLTV
ETVRNLQKYVLLSETKAGKPAESAIEDAKKWSEQFLAELSAMPDAKLDEIVSDALSIAITADVVDKLLDPLVQVSTWL
LDRVHK

>QLI66652.1

DKSTQDASGIGERAADAQFDPNTWYRLTNTYLGYSQSLDVINDNGPNSSGTLTMSATARYSGQFWQLIPQSPGV
YKLRTLFLGPNRVLDVWGDDKTKPHLAEKGNYSGQLWTITSWGDGTWRLTNAYSGPGLHLDTYSGTHEPFMGS
GDHTGQHWHITAIERIS

>QLI66658.1

IEPREGHCGGDNCARQVTGTRDGLTAITSRKNDCSNFMKTTVVPEATTVTVTITVDADEPASVTKRDIEYRAATEA
PTAVPAYASSCNNPGKYSSACSCWGITAVTVTAPVPTKTATVTSTADSCEDL

>QLI66660.1

NPQTAEISIQPISSSHPQAPLPFASISYDLAALDSSTITSYDAPDIPDSSSLVRIGVYDPKVKSWLSGTTVASTENFAKG
YSPTITVSVNSRGDVLSAALKGVQIDAGQTRDFGPKVVVLTEGRGPQPVLNKPVVVSKDGKKVEEEVEKTFLQKYW
WMIGIAVFIALSGGGGEQGK

>QLI66671.1

RTDLVGCTYFDTVSAYPGGQPFATRIWYVPGSGELCEFLDCGGGRAPPKTTVPGCAAYEGTETYSPSFINPKTLGHA
PATVSPAKTTSSSAAGTPTAASSPGITGTEVPATTEASAWSETGSKTAMVTSTPSGSGSRGAAMTNTTKPTTISSTG
AAAGLPTAGPVVLGSWMAAGVVAGLGLF

>QLI66676.1

GPIRRAYNVIPRDETTAPTGVPDSNRSNPLTTGVPVASTPGPNSGPPTPTPSASASLPDSLHPTTESTAHPGQSTDT
PTTDPLGAVPTSGSTTAVLGSGQTTHAAGESSTNAATGPALSSGGHSISESTGVATTTSTPGAAPTTVPGTSPSSVF
GVGPTNTIASSPATTASTFPTAVLGTDPTATGAVGTSPTAILGAQPTSVLGLEPTTTTSIAPTPTAGTAQTAVVTTPTT
TATASSTITAGDVGATGLPTTTAPGLLATPSTLPTTEAASRTTTTSGGVTTPTSNMIFETTSRATATTGTSSVPSSLTIP
GSGTDTGTGTGGSSSPNSAPTNVLGPGTATAISDLTSQTSSQTGAPGSGTTTPATGTLTSQSYSITGPTTTPIVTTRQ
FPLNTTVASLVTGGGDLTTPLGASATTSGAQFITSRENNSISTTQSDLFNTATTQSTTDQYGSTGNTLGSSATTTSVK
EFITSSQSEPTTSASSTDFSTSSAPSNGQTTQNVPGSTTIGTSEPTATVSTLTSGPPQTSNTGDASNPTGLPTVTLPGL
TTTSSSSTEQQGSQSTGTATSSPTTTITVTPTGQPDSKVPTAFSSFPTATTTIPASVYASNLAQAKDLNSVFSSLTPQS
SCSGSQTACISGQLGSCSNGAFNLDACPQGQKCYALPMTNVRGAKIGCYDPAYAQGILGPEQSTGPRSMRTTFITE
TRRPVVTRTEVRTVTNKSSTPTLVLSPQPEVGGSSSAQSESQPTPTASSLSAPTLISTSTSTLIEYTPSAPSASTTTQSSL
PVTTIPILSPSTTTTTPLPEATTPVLPSLETTTPLPTPTTTPSPSPPTTSSSIQPTQVVITQTFDKPTSLPPPNSTKLFLRPL
DDGTTSTTASAPAAVTRNAGGPDADPSMTTIVNNGTPTVSVFVTVTVTDTQKETVTVTAHN

>QLI66678.1

ANCPSFPSSLVEYSSEFKQPTPPAVKPEFQTHFVQHKWNQNLSHIQTGYMYNSPAKNLVRVDETFEDGLATSVFNF
ANVTEDARVDNTLTSVFKDFAHPQVWRGYVNTNYPLIGADFLAKAGAVFSGLVERDFIPGRVASWSIMYQGAIPV
TVYVDGCNVVQGYDYFAPIERTRVTTSFFNTRVGKVDI

>QLI66679.1

ESDSSSSLTSVITKLPACAAKCFGGALTQTKCSLTDQKCICSDQVLLETATACIMESCTAKEALTVKKLTSEGCNAPIRD
KNAAYIRVSDVLGVISGLFIFQRFAFKLWAKQTFWWDDWFALATVICGSPSTIINAYGVGVNGIGQDAWTLNFDQ
LYNFGKFFYIMEVLYFFEIALVKLSLLLFFLRIFPAPAVRRLLWGTAAFTVVYGIVFVFVGIFTCSPISYFWTKWDQEHS
GKCMDINVIAWSNAGIGIAIDVWMLAIPMWQLKGLKMHWKKKISVAAMFMLGTFVTVVSILRLRSLVKFGTDSL
NPTWDFFEVGLWSTIEVNVGIWCVCLPSFRLLLVRLFPTLGGSTARSYAGYDSSNNRPSEKNNRSAGLGPSATATS
GVDDWQNRSSPVGNQIAYHKSYAVEVSDLDEVALMTLRDHESRTAASSSRGSA

>QLI66683.1

LYPGGPRLPGSVRPVPVPDEDEEPAAAQISSNIKAYNMSVPIDHFHNETKYQPHSNGSFNLRYWADVSHYKKGGP
VIILHSGEFSSEGRLPFLDHGIASILTQATGGVGIVLEHRYYGTSWPTDSATTENYRFLTTDQALADTAFFSKNLKIPG
HEQLNLTAPETPHILYGGSYAGGFVAIARKLYPDVFWGAISSSGVTVAIDDYWQYHESTRYFAPGECSPTIQELTAII
DHALLKGSPRDQLEIKEIFGLRDLFDDEFASYLSDQLPSLQGTNWDPDLDDLGFGTFCAIITSDSLLFKSTEYLLERVRY
HVEAAGHAHDSSKPLTMRMLNYIGYIKDNVKRDTRRCRGKSLRECLSVRYEKSTTEINENTWQRSWLYQTCTEWG
YFMGGASTPKDRLPMISRALTAKFASYRCESFFNIKSRPNVGIINKHGGFNFSYPRVALIDGKQDPWRSAGVHAIGL
PSRQSTPSEPFELIDWGVHHWDENGLDVKSLGPDRWSVRPKAIQDIQRDEVEIVKQWLREFEKPRHARSGLEL

>QLI66687.1

QQVYVPAGGPTPRPQCPCSKVKEPHYSYQPFSFTLTETVRYATSVPAPTATTTYAPPPDAVSALVPSLSYATWGNW
NRNAPVKANDTNDPYGQAAWTALWQHADPPNFTERALYSTTVSPTPVATSELVLPPRDYFGPTDCYDFPANFSF
GVASSASQIEGAAADEGKSPSLMDILVRDSRPKSYTTNEHYYLYKQDIERVAAMGAKYFSFSLAWTRILPFALPGTP
VNRKGIDHYNDVINFILEKGMIPVATLLHFDTPLQFYGGNLTTAADKPEIGYVNGAYQNESFPDAFVHYAKVAMTH
YADRVPIWFTFNEPLLYSYNAKSIDHVIRSHARVYHFYKEELRGTGRISLKFNNNFGVPRNPNSEADVYAANHFNSI
QLGPFCDPIYLGKDYPESFKKTFPDYIPLSKEDLRYIGGTADFLGIDPYTATVVAPPVPNDSNSVLECAANSSSTFRPY
CVNQTSVTVHGWDIGYRSQSYVYITPTYLRSYLNYLYNTWKTPVAITEFGFPVFAESEKALSDQLFDSPRSIYYLSYM
SEVLKSIWEDGVEVIGAYAWSFADNWEFGDYSAQFGLQVVNRTTQTRYFKKSFFDLVDFMRARGA

>QLI66689.1

VPVAGGVDAGIWEFPEAVGFAPSCGGTLLNANTVLTAAHCVISSIAYYKGTDLSKSSVIAGTIFPKDLQYATIVGISSF
HVHPEYNRTGIREHDVAILKLSTDIAEDEWGTIRYARLPQWQSDPQPGSDTTVVGWGSTDTKGTRPTQLQKVVVP
VVDRGTCSKMLGRVPESTFCAGYKDGGKDACGGDSGGPTYGPDGTVIGVTSYGGKCGATYGAYARVDIDLAFITQ
YM

>QLI66694.1

APAGKVRSHRVRNFIYVVPDGFGPASQTLWRDYISIMNKNGTELRPNSTATDLDGIMIGTVRTQSFDKFVTDSAAA
ATAFATGHKTHNTGIGIDSDVKPVASILEAAHLEGFKTGLVVTSRVTHATPAGYCAHVMNRSLEVEIASHQIGQKHP
LGSVVDLIMGGGRRHYQTKRPGGNSTIDLITWAKGQGFSYAGNKRELEVFSRDLGKVPLPFLGLFADSHMAYELD
RDNEKEPSLLQMTQTAIKTLSDATTAGRDGRDGVSRPKGFFLMIEASRIDHAGHANDIAAHIHDIAMYYQVMSFL
RKFVTENPDTQILSAADHETGGLTLGTGYNPAMLERANHTSEYLERAFALYQGPDRATYLRNVILPQYGLVSASDK
DVKKYLDVLKRDGISEMGSVIRRDFAKSAGITWATDGHSAVDVPLYGFAVGDKAYSQMKEYLGPHTDNTQLALY
MESALGVNLNKATEALRENWVKFEGYGGKGGR

>QLI66721.1

TSADGVFEFTPNLVPLQENAGTDKLFPMGDCFGFKLEEATIDGMQEAMRKGTLTSVKLVTCYMTRTFQTQQYINS
VMQINPDALAIAAQLDDERRQGKVRGPLHGIPFTVKDNIGTKDNLETTAGSWALLGSRVPRDAYVVAKLRKAGAV
LFGKATLSEWADMRSNNYSEGYSGRGGQCRSAYNFTVNPGGSSSGSGVGVAANCIAFSLGTETDGSVINPAMRN
SIVGFKPTVGLTSRGGVIPETEHQDSVGTFGRTVRDAVYALDAIYGVDCHDNYTTAQRGKTPKRGYAQFLTTKETLK
NATFGIPWNSFWVHASAEHQRTLVSLVKLIEDAGATIVNNTEITNYETIVSPDGWNWDYGTTRGFPNESEYTYIKV
DFYNNINKYLAKLRNTNIRTIDDIVKYNLDNDGSQGGNPWPLGNPAWFSGQDGFLASLETKGIQDETYWQALNFC
QSTTRGGIDDALRHENKMLSGLLVPSNVAQSYQISAQAGYPAITVPAGIDDESGMGYGLAILHTAWAEEELVRWG
SAIEDLTKGTQYARTLPKWRGYMQRNLPVPL

>QLI66728.1

ARPLGPGEQTPETLLKENAQLDINCKLENGGVQCATDRGSNPRKENLLQLCDQIGGCVCGPLLGNRFGRKRFGGN
DSWFDFFCSSSQKEQDI

>QLI66754.1

TPQAGSAANLVPDKYIVTLKDGISANDFNFHLNWVRDVQVARAGRRRGLNFRGVEKTYGVGNFNAYAGHFDEHT
LEAIRRNADVESVEQQQVYHLHELTTQKDSTHGLATVSHREPGATEYVYDSSAGEGSTVYVLDSGIQVDHPEFEGR
AFRGYNAVKDATDEDVQGHGTHVAGIVGSKTYGVAKKTKLVDVKMFHDAGSTNAIILDGIEWTIKDITAKQIQNR
TVVNMSFGGGNSTALNKIIKTAYDAGILCVISSGNMGVDASDWSPASSPDGITVGAIDANWRLWDHSNHGPVVH
ILAPGVDVLSLAPGNRLREGAELLRRLLMLLGWPPIWQLLKTSTLQRS

>QLI66759.1

LKDLANCDPLRKDMRNDPIAQKSCPKKTRTEPSKLTVASDCPPDAAFAGKASFDFTTANWDNVLDFWAVDEATA
NDKKRLDFDTDGNGVAMGMWKPGDAPTLVSSKYLLFGKVSVTLRAAKGNGLITAVVLKSDSGDEIDWELLGAYD
NQAQTNYFYDGQALFNTYNDTYDLAASSFDALQKYSLEWTDQFLSFSVNDTIRKVWYVGEIPAAKWPQTPMQVK
LGVWSVRNDSDRGEIAWAGGVPDWGSAPYRGYFQSIEVEDYTGFCNQTDGYVEYQYDERTAGWQKIRIAGCQS
RPGPELPAPSPVHTGDATATETGETGGPETTAADDDNGGAGLSAGPSSALAAVLCLVWFLVL

>QLI66763.1

APPAKPKTAAQSKAPWNLQAISHRSAPTRLNMFRNSDYLYTPWPKDKTLYAYVLDTGIRTTHQEFGGRAENFWT
AFKTADNQDDFEDSSGHGTHVAGIIAAKTYGVAKQARVLSVKVFGPNGQVLTSQAILGFTFAMNDIIKKGRQNSA
VINYSGGRKFSMAWNTIVERAFNRPNGPILTITSSGNDAKDAAGASPACADEAITVGSIRSDWSVAPSSNFGCKVN
ILAPGGKILSLSNTSDVATKTLSGTSMAAPHVAALALNAMAVFGKSSKDVLEFLTQTATKDKVKGDLKGSPNLLANN
NNARQRA

>QLI66779.1

NNIYPRQTAAPSPTSAAAPQITAVTDCHLHGSQVFCLAGATEYLVHTTPTQTTDIPAQFTGCHSHGAETFCIAPNG
DDVQVNVPGAEGDAHQGGDQHGSDSKDSGHQHCHFHAGVEHCVGGDESENAAPKCDTTPRDYNIGLRVGLLF
VIMASSALGVFGPIFLHRVLPRRLSTIFTLLKQFGTGIIISTAFVHLFTHASLMFGNKCIGELGYEGTTAAILMAGIFLSF
LVEYIGHRIVLAKTRSTALLTREKQAEALLSTEVVSILVMEAGILFHSLLIGLTLVVAGDSFFITLFIVILFHQVFEGLALGT
RIATIGSSADVHLLPPAVNHSDRAVENDTDKSVNSPTEETADASSTFEPPTLSMKKKLGLASLFAFVTPIGMAIGIGV
LQKFNGNDRSTLLAIGTLDALSAGILVWTGVVEMWAADWMTGSHGHKAELADADMLTVGLGLFGLVAGMVLM
SFLGKWA

>QLI66780.1

VANWTLQSWDVIVVGAGTAGIIVADRLSEAGYKTLLLQQGSQSYGITGGRERPGWLDNTTLSRVDVPGLYSSIFAG
GSSLLCGPGQVDAFQACTVGGNSAINAGLYFQPPASDWDDFHPRGWHSADVANATARLLKRQPALTSYSQDRK
YYLQSGYEAVKKWIVEDAGYQDVSLLEQVNDKKRVFGRPVYNYIDGQRGGPTRTYLQTALRRNHFHLQTGVQVE
HINHVRGVASSVEVALDSGEKKAIQLTKRGRVVLSAGAMLSPRILMLSGIGPQDALEKLAAKSATPYNASSWMVQ
PDVGRGLFDNPNTFIVLSSPDVKSYVYKYDDPVPADRDLYLESRSGPYSFASQTSVFWTYIEHNGTRSGVQGTVSSS
GYRDFVGNNTITLNIYGTSGLLSSGRVELADDGNFAAKPSAGVYYGHPRDADTVAEFIHSLFQHLPPSTPTSPAKEG
LTPLNLARNSTLQEIRRYITDPNSPYAVGSVQHWSSSCRIGKCVDVDTKVIGTQNIHVVDASILAPLTVNPQFGVMV
AAEKGSERIIASMKNVTEGCRGRRRV

>QLI66781.1

TQVDPLTYVNPLIGTTNGGNVFAGATLPYGLAKVVADVDGQNTGGFAMDMSNVTGISSIHDSGTGGNPSLGNFP
LFPQVCRSDELNNCQFRIGDRKVHYRASSVVAEPGLFGIELASGIRVSMTISEHASLMKFTFPKSKGNHPLIMLDLTD
LWQSRQNASILVDEKSGRMVGNGTFLPSFGAGSYQLHFCVDFAGAKIHDTGVWVNNRAGTEPKQMYITRGFNLF
YLEGGGFARFSPGPDNTVTARVGLSFKSSEQACGSAEREIPNPLQDFDRLVKTAKDAWREKLSPISVKPGGADKNLL
TSFWSGAYRSFISPQNYSGENPHWDTGRPYFDSFYCIWDSFRAQHPLLTILDPIAQTQMVQSLLDMYEHEGWLPD
CHMSMCKGWTQGGSNADVVLVDAYAKNLSSDIDWELALEAIVTDAEDEPLEWSYHGRGGLQSWRKYKYIPYLDF
DPLGFGTNSRSVSRTLEYSYNDFCLATLAGGLGKHDMREKYMHRSMNWQNLWKEDQTSVIKGKDTGFKGFFQP

KYMNGTWGFQDPIACSALGSFCSLTSNPSETFEASIWQYLFYVPHATSSLISLLGGDDAFISRLNFFHESGLADISNEP
VFLTVFLYHYAGRPGLSAKRAHQYIPSSFNNTNGGLPGNDDSGAMGSFLFFSVMGLFPVAGQNVYLINAPFLEEVS
VKSPVTGKRATIRALNFDSAYKNVYVQKVTVNGKPWTRSWIGHELFTEGWTLELTLGNTESDWGKKAADRPPSW
TS

>QLI66786.1

DVPSDCPAYDNYAAQRHPPYSGGKWKYPYQRPEATCRSYEVPEVEMTLEKAKGMIEDGDLYRLFLNTWPNTVDT
TILWHGRALDNADEELAFVTTGDIHAMWLRDSANQLQSYKPILNITSHNATNNIASLYRGTINLQSRYIRKFPYCNA
FQPPPDSKLPLANHKRGLLAKRGDTVNPPYDPSVVWECKYELDSISAFLQLSWDYYDVTEDTEFFGKYGWADAVK
TILKLANDMMTGTYTEDGHVNKSPYQWFRDSNSATETVSNGGTGNPVAGNIGLVRSFFRPSDDSTIYQYFIPANM
MFSRFLEACAEIMQTINKDTASEMLTMAWGIESAIEKYGIVKHPKFGDIYAYEVDGFGSHNFMDDANVPSLLSIPHI
GFKPNTHTVYRRTREFVLSRSNPYFGFGPVLNSTGGPHLGPGMAWPMGVIMQTMTSSDDDEIVHGIKQLMGAT
SGLGLIHESVDTHDDQRWTRSWFAWANGLFGQMILDLLDRKPHLLAKSYQ

>QLI66796.1

MPARRAVTAPVADHDKAHDIKEAFEISWNGYYKHAFPHDTLHPVTNSYADDRAGWGVTVVDALDTAIVMNSLKI
VQPMLDHIAKIDFTTTAEANDAISLFETNIRYLGGLLSAYDLLKGPYKHLGADAGKVDALLKQAQSLGDSLSVAFDTP
SGIPDPTIFLNPNKKNSGTDRNNIAEIGTLVLEWTRLSDLSGNKTYAELAQKAENYLLHPKGAPEAWPGLIGTWVST
EDGSFLDSNGGWSAYDDSFYEYLIKMYVYDPKAFGEYKDRWVAAVDSTMKHLVSHPTTRKDLSFLSAYRGQQTIP
NSGHLASFAGGNFILGGIVLGEEKYKQLGLEITDSYYETYVQEAAGIGPEGFNWVDAGLSAGNPNNRPPPANQTEF
YEKAGFYNTSPYYILRPETVESVYYAYRLTGDKKYQDMAWDAFQRIRRLCRVNDAYAELTDVSKKNGGSFVDEMQ
SFWMAETLKYLYLTFAADGPVHVQGQGATNQFVYNTEAHPVAVRGR

>QLI66806.1

HSVKRNPVSRISLVQEPVIQAPSHRVHAFSKFDLTFSLQHGQQNIRLALEPNHDVLHEDFSITHLNEDGSVREIVKVP
RSEDKVYRGDAFIERPGIQGWSKAGWARVTVHEDGEKPIFQGAFRIDGDNHHIVPRSDYQSMKDEGDPAVDSAD
DDVMVVWRDSDVVDYYDGHGELKRDLADSSKCNADTLEFNPRFWQDVDETGNSLQSTSLPSLFGRQIDSGGGG
TGMNLLNSIGSSAGCPTTKKVALVGIATDCNYFSAFNDTKVIKRRIIGVVNSASEVYESTFKISLAIKNLTIFDRGCTGT
TPANTQWNVKCSDGVSINDRLNTFSKWRGESKDENAYWTLFTTCATDTAVGLAWRGMLCRAGASDQGSSAQN
ETVAATNVVVKSETEWQIFAHETGHTFGAVHDCTSQTCPIKGPTQDCCQLSREQCDAGGKYIMNPSTGSGITSFSP
CSIGNICSGLKSNQIKGNCLTDNKNVQTDVDIGSQCGNGIVEAGEDCDCGGEAGCNGNKCCDHKTCKFTSGSVCD
ASNEDCCTDQCQFASSGTVCRPSTGECDVLETCPGDKAACPVDKHKNNGDSCGNGLECASGQCTSRDLQCQHM
ANSLTGRNNTKACPETECMLACTSPDLPSNQCVTYNQFFVDGTSCGAGGHCSSGKCDGSSTIKEIGQWIDNHKAIF
IPIVSVVGLLIIIAIISCITGSIRRRMHKRRVRKQAVPGMNGSSWPPSSFSGQQQWGGPRPWNGPRQTESSGALFPP
EQQHQQQQSGFYDPPPYPPPPAANADGRWGNQRSLRYM

>QLI66818.1

APVEVEKRDGECPDTATIAYCPYPDGGIINVNIGDCEQIL

>QLI66834.1

DDSAKALETRWGARISNNDFANIRFYAQHSAAAYCNIGVPAGQQIACGNNACPLVARNRVKVVASVTGDLTGTG
AYVAIDTFRREIVVSIRGSNNIRNYITNLIFSWTDCNFTKQCQVHAGFAQAWDEIKVAVNRAITNARRRYPQYAIVF
TGHSLGGAVATIGAANLRRSGLWVNLYTYGSPRVGNDWFASWFSNVQGGQWRVTHEDDPVPRLPPIFSGYRHI
TPEYWLSGGNGGNTYKTDYTIANIKVCEGIASTQCNAGRDVTDINAHLYYFGAIASCAPSSLQLRDADAQDDPLPK
DLNERLTDWSRKDQEFVKSENV

>QLI66837.1

QLFTVNCAPLTFYRGDPIIFPGVISPHVHAIVGGTRFSLNLTNEQASQAKATTCDKHLDKSNYWQPQLYHQRRDGK
FELVEMQGTAAYYIDRACDYAPGKQNCKNAPHAKAPPKGLRMVVGDPTLRTYNKSNPEQRAISHVCLGADGGET
PHLPAKPCPRMRAETFFPSCWDGKNLDSRDHKSHMAFPAVGDYNTGVCPQSHPVAILSVFFEFFYNTGAVKDFN
RWVWAMGDPTGYGLHGDYLNGWADQAKLDRAIETCTGPNGVNDAGCSLNVGPDGPGRSSRQPVEVPPPNEEI
GFNGTLDKLPGDNPVTGQPVQG

>QLI66838.1

QNETCATKGRPAGKVLQGYWENWDGAKNGVHPPFGWTAIQDAQIRQHGYNVISAAFPVILPNGTALWEDGM
DANVKVATPAEMCQAKAAGATIVMSIGGAAAAIDLSSSSVADKFVSTIVPILKRYNFDGVDIDIEAGLTGSGTIGTLS
TSQANLVRIIDGILAQMPSNFGLTMAPETAYVTGGSVTYGAIWGAYLPIIKKYVDNGRLWWLNMQYYNGNMYG
CSGDSYSAGTVEGFVAQTDCLDKGLVIQGTTIRVPYDKQVPGLPAQSGAGGGYMSPSLVGQAWDHYNGSLKGL
MTWSINWDGSKNWSFGDNVKGRL

>QLI66842.1

APPVVIHTWGGPFTVAADAAHDALTNARSVLDAVQIGGAACESNQCDGTVGHGGSPSENCETTLDAMIMDGNT
LNVGAVGALRRVKHAIAVARHVLEYTGHTMLVGDSATRFAAQNGFKEEDLATDRSRDMCEQWKRNQCQPNSW
VGVVPDPKSSCGPYTPLENGASETAPGQDGGDIKGRGHDTISLVALGKDGSMAAGTTTNGKAYKIPGRVGDGPIA
GSGSYVDSLVGGCGATGDGDLMMRFLPCYQAVENMRRGMSPAAAAEDAVRRMVKRFPDIQAGVVVLNNKGE
HAAAASGWHFTYSFRGQGMNKTHVVQVDPINENRNMSIEL

>QLI66844.1

KLPPGPSAIQLLASMLLSRNYIWKQFQQWHKNYGPVICLRIGQVTIISIGDRKAAHAILNRRSPIYSSRPRMVVAGEC
ITKGLAPVLAPYGPQWIQFHKIHTTLLNARRCRLYRPLQELESRHLLFNLLSSNDFQTEFHRYASSLMYSLVYGKRFVS
ADDPELKEIRHMVDVTSQAISFGTWIVDIFPIFNCLPRSLAKWKRVGDDIHNRQTELFQQNTTAALNRPSWNLTKH
CMLEPPVPVSPKEYMFVLAEFIEGGSDTTAAALMVSMLACVTRPAAMHMAQEELDKVVGDDRLPSFDDLPNLPY
MTAFVEEVLRWRSLTPAGVAHAPIRDDTYNGYSIPKGTCIIANHWSLDMDDDAFANPQSFVPERWIDNPKLQGH
SAFGFGKRSCPGQHLARASLLLGLSRLFWAYDITWKQDQGCSPEAVNMINGAVSRPASFEAVFTIRSPARRRVVEE
QLPTETELEPVLDSIHQSICHE

>QLI66855.1

APASGSNASSLQHWLYSSQIDDKAIELLDSPDLIGVQALYSWKSLEPSQDEYDFSTIKNDISRVQAKGKKFWVQLQD
RTFSATNDPVPKYMHTPQYNNGSAPTCDGEACDIDFKVDGWMAQQWNPEVRRRYQALLSALSEELDGKITGLN
LPETSIAVNQSQDNFTNEAYFRGELENAGFAAKVFNKTYIVQYVNFWPDGWNDANNRFTESFDFYAEHGVGVGG
PDLIPFKKAQVSNSYPFIAKYHDKVPVAVVAVQEPDLEELNPRTGMKFTKEEFVDYAHNVLKVRIIFWATSSPWLQS
N

>QLI66861.1

QSISDIPSCALSCIQRAVLSTHCEGTDLKCICKKENFSTIQGAATPCVIEQCGTETAVNKVLPATTNLCAGNADGGSSE
AASSAADPTEMATQMTEAHPTEMAEPSESEAAGDTECHATMASQPCPATPSSPASNGTAPPAPSTPVTAGAAGL
APIGGLAMLAIGALAL

>QLI66867.1

QGGSQKPIQVDGTAFALNGDDVSYRFHVENATGDLIGDHFGGPAEGDTIEAEIGPVQGWVDMIGRVRRELPDLG
RGDFRTPAVQIRQSEGHTISDFRYQGHTVLEGKPALNGLPSTFGKDDDVSTLVVHMYDNYSSVAADLSYSVFPKYD
AIVRSVNITNRGKGNITIERLASFSVDLPWGDYDMLGLKGDWAREGMRVRRKVDFGIQGFGSATGYSSHLHNPFL
SLVSRATTESHGEAYGFSLVYTGSFAAEIEKSSQGLTRAMLGLNPSQLAWPLGPGESLQSPECVAIFSSTGIGGMSRK
LHSLYRQHLMKSKYATETRPVLLNSWEGLGFNYNASTIYRLAQQSAELGAKLFVLDDGWFGVKHPRITDNAGLGD

WEVNPKRFPDGLEALVDKVTALPVGNSSTKLKFGLWFEPEMVNPDSNLYEVHPDWALHAGRYPRSLTRNQLVLN
VALPEVQDFIVDSVSNILNSSNIAYVKWDNNRGIHETPAPYTDHQYMLGLYKVFARLTERFPDVIWEGCASGGGRF
DPGVLQYFPQIWTSDDTDAVERIYIQFGTSLVYPPSAMGAHVSATPNSQSGRNETIEFRAHVAMMGGSFGLELDP
SHMPEQDKAKVPDLIKLAEKVNPIVVKGDLWRLSLPEESDYPAALFISEDQKQAVLFYFQLKPTINNSWPVVRLQGL
DAKAMYKVDGIQTVSGATLMNRGVSYNFEAGYSSKVVFFEKQ

>QLI66871.1

ATINHDFNVTWVRANPDAAFERPVIGINGKWPIPRIECNIGDRLIINVNNQLGNQSTSLHFHGLFQNGTNNMDGP
SGVTQCSIPPGSSFTYNFTVNQPGTYWYHSHNDGQYPDGLRGPVIVHDPEFPYKKEVDEEIVLTLSDWYHDEISTLI
PKFISKTNPTGAEPVPNAALMNDTQNVTISVQPGKTYMFRVVNIGAFAGQYLWFEGHKMRIVEVDGVYTKDAEA
EMIYVSAAQRVSFLLTTKNDTSANFPIVASMDTTLFDVLPPELNYNSTGWLSYDKSKDYPAAALVDELNPFDDMTL
EAYDGMELLPEPDRTVELNVIMDNLGDGANYAFFNNITYALPKVPTLYTALSAGEMANDPRVYGEYTHPFVLKKN
EIVQIVVNNLDSGRHPFHLHGHNFQAIYRSNESAGTFADEGGETDKTFPRVPMRRDTMVIWPEGNMVLRFKADN
PGVWLFHCHIEWHVVSGLIATFVEAPTELQKTLSIPPTHLQACQKGNVATEGNAAANTRDLLDLSGQNEPPAPLPA
GFTPRGIVALVFSCVAGLLGVAVVAWYGFSQAVEEAPGAVTSIIRNADISESDNSQIVSSSTNAGRTTT

>QLI66874.1

MSIATEPLITTELPIVTSESQFTATVVSPPLFTETVPEDNGTAVTISGTSVPFPILSTASGSPVPSGNSSGILPTGTGGTG
GTGGAGGTGGTGGTGGTGRPPVPTSSHTTGPTAGAALTTTQNSWLAMGIAVVMASLIL

>QLI66885.1

RNLVPKQARATASVALADDGMSPRPTPPPGWDRFEPKHLFGRATSTQTPLTMMIAPDNTCGWISGSYAVPYSCG
LGATCGLVQAQKTFSGMIMCYNSVAFNFRFACVDYNMYQSSSCDHLCAENTQIVKCTNKVYPYCNTVAFPGNITD
YWCNSVSYSTAQGAYTTYAGQTEVRSYSRFVQTFSSTLPSTATLGVPTATGADAGASASATQTGNNGNNNNGGG
GGGDSGSKTPVGAIVGGVVGGVAAIAIGLLALVFFLRRNKKNSGKSQTTSTTDHSPMPPNATPGAPMAQSYYGGP
PLPQGYPPQKPYPQNTPSPPVGYYSPGGGVDPSSPTQSQMTDPRMSHLTTSPAPTWTNSYGTTPPVQQQPAGF
QPMGGTPPIQGHPQSQTQPQSQPQPQSQPQGVVHEVPAQTGENHRGHMHELA

>QLI66888.1

WGKLGHATVAYIAQHYLSPETASWAQGVLGDTSDSYLANIASWADDYRATAAGKWSAPLHFIDAQDSPPTSCNV
DYNRDCGSKGCSVSAVANYTQRVGSKSLSKDNIAQALKFLVHFTGDLTQPLHDEAYQVGGNNIKVTFDGYQDNLH
ADWDTYIPEKLVGGGALTNAQSWASDLIQQIASGSYQSQAADWIRGDDVGDAIATATRWASEANTFVCSVVMP
NGSAALQQGDLYPKYYDAVIDTVELQIAKGGYRLGNWLNNIYKSTVAKRDVGGVGGAEVKPAELPDLMGYDFLPE
PRPLSRAQLARAAMEGDCCGKGKGREGHQH

>QLI66922.1

DPKPQYQPLPSLRDQAALQDEWTAQRKASIPRLLQKHKIDAWLISQREYAEDTVFWTLKSATQFSARRRTTSLFLAS
TPDKSPTAYTWIDNTPRVWDELKALLEKHQPSSIAINAHPEIAFSSGLHAGEYEAISTALGEEWTSRFVVNPLLGVEY
IGTQLPARISWYRKLQETAWAIISEGFSSSVITPGKTTTADVEWWMRDKIQSLNYTTWFQPDVSILSEDSPWPQDN
TDERAIQHGDYLHVDFGVTAMGMNTDTQHLAYVLFPGQTEDNVPQGLKDGLKKGNQLQDMTRKHMKPGATG
NQVLKAIREEMTQGGLEGKIYCHAIGDWGHSAGAVIGMTNLQDSVPDIGDLPLLDHTWYSVELLADHFVPELNAS
LKFPLEEDVYWVEDGKGSGSFEWVYGQQTKFHLIHTTVGDKNGVTEEL

>QLI66927.1

AVSLRRQASVEQGSLALVSTSPLEFKYTTSEPNPRNWVGLYYAAGGGPDNGVFNQPSIRWSYAPEAQGSVKFDND
GLGPGEYKAYLLADDQYKSLAAPVQLALGESAKYSGSISVDYSRTPINIKYTTSQPNVKNWIGLYFAQGGGPVNQVE
DQPSLTWEWAPASVGEVTLSTKNLSPGEYKVFLLADGGYKWLSEPVAAQVRNAEPFSFIVKDITTKNARQGDKFEA

SLGNLVSQPGDKDTKFNIVGGGDWAAINSSGVITGTPSSSAKDTTLSVEAQDKNGVTSSISVHIPVRPAGSSLVDNL
RVLSFNLWHGGTQVSNYHEKQVRFLADKNVDIVGLQESTGGHGTRLAHALGWYSWQGPDVSIISRYPITQVYPAT
SVSGSVRISLDGADSDIIFWNAHLGYDPYGPYDFCFDKMSVSRVMEREAQSGRTPQIQEMTGKMGDHLANADN
VPVLLVGDFNAPSHLDWTEASKSQHCGIGQVAWPTSKYPTDAGLIDSFRVAHSDPVATPGITWSPIYLDNNGRPE
PLDRIDFVYHKGRKLAVRDSEAVVVGNPTAQPNHGNNEWTSDHKAVLTTYSVQA

>QLI66929.1

TTDFDYVIVGAGTAGNVVANRLSRYPNISVAVIDPGADQRSNPNVTNPMIWLNNLGTSTDWAYKTVPQANASNR
VITFDAGKGIGGTSLINGMTYIRGHKAQFDAWEQLGNPGWNWAAMFKYYKKVETIFPPAPAQIQVGASIEPRYH
GTSGELHVAFNPALENGPLYDTLRDSWAVMKEDVNRDVNGGTTK

>QLI66934.1

KPGGKNYTYSIASRGESSPSESFGISYTPYRSDQNCKSQQDVDDDIQRLAGYYSFVRVYGTDCDQVPLLYSAAKKHD
MKLFLGIWNPSSVEDEANKIISGVDGDWDMVHTVSVGNERVNNGEASPQDLIWSMSKARSILREAGYDGPVVIV
DTFTAVLAHPELCDESDYCAINAHAFFDGTIAASQSGEWLKDTVSKIQSRIAADKKVVVAETGWPMQGGTNGLAV
PGLDNQKVALDAIREEFVDRPQDVILFSAFNDLWKQKNSATFEADPFWGIGGAVSSCDE

>QLI66942.1

GPTATEQVFHAIKRDNNPLGIDWSPAPAPEDGPAFSARALRDTKYLPAQIGGIVAAYGVSLVLVAITLLSLAKKRRQ
HIQAGNDEADFDGSKAVYDSDSPSASFPLNQFNPQSHLRSSVVPNFSYPSPINTQFNDNTLPPLKPYIYPSPISSVGH
PGVNPSVDQSVVAQDRKMAQNQLEEMYKHVMEHEDAKQRGIVLDTPIYPNQQRTSTSDKSATTLSGRDRAKPA
SLNLSAEHEGKTQSRTSSFFSSLRSPRKKPIKGVHISPPIMTPQSATFPRYESREMNTIPHRNYAPPPPPPIPTDQTAF
GAQVRSSGAPPTPSMSPGSVQSIDERINSQLGPSNYPNTARSEVEPESATSQTPLVGLPSSPKAGATFPSLPSSPKP
GVRFQRANAPSAVRTGGNLPLRAYEGALASPSATPRTTKQTVFERRGPLSPTTGKTPMTAGAVPYSPYQPFTPLVPI
TPSLVTKEDRKRMRRMVPKTPTTELVKSSEDMW

>QLI66945.1

MFTNTTDGQETAGKAVSNTLPAVVGDYELLGCSAANDDFKSFVQVASTDHMDLDFCSASCHSKFMAVSGKDCFC
GEKADAAQKLDAGMCNSPCPGNQAQSCGGNGGGNGRRDGAAPMKAVSMYVRSQAAEAGGAITKTITTTKVAT
VTKCSSTVTNCPVGKPTYVVTKVTETCPRPTEAIEWHKKKITCYGGYCAPEIPRQSEKQRVLCDGAKCHAESCYNKD
WSSLVLCKGDDCKWSTSDDDRWFEKKIVCFDSKCAWENCHGDECHKKFVCRGEECKHESCSGDDCHKKFICDHK
GDNCKLAPPCNGKDCPKPPPPCHDKCRKAQPPPPCNGDSCRAVPCQGNDCVKPVRPTGISRGTGTGTSRNTGTG
RSTGTGTIYPHPIPTGPPIVAGAGNVVANVIGAAAGLLLVL

>QLI66948.1

LSLHRHGHKLQKKQDVVDWVTVFTTVYVTEGAPAPEPTVQTNDGIVTSQHVVVTPTPKIDAPANFQALLSSSAPA
PAPSPTSLVTQVKPAPSSVSQVKPAPSSGSGDGSGSSTGAPFSGKRGLAYNDPNLANLFGKECKSGACGWAYNW
GDSPGSLDNSYSYIPMLWGNKMDFFDRWGTSCSKANGAKAVFSFNEPDNVGQADMSAADAATNHVKYMNPC
DGKALVGAPAITNSNVPGEGRDWLKQFVSECETKGCKYDFCNVHWYSPAAQLDQFFEHIEEAHVICGKKPIFVTEF
APTGSDSEIDDFMTKAIPKLDSLDYVYGYSYFMIQPNSLLSSATSLSSVGKIFASA

>QLI66950.1

LRATILADTNRDGRVDVTGKTDVEGKATWTEERGALFLPNIVDTNRRCSQRITSGTTENGLAECHDASDNILRNPK
YLAPLRTVPINNLTSSATGTITISGKYAPEKVRVFHKDNGKWTYINSNYEFRGDALGTGLELGIDARDVRRPNEWDG
RVHVHFNVTDNGETASDQVALRVAPILTHHHLQAPDQVFTVAGRGRPAQAQFAAFIANYTAQAGVTKPVYMFD
ESDVWAQDYFEPGYTSIPGPEGPVYLRVNIRSSQPWRTAGRRVFSELRSDSVGAVQHFAAGTPEAPPTIDSTGNLE
TIPPYSYNGKRYPAGRAVMGRHDNKTPTMMSFLKAQEAQHPIDDLDHDWLSVGHTDEYMQFLPANNSRGWIM

TADDPAYGLSLLKKARADGHGKVQAMSRRHEPYDTEACLPANDIDGVLGLANFDSVNQHCADRIQHNIDIIKRET
GITDEEIVRVPSLFYYNNIEGFDCNRYVARARGAGGRRTARAQTKDIIEAAEAGSHLHSRQEQQAKPRMLALYPATI
NGVVYDDRRYMAPNPWGPVIGGRDILAEAVRAAYDKAGFQLTFMDDWFDHHVLNGETHCGSNVARDASQKW
W

>QLI66953.1

VPTSHDPTLDLSPYPGVVGTHESDFLLRKRQSSSDILIALYARGLNAEARKAAGIEAFHWGIHITPEGALKSEKTTSLF
HVINQEENIKLFQYEKRTINPFRQRVIFARVKVGTLPATVSVDKVDELLSQVQAPSKLENPGDSCVTWAVCGIKKLQ
ENGIIENFDTAGFADKVLEYGEQQTRALDDDEPEFEADEFDIGNYDAKEGKIIRQESVPCKRAGEPCINPNPKEKETE
PAVEKPEDAELIAVAKEKSKENFDGLLEEFNYGSVVKQDKLYGELNARLPEFSAPRVERIAGFASKLGEGALAIGGLVL
YGKAVADVFASEDASVLDKAAVVTSILPGIGCAVQLADDEQKGQVDVTHTALCFTEDALLVSGFWEIALVMQVGE
EISNWIQAENERSKFWDGDLLAQKGAEGWLQNVKRLINHIKGDEFFVNATSQFATYQILTLYQASQLTGDLHATA
KTNPQGIEADIHAHVQPELKRQICSVISDSKRQLQAKLEGIALNHTAKLEREFKNQFLDDWLKAATTPKPIFGITLPDF
ESNTKLIHEQVEKARNSPLQLYEKEVKAAIREVIERLPTPAPCQCDQGKKGGKCEFGGCQSTKPEGHPQDAGGRIYT
ANVQSIEVAKRLRLTDTCQALFAKCQGGEAGRALFCTPGK

>QLI66957.1

WPYERECKPATYRCEHSLRGWDVCSTSGQWVYAGRCEHGTKCKMNHQNGSPYCLPPRHHHLYPEEEFEI

>QLI66963.1

LPSCKNNNNIPPGKMPNPAVSSVAKLLITSPGNISIAEFDGTKFDIKSTKNVSGNPTWVAFSQPLLYAVDENSDTTSL
LNVDLAGNKINQLSSTKGSSGVVHLEFTKDKSQLVGAGFGSSNIDIFDISNGGLKFSNAIKSDDKVGPNTVRQKEPH
PHQSVLDPSGRFMAVNDLGTDKILVLDTQKAFNIVNRVAVEPAGAGPRHGAFFPAGANKATHYFVLCEIKNLVLVY
ALQYGGAKGIEFKLTQTISTFKSVEATPAEAAAGELVLAADNKNVYVSNRLTGGQTDNIANFRIAQNGGELKLELVG
FTSTGGVRTRMFSASLDGQHLFVGNQGGALGVVALKRNADGTLVEKPVASLDMSLFPGELAGPAFVQQVA

>QLI66972.1

TPKPNTRDRLRECPGYRAVKDGQISDGLWRFLEIHDDRARFIDLELAGKPCNVYGKDLPFLKVTHVVETPTRLHVQI
ADPKQEAYQIPELVFPRPKFSKKRQSKKPLLEFEYTEYPFSFRIVRTKDTTILFDSSAAGLVFEDQYIRLRTSLPVNPNLY
GFGEHSDSFRLKTNNYTRTLWNADTPSVPAGWNLYGSHPMYIEHRQKGTHGVFLLNSNGMDVVIDSDPYSAYLE
YNILGGVLDFYFFAGETPIDVAKQYSEVVQQPALVPYGALGLHQCRWGYQDVFNVAEVVHNYSQAGIPLETMWT
DIDYMDGRAAFSLDPERFPLQKMRQLVQHLHSRNQKFVMMLDPAIAVKDYGPYNNGKTWPMSFLVNSSGLPYE
GVVWPGRTVYPDWFAPAIQEYWNKEFDTFFNPATGVDIDYLWIDMNEPSNFCDFPCNNIDEVALLYPPPPPLVRS
PPRELPGWPCDFQPPGTKCDDNGIKPLASPKKPRAVYLYFPEPMLSIPPLYFPLMEEVKPGYRPFFLGFDNRDLINP
PYTINNAWGMLPQKSLNTSIRHSNGLTLFDTHNLYGHMMAAASRRALIAMRSHKRPFIVTRSTFAGSGAHAAHW
LGDNDSSWEHYRLSIRQMLQFNSMFQVSMVGSDVCGFNGDTTEELCARWAMLGAFQPFYRNHNAEGQIDQEF
YRWPSVTQAAKKAIDIRYRLLDYFYTALMTQSSDGTPAINPMFYIYPKDANTWGLDMQYFFGPSLMVAPVQEQG
STSVKIYFPNDVFYDFHTHEQFFGIGQYATRTNQTITDIPLFVRGGQIIPMRARSTMTTTELRQQDFELLIAVGSNGR
AKGVLYLDDGETLQKPPHSYIEFNYKGGRVTSKIRSMDFKTGAKITKITIMGGKRCGPGKSSCSKDLHHPLKKRLSVKI

>QLI67003.1

APAQNTGLKFSAETTHVSSPHVVHDMRRVVRKFLDQATVKALQSAVLSEREENGTVVTIPEPNDEGVDNLYLTEVS
IGTPPQKLMLDFDTGSSDLWVFSSDTSASQVKGQTLYKPSGSSSAKRLDGQKWSIHYGDNSTSSGIVYSDVVTIGG
VSVENQAVESAQQVSESFSSDKQNSGLLGLALDKGNTVRPSKQKTWFSNIMPRLSEPLFTVRLRHQAKGSYNFGYI
DESQYSGPISYTPASTDELGHRLFQSTGYAVGNGNFKKHTITGTADTGTSLLILPLEVVRTYWAAVPSAQPARLPQN
AGYVWLFPCDTTLPDFVFAVGSGRVTVPGKDINYAKNDGSSCVGGIAYYSGLNGLAIFGDVSLKSGFVVYDDGNKR
LGWAKGL

>QLI67017.1

AVVQDLPRQHDASGSADVVVRQTSLIQRATDQSPKGYAPSPVDCPTSRPKVRDGGGLSSQEREWLPKRRNETVA
PIRELLKRIAIPDFDSDAYLKNAASDPTALPNIGLAVSGGGYRAMLNGAGAVAAWDSRSTGSQTKGNLGGLLQSAT
YISGLSGGSWLVGSMYTNNFTSVQDAVNAPQIWQFDDSILKGPEQYSLLQYYSEILDDVDAKDKAGFDRSITDYW
GRMLAYQLINATNGGPGFTYSSIANDPDFSSGKNPLPLIVADGRAPGQKIIASNSTIYEFTPWEFGSFDPSLQGFVPL
QYVGSNFTNGSIPGNQKCIVGFDNAGFIMGTSSSLFNQIIMYIKDGNSRYVPEDIPKFVVDALTTFLNALGDESNDIA
DWTPNPFKGWNTAKNPSANETRLTLVDGGEDLQNVPYHPHLFRERRVDVVFSIDSSADTDSSWPDGASAIATYE
RSLQPSVANGTGFPAVPGKDTFVNLGLNSRPAFFGCDSSNLTAPSPLIVYIPNYPYIYESNISTFQMAIKSDERDAIVQ
NGWAVATQLNSTRDPDWAVCVGCAMLARSFERTRTAVPDKCRQCFANYCWNGTLNETKPAPYVPSLYGKPILSK
NSGAAGQELSAMMTTVGLVVMAVTAFNL

>QLI67030.1

APVPEPTPPGIPSASSARTTLASLQVSTPVDDGTYNRSLFHTWDIIHGKCNTRHVEIPDVVLIRDGEDVKTNRNCVP
QSGIWTSPYDGLVFTEAHKLDIDHFVPLKNAWMSGASEWTDEQREAFANDLTHPQLWAVSAHANRQKGDKSP
DRWKPPLTSFYCTYAESWVDVKGYYNLTISDSEKDALGSMLDDC

>QLI67031.1

MPASQRLERRYDITEKQWSSLQEDCVKAQGTNAVQACRSEGRGDCEGNDVRRKAVTECMVDKSKPKVTEQSRH
DECKPLAIQRVEDCVKNNTEIEGQCQRKAVESYLTCTYTGPGKPSTEQDREYTQIWADKCSKRTQEADEECKKKQD
FGGKCQRQRREDDMKCLLDNKKGHSDNDFIHNVCVAAAEFGANDCFITNNTRRMTLPQCQREQWDAYPACVK
KQEAWISGTKPEEDIAPKPEEDECTAEEDPTPKPEEDECTEEENTKTEPEEDECTEEENTKTEPEENKKAKLEEDECA
SEENTKTEPEENKKAKLEEDECASEENTGIKAEEDKKTMLKEDTATKQDAVTEPKEVTMAKPKGDGVTKLEEDTAT
KQDAVTGSKQDAVTGSKQDTATKQDAVTGSKQDTATKQDAVTESKEVERTSSAFKRWFSLWA

>QLI67070.1

ASITLYLPPVPNPFTLPASTHATLSTLGSAFSAPISALNTFVFHNVTPGSYLADIHCKTDGFRPLRIDVARDADGKETF
QAWDTFRGNEWGNKGEALPVKDGSAGWGVEAKSLGKKMYFVDRPQFSVLSILKNPMILMGLVSMLIFFGMPKL
MENMDPDLKAEFEARQREGPMAAMSGQQQNPLGNFDMAAFLAGSGKKEGGAGAGSVSDGRNEPVRR

>QLI67075.1

APRAPESGALADKAQPRQADGPIYSDDFVFPDDQRLKPKPTSTPVPDDAC

>QLI67077.1

NQRRVIQQPGLIRFPITVSEAPAGTFRRLRRQNNADLTSQSTGFFYTIDLVIGTPGQAVPVNFDTGSWELWVNPSC
ANTSETAICESLPRFTGSTTLVDTNRTGQIAYGAGYAKFKYMYDYISVGGAKINQQIFGVAYDTSVVAYGILGAGPW
VLGWQSPYPHVLDSLVSQNHIQSRAFSLDLRPIESPLGAVIFGGIDTKKYIGGLEKRPIATGPDGSVRYWIYVDGLSV
TREDGSRIPVFDQTNGLLALLDSGTTLTQLPRTMVTEILKAFPSARQDPNDANQYIVDCAAASLAGTVDFKFGNTVI
NVRYDDFLWKQPQHGICVLGVSPNDCE

>QLI67084.1

APSVKERDIFIRQEFVVDTETPSMTDAQGNVVPFDASNVNLANTNAGF

>QLI67112.1

APQTTPVLTMADMGTITPQRVLEIAMGEDLLCKEEQRGRISCESFIRPNKDIPAKDGLSPLCQEKERDCECQINAGF
KDRGSYTCTFDPFHQDLTEELIFSD

>QLI67126.1

AKVEKTLRFTWDKGAPNGQSREMIYTNGQFPGPSLLLDEDDDVEITVHNDMNRNATVHWHGLAQEGTPWADG
VMGLSQKPILPGESFVYKFKASPAGTHWYHSHERMSLVDGLHGAMFIKPKQNMKELWSKISQDPEDIEAMDKAA
ANPQLMVLSDWSRLTSDEYWRVIEESKILIFCVDSILLNGHGELYCPSHEFLISETQPMPQKFTFPDYNVTDKGCFPI
QAEGIQGPWVNESLPEKIPDHMQWGCVPSQGSNYTVQVDAADGWVSMNFIAAAANKQVDFSIDEHPMWIYEI
DGNYVEPRQIVAAAITAGERYSVLVKLDKPPGSYTIRLPDSGATQVISGFANMVYKGAEEVVPETRPYVTYGGLNAR
EDTATRSYTPYNISSDNMPPWPANAPAATADEEFLLVMGRAGAPHLYTMNTKYLYPMDFKADRPLLFYPNQTLG
TEDDNLVVRTKNGSWVDLILQVAVLPGDGMAFEHIMHKHGSKTWRIGNGAGMWNYSSVAEAIAAEPESFNLKD
PGYRDTWMTMFSPVPAGGYWSVFRYQVTNPGPWLFHCHFELHAMGGMSIALLDGVDVWPEVPAEYAIEKSHS
LTVTTDTVQKPLYVKIWEYVKGMLGFLPVSDEMRR

>QLI67131.1

NNLDTLDPCILSCMLQVLPLVGCTGNDDEVVLCGCKIIDRYTIVAPIADCTKKACNIESNDFPQVLKSTQRCSAAFGP
STRLSDITLPRYIPTIRIFSSSSPPGSPVSSSDPSIPPASTTPTRTPNSTPNDSEGTLQTITPTPSSLTTPIRTLVSTVTSNAP
IGGQTDGLTATPTASPAPSSDGPGLSPGVIATIVVSTVTVIGAPLWLIRCYRRRKPRATSSVEENASPKPAEKRNESVI
MGYTELLGSTQMRHEMLSHTPLNAPLPLYINTIAEIDSNPLRKTPEVSPEDVSPEDNSSQAMSFSTTTAISRPEPPQA
ADEEEAQEKTHTEAPEDSSGASDTEAELDMLRKRQKELERKRHFLQQIQEIDEEEARLQERIDELQRQSQSHEK

>QLI67138.1

KCGQTCANNMIAAGKAQELGCKENDVKCLCSNPNFAYGLRDCSRAICSEQDVNQVIEYGVKYCEGAGVAITGGTG
GPSQTGSNGEATQTGSNSAGASQTGSNSAGASHTGSNSGEASHSATGGGEATQSGESGSGAQVTTLYSTETGTD
GKVITTPVATSTIEGGNSGNGDASGSVLTYTTNGSQVVTTLATAVSSPTGTESSGATETGSGSESSTGGSGSESSTG
GSGSESTSEGNGGASSTSEGGAGSQTTGNGGATSTSKGLAAQITAAPGILAVAGLAALLI

>QLI67154.1

SSCIAARCDVANVTCICQHGGYTSAMGTCMFQSCGLSDALFSRNLTSTLCNEPTRDKGLEFRVVNFLLGSVALGLV
VMRLFFKKYLSTSKRLGLDDWTIVAGLILGVPSIVIQVFFLTPTGLGRDIWTLDIPTLIAFGRYFYVMEILYLTLMMLIK
VALSLFYLSIFPGTVIRRLLWVTVAFHVACGLAFILKTVLQCTPVAYNWEKFNGDASTTGHCVDINASGWVNGVIGV
VADIWLFALPLTQLKRLTLHWKKKVAAAIMFLTGAIITIVSMLRLKSLVHFANSYNPTWDQWSVVFWSTIEVSVGFI
CTCLPALRLILMRMYPQTFKSDSYASSSFTSRNLYRRSSISRIVDEGCENDLVDLSQSNLIPAHLQDTLKDNEDESGAR
RGKIRIQSLQTLSLDENIMEGTARDQTAARRSSANN

>QLI67155.1

APYKYGHDQAIISVPVPEHETTQSDNRPVTNVFTAPNDLAPPHEEPYWPAGSNPRKYEEIEGWILFTEQKLQARIH
GNTFWNVKGKLDDKFPVGSFEFDPVTFTLRLPEDYNGRFTGHAGRGEIVLRWEKSGATIAGRSPIGDFSFKGESYID
YSP

>QLI67163.1

VDTVYVTDIEIFTYLAPCASSAISYNVALETMSTRCGDGQAALQSCICRNTSEFNHVASRINSDISSGCGTAAGTSDA
WSASRIMDKYCHPESAVAFATPTANKVYASITEIPQISYLPSCAQSGLSYAVIGEFLSKCPRDASLYAPCVCNTDRAKL
VSETMSRSVRSSCSNDEDVTAAQGFFNDYCAMNSGTTSFAGPPRPPGDMTYYVTALPQFKSLRSCAQSAMSYVV
QGQTDWLCGSGPQALASCVCIKSGMRGKISSTLTSSVKGYCSSTAIDDVTSAVNVLDYYCSAAESKVVATVSESISES
AAASVPSRTQAGSSVPLETGGAGGGGGASNGDKSSSGNDGNKVNKVAVIAASVLGAVVVIVLVAGLVWFFKRRS
QRKQRGQQLPGGDTDARPGEFDPSKYLNHGVAELSTPSHTPRPELQGNAAPLPSELPPSPQHWPKSELQGDAVY
RPDLRPAQPPQELLAPASGYQAAPPIYSSPTNASPHQGIHAAYGYGGPQQTESYELGTRVPRS

>QLI67174.1

DKVPFESMTSPIGDLKALSLRASAACDTGTACGDSCIDIGAQCCSQSEGTFCRVGYGCQRDGCCPLGHTCYGPPSN
TCEGDRVKCGKSCAPGGSQCCSQSNGIFCIAGTSCAGTTQCSGPQRPASGSVSGSVTTTSGSVAATSDISSRVTTTQ
GSSATSTYSLHVAPSIKTTDSGSGSASGSSSATASTSGSGSSSGDSTPTASSASPTDDKKNPSPSPTGAGAINSPSILLG
LLAAVLLL

>QLI67181.1

HMQMSDPPPFLSKFNKFTEQGKQDDSMTSPLLASGSNFPCKGYHSLVGTPQGQSVATWTPGQSYKMTIAGTAT
HNGGSCQASLSFDSGKTWKVIHSYVGSCPLQDGAAFSFTVPSDTPAGDALFAWTWFNQLGNREMYMNCAAVTI
GGGAKIRKARGDTTPFSSRPAMFTANIGNGCSTEESKDVVFPDPGSELDTKSDKPAPPAGSCGTKGSGSGSGSGSA
SAPAPAASGALVNNSNNNAGNPSAPPASTPSPTPVSTAAAASTPKPVGSGGSCRAK

>QLI67185.1

MGWRSPEQKYVDKPLEEALRSPPPSPYASGANPSIFAAALSELQELESEPFCHRIAARLLVNNCQLLDGRNDATVM
TNTGRAARDFVDFFAASLAICDLERANFAIPSTCHKFREPVLANLPAPSKPELHVITREIDDCLEGLARSDSAWSTWV
AYRHKALGFCEAARADGEKDHHIFLHRKLANILERLTTDAEVQVQNRLNELDRMFRESSDNAKTLTAHVAGLNASL
LYFEQVITHSILSKSKETEMVVQKGLNEARSLQQLMEEVLGMMSLREEKHARTLETALEVAVTQINRDAHEVMKM
LTAVAMSSLSLQEQLKKSESQLSVVMRKQERVQEGMEELSLLADLVADKHHSHQEMLQSAQNETAYLLASLEAAS
FSMGNLRASFSDLGSPLAPTDNFARAEAYLRDAASQGANMAVLPEYHLASWVPDAVEFASIAAQSAPYLEKYRAL
AKELNMAIVPGTLLEPETSSGGGLANVAYFIGPDGAVLGRYQKKNLWHPERPHLAADIESPHTAFDTPWGRMGM
LICWDIAFPEAYKALVADGARVIISPSFWLADDGGEGSDLNPNCEKMFLDNVCIARAFENTAAIVYVNSGAPKGST
DGKDGRGNTFCGVSQVAVPIVGRLGGGESMGPAEEMRIFEVDMGLLDMAESVYKVRGDMAREGWHYGPYRS
A

>QLI67195.1

AVSIAEINGNRFLSSFKDQNVTGVTGLVTAVASNGIYLRSTEPDDDPATSEGLFVFGSAVGKQVQKGDVITLGGLVQ
EYRSNNNYIYLTELTKPANIVVVSSGNQVKPLVIGVDTLQPPNKEYSSLDKGGVFGVPNAVTTITASNPVLDPTAYGL
DFWESLVGELVTIQSAHLVSRPNQYGDVWVRGNYTVTGINGHGGLTMLDGGESLFFTLIRSSSCEETATAAMDM
ALTRQKTNHVDANPEAIVVGSPLDGSKNPTDTKMGDYIGDVTGVVYNAFGTYRVLPLTAVKTITPSSPDYPATSLAS
SAHCSGLTVADYNAENLAPNSTHLPSVVSQIVTKLRTPDLIFLQEVQDNSGPVDDGVTSANLTLSTLTQGIEKTSGVR
YDFVEVAPVDGQDGGQPGGNIRCAYLYRPDAVELYKPNPGGSNDENAVVDGPSIKYNPGRIGQSDANFEATRKPL
VAMWKPVKGPDKVFFTINVHFSSKGGSTGLHGDPRPPVNKGVEKRTGQMELTANFIAQILAQDPKARIITAGDFN
EFTQVQPVTTFASKSGLLDADEVAGLAAVERYTYLFDQNSEALDHMYISRGLAKNAQVEHLHLNTWQNYDGQTS
DHDPSVAKLNFCAC

>QLI67197.1

LPQQPGVQAVGENRLPLTYPRAKAPHKGGSCKPKRPTPVGPDVPKNTTLPGTEPGDNPAKPSPPDETGGRDPGE
MKDDYPDKDKCGQIDSNDLTACQCTGFAAFRAVNRMGVTNFTQYGQWGDAKNWASVARQGGLTVNDTPAP
GAIACSGKGQYGHVAFVHAVDGNQLQIEDYNGIGGNERYGNGKQPKSQYDAFIHF

>QLI67199.1

APIDVIVPMARKTPALTIKQPAPGIPHDLYDPLSETTYRPAVFRIITKKSENGPALDASTGPSWYPPAKFGKREDGSK
GNNPELYSPLAHVQESQTGF

>QLI67205.1

WPQVRNESRFFGEEIRAENIVADMPSRELPVVGDTLSVGTLPMPQLSQRHNAAGSLEATQDGQDSSFNSFQQDP
KQGPPDGDISPGHLTLPVLHVETRLDKRDVEVRLENRSDVAYYVQLNIGNPIQKVFAQLDTGSFELWVNPDCTNLP
ASADNRFCQAVGQYNPLRSSSSKGSNLSTELRYGIGSAQVTYFLDDIALVNSRQMKQVQFGVAQSTKDQFSGILGI

GHGVGINTGYKNFVDQLADQGITKTKAYSVALGSKADRAGVVVMGGVDTAKFSGRLAPLPLIPGDKSPDGVTRF
WIKMKSVQHTPESGRTADLTGATMPVFMDTGATLTLLPPRLANDIARQVGARALGDSGLYTVDCGLASKNGSLD
FNFDGVTIRVPYSEMVREVAGLPPSCYLGIMPSTSFALLGDTFLRSAYGEFFPVCAEYWNWCLTKMDRGAVLFDIS
NNMTYMAPYANCGSRTQSIDSSTDLTSIVGTCSPQQVNELSKPVGSGSPDGNSGNNNKENGAGSARMSWSWF
GVLGVFAMGVLTL

>QLI67208.1

HTYPNCEHDNCYRALIKDGLQDKAKAFCFDWLAGTTTAASAIPTDFSNCNVQAASSACSCVTYTATHTTGPTTTST
PPPVTTTTSEKPPTTTPTTSEKPPTTIPTTTKTEQPPTTTPTTQAPPTTTATTEKPHTTSQSTTKWTTSTIYTTKTNTITS
CPPEVTKCPGGGHTTVVTETIPVSTTVCPVTETEAPPTTTATTKWTTSTIYTTKTNTITSCPPEVTKCPGGGHTTVVTE
TIPVSTTVCPVTETHAPPPPPPAGNQTSTLYTTQSYTITSCPPSVPSCPVGKVTTTVYPTGTTTIKGWTSIPAVPTQPT
GVPPVPTQPTGVPPVPTQATQPTGVSPSKTNQNPPPVVTAAAGQLVGSLEFVAAAAGLAALFL

>QLI67211.1

SLNNVADVGDETDAATGRKCGTILGIPCPEGYRCMNPTFCADCEGKCEKIEPEEDECDL

>QLI67214.1

ERFIAPNAQSLAAVDAPLAVSMEEMKSVKIAEREQDIAKGVFDEGRYKLQLASPCSNGKAGEYSCKSVDLKGFLRH
EDLGSQTRAGNDVWGWTSSTGREFGIVGQTDGSAFVEILKDGSLVSLGRLPTQTDSSTWRDMKVIGDHVYIGSEA
PNHGLQIFDLTKLLTVDPKNPPTFDVKKDLAAHFNGFGSSHNIVAHEENNIIYAVGTARNGKCKAGLWMVDVSDP
KNPKDVGCAGDDGYVHDAQCVTYNGPDTAHKGKEICFGYNEDTLTIYDLSVRSSPKILSRTPYQGATYTHQGWTT
GPDHRYLLLDDELDEQRQNGAAADGRTTTYIVDAADLSKPVFTGYYKSPAKAIDHNQYVIDGLSYMSNYASGLRVV
NVTSVAQDNTGAGFEEVAFFDVRPEDDAVGGETVFKGAWSVYPYFQSGHILVNSIERGIFSVKLTL

>QLI67218.1

TKAPSTSSVHDYIVECESSVCVEQLANNVQEKGGQVRHKFNSDVFHGVSVQLESISTAEQTMAELEELEGIRGVWP
VQASTPAVKVGQENQKLGAAGNLHSGDTQRHGAGKRAADDLWPHLMTHVDKLHKKGFSGQGIKIAVVDTGIDY
THPALGGCFGKGCRVAFGDNFSKDGEKGDPMDCYGHGTEVAGVLAGYSRDQGFVGAAPNATLMAYRVLDCSAE
GTEDDMMAGWLKAYEDRAQIIVSSTGIQGSGWAQGPLAMVASRIAARGVTCVGGLGNMQEQGLFYAMAPAT
GDGVVSVNSVASDYTVPYLSAYGPTWDLGIKPNVIAPGQGIWVTEKHGRYTYTSGTSYATPLVGGIAALVAEARGG
SFNGVLINNLLMSTAKPQRDNGAFMSVAQQGGGLVDAWEAAHATTLVEPAGLEFNDTEHRVPISLKITNTAKSEV
SYELSHLAATTLYTFSPGGTKPGQGEHAQATADIKLSLRSFTLGPGQSATVDVSAEEPLGLDASRLPLWSGWVAITG
SDGTNLTAPYLGLSGSLRSATSLHPNTLRVRWADPNNVTPLDKNASVVFLDPPSGQRPGSPASIGELTQIFSGNLFF
RLSLLASPQVLMDIVPLDLCPLGTDTSTVQGPPDLSMNCVPSSMVKDSRGLKSIGQVAGFPRYYASRNAVGVEGE
WDGAYAKGQYAPPGRYKIVARALAVFGDASNPSDWQLTESPAVFILYLHNYIPVAQPTPEQA

>QLI67220.1

DANDNTNNNEPKPDKDGKYWIYGDGISAAFVAYGASVSNFMVKDQYGIERDIVAGFDNASYYGIDKQHPHFGGV
PGRYANRIKNSTFEIDGKKYHVKANENPTKGHPDGVDTLHGGPDGWDWRNFTVVSHSNHSVTFSLVDPDGKEGF
PGEVVTVVTYSLNGRDWDLKMVAEATTKKTPIMLSSHTYWNLDGFANNQTSKVFNHTLHMPYAGFRVGVDNILI
PTGELLPNKKGGVNDFWSEPKQIGASFGDEGMVNNCGFNCTGYDNCFINNRCAIGPYDWRKQGPVTTLSSPWS
GIQLDVFTDQDAFQIYSCNGQNGTMALKKTQGVTGGNKDKFPRTIPKYGCVVLEVQDYIDGINNPEWMRGKKQI
YEPGGDPYVLQAKYRFSLVSDHEEKKGRGPSS

>QLI67225.1

IPSKSTEPFWYSSKVDETDSRPSLPEDSKTVLRRASGDQADLRIASKRAYYDNDKPHGESEVITLYIYNAGPNRAVAP
EIRAGYGGSMDQDRATATLRQVNEWRQPSQGIKHGSLAWAGLPNPSSRVDERDMVITLPDVLAGVLMELNIEFP

MTHETSYADHTMKASVSSSTTDPRKGNNEATYIITPNHHDDGQDYWNTPGNLANLDFNGLKAEPLSQADLRIAS
ARERYNNDLPYGENEVLTFLVFNDGPTIDTRPVLTAGYTSSMDEDKMSSSAEQVWVPDGVDPSWDLEHHVWQP
LDGANCRIDESNVVCDLPDIPPTVLYRVNITFPMSHETTYKDHTATAKISSQATDPNPDNDSTSYVITPNHDDDGDE
YWRAPGNIAQLNGPSAPVGGRPCSTGWLLDTPIIMSNSNGRAQLPLNELEEWVNSKVGQVMSYDIGEKKLKPKT
ATGVEYGTSGEVTVVSFVSDGETQLSWSAHPDTLVYLYPTTIQVGQDKVRIGYWIPVRMLWVGATVRGMSPDGG
ITSSQVTNIQQAKGSEFRSAQPHMVSQSHSYVVGTMGAGGILTHNPNDNRLCGDANGPARLALLAVVANFLIARP
PLPNVPNVPQQGYHLRHRRPTDPPTPRPAHGPPVLPADVPRRDEHGNWLVYLYEEDTPAAVENARYAVNEAGH
PWQLTYDPAGAASRRRQSTGAHPSQRELLQLTTAELTGGVGTGQLDRDEYPPAIAREGGTGAVVTYIEAGDNRGA
GSLMGRQFANYRTNPQPDGHAPFGPGDTFRYAIIHENLRAIEYLGDGVVDETQIDAPPTP

>QLI67226.1

VPVTSGTPIVSIVPARGLDNWQNTLSSDTRRRLDVGLQHLDDESYPGQDFRNDTIMRINIEREALDLSLIDLLQAFG
MTLPAPRDTVSKMVVNKRGNAEDFREEVYTRAGTMAYPKVVEGRSIDVYSSLLIRDNLALGAIERINIAHRILGKPEL
TIEQAKEIGSHGDEYLTGYWNSSMKHGDFWTSDQAIGKPNFIKGGQSNRFGFTQSTFHKLYCLANLRMILAWHIT
GSGNKMTRDLNAHTIHCLEYIRWRELAHPDLNEEPIDTVDYEGMGIH

>QLI67259.1

APAALGKFENLVVFGDSYSDEARYAYIKQNGKLPPAGQMLPEADSPFSDGRIWARLVAKDTGATLYDYAVGGAM
CTHTNFDQRTFNGIANIPYPAVLDYELPLFDTDKKLGLYKNLNPDNTVYTLWIGTNDLGKDGIVGAKAGMHHNVD
ASSTVDSYLTCVWAALDKMYSQGARNIVLFKLAPLEKAPLYAVDYPDLKDSLSADVQNVNAKYDQVVAETKSQRW
AEATITIFDSYKFFSDVIASPSIFQFTEVSKPYKTCVSENNCQTASGPQSSYLWNDALHLSPAAEQLISQEFQRAVKGA
SSYAKNY

>QLI67265.1

GTPVESGNWVEDHPDYSQQQCAGGRVNGNTFSIPKSPNGDTSGSGCSNGHLRAERRYKDDYSSGVHQFGGEFKI
NSMSGSRISIKQTFNGNSGPYFIMGVEQGGRLYNVEGGKTIADGVAKVGATVRINTVHNANNHRFSVYVNGKEM
LRDENAPGGSFYDKIGAYATNSGTGDLSITWNDVQFWHK

>QLI67271.1

HPQRHHANEASLGKRGVDVSQFRMPGSSEYTVSTQAESDPAISSIQKRGDYVSSATQLVKTVLPDAEFRVVDDHY
VDTDGLAHVNFKQTVHGIDIDNADFNVNVNPDSTIFSYGNSFYNGEIPPESPIQKRGFTDPVNALKAVVDILALPVN
PTDATSEAMEGAETYTFKGTSGAVSDPAAKLVYLTKDDGSLALAWRVETDVMDNWLLTYIDADNSKDVHGVVDF
VSEFATLQVYPWTINDPTEGARSVQTDPWNVDASPFTWFGDGSQDYTTLWGNNAVAQTNRDGHNNTNDFAN
SYRPTSADRKFEYNYSPTQSNSTEYQDASITQLFYTANTYHDLLYTLGFNEAAGNFQTNNNGKGGKGNDFVVLNS
QDGSGTNNANFATPPDGSRARMRMYMWTLASPQRDCAFDSDVVLHEYTHGLSTRLTGGPANSGCLNGLESGG
MGEGWSDFMAVAVLAKANDTRTKDFPLGVWVSNKPKGIRSFPYSTNIQTNPYTYASANEKNEVHAMGEIWANT
LYEVFWNLVDEHGITADKYPTFDDKRVPKDGRFLTMKLVMGGMAIQPCNPSMVSARDAILDADKKLTGSANKCL
LWKAFAKRGLGENARYNGGRNRTEDFTVPAGC

>QLI67287.1

RAVEKEIRAPAAQSWRGLGGLFSGHPGVVSWSANRTDVFVRGTNNAVYHKWQNGDSGSPWGPSQTDYENLG
GVIYGDVTAVSWGPNRLDLFVLGTDNAVYHKWWDGSSWGGWESLGGTIIGEISAVSWGANRLDLFVRGTNNA
VYHKAWNGGSWSSWVSLGGVIVGNPQAVSWGPNRIDVFVRGTDNGVYQNAWSGSSWSGWYNHGGVIVDD
VTPVSSASNRLDLFVRGTNNALYQKSWSGSAWTAWISLGGTIVSRPSAAAWDGRYVAVVAQGTDNAVYLKEYN
GNSWADWRSLGGVVTDAPVINPRGGFGAAVFARGTNAALYAYE

>QLI67291.1

ASSNGTMFAAAPGLWNDPCALGCLAGNTCPGRDKNCLCDNVDLVTSDASRGCLRDGCPNVKADDVVAQLRGE
CSGRKMKTPTGSPVVPLQESTSGAAAGSSPTVNSIDSVGGSSLVPSSPSPPSPSATEGTSSTASAGSPVAPTAASTG
PWTGVPTVILNTYTASSLLSQISGLVVATSTPHTSTASTTGGGVVVAAGSGSNGGRSTTPPSPKGTTSPTSDQDTDS
HDSQLSAGAKAGIGISIAVLCLGIISAVFFFAWRRRRGQRNSGLREDTISWLGHAGEKHRTLQAIPAFGPTRKIGGG
GGGGGGDGFGSGSLAMSLEPSSHLTYLSLGSEPSPRDEMGGIGMALSSDSHDLLDSQTLVAEGDSETPLRDGDLD
SPVIPRFVVPHGYSLA

>QLI67299.1

LRASQELGYIPPIPEDAYHAGHNGLARRATNATNPNIKAATFQQLIDHSNPSLGTFTQRYWYNAEFYRGPGAPIVL
NAPGEYASDRFVGYTTNLTLPGVFAQTNGGAALILEHRYWGQSSPYPNLTAETLQYLTLDQSIQDLVHFAKTVELPF
DPDGSSRPDKAPWVLSGCSYPGALTAWTHHLAPGTFWAYHCSSAVVEAVADYWQYFAPIDEALPRNCSADLKKI
NTHFQRVLTNGTAEAKNGLKRKFGFDNIADDDFVSAVDGALGSWQSQQFYSGYTDTFKMCDYMETGNANGTN
GTIPGPEGVGLCQSLKGLARFWREYVLPGDGACSGVWDDYPPQACYNTHNASYPVFTNTTVENPYNRQWMWF
LCNEAFEYWQVGSPDSTVGYPAPLYNIDYWRAQCPIYFPEVNGHQVGMVKGVRADDVNRRTGGWGNVNTTRLI
WINGEYDPWRSASVESDFRPGGPFGGDDERPSYVIPKAAHCNDMLMRNAEANPGARKVIDAEVAKMKEWVDA
FYKNKL

>QLI67302.1

DDPNTQTCISPGANGCNLDGKVCCNSNEFYCVMQPGWKEGFCKKKE

>QLI67319.1

QKLLPWKQPATLTGSIDCGFKVSDEEQCGTELYCNLFDNQKPNSNYKSAFQCFQSHEAEPKLLPWKHAATLLGFM
ECGFKGGDEEQCGTERYCNLFDTQKSSDYKSAFQCFKAHEEKPNLLPWKQPATLLGAKDCGFKGADEEQCGTGLY
CDLFDNQKPNSDYQSAFQCLQAHEPSPV

>QLI67340.1

AFREGCADAPWDSDTDFFLTKFEKSPTNPFLAEYNKTYVTIKNRQSRYVVLYCSKEPPPTSIVGESSLFVKAPVKNVA
ALDGFSQNLIEMLGMSTSIKRTGVYSDVTSSCIRGNMKDNITFDDDEWDKAPKVDVTFYGDTATSDNKKVLIYNV
GNYAPLAQLGYIKFVSMFFGLEELGEKLYDEISANYRCAAAQVQQAVMAGTYPTGALISPIRKDGDKFTVFQSAW
WNSILSDAGSALVNVSADGQAAETGNPTKPALVTIDANSAGNNFARNSWAIIDTTQYDQLPGKQAPKTLPESTRIT
ADTYTSRSGASSASYAVKNNNVWLTDKAANRNRRHNFFDRGSARPDQVIRDIISVVSPSFLPDYSNMFIRSVSKPD
DEIALRRFTNTCAEKGKELETLSLTKCDAPAWVAGYHDSGLKPNAYRSADQAESLALRASGSGLSGGQKAGIAVGS
VVGFLLLAAAAGFGIYKCRRARAGDKNAKRSEMDQVEKGSVSSRSTH

>QLI67341.1

AVVTITAPPAIPSDEPQWKTADTFTSAILNSTNFYRGEHNATAVSWNETLASFAADHLARSGCRFEHSGGPYGENL
AEGYPNATASVEAWGDERDRFDFGDPGFAHETGHFTQLVWKSTTAVGCGRRLCGESGWYLMCEYWPRGNVIG
QFGDEVGREISAAALLRPSIVSVVALGICAVILVAA

>QLI67346.1

NFQPPNSGILQERTLGLDEILGDISSGGLLGLKAQKIAFLCKLVDKAESIAGFVGGLKLESHLKKKLEFFLKDKCKKTTQ
TQPPPSTSVPPPESQTSPSTTSSPPETSSPSTPPPETKTTPPETKTTPPETKPTPPETKTTPPETKTTPPETKTSPPPETT
PPKETTPTPETTPPKETSPPAPPTETTPTPETTPPKETPPPPATTPETETSPPPGTTPPEETSPPTPPKETTPATPGTPP
KETSPPPGTTPPNETTPGTTPPNETSPPPDTTPPKGPESSFITKTTPNNSPPPVTLPPPTPTTAPPITPTITECIDVTTAI
PPCATGCFSSAGSQVGCSDKDFGCFCKPGSQEQLSMLVQQCVSTACPPESATSVSMGVSSVCSCFPNPPVPTNTG
VPPTPCTPGSGDDCTQTKPQPEPTTPGVPPGCTGGEGDCSASPPKPGVTTTPSQGMPCPIVNGTGIDCTRPTSPP
VVTAAADRFSFGLGGCVLGGAAWLALAL

>QLI67350.1

QQASPLPTGQPPLHMFPPVIHTTEPVPSGASLPTTPGTKVTGASSGSGSSATDTTAASASGSGSAATTSGTRTSTGS AAPTAQVNIAPILGIAAAVMVL

>QLI67356.1

ENKWAIVAAASIGISVAICLPVIRYRWYEMFVRAHQCLAIVSVYGIWVHIAPQRLLLRLHVYVLVGIAGLSVMLLGLL VIYRNGIFRSRLPRADIMHAKGAVLVRVVLSRPVRVKAGQYICLWLFIPSTSFHALIEYHPFVVASWSDAPLDTLDLLI EPRTGFTRHLLHRSQTRQDLCRALFSGPHGNSIPVGDYEVVLMVASGYGIAAQLPYLKELLHGYNSRKARSRRIHLV WELKTIDLAIAIESLLNNALVDDTLDNGYVSKFALLSRFSAYWSEDSADICGLPDWCSIMREEVEGKYIKRVQAEAST RGDMIVTGK

>QLI67385.1

CPTRQIKTASKYDYIVIGGGTSGLVIANRLSEDRNVSVLVIEAGKSVLNNANVTDVGGYGLAFGTDIDWQYKSVNQT YGGNKELVFRAGKAVAGTSAINGMAYTRAEDVQIDAWQTIGNEGWTWKKLLPYYLQSEKLTIPSQSQVSKGASY NASVHGKSGPLDVGFFDIPDNDLTGVLNTTMNGLGIPWVEDVNGGKMRGFNIFPSTINVAANVREDAARAYYW PVASRQNLHLLVDTFVNRIIWQDKANNSDHVTASGVEVTLANGTTSVVSANREVIVSAGALKSPGILELSGIGDATL LEKHKIPVQVNLPTVGENLQDQTNAQSGAAIKANMTSATHVVYPNVYDIYGNQTDSLARSMQKKIKDYARATAEV SNGIMKASDLEALFQVQYDLIFKQRTPIAEILYSARGDNAISSEYWTLLPFARGNVHISSSDPTAMPIINPNFFMLDW DLDSMIAVAKYIRTSFSAGPLGKLVEGVTIPDPAVVGDDASDAAWKEWLLDGHYRSNFHPVGTAAMMPREQGG VVDNKLKVYGTSNVRVVDASILPYQVCGHLTSTLYAMAELTAELIKHNSA

>QLI67399.1

HGLIRSIEGANGVSMPGLTVADGTPRDCTNNPCGSQADTAIIRDVQIKLGKCGPLGQTQGHGSVNASAVVANFM GTGGPAPVNKRPDGALGTIGVVPSEDNSFMTPEQRRQEYNRQAAELFKGESNDVVSPMTPYDGAGKSKPKNKKP PYGVGKAKGKGKAKGKAKGVNKKSSKIYRSETLNGDMRGQGAETGLPTCNDEGVINLIYHQINEDGAGPLDAAID CTSGGSDASAFQPASVIRDIPGSAGISSITNTDHPVQVQMPAGMTCNGEVAGVKGVCIVRVRNTAGAGPFGGSA AFTQSQESRKRAIAYRLQKRMEIGSSKSH

>QLI67413.1

APANIESRDIPLLGGHSGPLQGLGETLDSIEKLVPGLGQ

>QLI67430.1

GVHKMKLQKISLEEQLAGASIEQHVRALGQKYLGARPASRSDVMFNTKAPQVAEGHPVPVSNFMNAQYFSEITV GTPPQTFKVVLDTGSSNLWVPSQSCNSIACYLHSTYDSSSSSTYKKNGSSFEIRYGSGSLSGFVSQDVVTIGNLKIKD QDFAEATSEPGLAFAFGKFDGILGLGYDTLSVNKIVPPFYQMINQKLLDEPVFAFYLGSGEEGSEAVFGGIDKDHYT GKLEYIPLRRKAYWEVDINSIAFGDDTAELDNTGAILDTGTSLNVLPSTLAELLNKEIGAKKGWNGQYTVECAKIASL PDIVFNLAGSNYSLPASDYILELQGTCISTFQGMDIPEPAGPLIILGDAFLRRYYSVYDLGKNAVGLARSK

>QLI67445.1

SPVPEPDPEAPLTTTIVHPQRADTRYENQGKIAKRTIDK

>QLI67477.1

DLTKVKKASTYQEAIDRRPDSFWDHIVKGAEIQDASTKPGAHKRLDGELANYQLRVRQADPAALGVDKVKQLSGY LDEEQEDKHLFYWFFESRNDPAKDPVVLWLNGGPGCSSFIGLFEELGPATIPNEDLVPVDNPYSWNSNASVIFIDQ PVNVGYSYGTKITGSSQAAAKDIYAMLTLFFHQFPEYAERDFFVTGESYAGHYIPAIGAELLSHNNSNINLKGLAIGN GLTDPYIQYLYYRPTACGQGGYPAVLSQSDCQAMENAEPECQRQIGVCYNDPSARVCRQATNYCNNFLLGIYQSG

TNNSVYDITSPIGTGKTSYASQFLSSTKTKQALGVEASRAYEECNFDVYNDFVNNGDWMTPAHRVVPGILEKIPVLI
YAGDIDYICNWLGNEAWTLALEWPGKSALNAARSQELHAKSGKNYGNVRAAQGLSLMQIYKAGHEVPQYEGEG
SLDFLNRFMGGEWSK

>QLI67481.1

YPSQDAASVSVSNTTNLTRGGYVPDIVPKPFYAIANRVLTKQGAFDALQMGANGLYIPVWGWIHTGWWADYDG
RGRSAGDKIENLFKEIVKFRIEGRNICLVWLDLRSPDWCTPHYPRCIFDRLVGFSRNILQSQGIYVLYGFHLESVNGSA
YASLQDDLNDMEAISIEGELAEVNASFWKYGPSAPDSRVMSYGYFNLATPRFGHCNEERFYVCSELKRGAKSRDDV
GKTYAWTVSGDQSVYVDRLMDAGVDGLVFGFRTIDFDEHDAPFFAFRNIIDWIDSHPYRYLANIYDKPWDRRLNN
ESDNK

>QLI67486.1

DVSEYGTVIGIDLGTTYSCVGVMQKGHVEIILNDQGNRITPSYVAFTDEERLVGDAAKYQAAGNPERTIFDVKRLIG
RKFSDKNVQADLKHFPYKVVAEDGKPVVQVDVQGTPKTFTPEEVSAMILGKMKDVAESYLGKKVTHAVVTVPAYF
NDAQRQATKDAGTIAGLNVLRVVNEPTAAAIAYGLDKTEGERHIIVYDLGGGTFDVSLLSIDQGVFEVLATAGDTHL
GGEDFDQRVIDYLAKNFNKKHDVNITKNKKTMGKLKREVEKAKRDLSSQMLTRIEIDAFFEGKDFSEPLTRAKFEEL
NKELFKKTLKPVEQVLKDAKMKKSEIDDIVLVGGSTRIPKVQALVEEYFGKKASKKVNPDEAVAFGASVQAGILSGEE
GTEELLLMDVNPLTLGIETTGGVMTKLIPRNTAIPTRKSQIFSTAADNQPVVLIQVYEGERSLTKDNNLLGKFDLTGIP
PSPRGVPQIEVSFELDANGILKVSAHDKGTGKQESITITNDKGRLTQEEIDRMVAEAEKYAEEDKATRERIEARNSLE
TYAFSLKNQVNDAEGLGGKIDEEDKETIIDAVKEATEWLDEHGADASAEDFEEQKEKLSNVAYPITSKMYGGDAPG
SQEEESDFHDEL

>QLI67506.1

TKEHLDGLPAYHYNAPIKVECMNRSSETGEHIEKNDEIQWIPFPVCNETGKPLEFHYGYEGELNCTLDFISDPFFHLL
EFYIHSDAPMSCRLPARPPAHVEIVGEKPPAQEYVPLVFALAGTLQMSHMHISTHMNVLLHSMPKHHQRPHDSG
VLDSAIAYSTSPLSHMEGTETARIIPGEPLPLTFSVRWFPTPALPKTEGRVEWRGLGGHIYATTVFYSIVSFGAGVVV
AGMYTLGVVLPRRLKGRSMGGATPLGYGLSSGGVGNGWGYTKKD

>QLI67511.1

GRIAPRQNDASTTGPSPAASSIGGGSEPTTPTSTSTSSSSSSSSISTSSSSAGGGDTTVFVTATVTSAGGGSTVSETTT
VTASSVTTVTITTTDIATTTITMTDGETSTRTVFVTSTQTVNMKRRSVGNVLQFYGNGPAHVDAQPTAATTATGIPS
RDLSPERIGIEKRAVVTSTVTVTVGGGGSATTVTQTVTKNVRSTTTTVVHTTSLITETFQANAKTTVTVTSTIVTKLTV
VSTGVVESTSTPTSSPNPPNNNDNNGGLSTGAKAGIGVGAGVAGLAIIGALLWFFTRRRRSPKPDPDDLLGPSSEV
PVGVGGGGSRPMSEGLASTPGSVPRRSPVLPNVQPEGYRGTAKGDGRAGYAKPEPYGAAYAPTRSTTNSSYPPRS
GTLSPGDQLPRHPTPDTTTMASVSPLSARPQTAELGNDGAGARWHTSEAAEMASDNPAAAKWHSDNAHEIDSH
AVMSHQSGPVYEMPTEHFK

>QLI67534.1

DSLKDVEHVVLFMQENRAFDHYFGTMAGVRGFNDANLQTNNGVPVWKQKTSPKLTNSTDYVMPWYLNYLGG
NWSEATQCMIAGSNGWYQNHAAWNGGTNDHWALNNTPYSIGYYKRDDLPTQWALAENWVVGDMYQESVI
ASTSPNRVMWISGSINVPGGNLSPDLGGNPYIDNYETPGCDDNGINCYPLKWKTAPEYWEEAGVSWQIYQDGTD
DYDNFDDNPLAWFQQFQQAKQGSNLNTKGMQGRKLQEFYDRAANGTLPEVSIIIGYRELAEHPPYSPHDGAWLE
NKIAETVIKSPKYNRTTLIISFDETGGWFDHVDPYRSPNGTRGEWYNDKAGIGYTFAGPGFRVPFYIISPWTRKGGV
FVEHADHTSQLQFIETWQAAKSRAFKSREIVPWRRENMADLLNAFDWDNPDYSIPELPKAPEPHRNSRGKFDGA
SHCMGLYGDPRPPVPYSGSGATTDMAGLVEHGFKPIRGNLTEGRYLVLEMGRYALANAEKVVTGKACGRHDSKS
QRWVVHVEELGGTRFKVSSGESGKYVCAGLALCSDKCKAIVFDVRFKPGRGYSFAVRDGEYLVADDRGELSLNGSS
AYWTIYSVSY

>QLI67542.1

EELDANDVPPACAVICRPIVSLTNMCDINPNEKYDDDHMGKRQLLDARDADDPDDRIEAECICKNKSFNVPTVMA
LCASCIAQNGRKTEDINEIMSQCSFSSVSYAPLSTGIVSGIQVQATKPAGGNTPAQTTSAPAATKTSGAGGVGKLAN
SAMAVVGVVVAVVAAGLL

>QLI67548.1

APTPTPDTAVSWNQNFSCGADFRTKFCHAVNFHSRCVNGRFYSDAGDSCANCWCTYRDERVNAATPMWIAN
WG

>QLI67552.1

SKWLTDASSKYVVNGTAIPDIDFDIGESYAGLLPVTIQPDETDHLYFWFFPTSTREFRKKKKEIVIWLGGGPGCSSLFG
LSHQNGPFLWKPGMKKPLKNPWAFNTLTNVVWVDQPVTTGFARGKATAKNDDEVAKQFLGFWKNFVNVFDV
RRYDIYIATESYGGMYGPHIAKHFIKNPFYIFKGLLIYDGIMFDRHIQTNVVVSSFTDMNRDNLPVDEETRQQWHN
TAHDCNFTSYEKKYLQYPPHGPQPTQLPGYEEGAEGFVFPRQSCVNLFTTVFDKITELNPCASMYNIMSKCPPQFD
PIKGSGAPWFDRQDVKAAINAPMDVQWVPCRDKVFANARDESPPSGQDVLPFVIDATKNVIIAHGAMDLYLPM
NGVLLGIQGMTWGYEMGFQKAPEEPFIVPRYNDGPASSSYAHDLPSGTGIIGTTHHERGLTFVATKLAGHEGSGD
SPAGALRHLEKLTGWVDTLSSHAPFTLPEIRIVPQPPGEVSKGIVPIPCLSSSC

>QLI67555.1

GYAEVCLNKAHGCIDYRVTKDCCAAVGGQQHFEEGSHICISNNLCGGNGVNIGAMVNCCEARGAGSRELPYDGF
CSVTDVPPRC

>QLI67563.1

LPAPAPAPAEAISTYPNENPGQSNRWPPPRS

>QLI67576.1

ENAAPFQAPDTYSLNGAGLANGFDGLAARDDECLASQVACGTGFCMPRGGSCCDRVRGTYCDVGFYCYPQGCC
PSGKVCRGSPTDGCASGKQECGSVCIAGSSVCCNPGASSGNVWCEYPKTCGAGGKCVGRALVTSASAVPSSTLPSL
GIADATETGGSSTKATGDSGDGGSSSNDNSSSKGDKKTPVGAIVGGVVGGVAAIALVGVGILLLLRHKRKQKDAAP
SQNMQPPPHQPLMQQYPPQTSPYAANISPAQGMGYPPAPPGSPPPPQGYYNASPPPGQYSPSMATGQVSSPT
GTYSTSTDPRTGAPTVSPVGFATVSATPPPPPPASQGRNGNEKPVVYEMSAKPGDDHRGNIHELA

>QLI67636.1

VLSTDSYRSSPGSVVGGGSAYDYAPSIMLDGVYKMWWCGQVPGQPVAGDSILYAESSSLDGPFHARGSAASHQV
VFGGTGSGSFDNEHTCDPSVVRVSGTYYMYYGAERHDGEPTTIGVASSPDGISWARLNSGQPIITPANQNTGNT
YGAGQPSVVYRGGRFHLIFTDTTGAGALGNGAGQFAWRSPDPTFQRDVDVFTASGWQPKTDANSRGFSVANAF
SADWQFSDALDAFIIAHDNGAGETTLTFLDAENPAVQKYSQVGIPGPWSEGPGIVSRPDKHSVVSASNECGRVPV
DVIRSTTGPPPRELGRIGVDLVSGASCGSMPDRVAAIYEGYGMQVPGLPAAVVVDGLRLQIQSLAVYTDLTHNAIQ
VPPSVYYAVPYGASLHEGDAVLGASGLPGAFRLDNNTLWPVSCLKIVTDNHSQITMIDPGAWQSYRKGPSLFCLG

>QLI67637.1

LPSNNHVLHEARNPTSTRQNHLWKRGNRVHPDAVIPLRIGLAQSNLHEAYGKLMNVSDPDSEHFGKHLAQDEVH
ALFAPSDETLTAVHSWLVDMGISKTQIRQYTNKGWLALDVPVSKAEELFQTQYHEHEHDGAIKIGCDEYRVPKHLS
QHIDYIIPGVKLSPLMVRRSAKARSPAKRITAQNRKNWEPKKVSRKKPSCKTPPPSNLPPELKDCARNFTAACYRALY
QIPLANTPVPGLEPAVYEIGDTYSQEDINSYLHKYTPYIPNGTHPTLHSVDGAQAPVPPQSPQNTGESDIDIDIVQSLI
WPQSMLLYQVDDIYYSTQSNTSGFLNTFLDALDGSYCHKTDFNITGDSPGIDPSYPDSHPGGYKQAEMCGAYKAA

PVISISYGESELDVPKKYMQRQCNEFLKLGLQGTTVLVSSGDYGVGIGPGANACLNGSGQTNTVYNPGNPVSCPYL
TAVGATQLNPNTTVRDPEAALQTPLPGAELFASGGGFSNYFPVPEYQRAAVRSYLARHDPGLRSYVADADASNIG
AGGGVYNRAGRGIPDIAANGANFRAFTNGTDQHVFGTSLAAPLWSAVITLVNQERARAGKTTVGFINPALYKNPA
ALTDVTRGSNPNCGSSGFSAVRGWDPVTGLGTPMFEELRRLWLRLP

>QLI67640.1

YDELDILAGQHVVYSFPSSSQPPDELMRLTRAGLVGGVILFGVNVDANTSQAMSALKHAYDSSPAPALLKKKTGKD
ARFLVTTDQEGGQVRRIKEGEPKLSAKQIGASADPAAAGEAAGAGAADSLLRYQCNSNLAPVLDVFRQPGDFTDY
YGRSFGNTSRQVVRAAVPFITAQQGRGVSACAKHFPGLGAASHDANTDNEPVVLNLTLSEMRAVDEAPYEAAIAA
GVDMVMASWAVYPALDRRPAGLSAKWIKGELRGRLGFEGVTITDALEAGSLAAYGDAGARGTAAAAAGMDILL
ASGKNVTQGEAVRTALVQALKSCKLDRAEFDAATGRIAAVRSRIAA

>QLI67676.1

APHEQPRPLRIRTNTISPGEANQIHTVIVGGPQDLIVPNFIIAAIGDVIQFQFSSGNHTITEGIEGSGCQPLQKSNPLAI
HSGHLPYNKGDQNVMVFNVPVNDLNPRLIYCATGPHCQTGQVAAINPRNGQQLVNYMKACQQAKENVDAGSP
SGGIVAQIPLEKAAFIPAEPEGA

>QLI67685.1

VPTLNPRQSEIDEADKICKSRGITKDQCHVYKQHCQFIEGITEPDQLNYCVKVLGTDCKDDGQCGQGYECREHRYR
PNQRTCYPKKKQSQAGKQESASSTKEPTQDNKQDSTSPAKNQTQAREQELASQQTEINKIEKICQKRGLTKGQCLT
YRSYCKENMGIVELDQLKHCVEVLGTPCRGDDQCGHGFECYKEASAPYKDGVCDTKQSQAGEQESASTAKKPTQ
DTTTNIATPTKELTQDTTINIAAPTKEPTQDTTANIATPTKEPTQDNKQDSTSPETDETDKICKSRGISEDECSYLTRIC
QDVVKGKPDQFNHCMKVILTSCDNDNECDAHGLACLETSLKGSQRGACFPNIQPESQAGKQESASSTKKPTQDTT
TNIAAPTKEPTQDTTKNVATPTKEPTQNTTTNVATPSRPCTKKPIKSSEKNSVVGAKSTQA

>QLI67693.1

QLTAIDHTKALVTRAGTERISNRDFSAIRFYSQHAAAAYCNFNAAPGTRITCQNNACPLVMRNQPVVVASAIGDAL
GVGAYVAVDYVRREIVLSFRGSNNIRNFIADLAFAWSDCNLTQGCKLHTGFAQAWYDISDAITKAVRSARSSNPNF
RVVATGHSLGAAVATLSAAYLRRDGLAVDLYTYGSPRVGNKNFATWFLTQRGVQWRVTNGDDPIPRLPPLVFGY
NHISPELWRPGGDVQTVWQPSTTAICKGVDNTDCNGSGLSPDPWAHRNYFGSVDACAGSSLTLRDARGLPEDLI
NRLTDWSRQDRQLSGSLGHHGDVEFLLKPLDESPASAPGKHEYTIV

>QLI67710.1

IPQGPLKKRAAAAGQDSTASSGPSFTVSILADTNRDGRVDVAGETDTVGKETWTDDAGALFLANIVDTDRRCSSQI
TGSCASGLGDIFNATEPPETPVPDPRLPDTPGDGGSMEQWYNSLNEGQRALYYDYLFKGPDYQKINEVDRKISAC
NDASDDILRNATYLAPLRTRPIPGLGNSATGTVAVTRMLAASKVRLFHKQQTSGQWVFITSNYTFKAAELKAGLEL
GIDARDVRRPGGWDGRATVEFTVKDGGHEARDSVALRVAPVLTQHHGQAAKQLLTSQVDGPKGRDSGHERFV
QELGEMSSSLGLESPMHAFEGCGELWTQDFFEPGYMSIPGPNGPVSLHIMLRSAQDYREAGRKVFQDLRSNTVG
AVQHLAGGDTVDSTGNLETIPPYSHRGKAYPAGRAVMGTQKTKPHMMAFLEAQETQAPIELNTSWLSVGHVDE
FLQFLPADNKLGWVLMADDPLAGLDILRQARQAGHGKEKAVSRPRSPADPEEWHVTTTIDEVLDSTDFAAVQNK
SAAHIAGNIEILKRETGLTDADVIRLPCLYHLEPDPWGRFVSDIWTYEKKAGNETRRMSRRAVRVKAANALDAGTP
PTKLADGNGASIERRQSFSWSEFETWPVLALYPGIINSVVVDRKHVIAPNPWGPVIDGRDVLAAAADEAYAKAGYT
VRYIDDWFTHHVEMGDVHCGSNVIRRVPADSKWW

>QLI67753.1

LPSAQLDERQAGSSWFLPHLDHTSGPVRAYVPNLVNGAGQPNYTYPVYKAVNSGDSQGLISAISSDGPSGGQRD
NCWLAGQPRVIYLAPGTYTLSSTLFLNTDTVIIGDASNPPTIKAATVFNGKYLIVGGQGDGENHPCGGFAGETHFSV

MIKNVVLDTTANSGSGNFTALSWAIAQNCALVNVKINMPQGAHTGIVMGGGSTISVSDVQFNFGNVGLHWSGH
QQGQVKDMTFNKCTTGILIDSGFTISIFAPVCNTVGNCIVLNEGNPWVAVIDGQSINSGDFFTSRVGSPNFMLENIS
KDTTNSNMVTVGGSVKVGGVSSLGTYIYGNTRDSNPIYQSDPTSKPVSRPAALAPGGKYPVINAPQYSGKTVSDVV
NLKDARQNGGFNLHGDGSVDDTAALQGALNTAASGGKIAYLPFGIYRVTSTITIPPGTELYGEAWSTISGSGSAFSS
ESSPTPVVQVGSSPGQKGTARIQDIRFTVNEALPGAILLRINMAGDNPGDVAVFNSLITIGGTRDTTLSCSDEAKCR
AAYLGLHLAAGSSAYIDNFWSWVADHPSDNSAKGIHTVVKGGVLIEATAGTWIAGLGSEHNWLYQLGLHNAANV
FISLFQSETNYFQGNHGAPLPGQPFNAIASDPNFSWCSSSDNVCRMGLAQYYLGSNSAIYHYAAGSWNFESLTGL
NQGLMNYIQSPVSNGHLHGFTTGPNVAEAMRLPNGNQFGRGGKDGFSGSWGTLIADIASQY

>QLI67764.1

KNKISLQWAICDSTPQETLAKLGLDPTTPPYKENPIAYYDEKPPIHIFSGMMFRTKTNKGRPLSTIKVSFDEETANVP
DFVECGWDRYGKNDPTYNCEKRCPLDPVSPENIWREEQVQFAERYQHVNWSALVKYGPYPDGKWKIRINGHKA
KFDDVVVGGLHLMEIETRVPEKKARKFIRETWQYLSDRGVTLCNPQEGKTMRLFKAMGYTINEEGEL

>QLI67765.1

SGNIVLVCTKDALYSSLYQNGRQFCSSVLGKPPCEVVTPTAYATYDPAVISSRCSCVLMDKCLPRPTDSSSTTTRAAS
STVSSTSSSHLSRNRTCSDHATSTAAGGTSSTKCAASHTVSSSSNRTTVDIDSTAVYTSSSVSTSYMYTNSSSSISYSNT
TSSVSPTATVSSVLSGITSSSSSTHSLNSSTSSVTHSTDTQSSFKPWSSSQSSSTNGTSSKTSISSTSDSVTDSWLLPTIT
LTPIPSANISTVHLTLPGSSLLSTSTNATSTVNRTVPVSLLTHDSTSGSISSKTSSLLVSTINTTTTQTLSKLSTSAYISTSAS
TTSSPSLSVANFTLVTRTAAIAEETCYQFPQDPDAGNKDRSATLHNDQVAERNITLPSPYIESVFFDSAGIDPIFLTLW
DALNGTYYMDVSNRSRVSIMDSQKNSMLIDANGIHFSTNTCAFDVSITIQDMYEQLESLSGEVCAKSISNKRSMEE
SSFEQVLFLKDQCGNGIKRALRTYPTLSVGDSDCMDTEVDSSTGKWTFLCPFPGSDSGNSRCRASVNNDIVRFLFT
DPFGEACPDLSTVATTLAATARDFLNEHSLEEELYQLPLSGTQKSQVDATVKKYGQLWNVFKQALAKGTAGTPGQ
GSSTLEQYINMYNKYRSFEGDICNDLHAGDLPLNMSLRAGVTTIDSITSLKAAPENPKPFNITVQDSNQIACCKNGS
KSSLNRPRGTCSYPENATVGDSDCVCGQTPGGDAIAFEYMECANFVSRCTSDDDCAKAGYKTYKCLTGSCCGGGV
CFDPYACSQKGVPLI

>QLI67810.1

LPPPTYVYHVTSKKPNFMGRGGLPLQRERPRVWGQGLSDYINTGMRTANGRLLYAPLGYQEVRQALRAWADPTS
SYDQLLNIAVGGWPYLYRIRPGPSTVNGPQDGSFYQTTGWPTDQIEWYSPLPDISGSPPSQQAIHDMSDEALDQY
IGRLKWAVVKSHIDHCMRVAAAATRPVLRNNDPNEQRRRIDEIVNAALEGCLCCSTHNPNPGPPPTGDQAAHPG
PSTRGDCERKSAEIVKGLTPDPDPARWEEVAWQLVNVCDPSQSEEPVAGPSGLQQRCREAVDKKLKPVIEKSEAQ
RRPVPEVIAQAQQAICSPSASDQAPESSSDEPPPKKSKDATERCNRDVSNVLAVFNDVGPGQEQGIIDYLVGSDHA
PPRDLCFSAGVYEYFSGFPWDMLDDQGQDEAALPAEPEQQQPGQEPQQEQDQDEQLGVQQWPDFLNLVLQR
FHVDERRLVVYLDQNDGLSSLQQEHAPIYSLLQQTFPSLLGVLRQIAEAGLTFGSCLPGPPSSPKARATQGKKDELCA
KVLAAIGESAKAPGSKQKENDKAPSTSPSTPPSAPQDNTSPSALKTIAAIGVPLAGAGLGAFAASAAGEGLLASTAAS
LGIGAAVGTAEGVEGAVSTVVYSMPQRVGQILTRALTRVSRLGHRLSNPLLRRITSDAVRAATRRAAERIPLLP

>QLI67826.1

RTLQPWQYAPLPLGDIKANGWLWGESRAMADGLFGHEHDFYVYVNLSSWLNEPGTNGVEYSNLNEALPYWFNS
IVPLAYTLDNHRLREQAEQVATQVLGHQAGDGWIGPEVGELRNFWARTPMFLGLIQLAEADKAQWEKPVVDSLR
RFMTLANKMLNNQSEGFARCHRDVDCTWGQVRIHDLIITIQWLLENHPGSPEEDSVLWENMDMFYSQNPRNW
DAWYANVQPANPADSRTHPFIHGVNVGQGLKASSVLYRINGSQAMIDMTHKAVDWTFTNYGSASGTILADEHE
HTSAPYAGSELCTAVETSYSLAYLYQILGDNVFADRAERTIFNAFPAMMTGDQFDGTEEERRRSRGKWAHQYMA
QPNQPWAINITKVDGHVPNVFTTANSGLATTFGMEPLYPCCTVNHGQGYPKFVSHSWVRVGENGLGHALLSPTK
VMTTVNKDPVTITCDTTYPFGDTLVYTIDAEAEFDLYLRVPDWATGYTITAVSPSGVSTTDAGTGKGMQKVPIQAG
GSKVRFTISAAVRTEERPGDSVAVFFGNMLYALDVGYSNTSSFPHAFYDTKGEGLDYLPYRELRDYYIKNTQPWNV

AIDPSTLKYSDAGNKRLVDPIFEYGQPPNYVTVDGCEIEWGLYMDTIPDLPPALADRRCIGDKKTYRLIPYGAAKVH
MSDLPTIKL

>QLI67831.1

QTANSTVRAAVVFVNHGETTPNLVSDHVILTPNGAQQMQRLGAAFRSRYLGGNASNTTSSNSTDTAPIQSLSTDSI
DNTQMAIMAAMQEWSTSSAAAFMQGFYPPSPDTSYVGHNLVLNSNTTDYPLNGYQYAQIITHPNSDSNSVAIQ
GYEACTAWQNQMSQNLSQNPEIAQRVHDKAYFYTKLFSSDPMQGSLPVDQATYLNAEEIYNFVDFNYAYNGTV
HKDLKDPNGTLSVLQNNAFSLERSKTSYSGNTNKDDPLHVLYSIAGRTLANKVTDQFSTFLATSGTRGKLTIMFGSS
RSLMAFFGAAGLMNDQNAAASPFSRLPKPGSAIAFELISESDPSSSTDSFQVRFSYRPSADSGDQFSTYPLFGSGFG
GAAMSYASFLRKMNEISTTASEWCTICSPRTTSTFCLTPNVLGSQKSGSSSSAISPAVAGVLGAVLMAVLIAGVTAG
LIALGGWRFSRNGRQDGGPAGSAVGGFKGNDKMVSDADMTVSNAGRNEERVGSWELRDGGRGTHTGPFGSG
IVTNEFSQRTRAMDEDGVSVTGAPVNARESV

>QLI67839.1

AAPSGDPFRVIDPQHWVNPDNMTCADYKAPPGTQWNDPSKNGQERNFNIALVTVDYSDLNFTITGPPNSTIFGT
PSADAANIPREDVPVWYRDFLNKPGQLNRGHTLHEYWMEDSHGRYGVDLTAFGPYRMPHLSYQYGISDMNPG
ACLPGKECSLDIRDGALSLWREDVGNSTASKYELVFILTAGQDESSTWQEFGEMKFNSKEDVPDAFGPPKTTGNAT
LPNYAATRYVPWTSWAAAASVWPYAEPSLSSSVQCESSGMATFAHELSHLLYISDNYNNPYGTPLRRTFTGPWSM
MSRGSFNGPGGPHARWHVPPVQGSSMGSLHTVRDKLQIGLIDEASIVNISRESLQTTGPLVTTVTARSVKADRIGV
RVHLGTDMSPACDVGKDLYCDGGGYDNYDVEVVDRMGADSFQADAGVMISKTKNSDSSEPFQWTIDANPQDI
KLTDFVRPNGTEAMVTMGDYRQLLDALFHAGTRSGSKAEYKDEANGLHFYILEKIRDASGVLSYTVGARALNSTSK
SRFGVRLGEGRPEPSGNTPTGKGIFCSFDLVNDGKYTQESDVTSALEPHVGFDIYRLQAEVKGKGWRTEVPNALVA
AKFGETVVANIAVGATANADDVGVVTLTVTSESDPEARTTATCEVRKA

>QLI67847.1

TEQTSSSDGAQPLGHIVVLKPGLESKHLERHLEWVKSVHKRSPDNDGENHHGVKHTYDGEDYGFLGYAGHFPPSV
LEDIKRHEHVDFVERDQAITLVLPGEETPESQAVSQDNNKQSSRIVGRGGLTMGQGYNTFLDKGAMHNAVLFSEG
MNKRSENSTAELFNQDMMTRFNFTPPSADLTDIDMSYFDRPDPRALIEQALAGLARDGKNTTDLTKRHTRTASQ
DECNGSMRFTSELVENYEQVPPNVPSTCCFIFMTQASTTSDTNQVLLTRLARSYLQTLEISGGATLSGWGQSASVS
GAYLNKAEFSKKSVIFVAIINIERQLDSPTGFEFNTHNYKPKTFNRDFGDKWIQGFHEGGKMIARVTFTAKNQIAKN
DLQAQAKASLRFWGVTGDIEASAKKSMEDVNTNADVEIKLFYQGELGRFMLQSGSPKSISEGTAQASFLQAKSWA
DQFIQNACQHRYAYRPLLDEYRNIEGFPEDQGVPDYYVAHRMSYMILSQIVVISDMKDYLLSRTDLDIKLKYSIQVD
EIKMVQLGRDWVQSTVEKPEDAITTAGDLLEKFDKNFRAKYEMLMPQKPYIAGVKVVYGGYPSKDPPAGRVKEAE
GRSDDINHGRGGDFVWLVPIRTDHAEDACTSFELVIDQVPDEFGNLVKGSKDKSRYLRCKKSSSRDKIRRLVLSRLE
AAPAPAPRVTKDRLAFIKSLLGRSWVEKVRFLRAFTGKTSNINQGRHGADELYLLWSPRE

>QLI67850.1

HPGHDLTEEILERRSFKNSVRRASLSHCAEKLKARGVQARNLERRAATAALARLSRGVSTRDADSALGESHNKTSLG
YSENTSISEIFSGNASCVLTPEVTQGPYYVGGERIRKNVTDGQAGVDITLDYQVIDVDTCDPVPNVYIEIWHCNSTG
VYSGVVANGNGESSDETNINNTFLRGIQKTDSDGVAQFDSLFPGHYTGRATHIHVMVHTNATELSNQTLGNQVY
ASHVGQTFFDQDLITAVEKVEPYASNTQSLTENSGDSILSEEADTTDPLMAYTLLGDSVADGLFAWLAFGINTSYVN
EVQPASFYYESGGVENPNAGFGGPGGNGGGGPPPSGAPGGRPGGSNGTGTSGGIASPTAISMGNRYTIGAVTFS
LVGLLLPLLWL

>QLI67851.1

ESGVANGQVTKTLEPKPIGTATENGALLVSMNFCSVVVNTQCQVSVMTSAPASGPTFAPSSDLGLSRASAAAPVP
APVPAPAPGSAPPQAASVDAAATETSSPVETSDAVQPVVPIVPLPTRFSVPASAPASAGAESTEAAMPTETEPPNQ
PTIEMTNSSGTVTRPSSTGAPAITSSALAAAAADIKALPSVAWGALILAMAVVL

>QLI67865.1

HPETEEERQAQAATLRRIAAHSKRSLANCADSPAAIALKERAVARRAAWADELRVKRGLKTRAERRGPKELEKYLDL
SHNETAKGYNLDTPLDVLFGSNASCILVPETIIGPYYVEGESFRTDVTDGEPGVASHLDFQFIDINTCEAVPNLIIDLW
HANSTGVYSGVTAELQGGLKTNFGRGIQQTDKDGVVQFSTIFPGHYIGRTNHFHVMSTDNATILSNGTFEGGTVS
HIGQVYFDQSLIAEVEKNEPYIRNQQPVTNNRQDEFTGDEATEDYDPFMKYTYLGDGAEEGLLLWLTIGLDITTNY
NANVSVASKWHPGGGTDEGGRPKEEEKASFL

>QLI67894.1

RTSESHRQLLQQLHGDDLADISDYGEAHIPPSVYTRTSPDSGPHRFLNNKTEKFAVNGTGLPEVNFDIGESYSGLLPI
SDKKDEKDNLFFWFFPTDNDEHKKKKEITIWLNGGPGCSSLLGLLQENGPFVWRPGTLKPVRNPWSWHHLTNIV
WIEQPVTVGFSTGNTTIHNEDELAKQFLGFWKNFIDTFSMQGYKVYVVGESYGGYYGPYISSHFVNANDTKYYNLK
GLMVVDGISFDGDVQSEAIAETFVEQNYNLMPFDDRFRDELHNTSKRCGYRDYVEKYYVYPPAGKQPSLLPWQER
LPNGTLQYKDGCGNLWNSIYRQARKDNPCFNIYNILDHCPDLYDPLGDDPYFNREDVKKAIHAPLQVEWSVCVNT
AFVKYDESLPPSKYELPNVIDKTKNVILVQGGTDFILPANGVLLAVQNMTWGGKLGFESRPTDPFYVPRYSTGRGG
GYGTDLPDKTGVVGTSHHERGLIVVVTGLSGHEGPQYAATSAFRQLEKLLGRVHSLSDTTPFTLPQLKNVTQDAKP
LGKGTFPIPWVYAPAEA

>QLI67895.1

YVHHFTPGIMDRNKNGVMGIFWKCARIGERLSPYISICCVFMAASILMN

>QLI67906.1

IDVDICASLNTADMNRNLSVFQTNGLCHDLCTPQGFVYAITQSNSCWCSNYTPAKSAQVATSKCSLGCPGYPDEK
CGGPGVYGYVFLNIAQPSGTKGAGSSTTSESTEATSKPTSAISTLTADGTVKTVTIIPTETGHTAGDNQGASVQDSGL
NTGGIVGIVVGVVGGILVLAGLIIFFYLRRKKQQQENDYQDDPSIRGSSSGMMGSGRPEMSRAPGSPGSTGNRSST
LQIDPRMDPFQQTLYARSGSRESVNTLRDDHDYSRRIQQPKVLRTTNPDPA

>QLI67921.1

ATTFNFCDEASAKEYAYNPPPERLIAPRRNCNRGKHSSPQRCIYRPPGSRLASIHVGSTGKLAVFMPNKMDAATVE
HAVIIVAGKLSNAGQYWHRLQNVTALHAARPDKTHRKTISVAPLLFSDRYTPGLHGDRHLSWSGPRAWIAGGAA
NSPPGSNATSIDALEALADEFACSGKYPLLTNVTFVGQSAGGQLVQRYAALARDRPSCRAHVRYVQNNPATCSYFT
ADRPSSVQGKALPPARSCAESNLWPFGFDDFPGTAAGTLAPVDYFRQYTSRDVVATVGYRDTNPCSGDQACQAV
LQGGHKRRDRNLIWYRYVHELARTGEDLTGFPGTFGDLPDWGPASENRVGLRLALAKDAGHEFAQIFSTPVGQSA
LFDDLDVLEGWRPGK

>QLI67923.1

APRLESRDEDKFAQGLPISKTGKGGPISGGTNHELDLQNPDNLGRQSTDNGIVPNLKWSFSDSKTRILKGGWVREQ
VVQDLPQSHDISGAQQHLKKGAIRELHWHRVAEWGFVYTGRVLVSAVDENGKYQAEELNYGDIWYFPKGVAHT
VQGLEDENEFLLVFDEGDFDKVGTTFNIDDWLAHTPKDILAKNFGVPESVFDNIPTPNPYILNATVSEKNVTGAVTP
VASGNSSFVYRTLEHPAEKIGGQGGVFRKIDSTNFPISKTIAATFVTLKPGGLRELHWHPNAEEWLYFHQGTARATA
FIGNANARTFDFRAGDTAVFPDNSGHYIENTSKTEDLIWIEIYKSDRVADIPLTQWLALTPPDVVSQALKVPIEFVEK
LKKEKQVFVE

>QLI67948.1

AITNLPAWSLSTNSNGAPGNGIVQKYIVETTGGDSPQFLSRLFDNLHAGDHEQFECEDIFTGATVTTYAENIDLIQAI
PGVVNVWPVTTLSTPRSPDVTNQINENRLVNYSVHHWTGVDKLHKAGMRGKGVKVAVVDTGIDYSHLAVRAFT
GVAPGAELLIFKVFSDAPTPSDTTEDVLIQAFCDAYTAGADVITASVNRPGGFIDSPWALVASRIVERGVFVSIAAGN
EGTRGPFYSGVGSNGRHVVSVAAINATGNPLLSDSDSQHRPIAAYFTSWGPTNELTMKPDIGAPGYNILSTYLDQG
FETDSGSSMAAPYIAGIAALYIAHHGGRELHGPSFAQSLAQRIVASGRNVAWSAGEVVLNESAPPFQVGTGLVDA
WKVLNYTTQVSFEPISLLDTELFQAEWDIEITNNANQTVKYTFEHESLPGVEIYDGVSDIRPLSQLQPLRIVPGVSLPP
DTTLHSGQTKTFRVRFELPTGADDDMLPLYSGKIWMKGNNGEQLCIPYGGAAYDTEKAFDTMFDGAPIIDGWHG
GATWSFDPAKTPSDFADVSSRLSYPCFHLRWDIFEAGWTESQWRYPLEIGKKQYIGSATSVRDSDKFLWFDASDV
DINDTVSFPLTRIPRGYQRYWWFGKLSNGTQISPGNYTMRIAALRPYGNPKISDHWDVVDLDRHTIQKLVQRNEP
QRDDFHNVITDVIHLSNVLMEKHNFQLL

>QLI67949.1

AANLVQHERVPQLPQGWARMDASLDPDRPFRLSIALRQPEIHRLASKFARGSGHLSLDEVRTLRAPDPKDAAAVV
DWLRENGVHDTVTKDDWIHVNTTVSTAESLLNTQLQRYSFDGKVSMVRAREYSVPSHLSDAISFINPISNFMTPLR
EPMTPLPAQDARDRRATAACSGTVTPKCLRQLYNMHYKAPGNSSEVRLGVSGYLEQHSNHADVRDFLRQQVPEL
PRYDFKVELVNGGTDPQKPPTAGSEAQLDLEYVMGLGFPSQVTYYATGGRGEKIGDDGKPVTGKKLDNEPYLEFIQ
ALLDKPDDEVPHVLSVSYGDDELSVPRPYAERVCGMLGLLTKRGTSIIHSSGDGGSAGGRVGNCRTKDGTNKKVT
MSTFPASCPWVTAVGATSNIAEPPSGASFSSGGFSQYFERPAWQAAAVDKYVEEINGHLDGYYNASMRAVPDISA
VGTNFRVIVGARRKLIEGTSASAPVFAAMISLINDARLRKGKPSLGWLNEILYSDKVTSLLQDITKGQSYSCTWDKVS
PGGWPAKQGWDAITGLGVPNDFAKFLEVLEDV

>QLI67973.1

SPESTHSSGDSLIKSTVLGSHRDVVGMLIDRMANIRHPDGLGRTPIRAAVGRIRNAALVRLLADSGANLSETDSDGR
NLLHVAVNGHPKS

>QLI67983.1

FDPDFRFYASNAQDCLYSASNASKCTGTDAQDLNSCLCNNGGGFITSAAQCLGQNSAVDLVGMYNTMKGACSIS
KTPISVSQNAFLNAANGAASSTSSSASSTTTSTTSSTATSASTATSTLTPTTGTPSPTQSNASKGADVDNKSSGLSQG
ALIGISVGTSVAGVLAIGGMILYFRYRQKKKAEDETSPMMAQNDYYKRDTATTFPPTEPSPSLGLSGADTKASWGS
SPSPAYPSPNLDKSPAQYAGPYAAPTPQDSPAMHGVASPAFEMDASTTSTPAPGEGPVEMEGSAPSTHLQIRR

>QLI68000.1

AQDEKIPNGPSGARAPQETVVEIRHVDLTLEQSFQVAEASRHDIEKERLLSIMARDHGTWSPNHPRYRLLDALHGF
SKYYERQRADVDRLRGLYKYASKSQKQFLEKHLAYSSKFKTVEQKLATNQHVCDDIVHAAMEFYNVGTSELKRHM
ADREAEGKHADKISVSQSLKHIVRDWASEGLNERNATFACLAGTLRRLFPDRNLLKEDVRILLPGAGLGRLGHDISQ
LGGFEVTVNEWSMYMNAVYRFIEAQNTPLSQSVHPFVDGWSHHVSDDNMNRAVPFPDVPIDSSRVLMVEGDF
TTEFKNQSAYYDVVLTYFFIDTARNLMSYFDTISHVLKKGGIWINLGPLLYGTSPFVQLSLEDIIAIAKEMGFQFLETD
DFCGEPTFSEPTVRSMEAVYSFDHMALTKNAYNAQFWAASKL

>QLI68007.1

QFPVSDLDSLNLTVARSPADSSITISYKEPRGACNTAFGQQKQYTGWVHVPGEQSTNLFFWFVQGRQHTDSLTIW
LNGGPGSSSMFGFFAGNGPCEVIEKGIDKYDTIAREWGWDRASNMLFIDQPNQVGFSYDTPTNGTLVLPNESMH
QPPIQGKNNSLPSWTYMNGTFSTMNKSTTANTTQTAAIGVWHLIQGFLTTFPQYQPQPNSSIAVNLFAESYGGRY
GPIFAEQWEMQNQKRTNGQLDANSTLEVRLSSLGIVNGCIDQEVQVPFYPIFANDNTYKYKALSDEAAAYYGRRFS
APGGCKDKVQQCAAVAMAQDPAGQGNQPEANRLCDAANTACFALQEPYSNSGRSVYDLAAPSRDPYPPLRFLD
YVNQGHILQAIGSPINYTMTSNAVFQAFQDTGDLARGGNIKRLADLLNQGVRIGLMYGDRDYICNWLGGQAVSL
AVANQTGPNYATGFPAAGYAPIIVNDSYIGGLVRQYGNLSFSRIYQAGHSVAWYQPETAFQVFARIMMGTSVSTG

ETISLSSFNTTGPNVASHDEKLPAMPSTTCYIRAFASTCDYDAQDLAFGGNGTVINGILYSKSADWPLATTQPPSSTS
GQPTSTTMPLTGVFTATKVPDSGSAARLLASGMLWIMISSFWVIQGVQFFF

>QLI68016.1

VPVPAPPGIPTASTARTLLAGLKVATPLSGDGYSRTLFPTWETIEGTCNAREFVLKRDGTDVQTNTACVAQSGNWV
SPYDGVSFTAASDLDIDHMIPLKNAWISGASQWTTDKRKGLANDITRPQLWAVSAHANRAKGDSSPDEWKPSLK
TFWCTYSKSWIQVKSHYSLTITDAEKGALSGMLDSC

>QLI68017.1

LDLDSIFAEVSAVNRDFENILRVLLRQALRHDDPQTIKEVCKFIESATDNLNTASDKIGFDSNVDDPSDVVAAFCDD
METFTNTQIKAADILVFKAANIWDAMNGAQLPDGIVRLSSAYDKYAGNMIPLTEHCTTKHMQNIYLVKLTLHRVL
VDLEPNNGHHRRGTHHG

>QLI68025.1

GPSTGGPAASSPSPSPSPSPSPSPKSSASIIIAKLPPCAIPCIASAVANSTCSPDDIPCLCADKAANEEGTTCVEARCSFSD
GLSTKNATQTACQAPIRDRSAEFNNTVIALGVIAIFITATRLAFKQFAHPTKALGADDWAILGTLVICVAGLVIVLRG
MTANGLGRDVWTVTPAQISRFVLYFYVMEILYLAGISLVKLSISLFYLRLFAATVRPAVLRATVAFNLLYGLIFVTGAIF
QCIPVDFYWEQFFDPAEGACININLYGWLNAAIGLAIDLWMIALPMSQVLPLRLHWKKKVGVAIMFLLGTFVTVV
SALRLQSLIHFAKSDNPTWDQWTTAYWSIIEINVGMICSCLPTLRLILVRLAPETFGSANRTPHLRELRTTSRTEASRT
KEETDLQQRYVVDLEEG

>QLI68028.1

VPFDQYKVHEQRGPLHQQWIKGQQASGSTVVPVRIALKQSNLEKAEDYLLQVSDPASPKYGQHFTTQQIVDLFAP
SEQAINQVKTWLVSSGIPAGSIALSNSKGWINFNTTASELESLLKTKFYLYTNNATDGVYFGTEAYSLPHDVSSLVDF
VMPGISFTQMQKKTARIAHPSSGKLQGASIDPNGTDHCDTYVTPRCISALYQIPPAKLANPNNTLGIFETEGDVYSQ
EDLNQFYAAAAPGIPKGTGPIIHLINGATAPNPPSSAGAESDLDFEIAIPIIYPQKTSLYQIQYPGNAGYKNFNYIFNDF
LDAFSGPYCHDNGDEGSGKECNDLTPPNVLSVSWGDSEDPSLVSFHKRQCTEWMKYGLQGTSVFVASGDYGVA
ENTCLGPKQNIFVPDGLCGCPYITAVGSTYLPKGAKVGDPEVATERFSSGGGFSNIFATPEWQHSAVSDYLTKHKPS
YKSYNTTDGKLPSSGGIYNRGGRGYPDISAIGDNGVVVVGGSQGTIGGTSMSAPLVAAIFNRINEERLNIGKSPVGFI
NPALYKAYSKKAFNDITKGDQPGGGGCGTTGFSAASGWDPVTGLGTPKYTQLLEYFLSL

>QLI68068.1

ISVLGVPANNGLTSSPLPQLDVGSQEAPSFLKTKRQSNSDILLALYYRDPLALWKKKLDSFHWGIHVTPENVPGKST
TLFHAVNEGEDLKVFEYEKRLVNPLTQKKLLGRIKIGTKPSTVSVEDIDNLLSQVPVPNKHDASSESCVSWALCGVRK
LQEGGVIESFDTETFSDQVLQYGLEQFRATLYAETGAENEITHKIARYDVQQGKTVVEEVQGNQGTPVENPENPEE
APVLCERSALDCMGRPAKEKTPNTADEGELVPVAKQSSKENFDSLLEEFGHDGLVKNDRLYTELNVRLGELSTLPRA
ERIAGFAKIGEGALGVAGLALYGKAVADVFTSDTSVMDKAVVLTSVLPGIGCAVQLAQGIQNDHVNAGHIALCFAE
DALMLSGFWEIALVLQLSESLVEFFKEDAEQRKLFDTELFRQKGSEGWAHNVERMLSHIKSDEFAASAKTQFLSYQL
LVLYQASQLRGDFQASHQAISASVNASASQPDHGTNVDAHVKFELNRQICAAMALAKRQLRQKLESVALKHTEKL
SNDFKEQFFSEYRKAATRPISFLGIPMPQNSWNIAELDRVINEARAFPLPLYKDRIDHAIQEVMERLEMPDRCKCLQ
GSKKKVHDYTKSIKQEASSIIFTAQRFLSPRPAVAIAIMRAFPTTLLLATLAPCIHAGVIAARKDHGTFNTYTDDHCRD
FEQTIYAWDGIERGQICPHVKSVQANIANDTPFG

>QLI68091.1

FGAGNIPSIAQVEGHNWRHGDIEDMLASVAFLHGKKWTSMLIKRTYFGNWLRDYSQAVDVGSLKGVNAATIRIL
VWVLSFMAFGYATEEFEVTEERLGCYRPEEHIDNPLGYADGVDARTFDPRLRGPVQPVETEIDMKTGMKNYIANE
TGQWATSTGYIRFSFARSIHFGRLYSCGERGKGKDENLCEALRCLGQALHCLEDFGAHSNYCELALRELGYHNVFPH

CGTQTEINIQGKRIYPIVTGTFGAVDFLHSVIGEANDHFTQSEVDEVDVALKNAEGARSGPVATGDRGLFDGSGPS
DFISLLSKLPDVGTGFASEAKDLKEASEAQERENRQNQQRTRANNANVVPGMSPDFDPVKTAKRIYPILVFRDKIVK
AISRGIAKVPGLEKLLEHISETLTAFVLGLLAPFIRPIINSVTKVLKDGSSGVITASSKSQLEPWENNRCSDPTHSMLSKD
HFTNVLNSCAGRVAATILQYAVPRVIFAWENPNVPVDEVVNDVIRAFHHPALRSENIEIQRDMFQTVRMWADQH
PRRHELERILSSESVKTGKNHILNGTKSGSSGGGHSHGAFDALSQLGHGKVAGSLWSQVRTRDLDSMSGNDGEQA
SNYVSNSPAPETSKMPTPPSYTSTSGGGEAASYMNEPNTFQAGYNAPPPPGQYYQGYGQQPPPGGPQGYPGTQ
QYGAPPPGQWQQPPQGYGGPPPQGYPPYGQGQPPSWNQYPGQGRY

>QLI68097.1

QYWDVAAVLWNEQGQSFMVGRVDQCVDIPPPIRGHVQWFELGPRSSGCIFFLENGCLVESEVATYDGNVDPVP
DDVNQSRSIFCDELLSSLDILDYDESRRKLETSCKKLPLYSKCPTRDILENTNKQWSPVCYRQAGRTPAKESSRLSPSY
EQNPENWNLFVNPETRMQYSKQGGFVEWTNKCKDGSSTTAVAVRSIMMEELR

>QLI68100.1

KDILPVQVEFDLLGTVLTNFSDICLILAISGLDEGILVAHNTEPSHGDGAVMPGTVQLADKIFHFSVYGLAGVLSILSV
VAFAIGQHLYSAFGLASSRIGYDHSYVGSSYSQLELGKTSQLLSLTMYCMALAVVVCISVKSIITKVRCRGDKRVAMA
STYLIVCDSLLVLRGSYNVGYAIAFKPRDGATNTPPSVFTVIDVIADTWPALVIFCVLFALGCKKRGLWSTKQPYTPGL
LDISPQQSSRSYSRGTSQPSEADLEPNMQEPQMHEMLQVTSREHILPAPMPRHEIPRRPVHPPPLQGEQQQIRET
LEAEGEDDSPPDYYSARHQVPPQPVPQHTLAFTPVGAGEEAQAGPSFALPVQQGYDGRPLSGSPPPHADAMGLY
HQADGRTPQSQPLPYNEKS

>QLI68104.1

QFIPSILGSRDVSGCPNTLAVSYPAPVAAKGWTYRLVAKGFKKPRSIVFDDNGGLLVVDAGVGLYRLTVDQDKGET
CVVMSSPKTLVNSTELNHGLALSNDGKVLYASSSSRVYAWTYDSKAATVSDTNYTVVANMSDTDKTTRTLLMSQR
RPGMLVVSRGTVNDQDNKARNPNSGHAQIRAFDVSSQRKGSEPHDFMDGVLLGSGLRNSVGVAEDPATGGIW
SVENSVDELARNGRDIHAGNPGEELNYHGVLDDPSTDKDRGGNYGFPMCYALWNTTGFPDLGGLKPGDQFPGP
DAETFTDATCAKDYVAPRLALQAHAAPLDIKFTKDGAKAFISFHGSWATTNPVGYRISSVAFDTSKGEPSAQKSSTD
AAVDVLSTPDLAKCPDACFRPVGLAWDSQGRLWFSSDSTGEIFVLHQNGTSSGGGGSTGSPSLVADKAAAWAVV
LAAIVAGLFLA

>QLI68105.1

FDKWNVWLDHSDVLGDQWLGDTQLGLLALRIAEQEGRKLCILRPKSLKRDNAPMIMKTLNEVCREKSLVYMPGE
VYNINSPMVTTNIVDVKIILTGRLQWSGNIDHWLRVSMPIGFQNQSTVWHFGGHNVLFDGYGIGTLDGNGQLW
YNWAVSEGNLPHRPININFKGLSNSVIRGLRFVQSQMWTMAISHSRNLELSDIYVNSTSNSRWSTLNTDGCDTISS
DNITFRRWWVANGDDAIALKGNSSFIYIYDSIFHGGQGIAIGSMGQFDGKYEYINNLYARNLTFVNTAHVAYLKTW
AGVSRGVPPNGGGGGIGLATNITMEDIKIRRLRQQPFFCWQCENYSGWAGRDCNSSKFKMSRVTWKNVTGTVN
KDVKDVGSFQCSSAAGGCDDFEVSDIDVWVDGGDKLDNWHCENVHGNKGFTCNDPPPQKSG

>QLI68133.1

VAPDYDTSTSLFFDRMYGHNITIPTIAARLTRWDALYFMHSTIRGYTYEQEWAFGLGLPTTVGAISRSLSSLTPANYA
LEPLVAIALAHASHFVAMLALHRLTMILSGNAKLAFVSSALHILSPAGLFLSAPYNESPFAGLSFMGNLLFAVSLKSKA
NGVKRNVTMLASGILFGAATAFRSNGLASGLLFAVQAVDALLLFARAPSLCTLLSMIAPVLGGLCVAAGSVVPQTV
AWMRYCGSDGVGRPWCEKMIPSIYTFVQDHYWNVGFLRYWTLNQLPLFLLASPMLAILIKSGLDLISKPQRITTAS
RNGAAESDLNTFVRALAGSQALIAILAITTYHVQIITRISSGYPAWYWWVGSCLMDKRRQGYGVAIVMFMVMYGS
IQGGLFASFLPPA

>QLI68151.1

QSYKEFRSRCSFDADISYWPDWVSSLPDVMNISSLSIPGTHDTMTYTISNSTLQCQNWDLKTQIYAGIRYIDIRARV
KDNILTTFHGDEPTGVTFERILLTIYDWLDTYPREWIIMRLKEEGAPLGNNNMTFQEVFEDTFNNYYFIKERAKKHW
FAYNTSMPIPSVAQLRGKILFLQEFPSTPPWKYGIEWNGPQMILQDEWIVKDVNHLDDKWKAIEEHFEITNFRPN
DNSYLYVSHLSASVGVLPVEAAAGPLERSIVGMNDRSCDYIEWHYPEPDALRLGTVISDFPGKVLIFDIIRYSRRSYND

>QLI68164.1

LVAAPPPTRVDRSGPTLVDRDLATATSVLADVKNGFGSLATAAKDFNGDPGPLKTSASSLLAKVDSGTTAIKNMTP
LSFFECLSLLTPAQDLQKQGKALSDQLKAKKALIQQYNQCVTTYDFLDQGVTKSATLITAVVSKVDSSFKETVQNEG
NKITQQLKDLRDSFSPGNCSDS

>QLI68175.1

AAPTPSNDTVVVAGMLQLTAESIHNATLDLKSKFTDLGGKDASDDAASQELGRLLQLATNTMSNGSAVIEAISKDS
KSLGNLKYKRDLLGGLVQSAGDFFGGIAKAGASIVAGFSKAGGNALKGNLLGAGADILGGFVQAGTDFSGGIVAAG
ESIINGAVQDVSHTISSLVDVVAVPAISTLINLVANTVQLFISSFLNLGLDVLSRFNHDSFIQEKFAGLTEQLVNFLEGL
KPFSTLPGFYPLVDDVTKVIVNIPF

>QLI68176.1

HDHGDSHIPEGQTVSLEPLDTTLWIHIFINMFAYGVIFPIGMVLGITKSRWHVPTQVVGSALALLGFFLGHAHSGRE
FVSHNIHSVFANILQLLLIGQVVLGLYLKGHWEKGVNGKIRKLIRPCHSIIGKAMPLLSWAQMIFGGITALGFCQGEH
VGQCAAHFIMGGAFIAYGVILTIILLVGQIWIQRTGRSQEFFDSAVIAAWGCVNTFTEHRWGTAWVKNDWQHTT
MGIIWWCAGLAGMWLSRDRDGNPKRNFIPGFVILVTGWAMSAHPQELMVSAMTHSTFGKTLMAAGLSRIIEIA
FVLKDKQSLSESGRSWNSFQYIPVFLLYAAGFLFMGATEEQMNLVAQSSMDGVSYILIMYSLAFLVFLFTNMLVHL
YDRLANPDINTKDFPNGHSRASGHAAEEGQLRDAEQFELDGLMSDDEGGEGQAMLRESLDRPSIDTPSTLGKNN
HGRV

>QLI68192.1

AAHAFPYVPTQILLPTACVSEATCSGADLAYVFSQGDDGRVRFSALNYSADMTAGAQPAPLTTDLPFLKDQPATTA
FGAARTSNGSVVVYSGACDGAAGSLWSFSREANGDGGGAWSKQTTTPGRSQQGSPRGPYFLGGTLAFSSKLSPA
MDQPTVYTYGGMCRAPEASAETWQSDANYTKAMVSLTPSTQAADTTYSLGVASTAGPRTPIAGFTLTQLPASVT
NISGAVTQQAGFVLLGGHSQKAFINMSTAAVWNLPEESWSYINVGGPDASAGGENVESRSGHTAVLTEDGTSIVI
LGGWVGDVSNAAEPQLVVLQLGQTYSSWKWKVPREQPGGKGVYGHGAAMLPGNVMMVYGGWETSASGGSS
KRQASSGGALRFFNVTSMSWSDSYSNPRPGKIPTGTGSHDAPGAGSDAVQSRKLGLGLGLGLGLALLLALLVLAFC
MWRRRLNKRRDSRRDEAAQVMAQDARYFSHDADEMTEREDAFSWHVAGHSGYQGASEKSMGYESLRGARAT
LDDGHGRAITRKPVMSRSMRGGYVPAETRFNALASPPGTIHPILEDEEDEYHQAPQHGTEPLTPTSEEHSDPFVTP
TATATAASVLSPPSNRSSTTASPEERRRYDADVQDWVCDVDAADSLLERYNSSRQSRRSPTRKNSTRSAALRDDES
RSGSNLSESNRSAADSLRRSQSNRKSGSFLGGMLLGGDHPKPSSSSSSSYNTAKSGFGALQAEGPALLGRTTPYTAA
NAHDDEDLTAPSSPSKSKPRRSWLGSLGRVFSQSGGSSPRASSENSAMRPEMEQYGGDYEPRAGLPGELLRRKQG
RSDWEDGPEAGPVAGGGEQRPENDWDIERAVEQRLVQVMFTVPKERLRVVNGDAESQTDMEADDTRQKGAR
FKETDELQEPQVAELVDPDKNSSLSTVERDERGRDDRPYSEVEDPDLLHVDFAEPRLSHSTDGDSERRSSGAVFTAE
AIRFERPRTRVLQMVDTIESRSQSSSPTRGQGHGEE

>QLI68212.1

GITVVWQGGSCSNPTGPTIDISPEGVNTCTLYNFFPNNDPRGHVTVTTSGGPNGVAIFQLPQSQEDQHIQCGSLQ
GIFHQSGCYDSVTPALDVVTAECDQTGCNNLAGIPSGKLVASIPSTPAKRAKRGAEGSFTSRRRDSCSGSTSVTCDD
CVSCLAQGARVSPFISCPGQGNSCSVQLSHSVTVTDSFSMSVGAADVFTASVDVSHSIAVTNGDSETFTVSPGQAG
FVQYEPLAQCGTKTVHNGDGSVCAQAKGTFILDNQGAGGQLSFVRSN

>QLI68234.1

ESISVRSELWGKTGVKANTVHEQKPLEDPSLPPSVPGDYITGISNNFNLNCSADMPHLRNIREKYSLNERFQYMKRY
IRFTRVEGLERKRMTKISQNLLNGAFKTIDVNKHYGQDACEKPLEVQVPASGLPSSVNASDFMFGVSTTFKRFMDP
ETTPINEWIYWLTDSRGHSNGGKLVLMLLDASDDELQEVANLLGDVGIDVDVYHSDSSLEMAVRYLYLVPTLYSHP
EAQRKKWLVICDDDTFFPSMHELMEKMSKFDHTREMYIGTLSEDVGAIERHGSQAFGGGGVFLSLPLAEKIAELFG
SCTTEQKVLESNSGWGPQGDIILRKCIYENTDTRLTTFWELWQLDILGHPAGFYEWGIKPLSLHHYRSSGWHKAKP
GMYTKIAHTCGEDCTLMRFQTKDDFIISGYSIAHYPEGVTFDTNQMEATLHAAPEDKGWNLDFMMGPSRQNLER
TGKKISWELEESEVQSDGSVLQTYTRKKNDERWVHPDRQPMSNIDGIIELVWIPS

>QLI68241.1

LKYPSVGDIILHARVCEKAENIDVKQCKRAFVKYVRQFVADHQHRSVSDEIFWNTMKQWSLLRTPVNYALYCDDG
DKKFKCEPSELNTNKLDCQSVSTRAGQNCPDEDCLPFSNLTSFCDEKGGCETCRDAGTVKNSRSIAYKQEPANSATL
KKRFGGALLNFSCTLKQGDGSGNMTRAQ

>QLI68248.1

APVEVRDPQPVRDRIDLDRGGFATGEYYNGVDLPPSNQGEFFSGTQRPSYDRGDFADEESTDEELYPNDFTLGRRK
TAVIS

>QLI68250.1

MPQGPLSASPTVQELTEKYNGNYKVQCQAANGRLECKTYELVDQYHDIPYFYLTPICNQHGGCGTCEYGPGPKPE
DWFLEQFHCELKH

>QLI68274.1

ALNCRPEGPVLPKPKLSGSPIFKSAGENLTKTLDDAVKGVIKAGWPVENVSFSLAVVSTDQESAGVPIWEYHHRAE
RNDRGVKNITRDSQYLIGSVSKVISDYILLKSGVDIDRPVTDFLPKLNSSRSKVRWKDITLRMLGSQLSGAPTNNGFS
EYYYLKELFVQSGFPSIKDSDYPPCGVIGFNKGCSANEILEGMISQYPVTAPMERPAYSNIAFVVFAMALQEATGKN
YTELVADIVSKPLDLRSTLPSPGDDGKAVIPPGESSWGADYGYNAPGGGLVSSVSDLCKFTHALLTRSLDLTPTQIRK
WLKPEDWTGAYSAVGMPWEFFRPLTLTPSHPHPVTVAGKGGGAQLYSSQLNVVDEYGMGLVMLSAGNPGASI
ALSDALLATFVPAADEVSRDQAEKQYARTFKSERTNTQNKLIEATFKLDNDSLVISEIRDGGNDVFGGIKKIWGLTM
GQYTATFGSTMRLFPTDLYQTTQMEGRNVTAEVWRLWPEFGEPLESDMPGSNLGFENCLQWALGDWIHYGKE
PLDRVVFYKDASQDVVGFEMPFLRSGILKPM

>QLI68282.1

KNLFVPPSNYGIGQVRPIMTLPEAFRVAKGSGRSKIVLVNNGYAGGDIDRVLDKIANDTTTTGAPDVPMDIVRVSH
EDDLVTTCRSTLRGVTTCFCAVVMRSSPTEGPGGFWNYTIRTDAAFWREPVKFDVRVSTNVEQVYVLPMQRAVN
AVIAAFNGSSSDLLAKTEGLPFASISQKERDTKVLQAYHKSIINFMAVAFIANILWITYHLTGFVATERESGMSQLLDA
MMPTDKPWKAQAVRIVAHHLSFSLIYAPAWIIGSIIIRFGVFVNTSVVIVLLFNLFSGLSFASMSILFASFFKKAQLSSI
TATITILLLGILAQALTRPAIGPVATLSVLFAPCNYVYFFTFMAKFEKWNTAADLAKSPPMSSWEMPGIVFFIIVVAQI
FVYPLLGAIVEKKLHGTTTEGRKIQLQDDGESSLVENAVELDKFTKIYYPSFVSRLFCSRLKPREPVVAVNELSLQAGR
GQIVALLGANGSGKSTTLDAIAGLHKLTSGSISIDGRGGLGIAPQKNVLWDDLTVQEHLVTFNRLKAPGAPASKDDI
LELIRAIDLFPKRNALAKTLSGGQKRKLQLGMMLTGGSAVCCVDEVSSGLDPLSRRKIWDILLAERGRRTLILTTHFLD
EADLLADHIAILSKGTLRAEGSSVELKDRFGSGYRVHVRGDTETAHLPSIPGVRKQQEFDLVTYMAPTSNLAAQVIKS
LEETGVTQYRFSGPTIEDVFLQLAEEINDEEAFKNVNAGVPREVDSANEKDLSSSNGSSTLQGFKLLDGQRVSYVKQ
AMILYRKRLTVLKRTWILYLIAFLLPIFAAGLTALYVRGKKQVGCTPADQDSSFGTEDAFTQVRNNMTIVFLAGPTSK
LPITTVNSLLQPILNGSRGGASVGSAALQNLKLVDTFDSWKQHINDEFRNITTAMWLGDENSNPAVGWVANVFIS
SSITAQQLLDVFLTNTTIATTWSSFDVPFNPGIGDALSLVIYMGLALSCYPAFFALYPSNERRRFVRALQYSNGVRPFP
LWGAYLLFDFTVAIISTALLTALWAGMSNVWYHLEYVFVVFFLYGLASILYSYFISLFTKSQLATFAWSAASQAVFFL
GYLISYICVVTYVQVDRIDSSLLICHFVISVFSPIANATRAMFLATNLFATACDGDKISKTPTGLVQYGGPILYLIIQCLVL

FGLLLWFDSGNVGSSVRGLFDRGKPVTVAEELDAETADELTRVTSSGQMGDGLRVIHLTKSFGKNTAVDNVSFGIK
RGEVFALLGPNGAGKSTTISLIRGDIKPSRNGGDIFVEDISVSKDLAAARRHLGVCPQVDALDQMTVREHFEFYARIR
GIPDIEHNVSAVLQAVGLEAFASRMGYALSGGNKRKLSLGIALMGNPTVVLLDEPSSGLDAASKRIMWKTLAATVH
GRSILLTTHSMEEADALAGRAGILSRRMLALGTPDNLRHRFGDILHVHLVSRTAPRTTDHEMQRVISWIQTRLPAA
NVDTKTYHGQLRFSVSASEVLALQKQSANSEPEDIKSHDSDTAHAAREGSAIGQLIVLLEENKEVLGLSHYSVSPTTL
DQVFLTIVGQHNVKEENYEEKKESRWVKMIKPKHPKK

>QLI68289.1

DDPDDACKGLSDGFGCFWTQGDCNRGGTCVRGTCRGPACDLPNNGMYTPQS

>QLI68297.1

GPTSFHGRSGSNNMENLVTFGDSYTDENRFNYFIQHHAAPPIGEMLPPSSDTWSGGYAWGRLVANATGAKYYN
YAVAGAMCTNNVDSRDLDAINGPFPSVLEYEIPAFEQDTRYPGLYPDRRPDNTVYALWIGTNDLGIVGILADKQKA
GTTITTYVECIWTVLDRIYSAGGRHFVLLNQAPLERAPMYATPESGGLGDTPGWSNKTAYNTTEYANKVLEYTTSV
NTMYDYGGPMYLLAKRRWPGASLSLFDTHSLIMDVVNSPASYLDAPADVVAPFRTCLLNGCVDSKHPRSSFLWFD
ELHTSERMQEILSKNFIDVVQGKSKYGTYYK

>QLI68305.1

EQLVPMQAVGSVSARQLFAKSEPVFDFSKRATCDIGQVACHDGCIPIGGRCCASSGWCKVGTVCDDKLGGCCPLG
KTCTSGPTGGLTCDTGYTPCKDKCMPVGGDCCSNGNFCKAGTKCDGNGACTTGGGGGGGSNQCLSTEASCNDK
CMPKGSVCCGNGRYCQSGYTCVNGGLSCSPGGTGGDGGGGGGGNQCLATQEACNNKCMPKGSVCCGNGKYC
RAGYTCVNAGLNCSPGGTGGGSSAGGDAGTTSAALGTTALPTYTLTSDDGSGASPTNAQPTDTQSGPTPLGGTTA
AANPVPTDSGAGTAAGTNAPTQTRAPAAAGVVQVPLALGAVAMALPLVL

>QLI68306.1

HFQMAPCARSCIQKALPNVGCTGNDKDIAFCLCRPRTKSKLVDPVAQCANQSACSAADLLRAQSIIDWRCQNVHP
ASFSEATFDEPESASWRQATQTPQPVPSSSSTSTPSSTPSESSTAISTASGSSGLSTGLVLAITSAVVAALGSLAGAFW
CYRRRHRRRASSGQVRNGPSEEEKDGHPTRSGHNELLGSTEMAHEMDIYVPQQPTPAGLSKQVYEIDSTPLRMVP
GSSALEAGQIVSNAAPPGSPRGPTRADGMKSRTLSDMRTAAQENGDQDTPVLGELQKRRIILAEERRYLQRVHEIE
CEDKRLEQQISDLTQQQPSSPLEK

>QLI68331.1

DDVLYSSRFSKRGLLPNGNYNLSFFHVNDVHAHLDQYTKAGADCTDATKGCFGGYARIKTKVHELQKQNPDHLW
LNAGDEFQGTLFYTFYGGEKIAETINDLKFDAMTLGNHEWDGGDENLGKFLKNLTFPIVSCNVKSTVKDLNETIKNY
HIFEKHDLAVIGATTETTPNIANVGKGTTFLDPIPEIQKAIYEIRNTTKVKRIVALTHLGYEVDQKLAQQTEGLSLIIGG
HSHTLLGNMDKAEGKYPTIVKDKGGNEVFVVTSYRWGEYLGSIEMTFDDNGRALSYHGAPIHMDNTTSLEKNLQK
NITAWRGPFEKYAAEVIGSTKNVLDQTACQKGDCLLGQVMADAMLEYRQNQTNGTADKPDFAIINAGGIRATIDE
GNITRGQVITSFPFGNAVTQLKYSGADLRKILEGCVSRVSQFNQKKTSSWFQVSGNIVVEYNESRDAGSRLVRATV
GGKALDDKTDYNVVTVDFVAGGGDNLLKPATEFVTLESLDQVLVAYIGAHTPLQNALQKRVVSSNGTAGGGAGD
GGKASNGESKGASLRAPRLLVGLSFLEALVL

>QLI68346.1

HNIEVPPCGQECKTLEYVGCVKDGSPSALIMRSNQDQGQMTVEKCGAVCKGNGFRYAGLKYYGICFCGNTLGGSP
ADEGSCSYPCSGNNAQKCGGDSTLSVWEDKTYPKKPEEVSGDDYKAIGCYTDDTNKGRTLSWPVNVDSASITPSS
CIAACKANGFAYAGTEFGGECWCGSFLNPATQAVDASQCNIPCHGDSSVSCGGRGRLTVYEAKVLISTEPCIPVPP
PISTSVAPPASTAGPGTSAASSAPGSSGAPGSSVPASSNSGVPTLPGTGVPSECSAPPTVTVTVTPSSTGPVPTGPAS
SGPESSGPGSTGPGSTGPGSTGPESSGPASTGPQSSGPETTGPASTGPVPTTTPATCTTVVTGSDCGPAATTAPNQ

STGPYTSGPVLPSGVVTCTKTLTGSDCPPAPTGTGPVPTGPETTGPQPSGPETTGPQPSGPETTGPQPSGPETTGP
QPSGPETTGPQPSGPETTGPQPSGPETTGPQPSGPETTGPQPSGPETTGPQPSGPETTGPVPTGPQTTGPQPSGP
ETTGPQPSGPETTGPQPSGPETTGPQPSGPETTGPQPSGPETTGPQPSGPETTGPVPTGPQTTGPQTTGPQPSGP
ETTGPQPSGPETTGPQPSGPETTGPQPSGPETTGPQPSGPETTGPQPSGPETTGPVPTGPQTTGPVPTTTPATCTT
VLTGSDCGPAPTTAPGQSTGMGSSPSGPAPTGPALTSTVITCTKTLTGSQCPPAPTTTPNQSTGPSTTGPAPTTTPE
TTGPETTGPPETTGPETTGPPETTGPETTGPPETTGPETTGPPETTGPVPTTTPETTGPETTGPPETTGPETTGPPET
TGPETTGPPETTGPETTGPPETTGPETTGPPETTGPETTGPPETTGPETTGPPETTGPETTGPPETTGPETTGPPETT
GPETTGPPETTGPQTTGPVPTTTPTICTTVVTGSDCGPVPTAPGQSTGPNPTGPAPSIITCTKTLTGSQCPPTPTTTP
GQSTTPETTGPAPTTTPNQSTGPVPTTTPVTTCTTVLTGSQCTPAPTMPGESTGPVPTGPATIITCTSTLTGSQCPPK
PTTTPGQSTTPETTGPAPTTTPNQSTGPVPTTTPGQSTTPETTGPVPTTSPVTTCTTVITGSQCTPSATMPGESTGP
VPTGPATCTSTLTGTQCTPTPTGPVSTVPESSTPATTVPASSTPATTVPETTGPVPTTCTSVFTGSQCVPSATLPGEST
GPVPTGPATCTSTFTGTQCTPTSTGPVSTVPESSTPATSVPESSTPVTTVPASSTPATTVPETTGPVPTTCTSVFTGSQ
CTPSATMPGQSSGPVPTGPATCTSTFTGTQCTPAPTTTPATSVPGSSTPVTTVPESSTPATSVPESSTPVTTVPESST
PATTVPQTTGPVPTTCTSVFTGSQCTPVPTTPGQPTGPSPTGPIPTGPVTCTSTFTGSQCTPTPTTPVTTVPESSTPV
TTVPESSTPVTTVPESSTPVTTVPESSTPVTTVPESSTPVTSVPESSTPVTTVPESSTPVTTVPETTTPVTTIPTIPATTVP
ETTTPVSTVPVTTPTTPVPITSTPTTPTTGFSTTTLCTTTATTTPDCEYQIGDWCAPPVPDWHDKLGCIKSAAVCKLQ
WASCFKKAGLTGAIGCFKYVEFCKSIELSCLKCVLGKCDKSDCKPPTVSVTTIVTPCEPTATNPPTPPPTVCPPKPTNI
CKDTGSICGVPLPIVSCNDDKSQFAAAPFKLYNDADTSKCPIFPHPEVPNACAEACKEQYDQCTKTSLWSCHGLKRS
ESAFEDDFVAPTANEGPHRRTLGLLTQLKCKKQYSACVAANKYIDTSNHCSSWEGCY

>QLI68359.1

AVVRNSECDFHMNAAGSHGGPLGELNGGQIRFGNIPQTTFHIDGDKIWDRNGRGCWWTPPTRVLQCDTNQPP
DSGFTIGCDGRVSYHGQTTFYQCETGDDNQYNIYLGPNEGINCGEVTLTADGCHIACPPPPQPAKSCPANLNGAYE
FPHLIVSVDKSNPDKAAGTSFFGEVTSSISSIFNFDIPAADAGKTCTLVFLFPRQDQLTTSSFNFSGDGKIDFSLLNSPA
TQGTTWNNQPGKKTDYGITTVAPGNSYTIASFPCPAGQAIGFEMSDCGDTNFRYFQDYNPSPIGLYITKC

>QLI68382.1

LPSLQSPRADQASNPYPRCRFVADWSQEDVLKDPRGFEQDLLFWEGKFHQNNVSYNSANGMTYDGTQLNWTT
GARSNKHPFSAASKEALQIMLYAHAVAGSKKAARFLTPDDPSKARKLAASIMKTKLQTYLQFNESNPGFGGFLPW
MTTSERQLSPTWDWVNRVPALDNGELVWAVYAYISAAEQRQDQSIRDSARGWQNWLDYVKTTAVTVFYRGGG
HVCAVTKMNNQTLPPKHAKQGYQCEGTNYLDDPYEGELFTHFLNAFGGLSREDKDTLWEVKRAKLVSVEYNMG
GVGPITVEQGYWFSSHEPWKVLELPYYDVDLVRRLYHNAERARTCNSVVTKVPGLFASVNNSTDPNTDEIIGYISPA
GIPSIASQKEQEHDVITPYGVWPTMLFDKAVGLAWWRNMVVAKKMQNLYGSTESTRVDGKLVSALVTWDSKIT
TVVALLGGVGDLVRSKMKMDRIYKDFIAITKKEYGLVFKDLKGEDIALCLPNETVPDAGLEDFTLCSA

>QLI68386.1

AATSYCPVPGQVCFQWGVPEAAASSGSGSVYIQIRATTSYEWVGLGIGEHMSGADMFVVYADGAGNVTLSTRRG
LNHVMPVYEANAGVELLAGSGETDGRMVANVKCGSCSQLMLGSSNAWLSAWKHGGSLGSRDLDAQIGYHDGH
ALFSVDFAKATISSDRNPYVGGGDDAGNSTSGSGAGPAPGGGVVVVQADPIQTLVNAHGIIMSVVFVLGYPIGAM
LMSLVGRWAIHAGWQLVVFLAMWAGFGVGYTLARRTDLFFRQCHSQLGIILVCLVSLQPLLGYLHHRHYLRHKQR
GIISYLHIWYGRSLIILGMINGGLGLQLAGLTTDNHAFIVAYCVVVAVVAAARVSRIRCDGDQPCHGCRIRNRECSYT
PSRRGGSRKKRNVAAKPTSNEQPDLAFPSPPLSEIDHLTSPGAGLRLLSFDELDLQNGAGSLGTSESGAEDFVQHAV
RIYDSEQAILNAYYIYLHQYFPILPPPVRPVTPDNPIRITSLEGCFPLSLTSPLALAISAMLALIPFPDQEPTPVMSTEEPL
RRSIAHKLAQLALECVESDSELIDDTPGGHPSPIPFKGQPIPRFPLHPRTPVMLEGLLALLLLSNYEHTQRGNMLKMT
TRASQALIMARNMSLHQLDGFEDEYSEAKRRAWWMSYFRAQLCSVVRQSASVIMEDDSLYTTPYPTIQGDPEV
WPLYIASLQVSFATVRLAAAGVPPCAKPDPDLLRSKSEKIAALDARAVQLIERAKTGPGSKGYPTNDDPASEPLVSKT
MRSIAAIRSSSARIRLHRYHAFSDIPLFSMNHCDLSSPDFVPDTDNVIPQTNPPESSPFTFDEAAEVCVESALEVSRQL

KKLPYPDKTLTTVPARMTPTILCCAMHASYVMMMQFYRLYLRHQQESGDMMTTSFERRVDELRHALQDVIATLDGFANVLEAIRGMKIDVEAVFTVAFSDMMT

>QLI68388.1

APAPSNTSPSDATKTTTLTGVTHSVVAGLGGLRFDPDNVVAEIGDIVEWHFLPQNHSLVQSSFAEPCKPLADGTGFFPGFEFVTSQGQAPNVFQLQVKHKKPLWYYCPQQKGNHCQQGMAGVINQNFDNPRVSLQRYKEAAALTGTSVIPPVNNVGRVIPNPNPNGGF

>QLI68395.1

QDIPVDLPVSTLLTSAQSHLARGETSEALAYYDAAIAKDPSNYITLFKRATTYLSLGRSNQASEDFGRVLELKPGFEGAHIQLAKIKAKVADWEGARSEYMAAKRNDESTELVELAEAQGAADLAEAAERDKNWEECISHAGVAILVASRAPSLRERRSRCRFQRGEIEEGMGDLHHVLQLRPGNTDPHILISATTVYALADFDNAIAQAKKCLHSDPDSKICQTLHRQERRLQKAFQKVESQLNRGQNTLAGRSLVGSADEPGLLPSIREQIDNLRRDGWIPMQARTKLYDRVVEMLCQAYSESNHKDASKYCEEAIELDPESFWGLIYKGKSLLKREEYDAAIQTLEKAAEIRPDKSDKVHPILQKAQIALKRSKTKDYYKILGVANDADERQIKAAYRKASKQYHPDKAEKQGITKEEAQKKMGSINEAYEVLINPELRARFDQGDDPNSQERGSPFQGSPFGGGHPFMFHQQDGGGANFKFHFPGGGGGPFGF

>QLI68411.1

QTFSKCKPTERDDCPADSAFGGKSDCDFTKGACSIFSEAEGTKLSYKDNGAVFTIQTETNAPTIETGKYIFFGRVDVVMQAAPGAGIITSAVLQSDDLDEIDWEFVGSDDVQVQTNYFSKGDTTTYDRGKYHRIGNPTGSFHTYTVEWTSKQVNWMIDDQSVRVLDAATVGAKFPQTPMQIKLGTWCAGGKNSPEGTRQWAGGFTDFSKAPFNAYYKSVSIVDYAGKDSPANGGIKEYIYGDKSGSWESIKVVKGDSSKDPSPSASASGSASASASASVSASASGSSKASATQTETDSSKPSTTTAPESKTSSANSTITTVSRSASASSNSTASATLTSSRGSATSAPTTVPTTVVPGAAPRSAAAIGGSLLVGAGIAMAQLFL

>QLI68413.1

HGNHGQKPLVSEDATWMEKHMAEEHHIDSWDTGAFFALHDYNSDGAWQGEEIMRTYGLMDPSNRDMSHDKKLEVLQHLMGLLDKDHDGEVSGKEFQEFIDGGETLPDMGTGPGHHGDDEYEYEIHHWEKYHDENTKLEDLTHPEDIEHFKHHEELEKAQEEQEAMDKKSIIEENIPAKFRRH

>QLI68417.1

QDGAGWYAAHPGMARVQRVNQDTHQIVDEFGRARFFHGTNVVMKEPPWHRPAEWVPGVSSFGERDVRNMRDLGLNVVRLGHSWAGAEPVRGEYNETFLDIMRAQTSLAEAHGIYVLVDVHQDVLARQLCGHGVPDWFVKKDWVPAWERYPFPLKLSPFSVDTAGFPSPASLCGSVDWALSYTSVAVSNAFGRLYNNFDGLGDAFAAYWRKLAAGYAATANVVGYNLLNEPWVGDSWADPSLLVPGVADHKALEALWNRAATQIRAVDNDTLIWFEGATLDILSGFNNVPLGDGARSVHSYHYYKPPQLGSIADTLANRRRDSERLRTASVLTELTFWMGDDQQMRDLADAMEATDAAMVSWIGWAYENLYNGTSGRPYPELAGHYARAYPAAVAGVPRSFGFDEKSATFTLRFTADPAIDAPTEVILPPATFPGGYSVRISPEGSLLQHKPDERTLALFTSPGVKNATDVSVTITRT

>QLI68435.1

AAADYHELLTLRPLPFSQLLASFNFKSNSSIADFEAHNFRLFPRSLGQILEYAGTRELHLRFTLGRWDAETWGARPWDGTKEGGTGVELWAWMDAETDQQADENWLTLTNALSGLFCASLNFIDGTRTSMAIDVKPICDQSGDCVLQMEQTIDMVMDVNRSKRPRDNPIPRPPPTHELVCDTSKSYHDDDHCFPADHLNGQDWTLSQIFGRPMKGTCPLADAQVPPVCLQVPDSRVVYASEGSAEIKDENGMSRCYTIPPQSEFALVLPKSEAKTPEEAAKELVEPVQPLLYAERSFTGHGQEHGGVQAILTNPNNVEVEFVYLESLPWFMRIYLHTLSTRISSSAAPTTNSSELIKQIHYRPALDRSRGTQLELLMRIPPRCTVFLTYDFEKAILRYTEYPPDANRGFDVAAAVIRTLEPQVMNLRTTSLLLYLPTPDFSMPYNVIIFTSTAIALAFGGLYNILVRRLVGADEVASPMAKWKLRNLLSKFQKRGIGRQ

>QLI68444.1

SPQGYPSFGGQLWQNPTQQICAQSGDSCHPNASNCCHGHCCDLPSGNGAVCGGNC

>QLI68463.1

HLTTRQKSSSNWAGAVHTTPSSVSFVTGTVTLPEYKRDPGLGASFWVGIDGATCRDAILQTGVDYLNDSVYPWYE
WYPADARLYAQPLSAQPGDRIRMSVNATSATSGVATLENLTTGEAARGEMKNQKRLCLRDANWIVELLPTKGLID
FGVVRFEAVEWHGGEGSKGASAARVYDIEKAGRKSTKCSAGKDVVCEFSQHP

>QLI68466.1

ASSVKVGMNAAFDAAPYLLELVETAAGENSTAYFPLLDRIASGYFLTVSSESELYSRFLNIIHEDGHITRPDALSTFNLA
LSLRSAAPRIEAHYQHYSTAMEPLKELRDCDIWVLLDGRQYCTPDFDAEAKNEPVSAVVKPLAFDRTMGIGRDAILY
ADPTSDRFADYHQSLLKAARTMKLRYRLRYRRSKSHISRPLPVSGYGVELALKKTDYIVMDDRHSSQSTEESPKEPVE
VLDGTDDVADLNPLSTSQLSSIGMKAASFIKDSDHPFDTLVKLTQDFPKFASSIASRNVSITFAAEIKQNQAKKMRG
GINFLWMNGAQLTDREIQPFALVNMLRSERGVVDGIRDLGFDGEQAVALLSHKAVSAAKEDDKPFRYDWTDRLE
AGRVILWLNDLEKDDRYASYPKSLSSLLQGTFPGQIPPIGRNIFNLVIPADLSNTEDLAFISEVESIRERGIPIRFGLVPL
QLSDEAKTRAKIAYFLTENYGIESTISYLSQLAKAHQKTADSKTLLSTITENHTLLPGGEDMSLSMILQASDFTERLVKA
EKWVKRLKADTVVRPLFVNGVLVPRDQSWMQSLSMTVSQDLQTVQRGIYHGVFDDDTWTVGVFLEGAASRRN
LYISETNEKTLRVLNIAKVYRENADLFNAVPVFDFYAESTQENWAVVTVLADMSSRAGLDLILSALEFRRNNPAIRLD
FVDTQDNTKISSQVNKALKANEAKLKDIETIQDLEQLLSEASSYDASDDFAVFVARFLADTKMPTSSQVVIMNGRVI
GPIEPEASFDAEDFQQVLSYEQTRRILPVYAAVADLGLNEKVSDCMAAAKLSSIIALSTLSDLPEGIFESSSATRSSIYTS
WNSSHTVIESGDPKKSNIHIVGLLDPVSEKSQKWAHVLRLLAELDGVYVKLFLNPKVQVEELPVKRFYRYAVEPTPKF
DENGSVKALAASFSGLPLNALMTVGMDVPPAWLVAPKLSIHDLDNIQLSAANSDVEATYELQHILIEGHSRDDEGS
APRGAQLVLATESQPLLTDTIVMANLGFFQFKANPGVYNIQLKEGRSAEIFTIESIGAQGWEAAPDEEGSELALMDF
QGTTLYPRLKRRPGMENQDVLEDTSNPSQGNIVSKGLKFAEGLLSGGKGKSTSDVQHAEINIFSVASGHLYERMLN
IMMVSVMRNTKHSVKFWFIEQFLSPSFKEFIPHLAKEYGFKYEMITYKWPHWLRQQKEKQREIWGYKILFLDVLFP
LSLDKVIFVDADQVVRTDMMDLVDLDLNGAPYGFTPMCDSRTEMEGFRFWKQGYWSNYLRGRPYHISALYVVD
LRRFRELAAGDRLRQQYHALSADPASLSNLDQDLPNHMQFQIPIHSLPQEWLWCETWCSDESLAKARTIDLCNNP
QTKEPKLDRARRQVPEWTTYDQEIAALDLKRKQRLQSDAAETDRTTSEDGKVEDGRSKDEL

>QLI68479.1

TLVPTSHAPVQKQQVTPFGGGPAVTTAAAAYSGLLHRRADDTCGYIDGTEAAWCNNPYECFYNDTASAAGCCTA
STSCYIPTACLPSTSPAPNDTGVTKHCSYSASPSCLSLIYSNGPCLGCTWFICDSAGGRMAILTTRTKGMSTTPPPTTP
PPSPTSPSPIPTPPPSPPAPTGAIVGGVVGGIAACALLGNLFYFLKRRRKPEGSGSEQELAPEAQGGSNDTSQDFESS
RAKSVMANLSTGPNSPSPPSQYQQSFARASWSASPVPAPVYPSSYLNTQILPQQHFQGLQNWYPAAPQEMANT
QPERPVREMSEMSGSPRT

>QLI68480.1

SASASSCQPRTQHLSDPPYENYFYSDCNVDAQAVVTSPLPDSNLSIIGPRLIVAFPAGNSGICTFFQPHKDKNGTLAV
ALVNSTLGAPLGAVTSQTNATKYPHVGVQGILSFNNSAEMAVAILGSVRNIRDFTEGPSLLSPVIQDAVKVARYNGT
GVQLSRLWLDNVTTTTLTLVPWRNDNASIRISNKTVVFGAGFYHFSTTFNYPQLKQLEPRQILNNQSQGLAQQDP
AGQVSSLSFFSYTEKLLAGGWRFLTYFGRDSLISALLLQPVLSTGNGSAMEAVIGAALERINRTDGSVCHEETIGDYA
TFLNMQGNLSSTAAGFTYPMIDTDYYLPIVMDKYFQAQPGRIQPLLATRAGAVDADNRNLTWGQLAYISAQKIM
NMTAAFEKNPSVANLIHLKQGQVVGQWRDSTYGIANARIPFDVNCALAPAALYSISRLAGMHGVYPNTPATSGW
ASAAARRARVWEAETLRFFQYNTTLDTARARLADYAAKNTFYKGPTNADSLGRYSSAGGLIVDYGIAVHSAAAPDV
VPVSHTDTAFRHFLLNATGDDAQLTAFVNASANAILRPFPAGLTTPVGAVVANPALSGRDVLIANFTNAAYHGTVV
WGWQLALMAKGFERQLARCGTPAPAGPPGFCRDQAVLGALRAAYNRLWDVIEDNEGQLQSEVWSWTFGASG
NGTGGAYKLSPLGVLPPPPGVGAGTESDVRQLWSLTFLAVKRNKAYQ

>QLI68483.1

AADIEPIEVKGSKFFYKNGTQFYMKGIAYQQEVGAGGSSSSSSDTKKNYVDPLSDETVCKRDVPLLKELGTNVIRTYA
IDPKADHKACMELLQDAGIYIVSDLSEPDKSINRDTPQWNTALFERYTAVIDELAQYPNVIGFFAGNEVSNAKNNT
AASAFVKAAVRDTKAYIKNNKKITRWLGVGYAANDDKDIRAEIADYFNCNTVEESIDFWGYNIYSWCGKSSMQKS
GYDQQVKFFENYSVPVFFAEYGCNNDGAANRIFDETTALYSDDMTGTFSGGIVYMYYQEANDYGLVKVSGGKAT
KLKDFDALKKKVNGATPKTLEMSEYKPGGKMNECPKLTDNWQANKALPPTPDKSLCSCMAKSRACVPKSGLAVK
KFGDIFGFICKASPESCAGINGNATTGVYGAYSMCSDEEKLAYVLDAYYSSQKKAKDACDFDGSAETQSANSDSSCS
DALAKASDTNKKAATATSPIGGGGNAKATSSSDNFAVHGAPVARIFAIGDFAVGLYMLVALCVGAGMVAL

>QLI68484.1

APASKRAEPAPLIVPRDDSGVFHGQYTVILKDDSDSQALTNVMELIPGNASQVYGNLFKGFTAELDEASLGALRDH
PAVDFVEMDQKVSLPDEPQGKVDAVEADPNATAPVVGPQSPVPSHPDQVFRRYNGLTQYLNNPNGGEGVCAY
VVDSGVDVTHPEFGGRAHMVKSTVDWHGQDWVGHGTHVAGILGSNSYGVAKRVTIYGIKALSERPDASGISNM
IAGLDYVAQDAPHRHCPNGIVVNLSAGIAERIYALNRAARGLVERGYFVAVAAGNEGHDARFNSPASEPSICTVGD
YRYRDSNFGPAVDIQAPAVNVLSTVPGGRIYRLTGTSMASPFIAGLAASIASAHHQRAGPDLCAWMVQRATPQ

>QLI68510.1

APTFCKCTCFKNSTIIPLGPKGESSGSPSNSFLSFSLFSESNPSGAAASDDATDDQGLSRRSLSTSCSECTKSFCLSQGI
DFCKEAKEENVVTMCFQRDSNKDKIIVWGFILGTVGLLGWAAFKRVVEVRGKRAMSRQDISYAPVSGERR

>QLI68515.1

ACGSAAATGDKYLIGVGKADITGPVVEIGFAGYANLDQKGTGLRQRLHSRAFIVADKNNPDDRFVYLVLDSQSGDT
ATRYGVLDGLKNLGAEYSIYGHNNIALTGTHSHAGPGAWFNYLLPQITTLGFDKQSYQALVDGAVLSIKRAHENLQ
DGYLDAGTTRVKDGAINRSLYAYLANPESERKQYEDDTDTLMTLLRFKRASDNKDIGVLTWFPVHGTSLLGNNTHA
AADNKGVAAWMFENAMKDNSNAADGFVAGFSQANVGDTTPNVLGAYCDDGSGQQCSLENSTCADGKSQSCH
GRGPEFRALDLGVKSCYEIGRRQFAGAQTIYNSLGTSGTPIEGSSVKAFHFFHDMRFWNFTLPSGKQAQTCPAALG
YSFAAGTSDWPGAFDFTQGDSGEPNANPIWRVVSGLIRTPTEEQKACQGSKPILLDVGEMDEPYAWTPNIVDIQV
LRVGQFLIIVSPSEATTMSGRRWKAAVATEASSFLPQKPIVVLGGPANSYSHYCVTPEEYEIQRYEGASTLFGPHELD
AYINLTVSNMHYLHPDSKDTPDQGPLAPDNRGKALSFITGVVVDGAPIGSSFGKVVHQPSASYQIGSVVNVTFQG
ANPRNNLRQEQTFAAVEQQGSDGSWTRVRDDTDWFLVYSWRRTNFILGYSEVDISWETYGNAQPGTYRIKYYG
DSKPPIGSVNPFDGTSNSFKLTGGLTRQLRP

>QLI68516.1

VPQGVQRTREEHVRHQGDDIKKALRRQDTAQCNAMPQECMSSINNVFNGFMEAMASGNLIAASGVAAGFQAE
VNSNGCMPKLSACASNMPNIQPSSPPSAAPSVVPSAVPSAVPSAVPSAVPSAVPSAVPSAVPSAVPSAAPSVVPSV
APGGVGRGVSW

>QLI68551.1

KPTADERYPYNGPDVPIGDWVDKTVEGNGKGFPRLVEPPAVKPARANPSNNVNVISLSYAGNGVNIHYQTPFGLG
ASPSVAWGTSAGSLTSIATGSSRSYDRTPPCSRVAVTQCSQFYHDVQIRNLSPDTTYYYKIPAANGTTASEVLSFKTA
REAGSKRAFTVAVLNDMGYTNAGGTFRELNKAVDEGVAFAWHGGDISYADDWFSGILPCASDWPVCYNGTGSE
LPGGVTPDYETPLPAGEVPNQGGPRGGDMSVLYESNWDLWQQWINGISMKVPYMVLPGNHEAACAEFDGPG
QLLASYLNRNEPNSTSPKSNKLTYYSCPPSQRNYTAYQHRFRMPGRESGGVTNFWYSFDYGLAHFISFNGETDYPY
SPEWPFSRDVKGGESKPKENETFITDSGPFGAVDGSIYTKESYEQYRWLEKDLASVDREKTPWVIAMSHRPMYSSQ
VSAYQKNMRDAFEGLFLKYGVDAYLSGHIHWYERTFPLGNNGTIDKAAIINNNTYRTNPGKSITHIINGMAGNIES
HSTLSKGQSPLNITCVLDQSHYGFSKLTIHNETVLTWSFVKGSDGSSGDDFTLVKKGSGSDSRNSTMTSQSGIAATD

KTTSTSTRTAAAGGITTVTEVVNSYTTYCPGSTTFTQGPRTYVVSEATTLTITDCPCTLTHTKSLPSAGISSAVISGNRT
AAPSGNVPDKTEGGSSLAHLTSGGQQPTPAGPTSSPVGAAGTTASVLVAQGGRVGADASLVGFVALAAAAAALM

>QLI68555.1

APQRKQGKQSGNKGGKGGGNGGGNGGGNTKQTLQQQAAQKPGGISKAQDGSVILDKTVKINGLNIRYKVSAPA
DQFKAASGVKSGNAARAADTEGTIGMNVLLHGDGGDSFFDFPNEGVNANLMGVAVLAPDKNLKWGGANQGK
QQRSQGVPHSKAVADLITQELPKMVAFNQSNVWFTGVSGGALTLSGFFIPAHMGQFPNTGVLLNCGGLPPQVN
FTPEAEAAISNTRIHTQSSQKELSSLQKTIPAAVKAYEDEARKARLNTQQINALQTVDNTPSGGHCDFDGKGFTSGV
KSVVTKFDQIMFPQGNGQVNGVDVKTGVIGNEALRFTGSGR

>QLI68568.1

TVPVTVTTTVTECAASCYSTGYATTGIPAYSTPPPWYMTPDPVKSPDSPVTTGYGPSAQDPNASSTKDSCSSSGGST
SNVYSPTPQPYSETTPPSYDGTATPTYQSPSGTTYTQESPIPSAPSTPPSYVYPSPSSESSAEATETSLQTTTPTTLATST
RERGSSSNASGTRGPPPVYETPTDEPPSYDDPPSYTDPPSYGEAGL

>QLI68584.1

APASDVTTPKRQDINTVTDQLLFSSTLPQFEARRNAKNPPSLDWSSDGCTSSPDNPFGFPFLPACHRHDFGYQNYR
IQKRFTKAAKAKIDSNFKSDLYYQCQRELAKGACDRLADVYYEAVKEFGGGDATKRDRSDYDRAVAAYNAAVKEA
QEQGLLPVLD

>QLI68602.1

RNCIPGVVYCGQTLIDIGAYHEQLFQANHCAGAPRTDHDIRSSLYYCVGGDGGVVNYMKVCSTGCKDNGPGNND
SCR

>QLI68610.1

QDPAALLNSVCFPSGQLKNTIPYPCPGIANAKQQCNQFLPGPNASPDQIKKHRDCLCFDEKIIQFEKGCTACKKTHA
FLSKRGEGYWNGILDRVYSDYCQAENPTAAYDASWASASQAAGPIPTGGPDDVLTNYAKDLPADEKTKVELYWK
GTTEEKRPSATATPTSVSTSAAATPTLVDDDDEWCDDEETTSASAGSAAATTASPNTAVSGSTQPTPVLNDDWITA
SAKEDPSASTVRPPQPSQSGWTKQNETSPAPATETVYIDRVVIVKVCQKCVYAQSDNGDFKLACSNPMEIEGTKK
QVPVEEAKHLPAVPVALPKEPVSVEQKKKIVVPDTIIQQVCGCSYTSGPGSNGNGANGSNPGSSKNPGSGSDSPKN
NGATGSNGKPGSGNNGANGSNPEGSKTPGSGPDSPKNNGSNGSNGPNGSTGSPSGLGNEGPNGSNGKPGSGN
NGANGSNPGNPNAPGSGNTGAGSDAGSPKNPGSNGSNGPNNAGSNGVVAPSGSNPSKPTRVGSAGLPSGTNQ
VPIVSSASGNMPASVAMLFLAAMALLI

>QLI68624.1

QFSITGAPVAANGNVPLRRNVNDLYSEGGPQWDLYIQALASLQSMNASDPLGYFQVSGIHGLPFIEWNRGGARN
NNGWGGYCPHGEALFLPWHRPFVLLFEQLLVEHAAGIASQYPARYRDQYVAAANNLRSPYWDWSSDSNVPPCT
VPERVFVNVPNGQNLGRIEIKNPLSTYNYPREALDGQFGYFTQSRQMVRCPAPERYPDTASQELQDYRLKQATYD
VFSTANNFYQFAVRGRVHHLEELHNDVHYSAACRGDFFDASLSGFEPLFMLHHTQVDRLWAYWQFINPSQASFS
GRYRGQSRFSTPEGTIIDQNSPLQPFFDSKRKYYTPKSVSSIKGMGYTYEGLEHWRKSPAQLRQDSVQIINSLYAPAS
AFAKRGGPQTKTRHFAHVELDRAQVERPCSIKVFVGGKQASTIPVMLFPANGNLRSSLAIDKFLTKGSSSNGTLESIE
HLIQVEISKSDGTVIPLDKVDSLNITLEQASITPAASRNEFPKITDAKEHAAKIQAYSKS

>QLI68659.1

IPEAMAPISSNNDGKTATGSFIIECKNSHEVEKLANNIKQAGGEVLDKFNSRFFYGLSVGSVPSGMDRKAPAEDYDI
SPNAEIKSELERDAGGQLRPTGRNSEPHQSRDTGNDAWHLAMTQVDKMRKEGFTGTGIKPDPIKAESVNGKADI
KLGKDSFVLGPGASETIEVIVNGTDSTSLSIPYLCLAGALRSAGRIYPGKPVLKIAGADGESTYPSTDAFVFNDPQSGQ

KPGPSNSVERKDAGVSLDKVFVAIHLPLASPKLRLDMVPLTLCSSSPDFKPQNTSQAGPDLSQACVLDEMVTKFAG
TKCIGQLSGSPFSLFSRQSDTINITHWNGSFAPGQYAPPGRYQFALHALALFGNPDNESDWHTSLSNDFSIAYKHKI
KL

>QLI68664.1

INCRGSGVCSFNDASLQVVHDQIGNLIAGGGGDRHFNQGQQIACSHGSQGSVCAFYQNGASGSAKDAYNQVQG
LIDHKCRQCGSIPTQPGNDVSKGELTVNYVGKPCCEGDCHC

>QLI68680.1

LDYVFLVKYPAEIPTIRYSRGFWSHVRSNIGYFTSQKRWIRDGYNEYNKKGLPFLVPSGFSRPYDVVLPRTMLTWLR
DQPESVVDARLAHNVSAYGDYNFLDSEIIRSPFGMRAVQKSMNRSLPGLVSAMDKEVQHAVDLALKDVGNDWT
SINLWGMWQAIVLFVTNRMLVGSTLCRDERFLNAMVSFTHAVMRNCVLLRFVPLILHPILGRMLAISNWVHWRR
AYCRVGPVIKTRIDSMSRKAKGDPELQNWSPPEDYITWLVRLALEENRDQELDPIVISKRLLPIEFAAIDTTVITGVL
WIQDLLKTPSAVEDLTAELRAHQPAPGESWSAKALQSLLQVDSSIRESQRLSNFHLTLVERVVVASDGLCLPGLGW
KIPKGAHLTVNADGSHHDGDLYHDPYTYDALRFSTMRKERGEHHNASNDAAKPLGMVTMNDHHFPFGHGKHA
CPGRFFVAHEMKLIAAHLLLNFDLKMEDSTSNRLWVGPGMMPPFGGRIKAENPAAVPTAEPDEEPPGVYKIPGQ
QTLKIDEILGLRYSPDICRWGQHPPGRTVFESADH

>QLI68699.1

YPITGTTVNCRSGPSTHDKVIKTYSKGNDIKISCQVAGETVSGNNLWDKTQDGCYVSDYYVKTGSNGMVTGQCGG
GGGGDSSVGGKITRKEIMDRGQYWVSKHIPYSMNKQYPDPQGRNYRTDCSGFVSMALHAASPGYSTVTLGQIAN
PISYSDIKAGDMVGTLAAGTGGAAGHVVLFHSWTDSSHKKYNTLECKGTDGCVKWVRSVGWGVGSVTAKPYRY
KNVVD

>QLI68716.1

ADDLAFRPRVSRHLSRRQFQQRPGGNQGAVKPPATIVVPPQNGGNKGQNGGNKGQNGGNKGQIGGGNGQEQ
QSLTLSRNSLQRTPNGLQNADAGTDEALQSQNNFINFCSGKPLCNGEQVKAGQSCCNPIIMGEVPAPEKMATVVF
SQPRLCGNISPKDGMKISAVYRNINLGTFTNPENTYNGNPQRLDGNGVVIGHSHVTCHLMENDELQGAAPIAAAA
YLQDRERAKSNNNRAPQKTCFKGFNGEGRKANGGLTRIDLDLECGTNLAQGCWTCSTMTGASTHQAAIMGNAD
RPAQDASTFFSVSNDPNADLCGCGRGNGGNNGGGNNNGGNNNGGNNNGGNNNGGNNNGGINGGTNGGIN
GGINGGINGGINGGNNNGGSNNGVDKNGVNKNGANKNDGIKGGINGGTNGGINSGNNKGGSNNGVDKNGA
DKNGANKNDGIKGGINGGTNGGVNNNGGIDGGINTGSNSGNNNGGINGGVSGGISGDINGAINGGTSGGINNG
ISGDINNGINGGSKGDKKGGNKNNLNSNGGIGGDINGGSKGDINGGSKGDKKGGNKNDLNNNGGIGGDINGGS
KGDINGGSKSDINGGNKNDLNNNGGINGGINGGSNSGNKNGGISGGISNGISGDINSGIDNGINGGSSGGINSGG
DRGSNNKGGINSGFNGGINGGSNRGTR

>QLI68721.1

QPKNSTGLIANTRQGPLSGIEVHSKVNAFLGVPYAQPPLGSLRFEPPQPPLNRASDGHSNVLNATKFGPVCHQFHY
RTIIGDSLVETSGLSEDCLTLNVFVPRHAFRRKNGLLPVFVWSYGGAFGEGGASMPLFNPTQFVAENKDVIVVTWN
YRLNIFGFPNTPALGAQNLGLRDQRAALEWLRDNIAAFGGDAHRITLGGQSAGADSGSAMIYSHMDDPIVSGLIL
QSGTVQIIGAATQNVDSEFVRVATSVGCANSADRLQELECMRTVNAEVLMRAISNKTLNAFGAPSGGTPMADNV
TLFTMREYVHRGSAGKFAKIPTLMGHTKNEGDSILNWTEKCGVNKTLSDLATALIFNCNMALEAGFRYVHRVPTW
RYRYAGVFPAVTPFPWARAYHQSDVPILMGTYNLLAQNKTREHHAATIGASRYLQQVFGAFIRDPSHGLEKMYQ
WPTFVPGLATLIDLFPNNTASAEFKISTQDEMCKDAPPFPWIEVLKAPPRC

>QLI68722.1

ATDQVETASNIVADAFIIECEGNELKSLADAVQAKGGEVRHTFDSEFFRGISVGLANVRSNEDKEALMDRFKGFTT
WPVTEIGQPVDSEATQQPGRNKRRHLGSRDSENATDGSWHLAMTQIDKLHKEGFTGNGIRVAIVDSGVNYTHEA
FGACRRVGPDCRVVTGDNFAWEGQKGDPMDCNGHGTALAGILGGYDAGKYVGVAPNATLMAYRILDCKGKGS
ADSAMKGWEKAALDGAQIIVSAFGGETNSGRDPLAMMVSRIAATGIICVGPAGNSPEKGAFNSASPSAGRGVISV
GSFSRSFNIQDQSSYGPTWDLDIKPSLGAPGADVPTPNVGGGYGLSSGTSIASPFAAGVAAVVAEALGKNLDWAK
MASRLVSTAKPQQDPSGTGLVTVAQQGGGLVSAWDAAHATTLVQPSHLAFNDTDHRVSTIRLSITNTAQSEVTYR
LSPLHATTLYAAKRNSFDLDPMKPEPVQAAADIKLSQSSLTLQPGESGTIDVSATDPSGLDPIRLPIWSGWISINGSD
STVLTIPFLGLAGSLHSAAREYPPFPDLKHFTAINGTQHLAKSETFIFRNPTNGQRPGSSKFIKKKYAGEDVNGLIFELH
LGIGSPRVRLDIVPEILCSDVDPSISNPRVPELSKYCVPDSLVTEFAGVMSIGQVPGFPFNFPIRNPGRFVGRWNGEI
AHGKYAPPSRYRVALSTLAPFGDAANESDWQTGFSGAFSMAYEHNII

>QLI68727.1

LQVRSPPPAGSEQARELYELVKKGMRSWGLAGAIHPPSGHISAPSANLPGGLPELNRPRPPKPNGVPGTPGHPLG
PRKKQGRIYERRKSCVAQKRSGIKCSAGTRVTTRASRFPKLRLIGQGGVMVAFSILSPHAHEILEAVKNWDNPIGTA
VGWFDEAIKGLQEAIGGKHVPEIDGNELKLRLICLFRANSPKHPDVIDDLCKRHQDESLEIKKQQQAIDGLNQISDLC
KKVEEEGPPIDVKIKRDVLALCDKYSKTIEGMVDANAGLILLGEWARARTLNNQDLEESDVAVARRFIREGAFGLAI
ATNETEASAIASLYMAHMSSYTIMEIDEDGNEELIYHSKPPVWLELLQTGASFVPEDREGINQALKLLDGTPYLHHF
DNEGREDRFEKVQTCWSHTNLLMFQPPRWDLIAAAMTYLEGRVRDLGLEQILACTPCLVLSDFWVLRCGSAVP

>QLI68737.1

TDPGETPNIGVTLYEHRDWQGLSTTVPYLDKCYAVPKEFNANCLNPALYTNVSESIRDLHETDDGDQIRSIFCTRIRF
ENGEWDPHD

>QLI68744.1

ASLVTSSALVSPQEVPATAARACGPECDTARYCSVPQGPFSYQLESGGIWASSQLGDAQPAGQTAQAGGVLTSQP
NAFFDQESKMVMFSGRVRDMSPKLAVTGCEKGRSTARNKDLLS

>QLI68757.1

QSNNPNSEQQDALFQQYETQVAQVLEQASHGVKCYTDYVPRVEHESPTLPEVFSLDKCHEAHDAHTIYLRDARNT
ARQHNFTRDQLLDKKLPYVFNTQQGGQDEYTEAYQIKLQQLRDAANSAQKVYRAKRDVVMEATTEAASKLKEESS
EHAKEIAGAGIAVGGVAVTGGVITFGPFLAKATEVADHVEKIEASATTAHSALTPVLNNPVSQITIGDLAQMRFFAS
AVVVGACYSKLDQITPLSISMSDHGTKQFLFYIDSQSPDITFNDGLKPNRGRHLLDDFETGVNEQPQYIYVLSLEGSN
HTASYGAKTHTGSTETFVPAINGSVKPETIVGAYPFGNLHGGRHVIREWIANPKYEHINTSPYIAQGNSCLARACPG
ARHLASKTCHSIVSCLSIGGKKLQARGNSQGLQCHLGAVNNLCALGEALDPVVETPSTLKETSIGEVADIAEKVSQDE
FAKLVKKFKLTRVVERWLMSIPDARKRLLNYKPLGPKSPKLRAGSGGTAGSLAIGAIGAIGAAAWVGGVVAAFVRD
ASALDRAAAVTSIIPLAGCITGLAAEENRGEISASSGVDSTMCFLGDALILGGLAPIGVVVHLARLLVQTFTPPPKPPT
KEEMQSLRDTQWQGFTKNSLYTYIYSHAYLYPSGSFANKLENALAIEAMMVISEGAQSIGALNASSRVPDSNSDQE
LLQGNSQEAAEKIREAISVEITGRQQRFLLGLPAVLRSNSTALLKTTAEQFNDNFVKKVTSRETVDRYRSFQENPSPG
GRLPGRSRQQFLVKLMDGIGKHLRETPLPMPDLIEVAFILGQSKGLTNIDPLVLSPREYMKQLANTASEEDINRVTIQ
HAFGVLHLIQGKIKQHELPTIFPVQDSQSRQGLQVLLAMQIGKAYEEAKIKRADKFKGEYLPDSDRKVLIHPLTIPLEE
HPDAPLLIGLVLGISKDLVEASLAESSS

>QLI68771.1

HPPEPGRSIETTLDRRQLGDVDAESPSHVYRGEIGRTPEDVEKDGGFYSRGLQKSQSYGGSSLSDVEMQEGSSLFR
HAAGDTAEYTRYVSTSTDPGVSLTFAINDEKPEEKGFVYKIHADKKLVNVNKSLGKYSPYPAQKEHAAVGFIPFEQIE
GWWQVTYLDDFSDPKIGKVTQERLRNEKFKAFQKNTKFSKNTFQNQRGAGVAPQLAGFPRLSTAWEEDPWKQF
KAQDVQKNLDDLITSACAGKDSCMTQLGHSSKQKSAKVPWTANAADGPDARPAKPKPKPAAVKSGFRVHGRAG

TLVGFSILAPYLQDVLRRLRDWDHPIGQTVKLFDDGVNAAQEAIGGPPRNDISGNDNQAALINFFKRVFWALQSV
TPGQERPADLALLSYGEKSRRRLDSVNDVLRTCERVDAVPPDEQEQQQQQQQQQLGAKVHNTCADLRKRAMEI
EAPAAAKLVTGQAACLVCGLAWDVTGSRCLDTTGAVRWPPKSPESYSPETPDASSKCRDATGIIPCGGGQTAAGL
KIGRAVCAICGFAWDAEGAKVANLDFEARVPVPFSIFPSTYRDSESQTAVQTHEEVDVKLPQRQEPAGRQDQYSSY
SAHVDLPPRGEHREHREHRFSEEEVRFSEERHRRPGFQQEQFVKEDFRPGRTEYTDTRIEIDTHQQPYSSPIDVAER
EYRERFRPVYHTTVDAPTRPQYHQTEDVRVNQYTVEERPVQTSVHQQVKFSDETVEPARFSQAQQKSNMGYYDE
DGHYHSFRQGIHKLADKIAHPHHHHHDHVDVDVDIKEDIRVTGTRPRPVQSGGSYVPNTVTIPCHHIRLGDFLML
QGRPCQVIRISTSNATGQYRYLGVDLFTKQLHEESSFVSNPAPSVIVQTMLGPVFKQYRVLDMQGGHIVAMTETG
DVKQSLPVIDQSNLWARLSTAFESGRGSVRVLVLNDSGRELAVDMKVIHGSRL

>QLI68829.1

INPPRKPQQPTGNGDRLLTYNETTPYARHNPSRMSVRWVSSVDDGQYITTNENSDLVLVDIVSEKETTFLPAKSKP
KNLHAFWIRHDRQAVLAAANFTKQYRHSYLADYFVVDVNSGNNTPIVDDQAGDIQYATMAPVGETIAFVRGNDV
YLRDEHGQIHRITDNGSADIFNGVPDWVYEEDVFGNRLALWYSPDAKFIAFLSFNDTGVGTFTIPYYMAGQEKAPS
YPQELELRYPKAGTTNPTVELNIVDVETKKLINVPIDAFSKNNTIVGEVKWVTDKHSALIYRAFNRVQDRDKHVLVD
VQTMASKTVRERDGTDGWLDNHMAISYVGEAQDSGNNYYIDLSDDSGWTHIYLFPVNGGDPVQLTSGDWEVS
TILAVDTSRKLIYFESTKHHSTERHIYSVSYTTLEITPLVNDTVPAVWSASFSPQGKYYILSYNGPDVPYQELYASNNTD
APLRTLTSNDEFYKLISEYNLPNVTYFELQHPDGFSLNVKQQLPPNFDPTKKYPVLFTPYGGPTSQSVTKRFQPLDWS
AYIASEPELQYVVVYTIDNRGTGYKGRRFRSSVVKYVGNLEAQDQIWAAQELVSRNEFLNPHKVGIWGWSFGGFLA
AKVIEADSGAFTFGLSTAPVSDWRFYDSAYTERYMKTPATNADGYNKTAIRNAEGFKNVAGKFALMHGTGDDNV
HYQHAAVLADLLVAKGVSPDKFQMVAFTDSDHGISFHGASEWIYKFLTAKLWEEVERKDGSLVHQWSRRRLVVG
EGTKGAVA

>QLI68832.1

HMQMSSPPPFRSKYNPYTTSVDYDMNNPLPDSGSAYPCKGYHKLLNTNQGRSVANWTAGEAYSISLEGSATHQG
GSCQVSLSYDAGASFTVIRSFVGGCPLTKDWGFTLPADTPAGDALFAWSWFNKIGNREMYMNCAHVTVQGGG
QGNYSSSEAAWASRPSIFVANVNNGCGTVEGADVMFPQPGPDVSNISKKTAKPVGSCK

>QLI68839.1

AYKIDTVDGIKETARSMAADLRSHYHGDEYGQVPGILPGPPSENKGPYYWWQGGALMGTFIDYWHLTGDTTYN
DLVMQGMLHQVGGSADYMTENYSLSLGNDDQAFWGMSALLAAETRFPNPPADKPQWLALAQSVWATQADP
SRHDELCGGGLHWQIPPTNKGYDYKNTISNGCFFNMGARLARYTNNDTYAKYAEASWDWIWEVKYIDHESWLV
YDGGYGATNCSILTRATYSYNAAILLQGAAFMYNYTNGDEKWKSRIDKLLAASLKNFFPKGVAYEPNCEMKQGQC
TADMLSFKGYVHRWLSVVTQVAPFTRETILPVLKNSTEAAVAQCTGGSSGRVCGFYWSSGKYYDPEVDHTSGAGE
AMSALAAVSSLLIDGSSPPVTNATGGISKGNDGSGHANNGEKKLPDISTGDRAGAWILTVLCFGLFVAGFVWVSM
AD

>QLI68842.1

AAPYGNVLASDVARTYIDDIEKYFKIAPDNPPAYKLRYVTEPTCFAYWGSCDTTYFHLKNTFVNGTAKAFYVPYTEYE
NANVFSEKSQMTITQSTAIVLGTSKGWNAAAKWTVSGDARGQKAALEVSGGYSSTTTGTTTTTKTVSTHAECRYG
YICEIQTWTFHVLIDGMCKTRPYLNCGSEKDACKRRDRIRCKQQRTYIDKFCNHHRLSTMTPCSASMVVRNAAGEP
FTTLALVSSRINSDGIPATAKRDNLEDLIVEILN

>QLI68852.1

AVAVPTPSEPPVLRRDYDNNDNLPAAPWVTVDDNGNPSKTFTPVFTTISGTPSGIDPAPHDLTASVYTLTSFGHLST
STGLPPNPTATNAGTNQGSFSRCYNKNGPYAPFCRPSYNSSIYTGNTYYVTWDPDYYNKSAATLNSTYFVSVRLDYL
NTTSNEYVLLETIDKSVPAKWGYFPLAVDGKYLKGQKHTNNITITLMGHDTENSIAYNNKSIPLPVVVSNPPLDPTA

PSNAPKGKTLTIALPVTLGVIALILVGGCLWNRKTRRIELGNIMSRSRQGYTGRRTRKLFNRSRKDNGIQLDTAPLSP
PPFDYRDTVPERARRDSDALGSLTGSPVRGTFEEPGSTGGRNTFRDEVRRQERERRGDF

>QLI68867.1

QFGFFDQMFGGHDAHEQQHQHQHQQRNNPSDAGHYRSQFEQSHCDNYLCPDTLACVHFPHHCPCAWDAYEE
KFELGEGKKVCVSKGGFKPGEAARKIELARKGLL

>QLI68881.1

QLESTRRTEIALDRRQAGDFDDEFPEYVYRGETGRSPADVEKDGGLYSRGVQKQRAGAALSAVELQEGSSLFHHAA
GETAEFTRYVSTSADPGVGLTFAVNDDVPEQKGYIYRIHTDKRMVDVNRSLGKYSPYAAQKEHAAIGFIPYDQIEG
WWEVTYKDDFSDPKIGKESQEKLRQGKFKGFKKNPKFNKGSFQKLRGTGAAPQLAGFPRLSPAWQDDTWKAFKT
KAVEKNLDDLISSICAGKSTKRDAGCMTRLGHQETTGLTRPKPPKKPSTASNPDGKPPKSKVSSKRPIYKSSKVRVTK
AVGKAAAFTLILPFARDLLEAIKQWDNPIGAAVRWFDDAIASIQEAIGGPSRDDIDGNDLKASIICALKGGRESETIQ
GRKSHFCTPTKDEFTESLQRDFKQGKIDELIRGCKGVDVYNGGDPHVWRWSQRRCEALQRTDEYAERIWEMGLT
GLLDSCSELESNPPGNEDLRIKLEDHCTAFQDEVERAEKPVKKPVVGIPKITAGKCKCDAYHLQPFSEHCGRLCRASY
VLGGWGAL

>QLI68883.1

APYAVPDDDGVRANAESPDGTRVTRDGETGAEPDGTRVTRRDRVDDVGPDGTRVTKRGGEEKANTM

>QLI68891.1

GPVKRVDDGPVWDTAPAPSNTPAVDFTDIFGGPIDPKNMTAQEAIKYYSWNKETPRDCQLRAEIDDLVDPSGSEV
NSLTSACEKNGGCGSCHETDDQVWSKFFCLLAADKKQ

>QLI68894.1

FSICSLCQSWVQPISSPTNMNEVNLISDPAWLTALKAIQLFLALVSNMFLLLDMTRKVRFTIAEPITIIGWYISAICLVC
LHAIASGPLLNSLGISADEKIWSQAFYYGIWSAILYFVAASILVITFWGSLHGHYEEDFNLSHSQRTLMLQTMTFLTFL
LLGALLFSKLEDWNYLDGVYWANVTLFTIGFGDIAPNTVLAQALLMPYALIGITSLGLVINSIRSMIVERGSQRLDAR
AEEQSRLNALRKLVRKGKGDMLTPLECGDSPADVSIRELERRYLEFGLMRAIQKRASSRRRWTALIISAVSWLCLWL
CGAVVFYRCEKTGQGWTYFDAVYFCFVSFTTIGYGDLVPKSNAGKSFFVFWSLIALPILTILISNAGDTVIRVINDVTIR
VGSITLLPGRGGFGHNMKQLLHQLSCGYLYSGSEAEESFDLEKRTARPRGTPEPRVSKRKGRDGQSHDIDNSTSKTL
ASWTGSPGSSRSHAPRQVTPAQGFAATCNAKLENLPTGDDLHFLLISEIQTVAKTIRDDRHRRYTFQDWAWYLRLI
GEDERDPDAHREVRTKERTRPINKIGSAGTKMLHRQPSVQRHDEDDENQTERNQQPTRNNASPLKWSWVGNQ
SPLLSRKEESEWILERLLERLRELLWETSRQHQDNVVESS

>QLI68908.1

LPGSFPEAHGPDWSKIHSRFAARSQKNSTQDKYLIGTGKADITGPAADIILTGYANLEQVGGGIRQRLFSRAFIIGDV
NNPEDRIVYVVLDNLVGDTAIRFGVLDALKGMGAPYSVYGQNNVALAAAHSHSAPGGWNNYLVPQIPCLGFTKE
SYQPIVDGAVLSIKRAHESLQEGYLDVGTTEITDASINRSQWSYLQNPAEERARYSASTDTTMTLLRFTRASDNKITG
LLNWFPVHGTSLYRNNTHVAGDNKGLAAWMTEQAMREDSAFASNFVAAFSQANLGDATPNTEGAWCEDGSG
KQCDFETATCADGTVAKCQGRGPHWQVQDQGASSCHEIALRQLRGVKDILTSMSKSSTPVQGPTVKSFHFFHN
MEYWQFTLPNGEAAMTCPAALGYAFAAGTTDGRGEFDFIQGDNGKPHNREWDFLTHLIKNPSQRQTDCQKPKH
IFLSAGELKDPYEWEPSIVDVMMFRVGQLVMILSPSEVTTMSGRRWKEAVGKQATSFVDDPIVVLGSPANTYAHY
VATPEEYDVQRYEGASTIFGRHELDAYINLTVSNMHYLKPDATEKPEQGQLPPDNRKKSLNFVLPVIYDTAPLFKQF
GQVLTQPQARYKRGDVVKAKFQGANPRNNLRLEGTFAAVEKQGQDGTWSQVADDADWYLVYTWRRTSTLLGH
SEVDFTWDTSGNAVPGRYRFKYYGDAKNLDSGIAAFTGTSNQFDIS

>QLI68913.1

KPVAKLPGEDGINDIDNNIPDSTGPPSGGSNGQPLKNPHNNLGAGASTVPYSDGLTLGQGYNTYLQEGCMNGAV DVTGPTNAPGQVQIEYQATQITEYSQIIKELGISASLGIQYLDDSVEVSGKMLDKSTFENSALTYIVRADVKRQPSSDL KYKFMWNEAKNPTERYYFVEGGALFAQVSIQSTESSKKHELEAKAKVAFGMFGVGVTVSTEMKQASEFLSKNSKVI SEMFWVGAPPKDTKSGVLGEIKALQSGDASLLDIKSISDKFIAEAGEHHALVERYDQVFNFKDEFKPLDYSDAIEQT WAVFNDYADYDSMQRMLKTIKLPESLQQKQVELRNAIIKAKDGVKTWITNVSKDPSKAASKPSYQSPDAFLGTVM DAFSTTYIVQNIEYDWTKPEYKDHPKNSTLIDDKLRTDIGKTTPVFNIQGVPFPGVTGTSKLTFGHQDGEPWWNIIA DGTLPTNYKEQSHVWLLTKKTPTYSQPIYVYEVLDKPNYWWNRIDVNKALPSSPTNIGLNLRFFTKAE

>QLI68914.1

GPVTRADTVICEPRQDTETGKIKKFTVDVGVAEDQAKKAGLTTGKSGDPHRYMNGDNINFGIHNCDKEGAILWEY PIYWVGKKAEWKKDEKTDRQPGGPTLIRVVYANNNGNIVYCGVMTHAVVNSNNQGEKFFLKCT

>QLI68917.1

LPQTLQVPGTYTGCAPLINNCCKMGLAPPNSSTRFTECSVVTGPLPNYVYFIQSCAPSLKLPSCCTEKKSNLIEGFFN NVLGTSLGESQCVLPPQP

>QLI68923.1

SVIKRQVLKDKDIAGRIAGGAKIGDQCHPPGTYALGGEKVIPPCLAEQAIALKCEIVTHLSSNSSEANRIAYHKCLVGH GSSYFLDIQGCLACKKTHGHLSKEQYDWYLQRWTAGYEAFEKDVVPKTNMWTYVEGAIGGTTCQNRNETLRGW KCWDQLPKGSGNTNKTVPVEQYYTNRPKTQNIGSFTLNGKKYPESTTMEVDLAQYTYEITGHLGYYSSAKLEDGTV TDTRVEFEVQVVTEYREIKSVCNFTTPDVFTIVSAISAPVPLKDSVATVPKKEATTLPTLDCDGTCIASALSIKELEVVVI QGSKKADPVVADAATPALTDLEEHKSVSTNSKISFLKRVEVLFANVKKYPTPSGGSPPSTDDDPCPARRRR

>QLI68943.1

SSCPGGNFKPNAESQTIVAASYVDIWNGDFSLENRTFAPDVVLYQDRLPGPGGSEDLPVGSSAEFIEFMRLARFGW NHYKFDIRNLAFDGYNVVIRWALNATVGPRDKMLIPINKPEGTDITFNGTDWLYLDECTFKIKQVDSAQDYIRELYL EGITEIKI

>QLI68953.1

EKVPVYYLIRHAEKNADGTVSVQGQQREQCLVKLFGKSSKYNIQHIMVQTPYPGDTAEDHTTQRPYNTTLPLAESL GITIDHPCNYTDTSCAGQAALNYAGPGNVLIAWEHVHLPAVSKAIGGQDVPSYPDSHFDLIYNQPLPYTKVTVTSE DCAAIGAASMTGSSTTSATSTTATASSANVAWQANTPKWTCGIVAAGIAALISL

>QLI68961.1

FPTGTDAKAETLVLSEPREAIHLQKRTQEIIGYRRVHPDQAQIYKDAGDTLTPSNNRGGGQIGKGVYASDDQGAW EGDPGHTYCVILADIDAFNKLDKAWVPRSHWWGDEAANVFKADAYVKDTLKLDPTKTIRMSIVDNPGKQTLQM MIPNDLLNQKGGGLSLQVNCQEPNKKKKPNLPNHKVDYNSWTNVHGEKYEQGEIEVHRVKEPAEKFLKDSEAAV AEAEAAASVQAAEAASTKAKAALKGLQDIAQQVVEHTLKNNEWMTIYQNRDFFELFNQVRANFKSVALKVDEKL VEGYKAKFDEVAKTPASPNEVLSEAESLGEKIRKVVAKVQNDLDAKTALKSNELKSPLYLGDINAIDVADLNELEGEAI ALEVSVLEKSAAKLAEIQSQVDAQLEKLRPNAEANPGEKDMQDKDKDQDKDKDKNKDKSNKGQDKSNDNKEQD KSNGNKEKEQDKDGNTKEQGKANGEKVIEERKKVADQVATTNGNSWVGKVLGGIGATVVTLVGATGLTLAAEA GATGGSFTVGGITMFSAPVSLQVSDAEVAAVLQEVLPEVTNDAITVAAETLHPPTPIGNVQIPVLARKRGQGQDEI PEVKRWRVAALALVAQQAIRDSLEEAFKQAQQEVGNGAKAGGN

>QLI68974.1

QFPPPSEGVKVIKSKLHENVTISFKEPGLCESTPGVKSYAGHVHLPPGLLDDGSGEKQDYPVNTFFWFFEARHEPEN APLAIWLNGGPGGSSMIGLLGENGPCFIGDDSKSTIHNPWSWNNYVNMLYIDEPNQVGFSYDIPTNVTVYAGSEE

AIIVPTNFSTETPQTNLTTRIGTLSSQKLSHTTNSTQQAAHALWHFAQTFFTEFPHYKPNDDRISMWAESYGGHYG
PGFMRFFQQQNEKILNGTIDVEHAHYLHLDTLGIVNGYLDAVIQEEASIIFPFNNTYDIKAINKTVYDGLMHNFTRE
GGCRDQIIQCQKELVGIDKNALRVARDFPSELCPKMEETCAMSGEMAFENSNNARFDISHPKNDPFPPPHYIGYLA
QEKALGALGSPVNFTMSSQTVALNFFATLDEIHGGFLDAVGYLLDSGVKVHMMYGDRDFACNWIGGEMSSLAIP
YSRAEDFKKAGYTPLITSEGVGGLTRQFGNFSFTRVYQAGHMIPVYQPEAAYEIFMRALFNRDIPTGLLPVHDELST
VGPPDTWHVKQAAPKAPEPKCYILAPETCTPEIWEKVNSGKVTVRDYFVVEDKEVGDGEL

>QLI68981.1

TGDLSVLTGEIGRQNNESLFWGPYKPNLYFGVRPRLPQSLWTGLMWGRIEEYNDVKDGLRYTCEQGEDIHGYGW
DEYDARSGGIQTIHDKGNKIDITTSFSKIPGGSNGGSWAVRIKGQLHEDAPADHKTVVYYYIAQEGKGELAAQGEG
DELGYKGDTTFIGNSEALGDYKIVVTEGNGKYPAGDHEISASRPGDVTLVHSAEVSEDAIWQAVPLLFQQLQPAAM
AVQEEYGMENPPPAWQVYRMAHQPGKGNSHILQRTYEGSFEFDVIFSSASAGKELTKEDVTNTIAATSSAFADRF
STIFELKTPFDGEQYQQFGKSMFSNLLGGVGYFHGKQVIDRSYAPEYEEESEGFWEETRQARERQAQALEGPYELF
TSVPSRPFFPRGFLWDEGFHLAPIADWDMDLTLEVIKSWYNTMDEDGWIPREQILGPEARTKVPEEFQVQYPHYA
NPPTLFLVIEDFMERLRKTNGTQPSGKEKLAQERKNPLHTAHLNNMELGEDYLRKMYPLLKRQYDWFRKTQRGDL
KGYDREAYSSKEGYRWRGRSESHILTSGLDDYPRPQPPHPGELHVDLMSWVGLMTKSLKNIAEALDMKEDVAELA
KTLDAIEHNLNDLHWSEKDGCFCDATIDDFEEHQLVCHKGYVSLFPFMVGLLKPNDPKLGKILDLVADEEQLWSPH
GIRSLSKQDEFYGTGENYWRSPIWMPMNYMVVKQLQSVAKQEGPYRSKARDMYNRLRKNLVDTVYKSWKETGF
AWEQYNPETGAGQRTQHFTGWTSLVVKIMAMDDLSEHGRDEL

>QLI68985.1

APADQKIKCPAGPRLAHWPPEAAKALNQMIARNAHKGRYAVFDMDNTSYRFDLEESLLPFLEAKGVLSRDKLDPS
LKIVPFQDTASYTESLYSYYLRLCEIDDAVCYPFAAQVFGGLPLRQLKAYVDELMAFNGTIPVQYYEEGVLTNATVSP
PKVFRGQAELYNRLMANGIEVYVVSAASEELVRMVASDPKYGYNVKPQNVIGVTLLMKDLQSGNVTSARKQIKEG
DYSESKNLDLVMTPLLWTPATWKEGKWAAILDYIDPWKRPILAAGDTPVSDGPMIFQGVDVGRGGIHLWVNRK
DKYMEQIDKMKKDFAAAQEREGLPVWADKNWVTVKPDDIL

>QLI68997.1

TGNSILHKVEHQGPHLYALQSDEPLIVWRGDTRSPDNVRNIHRGFSPPNEIEHLTKQQAEDACSLYQHPYGGTRSF
TKYVSSSTDPKVARLFASDFDDPANRGYIYEIQADEKFIDVKASLGKYGRYPEQVEQAASKPIGWDQVEGWYDLAE
FLPHEMAILEDAASGPLKDMSQFVHRFHENPDWDLHKYYGRKGAGARPELAGFRKDSPAWDEEPWSQYKAKPV
SKAWKDYTERVCASPVVTTSQGVGICPDDVLPQVEQEFTLADPLGARDLTGQLQSADPAQYKLDVSFTKASEAGE
MIEADEAAQAAEAAEMAEAVADAAAIDAIELAELLEGETLVDIILAFLLL

>QLI69005.1

VPQCATDADCLSGYICGPSDYSGSGNTDNVCVQLQSCMNTPDPEFPQNGPKCGASTFCNVGGFCGGGYYDVNG
NRVGTEVCVNQATGVRCAAPST

>QLI69009.1

TGIILPLYLYPSITWNDGAANWSPAFNAIAAHPSLPWLAVVNVATGPGSTYMPGNNDVNYIAGVSKLNSFPNVKTL
GYIRTAYASIPMDEITKNITTWANWASYSASNISIHGLFIDESSNLAYITNVTSFARKAFSGNITIFCHFGAAAPADFYK
ICDAVGSFESYASYLSTATMKNTIPAGYEKQAGIIIHDFVGKTADGIAADTNSLNSYIQGMVKGGLGWLYFCTGYFN
SMSTGPATVGQVAQYLASDTLLAVGSTGTSTQWTVPQCAQTMRFNITGGAGGQIGYQGGYGALISGSITVSPGQ
TISAVAGSAGGINTAGISAYGNGGTASQGGGGGGAASALYLGGILVAVAGGGGGGGSITVGTYPNSKAYQSDSNRG
SGETPGVSRVITPTGANMANYFSKAAGGSPGSASSPGVGGQYYGYATTAYVGRPGSGTAGGAGVGNPQTTSSGA
GGSGGGGGGYKAGGSGASVYWNYGDGWYVIPAGGGGGSSYVSGSVSGVSQGIAGSSGGSVVVTYRTPSGSECV
H

>QLI69016.1

AEEHVAKFGDTRPISNDPRPVSGTVVPRQQPDGTEEVGFDVYVHVVGRPEETKANQTEFLLTRDDVKSQMEVLN
RSFKPVGISFKLAGVDWTAISTLAPYENPFSNILYNKELEGIYKGDDKGDNTTLNLYFLNGTNGHGGISQNFFYLKRF
VFVNARTVPGGTEPSFNMGLTTVHEVGHWLGLVDVYKVKPSWGTAEDFSKARAACLKLDGPCDTQVECLNYMS
YASDTCKNEFNPEQIRFMKTYAKEMLAGGTPQPIEIDL

>QLI69017.1

LIQQCSSREAVDEGGNWFCGAVDQILYEGIQGNGSFKAVTKMSDAGECLQKDQSYRGPLAPLDQDLSVHFRGPLE
LRQFAVYNLASNQKRNQIDLEVGAGKSGTDARVQGGEIEAGPETSKISARHRHGHRFLHRAQRDKRGDIVTATIDG
KVVTWENDYLPTATPNPVPAPAPKVQAGAGAAAVKDKDADADTTATTSKKPADKKPTDKKPSGKPAPSGSDWD
RVAYYNAVKQVSENIVFMGNHGGQGSGVFDTVWGLSLSYLNSTGTGGASSPEILENIRIPSNTEFSIFSKEECNGSC
GFSRVPSIAYKGFGGANKVFLFEFKMPSDGTRCPAGTRPGSPGCANPDMPAVWALNAAIPRAAQYKGCSCWGT
GCGELDIYEVLAPGDSKCKSTFHMANGAGSSDYFKRPTDEYIKVATVFHEETASVSIKKLSDDVDFAKGLDDETVLS
WLNRPTDDKALKLSSLFQLSS

>QLI69018.1

RSVPYGDLMTVGEGYNTFLGKGVKHGAVQFSSVKAAPPENIIPKRSASNTPTRRETNPFQGLSVDMFDVDLDSYFI
APDPKMFDEPMYKQNEDNQNGINHSVAHVKRNAAASCPAEIDASVEFISDYESYLKVLDVSASATISGYGQTASAS
SSYLDKSRFASNTLTYMAIINIKKQINTGEEFAFNTNLYSNSSFAKTFGDRWIRGFQMGAKLVARISLTAKEKSNQEE
LKATAVASLAFWGVSGQVNTAVTSSMQKLNTQAHVKVDIFYQGEIGKQLQGQSASTSGDQQPAQQVFANAKS
WADLFLTEACKHNYKYQALLDEYPNIKGFPENQAVLDYSTAERVSYRVLSELVKISELAQVLRKSKVLNQADGDQIL
WDELAIVEACKTWVQKTAATPNNGTETAKELIKLFDTAFYEKWRPRLKDIKGDLTVQNTTEFVRVWDDRGSGAAR
GASFWLPRAQGELRPLGSMGVANYDDINSHFTAVLVAPTPGRTPSKPVVASPVGFNRIWRDLWSGANSDGSFW
RPTAPEGYKCIGDVVQNSWSEPNKDAIWCLRADLVKPSAYESPSLWDDKGSGSAYGVHVYNVQPRSDKRLNVLR
AFSDPDPDKNIASQLIAPSGANL

>QLI69027.1

LPTTPSSTPAGNASVTAKHNANFTRSGPAALAKAYRKFGKPVPVDVTNALNRQSSKRTTGSDPNSPQQYDIEYLAP
VQIGTPAQTLNLDFDTGSSDLWVFSSLTPSSQVNGQTLYNPSKSSTAQSLSGSTWSITYGDGSSSSGVVYTDAVTV
GGLTVNAQAVEAAKQVSAQFSQDAASSGLLGLAFSSINTVKPTKQKTFFDSAKPALDSALFTADLKHGADGKYNFG
YIDSTAYTGSIAYTPVDSSQGFWGFTSKGYAVGGGSLNTASTSGIADTGTTLLLLPSTIVSAYYAKVSGAKYNSSQGG
YTFACSQSLPSFSFGVSGATVTVPGAYLNYAPTDSTGRTCFGGLQSSSGIGINIFGDIALKSAFVVFDGGNNRLGWA
AKAL

>QLI69029.1

YHIQRDLSESTASRTLAETRDTAAEFVHPGIFVDSSQLQRMASKVASKTQPWTAAYDAMMKHPYAAIETPTPYETV
ECGPYSKPDIGCADERKNALAAYLNALAWATTKDQSKATRAISIMNAWAKKIKSHTNKNAPLQAAWAATVWARA
GEIIRYTDAAWSSEDITSFEGMLRNVYLPIVKNGSKNPNNWDLVLMEASISIAVFLNDRATYDASLARFINSTSYYIYL
KSDGPEPRGPYKMPRKTLLEHWWEGQKEFNEDGMAMEVCRDLTHTAYGLASISHVAETARIQGRDLYSEDTGTR
LRAGLEFQTKYDKKGGAQEVPSWLCKGNLKLHLEDVTEPGYSILGGKYDMPYTKKYTAAARPAGANTLFVGWETL
THATGEL

>QLI69034.1

TREAHVRITGLTLQLNNVPYYVPPQSIGVIPQPANNSKARDFDLVPITVVTSNYGKFGQDELENTTTEFSGIDDVFQ
DGFLQGIYIQSTTTEGSRFCDAALRNCVDVSASTLDDSSPSIPDGPYFMSSSGSIFQGYRLYSDTQGSFSETTVPDGN
GGFTVLPANIPGQSLAVAVPSRLYFQRTPEKPLAGVRLGVKDIYDLKGVKTSNGNRAWYNHYEAADATAPAVQNLI
DAGAIVVGKMKTSQFANGETATADWVDYHSPFNPRGDGYQDGSSSSTGPASGEASYPWLDITLGSDTGGSIRSPS

QAQGLYGNRPSHGLVSMNRVMPLAPEYDTAGIFARDPRLWAATAQALYLDNITLTSRYPSSVLAVGFPTNSSTRY
DALLSNFLQNLTRFLSASVTPYNVEESWKADMPHEPPLPTLLNNTYELLTAKQQAKLVRDVFVPDYARVHDGRLPH
VDPSPLQRWALGDSNPSTTEQVVAAKTKFMDWFNAKQLPRDAATCSRHILVYVPRRPAPKYRDTYLGGPSRPFPF
SATRLSVYSGAPDVVLPIGEVAYESAVTGRAEVLPVSVDVMAARGCDGVLFSLVRDLHAAGVLATVKTGRSIVDGG
QILY

>QLI69036.1

APAAVGPAAPWPTDDKTLHKIDRYARYSVAAYCKKLNDNSANNKVCTNDKGAQYCGDLADAVTVHQFHATESIS
GNVAVSNKSQSIVVSFRGTASIGDILKDLHVNLKDPKKHLERMAAAPQAIGAVPPAASPGDADPALPLCSKCKVHA
GFWEAFRGIKDVLKRVLKEQCERHPDHQVVVTGHSLGGAVASIAAGYLRKSGIDVDAYTYGSPRIGDPAFASFISSQ
KNGVTTRVTNGRDPVTVVPGVGFGYAHTTPEYWFPSRVEQPKNVKICEGVQNFSCSGQFNISLWYVGDHSSSKY
ARGFEACPEQKKLETEMLQAQTFTEADVEEWERVGFVDDADQIKAAQL

>QLI69039.1

APPIIVGYYPTSRHTVLKDLDLANYTHINVAFALPDEKANLAFEGDEVMPEIVPRLQEKGTKVLVSVGGWTGSAFFS
NITTEGMRETFANNVIELMKKHNLDGIDFDWEYPGQAGSPCNFFDEQNDTRNFLAFLQMMRSKVKALGEDKLIT
LAVLTRPFAGPGGEDVSKFAEEVDFANLMQYDMNGAWGNETGPNAPLNFEPGKATQGSFATAIEAWTTAGWP
ADKLTSGLAFYGRSVTTDVDMLAQDPVSQYANFSKQVPKGDDLDELERETCPPNSAVSVNGTEEAWSGAWRYAS
LRGQGVLTAPTTPSPPWVRTFDSITMTPWLFNPETKTYITYDDPESLDAKVKFALSKGLAGTMAWEIAGDFNGELL
GAVRGALGV

>QLI69045.1

SIAPNSSTTPAPTPPDGTYCECGYTYCASVLMAMKKPWTTKQLAEAYCATQNAVCNSGKPSTSINSALYICLCEDPG
QKYGKQLDLLCGCDECLVVGPDFRGRCETPCHAGQCKA

>QLI69048.1

LSLGGRPYQIIHEVDRRSGALQDFVTWDEHSLFIHGERAMMFSGEVHPFRLPVPSLYLDVFEKIKALGFNMVSFYV
DWALLEGKPGEFRADGIFALEPFFEAASEAGVYLLARPGPYINAEVSGGGFPGYLQQLEGILRSQAPDFLNSTDNY
MAHVCDIIAKHQITNGGPVVLFQPENEYSSGHNIPFPNGQYMQYVINQARSAGISVPMINNDVGPAGNYAPGKG
VGSMDIYGHDSYPLGFDCGNPTVWPPNGLPTSFHQLHERQSPKSPYSIIEFQGGAFDPWGGWGFEKCAALVNHE
FARVFYKNNLAAGVSIFNIYMIYGGTNWGNLGHPGGYTSYDYGACIRENRVIDREKYSEVKLEAQFIKVSPGYITASV
GLASTTAYSNNPGITITPLTSNKTGNFFVARQTDYTATGSVSYTVTLPTSNGILTIPQRGGSLSLHGRDSKIHLTDYPV
GDYVLLYTTAEVFTWKKYADKTVLVLYGGPDELHEFAVKSPSATRILHVEGSSISSHEQASSTIVVQWTTSPERQYIQ
VGNLAIYLVGTPTPITNLYMTPKLQFHPLTTETDRNSAYNYWVPVLPGSNSSPYGTSLMNPDAAIVNGGYLIRSASI
NGNTLSLRADFNGSTTLEVVGIPDGVTKLSVNGRPLKHTTSPTGTWLAQPDITIPTVQLPSLADLTWHTIDSLPEIHP
QYDDSLWRTASQSPGNNRPQAHHKTSNATLFAAPPRASLYGSDYGFNTGTLLFRGHFTASGKESLFRTWTSGGTA
YASSAWLNDRFLGSFKGNSAAENQNSTYALPSLTAGEQYVVTVVVDTMGFNENFNPGYEDMKAPRGILDYALSSP
DGAPTAVAWKITGNLGGHDYADSFRGPLNEGGLFFERQGYHYPRPPVDDAPFAPGSPFDRADRAGVAYYAARM
PLDLPADAYDIPLSFVFANASRGGGDYRALLYVNGFQFGKYLSNIGPQTEFPVPEGVLDYGGDNWVGLAVWSLDS
TGARVPGLALRAGTPVQSSRNKVRLVTGPAYSIRPGAY

>QLI69049.1

GLADAPIPGYDVVDLTWQVEVFPGHFENLTGTAEQVHRAARAMNPGWKPRTASDPRGSNLAKRVPLFNWAKVI
CGAGALGWHPCKVRRIEQGIEYLRGLAGVPTNGPGPETCGRVSCSYQAAIWWCNSAPFARALGSWDDIADSAQL
MVWKCSTGRPYADKYMTGQAFSTENWDVIIRQDEC

>QLI69060.1

YAIEADGVNCRSGPSTSDKVVRTYNKGNDVKLECQTAGQAIDGDSLWDKTTDGCYVADYYVKTGTTNMVTGQC
GGGGTINGKISRQEIIARGQYWVSRHVPYSMEATYPDQHGTRYRTDCSGFVTMALHATPPGYNTVSLPEIARPIT
WAELQPGDLVGTLGPGTGGAAGHVTLFHSWADASRNSYNTLECRGGTGCVAYKRPVGWTDGPYTAKPYRYIRV
E

>QLI69063.1

HNPGEGVEICANDPLNPPCTTVEAPSGLCVAIPEEYKNKVSGVRANDTASICRFYLEPECKGEYFEAGTEAVNLYTG
RPEFNDKVTSFICDTAKLIAAPKSSEWTFGRQRELCTHLETLSLQFKLGNNIGSGTYDKIKLGFEHAGQTVHVIAEGPF
HGYEVSQDINIQDVFGMETVALSKINRLRLLDEQPGWTFGADKWEIAGFTLKGRCANSGMTIALEKFSSLNEWLQ
APGWFPYQVELEVWAGDVQPEDWVTKSLCKQFPSMKTSICILPMKTGTGVSPIN

>QLI69064.1

DAFKQEQDSKLSPSIIGKSSPVPPSSPGTKSTAQEVLSGHVNVDDQCRSPNNDGQVDESSWDDAITDQVPEVDQ
MVSTLIHHPETEQNFDTNTAFSVDLTIANLFLVDNIIPYAGPQRLDPATGQVRGHVYVVIQKAAGGSGALLGDGSV
VYSARVPGRGNNVVYTGGGAMKTMNRLSVEVDDGLHEPGRYRVCTVATAENLQRVVMPPVMRGPQDDCIWF
TVGDGVVDREVGIANQPKDALPFAGSLPTTSYMPSLPPKPPILEATEQLPWIPFIDNFEFFVSNLENEETVLRLYTKYL
VSYDNLLETLQLGQNFTPYVLFVQYASKSLQPGLHALLTLMDCIIISLLYYYSGISGTLKFLGLCYCRGISVPRRQRAIGT
LVKSYDTLDTLQRTPSFVLYSVLTASIVHLANTISFTHTIFVAAEREGPMPAKRAGFSALGGVEEDVTSLEEMEQCHP
FAKKVLSILSYLMKAWNVQVEVEAGEISLQDCIEICRPYGQSPV

>QLI69082.1

QTVQSKPFNLLVQSDSKELDGRALGTCHTGAAIESICLLSDAKAVFNLNTTEGAQPGPGGLSGVLAWTLPSQPPIPS
SMSFYTDPSTNVALPLFYPGTNNVQYVGFDGDDLMNIVSYLDDTKSPPTGQDPRVLKSWYICTTYNVGYTYQTLT
WVLGGENPQNPSCVKVDVKRKFVEE

>QLI69083.1

DGLKIDVTHSVQCDRKTQKGDKVAMHYKGTLGDSGKKFDASYDRGQPLQFTLGAGQVIAGWDKGLLDMCIGEK
RTLTIPSELAYGDRGIGPIPPGATLIFETELVGIDGVAPPEKETGKDEAKENKEADKAGEKVITVVLKATEAPKTFMAD
NR

>QLI69094.1

ANFNVSPAVANAHSCGQDCQRRLNRTIEADISVVGLEFDYPFYETASNFSSSLGPGELLKLQPLDPRNLTINGGATV
FRFQYTSLDYDGSVVPATGFIAFPYTPRYSFAQELASASNTSIHKYRLAAFAHGTIGISPGCAPSNGPALYDYSTWQP
VLERGYAVVATDYAGLGNNYTSHKYLSLPAQAGDVYYSVVAARKAFPASFTKEWMSFGHSQGGGAVWKLAESRF
VRNDTTYLGSVAIAPATYFIRQLVDSLLAANSTSSGAQKGTGAGFLPYVLLAAQRAVPSYRESMLSPVLRNRTQLAV
EAQLCLESVIGISVDLDASQLVSVAGAEKDIPTLLEWEKMVAPAQGDPSPAPVFVVQGQNDTAVSWKTTVQAWN
SSCHDGNELHLRLFPTQGHRPSLTAGAAEWMAWMDHRFESKENKRSKNKCTKITRMPFNLQYVKAPTDIDLKPF
LS

>QLI69102.1

QDCPSSIINDHGGASLNSGPRGAASFSPFFDLVNLTTHSQKINGRIHDNTTIFRHDPSPAVDEAWDHLSTEGYEVIL
VDETTVIKSGKSPHLSVKAPLSWGFGDGKYLAQIDVFHQIHCLNELRKEIHFDYYYGANWDKTSIPPEHAAHKKHCI
HILLQNIMCHADVDIITHNWVHYENMDNRDRPYAEPLADFNLIKSCRDFDALLDWAFEKAVDNLTAKWAKWEM
PKDATLVHGDGYLS

>QLI69104.1

TWLDLNKIDVQALAARQLSDLVGGTSASESSTQSTPTTESTPSSTSTPSSSPTSTPSSTPATPTTPSSSSSSTPSSTPAS
ATPSATPEATSSSASTPSSTFVPVQTTSSSSTPTSSTPTPVVQTSVEVWTTTNTNGSKTVLSSTTRTTSTPGLTSGDGE
TTGMSTKTRNTVIGVVVGVGGAIVLGALGLVAWRIWGRKKHSEENDGLMAYDMSATGGVEKSERGSSAGGAHT
QRSPFQSTLENYHQPSQVTASSNF

>QLI69114.1

HLCNPDRGRPRDRRRQHLACLRAHLLAPRVQLLGRPLLRPLPVPLRANITTFSLGATWTGLIFAADTVRRSLRSSYGF
CDNYKVFGGVVAGIARLGAIEFLLFFIAWVTDAVVSYRHRRGGGRARARRVAAAGSGTAPYQLQMQRRTFYPKA
QHWAPAYGQHLRPDACASSSRGLSRARASSSPACGSLPPDLLS

>QLI69121.1

APVVGADKSNLEARNPQGSFIWDWAKSEAHKNEKRTPQGSFIWDWAKSEGHENEKRAPQGSFIWDWAKSEAH
KNEKRTPQGSFIWDWADGTSADHKSTEE

>QLI69129.1

AAVYPGSGPPQAFSLVERATPQAPDGYAPAAVDCPSTRPEIRVGSSLSPQEKEWLPRRRAETISHIRDFLQRNAIPG
FDSEKYLSGATANSTALPNIGIAVSGGGYRAMLNGAGAVAAFDSRSPGSTSKGNIGGLLQTATYISGLSGGGWLVG
SIYTNNFTTVQNAVNSGTIWQFGQSILEGPKGFKDYYKTVFDEADKKHDAGFNRSITDYWGRMLSFQLVNAEGGG
PSYTFSSIADDSDFSAAKAPLPFLVADSRAPGEKNTTIDDALFEFNPWELGSTDPSLNGFVPLKYVGSKFNNGKLPDS
EKCIAGFDNVGFVMGTSSSLFNQIILRLKDSNGSKYIPADVPKFVVKIILEILTALGDADDDIADWTPNPFKGWNKAV
NKAATSDRLTLVDGGEDGQNVPYHPHLLLERKVDVVFSVDSSADTNAWPDGAAPMRTYERSLSPVSNGTSFPVV
PGRNSFINLGLNTKPTFFGCNATNTTNAAPLIVYLPNYPYQFHSNISTFSLSVNNSERDAMISNGWAVATQLNGTR
DADWPTCVSCAMLQRSFERTKTPVPDKCNQCFSRYCWNGTIDERPPAKMYEPQLYGTAVDVKGTSKSAGLRLLG
STSAALATVAACLVIT

>QLI69131.1

IEEVGVASCMFGTDFFTLSYTIRSGATTLSRCFADAGEMEMSQSDVVSYSSGNNKGWFDYEPGDGYLYRHSFNKS
NTIFIHGVDTTWGRVVKIHID

>QLI69137.1

DLLTGRVEISTPVTSFKRLQEGLFLYNHNVWPYDGGVYHQAPLLLPLFSLLPSVKTWPIFTSVLYILVDLLSADALCTIA
NSGEAGQSRLFTSPRRAKRACGLAVAAAFLFNPFTIASCIGRSTSIFTTCAILHAISKAIQGSAFNAMIALSFASYLSMY
PILLLPPLVLLSFDRQPEARRTASAVAFGAKCVAITAVCLGLLLGMSFVLTGNSWEFLSRTYGIQLTLSDLTPNVGLW
WYFFIEMFDSFRAFFLAVFWLHLAAYVGGLTIRLRTQPLAVLTLLLGIFSIFKPYPSIADASLFLAMLPLFRHVFPLMRY
TYVASATLLYATFLGPAFYHLWIYAGSGNANFFYAITLVWSLGQSLLVTDLTFAVLRDEWEVERPDMIGKEVKQI

>QLI69138.1

TTTSDAPRRSQAPSDGLTTPQYGDGSCDTQGMINIETYITLFANNETHEGGWLGDDIVEKQIKALNEGFSSCRIGFT
LKGLQRSIDAMPLGFDPDEKLAFGFYGKYRKGSYKSLNLYYSPNQTGGVCTHPGMRGALHIGTAFNVDGCQMGT
NTMPGGTPPYEMGKTTVHEVGHWLGLLHTFQGGCNEVKGDFVSDTPAVNETVGRVEGTCPRGQNSCPGLPGL
DPIHNYMSYSSDECRTEFTQAQCERMRSIWRYIRIDPEHS

>QLI69141.1

APTINKRIIGGTDAEIGDFTSMVSIQMGNDLCGGTLLSKYTVLIAAHCLGAANNDTVAKAGVADNKKDGEYSSIREI
KVHPDFNTYGHYTLDDIALVYLKEGFDEGETIAYANLAVNGSDPEVGSEHIALGWGTQNYEKFKDRPDPTDKLSKV
GLEIYPRGHCWSRLLGAGKTGAETIVCAGGRGKNVCKHDDGGPLIDQKKRVVGIASLVIKDRGGSYCNLEPSVFTR
VGSYSDWINENLVTEPPTSTEEQTDFSFEFPKLINGDVEAKPPASTEDDVYVEFPSPTKPVPTSVPTVSNFPPNLNIV

VTDVCDRRGLGSDNSCPPVVKYCIFKFKEDGYSSTNACVGAFEKAGLMTATKNKEAGLTPGTENQGTENQGTANK
GTENQMCSST

>QLI69143.1

KVVSRQIQLGQHEYFMPPSSSWKVPSWDSNFLQNATVDEFVPITVMNLNGTKNDANIRAMLDKFNATDDVWTS
SFTQNRIVPSGPYFLHTSTGNAYQAYRLFADTNQAFIQSSYQDPQDTHHPLRAAISSDASLTVAVPSRLYYAPTKDK
PLAGVRISVKDLFDLKGLKTSGGNRAFYARRSYFTSHIDYLLPFNPRGDGYNLPSDSSGGSGASVASYDWLDASVGS
DTGGSVRGPAQQNGVHGNRPTHSAVDLYGAIPLSPAMDTVGMLARDPLLWSKINRVLYAGSVKEFAKFPKSILLD
PSSAKKLSAADQEYPKIAAAANNFLNHLSKILAANVSTFSIDEAWNKSTPVAFNTTPIVNAVDRIYSDLTRYEQWND
FGKAIVSEYMETHNGEFPHMVPDVRVGWLLANASLTEDDYKGDLKDKSGVAEWVAKKFLTRDEDTCSNAIYVYF
NMPYKSYKPDISGEYVLRHHYN

>QLI69147.1

HRGNFTYDRHNFLLDGVPIQLIGGQMDPQRIPPAYWTQRLQMAKAMGLNTIFSYVFWNNIEPTEGSWDFEGRN
DIARFVRLAQQEGLYVVLRPGPYICGEHEWGGFPSWLSQIPGMAVRQNNKPFLDASRNYLEQLGKHLAATHISQG
GPVLMTQLENEYGSFGKDKAYLRAMADMLKANFDGFLYTNDGGGKSYLDGGSLHGILAETDGDPKTGFAARDQY
VTDPTMLGPQLDGEYYVTWIDDWSSNSPYQYTSGRPDATKRVLDDLDWILAGNNSFSIYMFHGGTNWGFENGG
IWVDNRLNAVTTSYDYGAPLDESGRATGIYRQIRDVISKHVPAGTIPEVPHVPDLTTIDDFALEPAVALFDTRSDKPA
VQAGSPVPMEKLGQAFGFVLYEHRVSAAVSGAIAPGDGPRDRVMVYVNGARVGVVDKTHAAPAPVSVDLKQG
DVLQLLVENLGRIDYGQQLREQQKGIVGNVTVGGDAVLEGWSAYSLPLTDLPAALADENSETPEIKDGGAPVFYKG
TFGLPAGAGNDLSGDTFLSLPNGVKGSVWVNGHHLGRYWVVGPQQSLYVPGAYLYGGNKPNRVVVLELEPKAG
ADMVARGLATREWANHPDPDVA

>QLI69152.1

DTESILTGAVLSTEAILTGATLSTETVLTGVTRQTSEGVLTGVTRETSVPTSTGSILTGVTLETSMSVPANTSAAGPTGS
QTGVPVAGAALGSQNSRLVLGAAVLVAALAVS

>QLI69158.1

ATTFNLWAYGSSHGIGAAGFCQGNGIIFLCNGTSAGHLTPIYFSNTDDDTNESWTVHSNATNKPVSTLGKIALPNS
NSSNTVAIVTESNSTYGVRDFFLYDSTVLAKNGGQWENHFYALATNISGIWKLVWDPENLDNHNSQPVVLRNLSP
EH

>QLI69178.1

MRNGQHGLNRDVEKRSGYLKGLGSRVGLKSGGSDSQYDVNQVKDDTSRGFWTIVGPDGVVNTANAHMLPAAS
PTGRRKAVDASSNGRQDGQQHSTKGQQKGQRPKQPGMEQQHRNRTQSNNQQPAPKFEGIQITFVTVTVRPTV
SKGPVRTGTKSPKTVTTTVTVAGGNPQNSSQKSNTGEKPTPTAGGNGVGVKPVPIDKTALPTTTYSLNPPKPPPPA
PPKPESSSSLPPVPAPVALPKPPQPAAPKPDAQKESGTSPSSTSSSAPSSSSSDSQAPPPTAPLDSVVAPVATSPLGV
VAPAQPSVNLSGLTLNSIVDLGNLPKQAGNAPAPTTVAI

>QLI69179.1

QDASHEDEVRKAFDVRLYAGSFADLGEIMANSLDAVKKLGQDMTALRIAGANSMSEEDKDALEYAVDFADEIRN
NQDFEYLAGRARSAARVSRDVSDKANQRVNQLLGGKKN

>QLI69189.1

VYELHFSRLSHIPGPKLAAFTRLYELYYDVVLRGKYTHKIAEMHSQYGPIIRVGPREVHINNPDFYETVYALRGKRNK
DPWFTSNFAVPKSSFGTLSHDVHRSRRSLMAPYFSKARVQRIGSTILAKVDKLVRRLEEFVHSQQPLKLDPVFSCLLV
DIVSEYTNYKCFNYLDSPDFQPIWADTTKDLSECAMMSRNLPGVFGILACLPRRLVKQIYPKLITVMNFRADCIKEV

AHMFHNTSDSKEMAEVKFCQEPTLFHDLLKLESSPDAEERVLHEFISIITAGTETTSNTLTAITFHVLNNIKIKQKLRDE
LYQAFGDGNAMTWTELEQLPYLVGEEVPKIVATATNLSYAFQTGVIYEGLRLSYGLSTRSPRVSPDEALQFGDTIIPP
NVAIGMASVIMHHNETSFPDSHKFMPERWTDPEERRRLSKYLVAFSKGSRQCIGRHLALANLYFTVSTMFRKFEM
QLFETTEDDIKLEHDVMLPRPKLTSKGVRVKIKRPIV

>QLI69192.1

TYGNPDDGNDTENSAGQVDSPQSTVTVFDNSGENSNGFPSGQSCQMQGTTTVFVTVYPTGPVSNGNESPAAIT
GTDGTPDHTGYRTIQVSPLDSSNGNRPTVQVFTTLTISDLWSGGSDSGNNPTSVASDFGHVTSGSLPSDGSSPSSS
VTYGNPASASDKGPVETDDSGDNGSATPGTVTVQNVPSNSPYSTGNTGQVTANPNSGDGDNRGTVVHPSNLYTI
VTDTNVEWVTGSDGGPSPVTVISEHTITLGTAATEAISGAGPAVTCWTVTGTDGKETVIESTLNTNQVNGGAPTVT
SASVTGPDGNISPQPVSTAVGQQTPVTTITAGGAIPDYTGPGTARTTAITVLGTDGTATVRYSTWVIETGLVTVASA
ATLPTGVSVSPGATQAPSNDRGITSCTSYTVIGPDGRPTVIESTLVVPASVVLATELPQNLPNGISVQATAVPGSLIPG
AGAITTHSSYTVIGTDGKLTVVETSFLIPGPSATPVATIAPSGVITGVPGQVTAAPGQVGSVEMASQGLTTCLTYTVL
GTDGLPTVLESTVVMPNSNVLPTGTIIGLPSLVPEGQTNGLPQGVSIPAQTGSGYTTCITVDVLGPNGVATPVVETI
VLTTQESGLQGATVLATSLPFPSVVPPGFSDLHQGVTPSETVSVSPITTAVTLTVVGPNGVPSPVVQTIVITPQPQAL
TSGVTNAVTIPEAASAVSPALEEYGTGSPATPTVLSPPVVSSELGGLPVGTDVGAKPSIFTIVTGPGGIPVLSVVTAIPL
SVYNSPGNADNKGLPVSGPAPQGTPGEYGWQPAGASPAGYGALSSLFNPQPSPIATSVQTSTWVNVIPEPTTTYT
MKFALTTLTTVTVPSKVSVVKRAVQPLKKFVSPGAGWANSTSTSLPPLTESATLVPPPLDPTPAPSTLSVAAPPPAN
VMCPAGGKVGNTTVNFDTLKPGPLFNPAADFWFSEGFLVAPLSPQSVQGYMASSGGQLVEFVPPALTSPASTGSS
DTAEIGVGPNSPNPCFRFNLYSANLGCAALGTEQWCEFEVSAYTYNQATSNEMSIAWSEVKRVPACPSFPNVPCP
LTPVTFDGFQNITSVLVRLHVGLELRTWWADDLQFGWTDNSCEAAQCRQAVTPQPVKREVVESALRRGVWRWT
PTGHERMGEEYVWDALN

>QLI69204.1

APTKDHGGDQCKYVPCVDKDLIVVDANVDVDLGIIDLDLDLDIGVGLFKKECKAYCCHKPCNKGERIPNTCWVRH

>QLI69214.1

AAAASSQESRDLFARDFEMLNSRDLEQLSPRDVELLTRALVERGLLDDIWQKLKDATTCAGGELLLGALKVLAFFGD
GAFVKVIQGICKLAKVQPGDVCDGAVALEGPIIAADIRKISVGSRTSKAFCTTFLGVCGYPDVQTWDIAYPSAQPAG
GRPKPGGKDPIKIVHYSDIHIDPLYVPGSSTQCDGRPICCRPYTKDDQPGNTKFPAGPNGDHMCDVPFTLERSMYD
AINSIVPDAAFTIFTGDIVDHAIWNTSQPYNTNLIQHAYDTMNSSLKLVYGTAGNHEAHPVNAFVPNAIGHDSQW
VYNLLSSDWEHWIGESSTAMVEKIGAYSTKYPKGNLRIISLNTNLYYRHNFWMYQSYEDKDPNDQIAWLIRELDAA
EKAGERVYIMGHMPLGEADALRDGSNYLDQVFKRYQNTIAASFFGHTHVDHFEVSYSDYAHRSASNAFMTSYIAP
SLTPTSGMPSFRVYTVDPDTFAVLDHTTYMADMTNPSFQTTPVWTKFYSAKESYGPLVSPPLTDPKAELTPAFWH
NVTVAFENDDAQFNAYMARKSRGWQPASCTGDCKTDEICQLRAARSQDNCWKPKPGINLTKRSGVQDEKGEH
DECGVPITLKTLSAVGADDAALQELKKIMDEVIAEAKAKGIKGAEDA

>QLI69235.1

IPLRPSAEPMTLPAVFERSEPSEGCLLKRSEFKEHNNTAGGLQASCLQYQKQINEAFTKCSERAENVINALESKDFPS
KDKTVQETGQPKQDGKETLQRLLEDWFDVGINDTERITEIKKEYKSIKEECDKKEKTRFGIYCEDCPENILGQAYGGT
GPIRLCKLALERDRRSTNIRDVDLGGTLMHEMSHAISNTNDTGYGVTKCKNLKTRAAIQNAESYMFGALAATLGD
NTGPSGEGG

>QLI69247.1

DDSKDLGSVLAANKNLTKFYELIKKYPDVLLELPSDNGVTIVAPSDKAMANIAYTALQPLWDDDDKDKTTAILQYHV
LDGAVSAAALRDGPTYLETSRLTSPALTNVTAGQGVLVTRQRDDVVFTSGGGSRSSLVEADIPFAGGLVQVVDNLL
VPPAQLAGTADAFGARSFLGSLYAARLMPALAVRRDVTIFAPLDDAYGLVGGTLRGLNASRLARVMGYHVVPGRV

LSSAQLANGTGYDTLATDAAGKPARVLVRQEGNSKFVNAARIVQPDILLANGIMHIIANVLNPDAERAAPDPSAAT
QAPAFAVSTVSGVFTSDLPCTADCPATTTAATATGGADSSGATPTTTSSLFSSTSRGGGARCTAHVVGAAVGMVG
LGAGVAWM

>QLI69248.1

FDSFYARNNCTDAAATFVSPCKFFATTAVGIGSCVYKDGYFAHAASRAPVPLLSYALFTARDVVTLFASCTLPTMIAP
ELAVFSGAVASRASEWFGSEESRLQVAGVMAPVAAQIVGTPIHLLGLDVHHRRDGFSAAARFKSVWRHFRVCAPL
RMVRIMPAFGIGGAANTDCRGMMLGRLTG

>QLI69253.1

RSLWSTQPATYAPQSSDETILKTTYPVGNGKLGAMPFGPPGSEKLALNVDSLWSGGPFESSTYTGGNPPSSKAGAL
PGIRDFIFKRGSGNVTALYGSGDHYGSYRALGNISIAIGHGTLYSDYNRTLDLDRGLYTTTYVVDSVKYTTNLFCSYPA
QACFFNIASTGTVPKTTVKFEDLLVDTILAKSSCSNEFARLSGTTRAGPPAGMRYEARAKGGNGANTSCSDDGTLTI
TPSGGSNSLTIVFSANTNYDQKKGNAQNNYSFKGEDPGPGVEAAVLKAAKTTYEDMLSQHVSDYAELFGTFTLNLP
DPNGSVKKDTASIISQYSVDGKGDPFVEGLLFDYARYLLIASSRDNSLPANLQGRWAEQLSPAWSADYHANINLQ
MNYWAADQTGLTKTQPALWNYMQDTWVPRGIETAKLLYNASGWVTHHEMNIFGHTAMKDEAIWANYPASA
AWMMQHVWDNFEYSRDVSWLKTQGYPLLKGVAKFWLSQLQHDAFFKDGTLVVNPCNSPEHGPTTFGCAHFQ
QAIHQVFEAVLASGEFVSETDTVFKRDVASKLASLDKGLHFTTWGGIKEWKVPDSYGFDTKNTHRHLSHLVGWYP
GYSISSFQGGYTNSTIQKAVAETLKSRGPGNAADANSGWEKVWRAACWARLNNTAEAYYELRYAIDMNFASNGL
SMYNALSAPFQIDANFGLAGAMLSMLVVDMPQKHGETGDRTVVLGPAIPAAWGDGNVKGLRLRGGYSVDFAW
DANGKVNKAKLVGSGKPLKLYNVDGKVL

>QLI69256.1

RITGISVPETIRPGDTVNATIISENYIQAVYDVAIVFGYAPGHGTPESLGLVAGSIYLGPGQSNQLHSFTQQVAIPESA
PRGRGLITASLMSLYGALHMPTLSNFNVTVTFGEETSTKYISSQS

>QLI69263.1

FTTPGHVRDSPCGVEQPSAALLAAHREIAATEKNHLLRRNETTDTPPAIAVDVYAHIISTSETAKDHHDNNTIHEQIA
LLNTYFNPGNISFVLKNANWTVNPYFSEGNNTMRMKILHHKGDNRHLNIYYGKNPLGATNSRAVSTTPDDLVGQ
PLGLEMDGIMIPAYWLYGDRKQTVVHEVGHWFGLLHTFNDDCNGNGDEIDDTPAHADASEGCSAMPPLDTCPG
KPGNDPVHNPMNYIPE

>QLI69271.1

APTTTYTYTNTMTMVKTITLAQVHTVTSTWDTNSTTTTFAPTGGMTSFTPPAATTAPAPATKGPDNAAGVLDATK
VAFAGVAGMLVVALM

>QLI69277.1

RFYMSARRRPHNDLWNKIDIVGVPEGGGILSWTFAIAKSVTSMQEAMRDGYKRFSKFDKPFGLPTMWMGGALV
VLPPSMLHLLNRPRDELSSFDALLENAQFQYLMTDKDVWGNTIHFDIVRKNLTQKDMGPLAGIMAEEWATAFQK
CWGDSRNGTVVNAWDSMVRITSHVALRIMVGLPGSKDETYLEQSRLYANAVLVDACFINCLPPALRPILGRVLALR
ARYHQRKLLKMLLPMVEEKMQQFEDRGGDEADSPGDVVQWLIAVAKSHGPEQMTANKIAMRILALTSMFVFAI
GWVFAHAVIDIYSSDKRDEIVSTCRDECRRVSSEYQGLSTKESTDALYCVDSAVRESMRLNDVMVHLLPLDVISGKG
IDLGQGRRITARSGVRTVFPAQMVHMDPEIYHHPEHFDAFRFSRNFPAQQQTDRQHGTKRQLMTTVSTSFLPFG
YGRHACPGRWFVAQMVKQAVAYVLLNYDVEVVKRPGKRTSMLNFMLPPQKVALHVSRRSCNTTANEKTGTGLQ
DV

>QLI69340.1

VPIFGALGNMVDSFLSPLTGAVGGGSGSGQAPTSEQIDTAARAWRNDTGTVSNFLSNAESMDPQELQRQGKIAL
DAENDELLHKAVLDRMFLNGTAAARDAGVADANDVLVNQDTFQFIVDGLTLMSKRGSTMKPDEVKTLVKAMN
QDRCPHVLPAIDKYMAAAQGAVPGGDKSLKAYRPTNC

>QLI69355.1

INCRGSGLCPSDGAAGNLINLKAIVDGIQPRDRRYNTGQQIACTGSICAFYQNGATGTAAQTSGFLQALLDHGCKK
CGSVPTQPGNDVKNGELTVNVVGDPHCQGAC

>QLI69366.1

SVYTSFTGSGHTLLATDDPGLAFPLDPVPRRDFTTLAKYPAHNINQSSKFAFATLYCSRDPDTRGPYFESTQSIIWRLL
WSDYRSKYPVIIFVCPFIPKKNRDIFRGQGAIVKEIELLDNIIPDEKISTKRWIDVLSKLNLWKEIEWNRLVFLDSDAFP
VRNIDDIFDLVPEQQCKKEALLPEDQAVIDKGGDDMCNYVYAGVPQFTIDNINAGMFILKPNLDMHAKLIRAAKRT
GDYDVRYMEQGVLSSKNAFAADGPFPVNRLSPIWNTVPEYYKEHLAKAAESPEPPTIRILHAKMWNRFWGSWN
NLTHLNDMWDLDWMNMCRFFDSDEFVKARTTGVYVTPWERYLKAQETAS

>QLI69373.1

VLSRAPCTGNTATTRSQWCDFDINTDYYDIVPDTGVIREYFFNLEQVTTAPDGFSRVAYAINGTIPAPTIIADWGDIV
VIHVTNNLGIAQNGTSIHFHGIRQNRTNEMDGVVSITQCPTPPGSTITYTWRATQYGSSWYHSHFGFQAWDGAA
GGMIINGPATANYDEDKGIVLLSDWNHRTASELYSETLTKGPPGISNAIINGTNIWKDESGTTVGTRFNTSVTAGKS
YRLRLVNGGIDTHFKFSIDNHTMTVIAADFVPIVPYETTLLNIGMGQRYDVIVQANQHSVADNFWIRAEPQTACSS
QNSNQGNILGILYYGNKPDTPTTKGYAYKVAACEDESLDNLVPHVARSAGKEHWSDLELADLIQQENVFLWALNE
TSFYVDWANPTLLQVYNDEKEFTHRSHVIQLDEKNQWVYVIIQAQNAASHPIHLHGHDFYILGQGTGTYNSTQQR
SLSNPTRRDVALLPGSGYLVLAFVTDNPGAWLMHCHIGWHTNTGLALQFVEQYGAARGLINHDFLNSTCEAWSR
YVSRTSIKQGVYEDGI

>QLI69375.1

LPNSNFNVTPEYAAAHGCGSKCQQILRLGNAADLDAVGHDFAFDFFATAGNFSEATRPGDVLKVQVLDGRTLNV
DSGTTVFRIQYATRDLDGRTVPATGFVAFPFTPDFSYAHNGTARYRLAAFAHGTIGLFAGCAPSNSPDLYDYSTWQ
AAVQRGYAVVATDYAGLGNNYTTHKYLTLPAHVHDVYYSVVAARKLFGRVLTREWVSFGHSQGGGAVWKLAESE
YVRNDTDYLGTVAMAPATYIIDMLRGPGVDFTGYLSFLPFAAKRALPGYQPAFLSDVMSRRLELAETAQLCISGML
GLTLDLEKEQLFSAAGLAKDMETLEAWQRMLAPAQGDRSPAPVLVVQGLNDTSVLAPTTEAAWRNACRYGNEV
HLRKYRGQDHSPLMQASAPEWLAWMDDLFASRRDGRSRPARRCTAETRTPFSPEFVKQPPEAGDELMAVLEGIL
GE

>QLI69381.1

TKSPWKVSVRQLSSSAMQQNIGLQNNTDGNMGWEDIVKRKRAERDALLPQEWKLKAPPNTTAFSPLNQVINSG
LLSSEELEWTDTKKYDATTMLQRLSSGEITAEKLVTAFCKRATAATSLANFLTEVNIADAINRAKELDRILNETGKTVG
PLHGLPMTIKDTEDLKGFDTSCGITGWAFDPRESNGPLIQILIDAGAVIIGKTNIPQTVLAADSDSVVWGRTLNAHR
NTFGAGGSTGGEGSALGTGSTLLGVASDGAGSSRMPAMANGVVGYRPSGYRLPPGGREVFTDGRSGLSMTGPV
AGMGLMGHSVRDIRLAAKVVSDAKPWEAQTPFMYPSPWMNITAPEKPRIGVWNVESPNTYLHLFPPVLRGYQT
AQSRLRAAGFELVEFTPPDMSQVWDLCKEFLIFQGIETLTEMISREPITKIVRDTGIFVPDTPRFPVSVDTLYQLNTRL
VNLTVAMDTAWNSSGRPLDALLSVTAANTALPWDTWHDTTYTSIYNSVDWPAISLPLGLTVDKNIDHKYSDFRPF
SKEDARLEALYNPETFHGLPLSVQLAGRKFEDEKLLAIAELLHPVMKGE

>QLI69384.1

GDIEARTVEVFATTYLDLGHCHNACTTKKWHEWETPGHESPPYATHTMPAEEGCVTTSISTKHPECQKCKGTVYV
YVPKTEHHAKTTTVTKSVHTSTQHTTKPTTVKTWTRPSSSSKPVTESKSSTTKREPTTTKKETTSTKKEPTTTKREPTT

TTKREPTTKRTNDYQDMDMGYKQTTIFDQDVHYDERANFIFNEDMDMDMDFNQATLFYQDVNCDERANDYKE
RANDYQAPLSTKTSTATKEPTSTKREPTTTKREPTSTKHETTTTRTWTWTSKPLSTKTSTATKEPTSTKREWTTTKRE
STSTKRETTSTKRYPRPTPHSGPTVGDANAQCGNHQVLSCCRSGNSAGLLNNVLGGQCSPINVLIALVPLQNACSN
QVACCTGSANGLLNVECTNLNV

>QLI69390.1

AWLQGAWVRDAWVRKYPCGPLRPDFPAPDTPFWIDSLRGRLDTLDGSTQLSLSILGVHNTSTFSCLDLDVARFEE
ALGLEVLGYPVGRVGRFGLTCPLPIDDALTPFDGYRFSSFDMVYLLNHTHQLQSLVAEFRVHHVESGREMDCAVAK
ITPRMGNPASAALAYLPAGVMALVGVASWQTHLGELATNPLFEYGSAMAGRSLVWETVVDVADYLRHLQFVFLS
ASLSIDYPGFYQPVVSKIAWSSLLFWKGPINHGFTHQSVQEGMYINNASYGLDYMAQVLGFPRVPDSMLNAFINL
LLVASVLFFILAVLYLLTSRQGQGTPWPELLQMAGGMTVGAVLSLFSFPLVTYMSDEMIFVGYLPNYRIALAVLTLLI
LVCANYIISHRFGNRRRLKTTSHAESSRDCPIVFDSSRLWAYVSHNLPQSIPLLQAIAIGALQDFGTAQLLVLTGSETV
FLLHAILQRSAKCKFLCSKTVWCSVVRLATISSLSIAVISAASEATKQWVGYAMLCLHGILIVLGFSCHAIWQLYRANK
KGDHLSQEAYLLGSHSSHSNEQTLNLDDLGSHFVCVNRQLQRPETPDIPDERLRSLRNYPRPVLKTSPAPLEDTEGQ
PTLPSAAADADLQLDFSPFYRKPRSRNATPLSHLDSSSQSSRSTAPSPTPTSESDPESGASSQPGRKSHETLDALLEAS
LPPDVDYSVRESDRFYGRPVSGSANPAVTPGAAGCPSSRPVHDDWRHMIPEVFKPQKKEKGFQVARPPRPPQ

>QLI69409.1

KQYYVDCSRPDAGEGSQHKPWNSLQKVNSPTFAPGDVIAFKAGTTCTGTLSPKGVGTADAAIRITKYTTAGAADN
NPNPIINGTGAAGAAVTLTNQDHWQISNLTVTNPASGLAARQGIHVTASDGKAHTGITIEHNTVHHVAGQTNKR
THSKDFILSCGILVDTSGAGSRYDGVLVQHNAVSDCGGGGIKVRVGAADNLGHKARVTRNTIRACGGDGIIISYSDS
PLMDYNVASDLGTGAYPFTGGNFAGMWVLGDHNPTISHNVVYGSTMSDIDSEAFDCDWGNTGNCTVEYNYSR
ANAGGAFLNCDGCGPPDAPPGGADQIVRYNIFENDCRMYSNGRRPTLYFYQNVMYCPDEERGFDIAVAHNAYFT
NNIFVGNGKASLPARSDIRWRWNVFYRVPHPTDNGIEADPGFVDPGSGGNDLASVGGYKLRRGSPALGNGAVIP
DSGGVDFFGNPVSFTQKPNRGAYNGPGL

>QLI69420.1

GSHVSGHYVEAPRCNGPDCDAAMAKRADGQPGAPGSAGGNGTPGAPGSAGGNGTPGAPGASGGQGGAAMT
GTATSAATSTSVTPKAGANGADGSLGNNGGAGAAATKASTAASTTSASVTAKPGANGANGANGANGSSGSKGG
NGGAGSATKSGTSASARSTSQTSGADIKTISVAAIALAGFAALMA

>QLI69431.1

APKESFPSKNGKFSITAKHNVNFERNGPLALAKAYNKLDKPVPQDIADAVTRIQQKRETGSVTNTPNKHDQAYLAP
VQIGTPPQTLNLIFDTGSADFWVFSNETASNEVKGQIPYDPKKSSTSKRMSGASWSIEYADNGTSVSGDVYTDIVT
VGGLSVKSQAFASAKNISAYLSRSLAASGILGLAFSKANRIKPQKQQTFFDNAKATLDAPLFTVDLKHQADGKYNFG
YIDSSAHTGLIAYTSVDSTIGGWGFTSPGFAVGDGSFTNLSISGIIDTGATLLLLPDNVVKAYYSKVKGASYDESERGY
TFGCSTTLPSFSFWVGNSTITIPGSYMNYQATNDSGKTCFGGLQSSSGYGVSIFGDVALKAAFVVFDAGNNRLGW
AAKNL

>QLI69436.1

APAKNGKFIVGGEAAAPGDFPYIVALLSSNFQFCGGTLVNNDTVITAGHCTSSDVSGYEIRAGSLASGSGGTKVKVR
SATRHPNYNANNLDNDVAVWKLATGIPESDTIKYAKLPEPGSDPTPGTNVTVAGWGRLTEGGATPDKLQKVTVP
VVDRAICKKAYSTPTPLEITDNMFCAGLEKGGQDACQGDSGGPIVQGDVLIGVVSWGTGCARPNKYGVYTRLANY
DSFLKKYL

>QLI69439.1

ENFEYWPGDRCTGNIVGRGTLACGANRLPVFRGIHSINLIDADGFHTRFYESRDCTGTPWFTDYGEGGCVTDTSAR TNCIYVVCWVHLQKTAQ

>QLI69448.1

AIPAGHDTAPTATLDSGLIFGVATQLPGAPGPVNKFLGIPYAEKPERFTLSKPPKRWRSPKNTTAFGPSCYQLVPDS DVGPSKDLLHGLFNQHPPQSEDCLLINAFAPTPSGPASGRPIIVFIPGGGWQMGNGQLDLSGFAGYEDIVAFAFNY RTNIFGFPNSGELPVQQRNLGLYDQQLALEWVQKNAKAFGGDASKVTIWGESAGSLSVDIHMHAYTNVTKPPFR GAIMSSGEFSFGLLGTTAAPNNTKAWDSVVKEAGCKGASTIDCLRTMPAEKLVNITQKAGTIFIPIEDNRAVPAGRA SAWRQGNLAKVPILAGTIAQEGRALVNRNISLDRFNEAYLFGPFFSNEQRDEIYAHYRKLPGLKTDFDLAAAIYTDFL WQCPMQKLTQVSASIHNPTWRYYFNMSIVDRLPSEYGYLGKFHGSDLLLLVISPTYDDSTAAGALLTPLLYTFVNYF RGAIGRFVRNPGGGPGWPMVGSSFRPWDVVTLGDVGSAHAAGATPVNQTVLDENCVLFEDALDMYEKYMGS A

>QLI69456.1

ISLTRHNHQVVLVESAPQLAELGAGVQLTPQAIKYLFKWGLKDDLLAESIVPDCMYIRHYQDGRLLGTIPVDQMAA RYGAPYIVVHRAVLHSILHKHAADGGAEIRTGARVVTYDFAAGAVELHTGERLEADIVVAADGINSLARQQLLGAS DPGSRPTGWAAFRMSVEMGRVRDDPVLAHVTGLESGSSNFWIAPHRSCMTYLVKGGTMLNIVLSHRDDVDTRG FSLEQYKQTVDEQFRNFDTPGGASVRRLLDLSGPEISNYPVYEVPPLPKWVHSSGRFTLMGDAAHAMAFYMSMG VSLAVEDATALATALNFVEAKANSKAHGSAPTLASCAGKEILRKVMHGFQKVRMRRVLAVQRASLYAGDTLHVED GDKRVALYEALRQSDKAFLQPPMDPNQIVEHTIQIVPGEPERCGLGGISDKATRDWCYDFDADGDVLSAIEMADV

>QLI69459.1

IPTGHDVIHEKRDFDAVTSQVTKRAAADTLVPVRIALKQRNLDKGMEYLMDVSDPSSKNYGSHYTQDKVVDLFSP APESIETVKRWLVDRGVPENQIKSPKSKGWLDFETTVGQLEDILKTNYHLYENKKTRSEHLGADSYSLPSEVSQHVD FITPGVVPIKVKSARAAPGQPRKIPFTPFPPGLTVNSQDASNCPTQITPACIKALYNITDSTTAVKGNQLGMFESDNE MHKQSDLDQFYRLYASKIPKGTGPKIDLIDWGSTKPDPNQAVGEAALDFDVSIPVIYPQGTELYQTKSNFDGRTHL GFLNQFLDAVDGAYCTSDGGDDPDVDGVTANEACGTFTPANVISFSYGLTENIWPTKYLKRQCDEFMKLGLQGSS IVFASGDGGVAGGHGGDCLGSNGDIFNPASPSSCPYVTSVGATLLPPGTSPGAPESATERFGSGGGFSNIWPSPDY QSAAVASFFASNDPGFPSYNTSENKIPTDGGIYNRAGRGFPDIAAIGDYGVIVLNGQPGRTGGTSMSAPIVAAIFTR VNEVRLKAGKKPIGFANPALYKNPAMFKDVTLGSQSGDAACNGNGFSAVQGWDPVTGLGTPDFPAVVEYFSNL

>QLI69480.1

GVHNNSTATGHIVYTTEVVTALTTYCPAPTTLTHGDKTYTITSATTLTITDCPCTISKPVQPTAVPTPAPGCNDKCQA TYDQCRGKPGANLSTCVSDFEGCKASCTGATTAAPSKPVSPTVVPAPAPGCNDKCQATYDQCRGKSGANLSTCVS DFEGCKASCTGATTAAPSKPVSPTVVPAPAPGCNDKCQATYDQCRGQSGANLSTCVSNFEGCKASCTGASAPAPP PAPAPTAAKPSGNSTTPVTAGAGALAPAKVLLALGAIALL

>QLI69482.1

SDPLFDSNYAKQDIIFRDVAVIGGGATGTYAAINLRKLNQSVVLVEREAILGGHTNSYTDPATQTTLDYGVQAYWN LSVTRDYFAHFDIPISNWEPEPKTTVYIDFMTGKQVEVRTSSNYSAYIQQLDKYPWLEYSWDQGHPVADDLVLPFR DFVAKYNLVDIAYTTYFSCQGFANVLDQLTINVIKFFDKSYIGALTGDYVATKQHKNDEIYVRAKAELGQDALTSSTVI ASKRSKTGVQLVVKTPSGKKLIRARKLLISMPTRMSDMKPFDVDGKESKVFSQWKYSAYYVMLVHKTGLPAGYKFL NADPSTTRFNIPQLPAPYQITETRVPGLFYVWYSAPKDMTQSEVQADVTTIIKRLQDTVNGATKISPEFVRFNSHTP FKMVVSAESVINGFYSELFDLQGYRSTWYTGAAMMSHSAGVLWNYTSHLLPEMIAAP

>QLI69490.1

RPFVPDAVEAAATAVQCDAKDFCSGVPSPAASDQYICGDNRLGPIDLQNREILTNSVLGPLLTSYNPFAGSCPGAFL
SEYGADRGLRYPQKDGFQLDANGDPVMTNTTLTPGTLIDRFGADRGFFVAPYGTPYEQRSLPPMNLANNPKYSD
GTPYNFHVYQVIKELVVREGPIAPWFGQPGKGTQYVLSTSVKQAVADGFLVETCSPGIHGCGNAPGTNVPTQILCS
SSGRWETTGECGSGQFCRLQPTNQMPSCQAKSVVAR

>QLI69509.1

QYDDVQYDYDEAQSIPKTAAEIYENETCSGSPAITLFTECSNLDLR

>QLI69523.1

TCSSTGKNQSIIVHEAPQTIRPYVMPKYKGRAVLLSKTEVVRFAITANSSDGAFSMIQHNGKLTGYASARFHTHRHV
HEHVYCARGRVELWAQKNTTDSIQEARVATLGDYGNLPIGSIHTFQLIDPDTQLTHIFHPAGFEHLFDFYSVGDFES
LGVGTPYVPHIEDEAPFGPLTPELKTELASLDLYESEQFVPRRDLINGTAGDSKLNWHNGPNDLPTTYGEPYFIAKDH
GKKFLHTETGYKIIQPLTNEATEKNFTIGTVIMSQKLANESATTTTLPHHFALQMEDGQLILKVEGYESTAMLHGDV
AFIPAGTKFSYHAAVPFTKFLYMNDGYEGLDHQLLENAMPWELPAYPPYAGFKAKA

>QLI69530.1

APSAANPSNQLPNFINQLQQSLRTVDQNNPPFTDGSKLNEYTCPAHAFECTTTDFWQRQIIASAPDRVTITTNIHT
DATLINDGTKDATITSSFSTAVAIGTTRGWTIGAKVSLSSPNKVGSSELSASYSDTSTSTTTETKTVQYGAICPAGKTC
RIQTVTFQARLHAYCRHESMLDCTGAVNVCKRPTGVLQCQQYVDYYNRNCVNPPSDSPCSVDVQLRADDGKLLT
LIIISEE

>QLI69535.1

WIEYAYVLAPNGTMVGNIGYPRGYQPRSTSGWTDKIPQWILPENGTAFYTGEEILNKYPFEQNPKQPVLEAAPGD
HIALLHLENGHVTLPQTQANKPKNRGTIYLYGTSEPSENEKLFDVHLLWNKDGTGGNQKGVLLATRNYDDGRCFQ
PNTADITNQRVAEWKDDGAQNSQELLCHSDLKLPDNLKPGSFYTIYWYWDWPALNPAKINMDKTADGRFPWA
GSFMRGDKVPNGWTMDTIARNESYSSVIDIKIVEKPKGFAGKDAGKEAWVSKQNVYSMGVQDQMANNFQVNV
DGLENSGTGSAPTSAPASAPASAPASAPAPTGSSPAASSDAGGVATVTQYVTVPPTTLITTVFKTVGGGYKKVRRSE
PAPEQSMLRLTNHGTDEAPFTTASPTDTPDQHSAAKQVSSAAPYDRRRARWF

>QLI69546.1

AAAFNHHRRATTCDFSNTFALSVEEQNLNQELPNYGLFGPTLIQKAGFVPQVDQISNNFCGADSFDKAKQSVREA
GKLLFRFAVGRAQNDNTGGEVDRYDDRPLYWARLTLTKAIKQWKSGFEMSDDARKELVTTFDLASRGLDSVSFPT
GNSARRVMVAGFDPFQLNADIRTANPSGAIALQLSGKTFNTSDGPIVVHAVVLPVLYGENGFDDGVVEAAFGPAL
QDAERRPDGIVTISQGLPEQFDIERWAGGWRGGFPDNNNVKSTGPVPPAAGWPQPNINFIETTLPYQQMQDAA
TGPFKVRYNTEFCEWPKGSPTTEPCIRHDSGVPAADSTPAEGGGGDYLSNESMYRANRLRVGLELANVRGGHLHV
PVLGFPDGNAPSDQKFEKKRLDIISQALQLVEAVGKP

>QLI69562.1

KIDGSSVNDIDNNELGEEPEPVAQDLLYGLNPVLDIAKIKDNMTNRDVGYLFVKHEENGF

>QLI69571.1

SHLSPIDRDAVVGQDVFLPPAIVTPSNPESPRPADHTFTLRHIYHHGTDRHPRLHRSMDVTHDLSSSRVFLAAEDG
HAEHDLPHLSARSRGNTIQRLVDRRPSVVDPMVAHARQEGYVAVLDASAWTVDDVPSPDVTDKGTVLSLAYMA
ANAYVETEDAADWLDIGEPFNRSADFGWQSDGLRGHIWADDDNSTIVIGLKGTTPAVFDGDGTTTNDKINDNLF
FSCCCAQQGQWTWHQVCDCATSTYSCNNTCVTKSLHEESRYYAAARELYSNVTERYPHSTIWVVGHSLGGAVSSL
LGLTYGLPVVTFEAVPEAVAATRLGLPIPPGSDPKAPQTRENTGAYHFGHTADPIYIGTCNGATASCSFAGYALETTC
HTGSECVYDVVADKGWRVGIGTHKIRAVISDVILKYDDVPECSMTPECRDCAQWKMYESNGTETTTTSTSTTTTRT

RTRTSTCKTPGWWGCLDTTTTGVTATSTKTTSSTSTCKTPGWFGCKDKTTTTNPITTTSDVTTTTHTPTTTCETPGK
WWGCWDKDETSTTTTPLPGTRTQPPITKGPTSTTWTAAPTSSAPSTEKCLRRNWIGFCKEWDGGKGVDAFFPDE
M

>QLI69573.1

AMDKRIVGGDRAKLGEFPFMVSLEGPRAVNRTNGGGVKVKVASIKQHPDYRLNPTKVGQTGPDFAINDIAIVKLA
TPIQESKTIRYARLPEDGFDPAPNSTAIAIGW

>QLI69576.1

SPAKIDKRETDYGGEPLKEYCAIIGLEADTIKVTDEGIAATCRSDPAVPPKTLQSREDGAKFCEKLGMQFSTSGSLDTA
MKSPQVWCKYADDGDSNATELIAKYADENGNVNQENLKDFLMGLSRDAPGTVTGLYNALKAAPSVTKSKFGLGG
ASGTAGGAATVAYDFIMANMKPGGLFGADTSFGRWLRTNPIYGASGTRSSPFDTTGNVMVYYKPNTKLCIPFKDE
SSVFWSTSVKRCKFWHDKKDCDTSYAYDAKTDIGKELRERCTNELDRNGLPEKEREEQMRREEAACIRPRWVCGS
LGKRLGDPLNFQYCMDDSEAAKKACDLAGWTYRIGRMKTKAEEEQEREERLQKEESECFHPRQSCRNVYSKFLYC
MRNDWEEKEKCRREDWRPTEQPTKSSECVKIKSHRVPGGPYYTAVCVPEGEGKA

>QLI69577.1

APLEARSEPPSGSVYIAPTALYVYDVFTGAIGPKTTNAVVEKGRAVHEKTTLMTFEYPMATANKKCALYFKYDSVSW
SGTDKLAIFSSLKPAPGGATSDWGPGNQRNNQLGIWKRPSPSSSPSWADWEATYGGLSVPQDCKPGKTEAFEIV
GQGDETWISYANAIGGVRIAYW

>QLI69579.1

FPFEAAEDTGVTLYEHVHFAGHHYTIPSINRCWPVPQTLNRFCMPPVLYAGIRETIPDLNLIGIGDEVGSVFCSGINR
DDALNEQWVDEWL

>QLI69590.1

IQKLESTALASCQENSRFSASLFNVVFTPNNNSASFQIVATSTVENYVTFDIRAYAYGYQILQRVVDPCQLDVGLCP
MQASRLTNKFNQKVDPAFVKDLPGLAYSVPDLDASVRVFINVTGTNESIACLEAKISNGKTVNQLAVKWLTAVIAG
LALTASAVIHFLGQFNAAAHLAANALSLFGYFQGQAALGLMAVHLPPIVQSWTQNFQWSMGIIRVGFLQTIATW
YQRATGGTPATLLDSLASVSVSVQKRSAHVRRKEDESGMHIVRGIERVAFLSNIETTNLFLTGVMFFVIFVFGTVIAV
AGFKGVCQLLVQKGIMKSHRFTDFRRDWRVILKGVLYRVALIGFLQMATLSLWELTRVDSGAEAALAVLFLVGLTG
FLGWGAFMVIRTAQRSVAVYQNPAYILYSDSRVLSKLGFLYIQFRASAYYFVIPYLGYIMLKSLFIALAQPRGVVQAV
ALLVIEAAAVISASVMRPWMDKPTNSFNIAICVVNFLNAIFLFIFTNVIGVPGVVVGVFGVVLFALNAVFACVLLIMV
IVSTTLIMLRKNPDARYRLMADDRASFMKSEINLDTVKELDALAATARGDKSRSSLQLGQKRSQSPIAPSVPLLPASS
SRLNSSQSALSTHTS

>QLI69595.1

LTTRRAYVQILNNTTQPIRAATVIHKYSDIYQHHGYWEVIQPGTRSGNMLRVEYHTGAFATGNDWWLVSWKNPE
GTRYYYSNPGNGRIFFDTVEWIFAGRGGHRLESTTGFKQHTLMKTDANRVTQVVINTDNTIQFSSKSGYSSTTSSST
PIFRQYGNPQPFYAIAHRVLDKAGVEVALRHGANAVEIDANAWKLVHRGWWADHDGTLPSRGDRIRDVLIAAAN
ARRAGKNLGFVWLDLKNPDRCGQLETGCNIEALRDMARQILAPVGVKILWGFTGSDINGRASGVVREDLTPSEAIS
IDGLSGTSARYAERIFNTSGPTNTAQRVWSKGLFQMALNFGSCEDKAMSASSGQICPEIRLGVMSGLFGKVFGWTI
TRNDGSEVNKLMHAGVDGLIYGHMSSFYEDTKEARDALQIINNWLKANSNHRYLANLDDNPW

>QLI69596.1

KDWQENAKFQTLPRLAYACAKAGWYQGSGFFIMNALINYAWSKNPALLRDPVQKAVAGAMIAIMWASGWWY
AKNGVTSNAVAVGAIGALQGYSAFTI

>QLI69602.1

RYASDGFKYVGCVQAKACDFPVKMDLGDSFTVAQCQQACGKNGSYAAAGVNGCYCEDSVSRGAPEYEVTDDSS
CSKACKSGDDKAGRCGGPSPADGEQLYNLYQRIPAAPTVPNVTTTRLADKPAAAAGDATPAALKTIATHPTEAAST
PRTGADEPHAASFREPTPSAAESRGVAYSNDTAPAPPAPATTAYRQEESKPSASPPSGPCRGRECPEAEEVPSEFTS
KFAPGTTANNSPSSPSHSVIVSEGPSRPASVITVALGMAALVLAMGMS

>QLI69603.1

QPVEQTARASMQERAAGGGGGGLATILADHVKYVSNPASAKDPIPPEEDNAAREAHRSVHAALFDAIGGGGGAG
GQKGAAGGAAGAGAAAGAGGRGGGKGKAAGDKGAKEQ

>QLI69605.1

LPPSKDPFYQPPAGFESKEPGAILRQRLVVASFFGLIPDPVETWQLLYRTTAINGSPIATVTTIFKPLFAKKDRFISFHT
AYDSSASICNPSYNYQLGSLQTDLISSSEFFLLQLYLLSGYIVASPDYEGPDAAFGPGRLEGMGVLDGMRAVKNFGN
NLKLSTNNPMVVGVGYSGGAIATGWAASLQPTYAPDLALKGWAHGGTPANLTGILTFIDNTLFSGFVPAAINGLA
KPSAYGAQLTPLLKSIMTPRGQRVLDFAAASCAIGDLLAFPEQSVLSTSFQNQGPGLLYNPDLVSVLEQNTMGVHK
NETPTAPVLLYHATKDEIVPYTNASTLADAWCSNGANVKFITFANGGHITTEVLAILEVLEFVANAFAGKVASGCSR
TTVLRNTLNPIALGVALEPVLVMLIEVLATAGKEDINIVKNLSTLNKTIS

>QLI69614.1

GTLSVPLERRGPSEEHYRLLGRAISRRAADNGTAELTALNNITAAGYYANFAIGTPGQNLTFQLDTGSSDTWMNSP
QTRYCRSATAQSEAGYCTTTFNPRDSKTYTLVDQGGFNITYLDQRRIAGDYFNDTVTIGGHEIANQQLGLAISSTRP
TGIMGLGFSINVATPNKYPTIIDNMVSEGVIAHPSFSLYLNDLDATSGTILFGGIDSAKFLGSLATLPIKPMPNAEDTN
VTSYAVAIKSVSATGVKVPSVAAQSVGILDSGSTISLIPDSLVKPIQDKFGVIVVQIQGQAAPPMIDCAWKGSKGDGI
LISFEFDNKTIKVPIADMAIDSLPDELQQILRSSDAPSGFRSWTRACLFGLGGSSAFGVRDDQFYLLGDTFLRSAYVAY
DMANQQIGLAQSNPNATGSNVVEIQKDAKSFPNVQGSDAPGPGEKASASGRLAPSSSVAATLLLGVAVVFSALL

>QLI69629.1

HPLVERAGPTLPTQDSFYYVPDDIEQYPPGAILRERKPPAPIAAFGLLKANLKDSRQIFYRTTDSFGRATATVLTVLVP
HNPDYTKVLSYQIAEDAPTVDCAPSYALQLASATGGFLGTIITQAEIILIQAALEQGWIIISPDHQGSKAAFCANELAG
HAILDGIRAALLSSNTTGISKDPAIALWGYSGGSITSARAAELQPSYAPELKILGMAIGGTEPNIANVINDANKGPAA
GLLPSGFLGLGNEYPVVEEAINANLKPEHRQAWAKVETQCLLANGLDFAFKDVAGMINDLPGLLAQPNISRVIAEN
NMGYRGVPKMPVFLYKGVFDEVSAVKDTDDIYEHYCARGASIQYVRDALAEHGIAAVTPAPKALLFLKDVLNGRK
QPQGCSKKTVVSSLLDPEAARILPVTILRALLDLIGKPIGPLAVG

>QLI69630.1

APGFHSNLKRENSTSNAMMQLSADPDFHYELLRVISLAPYQGADVGEVLVAAQKIKPKDFESFYNVFNDLATRVDS
TARAIDVRKNPISARQHFFKAATYYRSADFFLHGNWSDPRIYSLWDRHLAAFNSAIALLAVPGQRVNLRAKNDNFT
IPAIFYGSGMPGPRPTVILGNGYDGAQEEMYHVIGQAALERGMNVLSYEGPGQPTVRREQNLGFIPDWERVVTP
VIDYLLTRPEVDSRAIGLLGYSLGGYLAPRAAAFDHRLAAVFAVDGVYDYGAANLDMYPESLRAIFRSGNATLFNEY
VEQGLADPAAKTAAIWGIQQGLWSFNAQTPFEWMTKVEDFNLASVVHNITAPVFVAQAEQDDVIPGQSKILAEK
LGKLATHHVFESVDGAAYHCSVGASVLQNHVLLDWFEGVLEQRK

>QLI69635.1

VLRRQEAELAPDSVANSEQVTACKKPSLELQRAWQQNGCEGTNDGGQDGSTASESHSPACDKAMVDFVQAWA
DNNCEEALNAAINNLSTPAGRSDSSTPTSEIIN

>QLI69642.1

HIKLRAPVPFEFPDPAHSLDPLLPDGTDFPCKNARYDAPTSGNEFAQGSEQTLKFIGSVVHGGGSCQVSITTDLKPS
KNSVWKVIKSIEGGCPAKNLSTNILPLNPNLETPFEYSYTIPKDLAAGKYTLAWTWFNKIGNREMYMNCAPISVTGS
GGSKDALGTLPDMFVANTGNGCGTLGNTDLKFPNPGKDLDQFGQQTPAALTAATGNCAAASGPQPTGGAQPT
GAPQPTSAPQPTSSAGVAPPADPNGTIPGGVFITKSQGQQAGQPSVTSQPQASQSDDTCDEEVSQPAVTSQAQA
SQPAITSQVQTSQPAVTSQAQASQSSVTCEEEASQPAVTSQAQASQPAVTTQAQASQPDVTNQVPVPTTAGGAP
PATNGTVGGDAGAFATGTACSTEGMWNCIGGNSFQRCGGGQWSATQGVAAGTKCSPGQSPELKMDTVQGNK
RSFRRRMRSFA

>QLI69644.1

YTTTPRPGKLVPGRYTVKFKEGTPVSVRDNLLSQLDNANEHVDYNDIFVGFTKAMSETEVDLVRNDPNVEYVEQD
RDVYGFGIVEQPKASWNLGRIANRKRGIDKYVYDETAGEGTCAYVIDTGVDDTHPDFGGRAKQIKSFVPGETTDG
HGHGTHVAGILGSTTYGVAKQTRIFGVKVLDNNNQGYESRIIQGIDFVVNDQKKRRCPKGIVVNLSTGAAKSNIFN
AAAAALVKTGVFFGAAAGNFNDDASNYSPGSDPSVCVVGGTDKDDKPFFIKGRGGQPDFATNFGARVDIFAPGQ
DIVSTRTGGGELTMSGTSQACPHVVGIAAYLASLEGITGTKPLCDRIRKLSTKNAIINQHPNTPNRLAFNGATLSSNR
KPLCTEIKQLEFGIALSDDMFAGTNDEIGAILEGPAGKAEFSIVTDASRGFNTRVPVDMKASFGSDTIKIDGINSISLTA
KGPWISLLTNDKWKVKDVTLHAKCAEPGLEVADEKYISLNAWYQHPDASWLPFTGHSKQIVAKLGVSRADWTMK
PPCVEVKDITYWFQLGDKWLGGADGILSFKLGDGKRITVGENLDAGFFKSGTMDLKDIYGRDTMDLRDIKKLQIFD
NVGYKGKTDEWFLQGIGFGATCAEGGQKVKLSKFGNEDEWLGDDHHYDDLVYARDIIPSDWVKAV

>QLI69649.1

ADRLAASLNIIAHDYDKLNQGITAWPGDYESCVPLVSEADAITKALTTVKPPVTISPPDAATVKERQQAAQALCKIID
AYLKIAIEVKHKVYMSQPSVKPMVVAAIEELKAAFLALSTACLPVMTDTQSRDAFDSINKDMLLVEKLYKEAC

>QLI69669.1

ALCVLSWNISYVPPGGLRDISFPINLSRAPHVAGYHFAQRVKFYGQTSVAYAGLQPRQDTSSGATVLRGVFTSFING
TTTTDPNCSEGVDDGPGVSCAHDFSGSHADTWDLIMTNTAGTTWAGKAVNYKNGTAFHIGTWTLPEGTGGIQG
SQVGFMDYFPWNTNFSMTCADLPPTECTFSPPTTGSSWNGYSVGSISGSVERCENNVDHGPSLNGGWRFAIF

>QLI69681.1

QVLISELSFGHSGRLGADDGKIPHFTITGQPQQPQLLSNKIILTPMDPGNQRSSIWSDSPLTRSTWVADVDFRASGP
DRAGGNLNIWFARRGKEEVGTNSVYTAGKFDGLALVIDTHGGSGGMIRGFLNDGTVDYVSQPNVDRLAFGQCN
YFYRNLGRPSQIKLRQTASSFKVEIDGRTCFETDKVSLPPGYYFGITAATPETPDSFEIFKLVVMSDSTVSGDNNQFQ
YNSRQHQPDNNQGQKAQENKNDDFGNAIPDQSADIFQTSKEQFADLHNRLQAATHQISGVYNAVSKHHQMDE
VRHEEMKKAFDSLRHELAALRQIGDLQSKIRELESEIHSMHHDMRQKLEAHGESFETNLRNHHRSLSAALSDSIPGH
GKLMVFFVGTQIVLVAGYVVYKRRRASSPKKYL

>QLI69686.1

LPAYTNHAHVHGHQKRGAIDDMDRYTLYTGDGSTGAGWPSQDAWASSEDLWNVNVKLMRQTCGWNGWGA
DNSVDEIRAIESAIQKLARQTEVDDRFILAVMMQESKGCVRAPTTNNGVTNPGLMQSHNGNGTCADTNPCPDSQ
ITQMISDGVAGTSSGDGLQQLLDQARGVTGQNGTRPFYAAARLYNSGLIDYNNLDKAMGSTACYVSDIANRLTG
WTQADSSCEI

>QLI69693.1

RGYGTYYYDVEQLQACNSDFHKDNQGPVMCSFTDFLPLNDVRSNYLVAMNNTQLRGHLDKYCGKRVVVTVNGV
RSPLPFFIGDGCERCGIGHPDGGWNSEGAPGLDFSYTGLSELGPQACAAGHIDLSWEIVDENLYHFKTG

>QLI69712.1

QNGRNSPFNGKVGDKLVGGSSNKDIEAACSAPECFEGKKLSYYIAYSPECTKNHHTEEVCLGSDAWCGHSNRTELY
GSKQKCLELRPTPQGKAPWQPEGKAGCTDETEACLGTEVVCSILLNEILVAQYIEFDIDNFKKLCLEQRSKQGNSPPK
EDNSPPKESNNPPKQPDKPFFQLPNSESCSKPGADEEPCLGTIEWCDKHGQQQGLGDKYACLLTRGLDLKAFEQIF
RKRLIAPVKEGILTWAQNVTKNAAIREILLNATTETTQRAITTDLSGFMDKIKGSLQRQTLEGVRKGLEKYAGQKLF

>QLI69713.1

AALDDFESRPVFIPEWEVEVTPGGDKVRLNGTIQEVHQELLSLNPNWDKDFGLGDKVEAPSDETHGQLAKRTDFS
NSKYFCGGRWEYCNGGAIAEGRAYLYRVPGRPTNGAGPGACGRVSCSYNAAIWWCNDEKTPKTLNSFGSIADGV
YELEKHCRKYFSWNPSGVSIAGQIFHETNWNVIVRKDKC

>QLI69718.1

SNDGIDGTSADYLIIGGGPAGLVLAEKLSRNPRKHIVLLEAGPDSINDSLVNTPAHYPLIKEQHWNFTTEPDSNLGGH
APGIAQGRTLGGGSAVNGMAYCRGASSVFDEWAQLSGNPGLAWKSMLQEFREVSHYQDPPHAEYEQYVNISGY
GNGPLEVSRSSGLTGFEFPFKEAIQGQLGLHEADLTDGTGIGIDMGVATIFAKNRTRSYPRNTFGLIAERRQNVRIIH
DAWVSKVDFKGQTAVGATYRFNGKDVKIKAREVIVSGGAINTPKLLMLSGVGPKDVLSKWGIPVVAESPEVGANL
RDHPVSIVELRVTPEVLTLWQWAFNETEADIAKKQYAANASGPLGWNNGLVFATFRVPDSVWDGMDGSHFRSL
PQDRPHVMIEFSTVPFIPSPNASTITAWASLVQPEASGRVSLRSGNYQDDPLIYTNYYGSVADKAAILWTYKKLREIL
HRPEVSPLIESEHYPGPGVTTDEAIWAAMGNQTYSFRHPVGTVAIGKVLDRNWRVKGLKGIRVVDSSTFPYPTTCH
PQAVVYALASRAAKDILEADCKR

>QLI69723.1

QNLDGLGPCVSGCIEKALPSVGCTGSAAEIAACACKPETQAKLITPVSSCAAENKCDLSDLAKAQSIAKAQCDAVAS
GSASASGSASASGSASASATATGSATESQSASASGSSAPVVPTVSTGTGGVPTQSKNQTASATSGATGTTKAPTSS
QGSGSATRSGTGSVPTTTGAAAAGPVVGALAAVLAAALAL

>QLI69727.1

KVVPGAFIFEFEDDQDTAPALDTVRKNGDVRMDLDFELFRGISVQLHDLEKANKLVDELAALPSIKRWWPVTLHN
VPDAQVHWAGNPDREKILQARDNSTVTNNFSPHFMTQIDKLHAKGYTGKGVHVAIIDTGIDYKHPSLGGCFGKG
CLVTKGFDLVGDNFDGKNAPIPDDDPMDCQGHGSHVAGIIAATDEKFGFTGGAPGVTLGAYRVFGCAGAVSSDV
IIAAINRAYLDDADIITMSIGGPNGWKQNAWAVTASRIVAKGVIVTISAGNEGSRGIFYASSGSSGEGVAAIASYDN
MHMPTLVYYGNVTVGADKPQEFPYVLGNPDKFDITLPLWVDTFNTSVAADLCSSLPDDTPDLSKYIVLIRRGTCSFA
EKISNAAAKGAQYVLFYNSVDAHPTQLNVKKDIPGSVKGVGFVDKKTGKDWVQQLDAGSKVTVALGSRLDTKRIV
KDLNNTASGGAVSAFSSWGPTWTMDVKPQFGVPGGHILSTYPRAKGSYAVISGTSMACPLTAAIYALLVEVRGTR
DPVLLQKLLSANSKPQVFNDGALFYDRLAPVAQQGAGLIQAHDAAFATTLLEPSSLSFNETTYFAPSKNFTLTNQGD
SKVTYEISYVPTLTAAALKPNAKSVTVFPGEFYTDSAAIRFSESKVTLAKGETATVEVLATPPKTLDASLLPVWSGYVR
VNGTDGTSLSLPYQGLAGSLRNHTVLSPGTTYIAGTNDKYRRPVAANAVFSIPRPGSTNNAALPVIVYAPHLGSRLL
QLRVARVSKRGTLTLIGQIKGSPVQYVSRARDSLIWDGRLNDGMYVREGTYMIIVRMLRLYGDEKDATAWDESAT
QPFVIRYA

>QLI69730.1

APALDPRQNVDKLNAALKALNEKNADVEERIGTSVDDQAKRHKQWKDRSTKQVELIKTAKVVVDQLAELINIAAQ
KADEAPGRLDEQEKRREADNQDYWNIAKDFKVAIKG

>QLI69734.1

HPANQQHTHGSSHKPALLENFDAIGAWFEDVAAINGTSIARRPNVTIAIVGAGISGLATGLMLDSIGVHSWEIIEAS
DRIGGRFRTEYVGGTQEWAEMGPMRLPYSVKYRSDNTTHEYTDHRMTFQLAEWLNKLNKNDTKWKVDFIPWL
QHHPNELLARGTGRHPDGRIPTRGDIAANPKLATPAPMNSAEYNGTKSAMNGILKNETRLREIQRDIWRAHEKA

MEENLDDWSEQGMMRHRFHASENVTDAIATDTDYEVFWDEMVHNSNLAQDGGRGAFGETEWKCVDGGFNR
LSDAFTPHVQDRVIFNRKIRKLESVGDGKGGLRTRLSWYPSAANHTYESKEYDYTIMTLPFTMTRLMDLPKFSSTLR
RAMGEHGLRFKSACKVALLFKERFWEKGERPIFGGYSQPASAAMGALYYPSYGLNESRPGLITQYRGGDWSDRFV
SLPDEEYVGMVLDAIVSLHGEEARRLYTGDYEKLCWLQDEHSATSWCRPNVEQHRLYIPSYHRTEHNTIFIGEHTAP
THAWISSSLHSAVRGSVQLLLELGMVDEAKKLNKVWMGRWIHGA

>QLI69746.1

SSTINSRDPGANICGSPLAVKGTSDFSHYSGVCGDLPGQDERTVHVPRYVGPSNEQHVLAPSDDGRDSWPSQASA
GPRKDLINCKRLLQKPYIDCGSMKKRACGLLTWLYAGCQKAAQCQGKSGLAAKQCCDYRRTLPFGGGAYYEGYM
RACAPQYKWSQFDRPVFNRLSCPALGDFECEQWMDRAWCDMRDWENARGSALGLPSKRGMPKLGSRSCQML
HGTRYITGSKHGPYKSSVPRAERREVANGRVASKGRFDAFFLYRYNQSCMTPIA

>QLI69750.1

APVLKGNEVKALRKPSFAADYGEIIQRDVDVAIDGKPVHIEGGYIRNSDLKRLGWDIRDRDEKRQVDVDGVATRSG
WGYIKARDEKRQVDVDGVATRSGWSYIKARDEKR

>QLI69753.1

IPQSYPNKCGDQVCPSDKPNCCAVNVNGVEELGCFAVCPNPPPPSQQLQQRQSQGVECGDSIFCKPNQLCCAVIY
NGVEERGCYDGPQCPPLNPATTMTTTTAASPAATTGPKCGDSFFCPVGKVCCPNKLYTCADTVDQCPQ

>QLI69754.1

VASLPYPTRSNYHIKGIQPDFWPNQDEISGNNAGGVSMNLLWSQWEPRVKPRPCGAGEQEYDGRCFLIQAQVD
SAIKGWTDRGLVVTAIVYGTPPWARGKRRCSPAAPGMDIFCVPDNPADFGRFAGMLAQRYNGLRGHGRIADMV
IDNEVNTYQWFNIGCGQGVPCNANEWLDLIAANYNAAYDRIVAEQPTAKVLTSLDHQFGREFDNPGQGLLSGMT
VIEGLAARAGSRQWRVAFHAYPPNLFIPAFSADDYPKVTFGNVGVLVGWLRQRFPNNPHAWVVQLTENGINSGS
QSSEASQAAALCQSFRNILGTPGIESYIYHRMQDNQAEGGLMLGLRRTDGSIKPSWTTWALANRNDISPAQLSCG
FENLPYVALTRGYNPQRGHVASTRQLPAGFHAEMSWRLLRGQRGGTVMLYECKVGGHSMLSRDPGCEGQFPW
GPVGYIYENQAAGSVPLYRCYIPQNGDHFVSPRSDCEGPYTRESLLGYAIQ

>QLI69755.1

ATTVTSTALIIAANDADVAKASLGLDAYGIPWTKALIPQAGGSLPALNSTATNGNYGSIVVLDSVAYDYNGTYRSALT
TDQWNQLYSYQSAFHVRMVRLEEFPGPEFGTTALGSCCNNNQEQLVSLNSSTPFPGANLKTGATVSTVGLWHYP
AQITDSSIATAFAVFAPATGFSTESVAAVINNISGREQMVWFLDFAPDWSATSSFLQHTYIHWMTRSLFVGKRKVY
LNTQVDDIHLETDMYLPANTTFKLRPGDLDAHVTWQKSINSRLPAGSDYRMELGHNGNGDIDSSIDEDTSTPRKC
NPNQAVDYVQPPDPPLEFVKPPGTGVDLWPSRFVTYTWSKACASIDPLAAWFLTAANLNSFAHVSHTFSHEELDN
STYHDATREISFNQAWLAQMGISQAQRFSPQGLIPPAITGLHNADAIKAWTDNGIKYAVGDNTRPILVNQQNQY
WPLASTVAVNGATGIWIIPRWATTIYYNCDTSDCTLQEWKDTSAGSGTFSNLLDNARTTNSRYLLRLQADPYMFH
QANLRQTDMPSITVGSQTGKMSLIMSWVETVAQEMVRLTNWPITSLKHDDIATYFINRMTLDACQPHASYTYSA
DGTSITAITVSANNAACSVPVPVTIPSGTVSASGGSPKSDNLGNEPPIVWVTLSGSPVTLTLSTPVKLG

>QLI69768.1

TPEVNNNVKRDVWPGCLDQHTTRALTDSKASVTTPSPVRSPMASNCNKFCWVPPGAWCESVAKNNGITTEKFV
AWNAYVGLKCDGLWANYYACVGVGE

>QLI69777.1

STYHGYTSAYSFDANLSHQPDWMARVPDAANITSLSIPGTHDTMTYNLDSRVLQCQNWNLTVQMEAGLRYFDIR
ARVKENRLHIYHGKQNTGFTFKQVLLQMFEFLDKHPSEMIIMRLKKEGAPVGRDSYSFEAALNYARLQDKDTKDG

AKKHVALYADSRKPIPTLGQLRSKIFILQDFKCAKGVTYGLTWDGPQMVLEDNFAIQGERRLATKWAAVDMALNR
ANQGPLKNDRLYVTHNSAAIGVLPIQAAAGVLNKAQVGMNYRTGEWLDAHIDDKTSMRTGIVIFDFPGKKLIDSV
LAWNKHVGAKLPL

>QLI69779.1

DPSVTSEPPASVFTVKKPYGGNFMPGNHPQITAGTASESPVSARMERRQKGERVRYGNLATHKSRNVHDGRGNG
VDSYTMYWGDGSTRQGWPPRSRWVSFENMFHNYKSQMLRSCGNQNPPQPNNSGKEIGAIYDAIQRAADASG
VDHRFILAVMMEESHGCVRAPTTNWGVRNPGLMQDHNGYGTCNDNGRVQSPCPASTIYEMISEGAAGTDSGD
GLASCMNESGRGDISAFYRAARIYNSGSISNTGQLQNGIATHCYASDIAK

>QLI69788.1

ILGGEVQAVQISNTTHICADNCVYDGIYLAQKGYFCPVKEVVRQEEGGKLREWYSYDCRGAKPAPGVVHAPLKGKE
APRNCTLFPGDLTKVSPSCIADKEVGGGGGTKILQLSELTEMLKSATANTADNKKPASPKSKKECAQIARNKFTECRE
KIDDFNECNNQGAKTFQNCQSGK

>QLI69793.1

LPSEKQESPAAAGVLHLPLVYVPSAVDTDGKPATRRQLSTGIDNYQYNKDIAVGAVIEVGTPPQKVIVEPDTGSNNF
WVLGLQPGQKRAGDESTFFDQNSSSSLQDLGRERLNSYGNEQITTELYTDNVSFGGRSVGKVTLGVGDLDRPGTN
LGRHVGVLGLLPERGNENSKDFILQSLLDQNVVKSKAFGLGVRKHGQGALTFGGYDTSKFSGQLEKLPKKDNRLGF
YAFEIKSLSFRPGSGQGAVELSTQDSRSQPLALGVDSGSLGFYPVRGTRRDFVAKTGAVVEGDAYNFPCDVVDKGA
AFDFQITDNTVVSVPLSDLVRERAPDGKTCKTFIGATNRRPTSIWVGRQFLRRSYVVHDHAGKTMYVARGADCGS
TVVAIDGTIPDNVTGKCAREEPEADQPPPAAPGSVPA

>QLI69794.1

QDVDETKPAPPEAAAYEIRIPIGNISSHPSGTTPLGSSGIFDVLTSKSWSRNSNLAAQYYQDGTVHNWLRNEEKPK
MSIIKVCGNYFYAPNIYWKGGLTGKHLHQDNKRNQKQFYAGVTQATHEVNGEFVTRDKPIEIMTERTSSACGSISF
SWKSKGNWGRLGDIMDHFLRIWFAPQDTCAVSPSHGGGNGLLCALPCDPISPMWFQSTNLYVRQTRWVNGKQ
LYSSVYKLRGQFNSGDGNGGLKNPTWLNAQCDGDRLYFKDTPNDATYVVFW

>QLI69799.1

HPAPSPPSAETTSSYTQTIASQAQATSAEMEAEIQRYWTTERILAIDHNPYSPSDPPEIPPELQPLGQEYSGKGAVPS
TVGRLFYSVHMANGSLHDSTCTATLLRSQNKATLVTAAHCIHTGRQGPGSEMSLSWHANLLFVPGYHDDVPRAN
FTIARAFLSSRWIDDRDYFVDDRAFIVLNRPDATAPAQDIQFKKIDPPYGTRYIMGYTRSVTFGPKDTYRYGTPAFTG
RRLAVSHGTAEDWWRFDHETTGIPSVQSGGSSGGPHFAEFNEETGVGTVVAVNSIEDYKEEDGVRATWMLGAP
VHDEFSKGLYDAAQVVKPALS

>QLI69809.1

GVVDPAAAVVERDINAITDVMTKVGQGLEKLDAQVKGFTTDAKPVQDAATAVIQTLQEGKLTVDAQGDLTLGDA
LGLQGPVKAMKAKGETLLADLKAKKDVIQNGNYCPLVREQTININIGSNALIAAVVSKVPTIAQLIAKNLAAPLIKVL
QEAQDEFNEKNCIDKGTPPQTTTKAQTTETTTKEPHTTTSQEPCTETTTNAHHTTTEPQHSTTSEEPCTETGTTEKP
HTTSKHHHTTTSEEPCTETETSTQGHHTTTATRGATTSSGAGISSTPSTKTENTTAPGSTSTGSGSATVSAPSTKTAT
TTAHGDTTTSLAMTTSTVYTTRTYTISKCPPAVTNCPVGHTTTEVVPVYTTVCPVTETAQPPKPTTTRAPIMTTSTVY
TTKTYTISECPPTVTNCPVGHTTTEVIPVYTTVCPVEETQTQTLVPTKPTGGQNPPPPPPPQETTFVTIPGKPQPTGG
NGGNPQPTSRNGENPQPTGGNGGKPQPTGGNGENPQPTGGNSQNPQPTGGNGVKPQPTGGNSQNPQPTGG
NGGKPQPTGSSGENPQPTGGNNENPQPTGGNGSNPQPTGGNGGNPQPTGGNGGNPQPSGGSGGNPQPSNT
PVTAGAAFVGPATAFALAVAALVVV

>QLI69812.1

ALDEEHKKPYHLSRNDSNDYTLGIIGHHNVVIACSPYSEYKTTSAAAVATNMLRSFPNICMGLLVGIGGGVQHDIRL
GDIVVGIPRDGQGSVFQYDFEETVNTRAFRKTRFLSPLPFSVLKEVTSLRAEQEHKGYTIGEVENHILQKIPTLQKIYK
KPDLDTDLILGPEVSHILTPADFYNKSNLRRPQNTPTVHYGVIASAKQPITDAALRDALAAERNVLCFDTEAADFQN
PFPYLVIRGICDYSDSHRDIEWQGYAVLAAAAYAKALLTRIRSRPIPLKDWTRVIHILSGTAKRNDLIGRTYSKDEREM
MLNWLTLFDYAHQQATYLQERQPRTKLWFLKTSEYTQWLNGNDFKTLFCPGGPGVGKTILACSIIHNLLLQFRHKE
DIGIAYIYFSYRPKDKQEIYDLLANLLRQLSQARSPLPEAVSVLYHKHNHGSTIPSLEEISKALHSVAALYRRVYIVVDAL
DECEESGTRARFLSELFSLRDKNRVQIFVTSKFISEITEQFKAGPILELPAGRDIFEEDQFFEIHTSTEEISSYLGSQLWKL
PSSLTRNSEIVARIKARIIESACSTIFLAQLQFNSLLDEVGPPRDMQAALNIRAALKGLPTGSDLSHRTHADTMKRIMR
QTLAATELAKQVLSWLTFTKRPLTTLELRYALAVEVGQCTLYEENLPQIENIVAVCAGLVAVDQQRKKIRLAHYTTQ
DYFKAEANRWFPNAHFDMMNICVTYLSFSVFQGGPCQTDAALANRLQSNPLYDYAANYWGHHARNASRLSLEV
MQFLHSKMAVEASVQALMHVDQYSPHAPRQMTGLHLAAYLGISSAVDTLVRQGRKPSVKDKCNRTPLTYAAEQ
GHDSVVDLLLGIDTADINSKDEDGSTPLLRAAANGHEACVKLLLKRHADSNSKDENGQTSLHWAAKSGHRKIVQH
LLQNGANIDSIDNRGSTPLHESIRNIQQEVQEFLIESGANLDITDDCNQSAVELAWSTKRLDLSAYEVDKEATINQGA
QANCAVLRNNYAIGTQRLIFRKTFTWSKSAAGNKIRRYFLRENRILQKLNHPFIVSYLGYQENIRLQEASVYMEFCEG
GDLESQHVYRPDSDDDSDSTSQLDLSHPVPLREEEVWVITFQLVAAMAYLHHGLTIRGWGTFSFARHWEPVIHRD
IKPANVVLKPVVGKIPIAKLCDLGLAKAIVGEAKQTRKVGTPDYSPPEVRLGKGWTIKGDIYSFGATVDDLYKEAEPQ
PELRALLDSCKSLDKSARPSSLSILEIAQEHVAKHEKEMCFYLNQFFKSGSGGYLLKSLLEIASGLDEIAAFGDEVRLRR
KRILERLRLLCDDGAGEVFDKHSKSLHLSVLLNHLDKLRELLATGKDIDVDEKWQNSGWTPLHLGFQEDNQDVVAL
LIKYGADPDMNDKYNRRPDYYKE

>QLI69814.1

IRNGLVQMISDNLLSSRPILPGSDIPLRTHWTGLGLLDLDFSIMVTVFSSILNHHNLSLFIQGNHFFGLWMTSWILILL
ESHNNGPQSKTTSRPYKYSYVLWGLVMEFAGVAVGLPAWCAFSLLRMRRASSSAEFTTITLADLESLPFAFLLGAGL
PTLLMLVGTWSPEPPLWSRQTWIVVRLFHPVLLAIAHALLRSIGPAPPADGRQQLERRMKRLGRLYSVAFWITASS
HILTVSAVLFAYSFPHLLPAHIVSSLSLASLWPVPSIWRGFVTKTDTIALGTSTFMTANEVISTVSLLVWAWNMNRM
ALQSSSHTDHLGLSTVKAGVFALVFGPGAAAVALIENRDHVLHQRFTVTKGAKTC

>QLI69815.1

HSDWPPFNSCICPGKMWCHGDTPQCTPDNTNATLTQCCASGQIALYGKCVSRGSQACPDGETICSGSESQCAVD
NNDKSICCAAGQIALNGKCVSRGSNLCSDRETICSGTESQCSIDTSGKSICCARGQVAVSGKCAEPGSNACSDGKTV
CSGKNSQCTVDVTVFVGKDGGAVADTVCCEPGQKAINGKCYDGKAKIMPCYRGPCDWSQNVYCAWNAGNGDS
RCCKTNEYYAGNSCVRKR

>QLI69817.1

QWTGNYNTTPVADNPIAPPGCQLKSLQGLDITAQTTVYKCVCTGGQVFPSCNWLLSPRHQLYNSVRNATCKCAA
AKLTGGDPRSLNTYGRLPNNMGFDKPGVVRTCICDPVAGKGDDSTDKLVETAGSCWCPADRFLWQTSLSDAKES
QAVPEMPPSVGEQSTGKQTEPSEAKESQGVPETPSSVDKQSTTQQPGEQQSATASIDECRVDPSVEPSTTQQPGE
KQPAEKQPATGSVQWTGNYNTTGAVLSDNPPRGCELESRLPRTILAPCVCTGGKVIASCNWQSIHGSYNTVRDAN
CKCQRAPAGSTWQYAKDLVAYGLDSNGQNLPGAVRGCGCDPAPGKGDNKPDESGLVVPGPNCLCPTRNLEEYS
GLSEAEESQQPGEQQVPTGSFQRCRDDLNKGKLRQNILCKQLMEVKPKELPQRMQSVLPNCSISSLTEICKDIPQD

>QLI69829.1

APAWHQGPVMADDDNLMERRDVVRVQVVQAHTSRFTNTMITKITRYAADAASDVQGFLSESFRGSVADGSGE
VMTIAYGKKASPFVVGSGIGSGRGKGKGKGLGRHSRHRHRISLLGFRNRLAVFILAFSLVLAVACSYLRRVYIASLQG
PVEDPVDSALAEKALLSRSTREDETRQS

>QLI69838.1

AAGLGLAPTPTAKQHVAAIFARQTGRRALDDCSSVLDNFLDLPTPPPDLSSYAQQVAATQTGAAAIRFPDSLSSRAS
KYQSSVLSWCSEQRDALTKCTELPALQSQYSGVCQAAGANAAATGANGGTDLGINIFNNIDTGGGAGGAGGAGS
SGGGGGGGGGGSSGGDGASGGGGISSGAVAGIVIGVLAAVGVLAGIAAFVIWRARRKRAAANAMDVARQQPM
SGQQTPPSGMLHNFGQAYDKDSPTYSEASYQTLPHERSVHEMPGVRAPAELMGSTEAVPVELHNPEPVYEMLA
DYRPNKAAVANVGYKS

>QLI69845.1

QLGQIPLDTLFSENWQKSLKYHGQYPSPCQVSRSTAFEQTEGPVEFSESPTEHLETPKLSTINATAWEQWEFDGTA
DSGMSGIILAFSRDASYHFFGQGNLRVEFYMILDDGTITRELDFLEQSTVITCQDSVTGIWNSTGHTYGFQVPRNM
SRARVWWSTPRSKGNFAIESDTLPHLAEGKLWPDEKGSVQMSASLFLNQPIAGGRVVVEYTLGKTSLNFTGHGGH
GRVWAKDSWFKVCDAWNIIRGFAGPYYMAHWRPVSRLNKGVPYCCAQLFKDGELLFGTAKGEALDTDDYVLFT
NNLSGNVSGSLANRSTGHFIEFVSPQKGKTWRFKVEHNRVKFNMGLGGGLGLSGFTARGDVDVFVPNPQSGLLL
QLQGIKVTPLSERSPEKDRQLFCKQIWAPALPDGLLAADNRASAEDVQLAADLERISLYYMNQVSRDTPEDKRDTL
PWHRKAMFDCFGHVIHHSRIGRQRFTEREWLNDTWEDISPIMDRYPDSIEIKLTRTVGEHLTAAVRGETEILQHLL
DDDLLNRYYVEAMGLKDATSFLSRVIAQIAHRFPHMDILDIGAGTGGATKTIMRDIGRSFASYTFTDVSSGFFEKAR
EVFAAQYESEKMTFKVLDCAKDVVEQGHEEYSYDLVIASLVLHATRD

>QLI69846.1

TDDKFRFERLEKNNSMLLVVDHQVGLYNTARDFDPNLFRDQVLAHSALGKVFDIPVVLTTSTQAGPNGPLPREILE
MHPNAPLILRGGEVNAWDNEEFRNAVQKANKKQIILAGVTTDVCTSFLALSLREAGYSVWANAEASGTTSALVRD
ISNDRMTQAGVHVVSLFSIACDLMRDWRHTPGAKEMIPYFDKYFPVWAYIARGHQAAVTNGTVLAGEEKLNG

>QLI69857.1

QVDSSDRQPGGKHFGCIRAVQQWEDCVEQPRASNRECYEAHYNSIRAACPAEVASALWDADVGGVWQVVNEL
TADEQ

>QLI69864.1

ALINARQSLAGGCGSERPSAHFLQISHHLATNSSLHSSRKRADIEVATFAHVVYANKSVEGGYLSEEIIHRNIDIINHQ
YAPAGISFSLKNLSYTENADWALGKDEFAMRLKLRGGGYNDLNLYFVADIPYKSEQDWGVRGICSLPTTRPDSLRW
TLDGCSLLSRMLPGSNGTAENISNSTATHEIGHWLGLLHTFEGGCGGEGDEIGDTVAQAGPTKLCPTGDPPHSCNL
PDPDMIKNFMDISTCTRSEFTAGQIARMKDWEDIRRDVSTDKPTPWLESDEGLVKEFCDKRALSASNSCLAARKYC
FAKGKADGYDTLESCVDAYKAGKITSFAWILPTPAYLQGDIAS

>QLI69865.1

DKAPRVLPGTYFVELNKGADVAGFAQRVTTEANARIRRQIDFTPAKGKAAEPYKAVSIVLDEAGKINDVASMDDVF
KVHQVEVHTMGGIAPSSDRPKSEKQESFAGKISKRRAMRRALPETNTTAYPPHVMMQVDKLRSKGITGKGIKIGM
IDTGVDYNHPALGGCFGPGCLFSFGADLVNNDPTPMDCNGHGTNAAGIIGTRPNAMGFTGAAPGAQLGMYRIT
CSGEFPTDVMVDAIYRALADGVDIITSSAGLPGGWPDSLLSSAATRAVESGVVFVQGAGNDGTLGLFSHLDPAVG
NGVISVGSVNSRVYPQLINEAKYTIGNGSEVRFHFFPTTPSDNFTGTPMEVYALPVNGSSAGNGTHACGPLRPDTP
DLSNKLVLLNFHGGRGDCSLRNMTKAVHAKGAARILAYVASEDWLPDLYYVDNLPEGVLALGMLGFDTATEMLK
ALQSGRKVLATAFPWNDAPRVYVEEPNDEIAGSVSDFSSWGPSWNLGLKPSLTAIGGEIISTDFREKNPSGYTITRG
TSFSGPLIAALVALIGEARGSLDPATVESLLVSHSNPQLYHDGENFLPYLAPVAQQGGGLARAYDAAYATTLVQPAG
LNFNDTEHMAASLNFTLKNVGQGAITYRLSHVPAVTVYTFTENGTVSSYPNKLEAVETPAAITLSETSVTVDPGNSV
NIRVSASIPEGLDASRMPLWSGWIAINGSDSTSLSVPYQGFSGSIRKHQVLRPDGASLTYRNASVTEGTTVVLPAPG
SITPSQLVLNINATMGIPLMRAEVVPANGTDATDITTSIGQVQGFPVQWRPRNLQQDSNSPDLLQFTQFKWNGQ
LDTGSNVPEGYYKLVVRALRIFGNPSNDEDWDVSESPRFQITYCQKGNSSSMIRRRAERGHN

>QLI69894.1

ATTIDRRVIGGEDAKDGEFPFIVSLSGRGGQCSGSLLDSTTVLTAAHCLEGRFTSNRRMGGVIADIASAKEHPSYIKG
HGVHDIAILKLSTPIERNDTIGIGYAVLPENGSDPAPNSMAITAGWGVQGNGNPGYNHVVNKLAKVVIPVREREKC
RLQFNPPEAHPDTICAGVNGKDACHGDSGGPLIDQETGQLIGLVSRGQCTNPPTVYTRVGSYIPWIEDHLGGVGS
GPIPISTTSSVLPKGTPTAGQKRPWIELVNYGIVASCGFTENREEECGTEIYCGLFDWSPSHDGAFKNSKECLDAHVP
KPKAARASIPRQ

>QLI69903.1

APAKRSFPAPVLVPRGAELIEGKYIIKMKAKAEVSAVSSAISSIAAEADYTYNDWNGFVATLTPEELQKLTDDPNVDF
IEQDAIMTMYATQPNADWGLARLSSKTPGTTTYTYDDSAGEGTCAFIIDTGIEAGHPEFEGRAEFLKNFANDGQDT
DGNGHGTHVAGTIGSKTYGVAKKTKLFGVKVLDAQGSGSNSFVLAGMEYVTQNAKTKTCPKGVVVNMSLGGTKS
EAVNDAARKITEAGLFLAVAAGNDGKDASGYSPASESSACTVGATTKDDKLATYSNIGSVVDVIAPGSEIKSTWING
GENTISGTSMASPHVAGIGAYFLGKGKSIQGLCDFIKGQGVEIQGVKGGITNVLINNGEGSNSTTPIRF

>QLI69905.1

IPTQQPSSNKIECNNTVFCCDKQTVGQAGIPIDALNPTCTNLNVLAVDALNPPCPGQTVCCNNVQQNGVVNVAC
TPISA

>QLI69909.1

THDAPVVLNNPHVTYQAVFPKDAFYHGNIHGNVCGSVRASRGPHGRGVRFDVRLENLPKEGGPFLYHIHEDRVP
ADGNCTKTLAHLDPYGRGEDPPCDSRAKDSCQVGDLSGKYGQPKRGLEIWYFDNYTSLAEGTPAFLGNRSIVVHF
ANKTRITCANFEKLSGCPA

>QLI69912.1

APNEPCYADGQAGVCTTEAACAAANGTTATGACPADGADIKCCSKARCGPDCASNCRWQSDCAGSSTANLCPG
PAQMQCCSSRDSGFGGYAAPATPPVGDCKPSSVEGAKKIVAAFPGRVWDVGCKRDCECPGTSDHCCGLASDM
MCSDGFGVPTLSGKQIAEWVMHNRKDLKLKYVIWGQKIWNPTVDAEPNRWEHWRTMNDRGDVTQNHWDH
VHVSYEEFEYQGI

>QLI69913.1

TNPLTSKAGQVGIIPAWDFQSSSSVSKDLASLSKPGVDTSSWNHVNDSRCTILGGMIESGAYKDDELWFSDNLYKI
NWGQFRVPWVYRNEFSLTPNQGQHYFLQTNGITSRADIFLNGEKIADKEEQAGSFGGHTYDITGHAGNENALAV
NVYPSDFNLDLIYTFVDWSPRAPDNGSGIWREITVKQTGPVALGPMSVSIDIESPVESNPAKVTVRAKVQNLEDHE
LQVDASSVIAESSGCKVGSQKTSLMLGPKETKLVEISLHVKKPKIWWPKFWGGQPLYKARLALHVANELSDVAEET
FGIRTVTSALNSHNDTMFTVNGYPFQVLGAGYSPDMFFRWDATRWANIMKYSHDMGLNTIRLEGMMEHPELY
RMADEAGMMIIAGFVCCSKWEAWSYNQDINPNVPWTDNDYETANATMRHEAAMMQPHPSVLGFLIGSDFW
PNDRATKIYVDALKDAYWQTPFIASASKRGYPALLGPSGMKMSGPYDWVPPNYWYDTESSEDRLGAAFGFGSEL
GAGVGTPEVSSLRKFLTQSEMEDLWKKPDANFFHMSTNTSTFYNRKIYNQGLFKRYGAPTSLEDYIFKAQLMDYEA
IRAQHEGYSARWSQVRPATGTIYWMLNNAWPSLHWNQFDHYLHPAGSYFGTKVGSRLEHVAYDYVREEAWIIN
HSLDKKGTRNIATELIGLDGKSISKDSVTVDSVPNKSSKVAKISGLNNMTDVGLLRLVLSDEQGQALSRNVYWLTKS
VDVLDWPNATWYHTPVTKFSDYSALSGMETAELSVTASRNDGAHSIVLENKSDVPAFFIRLNLVDKAGEDVNPAI
WSDNYVTLWPREKLALTVSGDGREAKVLVNAGNVQALEVAL

>QLI69917.1

APQGANNGGDQIFDFNDKPLKGDKKTKFLDLEKQGDAKFEELTKTYTPEQQQLENDLNSIHSQMDDILGVPKQQ
QK

>QLI69936.1

QSPSSCKGGGKRNGADFVLVDQGVNAHLGPLSKMFANANKQVNVSKVFDDGNHQMSGATTQLSWEKTKDFN
DVDTKKWYPQGISSTADAAEVGTYDGKDGWLVSWYSDESHQVRISFVNKATKKYRHALLVNPSADDDFQGVGIH
AGGIMWYGDTLWVVDTSNGIRVFDMANVWEVGSGDKVGKISAGKYAAAGYKYVIPQIRWYKWTPSFPFRFSYIS
LDRTASPDRLMVGEYQPDEKNPIRMVQFELDYTTRTLRTGADKKTAKGVWASCHDILRTQGAVQANGRIYVSRSN
GANTNGDIWGWIPGKGAAINKGMVPPGPEDLSYDKRGKKIYTVTEHPGKRYLVTLDSTRIKFP

>QLI69941.1

QDSSLTQTLAKFPGCAIPCLGQSIPKSHCSMTNQTCLCTDQVFKDGLTNCVMSSCTIKEAIMVKNLTSTSCGDPFRD
KSGQYAVVSKVFAIISSMFVIQRFAYKFWAKLEYGLDDWFVLLTTVVGIPTTVMNVHYVAPNGIGRDAWTLTYDNI
INFGRYFYILEIIYIVEVSLSKLAILFFYIRIFPGRRVRRVLWGSVAFVALFGIAFTFSAIFQCTPIDHNWLKWDGIHEGRC
VNINAGAWSNAAISILLDGWMLAVPLWQLKALNLDWKKKIGVGLMFGVGTFVTIVSILRLKSLVKFGSDSTNPTW
DFFNVGIWSVVEINVAVICACLPTFRLVLVRLFPRILDTSQRFYAKHGTKNGYKSPTRRGSRPMGTTSVSHVERSQN
GREPLSKHITFHKTFSIEYGETDPEHDAAQLVYMKDLEMKSHQPDSLSGGSSTS

>QLI69968.1

CRTSSECPKGKICDQQRGSPTFGKCI

>QLI69972.1

LPEFYWGVSKASPGQIIVTNNMNRPVRLDKVAQGPSVDGVKIIRDQEIILNQGQTVEVSATEISADLKFNEQQAHN
QVELSYTSGDQDTYNFAIKPIEGSGFPGAIEVIPLSPRPSPQCHPLVWYPGQGNTAQATCQDHTPLRVFLREAHHP
PEFNKFDKFDGWY

>QLI69978.1

YYATRNAEDPATSSTLPLSNVAEIQHDHSPRNLRDALGEFTNIIGKEHVSTSDKDIENHATSSWSSHPAKSTERSFCV
LWPGSTDEVSQIMKVCHKRHIPVIGYSGGTSLEGHFTPTRHGVSIDFGRMNKILSFHKEDLDVVVQPAIAWEALND
HLDQEGLFFPPDPGPGAMIGGMVGTGCSGTNAYRYGTIREWVLSLTVVLADGTVIKTRQRPRKSSAGYDLTKLFIG
SEGTLGLVTEATLKITTKPESTSVAVAAFPSIRNAADCVTKVVGKGVPVAAVELLDDNQIKCINEAGLTSRAWPEATT
IFFKFAGTKAGVKEQIAQVQALAQGAYSTSFEFAKNQEEQDELWSARKEALWSVLAVKKPDDRVWTGDVAVPMS
RLPDLIEATKEDLTKSGLFASIVGHVGDGNFHAMILYNDQQRTQAEAVVHRMVKRAIDMEGTVSGEHGVGLVKR
DYLSHELGQTTVDAMRQIKRAFDPLCLLNCDKVVRMQDIGEGSTQKW

>QLI69986.1

QVPPGWTNVFSPSNLYIYDVSNGAISDLSPTALVFKSPTNGGRDKTTLLTFKYPAGTNGKQCQLFFNFDQAADATS
RWDGSGRLDIFSSNSVAPGKTSGWPPGNQRNNHLGRWKKSATPWADWEATYGGLSVPQPCKPPGTVQGFEIV
GVYDNTYVTYTNALSGVRIAYR

>QLI69999.1

APSPAGGGTSSTPVPSTLGFVWRGETGRTPDDVRREGGIWARGFQLGGLTAEQLEAGSSLYAHVNGGTREHTQY
VSTTSSLQAAINFAVRPSPEHAHEPGFLYRIRTNWAMVDVNLSLENIAPFPWQHEHAVVQGIPWNQIMGWYRIT
TADVNRILSADDPVSEMGPFTRNPDFHPDATYPSGAQPQLAGLQLPVEGRHGAWEAFVGQSATEHLRAFISNHH
PQADTTTLRTIDWSPIRIPDHLRATIAQGTASSAQCALAAAAIIVSFRRHDELKRSIPGGQLTAREETGNPGYDSCAR
LAAVAKAKIDDEKPTVKICDSPSSGGQCTDLEAPAEKCVPLPFEWNDKASTVRPSVAAGVCKFFRDYGCVADAFEV
AYPGNDNLGEFSDGHFNDMISSFRCEGAGAGEETQIERPPTESREISGVHVCESDRFSPSCRRFPVSNGDCANLPE
PWDSKATSIRPEKDAGACKFYRQHGCNGDDFETSFPGVDLFSDKKLESFNDQVKSFRCDSSGKKPENECEQGREP
VKCTPSLQSPVAPPDEGLYIRGCCAKSVQTEEECCRSQLQWLDIRTQSQPYIAPDSWYKGCAIGKLMVNVQLANID
WAGTDDRLFLEIGHSSKWDGGQPHYLLKASPDKGDKMTKEINLEDAFDKSPVTPGDIDYVRLADRVDNGGVGTD
ELKLQDITLTLECAGTTVTAQARKTHDSWFTFWKADIKPADWAWNLGSQS

>QLI70001.1

QFFNPKSGAIFTIGGQISVSYKTSLEAYNIALWQQVPDGGAAILGPVILNVTNGPASSFDWTIAVDNLDLTYSKRFFL
WLFPNAYAQGNQSAPGSMSSGYFTIVDKTPGPASTSSSLTTLSTTTTASSSSTAATMGTPTSGPDSQQPKTSDGGL
TTGAKAGIGVGIGVGALAIIASGALFLRYRSKKDAEIRELRHEMQNKSFAMQSSAYRPAELMHQPRPVELG

>QLI70008.1

EKMLISNSLNSCQTNSKFEASLFNVVYTPSNNSATIDVVATSSVQGKVSFHVVVLVYGLKIIETDFNPCGKKGLEGICP
MVAGKTKFTWTIPVPAESTKDVPGIAFGIPDLDAMVRVKMNLIDTKDQIACVEARISNGKTVDLKGVKSATAIIAGL
ALLSSAAVSGLGHLNTAAHVAANSLSLFGYFQAQAIVGLTGLRLPPIVQAWTQNFQWSMGIIHVSWMQKIFTWY
QRATGGTPSSLFSTLRTISVQVEKRGLDVAEAAESLAKRGLSMLPESVSSTAALILKRAGNITNDSGSYLVFGIQRVAF
RAKIESTNLFLTGLVWFSLFVIVTAIGVVAFKGILELFASRGLVSKDKFLEFRNGWRIILKGIMFRVCLIGYPQIAILCLW
ELTQIDSPALAILAVVFFAAVSIILGWGSSKVIRIARRSIAMHRNPAYILFSDPQALNKWGFLYIQFRASAYYFIVPVLG
YILIKAMFVAFAQKAGLAQAIGFLILELAALIACAVLRPWMDKSTNSFNIGIYAVNFVNAICLLIFSDVFGAPGIVIGAV
GVVFFVLNAAFSLILLLMVIISTIFTFFRKNPDARYQYMADDRASFMRSQTHLTTTTELDALAATARGDKGGYKAQL
DLEDDGHLHPMHGGNRSASPSSPVNPSMPLFPSSREQSPFRTPSPTPSVGPYGGAPPSYRQVNNSSNQSMETGG
RI

>QLI70020.1

AINCRGNSACASDPGASVETIQAQVHALVAQGGGDGIFDAQRPIACSQGKDGSFCAFFQNTSGTGKQAEALVQK
LLDRGCKRCGSVATHPGNDVSAGALTVNLVKEPCCKGDCFCSGRGARIRKVE

>QLI70023.1

QYYKDNHAENTLWKEPKNHGLGPNTDVETEHVQPPVRQPGAEFVDAALSQLNKASRSSQHRRQQSSGIVGTVV
RYILNTLPTGPATAPSQEQQQQSKSAATGPLANAVDLLEKAANQNNSDALYLLAELNFFGNHSHARDLHAAYNYY
NHLASSYGNTTAQYMLGLFHSTGLGDVVPRDQAKALLYYTFAALRGDTRAAMATAFRHHIGIGASKSCEVAVKYY
KRVADKAIQWHRSGPPGGVTWIYQGWRIADDDGGVYGEGASAASSGLNARKVSVHSDANAAISDVIEYLDLMS
QKGDVKASLNLGRIFYDGQRGLDHDYGLAKKYFFLVASRYWKRDGTLVDNAKPGTDKIAAKAAGFIGRMYLRGEG
LPQNFERAKVWFERGTKLNDAQSQYGMGLILLNGLGVKENVKRASELFQLAAAADYAPAQVEIGRLYLDQGEAED
LRVASNFFELAARYGNIEAHYYLAEMIYNGVGREKACNMALGYYKNVAEKAEPLVSSWADANDAYDAGDYEVAFL
EYLMAAEQGYERAQNNVAYMLDMTESEKKSLWLGKSQTEGTLLNNPSLALIHWTRSSKQANIDSLVKMGDYYFY
GIGTKADVGKAVQCYTGASEYSQSAQALFNLGWMHENGVGLEQDFHLAKRFYDQALEVNEEAYLPVTLSLLKLRI
RSAWNTITHGEVHSIQDEPSKSKDWSLAEWIANFMKDDQYYDDEALYGDYMDDDTLDVGDDDYLDAGGVVESI
LVVGITFSLVLLLWYRQRMQQAYAQAEEARRQGQGQGQGQGQGQQAQAQQPIPGPAGNPANRFDGFGGWAAG
GMGL

>QLI70026.1

LLEFPMDNRMVQEPGLIRFPLDIAAGAPTKNRMLRRQNEIGVNAEKSGFFYSIELQVGTPAQAVRVNFDTGSAEL
WINPVCSKSTDPGFCDKLGRFNGSQTFVDTNTTHEIRYGTGFAKLEYGYDYVQIGSAKISQQMFGVATDSEFASAG
ILGAAPQLKGWKSDYPLVLDNMAAQGFIKSRAFSLDIRTLASKRGSVVFGGIDTKKFSGHLEKRPIIPAAESPDKLTRY
WIYLDGVSVTKEDGSKVVIFDQANGQPVLLDSGYTVSALPTKHFNKIKDAFPGASAPPEGDKSGLYRVPCDVGSKN
GSVNFKFGKTEIVVPYDDFIWKQPDNNLCVLGVTPDDEFPVLGDTFLRGAYAVYDQDNRNIHLANNEDCGSSLLAI
GTGPDAVPSVEGDCGRPKPTTTSTHSKTTSAPIPTGHNSTISSTQSTPSSSSVASPTSTHSRTTSAAIPTAHGSTVITSA
KPTASSSSTPSLTIPTAKNSTVISAKPTTTSTLVPTSHNNNNNNNTILNTSKLISTTSKLISITSNTPPTGTEQITTVPVTYTS
TFTTTSVHTITSCPPYVTGCPIGSVTTETITGVTTWCPDNTTPSAKPPQTTETKPPPTTVITAQPTITSAPSITSVFTTIK
THTITSCPGQASCSKGHVTTEVITSTTYLCPESTATFTIPRTHTCREGDSECKPGDTVTTVFTVTVEPQTTADQPTPVP
GCGDNCVLPPPAQTQPPAETTAAETQPGITAVVTAPGSVPTTVSGTNGTITTVTKPSDTPCSTCSPETTSPPLTGGA
SGSRVPIMAVAIIVGALAVTL

>QLI70042.1

DYVDTWEDEMPLGQFIGTFDTFSNNQCSEGGKGITVTADDRKGPLQSSVKSVKSYIQNQYYATRSVALPNFGKFSR
IKYQMR

>QLI70065.1

INCRGSGGCTFNDAKLSDVLTQVKQIQAQGNGNHHYNMGVQLACAQGQYSSICAFYQNGASGTANDAAGQLQ
GLVDHKCSQCGSIPTQPGNDVSKGELTVNIVSAPCCKGNCACPI

>QLI70068.1

QELGRIDPLQYVDTLIGASNGGNVFPGATVPYGMAKAVADTTSGSNQGGFTLDGAPVSGFSLLHDSGTGGAPSLG
NFPLFPYNTCKGGDVNNCAFPKKTRATFGKFANESVKAKPGYFGITLQSGPQVEMTATKHTALFRFTFKGAAAGD
KPLILQDLSDLSDSRQDNGTIQVDPETGRITGSAIFVASFGQGKYQPFFCTDFHGADIADSGVYADSRASTDVHQLKI
SRSINGYPLPGGAFVRFVNGDKPVYARVGLSFMSREQACANAEDEVPAFDFDKHARDAQDAWRQKLSPITVDPR
GVDESFVTNFYSGIYRTMVNPQNYTGENPLWQDGEPYFDSFYCIWDLFRSQFPFLTIVDPEAVAQMIRSLISTYEH
DGWLPDCRMSLSRGYTQGGSNADNVLADVFIKGITTGIDWDKGYEAVKKDAEEEPYDWCCRGRGGIDSWKRLG
YVPVQDFDHKGFGTLTRSISRTVEYAYNDFCIAQMAKSLGTKDEEEKYTASSGNWQNLYKADQPSSLPDGTNTGFT
GFFQPRYLNKTWGYQDPLKCSNTDARSVCSLQNSGPETFESSVWEYGFFVPHDQAQLIALYGGPDQFVKRLNYLH
DKDITYIGNEPAFLTVFQYHYAGRPAESARRSHFYIPKYFSPKPEGLPGNDDSGAMGSFLAFAMMGLFPNPGQNV
YLITPPYFESVQIRHPLTKKTATIRNVHFDPSYKNIYIQSATLNGEPYAKNWIDHSFFTEGKELVLTLGGAESSWGTAV
EDLPPSLSSYTGFNGTHGKRSVRAPRGGSGYRGEFRSGALGI

>QLI70091.1

TPVNEGQVSQPALDHRTANASEDGFKYLNFKTRKYLVDGKSIPEVKFDVGESYAGLLPISPSSNSSLFFWFFPTSNPK
ASDEITIWLNGGPGDSSLNGMLLATGPFLWRPGTDRPIPNPYAWNNLTNVVYIDQPAGTGYSPGSGTVGNVIDIA
EQFTSLFKNFATTFGLERRKVYITGESYAGHMIPYIASRMLDEKNNTYSNVKGVQIVDGVINSFSVIQQAPVVAAVK
HFNHVMNLNDTFISDINTRSEQCGYNKFLSEVLTYPPSKKVFATPDKDQPGCSIWSDILTAAFKVNPCFNTYHLTDK
CPTPESVMDDGPNSYFNREDVKKVLHVPPKTEYKAHGGFAWSGFPGPDGLAPRPSALGPLPRVIEMTNNTIIAHG
LQDFLLLANGSLATIQNMTWNGYQGFQKAPTEPLVVPYRHQRRELDYRKESAMETGVRVEGNAHTERGLTFVSID
TAGHAMPQYTPGAAYRQLEFLLGRIENLQQE

>QLI70094.1

VPQRPDMLYMVTGPCTDLRNTICGVNDMVEDPNVKGNRISCRFYQGEKQDDLLCQVNQLSKGQQAKDDASEIC
GALGGCTCAWSTTERKIRKRIGGRIVNFNCLPNPKSKNTPAAREQFLKSIDEAAAEACFWEKEMDESTCQSYKLDCI
HTLSEGEHEDPITPDQVERCVRDRIRENRAPSTAR

>QLI70099.1

SPTLNNNKEITGNWPMCVPAPTKVSIKRTPTEKRADSVKTPLPTQPGMVDNCDTFCYVQKDNTCVQIAESNHITT
NQFTQWNKGAGPDCLTLWAYTYVCVGVMKS

>QLI70103.1

MLGIKVDGVEITDKEYVCADDCTFEGVWFGENGNVCAAKELVVVLHDGKVNPKNIEYACAEAANPEWSTRPPM
NGEEPESCFLDKIDPLSPVKYNCEAKRISKDKKVDVNADPVLEQWSEVRKMLGLPPSQEAQGCGQATDGATKIDQ

>QLI70113.1

NPLLRRQDQQRADDALTLRANFCLHLDKPECKGLFEKCKAEGKYVEDCIPQHHPTCMQDESSPCSKAAVQCLADH
SKEEEAFKDCILEKVMPAPDTVDDADNEQSAPSGSGQAELACESASVDFQDTWVDNKCGGTTDAGETTDAGESN
NVALENEPAACKEARDAFDKVWDANNCEAVLNAALGTPQGDSDPNAVSLDDSD

>QLI70114.1

ASCNLPSPSAVISQGAIRGFRDGYCNAVYLGIPFAASTGGKNRWRPPQNVPKSKSVFKAVSYGPTCPQAIRNPRYS
RQSEDCLNLNIWAPPKGNNLPVFVYMYGGAMVTGSNSNPQLQGTNFARKDVIYVNFNTRESIFASPHSAELRNT
HPAASQNFGILDVDKALDWVRKNIKAFGGNPDHIVFGGHSSGSVQVDHYLWNHPDTWLKGAVQMSANAMSG
PPYAPVNEALDVVAAEVGCPPSSHGKGQLDCLRGVDVYAFQTARFNSTFNTWFTPVIDGITRHSNYLARFKARKYA
AHVPLLTGNADGEGTIFSLVYAAENSDFRSWIKTFDADSSHIPECALVRAYKPSDFASGPLRSGVQYGDARFNCPVD
YLVDMRSARQDTWVYRFFGAYDNVVGPPNTAPTHGTEVPFFHGGNECFQSLNNVTKEQQALADSIHDWFVAWI
KNPAAGPGWGKVSPKSQNMAKIGVPGDELSLVKGFTGTYNSRCKSVYAPAFPKYPVVQSIKPVLEALL

>QLI70123.1

IPDDASEVASGLVPAGELTARACSARCYVKVDGEFKRNGCDLGPVSYGVVSNLANSQLHSVHGSIRHNTQATPGG
GSANLNAERVKITAQGDITITVHNNFARNCAALLNIVDQSFSGNTVEVYVGP

>QLI70131.1

KIQKASCDGGKTSSIGMGTNPNIPIDGEDPTPFQLDSPIIRTKEKDNLLVNGCGRTKLQTNIDVANETEIAIQNNQIA
NCKPGDTLTMTVFVVNTDGSGPYFADIDEKSNVGIFRELGKVDGIDGKNGIQRNAFTAFDVQIPLPTDMKCEGGK
DLNACTLRIRNLADAGPFGGCVALQDVTKGGAGNGGAGNGGAGNGGAGNGGAGNGGVGNGGNGGNGGLG
NGDQGNNDLGDGGLGGNGGQGNKGQGNKGQGNKGQGNKGQGNGGLGNGDQGNNDLGDGGLGGNGGQ
GNKGQGNKGQGNKGQGNAKNPINRRGMRIKRAENAKQGGKGGDGSKNTGTGNRKPGNGNQNTGAGGNQN
TGAGGNQNTGAGGNQNTGAGGNQNTGAGGNQNTGAGGNQNTGTGGNQNSNNGGVRSPDDITTADTLDIIL
KEQQISLKSLEDFLKNGGKNQGGNPQNGQNNAGNGQTGGNQTGGNQTGGTQTGGTQTGGTQTGGNQIGGN
QKGGKQKGGN

>QLI70138.1

GVIPKRGQGISVTPHDQYSSSIGVIGGKINTNRVAYWPEAITCDNICVKVSYEGRSLNLLKIDQSGGAHDISYDAWN
YLAFGKSATDSPQMGGGIAMEYETVSVDECADLLTDGKLPLSAANSMGYVGACIGEPNSWVAKNYELVNILDPVG
KNGWNEICTLDLTVSNQPKCPHTLGEQHSPTGLEVVNIQYGTGKQVTV

>QLI70151.1

DPIKRQPATPTPAPKKTIPGHLAYIPWKVDKAPADGLQDVTFPISMKDSIRERGWYFAQNFQFHGKSGNAYIGVQ
PRPANSKGPVDHAAFSSFMPGTKSTDRNCSPGADGGPGVSCSVQITGTDDHSYDLRVENIGNTTWKGTMVNTV
SKVETHIGSWTLPAGAGGMKGNYIGFFEWWPFNDRSKPPTCNLLKRTTVAFGPPRTSTANAGAGSLGNPYEVYEC
KGKTDYKTKKVGDNVEISLGF

>QLI70155.1

IPSHLQDKRAEADDGAKLPDETDVYGAWVGGCGSMSTPYCVNYVWPECIYLYIDANSNAQSKQLSKAGLDKFEKD
VKACIKLRDPAANEGLCYDLKLSKESCQKYRQECRQENEHKLNPEQGFTEDSLIASIARCIREKPQDPSQEQSTTTSG
TQESTKSLEQQPQKTSRLLPFIGEDNLPDICLPGQFENCMGTKKYCELAVWNREEEPKQYSSTEECRADRKPQES

>QLI70157.1

QTVSYSATSELTFLTGTRTERPSFQTGPPTGPYQSYGSKITLTGANSSTTAVTSSSGTMTTGANFTTTTSSAPTNTQP
CNNHVELCTRRYSNITNVGCHNSPFVRPGNSGSNQEVDVLTQLDDGVRFLQAQIQWPPNSSTPHFCHTSCDLLDA
GPIYNWLGQVADWVDAHPYDVVTILLGNGNYSDPSLYVPFIERSGITKYVYNAPFLPMALNDWPTLEDMIIRGKR

VVMFLDYQANQTKYPWLLDQFSQMWETPFDPQDRAFPCTVQRPPDLPREAAMDRMYLMNHNLNVEFNVFGV
QLLVPAVSLLNETNGFNGTGSVGLAANNCRSDWGRAPNILNVDYYNYGSPKPCSVFAAAAAVNNVTYDYSKPCG
QPSAASRIIVQSLWISVAAMAVASL

>QLI70173.1

DLVVVEHLDQPPDGWTKLGAANASQLIRLSIALESQGHDVFERTLQEVSDPTHPRYGQYLSRDEAVALVRPRPRSV
AVVREWLLSENVTQGQVQERGLFVDAVMTIGTAENLLSTTYGLFQHGKQRAIGALAYSVPAEVRPHITSIQPTTFFE
SGNLFKKSPRMSGVGIATARRRNPGVRARSTGLQDCENYNTPECLRGLYKMSNDYTKPHRSSLLGVAGFHGQAA
QYDELEKYLDTYAPYAKGANFSVELINNGTNPQGEYPGFEANMDIQIAVSMAFRVPVRFYSTGGEGHEFIPDLDVY
DPEKQHLEPWLQFASYLLDLPDRDLPQVMSISYGGNEQAVPKPYARRICQMFGLLTLRGVSIILASGDQGPGVSCQ
SNDGTNSTKFLPAFPSGCPYLTVVGATERNAPERAINFSSGGFSEYWPRPAWQEAAVSRYLDAHGDKWKGYYNR
AGRGFPDVSAQGIGYPFFNHGGNASGGGTSASAPLFASMIAVINDSRMKRGKPPLGFLNPWLYLGASRAFTDITH
GRSDGCKGTSFSGAKAPVIPGAGWDAVEGWDPVTGLGTPLFDELQELAMG

>QLI70189.1

VPAVKDVAKDVSPAHKEFVLFHSSNMKGADKLPFAVRFHSFRVPALVTTSTGRVLAFAEGRRRDNRDVGDVKVVI
KRTKEATSHGGNPNDWEALHVVTRGDGVWTNPTPVVDGNTIYLFVNWHDAKVSREGDDKLHNGKKTKKVDGK
SRRHVYVTQSTDDGQTWSDPKEMTKQLTPKGQAWDTVGPGNGIVLTTGEVVVPAMGRNIIGQGTPGQRTWTF
KTIKGAGDEGTIVQTPDGKLYRNDRAGKDEEYRKIGRGTLDKFGKFSLDKGLPDPDCAGATLLYPADGKGPARVVFL
NSADKNSRRAMRVRISYDKDAKGYTNGRKLADAPVAEAGNEGGYASLTRTADGEVGALVETNFDKTGGSKDDHY
AIIWRKFNLSWVLHGSK

>QLI70193.1

VPTGAPIAEQTFVANVQQRAPSPTPASALDQRGLGDDIKSYVGSLASGVESKVSSLVESGILNFPNGFPAGTAVEKS
LGVSSTELDVEPTQVLNLPSYGNWTENGWSLRIHGNVYKIPNVSQSKIDDLANVFLIGMSVKDLNATEQAQARNV
TRSIFVVQQGNRNVTMNLRNNVAVQSNATGGAINAKGGAQTIHMPYNTTIEGDFDSFVDLRNTTGLNGGYMIP
GNETSKIQTLNVYAEGTDSGNATAYLVPRTGLTVVSDIDDILRVTKIYQPKEGLLNTFARPFTPWMNMPSVYANWS
SSINNMHFHYLTTTPEQATRNYMEFIYQTYPLGSFDTRPLNFSDASATLHIRRFLLDKIFQTFPKRRFVLVADTSNHD
VMVAYPQMFKDYPGQVACIFLRNTSATDSGDKFPYDTSGFKGIPKDNYMFFKVPEDLAHLDVENGRCLNSTIPQN
VTFGEQGLPLGLGDKGSLAGRIQIGAPTALFATLVTMTAAAIL

>QLI70195.1

INGGQIVPFGTFPYIVSMHRQHTQIGQATPVAPGKTRDRDRCTGVLITKNLVLTAAICIPNQRIGQTMVTLNATVQ
RRPKKYGNVVQLKDYFFPHEYLKGDGEYDIAIFEVQDTHNVTQFAKLPSRGQVPREGELAMILGAGMVLWFPIPD
VPVKNLTVPVISLESCRARMADVQIVGTDKFCTRQCYDSDYYGLCEGDWGGPVLINDTVVGVIAESPTCTKRYRPC
FPGVMTLVAEHLTFIEQMMSIYADSFINDGHLWTKRPFSKTEGDLRW

>QLI70203.1

ATGSDVTVTWSHVDTDPAKFDLNLWNGAAQSPAVNRLLASGVPVAADAVRVSVPCGIPSSGAYQLLAVDGANSS
DVYARSGQCSVAVVGCTSSATSTARTTRTSLRGVQTDATTASSSALTNVTSTPSGRGTSIPMAGAAQCTAQWLSIA
GAFMLAVVAFWL

>QLI70208.1

QTSPFSIDSTAAIKQSSSTLAWDMLQYYKGNLTGQTPGILPGPPPAGDYYWWEGGAMWGTLIDYWYWTGDSTY
NNEIMQSMLFQVGENKDYMPRNVTASLGNDDQGFWGMAAMTAAENGFPDPPSDKPQWLELAQAVFNTQAS
PDRHDSTCGGGLRWQIPFANNGYDYKNSIANGCFFNMGARLARYTRNTTYSDWADRTWDWMWNIGFIDNKN
YAIYDGAKVTNGCKDINRAEFSYNNAVFAEGVAFMYNYTNGNATWKARLDGLIKHGMEAFLPKGIAVEISCENAG

TCTTDMLTFKGFLHRWYSTITQLAPYTAETIRPVLKTSAEAAVKQCTGGALGRQCGFKWASGVYDGKTGAGQEM
AALSAAMSLLIPQAKAPVTEKDGGTSKGNPNAGGSGDDAQKKSKPITTADKAGAGILTILVLGSACGIFGWMSVG
V

>QLI70217.1

AVLGIDIGTEYIKAALLKPGTPLEIVLTKDSRRKETSAVAFKPLNSGPKAAQFPERLYGAKAMAVAARFPDEVYPNLKT
LLGLPVDDASVQEYAARHPALQLQAHSTRGTAAFKTKTLVPEEDAWLVEELLAMELQSVQKNAQATAGHGSSVRS
VVLTVPSFYTVEEKRAVHLAAELAGLKVLSLISDGMAVGLNYATSRQFPNINAGEKPEYHLIFDMGAGYTTASVLRF
QSRTVKDVGKYNKTVQEVQVLGSGWDRTLGGDSLNYLIMDDMISQFVETQAAKGISATAQGVRSHGRTMAMLA
KEAERLRHVLSASQNTQASFQGLYEDVDFKYMVTRDDFETMSEAHAKRIEAVINDAIKMSGIELSDMTSIILHGGAT
RTPFVQKALEKAAGSADKVQSTVNSDEAAVFGAGFRAAELSPSFRVKEIRIFEGPMYAAGLKRANGEKRQRLWTAI
SPLGGVAKEITFNDHDDFALSFYQQVGDDDRDIASLSTKNLTATVAAIKEKYPSCVESEIVFKLGVKLLGENGEIQVV
RAAVECEAEVTVKERIVDGVKNLFGFGKKDQKPLKGNEKNADSKKPEGSEEAPKAEAKSSATSSSSTTDSSTASSSSE
SAAPSEEVREVKQKQTVSIPVEVVLEKAGAPTLTKSELVKAKDRLKAFAASDKAKVQRAEALSQLEAYTYNVRDLLD
GDSFIDASTEKERADLAKQSSEIGDWLSGDGADATTEILKAKLKILQDVVVPVQTRIEEAEKRPGRITALKDSIKRISEY
LDSIHKQIAEYEEWEATASSASASGSSTTSASAETPTGEFDGLEDDDAKDTKNDNKPEEQPGPIPPLFKKEELKDLET
LVKTTSEWLSKMQPQQEKLPSNANPVLLVKDISEKIQKLDKVSMDLALKGAKGFGGKAKKAAQNLKKGKSKKGEG
AEDFKGQRFTEDQLEEMLEKIKAMEKEKKPEKTEKTEKAKESSEKPGHDEL

>QLI70220.1

APVPQTNGAGNLLSGIPLVGNLLGPLLSGIPLVGGLLGGGGGDAGAGSALSGIPIVGGLLGGGGGGAGAGLGGLG
GLLGRDGDAIPREENSASS

>QLI70230.1

LYFFVDGAAPKCFFEELPKDTLVVGHYSAEEYDDRVNSWQQHNGITIYISVDEVFDNNHRIVSQRGSASGRFTFTA
HEAGDHKICFVPSSTSGRSAWLSAHSPNGGIKMKLDLVIGETGQIESSDKDKLQDIASRVKDLNARLHDIRREQVFQ
REREADFRDQSESTNARVIRWIIIQLIVIGITCAWQLSHLRSFFIKQKLT

>QLI70250.1

RDEMLCVDKLEDRTENPAGRNVFDLSGHMLSTVQDLQKAVAYISDEGKKAQLERIGDELFYLGSQINGIAGGDML
MYSKPMGTCLNSTV

>QLI70258.1

TPLLEKQSKRAELLPYYLGKPHRFRNLQCAATGLGGRVLDEKICGTPYFCLYTAAAIDWELGHNQSYPTQEACLKDR
EPAPDGQQNAEGPQEGLIPWMERAVHDGHSCGRYGLRRPYNAVIYCGTEKYCEKVVEKGTATLDDCLAMFEKRP
VGPEHPEDPKSDTQLF

>QLI70260.1

APTVSVPTDGDKDCLANFIVRQCLQSENEKLAACSSTDHECQCYASQAIATCYNNCPNDARAPGATQAMNGAC
MAASAYATITPKATSTAEAAATTAAAAAAADDDASASASSPSVVPTRSVTSSAPSASKTNGAESLAGNAAGMLAA
VAGAVAIVL

>QLI70276.1

ATTRRPKANSKSAQPSYDDSDNPLPLVIWHGLGDDADSEGLAEIAKLADDIAPGIFVHIINPTPDGSDDRTSTFIGNV
THQVQIVCDQLAKNRVLSTAPAIDAIGFSQGGQFLRGYVERCNFPRVRNLITFGSQHNGITEFKQCGALDYICKAA
MALLRFNVWSTFVQNRLVPAQYYRPVEDSDYALYLDGSNYLADINNERDKKNETYKENLVRLDNFVMYLFENDTV

AIPKETSWFGEVQVNGRHTPLEERKMYKEDWLGLKTLDRKGALSFRTIKGDHMHIPEHVFKDVMTEFLGPFNKTA
VSVDGFESEEL

>QLI70296.1

QQKPLSDGHVPGRNPFDDKFGAFVKDTLDEWHVPGLSIAVIDHDQVFAEGYGIATFPDTPATPETIWYGASTTKA
YVAAAMAAVIDSKNYSQLTRGWSTPVSSIIRDDFVLQDEWATAHVTLEDAVSHRTGLGSLHFSSLRIENGVQVTPR
DVVRRLRHLPLFAEPRTTYAYSNSMYVALGYVLEKLTSSPLAKVLGNLIWEPLGMRSTYFDLDDAIKAPEHLASGYR
WDPDHGNYTEMPYMVVTEVGGAGAIFSNVLDYAKWVKCLLYESAPFSKAVHKDVKTPRFITSPLPGEGFDSVLYG
LGWERTLMYGHVVYQHSGGMHAYGAYVYWLPEIKYGVVSFANTAVTSNAVEIILATRLIADRLGIPEEKRFDYAGS
ERDQLEEEIEWLEHALDNLYPSRPNPPLAPTLNTSQLAGTYYHPGFGPIRLREVINPKNPKEKVLRSDREEASWDHK
FTLHHVTGDNWMIRTEMYTTVRNTAFRSQFKIGVDGKTAGLEVEFSDRGAEVAEAVVLFDRLKE

>QLI70299.1

MVGYHTAGLRYNSTHSFCASNCRADDVYYTDQGEACFMPDAYVAYDDDMGYMYCKGGKFELPSGSVNIAGSDL
LKSCWFLPDEQFNKVEIDGKLVISCSANRELAAVDTSQYRDFETHQQVVEALKALTEEKTEEPKTEKTKAAKPTQLCY
GQDNCKPVWD

>QLI70306.1

ASIDRRIVGGEDAKDGDFPFVVSITLTNRICGGALLDSTTVLTAASCLRGSVSVRAGSLQHRAGGTEAKIASRKPHPD
YKLNKRNPALPFADNDIGIIKLSRPIEIGNNISYATLAEDGSDPVVGSMATVAGWGQQEPMFALLAEAAEFMANVS
IPIHAREVCAKMDADVGDRDTVICAGGKGKNGCLYDGGSPLIDEETKQVIGVTSWGIRDKNGDFCGQAPTLFTRV
GRYISFIHENMGGSPVDAKF

>QLI70311.1

AVAPVRSTPLNPHTSHPFDRYPGCSVDCLKSSFDEAGCAYSDSKCGCIHKRVITKRSKPCLWARCTMDECIKIKNMA
METCDRIEKGI

>QLI70313.1

APLVTSPHFAEQRVVQSSDGFQNGSAPVGKTCFQVSPNHNTTFNTHNLVLRGAILKRQASPKHTASRPVKLPTIEE
HPVISAEEASRIKLPGGGKPGVFYRGDSRPPSQVFESGFAPRGHDLSLQRHLSFVGESGLVSLSRSRRSAEGYAFGRT
GAKTGRGYVYVVSPTDVPDGYWVPGLFPPEKNPAVGQNQEFAATGPIPGSSISHAYEVYEDNPSDKSRKIHNDNY
ALRGAPGCAQMKRSMTMCDPAKLMDDEVWLAKSVRPGRLGPPQPPGTTKIKIPGASPEMIGTVPGPDRPAGGK
GNSSPHASRPFKPSTKTVTRLRVAGQLGFVATISLLAPYAHMALNALKEWDHPIGHAVAWFDDAINSFQESLGGK
QVPEIYGNELKLRIICWIRGEQRFPNAVDRACQRLWDKDAGRTEQDDERDWQTGLAQLREACGKAAVTPPAEAG
LRDAILGHCEALEDSILRLNEARTQLIAHRERVQEKVDAGGRVEPEDIDKAAEYISDGAFPSFPSDDGDKVWELAAW
YMGDVKAQNMDMTLETESDTEPEPMEVSPETASEDSHSAPADPPAWLAASRVVAGAIALDEAVGREVLALVDE
GPYVGLFDEAPPHANETCYSPAGLARAQPGEVWDRISAALVYIEQNVAEGMKRVCKGCYRGRDEWALACGSTSA

>QLI70314.1

SPAPQRVPKDGSQNVIISDWLTATKTVINYYAPDPKCGGHRPWVGMWPADACNPYAADFKAWAYVKKTQGYD
DIATVEFDNAQLGPGEFKAAFVCEDGKRQPWMLSNTYKIDEAPPSKPGQCVHRQSSWQTTWKYTACDKVENDL
CYDSPLTYIACSDCREQCGRQSG

>QLI70332.1

SPLGRPADELIPTIDASAKASPTPANYQWSSGWTATIPIHESCNSSFRAQINQGLDEMVQLSEHARNHLLRWGHKS
TFTQRYFGNGSTAHAIGWYDRIILADKSDMLFRCDDPDSNCDIHKDWGSYWRPNATSEVNVCLFAAEYRLLSSVC

NLGHTVAGSGLELFWATDLMHRLFHAPIVNEDIVNHFSDDYSDLLELAKTEPEKSGIDMDALQYFAIDVWAHDVA
APGVGCTGKPPPKKSPAESVSGSPATATATAASSCHTHSDG

>QLI70334.1

HIKMTSPSPFDNENLSNGPMEKDGSNFPCKNANYRNRPVKENVYAQGSTQTLEFLGSAVHAGGSCQVSITTDPSP
NKDTVWKVIKSIEGGCPAKDQTGNLEGGSATGKVPYTYDFKIHEQIGAGNYTLAWTWFNKIGNREMYMNCAPIS
VTGSGGSPNYLDTLPDMFKANIGNGCGVPEGTDVQFPDPGQDVDQFGLKTKSALGAPTGNCKTGGTRPTSINTS
ATSQPTSGSSPPTSASSKPTSAVSKPTSAPRPTTGTASRPQPTNTNTPGGPRPTTGTPTRPRPSCSKRPGGSNSTLVP
QPTRPAGDSTVSVSQDETKVPAHSPTPSTEAGPAPTPGTDGAFAPGTKCETDGWWNCVGGKSFQRCANHKWSE
TLTVTEGMKCTPGTGENIEMSAAQPDLSPCDIRRRRRLRRHAF

>QLI70335.1

ESSITAPLESRTMLTASVTIREEPTSVWWDEAFTETGAAAETTSVMVDEDPTGSFSSAETTTVVVLKEDPAVSFPSA
ETSTVIINEDPTASSSPGTTTVIISEDPTVSDPAEPAPETTTAVVNDDPTASFSSAETTTVVFIEDPTASPSPGTTMVIV
TKEPTVPGPAPETTTATVNDDPAAPFPSRTSTTTVVLNENPPTTPPPSASTTIVVLNTEPTPVSTDSDEWTEPGNPW
TTRSTTLPVLHPYIHYDFGIGTAWPRGTTKPECTRWTRVGDTHNRWTQDVYTSTITTTLLHKCGACAIVWTTPHKG
YAWAKVYDSSTADYPFTVTEVACRAPSPDDAGYVPPPHTPTDPWSTLTKTQEFMSTIYVTYPDRGPSTITPPGVTS
PACTSSTLVYGGGALRANKQLKWKFSTERTVTKDCGTCALAWSTRYYWQNVKGDFYHYYTVTDEAFVATTLECGR
AVPTEALGPGPRTATAAVTSTLTLALTLS

>QLI70342.1

APQNDPVPEELAAKVEEVLRQVEVKNKEADNGGIGGTINDVVKQIVPQAAGNLYPPIGQGASIVALLATSEYKFDE
ANGKEWLKNLGGLALSFLPKVATLGNLPGFIGMIKTIATGIEAAQFPELTAKRAAAAKCFADNSQKTYNDVCANCK
PELAMSLLARLNKCTDENVVSRNAYEDNKFAQSFCGTVLCSGHKEGDIDNIIKQGFRYDPVRAFWCKEKEEIIGMI
MSPWIGQPLLENYHKLDHEDLREALTEPCQQLFKVQGLQIDGVCPTRAEFDEANTRSVPQSFCAPGKKITPQS

>QLI70345.1

APTGPSPDASGLSKGGLPADSIKARMSDDAVKSARHSARLNPDVIEAPYYLTGELQERSEALNPDVIAAPLYLVGEL
QE

>QLI70362.1

DGCNADNCLRALRATHIPGRLESAKAFCATYTKATGTVAPTAVPSYAADGCKDNLNGPMALRISSACSCIAPGPSTT
AIPTAPTSAAPTTAPTSTATGHPCAKVSASWAEQRKTATAIPTVAASLAHECLKTVPLGKAEALKLIDAIEPYLEWQS
DAGYKKDPPKSYFYPGFDIFGNLASVRSNVESGKYAGEFDFQTDLYKQVWAPGQDGHYVFYPDLLAKAFRWRRA
VPPIVSISEDGESLPVIKLQTDVIANPKTAQAITKINGIDAAKYVEDTVNAASFLQDVDSSYNTMFWSKSTAANGGV
GNFVSGGRSALFYHGDTTKLTYANGTTVEIENKASIVGDMNAVVDGPSMYNKFCTPVPLRSDSASSAALPSAAIPG
YPKPVIASSDGVVSGYYLNGPGLDDVAVIYLMSFEPSVPAEFQAVVSDFLREAKAAGKTKLVVDFQNNGGGYILLGY
DFFRQLFPSVVQDGNSRWKESKSFVAMARLVSDAVKNVNPATETDENLVSLSQTWWNYRYDMNITNENFATYE
DKFHPHVYKDTDYTNLMRWNLSDPLTTTNTTYGIGVEISGYGTRANLSQPFKAEDIILLYDGACSSTCTLASEMLRL
QGGVKSVAMGGRPKKGPIQGVGGIKGSQVLQFLNIFSFANFLSQQSNDEETKTELRRFTTLPMQRSAGSAVNVRD
QILRDNVNDGVPAQYIAEEADCRLYWTAPMISDVTEVWKSAANAAFNGAKCAHGGIAGSKANRRSRVAPVLGAR
RSARLSDAVDKTPIAHSLNWEAQHLQVAIN

>QLI70365.1

QDVPTHRYNNSGIGPPRDARQKFNDTGFVLPRGEGQKVNSTGSVLPRGECQKVNGTGSVLPRGEGQKSNGTGYI
QPRGEGQKSNDTGFVLPRGEGQKSNGTGTVLPRGEGQKSNGTGYIQPRGEGQKSNDTGYIQPRGEGQKSNDTG
TVLPRGEGQKVNSTGTVLPRGEGQKLNNTGRLFIDDEILTPTRSSHIRKPTATGTPTETGKANKSGKPTVTDKPTLP

WPSFSWTTIPGKPTHARRANDDGKPTLPWLSLSWPVWPTQSEEPISTGKPAYTHNRPSQTEEPSHTKKPAYTHRP
TLSEEPSHTWEPTETDGPTHTELPTHTWRPGHPHKPTHTEEPTDTDMPTHNTEEPTHTGRPPYTHKPTHNTEEPS
HTVKPPYTHKPTHNTEEPSHTVEPTDTDKPTHNTEGPSHTGKPPYTHKPPYTHKPTHNTEEPSHTVEPTDTDKPTSP
GKPTGTGKPTGTGRPTGTGKPTRPGKPGKPTKTTKPGKPTKPTSPGKPTSPGKPTYTGEPTYTGGPTYTGGPSGTD
KPIYSKPAYTTVPIKNTHSKRDLEEPECAHDPHCTGDSQSDCPKPTLCEREPHHPFCTETPPHTHGGKVCDKPHGEA
QHADDGTPCDGHGHEGHTCTKGCHKPMQAHNGTDELAPSGSMIGGDGSMPVGTYVPISAGETTAVSDLALGIV
ALAGFLLF

>QLI70374.1

SGRPGEGFRILRRAEIPDGTCIQVVGTTRLKCEKERNKGGSEDCYLVGQDAGIKCLVDSRKVPETSDAVHMQCQAE
AVKSSKVCYTKGSQTRWRLCSKVGVKAYNDCVKKDKVTTTSGQSQSPAIWDDFCSQAYDDAISKALADMNGSWT
TRFNPSAGQDASLECLVKYSKVPEASGAVYMQCRAEAFKGARDCIEKGKTPWKICFNEQSKAYPDCVKKNKVTTTS
GQSQSPAVTASTVASTSTQSSSGVAQPPAKVQAPQDPPRDPAQVIEDKLCIPKKAPNGFDDMQSWNKWGLNEG
ACRRTIAQCVFEEQKKNPKVKNFDGVIECMDKRQRSKDFDYGF

>QLI70383.1

LKFDLIAHTGGESTKKERCIRNFVGQETLVVVTATVDGFKGDGMVVNMHVRDALGNEYGRPRDVVGESRVTFQS
HADAAFDVCFENLFSGSRRPNPASRHVELDIDIGADAKDWSAIQATEKLKPVEAELRRIEELTEEIVQEMDYLRQRE
QKLRDTNESTNNRVKWFGVTTTWILIGLWAWQIMYLRAYFRSKHLI

>QLI70399.1

SDIYPPKPVDLTTPVQQRIAIYGPNHVSIGWNTYQRLSKPCVQYGTGNDALTQEACSNMSETYSTSRTWSNTVIME
GLKPATMYYYKIVSTNSSIDHFTSPRAAGDTTPFAMDVVIDLGVYGTDGFTTDKRDTIPKIEPALNHSTIGRLADTID
DYEFIIHPGDFAYADNWYERHKNRLHGEAAYQSILEQFYQQLAPIAGRKPYMASPGNHEATCDITRHVSGDCPLG
QTNFTDFMHRFGATLPTAFPSSSSNATARARAVTAQKLARPPFWYSFEYGMAHVVMIDTETDFHEAPDGPGGST
GDNDGPFGSPNQQLDFIEADLASVDRTVTPWLIVAGHRPWYTTSGGEACLPCQKAFEPLLYKYGVDLAIFGHVHN
SQRMLPVYKGIADPNGMRNPKAPMYIIAGGAGNIEGLRPIGKNVSYNAFAYADDFSFAKVSFKDKQNLQVDFIRS
RTGEVLDTSVLYKEHAETAPFSDDESTSLAGRSMAWLEIVVMVILVIL

>QLI70403.1

ASQPAPQDQLSQESEALSKRARQPSAQAIDAAARSWQRDTGVVSQFLSTAESMSPQQLQQQARGALAAENDEL
THKAVLDQMFLTGARRNRDATVRQANNVLETQGTFQFVVDGLQTLSRRGARMSPGQVSAMIRAINRDRCPQVL
PAIDAYMAAAAGAGAGQTGNALTAIRPSNC

>QLI70407.1

IDIAQAPRYMGQLSSGTLESVSPSLQFKQSISSAQEPDSDRLNKRQGGRIEKVETKYIDLSFYSRSQYLETHPTAIADK
DFKSFHWAIVSRSRNSELADAFDASDSLSVQQGPKVVSNPLGEGGQKNFLFQYKYGVKPEKASTLSARVTLGKTKT
STRAITEALEEVTIPNCQESCVDWAVGAITKLQEKGLLPTFNVGDLSTRAFDYALNTRTALKLMLDRGETDRFFQRR
LEIAQYQNEGVCKKRTK

>QLI70408.1

RPATPTSATSAVTSVAAPTSTDVVDTSFANIFNLGILTERGADLLHELLNEKHESKPKRSVAVRELALNLRDQLNSGD
VDLNKALDDGLKKLNSTTAEVDLNDLKTQGFQLLRERIEAEDFNKLAQKLFHAGTAKITRRETWSWAGFFNGPILRT
AAKMGVSVASNLLGRVDLNKVAQNGLSALGNMVGRVDMNQAAGQGASLLGSMLGGIDINNVVKGALGFLFP

>QLI70411.1

KPVCPEPKDLLPWLEKSLTTEECFELESNKPSFKELEQKCGTTWSCNVLSNSNEKWLIDNSGFANEEACLARHVRDP
GQKIPWYPKSSTDDECLAFKMCKPALEELEEQCGTDWTCSLLEGRGERNRLDNIWLAEKTGLWSKEDCLRHHMP
DPSL

>QLI70427.1

LDPASLRDRLPRCSLLCLADGVTRHNCSLADVECQCDKIEPIIRTVAPCLVQAGCDLQNITATGRVVLDVCKTLPGG
NETKPGGEGEVTTGTGSAAKTDGVGWVLVAIAAAATVLL

>QLI70437.1

ASKAGGAGSGAGAQSAVESRTIDEIYKAAVAEGGQVVLWHGGDEVNQMDFIKDAFEKRFPDMKLNITVDLSKYH
DGRIDQQVAAKAPSQVDSVYLQTVHDFPRWAKEGVLLDYAPLGYDQILPEFKDKSTASWYALEVLFWQNAWNT
KKLPNANFNNFDEFLKPEYKDKLVLTYPNDDDAVLFAFDLILQKKGNAWLDKLLAQNPTWVRGTATPFTLIAKEDS
PLAATFTTAVGFANVTNIRTAFPNDAQFVSWGQRGGILKNAPHPEGAKLLAAFMLTPEFQKEYGWSVRGDVPAP
AGFPKVTEMQNTDVFAFNTWMEDRARVERLRFWYEDRIGTAQGVSPLVDNV

>QLI70438.1

VQVHKVDVGKNMATNETGLKFYPDKIVAQKGDMVQFQFWDGNHNAMQTSFDQQCQPISRSNANVTGFDSGF
QPAKASIEKGMIPVYTVMVNSTAPIWVVCVQGRHCQNGMSMVINENTSANGSRSLENYRLLAKSAQGVTPGGG
PNGGSGSGEGGSTTPGSENIPTPVGNQSPTQSAPLSAGINLAVPSTLLIALGVGFMFL

>QLI70445.1

IPKAVNGLDSALYERNNAYIVKWFEAIRLYA

>QLI70450.1

LDAANVTKRKPKCGGFRPSQELPVLDITGDTATIVGHTPNFTDHSLNVASYSPNITSETDDVTSQTNNITNETDNIT
NQTINIVNNIMQCGVSSLSSQEPAEISETIPLGHNITKRQSVRRHSIPTYITVVAKDYTQAGGYLTDNQIERKINEMRK
YWSFAFKFDTSVDQIYRYVRPDLFDADTGFLRESWAETMVRFRRGPIDYTSLNIVFVYRYQDSHKFWGNPENNQQ
WGYSMQGTLGTSSIASENIAHYDNWKELATKDGIVISSGTIAEVAHKRAENVENTLPHEFGHWLGLRHTDSEVQV
PDNYVGLRPNPTEANCERVNDKISDTPPHVLNAYAQEVWGRCLRREPIDPVYTCLHLKDWRERLVPDPIFNLMVSI
SGTRCPIDGLTIQQKEKAIHGYETWRLPAKQAIENKRIGDGNRQQANNQAQPQPNQHQQENIQPQPNWHQWN
AQREHCLRQATDKFDYNVGIALNEKYKELRANYDREVWRPNRYLNDWQYSGWARQEINNAAVRES

>QLI70461.1

HNPKDNPEYYVFRGDGRDPDEIREAGGFVPDPEAAIYTNPTTFSLDNHVNGRTGSSAYVSTSAEFGQAARNFAGP
GNYVYRIHVTPNMIDVNAALAGGHPYPRQEEASALGGIPWTAVEGWLQLGEDREFPEDYEFLNDDSAARLTGRY
VAEFADQFEPNRAYDDRGLGGPARLTGTNPQVALLAAQLPDEARLINAATTFMNNYALAIGWTENQAFPYTPPTP
PLSTEPVALPEADVVSIPEAEREDLAEGRFDNLQCPALAVALGVTLLQPKGPNSKRYVLKSRDDKKEQCEQLRDIVSR
LTSRKKQTKPAGKKTTVDLCGNGPLNPPCTTVEAFDDKCVAIPQGYQNTLSGVKAHEATSICRFYLEPDCKGRYFEA
DGQVVDLSTARPDFNDKVASLICDTPKPHPALKRWQWSSRSHYRYCTRLDKVSLDFKLANNDGSGTYDKIKLAIDD
AGQKVHVITEGPSPGYKVSQDFNLRDMFGMDTVALSQIKRLRLLDELTDRTFGGDAWELLGLKLKARCAGSGINIA
MEKFSNLNKGLQAKPTEPGRFQYNRDWEVWADDIKPQDWVAKPLCSHFQRMSVNLHVADANWAGTNNDIYA
KVGEGSFLIARHPSRREVFTTDIDVDKAYKTKHVPVTNVASVGIESKGGNDAALPEMVTVYGVCSTTSTVLSVKQEI
TDWLYDGQTMTIKLPPENWVRA

>QLI70471.1

REIRSLHPVGVTVVSRDEDLLEKYRGQVTRLHDAISNATQCYTDFIDNLHKAEPDTLHTINACGDKNEAARAELAAT
HEIARSPAFPSDKLFSAQLPDVLDSRTSLQGPWTKDLQDRVHQLTLESLNAVEASKRAALLPEYRKHVTALYDHLSK

AVTCYEAYDNNVQKPGANPDSIDACDTEYDAVARSLRDARSTAYQPDFPDGNWLFEAKIPDMRDNRNQKKLDEL
RDAAKHAFEIKDQAKLVKNYHEEVTDLQRRLASATKCYVDYIDHIHREDITDEGYKSRADQACNEAYRQIQAKLTEV
RAAASKPNFPGASWLFNAHLPDITDGRAELQSEWTKNLEGKLRELTQAADRADRAGIQADDKLTQFNATVLQEV
VPLVNKTAMETAEVLVKYVALNLLTQGKTAEDVKDAANKALLGFVKGLGHDMVDDEYILFGAEDLVPLKEHDGNG
HWAVREPTLRTLNGEEYSVKRYRPRAEGLFSVIEHWTKNTGEIHWRSISKHNVTTLYGTNNESRVFDPEDPDSSKP
TRIFSWLITESFDDNQPCEHNRTETARYAAWYLKRIKYGNRQSRFVQPIPADTNWMFEVVIDYRDHDFDRPTPTS
QNPWLCRNDPFSVYRAGFEVNVRHYRLCQRVLMFHRFPDEVDIGVNCLVSLTDFECRSTRGVPSDIKLGNPTASFI
SSVHQSGYIRTELSLQSLGNLPTGLGSDKYHWVNLDGEGIACVLTEQGNTWFYKRNFGDAELGPLEIVAQKPSSIPL
NGKSQLLDLGGNGQLSLVDFLVVTPGFFKKIANEDWSSFTPFKFLPNIRWDDPTLLVIDLTGDGRADIITSDNQVFT
WYSFLAEDGFALIEL

>QLI70476.1

IPNGGIPIDAKIACEKPNVNFCLDDTTLVVCDANKVGTRTFCRESLIPSPPASGIGLCWQSSKDADDAACQKACVVY
HPTQYTIPAALCNPTYIPTEVPRSPTFGTPTHTASISVGTSTTQGPVSSSTSTSGVMSIPEGTSLGTATVPHTTHSEPT
NPTVPTRTSTGTWSPTGSDDCPAPTGTATPTGRPSSNTTSHTTSDTAIPTAGASTNHVIGALAAAALFAAFLF

>QLI70477.1

ARLPTIPSPDNNQVASTDVDVKGSRDILTSQQNAPGNLIRLSHYQTENVRTYEYDRSAGQGITVYVLDGGIRLTHEE
FGGRATFGAGFAFHQGEGDSDGHGTHVAAIIGGAKYGVAKQVQIVSVKLQPKKPQLEKALDFVLKDVEDKNITGK
AIISMSMSFHASDDIDKMFKRIVDSGIVCVVSAGNGNSDASIASPGRDPSVITVAAMNHRSDSRWEESNYGPAVD
LYAPGADITSASRDSDSASVTLAGTSQAAPHVAGLAAYIMSLEGITQPSQVAARLKDIAEQSGARVQWNAPDTTGL
IASNGLDKGGPNSLFPPKRIPWTLEPKKSGKCGDPEYSEWKCGSQKYCNAFDAAPQEPKTGFFKNAKECFDAHEPA
PKLPWIKAPSPKTGPDSCGSGFGGNPAWAIYDDASCGTQVYCEAFDKIKPRPDFLFGFKDTKACLEAHDPPPSG

>QLI70488.1

NKEPQLPIHNTADACPDYASYASYPHKPLSTGPLALPFQRPERRCRTFHSDEIEKVITDITTRMKDPDLARLFENAFPS
TTDTTIKFHNKGRDTGFVRFGGSRTVLDDGAWQGHHSFIITGDIIAEWLRDSTNQLRPYQTLAKKDPAIFDLILGAIN
TQAEYVIEAPYCNAFQPPPISDLPITSNGQDDVVHPAYEPSAVFECKYELDSLAHFLALANDFYEHTGSTDFLNNRW
YLAVETLLQVLEEQSKPTFDPETGDHSRNEYTFQRRTSAGTETLSLQGNGNPLNSGTGLIRSAFRPSDDATILGFFIPA
NAMMSVELRRTSKFLKASNKASLAEKLEKWGETLRSAVWEHGVVTHAKYGQVFAYEVDGYGSAIMMDDANYPS
LLALPLMGFCGIEDPIYKNTRKMILEKLGNPYYLKGKDFEGIGGPHIGLRNAWPMSLLIQAQTTGDDDEIKACLNLVL
RSSKLGLVHESVNVNHVRQYTRSWFAWANGVFANTILDIAKRKPHLIFGPGSSPYEP

>QLI70491.1

ASVQSKDEMPTGLCPKDQLVIVDGLGYYLPQAECLVLKERCGQRNTTKPAELKKCITNTRISEIKEAQKAVQDTKRSI

>QLI70497.1

VAPQGSTLPEGPLPSVVYRGDDLPPEYYKNLGGIPHEFEGKTDNRSYSLWWHNVGLDGWVGKMRGKNRHFNSA
YAATSSRWSSALYFAVLTREQEFGWMYQIHATPNMIYLEGSGFAPPFTDEVEYAALGGIRWDQIEAWLRVPKNVT
SGDRKTFKSDGTLAKWALLENFTKDFPDLKWVKNPDYNPAYNQYHGSEGQPQLGGRPIKVKGSLQLDIPREFEGK
SMEYHAIEFMNKVGGPVGWIGAFPLNFSAAADFSAAEI

>QLI70499.1

RDVPANVKSFYNSIRGQGQCRNVLAGGFHSIDGDSGNRSPDMKPIDFDYCGDHISDYNVIYLQGKNGQLANMDI
DCDGIQHGPADDGRCGSSGDTQSVTSFADTLRGYGTGQRDLDANAHPYVVFGNTGSRKGFANFDPRKYGVEPLS
VMAVVCNNKLIYGVWGDENGDDGSESMVGEASISLATACFGRGINGNSGHDDNDVLYIAFPGKDAVPGAKGAK

WNAQNYDEFENSITGLGNKLIQRIGGGGGGGNQPPPTGSCSWEGHCEGAPCKSENDCSDELVCNAGKCSSAGGST
PPPPPCSWEGHCQGASCRNENDCADSLVCRGGRCSQ

>QLI70504.1

AAAVPDSMPALQPRTTSGIDMNAACRWQYDDSYTSGTIGSGCYDWMCWKSGEQSGGLDLNAWCRHMHGPQ
SYASCSGGRYNWVCNY

>QLI70507.1

APSTLDLRAEETCTDKSIKTTQWTVGDFDFHASYIFTTPAHQNSWGYVNFTLENPNVAFKPQCSAASNQLNDFFYG
NLIYNCVQPESGDPATFTFSWPEKELKVNQTWACPEEGSRFWAQGGSKLDLTCTDETWQNDNWTIGQIYSSRTIS
CNHINAPVPIEAMQAVA

>QLI70517.1

DKITTQLTLCKQPNLQDCDDQFVPIDSCYKVPECEVQSLNTGGHVCDFYSHDGCSGNKYQYAGIQQNLPPGTTIRS
VFCW

>QLI70518.1

KRQPTPTRGVPNGQHGGDEVAFANEAFEQFILGQMNEWHVPGLSIAVIEGNKTWAKGFGYATLGSEPVTPSTLF
YCGSMTKSVTAAALSILIDEQKNGSNLQWTTPISSILKDDFVLSDAWATDHMTLEDALCHRTGYPRHDFSGPFNSS
VDMVRKFRHLPMSQEPRVKWQYSNMMYGTLGYVVERTSATRLADFFRDRLWRPMGMLNTFLHPDDALASGG
QLAHAYYYNNDTRQFGELPWNDEGSVAGAGMAISSVLDWSRYLRHMISESGPVSAAGHAALKAPHMVSEQDRR
IYSGTEFYGLGWGSNMMQNEAVWYHSGRVSGMLSYMAFVPARKFGFVIMLNTESVAALDSIFSTTLFNYFEVASS
NRYDIRQGWYDLLRDLDDGLRNCSARLYPGVGGAALPSGMALGDLVGSYYNDGYGYANVTVRCDDWEAAHGSP
SVLSLTSDGCRIVVPRAELFGKNVSFQLQHVAGDKWLAWYFVDDYKTVTRPAGCYRAQVVVGPYGKPDMLGMD
MRMEGDDLPLTWFKRI

>QLI70522.1

SPISHSNTTSRSLMNITDSIMFNIPLPEFTIRRDNELPNKVDWTSDGCTSSPNNPFNFPFLPACHRHDFGYANFRLQ
TRFTRTNKLKIDMQFRTDLHYQCEHTFAQGVCRALANVYYSAVRVFGGHDQTPGKRMNNGLLWEYHALVDIYEE
EVRKAQAAGDLPLLQ

>QLI70529.1

AEDYYKILGVDKQATNKQLKAAYKKLAVKFHPDKRNGDEESAHQKLVELSEAYEVLSDDELRQIYDRHGHDGVKQ
HKNGGQGGGFHDPFDLFSRFFGGHGHYGHSSQEPRGHNVDVKIKISLRDFYNGATTEFQWNRQHICETCEGTGS
ADGQVDTCSVCGGHGVRIVKQQLAPGMFQQMQMRCDACGGRGKSIKHKCPVCNGQRVERKPTTVTLQVERG
AGRDSKVVYENEADESPDWVAGDLVVTLAEKEPAPEDNPDKVDGAYFRRKGDDLYWTEVLSLREAWMGGWTR
NITHLDSHVVRLGRTRGQVVQSGHVETIPGEGMPKWHEDGESPGHQHEFGNLYVTYEVILPDQMEKKMENDFW
DLWEKWRATKGVDLHKDSGRPEPAVARDEL

>QLI70536.1

APVTDTKPKIDYDAIIVGGGPSGLAALSALARVRRNVLLVDSGVYRNGPTRHMHDVLGFDGVRPAYYRWAAREQI
SHYDTVSMTNGTVTNITAQENNTYFTVTGTYQDNEEKTLTARKVVLGTGLKDLLPDTPGLIENWGKGIYWCPWCD
GHEHADQSIGLLGPLTSVPGTVREILTLNRDIIAFVNGTDTPSNREATEKDNPRWQDYLNLHNVTVENRTIASIERLK
NGSNPNADPSLPSYPEYDLFRVHFTDGDSVTRAAFLTSFKHKQYSNVGEEAGVVLYGEKLGVDPTKGLVTNVPGIY
AIGDCNSDNSTNVPHALYSGKRTAVFLHVQLERETANAELAGLNKTLHTRSLHEEVRSLWDHMNGGKDDLLYAGP
FEE

>QLI70542.1

NGPGNAAAENLQRRQEDAAAPPAKVPSDGEVTKKIRKEIKDIMGSIDLILDGLDDDKEE

>QLI70550.1

HKRYGKVKYLETHPLRVYGDGTCADSIRISQGLIEKAKTMRQQCQDSASPLRTYGSWGARIATVTTHFSSPDDPQR
FYQKAIETHILHNLVHGSQLHVLCTPIIDHMWNKQAFVQFVLLSELAKPPQERLEWIFWADRDTIVLDYCRHPASYI
PAKLHRSYNPGTEPSKEQNINLLITQDTRGLNAGVFMIRVSEWSVNFLSDVIAFRHFKPDVELPFDEQTAMEQLLLE
DRYKNNVVYIPQPWLNTYAWDNAQDFMSRKDVEGLDDWASRRGDFLIHFAGNGDKELNIVEYSQVGDKIFNIW
ETRDMLRDVSLDIERFWGNRSNGPG

>QLI70551.1

SPNEAAWPETTHARGFRLVVNVTHPSRDFNPPVHNTYITGVHYGERTDAVVPGQDFKNARIFFVNGTQGEADAD
VATTIWADWDVYESWRLSPEPGSETLSEVLMSGADGDKGIGIRHYKSPFVFLTPETYVACRQVLPIGGEQVVIKQA
KTTFPPDGSPNYNIPRDCAPVRLLPECAELLPLSPRARFDYRFAVQSQCYKNVTGIDWAKYQS

>QLI70559.1

VGNLTSNGRADAPGFQSCQNVANEKIDACFSKTKGDGVAQQACACEDYILNYNCYAAHCWNRVWECEYQEYLV
SYLMDCPIAKLPVPYFPIPDNAAGACSCNVGKVYKAITDSITQGASCMKNVHGADTILRGDKIGACGCCEISGAMSS
IFEICPDTKPDLVGMASVSKIQTQLDINFRECQPMTKINCASDLNFSLAGVSTYFPPSSLPTSGTATLSNNPGSVTAP
PSGSVFSYTNGGDKVVYTITAASASNGKDSESGASGSAAAATASKKSAAASVMVPKGLSMAITGISLVAGVMERW
SLFA

>QLI70561.1

NLPPIPSDLTTPVQQRIAVNEPNSITVSWNTYKQLDKACVKYSASNCSLTEQVCSTTSAATYPSSRTWFNTVTISGLS
PATKYCYQIVSTNSTTASFLSPRLAGDKTPFSINAIIDLGVYGEDGYTIQMDQTKRDEIPNIPPSLNHTTIKRLADTIDE
YELVIHPGDLGYADDWILRGHNAFDSKNAFQAILEQFYDQLAPISSRKPYMASPGNHEAACEEIPHTTGLCPSGQK
NFTDFMTRFGGSMPTSFASTSHDPAAKVNANKAKHLAKPPFWFSFEYGMAHVVMIDTETDFAGAPDGPDGSAG
LNAGPFGRPDQQLQFLEADLASVDRAVTPWVVVAGHRPWYTTGGEACEPCRDAFEGIFYRYGVDLGVFGHVHN
SQRFWPVVNGTADPAGLDNPKAPVYIVAGGAGNIEGLSAVGMRPAYTAFAYADDFSYATISFLDAQNMKIDFYRS
ATGELLDTSTLYKVHDRQFVEQ

>QLI70564.1

SPVQLDGTTLGLINTYSRYATAAYCPDLQDLSLNSAVCSNPSAHACGATADATTVEEFGNAHSISGYIAVSKSRPVIV
VSFRGTDIWNVRDVMSDVLACLKDPKLRWTFLGVFTDAICALLPSQAADKADKLLPLCDGCRVHQGFWAAFTGV
KDRMMAVVQEQLARNPGYSVVATGHSLGGGVATLAGAYLRKGGVRTDIYTYGSPRVGNAAFAEYVSDGRGGRT
VRVTNRHDPVTAVPGDRSAGYAHTTPEFWFPEGLGRPSRICEGVHNLSCSGGIFDLLCLGDHDGYGYANGVDVCP
GKGGKTLGPTLRDIIRQEDVDEWVRFGILDNTTAPDAGA

>QLI70586.1

EPMVFLIRHGEKPADDDEPGLSIKGQQRAQCLRSVFGAGSNYHVGHIMAQAYKPDGSRKRPFDTVSPLAQDLGLE
VDTSCDRNDSKCVKKVVKNYKGAGNILICWEHKALRDIAEALGADDVKDYPKKRFDEIWIDPYPYSEITDIVSENCP
GLDN

>QLI70594.1

EGIFQSLAKQPQYQGKIGRIDSCGTAAYHAGEDPDSRTMSTLEANGIADYEHAARRFKTTDFEVFDYIFAMDRSNL
SDLQRLHKGNPDTKAKVMLFGEFSGTGKAEVVSDPYYGGRDGFEKAFEQCSRFSKNFIRDVIGE

>QLI70598.1

PVINNLSIMKVDLGRNRAGADQVTFGTFGYCLRGIDDSDECTHSRIGYDPANLMNRLDGTDFGDASATTAKGLTR
VMVLHPVATGLCFIACLLCIFTGTVGSFLASLVSLITFVVTLVAMVCDFVTFSIIKHDVNQNGVSTASWGSGIWLILAS
AIFTLFGAAIVFVTCCCARKKKSSERQKENWNETTTTPASGRKRRFW

>QLI70599.1

NKAEDNSAAVLFGNDYEFSVARGPCVNLKKGQPTFHEIKVTGHDVCTVYANLKCDTEVREFRAGVHEITNVVFKSI
KCSTSEEL

>QLI70603.1

APASEPGKREMNLGGIPGSGLIGGSGSGAANGVTDAFQQALALLFGAGDAVLNNPLDAVTKLFTNPTQAPADIAK
NLMGSVTGLAKGAGNVVTAIPKGVGQDMAKASGKTN

>QLI70618.1

DNRPFPIAIKKQSPGSGEKILREHLAFAPLLQVEPGVVSSFDAPSDEPHQNVDGTSRFYPPFAMSLVALMAQHAAE
ELDRVRPARQVVQRLWAAVAAFQDMFAKVRSKRIGDSYPTVPAARNRHIDENNNHWRESDHSHRNSYSNCFAG
SDDKNYHTKYHTFRHKHQRWY

>QLI70642.1

ECSSFETAVTVTVTAKTTTATTNATTTTTSQTTRSTSASATTTAPTPDVTPTYPRLPMFLPDGSSTNSSGVPWGDM
NLMTNNYLENPKTGVVRSYEFTVSRGLASADGHPTGVILVNGQFPGPLIEANWGDTIQVTVHNNIFGPEEGVSFH
WHGLPQRNKPWEDGVPAVTQCPITSGKSFTYSFEAEFYGTSWYHSHYSAQYSGGLSGPMVIYGPAAKEYDVDIGP
IMLSDWYHKPYFTLVEETMAPKAAPEAPRSDNNLINGRNSQDCTGNTNSSQTACGNMFKLSRGKVHRLRLINSGS
ESIEKFSIDGHVMEVIANDFVPVKPYKTEVVTLGIGQRSDVLVTADGKDSAYWMRSTIPAAACGLNASQLLATAVV
YYDDLNTTAVPQSQPWNVTDPQPCLNDLTNSQPILALDPPQPDLLYKMEVQAFQNESKIWLFSFDGQAFRGNYN
KPTLQSAAMGNSSFEKEWNVKNTNNAKAVRVYVHNATPLPHPLHLHGFDSYILRQGDGEWDGVTIDFPKNPPRR
DVVMVRALGHVVIQFDAANNPGLWPFHCHVAWHASAGFFSQFLVGNDALQKMNTQKTVLQVLNETCLPWGE
WTLTNIPNQIDSGL

>QLI70652.1

VRDELAAAHRELAAIERNLTRQGQSPVHPRVYIDAYMHVLSPPGEEILSLDTLKKQFDVLNTAFQPANFSFILQGIE
WDSSKSFTSFSYPWGGSWYHKGDKSSLNIFFVKHISWQADLILGGESSGPDLLEGYAKEDGVRIAAGTVPGGSFEA
FSGGLTTVHEVGHWFGLEHTFEGGCDGEGDFIDDTPASANASRGCLIGRNSCPGKEGLDPIHNMMDYSDDACKT
GFTRGQIDRMHGLWNKYRSFENDTQPRPWLLWIPSKPVRQTHKLPFFPDPWNIDLAKQRCRRNTEGSAEEHRES
YCGTNLYCQERLYEFGDGDKYADTATCLSVRTDPQSNEPYTERLPWTTGTIPDEGCQKLSSFNYFPERMCGTGVYC
KGFDSPSQATISDADNRDARGRYNSSTSCLEDHDPAPAAAPKPTVEPTPVLPTVQVKQCRRHQYKLEVLEGQACP
DGTSAQEWESGMPQPTKDASGCYQFTANCR

>QLI70656.1

APASVGETFSVKAKYNQDFKPDTTVELVRTALRYGKLASRDVARILERRQDTNGTVVASPPYKFDREYLAEIEVGTPP
QKLLLDFDTGSSDLWVFSTETPSAQSTGHTSWNVTASSTAKKLDKYTWSIAYGDKSTSSGDVYTDIVRIGGAEIQN
QAVESALQVSQQFTLDTASSGLLGLAYDKGNTVSPTKQKTWFSNILPRLKEPLFTVRLRHQADGSYNFGYIDKAQYT
GDITYTPAFTDDLGHRLFKASGYQVGSGSFEKLSITGTADTGSTAVYLPNSVSDAYWSGVKGVTTQGGFLDSKAYN
FPCDAKLPDFTFGIEDAKFTIPGEFINYAPTAEGSTICQGGILGIGTIPEWLGGISIFGDVALKASFVVYDDGKNRLGW
AKGA

>QLI70666.1

QEDPLAQYKAQFQTFMDKMSGYIPNPGKHDPVAALEAKLGSMKMSTLTLENWKETLYEPVAPGATVPTEWWVL
ISGRNKTCFGHCGKVEQAFNETAAKFAILPGSPHMGMLNCDDQPILCNAWSAGAGSVWSINMLPAPATVDIYKK
RLNLTSTTSEDLVKLNGAESKQAAGFVPLDSWFHPFNGKATELGLSVPYGYVMWAFGLVPNWLFMLIVSFASRSF
MGNRMQPGAGAPGAARPGAAAGQGRPQ

>QLI70672.1

FPNPYPDEATDIPPTKESLGKRDGEFSYLISGADVKASQKYGGHLVNYKLRANAVDPSRLGVDTVKQYTGYLDDNST
DKHLFYWFFESRNDPKNDPVILWLTGGPGCSSMSGLFMELGPSHIDKNGSLVRNEYSWNNNASVIFLDQPVNTG
FSYSNVPVDTTAAAAKDVYALMTLFFEQFPEYSEQDFHISGESYAGHYIPVFASEILSHPARNINLKSILIGNGLTDPYT
QYAYYEPMGCGGGGYKPVLSNYTCQTMEYALPKCQAAIKACYNGEDAACVNAGDRCNTPFLGAFASTGLNIYDIR
KKCVGGDLCYEEMNWIQDWLNRKDVMGALGVEVANFKTCNDHVNAAFQQAGDWFLPIQKHVPTLLEKIPVLIY
AGDVDFICNWLGNEAWTKALPWPGQTDFNDASMVELTASSGKAYGSLRHARGFAFLRVYKAGHMVPYDQPEG
ALDFVNRWVRGEWTD

>QLI70678.1

KPVEILSRNVSNNLRGPEDQPGLIDTNTLGSLDKPTLSPRQDFPPPQILPPPQIVYRGDKRSPDEIRATGGFLPSSDLA
PTDENNGFSLYMHHTSARINNKRVTSYVSTTRYFGTGLAYANQASREGGWLYQIQALPHMVDSDGTLLQGRKYR
NEYEFSALGGIRWDQVKAAVQVPGAKTSTDYKSGSKTWTFVTMEDFNKTFPEKQWVVNTEYNSSYDQFEASPG
QPQLAGWFQYDYDEDKYKSKEPWSQYQAQTVRQYFIDFMNQVGGPVGWTGTYPLVLSEDKWMDRARL

>QLI70682.1

CVKRSNDSMGNELDFQLGSDAPSDPATVGYLLNHLSLNVANLSASIAFYEQVFGMRHMFTIQVTDHYSFAYMAH
SHGGRNGTGYQTAAELNRERTNSAGMIELWNLQVPRRDLPESGDAISRVGHFGMIVPGIKAAQARFDTFPDLKIL
KRYGDPFPTQGRIITANSLSASSLGQLDAEERKNIADSLTSFHRTFIFAEDPDGNIIEIQPQD

>QLI70693.1

TLRGGRDVMDAPNQKGLLDSRAAAGYPDEPLQCFEVTNPVLSSDGIVDGNEILDSYPGSVPPKACKIQLMDHVFG
NSYGQPFVSDYTPPDETKCPFNRVIMNFTVVSEGHQFDRLAIMWLGDTEVWRTSTAEPNAHPGISWTYWKDMT
TYLSILKRPQKLIFDLGNLINSNYTGSFNTTLTATFIQDQNLTGPAAPPADEIVPFSAGNGASGKGSAFTYPDEKAEKSI
TLPRNIKRAVLSIAATGQSDEEFWWTNVPEDAINNWEGMTLLGKSSFREVRLRIDGQLAGLSWPYPVVFTGGVSP
PLHRPVVGPQAFDLREQEIDITPWLGVLCNGKDHTFGLEVVGADDAVVNRYWLLSGKIFLWLDKRDAITSGCPPK
VIISKPNYNPHELAVQNKSLRYDQTISRTLQIKSKIKHTGGKTFESAWSQRFAMQNTGYMLEAGNVQQVNASYEG
EDRATKDSASYYYAGYSYPMQVRLVQTAPDDNYTYTLDTNLTQTMQLAVTGNTVFSNGLEPFLARITGRVSGSVIQ
TTKKGRAFFFKKKDGGSSGFGSTRQTYSFGAKTLANGDGIGFADAQSLYSREVMVNNETTTLDNVWVFGKDLPKT
KPQGPPQQPIESNEYAARLIGGKRPGTKIGIQWAKEAVLHGNEKD

>QLI70702.1

HPSGFAHKNIHRQLDKRIDFYMNAKPDGAAVNNKAAVVPSPASPTVAPPPPPPATTSAAKSTPSTGSGSGGKKQF
CGGVSKRASIADIAYKGNVGAPNNYGCNLMLVDDAKDYEYTATIENKSGKDQKCAVWLKIGRNGGINGFFKGNQ
VMTFDLPAGGEKILAAEPNSQGGLACGVGEVPTNDIGLYTATWFEFDYASEKNGAWSGADASCLTAADSNRPIPA
LSVCAEGHKCSTIKEGGVGDNAYVKGTNALDGVGLNVSPGPVNFKVTVS

>QLI70711.1

APAKRAEPAPLLVPRGDTIPDKYIVKYKETFDFSTADSTIKEYHAKAEKTYSHIFNGFAGALNATAIETLRNHSAVDFIE
NDATVKISAFAEQSGAPWGLSRISHRRPGGSSYAYDDSAGEGTCAYVIDTGVEASHPEFERRALFLKSFVAGENSDR
NGHGTHVAGTIGSKSYGVAKKTTIIGIKVLSDQGSGDYSGILAGMDHAIEDSRTRSCPKGVVANMSLGGGYSAAIN

QAAAKMIQSGVFLAVAAGNDAKDASQTSPASEPSVCTVGATDSSDRLSSFSNYGAAVDILAPGSNILSTWINGATN
SISGTSMATPHIVGLGAYLASLEGFPGAQALCERIRSLATKNTISSVPSGTVNLLAFNGNPSG

>QLI70712.1

ATTCSKDIKVTQPTPVIDCDVVDADIIVDSSVSGSLSIEGPKQIKGDLIINNATQLVGITSSSINAIGGTLRLQGLQLLSS
CNLQSLKSVENLELINLAQLSGLTLGTTGVTKASSIKIQDTFISDLSGLNVASADNITIANNGRLNSFESKIENITYTLSV
VDNAGSMKIVMSQLQSAGILDFRSIKSFDAPLLETANRLSFQESPDLLSVSANNLTQIKDSLTLDNNKKLANISFTSLE
KINGDMTIRNNTALLKINQFPKLKTIGGAILCAGSFDTVEMPELNDIKGAVTVTSTTDISDFCGFFDDLAKKKAIQGK
ESCTSNNAKANEGGKGGTSGGNKTDSAAMSLGVNHALLGFAAVAGFAQLI

>QLI70713.1

LNPWQAPGPDDSRGPCPMLNTLANHGYLPHSGRNLTVQQFGDAMAQGLNADPLFGTAPATGFTLVWGRSTFD
LEDLNTPGLIQHIASLTRDDVTPAEANIGEVPARVSALLDDSPTDYLDVASLAKSRVRVEALSAPQRIPPQHEVLALTE
AGLLLMMMKDGPVPSFFSAPSVQTWKAPKDRVKVWLTEERFPEELGWKRSERTLSVLDLAPVVTAVTARKAASA
VMGQ

>QLI70727.1

SSSWKPAKNQLCFGRQSCHYASDEANKRQGNLMSHIFPDEVCIRGKINPESWVDRIPKCG

>QLI70731.1

GPVQSKEIVINSYQLYPENIDYDTKTRLAYISVLYNSTVAVYNPFTNKVTKTIAFDKLSYDPVLHASGVQVDPLGRLSVI
VNAGAAFDTRGANISGDNFLVKYDLARGQELWRANLTAVTDGIYSGYQDIEHDACGNSFAVGTWPSSIVRVSKD
GKTAAPWYLTNDKDHTKKGLTGLASKGDILLATEHTGSRLLRFDMKADKGVPDVVPVGQDGIGERPDGIYLPSKFE
GKVLLVSSQLEGTVVLRSDDGKWTSAQRLGVVPNKFADEGGSTTASVEIEGRIFVSTEWFGDAANKVPDTLSGNR
TEFPLYDITSEVVKLLV

>QLI70736.1

DSIIELALVTNMVSWLHGTTSATFSIMSNGTTFDLVGVPRNLAVDQGHSSNGAAGTAFVIVGLGGVLALWLQGRS
MHRGQKSSNLIYRTWLLFTVLAAVFTLATLAYVFAVTNSLKGQVIDVDLAATLVDTRYPRDNWTPQGWFGAVLRL
DLASASERRDVILHLRIMHGWQYNLIPMFLLQLILTVLAVVDATEVRKWRKVESVEDYN

>QLI70755.1

QTAPACVETCTNEVRNKFADLKCSDANAAPCFCTNPTFSAAILECSKPQCQATADNVFTYLTSHFCVGQPLPKPDN
TAAPSSEASQTPTPSAHATTSTSAVPEPSSSAISSSTTATPAATTSMSTESTPTSATVETTGTPTKSSSPSSTSDSAASA
TSSSAATEGSSSKGLSQAAIAGIGVGIGAAVIAIAGIVICMLLKARRRKPGRNGHNRDISKPLPGPDRMYAQRNTSFR
RNRDHSMEKFGNDLEMTSHRYEDMVPRTQPRTLV

>QLI70775.1

GDKSAADYYVRELPGLPKNSPPIKMHAGHIEVTPETNGNLFFWHFQNNHIANRQRTVIWLNGGPGCSSEDGALM
EVGPYRVTKDNALTLNNGTWNEFANLLFVDNPVGTGFSYVDTNSYIHGLNAMATQFITFLEKFFALFPEYQSDDLYI
AGESYAGQHIPYIARAILDRNKSKSRAETWNLGGLLIGNGWISPQDQSSAYLKFSLERGLIEKGSDNAQQLQQMQR
ICDKEMSINPGHVDYPECESILNKILELTRVGSGDQECINMYDVRLRDSAPSCGMNWPPDLKYVGPYLRQPQVISA
LNLDKQRNTGWQECNSMVNANFRNQNATASISLLPDILKEVPILLFSGAEDLICNHVGTEELISNLAWNEGKGFEV
TPGNWAPRRQWTFEGEVAGFWQEARNLTYVLFHNASHMVPFDYPRRSRDMLDRFMKVDISSIGGEPSDSRIDG
EKGPDTSVGGAKNNTQQHEEETKQKLKEAQWLAYQRSGEVVLVIVIIAASVWGYFVWRQRRKGTAYSALQSDET
AGQSRTGLAAFHNRQSDRDLEAAAFDETTVDNIPLQESIGRGESKYSIGDDSDEEEGETTKT

>QLI70793.1

SRAPDLLSSLPKSNAAIAGPATSLAVVGNTAFHHIIPNHSNLATKHGAASSRDMIKRQPTNPPKISAEEASTKSVPGG
GKSGVFYRGDSRSPAEIFKTGFAPQGADKSLQNHLSFVGGSGLVSLSRSRKSAESYAFGRSAENNQKGYIYVMAPK
DIPNGYWVPEIYPPNKNPAVGANQEFAVDGSVPPSSISYAYEVTREKPSSKSNKIRNNDYSLKSSLPCSTKKRAICDA
TRYSAKPYKSSKIRITKAVGKAAAITLLAPHARRLLEAIKQWDNPIGAAVNWLDNKIADLQELIGGPQRDDIDGNDL
KAKLICALKGGQAKEIIAGRPNNICKPSSEKYADNLRRDFESGKLDRVIEMCKGISVYPGGHPAVWEWTKGYCAAL
HRTSEYAARMWELAVSGLLNSCSELEDNPPENEDLYDRLEEHCSKFQAEVDRAENPVAKPVASSVTVMKCKCGTK
LPAFFFLCGRICRATLFQQRAKSSPAVKPAAKPAAKPAAKPAAKPQAKPPAKPAKPLPELTVGNCKCDALKLPQIFLP
CGRLCRASIIQQRKPSSTAKPAKPATKPAPKPASKPASKPVAKPAVQFTAERCQLVFVPMDEPRVVTWKHHTGGQ
LAFFLTRGPESFLSGAAHQLFVDSRINMAMLAIGTRARSPFSSHEWKTIPWGINPKSAKDRLVDIMLEIPTLLEQIAQ
LQSRSCPTAAGIVQEKVLSRCVQLEGAMRVWAAKMGTDILRFDYTFMGDCVPVPQKEAEFGLLHLSIVYWFIEM
MLVSVKMFVSELCAMETAEAMRQLQMAARKSARALSLLFASSGGLARRISGLLALSIALRYFLIVEAPGGISDESCM
LQSLLDRDLNGSTIRTLLTRMRGGQDPLLVGTDYAGSHWPENVLHWF

>QLI70800.1

ETQTPLSNEFECVHPPYKVYVASKSPLVMYLEGFITPEERRHLLELAYGNDTFKHSYVTSDSSQTIHAVRTSQSTSVRR
SPTVRCIERRALDLQGLDVPVSNVEPIQLVKYAPTERYHFHTDWFTDAQHAVASLGGNRASSIFGYIKAEGVVGGG
TNFPFLDAKSGEWCRFVDCDEEYEKGVTFRPVEGNAVFWENMVSGRGDERVLHAGLPVVRGEKVGMNIWVRE
GKVPDELRGL

>QLI70801.1

LVGSGTSLQLNGIDYFVSPFSQGKVTNGSVAINTRQNQLGFVPATVIAGDLYTESTLQSLFLNWSTVDDVWQPAFL
ETIFVFNFAKLTNKNHNYHDGVSSSVFPLQVTHKIPSGPYFLNVHTGEVHPAYRLYDDFAGAFTQSLLQRPDGRFQT
LSAQVPAAASITIGVPSRLYFTKTEAKPLAGVRIGVKDIFSLAGVKKGCGNRAWYHLYPVANSTGTAMQNLIDKGAII
VGVQKTSQFANGETPTADWVDYHSPFNPRGDGYQDPATSSAGAGSSIASYEWLDLAVGSDTGGSIRGPATVQGI
FGNRPSHGLVSLDNVMPLSPKLDTPGFLARDPCLWNAANAALYRDKYTFFGHQAPRYPKKLYLLDFPAGNTSHAPI
LQNFVTKLAKFLDTSPTNIDLNKEWERTRPTSAGDQSLAQLLNTTYAAIISKDQAKLVREPFYRDYAAVHDGRLPFV
NPVPLARWTWGDSQPSSLLSDAVRNKTLFMDWFNGNILPPSSDPLTCSSGLLLHVNGSADFVSRNRYINPPVPPF
GFSNSQISLFAETPDSVFPLGQVPVFSSITNNTEYLPVTIDVVAAKGCDGLIARLAEDLVAAGILTMPRVGAGIEGGEI
LMRRYFV

>QLI70809.1

APGSAPRAVHAPLLIPRGDDASLVADRYVVYLRSGASEADHKDAIKSFNIKPRHEYKHLRRGFSATLDGNTLAGLRR
HPAVDFIEQVTTSSISSSEPVNRALQNSTGTPIEQKGATWNLGRISNQKSTSEGESKYVYDSRAGEGTCTYVIDTGV
DDTHPEFEGRALQIKSFVANSTVDDSADGHGTHVAGIIGSASYGVAKKTKIFGIKVLDSNGDAEGDRLIAGLEYVPV
DAANRTCPNGVVVNYSINSKGYAKSINVAAAELAKKGYFVAVAAGNKPRDVAQSSPASEATVCTVGSIDINNKPAV
DTGYGPGVDLMAPGVDILSLQPDNRTSLLSGTSMATPHVTGLAAYFASIYGKSAIPNMCQYLKDVAVKGAVKEQR
LCTANLVATNAVVDV

>QLI70861.1

ANLQPRQTDLGAATQCLDLLKTIPTPPPDLVKEWTTNPPKDYCSISIPASLSKDWSSYTSSASSWVKAHSSDLAKCP
GAGQVTAKGPLDCKAGSGSATQATATSGSATGSATGSATGSAAGASQTTKTGAASRETGMAFAAVAAAGFALA
AL

>QLI70863.1

TDPPHDEKHCEKEDIFPDFENYEKWAICKEQITTKRFPTL

>QLI70865.1

KPVDIQPKIIGGWEANQAEWPWIVSIRLEYPGGRHRHICGGTLVSPDTIVTAAHCNQDRLRPGLFSVLAGSNDRES
REATVVGVAQIIDHPRFSTETMQNDISVWKLSRPIPESNTIKYARMPRQGEDLPRSAAVKVAGWGAIRDTRNKPQ
GAQGILARDAPQEPSPKRWVPVITPGPQPRPPVDETMSPFLLREASLSVVDLNSCIEAYISASKSKRGQFSKSFVPQF
AQTMMCAGVYGGAQDSCYGDSGGPLVDANSKALVGVVSFGLACGHPKAPGVYTKVSSYLDFIRRVAGNIGGNG
DNGNGNGDNNNNSDNGDNSSGNGDDDGVTQVPPNNYNYCRHLIGQFLLTAGNGVVIQYVASADYQVRNYISS
VHFIGNVVQFDRPLLGDTTGLNCVHLQCHIGTDMLGLARLGAASVTGLDFKFWGSNYSGAQTGRFDGWRRRRE
AQGLTVLPRDTFDLVFISIGSVCYVAKIKEWARVVSRLLKPGGRLFVREFHPVLLSLDDGKPDEMVFNLPYFEREEPG
IMDKQGTYVSSGDCIFTLTKRALFNHGIREVVQALLDEGMKLTGLREHQRAPLTGAQAELEVDQRGESQVKEKPLA
RRIYLHRRLLFEASTVAIALSYTLLAVKE

>QLI70876.1

QQCVCGYANQDACASAPAERADSYTGVAFKGQQGSTDERSEYAAAALPNGKQTGPHEYPFIAAVVYKPAHQRIL
CGGAIIDEHHILIAAHCVHGLKVDSLGVYIGSNRFNTTGAKLETVAAIHTHPQWDPELLVYDVALLQLRDALTFDRYT
TGPICVLDQRKSLDNQKAVAVAWDSATDGGPAVSSLLQATFETHDMASCGAQPSGGPQFCAYSAEAAGCHQGS
DDPLLLLQDQSSRRYFAIGISSYGQSCRQYPEVVTDVTQLRPWMQSVLQAAGGKLCK

>QLI70879.1

APGSETPERCKPATYRCDPNVNGWDVCNTSNLWVFAGNCPPKTICKFFQENGSPYCVPPNFTIP

>QLI70880.1

LNEDERTAVQEACRELAKTLPENVHLPNSPGYNLSNTYWSARQSALHPQCFVTPRSTQNVSHVMKTLTSLKAPFSV
KGAGHTAFPGGSNIQDGVTIDLAHLDQVAVSPDRKTVAVGPGNRWINVSSALDPLGLAVVGGRAASVGVSGLTL
GGGQSYFSGLHGWACDNVRNFQLVLSSGRVVDANASSNADLYRALRGGGGSNYGIVTRFDLASFEQGDLWTRSL
VFDGGEANRTLLPRMTGLAAEGLAADPGAHAYFVQTYDSAERRWLFLTSFFHATPPAANATPAVFDGFESVPGLV
LDNALVGSVSTISRLIDEPVGQRATWWDTTVAVGSVPLFEEIGEMYKDWVVRVKEAAQTRQFTPYLVYQPITDNIL
EKMQQNGGNALGLYPQDGPLMMVQVSARWADAELDAVIESSASELLCEIERVARENKLLRGYVYINYAGRSQEV
LRTYGNNYPWLQTIAKKWDPDGLLQRLWRGYFQLR

>QLI70899.1

ERQVLTDPGIYGPPLEIAHYYTGQMPTGIAVSRESRLFSTYPACLDANNTNTIQTPYKFQVAELMPDGSEVPYPSVEI
NTPPGGALNMSTNPPTSANYADYFIGCQSVVVDNKNVLYILDAGRAIDPQTSVLLNAIPGGPKIVSVDLSTNKIIRTY
TFPGSVVYGDSFLNDIRVDRTPGLSGLSGGAEEGVAYITDSSFEGRNGLVILDLTSGESWRHLDNDPRLRPQQQFLP
FVHGSPVMFGSSSQYTRATVGSDGIALSADGKDLYFSVISGRELWSVPTAALRARDAHSELLVQASVSAKGQKGVG
DGMETDSNGIIYTGHVEQEAIVSYSPGNATVQTFLRDPRINWVDTLSVGWDGSLYFTVNQVHLMPGFYPGTERR
QHPYVLFKAQLPDGGKKVGT

>QLI70906.1

VPFEGNTLDCFSLEAKSRPEGRRDFLSDWQAAHNRWGKTKPVPGSNMALKGDGSVTAEPLDNDQAYVTEVEFG
TPAQKLKMILDTGSSDVWVQSSDSVYRVNENGPWAPRYLPKNSSTSTRVDKAFWGVEYLDGTTATGIVYRDTLRL
GGLEMKHVAIESAQIMAPRFERETSVSGIMGLAKRLPNNITPPTPSFLSLLRSRLKKPVFTVDLRRNASSRFDFGYINE
SMAADNITWLDSNPDSPHWDIELELTAWRGKNPMWMYHKFQATVDTGTSLLFLPDPLASRYWEDVPGVQKSG
LLSGVYKFPCEGSQDLPDLLFKLPTEHVIRIPGPYLNYGPLDTEPSLCWAGMQSAEDMAGTILGNVMLKAVFVAFD
VGKNRIGLANKILHDA

>QLI70913.1

QTVTITKDIPSDAHKCVRYCLIYPGFQDDLGGALACGIPYKNDCYCATEVASASKASSWIERCAKEQCLVGDLNKDR
TSMQNIYAGYCKDAGLPQPSPQSVSATATAAAAPTSKTQVSSWPPSTSLNSSDGGTQPSKTDNTGSASVATTQTT
VVTQTKASSAGVGIIPQVTVQATSTMYVMASDSEGTPTAVKAGLGVAIPVVVLAIAGVGFYVWHRHRRARARAN
HSQSQPGYDEKIHTTSPGGRVPLQMAASFAGGHELHGDDGHRHVQMPESAARRRYEMGAAAAGNSGHAPCEL
EASGQIR

>QLI70916.1

SPAYYWSMQQAEAGEAIITNDMNNPVDVVKVPGGPEIQIAQTGGEARFPGGPSADLKINGQVEVSYVGGNEDTF
NYQVKPIAPGFVGCVQVSTVGCNQDYLEWCSQPGPSTIITCPAGTELPISLTELPNLWNFYDY

>QLI70917.1

VLVPDSFSDTISRLPTCALGCLIPAVSNSTCSIADAACICATANYDLIQGLATPCILKSCSPPEALFTKNLTESACGHPTR
DRSSEYSALAISVGVTSSIFVGVRIIYKVLFCHNNRLGYDDWAILVAFLVNVPSITINVHGLAAYGLGRDIWTLSPDDII
TFIEFFLIDEVLYLAMVSLVKLSLSLFYLRIFPGTTIRRLLWATALFNVAFGIAFVTTAIFQCTPISYTWTQHIDHLLPGHC
IDRNVWGWSNGALSIALDAWMILIPLSQVPRLKLHWKQKVGVAIMFFMGTL

>QLI70921.1

EPVTYCYKSVRTHDEERASATCFSVQGGVFSRVWSHDERTPAKSGESSSETVINGHVMPGLWDGHGHLMAYGEF
LHSVDLFGAKSLSEAKARIKAYIKKNPGVGTKDRWIRGVGWDQDVYGRMPTAADIEHDPDLKGTYMMLDRIDVH
CIWVSQSVLDLLPPDIPDIPGGEIIREPGMGVFCDNAMDYVVKLWPKPDAKSKAAAVKTAISKLNQVGLVGMHDA
SSLPDDISIYRDMASSDDWTLRVYGMYECRQRNTFCPSETTQIERDDSKFTVKSVKLFADGALGSWGSAMISPYAD
HSWTSGSLLVNASALTTLTKSWAAAGYQVNIHAIGDLANRNAIDAFVEALKQQCPLATTDADLAACQRAHRFRIE
HAQIIHPDDQARMHSLGIIPSIQPTHATSDMKYALDRLGPERTDNEAYRMRSVLDIHPILGSDFPVEPPNPFHGIYA
AVSRKNPDTGLGVNGSHQGWHTDEALSLDQALWGFTGATAYGAFLEGRAGLIKEGAFADWLVLDEPIGALDIEKL
RTLQVRETWVAGKKVYARDG

>QLI70924.1

HGVIRSIAGANGVIMPGLGVADGTPRNCVVNSCGAQADTCIIRDNEIASGKYGPLGWTQGSGNVDPNAVIANFM
GKGNAPPRNKGASDSVGVEDNLQGLLTPKERRDNEMNMMGNLFQGIANLPIVGTLGFGGSRKTYPTETMNAD
MRGQGAKSGLPTADENGIVNVVYRQINQDGAGPTTAAVDCESGGKDPKKFKTARVVRDVPGSGISGLSIATNTDY
AMQVQIPQDCACTGEAGGVKNVCILRIRNQALAGPFGGAVAFTQSKASHKRALEYRLQKRDE

>QLI70948.1

SPAPQRVPTDGSQNVIISDWLTATKTVINYYAPDPKCGGHRPWVGMWPADAYNPYAADPKAWAYVNKTQGRG
DIATVEFDNAQLGPGEFKAVFVCKDGKRQPWMLSRTFKIDEAPPSKPDQCVHRQSDWKTT

>QLI70949.1

DCSSYTAYPDDGEIALYRNYNCHGKYSNVGAMNTCVSKVDFDACSAITRKGVTCDIYKSNDCDGGYIATIDSDGYR
YFCGWVVDTVKSVRCRSA

>QLI70952.1

HPTADTGVNLHAIRQDDAGSYARSMLTDLEAAFTVNNASLPELGFTKEWQYICSPNIWTADQVAFWHTGTIRHD
DVRSLTAYYVPHVETTATNTGHKKDVKMVVTKSTTTLQVETKGWTVSAKLSGTGGKKDVAGGAVEVSASYSNTKS
NTHTETNTISHEAFCEPGYACRIETWAFHLGLYAKPAHWPYYQLWSLVYGYDDERYLCDMDVHLRDCDQFTDRL
NKWCDPKDGGLLDDKGGYWVSMQPKYYDVEIKLPVYEENGFQPMSRVVLVSEPISKRSRDVSEVAKVGTKKDITE
AMAKGIKYQFLD

>QLI70973.1

QYKVDTRDDIIASSKSLAYDLMKFYHGNESGQIPGLLPGPPASGTGDYYWWEAGAMMGTYIDYWKLTGDTTYND
VVTEGMLFQVGPQRDYMPPNQTLSLGNDDQGFWGLSALLAAENKFPDPPADQPQWLELAQAVWNTQADPSR
YDETCNGGLRWQIPRTNAGYDYKNTIANGIFFNMGARLARYTGNDTYAERAEKAWDWLWGVQYIDHETWAVY
DGASVNDNCTDIHKTQYSYNAGILIQGVAFMYNHTSDDKWRTRLDSLLDACLASFFPEDIAYELSCEFALGGSVCKT
DMLSYKGYLVRWLGVVTQVAPHTAAKILGPLRKSGEAAARQCTGGASGRECGFYWTEGKFIDPSVDKTSGAGEA
MDVLAAVSSMLIEDVAPPVTNDTGGTSKGNPNAGGKDNGERPVKPVTAGDKAGAAILTILLLVGAGGLFVWMSF
FDPMVS

>QLI70980.1

ERDAGVDESIQRIRHGPVLPPDPTGTGYKRAPRQDGTHDPLQRRSELGGNHDTRAGGNHQPHGLDAQGRGDPD
ARRAGRRDAPGPAPIPAYTPVVTPAPDQDAVLASQGYRQLTYYTCNTVGGNEHCGWHVPVVKAQGVRGDSATV
WVVVGCLAGVFALGLM

>QLI70982.1

HPGEDHAAELKARGEFLQNHVTNLDHCAQELEASGVMKRGIQRRLHMVNRLREKRGLEPQRDDKQSRDWGAM
EASHASLFKTTASCVLAPEEMVGPYYVAGESVRSNIVETQQGIPMEIDVQFIDATTCRPVEGKYVDVWQANSTGVY
GGVINRENGNGPADPNNLKKTFLRGVQKTDNDGVVKFSTLFPGHYAGRTIHIHVMLHPNARPYPNGTIIDTTAAY
VGQMYFDQDLIYSVENLHPYAQNPNPLTLNDQDFILRQDLWQGGNPYMQYKMIGNRLEDGILAWLSYGINPAK
RSRVTPVATRKGNLPRETTDMQRNEM

>QLI71006.1

EPSNSPSASIPTEKPLARSTSISAVLQSGTTLGRSRSVNATTPFITTTSPTTTPNTTTSHTAELTGGAIPQQMQASMA
GLLGFCIMGLVML

>QLI71014.1

MNQKMSAIVYFQEDSTPDSVIEKAKNTLIEAGGKITHSYTIIKGFVVIAPEKALQALQKVQAWGTDYGMTVEEDKE
ATIQ

>QLI71016.1

RRPTAAKKGCTFLTTTTFLRQTTSPHWPQCSFDGTERIYSSTVTMNTTVDCHGCSHIRVISKPNVRCPAKI

>QLI71018.1

SVLFPSSGPVLQTAYIVDDGRSVCDSGRGHWVEGSQWRISCIGGYGMRIATDGVDVWYDTPHGSFRWQHTGGR
SDSAFAWDNWKFC

>QLI71021.1

SAVYARQNEIILACAEEAPSPDFLELSKLLQASNSTSLTRRQSQQDFNFNVFAHVVYYDKTARGGYVEETDIKKAIEA
MNRNFDGSGISFTLVSVSYTENQDWATYRDVEAMKDDLREGTYADLNLYYISSIAEDRGQITTSGRCSMPVTFTSN
GKPVDITTRKDIVVSEEYLVGDGCVIVARAVDGDTVTHEVGHWLGLLHTWKSDCSDDGDMIGDTAPQESPTWRC
SSVTDQFSDGTPANYACGGWRESNMYNFMDYSDCTSTFSAGQKKRMAYFAEYREILGSRGPTHHSSRPAAAAAA
SAPAPAPEAPPEPPSDANEDNTQRLRWFKRSIFEQEGGYDLCMGLAYFKMSGSSSLSADHVDRACGTEIFCKIIEW
DWQGLGPQTMRDRLGFASHHECIAGHEAEP

>QLI71022.1

MTLPDKNLQVKKSDAGHARLMSELGRRYKRTMLASLTGTCSDKNVVLRKEWHALSAGQRRDYIKAVGCLQSKPA
TAPAGPARTRFEQFVLAHADQSLLIHFSGLLLPWHRYFLSLYEKALRDECGYQGHQPYWDWCRSAADPLNASIFD
GTDTSLGSNGKAIPHAGINLTNPVGPPPVSFFLRPAGSGGGCVSGPFENLTLHLEGSNAGASTPLKLRDRPRCLTRD
FSWPILDESNSYPRVLDLIINSSDIHSFLSAVEAPDGVHPGGHSFIGGDSLNLFTSPNEPLFYLHHAMLDRVWAIWQ
SRDWSARQNALDGTLTAKNYPPSANATVDDDMMVGDLGESRRIGEVMSTVGGYLCYVYD

>QLI71050.1

SPVGLRAETPTLAIPAPTESPSSAHQWAAGWKPSFNIHESCNSSLRAQLQQGLDETVELAQHARNHLLRWGHTSK
FVDKYFGNGSTAHAIGWYDRIIAADKTEMLFRCDDPDRNCETQKGWAGHWRGSNATTETVICPLSFAIRRPLSSV
CNLGYTVTGSKLNTYWATDLMHRLFHVPTISEGIVDHYSHGYAGVLELAKKDPEKSGIDTDALQYFAIDVWSYDIAA
PGVGCTGKPPAESASPSGTSTTVPAATGTTSSAASCHTHDDGFIHCT

>QLI71060.1

AETFHRKSNLTVALADVVPACAEDCFISFLSANYGLARGEEIPSLEELCSTDGNTGFTVGEGAVQCIAAEQAVGGCS
DKEADSPAMSKAYYMCRDEPGALTPTHGVITATLAVPPSGGGPISYPAPSKTKRPFTRMHPPTTLIIETHSPTFSPS
WSPDTTFSTAVVSTTTEITGTSSSTAEPAAAASGDSITPVQKAGIAVGVIGFAAVAIGLLLLFRLYRARKKRGGHTPLA
SSPRRRDSWGYKFDKSRRESSSEPSWMANPVHPPNDSAQGPSAAAMTGAIAIGGQAGARTQQQPQAAMPKSP
PPGAYNRSSWRPSLIGLALSPSHSKNTGQGTATPTRPVSKLLPAKPILSLDIPKKPPTPPPKHTPPPPGSSSRKQSATT
PSREYTRIASHPPVLPKLQIPQGHDFLHIPKSTNKPRESTMTEFEEDGRESSTVFSLESQIWSAPSAAPSSAAAKCHA
VDRHGNWMPSDSNLVQAAELEGTTPLSAYPKLATSMAVLPVGTNGRGEQPGQGTAKRPSHRRSSSAPMPPNPA
QQSIRVVTEPLATLKDGRDNVIPRPLFSDMGGNPRNASATASARHSFGRSLTRPRATSGDSGITNISVSSDEEHELQ
PPRRMSQANLSPVTESPRSGNGKSPVSYPKIPGRENGLSGKERSAQPPRMPILYKSAHHRTASGGIGNSVAMLTVG
SLAPNPNPNMPKDTKNATSTSATMAGPPPNAFKPIIPTTKVTTASPHQWASASRGPRPMQNPGLPRTGSPTMRI
VEPSPEPDDDRAMLPSSARPRSVQSVQQPQPQRQQTQLQPSPFPASQQRSQQHRYKVNRILNPINIRYSLNYSLG
LRTNLVRSHNYGHSSSSSSVCNKLLKLRLKLEQARYDKVSNGKLLFHTHLTSVNTKFTRSKKLSHINNMDMQRNLTF
RFNQPLYNQPLYNQPLYNQPLYNGRTKNSPYPKSLLLKLGQKGELPNCNKNNHLNETLGHNSHRETTTHHKSKRN
NNPQCNKPNGQTLPTGSPACPHILTPSAGPNTRAHSGYRRKPASATPARRGRRSRQSQTITSSLPCGLCGDNPYTR
RHNRNSKPGSRSKPLHSNHSRTTNTNTLQFHPDPSQQQMYIHRPQQCAYPYPDPRQYPNQGPGHHYPPSYPRQ
QQQQPQPQGQPRQQFQQPPLDQYFLQQQQQQQQPPQVSIVAKRLGRDRAVNMSISTDCTSSASGSNNRHR
ASGGPMYLSPEVVTPRGSSGDLPSTPTWLPRLTPTRRGADLYLNVQ

>QLI71063.1

MVNGLPGSDYRAPEGHEFIPAGHNDSRSPCPWLNALANHGYLPRNGRNITRKLVEEGFNKTFNFVPGTLEGPTAG
AITAGKRGNGTFDLEDTVKHNVVEHDGSMSRNDEAIGNSLVFDPVIWNRTRSHFKSDTISLRDIAVARLDRFATAK
ALNPQFDMSGDSLANTFVNHAVWQLSFGDRVEGNANTLFTRVIFEEERLPFKEGFVRPCQPITGSDIANMVGKIQ
KAAVEVGMSTILNLAYCS

>QLI71064.1

DPNLPCSSAYLIQNRDTGSGLNELYEVDLASGKTTLVNGNVNAGPPGSAINAIGFNSRDSFMYGTATNAANGTRN
LIRVGAGGKTTVLPIQISFDTVVGDVAANGQYYIRSTAGVGNVPWAQLDLSDSNSPTFGRVVASGTSDSLGLAMF
DWAHVPGAGDYLWSYGFMYPNEDKNVLVRWDIQSHAYVKVRDLGISGRYGAMYASADGYLYGEESGSGSIYRV
KVADSTAPVKFIAKGPASSGNDGAGCGGPLAPLPPTGPLSPLKCSSSGYLIQNTKPGSDLNWLYSVDVSGMATLVN
SNVNAGPSGSHINAIGFNIRDGYIYGTATNSANQARNLIRIGDGGMATVLPYKIPFATVTGDIAPDGQYYILQAFQP
DGAATSWAQLDLSDSKSSTFGLVVASGTSDQILPIFDWAHVPGTGNYLWSFGVTNTGNNFLIRWDMSTHIFTVIR
DLGVSGRFGAVYAAADGYLYGADSISGNVYRVKVTNNTSRGELVGKGPASDGNDGASCPVAN

>QLI71066.1

APAGTSVRNQTELGGYPAETSSSVPDRITGFPGGKPAPAVPERTKQEEEEFQNGLDKEFKENKQK

>QLI71074.1

QPHYSGPGTSKHANKHAHNNHKHARNNVNVPRAVVTVNEVVTETVYVTEMIDETTTVWITPGQEAKPTPTSAG TPNAPGNFYETPSAAAPPPPPAPEAPKPSPSPSSQAAAPPATTTSTSVYVAPPPAAAPSTTSVNVPTPHVDVAPPPP VSNPQTSSAPSTGGGGPGSDTITGDFTWYDIGMGSCGEDDEDKALTECIVAVSHIRMGQQSNGNPMCGKTISMS ANGKTITGTVKDKCMGCLANDIDVSKACYTQFFDLGVGRTKIEWWFN

>QLI71087.1

SSLPELPSFLSNTSQSVFFPPLKDIAVEIFNYPELGLNEYYAHQLVVDYFDQVEGWEVTPHAYGMDTAYTLEFEHRPQ GYDGALKSIGFLSEYDALIVGSDPLVGHGCGHNHIVLNGIAAATLASRALVEYNVPGRIKVVGTPDEENAAGKFKLK VAGAFDDADIWLIAHPSSVNTIQPLGSRINISPHFVGKSHQEAVRKAYEAIVAVDKIATSLPGMRSSVTKIQNVGMY STNVLQSQVNFGVSGSDMATVNRTVSDILDDTFPRVSFTTRQDPHGIAIKIHGPGGHASLTEKTPLDLSVATFQAFS NRSGVSFYVPGNTSATELDITFDVRSRYMVDLPAVVDAVRSAVGKLGSRVTMDLRYPNVEVPPFLPETWIDLVGRP AYNLSGWQITDQALADSDIAWVQGAHVDPQTHKLVGMEKVVFQPNYNICEPGSKSCPFNHEPGFLRLAGSEYSY TQTEIVARAQAQLAVQLLTDETMYNSATAILAKNRVIEE

>QLI71088.1

VPSCYSLFRNYLAARKIGLRFHVIPISHLNHFWMLIDKKVLGYVKLVFGESAFTRYNWMGWELHDRYYSHHELGDA FMLVTPGRNWLYVGHPEIVMEIVRRRDDFPRCVELTQVLDVFGPSVGSVEGQRWVQQRKLMASCFNERYNELV WSESISQATDMIRYWSSRPSVRSTADDLRGLSLGVLAKAGFGKSFSFQGYEETSHADPAASYKDSLQLILENCILLIA MGPKFFTNTPWLPFKWRQLGEAVKAFQRAMTDMYESEKRKVAEGTSDEGTRRTFLSSLAKASLDAKQGEGLTER EIYGNIFVINFAGHDTASHVFTFAVYFLASNPAVQDWVSEELRHVLGDRPPHEWNYTTDFPRLKRCLAVLYESMRL YTPVPVTKWTRDKAQTLDVGDKTLVLPPNTMICLAYSSLQTDPRWWGSDSLTWRPSRFIKGEGTDLDAEVFVQPR RGTFIGWSEGARDCPGRKFSQVEFVATMASLLRDWRVDPVVFEGETMDGARRRVLDLIDKESAMVLLIQMLHPE KAPLVWSTRES

>QLI71090.1

GHEQKSLAGPHQSLWYNMLPGDGGTQADSVFSGISTFGRLPYEPCLKNRDAKYDIAFIGAPFDTGTSYRPGARFGP SGIRQGSRRLNLYGGYNVPLKTNPFNSWATVLDCGDIPVTSYDNTWALKQIEEGHFEILSRSPTTDADKRGPAIKGR TLPRVITLGGDHTITLPLLRSINRAYGPVSVIHFDSHLDTWKPKVFGGSPSEVASINHGTYFYHASQEGLLANDSNIH AGIRTTLSGPSDYDNDGYCGFEIVEAREIDTIGTQGIIDKIVSRIGTQRPVYLSIDIDTLDPAFAPATGTPETGGWSTRE LRTILRGLENLNLIAADIVEVAPAYDTNAEHTTMAAADVLYEVMSIMVKRGPLSNMMSAGESYEL

>QLI71095.1

VQWYLEDTYNSTNFFDKFDFMTIDDPNSGYVNYLGRTEAINAGLAAVQDKEVVLRVDSKSSFTTAERRRGRDSVRL ESKARLNQGLMIARFTHLPQSACGTWPAFWTVGDTWPQDGEIDIIENWNLRGVNNPAFHMANAASYGSCRIDS ADQSGDLITPNCDNYYTDYNTQWLNQGCVVKDNGPSGGSGGVYAMEWTSDHIKIYSWFPNQVPSNIGSSSPDT STWGAPTMHLRKDLCNIDKIFKPQRIVLNIAMCGNPVEFSAWEGTCKNTHGDSCRDYVGQNPDAFKDVYFQVQ DIRIFNQDAPKTTATSTTATTMASSSTSKSEISTASATPSTKASSTSKSEISTASATPSTKASSTSKSEISTASATPSTKAS STSKSEISTASATPSTKASSTSKSVKNTSTWKSSNSTLSTVSKSAGSVSVSIASKASDSTVPTVSTQVPVTFSTSVPSLST RWPNSTIASSVEMTTSTVYTTSTRTITSCQPTVTSCSVGKVTTVTLPLYITICPVSAVETKTPAPKPTKTSANGGNGG NGPEKTTITTKVTKTYTITSCAPTITNCPVGKVTTEVFTTTYCPGEETAVPTGSNGGNNPNGGNGGNNPNGGNGLD KTTITTKVTKTYTITSCAPTITNCPVGKVTTEVFTTTYCPGETAVPTGSNGGNNGNGGNSGNNGNNGNGGNNGN GGNSGNNGNGGNNGNSGNNGSNGNNGNGGQNGGNNGNGGKGPDKITITTKVTKTYTITSCAPGVTNCPVGK VTTEVVPTTYYPGKETHPTEATGPKGGFTAPPPFHPSKTTIHNTQTIVSVPQPAKPSGGNNNNSTTNGTSIAQPQP GPSGSQVCVGPSCGAQPSSPAPNTGNGGCTGPNCPPTVVSGAAKQSLSALVVLGAVAAMML

>QLI71096.1

AISPEEKQEWESCTDDLIYAYNHGAARVEKPCVFWECLDTNTNKYPRGGGITAPPTLSAAKDNLFLNLIKTFDTPAS
GDEAACGMWACLHNDADEYNRGGVVTKTSMMLSPLCNVRGRTPARIKAKEAGRLWDSEGAGWWCMM

>QLI71103.1

YTPGRHHHLHFPRANTTSADGLTTVTIKTTQVHTITSCAPTVTNCPAHPTDVATLPESQKTTATVTDTVVLTTIVCPI
SEVGKVSSSIIDKASTGGLTGTTLSPTTKASPAPSTLPANSTATSAQPVVTSKTVTLTLGTGTSASVVTSTILVTASQPS
APTSEATADPTTLAPTGSDQVTTLTSTTKVTRTVTVSRTHPTTSAGGNPGGNPGGNPGGNGTCAPSTVTVTEAKET
VTLPASTVYVTIGGPTTTNAGNPKPTSAPGDGDQTSSDCTDETTTLQKTVTVVPYPTGNGTHTSGSPKPSGGYARL
R

>QLI71113.1

YESLTPRDEGLAPRDDQCKKNGEKCRREEFCCSKFCEVWENQTEGTCKDMPHKN

>QLI71115.1

QVATLDPAEDIIPIIPPDVLTNVQEERKYDACHKIDTSVAGGLIWPCASQLKILESCPKSAKTAEERKAQRDCICGKGS
SFLQDAVACSECKIQNGLQPDNQRDFWKKSFGALDKEYCQSDDVAVDYKEFTKDWTPPKGVIAGNSLDGQIGNP
RDYYKQAGVAVPEKQGPGKSTPAALHPGVNDNTVNADISDPLAGQVVVPAYVAVEVNNKPSGANTAPVTTTPSA
AGSQPTTLVRVTSGDSTAAATASLSPSSSLVPASGSNGGADVAHIIMINGKACHVYIIWVIIDCAPKKDAQGNLYIEY
KNNGIDNNKPVSMLENKKQFDQIKDTVHDASKDDNSAKELKDIVGTEADVVAAPGNKKPLPPPEKPASGEPQAP
NGAAGSGTSDEPVTGTTVNTPDVSDDKDECVNEDLTGGANTPDNGSTNTESIPSGSTGTGAPNSKPAADINTPA
MSDDDECDNEDLTNGANTPDNGSTNTHNPENILSGGTGTGAPNKKPATDNNTPAISDDEDECVNEDLTNGANT
PDNGSTNTPNTENVPSGSTGTGAPNNKPAADNSTPAVSNDEDEFVNVPSGATTPDNGPTNVPNTQNTQNPPSG
GAETIVSSLEDTMKNPNTPQGPKKTPSTQNPPSGGAETVVSSPEGTMKNPNTQHTENPPSGGAETVVSSLEGTM
NTPNTPQGTEKTPSTQNAPSGSADTVAPGPNGTKTTPNAPAQNSAGTPGGQTGSNTPSNSGSQSETKDCECASG
SKAPERKDADEICAKREKTETDCNKESGENMRKCFCSEGSFQNQRFFEEAITCSRRSDNCRLGEYGAQVFFQIQHLY
CDLKLFGNDYAAAYKNVLDSWGRERAPTASLM

>QLI71131.1

EPTISFPFNAQLPLAARIDQFFSYSFSQYTFQSDSKITYSLGDHPSWLSLESGRRRLYGTPREGDVPSGQVVGQTVDII
ATDDKGSKIMKATIVISRQPAPEVRIPLEDQMANFGNFSAPSSILSYPATKFKFTFDQNTFSSSGLNYYAVSADSSPLP
AWIQFDAHSLSFTGRTPPFESLVQPPQTFDFSLVASDIVGFSASSLTFSIVVGSHKLTTDKPNITLNATRGTAVSYDGL
ENGIKLDGKQISPGDLTVTTKDIPSWLSYDDKTGRLQGTPKDGDHAANFTITFKDHFSDNLDVLVVINVATGLFVST
VEDMKIRPGSKLNLDLTKHFKNPADIALKVSTSPKKDWLKVDGLKLSGEVPKTSTGSFKLAIDASSKSSGLSEKEVVQ
VYFLALDGTTTTMTSVSSTTATTTARATATGSDIPDDRQTQPGHMSTGEILLATVIPVIFVAVLLMVLVCYFRRRRSG
QGYLGSKYYRSRISPPVQSTMPADFSDPSMREAAAMGAFVHTETEVFKPAKSAFAEESSPISFHRRSSETLGGLSTS
EMPQSIMVDAARTTTIRSVSNVTSEDGRQSWITIDGAPGGIAQSDRSSQSEVTFPEATRQIFPGADYTPRRDTGLEI
TLPTLNELPSLQPTPLLSHDSMSLFSQHYLGHQSAITSSSAALPIQDDHQYTTAPLGKWPTGSTSIVEGSEPNWVTLA
KSETGRSMSEIRKPDAVAVKPSQPWNEADSLDGGKSVTTEASFASSENWRIVGRLGPTKTERSGKEIVDDGPVHP
DRPGTSRGAAQQADHEPSTELASPNRWGDVPSPLASGRPAPSMSRFSKMSGVGDEATHMSGGRGLDEAPWIR
DHSGKMSDGSFKVFL

>QLI71135.1

VRVSWDSGYDRADRSLTEVSCSDGKTGMMPKYQKQGDLPNFPNIGGADAIAGWGSPNCGSCWRLDYDGMSIK
VLAIDHAGSGFNIGQTAMNALTNGRAVEFGQVDATATRLTPGDCGL

>QLI71138.1

RTIATRQVSGVLDSPEQFALFAQYSFSSYCKQLHDTSLNTPVCTNSDKTPCPNFENATTVAEFAANDRFQIGGFVAK
NPGQQHIAVVFKGTDSAGDIATDAAIDQIDSDLCEGCKVHKGFGRAFNEIQGQLEQTIKTEKAVPGQENWRLVVT
GHSLGAGVATIAGSSLRKQGMALDMYLYGSPLVGNDKFAKFVSSQGGGFTARITNARDPVTAIPKNPLSPKTYKHI
SPEFWFADGVEGPQGLYTTSRQVCSGEKDCTSASCAKLSKGILLSGCNFTDHTRYAADLKPCDGLQGSTPLDIIPGL
NDTLEAAKENKDKKKDKKKDKKKDKKKDKKKGKSRERIVHLE

>QLI71144.1

AAATTTSGISELADRLFNGQGSAFEFVLTTRPEDWSRWNPPVNDNYTVQGARGKIRVEGTSLNALARGLRHYAND
VLQMDEFWFVHTYKTAPQRLPAPKEPLSGASVVPWRYNLNTVTFSYTFPWYQWEDWEKLLDWAALRGVNLQL
AWVGYEKIFLDSLRELGLSNEDILPFFSGPAFQAWNRFGNIQRSWGGKGDLPLAFIEQQFELQKQIVTRMVELGITP
VLPAFPGFVPESIKKVRPNANLTVSPNWFAPAPDKYTRDLFLDPLDDTYAELQKLFVSKQIDAFGNVTNVYTLDQFN
ELSPASRDTAYLRGIARNTYAGLTAANPAAVWLLQGWLFFSSRNFWTQPRIDAYLGGVEDDQGMLVLDLYSEVNP
QWQRTNSYSGKPWIWCQLHDFGGNMALEGRVQTLTSAPIDALAQSKSLVGFGLTPEAYEGNEVVYDILLDQAWS
ATPLDTQAYFASWVTKRYAGISSIPSELYRAWEILRTDVYSNTRTDIPQVPVATYQLRPALSGIANRTGHFPHPTALH
YDPLVLQGAWKLMLEALTRQGSLWKVPAFQLDFVDVSRQMLSNQFDVLYADLVNAYKCSTGAGGGRELRSNTPS
CDVKAAGARLLSLLSTLDLTLLTSRHFALQSWVDAASAWGKAAVNEDLFTFNARSQVTVWQVNATNLNDYAAKA
WGGLVGSYYKGRWSIFVDALVAASKSGSLDEGALARKLQVFEAEWQAGEQAVEQATPQDFRAVLAGLQGSWPE
LFLKVQ

>QLI71149.1

DSALPDGGLGNMHSYLSQHFPAAHRIVLNTVESSAEDVMITVPDLRSSARNLLQSLDRKKDKLEGERAVDVKNHG
DGPKQQGPNIPAPKLVFLCHDIGGFVVKQALLLANSEPCFEWVARATAAVLFFETPHTVPTHLSWERLMVRMLGQ
TESVLDFASFIRYLSDYASQLETEFEGISGTHHMINFLSRESHYAVVTRENLPCPIYSCDQIHCLDCDPKEMWKVSDQ
VSWADTIRRVISTHLSLDSKGRPTRSYAQFLDVFGAAESKIAPAWNASTPLDPDKEGIFTKDTFRGLLRSILSGCAGK
ATFCIINGLDHYTAEARDELLRDLCEMRTSSQAEFKVLTVGSASHVQYGKPGYPLVVYSQERILAAGELERLAKTRIRS
MAAVNPAWKTLDLESEQVKRLWTGSATLFELCQKMDFLEKGETLSTTDEAIAMVATLPADFTVFFRTLATKYDFLR
DGGLGAAVLPWLTRSVRPMTVPELAVCAALARTSTETLTLQHLQKSILWDFHRDLENELGPIIRISGETVEMRHRIY
QDLVLADSNALGKANVHLDILTTCLIYLRQISPSWKHGTEFRQEHRLAEYAALHWPQHYHEVRDKATAKPVVLSLL
QNEEQFTVWLDIYSHYATRSAEEESFPKTPIQVAAYLGLTEVLSELVANLHPSNEDTELSKAMEYAASKGHANVIRLL
AELGVRAEGALLRASWLGDNATVTELLKSHRTYINSRGGPDGFYNPLLQAARCGHVDSFAKLQSEGADTNAVAAH
TNLTALHLAARIGQRAIVQILITAKVPLASVDEQGYGALHYAAEGGFEEIVRCMIIAANDPAILADVSRVSQVLLAND
QTRDSNTPLHLAASNGHLKTVETLLEMKAEPGILNDRKYTPLHCAAEGGFPSVVKALLKVGTVAENQGEQSTIEVQ
PSPVELAVKNGHLGTVRELRSSRFYDDSEVLSLAFATACREGTNDCALYILHWYSEVSFAGGPLLDVDGNTALHLAA
RDGNVRLFQKLMDSKYVPIDSLNKKGLSPLHIAASSGSLAIIQLFKDDSALGGTTANGTGRTLLHIAAEAGYLHVVE
WLLDYTSLKKETATAVKDTAIMLAAVGKHEPIVKLLLDSKYAVPTGNLLHVAVLNSWKDVTKLLTSCDFSVLNWIDD
TTSNAALHLAVVIKNPTW

>QLI71150.1

SALPEVVFRPTTDGSLGAVASESAECSAIGRDLLARGGNAADALVGTTFCVGVIGMYHSGIGGGGFAMVRDSAGN
YEAVDFREAAPAAGHEDMYQGNVRGSIYGGLAVGVPSEVLGLEYIHSKYGSLPWKTVMQGAIHVARHGFRVSND
LVRYVERAVKERPNFLVEDPNWAQDFAPNGSLLQVGEIMTRKRYANTLEKIANQGSKVFYTGELAETLVNYIQQTN
GTLTLSDFKNYKVISRPVKNVTYRGLHLYTMGTPASGSITLNILKIMEQFDVADSKDTNLTSHRFVEAMRFGYGARA
ELGDPAFVEGLDEYEAHLLDAAHAKQIRERISDEQTLPVREYDPKGVALPESHGTSHIVTADRSGMATSLTTTVNLLF
GARIMDPSSGIILNNEMNDFSIPGVPNEFGFQPSVANFIRPGKRPLSSVAPVIAAFPDGKLFATVGAAGGSRIISSTTS
ALWRTIEQGMTMKEALREPRLHDQVMPNTLLLEYGFDAETAAGLGERKHNITWVGPGLSAVQGIRRLRDGSFEA
ASEPRQKNSGGFTI

>QLI71164.1

DNCKPGLSYCGSTLLQIGNYSDQVDAALKAQSLNTSAQVTANTLFDCIGGPSGDIKVISYCYYGCTTVSVGNSDYCK
NTGQAANSTSSATVLPSPLSTTTVVTYPSASPTTPTTSSNDSNGSYAAPIVGGVIGGVVALALIGTMVFLFFRNKRHR
QERSDKVLVPSVGHRPIGQQKPLHVSRPPNSRDFGSTAFPIEDERPVYEVPGFSEPRIHEAPTDARQELY

>QLI71171.1

WDVTSYRYISDCRVHDDGAYRYYEGSDSKTCHNLAGGDSGASCTQYTNGGDHHANCDGERVGREATGFVLAEH
SSLLPATFDGLRYAYITRLKLATKQVNSGIYRLWEAEA

>QLI71174.1

SPTPTEQEPPTKRDSFPTVTVSGNAFWKGKERFYLRGIDYQPGGSSANEDPLGDTDVCLRDIANFKDLGVNTIRVY
AVDNTLNHDKCMQALQDAGVYLVLDVNNPKYSINRGDPGPSYNAKYLQSVFATVDMFARYPNTLAFFSGNEVIN
DEKDTDKSAPFVKAVTRDMKNYMNTRGLRKVPVGYSAADVSSNRLQTAQYMNCGSDDMRSDFFAFNDYSWCN
TNFKQSGWDQKVKNFTDYGLAIFLSEYGCIDNRPRKFEEIEAMMSSEMTGVYSGGLMYEYSYEDNKYGIVELKGGL
KAKTVDKLDEYDAFKSALKNNPAPTGAGGAASTTHSVSCPTSNAGWQVNPSLVPQMPSQAEKYMKDGAGKGPG
FDLSGDGSQNAGDSGTSTASVTGGAASPTGSGAKESDSAGVTTFGPVEKAPFIITGMVVFFTLFGTLLL

>QLI71178.1

HMQMKYPPPLRSGFNKFTTNQDYDMTSPLDPRGSNFPCRGGLKVIDSPQGQPVDEWTAGLQYNFTVTGGAYHK
GGSCQASLSYDKGSTFKVISSMEGGCPLTPGDTSYKFKVPEDAPAGDCVFSWSWFNFEGNREMYQNCAVVTIKV
DPRRKKRGTAMAMSQRPDMFVANVGNGCSTTEGTDLKFPQPGPVVTIEKGAKLKGPVGNCQKPAPGGDNPTEP
APSASSAPAPTSDRPTVPSPSKPAPTNDKPTVPSPSKPAPTNDKPTVPSPSKPAPTNDKPTVPSPSKPAPTNDKPTV
PSPSKPAPTNDKPSIPSPSAPSKPTPTPSKPSPSKISCTGGKFPGDQNKPAQPPSTGAEPPVQPEPTSTSTPGDNNDV
CTPGAYACTPDNSAWQICVVTQVWIRAGSCPEGTSCQFDESTNTPRCR

>QLI71185.1

VPQLEVKGNEFYNSKTGDRFQIVGVAYQPGGSAGFKLDSPDPLSNPDACLRDAALMQVMGVNAIRVYNLNPNKN
HDLCASIFNAAGMYMILDVNSPLVGQALDSGKPWETYYSAYLNHTFAVVEAFANYPNTLLFFSGNEVINNIESAKY
VPQYLRAVTRDLKNYIKKNLKRQIPVGYSAADVRDVLWDTWNYMQCSTDGKSDDMTRADVFALNSYSWCSIKAT
YESSTFDKLTEGFAKSSVPVFFSEYGCIEPPTRHWNETRAMYSDKMAPTFSGGVVYEWTLESNNYGLVSINGTTLNI
LGDYNRLKAAWATIDWKSVQTQKAAKKDITPPTCAKSLLTKNGFDSNFTAPVPPPGAQQYIDNGISPKPSGKIVKIS
DFNVKMTVKDSEGKEITGLKVVPVPDDQFNMAGSNKAETGSIDATSNNTGSSTGDKKDSSAVLHTPMMLAAVVP
LIAMLFV

>QLI71188.1

TAIDKRIVGGEEATLKDLSQYPYLVSVHPMGGSYHFCGGILLDSTTVLTAAHCLYRPVKCREKPEDCQGDVFLVRAG
SLDRETGGTVSEVASFKIHPSYDGHVNNAYPHDIAILKLSTSIQESNIIRYGYAKLAASGSDPVPDSIALAPGWGLLEF
SGPMPDKLRQVYVPIRARDSCKGATEAMVCAGGDGKDTCTGDSGGPLIDRESGQVVGIVSSGVGCGGTGLYTRV
ASYISFINENLGDKGTPTREQQLQDHCGRSGNDKDACMFAARRCTGQVKPDATMLEFLQCVDVMQVCGEQDVA
DKTNQCIANAKVCREQEKLPLGDLDKLSQCAKKDL

>QLI71189.1

SCAYGTILQPREEGGAVKVNTFGYIGMKGPTNWMALDPGANSLCANGTSQSPVDMVPGSFSMIPGAELGLDIPD
MPEGTEFENLGTTVEVVAKGGNMSFDGVQYRLRQFHFHLPSEHLDNGTSMAMEIHMVWQGEAEQVAVVGVF
VDLDEGPGRAAKSTAKTRIGGGRLPAMKNGFFHVTAPATAAKKPSALLETVLSSVDGIAEPGTVVKTQPLVMSDLV
STLSSGSFQTYEGSLTTPPCSEGVRWLVSDQKLSIQPGTFMKARSVIGFNSRFPQNIPGQRNILAVS

>QLI71193.1

APYNVERDLNGEQHDLADRDFDIRNALPLEENSENTGGVANNNANANGEAATQVAAVSATQTAGAGAAQSSAA
AKDGAAKKKGKGKGKAKGKAKGKGKGKAKGKGKAQANGQGNEAAAKGAN

>QLI71195.1

INLFSPALYNVRLGFRWKFIDGPESGLKDITFPVNMAKTSREQGYYLSQQFSFHGVRRQGHIAIQPRPDNSKGQAM
LRANFTSHQAGTATCHPACQAGPGRGGAEGSRDGVTCAIDVPADYAHTFNLTVENVRDANTWRGLLVDTVTRK
THEIGVWTLPTGAGDIQRSRFGFVEYIHQPGSRGGCENYPKLEATVYHPWSNSAGAGELHNVGSMDLGLCGKNIF
KKIKVTNGFTVYYGRG

>QLI71198.1

QFTSPAAGAVLSAGDKVNVSYTTDLKNYTIALWQRAEGGGRPTLGSVVYAAAHGPSNFTWTVQTYALDLAASPTF
FFWLFEGGPSRQGSDPHQLSSGYFNVTDKSASSPVSVSAAESALTTAAAAAPAESAPRDPLSVGARAGLGAAVSVF
CLAVAALVFLFFWRRAKRRARKDGGGGSGGAATDYTESVAELQSECVSAGGLGKLPCRVYRPVPGDASGRPPALA
ELPA

>QLI71203.1

LPRHDVRPDTCCFTLHDSSTGEIVRQETNSGFLYLGGSQPTGWYCIKLSDPNKILWDAFNNACFVNPDKVFQCLDP
TPSNDSWGMEQSGSDVLVVVNRDSSFKVCPTDSGNMIYTSAKKDEGCRSLTLKAEGLKGSCGSFQG

>QLI71206.1

MRPWKAAGPGDSRSPCPMLNTLANHGYLPHNGRNLSVKHFGDAVVEALNAAPSYGTLPARAFIKSWGKDFFDL
EDLNTPVILQHRGSLTRDDVTPTERNIDVDVARVSALLEDSPTDYVDAASIAKSRLRVEALSEPERLSSWDQLLAYM
ESSLVLLMMKEGEVPSAFSFPSAKTWTAPKERVRVWLTEERLPDELGWKRSERKLGSLDLVPIMKAIFDEKRAQSG
KGRLWKSFLSLFWGSRDEL

>QLI71210.1

GFNGGSHKNIAVYWGQNSFGQGSGPNVQQGLAHYCENADMGIIPIAFMNGISPPITNFANAGDKCDKFPDNSN
LLKCPEIEKDIKTCQTKFKKTIVLSLGGATYSQGGWSSTRDAEKAAQSVWDMFGPVPSGSKVDRPFGSAVVDGFDF
DFESTTNNLPAFGAKLRSLMDGGGGKKLYLTAAPQCVFPDAAVGSALNAVAFDFVMIQFYNNWCGVSNFKPGSD
TQDAFNFNVWDKWAKGSKNPNVKLLLGIPANKGAGGGYTNGEKLKAVIAYSKKFTSFGGVMMWDMSQLYANE
GFLGEVVSDLA

>QLI71214.1

GPTQLPSGSRITIGYRRVHPNQAAKYKEAGTLTDDPITGHTQIGEGVYLTAGYGQWEGHAGDVDCAVTADEEEFR
TVPKVILPDEQLLDTTHEEIDAYIKSQDSSWDPLKTIRISHIPKYGDGTVAQMAIPQALLNSRGGSLQIQVDCEDAQG
PTVDYGQWENTKGDASIMPARADSNKIFDLSNELDASRDLVEQVSSPEHIAQLELRATNAMYTAREGVRHYVKTA
EECKGDSVLKGLCKDQIKYYLQDRQAYARFLIRAAERSFEMLTTPLNAVIDDAARQRAIQALQEWATRLQRQVDAR
AKNLEALKKGEPVKGLDDPKERDIEKTALKRELKAMKKKLSKLVDKAATFGAKAEQGSVPPKEGQKQTKLQQLREW
LQKNKNRFFPSGDGDKDEYEPTAGKDDKDPSRREACVKEQYSGDASQAFETAYTVIEERDDEDRICPEGQEGAAA
CAAAKFKGDGQAAFSDAYQLLKAIDSLELVCPPPDTAGDAPGGSSPAADLAGLRSEGWRLFVQQALGLGYTRERL
VHILDRGVGLLQLDNPRLYDLLRQHPAAFNVLLGLRRDGVRLSSCLPDGRLQRRANGAGQSSDHCEIVRAMTSGK
QADNATPNDDGKQPPTKQEPPKEPENKFHDQVKKLQHVSSGDGSWTGGILGAFFAGPLFAGSILATSPEVVALIG
ELGASLGLAGTAAGSFSESSAVGGALSAVRSAAARSVGSILRRTIGRLNYLAHRAINPVVRRIVSDAVRAGTRAAERA
PLLGAGSLVH

>QLI71226.1

APNPNSPSKVKLHGVSLLGSGCPAGSADIQVDATGTLFEATFSQYEVQTGPGTKAADWRKNCKLTLNMEFDSGFQ
FSILDTDMIGFAEIPKGANGQCSNVFSFTGNPQTVTSAIKLPGHYSGNFDLHSGVGIESWSPCGGSTAILNMNTAC
AITPTHLPALIAVDHISGKLRVKFAVQWRRCH

>QLI71248.1

NPSHYRIASWPRAVQSRWAMMRDERVSRGHLTAPKTDARTHGHDDLSCGIPDESQRMWFTQGGCSIASQDLT
YVDTSGEVPVVRTAAEGHLVKLTCEDVFCPIPPRLDCYEQPGICSANEWCMIDIHERWGPWAMNRDGSTPQWEY
CYKAADFVGNSSDQALIDSYYSECVASTIGDYGIKLGPKIEAWKPIRGRCVKFRQAEQSCIGNPLEFGAYEREFGLNY
RREQDGAPFPRPLVCGPELTCTGPDFDVRPSTCVHQRPQDICFAGPWWDSTQCPRTEPEAPRGGLTREQTVETLR
RAVLLYPGEIATAADCAYWNRSSAVGISVLATQHRFYNIAAVLWPTDLFGEIPSFDELMALIPDPNLFGSPADCVAQ
ADIPGSEINEALAEGGTLSNQPNQVWSLVHFLMHNQPIVLSSKKIAASRAMAAHLSESFWCDDCRGFFSVGVIERY
GLPPESSNPDDQARWWWWGHNVASEHVASTRGGHPWIHELGDEGVAYFQNPYFMTWEDAVAEWTYTI

>QLI71271.1

KNVTYVTEVVSAYTTYCPEATEITHGTKTYTVTEPTTLTITDCPCTVTKPVITTSAVECHSCTAAVSTPVNFPNSTVASP
TGGVTPTKPGNPVPTAGAGKAAALSGAGLAGVVGLAAFIL

>QLI71272.1

ANSPKAPALPQTDGASNLPQPPEGSKLLHIALGFGIQNYTCASVGASPAATGALAMLYDITSFYPGQSSQSLSQKD
WDSLTSRVLQTQDVPLNFNSSTEGRVEPDSPGASQTDPFPKDAPLDLECSKAPFLGHHLFNSAGVPEFILDGGKIN
VLAGKDAGVDAPANADKGPDGTGAVAWLKLSAKEGTVGDAKLVYRVSTAGGNSHGCGNAAGQDSTKYTAMY
WFYT

>QLI71281.1

DALVPRRDTNNTSGQNLLTSEVADFVKEHMDFWKIPGMAIAVVDKDDIFTQGYGFSELPDKKVTPDTLFYGGSTT
KAQTAACLSVLIKNGSYDALANGWSTNISSILKEDFVLEDKWATEHITLDDAVSHHSGLPRHDRALMREKNGVPLS
TADIVRNLRNLPYIHEPRVASYYNNHMYVVLSHVIETVTKKGLQQVMRELIWNPLGMNSTYLGLADAQKAHKDLA
TGYLWINQTAQTNCTRQANQTGQFKPVTYMPMTDLSGSAAVISNVRDYAQWIRGLVNHTHIFSEDVHDDIRTAR
FIFSPAPQFGYDLDLYSLGWFRNLLKGHLFYRHDGVMLGFRSMVYWFPEEKFGFVIFANSDTAATANSIIARKLVA
MKLKIPENEVFNVTASREGMKTPEQEFEVAVKKYYPNHTISDPSFSFKEMEGTYVNAGYGTMTLRSAPGPKNETVL
IAERSDMSWDYTYTFRHIFGDKWISGVSWSEERFNTDSFGPAEFIKGTDGKPAALKMTSMQGTTSEGNITYSKTG

>QLI71289.1

ADNGIESTKLAADDLILECESNQACQLVANAVEASGGVLHYLYESDVFHGVSFQLPKSATAEERRALVAQFKCIKAS
WPVEDVNLMPEDIAKDQPEDKLQVLKGGEEKRAPFGRRADDDRVETPWNHLMTHVDKLHEEGYLGTGIKIAVID
TGVDYNHPALGGCFGPGCRVVTGENFSNQGNRSDPIDCSYKGHGTIVAGILAGYNKADGFVGAAPNATIMAYRV
VNCEVQGREDDMIAGWLKAKEDGAQIIVSSVGFQGENWAQRPLAVVAARIVASGVPCIVALGNNKDKGLFYALN
PSTGRGVTSVNSFGRSLVALEHRGEYSVGTGSEPVDFAFEPGTGLHDWDGEWRPVHDVDADFGDKPDDELTGAE
EVPDYVDEDTVLGDNCKLSPGNSSTGFARDLAGHIALIRQTPETNGCHFDDRVRNAVARGAAHILAWQDAPNYI
MIRRQDAHGVRAIGITQTDDGRAMARALASGQPLTARRAGRVRIDTGVVAPMSAYGPTWDMDIKPTIGAPGHQ
VPVTRKGGGYDSDSGTSFAGPLVAGVFALVAEARGTFDPALLNSLVTSTAEPQSSHGRLITVAQQGGGLLRAWEA
AHATTLVEPSTLPFNDTDHRPDSIGLRITNAAKTEVTYRLSHLAATTLYTLEADSIRPSEDPAGELDKLAMMDDLDEV
DEAVDAAAHVEMSQSSLTLGPGQSATVDVSATDPSGLDPERLPVWSGWVSIQGSDGTNLTVPYLGLGGSLRSAA
VLDPASKLSTLAGSDFILPNPPEGQTPGPAQGIAEPAAAARSEAISTSFDLVLGSPQVRVDIVPLDVCPTPALVGTRA
NATEPDLPQACVPDSIVTEFAGAKSIGQLPGYPRHYVKREEIQIRWDGAFAPGQYAPPGRYKIVARALSIMGDAAD
EAHWQTVESPVFSILYEGNVKRAPENQPAQPAQTPEEEDAPAANAGDALAADEVKPAPES

>QLI71290.1

FGVTNTHDGAKGFIGYGITLYEPLCAWPCLSAITAPINCTDGDLANTARKRSERSVPDAVPQRREAADAGEPVYPS
GTGWQVTAEPTRQCQSRNEYFMKTAAFCLQTRCRSLPLQDLETFYEKQFPGVNKSALAPEYHSYTTLVAAITSVPT
QPLKASVILNYTAYIPDETYNIYYSTMYTYAKTEKRGSWYAMVIFVSGAIIPIGVSMLRFLPWPQRWLTKFHAFIIDPP
LLGTKHETTVWGLGTVPTRGQAILLLYMWVINVVATVQGIDYAFPSSRYSTPAEAYKTYVANRSGVMSCALFPLV
MLYAGRNNLLLWLTNWSHATFLLIHRWTAVLCMLHAVVHGALYLNSALLNYHGWSYVEQSTQRYWQLGAAALL
ALVILCVISVQIVRRKAYELFLVLHILLAVVALAGAFFHINLKYTSDYGYENWLIMALAVWAFDRAMRLARSLRHGVK
RAYLSPIDDEYYRLDIPNLSASGHVYLHFPTVSSWRVWENHPFSVATVSYRKSKLESTVQMVETKEKAKNESTIHTSR
GSSSAASISEQTQAEQLGSSGVVLFVRKHAGMTALLAKRRAREKGVPVLVEGSYDIGSTFLQDQHPKPTHDYPNLIC
IAGGVGITGVLSALDHFNNTAKPCGTRKLLWGVRTMPLVHAVESMLGYDCGRGLERRWGNLEVTLSVGQRFNLR
HVLEKELRVQRGGTTVVVCGPTSMADDVRYIVSALARHSGDNGPILVKLAIESFSW

>QLI71302.1

SRTVMRDGTDGKGMGNPAKVCYPEVNGSGVVPPCISISNIESACQPNGTESIDFNAHAQCMCQGSYFVDWRGC
QNCLFVHGFRSARDHVYWERVLSVASDSLCQGTPTAPFSAIFSSVQANTRDAPFVTTGDTISLDKFPGKTDVSLYYT
ATGPQGPGAITGGAATATRVSHPSATNLTASNTSGNSNKNTPSRTGSGSVSENASITRPSSSTSTGGAPAQGTGM
AMAIAGAALVLAL

>QLI71314.1

LPTEAVDVQGGLEARGNLPDLNALQSKYARGIIAQAKKDGVGAHGCQAGIATALTESSLVMYANSGVPASLKLPH
DRVGSDHDSVGLFQQRASIYKNVQCDMDAACSAGQFFTEMKRFKGWQTMAVGTLCQRVQRSAYPDRYNKFVP
TATKVCKAGGL

>QLI71336.1

LPNLTGWNRAKVFDMTVTWQKYAPDGLVKQMFLINGKSPGPAIEVEQDDWVVVNVRNKSPFGTTVHFHGIEM
DKTPWSDGVPGVSQRPIAPGRNFTYTFRATQYGSYWYHSHSLGQIEDGLYGHILIRPRPGTMKPFHMISHDPGAIE
GMERAERAVHALIVYDHMHITSGEKEDITPAAGVEITCYDSILFNGKGRVRCLPEDELMSHLSPTQKADLALVPGQK
LTDKGCLPPIVLAAFAGDVTKYNASAMPATIYQGCEETNGHTEVIRANLPGRNEWLALDVVAAINFVSGVVAIDDH
DMWVYAMDGSYIEPQKVQALGMSNGQRYSVLVKPTRPGDFQIRFHANSAPQTIIGNAILRVPGVAASPQEPKPYI
DLVGNPAFPGVVAFNQTIAYPYPADPVAQTADALYVLHMKLDGLSYLWALNTTGLKPMVVDTTLEPILFSPNATH
FKNAMLTTKNDTWVDLVLFASEFPMPPHPIHKHGVKMYQIGSGTGPFRWTSVEGAMREMPDQFNLVNPPKRD
TFTSMPTRAAVNWVVVRYHVTNPGAWLLHCHIQNHMMGGMMMVILDGVDAWPDVPSHYLHHS

>QLI71358.1

VEVVQVGPNANIDHGKIKYTRPGSDFEESFGCAEGSGKGLTFSRDKQYVACCAPGQHLGGSQSTQFQCCGEGHD
VAGSGAHGYHCCPTGMSYDGKMCHEGQGQGGNHDHSHDGHDQPQAILTRGGDGGGGGGGGGGGGAGDKS
GSGGGGGGGGGGGGNNGGGGGGGGGHNGGGGGGGGNNGGGGGGGGGHNGGGGGGGGGDGDEGNGN
GKNPHNGTQGWCQSPETCSSGVDTGKCYLFKMENGQYLGYDSRPGVKAYTAGQDSATHHMGKFRFCKDQNCT
RGCGVNPGEGFAILDIHGASNTGAEPNNFLDAKPNGGHIGKTPDYSAAGRFTITKWTCGKYCLGGAGNQGVGPT
CPSLEPALTFNTLDAQSCLPLTLVEVPCDVRDRRNNCRWDKTPGSCGPGDSSHCHCNK

>QLI71359.1

EDMLVSNSLNNCQDDSSFTASLFNVVYTPNNNSATVEVVATSSVQGYVLFDLAISAYGYTFLRKTVDPCEMKLGGL
CPMVAGKIPFAFNLPVPADAAKQIPGIAYSIPDLDATVRVQINLTKTDNPRSIACVEADISNGKTVDLLGVKWATAII
AGLALVSSAVVNGLGHSNTAAHVAANSLSLFGYFQGQAILGLTGVHLPPIVQSWTQDFQWSMGIIKVGFMQTIFT
WYQRATGGTPSTIFDTLTTVSVQVEKRGLDFTGHSLSLFRRGAAMMPRPVVAAAGNLIKRGNIMTSSGAYIVYGIQ
RVAFRSKIETTNLFMTGLTFFCIFVVFTILAVASFKGICELAVKQKWIDSDTFLEFRNGWFTVLKGILFRVALIGFPQM
AILCLWEFTQVDSAAEVVLAVFFFFGLAATLAWGATKVIRIARRSVAMHRNPAYILFSDPQALNKWGFLYIQFRASA

YYFIVPVLVYTLVKAMFVAFAQKSGVAQAVGFVIIEAAALIAASVLRPWMDKPTNSFNIAICVVNFLNAIFLLIFSNVF
GAPQLVVGVVGVVLFVLNAVFALILLLMVIISTTVSLIRKNPDARYQYMADDRASFMKSQTHLTTTTELDALAATAR
GDKAGGYKAHMDLEDDNESFSSEDLRRRAAGQNPSAAASQHSFQGQQGPRSPVNPSMPLFPADPRPESPFRSG
SPSPYNRSGSAAAQRPNQSSSPAPGYRSQNTSSPWQRGAGYE

>QLI71360.1

QITVSVSNVEPLPLKSASAVTSADELFRQSCPEEVTNSNPYTPNLLLSSYSEFEESGKKVFGGNVYPSSDSFVRGAIVA
WAQHQSLVLRPDVIWFEILAQLNFYMTEHAEEIRHLFVNFQGKKEIVVKETSWTNVIAAFATEIQKRVKTNWLLG
WVAPGFSTSTRNDNMTATVLMMGLMQHYFEFTGAVICGIPSVTLLGTKRDWVQLYKKLDRLADFGAEPAQFAH
NLKPILSRFVQTWDEPNSPAVKSFWNQIVRANKVFLCGAGPTEFDISGWITGFMHWREDGTLVAPNGTRPQPDD
VRFDGVAYTRVDIDHIPVGYAKAPLKMLDYPNRGKDTQAYVLAGNIGIKRTISNPTGKGAKPQVLAEPMNSWFLY
GPVDANFTTGPEYGNLSEVKSTATSLRDWCPAAYAKRY

>QLI71362.1

QQTSGDGSSQALGHIVVLKEGLNDGHLNEHLDWVKSIHKRSLDTSNDNENQENGVKHTYHGGAYGFHGYAGSFS
RDVLKSIKEHKHVDFVEEDQVIILEPTRRDENTDGDMLPEGGTRKKNKGHSLLSKGQGYNSFLEKGLILDAVILHEGE
KRRDIPARSAEELVSQTTMNFNFTEPSADFAGVDVESYFYPPDLDAIMEDILADLENDEQDVSASKVLFTRAADRPD
CTGALKFSSELAEDYNSYLKALDISVAATVSGWGQSASVSGSYLNQAEFSSNALTYVAKIDIRRQLDSPAGFQFNMN
KYTTTTFARNFGDRWIRGFHTGGKMIARLTFRSKGTVSKVDLKAHIEASLKFWGVTADISASVKKSQEEVSKHANV
EISLFYQGDLGRVMGQSGSPDKITATSADGAFHQVKSWADQFMDNACRHDYEYQPLLEEYRNAEGFPEHQKILD
YRTAHRVSSMVLKQLVRISEMKQYLLNLTTIDDEIKMNVEFDEIDMVKQSQDWVDSVAENPENSQSTGRDLIKTLR
DDFFKKYAPYISNSFTNESPRADSYANGNRYISGVKVERGATGPANSLGNINYGFTGGSVWLVPTYTSNPKKACTTI
TVEQTRIDYADAVKFLSKDGKYTARYFRCVTSSERKIRRLALSRGSGIINYDKAHGFVDATSNINEGFDMSPLYLMW
SFDETDPAPEDDFPEPQN

>QLI71367.1

QNSTQYVGAHVPSGTSIVGSYGGQYRPQVHFSPPSHFMNDPNGMFRDHNGTWHLYYQYNPTKSVAGNQHWG
HATSQDLYHWTNQPIALFPPKKNVYIFSGSAVIDKNNTSGFFGNQSNGVVAIYTLAEYLDDGSPGPQSQAIAYSYDS
GFTFTPFDGNPVIPSNSSQFRDPKVVWYEDHWVMVVAYAQEFAIGIFTSPDLIEWTHASNFSNQGLLGLQWECP
NLVRMPLYTEDGQRRDDMWTMLISINPGAPVGGSITEYYPGTFNGTHFEAVDSVARIADFGKDNYAGQFFFGVP
AEEDPVSIAWASNWQYTQTVPTAQEGWRSAMSLPRRNYLTRIDRVGWKLISSPYDMQPIMGRNLAYNQSLVNG
SLTVDFSDIASNAVYWEVNVTGLPKKGIPEMATLNFTLLSPITAEYIRGGYYFGGDNPFYLDRGGAKGFDNVFFTDK
LSTNSLFCNGSWSMKGVMDRSILELFLNDGVDSATATFFATQPLTFLIISTSSLPHGTRVSTRVNALKSTWQQMED
PGDGLVHGNISMQETASEAMKHLLQSM

>QLI71368.1

ATVGEGVKPATKDSSALSSVPQNPINIAASGSPDAWCGGFTAYQCSVLCGNYGYSCYYCSSSYCNCLNSGC

>QLI71374.1

SVFSLDSDDEIKKSASILAWDMLQYYHGNESGGTPGILPGPPPAGDYYWWEGGAMWGTLIQYWALTGDSTYNNI
IMQAMQFQVGEGQDYMPRNVTASLGNDDQAFWGMAAMLAAENKFPDPPANRPGWLGLAQAVFNTQASPQ
RHDSTCGGGLRWQIPFTNNGYGYKNSIANGCFFNMAARLARYTGDTKYSDWAEKTWDWVEKTGFIDPKNYAIY
DGANVAHQCKDINKVQYSYNNAIFAEGAAFMYNITNGDPMWGDRLDKLINYGLKTFFPHDVAVEISCELNDGCK
TDMFTYKGFVHRWYAIITQIAPFTAERILPVLQKSAQAAVAQCTGGANGRQCGLKWADGKYDGRTGVGQEMSV
LAAVQSLLIGKARPPVTHDSGGTSAGNPDGGQGDGSVMPNQKTVTAGDRAGASIITILLLGGACGMFGWMSYE
ASGP

>QLI71382.1

LPQAYPNKCGDQVCPADKSKCCEVIVNGVAEIGCFAECPPVQALQRRQTYPNKCGDQVCPADKSKCCEVIVNGVA
EIGCFAECPPVQALQRRQTYPNKCGDQVCPADKSKCCEVIVNGVAEIGCFAECPPVQALQRRDQPSTTTAASPSTP
TFGPKCGDSFFCPVGKVCCPNALYHCADPDKVSQQCPQ

>QLI71384.1

TPVSAPDTVIGKHAGGYVNAVYFTNWGIYGRNYQPADLPASQISHVLYSFLNLSNNGTVYTGDSWADTDKHYPN
DSWNDVGNNVYGCVKQLYLLKKANRNMKTMLSIGGWTWSTNFPAAASTAATRSNFAKSAVTIMKDWGFDGID
VDWEYPADDVQATNMVLLLQAVRDELDAYAAKFAQGYHFQLSIAAPAGPANYNKLHLGDLGKVLDYINLMAYDF
SGSWSNSSAHNANLYANPGNLNATPFNTDDAVNDYIKGGVPASKIVLGMPIYGKSFQKTNGIGKLFSGVGDGSW
ENGVWDYKVLPKAGATVIYDDVAKGYYSYDNRTQELISYDTPDITKEKVTYLKSKGLGGSMFWEASADRKGPDSLI
GTSSNKLGEPDATENLLNYPDSKYDNMRKQMA

>QLI71395.1

DGAALDTQVILPGRDNGNDDKDNRKDDQQQIPSSWPPPQHISRTGGWVSLNRAVTIVADNGTDAATINAVKAIV
SSAGGTATLSSQLSGHGTQILIGAEKADGVAVAAANALTGKSANGLAAEGYVLASGKYQNQDTVVLNGVDARGSF
YAAQTLRQLVDARQGIPGVKVRDWPLMPIRGSIEGFYGVPWSHQARLDQYVFYGKHKLNTYIYTPKDDTLLRTNW
RDLYDSSGLAQLKELVETANTNHVDFTFALSPGLSVCYSSDSDFNATVAKFDQVRALGVRSFYVALDDIPLAFHCDS
DKQKWPNQGNWHWLADAQAYYLNRIQKDYVEANGLMDLETVPTNYAGSAPDPYKGEFGTQLNKKIRIQWTGE
GVFSASVAVESVVRADSTYVTDKLFFWDNFPVNDDKPNRLFLNPLTGRDAVLYQHLLGFTSNPMVQPYASMAAL
ANYADYTWNGPAYNATASMAAALRELAGGNNTVYNAVVAFADLNQNWPYRTPAINAPQLSKDMAAFWAARN
ASAKHADGTRALRDRLQLLTSLPDVLPRMAVGGFAADVAPWSTLAMQWATACQHLVAMLDALDKGQGDEAAR
EFAAAQKWVGKTAAKTVTGLDGKGQVVPNFITPTTGDGVFDQFLANATAAYKGR

>QLI71398.1

LDSLSGYTKDIPKCAYSALVDGMKQEGCDISNVTAEDFDCLCKHIGAISIIVAKSVDSTCTADFTQAAGSMCGRWN
VDSTTATDLAAATSALANALNGKGSDAKQTATGISTSATKNIAMVPTGGAIGVVGGLAAAAAGIML

>QLI71416.1

AGLADIDHVVLFMQENRAFDHYFGTMAGVRGFSDANLQTNNGVPVWKQLTTRQQTTKTDHVTPWYLNYLGGN
WSEATQCMTAGSNGWFENHAAWNFGSNDHWAMNNTPWSIGFYKKQDLPTQWALAENWVIGDMYQESVV
ASTNPNRVSWVSGSINVPGSPQKPDQGGNPYIDNNETPGCESGGVNCFPLKWKTVAEYYQDAGVSWNVFQDA
DNFDDNPYAWFDQFRRSTKGSALYDHGLKGSSLDSFYDQAAKGTLPEISWIVGPMQLSEHPPYSPHDGAWLQDT
ITRAVLNSPKYNKTALIISYDETGGWFDHVNPHHSPDGTPGEWLNDPQGKVGHTFAGPGFRLPFYIISPWTRNGG
VYTEHSDHNSQILFIEKWQAAKGKNVQTREMVPWRRDNMADLTSAFDFQNPDYSIPSLPTAPKPHVNFKGQYD
GSSYCQSRFPATRPPVPYTGDGVIDDLTTMVEHGFKPIRGKLTEGRYLVLETNGHSLTNAGSKTATSATVTKSTPNH
DDIANRWIAHAEQIGGDVFTLQSASDEQYLCKDGSLCADAKNAEAFTVQFTASKGYSWKQRDGGKYLSGDGAEL
DFSGDAKYWKIFSVSY

>QLI71418.1

ADLPSIQMKGSKFFFPNGTQFFFKGIAYQQQTGAAGSTPTNSSFIDPLADTKRCQADVPNLAALKTNVIRVYAIDPT
KDHSTCMKLLNDNGIYVVADLGEPSLSINRDNPAWDTALFARYQQVVDEMAKYSNTIGFFAGNEVSNANNNTGA
SAYVKAAARDTKAYIKSKKYRWMGVGYAANDDKPIRDQIASYFNCGPSEDSIDFFGYNIYSWCGSSNFEASGYNRLI
EFFKNYSVPMFFAEYGCNLPNGGDGRTWDETTALYLKNMTDAVSGGIVYEYFQETNDYGLVEVSSDGSISKRKDF
AALQSKISAVDPKGVTMDSYNPTAKVMDCPAVNSSWQASATLPPTPNDSACDCMFKAASCVPASSLKTDDFGDI
FGYICGSDRSLCASIDGNFTTGKYGTYSMCSSKQKLAFVLDAYYKKNKNAASSCDFKGQATTQSASGTQSNCAKLA
PSNGGNSTSSGGSGSDSFAVMGAPLTRVGNSAVGLYLAAALFVVGMVAW

>QLI71439.1

SPTRKRSEPAPLIIPRGEAFTLVPDEYIVKLKKGCAKAALDDAMKIMPGDADQVFDSIFKGFTGRLDSSSLDALRDNP
DVDYVEQNAYYEAYSVTTQQQAPWGLARLSHRRPGASDYIYDESAGEGTCAYVVDSGLDAAHPEFEGRAHFLGTF
VGDQNDNCLHGTHVAGTIGGRQVGVAKKTTIYGIKVLDMNREQKCGADTSVIIAGIEHVARDAAERHCPNGVVV
NLSLGGGWSQAMNEAAAALVRRGFFVAVAAGNGDQNHNPMDAASVSPASEPSVCTVGSVDSRDRPARDSNY
GDVVDVQAPGVEVVSARAGGGYITMSGTSMAAPHVAGLGAYLLGLRKASASNLCSYLQESALQNSISGLHWGTR
NLLVQNGVGA

>QLI71469.1

TEQSTEERFIKFNRLARLSSPLQLNDVSYKSLTSTPRDYSVAIVLTAQDTRFGCQLCRDFKPEWELIAQSWARGDKQ
QESRLFFGVLDFTEGRETFLSLGLQTAPVLMFFPPTTGPHAVASAEPIRYDFTTGPPTAEQVRNWLARHLPGRPHPE
IKRPINWLKWASLFTFVAGAVTAMVSASPYILPIIQNRNLWAAGSMIAILLFISGHMFNHIRKVPYVAGDGKGGITY
FTGGFQNQLGLEVQIVAAIYGILSFCTIALATKVPRMTSPKSQSVAVLVWGAAMFFIYSFLLSVFRVKNSSYPFSLPPF
M

>QLI71476.1

KVVLGFGRKQSSTVVANANDFHTIPDTVHCEDLHYHEPSRLIFTACEGVEATRYAWFPALGHFDDPNVGLKAQGSI
EVIDPSTMKAKKLKFTNFNGPFVTHGIDVIDDPDKPKGKAVYLFAVNHLPNPAFAEDASEPKARSIVEVFYYDIGSDS
VEHVRSVWHPLITTPNDIVAVSPTSFFVTNDHFYRDGIKREIETLYFGAKWSNTIFVEFTELTDGSFRDSDVEVKASV
ALDGVHNNNGLGHGRTPSEVLVVSCASGRLHIADVVSPKSDGESPRIAIRQSVAFDSTLDNPSWFRDPYANSTYDA
SGLVVAGLPRAVDLAKNQHNPRGTDGAIVWKATPSRDETAGNEESWVNRLLFEDDSTRIRTASAAVLVAIDPAKE
KGERKAWLFVTGFISTNVVAAKVAL

>QLI71481.1

SPADASTAMLDDRQMKAIHILHSNKAALGLTREETEKFDKFMTSMRKRTPQGLLGALGALGNLGDLGGKKEPKNP
ESTAAPAVPSPTPAEADMDSEENPVPTPTAAAAPSETPAPPKKGGILGGTVLSGLV

>QLI71486.1

SSIPDRPTTLPLTLPIVERSEQPITCLTGIKRSPEPINGKRGIEQNCGRSQRDVTRAFSSCAAKAQQGREAALKGGELM
KTLFKNDDEDTRKRVADHFEQIASQCGARENSGVSVNCGQCEQGIAGKAFKGSGPITLCNVALADTQSTTQCGGQ
DLDDVLLHEMSHVLGSTDDLGYGLEACKQLDAQRSLQNADSYTKFARAATLNCSVGGPSGPGSGGPGSGGPGDD
GSSFPPGPNNPNNPNNPNNPNNPGKPGRPGRPGGPGSGGPGSGGPGSGGPGKPGKPGRPGRPGGPGSGGPG
SGGPGSGGPGGPGSGGPGSGGPGSGGPGGPGSGGPGGPGSGGPGGGDGGDGGDGPLFPPGTGGPGKPGKPG
RPGGPGGPGSGGPGSGGPGSGGPGGPGSGGPGGDGGPSFPPFDGGNGIDGGDGGPSFPPFDGDNGIDGGNGI
DGGNGIDGGNGGDGSNPFPFPPTTGGPGGNLGPGGGQPPLPPFDGGNGIDGGGGDGGFFSSSGNFPPQGAPP
PGGISAFNAPTAPQVLPIQDYDRVESS

>QLI71498.1

AELKPTKCATKVTIVTKLTGTTTIYKSVAAVPWEVDCRDCDLIVVTKTLGSLKPTITVTSDFILMHPPICTWFPEAPSQ
SPDLS

>QLI71503.1

ANFVRGNRVSFNTFKYDIDKCTDEKPCSLYLPKSAPHAGLQIRPRVTDCLERAFSLINGGTIENQAGTEFIARIQNVPS
GTYIFQATGGEDGKTAITTQFTYNTNLATIGTGTGTVTPPSPTTSSPGTSLETTSSPPGPTAASSDSGSTGTSAGPVT
GSLTGTSTAGRTGTAIISSTGSLTGSSRSLKSSGGNAGNGGAVVDENCNGAIDNNDCAELGGSCYF

>QLI71506.1

FGAGNIASTSKVEGQNWRHGDIEDALLTLAMARAMKHKKFNKIMVSRVYFGNWLRDYSQAIDVGTVKSVSAEAI
RLLLCVLGFLTFGYGSGEFEVTADRLGCYRPEDHIDNPKNYADNQDARQYDSRLRGPIDESRELSIDPESGMKNYIA
NERADIMTSAKHVRNLFGRCIELGRNYKNGGRKEDLYESLRLLGTGLHCLEDFLAHSNYCELALIEMGERDIFPHVG
RDTLIRLEGANGDVYPIVTGTFGGVDFLHSVVGEVSDKMTQNEIEELEGTLQDSKNSDTSLLRQLLDKIPEGLLGGK
DQKSRIDQIQENASASQVENMSVSPRNPEEFTAYVQQVYKQIMPAIEFHDDIMKGITSAVEKIPVLPKIIEQLEEQLS
RFVFSIMAPVVVPLIRQIKSELATGSGEVIKSSENEQHVVFNDDRSTDPTHSMLSKDHFSNILNEIAGRTAAKMISW
AVPQIMDAIDDDSTDVNRLCDRVINGILHHPAQRDMGRDGVSEARNTMFKEVTSWWQELRSEQDSYRSKLSRD
GVMRGENHKEGVDDTGHGHGCAGKLKMRKLYGEPDTLENKIAGAAADAIVSGATGLVAGIVEQNTGYKLPAGQ
SSQHNQQEEEKGGLEGFLSKAGSILGGAFGKDEDERKDSGRNDNDSSYSQSQSSYGRQDSSYGQSQQSSGYGHS
QQSSSYGQSQQSSSYGHSQQSGRHDDNNRRRRDDDESGGYGGRQESSYGGSSGYGRQESSHGSSTHHGRRDD
DNYGRQESSYGGREDSYGGSGGYGGGRQESGHGGRQEDYGGNSSYGRQESSYGREDGGYGRQESGYGGNGGY
GRQESGSGGNSGHGRRNEDSYGRRDDDDDNEGYGRREHHGRRRDNEHGY

>QLI71524.1

ATPNCFPYGNAELPGDLTAPNVKLEDWWCPQSMAYGFQGFSYPLEDDNCNSYTNSFDRMNQDFARMKKDFGA
SIVRMYYPTCTQPGVFENAIRAAAKNNMALILQIWTNFGDGDVWKQSQQAIYDTLAKREFAAIAPYVVHSADWG
SEPVGDGMDSGNFVNDLGAFRKRMNQHHVKAGISEDWDRPGSLRNGDGLTDLGRGIKSNSDYAHIHAMPFYH
GNNPENQAWAYIQQATQWVLDHVKLPTMITESPWAWGKTDHNPGKTDVGVAQYTRYWKTFDDNCEWFKQK
NVGWFLHAWQGEDKFDIVKPDGPGYVIPGWRPRKC

>QLI71528.1

QKLSINKPAAISRQIQQAQVMEKPCGETPAEARTRGCYLDVISFCWLPERCYDAELSQHLTG

>QLI71550.1

YQLPPKLRAIYDAHKNGSCSSPLSSPMTNGTVYCGDLANAIFLKSSDGYDDMDIDCDGANNSTGDCANDPTGQG
QTAFMDEVSQFGIPDLDTHIHPYVVFGNVDGSPTFVPQDRGMRPLSVMAVVCDNQLFYGVWGDINGGNLTGEA
SISLGKLCFPKEGLSGNKGHDTKDTLYIGFPGDKAVIGKDGANWRANSTLSFMDSIKTLGDELVAGLPGSDKRIRCG
FTEEQ

>QLI71552.1

QYDDRLYNEAAYGGAFDYTNAEEVAVAYGAPGQEEEILAPNQCKVLKAGVGGQVHSVKVLKHGYCQLFLDNNCN
PTYLRGTYRWHHGNPRQPVTGEATQSHTIRCRPGSPH

>QLI71556.1

VPQLELVPIFGRCADPSVICTEKDIVENDRVTGNKIFCTLYEGEKVDNIYCTVNQREKDEMEIDQASEICNRLSGCTR
CQWQPVERNQKRIGGRVAEFSCNLRPDAKNTDEARKIFREQIDKGAQAICELKGVDRSTCETYKLDCINKLSKNGPI
NPDQMEWCVGNKVRI

>QLI71557.1

TPAIDRRDNQQHCGGSLLDRKTVLTAGHCLLKSASVKAGTLNVQMGGVDARVASYKLHPKYIPSPIHPFNDIGIIKL
STPIVNSSTISYAILPESNAVPPVDSIAAAAGWGRQAPGDKSRVDKLGKITVPIKSPQYCVDHDPEGRLVHSITGQDT
VCAGESGKTLCYGDSGGPLIDQQTGTLIGVASKVLQDAKKNYCGESTVFTRVSSFIPFITENLEPAPLTDAEIDDFFN
QD

>QLI71560.1

INEPHTPRGANITNSVPLSVDPRSTLDVCEARTINYITHALPQSCLTISWRSPAPTSTASAGSQHGRDDGSDHGTDN
QPSWPPTPTLDHNATEVQATEPAATTFMSFEDWKEMMLRRAGQDPQDLRSRRPSEHNTDDRYSPESGHAGLG

EEGEISLNFEDYGNGDHQKLTSPRSTRGDGVDEQPAAADALLYEEGKAATVHLSKDAGKTCKERFSYSSFDAGATIL
KTSPGAKNARAILVENKDTYMLLECDAASKYVIVELSDDISVDTVVLANFEFFSSMVRHFRVSVSDRYPVKMDKWR
ELGTFEARNSRDIQPFLVQNPQIWAKYVRIEFLTHFGNEYYCPVSLLRIHGSRMLDSWKDSEGGRDEEALIDGDESA
GADSHQDENHVADAAASRDTNIHIMASNGTSSRSLSYALEIFTNMDATCPAPSSAGANPPTRDPKLSSVSLGTSQE
SDLVSSDVSQARSASPENSGNSSIAQAKSILATGSINYSTIMPTSQDDRLRNHTGALNGAALNVNTTTIDRENNAAT
PTAKLSASSGQNGKPRSSGTTGASAASPTMQEGFFNAITKRLQHVESNLTLSMKYVEDQSKHIQEALQSREQRQH
AKINYFLDELNKTVLAELHTVREQYDQIWQSTVLALESQRDRSERDMMALSSRLNLLADEVVFQKRMAIIQAIILLSC
LFLIIFSRGVSLPSLAPLLDQPSNSPCATPTSPATPRQSSYQLSKRHHREGQSSFQPPDPSCQVQMLYPGEARSDTSA
EALPFEAVSFQGTDEPKSECSAFQRLSPPPTPNLLGEMSLSSDPNSATGSNHTLRRRLGGTSHISSRKPLPSLPEHPRS
LDEE

>QLI71571.1

DYLAQLRQDTVDMFYHGFSNYMKHAFPEDELRPLTCGALTRDRDDPTRIELNDALGNYSLTLIDSLSTLAILAGGPQ
DGSYTGPQALSDFQDGVAQFVRLYGDGRRGPSGVGARARGFDLDSKVQVFETVIRGLGGLLSAHLFAIGELPITGY
DPKPAETLAEKDPLELAPIPWPNGLKYDGQLLRLALDLAERLLPAFYTKTGIPYPRVNLRTGIPFYVHSPLNGDADRS
KDEEGRLETTETCSAGAGSLTLEFTVLSRLTGDPRFENVAKRAFWEVWRRRSEIGLIGNGIDAERGFWIGPHSGIGA
GMDSFFEYAFKSHVLLSGQEMPNVTRSRRQSTTNWLDPNSLHEPLPHEMHSSDAFLQAWHEAHASVKRHIYTDR
NQFPYYSNSHRATGQPYTMWIDSLGAFYPGLLALAGEVDEAIEANLVYTALWTRYSALPERWSIRENNVEPGIGW
WPGRPEFIESTYHIYRATKDPWYLHVGEMVLKDIRRRCYAPCGWAGLQDVRTGEKQNRMESFFLGETTKYMYLLF
DPNHPLNNLDAAYVFTTEGHPLVIPRGKNSKPVRRKRQSSNLKDVSVSSYYNEKFTNTCPAPPPLQDPLTGSSTAAR
PDLFGVSRFTNLYNTPNLQGPVEEVLVHDEKKGLITKYRATSNHTIFPWTLPPTMLPANGTCVAPPQRVISAIEFPTT
DAASSLISRLGAHIAWYSYLGPTATNLEGLRLQLEQEFSDDRGENVWRITHVGNTQLGRHETVFFHGEHVRHLKDE
AFTCLRHKDSVNIQVLVEAPRPSNFTTPPTSENTSPPSSSSAILSSNIDTDLDAADLGVIPSESLFKHLLRAVTSVFDPS
ETDAPESETRDSVSASFHTWQAFTSVGAGSYPVPSILDSPIPGSPTYNSHDPMANFPWKAIYIADEACSGPLPDSAP
RDHQVIVIRRGGCSFSDKVANIPAFAPRRSSLQLVIVVDEADEHGQHRDELPRPLMEVEQLTPKGMKRLHGVPLVL
MRVARGEYDLFHSALAVGMRRKYTIVSQGLIIDNAVVL

>QLI71575.1

LNPVILADTNRDGKVDVEGSTDLAGKETWTEQTGALFLANIVDTNRRCSDKIITSFDFGDGPDPSTPNQELDKCHD
AADNVLRNSKYLAALKTAPMADVSSSATGSIAVLGEAAAEKVRVFHKSGSDWLYVAANYTFAADEIKKGLELGIDA
RDVRRPGVWDGRATVQFSVSDGAETVKDAVALRVAPVLTHHHLQQAREIITMRPTEKGLGARFAKELQDHITKA
GIQKPLRLVGADQFPQDHFEAGYTSIPGPDGPVTLGVMIRSAINNTEGRIAFSDMRSDSVGAVQFLQIDNHDSDV
DHTGNLETIPPHSHNGKSYPAGRVIMGSRGGQKANIIKFLEAQEMQKPVDIDTTWLYIGHTDEFMQFVPADNKR
GWAMMVDDPLAGLDLLVKAQKAGHGNVRALSRPSFPTDSADSRKPDDVSLPAMTIDAVLSRANFSRAQEYAAW
NIERNINIVKQEVGLDDGEIFRVPALYYNEAMAGPRPYQPKKYLPEPGQEEEHIKDRDHAFQVYAFYPGAVNGIVL
GKGKYLSANVWGPVIDGKDILKEAVNAAYAKANMTVDYIDDWFSHHEGVGEIHCGSNAIRDTDFPWW

>QLI71587.1

LEWPRWLPERDALIVRADSSSDSGQASVAAEKNPSQTVVPEVSASATDASSQAGGKTTGDSNTAKATKTGNGKS
GTAGTATKKASVTRTQFPPDAPPAGVSMQTPNTGLQPSGLYKISDYVTWSWNYTSLLGTPTAIDVLVSCSAASET
WTLTGNMSFQTAVNYVWDTKQQANDIQSPLGVQQYTLIVKDSDASITQAPEPGYLGAYASYSFGMYTGQPYTAY
PDWTCPGTCSAAASAFDRQAVGLAIATSIVTFLSFTWFVAGLGLH

>QLI71599.1

IWPVPQQISTGQDVLFVDKSIQVTYNGEPVSHDGAGETKESPSSSSQVVHGAVARSLTAIFEHGLVPWMLNPPGS
DFEPALDGGAGKVTSLTITQTGEDNATVFKPLAGQVDESYSLHLEANGEASIEAVTSTGLVRGLESFTQLFFKHSSGD
AFYTKQAPVSIQDAPRFPHRGLVLDLSRHWFAVDDIKRTIDGLAMNKMNVIHLHITNTQSWPLEVPALPKLAEKGR

YAPGLTYSPQAIQEIQEYGVARGVQVLLEIDMPGHVGIDKAYPGLSVAYNEKPYDKYCAQPPCGALKLNNTDVENF
VSTLFDDLLPRLSPYSAYFHTGGDEYKATNSLLDPDLQTDNMTLLQPLLQRFLDHAHNNIRGHGLVPIVWEEMVEE
WSADVGNDTVVQAWLGSASVAKLAAAGHKVIDSTFDVYYLDCGRGQWLDFKDGPSLDAAYPFADYCSPTKNWR
LIYSHDPVENMTAEAAANVIGGEVAVWTEMIDPVSLDTLAWPRAAAAGEAWWSGRKDGEGNLRSVFTARPRLE
EMRERMLARGVRGAVISQLFCGQSPLEDCAA

>QLI71607.1

QPIGSLPPDVRGELRGPSELLGALNINDLGRLDERALGARRENRPPKILPPPQIVYRGDKRSPEEIKANGGFLPSSEIK
PTNENNGFGLYRHHLGGHGLVNGKRVTAYVSTTRLFGTALGYANMASEEGGWIYEIQALPHMIDSDGTLLQGRK
WTKEYEFSALGGISWAQVKSAAQVPGFKTPQDYGVLSWSGVDPEGFKKSMPEKQWFNNTDYNSTFDQFKASPG
QPQLAGWFDDREKYKSQEPWSLNQTKPIEDYFMDFMNQVGGHVGWRGTYPLVLKTDDEYADNFIKWILDGY

>QLI71612.1

REPVYIKGLGIDGVNRDLEVSRYPALYTGDFDDCLGGESLFNVTKFDAAYYADNLTVLFHLDGTTNIHKENLILHISIE
AYGENRFNMTYDPCKANIHSMCPLNNSVPITAFAAIPIAPHDVSGIPSIALGIPDLEGLARLQIFANSTQTQIGCFQA
VMTNGNSFSQPEAVSSFLGVMVFIAVLASFATAIYGLSIPHMRVHYAHSFSVLVIFETFQSMFFSGALSVNWPSVLP
AWWSNFAWTAGMFANKQIVRSVSSFTGNNVNITQVGGAGSVQINNGGGLLQQIYGRSLFAASPFQNTAGSLKS
FAQRNAYNASDPYDYTWGGRPRMPGMPMPGTWPGFSGTLSMVNIPPAEAFTISLIWLLVILAAVALFVGAVKLS
LDLLIRLRWLKSDGFDYFRSHVWGYTASALLRTLFIAFFTVMTLAMYQFSLRGSAGPTAVAAIVWLIFLLGIGGITAY
ACYFRLRHGKYETGPDSLRFERGTVFGKVPFIATTRQSRIGEEEPSRRPYLFGTMPFVKVKYVDDDPNRDPVHLDEG
YVKRFGWLSARYRRSRWWFFAFYLGYQFIRACFVGGAARSPLAQVYGLFIFDIIALVVIMKLNPFESNRNTAVAVW
MLSISKIVTTGLSIAFLPAFSINRIAATVLGIIIIVVQGFVAIAVLVLILLGVLSTWMSLSRNREDFPQVLEPVRIKYFEHA
KACSDDFPPRPKKSDTKEETSEGPREAMFEVRDVRRAPKIEDEGVDKFPDLDPPSISLTTVGASGRRSRANSASSRFS
VNSLPRTARVHRASWSSKDIPLWDSEMNRGDQQRVGHSRASSLRLSGYVADAPGTSTPVRRPMTPQQEILEEPQ
MQQSDGSPTEYTESSQASNPEETKTKDSEEGVSTPATDTEPSEAEEKPRVEPEAKEAS

>QLI71625.1

TEQWERIPGGWNVASKPTPDTIATFTLALNMENIDFLASELLDISDTESPNYGKHWDQADVYSRFAPSNATVSTTL
DWLVGGGVQNYTVDWIFIDFTTTIATADSLLNASYHYYTNNVTTELRTASYSVPERIQNSALLISPGTYLGVPNSVPL
SLRPYGAREPLQRSVVSKDDNPCLQAISPSCLKHMYKVANYTPHEGSGSTIGFSSFLNQSALYNDLFEFERHFAIPGQ
NISVELVAGGIDNQNESTAQFAEADLDAQTIVGIAHPLPVTQFIISGNPPFIPNIDHKTENRNFNEPYVPYYRHLLSRS
KSDLPYVISNSYGEQEDSVPIRYALLTCNLIGFLGLRGVTVVQSSGDTGVGSGCLAPDLGTAEFYPIFPATCPWITSVG
GTVGFSPESAWKGSSGGFSRYFSRPSYQDATVCRYMDMVASETYAYYGKYTNWNGRAFPDVAAHSLSPDFQVV
YRGLVAMSGGTSASAPVWAGIVALLNDARLRAGKPVLGWLNPLLYARGFLSLNDITEGFSEGCHGINPGTNATEP
DGAGIIPGARWNATTGWDPVTGLGTPDFQKLKHLVLSL

>QLI71646.1

APAELEQRPRGNFKDEMLAAHNFFRGQHGAEPLSWKGNLASKAQDWADTCRWSHDSAGENLAAGTGLASWG
SFVNLWGAERTKYNWADPGFSPDTGHFTQVVWKATQSLGCGWNTCRGGKGKASGVYVVCKYAPVGNYVGQF
ADNVGEQTEGEASDVWHK

>QLI71648.1

APTPQDLSSIPVIGPLLSGATGAAAGSGASNPLSAIPVVGPLLSGASGSANTTAATTANPLAGLLSSIPGLGSLLGGAG
GATASSGASNPLSAIPVVGSLLSGATGAATGSGAGNPLGSLLSLGTGLLGRSTDSSTAA

>QLI71655.1

DGSGEDCYPVDYTPRCAPGKLCSQVAVPGLACPWTCGAPLPDACEERCKPCREEVCPTACVCEGVCPMGSGGTG
AQA

>QLI71694.1

WGSLGHITTAYLAGHFVANTTEAFFKDLLRSQDDDYMAKVASWADSIRYTKWGRFTKNFHFIDAHDDPPRSCNV
DFDRDCKEDGCVISALANYTKQSLDSSLPAWRRAQAAKFVIHFVGDLHQPLHNEDVALGGNRIHVSWDGKSFNL
HHVWDTSIAEKWIGGMRGKPYPLAEKWANQLAGEINDGKFATEKGTWLKDLNFTDAIETAMAWSREANAFVCT
HVFPEGPDAIVGQELGGDYFKKAGPVIERQVARAGFRMAAWLDNIADGFNAGTKDEPVSLEL

>QLI71698.1

FYVGKNLTRDGSVLVGGTGEEVSSHWLQLFPAADHAPNETVTVGVTGGANIPGELMRIPQVKHTFRYLSMEYSDY
MGFPAPLTNGGLNEKGVAVRDVWATNRAELIRMTPTPQRGVQYSDMARLVMERASTAREGVELIRDLIRDHGE
ATYGGNTHLIADKDEGWVVWELAGGRGLWAAERLGASQVRALYPGYIGDFPVDFANSSDFMGSDNIVTFAVEQ
GWWRPGSGEPFNIFKVYGPQGPGYTERDGGFKYMSQAALENATLAMAPVSEAGLMERVRDHRIADDEAGYGQ
VVSLREGVDPDMLRVWIAPTGSVASPFIPWWLGGQSVPPEFGQHRYLTTGASSSFLAPDFQLQEASRFAGRIFKR
VLYYMCSAPALFFPIVTDALTAFESESARDLEWVERSAETLIASGERDSARRLMTYYSHSRAAKALDMGNTMNDAL
DMYIKLRGLWRDPVGKDINDAGEGAETVNCLVGFNPDRPANQQVVPGSGRFVVQDRF

>QLI71704.1

GEVAGVLISDKEFFCADECAYKGVFFEQGGKWCTRNDTTAIVNSEGLAHPRYPHACAGSRVPEGWQERALKNGE
EPKQCYVYEPTEPFDYFCEGDKSAGGNAKNVNVQWSELEKKLAPASSTNSASREGEQGATEKSKPASPEKLKQCN
DLREKNFWECWNKFKDDFQKCVDDAQQVREDCRRGE

>QLI71710.1

ARQSPLPPLHVEPSGGRTWTAVSHGQRPRQHILNPPALVSVEEHDINFPHRPFRPASGFIALGDSYSAGIGTGFNG
TENNCRQGLHAYPVLVHRDLNRARGHHDNATEMQFLSCTGSTIGDMLAGSDHSQIDGLNATTDADFALLSIGGN
DLGFFNIMNSCIFRFYSFYSGTCEDALRNSEEALNGPEFEHDLRLAIMEILDRVQWEKRPWFTITVSGYARFFNEDTE
DCDERSFGVWWRGPKLKRKLRRRMNKMVLSVNDKIRRSISAINADFIEPRVMFVDYDDAFEGHRFCERNATEPDY
SRNETWFFLVGGKDNHHGFQSQLTGSDHSELLPRSSPLINPDTCLNPAEKSGDWADDPINKVVLKKESKSGTIKLKK
PPPKHNKPGNWRDGSVVEDEKKKAVSSPSTSVASPGPVVNQLDDTARETFATGRPLEDSPDLQQCKHCKKSILKT
AAKAHIAQCLKLKKEKAQRKKEAREARERAKAREEEARKADEENGEDESDDDDKKGNVAGKKAGKKPEDEKKGSK
KRKADGEPDKGPKAKKKKDEPKAKAPKPKGPVDVERQCGVILPNGQPCARSLTCKSHSMGAKRAVAGRSLPYDM
LLAAYQKKNQAKQQKAALDANAPVEDEDDANNGPVDSDEETGAVMSALSHWHPQPLIPQPVFTPIKRQYQLSRL
HEQLQLATNGGRTNIFHVVGYGAQKLPEGHPGLVEGEDAPGEPDIGALGFPASARSSNFGAATVSQRRSSVTSRG

>QLI71714.1

IPVTKFVPLGSFGDVKYGVFGYCQGEECTNIAIGYPRDGALTDETQKFDLPSSVRHTLSAILVIHPVAALLTLIMFCLAI
AAHLHSASHSSRYLLVVSVFTLITFLVCVAGFVVDVLLFIPHLAWGTYMVLAASIVLAICTFGSFAMRRTTVSRKARQ
KRIAENGDMSGESYYNRDGQVKAAAIFASQPTFSAVGGGNLGTDSLPAFAVFESQSQRKDDQVSDEKIPLTQRSP
VERSATTVHNDMANAREATSLANSRQSPSRDRYGNPINGPDAYGRERGGGYRGGRGGAYGRGGFDTYGIAGRG
RGGGGRGGYGPLPGRGGARGRGGYGLPPRGGRGPPPMGGYANMPPSQHNQRPPENEYAPYGQTQPVQNSLD
RGWNGSQPSLAADNNDLPRAESPPPLPGQVAGGSRAPDTDTTTPLSPPPPNAYGQHNLLRDSDVDVAGMVGLQ
QGRPMLGGGRETIMSDGSKYSTDEPQQYVPPRAAWNQNPGRNSPGAPSPGGQNMLPTTTGSRSDYYEDTNPR
FDTTPPSGPVQSHHPSAYPEPLFEDAPATGGRARSPAESERSNFTSISQRGVNPRWEPHPPMPNQAPPNRRPVQ
VKHQRQDVLLNNNPDFQLPSSRGPGPNRTGTGMIPGSAYPGGAI

>QLI71720.1

RPFSLMGSMAGAAKGVEGAAKGSIEAVGNAAGTVTKGMTDTLCDISVGGVVQTSTGPMCAAVESAAKDSMNA
VGDAAQNVAKGTAQLADDTVEAAKSMAKGTADMAGEAVDAAGNMVKGSVDTVSNAMKSTTDTLCDITVGGD
VNVSTGDLCKAAQSAAGNMVKGSVEAVSQAAGTAGNMAKGAADAVGEAAGTAGNMMKGAADAVGEAAGA
AGNMAKGAADAVGNAAGTAGNMVKGAADAVGNAAGTMAKSTTDTLCDITVGGDVNVSTGSLCKAVGAAV

>QLI71731.1

APSKRAGDSGADINAVIPPVVKVNTRADNVDLIAKLMTAPTQADRLKLLDQPGDFVFDFNVDDTVAGSEAKGKG
GATVAATSKTFPALVGNGAAMTVGFLGPCGMNTPHVHNRATELNFVAQGRLVTNFVEENGATPVSNTLTKFQM
AVFPQGAIHVEFNPDCTDTVFVAGFNNADPGVQTIAQNFFSLRPDIVSSTLGGVQSLNGADIESFRGTIPANVALGI
DTCLNKIDPAIRLRRAIHVGDSLLAIRILKSHPPLLHNPDSSLPGLSNSNLHLAASLGHKDICEALLKAGHEDPCPALND
NHQTALMLAASAGHSEVVHLLCEYDRSCILRRDVRGRDAIMEASMGGHDTALQILLTYVPGGPREAVQRADLEG
NTALHFASSNGNLLGLRTLLAAGADVERRNIWNWTPAAYSATVQSEVYLKGLVTEVERRQQLKKEIEATRHKKAG
AVRVVTADSDDD

>QLI71750.1

HEARSPLGLDLNVGNLVKVDLCLGLDLKLPLGISIETDDCPKKGPPAGCIDVWHPPHHVPMDGCDDNDNDEWHY
VHPCDCKPEAPHTWTTSTVTQTQVKTLTSCAPTVTDCPAGPHVTTVVVPATTTICPVPVHSTTMATVPAGNTLPA
TAPATMPGTVPATMPGTVPGTMPGSAPGTVPAVVPGTAPGTLPATAPVATQAPVWTKPANQSMPATQVPPPA
ITTPVVVAPSPATTPSEASSPAVVVLPPVGTGAPPTGNWTTPPVMAGAAQNSQKVGAVVAMGLIAALLI

>QLI71764.1

APQDTGKDAGATQEMGHIVVLKQGLKDEHLDKHLDWVNEIHKGSLNSRDGGNGQEKGVKHTYRSESIGFHGYS
GKFSDEVLKQIKDHEHVDFVEEDKRNTLEIDKREEVQGDKPDKVDTPPESVGNQNKNNGVGLLTRGQGYNTFLEK
GRIVDAVIWPENKKRAEADAPAAGAGNDAAQNEIVFDFTPPTTDVGDFEGVDAAEYFKTPKPDEVKNRIIEGLKKL
KEERKKQREEKKKLLESNNLQSRADDPNCPGTLRKEYKFVEDYNTYLQTVGVSGSAAISGWGQSASVHGNYLNQA
KLNKNSLTYVAVINVERQLSQPGGFQFNTARYKPGRFAKDFGDRWIHGFKTGGKMVARVTFTFKDDTKATDVKA
HAEAALSFWGVKGDLSVDVKKGMEEVNKHTNVDVSLIYEGELAIFMDDKEGSPKSISFGSAEAVLSQVKSWADKF
ESYACKHDYAYGPLLDEYDVVPGFSDLEDSPEAPDYDIARLYALEILALMVKIDEQKNILSSAKDLDDKKKREVSAAAI
KMVSAGKKWVKTAEQDPGKAEEQAEELMNNLSANFIDKYKGDVASALKVTDPAGFAKCKKLANDKFRECNHTQ
KDNHDGRDVVEFCHKEATDAQNQCRAGTL

>QLI71765.1

SALKKRQVLKDENVAARLAGGANIGDQCHPPGTYSLGGDKVVPPCLSEQAIALKCEIVTQLKKNTTSEANLKAYGSC
LKDQGSSYFKDVNGCLACKAAHNHMSQAQFEWYSQAWADGQKAFLNDAVPKATAWEYVLGAINNTTCQKNG
TNEKLTAWDCWNQLPAGTGVATKNLTIEEYYKDRPKTQNIGSFEFGGKSFPESSSVELGLLVPTFEIKADIGYFCSVK
LENGTITGSVNATAKVEILTQYQEITSIFNFTKPDEFTVLPVINAPVPVKDSVTTVSKDEAAVLPAVVCNADCRDKATT
IDKIQEAAAQGPKEADKAVVEASKPALQDLKENKPLSVTHKVQIITEIKTEIKAPVNFQGQGQAPPLQNGPGGSNP
NGIEAPGQQGQEPAKQQGPGPSDSKGPKSPILNGPEPPKQTGSAPASQKGPEDDKCEEDSEAPAAPPVGGADKG
ASKEKAKEKPKETTKETANATCEQGDPAKASVKVTICQMPGERDCKTYYQ

>QLI71769.1

WDWEPIDERDYSAKQVVQKLGLVPNPEKGYFIQTFEDPVRVDNRTISTLIYYLLEGDAGESVWHKLDAYEVWHYY
AGAPLTLSLSWNNGTACERHVLGPNVFNKRVEERPQVVVPKDMWQSARSHGDWTLVGTTMAPGFSPDGTILEK
PDFVPKGCCKQKKRKRSAKSIRGGR

>QLI71777.1

VDKPLDVCGTPPPSEGLVDAVKEFRLQEQELRTQGLVAQANVVVDTYIHIISSDGTPQGGNVSDDIVRRQIDVLNQ
GLSGTGFSVTLKGVDRTVNAAWANDRSEVAMKRELRKGSYKDLNLYYLNAPQGSASIGGYCYFPVQGARPGSDSF
IRDGCVMQFGSMPGGSLGSNNLGKVTVHEVGHWFGLFHTFQGGCEASSGGDQVDDTPAHVAPRTASDYQCPA
NSNIDTCPSLPGSDPLHNYMSYRQDRCYTEFTPGQISRMPETGFNQASKSLYSAVVPRPPTLLKSTMLTSAILASLAA
LALATLPTSEPVEFACGNALPSKQFLDTSSKLRAAEYSLTSQGELAAAEIVIGTHVHIVAANNTADSGYASMDALNK
GFASSGIRFVLESLDHTINATWSSGVEEEAMKKALRKGRYHDLNLYLSKQPIGRFGVLGCCTLPQWAPRGSDAFDS
DGWARG

>QLI71791.1

APQSDFMPGDVMTVKQVVKDADGGVSCTEANSNQNELSCSSTRDTNDGLTPLCEVKGCTCTKGFRDFAASSFHP
ESRTISLTSRSIIATLYVQIPVLKSDRTTLRWDICAQRLMLYPGRDGWTGGKLSFSWNGK

>QLI71804.1

IESPEECADRCTLAWYECKGIHGGRWPCDGAYSVCLGFNPFTRGYDVPIACRGKNKPFDPYAPPPPSPPPPPTPTV
ATDVHTQECLWAYKKCANKPDANKSTCREKYHACLKHKSLKAMIEKRMMVDSETTEPSITEPPSPCATDDLECLG
HRISSSTTENLGKTMTTDSETTEPPITKTSSACATDDVECSEHNPAPTTTKDLWETTTTDSETVEPSVTSVPSVPKGP
SPCAGRCLLDYYSCLLVPDFNHCPTMLLQCLGYNPFYSGKLNLPIATCANEPTSTSTTRTPTPTPTLVQDKCVKECTDI
LNACKRLPNSHEAVCGLHFMKCVGYNYFKEGLKFPTTCRTPTSTPIPTSTATSTATSAPTSTPTADLCAQNCLDEWK
SCKKSTRKARAHCKSTMTECLGYNPFKKDHKKPTACQRSDAPSPTAQPTQAVRSKSCTDEWQACRSRPGADQSLC
SFNFANCLGYAPFSNSTNSTLPVSRSSPIPANITTTAPFPFTNLTTAVSTTTAVTEFPMSTSFSSSSQTHVGTILVPTTT
MMVPTTTTNLTATVTAAAGHLEPATMLLLVLAVALL

>QLI71808.1

HVGGHCPPAGPVLPPPELSARLDLSKLDSQLDSIVRNASRSFNATENSFSILLTSRNATVYQYHHTAAVRDPSGVKK
VDGDTVYRLCSVTKLFNVLTVLLNAGDLLDTCITKYVPELAGDQVYEGITLRMLSSQVSGLPRSGDAFDLASTDAKS
WEDDGFPVPPKGDMPPCDVVGGEVCNRAQFFENLKSNQLIWLPGAKTAYSNQAYTLLGMAMQNITGKTFAQLL
RDSVTTPLGMSLTGLNTPDNSRGIIPNGAGKVLWDQDMGNYNATAGLFSTPNELGKFVRGIMNHELLSAANTRE
WMRPASFAGSYSMSVGAPWEIFRVAHLTPDQRPIDIYTKSGSMPGWGTYVFFVPDYNVGGAIAVSGDDGDAAS
LALLDMVAATFVPAVDSLARQQAKAAYTGQYGASSNDNKPMNETAHLELVIDQGPGLKVKSWFNNGKSIIKAIAD
HKGVKPEGTDLRLYPIGENNRWQLSVETLKRRVDVARKPSDACSNWFQTDSMRWATLPVDEFDFEVTNGRVVG
VRNLGLRANLSKIH

>QLI71820.1

QKKVTATFTDPRTGITFERFFGVKTSFSFGIALPETANNSFIGQLSFPLNDGAGWGGWSLTDDMEGPLLMAAWAD
GGKVVSSFRQAFNEDDNPPEVTGNFRARPIAAGTSANSSLFTYTFLCEGCLDDALGLGAAATSGTVKMGWAFGSE
KVGNPASPAAILNFHNKGFGGFQARLAQAKNGKFDEWAAMAGAPLAPATNASPITSKKKKNKGNKGNKGEGSG
NASDDDSDDDD

>QLI71821.1

APVQTEEYEYVVVGSGPGGGTVAANLARAGHSVFLIEAGQDQGENLIAQVPAWFNMAGETPGISWQFFVNHFQ
NETQARRDNKYTYRLDNGSYYVGLDPPAGAEPLGIYYPRGATVGGSSQVNAMYFVVPPDSDWTHIANLTNDDS
WLPEKMREYFVEVERNGYLPPDQLGHGYDGFVSSAQINDTFTTSRPGVTKLVKEAIRELEGVDVQSEQQLVDLLRR
DINRIEPDRYEKSQVFDIALHIDSRRRRNGARNYIADTLSALNEDGSAKYPLTLSPHSLATRVLFEDAKCGRKPRASGV
EYLVGEALYSADDRYNASNSGELKRVAASREVIVAGGAFNTPQILKLSGVGPRAELEKLGIPVVVDLPAVGNYMQD
NYETGVSVRANTPWENNPSENCKMNPSLPPSDDPCLELWQSQGLGPYGEGGAPLSALFKSSVSETPDCDIALFGG
PNADFHGFFPGFSRVRSPDNAFFWSLVKLQTGNEAGTVTLRSTDPRDTPLINFNWFEQKAERDLQALEEAADMV

KRVFNATGAPYAPFEIIEPSPEIETKQSIKDNAYSHHVTSTCRMGPKDDPDYCVDPDFNVNGVEGLRVVDASVFPRT
PGAFPVVPVFMISQKASHVILDGLSEKH

>QLI71827.1

TLLTREQGEFCDSQPDADILALHEELSKDESRLTRRDTGERISLETWVHVIATNQTRQGGWLTKAEIDSQMKVLNN
AFAPSNLTFNLNGTTWNQNASWAMHPKDFEMDYKTKLHKGTSRTLNLYYLPGIYDGLGGFCRFPGIYTNRGEELD
RDGCVIGSFTLPGNPGVLGMVTVHEVGHWFGLLHTFHGGCNEEGGDFVADTPAEASANNLDECPVGRDTCPDLL
GLDPVDNYMDYSGDSCLERFTPGQTVRMRGILSLVRLGGWKK

>QLI71830.1

IGNAVVENNCDFPVYLRSVGREVSPAFYLRPRGGRFSEPYSRDPTFGQALKITLGPDDVFDPTRPQTVYGYTLSEPFI
YYDLDDVRGNIFAGKRLVQRSTDDSCPSNVWPQGVPVEPHVHNCRDSEADIILTLCSAQRGLYYNIWEL

>QLI71838.1

ETYNIPSNPTVSGQPFDSFVSYSIEFSSFPDFAGNHSHPNKYSYNLINNIHAISNNYPVIRVGGNTQDFALYNASQPT
SLVGVVDPDKSPDYPTTITIGKSYFESYRTWPGVRFSHGFNMGLGGKTARGRETLVDTAPLACEALRHGNLYTWEY
SNEPDLFTSGTYAPRPKGWSETDMVAEWLAGTDEIRNQVKKQCPEIQVSFIAPSNAGVSNALKASKMWAAGLNK
RGDIAMFSTHNYIDGAKVPGVTLQGTLMNHTRTKLSVNNHVAEYNSIFRAAGSPPPLIFGETNSLYNQGRPGLSNT
FGAALWCVDFNMYSAAVGFKRVHMHQGTNYRYQAWQPVATDLAAIGTKAPYYASITAAHMTRRAARAPVSISH
VPLSSDAAESAYAAHCASRSGEKHLARLMVVNMHGYNTTVGGAGLEPLPSPPPRTVRKYKFRVEGVRDGVRASV
HRLMANGSDAITGITYDGWSYNYDLDEGRGVRLTNVTVGEKIIVEGGEVVVHVADSSAVILSFATGC

>QLI71844.1

AQLPSAKGVDESSYGKRTSDICLETEAKVVKKEATFEEWKKVCGAEGLCNAMHSEPLLWRTIPELYGWAFARACPP
SPAESEAEAEPAPSQLGCLETEVKAIKKEASFDEWKQVCKTEGLCRALHNEPLLWQTIPELRGWAFAQACSPLEPSP
APVQSGCLETEVKVIKKEASFDEWKQVCKTEGLCRALHNEPLLWQTIPDLAGWAISQACSSENRA

>QLI71848.1

ADTNAWKSRSIYFALTDRIARSSSDNGGDPCGNLGDYCGGTFQGLEGKLDYIRGMGFDAIWITPVVANAAGGYH
GYWAQDLYAINAHYGSANDLKKLVDAAHEKQMYVMIDVVANHMGPSALASRQPSPLDQASSYHPPCAIDYSNQ
TSIENCQIANLPDLDTQSPAIRRLYRNWIQWLVNEYAPDGVRIDTVKHVEHDYWAGFASAAAVYTLGEVFHGDPA
YVARYAGSMSGLLNYPVYYPLTDFFQQRGHPQALVDMHDRVGAAFPDPSALGTFLDNHDNPRFLHQGGDAALL
KNALAYVLLARGVPVVYYGTEQGFAGAADPENREDLWRSGFGTGGDLYGFIGKLTGVKKAAGGLAGDDHVHLFV
DETAYAWSREGGRVMVLTSNIGRGHTREYCFFTRRPGGRWEGVLDGETYVADGEGRLCATVRDGAPVVFRGLM

>QLI71849.1

CGYDDCYGPIDKVEHVRHVKRMQPGAPNATYGPKAPLEWGQVNFLHTTDTHGWLQGHLKEQNYGADWGDFV
TFSRRMKQTAGNLGVDLLLVDTGDLHDGTGLSDATKEDGTKSMPIFNEIEYDLLTIGNHELYVSEVAYQMFNDYSK
KWGDRYVTSNVKVLNHKTGEYEYVGATHRYFTTPNGLRVMAFGVLFDFTGNSNASQVIKANDMVKESWFTEAL
ASKKQVDLFVLFGHNPVRATDKYSTFKVVFDAIRASHPQTPVQLLGGHTHIRDFAVYDDNAVGIESGRFCETLGW
MSISGFDPSNSGFHGVRHPQGVPNPTRPARDGAKSPFVYSRRYLDWNRKTFLYHSKQTEKTYDYHSGLRVTGDIT
RDREELKLGQVYGCAPQDYCIDCAPFTDDKKNIFPGVIHPAVSAIVLNETRKDKSRIILGNTGAIRFDLHKGPFTYDD
NFIVSPFRDVFFYIADVPFDKASKVIGQLNNGVVDKRFASTRIPSDECTNPTLGHMSRRDMREPRGIVRRQDAVVP
GYTTEDDWGTDGDDTQHSDVPSFSIPGYWEARASFPKDGSNPDKVDLIFFDFIQSHVLADLGAGFTADMVHCYIN
CTFTSQDFMLPYAKLAWQENVDNCPI

>QLI71855.1

NPTRSLLNRGAMEADTSKASQVQDVFRTAWNGYYDHAFPHDTLLPLTGGNYDDRNAWGVTPIDSLSTAIIMEET
DIVNQILKYAATVDFTTTKEVNSSISLFETNIRYLGGLLAGYDLLKGPMKNLVDDTNKAHVDALLKQATTLADSLSIAF
DTPSGVPDDAVFLNPTRRIGGSTSNGPAGFGTLVLEWTRLSDLTGNKTYAQLAQKAQEYLVKPKGKPEPFPGLIGY
TVSIADGTFQDQSGGWGGGTDSFYEYLIKMYLYDPKEFSFYKDRWVTAADSTIKHLASHPTTRSDLTFLAGYNGQT
INHWSGHLASFAGGNFILAGVVLNEPKYTDFGLALAASYYETYKQTPARIGPEGFQWVAADGGSPPNGDQADFY
QKAGFWSFSGGYILRPETIESLYYAYRVTGDNKYRDMVWEAFTAVRDLCRAKHGFAGIRDVMKKDGGGQDNFQ
QSFFLAETLKYAYLTFAGDSAVQFHGKGGNEFVFNTEAHPFRVRH

>QLI71863.1

WPEPWLSHWHEPNSDVFPGQQIPLAADDNDTRAGCRWEFDGVSDDGMQSFVFGFYRDANYAILGTGNFRLSIE
FGFADSTRFYEVYYPQRSVVETCPHGTRGLWIDEKTGHQFSFQVNADLTEAIVTLDSDTLKGKVYIHSRALPLTADG
NVWPAENASTAPIPYWHWSQPIPAGTVETDVEIKGNPIKWTGMGGHERFWSAFSWFTCLRKLQAVRAMLGPY
VLSYFAFTSEINPEHSHQSVVLFKDGAPVFRSTANTPSDTEDYALVTKTYGGAVTGTLKDKVTGFQLELVSPSNMHH
YTFFVEHLNLGFEYILGEGVGGSGFSGRSRGGHVGLGQYDGVALTEALTFPQNSPLFKSNYVD

>QLI71872.1

AAKDDNSKSISSSSRNADDATNVEAAAADRPFISTRDGPVEDGKPHLGPFVETDGVAVDADGKQLPLLKGRPNDP
TRVGDQKIPETNSGVMFDKNRDRPEKGISTGTEGGVTEKSKARKEKEGKTGEKVLTQPESPKEKPPLPHSEERKLHG
GKDEDTDKSKLHKQDGSKDYTGLDKPDDLPDTPNKSKSSSTTTKDPLEPPPPKTKDTSQSKSQVPVEQKDDSIIQPF
HSWMLSFTMILVSEIGDKTFLVAALMAMKHDRMVVFSAAFGALLVMTVLSAVLGHAVPTLIPKRLTSFMAAALFF
VFGAKLLNEGMKMDPNEGVSAEMHEVEQELAEKEKEMGNGHNGVSAYNLEMGLGGRNSRSKSRFPSPPRSPS
QSPSRSPSRRSGSMGGFMQGVGNLCSLLLSPAWVQTFAMTFLGEWGDRSQIATIAMAAGQDYWWVTLGATCG
HAICTGVAVIGGRAIAGRVSLKIVTVGGAVAFLIFGFIYFLEALYA

>QLI71879.1

QDNSSTLFSASKLFSGGPSPIASLSYAQYRGVQDDKTDTSNYLGIPYAKAPRFDHSQIFNQNLGGVQEASDYGPACP
QHNIVSAFAPSDLGLGLVGGFIESLPFFQKILKQDEDCLYINVQRPQNESLEGLPVLAWIHGGGFELGSSSAVGLETT
PVTGIFYQGANIVKRSIDMGQPVVFASFNYRLAHFGFTASREFDEAGLLNLGFEDQRNALRWIQKHIASFGGDPNK
VTIMGESAGSWSVSGHLVANNGDNEGLFRAAMGLSGGPLKVDGPERQQGMFDDMVRFVGCNSAADKINCLRK
APYEKIYQHMQTINSLIGFRSLASAWTLRPDGKFFPQSPDKLAAAGRIANVPVLYGDMRDEGTLFSLINQLNLTTTR
DVKEYFKTYWWPAATEQQLNRLMELYPQDPTKGSPYGTGLLYAIPPQYKRLSSLIGDYSFEAQRRALLQKVAAPKW
NYLIEASIPLRGAENTILRPLIGAGDIPILGSFHGADVALYWFNTLPDTISLNSHHLLGVLVSFVNHLDPNMHKMDGL
PNWPQWDGQNKKTMRFRERGPDIVTDDYREMGINYINEIGDSLRI

>QLI71886.1

RDPPSDDELNRQAEKICGRLENINMDKCKSDAKDCVTSALDRKSPSEIENGDEVFWRHIRECTVSRNINGNGDSPK
NFVAAFDDSMRFICEPPAKGNKMDCVFIFRKMVEKDCANRGCRFPRELPHFCAKLGGCEECEHGAGDKTNGGLE
GKFPGAGPFFCTLRQNKVDTTQPAPLNSNQCNEIRMQKFTECRQSNPYDFDRCFNEGQDAFTKCGGLQ

>QLI71892.1

NGLSTSDHSVSHTSLLPLISSLPASETTYDVVPRRILENEIRDAAGSLTTPALLPRHTSMRMVGTAANAWFNPQSER
KDSYGGICLVVIAVGVAASVVVLVVASVAVFCIRCKRCTTNVLSAVEEQKWPKSTMVYEAYPPWLLKIPKQPEQPQ
VPVLQRGQSWTSTSTMANSQNDAVSPMDPQTWDGAQHTCQVSPTATYMSPVGYPTMQGHQGAPQWQPPA
ELPTTPDPGNTPLPSYPATMADAHLPPRFSWTGEEEASYRPSSWMKR

>QLI71901.1

PVVESQIDQPESVINDNTLAPLELALGLKNHVKLAKRSLQTQTSAPWGLRAISHRSPGDFYEGFPPPESSKYYYDDK
AGAGTFAYILDSGIRTTHEEFEGRAKAAHSIYPADQTIHGDHGTGVAGIISSKTYGVAKKATLISIHLLGPDGCTGSEAI
NALLWAAEDILKNSRKDSSVINLSFGIPKLQALNTFVERLIIETDVPIVVAAGNEADDASNHSPGSADGVISVGHINQ
QWAISETSNFGSAVSILAPGVGVETTGAGSDTNIIRETGSSFATPYISGLILNAISIHGIKGAANLKKHILETAIKDKACI
PPEKEDKNNRTPNLVGNNNNAEQDKEKQEDEPSSSRMFCCNGLLSKLACGRNRPMNV

>QLI71906.1

SAIHVRQEAAPPCQEQVPSEEFVEISRKLRLEASNSSLTRRQQENFNIQVYAHVVYDHTRGNRGYVQEHLIASQIQI
MNQHYRNVGISFTLADVDKTRHPRWAYGYDEFAMKENLRKGGYADLNMYFVASIPGGAAGGSRTYGICYYPIPR
QLQFVEFIRDGCTVIAQSVPGVDGWVKQNHIATHEVGHYLGLYHTFEWGNWGERICSPGDDVGDTYPQEQPSH
YNTRPMVWACRGWQRSNMYNFMDYS

>QLI71910.1

SPLRKLPGPTISLFTSLVLKWKEISAERTLYIHELHQRYGPVVRISPNEVSYTSWPALKEIYCSGGSGYGKSDFYNLFTIY
GRRTMFTILNKADAWWQHAKRKRILADRYANTNVMRSVSLDGIQARSRAFLHRCTQAGDSANDIFLPSHTANHH
QALHAYACDCITHHLFHPNGSDCIGTRADEDMMHQVAADDSLQNRLISHYSPTLYRLFAGILSLFVKPRSVPLADN
YVLETSKRTDAASFTLMSRLEEKSGDLDHIDMAAECLDHMVAGIDTTGDTLCFLMWELSQPRSLHLQRRLSEEVRR
NPDAGIDQLPFLDAVLCEGLRYYPAIPMSLPRLVPQGGRTIDGFHVPEKTIVSCQAYSVHRANTDVFPDPDAFHPD
RWMAAAGDADRRRLLFAFSNGGRGCIGKHLAIAEMKTLLRDVYSRYSTTPDPSMTAESMAMSDQLISTRPLGKRC
LLQFHRLPDDVKATGGSE

>QLI71919.1

LPATKRGIQDKYTYYQGDGSVAAGWPSMDSWGSWDDLWKANSGLMNNTCTWNGWGQDDSSDEIAAVGSAI
QDVANQTGVDERFILAVLMQESKGCVRAPTTNNGVVNPGLMQSHDGSGTCAGVDPCPSSQITQMVHDGVAGT
SSGDGLQQLIAKAKGTVSGDSQVYYAAARLYNSGSANYANLDLGMGSTACYASDIANRLTGWTLATTACH

>QLI71924.1

RPASNAVFIVGGSPAEAGEFPFIVSTLRNGRHWCGGVLLNANTVLTAAHCVESQPAISQVRAGSLAHASGGVVANI
SSITPHPKYEGLGYDMAIVKLSTPIEANGTIGYATLPEAGSDPVSGADATVAGWGDLEYAGQAPEELQKVTVPVVD
RATCSAAYQAIPNMPNITDAMFCAGLKEGGQDACNGDSGGPIIDTETRVLIGVVSWGYKCAAPNAYGVYTRLGA
DIEFIKSHL

>QLI71943.1

GPVETEQGKKPFIDPLYNAFGPKCLADNAEFPRDSEACLGTRDYCGWKKYEAMGEHFDSPKSCIDSRQPPPFKEPA
SEENQANFCEGNPTFKGDAWRATTEPCLGTVAYCNKKVYEGTGETYDNPDECITLRLKTLTKNASLFQPLLDGIHQI
SSAKVQQGLIDAIPKCKTVDGGTMERPLVEIECPKVYY

>QLI71945.1

AKTTPRPTTLDNGEFVSPLETCDDSCKSSFARPSEHVKTIKSKFKTKTKATTVSPSAEPDLSTALDLGPGTAFPPYVSSP
DCTQTTTVVGATHPAVVTPLSTTSSKQGKDKDKDKGKGSKTEHSEAPSPTSPLEEEPAITTTSVLWKDCGTCALFWT
TAQSTYVPRVTVANPTDYTYTITQVSCLTIAQAESTGTMLTELPETTEAASTELPEPTDEVPEPTDEVPEPTDEAESTE
LPDTTVPISIELAEPSTTA

>QLI71959.1

VNSTPIEQTLQQLQRGNQVLDLVLQYGFSPKQIADQAKAQVNDAVFAIRSAVEEAARAVHYANTSIRMDANDLEI
ARRNLGWAAEILESDRHHY

>QLI71962.1

HSVISYPGWRGNSLITNDTFPYGMQWMYPCGGIGTTKNRTYWPTTGGALAFQPGWFRGHSLAVIYVNLGFGTN
GPDHGPENMSNPMVHPFTIQGPTNNPYPGTICLPQIPLPINASVKAGDLATIQLVELAQHGAALFSCVDIIFADPG
DPKIGEVNETNCFNSTDIGFAEMYTIATKMSGTDAYITSAAASLRALSWMGYVPLVVAGLLTLL

>QLI71991.1

AELQPWKSGPGGTEQECKKLSLQKARECFDSHEARPDTEPKLPWQDGEWKANPRQFKLCNDTHPNPNFKFRPIL
EQFCGTEIYCNLFDQDKTRVDGLFKSTEHCRQSHEAAP

>QLI72012.1

EEDCGSLGVMEVDEAKLPEGVDASQVRKCLGHPEGHGTDDSGKALFARECEFRWALGCSRGGYCWKTCDTDNN
GKWCWAAEASGLGPWIRCSSYKDCERIASIAI

>QLI72027.1

LQAIILADTNRDGKVDMNGNSDDALKSTWTEERGAIFMANIADTNRRCSARNNSHHLDKCHDASDNILRNSKYLA
PLRTVADGALSDAARGKIFVPGKYAEKNVRIFHKSGGNWNYVNKSYVFSPPDLRAGLELGIDARDVGRPKGWDG
MAMVNFVLTDQEQSANDTVALRVAPILIPHDKRRLEKMSVATHKRYPNWELFAEHLQKYLVDRRFEEPVTRIDSL
HGWAHDHFKAGYTSMPGPDGPISLRVYLKGCLAGPEGERVFEDMRGDSAGAVRHHETSELDSDQLGNIEIIPPHS
HNGANYPAGRAVMGFARFMSGHKTPKMLKFLQAQKFQHPFTLDHSWLFRGQIADYMQFVPANTSRGWAMIV
ADPVATLKLFKKAKKDGHGENIAISHHSFLRPEPEKDCDKMTISQLLRLPLVRKTTEWSGKSIAHNVNVIKNETGITE
DDIVRVPTLFYSRDGLTTLRQMYCMGNRYYVPPKSMALKTKRWFHYMWNMAKNFFELARTTPWPSKPLYPTAT
NGIYFENFQYLSPQPFGPLIDGSDIFVEAIRQAFGKVGVNVTFFENWVIHHVRDGGIERAVNVERDIFQKWWTMD
AADETPQREVAPENHTEDQRA

>QLI72036.1

AKYADRTVAITTSHTHCTPVTLKPSITVTATPIKAYTTTSSSSYYDGHAYLIGDGSDDVVYVQRFYSPGAIPASNLTSST
RDYNQTDFTSTDFFQTMELTAPSSCPTPFTVTASSQVLVPEEVIDQVSIKSATTIRTTIDYDDTAYTYTLVSAFLASTSR
PTGAAATRNYIYMPYITDCRNPTSTPRPTSDGRGGPSNGDDSGNSINQVRHIPGSCAWSNCGNTPYWGIIILTVLP
AVFLLGFLESYH

>QLI72042.1

APEGPKATVQNRAPWNLRAISHRFPHKLPSVTVFRNFEYYYDLWTNGKTYYAYVVDTGIRTTHQEFEGRAENLWT
AVKTATGEDDFSDGTGHGTHVAGIIASKTYGAAKQARVLSVKVFDDKNDATTSQILAGFNHAANDIADKGRKNTA
VINCSMGSASSPALKLAYERAHHRGILTVTSAGNNAQSVGAASASSASGSITVGSINQDWSIASHSNYGPHVNIFAP
GADILSLSHKSDSATAIMSGTSMAAPHVAAIVLNAMAVHSQESSLVDFFLETTATRDKITGDLRGSPNVLVNNNND
RQESSCGQQDDRC

>QLI72047.1

SPVPQPEAALSNLPSLNAAQSGYAKSIIAKAKAAGVQRHGCQAAIATGLVESKLLMYANNAVPASLTYRHGAISSD
NDSVGIFQQRASVYKNIACSMDAGCSAGQFFSQMKRIGGWQTLSVGALCQKIQQSSFPDRYEKQVAAAASICAA
GGL

>QLI72050.1

LSIPDRQDGSPRVVGLPLHRSEIRDPVTHDRNRMRKRDGTVNATIDNLQTLYFLNASLGTPPQNVRLHIDTGSSDL
WVNTRSSQLCSMRKRPCAESLVYTANSSSTYKYVSSNFNISYVDGSGAAGDYVTDTMSFSGVNLSSLQFGVGYEST
SEQNVLGIGYPSNEVQVVRSGLKPYDNLPAKMAADGLIASSAYSLWLNDLDSSTGEVLFGGVDRDRFRGSLISLPIQ

KVGNGYNEFFITMTGLDVGSKNVGKNMALAVLLDSGSSLTYLPDSMVESIYGMVEASYDPSQGAAFVKCSVQQTI
ASMTFNFSSPASVTVPMNEMLINVSDTEGEQLIFGNGEPACLFGILPAGKASSVLGDTFLRSAYVVYDTENNEISLA
NTIFNATTSNQVEINSKSTAPFATKASNPITATSGLPGGINSKGNTQQPGNAASSLAPSLTLLFVCMMARALI

>QLI72069.1

ADAQDDKVVKTIVPGAYIFELEAGHDPAAFEQTVGKDGTTRMKLDYELFKGVSVQLHDVDKAHEKAAKLADTPAV
KAVYPVQLFNMPKPKVEWIAQDGTKAPGGLLSSRADDGADTFSPHVMTQVDKLRAKGITGKGVKIAVVDTGIDY
KHPALGGCFGEGCLVAFGTDLVGDAYDGFNTPHPDPDPMDCGGHGSHVAGIVAAQPNTYGFTGAAPGATLGAY
RVFGCKGQAGNDVLIAAFNQAYQDGANIITASIGGPSGWSEDPWAEAVSRIVDKGVPCTVSAGNEGAEGIFYAST
AANGRRVSAIASYDNVQTPTLIYSSQYQVDGDADTKFGYVPSDPASWDGVTLPAWSNSLDPTIPDDGCDAFPANT
PDLSKYVVLIRRGSCSFAQKVNNAVAKGAKYVIVYNNNAVGAIPMDLTGVPAGSIKAASMIDGTTGATFINALKDG
KKLTLKMVSPQKTDSEVSTSNNTVTGGALSTFTSWGPTWEMDTKPQFGAVGGNILSTYPRALGSYAVLSGTSMSC
PQTAGIIALIHEVRGTYDPELIQNLLSANANPQLFNDGTKFYDFLAPVPQQGGGLVQAYDAAYATTLLSPSSLSFNDT
DHFVETLSFKLQNTDSKGITYKITHTPAMTMYALGDGSPSVQQFPNVAVQAVATIKLSETSVTLNGGQSKSISVSPT
PPQGLDAKRLALWSGYIVINGTDGTSLSLPYQGLTGSLHNSAVLGANNTWISKSTDKKSNPVPPNSTFVIPAPGNA
GSNDTLPQLTVELYLGSRKIRADIVPLTTCPPKNLTTEFQGIKTIGQPYNFPALWGTRGLNTFPWDGRLDSGNYAPP
GKYKFVVRALRIFGDEKKKDDWDVSTSPALHIKYQ

>QLI72089.1

APSTKTTLATVSLTIAGFILFANLSYLEHTRSLRPSTPITIYLGISILLDLARVRTLFFIPGSQSVAKVNLASFCVKLIIFLLELT
EKRRLLLPAWQDASPEAVGSVYNRVLFWWLNSLFMKGFRNLISINSLPVLDTELLKTANPTELVEKWNRVDASKKN
ALLWTFLLHYKWDLLAGVVPRLACIGFTFAQPFLLERVLDFTAEPVESSRKEFAYGLIGAYAIVYIGKAATFTAYQHKT
YRLLTMFRGSLISLIFSKTLRMSATKISDAEAITLMSADIDRIALSLELVHEFYSSFIEIALSMWLLYRLLGVAMAAATAH
VVGIPIAAAAGNANVPWLEAIERRLAVTTKMLGSMKAIKMTGLTNVMSNVISSLRSLEIGASRRHRIFTVLEAVTSY
TSDAFSSVWGFGVFILLARSNNTTTLTEGIAFSALSLFSLQVQPLTFILNGFEDIQTIINSFNRIQEHLASEEREDPRDTP
ETRKNLERDSSSETSLGFGEPKAVPLGSVSALGDTNAAIVIKDATTGYSAESSILNGINLEISRGKTTVVVGPVGCGKS
TLLRLILGEMPVTSGTVSTSFSRAAFCPQSPWITWGTIQNNIIGMSRWDKPWYNTVVKACALSADFEQLRDGDQT
NVGTRGSRLSGGQQMRLSLARALYSRNQVMVLDDVLTGLDRATEASILDAVFGPSGLLRSSQTTVVLTTNSINHVQ
YADYIVILNKDGTVAEQGTRDSLSTSVGYIERLSGIVQGTTVRPEFELAEETLQELGLPDEDEEQDVTSRGTSDWRIY
AYFIHIAGKWTFLLYLFGCACFIFGLNFPSIWLQWWTNANAKTPNDKIGYWLGIYGLLGALAVLGCFASDWIFNIILL
PKISRKFHELLLATTMKQVLPFTCLLLFNFLTSLLSAPTSFLTSTDAGQIANRFSQDLQLVDNDLAHALDQTVVQLFTV
VISAVLVFTGSGYLAATVPVCIFFVYMIQFYYLRTSRQLRIIDIEAKAPLFSQFLETLAGVSCIRAYGWTDNYLQRNYH
VLNTSQRPYYLLWCIQRWLNLVLDLMVGGIAVILVAFATATAGGKTGYLGVALFGIINFSGTLQTLIAQWTQLETAL
GAISRIRSYVSNTPSENQGANTRHPPEGWPLSGTIEFSNVSASYQSSQEPVLKDVNLSINAGEKVAICGRTGSGKTSL
ISTLLRLLELDTGSVTIDGVDISTIPRQEVRLRLNTLPQEPFFLQASIRDNVDLLHLSDDDSIIAALRSVNLWQMLEERG
GLDEMISEELLSHGQRQLFCLARAIVKPSSIVIIDEATSSVNSDEEEIMERFLQDDFRGRTIIAVAHKLHTVLDFDRVVL
MDKGHILENGNPRELLANSESAFHSLYMSLSTVSEKKQGQ

>QLI72093.1

KCTDDEDCSLNGVCGRDTACRCDDGWIGDDCGILDVRPAKLDNGYNQTAKGTSSWCNSIVKDPLERNLHHLFVS
EFSHGCGLDYWAPYSRIIRAESRSGPAGPYEFAAQVQGTFAHNPTVVYSPADREWLMYHIGCPTDVADTCQSKHF
SCGAGNTNNGESGIAVMSSRDLRSWTPKGQILKGNNGRGWDADVTNPSAFPLFSKTSYGIHGRFDDDTPAVLLA
YRGCPYNCSGEELINVAISETGYAGPYEKVQQDPVFPNGNEDPFVWRDKRGNYHMLLHSLEPEGGFGDGPKVGR
HAWARDYKGPWTFGSRTLAFSTDVSYVDGSVITFYRRERPQLYFSEDGTMTPLFLGTGVQPRDSPMSYSVIVPVG
DAGVHAQGP

>QLI72101.1

CASCQGKAWTRRSGNDDNNNNAISPNSGAGFAQFACRTVPASGDDMGKRASVEGDCDQKAAIETALKSCADYA
NAAVEELKKKDSQLFEFFFKKENDGQRDKIAKRYEQIAAECGAAQGGKISVTCKEVIGSCTVPETGQDATAVADTKV
GDRLGTRVQLCKPFFNLREGGCGQFDRPSTLVHELSHSLVGTKDFKASYGLKAIQGLTAAQNLEHADTYGFFSQAA
SKGCKTADLEKARDSEVTVSPPGQGTQPSTGQLEGTQPSTGQLEGTRPSTGQLEGTRPSTGQLEGTQPSTDQGAG
PVPPQILQWFRSKWPGSTPEQGDRIVYGA

>QLI72104.1

APLADVDGKASANPSVATPSMSAVPSSPIPQELLVKADVIAKAKKIISETHTLGNTTGYEGVSASHSKSAWAGVGAE
IKAIINILDKIGENGKQNDELLKDEQHHISVVVAKLNEKLDVEPQPKPLQACDKHANATWTPQVSSVEVEIKSILSKL
QKTKVQGEAVAVRQVNLLVRLLSLLTAKVEAEIKAGAIRNRGVHIDVEVHELTLRLAKILTIKAGINTWNLKSTNASA
NVNVEIHSVLVKVLGLLRVEANGDAKAGVSHNQTISGDVSSDDSKIQLLEGLLKVLAEADVDGKAKTDVDVKTKA

>QLI72105.1

TTSSTASTSRSSTPAHTSSSSTARVTGRATTPSGSTRPTSSAAASTTAKKAGAGPNAVAPGAGLAALLALGAYVA

>QLI72143.1

ADVAAIVTSVTALQARVNEFQGAAQADALIAASKGVVAAIKKGTADARASPSLSQDEAFSLVPPLQNLIGVVNKTV
GDLVTKKHVFADSGRGCEVYPQLVEQKIDSQAFSDALVNKVPEALKGIAGQISAPILKSLADGAAAFKDVECAATST
GATQSSTISVSSTVSSAGSVTTTGGGTSVSSPASTSGAQTGSASGTATEPGSATGTKTPGTATGTTETGPKTRTGSG
TATATASESCGETTTVQVPVTETVPCTTTGGSGSHPTGGPGSQPSSGSGSQPTGGSGPQPTGGSGCPGCAPGQTS
PGSGSGPNPTGTLVPIPSGGEGGHGGSGGNGGSGGSGGSGAHPSPSSPVIVAGAPRGEIPLAVHVAMAAIVGAV
GAAL

>QLI72160.1

LVNSVSQNSIPARAVNLDAHEFPTGLQINKPNITSKWIDGSDLVQIVEVYINNTDPTNYLTEADNLVVEVLSDSVDT
VVRGTLKRLGPRQAAIVQIGIKNKPKAKAGSQCTGIIVANYGNKRGGKQATQPVSGTCGFIDYTATRSSVNPHLAP
DWFNDLKYGIFIHWGPYSVPAYGNVGKNENYAEWYWSSMQYPNDKTQTYQYHLKTYGKDVNYDDFFANFTDR
GFDPQEWVDLFSDAGAQYFVPTTKHHDGFALFNFSTAISKRSSIHYGPKRDIIKDLFAAAKKYQPHLRLGTYFSMPE
WFNPAYKKYGFSAWPGGPPTNPYTKAEIPYTGYVEVKDFVKDIQLPQMRTLADEYGIDIMWCDITSKGNNATIFA
SEWLNSARKNKRQVTFNSRCGIPGDFDTPEYATNGETVTRKWEASRGMDPHSYGYNAQTPDSAYLTGEQIVHTL
VDMVSKNGNFLLDIGPTGNGSIPQIMQNNLRDAGRWIKSHGESIFRTRFWTVKPGSDPFRYTTTKDAFYIHHIGKP
SKTLDITDPVPYLPGDKVTVVGGRLNGKTVPALWNGVGSLKLTLGNDVLSGDRYIWTFKIQYN

>QLI72164.1

VPTGSNTIHEKRDVSSGRWTKREAADANTKVPVRIALKQKNLDKGMDYLLAVSDPSSAKYGQHYSKDQVVELFAP
DESSISAVRSWLIKSGVSADKITFPQSKGWVDFQSTVGQLESILKTKYHMYDHIQARNAHIGTDEYSLPNEISDLVDF
ITPAVVMSQTSKPSNKVKRNDGRIIRPHKPLSAEVAKLLAAHPDSTDNCAKYITPACIKSQYNITDGTLHDASNRMG
IFEISEDAFSQEDLDSFYAKYATNIPKGTGPKVDLIDGAVAPVSPESAGGESDLDLEIAIPLIYPQGTELYEAATKNDDIF
NTFLDAVDGSYCKDPGDDPTVDGNTPNEMCGTFKAANVISFSYGTAEADYPTKYLQRQCDEFMKLGLQGTSIVLS
SGDDGVARRSGSCLGPKGDIFTPSQQASCPYVTSVGSTVLLAGGEEIATETFSSGGGFSNIWSTPDYQKDAVSSYFS
KHGPGYKSYTTANGTIPTTGGIYNRAGRGYPDVAALGDNGVVVVNGEPSTSGGTSMSAPLVGAILTRINEERIKAG
KKPVGFANPALYKNPAMFTDVVRGDQSKGGPNGDGQPSACGNKGFSAVEGWDPVTGLGTPKYPAMLEYFLKL

>QLI72166.1

QEIAQNAVAGAFLLECDSQSLKPLIKTVQEQGGEIRREFNSEVFYGFSAQLSNASVAGDELRHMPGVKKVWQVQV
SKHQESPPAESQATPESAHQRRQVKSPWNHVMTQIDKLHAAGFTGSGIQIAVVDTGVDYTHPALGGCFGKGCRV
ALGDNFAKDGKDNDPMDCSGHGTAVAGIVAGSDANYLGVAPNATLAGYRVLDCSATMEEDGLIAGWVKAYQD

GAQIIVSSAGWPGAAWATRPAAAVVSRIVDSGVPCIVGLGNDNNSGLFNTLNPSSGRGVTSVNAFARAPGAIDG
HVTDAPVAQFSTFGPNWDLEIKPTVGAPGDDVPGIKMGGGYEDITGTSFAGPLVAGILALVAQVRGTFDPVLLNSL
LTTTAVPQGKYYSVAQQGGGLARAWDAAHATTLIEPGSLSFNDTLHRADSRSLRITNTARVKVTYHLDTLAAKTIYT
LENGGGRVQHLDRPVDESADVKLSRRLLVLGPGESASVDVSATDPKGLDPERLPVWSGCISIQSSHGGSSKCSNSSS
VLTVPYLGVSGSMKEHQVLQPHGVVLSTLLDNGRDQVHTGGHRVDYESKNDGSVSIDLPVRITPVLGTRLVRAEV
VPLSPRKWLAARLADKNLKLDAFSLEALAHAYATRKTWSGRLESGDYIPAGEYRLAVRALRLFGDAAVASDWDLSE
DVSFEIIRPAGRKACERYESSKGAVPADALFTSLEECLQVHGDKVVDAPWVPAPQDKALRDRCANGELTDELCGTY
ELCRKHKDIELLSDVKSPFTSLSSCIKSHKTFPFKPLDYSRIQECMANQDDKSICGTDLWCNLHFGSPQPTDEYGSSEE
CRWAHGNYL

>QLI72167.1

QIDVLTGALSNVSSSVEALDKATTDFNIDAIKTNADTLISTIATGKDAVNASQVVTLVDSLGLNGPVLLQNKDFETLA
ADLKTKRPDIEKANACSDVHSRIASINTGSLGLTEAIIAKVPQAAQPIVRQVAAGASRVLNQAQYDFSEDNCKDASP
TASPTPSPTASSSADSKQTPVGAIVGGVLGGVGIMAVIGLSIFLFLRRRRQRRLKQEREPPVELEANDGPPPLLKDYK
EECAKAERGEQEAERAEMCADSTAIHEAPENARYELE

>QLI72172.1

SPLGEPSLSKRVDTAKIKEEVQQLNVLIQSFQEAKDKKQESKGEGWQQTIDLLKERAAAIEAAPEDKVAEEIGKLDEP
LARIRKSREGAIDRYTRNLFGFSDAKDRLTEAAK

>QLI72173.1

VIFKDCGSTAKNINIKIGGCPDAVSVCRFPSGQNASIEATFTPRSSFQEATIKLAASVGVINIDFPMKFPEACSHWGLK
CPGKAGLPQTLRGEVSVESSYPKIKTEVTLQLFDEKGETLICKSFPAEIK

>QLI72181.1

SQSKCSQSTLEAILDSIPGTSINYVEEVPQDGAFGDAATNIPFPRNATGLPALCAASFNTKTPGNTSYNFGVFLPTN
WNGRFLATGNGGFGGGINWIDMGIFSQYGFATMSTDTGHSSAPTDGKWGLHQPEKLINWGHRAMHGSVTAT
KQIVQSYYTGAIRFSYYAGCSTGGRQGLREIQLHPDAFDGVVVGAPAWWTTHLQPWTMKQGLTNAPANSPHHI
PPPLFRIIADEMTRQCDAQDGLRDGIVSDPDGCSFDFNRLLCAGNKTACLTAAQIDTAERLYSNYLDAKQALVFPGI
SLGADAAALSAQPSTLGIDFLRYWVHNDSAWDYTAFAYSDVLLSERVDPGAATADDFDLSPFQSRGGKLIHYHGLA
DNLIPARSSRYFYDRVYRALTPRGIRVGDFYRLFYVPGMEHCMGSAAAPWYIGGGTQSVTGASHSVPGFADPEHD
VILAIMAWVEGGAAPDKIVATKFRNDSVFAGVESQRPLCAWPLRAAYLGRGGVKDARNWECVEAGTDRAERGG
SGSIFGRLTGESG

>QLI72183.1

DENIFKALPIHAGPLSLPVPAESHKVSVPCKQCEGKNSHLQLNLDVVDHTRLLLNGFEVYPNADPWHGDLAAVVES
TDGESAKQKLGYSLAVAPEFMEQDSYIQILDVELRVIEVGNRFVDNVPVVNVKLVKAPSGEIAITDVDTTSTKSSSCN
SMACTAKEVMEEVFKALKSFKPFKGCHRRPYHDKNKEYLESYPIRPQSDDAPEAHHKHPHQNFGGKHRHEWGRLI
TNIAAQIFLPVLMGITAGVSVALLAMAVCSVFFQLTSCIRGKRDGRFGLCPYSRGQAVQTASEEAMTSEKVGLMVE
EAEAPPLYDDSKK

>QLI72211.1

NPFEYGTRPEPGHLSGVGPRDSPPDYGPPSYGYGYPPPYQYETSISTATWIISSGSSTFLTSVTTISTEALTVTNQPPST
SDKYTTLTYASTVTRIRLLGEKLGVGGDRGFDFFQFHRTGAFVFYRRRSN

>QLI72212.1

QPEHPRIYFPREIKREYHNSTITSKVSSSTPHVTTTTTTTSSSSTRRDLLSDLISEILGSDSPQKSSTTEKPPVPTVAVDST
PTPGGSSGSGLPKETVLPTSKINESKTSSSARARPSTEPGIVIGPTGIVSSTSEPKTTVEPSKKTTVDSPKVTSVDASAS
NSTVPETNPPTPATAKDPIPTGEPPKQTSVDPAVTNNTTPETKPTTPITVDKPTKTGEPATKSTTVDTLVGNNTVSD
TTPTTTSGGILDPIGTLLSSLLPGSSSASTSASTKTNSPEQTGTSISSTSTSSPEQPIPQNSTTQAAPTTEPSTGLLPSLTS
LLPLPGSTTTTSGSGSLPNATSTEVPVTATSSSSGSIITNSPTATTTDVPIGNSTSTSSGTVSSAPTSAPTATDVPITNGT
ISEPPTGTATSVPTTPISVPNDNSTVIVGPTTTSNIDTVVPTTKSDFTSTVAPSTTTGVEVTSIRPTATLPNTDNWLPTT
VIIEPTTFSFSSPTSNPTETSLQALPSTVPRVIRPDTDGDKKPPVLEGAVPIHIAFNYTLHYLFLANDTMAASQIFKYLP
DVLSSACGLPADQLPVTELAPYNTLATRGFVTTLARMNYTKALLPALAANLKTPSSAIYQNRDPVLHQFASYIDSTIS
PTGELNDGSSTDNGGPGSGNDGNSNNSNGNGNDAFGSGNQGDQTPKQKATTAGIAVAAVGFCALYGAAMFIV
ARRYKRKRQGHRRASSIVSGQGSPEMRYNDNASPALMGGALLSRDGSTYGGTGGRDSHASGGHSGRTANISAPV
ATENSLGWN

>QLI72220.1

GVAPREADGVLESREGDGVAHAIIKTYKDKSCKELTEEYVMEIPRIMRHAVRLDTKGIIAITPCDVEVETTKGKLSIKA
RDTTCHPIEEGSEAEFVVVDCSGKT

>QLI72239.1

LKVPGESPLEFCNSNRDHDAVQIDKVDISPNPPKPGKPLIVTFEGVVKKNIADGAYAKVVVKYGLIQLLSTTVDFCEQ
TQNVDLNCPLEPGKMVITKSIDMPSVIPPGTYNVLADAYTDDDENISCLKATVNFPRPGLLGEEL

>QLI72243.1

QEAKTVKVEVGADGKLLYNPSNFEATVGTKIEFHYFPKNHTVTQSSFNDPCHPLEGGFFSGFVPTTESPSKTTFTIEV
KDTKPIWFYCGQANHCQKGMIGAVNAPQTGNTFEAFSALAVKASNSTSPPGGPVGGVLSNGGSQSDSTSATQTT
AVTTKPYTSTWTSNGQTFTTTATTTVFAGGPAPEQTTWAYTTKPYTSIWTSNGQTFTTTATTTLVTTATVGADSTA
TTSASRGAAAAATGSFNIWHAGALVAGVAAMM

>QLI72261.1

TEPNIITQTSTTTRFLTVTHCGPAVTDCPGSKTSAVVTTLGVTSSVTSSTISSFVSSTTSSTSPSTTSAPVVKTTVKTSVV
PPAPSSSSFYPASNTTIPVGPTGSTTVPGTPTGPAVIPTAAANSLHVQTGAVAAVVAAVIAMAY

>QLI72262.1

AEVKQGLQTINSDTSAVTAAVNNYNGGGFTNAVPIVTAQQQLSKDVKETTETAKNTGVVNDADANDIINYITGTL
QPNIAASLAALKAKKANFDADGLTPIVKSSLTDLKTDTDKLGAALIAGTPAGLQEQAKAIQKKIDADFADAIAFFSS

>QLI72273.1

VRWANDDELAVMNANRGKVGTALAMPSALEMQAMEVERMFNITPGLLANSTHQAQDRRGEEQALAANQFIR
AGGFAMFIAFTSDAGGALGAVWSDCYGAATRDGTATGKECGDKVIAALVKASYSFVMGGAGYAAVLIQGQADP
NQPVYELQNQLPPDSEHGVQMRRSADQECANHNGDFTGLYGVNFHLGSHIGAKLSGYAPCGTSVSTPDEIAQGS
FGYQLNRAAGDNGAARIQFYTVQDGSRGNVMGGAIVYETRVTDTCPEYISFDGCEV

>QLI72274.1

QDQANCYTIENWGSKIHDCGQCDEGYKFSYTQSFCDIGFQCCKGTMQLGCYFGDGLCLALGESISRMGVEENWR
VLK

>QLI72284.1

ATNTTNTTNTTRIDLNPNAKGACQPTRSGPHACGPNGSQDWLNTGVRGNGWDPPSLDVRNITHIPLKAFYKGVG
SPCEKYDRFFKISGMKYDVDPAMLAFIAFHESSCNADAPGPAPGLFQCDPSNCQNGMKQCQRPIMENSDCGAH

VLRVALNRAGGNIVYALGLYNGWFTAGDRTGLNGGRGLTEEYPCSGEGRAYRVPPDLNYLHDMLNGWFQGYD
MRSEDASTGGTYYCHKH

>QLI72288.1

GSSVAQINLLAAPQSRGTLAYVRDSAPPEFTYTTDYAQSGNWIGLFEKGMGPDTGGDDKYLGWASAPASKGRVT
ADADFDQCLPAGQYDAYLFRQRGPRSYPVVGPVTFSYPGNPDYHECEGGICKPAAESCSCADGKWWCPARDECI
PTTKQCRGKCPKGWRVAGNSCVQDICENPLAGVQWDAYRMSIGNGLGQIPYPEPAPFLPQFNIDTLFGGTPVQP
TSGTVGSIGWRNGVAWITNQVLQPIPQDYFFMSWTGYLVPKHTGSYTFNMWWTDDVSYLWVGEHARSEFSES
NADLKVDYASIDTFGKKRFSYSAEQGKPVPIRVINVQAAGPYSLCFGVTDPTGQAVMNTCGEGGVLRESDGQIAYC
KDVKFGSVFRHVPVKRAFTPDMPSSRCTNLKSGAQWNLYKFQPGSGPGHISYTSAQPLLPAHSIDMVLAATPNAR
YTGQVDKIGWDSGYVEGVGSIYPPADTGPRGYFLMFWTSYLVPSREGSYRFDVWWVDDVAFLWVGNKAIADFS
ETNADLKVDYAFLDVFGKKYFEYHVKAEDVGKRIPIRVANIQGGGAFSVFMMVTDPTGKVIMNSGDRGSGKLPQA
SNGEIGYCP

>QLI72290.1

ASVDARDEFDSSAYAAKDVVERDFAIIGGGAAGTYAAVSLADRNKTFTLIEVSDRLGGHTRTFHDPVTGAKVDSGV
QIHVDTPIVRDFFARLRAPLAHADLKDFGKPRYYDFARRVALANYTRGAVQPDYVAELDKYPFVENLIDLPNPVPAD
LLLPWPEYVKKNNLSYSSALAGLSWPATPGDPLDTTALAILNDGNHWELAAFTGAAVRGANHDNSQIYVNALAEL
KPHVFLKSSIIAARRGSTRKCGVQLVANTPSGKKLIKARQLIIAMPPVLDNTKYFGLDRQEQAILGKLSGKYYYAGVV
NNTGLEDDVAYNNAGADRPYHVASLPGVVEIAPSASPGYHFYWYNTLQAQTRAEIEGAARSTIKWLQTQNNVKT
LEPKFVDFQDHSPFHLSPPTRDIADGWYSKMKGLQGYRNTWYISALFVVSSTQVWNNTQNILSDIINAAQS

>QLI72321.1

AEIGRRSPFSQALERRQRGGNGNGGGNRGGGNNGGNNNNGGNNNNNGGNNALVLSANVQQEGSESDGNNP
GTNGQAASDTSNNNFINFCEGQTLTNGKQIGRGSCNGIPMGQIPATQNMVSSIFINPKNGDNLPANQDFKIQVKL
LNLNAGSFTNATSTYYSAPQKLGGNGNIIGHTHVTVQDTGRGANPTEPLNPTQFAFFKGINDAGDGRGLLSADVA
GGLPAGNYRVCSMSSSSNHQPVLMPVAQRGAQDDCVRFKVSNGGNNGGNNGGNNGGNNGGNNGGNNGG
NNGGNNGGNGGNNGGNGGNQAGQGNQAGQGNQAGQGNQAGQGNQAGQGNQAGQGNQAGQGNQAG
QGNQAGQGNQAGQGNQAGQGNQAGQGNQQAGQGNQQAGQGNQQAGQGNQQAGQGNGGVNNAQQR
PNRQGNNGEQAGNGRGQAGQAEQAKQAEQAANGRGQAGQGANGRGQAEQAEQAKQAEQAANGRGQAG
QGANGRGQGSRGQRGQKLTPVTRMDLQVNGDTLMAGAQAVQQVCGQDPNVYVGALRRGPLPMAKRLIVMA
SSMLTQQS

>QLI72366.1

GIVITPIKDDQIVPRMGDDCAFGVVTPQGCGPKRN

>QLI72385.1

APEPQPDIWGDITSGAGGIISDASTFVDGVKSHASTWADGASTWADGAKSQASTFVAGVNSDASSWADGAKSH
ASTFLAGVSSDVAKATSEAGAKASSLSQFIATATGSAKSSAESELSKVTSSLAAATGSSASASRTTSSSTAGGAYGPAP
TAAMAVGALMGGAALFANF

>QLI72391.1

TDIPKIKGLPEIPGVPIFGNLIELGTDHARVAQRWARKYGPVFQTRLGNRRIIFVNSYETVKYFWITHQSSLISRPMFH
TFHSVVSSSQGFTIGTSPWDESCKRRRKAAATALNRPATQSYMPILDLESTVSIKELLEDCKGGTRPVDPINYVARFA
LNTSLTLNYGYRIDGSVDSELLHEVTHVEREISNFRSTSNNWQDYVPLLRLWGAQNSSAQEYRLRRDKYLSNMLDH
LKAEIAKGTDKPCITGNILKDPEAKLNLAEVKSICLTMVSAGLDTVPGNLIMGMAFLSTKEGQAVQAKALQAIEAVY
PEGDAWEKCLVEEKVPYITALVKEVLRYWTVIPICLPRTSIRDIPYKDTVIPAGTTFFMNAYAADYDEERFKMPEKFIP

ERFLEDTEVGTPHYAYGAGSRMCAGSHLANRELYTAYIRLITAFEMFPSENPEDAPCMDCIECNATPTSLTLDPKPF
KIGIKPRSEARLRQWIAEAEERTAHLR

>QLI72392.1

GRSGQTGFETRCLSFEPEKLVENSKRMRLEYVTNGTTLEFPDNDPSCNRASQLVAANLCRVALYIETSKRSGITFELW
LPDKWTEARYIATGNGGVDGCVKYEDLAYTTANGFAAMGTNNGHNGTTGVSFLNNPDVIEDFSYRALHTGTVAG
KLLTSQFYNKTPAHSYYIGCSLGGRMGVKAAEAFPGDYDGIVAGCPAVDFLHLQGARAMFYPITGPAGPSNFIRPE
LWTGLIHDEVLNQCDLLDGVKDGIIEVPDKCYFDPETLQCPMPWWPFGNKDKCLNSQQVLQLRKIYATYKYPNGT
LIFPRMNPGNEEQAIKKLFAGAPFSYSQDWFRYVVLNDSTWDAINYDSSLVAIADAQNPFDIRTFPDSLPAFKARG
GKMISYHGGQDNQITMFNTERFWDHMAKEDRHLHDYYRFFRVSGMFHCNAGPGAWAFGQGGGAPAAGIPFD
PEKNVLAAIVAWVEGGSAPETLTGTKFVNDTVALGVDFHRRHCL

>QLI72411.1

ATRTTVVASYPPAPRTSSKSVFGFMYYNTPFSLFPPNAAISLTDDNSTSFAARPAAFGPKLASSGLSGQLWVGSGFG
EDSLDTDGELGCSDLPGWGSSNTRASLKNSFKVGSTRLPSAKVKVPKKGGTLLDHDNREAIAKGKAQAKSPKNDN
TDNYLTEEVAGTPSRRDSSVGSSGHADIQSIQEAAEIEGKIVLLKRGGCGFLEKVMWAQRRGAIAVIVGDNQKGGP
LIQMFAHGEDVDNVTVPSVFTARTTAQLLSSLTQPGSFIEDTLDDNGNAVLKVQQTPAATHKKQSLARPRAATAA
KGPNGSKAKRSVEEKRSKSAAGSGKGLGGGIFSWGTTTRPVDSESRPPRSGRLDWVLVDDFNDEKDRLISDTMGK
AKRPMPKKDNFVIGVHDWRDPDLVGTGEQKSTAEQKPAKNAGSGKSKDGGASSGSEPKPAEPDSKHHGLMSKL
FGSDSSDGSKSAMPSVDEGEDSPTIPPPPDGSQPHEGLWVTITPTSSASPFFDTLLVLVISPLVTLTVVYALLILRARIR
RRRWRAPKSVVDQLPVRTYHTVALSSPSLSPRLPSPSSATPTTPLLQHNPSRPRPRSRTTTGVMESENLLSASAAVP
APQPRASRRTEFEKGAGGFSAEWRKYMGRQVECVVCLEEYVDGVSRVMSLPCGHEFHADCITPWLTTRRRTCPIC
KGDVVRSLARGKGNGPRYDAYREDSDDDDEEEDEAEASGSGSADRQSDLERGITSLEPRDAPQRPDRDEGWLGIL
SHSFNGMTRPHPQSPSPEDRNR

>QLI72412.1

WPTLAVRGLLANVPTFKDADFDTNNAIEILGKRGKKNAAKSATCPTAITKAPATITSAPSSNNTVLRIKGENNKKIKT
NGFFGSPNSVQGGFLFTKINMPSNAVGNLEVEYNGTEANTILVAPKMANIQPPAGMVFVDPMTFIVSTAKPPVT
GDTLKIDYIFTEAVKSAVDPSLVRVGKLDTAANQWVTDGLGEFEFEKEENEWSQEVSDLNGEWGIFAPVAAGQPG
QV

>QLI72431.1

LPLSMTLSQSQLRLISSIGVGVLVGTSLIVIIPEGIEAAASSPITSSHAHKVRSLSRRSPWKFGLEEREIIEQLAAVTTRHQ
VNRDDGARLHSEPMRIIVSQSTPTPDQKRDDQPSKPAPTDESHDSPKKEGEKHKEEEHVEIPAFEVGFSMILGFILM
FLIDRLPKHASDSLQSGPQPQHFSLDNLGGNGIETTSVDEEQGFLGSLAPTPKHTRTLATTIGLVIHAAADGIAMGA
SSASSNAKLGFIIFIAIMIHKAPAAFGLTSVLLRQGLSKRAARGHLAVFSLAAPVGALSTWLMIKLLGGDHLDGDAG
MWWTGMLLLFSGGTFL

>QLI72457.1

VPTKLSQRSLITQVGNVVLQLEDGVGVTAVEGTLDTLLGGALPKLEDALGVTFSEKLLGLSRSGAGPGAVQAVGEA
VAMLLQGIGLPQVDAFLDSATGGAMSSLEGALGVTDIEKALHLSGGV

>QLI72458.1

LGINCRGSGLCTSNKGILGEALGQLRGMDQSQQFSDGQHITCVKSSVTIGNPSLCIFYQNTNGRRWTVAQTASFV
QQLIDHGCAACGSVPTDPGNNVKNGQLTANMVTNAARRGLDMANKIAKREEAREETREPEPVTKADDSTAILAR
ALGINCRGSSTCGVGGIGHSPAGTLEQVRDAVAAGPDGSWTNGQHIACVAHVTGRLCAFYQNIGGRSFNKQQSV
TFLDQLRDHGCKNCGSIPTDPGNNVGNGQLTVNFVA

>QLI72459.1

ASNTKVPSAADLAASFTKIPPCALECLAQSVAEAGCGLTDPQCICVDEYVAIEKAGAPCILEACSLTEALSTKNVTETA
CLKPIRDSSRRYDTMNILLGVITALLVVIRLLFKKFFSYRRELGADDWVILATVVIGVPCTIINKVGLTANGLGRDVWTI
PVDQLIRFVMFFYIMEVLYLTEMALIKLSLSLFYLYIFPGSGIRRLLMGTAVFNVVFGFTFVTTGIFQCTPVSRYWTQY
VDPDSPGHCININLFAWIHAALNIALDVWMIALPLSQIKKLELHWKKKIGVTLMFLLGTFVTIVSILRLQSLVDFANST
NPTWDNWIVAWWSTIEVNVGMICTCLPTVRLILVRAAPRIFSTNISHNKSDPTHNGTHNRYSRNSKIMGHKQIEL
ASIETRIVEEGEKPRKARAFFGAEG

>QLI72461.1

APQPPTPLDVQLQMDGNSAVKATITNNGKDDLKIFRTGTILDKSAIQKTRITGVDGKAASFEGFRQRITTKGLKDDA
FERIPAGQSIEVTFNVGEVHDLSSGGKFDIHSSGVMHFANDSDNTIIGSVPYHSNSITANINGSEAGALLKSFQRAQK
RAMKRTNVQSDCTGSHRSVTDTALNNCQQLASSAQSAARSNDAKVKEYFKDSSEATKSAVADVFGKAATECGST
GGGVSKYYCTDISNQCNGGVLAYTYPGQSYQAYCGSYFEMTPLTSQCHAQDQATTNLHETTHLTQIKGTTDNGYG
YDNAMKLDTQKALDNADTYALFANAIRANC

>QLI72468.1

ADMVCTAASATTLLWRVTGFEYRDQRVAMGASRLEYGLANFTLQNSALAHKTTCTAVSTRSSPGFFAGDVAYDC
AVRPDAKGDLSRFAFDRASGLLTINQTWACDRDGSRFGGQGQVRLSLKCSEREQSSTLHSGTNYSDRSVVCDKVN
VPVNITYLQGTI

>QLI72472.1

KTIRIDVGKDGLTFSPDSVTAANGDMLEYHFYKQHSVAMGDFANGCRPAAQGGFFSGVMRTSGNGENDQVFQV
MVNNTNPMAFYCTVASHCQNGMVGVVNPSSTDSLDKYTSTAKSASTNQAPPAVFGGRMAQGSATSPSGASGT
ASGAQPTKSGAASHMQASLGGIGAAALGFAAFLM

>QLI72478.1

AGNSHKYDRRSHIPARSNNGTAAPKAGLFDQLIDHNNPSLGTFKQRFWWSADYYGGPGSPIILEAPGEAAVDENS
VNHSNSTLTGLFAQTNKAAAITLEHRYWGGSSPVGKNLTAQTLQHLNLDNSIQDLIYFANNVYLPFDSHGDSKPDK
APWVLTGCSYPGALTAWTNVLAPGTFWAYHASSAVVQAVSTFWEYFSPIEQAMPRNCSSDFKRLISHVDDVLFN
GTTHQKQELKALFNSSSNTSDSNFANVLSSPLGDWQNTHFTSGYTKFNQMCDYVENQWSGSNVTIPGPEGVED
QEDSDEPLWMWFLCNEPFEWWQVWGPGSDNGLVSKALTRDSLLAQCKDMFPTVGNSTYGLNKGRTVDQLNL
KTGGWNHVNTTRLMWVNGEYDPWTPATVSSKSRPGGPLKSTKQAPVWVIPKAAHCNDLNVGNRINEGAREVI
DGVVSQMQEWVADYYKQNGTGLADGV

>QLI72479.1

QTSPRPKLPEQLGNIERIPTEQQLQGIDPKDGLDACHKIDSITSDGWQWPCGMQRSFGPQRCVLKGETDAARKD
QQRCICESSTFFKDADACLDCKVRHALIGDEQDGYYQQLFAKLQETYCKAEVVNKNLGDYYNEAEALVRQAAPYN
GKTPGFTVPSNKVERQGHSAHPRDYYSRVGAPEKQGEINFKPSPEVEKLPAVNKTVVNQIPDPVNVPVMVIVALD
NSTAAQTSVLAATTTAGKPLQPTTLVRATSMNSTTRATPSAAVPSSISTDVSSSISTDVSSSIPTDVSSSIPADVQGQV
VQIGGKLCLVYVYFAAVDCVYKQSPDGKTTFYQYRKEINWFYGFQISSNKQFDLLKEVADACPDGNCKEQATAIAK
SKKVAVKEVVKKETVVPGKPQQLPPPEKPTTGGNSGVSGHDTPVSDNGAPVQQDDGGCGPEDSNDTPDNIPSQ
GRPGSPAQGNTKPSGPVTPQQDTPSGPAVSCNGPQCSPVVPEKDTPSVSGPPQQNTPSGPTGSPGTQSQCAPCA
DERKDGTEICRKKEETEGFCNNQSGAAKRDCICSKGDFGNKFFFNEAITCARRSANCLFAEFEAQVLFEAHFQYCQE
NKHDSISAALESVKNVWSARAPRQDLM

>QLI72488.1

KDIKNVLSAVGIEADDDRLKTLLSELEGKDVSELIAAGSEKLASVPSGGAGGAAAAGGAAAGGAAAEEKAEEKEEEK
EESDEDMGFGLFD

>QLI72501.1

APFNIVEVPTFVHEEPGFPLEGAAPPLEGSPEVPFLEGPEIWRPELETPPAFTPEGSPPPEGEIQPERQDAAAPEGSP
PPPPEGDAPPDSNEIPRYREEDRTPIDPKHRHAGIWDPLVPDVPEKDDAPTTRVYYTVKYTEGGIKNQGGSRNIAA
TIHGSNEQFIIDNMREWSGDTLEAKRSKSNPRIIVVSPVKPVKTMNEAIEVNKWAKNLVQSNTKGNGKGKGKGPK
W

>QLI72507.1

VGNAVVKNNCDFPVTVWSVGSQVSNANTLQTGQAYWEQFSRDPKTGGRALKITREPDGLYTGKPQTIFAYNLKD
GAVWYDLSDVFGDAFAGNKLVEGSADASCPSIVWSNGIPPAGSQVKNCRDSADVTLALCSN

>QLI72518.1

VDKTLLKGFSQSVSGDGFLAMPVGTVNRLPGVRRAANAFEDQLNNMDFFYATDVNIGSPPQRVTVLVDTGSSEL
WVNPDCTTTKTRQQANQCQQFGQYNPQQSNKSYGPFGKEQLNYGDPSDPKTQTSVLIHYYADTVALGDAKIDN
QTFGVVAESKGQAQGIMGLAPDLKGGFTTDEPYSLVLDSMAQQGVISSRVFSLDLRHSDDQYGAVIYGGLDRNKF
IGALEKRPIVKGVGGEWRLAVELTTIGVTMSSSSSFAVNKSDANVMLDSGTTISRMHSTVAVPILRALNATDDGEG
YYQVPCSAKTSGGSVDFGFGSKTVRVPLKDFILDLSGSSDTCYVGMVLTTDQQILGDSVLRAGYFVFDWDNQEVHI
AQADNCGKNDIVAVSSGTDAVPSATGNCKASDTSATVTGTSSAQPTSTTGSVSTKTYTTAYTITSCPSIDPACVTGV
VTTQTAQAVSTGGGGNKNAGVRVTAISSLLIIIGVAAMACNIL

>QLI72522.1

ATATRFEGIPSVGVLYTHSPKKHMCTAAVVDSKPGNMIITAAHCMRGDGKHLRFAPGYHDGSTPYGTYPVTGAYI
HNEWNKTFSINHDYAILTLGNATINGRSVNVQKMTGGNKINFSPSYKNVVKVFGYNLNEEKPMRCTTSTYRAGEG
QLGFNCGPFRAGTSGSVFMTKYRNKKMLGTIVGNIGGWQGGGCSDSTSYSAKYSSGLNRVFDEASNSTGKTDCG
WVVRGGAPKYC

>QLI72535.1

VEKPNVPLRSSSRWILDSAGKRVKLRCINWAGHMEANVPEGLNKKHIEHIADWIAGQGYNCVRLTYSIDMALNPA
LKVQDSFRAAATAAGVSEADMMRVYTAAVEKNSFLSGATVLDVFDRVQSALWNRGVMTILDNHVSKASWCCDL
SDGNGWWNDANFYVAATSRYFNTQKWLNGLKSMAQWSASRPGIVGMSLRNELRAHIAQIPWAPSTWLKYMP
RAGDVVHAENPRLLVIVGGINGGTDLSPLRNGAMKLGNWADKRVWEAHAYSFTVVTPSLGSCDIRKAEFGGLFG
FVLEQNKASTGPLLLSEFGVGMTGGPHDGLSNQDNDYLTCLVGYMENNDADWAHWAVQGSYYVRDKTVDYNE
TWGALDYEWSDWRNPKFKGMLGNMFAVTQGP

>QLI72536.1

RQETLKPFKQPGKKTNMALCGSGTFWGDAYKWNTEGCQEPHYWCLQSREPGSPVNQTKPTRDEVAGAPVTQK
RILEIIGHQNIACKEEEKKKIYCTASRKEEQVAVKDGLSPLCQEKGGCEECRLGTVNGFDTGFTCAVGLSSKNQKQDL
AEGLVFSD

>QLI72538.1

LVVRNMMSNRPESGQEAVRNLLYQMFPLTAGIANGAATLATFAFTLLGLMSPMRSWLKAGGYLITMCGLFTLCL
GVYLWIMTLRLKDGFFPTYLELEPGVQSLVQQSFQCCGYYNATTPAFVTDPTCPSPAAAALLRGCGTAISSFSNTFID
NIFTALFGIVGLDAILILSIACLLKERKERERYRHIDEKSGFRQF

>QLI72539.1

APLGKVTDYIFRGDDRDPTAIKKTGGFKPTENTYNDQLAFSLHAHIDHTQGDTAYVSTSKSFGVAVQFATTGGWVY
RVHRLGNMIDTNAALRDPPAEEQQEFAALGGVPYDAIEGWWQVPNTLDVPEFSEDQAKELDRDYAAKYQSNFIQ
NPDFNFDKYKNEQVQGPGEDVDVLASMPEEDPNKWDDTYWDGLRNTEFKKSALSFMNKHASSLGWKANQQF
PLALWEEGSGEPPAKKPKVEETAGPSGQTVPASTAPKYVFYGDYLWPAEAKRQGGFLTPADSLATIRNPPVTAYTL
NTHLNRDKLKLHPETYFVAVHQTFGAAAKEAVKKAAKFGGQFDPVVYLVHATPHMFKVGNELAVPGGMVWGQ
VRGWTQVPRDYALPEKTPTSKQELHKHFEKAYKAKPKVVFQKNPDYDSKFDQYTATEKEQPQLLGSRKASRELTNF
MKEHGSAVGFQEKFPLFTAPKVITGEASAAAKNVEPAPHEEEGVLEQVWDFVKEHAVAIALLPAVAALNLIPGVGE
VADAFEFAALSTEAAEGTGLVLEGTAALEEGAVIGEGGTVAVEEGATTVEEDVATVEKPDEELELPDVPTDPIEEDL
NLPDVPTDPIEEDLNLPDVPTDPIEVPEAVPADKVALKAD

>QLI72543.1

ATKAQYTREASTAIKALNSNWYKQDTGLWDDAWWQSANALTTLADFAVLQPGAAKSLGIANTIGNTVKNAPKKF
PGFVNQFYDDEGWWALGLIHSYDATRDASYLETAADIFNTMQTGRGTPCKGGIFWNKDREYVNAIANELHITVA
VSLANRIPTNRTYAQIAKDEWHWFQKSGMINSQNLINDGLDSNCKNNGSQTWSVNQGVILGGLTELSRDSLLGA
GVLDTARTLAMAAIQALSNKDGILVEIDKCETQNEACGFDGKQFKGVFVRNLGYLNQALKDGDIRAFILKNADSV
WNKDRDDTNKMGVAWTGPVIAANGASHGSALDVLVAAVRVA

>QLI72551.1

AGLHPQRPSNTGNRNTHLFPFLTKLRDSAVELLFGRHPTKNADQHAVPFSLRARYTNELVLRFNVTTPEEEIALAQA
AARLFLDIWAFTDEFVDIRMHRDEVSPLLSLLPPSLQVAQSTLISDLASAVYNSMPSYHEKPTVEAGEALSVSTSPLSE
ANGNLFFQDYQPLPVIVRWMRLLEAMFPSYVEYFSIGKSFEGREISALRVGLSSTIDPTKPRKTIVVTGGLHAREWIS
TSTVNYVAWSFITSFGKERMITKLLHEFDMVFVPVLNPDGVDYSWQVDRLWRKSRQQTNIRYCRGLDLDHAFGY
GWDGTQAQADPCSESYGGEQPFQAVEAMRLATWARNETLNNVKFVGLVDLHSYSQQILFPYTFSCQAMPPNLE
NLEELAAGIAKAIRLADGESYSVTSACEGATGRQDQFTPRVESGGGSAIDWFYHEMGAHFSYQVKLQDTGTYGFLL
PKEYIVPSGEEVFSAMKYYGDYLLGNNGIERLLEETTRDDQLESPAYDDVQISQELKRRRLRR

>QLI72562.1

SPFAHKCKPATYRCERSLRAWDVCNTSGDWVFAGRCPPGTICKFNKQNNSPYCLPRRTVEYDYTDEYYYPDEEIEI

>QLI72572.1

QNDGSYTLREVGARNTVDWRVWLQKDGYPVSPWHDVPLYPDNKPGPVINFVVEIPRWTDGKIETQRNEPLNPLF
HDTSKNKPRFVASFWPHKTYPFLYGSIPQTWENKNVKDNYTGLVGDNDPVDLFDVSSISPGYTGEVKQVKVLGGL
AMIDDNTTDWKVIAIDVRDPLANLVSSVEELDKYRPGLSKSFLDWFIASHTLLRFPPDTFFTNTIKYYKVARGKGLNP
IIGNEYVNARTMNAKLAESHQHWSDLVLGKEDRGKISIWQTTNPRACKTYVKPEDATKKFEIPEKSNILPPAARPAP
YDRWYYVDEKFTLITVPGDVIEVD

>QLI72584.1

RENPAAQLRPKLTQRGVRFDKWTRNGAVPRKFDSVSTRRFAVNGTGIPDVDFDIGEAYAGTLSTTKDINGTDRFYF
WFQPSPNPAARKEIVIWLNGGPGCSSLEGFLQENGPFLWQYGTFKPVPNPWGWHQLSNVLWVEQPIRTGFSQG
NVTARNEDDVAKQFLGFFRNFVQTFSMQGYKVYITGESYAGMYCPYIANAMLDANDTSHFNMKGMMIYDPVIG
DTRLQNDMVAVPFIDSNRNLFPLNDSFVKEIHRADNNCGFAALRAKHLSYPPKGRLPSVLPGLDPRTGEVLPACSLS
QRVQDAVTALNPCFNIYQIATTCPLLWDVLGFPGAFEYLPQGASLYFNRTDVKKAIHAPLNVNWMSCSDDSVFAG
DGADHSEPSSNRVLGRVIDGTQNVIIGHGALDFGLIANGTLMIIQNMTFGGKQGFHKRPVEPFYVPYHTDGADAT
LAGAGVFGTAHTERGLTYVGVSLAGHMIPQYAPSAAYRHLEFLLGRVDSLSSTRPFTTDSESSV

>QLI72592.1

HEVAIDDTQANHQYCSGMYSRQTWGGPVDPFILVKFFNSSIPEGTDPIVSLIIFQWRDSSLVGVPDPDGQRRLPICT
DEFVKSGHCNTTDIGEFVVSHNATEKSNALILTKAVHLKDAGPVHYSLKKTGYYCVVTQGYTIDKYNAVVEFRNAYG
ELQATQIPKLPFYGAMSIIYALMAGYWGFLYYQHRHDILAVQNYITAILVFLVVEMLITWGYYDFQNNGGSGVGSK
AFLIVVGILNAARNSFSFFLLLIVCMGYGVVKHTLGRTMIYVRWLAAAHFVFGLVYSITSLVVSPESAGPFVLLIVLPLA
GTLTAFYVWTLNSLNLTLKDLRERKQHAKEAMYRKLWWSILISIIVIFAFFFFTSFTFASINDPDFVPNHWKSRWFVL
DGWLNIVYFVDVAFVAYIWRPTANNRRFAMSDEIAQDDDGNFEIGDIGVPDDWDDDEEAEAGKNQPHPPPVTG
ITSSAPTRPDRHQQNSTRSIPRESIDGETIFAVGEDGDRFSDDGSDEENAKLVNKR

>QLI72603.1

KPTPPEKDTGRLIPVAQASTASGCYPWPAYEPCCIPAYCWCRNGWFYLLNTENEKAGGNGCEPPWGFMGKTLRE
YPGFCSKKLGCDRV

>QLI72604.1

LLTKYQDNQCVRDCHGKRPQLFEQPVLQTAQMFVGEMGCWLVIALMAAYRRIVSRPSPEERGYETVNTTENAEH
EEHRNRPKPDGDCPSVLRGFRVVLLALPAICDICGTTLMNAGLLLVAASIYQMTRGALVLFVGLFSVMFLRRHLHLF
QWLSLVGVVLGVAVVGLAGAIWPDEKQISQNGGSSLVDDGSSGGLSDAARAVIGVLLIAGAQIFTATQFVLEEWM
LENSTIAPIKVVGWEGIFGFSVTLLVMVVMHFLVGRTEAGRYGPFDMAEGIRQISQPKIWTSSVLIMISIGGFNFFG
LSVTRSVSATSRSTIDTCRTLFIWIVSLGLGWESFKLLQIVGFALLVYSTFVFNGIVQPPLESLRINEEVEELLPEEPIEHQ

>QLI72627.1

HTAAFVKGMYCEGGPDPNNYNPNANDPVNPLWMLSKNDWWMQRKSGCLNNPPKNGASVALPAGGQFTVEL
AHNQAQTSLSFDGKFATAWPDGKEHPEDWRGPGSPPDCIQDDGALHTNNQTMAAGTAWAISYESDLSKVTME
NLVVFSVLEHTPWKRIATYKVPKDLPACPAGGCYCAWLWVPTGCGQPNMYMANYRCHVTGSNSNRKLAPAKAP
VYCQHDRSKCVKGAKQMVAWNQAEGNNVKVPNGASPGYNQGMGWAPGAQNDIFQ

>QLI72635.1

VPQTNYTGLCYTDITNIDDNIKELTEKVQDFNGGLFSAAQQIPLALEATVATASAGLHAAFLDSPLLVDDLLRLADHV
NKTLAVDSPLAMQALVSKESVYEQIGLKGPIHLWLKAYLILFQQFAKNILDRVPAGAPNDRSEVLNSDLQIIMDAVR
KAMKVYE

>QLI72677.1

TFGLLGGGGISFNFGLDWSGAKTFPCPGNVVNKCTPEQENDWDWSDVATGSLNTYAGFNFGGGWSCESNFGK
RGDIQGRTFGLGKVISGSCNQGDEAGLSIGVGASAGIDAFSINSFDMSTEFDARLEFHYDMPDGSVCKQTSDCKRG
GSTIVNNQCGGAKKVRVIYPKQIIHKGISFSKKCKISCHKIKWHCGKPTPKPSTSVLTLPSTSTQVIQTTPVTTLQTYTT
PSQQTTPEKQTTSSKETTTSAQQTTPGKETTPAQQTTPSKETTPVQQTTSSKETTPAQQTTPGKETTPSQETTSAHQ
TTPGKETTPAQQTTPSKETTPAQQTTPGKETTPAQQTTPGKETTPAQQTTPGQQTTPSQPTTAATTTPATTFVTTY
DTTSTVFTTSTKTITSCGPEVTECPGKTGPHIVTVTIPVSTTICPVTETRTQSQGVPTTVILPSKSETTIKEQPTPEQPTG
EKPNPVTSQPPQSTQTPPCPPVVPRCLNTFVDLKAKCADNKDASCFCPDKDFVKNIFDCIYAHGESDNVISEAISFF
QGICGRYIPENPVIATGAETITQIITVTGTPHITQVPYTTVVVATTITENSSTQTISTEVTIPNIVMPTPTGGVPNQPAA
TASVPAGQNPPPVTGQNPPPAVTDQSPPPAITTGTGGVIPPKPTGSVPVTAGSGRVGAGLGMVLAVAAFVAAL

>QLI72680.1

APPASPEQSLATKVCPGTNPHESYPMIDVDGLGYRVSNKRCLELAGECRQDGTAEQLVECITKTIAAGGSSEARAEI
SCSPRADTMKAEGIKCKTDGKKADGKKARGLP

>QLI72701.1

APTFPTLDTNDNPLSALDNLSGYFNLIASKVEVAKGLSTAPECDLSKAHMPTGIDGLPPPDKGLTVRHVAVGRGTQ
NYTCDPNNADAAPKAVGAVATLFNASCIAALYPDVLSRIPAMAVQFKLDEAERLGPTAMAKSGVHYFDGSTPFFN
LDTPALAIGQVPCAKNSSANAPSAAAVGQTGEKAVTWLRLTAMDGTTGDIKDVYRVDTAGGSPPANCKDMPAR
FEVQYSAV

>QLI72707.1

GPQACPADSPLSCHNQTAVENTCCFIPTGQLLQTQFWDTDPATGPQDSWTIHGLWPDNCDGSYPAQCDGSRAY
TGIEDILTGNGAADTLAYMRRFWKDYKGDDESFWEHEWAKHGTCISTLQPRCYDGYRARQEAADFFRTTVDLFKT
LPTYRWLEDAGITPSSSRTYSLDRVRGALSSRHGADVTLGCRGKVLNEVWYHFNVRGSLQGGEFVASAPDGTKGK
CPSTVQYRPKSDELFSSSTSAWAPRSRSNCVTFVEPPAAAWDRVRPRSFGESTIGTSFEGICTDRLFMSAFCGDAM
AKGASYSSNEFTLASLLSMTCV

>QLI72709.1

APSPLVSQNPPAPGGQSPQSPSQDSPGLKFDWSKAKLFYPPESIKPMPTGSPRKLGRVQAPDDAFAHDDETKKRQ
RAVRDTFKKSYNAYRTYAWMKDELMPVSGGFKDPFGGWAATLVDALDTLWIMDLRDEFREAVKAVSGIDWSVT
KDNAANLFETTIRHLGGLLSAYDLSGDKVLLNKAVELAEMLYHGFDTPNHLPGFWFNYKEAKSGALVAGTNDPSAS
PASLCVEFTRLSQITGDPKYYDATDRVTRFLERTQNDTLLPGMWPITLDFRNEAARNSEFSLGARADSLYEYFPKMH
ALTGGVDPVYEQMYRTSMDTAAQHLLFRPMLPSEDDILFSGDYHARSAEKLVPESQHLTCFAGGMFGLGGRLFSI
DEHVSLGERLARGCGWAYSVFPTGLMPEIFRLIPCNDSKECPWDDAVWKRVGGKNLPKGFVNARDPQYQLRPEA
IESLFILYRITGKKELQDTAWAMFQAILGSTETKYAYSAISDVTTSGNTNKVDSMESFWLAETLKYFYLIFSPPDLISLD
EYVLNTEAHPLKRPTAEG

>QLI72717.1

GTGPKKSPNLYAIQEEFQKNLDAFTWTVREMSKSGSWAVGVKCHECLRFLKYMQGFATNTEAFIARGKEMCSMI
QRAKVDWKVEELCIESVERYGQAMAHILRNMNPVGESQGAQLFCNTWLGTCPAVKLRYNVPMNYTRNERPPPR
PKPSGQMPFKFVHFTDLHLDPHYFAGTQSQTDKCPYPLICCRAFTEKERDIIFQPRHKAHLDILIDPELDIGGPYGHH
GGCDTPHRLQQSFYRAMKKFAGDADFAIFTGGILSRQVWNSTAELNIGQIEKAYSNMSELFDYIYPAVGNDESSPA
NLFQAEQVTSKVSTQWLYDTLSSLWSRWIGDKDARRVKDGGFYSTKVPHRNLRIISLNTNLYNYKNLWVYQDPIN
HDPAGQLAWLVSELDAATLAKEHVYIIGHMSMGDPDILQHYSRSLNQIINKYGSTVAAMFFGHTFLNQFQLHYRG
FTDPQSPHWWRVGERLTAEDALVTSYIAPSITPVIGAPAFNVFYVDPETFGILDIVTYSSNMVDRYWEYDKGPKWA
KAYSAKTVYGIPQKLPVGGELSPAFWHKVSERFDWDRTFYRLYFGRQTAWNEKQTGESQRAKDQCFIRGGIARDC
LGIPGRGGLGGYLGITDG

>QLI72720.1

CPSRTNNTATSRKDVVAVPAVWNGFGYLFNISVGTPPQPLTVLSDWTWISLFVRSGRCMNQWNIPMCIGNNGQ
AFFNERKSSTFYNRSLPQLKWDITAFAPDFTVDYASDTVCVSNICTTNTTLQVSDLPYGGAVIPKVPFGGIYGMAPV
TPDVAESFYTQNYQAWKAGVFGPKVGWHSCASLSSRKSCLGGEAKFVLGGTDQAMYDASRMQVYNIQNPSWL
SEAFYPYNPPKSNYWSTALTGVWISGEKESTNFVYNFSGSNNVSMQTLALLDEGSEGLGAPLSLNAYKWLVKQVN
GTLASNATVKAIMAQGSSGYNGASQDWYTVPCNSTGSRPTLFYELDGRQNYTIPESDYIIRLESAATPVCYLNVNV
WKYGRTSSGDSKVLLLGGAFLKRLYVQLDFENLAFGLAPLKKH

>QLI72726.1

SVPVQWPSTPANLSALEEQRRPSGQFAGASRRDAGNGTLGFSGIYFINMKKRYDRLDALALQCFLSGVEVKEVPAV
EPDMMSDAGMPPSKSPSSVKVGERGAWRAHANIWSTVLRNNLPPVIILESDATWDVNIRSIMLNMNKHFHKFLT
NINSTRLHNPRWRDDAGESVRRTEAADDTWQSDHWDILSLGHCHDSAANRNISLIYDDPYVPPGKEFGDTVLGR
QRVIRKAGGIVCTTGYAVSQTGAAKLLVKTAQNLDAPLDLIVRSMVEDGELVAYSVMPPIMAQWQYVDGIGMDE
RGANSDIQGAQEDEDKDEGEEKKDSWEEVDRSGSVWTTKPYHEDVAFQDMALEVAWRRIFGRNNEGE

>QLI72741.1

APVAEVKRQTTGGATAEGLSKALQATTDSFRKLTDGLGGLSALGGGLGASKRDETPAGDVTVRLDQPAVDLSIGEG
SGLVGKRQQGLGSLSVLAQTASHAVLNGLAGGVLKRDGKQDPSKRQLDTSLPNKVLNDVTSGKVMEDLPKVPNV
HIEA

>QLI72751.1

VAVDKRIIGGEEAKDGDFPFIVSIGSSAEGLSGHICGGALLDNTTVLTAARCRRDAYYVRAGTKDLSKSGVVRKLDFV
VAHPDYKRGPPRRGHFPSAVNDIAILKLSTPIEQSESNKIKYATLPEDDSDPVVNSIAVTAGWGEQVSLEGDVYQDD
KLQKIEIPVRPLEDCSDVTPDAANRDTKICAGEDGKNVFRFDSGGPLIDQDGRLIGVALAGSFDIKNPSIYTKVGSYM
PFIKEYLGSVSNPDPTARPITEAREEELQPGTSLREEIKKHCEKFPENVLDCISAAGDCKGLRKPDGNMAEVFQCIDK
KLDVTRQ

>QLI72783.1

AETPKRGLAANDGIPINNFWGTFKDHKSQINWQYNWDSTTNQKQPFAEFVPMLWGTQSYHTQQWHNNVRY
WIQRGTQHLLGFNEPDRPDQANMSHEAAVTAWKTYMEPYAGQVKLGAPVISNAGYDWLSEFLNQCQGCHIDFI
PVHWYNNYTLEFDLENWVRKICWLGRGRQIWITEFQGFGSPAQQGEFLRKAMPFLDKTACVARYAYFGTAKNSQ
VLLQSGGPALSPLGTQYAFSPYGSGTVYGRMLGFR

>QLI72801.1

IPASKVSIVQKISNAPQGWTKHGAQVDKTTSMVKLRFHLTQPRISEFHDLAMKIATPGNELYGKHLSKEEADKFLAP
KPESVQAVQEWLRGEGLGDHAEVDGSGDNILVHASVADAEKLLHAEYDPFVAQDSGEVIMRTLQYSLPDNLKQHI
DVVQPTTYFGVKSVRLTQISLQNNNTPSTQASCKNSNPACLSDLYSYGSAKAYSNGRMGIAGFIGQFASKSDLATF
MSKQATQNNTDQSFSCVSINDGGCPENNPGVEANLDTQYARAITEKIPNVFYSTGGSPPTNGSVPDNEPYLEFLNF
FLGLSDEDLPNTISISYGDVEYTVPNDYATKCCDLFCKLGARGVSVLVSSGDSGVGGSCPGGKFETSFPAACPWVTT
VGGTDGTGPEKAWSGSGGGFSEIFAQPSYQADTVHQWLKNDQTHQSQDPYFNSSGRAYPDVSALATNYPIVVN
GSTFGVMGTSASAPVFASIIQLISSDRLSNGKKPLGFLNPWLYSSAASALTDITNGSNSGCANINGKGFQAVQGWD
PVTGLGTPNFEKLLKVSQST

>QLI72802.1

AEKPKFLNSNFQIIEGQSFDLKFDGCEGGCTITLQDGPNTNLKDYKVISTSATGGSLAWVPEGVVSGTYAFKITNNA
NKEYNYSQQFSFLGTGATATVTPSAASTTGSSTDSATATASSTEASSTVSITASSTESSTESRTASTTTISTVTSSATAA
SGSSTTSAPSSATSHSSTSTRSTTAATTTVPNAGVRATPMAFVAGAVAALAYLG

>QLI72833.1

VPAQPGFDATPEVGREHGLFLRQEPPLAERPVVEPNPEEVKTANAELAVLLQNMLDVMGSINEDIKQGKVQLETK
TKLTPEEHKAALEIIQEGIFKSAQTLVEIGKPLIEGKGEDKAEPEPEAK

>QLI72842.1

LKGSRIERDLDAEPPFYKLACGHSPSFEKYAYAKYSDAVQAVNEAYLYMMTQPGLDVKKHFDPGCWGVFLKGTAH
IWICNKNHDVVNVRQSDLLPMATQIIEQCRQGDGTLGVAYTTKNWFLIVSK

>QLI72859.1

APGPGRPPDSLAARQVQQPWAGAVQQGQGLSYVTGTVRVPRIAGQPSRSAVAAWVGIDGLRCRRAILQTGLSFY
GDGAVVPWYEWWPAPSHAYPGPAFRASPGDLVRMSVHAWSATSGNATLENLSTGQVVARELYGQAPLCGTDA
DFVLEDFGDYTGGHVPLVNFGEIDIWNTWAYGSSGWVDAEGASIVNLEIDNRVRSSCGSNANGVRCDYLW

>QLI72864.1

FGCNSISYTTCQDRIVHWYDPDDGQICDPHDCGGGRAPPRKDVPGCAFYTGTETLKTEPSYLPCWKPSTAMAAPT
GSSVKSSVESTPTNTGVVASSKETTTGSSVPASTTASVAPSTTASSATGSVPGAAASPSTTLPATISTTTQSETATFGN
NTGNTSTISTTSAHTGDAGRLVGASKMALVAGIVGGFALL

>QLI72866.1

GQILKTSGFSDCGSDSTIKVDKINISYNNENKTVNFDIAGSSSKEQNVTAILDVTAYGNTVYSKTFNPCDKGTFVERLC
PVPVGQFAAQGNQQIPSEFANMVPGIAFQVPDISAHATLRLMSQNGDRVACVQSDVSNGKTTDIPAVSYVAVGV
AGAALIMSGVSAVGAALSGGSAALGSGSGGAVGTISPSFGEVVGWFQGMAMNSMLSVNYPPIYRNFAKNFAFS
VGLFPWSGMQTTIDDFRSKTGGNLTQDSLSYLQNATLVFPDGSTSSPNKGLFNLKRAVDTFVELANRAVEASVNPT
EPVGNGDSNDLQHKVKGIQAFAEKLLVPKSNIFMTALLIVAIIIAAIVVGILLVKVILEAWALFGSFPESLRGFRKHYW
GSIARTITSLILLLYGIWVLYCVFQFTHGDNTIAKVLAAVTLAIFTAILAFFSWKIWSVVHKLKKQDGDAAAMYEDKKI
WVKYSLFYESYKRQYWWLFIPAIFYMFAKGVAIAAGDGHGMAQTIAQLVIEGLMLCLLLWSRPFERRSGNVINIVI
QVVRVLSIACILLFVQEFGIKQTTQTVAGVALIAVQATLTAVLAILIAWNAINACCKMNPHRKRRKEAEQLKRDMD
NLTPLDARNSLLLDRAPMPEKSMFAVSSDLNEKGHVRNGSAERFYPGGGDIRPPVAPSGGHMYRPLTPTIANDSQ
SLLDGAAPIGTSDRQPTVPSVDREYRGAYAPSTTYGNGGGYNRRF

>QLI72874.1

IHFNPAPPANLDFSNLGRIAIAGDFNGISLYEYEGQISKPLPTNGSEALLARLPNGALAPVVSTDASIRAMCLLHNDD
GKLKGVVIGGNFTSIDGTQSTAIALFNPNTTEISPLKGLEGEVNALYCDDSRKTVYIGGNFKASNSTNAIAWTDADG
WSNLPFAGFNGQVNAISKTSNGHIVFGGSFTGLGNTSTPSQPDSQIINLSGGKVTATNGATTNGFSDPQNIVCSAG
TDGPGSTWLAADNLPATWEADFGFTFQPTKLRLYNTRQDGRGTKTFRFLAFPLNGIMNFTYVDPSTGKNASCTSE
CPLSNDPNVKFQDFRFVNRVGMDSFQVAISDWYGSGAGLSGIELFQDDMFAYAINNFNEPNCKGIQFPSTATAT
GPWTEAPSVQSSSDYLVAKLSGGDINDKSASVVFTPNIVESGNYSVNMYTPGCMPDGSCSARGQVNVTGVMSTG
TIDAGFSTTLYQTNYYDKFDQIYFGYIEKSSDSFKPTVTLTPLAGQNLQSLTVVAQRVGFTLTKSTGGLNSLFDFDPEQ
KTVDVSSLEKSPINKLGSEFGRSSAVTSLVTSGDTLFIGGNFTSKNFVNAVAIGGDKDTTSPLDGGLNGQVLDMHLE
GTQLYVGGNFNSTLNGDKALGHAALYDIKSNSWNVLGGGVDGPVEHVVPMRINITSDKTETVIGLSGTFSQCNAF
QNNSISPAEGFAIWVPSLQNWLQNIDQPLPTYNGVLTASLENVADVGDLFAGSLTSAQLRANGAATLSTRGLGPF
PFKIEATTRSASKLHRRESISNDTLRGVITGAFYSTDSSNITVLAGHFTASSANGSTVNNLMILDGKDNESITGLGSGIS
ADSTFITVALQGSVLYAGGMVSGTIAGKQIHGLLAYDLSSKSFGSQPAPISGRNSTVAAIAVRPDTSEVYVGGSFDK
AGALGCEGLCIYNSNSGQWFQPGNGLSGEVLGLMWSSKSTLVVAGDLQANSTEKRYLATYDAKQQTWSAFPGA
ESIPGPVQVMTAGSRDGNQVWIAGKSAKDGSVFLMKYDGSQWLTVNGTLPAITILRSLQVFSLTKSHASTQLLGE
NQALMMTGSIVIPNVGIASAAIFNGTHYLPYALTTNSGNVPGTIARIFTQKDDFFSTGGGSMPLGFVVLIGLAIALGL
VLLMVLAGMILDRLRKKREGYMPAPTSMYDRGSGIQRIPPHELLESLGRSRPGAAPHV

>QLI72878.1

KDEPSMTVNTFENPPLNINYFEDSDVVIFEDKIEGNVYRSADAGVSWKRVDSVPDGKVLELIMHPFDKKRAYIITPS
KTQFRTEDRGETWTRFNTKALPSKFQPEVMVFNAGDPDRIIFNAMDCDGIFCDEQSMYTTDGFKKTQALRVSTTG
CWWAKSSPEFTTGEPETDKSRVLCIVRDSISFFRQEQKLVISDDFFAVQDKHIQEFEPNIEMNKGVTGFLNLAVVKK
YLLVATSSPHSDEMALYVSDDTKKWHRAMFPKDDSHDHSHRINQGAYTVLEGTNYSIQVDVMTSHPSRPMGVLF
TSNSNGTYFTENIPYTNRNTKGNVDFEKISGIQGIFLVNTVKNGAEVAKEKTDKIIVSHITLDDGRTFSEIKSGDDRLH
LHSVTELDNMGRVFSSPAPGLVMGNGNTGESLGRFTDANLYVSDNAGSTWKKALDGPHKYEFGDSGSILIAIKDS
DKPDIKEFSYSLDHGDNWKNVPLPKDLSIKPDVLITTQDSTSLKFMLLGERDRTFHMIAIDFEGLKERTCEDKDLED
WHARVDKDGNPSCIMGHKQTYSRRKKSADCFIKKPFEDPVPKTEDCECTDADFECDYNFQRDKDNKCVLAGPIPV
PSEECKDNPDGTFKGSSGWRLIPGNTCKRKSGEQKDDQVERKCSEGDTKPSQPPATGEIALKQKIFDFSHDRFEKIY
LEKGDASTSTDETVILWPASDGNQIWLSQDHGKEWKQVLKGENVHGIFPHHYFNDVVYFTTDDRKVIYTVDRGQ
SFHTFQAPSKPKTGAVPFFAFHPDRKDWLLWMGETCEKVGGKESCFPEVSVSRDRGDNWKTLRRYAEKCEFTGSS
AYKFRDEKQVLCLVNVEERMDSNLTVITSDNFFDDEPVKFDGQVINFATMSEFILLASKDPETGATRAVASMDGKN

FEVARYPHNFHEGHNDDYTVLDSSTHAVNLFVRSEAGAGRLYGSIIKSNSNGTSYVVSAANVNSDADMYVDFEKV
AGLEGVALINVVTNADNKKETTNTLQTKVSHNDGSQWGYLPPPAKDVEGKSYSCSSAKGDSKCALHLHHYTERDD
KQRTFAADTAVGFIFGIGNVGASLGDIKDADTFLSTDGGITWANVKKGHWTWQYGDQGSVMVLVQRATRKNDV
KTKIVSYSTDEGKTWKDMEFSESEVTVLDITTLKSGTSRNFLLWCRDNKDKMMTVNIDFSGLADKPCKYNEDSAAE
SDYTLWGPQHPLQADNCLFGHESKYLRKKTDRKCYNNENLKRLVEFKNCPCKREDFECAYNFELEDSDSQQCRLV
QGYQPLSGKEWCSRNPNATSYFDPTGYRRIPLSTCEGGRELDKASEEHPCEGHEDEFERKHRTSGTAIFFAVVIPFTL
AGAIGWWVYRNWDGKFGQIRLGDTSSSFDSDRPWVKYPVIAISAVVAVVATLPLLVGGLWRSATGSYSRVRGGS
GQSWFSSGNRRFTTRDSFARGRGDYAIVDDDEGELLGDDSDDDE

>QLI72899.1

APCAPQVKPITRIDVGPRPFFLVNNMTNGPLKSQLESCSEMTIKPSTFSLGHRGGACMQIPEHSRESNLAGARMG
AGVLECDVTFTKDLQLVCRHSQCDLHTTTNIVDIPALNAKCTQPFTPAKDGKPASAKCCTSDITLAEFKSLCAKMDG
FNASATNPKDFLDGTLKWRTDLYATCGTIMDHKEHIAMVKSLGLRHTPELKAPEVQMPFGNNYTHERFAQQMID
NYKNAGVPASMVMAQSFELKTLQYWLKSERAFAENAILLDESAEKPGGMEQAIQNLAQYKAEGVKFAAPPFHYLI
EAKNGTMTPSAYAKEAKKQDLGLVVWSFERSGPVALARANGDYYYTGLQDVATKDGDMYNLLHFLVKEVGIVGA
FSDWSGTVTYFANCLGIGLGNETRRK

>QLI72902.1

APTLDAAFSSVANGAPALVNREEQSTYTLQDKKAPFQDAASLPALNGADTVDLDLSGYTNMGYPETSDQFKCHGS
AGIVFRIWKTEQRCHHPYMVPFLRFAFVDCKDVQHFTPYPPVKGYKAWCKYFKWMDEIDF

>QLI72914.1

ATIEKRTAGGEDVKDGELPFMVSLQGQRGGHRCGGALVDSTTVVTAAHCVSGTYSIKAGTANISTDGVVVNVKSR
HEHPDYVLGNPGSPYAVNDIGVLKLETPVERSDTIDYVTLPADGSDPVPNSIAIGAGWGYQPDITPWPKQLSKVA
MPVRDREVCFNLSPEGLSGRTTVICVGGDGKALCDKDSGGPVIDQDTRQLIGVVSFGLGRWPCTPKNPMVCTRV
GNYISFIKKYL

>QLI72916.1

TTIPTNALIPALGKEFIDLSQPSDELLSIHQQLNREKFSSRSEFPLTVDVYLNIITSTNEGQDAVKNTTITWMLDTLNNA
YKAGQITFQLQDTARIINSTWAANEDDENMRSILHKGDKSTLNLYLVEALIDWIGGGNRDTIIIGSTTSPDELRFKNR
LSQDGVLLNIGTLPGGSFADKTTTLIHEVGHWFGLLHTFQGGCTGGDGVDDTPAQANPSYDCNTPRNSCPNDPD
MDPIKNFMDYGSDACRSEFTDGQMKRIKEMWNKFRDPAVTLVLEEDFLYQIRRIFNRIRFLPSLQLAFTDDATLKW
QLLTQLLAAQKDLTDNIGKPDFGTRVKQHFAGFRAIRNVQFSMDKDKTLKLELPLQFKEAEEGLLKMA

>QLI72923.1

QSLSGSASQRSGIVLKKALKNLQNDTTKDMEIIRSAISSLKAQSIETRNASSVKVVNDAFNRIIASVNHTTHELASGSQL
PKRQVSGFPPLQDASAIAKVLIDITTNIASLFVEALDVFNQSSILIAIGTVIGKALLGLLDVVGVIIRDVPGIQQAIQLVV
GILKKVLAFN

>QLI72941.1

IPQAQQSDAIARRAQVLCGRAENINMSQCKTDTEKCITDSLAEKKLDSVPSPADFIFWGNVKRCGLGKQIGSKNST
PQNFVALNNGVLTLIDGCEVQEGNVFYCNEWPKGNPGSCSSEDKNCYPMSMLPELCAEAGVCKTCEYGLSNRGG
FGKRAGFSPNTIYCKLE

>QLI72950.1

NGTIDNGPFPDELNGSNFTYPWPVKVFKFTSQQQDLQMAFMDVKPACRPNGKTAVLFHGKNFCGPTWQDTIRV
LAARGYRVIAPDQIGFCKSSKPDAYQFSLNQFAWNTRGLLNAAGVGNVTVIGHSMGGMMTARFGLQYPETIEKM

VMVDPVGLEDYVQKGVPYISIDQSIVSEAASTYQSIKGYEQEVYYVGQWKPAYDTWVNMLVNIYNGPKRKAYVK
NQAQIVDMVLTSPIAHYFGDIKPRTLLIVGNKDKTAIGAQWSPPAVAAKLGRFDILGPEVAKHLQNGELYQFPNLG
HAPQLSDPRNFHKVLLAFLKK

>QLI72956.1

DPTWPSSIDELEEIMYQINSFRARKFADTVNPCTNEASGPGRRNAAEWLRSAFHDMSTANTFFKTGGLDGSLQYE
LDNGENTGPGHRTTMNFMGPYVTRRSSLSDLIAMGVYMSVRSCGGPIIPIRAGRIDAAAAGALGVPQPQNSATM
FQQQFERMGFSTEEMIQATACGHTLGGVHQDEFPDLMPATGVVSGNVALDSTDAVFDNKVVTEYLSGNTSNML
VVGPSVKINKNSDFKVFNVDGNKTMETLTDANNFKAVCQKVLQKMIEVVPPTVTLTDPIVPYTVKPVNLQLSLTNG
GTGLQFTGFIRVKVTDLPKDSIKNVVITYKDRKGNANCGASSCTITATVQGVAQGFDDNFNFYPIEAVIPAASGISSF
TVTVNNADGTSKAYDNNGNGYPLQDDILFQAPQSCVTGSSGALTVVAAVRNDVASNGAQASITYKTPQTNSPVA
LLNNATVPLQKGACLGKYTLFSGQYTIPGGMPYQSRVDVISGSKADTFKAITDIGGTCTAFPNPGACDAVDPPVNS
TTTTTTPGSTATTTTTTSIVAPVTPTHRATVAGYTHVSCWSEGVGVRALAGTSFANDTMTLEKCADYCKAYVYWGT
EYGRECYCGNALDKTSAAAALTECNMACGGDPSAYCGAGNRLELYSTTSAPVTPTPTATLSHKPTVAPYTMVGCW
SEGNGVRALGQAATTSDKMTNEACATFCKSFKYFGTEYGSECYCGSFLADSSKTAPIGECNMPCSGDQYEYCGASS
RLELYQNPNITTGNPEQPAAVGDYVFAGCQTEGNNTRALAGPTLPQNNMTNEVCATFCNSNGNNFTYFGTEYGR
ECYCGNALDISSAVAPAGDCKMLCGGSDTEYCGASNRLSVYKKKQIPATTQKRRRNYL

>QLI72959.1

LPQSTPAGSAERNLLPWLPAGPFSHECGPFGWYSDAECGTHKYCDSFRDPEIPNDKKYQTTQECIDAHELPSGSGT
GKLPWARQGSDRACTSYIERKVYFPRIVMEENCGSQRFCDLFGPGSPLAAELPELKKRYGFSSVEECRAAH

>QLI72960.1

VTVKVNPLPAPQDVTWGDSGPKHVGWLNLRTNSNRKSESNTKLVSEAWNRASKAITSLRWVPQAIEQPIPKFEPF
PTAGSNDSSSSAKSKRNTGGSWLNEVNVQVSDWSADLKHGVDESYTLTVSASSSTVEIAAKTVWGALHAFTTFQQ
LVIFEAGSLIVEQPVTIKDHPNYPYRGVMVDTGRNFISANKIKEQIDGLALSKMNILHWHITDTQSWPIHLEAYPQV
TKDAYSGRESYSAKDVQDIISYARARGVRVIPEIDMPGHSASGWQQIDKDIVTCQNSWWSNDNWPLHTAVQPN
PGQLDVMNPKTYQVVGNVYSELSKKFSDDFFHVGGDELQIGCFNFSKGIRDWFAADPQRTYFDLNQYWIDHAYP
LFMSEENSGKKDRRLIMWEDVVLSPDAHAHNVSKSVIMQSWNNGVANIDKLTKAGYDVIVSSADFMYLDCGNG
GYVTNDPRYNAPQSNPDATGATFSFNYGGPGGSWCAPYKTWQRIYDYDFTANLTSDQAKHIIGAAAPLWSEQVD
DAVISSKMWPRAAALAELVWSGNKDPKTGLKRTTYLTQRILNFREYLVANGIGAAPLVPKYCLQHPHACDFYYNQT
AVQ

>QLI72969.1

APTNAGSLTNGAEHRPDANPSLPVVVDPEQGGLSDGVKRDATRSLLDGLIQDVPIEGGDGGLAGAASHVTKAAP
GVGKREVADDVAGIVTGLTEGADIAGVTDEALAGQKRDGTVGSPNGITDKVPIGQNLDPTGIAENLLHPGGFPGA
GAGSLVGKQKRDGIGGLGEGLPLGSPLGSAGGITKPITDIVGSLQSLDGNSLGGLTQ

>QLI72972.1

HPMSENKPRALPPSIDPFYRPPSGFESKAPGSILRQRRIAAAFFGFIPQPIEAYQLLYCTNAIDGSPIAAVTTIFKPLHA
KKDSFVSLQTAYDSTASICNPSYTYQLGALQTNIISSLEQIIMQAYLLSGYMVAAADYEGPDAAFLPGHLEGMGVLD
GMRAVVNFRTKLGLSDNPKIVGTGYSGGAVATGWAAGLQSSYAPDLNVKGWVAGGTPANLTATFMNLDGTIFS
GFLPAGLAGFLKPSAYGAQLQPVFDEI

>QLI72976.1

ASRSYNFTIHSETRAPDGVSRQVYLINGQQPGPLIDVDEGDSLEIFVQNNLPVDTTIHWHGILQRASPQMDGVPGV
TQVGHYFYLPTWNLVDHWQYPIPAGGNFTYRFNLQDEYGFFWYHSHLRAYYNDAIRGPLLVRPSPSRGRPFEQLA

STEGEKTLLMNAERDAPSILLNDWTHEPSDVIWSRYFTTGAFPNCVDSILANGNGRVQCLPEFQNGMMPKANQT
SLLTSSMAMIMSGMNKRMNMDGSSMDMGTTTMVMEGPTRTTVPDTATTASTTMMMASLGPRGCAMPMM
FNPGYNASSLPPETCTNTTSSLLNISANSTTGWLALNLVNSGAVSALRVSLDGHSMFVYAADGSFVQLHEVKVLHM
ALGERYSVMIRLNQMPGNYYLRFATYPVGDMQQVMEGQAIISYNISQTTSNISTDVMEDSSIVWMLKNGSAKSE
ASLLNPSKLAPFESNSPPSKAADRTQLFAINQTGVTAWVMNKAPFTEPRVPILYGAQSEGWLANTTIRMPSNSVIDI
VMNVANESLDTMGHPMHLHGHKFWVLGTGTGTFPYATAQEAPKSLINLQNPPYRDTVELPSSGWAVIRYITDNP
GAWLMHCHLQWHIVSGMAVVLIEGEEKLPGLLGSKAPPTKSTAVSFRSGYKHIAWATVAMFLLF

>QLI72992.1

APSIVPDDFEKRDVDAAARDSAIADAVQARQVDTTVTLDQRDEDVEMVARAPGNANAQGANKAGQKAGQKAG
QKAGQKAGQKAGQKAGQKAGQKAGQKAGQKAGQKAGQKNNGANNAKQNAASDAAEAAKPRVTSSFDAPGV
VL

>QLI72998.1

ARQDLAKANEALNAFDAKNAQVKAYFEQKKQNNPEGKGEKWKETAGQQVDLINTLKDQLKDSPQDVKDNINKL
SEISQNSLNRANEKHPEGAPGDQKSKPEQINEEYTKIASDLRAALN

>QLI73002.1

APVSDVEYTAIEKRDLNLNAFLAAIVEIFPVNVPVNGICTALGIGETALAAGFGLKINANRRGCADVTVVFARGTCDP
GNVGALVGPPFFRALEAAIGSSKSVAIQGVEYPATVDGYLRADPAAGVTMAKFITNTIAECPKTQFVLSGYSQGGY
AVHNAAKNLDAKSMSKVKAVVIFGDPMSKEPVRGIDASRVSIVCHEGDDICDGGVFITPQHITYAADAESSADFVV
SKL

>QLI73005.1

APQSEDLTAQRPGIIQLSLKAPRARDKRQVDIDLTYTSNFKYDVEFSLGTPGQKIVGSFDTGSVYIWVREKAEASVNT
AVAGARSYFDKSSSSTLVRTGESKTTGYNIGGNSSHQIDLYRDNMSLGDGGVLLGGVNRKKFWGRLGKQKMTVH
PFHGKDYVISFNEVSATAENGSKSSLSRSDEGIPALLDTGTLLNKFPPSVFQSILKRFPGAKPMNKYPLYIVDCSLRDV
NASIDLAFGKTTIKMPYSNFILSIEKPECVLVHTVLGQVFMRGAYIVMDQDNSEVYLANAVSCGSEPVAIDKDVSAIS
SLTGKCQPPVVEQNLLPWKRPGIDAENCVDAASEDVCGTAEYCRIFPGAFGSDFETEDDCLAAHEIPLDD

>QLI73014.1

KPANRRQLYEAENRISEQAQIDGRTVISYYAPNPGCADGVRTWAGVWPADASNPYTADYKARLYVDIINDDEIRV
AAFNNAELGIGEFKAAFVCDDDKRQAWLVSENFKIDDATPAKNNSTCIYAGPEWDPGFASVSCDEVENRFCFDCT
NGCAPCEYCKRQCGQANTGDDKQKA

>QLI73020.1

TPVSNSISNIDGTPDDVAKVLERGAPGDFFKDVAGIIKILEKGLPADIPSEAAAIAAVLEQGPPRTQASSDVYEKLSYY
AQFPGSATACVHKPAEGVVEQLYVNETTTDTQAMIYRLDSRKELILAIPGTQSQQDWETDENWRLVDYKSCQSCK
AHHGFLTAWDSIVDQVERGLESALHSYPGYSVTIVGHSLGGALAELAFGSLKPKPLNVTQVITYGAPRVGNAGFAN
YLDKLAGASNSNAGIAYRVTHYDDIVPHLPPFFLGFTHPRTEYWQSADKPTEATTYRCNGRESLDCIFGKAPSSKSD
AHGTYAGMKVFC

>QLI73037.1

TELLTPDKVEAGIQKDRLENILWNLNKIGDDHGGNRAFGTSGYKASLDFVLERVVNRFGKHLNTFVQPFDHLFHQV
KKIQVKGPDGAQVYYVISLQYNPGTPLPDGITGELVHVPYEADRGSGCFEDQWKNIDAQGKVALLKRGGCAFADM
TLLAKAAGAKAVIFYNNTPGKNYSTATLQAKNVGKLIPSGLVSLEDGEAWAARVAAGDKLSVTLVVDAVSETRETW
NIISETKAGDKNNVVMLGAHLDSVLPGPGVNDDGSGTAALLEIIEQLIRYDGIKNTVRVAWWGAEESGLIGSLYYGS

NLTEAEADRIKFYFNYDMIGSPHPEFVVYANDEGHKFGGAPLFDYLQAQGKPAEYKKFGSSSDYVAFLNLGIPSSGL
FTGASPDTDPCYHQACDTVNNINWDALTVNTKAAARVAAQFALSLEGVPPRNKTTINPRSRRGVRRAFDDWAEA
VDVAEKTHNCGSGDSLY

>QLI73046.1

ITVGSTVLILARDDAEAKGAAMGLDGYGIPYQKVIFPAGGTNLPVLNSSATQGNYGGIVVISNVAYDNNGTFASALT
PQQWAQINSYQSTFKVRMVRINEFPSAEFGTIVANTAEPGCCGSNTEQKISLTDISQFPGANLKANAGVSTQGLW
HYPATITNTTTTKEVAGFEAAAGFASKTTAAVVHSANGREQMVWFISWDPTWSQTSSFLQHAYIHWMTRSLFVG
KRQSYLSTQVDDVLLDTELYYPPNTTFRLRPGDLDAHVAWQKNLATRLPAGSNFWLEMGHNGNGNIIGSTATAS
GETTCKPAYAVDYTSPPDTPLEFMKPPGTGVDLWPAEFVSYTWSATCSKLEPLGAWFTNTNNLNAFAHVSHTFSH
EELNNATYHDATREIFFNQAFLKSVGIDRAARFSPKGIIPPAITGLHNADAIKAWTDNGIIYVVGDNTRPVLRNQNS
VYWPLRSTVQTNGREGITIIPRFATTIYYNCDLPACTVKEWIDTSGGSGDFNSLLDNARAVNTRNLLSLQADPYMFH
QANMRQTDVAPLTIGSQTGKQSLIMAWVETIAQEMTRLTNWPLLSLKHDDLATYFLNRMALDACQPKSSYTYSA
DGKTITKVTVTANGNTCSAPVPVTFPGGIATTTLLGPLKSTKVGSEPPILWVTLSGRPVDILLWTPVKL

>QLI73059.1

DHSDSSNVPKPDLICHTSNPDECYPRVFQPTEEFQIVHDDQELPKGLHVRLNIWTGLKEAKINVPDEIDPSLEGMPV
DQAVVIVDHEEQLGAPKIPKGAPEYEPVGKVKGPQHESAALVEGLNMLKSGTSKNNQAFDDALETLEDLSHDMYY
GLNIAEDTEAMEALLCLMSGQSAPSTNGAVPHDQQAASILAGVFQNNPAALKQVTGFWPQLLVESRCPNTGKSL
RQSLYSSVEPSRNGEGANAQQAAARVKPKVSVINGLIKDGSIRTHFLREGGMESLLKVLIPEDKAWAGAQRKVGQL
VLDNFLDENTGATLGEWPYLPKLGDELCQTEESGTAEGCWDYHVERIMKSSKASKEDWSWQLNDRLAAARQEQ
SNERKHEEL

>QLI73062.1

GPCDIYGSHGTPCIAAHSTTRALYAKYSGRLYQVRRDSDQATTDITTTSAGGVANSGHQDSFCARTTCVITVIYDQS
GRDNHLTQAPPGGAASGPEPGGHDSLSSAIGAPVTLGGEKAYGVFISPNDGYRNNAAVGTASEDEAQGIYAVLDG
THYNSGCCFDYGNAERDNRDTGDGKMETIYFGDGGYKGSGNGPWVQADLENGIFSGSTAGKNDGNPTVSSRFV
TAAVKGKPGHYAIRGGDGASGTLSTFYSGGRPKGYEKMHKEGAILLGIGGDNSNRAQGTFYEGAMTSGYPSDEAE
AEVQADIVRAKYAVTSLTSGPRFTVGSHVSLRATTPCCTSRYVAHSGGAVRTEEVSSSSGDQLKREASWVVRAGLG
PGDCLSFESVDAPGSFVRVSPEFRLVVGANDDSKEFREHATFCPQEGIAGKGSSLRSWKYPTRYWRHFNNDMFAA
RNGGPVYFDAQWVYHEDVTYEVGDGFA

>QLI73069.1

INHSVFFPKSSPTAIAKPEPLFCAAANYTNYVVGPRPTGTLAVAMASHTSELFQTCTLPLEEWFDCTFPESSRLCAFS
TVGPSEVMSAYSDFGSLALSWWTAHSLVAEQLASSCHFRWYHEMKLIPFASYKLEETISFAECFIPEVATASSEGVSI
VASTPPSTAPKTSAASAAKETAATASLAARWRQDKRISFGTATVATWMLF

>QLI73075.1

QSSTPSATPPATTSASAPESPADRCLKACAASDVNCKAHCITVPSPNEQNIENTNKCVAACPKGDGSPEQSQKYSD
CSQKCITDNYYASSEGTPNPTGSAGNNGNNGNNGSNGSSASGSDAAPTATGTGAGASPSGTASGTGSSGSQTTG
SGSASGTQTGTGASATKTGAAAGLTLGSSGAFVGALAALLAL

>QLI73080.1

DTSDFDWNAVEPSWHLHYSPCYDGFQCARLLMPLDWLNTEPSNETVALSMIKLPAAVDCSDASFGGTVITNPGG
PGSSGVRHVLKNGRYMQTMMDGEKHFEIMSFDPRGVAHSKPAADCYASEPARTAAAWQSRGFTNFDASAENL
KYQKAFAAARGLQCAKPGPHGYAIQEYMATASVARDMVRIIDEIESLRQKTLAQKLQHEAQKPVTSKPANVARLQ
YYGTSYGTFLGNTFMSMFPGRVKRMVLDGVIVPEDWVAADWHNSLLDSEKALEYFYRSCFEATAKCPLTKSSDHS

WHSIRDRVRTLLGELEASPRPVLTQGGTEAIITAGMVRSSIFNALYQPVDKFEPLAESLASALQGNYTLLVQNTGLG
HPADGCTPKKPGEYNWLGLSSSAVVCGDAQDVTHHDERYWQGYFEKLGDRSPEFGHHVAKIPFTCSGWKSRPKY
RFTGPFSSPQADTSDKQERPSAPALLLSSWIDPITPLQNAHRVSKSHPGSRVLSQRSVGHCALYSAPSKCTNRVVRT
YMATGALPDEGAQCEPDCVPWEQCDDERTSLPR

>QLI73082.1

DDSDVHQLTEKTFKDFVKANPLVLAEFFAPWCGHCKALAPEYEEAATTLKEKDIKLAKIDCTEEAELCKEHGVEGYPT
LKVFRGVDNVAPYNGQRKAAAITSYMVKQSLPAVSLLTKDTLEEFKTADKVVVVAYLAADDKASNETFTTVAEKLR
DNYLFGGVNDAAVAEAEGVKFPSIVLYKSFDEGKNTYTEKFDVEAIEKFAKTAATPLIGEVGPETYADYMSAGIPLAY
IFAETQEERDGLSKDLKPIAEKYKGKINFATIDAKSFGAHAGNLNLKTDKFPAFAIHETVKNLKFPYDQDKKITKDAIA
KFADDYSAGKIEPSIKSEPIPENQDGPVTIIVAKNYDQIVLDDKKDVLVEFYAPWCGHCKALAPKYDQLGEAYKKSEF
KDKVVIAKVDATANDVPDDISGFPTIKLFPAGNKDGAVTYDGARTVEGLIEFIKEKGKHSAAISFKEESTEEAAPAASE
KAEKEKKEEKTEKAEEKDDEDHDEL

>QLI73106.1

DRPNDQSICDYYASQRYGASNSTTQLRLMQGIVAYAYAGGNTLPNPATNSTGIFNPGQFNGYNVYLRPWFDGSK
ATTNLNNQAVGVDWLDGGGTEPLIAFLNGSTKSADIKNGTNQYKLFTHWYYVFGKIYSCSQYKTFLDSSFTPLTPAY
VHRFMDLNQTEVGYFIEQLIAASKYYGFSDTDAATLSTFMNARYNIRCAPPANGELYSICLANECPLAAPDANCDVY
NNIQPYGLSNATTQSSTPTPVLPTTSTASSPSSTAAPTAPAGGSSSLSGGAIAGIAIGAAAASLLTAGMWLFFRRKRR
AKAETPLTAEVSDARSSSGFVPQSAYGANRHGSYSSHSPYVFAASTTDSHPPTAVEWAPPQELDAENPPRVPMYY
TKYAQ

>QLI73114.1

QTTYNYTSELDMTIDPNSVSQTDRATWCQGQTNTCRVLCNADASANECTESDLKWKCTCTSNSSTPGIQYYRQT
MPFYICQEVFKKCISQNAGNAQGQDTCNNNIQPLCASIDPPKSPVKDSGSDGSSSTATTGASQPSKTSGGSTQVTS
TSSKGLAGPTLAPVGNGAFVAAMGLMAYLL

>QLI73154.1

DKRQAKEGADNLKAYCAEGGYEAGTVTEKQDGYERFTGTCVKKDGSNAEKFSVQLGVQFCEKLSRRFRGSGGPH
NQGDYVVICGPKEDIKKPDESNGRVLLNQIIADSQGDPEKIKIFLEGLSRDAPGTLTSLHNALKAPASSTPGGLGGGT
GAGIGLLTAIYDFITVNAAEGGLIGPETKAGQWLRTNPIWGRSGTQPSPFGFDACCATVELPPTQLCVPFKDESHW
YWREDVKRCKSWHDRQKCDPAYAYDEATPAGKELARACREAGTKDAASCVRPRLACESFDDKAGKFKFEYCMDD
NNSSREVCKANGWVVGSGRDKTPEEIEAYKEEQKKQEEANCVSPRWVCSNIKGQFVTCIDGSEQSQTECKSNGW
FPEPAPKRSDKQA

>QLI73156.1

SPLAESQQNRLKWFPCEIANATVPMECGTLAVPLDYTNKTANATLQLQLQRAPAKQKSKSTKSILLNFGGPGADG
VEDFAYFAKRMQAATGGGHHLINIVPRGTGKTLPFSCFGNDLLRTASNQALAGNASNVALGYVWQRAGFFASDC
HAAQTNTGNLIGTAFVVRDMMQIVDALGEDGMLRFWGVSYGSLLGSTAVAMFPDKVDKIILDGVANPFEETELF
ADSDNVMKGFVKGCIANPEACVLAKNQTAAQLEESIYQLFQKLKSEPIGVPVPGYPGGGLLVDYSFVSAVLFRQLYT
PFIWQYTAKCIAALMTGESTDPGLQHCVPGLTQTTAAEASAKADQEAQFGIKCSDVRVRTTNLTQVLPVLEERRKK
SRFFGDHADTVLAKCAHWTMPAKERYMGDFNVSSKNPVLIIGNTNDPVTPLVSARNVSETFKGSVLLQHDSYGH
DSLMQASLCTAKAIRSYVVNGTMPKKGTKCGVKVPLFSGEDGWDEVIEQLERS

>QLI73189.1

TLDKRIIVGGEAAKDGEFPFLVSITNTLKRTCGGSLLDSTTVLTAAHCVVETQEWLNRTTGGQVIAAKRIVYPGFLRH
RRKGYPKHDIAILKLSTPIQESDTIGYANLPAAGSDPVANSLATIAGWGQVHNDGHPLDQLHKALVHIRAREKCTNE

SWNATGIEDEVCAGGDGKDTCGGDSGSPLIDQGTATIIGITSRSSWYSDCGQYPGLYTRVSSYLDFIQENLGVSQTT
SSVKS

>QLI73198.1

QQLGEIPSCAQSCVTSYVTGTNIAGCKPADIVCICQNKEFIQGISCCLEKVCDQADIDKTIKVATGLCAASGVDTPKQ
LVCSSGSASASDSTATQTGSSSPTASGSQQNTSAPSGSATAATTAATSTHTGAAAPAFGNPGGLLGAALAIVAAL

>QLI73207.1

MHYQRPQLNIQGLIKGKLEVMEPTMGVDSSRKAQEAMLQGQVAATLL

>QLI73223.1

SQHHVIDSPSPNNESSPSSISTSLFADLERLSRLVDVSYCIGSTGVHKPFQCLSRCKEFPGLSLAMTWSTGFLMGDSC
GYIAVDHGSRWMENQDGGVSGERNGAIIVAFRGTYSIANTVVDLGTIPQEYVPYPAPDDDDSDFLENCRRKCDNC
TVHMGFLHSWRMARGTVVPELKALRKKYPSYKIQLVGHSLGGAVACLAALELKLSLGWDNLVVTTFGEPRVGNY
QLARYIDKAFQLDGTRNLEHRTYRRVTHNNDPVPLLPLEEWGYSPHGGEIYISKQELQPTEEDIRSCVGDSDPECSA
GAETSLLEDMRRLLHFASASPSIEEYIVARTLPTRLKIWQLFFAHRDYFWRLGLCVPGGDPANWGRGWSGGIESEE
L

>QLI73228.1

APAVEDNNDVVKRDSNWQSHAKYYAMGGGRGNELYVDGTWSKPHSFTEGTPVGRCSARPLGLYQAPPGSDFQ
PISDVSCRCWISSNDPIGSKTRCVAGWKETDPADASKMIKFKLRFECSNWFGDCTSFSVLTDEGICESTPTNLCDGF
TVTV

>QLI73234.1

HNIVLPAHGIECFHESLHRDDKMTVTFQTGDREFGSAGNLDVDFWILNPSGGYEVNERSVSTGDHSFTAHTDGKY
TYCFGNQHWGANTKEVSFNVHGVVYVSESEMPSDPIEAEVRKLSDLLAQVKDEQQYIIMRERTHRNTAESTNARV
KWWNLFVIGVVLGESLFQVWWLRRFFEVKRVV

>QLI73237.1

VVFNSARLGSALRPGNRDAWLRERARHPVHLASEVGCLIKTGYGTRHRVREQLAAFAVKGGVLGDEGRDYLVVG
DWEAANGTAGGDLGAAVVDAVGLVMRDERVAEFADHVRFGKYRSLSAAVGAGDEVRAGELGAKFGWELDALK
FIMGMELSYKRMPHKKWYLILDDDTFVVKESLELLLSHLDPSKPQYVGNAVGDYRARFAHGGSAVIISGEAMRILF
NRPDIVREAYVRSLDETWGDRLVATTLQRIGVYIDERYSHYFNGEAPDMTRIREDRVCSPIVSFHGLRKPGAMVHA
GTTLGRMRKPVLWGQLWELFGQTPLESFENSPLRGGDHVGPGEEEIKVWQGIKKAQDCQRKCKSRCLAWTYDS
QSRQCRASPWLIVGPRNSAGEVSGINLSAAKSAFRKCSPVG

>QLI73247.1

ATTNKRIIGGEVAKEGDFPFIVRLQYGDDTSILCGGTLLDSITVLTAAHCNLKDITSVRAGSLDKDTGGVVAQVGSRL
RHPDYVLNGHHNDIAILKLSTPIQASQTIGYAKLPASGLNPVIGSTAVAAGWGSTVELLGTVSDKLRRVTLPVDAPD
ECFDLEIDAGKGFQNHLDTRVCAGERGKDTGRGDSGGPLIDQETRQLIGVTSSVPLGAQYGRGFYTRVSSFIPWIN
QNLGDSGIDPRRVTGYM

>QLI73260.1

ILSEDRFLARINLSPASYDPAFGTGADASIKAKIIHLIASIRTIMRSTAFTFFFFPSRLVGSG

>QLI73284.1

ASSKNSQPAQFKPPQVFRNANLVHIISLEKNYAKEQINVLIENVSNEPQSEYYLPFTAEQIARVGGFEVKDRKNANA
GPFVSEAVEYDPNSDAQYYRIHLPAPLKAGGQQTLGISFYNLKAYRPLPASIAQDERQYLVHDFSVYAPSAYPTLKQ
KTEVKAASSTIPDYTKITEGKEELPQKQGAKLIYGPFGEKPAGAISPAEVRFEFTKPVTHVSTLERDIEVSHWGGNVAF
EERYALHHRGANLSSPFNRVKYAQSAFFSPASSALKELRVPLQVGSVDPYFTDVIGNVSTSKFRSNKREALLELKPRY
PLFGGWNYPFTIGWNSDAANLLRKTAAGGYVLKVPFLEGPKQAEGVEYGQVIIRVVLPEGARNVKYYTGIPESSIVK
TSVNVHKTFLDTLGRTSLTIKAQNLVDEFRDRDVIISYETSTFDTLRKPFIVFASMMAVYAAAWAVGQVEVGFTKK

>QLI73286.1

ADFDPIYAPKPNEIITAGSTFTITWKAPAKYSEGTVKIELIGGATQNTQVPLLDIASGVKNSAESYSWKVDASLGDKAI
YGLVIRYESDPTVTFQYSNPFHITASGNQPSGSSSGSVTKTVTLSSLSSSTAPVTSTAAASSTSVATTSAPATTSTHSKT
IKSNTTLPANTTLVVKTSSSQSAPILITSTAVFKPTTAAPTTTAPSTLATPTAAANIVRVGSLTILGVVAAVLAL

>QLI73318.1

REAQEEWQSDEFSRENEQAAFKPIVTPQHGAGIWTVVFSYYCLFIHGLVFVFPVRACWSVWTMTQSLKKAAQSK
AIEDYKRTMVRRRVSLTSVSSSATLTSETLATESNACSSMFSETSDHELENYTDATEYMEDAVIHAIIIPNYKEEMDTL
KETLDVLASHSQARNCYDVYLGMEQREAESESKALKLIHEFVNKFRSIDFSIHPADIPGEAAGKGSNLAWAARKLSV
KYSMGMRQNVIVTGIDADSHLSSSYFTSITSMHHAHPDTASTTLYAAPIIFDRNAHAVPAIVRVADILWCAAGISGL
YKGSVIAPPTSVYSLPLILVDRVGGWDCDAEAIGEDLHMYIKCFFALNGNLTCRTVLSPVSQSNVTGGGKGGIRGAV
KDIKARYKQALRHMWGALDTGFALRKAAEMWKERKQTTRTFRPLHSQGNESSLYIPDIEFTSTADVSAANGIFSDL
TQDTIKEPHLEKIFYLFHRLFEAHFLPVHMTILVFASALYVWFADGNGDPHNVAWIFAVSNMLRTMGFMGVACYL
FLYESYHRICVAAREKEMTRAKLADGMCFSHRSVKKNFVDYILVPVVAPLYGAIPCAQAEICHLWTVNLVYAVSKKV
TRQRATSKAAMDMA

>QLI73322.1

APAGTLRRDVGESSTGSVAARAGILDAFTGAVQGVVDTASGVAGDAINLGARAADEPLEDFERKEEEEEEEAARTA
AKRAVRLGALQDFEKEEEAEDAAAGFGTRDLEDFEKEEEAEDAAAGISTRDLEDFEKEEEEEEEARTNAKRATTANGL
EDFEKEEEEEEEAREAKASGLDARAEPEEEECVNEED

>QLI73330.1

AKGPKITHKVYFDIEHGGEPLGRIVMGLYGKTVPKTAENFRALATGEKGFGYQDSTFHRVIKQFMIQGGDFTKGDG
TGGKSIYGAKFKDENFKLKHTKEGLLSMANAGPDTNGSQFFITTVVTSWLDGRHVVFGEVLEGYDIVKKIENAATE
AGDKPVKTVKIAKSGEIEVPSEGLHGSAGWADGVAPVENEGIVGDSPSEGWSLLQKVGLFGFVVVAAVFYIRLRKS
SNGGYTEKLTA

>QLI73356.1

SSASMPETDAAKDPLRIMPLGASITYGQGSSTGNGYRNDLHKLLTDSGYTVNMVGSRKHGSMKDNDVEGWPGF
LIDEVQSKAEAAVPSRLPNVFTVNAGTNDCGRNFQLDGAGRRTDDLLEYLWRASPDSTIILSTLLINLYPVVEARVEK
VNAQIKEVARQKAAQKKRIVLVDMHAADGPQKADLADATHPNDAGYNKMAKIWFRGIQEAASKGFLKNPKPVP
GA

>QLI73367.1

HVIQQHASDSTEVNLDSRAPGMKFVHPGVFVDRTQLRRMRSMVKDGQQPWSNAYTAMMKHPYARVTDPKPR
ENVECGAYSKPDNGCTDERRDAMAAYTNALAWFTKKDQAKADVAISIMNAWANTIQEHTGANAPLQAAWAAS
LWARAGEIIRYSGAGWEEEDIATFSTMLKKVYLPIVQNGSDKPNNWELILMEASISIAVFIHNRAVYKSSLERFINSAS
YYVYLKSDGSVPLLPPGMSRETLLKRWWWGQGTFQEDGMVMETCRDLMHSSYGIASISHVIETVRIQGRDLYSED
TGNRLRYALEFLAKYDNKRGTEQAPDWLCNGNVTKNIKDMTEPGYSILSEKYEMPNTKQFTAAARPAHADSLFIA
WETLTHATGEVDL

>QLI73381.1

QQSSAVQTYRESKTYTYYGCYNETTEIDGSDHSRALSGGANEVKKGEMTVPMCLDFCNSGENGAHYRYAGLEWA
RECWCAQSIAGISAKLDDGECNFPCEGNTSLACGGSLKLSVYRMSSAGAQASPVLLASFLSLVAAFAWL

>QLI73408.1

ATIDKRIVGGEDVPAGEIKFIVSLRDQNGTHVCGGSLLDSTTVLTAAHCLIDDEIQVVSVTAGTVSTKTGGVNATVAS
VEKHPNYTPGAPSDCPADLPLHECGRRADLPGGREENDIAILKLSTPIEKNDNIDYAGLPPAGGDAVVNSTGIAAG
WGAQIPLESTVPKDGEMKPIILAEKLSKVVLDIHAREDCAAKYKNQQVGDRDTIVCAGGQGKNPCKGDSGGPLFH
PETRELLGVVSWSISDRYEDDLCNKTPTVFTRVGSYINWINDNLGSRPSSRLAAVKHCTRPENDVMQCFNALLFCH
ARELSPDAPVLEHLECIDRIQICADQKTRLDQCVANAKVCKEQEKLPVGDLVNLARCAKKDL

>QLI73417.1

TVRRDDFPSLLDATLDELRHGLDAGLFTSVDLTKAYIARIQEVADELHAVNEINPDALSIAARMDAARKNKTACSRG
PLYGIPVLIKDNIATLDKMNNTAGSFALVGAQPKEDSTIAAKLRKAGVIILGKANLSQWANWRSSNTSSGWSAYGG
QTKGAYLRDQDPSGSSSGSGVSSSIGLAWAALGTETDGSIISPSYVNNIVGIKPSVGLTSRYLVVPISSHQDTVGPMA
RTVKDAAYLLSAIAGADKNDNYTSAIPFKERLPDYVAACKDDGLSGKRIGVPRGLLTPSQDTSPLLQAFEKALEVLRS
GNATIVDDFEIPGSEKIGKFIEPILGADMLTDVASHYFNHLKTNPYNITTLKQLQSFTQKFPKEEWPVRDTQLWQDA
IDRGFDNTSPEFWSMYTEQLEFAGPQGILGALKNNSLDAIVLPSEMLNSLPAIVGSPIITVPLGKRPDDWPESKNGF
GNLIGDGPNLPFGIAFAGDLFSEEKLIEIAYAFEQKTKVRGTIQPLVKPKTELRDVVGTGS

>QLI73422.1

LSVASNPYTGGDTSGCGKMHTAQVIGLPIYRGIQSSGVHRSYSVHLPSQYDKHHEYPTILGFHGSSSIGLFFQADTKL
DEARFTRDKIMVYPNGLGGAWAGANYSQATVDQDLQFVWDLLADLRQNFCIDSSRIYATGLSSGGGFVDTIACN
STVGGEFAAMAPASGSFYTNNDANHHLCEPARVPMPILEFHGGADADVKYGGGQGEGGIEPAIPNWLGWWAA
RNKCDTPHHQEDLFDGDVHHLSWACGGQEGVVQHYKTDDQKHDWPSTEPNFSQLAAGDKPTHIQASAIIQDFF
MRFTRPEV

>QLI73423.1

ATQLFQFANTSQFVENIAVRPNGHLLLNTFDNARLYTLDPSAKEIVPQVIAQIPGSTALTGIVDVAHDVYAVSAGVL
NFTNLSFEPGTSKIVLVNVGHCAHGKPASVEVAAQIPEAGLLNGIAGLPKHRHIVLSADSKTGRVYRVNTLNGEVDI
ALQDDRFTPGPRPNAVPLGINGIKVFNGYLYLTNSGQGFVARIKINDFGDKAGDLEIITTLPLDPPKTPDDFSIARDG
TIYLGAHLDTLVKITPDGKWVSLIEGSSAGVYLDGPTSTALSKDEKTVYVTTGGGGQSGKGGQIVAVKL

>QLI73466.1

VPHFPQEDAKLRATNLTIRRGGNCPNSLEVLQQLVTDNDNTQLHLVSCLPRRDAPSTRRIISSFGPDTRVDFRHCIY
REGYTEAASSYIIKSQQSNSRTIVNYNHLPEMTCHEFAQVVDGFDGHEPTLWHFEVSAAFAFLFRLLNLALRGRHLV
LWYYPDVRQPSLGRIPETTLACMRLLRQKLPEANISVEVEKPGREGLSQLAQEADVVFYSRVWAESRGHRSATACL
TKERRHQGSLGLCTWGADGATIMSQATGACLHCPVESESGQISVVDPVGAGDTFIAGMLLSLIRNDWPLSDIGKA
ASFAVRLATLKVQREGFGGLGLDIASR

>QLI73481.1

QGPPYKETPPGKQLLDQAIAGIPNWGGPGSLQGKPNKSPKPPIKNMIGGLPELFKPKPGSSDWREDTLKGPRKKP
GVVRPKNPSPNCRKRDLCGPGLGKLPTGNKVPTGGGIPAVGSTLTNIFGGIAGGIAVGIGLQRLFSAIPGASDKIQEV
IDAITRWQRETFGPKNIAYPLDPGKYSNCIAARRHDIHANPEYRKELIIINCPWGTELEDTPKTPPEPETQPEPSRQCF
DSTGPIPCGGAQTEVQLDTGRAVCGSCGTFWDPEGGKCRDIKGALIWPSLTPITERTPTPSGQCSNSTSSFPCGGG
QTEAELDMGWAVCRVCSSVAWDPEGGKCVQKDGKLLWPRKS

>QLI73488.1

ATCPGDGELCFQWGIPAGSSRTSPGNLYFQLRGPSSLSWFALGTGSAMMGSSMFVVYSNGSGVTLSTRPGKGHV
MPEYQARSDVELLPGSGVVNDNLVANVLCKNCSDIAITSSSSWLYAWNMGAAIDSSSPSEPISFHDAFSGFSVNLE
QATIASDSNPFLTPATNGNTKPNPSGVETDEGNGMAMLYAHGIVMSVAFLIGYPVGSALMPLLGRWLIHASWQL
LAFVGMWVGFALGCVISSRAGSLFQDAHMQLGLAVSILMTIQPVLGWLHHTHFLKHGQRGPISHVHTWYGRIIM
ILGIVNGGIGLHFAGASSRFVATYAIVATLVSVMYLAGMGFGIIKRRRQQTRHYQSSSQTELAKL

>QLI73494.1

VQIKYTNCLPDSVQSQQPPFLQWVPVEAHAKFDTQNDAHNFQFIVWGNVTGSVNKQMLPPPTSPDWTNDNKT
DGKIVNSENDENGTTVKSSIGMLTYTPWSSRRFFCEQALVGGHCPLAPVFNTTGKVTLADLPSMNITNDMRSSYQ
LASFATTMLIIFGNQRGDNIGCISATITPDLGNLSWVLKIMPLIVLIFCGFAVVFAAIFSPWGSSNIFHWTSNYGRDA
DLLRLVTPGFGDCLQYIQFIALSGALSLSYPGFFQPVVSQVGWSALMFNESFVTHTPGWQSVRDGIYITDPQDGYG
LHALGQLVGMSESADIWAGLMVWLCVIIAAVFVLTQAGFLVQWLYRKIQNIPEEDLRAKNIPFSIGNVIRIVFNFLL
MPVVALSCFQLVVAHESPAFTVVLAAVTIAVLLAFACYILLLIIRTKPKSVLFDDLPTVLRYGPLYNTYSDEAAAFALIPV
LLNFVRGVAIGAVQPSGISQVVLLAICEVIQMFTIHAFRPFQSSTSMNAYHTLFAILRLACVLLMVAFAPSLGVTEGP
KGWIGYAILLIHGSVLFFGFFLSALSTIVEVVARLLGAGGDNITGLTRGGLSKIFGMRQLSRRETHRTAPSRASQLSSM
AMLHAEEGTSRTGYSVPGGRLRSASGASYGGIAAHNQRSSSVLDSADMWSAGHRHVDTNSSYIPGTPGETSTFSF
VASPTVGRPILAGGHMEPSDPYYRPPRRTANNNANLRDSTHSDTPTSNALGLDPKQAALTPATVGESGDVSGEDT
TRAATPAPPGAGGGIMNLPANRPDYATREVDFYYGVRGPALNSDNPGRRLGTGPADPTGPVATATGWFRTLFG
GKSKEKGKGFEVVRSSRMPPDMVRNGGFGDETPPEGIPVAMGVLRNGPIDSDDEGDPPVKRPPRSPQRSPGDLL
TDDGTPRGSDSDSDNDEEEEDIDHSKLPRGSKQGELLGLSLPEIPRKSSKRISGLVDDRHLPVLSAISSSSPAPSDHAS
HDGGPSARGHRQTLSASSALPFERTSSRQQRLSSKSSSEFPGELSDIDLQGSRQDEKHASYGRVQQHEINRVDPPQ
LPPLDLLGSSAELVDDFGSLGRKKSRHS

>QLI73502.1

QDSLDYDGILRCSTETSTLAINSSSLMEKRQNQALQPISIDLYLHFVAPDDGAPKKLLRKAAKRQLYVLNNAFRPSGIS
FTMRHVLFWNDAFHATIQNHQDLASMIARYHRGDANTLNLFVPKFPSQSLGIGACISFDNLFIDQTVPLSSDGCFV
SPTTFPGTATAGYNQGKTAVHEAGHWLGLLHTFEGGCDGQGDHIFDTPAQAKATYTCDEWQDTCPFSPGLDPIH
NYMGYSYE

>QLI73504.1

QSPSGQKLPFKPAPEKSGICPSSSRFNSKPLWDTTEACFNAHEQEPKENGKRPFHFGFSAGPDCADRHGGLMTVT
SCGTETYCKAIDEWGSEDGRLQEDIRWSSTKECLDAHVPEPRNLPWKRRDKANFCGSRADYSENTCGTEEYCRLLE
KQPGLISTPPYIGTKTQCLAAHVPDPDAIWFPVD

>QLI73519.1

EQPLSPEITRGRFKRSIVPSIPRSDSAKHVETSWQLNDRALDDGLPDGASENTAELQKFKIKYEEIPFKLDEAWTKLEH
TKGFSLRGEKWFSTGKGIVYPAELMKQNKGLWPRNYDPTTGKPKPFPEAGRGSDEFKSTSLWEHVNGQTKYTTR
WMSMTKSVHKSIEFSANWENGKPSKVLTQPEGRDGLDLNVIWKIQDAPNVVDTHGSLANKGANGEFVSNEKFIP
SDYLDQVEYSAFEGIPTEQIKGYFRTELILRNPGLAEKIANGDEPEGVFVKNIDYNDKFDSLSHAGVRPDLAGFPPGGI
NQGTMKNEGWERQPFELEPWNKFDQGPQGEAEGQKIQRRVQSHKDRARDFEDKSEREILASVCSTNLRKRAGSL
CNSDTSAPAPGNDRPEPGKPIEEEVKPTDPNGEELPKAIDDAKGVELTRKTSDDVFLDLFSSQKMGEKAAKLGVKA
GEARTRLSGYEPITAESPKLKLSGLKLAGEGAGVALWVTGMVQAFTSHTTGLQRAAAVTAIIPFVGCGFSALAEADS
QEGVDVVDKMMCIYADILLLTPLAPFSFVIQVFRGIMSLYKPKDLPKAEDVQDNRDKRWARFLDDRVFTYFYSDDS
VSKNRVFRDKLNGTLFADHLGVVSDAADFIAISNTSARVALQAQNADKPKIQAGALDVANQITAEISPMIVRRQRE
FLLKLPQLVLRDTKLSLQPIAEQFNKELIEHMTSESMVRQYTPLVPAIDGQPLGDEENLKDQTRGSLHAIGEHLKKNP
PPLPKLFDIAYVLGQSKGMLDINPLSLSPGEFIKSRAPDLSQDDVDFYSLHHALQISLLLRGKLTEDKLSKLWPSDDAS

TVQQLQLLLAVKFGKIYDEQKVLWVKSQSGVTGNFLGEKEVRWSTHPNIPPHGRDEESMAYLGLILDLSEERIKNIP
RHKEVIGFKDLGTDAKLITAMLEHAKELYIKKLDEEAVAMQDDKANDGEKPKSA

>QLI73520.1

GTVPRNPSVRSDLLQPSQDPFYAVPDGLDAIKPGTILKHRAPPSPIAAFSVAALNLKASHQILYRTTDSLGAATATVL
TVLMPHNADTSKILSYQIAEDAASINCAPSYALQLKSATGPLLGTLVTQAELLLVEAALQQGWVVIIPDFQGSKGAYL
ANKLAGYATLDGIRAALNSASFTGIMANSSNLKIGMWGYSGGSLATNWAAELQPTYAPELRIAGAAVGGTIPNITT
ALTTINGGPFTGFIPAGILGLAAQYPEIQQVLDQHLKPQFVDKFHAVLNQCLVADAADFLFQDVIGMLDDRNLVQT
NPIAVRILGENALGRATPKIPFFWYKSVLDEVSPVEDTDALVKKYCAQGVTIEYQRDLASEHGSCAVVGAPKALSWL
KNVMNGKSPQPGCSTKTVLTSLIDPATVQVVPGYIVKLLLDLLGKPVGPLLFG

>QLI73529.1

SNRKESIPSARCWPSFFPKVFDRLSYNSQAPQLIKHGYQKFRDQPFKLHKMDMDLVVIPLKYASELRAVTNDKLDPL
TASFDDNAGAVTSILLGSELHTHAIQRRLTPRLPKIIPVMMDELRLAFEQVLPMQDDSWVAVNPYEMVLHLATRA
AARVFVGEPTCRDEIFLQTTASYSRNVFESISTSRRFGSLLTSFFGTWVPEVREARDQLQYIQQLLGTEVKRRRESPN
EEHDDFLQWCMDLARTEEESQPEALAHRTLGILSMAVVHTTAMASTHLIFDMIADKDLTNSLRKEQAAVLKEGW
MSISQQSMLDMKHLDSLMRESQRINPVGEFTFRRLVRKPVTLSDGYQLERGQQIALLARSIHMDEENVPDAATFK
PDRWLKQQNSAPTSFSNSSTANLNFGLGRYACPGRFLASYTIKAIMSRLLLEYDFQLQGQFPSGRPPNMLHGDRIF
PHRTAVVLLRRRNAVASKA

>QLI73538.1

GPPTVVRTSSAGALETLDTEGNKELEEWLAANIRTPPSMLKPCPLPCSEAPDSINGGGWFLVPDATSFAQCNKTLLL
DLVVKSDDPPALRACTADYGSNYQLSKIHSPDDGNAALCSTPNHEMVKTSIRLLEVSTPTQNNDTFATQHLVSASR
QVMHYLASQKPSCSKNALAFGYSQTSVVGVFAGAELHQHGLAIDVINSLLSYATERSISQTTVLQLCQEHGLGADYS
FGLVATNANNLKFAQEAVRRWASGKCVGPDGGRDWKEVTIRVPSIVRPTNTTASSNMTGSRLSYRSSWQDLARV
HCKTLTVRSGDGCWALAKICGVSQSDLTKYNRRANFCDTLVVGEKVCCTRGILPSSIPDSKSDGTCVTKEVKSGDSC
GSLASKCGLSASDFMRLNTKEDLCSTLAEGQQVCCSRGELQDRRPKPESDGICASVKTNRGDSCASIAASRDLTVRD
IESFNEDTWGWNGCKVLWVGFRLCVSEGKPPMPEPVSNAVCGPTVPGTKAPAKGFDLSRLNPCPLKACCNVWG
QCGLSDDFCIESKSETGAPGTSGVRNGCISNCGRAIIRSSPPPSVIKIAYFEAWNHKRSCLWMDIDDIDTTKYSHIHF
AFGEVTKDFRIDISKVQTQFYKLKAMVGVKRIISLGGWDFSALPGTYNILREAVQPRNRDKFITNIVDFLIQHDLDGV
DLDWEYPGAPDIPDIPADDPQNGLNYFKLLDELKYRLSNAKSVSFAAPSSYWYLKAFPISLMAMSLDYIVFMTYDLH
GQWDYGNKWTSPGCPTGNCLRSHVNMTETKDALIMITKAGVPSNKVVVGVASYGRSFKMAEAGCYGPLCKFTG
TPRISHAAKGRCTDTSGYIANAEIEEILASGKVTKQWREAGSNILVYDDTEWVAYMDDKLKDIRTQFYLMNNFAGT
TDWAVDLQKFLPGDGIPYDDFEDEYEPYIDPDFYSDCSGKYSTLGDVEKHLTTMPTHCTEKYIAQAESAMIKEALTK
HLELLQDDYDSKFKTYERFVKQQVPVQINNFMASDKVHQYFKCSEYKKVICCSDCTYATCLETCANFAGCKSGYQTL
DIKCPQQKDELDMISMETTPNATFHLKDEEGFWKEIGSQYGIEEAWVAFGRRHMRTANGCQYAGKAIHECQNQ
NDRWWYNYPIAADDKIKVYNPKKIFGDSTDTIASLAENLDIIKDLNIYDTLMPWSDVVDAASLPALTLQAAVDNMK
SIIDKAEEIEKKQKEEFILNMVMGILFFIPVVGEAAGSAGLTAARSMLRLIGAAGDAGMTVYDIVKDPSNAFMAAFA
YVLGAGVGRSGFRDAANSRRAVRPRELAAVGSLKTDLQRIQVSRKNICYL

>QLI73579.1

ADAAAPAADDSASPKTPDLAAVVKTTFPDSDILGVRLINGRPTKALVEITNKEDAPIQISVLAGVLATAKTLPEGTPA
YQGIIRNLTVVQYNHAIEAGETKSFTYSFALDMQPQDVKLQIAAVIANANGDMYQVQAHDGLAAIVEAPTSFLDP
QIIFLYLVLSAAFGGTLYFVYKTWIEALFPQAKRSKSSSSGPKKAKKSADADAALSGSESAAATTGSKTYDESWIPDH
HINRPVAKRVKSTPKKKVVE

>QLI73590.1

LPQLDMVPRAAATTPVKPKYSVVPLEPGDDDPQSGNGGNGENSSKGSDVTKTVVETEDPVTHTVTKTGQPVTVT
HPAPTTISIIPIGGNPDVTITVTAEVTPTVASTTASIATPATTSSVIAPTTTSVSDSTTTSVSDSTATPVSGPTTTPVSDPT
TATLTTTSTQANSTAATPISPISSTTSIVVTPTSPTDTIPSIPSATPPAPMSTTPWSVPSSNHTTTTSNPFSETTNTAVR
ATTLLGTGSRPELSATTPAPIATTTWIPTTLSTVFLTPSSSSTSTNTYDDGKWHTTYPAWNDTATHRFRRF

>QLI73596.1

SNGKDRVAKCLARYSGQDRGRNRTTVYKTDFPGVTWDDDNWLLSTTTLEQGRYQSRGSVANGYFGISVASVGPF
FELDAEDEGGDVINGWPLFSRRQSFATIAGFWNAQPETNGTNFGWLLQYGYESVISGVPHWSGLVLDLGNGVYL
DSTVDNKTITNFRSTYDFKAGVLSWSYTWSPSAGSNGSYDIRYLMFTNKLHINQAVVDLEIIPSVDANATVVNVLD
GYSAVRTDFVQSGEDTGAIYSAVRPTGIANVTAYIYANMTGSDDVGIGRKTLVSNKPYIRKNESSIAQAVPVTFSAG
KAVRITKYVGAASGDAFEDPQQVAKNAASSALSRGFYKSLRSHVQEWDDVMPDHSVDSYADPDNGTLPQDNYII
DSAIIAVANTYYLLQSTVGPNAQSLVKDAPVNVDSISVGGLVSDSYAGLIFWDADLFMQPGLVVSHPQSAERITNY
RVNKYGQAKANAQTSYTSSQNKTVFSKDAAAFPWTSGRFGNCTATGPCWDYQYHLNGDIGISFVNQLVATGDTR
YFNESLFPVYDSIATLFSNLLAPNGSSWTVKNMTDPDEYANHVDAGGYTMPLIAETLQTANTFREQFGLEKNATW
DSMATNVLFLRENGVTLEFTTMNGSAVVKQADVILNTFPLSYTTNYTTQESLNDLDYYANKQSPDGPAMTWAFFS
IIANDISPSGCSAYTYSQYSYKPYARAPFYQLSEQLIDNATTNGGTHPAYPFLTGHGGANQVNVFGYLGLRLLPDDAL
HINPNLPPQLSHLRYRTFYWRGWPFAASSNATHTTVRRARHVRPLPSADPRFANATITIDAGADGNATAYRLPVD
GAAVVPNRNIGRVNTTPGNLLQCRSADSPGAHRPGQFPIGAVDGATSTKWQPQYAANLSAVTVMLAPGDVGA
MVSGLHFNWAQAPPVNATVVFHNATIASFADVDVSAQSKGADYTVVTHLAGVPLSNPYSVQATNLDAIAIPVGN
TTNVTLGEAVPAARYATLLIVGNQGLGQRDVDAKNGTGATVAEWAIISDERRRMDGEAAPAKVRRSLTWREKG
MLMGRP

>QLI73608.1

TVLGVNANANVNADVVVGSHSDLNAQVSVNIDDARARHEGYTCPHEMTYSPWTRCCGCDPGQWLDLGNNTC
VGEKMHGAWPMPNVAVYGSVNIQLGAFCAAAPNKIVAYDEKHEWCQASPLTIVFLADITIALELIAGVDIDINANI
SVALKEVCAALSGLYLESVVDAVIAFNTDFLGLAVIQADIEASLGLSLFSIIKNLGCKLGIGKCNFDCVAYCTKGCPNYI
DVVGELGGRITGLVGLCILPKVILVVNSLKVVVNVVVDSLLCLVGGIIKTVLSTFDCHCK

>QLI73622.1

SPVRRDARSPKTYAPNNSSRSSRRLSFNQDGTFQISIFEDLHFGENAWDQWGPQQDINSVKVMDAVLDSERPDL
VVLNGDLITGENTYLENSTAYMDQIAGPLVARGLPWASTYGNHDHNFNISGAGLLARERRWPNSLTRSMVAGRD
AGVTNYYLPVYAADCAPGDDDCAPELLLWFFDSRGGFYFRERGPDGRQVGQPDWVDASVVRWFEATSAALRRR
SGRVTPSLAFVHIPANASLAVQASVDAHRQPGVDDDVPLARQAQGWCADGSNGPECVYGGQDEPFMRAVAAT
PGLIGLFSGHDHGATWCYKWDRLVPGMTVAGTGLNLCFGQHSGYGGYGNWIRGARQLRLSADALRRRRWEAD
TWIRTEKGGVVGRVSLNATYGKDWYPATPNEKTYCPTCNYTVITPGPRRR

>QLI73624.1

QTYQRLGTCPTLGCLLPPDQSDFLPGQLFDLRVEVHAPVNGSEAAHDGKPDEKFKVTIAKDGKEAKDITNFFGVKE
PELEKWTFKWYEDLFAEDKKTPSIVNVASKAYRKLSLDEPGKYTVTLQYYGGEKTTAEWVVRPIVKKRKAKNVIFFIG
DGMTTNMITAARLLGHKSINGKYQTRMQMDEFPVLGHQMTHSIDSYITDSANSASALYSGHKSTVNAMGVHAD
SSPDPFDDPKVETIVEIFRRITKGAWGAVSTAFLADATPIALTGHTRRRSEYGPLIDQALNGLTNYSWTNHEGPDVY
FGAGAEQFFAGKGSYKGKDYYEEFSKKGYSVSLNKTSLEMIDTSKKALGVFCQSNLPVWLDRNVYKDNLKNFKNNP
KGGNDSALDLPGLKEMTLKAVEVLHKRGGDKGFFLMSEAASVDKQMHALDYDRALGDLLELDDTVRETIKKLKEL
KILDETLVVVSADHGHGFDVWGSADTEYIAEHDDERTKRNAIGVYEKSGLSQYTEKNKNIQYGTGVNFPSNWEPR
YAIAGGVGAAPDHREDYKVHKSGPREPAVKGADGSYIVNAKDSPNGIVINGTLPTNEAQGVHSLTDVPVFAMGPC
QETFGGTYNNVDIFYKMATCLGLARPNTEGKKPKKQ

>QLI73625.1

STFSLAAEPIPGKVNEFVVEVELGTPPQKLPLVPDTGSTDFWVYSTDTESSWNNHKLWDIPKSTTARNLTGYTWKIT
YGTGYADGTNIYEDVVTLGGVPFPNQAIESASRTDYKVDPKISGIFGLEFRPDQTGVNQAGEHVKTWSSTHIKSLD
QPLFTSHFKFNGGGSLDFGFIDNTKYTGSITYTPVTHDQHWMFNSTGYKIGDDPVQVEELQAVADTGTSGIFVPEN
VARAYYRKVPTSTGGGNFQYVFKCGNPLPDFSFNVENTVITIPGKYLDLGPTGLPQNGFCLSAIQRGKTTIFGTPALQ
AAFVVYDVGNRRLGFASPA

>QLI73626.1

AELPRDSSLWMDLTIPDTDRTELQTSSYEPIFSFFTRSILGRAQDVTPLENNRPLGLNVGPGSQPVCYLVKKGSLGAG
RDPRKIYISANTCLQPTTKAGKKAMSPGQLTLFVSNNTDAGCPQITSAGNGVEAKGFTSKVFTEGAVTFSVNSTND
VYVAVYAPKLSDDFEGTYNFEIAASNTTYFHRYKASGGAELLWMDSDSTSALLVTRNLTDDVSNLRHVMSEDPPYQ
LYVSGKDTTSLDGMRRSACGFQKNALIGANNQGNAKNNAMVKTAMTLRGPGGLPKQQFYVVGLNATSAYSGVL
VKPANVTVNSKRQVGGSDVPTNPGSIVFQATSFQTNAAPNCKVVTDLEFCDEIQYAVPGNDGKYNNTELAKAYD
NYAKSMYDNFLKVMMQVQCETDRTSRYSLARTCDDCKNAYKRWLCTVSIPRCEDFQGESRFSVIRNVGQPFPNG
TMLPTDVQTNLALVPSQNASRNAFIDTDIQPGPYREIMPCEDICYQVVQSCPSKIGFKCPQEGMYAFNVSYGKRD
NDNSTVSCNYPGEARTPVNGAAAFIPNLMFLAVVLSSGLLLLG

>QLI73631.1

NPLAASTKTARGYITSDEFKYLRVALVSCLEANPPQDAAVSTWATSIASKYTTMGEIVQAIVECEAKETKAKQA

>QLI73646.1

APWSNPTLDEIDGAPMTQQRVLEIFGHRDIFCKEKKMWKRIDCEVQQGSSEPPTDELSLICQQKGGCEGCELGIYD
AVVSLCTATNLGMIL

>QLI73656.1

VDPSQDSHTIQDFYHKTMDGMKKGSPPGQAIINTKTGEKAGHIPADKDADGDVDEDDRKITAEMQLRLKQAEQD
AKDKANIKGGLKPDIPSEIVGKGNSAEGQPKKDKNAAADKGVSGNADKTTDKTKTKTKEPKEESEAEAELSSILKKA
PVVIFSKTYCPFSKTAKGILLDKYHIAPVPFVVELDKHAQGASLQDVLLEKTGRKTVPNILINGVSIGGGDDIVELDKQ
NKLADKIRMLGNKRVKVSERFAPDEIKK

>QLI73661.1

QFSSPSKESALELDKQIDITWNTAGLEEPIAINLVPAGVAGRTVIAQQVAGLSAEPFEMISTNGNTVGIQNSGLLQW
APDLSIAAFPSFNMVIVDSKARVVVSEEFTILSLVQQPAIVTRLIPIATDVVATDSEGVVATQRVTSTSQMLLPLPTEPI
KLHMSGTQVVDAMVTGVNEQAPKPTEDVNKDGQKEGLLPLPGVEGEEEPVAGLKPLPNVSESTTTSSAEPEQTEA
RKGSGNGDAAAAGKKNQGSNKNMQGKQKEGGGSKNNDKQANSGNSGASKSGGGKDGNKSNNKNMANNN
NNSNNNNNNNNNNNNNNNNNNNSNNSNKSNNNSNNNNNNKGKMQVSKAKKPMA

>QLI73665.1

ACAPGAGGAAASPQNMVVSQAPDFVRPYVLPKYRGRSIMLSQAEVLRFAITTNSSGGAFSMIQHNGQFNNNVSA
RYHDHHNVHEHLYCARGRVQFWAQKNTSEGEQEARQATPGDYASLPTGTIHTFQLVDPDAQLTHVFHPAGFEHL
FDVFYGEGKYSSSVSAPYLATAFDESPFAGTLSPEAIASLNSLDLYLHDNFVPRRDFVNGTAGDSRLNWHNGPNELP
SKYGEPYAIAKDYGPKFLNAENGYKIVQPFADKNTEKDFTMGTVILSPKLDNETVTTTTLPHHFALQMEDGHLILAV
DGYESVALLHGDVAFIPAGTRFSYHADVPYTKFMYMNNGTQGLDHQLMQKAVPWGWPTYPHYAGYKA

>QLI73681.1

FDGPAPIDHGRRIDKRDREQEFGRRHGANIPGLSFMEQREEEAAKV

>QLI73683.1

LTPETKAVCSGLHAKFPGQLVWDPIGPQATDTISHASTYNTALFDYWNAASSNNRAGCAFFPSNADQVSVAVKLL
NAYPTVRFALKGGGHNPNLGHSSVDQGVLIAFRPNSQYAIPSADGETVEVGAGCKWEDVYSALEPLGKTAVGGRL
GDVGVAGFLLGGGLSYLSAQYGFACDNVVSFECVLANGTIATASSTSHPELFFALRGGGNQYAIVAKFVLKTYGIGQ
NGTIWGGVRTYTADKRKEVLSAVANFTANNADSKAAIIPTFNFFSTLGVNVPGSLVFFFYDGLEPRRGVFDEFDAIA
SFSDSTKRRSYTDLTKEVLAGDMKGLRFQIRENTFPNMPAGDMNSFLNSHYNLLVKKSTEAALGDVLDFKLFSFAV
QPMPRGIARASLENGGPNALGLVPEHGDRVWMEYDIAWLNPLCDDKCPGFFEKLVQSQHDLHREKYSGIYPTNY
ESGDLEWLSYNPIFMNDAMQGQDVLQSYGNETYSRLLSIHKAYDPRGFFSDRQGGFKFTT

>QLI73685.1

SPILGDLPQKTLQYESQPHQELQSRKLNGRFLHITDFHPDEFYKPHTSTEDGIACHRGNGMAGTYGAEKTDCDSPF
SLVDATFQWIQENIKDDIDFVIWTGDTARHDSDEAHPRTDKTVLDSNKVVTDKIIKTFSSPEGKLEVPIIPTFGNNDF
LPHNIMYPGPNHWFAAYGEIWDRFIPEEQRHSFQFGGWFHVDVIPGKLTVFSLNTMYFFDRNAAVDGCALPSEP
GYKHMEWLRVQLDLMRKTGTKAILMGHVPPARTDSKQNWDETCWQRYTLWLQKYRDVVIASLFGHMNIDHFL
LSDTKDIDLDVAAGQSPAARSIFRKSINESELSAQGKEDYLLELRDIWGDIPGSAIEVLNEEDDHAEVDTEKKKKKKN
GFKKIGGKYAERYQLSFISPSVVPNYFPTMRVFEYNVTGLENATVWKEKNATEKVQSTENLELRFVGIEKKKKGKKG
KKGKEGKQKPKDPNLIIPDDPPKTALPGPAYYPQQFTLTGYTQYFANLTHINNDQPGLDGVEGSKWHDGNHGDK
KPIHKPAQPRKFQFEVEYSTFDDKQFKLKDLTVRSYLHLAYRMGQRSKNNEIAEGDGASGDIDADKKKKKKKNKEK
NKTWLHWLNFAFVSAVSEDDLEEM

>QLI73692.1

EIASQDFLHNITTYARYAAFGYCDNLTDGRDINSGTKVCPSDGGIPGGCGDLADSVVVMEFPSAEGVSGFVAVNKK
TEKIVVSFRGTGSAKDVVADLKTCKTRAGRTLFPWLNEQREKFGNALNKGFRNVVVNIVGAACSLAGQPPAGDRD
DLLPLCHNCLVHTGFFEGFMGIKDKMLTTVRQQKKDYSNFEVVVTGYSLGAAVATLAATYLRKASFELDLYTFGSPR
VGDATFAEFVTNQGRGKNFRITNADDPVTNVPWNDPGFAHVSPEYWFPGGIATKEMQVCDGVNNVACSGQFE
FKLGNVVKGKDGMRPHLWQNYAVGFPFTGATACPGRGGRELEDAPFTPEEIAEMKRLAEQSD

>QLI73693.1

SPIEPVENNTVLPLKHVSSVKSVKGIVNKGLARIQKFNGVKALESNDASSGSASYPLAETHNTGDYDQMAHQASDP
GSFSGESPIAVGQPSRRLIY

>QLI73706.1

DKPEESPVVPELPTFTPTTLKAPFLEQFTDDWEKRWKPSHAKKDMKGSANEEEEWAFVGEWAVEEPYQYKGMD
GDKGLVVKNPAAHHAISAKFPKKIDNKGKTLVVQYEVKLQKGLECGGAYMKLLRDNKALHQEEFSNTTPYVIMFG
PDKCGHTNKVHFIFNHKNPKTGEYEEKHLSSPPTAKIVKTTELYTLIVHPNNTYAIKQNGEEVKSGSLLEDFTPAINPP
AEIDDPKDSKPDTWVDEARIPDPEAKKPEDWDEDAPFEIVDEEATIPEDWLEKEPTTIPDPEAQKPEDWDDEEDG
DWIAPTVPNPKCADVSGCGPWTKPMKKNPDYKGKWTAPFIDNPAYKGPWAPRKIKNPAFFEDKTPANFEPIGAI
GFEIWTMQPDILFNNIYIGHSIEDAEKLAAETFKLKHPVEKALAEADKPKEEDKPKSPSDLKFLDGPVHYIKEKLDLFLT
IAKNDPVEAIKFVPEVAGGIGAIIVTLAAIVVGLIGLGGSSPAVKDTAAAAKDKAKEAKDKVAQATATGAEKAKGEAS
KRATRSQS

>QLI73718.1

LLIDTPPEATRGSPLNITWKLDPDQVVFYLSNNNESYWPIGFANIHDGSVTAQVPTTYSYNTGHIIAFTNDGLGPVG
ASQQFPVN

>QLI73761.1

AEERVLGVYIFHRHGDRTAKAWAPVNLTALGAEEVHSSGSFYRKRYVQTDASVRIAGVSSNNPVLSQLSVIAPQDA
VLYNSALTFLQGLYPPTGQSETLANGTKVEGPLGGYQYIPINAISNAATANKAENQGWLQGASGCDNGVKSSNAY

FTSADYQKTYDESLDLYQSILPVINTTYNKDAANFKNGYTIWDYINVAKIHNSSIPSENLINSQTFDRLFNLASVHEW
NLAYNASEPVRAIAGSVLAGQILDSLQAIVDGTKGAARFNAQFGAYGTFMAFFGLAQLPKASQDFYGIVDYASSMT
FELFTTSTAAKPSTDDINVRFLFANGTAANNELKPFPLFGQDKTSLSWSDFRSGMTKFAIADTKHWCTLCGSTSSTC
AANSTSGSDDPSAQSNASSDNGVSKPVAGVIGALVTLIVILGIQAAVMLLAGLRLVRKSTLARANNGPETAGIKGQ

>QLI73777.1

APQNDPSTEGLSKELADEMQKVLKQLEEDNKKSIPGTIQAMAEQLGWQAAGNLPGKVGQGATLLSFLATSDYSIT
PENGKEWVKNLGGVALSFLPKVGTLGNIPGFLGMLKTIAIGLERAQFPELAAKRDAEAKCFADNKVRTYKDLCAYC
KPHLTISLLGRLNGCTDELVANRDDYVDHKRAQSFCADAVLCSGYNDGGPSKIIKNGFRYDPLRSFWCKDKDVQIG
MLLSSWIGEPLRDSLYMLDAEDFRNALTPACKEITKDLNVEGTCPKREELEAADKLAPPVSYCQPGQSVAAGGPDK
PFVPKA

>QLI73798.1

YPVADHLWARACTPGALMCNGTTQFALCVAADSTPQWMNVSEGTQCVCSGSECSITSTNGTLASAPAPGASPT
APLTSAPAPEGTPAQTASVSAAVPTSAPPAGSSLVPSSSPVAAPAPSSDPAPVPSSAPAPAPSSDSAPVPSSAAAPV
PSSGPAPAPSSAAAPAPSSAAAPAPSSGAGPAPSSPAPYKLYLGNGSPADGWPTESKWPSFEALWTINLPFIKTGC
NWQNFQPNSEEETAELKSAIESVGQSGGVDPRFILAIVMQESNGCVRVGSTANGVKNPGLMQSHMGPHTCYNK
STCSKDEIEGMIKDGTLGTPSGDGLKQVLVQAGPGEGQFYRAARIYNSGRVAAGGALQLGIGATNCYSTDIANRLS
GWNSGVTPCHV

>QLI73803.1

AVINYDGAEYDDGQYDDQFLIGPPAELSHKENCHQPDIRVLRNEECVQLSPSAPPPFPYRSIRVNSRHCTLYRDNKC
QTQLARVQHTQCHNIPPFARFSSIRCSNKD

>QLI73807.1

TPCKSQDGWETRYTATGTADVAKAAATAKTSSPTSHVKGKAFDRLAIIYFENQNYDKSFGDPNFNWFTKKGITLSN
YFAVTHPSEPNYMASIAGDYFGMENDAFTRSPRNISTVIDLLDTKGISWGHYQEDMPFSGFEGNAYRNQKNGAN
DYVRKHNPAVLHDSITHSEKKLSQIKNLSLIDTSRSMFHKDLKDNKLPQWMFITPNMTSDGHDTSVTVAGVWCRK
FLEPLLEDKNFMQNTLVLVTWDENESYAARNNILGILLGDAVPKELVGTEDKNFYNHYSEIATVSANWDLPTLGRW
DVGANVFKVVADKTGDKLRQWRSQEQLQSMYWNYSYAGQYNDQGGNKIFPKPNLDLDKAYNGRPILQSVKDT
WKNSKAPTYYEDTIETPDGLHPPEGYEPESE

>QLI73815.1

AVDCSSNKLLFKQVTPHEIIMGYKTKNTGCDAGVKAWVGIWDANAALKTNFKAWKYLEANDGEVRFTKTELGDG
QWKAAFVCEDGWRIPFLLSDNFELGSRVSSTGTCMVRRDIPGSEWETWSCDEFDEDCSFDCGHTLSCGLCNDCA
RQCNMPADYEDYSWGDDSKWSVN

>QLI73816.1

GVGAVLEARAPPPLPSADDFYKVPDNLDSYAPGAIIRHRPPPNPIAVAGLIHSQNQYPVHVDASYQLLYRTTDSLNN
ATATVLTVLVPRNANFNKVLSYQAVDDASNIDCAPSYAFQLDSGPSVAQGEVLLVEAALEQGWVVIVPDYLGPKG
AFLANVLAGQAVLDGIRAARNSGAITGIGKNPTVTLWGYSGGSLATLFAAELQPSYAPEVVIAGAAAGGAVPNITT
VLGKVDGTGAAGLIPAGILGLAHQYPEVDALIQKHVLPQHKDEFYKADKLCLLGNINEFSGKNLSQFVDDPSLLYAN
PTVVRIAGENDLGQHAPKAPLLVYKATKDEVSPVAETDALVDWYCKQGTTVQYLRDESTDHESLAVTGAPKALAW
LRGRMNGEDRQTACETKTVYSVLLDFGALAILPKVLLDAFLDVIGKRVGPFIKW

>QLI73834.1

GGSDQQAGLLARASSPRFPFDENTSQYCTFWYENDGSMPCTEALDLFSVSLEDFVRWNPSVTSTCDNFLESRSFC
MEASGEPVITTTSTSTEPTTTTKPFNGIQTPSPTQPDIIDSCNKFYWVNNGESCENVASKNGIPLQDFLAWNPKAG
NQCYGLWANAYSCVSIIGYIPPTPSEPTNGVETPTPIQPGMVADCNKFYYIKSGDSCGSIASNSGISVSDLINWNPG
AGDVYNDVLVRGGMCINTGCSVGSLEIAAEGYCPDGQVQISYWEQPNCVVQVVISTVRQGGR

>QLI73842.1

IPTTSPTSPSTSSHNDFSCKSSHNPVVLLHGLGATSCQDLNRLEAWLRTKGLCTFSLTYGAYDGFPLVGGLKPIRESSP
QIASFIRNVHRATGSRKVDLVGHSEGAFQALYTTKFGSVNHIVDALVAIAPPTHGTSLAGLYSLAELLGVERGAKDVL
GAFGCKACADLVTGGAAVQRLNDGSPIVQPGTVVTVITSKLDELVTPTETSFVHEPGVNNIYVQDYCPADLVGHIG
EAYDMNVWNLVLNSLEKKLGRDFSCVLASPGR

>QLI73852.1

DCPAAGSTDSQGRYSCNLAHQYPNGQTCAVVDGCPFLVGSDGKPVNNSPAQPSETAAACPAAGSTDSEGRYSCN
PAHQYPNGQTCEIDDGCPYLFGPDGKPVNNSPTTAAQPTETAAACPAPGSTDSKGRYSCNPAHQYPNGQTCGQV
DGCLFLLGPDGKPVINSPAKPSETAAACPAPGSTDSQGRYSCNPAHQYPNGQSCVAIQGCYFLCGSDGKPVDNAP
APTETAAACPAPGSTDSQGRYSCNPAHQYPNGQSCVAIQGCYFLCGSDGKPVDNGPAPTTSAVQPPQPSGAACP
APGSTDSQGRYSCNPAHQYPSGQTCTVVDGCYVLTSGDKPVNNNNGTTPAQPPVTAGAASLQAAGALVLAAFGL
LL

>QLI73860.1

DTINPAPDQKHDGDKTAATWKPESKQPAPTWNPESQKPATTTLKHETSKPPPTSKHEPTKPAISSRHESSKKPPPST
KHETSKKPVSTTKRESSRRPLTSSKHQTSKPTARSTSERSKSRTRSESVKSRSTRTQATTERKTRVTSPGPTQAQTTAA
PPPESSEAVPQPPVVPQPVPQPPEGGPPGAVPPEVGGTPPESLPGPVGGGGPGGAPGAPGAPGGGGPEGGPGA
PGAPGGGGPGGGPGTPGAPGGGGPGGGPGAPGAPGAPGGSAPGGGPGAPGAPGGGGPVGAPGAPGAPGG
GGGLPQPASGQPGGGAGQPQPVNQPGEAGPANVQQPGQAGAAPAIAEQPGEAGAAPAGAQPPGQAGPANA
EQPGQPGQPGQPGQPGGQGGGAAQQSPGGEQPTSPPSPGPADKAKVGLGAVLGAFLAILLL

>QLI73861.1

LVGNDWSFPGRPWDGSSDGLRDITFPFNMAGASHDSGYYFAQQFNFHNVPDVGYCGIQNRPDTNGKSVVHAV
FSSFQEGTTADINDENCHNGADGGPGVSCAVQVTGDYSHTYLLVIENNHGTTWTGTVVNTVTGNQTRVGTYTLP
YGAGGIQPRQVGFVEYYPWNVGGPDCSRLPKTEVTMYDPWSNTPGVGNGTMGRPYEYGQCVGRVRFSSQKLP
DGYNIDVGFERL

>QLI73864.1

NPLDLQPREDVKEDNFDLCRLLGCHRPRTVTWTLVYTITSTATSSATATATATITTTLDCSPSASTTLRTTATTSDFTS
TGTSAPTSTSPDTTASIDTTSADTAAPTDITTSTDITTSTDITTSTDITTSTDITTSTDTTSADTTAAPTTLTDASTDVSGS
TGATTSTGVTISTNTTSTGVTASSDITIPTYTASTTDFTTSADTTTSTEAATSGTTTAEATDTTTAAASTSSSSSSTTGLQ
SPLSTLVPFNCSADAFFIQDRHLLSVNLQTGEKNTLSSSVGGSSARVSAIGFNPLDNFIYGLQNKTVVRVGHDGALQ
RVAEGDMAANAGDVDSNGQLYYTAGGRAWARVDLKPGSARYGQVVARGTSDSSALPGNLAAGDWAFTPAAP
GYLHSVAVDDRGLYLVRWDLASHRWEVRHKGLPGFGIRDGVFGAVVATSDGVLYASHNRSGVVLRIPLDDPEDTR
KVKVLVPRAGFSDGCRCASMPDVAV

>QLI73878.1

APLAPRAPRAPGAQNVVYWGQNGGGVVENNDLAAYCTPSAGIDIVVLAFLYQYGNGHTIPSGTIGQSCYIAPSGE
GQNCDALAKAIDTCRANGVKVLLSLGGASGAYSLTSQQEAEAIGQNLWDAYGKPGGGSNAAVPRPFGNTSVDG
WDFDIESNAGNGFYPSMIARLRANFASDPGRAYLVTGAPQCPIPEPNMNEIITRARFDYLWVQFYNNPGCSVDGA

INFADWKSNVAGTPSANAKIFIGVPASPLGATGTSSGARYYLEPGKLAALVGRYSSDPAFGGVMMWAAGFSDAN VHDGRTYAQEAKAILANDTAVPTDVDASAC

>QLI73901.1

LTLSEPRVTNTTTNTTATLRARSAPPSRTSICGYSTGDPSKPWSAPDGYDCRVDTSHGLWGFCPTTIIAAPDCSLGG YCFDDTSCQTGCGRLRNNTRISTWTCTPEDPSSRYCSFAYLTFGVDQTYSYYHCGPDPGTAHFMAQPTAEIPATGT DSSSPSTLQGRPLSSVAAATSSSGVSLATSEPTAKSNSTAAEDNTNAIIGGTIGGIALVCGCVVAVVYLRNVRAKKTP GLPPSYTRGASREAGTASLRVKTGGWGPSELPGLEARI

>QLI73905.1

SPFPRNAVNDWGFSFLLRRDDCATYCGSEKQFCCSSNQVCTTLAGNVATCAPGGGGSYGAYTTTWTETRTYTSTI MTNWAPAPTPIPGVDCKPQSPEQEACGSICCAGWQTCAFKGQCSSRPGYQEPSTIVITSDGVVTTRYSAPYRVTG TTTVIGSGTRSDQSFFTATATTTSAGATSTSDGDTIGADGSNKGGTGGGLSGGAIAGIVIGTLAGVALLMLLCFCCIA RGIWNAIFGRKKERSEKVIIEEDVRYSRHGSQAPSAYSRRDRHSGWFGGRPTSVADRRPKKSDGKWWLGLAGAAT TLLALLNLRKDKKPARRPQSSRYTDSSSYTYSDVTYMSPSSSSSGRRTHRTGRTGRSHRSDSRGGASYYSRATTRRP

>QLI73907.1

QNRKDYFPECSLKCLDQATKQATDCSMDDAVCMCVQKNYEAIYNAGVNCVLQACGPDEAVGKVLPAGAKFCAA ATAQAKAGGASKTTAPTATGPATGVVTGDTTSAASTRSSAGQATSTSAPGAAATDAPGRALGLLVAGLLAAL

>QLI73909.1

SPIIGSRDMPTATPPTSKSQGFQLVVNVTDLSRDFSPSIHQTYINGIHVGAGLSLLGVGSKTDHPRTFYINGTALEFHF ANSTVLSDAGTPPTPWGLRLARDQGSDTASTARLDAGPGARGVGLTRWSVPYTFMYPETYAVCNETVPYYAGRP FLLVKQFALGLRGPEAIPGNCVPVRLFAQCAELDDLPEGSLFSHEFAYETECYEDVAAIEWPKYRPW

>QLI73916.1

STLERRDLVPPTDADLGNGWAYQGCYVDVGRTINSADTTDAKMTNEMCTQFCFGKGFPYAGTEYTSECYCGSSL ATGGVQAPAADCGMTCGGNATQPCGGPGRLTLWKTSQVAGPSVNPGVNGWVSDGCYSEGTTGRALLQGAGS VPGDQMTVAKCTAACAAADATNTLAGVEYGGECYCGKRISNGAQPAPASKCSMVCNGNSTEYCGGPGALNVYS LGGTLPTSSTSGGADPTTTPTGTPSACPVQPATVDMWFSFGCYTEATNERALSDKAYADDAMTLATCAKYCAGYT YFGVEYSRECYCGNKLNTGSKKAPAADCSNACAGSSCELCGGGQRLSVYTLGGGGVDPSTSSSVSDVFATSTGAPA ATGLPTGWSAYGCWVDGVNGRILNKQLPDDPNLTLESCAKACSDQGFTIAGAEYSRQCFCGNSIVNGGVKAKSD TECNTACGGNSNQVCGGGGRMSILSMGEPQVVAPPAAIPTVGQWEYQGCYQDNVNQQRTFFWQNFMNTDM TPKKCLDLCGSFGYMAAGLEYGKECYCGDPANIAVQGSQKVDDKQCNIPCVGNASAYCGGGSLLTTYFWKGDPFY SWNFPAAGSPDAGSYEFLIGGVCVPLITSQAITGKVTFLEKWGTGPPNSTGAYELDLSQIDNFKAAWRQMHVKTDI FCAGGLTLPDKAGRQLNVGGWSGDSTYGVRLYTPDGSPGVPGKNDWEENAAVLKLQQGRWYPTAMIMANGSI LVIGGEVGSNSAPVPTLEILPYTGTKPLYMEWLERTDPNNLYPYACVLPSGGIFVAYYNEARILDENNFNTVKTLPNI PGAVNDPMGGRTYPLEGTAVLLPQHAPYSDPLGILICGGSTNGVANALDNCVSTYPDSANPKWELERMPSQRVM TCMAPLPDGTYMIMNGAHHGVAGFGLAKDPNLNALLYDPTKPLGSRITIMANTTVARLYHSEAITLLDGRVLVSGS DPQDGVNPEEYRVETFSPPYLKRGKPRPTFTLDNKDWSYGQQVTFSLGSAAQNGDIKVSLLGSVSSTHGNSMGAR TLFPAVSCSGTSCTVTSPPSKYIAPPGWYQFFVLDGGIPAVGVYVRIGGDPAGLGNWPKGTGFNPPGV

>QLI73923.1

SPINAQANDDVGCLSDWEQIYQIINLYPLAVDFNSTHELFPLIYAENAFANYSSPDGKRQNLRGLQTIIDTFAPDGEH YDAHHLLGSIRINLSKDQPDKANGTFYALTNTFGKGRFAGQLATFYGIYHDEYERIEEQGWRVTTRLFSQVGPASG NVSLLEGP

>QLI73930.1

QSLCAVDCFQSVVTEHPPLSCKEASMYLCFCKGKDLQNYFAECAYSKCGDNAQDAVNFGVSLCKDMGVPIDPPTR
PVNPATSQAPAVTTPATTSNAPVDTAVKSSPENDVTTILNAASTATSVAASAVASTVVPPVQGHSSQASSTIATVT
QQPSQTTNQTAAQPTSSGVVTAAGVNLKLGTAMLGAMCVGAAAVQLL

>QLI73935.1

AHHRHAHEHLFKKGLNDTAAVCTPGCTTIWKTMTGEPTLVAPSTKTQTQILTVTPSPKTSSIQAPPPPPPTTQAPPP
PPATTEIVVVPTAVVTTCPTPGTYTFPATTVTIQKTTTVCDATSTKVPPGTHTIGGVTTVVVTATTVTCPVATIHTSGS
VTTSTIVQTTYVCPSAGTYTIAPITTTVSKETVIVYPTPATYSPGTYTAPQQVVTVTKTGFVYICPLTSKGLTTSTPVSTS
TPAPAPTSVKTQAPPPPPVKTTEAPKKSETPPPPPPAKTSEAPKSSQAPPPPNNNGGDLVSNNDHLGMTYTPYVGS
TGQCKTAGQVDSDISAIKNAGFSVVRVYSTDCNTLENVGNACSKYGMKMIVGVFVKSSGCSINTPDIKEQVDKLVA
WGKWDIVVLLVVGNEAIMNNYCSPEQLKQLITDVKGKCSGKFNGKVTISETLNIWQREDVSSNLCSEVDITGANIH
PYFNNGVAPSEAGQFVRGQISILQKICNKSVITLEVGWPSGGLDNGLAVVGLDAQSIAVKSIREECGNEVVFFSLEN
DSWKQKGSCGCEDSWGLGKAFNILTSS

>QLI73958.1

TATPYKVQTPPLDTDWTYAVGTNPWPEHPRPQLRRDDWRSLNGLWTFGDAAASDASPDRPPTAPLPREVLVPS
CVESGLSGIQSLNSTHMWFGRAFPVPDDWRGARVLLNFEAVDYEATVFVNGARAGHNVGGYFRFTVDVTEHVR
WGADNELFVFVHDPTDAEVIPVGKQTRNPSHIFYRSCSGIWQTVWLERAPANHITQLDVAAGMDGEVKVTAHTS
GKQQGAEVTIAVVDGEGRTVGQASGPSDAEFAFRVASPRLWSPASPTLYNLTLTLGDDRVHSYTGFRTVSAGLVD
GVQRPLLNGEFVFLFGTLDQGFWPDGLYTPPSREAMVYDLRMLKRLGFNMVRKHIKVEPDLFYRACDEMGLLVIQ
DMPSLPADGNRPPSPAQQAEFQRQLEVLVHEHKSYPSLVVWVIYNEGWGQLRGPPYPEERLTDVVRRLDPSRLV
DAVTGWHDHGFGDFSDNHHYANPQCGTPFYSILSSPYDPRRIGFQGEFGGIGHNVSIEHLWNVQQAIDTINQTYE
VSADLDAYNYRAGVLFRELTEQVERYACSGGVWTQTTDVEGEVNGLYTYDRRVLRANVSQWQADIRGLYDAAH
GRGGARP

>QLI73971.1

QSPGFAALMQLPQCAINCMNTPGFNLSACNVDDLPCLCHLLSSDAGKSVSTCVVTDCSVKDSLAARNLTQQACGP
EPRNQSTTYTTVAVVFTILSSLAVIQRFAVKLWFPHISIDLDDWLVLATALCQLSSAVLGVAGGVPNGVGRDIWTLS
YRQITNFGVCLYIYAILYFANVATLKLAFLFFYLRIFPSMLIRRLLWVTIGFTIVYGITFILTSVFQCKPISYYWVHWDGE
HQGTCANISEIAWANGAISIAIDIWMIAIPLSQLWRLKLDWKKKVGVFIMLSVGFLVTIVSIIRLQEIITHAIDDPNAT
WAIMNLAVWSTVEINVGIICACMPSMRLLFDLVFRATSPRLAASATNEANVHNSGRLFSRGRGLGVTSHSVFEPYF
APLSRQTGVITVQRTYNIEVETLNENLEEAQLVYMTNLSNDKQKHAEG

>QLI73976.1

DVLPQLGWDPETVKSCAGWYDNAGDVTCEYVRQLFGIPAEMFTQWNPSINLDCKPWRIQSYCIITQERVDEYERT
HGTSTATSTSTTKSITTRTSTTSTLGPSPTKWTELGCYNQGIPSILEERITTEGGDDTLTIPKCQDICYHLSFYFAALKSG
NECWCSSYIGGEYAKNAAECNRPCAGDKTTICGGKDRFNIFEAKIPEPWESMRKSTLSSPAPTEQFVAETTEPSSTIK
EMPPAPRTTQSTSGGSRNIGIF

>QLI73985.1

DLPSCSLSCLNDAVKQGTTCSATDLPCICSKFDVIQGAAAGCILSACGQDVALNEVLPATQKLCANAGSGSNTGTSA
PTPTKRPGQTATATVAPVPPPATTVEPVSPPPATTGGATGFAPMGGLAMLAFAALAL

>QLI73995.1

VSPPVYPTPQARGHGQWADAYVQARAFVALMSLGEKVNITRGFADPSNTCAGNTGSVPRLNWHGLCLMDAGN
GVRATDMVSAWASGLHVGASWDRNLTYERGLWMAREFKAKGVNIALGPNAGPLGRTPLGGRNWEGFSVDPYL
AGALGAETITGMQEAGVMANLKHFIANEQETYRRPYFGVEAVSSNIDDKTLHEYYLWPFMDGVKAGAASVMCSY

NRINNTYGCENSKLMNGILKGELGYQGFVMLDWNAQHNLNSANAGLDMLMPLGGSWGDNLTEAVRNNTVKE
ARVTDMATRILAAWYLVGQDEGFPTPGIGMKKLTEPHEPVDARDARSKPVLLEGAISGHVLVKNDNHALPFTKPL
RMVSVYGYDAAVPATKNTDVLFQLGYYSSKEMGQAVLGKDYHFDQAARGGTIISGGRAGSNAPAYISDPLSAIQQ
RAQKDNTWVNWDLASDNPDVNGASEACLVFINAMATEGWDRDGLHDDPSDALVNNVASKCANTIAVVHAAGI
RLVDRWIEHPNVTAAIIAHLPGQDGGAALVKLLYGEANFSGKLPYTLAKNESDYAVYAPCGRGPDNTTSPQCDFTE
GVYLDYRAFDEGNVTPRYEFGYGLSYTTFSYGGVELWQDEGLVESAGDAGDLWDVVARVSTTVTNTGSVAGEEV
AQLYLGIPGAPPKQLRGFEKARLAPGQSATVEFGLTRRDLSEWQVARQRWVVQRGEYGVFVGASSRDIRSTASIV
VPGRMERGRAETPSRIAAPFIMKSNGRY

>QLI74002.1

ADLLPEITDNPLDVIYTATLPQDPFFHAPELDGNIRGFISAAAPPDGLGVRFTVRFENLPKTGGPFPYHLHVNKASNG
NCTATGAHLDPTNRGEKPPCDAGSLPSCQAGDLAGKYGKITSDPFIVEYVDKFVSLKEGDATFFGNRSFVIHLANST
RITCADFVKNDPDIPYGDDQHIHYIHYTNKIYSDRHGGLF

>QLI74016.1

VQHYTHDESFTPDAVLTVTRQNISIAGVNKFATLVNDSIPGPALHMSENEVFWVRVYNNIEDDNLTMHWHGLTQ
GAYPFSDGTPQASQWPIPPNHFFDYELQSPNGSAGTYMYHSHVGFQASTAAGPLIILDPSAAPYKTDGERTILLQEL
FNKTDQEILAGLESTPVHSAGDPNAWLINGKGISDYGITHPVSANLDVTEVEPGLTYRFRCVAATSTSLAMFGFEDH
AELDVIAADGGYTMPSRVQQVQIGSGQRYDLLFRAKTCDELRRLGKLDYYIQVERREDTNITSYGLLRYKNSCGPDS
QFASRVSTSKNPTKPPVNLPQTIQGYLDYALKPLRNDTGFPPADQVTRRVIINVQQVKRGYQLWTLNNNTWTEDG
ADPLPHTTSYEPYLVALYKNQTQYLPDYNASLRNGGLDPGTRTYPAKIGEVIEIVFQNLGSVDYSGTSNGSLDVHPW
HAHGSHYWDIGGGDGAWSPARAEAQLAGSVPVRRDTTMLYRYNETTDAGAGSGWRAWRLRIDQPGVWMVH
CHFLQHMIGGMQTVWVHGDASDILKVGQVDVQGYLTYGGDVYGNSSHAPRVLHFHELE

>QLI74020.1

QYYKIDTEAAIKESARTLAYDLMLFYKGNQSGEIPGILPGPPADGKGPYYWWEGGAMMGTYIDYWKLTGDSSYNK
VVMEGLLHQVGENKNYMPNNHTMSLGNDDQGFWGMSALLAAENKFPDPPADQPQWLALAQAVWNTQADP
SRYDETCNGGLRWQIPFSNQGYGYKNTISNGIFFNMGARLARYTNNETYAKRAEKAWDWMTGVGYIDPKSWHA
YDGAHVNKNCTDVNKATFSYNAGVLIQGAAFLYNYTEGAQKWKTALDGLLDSTLKTFFPNDIAFELACERDNGSG
TCTPDMLSFKGYLHRWLAVTTQVAPYTKDKILPVLRKSTEAAIKQCTGGPTQRQCGFYWSFGRFVDPAADKTTGA
GEQMNVLAAVSSLLIENAEQPVTNTTGGTSLGNPNAGGKDNGERPVKPVTTADKAGAGFLTFLLLGGAVGMFV
WMSAFD

>QLI74036.1

AQGGKKWQLKAEPEIPEDGLQIKLDRDLYTHQGSIVYTSHLKFDEKPEISDSQLYDIATDAFIEMQKSAEANGLDKK
VPNVMTTLLVGNELIFGSSAKGPNKPYDRESQVQGDLKVCEDENEGRAHKNGGRCGEVIAFHTWYESHVRSEKLQ
PGEGTKIVAISSQKNADGQEEPGILAPCGNGEQWGCDKFVNGIYVLNKGRVDADKAANPDRESFDYKTLIKNKKLT
PAPTRPDLPPPNPLPPPPRPNTNPGGSDGGPPFNPLSPPGGKPKPKPKTNPGGSNGGPPFNPLSPPGDGNTDTNP
GESTDNQGGGSKPVKPKPTRKPKPPKRP

>QLI74041.1

AFDQKVLGQTYPFPENLGSAHAVRTASSYISHELQNALDTGLSKFGNITAKTNSLSATMISAQDSQPFLDFHYSAAN
LNVSGGSTNRVTGNSVYRIGSISKLFTVYSLLLHGGEKIWDMPVTNYLPELRKAVSQTGANSAIHHVQWDKVTIGA
LASQLAGIGRDVNNADMASQPFPHQAAGLPELSLDEIPTCAGNNTQPPCTRREFFDVLLKRRPVSLPFNTPTYSNA
AYRLLGYVVEAVTGTSYTEAVAKSVFQPLGLQNTSTSSPRGTGVGVIPPGNSGWGRPLGDEVSTGGLYSTSRDLAQ
FGRALLNHRQLSPLQTRRWMKPHAHTASPFFSVGAPWEIWRTRSQISSGYTIDLYTKSGSDDQYQALLILVPDYNI
VASFLCAGPNAGPAINMAAEVALQSILPVLDRVSQSQAVHRFGGRYVSSDAKNSSLLLTTDHQGPGLLVKEWLSN

GVNVQEAAQAYSDGTGGGTIKSIRLYPFITNTEDASSTGSSGTQVSFRAILETEPVNYDPGVLRILNADAGQWGRID
QLMYGEIAVDDFIFQLDGHGIATAVQPRVMRDTLKRV

>QLI74061.1

LLTPTDRFSIFEQLSLHQSYIDHSLTCDNAKLYASLYWPEGSFRVIDPNRDAIMTGEREIRSNYDYAHSVFPLSRWRH
SVGAFEISDGPLPNYPSPPSNTSAGGNERAYVHWNWRVDWRANTTGVVSTGTYDDVFEKRNGEWKVLAKVSRD
DANWPLYLFEPYVVSQAETYQSSCGDTSL

>QLI74064.1

DTPSHDAFASCLSDARVPIATKGTPEWTQHTTPFNTRLQYEPIAVAVPTQISQIAAAVTCAKKNSIPVTAKSGGHSF
TSLGLGGEDGHLVIQLDRMYNVELAQNGTARIQSGARLGHVAVELYNQGKRALSHGYCPAVGVGGHAAHGGYG
MVSRKFGLTLDWMKDATVVLHNGTIVYCSESEHSDLFWAIRGAGSSFGIVAEYGFETFPAPEKVTNFGIILDWDPE
TAPSGLLAFQDFAQTMPSELSCQIDVRSTGYTLNGSYVGNEASLREALVPLLGKIGGQLEVHEGNWLEYVKFWALG
QPNIDITPPADNVHLSLYTTGALTPSLSANQFRSFADYIATDAIKRGNSWSIQMFIHGGQYSAIGGPKVTDTAYAHR
DKFLIFQFTDFVWPSQEYPEDGLALGREFRDIITNSFTNGQWGMYANVPDSQLSSGEAQKLYWGKNLERLETIKAK
YDPNNLFRNPQSVKAAARCATRPLPLQGQSLLF

>QLI74073.1

LPCKECDGCGPTPGPAPGPTPGETPGDNGGNVNVCSNDQKNVCCSGSGGFMGGFFCNFNVLGENCSGSTYCCA
GDKVEQNGFVNMNFLNNHCGRVF

>QLI74100.1

APAVTDADIIVNSFQLYPENADYDFKRKLLHFGALYNSSIAAYSPSKNTIEKITEFPPLTNDTSYHASGVKVDPFNDRL
SVIVNPGAAWTTAGENITGDTWLIKLDLKTGDELWRSNLTTALDGKYGGYQDVAHDAAGNSYVIGTFPGSILKVD
PIGKKITPWYVSEETNTTVTGFTGIVYLGTKFLTTYGQNGQLQRWDSCQGQGKPTPVPIGTTGTPPTTDAVLQNIE
AITLPKKYEGKVLLVASNSKGVYVLRSKDGSWDSAENLGAIPNNYESDGVVVTTVQVEDRLYAVLEYFDDIKNPVPE
TVAGNRTEFPLQDITSAVEEYLQK

>QLI74103.1

RISRIHLNGLPPPPPNDGSLTKRSYGPTHEGVFQQLIDHNKPELGRFSQRYWYNADDWAGPGSPIILNAPAEHEAN
AFHATKNSLVGRFAQTNGGAAIVLEHRYWGKSSPFNNLTITNLQYLNLDNAIQDLIYFAHNVELPFDLGGTSKPTKA
PWVLTGCSYSGALAAWTHHLAPGTFWAYHCSSAVVEAITNFWKYNQPIKEAMPKNCSTDMQGVMKHIDGILSD
GTKDEKHALKKKFGLESLTHDDDFGAALAGGLQEWQRTVFFKTKKPNALYQMCNYLENVFPRKSSRIISDRPAVPG
PEGVGTSKALDGFAKWSKEVYLPRACAEFGYWADNNTAACMDMNNKDNPMYTDLSVNNTANRQWYWLLCN
EPFEWWQVSGPDDITGLASKHVGLDYARMQCRNMFPREGNRTYGLALGRSARETNRRTGGWGRVKTTRLMW
VNGELDPWRAATVSADERPGGPLTFTPEAPVWVLPGGVHCSDMLIRNAAANPALRRVVEDILGTMKRWVDEYY
K

>QLI74108.1

AGTWTPRQIRDLNQTLGGRVQILEPFARPCFSIYEGRPAGRDADECRELQDKYASPTFRSQFAGAYMYEESSMCAS
NASSTDKCLLDAANPRDEAAYRGVSCNQGNVPAYYIEVREANDAVEAFRHAGRGGGRLVIKNSGHSFQEDSSQK
GALMLWTRRLQSLVRDDKFVPEGCPAGDTHDAITAGAGVNCGEAYAFADEHNATMLCAYSPTVGVSGGWVQN
GGHSVMSTTLGLGADRVVQFTVVTPDGRLRVANRCKNADLFWALRGGGGGTFGLVVDSTHRVEPRMPVAVAS
VAVDAGNRDHVYGFMETLVDSALDLARDGWGGHIYGNRIVYLTPAIQSLARARQSVDRIVRFAEDHGGSANVSIA
PSFYAFFEQYVLGGAFSVGTLNILNTRLVPATVFATPALAARFKRHLRDVVDAGRLPYVPVVGPYMYGAATEGTSV
QPAWRTALWEYGNPAAWTWNSTLDERLAVVRDMQRQTAELVSLTPGGGTYRNEGNPFNPDWRHEYYGAPYD
RLLRIKNKYDPAGLLKCWKCIGWTDEDAKQSCYSAFDGVKA

>QLI74110.1

MVIQRDNPLAKCINSVTSSLNKLDVATKSFNGDIQPVVEAADNVIATIQHGQMVTDGTPSIGLFSAAALLQPVQILD
NRAKVLFVDVKGRVLDVQKAKKCDVTRAKLSTLNFVGHKLIDTILNKISSGFARNIARPFTDDIKGLLAKSLDLFEGAR
CVDAY

>QLI74112.1

QNRTEIVTITDTAYTTYCPVPTTIPIGNMTYTVSKATTLTITNCPCTLTKTGWFKPTATPTAQPVCSGGEDCGHGGN
GGGNNGGDHGCDGEDCGSNGGNPGGNGGNGGGSECNGKDCGNNGGNGGNPGGNGVNPGGNGGNNGNN
GGNECNGKDCGNNGGNPGSNGGNPGSNGGNPGGNGGSNGGSNGNPGGSGNPTPAPQGGCDEKTGAGCPA
VQTATGAQPTGTSAVVAGANVNGLSAGAMAVAGFAAMML

>QLI74113.1

WVERLMVIGTNGTMIGNPGYIRGAVSRLDPNFNDFKMQHLLPTIPEGLLTDKLCKNTQRNRTYTTDLPALQAAPG
AFIALQYQENGHVTLPGLTPQKKNSGTVYVYGTLYPRDDELLSSIHNVWNTDGTGGDGRGRLLAVRNFDDGQCY
QINTGPLSMQRQAKFHKTAMNPQGADLWCQNDIRLPVSIPFSWYTLYWVWDWPSSPSDHLPGGESEIYTSCMD
IEIQPGVQLDEMNFVDGQDLNMAGIKEQLE

>QLI74119.1

TTGGDPASLGAITATAQATTGGDSASLEDITPPAQATTGGDFNITGAQLYPEKCVFDSKRDVMYCSELYESKIAVVD
LKTKTIVKTIDFPGLSGDADYHASGVLIQGKDRLISSINTGAAFETSGGNITGTNYLLITDLETGEEKARVNVTDVTNG
TYGGPQDFATDTCGNIYQVFTYPGAIIKVTPSLKVIPWYLSKETNSTKAGFTGIASRGNMLLTSNEQQGGKIIRFDAH
NKTGDPFEVPIANNGMLGRFLDGILLPAKYNGTVLLVTSSQNGTTVVESKDGEWNSAEILGIVPNPYWENEQGFST
QTFDREDRIYQMFEWFLDSRAPANLNGLSGNRTVFPFQDITDAVDKLPIRLIGDVKEVTVLPPSLAQEIRNDKRLSF
NQWTFKAFHGHLPGFEAFAAGTGGSNLVQTVVTKDLTKFLNKITEPLAEETAMALEELLTNKTEWHTIAMRDVVL
QLVARISSRVFLGTELCRNETWLRVTRDYTVTGFLAGEELRLWPEFTRPLVHWFLPSCRKLRREVNEARCAIQSTLA
RRQQLKQDLVAAGKDVPEYDDAIEWFEKAAKGAPCDMTALQLSLSLAAIHTTTDLLTQVLTRISQNLDILGPLREEIT
SVLADEGWSKTSLHKMKLLDSVIKESQRMKPGEIVSMMRLAVEDVQLSDGTFIPKNTGVAVSSHRMWDPDLHH
DPNHWDGFRFYKMRDDPGKQNVSQLVFASPDYLAFGYGQNACPGRFFASNEIKIALAQIITKYDFELQEGSVPQIH
KHGFGLRGDPLLKLRVRRLAKGI

>QLI74121.1

APTQAASLHPQILEAMKRDLGLNAEQATARVAREIHATDVIEQLRSSVAFAGAWINADVLYIGITDQALADEVTAA
GATPIVMTNSLSKLEKAKEDLDKIFIGRANALETSSDTSSGIASYFVDVAANKLVIEALADSHGYAEQLASQVGLTTEF
QVRTVETMPTTMATVQGGDVYYINRSSRCSVGFAVTTGFVSAGHCGGSGASATTSSGEALGTFSGSVFPGSADM
AYVRTVSGTVLRGYINGYGQGSFPVSGSSEAAVGASICRSGSTTQVHCGIIGAKGATVNYPQGAVSGLTRTSVCAEP
GDSGGSFYSGSQAQGVTSGGSGDCSRGGTTYFQPVNRILQTYGLTLVTA

>QLI74125.1

FKATILADTNRDGHVDVTDHEGKAYWTEKQGALFLPNIVDTDRRCSSQITENTTESELAMCNDASDNILRQSQYLA
PLSTLPIHNLSNSIMGRITLQDARDKARIFYKDHDEWIYLGKDHVFNASALEPGLELGVDGRDVRRPDEWDGRVTV
TFELFENGKNISSDAVALRVAPILTHHHLQDPDQVFTVAGIDEMEDWKEAQAQFASFIVNYTAKAGIKKPVHMVE
HWDIWVQDYFEPGYASIPGPDGPIYLRVNIRSSQPGRHAGRMIFSEFRSNTTGAVQYFTNDTLPRSTIDSTGNLETI
PPYSYNGKDYLAGRAIMGRHDNISPTVMALLKAQEAQYPIDTLDHDWLLVGHTDEYVQFLPANNSRGWVVTMA
DTEYGMKLLKDAQANGHGTEKAMSRKPESYDPESPCLPSITINGVLELPEFEEINKHCADRIKYNVDIIKQETGITEGE
IFRIPSLFYYPDKGLACNATSTRRAKGQAMNIIEAAGGSDELNGSDEPKKRMVALYPETINGVVYPQGQYMAPNP
WGPIIGGKDILAEAVTKAYAQVGFNVTFMDDWFDHHILHGETHCGSNVARDASQKWW

>QLI74133.1

LAIKATDLDGNPIKARVFQVASCTSPFGWSPADINDAVGARNKLQIGPDPARSTVTILTGANKCSDISCYGEAKIVVC
NNSARDLSLTYYTILQAATESMGPCLNAQVQGKVLTQGFYSEDWNVIVRKKDGSERCVL

>QLI74136.1

QESSSPYLRPDQSEERKVDLLSKLKPGLNLILSNARYESDFKEATCEPHWNFWNLLHTDFLNVTVFEDISKVDYDLAI
NTEVIRRVDNDQRSGTMQLNTQKSTINIDSTTWGWNIGGQINAGFWSPYTGNMAGITITGGYSRSNTKTSEKTIA
EGTVSPCEAKYSCWTEAWTVKLILRGACKTEEIMRNENGDKLWSTEKCPTTIFDEGPFECAQWAHWKDESCERVK
EKIDCTVETPLLDSDGKTPFHLELAFRLPILPLWEKPQIIGYKAGRYLLKAGEQGYTEYDPDQQIAKYMDASKRWHYY
EAYPTLDDQVVKYEHPHPVIKSVKNRCYDLDSAEWYCPDRTGEDKFYTQDKGYYAKPGAPVPSFDEMDRADRRQ
WHVSQSREVCHDKGRKCGWFGRPSACGVDHHRVGAVDSFAEGDTSFDMTYIGKFKSNDRVQCEQWLGKGSSC
CDTQRVPCESGFWRLWCVEDAKAESEEMDNDINSV

>QLI74141.1

ASGESHDKRGNDKALYEAPSWELTNVTRRIPSDGEIWMRFDISSPNLDPMYCYMLVHVNGDARHASFSDQRCVG
SSFTMSWGYARDDDAGIMTIVNPGRTRRCWFGWRGVNAKEHLEDAGPNNTAEMPPM

>QLI74152.1

DAVIWEGEGWYKVYLLERVLFPDDPNSWTTCEGCKRLGKRRSTNDAFTKAFAKELEARGLPTSTVSVAEFFVGIGT
TNGHFRRFGSEDGGSEVKKKFLTLNYKMTDLLDEMRLQRAIKGTNNFKFAASKIIDHSNFPEADFNDGELFDVAKM
QRLISARAEQLRTMAKEASLIESWVKDRANALEVENMYRNADQVRWKSDWVDKELKYGPSNSYKQIKTPRLVIFP
NGTRPEKDLHPNRPPNTPDLEIWEEVDMDKTLAQGGQSSIVKDKTKAATKKAGTYGKWQGDPDPQKKATIHME
TIRANSEMIPFHRNQKGEFLKDFGLYAPGLVSNKDRPGLGAWCTSWVPPGTHAALTRYYTDGGSSLIDTTLGTAEN
WLVQATAFDGIIATNVPTSTSTSSSSTSSKTVSTPKSTPATTTTTTTPTAKPSWTGPTAVSDLPQCGQTCINNMLAK
HRELGCGDKGVACMCRAPNFNYGIRDCANGACSNEDRASTVISFETSYWCPSFMATGTNSPTQTPTPTPTATSDL
PPCGQTCMNNMIAQYSQLGCRSLDSACLCKNANFGYGVRDCANGA

>QLI74154.1

ALLSCFSSPRKGVGGIPTIPFWVALMPLVKDVDQQDIFATYIEKPLRKHGAASIFFAGQWNVLIHKPAYLAEVFRRE
DIHQKSGNYSKIPHSVLAALLGDNIIWSRGETWKKYTRVIKPGLQASPNPDVLLRNARQLSTILVESQALPTDGIQGS
IQRHTIACFENTYFNVDLNNKLCTRLLSARDSGQLTEKQFRDNLAVLFVAGQENPQLAILSTMYLLAKHQDIQDRLF
DDLNQQPQNPPDAEYLDSLPLLTLAVHLGDAIFLPEGTYVGYNCYSTNRDPDAWGQDADAFRPSRWGCSRRDIH
REYRRRRTRAEFITFHGGQRACLGEPFAVLQLKATLYTLMTSLRWRLDPTWLDRMTPRAEADQGEFLIGWAVVPK
KLALAI

>QLI74159.1

ATINKRIYLGEEAKEGEFPFIVRLHYEDPTLLCGGSLLDSTTVLTAGHCQPKRVTSNKDSGGVVAEVESVVVHPDYKR
FIGEVNNDIAILKLSTPIQESEIISYAKLPASGSSPVIGSIAVAAGWGTTGPLSKGDVSDKLLKVAMPIVDINSDSCKASS
AVQLPDTKVCAVGGGDTGKGDSGGPLIDQETGQLIGIVSIGPRYTRISSYIRFIDDVNNGINGVDPRHVTG

>QLI74162.1

TIPATDPGDDAKGCASGLYIISVRGTGEEQGIGVAGTLLGKKVAEQVKGSKIVGLQYPATLASPPYPSSVGNGTKNLT
ELLEDYHKSCPDGKVAILGYSQGAQVSLDTLCGTDEAGFTTTDAISSSAVDNVVAVALFGDPTHIANVSYDRGTSKK
NGLFPRNNSASCEKYASKIASWCDTGDVYCDLGEDTAVHGQYFSNYGPEIVQFIVDKYNGKSGGNSTSVPTGTSTP
TGISAPTGGNSTATTARPTGSSTSSGGAAPSTTPSPGAAAGLEVASKGLYVALPLALMAMFQML

>QLI74194.1

SPIHQVVRAVAELPRLVVYFQTTHDSSGRPISMLPLIEEKGIALTHLIVCSLHVNENGQIHLNDYPPSNPMFYTLWNE
TAVMKNAGVKIMGMVGGAAPGSFSSGTLDGDKATFNKYYGQLRDVIKKFKLQGLDIDVEQPMSQAGIERLVDKL
RSDFGPSFIITLAPVASALSGGGNLSGFDYKLLDSRKGSKIHFYNGQFYSGFGTMSRPTDYTTIVSSGFSASRVVAGQ
LTSPSNGYGYIPYEQLNATVISLRDKYGQIGGVMGWEYFNSAPGGTDEPWKWAQIMTQILRPDAVPKLAISTEIAQ
KLATAFNDSVKSNPVWAAKSGGALGVDYHRMINV

>QLI74200.1

CVLPPGALPNNIAEGFAIRVQNASFPVIHNRLLNQWAAGGGDQHLYLSPAGASARDLTLVDGVITQPSNGKTIRAV
INGEYTAFDNTTKMFMTERGDPRAVYGVVRGCNPDTDEVQMELAFKGRADVTGGHLCVRLASGNRWEFRYSPP
GNTAVDNPDRLCIKVTLAVVRIGDPVVQKLPPNVIRSSS

>QLI74204.1

IKRAALEPNLDLSVDITNDINIDPDIIGKALFEIIPDEKARNEDNPISRRDVFSRQEPGNPTLRSAEEVSKIKAPGTSKSG
VFYRGDSRPPEEIFKSGFKPQGENTNLQNHLDFKPNSGLVSVSRSREATYGYMFGRSADKNPKGYVYVIAAKDMP
NGYWVPGIHPPEKNAAVSRNQEFAVNGAIPATSISHAFEVTQANPTDKARKIKNEAYSLRSASKCTIMKRRAGGSC
DPAGWVDIEKNEPKKPASGDGGKGKKPVGQKISQSKFLQFITELEPKSIGGLIKVLKDGEATIADVQLGFKRALSEA
MDNRWADFGDWDKAAESLKNIVVDVASTVRYATPAGYWSDVIKRLPDIAKEISKGKTPKEKLEIANKKVNGAVNA
WAYTPVGFVNEALMREAAMRTPAVQAVAISINKLWSYTPLGWLINQIAPIESLRRKINAREEVLSLGY

>QLI74207.1

APAADENAVTSLDASTGDGLVARAGGSTLAPLLDQLDQVLHPEDNEDEDKPDEDEEEEKKKRRRMIGVAPRADA
GKNDDGETQADVAITFLPRRSEGKCKFMIDRDDVPADTPPGIICLNVQKERKIRHRDIYTVVSRILREYDLKCTDTSIK
EAYLQDGNRFKLDQQQVPAGEGETIQLTLRDVLTVKTECGAMLQCVQAPLNVMDAGADPFKCTLEYNSSGDIPLI
EEGGAGLK

>QLI74218.1

DWQPRKPNTVVGILMPYAGEDLGILAKNSGGNLPITLQQLQDLVRGVRELRKYELFQGDIRPWNTLLQPSTTASKP
RLMLIDIDMELPGYPGDAKALGKLLQWCLENSMALSEDKQAKVKLINAVKALLSENFDMAIDCLSATEQSMRRGP
PFYTCQQGGLDWV

>QLI74223.1

YNDFHFGFNFHDIGYAPQGVNISVDPRIVRKTLQRVRDYRPVTGLFDDWTVEGPSKDNITSVAKYWADEYSWEDT
QEQINRDYKHYITKVPGSGNYTSDIPLHFLHHESENDSAIPLLLLHGWPSTSLEWSKVIRPLAESSELPFHVVAPDLPG
FGFSPAATQDGLGTREIGRAVDALMKQLGYSTYGLATTDTGWFIGMWMVHDVADSIIGHLFDFWYPNPNSTDM
ERFQANQTTAEETAVVESLHVWDDFHNAYSMVHSQKPIAMSLALADSPVGFLGWYWDLSYAISDGIPHSMDELI
TDALMLWLPGPYGNIRSYRQIFRPDIVDFPKTNVPSGVSQWGWGHGPFPGIANFPFTPRNWIERLCNVTYFKTHK
RGGHFPARTLPEYWVSDVREFFKGL

>QLI74226.1

HGGHDDDDDGKEWTKEELAELQAKWGHEWSFSGIGSFAHLDYVKCLTSPQEKYDIAIIGAPFDTAVSFRPGARFG
PRAIRQASSRQTSLRAFNPRANVNPYQNWARIIDCGDIPITPLDNAIAAEQMTQAFRRLGRARPTSALSAGKPRLV
TLGGDHSIALPALRALREIYGRPVRVLHFDAHLDTWDPAAYPSAWGSTDFTHGSMFWMANVEGLLSNASAGPSV
HAGLRTRLTGTDWADHEADTAQNWVRFAADDIDEVGTQGIVDGIMAVLGTDDPVYLSVDIDVLDPAFAPGTGTP
EPGGWTTRELIRILRGVEGLNLVGADVVEVSPAYQGRGEETALAAAQVVYEILSSMVKRGLEDGDGETAAGGKDEL

>QLI74236.1

RLPSFTPTSENLPSVVYRGDLIPPEQYKKDGGIPPEFTGNMTDSSYSLWSHNKGTDLSPGRDFQSAYAATSTIFSVAV
AFAIKDNPNEAGWIYRIHATPNMIDLDHSDFTPSYLEREFAATGGIRWDQVEAWLEVPKNVSTNKSADGGIGREE
KQRWRTAEGFEKEFPDLKWVNNPDYNSVYDQYRASQGQPQLAGAPERRLGPLASEFAKENKTLKQHAIEFMDKV
GAPVGWKGTLPLDLKAPVRCRA

>QLI74238.1

TPGAYERLLYYYAYKLDNMGGGKTIAGGCPGPESGCSFDQFVHFIEGRGPSEATVKITSEEFPPIEPTVELLVKAGKT
GTIAESRIISNAKDYPDLFKKLGTNLSGILQKTQGFKHGTLNGQALEDTLREVRANIVLSMKRVFDGRVRATIDSFKP
YEGLKVVTNGATAIDFDQTVAENKGSITKEKLQELWDQHLGGRQVTEKIVAEFKPLAEYTPTIRENAEGQINLEETFK
TAVEENPWMKASWFKKKYLHSVRGNHQTNIKELNTALESIEPLVCATSKARKREVARLDKRQGVNACFSWEFPEIK
EIIDLPPVAEGSEGVIDAETLATGRFVERANRANQVSSEEFETAIGERLPKIPDSWLKNGVKTFHEAREQIGYKPMEP
TSADLVPGRGGKLSGTLGKLAGAAGGALWVNGIVQAFRTNSTDLDKAAAFTAIVPFIGCGVTTAAGAEKGHDIDLV
TVDAVLCGIADVLLFGPLAPFGFALHFVRFVMSFFPHPPEPPKFEEMRESRDDSWAEALSKVYGTVYSNSRTDPTQ
SFAAKLESVFAIDAVSVLSEAAGRIGALNASGSPDLYHPSALGEGETVDLAVLEKGTLQAIHKIRADRWNVVARRQR
EFLLDIPQEFANGTAFSLSELSKKVNDGFIEHINSDKFIENFRDQSRLDDIADAVFAGGPSVKPIDHYSEARTQMKEV
GRRLSESLPPLPQALNVAFVLGQSKGMAFAQNETLDLRSYFDTEVREALVEDIPHLYRDLILIRHTHQTALFLQGRLK
EEEFDDSVFPVDHPALLKGLRLLAAMKFGKIHEAEKIEHMDKVLGRDSMGRHPTAHPDFTNPSIPPLTALKPRKHKL
LVSLAIGLLEVVRDSQLNDDMAQYLRDSLAETRSKNWEAMQQRFKAMQAVLKKISQRDVGGPPTPEEFLNKHLKS
CVEENGSKTETCRHVVESCYSADDTSDGFASECIRDAFLPDEEAGRKMRTKQRELCSKFPGPRECTRALRSCGGTW
PKSRIQVIWACAAKALPKTDGADADAAVMPGSTS

>QLI74246.1

QSCGGGGSFSVDNVQENFGSVTLQPRSALRFSQGNAQICIVNRAPNTVVTISGNQINGAISAVLNKCCTNAGSCG
GGQQKITGPNGNVIDLSVQAQGQNCTP

>QLI74250.1

KQSIQDLTSHSSSIAWIDLFTASRSRKGD

>QLI74257.1

GQQKEPEARAANAYTKDNHNLKYVNNIPMDKTSEQVKIENKQSS

>QLI74259.1

LSLTARQGVTADDYAVQTLKSIESNFKMDDANLPKFPVNKEWQHVCPSNLNYGEKVTFFKTGTIKRNDIIDAKAYYI
AHVETTAVNPGPGDTSSKMTMSSSTTTTQTDTKGWTVGVKLSGKVSGGAEKPAPQGEVGVEISASYSDTKTKTDS
NTKTVTREEQCEAGYECRLETWSFHLDIHAKPRVDGFFQLWDSVNDIGKEIPMCSMPKKARSCEQFKQRIDEWCT
EKALPGGALYIPAKQDELHIKTPILETNGYQTFTRIVKVFNPIVKSQKARSVEPTVGTKETIMEAMKQGTKFKFLD

>QLI74261.1

NVRVKWSFPSAPATGLEDITFPMNMANAKHERGYYFAQQFNFEGISAVGYIGFQPRPDRNGKPIFLAVFSSFQKG
ATSTHAKCKSGADGGAGVSCAIEGSGDYADTYNFSVEHVSDTTWRGVMTNTVTGKKDEIGVIKLPSQAKNIKPSQ
VGFVEYFPWNANGPDCPKQPKTEITFYDPTSNTGGAGSITAIEDYGDCSGKANIKYTKITGGYTVNYGNV

>QLI74270.1

APAKRAPSGDFKGEMLAAHNFFRSQHGVDDLKWSDALASKAQNWANDCKYQHSNGGGENLAVNSAAGDWG
SFVNMWGSERKKYNFDNGGFSKDTGHFTQVVWKGTENVGCGQKSCSGLGVYVVCNYDPPGNYNNDYQNNVL
KQTKGSDTDVYQAGKQPDKEPPKEPEQPPKEPEQPPKEPEQPPKEPEEPEDDCENEDGNQEGNEDGNQGGNED
GNQEWPEDWNQDWQDQDWNQDWNHNDWDNKDWSHKDWDNKDWSHKDWSKYWNKE

>QLI74281.1

QSTTTEIDLVFPHANETYQRLYPFPIVFAIQNPAPVWPHNFQLFWQTGSVGEEPSRFDCDRLPLRGADLWTSRNYS
GPQTMTKIYSAGIYINSTVEQWYLAWHMTMLRNCTPDNNRVTLAPKRPVAEGKIFFNVTRGGKPPDVFQGNEST
ACPLPVWTVNIVDRVDSHAARNQSQYGDSTNTMCPVFDPDDAHPRPEPCQAKATEELVRNVTRGMLSTASVPIC
ILGRMLPSRGRATIRIGSRA

>QLI74282.1

VDLDHDPIIGVDGGDHLNPPPCHTTITIPYIGSTTTSTTVVPCGTGLATLVIQTPLITETCSPTHTTTRKPPPTSTSQHT
TTQTTTIRTTTKKPVTTTTTTTKIPEPTPGGPCPIIKPRCRPEGFDIDYYANPFAGYSRENSLPWSYYITQNLKPLKSSIT
NVTFFPQDFGPPANLPKVYPRPDLPGVWYAVGWTKQTNGGIVVDANNFTLVYSGFYRAPETGKYTLCTTADNEN
DVFFGHGNAFSCLDGRVDTNVKPLVVSTGGNYINGIKCADLDLVKDAYYPLRSVMGDWQGPSAFNLTIKTPSETFE
NRKNDYTGNAYPLSCRFGSV

>QLI74284.1

LPLSGEGPTVKIVNGQPAAPGKYPYIVALLPREGAPNFCGGTIINQNTVLTAAHCCQGMPQNAVVRAGSYNRLSG
GKISRVVSQSIHPQFTPEPLDNDVCTLKISPNIQESQSIKYAKLPQQGSDLEDGTPVMVAGWGNLEQDGQSPDDL
QEVALTTVGRAQCQNMIQQQVPQAQPITQNVVCAGAQAKDACNGDSGGPLVQGDTLVGVVSYGYGCARQGV
PGVYARVGNKVNFIQGQGTQSPGDGGGEVDDSGDTPPQGPGGQPRPPPPTLPTGPGGQPRPPFLGPGSPPINP
PPPTLPTGPGGGQPPFLGPGSPPIYPPPPTLPTSPGGGQPPFTGGNVPPILSPSLEGVSPFLPPGFLDGAEEVTVTTA
PISEIA

>QLI74288.1

GPLLSTATVNPGNWQQVPSNLTWAKHNTSTSTTALSSIVPNSPRPTIGVQEFLTLSQSFKFEIRSGRCEITNLPGSAA
GFFWRVVYDLDLKGSFRFETCEREQHGRVFTKIQGQYVLGYAVKYSQVTSGAELICDSGVLSYVNVQSSGVVVTAV
TESTVIEKYSNTMYACTEAAQCVPSKCENGCKKGVKDDKDTSPSSCGGPIPKPGSSKRALFFLDSNPEGASIIGLPILQ
DGSLDKSRAVRTPTGGRGSISRNMNGTVSVDPLFSQDSVVVNGKYLYTVNSGSNTLARFEIPDADPASPKLVGEPV
HTGGEFPNSVAVSDKNDIACVTNTGRRAGVQCFKTLDRLQPLGDYMPLPINQTTPPVGPPNSVSDIVFNPSQTALF
VTIKGNGMGPGYVYAYQVVNGRINPQPVISRPPGLLLDFSLTLLSDSAGVVTDPAYGASYISIARNLSVAVSNKITVP
GQGATCWSVFAPEFDAVYLSDGASPNITALDPVSGRTRFVITGDSRSTGSFDSAVDRSSLYVLQGSAAVAVFHLQG
SKTGDRTPSLSQYLDLSSFGARSGWIGMAVHSG

>QLI74290.1

GAVAAPGLFENKASQQRGHIVILAEGLHDRYVDTHLDWVRDLHKRSLKKRDGKDHRQGIQHTYRAKSVGFHGYA
GSFSDDVLDEIKRHNHVLSVEEDGFITAEYNKGEDA

>QLI74297.1

QTCMCPEVPSMGQPMKLARKIELWATECTYNLAYGEKDYHLRCNFHRNHQKPTEEEAKASGAKWKVLATEL

>QLI74301.1

SAVPRQNTQNTGDINLAEIIRDSPKVQAHMKSTMSHLGKVFPFSEGQMSASKGGVDLLNSAEIPEFDRKCLFVRNS
IKETGWQSSLDVNPHPNGDDVVTGENEEPMTISTSTVKTNSYRLGWNKESSKETGQSVTAEVSVGYGPFSASLGT
TVYGNQRMTEGQNAELSKQEEVSVKVDRPYTCPAWSICRVVTWTYIRTITGSCFLTPYYNETCGRGETGKGNLYSL
GLLRSCSPADKIANNFYEFILGQTGYGPDLEGIKMHITEGVIPRYQDNCSFSYTLRDEHGTPISAIANIIEKYPDPNAKK
IEVTSVPKALVLDAPNRFYISSTDGGDGKWHNRDDLPEPEGCMETRPDTESKSKRAEAELDNNSLEEEPPAYDGVK
VEILQDGMPAFLEKLGQSKSEGFIANRIDEAKPSSREVHGRAADYIPNVRDCLADFVKKNTQG

>QLI74302.1

IRIIQTNDDGWAEGNLRVLNDALNALGHQVVLSAPAENQSGRGSLDRDPQPRKDPCMYDSCPANSGPVGSNAT
RPDLNWVNSFPATSARYGIDSIGPKLWSGAKPELVVTGPNVGANLWLVDWVSGTVGAACYAAHDAGIPAIAFSG
ANTDTHPWNTPASLESAVFAEISANITQSIIDAGEPYLPKDVFLNVNMPKIVAGGSCSKASDVKYVLSRITSGIFSTPD
VNWCGGSRLPSELHVHQSTPCSVSISVGDASDKTTADADAQQKVLDKLKPLLSCL

>QLI74314.1

TIDDGPSSLMTNGLLSLASFCSGLREITSTSVRSGVMEPRSNAQTVCDFSLLLGFTMAANQFRQMNPRAGSTPMS
CEEDARFLGQVFKLSFAFIRLESVVRLVLCTIGFVRNQHF

>QLI74316.1

LQVTPNSPCSSACIDSAGLDASDPNSSNTRAGDIVCADGQFDADADAAGRKLRTCLACLQGSTYTQGGEGDQAW
FLYNLRYALDRCVLGYPNATAGATSSPCVTPESCGALAAALKSDIARPAAVSAFAYCDVDAGAATGMYFESCLQCV
QADGQHAYLSNFLVVLEAACLQRPPASLLVGVNETVFSRHTIAVTQPSPPASSSPPASHAALSTPAIVGVAMAGVA
ALALLSGCVYMQVRKRQNKRNRARRTLSFRCRTAAAAPAPAPAYPYQDDDYDDERFFEKRRGSRDASVVSMSPV
ARSKPCAWPLNITTTIADMPAPEAAYVSPVSAGGASSPDDATPRSSAPLLSPARAFASHLHFAGGAGHAAARPPGS
PVQGARIQTTFDPPPTR

>QLI74318.1

STTPNHTRFDVLDLVDPLIGTANGGHVFPGASLPYGMAKAVADVNKELQGGFTSNDGEITGFSHMHDSGTGGGA
SLGNFPLFPQTGCDGDVLNNCYFPKAARASQRVNGTVKARPGYFAVTMNTSVHAEMTVTSHAALYRFTFPTDGT
PTKSSGNATPKMPYSPLILADVTDLADSRSNGSISVDAQSGRITGFGTFNPSFGIGTYNAYFCADFAGARIRDTGVF
MNNRAGAEPKSLQLPTDGNTVPGGAWVQFHAPKSNQVQARVGLSFISTQRACDNAEAEIPDFDFTRVRRAAETA
WRSKLDTVRVNSTGVSKSLQRTFWSGIYRAMLSPQDYTGENPLWASDEPYFDSYYCIWDSFRSTHPLLTLVDPRA
QARMVRALIDVYRHEGKLPDCRMSLCKGFTQGGSNADNVLADSYLKGLGDGIDWETGYEAVVSDAEDEPPIWTV
EGRGGLHSWKTLGYIPTDDFDPYGVGIFTRSISRTVEYSYNDFCIAEMARDMNKTADAEKYLERSSNWRNMFDPT
SRSRLNTTGSQDPAGLVDSGFQGFLQPRYLNGSFGHQDPAICTALFNFTSCYLNPGGHETYEGGSWLYTFQVPHD
QATLITMLGGTGEFVRRLEFLHNTPGLFYIGDEQSYLLLYLFHYVGKPGLSSQYARRYIPSAFNDSLAGIPGNDDSGA
MGSFTALSMMGLYPMSGQDVYLIIPPFFPQVTIHHPGTGKSATIRNINFDQGHNNIYIQSARLNGRPYTKSWITHD
FFSTGGVLELTLGPSQSHWGSGENDIPPSASTHFWK

>QLI74319.1

DSSVMTPGSSATTSLGTTPTSTATCNPCAKISAHWAEHNRQNKTTLPTVKASLAHECLKSVPLGQHEAVQLIDAIEP
YLEWQSDAAYKKDPPESYFYPGFDIFGNLAKVRSNVQAGKYSNEFDFQTDLYKQVWAPGHDGHFYFKPDLLHRAF
RWYRNVSIVSISENGEALPTIKLQTDVLANPKTAQALTKINGINATKYIEDTANAASLFHDADASYNSMFWSKPTAA
KGNVGDFFGAYSFLFYPGDTTNLTYANGTTIEVENKAVIDGNMTGVVDGPSMYKRFCSPLPLQPAAASANTNTTI
NTITNVTIPGYPTPVIISPDRTISGYYLSGPGLDNVAVIYLQSFPVSNFAEFQTAISDFLRKAKAAGKTRLIIDLQGNKG
GAVLLAYDFFRQLFPSIEQDGISRWKLSKTFEHLPRVVSELIKDIDPATETNLELRSLYYTPWSYRHNLNISNHNFEKFE
EKYSPHTYKNTNYSNLIRINVGDPLTTTKLLGIDISGYGTRTNLTQPFEAENIVLLHDGLCESSCSTVSTLLRHQGGVKS
IAMGGRPNQGPMQGVGGVKGAEGLYFNQIYQYINSVSRFTTNSTVKSGLQRFKTLPMERSVAAGINAQDQILRD
NINDGIPSQYITEKADCRLYWTAPMISDVTEIWKSAANSAFNGAKCAYGGISTSKAKRGNKAPPVHGLN

>QLI74324.1

AREFRALVYRGPAVCQNCPGALAQLLESSPQKVAVTYAGPGEEVKVTAESLRNVDVFAYGGGPDLDGAWAEIQD
AAPAIRDFVSRGGRYMGVCLGAFLAGFSPGLGLLPAGADVDREIDQRRAQVANDSDTLIQVDWTFQSAAAGHAR
GQTVSDRWAYFQDGVAIKGLPGARRRARDRAAGHAVLARYSQSGDVAASRTPHGDGWVVLVGIHPEATSLWY
NRYNLTNPDGIQFDIGYDFVSAALGSADGTPGNQTQKSGASRGERGAPHNPLGLVVQMVKKLFWGR

>QLI74325.1

MPCNSPSSSQDKPATEVNSVNLDLCEKRMLEYLQTNSDGCKEIPCADSDSTALVLTAMIDCLIRKEPNSLQDGDPD
FDRDFDCVIEASESAAKCIQEKPPSNSSTYRFDPSGKQVATDENLRVARQCSQQAIPGYKQCLAAGEQL

>QLI74345.1

APQFFKYENEEVLVSVKVPEEETKPSTQAAVVINVMTGSDDPAVWPNCIPNGTKCKFNDKEKESL

>QLI74347.1

ILVPSGSPCAANCGNILDKTAPEDLVCSQGSFSSDPTGQQFASCVDCERSSTYHSGNDSDIQSMLYNVRYALSYCV
WGDAPAKNPKVANTPCITTKACGPFKNAVQFKNLSSTYDAYQYCDLWQTDDAPDFQGCTDCLQAEGRQYMAN
FVIALQAGCQQKPPAGLYIGLDGELFSTTAVQISTPSPTATVNPAWFDHGPLNLGAKVGIAVGALVALLVIVGCCII
WNGKRRRKAYLRDRDAKIAQRGWPSPTQQREMGQAPAPQSFRGYDDTPVSQRPLRGNGWDDTPVSRQGSRG
WDDSPVNANGEKPLPRYFSPYSSQYNSPVSARDGQAMPWPQGALPLNQHAGVAAGGEASGEPRSAVSDDKGK
ARVEAYEMHLVDTSESNSSRSHPPRDRSEAPVLNHPGYGRDGNVPAPQLALTDYDARCGNVV

>QLI74350.1

QVIINGKTMGGGSSSGVTCPGVLHATANSQGSNTYCCVGGTLNLSNCPGWPVCTGPTRFDPKSTTLSCAATVPLT
ASDYNARVSSASDSYYNGIASPTKPAQTSASPTGRGGGVNFSGNVTVSGADMMRPAWIPVIGGVVALVAVGY

>QLI74362.1

LVNRDGDKALAKCPGYKASNVKTSGSGLTAHLTLAGKACDAYGDDLKQLVLQVTYETDDRLHVKIQDKDNQVYQV
PESVFPRPGGSSSASANRLRFDYTASPFSFQVSRRDTREVLFDTSAAPLVFESQYVRLRTKLPRDPYLYGLGEHSDAF
RLNTTSYIRTLWNQDSYGIPAGANLYGAHPIYLEHRDAGSHGVFLLNSNGMDVVIDKARDGSQYLEYNALGGVLD
FWFFAGKTPTAVVQQYTEIAGRPAMPPYWGLGFHQCRYGYQDVFDVAEVVYNYSRANIPLETMWTDIDYMDRR
RVFSLDPERFPLHKMRALVSHLHARDQHYVVMVDPAVAYQDYAPLTAGLEQDVFLKRANGSAWLGVVWPGVSV
FPDWFARNAGAYWDAMFARFFHRDTGVDIDALWIDMNEPSNFPCNFPCDDPYAAAEGYPPPAPPVRAPPRPLP
GWPCELQPANATDCYRSAAAPPPSPEPPAIAPAPRAAAAAAAGDQKGLPGRDLLYPRYAIHNKAAYKDAWNGA
QGGLSNHTVNTDVRHQNGLAMYDTHNLYGSMMSTASHAAMLARRPGLRPLVITRSTFPGAGARVGHWLGDNL
STWAMYRASVRTMLAFTSLYGFNAVGSDVCGFGGNTTEELCARWAALGAFSTFYRNHNALGQAPQEFYRWPSV
AASARRALAIRYRLLDYLYTAMRRASATGEPALLPMLHAYPRDRAARALEDQYLYGPALLVAPVLEPGATSVQVYLP
AGALFYDWYSHRAVRGAGRALAVTAVDTASIPLFLRGGAVVPARLGAALTTTDLRRRPFELLVALDAAGRARGELY
LDDGVSLDQRARHSLVTFAFDAASARLEVGGVFDYPGPPARVSKVTLLGSEGRDPAPAQTHALGGSRVYSRSFDV
DWTLDRAATYTVGK

>QLI74370.1

GLVPRDASIPTANQTSENGSIECDKRGTRGTLVSQSNAPANLVRLSNNAIQGVSNYTYDESAGEGITVYVIDGGIRLT
HKEFGGRASYGAIFSTILPRNERDYDGHGTHIAGIIGGATYGVAKKVKLVAVRIDSKNASQMIKAADFVIEDVKKKGI
QGKAVICMSMHIDASNEVDQKFEEAVDSGVVVVVSAGNNNKDAGNYSPARHPKIITVAAIYDDTDFHWPNSNW
GSSVTIYAPGVGIRSAGPASDTDKRLYDGTSQAAPHVAGLAAYIMALEGITEPAKVMSRLISLAEETGSRVHWTDP
NTTTLIATNGLAGRLDPAIANKVKKLPWLSPEGYLVDRVTPCGSFYYSDANCGTARYCDSYDRGKPTTPKKGFFKSA
QECLDAHEPPPILPWIEKPTIVRHERCDETDISAKCPEACGTEKYDEEFCGTKFICEGFDEKPKPIWAIEYPNTKACFD
GHEPQPVSSNQSMPAAAA

>QLI74378.1

HLAIVNRAPPGPPYKETPPSKLLSDAVRAGIPGWGLAGLPKLPQDGPKPPMDKMPGGLPELHRPKLGSSDWRDD
TLKGPRKKPGTTYPKGPPCLKRGLSCRLRVPKAIGKAAAFMIVAPYARDLLEMVKAWDNPIGHAVTWFDNAMASL
QEAIGGPQRDDIYGNELKYKLIQFFKSVFRLFGETQWEMNERLREEAQAKEKARREEEENENQRIKGLEELAGICEK
MYNEELDSAIVTTQLKESCNKLVEELEKIQSEEEEEEEVPPERYVWFGKCRCNLFDLPPGGDECALQCRVVSIILSLNP

PGQDFESAPETPPEPEPIDICSNSIGEFECGSGQTVLELKTGRAVCGGCGFAWDPEGGICVSKTGDVLWPLESPEPP
DTPPEPVAGDQSIAELWTGLTVCSICRGGWDAESSTCMTKTGELLWPRKLPPNSPASIPPEISGTCSDSNGEIQCGG
GQSAAELQAGYAICGVCGFAWDPEGGKCVSKAGTRIWPPPEPEPERSCFDSTGEIQCGGGQTDEELEAGYTACRV
CGGAWDPEGGKCRSLTGAIFWPLAE

>QLI74385.1

LPTQTVDEFAVADRYVFYRGSGANWPSQKSWGSFDSLWNANVPLMQKSCGWNGWGADNSGAEINDIKNAIH
QVSGSTGVDKRFILAIMMQESKGCVRVPTTNNGIRNPGLMQSHNGSGSCAGVNPCPSGTIGQMIRDGVAGTSS
GDGLKQTFGKAKGAVGDNSRAYYAAARMYNSGSVDYNNLDNGLGSTPCYATDVANRLTGWTLAASQCRA

>QLI74386.1

QYDDGFYNEVQYDNFEDDFATSPKVGSLGLANNGIRGLTSRRQSSIASDIVRDHHWWPSTTIAAEIFHHTCQEIPIR
FDFSQAIVKRRETETMSDLINLDEFTITITSTYALEWMVEYIEREIERSELSYKCVFSINDSATFQMTTNAPKSREDATR
QHETLLGIMETMGFKKLGGNKGVRKEEEPANWIIEVALNWSG

>QLI74389.1

QNLCADINTSDSSPSADIYQSSGKCTSTCKGKYAYAVMQSSNCWCTNYTPDKASQKDGCSLPCPGYPFDMCGGKS
RYSYVLVDASLIAGTKGSSSSSSSSSSASSTPSSASDSASSSASSTPAPSASPQVTTVDGVARTVTVMPTQTGDTDSS
GSNSNDSGIKVPTLLGIVFGCIGGVAIIAIGIIWFFFRRKQDDKDSDAAASVSTPGRSGGTSRSSLDNLSNPFSDSRSH
GLYSWRNRSGSLEFLPEKDTTPTLRVANPDRT

>QLI74394.1

APAPTLEKENLAACGKGMTLVEGLGVCRPVEEQAAQKPDTCFDDKTPTKTILPEDIPTGIVPPSGNLAAPQPRCDG
GIELNGSCVCPAGTTRIEGGCTKNANAEADDEC

>QLI74400.1

TVAGFDISHYQPNVDFKKAYADGARFVIIKATEGTTYIDPSFSSHYTGATQAGLIRGGYHFAHPGSGSGAAQATYFL
AHGGGWSRDGITLPGMVDLEYNPSGSTCYGLSASAMVSWISDFVETYRSKTGVYPLIYTSTSWWNQCTGSSTAFG
SKCPLVVARYATSVGTLPAGWSYQTIWQNSDKAPWGGDNDIFNGNLDQLKRIANAS

>QLI74403.1

VRRQDTRLFSVPSQPTPNESTPTAVFTILPVSMSSASGPDSSGASQSSGTVAPTTMGHITPDPTTSGVTQTITSSVQ
VTTPGSSAPGSSSAPGSSAPGSSAPGSSAPGSSAPGSSAPGSASQSGTRSGSSTGTPSTTSNPAAAPTAFGSNLVAGVIALA
GLAVAI

>QLI74404.1

APAGSKSKAPAPGSSDNEKPKSKCTPQTIDEYCQVMQNTSNGLFDKQFCLDAFKSCGNPASLDDAKSKNCAGDIM
ADKYLGREPRPVSDSKKEDESTKQPNTSTQNRQ

>QLI74420.1

TRMLDSIMARQQGIVDSGAASSTLESGILAQAFEYAIAQYPAAKARYGGYLSSVLDKASASFANASYAATRPLDRFS
LATAIDAALASHAGPVTARSRAAYAAINASLALQPRNPAGGLWYYVYPEWSYLDGMFSLLPFMAALPRANLSDAR
HQVELLRDRCTQQNTSLLVHGYDWSRTAVWADPATGASPYVWGRSLGWFLAGLVQAWDQLACAAADDDAAQ
QPADRVALCSLVRNITVQVSGALVRHADPATGAWWQIVTLPGVGANYLESSSTALFTFAILKALRTGLLCGRTPDY
RGTALRAYNYTVGRFVTDTGNGTIGFDKTVSVCSLNSSASFEYYTTRSLVPNSLLGESAFVLAALEVERMA

>QLI74435.1

TPRGPPGECDTKTAVSKRGWLGHDVLDGNSIQPAHYSTWHWSNFGLCKGDWSNFGLCKWDWFNWWNWLG
SGDESCETDDDTPRPQKPTPTKPETPEEPPTQPSPSTTQAPPPGGGNGPEYINRVNKWMDACGLRHLAHDSTLES
NAHQTSVEANGNMVHNMLPGTMAQVMAHGDLTANFDRAFVGGWLCEIKTLFKDKNFCDESSLAEGWTYSGT
RHAEILSNSRYTKIGCGTAQSIVTCDVA

>QLI74449.1

IDSELSYDGHRVFRVRVADDGSHVQSVIDKLQLTTWQPPSRKGAFADIQVAPRQLEEFHKAMDGQDVTTMHDDL
GKSIKREGTFMTYAAGSANDTWFTSYHSYDDHVRWLNDLGAKFSKNAKTVTSGTTLEGNPITGLHIFGSSGGGKK
PAVVFHGTVHAREWIASMVVEYMTNELITKYGSDKDITAFVDKYDFYMFPIVNVDGFKYTQTTNRMWRKNRQTT
RGSNCLGHDINRNWPYKWDGQGASTNPCAEDFKGESEGDAPETKALSQFIQGVKDAQGLKLYIDWHSYSQLFLT
PYGYSCDAKAPNDAEIQSLARGTVDAIFAVHQVRFNSGPICQTIYQASGSSIDFVADVVKGDYSFAAELRDTGKNGF
VLPAEQIVPSGEEAFAGVKYLLQNMK

>QLI74451.1

GVTRGPEGLESHIAQLDSDIKGTNAAPLRRSLQNGPETSSAPGRHPMLAKRASKTQKGAPWGLRAISHRRAGAFY
EKFPPDPASKYYYDDRAGLNMYAYILDSGIRTTHEEFEGRAETVFTVYPGDEIDHRGHGTAVAGVLGSKTYGVAKR
AKLLSVKTLDDKGSCAASAALHALSWTAEHILSNGRQHSSVINLSFGIPKLQALDTFIEALVSQVGIPVVTAAGNENE
DASLSTPGSAKGVINVGHMDKNWVISPNSNWGPAVTMLAPGVQVECPSSGSDSNVKLESGSSFAAPHVAGLVL
NAISVHGIKGAAEIRKFLLESATRDQACTSRNTPNIVANNGNTAQKKHTKPRNC

>QLI74452.1

APNRVEEFCDWQPGQELNTVQDTVRRADTTSQETILEFDPKIEKLIQQLWPPEPVDDGRVINVGVHMHVVGKPQ
NNESEFLIDRTALDKQLDFLNESFKPANISFTLSSADWTKGGMLARDIFLRRPLAKSLHRGNFSELNIFFCDDDETIGG
STVTLRGYDLDDDDDKSDGCVVNANTVPGGPHPVWNLGVTVVHEVGHWFGLWHTDLTDPGDCEPNWRNATG
LSNEPCGARCDSNYMSYGADACLTEFTSEQIAEMRKFARKERGL

>QLI74455.1

INILLNNDDGFASGNLREVYRLLKTAGHDVWIVAPATEQSSQGGRSSFTELGNLTGPSQYDIIPAGAPSVGTDPHDS
QIWYYNGTPAACTFVALDYVLPRHAPFHVPDLIVTGPNFGANLGPFVWTLSGTAGASYAATERSVPSIAIAGSNKKI
AYFDIKNETNEATWTARVSVKVIEQIIRSAPDGGPLLPLGYGLTVNIPLLTANNTDPEIVQTRMTGNAHINEAVWDP
ATGVFHWANIKPYSAGLNACVNGDCRLPGETYVVESGRVSVSVYITDYDAPATEYTQSIRERVKPLTKNCSTAK

>QLI74462.1

AVVPRQAGDFDLAGVILQSSQVQRHLTSTKRFMGKEFPYSEGPMEASKAALGDKFKDDYEHFVESERKCLYIRNSFI
KEATQSFFDVVPNVDAPDTITEEEEETGSVTESKAIVDTQRIGWNKEESTEIGGSATVGIQSGISAFSVSLSATIYGNQ
RTTGGQYGEASKQTEYTVTETKNWKCPKNSICRHVTWTYTRTLRGKCITTPYYDAQCANSKHSKAKFSLALFGKCT
PSVRAGDQFYTYSDNAFGDFDGDLNTKIPGYGPDNQGIKMPKPDVIASHKFADDECTFTYVLRDKSGSPVRARGN
LIEKDSSPLSAQPKVTKVPKAVKWIKGQKDQSVCELEGGWYWMPGNQWYISPKDGNTAKKWARRAELPDPVDL
EKNCPENGQAKSKRDLPPANDLSDVPANDLVQIEITQDDMPAFLAELEQSQSEGFVANTIDDKAQPVRREGWGY
PPGRNFEHCLDQNIKQNGKA

>QLI74468.1

ADVGQQGSSTGDVGSGLYVLESDEAELTALANTLQQGQGIIRHKYNSSVFRGLVFQIPNATRAEEFHAELQKKGFA
RRTWSVTTSPEPAADDEQPGTQQKDERIRRAAKREDSKVRWTHLMTTIDKLHEKGYLGHGVGIAVIDAGVDHQH
PALGASTGIYALVDEARGTWDAAVLNSVIIGTGKPQIESPSSFVSVAQQGSRLVRASEAAGAITLVVTPTSLAFNDTA
HRVPSVSFRIQNQAKTTVQYQLSPISALTLSTLGQDNRRASGQAVQAPAKVQFSESGITLGPGEFATIDVSASDPEG
LDVKRLPLWSGWIGIQGSDGTRQTLPYLGLAGAHLKSKSTLLACASAGIPRRMQQSRRGSTSPERASPRLR

>QLI74471.1

RPAGFAPGLFPYIFEGQCYNISAGDEASSPTTIGFYTSGTRIKINDAEYDTPRVLGDYSCKFSELSVDGYATHIERSIALY
ITTEAGEGVRPSLPLRKAQPLVLQAGTGGDATFGCAGGQEAFRGSSADAWGQVRNKWLYVIDGELLNTEKDKCC
RKGSNPFTNAGSGRGYKSGPVCDPQH

>QLI74477.1

RPAADVAAESSADDKTYAISAILRYRQKEKKDVEDVVVRVAE

>QLI74483.1

LKVPYQDALTVGQGYNTFLGRGVIHKAVMTSSTDAATNTEKRSEGPGNQNSTGKFNFTEPGPVMPGVDVDGYFT
PTTLEELAKIIEEAQEDEADKEKRDGAKNMISDKKMTMQGCQAEIHSHIEFVSDYTSYLKALGVNAATSISGYGQEA
SVSGSYLDESAFSSNSLTFIASISINKQRRVSNEQFTFNTKLYEDSDRPFATRFGNRWIRGFEMGGKLMARIMLTFDE
ASDKEEIKAAAEASLSFWGVSGQLSTEVKNNMEKLSKKAQVKVKIFYQGDIGRQLQGRSESMDEKNSAQQIFSTAK
TWADTFLEMACAQDYSYQTLLDTYPNIGNFPENQSIAHYTTAGAVAYHLLGEMVKHTELRKTLQLRDALSQTEDR
EIQQHEIKLIDAQKSWIRDTALSPDNALETVQHLFWLSDQYYQKWKPYLTKQSSPVEIKAFNRLVSEKDPDTKDPTT
YDCSGWYEWYGKGEWTWSKLRFCLPTHTDVFSLSVFRERSQYLWGSAWYYEKVQYPADVTARVTLKRIDSKPRV
WTLTSGMTRRLSFNAVSEERLMPGRYKVQVNFYQDGPYWDDSPIGEVAEFEFTAS

>QLI74493.1

FAPSRFDRRSSSPCSGNTPADRSAWCSHSIKTDYTREVPDTGVTREYWLDIVDIIAAPDGVSRPAMAANGTIPGPTL
FADWGDTVIVHVKNSLHTSKNGTSIHFHGIRQLFNNQNDGVVSMTQCPTPVNDTITYTWRAMQYGSTWYHSHF
GLQAWEGVFGGIVINGPATANYDEDLGPLFLNDWDHATANQRSTVHRTDFLTPPLGTGLINGTNTYGNTGQRLN
IRFEAKKTYRLRLVNAAIISHFKFMIDNHDMTIIAHDLVPVKPYTVKILDITMGQRYDILVTANQAATARQFWMRSIP
LGLCGDPNWKFNDIRAIVHYDDNWTQPSTLPYLYSQLCGDQTMNAVPYIAENVQQPDHDPTLSKILRHETTFQKN
DSVIELPEANKTFYLVIKNPLFVAHPIHLHGHDFFVLGQGVGPYLPGISPLKTKNPPRRDTALLPGSGYLVIGFVTDNP
GVWLMHCHIGYHVDMGFSLQIIERANEIPAIINQDELNAGCAAWSSFQQSNNIIQDDSGV

>QLI74504.1

LPRNECFRVHSKALAEYTDCGNQAAVSRCLASLSSFEPSDLAPCYTNAGCSASQAALEARYITTRCQEYSTGNELRKR
FRAALEPVRATQAVVAAAHLAARATTTPAPVRKGADCFSASEFDTSSCDVATENGKAVTRTCTPSKGTSSDCLSGW
ICTVDSTHQDICMKKQPIDTGGIIVAIVFAAFFAIGLAYLTFACCRERKHHKRVAAKAEAVALARAATKKQRSQEARA
PLMQQTQEASSPNPFQDQPSHA

>QLI74510.1

EGVPGTLSPQELNLDEILAKHNLALVPRSDLTEALAELNGLLKKNHALQQRAALFPRANTTSSGSSSGSSSSGAQAA
GLLGGLGDLTGLNGLLDIVPAIKNIADLLKSIESLLTPEFLTGFHDSMIYLAATLKPPVPSQVGQLLNSTIPLVQLLGKLD
LEKLVGQIGNIDLGSLVKSILGLLTEKNIDNIGTLLTNGASLLTPTFVNQTQSLVGEVSPLLDALKGIDLKGVLEQLSPLL
TSDSIKKIVTLVDSAGTLLNNQTTSDLNDILKFAHNFIPYLSVINPTDILKAVAPLLDNLPAIVNGAVTLLSNQTVSEIKT
VLDGASPLIESLSKADLKSLIDQLGPILKELGGLDIAGLLKSLSPLLSDESIKGIVGLLGNAEDLLTKDFVNNTQSLVAGA
GPLLGSLKDVDIKSLVDQLSPILKEVAKLDLVGLFDALKPLLSSDGVKGIAGLLDNAELLLTKDFVNNTQSLVAGAGPL
LGSLKDVDIKSLVDQLSPILKEVAKLDLVGLFDALKPLLSSDSVKGIAGLLENAELLLTKNFVNDTQSLVAGAGPLLGSL
KDVDIKSLVDQISPILKEVAKLDLVGLFDALKPLLSSDSVKGIVGLLGNAETLLSSKFVNETQSLVYSAGPLVGSLGKLD
LQKLLDQIEPLLSELTKLDLAGLLKSLEPLLTPDSVKGIVGLLGNAETLLSSKFVNETQSLVYSAGPLVGSLGKLDLQKLL
DQIEPLLGELTKLDLAGLLKSLEPLLTPDSIKGIVGLLGNAEDLLTSKFVNDTQSLVSGAGPLIGSLGKLDLQKLIDQIEP
LINELTKLDLAGLLKSLEPLLTPDSIKGIVGLLGNAELLLTGKFVNETQSLISSAGPLIGSLGNLDLQKLITQIEPLINELTKL
DLAGLLKSLEPLLTPDSIKGIVGLLGNAEKLLTGEFVDETKGLVSSAGPLIGSLGKLDLQKLIDQIEPLLNELTKLDLAGLL
KSLEPLLTPDSVKGIVGLLGNAEKLLSGEFVDETKSLISSAGPLIGSLGKLDLQKIIDQIEPLLNELTKLDLAGLLKSLEPLL

TPDSVKGIVGLLGNAEKLLSGDFVDETKNLISSAGPLIGSLGKLDLQKLIDQIEPLLNELTRIDLAGLLKALEPLLTPDSIK
GIVGLLGNAELLLNKKFVEETQNLISKAGPLIDAVGELDLQDLLKQLAPILNALGQIDLPGLFEALKPLLTKQSVEGIVG
LLGNAEKLLSGEFVDDTQTLIKGATPLIAELSKLNLQDLIKQLEPLLGALGQIDLAGLMKALKPLLTENSINGIVGLLGN
AEKLLTGEFVDETQSLIKGAAPLIASLGKLNLQDLINQIEPLLATLSQIDLAGLLDSLKPLLTKDSINGIVGLLGNAEKLLT
GEFVDETQTLIKGATPLVSSLGKLNLQDLIKQLEPLLATLGQIDLAGLLNALKPLLTEDSIKGIVGLLGNAEKLLTGEFV
DETQSLIKGAAPLISSLGKLNLQNLISQLEPLLATLGQIDLAGLLNALKPLLSEESIKGIVGLLGNAEKLLTGEFVDETQSL
IKGAAPLIASLGKLDLQDLINQLGPLLKSLSKLDLEGILEALAPLLTKESIQGIVGLVGNAEDLLTGKFVNQTHTLIDGAV
PLVGALSTIDLPGLITKLKPLLDALGEIDLGKLIKQITPILNALGEIDLKGIFDTLAPFLTPKTVQGLIGLLANAEALLTGQF
VNETQTLVHGAAPLIADLEGADISGLLKQVKPLLNSLSKIDLAGLVDAVKPILDAVKKIDIEGIVDTVTPLITPKSIKGVL
GLLGNAEQLLTETFVSQTAELIGDATPLVATISDFVKAIFQSLIGGQN

>QLI74512.1

QKDTPSSKDDSASATPSPKDTGKSTPSPSPSSKEEPSSETPSPSSSGKASISNTGTPVTTDPTGKAVITGGPSATTGGE
LTGFPTLTGPGVIPSYPPPSVPPTNNAPFMQQSSLPDGTVFIAVGAILGALGLALLLWRSIVSLMLHRSVARAAMAQ
HNGDTKTGFPAPPAPFYKYSDHASTMSVGGGAGATSAASAGRGVRRTNRGPIPSATPSQSNLFFSPTAGTGASAN
RASTFLPSGFYAAGTSLPGGGGNQTNSINLSNLRPESRGHYANASRNTMDISPPDSPGVAARRDMSSSSLNLAPG
QRAPSAYLDDLLTDDPNALPPPHMPPSTGPRRSVSPGNRF

>QLI74514.1

APAPAEKSMLVQGIPEWTIEGAHRWCNKDNTECHWKFAINPKIYSKTAVDFVVRNAGSTPAAQSNGGAQNFGD
YTITSGWSGQFGPGNGFTTFAVVDNKHRIIAYPAYSDKEVENGQVVSPDKSYPAQGLP

>QLI74523.1

QTISGQFTHYDIGLVACGETHDNTEMVAAISKDRFGPSTTLCGRKLRIHYEDKSAEVTIVDSCQDCSSSSLDLSPAAF
TALVGSHEPGRVDGTWEFI

>QLI74540.1

EPASSGDGAVPALGHIVVLKSGLEEKHLDEHLERVKSSIQKRSIESGSSENQVNGASGVKHEYRGTSIGFHGYSGSFP
PDVLEDIKRDEHVAFVEEDRMITVEPSKREEVEEDTTPKDGGSNNQKKGPGLLSMGQGYNTFLDKGVIPDAVLLPG
EKKRDVPAALAEVLVNQTTSMRFNFTAPSANLTNVNVTSYFAPPDPDEIMNGVLDDLENEQNATALNVIHTRATE
DQQDCTGSLTAYYKLTESFDSYLKALDVSGAATVSGWGQSASVSGSYLNQAELSKEGLTYIAIIDIQRQVDLPTGFEF
NKAKYSASTFARDFGDRWIHGFHTGGKMIARLSFNSKGSTSKDDLKVHAEASLKFWGVTGDISASVKKSMEEVSK
HADVEISLFYQGDMGKVMAKSGSPDKVESASAEGSFKQVKMWADQFINNACQHNYEYRPLLDEYRNAVGFPSD
QKVLDYRTAHRVSYKILKELVRISEMTQYIVRLETLDAEFKDEVEFAEIEMVELSRKWVDSIVEQPQNALSLGKELIQT
FRAEFYDKYASAIAQDFYISGIEVQYGDKPPANRVIDINHQPEDINHDFGGEFVWLVPIYTTRRDDACTSFKVVFGSE
GAGLKDLTRNAPGPNRYLKCEKDMSKEKIRRLALHRGGENLEEFLSETSHGFVGKTTNINDGRGDAYHAMYLLWA
HDGKRDPSSKHQPEFIVPAN

>QLI74545.1

QQLSIKSPRPGTVVHRNTPTAMTVQTENNGTVQEVSVVLSILPCPDGSCPNAGEDLGTILSVGPFQPVGQPPEQTF
NITIPDSFPVGYAELVATHFVLRGDKHAPFLQIAGEIVIVV

>QLI74553.1

HLAHTPVVLEPQMNQHGSDFNKANGELPEMSSERNQPEGSLETRNDMTIEKRHGAPKTQTSAPWGLRSISHRLP
GVIYEGFPPSQNSEYYYDTNSGSGTFAYILDDGIRETHKEFEGRAKNIYSIFPEKQAGDYVHGTAVAGIIGSKTYGVAK
KTTLLSVKTLGTTGAAHSEVLKALLWTAEHIANNTRQKSSVINLSFGVEKSDALNKFIELLVGKYDIPVVTAAGNEGE

DASTKTPGSAKGAINVGYINKQWGLAPRSNWGPAVTILAPGVDVETTGSESDTNAVLKSGSSYAAPYISGLVLNVIS
VHGVKGAANIKKYLLEKATKDRACVFKRTPNLVANNGNAMQDKVKPGDKSALSKLMCCIKESLNKCK

>QLI74555.1

DSGDDFSNNLFSDLAPLLALFGERVTMQFMSQSTGWADSIILAMAPLGIITIIASAIRVGGPSWLKAIIGRARENLAV
AEAELMSSTSHEVCELWNGREVVRCMGSPSTAEFICLRPANMKCTENGANQVPDILELKEALDKQYLKELPSNDG
ASFWETILDSIGRNRESASAKVERGANSQTSVTIPVPKLTIVRNQVIDAPNISLNSHGYLSRRELRAVAVFGTILQLGV
LTYFGFATYHPALKFTKGDDAVANYAFPCTATGTITLVAGLLLCAHVVESSTEEKRYQPSKETKARLVWLQQAQTVG
DQSFQSFALSAGDNQTIITTSCRVNKKSQGIHLELKTIIGTAVSLCGFVVQFIGLRGMHWSASVAQLGAVIAMAALR
AWVRRGLAQPLQRVRLTPEHELEWFAMALGDFDGIPWQPDRSPKEYREQGQTARAGVNTMKNSGGNPTTHN
MMVTRRTLCELAGWQGPASAEALTLARAIKITMDALSSYLHPESDFTWSLGALSAGHEDKPPKFCWTRDVTVDTI
RKDIEAVLSLRLYYVYSQKRQYEDQPKSAQTQRRKYNARLHVTEMPAERGLRLLGPGTRALRRDLAWWMPPDTA
RILEIKEDENGTMKVENHRIVGYGGQQSIRVARYDGTEASLSPDDTYHCEIEESTLLASESYDSLELLYAQDMFTKFM
QSVAEKMARAIEGGSNVRPKDSSNMVAWKCFTLHHDRLSKMAQDIHNCGLGNIHQTYLSIIQPLSTEQKLPEPDN
VIALARKHAKPHERLQDFAEASNIYLWLFRTANTFPRKSSIFVKATAILMENVRMVTLTSKLREAQCYPETKIWQIRA
AKSNMENELKRVDRTILSGLMSLYKKQSRQWECDILQDAEPTIEGHTSHPGTFNCTPLHQICQEDRDHFLSRELAV
RYLDRKDIHGCTPLHYAAVKGSLDDTTFLLDFYPDVDARDLLDWTPLHYACNACSHSGVVQYLLDKGRPQVNAQG
IDGVAPLHLAAMNGNIETVQILIRAGAALDIQDASGATALHWAAFKGHEAMVEYLYEDSNKKLRDKNSRTALHLA
AIAGKENVVRLLVPCSDKQTNADMDTFDSGGYKPLHYAAMRGHEAIMRYLVNVAPFNREQATNYHGETPLHLAA
RRGHEAIVRYLVGETGANKEAKNKQGFTPLHAAAAYGNEAVVRYLVGETGANKEARNKQDSTPLHIAAAYGNEAV
VRYLVSETGANKEARNKQDSTPLHIAAVYGNEAVVRYLVSETGANKEAKNNIFNRTPLHLAAAYGNEAVVRYLVGE
AGANKEARDGSNSTPLHNAIFEENEAPSMYLAGEAGVDIEAKDHKQQTPLFWAALEDNEAIVRYPVHEAGADLEA
KDFKQRPPLRRAAKWSNEAIVRYFIEAGADLEAKDFKQRTPLHKAAYQGLQACVRYLVGEAGANKEARDRFNRTP
AQVAAACGFKSVVRILEQDEKAIRGAN

>QLI74563.1

ADTRLAERKDRAVNPSYNSINQLLSDMRFALRDIERMQYSVASATEELGIILESAGCKPALNGNIMSRIQSLGENAN
KTLESTKEALQTRDHLYGIVQGNAIHYSTPN

>QLI74564.1

VPLDTVQEIQNECCAGFSLGEAPVCPEDEDFPEDGICWGNQPKRIPRPPGRDPAVLGQKWLQMVNAFADKREIT
PEAKQGLLDIIPSCELNAGAHPGGANAGVRCTVDSEEQRPTPSCDDHRSDTTVLGRNWLQLFHKVKGGWKQKW
VDSISRCQIKVPTILPNTVLRLEDIKEVWCPLNPTSME

>QLI74567.1

QSYPSATEILEQLPPCAAKCLTAIVPGSACSVTDVQCICTSSPIQDAAEKCILRDCTSLADALSAKNITATACHAPVRD
RSGQYVAVSIALGTITVLLVVTRLVFKQFFAANVGLAADDKVILVTLAIRISCIVINVRGLAAHGLGKDTWTLPSHELT
SFAEWLYIMEVLYLAELSLIKLSLSLFYVRIFPGKTIRRLLWATVVTNALYGATFVVTAIFQCSPIDYFWTQYVEPHATG
RCININAFGWANAAISVALDIWMISIPLSQIPKLKLHWTKKVGVSIMFLLGAFVTVVSILRLSSLVSWANSTNPTWD
QWNIVYWSTIEVNVGMICTCLPSIRLIMLRAFPNMTRSHPTYGTDSLSHHTSGQSGVERRTTTRDHRLSDFEQSVG
HTWTRIESEAKQ

>QLI74568.1

NSHQHLHHHAKKDVASKVERRAADGIVWVPATETVYILGGEKISMEEALAGLNNGDFVIAGESTPIFTPPLAQPPK
PTTTTSLQDLGAQFLEKSSSAASLPNPTTTSQPPPPKSTQAPKPSPSPSTPPSGGIGLDSKFPSGQIDCSKFPSDYGAV
PLDYLKMGGWSGIQFCPDYSMGKSKSINNIVTGIHNDECTKGAMCSYACPPGYQKSQWPEAQGETGQSIGGLYC
NEGGKLELTRKSNPVLCEPGAGGVSIINELDEVVSTCRTDYPGTESMTFPAEAMPGKSVVLTNPDSSTYFFWKNLPT

TAQYYVNNKGLAAKDACLWNCPCDENKSQEKKTCGNWAPINIGVGQANDTNTYISIFQNAPTSSAKLNFNIKITID
GNTKCAYENGNFIGEGNGNGCTVTKSESAKAVIRYYS

>QLI74590.1

APEHTNLLPSQSNRVRNSGGFDIDPALSNKTALGIIPDNYDAVKRRDLVLRGDILKRQAPTSPSTMSADGASQIKVS
GGKQGVLYHGDSRPPAEVFKSGFTPQGGDTNLQHHLSFTGNSAFTSVTRSPQSAERYAFGWTGAQKHKGYIYVIA
PKDVPRGYWVPGIYPSDRVVINNQEFAVQGAVTGSSIVHAYEVPKRNPSDRSVTIKNENYVLKKSPGCLGLKSKPAL
CDPAKGENGRSGPKVSKSSRFRVAKSVGKSVAFMAVVPYARDLLNLLKQWDNPIGHAVKWFDDAITSLEEVIGGP
QRKDIYGNELQGRIIDALKSISLDSVSGGTPCPCSEWTSTKALSSWRALLRARTTPRW

>QLI74596.1

MMTSPPTLWSRIQGIRSRVKTCLKLASLPSEEGFFQPMSGSYGRPGCSLCWTARPSFTYRVDLSYEYADQGLVHTS
SALEPEPAAWGDNTKAARFYRTGDLGRYNPDGTISYLGRKDSQVKIRGQRFELGDAENVISSSPEVRDMSLSTKIHR
GRNELVAVISLADAQLPRGDGLRHLPSAYTRVVARHLQSIREFVRLRLPSHMVPSIWLAVEKVAQSVSGKVDRHC
MDNWFKAKDVSMAKAAMEEQADRELNPPVTVEEKLVQYVWASVLDIQETTTSWLLRNQTLQSAVEAALLANIE
GDEAAPVATRIPRDTSLGGLHDRLSRLSLSNPQFKHENIESIAPATDTQALFLIIGPPGVEGETGYHTSFTLDSTPALD
AAKLRSVCGQVIQHHAILRTAFVQHRHMLYQVVFKTPTSETITIEGGGSPATPAFFGKGSNLARFYLFSREELCNSIRL
NIHLALYDAASFDLLLPGLDAAYNNGQLRRGGPRYNPRISHLEALDGTAPRQFWIEMLRGCSMMHLTTPRIMPAQ
GYHLRDCSRLSVPLRNLQNSLGTPSTTLKAAWALVLPAALDTSDVLFGEISANRYLTMPGIDQVCGPCINFVPVRAIT
RHPS

>QLI74607.1

FPQSRPEEQTAPFKADLRVDTNRDGTVDLSGNTDSNGKNTWSETSGALFLPNIGVARRHCGSDYTCHDAYTNTP
WAPEYMAPMRTVPMPNISDSATATVSISDPVARKNVRIFLDYPGRIEELNFQDLIDIKPLPPPENDRWVYLENDTPI
PADKLRQGLTLGIDSRDTRRPGR

>QLI74608.1

QLSTLTTPYLNLTALTAADGESTLECWQLANPFRQSTESGITGSLQLSLGELANATYSVIPARFDGGFHHAPAFQYVL
FISGLVHISLYRGRDEAWIQGGKYGLIVAADTADRTAHGHLTRYPGAADTVAVSLPVRDRDGFKYSVLHGGACTQD
EMVGI

>QLI74616.1

TPFSLDSDDAIKQSASTLAWDMMQYYKGNLSGQTLGILPGPPPAGPYYWWEAGAMWATLIDYWAWTGDASYN
DEVMQAMLWQVGPNDAYMPPNVTASLGNDDQGFWGMSAMTAAEMKFPNPPSDKPQWLALAQAVFNTQA
SPDRHDGSCGGGLRWQIPFANNGYNYKASIANGCFFNLGARLYRYTGNTTFSHWAENTWNWMEGVGFLDSAT
YAIYDGANVGDNCTDINKAEFSYNNGIFALGAAYMYNTTKDQVWADRVTKLVNYGLKTFFPGGIAVEITCEDEGTC
TTDMITYKGFMHRWYSYITQLAPFTANTINPVLKTSAAAAVKQCTGGAYGRQCGFRWASGKYDGTTGAGQEMS
VLAAVSSQLINVAGKAPVTADSGGISTGDPNAGSGADNFQNKTRPVTTADRVGASIVTLLLLAGAGVVFGSMVMK
E

>QLI74621.1

ATESKHGTSASRSDSFLFQSRAIYPKGVKLNSTSEDATLAEALKLVQEASKQQGDYNAHRVANPKRNEEISKASAAR
SKRTANAGPRAPTLTPALRKAAAIIAEHQAKQNANKTQPEYPQPRHLKPRTGSSSGSLDARDLTSKYWLQDIKHTG
LAPMGANSSWPVFRDVTNPMFAGGAKGDGVHDDTDAINAAIAYGGNCGEGCLSSSVKGTLVYFPPGKYLISTPIN
TMYYSQLVGNANDVPTIVISKDFVGLGAIQSDVYIPNQNGDEWYIEQSNFYRQTTTKKTAAFHWQVAQATSMTN
VYIYTSTDPGTNQMGLFTENGSDGFMSDVYISGGKYGICTLAITLIVMEAD

>QLI74640.1

NAPGHVATQNLNRRQEAESVPSGGMNQEEDLNALLKELKELGGTIQSFADEMMNHSDQV

>QLI74661.1

EDALAKFGDVIPVLGQMARTDQDFVDGLALDLFSPRNRTLVVTNNSSPLPGTFVTGTTGEGFVNLSKYSWVVKLN
ETAEDLIAKIELPYDPVALAGQSVQVANTYVGKLAKDKKSWVISESQRNVHMTENKTRIIKMTSLDGEYMLLGRKSE
DTSNIFVQYGQGATRTVNITGGAEKIQEAEFVDGLRFRVQSAKSFALNADIPFGVNDKAMPEHAVPLNSFAWVIN
STASPGEGLSVDVHFPVNREMVEEKTRGQEEGHLNLLVARRDLKAPATAAFEPLKKQEYSKAKRLVEVRGMTAVD
GQYVLLVSKKKYLEASCGQGGNAQSCPGRNSTSASAGAASQARESELDGAQVTSRPAMVVSVASLSTAWGMNL
VVSCVFSMGVSLVVFML

>QLI74668.1

RSIERAASGDHVEKEKILQQTFDSPRPFFAIAHRVLMDYGVRDALNHSANALEIDMTAWSSQWYADHDGTLTSRG
DTAEHMFSAIAQERRAGKTAIFVWFDLKNPDYCDDRYPACNIEALRNLARDILQPAGVKVLYGFYSSQTSGRAYQV
ISQGLNSNEAIGIDGNVADANQVFNSKGPASIKNRVYTKGLFDPAWNFGNCESSGNQICPQLREGAQSKNFGKVF
GWTIAENNGKQADQLMGVGVDGLIYGFVATHYYDHADTRAARKILADWLSKNQDKAYLATLTDQPCHALSKSVK
IRSQPKLYHTMVLCDTPAKFSARYFAWDVIKAAVKFVSMT

>QLI74678.1

LALSDLAVDRLARYRDALHVLNTTRWGDFAPQNKTTDDGREAKHLNLTGFRETDGFAWDDLETFRQKGLKLSRH
AVPPVDGHQLWDVAQGEAMWTNASGTVHGDWVRKPGSAFRGYDSYNLSRSVPEVEWMAHKVEWARNVTG
NAGRMMLRLSGNKTLTHYEQLSMDMAPLSGGIIRSLRGTATIEDTIGSGLNWEMKLWGVHWPRQGVILMTTTS
EKFEGIFALPHLSPGPDFFQSSQMLLNQTLARVIRRKEKHVYVDQRMPWNSDIENPMYTAYPSPHCEYIMYAQVH
PPIPPKPTEGQDAAPGAVGDTINAIESELQYPVGAPIPRIPKLKMSAVVYSPDCSFFLETKGPPDYPLSEADHLVGMK
KEVQTHQIKTWLLLYALVVFGQVRLLRDQMKESFTPSTMGRISFGTISAMLIVDGMTFTAAATWVSSAAATFLPTL
ALMFASFLSMTIGGSFLAKIYEVQLPESRGRRDQSSSSTTTAQDSSANSIAATPTPGLLPGPVTASQRVETPIIIPSDQ
DISAEIAAAASAIPTANRSSPSTPETPTFQAIIGRFILFSLCVSFLAISSSTWYPRPRSVFLNTCAFIYLSMWTPQIYRNTL
RNCRRALAWPFVLGQSVLRLLPIAYFWVKEDNFLYATTDVPAFVFLAGWVWIQVVVLAAQQIVGPRFGVPLSWT
PDAWDYHPVLREDNLEGGGLPIGLVVPEERNSLDRALAGAEKTGVRHIDCAICREVLEVPVVKAGEEESSVSGVFA
RRMYMVTPCRHIFHTACLESWLRFRLQCPICRDELPPI

>QLI74692.1

MQLQTPLYSSYSFNPLQHLAGIAPYYEPADPPRDPNPPQGCTVTRAAYLVRHAAINANDFDYEQYLQPFLYKLGNA
TVDWAKIPQLSFLAVWEPPSFSEQELLTRTGKVEAGQLGLSISYRYPKLKLPQRVWTSTAERTVQSARGFVRGLEM
DDNTINLVEIPEGKEDGADSLTPYKSCKGYTAAAGAEQQRKYVDLYTAPIIARLKSLAPGFNFTSDDVTAMQAMCG
YDTVIRGSSPFCSTDLFSPDEWLQFEYGQDIQYHYNTGYGSPYAGAIGFPWVNATMNLLAADTSAQDMYVSFTHR
ELPPAALVAMGLFNNSLFSGGNDVNATMPLDQINYNRAWVSSRILPFLTNIAIERMNCSANHAVAAAQGRNMSS
SPSATTYYRVLLNRSPQVLPGCFDGPGQSCSAKVLPAFLQQRADTFRNYSAVCGNTYKNSTDAVTFYTNSNNGTVV
GKKRHLMW

>QLI74696.1

NGATQPFDSWSHGRVQLDDVSIHFRYAGSGPPMLLVHGNPQHSYTWRTIGPILANQYTVIAPDNRGTGDSSIPED
NNYSSEAMAGDLKGLLDFLKINQTLVFSHDKGSGPAVALAAQHPSLVPALGVSEYLLPGFGYEESSCPSSTWDLYSN
WQLAFFSVPDAAEFFIRGKEKEMLAWYFYHQSYSGNEAIPEPVLQRYASSISKPGFLRSMLGPFSTASVRADNKFFT
EVLRDAPLQMPMIAIGAEASFAPESTIREVWGPVASNLTVDITPKSGHWIADENPEWVAERLREYFGGIRGNIQPA
DLSWLSNRTTLV

>QLI74706.1

RPADASINVVGGTEDDGSQVPFIVALQREVQGKPQFFCGGSLMRNQFVVTAAHCVNSLKTTKGLTIRAGSLSPDK
DGTVVPVASIFVHPQFDPERINNDVAVLKLQNPVGGNQSESQATLPADGSDPEPGSRASVHGWGAKKEGGDPSK
TLLTAEFPVISRADCQAASESLRGLVNENMFCAGLLEGGKDACQGDSGGPIIDSNNVLIGVVSFGDGCARPNNPGV
YTRLGKYVNFINQITGGSGSNGGSGSNGGSGSNGGSGSNGGSGSNGGSGSNGGSGSNGGSGSNGGSGSNGGSG
SNGGSGSNGGSGSNGGSGSNGGSGSNGGSGSNGGSGSNGGSGSNGGSGSNGGSGSNGGSGSNGGSGSNGGS
GSNGGSGSNGGSGSNGGSGSNGGSGSNGGSGSNGGSGSNGGGSGSNGGGSGSNGGGSGSNGGSGSNDGTGS
NTDSGLDPIIIPFNPNSGSGSNGGSGSNSDSGLNGPSGLNGAGSNGGSGSKGSGSKGSGSNGSFGSNSGSGSKGN
SF

>QLI74720.1

TPLAMGKAGSHPSVRMNPAADNLITGRATSSMGNIIAARFFARAEGEAGQANEACEENAKVSEEKVEEAKKKAEE
AEKKAQESEAKVQENEKKATEAEKVVEEGKKVVEEDEKKAQESKTEAQLAEKEAEEAEQRVKEAEVKVQEAEAKVK
ESEQMVEVAKKRAEDAEKQAEEFENKAKLDESNVQQSEKVAEDMKKAVEEGNKEVEEAKTEAENAKREAEEAEK
KAEEDKKKCEEGKEAPKGGEKEE

>QLI74722.1

ASIDKRLAGGEAAKISEIPSMVSIQNERGHHECGGTLLDSTTVLTAAHCLGSSHAVRAGTLNAHEGGVIANISSRKP
HPNYKKRASVYDTPENDIAIVKLLTPIETSAVIEYATLPEDGSNPVADSAAIAAGW

>QLI74723.1

ESIDGIKALATRLFKGHGGEFEFTLTANTERPSRWNPPKNDNYTVSSRNGKIHIEGTTLSALARGLRHYSVDSLQLDE
FLFAESHAIIPDKLPLPRQSLSRTSVVPWRYNLNTVTFSYSFVWYQWEDWEKLLDWAALRGTNIQLAWVGFEKIFL
DSFRDLGMTDEEIIPFFSGPAFQAWNRFGNTQGSWGGVGNLSSSWIDSQFELQKKIVARMVDLGITPVLPAFPGF
VPPAFSRVQPDANTTKAPRWTGLPDTNTRDTFLSPLDTSYARLQQAFISKQIEAFGNVTNIYTLDQFNEMPPTSNE
PSYLSQVSTYTYKALTAANPAAVWLLQGWLFLNSGLWTEERVTAYLGGPEGHNSMLVLDLYSESRPQWQRTNGY
FGRPWIWCQLHDFGGNMGMYGQISDITVQSMDALRTSPSLSGFGMTPEGYEGNEVVYQMLFDQAWTTTPIDT
SGYFYGYVVRRYAGVSQTNSLFQAWDILRQNIYDNKDRQVPCVGVGIYQNAPSLSGLVNRTGNWPPPTKVYYDP
ATLKKAHSLLIQAANEIPQLWEIPTFQLDIVDVTRQVMSNAFNTMYTDYVQIFNSQLSRQKRQIGNHGELQRRDDF
ATKGKQLLDFLTDLDRVLATNQHFRLDSWLDAAQYWAKQTGANDLVAFNARSQITTWIWESEALNDYAAKEWS
GLTRSYYRGRWSIFVDGLNKALETKKLDEAAIHNQIRTFEKLWQYRGFQSEEASTARAAIKDVLPGMMEKWQSVF
K

>QLI74729.1

HPLAGSTDIRGYSDTANTIQEVRNKLMRCLERRKPIGTKPNWSIVIASQYTSRDEILRALAECEGREQS

>QLI74735.1

QDSNGQKDKYIVTLKSGISTRDVESHMNWARGIHDASLGRRALDLPGIQKRYDFGDFHAYLGSFDKETLEKIMGNP
DVLGVEPDGITMPLALTTQQNPPWALSAMSSRTPGPQPYRYDDSAGQNTFAYVLDSGVHDKHVEFGGRVTPGW
SDYEQDFPGRPHGDVTGHGTMVAGIIASNTYGVAKKANIIAVQTDQTVSGLLGSIAWAVRDIQSQGRVGQAVINY
SGGLHTVSNSAGYPYQPGIAMAKSMDIAFNEGILCVIAAGNDGKVVEQSTTPYQGNSTTALVVGGINQQWDFMR
LSNHGPSVDILAPGENVITITRDSDTATTVQSGTSLAAPHVAGLALYLIAAEKIKTPTELRARILALATKDKITNVPANT
VNLLAFNGVQ

>QLI74740.1

AVDVAGQSKVPETVEVDLVFPHNDTYAPVALMPLVFAIQNFPTSRPLFLQIDFDIFHTPSWNTTVQQGIIFLNHAN
YSNNASTIHFVYDWTTRLNNTEGSWAMCWGVYSANCTDTGLAPGPLKLDPNYRRNLVHFSTKHGAQQPDLVAA
SKDGVCDETTGVIFNITEVKEVSWFNRHSVDHDVCPILAPEAPKPNPCLAKVNTNFLILELLIYILE

>QLI74745.1

APVSLLERAVPVGHEGPPYEVGGGGAHLPLEVNVPFEIDLLPDGERLPIENPETTSPEGLPPAEGELGNQIPGEHTPE
MPVPEGLPPAEGNGPQLPGVENPETSVSGGAEGLPPPASEAPQERPSVWKGKSLEDIKTFWLNGLPETRVANWE
NSPVHEIWNLLSREERFSLVFENPDAKKWPNLTPEQRLYLWQERPILKARDNQRLWALLTPEQRETHWKQDPKR
WQNLTPQDQETVVQSVRQNPIFEGLANEEIRRIVAKIDKLAPKDRLALSNSRIEKMKEAVIEALAKESKQLATEAGK
AATAEEEENSVGR

>QLI74754.1

APVDGQEGTKNAAGQNGVLHLPLVHFAKEVDVRNIEKRGFSTPLDYFKYDGKNAVTAVGIVLDVGTPPQKVILEPD
TGSSKFWVLGLKPGDTRGNEPSAYFDQKQSQSLQDMNKEDGAAYGSGERVTYNMVTDKVSFEGKSLDQMKFGV
GNLTAPHTSLGRLVGVMGLIPDPNVKDFILDKVLEKNLVKSRAFSLGVREKGKGALAFGGYDTKKFSGPLEKLPIQP
AKKTGTTSQYIVEVQSVLFNNGTGNSAVAMDKGDIENGKPLTMGIDSGAPALGISMALGRLLMERTGAKFKLDPR
VLEFACDVVDAKASFDFKMSDKTVISIPLSDFVRSRIDNDKTCQLAVQPFEGPADLWIGGHFLRRALVVYDPDNKN
VYVARGADCGSNLVAIDGKMPDAAVIGECKEEAPVSNAPTDEPPNTQPVLDEDDIRDA

>QLI74757.1

QAPAGPKKERVPLPALPTDEPLPAKDSLQIKLDRDLYAKGSMVFSSHLKFENPQTITASQMKRIAVDAYDEMRSMA
KKNGIPEGDMPRVMTTLIIDNELIFASSAKGPQTPYGKESQVQADLDACRGPGDNRHKHNGRCGEVMALHEYYQ
SHGVTKRLEKESGARIVAIAGTRNAKGQLEKVIYPPCGHKDDWGCDRLVQQLDVLDDAKVDKDKDTPAFRYKNMI
NNPKPELKPVDPTSLSKEKGKEKPKPGGSNDDEFEEPPKYDESTEKEGKTRRPQQPNRNKGKTGTQDNKPSQPKE
QDDGGDFEEPPKYEEPTSDDRKKGKTSQKDRNKSQTNAQKKKTGNRPRHFQG

>QLI74758.1

CPGRISCYSPPPGDVNISSFQLYPENACFDPKRCVAYLSVLFNASVAVYDPAKNDVTNVITFPGLTGNPALHATGVR
VDPLDRLSVVINAGAAFDTLGKDISGDSFLVKYDLKTRQTVFKANLTAVSHGLYGGFQDVEHNKEGDSFVLGTYGSS
IIRVSTDGKHIVPWFVGTTPTSNVGFTGLATSGHNLFVADQTDGQLYRFDTRRERGEPVRIPLGHGNETIGKDLDA
VYMPPRYNGRIILVSDLNLGTVVLRSRDASWNSAERLGSIPSPYHDQGGISVATVQIGDRIYSLNLFLGETNTVPDG
DDTNRTDNPLQDISAEVERLSLL

>QLI74760.1

APLPQNANGLTSLLSNIPIIGTFLGGAGGAAGSASPAGSTNNTGAGAGANPLATVQEHLPAPGSGIEGILSSGQILG
DHIKYTSNPPSALEPIPAAQNSPPREIHRQAHAALVPASQRPASS

>QLI74765.1

ASTLFWGGTIIAFDSENESLRVIRNGSLLVVDDRIAAVHDDSQSPYNSVPADTVKIDVTGQIITPGFIDTHRHSWQTA
YKTIGSNTTLLEYFSRYGEYAAASSYRPEDVYLGQLMGLYEALDGGVTTLLDHAHHTWSNATAYAGLNATVESGAR
VFWSYAFHNISSLNYTISQQIPNFREIAESGIFDNTTTELGIAFDSWGPNPDVAEAQQIVHLAHQYNVSVVTTHCLS
GPWGFNNLPEDVQRFDMLNGTIPVVFSHASFITAQGAELLRSTNQYISITPESEMHYGHTHPHSYMLQDQAALGV
DTHFTYSGDILTQARLWLQSVRYYFSSGVLKDWGLPSRNPMSADQAFKLATRAGGLALRRPDLGVIQVGAKADLV
VWNARKSLSMVGWADPVAAVILHANAGDVLDVMVDGNFVKREGEILAENFPDTRARFLSSARRIQQIWRETPYP
HFEGKSANGFPYKEPIIADTSRGDGDGYGDLYVN

>QLI74766.1

LEVTPGSRCAVECLDSPGGNDFSASDSTTTVDDISCKDLDYSTTDAGIKFRKCLDCLQHSKKVDKTESDLKWYIYNLR
YTLTTCMYAAPKAVKNGTVAAQCNVDKACMAMKEPLIEPGFNANPNTTWDYCTANNGAFMGPNLSPCISCLQA
TEGEAYLSNFLAALEAGCKQAPEDGAVLSLSGSVFTTSPINTTDPSANDQANQSSGSLSPSAIAGIAIGVFLIFLLAAAL
LVVHFRRERSWNEWEQSCYYSNFPPPAAYTEQPMSQAYRRYYTGNAFSEKAHPAQPNSSGEYYDRVEAELAAAA

RQNKQANSAPHSHVSSSPTIHNQDETLPASSVCRSRSFGHVSRARTPSPPAETHTHRRSNTPDSFAVQAYLNAAED
SARLAARKSIHPTSYAPEPQSKRTPAISTPYLPYMPKLRIPKIFINREGPRDVRQMQISPPLMTHDPRFHDIPMSGPV
AMSRDRPPPPLASVARQDGYIEVPLRSGKSTLYGY

>QLI74769.1

KGVVYDCEKTPQICLNTCWAIKCQKNSATLHGGGQTKANAKKYGDGNRKKWGYDKDPCKKKGWAWKGGNSP
DEYPYASSKEGGQSDFAKKVALRCVPGKEQQRQGRKVRGIATSKNGEQWNTKWDKISKLSEDWCGPKPKCQND
GHQFVSGGPNGPWTLAASPKVVRDIGSYSEDGDSAWVSSRDIPDDVELPEDFDEDDGEVEARDVDGEDDLEIED
DE

>QLI74771.1

KTVKETLRITWKEGAPNGQARELIYTNGQFPSPTLVWDEDDDIEVTVYNEMAKNVTVHWHGLDQKDTPWSDGT
PGLSQRPIQPGNKFVYRFKASPPGNHWYHSHEKMSLVDGLYGAIHIRPKGDRTGLWSQISQDKDDIKAMENAAY
DPEYLVVSDWSQYTSEEYWKISTDSGLLVFCLDSILVNGKGEVYCPGQKFLQAELAPGLVEDAFPPGTEVSDKGCFP
ADLDQVQGGPWNITKRPDLIPPRVQEGCVASRHENATIVVDPSKNNGWVSMHFVAAATIAQITFSVDSHEFWLY
EIDGNYVNPRKFVSAVMSAGETFSVMIKLDQEPGKYTMRIPNSGASQVLGGFAEMVYKGCEREEKAGKAYLSYGG
NPTSPDVEKNSFFPWQLDTDHMSPWPPNKPRPGNADEEHLLVLGRVGAPYNYTMNTKYLYPVDFQNDDPLLFY
PNATRDTENDGLVLRTKNGSWVDLILQVSTLPGDTSSFEHFMHKHGSKTWRIGFGTGVWNYTSVEEAIQERPQD
FNLETPGLRDTWITAFSIGGEAYWSVFRYFVDNPGPWLFHCHIELHLMGGMGIAILDGVDAWPEHIPEEYQVC

>QLI74775.1

APAELVARTGGACSINGNNDGKITCCNAGIPILGQLLCNIAVLGSNCNAGQSTYCCNSQSSGGLINVDVSCIKL

>QLI74776.1

RRCQNITIPISISSRNGVFSLATPANDVDVTDFVLNNFRAGVNYTDDVLQGYKTIQANYNLAATYCTPESGHGKSLQ
VLTHGVGINRYYWDFSYNNYNYSYVDRALAQGYSTLIYDRLGVGESSRGDPIQELQASLEVSALHQLTKLLRQSKVP
GIKTKFDKIFHVGHSFGSILTYGLTTKYPEDSDGIVLTGFSIDETFFAWFGYASNWVVANSNPALKQFPNGYVALGTA
QAFQAVAFAPSNFDLALPAAANSIAQPIPIGEILTIGSPQEVSNPFKGPVLVIDGDRDLIFCGGNCSSSTPSAAELVRP
KFTKVTDFEAVIVPQTGHLLNFEYSHPATYSAIINFLNRHV

>QLI74777.1

SSWFPGTKAVYNKWHQTELERWLSDNDIPYPKASDRKDLETLVQRHWNNIIVQPYRSWDTAQLISYLRERGQDF
QTATEESKDSLISQVKSNWYESEEAARQSWTNTKEWILDTWSESQLKAFCDEHGIPVPQPRHRDTLLQKARLGYEV
VAKKMGETAAYPGDWLYQTWGESDLKAWLDKYGIPVPQPSTRDKLVAAARRNSRLAYLKAQQEAASARASARA
AYANLTDMIIDAWGESQLKEFCDRNNIRVPQGTKENELRALVRKHRADILGDNVQAKASSGFGAATSNARNEYAR
ATDGASLAAEDAFNQATSKWSESRLKAYLDARGVPVPQSSDVDHLRALVRKYSRKAASGWQAWSFDDFSYSNLK
EYLVRNGDSIAKQAAKKKDASREELISAAVSAYSSASSAGGSQFASVTSYLSSATASVEKTAFDSWTRSELKAYLDNY
GVPVPQGSKLEELRALARQQSTYFKFGTSSPSGTVLAKIGDAAAQGWRWTLDQLRLGSEAAQEKVRDTKEEL

>QLI74786.1

APISPVPGLTELSAWETIFSDPNVNPEDYGLSQAEQRERYREDCHKVCGKVIQLIAKDGCDSDWKGYEKSCETCFYN
RFIDGSEFREKWGNGLKKALGICGITVPEEIPKEMDADGCLVEKPSPAKPDAEKTATETPSAENPDSEKPDTVKPDT
VKPDTVKPDTVKPDTVKPDTVKPDTVKPDTVKPDTVKPDTVKPDTVKPDTVKPDTVKPDTVKPDTEKPPAGQSNTESKEG
GVGQSTNPQEETSEYISEYNCHAACGGIITKGREGKCKELKEQFVPVCEKCAIRTGIWGIYKCGARAGARKCGVQV
NPQ

>QLI74805.1

GTIRQRDSGPSLGHARDLFLRRILADSPEYGNFRNHAPTNHDELKVGIIGGGVAGLYAAILLESLDIDYEILESSKRIGG
RVFTHRFDQKAWEASKPGQPNYYDYYDAGAMRFPGMDWMSRIIGNASNSLIPYINSRLKPGDQPVKLIPYVFQA
NNTFRLFNDRLVYNQDTPSARTFGVLGFEGGTIQNNSFADTSPGAVFSDAVSDLVHSLEVNFEHGFKKLMQYDQIS
VRQYLATKGYSSQQIDWIETVNDATTHYNTMSLSEAVIEEWIFSEAPLDSWFCVEGGMDRITRGMASIINKTVETG
KRVTAIKKAGTDALNVVINDTETRTYSHVINTVPLGAMQVMDMTDLNLDYRKKLAIRKIQYDASDKLSIKFKNRW
WEHLDSGSFQGGQSFSDLPIRRCVYPSYGINTPDAPGTMIASYTWAQDSARLGAYCSGDAKQNLVDVALRNLAA
MHNVTYDYLKSQYVDSHHWNWYLDENAVGAFALFGPGDLSTTMPDLMQPGAHGRLHFAGDALSSGHAWIIGA
VNSAYRTVAEVLAVEKMDGKLAELVDRWGVIEEIDIRWYANGTMSK

>QLI74812.1

AVMDLTPANFDKVVLKSGKPTLVEFFAPWCGHCKSLAPVYEELAVAFEHAKDKVQIAKVDADAERELGKRFGIQGF
PTLKYFDGKSDKPEEYKSGRDLESLTEFLTEKAGVKAKKKLEMPSEVVMLTDKSFAETVGSEKNVLVAFTAPWCGHC
KNLAPTWESLAADFVGEANVVIGKVDAEAPNSKAVATEQGVTSYPTIKWFQAGSKTGESYDGARSEDDFIKFINEK
AGTHRVVGGGVDRVAGTIAVLDALVAKFTGGAKLEDIVGEVKSAVEKFNDDAKYAYAKYYVRVFDKLSKSDNYVSK
ELSRLEGILEKGGLAPSKRDEIQSKTNVLRRFAEKAAEKAEELKDEL

>QLI74815.1

ADGGGHRGNTYAGPNNYIIECHNGRDKDVLIHRIKHRDGQVHHDFNSGIFYGVSASVPGLGGDEIRNMAGVKDV
WPVQVFRHEAKENSLKPRAAPKRQPYRRAVDTSWNHAMTQVDMLHSEGFYGTNITIAIIDSGTGRQVNYTHPAL
GGCFGRGCRVARGANFVRNEGKYGDPMDQNGHGTAVAGVLAGNDPRRNFVGVAPGATLAAYRVVDSKGYAR
EDDLIAGWLKAVEDGAQIIASSAGFDGSGWAQCPMAAVVARIAASGIPCIVGNGNDSKKGLFFSLDPSTGRNVLA
VNSFAHRMVSSAGGSRAVTAGMSWLSASGPTWELDIKPNVGVPGDEIPCPQIDGSYDNCSGTSFAGPQVAGMA
ALIAERREDFDPGHLMSLLMTTAAVQKDGHFIPVVHLAFNDTDHRAQSITIRVTNKARFEVTYQLSILPAVTIYARRL
PRDFKNPEYIQAPASIDMSKTFLKLVANQSDTITISAKDPKGLEADRLPVWSGWVAINSSDGKTLTVPYMGLAGSL
HKQQVLESDGVSLQGFNHDMHNADSNRGAKFSYTVKKGFTTLSASIIINLILGSRVYVEAVPLSPRKWMTDRLGKS
RGFPIKGYSPRALQRYTGIGLYDKEWDGQIQSGDYLPPGDYQLVVRALRVFGDPTMEADWDAAEPLPFQVMSGA
GQEACKAYQSGKGPKDALFRNLQECHQVHNKTAVDAPWIPRPQDSSKCDDDNPTEEDCGTYHYCKAHQERLDDI
ISPFRNKYEFGV
