# Supplementary material for: Telomere length de novo assembly of all 7 chromosomes and mitogenome sequencing of the model entomopathogenic fungus, Metarhizium brunneum, by means of a novel assembly pipeline
Source: BMC Genomics. 2021 Jan 28;22:87. doi: 10.1186/s12864-021-07390-y (PMC7842015; doi:10.1186/s12864-021-07390-y)
Supplement: Supplementary file 6 — Additional file 6. Metarhizium brunneum self-cluster. The 183 unique orthologous clusters that were formed between M. brunneum proteins. [file 12864_2021_7390_MOESM6_ESM.pdf]

>Mb|QLI63474.1

MAVLSGITDRLHQILTGHNTHDVEQATMEVWDKGVFLNELHRQGLVLATD

KDGNLSGDLAATRGLRRGTYKGTRLALTELYNLIQEAVFSQFDTQCYEPI

IPQRRRLAEKQAIYQWSKTADDGYPPHLHVPKGQQVTVANDDGPIFNQAE

LGLVSTISQALSFLVPQEISHQGTPYYGPTLADVEAYNREHPSPSTDIMD

GKNIGFLPDWFSDARFAQQHFSGVNPTTIAAAPAATVREYVAQAKKQGLT

HMVELLEAGKDLLIQDYSYFRKATGLGDNETFINVIYELDNPKDPKPTGR

TASRYGCASVIIFQLHDDGRLHPLAITIDYKGSLDNSVTIFNHRLHPDAL

GSVTEAQDWPWRYAKTCAQASDWARHEIAVHLVETHLVEEAIIVATNRTI

PEDHLLYELLSPHWFRTLSLNKAARDTLVPYVVARVAGLGPNSDPSKYTT

NRCFKFIQHAYKNFNFVDKYIPNDLKKRGFDVEGEAKSCKYRNYPYATDM

VFLWHIMRDFVKSVLGTKYKCAADIKNDPYLADWCLEIQTKGELASFPTV

TTVDELIDAVTMCIHIASPQHTAVNYLQDYYYSFVPSKPPALCTPLPTSL

AQLQSYTEKELTEALPIGTEGPKWKDWLLAAQLPELLSFKVDQKYNLLTY

AQSLYNVNKSRTQKENERGEHKVMKEAAAKFYSRLKDCEFVFELVSKFQT

PGTVEYKVLQPEFAAVSILI

>Mb|QLI63477.1

MVRMDRITLEKNYSWPSPTRRCSLLRSFVSAKESFAPPASSSPVNARHYD

SVPGSDPQNSRERLQSVLESGLGGSDLDSGDMTSRTVSPEADLEGATIRV

ADDEETTGRIRRSNQKRKNNTTRTTVSGEHLIQKKRAAVSRTQVFEYSRL

GAVREGVGGRIQFKICWKPTWGTLEDLWGTRSLKEAEELILNEYDEVTWD

EEMPC

>Mb|QLI63485.1

MSFGDSSFLPLRFNFSTTPCEGQLIAKFTTPAEVPNGEADIFWQCAGLAP

YCYQANITNGTSDPTMQLDREAQVGCINEVLRTTSVLVVKTMSTRTTTET

AVSVFTWTTTSFPRSQETSPTVTLPSQSWVTSGVATPSSTVMNKDPTGTD

TTAARAPAADSVRVEIPRSDMIGRTVRTTSPLPVTTDTVFDSGASKVTTP

LVTSAVTTFLTTTLLRTVTVSCAAGSATGKA

>Mb|QLI63583.1

MCGIQKSTERCTKCGTDISNVEERHWCQEALENGEFGKCKKRVDTTKVTF

KSHESCEGTNIEAEAGFPMGSVFDDFNPLEEFSANMARHTEAFCRNNRGG

RAGMEAWRELNRNATREREALREMARERSRRWRCQGWMA

>Mb|QLI63590.1

MAPITCKVVDANNRGRPGVYVVLECKDQLHRGIATLESLTDEDGGISLWF

PTPSPGRTDDVEPQIVDSSNIPRVSLTFFPHTVPSTCPGPFLSIHTDLYL

QGDECHGITLYLDPHPRLEHSPVPVASPLNRFAAAAVSTQEPQRDLSTPS

PLLLPPPVMAVVSLTNATRAITSMGFTAVIGQGVIYGWVIFGAAICGFFS

MVIVLVYNFRQE

>Mb|QLI63618.1

MTSIPPGGLLLVTGANGYIASVAIQVFLQRGYQVRGTVRSAASNIWMKTY

FGPKFELVEVPDIHSPGAFDEALKGVDGVAHMAMNMDMNPENQSIIDQTI

QSNLLLLETAAKEPTVKSVVITSSLAACAVPKTGVPYRIDSSTWNTEAME

QTAKPWDGKGNPRWHGIMLYGASKARGEQEAFAWVHKHKPPFSFNTVVPN

VNFGIAISPENMGYRSTSAVIDAVVKGYPAAPSILPPQWYVDVEDTALLH

LGALTLDDVNDERLFAFAGTYSWVQILEILHRRFPGQIMLKSVNEEAVDA

GDVDNKLSVWVLQRMGQKHGFTSLENTLIKAVNTIVEHKSKSVPKTRIDL

YYDSLSSE

>Mb|QLI63619.1

MVGLVFQSALAAVTMALAASAATPQCNTSADCLPGYICGPSDFAFSGTSS

NVCVKTGTCNNKPDPQFPQDGPKCGDSIFCNVGGSCGEGYYQSGGETVLT

QVCVNQATGQQCAASS

>Mb|QLI63742.1

MDVDWIDMDEAGVAWLGNMGECNSLLDEYSYQPNEAFGSTPNLFNTGDSG

AIPCIKEPGSEFGQEMDQFIRLPYPSLVSDLSLNHYQRTSISESSTCSNS

NDTSPPEEALPLKPIQEPCGQIHPTQFSNDQRNSSTSTAMDSTETHPTKD

RRPRTRRKRAARNTKVDDDDGCFKNSPAAGEKNHRLRGCHNQVEKNYRSR

LNNEFQLLLDALADCTSEKDMTSAGFADRGTKNQSKGSTLRLARRRLLAL

HTENRLLGSELRAIRHAWTEWQMAWSGNNPVTQEY

>Mb|QLI63761.1

MSDKDSTLFIGDSDPTCPAGVTPQPDGYVFGQSYQQTYQKFSCSRIDMYL

GNILPEWPIFTPDEVLAMRQKYLAGQGSDNDRLSMLVIIDLADTMNPIHS

QDIQELQFGLRYRRTIQTHALLALYWKRRGFHGFANEHITKAFALGSSSG

VQWKNSSLDDHFADIVALWCCCFIISEYPIHTDMNPVPPSLYKYLPLPSK
DDIVRKFGTSAGAFFELRVAYISPRLTSIPRPQLECLPLELVEVPTPLVT
LKERYINTHGLSINPQQSRFTSLESYIKMIQEFEPANRRWAFPTERFCLY
QLTAIYSDTNQRSICILLVERTLKFLDPVPDCLTTMDINSIWVLWWKLWG
YPCLPHST
>Mb|QLI63782.1
MSSNIVAESWTWYALVWFVVVCRIISQILLRGSIKKLRPDDGLILFAMMT
YTVLIVSINIVSDTASNLIDPEDHVVLTPESLRERRFGSKMVLIVEQMQI
STVWIVKTCLLIMYYRLTLGLAQNTAVKLVAVYIAIGWIVMEVLYFGVWC
RPFSGYWEVPPSNPQCATALHHLITNAVFNISSDLMIILIPIPLLLRTHW
PLKKKLILCVVFGLGGFTILSAVLNKYYSFTQPYGSQWTFWYIRESSTAI
IVANLPLTWTLIRRVFSVGSFRGGSGRRSSAAMRSTQTENERKHAAYNPH
SAVREPSSSNQSQEVDGTL
>Mb|QLI63802.1
MAQTAGVCSFLGRPWTLEDVISIPSDSESDTDSDVEVVEQDVPRVHLVHN
QHPDDSLPSISEIVASLINVRHDPTEAIGSSSEDLCLPDEWESPNSLTSN
SSREVISPSMALESPGVTADSNMLVSPPIQQHDDGASVVWPTPIRRPCVV
SAASECCSVQSTTALPPRDEALHHEKTDPPSRSATSLVQPSQSQNCSALF
SNRFVQAHSINIGHGRMGEVVPRSDNELPSVARPASVKTPPSGCSHPVSH
RGAVMEAIRKHQAPDDILPLGSCPSSSPTGQGRDDERQTSIDAGGTESQP
DEPCEETEKRPTSRRGSGKPYNLRRLPPKRQLTAEQQDGGINEKLMPQRK
RRRLVFRQPKKRNSQQRSDRRSDITRNRETRLLRSTTKSNDNESVANKKR
PVRSGVASHEAWPLGQPVLRCTRENGTAMFQLHFTSDILWNTLVAQNDPA
PKGHQTPNKLEDRRPKAGKVKYPSVCRDVYRVDCLLARWRRHTFLVKWSD
ATTTWEPRKHIIDQGMLNSFEASWRGFDAGVDVLKARQRAGRRQHLLRWH
GRPSKEDTWVSDEFLSPQLMERIEANERNGPQF
>Mb|QLI63803.1
MEWVEKQLKWLQEMDPHNKLLVVWCRYPDLRTGHDPFDLRSRFWMCLDSS
ATPSFEKLIPIKIKEERLRMAGRMTVFACGDAYFTALMRDGTIKILKDKP
VKFLESEYQSRSLVRVSSVEGPRRGNPRKRKKLRGHTERLELQNMANNRQ
TTYSPVSEGSFYSVKL

>Mb|QLI63826.1

MESKYLEGKARVPLSSLKSEDLDVDSSSFPINIIEHLRDPGHAISVRITP

RTRDDILRDLSYSPEQLYATLELESSPLINDHQVFYAAKDFDLDRAKAAL

GRDHICNVRLYCIPVQPRKICDGAVFQNVRYYMAGSWKKNRVSVAQAWMD

KLSGSKRKILGSLLKHPDIIAAMDSMLCFPGYWDGLQLGNWAKHLAARIN

PLIINYWVHIKNVALKIMAGHEDKLHLFDANTVAILQYRAPSWNSQDRAQ

ICDLFEDGTLFPGILSKSARDRIKDNILELPDSIPSIQTFHENMRYLTIG

AKILEKHIENRPSESRADTIEPMCSSLIENLKKDWNSRGASMEVGHGRMI

QVNEPTADAAVIQAFLAALRYFPYLSPEAPLRDFDKRVWMAGNFDNSVLS

RLCVTLKDLGFSNANIEKGRLHPIENRHISYTQPKRRRDWRSGKPCLAGY

HILLQSSFFPQLFAEPLDDKVPPELRVQSDILQAFFGGKPKVPSLASAAS

LRAEMDVDTTEPNEAWRSEAEINEDTRLGEVLKLWPRGNNETRPKKKGKD

KMPRGIARSATSRDKPRVRGKGPKFTFRQQNVPDIPATRASDLYVGPPVD

PAGIPTQALAHLTPPTKNPNRAKEKKRKHDEVYEAAEVPEKRNRRKDQVG

GIQAVGEQAGAEQVTQIQPAEEPGRAVVDDLASTSQDVRWEMKAPLEAPV

ATPALPPSMNSQNSTPVVQRGPEPLSKPAQHVHPENNPQIPEINYDEAFS

PPHLEQEAELFAIGD

>Mb|QLI63869.1

MACNNALDNPDSYAVAWITALPVERAAAEAMLDEEHATPAGFTRHQTDEN

VYTWGRVGEHNIVIASLAAGVYGTTSAATTASSLLASLPSIRLGLFVGIA

GGIARPDESHDIRLGDIVVSQPDGTTGGVCQYDLIKAKSGDKRERKGFLG

RPPTVLLNALTKIQAVHERETPKIPCFLQEMLKKNPKMGKRSKKNPGYAH

QGADNDHLFKASCDHVPGPDCRGCDTAGEFQRDPRDTTDPEIHYGTIASG

NTLVKDAAARDRIVADVGEDCICFEMEAAGLMNHFPCLVIRGICDYADSH

KNDRWQRYASATAAAYAKELLAYVPAAEVQETKRALEVLQLVQQQIDGVQ

QTTIATKAATDSIRSDLRTDKIERWLRPPDPSTNANHARKLRHEGTGAWL

LENPIFQQWHSGARRHLWLKGLAGCGKTVLSATVLDNLTKGNDRLILSFF

FDFSDTTKQTLDGMLRSLAFQLYQGGAGSVGLLEASFQAHQDGRHQPATK

TLEDVVCKMLAVQKKGSIVLDALDESTTRDELLRWMKDIISRPELSDVQL

ICTGRPEPDFGGNIPSLIGEGNCLALDKESVNADIRSYVAAQLSQRSGFR

EKRLSQDLLERIRRKVGDDADGMFRWAACQLDSLAGCPSPKAIKTALYHL

PQDLKETYERMVRSIPKDCRNSALRLLQFLVHTHRPLTVPEAVEIIATDI
EEDPPCFDVDGRVFGEEQVLQHCPSLISIVLAYSHGRPTKELHLAHFSVK
EYLLGENQFNITTASISITRTCLTYLTDISGSHIEIKQDFPMARYAAEVW
TGHAALAQASEDIVRATVRFLEEEATFQRWARLYQADRDWDDDPGPPRGS
RLYYACCHGLVAPARDLLDKGADVNAQGGRFGNALQAASQDGHQEIVQLL
LDKGADVNAQGGRFGNALYAASFRGHFEIVQLLLDKGADVNAQGGRYGTA
LHAASWRGDQEIVQLLLDKGADVNAQGGKYGNALYAASSRGHLEIVQLLL
DKGADVNAQGGFFGNALSAASESGRQEIVQLLLDKGADVNEQGGLYGNAL
YAASSQGHLEIVQLLLDKGADVNAQGGRYGTALHAALGRGDQEIVQLLLD
KGADVNAQGGRYGTALHAASWRGDQEIVQLLLDNGADVNAQGGDYRTALY
AASDEGHLEIVQLLLDKGADVNAQGGRYGTALHAASSSGHLEIVHLLLDK
GADVNAQGGHYGNALQAASSSGHLEIVQLLLDKGVDVSAQGGEYGNALQA
ASSIGHLEIVQLLLDKGADVNAQGGEYGNALQAASSGGYFEIVQLLLDKG
AGEYVNALCAASESGRQEIVQLLLDKGADVNAQGGEYGNALEAASQEGHQ
EIVKLLQERALSNKSGEETSSRVPRTF
>Mb|QLI63886.1
MKILAHVSSDDGRYTEHWAAGRRTISNLRGFASVQLHKIVDYGAGEDGQN
TATKVENGLSTANPLLYQGGDSRPDGSGGALAAVFEKLGYGFENATWRNV
YLTMTPELRFKDPAAPKKPFSPPLNRLGSAERCLNGVKIGIDGPEATEED
LTIHIRDSEGGSKWTIQVRNGALTYRRLEREHEIVV
>Mb|QLI63897.1
MALTWTFWTFAILSVYLRLYVGLKCRHRVALDDWVMLLALACHTLFQTFL
TLACVTGLGYPVDTMTLEEIIAMSKWQWGTVPVNILANTISRVSIAIMLV
QIFGVHKWFRRAIAAVTATLTTFGLVNFIYVFFQTKPFQANWDVRIKPER
RLQPFAHYILIFTQCVLFALSDFMYAFFPVILIWSLQLATRKKVGLIFIM
TGSFITMMAAIGRVVIVRDTFTVSADRSLDSDRIFVMFGITSLLASVEGS
LVIILGSLPKMKAATKLKSFEAISSSFSSLIGRLRTGGQSDAPSSATNYG
RAESDIELCASHGHGMVCCPACKHHCSARAVDMDSMASALPRGWENEFQA
VEGHAVIYGPEAKNYV
>Mb|QLI63927.1
MQPNDLLHIRSSAAGLPGPYLQENMAPYEILDKIGEAKPRTILLIQGRTE

QGDGLKGAMRFPYRKFAIALQRQGCNLVVMCDMHKQPGNAIPRIIAGPVP
GNYCYHLVQQPPATMTDLAYRVYCDVFALFSDIVLISVADFGGLERVLSF
VCSWVLRRQLQKPKLRTHFVVATDKYCLKDIQFELLATMMADQWTQSVAS
VKRTISDYTELSVINESSASPGLVVKLFGLRNHRQAEGLHFTGSDTRILL
RAAIAHYTAKPMETFNLVAASRPSWPVPEELGHHIGEFLAACPPEPVDHY
PIIASALVMNAFHPGLHCE
>Mb|QLI63938.1
MARRVPLDRPEEAIRVVKEDGGVILTGFTSAGQVDKVNQDTEEIMTRRKS
DEAFKAIYNGRIYCGHLYGLSETAREEWALDPNIQSIVNHFLRTVNPPEV
DKNSMRARSTNAILSQANSILTLPGGAGQALHRDDSIWQKVHPSQEESGY
CLGSDLGIALLVPGVQTTRANGATLFVPGSHLWGDAREATDDQVRAVEMQ
PGEAFLFLGSALHGGGANSTQIPRLLHAIFFCRSWVRPEANEFAWWTEEE
VQKWSVDAQRLAGYVTDKMLGICDDGDPIDALRRHRPA
>Mb|QLI63981.1
MSSITRTVEVGPNPTEWYGTMIVKGIKYSDGSQVAIDKFLGIAFTSPAAV
DKDAFNVSVSPWQDVKAEVINEPIDASTCLVTAKLNFEKSYKFNASDTIT
IGVEADLRDHPEPYTESVRLGADAVPNVNGTVVITTADAPNPALESREVT
LSFAQGSRNFTATALPGKTTSIQIPYGKYVVSSSELTTADETVVATSEVS
PSSLPVDADQEVAVKVTFGSVQTYGAIDVSIGDIPSLESEMVHVYLTRDG
SQFTDFYSKDNETTSLRRLPDSGNVQVSIHAITLNNVQYGFSTKSLDLAA
QLFTVTFGQSDVTASPVDTTGFVELPIVMQSDGVTSDQDVSLRMASDELI
YTQKVPAKAGTTPFSVPVAPGEYQLQDSSFVVSGTVYAVNGPSTLTVAKD
GSTQLNLELVKGANLHVKGFPDVLAFGGLADMTPTNADDFVAARASSVFK
YAGNDGAGDSGSFLTDDPATTRTIQLARDIEAKLGDGSSVLPIMISYTVN
LSLGDIYTKLQDTTGLTHSFANLILSLNICNTFIDQDHPVPAGFIVNADF
LGEGQKENLVDYAMPVREPLQGALDHWSVKATIPDSINDTFAGYTLAVNW
LVRTVAPAATFGWQVNIWGGGTSTWIYDSGPGDQQPLDQAKQTADYIKKL
GTYASGDYTPDFLAVDRYEADDWTVRAYVPGYFYGPYEWRRYYDFVRYLG
LELQVPVAPWQIPTSHAPLVMDAVASDFDSQHWGTGGSYLLGDADVGSDI
GNINPTVLDLELNATLIKAKTAREVWQRGEPFDLTQPAYQDFPVRGIFAV
LLGGGSTTGIISTVGNAGEWVAGRLRAYADNPIPLDKYPVEASGSGGARV

GAIARRRVLRK

>Mb|QLI63982.1

MKTFATLSFGLALAASAHPTVSTRADEQDYNATYLGQILLDRLEKIAINE

QSPPRFVSPQAWQHTCPDVLLSFQGEQVQFWHEASIHPNDTISKRAFYIP

HVETGDINPGPGDLKLSIAKSTMNSKMESKGWTVGARVAGTFGAKDGPSA

NVELSASYSDTTTTTKMQTVTTSHDASCKPGYECRLETWTFHIAVRARTR

LLPYYQTWTAGGGHGAKTETCQMKQSVATCEQFRQRLREWCDYSPEVNNH

AVAWDAWHGGVWPPAPLDRTEDIELKLPIAEANGNQIMSRIVLVSEPIMR

KKSGDAPEAVKEGTQQTIKDAMERGTRFQVLDSSSSSS

>Mb|QLI63992.1

MSGLDLLARYLRDGLFDDADFANSFVQDQESDSNWALRRSYSAQGHTIRE

HFNIQLFDQDDIPLVKCSLKLFIQSPDAPQDDTREKIFKPRSWFGPDLDI

EVIEQQVDSQHRPTTTTLLPASTSTNSVSQGLTEESVLPSTIPGPEPAPK

TLTGKVAIKKDRSLEHRMLTTAKAFVRGIQILVSKPQGNQAKRLRTIRKC

FDTSARNEHPIETGQLMRLWMRLKSDKTPIDQICARTTSKTKDLELSVAS

IKNVGNYIAGITELENARKARDNGSKYLCGRALDRILEAISRLVEYYGSV

AITVFTFLEVGGFGVARLGNTAIKGADNERYFYDGLVALIDLSLRRFVAT

ARETEADAISDAIPVLNPVAFHWWYEGEKGRYEDLCQSLKLPAFANATTN

IYTERLPSTILDEFSAQSQQARSHDGSHDNNNNVDYANSESGSNSPDKST

DFILHNAQRLAKYMSCNKETYLQSFEGDKEKHGMAKLASRILNGTSSQEE

LLTAPTDVVYMIVEMLLLDSRSKQGVKRKRASGNEQDKRSDGSSFHHDSR

QPVSPVDLLDQNTSGQAAPLTGGMDISQGLPPLDPQFPPGFFDIDFEGMD

TNDRLPQFDSQLSLDWANLMAMDSFGAQGSSSGGVFLQV

>Mb|QLI64015.1

MSELTAAERVLHDQANLLPRRKLIPVLCVLAIPSMISFIDQNGISTALPT

IAAELNAKDTISWAGTSSLIANTTFTMLYGRLSDIFGRKNIYVTAVALLA

LADLMCGMSRNVTMFYIFRGVAGIGGGGIVNLSMIIVSDVVTLEERGKYQ

GIISSMVGVGSATGPFIAAAFVSRYTWRGFFYLLAPLSALSCLVAIIFLP

SKPPTAGFTESMKKVDWLGLFTSSVAVIFLLIPISGGGAYFPWDSPMVIA

MLAIGTLFFIAFVIIEWKVAALPMMPISMFKNPVIVVILIQTFLLGLVYQ

SFVYYVPLYLQNARQFSIMTSALIFCPTVGIQSVSGILAGYWIARYKRYG

IVIKCGFGLWLLGAGLTLIYDRQTNPGAIVAPLIILGIGVGLVLQPTLVA

LQAHSPKSRRAVIISNRNFNRCSGGAAGLAISAAVLQVVLRNSLPPEYAY

LGHSSFDLPDVPGGIPSSVLDAYMSASYAVFVLQVPLVGICFLGTFLIKD

KGLAFADEPKVQEDQGVGETDLESGPSSSGSSVMEKGTNNVASTGRNTVK

RGESGVRVTVSSKDMTDKRP

>Mb|QLI64041.1

MRFTHGLALLGLAVMAAAYSEVGIYERFYFYYAYKLDAMTVGEKKIATGC

GANCNFDEFINFVEENKGANLRKVSTEHFPDIERTAELYANSGAATQVRE

GRVFNGVGNYGELFEKVRTRLEGLQHKTNYEYDKLSGEELATKVQEVKTK

LMESLKQVWQGRVRASLSSFKPYEGFKIVETADGKAVDFKATLEAKENKG

AITEEHLKKLWDEHVENFKGADAVKEFKPLKDWHPAIVEENGIISMRSSL

EGAVESGWIPKDSKWLQRNWKKSYDIGHSRNVDSVGDTYKALETKLCPGA

PSRRDFGLLRKRQALLCIRAAEIEKTMDIKAGDVDNGGKPPGEKPPNPGE

EGKPPGEKNIPASEEQVKRANSVSENSFEKSAEKRLGKVPKSFEEKYGTN

SYKELRTKALEYKPLEPNSPKIRSGGTGLSKVGKALGAVGGVLYVKGIID

AFRDHVSPLHFFAAVTSIVPFLGCAVETGASVEDKVPAGFIVAEGVLCNV

ADALLLTPFWPVGIALHLVNAVVKFFRPPPTPPTVLELAEKRDAVWAHAL

EDIYRYTYSAYETQQKSSFAFKTENALFFDSLNVLSESADTIGSLNATGS

AELFDPKLFPDQSAVPDAAELETRSKEAITNLEEEEWGAVLRRQRNTLLR

TAIATANGSLFELDKAAVDVNDKFIKYIESPEFKETYPNSGWQGEKILAF

FKNWATSIRKHPELGVAGPAGLLLAIPAGEDKDFEYPDIHTFINKRVEET

TEYVRSKPPVLPRALDVSFVLGQSKALLNLPKDTLSPLSFLEVEVSSASE

KPLSPLYLQTLVVRHTREIVDVMYGRMKESDLKDTDFAVQDPELLKNFRL

LIAMKLGAVHDADRVAEIQRRHPSGYDPELLNHRGLVRAITPYIPVVLEN

PTDPVPVSHYLTMVSGLEKTLVDEALQARVAKYVETQDEATKSFWEEQKK

NFNSLKAIAEKITGVLARPPSMPGDFLAKHKPTCVSKGQDETMCQYLVEA

CYFAENMGDEFVNQCLDAAVSLEKTREHKVDLCAKFPSYNSCELAIHDCD

KTYRNSMYTVLFECAARAAPDREAEYAKMAAE

>Mb|QLI64049.1

MSTIESIVAFSRSHVSSSGTLSCRSPLFRLLVTALIFFTLSTLFIFATGP

VADRSALWLPSTRTNGQNGPSAIDREHYPPVVPTADNDAGNSTLGFSSIY

FIYLPSRYDRLDAMSLQSYLSGVDLTEYPAVGPQLIKDVGMPPTRKPGKL
RTSEQGCWRAHANIWSTMLRQKLPPLLIVESDATWDINIRKIMPNLNNHF
RQFLREINSTQLHNPAWPAEAVARNESTRTSDDDPWLSSHWDILSFGQCH
ETDENNDIRLIYNDPFVPLGKDYQGRALTHERVIRRSGGITCTTAYAVSQ
TGAAKLLLRTAVNLDMPVDMVMQEMIVAGDLVSYSVMPPIMAQWQYAEGI
GMEERGANSNINKAGIFSGLFSLFARKKAWENVKASGSVWTTKTYHEDVA
FDEMSLQGAWKRVFGTAN
>Mb|QLI64094.1
METETSLTAPPNTPEKNPYEGVCQRKVDAQSRNLDPTSCKSFYLAHEIIE
ISSESDGNSDEATVPNSPRVIEEDINEGDQPSENASRADAAEDEVNDNTN
KAKNGSIVYSNGLLATECLPNVDHARFANTAEESSSKYSIITMRLDTPAS
EKYGAVGQKFGEGGYVGNREGSSECSNGKLKRAYDRYESPPDDVPEHDLL
SKRRRTAKRL
>Mb|QLI64201.1
MATYAVENAIPHGGAWGRSAPITPVPIPACVAQNAEPESDACWFWAGVPP
ALQDLKFTLPLQNTIFSVMHSTGKPSYWIEVEDWHTAGEPFRIVDKLPAG
HLPASPTVAQRRADITATPGHPLDELRRLLCHEPRGHGGMYGGFITPPDD
SGAHFGVLFWHRNGFSTACGHGTIALGYWAVFHRIVVVPDADGVVDVVVD
VPSGRVVARMAIEKGKPVHADFINVASFQLTGPLYLTLPSCGIDVLVDLA
FGGAVYASLDAAQFDLEIEPRSAQRFVQLAREVEAVLGTRARYGSYDLYG
VIFFSEEENGPNATHAIYQRNVTIFADGEIDRSPCGSGTCARLALLFAQG
KLGPGSSRLVHRSIIASTFEADIVSAVASPVDGFPACIPRVRGRANLVGQ
TRFFIDPDDEVFPGFLLL
>Mb|QLI64352.1
MKQADQRAGGNAPGFLTRLAILGIGIAPTLVPSVAASPAAFHYSRQANPA
NATGEFPKPDDPFHFIPCVSNQTIEGIPLLNDTNPQETWRKRFDPNPNNW
KWGAAPGNATTPGSSNSTQGRGLHLCGYLDVPMDYLNKSDPRIHRLAVAK
YQASGLEGKGARTIVMNPGGPGGSGVPFGLHGGRYSQILSNGVHDFLGFD
PRGVGLSQPAIDCYPAALQDRWNLAAGKDLRESPSPRQQLELVNAVNDAK
FKACFEELGDVPRFVSTASVARDVDAIRAALGEPELTGYMVSYGTTLGQI
YANMFPDKAGRLILDGVDYSREHRVTGGYASTAIHSTTDAWNEGFLGECV

AAGPSSCALAKPTNGSGVTLDSLKARMAKLLDGLAERPIPAYDEEKGPVI

ITYSLIIAVISSALYQPRYWPEVAQALADLESGNTTIAASMAHGQYVYQP

QAPEKPLGLASELLPMVVCGDASADGPPASGLDFWESLWRNLTEKSFLSG

GGNFATVFPCQNYNKYWPQGAALYQGDLNNKLKHPVLLIAETHDPVTPLR

NARELHKEMGMENARLVVHHGYGHGSSPDPSDCTNAIIRGYLLNGTIPDK

RETDCYANGKPYRNSTRTNHKRSLPMVGNVPMRLPL

>Mb|QLI64419.1

MKKQPASLNEVSRVDLDRRFDLAHVIDALTWGVSAGVSIYYTVRAPRDGP

TIGARIWDQNFQYETGLTIDPNMVIAYWVGMHAALFVHMLANIFAASNDI

SYITISNHFSINNLLHVVFLVLFVRSAFGWAECILILNFVNLSVLYFKHS

KLSHFTRISVICGPLAWNFIAILWNWAIAVTAGIGYNDTIEGFGLFFVWA

ILGYGVFSLLVFQDLAMAFLLAYLCASIAVAQHFLQRSDRMWIPPVVIAS

LLVVMMCAVAARCLWNLRAVRRKKVNESVYAEFDERKESC

>Mb|QLI64453.1

MEPCVTWKQVEALRDETQLVQLLPHLKEIDQKIFCKHLEKLWVEGGSGNL

VDILLRWGTTPESLPGSLPHKYNVFRELLTGNHEQVEKEAWLRRCRKRER

ENNAKKKGNNELNDDTETEDDVEATHDVEAAHDVEAAHNVEAADNAAAKF

DPEEVASKIVQFCPNLLKYEYKSTQLTVIDTASRESGDIRYFVRKTRRKR

SPLDTVVHRSDLSSTWDLVGLIDDMLALTDEVQIDKTMVDHAIENEKPEM

TRIFIQRRPESVTQGVVTDAIHRKDDEMVSDLLKNCKNLFYGNESLHLAI

TVGNTGVIDKILERCPELAIEVDKKGEPPMSYISKITDKTTREELRQSLV

PFVVRHVGTRPPSPHNSDLGPPSPPISVIEHIRSLLGNPQDNSKDPPLER

TESSQLIGSSQPSTSKSRFGTPTLRVDFESTLRYVDIPVPDLPPASRDSR

MITIRSEVEEILDWLRNKNGVTGIHELRVSDSLYIPHSEEVIEKCLTGFD

IEVLDWKRVDLSIEPLFSGSRLVCPNLKKLTLYASGWIALQYWTSQEVQK

SLRKFPKLREVQVIIEQDLIGYALSEEYEERCRSDFDKTLAALGQPQDFT

FNVLRDIWAVSPAKEAELHGPRKCTAVEVTRLESFLSAYQILQRQFLDEN

WRAQLLKDATPSYMPHIKVAIIDTGVDPGSIQCSHIQGSSFVSSGSGESP

WWFSQHPHGTQMAKIITELDPFCQLLVAKVGDYSTDMTGERVKKAFGWAV

MLGADIISISAAFFNKIDGLDLEISRAMRAGTVVIASTAGEGYNQANAFP

ADYDLVLKIAATTDMGKEAQESVKRNADFLFPGQNLVAETTFLGSDHWPH

EVSGASVATAIAAGVASLVLACHRLALSTRTAKDRWERHNHFKREIVIKA

FKEMWDEDARYVRPWIFFHERDTQRSWGEAGSILAWIRDKYEELDRS

>Mb|QLI64487.1

MSAEKPESLPKDMTPNKIEAQTAVSSDGESLQDHNGYLVDAHFASRNIQT

TPDGNTILIPQPSKDPNDPLNWTWGKKHLFLFIIAATSLLPDYGSATGAV

TLLPQAEIWHMDPDVVNHSQVGNVFMIGAGGIVAICLGAYFGRLPVMFWF

MILALGTALWCTAAETFESFMAARILNGFFSPVAEAGGLMFIKDMFFFHE

HARKINIWASFIVLSPYFGPLFSAFIIDTQKWQWAFGLYTIMTGICVAAI

FALLDETYYDRKLPPSEQPQRRSRVLRVIGVEQWHTRHLRNTFTEAMSRP

FIVLAKLPVLISNTYYLLAFAWVVGINTTLAIFLTPLYKFGPKQIGFFYF

TPIVAAILGEIAGHWLHDLVAKWYQRRNNGRLEPEARLAVVGISTPFLCS

GLILLGFSLERAYHYMLAALGWGLYVFGIMITTVGVNAYNLDAYPTASGE

VAGWLNLSRVAGGFIISYFQVRWAEDMGAIKSFGIQAAICFGALFLIVLL

QLYGKQLREWSGQPHFKTI

>Mb|QLI64570.1

MKASLPLVFSLITRLAFANSPITFQNTSNTILRVDTGSYGPAIEEAHYFY

QDWPVGFAVSSKGRIFVSYYPGNVSFTLGEVVNSTAEKAYLPQYNVPSAN

STQTIDGTLFGSQNSTGFISVQALYVTPKTANRSETLWVLDTGRPNDASQ

MAYGLPGGAKLVAVNLDNDTIVRTYLFPSTVSYPDSSVNDMRFDFRENIT

TSGQGVAYLSDESPEGRNGIIVLDLGTGKSWRHLDRHPAGLSGYGVVPSY

QGMPFYQETPSGPFTHLPQGLDGIQLDLTGSTLFFSSMTSDYLFSIETKY

LLDHTSPACIQAANSAVRNLGQRGGNGNGFEGDSNGFIYQAMPEQNAVYA

YDPTTLRVAPFIRDPRIIWPDGLSVSEDGYIYIIINQLPYQPMWNNGTDL

RQKPGALLRAKLPNNGAKVKTLF

>Mb|QLI64587.1

MKISITHLALLGLSCAQLRISGRAPQGPEPPSKQILDAIKDGMKDWGPAG

SLKPPKDGPKAPIKNMIGGLPELHNPKPGSSDWREDTLKGPRKKPGKTYP

NSNPCSKRDVACDYGPSSSNSPSDKGKVKGKGKVGASVLNIQGGKFIDFF

SELDPEAFGPLIKAIEDGEITVADIEMALKRAISETMKQRWADFESAEKA

AETLKNIAFDIFATARYATPPGFWHDVVKNLPPIAKEIHNAKTPEEKLEI

ANKNINEVVTIWSYTPVGAFNENIMKEVAKGTPTTTAVAVSVNKLWSYTP

LGWLINQIAPIDSWIRGDDDQVAEPKPKKRPDPPKKNLKPPVATWGKCKC

ILSRPPARGDKCANTCRAVRALGGWK

>Mb|QLI64638.1

MVWYAYAKNSKTDDNWRYLIIAPNFKILDQFYEEARKIVGVNTFWRVSDD

FYVYDRSEFDLGKCTTQKKQLEQFKNKLIFTLLNDLGGRVVPTFNNGSIH

GGATD

>Mb|QLI64677.1

MAKTIWDYIREACEQCNISIPSDGLPFTNNQDVKSSIIDAEGRNRVMLFP

GAFNPAHEGHLQLLQSVLNDMKKHLDIRGVVIFPHDDEQIRDKTREEPAD

LGLDKSKRSALWRNAAGFPADNAWIFTESRSALTRFQKQLQGNLRKENVN

LTFLLLVGSDWISTRAVYDPGQWNCSETITSDVSRPVDFRCEYTLRQIPG

CFDWVQMVFAESTTYFPWDSFQTPYGPHMVEGVSLWSTSTVSPPIRRYYL

VPCLRACSLGTPCPSSTSIRQRIFEACAERLPIDEAVLATALSPDILLGY

IRGNVPSFDGEEARGEESQSVL

>Mb|QLI64685.1

MPKLLDFRCAVITGGGGGGIGKAMARHLVSKGKTVLLAGRTESNLQSAAR

DVGAAGYYLLDVGKTADISPFVDRITREHPELDCLINNAGVQRPLDILQD

GDFVAKADQEIDINVRGPMHLTLGAAAAPADQARRRVAALLEHEPAHAAA

AGVRVVEIAPPTVATDLHREREDPDDNKKEKNARALGVDEFMGEVARKLE

RGDEMISAGGGSDIVEKWYGMYGALYDETASKK

>Mb|QLI64716.1

MVFYAYAKNSNDDWSYRYVIVAPNFNILDEWYYEVKDKVADNVFWRVSNE

FYVFDATKLKLGRSTAPGHEAPKFMNKLIFQLLSDNEGRNISTFINGHLS

GGTAE

>Mb|QLI64728.1

MPQKKLTSVIDIVDKLVDVSIAIKNRGKLKDPEEARVGDAFALLAAGRPP

PGCPGEANKSRYLEFLLRVKQFMGPAGVVISAAGLGVSAVAGMRDRLRVD

LPVKMKEREREFAKTELETIACIFSAKNHQPSQMAAGASQRGEPASLSST

STVRRIEAAATAASQFTGDAYELGWEDVREIVNSGQEVGRVYLIDTYAEA

VHSFVIIPISPELTNHFAMQRAKI

>Mb|QLI64733.1

MSYFTFNMSASTQGPSTPKNPQRSSVSNSAVPQLDHDSSTLPINSSATML

CEPTVSGQTALNPGAQWSGFNSSLLQVDGAFSGSALPPYDQSTLHAALTT

NDDLRWMLIGMNERMANLEQTATAGNRTMDQMGNHVSDMLSRLAVIEGMI

KVVEDLKQSLRDFTRGLVPHILGVNLEEDLEKQG

>Mb|QLI64738.1

MKHIELILATFAGLAFAAPQRVLTAEEWVGDRVITQQRVLEEFRGRNIIC

DDEEENNRINCEIGFVSIGIEEKSHDGLSPLCEKKGGCKYCRIESGFRNA

QHFMCAYKSSNKDEPIFFPVE

>Mb|QLI64829.1

MSFAEKSTALQNVMKNLDIGDACVLRKAFSYSPILKQCYESRADPVGYLS

RTFRYPLTLLSAMFDSGCVITGPRALGFFLPSSSEDSAWTFFVPGYKESV

LDMVNVLEICGVSWQLDAAEVARSLRPLGTASISSADLECLNSRAESLEP

AAAENLLGTELYGRLKAYKEMNCGNRLNSDACHLSDLEKPTLGIASEESA

FPHSQDTSSAREALSVLQGYIQTSSGSQGVELIIGSSYSGINSCMSFIKD

FYASHVQCFISGWCAGHMYYTQSKDKHSSMWRPWPGQKYKNAHLDVKKYR

HQGFTFHRAKRGGPVTRSLRDSQSFLLDYGALYRSFIRPSHHALLDAWLA

ERRDNIDGISWTEFDGRIFSVHDTFESCYRQSRMTFASHSVDLPLNRLRR

LSNLVALNLTEPDALRAESFRSSIGPPAVGQKWQLGALARTGKVFNNLRD

ATPWSWAL

>Mb|QLI64830.1

MRLLLTSALATTVSATGLILPLYIYPAATSDDGAANWAPVFNAAASNRDL

SWLTVVNPHNGPGDTHLPGNNDINYIQGVTKLNTHPNIKPIGYVRTNYAQ

FSLDQVKQDVAAWKGWDTYSASNISVQGIFFDESAPNAPYMSALVGYTRA

AFGRPITVTCNFGKAVADEFYDICDVVVAFESCLNCPGLPQYKDAATIQA

NIPANRMGKAAVILNYFTGTAFDGTFADATLVHRYFQTARNMGLAWAYFC

SQDYNNLLAWPATIWEDVKALS

>Mb|QLI64855.1

MKQADQQPGGNAPGFLTRLAILAIGIAPTLVLSVAASPAALHHSRQANPA

DTTGDFPKPDDPFHFIPCISNKTIEGIPLLNDTNPQETWRKMFDPNPDNW

KWGPAPGNATTPGSSNSTQGRGLHLCGYLDVPMDYLNKSDSRIHRLAVAK

YQASGLKGKGARTIVMNPGGPGGSGVFFGLSGGYYSRILSNGLLDFLGFD

PRGVGLSQPTIRCYPAALHDRWNLAVGKSLRESPSPRQQLELVNAVNDAR

FRACFEKLGDVPRFVSTASVARDVDAIRVALGEPELTGYMASYGTTLGQI

YANMFPDKAGRLILDSVDYSREHRVTGGYAGTKVHSTTDAWNEGFLGECV

AAGPSNCALAKPTNGSGVTLDSLKERMAKLLDGLAERPIPAYDEDKGPVI

ITYSLIMWWIRRALYNPRYWLTLAEALADLESGNTTIAALVAHGQFIHQP

REPERPLALASELLTMVVCGDASADGPPASGLDFWESLWRNLTEKSFLSG

GSHFVTVFPCQNYNKYWPQGAALYQGDLNNKLKHPVLLIAETHDPVTPLR

NARELHKEMGMENARLVVHHGYGHAFLSDPSDCTIAIIREYLLNGTIPDK

RETDCYANGKPYRNSTRTNHKRSLPMVGDGLERPVHNVHMPDWVNMQQGG

L

>Mb|QLI64874.1

MARALAMQLIQRGLGHEEPIGIWVTHGSHQVIAQLAIIYMGGTCVPLDPD

RPRPDTQTQLQVAGVKNMVVDDAFQHRHLPVTKIPLLQSPDGGHTAGNAA

TLPVETKADFRSHILFTSGTTGTPKAVEILARGIVRLARDPVYGPRSSDT

VGHLGNPCFDLSLMDIWASLLNGSTIAALDRREALLPKSLDRMLKENHVT

YLFMPTAIFHIVAPDEGNVFLTGATGFVGAHLLVNLLQCPQVRSVCCLVR

SDSHASARKKMGRNLEKYHLQHLATEFESKIKILLGDFSKPRLGLSEPAF

LALAESTSVVFHLGAQINYNEPYLANRAANMTGVLNMIQLAVASRPKALH

YASSMAAFGPTGLVADKVGELTEDAPLQPYLDTTVAYETGYGQSQWVSDE

MLCQLMKRGFPAAVYRMGAVVCNSKDGVGNPDDFLSRLTADCFRLGIYPH

LPDQRKEMIPVDYVAPAMRMMVMSNRNLGKVYHLTPGVRENISVNEYFRI

AQQHTGIALSALAYGDWVHALLQADKSGVELGLKPLFPMLMERVKNGRTR

W

>Mb|QLI64926.1

MSYFTFNMSASTQGPSTPKNPQRSSVSNSAVPQLDHDSSTLPINSSATML

CEPTVSGQTALNPGAQWSGFNSSLLQVDGAFSGSALPPYDQSTLHAALTT

NDDLRWMLIGMNERMANLEQTATAGNRTMDQMGNHVSDMLSRLAVIEGMI

KVVEDLKQSLRDFTRGLVPHILGVNLEEDLEKQG

>Mb|QLI64952.1

MKTSLVYLLALAGVSAATPVGWGRAPQNSCLHEKLSPGSQVLNLTTKSIG

GQNLGELQASICESEKRDKGILQSPEAVSKDPKNMLTGTRLQPNLIVQRS

KTLPELAAEALLKLGKLSAYKQTRSLVTLDIILPLSQSSFDRLKQWDHPI
GRAAKAFDDAITSMQEAIGGEQVPEIFGNKLKLRIICYLRGEQRYKSAVD
YACERLHGSNEQQALEQENEMRDKLVQLFEDCGDATKTDEQNDLLQVCDE
LSRKTLDLEQATEELIAFRKQLQQKFGKAASVCPEVRIGLQKWLHGIWDL
>Mb|QLI64991.1
MAGPKDGDTVDVAYTTCPMTATEKTQSPAAHDSAVVKKPVSPWTRLGMAS
MLLDLVSPAAISVGEAQHVRTIGSNDTAGSPWTMVGNGTDAPVLRVTACM
ANLGVDTFIADLHSNWDGLEPPVSWDLSAERYNQHHGRARSAGPRSDWQS
FLPKYELPSNISSIVNFTVLPPGGVWTFTRALASSLQNPQSTDFTSVHSN
QTADRGVILYRYENNAHEAHSSLFQDTLNQTQSPAVALQALLTRICQMVY
YEKLPRLNESGHAETAFSHVTVLPTRWTGFGVGMGLH
>Mb|QLI64996.1
MHSSTIYVALAAFTSTLVEAHGKISVATGDAGGNTTALGIMGGVVPGQGP
NRQTEPDTTIFRSRNAASDGLGRTKGNGANTLEAMSRVVAMSGSTLPQVS
SNGGYISATYHIVTTDGAGPVRAIIDPSGTGQFSQGTEAEVMTQVPGRNG
NIAPGPKSNNRPQNGQGGGGGGLIGNLLGKRASNVDTDHPLKVAIPAGTT
CQGSMGGMSNVCLLKVANPSGAGPFGGVIAFQMAGSGANSTATDGEIAGG
SGNASNSNGGNANIGNSNNSNNGGNADVGNSNNSNNGAANNGAADNGENA
SSGNDITSTNEAKKGNSFSSKGNNREKRAVMFQS
>Mb|QLI65027.1
MKIKTQTQTQNHKNKSTQLTTITANGNMCGLQKIKEGVEDWFETSEKRYW
CEEAWENGTFGKCTTGVEKTERIVGKSHRSLAGAQIPADEVPRVGMIYAR
EGTKEGHHQSGEGTKTAADEVPQVGKIYARDDLKKQESEEQTWPDSEQQF
EQTIFTISSENIKGDLQAFVNQKE
>Mb|QLI65032.1
MLVRQIAVFHALIGLVCSHSWVERLMVIGTNGTMIGNPGYIRGAVSRLDP
NFNDFKMQHLLPTIPDGLLTDKLCKNTQRNRTYTTDLPALQAAPGAFIAL
QYQENGHVTLPGLTPQKKNSGTVYVYGTLYPRDDELLSSIHNVWNTDGTG
GDGRGRLLAVRNFDDGQCYQINTGPLSMQRQAKFHKAAMNPQGADLWCQN
DIRLPVSIPFSWYTLYWVWDWPSSPSDHLPGGESEIYTSCMDIEIQPRVQ
LDEMNFVDGQDLNMAGIKEQLE

>Mb|QLI65037.1

MPDSRLLPLRPAQTKQDVIIGSSDPRQSPSSSLKLVLEQANAGSSNTCRA

IHALWSQDVKEVTSRMYLVEASLSASQDEMARCDEQHEEDMHKLRTKMYV

LEEKVHVLQEDMLNSRQEHYDFSQELRAELHRTRERLHQITGDIQVLKDN

EVRIYVSSFSWHISYLHVESPGLVEVANSSEGFDSASSPATPEAIDAWGT

K

>Mb|QLI65038.1

MLKSSLLYGLSLLAIAPQVCNGRAVEGEKRAAAALTWKALGGSIVGHPGI

ISWAPDRTDIFVRGSDNAVYHKWQLGNPGTAWGPSDTGFQNLGGSIISDV

TVVSRGYDRLDAFVVGADNACYHGWWNGSYWSTWGTLGGAFSGDISAVAW

GNGRLDLFGRGNDNAVWHRAWDGAAWGAWESLGGAVIGSPKVVSWGANRL

DVFARGTNNEVYQIAWNGSSWSGWYNHGGTVLDDITPVSTAPNRLDLFVR

GGNNALYQKNWNGAAWSGWISLGGVIMSRPSATTWGGKYITVAAQGANNA

VYLLEFNGNSWGDWRSIGGVVTEAPVINPLGSVNAAIFARGNNAGLFVYE

>Mb|QLI65057.1

MVRKAAITLAVALSAVLAVAATIDKRIKFGEAAKQGEFPSIIRIHYNTTS

VLCGGSLLDNTTVLTAAHCWFEGLRTHHSEIVSVRAGSLNKNTGGEVAKV

KSIKVHPEYKPHWNRNDIAILKLSTPIQESGTIKYANLPATVLDPVAASV

VVAAGWMTRAASTVVAAGWGMTENNILPDKLLKVVLFILDPTACLEDGED

DPEYTNYLDTKVCAGYAGKDTSVGDSGGPLFDYNTTELIGVTSFGGLSPL

GGFYTKISRYMTWINENLGDVKSLPSGVAG

>Mb|QLI65093.1

MEEFITSNLDRYQWLLDRFKMQPKPKVTPNSDSSLDNLVCARIRPLAEEE

VAAGLPASIFIRPREPGTFDAHELRHAVRGKPVLRSSAYTVDRSYGPDIS

TETIYEDAVQHLVPWVWGGGIGTLFAYGQTGSGKTFTISGIEKLAAKDLL

EGNLQGDRKMFISVTEFAGNSAFDLLNGRRAISVLEDAFGNTQLAGALEH

EVKAVSEVLAHIEYATKFRLTEATKRNDTSSRSHAVCRIRIEIHDIPGAE

DGILYLVDLAGSEAARDRDTHEPQRMKEAKEINVSLSVLKDCIRGKVEAD

ALIGTQTKKKPYVPFRQSALTKVLKHVFDPAGTRVCKNVIMACVNPCLLD

ISASRNTLRYAEMLRVFVPQVKGVKYRSDMPTTWNNEQLREWITANSGSP

SVDATVLAPHEAGAQLLRLPAPEFETRCMKTPGVIHEQAFAFRQKLWQLH

VDTRRSKQKAQVEEDDGPDTVLSHLNKFDRSSSREPDALTQSIPFKDRIR
PGMAVSWTPTKEYDMGCSWEKLNIVLILCPAAAVGPTTQDTMGRRVDAGS
ASNKMVDGKYKRYLCAMVSPAVLPGAFEVSMWRYVVVDVDDMDKEVILEY
DSATRFYHLAV
>Mb|QLI65100.1
MSEILVITCPGGKQCSRLLPLLYNKGKFQLRLAAHSEQSAQKLKNLYPDA
EVVTVDLQSLAECTKLLEGATAINAVLPSLHSHEKVMGFNLIDAAVIESR
REGNVFKHFLFSSVLSTQHRTLLHHDLKSYVEEHLFLSPITCWTILKPVN
FMDTFPLARLASQEKPVLDKWWSPNYASSLVSLKDLAEVSAKVLNERERH
FLAEYPLCSTTPIAETEVVKLVEKRIGKQIEVRVPSLEAGSDKLMEFLYG
DKTGNTGNQGDPRGDLVRDTVERLILYYNRRGLQGSPNVMRWLLEREPTS
VEQWIESVLSSSA
>Mb|QLI65158.1
MPPNLLLLKRWLVGAGIIIVLLLLFREELPAVTDLRGRQVDRDGFVSGTE
MMAVVREWQKREGIRKIVGLVFYQKRQQAAILDCYLKRDLAKNGGVLDQV
IWLRQTDDARDVEFLDKLVRSEAHYSWRNQEGSDASAYDGIQDDLLYIQI
DSGIVYMEDGTILSMAHTRAMRPDFYLVSANVVNQPLSSWLHLSLGAVRP
YLPDNETWRPVEAESGVMNWRPSRLPSWRGPPDFDVAKWNPPADRQHLWL
PVTGKTDHLLHNTPIVHTVYDAYKDQGRWKWMAAAQQHYSLLENLERGEL
SKYKFHLWNYQELGMGTQLVAMTGKDINAAKPIGAAAERHFAVTMPRKIG
RPAVADGRGVAAYYSSKDQSEGLGQTDILERYRSFAQEHVCKGRMLWTPS
ADHV
>Mb|QLI65243.1
MSAPNEHVNLVTRSVMPASQDETVAESWRMRDGSKSNKKGCSLTEGQQTD
GKEATEAILSTPPHQGPENFEEAPAELASTIHELDIGSAQIYKPHTDANP
DMGDLIPGQSSGPYSNTSTSPLGIWDTSKDVPASSLLEMWTEEDLLKFGI
HEGMDISYGLPLFEENVREYHTAKTALGNRTESYGGINVTFGLPPWIQTN
FSDTGETPEKNSNRYHNEHLGAPFAQVSGLSPTPPRKDTDCTALRSVSLS
AVGTPTVAESRTLPFSEIKDSAATGDTDIDFPCSRKIPSASERPESSFAL
RDVSVISWTDCYPEDPDKARELATSTGVPGLQMYSVDVFTHATSDTQVLD
EVDATQDSHSFRVEMEPQPNDSVRASSVTDSEGADGSGWVKVEIETENPG

QTDNV

>Mb|QLI65244.1

MSPLNSPEGQYLSLAGSFQKSQKTLANNFKTWGLKPSSKNQAPTILAATE

AAYTNSVKQVEDARNRQILILNEDWVDECCEQKSWIAPEDKHIWEKGGTK

ESLAETGSTASGEPAGKPTEDDSAKAPNPPPNPPENPQPQPPNPTENSPP

QSPNPPEIPQPQPPIPPENPPKHESDNESMEVALLKKQIEDLKNSKTNGK

DGPSDRYDQSEDQKECFDVFCEWSRRFDDTDDPNTNRVEVDCHPADINKP

LSDRRKEYEMEIMLITQFRSYVFVIDPEGKIEGYDCLRPGYLIPRSDCPS

AARKYEDNGGEIVRAKQRENLQGKQMTDFIWWSVATDPEGRFCYCLGAFK

GDKRPSLYSRSTFKAKWGKLADDQINEIRRKFSQAELNKQAKARSQKLLK

LN

>Mb|QLI65248.1

MPDSRLLPLRPAQTKQDVIIGSSDPRQSPSSSLKLVLEQANAGSSNTCRA

IHALWSQDVKEVTSRMYLVEASLSASQDEMARCDEQHEEDMHKLRTKMYV

LEEKVHVLQEDMLNSRQEHYDFSQELRAELHRTRERLHQITGDIQVLKDN

EVRIYVSSFSWHISYLHVESPGLVEVANSSEGFDSASSPATPEAIDAWGT

K

>Mb|QLI65308.1

MDSAPWDTKPLSQSLVTNEQLGEGTRLLEPTSSCDEGGFECFPRRCGAST

ESQTYSSVASTAQQRNLSPIPLRSRVRPVDRDEDFVCSENPARPGGSRKH

RGQYFDAASGSRMLLQPDTRSISSDQLAAEVNGIYAGLVLLESKSIEYDS

TQKETDLSQEQYHALISLHRSLLHEHHDFLLASQHPSASAALRRLASKYF

IPARMWRHGIHSFLELLRRKLPGSLEHMLTFIYIAYTIMAQLYEAIPSFE

DTWIECLGDLARYRMAVEDDDIRDREIWTGVSRFWYTKASDKIPMTGRLY

HHLAILARPNALQQLYYYAKSLCVPVPFPSARDSVMTLFDPLLNANPSAS

QRLEPVDVAFVRVHGILFSGTHEDQLEPAVKQFLELLDNRIGREHGNWLE

SGYFIGISLSCLLLSFGDASNVLMNAVLKSQQTDDIIMLPDPVLTDAFKT

AVRFTARTYEIVIARWGDKNTFPCLHTLLVFYWFMMDFDVGRQYLEGSLP

WEQTALLLNHLLRTSEYTPRLDTPEIPWPEVGKAHPLPEDYAMRGLIYTG

TYFPKNWFDNTAIDDEEKYFEPASTVSKRCERILWLGYSMAMRKRRLHWD

KNTKQFSAKSNESNDDN

>Mb|QLI65323.1

MEARNREARNRRRRALRWTARETDAATRDLQLILGRGTDLGSSQKYGHDI

FGRYRLDPPAFRRHGTRIRVYNMDTLDAAAELRRQAMADPRWRRTRRPAV

LNFANADQPGGGWWNGAVAQEEAMCYRSTLAYSLHRRDYPLDSLEGIYSS

CVAVLRDSMSSGHAWITNRRLLSPDTVRELLPWYSALTVAAIYKPRTRYE

TIGWLRRQTKVFAYNADRQLAKDKMRLALHMAAMYGHDMLVLGAFGCGVY

ENPPWDVAQCWLEVLREYGPRWRCVWFAVYDPQGQGNFRIFKDVLDGRTV

>Mb|QLI65333.1

MENWRYADSTDVPSVIMDRMRREESSSRIATEAWKKTDSQVSMNYLPQSQ

CTLSLTVDWLRKAEAEIAAIFFHREVSLFFDIYVRASEESVFGKISRNYA

TVLTAVFDEESNFIAFCCQVHSHTYTCLKYSLKGLVEQGAENYKRTACRF

KAPWKIVEETGFTEEGLLRIRRDHSLVNRYDKSLVVGLWHNHDVSIIVTR

TKGLAMLYYITNYATKLDTPMWKRLALAADVFSSDSFANNLG

>Mb|QLI65349.1

MPRRQRSKVNKRPEIGTLNCSWGMFSKANALFRQYDVDVVVIARRPDGGI

GGYQSRPGLARDFLQINEQDLLGPHEVDPYMSKPSKGVAVLRAMSSSRSS

SCLDSTENRSSRASSSCVDSIGTETSPGIMEEAEPGVALLNIVRTPHCEE

GEGFMEEPLHPWAEVHSIPNAQRCASPETICPIRITKPAKEDIRQPTPLS

RIKREEILALIKKFE

>Mb|QLI65356.1

MSSLAAESWSWYGLTWLVVATRMASQVLLRGSVKKLKLDDFLMVMAMCTD

TVLIIATNIIATTNSNLIDPNHPASLSPEDIRQREFGSKMVLLAEQMQCV

TIWLVKACLLLMYHRLTLSLKGNLVVKIVACYVAVGFVLMEILYLGVWCR

PFSQYWAVPPDSTQCSAATNHLITNAVLNISSDIMIILIPMPVFLQSRLK

WRKKLILVGVFALGGFTILSAILNKFYSFNEPFGSAWTFWYIRESSTAII

VANLPLTWTVFRRLFHLRSFDNSEYSSKTRSGQPSSTLRSHGQAHGRTPN

RSYIRQGDANDDDLAGSQEEITKGHGISLRIYQRHDVQISSEPVTNHHAR

QLSNESLPDGLTTTIKGGASGSFLLDAETASDKSYPAVKVNSSV

>Mb|QLI65363.1

MGAYRGCDNCRRWKKKVVIPCRHHNPRPKKKANRGLAHRANQCDCARPKC

SLCARKNLPCEGVGQQRFRFKHQPASLSVVNKQHAAAPSRVASPPSNAAS

KLADSIVGLFEARHPGYNLSAIVRKSTVQHIGHSPVLDASLAALVGIVES

LAPVESSSSLSRLQLCRSSTGPLHKYTSALGALRESLDNPVIRYQRDTLL

AVFVLSSCHMWLVDGADESRGHMHGLAHLITAVLNKDVQLQDQAQFSDDA

LKLAAIQLLRSSVFNSDIHIYPWIFKVFDATAEPRHVAPRPWHKGETKKP

LWPYDFYCLDISVYLIIPQLLRRPRENMQSIELIYNMIRRAYPRCVKDAN

PMHELAVEDKIEHDAFIQAIQVEAQSLHIAIGLNFNAFLRASGRDFDDRQ

LAEDRAAFCADAATLAEKSKSQLPLSAHHIPLSTIAAWCTADQGSDLKIR

LRRLLEDYRASYAMIHVLHSAPYWREAPEKLSREIPWFPKYIGSPRERTT

IVTEDKSDEFDEEMYEYCCIL

>Mb|QLI65422.1

MESSRSAPKIAIHGHIARPGGKCSAVVYDTANHDWHGVEVEVTCRGAEEK

TTDITWLSCIIEEHLRRSYVDIREDPDHEWSADETDCQNNSYTIRIWPRS

RVLFYDHRTDIFQPTALDIKSPQPMGHRVYRCSWKGQECVLKYIETDTDV

GAIELEIEKRKDLINKAQIPANQVNSEMSRRFCLVPILAVVVADWQPRKP

NTVVGILMPYAGEDLGILAKNSGGNLPITLQQLQDLVRGVRELRKYELFQ

GDIRPWNTLLQPSTTASKPRLMLIDIDMELPGYPGDAKALGKLLQWCLEN

SMALSEDKQAKVKLINAVKALLSENFDMAIDCLSATEQSMRRGPPSTHAN

KVG

>Mb|QLI65458.1

MATKLGVDPTTWGYAGKNTCEKQESSQYCTRPEQGGWHPGPETCDYFKSL

EVKLFLGNGISGFDGAGTFDDLVIEFGNSHQTILVHPSKGEESTKPIDLE

KAFGLKRVKVEDVKSFKVYSVRDERTNPDSWEIGGISITATCDESSRKVI

INKYDDIYDWFNRFGDITGNEPSHFTGSIAVSDWKWQHPDEKTDTIAVMP

IGQSNACTKFKSMEAHLELGSGISALTAGTNDELVLDFSNEMFPKHVILL

ATAPSHGDVIDKTLDLQNEFGSSPVSALDVRQVMLYSREGKSHWADPWQV

ATFELRAKCEGSSRSVVVTKWRNIDKWFDRFEGPSALSGDLALKDWHWA

>Mb|QLI65504.1

MPSLSTPRNDMLDQRQLETGVTLNLFEASLDEGTIRQKNTPTDQIQRMVI

TDRNPTSPYKIQVLLRTCIHGTMDIQTKQPASLIIVDYSIMITKEGARFS

TVDTSFEFSEYLTPAEQAEAAGSFSRGTCPSVIAYAPFEEPVRFNYMASE

ESKKTQLEVKLSPEVAGVKPGEVSFGQERESTYSRRYFDQGLGGRNFDQN

GCAYRVWWNLIQNKKDNAGVPPKFRVAILLQRTGTGKFQAKFGFAARGGF
GYRLEELTNRWLRRTAVDDPIIFDPSADPIGSDLDGMKIDQQELRKLKVG
SELANLGRVWGLNQLQKP
>Mb|QLI65595.1
MSLGAINDIYLSHRDAIAAKLKGHSANQAIDGKSLDLATVIAIARYGISS
HLSDGSIEAVAKSAEMVQASIDKGEVVYGETRLSQTRTLNLTIIPGVNTG
FGGSANTRTNQVEQLQSNLLRMLQYGVIAEERAVTAPESRHDTDLNDIIS
QSALPLDNPLASTSMPESWVRAAMLIRLNSLASGFSGIKETTIRTLHQML
EEGITPLVPVKGSISASGDLSPLSYIAGVVEGKPGLNVWTRNTKGERCLK
RADVALAEKDIKPVSLGAKEGLALVNGTAVSCAVASLALHDAMGQATLSQ
ILTAMSVEALLGTDESFDSFFGEVRPHPGQVETARNIHAFLSKSSLVQHS
DTEEGELRQDRYSVRTASQWIGPVLEDLHLAHQQIGIEMNSVTDNPLIDP
ARDGKMLHGGNFQARAVTSAMEKTRQSLQTIGRMLFSQCTELINPATNRG
LAPNLVAEEPSESFIWKGTDIMIAALQAELGFLANPVGSHVQTAEMSNQS
INSLALISGRYTLEAVQTLSQLSAAHLVACCQALDLRAMSCKYLETMAPI
FKDMTSEALSGYLLTPETGDVFLPATWAAFQKALDTYTHFDSPRRFAMTF
ESLQPLVLKHIRTTAEAVRALQAWTEKCCAVALDHFKMNREAYYANPEAT
PYIGSASSRMYKFVRHQLGVPFMSNKFISTPREEGGDFTWGEPDGDSNRG
VTMGGMIAKVYESMRTGQLYHHVALCISDVHEYSSAANKDDEMQFSYALN
KAGELTGWGGNSPGSLSGDEKNDATQAQNISSSATVVDDTLYLLETDAGT
NQKLKEDMVYLTLHVESTRRQGQLSPHWG
>Mb|QLI65631.1
MRSVSAIALLSAFGAVVNAAPATMNSVSSRQAGDQGTFTISQKAPFQDTS
LLPALNGASEVDLNMAGYEMGYHPNSDQYKCHGSCGIVLRISRLPASEKC
THPYAQPFVNFCFGTCKNLAGFSPYPPALVFKNQCKLNAQEARSTTVEQA
ASTSAEAPASTSSSQPASTTGSPVSRPTSSAAAPSGSSSQAPPASSSAAS
SPSRTSAPHTLPTGTTSPRPSHPSGVASASGTAPKATPTNAGPVTAGAAS
TSFSVIAAITVATLAMIIA
>Mb|QLI65633.1
MPLQERAIAYFLYHYDLESPSQNKIGDMGFVILSKSDKEWEHYRLAFEAC
AMASFINETGGQLEYKSLTIETYNKALMAMHSALQDPGVVCEDATLAAVL

LLALFECLNPTTGEQESWRNHVQGAIELARGRGRKQIDTRIGQMLFRATR
TLMVIYSLATLEDEKEKELWWSGDDECTSTQKLCVGVASLSAKATVLLGP
SGHKEEVEVEVMLKRCQAHDRACKARWEELSKSTQGISSDNTDPWLLMLL
NMLTCARILLNSIIMRCATWTYKVPNYQTSEEYDGACSALAEIILHSAKV
NEQQLCWAEEAQSSFAELDVQLQPGRDSSRCAINKWIEDNRSLWEEKPLV
GLQPLAVIWSLECLNDDQKIAIDTQLRRIVGCTVDQIRRSPSYTTEACTR
PSPSLSEPATDTPEAGSRDSSPASTGRFRYTQQDDDTLLELSNEGIPWRE
IRDRFPKRPDSSLKTRLWTLRNCQLTADEDRLLIRLKEEEHCSFRDIAPK
MRRHRALVSNRYNQLRRNRAMNN
>Mb|QLI65637.1
MHPDYSDIVKELRDELLWEENDLEQAVKNARKWDIPELDKYGIISAKGFL
DFADWLVKGWVPTESTQGRDIYYILCIFYFVLSQKPLGGRQTKIHPLSID
QPMKPLSEWVVNFSKVVGQRMDEEGSINEASITTFRNSPLFRVFESEKHA
KGWTTFNKFFYRELNEGRKIDGPEDDKIVVFPADSTFTGAYGINEDSEVV
LKEEEWREVKEEVILKNVPWKVADLLGKHGSESVPSEPGSKSPPPTYGDL
FKGGIWTHAYLNVFDYHRQHAPVSGTVEVVDVIPGTAYLEVLVKTDEQVG
HNYLEPSRRIAEGQKNPSGINTLDMPDTPGYQFLQTRGLVIIKNDNLGRV
AVLPIGMATVSSVVMKEGLKPGAKIKKGDEISHFQFGGSDCVVMFEKKAR
VSGFPDSDPASREHFFYGEKLCRAYYKKLQPI
>Mb|QLI65689.1
MAAKAFGPNEASARTAQPFNAWDIIRPSLHASGGPWGWTHYGIYIPRLPQ
PYQYLNVLILVGLVDLTIFNHAEFAPGGPKAVVHDAEHLTTVMTSTASKG
HHFVRGYDSSTDCEFPDSGTPLRWGKDFSMEFTFPHKAVVTGAFETHGWE
YRLEFDMSPQAVWFIATPFYEHLSLPISGTAILSGERIPISGCIEYARCK
PPPGLPLEMHAAVARSTNYFIYHVVELRDSRQILWGEVRNGFMPPRKFVY
VRTVPDGRVLDEYTTELRVELLDDELVTDSLGRQTRMPTKFTASVVVGSG
RKVLEVAGRVNTPLRDGAGRGFVGGYDAVVTYKGEETRARGMFEWVDLEL
QEQKANL
>Mb|QLI65743.1
MATGSEDDPTLTPTPTSTVKPGLTLNLGPLTTTFVPPENCDALTLGMYED
LPQTTETSTLVRWNRGYECPTTYHGTLQLISSCYPQRYGPAYDNIKDWMG

PNAVWPFYSPGLLCPSGYSTGCTMIYSPPGATESLPTAYPTAFPTSTSYA
TVSTWTLLSKGQTGYGCCPIGYRCDTPYTCISTPSLSATMTGDMTQGCPE
KSIKTRTTSTFVISSKMWPFVYAPQVVLIQGNDTSTAAAAASSPSGPSTT
SSASRDGDAQAGLSTAAKAAIGTAVPLIFIALALAAYIFYRRRNRSKARA
EAASEQGSGFPGMNKPELDATPSPFALSLGPGSELDGTPRPSPPGDDSTA
ARGRVSELPGPGIARFVGPMCELPGDEKFARRPAQPKGSDGVVAADDEGG
KIGHGASINGASEPKAHDTKDMDVDKSSRGGKDK
>Mb|QLI65784.1
MSFRTAASLIALLGLNAGLSVAAGGAEGDAGRTTIGFRSVGQEEKDLLTK
AGSLIRSRNTAATHIGDGVYLGNSPIKRDGESLFIVTADEAAFKAASKVW
VPQEYFTLPVERENDILGEREAAISRDQLEYYIEDQGLHSSRTVKLAGIH
GSVSDDIQQMLIPNEMISHDQNGVPIETESKLDTKIEEPSGPMFWQVDYE
EWEGIQGSRVYTEDTLRARSASLAEKATKAVTEAEKLVESDSPAAAEAIA
EAVATAKRCAEGVGHFHRKRPDWVDFDDFNTVYKQYSNARKAEAKLRKLK
LTKAVEAQKSKLTEAAEKVTDDATRASGIAEAEQAAAESNKLLQTAADEA
EGVKKAHEKLESVRKQLDGGEQEEFIELLAKNPRAVWATLQESFTSMNGK
PAASLDAAIIDEADITQEEYSIFQEGVDEAKAMATEIGEGVRALQMTVGH
PEILCKRGDIDCILVPSDKEKLPTAEKPAKIEPANEGELIAIARQRSKES
FDDLLTDFKYKSVVKHDQLYKELNERLPEFSPVSRTERIVSLSTKFGEGA
LAVAGLALYGKAVADVFSSDTSSVLDKAAVVTSILPGVGCAVQLADSVEH
GNVDAGHTALCFAEDALLVSGFWEIALVMQLTESLASWIEAGNEQDKLFD
TEVLRSKGLAGWEANVDRMLKHIASDEFAANTTTRFATYQILVLYQASQL
TGDLHASHKAVSGKPGNQTDEIVPHIEPEFRRQICAAMAQSKFQLRQKLE
AVALEHAKKFSEEYKDKFLKDYREAATKPIPFLGIPISFGAGNLDEVLED
ARRWPLPLHEGRIKRAIREVVERLETPAQCKCLQGSKKAPCEYADCSTPK
PPRGRKDAAGRVYYVTNVQSEDHAKQMRLWDECVSLYTTCPYPGLTGEVGR
QLWCTPAS
>Mb|QLI65897.1
MSLFKILVAAATVATALAAPHEHKKPHPSYGDANAQCGNHQKLSCCNRGD
SGGVLDGLLGGNCQPINILALLPIQNQCTNQVACCTGNSNGLINLPCTNV
NL

>Mb|QLI65908.1

MVRVITITLAAVLSTFLVSAATIHKRLIGGEYAKGGEFPFIVSIRQSGSH

ICGGSLLDSTTVLTAAHCIKCLDFVKAGTLDSRQGGVEVEVVSAALHPRY

SRIRHHDDIAILKLATPIEESDTIRYATLPASGSDPELNTTAVVAGWGLD

GRNTGPADKLSKVNITIHERGECSEKLKLGAVEDIICAGDDGKDACEGDS

GGPPVDPVTGHVIGLVTWGQCRDPPTAYTRVSSYIDFINGFVGGSDSSPL

FNLGRKCRDLVQSVGNATGPGDAGIPSTPTGEEEEEEEEEEEEQGPTAFD

VLIAKIRELAKQCGLKENEKMTPEIQKCIARLHEEGVTRR

>Mb|QLI65918.1

MASNGNLARQDVDRGFVILAVGWIECGITALFVTGRVYSRFQKRGGLGAD

DWLILISFILAVAFMGVTTPEVQYGTGRHLEFLSEDEKIQSVKLDWVSQA

FHIMSTAVAKISIVLFIRQIIGKGHSRMWFLYIMVAMLFVISVVCVAFIF

AQCTPAAALWDPRLSEVGKCWDPKVQQGYGYFTASFSVFSDFALAVFPVT

VLWNLHMEWRLKMSLMFFMGLGAFAGIASIIKTMKLETLSSRGDYTYETV

DLIIWMMTEQFCIIIAACIPPMRSLFVNIYRKVFNIQSTHDASTNKTGGT

KSRQGYVNQPSQNEEHGLSRMESGYWRNETEISAAAYRSEPDGDNSSGHA

DRRSDSDELLREQHRGIVKRTEVMVSEHRILDADIIPKKGRPLAPAFNCG

CRVEKHVSTMIKPVNGYDDMTTTTKHEG

>Mb|QLI65976.1

MVRKAVTTLAIAFASATATAAAMDKRIVNGEEAKPGDFPFIVSIGSTPSG

KSHFCAGSLLDNITVLTAGHCVASAYYVRAGTQDLGEAADVAEVAYAKSH

PDFNLTTKDANYYAVNDIGIVKLSTPINRSDKIEYATLPEDGWHPAVKST

GRTAGWGPTISGKGSPKAVDHLRVLDIPVRPLEECLQYSLVPDNKDTKIC

AGGDGKITTRYDGGGPFIDQDTGYLSGVVSQAMSDAKYPGTFTNVGSYMS

FIEEYRGSNGRPDPNAPSRAKLLQEAAKKFISEDEVFEHCDRTGQPRLDC

YEAKKPCDTQRRKKPNQTHEEYFQCIDEEVIKWQKFLEEYDNQGNKKVQG

>Mb|QLI66000.1

MHVDHHTRLTLATWKRLKSLLRDHGSPVPVDGESLSIADVIAISRYGHKA

RVDDSPVAIAKVNESVDCLKELLDQGQVFYGVGTGFGGSANTRSADTAAL

QVALMQMQQCGVLSGHQSHGPSSTSMPEAWVRAAIAVRCNALVRGHSAVR

YQIIQMLERLLNEDYIPLVPIRGSISASGDLSPLSYIAGVLEGNPKLFLW

TGQSDSRTLVNAAQALTLLQLDPITFGPKEALGLVNGTAVSAAVATLALH
QVHQFTVLSQILTAMAVEALLGTTESFHPFLAKVRPHVGQIEAARNILGF
LRGSRLATQQVSVTGMEHSDDLYQDRYPIRTSAQWIGPLLEDLVLADKHI
TTELNSTTDNPVIDVQTARVLHGGNFQASTVTLAMDKARSALQMMGKMLF
SQCTEMMNPSMSNGLPPNLVFDEPSVSYAFKGIDIAMAAYTSELGFLAQS
VAPHVQSAEMANQAINSLALLSARYTHTAIDTFSMLTASYIYSLCQALDL
RVMQVQFEDGMKSVLPPLVEGFFAAVLPDDSLAELKLLTCRHIIDQFKKT
TAEDSTSRFRNIAVSTQAVFMSFLSKSPSLSSGMLLTGFSMIPVWVTSVE
ALMQSQFDEIRHSALTKCDTPSYLCDASKKIYELVRVTLGIPFNRGLPDH
PSVASAKRTREQIDAELTPFSDGYLIGDCVSRIYDSLRSGEMMDTVMECL
AEVVTCHDSPNHAGGPTNSFDCVR
>Mb|QLI66047.1
MPIPGVDLPLRPANAPAIPEPNEILKHPSLDARKRELETDIKDVPFVDID
PGVFDTELVKLKLYAKPNPEHLEKPPVLGGGNITKGTYSGTQAALTHAYS
RIERSYESYFDVMQVESTLPRYTDLSTKRGLFQYSPYPKNADGTVARYPP
HLQDIPKDNQVSLLKIFNALGLAETEILIKQVIPDSFLGKTATWLLDLAN
GNVSDAANQGYSIKAYETYNKLHRKSGTDIEQGANLGLLADWYGDRRFAD
QSFTGTNPTTIEKIPKDLLDEFIAEAGRGGYDDWAKTLGATDPSSLFVQD
CRYFRKAVGAQPNEELHHKERSSADSWACAAVTLFQLHPDGQLHPVAIVC
DYKVNMASSVVIFNQRRLPSDPTDRQESDWPWRYAKTCAQVSDWIRHEVG
VHLTRAHMIEESLIVATHRTIPMNHIVYKLLEPHWYKTLSLNAAARSTLV
PQIIKDLVGLKPDYLYQFIRYEFENFDFVQSYIPNDLKRRGFPNTTQGLS
DAKYKNYAYGKNMVSMWSCIREYVMTMLRTYYKDDKMVQQDQYIMDWSKE
VQTNGFIKTFPTIGTLDQLCDVVTMSIHIAAPFHTAVNYLQNFYQAFVLA
KPPCLCSKMPENINDLNKYTEKNLVEALPIGRQRQWLLSVQIPWLLSFKV
PSDRSLITFAQSQWRAHYGNDRADQEIRAISERFYNELRKLEVEFLATSR
DMDDGSIPYMVMDPTNTAVSILI
>Mb|QLI66095.1
MVRPVVSALVVACSAVSAAAAATIDKRILGGQPAEKGDVPFIVRFDNNCG
GSLLDKTTVLTAAHCVSDEAGKLSVRAGELQRAGEGGVVAEVASIKRHPE
YIRGRLPNGIFHPNDIAIVKLSSPIEKSDTIGYARLPANGSDPVVKSMAT

VAGWGAQGHSKNDGNLHKVDIPVHKRDDCSDIDKGAVRDTIVCAGAYGKT
ACDGDSGGPLIDRWGQLIGVVSGGGSACHEQGRETLIYTRVGSYIPFICE
NLDAPCPDTLYEPAAQPPAEPIPESEDPFWREVNSQAQQVCNDRGLTGEQ
AHLCEEYKQACVFDNLATDDANVIVDCVKNKA
>Mb|QLI66122.1
MKYSMLSAVAVLAATSLGFPEFYWGSRSSPGRISITNNMNTPVQLDKVTG
KSLANGIKVSRDQETVIPAHETLQIQAIDQSADLKLRVKGATHNQVEVSY
TSGNQGTYNYAIKPIEGGGFPGVVRVEPDRLQCRPERWLPGHPATPQVTC
RDGVELRVYLEGPYPRVFEAEYDDEYYDGWY
>Mb|QLI66222.1
MAQYDAIGTQYDVIKKTPFNKLEQFNFRKHIEPFLQRHGTKVLDLACGTG
FYSSLLLEWGASFVVGVDISSPMVNAAEARIAETPYASQARFVQGNGLIP
QEYSAENNGFDVVSGAWFLNYASNLDQLASMFRTISANLNSNGVFIGICM
HPTDDLESFASGVNNSAWAQTGVHYKFGNVLPRGIGFPIRVFGSVSPDSK
VEFGTFHLKKSLYEQAARVGGMAGRIVWQNCEFLGETWREEIGLQGDDDG
WRSLQEYPLLCTLLVWKE
>Mb|QLI66268.1
MSFGDSSFLPLRFNFSTTPCEGQLIAKFTTPAEVPNGEADIFWQCAGLAP
YCYQANITNGTSDPTMQLDREAQVGCINEVLRTTSVLVVKTMSTRTTTET
AVSVFTWTTTSFPRSQETSPTVTLPSQSWVTSGVATPSSTVMNKDPTGTD
TTAARAPAADSVRVEIPRSDMIGRTVRTTSPLPVTTDTVFDSGASKVTTP
LVTSAVTTFLTTTLLRTVTVSCAAGSATGKA
>Mb|QLI66367.1
MAPITCKVVDANNRGRPGVYVVLECKDQLHRGIATLESLTDEDGGISLWF
PTPSPGRTDDVEPQIVDSSNIPRVSLTFFPHTVPSTCPGPFLSIHTDLYL
QGDECHGITLYLDPHPRLEHSPVPVASPLNRFAAAAVSTQEPQRDLSTPS
PLLLPPPVSQNSRPSPMLLNGMHCHNGSRGQKRKAEDYPQSPNKRR
>Mb|QLI66414.1
MTFDAYYKANIGSENLSNSEARIVAIMKLKRPGPNGEEYIIQPPCGDGDN
WGCNRLVKDLNVLTDDKVAAVPDTTPFNWRQLKAAGKVAVTQSPTPADNE
SDPGTSDTGQVKPPPKHPSQGKKPGTSHDEQGTGNPGDDEIEPPPKHPST

RPDRPARPATQGDGEDHPGQGGDDDDNPSTGTKPKTKPKPKGNGRTRRHF

YA

>Mb|QLI66422.1

MGLLSIPFLYLYGNISAIDAYFMGSSASTESGLNVANLNELKLYQQLYLY

FATVFTQMGFVNILVVVVRLYWFNKHLNSFGPALLHARRRGGTDSQEDSR

LEYGEPKASPPDTSGVAETRASGKGSEKDGVQLAADNNDAIRGPYQDLES

EARVSCSSFVEEAIDASAPHISFDSSADKLRHPRSDSVLYVPGPRDRDLG

HPLVELTGPRLKSKEELTRYGEDDNGQHRLQTLGPLRESRSIDRAAALAS

SLFVIGSEPTPSSISRELTRRRRASDDMPYLSTGASIGRNSRFYNLTRQD

RNELGGIEYRSLKLLLKVVVAYYFGVHLIGAIGLVGWILHADSKYVAHLD

EFAQDKIWWAFYTSQTAICNLGFTLTPDSMVFFQDSPWVMFWLSLLTFAG

NTLYPVFLRSILWTMSKITPRGSSIQEPLQFLLTHPRRCYALLFPGGTTW

ALFGIIIGLNFLGTLILLVLDLHNPEFTHLTPGQRVAAAFFQSAAARHTG

AASFTLSNLSPGAQFTLLVMMYISAFPIAMSIRSSNIYEEKSLGYYAQDP

TYNEDRGASYLFQHMQKQLGFDLWYIFLGLFYLSVSEATKLADPNQPAFS

FFAIFFEVVSAYGGVGLTLGYPDITSALSTKFTTFGKVVMCAMMLRGRHR

GMPYGLDRAIMLPDERLVERSA

>Mb|QLI66442.1

MAADPPYLCFRAFVDALKADNDLVEIDTPVDPHLEAAAITRRVCDTNDKA

PLFNNVIGSQNGLFRILGAPGSLRKSPKERYGRLARHLGLAPTASMRDIL

DKMLSASDIPPIPPQVIPTGPCKENFLDESQIDLTKLPAPLIHQADGGKY

IQTYGMHIVQSPDGSWTNWSIARAMVSDEKHLTGLVSEPQHIWQIHQMWK

KQGRDVPWALAFGVPPAAIMASSMPIPDGVTEADYVGAMTGSALQLVKCD

TNNLLVPATSEIVFEGTLSITEQGVEGPFGEMHGYVFPHDAHVGPKYHVK

RITYRNNPILPMSNCGRLTDETHTLIGSLAAAEIRKICQQKGLPVTDAFA

PFESQVTWVALRIDTARLREMKTTPAEFRKKVGDVVFSHKAGVTIHRLVL

VGDDIDVYQGKDVMWAFSTRCRPNMDETFFEDVAGFALIPYMSHGNGPPA

RGGKAVSDALLPCEYTTGRDWQAADFENSYPEPLKQKILASWTELGFQDD

>Mb|QLI66495.1

MAQTARACSFLDRPWPLDDVISIPSDSESETDSDAEVMGQDVPRVHFTHD

QHLDDSLPSISAIVASMVNVGHGPTKDVSEQLQTQLQGAMAISSLGSSTQ

DLCLGGEGESSDSSALNSPRGVIFVSRALDGPGIAADSNELVSPAIQQHN
DGVNSVLPTSIPTPGVVSVASERRSAQSAPVLPPRDEALQEENVEPPFQP
CLASTTIPVQHSQSQNCIVVSSNQCSQIHNVNVDHGRISGMVSRSDDELH
SVARPASAQTTPSGCSRPVSRRGGLMEGVQKHQTADNTLPMDSRLSSSPT
GQGWDEECQTSISANHTVSQPEEMEKRSSSRRGFRKAYNLRRLPVKRQLT
AEQKDGGSNEKLVPQRKLRKLICPQPKKLGSQQGSGPRRNRKAHFLRKNT
TKSSSNASVANAKRPAHSGVAGYEAWPLGNPVLKCTKQNGTVMFQLQFTS
DTLWNTLAAQNDPIPKDGQAPNKVEDRRLRARKEQSNQQHAENIAQQSIQ
AAYPSVCPDIYRMDCLLARWRRHTFLVKWSDATTTWEPRGHIIDKGLLNR
FEASWQGFDAGVDVLGARLRAGKLQRLLHWHGRPSKEDTWVRSELLSPQL
MRRIQSNDIYGAHI

>Mb|QLI66557.1

MSDQQKASELMAKVTSSFTPRWNDAGSKASRDGGFWHPDSDRDFRPVGSV
GVGSHRDINGSYSAVLIAPNCAAMQKINAEGMCESPVASPTGYTQIWTDA
GSKAKKNGSFWRPIPPNGYVALGDVAQDGWLPAPSVGDVWCVRRDLAVAG
SFGSPSVWDDKKSGSKKDVSVWEIKGGYGNIQPAVTRLGAIRVSETYTAP
SLSFAVVPNV

>Mb|QLI66630.1

MQYPLLLVAAFATASIAAPAAGPPASTQGKEDVTKEVGKLAEEACPRDQL
VYSSRGGLLLRQEDCKRYADKCLQGGIIEPAKLKECINDARIKSKKPAQC
PKEPAKEA

>Mb|QLI66637.1

MTMGSRSDFPPGTVELLSRQKSGKEEITRRPTPSDDPNDPLRWPMWRKLF
NFGLLTAMTMAIFTGLAIQSLFFKPLRQELHVTTAQLLTAKAILLSGEAV
TCIVFIPLAKKYGRRSLYIVSTAVFTAAVWWTAYMQTATELYLTNLLKGL
AGAINETAVQMSIRDMFFVHQRGTANGFYFAALKFGFTLSPMAAGAQATA
FGWRSCYMTLAVFMTVLTLVFAVGYEETKFVRPAADDKGSAEDEDAVDGA
LEKDLDEKRTPQVPFPHYLRLQLLTPTGESLWKTLYQPLFTMAIPQVMFV
AALYGVDLCTITVSGSMKSIIFVEPPYNFTPRELGLMHLGPFVGSILGTL
YGGFLIDAAIVWLARRNGGIFEPEMRLYFLPIPALAMAAGMATFGVAADR
GMHWIYPIIGTTLSSFGFGGVADIVFTLIIDSYPDIVSQTFVVITFFRNA

IGVIGAFAVEPWRQAMTVSGMFVLIAGVIILVHLVAVPMAIWGKRARAAN

AERYYRLSQATW

>Mb|QLI66644.1

MSKLETSQDPHNDLSDQIERAASPSEGEVIDTEVKTSDAVFGNITDGGPN

YRNVGWLGTAALMMKTQIGLGVLSIPSVFDTVGLIPGVILLLFVGTITTW

SAYMIGVFKLRHRQVYGIDDAGGLIFGRIGREVFGVAFTLILTFASAAAM

IGISVAFNALSTHGACTAVFVAVAAVFGFTFASIQTLGKISALAWIGVTS

IVIAVFTVTIAVGVQGKPSSASQDYEGPWRSNYELFKNPSFSDALGAITT

LIFAYAGTPTFFSIASEMRDPKHYTKALMMCQSVITLAYIVVGVVVYYFC

GSYVASPALGSAGPLIKKVAYGIALPGLLVTDLLLIHLAAKSIFVRVLRN

SKHLASNTPKHWAVWLGSTFAITAVGYVIASAIPIFGQLIALVGALLGPI

LCFHPPGFMWMYDNWKRSDGRKQARWYSGVASSVFVVVTGTFLMIAGTYS

AIYTIVNTPGSSKVWSCADNSNST

>Mb|QLI66646.1

MKLSSGLVALLAGLAAGNPAMLRKRQADQGAKKALETYCGNAGFEAGDVL

EADEKLQGTCRKKDGAKKEGPSVAAATIFCDSVGREFNGAGASYMKADYV

IFCGSKVANKPDAAKNSGLALFNQIIADAGDDEKLLNKTLGQLVSEAPSK

LVDMYNIIRAPPSTAKLGLGGPARFAASAASFLLTAIPDNAKPGGLLGPD

TALGRWLRINPIYGKAGTQPSPFIDTDPSGVTLLPQTQKCIAFKDESRWY

WSKKVKTCESWHDVDPCDTEYAYDVAKNEGKTRETRCKEEANPPKEKTPA

ELEKEKEDKLREEEKNCFAPRMTCSNANGKFLYCMDERDSEECKRNQWHP

GQGHPRKE

>Mb|QLI66695.1

MATQRYTEALTLFGPAPATIVSPTRPITKRYDDIKSLVTDFATATMNMEL

ELPHFIDTLVKGNSPPAGTRDGTLGFVRSTYFSIPANPTMQALRDVIGGR

LYNIRHELDIDGNPRRLPLFDPPLDPGQLIAARAGGASLASLAGGRTEGP

MPNCRAKFLLQKAQDLRAELRSLSESYLSIREWRDAEAMARLRARQETGV

LSLLNDVRQHQVLEATSALGTLAETRKSHMMRLEYFLALIGESTDMVPSS

DGDWVDLDIGIRQPNKDELRVSAEEYLESAATEWAAYTNKIAAGIQQNAG

LLMTLPSLALQAQLMGVGISMQMDASIIAKALLVGASVIQAATQASTDMA

SRAAREGQLVRQLQEQRMCILLPRLPRRPGIAC

>Mb|QLI66763.1

MKISILLAAFSCLVSAAPPAKPKTAAQSKAPWNLQAISHRSAPTRLNMFR

NSDYLYTPWPKDKTLYAYVLDTGIRTTHQEFGGRAENFWTAFKTADNQDD

FEDSSGHGTHVAGIIAAKTYGVAKQARVLSVKVFGPNGQVLTSQAILGFT

FAMNDIIKKGRQNSAVINYSGGRKFSMAWNTIVERAFNRPNGPILTITSS

GNDAKDAAGASPACADEAITVGSIRSDWSVAPSSNFGCKVNILAPGGKIL

SLSNTSDVATKTLSGTSMAAPHVAALALNAMAVFGKSSKDVLEFLTQTAT

KDKVKGDLKGSPNLLANNNNARQRA

>Mb|QLI66848.1

MSSDKQMTNAADNDVIEQPGSSRQVRYTAADGSQYATTTDDIAGAERDVA

LPSRAPTADKWSVQYHNMSRIVSRGKLPPRLIPATPMIPFGPPSARWAQP

QEQVPGPQEAARRPGPGPLQGPGPAPQPPQTQNAQRPQQKRARGRRSRGA

NRGGVQKSGQQGTSSSMSASELMARIDRDPELLRQVEELALRKEQETSRA

AVERAKRAAICGNCLKVGHTVRDCAQPADDGYVHGCPICNGSDHESAQGC

KMHWPHRLERRLYWAIEQRARRPTLAAFPNWLDVFTEARNAGNDMEALPQ

SFPWTPLFSRTLIREYPDTKHPWSNFDYVANNSSALPVDPLVTDQAAVIA

NDAVIRSLLSRPVISNTGGRP

>Mb|QLI66865.1

MASTPQTNVLVRGITKLLCAYAAPLLDSRIEESSQPFTVPDIILPHDNSK

WWGWTHYGVFITDLPEPYRYLNTMTFIGAPGVLCFDNDYLSAPDARNTAT

VLSSTAYGDTHHYEAYDAASSCDFAADGSRLAWGNDLVITSNYPNFTVAG

RYRHMQVKLQISATKQVSWFVRSPVYDHLSLLATYTGSITDDRGTTEIAG

MCTVEYARSMTPQALSRHPLPPQLKIPVHFFTYQILHLDKRTQLLLTDVR

ADGMTLCKLAYVRNLDGEALVYQDVAFEVLSYRKQHVTDPRGRSMRTPER

MQWIVRDEDQEIIKFVASVDSPLRYGHGQGYVASYQFTGTWRNKDVTGIG

YLEWIDLE

>Mb|QLI66953.1

MTILSILLHFLLWSLQSCALSVPTSHDPTLDLSPYPGVVGTHESDFLLRK

RQSSSDILIALYARGLNAEARKAAGIEAFHWGIHITPEGALKSEKTTSLF

HVINQEENIKLFQYEKRTINPFRQRVIFARVKVGTLPATVSVDKVDELLS

QVQAPSKLENPGDSCVTWAVCGIKKLQENGIIENFDTAGFADKVLEYGEQ

QTRALDDDEPEFEADEFDIGNYDAKEGKIIRQESVPCKRAGEPCINPNPK
EKETEPAVEKPEDAELIAVAKEKSKENFDGLLEEFNYGSVVKQDKLYGEL
NARLPEFSAPRVERIAGFASKLGEGALAIGGLVLYGKAVADVFASEDASV
LDKAAVVTSILPGIGCAVQLADDEQKGQVDVTHTALCFTEDALLVSGFWE
IALVMQVGEEISNWIQAENERSKFWDGDLLAQKGAEGWLQNVKRLINHIK
GDEFFVNATSQFATYQILTLYQASQLTGDLHATAKTNPQGIEADIHAHVQ
PELKRQICSVISDSKRQLQAKLEGIALNHTAKLEREFKNQFLDDWLKAAT
TPKPIFGITLPDFESNTKLIHEQVEKARNSPLQLYEKEVKAAIREVIERL
PTPAPCQCDQGKKGGKCEFGGCQSTKPEGHPQDAGGRIYTANVQSIEVAK
RLRLTDTCQALFAKCQGGEAGRALFCTPGK
>Mb|QLI66957.1
MKFVTVAVAALASVAAAWPYERECKPATYRCEHSLRGWDVCSTSGQWVYA
GRCEHGTKCKMNHQNGSPYCLPPRHHHLYPEEEFEI
>Mb|QLI67041.1
MASHNALDSPDSYAVAWIAALPIERAAAEAMLDEEHAAPTGFTRHQTDPN
VYTWGRVGEHNIVIASLAAGVYGTTSAATTASSLLASLPSIRVGLLVGIG
GGIARPDEDRDIRLGDIVVSEPDGIMGGVCQYDLIKAKSGDKRQRKGFLG
RPPTVLLNALASIQAGHERKDAKVPFYLQEMLEKNPKMGKRSKQNPGYAH
QGSDNDHLFKASCHHVPGPDCRGCDTAGEVRRDPRDTTDPEIHYGTIASG
NTLVKDAATRDRIVADVGEDCICFEMEAAGLMNHFPCLVIRGICDYADSH
KNDRWQRYASATAAAYAKELLTYVPAAEVQETKRALEVLQLVQQQIDGVQ
QTTVATKAATDSIRSDLRTDKVKRWLCPPDPSTNANQARKLRHEGTGAWL
LKSPVFQEWHSGSRRHLWLNGLAGCGKTVLSATVLDHLTKGGDRLILGFF
FDFSDTTKQTLDGMLRSLAFHLYQGGTGSAGLLDASFQAHQDGRDQPATK
ALEDIVCRMLAAQKKSSIVLDALDESTTRDELLLWMKDVVSRPDLGDVQL
ICISRPEPEFMRDIPSLIGEENCLALDKKSVNADIRSYVAAQLSQRPDFR
DKRLSEDLLEGIQRKVGDGADGMFRWAFCQLGSLARCPHKAAIKEALASL
PRNLEETYRRMIQRIPTELKTDAMRLLQFLVHSKRPLTLAEAKEVIATRI
EHEPRGFDAKRRLFCETDVLSYCPSLVTVVHATDKELHLAHFSVKEYLLG
EDQFNSTTASISITTTCLTYLTDIKGSHDEIKRDFPMARYAAEVWTGHAA
LAQASEEILQATVRFLETEATFQRWAQLYQADRDWDHDPGPARGSRLYYA

CFDGLVAPVRDLIGKGADINAKGGKYGNALQAALETGHLEIAKLLLDRGA
DVSAEGGYYGNALQAASFRGHLEIVKLLLDKGADVNAQGDYYGNALQAAS
LGGDLEIAKLLLDNGADVNAQGDYYGNALQAASLGGDLEIAKLLLDNGAD
VNAQGGQFSNALQAASETGHLEIAKLLLDKGADVNAQGGHYGNALQAASQ
KGHLEIVKLLLDEGADVNAQGGHYGNALQAASQKGHREIIKLLLDKGADV
NAQGGHYGNALQATSLEGHLEIVKLLLDKGADVNAQGGAYGNALQAASLC
GHLEIFKLLLDKGADVNAQGGYYGNALQAASLCGHLEIFKLLLDKGADVN
AQGGYYGNALQAASLCGHLKIFKLLLDKGADVNAQGGQFSNALQAALEEG
HLEIVKLLQGRGAVTSYSKLSVSRTPSNLAKKTSSHGP
>Mb|QLI67090.1
MDSAATNTAADATITTKPALAARSTLPTTNLGALTTTFTPADDCMSLTNR
YFNSEKPGTYYYNYGFTCNVNGVNGPIFGPMAKPTCFPESFASAFTCEFE
LSDMVTPYPVYSPGLMCPHGYGPSCTISQPAGTASDAANATSSPNLAERK
IQTTMLTQNEFVYSGYACDTSKPHGCVSTVKLGGQVTAVNGARGVCFQST
ALVTRTAISDTRAIAWGPRVLIVQPTPGPSTGESSKDDGTAKTSLSPGIE
AAIAVSIPLGLTIFGLAIYILVYWKRKRAKSNGKAVSARLGGHDCGIPEL
EGKGVILPSNSPTDQSDVSYELPGDHRYLSQELESITKPVELGMGENN
>Mb|QLI67131.1
MNLLLVILSAVSIIIANNLDTLDPCILSCMLQVLPLVGCTGNDDEVVLCG
CKIIDRYTIVAPIADCTKKACNIESNDFPQVLKSTQRCSAAFGPSTRLSD
ITLPRYIPTIRIFSSSSPPGSPVSSSDPSIPPASTTPTRTPNSTPNDSEG
TLQTITPTPSSLTTPIRTLVSTVTSNAPIGGQTDGLTATPTASPAPSSDG
PGLSPGVIATIVVSTVTVIGAPLWLIRCYRRRKPRATSSVEENASPKPAE
KRNESVIMGYTELLGSTQMRHEMLSHTPLNAPLPLYINTIAEIDSNPLRK
TPEVSPEDVSPEDNSSQAMSFSTTTAISRPEPPQAADEEEAQEKTHTEAP
EDSSGASDTEAELDMLRKRQKELERKRHFLQQIQEIDEEEARLQERIDEL
QRQSQSHEK
>Mb|QLI67151.1
MSDGIDEAIRDGRIPPDVDMKLLRNNQNIPGIASIASVTFLATLVVVCRL
YSRKFIIKGYGFGFDDGVVLASLVVFIPFTALCIRLIVMGAGQNPMWIYF
MMDDETYTQIQALDTIAHLIYSTALVLCRISGIAFYWRLCYMDKKFLLAI

KGIAAIILAGYVAQICLLIFHCLPVSMVWVFYTKEDVIGLSASRVVLVIN

YGWESDQKIEFQFSFLKLLCLEVAEVSATIIAVSVPGVKPLVDKYILRKD

KETESSSWKSNGLSMSSSDDVTKPRQAHVDIMEWQRENATRLPAGKGL

>Mb|QLI67169.1

MGAFPPPIDPSYIPSVSLTRLPATAPTADIIATLERDGALILVDLVSPQD

VAAINAEIEPYIQKARAESHEAYDLIPKQTVMVPGVVGKSPTMARMAELD

VIDTLRTRVLQRKCTATWEDRTEDFSIDPLLNSSLTYHISYGGPRQRLHR

DDMIHGIYHRGGEYSLSDETMLGFMIAGSKTTRENGATMAIPGSHKWDHA

RVPRVDEVCFAEMEPGSALVFLGTVYHGAGHNSVPDQVRKIYGLFFIPGT

LRPEENQFLAIPRSKVLGMSDKMLSLLGYKKPGTWLGIVNNGDPAENLAE

VLGMANS

>Mb|QLI67212.1

MASQSSKTYAQRGHAHQHPVARKLFEIAELKKSNLVVSADLEETESLLQC

ADDLGPYIAVFKTHIDVVRDFGDATIHGLKTLAKKHNFMIFEDRKLVDIG

NTVQKQYHNGALRISEWADIVNLSILSGDGIVDALTQTVTDPGFPFRGER

GFLLLAEMTSRGSLATGSYTSQCVRLARRHPESTFGFVATRALTAVQPRD

GGPDEDFVVFTTGINLESRGDKLGQQYQTPAEAVGRGADFIIAGRGIYAA

GDAVAAAKRYRDEGWKAYLQRVGG

>Mb|QLI67221.1

MSSNKQMSKGVEDGVVEQPGGSRRVRSLNDERSSFVATTTTRDLSGAMED

IARRSEAHDDDDWVIYANNASRMLTGGLLPPPPAPPRDLLGITASPRTPA

SVQRGAFRGQARDARGSQTPRSGQGRRGQDRRGWQVQGQNSRGQDRRGQS

RGPSRGGIQKSRRQGTSSSARSLGARLGQDPSIIQDANKWRQQEVSRGRD

TARAAQEKAQNELFCGNCLKHGHRVRDCVHPAEDGFVHGCPVCNKADHES

AKDCKQTWPQRLERKLGWAIEHRANRPTLAAFPNWAKLFMEASEKGDITL

PSKFPWTPFFAQELMERETQPWTDFDYVANDSSKLPLDPMTAGQETVIEH

AESLFQIPLQVIGSTGGDPWYPRREEDEDMADPRPFHRPASDKLDERMDE

DLS

>Mb|QLI67287.1

MLKHSLLYCLSLFATTQVCNARAVEKEIRAPAAQSWRGLGGLFSGHPGVV

SWSANRTDVFVRGTNNAVYHKWQNGDSGSPWGPSQTDYENLGGVIYGDVT

AVSWGPNRLDLFVLGTDNAVYHKWWDGSSWGGWESLGGTIIGEISAVSWG
ANRLDLFVRGTNNAVYHKAWNGGSWSSWVSLGGVIVGNPQAVSWGPNRID
VFVRGTDNGVYQNAWSGSSWSGWYNHGGVIVDDVTPVSSASNRLDLFVRG
TNNALYQKSWSGSAWTAWISLGGTIVSRPSAAAWDGRYVAVVAQGTDNAV
YLKEYNGNSWADWRSLGGVVTDAPVINPRGGFGAAVFARGTNAALYAYE
>Mb|QLI67293.1
MTRRVIPHSESAESVDDAQHVSRRIYSTGFGIAVRTGHPQSQDNDPEAGV
KKVFYTIVTTKLLIPGDGEPIKDAALVVKNKIIDWVGRQADLPNEYTEKP
HKLHNVPYLMPGLWDCHVHFAGSNDEREAEEGSAGLSFLADHPTTAGARL
ARGCWDAIQRGYTSMRDLAGFGCEISKAIEDGVIIGPNIYSAGACLSQLA
GHGDVFALPAGDALLNLGVASVKAGQFGAGMSCLVDGVDECRRGVRLQIR
RGAKCIKVMASGGVLSRDDNPLYAQFSREELDTIVSEAKRMERTVAAHVH
GKPGILQAVEAGVTSVEHVSFADQECIDLIKDRGIIFVGTRTIVNLLLDS
KGEGMPKKMWEKAKLVGTHSLEGYKKAIEAGCTIALGTDTEPGFNMAIEL
QYAVEAGMSSLEAIKAATANGPLTVAGQAPLTGQLKAGYEADMIGVCDNP
VEDVKVLQKKSNIGWVWKGGKLFKGPGIGPWGEEL
>Mb|QLI67312.1
MDSSEEAYHRRRCLVQDIICSFGLKSESVTPVEYVGYNPFPVNNFIYKVA
LSSPATKEHFTRAGHQTCAVLPPLTGESTVIVRLSNPTSMGMNNTNRVEN
EVCAMAVAREAMASVEGGRYDAIVPRVYAWKSTRMMAGDAPEQGFGWIVM
EYMEGEVLDMLFGDMKWDEKKRVIGEIACIFAAFQKAKLPQRVELHGGMT
VRDGDIVSGQATMHKGEPSGYVGHWKARIDHALEQADESTLINGWKGGVR
ERIEKFKNDKLGVLIHNGEVDTTLLGLVHNDFTVSNMLYNPHTKRITALL
DFDWSSVTHPAHEFFTSFRDVHGRVDEASEKLRKAIFSGDFGNHPDEDED
QEAWKLAKTWDDALKQHGGMRPCDFQGIELLQGLWNFVYSICPFELGSKV
MLFRRSKKESEAAKERVEGQISKMLSGWGV
>Mb|QLI67423.1
MDQPPELFPTNYKPSIRIAELSANAPIEEILSVIDRDGGVIINGLVSSEE
LESIEKEIEMYTKADRSEDEGFFKDLVPQETILIGGLVGKSDTMARICEH
PVLEELRTHILTDVGTRRVEKYDLPYHIDPLLSVSLSFRIQYGAPRQRLH
RDDGTHLIEHESKPYKLNNAAQFGCLIAGVETTQENGATMFIPGSHRWDD

KREPRLDEVTFAEMKPGAALIFLASCWHGAGHNSVPGFTRVLHGLFFCRG

TYRTQENQFLAIPRSKALSMSPKMLNLLGYKQPGAALGMVENADPMLDLE

HFLEEANL

>Mb|QLI67481.1

MVVISKFTGCWALWALASLGRLTPVAGYPSQDAASVSVSNTTNLTRGGYV

PDIVPKPFYAIANRVLTKQGAFDALQMGANGLYIPVWGWIHTGWWADYDG

RGRSAGDKIENLFKEIVKFRIEGRNICLVWLDLRSPDWCTPHYPRCIFDR

LVGFSRNILQSQGIYVLYGFHLESVNGSAYASLQDDLNDMEAISIEGELA

EVNASFWKYGPSAPDSRVMSYGYFNLATPRFGHCNEERFYVCSELKRGAK

SRDDVGKTYAWTVSGDQSVYVDRLMDAGVDGLVFGFRTIDFDEHDAPFFA

FRNIIDWIDSHPYRYLANIYDKPWDRRLNNESDNK

>Mb|QLI67561.1

MNGNGDGTNSDLACFISTLKRRRIQAEHVNGDALPLEAMMHDTGAVIPAA

VVSPRSEWGVCQTVKLLQEFKLYDGFTISIKSGGHGYLHRDPSPHPVILL

NLGAMTNQYISNGTLVLEPGCLLGQVMHTLATNRKAVPHGDCFGVGVGGH

FTTAGWDLMLTRRYGLGCQSVIGGRVVLWDGSVVSVDEHSHPRLLYALRG

GAAAGAGVVTEIRLRLIDEPKVTTWCNIRLNREQVAVCVTSSLMDKTQNL

PLDISPSFKFYYELDDPEPVCAFRIASLLRKEETIACLREHLGDQIASWA

ADLSQWNEKPLIDYRLQVASEFLAANFGMLAEASSLAMLKDPLVFWKPAN

AAHEMTRSFSVQTSSWVVPDCEAVLPKLYDAFEFAKDHPVHNRMYALVIL

GAGAMSELPERCSMPLGKVLIRFESHWDNEEEHGPWCRELTSKISDIIQT

KADLNVKRPFRGDIWLREQGVDAELNGILETYDRRWPKPAILNRRS

>Mb|QLI67622.1

MKVAIVAAGDLARYFIEELRAQGHEVITISRSKKDHLDKLGISQHVSDYS

VSSLTSILNDCDAVICTIRAGVPNFTTVHKAILQACQTSPRCKRFIPSAW

SGNLEGFPDEPLEWADELQPTLRALGAQKEVSWSAICPGWYADYVYPAKQ

RYLVDIGEMWPQNYKDKEFTLYGKGSQLVNFTSVRDTARATIMLLQHDRH

EWDQYTYLSGEQRTWKQLSEFITARDPAYTVKSKSLASSIRQYIARESEE

STTAAIFEIWGHSESLMFPWEKAQRHREKFFRGLKFRTIAELADEAAAAP

ASFP

>Mb|QLI67638.1

MAPEIDTPAWKASILEGITAFPHVYTIVTCADTTHDLSPVFEADKDAYYA
RQVQEYCEHIQLSLHAKASALHEEVQTLRDENRDLKKEVATLDKVIDRVG
APKHTTGQLPHRQTRDHPTFNATEKDIEKRQEEYVGWRSKAMRNLAVDKT
IYNTEFRRLQYIGSMLDGSAYTLVRQSLDVITMNPDETGLWEWKTADDLI
EFLNSQYETIDLDRTASRNFDNYFMTNKPFENFIAEFNKLATLAGKTDKQ
KVEALQLKVSNEVIDEVMHRSGKPGNDDWPGWRKLCQDVYNDLEQSKHIR
RMRSRREPGPRSASDNPRPNNPPAPAAPIADAGELMQLSASSTSEARRRE
RQERGLCYYCGGTHRIRDCQEKKDNDAKYSLQNNTPRSDAARGRGHGRGS
YYSPRPSPQVPNATYQPQPQPQYRQLQGFPPTQQHYVQPPYLSPTFNRLH
SMDQGFIESDTSSLTDARSTPDTDRSQGNV
>Mb|QLI67741.1
MSLPVDTIDTDNHDFEFAQRGLVAKLNNPRIYDSNNNVVWDASAYDFMQQ
DCPPTANKSLWRQGQLCSATAGLYEVVDGIYQVRGLDLANMNIVQIPSDK
NKIIIIDCLTSVETASTAIQLYQDYHKGRFQRQAEIVALFYTHNHVDHFG
GAQAIVDLAGKDLRIIGPDGFLEHAVSENVYAGAAMSRRSIYMYGEALPK
SPNGQIGCGLGQGLSTGRSSLVAPNQSITQDGALNPGIKELEIICQLTPG
TEAPAEVNFYFPAYNALCMAENATHTLHNIQTLRGAPVRDARLWSRYLDE
SISLFGHKSDVVFSSHHWPTWKHDSAGSGIQDDHVEKPHNLIVTFLSEQR
DYYAYLHNETLRLLNNGSTPVEIAEQIKTPPNLSLRTNLRGYYGSVSHNV
KAIYDKYMGWFDGNPANLWKLEPTDEAVEYVKCMGGPKVVLEKAKAYVSE
NIDSKLRFAATLLDKLIFAYPDNVDAKNELISVYQKLGQSAENGTWRNIY
LTGAYELQNEPKPAVNTMTPASLMALNLDQLFDTMAIRINGPEAFAFEGK
ITIDFMVEDMPRKSVQGKGQIGWHMRLSNGAMTGHEIPYVAFKDLKDSKS
DLTVWLAHETLVTAVGIAAAGKKPEITAYQVVTSGDVGAWEKAIAFIQVP
KSAFNIVTP
>Mb|QLI67767.1
MQRAAENPANLGNATGAGDALDFYVGTFNLPYIFSLRFNPNGNQLHVLRI
SEATGPHSWLSLSPDQRTLYTTAWTKPTCSVAAYRNHGSTQQLELLGSRP
VRNKPGYVTASSSHIFSVGGPTGEVFRVEHGGGIGPLAQELDFAGGQEAD
APKAPDAVPHGDFGGLRHGAHGCDLGPDGRTLYVADIGRNCTWTFSVDGT
GRRVLHDQRRHAAPRDDDGPRHAWPHPNGDVLYVVQEHSSIVDVFRIQRD

AQGAVAALRHVQAASLLPQGKNARDYWADEVRLSGTPGGAGPEYLFASTR
GLESRTRGYVCAFALNRDGTLKRTAAVDIWETPTSGGIANAIEPAPWQVR
LAHSDLPQHIMAMTDSEAGKVFVLGFDGAKISMVSSITLEAPAGEDDHRG
LVEPATAVWVRPR
>Mb|QLI67801.1
MAVAENENTPQLAASEVENERLQINHHVTKLAMGGSILLAPFDVTRPNLK
ILDSGTSDGYWLTEFRKTLTRAETCTMIGFDIADERFPDPPPEGIELRVQ
DVMGPFPDSWHGTFDVVHQRFVLAATATKGREAVLGLCNLVKPGGWIQLV
EMQSRVGEDDGPAVHQFVELINELHIKIGAPRNLADVAVLEGHIMAAGFQ
RVGTMQAPGTLGAKVPDPNIRATAIKSTLESAASLLGANIGLPGGLQSMS
GDEASSFLERLEMELIEYGGSWPISVVWGQRPVK
>Mb|QLI67807.1
MVMGRKESSPLAPTFMVSAPGKVIVFGEHAAVFGKPAIAAAISLRSYLLV
TTLSKSQRTVKLNFKDIGLNHTWKIDTLPWGVFHEPEKKKFYYSLVDSLD
PELLEAVIPHAEAVSKHLPETQRKIHVRSATAFLYLFLSLGSPLSPGFVY
TLRSTIPIGAGLGSSASVCVCLSAALLLQIRSLAGPHPDQPAEEAQVQIE
RISRWAFVGELCIHGDPSGVDNAVSAGGKAVIYKRNYSGPPSVTPLTKFP
KLPLLLVNTQQPRSTATQVDKVRALKNNHPVVTQSILDGIGHLTTSALEL
ISSADLDSSGLSDTLERLGTLIRINHGFLVSLGVSHPRLERIRELVDYAD
IGWTKLTGAGGGGCAITLFRPDAKDETIKGLETKFTAEGFQKYETILGAD
GVAVLWPAVFRNNIGGEGGEEIDQEIFENVVGVEGIEQLVGVGAREDREG
WKFWARAV
>Mb|QLI67814.1
MAATTNIHTEGLVVDKPGADFVMRPVILDEVRDDEVLVEMKYSGICHTDV
LFQNAKIPGPKYPAIFGHEGAGIIRALGSNVKDKSLQVGDHVLLSFNVCG
KCKQCLADNPSCCHIHSPVNAGSVRVSDGSTPARLADGTPVSSQCFGQSS
FARMSVVAERCVVKCPYPESLSYYAPLGCGFQTGAGTLLNVLKPDKTKSV
VIFGVGSVGIAALMAAAYLQVRQLIAVDVVDEKLDFAKEFGATHVVNPTK
HAEEGVEAAIRRITDGGADYAIDCTGRIPVIESLFGCLAPRGTATTVGVP
PPDSIVRIEPQTFLMENKSYIGVLEGGSHPQKFIPQLMELHQQGHFPVEK
LCKVYSVKDFETAMSDLREGKVVKPVIHWD

>Mb|QLI67847.1

MKCFHWTVVLAALLQTAATEQTSSSDGAQPLGHIVVLKPGLESKHLERHL

EWVKSVHKRSPDNDGENHHGVKHTYDGEDYGFLGYAGHFPPSVLEDIKRH

EHVDFVERDQAITLVLPGEETPESQAVSQDNNKQSSRIVGRGGLTMGQGY

NTFLDKGAMHNAVLFSEGMNKRSENSTAELFNQDMMTRFNFTPPSADLTD

IDMSYFDRPDPRALIEQALAGLARDGKNTTDLTKRHTRTASQDECNGSMR

FTSELVENYEQVPPNVPSTCCFIFMTQASTTSDTNQVLLTRLARSYLQTL

EISGGATLSGWGQSASVSGAYLNKAEFSKKSVIFVAIINIERQLDSPTGF

EFNTHNYKPKTFNRDFGDKWIQGFHEGGKMIARVTFTAKNQIAKNDLQAQ

AKASLRFWGVTGDIEASAKKSMEDVNTNADVEIKLFYQGELGRFMLQSGS

PKSISEGTAQASFLQAKSWADQFIQNACQHRYAYRPLLDEYRNIEGFPED

QGVPDYYVAHRMSYMILSQIVVISDMKDYLLSRTDLDIKLKYSIQVDEIK

MVQLGRDWVQSTVEKPEDAITTAGDLLEKFDKNFRAKYEMLMPQKPYIAG

VKVVYGGYPSKDPPAGRVKEAEGRSDDINHGRGGDFVWLVPIRTDHAEDA

CTSFELVIDQVPDEFGNLVKGSKDKSRYLRCKKSSSRDKIRRLVLSRLEA

APAPAPRVTKDRLAFIKSLLGRSWVEKVRFLRAFTGKTSNINQGRHGADE

LYLLWSPRE

>Mb|QLI67877.1

MEKRLPLLLPSPSTVIIHSGKNNDKFSIVRQKRRQVASACANCRKRKEKC

DDKRPTCGACARRGVTCNNDIKEDEPAGTVALRHRNISLRQENDQLRELF

KLLHKLPTEEGQEVISRLKIADDPIQVLRSVQDASLLINNPNSSSYSALL

DSRLERLDLLALRESAIRVDAKPWTAVAGDGIVSELVSSFFNWDDAFYLP

FLDREAFLEDMRAGNVATGKYCTPFLVNAICADRSYTCRRTRAFSGISKN

DLADEFFNEAKKLLHLENGRVSIPTVQGLTLLFSIACYRGTDKLGGLYRR

SAYDMFHQLNVDAMYARIKDDPLAARERRVLCRLAWGLFLFESIVGYMYL

QQSLIPPPQVPRCFDPPPSAKDAPVPNIDLFGNPHTSESRSPPFVTGALY

LACDITVMLYESMDWNFRSESIWGTEQDLYRRRDMIDEVRQWRASLPPNL

RDDTNFTPQTCYLRTYMNEVLFSILRPLSPQTEIEPGWTVKDLQLSFCQI

DIDNMERFIKVYTLRDYACINIAGSYNAILVLVYHLADPSVHHLFAKAAY

LIGETGGDFPMCRYILQAIKAIAWQAKTSLPQMAKRYFENLDGARNSFRD

ISFALPEETGKRMFAGGPRPRFKGDDMGSLLFKWSAMSIE

>Mb|QLI67932.1

MSNATGLPPDNNPAASVNSGMSKEAFYAVAWTATCLPLPFLLFRLAIRIR

SFKTLFVDDALVIAAWLMLLVSTILWHTKAGTLFWQLRIVTGKAPQSPEF

ISQWSRLSSNIVVWNILCHTSLWAVKYSCLVFFRRILGPQAPRAKKIWCW

VVGILLLAGWVSCVAIIDYKCSANDIKYIMAHCAGRSHVEYQFRTFYGNL

AADIVTDLLVMSIPLWMLWHVRIQWPKKLLLAGVFSVTVFVMIAAGIRVA

LVRTSTTGVKHTSIDLLFIWSEVEMGVAVIVANLASFRQLFVFSRNRNPS

GGKGYDATSNRHHQFQPFESSNPSLQGRNRSENDLIGLTDFQAPQIYSTS

QHNPTAPDSVYVSHAIKVSSHEWTPEPQDSRRCHNFV

>Mb|QLI67981.1

MDRIVRGPWLNSSLEEIANDPDARNRDMARFNQPPPPYTSPCRSAATTEF

EVPDFEEPQAPLTRSELRLKLQHERCASCPYHQFMDQWNDERKEVENEVN

KKYAQRTVKERWVEQGIWRDNWSEIPKYDWKHEEPPESDSCSDSEIESET

PACAAPFSFSSNPPRPRPRPRPRPRPNKPDQEIWEAAERREIRRGIREKERN

ASRPFEQFLYQLRRESKQLETHSRDTVTMNLDINALAFQNVQRAWVKRAI

WDDEWREFPGMSWKYERPLVMPPTRDPSPDHAQSVENQLEPRIIPTYGFG

HVDAHVSHSQEMQTPLENGIAGTIEPRNSSAAAPPHARMIESIENEQHDA

AENHLEHDSSRIRDKSSSSLPSNPAAAVSPQRSNVDQNAATGFASNTIAV

LPDRKIAIVEEDPVSSPPGGANDSRTRLKTLQLYCLYLSRP

>Mb|QLI68068.1

MSVLSILLYVLVWSLNSRAISVLGVPANNGLTSSPLPQLDVGSQEAPSFL

KTKRQSNSDILLALYYRDPLALWKKKLDSFHWGIHVTPENVPGKSTTLFH

AVNEGEDLKVFEYEKRLVNPLTQKKLLGRIKIGTKPSTVSVEDIDNLLSQ

VPVPNKHDASSESCVSWALCGVRKLQEGGVIESFDTETFSDQVLQYGLEQ

FRATLYAETGAENEITHKIARYDVQQGKTVVEEVQGNQGTPVENPENPEE

APVLCERSALDCMGRPAKEKTPNTADEGELVPVAKQSSKENFDSLLEEFG

HDGLVKNDRLYTELNVRLGELSTLPRAERIAGFAKIGEGALGVAGLALYG

KAVADVFTSDTSVMDKAVVLTSVLPGIGCAVQLAQGIQNDHVNAGHIALC

FAEDALMLSGFWEIALVLQLSESLVEFFKEDAEQRKLFDTELFRQKGSEG

WAHNVERMLSHIKSDEFAASAKTQFLSYQLLVLYQASQLRGDFQASHQAI

SASVNASASQPDHGTNVDAHVKFELNRQICAAMALAKRQLRQKLESVALK

HTEKLSNDFKEQFFSEYRKAATRPISFLGIPMPQNSWNIAELDRVINEAR
AFPLPLYKDRIDHAIQEVMERLEMPDRCKCLQGSKKKVHDYTKSIKQEAS
SIIFTAQRFLSPRPAVAIAIMRAFPTTLLLATLAPCIHAGVIAARKDHGT
FNTYTDDHCRDFEQTIYAWDGIERGQICPHVKSVQANIANDTPFG
>Mb|QLI68132.1
MVAMHTFFKSAFFNFEYLRLLAMAPHEGAEIGEALEAAAKIRDLDPESWF
NAFLEAGNKAECIAKEAEQAGDVVSARRAYLRSSNYLRAAQFMLNEGKIG
QDRRVLATLERAIGNFRKGVQYRAGKTFFLEIPFENGIKLPGYLYLPEAS
RRIPGRKIPILLNSGGGDSTQEEIYFVNPAFGPDIGYAVVTFEGPGQGIV
LRRDKLPMRPDWEVVTGAVLNHLFDFAASHPDLELDLDNIAVTGASMGGY
FALRAAVDPRIKACISVDGFYSLASFVGGRMPGPLFNGFMKGWLSDRTFN
AILGFLQRLDFQARWEFNHLKWATNSTTEAEIMRSFGDYTLLKPDGTEYL
ADVKCPTLVTGAGASWYFDPATTTDKIYDCLTSLKDGVDKEKWIANDIAY
GGLQAKIGAFGYSAQRTFQWLDMKFGIRRETLNATSDLGSLVKNTAKNTS
LL
>Mb|QLI68135.1
MINSDAQSAQKQVEVEKPDEKYSAPRLLAPIPDSYQPAKAITKIPATSSL
EDILAILERDGGVILTDFVSLQELDKIDQELEPYTKSSIVDDDSYNNFIG
KKTLVIPGLVGKSDTIANILDTNETIDKLLKVILEERYPAVFEQHTEELV
VDPLLSICMGFHVGHGSPRQALHRDDMIFSSKHRPNMKINEVDGFSCFLA
GTRITRENGGTMVILGSHKWEHDRRGRPDEVSFLEMERGSAFIFLSTLAH
GAGYNTIPGEVRKITNLVFCRGTLRTEENQFLCVPRSKVLKMSPKMQTLL
GFKKPAGSWLGMVENEDPAKDLEAIYEKMLK
>Mb|QLI68193.1
MLGLRQLSWIDDRLREAFPNKNEEFFGGLNILLVGDFFQLPPPVLQKPPD
YDKEVQGVEIKGGNTYRRFDKSVFLKVVQRHRGDDQKAFRTALGELRLLQ
LSVESWKLLSTRVQAKLDDQEVARFANALGVYATKDRVNEYNHYHLDRLD
RPVIQVKAKNVGPGAAAAPDDNAGNLAKLIPIYIGARLMLTYNLWQPVGL
CNGARVTVYDIGWAPGADPVHDSPCVIMMEFDKYSGPVFLTTADGRKIVP
ILPAERDFLIGATCCTRTQFPLIVCYAITVYKSQSITEDLIVTDLSCRDF
QTGLSYVAVSCVKTLQGLMLDAPFDRSHLFYESPPDGMKMKMRDQRHRRQ

QVLTRNPYKTDQSSV

>Mb|QLI68254.1

MAKLGTMPPQHAAKPVDEHAAHLIREDNVPIQTQKPFTTLSAIGIGYGVT

NTAVGIPLILSTAMAAVGLAMAAVGLATATTLAELVSAMPHPGGQYIWVN

ALAPKRYRRGLSYTTAMISWVAAVATGSSGNLSVPLNAFSIVTLLQPDFI

YRRWMGFAAFQAINVVTCFGACFEHALPKLSKAFLLFNIVSVGVIIVALF

AMADARTSAKDFFTTVNTSGWPDGVAFIIGLNGANWCFSCLDVATHLAEE

IPSPGTNIPKALLWTIFIASTSGLLVVLAVLVNLGPVDVSDYSGIGIFYR

ITGSKAAAIGLWIPVLILVLASVWSIQTWQSRLAWTISRESGFPLHRHFS

KIFPAPFYTPIWSLVGSAVGTALFGCLYLASELAFNSLIATGILLQYISY

SIPTVLVLWQGRGNFRHGQFWYPKLGLVANFIMLAWTVVAFIFYCFPANA

QVRPSQMNYVSGVLVVIATFIAALWILYAKKNYRVMEI

>Mb|QLI68264.1

MAQPVKVKVSLPMLAAHIDHSLLHPTLTDAQITTGLLLCKQHAVASACVK

PCSVPLAAEVLAGSPVKVCAVVGFPHGSSTAATKMAETVEALDAGAAEID

MVVNIGKALGRQWHYVEHEVNAINRIVTTRGAVLKVIFENDYLQHEHIRK

LCEICTDLGVGFVKTSTGYGFVRRDNGMYSYEGATTPHLKLMRASAGPGV

QIKAAGGVRTLDDVLHVMSLGVTRVGATATEAILEEARARGIGDEEIEVE

VRRSDGTDGDGASC

>Mb|QLI68274.1

MHICARLCVLLLSLAGPTSAALNCRPEGPVLPKPKLSGSPIFKSAGENLT

KTLDDAVKGVIKAGWPVENVSFSLAVVSTDQESAGVPIWEYHHRAERNDR

GVKNITRDSQYLIGSVSKVISDYILLKSGVDIDRPVTDFLPKLNSSRSKV

RWKDITLRMLGSQLSGAPTNNGFSEYYYLKELFVQSGFPSIKDSDYPPCG

VIGFNKGCSANEILEGMISQYPVTAPMERPAYSNIAFVVFAMALQEATGK

NYTELVADIVSKPLDLRSTLPSPGDDGKAVIPPGESSWGADYGYNAPGGG

LVSSVSDLCKFTHALLTRSLDLTPTQIRKWLKPEDWTGAYSAVGMPWEFF

RPLTLTPSHPHPVTVAGKGGGAQLYSSQLNVVDEYGMGLVMLSAGNPGAS

IALSDALLATFVPAADEVSRDQAEKQYARTFKSERTNTQNKLIEATFKLD

NDSLVISEIRDGGNDVFGGIKKIWGLTMGQYTATFGSTMRLFPTDLYQTT

QMEGRNVTAEVWRLWPEFGEPLESDMPGSNLGFENCLQWALGDWIHYGKE

PLDRVVFYKDASQDVVGFEMPFLRSGILKPM

>Mb|QLI68278.1

MSAVSKLSTASLFDASSTVARAYAGEIAELVQRLRLCGIQVDETQGHGLP

PDAQMHDRAATVPAVVVSPRGEWGIVQTLTAMKDLDLYAKMPVSVKSGGH

GYFNGATCPGIMVNLAHMADSRVDGDVLTLGPGCVLGQTIATLARHGKAV

PHGDCYGVGAGGHFTTAGWDLVLTRRHGMGCQSVVGGRVVLWDGTVLDVD

DEHHPELLYAMRGGAAAGVGVVSEIRLRVMDEPARATWRLTPLTRDQLRR

CVANRAFARAASLPEDITLSFRFFFEPHDENPVCSLNIFSLLTVSETIEH

LRRQLGAEVASLVDDAAAWSEKRLLDLRLLPASRALSADPGMLSELSSAR

LHDSPHVFWNGAMVRRETGSSFLDTSSHWVGTDCDAMLPEIYARLEAVKH

LPMRDRMYLLVVLGGGESLRRRRDCAMPLGRALARFEVHWDHEHEAGECR

AFAATVADVLRHHQDAGVDRPFRGDIWRPDQAYSEDDGLHAISRRFDRRW

GRGPRRVRL

>Mb|QLI68287.1

MPNICNYASLLQQLRNSRSYKSWEQASITIVAQIDQYYSVLFALSILELV

KWAANPTSNARLRVLTLSAFEESDDLRFQKSERSEGHDTCFF

>Mb|QLI68306.1

MKAVSVVVAAVAAGIALVQSHFQMAPCARSCIQKALPNVGCTGNDKDIAF

CLCRPRTKSKLVDPVAQCANQSACSAADLLRAQSIIDWRCQNVHPASFSE

ATFDEPESASWRQATQTPQPVPSSSSTSTPSSTPSESSTAISTASGSSGL

STGLVLAITSAVVAALGSLAGAFWCYRRRHRRRASSGQVRNGPSEEEKDG

HPTRSGHNELLGSTEMAHEMDIYVPQQPTPAGLSKQVYEIDSTPLRMVPG

SSALEAGQIVSNAAPPGSPRGPTRADGMKSRTLSDMRTAAQENGDQDTPV

LGELQKRRIILAEERRYLQRVHEIECEDKRLEQQISDLTQQQPSSPLEK

>Mb|QLI68322.1

MAKNFGIEIEFMLAGRPPSAILHPRLSGDDRWPDSESHHDIYETYSASLD

TLKVCEALTSCNLPVACRINPGKLQDPLTKLPEGDLVEDIEGTYVTRTGN

LEDPEHPLRKAEIGHVFRVWNKNLAKENFPGQQYHLGQQHQFRFWLVDGE

HIPNRSNERPPTDHSWNGMEISLPVISDQKEIDAGLPTLTGAFEALRNSL

LINLTSDCGLHIHSSPSSGSIDLPLAKKVIAVIRLLEEPFIQRICHPVRR

NLPSVEPIGSMSILGRKGKGDETETAPSVGGFIQTIQGIRSKLQNRSPDE

PDAFRFMQFLFASKTIDSLRKDLKSGTSGTTTPHRCGIAISLLGTVEFRY

PQSSFDPEWPAFWVKLMQKIFQICAQPDAAFSASFERLYELGTRQQALGW

ESWLEELGLSSQYREVCARHMDDANSPRKNEILPKVSGV

>Mb|QLI68365.1

MDNFMNMTKKAGHTAELQRLVDLHAVFLDAMDGKLVLAPINLQEPGKRIL

DSGTADGIWLRDVRSPLSVQHEYFGSDIEPELFPETPDGITYFKHSFKDP

WPEHLLNSLDLVHIRGSLAGSAPGKPIEVVKNLISMVKPGGWVQLMEMNA

FQPPKGTLGPAMTDFAKMTSEVWTAIGVGNFANEMKHILEEAGLKNVQER

RILCDIGKLAKPEMRARSANGITGPVAPLTAVARSVSTSFSAEQLDALPG

RVKAELENEGARIEEIVVWGQLV

>Mb|QLI68406.1

MRSTKTGPLLTQDEARVSSGYQTLDPETQSVGSDGDETQSSSTLTLPCGS

GPDQLDDQTQRLPFPRLMAAYLCLCLCYFISYLDMNAVTTSLPTISDALH

AGPGITWVGTAYLLGQTSCQPLYGRISDIIGRKPILLFSVGCIIVGDILC

GFAQTPIWLYTSRAVVGIGGGGISSLVAIIVSDLVSLRSRGKYQGIISLA

IGTGAASGPFVAGGLIQTGAGGWRWVFWVPSIAAACCFGLLVFLLPLKPV

SGNWKDKVGKIDWLGVVVSVAGIVLIMIPLNSGGSMWPWDSAETISTLTV

GVVLVVGFIAIEAFVAKMPIIPLRLFRHRSSSLLLVMGILHDFVWQATQY

FIPLHLQTVRGYTALQSATIILPFLLAQGLAGAASGPVMSKLARYSPVLR

TGFLLWTLGCGLQLLFNLHTSTGTYVVVLAIEGAGVGWVHQPGLVALQAN

CRDEDRAVATGTRNVLRSLGSVFGVAVSTSAYYAVLDKALRRSVPDSLRV

RVLNGTWRIGETGTERFESDILNARMQGFRVVFIVLVPLMAVCLLASFFV

ADVVLTGDAEKQEPKETDEESPSDDSTMMPRPGVSGILQAVRGQEQRQD

>Mb|QLI68430.1

MLNPVMRRLLLGIALALVLLSYFFFKERLGEFGITAADRPFQDAVKPDRG

MLSDIKKWQRESQIRRIVGLVFYGRRSQASILDCYLKRNLVKNGGILDEV

IWLQRTQNQADLAFLDKLIHSEAGYSRVDVQHSDGNYASAYDGIEDDVLY

VKVDTDIVYIEDSTILSMVHLRATRPDYFAVGANIINQPLSSWLHWGLGV

VRPYLPETEVFYPKDDEKQGHQSIGWRASLLPKWNSPNAFNMSEWSPPEG

RKHRWLPVPHGVDHILDGTPITTTTYDAYTSPGWWNWIVGAQQHYSFLEH

LETDQLWRYRFHSWDYRDLRMGIQLIAMTGKDINNVKPIAADDEDFFSVK

MPRKLGRSVVAAGGGVAAHYSFGAQKDGMAKTDILDRYRSYAQEKICKGP
ILWSSEADDPAK
>Mb|QLI68443.1
MSKLTLTDWANQPEAVVFGHCNPNNLAPYEQVQLLFEGMDMTAEGHVKLY
VVADDTESQIMSAELKQRAALSPDFAIPTICDYALLLQQLRKTKVSKLWE
QACVTIVAQIDQYCSAPFALAILELVKWAANPTSQTRLRFLTVSTFEEPE
ESVQRALEYFCPKLTVSLVQVPTRVATTRPAPKRLLWSDPDSVDEVVKLV
KAQVDGQRTSIVLCHPDEIHAIITGLLRMRARVNNLVNSSRFLAHILAMR
IEPVKPGSFPAVLFCTEKSQIPMEIHNLGAIIISRRRQAAIWECGRIVYK
PEQTSQWEVNQAVSYVWQTSTPTTGITILAPEASKLAELLPRRRVDNDQS
IPFLLDLLANFDGMSMDDMLGCFVTDFPIVYSNLSQLKLMKCARECSDDL
RMFSLVPGNAQELLLELLPQFEHQFLPAWFLATGITFPGATVSAKKAMIR
LAAIVHEGVGFIDRGSIFWQEVPEIEKAEHLQTLGYKISGQFKMPHGLMR
QGGLWVALAAWHSASVTLQGFKDVSKAETDADCVKDIANGSRIVLIQTQL
AKRIADLVDKLEDFIGISPQDKGNQPLSLDENDCIVIQTVMVQTWMHRTI
GIAKQPRADGAGHTILCTDMVSMDTPVASSGTDLMPFAAIMESPGTGDAS
MLVAALHLQRHLRTRTPVLFGSCVILPIERIWEWQRNEGGKDFLLHIRCH
HPPVGEPEV
>Mb|QLI68461.1
MSSAEDSKLPNGTSPQSTKPFEKVLIVGAGPAGLLLAILLARSHIPSTVL
ESWDRVDERLRATQYGVPATRVFRRAGILDDIRSESITHFPYICWRSVRT
GQFLTGIDLSVVKADKDRMTILPLNEILQIMLRHCREKYSDYVTLLFNHK
VININQDSRSATAVVEVGGQDEVKRTVTFEASYIIGCDGGQSTVRKTLFQ
RNWPGETFGSRLLVQNVYYKGFADSGWDGGNYMIDDEFWGLIAKRGKAKG
PEGELWRVTYGDSVANLSEEEYLKRRELAFKKMLPGHPDPSQYKVTQTDQ
FRIHNRCVEKMRVGRVFLAGDAAHVCNPFGGYGCMAAVLDVAGLADCLIG
YYEGKADEDILDAYAEIRREKFLQFIDRRSRKNLNRISKTNADTALETDP
FLALLKGMEGNADETKKFLLKVSSIEFDFTTLYKNPVAA
>Mb|QLI68527.1
MAADSTTVPTWNDAWAQWPEWHPSSYSPHHINPDQTPVSNTILPNDYTSY
YPPCNVDSAPWDPYNLDAFLSWPAEPEVRKGHASSLMNLPHYSPADSAPS

DPQLMQMPEASRPAPPLETPRRPSASQTPSSFGLTPERPWPPSPSASGAS
SPAGGAGNRPSGRTPSKRGRASRRSKSDGGEACPRGTPAAINRHGKAHFR
VCHNEVEKNYRSRLSHDFGMLLDVLVDCADDQDLSSVGLTAGTEQSWSKG
SILRLARRKLLELQVQDC
>Mb|QLI68529.1
MPNEMNSITVTRQQVAKMIDHSLLHPTMTDEDILSGLGIAKKYNVATACV
KPYLIPMAKKELEGTDVLVCPVVGFPHGNSTTQVKVLEAEAAAKAGGKEI
DMVINIGKALSGDWDYVASEIKQINTVVTQSGAALKVIFENDFLQNEHII
KLCRICSDVGVAFVKTSTGYGFVKRPDGLYSYKGATVAHLKLMREFSSPQ
VQVKAAGGVRTLDDLLHVMSLGVTRIGATATEAIMTEAEKRGITDEPTQV
TFKPMDEGVAGAY
>Mb|QLI68637.1
MSQSTPSPSAAAMDDASGGSRAFVIIVTIGSITTISLRFWSRSIQRAPDG
LGGSHVSRFWWDDWAALAAMPFILGLCGIIFAMLYHGLGRHIQFVPPDKV
AIFLRLLYALYYMYNFGLFFTKCSALLFLSRIFSWHANTRCFNYAIATTH
LLNAAWLLGVTFGTVFMCNPVEKGWSQPLSCGPTSALWISSAVPSACIDF
IILVLPLPKIWALKMTRARKVGVTGIFVLGYW
>Mb|QLI68640.1
MGSSESRATTPPASHTQRARVPRPQNSAVPSSNNKAMQQREILRETARET
LAAVHSVQSQLPSVDLGMSTKYSFNSLRRLGPNQGVGLPQRTTIQVVNED
TLNAATKLSASARAHGSRPPIVVNFANARTPGGGWLNGAVAQEEAICYRS
SLAISLNPHHYPLAADEGIYSPSVLVLRGDMASGHQLLVPQTPLADLPLV
SAVTISAIRQPAVRTFQLGRGAARLPAHQQVQRVYARDRDRSLTKAKMRL
ALRMAALHGHDMLVLGAFGCGVFGNPPDDVAHCWLEVLREHEFTGNRWRE
VWFAVFDPDNRGNFETFRQVLSGKKV
>Mb|QLI68651.1
MAPIQKVIVVGAGPTGLLLALLLAKKGITVEVVEAEAEVDQRPRGLAYGP
SATRILRRAGVLEEIRNITSDITSTSWRKLGGEILVHIEGDGQSPDKPLL
YPVHLLSNLLLERVLQQETASVHMSHAVIDVGQSDDAAWVRVKSGDEVKR
IEGDFVIGCDGAKSVVRKSLFGDDFPGFTWDKQLVVTNVHYKMANFNWSD
VQWVVDPQYWAMVCCISRAEDTELWRVVYGEPIGLSPEQLRARLPEKFEK

ILPGHPKPDDYEIVRFSPFTVHQRCVEKMRVGRVLLVGDAAHLCNPMGGL

GLTGGISDVGSLAECLYGIHEGKANMDILDKYDEARRQVFRDIVDPVSSA

NLRRIWNEPESIQWTDPFFNKARRAATEPEILEEMKLMNINCDMTQHYNE

KA

>Mb|QLI68693.1

MGEVVVPGKEDEDYIPQPTEKQQKILSTPDFGIGDAVDDSPVSGFLSAYG

YLESQDADTGKVGEKTVSALKDYQKFHKLKVDGKFGEKTRHLMAQRRCSF

NDCSVASEVYVRGAWPDRTILYAFGTLSQQVDKKKCMQAIQSAMATWAKE

IPGLKFIENEAHRDHEVKIEFRKVPDTDCVESLEGGELAHASLPPGFGTK

FPGMPKPVHFDETEVQWVLGKQKGKYDVESVALHEIGHLLGMLHNGNQES

IMYPRVSDNKLARKLSEHDKTGIRRLYPEWKRVGGSFTSDPVLVSWTTGN

FTNLACIGSDGGLRHRYQREPYGTWHPAGESFEHLGGKLAGNVAAVTRAE

EHVAFFARGQDGKCHSKWYSGGWSDWMSRGGDVQGDIAAVSWGHAGTRTD

LFMRGADNAIHHKYYDGTAWTPGVETWTSLGGNTTGSPKAICWGAKRIDV

FARSAADKSVLWKAWDGDKWVPEGADWTSLGGTALEDVAVVSSRANHLYL

FIRGADNTVYSKSYLDGKWEPSITEWGHHEEDFANEISSPISALAYNDYW

DKFMYKRIMLAALSSNKMVRLKMFDGTKWRKWFYLGDKVMAGAPVLHPWR

DNTPVVVARGTDGALWKWE

>Mb|QLI68699.1

MQLKLLAVTLSAALPMVSAYPITGTTVNCRSGPSTHDKVIKTYSKGNDIK

ISCQVAGETVSGNNLWDKTQDGCYVSDYYVKTGSNGMVTGQCGGGGGGDS

SVGGKITRKEIMDRGQYWVSKHIPYSMNKQYPDPQGRNYRTDCSGFVSMA

LHAASPGYSTVTLGQIANPISYSDIKAGDMVGTLAAGTGGAAGHVVLFHS

WTDSSHKKYNTLECKGTDGCVKWVRSVGWGVGSVTAKPYRYKNVVD

>Mb|QLI68727.1

MKIISLALLALAGINFASPTAALQVRSPPPAGSEQARELYELVKKGMRSW

GLAGAIHPPSGHISAPSANLPGGLPELNRPRPPKPNGVPGTPGHPLGPRK

KQGRIYERRKSCVAQKRSGIKCSAGTRVTTRASRFPKLRLIGQGGVMVAF

SILSPHAHEILEAVKNWDNPIGTAVGWFDEAIKGLQEAIGGKHVPEIDGN

ELKLRLICLFRANSPKHPDVIDDLCKRHQDESLEIKKQQQAIDGLNQISD

LCKKVEEEGPPIDVKIKRDVLALCDKYSKTIEGMVDANAGLILLGEWARA

RTLNNQDLEESDVAVARRFIREGAFGLAIATNETEASAIASLYMAHMSSY
TIMEIDEDGNEELIYHSKPPVWLELLQTGASFVPEDREGINQALKLLDGT
PYLHHFDNEGREDRFEKVQTCWSHTNLLMFQPPRWDLIAAAMTYLEGRVR
DLGLEQILACTPCLVLSDFWVLRCGSAVP
>Mb|QLI68742.1
MLEKIRQGFGYHHLRISDPVSSAIFFHREIKAFLKHYVRMGEPSVFGKVS
KYYATIETNKWGALYLHGLMWLHGNTYLPTLLEDAYREEEEEYQRKIYKC
IDDVFYEVGAKYAGAQ
>Mb|QLI68760.1
MAVGLRHNHDISFIATQCKTLAIVYYVTNYATKVEDPVWKRVAAAADVFR
VLKDLTKGSQVVVAQVASEDDSRQNKTGQFLMRVANRISTERPLSQVEVV
AYLLGYPTEFVNNDAWTFLNASSLYWHIFRRWSHLRSASGMEHVDEPMDK
TVLLEEAGERVSFVQAYPHRGRLL
>Mb|QLI68764.1
MSSRLGTSMGLEVLLLRLQGTCNEQPLLVASSLVAGLAITWLAWRLAWAL
LLSPLRKVPGPFLARLTSNRGDINNFSGAVALQAQKDTARYGPVYVFKPN
AVCISHPDDVRAVLGSDDFCKAEFFDIFNDGRVQNIVSQRDPALARMRRR
QIGPFLNYAYLTRMEPVIQRHGYLAIRSKWDRRIMETKTDKDEPVEVNYR
HDTQLATFDIMSALAFGRDPDSISKGSSSISESAAVIMDILDYSLVLGLL
SLLPFSLIMRPWKTMYRELAAYSKTSAQMRKEHLANGGEPPADMLQAFIE
AEDPDSKIKMTPSEIQAECIMMMLAGSETTSSAIMWTIHLLLLHPDMLKR
VTEEIRSAFGPRHLISHKDVLTKLPFFEACVYESLRVSPTTAGLTPRVSH
KRGIVLHGGYYIPPGTELYVNLRSVNMDDEFWHEPQRFRPDRFVNCDAAK
KNLFTFSYGPRNCIGRNLAWVEMLTITANIFKDYDLALTPDSRYGPDNVD
ENGVPHLLPAKCFIASFPSKPDRDCRMLISRPALG
>Mb|QLI68796.1
MATTHHPLDPLSAEEIEAAVAIVRETHQNVKFQIVSLHEPRKATLSKWLA
DRSHATKPPRVADVSVITPGGNVGDGLVDLEKKQIVQWEWINGQQPIITV
EELQRVEQVIRSDPNVIKQCEISGIPRDEMHKVYCDPWTIGYDERFGSNV
RLQQALMYFRPHIDDCQYQYPLDFCPIYDSDKGEIIHIDIPQTRRPLRRE
TTINYYPADIEAKGGYRKDLKPLEITQPEGPSFKLNGREMEWQNWKFHIG

FNYREGIVLNDIRYNDGGTIRPIFYRMSLVEMVVPYGNPERPHQRKHAFD
LGEYGAGYMTNSLELGCDCKGCIRYLDAEFPTREGAIRRIKNAVCVHEID
GEVLFKHTDFRDESGIVTRARKLVIQQIFTAANYEYAIQWIFHQDGTIQP
EIKLTGILNTYSINPGEDTNGWGTEVYPGVNAHNHQHLFCLRINSSVDGP
QNTVYMTDAVPSDEPVGSPMNPYGNAFYAKRTKLATTRQAITDYNGATVR
TWDICNTNKIHPFSKKPASYKLVSREVPHLLPKPGSLVWKRAGFARHAVH
VTKYRDDQLWPAGRHVPQTSGETDVGLMEWIGDGSESIDNEDIVLWHTFG
VVHFPSPEDFPIMPAEPMTLLLRPRNFFRNNPVMDVPPQRALTASQALRK
TNSGIGSSKL
>Mb|QLI68842.1
MALINLFLGLLVIPPTIFAAAPYGNVLASDVARTYIDDIEKYFKIAPDNP
PAYKLRYVTEPTCFAYWGSCDTTYFHLKNTFVNGTAKAFYVPYTEYENAN
VFSEKSQMTITQSTAIVLGTSKGWNAAAKWTVSGDARGQKAALEVSGGYS
STTTGTTTTTKTVSTHAECRYGYICEIQTWTFHVLIDGMCKTRPYLNCGS
EKDACKRRDRIRCKQQRTYIDKFCNHHRLSTMTPCSASMVVRNAAGEPFT
TLALVSSRINSDGIPATAKRDNLEDLIVEILN
>Mb|QLI68866.1
MPLQDKLRLVQDDGFRLILTLSFLWILAKLLYAFTLSPLRSIPGPKVARI
TALRAIRNRLPKNVIRYALDDFHTYGDIYISKPDTITLSNPAHARAVLGA
LDSRKMDIYKSLSDPVMKNLVTFSEPRLASQRRRQIGPYLNSPSYLARME
QVVLQYGAVSMMHKWNEQLDSAASGDKDRDKDTRTIELNYRNDTQLATFN
IMSALAFGRVDKATSDSSTVVDWIAATAVYIGISINFRILLRFPLSLLVR
PWLRKYDDFVGYARESVRQRKELLDENPDDKTPADMLQAFIDAEDPDSKI
KMSPLEVQAESVGMQLAGSETTSASLTWVFHLLTLYPDVLDRAVGQVRSR
FGADHLISYTDCKQHLPYLEALVYETFRFAPITSGFMPRMCSKDMSFQGY
RVPAGTHIAFNLMAMNNHPDVWDQPDRFWPERFLGNEDAKRNIFAFSYGP
RSCIGRNLAWMEIMTILANVLNTFDIRLPEDAMYGPHNVDGNGTPVMMPS
HCHIVFAPQRPDRDCRLLVSRRPTPRKA
>Mb|QLI68892.1
MSQLPVPVNPDDQGRGPLVMGLTWTFASVAIIAGVLRFYVRTKLAIGLAL
DDWLMFAAIACNIVSQSFVTIAYHHGLGKHDASLRPDQVINVLKWMWLAN

TPGLIVSILARISIAVLLVRLFGGVHKWLKWFVIVVTGLCTILTLIILPC

TYLQSTPVSGNWDPFIPAEHWNPKIYISLAYFCQALWTFTDLTFVLFPIL

VIWGLHMAMRQRLGLVLLMSVSLFTMVLSILKTVGLKHIADQQADPTATD

VLYGASLEILWSCLEQAFVIIMGCVPPLRSVVKLQLVRSISDSLASIIRR

KKASKSSVDSPKYVSRAEQYENLEMSHGRLGRIDENDGLPYMGPRHAGSQ

QSLVGKDKMHSRPVPHVQSKQTWMDVDV

>Mb|QLI68916.1

MATPKDIHTEALVVSKPEGDFVMQPIILDEVRSDEVLVEMKYSGICHTDI

LFQTAKIPNVEYPAIFGHEGAGVIRALGSHVKDKSLRVGDSVLLSFNSCG

KFRVSDGSTPARFAEGGTPIRSQCFGQSSFSRLSIVTENSVVKCPYPDSL

SYYAPLGCGFQTGAGTVLNVLKPDKTNSVVIFGMGSVGIAALMAAAYLQV

KQLVAVDIVDEKLALAKEFGATVINPTKIGDEGIGAVVKRLTEGGADYAI

DCTGRLAVIESLFTCLGLKGTAVSVGVPPPNSEIRINPQTFLMQNTSYIG

VLEGESNPQKFIPQLMELHQQGHFPIEKLCKVYSVKDFDTAIRDLREGKV

IKPVIHWD

>Mb|QLI68923.1

MLLNSAVLTAALAILDVASASVIKRQVLKDKDIAGRIAGGAKIGDQCHPP

GTYALGGEKVIPPCLAEQAIALKCEIVTHLSSNSSEANRIAYHKCLVGHG

SSYFLDIQGCLACKKTHGHLSKEQYDWYLQRWTAGYEAFEKDVVPKTNMW

TYVEGAIGGTTCQNRNETLRGWKCWDQLPKGSGNTNKTVPVEQYYTNRPK

TQNIGSFTLNGKKYPESTTMEVDLAQYTYEITGHLGYYSSAKLEDGTVTD

TRVEFEVQVVTEYREIKSVCNFTTPDVFTIVSAISAPVPLKDSVATVPKK

EATTLPTLDCDGTCIASALSIKELEVVVIQGSKKADPVVADAATPALTDL

EEHKSVSTNSKISFLKRVEVLFANVKKYPTPSGGSPPSTDDDPCPARRRR

>Mb|QLI68927.1

MVDAVPDFTLEAHDVSSLSMLPFDVFPDGEDRFLDSHIFAPPANSIQLAG

LTLDVDLTACLGQTSAWSTSITDQGLSTWNQNHLSTTPLAPGIPQSPRWT

EGPDANLALIESWFEQVCPAWSAFDSTVNLNRTLANSLWHRSASVFNSLQ

SMSASFLSARLPHLRRQALALLNTASVCVQAEMDNIKGNPSIETFPTGLM

FSLLCLGTSVCWLDARLLGLPFLKEAKALLGRLSDQCLDTRDDAAQDFLF

FRNSLVYWEMLLAVVNGGDIAGDTDELVSRERNKSETITDLLPHPWTGIS

SMTARLFSRSVTLCRAYRRILTRPTGRVISLSAAMEAFQGAQKLEEQLLA

LDYSSMSRMNDTGDQKTPWLHLACAAEAYQLSALLQLYITFPDLVSMRLQ

LASALPDEDHASWDKLITPLACDLVKVLKKIPPDSGSRVIQPILYICAST

GLRYGRLDTPAPSAPHGLETGIPNGAAYLDCINSNILDYVDGIVATSDKQ

TDSSRVPLEDVGIGASREFIMKRLDILENTLQPRPIRVAKDLVKAIWTAY

DGEPPDCISVHWFDVMEAHDLRSLFG

>Mb|QLI68971.1

MKPSLTFVPVRLGLAAAAPQSNPDPSKYGLSKDFPDKIKKTLEDFKAQKQ

DVEKFMGDVAQQVRWQVTDSPPGAVGKGATMLSLLTSSKDLNNPPTEEQA

KDVLKKFASAAIAFVPTLGALGGLPGFLAIMKAITMGIFNFESPRACGQG

GHEGKALQRRGGIRGSLQQLPAATP

>Mb|QLI68976.1

MAMTVLSSPTTISAHASSPSRIDEPYASRPLEAAADGIRLVTIDPELTPD

GLLSCQLQTTTFARRPRYETLSYRWGDESCRCSIVVDGAELYVTANLFEA

LQYFRTHARQAALWIDAICINQRDTPERSSQLRIMPHIYARAESTLVWLG

GRYINLPIDLGPDSPGAEPNADIRDRVMADAYWQRVWILQEIGKARRIHL

CFGREPAEWDAFISWIRRHNGADQDDGVGPLRLDRLRRHKYDGSCSLRQL

LESHASALSKDPRDKIYGLVGLSTDGRGFPMDYSKTLLEVWCDTMHFMSR

HELLPSDCDARVRFCRLVRDLLGGEAALGSVGGVVQLRNDAGDESFHDSH

GRAPAGPLALSALSFFAEVYGVVVSLGPSASELLSSLDLVDAWEAELQRL

YRGDLDSAHMENDGLMRRILDSPDGRLVALSGFQNHRVVFHGPEMYGAYW

PFMHRRSPGPPPGLQGAWAHETATPDEPRLAMLKMSSAPWDQTPYKLAFV

PPDTRQGDLVCRVDGHPMKRVVVRPAPEDHSNDVRMHICGTALTVRDVLA

DGGFDDGSVHMSYKLDLMMDARTLYALIFGNHDERLETLLETLQVQ

>Mb|QLI69004.1

MALLHQLASWLMGRKFLTASGSRTMAKRQSAIKPSFSEVESENVCLQDIL

KARAISNRGAVICYFTGDTTTAKEFSCLELYTEASQYSRIIRNLPGFQDG

KPVVLHLDEQWDAIVWFWAVLLANGLPVLSPPLSHIDDHRHKHIRGLSDL

LQSPICITRNKLSNLFDGAKHLLGLHTIEALCCDTAWTQQHGSTTMTASA

TQQSSYLQYQGGQGLAMLMLTSGSTGNAKAVCLSHRQVLAAISGKAAVRP

LPTDGPFLNWIGLDHVASLVEIHLQALWLGVDQVHVNAADVVSSPQVFLD

LLTRHRICRTFAPNFFLARLVSVTEQESDLEQKDTPWDLSNLKVVASGGE

SNDIQTCLAASSLFRKYGASHNVITPGFGMTETCAGSIFNLSCPGYDITQ

GYASVSLGKCMRGIEIRVMVGSRLATTDEIGDLEVRGDVVFNGYYRDAEA

TAEAFPWKNEWFRTGDEGCIDPAGNLRLFGRKKEVININGIKIVIGDIGS

LLDQALGNRTSRLVVFPSRAVHTEQITIAYVPRDWPQKSDEMAEIDELAI

QTCLAGFYARPLVFSISQTSLPLLPLSSLGKVSRSKMTSLFEDGVFDEDV

TIYRQKLAAHKKDKIQDASPSMAATESEILLLDDFAKVLGVDLNTIGPET

RLYDVGFTSMDLIRLKRRIDTRLGMAVPVITLMKNPTARSLARALDSEPC

LDNWSEKPKQSTTDYDPVVILKPSGTKTPLWLVHPGVGEVLVFVGLAHHL

ADDDRPIYALRARGFEPGQGRFSSIDETVDTYVKAIRQKQPQGPYALAGY

SYGAMLAFEMAKRIEAGTGSTADSLVRFLGSFNLPPHIKSRMRQLSWNMC

LLHLTQFLGLTTEEDVEDMARDPGFSTAAREDSLAQVLGASDRHRMDDLG

LGDAALARWADVAYGLQSMAREYEPSGRVDGIDVFHAIPLKRAAASREEW

VDVHLSRWKNFSRTEPRMHAVGGAHYTMIAPDHVVGFAAKLKEALRARGI

>Mb|QLI69005.1

MAQFTIKSAIVAMGMALTATAVPQCATDADCLSGYICGPSDYSGSGNTDN

VCVQLQSCMNTPDPEFPQNGPKCGASTFCNVGGFCGGGYYDVNGNRVGTE

VCVNQATGVRCAAPST

>Mb|QLI69009.1

MRFTASVAIALTTSLAALVSSTGIILPLYLYPSITWNDGAANWSPAFNAI

AAHPSLPWLAVVNVATGPGSTYMPGNNDVNYIAGVSKLNSFPNVKTLGYI

RTAYASIPMDEITKNITTWANWASYSASNISIHGLFIDESSNLAYITNVT

SFARKAFSGNITIFCHFGAAAPADFYKICDAVGSFESYASYLSTATMKNT

IPAGYEKQAGIIIHDFVGKTADGIAADTNSLNSYIQGMVKGGLGWLYFCT

GYFNSMSTGPATVGQVAQYLASDTLLAVGSTGTSTQWTVPQCAQTMRFNI

TGGAGGQIGYQGGYGALISGSITVSPGQTISAVAGSAGGINTAGISAYGN

GGTASQGGGGGGAASALYLGGILVAVAGGGGGGSITVGTYPNSKAYQSDS

NRGSGETPGVSRVITPTGANMANYFSKAAGGSPGSASSPGVGGQYYGYAT

TAYVGRPGSGTAGGAGVGNPQTTSSGAGGSGGGGGGYKAGGSGASVYWNY

GDGWYVIPAGGGGGGSSYVSGSVSGVSQGIAGSSGGSVVVTYRTPSGSECV

H

>Mb|QLI69016.1

MRLYFLALALTAAAAPKSLAAEEHVAKFGDTRPISNDPRPVSGTVVPRQQ

PDGTEEVGFDVYVHVVGRPEETKANQTEFLLTRDDVKSQMEVLNRSFKPV

GISFKLAGVDWTAISTLAPYENPFSNILYNKELEGIYKGDDKGDNTTLNL

YFLNGTNGHGGISQNFFYLKRFVFVNARTVPGGTEPSFNMGLTTVHEVGH

WLGLVDVYKVKPSWGTAEDFSKARAACLKLDGPCDTQVECLNYMSYASDT

CKNEFNPEQIRFMKTYAKEMLAGGTPQPIEIDL

>Mb|QLI69018.1

MMEPRWCLVVLVLSALHIGPLLARSVPYGDLMTVGEGYNTFLGKGVKHGA

VQFSSVKAAPPENIIPKRSASNTPTRRETNPFQGLSVDMFDVDLDSYFIA

PDPKMFDEPMYKQNEDNQNGINHSVAHVKRNAAASCPAEIDASVEFISDY

ESYLKVLDVSASATISGYGQTASASSSYLDKSRFASNTLTYMAIINIKKQ

INTGEEFAFNTNLYSNSSFAKTFGDRWIRGFQMGAKLVARISLTAKEKSN

QEELKATAVASLAFWGVSGQVNTAVTSSMQKLNTQAHVKVDIFYQGEIGK

QLQGQSASTSGDQQPAQQVFANAKSWADLFLTEACKHNYKYQALLDEYPN

IKGFPENQAVLDYSTAERVSYRVLSELVKISELAQVLRKSKVLNQADGDQ

ILWDELAIVEACKTWVQKTAATPNNGTETAKELIKLFDTAFYEKWRPRLK

DIKGDLTVQNTTEFVRVWDDRGSGAARGASFWLPRAQGELRPLGSMGVAN

YDDINSHFTAVLVAPTPGRTPSKPVVASPVGFNRIWRDLWSGANSDGSFW

RPTAPEGYKCIGDVVQNSWSEPNKDAIWCLRADLVKPSAYESPSLWDDKG

SGSAYGVHVYNVQPRSDKRLNVLRAFSDPDPDKNIASQLIAPSGANL

>Mb|QLI69027.1

MQTFGSFLVSLVVASSLATALPTTPSSTPAGNASVTAKHNANFTRSGPAA

LAKAYRKFGKPVPVDVTNALNRQSSKRTTGSDPNSPQQYDIEYLAPVQIG

TPAQTLNLDFDTGSSDLWVFSSLTPSSQVNGQTLYNPSKSSTAQSLSGST

WSITYGDGSSSSGVVYTDAVTVGGLTVNAQAVEAAKQVSAQFSQDAASSG

LLGLAFSSINTVKPTKQKTFFDSAKPALDSALFTADLKHGADGKYNFGYI

DSTAYTGSIAYTPVDSSQGFWGFTSKGYAVGGGSLNTASTSGIADTGTTL

LLLPSTIVSAYYAKVSGAKYNSSQGGYTFACSQSLPSFSFGVSGATVTVP

GAYLNYAPTDSTGRTCFGGLQSSSGIGINIFGDIALKSAFVVFDGGNNRL

GWAAKAL

>Mb|QLI69029.1

MVSTKSFLAFMALSVGLHGYHIQRDLSESTASRTLAETRDTAAEFVHPGI

FVDSSQLQRMASKVASKTQPWTAAYDAMMKHPYAAIETPTPYETVECGPY

SKPDIGCADERKNALAAYLNALAWATTKDQSKATRAISIMNAWAKKIKSH

TNKNAPLQAAWAATVWARAGEIIRYTDAAWSSEDITSFEGMLRNVYLPIV

KNGSKNPNNWDLVLMEASISIAVFLNDRATYDASLARFINSTSYYIYLKS

DGPEPRGPYKMPRKTLLEHWWEGQKEFNEDGMAMEVCRDLTHTAYGLASI

SHVAETARIQGRDLYSEDTGTRLRAGLEFQTKYDKKGGAQEVPSWLCKGN

LKLHLEDVTEPGYSILGGKYDMPYTKKYTAAARPAGANTLFVGWETLTHA

TGEL

>Mb|QLI69038.1

MYSFLQSSFLDFELTRLLGSTSSGGCDVAEFLEAVGQIKKNDPESWFSAW

HEQSRRAERIAREAAQHGHASAAQRGFLRSSNYARASGYMFMAGDERVLE

TAERAVSLFREAFVHMDGQVIVLDMPYRDDVSMPGYLYLPPESRRMPGGK

MPVVVNCCGADSTQEELYFALVCAGAELGYAVVTFEGPGQGMLLKRDKVS

ARGDYEVVTRKVLDYLQRISEQKPEWGLDLERIGVAGASMGAYYSLRACV

DPRIKACVAIDGFYSLWEVAMERMPGWYSSLWLSGWLPEWLFDALVRFGM

RMDFTTRWEFGLGMAMMGTATPGNTLRRFREFSLHREGDEPIVDRIKCPV

LLTGASRSLYASAQDGTVAVYNALKMVPESEKEVWIPSSIGEGGMTGKVG

AWALLPQKSFQFFDKHLRVHRDVAVSGVISQEHA

>Mb|QLI69060.1

MKFVALALSLATVVSTASAYAIEADGVNCRSGPSTSDKVVRTYNKGNDVK

LECQTAGQAIDGDSLWDKTTDGCYVADYYVKTGTTNMVTGQCGGGGTING

KISRQEIIARGQYWVSRHVPYSMEATYPDQHGTRYRTDCSGFVTMALHAT

PPGYNTVSLPEIARPITWAELQPGDLVGTLGPGTGGAAGHVTLFHSWADA

SRNSYNTLECRGGTGCVAYKRPVGWTDGPYTAKPYRYIRVE

>Mb|QLI69093.1

MADRTKPWVVMPLYYYPLDEATWKPLYDAAASYPDVNFLVVVNPNSGPGT

EPLPGKDYAREVPRLNAFPNVHAVGYVRVDYCRKPLADACAEIERYARWR

RHPDAPPGLHVEGIYVDETPNHHSPSAARYLDHLRRLVKASDGLAGARST

VVHNPGTPPEGELASFGDPDLVCVCEEPYHLFQGDGLQRRLVDWAPEHER

CVYQISGVPQGELAAAVQDLCRRGRYVFATDLVDDFYESFGPSWPGFVAA

VSEAAPRG

>Mb|QLI69094.1

MNMIKMPLSPLLLLLLPTFTAALQAANFNVSPAVANAHSCGQDCQRRLNR

TIEADISVVGLEFDYPFYETASNFSSSLGPGELLKLQPLDPRNLTINGGA

TVFRFQYTSLDYDGSVVPATGFIAFPYTPRYSFAQELASASNTSIHKYRL

AAFAHGTIGISPGCAPSNGPALYDYSTWQPVLERGYAVVATDYAGLGNNY

TSHKYLSLPAQAGDVYYSVVAARKAFPASFTKEWMSFGHSQGGGAVWKLA

ESRFVRNDTTYLGSVAIAPATYFIRQLVDSLLAANSTSSGAQKGTGAGFL

PYVLLAAQRAVPSYRESMLSPVLRNRTQLAVEAQLCLESVIGISVDLDAS

QLVSVAGAEKDIPTLLEWEKMVAPAQGDPSPAPVFVVQGQNDTAVSWKTT

VQAWNSSCHDGNELHLRLFPTQGHRPSLTAGAAEWMAWMDHRFESKENKR

SKNKCTKITRMPFNLQYVKAPTDIDLKPFLS

>Mb|QLI69148.1

MSLVKLKRKGKRPATWGELELDSVWPLSQMWVQALRRPGRHAVVCLDGYL

CMDFGEEEELYHRRAAVQHLAIFVPWESFLSETSGDINTIWERQKQGLAR

RVSFLVDNVQLLRRSAEDVKRDARQWAAMSGETDPMADVTESGMADGDDE

LGIGYRSDNIGNAARLIDVFRNAVGSGQITTGSKEISTMVQELCRFQLAS

LCSTDDLGATMVIERRPRTLGLRGHPSSGAEIPRQEQVRSIKSQQISASK

ERERMIQGVQSLGSNNTTGHSRAAYSVLHGFGEDDISITAADSETVAGAT

GPSTSIRFGPATSFLEAGRQFAVSFTLNQRQSIALRLICRQLDRVRRDER

GTSQPCQFVGGEGGTGKSQVIEAIAALFASKGISHRLLVTATSGTAAAQV

NGITIHSACNLSKGISRTSLYTHVDGIRSSSSGDLYIDGQARMDWHEKWL

LIVDKVSMLGARTLHAANKQLCKLRGCMEDFSGIPIIVFCGDFHQFRPI

>Mb|QLI69155.1

MPETLYLPKDKLGLSGEPYSMSDDLVRAIIAIRCNSLLRGHSAIRLELIK

VLLQFLSKGMLPMVPLRGSISASGDLSPLSYLGGLLEGNPDIWVRYVKDG

VPQLVTASAALSTLNIREIHLQPKEGLAIMNGTSVSVALAAQVVFDSNNL

AVICQVITAMTAEALLGTPENYCEFISACRPHYGQTEVARNIRAFVAESS

LCREGNSRGGLAQDRYALRTASQWIGPLVEDLQAATSQIQTELNSTTDNP

LIDSEQSRVYHGGNFQAVSVTSAMEKTRTALVMLGRLLLAQSNELVNPYL

SHGLSPNLVADNPSTSFTGKAVDINMTAYFCELAFFANSVASHVQTAEMN

NQSVNSLGLIAARMTQKSNDLVSMMAASIMFLLCQALDLRARDMDFFLKA

RPVLLGLFHARCSAVITSECTQNQNLDTLFDALWASIQKIWAMNSRLDIQ

ERCKQTIIHAADDLLNRDEYISVWNCNISLVTSLHYWKSEAAILFHSRFL

DVDAEFCKCQSTSRYLAPFTSKIYQFIRNDLCVPFHLGFSDHPSIAQQRD

GGSKPTRTIGFSVTKIFSAVQDGTLSSRIQVAMPVPISPAST

>Mb|QLI69235.1

MGHNLVQVVLTLAAVSALSIPLRPSAEPMTLPAVFERSEPSEGCLLKRSE

FKEHNNTAGGLQASCLQYQKQINEAFTKCSERAENVINALESKDFPSKDK

TVQETGQPKQDGKETLQRLLEDWFDVGINDTERITEIKKEYKSIKEECDK

KEKTRFGIYCEDCPENILGQAYGGTGPIRLCKLALERDRRSTNIRDVDLG

GTLMHEMSHAISNTNDTGYGVTKCKNLKTRAAIQNAESYMFGALAATLGD

NTGPSGEGG

>Mb|QLI69242.1

MAFVFTGRVSRPSAHLRIPRPFARSAYRAQSTTSSQPDHVAAQLDFRTFV

DVLRADGDLAEINDQVDPHLEVGAIVRRVSEVNGKAPLFNNVKGAKNGLW

RIFGNAASLRSREEEKYGRIARSFGLPPDSSWKAILQRSQEAKRRPPIPP

RILPTGPCKQHKIFGDDIDLHKLPAPKLHQGDAGKYLQTYGVHVLQSPDG

KWTNWSIFRGMIHDSRRLVCLVGSGQHNSVIRDMWLKKGKTEVSWALAFG

VPPAASVVAACPVPQGVSEAEYVGAMVGQPLDVVKCELSDLLVPANSEIV

MEGTFSFKDKAPEGPFEDYLGLHFSDDQHMQPLFTVNAITYRDGAILPVS

VPGKITDESHVTASMASEELLELLRQHGYPVIDAFAPLETYATWCALKVD

VKKLAEMRTTPEEFCNKIGNLAFNDKSSMLMNRIMLFGHDVDVGNFRDIM

WALATRCRPGQDEYVFDDIPVLPLTPYMSHGRGDPTRGGKVVSDCLFPME

YEGKISFRGCDFERSYPEEIKERVGSNWTAMGFDEVESLSRST

>Mb|QLI69255.1

MSKTLLSNSEATVSPEVLVKSRQGSNAYRPARLDVELLAMTPSSLLIRKP

YNTGHTNHDVPVMKQYSSLNEADILSKCVAEEIPLHALEHYTQGPDQAVA

LRRRVLAKILAITGLSGQILAKLPHQNYNWDLVIGSCCENVIGYMPVPVG

VAGPVLIDSKNFFIPMATTEGALIASTNRGCKAINNAGGVTTNLTDDGIT

RGPCVQFAGTERAAFAKNWISSHEGQVILRDAFESTSNIAKLRRVNASIA

GRSLYIRFKATTGDAMGMNMISKGVENALRVMSQDPKFEDMRVVSLSGNY
CTDKKASALNWIEGRGKSVVAEAVIPHQVVQSVLKCTAEAMVRINNSKNL
VGSAMAGVIGGYNAHAANIVAAIFIATGQDPAQVVESANCITLMECNCVD
NSLRVSVTMPSLEVGTVGGGTVLEPQSSMLEMLGVAGAHPTETGRNAQCL
ASIIAAATLAGEVSLCAALTSSDLVNAHLNLNRASPRKWASP
>Mb|QLI69278.1
MAISYRCEYNVSEPTSTTATKASGVSNDAASQIVIGTLAGVAALLAMAGL
LGYYFMRRSRARHVQQTRDSLQRRKGAAFTGFMKFYLLQGAHVGWGQIRK
DFEEMQSCFHEVVRAGFDHQKVIPNSISLNKSLEPLGLSEDSRNLICELV
TEPRTRHIAIRHLLAWAIFSNLDIRSVGPLSLLHPAIKNFMLYVPKNKRE
PKEYSDPMDHEDVIQNAWRQLSAVLLQERPFVSMNHHFYDEFNPHLDSQV
ETLVNALSGFFDHLPRLDGVFVYGWTPALRSMISRSAHLCYIIASDPGNW
EITIPPPSPNYGICVFPGLTLLSDNDRDPLDRPQVFIMPEVEKVDLVGKA
DRGNNAEVHTDTSTLGRDE
>Mb|QLI69294.1
MQADDIILLTLAAGKQMSYLLPLLYGKCRLRLSVHKDSEKELLEKKYPGS
EVVKGDMERAEDVSSMLSGVTTVIYIGPTFHPRETEIGYSMIDAAASEAQ
NGKLKHFIYSSVLHSQLRKLMNHDCKRYVEEYLMESGLNYTILQPTHMLD
PFPVEDLMRQEQPVWHADYDPKVTFSFTVLRDLADAFAVVALEREKHYLA
EYPISSTGAVSHVAVVEALSKEIGKEIAIKQKGYYEAVGGLHGLLVRANG
RLDQRVSRDITDRLILYYNTYGLKGNTNVLQWLIGRKPTSIEEFFHDKVS
SIRQSGK
>Mb|QLI69302.1
MSSKSGSSGLEGEERHLSVVGGEHLGSDGDAGVRDAVFGNLTEKGPNYRN
VGWVGTVALMMKTQIGLGVLSIPATFDTLGLIPGLICLLTIAGITTWSDH
IVGVFKRRHPEVYGIDDAGGLMFGRIGREAFGAAFCVYWIFVAGSGMLGI
SISLNAMSSHGACTAVFVAVAAIIGFILSSIRTLGRISWLAWIGLTGILV
SIVLVTIAVGVQDRPAAAPQQGPWVSGFELFKTPSFTSAVSAISSIVFAY
AGTPAFFAIAAEMREPRHYTRCLLICQAGVTVTYVSIGAVVYYFCGSYVA
SPALGSAGAVVKRIAYGLALPGLVATTTIVIHFPAKYAFVRILRGSRHLT
ANNLVHWATWLSCTLVTTVIAYLIASGIPVFGGLVSLIGALLGTLMSFQP

MGCMWLYDNWSKGKQDRTVKWVLMVIFSVFVVVSGTFLMITGTYGSVVGI

IDSYKKSGGSGAWTCADNSNSA

>Mb|QLI69329.1

MPSLSLSEMASPQPTSLRGATPVIILFTLTVVGIFALLAFPISNGLFNAL

LLQASQRKIGAESSPYDTTLTGWNWLDALLDGFTVFFYDLVDGSRADMSL

LMIPFSGSAVGIWILGMIESRRNGNKDRIPAFFTTMASLGQILGLGIVSP

LFYGLSLNERNSSWRGSDYDVAPEVFYTTPVSIVIGMALPWALAALPAPS

VLSINQKVNMVRLWEIFPITTYLAQRGLDPLARRFLGSTRQAGGRGNGAM

HRVYGIGLLWSTATYWYFMSMVFSASVLPFIFRPEIAEAWSLRHMCQLRN

PFALGSPLPSVSQGQLWFVQWDFWLISVACFVWALAVRLELLEDSARHLK

ATWKIMVGGLMSALILGPVGAAVVLIWRRDTLVHENNHRRKIV

>Mb|QLI69343.1

MAISHLGLSAVGWHVMIDSWWELLLAGFILASIVLGSSYLLYTGCRFIIR

RAKTKRPKWSLPRPVARYFAKLRRSSKSLQTSFTSEKNTEEELNSFGVFL

GSFSQPPTAVQHRLLSRWDILVVDPFQDGVANALKTCQPTAKHVLGRIDV

HALTKNHDAGDKPAGVIRKLTMVTQALGSHLRDGNQEPPAYRGVLLAGFR

EHFQPPVMNELCRHIEDLGLDVWLELSCPEYLTEHEARAIDMKHVKGIVY

RNGTIRPDGDRQNFFQMEAMRTAMRAVAAQRVAHGPALVIWETVDDDRQM

QFAVVMRAYSWCTYNSALCWIGHADALVDAERAPTRSMATKPLGALMWLK

NEANMGAHDAWRANDKISPAWTSRDAVYDSISAFLPGLSAQLRLLPPAVD

VSQTGPPVSRHGAPLRGALVASDSPFDTPRTRHALSVVSRNSSNLDLDLD

LDSVGLGCFQLGHKATFDDFAQIRLNQSNLAELDLLAKLKPDELSNVRSQ

IETLCDDPDLSPRTLRAIHDLVDLLRTCTPDGEQHARIKVFSGLHSGFQT

DSETQYWGVYELGHEDSITLYLSNKAKDRTGAILHTFLSSRQLTCAECFL

VEQALATKTGRLDNPWQLCTRIVHDLERLSPAEAILFLRRLQSSTASPQF

LARVRSCLEYQLLDIPSLTQQRVLSSVDYLGGNISAEALVDARLAWLAAK

GCWTPDRTKAIQLFQNVGDRLYTVLMQGETSTLEQLSRGIQELVQQGQVD

AGVDIFALAVFSAFRKLALDEVYLEVLDRNVYPNHAADQAGCFAENFALG

SRCDSFFDATPRAIGRIIADRYRAYYMKYQPPMRDETFTELPTAYAAMQV

DFDPDDGKEKVTVAYRITFFAIFAVPALIDVMLLTTIGRGLYLTTYMSSE

QKTMATTALMLALLLSGAFGGWICSGGSYYFYANTFPAMNLFVLTRWVAG

FATTIVAAIGGFIVVAFVQRLSAALIFLFYFLMLSTYLLVLNALSIYQLP

GSSFLSGRTVIFSCMPILCISPIVSMFTDNDIVVYLPVLSCFLIALLVGA

RSTVSQWSSWYLKIPFVTDAEVISWYRQSANAPAADLANLDDKDMMPIAR

SAIHAAVLKECNRFYFSKRSPDPLIRKLAEGYQSTMFLMRWYCRHKRSRL

PFPYSTTWNLTLKAGMENLTNMQKGLKLHSAFLHWRATGRDIWSGVLYFI

VALLDKWVALLTGGGLVGLSAASSEEFRLGIGFGLCYYLVGAVSLDIVSQ

PLWTAANEKSTKPITSLASLKVINVEEAQARRRLYWRSLCKFFFFHIWGA

AIFAALMWVFQSSRDNTIMFMGYIVAYSGLLGYQFNKIFCGTNSAAALAV

GALAGLPTGLALHKAMPGFTYSGLICLGVATWVSCIWSFFSANLGWPTFF

KSAPKSEISTEKHNDESSKITYSVSALEPLPELSQGTLSKMFESTRNLPA

DERYFLDPTEHPGQRVLELMSSRVISRNPEILTDAFPSANQLLQVAVQNW

ENGKIVVELVSSHQFSQSDVEIRSISRHCDQTLHILIILDSSPVDNHRTL

TMYRHWKVIAESVIRAAAQHQLGLSYHDSILAELLVVDHFDDIGVSIPEG

VKRQLKMSSMERTRVIREGNQTLLRYTLLGVDLEREWDLLPEHIRRFLLQ

RCDGWIGPLSNSEENWLYHRLVSQGSSGNVGSHIARCKLGAALTTTILTY

AHDLEADGYLESPPHADIEDHGDSVQFDDETPSTRFASLIKPFSSRILQK

VKLCIKFLIISLIADPEYQRELDFVIQDKPGFIRWPLVFFLNTIWSYCKF

WQNVLIPLVLFHGREHVARVQKHIKGMTTVLEKRKIVTESFSGPSTWFWT

VRADGSLRVSQYQGRHDSEPSNIQKLMAVNMYTNKLILEQRDVYHKGVVS

SSYQYDYENGDSRLPLQRQCLSGARQGEIVQYDRRGYVTSGSAMRGDNRV

TWKLWYRKNAKHEDELLWAEYTFPHITVKVLWSMPPRNPQKRLEEWIPFS

TVTEATFIHGHDVYHASWGFEHKLHPELSVTLNGNPIPTPPMIKDDWFQV

LQKPEKCSFQSENPLLPFSSIKTNPVSRLLRFNVKRYPIPTSVARTQLWK

AWKDGREVDAISARWLDEKLLRSDYIMRPYWRKRDFGRLEAAKKYLDASS

DTIMARCDLDSSISSWVHIAYKIADLYSFGPGGDARINTRKLESQLHDTP

EELHVLAMDTSTWPNDPGGVSACRRDMVNDLKTIKWHVLAESANDYGVPR

FQIERNVQSLTILPLWGLDFLNPTHGVLETNLDSAVVQRSQMTRTADITH

NFLPILSSLVKCSRITDLNRQHIEEATRALVDLNTYFEKTRNWNDVWQHP

VVKEKWRELWLTESLEGAFNISQWWDFEKPSLKQLDLALDLWCRYLFIFS

LPVPEEIPDVFQASHHFTGATYGIVCKAKRNCTLHIWDHCISYREFTTFM

SSAVSYDAPFVNSSLISLTHLSCVLLEHHADVVLPCCDYFNPGWEVELGT

AEGVVEHRRTFERKIDPVVNGICNMEKFEPIKTIKTDQPTVVMLSHVQYA

KDIKNAIMATDIIVNKWGIKDYRLHVYGDQERAATIATECQELIASKNLQ

DYCILKGLGNPSVVLQDAWLFLNSSISEGLPLAMGEAALTGVPVVCTDVG

ASYCVVTDRATGDRFSEVVPPNDSESLARAQISVMGLLGRWAEYADDAPG

SEVPVLGYPMPTPEQVEQISKRIYAKTEQRRALGMRGRQNVLNNFSSERY

LREHEQMLWVGKYRSQAHRSRVAITGSSTPYSQQPSSHWVSRLTPESWIS

LSTENLARPGWRSLSSSTLSVHKLFAQKEKQIV

>Mb|QLI69366.1

MLTRPLRPRIAAAVACCLLFVTYLAYSNASVYTSFTGSGHTLLATDDPGL

AFPLDPVPRRDFTTLAKYPAHNINQSSKFAFATLYCSRDPDTRGPYFEST

QSIIWRLLWSDYRSKYPVIIFVCPFIPKKNRDIFRGQGAIVKEIELLDNI

IPDEKISTKRWIDVLSKLNLWKEIEWNRLVFLDSDAFPVRNIDDIFDLVP

EQQCKKEALLPEDQAVIDKGGDDMCNYVYAGVPQFTIDNINAGMFILKPN

LDMHAKLIRAAKRTGDYDVRYMEQGVLSSKNAFAADGPFPVNRLSPIWNT

VPEYYKEHLAKAAESPEPPTIRILHAKMWNRFWGSWNNLTHLNDMWDLDW

MNMCRFFDSDEFVKARTTGVYVTPWERYLKAQETAS

>Mb|QLI69368.1

MASHLPHPFDPLSGEEIQAATAIVRKAHGDELHFHVVSLHEPRKAEMLAW

LADPAKAPRPRRVAEIVVIDPRNLKGHGQVYDGLVDLQSRNITKWIKASG

QQPILIVEELLEVEAACRRDPMVIEQCRISGIAEHEMEKVYAEPWTITHD

PRFGTGTRMIQGLMYFRPQVDNCQYQYPLDFNPIYDASKKEIVAIDIPKV

RRPLQRHPAIDYHHLHIQKNGGYRTDLKPIYITQPEGVSFNMTGTGREIE

WQNWKFHIGFNYREGIVFNNITYNDKGNVRPVFYRMSIAEMVVPYGHPEP

PHHRKHAFDLGEYGAGYLTNSLSLGCDCKGAIHYLDVDLPTQSGEVRKVK

RAICVHENDDELLFKHTDFRDNSTIVTRARKLIVQHIFTAANYEYAVQWV

FHQDGTIQPNIKLTGILNTFVMNPGEDLEGFGTQVKKGVNAHNHQHIFLL

RINPSVDGQQNTVHMVDAVPIDAPVGSAENPYGNGFVAKRTRLETTGQAM

TDYNGSTSRTWDIVNENKINSASGKPVSYKLVSRDVPNLMPKEGSLVWNR

AFFARHAIHVTKYSDDELWAAGDHVVQSSGIPSRGLGTWVGNGTQNVANT

DIVLWHTFGITHFPSPEDFPIMPAEGITLLLRPRNFFTSNPVMDVPPSYS

ITPSEIAAKRTGFDTTDKASKLIAMGSQSCCKPPKL

>Mb|QLI69375.1

MQFLSVAALLAALGTATGLPNSNFNVTPEYAAAHGCGSKCQQILRLGNAA

DLDAVGHDFAFDFFATAGNFSEATRPGDVLKVQVLDGRTLNVDSGTTVFR

IQYATRDLDGRTVPATGFVAFPFTPDFSYAHNGTARYRLAAFAHGTIGLF

AGCAPSNSPDLYDYSTWQAAVQRGYAVVATDYAGLGNNYTTHKYLTLPAH

VHDVYYSVVAARKLFGRVLTREWVSFGHSQGGGAVWKLAESEYVRNDTDY

LGTVAMAPATYIIDMLRGPGVDFTGYLSFLPFAAKRALPGYQPAFLSDVM

SRRLELAETAQLCISGMLGLTLDLEKEQLFSAAGLAKDMETLEAWQRMLA

PAQGDRSPAPVLVVQGLNDTSVLAPTTEAAWRNACRYGNEVHLRKYRGQD

HSPLMQASAPEWLAWMDDLFASRRDGRSRPARRCTAETRTPFSPEFVKQP

PEAGDELMAVLEGILGE

>Mb|QLI69384.1

MPTHRGFLTASLAKLALIAPFLAQQAIAGDIEARTVEVFATTYLDLGHCH

NACTTKKWHEWETPGHESPPYATHTMPAEEGCVTTSISTKHPECQKCKGT

VYVYVPKTEHHAKTTTVTKSVHTSTQHTTKPTTVKTWTRPSSSSKPVTES

KSSTTKREPTTTKKETTSTKKEPTTTKREPTTTTKREPTTKRTNDYQDMD

MGYKQTTIFDQDVHYDERANFIFNEDMDMDMDFNQATLFYQDVNCDERAN

DYKERANDYQAPLSTKTSTATKEPTSTKREPTTTKREPTSTKHETTTTRT

WTWTSKPLSTKTSTATKEPTSTKREWTTTKRESTSTKRETTSTKRYPRPT

PHSGPTVGDANAQCGNHQVLSCCRSGNSAGLLNNVLGGQCSPINVLIALV

PLQNACSNQVACCTGSANGLLNVECTNLNV

>Mb|QLI69431.1

MQTFGRFLVSLVAASSLAAGAPKESFPSKNGKFSITAKHNVNFERNGPLA

LAKAYNKLDKPVPQDIADAVTRIQQKRETGSVTNTPNKHDQAYLAPVQIG

TPPQTLNLIFDTGSADFWVFSNETASNEVKGQIPYDPKKSSTSKRMSGAS

WSIEYADNGTSVSGDVYTDIVTVGGLSVKSQAFASAKNISAYLSRSLAAS

GILGLAFSKANRIKPQKQQTFFDNAKATLDAPLFTVDLKHQADGKYNFGY

IDSSAHTGLIAYTSVDSTIGGWGFTSPGFAVGDGSFTNLSISGIIDTGAT

LLLLPDNVVKAYYSKVKGASYDESERGYTFGCSTTLPSFSFWVGNSTITI

PGSYMNYQATNDSGKTCFGGLQSSSGYGVSIFGDVALKAAFVVFDAGNNR

LGWAAKNL

>Mb|QLI69478.1

MEKFYLENADFYRALLKSLNKTSPESRTPQQLATETNSPDVVVCARIRPL

LEEDVAAGFPCAVFPRVARPGIIDIHDLYNHPRGRPILKSSTYQVDRTFT

SQADTGEIYDSLVADLVAFASNGGVGTLFAYGQTGSGKTFTISQLEKLVA

MSLMEGNDTSQRQVHMTIIDLAGNSAHDLLNSREPISILEDSFGATQLAG

AVEHQVRCRDDMMELIERAASFRRTAPTLKNDSSSRSHGICRIRITNPAA

GPDSAGLLYLIDLAGSEAARDVATHGADRMRETREINMSLSVLKDCIRNK

AELDALASAGRGRKSKQRKPRVPFRQSALTKVLKHVFEPSVDRACRMVVI

ACINPSIADVGPSKNTLRYAEMLRVMVPTTSAKVNQNHMDPMTWTDEQLK

DWIIDKSGACSVGSAGVPWAKSLAQLVRLSKEEFENKCPGMIGFSSEQVN

SIRSKLWQMHLDSQHGSPGTRLDDPAGVDGANLRGQRPTSRILTRELNNP

TTVPLRKRLRPGMIVSWNRSPSSDVGASISGGSGMAVILCPVQNLDGTFI

DGAANVVNPTRSVETGTQARNSHGQGRYLCGQLTPGQVPESYELSLWHQI

VIDVDAMEKEVIMEYDAQTRYYYVSL

>Mb|QLI69482.1

MKLITFHASWLSIVITISASDPLFDSNYAKQDIIFRDVAVIGGGATGTYA

AINLRKLNQSVVLVEREAILGGHTNSYTDPATQTTLDYGVQAYWNLSVTR

DYFAHFDIPISNWEPEPKTTVYIDFMTGKQVEVRTSSNYSAYIQQLDKYP

WLEYSWDQGHPVADDLVLPFRDFVAKYNLVDIAYTTYFSCQGFANVLDQL

TINVIKFFDKSYIGALTGDYVATKQHKNDEIYVRAKAELGQDALTSSTVI

ASKRSKTGVQLVVKTPSGKKLIRARKLLISMPTRMSDMKPFDVDGKESKV

FSQWKYSAYYVMLVHKTGLPAGYKFLNADPSTTRFNIPQLPAPYQITETR

VPGLFYVWYSAPKDMTQSEVQADVTTIIKRLQDTVNGATKISPEFVRFNS

HTPFKMVVSAESVINGFYSELFDLQGYRSTWYTGAAMMSHSAGVLWNYTS

HLLPEMIAAP

>Mb|QLI69491.1

MAVGVCAYLFQAYIWKKKAIGHGETPIAGNAKPETAPDSDYSKIFPPSQR

NTIYDLIPTADNNIGPLSVSKQPLLKLECDYRLADPSTNLYSGFTVGDVR

ALGSFPDYAKLSGVPAPTPLKNFTIDTARPRPYRPFRWPYHQTMSFTKLD

ADYWLELESTYRERITQRRDLYAQHGKEILQALPGSELACKELMEMALQY

LGTRYPQHFQVKDGHFVNRILGTTRDVSAAEPLHVLLENLPEDFGIMMRD

EKTGRYHLRAGVICSSLGWKLGQKIGMGLPGVHRAVPGYKEKLAFSMDRF

FTKMPASSPIQRGSWGLEIGQPLFLPDDHPDWGNRGSRSDCLREEDVYLR

VDWQTLRRLPLSGAVVFNFKALYTPLCEFADEPYIPSLVLKILNEGEEGI

MKYKGTWHVEHVVKPALVRYERAQLDMGLIEKDWVVQTLDESPFFPGWQR

KCS

>Mb|QLI69513.1

MSGTFFLPPSLAELGWIRLLCLWAIVTFLCKVTYRLFLSPLRTIPGSPLS

RIFSSYSVVKRVVAQGAKSVRDDYEAYGDIYVNKPNGVSISNPKDIKIVL

TTYEFRKTDIYQMLDIHDQPSIFTNRDPKQASQRRRQIGPYLNHTYLSRM

EPLILKYSIMAIKQKWDRLLDEAEAKNTKIGGAASSVTVNFRNDTQYATF

DTIGALAFGREFKALETDDATIIRWIEATGFYLGMTKNFPFLLFWPLDKL

IQKKKDMFESFVQYSKESVLQRRQQLNSSSSSSSHHDKEKPVDLLQALMD

AEDPEAETKVRMTPNEVSTESIAMQLAGSESTSFVTSWVIHLLTLYPAYM

KRAVDEVRSQFPASHMVGFDECRKRLPFLEACIYETLRYSPITSGFMPRV

NPVNGVTIQGHYIPAGTEVAINLHGAHVHKDVWDRPYAYDPTRFLDNDEA

KRNVFAFSYGHRNCIGRNLAWVEMMVILANLLKDYDLTLPEDSVCGPHNV

DEFGRPRIMPTRSTLFTTPKYPERDCRIVVGRAPARNA

>Mb|QLI69530.1

MFALVSLSLLIGACSAAPSAANPSNQLPNFINQLQQSLRTVDQNNPPFTD

GSKLNEYTCPAHAFECTTTDFWQRQIIASAPDRVTITTNIHTDATLINDG

TKDATITSSFSTAVAIGTTRGWTIGAKVSLSSPNKVGSSELSASYSDTST

STTTETKTVQYGAICPAGKTCRIQTVTFQARLHAYCRHESMLDCTGAVNV

CKRPTGVLQCQQYVDYYNRNCVNPPSDSPCSVDVQLRADDGKLLTLIIIS

EE

>Mb|QLI69576.1

MKLSITLVTLLTGLTAGSPAKIDKRETDYGGEPLKEYCAIIGLEADTIKV

TDEGIAATCRSDPAVPPKTLQSREDGAKFCEKLGMQFSTSGSLDTAMKSP

QVWCKYADDGDSNATELIAKYADENGNVNQENLKDFLMGLSRDAPGTVTG

LYNALKAAPSVTKSKFGLGGASGTAGGAATVAYDFIMANMKPGGLFGADT

SFGRWLRTNPIYGASGTRSSPFDTTGNVMVYYKPNTKLCIPFKDESSVFW

STSVKRCKFWHDKKDCDTSYAYDAKTDIGKELRERCTNELDRNGLPEKER

EEQMRREEAACIRPRWVCGSLGKRLGDPLNFQYCMDDSEAAKKACDLAGW

TYRIGRMKTKAEEEQEREERLQKEESECFHPRQSCRNVYSKFLYCMRNDW

EEKEKCRREDWRPTEQPTKSSECVKIKSHRVPGGPYYTAVCVPEGEGKA

>Mb|QLI69577.1

MLTKTILAVTALATTAFAAPLEARSEPPSGSVYIAPTALYVYDVFTGAIG

PKTTNAVVEKGRAVHEKTTLMTFEYPMATANKKCALYFKYDSVSWSGTDK

LAIFSSLKPAPGGATSDWGPGNQRNNQLGIWKRPSPSSSPSWADWEATYG

GLSVPQDCKPGKTEAFEIVGQGDETWISYANAIGGVRIAYW

>Mb|QLI69595.1

MRTACFKLLSCLGLLQLAVALTTRRAYVQILNNTTQPIRAATVIHKYSDI

YQHHGYWEVIQPGTRSGNMLRVEYHTGAFATGNDWWLVSWKNPEGTRYYY

SNPGNGRIFFDTVEWIFAGRGGHRLESTTGFKQHTLMKTDANRVTQVVIN

TDNTIQFSSKSGYSSTTSSSTPIFRQYGNPQPFYAIAHRVLDKAGVEVAL

RHGANAVEIDANAWKLVHRGWWADHDGTLPSRGDRIRDVLIAAANARRAG

KNLGFVWLDLKNPDRCGQLETGCNIEALRDMARQILAPVGVKILWGFTGS

DINGRASGVVREDLTPSEAISIDGLSGTSARYAERIFNTSGPTNTAQRVW

SKGLFQMALNFGSCEDKAMSASSGQICPEIRLGVMSGLFGKVFGWTITRN

DGSEVNKLMHAGVDGLIYGHMSSFYEDTKEARDALQIINNWLKANSNHRY

LANLDDNPW

>Mb|QLI69598.1

MINPGLAESEALALGGVHWRQIRRFGLMSTAEGGRVAQIQWIDNPEYDRD

LYENSQYAARCVVDNDVPPDLDDDWSEDEMDSLDVTREDTDSDESDGEGN

DLYPRYRSAATHVSGPAMQALFGRFPPTFTQYPPRDDIPGPSGVGAAASS

EDDVIRDAVMRDLERFADLTPEQLRQLYPDYDEMLQELLDNLDRDTCSPP

KRPRTQLRARAGQSKNICCQSLYKIRGLVIDQTRQNRKNSATAKSAGITL

DELKRLRAGEFDGLNCAAIVKKLEEGVFGPKDGDDGSTGASPAGNQQDCK

RARKIVTPKSRSKGPKGKAPPAPAKVVFYGDFLWPAEAKTAGGFLPPRTR

PPGPTYEVEGGPTPDPATVVDWPSEVLQTRLIFGPAAKDAAGKASHATDG

FHGVVYAVHATPNMLVSGLDSIAVGGIRWSQVLGWVQVPLNYTLPEHDAQ

ERANIQQHFERAFKAKNDLFPGSNLFQKNDDYEHKFDELASTGEIPNFDL

PQSLEEFMDRNGQAVGWRGGFPLFNPPGRVTGESSTAAKAAKAVAPPHEP

GFWEKVGHFMESHILAIALLPAVVVANLIPGVGELADAAEVAALSTEAVE

GIELTELSSEGASELTPLLRSVAKLKVD

>Mb|QLI69641.1

MGWPTCKRNSGKTVNKILVYPGSFDPPHRGRLELLTHVFHHRDDIIAVIV

IPLDDDDIRTKCRATGDKLLFTKQERVKIWRGHGPSDWLWVFDRSATDWS

PFRRNLIKATAEDGFRLDFVLLAGPDQINPKFDVR

>Mb|QLI69674.1

MALHQVDPLVQDFINVVNGRKGWKKNFETAITTARERAEEDMNRQEIYNL

PDFFRYVNDFLHWVPRVDGPGDEVLQKLCVFYWVFEQPSVRDYQSELDPD

NTSGPLTFMSYWLVNYAQQMGSFLDRPESLTTETLETFYKNPKYNDGASQ

WLEPRGGWKSFNQFFARHLKPGSRPIAAPNNSKVIVMPADSTFDNWANIV

NGVVTFPDGTAEVKLKGISWKVSDLLQDSDYRDRFQNGVFMHSFLSTNDY

HRQHASVGGKVLEVKNIQGQVYLEVDVSSERGGGLVPIRPLPLPRRRSGP

GSGPERGIVPPDRAGYQWCQTRGLVVIDGGDVGFVAILPIGMAQVSSVVM

TAQVGTTLAKGDEISYFQFGGSDVVVVFEKHVTFVENPSRRHFKMGEKIA

TFN

>Mb|QLI69697.1

MWHPPVNFITLHYAWILSMGLLSFPVLYTYGNLAAIDAYFFGASASTESG

LNTVDIKNLALYQQLYLYFIPILTNMCFINIVVVVVRLHWFNQHLDRFGP

ALIRVKRRKSGNGGMHSDPEVAEPTEASSYTASAALSNIPSHVGDQLNNQ

RTNPPSANDDGGGIGEIRPEPETHNHALESSLEPTEEPTQRIAFHPSVWD

KPLNSRRDSTLYIPGPRERDRGDPIVELRNRRHSGIDATPTRRPSVADAE

DSRRRSTRARSFDRAAGAAGSVFIIGRTATVPQLSRAPTLHRAAADQMPY

LSRRATIGRNSRFHNLTSRDRQELGGIEYRSLQLLLKVVVIYFFGLHIFG

AIGLVGWILHANPKYDAYLQQCGQNSIWWAFYTAQSMMDNLGFTLTPDSM

ISFREAEWPLFLSSFLTLAGNTLYPVFLRLVLWTMSKTVPRKSAMQESLQ

FLLNHPRRCYTLLFPRGTTWALFGIIFILNVMDAFFILILDLNNAEVASL

PPGPRVMAAIFQAGSSRHTGASVFNLANVSPAVQFSLLIMMYISAFPIAM

SIRASNTYEEKSLGIYQVDTGEDLDENSGRSYLVRHLQSQLSFDLWYIFL

GIFVLSISEAGKIEHLAQPSFGLFPIFFEAVSAYGNVGLSLGYPGINASL

STEFSTFGKLVICAMMIRGRHRGLPYTLDRAIMLPDEHLIGAATRERITR

EDE

>Mb|QLI69704.1

MTRPETICKMDNYSVQEESARIFHEHLINNEQLGLPASIIEAAKHVQFVG

IDPKPYMPTCCKMTESASALSGFVAATANAICAHRYGTEYQDVQVDTDLA

TLFLQSFFLPEINGKPFWQHAPLAAELDKGDLYDIQKPIHQQATNIYQAK

DGKWYQLHGSLNSKPTMDMLGVQEQHVTHEEAFSIYARKVKQLDAATLER

MANDDFSQPGVTCLTHEEFLASEHGRIISKEPLWTMKSIPAPSTNWPPNP

HAANLKPLAGIRVIDFSRAMAGPIVSKLLALFGAEVIKISHKELPDFPIT

WVDLNTGKHDAEINLKDAEGKLKLAELIAGADVLVDCYRPGVLARLGFDS

KSMRKINERLIYLRENCYGFQGPLAHRAGLQQISDCLVGHSWLQGQFLGL

DEPVLPPLPNSDYQAGLVGAAVAMLALLERSKGDTAFDINISLTQYNIWF

YRLGQYTDDQCRRLRARNPSFQARHYDGMRTLVARTHAAMIKARPELCKT

PEYFTKMSGREWGLDDDISILEPPFRFSRLKLEYEVPSGARGRSKPQWSG

GGELGTGNMQSVHGR

>Mb|QLI69730.1

MKASSVLLAVFSGLAIAAPALDPRQNVDKLNAALKALNEKNADVEERIGT

SVDDQAKRHKQWKDRSTKQVELIKTAKVVVDQLAELINIAAQKADEAPGR

LDEQEKRREADNQDYWNIAKDFKVAIKG

>Mb|QLI69753.1

MASRILQITVAAFAFTTAVSAIPQSYPNKCGDQVCPSDKPNCCAVNVNGV

EELGCFAVCPNPPPPSQQLQQRQSQGVECGDSIFCKPNQLCCAVIYNGVE

ERGCYDGPQCPPLNPATTMTTTTAASPAATTGPKCGDSFFCPVGKVCCPN

KLYTCADTVDQCPQ

>Mb|QLI69755.1

MRHHASSRYLALLGVAVRFVAAATTVTSTALIIAANDADVAKASLGLDAY

GIPWTKALIPQAGGSLPALNSTATNGNYGSIVVLDSVAYDYNGTYRSALT

TDQWNQLYSYQSAFHVRMVRLEEFPGPEFGTTALGSCCNNNQEQLVSLNS

STPFPGANLKTGATVSTVGLWHYPAQITDSSIATAFAVFAPATGFSTESV

AAVINNISGREQMVWFLDFAPDWSATSSFLQHTYIHWMTRSLFVGKRKVY

LNTQVDDIHLETDMYLPANTTFKLRPGDLDAHVTWQKSINSRLPAGSDYR

MELGHNGNGDIDSSIDEDTSTPRKCNPNQAVDYVQPPDPPLEFVKPPGTG

VDLWPSRFVTYTWSKACASIDPLAAWFLTAANLNSFAHVSHTFSHEELDN

STYHDATREISFNQAWLAQMGISQAQRFSPQGLIPPAITGLHNADAIKAW

TDNGIKYAVGDNTRPILVNQQNQYWPLASTVAVNGATGIWIIPRWATTIY

YNCDTSDCTLQEWKDTSAGSGTFSNLLDNARTTNSRYLLRLQADPYMFHQ

ANLRQTDMPSITVGSQTGKMSLIMSWVETVAQEMVRLTNWPITSLKHDDI

ATYFINRMTLDACQPHASYTYSADGTSITAITVSANNAACSVPVPVTIPS

GTVSASGGSPKSDNLGNEPPIVWVTLSGSPVTLTLSTPVKLG

>Mb|QLI69758.1

MDSSQSMPRLIQLATTINLSVARIQRVLDSQQAPSPSFDEDSAPLPVDIR

EAQDVVLDATAELHDLLTDPLNLMHRFARGDKTACLQTIARFDIASLVPP

GGQISFKELASQTPLTEQMMGRIIRHAVTMRVFREPECGFVAHTRASRML

ASPEMRDWIRAGTEELGPAGSKLAEALEKWPGSQEPNETGFSLANNTTGS

IYDVISEYPQRAVRFANAMKVMTSKPEFDACYGTDFYDWASLGAARVVDV

GGGNGHFALTLAKRYARLDVVVQDMAKVVENATSGDLRERVRFMAHNLFD

AQTIAADVFFFRWIFHNWSDRYCIQILRAQLPALKKGARLVVQESFMPAS

GSVSQCKERDLRAMDLEMAYTFNSRERTLADWKALFREADSGFVFKSAIE

PKGSAMGILEFEWDGADGSAT

>Mb|QLI69788.1

MLALTILLSAAAGVNAILGGEVQAVQISNTTHICADNCVYDGIYLAQKGY

FCPVKEVVRQEEGGKLREWYSYDCRGAKPAPGVVHAPLKGKEAPRNCTLF

PGDLTKVSPSCIADKEVGGGGGTKILQLSELTEMLKSATANTADNKKPAS

PKSKKECAQIARNKFTECREKIDDFNECNNQGAKTFQNCQSGK

>Mb|QLI69795.1

MESSESSPGTPSRDSADSPPTVATWTTSPDVSLLDVSLDDGSGASPAASL

SARREEYVLVTGGLGFIGSHTVVELVKAGYNVIIVDDLSNSYRSVLDGIL

EIARRHYEDRRQGGGARRRCPMVEMHQVSYRDLPAMRSILELHSFPSPAG

TPARSNIVGVIHLAAYKAVEESIRHPLRYYQNNINGLVDLVALLGEFAVK

TFIFSSSAAVYGSLSAGGGRPLREEICAHPAERAGTGAPPSRSAGGGGIT

NPYGRTKLFAEAILSDVAAADASWRVVVLRYFNPVGCDASGLLADNPRGL

PSNLVPVVTQVMTGQRAGLDIYGGDWDTRDGTAVRDFIHVSDLARGHTAA

LEACRDGRVDASYRTYNLGTGEGSSVLDVVRAMEAVSGRAIPRETVARRD

GDVAASVAAVDRARRELCWETEKTLLDACYSVCSRLDLLPCRARGV

>Mb|QLI69804.1

MADQTRPVTSLDLGFWMRHSISPLIAFLRAAGYSAADQGEHIRILCEHVL

PNIGPRPTALHPIKSSLTNSGSPIELSLNLCSGKPTVRYCAEVLGSTWSK

DDDIYAVETVQKCLTSLCAELGFSRRWSDRLLEAFTPTAEEAKRSQENLQ

KWMASLLPPGLEIKPVSRLPFAAFAFDLDGPRALPKLYVSPKVKEIGSGS

STNETIWNLLRNLEPPINSKAVEAISEFLLEGVVSPSIDMLSIELVDEED

LHRARVKMYIHTTTNSFNTVRQCLTLGGRRRDKTTMKGLETLRTIWHLMM

QEKDQVADDYEKPVNDAVQQAMKLFFSLEITPGKDLPEVKLYVPVFAYMK

SDADTVENFEIMLKQCNQEWASSGRYRDMFETALYVTFPPLLPLQSSRE

>Mb|QLI69831.1

MTSLSTQGVYFRLYHILEEKTRHRPSWIEQANYTAWLLCLVVAYATWKIA

YARLLSPLRHIPGPMASRLTSKRGTWAVITGKASDIALADYNAYGDVYVA

KPNAVFLCDPEDARNVLSVGEFRKTDMYRIFEYEGVPNVSTFTDPVEANR

RRRQLHPFFNYGYLAKVEHKILEKGFLALRERWNKAIDKAAAEAGHADTT

VVNYRIDTQLAMFDITGVLVFGRDFHALRDTNLDYTSWVNNTLTYMLLNH

YFPVLKRWPFSGLTRRLKKSYDDLARFSRDSITIRKQLLDRGDKKPVDLL

QALLDSVEPDSKIRMTSREVQTESISMLVGGSESTSSVISWVIHFLLLYP

NYFTRVVNEVRSEFPPDHVITQAESRAKLPFLEACIYETLRCIPTASTSF

PRISHQRGITVKGHYIPPGTEIVTNKCAAHVHRETWEDPFDFKPDRFIGG

NNDSAEAKRNMLSFAYGTRVCIGKNLAWVVMMVTLANLFNTYDISIPDDS

IFRPENVDADGRPKIMPTKLGVATMPANPDRDCRMVIKKRASA

>Mb|QLI69850.1

MNLQHHLPRPWRRSKWFWPGVALVAVAVIIVIVVPLAVLLPRRNRPQQPP

TNVILPLYIYPLADSSWSPVYDAVSRRPDLNFTVIVNPSSGPGSSPLPDA

HYDAAIRRLNSYPNIETVGYVRTGYATRNLSDVTAEVAVYSAWFSNASAL

AMHGIFFDEAPHEYSPEAVEFLRAADSFVKSAPGLQGRKTIIHNPGVIPD

ARFNGSDVDITVVFEETYDKWQVRSTSLAALPRTRGAYSVMVNTVPTMSN

GTLSQFVNELSDLAKYVFITSLSVDFYNSFANDWLDFVGQVPA

>Mb|QLI69853.1

MPQPFHGDGRGDCDAKKDEPMIIEASNSSTQYQSIPDHSDAVSKVDDCAR

ELEAAVKQLLKRTEHARKAEQCQIMMQKKLQTLELENAAVLHRIHEMEEK

YLKLEAITLEKDEQIRQNWQALQIAAQSYSELEQGRNLAIAVVEETQQTA

KAYETWAKAEVERALQSNVAAHDHAKRAEEEMSSAVNRERESYHAREVAE

GRAEDAEQARHEAEKRAEDAEQARHEAEKRVEDAEQARHEAEKRAEDAEQ

ARQEKERRAEEADRARLEAEKAKALAEKHKEELERNQVWMITSTRQAEAR

AEKAEADLALAIESEKEVRRQLDTAAPVRHAIEPAVSLKRQKKGPFGPNK

KPRLSKSAAHNGTGLQSPEITWHDERLQYLAHDLDRVVDNWGAEEVRYAR

FTFSTDSVQVDGVEKNCEITESSHLVRCVRHAEEIVVTIFDVMSLARKVY

LTNLDLKAVEKLLHGNVAQTVSRRHAQKFKVGEETKRVTLFLWTNLADIL

NRVAEVYHVKDAVMVSL

>Mb|QLI69894.1

MVPNIAIALAAFSVVSSAATTIDRRVIGGEDAKDGEFPFIVSLSGRGGQC

SGSLLDSTTVLTAAHCLEGRFTSNRRMGGVIADIASAKEHPSYIKGHGVH

DIAILKLSTPIERNDTIGIGYAVLPENGSDPAPNSMAITAGWGVQGNGNP

GYNHVVNKLAKVVIPVREREKCRLQFNPPEAHPDTICAGVNGKDACHGDS

GGPLIDQETGQLIGLVSRGQCTNPPTVYTRVGSYIPWIEDHLGGVGSGPI

PISTTSSVLPKGTPTAGQKRPWIELVNYGIVASCGFTENREEECGTEIYC

GLFDWSPSHDGAFKNSKECLDAHVPKPKAARASIPRQ

>Mb|QLI69972.1

MKYSALSAISFLAVSSLGLPEFYWGVSKASPGQIIVTNNMNRPVRLDKVA

QGPSVDGVKIIRDQEIILNQGQTVEVSATEISADLKFNEQQAHNQVELSY

TSGDQDTYNFAIKPIEGSGFPGAIEVIPLSPRPSPQCHPLVWYPGQGNTA

QATCQDHTPLRVFLREAHHPPEFNKFDKFDGWY

>Mb|QLI69984.1

MTLPTFPSLFHGIHKADIGDSSRRLPGHPQTTLDRSAILQFLQNDFRTPQ

LDAFSKWLWLVSTQDSSHVFSLTHQVAVGREITVTENPELHLVWHDHRVF

LKPIPPFLLSYAFWSAVLEPEPSAGTNKDAVYKSILGFMRTYQHLVQHPS

DFALAEKHGLLPIDCNSIYKRADGESVAAELAFGEENSNITYTSFINFIQ

AFGPLAILDADVSPRYQYGQLRLGPLNFWGKFVLRKFVFFKVYGNYDAYF

SRFYGPLLFTFGVLSIFISSIQLGYSSQQGLYGTTKNSWRPLYKVGEGFC

VAVMLVVCLIGVCLIVVFVFMTAREITLALAALRRKKRKSNSHAAWQGQL

DSS

>Mb|QLI69986.1

MMTRLLITVIYLAASALAQVPPGWTNVFSPSNLYIYDVSNGAISDLSPTA

LVFKSPTNGGRDKTTLLTFKYPAGTNGKQCQLFFNFDQAADATSRWDGSG

RLDIFSSNSVAPGKTSGWPPGNQRNNHLGRWKKSATPWADWEATYGGLSV

PQPCKPPGTVQGFEIVGVYDNTYVTYTNALSGVRIAYR

>Mb|QLI70003.1

MTTRQDQSRSPSRPRRFYKRSTVACKACHGRKVRCNVALSGIPCANCAAD

GAVCEIAQRKRRMRRYAPFRNPDQLRATPDSQQGNSTSPAHSHDHPENEA

DDISGRRYPPGLQANEAASSAAGMVSGFAPGPPRDDQMNGTRSPYYLGDE

RGPLALAINICRDDGLKSSRHAFLPINNSTPLTPQDWQYLEQRGCFSLPS

RELQDALLRAYFHYVHPFAPVIDVADFVYRYTTGHVSVLLLWSIFAAAGS

FIDEQLPTNELGATRIEVKAAAFERAKALYDLEYERETIALIQSTYLMSY

RLGCLNDVKGPWYWIGVAINLAYTAGLHRLPPPDSPDSPAGNSRLWIQLF

WSVYCRDVWLCLAYSRPVRILLSEVTTPIPTRVQLASAPVEVPDDIYHRY

LPYEINELARLWLCLVRISATLGSVLSSFYTAKSSGPTRQQIDYMEHEIE

AHLADLPAGSHRSELVMIHVHQIRLYHEATTAILYIPQNHSLPYESPAKH

TDSLQSLCLRKMRASALRITHLINVMIGEGLTTHVHMLTVIAIIPSMQVH

LVDLMSSSQPTRQAGEHNLHLCMVLLENLRTTHVSAKATHCLFNAAIEKI

KNKPTSCPCPLLMGGGSASASAAAAASSGPASASGSDNRSIVTDDDSESV

FPVTVPGMSYLDMLPMMSPDGEFSLHD

>Mb|QLI70006.1

MSSTRGSPKFQCALVTGGGGGIGKAFAKYLVSQGTKVIIAGRTESNLRTA

VSEIGASAYYVLDTGKTEDISQFVRKIIAEHPELDCVVNNAGIQNPLEVD

KLGPDDFLRKADQEIDVNIRGPMHLTLQLIPHFLSKPSAAIVNVSSVLGF

VPLSIINPVYNGTKAWLHSWSMNLRTQLKHSAIRVVEIAPPMVATDLHRE

REDPDDNKKEKDSSTLTINEFMDEVTKKWEQGQDMITAGPGNRIVNKWHE

SFSNVYPG

>Mb|QLI70075.1

MSQIKCDETRPSCRQCATRGQDCGGYRLHVRWSTKHESRPVHRAKHQSSP

TWLYTARKSQPAQSRQLPAEPDATPEYPALSESESQLTSTTACSSSSADA

PFDCPRDETRSVLDLGLLGLVSRLAWDPFNTGSSSSNGAIEGDWAHAGPT

GFSGWDSDEPAPNNEDTALCDTEITWSTVSQESTAFSDDSGCFDTDQDAR

QSLVPISTVAHGPSMMVEYWFRDICPLWSQYDSESNFNRTIAAALWSNCE

AVQLSLQAMSTAYLASKVPTMKDMSFSMMKAAAEVITADLHTINSHQLFD

TVPVGLLYSLLCIGTSICWLNAGQLGLPFLSEAKALLYNINQHSDSLPAD

QRQLLQFFNKSWLYVEMLLSMVLSNCKSLQAVEGIGEADVRTCIGPDLPL

VPVDDCPHPWTGICSTSLRLFTRTMKLCHRFRHAARTRATFASAHVTSAM

RLMEQATSVEEQLLALDLSHLEAGGKTDDSKTPNQHLVRVGEAYRQAGLL

QLYQTFPDLLVARYSPQTTAENNAHFPWHTWITPIALQLVTLLKGLPGYS

GSKMTQPLLCITASSGLRLQRPLTQGLRQSQTADASDKHIEHHDMLQYIE

IARENSEREHGEQVQDDAESTVLSARQFIRDRLKLLKTVLPSKPVSVAEE

LVEAIWRAYDGEHSLEYVTHWIDIMERENLKSNFA

>Mb|QLI70076.1

MSSTSVRDGGIGAAVGVIIGILLGAAFVWFYSAKQRQDRPGEPVVYLREP

SREEEEFVKLVPKILTTMTHFRLCRNDGPDLRKALGMACAGFSMHTRHYY

HTDKLPKVPESLDENLRRLFLGEELTRALKELIMNPKTRYHALTHLQVRV

VLSNLDIHTIGPLSLLPLFVTEFFKSLPFTTSSYTAQNLSRGLMPLALWR

KLTVYSMAKHKGLLPGPIEPPEIVSLQVNRLVNALADVLDHFAKDIEDGV

PTHREELAKQIRWAVKFGYEIFSHGIEYQFQWQPEPTKDGIVVVPGLAEL

ADDDATPHVNPKVMEGTPEIRIPRKDPW

>Mb|QLI70094.1

MKLLAATIALSALAAAVPQRPDMLYMVTGPCTDLRNTICGVNDMVEDPNV

KGNRISCRFYQGEKQDDLLCQVNQLSKGQQAKDDASEICGALGGCTCAWS

TTERKIRKRIGGRIVNFNCLPNPKSKNTPAAREQFLKSIDEAAAEACFWE

KEMDESTCQSYKLDCIHTLSEGEHEDPITPDQVERCVRDRIRENRAPSTA

R

>Mb|QLI70103.1

MLALSFLLCAAGVKAMLGIKVDGVEITDKEYVCADDCTFEGVWFGENGNV

CAAKELVVVLHDGKVNPKNIEYACAEAANPEWSTRPPMNGEEPESCFLDK

IDPLSPVKYNCEAKRISKDKKVDVNADPVLEQWSEVRKMLGLPPSQEAQG

CGQATDGATKIDQ

>Mb|QLI70121.1

MSIFDNDSLLKKFYDNAAHQDEFASNAFWTKFLTHYVFNEIEWHVFQEMP

PKDHHDQTRFDIGVVYLELTCNVLAFRLTAEGKKNKSGKEDIITAERHAY

RKSLTYLRENQIPSLWVMTYFGTHARLWFCSLDGPGALELFYPLAGEMGQ

KSAYPEFRANEQGFRWAFNCVKSIQYPDSGKIAELYTQSESLMGLSSVGM

SQTTPSTTYQTYDYPGGDPTSQSKQASSSTASAQVPTTSFSITSFDQPVI

SQHYGTTPMETDVDEPQDQSASNNTAAPSQMQGQWFSSDRPPCEIEVLRR

RGHPIHNLVFKDRKKNPVTARREDFVRATHPDGRRVYVYRYSTKTTYWCD

NLDPKH

>Mb|QLI70148.1

METEISMTAPPNTPEKNPYEGVCQRKVDAQSRNLDPISCKSFYLPHEIIE

ISSESDGNSDEVTVPNSPRVVEEDINEGDQSSDNASRADAAEDDVNDNNN

NANDGSIVYSNSLLATDCLPDGGHARFANTAEGSSSKYGIITMRLDSPAS

KKYGAIGQKFGEDGHVDGSEGSGECSNGKLKRIYDRYESPPDKVPEHDPL

SKRRRTAKRL

>Mb|QLI70179.1

MKTPVDKQWKQRTSGIMKKAHGMHWTFGVKVALYLERDGELFVYRSHEDF

AWEAATFLAKQILTPNDFITLAQDMSSRRAQQSTPLPSSEQGPPQTPVSS

SIPTPVSTPGSSECSVTGSRAPRRTRSHATTLFNIAN

>Mb|QLI70181.1

MSTPSDNHDFEFAQKGLVATFVDPFIYSDRKQEVIWNNNAYNYLQEEAPE

TANKSLWRQGQLCAVEAGLYQVSTGIYQVRGTDIANMNIIQIPGTNGIII

IDCLTCVETARKSLNLYQEFHKGQFGQDPEIKALIYTHCHGDHFGGAQAI

TEIAGDDLRIIGPDGFLEHAVSENIYVGAAMGRRSIYMYGEGLPKSPTGQ

IGCGLGQALPSGTSSLLAPNQYIKQDGILSPSIEGLEIVCQLTPGTEAPA

EVNFYFPAYKALCMAENATHTLHNIQTLRGAQVRDARLWSRHLDQAIALF

GDKTSVVFASHHWPTWNEDDQNLVLPFLEEQRDYYAYLHNETLRHLNSGQ

TPVEIAESIQMPPNLSSKTHLRGYYGSVNHNVKGVYDKYMGWFDGNPANL

WPRTPVESATRYVACMGGSDQVLAKAKDFIANNDLRFAATLLNHLVFADD

KNAAARKELISVYTTLGYGCENGTWRNIYLTGAYELEHGPEAAVNAMSLE

SLMSVDLDELFDVVAINVDGPRACNEPGVTIEFMIGDMKASPKQPTEGAG

WHVRLSNGAVTGRAVEYVPSPEPRDPNVTLTVWLLHKALVELVASAVAGK
PTSIDSLDNVTTYGDTLAWDMITSLVTQPNVAFNIVTP
>Mb|QLI70220.1
MRFSIDSTLITAVLALSAAQTGLAAPVPQTNGAGNLLSGIPLVGNLLGPL
LSGIPLVGGLLGGGGGDAGAGSALSGIPIVGGLLGGGGGGAGAGLGGLGG
LLGRDGDAIPREENSASS
>Mb|QLI70245.1
MTISCQVNTLYDEPLEGIFVQLQCVEHPEYCYMGCSNHSGAIDEWFSLHD
HKVRPVRSSPNIEGLHWQMCFVVQPFFHDTCFPEIWNTVWVPKNTDSHTV
VTVDKDMYAVFKDDASSAALEGGTLPSHTVSVHPSHGPKVIHNTADSDDV
EIRVVTPLPYDRISGFNEPVCLSAGPEGPATFPWQERKPKKGHRGTAHPQ
QLRRSARLSKS
>Mb|QLI70271.1
MPKARTTRLQPNSILRRQYKKETQIIPDWEQTNDKFRRAEKRLPELAAEA
LLKLQKVSAYKQTRSLVTIDILLPLSKSSLDKLKEWDSPIGRAAKAFDDS
ITSVQKKIGGEQVPEIFGNELKLRIICYLRGEQRHKNAVSEACQRLHGHN
EQQAVQEENEMRNSLIQLFEDCGNPTKTNQQSHLLQICDELSRKTLDLEE
ATQDLITFREQLQQRLQDGGKVEQQDIDKAAKIIRKGGLGMPRSSDEDWA
TEMAAWYLGAVITSAEPNENNQPECQAMPTWATETWVTAQDIPKDRIASL
EAFYIISNGPYKDTYGAEDQLRGNEKCWSASGLRASKPIDWDKLSAALVY
VNKSIGTWRVTCKACESERIQEWQLRCGSTKASSAV
>Mb|QLI70277.1
MFGRRRRTPFLTAAVVAGTATVASRHGARKQSEMEAQRQFQMQQEYEFRR
NQEEREQARNQQAIDQAVNEALAKQKASQGAQQGSPAPAIAQPAPAIAQP
APGPPPMYMAGDEYASGSGAYLQPGAVPQRPRSTSAAESGICFCSCCGKQ
CGMQDRFCSRCGRSLQVESASYDQPEKQAM
>Mb|QLI70284.1
MGFVILSKSDKEWEHYRLAFEACAMASFINETGGQLEYKSLTIETYSKAL
MAMHSALQDPDVVCEDATLAAVLLLALFECLNPTTGGQESWRNYVQGAIE
LVRGRGRKQTDTRIGQMLFRATRPLMVIYSLATLEDEKEKELWWSGDDEC
TSTQKLCIGVASLSAKATVLLGPSGHKEEVELEVMLKRCQAHDRACEARW

EELSKSTQGISSDNTDPWLLMLLNMLTCARIFLNSIIMRCATWTYKVPNY

QTSEEYDGACSALTEIILHSAKVNEQQLCRAEEAQSSFAELDVQLQPGRD

SSRCAINKWIEDNRNLWEEKPLVGLQPLAVIWSLECLNDDQKMAIDTQLQ

RIVGCTVDQIRRSPSYTTEACTRPSPSLSEPATDTPEAGSRDSSPASTGR

FRYTQEDDDTLLELSNEGIPWREIRDRFPKHPDSSLKTRLWTLRNCQLTA

DEDRLLIRLKEEENCSFRDIAPKMRRHRALVSNRYNQLRRNRAMNN

>Mb|QLI70295.1

MESTTPFKESYELGYAQIQLRNLTFPFARDKELAAVDCLAAIFKERCDPD

AMENQIPVFVSPSELALILTQTPGIELETLAHGQPPFPRLKPTGNVRCLH

GRQRYEAAMRNFGPEKWWSVRIFRIPEGSDPEILLLRHIDHSSHQMKLSD

GDVFRHVFACRRMRQRENENYWHLHLSKDKKGALKRILEDEVMSELLYEL

TDYPGLRNGWQLGNIDKHFADRCPEEIYNYLRHIKETWRKITLNNSDVRM

ATDNETVKELTLLCPSASYADNDKVRLLMRSGKLFGSISDSHLRGQIEQQ

LLGIGVVIPSIETFHGNMKYLRVANNLFLPRMLRENEICLYPSTMFVQCD

FMRSFFAGFPKENSVIDQDTILRAHDLHLSSTSTPAASVEIQQQSAITDL

EGLDVSYSQLGSDSAVISAAQSALNRRRMRQPQSESPPSILVLDVQQASS

PSNCSSLDLTDCPLNEDGSTPSFGTPPRRTVTTSIGSQLTQDSATTGRTM

ISPGDIMEYMRIDKVGNM

>Mb|QLI70305.1

MFGRRRRTPLLTAAVIAGTATAASRHGARKQSDMEAQRQFQMEQEAEFRR

NQQDRERMRNQQAIDAAVNEALAKQQTNQGMQQAPAPIVMQPAVGPPPFY

ASRDEYMASGPGPYLEPGAVPPRPRSTSAVDSGICFCRGCGSKCGMQDRF

CSRCGGSLAGIAHDNSEKQAM

>Mb|QLI70318.1

MKLTRATSRQHTTEPYGGNVDSSAQRHAQRRLSDQSSSHAASKTPFRLRY

VVFSDGSKTEMDTAGYGFAVFHNGHLVDWGSGQLGRREVFDAEIHGAVEG

LRCAVLANLANEPITVSMGNTSVIDCIGATASNSSQAQFRAFQKIGDKYP

YQTFSPSKEPILQFQNTSSVSYRRRQVKGQIAVDYQRWWQGVERAGYSSL

GLAAELRKLPELALPRRLLGYLLAARSHHGDFADTTKNPTRARRL

>Mb|QLI70323.1

MTKAPSTMERLLKSHSSLILIIVLTLFCTITLLLHLLTVVEFFHQAGAQPN

DGAARPRPVYAVAHRVLTTQQVAEALRQGANALEIDVTAWKEGWFGDHDG
VWENIGDSVETLFRAAAKAREAGSNLTFVWLDIKDPDWCDPHDEDWRHCS
VAALQEQARQILEPAGVRVLYGFADDTAWGVGYDFISRHINKNEALNLDG
KGQDLQERFRNANMTETARRVLSYGASSVTWDFGNCYEDGVYTCTVLRQA
MESGNFGRVFAWTLHGGERRSVVDFLFGNAGVDGIIYGSENDPFRDNLDT
RAIYQDIREWVDRNGHGRYWAAANDSPW
>Mb|QLI70342.1
MKLSVTLATTLVGLAAAAPQNDPVPEELAAKVEEVLRQVEVKNKEADNGG
IGGTINDVVKQIVPQAAGNLYPPIGQGASIVALLATSEYKFDEANGKEWL
KNLGGLALSFLPKVATLGNLPGFIGMIKTIATGIEAAQFPELTAKRAAAA
KCFADNSQKTYNDVCANCKPELAMSLLARLNKCTDENVVSRNAYEDNKFA
QSFCGTVLCSGHKEGDIDNIIKQGFRYDPVRAFWCKEKEEIIGMIMSPWI
GQPLLENYHKLDHEDLREALTEPCQQLFKVQGLQIDGVCPTRAEFDEANT
RSVPQSFCAPGKKITPQS
>Mb|QLI70354.1
MELFVGTFNVPYIYTLSFDPLLAHLNVTHVSHATAPHAWLSFAPNQKTLY
AAAWDPPSVAAYKSQLDGSFHTLEPINTKPVANSPGYVVASDSHLYSAGG
ATGEAFHLDSDGSLGDLAQQLEFANGSPGGVDGLRTGAHSADLSPDGRTL
YVADIGSNAVWTFSVSQQANGSSALLTEQQKNKAAREDDGPRHAWPHPNG
HILYVIQEHSNMVDVFRVNRRGDGTVQDLEHLQGASVLPPGKNASDYAAD
EVRFSTGPPGHAPRYLFASTRGSDASPNGYVSAFELDSQGRLVRTEATDL
WETPTSGGGSNAVEPAPWVAETSSDVQYLALTDAAAGKVYILGFDGACIH
EVSEVALEIPASDGDKFNGTVQAATAVWLAPARGLIE
>Mb|QLI70359.1
MESTTPFKESYELGYAQIQLRNLTFPFARDKELAAVDCLAAIFKERCDPD
AMENQIPVFVSPSELALILTQTPGIELETLAHGQPPFPRLKPTGNVRCLH
GRQRYEAAMRNFGPEKWWSVRIFRIPEGSDPEILLLRHIDHSSHQMKLSD
GDVFRHVFACRRMRQRENENYWHLHLSKDKKGALKRILEDEVMSELLYEL
TDYPGLRNGWQLGNIDKHFADRCPEEIYNYLRHIKETWRKITLNNSDVRM
ATDNETVKELTLLCPSASYADNDKVRLLMRSGKLFGSISDSHLRGQIEQQ
LLGIGVVIPSIETFHGNMKYLRVANNLFLPRMLRENEICLYPSTMFVQCD

FMRSFFAGFPKENSVIDQDTILRAHDLHLSSTSTPAAPVEIQQQSAITDL

EGLDVSYSQLGSDSAVISAAQSALNRRRMRQPQSESPPSILVPDVQQASS

PSNCSSLDLTDCPLNEDGSTPSFGTPPGRTVTTSIGSQLTQDSATTGRTM

ISPGDIMEYMRIDKVGNM

>Mb|QLI70378.1

MVDVFMQVTPGGNSLYICNPDSLVEIFKRRSDPPRPLHLFEFLNVFGPNL

STVEGQQWKKQRKVRATCFNENNNQLVWLETIAQAADMVRYWASKPEIRS

TTDDTRTLSLRALSRAGSG

>Mb|QLI70405.1

MLNVVIVGAGIAGLSAAISLRRAGHCVHLYEKSSMNDEFGAAIHVPPNAS

RFLTAWGLDPVQWRWVEARHADLVDPFTLQPRAALYNDKSSASVGGLPLW

LSHRVDLHNALKWMATRSDGPGAPATIHLDSLVMALDPWKPSITLATGRE

ICGDLVIAADGVHSIAPEAILGRKVVPVDPVNANCCYRFLIPLETLEADP

ETKFFTEGHEGYCRLFAEDKSLRRLVVYPCRNNTLLNFAGLFHEPETKSD

RKENWHATVDIHHVIDTISDFDDRLLKVISKATDVKRWPLLYRHPLPTWS

KGRLTLAGDSAHPMLPHQGQGGAQGLEDGLALGIILCGAETPAEIERRLE

IYYTTRHRRTSVIQILSNVGADQADLVRDQLRQYMSDDEIPRDYQEAMSH

NFGFDVVRTTLTAMKEYDPSFRLPDDFFDGPVIGVPGRCKDFANDGFPSA

>Mb|QLI70411.1

MKSAVLLASTLALVTALPQAKPVCPEPKDLLPWLEKSLTTEECFELESNK

PSFKELEQKCGTTWSCNVLSNSNEKWLIDNSGFANEEACLARHVRDPGQK

IPWYPKSSTDDECLAFKMCKPALEELEEQCGTDWTCSLLEGRGERNRLDN

IWLAEKTGLWSKEDCLRHHMPDPSL

>Mb|QLI70465.1

MSREAASTPRPGVSPMHITEHHDEEQKIWCQVDVQRQNRYYGKLVVSSLH

TDGGELVRANQFLSVVFLSPTDVAARDFSSSTAVKITPQMINEQIDANTV

RVTARLQLVEAYVFQDHEMFEFGVYDDVGQNPDLYKESLVLGADRTPSGC

VIIHCEPAPDERLANCMQTICFCEGKRVQTVSVPLGQETQCEIAVGSYTD

VKALKLTAADDTAVGQPQASTHALRVEDGGMKHVDIRYDHVEHFSTIDVT

VGNIRPLERERFHIRIQRNGAEVASNWSPCDHKTRFSELPQEGDVSVSVD

ATTVNNYEFSFKPRDLKLSSRLDSASFPQADAVTGIDTTGFISLPIVVKT

SIVESGLTLFVRLRSETLIYTQTVPVRDGPVSLAVKVQPGQYTVEAPSFV
CSGIVYVVTAEASLTVLDCGSTKLEVRVQRGANLKVCGFPDVLGFGGYAD
LVDGNKDDFVEAKASSVFAYAGLDHDGDPLQRLTEAEERRATWETIQLAR
AVERELGHGKPVLPVMVSYTCNLQGFQVQDRLRNHEDWLANSFANLILSL
QITNSTMDPRHPVPGGYIVNPDFVSTCQREGLTAKFTMPVRGPLQSAVDY
YKVAAEIPDSISENLGGYVAAVNWLFRTVSPAVPFGWHVHLEGAGGPEWI
YRDDDDAASMAQKTGAYAASLGIFAHADFVAVGRHPGDDFTVQSYGVGYC
YGPREWQRFFAFCGALSLNQQVPVLPWQVSLSRTPLVDDGVAQDFDFQHW
GTGGSYILGDAAVGSSYHNVHPSILQLKFTLPYMGEDGRRIFIRAEPFDL
TEPAVKDLLLCGIFTVLVGGPSTTGIIAASFGNRGPWVRKKLKAYGENPI
RLTTIGTGVN
>Mb|QLI70473.1
MNLPPQSSTSTPCIQVSADVIRVTRDGRRTSYFTTNATENRLSVEKLNRE
YNLAARNAQNRVLRQSKFEDLVGNQAIVQTVRLLLGQLAQQENALELSGS
KTDLKAMMEKLEAVEVDIEKNNGLITLSEKAAYIEAIMEKLKADQTDTEK
KNALLGLSERAADIIARMQKLETAHVDTENTLRRMLAKQRCEAAIRTLGP
QAWWNVRVFLVPEGHHPENLLHRVINDHLFETPPSDGEVFRRTYAHWRAG
QMGHIDHVNLHLSEDKKTGLRRLMKKNLVAEAMYKLTDYPGLRSGLQLSN
MKKHLCERCPEEICNYLTHIYNTWGKITLCNPRIQQATDTVTVQALQRLA
PSASHVERGEIRNMMRSGKLWSDVSDANLRAEIELQLLNIGVIIPSIKTF
HENMRYLVLGMRIIRNHLLDALPAEKSVAEAMRKSWQPHQTLIEHAENQF
YQLTCSPSFSISYHVLFISAIRNFPKLSDEGPIHESGLPPINAGVDESCL
QRFLRHAQAQGFNSEKIKSKLREVPPSNAPPSGGQGYEGLQESIPKRRCG
RARTSSYNFISRSLFLPQMLVESEIGAYPTINFIQSDFMRSFFHEFAHDD
SGRVMGIVAAETSLPMATAILPDTPTRPELQQLQTDREITGSRSPNDELR
PDNTIRSADTIFYQECQSERSLLSPAHPAGQAHSDRPPLHSSPRATEMYS
RSRYSQGSTTTGRTIYSPEEVRRICG
>Mb|QLI70477.1
MKLRRALSGLLFSTCLAARLPTIPSPDNNQVASTDVDVKGSRDILTSQQN
APGNLIRLSHYQTENVRTYEYDRSAGQGITVYVLDGGIRLTHEEFGGRAT
FGAGFAFHQGEGDSDGHGTHVAAIIGGAKYGVAKQVQIVSVKLQPKKPQL

EKALDFVLKDVEDKNITGKAIISMSMSFHASDDIDKMFKRIVDSGIVCVV

SAGNGNSDASIASPGRDPSVITVAAMNHRSDSRWEESNYGPAVDLYAPGA

DITSASRDSDSASVTLAGTSQAAPHVAGLAAYIMSLEGITQPSQVAARLK

DIAEQSGARVQWNAPDTTGLIASNGLDKGGPNSLFPPKRIPWTLEPKKSG

KCGDPEYSEWKCGSQKYCNAFDAAPQEPKTGFFKNAKECFDAHEPAPKLP

WIKAPSPKTGPDSCGSGFGGNPAWAIYDDASCGTQVYCEAFDKIKPRPDF

LFGFKDTKACLEAHDPPPSG

>Mb|QLI70479.1

MEAALWGYPKIVELLLEAGADSSLKDRRGMTAGNLAEESERNDRERHERS

IKYLEDPFVKKRHRRLIRALLKHTPAASLSSGVQPNLADLTDAYFYKSLS

PGSISLVIPSQGIEIRTQSKTAAVLLRGEAFPLVAAVSGRTSPIRSEFLP

PEAGYIRINEGYWGGTENFAVAKDIGFSFESSPYDERGVAGSFNASHAEA

QLMCFFVRRNYIFRNIADGHKSGVDDFLQLFMLQERNRQVEIVVSKTPCD

SCRALRDCIRERLGINFAFRVLGVR

>Mb|QLI70491.1

MQYPLVLVAAFAAACIATPTAAPASVQSKDEMPTGLCPKDQLVIVDGLGY

YLPQAECLVLKERCGQRNTTKPAELKKCITNTRISEIKEAQKAVQDTKRS

I

>Mb|QLI70496.1

MRILAYIRQKRLKQPPGKSWVPVNIRIPFEVAGFALSFLSLSDRLSRIDY

KLIQNGLFVIIGILDVVTRARRLLKIKVEKGNEEMLGLSSHVIPGDASAM

SGGPATSARSRRNCSGPKTDADITFRHRYLEPFNMKDTKNKLPKTTSALP

PRYPTLQAVVSSRDETAASDPSPEGGDVLLCTSDAPQMTLQERANAYFMS

NYVLDSSNLSNICYTGFSVLLEINKESRHYRLAFEACTMAVFIQETADRR

DHRTFTTEEYDKAVVATNAALQDPAIMHKDATLASVLLLALFECINPTTR

DQRAWRNHIIGASKLAEDRGCQLRETKTGRALLMAVRTQMIMYHLINLEE

WDGHELWLVQDDIVDEYSPTQKLCIEVSSLGRRAKSLIGPQNENDVAREI

LEQCLDHDQVCKNWWEKMLDNTQRNMKRKRPCSEWGATSDNTGLWLSMPW

NMLTCARILLNSIIIRCAACIHGHASYTTTKEFLDAFAILKEILFDDFTT

NKRQLSRASEASSSSANLMEEKSLPGFRRHSMAWPIAFIWSLDCLTDDQK

TAFAEQIEHIGLQHELHSILLFQNEKTGLPPYMSEDLPYATSRAPGSCCA

TVITIASSPASVNSCDDFDNVEWNNRSNPDKPPSTHNHDNVSIESNVSTS

ASLQQATGQKKPETTPLPTPLATSRLDALLSHYENPVNEQVKSCQRHGSP

DKFVESPSGQPEPNHEASYSNMKPAGKPQRRISKYGVQGRGYNTRSAASQ

SRGKKHALAPTLKPFEYKGLGDARQTSNGSIEVEVLWAPSFLACDQLRGK

EAIEEAKDLVIKKFGHTVRRYKGTNEVECDVEKAIVHKEERENSYYTIPL

QMFDVYISSTPSSALWLQPGPKDKPVKLLLARAESFPEVFTISKTQQMHI

DPLVGRNGILLKTTTLIHFTYTVPATITKYQ

>Mb|QLI70501.1

MSRADQDIVREGQQSPRLARTRSGRTRSNLEDTAEEFTAGNEPPPPPQTR

GRRRASQTQVLPNQDSRRAPSRQTSQREAGPRPTTQRQASPQRDAASRTQ

RQASQREATPRQDPQRQPPRRQNTNGNSAESGDANEQEILIGLQTSGVDM

DRPTTEASSDTRDPSPQPDSRPGPSGSGGRPARPSESIPRDRGRQRDRQP

AADDNRSRRRGNAASDPNPQGLHSGTDTEHYDDGDGVPTFASEGEFESDN

GESDDGEPTYGTLALDGQLQERNGRQYFKTIHPDTGDRETARIEGKAPSK

NIVIFSTRPLKGHGSRPAYARRMADVGLSGVAIDEIQPMKKAEFAQFRRD

GRLGILLVASLLPVPEPLQQHPMTWIKLQSGDDTKWIARSTFVGGYKEGQ

AFINRHYTATGQDPPPQPLPVAERRRLQAEGQQLRRNPRRAARPERPPSP

PAGSSRQNTPPVVRRTPRRTARDSRTSQEPSTNAQRGNGSSQQPSSPRSP

SPNRQRSTDQGREESRQQPNTRRRPSTGQQSGTFPEEVRV

>Mb|QLI70513.1

MEGLHWQMCFVVQPFFHDTCFPEIWNTVWVPKNTDSHTVVTVDKDMYAVF

KDDASSAALEGGTLPSHTVAVHPSHGPKVIHNTADSDDVEIRVVTPLPYD

RISGFNEPVCGHSTSTATEALS

>Mb|QLI70545.1

MAPLQLKSPRGGPLSLLLFGIAITGFFALLAFPITDGFFGAMILQAHNQK

IGANHSHFDTALTNWTLFDDLTNALTLFFYDLVDASSADITLLMVPFGGS

AMGVWLLGMIESNRKGNKGRLPAFYTTMAMLGQLAGLGFISPIFYAMSLR

EQRASWHGSDVTVAPEALYTIPISIFLGMAGPTALAALPAPSVLSINQKV

NLVRLWEMFPFLVYLVHLALTPLARWILERSGQRDSHRRQRLQFVYSIGF

LWSAAPYWYWLAMVFSASAFPFAFAPKITKAWNFRHMLRLTNPFLLGSPL

PPIPTAEFWFLQWDFWLIGVSCFVWALSLRLETPKLDALYLKGGIVVEAL

AYAIALGPVGAAIVLIWQRDMLLIKDDDRCKQA

>Mb|QLI70547.1

MEPPAEYARLRATDPVSRVELFDGSLAWLVTKYKDVTFVATDNRLSKIRT

RPGFPELSAGGKEAAKAKPTSMVNHFFTPEYIKSLQPYIQKTVDDLLDAL

KAKGCASGPVDLVKEFALPVPSFIIYSILGVPFEDLKFLTEQNAIRTNGS

ASAREASAASKGLLEYLEKLVDLRAAAPKDDLISKLVVEQMIPGNISKAD

VVQNAFLLLVAGNATMVNMIALGVVTLFQHPTQLAELTADPSLAPAFVEE

LCRYHTASAMAIKRTAMTDIEIGGKLVKAGEGVIASNQSANRDEEIFTRP

DEFDMHRKWPSQDALGFGFGEHRCIAEGLAKAELTTVFSTLFQQLPNLKI

SVPIPEIKYTPLHKDVGIQELPVTF

>Mb|QLI70548.1

MQNEYEGSNAVFIDKARVQEGLILAKSGQQQLPQIPERKTLYYFTVSHNN

HCSVCEILITPIKHTQYPNFFFFPVRRYTKCCDSAPENELKQQHQTIYDV

LIRPNQWRLSNDDSDEVKLPQNASTIGICRDHSKLGLEYLLQTATPTSSG

AAKDVAFRTTSESESPCRCTDAADEANSSDTNGTKPRPRKRRKDGKTAAD

TISKKLLKAAEVKTPGSFNNVSETHSLSNVEQLDALPVLTTYDERFEFAE

HLRKLYSKTQDRAGQIIWANKRYSFDMRFLLRRERKIDGENEADRSARSS

MLINRIASKLTDNNPKCYCGAQVYDLFAKAGHLLTNLPGDQIERLKAADM

IAESIKELTPLYGPTTIFYAPAVIAALWKKDYATICKDLDAENLALHDVT

KHVVKVPYDDDLYTYGVYLEKGCPVHGGQSGNTASPNSLLYSDTTGVDDQ

QIQQDLHGVNIDTPFTNSQVTDPLEIFNNLDEDGAGSLDKFGIFPYAAEG

FWDFMNTIPDS

>Mb|QLI70550.1

MNIPRWAKISLCITGALMIYHASTFAHKRYGKVKYLETHPLRVYGDGTCA

DSIRISQGLIEKAKTMRQQCQDSASPLRTYGSWGARIATVTTHFSSPDDP

QRFYQKAIETHILHNLVHGSQLHVLCTPIIDHMWNKQAFVQFVLLSELAK

PPQERLEWIFWADRDTIVLDYCRHPASYIPAKLHRSYNPGTEPSKEQNIN

LLITQDTRGLNAGVFMIRVSEWSVNFLSDVIAFRHFKPDVELPFDEQTAM

EQLLLEDRYKNNVVYIPQPWLNTYAWDNAQDFMSRKDVEGLDDWASRRGD

FLIHFAGNGDKELNIVEYSQVGDKIFNIWETRDMLRDVSLDIERFWGNRS

NGPG

>Mb|QLI70566.1

MSRADQDIVREGQQSPRLARTRSGRTRSNLEDVAEEFTAGNEPPPPPQTR

GRRRASQTQVLPNQDSRRAPSRQASQREATPRQDPQRQPPRRQNTNGNSA

ESGDANEQEIPIGLQTSDVDMDRPTTEASSDTQSEPSPQPDSRPGPSGSG

GRPARPSESIPRDHRRQRDRQPAADDNRSRRRGNAASDPNPQGLHSGTDT

EHYDDGDGVPTFASEGEFESDNGESDDGEPTYGTLALDGQLQERNGRQYF

KIIHPDTGYRETARIEGKAPSKNIVIFSTRPLEGYGSRPAYARRMADVGL

SGVAIDEIQPMKKAEFFEFRRDGRLGILLVASLLPVPEPLQQHPMTWIKL

QSGDDTKWIARSTFVGGYKEGQAFINRHYTATGQNPPPQPLPAAERRLLQ

AEGQQLRRNPPRAAQPERPPSPPAGSSRQNTPPVVRRTPRRTARDSRASQ

EPSTNAQRGNGSSQQPSSPRSPSPNRQRSTNQGREEARQQPNTRRRPSTS

QQSGTFPEEVRV

>Mb|QLI70630.1

MYGNSSFMDSITASTNLSQKAMLMRQQCKDSLLDTRIATATAHFSDANNP

ETFYQQSIRSHLLHNIIHGTQLHVLSTAIIDYMWNKQAFIQLLILNELVK

SPQERLEWIFWADRDTVILDYCRSPASFIAEEIRSNNETTDTTQGPDINL

LITNDHNGLMAGVFIIRVCEWSVKFLSDVLAFRTFRPGVDLPYHEQTAME

ILLKEDKYKENVAYIPQHWINNYRGDTAEDFVNRSDASDMAYWVARRGDF

ALHFAGSGNKSGNIMEYAGVGEEVFDAIHGGSILRNISMDIEEFWGTYKK

T

>Mb|QLI70632.1

MDRHKTLPPVPTRQQDPEKLGSFSPETYIQLLEDENEQLRLRNQELIKQM

RALEAKLAAEIQIKASLSENLKETKGEIRRRDETIANIAGRIVQEFQRYA

DSVQGQSVEEGYIPIGYSYFDSSS

>Mb|QLI70633.1

MKLSYNPSHLPLRRQASDVPASPPTTRPTPPGISYELFIGFLWAGVAIAT

IFIASRLYARIRTSRRLYLDDLFISFAYVLVVVTAALWQWGAKDMYYILN

ANAGLVQVGPDFVTRMRRFLLVSFIVEMFFYTVLMLFKLSLLFFFKRLGS

SVDRFKYFWWPTLVFSMCTYLVAVGDIDYECLFGSLKDITVRCNSPSGTY

FLKVTLDVNCALDVLSDFLIMLLPILLLWNVQIRLGKKIALLGLFSLSII

TMAVAIARAADIGATQKSNGLPDSTYLWFWSSLQSFLCIVVSCAAAFRQL

FVSSTRSNSTPLRKPTRSCYERMASNFRPRRRKPNDTILYDMPSTRQTGK
GFNAFDCVAMRDGSEADSQHSSQSPVLVPRLGKPVAACYKAPDQNHTQPN
HITREVEFRVTQHTG
>Mb|QLI70661.1
MEKQILEPQLSALRLPAFNVPWPGARSPHAEVIEARMIEWADHYDLLVND
EHRSRVIRARYGWLAARCYPNAAKELLQVIADYFVWFFLADDLFVDRVET
VSGDTLRNLTAMIDVLDFNSAGLEPVWGELAWLDVCRRLRSLLQAEPFER
FAQGMRLWATTAGLQILNHIRPKSVGIREYQTIRRHTSGMNPCTALSDAA
NNGSVKPYEFYQPDVQALVRRANNIVCWANDIQSLGVEIRQPGQFRNMVV
IYAEQGGSLQNSVETTAARVDAEISSFLELADAVTARANVTLRGLVDGLK
YWIRGYLDWVEHDTLRYVDKFAAVDADDRFLSTPQVASRHSV
>Mb|QLI70670.1
MPQVTAQDNPPWNLRAISHRSPQRWPTALRKSKYYYNIWSDSKTYYAYVL
DSGIRITHGEFEGRAGNLWTAFKDAGNQPNYEDESGHGTHVAGIIASKTY
GVSKSAQVLSVRVFDSAGSAPMSQILAGYNKAVNDIIDKGRYSHAVINYS
GSKSRALYSAIDRAYRSPRGYILTITTAGNDNQKATGQPTGFVTNAIVVG
AIEPDWSIAPFSNFGYTVNIFAPGSKIVSLSHKSNTATMTMSGTSMAAPH
VAALALNAMAIYNKHPSAIRSFLKDTATKDRVTGNLHGSPNLLANNNNDQ
QKSA
>Mb|QLI70685.1
MDSAPWDTKPLSQSLVTNEQLGEGTRLLEPTSSCDEGGFECFPRRCGAST
ESQTYSSVASTAQQRNLSPIPLRSRVRPVDRDEDFVCSENPARPGGSRKH
RGQYFDAASGSRMLLQPDTRSISSDQLAAEVNGIYAGLVLLESKSIEYDS
TQKETDLSQEQYHALISLHRSLLHEHHDFLLASQHPSASATLRRLASKYF
IPARMWRHGIHSFLELLRRKLPGSLEHMLTFIYIAYTIMAQLYEAIPSFE
DTWIECLGDLARYRMAVEDDDIRDREIWTGVSRFWYTKASDKIPMTGRLY
HHLAILARQHALQQLYYYAKSLCVPVPFPSARDSVMTLFDPLLNANPGAS
QRLEPVDVAFVRVHGILFSGTHEDQLEPAVKQFLELLDNRIGREHGNWLE
SGYFIGISLSCLLLSFGDASNVLMNAVLKSQQTDDTIMLPDPVLTDAFKT
AVRFTARTYEIVIARWGDKNTFPCLHTLLVFYWFMMDFDVGRQYLEGSLP
WEQTALLLNYLLRTSEYTPRLDTPEIPWPEVGKAHPLPEDYAMRGLIYTG

KYFPKNWFDNTAIDDEEKYFEPASTVSKRCERILWLGYSMAMRKRRLHWD

KNTKQFSAKSNESNDDN

>Mb|QLI70708.1

MEGRDGRVLNIQLKKPIKARPGYFYIRAPELSPAMQSQPIHMLWWQPNAP

SIQHFAVLTDQVKRAPSLGRFPIRLDGPYQEDLHLGKYEAVMFVAQGPAL

SRVLPHLLYLTTRITRDKFAKVQSNRWYLDGLFSDKTRKVDLYWKMDQND

DICSVSAYFDELSDCGADSKVQAWIFYPEGLPRQARLPRPKGRQHWFPFD

GDFLRQVSSAIETQSRRTPGRAKVVICGSEVFKSTVRRDIRNYIQADNLI

SLSELVTMSSGTNEQGNKQQQKLRKTDVGDAGHREK

>Mb|QLI70726.1

MAAQYTELLRATGVDYLRTDAEEEDQTHLETMQAFDTIELDEEELVLARE

AAKQRFNRTQVRPNLYSYDWSDAHGAFWDRVKKRLTTRLDAWGASLDKVQ

TKAVSNEFEMEILIETPTPITRVHRRMWRHNLGYSLLQPGTAIGTRKEEI

YLLLAHFQLDDK

>Mb|QLI70737.1

MMKWADEHNLLVTDEYRNRVIRTRYGLLAARCYPNAGEELLQAIADCLVW

FFLADDLFVDRVEVATDETIRNLTAMVDVLDLNVAGSPPVFGELAWLDVC

QRLRRLLQAEAFERFAQGMRLWATTAALQILNHLRPTSVGMREYQTIRRH

TSGMNPCTSLADAANKGSVQACEFYDADVQTLVRQTNNIVCWANDIQSLR

IEIHQPGQFRNMVTIYAQQGQSLQDAVETTATRVNKEIASFCELADAVTA

RPISDELHGLIDGLKYWIRGYLDWVVHDTLRYADQFIESDADDRRF

>Mb|QLI70738.1

MPEVHCEVKLITIMFKSYDPGLQRLPKSKKVSIPKTYSKGFVPQENLEQN

EAVTEMSEDHGMTGDDAVGVTIRIEDINML

>Mb|QLI70750.1

MAYKNPTADIDNLAQQLLRDVSSAMTDDADSGSKSDSDFESDSPQDSNSS

LLHPTLVFAAHDIGGTVLKRALVMASRNLEYKDILQNTSLVLFFGTAHRA

SDALPWGHTLSRMLRICFTRKYGTWAPDFVRQLSTLHEKLAEDFGSIGGQ

FQIVNIFQNKSQSANYETIVDKFAATLDRDNEIQIGVDASFTMMGDACVP

ELRKVTTDLLINALTRKWKDYVTGMRLLSRGDLETGNRPNSKPIYRHLCD

WILSKPVLEDWVNSTTRHLQVLTVNPTVDQTSFLSSITASIQDMRKNQTG

IFITASYDQLGKGTYKRAKLLASLVSQILYRCPRLFLEITNYQDELRSAI

CRSNITWIEWLLWKSLETLLCCSATNQIFCIIHQSQQPRSLDCHESFIAD

LAALSELKEVPSKILIIGNAVSDTIPEKTTSHAEVNLGEAEAHAELRKDL

ERQLDLVGERNRGSFPLRSTILDRIFMGSIDLRRTRYFLRYIEHLPILTE

AAMKKALHQFLTDNSAFSAILELVPPSSRLLVKDALSWIVHSMRPLTCDE

LRVALSIPGCDDDDGDSRKTGRVSPDAAAGLENILCGVVEIDNNTIYLTH

ENFGAFLDHPHPEMSWCRMGHTAHSDIARRCLTYLSHHLGAHPHGQETSS

NTVGAKSAMETQKCPCWCNFGGVTGEIPFLEYTTQNWHKHLQLGPLEMDE

QVRIFLDIDKQWENWATSLLRHRGVANDSFRPNRVCAPTNLHGMLGIPLA

QAVDIAIRAISLPHFEQTTEWSLVFLAVVQGGDISTMLNVQEVMNEPNKE

STLMEAFATGSDDTLCELCKLQPDFVRARFDIILSHAAKLGNKRLMEFLL

GEKTHSWPRHVDTSGLTPLYEAVKWGHTIMSKRLFDWYRSSPNFQLTEPG

QDQSILHVAARHGDSGLIAMICTAGMDINGLDSANMSPLYLAARHGHVDA

VNRLLSAQANTDSADLNGDTALHAASRRGFCQIASALIRYGANVNMANIE

RNTALHVAIDSGHANIALCLLGLDENTADVQNDQTLCGGKGNAPPEEYAS

NAARSEDGASKSVDGDPVTLPAATTSTIFGETSLGLEETSMARPSTNGSN

TPCRSRVTMDVNIEPPLLLLATRGNHLKIIDALLKCKVSCDSRDELGRSA

LHIASEYGHYEVFRQLVENGADVNLIAQHHTSVLHEASGRGHTDIVEGLL

DKGADAGLKHITRVTALHFACKGGYIKTVQALLPRSTKHDREKALRVAAE

FGWVGILVLLLDSGTDKDAKDGQLNCALHLAAAFDYPRVAEVLLRRRARL

DMKNSYGETPLHRAARNNSLGVMQLLISADADMNVEDNAGRTSLFLAAAE

DCPEAVRLLLENGASLIIPRTSKYDDYKNILDLSLDRFSPNVTKLILDRC

MELNYRISTLVSSEMLQCLSTESGKDLAKIRIVLDSNLDPNKVIGDIGTI

LHYAAFHGSLDVVQLLLESADRLDLNIIAGESGTALQTAACSGTRHAPEI

IRLLLENQANPRIVGGPFGTALQAAAIASNFHGKDSNTIALNIARLLLPH

DVVNLVGGKHGTALQAAACDGSIEFAKLLLKDGAETHTVCGLYGTALHAA

VAKSASLEMVSLILEQTNLGPDQQDIEGRLPLHLAAAHCTPGLVKLLTKG

EANFRSVDKFGRNALHFAAGSGSMIVIKMILGSHPDLIHVRDNDAMQRII

TPSEEED

>Mb|QLI70773.1

MRIEATSWEVKAAAKRAGTWAKIPLSWRLSAGDLQRAKNQRDLTGPFIRQ

FLPENVNIITSKDSLELVESLRRGDLTAVEVTTAFCKTAAVAHQINNCLH
EIFFDEAIERAAHLDAHFAQHKTVVGPLHGLPISLKDQFHVKGVDTTMGY
VGWIGSNLGVTHPSQIHKLESQVVSELLSLGAVLYCKTSVPQTLLFGETK
NNIIGQTLNPRNQNLSCGGSSGGEGALQALRGSTLGVGTDIGGSVRIPAA
FNGIFSLKPTPERVSYRDVANTNPGQNTYRSTVGFLSTSLDGLELILRGI
LSTKPWLNDPAVVPMPFRQDFVDDYTCRVERNGSVKKSKQPLKLGVLWTD
GLVQPHPPVTRGLNTLVAALKGAGHLVVDWNPPSQKTGGRVHQSFLTADG
AHGIHGHLNLSGEPLIADLQEEFQLKPPIGLLQYQESTLEGLSYEAKYSD
YWNQTASDDGQIVDAVIMPVAPHAAVIPGKYYHTGYTKVINLLNYSAVVI
PVTKADKSIDAIDGSYCPQNGTDQRNWDAYDPEVYDGAPVGVQLVARKFE
EEKILAIAKIVVAVLEEAKNNGKR
>Mb|QLI70841.1
MSAWIDIVRGGNDFRTPVLQEFHQPYQSFDNRPDGQAKLLLLFCTGGCSG
AEAPRQVRMCRFAKTILLDCHLHHQKSYPAMKAGPCPPNLTRRQVESYGS
PEDLAHEFYARALSPLCEAVVFIQNDYWDILAIVGVLARWVDLSFASSIR
CRPQVLIVSEDEPKIALRDVERDLAAELMSHYSPLEGLSFSQAEKRWRSC
FAGIRLMRGCRSRTSADHVLEWTQATRNNDTALTFRNCDVKHLLRSTCSQ
FAEDHRKTFSFQQAWRTNPLPGQVSWGAEKLVRLTMHDATLYESACSAIA
NSLLLETYQHRRRVGHMLNPKLMELSSTDWFDEFYSPMLSSIEPVTLKAD
VRTLFIQFMKAEPSERQTFLASSMRTLSCSSKITLPTDLCLVCLCRFPAT
TLSCGHRVCDDCTETNGKKSEEGIDIYQMHFCVLCGAVNSVQTALKPPSA
GIRVLNLHGNVDDALPIAMFLKDLRSSLSGRLEDYFDLVLGSGIGAFFMV
MIFCNQATVEDCIYHLPKLKCVRIDDKSLFFGKGLRFPRSDLLDAKIKLV
LYNTDSRLAICQNYITKSSKWLQKFSILFQGVDNIAARAFIEANRIWPDG
RIDVITQCHNSNYPETLSMANELISALFYVQREGIPTFYDLFPARFVLWV
KCRLPAGRHLLDIAMRMRRRRVHIQGVEERKTAFATPGSPATFSKIYNHC
EA
>Mb|QLI70864.1
MADSRQVESWTWYGFASLFTLCRFVSRSIRLGGVRCFEVEDYVMVLAFGV
YTNVIVWVNIQEKHPHTNILPSTGTSGMSGAEMRDRVYGSKITFTIEESM
VVLQMLCKVCMCLLFMKLTSGLKRQLHVKLLLGYVLAGWVVTEAFFFGFW

CQPFRNYFRVFAGNTPGCTTSQSHLIMSFAFNLSSDLLMLAVPISMLLQS
QLPWKKKATICGIFGLGIFVILAAVLNRYYCFAHPESILWIYWYVREGST
AIVVTNVPHCYALPLKALKLKAFSSLAGSILKTRRASSGRHREDRGANKN
KVVSDKAVHHRKPESSESTENFATSVSKPASTLQIWQRSEYSVNTNNTSE
PRWEETELQQIWQGGLGTTAKVEAARAASYASPGNRDDREGIGQTV
>Mb|QLI70887.1
MVLYLAWPGGTHGAPQETNANQLRGPLSLLSGDGSISADFGLPGEHLAMV
KRQELLLCDFDFEDHEPVEGLDGERPITTDEEVLVTPERINVAEQAERVS
VKEFEEAATQRLLKIPNSWVKNGIDSFTKAREKLGYKPLEATSRELTSDL
GGKGISGLKAVKAAGGAAAAVAWVGGIVSSFVRNTTALDKAAAFLVVIPF
VGCAVNTAAELEKGDDKNFDLVVLDSVLCGVADALLFGPLLPFGLAIHFV
RAVMSFFRRPPNAPTLEQLRQRRDDSWDQQLEYIYQNIYSHPYLDPTQNF
ATKMESAFAIDTLSVLSESAKIIGVLNATSSADVVPSSADLVPSSDPDLE
LDEVQVRTEQAIYKIKQDRWDVVARRQREYLFGILDQFTRGTAFQLTELA
KAVNEKFIKHINEEEFIENYRDQSRFEEWIDSVTTETLRPIDRFSIARAQ
MKEAGTQLAKTPPALPRALNVAFIIGQSKGIDYAQSDTVSLFNYFEVEMA
EASTAAIPGLYRFYTMVRHTHQTVLFLQGSLKEEDFDNSIWPVEDLDTLN
KFRLLAAMKLGKLYEEAKIEYLDEEYRTNKYILSPTAPPIIVNPSIPPLP
EYPDNAYLISLAIGLVDAVSSDDFNDEVLDYLKTRTSEIRLKNWKALQER
IRAIKTKLDELAAEEYRGAPVSSAEFMTRHLQTCVDLGSDEGGCKYIVGG
CYYAQNTTDQFASECIHEAFQPIGQAQKTVQRQYREACDMFPSRQACMTA
LRKCAKENPKTRVKVLFTCTAKAAPKPDDSDTAAAPQSTG
>Mb|QLI70895.1
MYAGINIFVCGDFHQLPPIGATVMYSNLLNARNADFLAGQQAYRALDTTV
RLTQLMRQDGDDEETLQFRRALEELRVYQVSQQSWQLLNTRVQNELTHNE
VESFKDALRLYFRREEAHVHNHQRLRDCKQPILRIKSTHTGQGAEGANDN
EADGLDPHLCICLGARVMLTENIWVENGLVNGSMGTVRDIVWREGQDATK
DMPTAIMVEVDDYEGPKFPGTDYIPIFPVTRRFEYKKRDCSRTNFPLRPA
YAITVHKAQGLTLKQVVLNLERKDHAPGLSYIAIS
>Mb|QLI70899.1
MSLASWALSLAWAATAERQVLTDPGIYGPPLEIAHYYTGQMPTGIAVSRE

SRLFSTYPACLDANNTNTIQTPYKFQVAELMPDGSEVPYPSVEINTPPGG

ALNMSTNPPTSANYADYFIGCQSVVVDNKNVLYILDAGRAIDPQTSVLLN

AIPGGPKIVSVDLSTNKIIRTYTFPGSVVYGDSFLNDIRVDRTPGLSGLS

GGAEEGVAYITDSSFEGRNGLVILDLTSGESWRHLDNDPRLRPQQQFLPF

VHGSPVMFGSSSQYTRATVGSDGIALSADGKDLYFSVISGRELWSVPTAA

LRARDAHSELLVQASVSAKGQKGVGDGMETDSNGIIYTGHVEQEAIVSYS

PGNATVQTFLRDPRINWVDTLSVGWDGSLYFTVNQVHLMPGFYPGTERRQ

HPYVLFKAQLPDGGKKVGT

>Mb|QLI70916.1

MKYSMLSVAAFFAAFSLGSPAYYWSMQQAEAGEAIITNDMNNPVDVVKVP

GGPEIQIAQTGGEARFPGGPSADLKINGQVEVSYVGGNEDTFNYQVKPIA

PGFVGCVQVSTVGCNQDYLEWCSQPGPSTIITCPAGTELPISLTELPNLW

NFYDY

>Mb|QLI70929.1

MASLLADVGKWVAKFAAKEAWGAITGKQAVPTAELLDTIIAGQNEIARQI

ENLSIKLEILEAMRRILYWSKRMDEIMDDFKTLNGGVIDEADPAYAELLA

ALRNENHGVRYSTFSIYNAIMGLPGQGAGAIYVWHGQAFPKLKDDKNLYY

FMADYVKEMDDNLGPIAYLLRQGLVLSLFTAYSETDAERLRKECEDRVTT

IADTLCNTLYPPGLRFLKPSLDSAGNQGGNQWQRWQQKDKSDYFLVMNNF

YIPCLGSGKKPTQTNKTEAWNFALEQDSEPLGAMRFLSAWDNKGNKRLRY

SKMYQQGWNIDFSTSTNKDTSAILFKLIPLEESEAGKQPLFRFVPYLESS

KGIVTNNSKTFNSLFLPVNPQS

>Mb|QLI70952.1

MKIYVTLVSVLAFAASAHPTADTGVNLHAIRQDDAGSYARSMLTDLEAAF

TVNNASLPELGFTKEWQYICSPNIWTADQVAFWHTGTIRHDDVRSLTAYY

VPHVETTATNTGHKKDVKMVVTKSTTTLQVETKGWTVSAKLSGTGGKKDV

AGGAVEVSASYSNTKSNTHTETNTISHEAFCEPGYACRIETWAFHLGLYA

KPAHWPYYQLWSLVYGYDDERYLCDMDVHLRDCDQFTDRLNKWCDPKDGG

LLDDKGGYWVSMQPKYYDVEIKLPVYEENGFQPMSRVVLVSEPISKRSRD

VSEVAKVGTKKDITEAMAKGIKYQFLD

>Mb|QLI70960.1

MVPQNKESFERRMVVIKDGVEITPVRWADDQTFPYNNFIYKVELSSPALA
EHFTGSEQVWRRQPCTDLPPTEGVSTVIVRMSNPRAMGLNNTNRIENEVA
AMHLARGAIAQLGSRYASLVPAVYAWKAAAGPHPVDETGFGWIVMEYLTG
SPLHEQFKSFDMAEKKLIIPEIASIFSSIQRVELPPGVDCFGGFTINKEG
NIISGQETIQGAPGGPWREYDEVWRHQMLCQLKEADANELIQGWRVHGIR
ERIEQFLNVTLRRVLQDAGVDMSKLGFVHFDFTMSNMLYDAEEKRISGIV
DFDFAGVNNPAHEFFFTSLHDVHGTTREQKSDKLRRAILTGNFGASTEAG
EEEHADAWELAKLWDEVMGERGGLRPRDIRGMTTLAKLNTLTDMLFPWQL
GSVDMLRRQTREENAKMRARAEKAIDDMLSGWGV
>Mb|QLI70971.1
MDRHKTLPPVPTRQQDPEQLGSFSPETYIQLLEDENEQLRLRNQELIKQM
RALEAKLAAEIQTKASLSENLKETKGEIRRRDETIANIAGRIVQEFQRYA
DSVQGQSVEEGYIPIGYSYFDSSS
>Mb|QLI71029.1
MAPRRVATTSSHDYVSLVPLPPPTEEDDDGEDVGQRPPKPKKKKTWQRFG
LMSFIILLLGSAILLLVLLILWILWKSSMRAADGGPAETPWVYIFRANRV
TTLVALCTAGLRTVIGLQSSVATGMLAGIILETVGAPLLKAPFYSIIRAV
SVGPSNLLSTVRSRPKDATSFLIYTLVVLEVVLTVASQFLSAILTTDFAE
GTFANIDNSTDVRIFIHKRIRLDGINANYWELPPAASWTFAESSEPFTKG
ASFDDTGHTYRAFLPFEDEAQRTRLRKFRGPAHVIDNRVVCAKPSLLNLG
LIQISNAPRWALSGQIAMETTIYPILKEAEPENYINFTCALVTPTVYTEV
TAGESSLCAVDSKNWTILLEDPLVVPAVDDGINISPNEVGHPKGSRMFMI
LDIVSASPILRITHNQDCKVVRNDGPWVMAAGEEATETLRITACMTNLTP
KTFIVDIHSAWDNPEPRLTWDYEAKRYNTEAARHQLGASLIPESFETRRV
LTLSPRSQWQDFPNKTGMWDTTFYFDEVLEDTLRRSDLENGSTDAGIILS
EGNQSIDKNAYISHIHPFQDTLHDTKSPALALQVLLARITQMVYYVDFLR
LDNKSEALVSFSSTALIPMKWTGLIAATALTVTHLAVVVIVTILYLKCTN
ESLLGSSWQAISQVISDETLPILEHADRMNDEDIRRWAKGQFLDARNHQV
IRCRSTGRVCLGNKGEKA
>Mb|QLI71049.1
MHKPLKFKSYDPSLQRLPKSKKVSIPKTYSKGLVPQETLEQNEAVTEGSE

DPGMTGDDAIGVTIRIEDINML

>Mb|QLI71079.1

MASFFHDPRRPALSQVVEALQRLQNDPQQLAHEREGRYDDPPPPYPESGE

TTQPPTPGPPVVDETYQRELRRKARYKATPVNQFNSQANRELERLVHQLS

EKRFGRRQTLPWDGSSDLRANSENNVRSRWVEQGIWGNEWGPAWPEDSHP

MSKRWQQEGDGPFFGSYSSSTSKSLPGARWGHEEPDPEPESESESEQERP

PIFGIFGLGVGQPKRSKSPQRFRYIQTEQGQRLYIPNPTVRNPEASRPYN

QFLYQISKERDWIKDELDYKALGTSVDIDTIVYQSVKDMWIEDGIWNPKW

GELPGMTWIYEEPEEEEAVEAPASPDAVAGQHEDAIDDGRQHAPVPRTLS

IFGQALISNMASGGTDGSSAPALGDAGASTTDRRTGTREAPSDQTAVTHD

VQDPGDRPRRAARIERPPREERTPRLRKRSRGSDAVVDEQPPKRPRRSTR

LSALPHNASDSADANKPHGSKTVDTSGVEEGRRPPCDKRAAPASGKTKGK

KVNAVANPVRRSARIAERERKRMADMAEELPSKSRSPRAKTTRTARRK

>Mb|QLI71088.1

MNSIPIVLWACLFMLGAVAVPSCYSLFRNYLAARKIGLRFHVIPISHLNH

FWMLIDKKVLGYVKLVFGESAFTRYNWMGWELHDRYYSHHELGDAFMLVT

PGRNWLYVGHPEIVMEIVRRRDDFPRCVELTQVLDVFGPSVGSVEGQRWV

QQRKLMASCFNERYNELVWSESISQATDMIRYWSSRPSVRSTADDLRGLS

LGVLAKAGFGKSFSFQGYEETSHADPAASYKDSLQLILENCILLIAMGPK

FFTNTPWLPFKWRQLGEAVKAFQRAMTDMYESEKRKVAEGTSDEGTRRTF

LSSLAKASLDAKQGEGLTEREIYGNIFVINFAGHDTASHVFTFAVYFLAS

NPAVQDWVSEELRHVLGDRPPHEWNYTTDFPRLKRCLAVLYESMRLYTPV

PVTKWTRDKAQTLDVGDKTLVLPPNTMICLAYSSLQTDPRWWGSDSLTWR

PSRFIKGEGTDLDAEVFVQPRRGTFIGWSEGARDCPGRKFSQVEFVATMA

SLLRDWRVDPVVFEGETMDGARRRVLDLIDKESAMVLLIQMLHPEKAPLV

WSTRES

>Mb|QLI71120.1

MALGAQFLAKPWLPKKLRRIHEACVSFQPYMNDLYEQEKRLQNCQSVDGT

ARDGNLMTVLVQASTKEQKASSSTTGGWSEKEIYSNIFVFNFAGHDRTAH

TLTFMVAFPASNPAAQD

>Mb|QLI71132.1

MDRLKEVTDTSTAENEFADNPNSTSDLHEFKESEGYVTRTDTEHRGNLKT
ANNGRTILIPQPSEDPHDPLNWSPVKKHVILFIISFAAFLPDYGSATGAV
TLIPQAEIWNMSPDTVNHSQVGNVFMLGAGGIFVVALSAWAGRLPILFYF
LVLAAATAAWCAAATTFESFMAARILNGFFSTVAQGGGLMFIFDMFFFHE
RARKINIWAVFIILSPYMGPLLAAFMITSLSWQIPFWVYFVETALALILT
ILFVEETYYDRRIAPENQPPRGSRMARLTGIAQYPSRYLRNSFGQACMRP
IRVILKPTVFLSSLYYLLTFAWVVGINTTLSIFLTPLYDFGPKQIGFFYF
TPIVAALLGEITGHWLHDGIARQYITSHAGHFEPEIRLRAIWFSTPFMLA
GLVGLGFALEDAYHYMVTAVFWGLYVFGIMVSTVALNAYNLDSYPEASGE
VSAWINFARTAGGFIISYFQVNWAHSVGAKVSFGTQAGICLFAFLIIVLL
QVYGKAIRIKSGALHFPTA
>Mb|QLI71145.1
MEHKTGLNAFPRLWFKLVSLSISFGPLWHLMNVQGVEAAQAGAHVHVEHK
TTSQRFPKPNDPFNFIPCPNAIETLNFPLLNDTDHRGTWSHLFNPNPDTW
AWGGSGLNSSASPSTKRKGLFFCGYLDVPVDYHNKSDPRITRLAVAKYQV
SGVGKKSQRTIVLNPGGPGGSGVSFVLKSAENLAAALTNGTFDVLSWDPR
GVGSSQPQVTCFPYAGLEDRWSLVLGKDLKESACARQQLETVNAINDATF
RSCRERLGDFPRFTSTAAVARDVESIRIALNETQLTGYFASYGTTLAQIY
ANMFPDSVGRMILDGMDYARDHRAVGGYGRTALYNTTDVWNDGFLGECVA
AGPQRCALAKPVNGSHVTLDSLKERMEKLLQGLAERPIPTYSKKSGPLII
TYSDVVSLISATLYAPSSWPSAAKVLQELEAGRLASTTALVAKSWSYQPP
DLNLEADSGSELLLMVVCGDVSDTSQPEGLDFWENLWREMVKKSFLSGHA
NFASVFPCRNYKKYWPKGAEVYRGDFGQPLRNPLLLTSMTHDPVTPLVNG
RKLLREMGTKNARLIVQHGYGHGSWAHVSNCTDRLFRAYLLNGTIPDKAE
THCYADVKPYRPVTKAGSRPGLADLEAAYQAAIPRF
>Mb|QLI71153.1
MSRPLHNQKPTSPATRSATPDSQVEAHDEGLWMKRMRKSNYKKDPSEKDP
STSLRRGRGIHDISSNEQSAVASSDIAETVSRKTHTTDDLEKARARLVSE
VQKLGLRNDESHENPAHASNKLEDADTEGLVQVWDSSKDVPAGSVLEKWT
EEDRMKWAQGTGIDMNDLLDKLPIHKRCTSSTVLGDRSEDYGVMDVNDCH
PPWIETISGDEMSAKNMALCDNEYHGISIGRLSGISLTPPPCKDTYGKTL

QPVPFSAVGTPTVAVSDNPPLAKPDATWGTGVDCDLNDGSRKMTEEDIAT
LGLLGWWKESNVNTGLPPINKENFPGLENVRTLDWRCHL
>Mb|QLI71179.1
MAPEIDTPAWKASILEGITAFPHVYTIVTCADTTHDLSPVFEADKDAYYA
RQVQEYCEHIQLSLHAKASALHEEVQTLRDENRDLKKEVATLDKVIDRVG
APKHTTGQLPHRQTRDHPTFNATEKDIEKRQEEYVGWRSKAMRNLAVDKT
IYNTEFRRLQYIGSMLDGSAYTLVRQSLDVITMNPDETGLWEWKTADDLI
EFLNSQYETIDLDRTASRNFDNYFMTNKPFENFIAEFNKLATLAGKTDKQ
KVEALQLKVSNEVIDEVMHRSGKPGNDDWPGWRKLCQDVYNDLEQSKHIR
RMRSRREPGPRSASDNPRPNNPPAPAAPIADAGELMQLSASSTSEARRRE
RQERGLCYYCGGTHRIRDCQEKKDNDAKYSLQNNTPRSDAARGRGHGRGS
YYSPRPSPQVPNATYQPQPQPQYRQLQGFPPTQQHYVQPPYLSPTFNRLH
SMDQGFIESDTSSLTDARSTPDTDRSQGNV
>Mb|QLI71205.1
MNLQPQSSTSTPCIQVSADVIRVTRDGRRTSCFTTNATENRLSVEKLNLE
YNLAARNARNRVLRQSKFEDLVGNQAIVQTVRLLLGQLAQQENALELSGS
KTDLKAMMEKLEAVEVDIERENGLITFSEKVACIEAIMEKLKAGQTDTEK
KNALLGLSERAADIIARMQRLETAHVDTENTLRRMLAKQRCEAAIRTLGP
QAWWNVRVFLVPEGHHPENLLQRVINDHSFETPPSDGEVFRRTYAHWRAG
QMGHIDHVNLHLSEDKKTGLRRLMKKNLVAEAMYKLTDYPGLRSGLQLSN
MKKHLCERCPEEICNYLTHIYNTWGKITLCNPRIQQATDTVTVQALQRLA
PSGSHVERGEIRNMMRSGKLWSDVSDANLRAEIELQLLNIGVIIPSIKTF
HENMRYLVLGMRIFRNHLLDALPAEKSVAEAMRKSWQPHQTLIEHAENQF
YQLTCSPSFSISYHVLFISAIRNFPKLSDEGPIHESGLPPINAGVDESCL
QRFLRHAQAQGFNSEKIKSKLREVPPSNAPPSGGQGYEGLQESIPKRRCG
RPRTSSYNFISRSLFLPQMLVESEIGAYPTINFIQSDFMRSFFHEFAHDD
SGRVMGIVAAETSLPMATAILPDTPTRPELQQLQTDREITGSRSPNDELR
PDNTIRSADTIFYQECQSERSLLSPAHPAGQAHSDRPPLHSSPRATEMYS
RSRYSQGSTTTGRTIYSPEEVRRICG
>Mb|QLI71232.1
MSAWIDIVRGGNDFRTPVLQEFHQPYQSFDNRPDGQAKLLLLFCTGGCSG

AEAPRQVRMCRFAKTILLDCHLHHQKSYPAMKAGPCPPNLTRRQVESYGC
PEDLAHEFYARALSPLCEAVVFIQNDYWDILTIVGVLARWVDLSFASSIR
CRPQVLIVSEDEPKIALRDVERDLAAELMSHYSPLEGLSFSQAEKRWRSC
FAGIRLMRGCRSRTSADHVLEWTQATRNNDTALTFRNCDVKHLLRSTCSQ
FAEDHRKTFSFQQAWRTNPLPGQLSWGAEKLVRLTMHDATLYESACSAIA
NSLLLETYQHRRRVGRMLNPKLMELSSTDWFDEFYSPMLSSIEPVTLKAD
VRTLFIQFMKAEPSERQTLLASSMRTLSCSSKITLPTDLCLVCLCRFPAT
TLSCGHRVCDNCTETNGKKSEEGIDIYQMHFCVLCGAVNSVQTALKPPSA
GIRVLNLHGNVDDALPIAMFLKDLRSSLSGRLEDYFDLVLGSGIGAFFMV
MIFCNQATVEDCIYHLPKLKCVRIDDKSLFFGKGLRFPRSDLLDAKIKLV
LYNTDSRLAICQNYITKSSKWLQKFSILFQGVDNIAARAFIEANRIWPDG
RIDVITQCHNSNYPETLSMANELISALFYVQREGIPTFYDLFPARFVLWV
KCRLPAGRHLLDIAMRMRRRRVHIQGVEERKTAFATPGSLATFSKIYNHC
EA

>Mb|QLI71245.1

MSLPAVLAKYLREEIIGDVEFSKSIAEARSVGNQWILRRSYSKHPHTIQE
EFRIEVRDQEHVRCTLKLNVIGHDAPREDTREKIFKPRAWCGPELYVPMS
NATDRQLKYEHRDIEVSEQQTNLPQHLPTSNAREFASQANTSAPQTSIDD
TATPAATESELDKQSDGVADEQDDQKQDNKVARYILLMAQYFVKGSLVLV
KKPTKLQRTKLAGIRQCVKETQESDEAGDIVRLPALWRRWKKEGVKEDKI
FSTTFSHTEELKRSVASMHNVGYYLAGITQHDKLRPPGHPPPTKRKNTTR
YYCARAVERMLKLVSRVSQYYGTSAMTIPTFLEIGGFSMTSFGHTTISSD
ESFFYDGLVSLIDLSWRRLAAAQTETDQNIQTEQDAEIDHDAREAHSTMT
EQDAVPVLNPFGFHSFYTDADYNATCKTLDQSIFLKAPQTVSYELPTSKI
LRKGRERSDSTSNGNPTSGLSDESSAFILRYAQSLAKHITHDEKVERTKG
YKDNEENLELALRVLDGNALPEELATASIIVVYIIVDILLDRCPRPKRPG
QERRQKRKRASEKATYEDAGMSHDCLPDIRQNCTGQVSLNIGGYGNHTFI
PGHGTTFAMGSVDSTAFGNPAIGHCGQPATTPNFIGQGLGCESAHLDDFR
NAAGDNFGDFCESGGDSSLFNESDVITGLPFPDRENFRWDESNANIGLPT
MCWGNVQF

>Mb|QLI71258.1

MLNAWQQLALLRTAAWAKEVERPRLIARRFNDNIPTEAGGERPPKETAAV

EFQRWLEDKPPGYVVFSDGSKTERDTAGYGYAVFHNGRLADWGFGQLGRR

EVFDAEIHGALEGLQCAVLANFTNEPITVCMDNTSVIDCIGATAPNSSQA

CFRAFQKIGDKYPYQVSVKWCPGHSNIFGNELADLLAKQGGNLPVPEHLP

SVSYRRRQVKGQIAVDYQQWWQGVERAGYSSLGLTAELRKLPELALPRRL

LGYLLAARSQHGDFADYHERFHPGQATLECPCGRQKSPTHLFYCRKIPRH

LRARLTPDPEAAIGRFLGRSYKVYLRIADFYYTKINKRY

>Mb|QLI71267.1

MSFISTFDSDRTLSQLYHDAKRLPEYASTTLFGHWLRVYIFTEKDWVIST

ERPTREHHDRTRLDIVVQTLLLDPTYATIKSKFLRNTWIFWLTAEGKKQK

SGREDLRYAEEQAYTKTWNYLHENKDAVRSCWVMTYFGTQARLWACQYKG

SGALEAFLPLEGENGEKSSYLDIKENEHYFNIGWAYIKENLVPEPNEFEE

FWRLTTSPAGPSAEAAPDPSPTDDRPSVIYPAYYTFVVVVKLIKTAETAE

GLRTQVRHSDKREQHFPRQLWRSVKVMCDNNVHADGFVADDLGYWTWTLE

PELEDEDPEHIGTDKTKGKGKGKAKEKHHHKARK

>Mb|QLI71295.1

MDFTSHISDHDIAGLLSRNVVCHGNRNAIIDGSLSITYQQLDLKAKEISL

DLIRQGVAYEDPVGILTAFGLPHLIAQVGVIYAGATCVPLDSERSDAELA

AQLHVAHVNFLIVDDMHRDRQLLAEKVVLIRDPNIELLRDKTQDIPKPLQ

PTFRSHILFTSGTTGTPKGVQIPAAGIVRIARDPLCEAISGEDTVGHLNN

PCFDVSLIDIWCSLINGTTIVVMDRRELLSPGLFARSLKRQQVTFAFLPT

ALFHMVALACPTAFSEMNTVIVGGEGLSIGPCKAVLEHGPPRRLINGYGP

TECTILACGHQITWKGVQDAASGYLPIGRPLGKTVAHVFDDSLRPVTGNN

QGELYLGGAGLSMGYLEAPDAQRTGFVTVPGLGPREEPSRLYRTKDAVCR

DDAGNLVWVGRMDREVKMRGYRIPLDVIESEILCTGLVSSVVAAKVNLPD

ANMSLLVACVTYLPSRGGSVSPCLLARCQARLPKYMVPQLVEFGAMPMTR

HGKIDRKRVHEILVDGLIRRLSEQSSIEVMKSDQSMSSEAKLRSLWSQIL

VTVPRHDIQPETDFFGIGATSLHVASLIHGVHHVFGVDILARTVYECSTL

RGLADIIQQERHHRPAYQAKEIQDNLLMDAATLWRNIPIPQVEPVDWLSA

GEGRVFLTGATGFIGAFMLLELLRMPQVHRVRCLVRADRLETAYSKLFRN

LDKYRLAQLEEDLRTKIEVIPGDFSQNRLGLTESSFQELAEWASVVFHLG

AQVNYNEPYAATRTANVLGTLNLLRLAATGKPKAFHYTSTISAFGPTGLM
KNRPNLIDEDGSLAPYVENAIPYEMGYGQSQWVADELVSTLMRRGFPTAI
YRCGFVLCHGESGIGNLDDFASRLLTDCTRLGIFPLLPDQREELVTVDYV
ASAMRLISTSNDNLGRAYHLVPDLDSSIDMNTLFRLVGKAANVTMTGLPY
HDWLDRIRTAVENASKLRLTPLLPMLEEKVLGERTRIELYEGMARFKREN
TVDALQKTGCNGFLREAAVNEKDLQAYVRFLQSQD
>Mb|QLI71300.1
MAGTIETSFQAAIASGTINGAVICATDSKGDFTYNKAIGERTLLSGETRP
QQLDDVLFLASATKLVASIAALQCVEDGLLTLDGDLSPVAPELTAKQVLQ
GFADDGKTPLLEPAARPITLEMLLTHSAGTAYDFLAPLVGKWREIFSPPT
QGQLKTVEELFAYPLAHQPGAGWMYGSGLDWAGRIVERVTGRTLGDHVLE
RVLAPLGCRDAQFYPVSREDLRARMVDLNPDDPNGQGRAVIGGESDVNLR
CKGDFGGHGLFMPAVDYVKILQSLLANDGKLLKRETVESMFKDHLTPESA
AGHQAALAGPGGVFFRVGVDSDTKMGYGLGGLLTLQDVDGWYGSNTLTWG
GGMTLTWFIDRKNDLCGIGAVQAKLPLDVPAVSELKQVFRKDIYRKYAAW
KELPKA
>Mb|QLI71320.1
MEGQDGRVLNIQLKKPIKARPGYFYIRAPELSPAMQSQPIHMLWWQPNAP
SIQHFAVLTNQVKRAPSLGRFPIRLDGPYQEDLHLGKYEAVMFVAQGPAL
SRVLPHLLYLTTRITRDKFAKVQSNRWYLDGLFSDKTRKVDLYWKMDQND
DISSVSAYFDELSDCGADSKVQAWIFYPEGLPRQTRLPRPKGRQHWFPFD
GDFLRQVSSAIETQSKRTPGRAKVVICGSEVFNSTVRRDIRNYIQADNLI
SLSELVTMSWATNEKGNKQQQKLRKTDVGHAGHSEK
>Mb|QLI71331.1
MPSELDERRLKEAKKLLQGLVKDSSRLFAALDRTKSISQAIDSVGTGVGS
TPESQRRLALLADLDRAAAGNVELASLFWGQSQVRSMSDAARCYGLLLDR
EGAGVEDFQMRLFAEQPTATIYSLVRAGHLTIQPKTIQTAVVNLLDRAVS
SGFNIGRDPVDRLLSKVDALSAGKKKDVEALKSFIQKLQRLQALVRLPQD
LDALLKSSFMSAHQIAHTPRHSFVTVIGRHGIPAASAVRIHNHATLVDMR
NEQAWTALLASKHDVSLAALARPKAAPPVLAASAVASTTAPSPQVTSYTK
IFGDMTIGACDDCSSVSSPAAYFVDLLRMLKNTPSDATKTDSPSLLDKMA

TRRPDLLTLQLSCVNTNVLVPYIDLANEAMESFIKNVGSLLPPSPVPIQG

FNMTDQDTSEVSLSEPQHTDYSVYSNQIAAQAFPLTVFPYSQALDLQRLF

FAHLNASLADVMVLFGSASRLLSKTPTTDTLGLAQDVLQSAQAAEYLGLS

PADYVAITGASIFSLDFFKAVLDPSMQQDAYNAKIGLLSTAAYWGYEAAG

GQTAAQLMLSEVDEKGLPFVKAQLLRRADMTVVDLLDLLRARVLQRQLVL

ENADGSATFSGELQDLRLRHPTSSNAKAPLTELDCWQLQSYIRLWRRTGW

TLQDLDCAIVAFGGGGGTRVTAQTITAMAAVQRISALTGVDVFRLMPMWG

PMDTNGDRSLYARLFLKAKSGRPDPVFGPDAQGQYLTAGAGLMANRAPLL

AACGLTEEGFATVVAAAKITNDKLDLANLTAVYRIAVFCQLLAVDPVDFA

SLQAVLDPSQSAFVSPQAALDVIQQLQQLTDAGLSLEQLLFLTGNDAAQR

ARQSDTELPVQQVAVAASDVMVNIQSRMAALPAVDAHTTTASPADVAAAA

AALYDHETAQLAVTFIEMLPGTPITEDSTVYQSVLLPYFSGDAQAAHKVF

FEQPDPTGTDEEKAIARENILDARRVFFLQHVIGPLHARAAKDAVLQSIT

PLFPDVKAAVLGFLVETVQEPGSQEGGHQPASHTLILDVLGGLAASNAAA

LAKDSSDVFFRPPTADLYRLFVPDTTLTASPLVTLDGNTLLFDKGTDGWT

SKPVRLSNGQAYSLRRSDDSGLASCSYLTERSPGRPFALGALMARETVVL

AQTVLLLLSRAVHLSTSFELSLDEVQFFQGQSSSGSLFIDWGNIDYQAIQ

RAKSYRELARRLNGPTVLLDFLKWTQGSSRDGTLASSLVSVTNLSAAQIA

DYLGQRFAGLSEQQLIEVFAGTAEVARLLDRVAFMNRTGVPGLTLKLLFA

LATPVPPPTATIDFDNADQLRCLLQSTPASGNGADLATQVNGGLRQHQRD

ALVNYLVQHPYITSRGITDADGLFEFLLIDVQMGACLQTSRIKQAISTVQ

VYVRRCILGLEKGEQVASNTVNLDRWAWMEGYRLWEANRKVFLYPENWLD

ASLRDDKTDVFRALESGILQNDLDSAKIVDLVKGYVYGVDAIADLDVQAY

LWKKVEDYTGTFHFFARTRAAPYIYYYRSLDIVPSPSGHGIKMYWQPWSK

IDVEVPVHEVGADGKPLPVSGSYLTPALYGERLFLLFPQITLKTDPNPAA

KDNTIKTMEDKKPDEIKPTKYWQVQIGWTEYRNGRWTPKQISQAILEVRG

AQDTDFPVKGKTLPAANIWAEAQKLPDVASLKFWIRSRPSSSIPSGASST

VAASSPGPRSAPAPSTSILVIEVERWIDAGAGQTPRYQNYPLGRFEMRGS

QVILANLSALSGQNDPFLTPWRPTIPTVFAKMSYHMAKATDKPTPGITIG

RGASDKEPLLGIVSKLVDSTPKKDVVWTMSFNDSQVRNATGFIQDIITTE

STISYVTYPPIASTTTFASDVFQHTLDRDLVSMSTAYDGVDQVYAFLASV

PADDAFLAFGKRNGTVHELSTPYALYNWELGAHTVMLLMERLQVNRQYDL

ALQVAHLVFDPTVDGTSLDRCWLFPPFKELADGTIDSVEDILKRLQPSSG

AESEMKTSILDWRTHPFNAHSVARSRPLAYMRRIIINYIEILIASGDVYF

RQNTLEALPLAIQRYVEASHIFGSAPVRVPQLAKPTYKSYNDLASTLDDF

SNAAFDMELDFPFFSDPASRGTGTGANGAAGLTGILKSTYFCVPANPKLV

QLRDLIDDRLFKIRHCQDINGVVRSLALFEPPLDPGLLVRAAASGVDISQ

LVGNVVGPMPNYRFQYLLQRAFDICAEVKSMGALLLSIQERKDTEALSNL

HARQDKVLQGLMIEMKKTAKKEAEASIDALLEVRKSQVARLEYYLALTGT

DDHSAPDETQDWEDITQSIEKPTSDDLRMTSHERLEMEKADAAADLNQKA

TILDIAASIVKIIPDISEEAEPLGVGVSIDSVTKNISEAMLINASVMRFQ

AQYFSDEGARAARTGALIRQLQERRLEANMAGREIKQTDKQIATARIQVE

MCERDMQFQQQQADYARDTEAWLRTKYTSEELYAWMDGVVRDLYHQTYLM

AEDLAQKAQAALRFEKGDQSINIVTSPYWDDGRDGMFSGENLYLALKRLE

GAYMDQRLHDVEVVKDVSLLQVRPWALLALRETGTAEFDLPEVLFDFDFP

GHYCRRITSVSMTVSCVAGPHTGVNATLTLLEHSYRIKPDARNAQDYPRK

TPDDRFQTDRVPITSIAVSHGQEDSGVFDLDFRDDRYIPFEGAGAVSRWR

LELPTAVKQFDYNSISDVVLQVKYTALQGGAAFRKAASDAAVAFQKTASG

LSRTEGMFALLDLKNDFPDEWQRLVSADKTKPSTMPLASLQDRLPFFTRG

KSVKAETVSVLVAAGAAVNMTQDITLTASSKIPLQAGTAIGSYQVAVATG

QTVTVGDWSLTLSANAMNASVSRILVLFRYIL

>Mb|QLI71339.1

MKTPVDKQWKQRTSGIMKKAHGMHWTFGVKVALYLERDGELFVYRSHEDF

AWEAATFLAKQILTPNDFITLAQDMSSRRAQQSTPLPSSEQGPPQTPVSS

SIPTPVSTPGSSECSVTGSRAPRRTRSHATTLFNIAN

>Mb|QLI71362.1

MHGFLVLVFALLQRTTAQQTSGDGSSQALGHIVVLKEGLNDGHLNEHLDW

VKSIHKRSLDTSNDNENQENGVKHTYHGGAYGFHGYAGSFSRDVLKSIKE

HKHVDFVEEDQVIILEPTRRDENTDGDMLPEGGTRKKNKGHSLLSKGQGY

NSFLEKGLILDAVILHEGEKRRDIPARSAEELVSQTTMNFNFTEPSADFA

GVDVESYFYPPDLDAIMEDILADLENDEQDVSASKVLFTRAADRPDCTGA

LKFSSELAEDYNSYLKALDISVAATVSGWGQSASVSGSYLNQAEFSSNAL

TYVAKIDIRRQLDSPAGFQFNMNKYTTTTFARNFGDRWIRGFHTGGKMIA

RLTFRSKGTVSKVDLKAHIEASLKFWGVTADISASVKKSQEEVSKHANVE

ISLFYQGDLGRVMGQSGSPDKITATSADGAFHQVKSWADQFMDNACRHDY

EYQPLLEEYRNAEGFPEHQKILDYRTAHRVSSMVLKQLVRISEMKQYLLN

LTTIDDEIKMNVEFDEIDMVKQSQDWVDSVAENPENSQSTGRDLIKTLRD

DFFKKYAPYISNSFTNESPRADSYANGNRYISGVKVERGATGPANSLGNI

NYGFTGGSVWLVPTYTSNPKKACTTITVEQTRIDYADAVKFLSKDGKYTA

RYFRCVTSSERKIRRLALSRGSGIINYDKAHGFVDATSNINEGFDMSPLY

LMWSFDETDPAPEDDFPEPQN

>Mb|QLI71380.1

MPLQERAIAYFLYHYDLESPSQNKIGDMCFVILSKSDKEWEHYRLAFEAC

AMASFINGAGAQLEYESLTFEAYDKALIAMRSALQDPDVVHEDATLAAIL

LLALFECLYPTTGEQKGWRNHGNPYFNDFLIANFQQAIYSLATLEDEKDK

ELWWSEDDECTSTQQLCVGVASLNAEATALGPSGHREEVEDTLKRCQDHD

RACESRWEELSKSTRDISSNNTDPWLLMLRNMLTCARILLNSIIIRCATW

IYKVPNYRTSEEYGGACSALGEIILHSAKANEQQLCWAEEAQSSFAELDV

QLQPGRDSSGCAINKWIEDNRNLWKDKPLVGLQPLALIWSLKCLTDDQKI

AIDTRLRRIVEIRGALPFHEDRASMPSFCSMVEQIPRSPSHTTEACTRPS

PSLSEPATDTPEAGSQDSSPPSTGRFRYTQEDDDTLLQLYNEDSFMDTKK

LSVHC

>Mb|QLI71382.1

MMLNSAIVTFALSVAVSALPQAYPNKCGDQVCPADKSKCCEVIVNGVAEI

GCFAECPPVQALQRRQTYPNKCGDQVCPADKSKCCEVIVNGVAEIGCFAE

CPPVQALQRRQTYPNKCGDQVCPADKSKCCEVIVNGVAEIGCFAECPPVQ

ALQRRDQPSTTTAASPSTPTFGPKCGDSFFCPVGKVCCPNALYHCADPDK

VSQQCPQ

>Mb|QLI71403.1

MALLYETVPMFEDAWIECLGDLGRYRMAVEYDDIQERETWTGVSRSWYTK

ASDKIPMTGRLYHHLAILARPNALKQLYFYAKSLCVPVPFLSARDSVLTL

FDPLLNANPSTSQRLDPVDVAFVRVHGILFSGAHEDQLEPSMKQFLDLLD

SRIGREHGCWLESGYFIGISLSCLLLGFGDASNVLMNAVLKNQQTDDTTM

DDLPDPVLTDAFKTAVGFAASTYEIVIARSGDKNTLPCLHTLLVFYWFMM

DFEVGRQYLEGSIPWEQTALLLNYLLRTSEFKPRLHTPEIPWPEGGKAHP

LPEDYAMRGLIYTANYFPGKWFKNAAIDDDEKYFEPASTVSKRCERILWL

GYSMAMRKRRLHWDQNTKQFSAKSNESNDDN

>Mb|QLI71451.1

MSVEPPRFPFQRASGMEPPAEYARLRATDPVSRITLFDGSLAWLVTKYKD

VTQVATDDRLSKVRDRPGFPELSAGGKAAAKAKPTFVDMDPPDHMHQRSM

VNKFFTDEHVKSLQPFIQKTVDDLLAAMKAKGCAAAPVDLVKEFALPVPS

YIIYTILGVPVSDLEYLTQQVATRSNGSGTAREASTASQELLNYIGTLVD

KRLAEPKNDLISELVVNQVRKGIIEKSDAVQIAFLLLVAGNATLVNMINL

GIVTLFQHPDQLDQLKSDPSRAGAFVKELCRYHTASALAMKRTAKVDVEI

GGQRIKAGEGIIASNQSANRDADIFTDPDTFDMNRTWPAEDPLGFGFGDH

RCIAETLAVAELTTVFATLFKRLPDLKIAVREADLEFSPLHKDVGITHLP

VTF

>Mb|QLI71468.1

MSKTLLSNSEAAVSPEVLVKSRQGSNAHRPARLDVDLHAMTAPSLLIRKL

YNTGHTNHDVPVMKQYNGLNEADVLSKCVAEEIPLHALEHYTQGPDQAVA

LRRRVLAKFLAITGLSGKSLAKLPHQNYNWDLVVGSYCENVIGYMPVPVG

VAGPVLIDSKNFFIPMATTEGALIASTNRGCKAINNAGGVTTNLTDDGIT

RGPCVQFPGTERAAFAKNWISSHEGQVILRDAFESTSNIAKLRRVNASIA

GRSLYIRFKATTGDAMGMNMISKGVENALRVMSQDPKFEDMRVVSLSGNY

CTDKKASALNWIEGRGKSVVAEAVIPHQAVQSVLKCTAEAMVRINNSKNL

VGSAMAGVIGGYNAHAANIVAAIFIATGQDPAQVVESANCITLMEW

>Mb|QLI71484.1

MDFLADFIKAQKIKGPQMKDAPTFYRNLEEALDLRRSDHGMFTNGLKSPW

KSGKAVDFCSNDLLSLGKTGLLRKEFLNELGRNPDFPIYAGGSRLVDGNY

DYIEQVEQEIAAFHGAEMSLIVGSGFEANNAIYSAIPRPGDAIVYDELVH

ASVHDGMTHSLALTKVAFRHNDVDALRDALVDLVDSQPLIKDGSRTVLIS

VESVYSMDGDVCPLREMLEISREVCPRKNFAFIVDEAHATGIIGPKGVGL

VSLLGLEKEIAIRLHTYGKALASTGAAILSNGTVRTALLNFARSVIYTTG

PSFPSVACIRAAYNLMRNGETTKLQNSIQHLVKHFFETITSNPVWEKASE

MGILAIPLVDDWEGREFQTHIVPLWTRQRYNWWLVFHLHLAGISAVPIDY

PTVPKGQSRIRIMVHSANTEAEVEFLASTICEWAQEMIGIEESGEVKGKT

PRAAQKIYALMAAAGA

>Mb|QLI71486.1

MGHILVVAAFTFAAVTASSIPDRPTTLPLTLPIVERSEQPITCLTGIKRS

PEPINGKRGIEQNCGRSQRDVTRAFSSCAAKAQQGREAALKGGELMKTLF

KNDDEDTRKRVADHFEQIASQCGARENSGVSVNCGQCEQGIAGKAFKGSG

PITLCNVALADTQSTTQCGGQDLDDVLLHEMSHVLGSTDDLGYGLEACKQ

LDAQRSLQNADSYTKFARAATLNCSVGGPSGPGSGGPGSGGPGDDGSSFP

PGPNNPNNPNNPNNPNNPGKPGRPGRPGGPGSGGPGSGGPGSGGPGKPGK

PGRPGRPGGPGSGGPGSGGPGSGGPGGPGSGGPGSGGPGSGGPGGPGSGG

PGGPGSGGPGGGDGGDGGDGPLFPPGTGGPGKPGKPGRPGGPGGPGSGGP

GSGGPGSGGPGGPGSGGPGGDGGPSFPPFDGGNGIDGGDGGPSFPPFDGD

NGIDGGNGIDGGNGIDGGNGGDGSNPFPFPPTTGGPGGNLGPGGGQPPLP

PFDGGNGIDGGGGDGGFFSSSGNFPPQGAPPPGGISAFNAPTAPQVLPIQ

DYDRVESS

>Mb|QLI71525.1

MGDPRIDEAIAAGDVPPDVPIHLLRESRDTSAIAGIVVVLVLTTLVVVLR

LYSRKYITKKGALGIDDGIALASLLVFIPFTALCIELLVLGAGRNFLFEW

FIMTAEDDYKVQLIDSITHLVYSTTLVLCRVSGLAFYYRICRMNPKFLLA

IKIIFVVIILGYVAQLCLIIFHCQPITLLWAPWNKDDNAKYYCMYWYETY

SIISSISLFCDLLLFGIPAIMLKGLELQRKQKLQLAYTLFPGILVIGISA

GRIVLVTKYGYETDETFEYAFLNLLCVEVAEVGATIIAVSIPGVKPLVDK

YILLKDRDSTSSNSKSRGTSISSTEEIKQHRGHVDIMEFSEEKFGRWSRR

QTEGGTA

>Mb|QLI71538.1

MPLDIAIIGAGIAGLTAAVSLRRSGHRVQIYEASAFSGEIGAALNLTPNG

VQVLQHLGFDFERAKAVQMVNWDTVSGIDLGRIACQDLHAAADKFGAPFF

AVHRVDLHGELMRLANVSEARGPATGQQADGILLQLASPVTRVDAEEGRI

EFADGTVRHADLVVGADGLRSVVRDAVARGAGVGEPVGLVATGQSAFRFL

VPTEDLQATASGRKLLAWKTPGACLLADTKLVDKERHIMWYPCRDGAVQN

FVGIHPSLDTSEADAAPDVQAQMLRQFGHFDADVVQILRDAKDTKCWPLF
SMKPLSSWTFGKAVLIGDAAHPMLPFGGQGSNQAIEDGGVLGRVLTGVQD
AAGLPQRLELFDKLRVLRASRIQVLSSVRANNEHLVQDQIAQYMEPGVPL
PNSFLGRLVHDAR
>Mb|QLI71556.1
MKLLATIIALSGLAAAVPQLELVPIFGRCADPSVICTEKDIVENDRVTGN
KIFCTLYEGEKVDNIYCTVNQREKDEMEIDQASEICNRLSGCTRCQWQPV
ERNQKRIGGRVAEFSCNLRPDAKNTDEARKIFREQIDKGAQAICELKGVD
RSTCETYKLDCINKLSKNGPINPDQMEWCVGNKVRI
>Mb|QLI71648.1
MRFNVNSTLLAATLALSAAQTGLAAPTPQDLSSIPVIGPLLSGATGAAAG
SGASNPLSAIPVVGPLLSGASGSANTTAATTANPLAGLLSSIPGLGSLLG
GAGGATASSGASNPLSAIPVVGSLLSGATGAATGSGAGNPLGSLLSLGTG
LLGRSTDSSTAA
>Mb|QLI71704.1
MLALSLLLSAVAVNAILGGEVAGVLISDKEFFCADECAYKGVFFEQGGKW
CTRNDTTAIVNSEGLAHPRYPHACAGSRVPEGWQERALKNGEEPKQCYVY
EPTEPFDYFCEGDKSAGGNAKNVNVQWSELEKKLAPASSTNSASREGEQG
ATEKSKPASPEKLKQCNDLREKNFWECWNKFKDDFQKCVDDAQQVREDCR
RGE
>Mb|QLI71707.1
MYYESMVETWLKDHKLKTPEMQNEPAFYRNLEQRLDTLRAEHKLLTAKPR
WDTPMVDFASNDILSLSRTGRIREAFLQEISRHENWNLSAGGSRLQNGNY
NYILEVEREMAEFFGSETAFFGNSGFLCNAGITGCVALPGDCYVFDELVH
ASAHEGMKLGRASHKVSFRHNDVNSLRETLTNLRETHAEFKLGTQSILIL
IESIYSMNGDVCPLEEFVHVAKQVFPLGNAQFIIDEAHSVGVLGPHGRGL
VSMLGLEKEIAIRVHVCSKAMASTGGVILCNKTVRSLMMNQMRFAVYSGA
PSFPMVASARAGLQLFRSGGTHEEQERIQKTISYFFKRLTSKPDWQQAVA
AGLLSCPVAEGWEQRQFHAHIIPIRTRPRHEQYLFFHNMLANIMAYNFAY
PVVPKGESRIRIVVHAHNTEDDVDKLVASICQWAHEMLDLEKCGSNNTLP
SAASKVFASQAAVKA

>Mb|QLI71764.1

MKFMHWSLVASALLQSAAAAPQDTGKDAGATQEMGHIVVLKQGLKDEHLD

KHLDWVNEIHKGSLNSRDGGNGQEKGVKHTYRSESIGFHGYSGKFSDEVL

KQIKDHEHVDFVEEDKRNTLEIDKREEVQGDKPDKVDTPPESVGNQNKNN

GVGLLTRGQGYNTFLEKGRIVDAVIWPENKKRAEADAPAAGAGNDAAQNE

IVFDFTPPTTDVGDFEGVDAAEYFKTPKPDEVKNRIIEGLKKLKEERKKQ

REEKKKLLESNNLQSRADDPNCPGTLRKEYKFVEDYNTYLQTVGVSGSAA

ISGWGQSASVHGNYLNQAKLNKNSLTYVAVINVERQLSQPGGFQFNTARY

KPGRFAKDFGDRWIHGFKTGGKMVARVTFTFKDDTKATDVKAHAEAALSF

WGVKGDLSVDVKKGMEEVNKHTNVDVSLIYEGELAIFMDDKEGSPKSISF

GSAEAVLSQVKSWADKFESYACKHDYAYGPLLDEYDVVPGFSDLEDSPEA

PDYDIARLYALEILALMVKIDEQKNILSSAKDLDDKKKREVSAAAIKMVS

AGKKWVKTAEQDPGKAEEQAEELMNNLSANFIDKYKGDVASALKVTDPAG

FAKCKKLANDKFRECNHTQKDNHDGRDVVEFCHKEATDAQNQCRAGTL

>Mb|QLI71765.1

MLVNTAALAGVLALVECASASALKKRQVLKDENVAARLAGGANIGDQCHP

PGTYSLGGDKVVPPCLSEQAIALKCEIVTQLKKNTTSEANLKAYGSCLKD

QGSSYFKDVNGCLACKAAHNHMSQAQFEWYSQAWADGQKAFLNDAVPKAT

AWEYVLGAINNTTCQKNGTNEKLTAWDCWNQLPAGTGVATKNLTIEEYYK

DRPKTQNIGSFEFGGKSFPESSSVELGLLVPTFEIKADIGYFCSVKLENG

TITGSVNATAKVEILTQYQEITSIFNFTKPDEFTVLPVINAPVPVKDSVT

TVSKDEAAVLPAVVCNADCRDKATTIDKIQEAAAQGPKEADKAVVEASKP

ALQDLKENKPLSVTHKVQIITEIKTEIKAPVNFQGQGQAPPLQNGPGGSN

PNGIEAPGQQGQEPAKQQGPGPSDSKGPKSPILNGPEPPKQTGSAPASQK

GPEDDKCEEDSEAPAAPPVGGADKGASKEKAKEKPKETTKETANATCEQG

DPAKASVKVTICQMPGERDCKTYYQ

>Mb|QLI71791.1

MKYTQLIFATFATLALAAPQSDFMPGDVMTVKQVVKDADGGVSCTEANSN

QNELSCSSTRDTNDGLTPLCEVKGCTCTKGFRDFAASSFHPESRTISLTS

RSIIATLYVQIPVLKSDRTTLRWDICAQRLMLYPGRDGWTGGKLSFSWNG

K

>Mb|QLI71808.1

MMTMLPCLLFLAGLPAVLAHVGGHCPPAGPVLPPPELSARLDLSKLDSQL

DSIVRNASRSFNATENSFSILLTSRNATVYQYHHTAAVRDPSGVKKVDGD

TVYRLCSVTKLFNVLTVLLNAGDLLDTCITKYVPELAGDQVYEGITLRML

SSQVSGLPRSGDAFDLASTDAKSWEDDGFPVPPKGDMPPCDVVGGEVCNR

AQFFENLKSNQLIWLPGAKTAYSNQAYTLLGMAMQNITGKTFAQLLRDSV

TTPLGMSLTGLNTPDNSRGIIPNGAGKVLWDQDMGNYNATAGLFSTPNEL

GKFVRGIMNHELLSAANTREWMRPASFAGSYSMSVGAPWEIFRVAHLTPD

QRPIDIYTKSGSMPGWGTYVFFVPDYNVGGAIAVSGDDGDAASLALLDMV

AATFVPAVDSLARQQAKAAYTGQYGASSNDNKPMNETAHLELVIDQGPGL

KVKSWFNNGKSIIKAIADHKGVKPEGTDLRLYPIGENNRWQLSVETLKRR

VDVARKPSDACSNWFQTDSMRWATLPVDEFDFEVTNGRVVGVRNLGLRAN

LSKIH

>Mb|QLI71901.1

MHISITLALFFGLTSARVAHAPVVESQIDQPESVINDNTLAPLELALGLK

NHVKLAKRSLQTQTSAPWGLRAISHRSPGDFYEGFPPPESSKYYYDDKAG

AGTFAYILDSGIRTTHEEFEGRAKAAHSIYPADQTIHGDHGTGVAGIISS

KTYGVAKKATLISIHLLGPDGCTGSEAINALLWAAEDILKNSRKDSSVIN

LSFGIPKLQALNTFVERLIIETDVPIVVAAGNEADDASNHSPGSADGVIS

VGHINQQWAISETSNFGSAVSILAPGVGVETTGAGSDTNIIRETGSSFAT

PYISGLILNAISIHGIKGAANLKKHILETAIKDKACIPPEKEDKNNRTPN

LVGNNNNAEQDKEKQEDEPSSSRMFCCNGLLSKLACGRNRPMNV

>Mb|QLI72042.1

MKTSTALAVLFGLALAAPEGPKATVQNRAPWNLRAISHRFPHKLPSVTVF

RNFEYYYDLWTNGKTYYAYVVDTGIRTTHQEFEGRAENLWTAVKTATGED

DFSDGTGHGTHVAGIIASKTYGAAKQARVLSVKVFDDKNDATTSQILAGF

NHAANDIADKGRKNTAVINCSMGSASSPALKLAYERAHHRGILTVTSAGN

NAQSVGAASASSASGSITVGSINQDWSIASHSNYGPHVNIFAPGADILSL

SHKSDSATAIMSGTSMAAPHVAAIVLNAMAVHSQESSLVDFFLETTATRD

KITGDLRGSPNVLVNNNNDRQESSCGQQDDRC

>Mb|QLI72053.1

MPLSAKQFHKPKTPSGFRSGEFIPRFTPSQGALICGGCVVIDPTLRKVAL
IHDPSTGINQLPKGRKNIGEDVHAAALRETREETGLHVIPLPLKGLTRAT
PTAQMLGEPVNAETFAAGSAGEGEDDQDVWEDVDEEGTNAERARGLTGWA
HHCEPIGITTYRCEMTLAFKMIFWYAAQADSLEDPRQDAKERWEQQYELK
WVSAREASTQMTFKADGQAIEKALADMRRSGYEI
>Mb|QLI72166.1
MVRLSASALACLAAATVSFSTSAVVHAQEIAQNAVAGAFLLECDSQSLKP
LIKTVQEQGGEIRREFNSEVFYGFSAQLSNASVAGDELRHMPGVKKVWQV
QVSKHQESPPAESQATPESAHQRRQVKSPWNHVMTQIDKLHAAGFTGSGI
QIAVVDTGVDYTHPALGGCFGKGCRVALGDNFAKDGKDNDPMDCSGHGTA
VAGIVAGSDANYLGVAPNATLAGYRVLDCSATMEEDGLIAGWVKAYQDGA
QIIVSSAGWPGAAWATRPAAAVVSRIVDSGVPCIVGLGNDNNSGLFNTLN
PSSGRGVTSVNAFARAPGAIDGHVTDAPVAQFSTFGPNWDLEIKPTVGAP
GDDVPGIKMGGGYEDITGTSFAGPLVAGILALVAQVRGTFDPVLLNSLLT
TTAVPQGKYYSVAQQGGGLARAWDAAHATTLIEPGSLSFNDTLHRADSRS
LRITNTARVKVTYHLDTLAAKTIYTLENGGGRVQHLDRPVDESADVKLSR
RLLVLGPGESASVDVSATDPKGLDPERLPVWSGCISIQSSHGGSSKCSNS
SSVLTVPYLGVSGSMKEHQVLQPHGVVLSTLLDNGRDQVHTGGHRVDYES
KNDGSVSIDLPVRITPVLGTRLVRAEVVPLSPRKWLAARLADKNLKLDAF
SLEALAHAYATRKTWSGRLESGDYIPAGEYRLAVRALRLFGDAAVASDWD
LSEDVSFEIIRPAGRKACERYESSKGAVPADALFTSLEECLQVHGDKVVD
APWVPAPQDKALRDRCANGELTDELCGTYELCRKHKDIELLSDVKSPFTS
LSSCIKSHKTFPFKPLDYSRIQECMANQDDKSICGTDLWCNLHFGSPQPT
DEYGSSEECRWAHGNYL
>Mb|QLI72181.1
MRTSRAALPILAGIFLGGGCHGSQSKCSQSTLEAILDSIPGTSINYVEEV
PQDGAFGDAATNIPFPRNATGLPALCAASFNTKTPGNTSYNFGVFLPTNW
NGRFLATGNGGFGGGINWIDMGIFSQYGFATMSTDTGHSSAPTDGKWGLH
QPEKLINWGHRAMHGSVTATKQIVQSYYTGAIRFSYYAGCSTGGRQGLRE
IQLHPDAFDGVVVGAPAWWTTHLQPWTMKQGLTNAPANSPHHIPPPLFRI
IADEMTRQCDAQDGLRDGIVSDPDGCSFDFNRLLCAGNKTACLTAAQIDT

AERLYSNYLDAKQALVFPGISLGADAAALSAQPSTLGIDFLRYWVHNDSA
WDYTAFAYSDVLLSERVDPGAATADDFDLSPFQSRGGKLIHYHGLADNLI
PARSSRYFYDRVYRALTPRGIRVGDFYRLFYVPGMEHCMGSAAAPWYIGG
GTQSVTGASHSVPGFADPEHDVILAIMAWVEGGAAPDKIVATKFRNDSVF
AGVESQRPLCAWPLRAAYLGRGGVKDARNWECVEAGTDRAERGGSGSIFG
RLTGESG
>Mb|QLI72218.1
MLINNNGIRSRRNGIDGSKAIIGAISGDTVEGIAVIDIDILTIFHASQNR
EKGGKALTSVLKQKRDDLMSENDQYRELFQLLCTKPWDEALELFRHIRES
GEPFILFEAVRQAEVLFPNPSPSETYGNYQLERLDRLALDGSIIKVPARP
WTIVAGDGLVSELITNLFHFNGTYLLPVLEKDVFIEDMKSGHVIGSRYCT
PFLVNAICAVESNFSERAKQFGVIAKQNLSDRFFEEAKGLYEREQGCASI
PNIVALVLMYLTMAIKGRDKISRIYLYTAYALLSRLSLEEKFQALESRPG
SQKEKCIISRVLWGLYIMESRIAFYYSHPSILPAPKVPKPPDEFGTANMD
VLGRLYDEATSAVPLVPGINTINCNLTELWNELMQYVCEGAITGSDSDLR
IRKSFYCRLLAYVAQLPPRFHCWSNMTPETCMKRMHETEIIFTILRDVPL
DTPFHNLYDLPGTTVRDILRKRCIADVELIGVYMNKWQSLGGLAYRHIHL
CMYALIPLLDDSATHPAFSTACMIAQQGINRMKVLGYLLQGIQAFAWAMG
KTIPESARRYLHGWGVEAIEPDLPVSFVLPQPDNVKEALARNSGPLLEEV
EGQLGSLIELWARLGQ
>Mb|QLI72237.1
MFPPTRIRITRSRNGCAVCKCKRLKCDEARPSCSRCQRLGIACPGYQKPL
KWTTSLRTPGGTSSSPVASPSRPPPNWAAYSFAQPPAEPPALTTVDVSGS
ELLDQFTNPLVTGANGITQHDGLASDSPFDHLSGGTTPSFEQTPGPMSGG
MYQPFDEDGCWPNSLLSETRSEFDMPTLFESPSQPWGSRCEMPLAQALQD
HTSILVEYYFKEVCGMMSCYDSQMNPYRTTISNIWSGSQSLYYVTQSMAA
ACLSDVMPGLSSLGKQLRDQAALCLSMEARESQMETSSLLALVMLGMSLS
WHDPDSVGQLEFEILAKTVSSSGSGQINSTLAHKHKRVFFYNSLVYWQML
LAFVTDRDSNLQGMTQNQPQPAPFEGPETSESHIPHPQTGIGIQVQRLVA
KVASLVRKERNRIRNRQFASRNDIDQAKTAILDAERLHSELCAIQLPRED
EIIDSGDEMTPADHLVKVAEAYRCTGLLQLYRNFPDLLSAHASLCKQKPR

SVSSSTLEDSGPSSDAAFSQDVWLTGLALHILDLIQDIPTSSRSRSIQPL
LFVSICSELALGRGSVSLPDVLIPPVASIAASPRFKAPATDLDVLRARKF
VIARLSSFENILAAKPIRRMLLLVKETWKCMEEKQQNVYWMDVMMERGYG
TLMG
>Mb|QLI72290.1
MLTTLFCIASAVGALAASVDARDEFDSSAYAAKDVVERDFAIIGGGAAGT
YAAVSLADRNKTFTLIEVSDRLGGHTRTFHDPVTGAKVDSGVQIHVDTPI
VRDFFARLRAPLAHADLKDFGKPRYYDFARRVALANYTRGAVQPDYVAEL
DKYPFVENLIDLPNPVPADLLLPWPEYVKKNNLSYSSALAGLSWPATPGD
PLDTTALAILNDGNHWELAAFTGAAVRGANHDNSQIYVNALAELKPHVFL
KSSIIAARRGSTRKCGVQLVANTPSGKKLIKARQLIIAMPPVLDNTKYFG
LDRQEQAILGKLSGKYYYAGVVNNTGLEDDVAYNNAGADRPYHVASLPGV
VEIAPSASPGYHFYWYNTLQAQTRAEIEGAARSTIKWLQTQNNVKTLEPK
FVDFQDHSPFHLSPPTRDIADGWYSKMKGLQGYRNTWYISALFVVSSTQV
WNNTQNILSDIINAAQS
>Mb|QLI72392.1
MLSTLSVVLFLAAVSWVTDAGRSGQTGFETRCLSFEPEKLVENSKRMRLE
YVTNGTTLEFPDNDPSCNRASQLVAANLCRVALYIETSKRSGITFELWLP
DKWTEARYIATGNGGVDGCVKYEDLAYTTANGFAAMGTNNGHNGTTGVSF
LNNPDVIEDFSYRALHTGTVAGKLLTSQFYNKTPAHSYYIGCSLGGRMGV
KAAEAFPGDYDGIVAGCPAVDFLHLQGARAMFYPITGPAGPSNFIRPELW
TGLIHDEVLNQCDLLDGVKDGIIEVPDKCYFDPETLQCPMPWWPFGNKDK
CLNSQQVLQLRKIYATYKYPNGTLIFPRMNPGNEEQAIKKLFAGAPFSYS
QDWFRYVVLNDSTWDAINYDSSLVAIADAQNPFDIRTFPDSLPAFKARGG
KMISYHGGQDNQITMFNTERFWDHMAKEDRHLHDYYRFFRVSGMFHCNAG
PGAWAFGQGGGAPAAGIPFDPEKNVLAAIVAWVEGGSAPETLTGTKFVND
TVALGVDFHRRHCL
>Mb|QLI72432.1
MERKPSSPLAPTFMVSAPGKVIVFGEHAAVFGKPAIAAAISLRSYLLVTT
LSKSQRIVRLNFRDIGLDHTWKIDTLPWDMFHRPDKKKFYFSLIDSLDPE
LLDAMIPHAEEVSKHLPEKQRKIHVRSATAFLYLFLSLGSLHSPGSIYTL

RSTIPIGAGLRSSASICVCLSTALLLQIRTLAGPHPDQPAEEAQIQIERI

SRWAFVGELCIHGDPSGVDNAVSAGGKAVIYQRKRSGPPSVLPLNKFPEL

PLLLVNTQQPRSTATQVEKVRELKANHRHVTGLILDAIGQLTSSALADLE

TNGMSDTDRLGTMIRMNHGLLVSLGVSHPRLERIRELVDHADVGWTKLTG

AGGGGCAITLFRPDAKLETKRGLERKFHAEGFQTYETILGADGVALLWPA

LFRRGSEGEGDEEINQELFEKAVGVEGIEDLVGVGKSEDGEGWKFWTRTV

EDQCDQSLDHGPSQGNDDEAT

>Mb|QLI72448.1

MSSTASANATGQLPVVPSEPYASRPLSTSTDTIRLLTIERELSNQGYLVC

SIQVAAFSPPQKYEALSYRWGDESVQKTIIVDGISVTVTENLHAALQYLH

AHPRGCPLWIDAVSINQKDVPEKSRQLRIMPHIYSRAASVLVWLGTQYTT

VDVDVESHLQQAIQDVNGDEYWQRVWILQEIGKARKIQVCFGSAGPLDWD

CFVRHVKKHAESKERNIGPLALQAMRRNKYSGSCSLKRLLENHLDALCKD

PRDKIYGLVGLSVDGRDFPIDYSKSVLDVWADTVHFMGRKNLLPERNDEK

AAFCTLLRNLLGGDNMPSLGGVVQFQHKPGSQSLYDHLRAEGRDEWAASS

VELATTCLGRIISVGPAISELRASLDLTDKWEAELQRVYRNEYEDVLDAA

HQEHDVLMQHILDDTDSKLPGLTCFENHRIDFITTDHMHSLYPYSKYDPD

GSDEDDRISPEFEGSWNYASAISDEPRLAIVKESSSETLPYKIAIVPPTA

RLADFICRVQGAPMKRIVLRHDYGSWTTQHVCGTAVMMKDLMGETAALGA

EASEVERIEGIFTQIVRMDARAFYALIFGNDDGIQELKDKLGALAV

>Mb|QLI72477.1

MASTTILHDTALAALADGDVIYLYFQASSGYLCEATLTPGQPQVAYTITN

LRAADIKLYTPISATFGKNTEERYIFYIDTDGILNNAVYKNDTWCRGFLR

GQSIIPAHYSKIAAVKSCVCDDEIHVFYQTTDRNGAIRQVTGYENRWVLD

RRDLGSKVLTGTGLAAVHAELGIDITTGTSDSNKPPVVFFQQTNLGLTWL

QDAATRKVGGVVTKASPHTPLAATDAVSTNLKDVFLFFTSSANEIKRLAV

GSDGQEVVSTELLTTTPKGNLAAVVRKSSTNSTDAQVILFYQSTNPPKIH

QSTISDDDGGNCVCKTRYTVTEYSGVAIFSDPEVDHIAMRFE

>Mb|QLI72505.1

MHKLFPGSGFFDFEAIRILGTTVYGGADVAEVLEAVGEIKPGDPVSWERA

WRTQALRAEELADEAHRHGDRDAARRGYLRAANYTRASGYMYVSTSTGNG

ESLAQDACSVAEKVRTLFRKALPLMDGQVHRLSIPYDEYHLPGYLYLPPV

DRKIKGRKTPILVNCGGADSCQEELYFLNPAAGPGMGYAVLTFDGPGQGL

MLKQYEVTMRPDWETVVAQVIDYLVKFSSQHPELDLDLDSIAVSGASMGG

YFALRAAAEPRVKACISIDPFYDMWDFGTAHVSPIFISAWTKGWIGHGFV

DRMIGWLSAVSFQLKCEISITSTLFGLSSPAGILQHIKKYTFASGSEDGT

SFLSHVTCPVLVSGAGKSLYMDVDNHTRRCFEALTNVPPQNKEIWVPESD

GQGSLQAKMGALALCNQRTYQFLDKAFGIIRDPLL

>Mb|QLI72536.1

MQLALVSAFAGLALAGPLGARQETLKPFKQPGKKTNMALCGSGTFWGDAY

KWNTEGCQEPHYWCLQSREPGSPVNQTKPTRDEVAGAPVTQKRILEIIGH

QNIACKEEEKKKIYCTASRKEEQVAVKDGLSPLCQEKGGCEECRLGTVNG

FDTGFTCAVGLSSKNQKQDLAEGLVFSD

>Mb|QLI72539.1

MANRQWHFLSLCTALVLLLFSLGGEAAPLGKVTDYIFRGDDRDPTAIKKT

GGFKPTENTYNDQLAFSLHAHIDHTQGDTAYVSTSKSFGVAVQFATTGGW

VYRVHRLGNMIDTNAALRDPPAEEQQEFAALGGVPYDAIEGWWQVPNTLD

VPEFSEDQAKELDRDYAAKYQSNFIQNPDFNFDKYKNEQVQGPGEDVDVL

ASMPEEDPNKWDDTYWDGLRNTEFKKSALSFMNKHASSLGWKANQQFPLA

LWEEGSGEPPAKKPKVEETAGPSGQTVPASTAPKYVFYGDYLWPAEAKRQ

GGFLTPADSLATIRNPPVTAYTLNTHLNRDKLKLHPETYFVAVHQTFGAA

AKEAVKKAAKFGGQFDPVVYLVHATPHMFKVGNELAVPGGMVWGQVRGWT

QVPRDYALPEKTPTSKQELHKHFEKAYKAKPKVVFQKNPDYDSKFDQYTA

TEKEQPQLLGSRKASRELTNFMKEHGSAVGFQEKFPLFTAPKVITGEASA

AAKNVEPAPHEEEGVLEQVWDFVKEHAVAIALLPAVAALNLIPGVGEVAD

AFEFAALSTEAAEGTGLVLEGTAALEEGAVIGEGGTVAVEEGATTVEEDV

ATVEKPDEELELPDVPTDPIEEDLNLPDVPTDPIEEDLNLPDVPTDPIEV

PEAVPADKVALKAD

>Mb|QLI72562.1

MKFITVAVAALASVVAASPFAHKCKPATYRCERSLRAWDVCNTSGDWVFA

GRCPPGTICKFNKQNNSPYCLPRRTVEYDYTDEYYYPDEEIEI

>Mb|QLI72593.1

MEKVPNGTGRRGHTQLEHELASRGATLSNNTIPPSEFLVRCAPSAGLIVS
CGCVIVDPAARKVAIVHDPDTGITQLPKGRKNIGEDIHAAALREAHEETG
IPVAPLALRVATRATPTEDMLPLVARTPDGFPEDVTTAVRSCEPVAVCHH
RCPGTLAYKLVFWYVARGDSSLRPVDGTKEAWEEHYQVEWVGARAAAGRM
SVAADGEVIQKAIEDMVASGYDI
>Mb|QLI72667.1
MTRRVLEQRPAQEAPPCREPAAPAPFPRSFYSFSLGTTIRPRPGRRAVSR
HAPADDATKKLIYTIVKADLLIPGADEPVPDAALVAEAKLIVWVGKQDAI
PAEYTQKCRGTYSVPYLMPGLWDCHAHLLAGGDDEASWPAYMGFVAGQPA
AEGARLARLCWEVLQRGYTSVRDLAGHGCEVSRAVDEGAVAGPNIYAAGA
CISQLAGHGDVFALPAGDVLLNLGAAHVRPGHYASAPSCIAAGEDECRQA
VRLQVRRGARCVKVLASGGVMSGGDDPTHAQFSPRELAAVVDEARRLGRS
VAAHVHGKPGILAAVEAGVRTVEHASLADDECLALIKRRGVVYVATREAI
ELLLELGDDLPRVHRDKLRLVAGHHMAAYRAAVAAGVTIALGTDALAALT
MARELEWAVEAGLSNLEAIRAATANGPLTVEGQAPRTGQLKAGYEADFIG
VTANPVADVRVLQNEENIRWVWKGGRLFKGPGVGPWGEPAWA
>Mb|QLI72686.1
MAHSPGAMDVCLDIFGQQPRLNIHTQICLCFAMPNHVAQSAIVSTLTSGL
ERLSASFPWVAGQVVVEGSSAGNTGVFKIKELRDIPYLAVKDLRTDVSAP
TMARLRQANFPMGMLNESVMAPRHTFPTADDMSRNDPGSPVFLVQANFIV
GGLVLTFLGQHQAMDMTGQGQMMRLLSKACHGESFTSDEKFSGNLQRQTV
VPLLDNYHGGPELARHLATPATAPNRNGLDDGGEPARIVWESFSLDPTAL
ASIKSIAVKTLPSGSSYVSTDDALSAFIWQSIMRARLPRLPPDTETTFAR
AVDVRSYVDVPATYTGIVQNMTYTTYTLQELVALPLGAIASRLRAALEPD
TTILPFHTRALATYLHGQADKSHVSPVSSLRVSVDVVVSSWAKTDSYALD
FNLGLGNPEAVRRPWFTPLQSFVYFMPRKGDGEMALAVCLAEEDMERLKA
DEQFTTYAMHGG
>Mb|QLI72726.1
MRVSPEHFFRVLAPAAVLLLLSLALLKSSVPVQWPSTPANLSALEEQRRP
SGQFAGASRRDAGNGTLGFSGIYFINMKKRYDRLDALALQCFLSGVEVKE
VPAVEPDMMSDAGMPPSKSPSSVKVGERGAWRAHANIWSTVLRNNLPPVI

ILESDATWDVNIRSIMLNMNKHFHKFLTNINSTRLHNPRWRDDAGESVRR

TEAADDTWQSDHWDILSLGHCHDSAANRNISLIYDDPYVPPGKEFGDTVL

GRQRVIRKAGGIVCTTGYAVSQTGAAKLLVKTAQNLDAPLDLIVRSMVED

GELVAYSVMPPIMAQWQYVDGIGMDERGANSDIQGAQEDEDKDEGEEKKD

SWEEVDRSGSVWTTKPYHEDVAFQDMALEVAWRRIFGRNNEGE

>Mb|QLI72735.1

MGKLVFAPVDLGRNGLRILDSGTADGRWLVDLRAYLRCKDNVLIGTDSVE

RMFPPTPPGGISLQVQRVDKPWPQSWLGSFDYVHQRLVLPGCEYCPAATA

VKNLCDLVKPGGWVEILEQDHNPPTPGAFERAESMIREIFTVNGFGYDYP

LRIKKWLEDAGMQDVRQEVIDVPVGALNPNPEMAQKSTWQISSALAGFLP

MARALPLSMPRDQLESLPEHTAEEMKRVGGIQRIYIVYGRKP

>Mb|QLI72751.1

MRKALITLAVTLTAVSAAAVAVDKRIIGGEEAKDGDFPFIVSIGSSAEGL

SGHICGGALLDNTTVLTAARCRRDAYYVRAGTKDLSKSGVVRKLDFVVAH

PDYKRGPPRRGHFPSAVNDIAILKLSTPIEQSESNKIKYATLPEDDSDPV

VNSIAVTAGWGEQVSLEGDVYQDDKLQKIEIPVRPLEDCSDVTPDAANRD

TKICAGEDGKNVFRFDSGGPLIDQDGRLIGVALAGSFDIKNPSIYTKVGS

YMPFIKEYLGSVSNPDPTARPITEAREEELQPGTSLREEIKKHCEKFPEN

VLDCISAAGDCKGLRKPDGNMAEVFQCIDKKLDVTRQ

>Mb|QLI72761.1

MEAQLALLDPRALKNGIFAIKKGYQIKLNTFSQTHVSNNSNSKGSLILVT

GANGYIASNIVDLLLELGYRVRGTVREHKPWLTELFEKRHGKGCFETVVT

ANMEEPGAFDDAMQDTAGVIHVVASIVFMNPDPSAVIPNVVRGTESILTS

ASKHAAIKRFVLTSSSTAALIPKPNEKCTVTEGMYFAVAIASQEPVDPNL

QLDTWNDDAVKAAWSESTPAAHKPYVVYAASKTEGERAAWNWMRENKPHF

VMNAVLPNVNYGRVLSPQMTASTMGLARRLLQGDKTALHLLPPQWYVDVQ

DTARLHVIGLLDPAVRDERLFAFAGPYNWTDVIQVFRRLRPQSKLPPAPE

NEGRDLSDVKPAKRAEQLLMDFFGLPGWTSLEDSLADGIDGVGESDAPGP

>Mb|QLI72812.1

MPSLSHPESLPSSGGTSLSSSRPSTPDDGECQHGHDADQVVLVIGGLGFI

GSHTTLELLKAGYSVSIVDDLSNSFKVAFNRIRDLAVKHHASRGTKMPSL

QLHTLDYRSHSMRSVLDLYSESSWKASETDAQAPHSARRSRISGVIHFAA

FKSVSESITHPVQYYQNNVCGLVNLISLLGEHDIRNFVFSSSATVYGCKA

DLGRPLREEDLVHPESYVDDNGAEVTPASFASLQSPYARTKFFCEAILAD

IARADSAWRITCLRYFNPIGCDSSGLLGEDPRGTPTNLFPVITQVLTGAR

EHLDVFGSNWDTRDGTPVRDYVHVSDVARGHVAALAAQAVEPFRTFNLGS

GTGTTVAEAVRSLERASQRPIAVRLADRREGDVGSCVASNERACRELGWE

AKESVGQCAEDLWNFVSKAQAPTS

>Mb|QLI72824.1

MVARPFRPTSVAAIGLVLFLATCFLYASIPWHASLSRHGALSAAQDPGLE

FPNDPVPRRDFAPLSTYAAQNIHEPSRFAFATFYCSRNADTRGPYFESTQ

SIIWRLLWSDYRSKYPVIVFVCPFIPETHRQIFRGQGAIVKEIELLDTII

PDEAILTKRWIDVLSKLNVWKQVEWKRIVFLDSDAFPIRNMDDIFDLVPE

QQCNKAALHPEDQAVVGNGKGGEDMCNYVYAGVAQFQLDNINAGMLVLKP

NLDMHAKLIRAARSTADYDVRDMEQGVLKSKNAFAADGPFPVQRLPPIWN

ALPEYYTKYLGDEAERTEGPIRVLHVKMWNRLWGSWNNLTHLNDMWDLDW

MKMCRFFDSDDFVKARTSGVYETPWERLAKSQNQGIAP

>Mb|QLI72832.1

MTQYDSIGSSYLVVGELLFKQMELAMVRRAIRPLLKPNAKIVEFACGTGF

YTQEILSWTTLTITAMDISPVMLGIASKSLSTSDSDRVRFVLGDGTISTS

YAPDKSIGFFDCAFGGWFLNYTEKRSGLVAMFATIAHNLSHDGVFVGVVP

HPAEDLVARKKAYSRPPLDRMWPRLEYIKEIDEGSGWRSRIHLNEDVSLL

AAHMKKSVYEAAAREGGFRGKLRWIPEGIQELGFKDK

>Mb|QLI72851.1

MSSTATTVNPTTIGLELEFLVVNKPGTPTNEAASSEDDRWPPVPNPKAHS

DYYHVEPLLEICKLLEDQGRQVACILDNTKGQNPLITTQDSSILLRESKT

DGWFHLHRLSVAKDKDIGRLREKTDYRVWNTKFDESPKKLPLRFAISNFE

LERTIESTLDYPVLEMISPIISEPTQEKDILDLVETLRKKFKISINPNCG

LHIHVGYHSNLPQDSEDSEPSEASGVSDDLEDSEDSEDSEDFKAPKTSKA

PKAPKAPKAPKAPKPSKALIRAKKVAAIVFLLEKTLIRELCHPDRRSALQ

LKLIGDESALAEAAKKTPGRVVRHPKFNNLVENISQFRNQSKNRCPEEAS

VFRFLEVLFSYPMMENKEGRHFKKNLQTEKGERTSLAFRRDIKTIEFRHF

ECTFDTNMIKFWIEFVRQILSICSKSELEFEADFKDLYEMATRQEQKPSW
EDWLEKLGLSQYRETCASYIERCKESPLGESIILPESFDL
>Mb|QLI72884.1
MFMIFLNTLVPNTRGFSVEVCSGFNSIHQPQSQYTYSPEMWDYSTLQFFR
YNPPDGVSYETASAEEIPCRNTAAQRRKESLLAAIALHWSDSRVTVLCPF
CSKTHVHGISHFRYHGATGWRIQDDLGRYIYDGPSPTRCDSRVAQCEKKD
SDVDIEYVIIFPFENDCRVAGLSFEIERIPDDDDTKLEERFRTVGLKTIP
ADVLEELESCYPDESSEPSELADRLQDVALEDGDYDIISERDGVEEVMTE
RASVYLASAACCGSVNEMRPYLEGSPNPTALLQFKDKDGLSLLALAVPNG
HRKAVEYLLELGSNVNVTDAKGRTPLMEAALWSHPKIVELLLEAGADGSL
KDRRGMTAGNLAEESERNDRERHKRHSKYSEDPFIKKRHRRLIRALLKHT
PAMSTSTGIQPDLADLTDAFFYKSLSAATISLVIPRQGIEILTQSKTAAV
LVRGGAFPPVVAVSGRTNPVHSEFRSPEAGYLRLNEGYWGGVENFAIAEA
IGFSFESDRRDKAGVEGSFNASHAEAQLMCFFVTRNYIFRNFEAGQVQYG
DDFLQLFMLQERNRQAKIIVSQEPCPSCRALRDRILDRLGIDFSLSECSG
>Mb|QLI72902.1
MHYTSVFSVLAALGSVVNAAPTLDAAFSSVANGAPALVNREEQSTYTLQD
KKAPFQDAASLPALNGADTVDLDLSGYTNMGYPETSDQFKCHGSAGIVFR
IWKTEQRCHHPYMVPFLRFAFVDCKDVQHFTPYPPVKGYKAWCKYFKWMD
EIDF
>Mb|QLI72953.1
MTSFFQPGSNDPAIKECGNSTFAVYCNSAIPTHTLLREWYQQATLAVGGF
SLIFPILLFIVLLARQSWFSNTFKRLVERLRRKKSGYQSIPPAPNLVTFE
STRSFKVYSMMETRESVDVGTSTDRNYDLAIFPLTIGDLGGDEGWDGSSM
TGTACHEVTNLAYVNLQDMDPASLQPLEITSTTEYLVRSCVRNHLAGLAL
RPSSTIPTGQVEQLIQRLHHCDVSVLVLCHHDSPDMDSLSLQYASGVILE
NACILPNGQRRDYFKAKRLREVMVRCATERSQKLGFFIGFSELWETRPHP
SVVRRAVKLAEHFGAVVEHGFADPSMKLERPPRDATYTLSAFEYLRRGVV
TEVQKSWSSELRTVQVSTAPEQLNQEGGSGKGTVAEGLRCLSLSKVDAAL
PFASLLLQQEPLPSWLTVLQEQEVVMVQPPNYLQQAPKRTDFWKYSADGT
ELSTAGCFSILAEPSAQDYSAVLQTQCHLRELKLLSAVKGAEIHRWVKSL

QELTEPKECRGLINDLVTGLKSQSVLVFRGLDTGFKIPDGNSYFFGVSRT

RDERQYLALDIYISQSAPSDASAVLHTWLAHHGVSRATCFELEARLEGLA

GPLPPGGKLPLSIRSAVESGTHAEILQLLQQMRVAHLQDAWRSPIESLCE

ARLTTQTSKSSWLRAHARRILDGSITIQDMLLSRLESYVVAGATELPSLE

NLVALYEAMSASIRDALFYGDKEKLNALTHVLIDVFEFGTVGELAEKIDV

NADMFALMFFTAMRREAFEEVYIESTDRCPIFLCADQAAVFSELWVLGSQ

CEIFLGLLPRDLGDIIYDRYRGFLEACPPVAADRTGNEIMTMYSALEASS

PPGDAKANGENAQSAGPRLSTHETLQLWKRQLAEVGAITIFSLPAIIDVL

LLTFLGRGLFMTAFMDPAHIEAAAYALLISLLVTAGVTGWVGSTGSYYLN

HYAYDNMTHFHVQRLSGGFMLTLLIAVGGFVGFTVARSVTSGLVFVAFVV

AVTTYLNLLGIMATMHQHGSPMTSGRTVLWRTIPVLLISPVVTTFVNGHD

LAIYLPITYMTILLLLYQYRQLCHEWNGWMKNIPLVAEKDILTWYESKLG

SKKGSGAAEQSDGRSKMAQEALRDAVTCYVRRGRDDKAAELKNDAFVKRV

AAGMPYIDWLFKKTNPDGNLPETFSSAWYTQLGESKKQQEQLARGLKEHN

VVMLFRDAKFDLGQNLGLFLVALMDRWVGIIMSNGGHPGRSIYTDSRARY

AICFCVLYFLIGVVVLDLTLQKYWTLRFTLSKDKLVDYRHAQDVADQWER

VRHQKLGIALVELLARLLILFGITTLLMWILVDNPESMLLYYLYIVGYTG

VILFQFNRCFTTNVHGHVFIILGSAIVGLIVGCVLHAAPKTSGWLYSDVV

AQNIAALLAAFGTLLWTWKDWFAPSQATPALQFSKNKDAFYAQRKLEADP

GIETGISIRRFVKTPRPSFKHGDGSFLSEKIDDILVRSLMTPNATSKACS

WASEFIQTAFQAWEDGLVEITVVDRESFIDAGLQHLFSFSQRDGDVLKIT

AGFFGDYELRLPAWQTQLAQMIAESLMYHTARVEGELTHSQAVHAEHFLH

GTTPMSKRIQFELALADVKTLRQTMDKTNSELLRRLSLDLNVDLGWNTLP

PSVREAIVCRITGRQVKSSYEYEDWQFNQDVDVETIDFHLQLGLEIFRAC

KQRSNMATDLSPPMDFKDSLPPAELRPVKIPSGMSNGFVRGFFRRVTALP

VTFARWVALISGGGSNIERELYYSLGWLYFRRPIIWIILRLWKFCWAARN

FWIYYFLVYHRPSAARIVRLTQKGALRIIKKNSIVVELSRKTVTGFARDG

QDGNLVLEVFEGRLREAPEDKPPLFTATYDDKLRLASRTDRDGTTSTYNY

DSAKRRSWPISRAVFSSNSRALGFYDKQGRLESGTMMLNSESLVFSYHYK

TTPENSSDILRADFQPLDRSSNNKLSVFWGKPSSQGDEDYNWAPSEKIHR

IVRVIGDKTYMTEIEYHHRRDPKTLTYLVEDGVYKTIVAVVPKAFPDEDV

LLCRPRNLLFDVDDLLIHHGVGQINIMRQFASTKLSMMARIGSLAWWTRR

VHLPVATWRSRTELWSVWLKGDLDAVTACWMDEMILREEPILRTYWRARD

GGNLDGARAALDDIDQIVAAIELETDVSEVCLLPIRSADLYTMGLGKDAN

QVTNRPQDCYSDTKDRVSVIVNDIGCWPEAPGGVSNCRRDLVNGHGTIRN

HVMAECANEYGIPRFQIEKNVQSLKLLPLWGLDGKTANHGLIDNILQSEV

DDNIRDTDVQRDITRVFVPLLKAFVKGARTRDYTRATLATCTNVMLSMSK

YFESKDYNTTWSSKQVEEAWVEAWLVSYDDPNIQDPAECFDIARPTLFDF

REALGIYIAYFFIFSVRIPDECPRVFQSTHHGISSLLGIILKYRRGVTFG

IWDHAILWRESCLNISPAQCELPLSVQSMLLHGIRLASRLSYFHADVITP

CTSLFNPMWETEIGSDRGNICNRNLFSRKIDPIVNGISNMDSFEPIDKVR

TSKPTAVMLSNVQFIKGIKTAILAADIIVNRYGFKDYRLMVYGAKDRQPS

YALEMAKLIVKNNLSENVILAGFGKPKEVLKDAWIFMNSSISEGLPLAIG

EAALAGVPIVATEVGATALVMTDPNDAEQRYGEVVAPNDPVALARAQLNI

LCMVGPWTKFVDGEDQPQSLPEEIAPDDVERLYERMYAKTEDRRKLGLLS

REVVLQSFHGERYLREHEQMYWIQWHTARMRADMAKGNKRRKYTEFSSPL

PMRYVDEGVVGVENSAVLEDAELLEKSVKREDHGVTTEKMAPTPSDLESG

RVSQSSTLQVVHTGELYFAGQTAATGFTNWAPG

>Mb|QLI72959.1

MKFALVLASTAALVGALPQSTPAGSAERNLLPWLPAGPFSHECGPFGWYS

DAECGTHKYCDSFRDPEIPNDKKYQTTQECIDAHELPSGSGTGKLPWARQ

GSDRACTSYIERKVYFPRIVMEENCGSQRFCDLFGPGSPLAAELPELKKR

YGFSSVEECRAAH

>Mb|QLI72997.1

MARPFLIVPLYMYPLPTAWEPLLAAARTHPEVRFVAIVNPNSGPGPDALP

DASYLAALDAMHAIPNICAVGYVYCSYGKRPAADVRGDIDRYARWNQHHG

ITLGGIFFDEMPSDLAHLAYMAGLAEHVKTTWRQETTGRGDGLVIYNPGV

VVPRPFFAHPDYVVVFEQSHRHWDQDAAGQQRLRLAADEVRCKTAAVVHS

SRVQGGQVRDLTRGLLACGLAGIYVTDEQDGGYTQWPAVWTELTAAVAAE

CSES

>Mb|QLI72998.1

MKASSVLLAVFSGLAIASPALSARQDLAKANEALNAFDAKNAQVKAYFEQ

KKQNNPEGKGEKWKETAGQQVDLINTLKDQLKDSPQDVKDNINKLSEISQ
NSLNRANEKHPEGAPGDQKSKPEQINEEYTKIASDLRAALN
>Mb|QLI73007.1
MTIAPSTAPFAAEHQLSRDLDDGSSRRPVLPSHPDVSLHDRGQLRDHLTS
EFCSDDLDRVADKLWWMSKQDSRNISPLHRQLVKRRRIVITEDPKLHLVW
INDRIFIKPLPRYLTSHAFWTDYLADKDVQRIRRAALGYLRTYSHLVRHE
SDFRIAQDAALCLVSRDVTWEQFCAFASRLRRIPDRDVSPRYAYGEIRLT
RLNFYAPFLLGKSHFQRVDYQYGDYFARFFTPVVFVFAIVSVILSALQVV
VSVGDNASSVSARMALLGSVAAILVASCLLVTLGCLLVYKIAKEWRYALR
DRYRRVKGGGEQV
>Mb|QLI73024.1
MDSNALGQENLLNGFEFDVFEASLDQGQTRSDEPDADAQCSVITDRGRHS
KYKVQALLKTCIHGVLDATSKTPASLIIVDYFLSNLEEQGRYSSVTTTFD
FEPHVEDELGLGDGHGNPLDTPNITAYAPFEAVRWGQTTAQESSKHNLDA
SIEPEVEGAKAGKIGYAYESTTSHEQRYFTQGLAGRHYVPKGPARGVADQ
VWWNMQHNFSQHSGVPPHFRTAILLTRRSEAAEKKFIAHFKIAVRGGFWM
NMSALVDVFLRRSVQDRPVVFDPSIRFMGDELDGIEPNADGVYELGRLAV
GRELVKLTGIWGLSPLPKA
>Mb|QLI73026.1
MSHNAPTSGHEKLVQELLDMIKAKKWENDFASGIERAIAAAPVDLEKAHI
KKGATNDETINYFLDFCDRYLRWVPYTTSARDEPLWVLSLFYFVFHQKGI
IEKQTPIEPKYIGQHTELSLWLQKYARTLGTWMDTAASADIVSSFSENPE
YTVYQYEKPTGGWNSYNAFFTRKVNPVYRPVAPNNTVASPCDAKFDGFWP
ITSDGVVELKGLKWPIQQLLKDIPADLVNKFHNGIFMHSFLLPNNYHRVH
APVSGTVKHREKIPGDVYLEVTADSETGKLLLPPRRMEPMPYKDRDGKRQ
IDAQNHPGYQWNQVRGLWLIDTTGSKDIDIGHVALFAVGMAQISSVQWDD
TGKPNTQVKKGDELGMVSYGGSDYILMFEPGKVHFSSERGNESPKKDVLY
LQGAALVNKI
>Mb|QLI73027.1
MKVAIVAVGDLARYFVEELQKSGHEVLAVSRTPKTYLEDLEISQHITDYS
ETDLTAALADCDAVVCTLRVGVPDYVAVHLAILRACQHSATCKRLIPATW

AGNLEDFPDEPLGVADEIRLVLSALRSQQDVSWSSISPGWYVDYIVPEKQ

RFMSSLGDMWPQNYDRKTFTIYGDGTQLVNFTSARDTARATVRLIEHNRE

DWEEFTFISGAQMTWMELGRFIQSRDPEYAFQNKSLSQTIKQYIAQESLE

SYGAAILELWGHGAGVRFPWSKVERHRAKFFPDLKFRSVQELADEAASEP

NKVV

>Mb|QLI73046.1

MRSGIVDALTRAACALCLLGNILPAAAITVGSTVLILARDDAEAKGAAMG

LDGYGIPYQKVIFPAGGTNLPVLNSSATQGNYGGIVVISNVAYDNNGTFA

SALTPQQWAQINSYQSTFKVRMVRINEFPSAEFGTIVANTAEPGCCGSNT

EQKISLTDISQFPGANLKANAGVSTQGLWHYPATITNTTTTKEVAGFEAA

AGFASKTTAAVVHSANGREQMVWFISWDPTWSQTSSFLQHAYIHWMTRSL

FVGKRQSYLSTQVDDVLLDTELYYPPNTTFRLRPGDLDAHVAWQKNLATR

LPAGSNFWLEMGHNGNGNIIGSTATASGETTCKPAYAVDYTSPPDTPLEF

MKPPGTGVDLWPAEFVSYTWSATCSKLEPLGAWFTNTNNLNAFAHVSHTF

SHEELNNATYHDATREIFFNQAFLKSVGIDRAARFSPKGIIPPAITGLHN

ADAIKAWTDNGIIYVVGDNTRPVLRNQNSVYWPLRSTVQTNGREGITIIP

RFATTIYYNCDLPACTVKEWIDTSGGSGDFNSLLDNARAVNTRNLLSLQA

DPYMFHQANMRQTDVAPLTIGSQTGKQSLIMAWVETIAQEMTRLTNWPLL

SLKHDDLATYFLNRMALDACQPKSSYTYSADGKTITKVTVTANGNTCSAP

VPVTFPGGIATTTLLGPLKSTKVGSEPPILWVTLSGRPVDILLWTPVKL

>Mb|QLI73154.1

MKLSIRLVTLLAGVAAGSPAKADKRQAKEGADNLKAYCAEGGYEAGTVTE

KQDGYERFTGTCVKKDGSNAEKFSVQLGVQFCEKLSRRFRGSGGPHNQGD

YVVICGPKEDIKKPDESNGRVLLNQIIADSQGDPEKIKIFLEGLSRDAPG

TLTSLHNALKAPASSTPGGLGGGTGAGIGLLTAIYDFITVNAAEGGLIGP

ETKAGQWLRTNPIWGRSGTQPSPFGFDACCATVELPPTQLCVPFKDESHW

YWREDVKRCKSWHDRQKCDPAYAYDEATPAGKELARACREAGTKDAASCV

RPRLACESFDDKAGKFKFEYCMDDNNSSREVCKANGWVVGSGRDKTPEEI

EAYKEEQKKQEEANCVSPRWVCSNIKGQFVTCIDGSEQSQTECKSNGWFP

EPAPKRSDKQA

>Mb|QLI73189.1

MVRKIVITLAVLFSAVLSATATLDKRIIVGGEAAKDGEFPFLVSITNTLK
RTCGGSLLDSTTVLTAAHCVVETQEWLNRTTGGQVIAAKRIVYPGFLRHR
RKGYPKHDIAILKLSTPIQESDTIGYANLPAAGSDPVANSLATIAGWGQV
HNDGHPLDQLHKALVHIRAREKCTNESWNATGIEDEVCAGGDGKDTCGGD
SGSPLIDQGTATIIGITSRSSWYSDCGQYPGLYTRVSSYLDFIQENLGVS
QTTSSVKS
>Mb|QLI73220.1
MPEIDPDIIGKTVFDISRAYKGTQDNRSARSSKIRNKEYSLKSAGQCVLQ
RKRAGVCDPAGWSEEPKKTPPKQTGPGEDDGKERGKGKGAITGQKITESK
FLQFLAELDPEFIRPLVKAVQSGEVTVSDIQLAFKRALLETMEKRWAYFR
SGDKAAKSLKNIIVNIFATARYATPPEFWQNVVNVWPYTPVGVLNKNIIK
EAVNNAPPIQAVAVSLNNLLSYTPIGWLVNQIALVDSGLRQINVRDVMLT
LGQ
>Mb|QLI73242.1
MEFPDPAPSPPAAGASSDSEDTTVSDWDSEPESPRPASIVHEEETNDSPD
PIFISIASLSGELASLHYQQGTPTRREIEFSWAANAIVGEAATIKGIWTL
ANNLLQVALGAINSRRNIASPKPALIFAACDIGGTILKQALLIAASDTSK
FSSVLENTNLLIFFGSPHRESDCCSWEAMLSHLILTQHKGCPGSWIPSIV
SQLSSFHKDLTDKFIAFIEQYKISIISHYQVSSKSDENEMLAKKLNATLD
VHGEIQIGLPRGDNTLFNVYAHEHQTLIASRVENAKASSRKDYQEWLRLL
TRFGLDIPVRQGSKPAYKNLCDWILSDAGLASWADKATGASKETGAAVIK
SHEQQSLQDPDSEVSNDPKTGEVPKRSQHAVARTMTTLEIGGCIRDATIL
TSSLISSLRERQSWPMDMFLTLAHHKPRPFPLTKAQLMACVCRQLLTGHP
SLCVEIQPLLADARNALDSHNIAWIETTLWTCLAALLGGQVLGQPFLIVH
QPAEPDFASPFLQLIADLAGLVQTTEISVKILVVRNVASCPDTTPDASTC
INPNTARDGASSSSSTVCHVQKHANRVIIRKIIDPGDADVLKALEMDYDA
CFERLGPERAQVLSRKDGMEFLAASTDLGAMRYLFAVLEQHSFLSLQSVR
KSLFSAISQDVVTAILNLVPKSVESVVRTGILWITHALRPLTCAEFATAL
GMGADDVVGDGFSTITVSEFERLLCGLVEVEDGTVYLASSLLTQHLLQQT
SGHTKDSSHFLDLKPACSPDIALSCRRYILHHYRSGQPEQASEESEGGAS
EAQGFSDNAPGDVAAGQLDSGPLMSYAVEHWFTHLRLKDYSSGDTKLEDE

FVCDGGLVHCCLRLRSSGRAFQPPESNQQAVPSIVQIRDRLHVECAEAIS

LALHASDIECAPDQGKCPSTVALAACQMGIASTLHQLDQCGDFGDECTLK

RAFQIGSDSSLCILADRYSEFVKKNAHEILQDAVRLGNSGLIMHLMNKPD

LWADSDSGQYHQPLLHVIAALGGSLPPQALWERQEQYISHASEQYQEKTP

LHLASLSGQESLVSKILSKLSESEAAKASLNMRDLMGATPLFLASQRGRF

TVVRQLLTAGADHSVCDNQEQSPLHVACRNGHWNIAETLLVWGASPNAKD

SAGRTPLHLALENNHLGIAVMLLGDTTSAPTHERDFDVNSHASDGSTALI

IATKGNHLNMVRILVGRGASVIWRDGNGRDALQYAAQYGHDQVLHELLEA

AKVVGSDDVYQANPSLLSLSALAGHVQVIKMLLDRGFRDTDALVLACEYG

QTAAVKVLAPYSSSASRNAGYLEAASFHRQDTVKTILAAGADIDTKSSLN

LTALHHVAFSSNPRLVQLLVSRGAKLDVTDITKSTPLHYAAGQCSVECLK

ILVEAGAGLNVENEKGATPLYLAATAGSEDGVGILLAAKSSFTVPRPVAG

EYSTFLDLALATFKLDVFRLIVKHATDISGPGLCMSPEALFLFLHGQNEK

G

>Mb|QLI73247.1

MVPKAAVTLVVAFSAVLAAAAATTNKRIIGGEVAKEGDFPFIVRLQYGDD

TSILCGGTLLDSITVLTAAHCNLKDITSVRAGSLDKDTGGVVAQVGSRLR

HPDYVLNGHHNDIAILKLSTPIQASQTIGYAKLPASGLNPVIGSTAVAAG

WGSTVELLGTVSDKLRRVTLPVDAPDECFDLEIDAGKGFQNHLDTRVCAG

ERGKDTGRGDSGGPLIDQETRQLIGVTSSVPLGAQYGRGFYTRVSSFIPW

INQNLGDSGIDPRRVTGYM

>Mb|QLI73297.1

MTWQDLAANKKAHVSDAIPPEWRLKTTPSTDSVMSVPKESGILTDQELAI

TEASGADLVKDLASGKLSSVAVTTAFCKRAALAHQLVNCLHDFLPAFALA

RARYCDEYLDKHKKPIGPLHGLPISLKDQARIKGFETTMGYAGWIGKIDQ

RNSVLVDLLDKAGAVFYVKTSVPQSLMVCETINNVFGRTLNPRNKNWSSG

GSSGGEGAIIGFRGSVIGVGTDIGGSIRTPAAFNFLYGLRPSHGRLPYAR

MANSMEGQETIHSVCGPLAHSVGDLRLFTTAVLEQQPWNFDSKVVPMPWR

QGEADAVKAKVSAGGLTLGYYSCDGNVLPHPPVLRAVQTVVDKLKGAGHA

VLPWEPYKHPYAVDLANRVYASDGGADIHSALKLSGEPAIPNINDLVNPS

LEKLDINEVWQCQLDKWNYQMEYLAAIRNFEAKTGRELDAIIAPVTATAA

ILHNQFKYYGYATVINVLDFTSVVVPVTFADKNIDVKAAGFTPLTDLDAT
VQEGYDPEAYHGAPAAVQIIGRRFTEERVMAIAEEIGRLLHS
>Mb|QLI73367.1
MILGVFLAILALSVGLNGHVIQQHASDSTEVNLDSRAPGMKFVHPGVFVD
RTQLRRMRSMVKDGQQPWSNAYTAMMKHPYARVTDPKPRENVECGAYSKP
DNGCTDERRDAMAAYTNALAWFTKKDQAKADVAISIMNAWANTIQEHTGA
NAPLQAAWAASLWARAGEIIRYSGAGWEEEDIATFSTMLKKVYLPIVQNG
SDKPNNWELILMEASISIAVFIHNRAVYKSSLERFINSASYYVVYLKSDGS
VPLLPPGMSRETLLKRWWWGQGTFQEDGMVMETCRDLMHSSYGIASISHV
IETVRIQGRDLYSEDTGNRLRYALEFLAKYDNKRGTEQAPDWLCNGNVTK
NIKDMTEPGYSILSEKYEMPNTKQFTAAARPAHADSLFIAWETLTHATGE
VDL
>Mb|QLI73382.1
MAPTAFTSANKAVARLKSVSRAMQLRMCLKPAEEKRVREALTLVTSQITE
NLDPKQKKYRDFLQHILDIIGLYGVVISAAGLGISVVLRIKDLVRLEMPS
KLKESENDIRCTVLENITNKFSSEYLPRIKNKLTDRTPDNPQGTQSLEMQ
IIDHDTATGHNYPYAGDAYQLTWEEAETLVGSKRTVGQVYPVDTFMREKS
PFMLVPVSEEMTRLLAVQRPRG
>Mb|QLI73386.1
MTQYDSLSSTYDVFDTLPYRQMEWENVYRAVSPLLRPGMKVLELACGTGC
YTSRFFDWGVTDMTAVDISRPMLAEAAIRAHHHIEAGNLRLVAADGTETQ
SFAPDHSSCYFYLAFGAWFLNYAENKAQLRAMFRNMAQNLRPGAPFIAIV
PHPTDDVGARGEMYNKPPFNRMFPRNKYTEPLESGDGYGLRVLLDNNGVD
FMTWHMKREVYEEAARLGGFQGRLEWRREFLLDDVWKRRFDLTSDEWQIR
EANPHFGILIAWK
>Mb|QLI73481.1
MKVAITYIALIGLTCAQGPPYKETPPGKQLLDQAIAGIPNWGGPGSLQGK
PNKSPKPPIKNMIGGLPELFKPKPGSSDWREDTLKGPRKKPGVVRPKNPS
PNCRKRDLCGPGLGKLPTGNKVPTGGGIPAVGSTLTNIFGGIAGGIAVGI
GLQRLFSAIPGASDKIQEVIDAITRWQRETFGPKNIAYPLDPGKYSNCIA
ARRHDIHANPEYRKELIIINCPWGTELEDTPKTPPEPETQPEPSRQCFDS

TGPIPCGGAQTEVQLDTGRAVCGSCGTFWDPEGGKCRDIKGALIWPSLTP

ITERTPTPSGQCSNSTSSFPCGGGQTEAELDMGWAVCRVCSSVAWDPEGG

KCVQKDGKLLWPRKS

>Mb|QLI73566.1

MFAAAGNFVDEKLLTGELGFTKAELKAEASERAMALYDLEYEKETMTIIQ

STYLMSYHLGGINADRGPWYWIGIAINLAYGAGLHRLSPCELQTCPTCHS

RQWAQLWWSIYCREAWLSLAYNRPMRIQLDEVSTTVPTQVEVASICIDIF

DERYHKYLPREIDDLVSMWLYLVRISLTLGDILSKLYASESSRPTQQPIN

YTEDDVGAHLCELSDESLGSELIKVHLYQLRLYRETTVIALYHPHVQGLQ

DYQSEEHQS

>Mb|QLI73570.1

MANQASMTNLDLRYWMRHSVPPLLALLRAAGSYSEADQGKHIQFLCDHVL

PNFGPRPTDDSSSKSWFTQSGFPMDLSLNLNAGQPKVRYAWEFIGPNGPE

DGDMYAIGALRKCLASLSTELGFSTQWADALLDALAPTSEEATTTQQNIQ

QWQASLLPPGVEPKAGARLPFAAVAFGLDGPRTDTKLYISPQIKEMGSVK

SMNETIWNVARHLEPPISQNAITAVSEFLLERPGPPCMDLLCVDLVKEQD

LHRARFKIYIHTTSNSFNTVQQCITLGGRRQDEVTLKSLEVLRSIWHLLL

QEKEEVTNDYEKPVNDPAMLVMKLFFCIEITPGQDLPDVKLQVPIFNYLK

SDSETIENLEKIFQKCGQEWAVNGKYKEAFELAFGDPSTDREVPIHGHTS

FSCTASGVYQTLYFSVPRCLE

>Mb|QLI73574.1

MSSPTDGTELPPGSFDDRSPPLVALCIAMIVVISVMMALRFASRLIAESS

PGHFKSLWWDDWTALVSTIFMLVQLVMSLVMFDLGSGKHIWVVPPENVVK

ILRMLFAVYFIYDIHLAMAKASALFFLARIFPPNVSPPWFNNAVIITHAA

NAAWFIGIVLGTIFQCKSLRNWHPMLPGKCTDASVLFMGSAIPSVVIDLA

ILLIPMPKIWGLKMTPWRKMAVIGIFTLGYCSIVVSLGRLVTVIKMGDDI

TRDITYEAAPTFYWFTIETPAILISICIPAILSLSRFLDALYFKPLASKI

SLAWSSQSRGTLMTEQYDNAVSSRMASSKSKYVNDSELNASATNESQSRI

CLPSQQHGHYSVEVTGWEMQGQSPHRQSTNDAIRVQQDVEVRASPW

>Mb|QLI73631.1

MKSSVGLVAIMAGLAMANPLAASTKTARGYITSDEFKYLRVALVSCLEAN

PPQDAAVSTWATSIASKYTTMGEIVQAIVECEAKETKAKQA

>Mb|QLI73634.1

MEPSQLPARAVELIPRLCTIFAPHLALSGEEPPYRNSGAGLVLVSPEEIS

LHIADPDMIGEIFARWRDFVRPVQKYRMLAIYGPSIFTVGLDDWPRHRKA

VVGPFNDDLMSLVWRETLCHTRSMLHAWTTQFRGDIPSFENDLRALTFNV

LGATAFHERPQSQADTRPKDSEKIAEDYQDTLRTVLDNSILLMLIPYHRL

RGTITPRHLARVGLAAESFRSILLKVLREETAALGSDGSKQNGLLKPLVR

ALKPDTREQAKNSAAPVSAKAKKTGLSVDEILGNNFVINFAGHDTVRLTL

NFTLALLTVHPDVQEWIYQEIKMFSGGHPNDEWHYDMFPKLKRCQAVLLE

TLRIFPPNTGIPKTASHTAPSLRLGDQLLVTPPGDRGLPVADRCPDGRQV

LGRPIYLATISLDFA

>Mb|QLI73662.1

MDIQRFAAPRTTRHSPNIPEYRFGHVSNAIFENDIEELHRVLSTIRKMPP

ASVYQEIVPIWETKRNFVHAVLKWGDRAHKFQVLQAAASHIYRHDENENL

VHSSVAEEISICILTQSPDVIYHRHHDGQPTVLHIAAKSRSEALAQTIFS

SMQNNETFDQLLHVRNNQGSKTPLRIAVENDSLFFVRQILRYNTRPINDS

KLLKWVLNEGSSEALLTLIELRPEEMDESVLQHALKIKSERLINALKNAR

ECHDLFFRKRGFLHQLVKDGETLIVNTLLDKFPELALELDEEEKPVLSYN

SDESIRDRVAGIILRTLPSMCSEKRQRYYRLCADNSSIPSTSEIVRALID

DVPDKRKEISLSLGGFGSWVSHAESFLGLIQKSNSYTSPDDKPSNALLQL

MFEKSLIFVDIPITDLPAPRTDGKPTLIRSEVWSILNWLQKTKHVEGIYE

LSIRDSCYLPHAENVIRACLKDFNISVLDWRCPDLSLEVLHGGDESKTPI

CPDLETLRLYARGWPALAYWTSEESLYFLGRFTRLKQVEIFVLKEFVGMT

LCETYATEATERFNKFKTAHSNAITFQLEITPKRWSDLSPTDQRPVLRRE

TTAVEVTKLGDFLLAYESIHRDFANQAWRNDRLEKGILCNDLDESRKHTP

YIRVAIIDNGVDPESIHCHKITGASFVPSHTGESNWWYIRHPHGTKMARI

VTDLNPHCHLLVAKVGDSRSDFNAARIIKALGWAVAAGADIISLSLTLDK

EDVNLELAVNKAAASGAVILASVRGEGVNTEIKPIPAAYGNVLAIGSADG

TGAASSGTLEGQARHLFPGERIVAHTEYLGGLDDAPNVSGPSVATAVAAG

VASLVLSCNRFALFKKQNFKRPNYHHRLQVNVVKQIFEQMSDGKYVRPWV

FFKDEKAKPSWGEGDSVLDWIEGKYRGIRDDGRA

>Mb|QLI73735.1

MEQNPSNAKHNVVVVFCTAAAHVLGRLLIQLLQTLENLPWPVISIRRIPE

PKSTNYSNVFPPSQRKLVACIGADVGAGDASSSNPSKHPELVLGLDEDYR

LASPSRIVFSGFSVGEIRSLGSFPDYAALSEVPLPRAANDFDIATARPRP

YRPLRWPYNQTMAIQKMEPDYWIELESTYEEQIRLRKSIVEKHKTDVMQA

LPGSELACRELMEMVTQFLCARYPRQFMLVNGVLENKIRGTRHHVDQTDG

LHFLLENVPEDFAIMLRDPETGRYKLRAGIICASTGWNLGVKMGKGLAEI

HGPVPDYRSKMQLSMDRFFTKFPAGKPVQRGAWGFEVGKHLYTPPSHPRL

EGTKRQDAGLRPDDIYFRVDWQTLRRLPLSGAIVFNFKAFFTPVKDLMDE

PYVPSLCLKVLTESKANLREYKQVHHTEHVLTPLFSHYEQSQIERGLIEA

GWEPQTLEESPFFPGWMDHFSRSLSRI

>Mb|QLI73754.1

MDHTQPFESAISGGILPCVALLARRLDNSDVLSTYLGPKSIAAADPDPID

ANTIFTLASITKLATTVAALQLVERGLIQLDDDVAELLPVLCRQKIISGF

DDQGQPVLQDRKSPITLRHLLTHSSGAGYTFLDAGEKLRQYHRFHGTPPN

HGPTVESRFGVPLLYQPGEGWAYGAGIDWAGRLVEQLSGQSLEAYFTEHI

WKPLGASGTFTFFPGAQGRAGMASRGPGGPDKLAPVPGGLGLNAGVEACF

GGQGGHGRADDVLKLLHSLLANDGRLLRPQTADAMFRGQLSPASKAALKQ

SLEGSAWAVGDYYPGEEYDWGLGGLLIEKAGDGAPYARGANTLTWSGAPN

LFWFIDRANGLCGLFVTQLLPPGDAGATDAIREFQKVAYQKNKTQG

>Mb|QLI73777.1

MKFSLTLVTALLGLAAAAPQNDPSTEGLSKELADEMQKVLKQLEEDNKKS

IPGTIQAMAEQLGWQAAGNLPGKVGQGATLLSFLATSDYSITPENGKEWV

KNLGGVALSFLPKVGTLGNIPGFLGMLKTIAIGLERAQFPELAAKRDAEA

KCFADNKVRTYKDLCAYCKPHLTISLLGRLNGCTDELVANRDDYVDHKRA

QSFCADAVLCSGYNDGGPSKIIKNGFRYDPLRSFWCKDKDVQIGMLLSSW

IGEPLRDSLYMLDAEDFRNALTPACKEITKDLNVEGTCPKREELEAADKL

APPVSYCQPGQSVAAGGPDKPFVPKA

>Mb|QLI73806.1

MASKAAAEVIRAHNQQARQSLPFDDETDFQNATKGRIGTRQPNVITNAAG

AVIWDNDSYKFLENEAPDTVHPSLWRQAQLCRLDGLFQVTEGIYQVRGLD

LSNTTFVEGDEGLVIIDALTCSETAAAALQLYQQHRGKHRRIRAIVYTHC
HADHFGGVKGFVTEDQVHRDDIKIIAPEGFLEHAVSENERIAAGVRMVFQ
MAPDTEAPAEMLVYLPQFRALCAAEDATHTLHNILTPRGAVVRDAHRWSR
LLTETIDLFGGRADVVFASHHWPTWGQRSAVDFLASQRDLYGYLHDQTLR
LLNRGLTGPEIAEAMALPPALEGAWAARGYYGSLSHNVKAIYQRYMGWFD
GNPSHLWEHPPADRARRYLKLAGGAGQVVDRAREALGEGDFRWAAEILNH
VVFAEPEHAEARALLADTYEQLGYGSENGPWRNFYMSGAAELRGGKFGAP
TSTASGDVVGQLTPEMVFDSLAVRVDGPRAWGQSLAIDVVLVDAGERYRL
WLSNGVLVYSAARREASPDVTLSATRRQLCALVGLGRGPDALRAAGIEID
GDVSVLARLGGLLDPGDRNFDIVTP
>Mb|QLI73822.1
MANLNRITEELRRLEKEEELMEELLIERRRKAQLEIDEALNRLMRLRRMR
CYLREKGLEMARRDFASLEEMEDAD
>Mb|QLI73911.1
MLDSNLLDVLGNQPSLHKLYTQIASIYAVTGPEVHGHIINTLRNGLERLA
ESFPWLAGHVINEGASEGVTGTYRIIPTDKIPLVVKDLRDDASAPTMAAM
RQARFPFRMLDESAIAPCMTINMPGMPIGLVDDHGPVFAVQANFIQGGLV
LTMVGQHNAMDMVGQNNIIKWLSRACHGDAFSQEELAVGNMDKSQSVQLL
DASWEPGAEIEHQLRKPPVAGSEESTTSPGTPPRLSWSYVNFPPASLKGL
KSEATATKPANSAFVSTDDAVCAFIWTCLSRARANRLPAEASTTFARAID
VRSRLGLPATYPGALTNMTYNRSSVGAVGAQSLGSVASDLRKQLDSSVVD
VAYNTRALATFLSRCKDKSQVVITVCVDASSGVMLSSWAGVDLYDLDFNL
GLGRPEAVRRPGFLPVESLMYIMPKSPRGEMAVGMCVRDEDWQRLEEDEE
WSKFAEFIG
>Mb|QLI73928.1
MPSAKAVYNGSAPRKPDHKYTHHGATSGGQNLGNDRLANFLERGASEPQD
MAARHAQILACISSVDAKFNHN
>Mb|QLI74009.1
MEWVEKQLKWLQEMDPHNKLLVVWCRYPDLRTGHDPFDLGSRFWMCLDSS
ATPSFEKLIPIKIKEERLRMAGRMTVFACGDAYFTALMRDGTIKILKDKP
VKFLESEYQSRSLVRVSSVEGPRRGNPRKRKKLRGHTERLELQNMANNRQ

TTYSPVSEGSFYSVKL

>Mb|QLI74027.1

MNKPTFWRRRDAPNPNPQTQSQHWPSCKRPLDSSPESGVQAWWDASAPDK

YQSLTVKFLSSCPPELALPQTMLQQLLATRTHHGDFADYHERFQHNDVCV

TCSCGRRFAPTQLFYYRKIQPLCQMRLAPSPTVAINQAIGRDFDKIRQVG

EGKLFLREDLPAPLDEIIHHSTVVPPLLCLFLLRRQQAEGGLSTFCPRTP

LPAATAARTTDQSGPVDSITCTTR

>Mb|QLI74036.1

MELPKITGFLWFYLTILLSFSQVLTAQGGKKWQLKAEPEIPEDGLQIKLD

RDLYTHQGSIVYTSHLKFDEKPEISDSQLYDIATDAFIEMQKSAEANGLD

KKVPNVMTTLLVGNELIFGSSAKGPNKPYDRESQVQGDLKVCEDENEGRA

HKNGGRCGEVIAFHTWYESHVRSEKLQPGEGTKIVAISSQKNADGQEEPG

ILAPCGNGEQWGCDKFVNGIYVLNKGRVDADKAANPDRESFDYKTLIKNK

KLTPAPTRPDLPPPNPLPPPPRPNTNPGGSDGGPPFNPLSPPGGKPKPKP

KTNPGGSNGGPPFNPLSPPGDGNTDTNPGESTDNQGGGSKPVKPKPTRKP

KPPKRP

>Mb|QLI74059.1

MVRMDRITLEKNYSWTSPARHSSLRRSFVSAKESFAPPAGSSPINARHYD

AVPGIDPQSSCEHLQSVLKNGVGGSNLDSGDMISRIVSPEADLEGATIRV

ADDEETTGRIRRSNQKRKYNTTRTTGSGEHRIQKKRASVSGRQVFEYSRL

GAIREGVDGRIQFKICWKPTWGTLEDLRGSRALKEAEELIINKYDKDTWD

EEMNKSGRLALSTRSSR

>Mb|QLI74085.1

MASKSTVPYSSRAASHPHPVAKKLFRIADSKRSNLIISADFTDTESLLKC

ADELGPYIAVFKTHVDIIHDFSDKTVSGLKELAAKHDFLIFEDRKFVDIG

TTVQKQYHGGALRISEWADIVNVSLLGGDGVVDALEQVISDETFPYRNER

GLLLLAEMTTAGSLAVGEYTQRCVDVARRTGMGGAVIGFVAMRSLGPAGL

DPSLADEDFVIFTTGVNGSKSGNVLGQRYQTPAAAVGGGSDFIIAGRGIY

SAEDRVQAAKQYQEEGWAAYEARISM

>Mb|QLI74109.1

MARLTALASEPTASLEIINGKEMYHNDVFPILACLLWPATAIHSMCLTSP

IISLEAGQSVLSAVINAPQSTRCYVTMNFGDSSFLPLRFNFSTTQCDGQM

IATFTTPAEVPNGEADILW

>Mb|QLI74113.1

MLVRQIAVFHALIGLVCSHSWVERLMVIGTNGTMIGNPGYIRGAVSRLDP

NFNDFKMQHLLPTIPEGLLTDKLCKNTQRNRTYTTDLPALQAAPGAFIAL

QYQENGHVTLPGLTPQKKNSGTVYVYGTLYPRDDELLSSIHNVWNTDGTG

GDGRGRLLAVRNFDDGQCYQINTGPLSMQRQAKFHKTAMNPQGADLWCQN

DIRLPVSIPFSWYTLYWVWDWPSSPSDHLPGGESEIYTSCMDIEIQPGVQ

LDEMNFVDGQDLNMAGIKEQLE

>Mb|QLI74134.1

MDVAVFEYNVNLKADEIVASLVQNGGCIIRNMISDRRILDTIERDVRPYI

EADRPWVGVDFFPPETRRVMGLVGKSQAFTQNIPANQLYRDVCSRLLSST

HKAWLGYQLNTTVSEPQLSNTIVFSIGPGAARQELHRDDSIHHNSLPELK

HHNDYRIGRDTSVGLFVAGKKTTRANGATRFIPGSHLWGEDRCPSEELAY

YAELEPGDAFLMLASCYHGGSANTTKNEERLVYSCFMTKGFLRQEENQYL

ANTIGQVQQYSPDIQKLIGYSVSKPFLGWVNLEDPIKLLHHGSEIIGDGM

R

>Mb|QLI74140.1

MAQTAGVCSFLGHPWTLEDVISIPSDSESDTDSDVEVVEQDVPRVHLVHN

QHRDDSLPSISEIMASLINVRHDPTEAIGSSSEDLCLPDEWESPNSLTSN

SSREVISPSMALESPGVTADSNMLVSPPIQQHDDGASVVWPTPIRRPCVV

SAASECCSVQSTTALPPRDEALHHEKTDPPSRSATSLVQPSQSQNCSALF

SNRFVQAHSINIGHGRMGEVVPRSDNELPSVARPASVKTPPSGCSHPVSH

RGAVMEAIRKHQAPDDISPLGSCPSSSPTGQGRDDERQTSIDAGGTESQP

DEPCEETEKRPTSRRGSGKPYNLRTLPPKRQLTAEQQDGGINEKLMPQRK

RRRLVFRQPKKRNSQQRSDRRSDITRNRETRLLRSTTKSNDNESVANKKR

PVRSGVASHEAWPLGQPVLRCTRENGTAMFQLHFTSDILWNTLVAQNDPA

PKGHQTPNKLEDRRPKAGKVKYPSVCRDVYRVDCLLARWRRHTFLVKWSD

ATTTWEPRKHIMDEGMLNSFEASWRGFDAGVDVLKARQRAGRRQHLLRWR

GRPSREDTWVYDEFLSPQLMERMEANERNGPQF

>Mb|QLI74159.1

MVLKAAIPLAVAFSTVLAAAATINKRIYLGEEAKEGEFPFIVRLHYEDPT
LLCGGSLLDSTTVLTAGHCQPKRVTSNKDSGGVVAEVESVVVHPDYKRFI
GEVNNDIAILKLSTPIQESEIISYAKLPASGSSPVIGSIAVAAGWGTTGP
LSKGDVSDKLLKVAMPIVDINSDSCKASSAVQLPDTKVCAVGGGDTGKGD
SGGPLIDQETGQLIGIVSIGPRYTRISSYIRFIDDVNNGINGVDPRHVTG
>Mb|QLI74164.1
MSYFTFNLSASTQGPPNAKKEQCTSSSSELVVPQLDHESSTLPTNSPATT
LCEPALCGQPTLNPGAPWIGFDGSLLVDGPINGGALPSYDQSSFGLTTND
DLRWMLMGMNDRIATLEQAAATGNQTVDQLAKNVCDMLSRLAVIEGMLKV
VEDLKQNLRDFTRGLMPHILRVNLEEDLERHGQCAYT
>Mb|QLI74193.1
MNNDSEQTRQDRMAPITCKVIDTTNRGQPGVYVVLECKDQQHHVIATLES
VTDEDGGISLWFPTPSQGRMDEVEPQIVDSRVSPRVSLTFFPHAPLSTCP
APFLSVQTDLYLDREECHGIILYLDPNPRLEHCPFPVASPLTQVPSRQEL
PIPSPLILPPPVSPKQAITDTSM
>Mb|QLI74202.1
MDVDDRPQVVNDGDYTLVKVVKIFQDGDGNYRTQIRRSDKSEQHFPWDFW
REGEIRLNPNMPVQGFIADKARCWAWNLEVSASSDSSNSSDSESSEDSDK
GKGKTKARTKAKGKDKKLRAKGRGKEKTKKMPKEKAGRN
>Mb|QLI74204.1
MRAYQLLSLSAMLSSAASWGIKRAALEPNLDLSVDITNDINIDPDIIGKA
LFEIIPDEKARNEDNPISRRDVFSRQEPGNPTLRSAEEVSKIKAPGTSKS
GVFYRGDSRPPEEIFKSGFKPQGENTNLQNHLDFKPNSGLVSVSRSREAT
YGYMFGRSADKNPKGYVYVIAAKDMPNGYWVPGIHPPEKNAAVSRNQEFA
VNGAIPATSISHAFEVTQANPTDKARKIKNEAYSLRSASKCTIMKRRAGG
SCDPAGWVDIEKNEPKKPASGDGGKGKKPVGQKISQSKFLQFITELEPKS
IGGLIKVLKDGEATIADVQLGFKRALSEAMDNRWADFGDWDKAAESLKNI
VVDVASTVRYATPAGYWSDVIKRLPDIAKEISKGKTPKEKLEIANKKVNG
AVNAWAYTPVGFVNEALMREAAMRTPAVQAVAISINKLWSYTPLGWLINQ
IAPIESLRRKINAREEVLSLGY
>Mb|QLI74211.1

MVAQTPSPDEKPSMSTMADNLTAQSEKVLEYGEARLALLNSDDPGIDADR

FVINRYDLHQGKIIDQPRGASLPCKRAGVPCVAVNTNAKDAESKGPTVEK

TQDGELIAETSKKSKESFNCLLEEYNFSSVAKQDKLYTKLSTRLPEFTAP

RVDRIAGFTNKIARGALAVAGLALYGKAVADVFSSDASVLDKAGHRGIHP

ARNWLRRIARRRRVRHATYQILTLFQASQLTGDLHAAHRVLSENATISAA

AQQDQGNADVGAHVRPELKRQICATIAEAKHQLHVKLEGIALNYTVKLGR

EFKKQFLDEWFGHLPCAHLGRDYPRIAPCYKTLQEQVNKARNTPLPLYEE

QVKAAVKEAMERLAAPAPCQCEQGRKKGKCEFGDCQGPKLEGHPRDAGGR

VYITDVPSLEMARRVSISESCQAVFTTSHGPGSAGETGRPLWCTPG

>Mb|QLI74218.1

MSRRFCLVPILAVVVADWQPRKPNTVVGILMPYAGEDLGILAKNSGGNLP

ITLQQLQDLVRGVRELRKYELFQGDIRPWNTLLQPSTTASKPRLMLIDID

MELPGYPGDAKALGKLLQWCLENSMALSEDKQAKVKLINAVKALLSENFD

MAIDCLSATEQSMRRGPPFYTCQQGGLDWV

>Mb|QLI74238.1

MRFKHFLAIFGSALIAAAYSTPGAYERLLYYYAYKLDNMGGGKTIAGGCP

GPESGCSFDQFVHFIEGRGPSEATVKITSEEFPPIEPTVELLVKAGKTGT

IAESRIISNAKDYPDLFKKLGTNLSGILQKTQGFKHGTLNGQALEDTLRE

VRANIVLSMKRVFDGRVRATIDSFKPYEGLKVVTNGATAIDFDQTVAENK

GSITKEKLQELWDQHLGGRQVTEKIVAEFKPLAEYTPTIRENAEGQINLE

ETFKTAVEENPWMKASWFKKKYLHSVRGNHQTNIKELNTALESIEPLVCA

TSKARKREVARLDKRQGVNACFSWEFPEIKEIIDLPPVAEGSEGVIDAET

LATGRFVERANRANQVSSEEFETAIGERLPKIPDSWLKNGVKTFHEAREQ

IGYKPMEPTSADLVPGRGGKLSGTLGKLAGAAGGALWVNGIVQAFRTNST

DLDKAAAFTAIVPFIGCGVTTAAGAEKGHDIDLVTVDAVLCGIADVLLFG

PLAPFGFALHFVRFVMSFFPHPPEPPKFEEMRESRDDSWAEALSKVYGTV

YSNSRTDPTQSFAAKLESVFAIDAVSVLSEAAGRIGALNASGSPDLYHPS

ALGEGETVDLAVLEKGTLQAIHKIRADRWNVVARRQREFLLDIPQEFANG

TAFSLSELSKKVNDGFIEHINSDKFIENFRDQSRLDDIADAVFAGGPSVK

PIDHYSEARTQMKEVGRRLSESLPPLPQALNVAFVLGQSKGMAFAQNETL

DLRSYFDTEVREALVEDIPHLYRDLILIRHTHQTALFLQGRLKEEEFDDS

VFPVDHPALLKGLRLLAAMKFGKIHEAEKIEHMDKVLGRDSMGRHPTAHP
DFTNPSIPPLTALKPRKHKLLVSLAIGLLEVVRDSQLNDDMAQYLRDSLA
ETRSKNWEAMQQRFKAMQAVLKKISQRDVGGPPTPEEFLNKHLKSCVEEN
GSKTETCRHVVESCYSADDTSDGFASECIRDAFLPDEEAGRKMRTKQREL
CSKFPGPRECTRALRSCGGTWPKSRIQVIWACAAKALPKTDGADADAAVM
PGSTS
>Mb|QLI74248.1
MATQYTQLLMEDGVDFFPADVSENERTYPETMEEYDTFELDEQDLVLAQE
AARQQFNKTEVRPNMYSFDWSDAHGALWDRVKQKIITRFGTRGARLDRVK
TKAVSVAGGGGIRAMNGSITIAIPTQEAGFETEILIQALQPRWVPRKPWR
HNLCYALLQPGTVIGTQKDEISMLLATFELDDN
>Mb|QLI74254.1
MSSASVVSQLRGLLEKTGQLALKFDKIYPTEDQWNTLSDLSKSLVTAATT
ISDKIRLLRESRAERAWKDSQKLRQHAMACKGDVLGNGRLKHSPVFRRNI
ITIFEGPKESVFDSEDIKIRKCTTRQRCAQIRRLSPDGLISWAIAFAPSV
WAGGSMATDIFTCLLDDIEPQQQPSWPRVIDETLQMLLKDETALQNSQEY
KDFMEAGKTPPKEGQARQKKRPRLDIQEQSSYIEHEKRASFDSQHVKHKT
SPDLPPQPGVVYPPVISQPPTKENREMKHMCTNAPLRVVSQLPEPFRAAV
QNSRLFKWERSQGYATTNCLATLFPKDNAQDVSFTFWCGNFEGYDLTSLF
GLQHAMSSQT
>Mb|QLI74256.1
MPDSRLLPLRPAQTKQDVIIGSSDPRQSPSSSLKLVLEQASAGSSNTCRA
IHALWNQDVKEVTSRMYLVEASLSASQDEMARCDEQHEEDMHKLRTKVYV
LEEKVHVLQEDMLNSRQEHYDFSQELRAELHRTRELLHQITGDIQVLKDN
ESPGLVEVANSSEGSDSASSPATPEAIDAWGTK
>Mb|QLI74259.1
MKTCTALSMALASAASALSLTARQGVTADDYAVQTLKSIESNFKMDDANL
PKFPVNKEWQHVCPSNLNYGEKVTFFKTGTIKRNDIIDAKAYYIAHVETT
AVNPGPGDTSSKMTMSSSTTTTQTDTKGWTVGVKLSGKVSGGAEKPAPQG
EVGVEISASYSDTKTKTDSNTKTVTREEQCEAGYECRLETWSFHLDIHAK
PRVDGFFQLWDSVNDIGKEIPMCSMPKKARSCEQFKQRIDEWCTEKALPG

GALYIPAKQDELHIKTPILETNGYQTFTRIVKVFNPIVKSQKARSVEPTV
GTKETIMEAMKQGTKFKFLD
>Mb|QLI74260.1
MSYTILHDTGLAAVADRDKVTIYVQLSNGSLGEATSSPNGNWNVSPIQIP
NAPAPKLYTPLVVLIGKNQQRHLFYIEGNNYLSEAVYKDKDSGWSIGTLR
TLGIIPAQYSKIAAAKPFEGNDQICVFYQVPNMSGAIRQVTWNGTWWTQD
TRDLGDDVLTGTGLAAVGAELGTNISNTKTENPPVVFFQQSNLDLAWLQD
TSTRKINDVDPTASPHTPLAATGPRNENLDIGSDLFYTSDQNVIQSLAVD
ELGKKYGIDEVTATTPKGNLAAVVAKKRVGTRNVDQVIVIYQGPGTEPQS
QSVPDGSTKRDTGACLYQTSYIVTKADEGGKLARSEFEHALLRFE
>Mb|QLI74286.1
MAQTAGVCSFLGRPWTLEDVISIPSDSESDTDSDAEVVEQDVPRVHLVHN
QHRDDSLPSISEIMASLINVRHDPTEAIGSSSEDLCLPDEWESPNSLTSN
SSREVIFPSMALESPGVIADSNMLVSPPIQQHDDGVSVVWPTPIRRPCVV
SAASECCSVQSTTALPPRDEALHHEKTDPPGRSATNLVQPSQSQNCSALF
SNQFVQAHSINIGHGRMGEVVPRSDNELPSVARPASVKTPPSGCSHPVSH
RGALTEAIQKHQAADDISPLGSCPSSLPTGQGRDDERQTSIDADDTASQL
DEPCEETEKRSTSRRGSGKAYNLRPLPPKRQLTAEPKDGGIDEKLMPQRK
RRKLVSRQPKKRNSQQRSDLRSDPTRNREARLLRNTTKSNDDESVANKKP
PVSSSVASHEAWPLGQPVLRCTRENGTAMFQLQFTSDILWNTLVSQNDPA
PKGHQTPNKLQNRRPKTAKEKSNQCTANTARESIHAAYPSVCPDLYRMDC
LLARWRRHTFLVKWADATTTWEPRKHIIDEGMLNTFEASWRGFDAGVDVL
KTRQRAGRRQHLLRWHGRPSKEDTWVYDESLSPQLMERIEANERNGPQF
>Mb|QLI74290.1
MKKCIIWALALGPALVQKAVGGAVAAPGLFENKASQQRGHIVILAEGLHD
RYVDTHLDWVRDLHKRSLKKRDGKDHRQGIQHTYRAKSVGFHGYAGSFSD
DVLDEIKRHNHVLSVEEDGFITAEYNKGEDA
>Mb|QLI74294.1
MDAPANAYLWVPQRDWQNFQQQGVLFNTNPFRRDIGEFGLELETSVGEQF
SNGDTVIVELALGPHTIRSESHLSTYGVFFSQVRRHQSVEDREQANEAGW
TVNDRFNTSWNQFGAISGQANYGVPEMRQMDQRYLNGVAADHFTRALLHY

LTGASSILDANLRQSFRRLIGWGEEQGQAQLLPAVTNPLSMDSIVVQYLS
SLEADVRLARYGIIEGNLANGPCPALIRTIAAHLGVGPSLWGRDEDQATE
IPGSCENAVSVYCRQQAQAQDGLCEAVKESEQTDVETCNYFKSLEAKLTM
GNGLWGFDGAGTVDDIVIEFGGHNHQTILKHPSHGDEATISVDLEKAFGS
KQVLVEAVKSFQLYSVQDEQKNPDSWEIGGLYIYGTCVESSRRVVVKKYD
NVYDWFNRFGDMNPGYGSSHFNGHVALGDWQWETPDDKTKTIAVMPPGQP
HACTEFKSLEAYLELGKGVYQGSGAGTNDEIMLDFSDSAFTPSNILLMSS
PSRGDSVTKPIELESVFGKSVLSPKDVRQVAVYSREGTSHWADPWEVATH
CEGSAKTAEVTKWRDIYQWFDRFEGPSTLSGELSLRDWHWA
>Mb|QLI74298.1
MAQLYEAIPSFKHTWIECLGDLARYRMAVEDDDIHERETWTGVSRSWYTK
ASDETPTTGRLYHHLAILARPNALQQLYLYGKSLCVPVPFLSARDSVMTL
FDPLLNANPSASQRLEPVDVAFVRVHGILFSGAHEDQLEPSMKQFLDLLD
SRIGREHGCWLESGYFIGISLSCLLLSFGDASNVLMNAVLKSQQTDDTIM
LPDPVLTDTFKTAVRFTARTYEIVIARWGDKNTFPCLHTLLVFYWFMMDF
DVGRQYLEGSLPWEQTALLLNHLLRTSEYTPRLDTPEIPWPEVGKAHPLP
EDYAMRGLIYTGTYFPKNWFDNTAIDDEEKYFEPASTVSKRCERILWLGY
SMAMRKRRLHWDKNTKQFSAMSNESNDDN
>Mb|QLI74301.1
MVLTVVNAMLWATLPLSAAVYGSAVPRQNTQNTGDINLAEIIRDSPKVQA
HMKSTMSHLGKVFPFSEGQMSASKGGVDLLNSAEIPEFDRKCLFVRNSIK
ETGWQSSLDVNPHPNGDDVVTGENEEPMTISTSTVKTNSYRLGWNKESSK
ETGQSVTAEVSVGYGPFSASLGTTVYGNQRMTEGQNAELSKQEEVSVKVD
RPYTCPAWSICRVVTWTYIRTITGSCFLTPYYNETCGRGETGKGNLYSLG
LLRSCSPADKIANNFYEFILGQTGYGPDLEGIKMHITEGVIPRYQDNCSF
SYTLRDEHGTPISAIANIIEKYPDPNAKKIEVTSVPKALVLDAPNRFYIS
STDGGDGKWHNRDDLPEPEGCMETRPDTESKSKRAEAELDNNSLEEEPPA
YDGVKVEILQDGMPAFLEKLGQSKSEGFIANRIDEAKPSSREVHGRAADY
IPNVRDCLADFVKKNTQG
>Mb|QLI74311.1
MSASSVVSQLRSLFDKADQLIPKLNKIYPTDEQWESLGDFSAKLATAATT

IGQRVQALKESRADRAWKESEKLRSHALKCQGDVLANGRLKQSPVFRRNI
VTIFEGPKDSKFDTEDTRVRKATTRQRCAQIRSLSPDGIISWAIAFAPSL
WAGGSMATDIFKCLLEDIEPDCHPSWPSIVGETLHTLQADEEVLQRSTDY
QKLLCAYDTPLATNLESNRKRKRLDHKRPSTILGQKGEDIPCLRRNVAKV
LLEQSQYNNDKSTLSRGEACDPESRQGKQEDHSMLNPQPPNDGNREMKHM
CTNAPASRVEDLPEPLREAVENSRLFKWERSQGYETTGCVTAVFPKDNTQ
DVTFTLWCGNVQGYHLISFFGLQHAMSS
>Mb|QLI74315.1
MSDRRNDDSAVADQIDVPGTLLLLTTHNGGGGSEDVILNPVPSSDPDQPL
NWPTWRKSLNYGLLTSMTLAVFTALSIQPVFWNPMTQDLGITYQDLANAQ
AIQLVGLASSCVLFIPLTKKYGRRSTYVFSTALVAASSWWMAYMKTKVEL
YLTSLLYGLAASTTDTSIEMSINDLFFVHQRATANGIYAVAVMGGSFLTP
MIAGVQAEAQGWRVSYMTLAGFMSAIHVIFIFFFEENKWIPTIEGTQAST
YTTNGKIDLEAAPSATPSTWQRYRHAMRFLTQTDESLLKLAIAPVYTCFM
PHVVFGWLQLASGVCWLVVLSSVLTIVFAAPPYNFNPAQVGYMYTGPTIG
TILSFFYGGPLTDWAIVRLSRRSGGVFEAEMRLYPLILPAIVSTGGLIMF
GATADRVRT
>Mb|QLI74329.1
MSSDNTSVSRLTQLAASILNSVSRLEATLSMQACPLPSFDQDAPTLLPKD
AIGIRDSIIDCAAEIQDLLLGPFDMLYMHGSVNSSVSLQAISRFKIAELV
PLGGRTTFASIAQEIGLGERTVRRLLRHATTMRVFHEPEPGVLVHTKASK
LLRDPVANAWLLCGTEEMWTASVKMVEALEKWPESQEPNHTGFALSNNTG
ESIYEVLKQNPQRAARFGNNMKAFMEMQEFNSTHVVNNYDWKSLGQVRIV
DVGGGPGHISLELAKHFPHLSFIVQDLEIFTPDIPDEVGDRIKYMKHDMF
APQTIHAEVYFFRWILHNWSDKYCHLILKRLIPMLTPGTKIIINEICMPE
PRTISHWRERYLRSFDLLMGAGFASHERNLDEWKALFTEADPRFKFSMVN
ESNESALAIMEFVWTDLTG
>Mb|QLI74366.1
MKKQPASLNEVSRVDLDRRFDLAHVIDALTWGVSAGVSIYYTVRAPRDGP
TIGARIWDQNFQYETGLTIDPNMVIAYWVGMHAALFVHMLANIFAASNDI
SYITISNHFSINNLLHVVFLVLFVRSAFGWAECILILNFVNLSVLYFKHS

KLSHFTRISVICGPLAWNFIAILWNWAIAVTAGIGYNDTIEGFGLFFVWA

ILGYGVFSLLVFQDLAMAFLLAYLCASIAVAQHFLQRSDRMWIPPVVIAS

LLVVMMCAVAARCLWNLRAVRRKKVNESVDAEFDERKESC

>Mb|QLI74370.1

MKSLHGLVGLLLSASLASSGLVPRDASIPTANQTSENGSIECDKRGTRGT

LVSQSNAPANLVRLSNNAIQGVSNYTYDESAGEGITVYVIDGGIRLTHKE

FGGRASYGAIFSTILPRNERDYDGHGTHIAGIIGGATYGVAKKVKLVAVR

IDSKNASQMIKAADFVIEDVKKKGIQGKAVICMSMHIDASNEVDQKFEEA

VDSGVVVVVSAGNNNKDAGNYSPARHPKIITVAAIYDDTDFHWPNSNWGS

SVTIYAPGVGIRSAGPASDTDKRLYDGTSQAAPHVAGLAAYIMALEGITE

PAKVMSRLISLAEETGSRVHWTDPNTTTLIATNGLAGRLDPAIANKVKKL

PWLSPEGYLVDRVTPCGSFYYSDANCGTARYCDSYDRGKPTTPKKGFFKS

AQECLDAHEPPPILPWIEKPTIVRHERCDETDISAKCPEACGTEKYDEEF

CGTKFICEGFDEKPKPIWAIEYPNTKACFDGHEPQPVSSNQSMPAAAA

>Mb|QLI74373.1

MESKYLEGKARVPLSSLKSEDLDVDSSSFPINIIEHLRDPGHAISVRITP

RSRDDILRDLSYSPEQLYATLELESSPLINDHQVFYAAKDFDLDRAKAAL

GRDHICNVRLYCIPVQPRKICDGAVFQNVRYYMAGSWKKNRVSVAQAWMD

KLSGSKRKILGSLLKHPHIIAAMDSMLCFPGYWDGLQLGNWAKHLAARIN

PLIINYWVHIKNVALKIMAGHEDKLHLFDANTVAILQYRAPSWNSQDRAQ

ICDLFEDGTLFPGILSKSARDRIKDNILELPDSIPSIQTFHENMRYLTIG

AKILEKHIENRPSESGADTIEPMCSSLIENLKKDWNSRGASMEVGHGRMI

QVNEPTADAAVIQAFLAALRYFPYLSPEAPLRDFDKRVWMAGNFDNSVLS

RLCVTLKDLGFSNANIEKGRLHPIENRHISYTQPKRRRDWRSGKPCLAGY

HILLQSSFFPQLFAEPLDDKVPPELRVQSDILQAFFGGKPKVPSLASAAS

LRAEMDVDTTEPNEAWRSEAEINEDTRLGEVLKLWPRGNNETRPKKKGKD

KMPRGIARSATSRDKPRVRGKRPKFTFRQQNVPDIPATRASDLYVGPPVD

PAGIPTQALAHLTPPTKNPNRAKEKKRKHDEVYEAAEVPEKRNRKKDQVG

GIQAVGEQAGAEQVTQIQPAEELGRAVVHDLASTSQDVRWEMKGPLEAPV

ATPALPPSMNPQNSTPVVQRGPEPLSKPAQHVHPENNPQIPEINYDEAFS

PPHLEQEAELLLSGTN

>Mb|QLI74407.1

MPRRQRSKVNKRPEIGTLNCSWGMFSKANALFRQYDVDVVVIARRPDGGI

GGYQSRPGLARDFLQINEQDLLGPHEVDPYMSKPSKGVAVLRAMSSSRSS

SCLDSTENRSSRASSSCVDSIGTETSPGIMEEAEPGVALLNIVRTPHCEE

GEGFMEEPLHPWAEVHTIPNAQRCASPETICPIRITKPAKQSSHKTKEDV

RQPTPLSRTKRGEILALIKKFE

>Mb|QLI74437.1

MSPLNSPEGQYLSLAGSFQKSQKTLANNFKTWGLKPSSKNQAPTILAATE

AAYTNSVKQVEDARNRQILILNEDWVDECCEQKSWIAPEDKHIWEKGGTK

ESLAETGSTASGEPAGKPTEDDSAKAPNPPPNPPENPPPQPPNPPENPQP

QPPIPLENSPPQSPNPLRFLNHSRLFHLRTLRNMNLIMRAWSKTNGKYGP

SDRYDQSEDQKECFDIFYEWSRRFDNTDDPNTNRVEVDCHPADINKPLSD

RRKEYEMEIMLITQFRSYVFVIDPEGKIEGYDCLRPGYLIPRSDCPAAAR

KYEDNGGEIVRAKQRENLQGKQMTDFIWWSVATDPEGRFCYCLGAFKGDK

RPSLYSQSTFKAKWGKLADDQINEIRRKFSQAELNKQAKARSQKLLKLN

>Mb|QLI74451.1

MKTSAILAFFFGLTSAGVTRGPEGLESHIAQLDSDIKGTNAAPLRRSLQN

GPETSSAPGRHPMLAKRASKTQKGAPWGLRAISHRRAGAFYEKFPPDPAS

KYYYDDRAGLNMYAYILDSGIRTTHEEFEGRAETVFTVYPGDEIDHRGHG

TAVAGVLGSKTYGVAKRAKLLSVKTLDDKGSCAASAALHALSWTAEHILS

NGRQHSSVINLSFGIPKLQALDTFIEALVSQVGIPVVTAAGNENEDASLS

TPGSAKGVINVGHMDKNWVISPNSNWGPAVTMLAPGVQVECPSSGSDSNV

KLESGSSFAAPHVAGLVLNAISVHGIKGAAEIRKFLLESATRDQACTSRN

TPNIVANNGNTAQKKHTKPRNC

>Mb|QLI74452.1

MHLVSALSANVLALSLAVLSATTAAAPNRVEEFCDWQPGQELNTVQDTVR

RADTTSQETILEFDPKIEKLIQQLWPPEPVDDGRVINVGVHMHVVGKPQN

NESEFLIDRTALDKQLDFLNESFKPANISFTLSSADWTKGGMLARDIFLR

RPLAKSLHRGNFSELNIFFCDDDETIGGSTVTLRGYDLDDDDDKSDGCVV

NANTVPGGPHPVWNLGVTVVHEVGHWFGLWHTDLTDPGDCEPNWRNATGL

SNEPCGARCDSNYMSYGADACLTEFTSEQIAEMRKFARKERGL

>Mb|QLI74462.1

MISMMLWTALSLSAATAHGAVVPRQAGDFDLAGVILQSSQVQRHLTSTKR

FMGKEFPYSEGPMEASKAALGDKFKDDYEHFVESERKCLYIRNSFIKEAT

QSFFDVVPNVDAPDTITEEEEETGSVTESKAIVDTQRIGWNKEESTEIGG

SATVGIQSGISAFSVSLSATIYGNQRTTGGQYGEASKQTEYTVTETKNWK

CPKNSICRHVTWTYTRTLRGKCITTPYYDAQCANSKHSKAKFSLALFGKC

TPSVRAGDQFYTYSDNAFGDFDGDLNTKIPGYGPDNQGIKMPKPDVIASH

KFADDECTFTYVLRDKSGSPVRARGNLIEKDSSPLSAQPKVTKVPKAVKW

IKGQKDQSVCELEGGWYWMPGNQWYISPKDGNTAKKWARRAELPDPVDLE

KNCPENGQAKSKRDLPPANDLSDVPANDLVQIEITQDDMPAFLAELEQSQ

SEGFVANTIDDKAQPVRREGWGYPPGRNFEHCLDQNIKQNGKA

>Mb|QLI74483.1

MKAYPLVGKALWLAFSIRPSLTLKVPYQDALTVGQGYNTFLGRGVIHKAV

MTSSTDAATNTEKRSEGPGNQNSTGKFNFTEPGPVMPGVDVDGYFTPTTL

EELAKIIEEAQEDEADKEKRDGAKNMISDKKMTMQGCQAEIHSHIEFVSD

YTSYLKALGVNAATSISGYGQEASVSGSYLDESAFSSNSLTFIASISINK

QRRVSNEQFTFNTKLYEDSDRPFATRFGNRWIRGFEMGGKLMARIMLTFD

EASDKEEIKAAAEASLSFWGVSGQLSTEVKNNMEKLSKKAQVKVKIFYQG

DIGRQLQGRSESMDEKNSAQQIFSTAKTWADTFLEMACAQDYSYQTLLDT

YPNIGNFPENQSIAHYTTAGAVAYHLLGEMVKHTELRKTLQLRDALSQTE

DREIQQHEIKLIDAQKSWIRDTALSPDNALETVQHLFWLSDQYYQKWKPY

LTKQSSPVEIKAFNRLVSEKDPDTKDPTTYDCSGWYEWYGKGEWTWSKLR

FCLPTHTDVFSLSVFRERSQYLWGSAWYYEKVQYPADVTARVTLKRIDSK

PRVWTLTSGMTRRLSFNAVSEERLMPGRYKVQVNFYQDGPYWDDSPIGEV

AEFEFTAS

>Mb|QLI74515.1

MSFIATQSKVKAILYYVTNYRKPDPRHLRRTNIGDGSTTNSELGQDAVKP

GGDVALIQHLTTEPHMSLQWRASSSFYAEGDRVSETLTLNCRQRAAFLLI

CRQMDRIRQAQRETDVDQLCQFIGGEGGTGKSRIIEAIVQLFARRGAENK

LLVTATSGRINGITIQSACNLSVEQRSVWTSSKLNQTEWARPGLAQD

>Mb|QLI74530.1

MENYSVQQESQRILEEQLLKNEQLSLPREFVEAAKRVKITGKDPKPFIPT
PCKVTESAAALAALVAAEGSAISADRYGIGYQAAEVDTDLATLFLESIIL
PTINGKPAMQDDRVLAELKKGDLYDMAKPIHQQATNVYQTKDGKWYHLHG
SMNASATMGLVGVEEQDVTREEAIKIFSDKVAQYDAAYLEKTANEHLRQA
GVTCLTPEEFFASEHGKIMADEPLWTMKPVPAPRSTWPAQKSDKLKPLEG
IRVIDFSRVIAAPVISKVLAVLGAQVIKVSNKNLPDVTATWIDLSTGKKD
ADIDLKSDEGKNTFKRLVAGADVLIDGYRPGILAKLGFNSASLRTINPKL
VYVRENCYGFKGPLAYRSGWQQISDCLVGISWLQGKFLGLNEPVVPLLPN
SDYQTGLVGAAAVLQALFQRTKVDCTYDIDVSLTQYNIWYYRLGQYTADQ
GKAILARNEGFHVRHYDEMFSLIQKTHAAISKARPELFKNPDYFSTMSGR
EWGVGDDITILAPPFKFETSVLEYAVPSGVRGRSIPEWAA
>Mb|QLI74539.1
MSYFTFNMSASTQGPSTPKNPQRSSSSNSAVPQLDHDSSTLPINSSATML
CEPAVSGQTALNPEAQWSEFNSSLLQVDGAFSGSALPSYDQSTLHAALTT
NDDLRWMLIGMNERMANLEQTAAAGNRIMDQMGNHISDMLSRLAVIEGMT
KVVEDLKQSLRDFTRGLVPHILGVNLEEDLEKQG
>Mb|QLI74540.1
MTRMHWSLILVSALLRGAAAEPASSGDGAVPALGHIVVLKSGLEEKHLDE
HLERVKSSIQKRSIESGSSENQVNGASGVKHEYRGTSIGFHGYSGSFPPD
VLEDIKRDEHVAFVEEDRMITVEPSKREEVEEDTTPKDGGSNNQKKGPGL
LSMGQGYNTFLDKGVIPDAVLLPGEKKRDVPAALAEVLVNQTTSMRFNFT
APSANLTNVNVTSYFAPPDPDEIMNGVLDDLENEQNATALNVIHTRATED
QQDCTGSLTAYYKLTESFDSYLKALDVSGAATVSGWGQSASVSGSYLNQA
ELSKEGLTYIAIIDIQRQVDLPTGFEFNKAKYSASTFARDFGDRWIHGFH
TGGKMIARLSFNSKGSTSKDDLKVHAEASLKFWGVTGDISASVKKSMEEV
SKHADVEISLFYQGDMGKVMAKSGSPDKVESASAEGSFKQVKMWADQFIN
NACQHNYEYRPLLDEYRNAVGFPSDQKVLDYRTAHRVSYKILKELVRISE
MTQYIVRLETLDAEFKDEVEFAEIEMVELSRKWVDSIVEQPQNALSLGKE
LIQTFRAEFYDKYASAIAQDFYISGIEVQYGDKPPANRVIDINHQPEDIN
HDFGGEFVWLVPIYTTRRDDACTSFKVVFGSEGAGLKDLTRNAPGPNRYL
KCEKDMSKEKIRRLALHRGGENLEEFLSETSHGFVGKTTNINDGRGDAYH

AMYLLWAHDGKRDPSSKHQPEFIVPAN

>Mb|QLI74544.1

MPQSGSSQKSTSPQTRPATPISDKYVGGLWIEGKRQLNREHDSSGKDTST

PLDGHGQVFAPDGPQKTPVDLASYIHNLHIGNEQVQNPHTNASTDRGDSI

PGQSSGPYSNTSTCPVGIWDISKDVPASSVLESWIEEDLLNWGVGEGIDI

NDRLPPITNYVSVEEVEGTAATKKLQSDTGHVSSATVEVPGSLPPPLSAV

GAPTVAESHTLPLAETEDTVATGNTDIDSPCSHKVPSASERPESSFALRD

VSVIPWTDCYPDDPDKARELATSTGVPGLQIYSVYVFTHATSDTQVLDEV

DATQDGHNFRVEMEPQPNDSVRASSVTDSERADESGWVKVEIETENPDQT

DNV

>Mb|QLI74553.1

MNISAILTLLFGLASAHLAHTPVVLEPQMNQHGSDFNKANGELPEMSSER

NQPEGSLETRNDMTIEKRHGAPKTQTSAPWGLRSISHRLPGVIYEGFPPS

QNSEYYYDTNSGSGTFAYILDDGIRETHKEFEGRAKNIYSIFPEKQAGDY

VHGTAVAGIIGSKTYGVAKKTTLLSVKTLGTTGAAHSEVLKALLWTAEHI

ANNTRQKSSVINLSFGVEKSDALNKFIELLVGKYDIPVVTAAGNEGEDAS

TKTPGSAKGAINVGYINKQWGLAPRSNWGPAVTILAPGVDVETTGSESDT

NAVLKSGSSYAAPYISGLVLNVISVHGVKGAANIKKYLLEKATKDRACVF

KRTPNLVANNGNAMQDKVKPGDKSALSKLMCCIKESLNKCK

>Mb|QLI74560.1

MSVYLNSHFELASRLFEDTEAYKPGQKVIITGQDLEVSTVVAVSRGRCQP

QLTKDETTSRRIRASVSRLAEYLGSGNTVYGVTTGFGGSADSRTTETGKL

QLALLQLTQNGVLRESDKTADNANGNFEHHTEISCYPTAWVRASMFVRCN

ATARGHSAVTLEVLQAILRLLRHDVVPIIPLRGTVSASGDLMPLSYIAGA

IEGNPDVVVRTTLGTKSIIQSASEALERENIQKIIFGPKEAVGLVNGTAP

SAAVAALVMYEAHHLATLTQAITAMTVDAMTANAESFHPFIALVRPHAGQ

IEASKNILAFLQDSSFAKGIKTVKESNPSGLAQDRYALRSSPQWIGPQLE

DLLSAHKQITTELNSSADNPLVDGDSGDVYYGANFQAAAVTSAMEKTRIA

LQMLGRLLFAQSTELIDPNLNGLPANLAADDPSTSFTMKGVDISMAAYAS

ELCYLANPVSSHVHTAEMHNQSINSLAFLSARMTKKAAELLSLMAATCLY

IVCQAMDLRAFQSEFLSELQVAIDTLSKEVLAEHIEQPDLLELNLALRRC

IESSWKDASRLDVPDRAKAVGDATTLELIKAITLNQSSSSKFASLCIIES
WRHEVENITMRVYGIVKRKFFACPTTEKYIGIGPRALYMKIRHDLGVPFH
QGLVEHPVSQGKDERIQGKTARPRKTIGSWVSIIYEALLEGKIYDAIYRC
LPREAENAASNGGVSASDIISNTRPRSIESAAPSSSLYETSSIRKRKLSQ
DHLACARSQEHRPIRYRLNLVDSSNDTTNSASME
>Mb|QLI74566.1
MSFAEKSTALQNVMKNLDIGDACVLRKAFTYSSILKQCYESRADPVGYLS
RTFRYPLTLLSAMFDSGCVITGPRALGFFLPSSTSEDSVWTFFVPGYKES
VLDMVNVLEICGVSWQLDAAEVARSLRPLGTASISSADLECLNSRAESLE
PAAAENLLGTALYGRFKAYKEMNCGNRLNSDACQLSDLEKPTLGVASEES
AFPPSQDTSSAREALNVLQGYIQTSSGSQGVELIIGCSYSGINSCMSFIK
GFYASHVQCFISGWCAGHMYYTQCKDKHSSMWRPWPGQKYRNAHLDVKKY
RHRGFTFHRAKRGGPVTRSLRDSQSFLLDYGALYRSFIRPSHHALLDAWL
AERRDNMDGITWTEFDGRIFSVHDTFESCYRQSRMTFASHSVDLPLNRLR
RLSNLVALNLTEPDALRAESFRSSIGPPAVGQKWQLGALARTGKVFNNLR
DATPWSWAL
>Mb|QLI74612.1
METETSLTAPPNTPEKNPYEGVCQRKVDAQSRNLDPTSCQSFYLAHEIIE
ISSESDGNSDEATVPNSPRVIEEDINEGDQPSDNAARADAAEDEVNGNIE
KSKDGSIAYSNSLLVTECLPDGEHTRFSNTAEWPLSKYSIITRRLDSTAC
EECGSIGQKFGEGGHVGGSEGSSECSNGKLKRIYDRYESPPDEVPEHNPL
SKRRRTAKRL
>Mb|QLI74617.1
MVAVSLLVGVGKWVATFAVKAAWKSLSQHDAAHTTELLQSISAGQHEVAR
QIENLSIKVEILESMRRILYWSKRMDEVMDDFKKLNGGQINATDLAYAEL
VSALRDENLGIRYSTFSIYNTIMGLPGQDAGAIYVWHKQAISKLNDEKNL
YYHMSDYVKEMDNHLGPITYLLRQGLVLSLFTSHTETDAERLHKECQDQV
TTISNTLLKLYPPGLRFLKPGLEDSGNQDGSQWLKWQQADNSNHHLVMHN
FWIPVLSSDRKPIQTKKPEAWVFALQQNEQPLGAMQFLSAWENRGNKRLR
YSKMYQQGWNINFSTSKKSDTSAILFKLIPLEGERPEFRFVPYLEGSMGI
VSDKTRTFEKKFVPVVSK

>Mb|QLI74645.1

MSDKDSTLFIGDSDPTCPAGVTPQPDGYVSGQSYQQTYQKFSCSRIDMYL

GNILPEWPIFTPDEVLAMRQKYLAGQGSDNDRLSMLVIIDLADTMNPIHS

QDIQELQFGLRYRRTIQTHALLALYWKRRGFHGFANEHITKAFALGSSSG

VQWKNSSLDDHFADIVALWCCCFIISEYPIHTDMNPVPPSLYKYLPLPSK

DDIVRKFGTSAGAFFELRVAYISPRLTSIPRPQLECLPLELVEVPTPLVT

LKERYINTHGLSINPQQSRFTSLESYIKMIQEFEPANRRWAFPTERFCLY

QLTAIYSDTNQRSICILLVERTLKFLDPVPDCLTTMDINSIWVLWWKLWG

YPCLPHST

>Mb|QLI74648.1

MPVIGSYRGCDSCRKWKKKCDSTQPGQPCSRCRTRNIACVGVGQQRFKFI

NQSSSSSSSSSASSGGAAAAAAHSQKVVSRFVEHATPLATGSPKSPLRNG

NDRVADTLVYFLASTHQGYNLKPFRLSVFGELPRRIGYSPALDASLAAFT

SLLETRQSPSEQLNPRSLQLYASGLKALETSIANPSSRYRSDTLCAAYIL

SECHIWQGRNAKLSRGHAEGLVYLINNIACQDLKDLFLRGICYAITANLL

QDSVFNPNVKLNPWIQELYDMPEQPLRPLLDIQAMSRLPTCELRVFAMLP

DLIRHPSENKEQIHWIYSYILDQYLQVKVFISANRETSCWNAYDGIICNE

RHGLFIAIGITLNALLRAFNPSNILLVSQRSIFCADAIILAERAKQERPL

AAHHVPQAIVAAWCVTDDEGTKETLRQLIEDYRSTFAMAKLVQHLSCWPE

APAKLREIPWLTLPKKTEAAAAAAEAAAEADAQSVDTDDGTEIIDRETHE

FCCIL

>Mb|QLI74667.1

MDTWNCLQDVLRDRAESGSVLSFPLGDVSDPICISYKDLYFQARRNSGQI

RGIPRFKERHPIMLHLDNHADIITWFWSVLFANGIPVILGPSIRSNSSML

DLAVLLEYPICITTSDLTDMWDSGHGMYLHRVETPQKTASRAISSAEPNS

HGQYQGGTTTAILMLTSGSSGSPKAVMLSHRQILAAVAGKAAVRPLESPR

PFLNWIGLDHVASLVEIHIQALWLGVDQVHVHASDVVSAPTTFLSLLHRY

KVAKTFAPNFLLAKLLSALQDEQQDRNWDLTNLTMLASGGEDTDTSTCVN

LSSILQRYNAPANVITAGFGMTETCAGAIFNLDCPEYDVKRGRAVVSLGT

CMDGIEMRITNPGLDRSQVAAPDEPGDLEVRGSVLFSGYYRDARATAECF

TQDGWFRTGDQASIDAQGHLSLKGRVKDLVNINGAKFAMAPLQTATLDQAL

CHQAARLVAFPSRAAHTEQVTVAYIPRNWDMPAKDVVEIQHLAVQTCLLR
VGASPTVFSLRQESIPLLPVSSLGKISRTKMRTLYEAGAFSKDVDLHFSR
VEQFRKQRQKMDDTEANDAEKLLIRCLLDLKSIDRGAIGVNTSIFELGFT
SVDVIQLKIRIEEALGASVSVTSILRHPTPSALAKALCPESEKGRSLDTP
LNSSYDPVVAFNAAGKKTPLWLVHPGIGEVLVFVGLAQHIASDDRPVYAL
RARGLDPGQEQFVSIDEVVDAYLAALRQRQPQGPYVMAGYSYGTMIAFEM
AKKLEATDGHGTVQFLGCLNLPPHIKTRMRQLNWQNCLLHLAYFLSLITE
EQAEQIEDMSQDDALAYVFRLADADRIRELGLDKPGLTRWTNVAHSLPSI
AVNYEPDGQVDMLDIFYATPLKSVAPDRTIWVKEHLSKWQDFCRSKPRLH
EVDGSHYTMIGPDHVAAFSATFLSALHARGV
>Mb|QLI74668.1
MMTISQLLSSLFFFSAFLGWPLSIQARSIERAASGDHVEKEKILQQTFDS
PRPFFAIAHRVLMDYGVRDALNHSANALEIDMTAWSSQWYADHDGTLTSR
GDTAEHMFSAIAQERRAGKTAIFVWFDLKNPDYCDDRYPACNIEALRNLA
RDILQPAGVKVLYGFYSSQTSGRAYQVISQGLNSNEAIGIDGNVADANQV
FNSKGPASIKNRVYTKGLFDPAWNFGNCESSGNQICPQLREGAQSKNFGK
VFGWTIAENNGKQADQLMGVGVDGLIYGFVATHYYDHADTRAARKILADW
LSKNQDKAYLATLTDQPCHALSKSVKIRSQPKLYHTMVLCDTPAKFSARY
FAWDVIKAAVKFVSMT
>Mb|QLI74671.1
MYMALVAFSSSLVNAHGKPTTATGNAGGVSRALGLLPEGSVPDTGPNKLT
EVDTPVFGSRKIASKGLGRTAGGGKIKLADAAKSIQDAGGDPAKITPGGQ
LSFTWQTVTSDGGGPIRALCNTEGKDDFTQNSVELKIQDNVPGKGGNIKP
GPNASRKLPPGFQAKGQGKGGKNNARDEINDLFGRFIGKRAVNVNEKTNV
VATAPNDMTCNGVIGSNSQMCLCKIANNNGAGPFGGVMPVQMVGGNSTGE
ASAGNGATGGNAGGNSGDNAGGNSGGNSGGNSGDNAGGNSGDNAGGNSGD
NAGGNSGNNAGGNSGGNSGAANKF
>Mb|QLI74687.1
MSLPSQVPTAPETRPATPVSQVEAQDEGLWMKGMIKSDGKKEPSGSVLST
PLHRGHGISSNEPSVAAPSDVTKAPFHKISRPEIFEEAPAELASSIHELD
IGSGQIYKPHTDANPDLGDLIPGQSSGPYSNTSTSPLGIWDTSKDVPASS

LLEMWTEEDLLKFGIHEGMDVNHGLPPFDENVREYHTEKTASNKGKSDDG
IHDSSTPKPSASSVALGNRTESYGGINVTFGLPPWIETKFSETGETLEKN
SNQYHLGAPFVQVSGLYPTPPRKDTDCKALRSVSLSAVGTPTVAESRTLP
LAETKDTAATGDTDINSPCSRKIPSASERPESPFALRDVSVIPWTDCYPD
DPDKARELATSTGVPGLQIYSVDIFTHATSDTQVLDEVDATQDSDNFRVE
MGKQISPERPSVPGTKKPYDKFETKSSAETWDTDVDFDLNEGSRKMTEED
LRRLDLQSWWKEDLSDVTTGLPLISKEDLPGLENVCKESLFSVGGPAAED
SGENVAEKKTGIDRGHRRPPTANASSFLEMKVDPSIDVFGTTNRQESPGY
TLPSVSSDWQPPKATQESQHNDSVRASSVTDSEGADGSGWVKVEIETGNL
GQTDNV
>Mb|QLI74695.1
MSFGDSSFLPLRFNFSTTPCEGQLIAKFTTPAEVPNGEADIFWQCAGLAP
YCYQANITNGTSDPTMQLDREAQVGCINEVLRTTSVLVVKTMSTRTTTET
AVSVFTWTTTSFPRSQETSPTVTLPSQSWVTSGVATPSSTVMNKDPTGTD
TTAARAPTADSVRVEIPRSDMIGRTVRTTSPLPVTTDTVFDSGASKVTTP
LVSSAVTTFLTTTLLRTVTVSCTAGSATGKA
>Mb|QLI74702.1
MAVASQPSTINKGTSFQTQKAFTTLSAIGTGYGATNTAVGLLLVLGSTVP
MGGSPLFFWGFILLIVVAWATASSLAELASAMPHPGGQYIWVNQLAPPSI
RRGLSYSTAMISWLGAVATGASACLSASVGIFGVVSFLNPDFVYRRWMGF
VAFQILNLVTMFCSFFEQALPKISKALLFVSVASAAVVFVTLFATSRQHA
TPETFFTTISNTSGWQDGIAFLTGLSGINWSLCCLDLVTHLAEEIPSPST
NIPRALMWSIVVGFISGILVITSVFVNVPVVNADDDNSALILFYKISGSK
AVAVGIWIPILVAIVGALWSIQVWQSRLSWAISRERGFPLHRYLGRIAPE
PFSTPVWSLVWSAAFSAAFGCLYLASELAFNSLISTGLLLQYISYSTPVV
LLLVQGRSTFRHGPFWYPRLGLMANLIMLGWTVVAIVFYCFPYQIPAEAE
KMNYVSAVLIVVAFFICTIWFTFGRRNYQVNYFLRK
>Mb|QLI74705.1
MDLDCLESTDQAFQGDAGLSFTEFHDFGLWPMDFSTATVLGPDAESRLGR
NPEECLTPDLGAFLDVQHVQSVPNEPLGRMTDYATQLIAHWFHEVCPAWS
GFDSIKNMNRKLAEDLWHSSSAVFNSLQSMSASFLAARLPQMRPTAFRLL

KTATLCIQAEVEELKGKAHLDTAPIGVIYSLLCLGTTICWLDARRVGWPF
LQDAKSLLGRTMQQDRHLSEGDANILDFFSKSLVYWEMLISFIYDPEPGL
AQPNLVGASNFDSIPHPWTGISSFTSRLFTQSMSVCRTYRSRNSLSCIGG
TGQPPAREVGEAEELEKHLLQLQLSPSPLIEDTGDHRTPYFHLLHVAEAY
QLASLLQLYITFPDLVSSRVPREHHHSYEGNAMWYKWIVPLALRLTEVLE
RVPPDSGSLVIQPLLYVCAATGLRCDTVPKPGKYQDGVKESGRPSRPMEC
ILDYVSLLDRPAENHEREEYLVTQLALNVSQARNFIMRRISVLKDNLHPV
PVRVTEKLVKAVWTAYDEEPQGCVSVHWLDVMEQKDLRTLFG
>Mb|QLI74714.1
MEDERARSCIVFISEDQVEKGLVLAKRGQQQLEPVSEKKTLYYFTVSHND
HYSVCEILVSPIKVDHHKNVFFWPVQRYTKCCENASEDELKRLRQIIYDV
YVRPVQWSFGDDSNKAVPVSNSPTNGDCEEHPEVGRRYLQETATIITPAT
REETASRWLSQSPSQSSNATTETDLTDTDSTRPSQNKKRGSDNSLVSTIS
KKSRPNMKPGFECPFNPTTVITKLLRSLEDLKKATSHQKKVTGNVSNFHV
DSSTGFPLLTTNKEREEFANKLYKLDRNNHLTRMRAGDDERHDAADKIAE
SLREKLPALSGSTYIFHPPAVIAALWNEDYETLCKQLGADNLALQNPTVV
KLQYSTEPELYTYAVRLEHSCPLQHGAPDNGKIDFGNLADGKLFSPNTYQ
HIGCNSIARRQTQQRLDGVNCPVSVSGEPRAGSSSGRSSRSSSNGCPSAI
SPVTGSQEIDPLDIFRHIGNDTPATHTMERNGSDNFPDIEGLGINPFNVN
IFHEIDRLERNATDNFLDIEGLGINPFDVGIFQAS
>Mb|QLI74729.1
MKSPIALIAVMAGLAIAHPLAGSTDIRGYSDTANTIQEVRNKLMRCLERR
KPIGTKPNWSIVIASQYTSRDEILRALAECEGREQS
>Mb|QLI74741.1
MHSRLLSRPIRVVGCHAEGEVGDVIVSGVPDVPGRSMFEKMQNFQESTDY
LRHLLLNEPRGRASLNLNVLVPPCDPKADMGLIIMCNDSYAFMSGSNIIC
AITVLLETGIVSMIEPETVLTLDTAAGLVTATAQCQAGKCKSVAFDNVPS
FVAKLGFPVDVPGIGIVSVDVAWGGMWYGFVEAGALGLAVSNEHCRKLVA
LGDRVTKAIQSQFTPVHPEKPEMAGVCTVCITEPVARESGCLRAKNTVIV
PPSRLDRSPCGTATSARMAILYARGQLQVGEPSKHHSIIGTEFTGHIRYA
TKVGKFDAIVPNISGRGWITSYKEIVLDATDPYPEGFRVGDQWPVASEE

>Mb|QLI74742.1

MESSKNNMPSSLIIKAYDPSTQYQSIPVHSEAVSKVDDCARELEAAVKRL

LKRTEHVRKAEQGQITIQKKLQTLELENATALHRIHEMEEKYLKLEAITL

EKDEQIRQSWQALQTAAQSNSELEQGRNLAIAVVEETQQTAKAYETWAKG

EIERALQSSVAAHDHAKRAEEEMSSAVNRERESYHAREVAERRAKDAEQS

RHEAEKRAEDAEQARHEVEKRAEDAEQARHEAEKRAEDAEQARHEAEKRA

EDADRARLEAEKAKALAEKHKEELERNQVWMITSTRQAEARAEKAKADLA

LAIESEKEVRRQLDMAAPVRHAIEPAISLKRQKKGPSGPNKKPRLSTSAA

HNGTGLQSPKITWHDERLQYLTHDLDRVVDNWGAEEVRYARFTFSTDSVQ

VNGVEKNCEITESSHLVRCVRHSEEIVVTIFDVMSLARKIYLSNLDLKAA

EKLLRANVARAVSGRYAQKFKVGEETKRVTLFLWTNLADILNKVAEVYHA

KDAVTVSV

>Mb|QLI74757.1

MELYKTTSFLWYYLTILLAFCQVLEAQAPAGPKKERVPLPALPTDEPLPA

KDSLQIKLDRDLYAKGSMVFSSHLKFENPQTITASQMKRIAVDAYDEMRS

MAKKNGIPEGDMPRVMTTLIIDNELIFASSAKGPQTPYGKESQVQADLDA

CRGPGDNRHKHNGRCGEVMALHEYYQSHGVTKRLEKESGARIVAIAGTRN

AKGQLEKVIYPPCGHKDDWGCDRLVQQLDVLDDAKVDKDKDTPAFRYKNM

INNPKPELKPVDPTSLSKEKGKEKPKPGGSNDDEFEEPPKYDESTEKEGK

TRRPQQPNRNKGKTGTQDNKPSQPKEQDDGGDFEEPPKYEEPTSDDRKKG

KTSQKDRNKSQTNAQKKKTGNRPRHFQG

>Mb|QLI74768.1

MPLQERAIAYFLYHYDLESPSQNKIGDMGFVILSKSDKEWEHYRLAFEAC

AMASFINETGGQLEYKSLTIETYNKALMAMHSALQDPGVVCEDATLAAVL

LLALFECLNPTTGEQESWRNHVQGAIELARGRGRKQIDTRIGQMLFRATR

TLMVIYSLTTLEDEKEKELWWSGDDECTSTQKLCVGVASLSAKATVLLGP

SGHKEEVEVEVMLKRCQAHDRACKARWEELSKSTQGMSSDNTDPWLLMLL

NMLTCARILLNSIIMRCATWTYKVPNYQTSEEYDGACSALAEIILHSAKV

NEQQLCWAEEAQSSFAELDVQLQPGRDSSRCAINKWIEDNRSLWEEKPLV

GLQPLAVIWSLECLNDDQKIAIDTQLRRIVGCTVDQIRRSPSYTTEACTR

PSPSLSEPATDTPEAGSRDSSPASTGRFRYTQQDDDTLRELSNEGIPWRE

IRDRFPKRPDSSLKTRLWTLRNCQLTADEDRLLIRLKEEEHCSFRDIAPK

MRRHRALVSNRYNQLRRNRAMNN

>Mb|QLI74793.1

MANLNRITEELRRLEKEEELTEELLIERHCKAQLEIDEALNRLMRLRRMC

CHLRKKGLEIARCDFASLEEIEDTDQKKEEEKARKRELEQV

>Mb|QLI74795.1

MPSAGAVYNGSAPPKPDHAYTNHGIASGEQNLGNDRLTLFFERDPYSNSS

GPASREARVAAQLKAVSEAFQPTQ

>Mb|QLI74799.1

MQPNDLLHIRSSAAGLPGPYLQENMAPYEILDKIGEAKPRTILLIQGHTE

QGDSLKGAMRFPYRKFAIALQRQGSNLVVMCDMHKQPGNAIPRIIAGPVP

GNYCYHLVQQPPATMTDLAYRVYCDVFALFSDIVLISVADFGGLERVLSF

VCSWVLRRQLQKPKLRTHFVVATDKYCLKDIQFELLATMMADQWTQSVAS

VKRTISDYTELSVINESSASPGLVVKLFGLRNHRQAEGLHFTGSDTRILL

RAAIAHYTAKPMETFNLVAASRPSWPVPEELGHHIGEFLAACPPEPVDHY

PIIASALVMNAFHPGLHCE

>Mb|QLI74803.1

MAPITCKVVDANNRGRPGVYVVLECKDQLHRGIATLESVTDEDGGISLWF

PTPSPGRTDDVEPQIVDSSNIPRVSLTFFPHTVPSTCPGPFLSIHTDLYL

QGDECHGITLYLDPHPRLVHSPVPVASPLNRFAAAAVSTQEPQRDLSTPS

PLLLPPPVSQNSRPSPMLLNGMHCHNGSRGQKRKAEDYPQSPNKRR

>Mb|QLI74815.1

MIWLSVSALVWAALAFAPSSGAADGGGHRGNTYAGPNNYIIECHNGRDKD

VLIHRIKHRDGQVHHDFNSGIFYGVSASVPGLGGDEIRNMAGVKDVWPVQ

VFRHEAKENSLKPRAAPKRQPYRRAVDTSWNHAMTQVDMLHSEGFYGTNI

TIAIIDSGTGRQVNYTHPALGGCFGRGCRVARGANFVRNEGKYGDPMDQN

GHGTAVAGVLAGNDPRRNFVGVAPGATLAAYRVVDSKGYAREDDLIAGWL

KAVEDGAQIIASSAGFDGSGWAQCPMAAVVARIAASGIPCIVGNGNDSKK

GLFFSLDPSTGRNVLAVNSFAHRMVSSAGGSRAVTAGMSWLSASGPTWEL

DIKPNVGVPGDEIPCPQIDGSYDNCSGTSFAGPQVAGMAALIAERREDFD

PGHLMSLLMTTAAVQKDGHFIPVVHLAFNDTDHRAQSITIRVTNKARFEV

TYQLSILPAVTIYARRLPRDFKNPEYIQAPASIDMSKTFLKLVANQSDTI

TISAKDPKGLEADRLPVWSGWVAINSSDGKTLTVPYMGLAGSLHKQQVLE

SDGVSLQGFNHDMHNADSNRGAKFSYTVKKGFTTLSASIIINLILGSRVY

VEAVPLSPRKWMTDRLGKSRGFPIKGYSPRALQRYTGIGLYDKEWDGQIQ

SGDYLPPGDYQLVVRALRVFGDPTMEADWDAAEPLPFQVMSGAGQEACKA

YQSGKGPKDALFRNLQECHQVHNKTAVDAPWIPRPQDSSKCDDDNPTEED

CGTYHYCKAHQERLDDIISPFRNKYEFGV
