# Supplementary material for: Telomere length de novo assembly of all 7 chromosomes and mitogenome sequencing of the model entomopathogenic fungus, Metarhizium brunneum, by means of a novel assembly pipeline
Source: BMC Genomics. 2021 Jan 28;22:87. doi: 10.1186/s12864-021-07390-y (PMC7842015; doi:10.1186/s12864-021-07390-y)
Supplement: Supplementary file 7 — Additional file 7. Orthologous clusters formed between the entomopathogenic fungi. The 468 unique orthologous clusters that were formed between the two entomopathogenic Hypocreales fungi in the comparison test- Metarhizium brunneum and Cordyceps militaris. Proteins within these clusters may be involved with the entomopathogenic process. [file 12864_2021_7390_MOESM7_ESM.pdf]

>Mb|QLI63424.1

MACLQDQSTPETDVERSVSRIMSKNKHHESDKPDASAEDILALQDLDPGM

DKKMHLVNNTLDEIGWTNYHWKLFFLSGFGFSVDALQLSLQGIISVQAVL

EFQPSYPKGLTIALYTGMLVGALFWGLFADIIGRKTAFNTSLLICSVFTV

AAGAAPTWASLGFFVAAAAFGAGGNLILDTAVFLEHLPSSKQWTVSWLSA

WWGVGCTIAGLVAWGYMPAFSCAAASDCARGDNRGWRYLMYTMGGLILVL

SALRVTVIRLRETPKFLLGQGKDAEVVRVLADLAARYGRPCSLRVEQLEA

CGAVGFAHARSRFSLGEFAIHARALFATGKMAQTTVLLWLSWAIIGLAYP

LFNVFLPYYLASRGVQFGVAGTYQTWRNYALVQVCGIPGPLLGGHMANCR

VLGRRYTMVVGAVATMAFFFAYSQVASQEASVAYSCVISFTLEIYYGVLY

GYTAETLPSAHRATGNGIAVAFCRLTGAMSAVVATYANPATVAPVFICAA

LYGGLAVCAFLLPFEPYGKRAS

>Mb|QLI63442.1

MIGRTEKTASPTVLFISENDHYRKEARKVIKESGILKQHPGFKTAHISRD

PGWGGPLEQLGLCQETKGSIENPTGGPETPVFYEYSKRLHPTMTIYVRHG

SSTRVATANGIRLHGHLFYLAPSHVFFDRLDNSQTSQVDDDFEIDSESES

EYDDAAEASITSMGSQSTEALSDQDDLSDDELHSKTTLDSPQSSNIMASS

PGPRRLSKSSFAKEQIAHPPREITTPPWASLRQLGLLFDWCVDQDWALVK

VIEGTDPNISAFLSSFVFNPLDLSAARIAVLPKDGARVITHTASGGQMTG

SILGMPSYARLPRGKSFQEVFAVHLDGPLADGDCGSAIFGADTGELFGHI

VAGCRSTGFAYVMAARHVFPKLAKSALRIKDMERRHERCLISSDDDAAIE

WDDKDQEIFVVPPRSQHNTMVTPLTFGHKMAVPGTLVPRRVDRQDVQQPA

DHAERDGGSFELTQGLHLASQARAQSTWQERFRSSASDHSSVHPMYTAHP

AKYGPKPVGSQISKVYEHRRILLPCEFRKFSGCQKQFDLHDKDSWIDHII

DEHLHGRCPRVSICWFCDESVFRSGSNQKDEKLESFRQRMYHTATHFKEG

MTAADIRPDFYFLDHLRENNLIDESTFRSAKKLGELPGGYSTRPPGGNET

QSEVVVESSRSWCRRQSRDCQPGSSTKPNST

>Mb|QLI63449.1

MDSQEDWMSDERAAEEPEVDLLEPDECEPESVYVTLHRVRRLVLASIDDP

YTLDHFRQPSLNALIVRPLVDRLYETQNISIVYCLLANRVFFLKSQSGLA

TQSVNRARATLCELVATRVIRRFHEDNPGNEGLLLLAQILVEGFDPFDGA

PYEVEREGRYLQWPIQRRGGHERKLTALELAILSESKAFISSAACQRLVE

AVHMGKVVYTPLSFMDILPDHYKHRPIQLYDPRESRILNHRRLIVPRVRA

LIEMVQFMVLVLLYVMTMTNQQAASSQWELAFAVYTAGWVLQEFASVIEH

GWELHSQSLWSFLDVTFVAIYAAYVLGRAYDVLAGRLPNGYGLHILCVAA

PVLLTRIAFNVMPHNIVFISLHAMMRDFTLLTFLAVWCFLGFLLALLWLY

DAGQTDQTSTAAPPPAWPTVGKWLLWIWFGLDGTGIAESVRFNIVLGPAL

MIAFAFLGNTLFLTILVAILTNTFSHIVANEAAEIRFRRTVLTFEGVKSD

SLFAYPPPFNMLAVLAVLPLKFFVSPRVFHTINVAVIRVLNGPLLVAISI

FERRRLWAAENKRHRRRSPGLLFGWRFTGFSPHGDIQGVFTAPLPDDVSA

RIHLLDPVDDVPVLEDDVMSTLSGDVPRSRLRRPKLWAGRREYPRARPRR

RSPLRFQV

>Mb|QLI63487.1

MLAFATLILAATATLDTGGKLQTACANISSSYQSWVARGGTGTFAGIPGS

TAQGCLRSMPFHPQLALQFLKEYEKYLQFHSTIHTLKHAPPSYMSAHVDL

VAHLENIRQKVVNGLYSSQFDFDWDLGRSISKANDGHLTLSLCSQRIMHF

EHPPPLVSLSTDGLELPSIFTFQDAKLKLAGFPEVSPLVEINGVEAAYFL

EANYAIKLGYQDPDARYNHLFASPSANFSGKYSGGAWTSLLGLWPGASNR

LLFSNGTTVTVKTTASWPHSNGPMNYPDGKSLFEAACVPHPDSGSAFGSY

GGTYGISPQLEAPPSGASVFPDPIVGEQRDRARGYYLDGAGCEDVAVLQV

SDFRVGQNATRFASVVGRFVMQAAADGKRKLILDMSGNLGGDIAAGFNLF

RVLFPDKPIYPATRFRATELIDFMGRIFSETHKQENVTLDLPFVAPLAVG

IDGTTKMGSWGDVYGPHEIMGDQMSSMSGVFDFNLGSTSSDPISGYGDIP

LFPEVQLFPANNIILMTDGQCASTCSVVAHLLKEQGVRSIVFGGRPRHGL

MQAVGGVRGAQYWALDTISEYVERARHLALAALQTDSPILSRAEMQRFNE

LAPLPLRDLPLRLDTYEQSGINICNAYSPGDDSIPLQFMYEPADCRLFFT

AENYMDPATAWAAAAAAMFLNGSCVDATPAGRQGLSISPAKTMAESVSLH

SSRGGLRQKFGAERSPSGKALPLPRWAA

>Mb|QLI63543.1

MASKSPVASDITSPGSHNEDHATTAPAGTVVQSQGVTRMEAIYRSTKTNK

KTLWLVGGSVLVCAWAYSLDFATTNNYAVDVSSYYHQHSSVLSTLAIVTN

IISAVSKPFIAKISDITSRPYTYILALMFYVVGYIVAATSQSISAYVVGE

SFVALGSSGIDLVNDIIVADLTPLEWRGFLGALLSTPFIINTWFAGKIVQ

AMLSRNQWRWGYGMFAIIMPVAVGPAIATLIYLDRKAKKTGEVNLASSGE

AHRVEKEAADQAARGETMVDTVTMAKPEGSWLKKLRRGLIEIDAFGLLLL

GFGWTLLLLPFSLKTYARKSWANPSLISMWVVGGLLLIAYVIYEIKWAKM

PSAPRRLVFNKTFIVCVIIEASYFVSGNMRGLYWSSYVYVTKPWSYQNWV

YYNNSLTLALCVFSPLAGALQRWTHRYKTICIIGLCIKVIGMGIMLNGHK

ATVSTGALVMTQVLIGGGGSLAVVGTRVASQASVPHQDVALTISLLALWS

SIGRSIGSAIASVIWANQMPKQLRKYLPAKATDRDVQALFNDIKKLRTAY

AFDTDMKKGAIEAYQNALYYLLAPALGLAFVPLIAAFFMSNFYLGKQQNA

VTNVGVDGLPLDEQDRNPEPSHKPLKEKIKGLWKA

>Mb|QLI63575.1

MSESKSCLEFFSFGVEFCATCSSSQLSEEAFNERLENHYINPRWVLLPGT

RCHDTYTHVPDTNGFSLTYTDLSAPYEAGFKLGVEMYSPPYCDLTPKQAA

KKASEAIRDVERAANIRIMSHEGYGMTIHVAVKGGIRLMVARKAVTLVLL

LEDQLLSRLCAPSRRDPKNYHPIGSKSVIANLQLPDDPSALQRYSDAMLA

YLPWAGERLEKAPWNDHRTERFQNILRVLWDAQDITNIRDIVRYLWTEDR

GRCAFTFHICDRVPVDRRQVTERVSAPDYIRGLPSMFEFRYPESTFDQDF

ISNWIELSNRIIQIACCSPPEFRGITESIWKTLEEQAALHRPAWPALLEC

LGLGTQIDYWKEKLDSYGHHH

>Mb|QLI63582.1

MPLTAHGFSAAEIHSKIQLLINGLVNIKDDTGEFLMTLADGRVIDTKGWN

DWEWTHGIGLYGIWKYYQATGDARHLQIIEDWFRDRFAAGGTTKNINTMA

ALLTLAYVYDQTRNPTYLPWLDNWGEWAYRDLGRTKHGGMQHTTYLENND

QQLWDDTLMMTVLPLAKIGLVLDRPHYVAEAKRQFLLHIQYLFDAKTGLF

FHGWTFRDGGHHFASARWARGNSWLTIGIPEFIELLDLGRSDGPLCSHLQ

ATLAAQCEALKTLQSPNGLWRTLLDVSEEDGSYGESSATAGFAFGLLKGQ

RKRYIQGEYQDVAFRAVRGVLENISPDGQLQNTSFGTGMGSDLEFYKKIP

ITPMPYGQAMAILALVELLNIFI

>Mb|QLI63677.1

MELANLPTELLLHIVKALDRDRDIYALCRTSRGFYNLTIDYLYRHNATAP

NVERSAILWAAKHGRVLTAEKALLAGVDVNTPQAPSSTLPLVEAVRFGNA

IMVWLLLAKGADPNVLHGKAGTVLHTAAAQWDMGTATALLEHGADVHAQR
LDNGETPLHTAINSGAIMTGGATEMLSLLLDRGADVADADFVGATPLHCA
AACGNIAAIHALLDAGAGAILSSRGAHRETPLHAAVRVRNEAAARVLMRR
GADVQAPDARGKTVFWHAIKFGCPAILEMLLDKGGASPLHPNGNPALVQA
ARKGHAAVVKVLLAHGGGSDIKADPGLGESAMLAAVAKGHADVVQVLLDA
GVYICTKDSVGNTLLVQAAFRGHDDVVDVLMRNGVALASSREWPARAGRP
S
>Mb|QLI63682.1
MESGKRSSFDEISMTASAIRRFTRTPTSHRLSLGRSTTASISSSSIRSFE
SEKQPPCVYETTRDTSSDECALHRFIVWAAVKRNVCPKMTAEERRECPLL
RCRKRFPNHELLLQHLYDCKHLESGEYWCYDCGQAETFHEINKCRRCLGH
PSKRKKLLSLAKSVFSSLGRPKSPIPTPSPPNLDIEDDPPSYDGLVHEGR
PTEAHRGFELQSNEIHEIDSSEVVLAPIPEDGEASQQDYLSRSSSTSSAY
TACTQPLSVYSRNSMTGDKSHVSYEEYINWYSSLPIQTVSPADLAKPNTV
NTFAKPALQLNTRAALDFSRVRLRRRSRNLQPSSSVRSTSSTTSTNSTNS
TSSMASCTISPMSAWSGAWGNGIGFESALTSPADEVPNPDDVFPCSPPLP
THPEVDDVNMTFSHFVDGPTGLIGLTELPADIPMVLDLVDANHQPSAPST
VPLVPSPAEGLHPESLQNESQQLQHLLADLKSHAQKQASPHELLRSARDT
LNLHVSGSVKKLQPLNHDGKNHVASRFCSIPPNSVSLTGLETMSDIFQGQ
QVQSSAKMLCFLHVVYSLSLVIHEQDAANWLTKLFNQAVSYCSWMTDEDK
FAYLQVVEFLWKPGDVTDEDLVHIRQRCLPEPPSTPTARKGKGLATPIDP
NNDPLLFISQYFLDELEYAALRNTTKPEIQTSNLCTKHLKDTNLATIQDS
PFVDAAKSLVLAFSQQYHNAGGFVSGMKELLDRIESNHVSTVRRLELELL
QTGKMQLPPDLYFDVFVKQIRTRIDSLQIQNGLHYTPRLQYHKLGIELMA
SIMHGSDVPVLGIDLVESQPDISSHGISELEMQMDFAFDGLVSFQPGLPI
DYESLGSIHAADHTPVNEQEWLAASNTPSTLIPAPSQARTPSTPSRSGEA
ALPKKPNTDMCCEICGYRPKGNPQWFHGSMAKHKKLMHANTPPTIYPCPF
PGCNSQYKNRPDNLRQHQIEKGHFVDGQEESTKSRKRRKME
>Mb|QLI63685.1
MKLALFSAKPYDKKYFDAARAARQQTNIDVTYHEASLNDGTVALAEGADA
ICVFVNDSVGSSVIDQLASHGIKAILLRCAGFNNVDLAAAEKHGIAVANV

PSYSPEAVAEFAVALIQTLNRNTHRAYNRVREGNFALDGLLGHTLHGKTV
GIVGTGKIGIATARIMKGFGCKVIAYDPFPSPEFKEIGEYKALDVLLPEC
DIISLHCPLTDSTRHIINEESLGKMKDGVVLVNTSRGGLIDTKSVIKALK
NKRLGGLALDVYEGEGSLFYNDHSGEIIHDDVLMRLTTFHNVLVCGHQAF
FTVEALTEIADCTFRNIEEFVKDGTCKNLLTKRSVLNRSPSLPVRNV
>Mb|QLI63692.1
MAAIGSLVFCTDCGNLLPATQGSEKNSLQCECCDAWNKDTGSKVIVTQSK
PSDFPSFLRQKLQSNVQAVERHKLKTSSTVQERCPKCGREEVTYTNVQLR
SADEGSTLIYNCECGHSWHENN
>Mb|QLI63710.1
MQLPFTTTAVLLMACNALAGVVQPISVPAVHIRNGTVVGNYIESHNQDAF
LGIPFAQPPIGDLRFNAPQSISEGWKSPLNATAYGAHCINYLLGLPLDPA
DLATRYPQSEDCLTINVVRPAGTKPNARLPVLTYIYGGGFQEGGSADARY
NTTALVDKSVQIGQPSIVVTMNYRLQGWGFLAGDEARKQGLLNLGIQDQR
LALRWIQENIEAFGGDHRRVTIQGESAGALSVGFHLLANGGRDDGLFNAA
ICQSGGPYNALSFPSDAQSQKTYESVLKAINCTDASDTLKCLRAAPFDTL
NTAFASLSFLPVIDGTLVPEYASTALASGRFVKVPLLIGANTDEGKVFAG
MGVNTTEEFAGFIEKYPYVHTTTNATIRDLLEAYPEPGTNSTHGQSDDTL
PVSAPYGAQFLRAARYTGDVMFIAGRRYTCETWAHYGVPCYSYRFNTIPG
ATDPLYLGATHFEEVAFVFDNVLGLGMPSNAFDVEPAERKQSYKQLGDTM
SRMWMSFSATHSPNNHRVKSMRTLWPAYNLKNPQNMVFDGNITSFVEKDD
WRIDALKLIIERSSDFSR
>Mb|QLI63785.1
MFSQSGKQHQPLPESRQQLQSSTLQRTKHAFLYAVAGKGWLRSAVDRAIL
LCTSHFDEEKGAVLATDAADDGETLPDVEIMVVPVGSLPELYPGKSMFTL
QTCLIQPESTGTIEITSPDPGVDARIQLNILSDPSDWQVARKACRFALAL
AEHFIYQSGYPSEARVFAGPGSVGAREWKEGDWRVVSDQEIDTYIGMYVG
CGFHLTSSCPMGREEDEGVVDSQLRVHGFKNLRIADASVLPCVPPGHLMA
PTYMISERCADFIKHGRN
>Mb|QLI63795.1
MSLPSDFLWGFATAAYQIEGAVEQDGRAPTIWDTFCNRPGKIADGSSGTT

ACDSYNRTADDIALLKSLGATAYRFSLSWTRIIPLGGRNDPVNQPGLDHY

VRFVHDLLAAGITPFVTLLHWDVPDELDKRYGGLLDKTEFGLDFEHYARV

CFRAMPEVKHWATFNEPWCSAVLGYNVGQFAPGRTSDRAKSSEGDGTTEP

WIAAHTLLVAHGRAVKSYRDEFKAAAQGEIGIVLNGDAVFPWDPADPRDV

EACERKIEFAISWFADPIYKGDYPESMKRQLGSRLPAFTPEEAALVHGSN

DFYGMNHYTANYVKNRPGTPDPEDVAGHLDVLFYNKRGFCIGEETQSPWL

RPCAAGFRDLMVWISKRYNYPKIYITENGTSVKGENDLPMEKILDDDFRV

RYYNDYVRAMATTVSLDGVDVRGYFAWSLMDNFEWAEGYETRFGVCYVDY

DNGQKRFPKKSARFLGPLFDKLIRKEGQ

>Mb|QLI63817.1

MASNPEKQRGIHGSWIENAEWWDTTMGLDGNKYWQELQKPSLERLVPVQP

GGRALDLATGNGLVARWLAGKGACVTASDGSEEMIKHAARRSSPDEADRI

SYRVLDVTLPEAFEDLVKSESAEGGFDIVTCNMALMDISDLEPLADALPK

LLKRGGIFFATLLHPVFFTSGATRFVEVVTDEATGEYCNARGKIVREYRD

KAPWRGVAVNGQPAFQLYFHRPLDVLLGTFFKTGLVMDSLEELYFDEADA

IKERPESSANYTQIPAIMALRFRKLQ

>Mb|QLI63841.1

MATSIHTMAGDGTLTESVLKKFLAEGGRVDDQDDNGFTPLSYAARNGRLS

VVKLLIKTGGASANARNRNGSIPLYEALSGGTNRHNIIKELIPKTSPAEI

EGLSTGKNCLIRAIKQQDADAVKLLIQHGASLEAKDATGKTARQYATESD

SLEIQRAVLAPGEKKWVPELVNMLVSLVLFVLAYVDSGSLKGVARGTVSK

LYHIGSLQPDPDIAKEIQHPQTVDEFLFDLGDYVKSSNLGQFFPDDDNYL

TQVAERAIELKNDPRNKTGKPGDLRALTRLALYQPVFYCDDSGSMFGANS

ATDKGIRMDAQRALVKRMASIATRLVPDGTGAHLRFINSNNGGDNLTQQQ

QFDQKMPTEGQGGTNIGGSLHDKVLDPLIYEKLKSKTKLSRPFLIITVTD

GCPDPENVDVFKNSIITCLKWLDSYQYPPESVKFLVSQVGDDKESTKFLD

SLAGDAAVDQVLHRTAETLDSKYAALRDNEAGLEEWLIKTLMYPIMGDND

AQLGIRIE

>Mb|QLI63939.1

MRLLNTGTLTLEEFAEGQTPQYAILSHRWGEGELSLQDVEKGELLSQGVK

KGGYDKVRRFAFRAQCDGFEYAWIDTCCIDKTSSAELSEAINSMYLWYYR

AERCYAYLADVWLVDDMCESEWFQRGWTLQELLAPAEVHFFGKNWDDLGT

KGTRRQEISQCTGIPVGILTGHDDLETASIAQRMSWAAERKTTRVEDTAY

CLMGIFRIHMPLLYGEGQRAFTRLQEEIIRTTDDHSLFAWAHGDMSGGLL

AASPAAFKSSKNIITLRPSDAPSTVSSMGVHLELRFVGIGPRGLGLAVLN

CAEGNGKDGPIAIYVRDTSLTMDRFERVHSEKLEQLDMRNLRPSQISTRR

LCIQKEHVLATRQPNSVERSNGTAEYAIYDDETLSKLMSFGEPTALHDAA

KAGLEENVWLLLTRADATIDLEDKTGRTALMHAAEHGHATVVLMLLKNGA

DIEAKCRGWTALWWAANEGREAVVTLLLERGANIDAKDYDGETPLLWAAK

RGHEAVATLLLQKGANIEAADYDGETPLSWAAKAGQEAVVKLLLARGANI

EAEDYNSQTPLSRAAEGGHEAVVKLLLEKGADVDAEDCEGQTPLSRAVKR

GYRGVIELLRTKVDAIDTCRVGNGYIGA

>Mb|QLI63979.1

MAVGAFVEPLVVVALLFGGAWFNRNKEYDFREGRAAWESIDGTCKRSDEF

CQPSSSQESLLSADDSWTKHPSPSINDVPTLRRRKIQLFGYNKTVASPNT

LVFKHRFLSRVLQKFPFLVEAWYWALIYWVYQLGRAFTAVTLVEDTVNVA

RRHALQVIHLEQRLHVFWEVPIQKWFMQHPALVHWINRVYSFIHIPGTIF

FLVALYYITTTRKNRAMSHNVTAGPALYEARRRTMAMCNLLAFVVFTLWP

CMPPRLLSDPGFDGDGAAEAKGFGFVDTVHSSDGQSSVWTTNRFCNQYAA

MPSLHFGYSLLIGLTIATLPVSGVRATSWKRLSIIGLGMAYPALILTAIV

ATANHFILDAVAGAVVCGIAWKTNGLLLNLCVIEDYFLWMVRIHKPVNYT

DPETAVEAQFHSKIWGEA

>Mb|QLI63990.1

MPPARTDIESPNHDEKSVLRRNSSHGDNSANFTNERKPSTAALLRNPLIG

MTREQLIADADAFVDEKGLGEYREAFRKGALIAQVMNTSGGFEHVHLLNE

EEKTVLRREITNKWSQPFMLYFLCTLCAGSAIVQGMDQTAVNGAQEFYYE

EFGLRDPLLQGLMNGAPYLCSALIGCWTNPILNKIGGRRFTIFISCFMSV

VTGFWMAVADSFGNLLAARFVLGFAVGAKSSTTPVYSAESTPKTIRGALT

MMWQMWTAFGICLGFVVSVAFQHTDFLGRNSQWRWMLGSTSIPPLIVMLQ

VYFCPESPRWYMEKGKYDKALKSLLRLRHHPVQATRDMYYAYKLLEIEKK

EREGRNLLKEFFTVRRNRRAAQSAWFTMFMQQFCGVNVIAYYSTSIFQNA

GYDRSQALLASMGGGLINWIFAIPAIYTIDTFGRRNLLLTTFPLMALCLL

FTGFSFYIPGRAQLACVTTGLYLFMVVYSPGEGPVPFTYSAEAFPLHIRD

IGMSSSTAVTWGFNFIISFSWPSLVEAYGNTGAFCWYAAWNLLGWVFAYF

FLPETKNLTLEELDSVFSMKNREHGGYYLRKLPWYLNKHVLGRDVAPFPP

LYQFAEDQSLPNDKTESVSVTQHKEAPSGPAPTEGTVVTGK

>Mb|QLI64024.1

MSSYDDKPPNYSPGPVSAVSPNSPDPCYDHDANAPEVVELMSPLDANSVQ

GLHYGQQRFQDHTSAHYTHQNTNSEDNGGNATDKEVVPAEYATCQNLESA

IAPAGNTPSVSPHQPWQAQKAADAPLPPLPNKGGRILGLKRGVFFILLGL

LLAVVVAAVVGGVAGGVISSKSKSSSDANTASTPVPTPSTPTASSTASSA

SASATPSATFLYNETSFDQGFAFQGFSRNKFSGAYTAIVRDKQGGIDFDF

DIHSYDWVAKITNCCVSFCNNATREGWMGYTCDSRHREAAEESFARAFIW

CSDNHTTPYARGKCV

>Mb|QLI64035.1

MRPTIPVAYLLPFLCVTLFVTQLGLSLSDLPSLKLMQDIACKKHFGLVTQ

ELLPEEKCRDAAVQRILNRINIGISVSVTIGSALVAFPLGILADRVGRVP

VLAASLLSLFLSQGYAMLVCWRWEAMPLEAMWAMGVFFLLGGGQRMAEAI

VFTMVADVAPRSQRASWFQWVVGAVLAAELAGPFLSARLIESSIWLLLYV

SLALVAVGGILLALLSPETLCRKTDGVKEDLDGGSPVTTKRTIFAIFSRP

AVFLVPGAVLSLPMAATLSGILFRFMPVQFHWQLSKSALLVSLRSMITLV

TLLLLLPGAAYICNKKTTYSHRYRDGIFLRASALCFLAGSAFFVLVLSQG

FIIAGVVLSALGSGIPTLCRSMMVSALGEKSTGLLFGILALGEIIGFLCC

TVAMGALFDVALTSWIGYPFVLGVVLAGAIFITSWMAGTSKEKATPEKAD

VVLEPLKN

>Mb|QLI64037.1

MSRALLITGATGKQGGSVITSLLAKNADFQILAVTRDTTSPGAKKLAAAS

PKVTLVQGNLDDTEAIFKNAKKATEAPIWGVFSVQLAPMNKSGPSIEEAQ

GKSLVDSAIKHDVKHFVYTSVDRHGAKSIDNPTDVPHFKSKHNIEHHLID

KAQNTTMSWTILRPVAFMDNFAPGFIGKLFATAWRDALKSRPLQLIATED

IGAFAAMSFLEPDRFAGKSISLAGDELSFAQMEKVFREKVGTTPPTTFGF

IAWLVLRLSVELRTMFNFFEKEGYGADIAAVREMNPDVKDFGRWLEGSAF

VKSKRD

>Mb|QLI64053.1

MDNFLPNSRRTVRTLDFADFLHGDANKQSKFCRELIACLSTVGFVKLVNH

GLGDEELYEVFEWNKRFFSLPLAAKTKAAHPYGPNPHRGYSYIGQEKLSK

VKDYEKGTRNAAEVYDVKESFDQGPAHDELYPNRWPDEGDLPNFRVFMER

LYDRCHQIHQEILQALALGLGLPGFFRDICDQNTSEVRLNHYPGCEAAV

LHKGAKRISEHTDFGTVTLLFQDSVGGLEIEDQHVPGDYFPIPFGSRSEM

IVNIGDCLQRWSNNKFRATSHRVVLPPGLSDGWIEDRYSVAYFGKPNRSQ

AVGALPELLPEGVKSKYSNITAWEYNQEKLTLTY

>Mb|QLI64080.1

MSFLTWRRTTGSVEEIPGDTLTSLPWYRWWLYQQILSQPQRFLPLWHKVR

DLMLAEESVTIDGITDTLIENGTISVTDNYGYQSTRQLIFAILGWQTMLY

KPDLLSYVHGEFNIFDETDGFRGEARVCLVQPEHSGKQDLPSFLLGFGMM

LPPRQYCAFDDADEKKLFHRTKRITPKDLNADVLTKVCGIRLQWVDSLSC

HLELDRLSGTLFLYRYPSFCVSILQQRNCVSALQLRNMQEQAIDVIHRCG

SQSPGPTPWASERDIAELLQEILLSYRLLFGQSRRSRNLFRQLRPFQGIP

NEGHDKFLSSICGMKKFKCPIKLIEREEYDLSGDFSHFRSRMVQLNSYTS

SKKSRSILQLWRDKRDSTAWIAFWSVLIFGSVSILLGVVQAVFQILQFVQ

GSR

>Mb|QLI64106.1

MAMGEKLISTVLRADRQDILRYAPFVGIALLSILVINYFRNPLRSVPGPF

WARFSNLWLVYHTRQGRMHRKMIELHGKYGPLVRLGPHEISTADIDSLRT

IYGPGNDFRKSDWYMPWQGNRKWDLFAERNESIHRAQRRLVSHIYSLSNM

KKLEPYVDSAIVFLLKKLASMQGQEIDVGRWTRLFAYDVIGEVTFSNRFG

FMDKGIDDGAFTMIDNILQCANWVGHIPWFYYLSIWLAPIIGNHLAITQR

QGKLLHMSQQNIRERKKRGTDRNDMLEQLFEVQREKPDKLDDICVASMAA

SNIFAGSDSTSVSISAFLYHTLRNPEVKKKLMDEIDEHAAKNNIPRGAVF

DLEIINNMPFLQACMYEALRCHPAVGVSLGRVVPPSGLQIGEQFIPGGSG

ISANAWVIHQNEDIFGQDAHNFRPERWMEEPERVATMRRNFLSFGAGSRY

CIGRNVGWLEMSKLMPTLFHDYEVEFCDPSKDTREVAW

>Mb|QLI64153.1

MAARDNYILSRDLDASLRLDCQHLLARMYTGYTLHPQIPVVPSMKIAEIG

TGTGIWLLDLASQLPSTVVLDGFDISDGQFPHESNLPSNVKLSIMDSFDQ

VPPELVGKYDVVHLRFWVCIVRGNNVEKLINHAKALLKPGGWIQWEEANL

GRILTNGDEAKKFLQAAKLVLKSLEFDFGWIEALPNTLEGHGLEVVDFKT

GRIPPSLVPLIIKTGLAGWVEILDAAYKTQCQSLPPEKETKDILLRVVEA

LKDGAAYYWTPLSLLARKTAGTALEA

>Mb|QLI64160.1

MDSVLVIGAGELGLCVLQALAAHPKRHHVKVSVLMRQATLDSAAPAKKRT

VQRIKNLNVHFESADVVLAGVQELAGIFTNYHTVVSCSGMELPGGTQTKL

AEAALRARVRRYLPWQYGMRYDVIGEGSSQDLFDEQLLVRRMLRAQSETQ

WVIVSTGLFMSFLFAEDFSVVDLRRGVARALGSWDNRITVTAAPDIGRVA

AEVVLDPRGVGNEVVLTAGDTVSYGELADLLDEHFGTRFARELWDADTLR

GQMAEDPSAVVKYRDVFARGRGVSWDKDRTLNFERGMELLDVKGYLDSVN

FKVGEAWPSN

>Mb|QLI64202.1

MLPTDAASKTTMPSSKGIRLHDEDEKAKKANSRGKCLSAVAAFPGLIVAS

MGQFFAACCSCTRPTDDEAQDDCETLLDGKIIPFPRHAASSFLRTGTPRR

MREANHVL

>Mb|QLI64218.1

MQIKPALVALLSASLIDFGAAAYCNTSQLIHMDVIGTCDCSPEPKGCSTP

CPGCGVTPTKNIRNCNGGCEDSESDCAACGIWFHTLCNCLQHPLNCQNSG

TIQKYGAPVWVLLETPPGDHNLVTTTQFLPGIRQMNAGHDEAWLFAQQKF

DKTSEALAMNPVVVRTMEQVHIHVCPRNTTTASMLGKITAFSSSKLVQLA

DDKEMYCLGIDHSVDVKGFAGLVADFINSPPPNVCKDMVGAAIIEDDKSR

RWACATTNRQGPLGKVCFH

>Mb|QLI64247.1

MHHEGDEYDNNTTDPFRDDNDDMDDDDDLDVDVVQDGGRGSWWRGMVGRR

SSADDGESDEADEDDDEFGDFAMAEDDKSGGTSNKEQLVLRPLAVNPAKE

SSRGLSGLWPFGSRSESNKAKRDEEGHGLKDEVVVSADEKKGEDYRAIEV

REATSRTSIEEPDEEEIVVGK

>Mb|QLI64315.1

MAPDSAHDGASTAPGDPIQDLPRELVDQMTSMFGYHAGYRTTHAKGLLVE

GEFKPSNEAKSLSTAAHFNQPSTAIIGRFSVGGGIPNIPDADNQATPKGL
AIRFLIDGWTYTDLIAHSFNGFAANNGQDFLTFLKLFFAEGVAKKLLDDA
VKQGGSYSKEKADFEQAVKVFKTWLGTHPSAFRFVDSPKPNPFNYGTITY
YQPNTHVLTNANGDSVNVRYRLDPADGVHLFPENGAPKDPNYLEDDLLHR
FPTTPIVFNLQANVAGPDDILDDATIPYKSTKWVPVGTIEINKVAEDNAN
KQQKIAFSPVPESGGVHGIKSSNDPLIQTRKGVYHISSKQRQEAKREPGG
VAETWP
>Mb|QLI64330.1
MYTSLPPALFLALSAAAAAATIRVDVGEGGAFTFSPDTIKASTGDTLDFH
FYPLNHSVVMGDFSSPCAPAKTGGFFSGFMPVSSGEATDSFQVNVNSTDP
IFFYCAQTVLEHCKNGMSGVVNPSSSQTLSAYKNAAKSVSTASHPANSFG
GTLVSSGQTSTSASASASPTSSGGGTYGGGAGSGVRAPAEAVVAFAVGMA
AFLVG
>Mb|QLI64348.1
MLSSSLRRCKEDVGEGKEKRRVQLTHVSDTGSAHMVSISEKPVTSRIATA
ACTVRFSNKTAVSLIGENQMKKGDVLGVARIAGIMAAKRTADLIPLCHPI
PISHVTIDLGPGEEGDTVEIRATVACDGKTGVEMEAMTAASTAALTVYDM
CKAVDKGMVIDGVRVVLKDGGKSGRWEMD
>Mb|QLI64436.1
MSSPTKVTFPSFTLSISGELYAPAAGSPDRKGAAVVVSHPMTGVKEQTAA
DYARALSSAGFYALTFDAGYQGESTGEPRGLEDPRQRVEDNKAAVTYLTR
LHGKVDPERIGVLGICASGGYSSHAAQSDARIKALATVSAACVGRMTRNG
GLYEANDKESPEAIAGALKAAGDWRTAHANDPKAQAPRMFETEASAVPEE
APSFFKAAAAYYGSRRGHHVRSDQRVPPSSYDLMIGYDSFQLPHLIAPRP
LLMIAGAEAETLHFSRGAVEAAKEPKELFVIRGKNHFDLYDDLAESAPKL
IAFYAKALAH
>Mb|QLI64468.1
MAQTLETRDIQKLIDQVTRDGYVVIPHAFSASQVSQAKAEVARLSGTAEA
GPAGQAGRNAFEGLRTQRIYALLNKARCFDQFALHPAVLALNDHFLDDGY
LLNALHSVNIGPGEAAQRLHHDDQYVTVPRPHRPFGAAIMVALDDYTPTN
GGTNVVPRSHTWAGDRVPARSEAVAVAMPAGSIVYFVGTLWHGGGRNASG

AGRLALTVQYCQPWMRPLENQLLAVDWDKLDDLPPRLVGMMGYKVGAPFI
GYVDGRSPRTRVTELLGRWRARSKL
>Mb|QLI64523.1
MASGHYGSVLVTTLEEFKKNLSKRDIEEFKRVTKQEFDASIGNLQARQHS
QRRLQNMSRLKRFLETMEQFWNVAQVLCDHGEIIAFVWGPTRRLLQISSS
VAEAFGQVLDTYERMGDNLPLLGHYRDFFLTEPHMVQILQQMYLDILDFH
RIALQYFQQPQWERLFNTTWKTCKCRLSDKISKIARHRSLIESRATPSQV
EESQRVGQREDCQRDQEMDFEQESRFRAVCSWLNATSIETDQYYHSKIRS
EYPGSGRWLLDNATFKEWFDPQFPTIPPLLWLNGIPGAGKTILASLVVEE
AQKLPTKPTVLFFYCKDSNSERDNFLALGRSLLAQLLKSDKGLQPYFYQE
CCGSTGAVLTSPGVVENLLILAFKNCPSAYIIIDGLDECVREQRKHITQW
FRKLVEDLPSDNPDRIRCLFVSQDDRHARKDFTGLASIKMSPEDNKGDID
GYSQIQAVEVQRKFQLSDQKASEVSGIVNNSARGMFLLAKLIWLNLLGQT
SVSGLEEELEERIFPREVNDAYRRIMHRIKTQASRAELRDALMLLGWLVC
AKRPLRWHEVQGLKSINLDKLSVEYERQRFVVAPKDLCESLVEVRVDGTL
EFVHQTVKFFLIEDKHVDQPVEELKMACLCIDYLNLPAFIQPPTQDAVLN
GDYVFMDYAVLYWIRHLEAGVVHADAHQQLMNQLAESLEVFIHHHWSSPT
AKFAVSNGNRDRLQFFKGLPFYDKLEKTIISTRKQLSFFGQMKQGEIALN
LANIVVKVRAVMESILSSTIGVESPVREGLQEKYGSNLFKCYRFSCQSFT
AGFSAAHERDKHIGRHDRPFRCTDEACTGFLLGFISEKERDKHMKTTHAA
ADIQDGKFPTDEDVRNSVAEAHEVERESLESESSESSEFSESSEPQGALV
PRQSPKPKRPRQTSFKCPHCPATYNRRYNLQSHLYTHTNERPYEQELEGQ
GQISNT
>Mb|QLI64538.1
MDQASSPRSNNDKTPADEATHTNIDEPKFEARVRPETPQDCAIGDSDEDD
EELVEIQRTMTQSRIMRQKSPEELRKKVIPFHWAPMLSPLSPADIDACET
LENATMPDQLRHSNREQIEYRLRKCGSLCVGLFNTYRPRDAKDWWIQTMP
HARPVETGRQDGSKRVMFAHIIATLGKHPVVTDDDEQCPPNWRDSAASQS
STLGHQTSGRTICIHSFSVCPEVQGIGIGKAAMKAYIQMMNESGVADRIA
LVCNESLTGFFTRVGFQKAGKSQLSPAGPGLYNMVLELPGPKPLFRLLQD
LKPR

>Mb|QLI64541.1

MDTTLQSGTKRIPIQPAAGLAEALAPTEDWTGAASSAKRRKLQNRLNQRA

RRRRMLLPCPLPSTSAVGPRSLPTTDDGAHHTSDNGKPSSAPTCLLGKAE

TRQLLLQVAQRSYGRYKIGSPSLADLNGVVRFNVFQAFGLIAQALGFNND

WLTYEATSPFCTPGEYAPSLHAPPTRRPYFMNPTLLQLNVEHHPWIDFFP

CPRLRDNLLAALVPGREMLDEDHLCRDVVDGGAGAGVDSAALIVWGTPWE

PGGWEVTESFVEKWGWLLAGCVELKEATNYWRGRRGLEGIVFNV

>Mb|QLI64598.1

MCWALTSAAAALGASTVVAANPNNLGRDSGLKSTSYHDGAGVLKYVNPLI

GTRGIIPDSNGGMIPSISTPFGMTRWTPQTRENYISQVPYSDLDRRVHGF

QATHQPAIWMGEQGQMTITPGLLVSGDSIRTQFQKRGLEFRKSDEKSTPY

VYEVLLDADSIGDFNWNLTEQWAHEEYGDGCPPCPGGAGTVPESVEEGSN

GRVRKRGYTDRFDAFEERDLDESSSNSSDANLGGYEHSIKAAMSATSHVG

HIRFDFQTNSGSKSQIRPYVVVQATRLNWTGHVEIDPQRREISGSNTQRQ

DYAIGPDHPKNFSAFFVSRFSAPFESYGTSQGGKTERGHGSLHGKDVGAY

ATFDTAHDRIEVRTGVSFISVEQARKNLDIEIPDSHSFESTVEQTKKAWL

DQLGRITIEGVNKTDAEHDQRAIFYTGLFHSLQYPNDYSEPTGSEGVSKR

RFYSGYTDSVHEEHDSYYQSWSIWDTYRAEHALLTLFAPERVNSMMRSLL

RIFDWSGRLPIWANMVETNIMIATNADAVIANALVRGFNDFDIQKAWKAV

YADAYIPPERDTLLLYFDREPKTPHEARAGLTSYLEHGWVDNDRWAEAAS

RTLDYSLNDFAAAVVATYAGDHASAKQLMTRSRNYMKLWNNETQFMQTRN

SNGTFAREDWGWTEGDKWVYTFDVMHDAGGLASLFKGGKADMKAKLDQHF

SGGHNMHSNEPSHHVPYLYSAIGYPMSSAEKVRDIAWTEYNNTAAGLSGN

EDLGQMSAWYVFSALGFYPLNPASDEYVVGTPFFDRVEIRLPSMSGADGH

TLVISAPGAGTQGKAYIKSLKVDGKPVDKPLLKHEDIVNARKIEFHMSSE

PTGWGRKGTV

>Mb|QLI64617.1

MGANEASSTYRYNEKPIYTTSNGAPINNPEGWQRPGPMGPLLLQDFHLID

SLAHFDRERIPERVVHAKGAGAYGEFTVTHDVTDITSVNMFNQIGKQTPC

VARFSTVGGEKGSADSARDPRGFSVKFYTEEGNFDWVYNNTPIFFLRDPT

KFPLFIHTQKRNPQTNLKDATMFWDYLSTHQEAIHQVMHLFSDRGTPYSY

RHMNGYSGHTHKWTKPDGSFVYTQVHLKTDQGSKTFTNEEAGKMAAENPD

WHTQDLFEAIKKGEHPSWTVYVQVLTPQQAEKFRWNIFDLTKVWPQKEVP

LRPVGKLTLNRNADNYFAEIEQVAFSPSHLVPGIEPSVDPVLQSRLFSYP

DTHRHRLGTNYQQIPVNKPLNAFNPFQRDGGMVVNGNYGANPNYPSSYRS

LTYKPVTPSVTHEQWSGAAVHALFGDVTDEDFVQATGLWEVLGRTPGQQE

NFISNVAGHLSAAHVDTRKRTYEMFKRVDNGLGNAIEEQTEKLA

>Mb|QLI64620.1

MEHPPPATSSFGRRRAQPEMTQVTSQTWSSSSTRQHQEQPRQPQPEDTEG

EAEDEPYTNIQVRRAIRTMNAAFDWTGPDDPDNPRNFPLSIRLFSTVAVT

GLATVATVAGSMYAPAQDDVASKFNCSRELAVVPLSMYNLGLAFGPLIGA

PLSETYGRKAVFLVAGPIFAAFMVGSGFSTTLTSLIACRFFAGMFSSSLI

NNAPATILDSASGKLRGVSLSIYYTMPSFGAAFGPFVGGFIVQAGGWQWT

QWTAVSMAVGFYIPVCFTPETYKRVILRRRAIRLGLDTSSQRTSPGRAFR

YFATVLIQRPLHMLFTEPIVTLISLYNGFLYGLLYTFVIAVPWIFREYYG

FSNTGESLSYLGLMVGTLVASAPLVLIDLKYYQKRLAQWRESHPPNDDGV

EEPLPSEHRLIGAMIGSLLLPTGLFISAWTAQYKVHWIVPIMFQSCVMLS

SLLIYASATMFMLDAYGPMYAASASGAMMMSRYLLSTAFPLFALQMYKAL

GVGWATSLLGFITVAMAPIPWCFWVFGERIRRRSKYETSI

>Mb|QLI64672.1

MRYTSAEVIALTLAIAGIGTGLDVNRALQARHDTCPETKTVTETQTITER

NTVTVTTAGACTTATTPTTTTTTSKSTPTPTGCPAPPACDNLGFDWAYYN

NSAHNGDKTYSNFHPDTYKRVKPLYVGTTRYVGGLYGEKGGKDPAGRIYG

STKDLKLDYFALNHHAYFYACEAGTYSINIPYANDAVFVWTGAKAYKGWT

DDNADAKARYNQPDHIAGQASSKVNVPADTYVPIRFVYGQAQYGGGFYFT

VTTPSGQVIVSDKAHNSPYVVRHSCDGVKAPKYMPFGKEQ

>Mb|QLI64679.1

MSSTLTTRVSTTAYPPSVVTSFPRNPLTTTFSRPSDCDGIYLSGFLAMVD

LSSTCLPSNFKSDAYFSPGLVCPSGYVSACHDTTGVASITTVTCCPALKN

PDVTLGCLTTSTLSGSWSTLYCTWIAPPSSDSILPVTTSENGITTTQTFG

FHSPEGLNAFGIRMVYQSSDLSTKPTESTTHRSASRPSGGAATSNASQDT

DEPGGLSTGAKVAIGVVIPVVAIAALLGAFFWWRKRKHRYQVPPQAADKS

PTPRHAELHGTPFHELMAQPNNPVELAGSNPGNESGPGK

>Mb|QLI64694.1

MKKMLDNAKLLMSVQRDLFLGTGFYNTFEGKPTSASGKITTAISNLLLSD

ALGIPYTPFNPLPNIPLLRITDRDFVDSLMQEALPEDRSRFTEYLCNRPL

GLGIITAPPGFGKTTALAIGAVAMAQTLGKLYLSGPTHISVDNAAARLDL

ISDRVTNRLNDKLSQQGLSPKYHRKLIIRAFKIDDELKALKNALQNPQDA

SNATIESSWSRNSKWRLHLSLAYWVLVVIRSPAVRQLGPHDKESLHAIRE

KCDKDGSLAVLGKVAMGIIEWEKYKRRGLEVIDGRDSPVRRLMTSILSVA

DVIATTPYLACKKPFCDWRKREARGFIFDEAANMNRPDLLCVWGNTCKPC

LLAGDEKQLPPTVMTKQERDEDGNSLNRFAGDGAISPIHHFKSMGWPIYR

LRVLLRMAEGQFDLTYNEVYRDIPFTYGPGCAIDLPHHEIGRKLESYLCR

KFPRIKPAPAGELLPVFVHCEGSQSSQDEFSGSTRNPDQVTLGLKILVNF

IKSSGARPQNMVIISPYLANVGTIEKFRKRPEFAALKPMRPTATVDSFQG

QESDIVMIIMGTTASSGPGFTSDEHRLNVLLSRHRSGLIIVGDINVTGDV

LKPEAPFQGVMVIGRTGEKYFEPCRMLRNVHLALQGAGRVARIRLPKKD

>Mb|QLI64699.1

MAKDTTETSEEESSFIETIDPPLSPFPCPPPVIIYPNVPGLEHGIHDTTF

NIPPEGRRLAKHAMTFADYESARNTDLYMLHAVEFELCQGDTLIFVDLPN

IPKDTRKLADCDNIVFKSQKFLVHSKKLLATGSTKFAAMLGPSYQFRVQR

RRKLVNKLPDGVKYLLDLTPPSEGDELVFQMTEVSLTPGIMNWWTSYRFH

GAEIGLVAGHDDICCCKRQASSYLGFDSDEDDKYDSTGTRKPKSLGAGVL

LPPTPLTLMRMKAQDKDKLYETPEFRQIPDYCPIRHRNGIIRLLILLEGR

EISMDSAARMWTMVAVAKILDCTSVVRDKVAQWMLHDRNARFIEVLPEEA

LKIGYDLRLQEVTQTAFRILVNEMALEEAATDHGSLPRRARTTIFGRRIG

ECSDELSNLIQHASRTLVERVTRTNLELQSPDLFDRWAIGEWGKLRSLEA

LLSTQQPGENPGSSDFSNALDHLNRLMTQLRFVIGRKYSLSAESFGTSSP

FLMGMDIDRATYVHPMDFDELGQILVRFNPPQKLLCPFIYNEMSDRCHAN

FFTDPHPDKRDVQEPSIPALLDNFQASLEWLVSKHPDLIDRPEWQVIVNG

EIDKNASPVFRIRLPIVALLEMEQQVKHAMQPLTRSWVRHEIEPPANITR

HLLLTLDHEEMKFLPLWAGGLNDGTGGVFESCIPSTDMGANGPGPAYHTG

HTIPSAPPSISDTLVDDIFAMEVTSTPISGTIDFHGDMSIVYRDDEALAE

DVSIKTESFQTAESDYDGARFAVPASHQELAEAVNSLVEAVDDFEADMQG

TPIASDYESDSDGSVVMVKRTCGE

>Mb|QLI64717.1

MKVLVVANMLAMTATMVTAFPVTADSLNCRAEPNTSSAVKKTYKKTDDVK

ISCQTEGPSINGNSIWDKTQDGCYVADYYIKTGSSGYVTGKCGGGGGGGS

NPPPSGGFCKTLNKAGTDLITRWEGFVDRPKPDPIGLPTVGYGHLCQKKG

CAEVKYTFPLTKATALQLLNDDLPSYTKCLGRALDAGKVKLNENQWAALT

SWVFNVGCGAAQSSSLVKRLNRGENANTVASEELPKWKMGGGRVLPGLVK

RRADEVGLFKIASSRSAFPQCQ

>Mb|QLI64718.1

MPYCHRCRTLTAKELRENDVLFHPNLRSLKDSADRGCEFCFVCWTAFKKD

AAKHVDGLLRGESPFPKGSEWTPAIWLNGNQFGHPTSGDTIAVSCGRRGP

KSDFWPEVNPENIEAVLEVYEEPGTRSKYNIRGRRSTSYQNPELHVALIK

EWMATCRAHHPRCSLTRGSEMPTRVIHVGDPANNTQPRLIATAQHGISEP

YAALSYCWGANTSNVPKLTDATYSSFVQGIDESTLSQSHKDMIHLARALG

INYVWIDALCIIQFNAADWERESKRMAVVYGNAALTVIAGRSSDSKHGFI

ANNAAQNRIFCKLPHEASETPTIMVGLQRSHDIGPTSTRAWCFQERLLSQ

RAVVFGLEQLRFSCRAGSVYEDGFKTAGNAGSKLLGSAPIQPPSRNEQHA

AHEALKEWYGLLFPYTKCRLSNPHDAFASLSAMAQQAAAVLGSRYLAGVW

ECDIVRGLMWRPTYHVAPNWKPTTRPKPTSFTGSCQIVTRAPSWSWVSVE

GEVSQESFVPPKVALYRDRSNVKIRPVFLHPEKWSEDAECGVDKLHMPAC

ELRFVGRVVGARVLEESPRLRYTSLRKAIQKPVKTLAPGKYSVLLVGTGA

PAGELHEDELLGNVTALGHFDVQEERVGVSHVYCLQLIPRYGLMLKRNSD

GSYSRLGWFMLEQDEWFMSQVETDVRLG

>Mb|QLI64734.1

MTKFKQSNTAKYLTSIYSVSTPTECASIYDSWSESYDVDVVQGNVEYVAP

RATALRAKTEGGNMTGSILDAGCGTGLVGVALAQYGAKNIDGIDLSSGML

NEARKTGVYRDLSIVDMSKQIDKADESYDVITCCGTFTPGHVGPDPGIKE

LVRLAKKGGVIVATITDVIFDTGGYRAEIERLASDGLITIVATAKEPYRV

GSNTLAHMVVLRRSNLAIGH

>Mb|QLI64750.1

MLSTVVTEAPAANMTPDFEVRLQLDPDRVLHSNHQLMDLVRDNFSVKDTV

KMNVQFLDKCSNEIFKAGWNVRLRVVQPKPEIELTYKKRYDIAGGNIEAA

LAKANQDGFNSKSDKYEAQVEWGYKKQTLSVSRGKKVRYSGDGGTDLPGT

AASRDMLVDEAPGKFDNYKGKKWGTKALKEAVVFGPVHATRAIGSWRDHK

LYIEVWPIRSADGSGWQYFVEASFKAGRRDEAEAARGTLLKELQDKGWFL

PEDALKTSIIMERYACP

>Mb|QLI64763.1

MMKVFKLPRYTATVTKQLGLLFGIFLISIWWVIVPLGQHSIPPGFPLHVQ

DAWTKISDAPREVVGPFNASKLALMVESRPLPRLAPLILHTMAVVPPDWR

FLFIGSSKSVYSVGKSKAIQFHKNSGKLELMKVPEPWKLESPEDESRLLT

DARLYEEVLPDVEWILKFGHDGILCANSAVSLDEWLSWSWAGALRKAHGD

RLSDTGGLSLRRASAVRRILSFQRRRNNSEPEDVWFRKRIAALPGEKVAS

NFHGIISADHVFPRELMGYHVTAWNTWPDRGARTNRNMKQILEYCPELSM

ILDMSVDEGDIPASSVRERKKPSVEAKTKHDGIYVVKDDDKVKSKGLIYP

PQPAPLPLLHSE

>Mb|QLI64816.1

MSYPGAAPPPPGATADLDNPQDVLRTVNYVTQALTLLLTTAFVLTRLYAK

SRILGGGVSIDDYATYIAYVLMVGYCITAVFASMHGGGLNQWEVRREDIQ

PFFQSGYAATLFYAPMALAVKLALLVIIIRVFGAVHKKTLVGVYVLIGML

VAYYGSGFFIKLFICWPISAYWRGERDKCLNQSAIITSDAIISVISDLTI

LLLPTPLTWSLQLPRRKRLRVSGILCAGGIATAFSIYRLVLIVAERRSAN

QTVVFVQVILSGNAEVGIGLICACLPAVSALYIQKTRGSSYLKQSGYGRT

GSSNTPSRNNEIMLTRSFHVDTSSVHYEAQNMGNDELALVSKHPLSHKGS

RSDSVY

>Mb|QLI64827.1

MTPTREDQENGILCQNPPDEPPIEPREDQALLQQPAIAEWKPPRGFVWIQ

IALMNNVFLNAFDGTIMAATYAVISSEFDATNSASWLTTAYLIASTAVQP

LYGRTSDVFGRRACFFASTILFAVGSLGCGLSGSMVTLILMRALTGVGGG

GLHIMATIVNSDLIPFRRRGIYQAMQNGVFGLGAICGASLGGNIADRIGW

RWCFLLQVPVSVFALIVGSLVVRDQSSSMLLSLDEGLQVMWKRIDFSGAL

LLVVAVSIQLLGLGLGGNLLPWGSPWVISSLVGSLVLFAVFVVVEGKTCA

IPMIPLRMLKGRLPVATQISNACAGCAAYGFLFMLPLFFQVVLLESASKA
GARLVIPSLATPIGSVMAGIVMSRYGKLIAMMRIGSILMAMGNALVFSLR
FVDSSWKYFAYIFPANLGQGVVYPSILFTSLASFEHQDHAVSTSTVYLIR
SLGGVWGVSITSAIVQTSLSLRLPDALGGIPDKWRIIDQIRHSASSIHNL
PPEIQMKVRLVYYDGIRHAFAACTVISLIGVAAAFAATASKLRSTH
>Mb|QLI64828.1
MSSFSTQLEGKHALVTGGTKGIGRAIVELLLAQGANVSYCSRSATDNDFA
AFLDESKPSGTASGTSVDISSPSSIETWVEKAARKFGRVDVVVANAASFS
TEATAESWRTSFEADVLGLVSLINASTPHLEKTSGSIVVVSSVAGFETRF
DVAGSPYTTFKRAQATLAKDYARKLGPLGIRINSVIPGPIEAPGKVLPDG
SREPSRIQMLVEANPEYAQGILDAVSLRRFGTAEEVANVVVFLASPLAGY
VCGANVLVDGAMSTFL
>Mb|QLI64937.1
MSSSQASATSPPPPSGRKVNRSCTECTRRKIKCDGRHPCSSCVYYRSADS
CEFRQRSRRNAVSRSTFDKTSEQLKLQSKVLETLFPGSNLKDLLEKSQSE
LLELLSVNHVSPRSKVHQLPPHMPITPGPPPVLPQRREEDGQFASGSPAQ
GSASVSENGEAAEERQWDESLDQPATVASDDINALGLAADNHPARSYLGV
TSMSAVFRTIFQLCPAAKEHTNKCARSLAAVPSPAHPAVPVTGRDPALSV
LREQRFIDFYFEHVHPLTPLLDEQDFRSQYASGTRRDGSWMGLLNMVFAL
GSIASGSEGLHEQYYREARSFTGLDSLGSGNLESLQALCLLGGYYLHYRN
SPNMAYGILGTAQRVAIALGLHREPRRAMETPDPGEMESCRRRVETRRRT
WWSFFCLDTWACMTQGRPTCGRWESSTMDTYFPSCLYPDDQEATLLRANI
EFCLIFERAQHRYAQFSRLSNQEILALDAELQTWYKSLPQETKQVDNAPQ
RFRFARELLKTRYYNARIILARSLILYMAHDLKRKPAELLPEQAHILDHC
CSIAAEAIDSTARYWSPNRIHVWHSSWYLFQACTVPLLSIAINRNMEQRQ
QKQVSSPSSDRVVSWQTSLAKALDTFAEMRPWMRTSDRSPDIVSALYEAL
TAAGEVDGQTPSATDGSLDLFGWCDDQLTELDWGVFLGDEGLARGMYPV
>Mb|QLI64959.1
MSRDDYTIGWVCALPLELAASKIMLDQVHDLLPTLHGDTNSYTLGSIWHH
NVVMACLPISQVGTNHAAIEATNMKRSFPSLRMCLVVGIGGGVPSKADVR
LGDVVVGTRIMQYDKVKTNPEGEERTEVYKLIPRALGTALSHFAAEDKYT

PLNSRFSSILQEKLANENHRKFRPPNLEDRLFQANYEHISSEASCDRCDS

SRLMIRGQRKTNTVVHYGGIASGNKVMKYGRKRDEIAEELDVICFEMEAA

GIVDVIDCLVIRGICDYSDSHKSKEWQDHAAVAAAAYAKGFLGMIPMTEF

QSTTTNLSRSPQHIPREGQNAMLASLRFNSMSFRKHAIEKNHDKTGQWLW

NHPSFQEWLDPALLSRHPGFIWIRGKPGAGKSTLMKFAWLQTQREYQQAC

RDRKMIVASFFFNARGETLEKSASGMYRSLLVQLLEHFHDLQRVLDSRDL

EELLNLDSYPLVVLKDLLQDVVSELGNRAFFGFIDALDECHELQLMDMVR

YFEELAEHATQKSIPLRICFSSRNYPYINIQRGVELRLDYQSGHAQDIAN

YVRSELRIENPTLHEQLVFQILKKSAGVFLWVVLVVDILNKEERDGRPAF

TNRLSELPSGLSDLFESIIKRDNDNVDDLKLSLGWILLAPRPLELEEFYH

ACWHFKDPMLAKQYTKDRGEKYVLSSTKGLAQVAQSTPPIVQFIHESVRD

YLLKDDGLRKLWPNLPFKWKDMRHDQLKHYCCRYIQHAINSAHINSPDVE

NDDISKRLPLLRYVTQNVLHHAENAADSICQGVFLEKFPTAQWASLDNMY

EKYNIRRLSPKVDLLYILAQRGFPNLIRTRLQNHPNILIRGERLKYPLFA

ALANGHKDAVAALFNLPSTIYDGEDITAGVNYKKDYHGRTPLSWAAQEGK

VGLVKLLLLAQADVNERDNAGQRPLFRSQPKRHFPVAELLIKAGADVNEK

GYMYTNGNSAMRVTPLMHASAENDEIFARFLIQNGAKVDTICYEGEMGVV

ETALTIASRHGYEAMAKFLIENGALDGDWDKDINALAPAAENGHIGVVAL

LTENGIHPYTKRCGGFALVKALHYGHVAIASLLISRGADVEFLTDVWDKP

SSCSPLAAASSNGHESLVRLLISKGARIGRPCASFFVKSPLLGAACNGQE

AIAKLLIREGACVNEVAGSTTPLIAASKAGHGAMVKLLIDNGADVNQVCK

SYLCDPGTTTPLDAASKGGHEAVVQLLLEHGARTKQ

>Mb|QLI65025.1

MAPQKATQNKDGVSTASVDDRVVKDQTDYGRVDKELAKYISDARITITPE

KNHELRRKIDRRILAVMISTYFLQAIDKGTMSFASIMGIRDDTHLSKQDY

NWLTTCVFLTILIVEYPQNYIIARVPIAKYLGFNIMAWGTVLASTAACSN

FAGLVAVRTLLGLFESVCQPTFVVLSAMWYRREEQASRVTYWYMMNGAQQ

IVGGLLAYCFSLIKTGPLKSWQWLFLVYGVVSVLFGVFVMWWMPDSPMRA

KCFTEKEKHLMVERVRDNQTGMQNRQWKREQFMEGLTDPQVWGYCLIQLC

TTLPTSGLGAFQGIVIQSMGFSVLETQLLAMVLGFYIIIVLLGSSWLVKK

TNQNLYVMGAFVIPSFIGTICLMTVPLDTKSQKIGLVVCYNITMSFWAAQ

TLALSMLSRNIAGQTKKTVAVALNFIIWATGNAIGPQVFLWWNAPRYFIA

FATHLGCYSLLVIVIIALRVYLVRQNHERDQLAAAGVQEARDGRMLHAWE

DLTDKENPNFRYVF

>Mb|QLI65067.1

MPLPTLPSAGDCAPGGVCDCSRIPDKNSNEYFQCVTNPNCEYCWVGRSTT

STSSSSKTLVANSTSISITDYHTMSPGTYTITTNGTVINIVVTQEIATPT

SSTQVFVTMTETVVVTVSSASTWTAGKSSSTATVDDDCYGRCNCSKVEDK

DSKGFFECLTNPDCEKCHSTNAAALATETAASSRISKSSASSSTTTTSSF

GAPTDACPFFCDCSGIEDKDSEEYFQCITNPVCEQCKSRRW

>Mb|QLI65124.1

MSPLTDAGDVLRDAAHLAADQEVSRGRKRSRSTSRAATSKSLRPDESSTL

RGRSPRRATSPFPLPSRNTSPSILRSPTSQILLYNQLRRARREHCPSRTA

SPSDRSFRRRQRTRSRSRSRSRGSRRDHEPVRSPDILSSLRHEVFYEHAD

GTDNKAQP

>Mb|QLI65147.1

MEKSQTMVNEPKTVQLRLASGQEPVTRTILQTPPRDALPSEIPVIDISRA

FSPSIDDRRAVAREIGDAAENIGFFYISNHGVPDAVIAGAYETALAFFRQ

DLETKLRAKIAPSQGKISGYRPFASQRINPDEGADHRETFSWAYDATYDP

EMGDINAVPEHARKYIKADESPWEATSQMPELKAALVAYFQSCLGLARAL

TRSFALSLDLPEDHLDSKVKYPDVMMLLNYYPKKEPQPPAGARRQVSMGS

HTDFRLFTILWQDSTGGLQILNRDGQWINGLPVPGTFVVNIGDFLQRITN

DRYVSTVHRARSPSGEERVSIPFFWGFGLHERCGVLESCVGEGEGPKYDE

ISTQEYVASRSRQLMTWGNEADGGKSS

>Mb|QLI65165.1

MSNFGFETECHEVVNTFAQEVKGKTFLITGPTPGGIGAQTAISLAAASPS

TIILVGRSVEKGQPTVHHIKRANSSINVRFVEAELSSMQSVRKAAQCILD

DDGIPFINTIINNAGIMVPPYSQTKDGFESQFATNHLSHFLLVNKLMPKL

LAAGNARVVNVSSVGNKYSGMNWGDIHFRTTYTPQRAYSQSKTAQILFSV

SLNKKLASKGVRSFALDPGSVETGLSKYISLDIAEDMAQKIAGKSLVEAR

ASRRKTVCQGCATTLVAALDPTLGHGVYLADCKITSDPACVNAWALDEGD

AERCWTLSEECVGETFVY

>Mb|QLI65217.1

MAAVAASDPPNSSLEATLRESIARLAEAEKMGLQENKNSSIIEAVDQDYI

QGISKPPYANTALADPFDEKIARKWVDEVIMKFPRGPPADCHRRPLAKIA

IKNVSITYGSDDATFRVRVYVPAAGDMQSVPSRPALIMYHGGGWIHGYPE

VDEDLAIFFASELRAIVINVDYRLAPEHKFPLPLNDCYQAVQWTLDHAQE

YGIDTARIAVWGCSAGGNLAAAVALRDAEEHEESRIRHVNLVVPATCHPQ

LYPMTLKADESSSNKFGAGLGTAAAAILIGKYAGDKSLNPHVSVLSADIP

ANHPPVHITVAGRDFLRDEGIAYSLRIRDAGIDSQLDVVPGVPHGLLFPP

TTHVARQFFRNQARILDYALNSL

>Mb|QLI65225.1

MAFDPGEIDELVGRVQRSRDTHQFLGFGHGGGQYYPQELLIEEQRPLGEA

HTERAPSLATSATRMSSAFDAFDRSSVCTSATAPPPLYGPEMVGRHRPAV

PDPIPTNLPCEFKNFSGCDVVFPSNKVNLWIHHIIGEHLNWCCPKFSICW

FCDTYTFGSPSNQREEMCFRQRLYHIAGHFDEGMTAANIRPDFYFLDHIY

KRHLIDESTFEWNRSQREFPNHHLPRDLRFGVRERPGEVHVEHARSTCRR

R

>Mb|QLI65236.1

MDTSSSTRCSSSSRRPSHGAHPSQKETLDPNLDINLPYRTLTSGARMDEY

TIPSRTGEIPLDQAHKLVAFTPDDPGNPKNWSKAFKWYCTMVVALTCFVV

ALASSVVTANIPGVAEEFGTSEEVALLSISLGSTLLIAVVFVIPCAVAKN

MATLLVCRTIDGIAFSAPMTLVGGTLADLWKNEERGVPMAAFSAAPFIGP

AIGPLIGGYLSDATGWRWLYWIQLIFASVVWILITFTVPETYAPTILDRR

ARKLRASTGDNSHVTEQDLDLRPMSERLRIFLVRPFQLLFQELIVFLISL

YMSVLYGLLYMFFVAFPIIYQKGKGYSAGKTGLMFIPVAVGVLCSALCSP

LVNKHYLTLVKRHNGRPPAEVRLIPMMASCWFIPIGLFIFAWSSYPQLSW

AGPAMGGFPVGFGFIFLYNSANNYLVDSYQHQAASALAAKTFIRSFWGAG

VVLFTEQMYDRLGDQWASSLLAFISLACCAIPFLFWKYGARIRARSKYAY

GGEDAEGSGVDDVEKARKGDANGEQGLVRDDLEHDADLRRARSYVSNP

>Mb|QLI65278.1

MKTITRLVTSALLLGAQTAMANNAIPGNAFTNNNGLAAAEAAAPMWYMPS

GTCMPSAAEDGQGHQTNGVDTDNCNINKLSHNCPPQPQWQGTNTFYGNVA

GEPFSTIPTYWSAKKCANTWKIIYYVYFKKDTGHKSDWEGIVVSFRDTGG
DNWVRDTVKMEQDSNHKQINWGDVNDTFDGTDDWQAFRQRNRDHGKFYFG
KFHHSVHQDWHTTSFKDTCPPNSGDDFRNGDYQFWARNNLRHVSVLNPAW
NWGKASSPANIDLCTY
>Mb|QLI65283.1
MEDDNGRQTPGGPGPPRYIRSRIANACDGCKARKVKCDGKLPCGYCAGRQ
RPQACHYSPQRRRTTASRRVSKSVDDEGVGGRALRTRPAAVHDSAQGAST
PRTRDSAARTADDRQAAVNVNGPAEPSAEDDTEVPREARLLCDAQGKLVF
IGDCAPLSFFQSVRQLVTSRVGQHAFAPESSRYSVLENVPAGYSTRERAG
GGFGSPPDVGGVDFDAIVASYLATTTGLADLFDNAKLLSDLLLWANHGRG
SDELTSVVNYLVLAIGSLKDHEALSQGYFEYARSKAYATLSGNLSVGTVQ
AFILITIYMLCSCQINGAYLFFGIAARAAYSIGIHRTEVNARFGAHVHRQ
RDRLWKSVRIVDLFLSTSMGRPPSASDVDCTVPYRNVDGNGDETFDLLNA
SVQILLITETVVVEIYSRRKISLQLTEGISHQLRDWSGRWLQQLKDVIAR
TDSRSTAEMSGACQVLSSYYYAVMLVSRPFLMYELVRRLSDSQSAAGRSP
LTSGKSKLADACIDAASLMVDPILDLIDRGIFNSRAPLIVSWLFASALVL
GVGLLGGFGRILEKYCRMSIQALDHFAKADGHATQYSLIAQSLLMTALEY
LERRELQERLRRTENSSQLFGLMPPEPRTTPGESTPGRDSSQHTSRSPAA
VTFRGREPLDRSFLQHQGLQGVPSPRLAEIDSAFLGLTESLMQTPDDNYW
NGTVGNDGDAGSALNLFPLLEAGGGIDLAHYF
>Mb|QLI65287.1
MRKHAAPSSPTLQSVMPSLPPTPDRVSEESKPLVNRGMTIAAFDGSFAPL
VRPTVRRKTPWYKDRDYFLAGWLSPAIWKAAVVEAIATSCLSYVSLLAVS
TLVSYNTPRIGGYIGIFNTVLLTIFIYATSPASGGHINPMITFSAILAGI
CPLPRGVLYICGQLLGASLAGGILTGVWGEEKAAIIHGGGCFFTPSVTSA
GQVFLNEVAASFAILYLSYGVGLDPRQAVLFGPRLGPLLVGASLGLVSFA
SSGIADGYGGAQANPARCFAAAIARKDMSYQWIWWFGPAVAGVLLAVFYN
TIPPHHTDDQQQKERQHESNV
>Mb|QLI65334.1
MRKAIIIGGGPAGLSAALRLQQITDVTSTVYELRPEPTTLGGAVGLLPNG
LRLFHRLGVYDDLLARGSSHSMVNLRSLKGDIITTQDIVGRVREQNGGIG

YLRIKRIDLLQVLLKAAEKANIPIHYGKKIIEIEESEHEVKVTFSDGTTD

TGDLLLGCDGIHSAVRKLYVDPKHAIEYTGFSGLFALLPISALPEGSADE

LKGINATFTTEGVFLTMSCSAKDDEALWAFSKEVPLPKTGDTRDGWEVKR

EEEIAGFKNDLLDLISDAKGEWGNIMRAMVQETSVVRFYPVYRLASGGKW

YKGRCLLLGDAAHAMAPHAGQGVSMAAEDVFMIARLLENPDRSLEDAFAA

FDRIRRPRVNEITMQATKNSEVRRRSSEWGLWLKETAIWAYFWVSRLVGY

NSLANTERQLLYDVDEAEI

>Mb|QLI65362.1

MSKSLPLGILQDVLAETLKKKEDCLRKRGKVKIQGREIVIRDVLNKLSAC

ITKFISIGDIIVQYDPVHAALPWAAVRFVLQVAVDDPETFDLVLFYIERL

VYYITISSIFDVQYLQPKHHLLDVYPQLFEAIETLQTNVLKFLGYLLRHF

CSSTIARHLKSIVVSKKDIEAKFQPIEKAWRRVDELSRLATAEKNEEEFE

VLQNKLQELYRPIARSADHLEAIQDGLEREARVRILKAISTIPYPVHHET

ARKGRLERSGAWLLRSNAFIDGRSESLSYVLWLHGIPGSGKTKLALLVAD

ELMQTTKIAYFYCTRSPAERHRGQCDKMVASIVRQLASLRPDQPILGLVV

EQYREAIDGFSEFEDQAWSIEESQRVVLELLAEYPAVNIILDEVNYEERQ

LLMDVLSRWIQKSPNLLKIFISSRDNYDIFRWVELQIQSLLPLKVAADIR

NRLGKLPPTLEGSYWEIYNTILESGRHASKLAIFTFQWLLYGQKVITADN

FVAIASASLQIEEPDGETGRSDSLEAITTGEILDVCANLVVLHNGVVVLA

HLSVREFLEGLTARSVDTMIAARGNDAIAAACLQLFLTGTEAAYDELQEP

AVADSPRDTRNTQTPIEEVMDSPSGTKGPQSDDAAESTTAEPALKKDGND

SIDDSSTPGAEDYQPQTTVKAQPPEPKPIRVKIATKMINSDDSHALTYS

>Mb|QLI65367.1

MHSCGVSPFWTTLAPQLGHCDEAVQHALVALGAAYNLYKVGKGASSHLQV

SSPAVGTLERFTWREYNLAIRNLHRHLDKPEPASIAVVLVSCLAFISLEL

LRGDHYTAIAHMRNGIRILMSALDVRRLRAAASRRRARGSFLSDADLWDI

VTQFRNVEFALGGFSSDIPLLLGQQLRGDTPDARPIASVAQGYDARTQYV

NEVMVRCWELREHRGSQVFWAQPHMQQELHTLRQRGVAIMSALELLWAGP

QAPPLGTWLSYSSQMDWLLVNSARTMIQLVPFGIDSHLKMAQDPCFQDEL

SRGVSFAAKMLLAHNLEGKPPSDYTLETGLIALMYWSYVYSTRLGTKEAA

LRILQESTQREGPWDASLSLGFLSRDTKPRLLISFWREPQAITHPAIPWQ

LRVVEH

>Mb|QLI65407.1

MSAVTQAQDAPPWWSSFPEPKAPVHALEPEDVAQLLESHASAGPNSAKSF

LLVDVRRTDWEGGTIATSLNLPAHTLYQTRPVIYQLCKQANVKTIIFYCG

SSNGRGPRCAGWMQDYLNEMGEASMSAAILKGGIKGWQKKYSGKLMDWYD

EKFWTQQQSA

>Mb|QLI65414.1

MTEKGTWLGPLTTTWTMPSTCTLLYPWPDYWNSTMYAYRGQRCDTSVGPK

DGKDCWPPRRKEVQDKESAIGG

>Mb|QLI65487.1

MQLTSLALGLIGLYILRLTWQFFKLRRFAGPPWTGLSNVPHSRAMLRGDC

HRWYADVNRKYGPIARVAPNVLITSSPEVWTHVNNTPGYRRSPWYYNACR

IEYRRDNVFSETDNEKHDRRRKQMAPGYSGRENLDLEASIDECLTQLMHL

IRSKYVSSTGNAPHMDLATKAQFFTIDVISKVGLGTAFGMLQADHDIDDY

IKSSAEGLTIGNTALAMGFSWLAHSPFIGKFIAPSPKDNSGFGKMMATCF

RYVDQRAANPTDKRSDMLASFIRHGLSGDELRTEALEQIIAGSDTTATAI

RATLLHIMTNPRVYAKLQREIDDAVRDGRAPAPGDGLISATQAKQLPYLQ

AVMREALRVHPPVTNIFSRDVPAAGDMVTISSCDGTKIFLPGGVSIGYSA

YAMHHDKEIYGDDAEAFRPERWFEADEAKLHVMTATNELIFGHGKFQCLG

KRVAQVELGKLVFETFRNFDMAVVDPTRPWRLQNCLGLLHITDMWVQVSE

RTIA

>Mb|QLI65497.1

MSRLVDTSTSAGVWRQLRDNPYIFGLSAFASLGGFLFGYDQGVVSGVLKM

ESFGAEFPRVYLDSGFKGWFVSTLLLAAWLGSLVNGPIADRFGRKGSMLA

AVVVFVLGSGLQAGARSIGVLFAGRAIAGFSVGMLTMIVPMYMSEVSTPG

IRGTLVVLQQLSITLGILVSYWLEYGTQYIGGTRCAPDIPYTGGTESNRA

FDPRHDVGPDGCTGQSEAAWRVPFALQIVPAFILGVGMIFFPESPRFYLM

RKSEERALGALGQLRRVHPDSDTLREEYLAIKAEVLFDESVARDKFPGQH

GVSLYLSQYASLVSTWPNLKRLLVGSGTMFLQQFMGCNALIYYAPTIFGQ

LGLSGNTTSLLATGVYGIVNTLSTLPAVFLIDKVGRRPLLMCGAFGTFVS

LVIVGAVVGAYGPDLQSHKAAAWTGIAFIYIYDINFSYSFAPIGWVLPSE

IFNLGSRSKAMAITTSATWMCNFIIGLVTPDMLDKITWGTYIFFACFCLI

ALAFTYFVIPETKGKSLEDMDIAFGDTAAHEEKMRLFGIAATLGLTASVP

EEKIEAARGEMEEYA

>Mb|QLI65523.1

MAPQQETGRLPTPFKRASSESNIELEHLLRLMPASRRSKTVFHFNDRKSC

PMDIVQRPKADVEFLAEELCVPSLNDIHDLLWLAGRPMPPRPLTYQLTAS

RTIAVVEDINLHLIWEPGRIFLKPIPRFLLNAAFWTKHLASLIQHESDYR

IAASNHLLPADVDWESWRQTSWELLENSPCNMTRVNKRYRYGELRLSRLN

KISRWRSLTCSGDGLVGLMRGYKFGFATYGQLLEACLFPVVTATAYILLV

LTAMQVGLSTDYLKDNFAFQQASWGFTVFSILAPLILIVLSFVLVFLYVV

FNYRSTKAFNQRRMQFYDNLGRNGC

>Mb|QLI65549.1

MSQRDTSTRPASRRTSTSPTTPVEVGACPFGSSVGNHDAANDAIKLLPKE

NSGRNQQIPPKSLGMLNILNPSDPRPKDSHIAHDSSSPVVQQSASAYQSW

THGFPRQPGFGHSASASYPGIPVGNANLVSGPERQSPTVGYPFSNVNEPR

KALSPKVPRASSLSQSSGPPREFDRRQHGYVPSVSPAKRPYEADSAEEPR

QLPSFHQTPRIAQTAPQVPTPPTRSLSQSMNRVTEVSHVQGQPGPPQDIQ

APPSHTQFQLGQLSSVSQPPEASPWTEVMRRSAGSGSMEGQQAYMTLPGS

DIPIPVQVDYSQASRKADEKRQRNAKASTRHRRKKKTMQEENVRQLQELK

DERQQMSEEIDYLRRQRDFYREERNRLRDIVSRTPGIHQHAAGPRSPTPT

RSIDSHTDHSPVSQHLIPTPTQGYSSDPASIDRAAQRPKMEERAEFAGPG

PGTTPVGLAPPHGHLYNIPPRPTSAASSGSGDRLPPLRAMEGPSPTVQHI

GSGQAHEQDPRTANLSGLPGFSIRVRHGLRQVRRQAYTTRSSDIEADLAA

PNGKSWSQPLGLFIGNEFRNSAKGGRITTFNPYTETEICQVVAATSQDVD

AAVEASRLALKKPSWKSLSGTERGHLMNKLADLVDQHRETLAVIETLDNG

KPYSVSLGFDVPHFSEVLRYYAGYADKIHGSVIDVGHEKMAYTLKQPIGV

CGQIIPWNYPLAMAAWKLGPALCCGNTVVLKLAEQTPLSMLYVGKLIREA

GFPPGVINIINGHGGLAGAALALHRDVDKIAFTGSTATGKDVMRDPFEAT

TYQGPQVSATQRDRILSYIRTAQTENANIIHPCGAIPELPRTGFFVPPTI

FTDVNTAATIFKEEIFGPCAAVARFKTEQEALDIANSTRYGLAGAVFTRD

MARSHRVARELEAGMVWVNSSNDSDVRVPFGGVKESGIGRELGEDGLRGY

YSVKAVHVNLTES

>Mb|QLI65582.1

MRIQAGVVSLLASSALAARPFLNEPDTGIEDILKCSSNGTLPDIKAIVGL

PDFEYVARKYLPTRNFTYYRNGAAGEYSYRNNLEVYRRYRLRPRVLVDIS

NIESTLSTTILGHNFSAPFFISPCARADYAHAEAEINFVKGAAAGNILYM

PALFAQRSIEDIAAAKKPGQVLFQQLYLESNETFNKDLFERTEKAGAKAI

IFTVDSAADGNRHRAARFGVGSADSSYSAFSWTFYEQLRNQTKLPIILKG

VMTVEDAQEAVKRKVPAIILSNHGGRQLDGSPSALEVALEIYEKDPELFK

KIEVLADGGIRYGVDAIMLLSLGVKAVGLGRPFMYSNIYGQAGVEKVIQI

MKHEIAIDAGNMGIADLKKVSPDFVEWKKPNGWAM

>Mb|QLI65606.1

MQAAKAAHPTEFEFVIGSRPQELKVDTSRRLRSFLSKRAWREHRAQYEQR

SQSSSESSPSNSSSSGSKSEAQSSSSSIRDGNANPDRKPVATKASRRRRN

KLQTVTFEYIGAGPIAPPSALTPGTVQDAAGLEELYGLPGDRLLQLFAQS

LARFPPMDTHFGGGRVDPFRSYPGPWEPYIPALADHYIVQMARDIPELDQ

PGNKGLLRSRWFPLVLSDNAPFQVVMLLAAANYASVNNISTLGCHLLRMK

HDAITAINNTFKDDKTLASDCLIGAVAKMASFEAMHGDVLSYQTHMEGLV

RMLELRGGLDSLGLGGLLRRMVVWIDLNSSFLLNIPRYFPGTTFTGVERE

VTEVVEPNPERFIAV

>Mb|QLI65638.1

MCPIGRKSHKGQPEYVAAVEKPGVPAGYQPNIMGTQGQTPMGSDLPGPAP

HTAGPHRHDILNKLDPTVDSRSGGAQVLGPGTGASRGTYAPSHGQGVTST

VAQGYNAPLASGDTAYTAPPPGVSATAPMQNVPEGTYGPHSSRIANTLDP

RVDSDLDSGGAAYTHHRQGGVPVANTQGEVYGAQQPRPANTCTVDPNLRG

AAAAQGQVPRAVHVGGTKPGPAPNTSGPHRTDFMNKLDPNVDSKRVI

>Mb|QLI65652.1

MSMKAIVISAFGPPETLVVKEVPRPAPTPKTALIRIHAFGINHAEMHMRR

GEWAESVPISGIECVGTVETCPGGEFSPGTPVAALMGGLGRTIPGSYAEY

TVASVNNIVALGTPGDELPMSWADVAALPESYATAWTCLFRNLDLKAGQR

LLIRGATSSFGRAAVNLAVDAGAVVTGTTRSESKFGELKRLGVTNVVLEG

ANLGDRLQEQGASGKFDAVLELVGNSTVVESLKLVRRDGRLCLAGWLGGL

DPIQGPGPKPGFNPLLQMASGVHFSFFGSFVFGSDEFPVSDVPLRDIIDK

AAEGRFDARPWKVFGFGESGIRDVHRVMEAGEAGGKMVVAFEVN

>Mb|QLI65694.1

MATVPSRILILGATGNIGQFITKNILHARPNNAKVTILTSENTVSSKAAL

INGWKDAGASVITGDITKAADVAAAYRGIDTVVSCVGRAVLDQQKELIRL

AEESGTVQWFFPSEYGTDIEHNSKSPTERPHQMKLAIRKYIREHTKRLKV

TYVVVGPYFEMWVDGGTCSDQIGGFKVEKGEAFLIGDGQGRIAFTSMQDT

GKAVVAALRHPELSYGKALKISSFVVTPSQVLAEFEKQLGRKFAVKYIPL

ESLERTEAEFWEAGNPIATIATLRRIWAAGGTLYDKWDNDTIGLTSTDSL

EGIIKSYLEEKGHLSGSQGEKL

>Mb|QLI65695.1

MEEEEKKKTKAPIERLPPELLLNIGEWFDDRRSLNTLVRTSLVFYTALNA

HLYKLNIRNDPPSASCLFWAAQVGRLGTFRVAHRFGADLNVKRETKREPP

SPLHTAIKNLHPEIVEYILQNGGHVHSPPWIGKAKRYPLGIALTARNLRH

DASCVRKNQIILDMLIRHGANMVDDGEPALPHAAAMNKNRVVHLLLQQPT

HGHTELLHYLLNQPGINIHAQSTDALGFAVVNGHVDAVRLLVMWHGLTPD

AAFELRKTAFLRALETGQASICEFLITLPGINGALSLPRNCTALHWAARS

ENVDCVPVLLDKSSFHLGAVDDDGHTALHCLAMTLNSKGRTTRAEVVRML

VDRGAEVNPVSLMGYTPLHFAIARNNFDVATQLLHQEADPTIGAESDTGD

YIWTLLHESLLGNSWVPWNYEPRIRMLQALIQHDYANLNKESRVTSAEMR

RTKDAPFDGTPLLFAVLYDSEPGTIKMLIKAGARVDSVAMKRETAAPNSF

HSHSILQALLSSHLRPNYPVSYKDTNVSVSVEDVRHIKTRLEILLSNGAR

IDNEHGPQSVLEYACEVQLAGYPALLGALFEMAHPVNVSSGHVQSLIHSY

SGVAAEEYPPKFDLAAALQKWMDVWLIPEGRRMQLL

>Mb|QLI65774.1

MALHGKTVLVTGAGGGLGRQIANTFLERGANIVISDISEPRLTQAREEHA

SRPDRVLVLQADVTDEQSVAQLVAAAARKFGRLDVVVNNAGVMDRFEPAG

LCSKATWDAVLAVNLTGPFLVTRHAVAQMTEAQEPPGGLVVNVGSNASLR

GLAGGAAYVASKHGLVGLTRNTASYYGRRGVCAVALLLGCMAETNIHDAF

ADGLHKEGLDRMHHTQPDCAAVPTEHVARYAAFLSEGDTARSANGSCIVI

NGNWPEV

>Mb|QLI65796.1

MQSFLQYRRLRKAAHAQVCDANAAGAPGIPTPSHTCTLPTESLGVRTNFQ

GSIPGINVQTSGDRDAESSLLFIVTWASDQDAENPRNFSMARRLIATLLV

SILGCVVGVASSVYSGVAPQNAEAFGVSEVAASLVTGIYLLGFAAGSLVS

GPLSEVLGRNLVYVSSLTLFIIFVMASALAPNFGAQLAFRFLSGVFGCPP

LTCAGGTIADLWNPLEKTTYFPMYAIISFCGPAMGPLIASYIGQTDVLSW

RWAGWIVTIMSGAVLVLIVFFQPETYSPLLLSWRAKHLRAATGDGRFRCE

MDVDHISLVRRIGRAMKNQFLITIHEPIVLLISLYMTVVYIVLFTFLDGY

AYIYTDVHGLSQGLTNIIWVAMVIGIVSAAFLVPPAYIWTRRLLRDNTEG

RSSDGFHIQPEQRLWYAMLGAPLIPISLFWMAWTDFARISVWSPIIASAV

FGLGTICIFISSYMYVIDCYHIYAASALAFMTVSRYAAAGVMTVVGIPFY

RNMGVQWTLTILGCISAALVPVPFVFYCSGPTIRKWSKFAESQDSKV

>Mb|QLI65838.1

MACGLPRGSWVRPRQGKSAHLACLPPEIVCMIVRQLPLAGDKAALYRTCA

ALHIALVEEELFRHPEPGDVNDILNWAVERACLRTARRAVAYGADAEASR

RTDRETPLMTACRGGNLDMVRLLVEAGADANNRFSRDGSTPVVLSAGAAH

DEAVVFLMARGGDPARRNRYGETALHLAARKDNLGLVRVLLASGRVDVNA

ASVTRSTPLLEAIQVGNMQMVEVLLEHGASEEIYELNGGFAFTKALRSRG

TGLMKALLELRICPDVADARGRTALSWAAEYGHAMQVEMLLDSGARVDTQ

DVQGKTPLMYAAAEARIQVMETLLDRGASLAISDRYGMKAACHAVQSENW

QSVGLILDRGEDVLCGAGRCARLLLQMAGQKENMGIVC

>Mb|QLI65848.1

MALPVEEAAKQADAIQPTSSIPGYSETVAEAKPDVEQQHIGTLDRRLKSR

HVQFLALSGAIGTGLFVGSGQVLSLAGPLSAFLCYAVTGFNLYCVINSLG

EMAAWLPIPGAVPVFAARYVDPALGFTLGWNYWYQFAIGVPIEATVCGVV

VDYWPNALPKAALVTAFSATMVLVNCLPVRVYGEAEFAFGAVKLVTIVGL

IVLMFAITVGGSPAGGAIGFRYWRDPGPMRTYLADGATGRFLAFAKVFIQ

ATFSYGGSEMVVVASGETEDPRRNIPRAIRRVFWRIAVFYVLSVLLVGLC

VSSADPGLLDAIGRSAPGAAQSPFVIAIQNAGIGTLPSVINAVILTSAWS

AGNSFFYAATRVLYAAALDGKAPSALRYERFGVPYGCVAATAVLSCLVYL

NVNNRSAEVFFWISNLSAVSTLIVWASVCCTYLRFYYGLRRQGVSRDTLP

YKSPLQPFLAYFSVVFCLVVALFNGFDAFFPGNLSAKTLVPPYVDIPIFA

ALFLGYKFVVGTRFVSLAEMDLWSGKAEIDRLEGTWKVVEPRNWLERIWF

WLA

>Mb|QLI65998.1

MSTHYDAVVIGSGQGGTPLSMAFAQAKQKTALIESTHIGGCCVNEGCTPT

KTMIASGRVAYLTGRGPDYGVLSPRPDSARINMEKVRQRKRDIVDSFRSG

SERRLRDAGVDVIMGSASFQNETTIKVICQDGADKILSADKIFICAGERP

AVPKLDGLDAAKFPPGALLNSTSIQELDVVPSHLIVVGGGYVGLEFGQLF

RRLGAEVTIIQRNSQLLPREDAEVAEKMRQVLKQDGIRILLKTSPAAVRA

SGGNDPTKAVVVSASGPDGTSELTASHILFAAGRTPNTDTLNLDAAGIKT

NNRGHIVVDDTLQSSNRRVWALGDVKGPPAFTHVSYDDYRLLRANLLEKD

SHPTALTTVNRILPYVVYTDPQLAHVGLHEHEARAKFPDAKIQVATMPMA

YVARALETDESRGMMKAVVDADTQKILGFTCFGLEGGEVMSVVQMAMIAG

SKWTALRDAVWAHPSLAESLNNIWGSLK

>Mb|QLI66018.1

MAPIRTAIIGLSSTAATSWASTAHLPSLVTPAGQSRFQIVALLNSSVSAA

EFAIKTYDLPPGTRAYGSPEDLAADPAVDLVICSTRVDRHYETVLPSVRA

GKDVYVEWPVAATDADIRSLADEARRSGSTALVGLQGRWAAPVLKLRELL

DAGAIGKVLSVDVRAYGGTKDRETLPVGLKYFADRGVGGNPITIGFGHVF

DFVQSVVGDVVPETTGSRLQLQRRRVRVRDPENGEVVETVRSNVPDLLYL

HGRLAESPRNDADATLAFTFRRGQPFPGTPSLAWSIYGVDGEIRLVAPAG

ISLQADACDESVTIQVHDFDTDTVRDVEWDWTDVEKQVPVRGRSVQRVLY

AYADWKQGKSDGAGAWVGVDDAAARTSQIQKLLDGFEG

>Mb|QLI66040.1

MQLSRLIITASIPAVVADITPGSHPIVDLGYVKYRGSYNDTSGINAWYGI

RYAEAPVGNLRWQAPRDIEINGTGSGGQVIDAVDAPLPCPQGYPGWWFNS

TAGASTDLSKTPTGTEDCLNLDILAPAKPVNTSLPVIVQIHGGGYTLGSS

SSIGPGYELAGNSLVYQSSGGIVYVSIQYRLGAYGFLGGAEVAEKGVANA

GLLDQRAALGWVQRHISKFGGDPAKVTIMGGSAGGGSVTHQLTLHGGVSN

PPFRGAIAEYPWLQPLHSQSTIQRQYELLLTAAGCPDIQCLRALPESDLA

VATQKTYDTGYALLEYGFGDFYYGPYVDGDIIRGLPSHEFSQGHFAKVPL

LTNREGYEGVIFSNSSQTTSAELVTDLKTLFPGATQSFVSRLLQLYPRDA

FNSTFFQRQQIFGDFIISCFSQYMAAAVSDHGEPVYKMIFDAGGGLHGSL

VPFTETVNLNGTSNNATLASIIRSYYISFATQLDPNAVSYTDSNRPHWPT

YQTGGNANFTVLSITDSGIEAREDPDNSAQCDFFHGQSYVVRN

>Mb|QLI66078.1

MPSLIVTSTRITLALQARRAGPALARYTSSKSTMSSQPVASPLTQSATFL

VVSATESPDAIATIRAALSSVADITKNVSIRHPSSNLSCVVGIGSSVWDR

LTGIPKPKELHAFPEVRGAKHTAVSTPGDLLFHIRAERRDVSFEFENQLL

MKLGGSVKVEDETTGFRYFDARDLLGFVDGTANPVGSDVADAVCIAEADD

AAAVGGSYIVVQKYVHDIDGWRGLSTEQQEAVIGRTKLDNMELDDAQQGQ

QQSHKTLATIQDEDGNEHDILRDNMPFGSPGHKEFGTYFIGYSKKLWVIE

KMMERMFIGNPPGKHDRILDFSTPLTGTTFYAPPTSILEKLG

>Mb|QLI66132.1

MDLKTPGPDETADTSMFSQYTIGVPSGGPHRGVKASHKKSRYGCKRCRTR

RVKCNEQKPVCDNCKRHRSECIYDRVPPLTTPVISSAPFRPESAEARVAV

AIARNIENSHLSDPPESRERRILETRLMYQYSTSTGTTIAIDDKSKGLYV

NMVPRLALTSDALLYCMYSLAALHCKVVGDLMGLVSRDAHRKYLSMGLRE

HNLAIANISPETADAICLTSTLLRVCSFILLQDRLRQPYTPPVEWLMMNA

STKAIVGTAYELPGGKEKSVAAMMVGLSPLVSNEQMRFDPVQRRGFEPLL

QRDEIRDASEPWDPESRDAYESTLSYLGGAIETERVGDRHDGLRRLIIFP

MLVKGRFIELVQEGQPRATIFLAYYFALLALHKDRWWIGEAGAHEVRAMA

AHFTDQWRTLLNWPLRVVETGEVPAFG

>Mb|QLI66138.1

MHFVRVFVWMVVGFTTAVPVRGFADGLADQKAVSHDDQSLRVSKPDSSET

HHDTKFAYDSRYMKQLLNKTAHRDAARTLIQSYLITCRELQVQTWLMNGS

LLGWWWGKKMLPWDYPSNVQITEADLHFLAAYHNASVHFHRRKGMPKGKR

FLLEISPDFTNRAQTGATDVIDARWTDLGSGLYLNITAVRYSPDHPSGEG

VLYTKDGQQFHDTLLHPLRDTTFEGEPVKIPYRYKTMLAERYGEEALKKT

HYNGHKFDTPEMQWVMQG

>Mb|QLI66145.1

MALEALGVASSVIAVIEISGRVAAICFQYSKGVKNAKFEVNRLLREVNNL

GETTSSVQDLLDGPCGNRLKTSSKLLDAIEEARAHLSKLNDDLSPSSGRK

VFRRLRIGALKWPLKSKDVEKAVKQLGRCGQNISLALQVDQTSVLLSLDQ

KAVLSRLPIAAGATYDSFSEEGNPTCMENTRVSLLDSITQWVDDPYAESI

FWLNGMAGTGKSTIARTVSRILARTSCLGASFFFKRGEADRDNLRRLFTT

VAAQLATRLPDSATHMKDAIDADPTIFQKAMLEQFDKLILQPLSGIVPTS

LKSSTLVILIDALDECGGSYDDIRRVIHILSRTSTLKTLRVRVFVTSRPE

LPIRIGFSLVNILYRNVALQEMPEYVIQHDISMFLEHQLAKIRGEYNVTV

SEDRRLPLTWPGHETVRALVRMATPLFIFAATVCRFLADLRWHPDDQLAK

VLRYQTRTQQSKLDATYRPVLDPLIEGLDKVEKVELLQQFRAIVGSIIIL

ARPLSVLSLERILCIPKRSIEHQLDGLYSVLSVPKVSDAPVRLLHLSFRD

FLLDPDKKDDNPFWVNESTAHDTMASNCFRLMDDSLRKNICNLEDPAALQ

SSIDPERVNECIPDDVQYACANWVLHLQLSKSTLSNNGEAHEFLKKRFVD

WIEALSLMGRVRETIGMMKALRGLLAAQNSEELSDLIGDAYRFLLANMGT

ISSYPLQLYSSALLFSPKESPIRNLFYNSIPQWIRSWPDTQSRWGSCLQI

LEGHTNSVYTAKFAPVSARMVSASADRTVRIWDTNTGECLQVIHNPVGVS

SAMFSHDERLVVAACDDGIIRLWDVDTEECVQELRGHSGKVSSAVFSSDS

KLVVSASFDKTARIWDSLTGKCTMELRGHDHEVSSVSISRDSTRVLTSSY

DSSVRIWRTVDGKRIGELETDYGSNVCAISNNGSMAVAGLGSGCVELWCL

KSSELKMNLRGHIDQVWGVTFSPDDSLIATCEESATMRIWSTATGEPIQA

FYGHQSAAYGIDFSPDSSMVASASRDDTVCLWSVGDYGVQVDDSTTNFYI

APNSKYLISQSYRDNMVVWDIATQKRIHTIENGCCLSDPIVFSSDSTRVA

TQSGGSIQIWDINKGELISEFSSNIGRVASLAFSKDGQYLVCGSETLGVE

FWHLGKEESVRLRLQSLPRGFPRGEKVLVSPDMKYIAFGHENALEIWSLP

LQSPIQKILKPFPSGGPFQTAQFSPDGRLLAVAFTGGAYIYDTTSWGRVT

QLDGVRGAVEAALFWPDSTMVAFSQYRGEGAHIRICCTASGHCVQTISTG

QISNRLSLDATAQFLLTDIGSIPGRCLNFATDKTVNVNSEDVVSGYSISR

DSCWILWNGERLAWVPMEFRNSPRAVFDDIVVLGSCSSDRLGFIQFDANK

MPMRAKP

>Mb|QLI66160.1

MPSPASHTTSQNSDLETKEISKITPCQPCPEDTRKRKAFVSQFLKANFAP

LAKSNRSYIDTGALEHMAIKCLHVDFNTGRLSNGVAQFIDPIPPSKIEDD

GDDEDNPLENLGPASPQWEAASLYRLMNFYLVHDGQWNPGILQTQTLWRK

YPIPRGRTLCQLSHTQSPRNWRVWTIFRRFQYYEDIISETGESPLDPDKP

HVMAFLADGALIREDVLSTSELTCITYLVADGALKYKHHRFIPATLMSAS

LRTVRIVQGVLDCQKGTLEIRKSQPVTFSDSRIANWDKWLTLLGWMIGNP

ADGLKSSDNPLILHLGSGESVTPVELASRSYKRQLCMDFSPTVVDLMNQR

HAEVKGIEWKLMDVRDMVGVADKSVGVAFDKGTLDAMIHGSPWNPPETVK

ENTSGYLYEIYPVLKDGGVFLYFTFRQPHFMKPLLNPRSVWDMDIQVLND

SGPFDYYGYMLHKEGKR

>Mb|QLI66193.1

MIYTRIQLTNGWTFKQQEWPSEKWLPVSQAPSQIHMDLLAHKKIPDPFVD

MNERAVQWIGEKVWQYRVSFPAPKASSASAATDLVFEGLDTFATVLLNGR

EILKTDNMFVSYRVNISEHIKPDGNNILEIVFDSALIRGREIVREHSHEH

SFLVRQTEAGRVAVRKAQYNWGWDWGPILMTAGPWKPVYLEQYTARVDDV

WAQNELDSDLEAVTGTIYTNVVGDFHPDDRLAISLTLEGRKVLEELAPAP

RNGKYSVPFKIANPQLWYPHSYGKQTRYKLQASIVRSGAELHSTSKLIGF

RRAELIQEPDAHGKSFYFRINNVDVFGGGSCWIPADSYLSQISPARYHDW

IKLMAEGNQVMVRVWGGGIYEDDAFLDACDEFGVLIWHDFQFACASYPTY

PSYMNTLEIEVRQQLRRLRCHPAVVAWAGNNEDYQVQERYRLDYDFENKD

PESWLKSTFPARYIYEHFLPELVKQEDPFMIYHPSSPWGDGKPTADPTVG

DIHQWNLWHGAVNKYQEVSLLGGRFVSEFGMEAYPHLSTVRRMTTHPSQL

YPGSMTIDFHNKGIFNERRMTTYVSENFRLKYDLPSYIHLTQVVQAEAMR

AAYKTWRRDWGTSGDRKCGGVLVWQLNDCWPTMSWAVVDYYLVKKPAYYA

ISRALRPLDVGVSRTYHDWTQTGYYIDENSKLCTGQVDQTLPARESTFDV

WIASSKVQAVNAKVTVRFISIRSGRDVSESITASVSASANATTTVFSSKP

LKPSIPHHDDYTIPFDVTQYDPYVVYTTLSVDGAVVATDAAWPDPIKFLD

LSNRGISFAISENRVTVSSDRPVKGFVFEEVQGMKLSDNGFDIMPGEQHV

VTVEEPVSADKLRWTHIDADDASMEIKIRR

>Mb|QLI66256.1

MGELVNPHWDSKLFKPLKIANGKIELKHRVVHAPLTRNRGTPLSDSTPEK

PNRVWVPNEHVVDYYSQRATAGGLIISEGLPPSLEGNAMPGVPGLFRQEQ

AAGWKKVVDAVHAKGGYIYAQLWHSGRANIPQLTGTPIVAPSSVPWDDPD

EYFPYPPPHTTAPVKLADHPPIALTHEHITRTIGDFCDAARTAMEVGFDG
VELHGGNGYLPEQFLSSNANQRTDEYGGTPEKRCKFVIDLMDGLARTVGE
DNLAIRLTPFGLFNQARGEQRVETWAHLCRELKRRLPRLSYVSFVEPRYE
QIFSGAEKQAFLDSWGLSRVDLSVFRGIWGDTPFFSAGGFDGGNSWGLLE
AGRYDGLLYGRYFISNPDLVARLREGLPLAPYDRSTFYGPFADPVVGYTD
YPAYRDGAT
>Mb|QLI66365.1
MKNHVQPKTLQADSSATTSSAPSEVAESNFDHLPPSTGHQYGSVEPHVFT
SPARAEYWRSIYENAKYECRHRFDPSFQWSPQGEVIVKRKLDWRIMLWVW
VMFSSLDLVRRNINRAVSDSLLADLKVNTNDYNTGQTIYLVCFLAAELPG
GLISKRVGPFRYTPTVIILWGGLCMVQAAMRARWSFWLLRGLLGFAQGGF
IPEMVLYLSYFYKSNELPFRLSLFYTVIPLTQIYGSLLAGGLLEMRRLQG
LAGWRWLFLVEGLICVVIGFISYFVMAPSITEPAFAFKRPDGTNKWWTEE
EEKILVNRLLRDDPTKGDMNNRTAVSARGMWEALTTYDLWPLFILGIFVW
IPFQPTANYLSLTLRNLGYTVFQSNLLAIPGYALFAINTVVLGWLSERFN
ERLVIAAGSNIWVLPFFIGLICIKPDANPWVRYTLLTGINGIPYTHSIIV
GLVSRNAKAVGRRAVAAAVYNMTYQIGSIAAVNIYRDEDKPYYYKANKGL
VGLCVFNILLFVLAKLYYVWRNRVIERQHAETPASEKSRMADARFAH
>Mb|QLI66372.1
MACLEALPAEVLYEIAEWLVWDFKPPSDGRKFRGPSYARHHAALARSCRR
LYSILNPQLYKRNIQQDAVLNSCVLWAAARGLLGTIKIAHGLGADLDLLG
PQSRDFREKGWNGIGAPRGDASTPLHVAVENDHLDIVEYLLEHGAAVHIS
ADGCCLCHYHVGAYALHMALKHSRKPKETAAMLIRHGAYLVSGNTPAAGI
VSRMGHHDLFQLLVEQGRPESTKAALDHSLRSRNVPLFQDLLRRPGLDLS
PPPSSYPSLLGLAVYMGLTDAVRALLSRSDVDPTNLHESEHSPLQEAAQG
GHVGALHALLTMRPGIDVNATDDKGRSPLHHAADYGHVEVLKALTQYPDV
KVGVRDKDGQTPLHLAAYGGKYDAVRFLIKHPGVQPLAVDAAGLNVLHCL
AIGDLTRRKLELLKEEDDDDDDDDDNDDGYHWSNDLGHGYYYDFKDEHES
SSKSSNEGGEDKNKEASPDADEVKEAEQTIRELISIGIPIDQEAPTSETA
LRIAIRRLNFPMAIALLACGCDHTIGFEEPSGWTLLHECLKLPCRKLPAK
QSMQTELVKQLVGRGVNIEAAYSYDPDFVLMDMEERDMLVNESATPLYCA

AALAHNTRSMEVLLEAGADPNAEIYRFGRYSEPLLTALYPKLRYNPRQPF

SVFGVLVKHGAKLDEAGGEGKTALEAAIDCPKAAKEMLLHATSENICREH

VQSVIDDLSSRKKSRNGLELLGDLKDFRNRVFGGE

>Mb|QLI66415.1

MGSTAGTEFLGSIQNVFEEAQGQGFSSVTEFRKVTGSILKFLRACRKMTG

ARARASCPICLPDGLNVRTHVALPPTALKLNRETTRRLREAHSSNEFCGI

LLSQVHEQLHAKRSDLGKGGGNMTTSAGSRRPISKKKRASKRSPEPTPNP

PSAKKARRRHKTVQTPPGSGNHDHKAGASRTAGDGDGVAVSMTPVSAIGS

KQGPGAVVTKTQTGEKTGRAMHRQLTKSPTKAPRPAKSSASPENAEMVAA

RNYGPLTPVKRLRIVLPKRKREPEQQQVIQQQQQKQAAPSTITSQDDGPY

SDSSSDERDSMSDISEISDCMIPMDLDPSEDVAREKTKERGLQMAQKDQS

TQQTRQEPVQSAAATQFLRMVPHLRDGISSVTPTNWSRCTRRIQRYANSE

GIRGADFKQRLMRLVEIVYLPEELELCSVIAETLGPEDEVVDYETRNRWI

KEKNEDINSLKMLFEGIDFSA

>Mb|QLI66428.1

MENSEKLVEHHNSHRLGQDDKAEPVSFATPPRMRRLHDPDVSFQEYQHYA

RITRAEQDALPRPAGKKSLLYYLVPNLQKVETGPADVATRSDLNTSDIEQ

RKIISDEEWTNASRALRTAGTGAIFYLITTDILGPFGLPYAFATTGWGPG

TALYTVFGFMAGFSGYLLWDCFMGLDSFQFPIKSFGDIGFRVYGTWCRYL

FNVLQAIQLICNVGAIIISNGEALSEAVKFKLCYAICCLVWALAGFVLGQ

IRTLQKFTWLANVAVFINLLIMFITMGAAAHTPPLYSASASSAGYSIDPA

LVTPVNGTYPPVQHSAALPDSGNFAASLNGAMQAVYSYGGSMIFPEFMAE

MRRPRDFLKGMWSAQLFIYICYMLYGLFMYGFQGQYVQTPAYLGISAYGL

QTAGNSLAIVSALIAATLYGNIGIKVLYNNILVEFFKAPPLESKHGRYIW

FALVPIYWSMAYVIGAAIPDFAGFTGIVAAVCILQFTYSFPPLLHIGYQI

QKSAMEGEAGFDPSTGELAARDGGIRRWVRGFFGARWYLNVFNLLYFLGA

MALCALGIYSSVMNLIQIYAVPQLNAFGCKSPLDVSA

>Mb|QLI66504.1

MSSDGGSNSSPSSGSPELLTLEAPFLLQIEETEDHDASQQLNFSRSAGLN

ILLRVLTKQPPGSPSESQAIPRLWLTARIESYLTTFHTRWPVLHAPVINE

ITDSVQVISTIIMIDSWLHGDVKLRDQILQIHDCLVRQLHKELNQSDLDP

THPWPANLYQLSLLNVIFAFETGRDNIIRQSRMLFSLLVTALRMNGCLHG

DAVESQRLTHHPGDFKPFIFGTTERWKRIATCALKVDTYLSLLYGHPPLL

RRDELELGLPSTFAMWNSYGIMVFFDRSRSEPWSRDNYKMSRLNVRNPEE

VPSGILPEDIQLCLLGMWNDIRSLKKESMLNRDMAMLKKADLSQQLYLAL

EQLETIVAESRKPLVAGGYTATILKSYLGGEPSRGPNWRQEVTNRLDLCI

SNIRPLLLLLTIHLHADIHTLREIYLSPAPLQMEPTAASQTWQQNVLKIQ

EWALSADGRTAAIASLQTWFAYESSLLINPGQSAHALDPIVYLALSAAAM

VLWAWTMNDAEVCVCHPTTPKAEVTSSSMHLQPDVQNWIRGGGGGISFLA

QPVCKCTVAQRLSTWTEALAKAGRTWERAGVDANMFRCSWLGT

>Mb|QLI66638.1

MGENFKEPDIVLPCILTVTLHEAPGLPALYDKRRYALLNYDKCEVPVEPY

LVFANDPILVQKDYNVCKFYVARLAELTISFYVPDPEDAPAMLLGVARVK

PFSISRSANSRWLPVGHDSAARVRVSFEYQRTLQEPKFRLLGGDMIRSRS

RYVGVFKSEDIGQRYADGWFSNEHISSPDLISFLPSQINHPFIASVTHSY

ESEYELHLLSPYVSGGHLFHHIQNQRRFDPDTSRLYIAEILCALEYIHDF

HSIFGWLKPQNVLLDLSGHIVLCGFGLFILEAKNRDSTTPRVAEYPAPEL

LLGSDATRLADYWTLGIFLYEMLVGEPLFYDENPTNITEKILKQPLEFPG

NISTAAKDLITRLLDRRPQERLGAHGASEIKNHSFFKTINWQKVLQRNYE

PAFKPEYTPGSNPLCGSCNFSPNHVSGSFEQHGVPGSFEPPEPPSIFQGL

NRWAMAQGGRATENSETTIQADNEWDLIWQDSQPQSFHFHNRVTGATRPV

PSRAVDPCATQDAATYTNIGLDTTPGLTHKLDALEAALRAGYDHAVSELL

TYNMDLNVEIFGRDRRRPLHWAVKHKNLHLVCLFLEHGANADFGGPALIQ

AVEIGHLGIAEALVSKTSRVACTQSLGLAVDKQDMNMARLLLGYGAHCDF

EKDDRPHPQPTWDQSFMGCDISRPQEFKPPLIRAIQKHDIDIVRLLLLHG

ADPNVALHDTFDCGRAVQLAMKAEQLGIVQLLLDSGADISLPGDTWEGSG

HKCHISKRPVYQNVTARLRAAMAAKKLGKAANLYVSG

>Mb|QLI66652.1

MHISAIILSILFATLSYGDKSTQDASGIGERAADAQFDPNTWYRLTNTYL

GYSQSLDVINDNGPNSSGTLTMSATARYSGQFWQLIPQSPGVYKLRTLFL

GPNRVLDVWGDDKTKPHLAEKGNYSGQLWTITSWGDGTWRLTNAYSGPGL

HLDTYSGTHEPFMGSGDHTGQHWHITAIERIS

>Mb|QLI66816.1

MPTSPSQPAAANGVGMAPQNHEDDSISPPSLASTGRQSTLITPRVPLIPG

WMKLTPLDQIESRFILPLVLVFNMSSPALGRLVLRDLETSLASTIGEMPF

LAANVVPDCEEQGTIQLEISDDAGVWFHSQELPEIDYYALERREFPPGEF

PLLALMPEPRVHHAEQSPVLTVLATFIAGGLLLTFNSHHSVMDGAGMLTL

ATTFAKHMAALSGGRFIAPGDAFPEEALDRSNVFGCGGKEVGDFPNYRLS

QTYRCAMERELVEAAVSGHHPRLPLLQKLSLSHWFISAESMRAMRDAALP

PSEGLPLLTDNTILCAVLWRHISRARRLSCRGIVASSFVNTVNVRRRLDP

PLPLEYPGNAVVHAKTSAATADVESTEPGMLYKMARQITDAIEWWTSERI

WELVGAIESSAMVNKVEPSMDNFQGPDLEVTSTAAMGDIYRAEWGSGLGR

IRALRYAYLPIKDGWVNVLPQRTDAGLELLMCLEKSTLRLLRQDEEWLRL

ARESR

>Mb|QLI66842.1

MHNALLLLLFSPLAASTAPPVVIHTWGGPFTVAADAAHDALTNARSVLDA

VQIGGAACESNQCDGTVGHGGSPSENCETTLDAMIMDGNTLNVGAVGALR

RVKHAIAVARHVLEYTGHTMLVGDSATRFAAQNGFKEEDLATDRSRDMCE

QWKRNQCQPNSWVGVVPDPKSSCGPYTPLENGASETAPGQDGGDIKGRGH

DTISLVALGKDGSMAAGTTTNGKAYKIPGRVGDGPIAGSGSYVDSLVGGC

GATGDGDLMMRFLPCYQAVENMRRGMSPAAAAEDAVRRMVKRFPDIQAGV

VVLNNKGEHAAAASGWHFTYSFRGQGMNKTHVVQVDPINENRNMSIEL

>Mb|QLI66844.1

MALIYAGVALVLLLAYFYNAQSKLPPGPSAIQLLASMLLSRNYIWKQFQQ

WHKNYGPVICLRIGQVTIISIGDRKAAHAILNRRSPIYSSRPRMVVAGEC

ITKGLAPVLAPYGPQWIQFHKIHTTLLNARRCRLYRPLQELESRHLLFNL

LSSNDFQTEFHRYASSLMYSLVYGKRFVSADDPELKEIRHMVDVTSQAIS

FGTWIVDIFPIFNCLPRSLAKWKRVGDDIHNRQTELFQQNTTAALNRPSW

NLTKHCMLEPPVPVSPKEYMFVLAEFIEGGSDTTAAALMVSMLACVTRPA

AMHMAQEELDKVVGDDRLPSFDDLPNLPYMTAFVEEVLRWRSLTPAGVAH

APIRDDTYNGYSIPKGTCIIANHWSLDMDDDAFANPQSFVPERWIDNPKL

QGHSAFGFGKRSCPGQHLARASLLLGLSRLFWAYDITWKQDQGCSPEAVN

MINGAVSRPASFEAVFTIRSPARRRVVEEQLPTETELEPVLDSIHQSICH

E

>Mb|QLI66849.1

MADDGKMETVKDAELFDYSLHRLNIIRIQNDLIVTREHISHTRRENLEKE

KFSKLLDEYSEASNYDYVHEARLSIMKSLSEGNNASKFKFPVTTIHHHTR

PFESHYYYLHDNAKHAAVDALCNKLRHWISPQLSYSSQERLYRSKEFGDA

KPTDEISTLVDNLVRLLVALVAVLVVVIPMFIVLVHSLPKKSLIPSSVFM

IMFACVLSFMVKTSNVETLVTAATYLAILVVFVRANSLAG

>Mb|QLI66855.1

MHLLLLTLVSLAASTVAAPASGSNASSLQHWLYSSQIDDKAIELLDSPDL

IGVQALYSWKSLEPSQDEYDFSTIKNDISRVQAKGKKFWVQLQDRTFSAT

NDPVPKYMHTPQYNNGSAPTCDGEACDIDFKVDGWMAQQWNPEVRRRYQA

LLSALSEELDGKITGLNLPETSIAVNQSQDNFTNEAYFRGELENAGFAAK

VFNKTYIVQYVNFWPDGWNDANNRFTESFDFYAEHGVGVGGPDLIPFKKA

QVSNSYPFIAKYHDKVPVAVVAVQEPDLEELNPRTGMKFTKEEFVDYAHN

VLKVRIIFWATSSPWLQSN

>Mb|QLI66887.1

MAARAPDNNPKALSAKESDSAHGSAGGNRTTSGGANAAISGAESDDDDAD

ENITESTAAAGESGATEKKKKKKNKKKKKTKKAPTAQTEPPSVLVSQLFP

NDTYPRGEEVEYSNENRYRTTNEEKRHLDMLNSDFLSDYRQAAETHRQVR

QWAQKNIKPGQTLTEIANGIEDSVRRLVGHDGLSEGDAIHAGMAFPLGLN

IDEIAAHFSPNSGNKTVLQHNNVMKIDIGVHVNGRIVDSAFTMAFDPMYD

NLLAAVKDATNTGVREAGIDVRLGELGGYIQEAMESYECEINGTTYPIKP

IRNITGHSILHYSIHGSKSVPSVKTNDTTKMEEGDVFAIEPFGSTGKGKV

YDQGEVSHYALRSDAPKVDLHLSSAKSLLYSIKKNFSTLPFCRRYLDRIG

HEKYLLGLNHLVKSGIVEDYPPLVDQKGSYTAQFEHTAIRESSREVNATF

SRMFDLNPLLAIRQAIRQAPLLGVTAAAASLALYLQIRPTLRDVQHLPEE

QTITRARTRVEAGKGQPSADAKAPEDELPYSMDAFPGGRQFNTIYGTIQV

FEWGPENGEKVLMMHGLSTPCIALGDMAKEFVKKGYRVMIFDFFGRGYSD

TPNDLKHDARLYTTQILLVLSSSPLSWTGSSAFHIVGFSLGGSVAVAFAA

YHASMLRSVTLICPGGLIRTSHISPRSRFLYSSGIIPDWFRLRILHSSLE

PRNGAPSADVPEQVKDAEVDFDDVPIAADRHGVRIGDVVRWQLKNNDGFV

SSYLSSLRSSLVFRRHDGIWRVLADELRRRRTTNAPPGLPSGRICLIIAE

SDAIVVKDELVEDAEALLCRESVDMRVLKGGHEIAVSRGKDVASIAMESW

DRKH

>Mb|QLI66893.1

MDVPVAANVLGTLGAVCWSIQLIPQIVVNYRRHNATGLQSSMMMLWAWAG

VPLGVYNIVEEFNIALRIQPQILTFLSLVTWIQCYYYQRGWTVSRSMLTL

APIVVIMGGCQAALIVGLGVLRHQGVDWPMTVMAVLSASLLGAGVLSHYW

DIWVHRTVRGISFMFVGIDAAGDLFSLISVFFQPKLDVLGMVIYGTELAL

WLGVFACGGYYNFLPWARKKWLSQHGSPGESENAEPEDVNTASGSIYLQD

LPSSTSVFRTVSAASEGLRSRTVALGGIQ

>Mb|QLI67005.1

MESKDNYYNPLVLWVYDFFVQVLTNTFWWRCSTKSVLVPFFVDNASPNHM

EVGAGTGYYLRAKLDHEKRHIKLGDNTTTWPQKLTLVDFHTQCMEKAVNR

IEADCPVKPECVLANIMEPIPLKAQKFDSISLMYVLHCIPVSPAAKGRVF

ANLKPFLHDDGTLFGCTVLGKGVKHNLIGSFLMWLYNYIGMFGNWQDSKE

DFLASLRENFEIVESEVVGVSLLFKAQRPRR

>Mb|QLI67073.1

MAQGGAKSISAAPSASVSDCRPVIIIGAGISGLLLAQHLRQQGVPFRIFE

RDADMNTRGVGWGLTLNWSLPMLRSLLRPELLSRFAETYVDRQGIASGLV

PRFPFYDLSTGELKATTPPLPETVRARVTRERLRKWLATDVDIEWGKAFK

SFIETPNSCSDEAVNETGKGCSSVITVTFDDGSTCVGGLLVACDGSNSRV

RKALFPELAQENLYRIPIAVMGVKMQLSTVQIAGMREQDAYFLQGTSSEN

DSFVYMSVLDAPGNSPDNPPDSYNLQICLSWPYRDGYLGRKKQVCVPDTQ

EDRRKLFLEFAASYAEPFRSVMGAITDDTEIRSVDLSDWPPPKGLHTTGN

AVLMGDSLHHMAMYRGEGANHAIADVVDFANHVTPLLVADNESASTAGNE

RQTATSATADNLAAEEQAAHGLERGILRHALDQYEDVVINRARPGVLASR

RACLDAHEWARIGPESPLLTKRQMDIVYDEVADRVVG

>Mb|QLI67095.1

MVVVAVAGGTGGVGRTVLDAIAKSGQHQAIVLSRTTSISTAVDEPERFAV

DYNSVEQMKQILQENNVQVVVSALLLVDEAVAQAQINLIRAAAQSGTVTK

FIPSEYYIDFHAPIPGADLFTNFQLEAEAELARHAQLTWTLIRVGIFLDH

LTMPHNPKTTYITPFWVFVDIDHEQCVFPGDGSQPLVLTHSQDLAAYIER
LVGLPAENWPRESVVASNKLLVKDLESLVNKVTGKKFKVAYDSVEDIHKG
HITQLPSNTAVFQDPAKGEMFRDVERQVMLSMLSRAHDLPGKNLAELFPE
VETTDIEDFFRAGWTLKQSRAP
>Mb|QLI67107.1
MALGTFTKSVTQPINAGDPTENSTGRSKETRVSTRSLGGNSAVRIPTTLS
GQLIWSGQDYHHNNSLYALCLTSEDIAEVEQALEAFKALCLDGDEVSKAN
FPLPNLTERLEKCSKILHDGLGFFVIKGIDMSHYTVEDSIVIYLGIASYI
ADQRGIQDREGNVLRHITSSKMWDVPLEKRHGIHSNAALPFHNDMGCDIL
ALQVRHCAHSRGSTFVSSAASIFNQLITVEPAVVRTLFEPNWPVQCSGRH
AQHYLAPVMKWHHDRLMTSMDPNRFGPHPASSGSSIPVLTQDQRYALQKL
EEAARSTELELDLERGDLLFLNNWALLHRREAYDDDETTCRHLIRLWLRS
TRHGWHVPPEMLLPWQTAYNDKIQTRIYALHPSPTYSTPKYSVGSAAFLI
YDDEQEVDESLIR
>Mb|QLI67143.1
MSSSDLPDHRISFGPDANCTLSTCPIEASVYQYRPSLPANSVLLALFALA
LAVHAVLGIRRRTWGFMAFMIVGCLVEIVGYAARILLYKNPFDFIAFLIQ
IIFITTGPVFYTAAIYVTLSKTINYLAPQLSRFNPKFFYWIFIPADLVCL
ALQAAGGALSTQTKGSSDTGVNITMAGLVLQVVVIFVFVVAFADYMVRYT
RHPGTQALTPRVRLFLSFLGLAVVLILGRSVYRAYELSKGYRGSDLITDE
GLFIGLEGVLIIIAAFALCVGHPGLLFSPKANVAGSNSASDVEKQVAGI
>Mb|QLI67147.1
MASEDNTRQPLRTYRGNCHCKAFVYEVQLPEIKAVKQCNCSICHKKGYLW
VLAQETGHFKIVKGGEDTLSSYTFNTKNRIHKFCPTCASPVMAVAKQAPP
NRTIGINVHTLQNVDTWALQKERIDWAKVEPMYIVPEHKGAEPSANIEGG
KLYKGGCHCGAVTVAVMSKPLDETFEDGGLECNCSICERNGYVWMYPLDE
QVVLHAEEPANIGRYAFSMHMFNKTFCKICGVNLTNEYNADQNEEQKARR
PEQAEKWEAWARRHHPVNLRVLPEVDISKMSRRYGKGYTGIPLLYENP
>Mb|QLI67153.1
MSSQVAKAARRVAHELHGVVVSAGLMQKTVKVRVGGQKWNKVVNKWFPDP
KHYLVHDPNSSLRTGDVVSIVPGWPTSRHKRHVVKHIIAPFGAPINERPP

VPTLEERIEDRETKKAAKAERRLSRTNS

>Mb|QLI67174.1

MLASAVILSLAGLVAADKVPFESMTSPIGDLKALSLRASAACDTGTACGD

SCIDIGAQCCSQSEGTFCRVGYGCQRDGCCPLGHTCYGPPSNTCEGDRVK

CGKSCAPGGSQCCSQSNGIFCIAGTSCAGTTQCSGPQRPASGSVSGSVTT

TSGSVAATSDISSRVTTTQGSSATSTYSLHVAPSIKTTDSGSGSASGSSS

ATASTSGSGSSSGDSTPTASSASPTDDKKNPSPSPTGAGAINSPSILLGL

LAAVLLL

>Mb|QLI67213.1

MGWFDNDSDQAQAYDQVTNRPHEAKWSHELIGGAAAFEAAKAYEDHVARN

GHPDDHAKAKEILAGAIGFFVDREVETKGLDYIDREKAKRHAQQQAEQGL

SSEGRW

>Mb|QLI67233.1

MKGLILVGGFGTRLRPLTLTLPKPLVEFGNKPMIVHQIEALVAAGVKDII

LAVNYRPEIMEKFLQEYEEKYDINIEFSVESEPLDTAGPLKLAEKILLKD

DSPFFVLNSDVICDYPFQDLLQFHNNHGDEGTIVVTKVEEPSKYGVVVHK

PNHPSRIDRFVEKPVQYVGNRINAGMYIFNTSVLKRIELRPTSIEKETFP

SMVQDNQLHSFDLEGFWMDVGQPKDFLLGTSLYLSSLTNRGCKTLTPPTE

PFVHGGNVLIDPSAKIGKNCRIGPNVTIGPDVVVGNGVRLQYCVLLRGSK

VKDHACVKSAIVGWNSTVGCWARLENVTVLGDDVTIGDEIYVNGGCVLPH

KSIKTNVDVPAIIM

>Mb|QLI67275.1

MSARHAIQACRACRRLKRKCSRDIPSCSLCLRLSKKCEYRPGDAGSEPAP

NPDLASRLHDLEQLLQGKGLSDLMPLPSYANVNSSFPAAFFLDPDHYSKF

GQHDLSSEQLPWAEKAHTLVQDDWREVCERFLSTVYTWLPMISKKRLYSE

LTSSNPDTRAGNALFLLSMKLCTGRPLQSADNSSSYADNELYSAARQCLF

CAESGGYVSLRLVQSLVLLAVYEMGHAIYPAAYLTVGRAARLAMVIGLHN

TKLATQLFVLPDMWSLREEQGRTWWAIFVLDKYKVLPPILSPHVLVSLGS

PGLPFATPDPSVDHLLPSNDRDWNNGVISINEPVLAQSFHAANSIGSFAR

TCQAAHVLGKTMAHINRCKSAMTDVTEVVSEALHLHKALVALDTSLGSPL

DSFQGEGSSGPDSADVEDMNYTALSICCSARYLLYTQYACNESNRSAGPE

RIALEAEAQQLSIQGMEHLSGVLLPQIARHMEHMVASPAVRPSVSFVPGH
SMYYSAIDCACFYKEFGSRTMLDGIKQIVQGFKALRTEWRVGAEYLALLD
KEHVLELLE
>Mb|QLI67311.1
MTLYLKYSVALGNIYTQGSCLENFQNHQEHRLTIQSTGVPFAKSWIGELQ
LGTVDAIVAALLASMPNLTTLYLGPNFTIKSRLWGGVLQCALCQPKEYQL
PTLTQLRHVTSEYRAKEWHHRDTTNTADVLPFFYLPNVEHLSVSIDNPAQ
FAWPSDPPAPSSLVSLDLYRLRESRLAPILSVLRGLQKLHWNWLYQPDLD
ENVSQDVVELDTAAAALGHVRDTLTDLTIRTITRPRISVGDYDPPSLEIQ
PSLDGISSFNKLIRLCVPWVFLMGFSPSLSRKL
>Mb|QLI67325.1
MSQLKKRSVAVSFIFQFPKDDTTNTRPKVALFKRSDKVRTYQHKYAPVSG
SIESFDVNPLTTAWREIQEETTLTPCSLTLFRQGKPYSFVDESIQREWTI
NPFGFILTCDPSKITIDWEHDDYAWFDPADVADDQKVDGVPKLLESLRRV
WLEFDLGAAAPVLDASLTRLKGDHESGARQLAGIALGAFADVLKKSDISD
KEVWWANARRAGWHLWKNGRESMGAPILSVVLAGLDVVREKTSEGLSADV
VDDIVASLDELARQREASASRIANAFAAFLQQRPADGPIRILTLSSSSTI
ASCLTHALASIGHRLDLRILESRPLCEGAKLAYKLSSFAAEHNFSSRVDI
TVMTDAAAGIASQDVDLLLLGADLIDRHGNVSNKTGSLPAVLAARHVSNN
VKVVVVSEKEKVLPFEAPGHEDNDAGEVTRGWDSVGVVRGQRPGGDVKVR
NVYFEWVNNGLVDVYVTDEGISGTEDVMRWAADIERRAKDVFDGL
>Mb|QLI67397.1
MTTPLTHPAYSNNYRDRTFAIAKTRSANDRAVIENPLAHFTDAELEFDVR
NFAETFLPSVRYQELLRAARVGKDIQIYDEAARQPAGYEERNRLAVILTE
EEKIALRNEKDVAFSEKGMLAVIATVSLAAFLQGFVQSSVNGASLYMNQW
GLNPSNETSHQITPDNWKLGAANASPFFFAALVGCPLSLPINYWFGRKGG
ISVAAILIFASSIGAIFVTSWTQMFGVRVINGIGMGIKAVSTPILASETA
VGFWRGSAILAWQLWVAFGIMMSFVFNLIFTQAANPTTTFRLIQGAPLVP
AFALLIMALFVCPESPRFHLLKGPNYSVEKAYTVLKRVRNTKLQALRDLY
LVSKSIEQENMDFGDLDPQAMMSPGFFWVIRDFARQFVQLFQRRRLFNAV
LSASTVNLAQQLCGVNVCAFYSGIVFQQAGRSESNVITNMAYSLGFGAIN

FVFALPAVKSIDTLGRRRWLLLTLPFMAVFMLGAGLSENVRDDATRNGVT
ACFLFLFAVAYSPGLGPIPFTLASESFPLTHREAGTAWAISINLLFAGLL
AVCFPGINSALGQKGSMGIFAGLNIVAYVMVFLFVEETKQRSLEELDHIF
AVSKREFMHFKVTRYLPWVIGKYVFRLKRDEPKLYRDMVWGSRPVGLKKT
RIEPLGEARVELRPRRPTVFQTPKFSDTTRPNMAEMEEIYPPGVAVPYAR
EYQDGQTPRQGTRRNDSRFQ
>Mb|QLI67470.1
MPWITKGRHIKALWYHPGQSSPMRVEGNVKLYPGIVGSSNLQNFSPHNVS
SFQPSIFGSAVEAQQISFSAAYNPYPFVPEFSATTTSTPDNSPVQNSDTL
HVCVSSGQTAVTLTSEQAFAEMGGGEIHLPQPYNHDTYKHFAPEQGAQNV
SSAPLLPIVTKSLLDDLELPSTEPELGSDLLSSQPVVPAVLAMRPAQLVP
QSSNPETGQLLTTSHRQDDVEAASDSSRPSSASGKNAMHLVKKRGPFNLQ
KRKETAETRKRKACLRCRVQKIRCDADQDEVEGSCLPCQSFSKVSKKTLH
HVSCFRGKLTDVVLFRQGGLNLTQRWKGTEMKDVGDRVNTDIRSIQITLG
ICDTPIEIKVVRFRAAASDVVARFWTVREGERGDEIRKKKDLEPFCLVDI
WATATYFEKYVVDNAIATIVKNYTPHKMLQNTLAGQDVISRTYIAAVQYY
LSLEEKDEVMIPSGKIANPQKRLLGNLFIFWHANQHTAGSAYICGKETLN
MKPELKDETYPLFGKVSVPRMILAQFDSINHRKLLQRYGQKVLRDLEAFI
FRNQSVLWWPIYLTVFILLHDASKLSADRYRHARNNFGGRYRYSIPNFVE
ELQEGCNNILVHWHYYNCHPWPKPDDPWERHNHFMSELTSEQYDLVMDTM
TDRRVQKQLAAWAKCRQENGMMEKPPMRPMGKQATPYMGSQTNYDWDHPC
YWITQMFEERWQPRPTYQREYIH
>Mb|QLI67517.1
MSAVTANNEEADRVLRQKRQARGPSACYPCKKRKVRCDGSVPCQTCRKRQ
HPQICTYHLSMHPQRASVAAPVQDETARSALPVCPGEAGKVSCEPSSLPD
EASKNYVYSGDNSFVSILGSRASGANDSVAGELTSVLGLQNTFNIYPFMD
SKTPHDRWKLLLAVLPQRAEVLKFFHCYRATAYPFFNPIIVDIDQFESELC
TYLSTHAAGELHDPGKISDRWATNKSIGYISLLLATLAAGSHYSDVEYPH
RQELSIDFANFLFRPSLDTIQALLILGNTLQNMGQSDSAWAMLGTTVRLA
QAMGLHAQRNTVRWPECIRTRARALWSTIVWQDSLLSLCYDRPPIVSVTE
WPLDDSFFQRQNLSYTEIMHFICRLNLDIMRPETLAVAEVNRAIEGLQQF

DGVYQRGQPYLRAWENCTTIQQHLEHLALRMHSSFFISVLCRPAIKTSPA
QPPLPHTDILRTRAKASLIDASKAFLDFQALSVIPLRSWSMVHTVLSSTL
LLCAWEETRNDPECRGLQQRVIDVFSLSDSTAGDGALLSDNNREWLSARH
IRALVKLRNALDQEEGISTTGNENWAGLGANAISPLGTVPDGYLDMLDPA
AISPVAYLNSVLNVSLFDFS
>Mb|QLI67591.1
MPSTKATQNPPEQQPSSQRCKRIVVAITGATGATLGIKALIALRKLNVKT
HLVMSQWAEATIKYETDYHPSNVKALADHTHSIHDMAAPISSGSFKADGM
IIVPCSMKTLAAINTGFCDDLISRTADVMLKERRRLVLVARETPLSSIHL
RNMLEVSNAGAIIFPPVPAYYIRASSVDDLVDQSVGRMLDLFDLDTGDFA
RWEGWKTAKR
>Mb|QLI67606.1
MASQLNVEELIGAAEHHHEQYLQTLRSIQNVVGQRARERADSPPPRTLSH
PTFFSPEPHTPNLHLLSRLQRTRTSTLETTAERPSFYPSPKPVARSTISH
DSEFIPDEELSFIPLLDAPSAGARPAEELAPPPADITSRTQKPLLPLSFS
DDMLMRHLRETEFDGPTATVLEEVVRRRPDMDLAVPFRDFAAFERESYVS
TTFEVYEVTADAEAKKISVDIDVQADVKYGGEGVPYQSPDGIVDAPTVWE
AIKDVNSDGEAVGRITIVQEPTPLILAALHLTMTPHFDMGELLTHLLSEA
PNRGRTNAIMHRAFERTSSVSPYPPSPLIPPALDTRHQPASSYANVRQRS
FFFVFKYYTSVAEGLEPAPWQRFDKRPSDKRLGDHIDIAECSSVLALSLG
GEPTKPLRMRPRRERAREGFLFDTFGPWQLLSIQSFPDDQHTVRGEDFQK
KSFYNGPYAFLDLLTAEYRDAGRRNQILHEKITKLITPPTEFMFDRRLRD
KLLFEDKHFTYIRRYFWAYNTLAVINTGIKAMVGAYFDTFTDDFWPGNHP
VLWPHPSPSSPEGEAYLAKMALLRRELEKAVAELGEVLKRNERTRKEIEN
LRDQLFSGSSIKESRRAIDQGDNIRILTMISMIFLPLTFVTAITMVLVCI
PFMALIFLLQTRIFTVVVGELYRFILRVVTFPYRMFTGALEQDAPDNRSV
HLAKGRKRRLRMRVANEQPPVGSRWKWPWQKRWNGEGELGRV
>Mb|QLI67621.1
MSAHTHVVLNLVQVNPAIAGLATISILILSIIVLFGGSASKHRHGSISPP
SMPAYWLPFFAHAFQFFFNQDGFLASLKKRYPEGIFSLSMLGKRHHVIHD
PSIMINIWNRPRCSSGEKWTAARALTKSFGLRKQDQITYSNLAHETPDLF

KHMLSEPGLSEFVNGIVDHVKDHIAEFVTFNPSAADQTDWERSAKVDVVR

SSKGDEFVEADLMSLVRNFVAKTANPALFGTDFVENFPDFWDLLWLLDDG

FLSLATGLPGWIPWPKMQRAKAARRRMIAKAREFEEAMDKYLDGEDPGIK

WQDMDNASTFVKSRISLYRKHGLSVEARASCDVALAWTMNANANQLTSWL

LFELYRDPVLLETVREEIAPFVQVAQPQNEFGSAVWIAPELEKLDMDGLI

NQCPHLKAAYVETMRVYTGVWIVKWLGEDVVVEPKGKNTESYFLEKGHMA

HIAHEMHQFDPTYFPNPKEWHHDRFLKHTEDTGRPNLQVVEMGTLRPFGA

GPTMCKGRAIALREMLFYAAMVLSFYDMVPPEGKSWEEPELSKRAVTKHP

TKPIKVWIKRRNVSS

>Mb|QLI67624.1

MNTSEAAAWSDDDAERAKIENRGLSTTKYVRGSRLGLGGSSVVYKTLRVS

DGKVLAGKSSKAPKGLKREAGILRLLQHEHIVVFVELYHDPVCPVSGLLL

MELCPHGTLQTRIDRAAPHMARGEVLSVVRQIARALAYMHARGMYHSDVK

PRNVLIRDFDPVHVVLADCADVRMLGQPGELKGTPAYWSPEMKARRAHCG

PADDMWALGVTLLGMVGQWPQMVYTVMDLEVYPRRCFEHACRLGELNPGV

GIVSLLTGLLAWEAEERMRADECVLAVEELGAAEGGEELGIRSPEAFRPM

LFW

>Mb|QLI67631.1

MSSNEKSGESAAELSGNTYYPPPPPGPPPSQQGPPEQQQQQQQHLQQPLA

QQYQPHPQQQPDQQQYFPPPPGPADEKQPHVQQQQQQQQPYFPPPPPAPH

AVDETQSHQQHQQQQQHQQQQQQPSFPPPPPASADDKQSYQQQQSAYAIP

AYNPANPAFAPPPTAQHDAQTPTGHPGPASSYAHQQPQQPQHQQPPQQQP

SQTAPKKSGWGDRFKHFVSAGAAAPINSLAHKLGSQSFLPETLDKECDKS

AAILRSFCTKGVYADPGAGQPPTSTDPKAHESSSGVIDPTKEKPKNRVIV

TIPPKVISKAVGLAIFTTLRAGFQVSGATGSGVLIARLPDGSWSPPSGIQ

VHSVGGGFQIGLDIYDCVCVINSREALAAFMNTRVSLGSDLAVVAGPYGA

GGAVDFGTAVQRGRDDKDSKATAASDAQPAAAEAQPSSALKPDAGAASKD

SKRRSLSTSAFKPVFSYVKSRGFYAGIQVDGTVVVERKDANASFYGAPVT

VQQILQGQVPPQGPRDMWPVGARALLETLRGAEAGALGHHSSASASASAS

GAAGHPGSGAVPWANAPPAGAAPGEASSGAPPAYVDDGSHAHVGDAKYA

>Mb|QLI67637.1

MVKLSIAAVVALASASAWALPSNNHVLHEARNPTSTRQNHLWKRGNRVHP
DAVIPLRIGLAQSNLHEAYGKLMNVSDPDSEHFGKHLAQDEVHALFAPSD
ETLTAVHSWLVDMGISKTQIRQYTNKGWLALDVPVSKAEELFQTQYHEHE
HDGAIKIGCDEYRVPKHLSQHIDYIIPGVKLSPLMVRRSAKARSPAKRIT
AQNRKNWEPKKVSRKKPSCKTPPPSNLPPELKDCARNFTAACYRALYQIP
LANTPVPGLEPAVYEIGDTYSQEDINSYLHKYTPYIPNGTHPTLHSVDGA
QAPVPPQSPQNTGESDIDIDIVQSLIWPQSMLLYQVDDIYYSTQSNTSGF
LNTFLDALDGSYCHKTDFNITGDSPGIDPSYPDSHPGGYKQAEMCGAYKA
APVISISYGESELDVPKKYMQRQCNEFLKLGLQGTTVLVSSGDYGVGIGP
GANACLNGSGQTNTVYNPGNPVSCPYLTAVGATQLNPNTTVRDPEAALQT
PLPGAELFASGGGFSNYFPVPEYQRAAVRSYLARHDPGLRSYVADADASN
IGAGGGVYNRAGRGIPDIAANGANFRAFTNGTDQHVFGTSLAAPLWSAVI
TLVNQERARAGKTTVGFINPALYKNPAALTDVTRGSNPNCGSSGFSAVRG
WDPVTGLGTPMFEELRRLWLRLP
>Mb|QLI67704.1
MNNNNFGDSAYAHQGPGYQINAAGGTVNNTVYNERNGTPIQGKDCWKSLA
FPEMESRYYDIDAAQTGTCGWLSQHEMYREWVERNRGLLWIKGKPGSGKS
TLLRHALDDAKKKRNVKENPLILSFFFHGRGTELQRTPEGFYRSLLHQLS
KTPDAVSDVVSTFKEKCETFGEVGKAWQWHPKQLRELFDSAILEALKLRP
ICLFVDALDECGEKDATMLAQRFKDLLDSLRSTAYNSFHICFTCRPYPIV
ALDSAFEICVEEGNGTDISNFVGHQLSELKPTILPRIRNLITERANGVFL
WASLIVKQIVSLNLKATKLEKMEDAILRIPQELDDLYRQLIEEMGSDSVM
LIQCICVAMRPLSIADWQWAMVIKTDCPYSSLQECQNSPDYKPDDESIKK
EIQILSHGLIEVTSNKTIQFIHQTVKDFFVEKGLWLLKPLGSVPDGISES
DFAVGMAHHELSRICVRFLKMVPNTQIFLKSAVRTHDIPLSIYATIEWVP
HVQQSEQRGILQDTVLQDNLLDCFPWLMETVPSPFMRFYCTAFDRLFQYS
HPMSYLSHVASEFGLTGLLQAMLRRSKSVIDEKDTRGRTPLMYAAENEHE
ATARLLLGYGADIEANDEEGQTPLFFAAVSGHEGIVQLLLERGADVDGLG
ERRAHGNNKKHYEGKTPLMCAAEKGHEATARFLLGYGADIEANDENGQTP
LFYATTSEYVDIVQLLLEHGADSNTKDKVGRTPLFYAAKTIRGDIVQLLL
RYGADIDAEDDTGMTPLWIAYDNEKADIERLLFEHEAAENGEYDDI

>Mb|QLI67778.1

MCICTILHYHHAPPCPRPVIFTTHYLYCPDAIVNPVTNEILSPCSNTSYK

PSQGIDYPDPCSTGGCLISPLCSTGTCRLEDLNGLWVCCQCDRGGNQFRT

CTNRKWACPDTFCYHKICQGCRPDLGKVGNLPGGR

>Mb|QLI67792.1

MAFMDINILPAGAASLIVGLLTTVVALRLAAGRKVHPNEPTVLPSWIPFI

GHLVGMAIYGGRLSHPNEPIFTLPVPGSRIYVVTDPSLAASIQRNTVARI

RKNLDPEPGNPRGFLADVHNVVYSALGPGWHLNSLSCEAGQELCFQLTEF

AASFDKLGQTERELDLLQWARHLVTVGSARYLYGPRNPIAEDPGLEAAFW

AFDQGLGGLLMGFLPSLTASEAYQGRERMVTAFMKYFEAGHIKDGAQISR

DRVRLEEEYGMNKQMIARSALSFIFASIVNTTTTTFWVVLRLFANKKLLS

IARREVAEALNASTEREGSKRLSIGILKNRCPTLVAVFRESLRIGSENFS

VRLIKEDIFLTVRYFLKKDAVVQIAGGIIHAAKSIWGDDADEFNPQRFLA

PKAQSGGIHPAAFRGFGGGKTLCPGRHFATNEILLFAALIVHGLDMSSPD

GGDIRVPRKDDRVMPVHILEPYPRDRPKVLFRLRDEMKSLRDLTIVI

>Mb|QLI67798.1

MVQKRPVGLAVNKSPSAEKLAIIEKILEPFVDDIINEYFTKANECLPLLD

EQSFRDQYLEDKTHISPALLSCLYAHTLVYWRHSPTLSGNKCPDDRFIWN

LANEAVYSELYLSPGISIIKAILLNISGRPTTSIIGNGVLLGSAVSMAHS

LGLNHNPLPWKIPQSEKYLRMKLWWSLLLHDRWLSLAHGTPPHISRIQYD

VPLPTLECLCEGDSSEARVRTANVFIALVRLTDVLDQHLQRVYAVDGNRP

WDTTSLELALNNWVESLTGSCRLIVIRGSRLEIPGAANLRLAYLTTRLLL

QRMELEAEKRIYDQLNERIMNRYIQARRTAEEILLLLQELQVEQLSGFWL

PVTAFAFPATVNFLLRCALEMESSLQGLARSSSFRIAQDIVATLRKYQKQ

FEWDLADICVAQHADIVDRILGGVAREEESDNNMDIQEFSMPDASILDQY

FPSLWDPLQNAW

>Mb|QLI67808.1

MSELPDELLVRVARLEADLRAAQQAQVVTTTNNTTIKYDDAQRTAADAEK

HSSTTSPSPPMQPAPTWADVPSTAVGDAVESDKKKFSQLPPVPVLKSLAD

VYFSNCQNQPYCFFHENHFRRRLIAGDLAHYLLLAFAATAARYSSHDYFR

GSELEAVEGLARAAWIIILDQVFATEPSPDNVAAAQATSLLAIIDFTAGR

HRLGWVKIGLAVRLVQSLKLNAEPDPSLPPWQQEERRRVFWSVYLLDKFV
SCGRSHPPAILDMDCTLALPCSEEAFRMETPTKLPTIALVKDLPSNMAEL
AQLDDFAILVLMCSALSLTVRETFQQNASKIPPWDCRSNFAQISSILMTF
ENIHTTGADHLGPYISARFGTYEGFDRQRVGHFIWARAVYHLCGSLLCHP
VSLRRYRKSQGACFPTTFARELLDRCREHATQLSNMLETLRSAGCCARGS
FLGYVATCAASVHHIFALSPTPHIAARANASLEMCLGFLEHLPARWPNYG
MMASSLRALSIDGRSARLLIDPSPAAVEEDIEPVEIEQLWNSIDYGWMTD
RARQSETASSPSPPGLDVQIGDWTTFLEGQMGDNDMSSFYVTQAELMREA
DT
>Mb|QLI67817.1
MEDTYRALSERLRAKWAEKKALGQARLVVAIAGPPGSGKSTMASKVVEHV
ARSDNAPSIVAVSADGFHLSRRALRALPNAQHALARRGAPWTFDGEAAAE
LACRLRTEAGRSAVSAPTFDHALGDPVQNGVTIAPETEICIFEGNYLLSD
EAPWSAIGDVVDDRWLVRVDVEVAKRRLAARHLDAGIEESMEDAVARAES
NDLVNGEYVMAHSFGRHDVLIDSAEG
>Mb|QLI67824.1
MPKRTFEPESDGEAVCGPAKRRNTDYADTQTPTLVGVSPEAMDNDEYTVG
WICALSTEYVAAQAFLDETHRGPRFVSPYDHNHYTLGRMGNHHIVIAVLP
SGEYGIASAAVVAKDMLHSFPNIKIGLMVGIGGGAPSQKHDIRLGDVVVS
DPRDGIGGVFQYDFGKILQDQMKDVEFRTTGFLNQPPIVLRTAKIPRLQQ
EYKRPDPCSDRLYRCEFTHPVSGGLSCTAACGNDPSKLISRPERTRDRDT
PNIHYGLIASANQLMKNAQVRDRLALKKNILCFEMEAAGLMSSFPCLIIR
GICDYSDSHKNEEWQGYAAMAAAAYAKDLLSQIPSNGAENLRRTSSGISL
RPRRGENQSLNDEQRRVLLESLRFDQIDARQMTIKNAHAKTCKWLLQTPA
YLDWLDPNKFNEHFGFLWIKGKPGAGKSTLMKFALANARKKTKGKIIISF
FFNARGSDLEKSTIGLYRSILLQLLDRLPGLQEVFDSLPSAIRDSGYDQW
RVESLKALFEKAVQGLGETGLMCFIDALDECDEDQIRDMISFFESLSQTT
TPAGISFRVCFSSRHYPHISIKRGLNLILEGHEGHGQDIVNYVDSELTIG
HSRLAAQIRADLQEKASGVFMWVVLVVGILNKAHDKGRIHELRKILRDIP
GDLHELFRDILTRDCHKRDELLLCIQWVLFSRQPLKPEQLYFAILSGIEP
GALSDWNPDEITVSIMKNFILDSSKGLTEVAKSKNPTIQFIHESVKDFLL

KENGLREIWSDFGENFQGESHERLKQCCLRYMSLDIAAHLNIGSSLPKAS
SQEAAELRQSADTSFPLLEYAVRNVLYHADAAEGGGIAQTSFLQTFPQSD
WIKLDNLFERHEVRRHTNASLLYILAEHNMANLIGRHPSNLAGFEVQDER
YGTPIFAALATNSGAATRALLKAQVEINPEMSPIHSLFEQYCPNGSKRSS
LGRDFTFLRRRGVLSHVAEHGEEVITLAFLLAERCVDIDIGDRDGRTPLL
WAAKNGHEAVAKLLLEKGANIEAKNSFGDQRPLLVAAITGNKAVVKLLLE
KGADIEAKDDKGQTPLSGAVMYGHEAVVRLLLEKGANIETRDVYSWTPLL
WAVERGGEARADANIGLEDIYCATADRYNATLELLLKKGANIEAKDEKGQ
TPVSLAAKKGYRGVIELLRQYSS
>Mb|QLI67839.1
MWSSSHHVLATAVAALWVQTSHAAAPSGDPFRVIDPQHWVNPDNMTCADY
KAPPGTQWNDPSKNGQERNFNIALVTVDYSDLNFTITGPPNSTIFGTPSA
DAANIPREDVPVWYRDFLNKPGQLNRGHTLHEYWMEDSHGRYGVDLTAFG
PYRMPHLSYQYGISDMNPGACLPGKECSLDIRDGALSLWREDVGNSTASK
YELVFILTAGQDESSTWQEFGEMKFNSKEDVPDAFGPPKTTGNATLPNYA
ATRYVPWTSWAAAASVWPYAEPSLSSSVQCESSGMATFAHELSHLLYISD
NYNNPYGTPLRRTFTGPWSMMSRGSFNGPGGPHARWHVPPVQGSSMGSLH
TVRDKLQIGLIDEASIVNISRESLQTTGPLVTTVTARSVKADRIGVRVHL
GTDMSPACDVGKDLYCDGGGYDNYDVEVVDRMGADSFQADAGVMISKTKN
SDSSEPFQWTIDANPQDIKLTDFVRPNGTEAMVTMGDYRQLLDALFHAGT
RSGSKAEYKDEANGLHFYILEKIRDASGVLSYTVGARALNSTSKSRFGVR
LGEGRPEPSGNTPTGKGIFCSFDLVNDGKYTQESDVTSALEPHVGFDIYR
LQAEVKGKGWRTEVPNALVAAKFGETVVANIAVGATANADDVGVVTLTVT
SESDPEARTTATCEVRKA
>Mb|QLI67845.1
MSLQIPLTHRSKDQGTTKAIILVGGPSRGTRFRPLSLDLPKPLFEVAGHP
IIWHCLSSIARVPNKHIQEVYIIGYYDESVFRDFIKDSAKEFPSITIRYL
REYQALGTAGGLYHFRDAILKGKPERIFVLNADVCCSFPLAEMLKLYVEK
DAEAVILGTRVSDDAATNFGCIVSDTHTRRVLHYVEKPESQISNLINCGV
YLFSTEAIFPSIKSAIKRRLDRPSRLVSYPSSDNLESHHFPPGGDDDDDE
SSRKNEVIRLEQDILSDMADSKQFFVYETQDFWRQIKTAGSAVPANALYL

QQAWQSDNKELTQPSAKILPPVFIHPTAQVHPTAKLGPNVSIGPRVIVGA

GARIKESVVLEDSEVKHDACILYSIIGWGSRVGAWARVEGTPTPASSHST

SIIKNGVKVQSITILGKDCVVGDEVRIQNCICLPYKELKRDVANEVIM

>Mb|QLI67918.1

MGSILDAIGKTPVVQLKHVVPAGYANVYIKLEFYNPTGSYKDRMAKSMIE

EAEKRGDLQAGMTVVEATGGSTGSSLGFVCAAKGYRFRVLCSDAVAVEKL

RSMTALGADLDITHSPSGKLTPDLLPAMNKRAAEIGREHGHYFTDQFVNR

DALVGYEGIGHELVEQVPGGIDAFCGAVGTAGMAMGVGRVLRSKSPSTRI

VILEPASAPLLTQGVTGTHSVEGVAPGFVAQHLNRDLYDEVRAIDEEQAR

AICRRLAKEEGLLVGTSSGLNVVAALQLAKELGPGKTVVTVAVDSGFKYL

NGTLFQDAQSRAGGAAKL

>Mb|QLI67935.1

MSAEIALLVSNGWQAVQSWFQEQRSFAVWFAAIVCWASWWCWRFIITPRI

STLEVKEYPYWIPVLGHGLSFIYNSQKLFNTARQHFHNTNEPLQLTLFGA

SVYFVSRAEDVSEIYRNARTFTFDEFYHHIFISMGTSGASVQQIFAPLPA

RIKDPENTQGKPVAKLAKELQIAQLQPGSGLDALERAQLAYLECHVRPDA

VLESRYRYPDFLGGELPSQTGSVEMSLWHWCADLNVRASQRAFFGSALDR

LDPDLPQKFLEFDDLSWKLLYKFPFFLARDTIAKRDYLRTALKAFYNLPR

EMRNDAEGWALDSTEDRLRAIGIPTEDIASIALILYWGANTNTRKAAFWL

ICHLLSNPSLIDPIRTETAGAFGPEGTITNLVHLHRHCHQLDATWNETLR

LTGVAASLRTVTADTVLGGRMLRAGNPLIIPYRQLHHDKGIFGDDIMTFR

PERFLNTKLKKGKRVCLEHFRPFGGGSTICPGRFITKRVVMILVALLLRR

FDISMAGGSQDMPQADDWSPFLGIAGVKPGHDMRIRLTPRKAAAT

>Mb|QLI67988.1

MHPFSILTLGIFVAGYITARWDLVTRLYELAIFAAEYGVVIRAAKGVVAL

TALFLTIFIPVVWVARKETFLV

>Mb|QLI68031.1

MSTRQDEKMPLPPRIARARRDARAKKAKAFLNKTVPLVLQSNARARRGVE

AACLVVDPPKCNSSQEAVRRDGPSGKTLATDAGPEPSTLAIRITLRVTDT

LEAASRLMPRVAVLNMASPLRPGGGILTGATSQEECLCGRTTLYPSLRED

FYRLPDVGGVYTPDVLVVRSWDAQGDDLPVSKRFFVDVVTAAMLRMPDVE

GNVYVEDKDKEIVLRKMRAVMRMVKGRGVDRLVLGAWGCGAYGNPVREIA

RAWKKVLCGRKGKDKDDGEGWDGLEVVFAIKDRRMADIFATEFGGGLIVE

ENDAEEEAEEV

>Mb|QLI68067.1

MARPTGLIASKGIELLTWSTPNGYKASILLEELKEAYGLQYTSQGINIGQ

NIQKEPWFTAINPNGRIPAIVDHDHGDLAVFEGNAILSYLSRRYDTERLF

SFAPDDDDYTRAESWIGWQHGGLGPMQGQANHFVRAAKEKIAYGMQRYVG

ETERLYGILDARLKDRDFVVGPGRGKYSIADIALLGWVNAVRGTTISIDQ

FPSIKAWLARCWERPAVQRGFQVPSAPASSALTPQVGEAAEQAKQLKQLV

DEAKKQYGYVYSSP

>Mb|QLI68094.1

MEKSSFKVIIVGCSITGMTLGHCLDRAGIDYVILEKHENIFAEPGVSLGL

MPNGSRILEQLGIYSGVDEIYEPIHKIYQFLPDGFGLPFCVIARQHFLQV

LYAKFEDKSRIHMSKKVVEISHGKSNVSVTAADGTTYEGDLVVGADGIHS

AVRSEMWRIGNSEQAGFITETEKSELSAEFACVFGVCKAVPGQGRWEHIL

RYNEGFCFMFFPATGTDVFFNVIYKLKQKYKYPNIPRFTQDDAFKVCESV

GDFPVWKDIKFRDVWEQRTSFAIVALEEHMFTNWHHRRIVCVGDSISKMT

PNMGQGANTAIETVAALTNELRTLVKANHPDKPSEYELNNMLQRFNRKQF

RRLTAVHGDARYVTRLEALDGAMHWIFSRYIMGHCGDVLLGNTARIVSGG

KVLDFIPLTARSGKDWPPCPWQHSFGISDMIDFCKKMSIAFLIALVAVVL

SAFGAGRH

>Mb|QLI68120.1

MNARVSSIFRAKKSPQPATKLAPRFRRLHLAPPFLLDDYTPRYQTLSSRD

EAKKRSLAYAHLRNCNLCPRLCGVNRYEKTGMCLIGDKVKVNVIAPHFGE

EPCIQGHSGSGAVFMSGCNLRCIFCQNFDISHQRNGMDLTPEELGEWYIK

LQEVGNVHNINIVTPEHVVPQVALSVLHAKQLGLRVPIVYNTSSFDSLAS

LELMDGLVDIYLADFKVWETSSSKRLLKADDYAATAKESIRAMQAQVGDL

CFTGDGIAKKGLLIRHLVMPGKEAEGVEIMKWLASDVSKDCFINIMEQYR

PDAHVGKKRRKSSDQDKEEIRYADLNRAVTGEEVSVVRQAANKSGLWRFN

EPPKHDGFAI

>Mb|QLI68211.1

MGAHTPDAQVSVFDSPPEELLHLLASQLPASLTLLRRLQFAVHRNCTGPD
ARIIVSSDTGQIGDGPTKPTCFAAAYAELSTGPDTQMVMYSSMEQGRPSD
EELPVHEGHIMNLVRTLAELRREYGGKLAYGNSLLVGNVHSDVHDILTKT
GRVTARRDYDKWLFRMEHVPELKETLDLAGMHWGTASLEDCRLVASRTDI
PRPPELLVQLPGLMLKLQDGTPIAWAFIGLHGSLISLHCEDGYRRRGIAK
LLAAKLLRRTGVEFSDAKYAGHEWSCAEVARDNEGSRAICKSLNATPPRW
SVSW
>Mb|QLI68216.1
MAHHSAVGQSEAQKGRHAPTSRSQSPDSALTAGDNQSIGNHAAGVPYGVS
VEQAEADFVQLQRELTGASRASRRRSAVKNIDPEKAAPVDSRGTDNESLF
DLETVLRGSLAAEEAAGIKPKHIGVCWDGLTVKGIGGMANYVQTFPNAFI
NFFDVITPVINLLGLGKRPPEATLLDSFQGVCNPGEMVLVLGKPGSGCTT
FLKTIANQRHGYTSVQGDVFYGPWTAKEFTRYRAEALYNAEDDIHHPTLT
VEQTLGFALDTKMPAKRPANMTQDDFKEHVISTLLKMFNIEHTRKTVVGD
HFVRGISGGERKRVSIAEMMISNACILSWDNSTRGLDASTALDFTRSLRI
LTNLYKTTTFVSLYQASENIYRLFDKVMVIDEGKQVYFGPANQARSYFEG
LGFAPRPRQTTPDYLTGCTDEFERQYAPGCSENNSPHSPDTLREAFRKSN
YQKKLESEIAEYKANLDQEKHKHNDFQIAVKESKRGASKRSVYQVGFHLQ
VWALMKRQFTLKLQDRFNLTLAWVRSIVIAIVLGTLYLNLEKTSASAFSK
GGLLFVALLFNAFQAFSELASTMLGRAIVNKHKAYGYHRPSSLWISQILV
DQAFAASEIMLFSIIVYFMSGLVRDAGAFFTFYLMILSGNIAMTLFFRII
GCASPDFDYAIKFAVVIITLFVTTSGYIIQYQSEKVWLRWIYWVNPLGLI
FSSLMQNEFQRIDMTCTADSLIPSGPGYTDINHQVCTLPGSNAGTTFVAG
PDYIAQGFSYYPGDLWRNWGIVLSIIIFFLILNVVLGEVVKFGMGGNSFK
VYQRPNKERAALNEKLLEKREARRKDKSNEVGSDLSIKSESILTWENLNY
NVPVPGGTRRLLNNVFGYVRPGELTALMGASGAGKTTLLDVLASRKNIGV
ITGDVLVDGFKPGKQFQRSTSYAEQLDLHEPTQTVREALRFSADLRQPYE
TPLAERHAYVEEIIALLEMEHIADCIIGTAEAGLTVEQRKRVTIGVELAA
KPELLLFLDEPTSGLDSQSAFNIVRFLKKLAAAGQAILCTIHQPNAALFE
NFDRLLLLQRGGRTVYFGDIGEDAAILRAYLRRHGAEAAPTDNVAEFMLE
AIGAGSSPRVGERDWADIWDESPELERAKKAIVEMREERKSVAQHANPDL

EKEYASPVHHQIRIVVRRMFRAFWRTPNYLFTRLFSHFAVAFVSGLTYLN

LDTSRSSLQYTVFVIFQVTVLPALIISQVEVMFHIKRALFFREASSKMYS

PMTFATAIVAAEMPYSILCAVVFFVCLYFMPGLDPTPSRAGYQFFMVLIT

EVFAVTMGQGLASLTPSPRISAQFDPFIIIIFALFCGVTIPAPQMPGFWR

AWLYQLDPFTRLIGGMVTTALHGLQVVCTSSELNRFTAPSNMTCGEYMTP

FFERGAPGYLVSNDTQNCEYCAYKIGDEFYGRLNMSFDNRWRDLGIYIAF

IGSNLIILFTASRFLNFNRR

>Mb|QLI68221.1

MKTSSETPTTGPVAGMSKYSLMSDQAISRASTEGVLVLDYKRGDVEICHL

QQLDTHVTLNNLGLEQQTKRDVGPFDILCVGYNICNCWVGLAATMVIGLE

QGGTVTVIYGTLVVTFAMACSAATMAELSSVYPTAGGPYHWTSILAPKKA

YRHLSYWCAASNIFGWLSICSGITIQPGQFISAVRLFYNPDLEIPAWEYF

LYFQATNLVFLLYNIFGLRRTRWIHDVGFFFSIFTFVIIFVSCLARAQSY

QSNTFVWTDFPKPESGWNSRAVVFLTGMANPNFMYAGLDGAVHLAEECAH

ASKTVPRALFSTIVVGFVVAFAFGLAMLYTLTDFGQVLENITGVPIYEIW

FQATRSGVAATIFMMLLLCIACFALNACVEVTARLTWSFARDNAIIGSKF

MGSIHPRWEVPVWALVANATVILIIGCIYLGSSTAFNAFIGTALVLQQCS

FALPAALLLWHKRSSSVLPPHRYIRLGPLGWIANAVTVLFFLLILVMYCF

PIELPVTGSNMNYTSVVIGIMVIFSAINWFVQAGRTFKGPRLLAEVARD

>Mb|QLI68388.1

MSLKAIATLLLAAAVSAAPAPSNTSPSDATKTTTLTGVTHSVVAGLGGLR

FDPDNVVAEIGDIVEWHFLPQNHSLVQSSFAEPCKPLADGTGFFPGFEFV

TSQGQAPNVFQLQVKHKKPLWYYCPQQKGNHCQQGMAGVINQNFDNPRVS

LQRYKEAAALTGTSVIPPVNNVGRVIPNPNPNGGF

>Mb|QLI68421.1

MKQRAMKPNSWSKKLSWLGYVAGTLARNVPHEPSGKLPTLGWNSWNAYRC

DINEQHFLDAAQALVDTGLRDAGYNYVNIDDCWSERTGRVNGHIAVNKTR

FPDGIDGLANKIHDMKLKLGIYSTAGTLTCAGYPASLGYEDVDAADFAKW

GVDSRTVVVVRHGLNRNSHSDNCYIPEQWQDEYIYCEEDGAQIGPNGTCS

RSQNPRLAPDGYDWSKSKSAERFNRMRDALARQDREILYSLCIWGTADVT

SWGHGTATSWRMSGDISPRWRSVTHILNMNSFKMGAVGFHAHNDADMLEV

GNGDLTPAEARSHFALWAAMKSPLLIGTDLRRLGRRDLDLLANRRLLAFH

QDAGHGGPAAPYKWGVNPDWTYNSTHPAEYWAGPSTGGHLVLMLNTLEAT

VRKTAAWWEIPGLGGGRYRVTEVWSGEDLGCLDEYAVDVASHDTAAVLVG

PRC

>Mb|QLI68431.1

MSVPPGLISAEGDPRLSVAEKPPQDVVHQPIHGNDSTITFEEFMYWAERT

RADEALTNKALLDKRGPRTVKNTLRSRFSKGHQTDPATSVADVYSSDEEI

ITGISEREWKQASRAIRTASWGSIFYLITTDVLGPFSTPWAFAQMGYGPG

VALYTVFGAMAGSGWIIWKAFLGLDSDRYPMHTYGDIYFRIFGRIPQILL

NIMLGLQLLLSVCSLILSNGQSISQISQGNNGSNGNGICFVACLIIFMAA

GFVLGQIRTLQRFGWIANFAVWINVAIIFICVGVVVHYPPNFKATQASFG

DNFGPGPIRTFAGTPPDGFASGGTGFVASLNGLNQAVYSYGGCMTFTAFL

AEMRHPMDFWKGLLCGQVFIYSLYMFFGIFVYSYQGQFAFNPVMQGLSSY

GFQTATNVMNLVTGLIAAGLYGNIGLKVIYIEVFERLFKFPPLTVRKGKI

MWAVTVPVYWSIAFIVAAAIPQFSYISGLIGAFFVLSFSYTFPALMALGF

WIHKDAMVPEQEKFDPQTRTYNYVDTGLVRWRRGFLKRPFFNLFNLVYML

GGLATTGLGVYSSIEGLIAAFSGSSVASSFGCRSPV

>Mb|QLI68440.1

MSDAFHPYADLYVNPSGPGDQRPTALRVVQDSGAKGTWAGRVVLVTGGTA

GIGVETVRAMHSTGADVYFTARSLEKAAATKEDVLKTTSGKGKLEVVEMD

MDSLDSVRKAAKDFLGRSSKLNVLINNAGIMACPYTKTKDGFERQFAVNH

LAHYLFTRLLLPTLISSSTPAFNSRIVNVSSSGHGMSPVNFDNYNFDQPD

SYDPWLSYGQSKTANIWTASYLDRALGSRGVHALSLHPGGIWTGLATYLP

DEVVDSWKSDEKVSTQMLNPPQGAATSVWAAVAPVWEGKGGKYLTNCSVA

SNAAGTVEVLDPGAAPHAYDVQAEDRLWELSAKLVGVEKDV

>Mb|QLI68465.1

MAPIRVGLIGLSTPQRNANFGIWAASTHLSALQKSPDHEDIALANSTIEF

AERSIAFHNLPSSTKAYGSAADLANDTDIDLVVVCVRVQRHLELVKPALL

GRKNVFVEWPLAANPDEVEYLTKLGKESGVQAVVGLQSRAAPITLKWKNI

VASGHIGRILTSLVWTLGPRTGLYYLDMNRGGNEFYIIFGHLLDSFITVL

GDFSEVQTILKSHYETVPIVDSDGQVVDLLYRKTSPDHILVQGITELGAV

ASLLVRKPPATADGVGIRWVISGTLGEIIVTGPGLWHVMDKEAHIQVKIG
NKPVQDIDFQSYRVPLANEVAPFGANVASLYDAIAKGDNTEYATFESAAR
THRLLERIRKASGPPFARE
>Mb|QLI68494.1
MSAPVQNNDTAAEQTYKEGNHNGHHLPPTTADAGPGGRVPKPYQSMVSQV
YDPQFFKIANPGPLGLIAFALTTFVLGLYQCGAGLPNSNPLGSVGPDQAV
FGLAVFYGGTAQFIAGIMEFRVGNTFGTTVHCSYGAFWLAFAMFMVPQLG
IKEAYGGDEHAFSFAVGIFLILWCFLTLVFFIAALRTNIAILTVLGLLVL
AFFFLSIAQFISTTHTTAAVRVNRAGGVFAVLCAFAAFYAGSAGLMTPAT
TWVVFPLGEFERPPAQTNPV
>Mb|QLI68509.1
MALTYSQCQKIRATVPALNARGEEIIASFYQSLLRGHPELRSYFNTANQL
NKLQPKAMASLIIQFANNVSHIYELIPKMERICQKHCSVGVQPHHYKIMG
AYLLEAFTGILDPSMTPDAKMAWNNAYWMLANMFIAREKQLYKTFGPWTD
WRPFDIADRVHDGENVVSFYLKPRDKKPLPPFMPGQYVSIRVAVPGKEHK
QIRQYALSEAPNPDYYRITVQRNRGAAAAADDDEQHQSACPYASPPGAVS
NHLIDSAVAGDTLELSHPAGHIFLDTKNLSNAPLVLFSTGIGATPMLSIL
NFTAKEQPARPVSWINASQGAMPFQARVDSLAARRRNLRNTTLGIAHDGH
ASADCDGQDDARLDLVSLRPDDLFLRNECTEYFVCGAENITVQFVEFLER
HGIDRARIHCELQTVARY
>Mb|QLI68538.1
MLPFRPHQLPRTTNPSQSRPVEEWINDIYLGSYEQGNDPAVDCMCLPIRL
APESRIRRFRNDESGTAFLMRRNVDGFVLASVMIVYAFPSISHEEMGREH
EYINALADHHGQATMYEIGFLWVAPEMAVLLAQDYDMQNIVAAIISPLTC
WRIMPHQTFSVIIFWDEEENLRAYIPDPQSMNGDDPGYIEATNLSEVFPT
CKQTLSSASQQERNLGTTLFGYQNASAGADDRANIGADDFFTDGHHMKRV
QSFFSTIRRPHQRRVKIAVLDTGLDINHPLLQKFVRSKQIGEELGRDFTM
HPLGEPDLKDNTGHGTACTHLLLKTCPTAVVYTAKISNQSTFDEKTAERI
SEAIQTAITEWEVDIISMSLSYEFEVEIIDEALKNRQRDKKRPVLFFAAS
GNFGKDKGEHSAGFPARHENVICASSSTYEGNKSDFNQGPDDMNRWKNFS
IIGENLCVAFSAELNRGNYEKRVSGTSMATPIMAGIAALVLEFCNIWKER

GGRPTLEKAATMQGMLQIFRGCMLSNSNSSSQQGHLNLVPWYLFDGSSYT
RDYASVGNHIAEALRRL
>Mb|QLI68588.1
MLTPTVANRLSFLYAIGNTPATSLTRSVPIGKDVDVLSLGCGDVRNILFT
SYVERGLAKRKIDVTCCDYDEKVIARNIFFLTLLLDGPGHVSPSCLWDIY
YHLYVDAAAVEQVLKHVKKLLPLLETLGAWKRGPYAKVIRVADQDTLDDV
RAVCRRIRDGVEQRDTASYARTFKKNLKKTSEALEAFSGKDGVNMSGVRS
AAPLSLNSHNDMIKAFQTYSTHGTVTPHDPGNTVPNPMFASLVSETNVFH
YGSDPTLGYHLAVAFAPLSENSPLKMHDEKTEFQTAEAARTQFTEWIAAF
RELASRGMMVRFMVADVFACCHTLQHSASTGKPSGNWYRRQWDSKPLRLD
GDEYGPAGKGPTAFDVVDSSNLSDHVGALNIIVAASSLLKTEPWATLYTE
TLLKQEKSQQEAFEKLLCGNIATVLLLLGISPVQYWTNAKCESHVDEVFL
TMLVGAMNAPQTQIHSRMAWKRDDQLSGTLGARGKLHIDPSALVRILFDI
YMRMFSGENFKISVNAALDRSSAYTHFHRGSYASFLKLVKHRVRTDWPAV
CSQLLDKISQDRTLALSTNQLQELGIQMHLQDVSTESWLLRESDNLSTGG
RFRMWKNIPLAVAVTVIVPRSAVERLYAGENRERSSPTFVGSLRAGASSP
NQWQNMYSDVHIVFGKVKNSPKDENTPIIVEQDDQGWSGSSALIASFVVP
TAALQVDPVDARVAICIPPSTQAAFLYMGILGMEMTVFETKLSAASSVFV
TKLMPGLTGHKSVCSRVNNFTDVVDDAGNGTSNKIMAEVPASESAITTIT
GHVDILSDKGKKMLQDRIPIELQQRDAFVIDVIFGNNALVCPLRFPIPVS
QTGSRLRVARKSGYVEVVAPVASSKDSDVLSDFIYPTRLDGKGFPAALNA
SHMNLDALPVLDLEKKDQMQWLVTLASLEFSPREKSMRDEADKKSGISEN
LRVNFKESLLTMFMLTSGLQGGQTGMFAINHPEKGGIHMLIFVSALKLDA
DCSSVVLDAAVVPMTNQLVASGSMDPFLVLIRTLECCSINVNDAELILWK
RVLPALAERCRTWSHRSDCEYKRKGATVPLSLVQGEKFLCSCGNGKLPEN
FVSVPEWETAAPNAVRIAISPTFSVPLVEDVVDPGDMAKGKEQLAAGDRC
RSCGSAAAKDGGSLKKCMRCQAVKYCSTECQKKDWKKHRMECSES
>Mb|QLI68631.1
MSTCGYQDGDPKKFRTAESGFNCRVDTVNGLWGFCPTTVIAATDCGLQGF
CVDSHSCSGGCGNGDNSQLTTFTCETQKFCSFAALTFGVDQTYTYYACGG
TPTTMHYLASPTAQATPTNTNNVSKTSGGSSKPASVNTSTASSAQPSSWT

IASRTTAQSSQANANTDSAAATSKSGSSRGGDSDGVKADSSPNTIGTITG
GVIGGIAVLCIFGLAAIYLLRRSRSNRYNCSPSPNLPVPLESAETPGVNG
YKYSNRRTGGWGPSELPASEYGRPSLHPVELPT
>Mb|QLI68670.1
MGSRKSCWTCKDRRIRCDGGFPKCQKCIGARRCCQGYGMRLSWPRDDDKR
RVTSSSPLSALISRNQKTDFSFINTTWSDMELYHHQESFLDPIPRLSNLW
KLPQLSASNMQLLNYFHDSAHLSLVTFDADLSQMRDVLLRMALENDTLSG
YAAFYALLAFASFHHSGLNQQTMRLKISALHFLAASAKEETRTWTQAARQ
VAASMLLGTLDIMLPSASSGEWLWYTRGAMDIVQTTCLKYQWHESDIGRL
LDWVYYHDTLSRFPLRHWQYKSLAREVSETKDFHFRVTTFSALTKYRPAQ
SSPNPTYAILNLLSEACNALIDPRDPASRSGEYRDSLDLLKRRIGNVHLR
RDSSSLSPQETLGVEVWQTATQVYLARASQSPGEEPANLESLIDMAFEGP
IRTCACPHFFPLFILACEAQSDDRRASILSLIGRTEDIPGLRSKVWLRHM
IRSIWVHQDLHADGDLLVNYVGAMRAVINMNDTIPSFV
>Mb|QLI68680.1
MTVLYWLLIPVLGFLVGFALDYVFLVKYPAEIPTIRYSRGFWSHVRSNIG
YFTSQKRWIRDGYNEYNKKGLPFLVPSGFSRPYDVVLPRTMLTWLRDQPE
SVVDARLAHNVSAYGDYNFLDSEIIRSPFGMRAVQKSMNRSLPGLVSAMD
KEVQHAVDLALKDVGNDWTSINLWGMWQAIVLFVTNRMLVGSTLCRDERF
LNAMVSFTHAVMRNCVLLRFVPLILHPILGRMLAISNWVHWRRAYCRVGP
VIKTRIDSMSRKAKGDPELQNWSPPEDYITWLVRLALEENRDQELDPIVI
SKRLLPIEFAAIDTTVITGVLWIQDLLKTPSAVEDLTAELRAHQPAPGES
WSAKALQSLLQVDSSIRESQRLSNFHLTLVERVVVASDGLCLPGLGWKIP
KGAHLTVNADGSHHDGDLYHDPYTYDALRFSTMRKERGEHHNASNDAAKP
LGMVTMNDHHFPFGHGKHACPGRFFVAHEMKLIAAHLLLNFDLKMEDSTS
NRLWVGPGMMPPFGGRIKAENPAAVPTAEPDEEPPGVYKIPGQQTLKIDE
ILGLRYSPDICRWGQHPPGRTVFESADH
>Mb|QLI68769.1
MSRQAKSCTRCRQQKVRCDRVSPRCSRCASSKASCSFSQQPCASVSPPPS
TPSASTESQDLFLSLPSSPSLVPEEPHGRRVEPEPAHPSPGGGRGIPAKR
KKRRRACLSCVRCHRLKIKCDKKEPCTRCRLSGWGRQCEYTHRVEPSGEA

ALPYVLTEEDPQVALATWHSPHRGLSHWRSLLSKLGCVAQLESLPVLATE
DAILRNLSATGGVMLPDNFPFNSPRASPFACIQKVRTLIGSHRGDCDAFV
EGYLDLYQMVHPIIDTDEFRARVSAYWDDPRCPDAAWLSQFLMVLALGQL
VVTGKTAPTVELCMAAEACLAKTPFMLRPNISIMRTMCLMVLAKLTTNAT
CWSFDACWNLLGFVVRQAICLGFHRRHPPTYPSPPVEYADWEAGRVIWTT
LLYFNIQVAMISGMPSCLSTDVIASHDLASDLGPLDTAALAWHSVIHTSG
PTIIKIISRVNADTNLPSYAEILEYSSQVRQCMSILDRVQGPRTLRMTLD
MFFRRVLLVLHRRHALDVDAPSKYPVSYWASLECSLALLVHQRDLYDQDK
APRGADLLVRLYMLDVFAAGMTATIHLLRKDAPLASGFAIPPRQTILETL
QACTELWANETIQSPCFKMGHALLDRVVGALLEGTVSLP
>Mb|QLI68777.1
MSPIQSISSAIANSLTLQLVVIAGLILAYPIGSIVHNIYFHPLSKFPGPK
SWAATYLPYIRGIVTCNLVQSFAEIHQKYGDVVRVGPNEISIASEEAWRE
IYGHRPGHKEALKDATWYIAPSGAPQNVFTTPDPAVRARMRRCLSSSFTQ
SAISNQSSIIESYADLLITRLREMTSAPDSAQKGAVVNMGDWISFFTFDV
IGDLALGETFGCLQNGEYHDWVRSLCTFLKGMTFAGVARIYPWVGSFVER
FLIPRSVMDQQKQHVEFVSERVNRRLNLETTRPDFMTSFLKDNHDFENMS
RGEIESNFAILIIAGSDTTTITMCGTLHCLTRNTDKLAVLEDTIRKRFQS
EKDITVDGTKDIPYLDAVIEEGLRLCPAIPALLPRVVPAGGETYSGHYLP
GGTKISVRPAILHTSARYFEAPHEFHPERWLPADQRPGQFASDNHAASRP
FSVGPTGCAGKGLAWAELRLIIARLVWAFDMSVDPDHALDWTKWKARIVV
EKGPQFLRLKQRPL
>Mb|QLI68783.1
MADPVTGAAIRIWSVNLVETRETKSSKKATELLARIDQLEKELASAKADA
FRSTISLQLPESKISASNQAPLRLGQNWYHRGMPVVSEDGFKWIKSITTQ
DTTRFENHLLGSAHLFSCISYPTYPSSGESLELPDISLALRTFNAETSFF
FRLGIGILDKSLFTETIGLAYADCNPNASQGHLAATASLLAAFTVASYLK
TSAEVASIDAQAIASRAQGLLGRIDRFANLDALQAILLLYKYRIATGQYD
AAGALLPTACRMVCTLDGHMKHPTPSKAAPDMRREHIRNLFWTCYIADKD
MSLITGQPPLLADEYMDLGDAECSSPAQELDEVIDSSVACKLLAFATGDR
RLGLLKAKVFRVLYSPTALHITDSELLTRIRRLDDELENWRVNTYPLLRP

KLGIARSSSGYGSVQPCLRAAHLQLEYNYLLTMVHSVVRRFGVAHDPDAG
LPEDIHRVMHSSIDLALGAARNTIHAISEPVTVLKGKTWCTVFHPLTAAM
SLFVNILIHPTSDQAVTDVEWLALASKTIHGLAVACSAEELKYLQQADDF
AAELLKLANGAIARRS
>Mb|QLI68817.1
MAIREAKYSDLREMAEAAAAAFQDEELFGELMHPRRKEYPQDFVAYFERR
FLRHWNDPNCHLVVGLDKTTGKVVGCAQWERQGVQPASWIGSFMQSASRA
YLQTLDYLWPNRAADPDKINILDETFPLFAHHWTEPRRQNWYLEFLCTHP
GYQGQGLGKALVLWGVNRAKEDKICASVISAAGKEAFYGRYGFVEVGRAN
VGRLGACGIRGGAILFCEGHLST
>Mb|QLI68844.1
MAHGTLRAAAGFCHFMVFASAVIVTGLVSWFLNGFRFRGSHIVYTEVIAV
ITIPIYLVAMIAPILKSYEGYFLPVNFIFSYLWLTSFIFSAVDWSGHLCR
QEPLGTNRCGRKRAVEAFNFIAFFFLLCNIVIEAFLLRAFHNDRRESNVV
HKERPVSEATRNSAASATPAAAVQNGTAPATQV
>Mb|QLI68899.1
MQLDAAPRALFERLPAEILDLILLQPSLCGEDIKSIRLASKRIASSATRR
LFRKVHISALHRDREAFLNIAQSPHLARHVQTLVWDELTGNFRLLKTSFH
IEYLGETMGWGDSLNSVGSNLVEDLISQVQPLFWLTSESAPLEDGEMGYI
GALPFWDEFVEAADKLPALRSFVSRPMQPDREVQSAADGYPLTVRVIKGF
FLCEDVYERQDAIFNLGFLFYLIPLLKLYTERGDQRPRQLFFSDEDSVTK
SSLEYLATSDAPAFRHLHHLELNVFERARWSRRETSGLEACLTEAVNLRH
LHLGYIQAQCRDDFIPGADIDRSPLYAIPTLPSLVSLCLDDVILEHNYSE
SPDYGVDLPRDHEQPAFVNFITRHAATLKRLCVVSSYVTRHSVCKLARVP
DLKLHRFIMANPDTNNTYYNIKEEDILNFINWKYEYDLEGNKLPGQAEGP
TLSSRFPKRTHVLPALEEYGKDESLTMVDDCREICRTHDAPSGLWVDEDG
IYYDPATDQEMTEPRQEYCRPKDWQGQVDKRRWDGDLGLWRDTSSCHSTP
LHKYAIDRVADTTFGDNLDKRGENDPGLQEERDRAIELPAYPPELCLLEE
FEHQARKLGHPKWAWGTDDLGHVWYWEVPNNGPGHPTRIWHFTHRNGEEA
YGDDPLDFWPDWEGGTSGDTVSATPFGGELRSFAEAASDKRRGDSHPPST
QVGTKYGRPARYSLLSDPWWAQKHP

>Mb|QLI68907.1

MAAKHSADSCVILEAGVFSLLIRARVGADTSDQVNDINSIARSYILLKPI

ARAEQAVKQTLANNNAARVLILPKKALPSKGERFMAFVTKAHASDDDVKD

ELTTSADYETKTAGIHHTPAATKVCEGTYTLSSAGGRTHLVYVITQPETL

GKFLREHLKVQVRGCFLISTRNPAYEGPANVQLPVGPEFPEEILSDFRSL

RWIPSTPYHLDYTNAQFLLIGEKSVVEEDGESEDVDHELEVKIEDVDMV

>Mb|QLI68911.1

MTATEKIHETKPRFRVLISGGGVAGLTLANALQHARINYLLLEARSNIAP

QLGASIGLGPNGSRILDQLGCYDEIMALTQPIHYTGSHYGNTGDYIRPKT

DAFQLVQARSNYCMCFLDRQSILAVLAKRIIDKRVLLNKKITVVDHYKHG

VRVTCQDGTCYEGDVLVGADGNYSITRREMWRAADQESPGTIPESEKHKM

SAEYKCMFGISAPIPELTPRSFDVTYNKDMSPIVIVGKHGRVYWFLIARM

PQVFKAGNIPRFSDEEARAFAEEHLDQPLMPGALVKVRDLWERREAYNMV

ALEEAYYDHWTYGNFALVGDSAHKMTPNMGSGGNSAIESAAALANSLVQT

LDGCKGTRPTRDEIQSALQRYQNARRLRASRTVIASNLVTRLHAVKDYLH

HFAAHHILPNAGDLLVDLASENWIGAVKLEYMAVPKRSLAGTMPFNPQQG

MGNNENILFRALAALPLLFPVALRYCLPLYTPKTLLPWQKLLDESFDVPC

LNVEVADFGTVYAVILIEAARRANLMTPLALPLLFGMGCQLAPAETFLSL

YFFTYAALVPISRFKAGDLRLTDLSYTRSVLPATLLCYYLPYILSISGPS

REIRQASIWAFRLSPLLTSLAQRMASKFIFPSTIQQDRLTNVRRDMGTIR

LGIGGLALLSASSWLRLFRHTPSLMSGNLASSSHAWFAGVSILWITYLYN

DLKGAGMVRHGWLSIVSALAVSTCSVGPGATVAMAWLYREHVLATKRHKG

AIV

>Mb|QLI68915.1

MGFLYSQLFKQLPYPTGSYNGKTIVITGSNVGLGKEAARHFARMGAGKII

LAVRNLDKGNNAKVDIETTTGCANDVIQVWQVDMASYASVESFAARVNAE

LDRVDIFIANAGIAPAKYAIAEDNEASITVNFISTFLLAALVVPKLKSTA

RLYNTRPMFSIVSSDVHAHTTLPQKSAPNGEILTTVNDRVFAEKHWNEQY

PVSKLLGVFCVRALAEKYPASTLPVTFNCVNPGLCHSELGRDFPTLAFWL

IKLVLARSTEVGSRTLVHAASQDAASHGQYMTDCGTGAPSAFVTSEEGKE

VQARVWDELVLKLESIKPDCMSNF

>Mb|QLI68945.1

MADSNPKVTCQCGAVSFRTSRPKPLAKYACHCTECRKQSSSAFGTSALFP

AKGMWPLPDDVRSKLGVWTRISDKGTTLECYFCNECGVRVLHRPLLPDGT

PKPTLTVKGGALEGFTLEGARHIWTKSAVMPVPEGSWTESPEGSDAETVG

E

>Mb|QLI68973.1

MASAGLPPPPSGIDLSQDKRPLIVAISVTTWILAFTTVACRIVGRRMRGL

QLWLDDWFIVAALPPSLGHVLGMAAYAVSHGLGRHVWAAKRDCLYAWALG

LFVAEICYTLTLVFVELSILSFFWRSFSVRDSIQWPILILASLVCIWGAA

VLLVTFLQCLPTRAIWERFDPANSMSANNYTCEVDLVKFFYANAIPTIVT

DLVMLMLPVPYVWRLQLPRIQKIALGCVFLAGVFVTIISMIRFYHLLSLD

LEDPDITWNFVTVGIWSFVEGNTAIVCACLPFLRPVINRIPCGNPFILAP

VPLNIVQQSEDSGRGFNQKNVFPTWGFNKSHAASTAPVQSLHHDELDNDE

YPFAHLADDVSETDTPARRDDRAFVDLEAVVAIPSARYIPMTRDVRQPHQ

ATP

>Mb|QLI69001.1

MEAFRALSLFQPSNFVAAIQSTRDSDSSGQKRLFGAMLSIPHMEAARSAS

VLGFEFILIDAQHTAIDAENLVGLIRTINFTSEGKTCTLVRVPGAESHLL

AYALDAGASGIVFPHINSRKDAMAAVNKVRYAYKGGERSLAPWALVPFLT

DQAPDGHTAETISDEHVAVICQIETTLGLENVDEIAATPGINALMLGPGD

MRVSLRLPVRGPGRKEDDAVFCEARDRLVKAAKAHQMALMTIAFRATPGI

EEWLKDFDLIVTSSDINSIVRFHLDGRAAIRCALRASSIA

>Mb|QLI69025.1

MSEKKTRSRRTFPLSLVEKYYPRAWDCRLPFLHPSLAATRPKLIKAGIVN

TIIIQLLFFVLFCYLFGALYQQGPRTHNLDVLWVDYDGGLVGQAVNAAYA

RLRSDQFPTLVARPVSQYPSADALRDAVCDADYWAAVYTAAGSSAKLGLA

IAGLNTSRYNESDVLFYIWNEAKYPAVMDSALSSNMVALSGAARIAYVAL

NGTAALATVPPGDPAAVSVFANPWTITSVNLKATAQGTRAVYNTIAIVLI

LLQNFFFLATINGLYVQFKIYNRAPPRFIILVRAAIACVYTFLGGLLITA

AIWAFKAGWDVSGAAFVLDWATFWLLGHVSFLVLDVFSIWIPPQYVPFAL

VAWVVTNVTSVIVPFSLSNGFYRWGYALPAHAAYEVLTDIWSGGCNPHLY

FALPVLFAYEVLGTLGTSLGVYKRCHLAVVAEEATRQASVLLEQTRERRQ

SRANGEALIAGGSGRVAGTRAEDAEESEREQREELDEEDEQAVEDIQRLE

TQATRIYSNLGPSFQLVGSDERQG

>Mb|QLI69062.1

MAEALAVLGAVAAAVQFAEAAAGALIKTIGVVRGLREVPPKLARLLSEVE

ASTSHVESLCSSLSPQHGGSDMYKQIQSQAHLDGLLETMTALYKATQDVN

SFLAPMAEFGQGSVAKGRRMARMWKSIVSLKMEKELPDKLERLNRLNINV

VRELSLVGLQVQVTTNTLAAVNNDMLLRGFHDVAQQLTTLSADMKGLHMA

VTQRQSINNAGDSSFALTFHETASSGNVTLSTSSSLANAPTAPVSRTRRP

ASVERRGTQVQLHQKQAALPCPSQGPGGFTDSHLDLVLFSIRTYYTPGGF

DPTPAMLQPRFWKDCSESIYFFKISDLPKARRLLESSTAAAAHSNDIFTQ

GATTALIEILSTLSPVNTAANPDVRKALLSYLHSLALKQLPRESPILVVL

SRLHQGMDSQEYTLTALQCIVDRLRASLEPTNPLRLHAQKRLIALLRRGR

DYERALRACTLALGEIRTTAGEGSLQERQLARLLEHIYMDQGDWVSALTV

CFDIVGQPVGEAFGPNPDPRFHDECAIWTMEDIAKIYESNGKLDTAIAWL

KQARISGGIFWGPSVSLEHIHDKLMRLLNGCGRGDEAGLWSTAFGPVARH

DCSGRLGQGDGRLLSLVKNGIVRRMECLLAAVLSRGQATSTGLVVGSSTV

SVIPTCD

>Mb|QLI69072.1

MSRPNRPAVVAANNPPPAARIARNLCAMVVGEPSRRQFIGGQKLGLDTDI

ECFVMFIPRTDAENWFGFSIQVPLSADTEALGLGKWHTFNETRAPVTTDS

LYITVKFPRESTLSEITPVDGVLLRAFPPALQPGKVCQLYVSVRDPDAIQ

IEGIGMPFANPGHKCESWMRNGAVFEGKTLLDILKSGTYTFVVNRPDQPF

RVDWNVTRLPPRFQYPWGTVHYWDEQRYHDLVDANKGPQFAPARSFDSDN

SHLAVLTQSLAQDIMWVDRAAKEIYSQFRMRVYFIGIGNVVPSHQYYVLV

PLEPGFRSRFAAAWRRLAQGILELHIFDEVNLEGGPIANWLARIVDNPRS

IDDLANHVVRNHELILHNCAALHFDSGIDDYVRKIDAVCQFAPDSRPSNP

ALFGVEIGKDGRSLEGNQWDVTRVKQRLEVARAAVRGRGFYGVDIAVAPI

AAGNPDHEGLQAAALGEKMPILPRVNMLNIASGPERGALMRQLLPHERGF

FSEYLESRPLGLGLATAEPGFRRTSLLAVAAYGMIRTFKSIYVSAPTDVA

VDNIAARMYETSRRVVETANGSMESGNPNRLRRLLVIRGHKPDDEMAAFL

RLLRNPNLGDQAAPESACRGASRWKLHLSLAYWALKILRSQAVEPLHQDD
STALYQFQARIDNVAELAVLRGVAAGDQPWRDYEAQPLTDNQVKCFLGEL
LLVADAVATTPTLSWQLPYKRWKDSNGHAVVIDQADNISRPDLYSVWGNT
MIPLFLAGNEKQPQPTVRPAYDKDDKGNALNRHAADAGISALEFFKGIGW
PVFRLNAGRVATFGI
>Mb|QLI69074.1
MINLHMGYFGGIEKYLRAFLISASYVAPAANQVGPFFIMLEIMFPVVMYR
VPPEPLGSARNTAFTLSLARVGRREGLWLHWSGDMNDGIKAVLRTERDGA
LEDRHGRMHLFVAPDSRLAGTIAGREVHARADSEAVFGQIASWMKNCIHN
HPNLAEDARLCQDMSQSYDEIGRQQDTQQQLFQVRDADAHPVEELPLLPT
RVIDVGPSDGSKTPFLLETERELHGLYVTLSHCWGASRPLVTDVGSLAAR
KAQVPESELPETFRNAVLITRRLGVRYLWIDSLCILQGTDARAQADWQRE
AALMDQYYNKSLLTISAAGAAGSEYGCFLPRGRTAGRVRLNLKQSFAVTT
KVPRRCLPCLSPRIQRNVMDGPFPGGSDVYVSEMPPNVPFIHSERGDGRA
WILQETALSPRVLIYGQDMVAWLCNTRHCTERGDDVLHDTSSHYVMAPRL
PHHLRHAPSYSSEDVGWRMALRWVTMEASTWWAKMVQNYSRRRMTYEADK
LPAISGCARAIQALNKDDYYSGLWKRTLTNDLLWQSVASRAASRPATYRA
PSWSWASIEGEVKCCITPKYHDMNEKLIELLSVEAEYVGPDVFGQVRTMS
LLVNGSPARVRLGPRVSGDAGESCAENPIHLLEAIDDACACSTCDQKRAS
LKSLQRNVIASGADATRPRIPNFSAVGAADVYLSQDLVTMRHTGLEGGPL
GNSGPQYTETTLNINGNGLMDGQILEHDGLDAAGSASFDVESERDMAADE
LCCLPINRQFGLLLVRNGPLVVQGEAVESYRRVGLVKLNLVWRPAHELSP
LALV
>Mb|QLI69075.1
MNRSNDGCWTCRIRHRKCDEKHPICRECADRSITCHGYGPKPKWIDDEWK
LQAELSRIKKTVNSNLRRKKKLQACKASPARPSEHQNDPRQGEEDGYPAP
PGAPTTPDMAFREAQLLVHYLDYIFPLQYPYYKDEPSLGGRGWLFWLLMK
RGPLHQAVLTLSALHHHTQSAGAPGNRESELIGYHTNALKRLRQVLQECD
MDKFAESRQQMVEFLACGSALISFELFRGGLDNWRPHLDALASVVNKILV
PHPSTGTRESGDGVDKAQPFLVTKVLWLDILASTATGKAPQTRYQKWLQL
DQIDMSRLMGCRNWVMQAVGDISTISSRKTRSGFSKSDELQLKALEQILD

EGIERMKLGGDDRQALVYAITMVFATAALCQIETLRNAPSPGMRLYTRVA

GAIEAMEGLPAGVTFRGLVWPVATVGAVAASDQQSFFERAMIDVLDTSGS

EFTNCGTVLNVLRRCWQHQRESGLVWTWQDGMTAMGICALLI

>Mb|QLI69092.1

MPQKKETFSVLTPIPGDIPRQLAIDILHAHDEVITLNPLVVHHRPIPAPR

TATADEYYNTWYEIEERIQYMPWVGKVGSGKISLIGCFHNMPWGLQTHLY

APMKIDLRNEYRITGNQMGEEPAETRDTLLDSLGAPADGLYLREDIEIQC

SIAVVSFVKAQLKAASEEMAQRIVRRAGRLRPDAGVKRYPNTPSGDSEQP

GIQYISEGSVSSSPGTDKEAKMAVAPTVALLAPSVEPRTKGPPVAEFQQQ

QQADAAMSTSQRVWHASYESLEKGSDTAKLVKSYAKILMAVLGDGQHDSA

ELNDPTRRQDCMKDLVRQGLARVSTSSRIRQGVSDIAEYCLSAKAMVDLA

IQNIPQAALPWAGVCIGLQILLNPAKATASNLSGITYVISRMDWYCALTE

HLLKKDSINIEDGSITDSACKNVLQQLEVKVVNLYKSLLLYQMRSVCSYY

RNRGYSLLRDLVNLDDWDADVKSIQAAEKSLRMDSDQYNKLREKEVLGEL

AKRAEGMQGLLGDIHQTLQDFVALQKTIRRDDIDAACQKDLRVVDPQHDM

ERIERSKDGLIDGAYNWIFRTREYTAFTNWYDGSLESSPSRLLWIKGHVG

TGKTMLMIGIIRELSCQPAILAPSLSYFFCQGKDTALNNATAVLRSLLWL

LILQQPHLGSHLRQRYNESGANLFKDQNAFYALSEVFRNMLQDPGLSPAL

FAVDGLDECEEGQSDLVQLISASLTLSDKVKWIVTSDPALEIKAPGTACS

AVELDAERLDAPVHEYITRKLSKLRTRKGYTDAVMAKVSDQLHERAEDSF

LWVALVFKMLDSEYGWNATRVLERTLPGLPNLYGQMMGKMEKDTMDRQCC

ENVLVATSLAYRPLSLPELGVVAGVEPGIDLPTIVEECGSFLMTRDDKVF

IIHQSAKDYVLESFKAGLQSAGDVPGHASIAQRSIEAMSVVLRRNMYDLD

YGLKPDDLKPPEPDPLAPIRYSCTFWVDHILSASGLECSRESVLEFLKAH

FLHWLEALSLMGRLPDGVLSVRRLLHAAQESGSCCELAAFLEDAEKFVSS

YGSIIERAPLQAFGSALVFSPMKSLVRQAHWQERLPFIQTVAGVRNHWDA

HRQILEGHGSEVKAVAFSPDDNTIASASSDGTVRLWDAATGTCRRSLSGH

CGNVCAVAFSPDSSMVASASSDCSIRLWVAATGACRCALEGHKYWVSSVT

FSPDGKTIASASGDHTVRLWDAATGAHQQTLEGHRRSVTAVAFSHDGKLV

ASASVDRTVRLWNVTTGAYQQTLTGHTRSINAVTFSPDDSIVASASGDCT

VRLWDATTGAHKQTLKGHGSWINAVIFSLDGKLIASASHDCTIRLWDATT

GVLRETLDGHHRVNGVALSADGKIIASASADGTVRLWDMASLAYRQTPTG

HTHCVNAIDFSYDGTMVASASGDCTVRLWDASTGECRQILEGHNGSVNTV

AFCPCSKMLASASSDRRVRLWNATTGSCEQILQGHISDIKAIAFSPDGSV

AASASDDCTIRLWNVATGAHQQTLDGYSGEVKAIAFSPDGKVIALRLSDG

IPWLWDVATGAQWQLIEEGDSAPMALSPDGKISASASDDGSTIRLWDEVA

GAHQQVFLGSVEKGRPNHAPCVCGEWITLNGRNLLWLAKDYRPTSVALHD

DMIVLGYDTGGLTFLKLNLDLDNG

>Mb|QLI69122.1

MAYASTHPSVLSVVIEVEFLVAVEKDGHKYNNTAAAGNDTEGTASQIHKW

ACPSQADDPSMDVLHQCKAVLSRDHANVIIRHNETHLTERSWQSARFDSW

ILQPCHRGSSASSGSPTNYDWSGVKLRSPLMPDTQLTGDPSPALRCVETL

RRAILIRVDASCRLSVSLRPGSNFTPVQAKKLATLVWLTERDLLVPLRHL

GRDVAAVACPRPVTTASLAAISRHPQTVDAAEPLDPLLEGIMNSHLPTGL

GDAAARSCLQRLWACPDLARLSGALRDRHRGPLAFALYVYDDDDDTEDSG

ASCCAVASFRCAPWHPRRGLDVSPLWTDLVLALGRATILAPERFKSLVAE

IDDVNCASRQGDAHGASHRTRLMKALGTEDARCAEWERIVADCRNGAP

>Mb|QLI69201.1

MFIQGQSTFRHFIYTLLNIPKTAKPVLRLALNPTTPAHVCRDFFAIHLLF

DRQHDPYLNAEALIHLWYSAKLPLALWRHIEVVMKRYYYDFDECFENAKR

DQQSVCDDGVGYDVTYQMSWGGGQVKYVGNLFEHQWRLISKVLKPTEQMS

TDQAAIVRVLDAEKSCEPLKVAASRMTPSRTAGLMKWRTDGLLLPFGHPT

DGFDMPNPIFFQGDGCYPHGATAEPIAEWPMEFLDFQAGPLQNDVYGKLF

YYLRDTLVRFQEESKRLSIMVGLTSVGMPMSLHRAPEPVMYDRIHMGDLW

DFNPACNLTIAAGNLRHQDQNPFATMLAMCRLSVTNSDAGLQEEICGEGY

QTFEPSSTILDDYAPPIKIEQGCETETVIRRRIGLLMWRNWDKFSERFMH

DAKLFAFHLSTDSETDKETSVFKTGFLGMEYKDKNTITRRWPNRLVHSKS

DEPSLRDFERHVGWFDTMPQRWLEWKRVADADDNEWEMARECVLESSWRE

MAEIQAKIIEEEAKSVDEQEDLEQRIRELLAEDAADREKSEKSAAAKTKA

KASKRKKGKKK

>Mb|QLI69243.1

MPAADSPPSTESPLRPTSRFITTNRDGKAVYSKTLSESPPSELSYGGSRI

AFCYGTNQTPPNFAGERDIATYQDQIENPPGIIIPNGCVARLVDFPPGYT

SPMHRTISLNYNFVIEGEVELILNSGETRRLLPGDMAVQRAVNHAWRNTS

STSWARITAFAVPAIAEDGAAETWDETWAGAGEGTSGKA

>Mb|QLI69253.1

MHWINLNTIAVAFLQATALGSARSLWSTQPATYAPQSSDETILKTTYPVG

NGKLGAMPFGPPGSEKLALNVDSLWSGGPFESSTYTGGNPPSSKAGALPG

IRDFIFKRGSGNVTALYGSGDHYGSYRALGNISIAIGHGTLYSDYNRTLD

LDRGLYTTTYVVDSVKYTTNLFCSYPAQACFFNIASTGTVPKTTVKFEDL

LVDTILAKSSCSNEFARLSGTTRAGPPAGMRYEARAKGGNGANTSCSDDG

TLTITPSGGSNSLTIVFSANTNYDQKKGNAQNNYSFKGEDPGPGVEAAVL

KAAKTTYEDMLSQHVSDYAELFGTFTLNLPDPNGSVKKDTASIISQYSVD

GKGDPFVEGLLFDYARYLLIASSRDNSLPANLQGRWAEQLSPAWSADYHA

NINLQMNYWAADQTGLTKTQPALWNYMQDTWVPRGIETAKLLYNASGWVT

HHEMNIFGHTAMKDEAIWANYPASAAWMMQHVWDNFEYSRDVSWLKTQGY

PLLKGVAKFWLSQLQHDAFFKDGTLVVNPCNSPEHGPTTFGCAHFQQAIH

QVFEAVLASGEFVSETDTVFKRDVASKLASLDKGLHFTTWGGIKEWKVPD

SYGFDTKNTHRHLSHLVGWYPGYSISSFQGGYTNSTIQKAVAETLKSRGP

GNAADANSGWEKVWRAACWARLNNTAEAYYELRYAIDMNFASNGLSMYNA

LSAPFQIDANFGLAGAMLSMLVVDMPQKHGETGDRTVVLGPAIPAAWGDG

NVKGLRLRGGYSVDFAWDANGKVNKAKLVGSGKPLKLYNVDGKVL

>Mb|QLI69256.1

MRASIAFATGLLALANARITGISVPETIRPGDTVNATIISENYIQAVYDV

AIVFGYAPGHGTPESLGLVAGSIYLGPGQSNQLHSFTQQVAIPESAPRGR

GLITASLMSLYGALHMPTLSNFNVTVTFGEETSTKYISSQS

>Mb|QLI69280.1

MAAYLTQRISHPHHGIPVSRSETPAPEKTAKCLAPIPNVAAKGVPFFTPE

QDPVAGSAVDPQPSGKPIPKLFTPLKIRGITMPNRIWMSPMCQYSAHEGF

HTPWHITHYGGMIQRGPGLMMIEATSVQPNGRITPEDSGIWLDAHVDTLK

KHVDFAHSQNALIGIQLAHAGRKASTVAPWLSSGATATDEVGGWPSDVVG

PSDAPFAEHYPTPRAMSLAEIDQFKRDFLSAVRRAVRAGFDVIELHFAHG

YLVSSFLSPAVNKRTDQYGGSFENRTRLALELVEAARAAIPKDMPLFARI

SATDWLDTNPNWDGGASWTVDESVKLAKLFAERGVDVLDVSSGGNHSHQK

VIGGPGYQAPFAKAIKAAVGDAMLVSSVGSIKTGEVARDIIEGGKDKDDT

PLDLIAAGRMFLKNPGLVWSWADELGVTIHVAHQIGWGFGGRASKKSGKS

TVP

>Mb|QLI69309.1

MASGLTGTDAGQRPSRQPSTPRTQSLNGKPSSSGKRKIPHRITVACTTCR

NQHLRCDGTTPTCTRCRQGNKRCVYKDIRPQRRRLPNCNDSTALRPDPSP

LGHAGPSRQSWSASQSSEPSRFHEDVCSLDDSAGLFSDPFATPQDENPHQ

STALDSFYASFFKGHPFVLPKDRLVSQFDKDPSSVAHVIKAMAFIGALYI

PNSASQAYRAAIEMAIANGLPRNGFSVQCLLLHAGALEWSGEQDYARSIL

DKAKSMALDIGLQSRRFARAHSDGCPVLAESWRRTWWELYIIDAIFAGIR

HQTTFALWHAEFDADLPCEEMDYIQGNIPPPRTLAEYDDRAFEDGDGLFS

SFAYLIDASRMLGTALPTVDEQGDDTTSQVKNAEANITSWHLYLPESKKE

PVQPDGSVDEVMFRAHMLLNTVTTHLHRPRSRLHYSTMEILCSRYAPPQN

ASVLEVFNERHTMQAVNAAKALVQLFTLSASPTTHSPFIMCMGSMAAATH

ISACEYYLKGSDYAHAKDRVRVFLGALRAFEDIWPQARKWSGEVKLMAKA

VFDSRSKNGELILQNQQDGVGSGVGAGVAGLDLALLRSCFES

>Mb|QLI69344.1

MSNSHYSSKHKHHKSSKSGRSKTTESSSNGCSWPVNGDVSFLFVVNELEI

RFRDEQDLDSGIRKDDWLNVLPPNTPELYSPETLQHVSQVMRFRNGQVTP

AGPAYYWARAGQFDEGCIAMAAGETQYPLQKYKSASVFSCNPSLPVVTLE

GDARTNPDPDFNVLQFVHQRNADSGSNGVSLAAFSMDRSSSVCPPPVKFV

AGRHASWIPSLVPGICRNPYMAGESSGLGGELPIVIGLMAFHASPDGGRT

INDVFLGRGENSGLWRNYRWQSSSSPAGYTLSEQDTPRGYLVHICLDPEN

EAGSTLHSLYMLEWNGVLVQG

>Mb|QLI69381.1

MKLLVLELILAATAVGSLATKSPWKVSVRQLSSSAMQQNIGLQNNTDGNM

GWEDIVKRKRAERDALLPQEWKLKAPPNTTAFSPLNQVINSGLLSSEELE

WTDTKKYDATTMLQRLSSGEITAEKLVTAFCKRATAATSLANFLTEVNIA

DAINRAKELDRILNETGKTVGPLHGLPMTIKDTEDLKGFDTSCGITGWAF

DPRESNGPLIQILIDAGAVIIGKTNIPQTVLAADSDSVVWGRTLNAHRNT

FGAGGSTGGEGSALGTGSTLLGVASDGAGSSRMPAMANGVVGYRPSGYRL
PPGGREVFTDGRSGLSMTGPVAGMGLMGHSVRDIRLAAKVVSDAKPWEAQ
TPFMYPSPWMNITAPEKPRIGVWNVESPNTYLHLFPPVLRGYQTAQSRLR
AAGFELVEFTPPDMSQVWDLCKEFLIFQGIETLTEMISREPITKIVRDTG
IFVPDTPRFPVSVDTLYQLNTRLVNLTVAMDTAWNSSGRPLDALLSVTAA
NTALPWDTWHDTTYTSIYNSVDWPAISLPLGLTVDKNIDHKYSDFRPFSK
EDARLEALYNPETFHGLPLSVQLAGRKFEDEKLLAIAELLHPVMKGE
>Mb|QLI69394.1
MASYRDESVERDGFASQYGRFTAHHIERIDGSVLQRMFVPKVTREGQREL
YHHGSSFIRSQLKHYGVKFDEAEFSGNGTVLMRKTLLAGRCDKVPDHITA
LQRQMHTEWLETQTPRQLATKPSWVMEKYFLRSGQPDSTKTATVVGIPFE
PSSKYRSDLVEAASKVPGLQCATGYGPKTQVVFMGWTSAAVKKAANEHPA
KEKKEVQALKQEHEDGRTAKHNAYLEALKEKRGPKKYSPVGSYMIDCEEI
TDNWPDDAKNMTLNIRTTEERGVYEAKFDFGMFIGAMVICDNKATLVQYC
AELDREAESRLSDGSMGFGDDDDADADIGNKSASSSKGKAPEAPRGRGRP
PKQAKSAENQKAQPRTFYLRLKGQETGEGQIYPDAEKGAIKFKDERMASF
LGKASLPFEGESIPFTGRKISDQPFPKARGGEWEDYSDRAEGYARRRRWG
>Mb|QLI69499.1
MHGKWSAYIVSGIMPPRALDGRYDVYVWFVRARGNTLVREFTKFSVEMWE
GEVPITSLAVVPEEYWMDDLQGQDGKKPRMPLRQKLIEEGKLLWELLKEP
TSKQYNGELVDEGIHIKDTQKGLVSGRVICDSPGFGEYCPDMKQQLQCQP
RLTRPRSPPPENQWDLNQLPQAPPRCACRHCMSNREHHPEEVMFHEFKPV
IPAIDETPTTDLFYVVCSGLIPGFIMASRRWGIFQVSNLSDIKQDKEAFK
YLVLDDKIKRTVKALISRFAASVNKKLSPWGNDFIKNKGEGRIFLLHGQP
GVGKTSTAECIAELSNRPLMALTSGDLLSSLDDVEKNLAYFLALGQRYGA
LVLLDEADVYLEHRTASDVARNGLVSIFLRALEYYRGVLFLTTNRVQSFD
AAFLSRIHVALHYKNLSNENRERIWAHSFERLVRDSDGKIHVSAAARDYV
FHHAGVQLLRLNGREIRNAMQTALALAESEAEDEGDEVVTIRASHLEAVV
EMSSSFKGYIARLEGRDGCEGDAN
>Mb|QLI69508.1
MDGPIHLVLVSGVAGSRAGEDGDPKKFLIERFPKCLLDEFCLLGGSVTRR

RLLYHRSSLLLEQLRNSRSSQNVSHNLPIVFLGHGLGALIVKKVNKHPGP

VRMNTNMAELVFFGAPHRATGQNTWEHMCSRLILMFVPPPEYPVKLVQRL

AGGLDGLDASFRTISSSFDVVNFYESSRHPSIDSKCSILGLQDETDVPCD

TTHEELWRFYPGEPVAEELYQRILATRNSRNLLLYDFFRHLSLLDSQIHR

LRPTFVTSTQVDWICDNNAVMSWRSDPSVGILRISGPSSSGPTAVAARIL

GMLLEKTDLKRTVYLSFSFDKNNIRARAPFDLYLSLCRQLLSSRPGYFEH

ISPVAKFLTKDGVCTTETLWVMVRCLMSHLLDDQSVSVYCIVDGVDQCST

FQKETINRMEGFIGPSKGRFKLLLSGSLVALQSTCESTKYQDVALESSSE

NMLFAKEQHVRGRINELALDNSAWDGLEKLATEMLGELPKDSPYLLVKLN

MVLLEWTGRHSTRKSLKKALKQQPTTLDGFYNRALSSIDEANCNWVTAAL

RWVAFAVRPLRPTELAVAVALDEIPGGYPWTTDFSSDDFSDLIRRDIIGD

LKQHMVPLLKVEDNRVYFIHDTFRAYLVETVFASSLPQTENDTPSQKEEE

EDLLFYCLEYVRGFGKRGAESFICEDGILTSLPADWDLGLLSYASLQWPR

HFRQTTTKSEAQSFLLQFLQGGEGVKTWQNLHHLLNPLHKRINTSLNSPL

KIVCNFGLAELVDRCIGLLAPTEDPKVQMRESLNLAARNGHDNVVRNLLD

KGVRSPEALGLAARGGFENVVETLLSVGFDLNYIDETRYAPLHHATCGGH

TQIVSLLLEKGADINVLISPIPSQELSKLIREQNRLTRQSSYPESDSDSD

LDVDDLKLSPTGTEANVLAHLAWSESSLHLAALTGQLGIAELLLERGADV

HIENSTGYGPLSYAAIGGFPELLYLLLRDRSDGLEKVSVDKVSTSDRNTA

LHLAVTCGHTEAAGILLRHSKDAPELAQTANIHHLTPIHIAARQGHVSLL

NLLVDKTDKKQDTESIDSTDKDDQQMSPIEFVIPPGNPSTSSRRHTRRRS

RRKSTSRRSPERRSRPSSAAPTPITVREDRPKSALELAAGNGHDQVVRAL

LERKTWSRPRDRALALNLAAMNGHSKVVKTLLETLAGDILAPAAVDSGGK

TAIHLAAEGGHSKILEELLAHPRSFPVNATTNEGIVPLHISARAGHVNAM

MVLIRHKAEKDFVDQHSKTALLLAAENGHLSCVDQLLEQGADHKKTDDSG

RTALHLAAGNGHMAVAKPLCAFKDIMWTQDECHYTAFDLLVRHEKIEYVE

DFIQMLDNTVGDETESNRGGIPLHMAANMGNIDMLRLLLDKGWRPDVRDA

ESLAPLHIAVMDAFFPGVKLLLEHPLCDIAAKDSLGRTVTHLAGTAELAS

YLLSSGAANDLKDSFGRTPLYQAAFDGHVGVAEVLLDSKPKPNIATRDSD

GWNVLHAAFDNPSMTKLLLKHDAEPNALTNEGLTPLALAIRENFIKTAEL

LLGAGADPNLVGAFEDPPLILAFEQENTLQLIKILASRSNRLNLFAKGPD

GDTALRIAARRGKLPEAQYLLKALEDSATPGLKAVCFSALRDCVSSLEFN
SELAEMFVKRDVEGMVNRTSEPRPTALHVACSKGTIGAVKWLLEKGAYVN
TLRGGYSALCAAVESDQDAQEKVELLLEHDADVNVTHENQATALQRASSK
GRTQLVKLLIDNGADVNLTGGDLDSVLNAAIRTGSDLATIQLIIKKAGVS
RAGRDGRLPIHIAAISDRANVLQVLVNAGANPLARDADGRSALIHEVANL
SDEAVEYLLREGYFDADEVDTNSQTPLILATIFGCKTTVKLLLEEKGFSK
PEVLNAQDYEGKTALVRAAALDHLEIIEELLERGADPCLVDCRGRSALYW
AARGARMETLGVVIRALEERDDQPADLWNVAVHGAIASDRPYALEKLLEK
EDVDVEFTGPDGWTPLYTAQRYDSGRIESILHQHAQISSKPAEGLQRPTK
WHPQDSYRSFEMGPDGNTLSVLGGTKFMYRTGLDSAIVRADYPMLPLYKG
RVYYFELRLTKVAEKGFVGIGFCEDKTSLDRALGWFQGSWAFYSDDGCLF
EDGNNPWTGTKYADACSEAGKVLGCGINFATGEAFYTIDGTAVGRAFTQI
RGKLYPAVTMKVAYGGWEVSVVFPGEDGKSDDFMYKGDLESEDLTREAVD
LSYRK
>Mb|QLI69514.1
MATRIPFDESFWQEYLEGQEAQLPQLRDVEDVTDRVVRIMGGNPGIMQLQ
GTNTYLVGNGKSRILIDTGEGLPCWIERVIRVLEDRQLDLSFILLTHWHG
DHTGGVPDLISHNPNWADRVYKNRPDRGQNPIADGQIFSVEGATIRAVFT
PGHAIDHMCFVLEEENALFTGDNVLGHGFSVVMDLAVYMRSLDCMVAQGC
ASGYPAHGAKIANLPAKMQEYIHHNEFRIQQVSSALSWHCAKGAKGGMTL
QEIIQSIYGNVPKEIVDNALVPFLSQVLWKLAEDRKVGFSPGEPKKRRWF
GLGVAH
>Mb|QLI69519.1
MHEKGKMLDTNHYNALSLAQPTNFKSMLHSGKLLWGTGCRIPHEEAARIV
ASTPYHFCFIDAEHTPLNATLLVSLVRTIQYHSNGSMVPFVRIPGCSPEL
VNYALNAGAGGVMMPHIQNAKQAEDLVRLARFPPMGDRSFPPAALINKKQ
QRTPESQTVYDVWNSHAAVICQIEDLQGLDNIEEICGVPGVDGLFIGTGD
LRMCMGLAVGSLDGDEPVFVSALRRIRDAAKANDLPIMGFGISPCTLERR
IDMGWNAFIIHGDIDAICTSAIRSLDTYSDAADRHLSKISDKNGNGSANG
LDDDLGSRTRGEGAVGSLNGSQSVGPQ
>Mb|QLI69526.1

MAPIVVPENKPEKRFYAGGARISAVRSNPPCSSHQPEDWCLLSQRNSLAQ
SIWRNMALAHLGRNHGKAEAWYILTPGSVWLVLKESIDSGELPQLVETGR
GTNPLDRMQKFGLLPRQTLHVPPGTLHAISNGIVVVKIQGPEDLSVLCEW
GGFAIHGKNGGHLGLEFPTALKAVDYVQVEQWVTSGVVAKSVLAAESTKH
FGLERIHVEGSARTKRGFAILVVLDGKLLSNTSHSDPLPLSKGFTLVIPH
EDDELSLQGEADVLIARPPQ
>Mb|QLI69536.1
MPGLLCFYKHRWARAQYSGALWLNVFSFTLPALYGTLVKLWVANIDPRMV
VTTDTYTYITTIAEVVNDGLPRAAWSTIGDTTAPYTHRLSLTYTLILAQS
ILGLLMSIVILVAARAFAKSFVPVEVRLGSITYVRISAFSALGSAIEAAV
NASARPLDRPDIPLLLSSIKFAVNIVLDMLLISTFHVGRHTPSVNMQAGI
QVTCNLTAAFSGLAYLLAVHRRRKQGNGEEYEEGEREGDEGGGRSTTPKP
SIQALLKVLGPRGMPAFIESAVRNALYLWLIANITSLGITYATAWSVFNT
IRWGLVMVPVSALEATTLTFVGHKWGIWKRRSAGVTNVSLGSLLGIARPA
CKSIFIALVFEVPICVFLSFWGARPFAKFLSRNNDVAKVTAHMWKTIDWC
YILYAVSTQLAAVLLATRPKWYLWQSLASNLLYVLPWAIVCQVKHLDESN
AWTYHALVFGGSLVFSFFCVPIVLWLCARDLKTGRAHLEPVE
>Mb|QLI69543.1
MALPRVPAGLLRNHIRPAASRAISPRVTAAQLRHKITTNDGPGGQQPPPP
NPGGPEAVKRNWVPVGGAALVAVAAYAYLSSSGASVDKARQTDARNPSQA
ELEDLTAVSKDAEQLGAKVGQQVAEQASKTMKEMSGRQKTDQGSFRHD
>Mb|QLI69548.1
MANDAQPTTKKVVISAFGDVSNVHIVTEPMPPPSPNEVQVGIIYSGFSGA
DIHMRLGRYPQQPSAPLTPGYCLVGRVKANGSACTKFRAGDRVAALTVYG
SQSELVNVPQGKLIPVPAGIDSQVACALVLDWATAYAMVFHTARVAEKQR
VFVHGLSGAVGQAIMALSQLRGATVYGTSSERNHGDLEKMGAVPFSYKDQ
RWIKEMQDLGGAHAVFDALGFESFDRSYSILCDREPARLVGYGANLPYLG
DDKGKYGSPVPSVAKMLLRNAAVWSNKSTRFYDISKDKDFTENLARLMQL
VQQGKLKVPIKQVWGLDEIREAHSSWGKAHGMGSVLIRVADEVTLPSIEV
SAVAESSV
>Mb|QLI69661.1

MELCSACNRFVQDNYRECKHHGTLRELKNCAETKTTPSCALCCLLWTGVQ
AQYSVSVSMSNVGSILANDYTLDLGYTRQGHLRWMYAKGTVMSDGTRPIL
GTLDIISEQESSFSQRTITTGQWDAWSHQTALTVKGWLTECHDKHPWCGR
PRPQKAPTRLVDIGDPSIYRLIETKGGQKELYVALSYVWGIKKKKGESIH
QLKLVKKTRSDLMKGIKPSQMTASHREGSMVARDLGYRYIWIDALCIMQD
DIADWKNAAVEVPDIYNNAVLTITAGRSHDSRDGFLTERRPVKLPRRTEG
SSRNLASTFEVSLAKNRAIGPADERAWCFQEALLSRRSLIFGMGQLIFKC
RKHKAFQDGSHDKFMFDEAAFHHDWVLPFPPEAPRTENDRSPPTQSHIVH
GWYRVAQKYAMRDMYDPFDCYAALAGIARRCESALAKASKDGAAPRYLCG
LWETDTFAHALMWRRCLDATLARDCRVLEEPGPEQDPISRAPSWSWMALV
GRITFHGAPVTTSGPPAAAFIRVPCCVPANGRGSWTRENWGIRTQLEARE
IQTSLPLKIEVKGRPRRVRATTGRVCGYMMFLRYGEMLANHSTIDPAESE
NLREHGVVLQDLRDQDDEKGIALALFDLPPEEYRELPPVPKPVSDHELPL
KPNYDSTYERYRGIFESASTMQLEIPVTPMAEPLQGTPTIPWIVEAPLNS
VEVLGAILKPSHPFQIHTAHHVQRRFDRAHGSKPLYSEYIRLQGQLERNR
EVSFIRVPASAEKILRHWLMGFFKPVVLNYISIPWTTGRIQCLAHPRALV
MSDRPVAGIYTYEIDLPTKFR
>Mb|QLI69693.1
MKLSLLFASTVSGAALISSGRGYGTYYYDVEQLQACNSDFHKDNQGPVMC
SFTDFLPLNDVRSNYLVAMNNTQLRGHLDKYCGKRVVVTVNGVRSPLPFF
IGDGCERCGIGHPDGGWNSEGAPGLDFSYTGLSELGPQACAAGHIDLSWE
IVDENLYHFKTG
>Mb|QLI69737.1
MSAQTSPRILDGDSMEQAMMLNVDLEAKTDELSGRKPSWEPANTLRKLPG
SLGLSNVKSWLSAWPRARRRPKSQHKTAYLDGLRGFAALIVYWHHHVLWA
HKEDIEIFENGFGYNQQYYFAALPGVRLFFSGGHLAVSIFFVLSGYVLSI
KPLRLIEKNDTSGLAEHLGSAIFRRWLRLFLPVAATSFIYATSWHLFGLW
VDGAEPHDSWLHEMWFFYCELKNFSFPFKDGGVPWLSYNVYLWSIPIEFK
GSMVIFTSLLAMSRCSLNARLWCQISLIGYFMYIADGWYCAMFIAGMLLG
HLDLLAEVDRDNLPSLLTRLAPYKNIICYHFLLVGIYLGGVPSANQDVDQ
LANTRGWHILSYLKPQAVFDYKWFYLFWAAVLLITSVSNIGWLKRFFETH

TCQYLGRVSYALYLVHGPVLWIIGDRLYTVVGFHGQSQREHIPRWVNKFE

LPQMGPFGLEVAYLLPQAILLPLTLCMADFVTRVIDKPSVQFAAWAYRKT

LPAVAGKLSNA

>Mb|QLI69800.1

MSSIKPIKVWGKGGPNPPKVAIVLEELGLPYEAVVVPLADVKKPEYLAVN

PNGRLPAIHDPNTNITLWESGAIVEYLVEKYDAGHKISFPAGSPESYEAR

QFLYFQASGQGPYYGQAAWFKKFHHENLPSALDRYVKEVQRVTGVLEKVL

SEKKVSPGQDGPWLVGGKCSVADLAFISWQNVIGIIFDKTDYNLEGYPHV

KAWQDRMFARPAVQAGLGKSEKLQ

>Mb|QLI69869.1

MRRDRDCHSCKSRNIKCDLNRPSCSQCLEANITCGGYPQRVIWAADRSAK

DSHAVPLAAPASASGSASSLASPSASSSSSNATKVSKRQRREPKSKLSDG

NALSPEQDLIKTPTSDAAAPKSESPVLELRHMPATDRNSFITSLIAFYQH

IISSDGSAADHGHYLSSEAVGLISKLHDLMKARIEGRLAGPFAGGDMWDS

IDTARHRLAALIGLNEALEAANPFAFLGIAAFAVLEVCDSPFGEWQRHLH

GAKSLLDYHCADPPALEQLSHTVTGLTEIVARLVWWDTLGAIARGSKGLI

FDDWHRQTLDQSIFQVVGCSADTFDLFSRVAKGEVATDALSCCILAMDQL

AKIDMDESAWVLSANVNRCASAIAVLAQLEDTAHDDAQRAMNSAVERACQ

LISRMTPSSIYYIHVAVSAYLAGMHATSTRQCQTLRAYWHHCNHAGVQRY

PDGLAKCEERWRARGLTGRVE

>Mb|QLI69896.1

MSMTTASDPRADIDPMGRPLCLDALKALLTANIAVVLEEGVLIQRFVYFR

RPLNVGFASIPPALLSARVSPFGKSSYKKPDLRLKKRASKSRVCSTDGYT

GPCLLLSLPVEIRLQIYDWVYKASPVHKNLPLTGYPLPMRQPRSTILIED

KDNLLQEGREICVSTLLRQDRLYNCLPSNLLVLNRHIYAEAREIPFHSNE

FVFENWFSSGLVTAASLLTDVFEPWQAGAMRFARIETKLTDFHDDSGFER

WRILSANWASYFRGLRVLIHVSNDALFEKSRLPDALDGIAERWVVDGCLA

QMKALEKLEVELVTPSSCPDRDKLDWCRELQAALRVHGSRALVASVKCHV

NSLKDNKTG

>Mb|QLI69952.1

MTTHACRSLIHRLSLLRPTRPAACFNTKLPYSTCSNDSATKPQQHANQAL

SPATHHHDGQTRPRRIVVGITGATGTVYATRILSILQRLGVETHLVISKW
ALATMKYETTASEADIHALASRSYVVKDLSAPIASGSFQHDGMIIVPCSM
KTLAAVRTGFCDDLISRAADVTLKEGRKLLLAVRETPLSDVHLDNMLFLR
RAGAVIFPPVPAFYTRPKSMDDLVDQTAGRMLDSMGLFTDGFKRWDGFER
D
>Mb|QLI70051.1
MPSVPGQHAGILREENIALRQILGYRLESLLPAPPYNLPPISHSLPGTHI
KDVLLVAVDVDTGGGYEVISPGQSFHIGVSILDTRHLITTQLRHPAAAIS
SHQFINTDSRPCRWAARSFLFGDTERIALPDFTTRFARLTAGRAYVLVAH
GTREEIKFLNNLDPEIAARAAYIMDTVKAAQHPLQLYYRYSIEKLLDEFA
IPYANLHAAGNDAHFALKALLMIAVRDGRMAPETAAASQELFRTLDAIAH
APVALPVWIDRPPLDANPNKTKLGVKAKRRLKKARREARRIFPELPYCGD
SDGQDAEVREEQHASLPS
>Mb|QLI70101.1
MEGQQTPAQPERHVFLREGTVILRWLFGYYDKRGLQGWTTSPHWPHTLST
WVPQKPKTRFREATLVSMDVDELKESEGIPVQFHIGISVLRTQDLHDKCH
SPLSHADSKSYIIRSYHWVVEDENYFSTHDNTFCFGKHRCLPLADLDKRL
KKLLARCFPVILVVHGGHREITLLQKLNINLNPIFTIDTTKAARYPLQAF
YDFSLKRLLKEFNIPFTGEHLHIAGNDAHFTLRVLLMIAVTDVRRELGSK
EVPAWVPVFEAIARAPLPPMPLKRAQKAARKRREERAALGLGGPNVVTRS
QSRLADATHL
>Mb|QLI70106.1
MSPAPTVLIIGCGVAGPVLGNLLIQKGYHPIVFEKVSELGDAGASLMLMS
NGLKVLELVGVADNITAESCPIQRFIDSTSDGKLLGSSDLPSTFKDKYGH
PLAGIKRTSINLMLKKTLLDRDIEVREGWELLDIEEKKDSVTAYFNHKRA
VTASFLIGCDGIKSASRRALLRSKGIAEGLPSYTGLTQTAGISKVPDSLL
TPAAMRNWYGDGTHVIAYPISKTHISWAITRRETNEAAETWRPYRQEELP
EQTSQLCKLLEGWNSVLTDMINASERVIKFGLFDRQELEPSQWYSERCVL
VGDAAHPTSPHLGQGANQALQVVSFLLLLLGIHDLLANLPSFVREDCFHL
SQALPDLASVDTRFEKAVAALGPSLSDAIFKPYAEKRQPRTSHLVRGARA
VGEQRTASGEEACVLRDGLITEKFADEALLASRMDELLREPFQRTQSPA

>Mb|QLI70119.1

MSFFARQQQPAYTKLSKNQIERFTTTDDFVVIAHLHEDDEELLSRFSTTA

EAYRDRYSFGYSTDVAQQDASRLTCYNNIDNLEHSEAELDQSGVIERFLE

TCKELLIPQLTRRTELKYRRPGKSLVYYFTGEESDRDLYVKRMKPLARTY

HEYLRFVTVDSTEYADMSRGMGLELERGLVVENTHGGQLFPYRGSGGDIG

IGEVENFITAISQGAVQPWTGHRDDSAKDTGGWSKHNVRDEL

>Mb|QLI70125.1

MSSPPPPGIYVPVPTFFASKSSSAYDAAIPPADIATQSAHAIYLAKSGIK

GLVIFGSTGESVHIHPRDRRAVLQGVRDALVHEGFRDYPIIAGTATASIE

ETVEQLTDAKEAGAQWGMVLAPGYNAAVTPQEGIVGWFTAVADRSPIPIL

VYHFPGVSNMVKVTPATFATLAAHRNVVGCKLSHGDVSLLTQIALNPAID

AARFHVFTGLGQQLLPVVGVGCVGAIDASAGFFPKSLVRLLELAGKTRPT

EAEARERRELQYKVSCMEEIVVKHGIVGIKEATSRLRGFGDVDGSRLPLH

GTIQGGENEWKNWEGVLAALEEVEKRL

>Mb|QLI70138.1

MISSIATVIVASAAAVSAGVIPKRGQGISVTPHDQYSSSIGVIGGKINTN

RVAYWPEAITCDNICVKVSYEGRSLNLLKIDQSGGAHDISYDAWNYLAFG

KSATDSPQMGGGIAMEYETVSVDECADLLTDGKLPLSAANSMGYVGACIG

EPNSWVAKNYELVNILDPVGKNGWNEICTLDLTVSNQPKCPHTLGEQHSP

TGLEVVNIQYGTGKQVTV

>Mb|QLI70209.1

MIQPGLKVIFDSVYDDHPHRVAKLDVVAVHGLSFKNTDEHARKTWTMGDK

LWLNDFLPGALSRPIREMMFEYNSSPAVGAAAIKLDDHAKNSLQWLNLRR

KTDPQKPLVFICHSLGGLVVKEALVSATLDVSYRPIVEATRLLVFFATPQ

QGGNYATVGDIVAKIVRKSMVKPSNDLLEALKKTSNEATKRSEQSRHVYE

KCLVVNFFEGYEYGKMGIIVDKKSAT

>Mb|QLI70212.1

MPLADLRYLYHMALVDGMLALSGTSNMCLLWGEMRIMIQLATSFGFVAHT

LAATSAERLAVLTKSQDAANDGARYRALALHGLSREIQLFSKSNADAILS

AYLGCSFIMADYRAVMTVTKSIVLVAARMEHWSEQSAFRHLFDYDRLHRL

QDIHDGPRPRHQDVATLLAEGVQALNRLSNCLRHNLHLAAVVRQLRDVLR

LVDDKLDADVPAATQYRLIHPFISWYNRNEASSYVAISQRDPAVLVFLLY
MYSAFVSLAVALPATNLPLFTAIRFRAIVEINRAMEERAGLPCTGCNVFH
QYHELAPFPLLAMQVYLSHGAVY
>Mb|QLI70231.1
MSRGPGASRLCLLSSLPDQRIGEKVRFLGCVTAYNTGTACLTLGHVYPKD
TNVLVSVNIELVLETLQPGLARVGEWFNIIGYVIDGPAGDAETTRSVGAH
VQALMVWSTGPLDIQQYEREIEAGFL
>Mb|QLI70262.1
MPRPTKLIIVCCHGIWLGGPSKGHNEAEWLLADFQHGETPTFIAHIKAGL
QCLADDRGHAVLAFSGAPTRRETTLSEAQSYANVAMQNACFGLLDPPVTD
ADMMLEERALDSYHNVLFSLSLFYARFQRWPRHVVVVSHAFKRPRMLEGH
CAAIGFPPDRVSFVGVDPPSLEGKPGVVAGVAEAEADWGRDPHGKGQRLA
AKRARRNVWGAWQGVFEEGTEDADKGGLQTAGEGASEVIVDGERPW
>Mb|QLI70296.1
MVAPRRHRLDSLYRFACILTLVQLGVAGTVFAQQKPLSDGHVPGRNPFDD
KFGAFVKDTLDEWHVPGLSIAVIDHDQVFAEGYGIATFPDTPATPETIWY
GASTTKAYVAAAMAAVIDSKNYSQLTRGWSTPVSSIIRDDFVLQDEWATA
HVTLEDAVSHRTGLGSLHFSSLRIENGVQVTPRDVVRRLRHLPLFAEPRT
TYAYSNSMYVALGYVLEKLTSSPLAKVLGNLIWEPLGMRSTYFDLDDAIK
APEHLASGYRWDPDHGNYTEMPYMVVTEVGGAGAIFSNVLDYAKWVKCLL
YESAPFSKAVHKDVKTPRFITSPLPGEGFDSVLYGLGWERTLMYGHVVYQ
HSGGMHAYGAYVYWLPEIKYGVVSFANTAVTSNAVEIILATRLIADRLGI
PEEKRFDYAGSERDQLEEEIEWLEHALDNLYPSRPNPPLAPTLNTSQLAG
TYYHPGFGPIRLREVINPKNPKEKVLRSDREEASWDHKFTLHHVTGDNWM
IRTEMYTTVRNTAFRSQFKIGVDGKTAGLEVEFSDRGAEVAEAVVLFDRL
KE
>Mb|QLI70372.1
MADAGSSFNASSVDLDNTPLVDIICYLNGSGNAYDNRLGLHIASIFVILV
VSTAVTFFPVLATRIRRLKIPLSAYLFARYFGAGVIIATAFVHLLDPAYA
AIGPNTCVGLTGGWSTYSWPPAIALSSAMIIFLVDFIAEYYVEKRYGLVH
AEVENIITDASGANGNGAHGAHGAHGSHLHLHSADQDDRPKGARRAPAAV

RSEKYDSDLEELTHLTGDSEEIHRAFQSQISAFLVLEFGVIFHSVIIGLN
LGVAGGSDFNTLFPVLVFHQSFEGLGIGARLSVIPIPTRWRWLPWALCLA
YGLTTPISIAIGLGLHNTYSGSSYTANVVSGVLDSVSAGILIYTGLVEML
ARDFLFNPHRTNNKKRLALMLVSLYLGCGIMALVGRWA
>Mb|QLI70385.1
MSSSSGWSDMKRKESSLHVAIVRGIGEGPKGWDREKVVKWVKKSSYRRYG
APEITEFANTLDFDGGITPESIKEAAVRLLERMRKEIEESGVFIETPHRA
TEFESWESISTKLLLRRKDQGHCPSSTASEQLGNTLMKLSKSFQVMARIY
PIVNLYECINEEALRSTPLAFSKDTTTMGVPGEVCDRLRESKNRGKYVKF
FTLMDLWEEGQKDTYFRCLMDLSRADSHHSLGEHVHLSSCTPDKFNWILD
RDACKNWIGPACRPLWLYGMAKSDVAASMDVLERKARATFPDANVIKYSF
DAQDDRCRSTYGMLASLSHQLLTLKPGLFEHVRSLYHACDMFLEVEFKIE
HLWAFLRSLLSCPPKRHGVIFCFINMIDECDPCSNKFLKDLLLLASSETS
AGGFRLAVTSHKAPDSDVEFAAHAINVIQERHEDPDEKKKEIESVVARET
RLLFEARPELEKLDGIINEKLSAVDDVFNISLIAKLLQARLPHWGDAELE
TQLNALPPTTFEIYRHFLDSIPADNQSWARDVLSWIVHAFQPLKVTELAL
AMALQDDTLSILELQRLPQGHIESKIKNVFGNLIQIQHDNVFFIDSSFRD
FLIQTQSSCQERSLKLVNHANCARLCLRYLSLVTSATEESKPIFDKKNLF
TPSEGIEYGFLDYAGQYWFRHYNLAKHDAFPDLQDEACAFLERPKSDIMW
CKLKVDYSTWQRGNPAHVAISLGCNDVARTLLEKPEWNAEVLDHTQGLRL
TTTRETAIDFLWEAGARHGVILYAAAMRGRDELVDQILKDQAQVEMGKEY
ISPYGRTALHQASSLGYHAVVRKLLNAGYNANVTNDELLVPLQLASRFGH
VDVIEALLTGKEKADDENMDTDTHPACAAVVGKSPWLVQESLMMAIDSQQ
HDAIKKLLDRGAKLEKNPKALSHAVASGREDIVGLILRYYEKLPANILHE
AMATRGENNYTPLGLAARGGYMDVVRQLLDKMESRMDTTETEDMKKKSPR
DMPLHLAAEGGHAHVLKALIEWQWPQDSEYRDKSNIRAANSSGQAPIHLA
VLGGNASLVVQLCLEHQTQKVSLRLLNSRLRSPLHVACDCGFVDMVDILL
EHGGWYGEADENGDTPLLLGCAAGDLQTVKRLLTHDSNLLIKNLQGRSAL
HHAAASGEPDVVQELLRASSDANDIRIYTNAKDKSGSTPLHLAAVAGNVE
VMDILLDEMADITQVDGSGHDVLYLASRHGHANVVTFLIQESRKGIEGDD
GDTFHFGGDIFQFVQEMLSYFPHNLPVAEETDIAVEIGPRENCAMVLANL

TSLANQWTPSQYGITLHLAAKGGIVSVVDQVLLKGQDPNYMDTMEKTALM
YAAESGCSRVVERLLRVPNIDQDCQDTFGYSAISYAAESGHLEVVKMLAS
GRKSAIDAQTLTAASRCMANMKGSSIMTFFLDNDIDGSLVDSSLRIAVSE
GFVEVVSLLVGRGANVNTTYESTSTPLHKAAGRGNVGICKILLEAGATVD
SETKDKETPLILAVSRGHQEVSEQLLHSGADPLAATSSGKTPYHEAICAG
PEIFKAVLSRVLELNKSAHTKDAQNRSPVLLAAAQGTQEAFMALIDIEGA
DVNEKDKNGYTPLCHAIFRQQAVIVETLLKKPELKRSMSAKDMLQRAAGF
EEDKIMKLLIQNGIDRDLEFDDLFELAMESENLYLVEIVLQTPHDGIRLD
KHRWSLECLKHVLMDPEYKREHMIRNSDTDLKPPLGWCHQGEVDVERAPE
DAWGFPGPIFPNLRLENGQRLRADHPIPPVGKFKFEVHLVDATLNQ
>Mb|QLI70515.1
MSTINITLPNGVKYEQPVGLFVNNSFQHASGDKFDVVDPATGQQVLSVAG
ASVADVNTAVEAARKAFEGPWSELAPAERGDFLMKLASLIDRDRKLIAAI
DAYDCGKPYSVALEADLEESYNVFKYYAGWADKIYGDTIDTSPAKFAYTV
QEPLGVCGQIIPWNFPFMMLAWKVAPALACGNVVIVKPAEQTPLSAMYFG
KLVQEAGLPPGVVNVVPGLGPVAGKTLAEHTDVDKIAFTGSTNTGRAIMR
YAATNLKNITLECGGKSPLIVFEDADLEQAVKWAHVGIMDNSGQVCTSTS
RIYVHEKVYDDFVKAFTEFTKKSTVIGNPFKENVNHGPQVSKNQFDRVLS
YIEAGRKEGARILTGGLKVEGEGYYIQPTIFSEANENATVVREEIFGPVV
VIGKFATEPEVIAKANDTSYGLAAAVFTENISRGHKVSRKLQAGMVWVNS
SGDSHFGIPFGGYKASGIGRELGKYALDAYTQTKAIHVNLAMKL
>Mb|QLI70602.1
MPPDRPPVHLVIHRDDTIVTVVGVYARLTDANTECIFLGKEAGMQLTGES
GEMAPDDRELMPIEPMRWDSVAGVSCWVETHHVKLARS
>Mb|QLI70619.1
MPADFEKQSYWHDRFASETSFEWLISSSEFVFIIEPFLEALDPSSAHILN
LGSGTSDLQNHLRSRGFHKVCNLDYEPLAVERGRQLEEKAFGNVVTHYTV
ADTTQLAYAGPEIGRRADGKFDLVIDKSTVDAVCCGGQAALLRMAQGVRS
RLADDALWISLSFSASRFDNHQLPFHVETIAKIPAPKIKTTDPDIYHWCY
LLRPIRDRP
>Mb|QLI70650.1

MPQSTDYTMRYTDVRAENKETTSPDKQNRFATDLHLPDPTGRNDIQAQLS
AQAQLSVKTEDSSSFLSYPSRHASASAAIWPPATKVAGYRNQKSFIDRMD
SSCGEDWSITQVFYSDELPIHTPSSLGDTKRASLICHGPTKSHDWSSETP
AMKREPKLYGSKYSDDMMEFIATQDELNYLQAFADGVGVWMDSLNSGNHF
TQVIPWHALKSSLLLNSLMACGAKHLSFSKPGLEDRAIELYNTATAQLVR
MLQNPERNVVDCTTASILLNVYEAMSHKPIHRMSHMVGARALIRECGWNA
ASTGIPAACFWLSIGMEVLSCLSMNWAVTWHPDDWGLDLDWSHDNDLDGG
DSQTWVHRSFYILAKVVNFRATSLTYPPSDPHKHQSRLSDRLTEWQALKQ
LCDDWSNCSPRSMRPVGYLAMSGSSEPSAFPKFWLAQTSAMLGRIFYHTA
QCVLAQVNPIESSEQSPEMKELQLHHARQVMGIVASDRDRSVMTIALQAV
NVAFLSLVSPEERQEASSILHEARTSGANTNSEAHKMACQWRMAVMELSE
LESASGVSLPCEQSWFRAANMSNSTSWEFTSHQSSRAEAPIDNPLESAYF
GHVDQPYKTWYQPVCGNDFQAFGFGM
>Mb|QLI70664.1
MDGKPWVIIGASRGIGLEFVKQLLESGQPVIAAVRNVSAAPKLFDLIASQ
GAQDRCIVEQCDVSSDESITHFINKMQQHVHKGIKLGNVVLNAGVLKYPN
RATEISFADFELHLHTNTIGPIICAQKLLDLSPDSPPPKMAFISSDSGST
ARFLDFEDGFGAYAASKSALNQMLRHMAAELRRCQQKKRETVILALHPGE
VETDMADIQLGWEVKGCISASESVSGMLAVMAEKGQGDTGTFWCWDGRSH
PW
>Mb|QLI70675.1
MRGRETARVSAEANGSCPNQLKYTMFKQSRHLEAKQVTGSSAEPHEHTYH
CPFEFELPTSLPCACAGPCALPPSIHADTPKARVCISYSIAARVQRRVFH
RITRTTTARREIQLTSSPCITALPASAIASLPAEKLRRDSGIGIGIHDST
PALPSGSLPRYSPSLRMEVILPQAPVLTRGRGTPVRLVMHTPAEMLRGGG
LYVRSVSMQLRMCVSVRMRNTWHHIVQSRRGCVIGGVVPIRAGHFELELG
DWGSLIVVHPDCKPSFASCLLNVTYSLEVAGGISKGVDGQIQYVNTSLDV
LVMDPPPLYEEMDSGETGS
>Mb|QLI70713.1
MSYSFLTTLAALTVAVQGLNPWQAPGPDDSRGPCPMLNTLANHGYLPHSG
RNLTVQQFGDAMAQGLNADPLFGTAPATGFTLVWGRSTFDLEDLNTPGLI

QHIASLTRDDVTPAEANIGEVPARVSALLDDSPTDYLDVASLAKSRVRVE

ALSAPQRIPPQHEVLALTEAGLLLMMMKDGPVPSFFSAPSVQTWKAPKDR

VKVWLTEERFPEELGWKRSERTLSVLDLAPVVTAVTARKAASAVMGQ

>Mb|QLI70732.1

MSLSDLANELLVAVAQFLDAERDIDAFAKSNKRLYSLLIAHLYRYNAHNS

SASALVWAAKTGRVETLRRAVSTGVKVHEFPLLPIASDKGHLNFANLLLR

TPGVDVNVKDEDGWTSLAAASRQGHVDLVNLLLAANADLQTNDAGWTPLS

VAANDGHLEVVRLLLNKGADVSVPSETGWTPLRSAACNGHFEVAKLLLAH

GADVHVTSERMWTPLHSAANSGHADILELLIDYGANTAAATADGWTPLAL

AADKGMVEAVNILVAKGADVALPCGNGWTSMTLASDSGHVPVVQILLEKG

ASVDAPCINGWTPLTLASGAGSVAMVELLLDHGADIKATNESGSSSLLIA

SDRGHCAVVESLLARGADVLSTSRRGLTALHTASENGHVEVAELLLAQGA

DIAVGNFSGWNAVHIAVQNKHPKLVELFLGTPGLDVDLKDNYGRTPLYHA

AMVGNADMVDLFLARGASPTMSDSYGSKPIFTAVRNGHEAVARRLLAVSP

YPSEQLDGFGHSLLWWARRSGRPGLLQLVRQHITMHDPGAEMDEEMEPET

NPVKFIENACWCKVCTRCTVLGTTSYECTTCDGEVAMIVDGGVVVACLLK

ARFL

>Mb|QLI70736.1

MNRLYLYIALGAVMADSIIELALVTNMVSWLHGTTSATFSIMSNGTTFDL

VGVPRNLAVDQGHSSNGAAGTAFVIVGLGGVLALWLQGRSMHRGQKSSNL

IYRTWLLFTVLAAVFTLATLAYVFAVTNSLKGQVIDVDLAATLVDTRYPR

DNWTPQGWFGAVLRLDLASASERRDVILHLRIMHGWQYNLIPMFLLQLIL

TVLAVVDATEVRKWRKVESVEDYN

>Mb|QLI70753.1

MEQFLLQAQHSGTINHAHTFLFPPGAAQFAREIRLAVTVVAVAWVTVKAF

DLIQRKETNSKS

>Mb|QLI70756.1

MSEPTRSSPPVMHELQSMYHAGIEALPPHLCQMCADMTGSARGLSALISS

EGYHHSTVPRVRQAAEGGCMFCSTILDMLLTGHLYSRTAGQAAKRLELDD

TVRAFITGKVSQRAQYDNPGLRAPVMLLEAIVVNWVYQGQVCDFHVLHKK

VGFYMDRPVVFGAFTNAAEPVQICLVEAAGNPVILSFKPQPLSISAVGDD

LVEGYMRLRKECAAEHASTCPPDVEHALPLRVIDVECPGTPMNIGLVVND
ASAPRHGRYAALSYCWGKPPHVFKTTTSVMQDGFLLDWSTAPSTIVDAIT
ITRRLGLRYLWVDAVCILQDDEDDKTSQIKDMARIYKNSAVTIVAASASG
VNQGFIKDRSVKSIAFPVNAIDATSHNGLRVGTLWVHANTPDEPEEAIDS
RGWTLQESLLSPRILYFGSKDIIWKCQAKPFQPVVGWHNLYKAEGMKRLP
STIFGLALPGTQSPLGHWPHIMSEYSRRKLSLDVDNFRAIGGIAEELQKA
SGDTYIAGLWKNDMAKSLAWFRTDRIVPKPANDDPLKRPTWSWLTTLFPV
GQIGIEADASGNETVQVLSWSVELADKTAPFGHVIGGALELSGQVIRASD
IPPAVISSMTVMLDAGVCDEDDEQAGRFVGDEYYYMLLGSCGEGKDRIIM
LLRELEDGTVVRCGIANLTANTALGIWETGKVRRTKVTLM
>Mb|QLI70758.1
MLRLHRHQIRLTPSLHNSAMNVQRAKPFWGAPTSNLNFCEEDYLVTRYIA
EFINTLSSLVYVAYGIYGLAHGRRNGSRLVSYCGLIGVGVCSAGYHMTLK
YHTQMSDELSMHLLSTPLLHRVLTFNKSERYTKTAGVVLFVLFTVVMAAH
MLMDEFLLHATTFGFAVYMIGTSVMKLIPQQVPDPQTRSNIKKIARFGTI
SFGFGFFVWLIDEWACGMLNGARQSVGLPAAFFLELHGWWHIFTAIGGYI
AVALVDEITTGQVTADPIPLLAWPVPLAAKYVLGSTKTEKANGVYGKTA
>Mb|QLI70850.1
MRLQEELARRDTTVITHLLDGSRSVVHVGKAERASGCSQKGRRADMCNTL
GLYALKHGWASWALDAAARELRGEDGTRTSRYMPRVDGHAAEDDNGPLAT
WCSCGNDTAASVSASGRAKSLSSEVTVRIGTARHKATPEQEQLLEFAYQR
GAWTGATRQILVDAMWLAGPGRREIVQAGGKASQEINKGSRGSVETVWLH
YLFINNHVNLLADGLEMLLLGTYLTALDHGRVRASDFTAYASEVARHASL
TRWVIASDELMRRWPGIMDWHAGCVSTLGLMRALRLRAWSFAAHWNVTMH
HELGSGTDGEEPASLWIYIMAALGHDTGDLCSDARRGCLDNCYYAVGAHA
GYEGVAACMDMVCDALEAVVLRDARGAMDIGVVAGSACATFERTGGNLCA
CPMRPSCEAIVVMASVCGHRRIAPDDAADLLSMRDAVRSRFSHLPPSSRG
MARLDHAAREQAYAEALRTLATGNGTQQAHSCLQVAYASAARAMCGELRR
RAASLRHRVASSGHVLDLSSDCMCGGECKTWC
>Mb|QLI70861.1
MQTKTVLAALAGTAFAANLQPRQTDLGAATQCLDLLKTIPTPPPDLVKEW

TTNPPKDYCSISIPASLSKDWSSYTSSASSWVKAHSSDLAKCPGAGQVTA

KGPLDCKAGSGSATQATATSGSATGSATGSATGSAAGASQTTKTGAASRE

TGMAFAAVAAAGFALAAL

>Mb|QLI70879.1

MKFIIASVAALASVAIAAPGSETPERCKPATYRCDPNVNGWDVCNTSNLW

VFAGNCPPKTICKFFQENGSPYCVPPNFTIP

>Mb|QLI70920.1

MNTQEIYDQVNRRYGSIVKSSTGQYEQKVAKAFGYTEEELSTLPEGANLG

LSCGNPIALARLREGETVVDLGSGAGFDVFAAANKVGPTGRAIGVDMNKN

MIDKANANKARVHASNVQFIESAITSIALPDEVADCIISNCVINLVPTED

KQLAFSEMFRLLKPGGRLAISDILARKEFTDEIKNNIALYVGCIAGASKV

SEYDEFLRNAGFHDVLIVDSKSDLNVYCTTAEVKPSSHPETDAVQEEQTV

PSCCSTGARAICCQPEDKESCCQDNSSSCSCQKQSTSMATQVTTLAGQLG

IADFNECAGSFQVYAVKPTAN

>Mb|QLI70926.1

MSEKTTPQGPFHPADPSSAASGEAAAARATSAADAWKPSFDRRQSWSKED

QKHALQMRAVEGVKTGLGFTEKTS

>Mb|QLI71015.1

MSNDTENQPAFGQICWLEIAVYDIKRASKLYSEVFGWKINEDAMAMGHHG

IDAMHMFESPGKKLGGGFLVMQEGYQMTRYGSLEKEVLPPLPTFCVKDCD

ETLKQVEGLGGSTQCPKTAIGGDMGHYARFNDSEGNIIGIWSQE

>Mb|QLI71016.1

MTRITTVVLAILALTTDARRPTAAKKGCTFLTTTTFLRQTTSPHWPQCSF

DGTERIYSSTVTMNTTVDCHGCSHIRVISKPNVRCPAKI

>Mb|QLI71087.1

MPCSIFVVAAVVAAAAQGSSLPELPSFLSNTSQSVFPPLKDIAVEIFNYP

ELGLNEYYAHQLVVDYFDQVEGWEVTPHAYGMDTAYTLEFEHRPQGYDGA

LKSIGFLSEYDALIVGSDPLVGHGCGHNHIVLNGIAAATLASRALVEYNV

PGRIKVVGTPDEENAAGKFKLKVAGAFDDADIWLIAHPSSVNTIQPLGSR

INISPHFVGKSHQEAVRKAYEAIVAVDKIATSLPGMRSSVTKIQNVGMYS

TNVLQSQVNFGVSGSDMATVNRTVSDILDDTFPRVSFTTRQDPHGIAIKI

HGPGGHASLTEKTPLDLSVATFQAFSNRSGVSFYVPGNTSATELDITFDV
RSRYMVDLPAVVDAVRSAVGKLGSRVTMDLRYPNVEVPPFLPETWIDLVG
RPAYNLSGWQITDQALADSDIAWVQGAHVDPQTHKLVGMEKVVFQPNYNI
CEPGSKSCPFNHEPGFLRLAGSEYSYTQTEIVARAQAQLAVQLLTDETMY
NSATAILAKNRVIEE
>Mb|QLI71094.1
MAFNFCDQDHGGAIDVLIHNANAFDQSYTLLQSFSSTAGYQSHGGLFDAS
IRTAIYGNLWSTNAFLPLIETGTEKKVVHISTIIADLDFIKSSGIENALV
YAVAKAGMNVQVTKYAVELAPRGIKVLALSPGWVDTFEGDASLTTIDLLQ
AELLHQFITITGPGIAAGSDDPGHAMGQFWAQIAPGIGFSNPHVLHLAYS
LAGYHLARGDSWDRNAQAHRLAVAKLNFTAGLAELNKAISVMDNGTCGAI
YISVMLLCFCTFAAGPVGRNDLLVCQVGVARPNPSVPLARGAHLVRQRFD
SATLFSGLMAPLAPTNSQPADSRATCHRQCFVRVDWIDQLSRLRELIVSS
DTREVSVAIRSFDTLRAIYEATYGDRDGLYEGPPMHGMVLRWLYVMEDDF
VASLQSKDAMALLILAYFAPLLNTMSKAWFLKGWAEHLLTSIRIFIDKEY
VEWLEWPMGVAEQYSEHIC
>Mb|QLI71099.1
MASQKPAELIRRHRSVIACQGCRLRKVRCSLSVTGVPCTTCTQDGTDCLV
SPRRPRQRRNQRGRTSALYSPPGDAPQTNFANVDPSSIIEPCSNMRVDVA
AADDAEQTASCLDCESASIYNEERSGIEIAEAALGVPRRVGQLPFYSGEH
LGLHVGSSVPRHFLIPSDTEAFLEEEDREYLKAKGVFTLPQNDTCAALLR
VYLSHVHPVYPVIEVEQLWDCYHNGRLGQYNLLLLWSIFSVAVNFISSQD
FEREGYKSRRHMKAVMYSRAKCMYNNGGDRDLVVRLQASFLMGFWSAEPF
EHVRPWYWTGIAINYCQMLGLHRNPDSAEHKSSISEPQRRLWRRIWWSCV
FRDRWLGLCLGRPQRINLDDCDVPMPTVDDVIQELEKLPQGAYTAFMPVE
MPRLATYWIILLKLSRLLGTVLHMNHQARDPRASSVEIRAMEDEVLQCTL
PDQYEANQTRLASFYVHHIHLHHQALLITFYRHNATECPDDIPSAQREDW
KRMLCLKASTAALRTNDILDLVVRDGLLGFAGPMTPPLLIPAMQMHLLDC
KSPGSLPRRLGLKRLEACLLVLEGLQKTYAGASIHRGIFLKAIQHMFPGY
ATGSAPSSETMQSVVNARSETIGDASASETFVDNVSAEVSAPTDDFLDTL
LDDGAFFPLWLDESYRTVNVDSDR

>Mb|QLI71105.1

MASPVPMKHIDREDLYTNLEARIQYLHSFLDFSSNDIEALISGAKYIKAL

VPAVVNIVYRKLLQYDITARAFTTKNTTQGGEVGEALHEDSPQIVHRKMF

LRAYLNKLCSDPSKMEFWEYLDKVGMMHVGLGRKHPLHIEYVHLGTCLGF

IQDIMTEAILSHPRLHMYRKIALVKALNKVIWIQNDFMAKWHVREADEFG

LLETDVVIEKEGYLHGKKILDDKLMADAGAGLPAPSKSQCPFATSLPIPS

PLRTSIEVDNEETSVPIPASPKVEVEGR

>Mb|QLI71136.1

MASQPHASERDALTSRVLPSMTLAQGDDSKYTINSPATHTHPRDSAQGRF

AVSGNAIVTGGTGAIGLKTARAMLQHGLAGLMLLDLNADASQRSIQQLRD

EFPGAKIEALPVDVTNEEHVSAAVAETVSRLGSVDVLVCFAGIVGCAHAL

ETPASQFRRILDVNATGSFICAQAAARQMVRQGTGGRIVLTASVSAHRVN

FPQPQAAYNASKAAVVMLKSSLAAEWARYGITVNSVSPGYMDTVLNAGEG

LADARRTWCERNPFGRMGLPEEVAGVVVMLVSRAGSYMNGADVICDGGGV

VF

>Mb|QLI71149.1

MAVPLTFLVLVTGVAGDSALPDGGLGNMHSYLSQHFPAAHRIVLNTVESS

AEDVMITVPDLRSSARNLLQSLDRKKDKLEGERAVDVKNHGDGPKQQGPN

IPAPKLVFLCHDIGGFVVKQALLLANSEPCFEWVARATAAVLFFETPHTV

PTHLSWERLMVRMLGQTESVLDFASFIRYLSDYASQLETEFEGISGTHHM

INFLSRESHYAVVTRENLPCPIYSCDQIHCLDCDPKEMWKVSDQVSWADT

IRRVISTHLSLDSKGRPTRSYAQFLDVFGAAESKIAPAWNASTPLDPDKE

GIFTKDTFRGLLRSILSGCAGKATFCIINGLDHYTAEARDELLRDLCEMR

TSSQAEFKVLTVGSASHVQYGKPGYPLVVYSQERILAAGELERLAKTRIR

SMAAVNPAWKTLDLESEQVKRLWTGSATLFELCQKMDFLEKGETLSTTDE

AIAMVATLPADFTVFFRTLATKYDFLRDGGLGAAVLPWLTRSVRPMTVPE

LAVCAALARTSTETLTLQHLQKSILWDFHRDLENELGPIIRISGETVEMR

HRIYQDLVLADSNALGKANVHLDILTTCLIYLRQISPSWKHGTEFRQEHR

LAEYAALHWPQHYHEVRDKATAKPVVLSLLQNEEQFTVWLDIYSHYATRS

AEEESFPKTPIQVAAYLGLTEVLSELVANLHPSNEDTELSKAMEYAASKG

HANVIRLLAELGVRAEGALLRASWLGDNATVTELLKSHRTYINSRGGPDG

FYNPLLQAARCGHVDSFAKLQSEGADTNAVAAHTNLTALHLAARIGQRAI

VQILITAKVPLASVDEQGYGALHYAAEGGFEEIVRCMIIAANDPAILADV

SRVSQVLLANDQTRDSNTPLHLAASNGHLKTVETLLEMKAEPGILNDRKY

TPLHCAAEGGFPSVVKALLKVGTVAENQGEQSTIEVQPSPVELAVKNGHL

GTVRELRSSRFYDDSEVLSLAFATACREGTNDCALYILHWYSEVSFAGGP

LLDVDGNTALHLAARDGNVRLFQKLMDSKYVPIDSLNKKGLSPLHIAASS

GSLAIIQLFKDDSALGGTTANGTGRTLLHIAAEAGYLHVVEWLLDYTSLK

KETATAVKDTAIMLAAVGKHEPIVKLLLDSKYAVPTGNLLHVAVLNSWKD

VTKLLTSCDFSVLNWIDDTTSNAALHLAVVIKNPTW

>Mb|QLI71206.1

MLHTFTAIFLAASAAGAMRPWKAAGPGDSRSPCPMLNTLANHGYLPHNGR

NLSVKHFGDAVVEALNAAPSYGTLPARAFIKSWGKDFFDLEDLNTPVILQ

HRGSLTRDDVTPTERNIDVDVARVSALLEDSPTDYVDAASIAKSRLRVEA

LSEPERLSSWDQLLAYMESSLVLLMMKEGEVPSAFSFPSAKTWTAPKERV

RVWLTEERLPDELGWKRSERKLGSLDLVPIMKAIFDEKRAQSGKGRLWKS

FLSLFWGSRDEL

>Mb|QLI71235.1

MDAINAFKLAMEAVYGPLDNSTAQDAEKWTPPASPGAGGHHGRYLWTDAF

GVINLLTLYKETSAVRYLNLAAGLVSSVHNILGRTRDGSHRLPGATEDAP

LAGGLRIGKMAEFGADGDGQYHHYLTIWMFALNRMSMATGEPKFNTWAME

LGRAVHPHFVAPDARGNKRMVWKISTDMRDVLVPSEGHLDAATGFAVYRL

LQQTAESYGGGDELSLRAEIADYSGIMARPGKLAPTSDSLDLGMGLWMCQ

FCLDEPWADGFVDTSLRMLRDGLDHSRLGGSGLQRHKRLAFREFGAYLGV

RCIGADEELQDRVDALIKSWDGTGGHVDEELKPISHVMYASALIPGGEFY

NVHCLNSF

>Mb|QLI71355.1

MEPSGADKGFFQKAPVLRNQFHDDASFQRCFKCKLPPLLSPSSLPIVFLS

AEVASKAGGEVSSFGQDVISDQIFTWVTDSEHNKPYLKGSGRDAFGKWKG

ELITGEGWRSLQDFGLSKGMVATGYDTPYGAFCRPLQFLRTHLWVGSCAN

VGCPSAMQDGAACLLRRHLSHPELSAALSAAEKKVFTDAYQRLTSRDPKY

AWTSGQWMTERTGGSDVSLTETVATYQPEGANGFASKQGQIPLGPWSING

FKWFSSATDSEMTVLLGRTAAGGLSTFLAPMRKHDPEATTLTGEPRDDGR

ALNGVRIQRLKNKFGTQSLPTAELVLDNMRGWMVGREGRGIHEISTILTL

TRVHSAVAAVGGVGRGLGIARAYALVRDVGAGGRKRTRLVDSPLHMRTLA

KISAEYRRLMLVTFYTSYVLGVSEHSLGARPSPALDALTPQEKLVEPLLR

IMSQLAKAYVCKPSVALLFSCMEALGGVGYLVNEEQEYLNISRIYRDTCV

LPIWEGTTDVLCTDLVRAMKHARGGADSLAALDEVVRKAWGFEGRAGRPA

GWEPVEKWAALKSKIEGTEQADLMGEAREVVWQLGDILASVLLYVDAGTD

GNPVAKEVFVRFVEDKFGVERRARGSTEDELNKDLGIVYGHGGDVAAKL

>Mb|QLI71364.1

MSSRTLVSENDARHAAFDKILHGASSESRGGLRAMMGKDRGANEAAVKEY

FQHWDNNKAQDETEAVRQARADDYASLTRQYYNIATDFYENAWGESFHFC

RFAHGEAFPQAIARHEHLLAAHVGLRKGMKVLDVGCGVGGPAREMVKFAG

CHVTGLNINEYQVQRAKSYAEKEGLAERLDFVQGDFMKMPFPDDSFDAVY

VIEATCHAPSLVGVYREINRVLKPGGMFGVYEWLMTEAYDNEDLEHCRIR

LDIEQGDGIPQMFGVREGLAAIREAGFDLVYHEDLAATDSGPAPWYWPLG

SDLSYAQNLWDALTVLRMNRWGRVVAHAFLQALEMARIVPSGTKKTAESL

GRAADALVEGGKRKLFTPMYLMVGKKPEVA

>Mb|QLI71372.1

MSDRRRINGPGGPTNPPVYDEDLIIKPVRTRNNNDVRPFYLKTGVTPSAS

GSAYLEIDQSTGSGFGMKFTCTVHGPRSLPRSAPFSPHMVISTHVKYAPF

ATRQRKGYLRDASERDLSIHLETALRGAVIADRWPKSGVEVVVTIVEGDV

SRQTTIDEGHEEWNTMSILSGCITVASAAIADAGIDSVDVAAGGVAALVQ

DEGIGKEPILVMDPSVSEHKTILAACCVAYLPARDEITNLWYKGSSCGSH

LATYRSLVGRAVLASKGAGKAISVSLNEAALLA

>Mb|QLI71392.1

MAARELIVTFLNHTNEELSLVPGSVRLDHGEWMTGTPESEPPREIRAGES

GMWRCKSRHVGSGMVGAVDYCICGYGEKDKISVSWDLRFVTPNKFEHKVE

SDEFAIRVLGGNGWPAVAVFVLAAVCAGFEHRAVPNAPDGYAPASVSCPS

PRPTIRAATGLSANETTWLNLRDNNTIPALKSLLTRFNISGLDTNEYIDN

IVKDIDGGNRSAVLPRIGVAVSGGGYRALMNGAGALSAFDNRTSNSTGKG

QLGGLLQATTYLSGLSGGSWLVGSLFVQNFTTVDSIVLSTSGFLSTLWQF

DNSILEGPAGLRVGQYYNELYQTVQDKVDAGFNTTITDYWGRALSYQLVN
STDGGPAYTFSSIANDTEFAAANSPMPIIIALERAPGQLQVPPNATVFEF
NPWEMGSYDPEAAAFAPLKYVGSDFNGGTVGRTAKCFAGVDNAGFVMGTS
SSLFNQAFLQIGRASGVPDVLVRALNRTLGNIGEENRDIASWPNPFYKFD
GGSNINANASLLTLVDGGEALENIPLHPLTLKARQVDVIFAVDGSADTQT
SWPNGTSLVATYERSTSGEETNNTAFPVVPDVNTFVNLGLNSRPTFFGCG
NASAGPLIVYVPNTPYTFFSNVSTFDLSYNDSDRNQIIENGYNVATAGNG
SVDGSWATCVGCAVLARSLERTGTAMPAACADCFSKHCWNGTTNSTTPST
FEPQKVIASSGGSTRGPWVPGLVVWSCGLAAALAV
>Mb|QLI71393.1
MPPQPKKAAPAKMKTTNRPDEWKIEQGLSGAVLPPLDMTKPQTKALPIQT
FGPLTKDEKAIKAVGDPNKLFSMERKGWTGFVEWEKYPEKKAAAHTILTS
QTFPPNPEFQLGPIPGTNPVLPGTHWKMWHHAVGGELTKIPDDSWATVLK
EKHPDMLHLLQFPYNGEPPKRLVTAKEITPNSLHFVRNHGGIPLIDKDHY
SFLLDGLVAKPKEFTLDDLMDENRFPRMEKAITIQCSGTRRIEQILKYPG
QGDEVPQAPWAEGAIGTARYVGISLKKVIKECGGLIDGAKHLEFYGADTY
FKDDKTMNYLVSVPWSKVKANEVMLAWEMNGEVLPAIHGYPLRIVVFGYI
GARSVKWLYRIKAIKEPSRAPVQSQEYLYFPQQVGKHNLRMTDGIQIQEM
PVSSAIMSPWTKQVCIHNGKIRCKGWAYSGGGRWPERVELSNDGGFNWYT
VPLGNLSKKRKWTWRTWEFDLPCDVEGWVEIVCRCWDNALNTQPPHVRTA
WNWGLHVTSSCHRISVYSVNKSRPLTKARLDEFEQKGIPFAPLTVPLAFP
SQTWEDYEKFWQNNDPRDAEDD
>Mb|QLI71409.1
MTLRLAWRKVAKQSFVLGDYFTAGAIACAFIRLAVIHVVLVWGTNSMSMR
YRLNHDFTATEIYQREIGSKLSIAGRFVYISYLWLQKLVLLDLYRRLILD
LAYEKIIIRGYLVVFCVSYVAVEVITFTECRPFRLYWQVVPDAGPCAKAP
IQLLALGIINIITDFMLLVLPIPVVASLRAPWRRKAQLYPLFTLGIFIIV
VTIVRLRINYANIGSQGNRTTWASAELLTATVVVNAPTLYGLWNKRRRDK
RELDRQRERQEQRRNGNGPIHPDTIGGSNESYEMQRKRTPTARGILVTKD
VMVTETREGDGRNSRESGRFPHLDAESVSRHSSQREILPQS
>Mb|QLI71433.1

MIVTRAISLTNFLVASSALGFQVFVLYPWHKELDAGFEELKKEHLKVLNA

VGSSLSEQQRRGFMDKLNDMKSQSAKRWWFF

>Mb|QLI71463.1

MKEFEYNVLPSRVIFGSGSVKKLPAEIQRLNVSRPLLLATPGKSNFSNQL

SDIIQAAAITIAGTFPHAKAHTPTSVTEEATAFLASVAADCVVSIGGGSV

VGLGKAVSIRTGVPHISIPTTYSGSEMTPILGETQNGKKTTRSDPKILPA

VVIYDADFTMTLPPAICSTSGINAIAHAVEALYATNANPITSMLALEGIK

ALAAALPQIVRSPESQAPREEALYGAWLCGAVLGSSSMGLHHKLCHVLGG

SFTLPHAETHTVVLPHALSYNAPAIPEQMAKLATVFPGSNGDALRGLELL

LQELGVPRALKDLGMEEGDIEKATEIAAGNQYPNPRPLEKQWIRELIRRA

WAGETAKADLCNE

>Mb|QLI71488.1

MDDIEALRRKLREAENRALEEQRRREEAENRALQDQHRRQVAEALTAASQ

PQNLQQYLETCHSLHLAIEVVTNRSLTTQGETTHPTGRIFPRRIIPWDDF

AMRQEEIWNDLSISELFCEPAYPSNHQMEYVRSLLKPFSSEVGLRDFERD

VVENAVQKLVDRAKTDPLLRVLSPMEHMSLDRDDTGDTAQGSAPKQAPAP

KSRRKARGMGNRADHFAPHKLSLDEIITGLEAEVQPERDVINKDSQGFVL

TARRLAAAVVTQLFSYMIGKGIQFGYICTGQALVLLHIPNDPSTVYFSVC

VPNQDVMDDDETSLRCTAVAQVFAFILQALHARPPPQMWHDAAETLGIWT

VEYDDILRDIPPSERKRKEPLAKGVEGADLVCLQHEKDIYDHLQPIQGEY

VPICLGIIVLAIPYYYDSGIWVHFMFLSWAGQPILDCVDPAMKAGIATAV

AMAYRAVHKLRVLHRDAQPRNILYNTTSRGFMVVDFERAEFHCRRPLVSI

EPNLNRKRKRRAVQKQKKNDFARELESAVANALSCVVNLPF

>Mb|QLI71524.1

MHLTSLALAGLPALAAAAATPNCFPYGNAELPGDLTAPNVKLEDWWCPQS

MAYGFQGFSYPLEDDNCNSYTNSFDRMNQDFARMKKDFGASIVRMYYPTC

TQPGVFENAIRAAAKNNMALILQIWTNFGDGDVWKQSQQAIYDTLAKREF

AAIAPYVVHSADWGSEPVGDGMDSGNFVNDLGAFRKRMNQHHVKAGISED

WDRPGSLRNGDGLTDLGRGIKSNSDYAHIHAMPFYHGNNPENQAWAYIQQ

ATQWVLDHVKLPTMITESPWAWGKTDHNPGKTDVGVAQYTRYWKTFDDNC

EWFKQKNVGWFLHAWQGEDKFDIVKPDGPGYVIPGWRPRKC

>Mb|QLI71539.1

MASLARDMNNVPMQGMGQYSSNAALQHEAMLKALPLLEKAAHQATLNKKT

PDDEPLTVIEYGSAHGNNSIRPLEQVLQSMTGSTVQLLLCDRPENDFTTL

SRTMSGWIDALDKTSAPKAIFLSMIPRSFYQDVVPAESVDLAFSLACLHH

LEHMPPDLDGAPDSERKRLLQRQSRRDLCRFLRLRAGELRSGGTLAMSFV

SQSSLGKENYAGLVDSCRRAMADMVRVGELPLGVARAFQIPTHDRTLDDV

KGVVQELADAWRLHDVFEEGVLHPAVEGLERAKQSDPHGRASEQYAKTVV

DWLMAVCSGYFLKAVSVAAPDVSAEQAAVLLSKWVHGTRESFLRDHRDEE

VFCSFIYVLLERI

>Mb|QLI71572.1

MQEDAQSRLGSNAFGDNNGNSIAPCDFEFTFESSMLMIEPDFDEMSDVAS

LSDMSLSESVQNYPELFGRTYHAFHAGSYAFPNDELEQERLAIQHLAMQR

LMGGKLFFAPVSAEAPPRYILDLATGIGDWPIEMADEFPDSQIIATDLSP

IQPVIVPPNVRFYIEDSTEPWDFPYKFDFVHTRLTGGCWADFETQVIAQA

FAALKPGGWFESQEVDCNISCDDGSLDPNGPIVTWINDLMVAAEKLNRPV

LLGPILKKAYERVGFVDVQQRVYKMPLNPWPKNRLLKQVGLLWGANILKG

LSAFSYQLLHHGFNRSATEIEVSLVDVRRDLNDTRIHSGIAASFGHRRSA

HHRFRRSILNDYFSKRSVLNLSRIISERTQKLMARFQESEMNGAVLCLEM

AFAALTSDVISSYCRCRYWKFLEDGHVRNDARRAAEDTLDFAHIHRFFPR

LTYIWHLVSLETFSKLMPGNSALFPYLESFLTYSTANTNANKLDKPGQGP

SPVPMMGTKVVAITRPSIPPEERTAKSTWE

>Mb|QLI71576.1

MKEKMTQPHSPNTHVLYSPWLLFQLIWEFTQSDVLTFVLPNSAFGILSAL

AAAGFSIDCPQPNWEQVLFRSPVVVAFNWYSVLLFTLANQRRPESIQEDA

INKPWRPVPSNRVQPELVRKAMLVLIPLSKALNYSIGLWQEGLMIQVLTW

MYNDLRAGNELIRDAVIAVAYGFFNLASLKIAQGPNCHISTPVYLWILTV

SGVILTTMQIQDLKDQEGDRTRLRKTIPLVFGETFSRVTIAAFTCVWTEL

AVKFWKLGWLDAIATRTIALTLVGRVLTKKGVGEDRNNWKLWCFWTVSLY

SMPLISHQ

>Mb|QLI71600.1

MAMKVRKARPTPKWNPRPGDIQAGDPVPIRQKRRYRPGTTALREIRQYQG

TTKLLLLKLPFMRLVQEIGMNCRPRGREFRWQSQAIQALQEAAEAFMVHL

FEDAQLCAIHAKRVTIMQKDIQLARRIRGIWAGLG

>Mb|QLI71602.1

MTLTFDVFFSRRPSATRTGPAGHDHLKWVPTSSTLIYGARDAILVDAQLT

IEAGKDLSDWVVAKGKNLTHIYVTHGHGDHFFGSAPLLKEFPNAKVVAIP

EVVARMSNEVTPERLRGGWDKLFPGQIPTEPPRVAEPLQEGLLELEGQKL

IVVRTGHTDTDDSTALWVPSLGLAVVGDAVYANTHPFLGESGSKEARLGW

IAALDKIAALNPRWVVGGHSDPDKGYDPTAIQETKNYLDNFERLSQDTST

AEELYHRMVELYPARLNPGSVWAGAVLVKDK

>Mb|QLI71625.1

MIFRMNLATFLAVTKLFLMACEATEQWERIPGGWNVASKPTPDTIATFTL

ALNMENIDFLASELLDISDTESPNYGKHWDQADVYSRFAPSNATVSTTLD

WLVGGGVQNYTVDWIFIDFTTTIATADSLLNASYHYYTNNVTTELRTASY

SVPERIQNSALLISPGTYLGVPNSVPLSLRPYGAREPLQRSVVSKDDNPC

LQAISPSCLKHMYKVANYTPHEGSGSTIGFSSFLNQSALYNDLFEFERHF

AIPGQNISVELVAGGIDNQNESTAQFAEADLDAQTIVGIAHPLPVTQFII

SGNPPFIPNIDHKTENRNFNEPYVPYYRHLLSRSKSDLPYVISNSYGEQE

DSVPIRYALLTCNLIGFLGLRGVTVVQSSGDTGVGSGCLAPDLGTAEFYP

IFPATCPWITSVGGTVGFSPESAWKGSSGGFSRYFSRPSYQDATVCRYMD

MVASETYAYYGKYTNWNGRAFPDVAAHSLSPDFQVVYRGLVAMSGGTSAS

APVWAGIVALLNDARLRAGKPVLGWLNPLLYARGFLSLNDITEGFSEGCH

GINPGTNATEPDGAGIIPGARWNATTGWDPVTGLGTPDFQKLKHLVLSL

>Mb|QLI71626.1

MATTTTVVEKPGGGGATDSSPPEDTPASSSTPSPSIPAVAPPWQLTGDVY

FFSWWSRSSQLPAHAYSPLEAGSEFAAAASGRPVGGLGMIQILRYRDSPV

GPYDEMLVVPGSFDWSRDTSDGRPAGVGRNPRISRIYVSQERSCYNGRLN

WNTPKHLARFDWHFGPGGAVTVKVYPHDTHGDASEAQPSAVPFFQATWAP

IRFVPAFPFATGWVNRLGLDTTLVMPPLPRGRGSRGELPGTDAWCSLAPN

QYSRRTRLGWFDIAQPEAAAAADDDADTAPEHHPNFWPGLRRWQLGLKME

NADLRFDLPIDVWRPQRANL

>Mb|QLI71642.1

MADVDKNPDEGLAHHDSAALPRKSAPADPELLEAIRAEHQMGFLEALKLY

PKAVGWSAYVSLGVIMLAFDPQLIGNLYAMPQFQKDFGYPSGEGYIISAA

WQTALSMGNPIGQVVGALCAAYPMDVFGRKRTFGVCVILVAGLVFIQFFA

RSLPVLLAGELLAGLVLGMFVVIAPAYASEVCPTAIRGHLTSFVNLCFVI

GQLLSNGVTAGTQRMHSHGAYSLPFALQWFWVVAILPGMLFIPESPWWLV

RKNRTEDADKSLRRLASPGVNVAATLAFIVETDRLEQEIEAGSTYVDCFT

KVNWRRTEISMGVYCTQALSGIYLVNYGTYFFQQAGLPTDQAFNMSVGFM

ALGLAGTIVSWFLMVRFGRRVLYNYGLAVLVVLQFVIGILDCVPGRPSGA

IWTESALMLVWNFFYDVSIGPICFVLLAECSATRVRSKTIATATAAQGLL

GIVMTAAIPYMINPGEANWQGKLGFFFGGLAGLCLIWAYYRVPETMGRSY

QELDLLFDKGVPARKFKGYHLEGALSAGFE

>Mb|QLI71670.1

MQETRLSAEAGSEQADCAALGIDSFWDATPVPTLVVSRCYRVKSASRSIE

TSWNRSTKDLIGRDIFDVLYGGSVLERFDRIPLASAIENAIASRALRLCP

NAYKEGETSWSARIIPLFKNEELQLLVMEFDQEETLFSKDASHDGSATDR

LNDEMFRLLVHAVKDYAIFLLDTRGYVATWNTGAELLKGYKREDIIGRHF

SSFYGDEDLRAGKPEHELMTCLRQGRVEDEGWRYRQDGSKFWANVVITAV

YRHGVHIGFGKVTRDLTERRENELRLIAAYEESSRLKNDFLANVSHEIRT

PMHGLLSACALLLDTTLSEDQRETANMIAESGQVLLGVINSILDYSKLAS

GTFSITPEVFGVGGLVASVVRTAQTTLMPGVDMKLRLAPDLPRLARGDQL

RFRQIVQNIIDNAAKFTDAGCISVSCSVREETETAYSILTEVTDTGIGVK

NSAIKDLFQPFMQSEGSINKRFQGTGLGLSIAKSLAVLMGGDLGYRPNPL

RQGSVFWFAVRLDKTTTTTGEQPDDADKTPSSHLSTGEAALGSDDAAAAL

ARWKKAGSTMRILVVEDNIINQKVLVGTLHSFGLSNIAVASDGAQAAAMV

NQAQGEFAMVLMDVSMPVMDGFEATASIRRLGSSVPIVAMTANALHGYRE

KCIRCGMDDYVPKPVGRNVLLQKLLLWLDPKKRPQPVRPAVEMDLLETPP

VTPSETSEIGSGG

>Mb|QLI71673.1

MDLSAFYNIVAGELRGSENTTTPRTAASLPACPAAIASDVEDSVAAAQKA

FPAWAGESYETRTKLLEKFADLYLSHGQEFCTLLAAETGRTAENAAVEVY

WAAQWLRYPSKYKIPEERIEDDKKVTIVTHEPLGLVAAICPWNFPLMIAI

GKLAPAVATGNCVILKPSPFAPYTCLKLVELAQQIFPPSVMQVLNGDNNL
GSLLVKHPGIVKISFTGSTNTGRQILRDAAPEMKRVTLETAGNNAAIILP
DVDIKSTCDQLAGSLWFNAGQVCIAPRRLYIHQDIFEEFVQELATVSAEA
AMEWRSKIGPLQNKPHFEKIRTCLADAEASGEKFAIGGSVACDEGAMYVH
PAIIVNPSPSSRLVQEETFGPVVTCHGYSTMEEAVKAANAVETGLSATVW
GKDLGLTARIAKQLDVGNVFINGPPQPDPCVPFGGHKKSGLGVEYGMEGL
LSFCQTKSIYMYK
>Mb|QLI71712.1
MTSSTFKLLVLPGDHVGPEVINEALRVLDVVEESRPGLKFERSFDVVGGS
SIDKHGVPITQEVLQKATQSDAVLFGSVGGPKWADASPNPESGLLQLRHK
LDAFANLRPCEIIVPSLLDASPLKPDIIRGTKFIVVRENCGGAYFGTKVE
TPDVASDLWVYRPHEVERCARVSAAVARLLGKSGDGKGGGGPAVVWSADK
ANVLASGRLWRRVTEDTFKKEFPDVELRHQLADSMAMLMVKNPRGFNGVI
HTDNTFGDILSDISGGIVGSLGTLPSASISGVPGEGKCNGIYEPVHGSAP
DIAGKGIVNPVAQILSLAMLLRYSCLLTNEAAAIESAVQLVFEPKEAGGF
GIRTKDMGGNAGTREVGDAVCQVLRELLSRG
>Mb|QLI71730.1
MKASIVGITHGKLAPDGELDFMSGLGSPRPLFGRQKVDAVRLIEIDAGID
PKKMDLTNMGRLDLITHANVILVTPIEALGVGLGGQLPLILIHGVATARI
EESAPSRGYSFLRARFPDAILHEYQFPYWSHEQDYGPSDFSQQARRILDE
VAGTRNKEDEYKRGLPLFFMGYDLGGSLVKEIVRLATGEPKYHPIMMDIR
TIMFFGTPHKALDVVPWEQHIIRLLSITDALSESAVQLLQQLPQALEDIS
TDFSTLSHYFRIVNFLQRNGIGSTRPTITAACARLDVAKENNLELDCDHI
QLWDFEDRDSTTELICAHLVDVTGIGFDPVRLQIDHFISALVSIDPNTHR
VRPIKALPNSLDWVVKHKSYIDWVEDSRASILHITGSSGSGTTVIASHIL
QMLLQKPESERAVVLSFSFNKQDIRARTLDSLLLSLCRQLLLARPSLFHH
VMWTCSFLILGGLITTETLWSVLRSLAMHFSLCMVGTPLACVIDSLHECH
VSLNSTLKQLSEFMSFTQGPVKMILTSDLSFTLKSPPKCSYRINLNNQRD
NKSAVENFVRASLSQLAGENRLWRAKNLQDQVIKKLCVRKPNCLLAVRSM
DLLRTAATQSTLSSVQAEIERVPRTLNESYNRSIATASMDDWVGCALRWI
VHAVWPLSERELAVAVALDRSKDQPFELLKSHISVSITADLFHTIGPLIR

VIDGQVLPFHRSLRDYLSEFYTPVELQDHDNRAKDPHLEILTRCIDYIER

LIPHRSIVLADNQPTLELADAKLALIDYACLYWPEHYKLVQPKSKIQATL

KVSQFLIETRHVSFWSREYQQRAGTLTAKGHTLDTRLKVVCYFGLDDLLC

DSLVHAKSTAGNSRELQDALNLAASQSRSTIVDILVREGITSIDALGFAS

ASGFDNRVNLAAEDSDGYDGLKLAAQAGYSEIVRELLDKMVDPLKPLRSG

DTAIHLAAEFGHPLTVGILLKKSPNGVYYMNNRKYSPLHLAAARGYVNIL

QQLLRVKEPHDIPEGGHATDDENHTTEGSFAITAHHNRTPLQLAAENGHL

AAVQELLEPKIDDSVRNCSIAFFLAAANGHASIVERLLKHGIRNTVVDEE

GNTPLHVAAREGHVGVILMLPDTPQFAVNPKNAKGWTPLHLAANFGNLRT

VKELLKLGAGIKLVTDNNDTPLLLAATGGHRLTARELIEKAPDAKSRNRE

GREAIDVAAKRGHVAIVQELLLAGLLLDRNQWTCMELDNRGSMYTPLHVA

VLGNSLASVKILLGASVDVNAKDSLKRTPLHLAAENGYGDIAEALILAGT

DLDAEDHEGCTALYTVCYFGKLDVVQALLKSEDAKYRADVRKRAALRGWA

PLHAAHDNADITNLLLEANAEVDCQARNDGLTALAMAVFEDYDVAKMILQ

HKANPNVADVDGETSVHCAANGYGGPEMLELLATYEADLDAQSLDKTTPL

HLAAKEQEEGVVRFLLEKGAKVDNVSDRYGTALRAAAEGGDVSIAKLILA

KCADVNATGGRFHTALQAAVSEGNSEMVDLLLKEGAAVNMRHESVGTALE

SAIKQGHVGIAFRLLDANAEVNAADKKKESPLQMAVRRSLGSLIQRLIQE

GADLDCQESEVDSPLCLAVMEGDGSKNGHSLLSYAIWYKLHSRFPVLLDA

GARDELALEDAVRASDATMVDALLGLLEGPVADVSGRESLVHLAIERAPV

DILRALDKLRADFHVNDRYGRGVLSHAIDLHRNSIVDYLIQRRDSYYFEQ

ADNHGRTPLTWAVIRHSDCLQDLISMTSTLDVTDNEGKTPLIYACMHDYG

EAVRVLLERGADPSIVDCRGRGALYWAARRARLGLFETICDSLFPLQCRS

SHFAGALSAAVASRRSSFIDRLPYKYPGIWDLPDAEGWSPRYTAKQYGTD

IAHLHTDVEFEEKGFSYLPKRPARWSDTDRSPNLSVSSDGKVITVSGQPG

GAGSAYAAIRADFPMVPIPELNNVYYFEVKIDKSQDPRSWGIGFCEEHVE

LDATVGWYNGSWGYRGRDGEAYGEGSKGSNSNYDAKYDQDAVIGCGINFD

QHVAFYTKEGVVLGKAFTDVKGKLYLAVSVDECMVGSQVSTKFWDGETTN

FMYRGSFTAPETLAWPEAALDLIADGLELGKEVTEAPDGGVVM

>Mb|QLI71743.1

MTSLNITFIGTATAVLEINGVNFITDPFFSPAGTEWDQGIIVLKIASGPA

LGLADLPVIDAVLLSHEDHPDNLDELGRRLLDGRRVFTTRDGAGKLAPRP
GVRGMSPWETTTASIGGVRFEVTATPCQHLPGGECVGFVLSGPEFGQTNG
KPNAIYFSGDTVYVEELAGIKDRFHVRAALLNLGAASVPVSDPPLQITMD
GSQAVRLIGDIDPDVVIPMHCDGWGHFAENGSQAAKVFEAANIKNKIRWL
KPGEKTRII
>Mb|QLI71749.1
MKHTDPESRDTNGDYPDGGREAWTAVLGCWCGLLAPMGWLNALAVLQARV
SQHELAGVPESTTGWIFSTYAFLMFSCGVQVGPIFDAYNVKLLIVPGSMG
MVVMAMILSICKEFYQFFLTFSVLGGLSASLLFNPCIATTGQWFGKRRAL
ATGVVCTAGGTGGIVFPLVILYAEPRVGFGWSVRIVGLICAVSALFACRL
VKKRLPHNKTAGARMDFGALLDLKYALATLAVFLAELAVLIPYTYISSYA
LHVGFEAHMALLMNSLLNVGAIPGRALPGFVADRFGAFNSFLVTAAACAA
SIFSLWYTAAGGRMAVVSFTVLFGFWSGATIALTPVCVSRVCRIEDYGKR
SGTTYLVASVGVLVGAPVAGAIIRAGDGSYCGIMVFSGAVYAASAATLYW
ARGVAAGWGPKVIF
>Mb|QLI71803.1
MSANTSTAFVEANGAQVFYRKAGPSSADAPTVLLLHGFPSSSHQFRNLMP
LLAAQGYRVLAPDLPGFGFTTVPDNYTHSFDNLGTTIDAFASALSLQKYA
IYIFDYGAPTGLRLALKNPEKVVAIISQNGNAYDEGLGAEFWAPVRKYWA
SGADEDRNALRGLLESDATKWQYTHGSPNAGKVQPEAYALDQALMDRVGN
KDVQLDLFYDYRSNVALYPKFQEYLRTSKVPVLAIWGKNDVIFVPPGAEA
FRRDVEKLEVKLLDAGHFAIETNEEEFAASIVSFFEKFQVFGSRERKV
>Mb|QLI71883.1
MSVITDPDYMPSFSEPTPSSITDEARAFVANFYRISDMQVEDEQWVEFFT
GDAQVIMGEDSGRGHCEIKELRRRMWINVLARKHQVLKVFTGAFSENSGT
EGEFEYDMMLLGRVQRTSQDSKTTIQIPWASHMVLKKGPGREASQWKLAR
YRAWL
>Mb|QLI71972.1
MPFIGIQESSTWSVALTANMNTRATGLPKTSAFYCPQPVYNPTCISDPTA
SLHLPHHNAHQNSREQHVSVWVIPEPRHVFQIQQHRYTFHVAIPIRIHES
STWQVALTANANTRNRAESYYYRLGNTSATSKRRSIRSLKKIKTSDLVFN

MYSICKAVAAAVLAFSLAATAAPAAGEEHMNHLAARNAGDFTYYYTGLGA

CGETNNDSQMVAAVGHDLFDRSRPCGRMIRAHYGGNSADVKVVDRCGGCN

DDSLDLSPAAFQQLVGSLGPGRVQGTWEFI

>Mb|QLI72083.1

MTFTVTSISTPHKTLLSLTSFETRFVGISATLDNEHSTTLALAFRDAVQL

VDFHVTYLKLTSATSITEYIIKRLESYMHFSDAKIIAAGLPSAMGDFCST

LCSQLWLQLDIIPFVITEENWTRTPWRDKNVDEQADSMARRCIMCFNPSL

TPALQIGWHSCVEVDAGGIIRLCSLQDYQTTCSRESWDVLMFYANKLRAA

GTKMAFFSATPQGGGVALMRHALVRLSRLLSVDVKWYVPKPRQAVFRITK

NIHNILQGVARQDQHVSDAEKSAIIDWITDNAKRYWLSNGGPLCRPEQGG

ADIIVIDDPQMIGLIPLIKHVSPKRPILFRSHIQIRSDLIDSNEPTQVGV

WDFVWDFVKKADVFLSHPIPAFVPRAVPRDRVAYLPATTDWLDGLNKTLN

AWDTQYYKQVFNTECHLHDMPELEWPARQYIIQISRFDPSKGILTVIDSY

AEFRRRLDQTGFGSPPQLVICGNPSIDDPDGVAAFDETTAHIHTKCRHLK

RDIIVVRLEANDQLLNMLLRNAHVVLQLSTAEGFEIKVSEALHAGRPIIA

TAVGGIPLQINHGLNGYLVDAGDWQAVATHLMELFTDVGLYNALSVAAAA

KTGLGDQVSTVGNAIAWYFLAWKFVVGGGMEGGGRWVSDMAREEVEKQL

>Mb|QLI72129.1

MAPTCPRRLPIDAYCKYLSIFRDRLYPVWPVVDVDDLIAKLILDVNDLES

YALAASVCAASIAQLRLPEHTELCEATISSYQFARDAQTIREQYDYRECQ

KLSSILTPFFLHIYFANASKIRTAAVYLRESISSVHWFGLDRQETYESLE

QKERSLKLRIFWILFISERTFCAQNCFPALLVPIDERPPCEETDSEFGFI

TQAFSSLTHLFSHLKSNIMETSSSHRPVLDPDKVAIAQSDLCALVLNSSF

TEVQQVDLFVTRQWIRLLIWEYTMRHYKMSRTSSNQAFSLLLPVMIAREL

LSLFLSVSADSIFAHGYGMELKVFRMADALLDILACSPGSTKEGGICAGM

GDILHGLKKVLLDIGGLKSRFLDNLQIRMSNSELAKRPWPYLSMRFPGNE

AAGQGGNAVREADSHLETESDTLKVVPIQ

>Mb|QLI72131.1

MSPALVHTRQPLRLDTSRSTLAVVQTKGIRDSLRRLSPSETFEIETLHIL

DNKDKSTALYDFGQKSLEQPIRFVHFPKNNFPISQTYLPMDKATLEPAME

TGPEKAKTSWQEYAYIKELPDDIAPFKELLQQYSKVPPDKVDELLLRTRD

RLWDVVNYPCIGLWAFTKLHSTTDSRFEAATQRLLAEDPVESGGTRPAIL

DIGCCIGQTLRHLAHKGVHPSRLYGTDLRPEFIRIGNELFGDEQRGLTFV

AGDVLNADDSSMKELDGKVTLIHAAYFFHLFTWDDQVRIGERMVRFLQPG

TSDAVVFGRHIGTLRPRELEVPTTRASRCYLHDGESFQRLWDEVGSRTGT

RWRVEAEMADRLHVRFPFFGDDEKYMSFGVYQI

>Mb|QLI72192.1

MTPNTSQPILSAPTHLLASPWLANDLSVKSLVLVTLAVLIFAPKLAKLIQ

NILTPVFSIPGPLINKISSWPLAIATINGSSHDFAQRLHEKHGPIVVLAP

GMVSVSDTTEIKRIIHTEDWMKSRAIYGNFRQDPERPTLLAFTDKKPYSK

RKRMVSSMFGIKYIRSMEPLMLGCVEVAVDVMGKTCDGATGDFAVVDVQQ

FIHALAIDIIGVTTFGQSFHVVENGSHPLPSSLKKGLKIAGLMQLIPWIR

KIPLLPTRDPYVSSFTYDIVDNRRRTFRETTTQDLLQKLVEVSDDSPGSD

FNTTDVQDESVVMLTAGSETTANAELFTLIMLLKHPEAMHRVLEEVDEWY

SPGDRSRPTDCGYSQAGMTYLQACIDEAMRLVPGQPNGSPREAPKDEQVL

GYRIPRGTTVFPNTQAAHLQDVFADRPDEYVPERWLDIYSQGNDGSIPYW

PFSAGSRVCIGKHFAMQEMHLTLVTLLRHFTFQYVRGQDESTMFRIAQQL

RSNSYSVEVQRRAVS

>Mb|QLI72231.1

MSTESPWSIAKTIQQSPPVDLTKRYDPSTLRNKTIVITGGANGLGSHMVR

RWASHGAHIIIGDVDSPSGESLVAQLRTLHPSSTFAYVSCNVASWDDQTS

LFEAAIRLSPYRRIDIVVPNAGIIQSSEGYNFENPSLVNGKLPKPSTATI

DVNITGVIYTTHLALHHFSQDTSTTKCLLLIGSLASIAPLPGQTHYTMSK

HAVCGLFRSLRTTSFMQSGKLRVNMLAPYFVQQSRMLPLLADVVFLAGTA

GGATIPDVVEAATRLVADESISGRSLAVGPPLKDAPEGEIPVAEHEGDGR

GRAAWEIYAHDYDEVDAFTFRYIHMMNRVTQLRGAFAFFLDILLKIFGR

>Mb|QLI72429.1

MAQQAMGCEKPDPPPVGIKTDPEQQPVADRHLDNSTVKNITWSGVTVTVK

DRETKKPKKIVDDAAGAVQAGEICALMGPSGCGKTTLLNVLARRPTNASD

VQAKVLINGNQVSQSAFRQLTCFVEQEDALIGSLTVRETLEFSSRLASTS

SLPRKERILRIDSLLASFGLTAQASTLIGTPIRKGISGGQKRRIGVASQL

ITSPRILFLDEPTSGLDSAASREVVSYLRAVARRHALIVVCSIHQPSTAT

FNLFDRLVLLSAGKTHFCGPVAAVVPHFRALGAAVPNYTNPAEFLLELVN
LDFSHDTARASARLDALHTQWLESPQAREAAALVADLSATAHPLDLSEMR
RPSPLRLTLTLLHRSFIKSYRDVVAYGIRIAMYTGLAVMMGTVWVRLSPD
QESIQPFINAIFFGSAFMSFMAVAYVPAFLEDRLQYVKDLRNGLYGAGEF
VVANFFIGVPYLFFISVLFSVISYWLSNFQPTAKAFFTWIMWLFLDLLAA
ESLVVFMSSLVPSFVISLALVAFANGLWMSVGGFMVPPTILNAFYKYVFH
YWDYQKYVFEGMMVNEFAERTYACGEGCRCMYESPLADRCEIAGQAVLDQ
YGYSTGNMGQNVGIMISIIAGYRIASWIVLKLRK
>Mb|QLI72443.1
MTTNIARYVCANVGCGEDGKLRCKKCQLVCYCGVSCQREHWTSHKLDCNS
PLLKSTWRPQWEAEGRKPAFMISDAPARSFNHHKKYLWGNMPAFDVLKLE
ANEGKGYKGDLDLLFAASGDLRNVLRTLADLPNDFCTRVSVVVNDRDFNI
VARNIIMLLLLLMDDDPQRAADHIIHIWYSAFITDELSRTLQGQILKLVE
GVCHETTQKQPDDLFPKAFSFTRGSVRLVLTKRSWDALQSILKPSNLTLQ
KAQDVRRAVTLAPSRADYRERAFFRQDPSSRFGSFRFRSDGILLPFGLPR
NAFNTPNPTLFTDLGAWPMMDSAEPLHGWSLQEVSRASGAAANDVLGKLY
YSLLDLIRSVHGRLGHVNISFHLCNLDAAALPQHLGAMRFDRIETSNIAD
TPYLGPERCVSLLGTLLKEPQTNPHATLITLFMNAVPETVTKRDEYASLR
EELSHVTKYLTPSFSIAPYDADRLRIDSCRDMVRNNDKFFDRYMNACRFN
EFSSRLKLEMKSHHTIIDKWPLRIRKRPNQAGAQEEFLRIMGSGHEGCER
YVEWRVGG
>Mb|QLI72458.1
MVTTNAILAFAAALVGTSSALGINCRGSGLCTSNKGILGEALGQLRGMDQ
SQQFSDGQHITCVKSSVTIGNPSLCIFYQNTNGRRWTVAQTASFVQQLID
HGCAACGSVPTDPGNNVKNGQLTANMVTNAARRGLDMANKIAKREEAREE
TREPEPVTKADDSTAILARALGINCRGSSTCGVGGIGHSPAGTLEQVRDA
VAAGPDGSWTNGQHIACVAHVTGRLCAFYQNIGGRSFNKQQSVTFLDQLR
DHGCKNCGSIPTDPGNNVGNGQLTVNFVA
>Mb|QLI72493.1
MKIIIIGAGIAGCTIYLELRKHLPKPPSGEAHKITIYEAYDTNIDTTADD
RGNGPTYSSTLIVGGGLGVAANGLNVIKRLDENMLREIVRGGYSCATMNM

KSKNGRVLARMQPAGKPALDGKGQGTMNTVACSRHGLWRELRKRVPDDDI

VTRRVSEVVARSDGLNVVSFVDGSPSAEADLVIGADGLKGLAKKALFPGE

KTDPFPPHYEGLCGVGGFAPYSLVKGHVEIGSMNFVMGGNGFFGYFIAES

ALDAPHRDSPYHVSPPGESVGWWSTYSVDECPDPKSVDKEDILKQLRERH

KNWKDPVICKVLETSKVTSMYPTWIVPPLPTWERDGVVLVGDAAHALPPT

SGQGSSQALEDVECFSLFLGHHLGKAYESGGHDPTDYKEVIRAAAKQYMD

LRQPHVKRILDHAQQSQNRKRDKTVIEEYIMYAFMWMIGWFPNVMTKPLK

QLVEYDIASEVYKVLHVKS

>Mb|QLI72576.1

MRQVFPSTFFNFEFLRLLGTVPYYGAEVGECLETASRIKDGDPETWYQAW

WDIGQEVLSQAEDAAKTGDKTAASWAYIRSANYFRASEFLLHCTPNDPRI

LVASRASVEAFNKGWVLLDAEVREVEIPYDNDKTLPGRLYLPSTKHRVTQ

KLPLVVQTGGFDSTQEELYFYGAAGALPRGYAVLTFDGPGQGRALREKKL

YLRPDWEHVTGQVLDFVVDKLSSEYDLDLDRLSVFGASLGGYLSLRAATD

ARIKACITVDGPFDLFDITKSRMPAWFINGWLSGWLSDKFVNWVIGCLER

VNFQLAWEFGHSKWVYGVDTPADVLRFMQKFTLNSTKNAVLSNIKCPTLV

TGAADSFYFTPDQNAHKIMEGLTCLSDTQKRLWIGNGVKGGGLQAKIGAL

ALVHYEMFAWLDKTFGIERENLINKV

>Mb|QLI72625.1

MAPEFHAVTPILRIFDVPKADEFYLEYLGFTTDWEHRHDANAPLYRQISH

GALTLHLSEHHGDGSPGVRLRISTSNLAEYHAALTAKRYRYMRPGLEDGP

AEGSQEMTVLDPFGNTLIFCQEAK

>Mb|QLI72627.1

MLCSLPTILLLATGASAHTAAFVKGMYCEGGPDPNNYNPNANDPVNPLWM

LSKNDWWMQRKSGCLNNPPKNGASVALPAGGQFTVELAHNQAQTSLSFDG

KFATAWPDGKEHPEDWRGPGSPPDCIQDDGALHTNNQTMAAGTAWAISYE

SDLSKVTMENLVVFSVLEHTPWKRIATYKVPKDLPACPAGGCYCAWLWVP

TGCGQPNMYMANYRCHVTGSNSNRKLAPAKAPVYCQHDRSKCVKGAKQMV

AWNQAEGNNVKVPNGASPGYNQGMGWAPGAQNDIFQ

>Mb|QLI72639.1

MADSEPHKPSSSNPPEGLMLRHVLPKLDKWWFQHGHLRSLNLLLLAGLLT

QATTGYDGSMLNGLQALPSWADYFDKPTGIRLGAMVNGTVFGTLVAMFVS

AWICDKLGRRWPIFGGSCTILVGTILQASSQNFVSFIASRWIIGLGLGVL

QVCSTLFLSEVSHPAHRGKITAIYEPAWPLGALIAAWITFGSSKMHGNWA

WRLPSLLQGVTSLIQACLVFFAPESPRWLISKGRHVEAFEMLTKYHGGGD

RSSALVMLEIVEINVSLEADKANKAVSWLAFFSTSGNRRRFYIVATAGFC

LQWAGNSIVSYFLPLVLTNSGVTDSYTQLVINACTQVWSFLWAVFFGLTI

DRVGRRKLLLSSYIAMFLTYLVWTILAAIITRPYFENKYMGHGIIVLIFA

FFCFYHMAAPVIPAYCVEILTFELRAKGMTIFQLGSTLASIFNGFTNPIA

LENLGWKYYVVFLCLLAFWTLVIWVGYPETKGLSLEEMTTIFDGEHSTLK

NGVSASLESNGKSGVLFQMEHAEV

>Mb|QLI72691.1

MAIGDIETALTELNGALRTALKTLGSDCRQELTQSLHSPDELPEKHLYDL

STEAVDLLQETKLLLEPRTVILADHFLGSANCKCLNAAVELDVPDILQQQ

GALTVAQLAQACGAREDRLKQVMRVLQSAGIFSYEQDTAVYSNNECSTLL

LKDHWTGWRNWVDLYGNEFYDMARGIPASCRQGEKRMPSQINFDTDLDMF

SWFAAQGSLDRVHRTLGGGAIAQAPGILADYPWEQVAGGLIVDIGGGGGG

LIAMLLRKFPNLRGGIVDRPEVINQAVANFCDPDGLYRDVGDRVSTENLH

AGDFFKEIPSYEVYCMKWCLHDWKDNDVVTILKNIRKAIIPGPKSRLVLL

EILLKKGRSGHLSRMADLSVMMAANGLEREEWEWDSLAAQSGWKIAHKYD

LRNAWPCAMELVPV

>Mb|QLI72864.1

MVKAALSVLLPLLLAAGAAAFGCNSISYTTCQDRIVHWYDPDDGQICDPH

DCGGGRAPPRKDVPGCAFYTGTETLKTEPSYLPCWKPSTAMAAPTGSSVK

SSVESTPTNTGVVASSKETTTGSSVPASTTASVAPSTTASSATGSVPGAA

ASPSTTLPATISTTTQSETATFGNNTGNTSTISTTSAHTGDAGRLVGASK

MALVAGIVGGFALL

>Mb|QLI72865.1

MALLRHSLNAPETAVKIAILLFYKRIFSTAMFKVCVWFGIATISVWGTVF

FCFTLTDARPVSEPWSGHVSLPYDATALGLAQVGTNIALDVIVLCFPLPV

IARLHMPTNRKMAVALIFWLGVFCCVASVARLVLLKKSLAVIIEDPAQHI

CINTDLQVDLHRQLTLQEGKQGRGRLG

>Mb|QLI72890.1

MSWIAVPSRLYYKLRRNTHAAPKPATRPSPSPAAVQPRRPPNTRLCHPPA

MNLALLPYTVFFEIVSHLSAPEALASRRISRDVRSALVRPDLSISLILLH

FPRSVEGRALRGHLRAGRHGPLEEEDWAAVFATLARRYHHLGSATPWRVD

KVRVAGGPGLRGVTPWNRFLRLDDKTAAFDYWDPVWTFAPRQGVLVYPAA

AAAGAATVVYRARDLASGDEAEVPFDAGGKVVRRVRLSHGILVVEWCEEE

QGRDEADGVGAAAHRHFATAFDVVPGSCGRGRRRRVQVSFRSEWKTHCLG

LPLSHQDRFFSTHNATHYVVYIWQPTRSPWGEHAPLERLVVWDISRPSPY

RPSLDPAGGLAPPSSSSPGPRIIRRIANDDLSGWGVRQADTPRLRGLALD

RNTWDADARSATGHVFLVEEEHRWCAGPHSSATPPRQHHLRTTGIPLVGD

GPRWVDDCGGRGTFADHGMEFCPRSCRASCADDGVDGVDAAEDSSAWPGR

TPCWRHDDFPYLTVSEAHDALAGVRITARHCFMMETLSVHVRPRLCVEGV

ARYRSGDRAHAAPDDDEVRFEDGLWSQLMGKGCIAGDERWVVGEDEAGDV

TILMF

>Mb|QLI72895.1

MTLDDEDLMLDNEDEFHLLSEGATDEDQVRLQFENDVEDARRLSQTKFQD

GDEEREQRTRFVLDRTPQWSRTTHDGRNFLHHLASYDYNRKPFVSLQWLM

SRAMNKLPHLMGAMDRSRRTPLTTALAAGNVWFSYAACKNQKDETRQQFG

AALASECEDLDNDRGSTCLHTALVCPMSGEALRGEIVKIMCGFVPRSMFS

AVDVKGRTPLHVAVEYERCCRVQVGIVDELLRRGPGALDVEVAAYSGRAM

SVYQYHEYTRRRAETRKNPARGRKEVRDGGRSAAANPRPDLRAAAAPERP

DKTVMGPPLPRDRAEPLPGLRRRVSMPVAAAPPDGKPPSLSLNTQHPCPP

AAGVDESPLDAPLNGIDAALQRDEERDQAAGQIAQLLKLFYLRTQMPERA

SRCLHVQDEQDKELWFDFGPPKKLTKTDFRKHFGHLQFDSALQYVAFPQS

TLDKGEDSRHVLRQGRTDMVFFFEWLAQKGVERIIKVLSAQPRYNMCSIL

SRRQVIVEDLKTPSHSDQAIEKSLKPFNVEMLDWRRADLDPVSLARIGQC

LREVHLYWSGRNTVLRAWSEKEGLALIPTLETIYIVQVEGLEPETRVREN

LDAFERRLGESWPTEAKPKVYIQLLGAGGPLPSLTQPSDLQPRRQRPVDP

HKWMQCMEEFASHFRQIRALNDKSADPALAPVKVALIDDGADITHPDLKG

MKFPGKSFHHYREGSSWRVSPFWDSSSGHGTLMARLIHRICPSAVIHVIK

LSTFAGEASAKLQINTDSAVQAIEYAVEQGAQIISASWTVKPPTEAGRKK

AFDDAVHNALNTKGALMFCAASDQGKSADLTYPHGSNPNSFRIGAARATG

SALDNVGDGHELSFLFPGHEVVVDSAYEDVPDKQFGRFAPHSGSSVATAL

AAGLAALVVECVRLGVLYTGETGPLDETVTIGRDDLVRICERRQMEYALA

SIGTSRNTDNKYIEVWNTFGAAAEKLKHSEGDRMSQLEIIAGLARLFLRK

GA

>Mb|QLI72896.1

METDSFKRQYADILRRYLCGISYQKLSCEYDPSIEETVVQHFRTLNFPND

FLKRMMPIIHASAWIATSTYPFTPRHVQEAIAVYTSLAIAIEDTSKESTH

DLKRFQQRLFNRQPQPNLLLQAMVDCLVSLRGIYGPFICDMVAKSTAEYI

SVCAFEAKYDGTLRPTPSSPDFPYYLRLKTGVAEVYAFFAFPEVLYPEEA

FLHEYILAVPDISRYFNLGNDLLSFYKESIVADERLNYIYNCSRVSNSTP

LESIWSTHLALITCVENIRKTLSASPQMRRNIDQLINGYVMYHFGASRYK

LSDLGIQEVDELRAKICCSTTVDNGVGAYKH

>Mb|QLI73033.1

MVAQKPSSRIRDWGYSPGRFPTGPRNSVLDVPGVRVGQVDINKGEDVHTG

VTVIFPRGIDKTTYIPCYGAVHDMNGIGEWTGVHQLREWGFSRAVRTIFA

FPLVVIPSEDLTNHTADCIHEYRQRGQSLRHPWRWTAQRLRQDGIADLLD

QVQYYGFPTVGETFDGLLNNIFKSAVEEEHVLAAIKSAETQGAAGTGTSS

RVLKGDGDIEYTVGVIVQNNYGSMDDLQIDGVPVGRMLKARKMAMAAPTA

PESGKAAEGSCLVVIITNVPLLPHQLRRIAQRAGMGLSQVSGHGVGRNFS

GEFFMALSTANTPESPSSWDGVSSSLPPLLETDTVETMKSMLIDSVFVAA

AEATEEALLNSMTEAETMKGFNGFEAKALPRKEVEELLRKHGRGYVRDLV

PESYTSQIGL

>Mb|QLI73120.1

MPNDFSVIIVGGSVAGLALAHCLQRLGVSFTILEQGEIAPQLGASIGILP

NGGRILDQLGIFDNIEKEIEPLELARIRYPDDFWFESRYPKALHSNYHYP

VSFLERQRFLQILYDTLEAKNHIYTGKKVLAVESGIDCAVVKTSDGSEYR

ADLVVGADGVHSVVRSEIWRHLKQTCQIRATEKENSGIKYEYSCIYGLSR

DVPHMKLGEQLSRLDDGVSIHVFTGKQSKFFWFAMVKTPQAGYAGKGACS

DQAARQICDGMRSKKLSDVLTFGDVWSRCTIYKMTPLEEGVFKQWNHGRM

LCIGDAVRKMCPNIGQGANMAIEDAARLANLIHKRLPLGKLSASDVDSML

REFTAAQKPRTNSICAQSEFLVRMHANQGMGRRLLGRYVIPFLNDAPAGL

SGLSINNAVKLEFIDAPTRSLGGAWEASWESVLRILTGLRPKMGIHYSVY

VLALSLATYLLL

>Mb|QLI73146.1

MGHKNNAEEFAKYRPIYRPGRAALTPPDIIYSGRDEHAALRRQLSHGFSD

KSMRGQESRIVHFVDLLIKRLHQWNNNGKTPLRMDSWYNFTTFDIIGDLA

FGDSFGCLENSDYHEWVKCIFELTRLGTYFQVEDYYPLLKRIVTRILPNR

ALEKQRFHDKINKAKLQKRIEAGSRDDLIDGVLKKKDEWNMSIEQLESNS

TLLVIAGSETTATVLSGVTYLLTSNPHTLKRLTEEVRSTFADEDEITIES

ASRLRYMLACLDEALRMYPPVAIALPRTTPKEGGMVAGTFLPKGTVVGVH

HWAVYHMEEHFQDPFNFHPERFLGDARFANDNRNIFQPFHVGPRNCIGRN

LAYAEMRLILARVIYNFDMKLASESSGWLSQRSYTLWEKNPLMVYLTPRA

DKEKPALWREAAASTGA

>Mb|QLI73165.1

MVAIQGRRYIIPGLILAALAVVLLSTDRSHVVIREQVNKFKQYQQGKKDP

AKEAKPPGPKYLPAPEYVPPPVVDPFVSLATSPPPSIPRWNVPEKNLHKK

YQLPYAPPLFIGFTRGWPLLVQAVVSYITAGWPASQIYVIENTGTQWANP

RGRLSLQNPFYLDYNGLKNLGVNIIQTPVLLNFAQLQNYYMHLSQEREWP

YYFWSHMDVIVSSHEGGHNGLPKAGEHGYRSLYESCLAELNTTLHSGERW

GTRFFAYDHLTLVNRAAYEEVGGWDTYIPYYITDCDMHQRLLMHNWTLDA

RDVGVISDVNTVLKDLRALYREPDAELDYRDPNPPEKTAPAAEQDGKGSG

GGEPVGEGGDPEHVEYWRRLQGVVDRMVEYKKGDRGRNTWQSSQKGGEGE

PFYYSSRGVQRAFDMLTAVGGQIYEEKWGHKDCALIEGGRLKLEDQWRVA

HDWD

>Mb|QLI73205.1

MPAQNSPRLLDRGSLEEAMLNADPEAANDGPLPTHWTSKPSGDTSTSLSR

IKSRLWPQPAHRPRALHKTAYLDGLRGFAALLVYWHHHVLWVHNADRLAQ

NPIFENSFGYASKYHLAALPGVRLLFSGGHFAVSTFFVLSGYVLSIKPLR

LIESDDLAGLVDHLASAIFRRWLRLFLPVAATMAVYATSWHLLGLWVDGA

SPQGSWLGEMWFLYCEFKNFSFVFKDGGVPWLSYSMHVWSIPVEFKGSVV

VFASLLAFSRCTLAARLWCQVGLVVYFMYVADGWYCAMFVAGMLLSHLDL

LAALGRLPRFLARLAPYKTVVCYHMLVLGIYLGGVPCENRELDQLARNRG
WYFLSFFKPQAVFDYKWFYLFWAATLLVSSVSHIGWLKRFFETRVCQYLG
RISFALYLVHGPLLWTLGDRLYAAVGFKGPGQLEHMPQWVDRFVLPQNGP
VGLEFAFVLPHLILLPVTLCAADFVTRAVDRPSVEFAAWVYRKTLPSVPR
KHAKA
>Mb|QLI73304.1
MAPKLSIHPPLLNTACPWATTPAHLEALLRCPSTGAITTRTSLIDGFPHD
DAVHRYAFLDPASTSGRAPEAGPSASPVGSINSLGYSPLPLDAYLAALSG
LSASLPPDVTHKTAIVSVAGPPSAVAECYARILAASSGVSFPLAVEVNLS
CPNIPGSTPPGYDPVCLGAYLAALPGDPLVPVGIKTPPYTYAAQFTALVD
ILALYAGRVSFVTATNTLGCSVFNEGEGEGEGEGASWMEGGLAGAGIHPL
SLGNVRMLRRALDGRGLAGVQVIGVGGVHDASGYRRMRGAGAEAVGLATA
LGARGVGVFESIERDVGSEW
>Mb|QLI73352.1
MAILKMLGSELFTGLDARLAARENEHTGPTAGISRGYIQSNMIVLPSRYA
NDFRALCGRNPVPCPLIAESTTTGSFSSIKSCIRGLEDKDMVSTGCDLRT
DIPRYMVYKDSELVASHVTDVKDYWTDDHVAFLIGCSYSFESELIRAGLP
PQHVLLGRNVAMYRTTVPLCPAGVFVGGTYVVSMRMYKVSDVGRVRELTG
RFGITHGEPLDWGWDAVARLGIGDIGEPEWGDAPVLRGGGDGEGEVPVFW
GCGVSPLEAVVTAGLEGVVLGHAPGHMLVLDLRDEDIVGVL
>Mb|QLI73354.1
MSLRAAVYIAPPVPFKNPGKGSGGGLWSPISCTLIYSATHAVLVDTPITI
AQTKELIAWIRRIAPNRALSYIYITHGHGDHFFGIPLLVQEFPEAKPVAT
AGTVQHMKQQVEENVFRFQWDSRFPGEIQQPFVLAEPLPRDNKFTLNQWE
LQAVEVGHSDTYDSTVLWVPALRLAVCGDVVYGQVHQMLMETNTPAKRAE
WIRAVEKVEALDPLHVVPGHCQEGEILGRWHLDKTKEYIRAFEDVLKGNP
KSARDVVGAMTKRYPDRFNVGAIILGAQAAVQAAQSRI
>Mb|QLI73369.1
MEKPPLMPSLRARLSPKLRSITLPKKMQKCLAMYLGCVFLLVLLLNLDVL
FLLPSPSSIMASPAPPPAKLPGSTFPQKIWQTWKIDPLLFGITETARAMT
WIKQNPHMRYEVITDASDLTYVEQHFGPDGFNRPDIVDFYRQLNLRIIKA

DLLRYMILYAEGGIYADIDVEALRPFHRFIPDRHDERDYDLIVGVEVDMP

QFRHHRILGQKSQSFCQWTIAARPRHPVMLHLIDGIMKWLKGVARRQRVA

LGKVELDFDQVITGTGPSAFTAAVLEEMNRAARAKGEPAVTWDDFHGMGE

SKVVGRVLVLTVEAFCAGQGHSDSGNHGSRGALVKHHYHASNWPSRHRRY

RHPAYGQVEECNWDAECVDQWDRDVAAFDKLSEAERKKIIVERLKLLQEQ

QQEQQQPQQQQQQQVKSP

>Mb|QLI73436.1

MTPKPVAIPPPNGNRRLEALPILLWSLLIVPSLLGVSTIAPVIRHMREAL

RYESNGATVPLLDRFTGIDWLDQIVGDLVTSFGLLQFRPDSPYYWHALDF

LAQFGSIYAALLLESCRRAHGSQLLLWTVLGSLLAQLTTAGLLLPVYFYF

LHGSTTLSKLVSSNGPEELRDSSALSVLPAVALSFYVPHFESFLGPDFSA

RHWWNWAWQLFGLWGAILFLGFCGMLWTLRQLLPMMPTSLSKRVKLHNGL

KATRLTAVCLGMVNVGTYWYVLMSSHYSWSEVYVPKYFWQSAADPGAALG

TLLQFDYICVFGSALTWLGYQFRDLKTAGLIETSWAKITSLGLIVGVLFG

PGSLWWVAWLMREEILTSLRPRREPRKND

>Mb|QLI73585.1

MILLSLLVLLGVVVFIVRLNKIMTRTPELSSGISIYKAEALTREYVRRVD

AKIKAEGIDFRKNHPPRLNRRYIVVGGSGWPPRLWDLWDLLDRLVGAQII

LDLLDGGTPPSAIRLVDIRPPSRDEFSISGRASRVLFAQADITSDASTAA

AFETPWPSSVARLPLTVFHTAAVIRPYERHPLFYTRCSRVNVVGTANSIS

AAQKAGADIFIFTSSSHVAARAVHWFSLPWRRHPRNLIQYLSDRDFFKPS

KSPAEFPSNYARSKAEAERVVCAANTPGFRTGCIRPGNGVYGHRDDQIIG

RMLRRGRVPTFSAPWVQSWVGARNVSLAHLLLQEALVGDAGAAVSGRPFT

VTDEGPPMRFDDLYLLLDELSVTGLQVDYPPPAVLLLVAFAIEAYCVLLK

KMPALGRLGLREPAEPMCLFQPGIVDSAVTQIADDGDARRPVGEGGLGYE

GVCSTAEGLCVLVGEWNTWVEEQKKRD

>Mb|QLI73823.1

MDPNNKPAPYQVPELLFHTILTVIDYSHDASGASRTTFVLGTHGTLEAAK

AFAAQSLETLNFKPDDFQKYNVRSSGEEAPGKTWIHGDGVLAFARSFDGQ

ELRVSIDTTPNNESLYASTEDGKMRLPEGAQFLHYMVQTMVDYNVDRSGS

LQRTEIQGVYVHRADAWTAAHKCLDRSGYAEYDCRGDAEFVEQWPFGENV

AVHAVSETGQNYLVAVNTPPQHKHDIKRHGRKKSAS

>Mb|QLI73848.1

MPPQKVAHAAVTGFDNASAYDVHRPSYPADAVEALLRNLDVANRPRAKIV

DLAAGTGKFTELLAAREEQYDIVAVEPVAKMRQSLVGKHLEHVEVIDGLA

TSINLGDGWADAVIAAQSFHWFANEEALKEIRRVLKPGGRLGIIWNIEDY

NQPADWTASTAWEKELKQLILALPPDGTNRFRDDKWRDVFERQARDPAPL

FAVPIGQEKIPFTVWLAKELLWDRIQTLSQVAVLRDADKDAFRAKFDGIM

SEGDQVWNEKEEVEFHGFTVYAWTRRI

>Mb|QLI73968.1

MEATPIGESIKTSDVAVARLAERHPEYDPKADESVQIKCDRKRPCSRCIK

AGTECVLQGTGEKQRPVSKSYVQALEGQISALEQVIRKLAIADSTERDEI

LLELSLSSAPEDGAPLDKKPQPNPTGDHGVAVARLRAGQLRRPRDGNATQ

FFGGTSSFQIHFSRDETQTTPRTSANSDGTMVQADCQVNSMLEMTDLALE

ESSSSGSGCFQYAPHDQTSQTLMAAFFTEQYQYNMVVYREYFLRDYDVGS

GRYYSDLLLYSMCSLSALLYDDFFKLSDVFSGQAQALLFATLDKPDLTTL

QALILLGHREIAVGQTSKGWLFCGMAFRLAHEMGLHLDPSNWDASTSSFR

DREILRRVYWGIFIADKQLSLYFGRPPALYPSESDVRNTIRLQYPHDWQN

LLETYICRGATVPEYENGVALVGSFIYQAELCKIIHVMIADLFENRRGDV

DPAIAAAKSRKIHVSLTKWLASLPGTLHWNQWTVGQVPSPVLHLHMLFHT

VMIILHRPPSNMFEKPGISESEDVEICYESLQAILRLMRSYSRYYRYRSL

PLDFAQTLSTATGAVMMKRFFQKSSWEDPEIERSLSLLTEAMDEIQNSLP

CVKEIRDCVSVARQCRETTLPPDVPLNAPDLMNGLELGGADATTGPLTSF

DDELGTLITDEFLSAQLQVQDGSGFDFEPFDFNNPPEGTIDSRQ

>Mb|QLI74035.1

MIYTTALYNRVFSTSARVHPSILPHLRQSRRPPFRWLKAALVLCGAGYIA

NVYIGRVRERRIADIVAAETKAAELERRNNALLDVYGDRSSLEELEKAVQ

FYEKK

>Mb|QLI74041.1

MLLYLCFFSLSCLTSATAAFDQKVLGQTYPFPENLGSAHAVRTASSYISH

ELQNALDTGLSKFGNITAKTNSLSATMISAQDSQPFLDFHYSAANLNVSG

GSTNRVTGNSVYRIGSISKLFTVYSLLLHGGEKIWDMPVTNYLPELRKAV

SQTGANSAIHHVQWDKVTIGALASQLAGIGRDVNNADMASQPFPHQAAGL

PELSLDEIPTCAGNNTQPPCTRREFFDVLLKRRPVSLPFNTPTYSNAAYR

LLGYVVEAVTGTSYTEAVAKSVFQPLGLQNTSTSSPRGTGVGVIPPGNSG

WGRPLGDEVSTGGLYSTSRDLAQFGRALLNHRQLSPLQTRRWMKPHAHTA

SPFFSVGAPWEIWRTRSQISSGYTIDLYTKSGSDDQYQALLILVPDYNIV

ASFLCAGPNAGPAINMAAEVALQSILPVLDRVSQSQAVHRFGGRYVSSDA

KNSSLLLTTDHQGPGLLVKEWLSNGVNVQEAAQAYSDGTGGGTIKSIRLY

PFITNTEDASSTGSSGTQVSFRAILETEPVNYDPGVLRILNADAGQWGRI

DQLMYGEIAVDDFIFQLDGHGIATAVQPRVMRDTLKRV

>Mb|QLI74042.1

MDVATEPVQVDSRQPSSITKTSSWTSLPAEIRQKILSQVSLSASGRGHKG

LGSPKVARFTAVCREWQVFFETCTFRRLVLNPDSLGEFDAIVRRHDARLG

YIRKLWLRVQLPRYECPDCDETEDEATQRCNNKIFTTCIRSLLGTLKLWD

PVRHGGEGLALMLSASSPSDSEHRFKRCEIRDDYPFHYTEDLYSAPGMAF

FHRVNIAGLFSRYFHRDRLPPWGDQHLKRVQGTPLRLERQRGERGRFISP

CKSFPAVPIVRSLVMRRQFRRDIHVRALSWLLARSFVALEWLHLERTVSL

EPQEEISFHQGFQLHLLPSLPKTLRRLSFTRWKIPKTERYYDVEQVGSRI

PPHDQDFLPREMARLSQRLQQLCPPWQMDTAAFLRSLVQLRESPGMLESS

LKHVILRCLLPSSQESRENFESLALLAANAALSLPQLQVMELWGTCLDGA

ESRAYIFQYARQDRRASIVWRSCQEAMVARARILRRWTDVAQKHSYSTLA

YKFVPFAETNAEIFWSDGACVYQHLLLKDLLFDPITRVIVENEPYQWRGL

SRQGDDALENLGTPNLLGGHL

>Mb|QLI74044.1

MRLESSELYTIGWITALHIERAAATAFLDDFHESPERFEQNASDQNSYTW

GRMGKHNIVIVSLPEGTDGTAAAATTTSKLLSSLPQIRIGLLVGIGGGVA

QPHKGQDIRLGDIVVGKPEGKYGGVVQYDHGKALRGPAWQRKGMLNKPPA

VLLYALSHLKAQHEMHGSDIPRLLDELWKSTPRMKNGTNDSPGYVHQGFE

HDRLFQSTYNHVPGSTCKDCDQSQEIKRVEREDADPRIHYGLIASGNTVV

KDAVFRDEISEVIGQGCMCFEMEAAGLMDSFPCLVIRGICDYADSHKNDR

WQRYAAATAAAFAKELLGYVPTAQLNATQRAIELLSSIDGSVKNIESCSR

QTNENVKMALDDVHESNQKQLLDRLPVVEDAAFDSHAEEHNSTCLKDTRV

DVLQNIDTWAVSSGTQQIFWLSGMAGTGKSTIARTIARLFAAKGILGASF
FFKRGEGDRGNSSKVITTIAAQVAERYPAISPQVAKALENDSRIVHKALR
EQFQELILKPLQANSQHVGKNSDPIVIVIDALDECDSADDIELLIFLFSR
TEGLQSVRLKTFVTSRPELSTRQTFSQMSGKYNDLILHTIPLPVVENDIS
LFLYHEIKEIREKYNALVSNHRCLATNWPGQSTVRTLIKMAVPLFIFAAT
ICRFLNDRRCGNPDKQLEEILRFETRSQESQLDATYLPVLDQLWNGLSKK
QRKNMLEQFRLIIGSIINLASPLSISSLSALLDVPRADINDKLDLLHSVL
SVPSSQDKPVRLLHLSFRDFLLDPDKQNRNDFWVNEKQAHSVIAKNCLRV
MDCLRQDICDIKDVGTHQFTISRRRIDDCLASEVQYACQYWVHHTQEAGT
YAPDSGIVYRFLTQHFLHWLEALSLLGKVSGSIDFLNVLKSVFKASKPSK
NPGMRRS
>Mb|QLI74045.1
MPTPRADELDAVMVDGIDKHDDAALHPLCDFEFTFESSMLLIEPDSGESE
VSSFSDASLTNSVLEYPKLFGRTYHAFHAGSYVFPNDEKEQERLALQGPI
MQKLMGGKLHFAPISSRDYPHFILDIATGIGDWAIEMADEFPDARIIATD
LSPIQPNYVPPNVRFYVEDATEPWDFPHKFNFIHTRLTGGCWEDFEKQVA
AQAFEALEPGGWFESQEVDCNISCDDGTLDPCGPIVSWISDLILAADKMG
RPTLLGPVLKRAYENAGFVDVQQRVYKMPLNSWPKNRYLKQVGLLWGHNL
VEGLSAFSYQLLHHGFDRSAEEIEASLVDVRRDLSNTSIHAYMPTFVVWG
RKPYPHEVGAQSLHRGKVVFGAM
>Mb|QLI74057.1
MDEIEELRRLLREEQRRREEVEELAKASLPLDLQQYLGACHSLDLAIDVV
TDRSLTTQGETTNPTGRIFPRRIIPWDSFAMRQEEIWNDLSISDLLPKPS
FPSYHQMEYVKSLLKPISSELGLRDFERDVVENAVQKLVDRAHTDPSLQN
SLGLQGTVTFESHTNLGTTDDAISAPMEHMSLGRDGADAAVQVPAPKPPP
APKARRRARGKGNRADQFCIYRTSDGQNCPAVAIEYKAPHKLSLDEITTG
LGAEIQPERDVINKTDHGFAFTARRLAAAVITQLFSYMIGKGIQYGYVCT
GQAFIFLHIPDDPTKVYFSVCVPNLDVMDDDETRLHRTAVAQVFAFILQS
LRARPPPETWHDEAETLGIWAVEYDDILRDIPPSERKRKEPRASPYKPQR
WKGFQRSPIRTRSSCQQPNTSAGRLEDDDEDPPSPTPNPSRRGEKAFSSV
DTSLSNRNQGRRRGQQKVSDPGKKPDIQSRPFCTQRCLLGLAYGGPMDKN

CPNAGYHGQKRISRLEFLQLIRHQLAVDRGRDADCVPLHRSGSRGSLFKV

RLTSHGYTLVAKGVEGLDLACLQHENDIYDRLQPIQGKYVPVCLGTIGLV

RPYYYDSGVYMHFMFLSWAGQPIFDCVDRAMKAGIGSAVSMAFKAIHNLR

VLHHDAEPRNILYNTTRRGLMVVDFERAEFHGRQPLASIGPNENRKRKRR

ISQKLKKDDFVRELEYAVARVSSCIANLPSPAALIVDETLHDVTT

>Mb|QLI74079.1

MGSVYDGATGSQSSPLDLVVLGLNSGTSMDGIDCALCRFRQATPDAPMHF

ELLKYDEVPLEPTIKKRVMAMILHNTTSPEELSEVNVILGKEFAQAVKIF

SQRHNVPLSSIDVIGSHGQTIWFASMPEEGNVTSALTMAEGCFLASMTGI

TTVTDFRVSDQAAGRQGAPLIAFFDSLVLHHPTKLRACQNIGGIANVCFI

PPDRDGKLNPEFFDFDTGPGNVFIDAAVRYFTDGQQEYDQNGRMGKAGTV

DEEMVDKFLSSFAYFSLDPPKTTGRETFRDTIAHDLIEQGLEKGLSANDV

VATITRITAKAIVEHYKRYMPTGYGPLCEIYMCGGGAKNPNITDYLQAAF

PAAKVMMLDEAGIPADAKEAITFAWQGMEAIVGRSIPVPDRVATRQPYVL

GKISPGKNYRRVMKRGMDFGAGQDELEPVTELVHYVDGKVFSNK

>Mb|QLI74082.1

MDTAFSDEASEHSATRSDPGNHIQAGCWNDGLKVISILGRRGMNPDDVTP

DIDIVHVTGLRRESDRLSIDSILKSSITTLRREILFPCDIAQLLVGDFTR

DVVHTTALRLLNSLACLADKDASKRNQKSKHAIIFVAYDLGALILKTALS

VAEKERYNWPDTFTSSARVIFYGAPQRSDSLFIMESKLYEFLHDDKNTPW

LHMLTPSSIRGLAQAALESTDEFLSSKFTLRGRIVSLYANSRTPRKIHPA

FDEEAATLGVPLEESLGEMDWDNADASSLLATKICHLVEQWVPHPDQVAA

EQTLLSLAVPDSQFGITKPMESHPILKDTEHQKWIEGRGHRLIYVHGTDR

NASIVAAEQIFAKCRQRMQALGIPQGCAFSFSFSSRDPLRRSASSMISTM

IAHDLTTHRKQTALHGLVLDQYHVQNGWTEQDTMLWLNMRWLGEPSSYRC

ILLHDFDECDKQSRTEFLNFFNTRADGCEFNAKILVTSRQPGALLDEVAI

WPEINIDRLCSPEEKFKGAGSLEGPGLDGFATAASTSRDPFRLLPDLDLT

RTYAARLERGRHQLKAMDYDRSNFILELLQAQSAWPEDISTRSLTKFANL

LDLISPTTTAKDMLVIILQSLPDTGAVQWILKWLVHSYRPLTRVELATIL

YLQPVKGAQPSSDASPAALAEALSNITNWLRGVVDIYQDQMVIRSEVWNI

FMEDAGTENRLLRDNSDVCHSTILDFCLAYLSEPDVQEKAMRIYQLYLSQ

VRRAAGDKISPPMMPDGRDPLFYFIVALPYHALQDPCRREKITTLLKNRD

KCLEVWSKLWWAMSNSLSRPREPPRCAYPLLAGLGLVDYRKLGTSSRERS

QILILAAANKKKDVVVDLLGALTEYSLSTLTNALEAATKAGDEDLALLIE

KVLGFWHKPSREQPLNVQQNISWPPSLLWRAVQLNMVRLMKVLLENGMRA

DPEIFNDYFFPSPLHIASVLGNAHAARMLLNHGADPMLVSNEQHEEIRFT

ALTRASAAGHGAVIKLLLSKEPLLLHNGYPMEVLGVLGRRQWQLSTPLSM

ASCTGHWKAVEALVQAGADPDPDLMYEEVEGGGGKEKDCSVSPLCFASMN

GWLQTCKALLENGADVNRLGYMGKFDALFCAAVFAASVPCCWALIARGAN

PNGQAQPLLVELVLGRSSLQPRPVTETIAVLDVFLDHRPRLDIDQTDSAG

LTALMHASKRGSLPLVKWCLEHGANIKAVDNQGQSSLFYAVDAGCFDVVR

DLLTWEVNLVTDAILQRALDGGNLALLERLLRAGKDSDSASESVNTLIRL

AIHARNADAVKMLIKFKPDINRLDEYGWSPLCDAVGYVPDTKIARLLIDA

GANVGQILEDSRRNLMHLAMGADADVIRLLFEFPKAVDIDHRDKFGNTPL

MVTDNTTKVECVRHLLKAGADINAQNSDGLTPLMNAIACDAPSEVIQCYL

SQPQLDPNIYSKIHGSALHVACRTLDKDIVNGLIRIGSDVNQHLPAPALS

PLIVVCIPFPDVSLKKRENTDYIVHALIRAGANVNSTSKMAQHDSHRALS

TASLWARTSTLEIFLYEGADVEARDPVGRQAIHFAAANGVGNLRAVMRFF

EGSVAIADSSGKTALHWAAQFGRLQAFKLLLSTMSPDERMRRVNEPDIDG

WTPLCWAMRTSPIVWNHMSEPFECKGLVQCLLQEGADPSIQCWIGEGQDK

ECFTLLHLAYLHDADEKMVALIRDALTKAIDTHQRRTEWEGKTCSLYKPR

DVLCDVCLCQIYGLEYVCKICPDFRVCKKCYGRIDRYHNRFEDDITPFLP

HIFEVPDHEEFMDTTDVVETEENTSTKMRKEDDEEIEENSRWGRF

>Mb|QLI74095.1

MAPDEVLLHGWTAVPVDAGQLFDGKTYKNTPTPLKVDCIEFPSDDPIVVK

AQGYAKDKLPPETFNHSMRVYYYATAIIRQQFPEHVKSFSPSTLALTALL

HDIGTAEENMSATRMSFEFYGGFKARGVLQDFGSTQDQADAVCEAIIRHQ

DLGTDGNITFLGQVIQLATIYDNVSDHPHLPDIKELVHAVTREEVIDAFP

RKGWLGCFAKTVQKEVGLKPWSHTTHIPDFDDKILGNALMKPYE

>Mb|QLI74112.1

MKASQVLAAGALVAPALAQNRTEIVTITDTAYTTYCPVPTTIPIGNMTYT

VSKATTLTITNCPCTLTKTGWFKPTATPTAQPVCSGGEDCGHGGNGGGNN

GGDHGCDGEDCGSNGGNPGGNGGNGGGSECNGKDCGNNGGNGGNPGGNGV

NPGGNGGNNGNNGGNECNGKDCGNNGGNPGSNGGNPGSNGGNPGGNGGSN

GGSNGNPGGSGNPTPAPQGGCDEKTGAGCPAVQTATGAQPTGTSAVVAGA

NVNGLSAGAMAVAGFAAMML

>Mb|QLI74150.1

MALSTRAQDLSKPDPKFFFWEVLQNIWDPQNNPNGFVSLGVAENVLMHDM

LSEHMHRNMALPNEDFTYGDGKKRLKSALARFLTRHFHPVVTVEPSHLTI

SNGCSSALEHTAWAFGNPGDVFLLGKPYYGTFVPDVTLRMGTQLAMVDFD

DADPLGVDGVPKYEDAIRDAQAKGKRVAGLILAHPHNPLGRSYPRKVLLA

LMRLCQRHQVHLICDEIYALSTFTNTVDQGVEIAPFESVLSINTAGVIDP

ALVHVIWGISKDFGANGLRLGAIISQNNPGLHRALVPPSLYSSSSSISDH

VVANILEDDAWIDGYILQNRTKLAENYEHVVQWARSNGIEYAAGVNAAFF

LWVDLGSAYRERHIDDGRGIDQAVNDALLRQRVFLASGLQFGSERPGWFR

IVFTQRRDHLDEGLRRILAAMNEVQTHGHRQ

>Mb|QLI74156.1

MTTALQQLKVLQVGKTPNGFNGPAYGWSSFGMSVHSPTFEMNESSIIQQC

DVLSSTLGNNYPYCNLDAGWTGPTDNFGRIMYNKTLNLPDLANHLHSIGL

FLGVYVVPGALKSDAKKTIYGTDVTIGQVCSGTEGLARCVFNYSRPEVQQ

WHNSVVAQFASWGVDLIKLDYVTPGSPPPPGKQSKLPANQSEAVIAYHKA

IENSGRQMRLDISWQLDLSEPSFAIWNQNADSMRVDSDIINYQGSSLTSW

KAIQRAIDNYRNWTIAALDLPFALDIYADLSSLAVGNNETLAGVNATQQQ

TIMTHGIASGSNLILDSDLTQLDGRLSQSLLLNASVLQLADFTARYPMQP

RNPGTGGQDAKQLQAWIAGPSEDSRAVVVIANYGPDQGQGGFNTSLRGPQ

QVTVSWTDLGISGCWQVRNAWTDEIEGRMDTSISVLLGEGESVLLDLTYV

GLSSRVGTCGFAARAGKFMSRFLFQTPG

>Mb|QLI74209.1

MAARCDSPDFSDFEQVDVPEIPQTPLLSSSHELSVSSSTGDGHPPWQGHR

DKLVGLVDMGSNGIRFSITDLSDPLARILNPVIVYRSSISLYDAQFDPET

GDQIPIPETTITAVCEVLQRFMVVCKDFDVPEEHIYLVATEATRLAKNSA

QLLQSIKEATGKNVELIEKEMEGQIGALGIASGFLTMEGLVMDLGGGSTQ

ITWIISRQGHIRTSTNGSVSFPYGAAALTKTLDDIRKGKDEKDADHAVEQ

LRKEMVSNFRDAYQRLQVPQAMVEEAQREGGFRIYLSGGGFRGWGYLLLY

LNQSQGKHYPISIINGYTVGREQFEDTKSIEKAAKAARDIFRISDRRRSQ

VPAVAFLVNVLAEAIPLGIREAHFCQGGVREGYLFRQLAARIRREAPLEV

ITRNFAPGSRLQIQELIRGAIPKPSKDATRRFPPDFGGHVMEAFTNVMYV

HMFMSKETASTAALYSTSSGILASTLGVSHQDRARLALMLQARYRGELPP

REVEFRDALRGIISPEDVWWAQYLGRVGFVITCLYPSGKVDPANLKVLFS

SSWSSTLGKKGTKEGLVLKISIRKVKHDPEKLKKLVTDNLKVIEKSGKKK

NWAGKDQSWGMKVEVRVAEEQIL

>Mb|QLI74215.1

MAAAEPKAAPSAENQTTQNLNVDATAAVISRWSDLHVSKPASVTTPGSEQ

DVISAIRLAKSKHLTIAVGGGGHATFIPVDSSTLYMDMKLFQSINIDKDA

GVVRVGGGVTTGRLLKCLAENGLYTPLSDSNAVGVVGSLLGGGQNSHVGL

HGYMVDNAVSFRLVTADEEVIDVDAQSTGGKATLFHALCGAGQGHGVVVS

ATMKVYPLAGLQLTDNKIWTRVMVFPSPALAHVVEAYLALLPVPGPLYPQ

LLFQRAPPGTPVAGSPTVVLSVTYFGPASEAEKAASVLFAADLNNAAVQV

DSNMIDASHLNDALEPLNVHGGLKSACGARLGKVNAKMITSSFAKWLAIT

DELPDAHRSLVFLQSFNPAVLEAKGKSDPGKHMFLEARDRGFFLVVVNWC

TTQSSLEKLQPYADDVLHVCRSEDGATPRTIPNTMRYKEPLKDIFTGERI

MELHHLKKSWDPMGVFWSPYVGAYRHHHEEVAP

>Mb|QLI74223.1

MLHHIALALAFSSHVNAYNDFHFGFNFHDIGYAPQGVNISVDPRIVRKTL

QRVRDYRPVTGLFDDWTVEGPSKDNITSVAKYWADEYSWEDTQEQINRDY

KHYITKVPGSGNYTSDIPLHFLHHESENDSAIPLLLLHGWPSTSLEWSKV

IRPLAESSELPFHVVAPDLPGFGFSPAATQDGLGTREIGRAVDALMKQLG

YSTYGLATTDTGWFIGMWMVHDVADSIIGHLFDFWYPNPNSTDMERFQAN

QTTAEETAVVESLHVWDDFHNAYSMVHSQKPIAMSLALADSPVGFLGWYW

DLSYAISDGIPHSMDELITDALMLWLPGPYGNIRSYRQIFRPDIVDFPKT

NVPSGVSQWGWGHGPFPGIANFPFTPRNWIERLCNVTYFKTHKRGGHFPA

RTLPEYWVSDVREFFKGL

>Mb|QLI74264.1

MGFIYIRAHGFGKVHGGEISCLQLQPKANIDKIHNGEPLTDSDCEHWLEA

ISEHVSDYEKDQVHQRRLIIICSALKRSYRELLRQSCHHAGYSVVHFVYL
DAPESELRRRAEAKHDDFPESNLVHSQFQKLERPGIDESDATIISVVPPL
EDVQTAALEAVSKTFIDKGVQK
>Mb|QLI74265.1
MDIFGITPEGINALARETVHDRIIGCLFGSALGDAVGLYTEFLSGDMSAT
AYPSRKFVLHPESEATLFRSDAHRNPHRPGEWTDDTDHALLILLSCLHKD
CTELDAQDFASRLSIWVRFGLLALDTLPLGLGRTVGAIVRTRTYLDDPEG
TARAHWKNTNYHAAPNGSLMRTHPLGLLCLDKPLYDTFETAAAYSAVTHV
DPRCIISCAIGTALIRGLLRQQVRQEDDIDTAIAEGLSWWTGYRARQMQD
PDRRDEPDLDLAEFRRHTKVENLDALQLDDALKMGYVYKTFGSGVHLLRM
AMRKSADSGHSLSAQSAMFEPLMTDLVMRGGDADTNACFAGALLGAYVGY
RALPPHWRDGLRHGSWLMQKAEGLCQLLGVAEGTYKGSEDKETSPDGGRG
FPTEKQMDGKVMLLQADMARRQQERTRMEEQGQKKGKGIGWLKWK
>Mb|QLI74272.1
MNPTDPRILKTLHDKRGGDFFGPSTPRTSTCQSSGGIIRCLKKQSKVSYP
MELDDELIQQQSSNPLNQLNMSPSSRRHSSRVYDESWISEWSFITDLYRV
LEHALTRFRGDSNRTGEVFHHVPPPCKALKASPPLPQV
>Mb|QLI74276.1
MKIRQRTICHTCRARKLACDGRSPSCSQCLKSSRECGGYQYGLIFAEPQT
TGRRTAGRKRITLSEVAVEPRLPTPGIGQPLSWPMNDILSMVVQNFIPRR
DETNAKSQGLTAPSRVCGSWIEVVPDLKTSGPNGHALTASIKAFAVAMTT
CRRPLGAPIVDALAAHGTALSSLQQALTRGRNEGPDELSAAIMFLFLSEI
MVPACGSYGAHIGGISRLMLSRGPAYYANGTSHRIFIGCRPSQIICFILY
RRACFLEAAEWCTIPFQTSPAEPLQELMSIGACIPSLLQAIDQLEATSKH
DHFSACIDKLLDIIRRLDGWRYSYSANLSSTYGRDFVAPPTSEDIWYPDI
TVANSLTHYWAFWIICAVYAIQLDSGGIWSGRLGEANVVQQEAIRSKSNL
IMRSVEYLTQKKMKLFGATSLGLPGKVAYEYSQQAGDLNSATFSNKVIKN
ISEKGYSYLNAFIQSDFKILPLSTPRHQNGDQRGRTISNSTALQLKAQEE
REIR
>Mb|QLI74288.1
MKYATSLAVLVGLAKAGPLLSTATVNPGNWQQVPSNLTWAKHNTSTSTTA

LSSIVPNSPRPTIGVQEFLTLSQSFKFEIRSGRCEITNLPGSAAGFFWRV

VYDLDLKGSFRFETCEREQHGRVFTKIQGQYVLGYAVKYSQVTSGAELIC

DSGVLSYVNVQSSGVVVTAVTESTVIEKYSNTMYACTEAAQCVPSKCENG

CKKGVKDDKDTSPSSCGGPIPKPGSSKRALFFLDSNPEGASIIGLPILQD

GSLDKSRAVRTPTGGRGSISRNMNGTVSVDPLFSQDSVVVNGKYLYTVNS

GSNTLARFEIPDADPASPKLVGEPVHTGGEFPNSVAVSDKNDIACVTNTG

RRAGVQCFKTLDRLQPLGDYMPLPINQTTPPVGPPNSVSDIVFNPSQTAL

FVTIKGNGMGPGYVYAYQVVNGRINPQPVISRPPGLLLDFSLTLLSDSAG

VVTDPAYGASYISIARNLSVAVSNKITVPGQGATCWSVFAPEFDAVYLSD

GASPNITALDPVSGRTRFVITGDSRSTGSFDSAVDRSSLYVLQGSAAVAV

FHLQGSKTGDRTPSLSQYLDLSSFGARSGWIGMAVHSG

>Mb|QLI74289.1

MVIGNHGKSHLEPGRDGACTDDAHISNEDIDAAAECRLVRKLDVIIIPTF

FIIYMMSFLDRINISNARIQDMPKDLDLSGKKFNVALLVYYASYILLEIP

SNMIVKKVRPSLYMSTLMFIWGVVNMCMGFVHSYQALVGLRFMLGVFEAG

VLPAIVYITSTYYKRHECQKRMSLLFCSTVVAGAFGGLLAFAIGKLGGRE

GFRAWRWIFIIEGAITALLAVVASFTIIDWPEQTKYLNKEEKELLRRRAK

NDVGDMVRMDTLNKFSMQLIASDYKIWLGGLMYFGVGVAGLSGAMFLPTI

LVEFHWEPEQAQVRTIPVYVFAAGTMLLGAYASDRLKHRFGFIVGGASMT

TVGYAMLLSQDGKTRDYKFAAVFLVFGGAYMIAPMALVWLQNNVSGHWKR

SFAASTQVMVGNVAGIVGSLIFVPDESPRYTTGYSVSLCLMWVGICAALA

MFMLMRRENGKRAAGRRDDRLALPEDVRANLGDWHPSFRFTL

>Mb|QLI74342.1

MEVDRYPVVSPMHPWVNPTLLNEAVIPEAEKPLVKTEASDAHALGSGSCN

SLKSFNGQYYSGMVVGGSHTWNYDGGVWHEVKTEPDLWKIKLRSLHMICG

RIRAGLSRRWRNSASTKKLAVRARPILLKRLLVGVMAKRLTGGICSIFLF

HRVYRGRNE

>Mb|QLI74344.1

MAISDSLSPEPQQLLCLTITAYRKPGMSEAAYREYMTKTHAPLVSGLMEE

YGIVRYNMTHNNSKSRPLLFQLYDPEFSKLSDYDCIVQFVFRRMEDFLRM

KSDPRFLEKVAPDHQKFADTARSTMTIGYFEEFLENGKVVPK

>Mb|QLI74355.1

MSFPGFLYSQLIQRLPYPSGSHAGQTVIVTGSNVGLGKEAARHFARLHAK

KVILAVRSLEKGEAAKQDIESSLRDAGGAGSTTEVHVWPLDMASYASVEA

FAARANSELSRLDVLVANAGIASGSYYTAEGNESMVTVNVVSTFLLAALV

LPKMKESARQFGTRPTLTITSSGVHAEAADLVARAAAAGELWPLVNDRQT

VEAHFAAQYPASKLLEIFGVRAIGEQSPAAGFPVTINCVDPGLCHSELGR

DYPTWGFWLLKLLLARSTEVGSRNLVAAGSMGQESHGKYVSDCVVAEPSA

FLDSEDGRKVQKMFWKELTQKLDEIKPGVSGNFL

>Mb|QLI74415.1

MAYSRSLMYQPAACLMTHGTVGPINDDQPSQQGGPWALIEDQDFISKVDL

IAPVEVYQTIQERILQHKPQPCFYRTTISLGQLLNSEQFIQLIENQEITM

FCEANSASASSYAIRNGILTIHMDKETFQRAGIPGKPLVAQGGSDGQPKW

AGLHILMEEYLSAWHMPVPQSSLNG

>Mb|QLI74438.1

MATKKTDIETAKSDSAFENIPIEEKDRFPPVDNIRNRMFFVVRETLRKEL

MRDALGELIRHHLPILGARIEKDETGLLVYRMPSTFSHGYKLFEWSENSI

DATLESTQLLPTSLPRDGSPIFGSLSVQDMENKWTPSDWPVERQFEKADT

PLLLAHITSYADATVVALSLPHIVSDGMGFGSLVTAWLQVMDGQTPAPFL

NLEPGTLNGPDDIPQEELRRKKKFRVTSKAETAASLLGFLPDLVKHPKEV

RRILFLPSTLVTDLRNRHNDDLKARYGKNTPALSDGDILIGILAKFAYIG

RKSAKMVTLTSAVNGRGLHPALPANKPYLHNCVTFAIGRLPISNKTPLLE

IAHAHNQAVWEAFKVENIERDLAVTREACRRGAFPYFCEPLEMAFFTSNW

SGAWHQANFAPAVVRKGADATGTIPDVVTPLVFGQARRLRNNFLMRYGSQ

IMCKADGGYWCDFTASTKGMELVQDLIRLDPKLHSL

>Mb|QLI74448.1

MRFRQSKLENYTQHARDPDHGSMAADIYHLDERGERFIIRLPKGPQPNKN

RPNNLQDAERKRNLAGGPSAEELRSSKRVRLDQDDQEAPGSPQSIRQNMA

SRLPAEAWHHVFTFVPPRTLGSLMLVNKQFNSYLDPSSGFKSTRVLGTSD

TRSLPRLESDAIWQASRRLFWPRMPAALEGRSELEMWRLCCSRVCQFCKF

RDDSAADISGDQWRRGPGAKGVSPVFPFFIVSCGRCLRAKSTTEIDLLLN

SSTPSALLPGVPMVFLSQENHVVPPHVLVSTTSLSHAQPIKVFWNEQIEN

IKSAFEDVKGLGPAAAEEWIKGLGQEGKRCLADVARWERWHIAAGVYEMH
RLSPRACVQTAANNQELTTRRSPDSGRLAITANKSVEMKARTNGEHREDL
PVHHDCLPASGLADLPAQENAPVESAQTTSIEDMTVSDCKSKPTSEEAEI
LKAARRGEIERRAKTLKPPILPNVLAHMPAFHEALQSTKPLNAKAWQVLR
PRFLLQRKEAEIKERELQANKRKKRKKKKPKHLKKIVSSTHSSDGALVAA
DPHGPRGVAIGTNADWDQAQGLLRGQISEFADEIIQNEWRIKTVTEENAP
QFAADILLYVRNKFYAKIANDAKADKASGKRLITKPPEAPWTRKLTLEDM
KWVFDEKIKPYTNAVRKNVFRCNGCARSTKFYKLYGMLWHYASNHTKALS
LGDSVIHWRAEWPKKPVFKADSQRSAEFRTEAKTEPSDNSLLRIPELPQS
VTAQPGLEYSPYTPDLGMSLSTNHGTDVQSAKAPTPNLEYLDDIVMVAKK
TWRKLAKVKGLQNSVRVCAVIHHIAKSFQTKHSEAAPLKLFTRALKTHNS
MSQLGSVPGLSCKPCKLNSHSSLKSKDYTLVKLSRHFDRTHSNQVVSSID
KPLDWQDMIWLPETSTLTNLRSVIGDDKEVLDVVSDALPWAFETPGCGHG
AGRNDNSYAAVSRSPKTEWQQPSALAEVTFRCSNERSAVPCLELDGFEVI
EQPAVWSRRHDRIHDESHTRLPQHGIPPPPSTPRHMEYRRTGVNQQGYTK
PVQTISAMSSRYNTHLGNGYENDHTHVRRQAHHIPYPQYESHALELYEIV
EVFDPYGSYFIKRPVRHPSQREMHYEDRPQLYAVPNEFQRQHNHDIRSGG
ERTMRSNPRSLDYEEYDPRFPAASWRG
>Mb|QLI74453.1
MSLPTTARGWSVRAIDKDSFDGLNFQESIPLQPLGDYDVLVKIEAVSLNY
RDLAIPRGRYPFPMNLPVVPGSDAAGIVLAAGPKATKFAKGDRVCTLFNE
LHQSNPITPEAVESGLGGAIDGTLRNYAVFTEQGLVRAPASLNAIEASTL
TCAPLTAWNALYGLQSKALKTGDWVLAQGTGGVSLAGIQFAAAAGATVVA
TTSSNDKIDFLKELGASHVINYREESDWGQVARDLTPGRAGFDHILEIGG
PGSIAQSLKAIKLEGVITIIGFLSPSDMQPPLMDVLNHLCTVRGIFVGSR
QQFEEMNRAIDSAKIHPVVDPNIFTLEDLKKAYQYQWEGKHLGKVVVKVA
S
>Mb|QLI74496.1
MAVPPLKHWPSELLLSLLELLSLNDLLSMSRVNKSIRALALPLVYSTVEV
SWTWRRTPPVILLLQSLLDRRELSGYIRNLRLVGNGFKDRPEVREPPVPP
LDAHLLSKATEVIESTRVPFTKLWIDELHSGSVDAIVALLLAMVPNLTSL

RLGPNFTVRSRLWGGIFQCALCGPPNTYNLPAYEDLRSVKSEFRTKEWHH

IDVCNAADVLALFYLPRIQNLSVSIDSPVEFAWPCSHPPAASSIVSLDIY

RLRESRIGPVLSVLKGLQRLHWHWSYQPDLDEEVSKDVLDLDTAAVALNE

VADTLTDLTIGAITRPRISVGDYDPPLLEFQGSLDGMSRLGKLRKLRLPW

VFLMGFSESPASKLLSALPLGLEELELTADLNDHEEWEWDDDDSIIHAVK

LELASPSLSRYTCLRRIVLPISSEYCEMTNEQREELSIIGARAGLELGWM

EK

>Mb|QLI74520.1

MPETSPLEVRAAAGDRLVFCHFMIGIVGDRTSASDYDDDMRRAKDAGIDA

FALNIGVDGYTDQQLGYAYDSAAKNGIKVFISFDFNWWSPGDAGGVGRKI

AQYAGKPAQLVVDGRVFASSFAGDGLDVDAMRSAAGSNVYFVPNFHPGQS

SPDKIDGALNWMGWDSNGNNKAPSNGHTVTVADGDNSYQSWLGTKKYMAP

VSPWFFTHFGPEVSYSKNWVFPGGSLIFDRWNQVLRKGFPMLEIVTWNDY

GESHYVGPLKSPHYDDGNSKWVNDMPHDGWLDLSKPFIAAYKAGASSIND

YIKEDKIIYWYRRTLRTLDCDSTDTTAGRPANNNSGNYFMGRPDGWQTMD

DVVYVVSFLTQPGTIIVQSGGQSKTENAPSGAHIFTVPAGVGQQKFSLTR

SGNTVLSGTSLMDIANACPCGIYNFNAYVGTLPSGFNDPLQPHGLASLTV

GLHVSTCEAKPSLGTNPPGGGTSTTTTSPGGQRPTTTTSSTKPPVTSPPG

GGQVCTGGTNADGESGNYSGLCGFSCHYGYCPPGPCKCTSFGSQISPPPE

TGYNGCPLPGEGEGYKGHFETYFNMEALPRVCVHSPSFLQQYQRFWDNPS

QAPLTWIALLFGIMSFAAQAQPGTPDQAHDPALPLPFLEQIVSCLVLGNY

THGPPYAIEALLHYFVLERRRARDTQADTWLVMGIILRLAQRMGYHRDPL

HFPGITPFQGEMRRRVWLMIHGIDITTALQFGLPRMISPRHGDTAPPRNL

SDHELREDMAVLPPSRPETEHTPMLHMVARNRFFVVLGAIVDASMGASKM

TPVEESRLLQQLYDARDAIPPVLRYTSLSDSLGDAASHNIHRIILVVLFY

KGLIVLHWHHVRLVNVRTRSEEPRPAAAREETRSSYRICTTAALKMLEIQ

HEVEEERRPGGCLASLGLRFSAAYNHEFLMATLVLFTHVYGIANGAPGSY

LDDGEKAEMHEMEGVLRRARRTWSLRSAHSREAAAVDKLLGKLFRILDGP

GCEAVLTDGVADDASMGQLDGVDWGFLAEFGLLSHLQDLSRHCIGR

>Mb|QLI74552.1

MSTTAQKPLPIIHLNGFPGTGKLTVAKCLQKMLGPHGRLVHNHLLINPAD

AVLHRTEPGYQDLRRAIRSAVFSSLAQSAATHDFAYLFTDFQSSDPVGSA

TCAEYLSAARARGCSLVSVVMSCDEAANVERLITSERQAHGKIVDRELLG

KFRSGVDIHRFDGPDHASLELDVTQLSAEEAAHRILQHVFRTCPEVERAV

AGGKAS

>Mb|QLI74555.1

MPAAWKLALAVVITLAATSAADSGDDFSNNLFSDLAPLLALFGERVTMQF

MSQSTGWADSIILAMAPLGIITIIASAIRVGGPSWLKAIIGRARENLAVA

EAELMSSTSHEVCELWNGREVVRCMGSPSTAEFICLRPANMKCTENGANQ

VPDILELKEALDKQYLKELPSNDGASFWETILDSIGRNRESASAKVERGA

NSQTSVTIPVPKLTIVRNQVIDAPNISLNSHGYLSRRELRAVAVFGTILQ

LGVLTYFGFATYHPALKFTKGDDAVANYAFPCTATGTITLVAGLLLCAHV

VESSTEEKRYQPSKETKARLVWLQQAQTVGDQSFQSFALSAGDNQTIITT

SCRVNKKSQGIHLELKTIIGTAVSLCGFVVQFIGLRGMHWSASVAQLGAV

IAMAALRAWVRRGLAQPLQRVRLTPEHELEWFAMALGDFDGIPWQPDRSP

KEYREQGQTARAGVNTMKNSGGNPTTHNMMVTRRTLCELAGWQGPASAEA

LTLARAIKITMDALSSYLHPESDFTWSLGALSAGHEDKPPKFCWTRDVTV

DTIRKDIEAVLSLRLYYVYSQKRQYEDQPKSAQTQRRKYNARLHVTEMPA

ERGLRLLGPGTRALRRDLAWWMPPDTARILEIKEDENGTMKVENHRIVGY

GGQQSIRVARYDGTEASLSPDDTYHCEIEESTLLASESYDSLELLYAQDM

FTKFMQSVAEKMARAIEGGSNVRPKDSSNMVAWKCFTLHHDRLSKMAQDI

HNCGLGNIHQTYLSIIQPLSTEQKLPEPDNVIALARKHAKPHERLQDFAE

ASNIYLWLFRTANTFPRKSSIFVKATAILMENVRMVTLTSKLREAQCYPE

TKIWQIRAAKSNMENELKRVDRTILSGLMSLYKKQSRQWECDILQDAEPT

IEGHTSHPGTFNCTPLHQICQEDRDHFLSRELAVRYLDRKDIHGCTPLHY

AAVKGSLDDTTFLLDFYPDVDARDLLDWTPLHYACNACSHSGVVQYLLDK

GRPQVNAQGIDGVAPLHLAAMNGNIETVQILIRAGAALDIQDASGATALH

WAAFKGHEAMVEYLYEDSNKKLRDKNSRTALHLAAIAGKENVVRLLVPCS

DKQTNADMDTFDSGGYKPLHYAAMRGHEAIMRYLVNVAPFNREQATNYHG

ETPLHLAARRGHEAIVRYLVGETGANKEAKNKQGFTPLHAAAAYGNEAVV

RYLVGETGANKEARNKQDSTPLHIAAAYGNEAVVRYLVSETGANKEARNK

QDSTPLHIAAVYGNEAVVRYLVSETGANKEAKNNIFNRTPLHLAAAYGNE

AVVRYLVGEAGANKEARDGSNSTPLHNAIFEENEAPSMYLAGEAGVDIEA
KDHKQQTPLFWAALEDNEAIVRYPVHEAGADLEAKDFKQRPPLRRAAKWS
NEAIVRYFIEAGADLEAKDFKQRTPLHKAAYQGLQACVRYLVGEAGANKE
ARDRFNRTPAQVAAACGFKSVVRILEQDEKAIRGAN
>Mb|QLI74557.1
MAASTSDWRARLAAVPVPRPSFSAQSLNYFNPSATAGIPITANDCGIFEP
PRCTELVLTKGLSALVYLTNNKVVEVIAFSITFKTVHSVVNHSLASFQHQ
LGTLGREPSPLRSRPTSRSPHSDRCVTEQHAKTDPCSAQIGFGDHVPRWA
PGSVLTYVICTETFPSPDDASYATNQLTEAIHMWMGVGATFKQVRRDRKA
TFRVVYRPKHRPDTEDVLANAFLPNGGRRNQRTLHIYALAFSDTHVDYQA
NILAHEIGHILGLRHVFAKEKEYKWSAKWGNGCQNSVMDYYTHSSLLRVQ
QQDLEELKAFYDSPMTEYEGKPVINFVPRSSVYPNVRRHRKRKAKRRS
>Mb|QLI74570.1
MSNYRENNVSGAGNTGRHGSDKATSGAGGIGQRRGPISGLVRAVAGGIGF
ASESIHHHKEKKDEQKKAAAAAAGTQSEVGESSRAGNGITSGSAVASGGG
SKPVSQEEPISSDSDQDASEEASTELDEAAWQLDDAQTELAPPPDYATAT
QDDADDMARRFIANHQIQEKPSSETSRLPVPVIIPQRRPGERARGFIHAY
APVLENVGIDQPTFTDFIKQLNLATKPSPWIHAINVASIAAQSVPEPITI
AIAISAQIATQVGLQAHSRSKTNAFLDKINADFFRPLGLIAVILTWKPSR
PGEVVSQARFDAALEQAADSVSGPGQGTTGRIGKRMQASNATTDFEWPES
APLVFPDLDKLAGTPEGKEAAEDAAKKPGSVKRGMLFAMEYMDKRGQAQF
AKDHPSSGLAHMGVEPEFHSRYSDPNHPAGSGSLIALLTGGAVTGRLSER
KMARRERRAERREQRTDRRSARGPTVLGTIGPGALVRGVRKFMHEDVLYL
MIANMPSAEQLAVAQTLLGSHPPTME
>Mb|QLI74599.1
MTIHNGKLRVKSTYSLSARVERRVVGRVTRSCRVKQELPFICNPVIQNLP
SLCAVAISADKLSRSGKFAPRESAESNYEESLPLYSPSLQMEIILPQPPV
LILGRGTPVKLVLHTPREIMQSATIYVRSVKLQLKAIISALPRSTWHSVT
DTRLGNTISGAVPIKSEHFQLELGAWGMFMAMQSRPSLNSCLLKVAYSLE
IVTGLSNGLEGPIQYLTASLDVLVMDPPPPYEAVVANVI
>Mb|QLI74622.1

MGLLSKTVFSSLVATTSLAAYLAAKNPVLSPLAASDPIWTSKLFKRYNPS
ANPATQDVCIKRLPLDRIRPELLKNPGDLVLEYCRGVWSGYGFEAQRRYL
EWKYRGPETSADLWTREQLSRSAYDEGTCIADHFEVVEKTATSITVRCGD
SPRVRGPRGGDGLFVIGAVVDDARAEVELTLKSCLFASQGKVLGVKGPMP
PWMEELHQWYARIWSESGSRRLLK
>Mb|QLI74623.1
MSALGVSPEAAPAPVPPVQHVTQRKTSKKGGYTRQRRGCLTCRQRKKKCD
QGQPVCGHCSRLNLVCAREKPRQLSSSWGEDAVQDARPPPAHPRRGSGSP
CCSWHSDHVSEVLSLASIPEPLDLVRPDDAVGRRDLSASRRTMMRYYTST
LAVMLSATAENNCFLSVLLPMAFDCPTLLDAMAAWASSHLALREPSFRDT
SLLHRGRVLANLGAALRDGSLSGEMCLAVAMAMCSMETISDATSSSWAHH
LSGAAAALQPGSSDTQLGPLRTSASVLGGDWLRSVEGKWLVRNFAYHDIL
MSVSLDRRPLMTGDYWMSADDAMADPYFAFASRIMLLTSEISVLRADCAE
CYNASLGGGGSNTSGLALDSNYDALLQRALGIAGDLREWQCPAPSTDTPL
TSLSETYRSAALIHLDRVVRKYLPQHAADVLPEGVRVYVDSVCEIAEKVP
AGSLAECSLLFPLFMAGGEAEEATHMERIRSRLYTMNKWRRFRNVDACRE
VLEQVWQQREEWARGMRRDKVDWRDVVEQRGWQLALS
>Mb|QLI74649.1
MNWPLSNATLGSAMRVFFESVAGKIAIAIIILVICQRMICRPKKHNLFPV
YATIEIAVASYILRGDGLGRRIFSSVRRYGGSLFGITSTHQILVNLPGLD
RFMSQSFHTLTVVPVQYTLLARVFGAVDSPDLEVKLEKSFKDLLAPVERL
FLNDAAVTAAVEKARVEERAASFVTFSTDPAHMKRWERAADIRVIAPGPG
KRAVVEANLQSLARDFGACMAIPLLYGEDFLNRYQQLLEDFWLFDNGFFP
LLMIGVPPWAPFKAIKQGRAAQARILREIEALYRRIDQYQRGEDVDFGAD
MSDVSSAALGRNRVYARDGWSFAERAWGDFGMLWGQNANTHPLLFWLLIY
VYSTPGLLGRIRAELAPHVCLSRANSPEIMSTDLAALSRNCQLLKASIFE
TYRLVNEPTSIRYVAKPTTLNDGGIKHKLPSGMFISAPLSLINRDPLVFA
DPERFVPERFLEVDSESGKSVARYGRLQPWGTGPSMCKGRTFAEREILSL
GAVIISIWDISPASGVWELPAMIPGTGVKKPKRDVRVLISRRTV
>Mb|QLI74653.1
MSQPPEPQAVAKELLSWIGFELISCNTLQTLWAGYGHICAIRCRPLPSAA

PAETTRATHSLILKLVSPPRGRKDEGHLRKVLSYQVEQHFYDHVAPTLRG

LPLAECLVSTSKFPHKAAAAGLQHVTASVLTDLRLDYPVAGEKRTALSPR

QVHAALGWLSRFHGSYWTSPRPDAQSLILPPLEEDKARQQQPGNSSARRG

LWLNGGYTYLATRRSEYNALAQDGGSEWSQTFCQAVEGTSMSVAELAAFF

LTPTGRDFETYLHGDVKSENLFSTESGDEVAFFDFQYTGLGLGVCDLAKL

FTCSVPVEMLTASTGEPRRLTMGAGERHLLLQYRRELLQGGGVIHAPVYD

WIEFQRHWETALVDWCRFQASWGFWGNTTWLQARVRSILHEGEWLWWINN

ARRCGLEKSR

>Mb|QLI74685.1

MPLTVIKVRFESNLYSYTSVVSAAADIRRRDGFRGFFSGFGATAIRDAPY

AGMYVLFYEVLKTELGSIAASTIRVEAGAACSVISNPFDAVKTRIQLQPR

LYRNVWQAGYKMITEDGLRSLLDGLALRMSRKALSSALAWTVYEELIRRF

GAS

>Mb|QLI74689.1

MSSLRCAVYVAPPIPIIDIFTKTKGKTWSPTSCTLVYSDEAAVLVDTPYT

NALTEDLIAWIDKIAPGRRLEYIYITHGHADHFIGIPQLLKRFPEAKPVS

TAATLRHMEKDIAEPRWTETWEAFFPGQLRKPDRLTDPLPASNEFFLQDR

WRFQAIEVGQSDTYDSTVLWVPDLKLAVCGDVVYGQVHQMLFEANTKSKR

DEWIRAIEMVEALNPAYVVPGHKQAEEMDGVWHLAATKKYLVDFGDVMDK

SPKDWTEVRDAMLKLYPDRFNPMVPVLSSLGAFKALEESRL

>Mb|QLI74696.1

MTVRTVSTLALAALNTFVTASANGATQPFDSWSHGRVQLDDVSIHFRYAG

SGPPMLLVHGNPQHSYTWRTIGPILANQYTVIAPDNRGTGDSSIPEDNNY

SSEAMAGDLKGLLDFLKINQTLVFSHDKGSGPAVALAAQHPSLVPALGVS

EYLLPGFGYEESSCPSSTWDLYSNWQLAFFSVPDAAEFFIRGKEKEMLAW

YFYHQSYSGNEAIPEPVLQRYASSISKPGFLRSMLGPFSTASVRADNKFF

TEVLRDAPLQMPMIAIGAEASFAPESTIREVWGPVASNLTVDITPKSGHW

IADENPEWVAERLREYFGGIRGNIQPADLSWLSNRTTLV

>Mb|QLI74699.1

MTTTTTDTVAETVQNIPLTETPHATTVDAPPHPSPAATLKLISVGFSFFV

AGVNDGSTGALLPYVIREYGISTAIVSSVYGANFAGWFSAAVANAHLCSH

LDLGAMLALGAACQVAAHSLRAWDPPFGLFVATFWLVSVGQAFQDTHGNT

LAAGVAGAHRWLAAIHAAYMAGCLAGPFAATAVAAASRWHLFYAFPLAVG

VANLALVLVAFRDSLRLRADRDRDRGTRPSRSGEAWRLIRATAATPSVWF

LCAFFFFYLGSVLTAGGWVVEYLVDVRRGSLSQVGYVPAGFSGGALLGRL

LLAEPTHRFGERGMVFVYCLLSTALQLVFWLVPNIIAASIAVSFIGFFTG

PLFATGISLGSKLFPRHIHSTALALVFVSAQMGGSLFPVITGVVSSNAGV

QVLQPILVGLLAATAISWLCVPRPKDSAHAGLHQE

>Mb|QLI74700.1

MTTISQAIINDHRELEQYYNEVINNENDHNHQQRFGNQFIWELARHSVGE

ELVVYPALEKHLGAEGKEMAESDRKEHHSVKQMLKEFQELSASDSGYVKQ

LKKIWGSLSDHIKEEEERDLPALESKLKSSEDESKSMAQSFERTKLFVPS

RSHPSAGEHPPFETAMGLLAAPIDKVADLFRQFPDKEKL

>Mb|QLI74737.1

MSDPAHPHPNPRHPDYETGLTDEAVIEGHDLIQHAEEEEERMHPDKPEPT

MPVANPPAGQQAKHDKKESTMDKVKHALHLNK

>Mb|QLI74740.1

MTTFARTIIHIGRLFLLLASCGWAAVDVAGQSKVPETVEVDLVFPHNDTY

APVALMPLVFAIQNFPTSRPLFLQIDFDIFHTPSWNTTVQQGIIFLNHAN

YSNNASTIHFVYDWTTRLNNTEGSWAMCWGVYSANCTDTGLAPGPLKLDP

NYRRNLVHFSTKHGAQQPDLVAASKDGVCDETTGVIFNITEVKEVSWFNR

HSVDHDVCPILAPEAPKPNPCLAKVNTNFLILELLIYILE

>Mb|QLI74743.1

MATTIVGKNYHAGGRFVRPDSTFRNFISRDPESQFPAEAGRYALYVSPGC

PWAHRTLIVRLLKGLESIIDVYQVHFVMGPDGWYFSGEGDSLPEDPLHGF

KKLRELYLKADPSFVGRYTVPVLWDKKADVLVNNESSEIIRMLYSEFDHL

VPENLREGNRPGGGLYPEHLRPQIDELNAWVYDTVNNGVYKTGFAKTQES

YEENLGILFKSLDKLEETLGHGKQFLLGDDLTEADIRLYTTLARFDVAYH

PVFLCDLKMIRHDYPRLHLWLRRLYWNKDEDGVFRGAFHRTTEPYIGKYG

YGYAQSRHKIVFGDQGPLIVPAGPEIKMVSLDVGDEGA

>Mb|QLI74746.1

MANLFTDSSTEKVHVPSEPSVSHAIEEINEKALLRKIDAKLLPAVGVLYL

LSFLDRSNVGNARIEGLLNDINATGNEYLTGLTVYFIGYIPCNIILKRTT
PRLWLPTLTIAWGIVATLLGIVHNLAGFFVARFFLGVTESGLFPGVVFYF
SMWYRRRERQYRISLFFSAASLAGAFGGILAFGIGKMKGIVWENGWRWIF
ILEGLATIVIAFAAYWFIENYPETSKFLSEPEKEFIQARLAADSDATQHE
KFTWSAVYDAFRDANCWLYGLGFHTMSLPLYTLSLFLPTIIKDLGYTAAT
AQLLTIPPYALAFVTTLAVAIYSERLARRAVFIAASAIVAAIGYIILLSN
TDPKARPAVSYLGTFFAAAGIYPATALVLSWPAINVSGQTKRATANAMQI
SIGNLGAVLGTQLYRSNDGPRFVVGHAVALAYLVANVAVVGVLAWKLRKQ
NLQRAEVADEVVDVGRAEDWKGDGDARWRFQY
>Mb|QLI74748.1
MADTKASRQPVTGSCHCGTIKYVAFLTLPQTHNESNPPTKQEQRIYRCNC
TMCHKAGFFHVRVANKTDDFLLLSPLDPLQELGDYLIHNKVLHWLYCKTC
GVRCFTFMGTGEVVDLDLAELCVPGYTDKGQKTRVWRAKEDGGHPEYGTY
LSFNGNTVDASSKSFDMREMVEQKCVQFYDYLAEGEKRQPVRYGRPHQGG
CY
>Tr|8076_t
MRLLYTKELERGGFELREFGEQDTPPYAILSHTWGDGEVSLQDMTLGHFA
EKKGYAKIQGCCSLARANGYDYAWVDTCCIDKTSSAELSEAINSMYQWYG
EAGVCYGYLADVPSTAEFAESRWFTRGWTLQELIAPETVNFLDEAWTELG
TRETLKQEISKRTGIPLSVLSGSDLKGMSVAQKMSWAASRKTSRSEDRAY
CLMGIFGINMPLLYGEGERAFMRLQEEIMKVTDDDSIFAWRSKGQNHGSL
LATSPDAFEHSGNIVHVRTAWLPDSKPWTVSNKGIRLELSYMGVGHQGLG
LGILHCAERKTKGNIFIAIYLKDVSLTMEHFARVGCDRFELFNLMPFRTS
QRPQRWLNVQQHRPTAIRARNHHQILGKPPPSRDHPDWGLFDSTIDFTNG
MSSTSPENWDESTPHLLIRMAEEGRVEEAKWLLAKRTTKPDHKDEQGRTA
LSHAASNGHTKIAWLLLLRRDVKPDDKDARGRTPLHHAAGEGHAEVVWLL
LTRGDVDIHSKDNVGRTPLFYAAANGRKTVISMLLARGDSQHHMRDNKGR
TPLSYASEGGHETVVEMFLDRSDMEADSRDKNGRTPLSLAARNGHLAVTG
MLIEQGADVDSQDKYGQTPLRWATQNGHARIVQRLLEDGANMEIKGQMDG
CTPLLAAAHFGREDIFQLLLDKGANMEATERGGLTPLLVAANDGREAMVA
ALIAKGAKIDVKDVGGSTPLAVASARGHLGIMRLLLDNGANIDAEDEDHV

TPLHRAIMERGNDAAVELLLARGANPRSSSVVALGSDYTTEQRDRFREFV

TRSSRGLGP*

>Tr|2956_t

MSTPPKGLPGTPGGYVVFGPQANCTLDVCPIEWSVYKYRPSIAANSVFIA

LFAIAISVHIILGIRWRQWNFMGLMIFGCLVEIGGYAGRLVLYNNPFSFG

GFMDQIVLITIGPVFYTAGIYITLSKTINFLAPDISRIKPELFYWIFIPL

DIVCLILQAAGGALSTISSGSSQNGVDIAMAGLALQVAGLFFFSLLFVDY

VTRYVRKKKSAPLGQRMRIFFGFLGAAILLIFTRCIYRCYELSKGYVHSD

LITDQGLFIGLEGVLIVVAAFCLCVGHPGFIFGRGETKDSSIPTDDETAN

FESKP*

>Tr|8945_t

MESSASAAAAAAPPQPSLVTTTLLAVAIVFPILGTIAVGLRCYTRIIKKQ

KLSSDDWVCVLAQVAAWGISIDIFVAGGLAGVDYTYSTLDPLSAAVIFLR

ALWIEGFPLVFSLVFVKIAILLFYSRIFTTSKFKLAVRIYAGILSAWCVA

MIVCQLLAANPIDAAWNPLATNPLRYNYNDFSLAFAGMSIVFDVIVLLFP

IPVIRKLQMDRARKLQVLGIFWLGLFCCISSAVRFYYLYSEISRTIAATG

SDRYLNMSAAFIWGTIEPNTSIVAACLPCYAPLFAKVGGLPTLLANFGSL

FSSSRSRSRGSRAKARNGSDLLASGGSSSSGSYQLQKANGMAGPWERYDG

SLPSYNVDVERAGSFQSDQEPLKDAYPSRIKVTTDFVATAGRDPAF*

>Tr|7258_t

MDVGHHGSADLDPLPPVRYIRSRITNACDGCKARKVKCDGRLPCGFCTAR

QRAQMCHYSPQRRRRLSQRHPQPQGLSQSQSQSQSHEKLLRSPPLSSRES

RDRSVRSPSPTQTPGSRGMSSSVAVSSTSASAAASAAALSPRMGSERRES

AATQPRDASVAEDETEVPREARLLCDAHGKLTFIGDCAPLSFFQSVRRLV

TNRVGQNAFAPHTSRFSVLENAPARQSKRSLRGADMPEVRPVHVAAAVSA

YLATTAGLVDLFDSERLLDDLMLWANLAHKPGNVTTIIHFLVLAIGLQVE

DEMLSQQYFEYARDLAYTNLSDNLGVETVQAFILVTIYMLCSCQINGAFL

FFGIAARAAYSIGLHRTEVNARFGHEIHQQRDRLWKSLRVLDLFLSSSMG

RPPSTSDLDCTVPYRTIGSDGNEVLNLLNASVQILLILECIIMEIYSRKK

VSMRLTEGISLKLRDWSARWLGQLKDVIAQPALRNEAQVTGACQVLSSYY

YAVMLVSRPFLMYEVCQRLGGGSAPSNRPALASGRSKLADACIDAASLMV

DPILDLVERGVLKGKAPLLVSWLFAASLVLGVGLLGGFGRILEKYTQLSI
QTMEHFAQNDAHAKQYSLIAQSLLTTALEHLERQDLQERMRRTESSSQLF
GLGAPDARSPLNKSPAPLRDVDLRVSTPDATMARYRGSLDRPSFLQHQGL
SPGGPSPRLAEMDSVFLGLSEPMLHTPDTNAYWDGIVGNNDNDPNSALNL
FPLLEAGGGIDLAHWL*
>Tr|4933_t
MFQFFKSEFFNFEFLRVLHAAPFHGSEIGECLVARTRITDNDPESWYRAW
TAEAERAVSVGEDAVKHGDRVEASWAFIRASNYYRSSEFFLHCEPQDPRL
LNAAQKSFDIFERGVRLLDGELHTLEIPFEGNISMPARLFLPPAHKMVTD
KLPILVQMGGFDSTQEELYFYGPAGGLPRGYAVLTFDGPGQGISLRRDRT
HMVSDWERVTSKVLDFLESKLAANHNIDMDRVAVLGASLGGYLVLRAAAD
PRVRAAISTDGCYDLFDVTKSRMPNWFIGGWLNGWLSDSFFNFVVNRLAA
ANFQLRWEFGHSMWIYGVNNPADVMRQMQRFTLRLEDGGEYLSKLKCATM
ITGASDTFYFVPEINAERIFRKLDHIDNSKKELWIGKGILAAAKVNGINV
ATASDYKHMVTNKTPEFLAKFPIGKVPAFEATDGTTIAESDAIAQYVSEV
GPRSVQLLGANAPERARVRQWISFTDNEVYGNMMGVVLSRAGFAPYVPEK
EAAAAKGLSFGLGVVEKWLSGREWLATDQLSLADLTLAAALYWAYMHYLD
DKRREAYPLVTSWYLRTIGAEGVKDVFGHPNLEANIPDLQSRVSWLVADY
ADATRLVDILQGVHTVLSFIVVAQDKGNLSQRNLIDACVKAGVKRFAPSD
WAGASTNGLPWYAGKTAIEQYLKKINEAGKVLEYCCFRPGMLMNYLAFPQ
KTTKYADIWGIHIDMEHRRAIILGDAKNPGYFSMTTMEDVANIVAKAVEY
GGEWPTVGGIRGDNISQQELILLGEKIRGGRFAVETIKSVQARAGRLTAT
WHPRMEHPTVPEELRVSSAKIFTAKTIVSIYQGSWEVSDEWNQIFPDYKF
TKIREFLEKWWTDAE*
>Tr|4045_t
MADRRRINGPGGSTLPPVYDETESDAASLRQRAPDAIRPLYLKTGVTPSA
SGSAYLEIEPREGKHGSGMKLACTVHGPRSLPRSAPFSPYMVLSTHVKFA
PFATKQRRGYLRDSSEKDLSTLLETALRGAMIADRWPKSGVDVVVTIIEG
ESTRQDAVERRVEEWDAMNVLGGCITVASAAIADAGIDCVDTVSGGVAAL
VADKNGGELSMVLDPVALEHESVLAACCVAYLPNRDEVTNLWFKGQLPST
GTHNYQTLVAKAVQASRGVHGLISASLSEVIENSQG*

>Tr|7231_t

MVSFTLTALSAIAMALTTSAAPTEPNTRATINRTGSITWYNTGLGACGQT

NNDGELVAAVSASLYDREHPCGRKIRINYQGRSEVVTVVDRCAGCAENDL

DLSPTAFNGVIGDLGLGRVTASWEWA*

>Tr|1422_t

MIKRSIVTGQQARLASRNGTHTTSTSGLAPGHLQANLIILPSRYASDFRT

LCARNPVPCPLIAESAAKGCSDAFISHLKKLGGDEILGRGSDVRRDAPRY

MVYKDSKLEKSHCEEVTAEWTDDHIAFLIGCSYSFETALVEAGLAPRQLV

QGRNVPMYRTTMGLCAAGVFRGGTYVVSMRPYRKGDVERVREVTRRFGTM

HGEPVDWGWDAVRRLGIGDIDGPEWGDGPLDEGGVMGFGRARRERLLREG

RGGDGEDEDEDEDEEEEVPVFWGCGVTPQEAVMRAGLEGVVMAHAPGHML

VLDARDEDIVE*

>Tr|7114_t

MSLSEVEDRSNVSEPLEDDSVTCPTTTLSTPEREFLDTPEQVRYMQIYVE

EVAAWMDSFNKGKYFGECLPYRALDSPLLLYSLLACGIRRLALRHPDMAE

IAAAYYSTANMHLFRYHENPERDVEDCSIVAVILKLYNVMFREGPQSGGH

SLAVLNSIISESRWNTDSDGIGSACFWLNAYLDIVGSLDVGYCLYVDSWS

INMDFSSEGGEGMAYRGEELWVQRMLFILAKVISYRLCVLSFTEIDLREN

RTLEDRLPEWKHLKLLCDQWNDACPRNMRPLGYMYPDQAENNSAFPKAWL

PKREAVFCRLLYHTAQFILTQTHPLEQIMLSEEMIALQLHHAREVCGIVA

HTEDRYLGPISVQSLLIASSTLTEDGEQMEVLDILQRMKAQIEYR*

>Tr|8660_t

MARPTGLIATKGIELLTWGTPNGIKASILLEELKEAYGLEYTYQGINISQ

NIQKEPWFTAINPNGRIPAIVDHNNNDLAVFEGNAILGYLARRYDTKNLF

SFPVDSDDYTRADSWIGWQHGGIGPMQGQANHFVRFTKEKIPYAIQRYVG

ETERLYGILDSRLADRDYVVGPGRGKYSIADIALVGWVNGLPFISVSHDQ

FPNVKAWLLRLWARPAVQRGFHIPSPPFFDPRQGLNAEQAARIAEDKKIV

DAAKEQYGYKYQSP*

>Tr|6243_t

MGGGPQFPMSHQQAPADGYPAQTPTAPVPSVPMTPGSAVSPSHTGSGTAI

GELDAQGQGSSSYGQPPDLSGNDEGDIDEEPQEDCIVCDFGLPDYENTAD

AMDADSNADADADADVDADAEADADADADPDVHDASPAGAVQQESSSATA
EEDAASSAASNKPRRSPLNPDQRKQAAETRKIKACTRCRMQKMRCDPDPD
DPMGACIGCQTFSRTSKKTIHRMPCYRGKVTDAVLFRSGGLELTKRWKGT
EMRDVTDRINPKDIRTIKFTLGICEEPITVEVVAFQPQSGDVTARYWMVP
DGELGVRKKKDLAHYCLFSIQKTADYFEEYIKKHAVNVMKLERAGGKGDP
RDILERTYDYIIKRYQDLSARFELTGNEEKEFKFLGHMIILWFAMRHSTG
SSWLLGQELLGMKPETRDDTYPLFGKVSVPRMILAQFDSINHTRLLSKYG
KLVLNELESIMSRNQPQHFFLVYACMFMLLREASWISEDRYRHARNNLGQ
SLRYSIPAFVESLHESCNNLLQHWHYFNCKAWPHPSEKDERYKGPLAGMS
STEFDLLMQARTDRAVQGQLDVWRRYKADNGHVTKPAPNDVGGRPYEGRQ
VFYDWDHPFYWVSQLFEEEWHPHATYQREYIE*
>Tr|21_t
MDQTYRSLADCLLCKWEELRATVATTNGTRSPHSPPRLLVAVAGPPGSGK
TTIANKVAEIINALSPRSNNPKAIVISADGFHLPLATLRKLPNASEALAR
RGAPWTFDGHAAVSLIRKLKSDAPRRPILAPTFDHAIKDPVSDGLLIEVD
ADICILEGNYLLCDEPPWDEIANLVDEKWFVYVEPELACKRVAFRHLAAG
IETTMEKALHRARTNDLVNGEFIMSKSLGRYNVMIESIETNS*
>Tr|1578_t
MAYFPALCIALGVLMVDAVLELAFITSMVAWLHNTASGTFAVNFNGSTFD
LYGEPKHFLVDQGHSSNGAAGTAIVLIGFGGIVTLWLRSRPSILGPRFTS
LLYGIWLVILVLGLMLTVGSLGYVFSVTNAHKGQTIDVKIASTTGNHKYP
LDTWTPQNWFAAVLKLDLADDSQRSDIENHLRVMRGWQYNLIPLFLVQLT
TTVLAGLEFLERRKHRPSAGEYGSVERNSGEQKFVATP*
>Tr|7691_t
MSTDFSGKLAIVTGASKLNGIGFATAYALAKAGADIVIHYNSNKTAANEC
LAKIKEVGVKAIAVQSNAASVTFGTDIVNATIAAFPGRKIDILINNAGHV
TMQESPAKTTIEEFDALFHPNVRGPHLLTIAALPHLASPGGRIVNIGSVV
ARTATKVATLYSATKGALNTLTLAWAEELGDRGITVNVVAPGPISTDYAP
PEEAELTQKFRVMQHVKRNGTPEEVASAVLFVASPGASFMTGQVLGIDGA
SYPPGTQASPLDQRAAPVAPDGYTPARVSCPANRPTIRAATGLSRNETDW
LPLRTNNTVEALKDVLSRANISGLDTGRYIDNLTNVGTGLPRVAIAISGG

GYRALMNGAGALAAFDNRSTNATEAGHLGGLLQSATYLSGLSGGSWLVGS
LYVQNFTSVESISLATDGFLSTLWQFDNSIINGPSGQSVISYYRQLFDDV
DGKSNAGFNTTITDYWGRALSYQLVDPSDGGPAFTFSSISNDSDFVNAQA
PMPLIVAIERTSGQLQIASNSTIFEFNPWEMGSYDPGLEAFAPLQFVGSN
FTNGSIPRDGECIAGVDNAGFVMGTSSSLFNQAFLQIGRVEGVPEFLIRA
INESLANVGSENRDIANWPNPFFGYNASVNVNANTTVLALVDGGEDLQNI
PLHPLLLAEREVDVVFAVDGSADTETLWPNGTSMVATFNRSEARVSSNDS
RFPDVPDQNTFVNLGLNQRPTFFGCSNGSNTPSGPLIVYMPNAPYSFHSN
VSTFDLEYSDAERNQIIQNGYNMATMGNGTVDPNWPTCVGCAILSRSLIR
TRTAVPAKCRDCFARYCWNGTTNDSLPNTYEPAQILEGSTQQADSSSAAR
LGGTLLLAVVALMVAL*
>Tr|7382_t
MASPLRPGGGVLTGATSQEEWLCSRTTLYPSLLDEFYRLPEVSAVYTPDV
LVCKTWDEDPLALTPVNQFFIDVVTAAMLRLPDTETNQQGELVYSEQKDR
DMVLAKMRCVMRILRAKHIHRVVLGAWGCGAYGNPVEEIASAWKRVLLGP
PARRTHGAEVYDDLDIVFAINDEAMSRRFATAFGPGAVFEDGGGAGDEA*
>Tr|6265_t
MAGLPAHLTEGIPTADMSYEKRLFVFTQGAGKREPYFAAFRGLHRLNIVR
LQMELAQLSKEVNEAQELPKAKNEELTRLLHAYTNAIRDYEYLSGLVPAT
AYQAQNQRLDIEQGFIEVGDVSDDPGAYRRLPDRSILASDPLRDVLKAVL
PKNLTYTKREIHRHTSEYLKGLPPTEVSPFVDRMARFIVAFIGGAALLVP
MLIMRLPRATLAKSLVTVSVAVLLFAAFLSVFMRASNTETMISTATYAAV
LVVFVGTTS*
>Tr|7355_t
MATVKPVLRRSHTKSKLGCQACKQRHVKCDEFRPSCHRCLSSRIDCKYPA
QSESEDEDEKAASALRWPDDIEESCAQWKESGRPPFMFMASSPSWHNMPL
KDLRYIYKTALVANVLELSKTADICLVWGDMRTYFQLATHYDFVAQTMAA
AAAQRLAVTTKSYEASQDAYHYRKQALCGLYRAMSCFSKDNADAVLATSL
GCSYIMPDYRSLMALAGDISTVVARMRPWLKRSAFHRIFTYEPTYPEAEE
QASSSPPETGDMEYRFVLVKKLLSEGIGAVNGLSFCFQSDRDLSAILRQL
RDVMRLVHERLGADISPEDQFRLVYPFTSWFVKNSAASYVALSAKNPFLL

VFLLHFYSVVVALTVALPRIDVPLFAKYRMRAILEICSLLREEPGFLCQR

CNGFHAYEEIMAFPLSSVSVYQQVGRQAGGFEGIVAERFSP*

>Tr|4958_t

MDAIVISKFGGPEVLEIQKVSKPEPVNGEVLIQVFAFGVNHAEMHMRKGE

WDEWNPITGLECVGVVATCPGGEVEVGTKVAGVMGVKTELPWEYLAAIPE

VYSTAYSCLFTVLDLQPGESLLIRGSTSTIGQAALHLAVDVGARVTATTR

REARFSFVKDMGAADAKLEHKDLRSELPSGFKFDKVLNLIGNSVLLESIG

LTRAGGRMLQAGWLGGLEPVANFNPMVEMDSGVHFSLFHSKVLGSPEFPM

SNIPLQEIVTKIENGAWDARPAFVFEYKDIQKAHEMLDSHNAGGKIVVKH

*

>Tr|4776_t

MEQFILQAQHSGAINHAHTFLLPQSTAQLAREVRITVAIVAFAWVAVTAI

RQFGWRHHGPK*

>Tr|6718_t

MAKRERSPTGYGPQPEEAIPSPSRPGILTVVLHEGTGLSIPGSYKEDQRP

DQQGLSKRNTPYAVLDYDRSQQQADSDEGTAEDPVWVSDIVPWRLDEVTG

EYRDANWKFDVCRPAELAIHLYLRDPHASPCVRSQDACIGVARIPIDVAQ

ASASEETPSQWVDVEGGTGRLRVSIKYDDTKDKTLEAADFIEQSKIQKGN

SGYTARVIKKDTRQRYTTRTIPAVRRPQNADHPFIAPLTLVFQSQEGLHL

LSPTMCGGYLFQHLQNQRVFSLKRARIYAAEILCALEYLHESRGIYSWLK

PRNVLLDSLGHVVLCGSGLYDPGVEDDGRGGYGLPEYPAPEILRDQNRSG

AADWWTLGIFIYEMLTGLPPFFHENADEIRRQILSSDSIQFPDDLPPDAR

DVIAKLLDRKPDFRLGARGGASEVKEHPFFADIDCGKLKQREYTPSFKPG

FSIGDFKQHGVEGSLRPVRQDIYKERYDMQQMSLDNPEPESNFDMELDDA

AIVLDPSHEASNAEADDHQDDGWELVWEADTAGPGQLFFRHQPTGEKKPI

PTRADGPSHEVNDANTFLGSTVPSTTQKLDALEAALQAGHDHIVSQIVLE

HGIDLNIRLFGYQRISPLEWAVDHENLRLVRLLINNGADVDFPGYEARVW

GQGGPLMVRAVATKNRKLVELLLTLSKTMNKTPDRVDLTRALGLAVDQRD

AAMARILLANGARCDFEDGDRPLPENGLEDGCNFYDISEPTEFIPPLARA

VKMGNVGMVRLLLERGGADPNVGYHDLTAEEGKKDIKFRCGRVIELAMEL

KRQETVKLLLAAGADLRVERPVWRVPGHTCSEVSRAFHQSITARLRTAEE

LLKKPSYE*

>Tr|2359_t

MASDDPKFDFTRVSSPTEHLLDKTEMMRDLPRHDDPPWSPHDLHGANAGL

GLAMAPKDGYEPSSSDGTSVCTPSAVDLGHGLIEVMSTTDLDLEDVDLGL

AVQNVADDLDLEKVGVDAISMMNNLSLDAQNGRSWISAHDAGNDRKPILA

YLRGGTQPTSDGIPGYYRAQIELDDDGDEPASLRLHPMAGERMATESPHQ

CPDLVPTRNTVYSSNCVTQALASVELRIGWNDRIPRWRKGALLTYVALVE

TFPPSLAPVVEDDMKKAISRWQGIGATFKQVARDSPATFALRFDSSKCKK

GYAVSFLPDTGPAELVVHEKSRNKAEYLVNILTHEIGHILGLRHGFAHER

GREPHSVLFGSDDDRSVMLYYDHPGELQVSERDLQGLRNFYEYDDAEYDG

LKIVDIEPELHDYNETSITSHAT*

>Tr|1567_t

MKFTAVAFAALATLATASPHPPPPPPPHGCKPATYSCLPHDNGWQVCSTA

GQWVFAGNCPPKTICKFDKENGSPYCVPPGFTIP*

>Tr|9141_t

MKSLLRYIILSLISSTTALTFHHKPSSTDYHANAGVLQYVNPLIGTRGYD

PNDLGGMIPSVSPPFGMTRWTPQTRENFISQVPYNDRDRRMHGFQATHQP

AIWIGENGHVTLMPGLGDEIQPLFQHRGLAFRKEDERSTPYVYEVTLDAD

SGGEFGWNLTEQATSELLGDACPPCPGGAAFVPNKDVTEGANGRVRRDYV

FEPDNQDELALQSVFANASNHGGDTDGKGYENSLQVALSASAHVGHLRVD

FRDTKERQPYFIIQASRLNWTGHVEIHPEAQEVSGSNSQRQEYLLGPDKP

ESFRLYFVSRFSAPFTSHGVSHAGDLYRGQRYIEDKFVGAYVTFDKSVQR

VEVRTGVSYVSVEQARKNLDIDIPDGTAFETTVENVKSAWLERLGRINIS

GVNDTDADHDPRTIFYTGLFHALQYPSDYSEPTTSSEGGMRRFYSGYTDS

VHEADDSYYQSWSIWDTYRAEHSLLTIFAPERVNSMMRSLLRIFDWSGRL

PLWNNMIENNEMIATHVDAVIANALVRGFDDFDVSRAWDAVYVDAYVPPD

NDTDLLYYSREPNTPYECRAGLTSYLEHGWVDNDRWAESASRTLDYSFDD

FAAAVVAEHAGDLEHAKELRERSQNYKKLWNPETQFMQARNANGTWANES

WGWTEGDKWIYTLNVMHDVGGLASLFKGGKKAMKARMDEYFTGGHNMHSN

EPSHHAPYLYSAIGYPADAARQIRELAWENYNATASGLSGNEDLGQMSAW

YVLSALGFYPVNPASDEYVVGTPFFDEVEILLPPGPGDDGKEHTLVISAP

GAGTEGRAYVKSLKVDGEDIHRPMLKHGQVVKARRIEFEMSDEPTSWGQE

GTV*

>Tr|4996_t

MSKVIAVAGGTGSFGRTLVDELKKSPLYSVIVLARKAPEQQDEKAPVIAV

DYSNVAETAQKLASNNVEVIISTISVMDATSGAAQVNLVRAASQSGTVKR

FISSEWGAPHTPASPIYQVREDTIIELRKTNLEWTRVANGYFMDYYGYPH

VKTYLQPLFFVVDVPNKAAAIPGTGDEVLAFTYTQDVAKFTVASLSLPKW

DEVTYIYGERSTFNKLLALAEEARGTKFDVTYDSVEKLAKGEITELPSHH

EIYPVFPKAMLQGLFALFSLWIVEGRLDVPEEKSLNAKFPQIKTTKLSEI

VGAWKGH*

>Tr|2299_t

MSSLLRAAAEADLFNVSKPVTSHIAANRPSMLTMLPCCITHIVMAGAEYE

YCPFMFWYPPAVCYYKRFGPGVDEDEENEGAATDGRDIKPHGDIEEKDSN

GATQPQNSRLLSLPVEIRLRIYYWAYRMSPIQPKELAAGYPIPMLCRYVL

HPLDPDLEKKIEEEIEAEEAVIELAKPDESGPVMEGSEGSQASQGKWEKM

KAEQERKKLVKERTKERMRELNRNTPGLLSSERPLAGIPTNLLRTCRQIY

FEAREVPFAENEFVFLNWFSSGLNAASAVTKSQRPWQRLAMRYARLEIMA

EDLARKAALSKWADLCSPGRAASWTRGLRGLRVKVVGQIGRRSTDDQKDD

DEFVDGLEEAGGARRWVEGGRLAEMENLERLEIEIIKNSWSTEDKIAWCA

AVQDALREKGSNAVVAAMGRIL*

>Tr|5074_t

MSTCGYQNGDPNKFRTANPGFDCRVDTMNGLWGFCPVTVIAATDCGLAGS

CVDHGSCSRGCGKTANSKLTTFTCGPKQFCSTALLTFGVDQTYSYIGCGG

SPKTDHYLITPIASSTPTTSTAKTTSTTDQSYSPTTATESPSSSSKSTDA

SSGTTPSANASPTKDSDGGSRSANNGGSSPNNTGAIVGGVIGGIALLCIS

GLVAILLLRRNRPHGRRTATKGAQRDTHQSWFDPSPKTKHRFTGGWGPRE

LDGSQHERYPDHPIELPS*

>Tr|8352_t

MASTTKPKGFYSAGFWTEYLSTQRTKLPDLADVSNVSPCVVRFLGGNPGD

MQLQGTNTYLIGTGNSRILVDTGEGFPQWAVNVTRYLEDHDISISHVLLT

HWHADHTGGVADLLAHDPSISVHKQKPGKDQEPIANGQRFETQGATLRAI

WTPGHTADHTCFLLEEDNALFTGDNVLGHGYSVAECLDEYTKSLRLMASL

GCAIGYPGHGDVIRNLPRKMVTYIAQRDSREKQVYAALLEEASSESNRSI

GSSSRSGSSYSGSDYGSGDDEEGDEMGTAKGLSTTDISLRLYGDVSKDPV

TFESALKPLLNQVLFMLADHGRVSSKLVGPDKTRHWFVKGSTY*

>Tr|1807_t

MPRTSKRLKQSRIEKRASPSPTQHDDSTHADEHKSPAKTSPMPSDEVDAA

TINLTDDSLLDEEEMPEDGQAPANKKAAGQSYTLGGGYNALKSFNGQYYS

GMAVGGSHTWNYDGGVWHETKEEPDLWKIDYKTTKRRARKAPEKSGAPVG

TEYHWLIVAHQHVRKIDANTYETHLEGSKYKLAHKGATSNTWSIPTVKGQ

RDRQLELLEDAKRRVQGLPPVLGSEKVKVKTDEKGQQKLEDLFSKGNTGE

GGGKRKRDQP*

>Tr|7663_t

MSKNEIQQNGWTSVPLDPSILFGGEPFKNRPGPLLVADIKFPNDDPIVVK

VQSYAQERLPIQTFNHSMRVFYFASAIVRQQFPEHAENFSPSTLALVSLL

HDIGTAEENLAATHMSFEFYGAFKALNLLLELGASKDQAEAVAETIIRHQ

DLGTVGNITFLGQVIQLATIYDNASNHPYVEKITDIIHTDTLVDVTKVYP

RLQWLGCFADTIDREETLKPWCHSTHIPDFSQVIRNNTFMQPYDA*

>Tr|383_t

MSEPAADAFAWDRSDARSRQPPIVNTIRRIRQACTNCRHRKTRCSGERPR

CMNCRRVDRVCHYEPYSATNPPSSTSSAGNGAKGRSPPATGSTSTLFPRA

GVVDPELLNRINTIESQLAQLHSQGLLGFLNGENRGASGAFPDLTAASRT

YQDLSPDFTEPSPPQHQFPPSLPETTEPVSDISNAMRFNTTPPPPVMAHL

IDTYFLHVHNQPYSYFHEQSFRERLNYGLVPKCLLFAILASALKFSDSEY

FRGSRREATEAYAREAWLALLNDHLTVENNPSLPVAQASNILGVVDFTCG

RTSSGWLKIGLAVRIAQDLQLMREPSSTLSPIEQEEHRRAFWSIYLLDKL

VSVGQSRPPAISEEDIHVQLPTDEDTFRRGLWKKTPTLHQLINWKSETET

VNSTFTQTILAATALGRCVRYVLHGREIDEMPPWDPRSDFTLLNTFLLLI

DHHLQIETTTIPQIVEQHRKPDGTLDFQSLEHVVFARIVFHTTHCLLNHP

FLLRMRLKKGNCKVPPKFLTRSFEVSCTHARSISCVVEDAIASGCHVQSS

FYAYASILAGGILSLAIHIDQEKGQVTDPELLNHYQKSIQILERVGRRWD

HASKMHLQLMLFDANYHSLAALLDSTTPDVDPKLESALWLMVDYVAMSAH

NDSAITPPPLSTDTFMGPLDTQMGMENNDAFGGMAPPNANPDPNVGFLGN

SVPL*

>Tr|1832_t

MFWTVPVTLALASGASAHVAAFVKGMYCEGGPDPNNYNPNSNTPVNPLWD

LPFEQWWMQADRGCNKAPPPDGASVALPAGGQFTVELAHNQAQTTLSFNG

QFAGEWPDGQPHPENWSGPGSPPDCIQDDGAMHTNNQTMAAGTAWAISYN

DDISKVTMDNLVVFSVLEHTPWKRIATYDVPKDLPACPAGGCYCAWLWVP

NGCGEPNMYMANYRCHVTNTTSTKQLAQAKPPTWCGGDSSKCTKGAKQMI

AWNQATGNNVQVPNGASPGYNINMGWAPGAQNDIFA*

>Tr|511_t

MSPLIDDEERDAGPLAPEEEVEKADSKAGDDSSEEESDSDEEEGEVTNRG

GKPVKELLEEVLKGIQDGTRDLTNESQLKAFKASGGDGHYLASNTADARQ

PTALHIMAAMDKKELPKLDGRMEPLIRFLVEHENDLLRIQDRSGHTPLFL

AIESKKEKMVQWMCDSHPRISDILDISSNDKDKMNCLHIGVDKRVKFLDL

LIDKAEPTTLAAKDGDGNTPLHLAVEYKKCKKEQLDIIQKIIAKSDVAVQ

RTPNGDFNNDGLSPYLHHKESCRKALARAKEKERKLAKDEKAREKSARAR

PDGGDADASRPNKEAIPKPQGPAGPAAPGEQGPDPSSRPDKRPNIQIDSS

RTKYGGKTQPIVNVNSPAVATAPQLVNRDDKTPASRAADTLAVKPTADTR

SITDGGHKSRIDEATLKGVERLLKLHYLRSRSYNAAMEILYGRNTTSDYE

LYFDLSGHGNITQTGLENLLSKLRFEDILQYVAIPRLNVEVNMNTANSKR

SRASGRSHKQDGEGRRDLCYIFDRLRKKGVKTILKVIIDDSMTPAHSDEA

IEDALKFMDVEIWDWKRTDLCSEVICRVAAKAREVNLYWSGNNAVLRGWS

EEGGLKKLGELKTVYLHIQQGLESHSRTKQNVEDFCERMKKLCPDVTVHK

EWPIVQKDAMDANALAAGDQGEHSTKHEWLQCMKEFKRLLFDAERSYYDH

GNIKVDESIEEPIKVALIDDGVDVKDLEFNFIGGRTFCTRDEEHNLNDPY

YVSSTGHGTIMARQIQSMCPRAQFYILRLEDHASEEGSRQITAKSAALAI

RAAVRKKVHIISMSWTIDPPEDEEERRFLDAAIVEAANADILMFCSASDK

GAKQNLTYPSKATTKIFTIGAATASGAADSWVGNLGNINFIFPGTKVEMD

GPRSGTDTSSREVTGSSVATALAAGLAALVLYCVQVRLYLATDQEKQKAR

RDFQLLKKHEHMMKALKDIGTTEESNHKFIEVWEVFGKKVEEKERYDQDR

WLDLIADVGTILCRKIG*

>Tr|4552_t

MRQSIGTSLLYLIGGANLVLAACENYSFTTCDDGIVHWYDLKDGQICDPK

DCGGGRAPPRTDVPGCPLYSGTILSEPISYLSCFTPSNAVPVTTSTPAGS

AGTTADVVITSAISTSDAGRTTQEPASETSTVDSSVASGKPSTLITTAAS

SPASASTKTPITSPATLSTKSQATHAASNTTSGSASTTSTTNAGNAVGGS

LLAVAGVALGAFALL*

>Tr|7352_t

MATEAIPTTQLRLASPNGIVTRTILKTPLRDALPSEMPIIDISPTFSPSL

EERKAVAQQVRAAAQNMGFFYIKNHGIPADVTRDAYEACLAFFRQDKDIK

MRADSRKAKWENGYRAPNTQRLNPYEGIDVRESFSYRYDPRYDDTVGEQG

LGEIPEEVRRYLRCEEYPWEKTRNLPQLKESLTRHIGECLRLARALTRCF

ALGLGLDEGCFDGKVRYPDASYGLNYYPPLERRTGDAADNGEDDDDDDDT

TEDDGVQERVSIGSHTDFQLFTILWQDSQGGLEVLNKQGQWLRARPMEGT

LVVNLGDLMQRITNDEYVSTVHRARNRSGRERVSIPFFWGFGLHETCGVV

VGGEGEKRYGDVRCEEWVARRIRDMKSVGDMI*

>Tr|6059_t

MSRSKLPFRSSSVKAMGSSLFSRNRRDDAQKDMMKPSGEPRRRRASISVD

ERRPSELQRPPLTKDSRPHRDDLDKSVVSSSSRRRESASRSSVSPHSRQR

PSGAAGGHHSRQSRSPPRRRYSSSEVPRHHDMPQSPVPSRSQRSDSHDRE

RRERPGQHGPPVAAGALPVSAHKSEWSASSREARQEARRYPPSASSNNRP

QPTRRGSSPYHRPSAASDRHRSASVDGYNANSHPNPQHNSAQPPNPPLRR

TRHERKGSDSFFSKFPTSFATYAGIRALSEHADKAKEWVEWFNNVNDAPD

EIRALTSKISTTRDTIQQMKECIEARPDLIEDETGQVLKSHIEEAIDSTS

ETLKKMTKLLAGFSGAAKQDGTALGRLEEFWHSYNYKHEGEEQIKAADAE

LQQQLLQLGTLMSNIYARALKRPSPSSTGTATPNAPSPPQQKSRIHVNSA

PQPPVHAPMQQPSTSIPNRSPPSPELSPRTREHFEPPVNPPKAATSPPTP

PHSEPSVEQQQPPSHASTSAPTAGPDESSTLPSPKDILLDAAWEGDVRTV

ANCMRQAPPNSCDPHGLTPLHLAVERDHMAVAMYLLDHGADVHARADGGC

MPLHLAARYASAATVEMLIERGKADPNCQTTDGRTPLHYAARSAEDGDAE

RREVIRALRDLGANPTLRNRRGELPRDVAQKRDFWDAAATLSRAELKWEQ

QQQQRLREQQLQLQRQQQLQQREQREQQNRQNLPKDTNVREKGAKKEGSW

LQRYGLRK*

>Tr|3860_t

MDRADLYCVWGNTCMPCVMFGDPQQLSSTALTTFERDSDGNFLNRFAQTG

SIPALVFLQATGFPVYRLTTQLRMANGLFDATSRVIYPGVPLVYASSCDI

ANDAFSAGRALESYIQEKYPTVGVCPENKLLPVFIHTPGSRVFTYATTGA

KMSLDQVKIALDFLLDLVKSKPDIKPSQITILSPYSANVHILEKKLRGSH

KYEALQGMSPPSTVHSFQGQENDIVVVVMGTVARSLGPGCTTQKRLLNVM

MTRHRSGLVVVGNINVAGDLLDGDGNRVRAGWTSSGNKRIVTQGAHGELR

YANVSTLLKVHKAFCQTGRVIIIPVATEETDVEVVEVEAGPAPAE*

>Tr|7860_t

MPSVNGENNPPNANGEQSWILFISGPTGSGKSSVAEFLASNLKARFIEGD

DLHPKTNIDKMHRGEALTDEDRQGWLEAINEQATAFNKEQSPNHHLIITC

SALKRAHRDVLRESCIKAGYSLVHFFFLDAPEAELKRRTEARKNHFAKAN

LVHSQFEALERPRIDEYDATIISVVPPLESVQNDTLNVALQLINRR*

>Tr|9222_t

MRDAARSSNQSRGPPLSSPESSTTSPGGDAAPIRDSQPPLGTPDTRVSAA

SSSSNPSTADEEQLGVEMVEAALGHPRREGRVPYYIGEQTGGPTSTMHIC

STDHVIPKHLLMPAAKQTILTDDEKAFLRSKGVYSFLSEASADSLIRAYF

HHVHPIMPILEADKLLEYHRLKRLNEYNLILLWGLYTIASNYMPAEVYEN

EGFASRKAFRAEMFSRAVCAYHNTGEKNNLILLQGALLLGFWNSDIEDHM

QPWQWSGKAINLCQILGLHRDIDSVGYNSSITEQQRSLFRRLWWTCFWRD

RWLSVSLGRPLRINLDESDTSDPLVSDIAIDLEGVPESITSAYLPRDLPR

LAEYWIQLIRMTKLLGKTLTTCYQLRRPKPTISQIDALEAELMRLNMPDH

PDPMLSRLATFYYYHLQLHYHVEAPSDLPTYRQREWQQEMQGKAMSAASK

TCNIVYALAQGNFFSYAGPMTPTLLVPAMQVYLLNCKFGDALSRRLGLNM

LNMCMMILEELQKTYSVASVCRGIFGKAIQQLFPDDAASVSLTHSLPEQQ

QPQQQQQQQLLLQGGTSSASSSSSLAVAEQQQPLPPVTATVTDPSLQLEL

FSVNATKADFIDALTAEASIFSFVDMLNFA*

>Tr|6104_t

MSVDDSQGLLQSVLGFFVRRRPEKPAQPASLRSRRAEAWLRTEAFLQQEA

AEFEEPDPERIARTLRAFEDYVQYNSDGESDPGFRHDMPYPKLQAMRNMM

ERRSHDSIPYPGTTIRVSKRKRDSDTVIQELSNMDREARKRRVLAREEPN
AADNVEEPDADFAVLMRRHLVSLDNALRKHWVCVCQKCSGLSVRLSLPQH
KKDFNAETSFEVFFGVRSPVETALQEAKITIKDVQNRMRSASEPAISGVS
EFAHICQSITESLGQRNCLHLALEGGIFQRLRPQPKTFGGEQMSRTVSLS
ALFQRQQELRGSSSALPLKGKRILAVILATALLPFLETPWLQPSFNHSNI
LFVEPLQEGELPDITKPFLAMEHIPIVSAGRSGSGVSTDSSKHMVHPNAT
VLALGILLCELHYCTPVQLMAKDPHGPRNVNSDYYTSLDMLKSLEVDAGV
DYYLATKACLQWEYYPPGQQADFESVDVQRLFYQNVIKRLEAEIFKSWGL
RIQNLGSFDPRQNELCWGSIGQEVVRHQTGKADHSDTNNGAHPPMPYRSI
SDAAPASYSSFHSDMALRIAGPTQSSPGLQLRGHPVESSTKSLYFFDASH
QTGSEQETPLSRQWMDSLLSSIYHHVDPLDAVNAGRGTFEPVRIAILDSG
FDPENPLLMTEDHQLDPRIKDARSFVHGTAQHEIRDEIGHGTHALGLLLN
VATCAEIYIARIANRETLDRNTYDDIAKAINHAVSEWKVDIISMSFGIRE
YNEPMKTAISNALHSQTLLFAAASNDGANLGRAFPAKYPSVFCIHSTDGN
GNPSAFNPTADDKDVNFSLLGEHVSSHWPTGKNGHNEPVKAMSGTSVATP
IAAGLAAAILSFVRQQEQHMPSGSELLGPWLKDVHSMDEVLKSMVKQRRG
AGYDYITPHLLFDKRATREEVYDKIKDIRRHLYD*
>Tr|9123_t
MKLAVFSTKPYDKRYIEAARTASSTNIDIVYHEFPLEEDTVILARGADAI
CVFVNDSLTAPVVEALGREGVKAILLRCAGFNHVDLVAAEKAGIMVANVP
SYSPEAVAEFAVALIQTLNRNTHRAYNRAREGNFALNGLMGRTLFGKTVG
IIGTGKIGIATAKIMKGFGCRVVAFDPYPSKAFEGIGEYKGLDEVLAESD
VISLHCPLMDSTRKIINDEALAKMKPGALLINTSRGGLVDTKAVIRALKT
QHLGGVALDVYEGEGSLFYDDHSGEIIHDDVLMRLMTFHNVIVTGHQAFF
TEEALKEIAECTLRNIGEFVSTGTCMNSLTKDLRLARSESLPVRAV*
>Tr|8169_t
MGKEEDVIEVRRDVERDFQQDEKPDFSGGAEEVVGMQDLDPALDKKMHLV
NNIGWTNYHLKLFFLNGFGYGVDALQLSLQGIIAVQAVLEFQPSYDKGLT
IALYMGMLIGALFWGFFADIIGRKIAFNISLFICSVFTIAAGAAPNWAGL
GVLIAVAAFGAGGNLILDTAVFLEYLPSNKQWLLSLLPAWFGIGCTIAGL
VAWGFMPNFSCSDASDCTKANNSGWRYLMYTMGAFIAVLAVARVTVIRLQ

ETPKFLLGQGRDEEVVNNLQGIAKRYNRPCDLTLAQLEACGTVNSTHSKN

RFSVGEFLVHLRSLFLTKKMGFTTILLWLSWASIGLAYPLFNVFLPYYLA

SRGVQFGVTSAFETWRNYALVQVCGIFGPVLGAVMCNWKPLGRRYTMVIG

ALITMAFFFAYSQVKSQVENIAYSCVISFTLEIYYGVLYGYTAESLLSAH

RGTANGIAVAFCRLAGAFSAVVATYADPSTTGPVFVCAALYGGLALFAVL

FPFEPYGKRAA*

>Tr|8579_t

MPEMLLASELTLTKEETLREAIEEGSPNLGNRAEFQVAPRDVPSAAAEMS

APQAVSQSDGVAFAEEPIGAPQNSTGEPLDSACELQNSICESEESLFVKD

ADAMEIDLPLPPPPPARTKRKRAPVAPMAPESQHEGDEMPEHLVAALQAM

VKNADPEGFLAKQVDITALVLRLKHLQDHQPPKVSFTPVIAASRRTRLEA

ALEVVKADFCAIPPLSWATYRKKLLRFSETESVSNMSFAERLYKVTEIVY

MPEELDALPKEDFKRLEAVVRICATAAQALTVDGEDSADELGERWVRAKM

KDLEFFKKMFASIRGTFRASIGVGVVVEMPWEKKSEHDGSGQ*

>Tr|8275_t

MTSFQPLHILAFGATGNIGKHIVNQLIRANPPFPKISIFTSANTVLNKPE

LLSRWKDAGVSVIVGDITNSADVKNAYQGVDTAISCLGRGALEHQFQLIK

LADESDTVRWFFPSEYGTDPDHDPSSAHEKPHQVKRRVRKAFAEEVKNLK

PTFLVVGPYIEMWVDGGPLKDAFGGIDVEKKEAALLGDGEQPIGFTAMED

VGKAVVAALQRPEVSYGKILKIASFTKSARQVLAEFEKQVGHKFDVKHIP

LDDAKRLEKQYWDEGNPLAVVGTLRRIWATGGAVYEKLDNEDLGLHPDQL

QSLEAAVRNRLNHVAF*

>Tr|7644_t

MENLEGVSKNEPALPSPMGVGEGREDHGLILIPALEATGEKPGAGKRTCI

DVVHVMAIGSAEDRLSLETLLAGPSTISAMKRREFVFQCDMASLLVGNIT

SQCVEVLAGRLLRGLTSFDEQESRRAIVFVAYDFGDLIVKKRLEAAKRPD

LPVVLDLIANISNWPREPSAEAFDMFASHLMAVAYEPEPEPAYVLHKALR

DVADQDGLRWALSWLLHAQRPLTYGELAMALCYHQREEWETFCTPPLTAV

RRSLSRLQTWFRGITESCSGQVRFRQIVRDCIDGNWTHNCAEPAASDAIS

TFLVNFLTSSETRERLDAIYGQYESLLRASEDGITPPLVPDGQDIIFYAI

EALPHHVSRSASALRLLGDDLRSVDGKLAPWARAYWAMSNPFSRPKFGTL

KTPDQILWSLRHLDHAPKGAFLFQNALKTETADTLQSYLRMSSEDCLACG

IRAGDEDLALSLASASGVVARGHDNENEDSANSGGCPRPVWPSCLLWRAL

WLNMDRLVALFLAAGVGPDPADCTSRYFRSPAHMATQLCRQSVMDALLKA

HEARPDMKEVGGESLIFAAARSGNMDCINLLIAKDNPLLDTRGLETPLFA

ASRHGNWKVVGQLLKLGTDPNAGIGPEINDRRAPLAVAAEYGHMETARIL

LEYGADPNIRGPGNQGTPLWFAAVKAGNRKMVSLLLANGAEPGHELLASP

LLVDLIGSPVSTDDKIKVLRRLTRSRCPNLVNVADRNGMTPLLHAAAAGD

LDIVQWLLRNEADVDARDSRGRCALYYALVNKHERIVRDLLARKPQLNVS

TTDGQTLLETAMEQVSMVRLLLDAGADTELGNKRNRTALIVAVLEKKTEV

VQLLVERRANVHHRDKDGFNPILIATAHSTDADIVRILAEAGANLNDAHP

ATGDTPLHLAVAIDADIARVLLRYREAIDLEKRNAVSRTPLLAAASWNKM

ECINLLVRAGADINAQDDWGWTALSLVSTDSSTAALGVCDLLLSQPKIMI

DAKGKALGTALMMACRSLNHGMVTKLLAHGADPNVSIMGPYSTAIAAACF

PLDEMKDGNMEKTDCIIRELIDHGADVNATGGFSIFNAICAAAFSAGAST

IELLLGKGASVHIPDPLGRLPIHFAAASGIKNFEAVASVYQDDLMICDKA

GKNVLHWAAQFGHVETIKAILERIDSSGRDRKEYINRGDIDGWTPLCWAT

RPFAQDLEPNRASERGSYVETVRCLLGQGANPHVKLRMGKGAVAETYTPA

QMAELYDAEDEVVSLLERNAASSESSWSAASGNTSNSVHPYVPRDWFCDI

CQTTIFGHSFRCQCCYDFDVCKKCFGNIDIYHARVVSDDCGEHLFEIREG

CEQEFEGCMVTPSFSNSHAGSKFADELEDGGPEGYLLGDWESVEMPVIED

Y*

>Tr|3391_t

MGTTTSGCCFPLRGSQAPTGPEVQLCENCKRLVLTEKEVIHMARTGMWEI

EGYEMTDSSPDFPKLKESAENGCDFCLFLRNTLLSPEVDFDADEIPRIRK

TYRDAGHQLLFHRAGYRMSIYDEGKLWLSFEIWIVHGENDTCLIFTVEDA

TGDMELLPSLSLQDPPKPRVAPSQNALQSTTNLFLRQAIERCDAYHDKCK

VGRDPAFLPTRLVDVRDENAVRLVEGFTIVGGDDASSSSAPKYAALSYCW

GPRAHADQQLKTTDENIGQHLFEIPMKGMPAVLKDAIRATRELGIPFIWI

DCLCIIQGNLQDWEKEAGSMHKVYGSAYITLCPTMSKSCLNGFLFRDPPS

IVFPYHSLNGQPAGMYRIRFTRSWIWKMYLTVPHHPFAVDLISGSWVRRG

WTFQEAALSSRMVLFGASATHFHCRTSRVTEGEDTETSVSLDDLGLFPLL

PSTLSGDKLDEAWKHIVASYSQRNFTKHTDAIAALAGLAHRFHSLREKDE

YCSGMWLSQLHEQLLWRRAMYEGISWGWFFNLLDPELPERVLLHAPSWSW

IQRINIDYQYIRDSVLPTEPEDDGSDGHGDMSITPSPGLTDETLRLEWRL

PGKSGDESSVVDRFMQFRGQELEITGFVARFPLANADKPLEVDELREADT

NLFWKWTISGQYFCHTNWDFLSYTSWDRIDKGEFQHLRMLLLSSGRGKSS

GEVSPGNAASSDGNISENGDVEEDDRTAFGLFLFPTIKESENGKFYRVGT

WHSFCNGKGGFVRFLQYATVESLVLV*

>Tr|7609_t

MSYQRPLSGGSIPRETGADSRLARTYSSSPPTPRIQSLIDEVTAHGYVVI

RNAFSPASVAAAKAEVARLSADPETAGPAGAKGRNTFEGFRTQRLYALAG

KSRVFDEFALHEDVLALNDWFMDPGYLINSFQSINILPGEKAQTMHYDHG

YVSLERPHRPLGTGVMIALDDYTATNGATVIVPDSHLWDSRRVPKPEEAI

PVIMPAGSILYFVSLLWHGGGANRSDKSRLALTAQYCQPWIRPMENLILA

VDWEKLDEMPPRLVDMLGYKVGNPFLGFVDGKSPRWAVARLLKKYRGEKK

EEKEKAKL*

>Tr|9263_t

MSSESDLETWKKLTYIDELPQDIGLFRKLLEQYSRVPPNEIDELLFNTRK

KLWEVAMYPCIGRWSFLNLRSMHDHHFLTAFERLKNSTRPGSASGSTDAL

LDIGCCIGQVLRKLVQDGIEPTRLFGTDLHPEFITIGKELFNDAGNGLTL

VAGDMLNPEDKALTELNGKITLVHAANFFHLFKWEEQVKVGTRITRFLQP

GTVDAMIFGRQIGTHKPREREGKLGSFLHNQESLQKLWDEIGTTTGTRWR

VEVGFNEERQVSIPGFGEEDRYIRFGIYQLPPQ*

>Tr|2731_t

MAVQPRTNTSLEELARSVEHHHEQYLRSLHGFHDVLNRYRRERETSDGGR

TTVQGLATPPLRAFTFASDANSILQVLPRFRRDTPDTHDRPSCYPSPKFL

PLTPNLQGSDAVPDDEIPFIPLLEQSPSVHGGDSRHLRSDTATAATIAVS

AAQSHVHQAITPMSFSDDMLFRHLRDSEFCPEFSSLLVDEDEQPLPPWEI

DSAQAFREAAAAEWERFGNSTFEVYEVGEDGRAAKLNANARVEVDGQCFV

KYGVEELVEPPDGIVEAPTVWETIRHVNTSGKAVGRITIVQEPTPLMLAA

LHLTMSPHFDMTELLHHLLSDTPNRGRTHAFMHRAYERTPSSQAYPSSPT

MPSHGVAPPVSFAHLRQRSFFFVFKYYTLLGPGLDPAPWQLYEKRPTDKR

LDDHIDIAECGSVLALSLGGEPTKTLKMRPRRERAKEGFLFDTFAPWQLL

SIQSFPDDEHTVRGDDFQLKSFCSGPYAFLELLIAEYRDAGKRNQVLHER

ITKLITPPTEFMFDHHLRDKLLFEDKHFTYIRRYFWAYNTLAVINTGIKA

MVAAYVDTFTDDVWAGAHPLLWPFPSSSQQSAEATAAAAAAADYAARMAV

LRRELDKVVSDLGEVLKRNERTRKEIENLRDQLFSGSSIKESRRAIDQGD

NIRILTMISMLFLPLTFVTSVFGITEFTIPVTDWRFPLTMVLVCVPFMIL

LYLIQTRPFIQTIQALHRAANSLALLPAKLLRLLLLRCREPDPSSSSSSA

QDSRSGSASLSPKGRKRRLRLRRHGGSAAAGGGGSSGGGSSWRRPWGWLR

KRRAGGEENDLGRV*

>Tr|6321_t

MPSTTTHTDFGPHTTALTVAAAFPSSISGKAILITGVNLKGLGYATAEAF

ASQNAALIIIAGRTPSKLQECADALAKQYPNTKTRLLELDLGTQKSVREA

AARVNSWDDVPTIDIVVNNAGIMNVPTRQLNEDGIERHFATNHIGHFLFT

SLIMSKIIAAAQKPGAVKGATRVVNVSSTGANYSPIRWSDINWEKPTGTL

PLVEQPDYAGLYRLAKLEEEAVKAESYIHQGAYGQSKTANSLFSLGLNER

LYEKYGILSFAVHPGVIITELSREVPHDQFQAGIEMLKSRGFVFKELGAG

ASTSLVAAVDPALTLPAKRPLQNEADLAKTGPEVKGEDGEWDGRGVFLSD

CQILENTKIWYTSWSQAEKLWAKSEELVGEKFVF*

>Tr|3574_t

MAKLSTLRLASLLSLVSVQVSASVHLLESLEKLPHGWKAAETPSPSSQIV

LQVALTQQNIDQLESRLAAVSTPTSSTYGKYLDVDEINSIFAPSDASSSA

VESWLQSHGVTSYTKQGSSIWFQTNISTANAMLSTNFHTYSDLTGAKKVR

TLKYSIPESLIGHVDLISPTTYFGTTKAMRKLKSSGVSPAADALAARQEP

SSCKGTLVFEGETFNVFQPDCLRTEYSVDGYTPSVKSGSRIGFGSFLNES

ASFADQALFEKHFNIPSQNFSVVLINGGTDLPQPPSDANDGEANLDAQTI

LTIAHPLPITEFITAGSPPYFPDPVEPAGTPNENEPYLQYYEFLLSKSNA

EIPQVITNSYGDEEQTVPRSYAVRVCNLIGLLGLRGISVLHSSGDEGVGA

SCVATNSTTPQFNPIFPATCPYVTSVGGTVSFNPEVAWAGSSGGFSYYFS

RPWYQQEAVGTYLEKYVSAETKKYYGPYVDFSGRGFPDVAAHSVSPDYPV

FQGGELTPSGGTSAASPVVAAIVALLNDARLREGKPTLGFLNPLIYLHAS

KGFTDITSGQSEGCNGNNTQTGSPLPGAGFIAGAHWNATKGWDPTTGFGV

PNLKKLLALVRF*

>Tr|5040_t

MDRLPAELMMKIVGDSQISHSDLRRLRLVSKHVTATATSRLFTRIYISKL

HSDRDAFFNIAAQPHLAVAVRSVTWLELAEDDTDLPYVANPPVRWLRLPQ

DQLPSAGVADDLDFLLCRLPRMARALFWLPFEDFSSYFTGTEKRMQAISS

FMPDFFAALDRMLNLREFASRPMHPKRELIQPLESFTSVSPTVLHEAEDE

LEEAIWLLKEAHAMSVIRRQSMFDGAFELGQAIAKRTVYLYTHFYAPSLL

RISCFDRHDHVLQPVVEYKTVKNCDDATYNSDQVDDDEIDTEEAAEADDE

LAKAPPGLLGNHDASHSNDSSTDSGWHIETAAEASIHKMLATAPRWELGI

GRDDTACCWKVTVAHSSHDSYPTRVWRFLHRNGEQAMGDEPLEFWDDWEG

SQSGDCYEPTPFGPELDSFMRKHGLQGVPEIPQDWEAREMSARLKEPC*

>Tr|7831_t

MLFKAAAFSSLLAVASARIAGIAVPETVAPGSTIKGLILTENYIQTVYDV

AVAWGFAHGEGYPQSLGQVLDSSYLGPNLSNTVNNITQYLTIPASAEKGE

ALISASVFSLYGAVYGPTLTNFNVSITVGDSTSSTYKSSTF*

>Tr|1127_t

MARHSIQLDKGWTFRQHQGSSPEWLPVEKVPTQVHMDLLANKQIPDPFVD

LNERAVQWIGYKDWEYQVTFTPEAAQVEDATRDLVFNGLDTFATVYLNEA

KILEAENMFVSYRVNVTDRIKASSENTLRIVFHSAIVRGEELIKEHPEHN

FLVRQTERSRVPVRKAQYNWGWDWGPILMTAGPWKPVALETYVARIDDVW

AQSDVSQDLKTVSGIIFARVAGRPSQDDQVSLTLSLDGKAVFQQTVDVAS

AKDGLIKVPFKLEDPKLWYPRGYGSQPRYQLNADLARKASDASQIDSLSK

LVGFRRAELVQEPDAFGKSFYFRINNVDVFAGGSCWIPADSYLAGVPPER

YHAWAKLIADGNQVMLRVWGGGVYEEDALIEACDELGILVFHDFQFACAS

YPAYPSYLENLEVEARQQIRRLRTHPSVIIWAGNNEDYQVQERYKLDYEF

ENKDPESWLKSSFPARYIYEHFLPKLVEEEDPGKIYHPSSPWGDGKPTAD

PTVGDIHQWNIWHGTMNKYQEAVNMGGRFVSEFGMEAYPHLSTTRRMASD

PAQLYPGSMVLDAHNKAIGHERRMMSYVVDNFRPRHDLGGYTHLTQVVQS

ETMRAAYKAWRRQWGKPGARRCGGALVWQLNDCWPTMSWAVVDYRLVKKP

AYYAIARALRRVDVGVCRTWHDWTQTGAWVDENSGLVTGQVDHTLAAREG

TFDVWVVSSDTQPVALDLVVRFISVRTGRDVVDPILHSRVVAAANSATDI

LQGKTLPPSIPNPEDITKPFPLAEYDPYVVHATITDAATGTVIAADTAWP

EPIKYLDLSDRGIAFEVSSAGDEVVVSAEKPVKGFVFEEVEGLELSDNGF

DVVPGEKQLVKVGGALKAGELLWTCIGADSASLKIEASSSLAPR*

>Tr|2907_t

MAFTNQPGTLLGPLTTTWTMPESCSVFMPPCSTCDQGFRGQSCNAVSGGR

VQDNTACWPPVKKGVASPTWPFVGWGFYSPGLACPAGYTTACTAVYGQRP

EWDTQFTLVSSETAVGCCPTGFSCANNNGNTCIATATSTAIPTAFCDGTD

LTDSATETFPTVVTITQTDTASGEVQVSTSTRTMMLFAPMFQLNFQASDL

PASTTASTTRPSSTPSSTSSGEQSTTSAADNNDGSTSKSSGGLSSGARVG

LGVGIGLGIAFLLAFSGFLYYYRRSRRVQLPLGSELPDTEISEAAEGPKV

VKSPAQVYEMGDYHAPAELPGDGHGGDQGGHGAARYV*

>Tr|449_t

MRFLHLLASTAALASAASVADLNSNYNKPDDKKVDQFDKPNFEEPKVEGP

KVEGPKVEGPKFVPIKADEHRDGNANNANNNNNNKGADFHNGPENDRKDG

FKQDDHKGGFQQDDHKDVKQDDRKGGFQQDDHKDVKQDDRKGGFQQDDHK

DDHKDDHKGGFQQDDHKDDFKKEGEHKDAYKPEPKKDEHKDENKWDEHKG

EHKDEHKGAYKPEPKKEEHKEEHKEEHKEEEHKWEAPKKEEEHHKEEHKE

EEHKWEAPKKEEEHKEAPKKEEEHKWEAPKQEEHKEEHKEEHKWEDHTVY

TTVITTDYTTYCPSPTVIPIGSITYTVTEPTTLTIKNCPCTIPVPPPPPP

PPPHTTPPPPPPPVTTVVPPPVTSVAPPPPPPPPPPPPPPHTIVPPPNS

TVIPPPPPPPPHTVVPPPPPPPPPPHTVVPPPPPPPPPVSSVAPPPPPP

PPVSSIAPPPPPPPATGTTPLVVPTAAADKTKAGIGALVIAGIAAMAL*

>Tr|7277_t

MADEMSKSHDPNAEIEAIGIDNERNERSLLRKLDRRLLPAVGVLYLLSFL

DRSNVGNARIEGMVNDLHMSNQYLTGLTLYFIGYVLFEIPCNVILKRTSP

RLWLPTLTIAWGIVATLLGVVQNLQGFFVARFFLGVTESGLFPGVVFYLS

MWYKRRERQYRISLFFSAASLAGAFGGILAYGIGKMAGVVWHNGWRWIFI

LEGIATVVVATIAYWFIENYPDTAKFLTKPERELIQTRLAADSDAMIKEE

FNWAAVLEAIRDPNCWLYSLGFHTMSLPLYTLSLFLPTIISNLGYTAAKA

QLLTIPPYALAFVTTVAVAVASEKLGKRAVFLAGSSLIAAIGYIILLANT

DPVARPGVSYVGTFLAAAGIYPSTALVLSWPAINVSGQTKRAIANAMQIS

IGNLGAVMGTQLYRANDGPRFVVGHSVALAYLCANAVVACYIGWRLKSEN
KARADISPEVENVGDAVDWQGDKDPRWRFSY*
>Tr|2054_t
MAKRSCDFCVQRKTRCDNGRPCRKCLDAQPPLECTYLKPVLKRGPKTSKL
TQRCNWKWRVDAQKLASDQVNPNLAGVDVHDASTAFEPARVPLSSLRQII
EAYRSRMYPVWPVIDVQGLLKRLEESETNESPVSDAYTMALALCAATMAQ
LNLTAIGDAQPRVTSEYLERECNRCRNLTNYREHPSIEGALTSFFLHVYH
AKADNRNASMLYLQEGISIARLLHLDRIEVETCQSSHDGINAQGAVTSHK
RLVYILLWVSERGYAIQHDLPISILETVQIPTANLSDFDKYGRGLIELAT
LFAAFDAFFYRTARYMYESYQAHAEQDRLIAVQRSLEAIAPKAPDYDLVQ
RADYNITKHWMRILIWQQAMSRSLLSSCANDDSMTFLFPSRIAQDFLTSI
TMNSKEHLVSLGRDQLIKSFEITNTLADVILCTTNAYDSPHIHDSYNSRR
GSYFATREYLECGPADFLHAMYQTISPFLTYDKRLYDMIRLKAADALLRS
PSRIIPRGVDGVSQEVAVRVSEDESHGMLVQDTWSTDLDGLGLDMDLLND
AEVGEVCAAQQLPDTL*
>Tr|3765_t
MPSRKVQAPSPAPTSPSELSQARDSWSQSPDWDDPRDCGHWNKEDESVSM
ENVPEVIPGKYLRGTVIGQGGWSTVIKVKRTEDGKLFAGKKSQTPEQLYK
ESKRLRKWEHPNIVKFMELYKQDSDEENNLLIMELCAKGTLQMRIEWYFE
PMGMLDIVSAIVQVADALVYMHGKGFFHADIKPRNIFVRRFDPIDVALGD
CSDCKVHGKYEGPRTGTSDFWSPDMVAYNRHSGKSDDIWALGVTLLAMMG
QKPRKDKTDVAKFQYPSRCANYAQELTRLNPGNALVELVSKMLIKRARER
PTAEKCLDMATRIMEGMSEDEKMAGRANGLGLTSPEKHRPVEAPQVYHW*
>Tr|1922_t
MSRYISLSTITTALTILPRIQAILSSPPLIINTWSGPFTAATDAAFFALT
SSLSSSSLALDAAQAGCQACQSNQCDTTVGFGGSPDESCETTLDALIMDG
QTLNAGAVANLRRVKDAVGVARHVLEHTRHTLLAGEQATQFAVENGFAEE
SLSTEDSLRVCHEWRANNCQPNYRSSVLPDPLSSCGPYIPNINTKTNVLA
DNNQHLTSQSTDINDPSHDTLSLIALSAAGQLATCTTTNGASHKIPGRVG
DGPIPGSGSYADSQAGACAATGDGDLMMRLLPCYQAVESLRRGMTPLEAA
QDAVRRIVARFPDARTGIVVVDLKGEHAGAGSGWEFTYSYRGGGMERAEV

VVVEALDKEADERLEL*

>Tr|6371_t

MPSATPNKPPSSSPLPPHDIESQQTTTTTTTTVTTPLLSRNASPSPSPSW

SPPPGFIPIQLAIMTNVFLNGFDGTITAATYAVIGSEFDAANSASWLTTA

YLVTAAAFQPLYGRVSDIFGRRACFFVSTAVFAAGCVGCGLAGDIVLLNW

MRALTGVGGAGLMTMATIINSDMIPFHRRGMYQAMQNGVFGFGAICGASF

GGTIADSLGWRWCFLLQVPLSMFALVAGHVVLKDEHLGATATPSSSSFSS

SSSSSSSSAAAAAAAATFTDASSSSSSSFSAMWKRVDFSGALLLVLAVST

QLLGLSLGGNELPWDSPWVLGALATSVVLFAVFVRVEERTAAMPIIPLRM

LRGRMPVATQVANVCAGMSSYGYLFMLPLFFQSVLQDSATKAGVRLAVPS

LAIPLGGLVAGTVMSRWGKLIPLMRAGLVLMTLGQALVSSWAFADARWKY

VVYIFPSHLGTGIVYPAILFISIASHAHSDHAVSASTTYLIRSLGSVWGV

SVASAIVQNTLSVRLPQVLSEVPDKWRVIDEIRHSVEALRTLPPDVQLRA

RLVYFDGLKLAFGATTAVAAVGVVAAFVANPANLRKTHAGR*

>Tr|1123_t

MAGSVVNEAGYTPLYAAADSRLPDEAGIRAFLSDFYRISDRPEENERWVE

QFTADARVAIGSGKATGTEELRTMREGMWSVVAKRKHTVVKVFPGRFDDD

GDDGRAAEGDGRCELMLYGDVAYTTKDGGSSTVAWAGHGILRKVRVDGEE

EVWKFAEYRVYMLK*

>Tr|2962_t

MGSANAVINYGEESLPLDMTVLGLNSGTSMDGIDCALCRFRQASPSEPMH

FQLIKYDEIPLEPKIKKRVMDMILHNTTTPEELSEVNVILGETFAEAVKA

FSDKHGIPLSSIDAIGSHGQTIWLESMPQGGRKKSALTMAEGTFIASITG

ITTVTDFRVSDQAAGRQGAPLIAFFDSLVLHHPTKLRACQNIGGIANVCF

IPPDVDGRLNDEFFDFDTGPGNVFIDAAVRYFTNGEREYDRDGEMGKAGR

VNQAMVDDFLQTHFYFALEPPKTTGREVFRDTIAHELIERGIASGMSPND

VVATITRITAQAIVDHYRRYMPTQYGPLAEVYMCGGGAKNPNILDHLKAS

FPDTKFVMLDEAGIPADAKEAITFAWQGMEAVVGRSIPVPSRVETRRGYV

LGKVSPGLNYRRVMKKGMAFGGGLEQLDPVRELVNYVDGSVYNNKV*

>Tr|1638_t

MAAPLIWINAFPGTGKLTIAKAIKALDESVTVVDNHQLIDPVASRISRDD

PRYQAERKRERERAFEKYVYDPATASKTVVFTDFQSDNELGRSVAKEYLM

AAKKAKRLFMPVYLGCDVDVNLKRAASQDRVSGTSTKLTDIALLRDLRSR

CKLFEFDDQDFSTWIDTTNKEPEGVAKMILGRSRVISEGR*

>Tr|1330_t

MADMSPHSAPPQHQGITHIQASLSNDLLTHSSDAPRLQHSDYTIGWICAL

HIEMAAARAMLDCVHKPLPVGAKDSNAYILGSIGPHNIVTACLPVAQYGT

NNAANVASNMNRSFPSIRIRLMVGVGGGVPSQHADVRLGDVVVGCRIMQY

DMGKVVRGGETLPTGIPRITPPDHSTHVASLRAIHELKPSRVPAILQEMH

QRHPGMTKYACPASPDRLFQAAYEHLPGAFDCSTCSEDSLVARPPRLSRD

PMIHHGGIASGNQVMKHAETRDKIAQAFDVICFEMEAAGLMDYCPCLVIR

GICDYADSHKNKQWQEYAAAAAAAYARELLEEMPPFETNSYVSGGALHVG

DTDVTSKVGQDNFVTRRQELLDSLRFDQINSRQSNIETAHEETCNWFLEH

PTYLDWLHAGGSPTHYGILWLAGKPGAGKSTIMKFLYTHLKQAPQDSSSP

SSSSTSAIISFFFHARGELLEKSTTGMYRSLLLQLLETFPDLQSVLDDFS

LVPKDKHTCPPMNIIRKLFRNAVLLLNTRTLTCVIDALDESDEQQVRDMV

SYFEDLGREAIKHNIQLRICFSSRHYPYISVPHALRLTLEDQTGHGQDME

RYVKSRFKPSPDLFGDVQAQILQKAAGVFMWVVLVVSILNTEFDRGRIFA

VRKRLQELPVGLSNLFKNILRRDDDNMQELLLCLQWILYAKRPLKPEEFY

VALLSGSPDDDALSECDPSHLTSDILEKFVISSSKGLAEVTKSAHPTVQF

IHESIPDFLLKDRGLLELWPDDPSTNIEASSHNTLK*

>Tr|3369_t

MALSSSVKALGVSILARGHSDVLAVAIMCLMISELILPTAVSSSAAHAAG

LSDLIQLHSPEAYSSGPSHRIFIGLRPAIIIHSIRNKHPTFLALPSWRTV

PFQYVQANSFHALMTEATVIPSILVDIDRLKYGLPGAKAPLAILGDLRDL

LEALANWNVSSPGLTNKPSFRVLGKIPNKAHLWFPDITTANAMTHYWTFW

IMCVIYIRKLRKNHPELLDEDLLINGEGPESPLITEMAIQMSTWVFQSIE

YLMQDEMRLFGAISTMLPTRIAYQFLRCNHFYDRELLSWCERVIDGIRDR

GYDYIAQYILDDDGV*

>Tr|4826_t

MALTFQQVKLVRDTIPALTEHGERITTTFYRNMLADHPELHNYFNTVNQA

NGRQPRALTAIILSFANNINHITELIPKMERMCQKHCSLGIQPEHYDIVG

KYLIAAFGEVLGPAMTPSVQIAWNKAYWLLAKMLIGREAQLYHDWQHWQG

WRRFRIEQKVEESDDIFSFYLVPEDGKQLPSFLPGQYVSVRVQVPNKDYK

QLRQYSLSEQPRPDYYRITVKRDRGSQTVRPPSYLTDSFGENPGVVSNVL

IDQKKAGDIIELTHPAGEFFLDTYNTSNAPLVLISAGVGVTPMVSILNSV

IEKQPNRQISWVQGSRDSIPFFLHIRKIARDKPNVKTNIFKTRLAESDLA

GVTYDHDARIDLDKVKAEDLWLGNASTEYFICGPEQFMMEMAKRLREFGV

GSERIKLELFSTGDTEFQVDALSIASGRSTGVTPCSSSN*

>Tr|3548_t

MFTTITAILAASAATVSAVAVPRGAGKSGSASVTPHEQYSSSIGVLGCLI

NTNRVAYWPMGVDCDNICVKVTNGDRSLHLLKIDQSGGAYDISYDAWNYL

GFGEGAQQDPHTGGGIDMTYEFVDASECSDLLHDGKLPLSASNSMNYVAS

CMSEPNSWVNKNHELINMADPLCKYGYDEVCTLDLSKSNQPSCPHTLGAQ

VAYSGKGVTNIAYGTGKTEAAQ*

>Tr|2201_t

MAPIRTAIIGLSGNAITSWASAAHLPYLLSARGREKYQIVALCNSSVEAA

KRAIEVFKLPPETKAYGDPQSVADDKDIDLVVVSTRVDVHHSSALPSVKA

GKDVFVEWPLAQDVAHAKELVDAARAAGGRTLVGHQGREAPPVVKVRQLI

EQGTIGKILSSEVRAYGGAVDRESLRPGLKYFLEKKVGGNIVTIGVGHLF

DQIQHVIGDLQNIQPHLQLQRPNVLLRDSATKEVVEVVKSDVPDLIIFNG

NLAESQKAVEGANVLFRFRRGQAFPGEPGLTWAINGEKGEIRLQAFSGAT

LHANSYDAPVTIEVHDFATDKVEKVDWSWQEWQEALPVVGRSIAVLYDKF

ADGPFEGQPTFETALVRHEQLETILAGWDPK*

>Tr|1580_t

MLPTKGPVKSRSKRGCWTCRIRHRKCDEGLPMCKECENRNIQCHGYGPKP

SWLDNEEQVRAELSMVKRAVKQNARLLNKGPVRVAGPDPQLRAISSKEEQ

EQQQQDTAASKPKETLWAPCPTRELRYREAELIMLYLDYIFYIQYPYYTD

NAECGGRGWLFWLLLSNRPLRQASLTLAALYQRTKFARGTEFMEAELIEY

HTTALSAMRHALSEQESDLALDAGEKLVNFISSGCCLISFEVLQGGSLNW

QTHLDALTTVVSKIKLPPFDDVDYLPSTQQDPGDIAAVRRGLKYARRFEI

TKLLWFEILSTASTGVPPRMPYRPWLDSQEILMADYVGCHNWVMRAIGDL

SMIQFGGDCPGIDQRRETLLGLEKMLREGIERLRAGSKRPGAPVSYYISR

VFATGALVLLHFILSSTHPAEADIYGAVGFVMGELRQAPAHVSLRGVVWT

ICIAGSLAQPDQQPFFEHLMTRILNKGNKSHPNFGNCESVQRILRQCWTK

RHEDPLGDWTWRDAMAQLGDFPLLV*

>Tr|6231_t

MAVISKPVIDITSYKGNIINGQFVPTAKTRHSIDPATEEPLPPAPLATKQ

DLDAAVKHAHEAFKTWSQTTFDHRAQLMLQYADAIEANRADLEKLNTMES

GKPLLVSKTEFDFTIRWLRTFATMQLKDEVIEDDDERTVYGTYRPLGVCG

AIVPWNWPVLLGLGKIGPALMTGNAVIMKPSPYTPYTDLKLCELSMAIFP

PGVFQALSGDDALGPWMTEHPDINLITFTGSVRTGKLVAASCAKTLKRCI

LELGGNDALIVCEDVDIAKCLPKIATMTFINSGQICMLAKRIYVHEAIYD

EFRAALVEFTKEHVKTGGGFEPGVLVGPIQNKMQFDLVKDMYRDVKDSGY

QLALDGRILDSGKGYFAEPTIVDNPPDDSRIVVEEPFGPIVPLLKWSSDD

EVVDRANSLNHGLGASVWCRDLVRAERLARRLSAGSVWVNSHFDVAPNVP

FGGTKESGIGREWGLEGFKQFTDHVSLWVWKKIFD*

>Tr|1307_t

MGWASVVLAAGVLCLVLYALRLNWVLSHTPQEAAACAGEPLTKEYIQDAF

ERVKRDGIDWVGKLPPRKDRRYIVVGGSGLLGGQIILHLLAMGTPPEAIR

ILDIRPPFTFSKDFSHEPASKVAFVQTDITDEAATLASYKVPWPNEAVAD

LPLTVFHTAGVIRPFERHAMFYERSARVNVTGALHSLQAAKKVGAGIFVY

TSSSNAALKQVKWLFAPWGSNQRKQQQQQQQPQGFVQLLDNADFFEPLRP

PSGFPTNYARSKAEAERLICCADERGAGAGMRTGAIRPGNAVYGHVRDNV

VGRMLGLKYCPTWVAGNVQSWCHVQNASLAHLLYEDALLGEHADKVAGRP

YIVTDRGTPVRFDDVYRILDVASTTGLRVSYPPPVLMLLVCYVVEWYALV

VNLVPALQRWAPAPSPAADPGVTATIITTPYKHLER*

>Tr|2025_t

MALPLQTRNESAPVMPVTQAKSVATVVLDAASAKREASTAPSPEGPDLVS

HPISGKDQQHLPCARAMDRAHQRVHGPPRGLRSLPDASVSQELLQQLDPS

SASHEGLQLVLEAVSRPSSSTSSSRPQRQPSPEVKKWSAACIDAYFGRFH

ERWPVLHAPTFEKEVVSARLRSTVLLIGSWLRHENEGNNHLIFDIHSILV

KRLLDELTDNVYDPAGVWPTRTLQSSLLNIIFAFESGRDKHMKKARLLFS

LLVTVFRHLGIFTTEAIDHQIGIHFSGDFPPWKFSMKEKWKSMFKLDSYI

SLLTKQPPLLQREELSLSLTSTFGQWNAHGLDVFFRRWPSEPLERTQYRI

CDLALGSQQPIPPVILIEDLQMRMMGVTNYVWILNKMRGSHNPVLCISAD

QNELISARLKRCKLQLDSMTSLWKEPEQHKMHIEFLLRAYSAKEEPFHQG

WEVAARNRFSSFVFSTTMLYHLLNMHIYADVHSYMQNLPVLSPAGTSTAN

LSSLTAAVNNAQVQEWASSPDARVAISHALFAYRVYGSTTSSPSDLKAEV

IDPIAHMTIAAGAAILWTWIQNSTSACTCMSTMPLPGELDFGFVPLGAGQ

SPEVDHWILTGGNVWLHGVHLCKCNVDVWLSPFAAILSHGAKMWEAGNVF

AEKLWSQLGLQRSV*

>Tr|501_t

MSWGPVPSKLYLLSQLQDVPVGDKVRFLGCFNNSVISYDTATACLELGHM

YPPDTNETVRVDIELVLETIQPGLTQVGQWVNVVGYIREGRGSPVHVQAL

LVWLTGPLDLGRYEKSLQDQMMERA*

>Tr|6224_t

MCQYSFVHYHHQLPCTHPASLVPRYSYCAAAAINPATNQYSPCAATTVFA

SPEDTGEDPCARGNCLISPACRSGTCRLQDLNGRWVCCKCKRGGNVYRSC

HHRKKGCPDTFCYHDICDKCTPDV*

>Tr|2211_t

MGSSGNPPGENSKRNSTLMDLKNFQFTFDCSDFIEPDSAFERKDDTISLS

ESVLDFPREFGRTYHAYRAGSYAFPNDIPEQERLAFQGPIIKRLFGDRLY

FAPLSQSNPPRFILDVATGVGDWAIEMGDMFPTSEVIGTDLSPIQPAMVP

PNVKFYVDDSSDEWNFTDKFGYIHTRLTAGCWSNFETEVAKQAFEALEPG

GWFESQEVEGVFSCDDGTLDPAGPMCTWLNEMRDAAEIMERPAVLGSILK

EVFERVGFVDVQDRIFKIPTNEWPRDERLKEIGRLWGENFSQGLNGFSIQ

LMNRVFGRSPAEIEIHAYMPVFVVWGRKPYVGEQVNTPMTE*

>Tr|4854_t

MTSWAIVGAARGIGFEYVKQLSANPNNTVFALIRSQATAGPVNELAAKRS

NIKVIETDVSSLDKLNQTAESISEVTSGKLDVLIYNAYSAGTDEGKLLPP

TAFHGKEDLFKREVNESLSVNLFQVVNTVSAFLPLIEKGQQKKIIYISSG

IGDIPTTRATELPNLLGYAISKAAGNILMAKYAVDLKAKGILTLSLSPGW

VETDASRELSNSPEVFQWMLSSFQKLDPTVKGMISTEESVSAQLSVIDSL

DEKLSGAFVSHRGNDRDWF*

>Tr|151_t

MPLSNKKAALAAEQQNLSSTGATHAGAAPPANTNMNMNNPNANANLPGPA

PFTAGPHRHDIMNKLDPTVDSGTGGMQVLASNAHTNNNAATGISGMPGQP

AAAGGMTTTGLGSNNPYNNTSSSTTAPPHNSRLANALDPRVDSTQPSSTN

YYTKGTNVVPGAGATPRHVPEGTYGPHSSRMANAADPRVDSDADRHRMGG

VGAGSVGGAAAGSAPNVKPIYPPGVGGPAPTTAGPHRHDILNKLDPTVDS

RKVANVDSTGHRVV*

>Tr|1825_t

MKSIVIILFGLALADGAAIHHHSLPTVDLGYQVHRAISFDEHYKTYNFTN

IPYAEPPLGPLRFAAPVSPRGRKSAVQDGRVGKVCPQANPDWLALGEMFT

AAYGLNQLPFNVTQAKEVLSKLPPPPEDDRITEDCLVLDVLVPQRVFDKR

RSIHQRGLKQTKGAPVLGGGYVIGDKSMFGTPSELMAASQANGQDGAIWV

AMNYRLGAFGFLSGPTLQQSGGSPNAGLLDQRLALEWVQTNIHLFGGDPD

NVSIMGVSAGGGSVMHQITAWGGSKPAPFRRAIPQSAGLVPVPGNREQEQ

TFNDFLALLNVSTLNEARALPSSALLAANLKQVAAAPYGSFSYGPVVDGT

FVPGMPSKLILQGAFAKGIEIFSTYNSHEGIIFTDPSAFDNESLLRKQLG

AIFTSFTSEDYQHVFETLYPPMYDGSQPYTDALGRAQLIIAEAALTCNQN

SMIKAAQKQGVAAYGFQNSVWPAVHGGDQPYLFSHGPTPGVDARIASIAK

DAIAGFVNNGLPIKSSVGDISFPPYGRDGFILNFVANSTSVIRDPTLNER

CDWWQKALYY*

>Tr|2950_t

MVEKTTEDDQHQQASAGEKHKTEAETPDPKRTKVESETSADQGKENDKEN

DKREESNTHQTKNKEPEESKSEVKSENGTTGRAKADKGKDAPASILEKGI

IYFFIRGRVDVDKPEEVDDIARSFIILRPIGHDAKLSKPPLADAGNTRIL

VLPKKVLPSSGRDRFLAFVDKTGASYDEIKGQFLAGADYETKTKGTRHTP

AATPVGEGVYAITSTGRESHLAYMLTIPQELGEVQKDLGLKEQGSFIIST

KNPEYPAPASARLPQAPEYPKKMHEEFHEYRWIPTRPHHLDYANTQILMI

GESSGIKKALGVEDDGGKQANKEEPLEEMEELEEQDLKRMQQLSGDDSAS

IYADLKAHAEDYPKLQTTF*

>Tr|5000_t

MGFSKVVPLLLLAAKSALANTPIAASDFSQNGGLAAAQAAAPMWYMASGT

CMPSAAEDGEGNQTNGVDADNCNINALAHGCPQQPTWQGANTFYGNVAGE

PFGTIPTYWKAGYCSGDSSWRIIYYVYFKKDTGHKSDWEGIVIKFTSPDG

GNTYVRDSVNMEQDGNHPSISWSDVNDTFQGDDDWEAFSQKNLDHGKFYF

GKFHHSVHQDWYTAAFKNTCPPTSGDDYRNSDYQFWAYNNLRPTSVLNPS

WDWGKAGSPANIDVCSF*

>Tr|3859_t

MKDEALGSAIFVIDPSLGGGSVVPADRIRLTVRFPRDGCSWAYEEASIPL

LDRFPDAKGREKGLTVIRVSLANEARIKVTGFGIPFANTDDPEVEGWMND

NKPIAGDATLLGFLRQRMFHILVPIAPAVASKRFSVERLPPPFMYPYGTD

QRWDVGKFKDLIKSKKGHQFREVYSHDDDNHHMTAVNQNNVQDVMWLDDA

AIEIATIKFPAYFVRPDPETPTADTDVFFVVTAIRTWFAKEHDKAWARLS

KKEPVLLHLYGRPENSEPDAIWKCKIVDRPTKEPALALHSIQKSDLVLEV

HRPKRADKYAGFVVRDFACREEANDALEQGIEQWHRVSLCFDAGLRDCKR

RVEAVSLFHPLAEPSNPFRWGLPDPSMPAKAQEAFKGKDKVTLTRERAQL

MDRMELHRALVRGNGFYDWMIAKHATRSNVDATSSPSVVDADITYRQLPC

ANFLDFGDAVRAEAIVNEALPHDRDRFRAYLSKRPLGFGIIAGGPGTGKT

TAGAAATAAMVERYGRVLCSAPSDAAVDNFAARLDTRTRAMTEACNRGNE

QSGSKRYRHRLVIRAYHLSAEVDAFNALLKDPKLGDEAAPKGWEPRWMLH

LSLAFWFLAVLGSPAVRKLHPDDSQDLWDLRQQVDSTEPDLERLRDVATG

VTTWEQYEQEGPVLDKTIEFYLLRLLPYADMLCITPAESEDVAMYRA*

>Tr|4883_t

MTETSASFKELKKRKCVLQRELRRRQGFPRHRKRFDHDEDEELFQDFVAA

ECEKREQECRDIQRECDEIYQRKLRQLARERDELYERERQKIDVRYEELF

DREYKRIERQIDQRSLQQPIREREPTRERDRLPQNLAQYLKTCHALHMRF

QKTPSRSLITQKEALSWAFPRRIIPWDGFAKLQESVWSQLPSETEFCTEQ

QFPSIEQLDSVRRTLTPVNSEASLQHFGRYAIENVVQELMDKLYEDGLLW

NGLGLSDDVKVTIETDKDLGQVYEPMLESSWHKDAGELKLSMRNSHRRAK

RAGIWVNRSCVYRHREDHGTKTPIFAIVHQLPHRLQLDEIKCGLASEIRP

ERDVIDKEGDDYTFASRTLVAATVTQLYSYMISRDIQYGCVCTGEAFIFC

RISEDDPCTIYCTPCVPEREVLNDDPTRLQRTAGALVFGLVLQALRARPL

PESWCESITYMDTWTMGYDDVLDRIRKTCTSRPQSPSSFKVYRWQGFKQS

PLYIEMKTTEPKESEKIRSWLDDVPGRDGVILELDRCLSGPRPPLMDQPY
CSQSCLLGLVQGTAPDLHCPNAEFHGPAHLDYSDFMRLLLRQLDSREGQD
PDCVQLRIGNTFRSPLKVRLTSHGYTFIAKGGKSSASMWMEKSVYDHMVG
LQGDCVPVCLGLLDMGRPWRYSENPWKELLLLSWGGRPFLSCYRQVDKEM
IAKMADNALNAIHKLGVVYNGGLSASNFLFDRSRESVMLVDFEEASLDFC
KTPAVMGDWQTRKRKRYLWQEEVRHQFMMDSSVAMATIRSAQRQSGGRFG
DDESDVDDSVSRC*
>Tr|3431_t
MSLPTTTRAWSIREIRSDSFDGIVLQGNVPLPKLGERDVLVKIEAVSLNY
RDLAIPRGLYPFVINLPVIPGSDCAGIVLATGSKVTRVSKGDRVCTLFNE
HHLANPITPEAAASGLGGAVDGTLRDYAVFGDHCLVKAPSSLSAIEASTL
TCAPLTAWNALYGLASNVLKAGDWVLAQGTGGVSLAAVQFAAAAGATVVA
TTSSDDKADELKKLGASHVINYNETPNWGEVARSLTPGGVGFDHILEIGG
AASIAQSLKAIRMEGVITIIGFLTSSEKEPAVMDILNHVCIVRGIFVGSR
QQFEEMNRAINSTKIRPVVDRNIFSFEDLKKAYQYQWERKHFGKVVIKIG
N*
>Tr|4930_t
MISISDHAEVKRIIQTQDWHKSEAVYGNFRQDPRRPTLIAFTDKAAYSVR
KRMISSMFGIRYIRSMQPLMLECINVAVDKLDAQCGKQPVEVDMQHLIQS
LAVDIIGVTTFGQSFNVVQNGSHPLPDQIKKALKLAGVLMLVPWIRNIPF
LPARDPYVDKFTSDAVENRRAILKASNKRDLLQKLVEAGDDSEDSVFRRS
DIQDEAVILLTAGSETTANAEIFTLIMLTKNKDKMAKLVAEVDKWYPPSD
PDKVVDCEYSFTGMTYLQACIDETMRLVPGQATGSPRECQKEEKVLGYSI
PKGTTVFPCTQNVHMDEKVWPHATEFLPERWLDIYAGGKVNEVPFWPFSA
GSRVCIGKHFALQEMHMTLVSLFRRFEFEYVPGQDETTVFRVAQQLQAHR
YMIKVKRRSF*
>Tr|6204_t
MSLSERAKQSEKAAQELTIWKVLQDLWDPDTNPSGTVSLGVAENRLMHDI
LSKHIHEKLALSNHAFTYGDGASGSKHLRKAMARFLNKHLKPVTPIIPAH
VIVTNGCSAAIEHLAWACANPGEAFLLGQPYYGTFIEDLTARTGAKVIPV
PFHGIDPVSGDAVVKYEEALKKAESEGTKVAGLVLCNPHNPLGRCYSREA

IVGLLRLCEKYQLHFISDEIYALSVWENTVDAGVPVESFTSSLSIDPTGI
IDPARVHVLWGMSKDFGANGLRVGALISQANPSLHAALIPLNLYSSASSI
ADHATANILEDDAWVEEYIAENQRRLAEQYRVVTSWAKENGITYRPGVNA
AFFLWVDLGSVFRKLHPNTETGDLNKTIATALLKHKVFLASGTAFGSEEP
GWFRIVFSHPEDYLQEGLKRVLTALAEL*
>Tr|8332_t
MDSSQIQDQQPQQPQQQHQQEHAPHRQMSFPPHVAQLDSPACRSAIQHTS
SWKPSSLDRRQSWSSQDQKHALQMSGIDLDSVRSGHQGFTERT*
>Tr|1839_t
MFGLVRRLGVGALVAAALSSLAAAAPANVAIRSLEERASSADRLVFCHFM
IGICGDRTSSTDYDDDMQRAKAAGIDAFALNIGVDGYTDQQLNFAYDAAD
RAGMKVFISFDFNWWSPGNAAGVGQKIAQYASRPAQLYVDNRPFASSFAG
DGLDVNTLRNAAGSNVYFVPNFHPGQSSPSTIDGALNWMAWDNDGNNKAP
KPGQNVTVADGDNSYRSWLAGKPYLAPVSPWFFTHFGPEVSYSKNWVFPG
GSLWYDRWQDVLRQGFEMVEIVTWNDYGESHYTGPLESRHYDDGNSKWTN
DMPHDGFLDLAKPFIAAYKNRDTDVAPYIQNEQLIYWYRRNLKGLDCDAT
DTTSNRPANNGSGNYFMGRPDGWQTMDDTVYVVALLKSAGTVTVTSGGAT
QTFQGTAGANLFEVPANLGQQKFALSRNGQTVFSSTSLMDITNVCPCGIY
NFNPYVGTVPAGFDDPLGPDGLASLTIGLHVTTCQAKPSLGTNPPITSGP
GSSVPVSTPPGSTTRFSSTPVSSRSSSSTPPVSTPPPGQVCVAGTVADGQ
SGNYIGLCNFSCK*
>Tr|6787_t
MTTSTFKDTNLLTFAHTIPDPNSSATALLSRWSDTLVSSPPALIAVPDTE
QDVVSAITFARSNNLVVLPVAGGHGSAVPITSRTLYLDLRNFNAIELDNL
NDQVVVGGGVLTGQLIRHLADEGYFTSVVNSNAVGVVGGLLGGGNTSVNG
IVGWMADSAVSFRIVTAAGDVLDVGGSSSSSESEERALFNALCGVGHGLC
VVLRATMRVYPLSSLGLSPTSSQDPTPSIWNRTVILPLDAIDSAVNTFLA
FAPPPEPMNMILAFSRGPPGTPMAGTPILVLTCTYYGPAREAEASPAGAS
LRRPSLVSKALKTDTVQIPFPGVNAYTEPLNAHGGLKTMFAARLARVSPQ
ALKDAYARYVAETGRYPDASRSAVMFHSSNPRKSTELGATPEHAGKFLEA
RNRGINVLGVLWCQELATQEALAVFYDETLAKLQKADEEDGLPPRTFPGA

MKFLPGRRDLLSEEKLAELDRLHAKWNGDGLFWNPYKA*

>Tr|3071_t

MFKLRRSNRKAFLAGVSLSKLCLTHRQAIQTAKELGYHGIWIDALCIMQD

DKKDWALQASLVPEIYNNADLTIVAGRSEDADKGFLEPTYSPDTPDAHLP

YECSTTFTSTVCRIGKARTRDIGPTNDRGWCYQELLMSRRAIIYGEQQLS

FQCRERHEFEDGECNMVGQRDTWYNLLFPSRHSVSLPEVKEIHKPCNQVR

SFRRSTRYLVPSFGNKRRPWELGAGSDPALQRWYAMVAEYSKRSFYDPTD

NHAAISGVARLFQTVLVERFGPGSDGYMAGLWEMDIISGLLWRSSRILDP

DLPALQRPTHEGRIIRRAPSWSWMALVGPICQGISTDRAGSRGFSRLGVP

CCVPRSDNEWPGPMVEYSKSPDSFELGIYGYVRRLRISQYSTRDHAEYSA

WGNIVPYPTESLNRHTFRLEAEEAKQLIRGIRDPSLDWPNIAATGIFDME

CKATNRPSSITALRLTSEEGLLLDEVYDSRGDTVGFRRVGVFVIENPVAF

YPAHSIAANAYGGYDIIEGRLPVESMTIL*

>Tr|1630_t

MAVPTLDFSKFISGTPEERKQFALDLLDSFERTGFAKLKNHTFSNDQLRE

LFHWGHSFFNQPLEVKNAIPNEPGPRPMRGYTPWRVEEVGKLHHEERVRI

MKDSKEHFDQGPSDDVEFPNKWPTDSSLAGFRPFMEAFYQQCNDVCLTLM

HALEVAWDIDDGSLVARCAPSATDMRLTHYPEIAVDELQTGRTTRIAPHT

DFGPITLLFQDSTGGLEIEDRRANAFVPLPPTDTTEMILNVGDTLTRWTN

GRITGGVHQVAVPEAMRGKSGLIIPSRMSMAYLFKAGRDTSVGPLPKFVS

AKEPAIYPDITALEFQRWRNSIVYNLDKEEDSVKYNGHVDKPAAAAGLQQ

VQAIRA*

>Tr|2964_t

MSSDQSPRDLPKPKQRRISRACDYCHRRSIRCRPAEDGSGCQNCRDFAQT

CTYHRKPRRRGVPARGSAGPAANRHAVEGVPDAESSRSPSQGGDLRIIPE

IQHPGPNANPSPQSSVAAAWRAPYIVSQATVVDLVELYFEMVYPIFPFFH

QPSFTRKISRAEYNTNQPLFATTMAVCALVGGRVRDGSVMNPRWDVRALQ

RETLPDTFYAEARRQLLEFSVESSDFNILRAHAIVAIAAIQNGKVRDMHY

HLGIYHTLMAMDGLHDESNWPSGIGIIEREERRRMFWSIYTLDIFTSVVW

SGVIRSREQQANVAYPAEVDDQYISDGGIARPGLSPSGLSPPIARLVLTA

QSDCWLSGWNFITDLYRVLEHALARFRERRRSFMSEIIDDQSTVTGTSVR

DKVLRMYLNLPDCFKNTPKMTFNQKQDLFGFQAANITASLQLLRITLFAA
GGASIAERCDIAADVVDAFNSIPVQYCLTISKPLLHHLGGIGAILGSVFE
EPLSEAEYSRVRSVMLAMARLLENLETIHRSSSASDKLRSQVARIDEYMS
AQRQQSRPPPEEAPVSTTVQQVVPVDGARGSAHAYEQPPVNSEMAGDWSF
QVPPDVLGDLTWNFDFGSVWT*
>Tr|1559_t
MPPRAQQLIRRAVPPFGLPSAASFGHSGQIQTQQLRQLSTSRALKTTKKD
DDEDEIMIRLGNDKGDKPANKARLTHVSESGSAHMVDIAEKQVTSRVATA
ACSVHFSGDTAIQLIQDNQMKKGDVLGVARIAGIMAAKRTADLIPLCHPI
AISRVSVDLDVVAGSRRIEIRATVTCDGKTGVEMEALTAASSAALTVYDM
CKAVDKGMRVEGLRVVLKDGGKSGRWEMD*
>Tr|5069_t
MAKKDEASPYGGDVEAQDAISVTAAPVTTFDNADQKAEHEQQHLGTLDRR
LKARHVQFLALSGAIGTGLFVGSGQVLSLAGPLSAFICYLITGFNLYCVI
NSLGEMAAWLPVPGAVPVFATRFVDPALGFTLGWNYWYQFAIGVPIEVSA
CAVIIDFWPNDVPKAALITVFFAAMVLINCLPVRIYGEAEFVFGAIKLTT
IVGLILLMFIITVGGSPSGDAIGFRYWHHPGPMNEYLEDGALGRFLAFFK
VFISATFAYGGSEIVVVASGETEDPRRNIKKSVRRVFWRILIFYVLAIFL
VGLCVSSKDPLLLNAIDSSAPGAGASPFVIAIKNAGIKVLPHIINAVVLS
SAWSAGNSFFYASTRVLYSAALDGKAPAFLKYEKFGVPYACVALTTALSC
LVYLNVNNESAEVFFWISNLSAVSTLIVWASVSFMYLRFFYALRYNGIDR
DTLPFKSPFQPFLGYFSIVFCLVVAFFNGFDCFFPGRFSAKSFIPPYIDI
PIFLSLFFGYKLVKKTKFVGLAEMDLWSGKAEIDRLESTWVKPVPRNFLE
RIWFWIA*
>Tr|5450_t
MNVPQPVQAPLTKSATFLVLTIANPSSPSSLKTIRSALASIHDLSKNVSI
RDLNSNFSVTVGIGSAAWDLLIPPSSSSHLPKPSELHPFRPVHGQRHSAP
ATPGDLLFHIRSDRRDICFEFESQLLRLLGASVTVEDDTQGFRYFDARDL
LGFVDGTANPSAAALPEAVLIAEQDDHKACVGGSYVVVQKYIHDMPSWRA
LSTSDQEAIIGRTKADNVELDDQAPGAQQPHKTLATIEDEHGNEHGILRD
NMPFGSPGSGVFGTYFIGYSRRLWVVEKMLERMFVGDPPGKHDRILDFST

AVTGGTFYVPPGWVLGELDED*

>Tr|384_t

MAPSATSPPPTTTNSQPIDLGSNYVQIINGKSAPTEHVRHGINPASLEPK

PDVPVATQADLDRAVDAAKAAFKKWSRVPYEERRAAVLAFADAIDAQRTE

FRDLLISEQGKPIPQADGETDAAIEWIRGMSNIKLPEDVIEDNDKHTVTT

RYTPLGVVAAIVPWNFPLLLATGKIAPALLTGNVIIVKPSPFTPYCGLKV

VELAQQFFPPGVVQSLSGDDNLGPWLTSHPGINKISFTGSTATGKLVMQS

ASKTLKRVTLELGGNDPAIILPDVDVDKVAEKVALYAFLNSGQVCLNLKR

IYVHDAIYDQFRDAMVRHVRAYALGDGAQPGVTHGPLQNAMQYDRVKTFF

DDIERQGWKVAVGGKIEAAPEKGYYIAPTVIDRPPEDSRIVVEEPFGPIV

PLLSWSSEEDVIARANNTSMGLGASVWSNDIDKAARIGRELEAGTVWINT

HFEISPIVPFGGHKESGIGAEWGINGLKSFCNVQSMFVNKHVVS*

>Tr|1752_t

MFRQLTTKVKGKIRSHRSRSRSPSVRDGSSQSLPNVQAIVSASSATANTP

QNAPTFTAEGHQSRHSLAIPTINEPAPEQEPIVQDPPAAEPGDDDEEGRP

AQKLSDELWDRAYDELKAEEPALVQAYERILTAHLICDNNGTTDATSGEN

RIEQQNKEYRRIQMQNLVKNGLDKISRESKAKSRIGDFLPLINVSKDIVT

NVVKGVPQAALPWAAVSLSLELLTNPMSETKANCTGVTYVIEQMNWYCEL

AALVFSDHNGSTARLQGELQTKLIDLYKKLLFFEIKSICSYYRHRGLTFL

RDLVKLDDWKGSLDEIKDAETFFDKHFHAFRSIDLGQYMKQLVLHAKDEQ

AYRKTEEDKKCLRDLYITDPRMDKRRIENTKGGLVQNSWRWILNHPDYET

WLHDDDKRLLWIKGDPGKGKTMLLCGLVDEITKRAAQSGDAVSYFFCQAT

MPTINNHLSVLRGLIWLLADQQPSLLSYIREAYGTSGKDVFEGPNAWYSL

TSFFRSILDDANLTRIYVIVDGLDECTTGLPELLELFKEVLPLQNVKWIL

SSRNWPDIQASLEDTPDAEGVNLSLEVNAAVVADAVDAYISEKASEVKLF

RGKDYLREEVRKVMKEKANGTFLWVAIVFQQLQRMKFLDGGFSALMERLN

EFPKGLTELYDRMLKQIKDLDSEQESELCQTVLGICVVAYQPLRLQELAT

LAGFKDGLTEPSALRTLIETCGSFLTVKDETVYFIHQSSKEYLTTSNGAQ

SAIFPNGTDKVHHKIVMHSLEIMERKLCRNIYKLHDPAILIGDISTPEPD

PLVSLRYPCSYWLRHLCMSMASFNLSDGKLLKFLKEHILHWLEVMTLMEN

SLEGVPDVIHFEDLVRVGSIFPMSEH*

>Tr|7239_t

MSFAQTDQVLQGPAFDPPSHASKSKLILQAVSEITNSLNEAISSGASPFG

SFNSSTTSFSLTAASRLDSSPIIDFHHTSGSLNLSAGSTSKVTGDTVYRI

GSVSKLFTVYALLLNNGTKYWNRPVTDFIPELRKAGHRSLYQDPSIIDHV

QWDQVTVGALASQLSGIGRDCNSADLAVQDFPWTKLGLPSLPPQDIPTCG

INDGQLPCSRKEYFASYTQRHAVFPPYSKAMYSNGAYRILAYVLEAITGR

SYEEVVNDEIFETLAMKHSSALPPIGKGKGVIPDGDAGWNRAYGDEVATG

GLLSSTKDLVKFGRAIFSNKQLSPMETLRWMKPNSFTASPSLSIGAPWEI

VRTRSGIATGRIVDLYTKSGSVGAYQSLVILVPDYQVTLAVLAAGPDASA

AVQVATETAIQVLLPALEEAAKEQACDKLCGEYFASTDASNSSLVITVDD

SPGLLINSWTHQGHDLIAAGQAYANATRGGQIQSVRLFPSGLQTRSRAAY

RAIFQRVLPDDDASVHKVFDPAAGTWGMPDQLMYGGIAVDEFVFHVDAGR

RAAAVEPRVLRAVYKRLEM*

>Tr|3072_t

MAEALAAIGAIAAGLQLAEGSARALIKTIRIINALREVPQKLARYFQEIE

ASTTHVEHLCRNLLQDESKFYQQLKSLSHTDGLIKTLATLHEATEEVNEF

LLPFAEFAPSPTAIAKPGRWLWKSITSLKMEKDLPHKLKRLERLNGEVIR

ELTAVGLQVQMATHKLMAANNSLSIEGFASIGAQLTELAVGVRTLSLAVA

QSQPVDIRSNSFDHVSATSSAVELLDDCDTWSNTSSETFSESSMYKSSWQ

KVPSQSSTSQTVAQRKEQLRLHLQQGLSSSLTVTPRDTPTDAHLHLALLS

IRTFYTKGNFDPEPQVLQPRFWKDCSNSIYFFKISDFPKARMLLQQSTAM

NSDDIFLKGSVTALIEILSVLSPINTVTNPDVRKTMLRYLHALALQQLPR

QSPIMVVLSRLHEGMDSTDWSMTALTCIVDRLCASLAPSNEVRLLAQRRL

IALLRRNKDYDGALRVCTKALDDIRKASGPMSLQERKTARLLEHIYMDQE

DWVSALEVCFDIVEQRFDETTGFNPDPQCHDECAVWTMEDIAKIYENSGN

LEAAIAWLKQARISGGICWGPDVSLGHIHDKLVVLLERCELHDEAKLWST

AFGPAMV*

>Tr|8325_t

MSLQHRQHPEPSPRQVEIDRVLRLRRLSQARSCYPCRQRKVKCDHEHPCQ

TCQKRGHPEICSYDVGVSLEQRRGRRVKGAAAGAAPSRAGRGRETVGGDS

EDEPRAESSLQPRPPRRRIRGAGAGANGPLESGPSVTPTHNTADSPSFLD

TPQTSHSTLFRQQDTEAADWPPARKELYQGGSSVLTMLHGSADSPAVEMR
RKAGPVLGLHNTLEFYPFMKLKTLQERWAALLKIIPQRQEVLRYFPSHGA
TIYPLNPVLLDPDDLESAFCDYLSALDAGELRNPDVVSPKWVSKVWISRI
ALLLASLATSAHYSVMQSPRKRAEHSLDFLQRAFDALCLANYVLHPSLDA
VQTLLIIGTVLQDVGQSDGAWVMMGTTVRVAQALGLHLQSVSEQSEDGAK
RKRSLWDTVCRQDCLLSLCHGRPHIASRQSLATRKILSNPDRPLNFAETL
YGMVSVCTGILELEKTSCESSMKLLAELDGYLSRALPYLQGREKCANIQQ
RYEALTVQVQLSWAASFLCRPVLTTSAVTSSDQAGVVQLLKARAKESLMD
TARAFIEFQALSNIPIRTWSMIHAVLSATILLCLWEETRCDQESRDVLQR
VIEIFSRAAQADDETGMMVDATGTNHWLSMNHTRALVALQAALQKAPAFD
ASPPSSPGRQQADNLVLQNEALPRAQDALVVDPAEGAMGYDFTALLGDGL
LLDGSMDSWDMSGLSPLMYLDSIMKAPYEAIDEF*
>Tr|8630_t
MALFDIEKWSINSATIIMGLLTLSFALRYTSGRKLHPSEPTVVQPWIPVI
GHLLGMVVYGGRYIKRLGLCYPQEPILTLPVPGSRIYVVTEPSLAASVQR
NTKTLSMTPLLPEITKRVLGLDQKTYHVISQNLDPEPGEPRGFFADVHDL
VYTSFGPGDYVNALSCEAVQELCFQLLETIDVTTDATGSVPGPVDLLAWV
RHMVTVGTAKAFFGPHNPIAEEPDLAHDFWAFDHGLGGLLIGIFPSLTVP
KAYAGRERLTAAFRKYLEAGYLEEATSIVKGRARIEKEYGMSTDMTARSA
LSFLFAGIVNATTTTFWVALRIFANEILLARVRQEIRQALEVSCQRSGPD
TLSITAVKETCTTLLAVYRECLRVGSENFSVRMIKEDTMLADRYFLKKGA
VVQISGGAIHGRSTIWGQDVDEFNPDRFLRQKGKGHGFHPAAFRGFGGGK
TLCPGREFATNEILAFVAIFIHGVDWVAPDGGRLSVPEKNDRVMPVHVLE
PATAPKGIVRPRAEEVKMLSRLKVVM*
>Tr|8298_t
MGSEKEDYSSGPEDAFTSANVIASTAIKPQHRPLHDASVTLEEYMYYAEK
TRAEEDSVAATAPPTTLMDMVFPSRGTSRRIEGKQPSEKHEGQKSNLNDN
KLLITDLEWTNASRALRSASAAACFYLITTDILGPFGVGFSIGTMGWGEG
IGLFTLFGLCAGGSGWILWKAFLDVDSYEFPVKNYGDLGFRIWGPWLRYT
INFLQALQLMISVGILVISNGLSISQVSKFHLCYIVCCLIWAIVGFFTGQ
IRTLNKLGFLANFAVVLNLLIMFISMGVMAHSEPNYAAAQAGSAGAALGG

LSVTPSANGTYPPIQRYGGIPPSTNGFTGSINGLMNGVYAYGGAQLFVEF

MAELQRPRDFLKAMWGAQFFIYACYMSYGSFVYFYQGQYANQLAYQGLSP

YAWQTVCNILAVISGIIAATLYGNIGIKVIYNNVLIEMFKAPPLTSTGGK

ILWAVIVPIYWVVAFVFAAAIPDFFGLTSVIAAICLVQFTYTFPAFIGLG

LFVQKNAMRGEEAFDPATGAVHHRDSGVKRWIRGFFGRFWWLNVILLVYT

LGSLALSGLGAYAAIEGLIDAFKSPQINSFTCTSPLEG*

>Tr|4601_t

MPMIFSEHMEHYDLETIFPYRSDVIPPYERIEVDIITVHCLNIEAKYFVH

KNTWSYSSKLWPRDVLPSCYQKRCRVMLFSWNDGLAVTEEDSRFVKHGDH

LLRLLMENRLEDPTRPIVFLCHGVGGLIVKEALAKAHLDKTGTIHIIDQC

TRMLAFFNTPHHNVAKSVRNIASAAVNPTAPDVLDKLDKACEEHVERMAP

VEARGRLYRRRVIINFHGTSIYRGTGMVLVEKEDTKFNARAEYWEKYFPI

YGDHTSMCRYRGSEDLTGETVLKIIGLNTRLALRMFPTMDIINRPRFRF*

>Tr|4969_t

MAAPFKVSLPNGLNYEQPTGLFIDNKFIPASGEKFTVYNPTNNEEIATFQ

GASEQDVDKAVAAARRAFEGEWSELAAVDRGALIYKLAELIGRDLKLLAS

IDSLDNGKSITDATGDLTESYNVFRYYAGAADKITGKTIETSPKKLAYVL

QEPLGVCGQIIPWNYPFMMLAWKVAPALACGNTVVLKPAEQTPLSALYFG

KLVVEAGFPPGVVNIIPGLGYISGKALASHMDVDKIAFTGSTATGRSVMK

YASSNLKNITLECGGKSPSIVFEDADLDQAVKWCHSGIMDNMGQVCCSTS

RIYVQDTIYEDFLARFTQKTKDNAAKIGDPFHEDTYQGPQVSKEQFDKVL

GYIDEGKKAGARVLHGGAKHGDKGFFVQPTVFADTTEDMSIVKEEIFGPV

VAISKFSTEAEVIAKANDTSYGLAAAVFTEKLAKAHKVARKLQAGMVFVN

SSGDSHFGIPFGGYKSSGIGRELGQYALDAYTQSKAVHVNLGTEL*

>Tr|7454_t

MLELHKTYGDVVRIAPDQLAFSDGAAWQDIMGHRKRGQGENGKDPVFWKS

NQHSVISADRENHTRMRRTLAHGFSAHSMMEQQPLIQGYVDLLIQKLREN

CADGSKPVEMTSWYNYTTFDVIGDLAFGEPFGCLENSDYHPWVSLIFDRI

RVGAESIALRRLPFGEKLIQICLTKRERKRFQEHFELTQQKVNKRLALPS

SRPDFMEVMTKREGDQKFSLPELWDNASLLIIAGSETTATTLSGVTYFLL

THPEVLEKVKNEVRSSFTSEDQIDLISVQKLNYMLAVLQETLRMYPPVPS

AIPRRAQPGGDVICGQYVPENTILGIWQWPMYHLSKNFTLPDSFIPERWL
GDARFSSDPKDVLQAFSFGPRNCIGKNLAYAEMRLILAKIMWHFDLTLHP
DSADWMERQQAFTLWQKPDLNVFIKPRATQSE*
>Tr|8022_t
MSGFNTSGVLNIAPARIVVPILLVFMWYLGRLHSQYEPIVSSRVTSRLEE
ARRMIPSVKLDWPIPPAKDPRAAFNSSKLALLIEARPLPHLSPLMMHMMA
VVPPDWRFLFVGSSESIMSVSRAYSIQHQQVIGKLDLVELPPPWSIASKE
DVFRLLTDPRFYTEFLPDAEWILKYEHDSMLCANSETSLNDWLDWDWTGA
ARSDDDGFSGYGGLSLRRVSVIKRVLSFQARFNDSEPEDEWFGKRLWVLP
GAKIASKLDGAISVEDVYMERPVGFHIREGGNNSNEDMWSDAARRREIFD
YCPEVSMIMDMKLERERCPDDDGSGVREDPGMEEESVDVEVPIEVETPVD
VEVTTAVLGEDGDAQTTSDNEPAMQPPQPPPLPQAWKASATTMERVLRTG
VH*
>Tr|509_t
MGSIGHGENYILGRNFVESVRLDGQHFLFRLQNGYTIDPRIPISPNTKIA
EMGTGTGIWLLDVASQVPSTVQLDGFDISDEQFPNQSSLPTNMSLSVMDA
FAEVPEKHRGKYDVVHMRLWCAVVQGGDPSALIRHATQLLKPGGYLQWED
CDPSPHHMVMKGVEVEALYSYFSRIHKAFNINHSWLENLPTHTNNAGLEV
VQFTMRPFSQSCVPIVTNSFLMLHIGLLNAAYRAGLPFLPPREEADNVLA
GALDAVKNGGSYHYTTLALLAQKPLSS*
>Tr|4166_t
MENKKVDDPGALALRRLVRSPTGGRLGPRRSSTIVSFSSSRSSSSASSVT
STVDAEMPASCVDECALHRFIVWAAVKRNACQKMTPEERRECPLLRCRKR
FPNHELMLQHLYSCDHLAGGEYWCYDCERPEQLSDVKCRRCLGHPSKRRK
IMTMAKSFFSSLGHKPKSHGLALSMGMGLGGMGSVGDGGIGQGSSSSSGG
GGNNNNSDNAGGGMDEPPSYDSVLFTPPPPPQAELFSTEIHEIGSSEVLL
PTIPECETETEPIQAVPAPRMIPCLPPSTFPISPPGSHPGAVPLPSTMEA
GFINWRPSPPSPQTVAPQSLMKPATARLVDRPTLQVNTHLDHYRGQASRR
SKALAPSSSVRSTSSTDSTDSTDSTASYNISPMSGWSSGWTKTSGFESAL
TSPDDLISPQSLLPTGPFVSCGHTHTTTNTADASSSSMRPVPFSDSEFQN
MVANSCPSELPADIPMFDDLPTTMDPQKTQAPMGLDHSAFSFNTNLPFQL

SLEHQLPPTNTVDDLSLHALPPPPHQPPPAPSSGHPHPLQSADDTESSMM

QHVGSSSLTTTTQTLARSALDALQMHIAESRSKLDDINENRLMVQLCQMP

LSSVATAGLETMVDLLEGRQASSPLHLLCFVHIVFSLSLVIHEQEASKHV

GALFKQALLYSDWFTGDDKMLYFEVVYTLWKPSAVRDEEIIELLKPTEAA

MSKGKQPERSLPELRSDPLILIAAHFLDELEYMALIDSNYMGQPFDSSLQ

HSKDSVGATHDSPFAIAARFMIESIFAHQYSHFPGFELGMEKIIGQLNSG

TVTSVRRLELELMQAGKTYLPSDIFFGDFVEVVRRQTDELYTLGPNLSTR

SEHHQCSIQLLLSEYSRNPIAVEEAVEQPGASGAAYGIVGVVPGGFESYF

DDMVNLNPGPGLDVDNMAGAPPLEVAEWTGEEPAFVSSSSSAAAAAGSAA

IATPVPQMDDEALISPVAATPSKAEPSASTSSTKVESDNCCKICGYRPKG

DPRWFAGSMAKHMRTIHSENPQIFRCPYPGCTSQYSKRADNLRQHQIEKG

HFVDDEGKQGRRPRKRKRPSEGGGES*

>Tr|8513_t

MEKSSSFVSQDGAENVEHARRHSLFGNTDNLAAVFENPLKQRTKEELLKD

VDEFCRIHELGDHLDDFRKGALAAQNPESIHSLEELSPEDKDIIEREQTH

RWSHPKMLYWLCCMCSMAAAVQGMDETVNNGAQALYLADLDIAGPNVTRF

SPSMQDNITGLVVGAPYLCCAILGCWLTEPLNRVFGRRGTIFLSCFIAAV

ASIWEGVANSWVNLFLARFVLGLGIGPKSSTVPVYAAECSPAPIRGALVM

QWQMWTAFGIMLGNIMGVAFGPSTGIDPSLGWRLMLGSTVVLPLIVCVQV

YFCPESPRWYIQHHRVKKAFRSFQRLRYSNVQAARDTYYTYVGVELEREA

NRGKNLFTQFVELFTIPRNRRATWATWILMFGQQFCGVNIIAYYSTTIFV

DSGYSTNSALLASMGTGILNFVFAIPAVFTIDRWGRRNLLLFTFPFLCIF

LLWTGMSFFIPNDDAHTKTRVGMVTTGMYLFEVFYSPGEGPVPFTYSAEA

FPVHVRDVGMSWATATTWCFNFILSFTWPSISRAFTVQGAFGWYAAWCAV

LWFLILLFVPETKALTLEELDQVFGVSTRKHMSYQIKNAAWHFRTWVLRQ

KLEPLPPFYRRAEKIGPAKGGM*

>Tr|2039_t

MANIMPTGGLQPVAIPDDLINTAVEKESEALEKPQAITERVWGFAARIDP

TVTFEEYQYWAKIEREEEYQANLEFKAEHGPRTVKSVLLGRFSKGIHHEN

KKKAEAAAAAAAAAADAGDASPTDEKSGIVGAPQKSVSLPTEAEWKQASR

AMRTASWGTMFYLITTDILGWSSTPFVFASVGFGPGVALYIVFGAAAAFS

GYILWKVFLGLDSSRYPMVSFGDTYFRVYGPFARHFINVAQAIQQFMTVA

VLILGSGTTIAQLASEKICFIACLIIFMVVGMVFGSIRSLQRIGWLANLS

VWINIVSFIIIMVACANYPIDYSAVTSSTLIKTIEPIKLFAGPPPDQYQQ

QATGFAGQFNGINQMVYSYGGALLFIAFLAEMRHPWDFWKGMLCAQTFIC

IVYIFFGAFVYGHYGQYSASNINNVIQPVSLQTANNVLGLITGAIACLMY

MNVGMKTVYVEVFQEILGLPPITTRSGRWLWYALGPLYWALAFVVGAAVP

NLNGISGIVGALLILNFTYTFPAFLYIGYRIKADAALPGEGFDPVTRVTT

RHDSGFKRWRRGFMVNWHVNTMNIIYFLGGLVCSGMGSWAAIEGLIEVFG

PGGTVATSFGCAAPV*

>Tr|4469_t

MVATACQGSLATNNSSLGTCRCVVSFLPISTNDEFDIDEDLMEVETIGFD

RISQFHFYEVCRSDDFLRSFPFCFNPKLSFLKPQHVGTAVLLEQFAFEHS

QRGFSSPLAEYRIRFSGNLSYGLFNICDMTERKYWWLATKMRGWNIDGCP

EDVRRLMFVHIIATLGCSPVVTDEDMDYPKNWAAILHGRDRYPSEPVGHR

PHGRTICLHSVAVCPRLQGLGLGTATLKSYVQRMNSLGAADRVALVCRKP

ETRFFERCGFRNSGRSSIKTLVGEYYNMVFDLPGPKDFIDWNSIADAAKK

M*

>Tr|425_t

MFGQYSLGGNPADGAFRVPKLNHKKSRYGCQRCRTRRVKCNEAKPVCQHC

ERHRAKCIYDRLPQKNREEETSPKSFSVAPAGESRTSETGSAKHSPLSAV

DPLDEDAIETKQRRLLETRLMHLYLTETGPSLPIDDKTFSVFAKKIPKMA

LDSEALLYAIYALAAIHSLICHIDDSFDGLDVHRRYLAMAVHEHQREVSE

LNESNFEVVCLTSHMLRISTFATLRFRSREPYTPPLEWILITGTTQALFN

KAWGIIMAHPESIVSGMMKSSPAIYDAQAKFGEAHRRGLEFLLERDAEDL

VAEPWDVEIRDAYETTLSYIGGVLHLLHPRDGVYDVDDARRRMVIFPMAV

KKKFLELTSEVRPRAMVILAYYFSILVIEKLWWVGDVGKMEVKGIAANLP

AKWRRMVEWPMRVVETGRILPLS*

>Tr|3472_t

MPSPNHLIIVCCHGVWTGGPSNGFSEDEWLIADFQRGETATFIEHIKAGL

RCLAEDYDNAMLAFSGGPTRRETQLSEAQSYANIASSNNFFNIIPSTKNP

SSKVLIEDRALDSYHNVLFSLTLFYTRFHTWPAHLTVVSHAFKEPRLVNG

HCTAIGFPLERTAFVGIDPPGMTNEENVDGMKGVGQAVDDWTRDPHGRGE

VLAGKRRRRNPWGVWQGIFKEKGGDKGGLVTEGEGEAETLVDGAPRPWK*

>Tr|9083_t

MAEKPLHLDLIDTLKDVSVRVDHVRPSHAKGVFVIGSFTPTKEAGQLSTA

PHFNSPSTPVVARFSLFTGFPDLPSNDPQAVPHGLAVRFLIGDGVKSDIV

CHSTPLFPANTGEGVLAVFKALRDNIVEEYLSTHPEAIPFFTEDRKTPEH

WGTQTFYSINAFKFVNAEGKSVFVRYRWVPVAGRHFLTEKELEAKGPNFL

FNELPEVFAKGPIIFKLVAQVAEEGDVTGDCMQRWPEDRKLVELGELKLT

RVADKQDLPPQKEAISYNVIPGVPGIEPSDDPIIATRGKVYATSGQTRRA

AEIEAEA*

>Tr|7065_t

MHSSTWTSLLLLGLANLVPDASARPSACPPPVKPGKAIYTITNEKQNSVI

ALPIGSDGLLYQGTSTPTGGSGSNALTASGKTTAPAAPDALVSQSALTVV

GNNLFAVNAGSNTVTMFAIDPKNPTRLTMLGKPIAIPGDFPTTVAASAKN

QLVCVGATGAKSGISCAPFSSRGIGKMDQLRPIGLNQTTPPVGPPNTISQ

VFFSADEETLFATVKGDPTANTKGVLGVFGVEPPCNGRGAATVSEEGKLT

QVDGSIVLFGSSAIQGSSDLFVTDAAFGAAVLSVDAKTGAASIKGKATID

GQGATCWAAISPETNTAFVTDVAVNRIVEVSLTDASIQSITDLNNGDPGL

IDIRAAGSFVYALSPGNGTTLPAVTVMDVVTKKQVQHFQLKGLGASKNAQ

GVAVLL*

>Tr|730_t

MAQSIQYLLLLLGAFATLIQASPQAKPPLPYCDMKQIHTMNVANCRCRPH

YKGCFTAARTCHMDIPNRHTNSYYPGCTDNDKLCLGCGLWFHTLCDCVKG

PSGCTHQGTVQPNGDQVWFLTPVGEKLVTTTDILPGILEMADNPAKYGES

WNFAQKYYDPGSEALALNSVRARTHEQFHIHVCKKPDVKKDAKVLKILDT

AKHNTGPTLEQVGNNDLWCRTVTKGNGPVRHFAEAIQAFLATGQLCKGLA

GAAIIRDAHENLWACVTGDQHGPLAQFCAGK*

>Tr|9203_t

MSSLREPLEKRGQKRKFSRASSTASGSGPEANVTVSQLPTGEFEQQDVAS

SLELHHDQTAATNVTSQEESCQQHISDSPSAPVLSPEIAKAPVIPPESIW

NDAYDRLAAKEPRLVEAYEKILSFRLSRETADSADLTAKQNTIERNNVLM

MMNPVSQTKSSPEAVDYVGTTIQWYWEQAARVFDSQEGDGDKTTLYSSFV

NLYEQILQFQMGSICDYYRTRGSVLLRDGLKLDNGEEMFKSIQQADDVLR

EEIKAFLVLESEPQLLNSHTKVQERYYLTKEDQQCLRDLRITDPRADRIR

IEDTQGGLLQESYQWILENPQFKTWRDSNENSLIWLRGDPGIGKTMLLCG

LVQELMQQSAPCLVTYFFCQATIPTINNHIAVLRGLMWLLAEQRPSLISH

IRKAYDAAGMSVFDEGNAWDTLSNIFRNMLADDDLPPVCIIIDALDECTT

GVAELLDFIQEASSLPNVKWMVSSRNRTDVEEKMHQGTCLSLEINATLVK

AAVEAYISQKMSQMPFLNQEYELRREVCRRMEEKANGSFLWVAIVIQALN

SRSYAHCDGISEMLDLLDQMPEDLSKLREIMLQQIFQLEGRDPELCRAIL

ATVTLAHRPLHLDELSCLVGFEGDPRRLPHVTELVNVCGSFLTIREDVVY

FIHQSAKDYLSGPSPRPVIFPLGEDKIHHNMVTHSLNAMARTLQEDIYRL

GHLGILIDEISPPNPSPLRRVLYSCTYWAAHICQVASRRDKSLFDDEKVL

GFFKAHFLHWLEALSLTRNLPSNIGHIKRLSELLKVNQARLAYRQQTPAR

DELQAFVRDASRFLQYHAHIMENAPLQIYVSALLFSPTESLIRTCCDGKL

SAWILMSPNVGRVWSPCLHVVEANESTTMDSSGTTLLIMNKKSRDVEIWD

IATGRRSQVLSHAETVHYARFLRDSGRILTFTERLSSSWDAVSGALISMG

GL*

>Tr|132_t

MSKPDKITRFTEAMKTVYGPFDQLSPDDAKAWTFPDDPGAGGYHGRYLWT

DAFGVVNFITLFKETGNAVYLTLAKRLVQTVHDVLGRTRDGSKRLDGATD

EEPLRGGLRIGKMDARGSDGDGQYHHYLTLWMFALNRLSIAADEVQYNDL

GIQLARAIHARFMVHCLSGGMKMVWKISMDMKTVLVPSEGHLDAATGYAV

YKLLQDTAARQGHREHVLQKEVDDYWDLMVKKRKGSLSPGTDYLDVGMGL

WAAHLWKHERWAERFCDRALPVAGELLSSTRMRAEGARLAFREFGTVLGV

GCWDVGEGLRARVDGLMAFWERWLDDEAEEELKPISRVMYASGLIVGAFR

RDYLEDG*

>Tr|6209_t

MPRLELLSLGLAITGAAAAPAASAATPLKVISYAQTPGGFRTPARGWNSF

ALQANPNAGPSFKFDQSHVLTQCSVLASDGFKGNYDYCSLDSGWSVGDHG

DDNGRLIYDSSLFDIPSLASSLHDQGLKLGVYVVPGAFISDRNKTILGTD

TTIGEVCTGDEGLIRCIFDYTRPETQQWHNSVVDLFASWGVDFVKLDFVT

PGSPDNGQNLPADQSGSVIAWHNAIKNNGRQMRLDISWKLDRTKKYFDIW
SANADSMRTDQDLNNSGSNVLVAWGTVQRAIENYRQWIVAGLQFYDQLTI
YPDLDNLVSGNPESISGLSNGQRTTVTTLWIAAGANLIIGNDMTTLDDFG
KNLLTNPQALQVAAFTAQYPIQPRNPGSGGQAATQFQAWIAGPSPSGEAV
VVLVNLGPDNGQGGFGTQTRGVQTVSATWQDLGISGQYNVQDIWNNKSLG
VVSDQVSAQLNEGDSVLLHLTRA*
>Tr|16_t
MFSYEVEQYFYDEVAPRLTGDIAVAQCFASTRRMQKQAAEHGIDHVIATL
MEDLRLEYPISGEKRAMLSSQQVYAALTWLAKFHRSSWAYKPQDLRHFLL
PPLEEALRRQKDSSTGGKALWLNGGYTYLATRRQEYQMLAQDKSSEWSGA
FCTTVDGELSLAEQVADFLTPRGRPFETYIHGDVKSENLFSTQSGNDVVF
FDFQYVGIGLGVCDLAKLFTCSIPMNMLTNHQPIPAEMEMDEREKRLLSH
YWRILSDGQHDRKIPLYEWNDFLRHWETALVDWCRFQASWGFWGNTEWLE
ARVRYIIKDNAWRAWLASEVGTDSVVGSRQEE*
>Tr|6513_t
MDSPIILPQGAISRASILGLLLCITTALLNRFLRGGKARKYPPGPKPWPI
IGNMFVFKGIVYDTEATLARLAKSFGDKVMIWLFSKPFLIVDKLEDAKEL
MDKRGSIFSDRPRPSNFVERVWPCLLPIKPLGDEYRIVRRIYNDLLGPKQ
SQSVRKYQDYESRMLMRDLYNAPEKFQVITERYSMSVIFSAVYGVRIGRL
DYPVIEELFSIMDTMANYILPGSLLIDYLPFLQRLPECFQPWLKFADTMH
ARESKFHNGFLNVLKEQIEAGTASYCFGVDVLKLQEKTGLSDEFTLDILK
GVIAAGSETTSSMLQSIFKALAMNPEAQRKAQEELDRVVGPSRLPHWDDA
PNLPYIRALIKELHRWTPLFILGVPHASTEEVVYRGYTLPQKTLVMPHVY
RLSRDPDVYENPDAFAPERFLGDDLDSYASAKQSDFRKRDHVNYGWGRRL
CQGIQVAENSIFMQVSRLLWAYNVAPLPDEPLRLEDRLHGIVKKPKPFRL
SITPRSEEAVQVVLQAAKQAVTSLPDADSVVY*
>Tr|7684_t
MIVRLLKGLDSIIDLYQVHFHMGSEGWYFSGEGGSLPEDPLHGFKKLKDL
YLKADPNYTGRYTVPVLWDKKADVLVNNESSEIIRMLYSEFDEFLPENLR
EDNRPGKGLYPPHLRAEIDAMNEWVYDTVNNGVYKVGFSKSQESYDENIR
PLFASLDRLEEHLGHGKPFLLGDSITEADVRLYTTLARFDVAYHSVFQCN

LKSIRHDYPRLHGWLRRLYWDQDEEGPLRAAFYRTTAPNIGRYALGYADS
RWRIVQEGQGPLIVPAGPLVLMEPL*
>Tr|8043_t
MAPNAIETAAPIAPAPASESEFEFVVGDRPDQLKAGSNPKLRSHLSKRGW
KVYLLQHNAAAGSSSSSSPADEATRQREDRSRRKKRRRLQHTITWDVRGP
HDAEFPGMATATNNNSLIQAMIHATAVQPFAIEYQLGGGRVDPFRSYPTP
WRPYIPHLVDHYIIHMAVDIPELDEPGKKGLLRSRWFRLATTEISTFQVV
LLLSAGNYISVKGGIAAEAGFNMDQLRIDALNSIGMAMDLPNNASDSIIG
AVAKMASFEAMHGDLDCFQLHMNAARRLVDMRGGLHNLGLGGLLRRMLIW
IDLNGGHLMNTERWFPGQTFAGSEDEVEVEPNPERFIAM*
>Tr|3368_t
MGESAFGVDSKVDLDEAVSLSASDEKEARDSKSASRWAEALQKLSKEDQA
QFELALKSLDDPKSVLSSVLAATDTRKEECKKKRWKLSIKGQTVIMRDVL
EKLSAWVQKILAVGDVVIQYDPSHMALPWAAVRLIMQVTVNDIEVFGHVI
NSLENISNLVAQCQLIEVIYLVDRRKSSDELFGQLSESITDLYAAILGYL
AGALHYFGLNTAVRILKSVVVSKDDVKTRYEPIESAQAKFRRLAEMAEAQ
DLGSVVDGIQGIEQHLQQMTKRDAVEMQSLKDAMKQLSQPINRIDSRLEQ
IQDGIEQQVRTQILKAISTIPYGSHHKTASKGRLEGSGRWLLKHYEEAIE
GFVGFDDQAWTSDECVEVLLRLVDEYPAVTFVLDALDEVDQEDRQDLLDA
LDRILKESSTLVRVFISSRSNYDIALKLSGAPNIYIEADDNAEDISTFIE
TQLTSAKLLHGKLAPSLRAEIARTLQEGAKGMFRWVDLQIQSLKRVKVAA
DLKARLGALPATLEESYWEIYQEIREAGDHAFGLANFTFQWLLYAQESIS
IEALAVLASSESTSESETAFSGDEVLDVCSNLIVTRQNSFDFVHLSVREF
FERLSNRGIHSYQLEACHAAIAAACLRYLNMAMVDNRGLDLREKVMQNIK
ELQVTILVDIKNSNASGVSDEQEKEGGEDVQDGKAGEAGEDGGDARENGD
ASSQDKGTNEVEEPDERSEDGDIEDPAGQETKKDADKTAKKIAKWSKNME
DDDLIGRSAEILAVVSSDDDYGQPSEYATTWVMYHIEKSFKHRLESPLAN
LIKAFVLEKSGDSGDLAYKVSRAFHVWSALMTKVVRDSEQSRELKWAARL
PPSPIWVVCQMNWIEVAEYLYNCSYPGINDGRKMHTSERELDVNPLWYAI
LSKKADLIDCLTKCNADANRVFIVDTYDEPIVTAARKNDTELITILSKQN
YGGQKAAEEAFIEATRFLLSQKIGFDVLSTALVIALSNSDETCADLLLEH

GAKKDGLALVRAIRDKTAMTALKLIEAGYDVNGRYLDKRRTALHYAALSG

QTRIVEALLGASADVNRVDANARTPLHLAALNGHVDCVQLLMKHGADVLC

EDNQGKIPLDLAEEKNHVEVERAIREEMETMLRKLMAARDGEGGAHKS*

>Tr|7563_t

MSNFEASSQRPAPAGILNEEQTLAQPKRQVFLREGTVILRWIFGFYDSQN

LPGWKKPPGWPQIGFTWQSQKPNTRFKNVTFVAIDIDELEERNDGMPIRF

HIGISILQTKDLHGLCHDPFPITGSQTNIIRSYHWAVEDSQYFTKNDSRF

CFGQHECIPLSGLEERLKELLKPFSPRVLVAHGISRERIVLRRLNIDPNQ

IFEIDTAKAARYPLQELHDSTLRKLLQDFDIPCEGGLLHFAGNDAHFALR

ALLMIAVRDARRELKDLPTWVPVFEAVARAPLPPIPLTRAEKAAIKRRER

EAARLQDELYQFRERWVEELERKKQPERRPSSTTSSSLDFLAYIANVRPA

DGR*

>Tr|8421_t

MTEVTDRNERRRINAEGDWTIVYGDLINEADVAELVISIPTGTVAGWVSS

QVDAQVQKFNQSLATVSDDVVREATQYLRSLVQNGGSGERDINGLGVKAG

ILTYHRKLKTPLGSVPLPNNFQPYIGIRITKPLPPKGAPAPVQVPPGVQP

TTQSNPAGLDSRSWYRITNPAKPGMAIDVVNDGNQQQDGQVQMAAEGNFS

GQHWQLRPSRTQPGAYNLCNMWLGAGKCLDVYGNDKTKPHLATAGNYSGQ

QWSVVRNGNGTWKLSNSYSGPLVLSADVSGGGLSLKDPQVSPAMMWTLQP

IKPITEVGF*

>Tr|1901_t

MTQLRECAVAVSFIFQFPDGDEERTPRVALFRRSGQVNTYQHKFAPISGG

VEVTDENPLATAWRELREETTLTSASLRLFRQGKPYSFADPSVGRRWTIN

PFGFVLKPAAEGGSGEEGITIDWEHEGYEWFNPDEVTDDESFQGVPRLKE

SLRRVWFDIDLGKEAGRALAASIRSLQNDHESGAGLLAIAAYYHLYEDVI

PKLDTSDRDKWWRNVRIAAWHLYKNGRESMSAAILNGMLSILDTIDQKLQ

SSIEGTPTKEDVDQLCDNISGIRLQRDMAGAKIARAFSEYLENIRSQPGS

DGGPVKVVTLSASSTISNAICRGLTSPPPFYVPIDLRVLESRPLFEGVKT

ARQISSVMQGKPHAKATSKLSVYTDASAALAAKGAQILLLGADLIDKSGN

VCNKIGSLPATLAAKHVSPDIKVIVLAEKSKIYPFEPPPCEENKEDEVVA

AWKKDAVIGSTAVWELRGNASIPNVYFEWVPAGLVDVYLTEDGALSREQL

LELAKRVEEKASRYFDTL*

>Tr|19_t

MGDVRQTIKQSLPVDVSNPYDPTTLKGKTILITGGANGLGAHMVRHWASH

GANIVIGDVADTAGEELVASLRTAYPKAVFEFRHCDVTDWESQVSLFDTA

TAASPHGAIDVVVPNAGVLLPGEATEFENPELANGKLPEPNTATLSVNVK

GVIFTSHLALYHLPRNKRSDRCLLFVGSVASLIPLPGQSQYTTSKHAVLG

LFRSLRGTASMRGIRVNMIAPYYTAQTSMLPTVAEALLLSGSAGPGSVPD

VIDAATRLVADESIVGRALVIGPRLKAQDIVEAVGEGSGALLKEDVDQSR

AIWECYAHDYDQVETFVKRYLWLLNATARARGMFAWILDILAIWKRG*

>Tr|5748_t

MATAVTLAAEDISITSYPDGGLQAWLVVLGSWACMITSMGLLNTMAVLQA

RLSENELKHLPESTIGWILSSYAFFLYFCGAQVGPIFDAYDVKYLVIPGS

IGIVISMMLIGLCKEFYQFFLCFGLLGGASASLLFNPAIAIIGHYFDKRR

ALATGIACTAGGLGGILFPIIILYLTPEIGFPWATRVIAFICLAMGCLAV

ALMKNRLPDNKNTSIRPGSAIDLAALRDPKYALTTLAVFLVEFAVFIPYT

YISSYAIHAGMPTQRAYLLNALVNVGAIPGRALPGYAADRFGAFNILALT

AATCAALIFALWYTAATNEAEIYSFTVLYGFWSGAAISVTPVCISRVCNI

EDIGKRTGTAFFIASFGVLIGLPIAGAIIKAENGSYNGVIVFAGCFYAAT

VATLYIARGVAAGWGLRVIF*

>Tr|4311_t

MAAIGSMVFCTDCGNLLPATKGTEQNVLSCECCSAENKDTGAKIIVTQSK

PSDFPSFLRQKLQSSVQAVERHTLNTESTVRERCPNCGREEVKYTTVQLR

SADEGSTVIYNCECGNSWHENN*

>Tr|3370_t

MSTFKSSVSVTHIGTATALLQIDNVTFLTDPFFAPSGTKWDLPIISLKVT

QDPAIKPQDLPPIDAVLLSHEDHPDNLDEVGRQVLEGKKVFTTKDGAKNI

GDKAIVTGMNAGDKATLVVNGKTFEIIATPAEHAPQGECIGFIVTSPDFG

TTNGLPNAIYFSGDTIYNRDVANSLQGYHISLALLNLGRAAIPVGEPEPL

AITMDGKQAAQLIKDANIEKIVPMHFEGWGHFSEGREEATKAFAEAGVSD

KVFWLPRGDSKAFL*

>Tr|33_t

MIGFVTSHEDAKDIFTSWHTRRRGTTHWKALITRLEVSALNLGQTFVHAE
DIRILHIDCSTDILLPSNFPFNSPGAIKYASSLDEVRNLIYSHRDKCDAF
VEAYLALYQPVHPILDPPRFLKEINYFWEDAAQTDVSWLSTYLMVLALGC
FAVTRDSPSTIELCLAAESCLARTTFMVRPSMSVMRTFCLIVLAKQLANG
TCWSFDASWSLLGIIVRLAVCIGLHKPPVASSKPMDSHAVTVSEWQTGHI
LWITIVYFCIQTAAITGMPSLLSSDDILRRTETHDALLPHIEPKFGPWLS
LSDSFPTICSLIARVNSDTEKPTYDEILRYNADIKLLMATTLEHPGCRNP
SLRAVLDIFFRRILLVLHRCHALRPDAPILHPVSYWASLECSLAILVHHR
DLCEHVGNPDNRDLLGRFFKLDFFAAALTAGLHLLQADAPLADGFSIPPR
QTILETLETCTEIWGRDKERCYEA*
>Tr|43_t
MSSNPAGGAAVQPKKGVFRQLRENPYIFGLSAFASLGGFLFGYDQGVVSG
VLGMENFGALFPRIYLDSSFKGWFVSTLLLTAWLGSLLNGPIADRFGRKG
SMMAAVVVFLLGSALQAGASTIGMLFGGRAVAGLAVGMLTMIVPMYMSEV
STAGIRGTLVVLQQLSITLGILVSYWLEYGTQYIGGTRCAPDIPYSGGTA
EKPTFNPQNDVGPDGCTGQSQAAWRIPFALQIVPALVLGIGMIFYPESPR
FHLMRKNEDAALRSLARLRRVHPDSDSLREEYLAIKAEVMFDESHTRENY
PGKSGVSLFFAEYYGLLSNWPTFKRVFIGSAIMFLQQFQGCNALIYYAPT
IFGQLGLSGNTTSLLATGVYGIVNTLSTLPALFLIDRVGRRPLLMCGATG
TFISLIIVGSIVGKYGSALSQHAAAGWVGIAFIYIYDINFSYSFAPIGWV
YPSEIFNLGSRSKAMAITTSSTWMCNFIIGLVTPDMLETLKWGTYIFFAA
FCLIGLVFTYFCVPETKGRSLEDMDRVFGDETALKEKERLFAIAASLGLT
APIPAEKTEAITAEAEEYV*
>Tr|1811_t
MTSSDDSIAVPRGQKSCLNYRKIACDRLMPACSSCKRRKKICSGYDYNLS
WPRDGDERRAMTAQVRSQKYPASAFAFLNTSNADMMLYYNGAASKCESDK
IAVWMPSIRRSPQLPRFGSDKHPIYDSEMSSAVRLITSRGDLRQDVCGLL
RRMSLTDNGVASLAVRYAMNAISYLYLRRSDEAMLHQMRAVSALQGAIDR
IQDPRCRAQAIAASLLLSLYEVLCPGGQTTVWSVHFDGCMTIVKSSYQGL
QVNEGEHATLLDWVFYHNVLYKFSLQHWQRRTPEMAAIAQRDMRISRIVS
TSHFVTIHSTLGCSLELLEYLSRSINLVKDRDDPDYLSKRHSHAIYDLER

NIRDIDQQLCSGIDREDYHVAGSRKRYLTVSRMYQLAVLIYLDRVVRGSF
AESRLSRASAEEAFGLLRDLGSCERPFIMVMLALQAENDSDRLLILSTLK
NAIHERPLSNLTSTERIIRRIWAQHDLHGSAQADALKILNTVISTNEIPP
SFT*
>Tr|7423_t
MTARKEYRELATREYWDKYYAAAKKSNEKGHEWFRTYEQLKPFFARNLFN
REGLQVKDNPMILHPGSGESDIPLWLAKEGYKRQLCFDFSKDIVETMNEV
ISKMKDANEIENIEYREMDAFNMEGIPDKSIDVAFDKGMMDSLIDGDPWN
PGPEVRRDTRNYQKELHRVLKDDGVFLYITFRQPHFVEPLLIPNDSEILW
DLHKEVLIDNAASLGYFAWVIRKKGAPVRPLVEAPSETKSDDQDS*
>Tr|7280_t
MKLTRAISLTNFMVATSALGFQVFVLYPWHKELDNGFEDLKKEHKKVLDA
VGKSVSEQQRMAFTNKLNELKAESSRRWWWM*
>Tr|8391_t
MASPFMRHCLLQRAPSVPRTARRRLHLAPPFLLDNYTPRYLGLSSRDAAK
KRSLAYAHLRNCNLCPRQCGVNRHETTGMCLIGDTAKVNVIAPHFGEEPC
IQGHNGSGAVFMSGCNMRCIFCQNYDIAHQRNGMDLTPEALAEWYLKLQD
VGNVHNINIVTPEHVVPQVALSILHAAELGLRLPIVYNTSSYDSLASLEL
MDGLVDIYLADFKLWEPSTSKRLLKADDYPQTARESIKAMHKQVGDLCFT
GDGIAKVGLLVRHLVMPGREAEGAEIMRFLASEVSTDCFVNIMEQYHPDA
HVGKKKRRTKASGAVEGAKGQTEPDEGDPDVRYSDINRAVTNEEVSVVRK
AAMEAGLWRFCDPPKHGGFVI*
>Tr|862_t
MHSSSDKPFWGEATSYLNFCEEDYVVTRYVAEFINTLSSFAFVVYGAYGL
FRPGRNGQTLARLLSYSGLIGVGICSASYHMTLKYHTQMSDELSMHLLTT
PIVYRLLTFKSSPQRTKLVAVLLTVLFTAVMVTHMVMDEFLLHATTFGLA
VYIIATRTLKLISQQVPDERIRKNLRNIALFGCFNFAFGYFVWLLDNWLC
SGLTSLKHSAGLPLAFLLELHGWWHIFTCIGGYVGVALVDAITSGQVRED
PVPHLAWPIPTAARFLGGADASQKQD*
>Tr|7500_t
MSQDQGQTQKRCWNCRQQKTACDKTLPHCGNCVKKGRRCLGYGLKLSWPR

KDDQRRSASSRQEHYIIPIRTSEGGFINATSDDVRASSQEQALSRYDATT
FGEVWLTRQPQFNMARLDPFLASHAWSPVARLVSSSQSGDDLYGLLVRMS
FQDETLPSLASRYAISALSYQHLAMDQTAVMHQTRAIRALQTAIETVAPS
ECMQLMAASMLLNIYETLNFDTSELSWSIFFCGTKRIVNLVTKTDDTFFG
DEALIIDWIFYHDVMYKFSIRHWREKNTDQILLASQRKVLSKAVFSPERQ
VIVPILGCSLELLDLLCQTIDAVYEPDDPNLQSESHLKQIRSLEIRLKSL
QQRQSSISPLNTPDSNQETTVAELYRLAAIIYLLRMAKGEPDNAKSVLQV
VDQAYETLNRLEYCERPWPLFVVALEARSEEHRRDMLRVLGKSLEKRPLG
PMALVNRMIPDAWTQQDLREAAVDPFTLYGMIISRHRVPPCFT*
>Tr|6040_t
MSDQPEPASQPQRLPYKGSCHCGAIQYVVFLNVPPPSYVNSEPPPRRGVQ
RIYRCNCSICHKIGFLHLRPVSELDDFFLLSPLDPLDSLGDYLCNSKRLH
YLFCKTCAVRCFTFQGEGEIVNVSLPEVLALAGGGSETEQDGGSVKAWRP
KRIELRDGIPDQGSYLSVNGQTIEPGQEGFDLREWAERKAIMYLDFLPDE
KDRLVERCERPHFGGAY*
>Tr|4150_t
MPDRNRYTVGWICALSKELVAARAFLDSEHKSPSYVAKHDSNSYLLGSMA
GHNVVIAVLPAGEYGTTSAATVARDMVHSFPNIRFGLMVGVGGGAPSSEH
DIRLGDVVVSMVREGKGAVFQYDYGKTMQDQAFQYTGYLNQPPTVVRTAI
SALESEGLAGGYQLEEAINDVLLKRQRLVPEFGRPDPSSDKLFRSDVVHP
PVAGSCAQLCLDDPWKLVSRHERTAAESNPAVHYGIIASANQVMKDAMIR
DKLVGQKNVLCFEMEAAGLMNHFPCLVIRGISDYSDSHKNKEWQGYAAMT
AAAYAKALLRKIVPSQVDEVERIGKRLAQRSAAFNSYDESHNSTCLPGTR
VELIQQILDWAGSKDGKPIFWLNGMLGTGKSTVSRTIAQSLYRAGRLGAS
FFFKRGHSDRQNAARFFTTIADQLARMQPAMADSIKDAARTDPSIGSKGV
EEQFYQLILRPLSALPKENWKGKQVVIIVDALDECGQEDDATEILRLLSL
AEPWLRVFVTSRPEFPIYQGFINIKGQVQDVAFNAIPRTMIKQDIKVLIT
YRLNEIRDRFNRTKQRRAPLDQSWPRQDHIDALAEMATPLFVYAAMMCNF
IDDIRCGYPNSQLRKVLGWKAENSKTRPETKLDEAYLLLLNQLLASIAKE
GRDTFLEEFRCIVGSIVLLGSPLPMPVLAKLLGLPFERVDITLNMLRSVL
DIPPSDEAPVRPLHVSFGDFLINDDKGKLSAFVGDALRILRANLQIIIDE

PRQLYSSVILFAPDESIIKRLFVGSMCIQVSPKPRVESRWNHRMQILEGH

TESVLCVAFSHDSLRVASASRDHTVRLWSIDTDEAVILKGHSRPVYAVVF

SHDSLHVASGAGDSTVRVWRTESGQCIQTLDSGGMKIESVAFSYNSTLIA

SVSQCGVISVWRAQTANDGLIYVKDYESTIFNYFTANCLAFTHSEQGDEL

HTALAKYTGSIKQLLSGRIIRTLDSHKGRVMCMTFSHDALLLASASDDCS

VKLWKTQGGDLIQEYADYPGVVTAIAFSHDASLMALASDDHTIRLHCTIA

MKCVQEFHNPGSEVTSVAFSHNSSTIASASEDHSIQLWQVNLVPELARHN

EAAGAVINPDATDILDHSGHIVSVAFSQDGSFMASVSYGHTVQIWRTDTG

ECMRRFQGRTVSDVSPRQEPCPMTCLAFSNDSLYLAVSLQEETVTIWDVE

RGEHEQELGDHEGSVTSVTFSHDSKLLASTSGAWIRLWDFATGEVYLKIR

SPPRPSTRRSRHREGLFNGVAFSHDSSLIVAASTTGLQLWRLDTKECHQM

WGGRDVCFDSVALSHDSTLVASASKSKIQLWRTHTGQRIQAFDTGNSSIS

QLSFTCDDLSIVTNIGMFTRATNEGQFHVDGYGFTDGLSWITHKGDKRLW

LPKDWRPTCSAILTDLGRV*

>Tr|2407_t

MDTNPRIRCQCGAVSFRAVLPKPLRVDICHCLECQKQSSSAFGVSAIFPV

DGMLPFPESIRDRVGMWTRKTDSGNTLECYFCKTCGVRVLHRGLLPNGTS

QPTVTVKGGCMEEGLDLKDARHIYTRSARVPVPEGSWPGPPDKY*

>Tr|3266_t

MKLCAGTAKRSIVAAVWAAAAWGISAAPSAKSKATVPKYFHENRYDVHYD

DRYSRILLEDPEQREAIKVLVQTYLATFRDLGVQTWLMHGTLLGWWWGKK

VMPWDYDADVQVTEADMYFLAAYHNMTIYYYKYGDMEKGRFFQLEVNPYF

VHREQDDKSNVIDARWIDMQNGLYIDITAARYALDHPEGEGVLYDKFGHE

YRDTYVFPLRDTTFEGVPCKIPYRYEDMLQAEYGRSSLTNTEYHGYRFDN

EAREWVLLEKKPEEEL*

>Tr|1192_t

MLPKDFQWGFATAAYQIEGAVDQDGRGPSIWDTFCAQPGKIADGSSGVTA

CDSYNRTAEDIALLKSLGAKSYRFSISWSRIIPEGGRGDAVNQAGIDHYV

KFVDDLLDAGITPFITLFHWDLPEGLHQRYGGLLNRTEFPLDFENYARVM

FRALPKVRNWITFNEPLCSAIPGYGSGTFAPGRQSTSEPWTVGHNILVAH

GRAVKAYRDDFKPASGDGQIGIVLNGDFTYPWDAADPADKEAAERRLEFF

TAWFADPIYLGDYPASMRKQLGDRLPTFTPEERALVHGSNDFYGMNHYTS
NYIRHRSSPASADDTVGNVDVLFTNKQGNCIGPETQSPWLRPCAAGFRDF
LVWISKRYGYPPIYVTENGTSIKGESDLPKEKILEDDFRVKYYNEYIRAM
VTAVELDGVNVKGYFAWSLMDNFEWADGYVTRFGVTYVDYENGQKRFPKK
SAKSLKPLFDELIAAA*
>Tr|1802_t
MASDPVSDGFASVGGHNSATSVPAYLLTLVALTVAFVFAYSRLQKSKREV
EVDFPPWVPLEVGISSALIQAGGTASRLFSLIWPRKGSRFGLTSRHQILH
NLDNIDRVFTKSPHILSADVLGLRLGIRVFGARLTKEISEEFLVINRKLT
QIIERYFVNDASATASIQAADIPGKVARLLSFSPDSQKQHQWERSAAIKV
LSTDSEGQSGAVEADLESLMRDFGACVSIPRLYGQDFLDRNPHLLEDFWK
FDNNAFSLLVIGVPTWVPIKSFREALAARTRLHDALEGFYIRLNQYKHGQ
KVDFDADMRDVSTAAMERNGFLEEHGVPIRARAELELGTFWGLNANMQPM
VFWLLLYVYSTSGLVDKLRKEVRPCMTLSTEESSPQIAAFDFSRLSHECP
LLKSCLFETFRLVEDPTSLRYVSKPMTVSDGNLEHHFEPGTWLSAPHALL
QSDPSIFPEPEKFIPDRFIETDKETGHRVARYGRLKPWGSGSAVCKGRTF
AEKEVMGIVACFITLWDMENAEGPWKLPGMIPGTGVKRPQKPIRVILRRR
SLE*
>Tr|920_t
MSTAASSQTTKSAAAAQHFFFGLGSGVASAVLLQPLDLLKTRTQQSGQHP
SSLLGYWRELRQSPQPIRAFWRGTLPSSLRTGFGSALYFSSLNAIRQFLQ
TSNAFSQRIHASSASSSLPTLTPTANLVAGAVARTWAGFVMMPLTVIKVR
FESSLYSYPSMWAAVRDIHRSHGLRGFFSGFGATAIRDGPYAGMYVSIYE
MLKKRLASLASGGSRSSSSSAEGSRAMTASVASSVNFVSAISAGAACSAI
SNPIDVCKTRIQLQPQRYRNLFHAGYRMVVEEGFGSLWRGLALRMSRKAL
SSALSWTIYEELIRRYTASYGGAGRSLKSV*
>Tr|7865_t
MTALLDCPAEILHAIFEHLPTSSLHALCLVNKRLHQIAEPLLYTEIELIW
MARRPPPIIPLLRTIQDQPRLASHVERLTLRAPFRRIARPDEGGGEIEPG
GFHYTQLPHSIPTDGTDLAGFIATIRDIDPPFRDRWIQELQNGTMDAFVM
LLLLYVQNITHLRITGVFARANHLLGMMLRAALCQDLHCRLPQLQFLQNV

VTEPELSCSRYNDPKNTADILPMFYLPSVRSIAAEIDNPAIFAWPTYTPN
ASHITTLDLEVIREGHLGRILATTKNLKVLKWRWLYEQLLRNEFNSDIIN
LDQIAADLTFVQETLESLHLSVMFDNDYWEDTLSATGSLKGLRNFERLQR
LEIPELFLMGFSLVDNVDCLEDLMPKNMHHLTINDDSIWLEGIAWQDRDL
FNKLQRWWEGNMHQTPWFTSFKLSLQYSDEQWCAGIRQELSDLGARLGIQ
LEIFKNHRDY*
>Tr|4865_t
MPQSSWQDIVARKRAERDSKIPLEWRLSGSLPPGTRPIDYLPRCGILTEQ
DLALTNPARDATKLLSLLSAGQFTAEEVARSFCKRAAVAQQLTNCLTEIM
FEEAIGRAQWLDAEYKKRGKPVGPLHGLPISVKDQFFIKGHDSSLGITSL
CFRPARSTSQAVQMLLNSGCVIIAKTTVPQTVLTADTDSIVFGRTVNPHH
AEFGAGGSSGGEAALIAMGGSALGLGTDAAGSVRIPAAVCGVVGYKPSAY
RVPIDGQRILGKGIMGTTALGPPAVSGFLARSVRDVRLAAKVMASAEPWN
DSPFIYPHPWMQITFPREPRIGVWLKTGVVHLHPPVERGLRLACDRLRRA
GYDVVELSPPPFQQAWDVHREFGEFMDLSHMRTLLESEPHTKIVQAAQVM
TPEKRRPEPTVEYLHQLNFQIIRIAMQMKRLWRPDGGRPLDGILFVNAPH
TAVPFDKFTWLAFTSIMNLVDWTGISIPLSEAVDKKLDVGMPLENYYSDL
DRSIQELYDAERFHGLPLAVQLIGQRFEDEKLLALADELYPILTQRGQSK
L*
>Tr|4255_t
MASTNGTAAPTDIFVPDQLFHTLLTVVDFSHDSSGATRTPFVLKSHGTLD
AAKAFARQSLETLGFAPEDFELYRVREPQDSSSSSSSTSSSRPWTHGDGV
LVFARAFDGKEFIVGIDTAPNNESLAASSSDGELRLPEGARFLHYVLQIT
IDYNADRSGAAQTTEIQGAYIHRADAWKAAHLCLDPAQYAEFDRRGDAQF
IEEWPYGEDVAVHAVSETGQNSFVAVKRPPEQRHELKPHNLKKK*
>Tr|8112_t
MDGEPGAGPHQLTVANLQTLNQQVKQFHLLRHRKAMASNILVLGAGELGL
AVLQALSRHPKRSHARITVLMRQASLDSAAPDKKKLVQQIRALDVRFEAA
DVVQASVSQLAAVFAKYDTVVSCNGMGLPPGTQTKLCEAALEARLPRYFP
WQFGMDYDAIGPGSSQDLFDEQLLVRGMLRAQDATDWLIVSTGLFMSFLF
VPDFGVVDLASRTVRALGSWDNRITLTTPDDIGRVTAELVLDSQGLRNQC

VYVAGDTLTYRQLADLLDERFGIKFCRELWDAEELARQMREDPNNGYVKY

RDTFAQGKGVAWDQDKTVNSARGIKMTDVKAYLAAMDVKLE*

>Tr|2869_t

MGRRLSRFFPALLALAVVGSAAGDHVKAAAAYYSHEVDARQRSEQKPFSS

EHKPAAATKENPLTAEFGDFVREQLDKWKVPGIAVAVVDGDEVYAEGYGY

ATLPDVPATPETLWYGASTTKAHVAAVLSALIHSGNHSALFSRGWSTPIS

SIIRDDFVLQDEWATNHVTLDDAVSHRTGMPRHDGSLRSVVDGDGNGNGD

GGSRPTTPKDVVRNLRNLPLAAEPRQVSYYCNLMYATLSHVVETVTGKWL

GQVLREVIWGPLGMDSTFFDLQSALDATGHMASGYAWDDERGEYKEIPYM

TLTEVSGAGSVISNALDYAKWIKSLIQQTGPLSEEVHKDIRTPRAFWGGS

PDKGYDLELYGLGWIRTLHKGHVVYTHSGGMHAYGTEVYWFPEAKYGVVV

FGNTATCHVVEEIVGWKLIDDKLGVPEEDRFDYAKKWKEGRDKLTWLYEN

AVDVLYPERPDPVLPSTLNTSELAGTYYDRGYGRITLREEPHPDKPGETI

LVADRPETTWKYSMHFYHVSADHWIAYLDAPIYSGTKFKDFYAAEFKVGA

DGKAWGVEVLMESRTDPMPEGTVLYKRVA*

>Tr|4156_t

MSTLAASSLIIQPSSIIVQPSSSIVQPSPAPQPRVAQQQQQQQRKSSKKG

GYTRQRRGCLTCRQRKKKCDQGLPICGHCSRLNLVCKHEKPRQLSSAWSE

DAVEDDPGPSSSQAHTTYMCWGSQQQQQQQHPVTARGDHVSELLRLTKIA

EPLDLVRPDDAAVGYDWSSSRRTMMRYYTSTLAIMLSATAENNCFLSVLL

PMAFDCPALLDAMAAWSSAHLALRDPSFHGASLQYRGRVLANLSAALQED

SLPGEMCLAVAMAMCSMETISDATSSSWSHHLSGAAAVLQSRTCSNAMIG

PLQTTTTTTTTPSAVSDYWLQSVERRWLVRNFAYHDILMSVSLDRRPLL

TGDYWMSRDDTMADPYFAFASKIMLLISEISVLNADCAEFKASLGGASTL

KQGGDLALVLEESPLDHNYHTPLERADNIANGLRDWKCPAETANAPLALL

SETYRSASLIYLDRVVRQHFPRRAAEILPEGIRAYVESVCHVAQKVPEGS

LAECSLLFPLFIAGGEAQDASHIERIRDRLCSMNRWRRFRNVDACREVLE

EVWAKRGESGDAERVDWRDIVRQRGWQLALS*

>Tr|4939_t

MGILKPTTKETEGSEPIVTQIANEDKVAWYRKPNLRNLYFILFPACMGIE

LTSGFDSQMINALQIVPSWVDYFDNPQGSLKGIIAAAYSLGAILSLPFIP

IVNDKFGRRWSIVGGSVIMIVGALIQGFSQHVAMYIVARIILGFGIPTCI

VSGSSLIGELGYPKERAVLTSLFNVAYFIGQIVAAGITFGTNSIASDWAW

RIPSLLQMAPSVIQIVFVFMLPESPRWLVTKGRVAEANDILVKYHAEGDQ

DSEFVRAEMAQITATIELEAEYSKKNWMSIFETAGMRRRTLISSFLGLFT

QWSGNTLISYYLGDLLAMIGQTESVFKQKINVAIACWSLVAGVTVSLLVN

RFKRRTMYLVCTCSLLVVYICWTVTMERSIAGNAIGHPNKAAGAATIFFI

FMYQPCYNIGYNALTYTYMVELWPYAERSRGISVFQLFGRLAGFFATFVN

PIGLDNISWRWLIVYCCWLAFEICFVYFLFPETAGRTLEELSFLFEDKTL

ADQAVMAVEKVVHHDEVDKVVEIGEVQHNEKTTS*

>Tr|6320_t

MSPIAPYANDHRDTNGPGDARPTALKIVRDQALDGQLSGKVILVTGGTSG

IGFQTVRALHVTGADVYFTGRENQKGKEAEEELRRDGKPGKVEYMEMGLD

SLRSVREFAAEFLKRTGGSVNILICNAGIRGYPKGQTEDGFELHFGTNHL

GHFALFQALKDALIASSKPSFQSRVVCLSASGHRQSSIRFDDINFDQEDV

YQPLLGYAQSKTANIYMALEIERRYGAKGVRGLAVHPGGISGTRLNRMTP

ETQLNALISDPKIARKMKSAEQGAATTVWAAVAAEWAERGGVYLEDCQES

EPWNGDETVLAPGYALHIHDAESASRLWEISLEMIGSVV*

>Tr|7969_t

MDVASFPAIRLYRQDGSVTRYRGPRRTAPIDAFVKRALKPSVQNVPGQQL

ANFITNDDYVFIAKLQGESESINSHYRDFAQEYSDRYSFGIITSGSVPSN

GVWCYNNVDGNQHAATDLNDPNALKKLLNLCTAEVIPQLTRRNEMTYLSS

GRSLVYYFSNNEADREAYVKALKPIAQRYAEFLQFVTVDSGEYPDMLRNL

GVRSAGGLAVQNVHNGHIFPFRGDAAASPGQVDQFIVAISEGRAQPWDGR

FDEGQEAHDEL*

>Tr|7131_t

MAPPTVLIIGCGVAGPFLAILLKRKGYQPIVFEKVRELGNAGASLMIMSN

GLKVFDLIGVADAIKAESLPLTTLWDAKASGEVLGQSNLPSTFADTYRQP

ATGIRRTTLNLLLKRKVLEEGIELREGWALVDIQEHEDSVTATFSNGQSV

TGLFLVGCDGIKSASRAILQRQRGVEEGLPSYTGLTQTAFLSETPATLEA

TAAMRNWYGDGVHVIAYPVGPKTTSWALTQRETQEREETWRPFTGDEMDT

QREALCKLLDGWDASIAQGVRAAERIIKFGLFDREELRPEEWFSRRCVLV

GDAAHPTSPHLGQGANQAMEDCYHLSTMLPDLSPGSQHEDDINKSLEDVL

RNNLAESVFRPFAEKRQPRTSTLVKGARALGEMRVAVGSDKGRERDEAVS

RSYEGDAAAIVAKFENLLRQPFN*

>Tr|6244_t

MSELQVAIKGPLAWSGADFEDGEAYTLQLHDEDIEEINAALQSFKALSLD

GDEVDQDNFSLPKLKARLAQCAEAIHDQRGFFVLRGLGGSKYSVEDSITI

YLGIASYVADKRGLQDRKGNVLSHITSSKLWKVPAEERHGIHSSQALPYH

NDMGCDILALQVRHMAEKGGYTYLSSVSTAFNELLSEAPWAAKALLAPDW

PVQISGRGAKHYLAPALAIHDGRLMASMDPNRFGPHPSSGPTDVPPLTAT

QLSALERISKAAYRSELRLHLKTGDLLFFNNWALLHRRDSYTDSDETSRH

MVRLWLRNSKLGWSVPNSMLPPWYAAYGDNVVRNRLYPLIPMPDYKVPKY

SAGSAAFVIEDSDASEDEDILP*

>Tr|7475_t

MAEKSSPFKVIIAGGSLVGLTAAVALEKAGIDYVLLEKREIAPHLGASVS

IHPHTQRVMEQLGVWPEIKAAVVPLETRQHYDENGFLFEDSSILKEISKM

TLNRWTTFMERRFMLSCIYNQVADKSRTRAQTGVASYTETEDGVEVITDK

GEAIRGDMLIGADGIHSTIRTLMADHIASTDPEAAKEMRSGFVSNYHCIF

ATSKNAKATADGKPFLPDGAVHNVYYSGFSGVIAAGVPGLVFWFLFVKSD

RTTYTPNTPRFTEEDMETTIAKYGDHAVGPGYTFKDLWESRVRANMLPLE

EGVLKPKWNSGRVVLMGDAVHKATINPGLGGNLAVEGVVHLMNELVPLVR

RCEADGRRPTKSEVTAVLDTYEAKQRPHANTIVTMSGYVTKYEAMETWWL

RLLRRVSPWVSDKTKAGGFVGYINEGPWLNYLPNPDNERLTEKITEKQ*

>Tr|4115_t

MTSRLISALVLGLGATGAVAAPNTKRTSSHAPQLKIISYKETPNGFRSAA

RGWNSFGIQANPLTTPGWKFDQEHVIEQCDHLAGLPGYDTCSLDSGWSVG

GNGDDYGRIMYDEDNFNIPMLADHLHSKGLKLGVYIVPGAFLQDVNKTII

NTDVRIGDVCHGDEGLVRCIFDYTQPAVQLWHNSCADLFAKWKVDFVKLD

FVTPGSPDNGQHLPADGSGSVIAWHKAIQQSGRKMRLDISWKLDRTEKYF

KIWNENADSMRTDQDLNNSQQPTFVSWEVVQRAFDNYRQWIVAGLQYFDH

LNIYPDMDNLLVGNNASVSGITDAQRQTVMTHYIGAGANLIIGSDLTHLD

KFGLNLLTNKAARDVADFTAQYPMQPRNPGTGGQDAKQLQAWIAGPAPTG

KAVVVLANYGPDLGQGGFGGTSSAEQWVSASWEDLGIKGTYRVHDVWNNK

DLGKQKSGVKAKLGPGESVLLTLTRA*

>Tr|2213_t

MPKLEDNSHASWPVESQSWDSFDIHTSDISLCRACKVSICWNGAQKCVGC

VSGLPRPLPSKFLRQLRDELASRDATVMLRWLDGTGRVVHHGAVEREPAC

VDKSQRADMCDMLSLYVLKNGWAAWALDLAARELRGKPVEKSHVSGGYKR

SRPIEDDAGPIAHWCSCGNRKLESSGQAQSLSIEVAIGALKEQAKALKAP

GKQQHQKPTTLTRQAGVTKKQAKTQKKLPPVSTRQLRSSTKQQLEASKNA

PPAASEAAPKSEEVVRARVPTREQIDLAYERGMWAGATPEIFADALWLAG

PGRRQIEKESGKASQEMPRGARGSVETVLLHYFFMNNHINTLTDGLEVTV

LGAYLAALDHGKVRDSDFTAYAGDIARHASLARWTVATMELAVQWPTILD

WHAGCLSTLGLVRGLTLRAWVYACHWDVLMHYELGSSADWEEQVPSWLYI

ASALGHDAGDLCSDTRLGCADNAYFAVGATAGYEGVAACMDIVCDALEAV

VLRDTRGAIDIGCVAGSACATFERTGGDLCACSSSSSSSSSASNSNSSSS

SNSKPGAASSRSCEANSVLASICGDRRISAEDAASLVGIRDGIRARSREL

PRFRGSRDMAHLSHTDREAVYGQCLAAIATGGSGAKQAHARLQVAYASAA

QSLCGQLRRSAAELRQRVGGDALNLSSDCMCGGECSLW*

>Tr|2214_t

MGQSTLSESGRYTSAAAAEAIISLLDFLQIEKVAVFGYDKGASVGSVLAW

KYASRVTSLSVGEYALPAFGHEIIQNPEPSRNLYGHWHLALFTIPEAAEF

LIRGREKEFLNWYFWHSSYSASAIISLEHVERYAAALAKPGYLRSMTEFL

NGNIWREVEYFKPLRDDPIRLPLTVLWGEASLVPEEVSQGLWGPTATSVR

TVTVPKAGHWLADENPKWLANFIDQNVASSAVEPTSVDLGYLEDRVTMM*

>Tr|8300_t

MSSNKALLLLTLTASASAAINYMAIPQVRDLLPAPTPVSHNLNLARDAAD

DAECASSALDLLQSLPTPPPALLSDLSKSAQQEQTDPCKLSFPASLSDDV

SSYSSKVLSWYSGHEAELTSLAKQCSAAQSYTGLVNVCTDDDSSGSGSAA

SKTADATTKTAGAGAATSTNVGAAHETGMAFAALAAAGIAAIL*

>Tr|2819_t

MHESQQRQSGGRPYRSHLRPACVPCRRRKSRCQTAGDAAVCLTCRAHQTE

CYFPGDSRESASSEPARRRRRVEARPSSSEPSGASPLRVAQAAASFREGS

KANTSAPVVGPVPNIPPRPIAGMSRTGQTYHDPEEESTLALGSDDDQHLN
LHIVGPAATNDSQVLSTYLSGIPGATRSTRMIIPEPASCSRPVLFTEVQK
RPFGVKLNRSPSAEKLEIIEKLLEPHNEAVIDEYFRKVNVCLPLLDEASF
RRQYQEDKTRISPALMACLYAHTIVYWQSSPILSRYRCPESRFIWNLANE
AVYSELHLSPGMSIIKAIVLNIPQQEKYLRMKIWWALLVHDRWTSLAHGT
PPHIQRSQYDVPPPTLEYLGETLTETNNPRSELRANVFISLVTLSEVLGR
FLQYVYCVGRDKPTTTDLEHALHQWIETLEGPCRRIVLRGSHLDVTGAAN
LRLAFLTAKLLLQRIQLEAEKQGDCVNEEGVMNRYSEARMTSEEILMLIQ
DLQPENLGDFWMSVSAFSFPAAVNFLLRCALETENSPEGLAQSPSFKIAR
DLITTLRSHQEQHRWDLGDVCLAQHAEIVDKMLTGAVPDEQGGNNSSLDL
QDFDVSILDHIFPSVWDPLQNAW*
>Tr|4900_t
MSATPIAQGHHIQAYTYWDSPVPANLAGQNFTDPVIFNPTTFTLITTPRE
AVLVDTPTVRSRAEPVADWIAEIIEGRKLSTIYITHGHGDHFFAAGVIQE
RFPEAVIRATQGTYEHMQEQLSPAFWDGFWVPTFPELQEGPKPNLTVEAL
PKGKDSFNVDGHEFRAVEVVGGDTASSTVLHVPSLDLIVGGDVVYGGCYQ
YLAENTTPELRHKWIEAVDEVAKLHPKVVVPSHRLSTDGFGLDNLEATKQ
YIRTWAKLDAETKTWQEMEASVLKAYPKRIGNYILRLSELVTKGDLQLP*
>Tr|7329_t
MAPPSIPPPPPPPPPLNIPPSRARLQLAARLAMHQKNNQAAAQASSATAN
DNHDDDDDDDNLEDPFGDGEGDLDDDLDDDLSNNLGRGAWWRGVVGKRSG
IEDDDSDDDEEFGDFAMAEEEKGGEGQDEGDVVLKPLAVHPPRESSRGLS
GLWPFGSRADKDKSGEGKEEQKGEGEGSTAEERDDEPERAIEVREAQRRT
SIEDDDDDDEATVGTEYGVGFKG*
>Tr|8151_t
MAVFSRKSQSNDASCHTAAASACRRVVNSLKRFLPKKITRRSRQKNDLPQ
EPEKLAAQVGPICADMLNQLHYPGAPKLKAEAVEGLLAYMHKRAIEFDVP
LNNALSAKGFRLGYAEGLLAHPNHPVQVQGYVGLFTWLVVQYDDIVGQGN
VAGREMLKEALEFHARFFRGEPQANNLLEGIAILLREADDHFDTVMSNML
HISVLKFLTSNLLERHDGFQNLEVTRAGFKFPDFYRDMSGMNVAYAVFCY
PKAQYPDASAYLEAIPDMARFIDISNDVLSFYKEEVGGDTRNYLHNRANA

TGKPIMEVLHEVNKETIDAAKRVEQILKGRGVYEQSWQDSVRGYMAMHTT

NPRYKMSDMGLGEEHPLAPFEYKIGELFDRINAAS*

>Tr|105_t

MMSARALRTLRPTLLASSSRPFTTTARVRAGSGSAFNSGKEDLGGPGGQQ

PIPPNPGGPEALRRNWLVIGGAALAVLVGYNWIYKNPDEARQQRDKTLGE

MSGRNKTEMGGFRHD*

>Tr|8543_t

MEAYRQYRSLGTRLEAQLQRNRNRRHSTTTTTATDNDNTTPSPHPNTDQT

GINDTEASANTTDIEDNDSSSSPPLDHSLSKIGTNLAAALTGIVHKPHPS

SSTDGSKVFIVGFEGPNDPLNPRNWSLTKRMLCTINVGIIALVVGMAASI

DAAVMKRAAEDFGVSEVAEALATGLFLAGFGFGALIAGPLSETVGRNPVY

FGSLAIYMLFLMGAGLSKSLGGQLVCRFFAGFFGESPLSTVGGSIGDLWD

PTKRLVAFPIFAATGLMGPVLGPVVGGWISQAHDISWRWTEWVTIIVSGA

LLISLLLFQPETYEPVLLQWKASHLRRLTNDTRYVSAAEIEHVTFRARMM

TAVKRPFIMTIQEPTIMLWTGYLTVVYLMLFGFLDGYTFVFQETYNLSDG

ITGTIFVGIGVGLVLSSAVLTPLLYKWAKQELSKIHGSSEPARIPPEFFL

WYALIGAPAIPISFFWMGWTAYPHISIWSPILASVLFGFGMFSVFVSTYM

YLIDTYEVYAASALTMITLVRYVASGGMIEISIPMYENLEIHWALTMLGL

IALVFTAVPYAFHRFGPWIRSKSRFAKPK*

>Tr|2674_t

MATPGDDPVEGQYNKESAGNVPPTNGNTSNGMGPGQQFPQQFQQYPGYQF

IPFSNTMNPQAGPHQAMVSQVYQPTLGKIGNPGPLGLLGFALTTFTLGLY

QCGAGLPGSNPLGDVGPDQAVFGVAVFFGGMAQFVAGVMEFVLGNTFGCT

LHCSYGAFWLAFAMFSVPTLGIQAAYQGDQRAFSFAVGIFLIIWCFLTII

FFIAALKTNITILLVLGLLTLSFFFLSIAQFVSTEHHTAAIRLNRAGGAF

AVFCSMCAFYAGAAGIMLKDTTFVTFPLGEIPYPSVRRERAAAKARANNV

*

>Tr|193_t

MFANLTHATLRFIAFFNHLMILASSAIVTGLVSWFLDKYDYRGVNIVYQE

VIATITLGFWLVGAVLPLVGRYRGHLAPLNLIFSYLWLTSFIFSAQDWSS

DKCSFGQPGEGHCSRKKAIESFNFIAFFFLLCNTLVEMLLLRAEYATPVA

AAHNKEISAGRPSDNSV*

>Tr|6029_t

MDEYQQKQQSILKKVPFRSSIMRATALASAAIIALASHASAETIKIDVGP

NGQFAFQPNNIKAKVGDVLSFNFHPLNHSVVMGDFANPCAPAKTGGFFSG

FMPVASGVGSQTFEVTVNNTDPIFFYCSQNKFSHCISGMSGVVNEPSSGD

TLDAYQAAAKSVKSASNPATVFGGRLVANAEATSSATTTAAPAATTTSCT

TGNKAPNGNGYKRDTCSGAGSVQASIGIVAASLGMALLLS*

>Tr|5042_t

MSSLTEPHSCALCQSRFTFTYGNYYDSNGWYELIDENYYATGEAYIFNIA

YHEAVSLADRGCELCAWLVTLRRREEEKDDDDDGTFDERCWLRGYFAHNS

REQIVLDWVGEVDEWIDGGTLERKVIVCAEESELAAKEVAYRLPNLCPSS

DETVETMKKWLRECITTHVECQQLQSSDKGLQASASGPRRLLHLSGSTDA

PQVRLVQNSGSGALILEYAALSYCWGGDQAVKLKDKLVDSWTRGIPYEEF

PKTIRDAMWMTMALGLEYLWVDALCIIQDSDKDKAEQISRMAEIYEQAYV

TISAARATAAPEGFLHSRYMPGERGFRMPFTCEDGRTSAVVLWQGREPDA

WEPLDKRSWCLQESILSPRLLEFGTHNVRLRCARSRADEAQLQCDGWVGQ

SKTFAQLGMAQPLVSSIDWQSLGEPYRFVQAWQMLVSHYTRRGLGWYQDK

LLAISAIARKMSRVTTGNNNKRYIAGLWAEHLGEMLLWHPNHGPDAKERA

RRHETYVAPSWSWASCSGEIDFWYGSGMHLLEFVGCEVRTAIPGDEFSAV

VGARLELKTLVMRTRMRKGGGGGWGEGEDGWFLWDEEAGEEVNGSLMLDV

EDEESGGEFVCERDEEVVLALVQTQDALVLKKRKKKGEEAAGVYVRIGIY

REGPRGTDPDRWAMEGVVIV*

>Tr|4586_t

MFYTFQGVQGKAADRSPQSSSDGNRSQDTPGSRAAKSLHACLECQRRKIR

CDGKQPCGRCQSSRSRRRCVYEQHRQRLVPSRKSLEELSRNLEECRSVLR

RLFPNHQVSHLLSLSRQELCSLLDQPAQQRTVDLSPVPPSPKDDQQHPTE

LEQMPAQNSQWDEERRDRDPLPAEADDVNALSLAMDKQASSYLGASSVKV

ALTVMLQIQPQLQLLLASQPSADSSSDDNTRSKVPLIRAASRSAKKQFPI

PWTYKGQALVDAYFKRVHVLTPLLDEASFRKHYSEGRRCDAPWLSLLNMV

FATSSIMSTPSSNLSHIKYYNQAIEHIHLSAFGSSHIETIHALGLLGGYY

LHYINRPNMANAIIGAVLRMATALGLHREPPPEEPQSDPAVVESRRRTWW

TLFCLDTWGTTTLGRPSFGRWGPAINIQPPKLVTENEEYDSAQFGGIMPL

LENIKFCKIATKIQDMLAVSPLLESEDRHHLDSLLVDWHNNLPWLLRSTE

PCVESLHLSRSVMTWRYWNLRMLLYRPVLLTLASKGQLPISEQDVAAVKN

CQEAARETVDSVSKGWMRQQMSGWNAVWFLYQAAMIPLVSILWQPDNPAV

VDWRAQVESTLELFEAMKDWSLTARRSRDVVSRLLEASSKISNTRQGSDP

QDALLKRASDEASGFWADEGADAVGSTGYSTGDIVNMLDQDWPWDLDLDG

LVWEQQIPLLEEDNLDNNACIDEGVIGVDYLSLATEPGDALPPS*

>Tr|7295_t

METKAPTQQKRARRIEGRVAIACNHCRNQHLRCDAIVPTCSRCRNLRKTC

VYTDLRRSRRRRNSVEKPTPSGHRRDAINAARDSPALILQSPAFEAAHSP

VTPFVACTHALPPAVLVDSHLLTSRPVDAFYKFFFPGHPFVLPREHLMSR

SERTTDVSYGLALIIAFIGSKYTHDSLSSEYRQGAERALSEQLVPNGFNV

QALMLLALTLEWSGENERAAAILEQAKALGLEAGMQNRNFAADHGCGDKV

LEESWRRTWWELYVTDALFAGIRHLPTFTLWQVDADVDMPCEEEQYIEGV

IPLPRTLAEYDDRGFDEDDVGFSSYAYLIDTARILGTALAAGDIANKSAY

SLVKSAEANLMSWELHLPRSKRDPVRSDGTVDEILFKAHMVFNT*

>Tr|2443_t

MSSQVAKAARRVTHELHGVVVSAGLMQKTVKVRVGGQKWNKVINKWFADP

KHYLVHDPNSSLRTGDVVSIVPGWPTSKHKRHVIKNIIAPFGTPVEERPP

IPTLEERIAEREAKRATKAERRMKAKEEQKQ*

>Tr|1256_t

MASAEYLQASSTGFKDAKAYDAHRPSYPPAAVESLLNHLGVAGKPHARIV

DLAAGTGKFTELLAARPEEYEILAVEPTQSMRETLADKGLRGVEVKDGTA

ERMDVGDGWADGVVAAQAFHWFANETALAEIHRVLKPGGTLAMIWNIEDY

NKPASWPAASKWEQALNERIFALPDFGPPRFRGNAWPQVFDRQAASAKPL

FSTPIGEEKFPWTVWLDKEALWKRVNTLSHVFTLEGEDKAAFRRAFDEDV

TEGNGEFNEKGEVGVHGVTFLAWSKKL*

>Tr|1836_t

MFFGKALFAAASVASVVNALGINCQGSGLCTGNKGALGNLLGQVKALDQS

KTFSDGEQIACVESSVSIGNPSLCLFYQKTGRTFTVAQTVWYVQALIDHG

CQACGSIPVDDGNNVDNGELTANMVAKFRRRDTGMVKVVRKAEGQTENVS

KLSKRLGINCRGSSTCGVGGVANLPAGHIDDLKDVIAGQGDGVWGNGQQI
GCVAHVTGRLCAFYQNVGDRTFTTQQTLMYLDWLTDHGCTVCGSVPTDDG
NNVDNGELTVNYVA*
>Tr|6160_t
MNETAAAGTDLEMNVTENAHLVNDTVTSFTWSNLEVIVQDRNTRAPLSIL
SNATGNVCAGEMLAILGPSGSGKTTLLNALAHRAAAANATTAGNILVNGH
TASLQEIRDLSAYVEQEDALIGSLTVRETVVFAARLSLPHTISRIEALRR
VDALIAAFGLQAQAHTIVGTPIKKGLSGGQKKRLGVASRLVTSPRIIFLD
EPTSGLDAALSLEVCTYIKEITRKNKLITIASIHQPSSATFQQFDKLYLL
SGGLTCYFGRVGEATAYFSTIGYPIPAETNSADFFIDLVNTDLDKNGEIR
RRTSHICQAWKSSANSRELDTAIQKTVEGTSSSSDIKIPRSTSWRTPIIL
VHRSWIKSYRDVMAYGIRIAMYLGLAILMGTVFLRLKTEQAYIQPLINAI
FFGGAFMSFMAVAYVPAFLEDLNTFRQERANGLVTPLSFMIANFVIGLPF
LFIISLLFSIITYWMSNFRPSASAFFTWVLWLFLDLVAAESLVVLVSSIF
NIFVVALATTAFANGLWMCVNGFLVPMDILNPFWKYVFHYIDYQAYVFQG
MMVNEFGRRSYSCDKIAKGHYQCSYPSDLNSIGKIRGVDVLRQFSIKPGE
EGTWFGIMVGIIAGYRLLAYIVLVLRK*
>Tr|1820_t
MAAKTPNDEHNVLNVQTSGKVANAAGAHGAAKANAAEQSGDDSDDDPDQQ
ITEVAVAAGESGDVKKKKNRKKRNKKKKSLVQTEPPSILISKLFPMGNYP
KGEEVEYNNDNLLRTTDEEKRHVESLGSDFLSDYRQAAETHRQVRQWAQR
NIKPGQTLTEIANGIEDSVRRLLGHDGLTEGDSIIAGMGFPTGLNIDEIA
AHYSPNAGDKVVLQQNNVMKIDIGVHVNGRIVDSAFTMAFDPMYDNLLAA
VKDATNTGVREAGIDVRLGELGGYIQEAMESYECEINGATYPIKSIRNIG
GHTILPYRIHGTKSIPAVKSDDMTKMEEGDIFAIETFGSTGNGWVYDQGD
VSHYALRADAPKVDLRLSSAKSLLNVIKKNFHTIPFCRRYLDRIGQEKYL
LGLNTLVKSGIVEDYPPLVDKKGSYTAQFEHASNH*
>Tr|5020_t
MSLSPKAVLVTGSASGLGLSIAKSLLANGFLVTISDINPSRLAAAKADLS
TTYPPEHLLALEANVASEDSVKALVDQSAAHFGHLDIVINNAGIMDKFDP
AGDCDTKMWDGILAVNLTGAFLVTKHALPHLLQAAQPSGGLIINIGSTAS

FSGLTAGVAYTASKHGLVALTKHTAGFYGPRGVYSVALMPGGMNETNISD

AFAAGMNVEGFKAVKEAHPKMNNVPVDHVARYAAFLCEDGIGQSANGSCI

TLTGNWPEA*

>Tr|4872_t

MESLREKKPQPAGNDATANRVQRGVNLPPSSSDSAAPGPRSAMPKMDLAA

FDGSFAPAVRPREVRLAPWYRSRDYFVGQWLDVSVWKSAVVEMVATSCLV

FLSGQITATIEGYGTPQVGGYIGISNIILLSTFIYATAPASGGHLNPMIT

FSAILTGLCSVPRGILYMSAQTLGGALAGGLLLGVWGHERATSLQGGGCW

YDPSQASPGQVYLNEVFSSFVLLFLSFGVGLDPRQAALFGPRMGPLLVGA

SLGLVTFSSSGIIPGYAGAQMNPSRCLAFGIARRNMSFTALLVAGSLAVP

AGSYPPPPPTYGDDPSGEVGHPPEYPPDYSSQYPEYPPGHGGKDDGDEDS

YTPPSTTLSPSYPTSVGGDKSSYPPPSSTPYPPPEADGDGGNNNSGNNSG

DNNGDDNDDNNGDDDDDNNGDDDDDDNDNGDDDDDNVPGAPEQPPTDGDS

DSPDNGTGGDGTVGGGDDGDGEDNASDLCPGILYASPQCCDTSVLGLLDL

SCEPPRSAPADVEAFNDICQEVGAKAQCCVLPVVYHDFIDIGSSFDNDIS

SIIFDPNFIDRPTSSTSSSVTSTTERSSTTEYSSTTEYSSTTEYSSMTDT

NTPPRPSALPRPNTLPRPIQILPHDRIPFHDQINIHDRYKYSSPTKYTST

TAPCPYTILSSHLYTPSLSPVAIDVVIFFIYTPSLMSFVNVTTAGDPLVL

TTFRNEGHTTLHAAMGRRSRLIHQMISAASAQSLPWPPQTSLVEHLVRPP

SWPETTSAELRFHRLRPLLPFGRISVVSTTAPSTEIRLHPLRCSRSIVAS

VGRASHPPLSFPIRQAFGMHWPQLESTSRTTLQLSLYPFTVSGYDSKEEH

PPHLSSGRLLRGPNKGSGDAKGTHPLDRVFGY*

>Tr|8457_t

MQAMGRDFLLEKGVTTPDADVGQILNVSKRSPFYQLKTLFLFTKSDFKTV

IVPQSIFAATAALSKTQLTTAGPEGPDATKGWGVVSRVPLMLAWLWLNLL

VEDIANQRLEGAIIEDAVNKPWRPLPSRRLTPEQARDWLIVSIMAAVGLS

ALFGGYSASITLMVLIWMYNDLEGSSSGIWIRNALNAGGLMCFSWGALAT

LSGGELLPRGFAWILVTGAVIMTTVHAQDLPDIEGDMARGRLTMPLTYGE

TAARASLAAMVMFWSVTCPLFWDASLWGWAASTGIGSTMSVLALQKRGQR

WDEVVWKLWCLWITVLYMLPAMEK*

>Tr|8019_t

MTAKRLALPARSVAFRHVLARRLSHTRRLHVASPRLEAELTAPNGTRWTQ

PLGLFIDNQFSASVSEDLITTVNPFTEQDICSVSAAGEQDVDIAVRAARK

ALKHPSWSLLSGTERGHLMNRLADLVEQHGETLAAIETLDNGKPLSVSRS

YDVPQFSEVLRYYAGWADKSHGSVIDVGPKKMAYTVKQPVGVCGQIIPWN

YPLDMAAWKLGPALSCGNTVVLKLAEQTPLSMLYVAQLVREAGFPPGVIN

IINGRGGEAGAALARHPGVDKIAFTGSTATGKEVMRMAAGTLKAVTLETG

GKSPLVVFDDANLEQAVRWAHEGVMANQGQVCTATSRLLVQDGIYERFVE

RLKAFTEETSILGDPFDPRTYQGPQIGRAQAERVMSYISSARGAGANVFH

PRGQSLPSTGYFVPPTILTDVGVDTAAFQEEIFGPVAAIARFGSEQEAVE

IANATRYGLAGAVFTRDLGRAHRVARDLEAGMVWVNSSNDSDVRVPFGGV

KESGLGRELGEDGLRGYYTVKAVHVNLTDD*

>Tr|2767_t

MSGEQPPNGESLPSTANSELAKDLNEQLDSTSVETHVEGQRSSNELARVQ

TGVSVEQAEAQFAGLQREFTGVSRASRKNKHQSSSDPEKGATVTAEDDAS

LFDLEAALRGDLDASTEAGIRSKHIGTCWDGLTVKGIGGFTNFVKTFPDA

FIDTFNVLTPLLSMVGLGPKFTEATLLDNFQGVCKPGEMVLVLGTPGSGC

TTFLKAIANQRYGYTSVTGDVFYGPWTAQEFKRYRGEAVYNAEDDIHHPT

LTVEQTLGFALDVKMPAKRPGNMSKAEFKEHVITLLLKMFNIEHTRKTIV

GDAFVRGVSGGERKRVSIAEMMITNACILCWDNSTRGLDASTALDFAKSL

RIQTDLYKTCTFVSLYQASENIYNLFDKVMVIDEGRQVYFGPAKEARAYF

EGLGFLPQPRQTTPDYVTGCTDEFEREYQPGRSAENAPHSPDTLLAAFKA

SRYQKMIEDEIAEYKANLEKEKQAHEDFLAAFKESKRGTSKRSPYQVGFH

IQVWSIMKRQFILKLQDRFNLIVGWTRSILVAIVLGTLYLNLGQTSASAF

SKGGLLFVALLFNAFQAFSELGGTMTGRPLVARHKAYAFHRPSALWIAQI

FVDQAFAASQILIFSIIVYFMTGLVRDAGAFFTFYLMILSGNIAMTLFFR

ILGCVSPDFDSAIKFAVVIITLFITTSGYLIQYQSEKVWLRWIYWINVLG

LAFSSLMENEFERIDLTCTAESLIPSGPGYTDINHQVCVLPGSTAGTTFV

RGRDYVAQGFDYLPGDLWRNWGIVMALIVFFLFLNVVLGEIVTFGMGGNS

FKVYAKPTKELDELNRRLLEKREAKRKDKSDEAGSDLKINSTSVLTWENL

NYDVPVPGGTRRLLNNVFGYVKPGELTALMGASGAGKTTLLDVLAARKNI

GVIYGDVLVDGVKPGKQFQRSTSYAEQLDVHEPTQTVREALRFSAELRQP

YETPMAERYAYVEEIISLLEMENIADCIIGSPEAGLTVEQRKRVTIGVEL

AAKPELLLFLDEPTSGLDSQSAFNIVRFLKKLAAAGQAILCTIHQPNAAL

FQNFDRLLLLQRGGRTVYFGDIGKDAAILRAYLARYGAEAAPTDNVAEYM

LDAIGAGSMPRIGDRDWADIWEDSPEFAHVKDTIIELKRERVAANQASPG

VEKEYASPLSHQMKVVVRRMFRSFWRSPNYLFTRLYAHVAVALITGLTYL

NLDNSKASLQYKVFVMFQITVLPAIIMSQVEIMYAIKRALFFRESSSKMY

STSSFVTAIILAEMPYSVLCAVGFFLPLYFMPGFQTTPSRAGFQFFMVLI

TELFSVTLGQGLSALTPSPRVSTQFDPFITIVFALFCGVTIPYTQMPEGW

RVWLYQLDPFTRLIGACVTTALHGLEVVCKSSELNRFTAPNGTTCGEYMK

PFFENGGPGYLVSNSTQNCEYCSYSVGDEFYSPLNLSFDHRWRDLGIFLC

FVVSNITILYLGSRFVNYNKR*

>Tr|4991_t

MISSLLSLAALAAVPALAAPATTAAAAAGNCFPMGNATLPGNFAAPSTPL

NQWWCPQSDYYGFLGFSYPLEDGNCNAASNGYDQMNRDFAQMKRDFGASI

IRMYYPVCTQPSVFINAIKAAYNNNMGLIVQVWTNFGGGNVWQQSQQAIY

NALNDPSVAAIAPYVVHWAEFGSEPVGDGMDQGNFVNDLGKFRATLNQKG

ILVGISEDWDRPGTMSGSNGQGLASIGAGVKANSDVAHIHPMPFYHFNQP

ESQAWSYIQSQINWVKANVGLPTWVTETQWAWGPTSHYPGHTDLGRTQYT

NYWKTFDNNCAYFKSQNVGWFLHAWRGEDTFDINDPNGGYIIPNWRPQKC

*

>Tr|6762_t

MALSLTDLPYTIFTTIITHLSPTQTILCRRLSRAFLSALTRADLCISLIL

CHFPRSLEGRRLRTYLKTGNLAALESGDWAAVFARLARRYYHLGNALPWK

VEKVAVLKDEEVLRGVEPWDRHLSLNGKTAAFHFWDPVWTVAPKEGLLVY

PAPSPSPSPSLLEGEDDDDDDKGASAGGAAAAAAARYCYRARDLQTGREV

DVPFDLRGRIVRRVRISHGVLVFEWCEREPLSSSRSLVGGGNEETTTTEE

RVHRHYATAYSVRRTGSWDGVIPRRHTDAGVAHDGSSEMPTCSWEFELCC

ELKIHPVGLSLDHHDRFFSTHNATHYAVYTWQPPPRSFDAAWPDDEPLES

LTIWEFNPPNSYSQRPDAASSSSPPTTSSPRIIRTLNNAQLRAFAILQHD

APALRAMALDDSTWDAARASACGHVFFTEEEHRWSAGPHSRPSRPRLHRV

KTTGIPLVGAGPRWVDDCGGGGGVNLRFCWRGRWRRDMTEMEEDEDDDND

GDGEASVGGTDDDDDGDDDKKRGIWSPERAWSHSQTWPGRAPCWRHDDFP

YVTLSEVFDAAAGVRVIARDCFMLETLSVHIRPKIRVQGVGPNGAKLVSR

RASSGTDTKKTTRTFTNTATPSPQIVKNSTTTTAITAGERKSTATITSNT

AARTSTSTSTTTTTQGPDGHEVQFADAMWSEMMGKGFICGDERWLVGEDI

AKGDVTILVF*

>Tr|5435_t

MASVPGPHTGILRDETLALRQMLGYRHEAVREPPVVHPKLDIKILARLPG

SSIKDVLFVSIDVDTGGGYQVISPEQSFHIGISLFDTRSLTNPMDDPAAA

IQSYQYITKESRPCKWAAKHFLFGDTESIQVWTEKPPKPKAAAAAAPKLG

IYAKRRLKAAKKTSKRLLQELPFAKELDEQDEHEFA*

>Tr|2048_t

MSPPLIRRLYPRAHEGRLPINHPELKAIRPKFFKAAILNLCLYILLFFCL

FCYLYGSLFQQGPRTHNLHILFVDYDGGVIGDAVRNAYSSLQSHEFPSMI

ERSVSQFPTQADLREAVCHAHYWAALFTSPGASTRLGLAIAGLNTTQYNP

SDVLSFIWNEAKYSAVMDSLVASNLHTLSDTAKASYIALNGTSAFPTIPS

SNNAAAISAFTNPWTLTSINIMPTTQGTRTVYNTITIVLALIEDFFYLAT

INGLYASFKVYTRISPLRIVLVRDAISITFTMLGSLLNSAAIWAFKANWH

VSAGQYAITWLILWLFAHVNFLAFDVFTIWLPPQYVSMALVTWVVINVSS

IILPFSVVSPFYRWGYALPAHAAYELLTDNWSHGCNPHLSYALPVLFSYE

VVGLILTGIGVYKRCHYAVILEETTKEAMRLRVEAVLKLEREHDILAQRE

KSETSSGEVSPGGPSRATTADMLGQLDQASREQRRQVETGLQQLDNEIER

MGTRASRASNIGPAFCLIGSREE*

>Tr|2072_t

MSWASLSTEIQWLILDYLRDSKCRQYEPPARRRARRETTGISQAAYASVC

REWKELFEKANFEKLILHQDDVTMLGEIIPRCGALVRWIWLRIELPSYDC

SLCDQPESVEEDKINKYLFTDAIWGLFEILSQLNDKHHPGITLELSAHSP

SLVDHYAQELGCMINDTAWHTLGGIQSPRSLNDRFHRWWFGQRRRITDAA

ALRMVGHPQGLRFDLRAPAVKRMDKTLPRVRAVKALVVRGQCYRHLSVSK

ALDPIIRNLTQLRDLSYECWEGYDTAEIKGRHIRLRENKILLSETLRYRR

SLRRICLYEGTSRCNEDHLRPSLTWNRSVIGFGSDLAKASRNLNELYVND

LAEADDFFRPFWGTDLQKRAPRQMVWRNLKRISLFAELVPPLSYDGRIQA

AGHAACRMPQLQFMELWYCNGQYQCVFTYDISQHAHDTHKLELRSSWGGH

LTPATVQVWRSAVCGRGLGMELEVRGQGEVCFLPRGRDLMCALVLRGDAL

TETSRRQILGQHGHSGLGRGSRQMKADY*

>Tr|8265_t

MTVTIERSPPFAVELQRQASDEATRDDHDTARLSRVLPATIRSRSDEILN

PISNMEEFLHQELLVRKINAVQDWLWACGRPMPPRPLHHQLVLSRDIIVT

ENPELHLIWGPRGIHLKPLPEYLLDAGFWRQHILPSSHPIHVEIEQCARG

FLFSYCALIAHYSDFRLAREKGLLPDTVTWEYWKILSSQILESHCYSAVN

PRYWYGELRLGRLNKVYRFRKGHLLRGYSRIAGYAIYGDLIRDNFASLVT

VLGYVVIVLTAMQVGLATDKLVSNSAFQAASYGLTVFSIIAPLISTVAIM

LFVSLWVITNWRATTAFEKRRFAEMGVEPYWREQYQH*

>Tr|5939_t

MKGLILVGGFGTRLRPLTLTLPKPLVEFCNKPMIVHQIEALVAAGVTDIV

LAVNYRPEIMEKFLAEYEEKYNINIEFSVESEPLDTAGPLKLAERILGKD

DSPFFVLNSDVICDYPFKELLEFHKAHGDEGTIVVTKVEEPSKYGVVVHK

PNHPSRIDRFVEKPVEFVGNRINAGMYIFNPSVLKRIELRPTSIEKETFP

AMVADNQLHSFDLEGFWMDVGQPKDFLSGTCLYLSSLTKKGSKELTPPTE

PYVHGGNVMIHPSAKIGKNCRIGPNVTIGPDVVVGDGVRLQRCVLLKGSK

VKDHAWVKSTIVGWNSTVGRWARLENVTVLGDDVTIGDEIYVNGGSVLPH

KSIKANVDVPAIIM*

>Tr|6037_t

MAVTETQTTVEDAIEIADLTQTGRQDAAGPEPEAESRSLDKAMYLKLISA

GFSFFVAGVNDGAIGALIPYFIRDYNVTTAIVSSVYGANFLGWLFAAITN

THLRQYLDLGAMLALGAAFQIAAHALRSWEPPFGLFVVTFWLVSVGQAFQ

DTHANSWVASAVPKGAHRWLAFIHAMYMAGCLVGPFVSTAVASAGEVSRW

YLFYTFPLGLGVMNLVLTCVAFRDTLGLQRKSSASESQGRTLGETAAAEP

SNEAVSRNKEATQLIKKTASTPSVWLLSLFFFFYLGSVLTAGGWVVEYLV

NVRHGNLSQMGYVPAGFNGGGLLGRLLLAEPTHRFGERRMVFLYVVASIG

LQLIFWLVPNIVAASIAVSLIGFFTGPLFPTGISLGSKLFPSDIHSTALP

LVFVFAQLGGSLFPIITGVVSADAGVKVLQPMLIALLTVTAISWLLVPSP

KTSNAELHQE*

>Tr|8324_t

MAPPRPAPKNAIKPPPHRKRIIVAMTGASGAILGVRVLMALRHLNVETHL

IMSKWARHTIDSEVIGWDSTKLHNYAQHVYDIDDMEARIASGSFRVDGMI

VVPCSMKTLAAIYNGLCDNVITRAADVCLKERRRLVLVTRETPLSEIHLR

NMLGVTRAGAVVFPPCAGVLHRGDFGGRSGE*

>Tr|7086_t

MAPHTAKALLLTLLLALQKATAISHENAPETIINNNSGCTSTMTLTQSRL

PTPPYWPQCSWDGTLSIYSSTVTVTRSVDCRGCANVRVTEAPVVHCPAKI

ISTRVRVGTPSTTYRTVCSVTPTL*

>Tr|2830_t

MGLFIFRRGKLHEDFAETPRSVKKPPQDDFSYDFLSVKESSLSNSRTTLC

QHCSVLQLRDVKIVQEDTSRGRKPPELKADVPGFEADLVYRRRDVVPHLP

LMANSADSGCGFCVLLRNAIIQHFRQYLLGRLPDDVIEIVRIRQYWNSGL

AAFTVHSPAFVRPGHRFGYLTFRVSVNSTDPCASMFDIDSERVPESVISP

AGIFTMKQWVFGKGEEKAALQTCYRPTRLICVESRKESGLIRLVESDDGD

KDEKPQPYLALSYCWGRKAPSLVTTKSNYAHLKCSIPINSLPKLYRDAVR

LAQVLGVRYIWIDALCIIQDDLDDWERESQMMADIFRNSLITVIPLRAVS

TDEGFLERNPNIKVLYHSDEWNVSGSFFVRHMPFPYENAESAIRNGPSFS

DRPVSLELQNSAWQTRGWTFQEDMFSTRKLYFGELMMYWDSLRPVDIMRT

EDTIIDDKLDRIDTAELSLIHSSGPWRGDYDYDGWYYPILQYSKKKLTYE

TDRLPAVSSYAKLIASKSGDTYLAGLWENNLHRGLLWKLQRRDRVTFDEL

IPRLASPSKYIAPSWSWASRRGALDLDNTDDMKLECGILEAKTKAHGGDA

YGRVRGGHLLVRGRVCSVPGGRLQKLPLGTPYMDMQCEWLALEDEQYVAQ

CALDWRLTNDRGTQLSEASGRSIEAIVMLLVSSGHSTTPSVISRNPQEWE

DKDGVPSLEVMHGLLLYPTGKADGEYWRVGLFHSLADEQGGRGYFDRCDE

RTLRIV*

>Tr|5862_t

MLQQNILNTSRDWEEIKADYDPESIKESKGYAIWKAIPYTPKHKRVIGRV

VNALSKIPVAFTVAEKLRPTSYLDGLRGFAAFLVYWHHHELWVHGWTKEN

SIFENGFGYDGKYYMAAFPGIRNFFTGGHYAVSTFFIISGYVLSLKPMSL

VQAGELAKLGDNLASAFFRRWPRLYMPIIVVVLAYITLWHMAGMWINGMK

RAESWSEEVVALYREFKNFSFVFKEGGVPWLSYNFHLWSIPVEFKGSMVV
YASQLALSRSSKAARLSCEAFLVFYFMFIADGWYCAMFCTGMLLCDLDLL
ANKGELPLFLACLEPAKDFLYYHLLALSLFLGGIPSENSDVQNLQKSRGW
YYLSWLKPQAVFDYKWFYLWLAATFLVASIPRITWLKRFFETKFCQYLGR
ISFALYMVHGPVLWTLGDRIYAAVGWRTDEHLQHMPHWVDKLPLPKSGPL
GLEVSFLVPHLVLLPLTLALAEAVTRWVDTPSVRFCSWLYHKTLGNSDPV
LAKQARA*
>Tr|4283_t
MASSEPSAKVPVLVTPITPPQESEAQPAKEKRRRAGKPKCDEQKPACQRC
ISTGRTCDGYQATTKPKATRKPGFSQQLSSSSSSSSSIHSSASSLTLLRP
LVTNFSGDVVESQYIRQFLPLAEDLVGITHIHNTFFWGHVVPEYCFTSKA
VRHAIVALSSAYHDFRLAGSNVTVLGPPTNSERYIIRQYNLSLNRMAEEL
NGLPMRKRYGIIMICCVSFFYIEILRGNWPTAMMHLANGIRLMSNLPDEV
EDIFRHPEIFSHDHDNSHARAVYMMKLLRRWEGSAAFMRTNSPPSLSLQA
YETRKSNAGGPKEFTSLEKLQEAVDDFFQDVNAFSWMCRQYKGDDSAWDH
GAARFQVDVLQQRSYHIRELLNKANSVYGDMERAPAPRDFLKYMGCLLRH
RAASIALDAMPLPLGMAYTPPDEDEHKFKEVASIADAIKQALLATLAGGS
PWFNVDFGVITTLYIAATHCYTRSMSEKLFGMMKSWPWREDLWDGPQLRQ
QLYELRKPIVEVPVATTPDAETPVPTTPHLMKLAHRPPSRAAVYLTDSDD
*
>Tr|2787_t
MSRSLLITGATGRQGGAAIRALLSKNADFRLLAVTRDKTSPSAQRLASLS
PKVTLLQGDLNKTDALFQQAREVTGSSPWGVFSVQALKPGPKGPPIEQAQ
GKALIDSALQSGVKHFVYSSVDRHGPESFTNPTDIPHFVSKHNIEHHLVN
SAAKPNSGMSWTILRPVAFMDNLDGGFFGKLFATAIKLKLSGDAAKPLQL
VATEDIGHVAAEAFLRPEEYSGKAISLAGDQVTFQQLSDIFREKTGSPVP
VTWDVVARMVLAMSKEMRTMFDFFQREGYGADIEALRRQYPQLKDLRTWL
ESTPYVKK*
>Tr|4780_t
MGSQVKVGKESKLFEPLTIGNGKITLKHRVVLAPLTRNRGTPLREVSTAE
NPNRIWLPNDVMAEYYAQRATDGGLLISEGIPPSLEGNGMPGVPGIFLEE

QAKGWKKVVDAVHAKGAFIYAQLWHSGRANIPQLTGTPIVAPSSIPWDDP
NECFMYPAPHSTTPVKYSEQLPLELTVDHIRKTIRDYCAAAKTAMEVGFD
GVELHGGNGYLPEQFLSSNINKRTDEYGGSPEKRCKFVLDLMAELASTVG
EDNLAIRLSPFGLFNQARSEQRLETWGHLCRELKKRHPGLSYVSFIEPRY
EQIFSEAEKQKFLDSWGLPDVDLSLFRQIFGDTPFFSAGGFDHTNSWGVL
ESGRYDALLYGRYFISNPDLVERLRNGWPLAAYDRSRFYGPFEDPTIGYT
DYPTYEEAKK*
>Tr|8285_t
MNPNARPIKHIQREDLYTNLEVRIQYLHSFLDFSSRDIDALISDSKYIKA
LIPAVVNIVYKKLLQYDITARAFTTRSTSFEGPLDDIPDENSPQILHRKM
FLRAYLNKLCSDPSKMEFWEYLDKVGMMHVGLGRKHPLHIEYVHLGACLG
FIQDIMNEAILSHPRLHIQRKIALVKALNKVIWVQNDLMAKWHVKDGAEF
ETPDSDIEIEREGYLHGKKILEDDDMKDTADSNGSPDGEESKSKVVRGTC
PFSGVSAAGASVKHDSEETAQ*
>Tr|1744_t
MPLSNVSSILIVVKMFENAVNEAASNRDGIEYVITRIRWYGNLSNVLLEG
SDLDGTAVSGMKNELEARLAELYKALLAYQIKSVCYYYRNRAYNFLRDLV
KLDNWAVNLQDIQKAEDSLRHDIGGFGNQQIRTRLEDICLRHLWSHDPYV
EKKRIEDTKGGLLQDSYNWILGNEDFQRWFQDPRRPLLWIRGDPGKGKTM
LLCGIVDSLQKLAPSSLVSFFFCQATDQRINNSTAILRSLMFLLIDQQHH
LLKHLQERYVRQGKSLFEPPSAWYALSEIMESLLIDLNDSQKPTCLLIDA
LDECVNEDLPRLLDFIVKMSKKIPYVKWIISSRNWQHIIGKMETLGPDAQ
LSLELNANSVTTAVEVYIHQKVLQLSENKKYTKEREKKILDHLLVNAEGT
FLWVALVCQNLQRVPAWKALEALEATPRGLEPFYRKMVQSISLSDDAEIC
ESILTVATVARRPLTLQELIALAETPGDAFEKEQAACELIGQCGSFLTIR
DEVVYFVHQSARDFLSEMLTKREFATGMIEARCHMVRRSIQLLSQSLKRD
IYQLSDLSIHVHAVEAPTPEPLAAVGYSCVFWVDHFADLVDGEYNETTGG
YANDALVPYIKTFIGTKYLYWLEALSLLRRVPEGVIAMRKLQDISVRSPS
ISTCP*
>Tr|570_t
MKLDAIPPELLTTIADFSSTHRDTNALARCSSRLHAILNPLLYRQNAKSS

HSDALAWAARTGNMATANKALAYGADPNASKRGEFGALRWAVAQGHLDIV

RLLLEHGASLRDSMLIVTAAGRGFDDIARLLLDNGADVNALGYLGKTPLL

FAAHEGNERTFDLLLRRGADPFARCSAGSSALFYAVEGGSVSILGKLLDL

GVDMLQTDYESRIAQSHAAELGRQDAIKLFIQCGIDVDHPDREGRTALSW

AARKGHHELVRYLLQEGASVHAADIWGHTPALHAAMTGMTDVVETLLEHG

VALECKASDGRTMLAWAAVNGHVKTAKLLVDKGADMESLDKDGWTPLVLV

ANAGTLLTAKMLVEQGADVNSSNGHDGSPLTLAVARGHIRMVDVLLDHGA

DLGARDEDGKTPLFLALGQLGGEMFHHLIRRGARIDVKGINGVSLLHEAA

RYGGVSYIEYLVDNGLDVNHTDESGRTPLHIAVLNDAKTCFPALLMHGAD

PELRDADGYSAIERARLEGNDADLLFAVEQVWDC*

>Tr|6516_t

MTQIRASIPSLPLPASNRPRIRDLGYSPGRFTPGPQNSILDVPGVQVGQV

TVHEGANIHTGLTVILPRGVKDTRLKPCYAATHDLNGMGELTGCHALAEY

GYINTPVAITNTLSVGKVYDGLFLWQMEQARKNGEDDIESLRRFSIPVVG

ETYDGLLNDISASVIDKASVYAAIEAAQTQTEVLEGNYGGGTAMRCHGYK

GGTGTSSRIVPGADRDYALGVLVQANHGQKPDLRIGNVPVGELLMAEEAE

ARGEAARADKGLPIGGKAAEGSIIVIIITDAPLLPHQLRRLAQHAGMGIT

QVGGHSAGRNFSGEIFLALSTGTSPNQLATSSDGFAYLPPLETQPVETLK

NETIDSLFYAVSETTEEAILNAMCKAETLVGFKGRTTTALPVDRVQELLN

KYGVGLRK*

>Tr|205_t

MAARGTHKHAGGNSKHKVSASSSNLQLSHDPRSFLFVINELTLHDDDRLH

RGDDWYNRVPPSEPRCFDREITGTVMRYHNGQVTEARGYQWLRGDFDITR

NAWPTGYLYRYDSADSPMLTVDGNFQCASVYKQLAVFSCNPLLPIAVLIG

DPLSASSLARVPLLFHHPDHQPGVSHAVHPDSRLRGGGVMCKFVAGASPS

WMPSLVPKTYRNPYEPGPSRGLSGELPIILGLMAFSEARSEGEDIARRIF

LEDNRWRNGEWRHAATPKGYARSALENPLGFLITVFYDPENEEYSNEERL

DDMEFGDVIVRDSRR*

>Tr|3165_t

MFHTKGFRISSQPSPLPPPLFYTPKPSTSWYSDVFESIKRLFTMCCGHRT

AYKTSDAAVRILEEGKNKYEEDCEPLIEGGVEEAEKKTKKLEMSRVTFPR

HAAASFLKTGTSRGMMEANWVL*

>Tr|7580_t

MQGLDNRSRPQAIAASLLLTLYEVLSQSGKTNDWSVYFDGCMKIVNSGFN

IHRRYAGDCAILLNWVRYHKTLYKFSIKHWQQRTPQMELMAKSEVLVSDS

AYDIDLITMHNMLGCSPQVLDYLSEAMDLVKSRDDPNYLSLKRLRAINEL

ELRLKNIDDLRDCFDVEKEGITISKATEIMNDRLFQYALIIYVDRVVKGA

FMSSTAAQSAAAKAFALLEKIRLYDRPFPLFILSIQAETDEQRLLILTTI

QRTMEARPMNNLGSTEEMIKKLWAQQDLQGPEQADALFLLNAVISASSMP

PTFTSPFVINAITGAHAVPFLSI*

>Tr|531_t

MSSTTEPTSTPASTGLPPLRRVITEHDADGNPIINKTLSQHPPSISPGGM

NFTLGYSLNESPAILEHDQDLRAYQDRLPHNVGLSIRGGTVLRIVDFLPG

TEAPMHRTATVDFGVMMEGEVQLIMGSGETTVLRRGDIVGQRGTLHAWKN

NGNREPARGFFVLVDADLPTVNGTKLEENIPNQQYM*

>Tr|1632_t

MMTEVQTPQMYLAQARACGSVQDCMSLYDKWAATYNDEVGDKAQEYVAPE

IVAQVALRSSQDLAHYSILDAGCGTGLVGQALAQAGAKAIDGLDLSPAML

KIARETGVYRNLFQADLTQRVQQPEGVYDLVTCVGTFTNGHVGPDPALRE

FVRLAKKGGNVIATVLQEFWETARFDAEVKKLVAEKVVEIVAQDVIDYVK

GHGDKAVVVILRKL*

>Tr|2713_t

MDSTLIIHWLFSWLALLIMGVRLWGRKYVRQTFNTGDYLTMAACACALIR

LGMIHVVLTWGTSNMTAAQRQDHHFTPNEIYQREIGSKLSIANRVFYNSY

LWLQKLVLLDLYRRLIRDMPYEKWLIRTYLLVFFATYTIVQVFTFSECKP

FHLYWQVLPDPGPCAQAQMQLIVLGVLNIITDFMLLIMPLPVIFKLKAPT

GRKAQLVALFTLGIFIIIITIIRLPINSSHPYSQVNRTTWASTELLTAAI

VVNAPTLYSFWNTSRRNKSTPREQEQGASDKAKDGIVLETIGGSTFSGTG

GGKRKPSGGILQTKEVIVSEYRHSGEYIKLADERDHTSQNSGG*

>Tr|8268_t

MSSSVKSLPLLAITLGLAYVMCVTIYNLFFHPLSRYPGPWLWAVSDIPYS

LVSISGDAHKRMLQIHMRYGPVVRVGPNTVFYSHPDATKEIRGHRKGNKA

EHLKDPHLHSGNQSNVIGANHENHVRYRRSLAHGFSHQAMLDQEPIINKY

IDKLLKELKNQSTKQEKIDIVRWYNYTTFDIIGDLAFGEPFYCLEKSDYH

PWVALIFSGIKNLSFMSVCSKHGQLGKIVAMFLVPKDLPKKGTQHRRLSI

EKVRRRLDSGSSRPDFMTAMMTPRGSSEELTFTELASNASLLIAAGSETT

ATALSAATYYLGLNPETFAKLAAEVRSVFCSEKEITLTNVQHLSYLQAVI

DEAMRLFPSAPGTQPRIISPGGDTIVGRYVPEGTVVGVWQWVNHHNPAHF

RDPESFLPERWLGDARFESDKRDAFMPFSIGPRNCIGRNLAYSEMRLILA

RMVWSFDIRLAEESVGWDMRSKVYMLWEKGPIYVYLTRRE*

>Tr|336_t

MATIYHAAALGRITDLAALDPYKSADSEFGIDKPDDKGRTALSHAASRGK

IDVVKFLISQKANVNAQDNSKRTVLWWASHSDPSVTRQERFVTVEYLLQE

HADPNIPASNGSTALSKFIEHREPTVIKLLRHYGASTDHKIQRGSTTVSI

EELARATKDPDVIEAVMLKPGQASSRDVVVTEIVNYLFRSIGYMNTAFGG

VVRSFFGISGDIRAPLQAAPRERQFSDSDSAIDISQPEEDKVAALSGRIH

DYSDSDASRITASTVGFNTAALDPVQGDHRDQEDQQDKKGQQDDEDKTPH

VSEISGKTTAKQFQAGMTQFIDDTGLGCFFAEDDKFLEDVAMKAVELENN

VDEVLNSKKDIQDITKLALYQPVFYCDDSTSMKSGTRARDQVELVRRVAR

ISTLLVPDGCGTGLQFINKRHTLDDNLKAEQVEEIMRSFEPRGNTKIGIN

LERKILNPLIYDVIDSGKKLERPILISCITDGCASGEPSTEFRDAIVRCV

GYLKEKDYPPTAVRFQISQIGNDRGAESFLNQLRDDPLLKDVLFCTTQRL

DEEYKKLNENEEDLERWLLQTLMGPILSLGRK*

>Tr|8358_t

MSLSWWSQWWKQSPSGGNEHAPKKAPTYRIRGVPNDWDKDRLETFLAERG

FISRPSVRSLAIEFHGRSQTATVLFDTTGHLPLKIPLPDSGGESHSLALD

PGFLSITSLFTPCQQDHKVDIIAISGLSGHAFGSFKERNGEYMWLRDSLP

HAMVDENGKNTARVMIYGYDSSLPNSDSFQNLEDLGTALHFTLRMLAVDG

KFKPIVFIAHSLGGLVVKQFLISLSNSSDELDKKLRRAVYGIAFFGVPHD

GMDIRSLIPMAGNGPNRFLLESIGSYSSQVLSTQQRDFTQALGGQGETEL

ISFYETRMSPTAIKDDEGRWSMTGEPAILVSKASATHCRPWESGPEHTCA

IHRTHSDMVKFADQDPEYDKVLGRIASLAKRAVHMRRSPIQPSLSQPEQE

CLKSLAFERMQSRGNDIDRAVKGTCEWLLKHETYMSWAANHQGLLWIKGK

PGAGKSTLLKFALSKQRDMPSATDRDLVLCFFFHGRGDALQRAPLGLFRS

LLHQILKQIPGALSDLVDSYQEKCREIGDPSEAWQWHPEELWHFLEASVP

RILGARPIWLFVDALDECGEADAVDLAMKFKSLLDSLPSSATQDIHICFS

CRHYPIPPDLDGVFEICVEDENGDDVSAYVRQRLSATFVREASSIADLVT

SRAAGVFMWARLVLERVLRLERQRASWGKIQDEICSIPPDMDSLYLDHVQ

RMEDKVASLRLIHWLCFAVRPLSLVEVYWALAVDADCPHKSLKECGSTED

YGTDEDMERRVIALSCGLAESVTSTSQTNIVQFIHQSVKDFFVDKGLLVL

DSSSATVNEAIGRAHFLLSRTCIHYVLMEDFCDVKTFSWYELAAQFPFIT

YAATGWTTHAHESDSLGVSQDDLLQLFRWPSSDLLNQWARVLDRVVTNGL

DRQPEKSSLVQVAARYGLTGLLSAILQDTSRISENVNLKDDYSRGPLAWA

AMSDQESIVEMLLDTGLVEIDPGDDFGMTPLALAAENGHEAIVKQLLATG

EVNINSRDCHHHSPLSWAAENGHEAVVQLLLDTGQVDINIKDKFGQTPLS

YAAENGHEAVVRQLLATGQVVVDAKDNDGQTALGYAAYRGHAGVVKLLLD

TGEAEAGVKDNEKRLPLSLAAANGQEAIVHLLLDRGLEVDARDAAGRTAL

SWAASNGHDAMVRLLHSKGAQLDSKEKTRRTPLSRAAASGREAVVALLLE

EGVEVDSRSLYDETPLLLAAEYGYAGVIALLLDKGAAVDAKDIDGRTPLL

RAAWRGHEAVIRLLLANNADVDAEDEEGETALSIATANRRQGAVELLKAH

AMTSTASPQ*

>Tr|316_t

MAVGAFLEPFVVVTLLFGGAWFNRNKDYDFWKGTQGWAGGSKRSDDFGGK

RISTETSSPRSPPWSSGSSSPTLGSDDGSTWSLTSRRRNVRFLGFKKTVT

TPNTLVFKDRFLSRVLQKFPFLVEAWYWALIYWVYQLGRAFTAVTIVQGT

VHVARKHALKVVHLEQRLGIFWEVAVQQWFLERPILLHWINRVYSFIHIP

GTIFFLVALFYLTTSRKRRAMSGRVRNDLVSAGPALYEARRRTMAMCNLI

AFVVFTLWPCMPPRLLSDPNYNGPDAPEAKSFGFVDSVHSATGESSVWTT

NKFCNQYAAMPSLHFGYSLLIGLTIATLPVNGVRPNSWKRLAIGVVGLSY

PALILTAIVATANHFILDAVAGAMVCGIAWHANGLLLNLCVLEDYLLWIV

RIHKPVNYTDPETAVEPDYHPILLSEEV*

>Tr|8380_t

MKKVAVVAVLAMSQTGVAARKFWTFKPADPGNVLMTAYTIGNGRQGGLPL

GIPGNDLLCLNDDSLWRGGPFANSSYTGGNPSSSLAHFLPGIQEAIFQNG

TGDESALYGGTEDYGSYEALANLTVSIAGVSHYPKYKRTLDLQTALHSAD

FTANGASFSTVQFCSFPDQVCVYHVSSNKPLPQITVGLVDNYRTNPPSAV

TCSTDGVHLSGRTVADEGEGLIGMKFDAQAHVLSSSGVKTSCNNNKQIVV

HANKAKSVTVVVASGTEYDPKKGNAAHNYSFRGVDPYPGVLKTIKAVSKK

SYNTVLQRHVKDHGEWFNKFTLDLPDPHHSASVDTTDLLTNYKTDKGDPF

VENLLIDYGKYMFIASSRPGSLPPNLQGSWAPDGNPAWSSDYHIDVNVQM

NHWHVEKMGLGGLTDPLWDFMTYTWVPRGTESARLWYNVSGWVAFTNLNT

FGHTGQMNDATWSNVAHDIAWMMAHVWDRYDYGRDEKWYASVGYPMMKGV

ASFWVDMLVQDRYFNDGTLVANPCNSPEQGPTTFGCSQFQQVIWELFDHI

IKDWDAAGDKDTTFLKRVKASYSRLDPGVHVGSWGQIQEWKMDIDVKNNT

HRHLSHLYGFYPGYVISSAHADNKTVMDAVATSLYSRGNGTEDSNTGWEK

VWRGACWGQLGVVDEAYKELKYTIDMNFAANGLSVYTTGSWPYEVTLPFQ

IDANFGLSANALAMLYTDLPKRWGDDSVQKVILGPAIPREWAGGSVRGGS

LRGGGTVDFGWDDEGVVNRAVLRGRRLPIVVVNKHGKVLARR*

>Tr|1993_t

MGHVDPKQPPSKGKPWTAISELDFVHKVDLVIPQDICDTIVQKMAEREAP

VYYRVIMTLGQVLETKFLTQYIKLGNIMMLSEGKTTIGNLFTLREGMLDL

YLDRETYERAGLEGKPYGNKGNRGSKPRWKVSYNLREESMLHGRKKFDRL

AYACKQVLNKPMTWLVCNASSSDLLQSLNATERTSSPRLSTDTGINQIPL

NIQPTILAQGDREALEYSATEIYEWLSLVRLESPRVVAGDNIDPYLSRYA

PPEADGEVAQSPVFVVRA*

>Tr|6071_t

MSHSVVPAPQDSKEDIPDEKAVFTSQESSVTGEVQERKDEAFGSTKDHPF

ADPAMAAHWRGVYEKANYENRHRFDPEFTWTAEEERRLVRKIDLRIMLWA

WIMFCALDLHRRNINRAISDNMLPEVGMNTNDFNYGQTIFLVTFLAAELP

SGLISKRVGADVWIPFIMMSWSLIAGSQAFIHNRSAFFAIKALLGLFMGG

FIPDIVLWLTYFYKSNELPVRLAWFWTALSTVNIVGSLIASGVLQMRGIA

GWGGWRWLFLIEGILTLIIGTFSWVLMPPGPCQTRNWFRGKDGWFTEREE

SIMVNRLLRDDPSKGDMNNRQAVGPVLLLKVLKDWEQWPLYLLGLLVYIP

PSPPSTYLSYILRNIGFSVFKANLLTIPSQFLFAVNLVIISWVSERFKER

AIISSISNIWIFPWLVALVTLDADSSPWIRYALLTGLLSYPYCHAIVVSW

NAKNSNAVRTRAVSAALYNMFVQSGNIISSNIYRDDDQPLYRRGNKILLA

INCFNIVLFYAVKVFYIWRNKVRERKWNAMTKEEQEDYSLNTTDEGMKRL

DFRFVH*

>Tr|2206_t

MVWILEKTRKPVDLTIVSVYPQHFKINSKPHRKTMVPHLATRISLHSLSL

ILLLSPTSIAATNLIPHKPPARLLARAPPTSSFQDFSAIVVAAQCRPGEF

TCNAGCMPQRAECCPDGLGYCKQGYACVVDGCCPIGHTCGDEITCDLGEV

PCGEKLDTDSGADLIFHVTAGYLFRVCHQHNFDASSDFIAPLSPRRVIKF

VTCNDDHFIYFALFFFFFSSRISNDDFN*

>Tr|3147_t

MAGRRIVRDYEPRIPLSRGDSREDSMSDGFEELGPRSMLPNPRLLKYAVD

GEVPDVLYTLNHVSQTRRFLGRQRSSKPFENVTSYEIKRDESDEEGELVK

KPVFEIVTTVCIEGRPDDGRRIVHPRRGHPQMERPITSAFDNVDYRSDGY

YTEEEQYYDPPGTPQAETPLMVIYSEYLINALRAVVRYYPNLNLDGKTVQ

ITAPYRVLYHHRQELSEYRENQPAGHSAEYAETTNHHIDVLLKFLDESLK

DEISKEEERHQLETPTATFEHFWLLLKPGEVIYTKKHDIWTPFVISSVGV

GNRSHGNLDNYRISCWLLESNGVKVGRFMESYVVTPWPGEQAISSLSVIP

AKFWKEDLAAQGNRTMREKLVEEGKLYWELLKRPTYMEYDGLLVNSGSGS

RLPGGPTGFMNGRVICDASGFDKYYDSAPDVEGRRYPLPGRRRVTQGVPN

KDFLPQNLPRCGCAVCDEGRVMENDGPYVGFEDLDPLCDAPPSNDLFFYV

LSKTIPGFILGTRRWGNLHVANLSPVKPDKEAFKYLVLDDDIKMTVRALI

GQYTTGVDGQVAPWGNDFIRNKGEGRIFLLHGAPGVGKTCTAECIAELTN

RPLIALTSGDLSVDAFRVESNLNYFLELGQRYGALVLLDEADVYLERRRA

KNISRNGLVSVFLRALEYYRGVLFLTTNRVRSFDTAFLSRIHVALHYKNL

GDEERERIWTHNFDRLDRDSRGKIRVAMAAREFVWSSQEVRALKWNGREI

RNAMQTALALAESDSNEEGLQTITIGEKHIRAVVKMSKGFKDYIKSVVPA

GSDDVDYDGDSDDADAN*

>Tr|6199_t

MPLGVVGEDIVVLNEHSLWAGGPFQSPDYIGGNPPAPVYTALPGIRETIW

KTQINNDISALYGDPAYYYYGNYETLGNLTVNIAGVSKYTSYNRALDLET

GIHTTEFKANGAKFTITTFCTFPDQVCAYNIQSSKPLPAVTIGLRDSLRS

NPASNLTCDANGVHLRGQTQQDIGMIFDARAQLINRPKRATCTSSHGLSV

PSDGRTTSLTVVYAAGTNYDQKKGTKASNYSFKGVDPAPAVLSTIKKVSQ

KSFNSMYNAHIKDHNGLFSQFSLDLPDPEKSASVPTATLMENYDYDLGDP

FVENLLFDYGRYLFIGSCRDGSLPPNLQGIWTESLTPAWSADYHVDVNVQ

MNHWHTEQTGLGEIQGPLWDFIIDTWVPRGTETAALLYDAPGFVGFSNLN

TFGFTGQMNAAVWSNYPASAAWLMQNVWNRYDYSRDTHWWKTVGYPLMKS

IAEYWIHEMVPDLYSNDGTLVAAPCNSPEHGWTTFGCTHYQQLVWEVFDH

VIEGWEASGDKNTTFLETVKETQSKLSPGIIIGWFGQIQEWKIGWDQPND

EHRHLSHLVGWYPGYSIGTHMWNKTVTDAVNVSLTARGNGTADSNTGWEK

VWRVACWAQLNNTDIAYTYLKYAIDMNYANNGFSVYTTGSWPYELAAPFQ

IDANFGYSAAVLAMLITDLPVPSASKAIHTVILGPAIPPEWKGGSVRGMR

IRGGGSVDFSWDDNGLVNKAKLHNHKEAIKIVDVNGKVLIHQ*

>Tr|1208_t

MSSVLPVELLLIIAQSLNAGDLYAFVRVFPFLAGELTYTRHLLAVPDEYG

NNLVHLFARNGEAASLELMFPERSIEESRSNDILRQRQELLASSANQKDA

TPLLLAAEGGHMAVVEKILRWPGVDLNHEDHEGRTAVDVAAKEGHTEIVS

LLLERPELTVDWNDGNRRANPLCLAIASGHVETARRIIERHGNRISVNSK

SAHGRTPLCHAIDRGHDALLALLLEQPELDVNLAVDFAATALHLAVRRQN

QAAMKAILAHPNVDPNRQDVVQQTALHEAVYLADESMLRLFLRHPRIDVN

VRNCHGATPLVNAVKRGYGISVLLLLQHPNIVPDRKDDVGMTALSWAAHL

GSYNVVKMLLNRQDVDPNARDDDGMTPLSIAVQGRRYQVADLILENPSVN

VNDTDDWGWTALAHAYHSPAGWDGPSVSLLLSYGATMTVYPEMTVRLETL

SDAEIALDLVEAMVKDRDLERYMIRRFGLERTELAFSQGERVVRALLFRQ

TPAAARRAHDQLGSSLLW*

>Tr|4998_t

MSPFSSNFTNKHVLVTGGSKGIGRSIVELFLAEGANVSFCARSIRGDEFS

LFKGAANGARAVGSAVDIGNPDEIKAWVDQAAKEFGRIDAVIANASPLIA

EATPEAWEKSFRADIMGLVTFIEASIPYLEERTKASENASIVVISSMAGF

EARHHVPGSPYSAFKRAQAVLAKDYARKLGPLGIRINSIAPGSIETPNIT

WPDGTVQWSQYQIVKRDNYPFYKSLLDSVPLGRTGEPKEVANTVMFLASG

LSSYINGTNIIVDGGLSLFF*

>Tr|3606_t

MHLSSTILMAAAGAAAQSTFDLSDLPAFIKSSSSTNWPTLLNIGQQIWND

PEVGLNETHAHDRIVKYFSSVGGWTVSPSAYPGLPTAFKAEFDNRPSGFT

GDLPTVGFLAEYDALVGIGHACGHNLIALNGMSAAKFASEALAHYGIPGR

IIVIGTPDEENAAGKFKLNNLGAFNGSDVWLMAHPTVTSAIQPMNARLNY

FARFVGGTHSEAVSKAYDALDIVQNLSGLPGTSSSAVGIENVGVYATNIV

QSIIKLGVAGLSLDTVNSTVSGILDSTYPNVTFSAATDADGVALTILGPG

GHASEATKSPLTLSVNTYNALKNSSGVSFYLPGNTSVTELDITIDFRTRY

TADLNSVANFASAALGTLPAGISHDVQYPSLEVTPFLGQAFIDIVGTPDY

GLANFPATTLAPASTDASWVQDPVLDPVTHELKSVAKAVLHANYGICTNP

SGAGCPFNHEPLFKGLANTDFAYQQTEIVARAEAQLAVELLADPSQMAQA

VAIVLK*

>Tr|4276_t

MDSRFNEPWPYSSTQRASDLIVPQIVDGQAGDSADEPSGEAFPPLTDNDF

HSILRQANGDPLTNSDYDSILRQASEDFVRDRQPEATSSVKIVFDSSDFL

NKSQWDKVMQGLMSEDEIQSSRFAEACKLRVVEQELNTGDTLVFVDFPDL

SRRRSRQTDCYGVPYSSQKLRVHSQKLLATGSAKFAEMLSPTYQFRIKRR

KKLTKDLPEGVKYVLDLTPPSEGDDLVSQMTQLSLTPGIIKWWAASGLHK

VTNWLVSGHDDVCSCGRQHIPGWGAPRQDGTGQEQDGERSVNGGATLFDN

KKPGGNTDDTVVTAPTPQDLLSKKEHGINQPYEIPDYRNIPDYCPIRHCN

SIIRLLMMIEGQDIMLDSANRVWTLVAVAKIFDCTSIIRDRVAQWIMFSD

NTKFIEVLPEEALQIGFDLELVQVTQCAFRILVNELALKEAATKAPPGGS

SPGESKPDLDWVTVFGRRLGNLDDELHNIVQHAARALIERVSVIPGIFRD

RETLDNWMVDEWAKLRKLEALLQQQSDEASTKASKSLSSLLMAMRRSIST

AFFRLSVAATTEGKQALVSMDEDRATYVLPEDFKLLEDILPKLNIHQKFL

CPFVYQQMSEMWHSDPSQSKWIAMAYQESPVSIKSNMDLTVDDVQRAVTA

NPEMASQPSWAMFMDPGEGPLRVKNPIVDLEALNRQIYDAVSPYINLWLR

YDIEPALNMTRHLLLTLNNNEMKFLPLWAGGCNDGTGGVFQEELPPAYWG

PSGPGPAYHTGITIPSASSTSGSLVDGFSTFNVRGSTVRGSTVVGSVDVQ

DGASTVYAPNHVIADDVSIASESFDVDGSESYQEARFAVPAEHQDMGHAV

DMLVESVDSDDDTASDTTDRVMISGSAMETGADDLDEDEDMSFDE*

>Tr|2216_t

MSGQDSHSRYLQPGNSAGGAYGNHYENDGNASSSQLSSHSRSHSQSSTGG

RRRKGIVGRIVGGVAAGIGLASESYHNHQEKKKAQAAAAVDESQAVNAPE

AYRDEGRHISQAREGDGASDSDDNDEPVRVDQETSRDLDEAAWQLDAAQS

ELEPPPDYATVTGQSLDVQAMAEGFIQSHALLSSQQVQRQGLPMPVILPQ

RRPGERARGFIHAYAPLLQDVGIDEATFNDFITQLNMATAPSPWINAINL

AAVAVQNVPEPITIAVSIAAQVGTQVSLQAHSRSKTNTFLNKMNAEFFRP

LGLIAVILTWKPSRAGEVVTQATFDEALEQATQHASGPGTGAASSFNNKM

QASHGTTSFEWPESAPLVFPTLDKLANTPGGRQAVEDAGKKPNGLKRGMN

FAMEYMDKRSQAQWANEHPESRLAQLNEKPGFHSRYADPNHPASSGSLLA

LLTGGAVTGKLSQRSQAKRDERAARREHRSERREAKREQRGPTLLGTVGP

RALIRGIKKFMHEDVLYLMIANLPSTEDMAAAQRLLQAHPVQG*

>Tr|2590_t

MDKAAAAAATPPAQNAGSSTYTPFLLKLYDLVVLSISNSYAWHCSTTAVQ

LPLFQSSLGRRHLDVGVGTGYYPAHALKSSSTNNTSTNDKTTTTTTPCRE

IVLLDLNPHTLDLAAARIAAAQPAVKIRSVVADVTDPATLLPALPPSEEK

FDSISVFYLLHCLAGPPESKNRVFDLLREYVADEGVLVGTTILGEERPMN

WLARRLMTIYNSKGIFDNWKDTRAVFEEGLRRNFGEVDVQIIGRTMVFTA

RRPIRASS*

>Tr|7662_t

MASFEYNEHSQRVLFGQDSLSKLPSELARLGCSKPLILTTPTKTSYVDTI

TDLLYGQLAGSFNRVTMHTPTEVTEDALKHCKAVDADCLVSIGGGSAVGL

GKALFVRTGLPHITVPTTYSGSEMTPIVGQTEKRVKTTHVDPKAIPAVVI

YDVNLTLTLPAKISATSGLNAMAHAVEALYAQDGNPIASMFAIKGIQTLA

TSLPRIMRNVQDVDARSDALLGAWFCGKCLAGVGVSLHHKLCHVLGGTFN

LPHAETHAIILPHALAYLAPSIPNEMKVLAENVPESEGNAVNGLNRLLSK

LGITYSLKDLGLKEDDIDVAVTTLLQKPFWSPRPLEADLIHELLRRAWEG

KPARMAE*

>Tr|1838_t

MAADGPMVPDYEVHLLMNPSAVLGSDFKLTPTVLSTFAMPTTVTKINVQY

LDTITEDFHVNGWSCRIRNVENKSGFDLTYKWRLPVNNGDINGALNTAFS

DGWNSGQGNYDAQIDWLYSSQTLSIQRDYSASSQGYSGTDDPSEKDSRIM
LEDNAPGMFDDWVSDGWGTDLLKSAVIYSPVLTKRSTGTWNGLELDIEVW
PIENSSGTGIEYLVEASFKTDDYDTASTLRDQLITLLQNNGWFLAESVSK
EDLVMENYHPTS*
>Tr|8557_t
MPADSTQPTKALADFFPNQRKNLPIALTTEDVKGKTYIVTGATGGIGYEL
AKHLVRLEASKVIIGARNEERGKATKATIEGDAGRKDVVDIWPLDLASYD
SVKAFGKRIETAERIDALVLNAGVSHAGWQVKEGNEAHLTVNFISNYLLA
FMALPHLQAVGKTHGITPRLVVVGSMGAFFAGESVKKFPSKDILDDLNDR
AKWESDFTNRYSVTKLLEHWAARQLASLRPVSDLGVIVNVVDPGYCKTGL
NKELSLWTRAKAWLAKVMLGRTPEMGSRTLVHAVKVGEESHGKYLTSCEI
REDHIPEWVTNEAGQAVQKQVWDELLVKLDKIEPGLPSRVIADWA*
>Tr|1065_t
MALKFRRPTVPTTWLLPLLVVNLFVINLGLSISDYPYMKLWQDIICKHHL
GLSSDELLPELECHDRAVQRELNIVDVGSSVSSTIGSALVALPLGMLSDK
VGRVPVLALSIISLFLSEACDLLICWKWHHVPLRAIWGSGAIMLLGGGRG
VAEAMVFTSIADVIPESKRATCFQWVVAAVLSSELFGPLIANRLTEISIW
CPLLLELGLIAAGGLVLVLFMPETLPARNSPVLVGEDTPIMSNSSNNNND
NNITTNNNTNKTTNTIDNITETPENSSSQSPTKSALATIFRRPAIYLLPG
SILAMPAVTSQYGIVMRIMPIQFNWPLSRASLLFSLNAAVTLLTLLLILP
ATSYLLYRRSSSSSSSSSSPLQRDRILARASVVLFVLGSLFLVVGGRVSL
VILGVAVSALGSGVPTLCRTLLIASVGEQRTGSVFGVIAAGEVLGMLACE
LIIGPLFDIGLRTWLGLPFCLGMVISTTTCVLTWLVRDIDV*
>Tr|4947_t
MLPFYTAVLFNLVWTLNAFSIPNVNATFSKSPKPFVLRVNKDFIDDTRQR
VLTARSPIFLETTTYEGPSRDNFTHVQEYWVQEYNWTRVEESINKQLQQF
TTIVEPTIGNDTSPIPLHFVHHRSPREDAIPLLFIHGWPGSFLEVSNIID
ELTHPPNKSLPAFHVVAPSIPGFGFSPAPERPGFGPVRAAHAFNKLMQQL
GYVRYVVQGGDFGGVILRFQAHLFPNNVVSALSNFWIIQPDQSDFRRLAQ
GLATPDETTYIATLETYINQASGYRILQQTQPLTSAFAVSDSPLGNAMWM
YALMAEVVDPTIKNWTPEEIITWSMMYYIQGPYAGMRFYREVANDGALDG

VDFGTIPWVKIPVAISQFPYDVAYRLPLEWARRGGNVIKRNVHDHGGHFA
AYEVPDLLLRDIREWFGDRVVSGTKAFLD*

>Tr|8958_t

MADTASQALLPANQARNMAFDRVLHKNSSNSQGGLRAMVNKDNEAHKAAV
NEYFQHWDDKKAEDETEAVRQARVDDYASLTRQYYNLATDLYEYGWGEAF
HFCRFAYGETFRQAVNRHEHFLASSIGIKPGMRVLDVGCGVGGPAREIVK
FTGCHVTGLNINSYQISRAKQYAVKEGLTHKLDFVQGDFMSLPFPDNSFD
AVYAIEATVHAPSLEDVYREIFRVLKPGGVFGVYEWLMTDAYDNNDLAHR
RIRLDIEQGDGIAQMLKVEDGLAAIQAAGFTLETHYDLAEDDVNDTSVAP
WYWPLGTDLRYAQTLTDVLTVLRMNRFGRELSHAFISMLELLRIAPAGTR
KTANSLGKAADALVEGGKKKLFTPMYLMVGRKPEL*

>Tr|4672_t

MSALPPPPAGINLHDDKRASIMAASIVTWALAVIAVSLRIVSRRMRKMRL
WIDDWLIIAALIPSCAHVFGIGVYAVSRGLGRHVWAGPPDARYAWAIGLF
IAELGYFVTITCVKFSILAFYWRAFRVMRSVLIPIYTLALIVACWAIAVI
LVTIFQCRPTSAWWNRFNPSHPLQPDQYDCTVDSVKFFYGNAIPTIVTDV
LMLALPLPYIAQLQLPRGQKWGLAGIFLVGIFVTVISIVRFHYLIEGNLT
SPDITWNFVNIALWSVLEGNLAIFCACLPFLRPLIIKLGCRNLSLSTATS
KKQKQSSSTPRMSLHPNDHPRTWQGDGRFGTSTCHSRAYSTTRETDGHPF
VRLTDNGSEASVPVKERPDEPQIELGNLPAGGQLGESNGIVITREFHLQH
QDIRDASTYIYHYGYGVRNHLLAKAVLADNRQDMALGLGSRRD*

>Tr|5861_t

MSSPLGSPRLMHSASPFRNKVPTQMRRCLPLYITVVCVFLVIVNFDWILA
IPNPASVLRREPKAPPLPGSTFPQKIWQTWKVDPLNFDERDLVTARTWTT
INPGMRYEVVTDANEMAYIEDRYGPNGFDRPDIVEFYKMINLPIIKADLL
RYMIMYAEGGIYADIDVETMKPFHRFIPDRYDEKDIDIIGVEIDQPDFK
DHPILGKKSMSFCQWTFVARPQQPVMMRLIENIMKWFKTVARDQGVPLGE
VQLDFDQVISGTGPSAFTKAMLEEMNRKTKGPKVTWDAFHNLDESKLVGG
VLVLTVEAFCAGQGHSDSGNHNARNALVKHHFHASNWPSRHPRYKHPAYG
QVEDCNWVPECVRKWDEDTSNWDKYSENEQKKILQDIENARLERERQQQA
LAALP*

>Tr|309_t

MGWFDDDSEQAQAYQQVTERPHEAQWSHELIGGAAAYEAAKAYEDHVAEN

GHPDDHAKAKEILAGVIGAFVDREVETKGLDFIDRERAKRHAQEQAEAQL

SNQYGDRW*

>Tr|5802_t

MNFSTKQYEEIPPPKVSCDVVEPAEVYEWLEQHKAAGEDAQKDFQLVDVR

LNEWEGGTITTSINLPAQSFYQAREMVYMLSKQAGVKKVVFYCGSCGTRG

PKCAGWFQEYLNSVGETEMKALILKGGFKGWKTTYNGQMVDVCDPDAWRY

AWRSPSK*

>Tr|7874_t

MTQITLTDQDLNLTFHATYTTTTITPFLISVKQPFLDETKLKASLTRFVT

TELKPPSNTQPTASFTDGPPADAARTIAAHWTNEYSWRAVEAAINARLTQ

FTTIVTTTTPETAAYKEPIPLHFVHHRSPRDDAIPLLFVHGWPGSFLEVD

RIIRLLTHPPKSEKGEEGSSSCPAFHVVAPSIPGYGFSPSPLARGFGYRQ

AGAAFNALMHKLGYPRYVVQGGDAGDFIVRYAALDYPEAVVSLHSNFWVV

PPSEDDRARLAQGKATQEEADIIRRLDGFSSQRWAYGHLHQTRPLRLAHA

MTDSPVGLAMWIYDVLVSCVEEENVGRIWTPETVITWTMMHWIPGPYAAF

SLYKHGAADGAISVRGIEALPYVKQPVAVSQFPHDIWYGTPLEWARRMGN

VKWSAVHEKGGHFPAVETPEVLVEDMRRFFGNEEESGTAVFRQ*

>Tr|7173_t

MLLLRILASLLFHLPIVLSIPRHTPSLSPPPSPPPPPHHRNHHYNRSLEA

IVVVAPLPPPPPPPTTTTIIEPIPFPLPPTTSFPLPPPTTTSDPWKDMCL

PGQECDCSRIKDKNGEEYFQCVTNPRCDHCWINITTTTTTTTTSSSSLPS

FSTPIILTTPATTKNLLSTDYRTLLPGTYTSSSHGTAHVVVVQQPASIHF

VTLTVTREVQVTPLCEAVGSGVV*

>Tr|8453_t

MDLSDFEDLGISSDAEDDNEADEPYMTPAVSAKSYREGDGPRPPWEGRRD

KLVGIVDMGSNGIRFSITDLTPDRARILPTVLAYRSGISLYDSQFDPETG

EQVPIPDHVIDHIVTVFSRFLIVCEDMGVEKKNIHVVATEATRKAINSAK

FLETIKKQTGLSVEVLASEVEGQVGALGIASGFDTLSGLVMDLGGGSTQI

TWMISQGGYIRISPMGSFSFPYGAAALSRQLHELRKGKKKDEGEAAVAQL

RQEMIEKFRSAYKHLQIPDSMVEKAEREGGFRIYLSGGGFRGWGYLLLYL
NQIHGKHYPISIINGYTVGRDQFENTERVKEIAKAAKDVFRVSDRRRSQV
PAVAFLVNVLAEAIPHGIREAHFCQGGVREGYLFRTLPPEIRQLSPLEVA
TQNLAPTSYRSIQELIKRAIPAPSRHETRKFPDEFGEHVINSFANTIYVH
MFMSKETASTAALYSTSVGMMSSTHGVSHQDRARLALMLESRYRGELPPR
EMEFREALRSLITPEEVWWTTYLGRVGYLITRLYPSGEIDTKKPRVVFSS
EWSWTLGKKKNKEGLVLTLSVQKKKDDPARLKHALEDNVKLIQKVGKKKH
WIGKEDPWGMKVKVVVVEEGILESSD*
>Tr|9268_t
MKMESSNPTGSMKDRMAFAMVTAAENDGRLKAGGAVVEYTGGSTGVSLAL
VCSVKKHPLHVVTSDAFSKEKLDHMRLLGAELTLIPSDNGKQTEKLTKDM
IREAHALAERTGAYITAQMDNTDQLAAYTKLADEIYKQTEGQMDVFVQSV
GSGAALRGVSERLRRLDNRIRFVAVEPDESAVLSGGPSGSHRIDGIGAGY
VVPLWYDGIADEFQAVSTADARAMVDRLAREEGLFCGLSTGANATAALRV
AERLKPGSTVVTVACDSGLKYMSSFRQHMEPLT*
>Tr|1421_t
MDEPLLGDSIRTSCERCRRRKIKCDRKRPCSRCAKAGTECVLQGIGEKQR
PVSKSYVQALEGQIAALETVIHKLALADDAERTQIISDLSLSSAAPSVPL
AEAGPADLKTDPSLAAARVKSGQLRRLRGSNAAQFFGGTSLLQIHFSQQS
SSSVAANSTAMESLAISTDSQPYSNSNSNSNSHSIGVANNGNPSLELSEL
YGEFDDLSPMGASFQYEPHHETSQKMMSRFFQEVYPYNMVVYREYFLRDY
DVGSGKYYSDVLFYSICALGALQCDDMLNLSDVFAGQAQTLLYSTLDSPD
LTVLQALVLLGYREIAVGRASKGWLFCGMAFRLAHEMGLHLDPTNWQGPT
HEETSGDREILRRVYWAVFIADKQLSLYFGRPPALYPSESDVRNTIRLQY
PPGWQGLLETYICKGSSANEWDNGVTLVGSFIYRAELAKIIHLIITDLFE
NRRGGADPTIIATKTRRIHVLLTRWLTNLPSSLHWNQWTVGQVPPYVLHL
HMMFHTIMIILHRPPSHMFENPGIADSEDVEICYESLQAILRLMRSYSRY
YRYRSLPLDFVHTLSTAASTVMMKRFLQKASWSDPDIERALSLIMQAMDE
IQNTWPCVREIRDVVLQAQQTQATMPPEDPLNAPGLMNGLEVDHDGFLAN
LGEDVDIGTLITDEFLSAQVPILEQGLEPFDFNQLSGPP*
>Tr|3575_t

MSAVEEKQPALTREHEMPKPHQTNGSDFSDAEIDPRAESRLVRKLDLIIF
PTFFVIYMMSFLDRINISNARIQGMVEDLDLTGNKFNIALFTYYISYILL
EVPSNMVIKRFRPSLYLSTLMFCWGIINMCMGFVHSYNSLIGLRFLLGIF
EAGVLPGIIYVTSMYYKRHEYQKRMSFFFCSTVVAGAFGGLLAYAISKLG
GQHGLAAWRWIFIIEGAITSTFAIFAAFLIIDWPEQTKYLTAEEKELLRR
RLAADIDETCRMDVLNKASFQRIISDYKIWLGALIYMGVGVPGLSGTFFL
PTILLEFNWKAQEAQVRTIPVYVLAGGTMVIGAWASDKLRHRFSFFVAGT
SMVTIGYGMLLAQEGKSRDYKFGAVFLVFGGAYMVTPMALAWLQNNLSGH
WKRAFGSSIQVTIGNIAGIIGSNIYLVDEAPTYVTGYSVSLAMMWLGMIS
ATILFLLMWRENKKREAGERDDRLRLLEDEKNNLGDDYPTFRFTL*
>Tr|6075_t
MSGHWSIGVCALALLLLSSLSPASAAAVHHQNAPSAHTVNGTYTGLSLES
FNQEAFYGIPFAIPPLGDLRLRYPVPYNQSWRGSRNATVRSDSCPGFDKP
FAQGFADGLTMSENCLTLDIVRPANVKPGDNLPVFFWIYGGGFKAGGSAD
PRYNTSFMVRNSMEMKKPIIAVVPNYRTGAFGLLASKEVAAAGVGNIALF
DQRLAMEWVSENIRAFGGDPTKVTIAGESAGGSSAGYHLVAFGGKNNGLF
RSAILESSSLLGATMNTVETLNLTYQGWYDNITTTVGCNTAADSLACLRT
VPYLKFFNAINGFQFKPYIDGRFVTQPPSISIAKGQIADVALIMGSNTDE
GTAEFFTPRGTLNNDSDIASLVAHLANGLSDEIVSNVLRLYPDDPIQGCP
FGTGPERFADQGVQYKRGAAITGDLNIHAGRRAYAVSHSQRSKHPIYTYR
FDQAPWDMKEVDVTTTAPVFVTHYSEIVHVFDNPDKNVNWIGPYPILSEL
ANYVSRSWVSFIHDQTPNNHGLQNKPVWPRYDASKPQNIVFRAGASWIEK
DDWRKEQLAYWSTIWSEVMT*
>Tr|6282_t
MLLLGSLLSFTALATAACAARFPKPQIKPWVPPGPDDSRGPCPMINTLAN
HGYLPHHGRNITASMIEHAFTSFLNVEAGFADAARDFAKAFGHDVFDLVD
LNTPGIIQHITSLTRDDYTPEQPQLKADPLRIESLLADSDTPFLTVDSIA
KTRLRLLQESEPRVLPDNQVTFTLFEAAMTLTMMADHSPDADHDPPPSAY
QGPKDRIRMWFEEERFPTEFGWRPSKRTIKLADMSPAIDAIVKSMEKQDQ
NGKVALKFHS*
>Tr|1647_t

MGASSHFRLAWAVLTTGLMTAMGDKSDESLVALPAVFEVDLLFPHNATYA

PSALMPILFAVQNPTLAPSSATITFDLWRGNDTRSPGSVGSVPLALSLDV

ATERQRSNEPLLLNAPVNTIAYPDGVWTLAWSLEYFNCSTSQGKARSETF

YTVFTVSKSGQAPDMKAGTSADVCGSSAALAYNVTSWDELACGMPGPSPT

TNPCSVTVDSAAVSSLSALARATGYVCSPNSNITCPTSFSSSGSPRVAAV

STLLTLSAALTVLIQLR*

>Tr|6174_t

MHFSYLATTALVSRALAAPPKWNTPTLQHWLYSSQVDEQSLDLLDRPDIV

GIQALYSWRSLEPAESHYNFTVIKNDYDKVMAKGKKFWIQLQDRTFSAGN

DPVPKYMHTPYYNNGSAPSCDGDSCDTDFEVTGWVAQQWNLRVRQRYQAL

LSALAKDFDGKITGLNLAETSIAVDEDANNYTNEGYFLGELENAGHAARV

FKRSYAVQYVNFWPDGWNNTNNRFEDSFNYYAKHGVGVGGPDLIPNKPGQ

EANSYVYIPKFHGTVPIAVVAVQEKDLEEINQETGQPFTKDEFVDFAVNE

LHVDIIFWALATPWLQSS*

>Tr|2229_t

MEAKGDSSKILDYVARSLNEEEDFHFLRLEFLHRLNIVELQIQLVQIKRR

IQNEHVSDAESLEKLRVGLKHYATAIRDYQFLRNMKNVEKASIPHRKLLL

QKYFDAELDLDDPYEAHYAYCHDVNANIDPIRESLLKYIPDNLAFSRSKC

LQKGDGDLPEGKPPRSVSPFVDRLARFIISFIGPVFLVAPMIIMTLNPSQ

TKSLVTVSVAVIIFSLLLSFGVRVSNVDTLVSTATYAAVLVVFVGTSTGG

SNS*

>Tr|4959_t

MTATKNIRMALPKSGVDIFYREQSPADPSKTILLLHGFPSSSHQYRNMIP

LLATQYRVIAPDFPGFGFTEAPAGFKYTFESLTDVLSEFIDSLSVASFAV

YVFDYGAPIGLRLALRRPHAVDAIITQNGNAYVEGFGDVWGPIKDYWASS

NTSDDRSKIADAMLSFDITKFQYENGTPNLQSIAPESYTLDYALLQRPGR

MDAQLDLFMDYQNNVPLYEKFHAYFRSSQVPLLAIWGKNDVFFIPAGAFA

FKRDLPEATVKLIEAGHFAAESDGPSIAREIIQFLS*

>Tr|4866_t

MADADLKHEVTRLDSVHDVKHGGATAEAVEAIRAEHELTFTEALKLYPKA

IAWSGFVSIGVIMLAFDPQLIGNLYSTPQFARDFGHLYKGDWVIQASWQT

ALSMGNPIGQVVGALFAAYPMDYFGRKLTFAVCVVLTAGIVFIQFFSKTL

GVLLAGELLAGLVLGMFVVIAPAYASEVCPTALRGHLTSFVNLCFVIGQL

LGNGVTAGTSKLNNHWAYKIPFLLQWFWIIVILPGMAFIPESPWWLVRKN

RLEDAEKSLRRLASPKVNVQATLAFIMETDRLEQELEAGSTYWDCFKGDN

WRRTEISMGVYCTQVLSGIYLINYGTFFFQQAGLPTDRAFDMSVGFLAVG

FVGTLVSWALLIRVGRRTLFVSGLAWLVILQFVIGILDCIPGRPSGAIWA

ESSLMLIWNFFYDISIGPVCFVLLGECSATRVRSKTIAAATAAQGILGIV

MTVAIPYMINPEQANLQGKLGFFFGGLALICLIWSYFRVPETMGRTFEEL

DLLFDKKVPARQFKGYKLEGAVSTGAA*

>Tr|9043_t

MSSQEITLGWELELIVHLQPGEQSGLALEAHKLRALALTLAEQVPNLPIA

AHCIHRPSASCIICEDAPAEHRLPHVRVLNPATPIQLPNRPIEDLYYFVK

REWLNVPTDDDGERLAHGFEITSPILSQTELRAGLPQTKQILGAIRKSGL

PISAHTECGLHFHVGVKSGMTLSIAKKAATLVMLLELPLLKKFASEERSE

SRYWFIPISKRSKFISFADRYHDEVTDAIASPELRQHVPDLASMKPAEWN

SNEPKRLHLALNEIWLTDSIWRLSMGLFSYEGQKASLVLCTREKNGETAS

EPAPGTTDNLEGTPSTLEFRYPPMSFDIDFVKSWAEIGCKIVEIATRDTA

AFRQVATDVLKELERNDASQWERLLKVLDLDHQVGFWRQQLARFDEDEPI

RFLDDDGFLIPQKK*

>Tr|9033_t

MSASNLAREALSTWEANAEFWDSTIGETGNKYWKRLQKPSLQRLLGPTLS

KKSCSALELATGNGLCARWLAENGASSIIATDGTFGMLQQANKYMDEDKA

GKISYRKLDVTQEGDFAPLVEKAAENDGFDIILMNMALMDVATLDPLADA

LPKLLSKDGVFVATLLHPVFMTSTYSRTVEVTFDPQTGDQIITRSKSIRE

YLHVKPSKGVFIVGQETRQFYFHRPLHELLGTFFKRGLILDALEEPGFTE

EDGDADKAYATANFPQLPPILSLRLRRGL*

>Tr|7479_t

MAPIKVGIIGLGATGPALGPGAWAVAAHLASFVPSPNYDIVAVCNSSAES

AQRSIDYHKLPATTKAYGKPEDIAADPNVDLVVVSVIVTSHHKVAKPVLL

AKKQLFVEWPLAASLKQAEELAQLAREANVRTIVGTQFRADPAVQKVKEL

VDGGAIGRVTSTQVQYSPSLGPPDMWMDSARYYLDFKSGGNEFHISFGHL

MDSFLYVLGDFDQVKASLSKQYPVMKLFDLSNGQVTNPGVPRTAPDHIFV

QGVLESGAIASINYHRPGTLLGKNFRWLISGTEGEIEFTLDGHLQIGHSE

REIRIKTVHEPSEVRVVEWKRETPKHVESVPFPGQNTAFVWEAFASGGEG

VADFEDGLRLHRLLDRIAREAGSPYAEYVEEM*

>Tr|3571_t

MKSQFFTSVGIFAASAFAQAVIDSGTGFGTYYYDIEQVEACGTSFANQNL

GFVECNFSTGLSLNEIDSNYLVAMNHTQIAGNLAEYCGKRVVVTVNGVKS

DLPLFIGDGCQRCGTGSRTNTVWNPNGAPGLDFSYTVLSELNSNACFAGH

IDISWEIVDETLYDFDTNAPGQPTGPVNQRRSVSQRSERTTRRRR*

>Tr|7594_t

MSALESFLAEFTLVLQDATAKLQGPLSDERISTITPSETLPENNKKLWDL

LAQTVNMADQIVHLLQPPAIRLAETYLAYLDTKALVSAVTHNIPDLLTNQ

TPLPTALLAQKANLQPLRLKQIMRTLHHNANIFAYDPLTDTYSHNEASLL

LQKHHWTQWHRWVTLYGEEFYDAARALPQAITADESRSAAQIAYGTDKPI

FTYFAEQEGLQEKFHKALGAGAVAQAPGMLADYNWAELGDAVVCDIGAGG

GDFIAALLRKYPRLKGAVFEIQPVVDMLKPKFDSTTPDGIFNDIADRMTQ

LHAGDFLTSIPTYEVYTIKWCLHNWTDADVLKILANVRRAIKLTPRARLV

VIESVLKEGRSSRVWRYGDLTMMSTVNGLERTEAEWRKLAGQAGWVVKDI

VPLRHAWAAAIDLRPFFTVSM*

>Tr|8121_t

MADEFNPQSVDLDTADPKDIICYLNAGENEYNGHLGLRVSALFVVLVTST

LTTFFPVLATRVRRLRIPLYVYLFARYFGAGVIIATAFIHLLEPAYEEIG

PNSCVGMTGGWAEYTWPPAIAMASAMIIFLLDFLAEYYVDKKYRMAHVQV

EGTITTGGHHDHQGLHSADQDRAAPPNGKAAERVPDAAISDKGTPSNDSF

DVEELKNIEGDNQQAAMGFQSQIAAFLILEFGVLFHSVIIGLNLGVVGDE

FKTLYPVIVFHQAFEGLGIGARLSVIPFPKHLRWMPWALCLAYGLTTPLA

IAIGLGVRTTYNSGSFTANVVSGVLDATSAGILLYTGFVEMLARDFLFNP

YRTQDKKRLTFMLVSLYLGCAIMALLGKWA*

>Tr|8998_t

MTPHSIDRAARPSVWSGLALLLSTAHAIVMPDGVTGKVPSLGWNSWNAYH

CDIDESKFLSAAEVIVSSGLLDAGYNYVNIDDCWSMKDGRVDGHIAVNTT

RFPDGIDGLAKKVHDLGLKLGIYSTAGTATCAGYPASLGYEDVDAADFAD

WGVDYWQDEYVACAPDAVQTGPNGTCSTALEPNLAPPGYDWSTSKSAERF

NAMRDALAKQSREIVLSLCIWGVADVFSWGNETGISWRMSGDISPEWGSV

THIINMNSFKMNSVGFWGHNDADMLEVGNGNLTAAETRTHFALWAAMKSP

LLIGTDLAQLSQENIELLKNKHLLAFNQDSVYGQPATPYKWGVNPDWTFN

YTNPAEYWAGPSSKGHLVLMMNTLDHTVRKEAKWSEIPGLSAGRYEVRDV

WTDKSLGCLSSYKTAVAAHDTAVILVGKKCRNW*

>Tr|1635_t

MAAYLTQRISHPHHGITVSRAVTPLPEKRAKCSEPIYNVAAENATFFTPE

QIPIPGTATEPQRSGKPVPKLFTPLKIRGIQMANRIWVSPMCQYSAHEGF

HTPWHITHYGGMAQRGPGLMMIEATAVQANGRITPEDSGIWLDAHVETLK

KHVDFAHSQNGLIGIQLGHAGRKASTVAPWLSSGATAIEEVGGWPDDVIG

PGNEPFNEHYPKPRAMTIVDIERFKHDFHAAVLRALRCGFDVIELHFAHG

YLVSSFLSPAVNKRTDKYGGSFENRARLALELVDETRRIMPKQMPLFVRI

SATDWLDTNPDWNGGPSWNVDESIKLAQLLAQRGVDVLDVSSGGNHPQQK

VVGGPGYQAPFAKKIKAAVGDSMLVSSVGSIKTGQVAQKLIEGSDEADDT

PLDLIAAGRMFQKNPGLVWAWADDLDVSINIAHQIGWGFGGRSKKAPVDH

TKLNVP*

>Tr|6299_t

MGDKPKSTDETIIPLSAKDQWRPINNIRSLLFIVVRDILDEPFMKLSLDK

LIREHIPLLGARIKTRPDGWLEYHLTTPFPSKHKLFGWSTSTVSSTLEQA

HLVPNPNPQRGITILPDVTTLEPRWIPSHWPILRCQDTPDTPLLLVHLTY

YKNATVVATNLPHCVSDQMGYGSVLSAWIDVMKGKEPLRFVDIPPDGLDG

DKDIPTKDLFRKYEYRLRTKRERAEVLMGIVPELVVRSKETRCILFLPVG

LVTGLRDRWRRELKGKYGAEASDISNGDVIVGIIAKFANMHRKRTKKQVI

SGPANLRGHHPLLPPTARYLHNALIFAVTHTSISRSHPPTSDLAYAFRLA

VNDALTPEKMDRGLAVSRELCKRRVAMHICEPWEFSYGTTNWCKAWHGLD

FSVASARRNRSEEDDAVGEKRQQQQESDGSSGDNAHTMNGTASSCSSAPL

IFGHSLERNHPNRLSAAIMSKAEGGYWVDFAAPNKGMAAIRALLERDPNL

ETI*

>Tr|5085_t

MSHPVGPQTPPLRPLVFNSRPQPPHLCVVDTSLSPDELHHDDLHDQPCSP

LSPVSASPWEDASAASTTASIPSTPTTQVSVNEPLPLPLPASSFPLLSKD

MSSKTMPPLQSSLLTSPLPSPILLSSSALPKPEPESETQAQTQTQPEAQT

VKIPPESMGGPIRHIPPKWALRGDIYTFSFWTPASAARNLPEHAYSPLEG

KTSFADEAYSRPVGGLSMIQILSYRDSPVGPYDEMLVAPGSFDWERTEED

GTKTRGSNPKITRIYVSTPNSCFNGRTNWNTPKHLAKFVWDHHPDGSTTI

QIFPHDAPSHDGDESRPSSTPFFTTTFKPMPLAPRFPFATSWVDHLGFNT

TLVMPPLPPGKGTYGELPSTSRWISLVTKQFCKRSTMGWYDVRQPDDGSG

DGRALCGGNENFLPWLGRWQVGLKMEDADLTFEIPDETWETGDKLSDGDN

KEGMGSAKTTTPTTTMTTTTTMNKTMMETTTTTPPYDEPQGEVLEKPRSW

GSGNFLYDYFT*

>Tr|3467_t

MASNSNFGAETTAEEVASTFCDQIVGKTILITGISPNGLGAATAHALARY

DPASLIFTARTVSKASAVANTIRAEYPNLKTQIHVVRIDLSDLESTRQAA

ATIQSLTPHIDIMINNAAVMALPNRVITNNGLEAHLATNFLGHFILTSLL

LPQLKAASPKARVVNIVSGGFYVQPFRFSDYNFDGGKELPEDERVDLEMA

EELGMGWVKDVGTGYVPFLAYSQTSTALMLYTKGLNEGLSGDKIKAFSAA

PGVVLTELQRHLPSGFRNPKMVYKSASQGAASFLVAALDPSLEGHPGAYI

DDCQIRQTPQHARDDAAARRLWSLAQSWMKAA*

>Tr|759_t

MPPKLHIHPPLLNTASPWSTSRAHLAALLRCPSLGAVTTRTSLLSGFPHD

PSRHRYAFFDPAAGAPLSVPGNNNNNNNTSSSSTTVTTTSSPSTTTFSGP

PEDSLVNDIFRTDIPTDKLPLASLNTLGYSPLPLSEYLSIISSFTHSPPS

SPKTIIISITGSPSQISSSYALISSFSHSPQSPSPFPLAIEINLSCPNIP

DLPPPAYSPASLATYLAALPTDSDIPIGIKIPPYTHAGQFDDFVSALLGS

HSPSSPPPSSSSSPPPSTSTSSSIPVPASKLSFITATNTLGSSLLIDTAS

SSSTSPGPLLPDAGIGGMAGPPLHPLALGNVSILRRRFDADPRLAHLDII

GVGGVYDGQGYKRMRSVGAMAVGIATALGRQGVDVFTSIEKDINSAW*

>Tr|7593_t

MKHLPPGPQGLPIIGNMLDMADTDKMMTLAKDWADQYGDVFYTKVGLQYF

IWLSSPTAVKDLMDKRGSIYSSRAASPMINMVSNQERLNFLPYGEKWRTL

RNILHSALNLETSTSYKPVQDFESKQALWEILHAKDDTEFSDINRRYSTS
TIMTITYGQRVPHLSDPLYQDILKIVRHFSLATAPGGWMIDTLPMLADIV
PEWLLQNWKTVARQWYEEDSRIYLRMYHKLMNDIENGTAPDCFLKDMARE
KIEKSLISDVTAAFAAGALIEAGSDATTTALNNVILACLLYPEVVAGAHE
ELDRVVGSDRMPDFSDEPNLPYIRGIAKETLRWRASTKIGTCHSTTQDDW
YKGFFIPKGAVIVLNWWAIHMDESRWKDPQRFDPTRYIADPLTEAESMAQ
ADATLRDHFTFGAGRRNCPGVHIAHNSLFINIARLFWAFNISKSVDAEGN
AIEPSTEAQPGFLLTPVKFPCRLEARSEKHAAIIERTWEEAQRQ*
>Tr|7560_t
MAEMKPIKVFGIHGPNAGKVLLLCEELGLPYETEVIPLTDVKNPNYLAIN
PNGRLPAIQDPNKGLTLWESGAIIEYLTEKYDTDHKLSFEPGTAEAYHAR
QWLFFQTTGQGPYYGQAMWFIIYQPLPEARARYVKEVNRVTGVLEGHLAK
QKPDADGNIWFLGGRLSYVDIAFFTWQHTAEPRIPDEEFNQGDYPHVKKW
LDNMLARPSVQKIVKIQEAK*
>Tr|3885_t
MSFKERLRRWFRRLIPQKGQISVQQKLEKQDGQNEPASTPTPPVKASIAS
SAPTDLTESLWDQAYDELKNSNAALVEAYEKILSRKLNGQGFATPVTAAD
GNIIAQDNPQKRRGQMLQLIQAGLDKTAGEAKIKQAIGSVLSAKDIISDA
IQAVPQAALAWTGLCVALEVVQNPIAATEANRRGIEIVLKNVDWYSELSS
VVLEKNDNERGLSGIRQELKIQVVKLYVLLLSYQIRSVCSYYRDRVLELL
RDIASLDDWKADIKAIEEAELGVRHKIKTYAELKTASDLDKLVNHASTKE
DKQCIQDLRITDPLDDKRRIEQTKGGLSRELYCWILENPEYQQWRDSDEN
RLLWIKGDPGKGKTMLLCGIIDELNQQPSRSGLVSYFFCQATDQNLNNAT
AVLRGLIFMLVRQQPSLVSHVRRRYDQAGGKLFEGANTWFALSDIFTTML
QDPALKGAYLVVDALDECVSDQQQLLDLIILASGTEARVKWIMSSRNEFR
IEERLKHAEQKVELSLEVELNAQSIATAVKLYIEDKLQKLSDAKGYNEVT
QERVRNYLLKNANNTFLWVALVCQNLEKHALLEACDMLEAFPKGLESLYG
RITQQVLKKDGRYANLCKQILMVMMSVYRPITLRELGSMAEINQEFSDDV
GYLTDAVALCGSFLTIREDTIYFVHQSAKDYLSKNEGAIFESRLKDIHQT
MVSLSLQAMTKILRKNIYALPWTGLLSDDGIDMPSPDPLSAIRYSCVHWV
DHLCKGSTVEAYLEDKGPVDVFLREHLLHWLEALGLLHQVSAGILSIVAL

KDLVAAKLPGGELSKFLRCTSVCPLQSKAYRDRPASSVFICARLQSYAKP

RAENFPSRDTIVDSK*

>Tr|2066_t

MKRGFTSGNHLEAAEGAGKRLKTRSTGRNWSEEDLPRKRNDEYTVGWICA

VVTEYVAAQAVLDELHGRPEGVSPASQSDFTLGRIGEHNVVIASLPFGEY

GTASAATVANDMSHNFPNIRFRLMVGIGGGAPNNKHDIRLGDVVVSAPCN

GHSGVMQYDFGKTVQGQPFQPTRYLDQPPMVLRTAMSGLAAQYEHRGHRI

EDDVAEILQDSPRLQEKYSRPHESSDRLYSSTCVHPFNGSADCEITCGGK

SLIVRPPRPPSQRLVIHYGLVASSNQLMKDAIVRDHLSSQYNILCFEMEA

GGLMNQFPCLVIRGICDYSDSHKNKAWQGYAAMTAAAYAKDLIKRVSPPN

SSAGQGTNNTSLSTQLQATQDRSYTFSSEERCQLLKSLRFDQIDARQMTI

KKAHVKTCQWLLQRPEYLDWLDPAKFQEHHGFLWIKGKPGTGKSTLMKFA

LRNAQRKRGGKIIIHFFFNARGEDLEKSTVGMYRSLLSQLLERIPSLLDT

LEVPGSIARSGEDHHWNTEILTDLLEQAVQNLEGFNVLCFIDALDECEED

HVRDMIRFFETVGESITAGTFRVCFSSRHYPHITISKGLGLVLESQEGHT

QDITSYIDSELMIGREALATQLRTELREKAGGIFMWVVLVVGILNKEYDS

GRVKDLRKRLQEIPGDLHELFRNILTRDSQNTNQLLLCVQWVLFAKQPLE

PQELYFALLSNEMYRWNTEEITLADMGRFILDSSKGLAETTNSKTPTVQF

IHESVKDYLLKEDGLKQIWSDLGANYVGESHNKLKRCCLDYITLGKRAYG

DAMDLTLKASSSEAKAQRKAANGSFPFLQYAVKNIWHHANEAQAGNVDQA

EFLRTFQLSDWVRLDNFFAKHDTRRHRETVSIVYILAENDAAHLTKLYPA

RSFVAEESDQYGLPIFAAFATDSREAVRAMLDAYVETEPSFRGVAQYFHR

NEAEQSGIGIGREFKFRSSLALEHISRRGATLAAVFYLTLKHRGLGLNTH

SIEALLYAVKGGQTSMVEYFFENGVQPEPGSKFIQELLTFAIAERREQVV

AFLLNNGATFDPLTKEGRSFFRVAAEGGLQSILEIFLRRDDKGNTPLIYA

TSYRRLETIKLLIKRGADIRAKNNQGSTPLRYAKARGLDSSIQKMLKGSP

KVDHGGGGKSAPR*

>Tr|2946_t

MASVEALWSHVKAGLDEKQASVAVAAMITFLVLVFTTRAITGSPATVDAS

GKHKIPPSPSYWIPFIGHALSMASAGFLPSLRDRFSEGIFTLKMAGKMHH

FIYKPSLAAALFNLHRPAAEEQMLANRLLTSTFGVSKKDAAIYNSIFPEA

LALYKHLSSEPGLSEVTNAAVSQVKQTINSFVTFSPNPMDQTEWERMADV
DVIEESGPDGPITQVDLMELTRNYVATTAIPGIYGTDFVENFPELWKWMW
IYNEAFVLMATGVPAWVPWPRLQRGKLARRRLLGYLYEFNEAMEQHMDGQ
ETDPKWQNLENVSELVRSRIALFRKHGVSLAGRASFDLALLWASVANSNP
LIPWMLYELYRDPVLLEQVREEIAPYVRAVQPKNEFGDAVWVPPVLEDAD
IDGLITKCPLLKASYIETLRVYSCGWAMKLMYSDTVLEGKGKGGESYLLK
KGSYVHVPQELHQSDPAYFPNPTEWQAERHIRETVDDSGNKVLTAELGTM
RPYGGGLKMCKGRQFALREMLLYTAAIITFYDMQPPKGGPWPVPRTRKLV
ANKHPKKPLKVWIKRRKLPAEGEAR*
>Tr|435_t
MDPAQDGPGERQHKGQDGAAQKQSPPEDERQLISPPPPDSPPPPDSPPPP
PAAAAAGTATSPPLSPPPVERAASPSSTISSGYGPTMGMYPAHDEGLPEV
VMPDETPQALSRLEAEYKRKYLEGDSAPQTVIPKDMDTTKIAVAGGSPYE
VTPDGTRVDVEGAGTAGKGAEKRILGLRKKTFWILVGIVLALVVAAAVGG
GVGGGLASKKDKGGASSVDEAATTSSGPPTSTSPSPSSTTSSSSSSSRTS
TSSTTSSASSSPTPTFLNNQTTSANTFAFQAFAANKFLGNATAVIDDEGG
TDLGFEAHSYVWLPALTDCCITFCTNATSKGMVGWLCSQRKQPESSDPFR
RVYVWCHQKHDKPNAICIDPKT*
>Tr|148_t
MGSIAAFSEKTLVRPSIPLVPGRTSVTPLDQYMVRVILPMMVFFKVDEPS
LRPIILDNLKKGLSRAIDELNILAADIIPLDPKDDTIQLEYHKDSGVWFH
VKELPDVDYEDLARRNFPFSALPASQFAPSPLGHSARSPVMTLQATLING
GVVLTFGGHHVVMDAQGMGTFVAVWAKHVAAVSEGLVVPDEQRMGNESLD
AFQLFGPRMERPLSEFPTYQVGPDRSYEDMQAKVLQKAVAGDHEGIAKLI
RVSHWAMSQAKLDALRDATVPRTKEEASVTANATLSALIWKQVSRARRLT
EKQVSQSSLITSVNVRRRVEPPLAPEYPGNAIALAKANATAEELEAPGVE
SLYALAKKVTASIDWWTADELWSLTGALQTCGDVANKLLPPLNYDVLVTA
PARLGDMLKAARWGSELGDIKALRWAFPAFMDGFVIVLPSIHDGIEIMLW
TAPDTTERLREDPEWTQWVTQLE*
>Tr|2701_t
MHPFSTLTLGIFVAGYITARWDLVTRLYELAIFAVEYGVVLRVARGIVVL

TAFFLAIFVPVALLARRETTLHPRDPGAGISAREQLKRRGSI*

>Tr|4874_t

MKIAIIGAGIAGCAVYLELKKHLPKIEAFAEDHEITIYEAYDTGKDVTSD

DREEGPTHSSTLIVGGGLGIAPNGLGVIQRLDEALLRDVTRGGYVISRSN

MKNKNGSLLMCIDINSPDASEPASPLKKLNMVACSRHSLWRCLRTRIPDT

AIVNKRVSQVIANPEGRHAIQFVDGSNAVEVDLVIGADGVKSITKRALFP

DAAEDPYPPHYEGLVGIGGFIPAAGLEEYVEKGSMNFIHGGNGFFGYFFS

ESDPSDPNRESPYDVSKPGDTLAWWSTYEIKECPDRKTLDLADISRRLRE

RHAQWKDPVIQRVIRTARVSNMYPTWTSPPLPTWECDGVVLLGDAAHALP

STSGQGVSQALEDCESFALFLAHQLARGDSSSPMTQRQAVTKAARQYMDL

RKPRVTEILESAKRIQNVKRDMGIIREYAMYSFFKLMGLFPALAAKPMRK

VYEYNIAEHVAQVIASDG*

>Tr|8249_t

MSTHYDAIIIGSGQGGTPLSMAFANAQHKTALIESSHVGGCCVNEGCTPT

KTMVASGRVGYLARRGPQYGIFTGDETDSTENDIKVRMKVVRQRKRDIVD

QFRSGSESRIREAGVNLIFGTACFKDERTVHVRTMDGPEMNLTADRVFIC

SGERPAIPKIDGLDVAAFPPSILLDSTTIQELGVVPSHLVVIGGGYIGLE

FGQLFRRLGAAVTVVQRGAQLLPREDTDVAEAMLGVLKEDGVRVLLNATP

TSIRPAGDGDSTAASAVLVTVSGQGSLSELQASHVLFAAGRVPNTEMLNL

GAASIETTARGHIITDAQLRTTNRHVWALGDVKGGPAFTHVSYDDFRLLR

TNLLEQGNLTVAERLVPYVVYTDPQLGHVGLHEHEARDSPPGPRSEKVSY

DGYHVYSVTTTDRSQAEFLATNLAAYPTEFTSRGFEVAIPPNEIQSFNKL

GLAAQLLSDDLGKLIRRERKAPTYNRGHHKAGELPDLTWYDTYHDYDDHL

QYWDDLVAAFPRNSEKYDIGTSYENRTIYAFHLFGDKGETANKPIILWHA

TVHAREWITTLVIEYWAWQLINGYRIKDKEITRFLDYYDFWLVPFHNPDG

FSYTQTTDRMWRKNRLPRGNTTCVGTDLNRNWRFEWGGEPGTGAASTDPC

DSTFQGLSPGDTPENAAVSGLSDKLGKSPRGIRSYIDLHSYGQKILTPPG

WTCNTTQYPATLPRMLEVGGGFANAVQAYDSRNETYQYGTGCDIEYYSAG

NGRDHHYGAYGADHSWTLELDPVTSGQGGFVLPPEDIWPVVKEQWAGVSW

LLNNVWHN*

>Tr|9278_t

MSPPKPSPSVNEIEDNVEAVLSHEIDKDHTDYRRVDKELAQYISEARITI

SPETNNELRRKIDRRILTVMISTYFLQAIDKGTMSFASIMGIKSDTHLVG

QDYNWLTTCVFITILVVEYPQNYIIARVPIAKYLSFNIMAWGTVLACTAA

CTNFTGLVVVRTLLGLFEYMMNGAQQIVGGLLAYCFSLITTGPLQSWKWL

FLAYGVVSVVFGLFVGWWMPDSPMRAKCFTEEEKRLMIERVRDNQTGIQN

REWKKSQFIEALKDPQIWGYCLVQLCTTLPTSGLGAFQGIIISSMGFTVL

QTQLLAMVLGFYIIIVLLGSTWLVKRTNQNLYIMTVFIIPSFIGTICLMT

VPLDTRSQKIGLVVCYNITMSFWAAQTLALSMLSRNVAGQTKKSAAVALN

FISWATGNAIGPQVFLSWDAPRYFIAFATHLGCYSLLVIVLLALRLYLSR

ENRRRDNLAASGVREARDERLVHAWEDLTDQENPNFRYVL*

>Tr|3613_t

MASNANASNTKDGAPPHHNQLQQQRSKPVGKPRGMRRDRDCRSCKLRDIK

CDLNRPSCGEVGVPCGGYPQRVIWVGSTSSAKDASPTTSAAPSRVQRQAK

AHQSTLSPSSDSSLTPDRPPDRPSESSLSPSERLVSSPGDEPINWAEADQ

NSFIRPLVSLCQQIISLDGDALSSNRYLSVEALRLISRLRDFVQARIDGH

PARASRDLWESETMARYRLNALMSLKDTLKATNPFAFIGIAAFAFFEVCD

SGFGDWQRHLYGAKSLLDFHCKSRRELDTLSESVTGLGEMVVRLVWFDTC

GSIIRGTTDLIFEPWHRELLTESFFRTVGCASDTFRLFTRVASGEVASNP

STSVILAMAQLLKLGQGSSDWDRSADAYRCAGVIAVLTRVRDEQSAESTI

SLAVDRTCQIIAATPSSSQFYIHMAVPAYLAGINASSMKQCDVIRAYWHN

CNHAGVRRYPDGLARCEDRWKMKELV*

>Tr|2523_t

MDNHVAANVLGTAGALIPQIIINYRRHDTTGLQPSMMMLWACAGVPLGVY

NITKNFNVALRIQPQILTFLSLVTWAQCRYYDKKWPVSRCLLVATPIALL

MAATQAALIFALRAAHSSHLTWPDTLMAVLSAALLAAGVLRHYWDIYVHR

SVRGISFIFVGIDAGGDLFSLISVFFQPELDVLGMVIYGTELVLWIGVFA

CGGYFNLLPWIKQHFETPSQSELHEGRSSHELSANPSSRSPRAISLRNIP

SSTSVFRTPSSELAVARARTGFHDTADESVTVSPR*

>Tr|5756_t

MAQVAKPQVKVEKTSNRPDEWKIEQGLAGAELPVLDMTKPQTEPLVPQVF

GPCTKDEEAIKSVGDPNKLFTREREGWTGFVEWENYPEKKAAAHKILTSQ

TFPPNPEFQLGPIPGTNPVLPGTHWKMWHHAVGGELTNIPEDSWSTVLKE
KHPDMLHLLQFPYNGEPPKRLVTDKEITPNPLHFVRNHGGIPLIDKDKYS
FVVDGFVAKPREYTLNDLMDESRFPRMEKIITMQCSGTRRIEQILKYPGQ
GDEVPQAPWAEGAIGTAKYVGISMKKVIKECGGLVEGAKHLEFYGADTYF
KDDKTMNYLVSIPYSKVKANEVMLTWEMNGEPLPAIHGFPLRIMVFGYIG
ARSVKWLYRIKAIKQPSRAPVQSQEYLYFPQQVGKHNFKLTDGIQIQEMP
VSSAIMSPWHKQVCIHNGKIRCKGWAYSGGGRWPERVELSSDGGFNWYTV
PVDNLSKKRRWTWRTWQFELPCDPEGWVEIVCRCWDNALNTQPPNVRTAW
NWGLHVTSSCHRISVYSINKTRPLTRARLDEFEKAGIPFGPITVPLAFPA
QTWEDYEKYWKTHDPRDAEDD*
>Tr|3030_t
MSSQATRQIGGVAPRVIYVITKPKDVADAYRNTESLSFNEFVQAVMRACD
NTESCIQAMYTPLPRDKAGFANPQGKSLATLARQMHIHQLYPGKGLDFLE
RTFLEWFDVRMNMEKLHSGCSYATKRDDGDVVLPLMEWCSDFLISAGQRA
YFGDELGQIDGNLVREFVKFDELSWQVLYQYPELLAGEMKRSRDVIQRAL
KKYIQLPQSQRSGEAWFTKAMENEMRALNIGEDDIATMLMTIYWVLNTST
RKAAFWLLSYILHTPSLILVIRNETKPAFNNSDGKLNLDYIHNQCPQLNA
MWNEMIRMSASAALVRFITADTVIGGKVLRRGNRLLIPFRQLHFDKSVFG
ETADSFDHERFLEMKPSLTQSGTWRPFGGGGTMCPGRHAAKKCVLLFVVL
LLQRFDIEVDGWQSLPVADEGKPVLGIMSTRENRDLRVRVTARKTFA*
>Tr|8399_t
MAIISPIKPPFDPEVAKGVPPGMVDGSLSKAMPTDAAIRVAAMRVAADQV
AQVEKNAILSDPTLEVEEVFIPGPSGEIRLSVIRPKDAASDIKKRPAIYN
VHPGALVMGNEYTAITSAISWMKEIGGIVASVNYRLAPEHPGLAPFEDSW
EGLVWFAKQADRFGFDPDKLILSGFSAGGGIAAAIAIKARDEGFPKLCAQ
ILVCPMLDDRATTVSHRQYFHHGSYTGEADEFSYEMVLGDLRGTDDVPVF
IAPARATDLSGLPPTYIEVGSAEPFRDSVVGYASKLWEHGVQAELMVFAG
GLHGFDFYAPEAMVSKDAVQARLNWIRRLMAQSK*
>Tr|4846_t
MSSISEAIKKDHRELEQYYNEVINNQGDHDYQERYGNQFVWELARHSVGE
ELVVYPAFEKYLGSKGKEMAESDRKDHHVVKEMLKTFQNLKPSDPDYVSK

LKELWAPLSEHIKEEEERDLPTLEQKLQEVEGESASMARSFGRTKAFVTS

RSHPSAGEHPPFETAMGLLAAPIDHIADLFRKFPDNTVSPNPSTK*

>Tr|6055_t

MEQTFQSVVPVLRIFDARKAEEFYLDFLGFTVDWDHRFDDNAPLYRQISK

GNLILHLSEHHGDGSPGVRIRVLMGQGITGYHEELIAKNYRYMRPGLEKG

YGGPDAQELTVIDPFGNKITFCQVDKDAQAARML*

>Tr|5216_t

MCAERAQYFVELYIDHDHSPNDINFVQACFLLHKYRIAIGQYETASYMLS

TAHSRVERLNGHLHHPMEHGSNVEPSLLERRRNHLRKLFWLCYTHVQDLV

LPSDLRTRSVTQKPDLRDPDRHLGHDVFLQMPGETVDIEIMGSIAWERIL

SFLPGDPHLGLLKENVYELLYSPSALEIIDSELLVRIRQLDDELEDWRLA

IPPVLRPKLSITPVQGILWNHTFSSYLRSVQLQLEYHFLVTFIHTPVRRF

GATQGQHTILPEELHSAMHSSIDLSLEASRSTLRLLREPIAMVRQDTFW*

>Tr|858_t

MAIRKTLVQALVASAMANLVQAHAIVAPAGQLDARTHPSPTCDPSQTVTQ

TKTTTVTSWTALLTITLGGPGIKTVTETTTTTATASPTFTLPSCPTPCPP

PPSCNNLGFDWAYYNSSARNADTTYSSFHPDSFKNTAPVYVGTTSYVGGL

YGQSSTETTGPIYDSSRVSFALDYFALNHHAYIYACEGGTYTVDVPYAND

AVYLWTGAKAYSGWTDANADAKALYDQPDHVAGRSSFRFNVPADTYVPVR

FFYGQAQYGGGFSFNVTAPSGQVIVSDRETFSPYVVRYSCDGTTAPAFPA

FGRET*

>Tr|6138_t

MSGQGYLENTSLHTQAKWRSALLTYPGNLKGALKEAQKDPKKTLFGVAQG

IPSTFLTKVFASTRPDFIWMDVEHGMFDRLVLHDAIQAAQHHSEGQTMVV

VRVPAEDEVSLSTALDAGASGIIIPHCETREQVEEFMKRVYYPPLGGRSF

SPWVFTPGISDASLYPNDAFNMKTANNHIAVIPQIESVKGIENVDDIASI

PGISALMFGPGDFSADAGLELSLGQPHPTLIAATESMSKASKKHGIPLFG

AAMNLEMVPALVEAGHGAIVVMLDVWGIANFTKSSIEKARGLIPT*

>Tr|3797_t

MSYTLASDDASGTGMRQVDDDTEATMHTGEEVDERLFADEDEALEPPDVF

TTIHRVRALVIASIEDPYTLEQLTSPRINTLVVRPLVDRLYDPDDMSIVY

CLLANRLQFLREQTAAKHQSVSRARATLCEIVATKILRKLHEHNHPDKAG

AAGLLRLANALVQGFDPFSGAPEDVALAGRYTHWPVQRRGGNERKLTALE

LAILSESKALMSSPACLRVVNAVYEGQVVYTPISFVDILPDHYKHHPVSL

YDPRKAPLLNHYRLIVPRWRNLLESMQFMVLLGLYVLTMVNRGNSRPLKL

YESLFVVYTLGWMLDEFAAIIEHGWAVHSQKTWSFLDVTFTIIFCAYLFA

RLYDVWVPVAQGTTGGTGSAGLHILCVAAPVLLTRVAFSLMPDNIVFISL

HAMMKDFLVLTFLTLWCVGGFFLALQWLLTAGQGPEPEWYTVAKWLLWIW

FGLDGTGIQESVQFHVVLGPALIVAFAFLGNTLFLTILVAVLTNTFSRIV

AAEAAEIQFRRAVLTFECVRSDAIFAYPPPSNVLALFFMLPLKLLVSARS

FHSVHVAIVRVLNFPVLLLISLYERNLFKTGESKRGVKSNQLLRWRFTGF

NPHGDIEAVFEADPPPVAVDEAGELDGLSELGFTDDGVSRSSREMPRPVV

FRLSNSRSRDETLTMLGRGSPGAEGIVASQGTEDS*

>Tr|5750_t

MGARDDGPGADSMISQGAGASGHLSQHDDDHDYEASQGRRDRSDAHGISS

SNSNSNSSFSHSGGGGVAAGGGAGSKDRDKDSAASMMAMSGGSLDTSLSA

SMMSMDSVGGGDAVGAGAEGANMNSNSSSSSSSSSNNNNSSSSRRTRNDS

ASAFVDGTTTTTTTGDANINSKTTTAPTETDSTIPPFSAADLVDLSPVPT

LVVSPSFRILRASQGVVDAWKCRPDELLGKDLFATLYGGSPLERFDRIPL

AAAIEVAVASRKFNLCHGAYAAHGVSYSARIIPILRGDQLLSLVLEWDEI

EPTTAETRGEIIQHALSVDEVLRLLIQTVKDYAIFLLDTRGYVATWNTGA

ELLKGYSKEDIVGRHFSNFYGREDLDAGKPEAELITCLREGRVQDEGWRY

RKDGSRFWANVTITAIYRNGVHVGFGKVTRDLTERREAELRLVAAYEESA

KLKNDFLANMSHEIRTPMHGMLSACALLLDTPLSDDQRDTANIISESGQV

LLQVINGILDYSKLASGNFSVNTDLVGVASIITSVVRNVQMTLLPGVYIK

LLLSPDLPKSAQGDPLRFRQIFQNIIDNAVKFTEAGSVQVAASVTSQDDT

YYTILTEVTDSGIGVPEDAVQNLFKPFTQLEKPTKKRYQGTGLGLSIAKS

LAELLGGQMGYRPNRERNGSVFWFTVKLKKIANLNQLDAPDKAERESEKS

RQDAEKERMMQRIKELAPRKRILAAEDNLVNQKVLARVLKSLGFHNTTIA

ANGAEAVSTLKASPNTYDLVLMDISMPVMDGFEATRKIRTQGIPIPIIAM

TAYALRGDSETCLEKGMDDYISKPVNINKLLQKLLHWLDSSEWSNSPTNG

PP*

>Tr|5147_t

MASQLTPPPGVFVPVPTFFKPAAASDDVFEPTIDIETQVAHTVFLAKAGI

TGLTILGSTGEAVHLSRQERYDLVAGVRAGLNDAGFANYPIMAGVLTNSV

GETVEWLQDYAKAGAQWGLVLAPGYFGKAVNQQNLIDWFTVVADRSPIPI

LIYNYPGVTNGLLVEPDTYRVLAKHPKIIGCKMSHGDVSYHVQVSTDPQI

DHDAFRVYSGFGQQLGPIVLFGAAGVIDGLAAFYPKTVVRLFALANQGSF

QADALSEVRRLQFAVSRAEEFIARTGIIGIREGIIRKTGLGALEGGRLPL

KGRLPEENWTALHKLYLSDIEQIENSL*

>Tr|2382_t

MVAVWGWHVSASAWLSTTALTLITYNLLRIIYNLYFHPLAKVPGATAWKA

SRLPFIWSLLRGTIVHDIERLHRKHGPVLRIAPNEVTFAHEDAWRDIFQL

RKDHQPFLKDPVWWKSQPGMPESLLSAIDPKRHARIRSLLAPGFTPKALK

EQEGILHFYVNLLVERFRELAGKDPESSGAVVDVVPWFNFIAFDIFGDLG

FGESFNCLQDSKYHPWIALLFNSVKAASFVAAARFYPIVQRLLLMCIPQS

LRKMQRDHYQQIVDKVDRRLNFELERPDIMSHVIRGTERRELPPGTIHTT

FMVLTTAGSETTATALSGTLNYLVNNPDKLETLTREIRGAFEKEDDINLD

SLRKLPYLNAVLNEGLRLCPPIPWVLPRVVPEGGDTTPVSIQAYSMNRDP

ANFHAATSFEPERWLPASRTDPASPFFHDRLQVVQPFSDGPRACLGQHLA

WAEMRLIMTKLLWTFDFAVVEDRRTRWEDLRTFLLVEKRPIEVRVRLRDT

AAGSVA*

>Tr|6023_t

MVSFNVFTILAAAVASVAAIPAGTATIGNRLPSRTFLTGVTHSVVAGRGG

ALVYDPDNVVAQPGDTVEIHFLAANHSIAQSDFAHPCTPLADGTGLFAGF

NFATSEGQNPNVYQFTVIDNSPLWFYCPQPKGAHCTKGMLLAVNQNFDSQ

NTLSKQRELAAKVEPVVPPVVQGGRLGGFQRANPNPLSGF*

>Tr|8102_t

MAAIDSTATQATTKPAVPVVTSFPFNPLTTTFTRPSDCDGLFASSFLSGI

DFSTSCVPKGFHTDETSYFSPGLICPSGYYSACHDNIGARTITTVTCCPT

YGSDVSLSCVTASTLRSVWSTLFCTWIAPDGDGTVLPMTVSNGGTTSTVQ

GAFTAPGGLNAYGIRMVYQKTDTETTTMATRTTSSPTRSKATTTATRTGG

QPGKTSEASSGLSSGAKAAIGVGVAVPIAVVGLALGLFFWWWRRRKQYNR

VRAGQTTPPTELSGHQRPSELPNEGVNKPVYVAEVPAHEPPAIELPAPLR

*

>Tr|5416_t

MAAPRQEPLAEDIYGFLQGHQEKRARSTATGSSEYTHLDVLPKPPRHAFT

LSEDSRSHPSETHVSSVASGESRRSSVFDAPGYSSSASSVPPGRQRRLPC

EFFWYGNCDEIFDIHDIDGWIHHVAVHHLNMVLPAKCKCWFCDDWVFIAE

PDSFQQRELCYTARMQHIADHYRGGARTIDVRPDFDFLDHLWENDLISRR

DFQYMKNYHEAPQPRNGINPIPTAPSARSPAAVAVAVEVRPRHRGRRESR

RSYYS*

>Tr|4885_t

MWPFNSYPERTAEDVDDETYDYVIVGASRLSEDPNVSVLLLEKGFVNDSL

IGRIPLLSLHPSMPGTAAVKYLSQLSTAVNRKLSLFWGEALGGSTRINAM

VLTRGPPSNYNDWSQEYGLKNWAWEKVEPYFIKSENAIGHPKAQHRGHQG

PVENRQMALPFSCYPFIEKAVRKLGFPVYDDCNDPAAGAQGYFSLDHTIN

GNGRRLSAYQAWLNKDIATTRKSHLTVCTGVTVSKLDINPVSRRVVGVRI

HRKGKTGNGDYSVRARREVVLCAGALGTPPVLMRSGIGPKDQLDQLGIPI

IQESSAVGSNLMDHIAVAIMSKLPARDTIHSLYNPLVFIWQLILWIFFGR

GLLAYSSTSSTIFLRTTAIDDTTLQVQTCRDGIDTMDSMNPQNVPDVEIM

VIPVSCYLDVNVTAHPLMTWQCTLVQPFSKGSLRLTSTNSEHLPEVFHPL

LRDDRDLVPMRKAIRMSLRLGQEFARSGYPHPAPVLYGPGMDLQYLDGIF

GYATQLPGKQQERKGGIKELPDWQTISDKAIDDYAYRTYQSSLHYACTCR

MSLDPTDGVVDQKLRVFGIDRLRIADASVFPKVTSGHTMAPTMMVAERCA

DFIKETWRDKESLGI*

>Tr|2850_t

MPSYAITGASKGIGREFVRQLAQSPSNTILALVRSPSPALLALAQSHPNI

HIIKNADTTDPSSVLAAAREASQILGDSSGGLDVLIHNANSVNLDTFALP

PSKVPFDLEATRTFYEVPARTAIWGGAWTTNAFLPLIEKGQLKKIVHITS

TMADTDFILGSGIDYALAYAIAKAGMNVQVAKYAAELAPKGIKTLSLCPG

WVDTWESPKPPQLVEAEELMLAQFRKVEPDLKGQISVQESVSQQLKVIDA

LDAARSGTVIKARDFKA*

>Tr|9062_t

MSIPNRKVVITAYGSPSTALQIVTEQLPPPPKNHVQVKIFYAGFSGADVN
MRLGVYPLQRSAPLTPGYCFSGRVSVNGPGCSKFKEGDMVVAMTKYDADA
EFINIEEKHLVAIPDGVDLQQAAALALDWTTAYGMVHRSAKVSAGQRVFI
HGISGAVGQGLMYLCLLQGATVYGTAAERNHEALKEAGAHPFVYTNKDWI
KAMKEIGGVHAVFDALGFESFDESYSILTLEEPSILVAYGNNLSSLTDDG
KPRSPFPAMIKMLSKSLKVGCNRSTTFFGITRNQKTYQPELRTLLDMVKD
GTIRVPIKAIWDFDDIKKAHEAWGKGAGMGSPLIRIASEA*
>Tr|7439_t
MPSYVITGASRGLGFEFLRQLSSDSNNIVIGLVRNKAATDQAVAHELAGR
SNIHILEADITDYEAIKHAVAETARITGGTLDYLIANAAYVSEFDAYDPI
GVLGENPQALEQDLLKSFKVNVIANVHLFNLYMPLILAGTTKKVITLSTG
MADLDSMNKYELHVAPGYAISKAAMNVAVGKFHAQYKKEGVLFMSICPGV
VDTGHYKNATPEQMEKLGDMFQKFVLNNPDFKGPAKAEDAVRSVISVWEN
ASIEGGSGGSYVSHHGNKKWL*
>Tr|6090_t
MADALGLVSSIIAVVDLFIKVGVQCSIYCSGVKDAPRDIRQILNEADRTT
ATLEDLRRLLASPTGARLSSSQRVCQSIEDARLQLQDLAFKLEGGLRTGQ
RLRWPLRKEEVAGIISQLQKCRASIALDLQVDQTALLLNVHQEAVLAKLR
TAKGAAFDSPSHANSSKCYPGTREGILRQIQTWSTKSDGQCIFWLNGGAG
TGKSTISRTVAQFFADNGILGASFFFKHGEADRGNMALFFPTMASQLIQA
FPQIAPHVRAAVEADPTIHDRSIKEQFDKLIADPIIMASKASQLPTIVVV
ADALDECDNDEHVRLVIHLLSQTRHFTSASLKFFVTSRPELAIRLGFADI
CGQYEDLMLHQVPRVAIEHDITLFLEHEIAMIRQDYNKSVSVNRQLPLSW
PGIQSFQRLVSMSIPLFIFAATACRFIQDRRIGGPKEQLAKILQHQTGHG
PTSNLDATYLPIVNGLLAGLSDVEKGFVSERFRRIVGSIVTLANPLCAPS
LARLLGMPRESVEDLLDLLHSVLYIPTDARLPVRVLHLSFRDFLVDPTKA
SAADKYPLWIDQQKAHRVLATRCLELLLEEGTLRRNICGLKLPSTSRSEV
CQSTLEAALPSEVQYACLYWVFHWRESMSKVEDGGLVDCFLNSHLLHWFE
ALGLLGHISECIGMINDLLDLVHVWHPRDSALILRPW*
>Tr|7540_t
MSAQNIYAEVGKRYSAAAAGVEAQYASSVAKAFGYSLEELSDIPQDANLG

LSCGNPLATATLREGETVIDLGSGAGFDVFLAANKVGPEGKVIGVDMNEE

MLRKAEELKVSSGKQNVEFVKSQITRIALEDSIADCIISNCVINLVPEEE

KQLVFHEMFRLLKPGGRVAISDILAKGPLPEDLRKSMALYVGCIAGASQV

GMYQKYLKEAGFHGIVIQDTGNDLNVYRSAAVDNSGQDPSDGTQDKCCCA

KSSTQDGFIMKDVDYNEWTASYNIFAVKQ*

>Tr|1357_t

MLDPTPSNYQEMLAFVQSHPLYLLIVFGSALLVASAAYVVYQCYFSPLAS

FPGPFAAKVTSLWRPYMTSRGQWHRYLDELHRRHGSVVRIGPNQLSIGDP

DAFRTIYRISGAFVKSSSYAVLQGSRPFDLAGERSEKIHAAQRRLVARPY

SMESVVHLEPQVDRLLDDLLVKFDAFALSPNPIDLGNWFQLFAFDVIGAV

SFSKPFGFVARGTDEGIFGRIERAFSSTSWLMHAPLVYKFHQKFILPYIG

NFLAANDRNGYFFEFAKSEVQDRRDKGGNDKDIVGQLFQTARNKPELTDL

SISFMMTSNVFAGSDTTAIGLRSIFLNLLQHPRVLAKLRAELEERKAAGQ

LSNPVTFHEAEACPYLQAVIYEALRVFSPVGTIPDRDVPKEGMTICGRFI

PGGTVVGTSPWVIHHSPEIWGPDYEEFKPERWLGEDSSHLKRFFFTFGGG

TRTCIGKNISWLEMDKLVSTLLMRYNFELVDEANITDVCSTLVFLKGLKV

KITMRDA*

>Tr|1985_t

MNPVTRSFSTAARVHPSILPHLHASPRRAPIRRWFKTAVVISAVVYASKT

YLDFTRYSSSSSSSSHPSSSQQQHSHTAKDIRAALQEQDNESLRRQRMME

DLYGGRESLEDLERGVAEYARR*

>Tr|3599_t

MSSSQNQHIQAPGEPYFPPPPPGPPPASTQQQQPYPQFPPPPQQPEPQSH

QAQEAHKSDDNQHQQQQLQQEQTQFQQTSQQQHFPPPPPGPPPSQQQQPH

YQPQYQPPPQQQQQPAQPQPSSYAIPAYDPANPVFAPPPSNALNASATPS

AYHQPPAEGQQQQHQQQPYQQAPPSPQHQTAGEQQGHHKLGWSERLSALG

MKAAAPINSIAHKLGSQSFLPETLDKECDKAATILKAFCRKGVYADPAAT

SVPTSTDPNAVESTDRVIDPTKEKPKNRVIVTIPPKVISKAVGLAIFTTL

RAGFQVSGATGSGILIARLEDGSWSPPSGIQLHSVGGGFQIGLDIYDCVC

VINSKEALAAFTNTRVSLGSDLAVVAGPYGAGAAVEFGASLEPKGKHGHQ

EGSEVQQQQSQQNLQPAEDSKKSHRRSLSASATKPVFSYVKSRGFYAGIR

IDGTVVAERKEANAAFYGQAVTVDQILKGQVPPQGPPGMWPAGAQTLYTV
LKGAEASALQASSGNSSPGHHQQQTAHGYDHPQPGHVEQQTSPPSYADSG
PRPIDGDIKYA*
>Tr|1723_t
MAILTHDVAPMLLLKPALVIPLLLVISGLLYVGYTIVYNLFFHPLRHFPG
PKLWAIHYGFYARLELSGDAHKLLIPLHQKYGPVIRVAPDHLVFCHPDAI
NDLSGHRKAGQLENGKEIARNLLVPHTIFSADRENHTRIRKSMANGFSNQ
AMLDQQPLIMTYVQKLFEKLDEESANGNKIDIAARYNWTTFDIIGDLAFG
EPFGCLENSMYHPWVEMVFDGVKNVSVDATFRRMPLLYKGLVMLTPKAML
EKLKQHSSLSEQKVRKRLDTVTDRKDFIAAMTSKKGKDEVTLQELIANMA
LLIIAGSETTAVALMGATYYLGRNPGPLKKLCDEVRSSFTSEDEIDLVSV
GRLNYMLAVLDEAIRLHSPVPGTTPRTINEKGDVIAGYWVPPGTHIDIWY
WTMFHYPEFWTQPEEFIPERWLGDSRFANDQKRIFTPFSVGPRVCIGKNL
AYAEMRLILARLIWRYDIELVDESIGWDARSKVYIAWQKGPLYIRLKPRA
RTA*
>Tr|7100_t
MPADFDQQAYWHRRFSTESSFEWLLSSNDFIAILNPLLNTLDRTSTRILN
IGCGTSDLHNHFRRLGFSDVTNIDYEPLALERGQQLERQAFGDVRMKYAV
ADATKSLVLNPSSSNQNDSSETRNEKFNLVVDKSTVDAISCGGEEQVRRM
AHCVRECLADDDDDAVWVSLSYSAARFELEGLPFDVEVLERVLTAKRAVA
DPDIYHWCYLLRPR*
>Tr|9114_t
MKRIDPFLTGMEHFEKVLIALKFEDVPKIMACIWGPLRFLLEITNLTDRE
IDNLLEAYKKIGKQLPRFYEYKIYKSAWRELNVTIDHLLGALEKHDEFIR
KSGSSFRSGSATPDMDSDSTTEQACSGSDRDRERIADDFSNYMINLRRYR
ENFEESEKERKEQAKRRVLAWISASKKTDSLHRKFQDTRICPGTGRWLFK
RYRNVSHWMKEEQPPESALWLQASRGFGKTILASLLIDELGELRTKEIYG
VPPGAKIYHFYCQEEDSEHRTHLDILRGILHQMVDANEELLPFCIDKAQS
TGNPSLSDTQTAHYLIEAFVEYNTRQYIIIDGIDECETSEVREMAKYFLG
QVTKRNKDTQESQLRLLFMSQPIAELTRDGFMPDGDARVQLKATDNAEDI
KFYVKKRLVDFSKGHATRIGFNLSESDKDQIESSVCHRSEEMFLYAHLAI

EYLLQQPTKEKLKEKMKEDMAPKELSQIYEKLLGIVRAELLSLTEGQAHW
DMAKQLLGWLVCAKRPLRWHEMQSILSYNPDQQKVDFDNRMLRQDANKYL
GSLVQVLDGGHFRIVHSTARRYIIQNRYINDQAVYCELAVICLRYLCLLT
ESNHYDDEERREKVKLGWFSFQDYACSQWHSHISTVIEMCSDLFYDTGYG
QKYGTMFGAALQEFMNKHGADMAKSQHQDIESQMPTALTRFSRLPFYDNL
CNLWNHIYTHQKGEYEVRNTVGIKCVDQALLGNRTFLERFNPQMEAYHED
TIEDYYGPNLFKCKRLLCRFFYVGYDKIDDREAHDSRHDRAFHCPLRCNA
APVGFRNEKDQQKHVRIYHPEQIKEPSVFEPMRPRNSKRTFRCSMCNKMF
TRKSTLIGHEYSHLGERPYKCPSCGKAFARSSDCKRHQKSRAHRAMGSGR
*
>Tr|3542_t
MGWLSRLSAVAAVATTALAAEASTSSKSSTAAEPCAQIAKLVKSGATSFS
SQLALACIETTPFKSDVAVSFVDELKKYLEWHSTTEILRNPPPTALSATV
DLFGGLANIRDRASANLYKSQYDFDADLFHLLSFANDGHLVILPCSFIFT
FRSPLRLASLSSDGIAIPRLYTLGDGKLLAQGNKDVSPVTLINGVGAEAF
TEQLSETMGFQDPDARYNSMLANVPIARDGSEFPGSYAGFVTIPGAHEFN
LTYANGTQQTLPLTAIVSSRLDTDFTFSNGDELWDAACKPKPASSDDSGE
KKRAESSTSAKEKPAPETYPKPIVKDPFNLITGYFPDDKGLEDVAVLTVP
TFETTDAASGIPVNEIANFVLEAQDFVSKAIAAGKSKIIIDVTNNGGGVV
NSGFGLVSVFFPNMTIFSATRYRSHPGTQFVVETYNRSPDTVADGLDQDG
FFVPTLVKPDQNTTFKSVEDFLGPFDILGVPSTAIVAENTFAFNNETLFP
INVYNEGGVLNGTTPPFAPENIVILTDGQCSSTCTIFVNHMIPYGVRVVT
TGGRPQAGPMQSIGGVKGSQVLAFNTISTYFNNAQVLVQNATDNKKPLFT
DKEMSVFSPYIPVALEDMPLRLTTASVNFRNAFSPFDDQTPTHFVYQAAD
CRLFYTAETLIKPETLWVNAANSIWGDGDCVFRSVPRKAIKSAGTKTESS
KSAATSAVSGSTDENDEVQQDETQAPAPKKKKTRAPAHKALGLLLAMAEG
LRG*
>Tr|4779_t
MSFPGAAPPPEGVVPDLAHPQDVLRTVNYITQGLTVFFVTIFVAIRFYAK
SRVLGGGFTPDDYATYAAYVLMLGYCITAVFAGAHGGGLNIWEVSPAEVE
QYFKSCYAATIFYAPMAMTVKLALLVIIIRVFGSVHRRTLIGIYIFIGMI

VAYYVSGLFIKIFICWPISAYWTGDSGKCLNQSAIVTADSIISVISDLAI

LLLPTPLTWSLQLPRRKRLRVTGLLCAGGVATGFSIYRLAMIVDQRNSPN

MTIVFIKVILSGNAEAGIGLICACLPAVSALYVQRTRGSSYFKNPGQSSV

TGRGEIMLTRSYHVDRSRVDKGVDESALELGHDEAGLVSNIGTEIKANAA

ESHHSNRSEETL*

>Tr|7237_t

MATSLPQEALEALAKETMHDRIVGCLFGSALGDAIGLYTEFLSGEKARIA

YLSQKFILSPPSEATPFRRDAHRDPNVPGDWTDDTDHAMCILLSYLHTDG

KWMSPHDFAYRLHIWVRMGLRALDTLPLGLGRTVGAIVRSKTYLEDPEGT

ARKHWRNAKCKLAPNGSIMRTHPLGLMCIHKTMEETFQVAADYSVVTHVD

PRCIISCVIGTALVRGLVRHEIYKEEHIDAVVEEAIAWYSGYRSRQIERD

PSCKDEPELDLDELNRHVKVNTLAELELDDMYKIGYTYKTFGSGVLLLRL

AMREVASTESRLATQKSIFERLITELIMQGGDADTNACFAGALLGSYLGY

KALPPHWRDGLKHGEWLMKKAEGLSILLGAGQGTYSGLQDKDTAPDGGRG

WLTEKQMEEKVMLLQADMVKRGQERDRMEEADKRRARTKRGSWLGGIGGK

W*

>Tr|6890_t

MVANDTPASTFRYNEKPVYTTSNGAPIDNPEGWQRPGTIGPLLLQDFHLI

DALAHFDRERIPERVVHAKGAGAYGVFEVTHDITDLTSINMFDTVGKKTN

CVARFSTVGGEKGSADTARDPRGFAIKFYTEEGNWDWVYNNTPVFFIRDP

TKFPLFIHTQKRNPQTNLKDATMFWDYLSTHQEAIHQVMTLFSDRGTPYS

YRHMNGYSGHTHKWTKPDGSFVYTQVHLKTDQGIKTFTGEEAAKMSAENP

DWNTQDLFEAIQKGDFPSWTVYVQVLTPEEAEKFRWNIFDLTKVWPQKDV

PLRPFGKFTLNKNPENYFAEIEQLAFSPSHLVPGVEPSIDPVLQSRLFSY

PDTHRHRLGVNYQQIPVNAPLKAFNPFQRDGAMVVNGNYGANPNYPSSYR

QLTYKAVKPIVTHETWSGHAVHELFQDVNDDDFVQAKGLWDVLGRTPGQQ

DNFVSNVAGHLSAAHQDTRNRTYAMFTRVDPGLGAFIQESTEKLVK*

>Tr|5763_t

MNPNTGITDEAAIEGHDLIHNAEVEEQKAHGDHALTQPDEGDEPLNATKQ

TETGAAPGHTQRRHSAMDKVKEALHLKK*

>Tr|135_t

MPPIILPANQPPNRFYAGGPQIAAFRSHPSSATHEPEDWIASTTCCSAGQ
ASSSNIGLTRLPSGTLLRDAVSSEPEKWLGPAHVAKYGSDTKLLVKLLDA
GQRLPVHAHPHVDWAAKHLREKHGKAEAWYILTPGSVWLGLKEDIDPEEL
LGIVREGRGGMELLERMHRIDVEPHQTVYVPPGVLHSIGEGIMVVEVQEP
SDMSILCEWSGFEIDGAKEGHLGVGFETALTAVEMKRRTREEVMRLVTGP
RVAESVCAEESRGYFRLERVLVKDRSSCRRGFAVVVVLEGEVVLRTEAGE
VLSLTRGSTVVVPYEDGGIMLEGEADVVIARPPE*
>Tr|1145_t
MAASSSSSSASTLEGPATDVVKEQGSHFQPSRRNSKSAPSRRGSTASRTR
SPQKETFDPNLDINLPYRTLTSDANFDEFRVESRTGAIPGPVEPPAVAGQ
GGEKRYQLVTFTPEDPENPKNWSKAYKWYCTMVVALTCFVVAFCSAVITA
DLVGPAEDFGVSREVSILAITVFVVGFGVGPMAFAPLSEVGSTLLLAVIF
IIPCAVAKNIGTLIVCRAIDGIAFSAPMTLVGGTLADLWRNEERGVPMAA
FSAAPFIGPAIGPLVGGYLADGAGWRWLYWIQLILAGIVWVLITFTVPET
YAPTILSRRAKKLRQTTGDETYVTEQDIDERPMSERLTVFMIRPFQLLFR
ELIVLLISLYMSVLYGLLYMFFVAYPIVFQEGKGWSAGTTGLMFIPLAVG
VLCSAACSPMVNKHYLKMVHKYNGKPPAEIRLIPMMISCWCIPIGLFIFA
WTSYPRLIWVGPALGGFPVGFGFIFLYNSANNYLVDSYQHQAASALAAKT
CIRSFWGAGVVLFTEQMYARLGYQWASSLLAFISLACCAIPYCFWIWGAK
IRARSKYAYGGDDEEVETSQNPNDLEKAQRVPTHGAAPRDDTDDDLDLRR
ARSYVSNP*
>Tr|997_t
MDGLPQRPEADASLPSPQRLDEERVLSKKPKHARRSLAPSAAASSSSST
MVHPSAADRKRKQAAISASSEDGAAKRVRIAATDRDLVVTGTAQPSRYDM
SQHVLAEVWQHIFSFVPPRSLGNLMCVNRLFHRYLDPASTFKVSAPDSDL
PSSLPKQSPNTIWRASRRFHWPSMPSPLSGRSELDMWRLACSRSCQFCGC
RADETDYAGDPLRWSRGPGNDTVSPVFQFFVASCGQCLAKNSIKEVDLLL
SPSKPSFIAAGAPTVFCSGLLSWRAFKQNLETPSDLVPQLLKNGSKA*
>Tr|1872_t
MPGQIASFNSVPEWLVKLLAAELPYSLPLLRRLQSTKFKHGTSPHARVVF
VHDTDSPLEAHHDFNKFTAAYLDFSRQETQMYIYSTLEHRRNKDDESDLH

LYEQQLARLVSEVVRLRKEYGHKLLFTNPDRILVGSLHSKVRSILERFDG

VVEPRPTGVYDKWLMRRDELPSLDESLPSGMYWDSASLEDCQIVVSRTDI

PRTAEMLVNLPNLMIKRDDGTPIAWALLGTDGSLVSVHCEEPYRRRGLAK

KLATKLLLEKSHQFGEDGWLCADVSPSNESSRAMCKSLNGKPNWLVSW*

>Tr|6732_t

MSPPSRDTSPETHETDALTSSSPTPNLIPPKPSTTINRLSSLIPLHRDRY

YGALTFNTASFILPALYSTLSKLWVSRIDSSMVVTTDSFTYINTFSEALN

EGLPRAAWLIIGDKASRSLPQRLQLTHTLIAFQAIVGLLLSIVFLAAAPR

FAESFVPQEVREASLTYVRITSFTVLSGAVETAVASATRALDKPDVPLVI

SSVKFAVNIILDLLIISNFHVGSFQPTVNTQGIIQLVCNLTAAFIGLAYF

LYSASLSTWRKQRHVPLNGLTTSSHNKNHSLSPSLRALRVLLPPGLIFFA

ESAIRNALYLWLVTTVVALGSVYATAWGVFNTIRWGLVMVPVQALEATAL

QFIGHKWGQWRQLIGPSNRRPKASWNELFSLVSPAIRSLLIALLVEVPIA

IFLSVFGARPFALYISGSSEVADVTAHMWRTIDWCYIFYAMSTQLATILQ

ATRPKWYLYQSLASNMLYVLPWAIVCQVAHLDQENAWTYHSFIFGGSLVF

SFVDVLIVVGVWMWTLRTGRARLEVIQSS*

>Tr|6125_t

MGIIRKASLGVTLGTAAALGYVRLSTSIIAPIPLSDALYSSNTFKKYNLH

RNPATNDICVKTIPLNRIRPELLQKEGDLALEFCRAVWKGWGYSIQRRYL

HRKYFGADTSSQLWTPEQIAASNFEPGTIVTDHFEVVERTPTEITIRCGD

SPRTQGPRASDGLFAIGATIDKDAQVARLTLKSCFFPSDRKVEGDKGPMP

GWIENLHQWYSRILMVSAARSLMR*

>Tr|6147_t

MAISDSPLPESQQLLCLTITAYRKPGLSESAYREYMTKTHAPLVSGLMKE

YGIVRYNMTHNDSTSRPLLFQLYDPEFSKLSDYDCIVQFVFRKMEDFLRM

KADPRFLEKVAPDHKNFADTSRSTMTIGYFEEFLDNGELVSK*

>Tr|2539_t

MLTPTVLHRFRWFHAFGNAPAINLARSIPHGQDASVLSLGCGDLSSILYT

SYVQQGLPGRKLDFTCCNDDENITARNLVILTMILAGEEGASYQALWDVY

YHMYLDEQTTELVIRHVRTMIPMLESLETFNNGLYGSIMQICDEDTLCDV

RREPSLDLAAVRAASPLALARSCAGPPHSHDYWNGVIAMINGKDHRTPNP

LLDGLVSDWKILHSGSDPVLSFHLASAFTSLTANSPLRHDEDDEEHRVVA

AARSQFIAWTRAFRGVKDGMCVRFVVSDPFALSHTLQYAGATGQLSANWY

RRQWDSKVLRLDEALYGQDGSAPTSFDAINTSNLSEDFGLLNVLLSAGPL

LKAKPWATLLTEDHVENHSPEKHRLREPLHGSSSTMSLLLGLVPLQCWTN

AQADSPLREMVTLTVPPQLAPSRFPVCLSWKRDDQLGDYNVERSQIHMNG

KEVIELLFRMYLDMFEAETEKGLASQSKNKPQNQPCFHRGSFMAMLKLIQ

SRVKTDWNAVIDGLLRRIEVERTLSMAGRQAQDLRLQLALFNIRTSIGVT

GVLKVPNTGLLSRWKHVPPEVAITLVVPRDALRDFFPEPHPTWPVPTVVG

TLKSSSIISKRRCNVFDDVHIVFGRVTAIGNLASGDASVRIEQDELCWSG

TSPLVATFLVPTSALAGQPVTDVVGLDLSTASPNTTFFTEMLGMCHRTMY

ETTVSNTTSVFITKLMPGTTAHKVTSGGVRPLRDEVTGVDNEARMKLMAE

LGSDKRDITTLSALIQVSSESFKNDSQNKPPFELYQNNPFSISVIIGKQK

FICPFRYPVPLSSIASKTGLSGVGRTSVYIKVVAPLVDPRQSSVLKDWLS

LTTITPGGLPVAVNMPHLSLDALPVIELSQPQRLAWLTTLTSLQWPRHDQ

RESTEDVRAKFKGTLSAMFKACSGTLKSQAGMFSLNGRRRGDIQALILVS

ALRLDGDTASVVLDAAILPLTSRYLASGDSDDFLKGLELLDPLTLQVDDE

EMAL*

>Tr|1876_t

MAIPELMGSVTSSQLWSFGALFLVLSFIVDFASHPQYPRQIPVMGKGHGI

LNAFLDSFRYVTNYVDWIRDGYNKFGKKGMPFIVPAPFSRPPEVVMPRDQ

LAWLFDQPDNILSTYDAHNAVLFSEYNFLGRRLAYDPFPNRVIHKHLARH

LSTVIPHVDDEIQHAVDTALGTDTDNWKSIKLWNLWLEIVPRVTNRLLIG

PEICRNKQFLDGMVKFADDIVRNGLLLQLIPKVLHPIFGRLLSIFNYLHW

RSSNTQAQPVIQKRLDAMLKKAAVDEEYKDYTPPEDFITWLIRQALLEQK

TDELDSTVISRRLLPIEFAAIHTTVLTGQLWMQDLLTSDPETGILDILRA

EVIANKPSSGPWTKAAISNLVRLDSSIRESQRLSNFAATLIERTVVAQNG

LYNPDLGWTLPKGSFVTVNLEGTHHDPDLYPNPHSYDPLRYSRLREAWDA

KSDEEKKRDAAEGTKIRGLGMVTTSAQHLAFGHGRHACPGRFFVAHELKL

IMAHILLNYDIKLVGKVPEKVWIGSSIFPHLKAEIQVRRKKTQA*

>Tr|7356_t

MKTTFAALIVSLTSIVSAYPITGDVVNCRTGPGTSYAIKTSYKKSHDISI

SCQTTGTSVNGNNIWDKTADGCYVADYYVKTGSSGFVTKKCSASGGGGSS

SGSYCKTINGAGVDLIAKWEGFVASPKPDPIGLPTVGYGHLCQQKNCAEV

KYKFPLTKTTAKELLLDDLPKYTKCLADVLNSKPKLNANQWAALSSWVFN

VGCGNAKTSTLVKRLNNGEAPNTVAAQELPKWRMAGGKVMPGLEARRKDE

VKLFKTASSKEAYPKCQA*

>Tr|8271_t

MGFSPRALLFLTASIAFINLSVQGLPSSNYVLHEIRSPTGTGHVNSHREW

KRGSRLDPHAIIPLRIGLAQSNIHLGYEKLMEVSDPSSETFGKHLSQDEV

HDLFAPAHETFDAVHSWLVESGVNASEIRQYENKGWLAIDLPVTHVEGLF

QTQYHEHEREGELKIGCDQYYVPRHLSEHIDYIVPGIKLSPPMVRRSLER

VPDTSSRKYRKGWRPKQMPPDLLSLVKKPPTTPKLPANLQDCARNFTAVC

YRALYQIPATSHPVAGLEPAVYESGDTFAQGDLDSYYHKYAPWIANGTHP

RILSVDGGEAPVAPDSEYNTGESDIDVNIIQTLVWPQTMVLYQVDDRLYT

TANNYSGFLNHFLDALDGSYCHSTAFGITGDSPGIDPSYPDNRPGGYHGT

ALCGAYKPNKVISISYGEGEIDVPKNYFQRQCNEWLKLGLQGTTVLVSSG

DFGVAMPPESDTATGCISGSGQNQTIYNPGNPVSCPYLTSVGATQLEPGT

TVLDAEGAMQTNLGPGAELFASGGGFSNYFPAPDYQKAAVSKYFAKHDPG

HPYYIANANASNIGENGGIYNRAGRGIPDISANGANFRAFNNGTEGHWFG

TSLAAPLWASIITLINQERTKIGKGSVGFINPVLYANVDALTDIKQGSNP

NCGTSGFTAVEGWDPVTGLGTPRYPSLLKLWLKLP*

>Tr|486_t

MEPSGANKGFFQPAPILPNQFYDDVSFRRCFKLFLPFSVITEVEAEVAAL

GRDVLSDEVFAWITDAERNKPYLKGSGRDAFGRLQGELVTGEGWRQLQKF

GLSRGIVATGYDTPYGAFSRPLQYLRLHLWEASSANVTCPSAMQDGAAAL

LRKHLASRRLTETERKVFEDAYRRLISRDPSVAWTSGQWMTERSGGSDVS

QTETVAVYKPEGAEGQLASKEGKIPLGPWSISGFKWFSSATDSDMTVLLA

RTSHGGLSTFFAPMRRHDPTAKTMTGHPKSDGTRLNGVWIQRLKNKLGTQ

SLPTAELVLEDMRGWLIGEEGRGIQEISTILTLTRIHNVITACGYVGRGL

GIARAYARRREVGAGRGARMKLTESTLHMRTLANLTAEYHGLMLLSNFCG

YLLGISEHEATPPTSALAALTPANHYIDPLLRIVVPLAKAYVCKAAVPLL

YSCMESLGGVGYLVNEEQEYLNIARLYRDCCVLPIWEGTTDVLCTDMIRV

LKHPQSGTRSLDALDNFIKNAYGLQTDAVKKPPEWDPVSKWSALRWYIDD
KTQGDLVGEGREILWDIAELLISLLLYVDASTDGNEAAKEIFRRFVQEKF
AKEKRQRDTTAEELKRDLAIVYGVEEAEAVSREISKL*
>Tr|4925_t
MLEALPLLRAAASSINVHSKQMGRLTVAEYGSAHGNNSFEPFSAIVHAMH
VPEDTQVTLVLSDRPENDFSILASNIAAFEVRQQQKKQLFTAMVPKSFYQ
QIAPSDSVSIGFSLACLHHLEHVPPLAEGESPVDVSRTSILREQSHKDLL
RFLNHRAAEIAPGGALVLSFVAQSSTGEENYAGLVDACRRALVEMLQAGL
IPIAAATSFEVPTYNRKLEDLGELIKDGVPGKWVVRDVAEKRVVHPAGAE
LRARHQTTDSIRDNGDKQKQRDEDSKWYAETVTDWLMAVVSGYFLKALRV
GLGVGYTEQGGHTLLAEWVQRTKDMLLAHHRDEDVSCWFIYLRLERL*
>Tr|7678_t
MKRSIRDTGDGSSAQKPPAKRAATAKRAAASKKTAAQATESPEPVVIAGP
APAASPSPSSSIPNTRRVPVGPNGEFFIPRTFPRESIKALDKMVVSKRWV
QIDGDPNHEFTLGNRRWWELNSPWLEANKKSIGLTAKHWKMREEAFAKGE
PRGDDEGDNPEDFVCISPPALEARDSEEDEDEDEDEEEEEDEENEEENEE
AAEEAKASKEKAKEEQCAMHKVVGKLASLHPEHKWVSTMRGNERSKWWIS
ELLKRDQDDFLMHVYNDFTWYGTIEVMENIFVNFEKVLKRKNHTVMELWF
ELEGLALVLNSGCVEFQMCDDADRCGQILELVGYMTITVIGSLQKNKLFA
KDSQIPNIGIMLALVLTYAHSMGTDYGWEDQVGWTPYVVQQATVAHITLA
GPKKFAETLQGINEWGAGKTAASQAKWAKGTFPSKLAAYGSRGGNQFDIT
TFSAAERKKPHNGRNITAQQLTDAVTEAINLDGKAVSKIANDFMKAVNKS
AIDLPELNRPGIIQHIASLTRDDVTDPDSLECAASPERIKHLLEDSPTDC
ITVSSLAKTRLHVEALSAPQELTLKESFFTYFEASLLLLMMNDGPIPSGW
SFPSPDSYCAPKEKVEIWLTEERFPVELGWKKPERKLGIVDFVPAMKGVF
EHKRLQAGKGPLWKALVPSFLQRQETHGEL*
>Tr|7830_t
MPDVATGEIKDKSKGDIFVKVIALGQILWSVFQIIARLVRDLPISPLEVA
VVAFAVCAVVIYGLYWGKPQRVGVTITILTYDGVIPSDVLAGLKEVRPAW
RLPTLGDALPGAPISVQCTRSVGYRGFVLTNVVTTLGAAVFGGIHVIAWN
FAFPPHTANPNRPLDTREISTKSSSPSSATMKLSLGLTDALKGPYLARDF

VRASLEIRGPAFQKSPRISAKLAGAMIVSYPTQINAASPGQITNVLFEQS
KTIEYRDLQCKEDPSTGESIYTTPVEFQFPAGDCHCDKAKKCQLPPTMDV
VEQGMRVRVKYTLSVTVGRSVLGPMTKSKSVAMEVPFSCNPSVSKLPRSA
FVLSVPMVEKPGHIAQLHSLTQDQERADGSCPLYNPKSVPSIKIEVILAQ
PAVLIRGQPTPVRIVIHTPTDLIEAGDVYLRNISMDLKSSVTTSTGALPR
TLTQRNRGLSMAGAVKIDSELFELDTGAWGNFFVLNTKPTTESCVLKLSH
VMEIVAGISVGLGSDVKYAGAAYEVIVMEPPPAYESTTKSDCISNGE*
>Tr|23_t
MTKDPCQEFHVSASLRRKSQAEKVVQAEGCFGLALTPIFIGISAVLSGYK
TYLVSLAIRDATYLIDFTIQTVTAPEGSNGQDYLAEYIIKSIRGYEHRTF
TKAVGAGLPSSLKTTSPSLCSRLWLELDIVPIVIQQPHEHTERIALWLSK
RVDEQADSMARKCIMNFGPSLAPLLQVAWRGIVEVDASFQAPLISLEDYK
TTCSFASWETVMHYAKTLRKHKTKIAFFSSTPQGGGVALMRHALVRFSRV
MGVDLRWYVPKPHPGVFRVTKNIHNTLQGVSPKDQFISEEEKSSIREWIT
DNGDRYWFSEGGPLCRPEKGGADIVIIDDPQMPCLIPLIKKLTPSRPVFY
RSHIQIRTDLIKDPNSPQADVWDFLWQNIKHADLFISHPMPSFVPHNVPR
EKVLYMPATTDWLDGLNKPMEIWDTGYYGHLYNIKCRSERMTELQWPRRK
YIIQVSRFDPAKGLPTVIDAYACFREQLEKHGGISAPQLVICGNASVDDP
DGTTIYDQVMTQLETEYPHLLADVSVMRLDPCDQLLNCLMANAHVVLQLS
TYEGFEVKVSEALHAGRPVIASFAGGIPLQVKDKINGFLVQPGDSRAVAG
HLMELFTDTKLHERMSQAAKTGVSDEVGTVGNALSWYYLTSKWAQKGPKL
ELLGNEQWVNDMARNEAGQPYNEGENRLPRSFTQL*
>Tr|3423_t
MLSYIEAVTPIAVSFPSFQLQIRGDLFPPEPGSPNRKGAAIVVSHPMTGV
KEQTSTTYAKMLSNAGFFALVFDAGYQGESSGEPRGLEDPHQRVEDIKAA
VSYLTALSGRVDPDRIGLLGICASGGYASYATQSDSRIKCLATVSAACVG
RMTRSGGVHEEQAARENPQAIAMALEAAAQARIMAAKNPTAKLEAPVMFE
TDPAKAADNPDSFFRDAAAYYGSRRGKHERSTQRVPPQSYDLMVSYDSFN
LQHLIAPRPLLMIAGSEAQTLHYSRTAIAIARGPKEMFVVPGRNHFDLYD
NLLETAPKLMDFFISSLE*
>Tr|9279_t

MPLTAHGFDSREIHSKIQLLIDGLVNIRDETGEFLMTLADGRVIDTKGWN
DWEWTHGVGLYGIWHYYQTTGDAKYLQIIEDWFKNRFAAGGTTKNINTMA
AMLTLAFVYEKTRNPTYLHWLDSWAEWAYHDLERTKYGGMQHITYLEEND
QQLWDDTLMMTVLPLAKIGLVLDRPHYVNEAKYQFLLHIQYLFDSRTGLF
FHGWCFKDGGHNFASARWARGNSWLTIVIPEIIELLNLAPDDPFCVHLKS
TLGAQCEALKALQSPCGLWRTLLDVPEEEGSYVEASATAGFAFGLLKGQR
KHYLPKEYQDIAFKAIQGVLDNINPKGELLNTSFGTGMGSDLEFYKKIPI
TPMPYGQAMAILALVEHLKIFL*
>Tr|3611_t
MAQTFHILILPGDHVGPEVMAEALKILNVVEECRPNLKFERTFDLVGGSS
IDKHGVPVTEEVLDKASKADAVLFGSVGGPEWADASPNPEAGILGLRQRL
DAFANLRPCEILVPSLLDASPLKPEIVKGTKFIVVRENCGGAYFGEKVEK
LEVASDLWVYKPDEVERCARVSAAVARILGRNGDGKGGGGPAIVWSADKA
NVLASGRLWRRVTQNTFTREFPDIELRHQLADSMAMLMVKNPRGFNGVIH
TDNTFGDILSDISGGIVGSLGTLPSASISGIPGQGRCNGIYEPVHGSAPD
IAGKGIVNPVAQILSLAMLLRYSCVLVDEAAAIEKAVETVLDSKEAGGLG
IRTRDMGGEAGTKEVGDAISVELRRLLQAS*
>Tr|2551_t
MASTGRITRTSTRNEAHSRGRRANGNGNVVIENALAHLTDEELERDVQSF
VENHLPSVRYEDLLRAARVAKDIRVYDEVARQKGYNFESTLPVQLTKEEK
YALRRERDVPFSEKGMRIVILTVSIAALLQGFVQSSFNGASLYREEWGLR
RDLGPDDTSADNWKLGAANAAPFFFAALIGCPLALPINYWFGRRGGICVA
ALLIFASSLGAAYARTWYHLLGIRAINGVGMGIKAVSTPILAGETAVGFW
RGSAILAWQLWVAFGIMLGFAFNLVFTKAQSSLTTLSLINAAPMVPSFFL
FMITLFLCPESPRYHLTRGPNYNVQKAFISMVKLRNTELQALRDMYLIYK
SLEQESMALGNLDAHAFRSPGFVWVIRDFFRQYAQLFQQRRLYNAVISTS
TVNLAQQLCGINVLAFYSGYLFNGAGSDVHSKVIPMAYSLGFGAINFLCA
LPAVRSIDTLGRRKWLLATLPFMALFMFGAALAFTIEPRNIRVGVAAFFL
FIFAAVYSPGLGPIPFTLASESFPLSHREAGTAWAISINLGFAGLLSIVF
PSINSGLTETGALGLFSGLNVVALVMVFLLVEETKRRTLEELDHIFAVSK
RKFMRFQLFEYLPWLVRYYIFGSKKPRPELYEDLIWGPSEGEDLATFRAV

DLVGYYSEPSVPEPVELAHSRSRDVPQAGDQYGSVRSKELGAKVAADPGD
DSEMQATNNDGPVSIRY*

>Tr|912_t

MPPRQSTSGTSQKVRSRPSYVQPGDPLPTRQRRRYRPGTVALREIRQYQA
NTKLLLLKLPFMRLVREIGLNCRPTGKEFRWQSQAIQALQEAAEAFMVHL
FEDAQLCAVHAKRVTLMQKDIQLARRIRGIWGGLG*

>Tr|1884_t

MSLHVPLPHRSATPVGAATKAVILVGGPSRGTRFRPLSLDLPKPLFEVAG
HPIIWHCLAAVARVKQIQEVYIIGYYDESVFRDFIKDSAREFPGINLRYL
REYEALGTAGGLYHFRDAILKGRPERLFVLNADVCCSFPLEEMLKLFMEK
DAEAVILGTRVSDDAASNFGCIVSDAHTRRVLHYVEKPESHISNLINCGV
YLFSTDAIFPSIRSAIKRRTDRPARLVSYPSSDNLDNFTMPRAVADDDDD
EDKKEVIRLEQDILGDMADSKQFFVYETKDFWRQIKTAGSAVPANALYLQ
KAWQSGSEELAPASANIVPPVFIHPTAEVHPTAKLGPNVSIGPRVVVGAG
ARVKESIVLEDSEIKHDACVLYSIIGWGSRVGAWARVEGSPTPAGSHSTS
IIKNGVKVQSISILGKDCGVGDEVRVQNCVCLPYKELKRDVANEVIM*

>Tr|7397_t

MSSTFAIKDPVSSSQDPHQRLPWTPVPFLTAGQCYRLGKTCEYAETAASE
STISSRLSHIEQLLQVEGVSPSRAFTGISAAAFFPRPAEAFPLPFFLDSE
YLTSQSVNALSWSRHPYIHQIVAEHLDDDRVTLCEHYLSTVHKWLPMISR
KRLFNEVNSDATEVDGCLAIFLLSMKALSAKDGECAKSSSWYLLARTLCS
EAESAGFVSLRLVQALVLLSVYEMSHAIYPCAYSTLGRASRLGVLLGLHD
RKNNQLFKPAETWTMREEQRRTWWAIFLLDRLMNIETNLPSTVPEPVKDE
LLPINDEDWDEGKIVPSEPLFTTSFSSLTAVGSFAKMCQAAHMLSKVLSH
RASKRSTQDVGSLLPEALALHSALSALHLSIKEYISGTTSSSSSISGIIS
LSLCCSAQLVLYNMYGCNEPLALVEQSRIAMETEMQSVSLQGIKSISCNV
MPAIARANIECPLMAQCLYHVATECAWFVREDHEPQMYSALEDVVRELRS
TGANWNLATQYLSLLQQGGVLDLVNVDTDASTTVTSSSG*

>Tr|7582_t

MRYHLLAALLASEALAARPFLNEPDTGIDDVLSYLPKGILPKLSDIVGLP
DFDWAARHYLPVKNYTYYRNGAAGEWSYRNNLEVYSRYRLRPRVMVDITN

IESTLPTTILGHNFSAPIFISPCARGTYGHPDGELNLVRGAAAGNILYMP
ALFASYTIEEIAAAKAEGQVVFQQLYLSSNDTETQDLLDRSKKAGASAII
FTVDSAADGNRHRAARYGVGSADSEYTYITWDYYKKITRMTDLPVILKGI
MTVEDAKLAVKHKVPAIILSNHGGRQIDGSPSSLEVALEIHQRAPEVFKK
IEVYADGGVRYGADVLKLLSLGVKAVGLGRPFMFANVFGTKGVEKAIDII
KHEIAIDAANLGVGDLKKIDPSYVQWTPNNWMG*
>Tr|5051_t
MIGLLSSPVEAAIKTPWGEPFKPIDPQEWVNPDDMTWDDYKAPPGTQWND
PSKKGASRNFNIALIAVDYSDETFVVTREPNSTVFGNPLPLVSGIGRDDV
PAYYRDLLNKPGDLNRGHTLHEYWMGDSHGRYGVDLTAFGPYRMPRLSYE
YGVDSGFNPGACPDDACDQDIRTDAFAAWRAEVGNDTSNSFELVFILSAG
QDESSTWQEFGEMRFSSPEDVPAEFGPPQSANGTSLPNYAATRYVPWTSW
ASASTIWPNAGGGSSTQCESSGMGTYAHELSHLLNIGDNYNNPYGIPLRR
SYTGPFSMMSRGSFNGPGGPHTRYQIPALQGGSLGSLHTVRDKLVLGLIS
NDSIVRISRDALAESGPVVATITARSVVSDLIGLRVDMDQDRSPACNIST
DVFCDGGRYNMYDIEVIDRMGADSFQPDSGVMISKSKTSNSQPFQWTIDA
NPQDIGLVDFIRPNGSAAMITLGDYRQLADALFHAGTGSGSKYEYVDEAN
GLHFYILDVHRDEVGVLSYTVGARSLTDKSASEYGIQLTSGVPFAFGGKA
TPTTGGVFCEFELTNNGTYAGSSEGRAQVQAEHVQWEIFRLETEVKGAGW
RAALPNAVIPVRFGETAKAYVAVGAEAHAKDRTGVVTLRARSESDPSVVA
TATCKVKKSSCT*
>Tr|5705_t
MPPTFRLRPGTASDLPHAVRLYDACLGSDKLVALLFPRKEEDPESYKKYL
YRLYAKRYWSVEWTFTFVVKEEAGQGEDDGEKEEDAIVGFSCWKRPSADV
SFRERWFTLFSWIAPLVRTFISLQDTLSPLNIDSHAAHAFDRVFPPIEKT
LFADKNRAEWWYLSTLAVHPSVQGIGLGALLMAEGLTRADEWQRQRLLQL
QEKPSQEGEEEEDDDDGQQQQHRMEGGETKVRGKVWLIGLRGTDAFYSRF
GFVEVGRANVGDLSEWDGGIVMFRE*
>Tr|8976_t
MDEQRGDSPDKAELDNSLESATTESTSETIQVIQLNPQAVERDIGPLQII
ALGFNIPNSWAGVATAFSAALTAGGPVSLIYGNFVSLAMYGSAAVTLAEL

ASVYPTAGGQYHFASIMAPKRFNRSISYVCGMMATVSWVICSASVASITS
LCIASIAEFYGGYKASNWQLFLVSQALNLFALGYNLFLMKRTGWLHNAAF
FLTLSTFLVICIVCLVRGDKQSSTWVWTNFEPSSGWPPAVSFFIGLSTHA
YMFGGLDSALHLAEETLDASRTVPKALMSTIGLGFFSGFVFSVAMTYCIP
SLDIFANDPVPIYKLWRIASKSDTAATVFLVIVIVIFTFILIAIQQTTSR
LLWAMARDQGLIYSTRFAKLSPRLGDIPVAALLLDAGLIALCGCISLGST
AAFNALVGVFSMMQMTSFAIPAALLMYRRRSKKVLPRKRAFKVPEAMGWA
CNIGTVVAAVIETVFFTFPTVLPVTGSNMNYAVVVLAVIAIVMALNWVLF
ARKHYQGPRIGVTL*
>Tr|1584_t
MARKKSRKKNKDSSQTEPDPERGSASKTQEQEKPSVLKCIDGAAIPNHPI
FDTGVFWGNHAATDILNLAENEGAEYDGFLRLLLLGPFGLRHLIYSVAAM
PETANPLINVVICETDFSQLLRTIISLHIMVSWRQDPEENTALIAEIVSH
VWYSLKWPQELHSYVKDTVGNGALEAREHVRSSRDSGTVRTPSVTFGEGR
LQLHAQLEPDVWDAIVNQIYQTSSKNERTARMARETDAMLYGEPLDRVHA
RMSPSRAAALMKWRQDGILMPYSSPTEAFVKPSPIFFNPDGSQPTGLTNE
PLSEWPMKGILEYAPYPAKGDVYGKMSRYIHGKIVAFLERVQRREVHIRL
MACGLTEMVGNLRNTYEEDAPYFDRIEVGHLFEFEPELCFLSCATLLRHA
DENPRATMLTMCRESVVSAESPKLDKYVAAEKDLIFHRGLEPLDAIIPPA
QAAGENYSAASLPRHMGILLFRDWDMFSYHYLNDADRFGGRDPGEGLSQQ
PKASFLRTGYMGVHLKRNNTVVSPWPNRLVHGAGSKPSKEEVMRWMSWST
TKPERWLEWKRTGDVNTKEWLRHIEKIRGPSVLANMKKLYEVLLELDQAR
DFDEEEEGEGGDEVMDVRVKKVGVREEETDRGSHDAKLGPVGRGSSDNQG
MASDEAAEVEGKYCPHMLSTGPRSRRWTAGF*
>Tr|2800_t
MTQTQTEPNRRTYRGSCHCGAFVYDIDLPELRVVVECDCSFCRRTGNLYV
PTGEDANFRVVNGSEGNLTSYTFGPGSKIHKFCPICATSLLSRMPDGPAF
MKLLLNARAFHDVDVGFLERKYIFNSKVGAQYQPPEHKGDMPRIVEGGRL
YTGSCHCGAVTVALSCRPLKEPSEESLVFCNCHNAYVWLFPKFESIVLSG
SKADIGRYAFSEGLTSKSFCRACGVGMTYLMNQPVEDRDLAPSEPSCRAY
REVNTSHPVNARVLHGVDVSESR*

>Tr|5778_t

MLNAEDEWKGTSNPNLRRRVQNRLNQRAYRLRRKRMLGTLEETPAKAVNE

TTAECLDLGHASSPSPAREFQVVLCRTPTGYADHDDSHHDCKQRPANRPP

NVDTRIEPAPWYTDPAAVATQFRALKLGRHAKPSPSNDHLLCIMQFNIMR

AFGTITSIVGLSPTDLLDDDTPSPFSPRASSLTREFPARHQHHHISLPKS

LSPTSLQRTTPHHPWIDILPFPEMRDNLLRLEAGSCAAAEQHRYDPDSLC

HWMVGLDGSQKESGFILWGEPWDLAAWEVTVEFLDRWGWTLKGCFELFQS

TNHWRRKRGLMPLFAM*

>Tr|9002_t

MSNSNDTSQLPGRPQMPTLKSKGFQLHGQDFTVGGIARVGGARLQELLNP

SALRLKREQKHAAEEAHRLFSKAFFAAQLKHYDIPFRSSHKRDELLSLLK

NAVSQGKCNHVPQSVLDIAAAMKTDYAPLYQKWQAECAAWDAAKKQRDDE

AFAKCKTPGERANCDLDRFMDMYFLTDGKPDKSKTPKPLVLSGFQDRWSL

HSRAERVPGLETCSGGPPSNRELCIGWDRSEVFALASRVSERAYEAEKAK

QKAEWEQQMARHRRYIARGLGTGSGGSRQPDTFDMARCLGSYIIQCDEIA

DQWPDVVRGHTLTMDISKGRGNTLLAAYDFGIIEGTMILSLSEDTLKAIV

GGNTSESEASRSDDFYSSEDDGDGDEEGIHTQQPDGGIKRKAGQSSSLAR

ATASAAAVRHPVTAKRRKTGAVPSLTRRVYLRLRGRETGEGEIIPDPDSG

HIDFLSNSCATFAGLVYHLTFVAKNVEFRGYKVSDTPRVKPEAWEDFSYE

AYEHARVGRWY*

>Tr|1587_t

MRPEYDDRLPQTTYSFEVEFLVAQELPGFNYDSTFNEYHGRREELPWACP

ADAKDPYETILNEVRGLLLKYGQPVAICEDSSDQQPYAAAYDPRKARQYL

TADDEWWHVEPSVTTYAKRDSPQMYEWFGVRVRSPASQSRTIVDNEIESP

TDWILGVLTKGLVMHLNSTCRFNVRVRPLSEDISPVHVKKLVTLVWVLER

ELLERLCSSSYGRPHPHVRTLSAHSRVASHIWHGSGEKSPPNDPLGSVVT

TLHLPQLHNKEVLARLQFLWQMQSLEDLAAALRTTTGEATSFAIQPTGGP

FGMPIFEFRYSLWHPFGQLDASKHWIELSVKLLQTSMWNSPIFKKNVTLL

DGMIYNFWGSNEPPTYRWKALLAILDLEKWSESWEVIIGQYKDGQRLAAR

SLDKQKLLHKELKADGQRKTTT*

>Tr|3363_t

MESVTGIPISVVLLASSLIGLFIVGRIIQFNRLRTFKGPFLTHFTNLPHR
KALFKERCHEWYAEVCETYGPIARVAPNLLITSDPDVWIHVNNKPGYKRS
DWYYTACRLEYGRDNVFSQTDNKKHEQRRKQMAPGYSGRENLDLEIAIDQ
CVQEFLDLIRSKYVSTDAKIVPVDMAKKVQYFALDVISGVGLGKTFGMLK
NDTDVEGYLQSAEEGLRIANFSLAIGLSWIAQAPVIGRFIAPSEKTQNGF
GRMIATCFRYVDEREANPTDKRSDMLASFIRHGIHGADLKTEALEQIVAG
SDTTSSAIRIILLHIITNPRVYAKLQREIDEAVQTGKAPREGAIITHSQT
KQLPYLQATIREAMRVMPPVANIFSRDIPPQGDTVKIKGQEVFLPGGANI
GYSAYAMHRSPEIYGEDAKAFRPERWFEEDQDKLAVMLRTNELIFGHGKY
QCLGKPVAQIEIGKVIFELLRYFDIAVMQPDKPWHAENALGLFHIKDMWV
QVMERAREN*
>Tr|3460_t
MAYRVPHDRDRRVYIILHQDGVTVTVLGVYEELQDANRDCLWQAAQAGID
LLQASPTTGPDKYHIQPVEPARWDTPDGVSCWVESHMVVPSRVAGSNLAT
R*
>Tr|7125_t
MANSHTSDREALTSRILPQMTLSSGLSPPPSSSSSSSSAHDSALDRFSIA
GNAVITGGTGAIGLVVARAILQHGASGLMLLDLNVDASSSQLAVQALRDE
FPQASIAARSVDVTDEDAVNAAITEEAVQSLGSVDMLVCLAGIVSCSHAL
DTPISTWRKVLDVNTTGAFICARAAARAMVSQGTGGRIVLTASISAHRVN
FPQPQAAYNVSKGALLMLKSSLAAEWARYGITVNSVSPGYMDTVLNEGEG
LAAARSIWNERNPMGRMGLPEEVAGVVVMMLSRAGSYLNGADVVVDGGGH
VF*
>Tr|8299_t
MASLTSFIFLFLLVSQSVSADDGDDFSNNLLTDLAPLLALFGERVTMQFL
SQSVGFSDHILLATAPLGIITVIVAAIRVGGPLWLKALFGRATENVAAAE
MEIMSSTSREVCELWNGRDVVRYLGSAAVWEFICLLPGKDVPKHPKIRVV
SMDEATSHGYIERASDIQIPFWEKMRKLLPFPSNKGQNGAPGAGGKQSIN
SRQNEVIIIRNIRPDSPNISHNRSRNTGRLELRFWACVGVFLQISLLVYG
GLITQYSGITSNFQKDSHAVESYSFPLTLAGTLLLRIGMGICSHVVGSST
EEEIFIPAESWRARLVWLQQQKTVGDQEFKSFALFTKDDQPNIITSSRVN

QDQYFNGKKQQGTNQSGILGLQFRTLLGTAMSLTGYVGQFVGIRGMHWSF

SVASLVVVLAMALARALARRGLMTPMFSQALPPEFELDWFADTLRNIGDA

PWVQDQEKSKDKSPVPSAKGSSQTSPDQEDIDEIQDYIMRRQMIAKLAGS

RGPVARQASLLARTMEAALNSLHYCFHQESLSWTFRVQSEGSAQREVKLS

VTKGAEGCWEVDEEAISAALSLGLFHVKAQRDAEPSTRIIPSFDSQPREH

MDIPEVGVSFIGPWTRALQQHIMWWIPKMPAAEEPVLYATEVQYKTAEEH

HSTSSDNIEQPPSSPAGVLEVEERFAVGLDPRQVERLNMNSGPTRSGRGV

AVECDLQIPWRYSGDSQPGRWKFALPQRDPKPEPLEGNITPSHPIVYVKT

TDTLETLYAKDMFATFMWAVVKSIDDTLPGLTRVKELRGFSYDDRISWKT

FMLENDNVSNWARAIESVGLCTRHEAFRSIIAPLSMQQKLPEAPCIVEMV

RRRAGAFEAQQRWREAAEAYLWLRDIMGCFPVQSLTYAIAAAVLTSVVVD

MAGASPKASLGLPGVQAAFVRLKQSIEGIDEVLIRALRSLWSISSMNQWE

ECYDDISDDFSARYPRLWINSRGIQIRASKSILSSIAPDIFYRTRLHYSA

MFLEEGDMFADSQSSDYMYIRSPGEAYHLWGSERNNTSTLDEFGIDTTDI

CGQAPLHYACRVGNTTMVRILLNKEALVNIQSRDGTTPLHCAAGSGHLEA

AEVLIEHGAEVDMADGAGITALHLAAFGGFVSIVKALRRRSSRSPRDKLG

RAAIHMAAIGGRPAVIDHLAIDVGVRSKEDETPLHLAALFGQVEALKKLL

SLPQVDISARDESGSTALHNACLVGHDESVQALLDAGSPLESQSHMSSTP

LLCAVEGNHEGTVRLLIERGANVEFRNKLGQTALHIAALEKNKAIAEYLL

GQGADINAGTEASKGTPLHSAISSSNPDSNSVTRFLVEKGAWLEATDLQG

QTALHMAVKQSAEETIKLLVEHGANMEAKDHQGCNPVYYAFWKPDILRLL

LELGAEVNEAYPDGSTLLHWAVMRQAKETIKVLVQHGADVNARDKAGDTP

QKIAESVVWLQKEDMR*

>Tr|3615_t

MGVLLGSDALGVEPPVKLSVALPLAVVALGLGYFVWSCIYNVFFHPLSKF

PGPKFAAISRIPYSRILLNGVGHREILDLHLRYGPVVRIAPDFLSFNHPD

AVNDIRGHRKAGQPEHRKDPFRQEMNVHNIIGANRHDHTRFRRALANGFS

HQAMLDQEPIINDYVEQLMEALEKQAEGGSKPVDMVRWFNYTTFDVIGDL

AFGESFGCLKSTNYDPWIQLLFDSVKALVWMGTIKHFEMIPRWVIKYIMP

KGLQRKYVENQRLSAMKVQKRLDTGSDRPDFITSMTAKRNGESLTFEELA

ANASILIIAGSETTATALSAVAYFLSLNLDVQAKLAQEVRSTFKSPQEIT

ITSVQHLTYMLAVIDEAMRLYPPVAASLPRLISEGGAVVAGEYVPEGTYV

EVWQWALFRNPNYWKQPEEFIPERWIGDPKFANDRRESFQPFSAGPRNCV

GKNLAYSEMRLILARVIFQYDLKAAEGTEGWDYRSQAFSLWSKGPVNVYM

TPRKMD*

>Tr|754_t

MCWLAIPVIDIERAVAFYSQIFPWDISPNGVPHQRPGVKELYFFNGGDTL

HGAFYVMEDGFHVINHSIDFQDGLSVHPSFNVRNCKETLELVEKLGGKTQ

LHKSDLGGNMGHYARFIDTEDNMIGIWSKI*

>Tr|9080_t

MGLLSFVGAIFSKISSFSRRSGWIAAQAGSFRLHHFPTDEEDGDDETRIS

ARKSRLHPRLKLAILLLGVAAAAIVLVKLIDPSRVTSLSRQSFSYSDSII

PMFSSNKERETTPVLGESFFDFGTYFPAQGNNFFPLVASYNEPPRHRPKT

PLFIPFTRNNAMMRQTVLGYIASGWPREDIIIVDNSGTADANNLKLLSQS

NPFFLDYDLFRYRYGVSILQTSVLLNFAQIQNFLIRVAMARHWPYFFWSH

MDVGILSHEEETPYLSFYERVLKILDDAESSRMSGESKWAVKFFNFDYLT

LVNVEAWRHIGQWDVFIPYYATDCDAYGRLKMMGYKIDGVNAGHIWDVAD

IVDNPEEKFFPPSPTKNGDSTNATDDNAPNSERFKALREELQKIQDEKLS

NSKGRNTWQNMQKGGKGEPWTYDPAGFQVAWWEMAEDGRKLYKNKWGVSN

CDLMAEKKTLDDMWSKVNKGDE*

>Tr|5782_t

MDSQNETISRVAIDTTEPLAQQHYSSEKQTQGREHGLYEEPRVALPATIN

AAYDWTGPDDPDNPRNFSASLRIFSTIAITMLAMIGTVAGSMYAPAQDAV

ASAFHCSRIVAVLPLSLYNLGLAFGPMVGAPLSETYGRKSVFLISTPIFV

LFMLGSGFSRSVAGLTVCRFFAGVFASPLINNAPATLLDFTPPRYRGVSL

GGYYAVPSFGAALGPLIGGFVLLVKPWQWTQWISIFITVAFYIPVCFTRE

TYKKVILKRRAARLGLRDSASQRTSPGRAFRYFFTTLIQRPLHMLFTEPI

VTLVSVYNGFLFGLLYTFVVSVPWIFRHYYGWSVESEPLSYLGLMCGTAA

AAAPLIVIDLRYYQRRLTEWQMSHDDEDEPLPSENRLMSALIGSVMLPIC

LFVVGWTVHFRVHWMVPIVFQGLVMLSSLLVYAGANLFMLDAYGPLYGAS

ASGAMMFSRYLLSAAFPLFALRMYEALGAGWATSVLGFVTLVMAPIPWFF

RAFGERLRGRSKYEMST*

>Tr|7275_t

MSTEVKIVPVGHEAIDTGNGHDALKPAVNSGDIQSRGVSRMEAVYRETRS

NRRLFWLVGASVLVCAWAYSLDSSTTSYYSVDASFYFKQHSSVLATLSIA

TNIISAVSKPFIAKISDITSRPYTYLLILGFYVIGYIITATSKSISAFVV

GEVFVAIGGSGIDLTNDIIVADLTPLEWRGFASSMLSTPFLINTWFSGKI

VDALEKQDKWRWGYGMFAIIMPIALGPAVAALIWLDRMAKKKGEANIAFS

NAARRTFDPPVDQECQSNSLNITIAPAANIDQSWTQSLRKHLEEIDAFGL

VLLGFGWSLLLLPFSLKTYADHGWRNQSLIAMMVIGGLLLMAYVFYEMKV

ARVPSAPRRLVFNRTFIMAIIIDSFYMLAGNIRSLYWSSYVYVAKPWSYQ

DWVYYGNTLTLALCIAGPLVGLAQRWTHRYKLIQIIGLCIKLIGMGIMLN

GRLAAISTIALVMSSILVGIGGSMSVVGSRVASQASVPHQDVALAVSLLS

LWSKVGSAIGSAIVAVIWSNQMPNQLRKYLPEGTSDATIQKLFGDMRSIR

TAYDFDHPIRQGAITAYRHALYYCISVALALAFVPLVAAFFQTDYFLGKQ

QNAVTNLGNDGSPLDEKDCNPEPLPRPRTKKEAFLRFWAGSP*

>Tr|6150_t

MVYQPEFNVCSPDKLANHDGNGPDSGLVFDVYNVPADMCVRPLFVLLKCF

GNLLPARSSSSTQFAVETSKTAASWAALYPSEVNAYLLHTLLGVRFRWVD

SIALHLDYDKSSRTLSIFCFPSICASQLESRSGTIFAFASTERNGVDPRA

DEDDIAHLLEEILLSYRLLFGQCEKSRKFFRLVFDSANLPFPQPDTLLHV

LCGQRQLSHDAYHANLLPRDRRVYYAARDFPVLYERVELLAKELSGGRPR

SMSGLLRDRRDTLQFWTFWLVALFGGLSISLATIQTILQAIQIAQQAGKI

*

>Tr|4277_t

MATTDSFHNIKMTVIPPQEHDGSRGRTRDRTMPISNSKNLRPDESSTLRG

RSRRRSVSPFSLASRGSSPVKPSANRLMLHNRLREKRREHCPSRIASPAN

SEVLHNTQTRMRSRSRGPRRDKEPHRPVDHLSSLRNEVFLSDEEQPDHGK

SS*

>Tr|2854_t

MTPANAQPLVLIIGAGISGLLLAQQLKREGISYRIFERDADLQTRGAGWG

LTLHWSLPALQSLLPSELAQRICDESSVDRWADRRGEASRFPFFDLDSGE

LKAQTPGAVKSARIRVTRQRLRRLLAEGIDIEWGKTLSEIHTSDNGIVSA

RFEDGSSCDGTLLVACDGGQSRVRRVLFPDESKLMVQLPVRTMGVKILLT

ADQIEPMRKLDLYFLQGASPSNSSFTYFSVLDVPGNQVDSDPSIFSCQLH

VSWPYPGSGTSVDVPTTNKERYELMHAFAKTWSEPFRSLVLNNVHPDTEI

KRLDVLDWAPPLGLRSKGQVVLMGDAFHLMSMYRGEGANHAIVDVHDFAT

HVVPLLSPDRGFSSRADSDSQQVIDQNQGLDFRRALRQALDSYEDAVVSR

ARPGVLASRRACLDAHAFSRLFGENATGISPLLSKREMMLQFDDESTELM

DVA*

>Tr|7615_t

MSTPSTPIAIIGAGLSGLTLALALHAHSIPSTLYESRPCPLDIGGAIMLS

PNALKILDTLGVYQHISPLGFHFEKLSFRSHDDHPIDDFDFGSRDKHGYK

ALRIYRYELINVLLSMVRAAGISIEYNKKFSHVTAETPEDITWAFADGTS

ATARLLVGADGIHSLVRKHLYPDLTPQFTNMIGVTAAVPRAQLKTEPNYP

LPVTIMSPQHGAFVIAPQRADGSEVLIGKQKRLEQEEHDRAGWNELMENN

NKSWCIDFLRQGSQDFPPIVANAVSDIPLKGINLWPFYFVPKLDTWTRGR

VVILGDAAHAIPPTAGQGVNQAFEDVYTFAGVVAKQREQCQSSQGDLDAA

VRMDKALKKWQLGRQARVDKVLELNDQLNRRRLPNAGEAGHLEPFDLEWL

YGVNFEESIAEWAS*

>Tr|3367_t

MATPQFACANWKPDSTDCKKYGRYRCKNCLLVAYCGADCQKAHWVVHKAD

CRSPLGKETWTPDWVLEKRVPAFIGDSDNGQAVFGVKKFLWGNIPAIDVL

QLGSNEGDGYKKPLNLLFAASGDLRNVVKTIAQIPSSFEEPVTVAINDRD

IDIVARNAILLLIALVSENMEEAVDCIIHVWYSALIRKSDLEMLQQRIRP

LVESVCEKIKDKPRTSVLAKTFTFGERSLRLVLQKSSWDALLGYMSIPAG

LTAQRANQIRTAVTLAESRKDYRHRNWLVQSPTQRVARHRFRQDGLLLPF

GAPRDEFQEPNPTMYQTANTWPMHDSADPLNGWSPKDVEDTSSGPATADI

YGKLFYHLRALVHSFLLHLSKLRVSFRLLQVDFSEVPKHVESNYFSRIEV

SNVSDRGYVGIHRTVAMMAPLLQGPLINPHATLITLFMNAVPETMTMEEQ

RAVMSPHGQAMRRLLKFLPMERMPMSPYDPAFVKMSYASSVVAAYDHIFD

RYMKVMAFSEMEEALGVAMKAKHTVIEKWPYQMKLQPGQPGAQEEFNRLV

RGDLSGNERYVEWRRTKMYREEMDGEAGKDLLENMSSLDLRR*
