# Supplementary material for: Telomere length de novo assembly of all 7 chromosomes and mitogenome sequencing of the model entomopathogenic fungus, Metarhizium brunneum, by means of a novel assembly pipeline
Source: BMC Genomics. 2021 Jan 28;22:87. doi: 10.1186/s12864-021-07390-y (PMC7842015; doi:10.1186/s12864-021-07390-y)
Supplement: Supplementary file 8 — Additional file 8. M brunneum singletons. Metarhizium brunneum proteins that did not form orthologous clusters with any other proteins. [file 12864_2021_7390_MOESM8_ESM.pdf]

QLI63423.1

QLI63427.1

QLI63430.1

QLI63434.1

QLI63456.1

QLI63476.1

QLI63480.1

QLI63489.1

QLI63521.1

QLI63528.1

QLI63539.1

QLI63556.1

QLI63577.1

QLI63585.1

QLI63595.1

QLI63602.1

QLI63603.1

QLI63608.1

QLI63616.1

QLI63621.1

QLI63626.1

QLI63631.1

QLI63636.1

QLI63638.1

QLI63642.1

QLI63644.1

QLI63650.1

QLI63657.1

QLI63661.1

QLI63669.1

QLI63670.1

QLI63671.1

QLI63678.1

QLI63689.1

QLI63691.1

QLI63694.1

QLI63698.1

QLI63701.1

QLI63704.1

QLI63705.1

QLI63717.1

QLI63720.1

QLI63725.1

QLI63726.1

QLI63727.1

QLI63733.1

QLI63739.1

QLI63741.1

QLI63743.1

QLI63745.1

QLI63750.1

QLI63753.1

QLI63756.1

QLI63759.1

QLI63762.1

QLI63766.1

QLI63774.1

QLI63790.1

QLI63793.1

QLI63812.1

QLI63814.1

QLI63819.1

QLI63821.1

QLI63829.1

QLI63834.1

QLI63843.1

QLI63857.1

QLI63859.1

QLI63866.1

QLI63873.1

QLI63877.1

QLI63889.1

QLI63900.1

QLI63903.1

QLI63904.1

QLI63906.1

QLI63911.1

QLI63915.1

QLI63918.1

QLI63919.1

QLI63925.1

QLI63928.1

QLI63932.1

QLI63933.1

QLI63945.1

QLI63952.1

QLI63957.1

QLI63959.1

QLI63960.1

QLI63972.1

QLI63977.1

QLI63980.1

QLI63985.1

QLI63986.1

QLI63995.1

QLI63997.1

QLI64000.1

QLI64004.1

QLI64007.1

QLI64008.1

QLI64010.1

QLI64014.1

QLI64018.1

QLI64026.1

QLI64030.1

QLI64038.1

QLI64039.1

QLI64044.1

QLI64046.1

QLI64051.1

QLI64057.1

QLI64062.1

QLI64064.1

QLI64069.1

QLI64070.1

QLI64082.1

QLI64083.1

QLI64086.1

QLI64090.1

QLI64091.1

QLI64100.1

QLI64109.1

QLI64114.1

QLI64117.1

QLI64128.1

QLI64129.1

QLI64141.1

QLI64143.1

QLI64147.1

QLI64150.1

QLI64163.1

QLI64170.1

QLI64183.1

QLI64186.1

QLI64187.1

QLI64191.1

QLI64192.1

QLI64194.1

QLI64199.1

QLI64203.1

QLI64211.1

QLI64214.1

QLI64217.1

QLI64243.1

QLI64249.1

QLI64254.1

QLI64261.1

QLI64263.1

QLI64286.1

QLI64297.1

QLI64304.1

QLI64305.1

QLI64312.1

QLI64313.1

QLI64314.1

QLI64328.1

QLI64331.1

QLI64332.1

QLI64337.1

QLI64346.1

QLI64351.1

QLI64355.1

QLI64358.1

QLI64359.1

QLI64362.1

QLI64366.1

QLI64368.1

QLI64392.1

QLI64401.1

QLI64406.1

QLI64410.1

QLI64417.1

QLI64425.1

QLI64433.1

QLI64435.1

QLI64441.1

QLI64449.1

QLI64455.1

QLI64461.1

QLI64464.1

QLI64466.1

QLI64471.1

QLI64478.1

QLI64489.1

QLI64492.1

QLI64497.1

QLI64502.1

QLI64503.1

QLI64504.1

QLI64518.1

QLI64519.1

QLI64521.1

QLI64524.1

QLI64528.1

QLI64532.1

QLI64537.1

QLI64539.1

QLI64542.1

QLI64554.1

QLI64562.1

QLI64565.1

QLI64577.1

QLI64583.1

QLI64593.1

QLI64612.1

QLI64614.1

QLI64615.1

QLI64616.1

QLI64621.1

QLI64622.1

QLI64625.1

QLI64650.1

QLI64653.1

QLI64667.1

QLI64668.1

QLI64669.1

QLI64671.1

QLI64676.1

QLI64681.1

QLI64688.1

QLI64690.1

QLI64692.1

QLI64710.1

QLI64713.1

QLI64720.1

QLI64722.1

QLI64723.1

QLI64726.1

QLI64732.1

QLI64737.1

QLI64749.1

QLI64753.1

QLI64757.1

QLI64762.1

QLI64765.1

QLI64767.1

QLI64768.1

QLI64771.1

QLI64778.1

QLI64779.1

QLI64780.1

QLI64789.1

QLI64793.1

QLI64796.1

QLI64800.1

QLI64802.1

QLI64805.1

QLI64813.1

QLI64817.1

QLI64821.1

QLI64833.1

QLI64834.1

QLI64835.1

QLI64837.1

QLI64839.1

QLI64854.1

QLI64856.1

QLI64868.1

QLI64871.1

QLI64877.1

QLI64879.1

QLI64881.1

QLI64886.1

QLI64890.1

QLI64894.1

QLI64895.1

QLI64900.1

QLI64904.1

QLI64907.1

QLI64912.1

QLI64917.1

QLI64918.1

QLI64920.1

QLI64921.1

QLI64924.1

QLI64925.1

QLI64928.1

QLI64933.1

QLI64938.1

QLI64944.1

QLI64947.1

QLI64958.1

QLI64967.1

QLI64971.1

QLI64972.1

QLI64975.1

QLI64976.1

QLI64979.1

QLI64989.1

QLI64993.1

QLI65005.1

QLI65028.1

QLI65030.1

QLI65031.1

QLI65040.1

QLI65052.1

QLI65061.1

QLI65077.1

QLI65081.1

QLI65083.1

QLI65087.1

QLI65092.1

QLI65103.1

QLI65111.1

QLI65112.1

QLI65118.1

QLI65126.1

QLI65128.1

QLI65130.1

QLI65132.1

QLI65153.1

QLI65155.1

QLI65162.1

QLI65167.1

QLI65172.1

QLI65176.1

QLI65179.1

QLI65187.1

QLI65191.1

QLI65196.1

QLI65198.1

QLI65199.1

QLI65201.1

QLI65207.1

QLI65230.1

QLI65235.1

QLI65255.1

QLI65257.1

QLI65258.1

QLI65261.1

QLI65263.1

QLI65276.1

QLI65279.1

QLI65292.1

QLI65295.1

QLI65296.1

QLI65297.1

QLI65298.1

QLI65299.1

QLI65306.1

QLI65315.1

QLI65324.1

QLI65325.1

QLI65330.1

QLI65344.1

QLI65345.1

QLI65347.1

QLI65359.1

QLI65361.1

QLI65370.1

QLI65371.1

QLI65393.1

QLI65397.1

QLI65403.1

QLI65404.1

QLI65406.1

QLI65413.1

QLI65415.1

QLI65416.1

QLI65426.1

QLI65431.1

QLI65442.1

QLI65446.1

QLI65447.1

QLI65448.1

QLI65451.1

QLI65452.1

QLI65462.1

QLI65466.1

QLI65467.1

QLI65468.1

QLI65469.1

QLI65470.1

QLI65480.1

QLI65485.1

QLI65486.1

QLI65489.1

QLI65514.1

QLI65518.1

QLI65519.1

QLI65520.1

QLI65521.1

QLI65524.1

QLI65525.1

QLI65528.1

QLI65529.1

QLI65530.1

QLI65537.1

QLI65545.1

QLI65553.1

QLI65555.1

QLI65556.1

QLI65557.1

QLI65572.1

QLI65573.1

QLI65574.1

QLI65578.1

QLI65580.1

QLI65586.1

QLI65590.1

QLI65592.1

QLI65598.1

QLI65603.1

QLI65605.1

QLI65608.1

QLI65619.1

QLI65625.1

QLI65627.1

QLI65629.1

QLI65630.1

QLI65632.1

QLI65639.1

QLI65650.1

QLI65651.1

QLI65653.1

QLI65658.1

QLI65672.1

QLI65690.1

QLI65706.1

QLI65707.1

QLI65710.1

QLI65721.1

QLI65727.1

QLI65733.1

QLI65739.1

QLI65742.1

QLI65751.1

QLI65756.1

QLI65762.1

QLI65773.1

QLI65782.1

QLI65783.1

QLI65786.1

QLI65791.1

QLI65799.1

QLI65816.1

QLI65818.1

QLI65824.1

QLI65830.1

QLI65839.1

QLI65844.1

QLI65850.1

QLI65855.1

QLI65868.1

QLI65872.1

QLI65881.1

QLI65891.1

QLI65894.1

QLI65900.1

QLI65902.1

QLI65904.1

QLI65907.1

QLI65911.1

QLI65920.1

QLI65930.1

QLI65933.1

QLI65953.1

QLI65954.1

QLI65955.1

QLI65959.1

QLI65963.1

QLI65969.1

QLI65971.1

QLI65972.1

QLI65973.1

QLI65985.1

QLI65994.1

QLI65995.1

QLI66003.1

QLI66019.1

QLI66034.1

QLI66044.1

QLI66045.1

QLI66053.1

QLI66055.1

QLI66057.1

QLI66063.1

QLI66065.1

QLI66066.1

QLI66069.1

QLI66070.1

QLI66091.1

QLI66092.1

QLI66093.1

QLI66100.1

QLI66105.1

QLI66113.1

QLI66116.1

QLI66117.1

QLI66126.1

QLI66139.1

QLI66149.1

QLI66151.1

QLI66155.1

QLI66159.1

QLI66162.1

QLI66164.1

QLI66172.1

QLI66176.1

QLI66178.1

QLI66189.1

QLI66197.1

QLI66208.1

QLI66209.1

QLI66211.1

QLI66212.1

QLI66223.1

QLI66234.1

QLI66242.1

QLI66249.1

QLI66253.1

QLI66260.1

QLI66265.1

QLI66273.1

QLI66276.1

QLI66277.1

QLI66290.1

QLI66293.1

QLI66301.1

QLI66308.1

QLI66312.1

QLI66313.1

QLI66318.1

QLI66319.1

QLI66337.1

QLI66339.1

QLI66349.1

QLI66351.1

QLI66358.1

QLI66361.1

QLI66363.1

QLI66366.1

QLI66368.1

QLI66369.1

QLI66371.1

QLI66373.1

QLI66379.1

QLI66383.1

QLI66389.1

QLI66402.1

QLI66406.1

QLI66408.1

QLI66409.1

QLI66412.1

QLI66426.1

QLI66429.1

QLI66432.1

QLI66435.1

QLI66440.1

QLI66446.1

QLI66451.1

QLI66452.1

QLI66459.1

QLI66463.1

QLI66473.1

QLI66484.1

QLI66488.1

QLI66489.1

QLI66492.1

QLI66494.1

QLI66507.1

QLI66516.1

QLI66518.1

QLI66524.1

QLI66527.1

QLI66533.1

QLI66535.1

QLI66537.1

QLI66538.1

QLI66539.1

QLI66546.1

QLI66548.1

QLI66551.1

QLI66559.1

QLI66560.1

QLI66562.1

QLI66565.1

QLI66566.1

QLI66571.1

QLI66572.1

QLI66578.1

QLI66582.1

QLI66587.1

QLI66588.1

QLI66589.1

QLI66608.1

QLI66610.1

QLI66612.1

QLI66633.1

QLI66634.1

QLI66639.1

QLI66640.1

QLI66648.1

QLI66649.1

QLI66661.1

QLI66669.1

QLI66677.1

QLI66678.1

QLI66680.1

QLI66687.1

QLI66688.1

QLI66689.1

QLI66697.1

QLI66700.1

QLI66703.1

QLI66706.1

QLI66708.1

QLI66713.1

QLI66716.1

QLI66717.1

QLI66727.1

QLI66728.1

QLI66732.1

QLI66733.1

QLI66736.1

QLI66740.1

QLI66742.1

QLI66767.1

QLI66769.1

QLI66770.1

QLI66776.1

QLI66782.1

QLI66785.1

QLI66792.1

QLI66802.1

QLI66803.1

QLI66804.1

QLI66818.1

QLI66824.1

QLI66825.1

QLI66841.1

QLI66854.1

QLI66874.1

QLI66878.1

QLI66890.1

QLI66891.1

QLI66892.1

QLI66897.1

QLI66906.1

QLI66912.1

QLI66918.1

QLI66924.1

QLI66926.1

QLI66929.1

QLI66930.1

QLI66931.1

QLI66936.1

QLI66937.1

QLI66949.1

QLI66954.1

QLI66964.1

QLI66969.1

QLI66972.1

QLI66978.1

QLI66979.1

QLI66987.1

QLI66989.1

QLI66990.1

QLI66996.1

QLI66997.1

QLI67002.1

QLI67008.1

QLI67009.1

QLI67012.1

QLI67014.1

QLI67019.1

QLI67020.1

QLI67022.1

QLI67024.1

QLI67027.1

QLI67030.1

QLI67031.1

QLI67032.1

QLI67034.1

QLI67035.1

QLI67042.1

QLI67043.1

QLI67050.1

QLI67051.1

QLI67052.1

QLI67056.1

QLI67059.1

QLI67061.1

QLI67064.1

QLI67075.1

QLI67077.1

QLI67079.1

QLI67080.1

QLI67085.1

QLI67091.1

QLI67097.1

QLI67098.1

QLI67111.1

QLI67112.1

QLI67119.1

QLI67134.1

QLI67144.1

QLI67145.1

QLI67146.1

QLI67150.1

QLI67155.1

QLI67166.1

QLI67167.1

QLI67184.1

QLI67197.1

QLI67199.1

QLI67211.1

QLI67215.1

QLI67225.1

QLI67226.1

QLI67234.1

QLI67235.1

QLI67239.1

QLI67240.1

QLI67243.1

QLI67244.1

QLI67252.1

QLI67259.1

QLI67263.1

QLI67267.1

QLI67272.1

QLI67274.1

QLI67278.1

QLI67279.1

QLI67289.1

QLI67291.1

QLI67298.1

QLI67302.1

QLI67303.1

QLI67313.1

QLI67317.1

QLI67319.1

QLI67320.1

QLI67322.1

QLI67335.1

QLI67346.1

QLI67350.1

QLI67352.1

QLI67356.1

QLI67357.1

QLI67359.1

QLI67362.1

QLI67363.1

QLI67364.1

QLI67373.1

QLI67380.1

QLI67394.1

QLI67399.1

QLI67405.1

QLI67409.1

QLI67410.1

QLI67415.1

QLI67421.1

QLI67429.1

QLI67437.1

QLI67443.1

QLI67444.1

QLI67445.1

QLI67454.1

QLI67461.1

QLI67463.1

QLI67466.1

QLI67474.1

QLI67476.1

QLI67482.1

QLI67485.1

QLI67487.1

QLI67488.1

QLI67500.1

QLI67504.1

QLI67518.1

QLI67527.1

QLI67529.1

QLI67530.1

QLI67534.1

QLI67548.1

QLI67552.1

QLI67555.1

QLI67563.1

QLI67567.1

QLI67569.1

QLI67570.1

QLI67581.1

QLI67587.1

QLI67604.1

QLI67605.1

QLI67609.1

QLI67611.1

QLI67617.1

QLI67627.1

QLI67636.1

QLI67644.1

QLI67649.1

QLI67650.1

QLI67657.1

QLI67663.1

QLI67666.1

QLI67680.1

QLI67682.1

QLI67685.1

QLI67689.1

QLI67692.1

QLI67696.1

QLI67703.1

QLI67706.1

QLI67708.1

QLI67709.1

QLI67710.1

QLI67711.1

QLI67712.1

QLI67713.1

QLI67724.1

QLI67733.1

QLI67739.1

QLI67744.1

QLI67748.1

QLI67751.1

QLI67762.1

QLI67764.1

QLI67768.1

QLI67771.1

QLI67773.1

QLI67774.1

QLI67785.1

QLI67790.1

QLI67797.1

QLI67809.1

QLI67810.1

QLI67831.1

QLI67834.1

QLI67837.1

QLI67846.1

QLI67848.1

QLI67855.1

QLI67856.1

QLI67858.1

QLI67860.1

QLI67861.1

QLI67865.1

QLI67869.1

QLI67876.1

QLI67880.1

QLI67888.1

QLI67889.1

QLI67913.1

QLI67921.1

QLI67936.1

QLI67954.1

QLI67960.1

QLI67962.1

QLI67963.1

QLI67966.1

QLI67968.1

QLI67972.1

QLI67973.1

QLI67987.1

QLI67989.1

QLI67992.1

QLI68001.1

QLI68006.1

QLI68013.1

QLI68014.1

QLI68015.1

QLI68023.1

QLI68025.1

QLI68026.1

QLI68030.1

QLI68032.1

QLI68034.1

QLI68042.1

QLI68060.1

QLI68063.1

QLI68069.1

QLI68071.1

QLI68074.1

QLI68081.1

QLI68086.1

QLI68092.1

QLI68097.1

QLI68099.1

QLI68100.1

QLI68106.1

QLI68108.1

QLI68109.1

QLI68111.1

QLI68123.1

QLI68131.1

QLI68140.1

QLI68150.1

QLI68151.1

QLI68163.1

QLI68168.1

QLI68175.1

QLI68178.1

QLI68181.1

QLI68182.1

QLI68199.1

QLI68205.1

QLI68206.1

QLI68207.1

QLI68212.1

QLI68214.1

QLI68215.1

QLI68217.1

QLI68222.1

QLI68233.1

QLI68240.1

QLI68241.1

QLI68248.1

QLI68249.1

QLI68250.1

QLI68251.1

QLI68267.1

QLI68283.1

QLI68286.1

QLI68289.1

QLI68299.1

QLI68302.1

QLI68313.1

QLI68318.1

QLI68319.1

QLI68325.1

QLI68327.1

QLI68328.1

QLI68337.1

QLI68345.1

QLI68352.1

QLI68354.1

QLI68368.1

QLI68377.1

QLI68378.1

QLI68381.1

QLI68390.1

QLI68397.1

QLI68398.1

QLI68400.1

QLI68405.1

QLI68416.1

QLI68418.1

QLI68432.1

QLI68436.1

QLI68437.1

QLI68444.1

QLI68449.1

QLI68450.1

QLI68451.1

QLI68458.1

QLI68462.1

QLI68468.1

QLI68469.1

QLI68471.1

QLI68475.1

QLI68476.1

QLI68478.1

QLI68479.1

QLI68481.1

QLI68484.1

QLI68491.1

QLI68495.1

QLI68502.1

QLI68505.1

QLI68511.1

QLI68516.1

QLI68517.1

QLI68532.1

QLI68534.1

QLI68545.1

QLI68564.1

QLI68566.1

QLI68568.1

QLI68576.1

QLI68579.1

QLI68596.1

QLI68602.1

QLI68603.1

QLI68605.1

QLI68610.1

QLI68623.1

QLI68625.1

QLI68630.1

QLI68635.1

QLI68636.1

QLI68645.1

QLI68646.1

QLI68648.1

QLI68658.1

QLI68659.1

QLI68663.1

QLI68666.1

QLI68668.1

QLI68682.1

QLI68685.1

QLI68687.1

QLI68690.1

QLI68696.1

QLI68702.1

QLI68706.1

QLI68707.1

QLI68712.1

QLI68716.1

QLI68717.1

QLI68720.1

QLI68723.1

QLI68730.1

QLI68732.1

QLI68733.1

QLI68737.1

QLI68744.1

QLI68746.1

QLI68747.1

QLI68749.1

QLI68754.1

QLI68755.1

QLI68757.1

QLI68759.1

QLI68768.1

QLI68779.1

QLI68780.1

QLI68782.1

QLI68785.1

QLI68788.1

QLI68790.1

QLI68801.1

QLI68804.1

QLI68809.1

QLI68810.1

QLI68812.1

QLI68813.1

QLI68814.1

QLI68819.1

QLI68820.1

QLI68821.1

QLI68824.1

QLI68833.1

QLI68836.1

QLI68839.1

QLI68840.1

QLI68845.1

QLI68849.1

QLI68850.1

QLI68860.1

QLI68862.1

QLI68864.1

QLI68865.1

QLI68872.1

QLI68873.1

QLI68876.1

QLI68883.1

QLI68885.1

QLI68890.1

QLI68891.1

QLI68894.1

QLI68895.1

QLI68897.1

QLI68900.1

QLI68904.1

QLI68914.1

QLI68917.1

QLI68918.1

QLI68920.1

QLI68924.1

QLI68935.1

QLI68936.1

QLI68938.1

QLI68939.1

QLI68941.1

QLI68943.1

QLI68947.1

QLI68951.1

QLI68954.1

QLI68957.1

QLI68958.1

QLI68968.1

QLI68975.1

QLI68977.1

QLI68978.1

QLI68987.1

QLI68989.1

QLI68992.1

QLI68994.1

QLI69000.1

QLI69006.1

QLI69010.1

QLI69011.1

QLI69015.1

QLI69020.1

QLI69022.1

QLI69028.1

QLI69041.1

QLI69042.1

QLI69044.1

QLI69051.1

QLI69057.1

QLI69058.1

QLI69059.1

QLI69061.1

QLI69063.1

QLI69064.1

QLI69069.1

QLI69086.1

QLI69090.1

QLI69096.1

QLI69097.1

QLI69100.1

QLI69102.1

QLI69103.1

QLI69106.1

QLI69108.1

QLI69114.1

QLI69119.1

QLI69121.1

QLI69126.1

QLI69127.1

QLI69131.1

QLI69134.1

QLI69140.1

QLI69141.1

QLI69142.1

QLI69143.1

QLI69152.1

QLI69153.1

QLI69158.1

QLI69166.1

QLI69168.1

QLI69171.1

QLI69173.1

QLI69178.1

QLI69179.1

QLI69180.1

QLI69182.1

QLI69183.1

QLI69184.1

QLI69188.1

QLI69189.1

QLI69193.1

QLI69194.1

QLI69199.1

QLI69204.1

QLI69206.1

QLI69208.1

QLI69212.1

QLI69216.1

QLI69217.1

QLI69223.1

QLI69228.1

QLI69234.1

QLI69244.1

QLI69245.1

QLI69246.1

QLI69248.1

QLI69259.1

QLI69260.1

QLI69263.1

QLI69264.1

QLI69266.1

QLI69268.1

QLI69272.1

QLI69277.1

QLI69281.1

QLI69284.1

QLI69287.1

QLI69292.1

QLI69293.1

QLI69296.1

QLI69299.1

QLI69303.1

QLI69311.1

QLI69314.1

QLI69315.1

QLI69318.1

QLI69321.1

QLI69322.1

QLI69327.1

QLI69332.1

QLI69333.1

QLI69337.1

QLI69347.1

QLI69350.1

QLI69352.1

QLI69353.1

QLI69360.1

QLI69369.1

QLI69371.1

QLI69372.1

QLI69376.1

QLI69378.1

QLI69379.1

QLI69386.1

QLI69390.1

QLI69392.1

QLI69396.1

QLI69397.1

QLI69402.1

QLI69407.1

QLI69408.1

QLI69411.1

QLI69413.1

QLI69415.1

QLI69419.1

QLI69420.1

QLI69424.1

QLI69429.1

QLI69435.1

QLI69439.1

QLI69441.1

QLI69444.1

QLI69450.1

QLI69452.1

QLI69454.1

QLI69455.1

QLI69460.1

QLI69461.1

QLI69463.1

QLI69464.1

QLI69467.1

QLI69468.1

QLI69473.1

QLI69474.1

QLI69476.1

QLI69477.1

QLI69483.1

QLI69484.1

QLI69494.1

QLI69501.1

QLI69506.1

QLI69509.1

QLI69510.1

QLI69517.1

QLI69525.1

QLI69534.1

QLI69546.1

QLI69547.1

QLI69549.1

QLI69559.1

QLI69562.1

QLI69564.1

QLI69567.1

QLI69573.1

QLI69579.1

QLI69582.1

QLI69586.1

QLI69590.1

QLI69591.1

QLI69602.1

QLI69603.1

QLI69618.1

QLI69620.1

QLI69621.1

QLI69623.1

QLI69635.1

QLI69643.1

QLI69644.1

QLI69646.1

QLI69649.1

QLI69650.1

QLI69656.1

QLI69663.1

QLI69666.1

QLI69668.1

QLI69669.1

QLI69672.1

QLI69673.1

QLI69680.1

QLI69689.1

QLI69690.1

QLI69691.1

QLI69694.1

QLI69706.1

QLI69709.1

QLI69710.1

QLI69712.1

QLI69718.1

QLI69724.1

QLI69739.1

QLI69740.1

QLI69742.1

QLI69746.1

QLI69750.1

QLI69754.1

QLI69757.1

QLI69761.1

QLI69766.1

QLI69768.1

QLI69771.1

QLI69776.1

QLI69780.1

QLI69784.1

QLI69789.1

QLI69790.1

QLI69794.1

QLI69796.1

QLI69799.1

QLI69802.1

QLI69803.1

QLI69805.1

QLI69814.1

QLI69815.1

QLI69817.1

QLI69821.1

QLI69826.1

QLI69830.1

QLI69835.1

QLI69837.1

QLI69838.1

QLI69840.1

QLI69843.1

QLI69845.1

QLI69852.1

QLI69854.1

QLI69856.1

QLI69857.1

QLI69858.1

QLI69865.1

QLI69867.1

QLI69875.1

QLI69884.1

QLI69886.1

QLI69888.1

QLI69899.1

QLI69901.1

QLI69904.1

QLI69905.1

QLI69912.1

QLI69914.1

QLI69915.1

QLI69916.1

QLI69917.1

QLI69921.1

QLI69923.1

QLI69924.1

QLI69925.1

QLI69926.1

QLI69935.1

QLI69936.1

QLI69937.1

QLI69939.1

QLI69944.1

QLI69949.1

QLI69950.1

QLI69951.1

QLI69954.1

QLI69956.1

QLI69957.1

QLI69959.1

QLI69962.1

QLI69963.1

QLI69965.1

QLI69968.1

QLI69978.1

QLI69983.1

QLI69985.1

QLI69988.1

QLI69990.1

QLI69996.1

QLI70001.1

QLI70004.1

QLI70005.1

QLI70011.1

QLI70012.1

QLI70013.1

QLI70014.1

QLI70017.1

QLI70020.1

QLI70031.1

QLI70034.1

QLI70039.1

QLI70040.1

QLI70042.1

QLI70043.1

QLI70053.1

QLI70057.1

QLI70059.1

QLI70061.1

QLI70062.1

QLI70063.1

QLI70067.1

QLI70071.1

QLI70078.1

QLI70080.1

QLI70085.1

QLI70086.1

QLI70091.1

QLI70092.1

QLI70097.1

QLI70099.1

QLI70108.1

QLI70113.1

QLI70114.1

QLI70123.1

QLI70124.1

QLI70130.1

QLI70133.1

QLI70134.1

QLI70151.1

QLI70155.1

QLI70163.1

QLI70165.1

QLI70169.1

QLI70170.1

QLI70177.1

QLI70184.1

QLI70186.1

QLI70191.1

QLI70199.1

QLI70203.1

QLI70207.1

QLI70213.1

QLI70214.1

QLI70219.1

QLI70225.1

QLI70228.1

QLI70236.1

QLI70242.1

QLI70250.1

QLI70252.1

QLI70257.1

QLI70258.1

QLI70269.1

QLI70287.1

QLI70290.1

QLI70293.1

QLI70299.1

QLI70300.1

QLI70307.1

QLI70311.1

QLI70316.1

QLI70317.1

QLI70325.1

QLI70326.1

QLI70332.1

QLI70335.1

QLI70345.1

QLI70348.1

QLI70360.1

QLI70363.1

QLI70365.1

QLI70370.1

QLI70373.1

QLI70374.1

QLI70376.1

QLI70386.1

QLI70388.1

QLI70391.1

QLI70396.1

QLI70397.1

QLI70399.1

QLI70406.1

QLI70407.1

QLI70408.1

QLI70415.1

QLI70417.1

QLI70418.1

QLI70421.1

QLI70423.1

QLI70432.1

QLI70436.1

QLI70440.1

QLI70441.1

QLI70444.1

QLI70445.1

QLI70447.1

QLI70450.1

QLI70455.1

QLI70456.1

QLI70457.1

QLI70460.1

QLI70462.1

QLI70464.1

QLI70471.1

QLI70476.1

QLI70480.1

QLI70484.1

QLI70485.1

QLI70489.1

QLI70492.1

QLI70503.1

QLI70504.1

QLI70517.1

QLI70520.1

QLI70522.1

QLI70531.1

QLI70539.1

QLI70542.1

QLI70544.1

QLI70551.1

QLI70554.1

QLI70555.1

QLI70560.1

QLI70575.1

QLI70584.1

QLI70592.1

QLI70595.1

QLI70596.1

QLI70599.1

QLI70603.1

QLI70604.1

QLI70608.1

QLI70609.1

QLI70614.1

QLI70615.1

QLI70622.1

QLI70627.1

QLI70637.1

QLI70641.1

QLI70642.1

QLI70649.1

QLI70653.1

QLI70656.1

QLI70659.1

QLI70662.1

QLI70668.1

QLI70679.1

QLI70680.1

QLI70683.1

QLI70689.1

QLI70690.1

QLI70691.1

QLI70707.1

QLI70718.1

QLI70720.1

QLI70727.1

QLI70730.1

QLI70735.1

QLI70740.1

QLI70745.1

QLI70746.1

QLI70751.1

QLI70759.1

QLI70762.1

QLI70766.1

QLI70770.1

QLI70776.1

QLI70783.1

QLI70784.1

QLI70786.1

QLI70787.1

QLI70789.1

QLI70799.1

QLI70803.1

QLI70804.1

QLI70805.1

QLI70807.1

QLI70809.1

QLI70813.1

QLI70817.1

QLI70824.1

QLI70825.1

QLI70832.1

QLI70833.1

QLI70843.1

QLI70851.1

QLI70852.1

QLI70857.1

QLI70858.1

QLI70863.1

QLI70876.1

QLI70889.1

QLI70890.1

QLI70894.1

QLI70901.1

QLI70902.1

QLI70904.1

QLI70909.1

QLI70912.1

QLI70913.1

QLI70917.1

QLI70918.1

QLI70925.1

QLI70937.1

QLI70939.1

QLI70944.1

QLI70949.1

QLI70954.1

QLI70959.1

QLI70967.1

QLI70973.1

QLI70976.1

QLI70989.1

QLI70993.1

QLI71010.1

QLI71011.1

QLI71012.1

QLI71014.1

QLI71018.1

QLI71019.1

QLI71023.1

QLI71030.1

QLI71035.1

QLI71036.1

QLI71040.1

QLI71047.1

QLI71055.1

QLI71064.1

QLI71065.1

QLI71066.1

QLI71068.1

QLI71077.1

QLI71080.1

QLI71082.1

QLI71091.1

QLI71096.1

QLI71098.1

QLI71101.1

QLI71111.1

QLI71112.1

QLI71113.1

QLI71114.1

QLI71119.1

QLI71123.1

QLI71126.1

QLI71130.1

QLI71134.1

QLI71137.1

QLI71141.1

QLI71142.1

QLI71148.1

QLI71151.1

QLI71155.1

QLI71157.1

QLI71164.1

QLI71168.1

QLI71171.1

QLI71177.1

QLI71178.1

QLI71184.1

QLI71192.1

QLI71193.1

QLI71195.1

QLI71196.1

QLI71198.1

QLI71203.1

QLI71209.1

QLI71210.1

QLI71214.1

QLI71217.1

QLI71219.1

QLI71220.1

QLI71241.1

QLI71244.1

QLI71246.1

QLI71247.1

QLI71248.1

QLI71249.1

QLI71253.1

QLI71257.1

QLI71261.1

QLI71266.1

QLI71270.1

QLI71281.1

QLI71285.1

QLI71288.1

QLI71291.1

QLI71294.1

QLI71303.1

QLI71304.1

QLI71307.1

QLI71309.1

QLI71312.1

QLI71313.1

QLI71315.1

QLI71318.1

QLI71341.1

QLI71346.1

QLI71354.1

QLI71358.1

QLI71366.1

QLI71368.1

QLI71374.1

QLI71377.1

QLI71378.1

QLI71391.1

QLI71398.1

QLI71399.1

QLI71402.1

QLI71405.1

QLI71406.1

QLI71411.1

QLI71422.1

QLI71426.1

QLI71431.1

QLI71432.1

QLI71435.1

QLI71440.1

QLI71441.1

QLI71442.1

QLI71444.1

QLI71445.1

QLI71446.1

QLI71452.1

QLI71460.1

QLI71464.1

QLI71472.1

QLI71479.1

QLI71480.1

QLI71482.1

QLI71491.1

QLI71492.1

QLI71498.1

QLI71501.1

QLI71503.1

QLI71509.1

QLI71512.1

QLI71513.1

QLI71519.1

QLI71528.1

QLI71531.1

QLI71536.1

QLI71548.1

QLI71550.1

QLI71551.1

QLI71552.1

QLI71553.1

QLI71557.1

QLI71574.1

QLI71579.1

QLI71585.1

QLI71589.1

QLI71601.1

QLI71605.1

QLI71610.1

QLI71611.1

QLI71613.1

QLI71616.1

QLI71619.1

QLI71633.1

QLI71639.1

QLI71645.1

QLI71647.1

QLI71652.1

QLI71653.1

QLI71655.1

QLI71680.1

QLI71682.1

QLI71683.1

QLI71686.1

QLI71705.1

QLI71716.1

QLI71719.1

QLI71720.1

QLI71729.1

QLI71732.1

QLI71733.1

QLI71740.1

QLI71741.1

QLI71745.1

QLI71753.1

QLI71755.1

QLI71760.1

QLI71763.1

QLI71768.1

QLI71779.1

QLI71780.1

QLI71784.1

QLI71785.1

QLI71786.1

QLI71788.1

QLI71793.1

QLI71804.1

QLI71809.1

QLI71812.1

QLI71818.1

QLI71820.1

QLI71821.1

QLI71824.1

QLI71829.1

QLI71830.1

QLI71833.1

QLI71842.1

QLI71843.1

QLI71844.1

QLI71850.1

QLI71858.1

QLI71859.1

QLI71863.1

QLI71879.1

QLI71881.1

QLI71886.1

QLI71890.1

QLI71898.1

QLI71903.1

QLI71905.1

QLI71909.1

QLI71917.1

QLI71923.1

QLI71930.1

QLI71940.1

QLI71943.1

QLI71945.1

QLI71954.1

QLI71957.1

QLI71959.1

QLI71963.1

QLI71964.1

QLI71979.1

QLI71983.1

QLI71991.1

QLI71993.1

QLI71996.1

QLI71998.1

QLI72000.1

QLI72001.1

QLI72002.1

QLI72006.1

QLI72009.1

QLI72012.1

QLI72019.1

QLI72023.1

QLI72027.1

QLI72033.1

QLI72035.1

QLI72038.1

QLI72047.1

QLI72055.1

QLI72056.1

QLI72057.1

QLI72058.1

QLI72074.1

QLI72077.1

QLI72079.1

QLI72095.1

QLI72100.1

QLI72101.1

QLI72104.1

QLI72105.1

QLI72107.1

QLI72111.1

QLI72119.1

QLI72122.1

QLI72126.1

QLI72127.1

QLI72128.1

QLI72132.1

QLI72134.1

QLI72135.1

QLI72163.1

QLI72167.1

QLI72169.1

QLI72172.1

QLI72173.1

QLI72180.1

QLI72185.1

QLI72187.1

QLI72191.1

QLI72197.1

QLI72204.1

QLI72205.1

QLI72206.1

QLI72209.1

QLI72220.1

QLI72225.1

QLI72229.1

QLI72240.1

QLI72241.1

QLI72243.1

QLI72257.1

QLI72264.1

QLI72265.1

QLI72267.1

QLI72272.1

QLI72273.1

QLI72274.1

QLI72281.1

QLI72283.1

QLI72286.1

QLI72288.1

QLI72300.1

QLI72311.1

QLI72315.1

QLI72323.1

QLI72336.1

QLI72339.1

QLI72344.1

QLI72351.1

QLI72354.1

QLI72358.1

QLI72360.1

QLI72367.1

QLI72368.1

QLI72369.1

QLI72371.1

QLI72379.1

QLI72382.1

QLI72385.1

QLI72397.1

QLI72398.1

QLI72399.1

QLI72403.1

QLI72412.1

QLI72413.1

QLI72417.1

QLI72419.1

QLI72422.1

QLI72438.1

QLI72441.1

QLI72446.1

QLI72455.1

QLI72456.1

QLI72457.1

QLI72468.1

QLI72469.1

QLI72471.1

QLI72473.1

QLI72479.1

QLI72480.1

QLI72482.1

QLI72484.1

QLI72491.1

QLI72492.1

QLI72494.1

QLI72501.1

QLI72504.1

QLI72508.1

QLI72509.1

QLI72517.1

QLI72521.1

QLI72526.1

QLI72532.1

QLI72552.1

QLI72558.1

QLI72560.1

QLI72564.1

QLI72570.1

QLI72574.1

QLI72577.1

QLI72578.1

QLI72584.1

QLI72586.1

QLI72594.1

QLI72606.1

QLI72607.1

QLI72609.1

QLI72611.1

QLI72615.1

QLI72618.1

QLI72621.1

QLI72624.1

QLI72635.1

QLI72644.1

QLI72648.1

QLI72654.1

QLI72656.1

QLI72660.1

QLI72662.1

QLI72680.1

QLI72687.1

QLI72689.1

QLI72697.1

QLI72712.1

QLI72720.1

QLI72725.1

QLI72730.1

QLI72739.1

QLI72741.1

QLI72742.1

QLI72750.1

QLI72755.1

QLI72763.1

QLI72771.1

QLI72772.1

QLI72777.1

QLI72778.1

QLI72780.1

QLI72782.1

QLI72785.1

QLI72790.1

QLI72795.1

QLI72799.1

QLI72803.1

QLI72804.1

QLI72808.1

QLI72809.1

QLI72817.1

QLI72818.1

QLI72819.1

QLI72822.1

QLI72826.1

QLI72833.1

QLI72838.1

QLI72842.1

QLI72846.1

QLI72849.1

QLI72850.1

QLI72859.1

QLI72862.1

QLI72868.1

QLI72870.1

QLI72881.1

QLI72882.1

QLI72883.1

QLI72886.1

QLI72888.1

QLI72891.1

QLI72893.1

QLI72898.1

QLI72909.1

QLI72910.1

QLI72914.1

QLI72918.1

QLI72923.1

QLI72935.1

QLI72936.1

QLI72938.1

QLI72941.1

QLI72946.1

QLI72954.1

QLI72957.1

QLI72967.1

QLI72969.1

QLI72972.1

QLI72974.1

QLI72984.1

QLI72987.1

QLI73001.1

QLI73003.1

QLI73011.1

QLI73012.1

QLI73014.1

QLI73018.1

QLI73030.1

QLI73032.1

QLI73035.1

QLI73041.1

QLI73069.1

QLI73080.1

QLI73086.1

QLI73094.1

QLI73098.1

QLI73100.1

QLI73109.1

QLI73115.1

QLI73123.1

QLI73143.1

QLI73145.1

QLI73149.1

QLI73161.1

QLI73163.1

QLI73171.1

QLI73175.1

QLI73178.1

QLI73181.1

QLI73188.1

QLI73190.1

QLI73197.1

QLI73203.1

QLI73207.1

QLI73211.1

QLI73217.1

QLI73218.1

QLI73222.1

QLI73225.1

QLI73227.1

QLI73228.1

QLI73236.1

QLI73244.1

QLI73255.1

QLI73263.1

QLI73264.1

QLI73266.1

QLI73268.1

QLI73271.1

QLI73273.1

QLI73275.1

QLI73278.1

QLI73288.1

QLI73302.1

QLI73314.1

QLI73315.1

QLI73322.1

QLI73323.1

QLI73328.1

QLI73329.1

QLI73335.1

QLI73340.1

QLI73341.1

QLI73346.1

QLI73348.1

QLI73351.1

QLI73364.1

QLI73366.1

QLI73379.1

QLI73388.1

QLI73390.1

QLI73394.1

QLI73398.1

QLI73406.1

QLI73413.1

QLI73421.1

QLI73424.1

QLI73425.1

QLI73430.1

QLI73434.1

QLI73448.1

QLI73451.1

QLI73455.1

QLI73458.1

QLI73475.1

QLI73496.1

QLI73502.1

QLI73505.1

QLI73507.1

QLI73509.1

QLI73513.1

QLI73524.1

QLI73525.1

QLI73532.1

QLI73540.1

QLI73552.1

QLI73554.1

QLI73560.1

QLI73576.1

QLI73594.1

QLI73598.1

QLI73601.1

QLI73607.1

QLI73609.1

QLI73625.1

QLI73632.1

QLI73641.1

QLI73646.1

QLI73647.1

QLI73652.1

QLI73654.1

QLI73659.1

QLI73661.1

QLI73666.1

QLI73668.1

QLI73669.1

QLI73676.1

QLI73681.1

QLI73682.1

QLI73684.1

QLI73687.1

QLI73693.1

QLI73703.1

QLI73710.1

QLI73718.1

QLI73724.1

QLI73726.1

QLI73732.1

QLI73734.1

QLI73737.1

QLI73739.1

QLI73744.1

QLI73746.1

QLI73752.1

QLI73758.1

QLI73760.1

QLI73762.1

QLI73766.1

QLI73769.1

QLI73778.1

QLI73781.1

QLI73782.1

QLI73787.1

QLI73790.1

QLI73793.1

QLI73801.1

QLI73803.1

QLI73804.1

QLI73811.1

QLI73815.1

QLI73816.1

QLI73817.1

QLI73825.1

QLI73827.1

QLI73828.1

QLI73836.1

QLI73837.1

QLI73839.1

QLI73850.1

QLI73853.1

QLI73859.1

QLI73860.1

QLI73862.1

QLI73864.1

QLI73869.1

QLI73875.1

QLI73877.1

QLI73878.1

QLI73882.1

QLI73894.1

QLI73895.1

QLI73898.1

QLI73899.1

QLI73901.1

QLI73906.1

QLI73913.1

QLI73917.1

QLI73920.1

QLI73923.1

QLI73924.1

QLI73930.1

QLI73939.1

QLI73946.1

QLI73949.1

QLI73963.1

QLI73964.1

QLI73967.1

QLI73971.1

QLI73975.1

QLI73976.1

QLI73992.1

QLI73997.1

QLI74000.1

QLI74006.1

QLI74012.1

QLI74014.1

QLI74016.1

QLI74023.1

QLI74024.1

QLI74025.1

QLI74032.1

QLI74039.1

QLI74061.1

QLI74063.1

QLI74066.1

QLI74067.1

QLI74068.1

QLI74072.1

QLI74073.1

QLI74077.1

QLI74083.1

QLI74087.1

QLI74090.1

QLI74092.1

QLI74094.1

QLI74097.1

QLI74098.1

QLI74102.1

QLI74106.1

QLI74122.1

QLI74126.1

QLI74127.1

QLI74128.1

QLI74129.1

QLI74131.1

QLI74132.1

QLI74133.1

QLI74135.1

QLI74136.1

QLI74137.1

QLI74141.1

QLI74143.1

QLI74144.1

QLI74147.1

QLI74149.1

QLI74151.1

QLI74153.1

QLI74155.1

QLI74157.1

QLI74161.1

QLI74168.1

QLI74169.1

QLI74176.1

QLI74178.1

QLI74180.1

QLI74185.1

QLI74197.1

QLI74199.1

QLI74201.1

QLI74207.1

QLI74212.1

QLI74214.1

QLI74222.1

QLI74225.1

QLI74232.1

QLI74237.1

QLI74240.1

QLI74241.1

QLI74243.1

QLI74244.1

QLI74246.1

QLI74249.1

QLI74250.1

QLI74251.1

QLI74252.1

QLI74257.1

QLI74261.1

QLI74273.1

QLI74274.1

QLI74277.1

QLI74278.1

QLI74281.1

QLI74283.1

QLI74292.1

QLI74297.1

QLI74300.1

QLI74303.1

QLI74304.1

QLI74307.1

QLI74309.1

QLI74312.1

QLI74314.1

QLI74319.1

QLI74325.1

QLI74327.1

QLI74328.1

QLI74333.1

QLI74336.1

QLI74338.1

QLI74343.1

QLI74345.1

QLI74349.1

QLI74350.1

QLI74358.1

QLI74359.1

QLI74365.1

QLI74374.1

QLI74386.1

QLI74387.1

QLI74388.1

QLI74394.1

QLI74395.1

QLI74401.1

QLI74402.1

QLI74403.1

QLI74408.1

QLI74410.1

QLI74416.1

QLI74418.1

QLI74423.1

QLI74425.1

QLI74433.1

QLI74435.1

QLI74439.1

QLI74440.1

QLI74455.1

QLI74456.1

QLI74463.1

QLI74465.1

QLI74466.1

QLI74467.1

QLI74468.1

QLI74471.1

QLI74472.1

QLI74473.1

QLI74476.1

QLI74477.1

QLI74479.1

QLI74480.1

QLI74481.1

QLI74484.1

QLI74497.1

QLI74498.1

QLI74502.1

QLI74505.1

QLI74511.1

QLI74517.1

QLI74518.1

QLI74522.1

QLI74523.1

QLI74524.1

QLI74528.1

QLI74529.1

QLI74536.1

QLI74541.1

QLI74542.1

QLI74543.1

QLI74545.1

QLI74548.1

QLI74549.1

QLI74554.1

QLI74556.1

QLI74563.1

QLI74564.1

QLI74565.1

QLI74578.1

QLI74579.1

QLI74581.1

QLI74582.1

QLI74585.1

QLI74586.1

QLI74587.1

QLI74590.1

QLI74592.1

QLI74594.1

QLI74596.1

QLI74598.1

QLI74601.1

QLI74606.1

QLI74607.1

QLI74609.1

QLI74610.1

QLI74611.1

QLI74616.1

QLI74618.1

QLI74620.1

QLI74621.1

QLI74631.1

QLI74633.1

QLI74634.1

QLI74639.1

QLI74640.1

QLI74641.1

QLI74642.1

QLI74646.1

QLI74647.1

QLI74652.1

QLI74656.1

QLI74658.1

QLI74659.1

QLI74673.1

QLI74674.1

QLI74675.1

QLI74677.1

QLI74680.1

QLI74681.1

QLI74682.1

QLI74684.1

QLI74688.1

QLI74697.1

QLI74706.1

QLI74707.1

QLI74709.1

QLI74712.1

QLI74718.1

QLI74720.1

QLI74722.1

QLI74723.1

QLI74724.1

QLI74728.1

QLI74734.1

QLI74739.1

QLI74744.1

QLI74745.1

QLI74749.1

QLI74752.1

QLI74753.1

QLI74755.1

QLI74760.1

QLI74763.1

QLI74770.1

QLI74772.1

QLI74775.1

QLI74776.1

QLI74778.1

QLI74779.1

QLI74784.1

QLI74786.1

QLI74787.1

QLI74789.1

QLI74800.1

QLI74808.1

QLI74813.1

QLI74818.1

QLI74820.1

QLI74822.1

QLI74824.1

QLI74826.1
