# Supplementary material for: Telomere length de novo assembly of all 7 chromosomes and mitogenome sequencing of the model entomopathogenic fungus, Metarhizium brunneum, by means of a novel assembly pipeline
Source: BMC Genomics. 2021 Jan 28;22:87. doi: 10.1186/s12864-021-07390-y (PMC7842015; doi:10.1186/s12864-021-07390-y)
Supplement: Supplementary file 5 — Additional file 5. Core Hypocreales protein set. A list of core proteins found to be orthologous between the four Hypocreales fungi; Metarhizum brunneum, Cordyceps militaris, Epichloe festucae and Trichoderma reesei. [file 12864_2021_7390_MOESM5_ESM.pdf]

>Mb|QLI63422.1
MATFSRGIALLPLPLPLRALPRPAHPVGSTRFLTTSLRRLRAPQSASPSA
KPAKKPPSSPPPAARGSPTTAAASRYAFIKSLSSKQTPTVLYEAPSHFWF
YFGCWTSGTAILAWTALTGPTVVEQPDGTPQWVGYVFGASYVLLGAMGFY
LLSKTPNIVGAIRVLPPTTGAAHPRLEVTVKRMLPVLKPRTVVAPLENVR
LKSRFSLPEEYVPELRRLELRMREEERRQALRKFDMEHLLTMPFRRLGRA
FANMFRGVRSAWTDMGFGVLSVDGKEYKVDVTKGFAHDGFKTLEQIVAVG
YTK
>Mb|QLI63426.1
MKSPLASLLWRSLRVYQVFGANTDVGKTIFTTLLARTAKKLWKDENVAFL
KPVSTGPEDEADDRYDCSLNKPYLRPQVISNLIEQTSIASGRPIPSEDEL
LAKCRAFAAQCATRNGWLFMETAGGVHSPGPSGKTQADLYIPLRAPSLMI
GDSRLGGISQTISAFESLRIRGYDVESVLLFKDDGYGNYRYLEEYFRKQH
GIPVTSLLPPPKKHDNPDQDAEVMENYYRRKDLAEITGQVLETLDRNHAA
RIRNLDTMATRASKHIWYPFTQQSLVGPKDIMTIDSAHGDYFQTLAPPTS
TSTSTSAPSPDTPVIRSSFDASASWWTQGLGHSNPKLTMAAAYAAGRYGH
VMFASAIHQPAMALAETLLDGIKNPRLNRVFYSDNGSTGIEVAIKMGLRA
ARKRYGWDTTQKLGILGLKGSYHGDTMGAMDCAEPGIYNEKIEWYQGKGH
WLDYPTVLCRDGKWSVSAPDGLQESLGPGRTYASLREIFDVAGREANGEH
EAYKTYVTSTLQRLKSQGRKFGALMLEPVVLGAGGMQFVDPLFQRTLVNT
VRESSHLFGESALSADEAQDPNEWTGLPVVFDEVFTGLYRLGRFTPSSFL
GVHPDISVHAKLLTGGLVPLCTTLASENIFKIFLSPDKTDALLHGHSYTA
HPIGCQVALESVKEMQDMENRGEWDWAKAQGWTQQHSGQSEAQIDVWSTW
PREFVERLSRQTGKVAGVWALGSVLAIHLRDEAGVGYSSNAALGLRESLG
LGRADGEGGPWNVHSRVLGNVLYVMASQVTSETSVRQLSELLMESLAGKK
>Mb|QLI63428.1
MGWTPTSDLWPPDLLPLDITPKLERRRRTRPVEFKQPRIPAIARYSPTGM
DYNAVPTPEACYADFCLIPVGTGNVSVAEEVAQVQRVLEASGLKYTLHSA
GTTVEGSWDDVMAAIGKAHAVVHQRGVVRVQSSMRVGSRTDKKQTAEDKV
KRVEDLLGNKS
>Mb|QLI63429.1
MSGLPPVYIVSAARTPVGSFLGSLSSLSATQLGSTAIKGAVERAGIKPEQ
VEEVFFGNVLSAGLGQAPARQCAIGAGLPNSTISTTVNKVCASGLKAIIL
GAQNIMLGTSDIVVAGGTESMSNTPHYLPNLRTGAKYGDQTLIDGVLKDG
LTDAYKKEHMGLQGELCASDNDFSREAQDDYAIKSYQKAQAATEAGLFKE
IVPVEVSGGRGKPPIKVERDDEVKNLNVEKLKAMRPAFKSDGSITAPNAA
PINDGAAAVVLMSEAKVKELGVKPIAKILGWGDAEREPERFTVAPSLAIP
KAIKHAGLTDKDIDFYEINEAFSVVALANIKLLGLDADKVNVFGGSVAIG
HPLGCSGARIITTLTSVLKEKGAKIGCAGICNGGGGASALVIENLQ
>Mb|QLI63431.1
MATSPPPRRLVGLSAKMYFSLSRNREVTKGFLDCLSRTSLESLSNVDIFI
IPDFVSLTETIGQLKSSPVPIWPGAQDCHWEDSGAFTGEVSPAVLSEAGV
KIVEVGHAERRRLFGENDATVAKKAAAVSRNGMIPLVCIGEQTKGDIGVA
VDECRTQVEAVLATVPSAAEVALAYEPVWAIGASQPAAEDHVLKVVQGIR
RLDCVKSRQGTIRVVYGGSAGPGLYERLKSGLDGLFLGRFGHDPERFVKT
ILEVAQA
>Mb|QLI63432.1
MTSTPSIKGRLAKAVGYDSDGRDDDVPSISNADLFIEEEPTIGEFLAEIT
PSLQNVINYFVNLFPFVRWIGKYNLTWLIGDLVAGITVGAVVVPQGMAYA
QLAQLDVEYGLYTSFMGVLIYWFFATSKDITIGPVAVMSQVTGNIVLKAK
DELPNVPGHVVASALAIITGAIILFLGLARLGWLVEFISLPAICAFMTGS
AVNIISGQVPKLMGISGINTRDAPYLVIINTLKGLPNTKLDAALGLTALL
MLYLIRGVCSFMSKKQPHRAKMYFFLSTLRTVFVILLYTAISAGVNVSHK
KKPSFSLIKDVPRGFQHAAVPEINAPIIQAFASEIPAAVIVMLIEHISIS
KSFGRINNYVIDPSQELVAIGVTNLLGPFLGGYPATGSFSRTAIKSKAGV
RTPFAGVITAIVVLLALYALPAVFYYIPNAALAAVIIHAVGDVITPPKVI
FHFWRVSPLEVPIFLAGVLVTVFTTIENGIYTTIAVSAAVLIWRLFLSRG
RVLGVARIRTAKADQELKHAGDSAFAEESDESLRKGFLPIDHADGSNPRV

VVQSPHPGVYIYRFAEGFNYPNATRYLNHLTEVIFAETRRTDPTLLGKIG
DRAWNDNTPRNQDVDKVDTRPTLKAIILDFSSVNNIDVTAAQALIDVRNQ
LDRYAAPEAVDWHFANIENRWTKRALAAAGFGFRSPRPAHGSGVANWKTI
FSIADLGGSDSAAKAAEAAAIGDKKSVREYDIEQAESDISRLSDKDVPQN
PGSARLVAIQGLNRPYFHFDLQEAVESAIANSERKR
>Mb|QLI63433.1
MPPEGIDAVTSRAARHDLARRLSHLARRLTYDGSDDVDEMMVGSQLEQLE
KVVGGARSAESKKLPYRTSFDSPGRSDAGSVLGSPVSSLFRSRFSDLSAS
LHREREAEKEPEDEAPSKKGMTGTQAKKVIAEMSKLNEELSTVVNNLKAR
QEESEHIQGLLIERAERAAQRIIFLQNRISYLEEELQENDGELQHLRICL
KAVEIQMPPHPDKELQRCIATFKDDYQALKRKRAHRASMASVRGFDPLQM
GTPLR
>Mb|QLI63435.1
MKVTFRDLKQQKFVLEVEPTDLISAVKEKISGEKGWDPKHQKLIYSGKIL
KDDETVASYNIEEKGFVVCMVNKPKEKPAPAAESSAAAPPATPAQPVAST
PAVPAAPAQPSTTQSAAPATPTPQRSGEAGGETGSGLAMGAERAEAITNM
EAMGFERSQIEAAMRAAFNNPDRAVEYLLNGIPENIQQEQHARQAAAAGP
TQATPAAPAAQEGGEDDGGVNLFDLAAQAGGGGRGGSGSGSAAAAAAAAA
GATQGGADLGNLDFLRHNPQFQQLRQVVQQQPQMLEPILQQLGAGNPQLA
QLIAANPDQFLSLLGESAEDDVPLPPGAQAISVTEEERDAIERLCRLGFD
RDQAIQAYFACDKNEELAANFLFDQPDDDEPPTN
>Mb|QLI63436.1
MSDDVDNSLLSRLQALRGEGGSPKIPASNPYGTSPLAHFNSVKMRITNGH
SIKFDVIERAKTPTKGDMLAARLKNLRSQADGPISPASAKTTERTTAQTA
DAPGSKKTPTSEDDVDALFETDDRALEEMLADLDNPDTPEPESKDEHVQA
LLEQLSAQIPKDSDDNKAKQAANSDDSDGEQMAQEVDDVLARLKDEIQVE
AQDGKQRQEPPQGETDIALPSVPANPDLPQDTSPKPRGLDDITARMAALL
APTAQDDLPSVPTSKPSKRVNRLTSRTSYTDDDADSWCTVCLEDATLRCL
GCDGDVYCARCWREMHVGPAAAWDERSHKAVQFTRDGGKEGKIALGA
>Mb|QLI63437.1
MAAEKEAPNELRPAHVADEKHGPGSINDFDIAREHDHDNEVAIMRKDYED
KPTDEEFATLRRVPGKIPTVAYLLCAVEFCERASYYGCAQIWTNYINRPL
PKGGNGYGAVAAGSQSTQGALGMGEAIANATTQSFSLLAYCLPLFVGYLA
DTRFGRYPMIFWGVIICGVGHILIVAGGAKELIANGTAKIPFFLGVYILA
IGAAMFKPNVTPLLLDQMKSHVPRVVTLKSGERVIEDPEHSTERVMLWFY
LLINIGAFMSTATSYSARYVGWWLAFLLPLLLYLPLPLLLLWLKPRIVLH
KPGGSDLPNVFRAIGHGMANGGIFRIGRAGWWENAKPSVRAQKGLSPETR
YSDEFVVDVQRTMQATGMFCFFPVQYWNDNGLGNSANYLGTMLTGNGVPN
DVIGNFNSLSIILLGPLLNYGVYPLLRKGRIHYGPVARICTGFFLSTLAG
IGYAVLTYKAYQTSPCGWYGSSDPYCVDNGLVSPISLWWEAIPYALGGFS
ELFINVPAYGIAYSRAPINMRGLVSAINLFNTGFAYIVNLAASAAIADPH
LIWDFAGPAILGAIVTVGFWFIFKHIDKEEYVLSTTQTSEAAEAEIIEDT
EAVPTDRR
>Mb|QLI63438.1
MKSSLLAVAACAVLAAAGPLKKRALTTEWVTEFVTVTVTADDSEPTPEIP
AVFVETEVVTLTPSAPPKVKPTPVFPDSPSVEIKPSTSTFSPPPKPTVIQ
PKPEVSTPAAPPPPATTSAAPPPSPSSGNSGSYADKMVQIHNECRSKHSS
PDLIWDPNLAITAEKIAKSCKFAHQMGVDGGSYGQNIAAASSDKNSNPTD
SGVNAVGDMWYGEVKNYTPAFYRSGPSQGTPLGDYGHFTQVVWKGSTKVG
CATVYCDAGTIFQMPSQFTVCNYKTQGNVGGQYPENVLPPKTG
>Mb|QLI63441.1
MVALRDGSKAQGELGDDSDLITPDPTETTHLLATGVEGNDNIVTAGGVKG
TNGGWDGLADFEGLPWWRRPSVFWLLAPYSVFTLAFGSVIVPKLNLILGL
VCQHYFASQSHLHPDLRVNPITIGADNPQCRNPEVQKRVATFMLVMNFCT
GLLSAYAAPRLGHLSDRYGRRRLLALASCGGLLGEIITILAAKFPETVDY
RWLILGSVFDGMTGSFTAGNILTQSYASDCTPPSRRAVAIGYIHACLFTG
LAVGQLLGGEIVRLTGSLVSIFYVCLAAHSFFIVFVAFVIPDSLSKKKQL
AAREKHRKEEGERRARRTSWFSSVQERNPFSSLSVLWPRGRGTSTRLRLN

LVALATCDMIIMGTAMAAGQVIILYAEYVFGWQTPETSRFVAAASFFRVI
VLMGIFPLINYFFRIRPAAKRRRESGITPVDKNSGADHLDIWILRIALLS
DILGYIGYTFARAPLPFVASALVTAFGGLGSATSQSVVTKHIPHDKVGRV
LGAIGMLQALVRVVGPVTFNGLYAATVGSYPQAIFVALAGLFSFALLCSF
IITPFVHWHEDHEEEEEREPLNGTASQAVGPAGTGFPCDEDEIRY
>Mb|QLI63443.1
MASYALATSYAGESLLSGFNWFHGADPTHGYVSYQSRQNAEALGLYSVDE
KTGVVRLGVDSTNTYPLTAGRPSIRLESKEAFSHGLFIADFLHMPPSQCG
VWPAFWSYGPNWPTSGEVDIIEGANDQHNNLISAHTGPGCTISKSLGGKF
SGAQRETDCNVGNNNVGCGYSSPAGDASAYGDGFNAANGGVYAMEWDNEF
IKIWHFARSEIPKDITNKTPDTKGWGVPDAVFGGGDCNVDNFFKDMSLVI
NINFCGDWGNAIWGKSDGCGKYAATCNEYVANNPKAFANAYWDVRYIEAY
QKTDVQTSSSSPSASATEPLTRVTRTTSITTTITIRRPHRSTRTVVEGRN
TTSALGVDAASSYQSVPGAIAADTTPVYRVNATSSYESTPGKATAGTTLI
YRANTTSSHEPTPEEATAGTTAPVYRVNATSSHVSTTEEAAASITPVYRL
NTTSSLWSSHSSRSANTTDAYLYATSSLRSTRRSIPTNGTALPRHTISSL
VSVPSGAITNTSQAARPQAASSPVSIPSEVPAPKNPVQLGDFAYLGCYGS
SDDFKSFRKVKDSKDMTIDLCIQLCRDSMFSGVYDTQCFCADSIDADTRA
ANTKNGDVCDHPCPGNDVQYCGGLAKSPEKLANTASTPANDRFENIASST
GVAASKSTAAGQRFRNSTTGGAGKNSTLATRFHVPSKRSPLSRMEKAILL
TVYGVVKDETPPPPPPSMTGNNITHTFTAYSTTMRVKTITVIPVQATAGA
SKADVVADKSPSGPPVADETVVSTQGTSVPSENKPVGHKPVGDKDRGETL
PTGENSDPEKDGFAPDRQPGSSYAGDKTVDRVLETAAPSHVEFVTDKRPD
GSPVGGKTVTKTVDQMEVVVEDCGCTEGETRPTCTPHPSSPPSAEKPNPP
HAVPSDVEVPVPAEGSKQSQPAPPAKATNARQPVPSDIDVPVPVEGLKHN
QPAPPPAQATNARKPVPSDIEIPVPVEGLKHSQPAPPPAQATNNPHQSAP
SEIEVPVPAQDPKHTQPAPPPAQNTKADGPESSGNDSPAPIGSPGESQHA
PPSPATPPPAQITYPYRVRPSGFETLPPAEASHGAKTPKPAVVTAGTGKT
WSEKPGVMLGLMTLVVAMLL
>Mb|QLI63444.1
MSSQQVPQLNVDRYVVIHVATTCDEHGVYVTKDSAEVIELGWILLDANSL
EEITHESVLVKPVNTPITPLCTSLTTLTWEHVRNAGTFRDAISRFDTFAT
DYLTSKNLDFVFVTLDAWDLRVQLPREARDKAVVLPPYLQHSRTFDLRTE
YQRWQQHHPESLPFGPSMLSNICAALEVDPVQSSAPIKHNLPFHLQALAP
ASPRRAMEEAITLARVLRGLIRKSQPPQDHPDVLTRPMDARADVRAFLSE
RSKVLHMSGLPHDTTQSELESWFTQFGGRPIAFWTLRTPEQHKPTGTGFA
VFSSHEEAAESLCMNGRALNEKAIEVSPSSSRVLDRAQDILTPFPPSKNR
PRPGDWTCPSCGFSNFQRRTACFRCSFPAAGSGPGGADAAGGYGYGGGYG
PPAMMPPPPHGGHHGPMGHGGRMGGGGVVPFRAGDWKCGNEVCGYHNFAK
NVCCLRCGASRAGAAVVADSGYPSPMDNPSQYSMAQGSMAGTPGPGPFNS
GNSFGSGAGYNQHFGGPPSHFLPAGIGGGAGGYPSSMNNQNFASTPGSHS
AGPFDSRAAEAAFQSASNGPASAGSGPSNHFYNNNNNGENDPFAFLSTGI
GGLSVSGNDARQNGGGAPPSKSPA
>Mb|QLI63446.1
MGSVNTETPALATNPKFIFFTDFDGTVTTADSNDYMTDNLGYGAEKRRQG
NKDVLTGKMNFRDSFQEMLDSITTPYNECIDILLKNITLDPGFKEFYDWS
RENNVPIVILSGGMTPIIRALLDKLLGPGWDIQIVSNDVRPREGKTINDK
GGWRIEFHDDSIHGHDKSIEIRKYSSLPNRPTMFYAGDGVSDLSAAKETD
LLFAKAGKDLVTWCENEKVPFVTFKDWTSITQTVKDIAAGKITVQDAAKG
RL
>Mb|QLI63450.1
MFSNWRSQPEGSGHSSSDDTRVGDNDQDTQVMEPDQGNVLSHIISQLRPG
ADLSRVVLPTFILEPRSMLERITNFMCHPEMLLQIPEIDDPVERFVSVVK
FYLSGWHIRPPGVKKPLNPILGEIFTCYWELGNNKRAYYISEQTSHHPPK
SSYFYMAPDYHIRIDGTLKPRSRFLGNSAASMMEGVAYLSFLNRGKNTAI
GEQYILTQPNMYARGILFGKMKYELGDHSFVRCPELDLTADIEFKVKGWV
SGTYNAIGGVIKRESTGEVLYELSGLWSDEMFIKDVQASPYQRDSSKYTK
PANNHCKKTGHKDMFFNAMTSKPSSPKVRPLAEQEERESQKLWASTAKAV

KEKNHEVATDEKTKIEDRQREEAAARAQDGVDWHPRLFRRVKPGDGVGDD
LEWIIHTPVDPTTSPEEQTKQFLSIYPVVDGQTLDGDFAIPPRKEANGQA
EKNGVAGDLIDIGGDDPPTQPITNNKSTEIENLLNSTGKPAEGALIDFAD
DMKKDLPAAGDKP
>Mb|QLI63451.1
MLLLLRPRCRSLQYLLPRHSEHHPNRSHLQKRTLLTLAIESSCDDTAVAV
LSHTPPSTTLLFNERISSDNRAFKGVHPVISVQGHNSSLAPLVQRALQYL
PDASPSTPSIRIPSNDGALLMKQRPDFVSVTRGPGIMANLAVGLNMAKGL
AVGWDVPLVAVHHMQAHALTPRLVRALETDAGRCSDSPGPEFPFLSLLVS
GGHTQLVHSTGLTGHRIIATTGDIAIGNLLDQTARVILPADVLDASPDVM
YGRLLESFAFPNGPSHEQYSFFEPAASRSDEINTLPTGYAWDIPLPFRNT
RRLAYSFSSIHTRVHRIAASNPSMPLDERRSLARHTMRAAFQHLCSRLVV
ALEDRPELRPAARTIVVSGGVASNKFLMHVLKETLRIRGYPDTEIIAPPV
KFCTDNAAMIAWTGMEMFERGWRSDLSVLPVGKWPMEMDGGDGDGILGVG
GWIKKGRAW
>Mb|QLI63452.1
MPQSESNSEPSFQQKTVVFPPSPPSTVGRSRRCSADGNSNDITFPVSPTI
ANNDALIYPLPPTNSEKSVRPEDLKTPDSHVERDARLIRLTGTHPFNCEP
PLRDLYDEGFLTSEDLHYVRNHGPVPRCDDGDMDQWTFTVDGLVENPFTL
SVRELIASYEQVTYPITLVCAGNRRKEQNVVRKSKGFSWGSAGLSTALWT
GVALGDLIAKAKPRRGARYVCFEGADKLPNGYYGTSVKLNWCLDPNRGIM
VAHKMNGCVLHPDHGKPVRVVIPGQIGGRSVKWLKRITVTEKPSDNWYHI
YDNRVLPTMVTPEASADLPDTWKDERYAIYDLNTNSVICYPAHNETLLLN
GDSSTYKVRGYAYGGGGKRITRIEVTLDKGKTWRLANIDYPEDRYRLAPD
GERLYGGRVDMWWRETSFCWCFWELDIDIGDLKTTTDIMVRAMDESLMVQ
PRDMYWSVLGMMNNPWFRVVVHDIGHALQFEHPTQPALIPGGWMERIKKA
GGNLTNGSWGEKLAGQTEDVMGQEPEQDVCMTNPRADRLVTMCELKSHSG
EQEPWFIVDGQVYDGTPFLEGHPGGSASIFGAAGQDVTEEFVTIHSENAK
AMMPAYHIGKLDEDALAQLSGETNECDPSRSVFLDSKVWTKATLQEKISV
SSDSKIFRFKLDHAEQEVGLPVGQHLMMRLRDPVTRESIIRAYTPISEGT
DRGWLDVLIKIYHGTPERRGGKMTQALDSIPVGHFVDFKGPVGKFEYLGR
GLCSISGKPRHVSRFNMICGGSGITPIFQVFRAMIKGADDPTECVILDGN
RVEEDILCRLELDQMASSAKHRCSLLHCLSRPSSTWEGRTGRIDKDLISS
EIGSPRLDKDDIVLVCGPEQMEKGVCETLRVLGWPDENIVVF
>Mb|QLI63454.1
MSPPRRRSARLASSVKSKATPELGSVAEAEETPCKSRTRNVDVDTTMESP
FPEPSTPASSALKPPRSEMHPSKYHFSTGDPSSALRLGFSDIPKEAPTPG
CQDRTPSKVNRVPSSSFTFRVAREPADTELSGEAQRMLKEIRNQAAKIKA
ELVAQREAEGGVNIGDRPFAKPKGKVGRYSAAHMAEFKKMDSIEGHASAW
RAQEGRFTPVKALKRTPSKANLDGTPTSQKSVCIQRSPAKTVTDETSNSR
PKPNLKRKSSAANLDSDNHAASKRHNESANPGKCSITAPENRTPTAKRVK
QRREDDAAIARPGHVEFSPAKPTSIFSRLATPRKAAAAPSPISAKIATKP
ATSSVQSPLKSSLHSVSMSVSGDNLNPPVSKASELKRRIISPRSFSKVKS
ILRGSKNDSEEGVSAIPHPQISRTPAPPRVNKELPPVPLTTPRRKLTKRV
TFTPEVVSAVNVQNSPSPQRRGNFKPRPALKSIETHYPALDAVLTESTPS
SSLYPDLSPLKHLMGSQSQRVVSESPSMAGTFTFRSDRTIKFGDAPSTGF
GASPGQSSVRHVSGPVVSSNMPGSFPTPPSPNSHPNKENKAPSLTKLLPG
TAHGMQNKKRHRATADEDDFETEPAAKKRKNENVPEGQALLAPRLAKVGS
ASATKNNRFTRTPVKAGSSRISGKTPVRTSVVASPKKKVTGLSMSRLNML
AQPKNRA
>Mb|QLI63455.1
MDSRSVYSAHVFEPSYAENGDTRLQLLQQLETFILDFRLDNNFVYRDQLR
ENALLKQFYCDVKINDLINFNEELAHKLASEPAEVIPLFETALKKCTQRI
VFPHEKEVNLPDHQLLLHSDAEDVSIRNLDSMTIARLVRVPGIVIGASVM
SSKATELHIQCRNCQFQEVIPVLGGFTGVTLPRQCNRKRIDNDPTPKCPL
DPYFVMHEKSQFVDQQIIKLQEAPDQVPVGELPRHVLVSADRYLTNRVVP
GSRCTIMGIFSIYQNKASKNSSTTGAVAIRTPYLRAVGIQTDLDQTAKGH
VSYSEEEEQEFLEMSRRPDLYNIMADCIAPSIYGNRDIKKAILCLLLGGS

KKILPDGMKLRGDINVLLLGDPGTAKSQLLKFVEKAAPISIYTSGKGSSA
AGLTASVQRDHSTREFYLEGGAMVLADGGVVCIDEFDKMRDDDRVAIHEA
MEQQTISIAKAGITTILNARTSVLAAANPIFGRYDDMKTPGENIDFQTTI
LSRFDMIFIVKDEHSREKDERIAKHVMGIHMDGRGTEEVAESEIPAEKMR
RYITYCKTRCAPRLTPEAAEKLSSHFVSIRRQVHAAEMEANTRSSIPITV
RQLEAIVRITESLAKLTLSPMATEEHVDEAIRLFLCSTMDAVNQGSNQGS
RELNEEVNRLEVELKRRLPIGWSTSLATLRREMVEGKGYSEQALNRALMV
LQRRDTIMFRNSGAQVYRHGA
>Mb|QLI63457.1
MEHGRSMGTSPSRRDVLSSSAQQRFLSSSPLAGRFLAEDIAACSDDDYDD
TVIEDQGEAERPSHLMYRRPSGVAYGGTRPVFNAQPIDEPILTPMERRQS
RHAERSLLRDNHVLPPKHASQKQKPSFARRLYNRLFSTKLPREAVDEESP
EYPPTETSPLLNGRGHGQGVGSDAGSGSADDGLEQQWEEAIASGRLQTTW
QRETKTIVAYSWPLIITFALQYSINVTSIFAVGRIGRVELAAVSLANMSQ
AISCLAPFQGLATSLDTLCAQAYGSGHKHLVGLQCQRMACFLMFMSLPVA
ILWLFADRILFHVVPDAETARLAALYLKVMIASIPGIVLFEVGKRFTQAQ
GLFRATTYVLLVAAPINIFINWLLVWRLGLGFVGAPIAVAITESLLPILL
LLYIVFVDGRQCWGGFSKRIFSNWWIMIKLALPGMIMVEAEWFAFEIMTL
LAGQLGTEYLAAQSVLVTLSSISYQMPFPMSIAASTRVANLMGAGLVDAA
KIAGKVTFVLASILGALNVTIFSSLRFHLPILFTNDPEVIKIVASVLPLV
AVMQLFDGLGAGAHGLLRGIGKQSIGGPANLISYYVISLPISLGLAFGLG
WNLDGLWIGLTIGLALVSVIEYTYLLHTDWHKAALEAEARNAAG
>Mb|QLI63458.1
MASPRGNAVPTTSKLASIADSSCSIDVHNVFGPTVAYACLNGFDFTLLFE
ESILTLLPLLLTVLILVPRAVILWNTAPKVNRSWLFTLKLVTFSVYIALQ
VAMLALWTQPNTPTTRLTIPLRAIIIASFIWFVYVSYLEHVRSVRPSTIL
CIYLGISCLLDMACARTVFFIPGYHIVAPIHLASYFFKLVLLALETTEKR
RLLMLQWRNTSPEATASFYSRVLFVWLNSLFLKGYKTLLTVDTLTPLDQD
ILDASRPTKLEERWGKADKSSKTALLWVFIRHYKWDLLAGVLPRLAYVGF
TFAEPFLVKRVLDFTVEPDGPNTRNFGYGLIGAYALVHIGKALSLALYEH
MTYRVLTMFRGSLITIIFGKTLRLSASAVHDAEAITLMSADVDRIGLCMP
VLHDSYASLVELALSLWLLYRLLGIAVFPPTGFIILCLSIGIPIASAAGN
AQVPWLEAIEKRLAVTSKMLGTMKAIRMAGLTKTIHAIVSNLRIKEIRSS
RAFRLLSVLQTGAANCTFTFAPVLGLGTFTILAKVNNTETLTEGVAFAVL
SLFQLQDRPVMSLLHGFEDFQTIINAFYRVQTYILSAEREDFRVIPVQKS
GTSSDENLPQKSDTNVPGNGILRGSESLDTSTFSAGASAVSLKDAAAGYS
ETMVVKDINLEIARGKITMIVGPVGSGKSTLLRLILGELPEVSGSVISDY
SKCAFAPQSPWITWGTVQSNILGMSAWDTEWYNTVIRACSLDTDFKDLPD
GDQTRTGTRGSRLSGGQQMRLSLARALYSRNPLIILDDVLTGLDRQTEAS
VLEAVFGAGGVLKKTKSTVVLATNSVGHLRFADHIVVLNKEGSIAEQGTL
EQVSASGGYVQRLASLPAVETSRQELLELPEEIYQELGLSNEEDELGSAR
KTSDFQVYAYYIKVAGTWTFSIYIFICAAYTFGLSFPSIWLQWWTDYNAK
HPNENIGYWLGIYFGLAALAVVGCMGSDWAFRMMLVPKTSRKFHQLLLDT
TMHATTSFLTSTNAGLTTNRFSQDLQLIDSDLPQSLDETVMNFLGVITSA
VLVFTGSGYISASIPVCAALIFLIQTFYLRTSRQLRLLDIETKAPLFSHF
IETLGGITCIRAYGWSAQYMDQSYDALDTSQKPYYLLWCIQRWLTLVLDL
LAAGIAVMLVAFATMVHNGSTGLLGVALFNVISFSGVLQSFVTQWTQLET
GLGAIGRIKAFVQDTKPENSDGEVDDLPDNWPSAGSMVFDNVSASYEASS
KPAIDGFNLTIKPGEKIALCGRTGSGKSSLLLTILRMLELDAGSIMIDGH
DTSKISREELRRRLNTLPQEPFFLHGSVRENVDPLHISTDERIIETLRAV
QLWDMLESRGGLDAPVSEDSLSHGQRQLFCLARAIARPGNIVIIDEATSS
VDSETDELMQRVLRQELDGRTLIAVAHKLHTVTDFDRIVLVNKGKVVESG
NPQELLANPTSAFYKLYTSGGSAAE
>Mb|QLI63459.1
MKRLRVSQWSSKQLLRARPGASSSVPIAIAGRQPFLQSLRCQSSAAIAPE
HLANDAAAAQHITRAAPSPPHQRSLESAKLAALHARLALSPKIPLQTLAR
ALITPSADANGSFNNSNLAFLGSTIINYHVLEYLVCKWPRLPMAILYEAL
RAFSGQESLQQVSRRWGVEAAAAPGEEVDAGLLQWKADGVQLVNTRWGYV

RSEVGKDSSYRRGMSSRVVLDDVFGDALSKPDNAGRQGVDSLQNEAFASF
VQAVVGSIYAHCGREAAKSFVTSHILSRQFDPSALFQFQLPTRELAMLCA
REGFEAPVARLESETGRLSKTPVFVVGIYSGKEKLGEGAGPSLDIARRKA
SMNVLKAWYLYCPGNKVRVPSDMMEEGARPWKAPHIDVGEII
>Mb|QLI63460.1
MASSVFFKFKSQKEPTRVEFDGTGISVFELKREIINKSGLGDGTDFDLFI
YTDDNSEEYDDDTTIIPRSTTVIARRQPALKPGAGRAARYVSGKMPVTAK
NAGRKEQSSKASSSKPSATAISEMNNAMTEEEKMAAMFAAQTEQWSAQQE
EMSHQAPVLKPGAKKPANVPDHDPPQGYICYRCGNKGHWIQLCPTNDDPD
FDNRPRVKRTTGIPRSFLQKVDKSVVLAQSDGDESKRPSGIMVNAEGEFV
IAEPDKASWEQYQAKAKSSTANKEAQAEDQEVQERGLQCSIDKKMFIEPM
KTPCCKKTFCNDCITNALIESDFVCPACQTEGVLIDDLQPDDETSKKMQE
YLKEKETAKSPPPVSPKGDVATPENGTKNVDAKTDDEEKGPEGEPSGNAQ
ESKTTQKSPTPTEATVKSPTVAAEGEMQVVKSEDKQAVGESNTQEINLKK
RPADDFLENPKIPKGPKAMQNQQSNMNGTNNMMNGMPNMGMPNMMLNGMG
MMPPNMMGMPGMNMNMNMGMPPNMMGMPMMGMPNFGNMGGGFGMNGGWDM
SGMNGMNGMGGMPGMNGMNGMSGMSGMGGMGGMGGMGGGMNGMNGMNGMN
AMNGMGGNMGNMGGVGGFQNGGQNFGQQNNQADEDAYFRKPVNPHRHQNK
QRRIRPSDYREL
>Mb|QLI63461.1
MATTSQDKSTRTIHSAACLIIGDEVLGGKTVDTNSAYLAKWCFSLGISLK
RVEVIEDDEGEIMEAVRRMSDRYDFVVTSGGIGPTHDDITYQSIAKAFDL
PLKLHDEAFQKMKVLSRPDTLTPNFDWATDSPALQAKLRMVRLPTDESRD
LKSQFLFVSDNLWVPISVVNGNIHILPGVPSLFQALLDGMKPFILPRLVD
PEGKGTCRILISTPMPESAVAAYLTELAARVEAKGVKVGSYPRWGKKRNT
VTLVGRDQAFLESLVAEVEENVQGRRVSQEDEADDL
>Mb|QLI63462.1
MSDLDKAIAQLRACRPIPEAQVRELCHRARELLIEEGNVVTVSAPVTICG
DIHGQFHDLMELFRVGGDVPDTNYLFMGDFVDRGFYSLESFLLLLCLKVR
YPDRMTLIRGNHESRQITTVYGFYDECLRKYGSANVWRYCCDVFDYLALG
AIVLGASNTLSPASGPADEETEIEVCDQNGSIMSRFPRQRQRQQSSQPSS
SQEHGANGSGPAGQTGPPGSGASGSSNGSVGNPAGAVLCVHGGLSPLIDT
VDKIRLIDRKQEVPHEGAMCDLLWSDPDDIDGWGLSPRGAGFLFGADIVK
VFNHRNDLSLIARAHQLVMEGFKEMFDASIVTVWSAPNYCYRCGNVAAVL
ELTEDESGTGVFSRSNGEVNRSDGGRGVMMESNGMMKSGPARRYRVFQAA
PQDSRGMPAKKPVADYFL
>Mb|QLI63463.1
MVAQEAQEAPPAAAEALKVPLEAPTPESTPEPRPELTPDQQTKYTTLLER
ARSISEVKCASKNNPDKSGPVTEHERAWLTRECILRYLRATNWAVGESEQ
RLRDTLAWRREYGLDAFTADYISPEQETGKQIVVGFDKQARPCQYLNPGR
QNTDPSPRQIHHLFYMVERVVDVMPPGVEKLNLMINFKPSAQRQNTSVPV
STAREVLHILQSHYPERLGKALIINVPWIVRGFFKLIQPFMHPVTREKLK
FNEDMRQFVPAAQLWSSDWNGDLDFEYDHAVYWPALNDMCRRRREERVAR
WKAAGSLVGESEDYLAGGTDVSVGGFKYTGGDGDGDVDLVQEKMAAATVE
DGGACPVAAA
>Mb|QLI63466.1
MSQSAGEDSPLEVAIVGCGIVGLIAAAGLARRRIKVTIFEQGQGLRELGT
GIAFNPAAQGCLEMIDPEVTMALRMGGAVGLSAANEDDPHDCLRWIDGYN
QRDKSDPYYQKYYLKTNAGYRGIQGIRRDHLLEQLIRVLPSDTVVFKKRL
EDIVEGGDDEKIVLKFADGTVTEADAVIGCDGIKSKTRNIVLGPDSTAGK
PTYTHVNSYPTVIPMEKAVSALGERKARAFHNHLGPDANILHYPVAHGTM
CNVTVFVRNDEEWPQDKPTALVGQRADIEHRFRDWNPAIRDLISLLPETL
PTWAVFDLWDYPLPFYNKGRICVAGDAAHASSPHHGAGAGMGVEDALVLS
VLMADASMKLGKGKLSKSQVISSAFAVYNDIRRTRSQWLVQSSRRICDLH
QQKEWADPTKWLKAESTLEELKDRTYKIWNYDFRQMVAEAAESYGMLISR
LEKQASATNGDVKASSLVNGNVSESIAKTKTVAHGIGDSKTLSIETHAEL
LAGPGQSCTTDVMVS
>Mb|QLI63467.1

MGRVIRNQRKGRGSIFTANTRLNKAPAKFRTLDYAERHGYVRGVVKDIIH
DPGRGAPLAKVSFRHPYKYKQVSETFIANEGMYTGQFIYAGKKAALTVGN
VLPLGEMPEGTVVSNVEEKIGDRGSLGRTSGAYVTIIGHNPDEGKTRIKL
PSGAKKVVHSTTRGMIGIVAGGGRTDKPLLKASRAKHKFAVKRNSWPKTR
GVAMNPVDHPHGGGNHQHIGKASTISRYAAQGQKAGLIAARRTGLLRGTQ
KTKE
>Mb|QLI63468.1
MPPKRKAETAVQGGSKAGRSSRVSTPGAATPRSLESTDDVEEDDGPVDEV
LERDIDKNIDLFSLDRFQKRHAVREPLPHIFGDRDFSYLDLKKDHQNRPL
WIDPQKGRIILESFNPLAEQAQDFLITIAEPLSRPTFMHEYALTTHSLYA
AVSVGLSPTDIINTLERFLKTPLPDEIRSFITSCTQSYGKVKLVLKNTKY
YVESPDPEMLQMLLKNPVIGPLRVQGTEEITTSAAPKIGGLVIPGTKNAA
GVKQANGLSQGAEQQNEQAVNEGDVLATLNEEDDDDQEVTHSFEIADKDV
ETVQKECLNLGYPVLEEYDFRRDEVNSNLEIDLKPGTQIRPYQEKSLSKM
FGNGRAKSGLIVLPCGAGKTLVGITAACTIKKGIIVLCTSSMSVVQWRNE
FLKWSNINPDDIVAFTSDSKGSVFTGSTGIIVTTYSMVTQSRARSYDADK
MMKFLTGREWGLMLLDEVHVVPANIFRKVTSSIKTHSKLGLTATLLREDD
KISDLNFLIGPKLFEANWMELSKQGHIARVQCAEVWCSMPTEFYDEYLRA
PSRKKNLLYIMNPRKFQACQYLINYHETRGDKIIVFSDNVYALKAYALKL
GKAFIYGGTGQPERLKVLENFQHNPLVNTLFLSKIGDTSLDLPEATCLIQ
ISSHYGSRRQEAQRLGRILRAKRRNDEGFNAFFYSLVSKDTQEMYFSSKR
QAFLVDQGYAFKVITQLANIEKTPGLAFATAGERRELLQKVLVENDSMED
EDITDDLFHSGSMGRKKKKGPRRTAGTLGELSGGQDMAYIEQNKRVNQGL
KKGKGKKDSNAFFKKIGRENARRAAAQ
>Mb|QLI63469.1
MSATNVEKATDAAVNDVANTLSNTSISQPADDKAAANEAASASAAEGRRL
YIGNLAYATTEGELKDFFKSYLVESVSIPKNPRTERPVGYAFVDLSTPSE
AERAIAELSGKEILERKVSVQLARKPEPAGEKTEGANGEGSGAEGSRRRA
SGRGRGRGRGRGGRAARGGRAGDATEEKKEGETAIAATDAAAAPAEIEGT
TEVQPLTDITNHHQNKTDSETKKNQTRPQRERRERGPPADGIPSKTKVMV
ANLPYDLTEEKLIELFKAYEPSSAKIALRPIPRFMIKKLQARGEARKGRG
FGFVTLASEELQQKAVTEMNGKEIEGREIAVKVAIDSPDKTDEEANAPQE
ENKEATEAATTTA
>Mb|QLI63470.1
MPKATTPKADRPARRHNPLENDILATGLLRNKPTKKKSKNIEDADESFVD
SKASKNILRIGRELAEEENSERASFAPEPTIDSFGYDSRFDDKLDSEEVK
TYDDDDAWGDDDEVVEEIEVDPEDLEMYKKFMPDEEDDLLKHGWDLKPSG
DAVEGESRNLADIILQKIAAHEAAEARKDAGVPVDDYELPPKVVEVYTKI
GEILSRYKSGPLPKPFKILPTIPHWEEILEITRPDRWTPNACFQATRIFV
SHKPIIVQRFLEMVVLEKVREDIYENKKLNVHLFNSLKKALYKPSAFFKG
FLFPLIGSGTCTLREAHIISAVLARVSVPVLHSAAALKGLCEIAAQEASQ
GSEGGGATNIFIRTLLEKKYALPYQVIDALVFHFLRFRSEDPASVQEGDA
MAGVSGEGDVKTKLPVIWHQSLLAFAQRYKGDITEDQREALLDLLLGHGH
SAIGPEVRRELLAGRGRGVPLEPQGEALDGDDTMAVDS
>Mb|QLI63471.1
MNVRPPIEASNPTAVLSVAFNNDSSCFAVGLESGICIFHTKSCLLKASRD
FNAGIGLVQMMGMTNYLALVGGGRSPKFAMNKTIIWDDMKGRVALEISAL
SSVRGVQLGRERIVVVLQNSIRIYSFAKPPNLLHVYETADNMLGLCSLSS
KTLAFPGRTPGQIQLIELATGNVSIIPAHSSALKAIQLSPNGELLATASE
TGTLIRVYSTANCAKVAELRRGIDPATIFSLAFSPSGAMLACTSDKSTLH
IFDIPNTRRQSIQRSQQLGTSDAEPGKWGILGKLPLMPRVFSDVYSFASA
PFEAGDETMIGGIPFSEGTVLGTMRPAKGIIGWISEDSLAVVGAGKDARW
EKFVLVDGENGKRLCMREGWKRYLGNT
>Mb|QLI63472.1
MAAMLEFRTQGYNPYAVKYSPYYDSRIAVATSANFGIVGNGRVFALGLTA
QGVQVEKTFDTNDALYDLAWSEINENQLIVAGGDGSLKLFDLGVNEFPIM
NFHEHKRETFSVCWNPVTKDTFISSSWDGTVKIWSPTRPHSLKTLPIGNC
TYSTSFCPSNPALISAVSSDSHLRVFDLRTPTSAKYHLVSTIPVHAAGAQ

PGVNPGLAAGSPPAEVLTHDWNKYNDTVVATGGVDRVIRTFDIRNPTAGP
LGLMHGHEYAIRRLAWSPHASDVLISGSYDMTVRLWNDGSAQPSGPLAGA
KPGHQLGVMNRHTEFVTGVDWCLFGAGGWVASVGWDERVLLWDSNMLMGP
GPR
>Mb|QLI63473.1
MALKSFLARVKGARGNHAAIVDSNGAKGYGTTTTTPLPGPADDSGFASNV
ERQDAAPGVIRTTPGPPLGYNDGHDPGDDGDDDHAGDLVTAGEGRHLKRG
LAERHLSMLGIAGAIGTGLFLGLGGAVQRGGPLGALLGYATVGLVVCAVQ
FALGEVAALLPVTGAFVRHAEFLVDPAWGFAIGWNLVYGNILSIPSEITA
ICVLFEFWTDLNPSVWIVTFVLLTLVVGLSVVRLFGEVEFAFAVLKILLV
VFLVVLGLVIDLGGVRGTPPVYFRYWRDPGPFVEFIATGHWGRFLGYWSV
MTGAVFSFAGVESIAMAGAETKNPRKAIPAACKNVFIRIVLFYMLAILIV
GMLVSSQDSRLDSESGDATQSPFVIAASAAGIPAIPSVVNAVVITSAWSA
SNQSLLAGTRVLYGLAVKKQAPRIFLRTTSWGVPYACVFLFMAFSLLSFM
SLSSDAITVFWWLVNLTAAGVLVSWISILVNHIRLRLAMQKQAIPITRLP
WSNSWTLYSSYFALFMCIIILLTGGFAVFTKGNWNASNFVSAYLDIPLVL
AAYFIWKFVKKTKIVALADIPLQRAFERADQKYESLAS
>Mb|QLI63475.1
MLIPRLSLSLLWAAVALASPTGTPPAGDPERIATRTATAVVPAGTILGSA
RFGVEAFNGIPYAEPPLGPLRMKPPVRLTESLGVFDATGLARPCHQQVVS
TDSKNFLLDTLGSLANLPFVQTVTGQTEDCLTISVARPEGTTAGDNLPVL
FWIFGGGFELGWTSMYDGTGLLNHAKSINQPFVFVAVQYRVAGYGFMPGK
EIMGEGSGNAGLLDQRMGLEWVADNIAAFGGDPDKVTIWGESAGAISVLD
QMIMYEGDHTYKGKPLFRGAIMNSGSVVPTNPLDHPKGQAVFDKVAETGG
CGGAADKLACLRALSSNDFLNAVTSAPGIVSYDALALSYLPRPDGKVITV
SPDILALSGKYAPVPMIIGDQEDEGTFFGLFQPNLTTTRGFADYLQSYYF
PGATTDQLVQLVETYGTGLSAITNGSPFGTGLLNEITPGFKRRAAVLGDL
VFTLSRRVFLAGATATHQNVPSWSYLASYGRGLPILGTFHGSDIVQTFYG
VPDNYAANSIRTYYSNFVHNLDPNVGVQGEFPNWPRWSEGNNLAHFFRDR
STLLKDDFRQTSYEWISNHIAELRF
>Mb|QLI63478.1
MDPPAEQSKSALKKAEKQAKLAAEKAAKAAKKASLPVVGGKKMDEIIGIT
HSKAENFSAWYQEVVLKAEMVEYYHEISGFFVLRPLSMHIWSTIRKWFSG
HIEELGVEEASFPLFLSKTSLDKEKDHVDGFAPELAWVERAGDKKLDVPV
AIRPTSEAVMYPYYAKWIRSHRDLPLRLNQWNSVVRWEAKQTTPFLRTRE
FLWQEGHTAHLTEELAGEEVLEILELYAGVYEQLLAVPVVRGRKTENEKF
AGGYYTTTVEGYIPSNGRGIQGGTSHCLGQNFSKMFEITVEDPHNKGQHV
HVWQNSWGLSTRVIGVMVMVHGDDKGLVLPPRVAQTQAIFIPVGINKNTT
PEDRQKHEDQIEDMRATLKKAGVRTQSDWREGYTPAWKFNDWEMKGVPVR
LEFGPKDAAKEVVSFARRDTGEKGTIPIADIATKIPELLETIQADMYKKA
ESSFAEHRKKITKWEDVLPALDEKNVVLIPFCLDGKCEDRIKELTTRADD
NRDVPENQKVPSMGMKSLCIPFEQPQGSELVPGETACLNPECSGKAQKWV
MFGRSY
>Mb|QLI63479.1
MDDNDKSPRDGTEDKHRGQVLPRYTADKSTHNFQLPKPVVAVRSRALPTW
TSNPSALSFVSVPVLAPNPHLIVISSELSLSAALLSHAAHLAVISCPALR
SRSLLLLSTAQPDPSPDRWSPAAAFAWRKQASQPNARWRLTRQPIRHSQP
RGLAFIKPLPTRPPENIGHLSSPFSTRRPSDPADRTLPQQPAPPPVAVVA
CASCSPGAQQNRTVAALTILSHETPLPELSLQQHLDIAPDLAATYRPLAT
EADSEPVMMAQQVPHQATDKKRVKVYELRDNDWFDRGTGFCTAAFATTED
GQKDPRVIVESEDQPNRLLLETKIQKEDGFQKQQETLIVWQEPGSGVDMA
LSFQEAEGCALIWRFVSNVQQTFQNTIAGDDGLSDDLAIEVPATISLPAA
ELGNLPEIESGMRFMSSSANGRDALAKFIMADDYIGKLIPLVEMAEDLES
LPDLHRLCNIMKTIILLNDTTIIEHAVSDECVLGVVGALEYDPDFPSHKA
NHRHWLDNQGRYKEVVRIEDEQTRQKIHQTYRLQYLKDVVLARILDDPTF
SVLNSLIFFNQVDILQHLQSNSEFLNELFGIFNIRRPDQKKRKDAVLFIQ
QCCAIAKNIQPPARQSLYNNFIAHGLLQVIHFGLKHGDVAVRVGATDILV
SIIDHDPQMIRQTIYRQMHENTPSLVDSLIELLLVEVDLGVKSQISEALK

VLLDQGIPPPGPEFARMNGEFPGRPRPQPAADPQQELFLTRFYERSVLKL
FKPLLELDNKTGLEFSVQQASMFTYLVEISCFFIRQHHRFSRYFVMNHNI
IQRVSRLLKNPEKYLRLVAIRFFRNIIGMQEEFYIKQVVEKQALGPALEV
LAETLPRDNLLSSACLELFEFIKKENLRDLVKYLVANHRDLMTSLSYLTT
FKDMIQRYDQTQGYTANMDYFLEENDEMARKPPPHTRLMEHITVDPSEEE
YWNTSDPEDDDEEHRPDVRDKILPSNGPLTPSKPLVDYPSDEESDENSNT
EGDTATKANNGPEESAATRRPSLPSVASPPERLSEKRRREEDEEDEIGKL
MHNKRRNSSSSESNFATGSRMTPKRKPFGGGSGNGKIAISLSTAVRTGGG
SRPDEES
>Mb|QLI63481.1
MRTFIKHVLAAACLAATASAVTPISDSDMNQMLNEGGVSLAMKAQPMFFF
GQAMKQPPCIPTFATTPDGKQVPSSKLCAFPDVGCECRNPGVGIGNPSPS
FPIYYSYQKCSDTSVRVAYNFDWERVIVVFTKAADGNWRPDQLFLSQHSG
YGRLQWAKIQNTINREDAAEPRGGKDGRQYLDHPKVYVEWSKHAHRDTRN
TGWNDPLSQLTDNAFRSNDWWYFPKRDDYIRADRSTEIGRLLGSFDWGDA
SSNPPSVHDGLCTA
>Mb|QLI63482.1
MAPSNQLGKRVKLTQVRRPFIVGTTAIPFSETNPKPPGTPDNHTHSWQVF
VKGLEDTDITYWLRRVQFKLHESIPNYVRMIEGEQGKPFVVNETGWGEFD
ITIKLYYVNDSGEKPQTMYHYLRLHPFGRTEEEKQSMVTKNGEVRSWSYE
EQLFNEPYEAFYQILTSGAVPKGWKPSGASGKGKGKGKNRAPPPLPAPDS
GEVWEHTAMIPNHNRPGQPFSRETEAAEVRKLKEAQTKTEEMAAQILSEL
KAKEELLAKLKAENQAAAAAAAKPA
>Mb|QLI63484.1
MADVDMDVDRPASPPARNESEMQTHTKATAVRSIEGWIVIVTNVHEEADE
EAIQDKFGDFGEIKNLHLNLDRRSGYVKGYALIEYATLEEARAAIDGAHE
TKLLDQTVSVDFAFVRPPPGKGGRGGRAGGGSGAGGRRQRSRSRSPAAKD
E
>Mb|QLI63488.1
MAGNARSGAGSPSHDLAGSSRLFSKRAREIQAQEGIPGLPLNPWGGPPTS
GNSTPLRENIPESPTDGFPDFGHLPTPESMPHTGRARAGTVPSRLPGGPV
NGLLNIPGLTVKSARVTPAQSPFGKSPSPNLDHSDGATGGSALLSRLRAG
SMPQRSQFSTLPGTTSPFGPSIFSSWNPAGVGRERGSTLASIASVGSNGP
SSPSQSQFSKEGGGDSDVHMRTLDYLGLAETPQPPRAQLATPYMANLDFT
RQSNRFRSYSVNNKDRYADEEDDYDVDGLPMESQYVALQDQLAATNAAIH
SHNLAVQAFVNQATATRPRARTAGVLEAPGNRLSRAHIPSNVGIPSSVAP
AEIRLQDGKEYDDLPQAIAGMNLGRSNSRNAGLLNAEEQGLEGPTSALWL
GGIPTSTTTSTLIEMFKCHGPISSARVLTHKNCGFVNFERVESSVAAKAS
MNGKEIFPGAGPIRINFAKPPSASNTPGHDGVFPSPSPDPFAKGQDNASG
PGLTTSTDGTLTVNGGTSTPTVPPMADLAEDMMEIVTQFGATDEDKAAVS
QYIQSATHYTAYVEEIPPIREPTHTRVHDAPKLRDIRKRIDNQLLSKPEI
ENIAVDMLPEIAELSSDYLGNTVVQKLFEHCSDQIRDAMLAEIAPHLAEI
GVHKNGTWAAQKIIAVCKTHEQKTTIVEHLKAYTVPLFLDQYGNYVLQGC
LNWGPPYNDFFFEGVVSRMWEIAQGRCSARAVRGCLESKLGTRAQDMVVS
AATAIHSAQLATNSNSALLLTFFLDSCSFPKKRTVLAPRLVPHLVYLCTH
KVAYVTVLKIINQKAEPEARDIILKALFFSPNDQVLEAILHDPNCGATLI
FKVLTTPFFDESTRTQVIENVKTVLLKIKAQPNQGYKRLMDEVGLSTRAA
PTRENSSTSEGRQRPTSRQANNRTINGQHAQGGQSNNQPFFNALNAQPSA
AGLETGYGNQRGDTADANVAAFQQYGQGIMYGTPAGPMAPAVSLQQLQYQ
QNMLNRPTPQMTYSYQTMQAPYATYGAPPAPAVDQYRQPNLANGSPIQPP
TSQMPQIPTGQPPYAPPGFGVGLGGYSYGNMGGMQSMAYMPQDQSNNRRG
GRR
>Mb|QLI63490.1
MAPDIPKTVTQWNVTSHDGTAGFDALAFSEQQVPRLGDSEVLVKMHAASL
NYRDLIIPQGSYPFPVADNVVPGSDGAGTVLAVGRQVTRFQPGDKVVTLF
NQEHIGGPITQDNAETGLGGTRHGVLRSVAAFNEQGLVRQPAGLSAVEAA
TLPCAGVTAWNALYGVADNQVAPGQWVLTQGTGGVSIFALQFAKAAGARV
IATTGSNDKARVLKDLGADHVLNYRENEDWGAEAKRITGGRGVDHVIEVA

GPKSMAQSLKAVKVSGLISIVGFVGGHTKDQPGFLECLTNLCTTRGILVG
SRKQMEDMCLAIEGNPERLRPVVDKKSFALEQLKEAYEYQWSGQHFGKVC
VTIA
>Mb|QLI63491.1
MDAPTPQIWGLGLKPKISLNLPPSFVIFFPAPDNGTSSYGKNSTGLRYTN
LSWVGVLRAPIPFQREPAWHRVPTEYLPSQNTNLTRIVMRRTCHPQPRLY
GTRFFVSGNSEIAATKRIAAWTEPTKCKMPPKKSLRLKSFKPNEFLIAFC
GGPESEAETTHFFMKAKVDIANGILEIDPELLRAELRFLQTQTHRSTIPA
KRRIAGNDCIETHGLWGGYKLITYTEVLHLTRVATNRGGTRITFLEGSIT
DVAYNFKEMGCYSTRERVAICGERPLPVIADEPEPLSMLELDKFQDESGK
SPTKKSPLPIEPLKASPRYLSDTVVKLMPATSPQVAISPVKSLGSPSTPF
VDSPCSIEAAQSPSPLARVIAHGAPSSPLADIPCLSPGPTKHFCAPSYDS
SFSNLNNTTNLCPSLQLSPTKVSEPIAQTPSKVSKAEPSTPLQTATPLQN
GKAGLGLVESIPTSTPKLDKSSLQPISSIDSAQLKFGPSTSCFNAPTWLS
STITSRAKQTTKKITRRNSEPVTRSLVRTQIARRQTISPRKYDFTSEGVF
VAQHEVASDGHNPVPMNLGRTPEKPAFSEPASTMTTPAISWGKMTGRKSG
DGVQSTPAPKRAGNKDGMRKIDVRRHPNIFRASGPSPVSSPLAINRLAEM
AVECCGGQAKVMVTEENGRLVVRFKLPTEYTYLFPNSQGMDESRFTTTPS
CISSSPRITFEEVNELALTNGQDSPRTEQAISPSSMHNEHIISAVSDLAS
PPVVTVEENQGPSIRTPSPETKLNTSFTPVNRSISHDEAVSQTSPASAHQ
EPHAASSGNSTQHGSQPAQQNHYEDSPGRDYMRDFIKRTHRNRLSTTETG
SPVAPAVRRAPLGIKSPNVESPQKGKRKADTENQDADSSLKTSIAPLPKK
VRRANNPGPEKKSLPDPKGKAPEVGTQNNDTKANPIAVTNGGTTTDKRSE
EEQAETGAGYRRSTRLRGQQPGYTAKSSIPTPINLRGRPNSGRAGFNRTR
NEEKEISNKTRKNTLKNRGNAEYPAQVLARYQEKGLGEESGEASRDPEEP
TKGANGRKCVGWKTPLEAHQGEDAKKSRIATMKGKNIAKASGSGIVKPTR
TMTAQKQQRTAKVAATLGMSQNGTPAKPSRVTRSSTRVRT
>Mb|QLI63492.1
MASNAAAMFSFAQTEPSTDLLNGQSWGSTSESHHNFQDFPDNGSSHSGEA
EDYLFTSGHTTPRGSRLEQAASMDSVWTNPRTTQSSVMAQAMARADSSRS
SGSSLSQHAQLSRGNVSAFRNVSHAAATTAGTMAGMNSCLLVAADAHAVS
SNAQMYWPEMGLDMNIAAGGGSAGGSGAFAVTQAGPMHMVPAHMHLGPES
VLPDNSSPGSWDCFSSSISRTSSPATVDDVWLPSALSPNSSPEIVGDSPR
YVEYFNANTEQTSYASLISSERKVSMASDKDTIVPKIEEGVAMPHGYPSR
RHGSDGESSARDHRLYKNVTPGPDGLFHCPWEGQTSCNHKPEKLKCNYDK
FVDSHLKPYRCKADSCEGARFSSTACLLRHEREAHGLHGHGDKPFLCAYE
GCERAVSGNGFPRQWNLRDHMKRVHNDHGSSSGSPPATANGQSAKGRKRK
TEVAEPQNGTRKATLKSMPVADSKQVSSKPLLEQWLDQRRAVEDLCRSLS
KPDDDRNLQQIGEMQKRLSIMAHMASSFHSTDIRPDTNRRNYTTG
>Mb|QLI63493.1
MSFKGFSKSLTRAPQQFKHKFNIGEHTKDAVYIDAERRFQELETETKKLH
DESKKYFEAINGMLNHQIEFSKAMTEIYKPISGRMSDPDSIKPEGNPEGI
RACEEYEAIVKELQETLAPELEMIETRVIRPATELLDVIKVIRKTAVKRE
HKKLDYDRHRAALKKLQDKKERSPKDEKALWKAEGDCEQATQDYEYFNDL
LKDELPKLFTLEQEFIQPLFQSFYYMQLNIFYTLHERMQRCDIGYFDLTR
EIEEAFEEKRGDIQEQAEALSICRFKTTGRPRPIRYGARPALEGGKQPGL
LTAGPSTSSGGTSPKISASGRPASDSEAAPPPYSPGNKTALSTIAAAKAK
PPPPKPKPKRLTAAPAAETVTAMYDYSAQAEGDLSFRAGDVIEIMSRTQN
ENEWWTGRLNGKEGQFPGNYVQLG
>Mb|QLI63500.1
MTHEQMLDNAQRVPSNSLRGPRGPGLDYAAELENRMPIRQEKVDRIEKLG
RRESKLGLRNIFGRSKAARDDQQPSTTPATLKKTNFRASMARVSQPPLVY
QYDSNLPTNFSWKPLAATAEDPDAEQSSHRNVPPARKKRSNSRVSGQPDD
SSTNWTLPPLFKAFPQAVHHATLPTATISADAILRINEKKNSLQTKEEED
LADGAGNDHLDADMDKHRKKNNKWELNAPATNFEWTTKIYVLVTSGYLLQ
YSRNGTFDRLPEKILRLGPSSAAFAADAIPGRHWVIQVSSVADDYDHQGL
ESRSIFSKLSFRLTERRNASNLLMVFENAEDMEGWLAVLRNEIEKQGGRR
KLSETGKPKPAGADPYVREHMSQRTLIVRDPSRFTSSRSRASQSIMSPPR

ETRSSETFTRQTSTDTARHQSLDDISTTNSMMSQDERQLESLRETSNRLS
FVSCGQRTFVTSTVSSPDGSPTHDKFDEGIFGHHESSGRGEVRTRPNASE
IVTRRESVQATAPFIDDGLAPKGKMRSLPTCVTDVHPDRVNSPLGVKPTP
NFSVPRSSTRRFSYARGATSTTDGSPSRRRDRDTSPRRFGRTLPTAMQSA
HRLSVVIDQGVSRAIATERLPTSPKSPPLPTLPTMDVGQRRLRSVSRGRA
GRRGDDQVSQASHVPDSIPERSSWRLSPRKQSSTGALRQGIPSPEPPLAV
VNRRPHIASMVVPTGPPVIDGPPRGHRTSFLDIGESTGDMIRSQSLEPSR
AAKRASMCSVFSERSSRSYEVHDSTVSSHHSSDSSATITSESQPTSGHYL
NVEKTPRTSPMNRRSMPQIAVSGPPPAPPPTRALPPIPQKMQIKT
>Mb|QLI63501.1
MASKNPTVAVSAPGKVLLAGGYLVLDRRHTGLVFGLSARINVVAGEIHTS
QGVQLNEIMVDSPQFPDAQWRYGYHLAPEGGGIQVTQLQVGSVICKNPFV
ETTLSYVLTYIHRLMSRVSSDHSLRPARLIVLADNDYYSQSTPRSGEPGR
FAKFTVPLGGANKTGLGSSAALVTALTAALLSHYLPWRLLDITSAPGKRR
LHNLAQAAHCAAQGKVGSGFDVAAAVYGSCLYRRFSPGVLGQLPEAGSPG
FAEKLVSVVDGVAWDVEVLTEGLRLPKGLALRMCDVDCGSQTVGMVKRVL
AWRGRDADVSSRLWDELQARNEDLAAKLRDGNVADIPTAVGKVRELIREM
GKLSDVPIEPESQTELLDALGALEGVYGGVVPGAGGFDALALLMKDDEET
KRRVEERVAEWSREKDSKVRLLDVKGEMEGVRCENLDVYSGWIEIHDKD
>Mb|QLI63503.1
MIRQPWRAASRRLLLLPPQPSPRFRFSSSTATAASQEQPSMTPAESAIAD
LLRAKLQPTELLVRDVSGGCGSMYAIDIASPAFKGATMLKQQRMVNAALG
DVMRAWHGVQLNTRVSE
>Mb|QLI63505.1
MAGRPTEGRHGASSGNRDDLLLDLDNDQPLYGGGQRSALNDDDLMRRYSH
DQEEPQTRPSVSYDDFVGASTSSATRSGARGTPPQGQGSTSPYMSRQYSQ
TSELGNYQRYADDFEDYPVDGDSYYQHGGSIPTNARSTAARSNARNRNSV
LSLGGGFFGRVKNKLGMGQGYSEMDLPLTEGGHERGASSGGSPPDQRSKG
FDMGNFKFGFGRSKPDPSTLGPRIIHLNNPPANSANKYVDNHISTAKYNI
ATFLPKFLLEQFSKFANVFFLFTAGLQQIPGLSPTNRYTTIAPLLIVLLI
SAGKELVEDYRRKQADNALNTSKARVLRGSSFTETNWINVAVGDIVRVES
EEPFPADLVLLASSEPEGLCYIETANLDGETNLKIKQALPETSSMVSSSE
LSRLGGRIKSEQPNSSLYTYEATLTMQTGPGEKELALNPEQLLLRGATLR
NTPWIHGVVVFTGHETKLMRNATATPIKRTKVEKKLNWLVLVLIGMLLAL
SVVCTVGDLIMRGVNGDSLGYLYLDKIDNAGTVVKTFARDMVTYWVLFSS
LVPISLFVTVELVKYWHGILINDDLDMYYDKADTPATCRTSSLVEELGMV
EFVFSDKTGTLTCNQMEFKQCTIAGLQYADEVPEDRRATGPDDDTGIHNF
DRLRSNLKNGHDTAMAIDHFLTLLATCHTVIPEMDEKGHIKYQAASPDEG
ALVQGALDLGYRFTARKPRSVIIEAVGQEMEYELLAVCEFNSTRKRMSAI
YRCPDGKVRIYCKGADTVILERLNDQNPHVEATLQHLEEYASEGLRTLCL
AMREVPEQEFSEWQRIFDAASTTVGGTRADELDKAAEIIEHDFFLLGATA
IEDRLQDGVPETIHTLQEANIKVWVLTGDRQETAINIGMSCKLLSEDMML
LIVNEETAAATRDNIQKKLDAIRTQGDGTIESETLALVIDGKSLTYALEQ
DLEKLFLDLAIMCKAVVCCRVSPLQKALVVKLVKKYQKSSILLAIGDGAN
DVSMIQAAHIGIGISGMEGLQAARSADVAIAQFRYLRKLLLVHGAWSYQR
VSKTILFSFYKNITLYLTQFWFTFQNVFSGQVIYESWTLSFYNVFYTVLP
PLVLGILDQYISARLLDRYPPLYAMGQSNSSFKLKTFAQWIANAFYHSII
LYVWAELFWYGDLIQGDGKIAGHWVWGTALYGAVLLTVLGKAALVTSNWT
KYHVLAIPGSMAIWYIFIAAYGSVAPKVNFSMEYHGVVPRLYTSPVFWLQ
TVVLAFMCLLRDFVWKYAKRMYMSKPYHHIQELQKYNIQDYRPRMEQFQK
AIRKVRQVQRMRKQRGYAFSQADESQTRVLQAYDTTKHRGRYGEMASSRT
PAR
>Mb|QLI63507.1
MLSLQEAAQSPPVGGTGGRSRYSKALPSVPVADSQGVADLPPLPLPKDVS
EFQFPLPPRKSSRANDNTTGGKSIPRKPVGSSASKPAAPPSGASNINSPP
LSIPPPGGSMAMPREAVARAPEPQAQPVLIVPPESSPTDSLSSLLSAYSR
EPDAPMSGSTYTTASTANSFHDSHLSPSLGKDGPSAGSNTYSQTQVPAVK
SEQGLEHKAQSRPAPPRKDDSSHPSSSSPKLLPVAPLSEKSSAPRPEIWK

RRPQTAEKNKELSDLKLSHSHGSTASASTIQSIETAVLLAYANIGSEIKP
AEEPKQDAIPPPPALGLPRHNVKPVSQDKGQETETQMGSIGSKLQDKVGK
AQKSRTPVDRKDLPVRRDSKRPPTPEYRKGDASPPSSRADTIAKPASPVS
ATGSPKEGALSTASKSPPPQPTAPSKSEPVAPSLVSVPPIASTSASRLPQ
AAPDLRLSTNLLEMKRSSPPAKTQSLTMNDSNRGISISSDSVFSRTSSGQ
NVVGASAEEPSRPATATGSFDPRIVTSDTHGPMYRGRDGTLYPEMKVVRE
PDPRAAYFPIQTDKPLQPGAIISSRPLNHTHYNCFQKHRTMNRRSNRNYP
LTCQTCDKADTEDRWVCTFCHLRICETCLQGLNGHQKVLRRLVDQLRADT
PVSLSSASPPGSALGIELPA
>Mb|QLI63508.1
MSSSKTAAKSAPPAKHGYEFFGPPGAFGISFGLPILVYVFTFVCNDISGC
PAPSLLGPRPLSLEQLKIEVGWPQNGIWGLASWEATGAVLAYYLLNLILY
RVLPATEVDGTVLSSGGRLKYRFNTFYSNTATLAALAAGTVAQGVEFPVW
VFISENYLQILTANIGISYAIATFVYVRSFSVKPGNTQFRELAAGGCTGN
LLYDWFIGRELNPRVTIPLIGEVDLKEWCELRPGMMGWIILNCAWCAQQY
RNFGFVTDSIICITIVQALYIIDSWWFEPAILTTMDITTDGFGMMLAFGD
LVWVPFVYSMQTRYLSVHPLSLGPAGLAGTLSLISLGFYIFRSANSQKNT
FRTNPNDPSVAHLKYIETNTGSKLLITGWWGIARHINYLGDWIQSWPYSL
PTGLAGYQIMAAGAGAAGQGAYIMKDGREVIQGNARGWAVPITYFYIVYF
AVLLIHRERRDDEKCHRKYGEDWDKYKKIVRWRIIPGIY
>Mb|QLI63509.1
MYGTGTGPQTGISTPRSSASLRPLTLIHGSLETSFLVPTSLHFHASQIKD
RFVASLPTATDELAQDDEPSSVAELVARYLGFVAKEVEDGEDDAQGSYEE
VLKLVLNEFERAFLRGNEVHAIAAALPGIEMKKLEVIRSYYMARAVSNRT
IKPHESALFRAAGDGSADIYTIFGGQGNIEEYFDELRQVFQTYSSFIGEL
VTSAAEQLRTLSTNPSAEKMFPKGLDIMNWLQHPDSTPDVEYLISAPVSF
PLIGLVQMAHYEVTCKTLGVHPGMLRERISGSTGHSQGIVMAAATSAADD
WESWREIVSSTLSILFWVGTRSQQAFPTTSMTPTMLRESIEHGEGNPTPM
LSIRDLSQQEVQKHIDATNQYLPPHRHINISLINSPRNLVVTGPPTSLYG
LNSRLRKVKAPTGLDQTRVPYTDRKVRFVNRFLPITAPFHSKYLAEATEM
IDADLKNISIDAEDLGIPVFDTNTGKDLRTEVKGNIVPALVRLITRDPVN
WEKATIFPDATHILDFGPGGVSGLGTLTSRNKEGTGVRVILAGTIEGTMN
EVGYKPEIFDRDEENALRYAIDWVKEFGPRLVKTSGGRKYLDTKMSRLLG
LAPIVVAGMTPCTVPWDFVAATMNAGYHIELAGGGYFMPGMMTDALLKIE
KAIPSGRGITVNLIYVNPRAMAWQIPLLGKLRAEGVPIEGLTIGAGVPSI
EVAQEYIETLGLKHIGFKPGSVEAIQAVVNIAKANPNFPVLLQWTGGRGG
GHHSFEDFHQPILQMYGRIRRQENIILVAGSGFGGAEDTYPYITGDWAKK
YGYPPMPFDGCLFGSRMMVAKEAHTSKDAKQAIIDAPGLDDSEWEKTYKG
PAGGVITVRSEMGEPIHKLATRGVRFWAEMDQKIFSLPKEKRVAELKKNR
DYIIKRLNDDFQKVWFGCNKQGKACDLEDMTYGEVVRRMVELLYVKDQKR
WIDPTYVKLTGDFIHRVEERFTSTAGKASLLQNYADLNEPFSTVERILSH
YPEAETQIINAQDVQHFLMLCLQPTQKPVTFIPSLDDNFEFFFKKDSLWQ
SEDLDAVIGQDVGRTCVLQGPAAVKYSTVVDEPIKDILDGIHESHIKMLT
RDMYNNDEGSIPFVEYFGGKLVESEIPLDVEGLTVAYDTHKNTYRLSFAA
SASLPSLESWLALLAGPNRNWRHALLMSDVVVQGQKFQTNPLKRIFAPTR
GLFVEIQYPNDPSKTTIIVREQPRHNQYVDVIEVKLVGKNEILVNLIKDT
TALGRPVAMPLKFTYNPEAGYAPIREVMTDRNDRIKEFYWKAWFGEEPLN
LDALITSTFDGGRTTITSEAINDFVHAVGNTGEAFVDRPGKIVNAPMDFA
IVVGWKAITKPIFPRTIDGDLLKLVHLSNQFRMIPGAEPLKKGDEVATTA
QVNAVINQEAGKMVEVCGTISRDGKPVMEVTSQFLYRGKYSDYENTFQRK
IETPVQVHLATTKDVAVLRSKQWFNSDELPSDIDLLGQTLTFKLQSLVRY
KNKNVFSSAETRGQVLLELPTKEVIQVASVDYEAGESHGNPVMDYLERHG
SPLDQPILFENSIPLSGKTPLQLRAPSSNETYARVSGDYNPIHVSRVFST
YANLPGTITHGMYSSAAVRSLVETWAAENDIGRVRSFQATFAGMVLPNDD
IQVKLQHVGMVAGRKIIKVEVSNTESEEKVLLGEAEVEQPVTAYVFTGQG
SQEQGMGMELYGSSPVAKEVWDRADKYLLDNYGFSITNIVKNNPKELTIH
FGGPRGKAIRQNYMAMTFETVAADGSIKSERIFKEIDESTTSYTYRSPTG
LLSATQFTQPALTLMEKASFEDMKSKGLVPRDSTFAGHSLGEYSALAALA

DVMPIESLVSVVFYRGLTMQVAVERDAAGRSNYSMCAVNPSRISKTFNEE
ALQFVVNNIAEETGWLLEIVNYNIANMQYVCAGDLRALDTLAGVTNFLKM
QQIDIEQMRSNIEEAKDALRKIIRGCAEATLKKPLPLELERGFATIPLRG
IDVPFHSTFLRSGVKPFRSFLLKKINKTTIDPSKLVGKYIPNVTAKPFEL
TKEYFEDVYKLTNSPKIGAVLANWDKYTQEDESKAVSDSSSSVGYDGPGA
AA
>Mb|QLI63511.1
MANQADFKNREFLAVIGDEDSVTGLLLAGIGHVTAGADAQKNFLVVDSKT
DTAAIESAFDRFTQDRNDIGIVLINQHIADRIRHRIDTYTAAFPTVLEIP
SKDHPYDPEKDSVLRRVRRLFGE
>Mb|QLI63512.1
MRSSIIASLALVPWATCHQQPLKDAKHRVPTSHHTVTTAAPSYRQELLSL
HRQLISIPSISGDENAVGNFLVDYLTARGYHADLQPVAARDGTPPNKQRF
NVLAWKGERIPSAKVVVSSHIDVVPPHIPYSISPGEITPETMIKGRGSVD
AKGSVASMIVALEQLHASKEIANRGDVMLLFVVGEEVAGDGMATFSDSLS
RMEEPPKFDAVIFGEPTENKLACGHKGGLFCDITARGVPGHSGYPWLGKS
ANELMVRALAKILDADLGSSELFGNTTFNIGRFDGGVAANVIPEKAVVKF
AGRVAIGPEDKGHEIVTSKIQRILDEVDDEAFDMDCTHGYGSVECNCEVD
GFEKITVNYGTDIPNLAGDHTRYLYGPGNILVAHGARENLTVADLEEAVE
GYQKLIVHALKQ
>Mb|QLI63513.1
MHIKDMLAEAEKGGQPSFSFEYFPPKTAQGVQNLYDRIDRMYNFGPKFID
ITWGAGGRIAELTCEMVLQAQSVYGLETCMHLTCTDMGLDKVNAALQKAY
KAGCTNILALRGDPPRAQEKWTAAEGGFHYARDLVKHIRATYGDHFDIGV
AGYPEGCDDNKDEEQLLDHLKEKVDMGASFIVTQMFYDADNFVRWVGKVR
ERGINIPILPGIMPIATYASFLRRAKHMQCKVPDHWMTALEPVKNDDVAV
REIGKTLVAEMCRKLLANGIYHLHFYTMNLAQATRMLLEELTWAPCAERP
LQQALPWKQSKALGRRDEDVRPIFWRNRNKSYVLRTQDWDEFPNGRWGDS
RSPAFGELDAYGIGLTGTNEANRKKWGEPTTVQDIAGIFVKYLNREMESL
PWSEAPLTGEANAIKDNLIQLNKRGLLTINSQPAVDGVKSSHPIHGWGPS
NGYVYQKAYLELLVPASIFTEMVKRVEDKPTLTYYAVTKDGELKTNAPSD
GPNAVTWGVFPGKEIVQPTIVESISFLAWRDEAFRLGTDWAHCFESETPS
RVLLDQIMNEWYLVNVVNNDFHENQTIFDLFEGLEVKGLDTPVAV
>Mb|QLI63514.1
MEHESVPLVTCGQADDGTETQMPKTQLLAQHFANPPAACSRPSSNRVFYS
SAQWTTDGTTIITGSSDNSVSSFVLPADLLDFDSGTRQLEPQAKIKLPEP
TQVIAPAPFFSLSEAASQTFLVGCRDHPLHIYHAFPDDPRSAPIGHYKLI
RHETEQYIVPASLVWQYPGTHFICGSANRLDYFDVSRHGSDGPVLTVPTI
PSKRHISKGHGVGMKGTVSALGVSPPDVNGHPLVAAGTWTRWMGLYDLHR
SDGAVANWRISESDLPGSSDSYAGQGVVQVLWSPCGRYLIINERHADGLL
VYDIRGSGRLLSVLAGRKNSSQQRLNCDVFPGSQYGNGGFEVWAGSQDGS
VIVWDQVGLTAAEMQPSWSWNPHNSPVCASILHHSGSVVATCSGGWEHSD
VNDEYDAVDAYAAQGRSCKILEESSLKVWSLGGGVVG
>Mb|QLI63516.1
MTTPASSAYPSTHASEAEVDDVGNIPIASLTLDTPLIPLRRKSRVPEKPF
RFLDLPSELRVKVYEHYWSNAEKVLDLDPGNHKRYHRALGLVRTCKQVRA
EATHFFFSSRAIRLFPTFPGKYFKSRKPLLARLKPSQRRCITSLELRLGP
GWNAPPRGWVVNDALGLKDCSNLRKLTVYVECDPSDGIYEGFRRSEGFYE
GFCRNLLGDVLGEIPGHVAIEFEGYPGVKKSGAMMRGLLEVAAQSKRKIL
WGPERGWTDGPEKEEKRNADAAAQLFDGIPREGYAPHNMLVVA
>Mb|QLI63518.1
MDSLPTEVAETIQAGHIRSHPDINFDIAPSTAADKREPVTLHSPRLSDDD
LLNDIEDDEEDIPYSVLHPTRKSHSLPPLPDLRFEQSYLRSIANADTWWK
VLLITARDQVMMPLAQGLIYNLLLCGWQHWNRTAKLHGNTAGSRVRRWWY
GVNNWTIQKRKL
>Mb|QLI63519.1
MAQAESVAYAPTGAGLSSTNLSSEEIFEIQQYRRIVQLRDAILSGKHPTL
KPPPGSNTSLEPLSLRKTALEPRSEAKQTRGSNGNGDGNGDISSAAFTQP

NSGPPELNPIFLEKSESLVRAELQIQRQRLERAIKEEVEQRRAAKNGSAE
LIPDLDLSDVLSRALVLAQTTSVPITHGENLTANAEATSDSLDDSTFYSS
RHDTPESHLTSRIRNQSEGIQTTNSQELDVGEAPQQAKNLSPRPHDDNGG
TTTGTCPPQLNEINHLPETASASRPPFASVVPGLNNYVQGASQTRSNVAS
QSGEKDHLDVEAAKDHEPSNASHHGNNEHLDSRPPSPLVRNHTLQPVAPQ
PTHPSALSALATASSTVHDTPIVGGRSGMGTLAQVAALRTESNIVISPDS
SSQGGKKKGKKKKKRKADRQAPEPEAAPYIKPEPRSPSPLTAPSYLRPTK
RPRHEQEPPVNAQHEPRYVQDDGVQYITRQYREEPIPVGYARPAAYHQPQ
PGDVRYHSGEYYDVRQVRGEDLGPRPAQVEHSTMSYPARATPVSRSISRV
MVTDPYPAPSRQYREYRDGPRMTGHTEGDTFIAPSRSAHTRILVDAHGRE
YIEPLHHSPSRISATSPSRHGEHEVLYERLPPPRAVSRHPAPGPYEDDGV
VYASSNQTYVVPRRVVTQPEYVSHDYRDVWKRETSARGPPPHGGFVQVLA
PSERRYIEETYGGGQSSMRPVETVRYQLPAEYGRVPSVRPEAQGTTDFRA
SAHPDGHHEVLQPYMREYRPGQIQEPIVQRGFSARPGDRPLNGQTRAGEE
IAFIERPRGATQEIVYADDMRREVYR
>Mb|QLI63520.1
MSWQAYVDTSLVATGHIDKGAIVSAAGDSAWAASTDFQLKPEEMKAISAI
VSGDAAAKDKAFAEGLYIAGSRYVMARADGRSIYARQFGFCFCGQGRLGV
AIAKTKQAIVVGHHGETGVAGNASSTVEGLADYLIGQGY
>Mb|QLI63522.1
MHIIQPPFDHTVAPKHAASSALDQDFSFPADSRLPNPPFVFPASPASSSS
PPVSRQRRRPVSMVDLQNRPPVSPTVESPSRAHRPAALPQFSFNPGAALS
TGLESTTALSPPITPNSARALPSPLRPAGHGHRRGGSEFVGGSIRDGSSI
AIMSTSPIKSESGFASPGLQPPRRGHRRGVSGTISTNELPILHPQPDLLR
PKGSSAPNSPTAFNPKEGALLPIDHQPPPLPEPANPIAPQPFNTTEKNTI
SNHSVTISSSLPRPEPSLRQSRPGRARVGFSDTLEFIPRPLSLISSDTSS
TMTARPGHSVSGSISSLVSAASPGGRESPASLARSPTRETVDSRPSTAGA
ILERTTDFKAASGGISTSPRRRNSIPTLLDMANSQPTSATPPSPTKTPKR
WSFFGLEPFMGSMPSSKQRSCSSSSSESISKTCPGGLSSDHESSSSGDEG
TVGDSSTSTTKKDRKKSSKKKRVKGWAGSILPLRNNRKRPKMHAVRPPTP
PASVGPVADDEDENEDCEITQKEMISAPAPPIVTVTESPPLEEPSVPQRR
SEEESSYPMIDLDAALGPFNTPLPQNPEWDAAQRAAGIVNKRRLHSAQGL
KGFSGPGMHYHRRTESAPDLPPFDPGRAGISRFGSSSTMADVFEEEEEEQ
GEEEEEGEDDLKEAEVALDEVGTQKNDDSDGEITPPATELVPADSLILGV
DELEAAAVRRKASSGTCNTDHSTSAHAVRAECSRTSLHEDVINEEPVPMI
FRGGPVFPGLPADQHGSTSSSPRMPPGQRDLSMVETNQAMPTSPYSTSHA
SSYPSPRSPMSIDAQRISTAPSSVTDENSFHSLLMGEPGPEVRISVDYDI
PSLTSSNSTMTRDSAFIPSARMSQPSLREQRPSSVSSAAFGRRRSSLVSL
SRLISSSHGERSKLSMEVTLDNETEGKRSRSKSSKTRRLGRMMQFWKPNK
ESSPT
>Mb|QLI63524.1
MKRLQRCEAGGLPCEYTPAKRRFTNVRFQNASDAAGSSSNANNAPEANLS
TSGTASRSDTVTSPASSTGSTNHNHFALITEGSNLTSEEMLVRRDILLRH
VDAYMDNMYWLPCQGFLHPDTTYKEIQEGTFDPVVAAAVCGITAVFVSPS
DSGGEFGVKCSAHVEMYLFQNIYKFSDDLLVLFALSITFNLIRGEFAKLW
QSFGVASRLMLGLRVNWDVLPQNQTFAQQECLRRIAWQLFYLDRMLAGGY
EEYISCRAENMQIALPCSEAAFRENRPINAERLYDKPGSHPSTINLHGYQ
LRLIDLRHRIQVTTKRLCSSSSPSQSQLDASKVMADVNRLQNELTRFHMS
LPPEILLSDQTVAKYMASPERAGYVFLHTHLAVSHIDLYRFSLPGQRDKV
SVEILRKLPREFIARSQKQAVAHAMCLGRFCDAIQNEVDQMHDTGKLKLA
GDHSTFQMSTQCVRVLLVALQYGLYRDIADVTTAPLWRSVEVDEAHIRFL
IDSVQRVTRPWCDILNVAQQAYNHNATLVEDFSKTRKVADQRAAVSMFGS
KSEGSTRLPGPDAILESIATGKPEEYHRRMASHSPSDSTRWLGSHPSSPT
PNMFATKAGTQTTEASQGDAAPGIPLFLAQARNRPGSATQDPLTYDAENS
LMPMGDLDAVLPDTNPLMNVEGVPMSGMHSGDPSMFQQAIPQPSAGFLMA
QQSVFGGDVFMEHYMNRQHGQGGYSQPPGGY
>Mb|QLI63525.1
MEEPNGHDMAAQQEAAKDYRPKLQGPLVGEKTPSEVITNEYAKADQVYVE

KTIALPQTYSHYRPIRGDGNCGWRAIGFSYFETLIERGDQAQIEGEFARL
KSLNHLLATVGEYNYFEDFADEAFDLIRELARIIDKPAAAHSLLHQKWND
VAVEGSLIFYFRLLAATFLKANAETYAPFIPGGQGVDEYCSHNIEVVNRE
IEHLGIVALANILLKPVNFVLEIAYLDRSTGSQVNQYRFPEEANDKDVSS
LGTFIYLLYRPDHYDILYRTPTVTASPPPVSLQVNRVTGFTHNTAFNTTG
ASMGAFSTVDFGALSMIPGLSTGSSDGLAGLMPMPAVTAPTNPIIGDAYS
SSQQAVWMPQYGTDIQSTKGESPAQPPPAVATVQHPSPSPLLTPTTPMSS
NAPMMPPSTNLGPQQLPSHMATPGATGYPIRFSTHQLEYENNSFPEPTFQ
VTTNTFKNSVWNRAHYGNPDFQPEEWNPDEDGAESRLAGKRKVRKDSS
>Mb|QLI63529.1
MAEAAKAVARQLPGFRLGQKQVFLYDTNPPSPIPKTRHTRPCLLKLIKNL
FPFAHSPNHVITFLRKEHLPPNEACFQVPLRFTKFDLRDYLWNLYNVEVT
KVRSYVKQQPLTQRNSHSRSWYRPQPLKMMTVELAKPFQWPEAPTDLEPW
NHELWKMREDLMEKRNEDQINQHKFEIAMRSKEEMSKERKELKGLAERML
RGEVKWDNGVALDPKWDSVLKEAQRKDAAA
>Mb|QLI63530.1
MASPKVFIVTGASKGIGAAVTRYLLDQSHKVVVTARSAEPLEGIKNAYPG
QVEYVAGDMTSPEMPDKLTSLAVTSFGKLNGLVINHGLLVSSRLENTSID
TFKNLYNVNVFSCLAMAQAALGELRKTKGCVVWVSSGAALKPYTGWSAYG
SSKAALNSLSSHLAFEERDVTSITVSPGRVDTDMQVQIRSSGQQSMDKAQ
YDTFIEAFEQGKLLKPEQPGNVIAKFVATPNRELSGKDLNWNSAELASYR
E
>Mb|QLI63531.1
MPPIPPHKHPLPRIITYYQTHHAPSGEHISVLPLLTQPSISLTHLILAAC
HINASPSQLTLNDHPPSHPRFQTLWAELRVLQASGVKVLLMLGGAAPGTY
QRLDSESDQFESYYAPLRQLIRDKNLDGVDLDVEEPMSLAGIIRLIDRLR
ADFGPGFVITLAPVAMALLDPEKNLSGFDYEALEVMRGKEIAWYNTQFYC
GWGNLTNTLMYDLMLQKGWSPEKLVVGVVTNPANGSGFVPWEMLSAILAI
LHRRHRNFGGVMGWEYFNSLPGDASRPWEWAGNMSSLLRAHSLGPVPTPA
GTDIQPSVDGEVDPDPVNNRPLEVPKSFDYYSDGTDHE
>Mb|QLI63532.1
MDVSVLPKRDGGSGGLSLGGLLAISLCGGIVLFTSVGLFMAWRVKVRQRR
TTKRMYTMPDEYSKTRLTKRPLVMTESAVGRLPSRLSSRFSLTLPPIIPL
PPMLSYNNLNFFRSSSKRRAERKSWLNGEEFHGPSVKKDSKDGWFSRDSW
IRQVPTVPNLRAAEEGGDGIRDAGRQNSQHRPSYEEYRKQQQQGEPYQFQ
NHPPYHQEPQAIHVQKRRSGSTLPTSQTTPNLSSQNDVPRGRQSAVRTPA
QAHVRPSVTMTEMGLRDILRSTDQRLREGSSRSPAKDTPQPSPTRTSPAK
TPSSRKSESGRAAGRHNRGTPSPSKSGNTASLPTQASAASIGSVANSLIA
EATQELKLPEGMPSPRRLRGREWEPQDHQILPPSLPGLESPSHRSDSPQR
SPQRSPQRSPQRSSPQKSPSKRQSADSDQSSSLSTLYSVGEPESEEQEIQ
RQYFLQQQRYRQEELQALTAGDDPFTDNGRARGRHVRQKQEQPAGPRPLR
RTKTMSPSVLTTRANTSPISQPLRPLSVNSKSGPGRGLDLQVAPPRPLTL
RASRTMGSGDVAAKRAPEQQPPMPEPQPDCSFTSDSVGSDDSEATALPAC
ETPKAEWVAAADRGHSPTEGSPLTPGARGDKDADLSSSPFSEQEILSMLL
ASGGNRRALPQPPAAVASADGSVLPTGLSPRPSVKSAASSPRRKLSAASS
CSSSLATPSRRAARRSSRDAAPALGATIAQLRRMNSVVSSYSAASVASTA
TVGAGAGEADSPTLPGIFTGQPATPRKQLSRPEASGSRHYLNVGKDSGGS
SGSLPKHHWSMADLDGGFGRRRRQDPMKPRGGSPGGKENTLEGVEEGGGA
ARPDVRGLREAWLSATAPSPAPPSPAARPAHSTFRLRQGPVADLLAREGR
AGWRDSAESLGLYDRDGFLLPSPEREARARRGRALRI
>Mb|QLI63533.1
MPAIRHSSKRKPPPEGFSDIEDDLLIFANKMKDAQNKPPPPGPKHQAQWE
IFQISHQRSRYVYDLYYEKEAISKQLYDWLLKNGYADAMLIAKWKKQGYE
KRPILTARASVESLESKSRRVETFNASTVDAEGAHRVTEMEYAI
>Mb|QLI63535.1
MAHAAQHITSPEELEALLSSTTYVVVDFFADWCPPCRTIAPIFESLAAKH
SKSGYLAFAKVNVDHVQAVAQKYRISAMPTFLFFKEGKQVAVNGQSMIQG
VDPKSLGAAAEKLAGLAAQRAEEAQKA

>Mb|QLI63536.1
MSKRHLFNSPEGLVNKALKGMIAYNPSLSLDETNRVVYDSSYDKSKVSII
SGGGSGHEPAWTGYVGTNMLTASVQGDIFASPSTKQILAGVEAVPSDKGT
ILVITNYTGDCLHFGLANEKANAGGHNCRMIICGDDVSVGRKGSMVGRRG
LAGQIGVLKVMGGAAGAGGSLDDVYDLGVAFSQQIVSIAATLDHCHVPGR
TEHGMLNDDEVEIGTGPHNEPGYKKLSPAPSPSELVRQVLTRCLDESDPE
RGYVKFSPGDEVMLLVSNFGGMSHLEMGALVDELLEQLQRDWNMEPVRIC
AGFLETSLNAPAFSVSVINATAASKNCRYSVEDIKGFFDARTNTHWESMA
GSQVKRRSRRAQLVHAPKEAPKAVDESRDLKLDPTVLEDMLRNACTQLVE
AEPDLTKWDTVMGDGDCGETLKTGATSLMAALDGGLAKSGSVVKVLVELE
DIVEGKMGGTLGGILGIFFVSLRSAVENNIGIAATEGPVALWAKALATAL
GSLRRYTPAKIGDRTVMDTLIPFADAMGKSGFNDGVQAAVDGAEATKKMR
PRLGRATYVGAGSSDGQELPPDPGAWGAMVAIRGLQKVASG
>Mb|QLI63537.1
MISLGRRRILSALLAAFVILTVWRARELSASGGGAAPERPMTGYSKYEPE
KDYFWRTIKHNFPINPSQMRSLPTSAAATLPPIQAKFPPESADEKKVRLG
RRNDIKESFIKSWNAYKKYAWLRDEVTPVSGSYKDPFGGWGATLIDALDT
LWIMGLKDEFEYAVYDVEKNALFLSTISKEINVFETTIRFLGGLLSAYDL
SGDKRLLVKAHNVGDMLYKAFDTPNHLPIARWDLHDAAYGNKQESQSTSL
LAEIGSLCMEFTRLSLLTKDPKYYDAIQHISELLAASQEKTKLPGMWPVV
VDTQKEYFDAGTTYTLGGMADSTYEYFPKMMALLGQQDGIYYDMYTRSME
AANEHLFYRPMTPTSEDILFPGFVDIDANDPSRKKELRPASGHLTCFTGG
MLALGGKLISSNDHLSWADKLTNGCVWAYRSFATGVMPETFYLTPCADRD
ACPWDATKWQADVAKAHGAGGGAKEAQSIIAKEHIPEGFSSIMDARYILR
PEAIESVFIMYRITGDRKWQDKAWEMWKAIDNLTSTKLANSAVRNMNPPE
GQKVEMADSMESFWLGETLKYFYLIFSEPDLISLDEWVFNTEAHPFKRLR
>Mb|QLI63538.1
MPGSSRMPVSLLGGFEKVKRRGPANPLDFLTVDLLRNIVFYYFVLRWTRR
AFWKLKGRGPIGAILELYHVIHRTLYGYFLRAPGVRGKVQKQVNESIAQM
SSKLIAQTDTRYLTLPKEGLTADTVRAELEMLANLDHTRWEDGFVSGAVY
HGEESLIGLQTEAFGKFTVANPIHPDVFPGVRKMEAEVVSMVLNIFHAPP
GAAGVSTAGGTESILMACLSARQKAYAERGVTEPEMILPYTAHTAFRKAG
DYFGIKIHLVDCPAPSYQVDVKRVARLINPNTVLLVGSAPNFPHGIIDDI
AALSRLAVRKKLPLHVDCCLGSFLVPYLEKAGFESELFDFRLKGVTSISC
DTHKYGFAPKGNSTVLYRTAELRQYQYYVCPDWSGGVYASPGIAGSRPGA
LIAGCWASLLTVGEAGYVDACVKIVGAAKKIAEAIQSSGTLNGELEIVGK
PLVSVVAFTALNLDIYDIADAMSSKGWHLNALQNPPAIHVAVTMPIAKVW
ERLVSDLETVIEEEREKERVRQVEGKGAKGKAMGNSAALYGVAGSLPNKS
VVVDLAKGFLDILYKA
>Mb|QLI63540.1
MTKPRVVYWFRTDLRLHDSPALKAALDLEPDVLWPIFTWDPYYVYRARGG
TNRWQFLLDCQNDLSSSITKLNAKSKLFVLREAPQTLFPKLLKAWKVTHL
VFEKDTDAYARQRDGVVAKAAKAAGVKVVIHSGRTLWDSDEIVAHHDGKP
TMSMTQLLSAAKKVGQVPRPIAAPKSLPDPGDMPLDFEQHVPSSVPDVNA
QTREKKDTAYKSIAGPKGDFAIETLEELGFPAATTPYRGGESIALKNLAD
IIKKEKYTATFEKPKTSPAQFEPQSTTLLSPFLHFGALSVREFYWRVQDV
VEAYGKGASTPPASLTGQLIFRDMYFAAQAAVGAKFTQTAGNPYCRFIPW
HLPSERDSQTGLVTDKYTVDSDDAEKWFQRWKRGVTGFPWIDALMRQLLH
TGWIHHLGRHSLACFLTRGGCYVDWERGAEVFEVHLLDHEPACNAGNWQW
LSCTAFFSQYFRCYSPVSFGQKWDKNGDLIRKWVPELKNLDSKYIYEPWK
APIQDQKKAGVRVVGDGLGDQEQGTYPKPMFDFGKRRETCINAMNIAYGV
GLYGDDERVLDGSWRELFEGKGGEMMEMVASDDGENADEGDEDVGDKRRR
RKGEGGNGVKKQKK
>Mb|QLI63542.1
MQVYTELVPPSAVTHSLSLPLTSASANNLVVAKGSLLQIFSTRSIPAELD
PQAQDAQSSVKEDHVHFDRRANDDDGLESSFLGGESLIVRADPSNITKLV
LIAEIPLAGTVIGLARVKVKNTPSGGEALLLAYKAAKMCLTEWHPQRHTL
ETTSIHYYEKDELQGAPWEMPFGDYVNYLEADPGSRCVAFKFGSRNLAIL

PFTQSEEDLEMDDWDEDLDGPRPVKEEPPLTNGDGPGDHDLVKSRYTPSF
VLRLPLLDPSLLHPVHLAFLHEYREPTFGILSSMQSPSAALGIKDHLTYK
VFTLDLQQRASTTILSVTGLPQDLFRVMALPAPMGGALLVGENELIHIDQ
SGKPNGVAVNDMAKQMTSFSLVDQSELGLRLEGCAVELLANDIGELLLIL
NDGRLAIVCFHIDGRTVSKISIRLVSAEYGGNLIKSQVSCISKLGSNTLF
LGSESNDSIVLGWSRKQGQEKRKKSRLLDPDLALDVDDLDLDDDEDDDLY
GNDASLAKPSHTINGGSKPGEVSFRIQDALLSIAPIRDVACGAPVLVPDS
EEATLSKGVTAELELACAVGRGPSGSVAILNREIQPKVIGRFDFPEARGF
WTMCAKKPLSKGAAVASDFDTTGQYDKYMIVAKVDLDGYETSDVYALTAA
GFETLKDTEFEPAAGFTVEAGTMGKQMRIIQVLKSEVRCYDGDLGLSQIL
PMLDEDTGAEPRATSASIVDPYLLLIRDDSSIFIAQIHSNNELEEVLKPD
GTLKSTKWASGCLYNDTQGIFQNNVNEQQADETDRIMMFLLSSVGALHVY
ALPDVSRPVFVAEALTSIPPFLSAAFVARKGASKESITEILVADLGDAIS
QTPYLIVRHASDDLTIYEPVRCQAEGDAELSASLLFKKCVNTSLAKTAPE
VSEDDAEPPRFVPLRRCANVNGYGAVFLPNASPSFVLKSSHSEPRVMGLQ
GLGVRGMSTFHTEGCDRGFIYVDMEGIARVTQLPSNANFTELGVSVKKIA
LDGDVGMISYHQPTGTYVVGCTKLEQFELPRDDDYHKEWAKETSNFPPTM
ARGILKLINPVTWTVIHELELEPCESIESMKTLHLEVSEETKERKMLVAV
GTALSKGEDLPTRGRVQVFDIVTVIPEPGRPETNKRLKLIAKEEIPRGGV
TALSEVGAQGLMLVAQGQKCMVRGLKEDGSLLPVAFLDMSCHVASVKELP
GTGLCVMADVFKGLWFAGYTEEPYTFKILGKSSGKLPLLAADFLPDGEDL
SMVAVDAEGDLHILEFNPEHPKSLQGHLLLHRTSFAVTPNTPTSTLLLPR
THSPSYPQASSSSSHMLLLACPSGQLAALSPLAESTYRRLLSVTNQLHPA
IVPHGGLHTKAHRYPDPSSVAVGVETAASSGRALVDGTVLARWSELGAAK
RTDVALRGGYDGVADLRDDLEGVLGWSAMAYF
>Mb|QLI63544.1
MAGRFVRASKYRHVFGKPTRKEFCYDNLHISRNAWDTNLVKVNPEYLSVN
WDSSGGGAFAVIPLNERGKLPDQIPLFRGHTATVLDTDWNPFNDHIIASG
SEDGKVFVWEVPRDFTLYTDAEDITDVSPVGKLAGHSRKVGQVLFNPAAE
NILASASGDFTIKIWDVGTGQASLALKHSDIVQSLSWNASGTMLVTTSRD
KKIRVWDVRQEKPVHEAAGHSGAKNSRAVWMGEHNRFATTGFSRMSERQI
GLWEPGRTEPIGGFTMLDSISGVCMPFWDEGSNCLYLAGKGDGNIRYYEY
ENDKFEFLSEYKSPDPQRGIAFLPRRGINVHENEIMRAYKTVNDSYIEPI
SFTVPRRAETFQADIFPPAVGTKPAVGAQGWLGGKTGIPAKIDLESIYDG
SAPKEVPSDYKPPAQPAPAPAPAPAPAPVKKEEPKQEVQKPAPVTRGPPP
TMNDQKTSISNLANKFQDTEGDDQDDQDETSSFEEISRPTQRVAGRNDPK
PLPVPVKTQPAPTPEPAKVASPAKTPTPTTASTAAAPRSSTPSVGVEASL
DQIKQLLENQAKIITVQNDKITQLVGEVESLKKKVNTSGSQDQSERIRQL
ELELEEARS
>Mb|QLI63546.1
MSTDPPIRRIPPQHVHHAQTAQMSSFQFAPQQFPQRETQKNYVFVDEHNR
HKRLKVMRACEGCRKRKIKCDAATTNTWPCSACIRLKLHCVRPNGYEGSD
TTTTYDTILDPSAQFQQMSLPGQAGHDSSKPIPDVYQGSFTDRDQTAFQQ
VSFDTASGQHNLHYTTVPAAAVLDHVYGGPDAFPTPPMHQGPRPESSPEA
QSVDSYQQQDFADLLGGLRLNEIGTAPYLRNKASFRREEQPAVEDDDDYG
SILPAVTAIPGHKIRIPPALMPDESTALHHFDLYFRHVHPYVPVLDKAAF
YQQWNTGRESISPLILEAIFTIGGRLAEEPAMGQQWLALASRHADSFMDV
PRLSTLQALLMMLKAREAAPKRGYFYRSWMLVVQCVQMGKDLGLDEHFED
HQAGISCGFTPAECRLRTRIWQTIFVCENMVGAPQGRHDLTVNHESVDFN
PPRPIPGGDESEYHVSRNFTYLARILRNIRKMSIAYAKLRRTKDWAVNPE
FQQLEQMISAYLPALPGDMTINFPPDSSPPYLPSSFLGNLHSYYYLLQIL
YHRPVLSFLDPTANEAQWKHHMMISYNSAKALCRLQEATLKQYGLVDLQS
MQRGFSFALYAGLSCIVIHLVAIVSPDPDLNSDAREYFERHMRLMETVME
AWPMPDLQKQVDAIREAFSADIGRPFVLKPSFPYGSPHPSNQSSSPRGTM
AYHIPAHRTGSMDHQLDPSNQHVSYINRPITPPISVGPMDSRSDSPAVQS
LVMMPQDGQVPDMQQTLGLANNPPTWNPARIFEHWNTSFGTPSQPGTVLG
SASQATSLNVVTAGVPDQTTIADQASNQSLSPGTQQMTHQQYSTTTSMPN
FITPAMWQESVASVYEGGLKRGWDYDGGMVLMKRR

>Mb|QLI63547.1
MGRSQTASLPIIPLARGTVLLPGLIQRISVTSSRPDIPALLAHVYEQAAA
KGPEGRIDSIPIACVPLSSPFVGPTGQLLINNGEEIDTSQLAEVNPGSAN
KADVFGFGVAAKIVGIDGRGAGEFALRVEGTSRVRVDNISRERPFFQGRV
TYFSDEIDMADKQLQDLFGLLKAQSRELVTILRISSLLPRTKNGPALSPG
LTKRLEMLIMRREIKEAGLLADFMSNLVEASHEEKLGVLAALDVKVRITK
VIELLERQVGGIKNNFKITTFTSVPVQILDRLNDNPNKRNSSGLPIPGVN
FLSGAGQMPSGNDQGDEQEANELDELKKKLQNAQLPAEVAKTVDRELRRL
QKMMPMNQEYQVTRNWLETLSEIPWAAVTDDRLGPETLNRARKQLDDDHY
GLDKVKKRLIEYLAVLRLKQSLNDDVEEQIRKAEAEIAPEHDSGNPPTES
AANPENSAKLQILKSKRMVDKSPIMLLVGPPGVGKTSLAKSVATALGRKF
HRISLGGVRDEAEIRGHRRTYVAAMPGLIVQGLRKVGVANPVFLLDEIDK
IGMASVHGDPSAAMLEVLDPEQNHTFQDHYIGMPIDLSKILFIATANNLD
TIPAPLLDRMEMIYLPGYTTLEKRHIAMQHLVPKQIRANGLVEDQVTFNK
DVVSKIIESYTRESGVRNLEREIGSVCRAKAVEYAEARDGGSIEKYKPLL
TIEDIENILGIEKFEEEIAEKISRPGIVTGLVAYSTGGNGSILFIEVADM
PGNGRVQLTGKLGDVLKESVEVALTWVKAHAYELGLTPEPTTDIMKERSI
HVHCPSGAIPKDGPSSGIGQAIALISLFSGKPVPPTMAMTGEISLRGRVT
AVGGIKEKLIGALRAGVKTVLLPAQNRKDVKDLPQEVKDGLQIVHVSHIW
EAIRLVWPDSHWAADSHYAGIESRL
>Mb|QLI63548.1
MSKSTLAMFAALGAGGGAALTAAAFALRTSPENKKPELPIATPPTARPNG
AASTTTALAIPSQHVYSTPPAVLGAPVNPAGLFEYGFPGPVSDIASRAAL
ISSYDRRTRNPHWVVEHITPESLCSRDGDRKHSVFLEDTAVPVQFRALLK
DYFRSGYDRGHQVPAADAKWSQKAMNDTFYLTNISPQVGDGFNRDYWAHF
EDFCRRLTGRYPSVRIVTGPLYLPRRDPVDGKWYVRYEMIGAPPSVAVPT
HFYKVIFAEDGRAGGNVAIGAFVLPNAAIPNEKPITDFEVPIEAVERASG
LEFANKLPVTRRKRLCAETTCALVIRDYADRQKAFGKGGQRAISPPPSPP
PSPSS
>Mb|QLI63549.1
MVTFSIPGDDVVEPTTNGTPRSRPPLPFAKRAYATASPFGSSSAKRLGTP
HTSSARKLLTTRDEAPASSLNQSSIATARNIFRASTITESPPTSSFSPSI
PNSTMKKVFAPGATPEPSRMYRNSIAQATPRGMAAKTTNKELFPMRIASP
PPELTGEVLTQKVPKEWNAKGSIYADQFLAHLCPPELDEEQRRQFFCVLD
LRRLKYAANEIFARKDWKLNVINFAKEFEKSRSIILLRYGLYEFQNVKPS
KDVLKRWRREHGLPEPEDEEESEATPSKTAPSKKRKATEEYSETPASKGK
RRAVGDDSEPVVASVATTPAPSVGKNKRKASVSEEQPSKMQKPTPSSAKS
LLEKITNNTKTAEGPTAAPAPKLKPFSANKPAASSSLARTVFTNLKPPGT
QAGHGGAGSNIFGYLSDASSAKNSGIDADAESESDSDQDGSQEAGQSDEP
SVVASGAGDTGSQIGASLFDRVTKGSDGQPIRIEEKIEVSEPEPESEAVS
EVVSEPVSEKPAVTDQTWNPSTTPIKFAPSSTPAAAGSFFGTSGANSGST
IFAPKATAASNFFATTKQEQPAAKESSTGANASEGGRDGGESDKENESQS
SKKSLFDTKPAAAPSPFGSSLQSKPAAAEVPKAAEAPKPVANLFAAKPDA
ARPAETTTATSASNLFGQVSKPAESTSSTLFGAKPTEPGKVTTAEGPKPA
ASLFGNTSSFTAAPATGNAGTLFGSKPASTASGIFGGGAATPTTQPQQSL
FGAAPAATTDSTAAKADTPKPFFNFGGSNTGAPSATASKPQFSLPQSPPS
SGNNMFGSPMKQDDPSPAKKKMFNGGNADGSSAPLFNFGATSQPPASTSG
IFAASSAPAQDGSTASAAPIFAGASSNTGSGSAGFTFNFGGASTDGAAKG
SFNNPFSSAGGNNATTAAAPGSGAAFPFAAGSTPAGTSPFQFGGSSGNAS
TSTGTGGLVFGANQGTNNAAPIFGGASGSGSAPSFNFSAGGAAPQGTGSV
FGSNQAVPAFNLQPPAGGSTTTGTNSPLNFGGGSSLATTPAAGTPEPSTQ
ADTAKDGEATNEDGEKHEQINLTDSVNDEEEVRHEVRAKVLKFVPASEKS
DEDKPKSKSPWSTQGVGQLRLLQHKETKLVRLLLRAEPRGHVALNRALLP
NLTYKADEKYVKLTTSNEKGDGLETWMLQVKTKDLAKELAESLEKNKEAN
KK
>Mb|QLI63550.1
MVDKKYEDLYEDPLERAKAWLTKDDDDHWKAGEWMSKLLPHLVVLAANPD
APAPSVTFTFTCLEDHCNRLGNLHGGAAATLFDLCTTIPLVLISKPGFWQ

FLGVTRNLNVTYFRPVPKGEEVIIECEALQVGKRLATLKGVMKRKRDGAI
VSIAEHLKANIDPEPKV
>Mb|QLI63551.1
MDHMNFQIPGMGPPPIMNQPPQIFGGYGPDGLPNAPQIPADLAAHMFPDT
HLLMEDTNEAKRRRIARACDMCRKKKIKCDGKLPACTHCINYKTDCVFTQ
VEKKRSPPKGAKYIEGLENRLGRMEHLLRLSGLLSEDDSGTDLGALEKKL
TEKSRQASTVATSPISPSQATSGQDGTASTPQSALTSPDPTKDKDKDKDE
KRKSTTPGDERTGDGEEEVEALSEMMCSLVTNQCGETRYIGSSSGFSIFS
PKGIQWVNGRTGDDSFAKMISEVSVDDHKWTNWKPEVFGDLFQRRVFRPL
PPKNEAMSLLQDYFENFNCMFPLFHQPTFMHLVERQYSSDPYEGSGWWAS
LNCALAIAHRLRVMSNLVPQEEDEKAWAYLKNAMAVFPELTMRNTDLLSV
QALLGMALFMQGTPNPQPTSLLIAAAIRLSHSIGLHKRGTGFNLNPIEIE
QRKRVFWIAYMLDKDLCLRSGRPAAQDDDDMNVELPDANPPDNIGNIPLA
DGKGKMNLFRVICEFTVIESNVYKRLYSTKATKQSDGELLNTIGELDQEL
EEWKDRIPVDFRPEHEIKASHTPLILHVVMLHFTYYNCLTTIHRMSIHHG
YWTSRLSNYAIQGLNSRPLNPRVFSSAQLCTAAARASISLLKYVPQGDFS
CVWLILYFPVSALMTLFSNILQNPLDPRAKSDTKLMDLVVTFLSMLGQEA
EQGGVHRMLGICSEFVRISKVVIERAEKEQSSRRKRKNVDSGSKNSTVTN
SSSKPSVQSTPGETASSETPRPTTATTPTPTPHNLSISSASRDALASLTS
TSPADRRGSFDPSAVMSMNSQSPSGGSTSWQQELQPPQNGDFDSYNNMNA
FGAIGVSPPPVPLTARPFAQPLLPQDLFGLPATLDWSWAEMSGGAYPSVE
NGNYGGEQPDLSTTGV
>Mb|QLI63552.1
MVFIGLLSSDKASASRSKPTQETELLPEYIVDEAQLPCVPPERPNLRELN
TSLEALAAVFPDIQIEVFRELLSNFDGESRLALVANALLKNRVDWVKGRW
KVVDKQEAAAASLPKSDMFRSQEYIQAVRTLAVHEFRGLSKSTVNAVLAE
SNHSYLDARRTLVTLSSKSWRFTIQSLLLRRKPLTTGEAEHHPLVVWRST
GQGSITPTVRTTGNAELDRELYDTLIRPLKQKMKDDREVKDRILAVLLNN
EEAEAASATHECACCFIDYSFEEFTSCSRDGHMICYKCVQSSIKEALFGQ
GWLSNINTDTGTLKCLAVDGGGCAGHISSDYIHRAFIEEKSGADILHKLD
QRLAEHSLVASKLPLIHCPFCNYAEIDDIYLPSRETRLRFKLRNLYNLVV
ISIVIGALIVISPFALVVALICVAVSVEQNVWRDIGAEWNKAVHRHCRRR
RGLRFSCQNPKCARVSCLSCHKAWTDIHVCNESSLLALRTQVEQAMSMAI
KRVCPRCNTSFVKNAGCNKLTCPCGYKMCYVCRADLSDEGYRHFCDHFRP
DGDPRPCTQCDRCNLWESEDIDTVLREAREVAEKKWRETEKRELSGAEMA
YLETGVASRNANSRIDQLLTNGTSPTLSDFLDLLVEAIYF
>Mb|QLI63553.1
MTEDEKKIQDIYKKIEREKNIINAAQAMRSQTTNEAVRSRLDTQLRDGRR
NLQFFEEKLRDIQMRQVNQDMGNVSLGAGAGADDGGPPPPPPKDPSAAWG
GDQGGYGTAQYSQGGQHGDLMPPRHPYAPPGPGAGMPKSRPNFTKLDLIK
YDTPYLGPRIQLMLSQIQFKLNVEEQYLKGVEKMVQLYGMEGDRRSKAVA
AGRRVESKQKIVLLKQALKRYEELHIDMDSADAQDDDSINTPNLRKPLSG
QLSIRVMAVKDVDHAATGRLSRGPETFVAVKVEDAVVARTKTSRTDRWDA
EYHTIDVDKANEIELTVYDKPGEHPMPIGLLWVRISDIVEEMRRKRIEAE
MNSSGWVSADQMGTPSSPPGHLPMSPTQTGFGSQGHGADQSSGYSGGPPP
HVPQPQINTGPIDAWFNLEPTGQIQLQMSFVKQNKDRRPVDLGLGRKGAV
RQRKEEVHEMYGHKFVQHQFYNIMRCALCGDFLKYSAGMQCEDCKYTCHT
KCYDKVVTKCISKSNAETDPDEEKINHRIPHRFQPFSNMTANWCCHCGYI
LPFGKKNCRKCSECGLTCHAHCAHLVPDFCGMSMAVANQILEGIRTQKRQ
TKASSMSERTLRPSSSKPGSTASPAPSYTTDASKPYTAPSTAATEAAKLM
YAQPTSPTQQRPPAPDRLPSTQAAKAAQAALGGTTAAQAQQQRQSDYGRY
GGYDQEQPDDHYSQQYGGQPQQPQQPQQPQQPQQRPYNPADYLGYAGQPA
TQARPPVQQLPPQQQPAPPQQSYQPPAPVPKPEVVVAHRPQSGPIAGQPK
QLPLATDPGTGQRIGLDHFNFLAVLGKGNFGKVMLAETKRSRRLYAIKVL
KKEFIIENDEVESIRSEKRVFLIANKERHPFLTNLHACFQTETRVYFVME
YVSGGDLMLHIQRGQFGTKRAQFYAAEVCLALKYFHENGVIYRDLKLDNI
MLTLDGHIKVADYGLCKEDMWYGKNTSTFCGTPEFMAPEILLDKKYGRAV
DWWAFGVLIYQMLLQQSPFRGEDEDEIYDAILADEPLYPIHMPRDSVSIL

QKLLTREPEQRLGSGPTDAQEVMSQPFFRNINWDDIYYKRVAPPFKPTIK
SATDTSNFDSEFTSVTPVLTPVQSVLSQAMQEEFRGFSYTADFE
>Mb|QLI63554.1
MASRSQLANPLLNPLLFVYQIIQWLINYALSPSPPRADAELSRPKIAVVG
AGITGITAAAHCVGHGFDVTIFEAGPEEQLGGIWSRVNDTSALQIHSVMY
RFHPSVKWERGYPDRKQIVHEAKKLWRRYHLQNKTRFDFRVDKAYQDDKA
RWIINNPSNGRFDGLVAAVGTCGDIKMPNLPGLGSFKGDVYHSSQLTGKN
AKGKKLAVIGGGASAIEALEFAARADAAKVTIISRSEKWIIPRNIVVDTL
LSFNILGQETVLSFIPEFLLRKLFYRDLEDIAPARGNGLFMDTPMVNSDI
MAKLRDGKAAWVRGDIEAVVERGVRVNRRARGVPKGGPGHDEVVDADIIV
MATGFKRPSLSFLPDDCFADPYPPPDWYLQTFPPPHPSVSAINCTYVSAI
GSVGNWHIGIYTRILLMFLVDPLARPSPFWMRRWIDMTRLLKRSSPTGAF
DFFTYLELVWWFVFCVAINPFRWKWAVFVFLGVGIDLPKAFVERERRLLN
GVEGYENRDAGRSF
>Mb|QLI63557.1
MSALQERELKQQGAIEAAEENLVSPEQAEKQMVEEAKNAGIPAFTFDPDA
TPEQKRAQAQAAIPPELQRRRRPKGAAIITDVDDGTGPDEVLPDIGKAGV
LDVAKDAQGKDAAGTIGGSQEAPYSRTGWAPKFGWGGGVIDAESLLDHST
WLEGRLPHNLYGDWYHNTALIIFACISSWLVAVFGGGLAWIMVIMAICST
YYRTSLRRVRRNFRDDITRELALKNLESDHESLEWINSFMVKFWPIYQPV
LAQTIINSVDQVLSSATPAFLDSLKLKTFTLGSKPPRMEHVKTYPKTEDD
IVIMDWKFSFTPNDTADMTARQVKNKINPKVVLEIRIGKAMISKGLDVIV
EDMSFSGIMRLKIKLQIPFPHVDRIEMCFLERPTIDYVCKPLGGDNFGFD
INFIPGLESFILEQIHGNLAPMMYSPNVFPIEIAKMLAGTPVDQAVGVLA
LTLHGAQGLKNTDNFAGTVDPYASISFSRRQELARTKTIEDNANPRWNET
HYLIMTSFNDTLDIQVFDKNEFRKSKELGVATFRLEDLEELNVHENERLE
VIGDGKARGVVSCDLRFFPVLESKALPDGKVEPAPESNQGILRFTVEQAK
DLDGTKSLVGLLNPYAAMLLNGKIVHQTKILKRTNNPIWDNGSKEILITD
RRKAKLGVTVKDDRGLISDPSLGMYQIKLDEILDCMAQGKEWYQLSGAQS
GRVKMMAQWRPVAISGVIGTGGYSTPIGVMRLHFQKATDLRNFESFGKSD
PYVRVLLSGIDKARTVTFKNDLNPEWDEVLYVPIHSPRDRLTLEVMDAEK
MGKDRSLGLVEMFTGDYVHQEETGEYMVNEKKSIRNEGLRLHGKGIAKGT
LTYTAAFYPCLNVADPEEEEEGEEEGEAEVTPKTEKANGKAAGDKSEETP
RASVDRSSNAGNVRASVDKPQANLEKVNSNGGSDKADSETTDRRSVERQG
PPKIRLSPEELLKHESGLLIFRILEAELPESHSRLEVFVDDMAYASYITS
TAPSKTYKFDEIGDCVIRELDFSRLTLKARKKGDDKDQTLAQLTGNTMET
LKQCLNNPTMLKLRTDDGKGGWVKVSLKYIPIKMQLNPSESINNMGNLRV
DVLDGTGLPSADRNGKSDPYCKFELNGEEVYKTKVIKKTLNPTWNEYFEV
AVPSRTAAKFNVDVYDYDFADKPDFLGAAVINLDSLEPFKASESKYILDG
KSGTIRLRLLFRPDYITRSIHGTSTFSGLGAPTRIVTGVAGAPIKGGVAV
AGVVGHGVGKGASFLKRGLLGGKKEKENGAVPENPEIPVIATTPSEGGPG
MEPGLRKATGLSESQDAAQTISTSQASSPGHARTKSIATSIHSTVPGGGP
KGTATFTIVGASGYPLSTDLFIQVSMLSPKEKVVGKTKHFKSSNGKWSFE
ETFKVTCGADAQFKVEAKGDHFIGSSDELGEHVYFVDESGSGHAKELTVG
DGTVTLKSSFQHAETDAPDTPKSNLRRSFLSKRESRTSRETTPNP
>Mb|QLI63559.1
MAENGQDWKREDEDDGDQEIDETTYKAQKDAIIMAIEVSDSMLKPPPPSD
SKKADQDSALEAALKCAYHLMEQRIISNPKGMMGILLFGTKKTKFYDDED
GRNGLGYPNCYLFTDLDIPAADDVKVLKNLVEDGEDDDEVLTPSSDRAIM
SNVLFCANQIFTTKAANFGSRRLFIITDNDNPHPSDKAAMSAAAVRAKDL
YDLGVTIDLFPITRGDSKFDLSKFYDDIVYRDPIAEANSTAVAISKSGDG
LSLLSSLTSNINSKQTPKRAMFSNLPFEIAPGLRISVKGYNVLHRQTPAR
TCYIWLDGEKPQIAAGETTRLAEDSIRTVEKGEMKKAYKFGGEYVCFTPD
EQKSLRDFGPPVIRIIGFKPRKLLSMWASVKKSTFIFPSEEDFVGSTRVF
TALWQKLIKDNKIGIAWCIVRSNAQPTLAAIVPSREPSDEKSGTPYLPAG
LWIYPLPFADDLREIKPPDGVTQSSDELKTQMRTIVQQLQLPKAMYNPLK
YPNPALQWHYKILKALALEEEVPETAEDATKPKYKAISKRAGGYLEEWSE
KLEEDAGTARTSKSLKREADDESSDRPAKQRRGASSGQLNPSSMSVAELK

EAITSGAIMKMTVSQLRDVAGSKGLSTAGKKAELAERIEQWVDDNA
>Mb|QLI63560.1
MRRPAALLSTCLSVFTLIHAVEVVSDSSCDCFLTNGTQATYFTNHLFFDF
RSLKDHASVPSVISDPEDTPNAPPTSDYFKSEAWTKTWALQTWANSKRNG
NGFSGDATVLMVNSPNNVFIEPNKDASASSATYLTMRTARLSNFQTAAEF
QTQNSDYHYLSLRMLARTVGPPGAVSAVFTYRDASKLADIQEADMEIVTR
GPGNKVQYTNQPGYTDNGDGIKANATRNATLPRGLQWSDWAVYRLDWTPT
RSVWYVDGEEVANIAYQVPRDPAAINLNAWGDGGSWSGNMSVGAESNLQL
QWIEMVYNTTEDSQRSRRKRRGAAAQLLTRGGKATLCGAVCSIDEGSQAG
AVAKLWGVKSSSARTAASSCSLLAVAWFCLALGVFA
>Mb|QLI63561.1
MGMQGPLTAQALGRSPYDEVVDGEGEDDPRHPTQQYDYRGRPINPETKRM
NRDLIRAHNEVMLVIGVAEPENPHPGSEYESQCRHEIYEESMGFQLRTPA
QICIESAGVFGVHGLRQRILIYKKYSQIPFWELFRRARTDFSLTRDVLAG
APTALLSDYIERMVAPLWVSDDDEDKKVLRRVIHEIWSYLRIHLELHVAM
QRLGILPNALWLPHPSFFIPFTKSSPIPAPPSLDDYSIPSLLKWVGGALI
SATPFLVWIMAQRMVRDWKHPIWSRIFRNLPSTLFKIHRLPFSLQFPLPP
LNTQVAPPPPPNPPRVVDTDAENRTQVESSGGDMPEQTGDTTPGGRPAEP
QTPRRASLFSTRGDEYPSDDEDNEGVSATLISFDVEASESQDAPPGLWSA
ELRPSAGSDSRSNGSPQPIYLDTLLTQLPAAMAAHVLVDAVSRVLMAPYE
ATALRLVARSFRSRQGLPCWDIYNANMLSGFTSTALVNFLGSQLLHLTLA
SEAWAVVTCVSQWFHMTDEEWKIEDTKTRESSV
>Mb|QLI63562.1
MGRADNHSPILQSILRLFSAKFNHGYEPVDTQDPDGQEPTPGARIMTTIN
DHPENMAPNKSGLTAHSARAWSGHADDPFSDRGSGAARDGEPSPETAEAT
QGLYILESKSVHWWSYLATADFWIVVAIGQVLALCITATNTFTSFLQQEN
FSAPAFQTLFNYVFLAIIYNSIFLLRDGPRAWARVAYRDGWKYLIMAFLD
VEGNYFTVLAYEYTNILSAQLLNFWSIVCVVIISFVLLKVRYKPFQIIGI
LVCCGGMGILLGSDYIVQRDSGTSGGGSKLKGDLFGLLGATLYGTSNVLE
EWLVSKAPMHHVLAFMGLLGMIINGVQAAIFDRDTIAGSAWSGKVGGYLV
GYTLCLTLFYTLAPLILRMGSAAFFDISLLTANFWGVIIGIHVFGLSIHF
MYPIAFVCIIIGLTIYFLSGSVLGDSKKPWLGDNQEAGVAGIGTAKLRAI
NAARMASVEAEYGAAPPKTQMSARIRAIFGKTM
>Mb|QLI63563.1
MATRYSADFLLHLRQSPLCTRPSNLPPAEEWMGQTLEQLQRQNVKPSNDR
PRTNDLSLLDQANRRPGLDRHVARNSANPDDIVFGPPRMAFSSARGNKAV
ENDKSGRDSDSPGRITFRNRGDNDGDRFRDGRSNPARRRGDGDQDSDGWN
MVKPRKSFGAEGAERFHGKMGGNFREDRRPSKDTISNKDDRENGRERPGR
PFDGFTRDKDAADTEGRPPRNGLNRNKADNWRTTESNEAPPMPEKRDRDR
TKSWRDRDRDAEPSDDRGAGRNNDRRWGRDRDQRNERDPEWLDEPADARE
AHTQQDFQKWMEQMKKAKSGSSSSAKATDTPAEPPKTSLPVAPVETGPDK
FFLAFGGTSASTDVTSPSEPKDSASSKSKTSGKSSRFTSFFSQPQGDSRP
RTEVSTPLAGPPVNGPGSTAGSAPVPPGVLAALLGSVPSQEAAHSGAPDE
ERQAFQSLLAKLQKQSVSATPPGPSPFAAPPQSKPLGNKNSMGSPEPLHQ
YGAGPRDTSLGRPPSQQPHQEILAPRPQQSARPEQLLQDLAGHHQRVSSQ
GSANRAEPNAARNNSNTEFLMNLMRMAPDAHRPDQGRVASQSQPQIQKHS
PLPPFNDREQEFPGRENRNNERALRPQPPPGFPMDESFHGLERESRQAQP
TQILQRPPPPGLDQMPPNWMAGAAQMPPPQQRGGPMLPPPGLAGGLGGPG
GPNRNMPMPPMFPPNFPPGAMPPPPPEAMGGMPPRNMPPPPPGFFSGPPP
HGFLPPGLGGFNGPPPGPEGFGGPPFETRGMPPSAGNNGRGAAYGRP
>Mb|QLI63564.1
MTGRGKGGKGLGKGGAKRHRKILRDNIQGITKPAIRRLARRGGVKRISAM
IYEETRTVLKTFLEGVIRDAVTYTEHAKRKTVTSLDVVYALKRQGRTLYG
FGG
>Mb|QLI63565.1
MSEVDAGTKPVEAPPVEAPSVEAPPVEAPPVEQQQETSADAAASAAEDDS
MIKTTAQTDHKDVKKNNKFDPSVREVTDDPEAIRKQVEFYFGDWNFPQDK
FMWETCGGSENKPMPITKIHSFKRMRTFQPYSAVVAALRDSKFLEVSGEE

GAEVVKRKTPYKPMPEGKAKAEAATVYVKGFGDESPDTQFDLESFFAQFG
EVKGLKLRRTNENLFKGSVFVTFMDEDAAKKFLATEPKPTWKGHELKMMT
KKAYCDEKSDLIRQGKIEPSSSGPRKFYEGKDNKARPAKKGGRDQDDWKK
RRDHDQKSGFRDGRGGRGRGGRGGRGRGGFGRGGRDGGRRDKDQVKDAMY
SESAPEKKEEKESNGKRAREEDAAGGEEPAAKKVDTKETTEA
>Mb|QLI63566.1
MSANYWESTQRRHWLFTKDQLSTMRQKLDDDNADLVRMFPLPQPRHLAIY
FNQQLLRLAKRLSIRQQAMATAQVYLKRFYIKVPVRSTNPYLVITTSLYL
ACKMEEAPQHIRLIVTEARQLWQDFIGLDTSKIGECEFYLISEMSSQLIV
HQPYRTLTSLRTELSLVDEDVQLARSVINDSFNSDLPLLCAPHVIALVAI
LLALVLRPNSTAPGQAGSGSAAAAGLAAAQAALAQAARGNGGSNIHGLPD
PPSASESKEKQQEARISRVQYFAAWLAESSVDIAAMVDATQEMISLYEFY
EQYNEKLTREQINRFVKARGLDK
>Mb|QLI63567.1
MAPAQPELKKYLEKRLFVQLNGSRKVIGVLRGYDVFLNIVLDEAVEEKEG
GDKVKLGMVVIRGNSVVMMEALDRIGGDDRQHHHR
>Mb|QLI63568.1
MAATSEAPNMDQQVDLSTIPISPNGKSESAEAKDGDKPVTVFHDKDNFNV
KHPLQNKWTLWFTKPPSGKGDNWNDLLKEVITFDSVEEFWGVYNNVAPVS
DLALKSDYHLFKAGVRPEWEDPQNKHGGKWSFQYKEKRNVDIDRLWLQVM
MGAIGETLEEEDDGEVMGVVVNVRKAFYRIGVWTRTIGKSIPGRGDGDVA
GGKGRSGEKSKDILMSIGRRFKEVLELPNNEQVEFSGHSDSAHAGSTRAK
AKYTV
>Mb|QLI63569.1
MSNCLSGPQQHKANLARIRDNQRRSRARRREYLQELEQRLRVCELQGIEA
SAEVQMAARRVADENTQLRELLNRYGVTNEYITHYLQASTENGCESGRAQ
PIRLREEAGVAMQSLSQAMLPRRAAHLEQTQQFAIPSQSSREDSIASAST
TNSSVWEPSQPSVSYNHHPQQLAGVSPTDHPHYSPSAFSAQPAITQSDQF
QSASGQMVNDSRHDLVRGQPMPIDNRLTMNYQFSMSPYNDPTNRYGPQGG
PF
>Mb|QLI63570.1
MAPKRPLESGDPSLEGKKPKRGFRKDLIQKAKVKKEYAKIKAREQQKDDN
DDGVNSQDEAPKSQEEEPEKMHPTRELMLKDEDKAQAGAGTGASADPTSD
GMRRRTRRPGYYDKQLKKAEERKTESEQRTLEIQQRREERERRLAERERF
KKAMAKTIGRDGKKKLGRESTILLDKVKRIMEK
>Mb|QLI63572.1
MEVSSPPAQNPVATTNGGGAPSPAFPTIDPERVVEHLAAVCQVALGATRE
DLEQPGNLLHNSRYSETVSRCTRFANDTQSVLYIQKDIAHSSTVENGTDA
TVPSSFYYTLSTEISSSASTVASLVLLKTPQPIDPARPLTSQIFITNLPG
PASLNTSVGEQGASLSPWEVLHSQVHHALVPYFDANTKSQQLANGSRGRS
DVDAKTGIPVTKKRLNDLELSLLHLQQNVDIPEISLIFHSVVQNVLDEAD
ARHSKAAIDAIPQNLLQDSTFLNRLQANVNTWIKSIQGITKMTKDPSSNA
NQEFSTASQEVNFWLSMESALEGIEGQLRSEGVLLTLEILKHAKRFQATV
SFTADTGLKEAMDKVQKYNQLMRDFPLDELLSATSLPKVQEAISQIFNHL
NKKLRICPYPIRRALPLVEAISADLDDVLHRLLPGTELVNLDYQQFQALM
RTCDGIFRTWEENIKEFTNVAREVTRRRNEKFIPIKINKKHSELESRIKY
VGTFRDNHEQLQRTIVNVLGPKATIPGVTETTGTNGVVVEEIGDVDAVEE
VKQAWEALQNVDLLDVTEQGKERWVRAENLYNERTTRVENSIIARLRDRL
ATARTANEMFRVFSKFNALFVRPKIRGAIQEYQNQLMDHVKQAINGLHER
FKQQYGHSEAHAMAQLRDLPPVSGAIIWARQIELQLDGYMRKVEAVLGPD
WTLHAEGHKLQEESELFKHKLDTGRIYEAWLTDVSRRKISISGQLFEIAR
VRSAGGILELAVNFDPQIITLFKETRNLTWQSYSVPHAVTTVSKDAKRVY
PYAVSLMESVRTLSQTLRQISTMGEESVLLNGYRNDVYKLIGDGVPLRWE
SFINSHELFYAEQRQARPMLPGGTEFTLSKSNESKHGMFIRGFAAAVSVL
QSKAVALNHIHAIVEQALKEISSCPYDSSAFQSRLNTIQTAVDQLNLEQY
VNLEFWVRDLNQKIQSILLGRLHAAINAWIAAFEDEASDEGRWKKTSEDE
GKPDGPVMKRLVLELVMRNQVIYLDPPLEYARASWFLHLHDWLGIVCDLR
KIKAARYQMSLNTTAAEEHRFTDLPTECADMLHRVYVSVEKKLQEIGAYI

DKWLQFQSLWDLQSEQVYDILGDHLPRWLECLQDIRKVRTTFDTQEVSRS
FGHITIDYDQVQTKVNAKYDQWQHEILIKFASRLGNRMRDVNAEIEKARK
QLEGQSSDTSSTAQAVQFITAVQSCKRNVKLWAPEIDMFRQGQSTLVRQR
YHFPNDWLHIEQIDSQWEALKEILEKKSRIMEEQSDAMRANIVAQDKLVN
ERITEIISQWNDEKPVSGTIQPDIASATLSSFETRIASLQDDFQQVVKAK
EALDLPVGGDSFLDATLEEVRDFQSVWSSLSTIWSSLNETREILWTAVQP
RKIRSKVDDLIKSTKEMPSRMRQYAAFEHVQSVLRGFLKVNPILSDLKSD
AIRERHWHKIYKQIKPQKRFSPSSMTLGDVWDLNLIATEAIVKDIIAQAQ
GEMALEEFLKQCRLIRGWDDLFAKCSENLNSLQAMKHSPYYKEFEEEAVS
WEDKLNRVHVLFDVWIDVQRQWVYLEGVFTGNADIKHLLPIESGRFQNIN
SEFLAVMKKANKTPYVLDVLNIPNAQKSLERLAEMLNKIQKALGEYLEKE
RVSFPRFYFVGDEDLLEMIGNSNDTLRIAKHFKKMFAGLSGLVMGDDETV
ISAFTSKEGETVQLKREISLAKTPRINDWLALLEGGMKSTLAELLSEAVE
QYTPIFETEAIDSDALNAFMNAFPSQIVVLATQVVWTTAVDKSLAAGGQT
LQTLYDREVQVLRVLAETVLGDLEIIPRKKCEQMITECVHQRDVIEKLIN
NNATTAGHYLWQLQMRYVVYAPEGNFLERLHVRMANAKLNYGFEYLGVPDR
LVRTPLTDRCFLTLTQALCQRLGGSPYGPAGTGKTESVKALGLQLGRFTL
VFCCDDTFDFQAMGRIFLGICQVGAWGCFDEFNRLEERILSAVSQQIQNI
QLGLKQGAEDSKSQIELVGRQLSVNENTGIFITMNPGYAGRSNLPDNLKK
LFRSVAMSKPDKELIAEVMLYSQGFNQAKQLSKQTVPFFDQCSGKLSKQA
HYDFGLRALKSVLVSSGGLKRARLIDGNLGAEEVVEPEIIVQSIRETIAP
KLIKSDVDILTAIEKDCFPGVDYVPANLIELEGAIRTLAAERHLVVTDLW
MTKVLQLYQIQKIHHGVMMVGDSGTGKSGAWRLLLDALQKVEGIEGVSHV
IDSKVMSKEALYGNLDATTREWTDGLFTSILRKIVDNLRGEDSKRHWIVF
DGDVDPEWVENLNSVLDDNKLLTLPNGERLNLPPNVRIMFEVETLKYATL
ATVSRCGMVWFSEDTVSPDMMVTNYLETLRSVPFEDLDEDSVATGQNPTK
MLAVQGEVADLLKAYMTSENFIHQALERAEKYNHIMTFTVARVLNTLFSL
LNKAVRDVIEYNGQHSDFPLDAEQIEGFIPKKLLLALVWALTGDCPLSDR
KAFGDDLCAFASFGSPPLDGSSSLIDFDVSLPRAEWISWQTQVPSIEVNT
HSIIQTDVVIPTLDTVRHEDVLYSWLAEHKPLLLCGPPGSGKTMTLFSAL
RKLPNMEVVGLNFSSATTPDLLIKTFEQYCEYKKTLNGVMLSPTQIGRWL
VVFCDEINLPAPDNYGTQRAISFLRQLVEHNGFWRTSDKSWVTLDRIQFV
GACNPPTDAGRTPLGARFLRHAPLIMVDYPGELSLNQIYGTFNSAVLKII
PALRGYAEALTRAMVRFYLESQQRFTPKIQPHYVYSPRELTRWVRGVYEA
IKPLESLSIEGLIRIWAHEALRLFQDRLVAEDERKWTDEAVRRIALEHFP
NIDEEKSLGGPILFSNWLSKNYVPVDREQLRDFVKARLKTFCEEEVDVPL
ILFNDVLEHVLRIDRVFRQPQGHLILIGVSGSGKTTLSRFVAWMNGLKVF
QIKVHGKYSGEDFDEDLRDVLRRCGCKGEKICFIMDEANVLDSGFLERMN
TLLANAEVPGLFEGDEYAALMTACKEGAQRQNLHLDSPEELYKWFTQQIV
NNLHVVFTMNPPEEGLSSKAATSPALFNRCVLNWFGDWSDQALFQVGHEL
THSIDLDRSTFQAPDTIPVAYRGLNLPPSHREAVVNSMVYIHYSLQRYNE
KLYRQQKKVTFLTPRHFLDFVGQYVKLYNEKREDLEEQQRHLNVGLEKLR
DTVDKVRDLRVSLAEKKTQLEQKDAEANEKLQRMVADQREAEQRKNTSLE
IQAALEKQDAEVAKRKKVVLEDLAKAEPAVEEAKASVSNIKRQHLTEVRS
MGNPPQGVRLALDAVCTLLGHKINDWKAVQAVVRRDDFIASIIMFDNGKQ
MTRALRNKMRNDFLSNPEFTFEKVNRASKACGPLVQWVAAQVNYADILDR
VGPLKEEVTQLEEQALQTRAEAKAVENNIAELESSINTYKTEYAALISET
QAIKSEMSKVQFKVDRSVRLLDSLSSERTRWEEGSKSFETQISTLVGDVL
TAAAFLAYSGLYDQTFRKSMMDDWCHQLHLSGIQYKSPNPVTEYLSSADE
RLGWQENTLPVDDLCTENAIILKRFNRYPLIIDPSGRVTEFLQKECKDRK
LTVTSFLDVTFTKQLESSLRFGNPILIQDAEHLDPILNHVLNKEYQRTGG
RVLIQLGKQEIDFSPAFKLYLSTRDPSAQFAPDICSRTTFVNFTVTKSSL
QTQSLNDVLKSERPDVDERRTNLIKLQGEFKVHLRQLEKQLLQALNESRG
NILDDDNVIETLERLKTEAAEISAKMSNTEGVMAEVEEITQQYGVIAGSC
SAVFAVLDQLHFLNHFYQFSLQYFLDIFQLVLHGNKNLANETDHNTRRDI
IVKDLFVNTFKRTALGLLQKDRITLGMLLAQASPHKMDKSLIDIVLDNRI
EGQDLSSTPELKDKAFIEAKKLSILKETLDGVADEDWNKFFTEELAEDVV
PQIWDDKTSPTDQSLLSLLLVKMFRMDRFVPAAERFVGRVFGSEIFDFVE

DLKETVAQVSATLPIALVSSPGFDASYKVDNLVERMNVKCTNIAMGSNEG
LASADKAISNAAQNGSWVLVKNVHLAPTWLQSLEKRMDALNPQKDFRLFL
SMESSPKIPVNLLRASRVLMYEQPAGVRANMKDSLSSLSTRSTKSPVERT
RLYLLISFLHAVAQERLRYAPDLGWKGFWEFNDSDYECSAFIVDTWLDSV
AGNRTNIAPQNIPWEMIRYLVTETYGGKIDDEGDFKLLGQLVTSLLTPNA
YEVDHKLVDGPDGGLLVPSGTSLQDFMTWVHKLPEREPPTYLGLPANAEK
LLLVGLGRSLISDLRRVTELLDQGEQLVTDS
>Mb|QLI63573.1
MRGTSTNTTAGLGAVHDLPVLSEPDLSVSTAPAFSAGSADDAGSSISSEK
SDSKPPRSAMRRSSSIPKLPQSPRRVRFDFMGEEVLPTTSPQPSAFISAR
ISSPDPMGDGANCTSRLATEPGEEEDEEEGEQAPRKVSSSDALRFLSRAP
LEEDGTVWTVVNSDPDEAVPDQPHSLPESQAVDTAPSMPTTMMTTPDTNV
LGPTRTATTPEPSHKHTFVGDIKRASSNSEEDSSDDDFLAMAKLKSPVSS
VSRAQALLSPTKSSQEGTSVQLGDQSGVSPKAKKSAANNARGEATHGVSA
TKEDENEVFHFEEEGLQLPERPRQTRNLAAHIDPDDGQSDDHDEESSGTP
TPQPLSLYATSPAVPITRPVNRDREQDPTTPSRMKFEPNTVGSYKGRPLT
IPILRNPEILSQLESAQPVNEVVGSVHHRGPADAFNPASFPEGAEQAMAF
SSPRSFSERLLIEDMMEAAKARSLGSGAGPTDHGQ
>Mb|QLI63574.1
MTLEALSIEHIPPSYKVHLGLFQNVHNADFLHQQLLSRNTDFEYAFVDAS
VVVSRRQLLSAIFKAVNAATNGSLKTPNVHSEIVVSLNPSNNITDSYRRF
GISPTTKNLAVVKVTYSPESSESNSAASKDSIQAHLAEHVKGTAVDVTDA
NIAATTDISKVRKYYKLNGLDWLEAIRDEQKKRAELDTLVVSAMALRGL
>Mb|QLI63576.1
MSQPKYTVFVRVPIPRGDFVDPPPVHWDSSKDEALWKILSGATQTEIDWS
QVAERFDVPVDFLLKQVAYLTERHASQVRAQVRKATAAAKGSAAPSPIPG
AEPTGSSYQRAPSALSGRRDSPMPRPEGALSGTPLSIPIRPNMSRDTSGN
TTILKDVASSSSPRHGTSFPGRAVDQAGRKRLSSLPIPSPTAKSPEPQAD
HRHNEAISPGPAESSSNESSDEESSPAQSRIIRRPPRFQQQEAAKTYPDD
GDDELEPAFQPFQASDQPGQDLASTLRGDGRASGRRNHKNPMREYLHQSQ
TSDDSSTGSPTAIPRPKSKESKIPGPLSPRRTAELAGRSPSGKGKASSRE
GSDGTPSMGSSFSDLDDASVTQSALEEALASHMNRGASSRFSISQAFRSR
YTQG
>Mb|QLI63579.1
MPPSPVLDEEDEQYASSQDSDFAPDEAPEPVSDQSDAEDAEEGEKKRKQS
QPDGQAHDNGYDNSGDEAIIAKGKKRRKRAEEKGRPVDEDEGGEGGLVKT
RAQRAAEKEERKYAVNNGPVTIDVDALWAQMISGEQIKPSAPPVEANGEH
TREPSDSKTAPTASSDDPQSDTIRIKRTYNFAGRVHTEEKIVARDSAEAK
LYLASQGQDTLDVDSSPTKRATRKAFRSAFEPMIDVRLGRADLNLGLAAR
IQAGKEARAKKLTTVEKSRMDWAGYVDKEGIKDELELASKSKDSYAARQD
FLARSEAIREDDARRARIAGKS
>Mb|QLI63580.1
MSSQNSKGSDAIRQGVPRMPSYPDSAHRNENDRLELPKRAHTFHNGSIPE
VQPNHDAPDAFDTAEHSENDDVTETARASVELDELPIELITLTDSFIESL
SAKVHSTPPNIEKLSRLFQDFYALASSHIATHISALATKQSRDISPAPSG
SSISSAASRLRSKAASLSTKDKPRVETERQMITAEELANRKRARKVLEAK
RGTLEEAVERRLCEGIFNKIYRHRTTQDEAQDEKLRSKTAALALVGITPV
DLGVDYGEEAAQSPEMAAKVTEQMRHSLGEARADMVRMSEARYPLAKTNH
LKAAHKSIVDTLAEVHPSASADEIMPMLIYTLITLPPENLHIISDLHFIQ
YFRWEQKLTGEAAYCLTNLEAAISFLQTVDLSTLRDDELPSGPVKPDSHV
GTPKTETFPPAYGITAPSQSATEASTDNVTASKPAPSPSGLKAANVLRNR
RLSDLVNTPAQAFGAASGAVFNTADQGLKTISNSLGDSYSFLLGKLRERQ
ESPKESIIVPRTLDDARKLVSTPPPDEDDNASTTSSVAGGSEDTEQPKRP
SAREDRVLSLISGRRDASVDSVRSGHSSTKKPGFSEDSRASSTPTSATPT
APAMLDSVRNLGSSLNPIGRLSSIGMIRGFGRNTATPSAKDSTDSGELAT
ASPEIAAALPPKQITVPRIPPPNKRFMEIQGPGELRLGEVRELLKDYRRL
AGVLKNIGAFDEK
>Mb|QLI63581.1

MRAQAAVTTVAAVSASVAPIVKATEPQPVAFYPTAWLGVDGNWSTFGFLV
GSNNVNVLFSTALSEFWAVGPGGCNRNDPHCSSNRGGIYYPSDSKHWSGL
GTWELGLPDLGTGGNGQYGFDTIAGLNPMTSVGYQMSNVLISAINSTDYF
LGFFGVGMRSGNFGDIVATPPMRQAVASFGWVSSYSYGYTAGASYRNTVG
SATLGGYDAARLAMHDKTFTLNQTEGVPRPLIRGIEVSSSNGSSWKPATR
ILLDYKSAFTAVIDTTTPYLWLPPALCDNFAKALNLTYNETFQLYTLTNE
QYRQYSSNSSLAFVFSFSSMDNRDNFGDPLDVTGVVNITVPITAFVSLLS
YPFMKGAIGYTDPAVPYFTLRKAPANSTAIIGNAFMQEAYLMTKYDSGVF
SIHQARFPSDPTGDAKLTKINRPNNSPYPPPPDPNGGNGLSTGEMVGIAV
GAVAFFSTLVFAFFCYRRHKRQQRERSGDDFDDGKDSASTLTPDSPRSPV
YRMLSRIVGRRRSRRAGTTMTAGGDPAEAPDHQIYELPAPVAPAELDAGG
SDDNSILGEADLGTDSTQHLSAYEIARRKLDRQLQGPVPEYTPPVDGTVV
QLEKASVPELRPTIQTDMIDQPSPVSPTRSRGADSTSNTFLVSEPSPVSP
RGDWNSAELPSPLTASMPPRSFSSGTRSGGQSTASRSLSSNSNTPVSQVT
DARPPPIPAAFQRTPIDPSKVVCLGPLPENVRLSGHTVGTGSRIVNSDGR
DIPASMFIPGSRTSEGSLGSNFTEEEVRMAEEKPRCTNHSSMGLSHGHHG
TLPSQWETDRQSLPSQPREHGSETGSPKPVHSDESSEEGRIDPGRDLIHV
PQMADKRYSWEEYRS
>Mb|QLI63584.1
MSKITVANVRTQVAELLEYSNETKKRNFLETVELQIGLKNYDPQRDKRFS
GTIKLPSVPRPNMAICILGDQHDIDRAKHGGVDAMSADDLKKLNKNKKLI
KKLARKYDAFLASDALIKQIPRLLGPGLSKAGKFPTPVSHSDDLSGKITE
VKSTIKFQLKKVLCMGVAVGNVAMEQEQLIGNIMLAINYLVSLLKKGWQN
VGSLTIKASMSPPKRLY
>Mb|QLI63587.1
MPTLRSQQRRDTKGQKRGVASSPDEITEDRQSTRPLKKQKRGHSYFPPPK
FWDGLSTIPLTRNAIRELDRRNQLSSHPPSRIQTLHTPRQPSTLQRFARQ
GGPDLSAIRGCQFPIDLGGNMSSSQSSLGRRKRGSQSPVKRTSQSSSKTN
ATPNTTSTRSTGPYDRAFQQNLIDHNIFPDGYEYPDGALPPEPENMNDIL
HAMAQPRPSLSPPRFSNDDFRRFKRADTHAFKEREITTNVIPLIEGTIAD
SKCVAGDVPFTNLDDLTDGTIVSGKPDLYHGARPEQLDIRVRREQSRKIN
PSPQSDLPILANFFLEAKGPDGTLSVASRQSCYNGALGARGMLSLQSYGQ
PEPEYDNKAYTISSIYHGGTLKMYTSHPIPPRTPEGKPGFVTTQIKTWGL
TGDADTFRQGASAFRNARDWAKEQRDNAIQQANERAKRVASFTASGLGLD
LADEGSGEDTITTSQETVLRPHSHSHKSRSYDSDTSADELSLDIGDWSVA
KRNKSRSRSPRKKQ
>Mb|QLI63588.1
MIRPQGLRRTAAALPPSSSIASRNLSSTTPTQADPQWPLLKKRASPPNQQ
DTIHSGGTSLIESLKNRQPQTPSAADPASSPASLDTTSPKPDSRLMLILH
GLSPHLNASDFYRLAPSDLSSWQSVIKKVQQQRNPTTLEPLGRYHISFST
STAAISYRDRLLRLHKLAHYKIRSPGGLWESSTPRHLTSPAGEDPAAELE
TYTVTSGTQRTVDAQRRRASGGNKWAQRLAGAVGGLGHGEKPPAVLVHVY
PPTLTAGDLAAHIVEDGLSRGCGWKVCPPQELQAEPAERATGRASSHGSS
PGADVQPRTQRDIDVRERLKSRFVVVCANDAEARRFHRHWNQRTLTAGPG
DAQATRRNIVHASIINW
>Mb|QLI63589.1
MPVSVPKTDRLVDLFSLKGKVVVVTGASGPRGMGIEAARGCAEMGADVAI
TYSSRKEGAEKNVEALTKEYGVKVKAYKLNVSNYDDVERCINGIIGDFGK
IDGFIANAGATADAGVIEGTAAAWDKVIQIDLNGTAYCAKAVGAHFKKQG
HGSFVITASMSGHIANFPQEQTSYNVAKAGCIHMARSLANEWRDFARVNS
ISPGYIDTGLSDFIDPKTQELWRSMIPLGRNGLAKELKGAYVYLISDAST
YTTGADLVIDGGYTCR
>Mb|QLI63592.1
MSSPPPLPPRTPSRLSPYPLDNPPLHTSDPRTSSTQSLVPSEASLSKRRL
LIVYIHGFYGNDQSFRSFPAHVHALLKELLAETHVIHSKIYPRYKTYKAI
EVARDNFSAWLEPHEGDGTDVVLVGHSMGGLLAGEVVLMPNTSPYKTQPF
KHRILGTIAMDSPFLGLHPGIVVSGIASLFQPSPNQGDLQEQQQPGQEK
LSASPDSEPCSSSERLPSIDSRIDPMDTGASPRPQRQPTDPHFDPPYYND

APFLEKPLVKRLIHFTAKHKREGLLNAVGRHITSHLEFGGCLADYRGLHV
RYNRLRALEDVDEIEKIAQGHPTGAYARVRFVNYYTLSPGRPKPVKAENE
QSQEIRAERNSVDLQLAELDIQDISASKSPGPQIPNTPEKEGEETIQLGA
NPGPQIEDTVETSTIQDAQNQGEEQHETPAEDTLELTSDNACRISMQDID
PIPFFEAETKECQSQTVTANTNTTDDLDLPPIPDLPRKPTFPDIDANLDK
DAKKQAERESKRLQKAYAQAVKDRAKAVQEREKVLERRRKKAAKEAEKQA
KDAEKTRAKLEKQVEKRRAREEKQASESVAAEGHGEQDGTEDAERPKKLK
KFCALPSKRDGARDPTWVDVYMDGLDEVGAHCGLFFPGPHYDGLVGDVGS
RIAAWVGDELTKRAIFEAG
>Mb|QLI63594.1
MSTALDSQRLGLIFQSRPDIIEGINRAADSPGRIALFNEIAGHVYDQLHN
GGEPALKKRKVDTPMNANGTQQAEVSSGNASDEPVLLHIKEISVSVPQRK
KFEICLTQNFVYARAPGTTVPIPGITYAWKDIEYLFYLPVPEKAQIQHNY
VLFPRATCLPSRTNPPTIEPLVFTVPATAPKEGTIGGTEASAAAAVSDTY
KSLFHWAFDRRLRAAGNAHDIISADPNKFHSVVRQPHRPNERAVHVSGFR
GSKDGYLFFLENGILWGFKKPLIFIPLDCIAAISYTNILQITFNMVVETF
SGEGEPNEELEFGMLDQQDYGGIDDYVKRNRLQDRSMAEQRKAKLQLAEN
RGPKKGSDDAEGGNAEGGTELEKAQLEAEQQLQDDEDEDEEDYDPGSEGD
SEGSGDSSDENGDDDDDGDGDGEEYDDDEEGVEEGEEGEEEEEEEEEEVK
PEPVSKPVQPVGAAQSKKTSQVPVRQGWATMASSRPTEDVDMEEKFEVM
>Mb|QLI63596.1
MTTTVEVATVPSTDTPGTCIYLHHDKRSYIFGQVAEGTQRAFGSRKIHMG
GTEHVFLSGSIGWNQMGGLIGYLLSVGGAIDAAKEHTVTENVKRKAKGQK
LLTPAIHGGIGVHGGDNLCHILAACRPVIFRQPISVRPHEFREDPRSIDP
TKTEPDWVDDAVRVWKVPAKRARSSSPPKRRHEEISNGNGGQSQDQTSKR
RSAISDPEVAGMIVERIMFSGSLNNRSVLLPRKIRDLKPTDKAVIINGKT
MRVYRGPFASDDVELSNPDLTAWVFPDPGDNAGDGKNEDVLAINHFPLPK
TAYGDTSMSYIVKCHDRRGKFNAVVAKEHGVQPRDFKLLTAGQSVEGKDG
KVVTPDMVLGERQPGKGIIIADIASPDFLESFLERPEWESPEMMEHICVI
YWILGSDLTSDARIKKFIEKRPHIKHIFCSQDTCPNMITHPGAAEIQTKL
RRIDPERFPLPKFDNTIKHPALPAGSPIEFGRSGKKFQLMPRLTFDDQAM
APFPDLLGAAKSVDEELITLAEEAKAEATNAEFLARVEETEKDIPNRDAE
IIPLGTGSSVPSKHRNVSATLIRVPGIGNYLLDCGEGTLGQIRRAFDAEE
TADVLRNLKCIVISHVHADHHMGTASLIKAWYEQSIRDGTSTTLAISCIG
RYRNMLEELSQVEDLGFHRLRFPSCPFPNGKDRDMTTAEDLGEENFGLAS
IKRIPVPHCWRSYGTQLELTSGLRIAYSGDCRPSSAFAQGCKGAHLLVHE
CTFGDDKQDHAKAKKHSTMAEALSVAREMEARRTLLTHFSQRYSKADSLK
RDRVHGEEHSVLLAFDLMRVKLGDFQKAACYVPAVQKLMEKLAD
>Mb|QLI63597.1
MQLTNARLLASICGVSAHVLVFRVGEWDLASPSIFVFHALLVVTALFASR
VQLLGLSVSELMTCLGYYVAGLYASMMMYRAYLHRLCKYPGPVLASLTNF
YITARSVSKFQLFKEVQKLHLQYGDYVRLGPSELSIADPKAVEAIYGSKS
PTTKGPWYTLLEPRTPVFMVRDKREHARRRKTWDLAFTTKALHGYDFRIT
KAITELLQVVDRHQEQPINMTTWFSFFAFDVMEDLAFNKTSHMLRHGRES
YIFKTMRGDMYSIAFFSHLPWLMPFLKRTPVLNSNYLKFWRWIQNQIDER
IKNTPDWPDIFSFLLADFNRGSKTQQERWDLHGDAQLIVIAGSESVSTTI
THVFFHLAHDAHLAHALQRELDMLPGLAHDNLLKIPLLDAVIHETMRLHP
AVPSGTQRVTPPEGLTIGERHIPGNTIVQVPSYTVFRDARCFVQPNDFIP
ERWTTRPDLILDKSVFIPFNIGTSACVGKRLALMEIRRVVAELFWRYDVG
IAPGHTKEAFLEGKQDTFTLMSPPLPLVFTPRSR
>Mb|QLI63598.1
MTSSQSTASQQVPAWKRLGLKLKQPSAATDAASGGTLSVGHPSSTKREAQ
SNKRKSEAPPATESSHAIKKARKDDQSPPSVDSKTSLKKETKKVTFGHTP
TKNGTASATPDSTKPKPASSKKNKGPTKKPPPATPKDIKPALDYLQQWKT
AREAWKFNKNFQSLLIDRVFNADDIPAADIATFYEYIRDLKGFVRTRLRE
TAMEVKAKDIADGPSGFPEGTKEADAKHETYMNLLSDFLSRQHIGKKRKG
YDEVEFVASSGAENVIIRRVVKRLRAEIILDELSDGEQTDTSQTTQSSDN
TFTTNDNHTLNRADGAKQLKLNDGTAKRRRKIRINADDSSSSESESDSDS

GSSSSSSEDSDSSDSDDDDEAVAQEDDGYESSSSSSSSSSSSSADESDSDD
DSADNDD
>Mb|QLI63600.1
MAALVQTYPQQTSTATMLQTRPSSTGGMITTNQPHGNHPYNMGGSQAPRS
AYSSNGSTGYRNGSVSRQPFAFSSTPNLGQTLSWQQYGTYRTTPSGAVPS
SPSHDSGVNYRPAMQSSPSANSMGHSNSFGLGYGGSRDDSALTPGRVTAP
TARPQSYFSVSTQPSFSSSTKQSGPDRYRRTSTVQPSHHSRSQSTTLSPS
PSMVSPGQAYQANRRYSASNMSANQGLVSVSSIDDLQQYKKSGQDDAMRM
RRRSVHTIDTREFSNTRPISRDSADGKQARLLQTNSHSRNGSSESVASTR
SSHSRRSSATNHNVSTPTSNPSLSGSDETAKSEQQKMLSVPPRGSSSDVV
KRTHSPSPLSKPATMANEATEDTGSASGAATNFESPAAKQLAALNQKGGK
TKSKTSRLRRAFSFGSAAEFRKAAEEAEKAEPSKLHKEPTAEEAYDAEQA
RIAQAQEAGGIGNSIYGGRFFGSTDNLSISSTASSASIMIRKMGRGMKKS
TRSLVGLFRPKSVIGVPAAEPLAPEASQATVSMITVEAEAQRVNVTADPH
AANGGTGFPRLERNSIDAAHVPEIGDERLGSSAGEQRKSIVGGDKERAEV
LAAVRKGILKRPGSPSVRVTDGCPSPDLVLPNIPAIADSPISSAPSTPND
ETLGHKRAGSIAIGSEDYFMSALRLRQDSQSGTPPAKRNATFSPRIIFYD
TWPSQEYDRRGEIATCNRLTPLLAQQIKEELNSFKMEMEVHENSKIYTHF
F
>Mb|QLI63604.1
MFSSARRWLRNNRTPIAIGVGIVGAGYVATQYVLNKINDARERMSSDRIA
KENLRRRFEQNQEDCTFTVLALLPTATSNVLAAMNTEQITYEIQQIKGST
KGLKSGGIESTSPPSIADTTMTEEDGKSMATSASIQSESGIHASQIASPT
PFSAAAGQDGQQDGASAPRVSRKTKRQLWDDLTISAVTRAFTLVYTLALL
TMLTRVQLNLLGRRSYLSSVVALATGSQQATISLENNDDDNPEQAYGSDF
DTNRKYLTFSWWLLNRGWIQVMNRVESAVRTVFGSLSPRDLVSFDRFSDL
TMEVRKLVEGSTADERRQTDWLAFLLPPRNEEEEVIRQSGILEDNSMQGG
AAAEQSPAALRRLLDETADLIESPSFSHVLTLILDAAFEALVDKKLATEA
FELPSQSPFPAPESLELRHSKLILLPKILSVLTRQAHVIGNGVPNEYLQE
METVRALEAFAAVVYSSNWENEIRDEGIMDSAVNISKGEVESQLIQTLEK
SQQTGVGESIVMVDSPGGFEDAWEKAVQQKS
>Mb|QLI63605.1
MDGDSSGSNIPGHVPGPAVNPQAHAQAQAQAQTQIHANARGSGERSEGVS
DLGSQGRPRKFVIQSTFGVPRERRSRKSRPCDACRRRKTACIITTEPPCA
FCKSRGIVCQSTSTTDGPLLRQQSGSEGGSTNSSKPAGADHSFPDSGPGD
SGGRDATDILASPNTIKSSPRPITSPSNVSVSAASDCSPRTGYEASSGSY
LSYASPRGRLRLDADASAASMDIVQPTLNSPESSCISGAGSVHTLEDNPG
RATYFLGRTAEQDPFVLDAFSYGILSETATVDANVVQLHRGGSDVDDLPL
HFLFLSMGHPKHTNETREQASDAIETKVWPHADVLVALYFKHVHPVLPIV
SKVRFLRRYAGNSKSIPGCLRGAVYALASVFWAEDPSTRRGDRFPLEQHE
IVDQAHRALRREIENPNFFVLQACLLLIHVQPPSIDAMEAPSTFTLSAQA
TACAQLIGLHQDPGDWNLEIVEKKLRKKLWWAAFVTDCWAAVSHGNPPHI
GETSYNTTMLDIDDLRCDEDVPEELQHLVDRSSWSFDVASGARFLETIKI
TRLLRGVIDSNFQVDTRTTDLHQRIASRNQLADIHDKLKDWPSLIPSCLV
VSRNREPATSCYNCPLHLTFYATKVLLYRALMHPATKAAKTDPNSNLRQW
FPFAIADFGWFTEFFISINPGDLRGFWGRHARSQLILCGNFLVYLFLMAA
EREHIEAAHRMLETFRVKVNNIADTDHVPTRALIRATTIRTNSFFSQAAA
VMRHGPEASITEPITICGVDGPIKGQQGTGQFP
>Mb|QLI63606.1
MADDGMLLNFELGSGPLQTQVKYKGGRWRDRKRAERSANLRHTRPSSDQT
QNDDSFRSSKRQRVNHDTSGIQPSYGQTSRYNRSGHDAVENDGPDQHKSD
PSAKGNRQVISRLFSFNPAQTTENKKEEEEWKAPQPTNAPLTDVANFGSL
TLSTRLVDALAKMNLERPTAIQQKVIPHMLSNSGDAFVQAETGSGKTFSY
LLPILHRVLMLSSQGDGKQIHRDSGIFAIIVSPTRELAKQTHTVLEQLIR
PFPWLVSTAITGGESKKAEKARIRKGVNFLVATPGRLADHIDNTKALNMG
TVRWLILDEGDRLMDLGFEEDLKKTIDALKNVPVAKITENGTSLESLPAR
RVTVLCSATMKMNVQKLGEMSLADATFLATEKSDDDKMNEDIVHKAPAQL
HQTYIIVPSKLRLVTLTSYLKSVFSRRGRTMKAIVFMSCADTVDFHYEML

RNPNETELPAAAQKEKELIEKTVSKAAYITSPASPDVILHRMHGSLSQPI
RTATLKSFSACKSPSLLITTDVSSRGLDIPSVDLVIEYDPAFSFADHIHR
VGRTARAGRSGDATLFLLPGTEEGYIELLKSSTPPSSQSYENILKTGLMN
KLEFPVETSATPTDGQSYHDKAESLQLHFEQRLLNDNKRLELARNGFKSH
IRAYATHIREERVHFDITQLHLGHVAKSFGLRDPPGGIGAGIDRKAKKKK
GSANHKDRHSSFKDDDTSSAAVDQRPAMDIIKQKSMMLMSGAADEFNIG
>Mb|QLI63607.1
MPHSSNPEGPLVKRQKVSSVASSVKDVANSSRIFAPFRTVGLVSPTSVPF
TSIPLGKTTFQITTSVGRALQTYDLRRGLNLVFVTRPQTPANITATFAWK
EKVFAAWGNGKDGEPQGFWVFQRGKKVSELGLPSDLAEPIQQILVFGTWV
VACASTRIEIFKSATLEHYTTIYTMKADKGGNEITGGVVSMPTFLNKIFV
GRRDGWVEIWNVSTGKLIYTLLPPAPDCGAVTCLEPSPALSLLAIAYSSG
TLVVTNVLMDRLVIQIEAGSPEAPVHSISFRTDGMGAGHDGRKDGVMATA
SRATGDITFWDLNKGGRVMGVLRSAHNPPSRDAKNVRGGISRVEFLAGQP
VIVTTGLDNSLKTWIFDTTPFSPIPRILHSRSGHAGPVNCLHFLPTDFDG
AESGNKWLLSGGRDRSLWGWSLRRDGQSTELSQGNLRKKAKKIGILASNP
LAHGPTTLLEDLKAPEVTSIAMSLNRDGGIGAIPGNQPIWQKGHQDSKKK
VDAEISGMTGWESVITAHKGDSYARTWFWGRKRAGRWAFPTSDNTNVSTV
AISPCGTFAVIGSEGGSIDMYNLQSGLHRQRFPSRLTPSQARQLRLQQLR
QADDVAQLQAGGGQKFLPGTGKHAKAVTGLVVDAMSKMVISCSSDGRIKF
WDFLTGTLLDQLDWAPMTYPIACRYHAANNLLAFACDDMSIRIVDMETKK
TIREFWGPEGCINDICFSNDGRWIIAASDDKTIRIWDLPTSHLIDAIRLS
KPCTALDMSATGEYLAASLEDEPGVAIWTNKSLFKHVSTRQISEDEIGQS
SAPTVSGEGNQGLLEGAFEDSRDDLDDAIIAPTVDQLSSELTTLSLVPKS
RWQTLLHLDLIKERNKPTEAPKAPEKAPFFLPSTSSSKMPEQQAIAESTE
NESKSRITKLDEARFEEKFTSRLRQGAQTGDYGAFVEHLKSLSPSSADLE
LRSLSGGNGDDESNELLHFIRALTSLLKARRDYELTQAWMTVFLRLHFDV
VMGSELILRELEQWKNYQEKECNRLDHLVGYCSGVVSFLRSPRT
>Mb|QLI63610.1
MIRKQARQRRDYLYRRALLLRDAEVSEKRAKLRASLASGKPLDPSIANDK
SLRKDYQYDESTPDLGVNEQLDLDDEYAQLSGIVDPRVLVSTSRDPSARL
SAFAKEIRLLLPTSIRMNRGNLILPDLVKSAQSAGLSDIVLLHEHRGTPT
ALTLSHFPHGPTVSFSLHNVVLRHDIPGSVRGTVSESYPHLIFDGFTSPL
GKRIVKVLKHLFPPREAITSKNKMGNRVVTFKNIEDSIEVRHHVFVRTGY
DSVELAEVGPRMTMRPFEIRGGTLENKDGDVEWHLSQYTRTAKKKNYL
>Mb|QLI63611.1
MAFSAASHLPSTDLTDDFNHALQKHNAAPTSGKAALDVDVLEGFVKEAYR
INSHITSLHKELRDVRQAYLSTAQPRRTPGRTAHGPPQVLTDREREDVDV
NAKQMIRELNASIRGLEDAEQLRRETESALIRKKYASGFSALSSWASGGS
ISSRSPEHAAAEAQAQQTDMHRDGILWYLRQRLELCCRTQQDMMETRLTR
EVEKSRGMLSPPVGDFAPFPPVSKQAFMAAAISEDPALAGETATQASALT
QGLTEEQLQMFEEGNQDMMQHYESTLDKVRTAEKSLVEISELQSLLVNNL
EIQSEHIEQLVTNSLSTAENIGGGNKQLEKATKRPSTARYTFFAASGLCA
FLVLWDLII
>Mb|QLI63612.1
MPGRTTRKTQDLSQDADMDDVPPSAQSATVEDAEMGDGVEGEEEEEEEEE
EEEEETEPQRVKILPGSTDTAASFEFTDEGHTLGNALRYIIMKNPDVEFC
AYAIPHPSEPKMNIRIQTYSGTAVDALKKGLADLQDVCDVVSDEFWTKRQ
AFNAENGIEH
>Mb|QLI63613.1
MPPSVAQDDVEHAPEDTTSTQQPGDVEMTEEHETTRAPEVEETTSKDEEP
PRMSFMSYLSSPMVTLVVGSDENQTLLTAHQALLCKSPYFQDLCQTFVDD
GSPRQLELPEDTVAVGSFLEFLYTGEYFPRKIPGQRVLEVDPAMPKVDDT
GVHLLKHARVYTLAEKFGVPDLKKLSSSKIHCIDSTAKGEIEYAKYVYAH
TTNDDTKVRAPIASFWATRSHTLRAEAENEFKSLCLEFPQFGYDVLTRVL
DDKLRRERIDKMHPATTSGRKRARHSSSAAGSVS
>Mb|QLI63614.1
MKTIRRILTTALALSLELCQGHGLSSNTTIDRRQTGGESTYGERAVMHVY

RGEIRRTPEQVKQDGGFYSRGVQRILSGNPPSVEELELGSSLYRHAAGDT
AEFTRYVSTSTDPGVSLTFAVDDERPTGKGYIYKIHASQRLVDLNRSLGK
YSPYPGQHEYVAMEFIPFEQIEGWWTVTYQDDFSDPAIGRRTRMQLREGN
LRGFHQNPDFDNSFKYARTGGMAPQLAGFPRLSAAWEDRSWQEYKTIPVS
KSLDDMIGAACAGKASCRLERITPKVASWRFNKLTTSKALASNGPIDPTK
QRKPFSGFRVYGKAATLVGIQALAPHMRQVLHWLREWDHPIGHAVRWIDD
HINELQQMIGGPPRSDISGNDNQAALINFFKYIFWLLQGSDRKPEQLDLL
SYGEKTRRRLVSVNDILRTCDRVDEQPPEDALLRDNLHESCSEMRDKAME
VEGLTEEEVVVGRNLCSSCQTCTWEPEHGLCRDKAGTIVWPREPLPEGDQ
YAPTLQTGENPCKASRKRARGAAEA
>Mb|QLI63617.1
MRSSILSLLASALTLVGAEASGASETEKDAVQSTIFNNQTVPPLVELTPS
NWADEVKKSKWLLVKHFSPWCGHCMAFAPTFQTLYEFYHTSKPKDDSTFE
SYYDFRFGVIDCTVYADLCSEHNVAAFPTTVLFENGAAFDRVQGAKGITV
VHNLIERGLEKEHPGSRPKTIDLPEPGATESPKKPTATTEDDVKKDEEPK
KDEEPKKNEEPKKNEEPKKNEDAKKDEDGKPSKKPLGNDWKVPTAGELEK
ERKPKKPTVTPNPEGVSIPFTAESFQKLVTQTQDAWFIKFYAPWCPHCKA
MGPTWEQLAKTMRGRLNIGEVNCDKESRLCKDVHATAYPTVIFFKGGERA
EYSGLRGLGDFVQFADKAVDLASGIPDVNATSFKAMEETEDVIFMYFYDH
ATTSEDFRALEMIPLSLIGHAKLVKTNDPTLYERFKITTWPRLLVSREGR
PTYYPPITPNEMRDKNEVLNWMRSVWLPLVPELTPSNARQIFNGKLVVLG
ILSHVDKDSFSSSLREMKSAANEWMDRQIQEFQLERKKLRDSKQMRIEEA
EDRGDQRALRAAKGIRVDMNNSGRKEVTFAWVDGVYWQRWIRQTYGIDVK
DGEKIIINDEDNRRYWDQTVTGNYIMVSRTSIMETLDKIVYGPHVIKYKL
TVSTIEKIFFDIKVAFVEHPYLSMGCVLGMAFGTLSWLRGRSRRARGGHF
RLDDAMGINQLKEGLLGMNGNSNQKAD
>Mb|QLI63620.1
MIDAENLRTASLYINHQLLSRGLLRDGDTIDFAGAGKCAEDGAALSGRII
SILNDLIIRRDRDAEHRESLSTAMRALRAENLKLTSDTARLTEKHAEAQR
KADIATATETMLKTQLKSAEANARALKEDVARMKSLVAQTRASCATEIRR
RDRQIDTLKKQLGEAGRSRGSRGNPAVTVITVTGETGQERDRTTLDGSLV
GRDNELQSETNTTLSNLAQHLTEENDTLLCVMQRAMAQLRDMSGCTEESK
GDGEVNKRPSCEEIAAELDSIMGHMRTILTNPSFVPIEEVMVREDEISRL
KTGWVKMETRWKDAVHLIDGWRKRMAASGKSVCDEELQMGLRLSPVRVND
VEETRDAVMGLSVVKEEEEEEEAERFMRSPCPADRVGNRPIFEAPGEGYDR
VEEGYESEMTDYEDDAPADERVATTANNGMSNGFEPEPDGQRLDESSESI
PLPEPPQLSPLRNSSSAGNRGFRNSEEMQSLPSEPSGVGDETGQNRMSQA
KSMRSLPVRPRSLARQSMLPSKIQRLVERPRSPSRTSLDDALLPKRDAET
VQRDEIEIPGELSSAKEEPRETGICPTTSAQRRVESRVASQPILPRTDEP
GPQQSPLTISNIAAKLAASEKEADAARVRAKLRAARNSTRGVSRPTIAKS
TKSDVAVADVQMQASTEREVAMDGDPVKQPRAPSGETDKPEKRKRDRKVG
KAASRRRSTLSPRELETLISGTAE
>Mb|QLI63622.1
MAIYSAVPPPEEHTNIPVHLQSPSTPVTNGAIDIDAWTVSALQSLSVSPV
ARGTGTPLSIPLDNEATTQKQTRVVAFNEAGGENALPRRPPSRRDSMKRR
DELLKGKEGSRQRRRWENDRLVGVPNVQPPAAADWEVRPTHPVQRVPYQL
AHFWDRGVRQRVEEKTTKLQAVRKKQQLKTGSATGLGVGEVPRDLREMAK
RTPAIRSWVHAIEDPVRQFLFEEQDRRLESARRQRGSDEISNESELDSDD
EEIVFVGRESAMRELREKKEARHRRAQRTVPQEQVDSGLVFDSFGEGESA
AFK
>Mb|QLI63624.1
MPAPPALGALGMTFTAMRALQAISLITIIGLASNFISEMVTASYVPPPAL
VGTLVVACITAVYTVITYILYWDSMLPLLISTGADGLCLVATIVVACVVG
KPVSYLSCPSLPDKGNTANFIHSLFLNLSRKNYFEWVDPDKATCFEIKAI
WGLSICLCVLYFMSAVTSACLWKRIKGGSRPPPKDLE
>Mb|QLI63628.1
MSADLFAEFSNPSTSTPPTSQSDRLFGQPPQQQQQIQSHVHIQDPFHVSS
RNNAVPVHSQASQSTPFQSSMPNNMDAWGQPSHVSQSSTVVPQQPDDDDG

WGDFEVAQTPQVNTRPLVSSNSGWGSYSASKSIPHDISYNAAAATHTPAD
GLIGGGFEDFGSLGGRVDHVPQLSWSLAEPRDRAQMKTTHHDPNVLFDAD
DFELQGGGEEHDDFDEFGEFEAVESTPRSKPVPATIPQLTPSLDLLGLDD
SPITPLRKEAKPRVAKEESGPPAAASSFGATSSSKLQPLQSLANQTQPPF
VERNVKKTTHTSTHKANPSSILMPSTKMTPVTKKVSPAKATANDDEWGAW
DDFSADNGDNDSSQETAETHKWPWDAGDGSKATKAMVDDSSPPPTNVPPP
SILLSAFPELFNTGNSLFKSVTGQSPSVKQQVLSNPKTIQFLQGYVLLAS
TAARVIAGRKHRWHRDKILAKSMSISAAGSKGMKLAGVDKQQSAREDREA
ADVVAAWREHVGRLRSAVATANSAGKAKIQVPELTETPLIQTAKMVPTAP
KPCIICGLKREERVAKVDLDVEDSFGEWWVDHWGHRACKNFWIEHEQLLR
QR
>Mb|QLI63629.1
MLRTVAKCVSRPSVEATKPAWKITPFKIFVAGLTLTSKPKLRDYQQECIK
SVLLSLRRGHKRVGISLATGSGKTVIFTQLIDKVHSRSDGGDRTLILAHR
RELVEQAARHCQLAYPDKTIEIEMGSIHASGAADITIASVQSITSRDRLE
KFEPSEFKLVLVDEAHHIVAPGYLKTLKHFGLDQKQRESPTLIGVSATFS
RFDGVKLGAAIDEIVYHKDYVDMITDKWLSDVIFTTVESSANLSKVKNGA
FGDFQTGELSKVVNTNEVNDITVKSWIAKAGDRKSTLVFCVDLAHVSGLA
RKFREYGYDARFVTGDTPKQERSEILHDFKKGCFPVLVNCGVFTEGTDIP
NIDCIVLGRPTRSRNLLVQMIGRGMRLHPGKKNCHVIDLVSSLETGIVTT
PTLFGLDPKELVDKASVTDMRMIKEAQREEEARHQEAYSNGTTSAASSAD
AVTFTDYSSVLDLIADTSGERHIRAISQNAWVQVGPERFVLSAPSGSYIR
IERIQEPVTTSSPTYRALEIRALPAGLAKSPFAAPREILTAATFNDAVHG
ADSYAANAFPHSFIHRHQSWRKLPPTQGQLDFVNRLRGKAKALTVSDLTK
GKAADMITKLKHGARGQFARIEAEQRRREKQTNILEARQSRERVMVGPVM
A
>Mb|QLI63630.1
MADPFEVRMRFSLQLQHLNASVVSAQKAAQYALKYKDMDEDLHSCILEQV
ERNNMNTRANIMYFIEHFLDLAQQGHAEYVRMMQRDIIRVVDAVAPDDGS
GATNVKVVRKVLQGLQTKGHLESQAVSQIEDVLKEREINEADLSPASPPS
DVEMTDKSDGQTVPKSAQKSAPHRLDKRQVEQRIEEDRERHKRQRESIWA
IPKTEDAEMNKLWEETSDFGEDDDRLLSEETEDCVKEMEMQHCPHRRSAN
GDHH
>Mb|QLI63632.1
MGPAILDIPPAKLLSSQDHLLPGQLKITTFFFQVPLDYENPSASSIQLYA
RRVVKHESPIFPPDEEDALKNTKPYMIYLEGGPGFGNRAPADHPVTRAAL
PRGYQVLYIDHRGTGLSTPVSTAMLANVGDADAQAKYLRLMRQDNTVRDC
EAVRKLLTAGWPEQKTQWSTFGQSYGGFITLSYLSMHPEGVRESFLTGGL
APVGKTIDQVYDATFRKTTERNEQYFAKFPEDARVLRQIATYIEGQGGRI
PLPAGGFLTVQRLLTIGIAFGGHGGFDTVHSTLTTLKASLDQFGFFTRAA
LAPLESFTPFDTNIIYAILHEAIYCDGPRGPSNWAANRVGRILGGPYSWL
NPDFAAAQSTGPLYFSGEMIFPFHFDTYPELTPLRDVAEKLATYTDWPAL
YDEARLRNNKVPFYAASYVEDMYVEYHLAKDTSDMVKGSKVFETNVMYHN
AVRAKADEVMHQLFSLRDDVLD
>Mb|QLI63635.1
MEQKVLHNDTNDQTPIQFADLIKQGDDVEIAELDKSGNVQRRWLHRPDNT
IAILESKTEPNGIIHSSVREWKRFLSDAFLPIGFPHSVSNDYLAYQTYDS
LQAFFSTITSLLANRALLQGLGVGDADSSATFAMLITILTDATSRIATIV
FAHRFGLRIEPDAKRFRFLADIFNDSAFFLELYSPYFGDYGKILALSVGQ
ALRALCGVAAGASKAALSVHFAKHDNLAELNAKEASQETAVGLIGLVVGT
LVVKLVQDHQSVVYLMVALVFAHLWMNYLGVRSVCMTTLNRQRATILFRE
YMTTGRVLTPEAVAARENIVLWLPVVGGRLAERVCKIEFAESYQRAAASC
NGRRVKVVDGEKYTLFVTEPRDGRIGAIKVLLWFGCEPRHAVLAWFKAME
TAWSMDKEGYHVETSAALTQRHADDEDLWKSLGRLGWDLEAQAFETSAPI
RLRAQSAARKDE
>Mb|QLI63637.1
MPPGSNDKSQHQQSMGSGFPGSNLYPNSNIWTASISNARERSIGSKETEG
SPSGSSALAANSEADVWGASGPWKTEHPARSVSTSPNRTRDAAISNGAAY

FDHTPSAIGVKKGSFGSGHTLPGDASAYSSTSAFTSQKLPAMDALYMEAL
AQFPGVQSVSREAGMPPSRQSQGSPAFQQDIYRGHTPSNSIHSQRAIGNH
APSFSTQSANQRAFNLNKQIDDELAYQTSRRLGLDNASSGSNTFNPTSQP
FQFNPGSQSWMTEPSSSRYDSSMDTDTMAAQYIKRGSIDRISGSPAFRLE
SGNSPRNYAVNPDTFPARPSSRDHRSNETERRGVPAQHFTGGFAPSFYPG
QYTFANLPPQYAPSYIDPYTQNFRNAMMSGYGIPPVPSAYSLAANLPPVR
PSRDQDPGKGVRSVLLDEFRLSNKSNKRYELKDIYNHLVEFSGDQHGSRF
IQQKLESANSDEKDQVFREIEPNAIQLMKDVFGNYVVQKFFEHGNQVQKK
VLAEKMKGKVVDLSVQVYACRVVQKALEHVLVEQQAELTKELEPEILRVI
RDQNGNHVVQKIIELVPRQCIDFIMNAVRGQVTALASHTYGCRVIQRMLE
HGTESDKMEIMMELHASAQILITDQYGNYVAQHVIQNGKPEDRAQMIDLV
MAQLLTLSKHKFASNVVEKCIEHGTAEQRTSIREQLTTVGSDGTSPLQQM
MRDQYGNYVIQKLLGQLEGREREMLVEEIKPQFYALKKNGTSRQLQALEK
LLGLGSGGSPSRNDAASAQTDLNSAADTPALTNETSSPQSSSPRSAHASA
VSIPAEESGNKTSDELSNMIDLQVREEEA
>Mb|QLI63640.1
MISMQRSLSSPVLTQSPASVPASPTRTPGSPSPSPAEDQKDVSQASCYRS
RTPPRSPEVSQQTLPLRHTDGGNRWSYQRTHSGLTTSRRPLVAQPDVAVQ
TNDRCNSNELLTGPQIEPPSERACRRPCTQPLIVDVTQYTNPHLTKEDEN
ITLQELAHVVRLSKYQERKRANTRVRLQRSLISTALSARLTRCGDIANRN
LVESFRKDDKDGFAMLYNAIQDVRKSCDELRRYALLEPDMEPLSSPALGS
SESLDTPTSSVVGVAPLRPITPFLHDISATSRDTILDFLSELRTNPDYLA
TRICALSSSELNAFLGFHKGMEPVESVLPYHGRSASRHHAGVSSSRSNAS
SDIERLLSFQRHDPLSILIHSCFANSAGPDSTEDQRRTDIWATTLARLIQ
QPKSSSEHFLVSVLNIWTGMRDWSGKSNMEWYLMKILEEGAFILDRAEDQ
HGTRFNLSDWNSSDEVAAKEFYDRAVQGLFELIDDEDATGIPEGLLELGN
AIVKKLDNKYVENTSRWLVWKCLFFGFLLGVIVHPESHGMLAEYHITPYA
RETILKKVAMKAYEYVSSMWSGKPSATCVPVEVPPQIKDHVERILARFQG
SRSKTLSAKLLPARSITSLRETVEVHPYLVLSPPDLATLANALFPERRPM
SSMSNTLRSGAASISGLSAISQQVPTPNLRNSNLETASIISTSFSSVISD
GTTAPREGSQDGQPHTPSQRHSPPSADPDAQRKLANYEDDGYRLGLAMHE
LSQNLGPEALKGTFHPCAERWIILFLSPDGKKLSTSMTYDPEDEADEDDN
SSSTDTDEDEGTKGPELDKDYHQIRDAILRLVEEYEIPRNLEAEGDRAPQ
LTNRASRLRRYKSKNKIITTEKSVSRRNPYRTHDGNSRSNNASLGDSAVP
DSAFRTAWEEDEEPVLIKMLRAASSQSKAQADFVSAHVYWKTLKNLCALE
SPSLRANGFAVLINIFSRGPRDSIRRSAAAIEEYDAWLVWLKQSQERHEG
SIDRMMRRVRAMRDKMWFVADVRNSKEYGHSRDICQALKTMGMPRRWSSI
QRSRANVGRAPGSSYLYRTESQIMDLLAASEEQGGPNKLSDDQAEMTSNW
LQQYGVENFCYGEERIHRFCCEVDKCISKLVGETIRDAPVLWSSDLYKRE
KVVYDRIRVRERDQSWSGDDTASILSDGERRLGSPSSRPSWMLREPERGR
FPNSPNGGQHALDMPRINFARPTASLSDVVENHDAFEKSSPVNTVDSMST
FWSPFKPVMTPGSGVSRAYSPTTSLTNVSTTFSGPLHHFPLPSSSSVSTG
RPGTSATSNETIFQQRAEDEKSRFLNELRQTLTSLLLSDLGNQVLARGSE
TDAWFHVLGQQLIDRKDAHDHARRRSERKDKEVGGRSSTRPRVIEKKKSF
KNLRNAGDNASERSADVPLVGMADEADTTGTGNGSKAKEATTDFPFKKAY
HRLLSMFSVNPNPFAKLNALNELENLIIASLMWHGSKKLRANLSDAGSST
ASDRGPYNHQTPLDGTIDNVKERRSQAIQSVFHPAGYGNQPRQANAETKS
ISSGSSSTDAITSELQRLFRDAGIRPKSLFRDLQLIAAFVPSAILDKPER
GKAFWNVGLAALKLKSEVCRTMVEIADNVIEVHTQHRKLVNDSHILSESK
PSVTGTPPPPPTIYNLHDVGKMCVITAREGYPAAQRELALLYMSNPECVE
RTTLPLSKPREVFKQAVMERYGRSERTRGSSGAAGPAGLANASMNMRAMG
PAHPAGDGPALGGKDSDARNDAGLLCIAIHWMKSAEQGGDETAARFFQQQ
DFKWDEG
>Mb|QLI63645.1
MDDLSGLDWSKPTGSQPKQPSMSIPMNSSYPTLRPTPPPAGSGRTTPSST
SQAPGVSSPLSKSAAKPAQDSFSNLMNFGPAKAKQNLSLAERQAQLEAEK
RKKQDERKKQAEAQFGSAQQWDALGSRTFTPSPSIPPSAVSNGAANDDDD
LFAAFNKDTKVDNSSHYPPPTSLRSTPLEEKKTLDLSNPSAWGASSIPTD

INNDANANGFDDDDDPFGLNDLKPSGQSTAPAQATAGGDDDFLGDLAKPV
EEVRMKQQQQQQSEPGKPIEDDDSTSSEDEHSRPRQQQRETRGDAGVSTA
LDRAVAQLVDYGFSPEDARRGLTESGAGLNVQAAANWLLDDAHRKAKENS
QGRSSSTSSGRIASPHERQAGGFSGRGGDSDLTKSAATVGSSLFKTANSL
WKSGQKKVQRAVADFQQDGGDPSQPRWMRDAQLGRGHDAGHASQDATDEA
MALETGGRTKPSRTNSRPAADRRLGLQQSRTSSPSATSSGNQSRNSPVPR
WQQTSSPSVSDARSRLNRMNIDDDDGFFVSANRRKKTTPAPTEEPRQPDD
EPNLLFSTEAPKPSQPLPQHPAQRQTPPFMKQSQATPPASQRVTPKPARQ
APPIGAAALQSSTRHRLEGSAHFKRGDYSMAHSSYSNSMSGIPDGHPLMI
ILLTNRALTAIKTGDTKQAVRDADEALKLIGPENGQGESIAVQGEDGKDE
TRNMKDLYGKALTRKAEALEQMEKWGDALNVWEQCVATGVGGVTATKGRQ
RCRDIAAPRPKAPAPKPAAKPRPKPSATAALSSGNSSEAVTRLREANAAA
AREDDEKFALADQVDARVGAWRDGKRDNLRALIASLDQVLWENSGWKKVG
LHELVVANKVKISYMKAIAKTHPDKLPQTASTEARLIAAMVFATLNESWD
KFKAENGL
>Mb|QLI63646.1
MDSPPPPSPQARQYWHQALKLKKHLMKQLERLKAESASGVDVAHFEAIEG
TLERFRFAFVHTIFLDFAFAVKENTEDALWALHTMINSEYRRIQGRLKQS
SHAVERRKMEKSYNNFLRVAQKFYKGYIQRLSARYDVPELRRVARGIDVE
PMSNGDRISPVPSGLSAMVLTSCHSTLLRLGDLSRYRTQARHKNSGYETA
LTFYSLAHHLKPQSGYAQHQMGIVHLDQGNHLDIVYSFYRSWAVDAPHPN
AKANLEAEFKSLHLPNSSRSRHNASPTAQDPFPMWFVRLHALFYKGETFP
QQAELEGEVLHKLEIACHSEKSAATLLKMTLVNLSAHHVASTIYRESMSL
AAFYQFTLRFNALFISHFCVAFESELKEAASEDMGSDGSNNVPTKITPIL
EALLPVLRLYSMWLTACRGEVSAAAQAFGGVIPRLVQNLAKVLTLLCIFT
YSQALSNCPYLLAEDLEIRGFRPLSEETIPQQCRCFCTEDGSPKTYLQDH
SSRLEPSQENLARILDILRCAYFLAEDSNFPMSYKVVDNSLVFEYCVKPT
SDSPEAAAKTSTSSPNTASKNPLGNEDADDSSLPSRVILAEEPRQLRENL
NTDANSQQPQSTQQERVQDEANLEDRDDALFEMLSPFLKPPTPQPQRQRH
STDESSYGMHTMTANNVLGSFQTERSPPGSVLSGKFAPLPWAWFNTPEPE
KPADSMTPNGSTGFGESASPYNSPRRPTSSGNHLDDPFATPGRNLHGVLV
NSHTAQQQNRRSFTGPVVGPVVSGPETAHRSNLLQAFSGTSVPRSSPFIR
WGDNQQSTLQNSGPAVSPWGSRALDSLPQSTGLSAFSHPSSLYQGTPANV
VGHGMSPISGRPEPQFSSPARAETAARYLQMDQTALNYNEAIMQTAYHGN
K
>Mb|QLI63647.1
MATNVAVLDDYQSFAPPHFDKLDANFAVTYIPDTLLPYNHPNTPQTVKDE
IVKRLEPFDVISTMRERTPLPADLINRLPNLKLILSCGRRNKAIDMDAAQ
KRGIPVTAATDTQAGPVDSTTEHVIAMILSVTRNIAKNDAAVKAGAWQTD
AVTGVSGKTLGLLGLGRLGGAVARIMNLAFGMKIIAWSTNLTQEAADAQV
KEQGLPVEQANGEKTFKAVSREELFSAADVVSVHLVLSDRSRGLVTEADL
SRMKPSSFFVNTSRGPLVVERDLLNILEAGKIRGAALDVFDLEPLPGDSE
WRRPDWGTNGKSQVLVTPHVAYVEERSINGFYEQQVDDLVAWSAGRPLKR
TMY
>Mb|QLI63649.1
MPLIMNADVDKLDGLAPRSCKLCHKKEGVARCSACLAVFYCGRECQVKDR
DFHKKPCTLIKKNRLRYKSEQKKLREMPSGPFLPDNVFEDHVGRFWGILG
TRPYMCARYALVDAMLITYGTAGGPVDVVQMSLDHLLDMMRLCRSDNLCL
RKLIPGLYIRLGRDQDAYDFMKWYATTGQKTDYNWGNMEEPFLDTKDADV
LEAPVKSWKGRVLDPNHVVAVVLIKVRIMLDLQVIQNARIADRGDNPEET
IKIIGGKLVSPIISSRAESLLAEPQETARLAGKIKKQIKQLYDAVGSYNR
HFWDLLVEDPDCGVLRRPASNPPQSKNEAIEVVKYSYASWYKTPGAVSVL
RNLSEED
>Mb|QLI63651.1
MTDQTKSSKVLHRLLLHRPETVTSASGSYYTLSTGQRILDGCTGAAVAVI
GHGDPDVKAAVVEQMSQVAYAHTLAFTTNSAEELAGTLLDGNPHGLCRAY
FVGSGSEAMDAAMKLARQYWVEAGQPQRRRFVARRQAYHGSTIGSMSVGS
HVARQKPYKDALLLDNVSYVSPTYAYRFQKQGEPEEAYAQRLVDELDAEF

RAVGPETVMAFIAEPVGGATVGCITPPRGYFEGVRRVCDKYDILLILDEV
MCGSGRTGTYFAFAQEGDVRPDLVTLGKGLGGGYTPIAGVLAHQRVIDVL
AAGSGAFVHGHTYQAHPVCCAAALAVQKVLRRDDLVARCAEKGKWLEQEL
RTRFEPRRYVGDIRGRGLFWALEFVKDKETKEPLAPDLHFASEMRDQAHR
LGLAIYPGAGAADGHAGDHVILAPPLNVSQEDLEILVDLLEKAYDVVESR
VS
>Mb|QLI63652.1
MPLAAPVVTTGPPRPTDRGVPVGLNEAALDSPTFRATASHFTEQIEAVEK
WLNGYVTSTSKFAHEIQGLEGTINTYLTKTMPSAADGIIENDYAFLALKR
VGDGQRECWQQLLSSVKRMDTAVIDPIRNFLSGDMRNFKEIRRILEQSQR
AYDSTLARYIGQSKTKEPSALREDAFSVYENRKAYIQASLDYCQLAPQLR
LATDKLLVKVCNEIWKDLTRSRDAVVNATRWTWEMDRVRGWAKEMEASEG
VFRTELQAMRRKLGETTLEGFKPSRELEDYSASTVPFLGSRGPLNLRPKD
GTAVISEKQGWLFLRTMSGKPIRYTWTRRWYYCRDGVFGWLVPGPQGVLQ
GDEIGVLLCNAKPAVGEERRFCFEVKTKNQTMMVQAENQRELMEWLEVFE
ATKKKAFETSMSNNDNSVAGGTDPAFSIHPPSAPEFSAKAFDNPTAAVDD
AGPGFDRSATLYIPGQDVHVGARHSVDVNNASLGRRSFSALGRDLAREDD
ESRREHAARIIQKLDLHRKSTFGPGVEPVPLSPGMPNNGIASLISASHNL
LPAHPGTQVSASTPKQPSGFLPSLDTPPGSLAPLTLAKPPATTSLSRAAV
LLTAEKRSVSNSRKLPTSVVANYWGSNVWATINTPEQPVLPRRDDDDPIG
VVLAQNSETSLPGAGSQGHTSRPLPRNYPTELRAQNAQFRLLFPNAPLGE
NLVLVFRAAWSSSKARDSPSDTLAGDGRIYVTPDNMYFYSQQMGLVTAYG
ISLDIITEVTSAAGKDCDFIFIHLGQDTNETGYTRITIKVFLDDLHLLHT
RLNLLVDNLQAEEPMDTQDIISALINVEKEEFERPSPGAESWEGLSPISP
IDDGTSSGLPLARSQREYGSHLHPSRSPLRLPGKLHLPSRPVVYEPESMT
QMAAERHFEISAKACFHVLFGDKSFVFPKLYFEHRAQQIAQGPWVLVDQG
QMRREFHFKVDCVDMLGRKKSENVIDYQSIDVYSDHVTYVVTHIKTAWHL
PHSGSFKLVTKIVITHVAKSKCKLAAYVKIDWSKTPALSKNLVERQALRD
AINDAEEMAELATDQVRKLGPRSRTNRAIQVYGHVGQQTQVVVFSPAAAE
SSKKQAIKPRTLTAMLFETVRSFGESAVSSLIMWAFAGLKTLFGIITAHR
VIVILLGLSALMNLLLTSAESSTWWKERSAARFMRRIGVAPNPMMSKAIY
FADLDEATGTHGHELKFPKNSSCFGTYKSLLDTTEIDIPWDEAGATLSSP
SSRATARRLRRTRQRLGSYRHDLVVAMRVVNSIEREMLQSEWENWLINEK
SLCDDLDLVLVEDGKDELAKGSSQNQKMMGSLTGSRKKALQEWRDDYCGS
CRRDFESVIKERIVTGVLRAAKRHVVIVVVVVGVNKIRGAMASHHLSFQA
LAPINWNDVPRQDFQSFLDSTFKDAHVIIDSVPSTVTPAAKPPPTGRARA
KTDSALVYTDIQRSLSLHQSPAAAALSAQLRKEWKEIKTNPKDNPLGITV
HRLAAKDGKGAWFARRSVHEGLSFEQWKSGLEREFAETMKVQGSPGSGNI
RGIGADRNVEHHVVDAGHVDVFQLSAQFPGPTAPRDFITLLLTSDFSHIK
SDQQQQPPLRQYIVVSKPCVHPDCPPRQGIIRGQYESVEIIREVPIHGDA
AANKRSFSSADLLADDSRKSMSPSRSSDDLQTPRAIEWLMVTRSDPGGSV
PRFLIERGTPPGIIGDAAKFLDWVSSTTHKDGTPRSAQETDQRNKNNENP
GNGSVNGNVPAVTINDNARTPPEMSFNDDQDSRDHEQGYGEWIPSSTGLY
GIISGAFGVAGSIASGLRSQFSNPLSFNSSQDSLADSQPIPEEDENEDDN
ASQSDTSSTRSFTSAPEKTLTREKASDSMTGSVSDESKSQRLQPTEKDLQ
RLVDRRQKLQRNYTQFMERMETKRLGEKEKDAASQAKHRERIDKEMAKRE
AKHNRDMRKLEEKREQEERKAEARRKKAAEREEKSSLTLALEKARADNDV
AMKQIELLRGQVGELQAQNTMLVAKLGRMGALNREDSSSSKTNSIKSLKS
>Mb|QLI63653.1
MNLNLPLRNTTTSRRNQDSRNYFTSYTPPSSTEAINGPVREQREPVPVSD
RLIVGVDFGTTFSGVAAVYTSTPDDIEIIKTWPGGNGITSDKVPTEVAYD
FPANAPPEADPTIKWGFQFRPEESRLRCIKLFLDRSQKLPFYVSPLDTAA
QLKRFNKNVVDAVSDYLTQIYKHTMDTLTRRYGESFMVSTKVEFVLTCPA
VWSDAAKNTTLLAAERAGMGSRSDIQMISEPEAAAVYTLKAIQPNHLNVG
DNFIVCDAGGGTVDLIAYKIMSLKPLRVEESAVGTGGLCGSAFLNYRFEE
HVRNRLGQARFDEMKAKKGRSWQMGLKYFEEFVKRNFNEDEYQEINVPFP
GLTDDEEAGLDDGFMVMSAEQVKDIFEPVVKEVCDLVQGQVEGLRAKGGI
VSGIVLVGGFGQSDYLYRRLKAHFTSAAPPPYSERPTHANANATEGNGSI

EVMQPVYAWTAVVRGAVLRGLEGNMVISRKSRMHYGTSYATVYDEEKHSV
SERYWSPLWERWMVSDRMQWHIAKGEALSPLSPIAFHYTRNFRPGQSLIV
TDDLIACDADEPPAAYTRDLINVCTLTTDLNAVPRSLFTRLTTTRGVEFD
NLDFTLEMIVDSAGLGFELKVDGVRYGRVEAEFHQD
>Mb|QLI63654.1
MADTEEPQFNTLAERIAALNKQKNFNGNGTTDLPRKRAPPPAPPVRTKSP
ARSEPAPSPASATASGHSHPSIPTRPVRGNAPPPLPRRDTQTSVASNDSL
VTKPGGGPPPLPGRPATSQTPPALPRRTSAQSTAYLSPRRNSASSEISQH
STLSTSSLVRTTSSNISYTSSGSAPRKIMAPATCDAASLPPLPPSKRDLE
ARAKEAAAQEIAAKDAAARDYRAKQAAKAQEARAVSNTSSPAPPARPSLP
PPLPSRPAKSPSPAPAQTSSAVEVEETKPAPPKRPFGTIKGFSSGKSIKA
PPIPVQRPQLPSRSSASAEATRDDAPPPVPLSSRPSAAQIHAASSEAATG
ASNGDSKGDSTTGCWVCRDWSGPDAVAAQFPRQSLPHRDPVGHLARGLCG
PFPSYDDKARAIFTWCHHNISYDTVSFFGNNVRHMSVEDTIFSGLAVCQG
YAETFKAVANRAGLECVVVGGHGKGFGHTPLKKGERPPPAKPDGHAWNAV
RVDGGFWKLIDACWGAGHICSAANLYKKEFSPREFTLSNEKFGLRHFPQD
SRLQYRSDGRTISWEEYYTGGLDGEPHQWYGTGNSEGIAEESVEPKGRDV
SVHSGQVVRFQFSKVCEHWTSEKAGLGKPALFLLSIHGLDGRKDEIIPTE
TNGYWNWVDVNARDLGAPGQSVEILKLETMDNRDARGVTPEEFESKRGRV
GMSWSYIAKWNLV
>Mb|QLI63655.1
MPVETRKRKAMAQAAIQSAQPAPSPPTSTTKRQRKLPVRSKDDAPEQKPE
ATTAASKRSNVITFDDDGNADREPVVPAKPVEASKPAQQEEESDGDEAPE
AVSTAMVAVEMKKSAQAEKKAAREQAAAEKQKRQQRDALFKQQASQRKAE
TRTEKVPASIQEVSARKLSGKMVIPSVLPAEFLTDSDSEEEDDAESSLGQ
DLPRKRTVSGVEKRMSRDGRGPRDEVIGSTVYRISKKVDGRLAPKARKYS
KSSKDILLKRGRAPVQGRSGFFKK
>Mb|QLI63658.1
MAYNPRMSIIPTNQQQGRSRKKEDEADAFMRLPDREIVGCITDIGIHFTV
ADLQKPNPAHVQQIFEWFAELLLNSTRETVEPAMRAAAEDVCGEFSDVVP
TDTRNLMGFYVSLRRLLYECGITDFTFNDLYKPTYERLVKIFSYLINFVR
FRESQTTLIDEYYNKSESTKSRIETLHAENQENEARLEDMRHNRKAMEAQ
VREKTMRNEELKKQLLELQRHQKTVAARLDEAKAKKGELTSRLEQKTQDK
LTLKQESNKLRPYMLQSPSALHDNLTELREILNNDKAHIDALDRRARALQ
TSTDSFTVVSTDVASCIKILDEIATELAKEEEEMVRNSKQRDALSERGNN
AREVERSEVLLRRQLSKWADRTEKLREQSHQKAQEAKEKMHELSAIHRKL
TEEHTDKGKEMEIRRVRIEQKEKKMLDLKENIENEVHAAYDEYLKMEAHI
KLYITEMEQSIS
>Mb|QLI63659.1
MLSRIARAPRLGMLAAQLSQSAASPALRSRQTAVFVSPWMRSFASNNKNI
GGGEGQSRPESSTQPKSDSKPSASQNTPPSNETEAESSQPSKEAEPIPFH
KLPDLTQGIPSTLDAELRKQSGQATSALEAVEQEESEGRGKRERAEYVST
SDRNRKWWARFMLAAAAAGASFGVLYMGRDWDDAIEVERHSDIPNGWSPT
LWWQRARARMGESVSYYQDPAFDKLLPDPDPSFERPYTLCLSLDDLLVHS
EWTRDHGWRVAKRPGMDYFVRYLSQYYELVLFTTVPFAMGEPIVRKLDPF
RFIMWPLYREATKFEDGEIVKDLSYLNRDLSKVIIIDTKAQHVRKQPENA
IVIDPWKGDPKDNDLVGLIPFLEYIHTMQYSDVRKVLKSFEGKHIPTEFS
RREAIARKEFNKQLEQKKKHSKGSGVGALSNLLGLKSSNMSMTMAPEGEQ
NPSEAFAQGKMLQDIARERGQRNYELLEKEIRENGEKWLKEEQVALEKAQ
QEAMSSMMGSFSGWFGPKPGDVAGAGASAGAGAEKKA
>Mb|QLI63664.1
MTVPKYKDEVQVILKAVDPKLKSKFCDRHIFLTKDNPKIFIGRSTKRDAR
LAAGAKNGWFDSAVMSRNHAKIVYLPANNSVALVDVGSLHGTYINDKSIN
KMQFRRLHQNDRIRFGIPIQKGSETFPPCEMRVSLCHGSQDPKERPVVFK
VPDSSDGEDETSDIDEAIETSVSLLQKAGMTLDGPQGIGAIDLTGEEHTD
LPDADPEVTMEVPEMSTPEDKEPDELQNRQPSVRWSVEAEELEGNEDTFS
QESDSSSDFESHDEDSAESIAAGEEVGNSDWDEKDSWKCSDALHVDASPP
IPEADGRGKLIEVLGDATSHLQTSSGRESDTLPPLVITQTSQPGDVHLPS

LFDAIPSSSYQSPHLGQNISTESLGAKSGKAEYFEAREHNRRVCFGPEEN
KENDFPTSFQQLDIAMESSTPYPKHGNVLTTGLLESCTRDSATSALLASG
EKFLRTPVGELVEMPPKEDYLDDTSAYTYELSKRGAELVEDVQPPETVNF
ATGGRKDKPVSDSSPEYSESAPRSVQRSSKRKSDAISELLPNETEPSSSQ
TIAEPSTRPTEFALNSATQPPDVPRVESSSMDSADQRPAKRFRRVAEAVG
YAALGGIAVMSALIATAPSL
>Mb|QLI63665.1
MPAPRRRKIGHRRRVEDEGDDDGRPDALDLDDDSLTEGSLTDDDDAGQDS
DTSNIDEASPTCPKASEATNGPSAKSARANTAAAANGNAKQASDTDMMLH
GLSIADQPAPPQEMQFDEVVVSPPKSPSAPVVVSSASAMRQQPDTHAERR
RQEHEDYKRRRDQDPAFVPNRGSFFMHDHRHAGPAANGFRPFGRGRGRGG
RYGVGGPFAPFNHMQHPSDPTTNSPWTHDMHDTVTDPVPRRPRQMPDDEG
PLNGNGFVPTCEPSATPINRTLSTEKHIGNAQVRVMLPGMVEPAVFPGMP
VKQYTKLPDHRPPLRRDKPVRISLPGHPPRYILPAPDRSFIFIPRAQRPN
QQRMRGKPRPTWSSMGGFSRRTSVFGGSYYGSAYSPSIALSRRSSVAHER
DYMFSPTGSVISRPPMPADNVRPVVRLPPSAVPQPPMPVQVPAPGPVHVA
APALGQEAVLLPVPQAPDHPAALLESSINELPPPQTYPLPQKPTVQENRP
NSIPMHQPRPQKNISVADIESPTMTQGVGSQAFQGAFHQQVPVQVANGYG
QEAHARRPSYPSQQSTGTPLSHIPERAIHAAPFQPNTYGQPQYYGQQVYQ
TQQQQGYFYAPGYSGPNMAPTAPAPGFVPAAQPGNVPGGFPPPGPPGQPD
QGSASHGHGPNPSIVNPYSTYSAPQGYQPSVMGMGGMVTPSPDGFYYPQH
ASGMVYYPQ
>Mb|QLI63668.1
MADQHEVDLDSIIDRLLEVRGSRPGKQVQLLEAEIRYLCTKAREIFISQP
ILLELEAPIKICGDIHGQYYDLLRLFEYGGFPPEANYLFLGDYVDRGKQS
LETICLLLAYKIKYPENFFILRGNHECASINRIYGFYDECKRRYNIKLWK
TFTDCFNCLPIAAIIDEKIFTMHGGLSPDLNSMEQIRRVMRPTDIPDCGL
LCDLLWSDPDKDITGWSENDRGVSFTFGPDVVSRFLQKHDMDLICRAHQV
VEDGYEFFSKRQLVTLFSAPNYCGEFDNAGAMMSVDESLLCSFQILKPAE
KKQKYVYGGLGSGSKPVTPKYKSK
>Mb|QLI63672.1
MPPKKKGNKKAQDDWEADLGETIPPHATEPNDTAADANADADANGDDATG
GGGLMAMLRKNKEKRKKKGLADDDLVQGEDTPGAVSAELNSKAPQEATLE
DEFALPEKKGKAAKGKPAAKKGEPADDDEDEDDVNGGRILTKAEKEKLKK
EREKQRKKEQAAKKKTTAPAKAAEPTKPASPPKDAIATPASLAPATGTEA
GGKKKKLPAHLAMLQKQQEELRKRREEGERMQAEAKARAEEEERLAEEEI
KRREEAKVLKKQKEKERIEQLKKEGKFLTKAQKEEKARNERKLQQMLAAG
IKVGPQESSDEPKKKVVYDSKKKKPRNEKVDEDAALAEAAERAQLQAEKA
LKEAEEKAAREKAEAEAKAAAEKAAAESDIEDDWEAAAASEDDVKDSWDA
DSEDEEHAAATKTNGKGDAKPRAEKDGEGDDDDDDDDDEDDQSESEDEEL
SAAKQAEMQRKKEAAERREKAHQAALAARSKDNLRSPICCILGHVDTGKT
KLLDKIRQTNVQEGEAGGITQQIGATYFPVDAIKQKTAVVNPNNEFEFKV
PGLLVIDTPGHESFSNLRSRGSSLCNIAILVVDIMHGLEPQTLESMRLLR
DRKTPFIVALNKIDRLYGWKKVDNNGFRESLALQNKAVHNEFKKRLEETK
LAFSEQGFNSELFYENKSMSKFVSLVPTSAHTGEGIPDMLKLIVQLTQER
MVGSLMYLSEVQATVLEVKAIEGFGMTIDVILSNGILREGDRIVLCGTEG
AIKTNIRALLTPAPLRELRLKSQYVHNKEVKAALGVKISAPGLEGAIAGS
RLMVVGPDDDEEDIEDEVVADLASLLSRVEKTGRGVSVQASTLGSLEALL
DFLKDCKIPVANVGIGPVYKRDVMQCGIMLEKAPDYAVMLCFDVKVDKEA
QVYAEEQGIKIFTADIIYHLFDSFTKHMDEMLEKKKEESKMLAVFPCVLK
PVAVFNKTSPIVIGVDVVEGQLKINTPIAAVKTNSVTGAKEIINLGRVTS
IEREHKQIPVCKKGQPSVAVKVEMGGHQPVYGRQLEEGDALYSLISRASI
DCLKEFYRKEVATDEWQLIIKLKPLFDIN
>Mb|QLI63673.1
MAPSPPADLQVLCRKLTSIPQAQLPHALPLLTRHIVRCKDTLAAPQEQKF
KDDGSQSAQLVHKLRTSITTLLKGRDREARFAAIGLVKAIIDVGGWEMLR
VSEPWVAGLLSIVQKGDSFPAKDLAVATLARIYILVQPYQTLVREIATRT
IPTFITACLQLIKPRSTGQDPSAPLSVVETICDAFSTLIPIYATTFRPFS

SQIRSAVRGYLAPTLSDDLLIPLSLQRAARKVVISLHCVAAKSGGSEEWA
KIVDSLLRELHATADQVLRAVDESWEGTSGYNRKRVNPEGESSGGSNSGD
QLPPWSGLSSGAERLVGIFAYLSDCLRYPTKAPVVIPVGALLDAISRVCL
IARQSPKCQTWDQAVETNAAIGREEKAELWAAIPDIHMSALALISVMLQR
FRQGMIPLIPEVLDHLVRVFKSGISNPAVRMTGYRLLNNVLPLAGPTMSR
ATVSVLEPLIAACCRDLQEDAGFLKAIPKAATASKNDSKKSGLANADLFL
QPQASSVETSVHLDAAHKGAADDLLAAFLSSLPQHHLKPTLRGLLDKTAI
LTQNRDAMLSSVLNPYKDQRGRMYPSILPHLSQQYPQDQGVEILRSNLRT
SSVPGSIDLVASVTEVEQEKQQDEEEEQMGGGDEPIDKEARATESLQDMV
KPAVPKVDLPIQSNPFEVKATASNAFNVATVTRHDSVTKRKHEDADTAPS
KRQEVNKSAPANLSEPVPAAEDDDDSDSDVSVHLNMELDDDEEEEDE
>Mb|QLI63674.1
MAKRHASESLDELLGIASPASKKSRTAEVETPPNENGVSFAAPTQIYDGN
NSSDNDDDDLDEAVPSAPLRQANPTEGYDDLYLDTIDRQVLDFDFEKLCS
ISLSNINVYACLVCGKYFQGRGPKSHAYFHALDEDHHVFINLETQKVYVL
PEGYEVKSKSLDDIKYVSDPRYTKREVMSLDRAHHKSWTLGGKEYTPGFV
GMNNIKENDYLNVIVQALSHVPPLRNYLLLEDFTKTTELVKRCSILFRKI
WNPRAFKAHVSPHELLQEIALRSNKRFTLTTQSDPVEFLSWFLNNLHLGL
GGSKSKPGSSMIQRTFQGRLKVESQAITARADATDRLRFEEAAQVKVDIV
RFLLLTLDLPAAPLFQDELERNIIPQVPLTTILSKYDGKSAQEHHAQRKR
YRLLHPLPPYLIFHVKRFSQNKFVSERNPTIVTFDARNLDVSPYVEPNPA
EWPVSEPIWYDLVANVVHEAVRTREDVADSGDERKTWKAQVRDRATGEWV
SCQDLYVEKVQSELLYLGETYLQIWERRREPKPAASGARNKAMA
>Mb|QLI63676.1
MGHDAASAAGDDHENRDEALSPTDGYFHASESSGAASSSHHVPDRPYQQH
PAVTWQQHHQLQRPQRTSSHVPVVPNVLVEDPTLRHESAAKAKEAEEERW
INNGASQASPEAAFTPHMGSNVISQGMHHGASASLDTYMGNNQTTTATPA
SPTVVTSPLHHHRRSVEEDGPLLYTGHSTRLPYSHTPFETAASATSTSLN
QPTPQSQTHHQNHLDAPPAYSPSPSSPSAGHSQDYRTFAPPSSGSNNVNP
DVNQDTMGIPEEHQALLPRQPESMGGSPNGPPASRWQRVKNAVHSPNTRK
NIRTLLGIAVVLSIFFAIFGSISLSSGGSQHSKIPKIIDNDPVKEPGMGG
ERDLEWRPSGSCRNTPHKFDKSITSLQYGSGFSLDIVQRLERNGYRGGRQ
PNVSGQLLLRPTKESGGSVEIEIISNDERLRPSTEIHVDDGHQSIEIISP
RALDWWDYSGATPCIQMRVTVYVPHNAYLDNLKLDLVHLDVDIIEGLVMG
VVDGPTIKTVVGDVNTPRASNIDDDVAPYSVQSRRIIIETVSGNIKGWFP
LYDLLQVKSSSGDVEAQVAPKASDKKSGNQATLKVESISGDVKLEEPLTS
TVRSGKLGKKIPPRDYQVEIGTTSGGITATVVMTSSAKIGSVSGDLNLNV
LPLVTKSSDKTSFSTDTKSGFTNVNIYEAVAMELSDNAKSHVKDFEIRRT
GRPALYNFDSKHSSISGDFKLYYPAAWVGKFRAESMSGDISVSGQGVRIT
RRSRGFGKIVEGEKGDGASFIKMGTVSGAVRLQVGKE
>Mb|QLI63679.1
MVATEVDTGVALFRCSEGLGQVADQPGEYMPRPGSGAVRTQISDTPREKI
QSTSHGSRRESVKANDTSRSSRKAHPETLKDLLSRPDEKNYHDVWDLYCR
LDESQKQDLRALVVIYLSASHGSVERGRAISILRQIPVQLWDEKLQAAGV
LLYLHAGDRTAAIDVFNTGLATKSSGAGFKSILEYAIINKEWPIALKVWL
DYNSHFTRSNMATSSIYADLPLLKLVPDLAKLYFSFEHYLAVEAADPVKA
IHLYEDTRQGLRALRRWLAQQVLRQPCSPREAKTILQIWNDETLYRSYLL
RMLRRWNDGLETRASLAMLPEIYRQYRTMYHAKPPRLLLRQMFNFYYPAD
AAGLAQIYSDWHQAWGDLDQWGYEKFLQFYSTTGDVPAVKDLWARYTNLF
PEAPKTPQAFRGPMNVYAQLGDVAGAERELRIMTDQYGVKPDIDTWNMLL
KCYSKTNDHARVLQCFEEIKRLHQPDSFTYAQVMAMAAKKGDLATTLDFY
NQSQKAGVHISREMAMALVLVYCHNDRFIDAEKICAEFAERNLSSTAVWN
QLIYFNGLRGKLNKCYSILQSMTKYGLEWDHQTHQYLLQALVQVNQVQQA
YRLLRNACDNSLFPLGPEHFAVVMTGAVRTGQLDLAETILSHMRSAGFTV
PLSAHVSLLEAAFRRGPSAQNTRALAKDLVERLYSMLQPTKTAVPSGCPD
VPSWDTLTGPVELKKQTNEIGRAIVLLVELRDFVAVERLVTAYLDVFPDF
KKQNYFPPNIASALMLGYLKEGRRDQVHNMWRHTLDAALASGKTPEGAIY
PALQYDLARPLNVVAKAYREVNDGPGLLNTVEQLTSAGFKLTRTNWNLCI

RYLAEMGLWERAMDWCEDYLMSRWRGWMPATKSLQERRDMKNTRVLAASK
ATVFSLQKEWLKLRKLAAWSGSISSKLKEIERRHPMLHHAFITTDYEHLP
ATWMFPRKQSMTKAIKEMLSPLSHDELKAMRKALEKRLRLEKQKTRKAVR
SPFHVVVGRSKTQGEVFTKAFKTGDLKTLDAVLKEELAANRGESSTSAQV
PR
>Mb|QLI63680.1
MKLTSAHLLLTGAVELCGALPHYDSPQQRLAKGLSLTGKYAKDNPYTPDY
SDPHDHAIDAVGKGLDPRPWRNGNGATVLGPYNRDRSRQSPDMIRPPSTD
HGNIPNMRWSYTDSHVRIEEGGWTRQTTVRELPTSIELAGVNMRLDTGVI
RELHWHKEAEWAYVLEGEVRVTALDYEGGNFMEDLKKGDLWYFPSGVPHS
LQGLGENGTEFLLIFDDGRFSEESTFILTDWLAHTPKSVIAKNFRLDPQV
FAHLPAGEKYIFQGSQPGAIDQERPSGKHVKKSRYQFTHRMLDQEPKQTS
GGLVRITDSTNFPIAQTVAAAHVIIEPGALREMHWHPTADEWSFFIRGRA
RVTVFAAEGNARTFDYVPGDVGIVPKNMGHFVENIGDEPVEMLEVFRADK
FRDFSLFQWMGETPRRMVVDHLFAGDEDNGRKFWDAVRDAEKDEVKQPGD
VDDDAAEE
>Mb|QLI63686.1
MSEIPTPTGLADMERFTTPSGFQSYHRPMRRGTVLSRSPPDNQYPSATGL
RGPHVNEADGTGIIPIGMALGSPTEERGSRGNVWQPRVETTVIAAREDSQ
DEQQTKDGLSRSKSRKWGIFGRSKSKRVKNNDRAMDSPDQLTSPSDTPTR
GTPTSAASRGPRYTDLRDTNVSPRAKALGRSLTEPTGSESKWSPQVGSPQ
AKTQTTPDSTRTPNREPVSEGSNPIKQEPILDVEIPNITLDRYSVMFANL
LERRSATSLLARRQVTQDKIRALREEVSSPGSQTEILRSGRKQSMDRDLP
PIPSLRLEPLQTTRKYHQSSRSRSNTSPAIMGTPSKETFTDSHLRDGEKG
KSPHIVRLASVKGSKPTVGSGISTQGDRPQLRSKFHIQSPTHQHSGSKST
IASSVDLSENDMEEGGLSPPPARSHSYKKGNPSQSDPSPLTLQKATRSTV
YTTSTTGVQGSLSLSSLSGEEPDEDEDRAVQDAVEISIARQISVSREQRR
MLGPLQMHPIEGRRIAETKSSTPRLVDPKLDPSSPSAGHRNSERVVLERV
>Mb|QLI63687.1
MADKEATVFILDLGASMAQKHSGREESDLDWAMCYVWDKITDIVAANRKT
LCVGVVGLRTDETNNRLQDGDGYENISVIQELSQITMSGLRALKASVKPN
ETMSGDAVSAIVVAIDLIGTFTKKLKWIRNIVLVTDGQGEMDADDIEDIA
KKINDSGIKLTVLGVDFDNPAYGFKEEDKPPTKAASEHTLKALINQCEGG
TFATMAEAIDELDTPRVKSVKPYKTYDGALTLGDPEKFPAAMNINVERYF
KTHLARPLTASTVVVKSDQATQSTQTLEDDPMDGIEFSAVKQARTYKVND
PDAPGGKRDVEFESLAKGYEYGRTAVHISESEHNITKLETEKSFSIVGFI
PWTKYEPFLNMGEVCVTHARKNDTKSELALSSLIWALLELESYAVARIVT
KDGKDPLLVLMAPQLELGLECFYDIPLPFAEDVRSYQFPPLDRVITVSGQ
TLKTHRLLPSDELAKAMSDYVDAMDLATYGQDEEGNPAEYASIDETFNPA
IHRVNNAVKMRAAHPDKPVPETPASLLRFSAPPEDLIEKVQPRIDSLIEL
ADVKKVPPKAKGRRARETVKPISGLDVDALLGEDTKRTVSEENPVPDFKR
ALGTADEMDQIEDAAKQMGNIVRSLITDSFGDSKYAQATECLGVMREELI
NMDEPEMYNTFARDLKKALLSGALGGDRRDFWFKVRWSRLGLIDQDQSEV
SKVTHEEAEEVRAHIVLLE
>Mb|QLI63688.1
MRARLVMRRQQAVLPSSNPRLICTRSFANQTKFTSTTSRPSLPFLLVPKA
SPSIARSFTSERKRWLKHEAKLVVRYTITVWGLAICGILIFFAVNEETSE
RESPTPHEWHYLTRKFLRDAQSCRDPKDGEVNWARCLELARGVVIRLEDP
KLGGGNVVKLSDKVDPSLEVPGEFINCDISAKSEEWRRGYFEAIMLAAKA
AEHVDGWLRDTTRNVVSAPEFVIGPSNPRPKPIPPGRPHAPREEDCEPAY
PAADSWYVKILATTGLSPRQKMEAALEYATFMEFKHRQEGSEALYSLALA
EASTGLDPSKLPYDPKTFIVKSNAPAPSMNVLDALTAIANYKARRGDVSS
ALPIYISLLKARRSLSDTPPPAIRTEPKMLSPYERFVRFFGPPAYPAPAP
DGTQPPWRTPYERCQEASLDLYIGEILYATSSEDEGLAWTRDGVDLAEEQ
MRALGSASKDKETKQICRECLGTGLENWTTMVSLLAKAEESRNIDAKSKL
FSFWGGPQDAEGRWAAEEAVVEERIRRTRELMEDVTPPRMGIWSYFKA
>Mb|QLI63695.1
MKFGVLVLASAASAHTLFTTLYVNGENQGDGTCVRMPHDSSTANGPIYPI

TGDDMSCGRDGNKAVAYTCPAPHNATLTFKFREWTDGTRPGVIAPEHKGP
CSVYLKKMDDMYADNAAAGPGWFKVWEDGYDAGSGQWCVDKLIEKEGLLS
VRLPEGLPAGYYLVRPEILALHNAPQGDPQFYLGCAQIYVQEGPDGPLRI
PDEANVSIPGHVSADTPGLTYNIYKKSQGEYPIPGPKVFIPEAKSFSPSP
GNQTEGAIPKDCVVKNANWCAKPLPGFSNEDGCWASVENCWSQDQACWNS
VTPSGDVNCKTWQEYCTQSETRCNSGDYAKGPVEFKGEDTMPDLPGKALG
MWNDVFTATG
>Mb|QLI63696.1
MASTPEQLALLGAFLEPMSIDVQKCHVLSERFLQNFTHLSAESLDQFLPT
PISESILRPVEDYGHGRHLAIDIGGTNLRVGFVELLTVESAISASHGATT
TSNGINGINGAISSSDEDQAVPTLPTGRLHRQLEKSWPISNHLKSDNADS
LFLWIGKCIAEVVEEGCKAFNLSIETPLPLGITFSFPVEQPSLEKAIISS
MGKGFALPPHVDLGIRLQAAYDKHRGDNLPTIYVAAIANDSVSTLISFIF
NYDRLAHRRAAMGLILGTGSNATIPVKLSLLHPSKRPKNVNVLPGEKVED
AKIAVNTEWSINGTAPPMRELGLISDWDDELSAKNEKPGFQPLEYMTAGR
YLGELGRIMLVDYMTSRLNVGRDLLPPKILEPDSLTTTFLSHFKPLQPTA
LLSTLKTEFPEPSGSQFVWTEHHAEALYRISKAIEVRAAGIIAAAILALL
TLGEEIPVDGASPIPTSEIRELGVGYTGGCIVHFQDYLVDCQHFVDQLVK
KRCGIHFPLKVTMNPCHDGGITGAGILVAAALSSQASQT
>Mb|QLI63699.1
MARRKKAAPKRKSKWTPDNILSDPKSPLASADLRSVLANPLAWTSLSIQE
RRDVLCLFPDMAHTLDADTDDARPNFETLMNDDSFRYDCAAYVDNLAMGR
HDAVWLEDAWSAHDRRKAGEFDEWLRDKFVEDWAVEPPDDSKKAKPEERR
VQDGQGEGVDGQKRGDETESTLAGCQPQDEADNGDSNHCMGEDSHRIAIL
>Mb|QLI63700.1
MVRLTVISLLTLAVTAYAALDPSELKNVGQGNGKQFITGKCLSNADCSTN
CCAGKNGGGVCSAVAVANADGKTGCGFGGGNAGNNGANAGNNGANAGNNG
ANAGNNGANAGNNGANAGNNGANAGNNGANAGNGGNQGVPTGDGAENFAN
GAGKQFITGVCASDADCASTCCAKTTKACAAIGAVGAENCGFVA
>Mb|QLI63707.1
MAPWPHDLILMRLCDGFGFGLGFALGHGVAIPSLARGKDASKSSKKNKDS
KDGTSSPSSRDSSNQSPVLTPTSSSTTLNDIRNKPLPSSTTGHGDHGVPN
QSSNPGQQGNVPDRFGPMGGASSPNGGNANARLPPTVVISPTPGHIPPPG
AAETMPHDLAPPKAGQKSLMIHRGIDNRDAIPEGLRTPKRQHSSRFDISA
HRELEKLPGFHEVPPNRRQELFMQKIDQCNVIFDFNDASGDMKAKEIKRL
ALHELLDYVANNRQVITEPMYPRVVDMFAKNLFRPIPPPMNPQGEAFDPE
EDEPVLEVAWPHIQVVYEFFLRFIESQDFNTNIAKAYIDHSFVLQLLDLF
DSEDPRERDFLKTTLHRIYGKFLNLRSFIRRSINNVFFQFTYETERFNGI
AELLEILGSIINGFALPLKEEHKIFLTRVLLPLHKPKSLSMYHPQLAYCI
VQFLEKDASLTEDVVLGLLRYWPKVNSTKEVMFLNEVEDIFEVMDPAEFA
KVQEPLFHQLAKSVASPHFQVAERALYFWNNEYFCNLVSDNVEIILPIMF
APLYENSKGHWNRTIHGMVYNAMKLFMEINPQLFDDCSHDYTEQQNSAAE
REALRERKWAALAEKADQRRVSGGVSDAPSRPQVGGVLSRVDEVDPVTED
NQKRLDSLKLQDAAGRRPGGHERQNSQSSARSR
>Mb|QLI63708.1
MDAQNAAESDQTRPAGASSQQRPSSETGKKSKSPTLVKAMSPARDPKPKT
GLATADDASASSLTARPDDAPEDRDSDAETIVLPGKDGHSPSKARKVRQE
DRSESDGDASKGNAANGNGKHTHDGEGAAASAAGSRLVEGARKKRPSVSA
GSSADKDRRPRNKEAASSGMSSSPVSPLHNLQQQNQNHHPNRRRRSDAGP
SSPSDSEQSHHKISSKASLRDKLKSGERLVPHKRKAPKIESDEEADIRKA
RRQRTTSAGVDTGRHKDSRSSHPKIEGRNRSVSPPYRQHRRSISTQFPSQ
SSNGLGIKKRRLPPPLQSTDYQSDDSSTGGSPHPRSSKLRSLATPASAEA
NVSPAKMGPHKKHLDAHGQTLLARACARGEYEGAKQRLQERPEDLNVADY
AGNTPLQIAAINGCEDIVKLLIDAGCNLECVNYDKDTPLLDAVDNGHLGV
VRLLLDAGVNPRKANVNGEEPIDRVSDDTDNAEEIRAAISEAKKRQGDRR
RTSEDRHSHGEGHDTRDSHAPDSPRHSPAATTSTSTGRRSGTVRSTKTRN
DLLYMPLDDKTLRQAAGRGDEETVARILQVKEGYDDPESMVAAARGGHDL
VIQLLLGLGAANADPGPVPSLPGEFATPILAAIGQENIKVVELLLEQQGF

DPTRRFKGETYYEIAKRRQGTNWKEEETKLKEAYDEYKRSHKDGLKMKSP
GGRRDKAARRDDAGEGPKSSRRRMSSPSRDVELKKKSSKLGSPGEKRRAN
SINHRGDDEVPKRGPGRPKKEHSAISDREISPALSYKTQKAKRTDSDVAA
MSSEGEAAKPRRKLVSKGELRGERDKHKRISSHKEPSSPRESRSDDVPEK
PRFSEKYHDRAKALKRDESRDRPSGDGSAKRHRTSVTPDHPSDFDKDGND
VPVKRRRLDAESQESRQKRVPSEDGRQRKSSSSRDGSSKASSTSSHKERE
DGKKRDSEMKNFGQCASGEKSIYVKSEDVDIPMPDLEPAKDTESRDMDST
KQEEEAEQARLKEAEEEAAKKKRAEEEEQEKRRLEAEEAKKREEELARKA
AEEKKRQEEEAKQRKEQEERERREAVQREEEEKKKRLEEEKKRKAEEERR
QREEEERLHREQLEREAAEEARRQREEEERKERERRERAHREEMERKRAA
REAEQRRQREEQERARLEKLPPLLRWLDMHPGPKTGAIAEKFKNVLGFRY
DTIRQDATGTAEGREQWLLNTNVALLLGEKDLDLSRYTAWERAPVTHLNK
VQIWRTEWRQYTLLSEDLWDLGRQLPGCYNGTDPSQLEFSTKQQLTEDAK
KKFETLDMFFVKLSDFLYTVPNIPHLRNLRIAVNYRELLENESQLFGWKV
TQKWKQDPGAERFHVFAPRNKYYINGVFVGEDLPTRHKTSKTPFPEKRVP
RRGLVQVFPEDPDYERICMEQGLEHLVKGLQTPSLPNGDHSSPTMQTATT
PSKPSVNGISGLANGAKLNGAHGTSD
>Mb|QLI63709.1
MSMDLDDAPVPLGPATQQTAATILCCNCGAPIDGTTATGALCFNCVKLTV
DISQGIQREATLNFCRDCDRWLLPPTSWIVAMPESRELLALCLKKLRGLH
KVRIVDASFVWTEPHSRRIKVKLTIQDAVQDGVLLQQSFEVVYVVATQQC
PECAKSYTANVWRACVQVRQKVLHKRTFLFLEQLIMKHNAHRDTLNIKEA
KDGIDFFFSQKNQAEKFIDFLNSVVPVTVKASQELISQDVHTSKKSYKFS
YSAEIVPICRDDLVAMPIKLAKQSGNISPLVLCHKIGTSVYLMDPQTLQT
ADIASPIYWRAPFRSLANTHELVEFIIMDIELTNTRRGKWVLSEATVARA
SDLGVNDKTYFTRTHLGNFLQPGDSAMGYLLTGTMFNNPEYEAIEESNTY
SSTIPDVVLVKKHYPRRRKNRRRNWKLRRMGKDEGELLPKKADQDRMDRE
YEQFLRDVEEDEELRAAMALYKNEKKRAEEEMSVAETEEDEDDGTPHVNM
DELLDDFDELTMQES
>Mb|QLI63711.1
MSPVFWPFWRRKPRSQLPDEHTTTTTTTAAANPNIPIGPLDTSAASTTHP
PRELELHVRGDAAQAPPPAPPSRFAATTSGSRPAPSLITPERPEHPEHRR
PRRRRADPDSPITTPNPFPPSSSPTQTPARDKPASRRLKAPSQQPAYPLA
SLDRAAEPAAAAVAALSPPQPSVHGQVYKPSLVQSPFRPRTKATTDPAHR
LRRLPAARLWNPTNSTPRLPQPRKRRPSSPPIPALPLKHPALDRSLDIRD
PVTTGAGDYPLLTLPEQRRSRYSQSTPVSFQLELGETSDHRISLPRSVRA
SYEGKRSVHRTSTHLEATAEQQVNDQHAQEASVAYLSAKSKGKQKATMAP
ESEDARRPYSKDLERGPDVMDHRASNISAGEGIGSALSSSNSSIMGDDVQ
PDAGQEWGPQHPCFPHLNPHVPLDSVEHATTRIIRVKRDWLVAGDLAPTF
SNLYPEILDPAGVSEQEFRRVIEKLNGELIPIFDPFTFRNMLDSVLGLVT
GWLWDDFGLTAAKSRLHNLEKWIEKWNQEMEKTMASEEGIIPPKLISLRQ
TGYMTLDIQIPDPEIAPAPSTIGAGESQTALPMEPAPAITA
>Mb|QLI63712.1
MGEVADPALQVIVLGSGGGPQESNITAFLVRSVAEKWSKGSIIAVDAGVH
LSAITRLLQESYPSTPPTNLPFTLSTGAFAGLELPYASAAANAAHVTRSL
VDTYLITHPHLDHISGFVVNTAGLPGTRPKKLAGLPSTIQAFKNHIFNNV
IWPNLSDENNGAGLLTYLRLVEGGSPALGDGEGKGYMEVCDGLLVKTWSV
SHGHCIERHPHRGSASSVSTRFGSHDASSQTPRRDIHYHTAMQPTPKATG
LLSQAAFSAVSSTQFAASNEQEKICVYDSSAYFIRDQSHRREVLIFGDVE
PDSISLSPRNLYVWQEAAPRIVSGILAAIFIECSYDDSQSNDRLFGHLKP
GFLMEELRVLANEVEVAQKLGTFDSKKRKRSSMDEIMNQRRNPFGKPGFM
YDDSVSPKSIKPTVPKQEATPDHADTPHLATPTDELSLHDFGPMDPILEK
KPLEGLKVIIIHVKERLEDGPQVGDTILQQLQDYEKEMNLGCEFIMSKSG
MSLYF
>Mb|QLI63713.1
MSSDSSDDDRPLARPNGHLSSAKVSRAEDQALDRSQPRKSSGMTGIVVRN
GQVDDAMDLDAPNGVNKRKSRSSISKVSYRDESDSDGEPLAKRPRPSARP
VDSDSDDEPISKVAKKKSTSMLADSSDDEKPLGIQLAKKKAAIEKKAAR

EAQTIRSKETAKSTPKKNIKEESDDEPLAKSSANKRQSNGASAKRKSNGV
KKEESDSDAPISKKTKAKGKTTASSKGKSTASTKDSKKAKVKETSEDEEG
YAWWNAPKPEDDSIKWQTLEHNGVLFAPEYEPLPKNVKLLYDGKPVTLSK
EAEEVATFWVAMMTPASSHHLDNPVFRNNFFTDFSEIVKKNGGATDREGN
KVQIKSLDKCDFSKIYDHWLAKTEANKTKNLSKEEKEAAKAKKDALEAPY
LHCIWDGRKQKVGNFRVEPPSLFRGRGEHPKTGRVKTRVFPEQITINIGK
DAKIPEPPAGHKWKAVQHDQKATWLAMWQENINGAYKYVMLGAASDVKGQ
SDYKKFEKARELKKYIDKIRKDYTRELKSEVMADRQRATAMYLIDQLALR
AGNEKDTENEADTVGCCSLKYEHITLEPPNKVTFDFLGKDSIRYNETAFV
EPQVFKNLKLFKKAPKSDGDDLFDRLTTSQLNKHLNSYMTGLTAKVFRTY
NASYTMSTLLKELSQDPRSKGSIAEKVKLYNDCNRKVAILCNHKRTVGAG
HEQQMQKMGDRIKGLRYQKWRTKKMILDLEPSYKKKKGASWFELDEDLDQ
EWIQEHQQFLIEEQRTKITKKFEKDNEKRKANKEKPMPEKELKERLQVVK
DLETKFRKENKIGKVEAEGRGASVDKYLKAIEKLDERVKVLETQAEDRDG
NKEVALGTSKINYIDPRLTVVFAKKFDVPIEKFFSKTLRDKFRWAIKSVE
DTEDWEF
>Mb|QLI63714.1
MDAELVARALEGPDRPPYYKVVGIALAIGSGLFIGSSFVLKKVGLLRANE
KYNEVAGEGYGYLKNAFWWSGMTLMIVGEICNFVAYAFTDAILVTPLGAL
SVVITTILSAVFLKERLSLVGKVACFLCIVGSVVIVLNGPQESSVADIQE
MKTFFIAPGFLSYAGVILVGSVITAFYAGPRWGKKNMLVYISICSWIGGL
SVVCTQGLGAAIIAWIGGKPQYKEWFLWVLFVFVIGTLVTEIIFLNKALN
LFNAALVTPTYYVYFTSTTIITSSVLFRGFKGTPQAIATVVMGFLTICSG
VVLLQLSKSAKDVPDVAVFKGDLDQIHTIAEQEQPESEPKADAIRGAAAI
VRRISNTRLRMEAEELKRLHEEKMAETMAPISEDGQSEYEWDGLRRRRTL
AASTRARAATSPQPRTPVPGPLGSPTSPHPPLGMSRFPSDEELEQAEQGT
IFSSIAGTIRGRPRTSTTLPSYEDEYPIKGRSPLHPIPLTHIQQVPTQKP
DDEAGAYYGHTREHVFSHQDTAYHGVADRNVQDIPPTPPPHSARRQFSFN
RVFRRNTGPTEEERVGLVKEGGTEGQPSFI
>Mb|QLI63715.1
MAGVATYNIAGRQVGGHYLAMGILGSLFGGVYYATSGPKKPAAAATPPIN
ASSSDEADFIKKFMEEQEKKQ
>Mb|QLI63716.1
MSKPLAFGLSLSKKSGAAKPKPKPTPFGKKSAFGGDDGSDNEEHSSATKF
ERIDGVLDDFTTKAPKSDTRKSSKQKPGALTQPPKLKSKNEPSAMFGDLS
GTLSSRRNAEAATELDSSIYEYDSVYDSLKPKKDDAKQDVERKPKYMKSL
LQAAEVRKRDALIAEEKKIAREREAEGDEYADKEKFVTEAYRKQQEENKR
LEEEEKRREEEEAKKNEGGGMSAFYRKLLDKDEARHTELVKAAAEKAKDG
PATDQGDEEEADREKAEADLARELNEKGASVAVNEDGQVVDKRQLLRGGL
NVGAKKKEEARREAERPKEQERRPTNGAQLGRKQAMRERQTRMMEEQLEQ
SLKRSREAEESQREEIERAAKSQKTQSEISSAKERYLARKRAAEEAKKQA
SEG
>Mb|QLI63718.1
METSPLHLLQARGPVTVVASAPTFPPYTSPGLTANLVCRVLLAVVANLVC
LVPLRLLYRNGELAAVVFILNTQIKNLETIINALIWRNDDIMSWWAGYGW
CDFDAYIHNISIGLFVTCLLAIMRNLAQQVGLMRANALTTRERRRRNLIQ
ALIIFPFPILQLALTWPLTAQRYLVGTLMGCNWIPHPSWPFLIFFVLPPP
IFALITTGYAGKCRAECLVKTWADIYTLFSSPFLVLIYKRFRQVHKTTET
ALCGNRIAQQRSQRTRRKLYLMVISILVPFLPIVIALSVLNIMDMHGIKP
YSYDQVHNHASPFPWNTIIFFPSSEISWTYMNNCYIPIATAIPIFVFFGL
TKDAINDYRQVLLFFGFGAIFPGLHNEYDPDRTVTTSGGSFGSGRLQQTL
TR
>Mb|QLI63721.1
MSRHHPDLVMCRKQSGIAIGRLCDKCDGKCPVCDSYVRPTTLVRICDECS
FGNYQNKCVVCGGEGISDAFYCFECTRLEKDRDGCPKIINLGSSRTDLFY
QKKTNRSANY
>Mb|QLI63722.1
MAPVDRLDQDVLEQQLKDVIQDLYQIMVQVSTYDTTGRPSRDVLSNEMKT

LSASLQALHATTSSNASLPSVPPELLEYVENGRNPDIYTREFVELVRRGN
QLMRGKMHAFGEFRDVLAREMATALPELRPDVERVVRETGGRPLPEVNGA
GAAAAAAASSSTGAPGNGTR
>Mb|QLI63723.1
MPGHGLQSCHNDPLPQLDVTGDCPHIPQRAKMIAGSETTSTQAQVDTTTS
GSITSSLTSNIVDELKSSAQLQQEERKSTTSGKSAGSIRGLAKRSSRTLD
DSFSPENAGRPEDTWDMAANPMPVHCEGDGVTYVPPPQLFKAGMYRDETH
GSLIPGGLGDSFSANSQSKIDLSCPLERSSSKNESDGRSIDSASGPNTAN
QNYTSQPARDEPSDTECFWMSDCSETVPILEDRHHFLNVKSGKVRDALVR
LDRWRSRWANRHMRQVSVQKNYTQHIATRSHDDDSSCGRKNGTKSGKKGV
THGPERTTTKGGKDNPGLRKDVFRKKELFEGMVETPTASRRSSRRQSSFP
PELLSDADNDSSSAGDVSTRTRSLRRRTAKFTESLNEEEEETKPQTNGKS
AHGEEVVSNGSPKKSTPKSTPKKQAAPKSADAGVVDGWREGQDPKVDYSG
EFEFGGSVGTLAMMILFPVLMWYMWIGATYYDGKFPSRAPGQSWASFGAQ
LVNYVYTGAFPHVKAWFWYWSYLIFQGACYCLLPGVWGWGKPLPHEGGKQ
LKYYCNAFATLYFTLAILATLHFTGIWPLYNAIDEFGPLLSVAILSGFLV
SFAAYFSALYRGKEHRMTGYPIYDFFMGAELNPRMFGILDFKMFCEVRMP
WYFLLILSLGTAARQWERYGYVSGEVMFLVMAHYLYANACAKGEELIITT
WDMYYEKWGFMLIFWNLAGVPLSYCHCTIYLANHDPSEYHWNRWFLAFLY
IAYLFWYWVWDSCNSQKNRFRAMERGKMVARKTFPQVPWQTIQNPKTIET
PQGTILVDGWYGLARKIHYTADAWFAISWGLITGFKSPFPWFYPVFFCCM
IAHRAARDIHRCRNKYGEHWLEYERQVPYLFIPYVI
>Mb|QLI63724.1
MASSHHVLLIGGHGKIAQLLTPLLLKRSWTVTSMIRTQEQAPAIEKLGSG
LPGKLHVLVSSVGEVSTQERAAAILNNVKPDYVAWSAGAGGKGGPEMTFK
IDRDAAVHFIRAAADIPSIKRFLLVSYGGSRRAGASWWPEGEWDEYNKKV
NYGVLATYYKAKIAADEVLYETSKKSSTLVGICLRPATLTDEPAGKVELG
KTAHVNGDASRATVAATADALLAADGVKNTWLDLQGGSEDLDAAVKRCIK
DGVDTAEGEEIFTSRI
>Mb|QLI63730.1
MSYPTSKISALRQLMSNSKTRSSGGCWTCRMRRKKCAENRPECDTCKALE
ITCYFQDEKPDWMDGGPKQREMADRIKAEVKKQASQRRDRKYLEMLEAGT
KMVSISDTDDPQANGGRGKAPMSGASDTDPSPRSHGIGSSPESSNTNGAS
PPEVPWHSQVFVRQEDIDDNGLDNDLHFLMIYLDYVFPYLFPHYRPPVLA
GGRGWILDILQRNKCVYHTAVSLANAFFAIVLADGKQEHEQCAMRMVHSL
ESQLELGLKELSKEMCLINASKTGFDKEKGLVVMQSIIQMLFFEVATSKK
ENWRLHLDAAIALFVQILSEPEQWTGTLHSLYSPRWPPPEIGARRPWSTN
QAALRFFTATLIYIDVLSSVTLGSSPRLLRYHDTVIPACAEFQRSIEPTP
AGPLTMDEFFGLPNWIIQLLGNVAALESWKREQKQMGSLSTDELASRGKV
LSDGIKTGLDLLENHPELRYPPQGVFPLLVADPVTGAHAEAQPRFQMIWL
LATLSYLHVVVSGWQPSSSEIRWSVSKATELLTSLPTGAWLRAVAWPMCI
CGCLSPPEDEDKYRQIVRRLGALQIFGTVKEGMEVMEKVWSIRGQIDESW
DVARCLNILGYGILLI
>Mb|QLI63732.1
MSAPQDVLSANGNDLVDHLDQLKMEERLGPDGEPAPKTDEEYAQAQLTLR
AIVSSKEAGVIIGKGGKNVADLRDETGVKAGVSKVVQGVHDRVLTITGEC
DAISRAYAIVARALLEGAPAMGMGGIVQSNGTHPIKLLISHNQMGTIIGR
QGLKIKHIQDVSGVRMVAQKEMLPQSTERIVEVQGTPDGIQRAVWEICKC
LVDDWQRGTGTVLYNPVVRTQAGSGSLGSNYNNGGGRSDYGSPRVMRTGN
GADFSNGGVRPFSRRSDSDAASRGPPTHDENGEEIQTQNISIPADMVGCI
IGRAGSKISEIRKTSGARISIAKAPHDETGERMFTIMGTAKANESALFLL
YENLEAEKMRRSQLQEPE
>Mb|QLI63734.1
MAQQNDSKVTNADAQSAPDPVNASSNPDPSSKDLGADMAAGYGTRSRNRG
GNARINYAEDKDIDMDVYDYYDKKDQDVPKKSSRKSDVAANGDGAARAVG
SRRAAAEEARAVGASNQNGSKSSTPSGVGVGVGGGASQGAPTSGAAQGSR
KRKATAVSTRKAGQMGQLEGTPMPETNMLTFENCNHRPDANGCMVADDGT
VLEPNDHVYLVCEPPGEPYYLGRIMEFLHVHAKGESKRVDSVRINWFYRP

KDIGRKNTDTRLLFATMHSDISPLTALRGKCQIRHRVDIDNLETYRRTPD
CFWYEKLYDRYIQKNYDLIPTSTIVNVPDKVKKVLDERWKFVLVEQGRGK
ELTSAVKLCKRCSGYCASNDSVDCAVCQNTYHMNCVKPPLLKKPSRGFAW
SCAACSRAQERKLEARNTPNGTDANGDADDDDALDDDDDDMPGIITDRTT
PADNEEHHHATAEQIYQASLWPWRYLGMHCKPEDALDYDDRIHPRASTRI
GPRHQANVGVWPGRPVEYVKPLETRKGRGVPKLSKEAQIAQEAEKALRTK
RPKWVQDEPPGYQVRGEDLDENDPAATSTRLWMPPPSDTVGDDDIVGYMT
KAQGMAKRFNIPERSTNLQDVALETLFRHDYDPAAALKALPDTKRDSFKE
PTLTPVENRKFEEGVAKYGSELHLVMKHVKTMTPGAVVRWYYTWKKTERG
RQVWGSYSGRKGKKLAKKEETAASKAADDVADADDDSAFDTEKALVQKRS
FICLFCESQDCRQWRRAPASQGPLSENGGRATSKDKGNQSVVALCRRCAE
LWRRYAVRYEPPEDLIKKAGQSGAKIWKKKQEEELLKEIQIAEDMGLMSP
YRSSTPATSVNGLEPPRKKLKGAPERDTDAVVSDAGSNTTVSRKKDKTAE
TTPVPDLPKPRTLPCAICDQMEPLGDQHLSCRECRLTVHRHCYGVMDNRI
QGKWICDMCSNDKSPQVSIQYKCVLCPVEYTEQDFIEQPKLTHHKKKMSD
KDRERERLEVQQARKAAEFYRKRQEDLNRPVNPREPLKRTADNNWVHVTC
AVWTPEVKFGNAKALEPSEGIPSIPRARYDEVCQVCNQQGGACVSCHQCR
IPYHVECARQAGHLLAFDITPVKSSRRDQFNIVTVKGEIGTMSAVVWCQD
HIPTKTMAHKMYDVVSESGLSALQLYVQHYKQADLGLTGTVRKANLMMAA
AKMSGLPLVPGARRTSAISATTAAAAPNGASSHSRNREPMDAATNAQQPG
EKVCITCGIDVTPRWWPIEKTQEQQLTNGHHGAIGFEAQKFVEQRKFQCH
KCKKNPRTPKSFAAAPKHPHHHRCEALHEYSLQIIEQLLCVQKSIHCCIT
HHPMVRQHLARQHLARQQLMGHHPEGILPHTRRRIRSALDLGQYLMAIPK
YRPQGQRTTIGGANTARRLDTLMEDRHHYHCTTLDRLQHRALQRCGHRLC
RGRPRWPQSLFHTSVHMAPRYMEAHCPPHRDD
>Mb|QLI63735.1
MPGASSLLASAFGLPRSRSQSRSRTASPNKRGDVTDATYSADWTTSSTPL
NSAPDSPSVPATPTFAGSAFGSPRHLPKMADSAVDVARDSARSSMEKLSL
TNYRQELDKLNEKSQRSSPDRTGDSSRSSMESGRTSMSADWPTHPYELNP
IAELDKLPLGSEVNFRARISTQRQLSKNLDFLLFRDQTNTVQGVLSREYL
DMVRWVQKLNSESLVQVNGVLKAPPEAVRSATHSGVEVEVHSIHLVNAAH
GAPFTNYKPPETLRNRMTSRILDLRHPSNQALFRIRSMVTRMFRETLEQQ
GFVEINTPKLQPAATESGAAVFKVNYFGRNAFLAQSPQLAKQSAVSADFG
RVYEVGPVFRAENSNTHRHLTEYTGLDIEMSIEHDYHEVICVIDEFLKNV
FTSVYAMPEVKEVQKRWPSKEFKWLEETLILNFEDGIQMLRDDGRDVEME
DLSTPDEIRLGELVREKYGTDYYVLDKFPASARPFYTHKDPENPFWTRSF
DIFIRGQEICSGGQRIHDAAELRENMQAVGISEDGMEDYLAAFDLGAPPH
AGAGLGLERIVAWMLELGDVRYASLFHRDPKSLPERAPGLPHPDADTTKP
LRDGAMPPVEKLIANYGDASNTSWLDERFKIWRHPSGAAVGYVKQDKFAM
ITGDPLCDKTQYKDVCAAFVKFVLSELKLTPVWMLVSYEIQKILAQQMSW
RTLSCTEEQRVDATKHHAINGGGPKARRVEREGVKIHEVKPDADFIRRAD
EAIVNWKANRKGKQVHLTEVRPWIDMEHRRYYAAEKDGKVLAMVVLAKLS
PKHGWQVKWALDFPGSVNGAIEVLIEYTLSTITGQVTFGVGVSEKLTPGE
HLHGIRARFLATTYSSIVDSLGLRRKAGFRSKFGALGEEVYICYPKHGVG
LRDLQQIIKFFQD
>Mb|QLI63736.1
MAPKPKSAEKKQQSLISFFTPKAVDGLAVHPKPQTEALAADAAVNHDAES
PPRKRPLEENTDNGNEGQERSSKRPRGDENHVNGEKSTFIATRKPTNSSG
AVAAPRAGRYLYNGSRTKPATQSTQDDDDEDDQVRDRNQDLHRQFVKKLG
HPDAMARRSRVAQDEGAAGGEDGDAEDADEDESPAPAKAKKKGAKSGKLT
PMEIQFLDIKRKHLDTILIVEVGYKFRFFGEDARTAAKELGIVCIPGKMR
YDEHPSEAHLDRFASASVPVHRLSVHAKRLVAAGHKVGVVRQIETAALKK
AGDNRNTPFVRKLTNVYTKGTYIDEVGELDQRAEGAPAGGYLLCITETKS
KGSGTDEKVDVGILAVQPATGDIIHDNFEDGFMRSEIETRLLHISPCEFL
IVGDLTKGTDKLVQHLSGSSTNVFGDRSRVERVPRPPTMAAEAYSHVTQF
YADKLKDTSQNETASALLDKVLKLPEPVTICLSAMINHLKEYGLEHIFDL
TKYFHSFTTRSHMLVNGTTLESLEVYRNSTDHAERGSLFWALDKTLTRFG
QRLLRKWVGRPLLDQDRLDERLAAVEELLNKQSTAPVDDLERLLATTKTD

LERSLIRIYYGKCTRPELLSVLHTLQKIATHYSSIKSPSGNPFSSPLLAT
SINALPQILNTVVSYLERINLEAARKDDKYAFFREDYQTEDIQDQQMGIA
HVEHELGEHLKVAAAKLKKKKVDYVTVAGIEFLIEVPNSDIKNVPASWSK
ISGTKKVSRFHTPEVMRMISERDQHREALAAACDKAFKDLLAEISSDYQP
LRDAVSSLASLDCLLSLSKVAAQPGYNKPTFLPSSSEPTVAITQGRHPIA
EHTIESGYIPFSTTLAHPSPLAHLITGPNMGGKSSYVRALALIVLLAQIG
SFVPADAISLTLCDAIHTRTGARDNLFAGESTFMVEVSETARILRSATPR
SLVILDELGRGTSTHDGAAIAQAVLEHVATETRCLTLFITHYQNLARVVE
GLPGVSNLHMRFKAGKGPDGDEEITFLYEVGEGVAHRSYGLNVARLARIP
KKVIDVAANKSGEMELDMRMRRLKGASRMLAEVMSDGPDQLEHLIASIEQ
L
>Mb|QLI63737.1
MSVTSASIASDGGTGAKFTYATTVIAGVASFAATLLSVVSIWLQTKNYRK
PLLQRYVVRILLMVPIYSIASFTSMVSLTAAAFIDPVRDIYEAFTIYTFF
QLLINYLGGERALIIMAHGRAPVEHLWPMNHILRKVDISDPHTFLSIKRG
ILQYAWLKPILAIAAIVMKATGTYQEGYISAKSGYFWSGIIYNISVTVSL
YSLGLFWVCMHRDLTPFRPVPKFLCIKLIIFASYWQGFFLSILVWLGAIP
DNVQGYTRDNLAAAIQDALICVEMPAFAVAHWYAFSWHDFADNSILSARM
SLHHALKDSFGVKDLIEDSKETFRGNNYGYRAFDSGDKVMAHEDSQSRLA
RINAGMRYERGGKAKYWLPKPGDVSATSPLLGGPSSSRTGDTLFEQQHGT
FDEPELDEDEERLYNSARQLKYGDWNYPVITASEPLAERYRSSMSSHNGT
SPAWSTRSARPPPPQAANSNSGSSRQSSSQRIEEPQPVVVVKKKKKTKKV
VKHPESDPAGLGLGKGAPAHSPDPTGTTDASASVQKPEPEEAGQGGDSAA
TDEGQQTHRYHVDGSDEFCNVWE
>Mb|QLI63740.1
MASLAVPSHQRPTSSTPTSYNLPSDSSDVDPDNDAPLPFPAALPRSDFLV
PDFDPAAYLSALPHRHQTLEDLRSDLRDRSSTISSELVELVNSNYTAFLS
LGSELKGGDEKVQDVKVALLGFRRTVEEVKVKVAKRRDETREFNDELRHV
RLDIEQGRKMLEIDDRLSALEERLSVDSLPAGAGQKEWDWDSSDEEEDEG
AVAGLLGSPPAKLLPSAKECGEIILLKEPLDQQHPFVLKLGERLNRCRNT
LLLDLGNALKEAKGAGVQGHDRVLKYLAIYRMLDAQREAIRALRGG
>Mb|QLI63746.1
MADFLKNVFGGGKANEPAVKPKADPDFAEFAGAPDPVAEPVPAATIGGPG
AAATSRPYTKWYNVHERHSLSEFKAEGVILAISALIFLFHMIGARANRSK
ARSWIRANASILRKEFALVGFGAVPTMDNEHIKPDSLLKEKSLFEFATYA
SGRQNTAFVDVKLTLTKKFNPIMNLAETAASFFSDVFTAPGDVMEAFLYP
FDGKESLTIPSLPGSEEARAKDAKSNYDGFVWALVNKESMKRLREDRYDV
TLTATKESPKLPNWLTIMSESAEVTDTLLTQDLIDAVVAAGDKFEYLIVS
DQPVDKPVKLDDAAPRKRIFLKYRLPSDNKYDNLLPIFSHFLRLPDFLVQ
NGRFRPEVLKKVRATREAMISHIKKAAEEEKSEERQFEKEKAKKAKRDAE
LKGLDAKAQKKYLEKEKEKELRRSQKKMTMRG
>Mb|QLI63748.1
MLNNSVRSTKGMAILVGAIFFVAAFVGLVYNHLDSWNLPVRPHTQHSQPR
PSTTDDNSTTPIQRPSPDSKTPAAATSSDDELRKFFLKQMYKPALTPDQP
FFKPYNAFEWRLPEAAHWQQPMGENLCIIDLDNRLFNESGQVFGPDVMDW
DSPKGVHGLSLGLLNHYVYARIHGYKYYYINTVDPGDRRASWKKPPVISK
ILKEHDVCIYLDSDAIFHYLDLPFEWLMNYWRLYPDTNSMALAVDPDTDW
NKDRFGKLYLNTGFIISQNNPMTYKIMDAWNKCPDDDSPYPECKEFRLNA
PGRPTDQGGFGTFVRYNYTEHIRELPCTEANGFPQTDSGCNGIFVRHLWT
GKDDQIKIDVGTQMPGPYLKLFHEQYLKEKPTFYIGQEDLLRLGPSQALK
GKSVMP
>Mb|QLI63749.1
MATTTTGGEAAAAAASQASRTSITILDLPAETQQEIVSHCSQGDLICLAL
VSRRFRELAAAQLYRSFHIIFPDDDDINIESPIDGLAGGLDTFTTSEYNY
AQHLLDLSMDTLSAGVKGEHSYQPYLYSASCGKFLNTLLYLTLKRAHSLE
SFRWNIRVELSRPVYAQLHRIRSLKRLHLRMQAGDSYYTPPPPLPVSIDT
QPHHQPNGHWPDAILPLQPLGPPIPTSLQPPGSPPPGPPPALLPPSSRPT
PKSKLGKRGSDNKEPLTLSGFKSLKTLSVLDIDNLDILSELRTCVKNCFS

TIKELQLSLSDSLAQQARRPLADSDADDSDVDDEYQVVPTSENATSSYDT
TGPAKVFRAQEERKIQEVILNKVFDLEEPHLLKKSPIQLSASLNLQPKSE
ANTQESKATRGDPREEYISSIRTVSTRLMAAMHGSRDFSISQQEILDTIE
KASRKYVESDVTGENSTNNGSGTVNEAEESTSLKPESTADMPRAGPSADI
DQNENPHHNAPIASEDFCQHQLVDNPPSSGAQGSRNEGELSPDDIDIEHL
ETLDEHIDEFEDQQPTEFSSSQTFALRQGPSGQHGESCSNSGTTSKQNLR
TMMPNQYHIGAESRPRKTDRTAITEKLAQLRRLVFEAGQRVLEIRDERGS
ESSSTVANALDSELALLTRGAIEVSNEIRILESEIEDLLDAPSIQKGKNL
KQNMDTYARKTRGMSIETLKICLIPVKASVLSQAIDVACIKELTLINVGN
QAPIWSMLTKEHKFRPIALRSVFTDHVSASFLTCMAQLPQLDDLFMLERS
LKHKPESFATRTTTSIDQIRRLVLKKHMPTLKRLMIKDESNGPNWDTNVK
TMILICTRGANLEELALSMNIHTVHAFVQYFSGLINLRAINILHFKNSDT
CIWVMRELLRFIVDNLSHHPELKLEWIAMEDDRVDRVVRPSECSADSSSQ
EPMKRTKNKGKDKAHVPANGSSSQEYPVFPLEGLDSESESEDEGMDSGSR
LRYKTVGPLPFYDVWGVKIFEKEIRSGRL
>Mb|QLI63752.1
MPPFVRSLRKSKSMYSQSMGSVSGYDNDDIGADGLKPLPTTPSTIQFQDR
QNQTKRYSIRRSLRAPKSMSYLEFQTIKPLVEGAENKHNGYSLPEKVMHS
KGSFRHLKSHSSMFFRSKHRQQESSTGLSRSLRNSSDNSAALSPAFLGNG
IPPAKHTGIRFTARRVSKTVRNKLSRLFGRSKSTDTSGNDIAVDASQDTD
SDSFHHRTDTPPIEEASMSRVTSHVPSLHAVPSYQQMRSRQGSLESISHE
ENMPTDDKSRVTSWTNSSANTVISFGVNEEHEYQRLSVIKENGMHIPSSS
RIPLANPTIPGMTINSQRVYSALMKKLTNTPSNPDEVQNHESSKQSPAES
VPLGHGSGDQGNAQPWSPPIIRCIGTSDDDVFEDTKEVISSMSRKTAYKA
YPNASAGDGKGLSPAKIPLPESATKQSSIRADRNSTFSPSSDNHLFRTTS
PYRRAIQRSMRDYQESDHTHALDTRYLSTLSALSLPSRRPSTVGSERDAR
LTYAESFYSFTTEELTTVRPDGLASPPLPDVLNAVAGDVAVPVESSANME
TPVRGHDASAASSVEWKAGLSANISKLEKPHTAHTQCAEQPSGAVLHAGH
VRESAEIESPGETPKTAVTGTGEELPGCVASLHALGIRPSPPAQTGSPLK
STEGTAGPNKRDRVHSASPSIPSSPLRHTPCSTTSLTKVDKGTTPNFNDC
RPGLLRMRSLNTVPTTAPQPHDGLPWKQCDQENSRTGPPIMSKTTPGLSA
IPQRAASGKGSSPGKLGTGASTSPRTPEGSMPVSNGLSETTKSEWDAQIR
GSRRMVDLFLSSRRKAIHGTMSRNGSENFSTAFL
>Mb|QLI63754.1
MAPNENSNVKPSPGSRHGLPVRYRCKVGGEWKSLQFFSKNQQQLLQRQTS
LRGDVDAANSGMTCIEHSVGFRGEIRCELCGLIKPIDMFSKSMRRSDDPE
IQEPGVTPMPLQTGHRSVEENEGDSKRRFTESTDFFPDPMPQVPITALSS
LGVCDIATSGRISPESGRKQAQPGGPSSSRGPSSVVSAPVSVASSGTVPP
HLESIVGQLMQNDGLRKSGNPENRSPESISSIRPVNIQGRPAENQILSVV
GSEVGSEMEGSDAGKTRRSNLHSQLPPHLQGKIPNMGSRATLFQYAVPGT
SSQGSDARTISTATTMRSEQDNGHAKTRVPYNAWDNAGKLHEAIKSHTAS
EGGTASTLESRPLLDIGHISDQHIDPKIDRQKRMNYCDSEDSRY
>Mb|QLI63757.1
MQHQRRKEPPTTTTTKAASTTLDHHRFSGGAISTGELSSGKHKNHDKSAA
SITTAAASTRRKPECGEPLLVLRLTSHRLATMSLNIPNAPNAGLFKQGYN
NYDSEDGAVLRNIDACRAIASTVQTSLGPYGRNKVVINHLQKMILTSDAA
TILRELDVVHPAAKLLVMASQQQEAEMGDATNLVIILAGELLRKAEDLLR
MGLKTSDVVMGYEKAQNFALETLEKLAVDKVEDIRDQEELSKAIRTVIAS
KQNGNEDFLADLVAEAVLAVLPKNPTNFNVDNIRVVKIMGGSLEQSRVVK
GMVFPKEPDGSVKKARRAKVAVFTCPIDTSQTETKGTVLLHNAKEMMDFS
KGEEAKLETAIKELHDSGLRVVVCGDKVGDLAMHYLNRFGILCIRILSKF
ELRRVCRVVGATPLARLGAPMPDEMGNIDVVETLEIGGDRVTVFRQEDEV
TRTATLVLRGATQNHLDDVERAVDDGVNVVKAITRDPRLVPGAGATEIQL
SARIQAQGEKTPGLSQYAIKKYGEAFEVIPRTLAESCGLDATEVLSKLYA
AHHKNEDGDTGVDVENNDNTGTLDANSEGILDLLTSKSWAIKLATEAART
VLSVDQIIVARQAGGPKPPGPNPNWDED
>Mb|QLI63758.1
MPSRLFALATAVSGAAAAFQGFNYGSTFTDGRPKAQTDFENEFKTAAGLE

GTNGGFTSARLYTMVQAGTGNDVISAIPAAISTKTSLLLGLWASAGQASF
DNELAALKKTIDQYCSQLDGLIAGISVGSEDLYRISPTGQAASPDPGASP
TQLVNYIKEVRTTIKGSCLETAPIGHVDTWTAYVNETNKPVIDAIDWVGM
DAYPYYENTKPNGIENAAKLFQAAIDNTQSASGGKPIWITETGWPVSGPK
EASAVPGVEEAQTFWREVGCPRFGNVNVWWFTLQDGAPTIPSPAFGVIGS
TLTTKPLYDLSCDKKQPTSSKAAQSSQTSAQSQAPTATGPAGTSVQGSTV
QQTTVPAAGTTQQGGQSNTVVPPPASTLTTGNGGNGGNGSNGGNGGNGSN
GSNGGNGSNGGNGSNGSNGSNGGNGSNGGNGSNGGNGSNGGNGNNGSNGS
NGSNGGNGINGGGAVNGSTTYIVPGQGTATGTGGLPGVTVPPNAGSKLSA
FAGVAVGAVMAVAAL
>Mb|QLI63760.1
MESISRMSSFLGSARALTLDVAQATRNVGSSSPSLSPRDIERNLESGSDK
DVLGALRLVISMMYRGQETLGFFKSVAKQVAATNTEIKKLAYIYLTHHAE
EESDQALISVNIMLKSSQSDPNPQMRALALKTVSGIGAESINPYVLVAVK
HGVADMSPHVRKAAALSIPKRYRLDPSQSPELIGYLTTLIGDKQYFVAGA
AVSAFLEVCPDRIDLIHPHYRSLVRKIVDMDEWSQLAMLRLMVYYARKCF
PRVKEPAATSPHRDTNADDFYADSPHAKKTQQVPLNSDLLLLLNSIKPLL
QSRNSGVVVSVTRCYLEIGTLEYVKLAIGPLIALLRGAQDIQQLALYNIV
SVCLKRPKDFVKYATHFLVRATDPAPVRELKLEVLTLIFPHSPLHIKSLI
LKELEHFSQSSNKALVLEAVRAIGRCAQSDAKTAPRCLKLLLSQTTSLDG
TSTAESLTVIRHLIQQDPEGHVGTVVRLAKNLDSASDPQARASIIWLVGE
FSGLNGEDNIAPDVLRILLKDFVNEPEAAKRQILLLGAKVYLHFLNRKSD
TEKTEETEENSQQEPDAHPITRLWDYLLLLVRYDTSYELRDRARMYKALL
EVPQLATLILLAPKPAPQAPSPSESRKGFLLGSSTLALAGGGGIHGLRGY
EALPDWVEPGREPDPRLRDHADGGTSPRHGEQSAVPAGHMLDQAVTSRPP
PNKLNGLGEGVGTKTLDDWLAEEESEETEEETEEETEEEETEEEEEDEEE
DDDDEQSDEDGEGDRLVKG
>Mb|QLI63763.1
MKIEEIVDRMEEASLEAEPSTEQAATPVQDVSQPTKPELPPGMASHRKQT
FDEIMADLNKSPLFMTDMEENDEIAALQALNYEGTPLENGTDFKMRGNEC
FKVRGYVDAREFYTKGIQILSLEERKRTRGEVTRNPEGVEDSAEEIAAQR
EVLEALYVNRAACHLELDNYRSCWTDCAAALRLNPRNLKACYRSARALLA
VGRVEEADDVCARGLALDPDNRGLKEVAGRIVKRAGELDAKNKREADRRA
LLKRRETLLRAALKARGIPTRTTAQPPEMGDTKLELVPDPDDPRSSLAFP
TVLLYPEHLESDFIKAFNETQTLEDHLSYVFPLPWDKEGVYTAAGVTCYV
ETMGGGVLKMGKRVPLLKVLGTGKVEVVDEVVRIFVLPNDKADAWVNKFK
QQKAVEMGKGGGS
>Mb|QLI63764.1
METFAHLNGHRQHASAALTPNHDQNHLSSSNTRDDIAPDPLVESPSSPSP
DLPPAQQPSRDNCDRRRPQEEDNTRLKKMASELVVKTVSPFLKEHIPGLY
APVGKDNTKTGAKSPATWTRIKDPNTKFCYRHRPDSKCRRAADETKMGFI
QSELNSLPTADQEAITHVWSLFSAAPSKQRELMLQGIITQCCFPQLSTVS
REVQEQLKIDFLAALPTELSYKILSHLDTVSLCKAAQVSRRWRSLADDDV
VWHRMCEQHIDRKCTKCGWGLPLLEKKKLMAWSRHQTAHHRHDQAERAGP
SVETNGHRADSPESNKRSNPSADDGPSKRPRLSGPSNSRSHLDNERKFRP
WKDVYRDRYKVGFNWKTGRCTIKTFKGHENGVTCLQFDHNILATGSYDTT
IKIWNIETGEVIRTLRGHTSTVRCLQFDDSKLISGSFDKTIKIWNWQTGE
CLSTLQCHTEGVLSVHFDGCTLASGSIDKTVKIFSFGNKETFSLRGHTDW
VNHVRIDSPSRTVFSASDDMTVKLWDLDSKQCIKTFRGHVGQVQQVLLMP
QDFEPDEIPSDDKADTISVHSNRSATPTPIPEQPDDSRASFGCGFTHDTT
RPLPPRYMLTGGLDNTVRLWDIVTGKCIRSMFGHVEGIWGLVGDTLRVVT
GANDSMTKIWEPRSGKCERSFTGHAGPVTCVGLSDSRMASGSEDGEVRLY
SFEGETLEERGTPS
>Mb|QLI63765.1
MAPPKIEAKEIETYWNIFSDRTGGGQFLTGEQAAPVLKNSGLRDDQLERV
WDLADVDNDGNLDFEEFCVAMRLIFDILNGEYADVPKTLPDWLVPESKAH
LVQATRAIAGKQPQFEQVEDDSDDLGLKDGFEWYMKPGDKAKYEHIYQEN
RDMRGEVSFNALEELYESLDVPDTDVRSAWNLVNPSAGSTINKDACLAFL

HILNYRHEGFRIPRSVPASLRSSFERNQIDYQLDSQRAGTNSRWATKADD
STSTGRKTKFGEQYLTRLGRSGFKTAGTDFSTEKTEDWEEVRLKRQLADL
ETKIQKVEDMADQRKGGKRDSKPALVRRELDQLLDYKRRELRELEEGRGK
SAVGSGLKSVSEDLQTVREQVEGLETHLSSRQAVLDDLRRQIDAEKA
>Mb|QLI63767.1
MTYGEIDQLAINTIRLLAADATFNSNSGHPGAPMGMAPVAHVLFNKFMKF
NPKNTKWLNRDRFVLSNGHGCMLQYALLHLFGYDLSIEDLKNFRKVDSRT
PGHPESHDTPGIEVTTGPLGQGVCNAVGLAMAQAHTAATFNKPGFDVVDN
YTYCFLGDGCLMEGVSSESSSLAGHLQLGNLICIYDDNHISIDGDTNCAF
TEDVVKRYEAYGWHVEVVHDGDSDLAAIEAAIKKCREVKDKPSLIKLKTT
IGYGSLQQGTHGVHGSPLKADDIKQLKQKWGFPEDAFHVPKEVYDLYGKH
SSEGASHEDKWNQLMSKYAEQFPNEAADLKRRQTGDLPQGWEKNLPVYTP
ADAAVASRKLSETVLGKIEGAIPELFGGSADLTGSNLTRWKNAVDFQPKA
TGLGDYSGRYVRYGVREHGMGAIMNGLAAYGTILPYGGTFLNFVSYGAGA
VRLSALSQVRVIWVATHDSIGLGEDGPTHQPIETLAHFRALPNCMVWRPA
DGNETSAAYYIALTSKHTPSIMALSRQNLPQLEGSTIEKASKGGYVLHEV
PGANITLVSTGSEVGICVDAAKYLEEKHGVKARIVSIPCFEVFDSQSKEY
RLSVLPDGIPSLSVEVMSTMGWERYTHEQFGINRFGASGAYKDVYKKFEF
TPEGISKRAIATIDFWKDVPNVRSPINRAFQQHI
>Mb|QLI63768.1
MSSTASSASPPGAQHLHSRPPPLPPSSTGPSTRRTSDTRRDPFPFSMTRP
FRPNPTSTSVAPSTAEGRAIPVDDTTAEHRTSALRELNSHYPSRHRYAKS
TGAQSSTYSEPVIVRSYYSPAPSRHDSSTRRRPGRGGAGSEVGGPVAGVT
RALPFSGRVSSAGKGMLSTMARVYNGKMPTVEAQQDTRLPPVEAFSFKSF
MANMPTHAGTADINSDLDRIAEICARSRYSLSNQYEVHYAPHGSGVSFLA
SAQNQEPQGPTLQAVSSDDERDMRNTTRRRRQAARRNSRAMGTLETIMSS
SRSSDEEGSKKRSASDLANEVRGRTARKGSGQSSPTPTTSSRSERDLSNS
QDSEQQPRTPARRRSTSLALIDSSRLTSTPEATPRTSATALVSEPALPRA
SNNQLETRTAAESESQQRTLENKPSPRPDRVEEHMARGSISSVQDGARRN
GILSTLAGWLPWASHPSKPKGRAEGSLRHLLNNTEAGGKKTEPAL
>Mb|QLI63770.1
MDSEDGDYFIKQLAAFVRNHEKALANALQFQRREVRHRSSQSTSAATIPP
SPTLPDRPSTSSSTTSSLAAAFSLGALNFTSHNVKSAKLALTPHHLFFLL
SRFEELGINVGPMKVRLENLHDSSSSTNYVSFLGNSQRARSRSSDVGSIH
SVSSMRSVMSGMSALWNSLGIGASISAARTERQKAALQADLKYLYSAFTK
IPCLRLAPDWRARLISGFEEFPFDSAVPIYVFKNLQALEINSIDFRQFFG
WDRLADQLRSLSLRHAGIEDPADILIDIVLDDMDKRRRRTSKSQSSPTTT
WAAGTNSPRRSPTMPPELIRAASAPGSPEHRTLLAELRRGSMTPSEPVLE
DSESSDTARPSISRDDTDDVPRCPCKASRPRSISPSRPTSSRNHSTQARS
SQQRIKRSGSGSSHSSLSESWHHHARGSSSNLLAMGILPASKWRFLRHLS
LADNAMTSIPPSSLAPLSNTLYSLDLSSNLFSQIPDSLASLTALRALNLS
HCMIDSLQSLTRNPLPAITALNLRANRLQSLAGVEKLYPLERLDLRDNRL
SDPLELARLTGIPDIREIWLDGNPFTRTHKDYRVTIFNIFRSAPGCTEDI
IIDGYGPSYSEKRLLIERAPIPESVPVVKPAVSDSPAVVDVSKPTIVYNL
PKEASVLRKERPVAKAVTSEVNTNSYRRRKVSKRRIVDLATGDGGSMPTP
TESLTEDSVAVTEFEGGNYRITRQPEKALLPPVILTDSKKTLLASTSSVA
RAPGASASTNKPLPPTPSSYDSNSTTDEPRASEDWDAGGEMYRRRIEALR
DRVGSGYLSVLSEESWDGTSNPFLSSGVPSVATLRTGHASHHVSPAQPIH
SGRTMG
>Mb|QLI63771.1
MTIGSMHSDPEPTEGPARPDGRCGRPAIRRDSQQRTTQESLEEKAEAVRD
ACKWKDIAKLRSLAESTGGLLTDELRRLAWPLLLGLSPDLMNDDVDASFD
AQEAAADWEQLEPHRDEDQVQLDVNRSFIYYPNDKSEAQLDKCKSELLVL
IVEVLRRHPYLCYFQGFHDICQVFLLVLQPGWRARVVARLSVLRIRDFML
PNLGPTTAQLRLLPDLLAKADSELRKHIATIEPFYALAGTLTMYAHNIEA
YGDIARLFDVFLAREAVFPIYVFAQIVMGRRSEILDVEEPDMLQVMLAKV
PPNMDLDSLITNAASLFDQFPPESLPSWRRISKSSTLKTARHIETCANQT
LEDGRAFFEEQAKEVRWAEKRDRISMAIWRYRRPIRAIGMTTAVAAVAFY

LRRNPSAIHYVMSLFRTLSKA
>Mb|QLI63772.1
MNRALTCLRCRGLKLQTRAVSARTFADNKRPPIAPKPIIDIKHIRQNPEL
YELTCLERNYRNQATYPARILELHSEWQVLQRQGRALRERSNLLRKFLAN
PATSSGDDDVEDIREMGREQVQDEARKLKGELAVIEKGEAEAVAEMEALA
LEIPNLTSPDTPRGDEAVLLSYVNDPPTAFDNSPENKVWRSHVHVGSELG
LFDFSGAATASGWGWYYLVGEAAQLEQALVQYALAVATKYGWTQVSPPTM
VYSHIGAACGFQPRDQHGEQQVYAIAQSKADADRGVPEMCLAGTSEISLA
GMKANTTLDPEDLPMKRVAVSRCYRAEAGARGADTKGLYRVHEFTKVEMF
AWTSPGEDEAQDVFEEMLDIQTEILQSLGLYCRVLSMPTADLGASATRKV
DMEAWFPSRQSINSGWGELTSASTCTDYQTRRLATRTRVDGKISFPWTCN
GTALAVPRVMAALLENGWDEETGTVTIPEVLRPWMDGLERMGRVGKRRNA
QEVTFKQKDGHVEEA
>Mb|QLI63773.1
MTDIQGLLNEAGAAGLTPDDIPRDGTGVVKLDPGLDPFSEALKRRYGKSQ
DWINRIKATEGGLEKFSRGYEKFGLNASDDGTITYREWAPNAVAASLVGD
FNNWDANSHEMKKNDFGVFEIVLPPKDGQPAIPHNSKIKISLELPNGGRV
DRLPAWIKYVTQDLSVSPAYDARFWNPPKADKYEFKHERPSKPGSVRVYE
AHVGISTPEQRVATYKEFTQNMLPRIKGLGYNTIQLMAVMEHAYYASFGY
QVNNFFAASSRYGPPEDLKELVDTAHAMGISVLLDVVHSHASKNVLDGIN
EFDGTDHQYFHGGGKGRHDQWDSRLFNYGHHEVLRFLLSNLHFWMEEYRF
DGFRFDGVTSMLYVHHGIGAGFSGGYHEYFGSEVDEEAVVYLMIANEMLH
SLFPECITIAEDVSGMPALCVPLALGGVGFDYRLAMAVPDMWIKILKELK
DEQWDIGNICFTLANRRHGEKTIAYCESHDQALVGDKTLMMHLCDAEMYT
NMSVLSPLTSVIERGMSLHKMIRLLTHSLGGEGYLNFEGNEFGHPEWLDF
PREGNNNSFWYARRQLNLTEDHLLRYQYLNNFDRSMNQCEAKYGWLHSPQ
AYISLKHEVDKVIVFERAGLVFVFNLHSSQSFADYRIGIEVSGTYRIVLN
TDSKEAGGFNRVDENTRFFTTPMEWNGRKNWTQVYIPCRTAMVLALE
>Mb|QLI63775.1
MAASKTRTIKNKHAAPKGASSKTGGSTGGKRSATDGISKSKKPKGPVVSQ
QVKEKNRAALLKKPKKKVYTEAELGIPTLNMVTPVGVVKPKGKKKGKVFV
DDEESMGTIMAIVQAEKNGEIESKMIKARQMEEIRESRKLEAEKKEQEKK
SKLEDAKDSLRKKRKRKSGGEEEDSVKTLASAGSKAAKSKKKVAFA
>Mb|QLI63777.1
MERHQKRQRVDDLEQNDAPTSLSAPISPPRKSQRREPERLASPWQLTWIR
DLPEELNYDAVTLKDLLGDPLISDCWEFNYLHDVPFLMDAFDQDTRHLVN
VHVVHGFWKRDDPHRLALTAESSEFDNVKLHVAPMPEMFGTHHSKMMVLF
RHDNTAEIIIHTANMIPKDWTNMTNAVWRTPRLSQLPPGLRQLQEYCDLP
FGSGERFKVDLLNYLKSYDSRKLTCRTLIDRLVQYDFSSVKGALIASVPG
KHDIHDLSGTAYGWSGVKRYLSSVPCKEGSSEIVSQVSSIATLGAKDTWL
QKTLFDSLATSKTKSLQRPKFSIVFPTADEIRQSLDGYASGASIHTKIQS
SQQAQQLGYLRPILHHWANDSPDGIASSPEIKTRNGGRDRAAPHIKTYIR
YNEEGSIDWAMLTSANISKQAWGEASRPSGELRVASWEIGVLVWPGLVGQ
DVSMVGTFQSDVPKKPKEQASPKAGASGVLMGVRIPYSLPLQRYGAEEVP
WVATMQHSERDRFGRQWME
>Mb|QLI63778.1
MRVIEVIIDGFKSYAVRTVISGWDESFNSITGLNGSGKSNILDAICFVLG
ITNMSTVRAQNLQDLIYKRGQAGVTKASVTIVFDNRDKKKSPIGFDEYAS
ISVTRQIVLGGTSKYLINGHRAQQQTVQNLFQSVQLNINNPNFLIMQGRI
TKVLNMKAVEILAMIEEAAGTRMFEDRRDKALKTMGKKELKLQELRELLK
DEIEPKLEKLRTEKRAFLDFQQTQNDLERLTRVVVAYDYIKCQEKLKQSA
ADLEDKKKRHRMLEESATRLRSEISHLEEDVKKVRAQREKEVKKGGKAGA
LEETVKKHANELVRLATVMDLKNTSLAEEKEKKQMVEKTVLEMETALKDK
TAAFDDAKTSYDKARDDLAKQTQEVESKEELLQTLQTGVASKDGQESGYQ
GQLQDARNRATAAATEQEQAKIKIAHLESRLKEEEPRAEKAKEQNAGLLR
DLDGLKGQVNQLEKELGKLGFEPGQEEEMYKQESHLQQTIRGLRQDSDKL
KRQVANIDFNYADPVPGFDRSKVKGLVAQLFTLDKEHTRAGTALEICAGG
RLYNVVVDSEVTGTQLLQKGKLRKRVTIIPLNKITAFKASAETIATAQNI

APGKVDLALSLVGYDEEVSAAMEYVFGNTLVCADAETAKRVTFDPSVRMR
SITLEGDAYDPSGTLSGGSSPNSSGVLVTLQKLNNITRQLKETEQALKEL
QNKISKEKSKLDQARRIKQELDLKNHEVKLAGDQISGNSSSSIIQEVENM
KTSIPEIQNSITEAKSRQAEANADIKRIEKDMKDFDTNKDAKLIELQKSL
DKLRANLEKNSAAVKSLQKVHQSAQLDLEQVGADLSAAREQLQEAEVGIK
AAQQDVENLSRQRAALKETHDTVQAQLDDERAKLNLFDDELRLSEEAMRS
KNARITEEGLEMQKLGHQIERFHKERQAAADNVAHLESEYEWIQDEKDKF
GRSGTPYHFQDQNIGECKATLRNLTERSQGMKKKINPKVMNMIDSVEKKE
VTLKHMIKTVIRDKRKIEETIVSLDDYKKKALHETWEKVNGDFGQIFSEL
LPGGSFAKLDPPEGKTISDGLEVKVCLGKVWKQSLTELSGGQRSLVALSL
IMALLQFKPAPMYILDEVDAALDLSHTQNIGRLIKTRFKGSQFIVVSLKD
GMFQNANRIFRTRFSEGTSMVQALTPADMK
>Mb|QLI63780.1
MASADELKALGNKAIAEKNFDDAVDKFTQAIALQPENHILYSNRSAAYAS
QKDWDNALADAEKTTQIKPDWAKGWGRKGAALHGKGDLLGANDAYSEGLK
LDANNAQLKSGLASVEKAMQNEAGGAGGFGADPTGGLGQMFKDPNLIQKL
AKNPKTSGFLADPSFMAKLQQMQQNPGNPQDLFTDPRMIQVLGVLMGVDM
DVRDTDPRGDTNMPDAPEPKKPEPAKAPEPEPEPEELDEEALEKKKKKEE
ADKEKFLGTENYKKRKFDEAIEHYSKAWDIFQDITYLNNLGAAYFEKGDY
DKCIETCTKAVEEGRAVYAEYKLIAKSYARIGSAYEKKGDMAQAVENYSK
SLTEHRTPDVLNKLRAAEKAKVEAEKNAYIDPVKAEEAREEGNKKFKEND
FPGAVQAYSEMIKRAPDDARGYSNRAAAFVKLFEFPSAVDDCNMAIKKDP
KFIRAYIRKAQAFFGMRKYSDCVDACTDATQVDQEFHNNANAREIEQQQQ
KALNAMYSARDNETEEQTRERLMKDPDIMSIMQDPVMQSILQQAQSNPMA
LQEHMKNPGVRSKIQKLMAAGVIRVGR
>Mb|QLI63781.1
MATTAPPKAKHDWADDDDVEETTDLPPPQTISNKDGTKTIITFRYNDQGQ
KVKTTRRVRYVTHKETVNPRVADRKNWSKFGLSQKDGAGPAPDTTSVGEN
IIFRPSINWRKDAKDESQDPNAQSMKDKLKDKKVKCRICNGEHFTARCPY
KDTMAPIGENGAADVAAGMGDEPAPSGAGGAAKKGSYVPPALRGDRAGAV
GERMGGSKYGERDDLATLRVTNVSEMAEESELRDMFERFGRVTRVFLAKD
RETGMAKGFAFISFADRGDAVKACSKMDGFGFKHLILRVEFAKKAQ
>Mb|QLI63783.1
MKPDPETSAAPSSFPTKTPATTPKKPPCAQSKDFTLPKLRLEIRDLTHPG
ANLALTSVNAAETITTAARNVLRLLYTSPASPTTTPPPTRSITLILRDMP
GVAYTTGTELDDDHKEVHFSLNHIAGTSKPRAAQEITGVITHELVHCLQW
NAFGTCPGGLVEGVADWVRLNCDLAPPHWKKEAADKWDAGYQHTAYFLDY
LERTRGAGLVTRLNEKLRIERYAERRFWMELTGQTVDDLFAEYVESLKAG
Q
>Mb|QLI63784.1
MSSEDKYETLEKIGHGSFGVIRKVRRKADGFIMCRKEISYLRMSQKEREQ
LHAEFQILSHLRHANIVAYYHREHLKASQDLHLYMEYCGNGDLGRVIKDL
QHKGQRAQESFVWSIFSQLVMALYRCHYGVDPPEAGANALGLTQGSANGT
PKVPAGAMTILHRDLKPENVFLGEDNSVKLGDFGLSKMIKSHDFASTYVG
TPFYMSPEICAAEKYTLKSDIWSLGCIIYELCAREPPFNAKTHFQLVQKI
KEGKIATLPEMYSPELNQVIRDCLKVNPDRRPDTVQLLDLPVVKLMRKEK
EVVDLNKSLRAKEDLLHRKEKELNERIANLERERQMMREEIDSQLRMEWE
VKARLEIDRLANVEIEQLQQRFEEEIQVRVDAELQKLKHSNLVQPEATLP
IEDYASAIAKPDIPQSSTGGSGDGFPSTTDPSEYSLNSPEASKEPKRAAR
TPFGRAQTMFAGHAGTPMDVEMASPSPMAIANLSLSPRRGGATKAPTSHS
GNIFAANANKSADPRWDLREPLSIDSDDEDIVPSPTRNIRSANNPFTSKN
RPVLSSQKSCPLNRLKPQASATGLVSKAVFGPQAEPRPRSPTRQISKIPS
MASLPTDTGGGLTRKHSVKKDINGGEALNKMAAKNNIKGRTLVELQQARA
GGRPMSAGGGTLENVSPKRAMFKEKVCERRVSGGSGSGSSSSSSGSESVA
VWDPEKDEMPSPFLVRQKRIVKV
>Mb|QLI63787.1
MKGIQVQEYVKSPTELKVSELADPVPKDDEYLIEVRAAATNFFDVLQIAG
KYQNQPPFPWIAGAEFAGVVISTPKGSKNPRFPRGARVFGASQGAYATKC

VAKEVSMLPVPEGWSFTEAAGLFITAPTSYGALVVRANVKQGDYVLVHAA
AGGVGLAAVQVAKAFGATVIATASTPRKLEVAKSFGADHVLDYKDPEWPA
KVKKLTPKGRGVDIVYDPVGLVDKSTKCTAWNGRILIVGFAGGAIEKVAM
NKVLLKNISLVGIHWGAYSINERETIPVVWQGITKLVKEGKLRGTEFTDE
EFVGLDRVKDALVALSGRGTWGKVVVKVPQEGGSRL
>Mb|QLI63788.1
MAPETPKGVSSRLLTMKFMQRAAASASSNGSPGSDVPSSKKRKLDYSPAP
GRINPNIDHALIQAALDDQEATRQAALAKHSAADTHWVLKANIDKSQDKK
QSHPLNIVYVGYGDIDASKDSDENEDNPAEGRTYHKPLKTEQSNERCNSD
KSGDESRRTSDDESDSIGRDHTTGSSDENKRMTTSRSRSRSQSRSRHGHQ
ASKAKEFRDKRKKKEVRLNKLTSISSVGNGNFSSQGSPSTKAIKCYNCQQ
LGHRASECSKRSTRG
>Mb|QLI63792.1
MDKLTRGWVQHQRKAARQEAASRSVSCPLCEAEVQHSLDEFKAHYTLKHP
TSADHSDIEEAFKNCTLQGNDATKARSSETRPPGDTTNAGAVTRKRPVSG
NPDFTDRAEDDFDRLNLAGDDSVSTRRGSKKICSPPASITQQRGSSPPTP
GRSRARLSERDDDFVRTKNPTGRQLWTADDARKTGSPQRPRTPQTRHGQL
MSSSSHHRSPQHKGQRNPGQHHGQPKQSADAAASTSELFRQPDTRSISSD
QLAAEVQGIYAGLVLLESKCIEYDSTQKETDLSQEQYHALISLHRSLLHE
HHDFLLASQHPSASAALRRLASKYFMPARMWRHGIHSFLELLRRKLPGSL
EHMLTFIYIAYTMMALLYETVPAFEDTWIECLGDLGRYRMAVEDDDIRDR
EIWTGVSRFWYTKASDKIPMTGRLYHHLAILARPNALQQLYYYAKSLCVP
VPFLSARDSVMTLFDPLLNANPSASQRLEPADVAFVRVHGILFSGTHEDQ
LEPSMKQFLELLDNRIGREHGNWLESGYFIGISLSCLLLGFGDTSNVLMN
AVLKSQQADDTMMDDLPDPVLTDAFKTAVGFAARTYEIVIARWGDKNTLP
CLHTLLVFHWFMMDFDVGRQYLEDSLPWEQTALLFNYLLRTSEFTPRLDT
PEIPWPEGGKAHPLPEDYAMRGLIYTGTYFPKKWFDNTAIDDDEKYFEPA
STVGKRCERILWLGHSIAMKKRQLHWDKHARQFSTKGGNHNDELEDEPVE
LAASVTDVASTEPANP
>Mb|QLI63794.1
MAEIRRKLVIVGDGACGKTCLLIVFSKGTFPEKHADSTAENRNKIVVTVI
DVTSDDSITILTPVAPPRQSKQSGSSGDLEDWPYWIYFADAEAQVYVPTV
FENYVADVEVDGKHVELALWDTAGQEDYDRLRPLSYPDTHVVLICFAIDS
PDSLENVEEKWIPEVLHFCQGLPIILVGCKKDLRYDNKTIGELRAIDQKP
VTPEEGEEKRKSIGAYKYLECSAKTNEGVREVFEHATRAALLSRTSRKRD
KKCLVL
>Mb|QLI63796.1
MSALRILVPVKRVIDYAVKPRVNKAQTAVETAGVKHSMNPFDELSVEESV
RIREKKRAPGGVEDICAISAGPAKAQDILRTAMAMGADRAIHVDVKEGDD
LEPLAVAKLLKKAAEEQKSNLVILGKQSIDDDANQTGQMLAGLLGWPQAT
QASKIEFGEGDSVSVTKEVDGGVETIRAKLPMIITTDLRLNEPRYASLPN
IMKAKKKKLDKKTLKDFGLENEKRLKVLKVSEPTPRQGGGKVEDVDGLIG
KLKELGAL
>Mb|QLI63797.1
MSRYLRQSSQLARLARLSQQTSLHRSLRLPAASRSIPTRTVTGAYGLSVR
YYSSSKPPQPDEDSKANKDSTPEIPKNQNESETKTGKEKDSDLTKLAPLP
EGWIRLSKDEIAQLDAFQKVLPEAQRATLKDILRGLQQTGAPAEVRDLLA
KMRRGPGNLSILDKGRLMRCVFMMAERLAEWEVEEQRKGNRMFDQNQRPD
QEQNDAHTGKSEPEFEARNKKPDEGGSHHQGPGGPKKPERNGWMDALQTG
LVLGITLWAVDSLSKPFSEKEITWQEMRRAFLDKGLVQKMVVVNGSQVRV
ELHPDAAQGDAGSNGKRTYIFTIGSVESFEKKLEEAQDQLGIPASERIPV
SYEAGGSTIGNLLLAFGPTLLFIGLILWTQRSMGGRAGGGGMFNFGKSKA
KKFNAESAVKVKFADVAGLEEAKTEIMEFVSFLKQPEKFEKLGAKIPRGA
ILAGPPGTGKTLLAKATAGESGVPFFSVSGSEFVEMFVGVGPSRVRDLFA
EGRKNAPCIIFIDEIDAIGRARMESGRGFGGNDEREATLNQILTEMDGFN
TREQVVVLAGTNRADILDKALMRPGRFDRHIFIDRPTMSGRKEIFKVYLK
KIVTNEDQDFLVGRLATLTPGFSGADISNVVNEAALIAARGNAEEVKMIH
FERAIERVIGGLERKSLVLKPDEKKTVAYHEAGHAICGWFLRHADPLLKV

SIIPRGQGALGYAQYLPQDAYLMNTNQLMDRMAMTMGGRVSEELHFPTVT
TGASDDFKKVSQMARNMVTQWGMSDKVGPVFFENDPNRMQKPFAEATAQQ
IDQEVHRIVDEAYQRCKDLLLEKKREVGLIAEELLKKEVLVRDDMVRILG
KRPFEDNNEEFEKYFGGGKEESAPPAPFPADETDTPKEEPPAPAPAFSQH
TDGSR
>Mb|QLI63800.1
MSFAGMLNRLHGQPESYDKKSKYKFGRTLGAGTYGVVREADGPSGKVAIK
IILKKNVKGNEKMVYDELDMLQRLKHPHIVKFVDWFESRDKFYIVTQLAT
GGELFDRICDQGKFTELDASQTIKQIMTAVDYLHDNDVVHRDLKPENLLY
VTREPDSDLVLADFGIAKTLDSKEETLKTMAGSFGYAAPEVMEQKGHGKP
VDMWSMGVITYTLLCGYSPFRSENLRDLLRECTAAPVPFHERYWKDVSPD
AKDFIMGLIVPEPEKRWTSKEALGHIWLSGKNATDHNLLPELEAYRRRAR
LRRVIEIVKLQNRIAKLKEHEEDPSESDMGDAQGASDNHKGDGSRLHALG
LFALREAKQKQESLQVEEELEKESRRRSFSNA
>Mb|QLI63804.1
MPDESPVSDTQQLDQSLHKGNLMMSFPITKRKRATRAKFAKASKNNVLVY
PPACPYTNTHHRRRHVKCDETKPACKNCIKWAGFCGGYEPILNQPKSPAK
QAFVTPRSPENDGTSSPEELDLSLYDCGYQLQLPENLSPPESIDSQASSH
GYTSYQPQPTGHYFTVPDTRSTFDDTFWTFELPRLAHDNPAIRYANMAVH
ALLFARGHTLARAGKVTERDHYGEALSCHGIALQEARQATTGYIDLREAV
ICCMFFVIFETINGDQEAAQAHLQSGQRILEEIDPEYNGAERFREELRNV
LQFLAQQARESGLDEANQRGGDEGGSILDTLMV
>Mb|QLI63805.1
MLPTAPEPSQTTANAPGDVSEPKDDAPAVRFASSIEEISPSAAAPATSQD
QQDYSFSEVAADELKSFTKSLQGRPLQERRMNTFQFEAFSLPPSRVPSRE
DESADSTRLPTPNSSSFHSPHGSPRLSALASPPLTPAGSGPSIVSEKRIS
RGPSDKGPSSGDPQIITPQASSSHDRSPVVEERKQLTHRPASSDNVMRRS
SSVEDHRSHRRGMFGVGAGSVPVSRESSPSRSSASNFYSKPVTPGGDAND
PYAKGKRPPQQSHPTRHTIDPRFVFSRKKKHGSPGSSKTSLTDKRASGVF
GKSDEHLTVEGAGAGSGHTPQGSMADLKRFFRMSGHNQHHHHKKRDQSPA
SGTRTPPTSRSANQQLPFSDDHGLSSRYGKLGKVLGAGAGGSVRLMRRKE
DNTVFAVKEFRARHSYETEKEYNKKVTAEFCVGSTLHHGNIIETLDILQE
KGRWFEVMEYAPFDLFAIVMTGRMSREEIRCCFLQILNGVTYLHSVGLAH
RDLKLDNVVVSDKGIMKIIDFGSAHVFKYPFENGTVSAKGIVGSDPYLAP
EVYDVKEYDAAAVDIWSLAIIFCCMTLRRFPWKIPRMTDNSFKLFAADPT
PGHDPTKLITHSKSTGNLSSTVAREFLTDEDLKEKGHRNHSHHSHHQGHG
HSHGKENAEAPSTAESHSQQAQNQGAAGERKEIIRGPWRILRLLPRESRH
IVHRMLDLDPKTRAKMDEILSESWVADTVICQQLENGEVIPAEDHTHVLE
PPASQQQQSKT
>Mb|QLI63806.1
MAEFNARAEALVPKFQLERVLNQDQAGRRTSMYGTIDNEPALLIVERAPF
PTSDKYLGRIPSSLARLKNLGANDIYSWSMARTGSDDEEESNKEKAGFFA
DLKINLIYPCTETHVQKYSKQQVRFVTETPEIYRASIRPYMQSKRDQGRL
NWVFNIIEGRKEVEDVIYRTKLGEAGDEGFLLLPDLNWDRSTLEALHLLA
IVERRDIWSLRDLKKKHIPWLEHMKNKLISATVETYPSIEPDQLKLYVHY
QPTYYHFHIHIVHVALEAGATQATGKAFGLDVLIEMLKVMAGDEEAGMDA
VSMTYTLGEASELWTEVYEPLKKSGRASR
>Mb|QLI63807.1
MTDKLPPNLLALFAPRPPLRWVEPPDYAPEKRKTAPIHGVAALLPQLQEY
KETDVYNPTESWLEAKDRKKHEKKEAVEKLLTEGPKNYAPNEDPNIRGDA
FKTLIVARLSYDADERDLEREFGRFGPIERIRIITDTHAHEKPNKKKKAH
RGYAFVVFEREKDMRAALDSCDGIRIKDRRIKVDVERGRTVKGWKPRRLG
GGLGGRGYTKSTPARPGGGPAGGFGGGFRGGFKGFEGGRGRGGFRGGFGG
RGGGFRGDGPRGDRNGFGAPSGAPSGPGGDRRNGDRGFGGGHDSRGGRSF
DDRPGGGYRDGGRRTGGNMEPIGRREGGFRDRDRDRDRDRGDRDRDRD
RDRDRDRGGRDYDRPPRDDDNRKRGYDGGGYEDPRKLRRY
>Mb|QLI63808.1
MGQSQSDEQPRRRSREDLAHDLALRFKDECFTSLEFYSLKDVFKSLADQQ

GSVRYLKEDTIARFLELPDILGASPVVFQMVSYLGAFPFLQDAPAVLELP
QMVMVIVIMTERYKRVLARGSTDWTKLIFKSLAVYDRKASDAGVSPPSDA
AKDESAATAGNGGGFALDIPGEDEPYDDDDDLVLTAYELLDIKEAVKQGS
APEFHGAIVPTDNFRKLVMLLLLAAPLDAQESLSQYSDRVTGKELEVLRS
TAECVLMSFVDVESSPGIKYRQFQTIMPVLFPNLFNSFNALFEHFLFSKN
LDFSKHKDANPNMQKQSAPKPAQPLLSDKGDILNDHLLAQISLFLPGSSL
FRRVRRLYSGNEAGFSMGSFESKVFNWRAPTILLVSGTLLTDTPHSGQET
AFADTLPTKRFQNGSRDASSRVTYGVYVREPWKHTHKQCFGDSETVLFQL
SPVHDVFPASTINTDYVAFTKPPGNQPCVSFGSPHPRPSKSSRRNTHYSI
GAVSLLLNDSFEFGVFNHDYTSRGGAFHTSISRKFDFQDRFEIDQLEVWG
CGGDDEAKAQADRWAWEEREAEARRKINLGSGDIDADRALLEMAGLVGAN
RSGGSMG
>Mb|QLI63811.1
MAQDTDAPKAADKGKGKAVDESKDEKPILNGKKEDDKKDAVEEELSEEDQ
QLKSELDMIVERLTESNTELYKTALEAMKTSIKTSTSSMTAVPKPLKFLR
PHYETLTKLYEEWPAGENKTSLADVLSVIGMTFSDEDKQDTLKYRLLAPT
SDISSWGHEYVRHLALEIGEVYGKRITADESTSDLIDLALILVPLFIKSN
AEADAVDLMSELEIIEQIPKFVDENTYARVCLYMVSMVNLLTYPDNETFL
RTAHNIYMEYKQFTQAIVLAIRLHDVELIKADFDKAKNPTLKKQLAFLVA
RQRIVLDLPESNDDEQVILECASNLKLSEHFKSLGKELNILDPKTTEDIY
KSHLESSRVAGMTNLDSARHNLAAAFVNAFVNAGFGNDKMMLVEGEKESW
VWKTKADGMMSTVASMGTLLLWDIENGLDKIDKYTYSAETEISAGAMLAI
GIMNSGVRFDSEPALALLGDTEKLHHSNPLIRTACIMGLGLSYAGSNKED
LLELLLPIISDSSQDMQISAMAALSCGLIFVGSSHPDVTETIVTTLMDDE
RKNQLTDKWTRFLALGLGLLFFGRQEEVDVILETLKAVEHPMAKPTAVLA
EICAWAGTGAVLKIQELLHICNEHKEESDDKKGDELLQAYAVIGIALVAM
GEDIGQEMVLRQFGHLMHYGEANIRKAVPLAMGLISPSNPQMKVYDTLSR
YSHDNDPEVAINAIFAMGMLGAGTNNARLAQLLRQLASYYHRDQDALFMV
RIAQGLLHMGKGTLSINPFHTDRQVLSQVSTAGLLATMIALIDPKEFITG
TSHYLLYFLVTAMHPRFLVTLDEQLKPLKVNVRVGQAVDVVGQAGRPKTI
TGWQTQSTPVLLGYGERAELEDEEYISLNGTLEGLVILQKNPEWENGQ
>Mb|QLI63813.1
MALITSQTLITSISLFHITLGFFFLTNPSTINDQILVQVLGQSMGIPFAR
GFDAQSHTLSFLAVVLAFFGFSDLLSLGMPEELGSLYYWGTQAPMRAFLS
MTLTFYTYTFGPSSPLYGGATHRTRGGYNASYSAASWGGDGLKNRVFFTF
VFIEMIAWFWVWVTLREEKAGVVERMRTGAAAKRRTHEE
>Mb|QLI63816.1
MGLKDRFGLSQRRMSGEEELPAGVMGDPTHELKAFRRQHKWDPFLDNEKL
DTIDDALASENVEKQAVVDETLIQEDSPYPEVRASVPPSDEQDMPVDTIR
AWFLGAVLCTVVAACNVLLSMHVSSANISSTVVQLIAYPLGVGLANILPN
KERSFLGWKYNLNPGPFNVKEHTIITMMTAAGSTYSYAIDILLAQEVFYK
QRFGWGFQILLIVSTQAMGFGIAGVARRFLVWPASMVWPANLVTCTVMHS
LHNHVPSDPASTNGWQISRYRFFIIVSLVTFVYQWIPEVFATFLQYFTFA
CWIAPNNVIVNQLFGHVSGLGILPITFDWLGVSSWLGSPLQTPLFAILNV
GFGLLICLIGAIGLAYGGPVEYKYLPLSANKNWDRFAKPYNTSRILSPDY
TVNETAYQAYSPILLGATFSISYGMSFATLMSTIFHCGLFYGPDIWRRAF
SSRSEEPDIHLKLMRKYKEAPEWWFAIIFVVSFAFGMIASQVWTTHLPWW
AYIVCILIGIVLFVPIGMIQAITNQQPGLNVVTEMIFGYMLPGRPIAMML
FKSWGYMTCANGLTYISDMKIGHYMKVPPRSMFYAQAFAVVWLSIVQIAT
YNFVIGNIKGICTDEQAQGLICPNAATFYNASVIWGVIGPKRVFGAGGIY
SWTNWFWFIGAACPTIQYFVARRYPRSFARYIIWPAIFSACGLVPPATLY
YLLPWVIVGVIFNGFIRRYFFGWWSQYNFVLSGALDIGSRLCVVIVALAL
GLSGKNFPDWWGNVVYTETLDYTRKAVTRKFIPNVTEPIGPSSW
>Mb|QLI63820.1
MSNGDPKYDDIDSESDSGDSIDQHNEVGDGLDEKPRKSALKKSAVALAPE
PVVQRPPLPPQTDPKDLDVSSLTPLTPEIIARQATINIGTIGHVAHGKST
VVKAISGVQTVRFKNELIRNITIKLGYANAKIYKCDNSECPRPGCYRSYK
SEKEVDPPCERDGCGGTYRLLRHVSFVDCPGHDILMSTMLSGAAVMDAAL

LLIAGNESCPQPQTSEHLAAIEIMKLDKIIILQNKVDLMREEAAQQQYES
ILKFIRGTVAGKSPVIPISAQLKFNIDAVNEAIVNTIPVPPRDFTMDPHM
IVIRSFDVNKPGAEIDDLKGGVAGGSILHGVVKLGDEIEIRPGIVSRDDG
GALKCTPIFSRVVSLNSEANDLKYAVPGGLIGVGTRIDPTLCRADRLVGF
VLGLKGRLPEIYSEIEVNFYLLRRLLGVRTADGKQAKVAKLAQNEFIMVN
IGSTSTGAKVAAIKNDAAKLVLVSPACTNIGEKVALSRRIEKHWRLIGWA
TITAGVTLEPSTS
>Mb|QLI63822.1
MASTDALTSNSLFKLQGRVALVTGGGSGIGLMAAQALAANGAKVYITGRT
KEKLDTAKSQHFGDDNSNVITIQADVSSKESIQSLVKEIESREKCLCVLV
NNAGIAADHHQTAEAKSASELRDSLFQQSDFDNWTDVYRTNVAAVYFTTA
AFLPLLQASSERHAGWSGTVINITSISGLVKTSQHHFSYNASKAAANHVT
RMLAAEIGAAGLKIRVNGIAPGVFPSEMTAGESDEAQKSQLPKDKYQGKI
PAARPGKDEDMASTVLFMAANQYVNGHIVVVDGGYTLSAGM
>Mb|QLI63823.1
MDLIKVHNSLKPGPPVLFVPMEPGKVSWYACGPTVYDKSHLGHARNYVTS
DIIRRIMMHYFGFNVKYVMNMTDVDDKIILKARRQRLLDLEKKKKYPEEE
LRTLGLAAFRAYASKNLPLLVNDASKLDETNYATQRDTAYGTVLSGGTLT
GEGKPGDNEAKIKMHVGNMDAAAGALKQNAIFPGADEVLLPYLDSLHKET
MDTSDQSIFTDLTKRMEAVFLEDMDNLNVLRPDVITRVTEYVPQIAKFVE
QIVDKGFAYEAEGSVYFDIAAFQKAGNTYARLRPDSKNNKALQEEGEGSL
STNLGGKRGAGDFALWKKSKAGEPYWPSKWGDGRPGWHIECSVMASDVLG
SRMDVHSGGVDLAFPHHDNELAQSEAYFCQHGKGEHTWVNYFLHMGHLSI
SGSKMSKSLKNFQTIADALATTYTARSMRIVFLMGRWNDGVEISPDMRSA
ADNWETTISNFFTNTKSWIREAGVGGGVRSLSINQDMPATGLLADLELAK
KEVEAALTNSFDTPRAMLALLKLVNTVNTFVMNNKDVDLAHVEAIARWIT
KMVGIFGLDPNATPPYDGLGWASSIAADIDPKKAIQPYVQVLQTVVTEVS
KLSLSSDTLSNLLSQDPSKEFESIVSSGTRDIEELSLPYIRVVSQIRDEL
RRIVSSQTAENKKAILAIADRIRDVDLTNLGVSLDDRPDNQPSLIKFIPA
AELIRVREEKAAREAEKAQKKEEARIAREKAEQEAKEKAKIPPEEMFKKD
ERYSAWDESGMPTKMKDGSDVPKSQLKSLKKMWEKQKKVHEELKAKGGL
>Mb|QLI63825.1
MQHWTVAAPWLDLHCSLAEGPFYEKGTDSLRFVDIKKKRVHTVSLSRGLS
SLHTIQLDICPTVTADIEGVDPRERILLGVKYGLAGLDRETGAYEMLARF
NDPDNERLRSNDGAADPHGLFWVGSMTDFGQGEFQPEGSLFRFSKDGKEE
MVKGLTIPNSIGWSPDNKTMYYTHSKARQIFALDYNAVSGAVSDHRLFYK
HPTSGEPDGFRIDVDGNLWTAVYGESKVLKINPEGKVIGQVTLPTRNITC
VQFVGTELIITSAADEDGEDKTSRDYGGALFRVDVGTTGLELFKFRM
>Mb|QLI63827.1
MSVEVINTISPNTNETILSRNGVSAAELDLMPEVATEAFQSFRKTTLKER
QEIVKKFLKELSSHEAELAEELTVQMGRPIAFTGKEITTAIKRAEYLLKI
SDEALKDTEGEPERGFTRFIRKLPVGPVLIIFAWNYPYLILMNSIIPAIL
AGNTVILKPSPQTPTIVEHVAQFFAAAGLPEGVLQYFHCGSPSAMESLIR
DPRIAHVCFTGSVAGGLAVQKAASDRIVNVGLELGGKDPAYIRDDVDVAW
AAEEIVDGAVFNSGQSCCSLERVYVDEKIHDAFIESVQKVLRGYKLGDPF
DKTTHVGPVISRRSKQAIESHIKDALDKGAKDATPDNESFNNPPPKGNFV
KPTLLTGVDHTMTVMTEETFGPVIPVMKVKSDSEAIKLMNDSEFGLTASI
WTKDTEKGYELAEDVEAGTVFVNRCDYPSPDLAWTGFKNSGKGQTLGRFG
FEQFVKSKSFHMKKYPQ
>Mb|QLI63830.1
MSEIVHPTIKDGWFREISDMWPGQAMTLKVDKVLHHEKSQYQDVLIFKST
DYGTVLVLDNVIQATERDEFSYQEMITHLAMNSHPNPKKVLVIGGGDGGV
LREVVKHDCVEEATLCDIDEAVIRLSKQYLPKMAEGFKHPKSKVHVGDGF
KFLDDYKNCFDVIITDSSDPEGPAESLFKKPYFQLLHDALRDGGVITTQG
SENQWLHLPLITQLKKDCKEIFPVAEYAYTTIPTYPSGQIGFMVCCKDAN
RNVRVPLRQWTPEEEEAKCRYYNAEIHKASFILPTFAKKALE
>Mb|QLI63836.1
MRRPRDDSEDDEIPLHHKKPFGSGLKRNKIEFVRATESDDGTAAVSKPRV

ATAIGDLYASIVLGDGQVNSVSAPATTTASPQPDVCPVCSLPITTSLKEH
EASLAHQVSMPHSHPPSALDRSRMGLRALKSQGWDPDARRGLGAEGEGVR
YPIKTKAKDDNLGIGAAELLERQKQEAREPPPRKLTGKELKQAMAKEKQR
AERLQAEIYGRVDVESYLRRQMDDE
>Mb|QLI63837.1
MQRMSSLLPSWDKRNSTSGSAAASTTLKPGGIFGWAGRNSNSNKASNSSV
AKSLAQINVAAANANATGHGGRVQREAFWPSTLDMECLKAARILKSFCVD
GYLAPIDGDTSARTSVSGPQSPLKITKKIPRRIIQNAAGIAVFTCMRSGL
WMTGSGGSGILIARKSDGTWSPPSGIMLHTPTLSFIIGVDVYDCVLVVTN
LSALESITRPRVTLGEDVTLNNGPSVTMDADHTNFNWKDLDNTVLAYMKA
RGQHQAVNLQGCILTERSNENERFYGADVTQMDILAGNVARHVEETRPLF
EVIKMAEGRTDYDKAIIEQTAIEPAPGDAVIATPKSTPASPRSVFGRPKP
DDPDPFGVLALEMAGLEIREAGTRLRPASNQFEINSNTRSPTLSKFGRQS
IDTFVSKSNRASVLSSRTVKSQMTDAGTQTGGATAETTPSPGQSEDGHEG
VSIDRIPEVKEDDEVDYTAVDFTPLRHISQEHSIDLSLPAEEPAIPPRNR
LRASVPSSRSSRDTEQPTGASIKEETHQMQDDEDELEYDTNDADDEDDLD
DSDEEPVVFEVAQVQPTRTRAVASRMIQAKGSVVTIPKRVPPPLPTRSPA
RNSGSMKGDNCTESGTALSPLRQAFSEADLRGEEDIQNDQTQQSPTHVIV
SNKSADLGQTHGNNVDDVLLGETLADKLQADEASKASPSTNEAVMPGSLP
VDDTDEEAKHSDIVKSVEKIVPNKSTIPENVPLPADTDPEDLTSRLEEAE
SDYGSVEERATEDDESIKTPVDNSDSTTSKKHTSSIYTGMTEDRWSIDQS
SLTTPNSERPLSVVEYSTEEDTPKKIAKEVELEVEKNAENRTAKAPVSAA
SEITVTSA
>Mb|QLI63838.1
MVSAKRDSVHGEATNGEQQITEPKGRNDALLAEQKITFLACFQGGVASLG
GFIFGYISGQISGFFLMQDFKERFGERQLDGSYNFSAARQGTIVGLVSIG
ALIGSLIAGKMADLIGRRLTISAAAFFTCVGTIIEISSSYHWAQFAVGRL
VSGVSIGALSVVVPMYQSECAPAVIRGVIVASYQLLITLGIWTAEMVNWG
TEAKTDSSSWRIPNGLSFAWALILGSTILFMPESPRYDCSRGRVDRARLA
IAKLSGLAPDSEGVNHQLADIQNNLHEESRSADQFRLSEIFTAPRMLYRT
VLGIVLQAGQQLTGANFFFYFGTTVFAATGISNSYVTQIILGSVNVFCTI
IALWVTKRFGRRNTLMIGAAWMMMCFLVYAFVGHFALDHDNPMNTPKAGS
ALVTFSCLAIAAFAVSWGPLVWAVNAELYPLRYRSTCMGLATASNWLWNF
LISFFTRFITDEIDYLYGLVFAGCCAALVVIVFFFVIESKDRSLEEIDTM
YVQKVNPITSGHTSGHWNTNDYRQDIRRRSEAQTETD
>Mb|QLI63839.1
MDFDDDAPPELVETAGQIEQNGEEITVKVPITIVTGYLGAGKTTLLNYIL
TAQHGKKIAVIMNGLCLEFTFSGTGLILVSCLAEFGDSLDIEKSLTVNQG
DDRVEEWLDVGNGCICCSVKDTGVNAIESLMEKRGAFDYILLETTGLADP
GNIAPLFWVDDGLGSTIYLDGIVTLVDAKNILRSLDDPTGKVEGHQDHDD
HGPLMTTAHVQISHADVIVINKSDLATEDELQLVKARIQSINGLAKLHVT
EKSVVPQLEGLLLDLHAYDQFNEANERLKGHSHLDPTISTMSIPVPCLLP
GKVAEVDKWLRSVLWDNKLPGSENEGTFEIHRSKGRIVLRDGTARMLQGV
REVFELTDAPETAQVQPEQGKIIFIGRKLVEEDFVRSFNNAIN
>Mb|QLI63842.1
MASSPKYLTGDKASINEFIDKFDTFLFDCDGVLWSGDHVFDRVPETIMML
KARGSSPLTSHPSCAFNSYSSPSGKRTVFVTNNSTKSREDYLQKISNLHI
PCEKEDVFGSSYSAAVYISRILKLPPGKTKVFAIGEAGVEKELAAEGIPC
LGGTDPNFRRDMTPKDFQALADGTALDPEVGVVLCGLDFHINYLKLSTAL
HYLRRGAIFLATNTDSTLPMHHSFFMGAGSIMIPLQYASGTKPLELGKPS
QAMMDAVEGKFQLDRSKTCMVGDRLNTDIKFGIDGKLGGTLHVLTGVHQK
EDWDREDAVAVPAYYADKLSDLAVVA
>Mb|QLI63844.1
MAEAAAPVKPQAPTAESQARPTRPDEKTFNEALAKAEKDHKASMDRLNAI
KAKIDIALPNKNKDQPNPTQKRRQELIAQANEIRQKQAGGKSARTTKLDQ
IKRLDEQVRSRIAEQKTAKAKVSFKSAEDVDRQIAALEKQVNSGTMKLVD
EKKALSDVSNLRKVRKNFGQFDDSQKQIDDLRAKIKEIKDSMEDPEQKAL
SEQYNKIQAELDTIKAEQDEAYKGISSLRDERSKLQAEQQEKYTAIRKLK

DEYYGQKKAFQAFEREAREKAKARREAEKEQWKKDALKKDADKALAEASY
PAYSDEIRRANSLLQFLDPTHKAETKGPLTADSGLGAQAQRTVDDSALKG
TKLVRKEDRDDEYAPAMKKGKKGKKGGASAAAAPAKFFNCPPSVVEDCAF
IGVDPPMASADVPTVVEKVKAKIEHWKNDQEAQTQRNIEKAKKQIEKLEQ
EGANGDKVAEITEGVKDASLNDKQES
>Mb|QLI63845.1
MPSAAENLPAPVADKLPRLPETKEELRETKDAAKSGLLSQLRALAAGGFG
GICAVVVGHPFDLVKVRLQTAERGVYSSAVDVVRKSVARDGLRRGLYAGV
SAPLVGVTPMFAVSFWGYDIGKQIVSATSAVGPEGLSIGQISTAGFLSAV
PMTAITAPFERVKVILQVQGQKQLAPGEKPKYNGGLDVVRQLYREGGVRS
VFRGSVATLARDGPGSAAYFAAYEYIKRALSPKDALTGKPTGELSLTAIT
CAGAAAGVAMWIPVFPVDTVKSRLQTAEGNVTLGGVIRELYGRGGFKAFF
PGFGPALARAVPANAATFLGVELAHQAMRKAFD
>Mb|QLI63847.1
MAITTVTANLTPMLTPILIMLMLMLMFTPTARAPAIAITIATVTVKATAM
VMMRVVTVDDLIVLHRQTECRYAHVANLLEETSRSAANGRRMTRPPKKKN
VPDGPATIPNIADRGAINDHEGSSRGAAALSIGLLSHVPYSVTKASNVFG
IGSEHPFANYWTCEGGLPEVISVLPDKAQADSLVARYFECVDPVYPMIHR
QTFYADYEHFWRMKTDERNKMDPSFIALIFVILALGTQFVSSTTPKDRKQ
TAEFYASASNQALRMFSYLSSASIRSIQAMVLITYFLINDNHASDGWAFA
GILIRQAYAMGLHRDPNIVTPNANPFEKQQRRKVWQAVLLQDTFLTVLLS
LPPSATHTDVSVDDLLDDSSSIASSDPTDTAYIRGSWMLANLVQETICSP
RSLDVPICTTVRHKSKLVADFRAVYRSFPDIFRSWDTDSLTAVASTNKRI
VRQTLFLTSNYFHNLMLVHASESPDVPVNVRGTLEAAHDAISAFFLMFSL
LESEARVWWVFNHRAFLEALCIANVLRETAKDAAGKDMLARDPLFVRART
DITRMIQIMELIGSNNDVARTRVQILSEFLEDTDTA
>Mb|QLI63848.1
MGDSNLAQAQAQLGSVALNGSSPKKVAYFYDSDIGNYAYVTGHPMKPHRI
RLAHSLIMQYNLYQKMEIYRAKPATRGEMTQFHTDDYIDFLQKVTPDNMD
SYMREQGKYNVGDDCPVFDGLFEFCGISAGGSMEGAARLNRQKCDIAVNW
AGGLHHAKKCEASGFCYVNDIVLGILELLRFMKRVLYIDIDVHHGDGVEE
AFYTTDRVMTVSFHKYGEYFPGTGELRDIGIGQGKNYSVNFPLRDGITDQ
TYKSIFEPVIESVMKYYQPEAVVLQCGGDSLSGDRLGCFNLSMDGHANCV
NYVKSFGLPTLVLGGGGYTMRNVARTWAYETGVLVGQEMDRTLPYNEYYE
YYAPDFELNVRASNMENSNSREYLDKITVAVIDNLRQTGPAPSVQMQDVP
RKPFGGMTDEEEAELDDLDEDENKDVRMTEHRWDKHVENGAEFEASDDDE
MAAANGATRSNGNKGTFNDFKSADAAEDSRSKSPREKQDDNTKEAVEAET
HDVNDDTIEDVGAMEEQENQAAEQEEKEDQDLKKNKVDADGDVGMTYSSV
ADEATIKKEEGEPESVPEDEKEPEKPTEEKPSVAEPDKPAEAEGATEVKA
SDKPAVDQVSEEPVEKEGEAQATTEAETEPMEVDDKEKSEDKAEFPSSKF
LL
>Mb|QLI63849.1
MPPAAIKSGLPPRPGSRGPIASQLPALTAPRLHRIAHQVPGSSHLRSSFI
ANVSVDLIFLSSPVFVSFLRPTDDLSRCIEDTGQAQRYEDDSKMQPTEAR
GSRLRSRPFIATVVVLSALTAYSVLLQTHRHARPELRRDHQAALFQRAKE
PECGDVHSAADQCAFVRKYCLDEDAGLLPYLEWYFCAFNGIKPIGFTLLV
IWLGLLFTTIGIAASDFFSINLSTIAAILGLSESLAGVTFLAFGNGSPDV
FSTFAAMSSNSPSMAVGELLGAACFITGVVAGSMALVREFRVDRKTYVRD
IGFFIVAVAFSMAFLADGQLRFWECWAMVGYYAVYVVSVVGWHWYSTRRK
RRLRREGEARSHYYSIPGHAGDELFGEPYRDEPENPQDDVGDDSRSGSAR
ADISALERAPRIDIENRDQDSDRGGSLDDGSDEDHERMVAAEVSRSMRVL
RTGRTKSINLTPIRPSLVGALEFRSALAQLQRESNLQLSDIPGRTYSDHP
TGRDRRGTTSVVPDSGLWETASTPGVSSRERAFSYGARAPLSAPLPISED
EETSSESSANLSPGHATPAHGATPSTAYVVGGNLAPPPVGPSGAIETGRP
SSGAHGHSKQPSGLRLIIPSRRSSYSDESSPSSPFPRYADSPSAMTPSQH
LERTEFMLPPPVAPHRDGHFPDMSGSIIVRRRPIHWWPYALLPPPDVVLA
TLFPTLQAWGEKTIWDKFLSTISVPSIFLLVITLPVVESETNESSVDDLT
VMESSGRRELGHETQSSPMEPQLIEPEGEWERYRRHSLSRSRRNSLATVE

DRSPRLVVTLENEDTVVTPEVEATNGQTVAKPLSDMPSVSNANDELSTWN
RWLVCVQFVTGPLFAVMILWANMAEDFSNPGKVLVKMILYTLLGSLILLA
FLFLFTSDHKRPKYHFLLCFLGFIISIAWISTIAGEVVGVLKTFGVVLNI
SEALLGLTIFAAGNSVGDLVADITVARLGYPVMALSACFGGPMLNILLGI
GIGGVLMMVQNANKKHAKHPERPIQYGPYSVKVGPTLMISSVTLLITLVL
LLIIVPLNKWILSRKIGCGLIALWTLSTIANVVVEVTGVWQES
>Mb|QLI63850.1
MANQDDESKADDNKLPTAKWGAACSACATAKAKCIRSNPGPSAKCDSRTA
QIEERLNGLVNLLQASGELNNNQSALGALSDVAAREHSAITHDTPSTNST
TSYMPHSSSWRIPETYNCYAIPACICRPEPGDAPRDAPPPPDSDDVLLEL
YRTQMLPVFPFVVIPPSVSASELRSNRPFLMSAIRMVTSFRSLRSMRAQM
YRLMSHIADYVLIRSARSLELLQGVMIMISWHQYHCLMHGQMNNLIALAM
SLVVDLGLNWAPGLREQARLMVARPDEPPGRTNEERRALLGVWFLTCNIS
VSFNRVESLKYTAYIQDCMNVLENSREYESDITLLYLVRVQRLTERIFEL
NSKDKAVEDIPGLPSAPMSAYIAAFQNEIETMRNTLPPNLQNDGAFLSYL
NTAMLRLYEPHAVDLAFVNSLSQSLTAGPLGRGTPLDKLYQSSAALRNWF
DSWLSVPASSYFRHPTTIMSQLVYAITMLGRWAQLATPRTVYEGGTPMPL
GEGSSAYNADSQMGTNGLSESKSFQGYNQGDQKPRCGESVDQDFIAALAG
LQSQLQSQPGLTINIPEILSELCSKCEQVNNIFQQTAPEEEKMDNNVWTF
SALKVRITRVKLERWAELVAAAAEGHDRLSKVAHPQEWRGSYPQTSSANM
GQPPNGMANNGFAQDQTQIQNFLDSTPWTSDLLDGIDPTVWFDGYLDWGT
LVMNSMGQGPVGQQPQM
>Mb|QLI63851.1
MAMSCRYATQSCARQLRSVSSARAPAQMLRIASTTRRFNSTEAAPTNPKI
AEIVDQISRLTLLETADLVSSLKSKLNIPDMPMGGFAAAAPAAAPAEEVE
EAAPAAAEKSLFTLKLQAFDAGAKAKIIKEIKSLLGLSLVDSKKFVESAP
KMMKENIPKDEAEKIMASMKELGATVVME
>Mb|QLI63852.1
MPLDIEQILRQKKAADAAAAKPRFIPKAQRERMAAEQAKREEDDKRRKAA
DEAQKRRDDERKWESRPNGPSSTSHRIPNGPTQPPTGPRAMNQRKGDSKK
SDKVNGDKKSAEDIEATLFRSRYLGPEVNQQSSFSAKKKRMRTTEKKFNF
EWDLEDDTSRDNDPIYKNQAVNRGGSLAGLGGEFDEEAERRARKRARLIE
ERDVENGKERAKGIMEDFYRARAKAKERAERTGLGRHWSEKKLGEMRERD
WRIFKEDFGISTKGGAIPDPMRSWEESGLPQRLLDIVDRVGYKEPTPIQR
AAIPIALQARDLIGVAVTGSGKTAAFLLPLLVYISDLPPLTEVNKNDGPY
ALILAPTRELVQQIETEAKKFAEPLGFRCVSIVGGHSLEEQAFALRNGAE
IIVATPGRLVDCIERRLLVLSQCCYVIMDEADRMIDLGFEESVNKILDAL
PVSNEKPDTDDAENAQLMKSYLGSRDKYRQTMMYTATMPPLVERIAKKYL
RRPATATIGNAGEAVDTVEQRVELISGEDRRKKRLQEILSKNDFAPPIIV
FVNIKRNCDAVARDIKSMGWSTVTLHGSKTQEQREAALASVRSGATQVLV
ATDLAGRGIDVPDVSLVVNFNMATSIESYTHRVGRTGRAGKSGVAITFLG
NEDADVMYDLRQMLSKSSISKVPEELKRHEAAQSKPVRGGARREKDDAAA
GGGGGGGGAGKGGW
>Mb|QLI63853.1
MVSDEHYDLCLPILQDTTLEDEDKTDKLEELLKKETSLTGSSLENVILDA
MWRFREGGGSSASPPPIRSSILRRPSPASWRGSPTPLSGSPRLAVSPLAP
PGFIPSTFQRAKSSTASPFSSPRASPRLALATPVIPHSPSLNAYEFASDA
SPATEILGDYQSENVEWLVNDDAASTSSSVGTPSLLNAAAPEFSSMSSQQ
IDMSPYDMLRSILGQTRTDEEIEAALASHGYDLSATIASIMESQSQDGGP
PLNSLDEPKSVLIGKSISSDGRPVTPNSQSKGSVICKFYMSTGQCLRADC
RFSHDLSNHLCKYWIMGNCLAGDLCVFSHDPSRFMNKLSLDDPSGTSKSQ
GNISLQDMASFPALQHGGESFNNFGGAGASLIASGLNPPQGPRSYHGAES
PRLRSRPGSRHQVKDFTTAAPAIDDNEAFPSLGSAPAKQGKKHHGKRGGH
GHHKENVIPNSLADIVKMSPSPTPGSPRLESRRLGNGNSGANRNGENSAA
AQAIQSPKHIPWLETGDRVNKAYLKARQEAIKHGGLRNKFLQSAAQAWNR
NDARAAKALSLRGQSENELMRKAHREAARELYEHRNKHMGTAAEIYVDLH
GLHPEEAVEYLEKILMDNSKESRPVYAITGTGNHSKNGKDKVGRAIRSFL
NEWRYAYREFSVPGDRNSMGGILGIDARSWDKSLAREAAAEAAEEKEEVD

ILSQGVEIGDGKVRLLVRDPPKGPSGRR
>Mb|QLI63854.1
MGAILSLPLLAVPSMSTVLSFAASCCGAATCSMVCSACGKCGNSVATRIA
YALLLLVNSILAWIMLTPWAIEKLQHLALDYVKINCPNGECYGWLAVHRI
NFALGLFHLVLAGMLFGVTSSKHPRAAIQNGYWGPKVIVWLALVVVAFLI
PNDFFIFWGNYISLVCAMLFLILGLVLLVDLAHTWAEYCLGQIEDTDSRF
WRFVLIGSTLGMYLGSVAMTVVQYIFFAQGDCHMNQAVITINLLFWLAVS
FISIKSKVQEFNPKAGLAQAAMVSVYCTYLTMSAVSMEPDDKHCNPLIRA
QGTRTTSVVIGAIVTMLTVAYTTTRAATQSLGLGGNVGGIHLPEDDEHGL
VTQQPSARREMRAEALRRAVEEGSLPANALLSDDEGDSEESTPHDDERSR
TQYSYTVFHIIFFLATAWVATLLTMQYEESTRDRDFATVGRTYAASWIKI
VSAWVCYGLYIWSLVAPIILPDRFEFS
>Mb|QLI63855.1
MDLFAAGFNAWNQLCFVPESGDVAEPKDLSTFTKVLDDDCLERPRAGLYY
TLVRGRAGYYRAGIGIETASDEDIQRSYRACRAGNGLTLIFDGPTSAENT
GMRLGPNKHFLTVHPCYDDFVSKSNPTSWPCNSQVREVASFAAGFIILYQ
NGQVATMGDPRYQDCLAREVTSDEPADQPGFVPDLINLGEPIKHVTAGGY
CLTALTQSGSIYIWGRRCTGQRQSGHSAFAELCGIPNYLGVDGDKDVQDV
ALGDSHAIALTTDGTVYVIGRNDNGQLGLRQGVDMAAKTWTKVKLGVPDG
CQVVGVAAGPKSSFILTAQKTGVT
>Mb|QLI63856.1
MVHEGTTTESLALAVQAHVKYVQSLDTKKDELDYWLTEHLRLNGVYWGLV
ALHLLGQPDALPRQETIDFVFSCQHENGGFGAAPGHDAHMLSTVSAVQIL
AMVDALDDLDARGHGKAKVEKYIADLQDSNTGSFYGDEWGEEDTRFLYGA
LNALSLLGALSRINLDKAVSHIQSCANFDGGYGAKPGAESHSGQILTCLA
ALSIANRLDVVDEEKLGSWLSERQTPSGGFNGRPEKKEDVCYSWWVLASL
AILKRTHWIDRDALITFILSSQDSENGGLSDRPGDMVDVWHTCFGLAGLS
LLQYPGMVPVNPVYCMPEAIVAKCVGSNR
>Mb|QLI63858.1
MVNFTIDEIRALMDKPSNVRNMSVIAHVDHGKSTLTDSLLAKAGIISTAK
AGDARATDTRADEQERGITIKSTAISLYGHLDDPEDIKDIVGQKTDGQDF
LINLIDSPGHVDFSSEVTAALRVTDGALVVVDTVEGVCVQTETVLRQALG
ERIKPVVIINKVDRALLELQVSKEDLYQSFSRTIESVNVIISTYLDKTLG
DVQVYPDKGTIAFGSGLHGWAFTVRQFAIRYAKKFGVDKNKMMERLWGDN
YFNPHTKKWTKNGTYEGKQLERAFNQFILDPIFKIFNAVMNFKNDEITTL
LEKLNLKLDTDDRQKEGKQLLKVVMRTFLPAADSLLEMMILHLPSPVTAQ
KYRAETLYEGPLDDPAAIGIRDCDPKGPLMLYVSKMVPTSDKGRFYAFGR
VFSGTVRSGLKVRIQGPNYVPGKKEDLFIKAIQRTVLMMGGKVEAIDDMP
AGNIVGLVGIDQFLLKSGTLTTSDTAHNLKVMKFSVSPVVQRSVQVKNAQ
DLPKLVEGLKRLSKSDPCVLTMTSESGEHIVAGAGELHLEICLKDLEEDH
AGVPLNISDPVVQYRETVQGKSSMTALSKSPNKHNRLYMVAEPIDEELSL
AIEGGKVSARDDFKARARVLADDFGWDVTDARKIWTFGPDGTGANLLVDQ
TKAVQYLNEIKDSVVSGFQWASREGPVAEEPMRSIRFNILDVTLHADAIH
RGGGQIIPTARRVLYASALLAEPALLEPVYLVEIQVPEQAMGGVYGVLTR
RRGHVFNEEQRPGTPLFNIKAYLPVLESFGFNGDLRAATSGQAFPQSVFD
HWQVLPGGSPLDSTSKVGQIVTEMRKRKGIKIEVPGVENYYDKL
>Mb|QLI63860.1
MKHEELDNLASNLSGDFRSIEPRLRDLDKHLTLRTYLEGYTLGDLEKKIW
QTLRGNKVAIGFIRRNGLPNLTRWFTFLEQTHPELQEDATKGKAIANKGG
ANYNLALQDADKGVVTRFLPEPSGYLHIGHAKAALLSDYFGHVAYKGQMR
LRLDDTNPAKESEEFQDAIVEDLALMGIKPDALTYTSDYFEYLYETCKKL
ISMGKAYADDTDQETMRAQRMDGIPSKRRDRSIEENLRIFEEMKKASEEG
LAHCIRAKISADDPNKAMRDPVIYRCNIENAHHRTGRKWNIYPMYDLACP
VVDSYEGVTHALRSTEYTDRNPQYQWFLDTLGLRQVHMWDFSRMNFVKTF
LSKRKLAKLVDSGRVWGWDDPRMPTIRGVRRRGMTLTALRDFIIRQGPSR
NVVTMDWTSFWAANKKEIDPVVARHTALLQANAVKVKLTGAEVPSGVVTE
QRPKHPKNPDVGMKDVVYSPEIYIDQEDAKSFKLNEEITLMGWGNAFVRE
IPSEDPITTMTMELNLKGDFKSTDKKITWLSAEGLEPVPAELWDFDYLIT

KDKLEEDDNVEDFLNPDTFSLVDAICDGGVGKLKKDDIIQLERRGFYRVD
KGLNDWAPGEAGLKGKRVVLFYIPTGKTGGK
>Mb|QLI63861.1
MVAQAQPVTVSLKDLTDGNVSFETLQRAFGPDSLGILVVKDVPQEFPQLR
RMALSYASYLGNLPAEELEKLENVKAKYLTGWSLGKETLKNGQADTFKGS
YYANCAFYVDPALECARPTPEFSPDTFPEYLSPNAWPPENVLPGFKPAVT
ELCRLIIDVAVLVARACDRFAEREIQGYPKGYLEHVVSTSNTTKARLLHY
FPQDVHSNDGTSEDDWCATHLDHGCLTGLTSAMFIDEHKVSPAVPGDVGL
TGASLPPLGELPASPDSSAGLYILSRAGETCQVKIPRDCIAFQTGETLER
ITAGRFKAVPHYVRGVRAAVSGGAIARNTLAVFTQPNLGDEVDLESHLTF
GEFARGVVAKNTVS
>Mb|QLI63862.1
MPQSPMISVPLKATNEIDWVSPLKNYIRQSYGDDPERYAEECATLNRLRQ
DMRGAGKESTSGRDMLYRYYGQLELLDLRFPVDEQHIKIAFTWFDAFTHK
STTQYSLAFEKASVIFNISAVLSCHAAFQNRSEDSPLKVAYHSFQASAGM
FTYINDNFLHAPSFDLSRETVKTLIHIMLAQAQEVFLEKQIADQKKVALL
AKLAAQSGYLYGQAIEGVQENVNKAIFEKVWLTMVQIKSNLLNSMAQYYQ
GQADDEAKQHGSAVARFEVAERLAKEAERLSRNFPATVPANSNLSAECGG
LLQELSKRQLSSVQDRLREAVKDNDFIYHQTVPAEASLAGIAKLPASKPI
PVSELYAGQDMQRITGPDLFAKIVPMAVTESASLYDEEKAKLIRAETERV
DAANGEMAASLDYLRLPGALQVLKGGFDQDIIPDEDFRQWCIDVADHEAP
AEIFELLRNEKETIVSLLDNSSKQLDLEESVCEKMRSKYEGEWSQQPSSR
LTTTLRSDIRNYREALDVAMRSDNQLAAKLRENEVEFDEMRHAAQEADVD
QLFRKAITQARSKGSHASSPAHVEPNLLDADFGESGPSVMDQVNMVEEIL
KRLNLIKRERNQVLKDLKEKVHNDDISQVLILNKKSISNYETQLFEQELE
KFRPHQNRLLQANHKQSQLMKELTATFNALLQDKRVRSEQSKYEGMQRQR
SSVINKYKRAYQEFLDLEAGLQSAKNWYSEMRETVESLEKNVQTFVHNRR
AEGAQLLDRMDQDRSASKSNQAELEQERLRGLMERMSVDPAKASPQTSTS
NRPAPAPLFQPGQGPRYAQNNFQGQYQVPTSPPPNQATYPGYSSPPPQST
FSAPMAYNPSQYGRTPGPTSPPPNQASFAVNRMPHSPPPTQTSFGQTHQH
QPHQTYGNYGASQAQQPAGYVPPGFVPPPPPPGPPPLGPQQTFHYDGHSG
HQQGAQTRVTQPEQPHDPWAGLNAWK
>Mb|QLI63863.1
MSIPPQLIRVKRKRVAESPVTFLQFDQDSKRHRSGSNWAYQRREVAGQQP
PREGKSTQPIIHVSAPDKVASHDKRKDEAHIPAKPAQPSETTPRPLEPRR
FHMSKSMLAKRAAQGPDSGVSKKTRYGPAVFVESTRKRRVPRRSLAASQL
PMQLENELHRAEQNSGSDIERRQLKRPGVTNKSRDTASQNVIPTRDPLPD
SLMNRHNEDMDKIANDMNAWVLNELGANLHSMEQDNRPLRFKPKSPAKRF
HERHPELAPPQSSGTSTDTTVSDLSDEGDDDDWVIEEYVRIPANSVALDA
GPADVGILVLEDEEESLLFFGSSLDEDDELGEDDEDENAENYYTADYPED
EVDSDDEYGRDAYVYRHGNNSDEEEYDNEYYEEQDEMILEGGIDDNDDAR
MARIKEFMKRNSAFP
>Mb|QLI63865.1
MPPESLVSTREETTRTAVTMVVSLRRIVMPPLPIRYGLNWSVHLQSSPAM
NLQSSSTGVLKQSGGSQSSQPSPSTASIMLATTIRSPPSVDDYTPLSEYQ
SQTPESFSGGKPVLHFHLKGAVASIPRAQCGTLAIFPADSPAAENAVAST
NGDAEGLVEQKVDVFVNSEHFTIFSEQTESGVSIPYPSISIHAVKQVSAR
EGASTPAIWMQLEFSDGGADDDDFNTVELTIVPASSEPPQTAQQLYDAIA
NCSNLHPDPTNGDEDGDEEDDYDRIVFEGNAEQEAVEGFTGVLRGTADGG
LPPPMPGSGGWITAENVHEYFDEDGNWIGGSNGEGEAEELGDGAGRTRPR
EEIDDDGVNGDDAESKRPRVE
>Mb|QLI63867.1
MDAPEPEQTPFAAVTAHTTKLQRHYQALLDQSTPFVLYRWVATGVTLLLF
FVRIFFIQGWYIVAYALGIYLLNLFLAFLQPKFDPSNDAVDQDMEDGAVG
TLPTKQDEEFKPFIRRLPEFKFWYWATRAIAISFFCTFFEFFNIPVFWPV
LVMYWIILFVLTMRKQIQHMIKYRYVPFTMGKKNYRKDNS
>Mb|QLI63870.1
MWIVNWFYDVLSSLGLLNKHAKLLFLGLDNAGKTTLLHMLKNDRVAILQP

TLHPTSEELAIGNVRFTTFDLGGHQQARRIWRDYYPEVNGVVFLVDAKDH
ERFAETKAELDALLALEELSKVPFVILGNKIDHPDAVSEETLRHELGLYQ
TTGKGKVPLDGGVRPIEVFMCSVVMRQGYGEGIKWLAQYV
>Mb|QLI63871.1
MPQYTSRDVGDPSQIKKTKQSMADLKLRRLTELNNRLREDLERERIPIST
AAKSIIAYCNGTRDYMVPSVWGSVPKGEDPYAPQQSGGCCVVM
>Mb|QLI63872.1
MNVALPQNGAPKSPLSRYRLLSPTASVRVSPLCLGSMNFGDAWADWMGEC
NQESTEKILDFFFEQVRRMNETEPDRTPEPDLQGGSLIAFFDLGNFIDTA
NSYQFEQSETWIGEWMKKRGNRDQMVISTKYTTNFKAGPNHPHIMANCVG
NGTKSLHVSVNASLRKLQTDYIDLLYIHWWDYSASIPEVMQSLNQLVATG
KILYLGISDAPAWIVSKANEYARNHGLRQFSVYQGRWSASCRDLERDVIP
MCKAEGMAITPWGSLGGGNFKTEQAQRSSEGRRTEVSDTDIKTSRVLEEI
AIRKNTAITSIALAYVMHKTPYVFPVIGGRKVEHLKGNIEALTIELTDEE
IRLIEAAVPFDLGFPHNFLWGRQVPNPLQDVWLLSTAGNFEHVEEARPII
PKKTGE
>Mb|QLI63875.1
MVVGTGAAALAGARTTKNVVNQKPNIQIKIEKHFNSKVYTSGSTISGQAL
VKTQKDTRFDDFDIIFSGIAATRLDFVQQYPSHSFRPFMKLRMPIDRSTL
PESHVFEAGKTYTIPFNFVVPHHLTIGACNHYCSSPAVQEQHLRLPPTVG
FWERDDQAPDMVQIEYSIKARAYRRASEAGGIPQKIMDGYHILKVLPALP
EDAPLDITFRDERYSLSKTKTIRKNLFSVKAGKLTVSAAQPSAVMLSADG
RHALGSVARISLEFAPTSTESVPPKINSISGKIVSSTFFGAAPSDLLPNL
GSRATYTANPSLSYTVSNNIFNNKPVEKVAWQVHNVSGRRDSGYSSLGNA
DEEQSEMECSDDASKTSKKCPIRHNAVVDIPFSIPMTKKLFLPTFHSCLI
SRTYTLHLNLSVGATNTTMSLAIPLQIGVETIYEPQNAGLPSFESVMAEN
EQAAVGGQLDVHNAQFPELMEHTNGMLPGYDEISRRAVRVA
>Mb|QLI63876.1
MAPHEPPHKQALRFALDVANGRHALSKLVPLVLWLADAVLCGLVIWKVPY
TEIDWVAYMEQVAQFVAGERDYTKMEGGTGPLVYPAAHVYVYTGLYYLTD
RGKNVLLAQQTFAVLYMATLAVVMLCYWKAKVPPYIFPLLILSKRLHSVF
LLRCFNDCFAAFFMWLAIFFFQRRQWTMGSIAYSWGLGIKMSLLLSLPAV
GIVLFLGRGFGGSLRLAWLMAQVQLVIALPFVAKNWRGYLGRAFELSRQF
KFEWTVNWRMMGEELFLSKQFATVLLVLHALVLLIFVTSRWLQPARRSLV
SLISPMLRGRSPFTPAEELAVSSRVTPDYIMTTILSANVVGLLFARSLHY
QFYAYLAWATPYLLWKAWPYTPVVYLLWAVQEAAWNTFPSTDLSSTIVVN
VLLMTVVLVYFGTLKPVRSGERKKAKASKKNR
>Mb|QLI63878.1
MQSGISASQELQAEFNSLLSSPSTFGLIVGIEKESLVPLTTLPSQGPSFL
ENLGSLQSHLEPNVPLYILLRRYDDIPRLTAISYVPDTAHVRQKMLFAST
RLALVRELGSEHFRETIFVTTADELSESGFKKHDAHSQLAAPLTEEERTL
GEVKRAEQEAGSGTGTREIHLSKSFAMPVAEDAVAALKDLGQDDGRVVAM
LKINPESESVELLPESPRPASIAELAKAISTTEPRFTFYRFKHTHNGAEQ
SPVLFFYTCPLTAGNKSIKKRMLYPLMKRAVLEIAGKEAGLSLEKKFEVE
EPSEITEQSVMEDLHPKTTARSGFSRPKRPGR
>Mb|QLI63879.1
MPKKRNRIKPFKPSLATSTTSSSSSSGSGGYKASKSVNELLANLRLAGAG
PSSSPSLPATSPSVPPAIREILQIPETPSPAPRRPLRQRFDSHGRRLPAG
PAPPRSWLLGGREDGNVSNSLSRSLRVSYSGVDRSTLPGAPLPQPGSLID
LTLRRLAADWDIHRVFNQYHLYFVPSHLKPALIRYVGVASDSGVSISDLR
VILQPPENVFSDEELDGQSGANSEITCLDLSASIGKSLKLKDVIDVLFSV
VEPNAIDEPQDSWETVDTSPSPPRLLLPNLTHLSLALDPRGPRDVSWKQL
LSLSSKLPSVTHLSLAYWPEPCLTPWAKFSTVSSPQGRNIPYAGTNYYSH
SLDHDWSEALLVLRMLSKNFYKLEFLDLTGCATWFKALHLKDGHDYIDWS
GSWGKLTLLWLYTGWAPGIDAMASDQAAYTSAIDTAKCIERHIVAMRAGK
GRFITVERDKTEAEK
>Mb|QLI63880.1
MPGISRKILICAAIDGLILQPLPTKGQRPFQPARIRYGDSSISAVPRDQI

PDTSGPNSSFEVFGVIGLITVSRLSYLITITRRQQIAQIFGFPIYVVTGV
AITPCNTKQEADESIRRTASLLRKQTPNGEDGDSESSDEDVEIPQSPLDE
VEDTIGEEDKTRPDSSRSSVAEDVMRRRGSYGRFAQRWFSNSGWTMDQRR
SLGLSIATAGSPTTLEASGEPATSKTPIEEDMSDQSQVTALLPKLLRTIQ
VFFESSRSFYFSYDIDLTRSTSQQAWVPNPDTPLHTQVDNKFFWNRHILG
KFIATGQDSISLPLMQGFIGQRTFVVDSDPPQMDEGASESMELADMSTSG
SLPVSPPSGRVGDHADLRPSERKCLITLVSRRSTQRAGLRYLRRGINEEG
FTANMVETEQIISSPTWDDSSPIHSFVQIRGSIPLFFTQSAYSLKPVPVI
QHSPESNYRACKRHFERLHSNYGSLQIVNLVEKRGVEEPIGTQYEANVSR
LNEELGENDKIPFEWFDFHHACRGMKFENVSQLLIKLRGRLEELGSTTQV
GGEITQRQAGVLRTNCMDCLDRTNVCQSSFAKHMLDLQLKGDGIDMSAQL
DQETRWFNTLWADNGDAVSKQYASTAAMKGDYTRTKKRDYRGALNDLGLS
LARFYSGMVNDYFSQAAIDFLLGTATEKVFDEFESDMMTKDPAVSIAKMR
QRAVELCQKRVVADASEEVHGGWVLISPNMADVVKSWPMEEVVLLLTDAA
LYLCRFDWDLDKVSSFERVHLGNVTHIKFGTYITSTLAPAHMDETKNVGL
VISYQPGKSNIKRTNTRTLSTKGYIAPNSASQGEAKTQVGLVSFFSPKAK
SPAVRRLAFKAPYMDSSTAVSGTAGLQQTELQQVITICAEIERLALEAQL
RKEGEGEKSFVEKGEIISLQEAKKNTGLLEQLGHSIKRLVWA
>Mb|QLI63884.1
MAPPKAALDFVDFVNDSPTPYHATQCAAERFEEAGFKLIRERDSWSTIVQ
PGGKYYLTRNGSSIVAFAVGRKWRPGNPVGIIGAHTDSCCLRLKPVSKKS
NVGYLQIGVEAYGGGIWHSWFDRDLSIAGRVLVQEGDNFVQKLIKIDKPL
LRIPTLAIHLDREPKFDPNKETELFPIAGLAEAELNKSSDDGAPAETDKD
ENFRPLKNMAERHHPAVLDVIASELNANVSDIIDFELVLYDTQKSCIGGL
KDELIFSPRLDNLGMTYCSVMGLISSVSNDKSLDDDSTIRMTACFDHEEI
GSQSAQGAHSNLLPAVIRRLSLLPSGRDSSSDGSYEAVHHEGEDTTAYEQ
ALSRSFLVSADMAHAVHPNYAGKYESSHQPAMNKGTVIKINANQRYATNS
PGIVLVQECARLAGVPLQLFVVRNDSPCGSTIGPGLAARMGMRTLDLGNP
QLSMHSIRETGGAADVEFAVKLFEAFFKSYGRLEPRILVD
>Mb|QLI63887.1
MLRQGFRHCAGLTSAVTRSAFFHSRTAIPIAPRGVVSLARTPATFKSIAY
SSRFYSAEAAAVETKPIEGTVPSDVVNFADLSQAGVHRNLLDAITKDMRY
EAMTPVQSKTINPALKGTDIVAQAKTGTGKTLAFLLPLLQRMIEEDPTLA
TRRASRNARSDDIRGIVLSPTRELAEQIAAEARRLTKYTGLVVQSAVGGT
HKGSMLRQTQRQGCHLLVATPGRLNDLLQDPQSGIEAPNLAALVLDEADR
MLDVGFEKELGEITSCLPSRNEKTRQTMLVSATIPDDVIRLARTMVRADD
FEFVQTISENESLTHDRVPQHVVPLASWNNVFPSLFELIDRESAKAAEDR
SLPPFKAIVYFNTTSLVEMGAELGFQRRRNGLMKTPTFSIQSQLTQNQRT
KAADMFRHARSGVLFSSDVTARGMDFPNVTHVIQVDTPRDRESYIHRLGR
TARQNKGGEGWLLLPPTSMGSARKLLRGLPLQPNKTLESFTFDAEEGAEK
LPKYHQEAQDLTQSLPRKMLSSAYTSIFGNSTQQREELVEDLNLWTTVGW
GWSEPPPVSHAWARNQGLLHSELNIQDRATRQDDRSGRREGRGPRRFDDH
ASADPFADMGRRVRRDDKGFGRSNRGNSHRGSRGFRGSRESRESRESSF
>Mb|QLI63890.1
MDAGNSVGGPYLPNPTGRNVHSHGPAHSAADPNAMLNQFAGLSLGGMSMP
SSSAPMPMGHNPPPYMVGTDTPFVLAHMSAHQIGMNHHHTENPYQNLGIP
GYGSPYVGVQMPFVPFTPGRAHIAQPRVERGHSDVPGLENRRGSYSTTES
TPATPFYGTVSQRDDGPRVASLDRSAYTTPSPQQIGLPALHADPTKPAIA
VVSDRTVDEYLQQEPAIPRAVPAVFTPPSQMKSLEQSLENRIPGNRNVYI
RGLHPTTDDELLYKFASRFGPVETSKAIIDTGTGACKGFGFAKFFDSQDS
EMCIRGFHRLGYEVGFARESFNSRLKAEGDEGSTNLYISNLPKTLTEVEL
GTIFLGTTILSSKILRDSMGNSRGVGFARFESREICDEIIKKYNGVGIGE
EGLLMNIRYADTPAQKELKRVTAERRQFRTNEYNIGAYGTPLVGMSPTLY
NQQSQWRRTLPVSRSGLSASSNEEGNVMRHSGTRRGLSEIAPNTITDVAA
AATMSDSDEGVTIHADSSTVAGGTNENKSPSTKGARRDEISD
>Mb|QLI63891.1
MLNAALTSSMLLTAAHAAANVLYVSSYSGKVTTLDLNSARSPSCRVADNV
PVLKAVSSTDGCAVSPSWLTLDHAHSTLFCIDEALPSDKGSLSSFKTNSN

GSLTTLNKAVTLGGPVSGAGAGALTTWDVSNPSNLTLVQSELYHMDKPGP
IADRQNAPHPHQTLLDPTGKFILVPDLGADKIYVYAVAANSLRLTVLPPI
VVKAGSGPRHVAFAVRDDKTFMYLVTELANTIIGYEVTYPDGGIEFKELF
NIDSHGAGKPVSEAVYAAEILVSPDAKFLIVSSRGDNTFSIPNFDRKNST
NITSDPLINFSIDGETGSLTPIQEAPCGGRYPRQFSINKAGTLVAVGQQK
DARVVLIKRDPESGKLGDFVGYADVAGEITSVIFNE
>Mb|QLI63893.1
MTAIVDEYIESPLDNDDVFPCKGCGEILEEGKAFELAGNRWHLDCFRCHT
CGTLLDSDANLLLLGDGSLICNNCTYSCSACGNKIEDLAILTGDQAFCAT
CFRCRNCKRKIENLRYARTSQGIFCMGCHETLMARRRKKSKAASAAKSRD
KENSPMITEKSLPALPPSAIPPNAFSNDRVDPDSDTPTELSPRPRGTHRH
NESSSRGSSRPSRSPERSETTSKPEGLGLPATTYRKNRNSTIYQSPNSGP
EEGPESFFIPVALDPSPALTGTPRSASDNAAENGRRKDRDYFGVSKGSGS
DKRDDSQSSTPHIAFQEKGRQPSSDLDVARLKSGKFTKNSEQGQRKASVS
GDEFKLQDAPVKKRGVSTRSNSSHSSAPQEGSSGKTSNGVVRKDGHSNLS
EARKVNEPRTSEDTDGAKSGSRSESSKAITRKELPASASMNSIGQRSSER
SSRSGQSEQNNLSDSYTQPRTAPPPPPTEQAQTPRKSSGSSKDPSEPKHS
PKLPRWSNGGDFSMDEDMARILGTDEGSSSILRRVSNVVRHGRTGSVEAG
HPNSGRINHSRSISETTRATGSPRWPKTPMGDDHPGMPHDISSPISLTPP
HDAVLLKRQLRNSEQRVAELERQFNTEKDLKSLNRKLVEKRKTVSVLDTQ
TEIMIRQLEVLAGYVERAKETKTPIDPRELEDSAIKDFVKKLDAVKETMS
EAIEKLHEERDQLVEEKNQAIADRDRALMEFEQLSSKNAQLADMNNDLTH
QIQERFKSQIGDLKSPNGLGIYGPTKSGGASSINLDTSSLNTGTTLMGDE
EPIVESGPTVVQVRKGQVKKFNWKKGSKTMAQNVAKGVNRAVVAFQQTDR
ERMQHQGLVGDNIGLPYNMTVTQVESAAPSGLNGQNRQQQHMDPGRQGFG
FFGKKNAMPKSASSNTVTLAAAAEPASILFGSDLVERTEYERRQIPAVVT
RCIEEVELRGMDQEGIYRKTGGNSQVNMIKDGFDKNENFDISDPDLDITA
VTSVLKQYFRKLPTPLLTFDVYERVLESNAIADETDRCSHLRKTFASMPE
RHGDCLEFLMFHLNRVASREPENLMSPKNLAVVFAPTIMRDLSIEREMTD
MHAKNIAVQFVIENTNRIFDDS
>Mb|QLI63894.1
MYRYAPQPPPSPPMEETKCSLPSISKLLGIADAGSPTSIASPDSTSESPL
TEDRAVPRRQETPFSNHESCINSSYAQHLSQSKGFPPTPPLGSDASFDNH
SSPITKPQRHFSIGTFSGGKYLEATPPPMESDARRFSLPQQSVTGTQGHP
QHAIPQQNLPLGAFHSLSTVGGYYHSALPSGAAPHPQISNLYYQQPLPQT
FPPMTLPIAYTPSSGSNPWQHHHYLNPVHGMSYTQSQDRYICPTCSKAFS
RPSSLRIHSHSHTGEKPFKCPHAGCGKAFSVRSNMKRHERGCHSFNINSG
RQPSR
>Mb|QLI63895.1
MKLDTRAMRHLASEDWRVLTAVEMGSKNHEVVPTPLIEKIARLRGGASGV
RRSISALAKVGLIARVKEAKYDGYRLTYGGLDYLALHTHASRKDLYSVGN
RIGVGKESDIMVVADQTGTQRVLKIHRLGRISFRTVKSNRDYLKNRASGS
WMYLSRLAAMKEYAFMKALHEEGFPVPVPLAQSRHTIVMTLVDAFPLRQI
SDVPDPASLYADLIALILRFANHGLIHGDFNEFNILIKEEKTVSESGEES
LKLEPVVIDFPQMVSTEHQNAEMYFDRDVECIKRFFSRRFHFTSTTPGPF
FKDSKKKLAKGGFTRLDATVEASGFTKKMLKDLETAIKDQVTKRGEDGVS
EEFDDDDDDDDEVGSEDDDDKSGDDEKDDGGILGPHSHERTLSTGAKVDD
EWSAADAQPLLNEDLSKLTV
>Mb|QLI63898.1
MATESAKQGKTHTRNSPEAMQDQNGSAECASAEPTIASKRTSSIVAIQHD
TTTVLSITTSVTPSIADTVRQYRSMLDVEEPPKLNPLNPMSFDELYTPPS
QQPPKSASEKPQYELTANGVCGTLANTADSQVKDLSSSNSSDSPMGSPAS
PPAGKPESAASAQDAHPSDPAPAVPSQSVPSTIQDARNIVRRKLTGYVGF
ANLPNQWHRKSVRKGFNFNVMVVGESGLGKSTLVNTLFNTSLYPPRERKG
PSLDIIPKTVQIQAISADIEEAGVRLRLTVVDTPGFGDFVNNDESWRPIV
DDIERRYDAYLDAENKVNRMNIVDNRIHACVFFIQPTGHSLKPLDIEVMR
RLHTKVNLIPVIAKSDTLTDEEIVSFKARILADIKHHGIQIFEGPRYELD
DEETIAENNEIMSKVPFAVVGATNEIKTTDGRAVRGRQYPWGIIEVDNEE

HCDFVKLRQMLIRTHMEELKENTNNALYENYRTDKLIAMGVSQDPSVFKE
VNPAVKQEEERALHEQKLAKMEAEMKMVFQQKVAEKESKLKQSEEELYAR
HREMKEQLERQRAELEDKKQRIESGRPLEKEGKRKGFSLR
>Mb|QLI63899.1
MLILRVPAPKAILPTLARVPPKPQPPWRRILQLGSPHLRLTRPPSQLRSF
AHQPSKSSSEAAKRQAKSAAQSAKSHGTSTRPPPPSLSTAKTRPDIPERI
LIYHAGAGRIAFLAVLKLTSLLLGAFFTLLAAPSYFRAGKPPPATAAVAA
CGIVPAVFVAWSTAPFVTHVHMHLPAAARASPASLARFVGALPPAARLTL
TTMSLVAAPRCTAVRAADLRAAPPRSRRLGLVNYVRDTAADNAARRWYMY
RAVGAFYVQDGGGGKQRVAAGHQARRNKAAAVDTWIWEAVRDKIARRDAP
GPV
>Mb|QLI63901.1
MAQQPAGNGLSYTIVPFNDISKLPACANACGPLWDANGACVPPALPTADA
GTYESCFCADSRVAPFSSGGGANVCPNACPGNPQGLSSIGNWFTSFCNGK
APKNTPQTTGTNTATETNAAGSSNIGGSNSGNGGDWLSNHWQWVIMLVIL
VVAIAGIWIGACIWRRRYLRKKDRQTSLGQKHSGSASHPSWGPTVTGTET
GTPTAHNAGRDPERGAATEKAGKTQPKKKWTVTQRT
>Mb|QLI63905.1
MVKRSTHDTDGAAAFRKRQKLTQDIPTGEQVTSSEQLRQLLTFDQDMRKA
RHGLQSFKKFLDDILSSDDDRKQNLAILQDYLEGVKPRDTSEEAVHLHDI
MEMWSFSIQVNNEGVMSSVAVVLALVLQVLSGSLHLVPFGLGICQTLVQE
AQLKCLSRNLGSEKGKGFIISPTLRLLREAVSFDGGAYAKKIVRARIYTF
TSLGRNLEIGHVGDMQEDVRKASVRTNAVRFFLSCLKYLHSEGRRELLSQ
RDQLSHLTYMMKNDPPYLVIEILESLQAHVLADSRIAREVKFKAFNTKIL
MRILSLYSYSHTTANADEKDIVCEKAHHLLTNACTTYSGGILYPYKGLYP
KEADDEFSALSAKNKNNEANGDPWEGKLREGIPVYNFVLSEFIGKLRPWS
NLKHCELLVAIFRAAPELISDYFHKNQSFTFEPKLSMTWIGYAAFLFNTM
MIPLPASFGDPSRYAAMPPPTPVLLDNIVPRPINQKVLIRCLSPKSHLTS
FFATRILVAALEKLSAALKMLDGQSRRRNAVWMEASRRLVDMFCQRIPDM
KEIVRCYKGIPAENILHRTLTSRLLRLYYEVIPRVALAANFDVSPFFAGV
LAGLNRDNIEAEPRALGLMELENLVLIASYSPGMRWFAKLEALGHGYASS
PFNALLQLLCGDKEGAPLGQLKAVLGDVAVESQVVTKSVSLKHLLQALRC
ARETLGAAGMETVWSFLDNCVSRCANSPIKYLDLVKVYSSKPSTLETDGN
MSLLNVVIMDQLPYALNAGDEKAANQLATFVSFYFSAVQASSKDVAPLER
LYKDIENLFSTASANLADLGDTKQVKTLKKHDGIQWTTRKPVSGGEGEIE
GSTVNQAALEEMLHVPFLSDDDATVLTKWVSKGVEDMVEDGWAAGLVRLL
ASEHINLRKEALTGVLKMAAQIKDSSYEEKTQIWLLLSELAESSRAQVDI
GPVPSAFIAFAIHALDVLKNTLHPLYPKVNSYLTRSPIWGLDKLPLVHDI
LHGTPSEDDKYYTELTWLFKYLLDSLRTPLDLSVFHKKRWFEKIFAVGSN
PYLRSALRTRLLKLVYRATCIESGSTTLITRFGILSWLDSQRASCDVDET
AAIYAALMKRAWETCDQDRVWAWSKGGVDQLLDKISKV
>Mb|QLI63907.1
MDSSTVKQSVMKQVLAEANLANARVLIEKLQENCFEKCIPKPGSSLSSGE
QTCMTTCMEKYMAAWNQVNAAYINRIRQEQNNPSQ
>Mb|QLI63908.1
MDQYFTYEARLASFQKTSKKRGSTAGGRSKALNWPHKQIAPASLAIAGFY
FDPYPENPDNCVCFLCGKGLDGWEAGDDPLEEHLKHSPQCGWAIVSAIEA
EIDEYSRQDPTLPHMVEARKATFAGKWPHEGRKGWKCKTKQLVEAGWKYT
PTDESDDMATCADEHYNRSPNCTFFALVNEFGGPKKRTARGKAAGVSKTS
RLSMQSVATTASDIASVADMTAEPEDSILTTTSTATQGSKKTASKKKKPA
TTKGRAKTKKAQGIEREEDDTVDEEPVSLPKATKATHGKKRTSDAIDDSV
LFLSEAPASKKQVTQNRESSQIDSSTITPEDVEFVDAQASKSAGRARSSS
VKSSRKPSALGASVASLRAPLADFPDDDEIERQLEADLERALTDDDEIAH
DSESERSKAMANKKSKGKLTDEQLEDYAMFDPAEVEHDEDALDDELRNLQ
AEMEVEEPNQKLHVPKKGRKPGTGIRKASKQTKAKKAKAPSPSPEPEPEA
IDPPSEIEEHEASIGSTDAVVKNTDTVDSPPSIKAPSRAVKKSSLASNAA
SKPAEAPARRGRRPSSTTAEPIQPSESTQPSEGHADDEVSEADGETHRRE
SAGTPQLEEASSPLEEASSPAVSRSRLGVEPPSTPPKFTSPAPSARQPAL

SPSPSPQSSDAENLPPSSLPPASAKAKRVVLAPLAATPARNSPSKRNMLA
AGLQSQIPWTAVDIDAVMGTPRCGDDKENAVSRLLKQGKELSSPETRMTV
EEWIHFNAGEAEKKLKHECEAMVTQFEREGTKAMNVLESLSIE
>Mb|QLI63912.1
MAPRTEFSTEQVRAKARKDLLYLLEGVRGKKNLVFDKSLVGPVGTIVKVN
TLQEYGVDKFFVLENDNVDASQHNVVFIARGECGRHAENIANQIKRVRRQ
SSTSHEFHIFWVPRRTLVSDQLLEEAGVLGDASISELPLFFFPLERDVLS
LELDDSFKDLYLSKDVTPNYLLARALMEVQKNHGLFPRMIGKGDNAKKIS
DLLVRMRQELLAGEDTSDSNKPGLTPSSTNESVIIIDREVDFVTPLLTQL
TYEGLIDEVFEIQNNQTKVDTTIVGAPARSSAATTQGRKETVPLDSSDKL
YDQLRDANFAIVGGMLNKVARRLQQVQSDYETKHKTKTLAELKEFVAQLP
GYQQEHRSVRIHTGIAEEIIKRTRTDEFRGLLEVQQNLAAGAEPSSQFDG
IEELIARDAPIRETLRLLCIYSCISGGIKPREYDQFRKLVLHGYGYQHLL
TLHNLEKLHLFLSRSSPLAGVIPMTGNAGATGTKTNYTYLRKQLRLIVDE
VQEDDPNDIAYVYSGYAPLSIRLVQCVLQKQYLLSFAKGGSAANAGVAAA
AATSQGWHGFDEAVKHVRGQTFYELQTGEDKAVKARALLSGGSAKQTVFV
VFVGGITFTEIAALRFIAKQDEGRRNIIICTTSIISGNRMMDAAIETGSY
ARESPSVEGERR
>Mb|QLI63914.1
MATNGNFAQQEQYKAADPAAGAETNNNSATLSKDEVGWYFVEQYYTTLSK
SPEKLHLFYSKRSQFVYGRETEVANVSVGRHAIQERIKALDFQDCKVCVT
NVDSQASFDNIVIQVIGETSNKNGEPKKFVQTFVLAQQPSGYFVLNDIWR
YIIDGDEDEEAAAEPSDAPAVIEEETAAPAVAVTEPEPVPEPEPEPVAEI
ITEDSIKFDADAVDEKLQQAASAQVDTPVNGEDAIETPKAPEPKVEPAVP
AQEVSENLTPEAERPKEPSPTPVAKKEPVPEPAMPPKPMTWASRAAAAAG
PRPVVPLPKTATPPAQSQSRAPAPAPAPAPAPAPAAAPAASAQPAAPAAA
APATEVPAKESSGWQTAGVDSKRQNRPQSISGNAAEKEGTLGYVKYVTEK
VQEADLRNALATHGELLYFDINRQKNCAFVEYKTFEGYHAAVSANPHVVN
GENIVVEPRRPKSTAYGGNNYGSGRGNASGGRGRGGFDGNRNTQGSSRGN
FSGQNRGRGGGARGRGGAQASSS
>Mb|QLI63916.1
MSTGVMGELKYLGVAMAGIDFGCDIDGSCPGDHVQVPLASLGGADSAGQM
KHFVDDDGMNTFRLSMSWQYITAGQPSGGLDKINFGNFDKLVQACLDTGA
YCMLDLHNFARYDGGIIGQGGPSDDVFAGLWKQLATFYAKSDKIIFGLMN
EPHDLDIKTWAKSCQAAVTAIRKAGATSQLILLPGTNFASAETFVSTGSA
EALSAITNPDGSTDNLLLDLHKYLDINNSGTHAECTTNNVAGFKTIAEWL
RKNKRLAFVSESGASMDQSCMTKFCEQNEFIADNDDVLVGFVGWGAGGFD
GTYVLTLTPSRSGDAWTDNKLMKQCIIAPFGKAASATASTTQAPTSTTTT
STSATSTEQATDKTPSATPGTRNTNANEAAPKKGNAADHVAISHIYIILA
SMAGLRLYL
>Mb|QLI63917.1
MATIALPRPIPPHGASSDIDGPITPPHSLEDVNRQVQSQRCPAPLPNKHI
PACPAGPPKVDDTDTPPASPKGAAGALCQQSLLFPPDSYVRLEMDSLCVF
ELDASQVNRAIDFGSRQPLPSPEVVFPWLHGLHPKNHLQQAFFMGRKRST
RNPPTCNRGILLVKANGDLLTARLKGAVAPDEFLQPGPCPRFIEADPEEG
FSVRNFQIQTAKAALVSDVIVYGENLAESKKVAWEVAAAQLLQRQSQTAQ
AGNFTEYNTFVCTSPFSDFEDANSDIVAIGSDGCATGKVLDFVQQERTEM
WDMTQASEISHNVFMGPTPEAGSQEEQIFDILIECSDLGHLDPGALQRLV
ESTDGPAGRSHFDFPSSGSILPPTWSHDEADGIIETCKCIYYLSHGMRLN
SGVHDENTPTGTTGLTAGTFEPRKVLIHCADGYTESTMLGIAYLSYSTGQ
PVSNAWLQLHTAKGRNFFAYPTDVALLTAIAPRLLKESPACVGYSLEKIT
NLLRDEPKWLPGLDGSFPSRILDYLYLGNLGHANNPDLLAELGIGQILSV
GETASWKDGDLEKWGSDNVYVVQGVQDNGIDPLTDEFPGCLEFIEDDDEG
RQLWCTVGLGYRVAPPSVSLK
>Mb|QLI63921.1
MTKPSHGSSLLQARDSRSHSHSHSFFHRFRHRPLHAHDPEQQSFGDDAAS
DNGFGNHLGANVELDSSNNALAKRDASETSQPVAVNAVGDEQTSLVTHVR
QTISLVQYVDPWGSTYETKTIYSPPNTVVVDPKTGETIDISSGPESTVAL

PGPTPSGSTYSSSSLDSATPTSLSPTLTSTPSSATVKPAYPSVGDIRNST
SSAFPSNSSFATPSNSSSIYKLISTSVPISTADSSTTDSSTTSETKSTST
SAHSTHLTSSSSFLTSSSFSSSVSSSSSSSSSSSSSPSSSPSTSSSSATT
TAPGNGYGTITVPGSLPTTQSEAGSSSGDSLTPQQKQVIGGVVGAVAGVA
VIGLLLMLFLRWKKRKGSSTILDSQSGGATSRTLGDGTSGDSGSGGAMAE
RAAAPGAVAATLASLTGKRAPQTSHSADSGERGFYRVSGRKLPSVLHSGG
DGYSDPRGSATSGSSDYHRGSQAFAPTSAGKGQFALGVPMRPISGVPIIR
SGPARIPITQNPFADPPASPNNLSTRTLGSRESPRASGSKFQEKI
>Mb|QLI63924.1
MGNGHSTPLQQCLNTICHGRSGCVGYPSDPFYQLSWVKPYNLAIKVTPVA
VIRPNTADEVAEAVKCAVQSQVHVQAKSGGHSYGNYGLGGQDGSLMIDMA
NFKHFTMDTTTWQATFGAGYRLGELDHQLHKHGGRAMAHGTCPGVGAGGH
ATIGGIGPSSRMWGTALDHVLSVQVVTADGHVRTASRDENADLFWALRGA
GASFGIVTHFTVRTQPAPGHVVEYTYEFRFGSQHEMAPVYSAWQAVANDP
DLDRRFSTLFIAQPLGAVVTGTFFGTRREYEASGIHGRMPAGGAAALRLT
DWLGSLGHMAEKAALALSDLPTQFYGKSLALRREDALSPDAVARLFNYTG
AADPGTPFWTVIFDSEGGAINDVPAGETAYPHRDKLFMYQSYVIGLPLSE
KSRRFAEGIHDIIQRGAPGANTRYAGYVDRELGRAEAQRAYWGDKLPRLG
EIKARWDPGDVFHNPQSVAPADGVGGG
>Mb|QLI63926.1
MSAEDTSGAKVYLVSNDNATLQVDRVVAQRSILIKHMMEDIGYDTISQDN
PIPIPNVNEAVLRKVIEWCEHHRNDPPQAQDDESDGRRRTTDIEEWDQKF
MQVDQEMLFEIILAANYLDIKPLLDVGCKTVANMIKGKSPEEIRKTFNIT
NDFTPEEEEQIRRENEWAEDR
>Mb|QLI63930.1
MLVGILLAVLGASPAAALAALTDDDLRSIPSPGDDFDIHKGKLLAPILIP
RVPGTEGNYKTQKHFVDFFKSSLPSWELAWQNSTSKTPLSGTKEVPFSNL
IFSRDPPGSHPGDVSRLTLVAHFDTLVKPEGFVGAIDSAAPCAMLMHVAR
SIDAALTKKWEHDDGGSGGLEVRQGVQILFLDGEEAFKQWSATDSLYGAR
SLAQEWDETFHPALSTFRTPLESISLFVLLDLLGSPDPSIPSYFLPTHWT
YQSMAALEGRMRALGLLESRPAKPFLYNADKQPHQFYSGYIEDDHVPFMQ
RGVDVLHIIPGSFPSVWHKIDDDGDHLDLPTTRDWARIVTAFVVEFMELG
GLLDKTAATSAEKTSATQSVHSKRTEL
>Mb|QLI63931.1
MDRQKDPPEFILDVFTDPRSVRDVVKGILHTIFFTRFFPSLTPRTRDVLD
LTLPYVDDDELETMIDQRASALERQLDAERSSGVGNHAATNGGGRGQVAI
QFFEKRRRKAWLSRGDEEVCWESWTLKVTVAEPRTESERAKVRRAMEQTL
LTTVMKIVTFANAHKDHIPPITTQGGNPFPFKINIDQKETGWASRMRIY
>Mb|QLI63935.1
MSSFLLRRATVASAGAARSFSTSSPRSVARISIIGNLADTPEIQPTSSGR
EILKYAVASNTGPKDNRQTSWFRVTSFAEGPRKDFLLSLPKGAMVYVEGD
ATISTYQDPNGQTRSGLNIVQRSIEVLKRPQNAGQSE
>Mb|QLI63936.1
MTRVYVYSSALVAFVAATGMIVASIFIPNWVSYSVTTPKGDVVEKHIGLH
KTCSNLNSPTCREFPTAELCQAGERYFCDMWKTVGFMASLAAIMCLASLV
SFLVVMKGGKYKRETGWPLVASMLTLVSVVEFAVISIVAYLFDHDEQFTI
PGWNLDVSWYISTVSATIALLAAAGLAVSAYLLSPEGGYEFLEDPIDA
>Mb|QLI63937.1
MRLVGARLGGLVQRRGFASTAGRLDHYAFVGLGQMGYQMAKNLQSKLKST
DKVSVFDINPQAMKSLESDVKAVSGGAKVELAPSAWAASKEADTVITVLP
EPQHVQGVYKSILTGTLPQKDRVFIDCSTIDPSTSREVARSVSDAGQGTF
VDAPMSGGVVGATAGTLTFMLGAKADMVPRVEPTLLQMGKKVLHCGEQGA
GLSAKLANNYLLALNNIATAEAMNLGMKWGLDPKKLAGVINVSTGRCWPS
EVNNPVKGVVETAPAGRDYGGGFGISLMKKDLRLAMVAAKEAGAKMALAD
TAFGVYEDAEKREECKGRDFSVVYRYLGGKE
>Mb|QLI63941.1
MSYQQNPYSPAPPQDAGRAESEDNRWGVGRYEMQPLNNQRPVSYQQPVSY
QQQPPPQQQHDGLSEAQFLDQVQGLRNDMKAFNQSLDELSKRHKRSLADR

DAKDQFGDYRFEVQNQREMLQDGVRRLENDLASTSPSDKARQTKKRHLQS
LKTPLQKVVEKYVKVEQDIRNGEEAERIRQLGLVHQDWDYSRLQEEARRD
WGNEGVFQDALMNNRSEQATQVLGNVRARHQDLQRLEKDLSEIALLYQEL
ATMVEQQDETIVATEQNAEYTVDNMEAGNQQVKVATDHARRARKLKWWCL
LLCVIIIIVVALGVALGICLNGNRCSGGK
>Mb|QLI63942.1
MYMYAVGRAATQSCGKAVRQTLHLQTRQFSLSAARRTDDGNGTALNPRWF
TDLRGRIKACLAANPQGEEGARLNKHLEYVDDNWLELSAGREGFLTDERW
RGLNKFAVAWGDMVGHVNNIMYNRYAESGRVNWVTSFAAHAPPEQRQQWV
DIMSPRGIGLILKSIKTDYKLPVEYPDKITVIHKLAQRPTYSSDSIFLDA
VIYSEAHRRVAARCFEDIAVYDYRAGKRATLKGFMVDELQKIYDLQERGR
KQVDGKVDELERDIKVMEDKA
>Mb|QLI63943.1
MSLKRILVLGGAPAVLGAACSKDYTRNGLVWGDAIRLNQIQVVGTHNSYH
VEAPDEEKALMLNLSADTQDLFYGHPPLETQLQEQHVRNLELDVLADPEG
GNYAKPLIKALAGQPYNTGPEFQTPSTKVLHIPDLDIHTVCTTFTACLGV
IKSFLDANPWAVPIPIMTEFKTANPLGQSQGGAKVIPWDNTTLLDGLDAE
IRSVFTSRQLITPDDIRRPGMTLEESVLKYGWPDLDSARGRVLFLMDNGP
EDPVNEKYIEGRPNLEGRVLFTNSAPGRADCAFQKLNDPVTDAYIQNVQE
QVKANYLVRALADSALSTVRDCSTIQRDGAFKSGAHIVSTDFPVAGMSER
YGGCEYVVELGDGKVARCNPVNGPRGCVDEELE
>Mb|QLI63944.1
MSTLSEPRRPRLLPQNRKLRHLKGLSLRNLTFALATLYSADDAVIPGSPS
KLGMLREAAHLHTSRSSDSLRRDGLRPEKRRPPPQLRRTSLSLSHANPST
RQKRLESLVQASVGDVFFSLHAAGCEEPIYISEVRNQSANFNFQFFDLSA
DKPLVSRSCTVSIRVWAKRPQQTSWVFLLQEFIDLRRLNFIGTLIDRQFP
PNAIILHLEDGVYSLDFPTRTADPKHAPPAAATSSYNALMKLANLESSIQ
DAIDTQHRITQQINQILEKSPSDPTPAADEEVSLAGKFVATQQRANKTAR
RRRDEMMESLRSRREAISIGREAQAAAEKDIANNKEKLAASIALLHKTEQ
QIRGQRRRICSELYDIFPITPIPNAPPLSFQICNLPLPNSTYDAATARTI
NEDVLSAALGLVATLTRHLQFYLSCPLPYPLSACGSRSYARDDISHIPER
QPQRREFPLYLPRGGSTIAQWRFEYGWFLLNKNIEALCASQGLKVVDIRH
SLPNLKYLLYVCSAGTDEVPERKKGGVRGLWAGRLKGRMGAAADPESGSS
AGGSRRGSVDSEVANQHGEALRSAAVQANGDGDGGWGADSGVGLLPFGDD
ARFTLRTKGLRENVA
>Mb|QLI63947.1
MERFRNLLGGGIGALGAAHGTDNTNLIDNSETVYISSLALLKMLRHGRAG
VPMEVMGLMLGEFVDDFTVKVMDVFAMPQSGTGVSVEAVDPVFQTKMMDM
LRQTGRPESVVGWYHSHPGFGCWLSSVDINTQQSFEQLNPRAVAVVVDPI
QSVKGKVVIDAFRLINPQLLMMGQEPRQSTSNLGHLNKPSIQALIHGLNR
HYYSIGINYRKTALEENMLMNLHKHVWTEALEMNDFHSEGDKNKERLQRL
VSLADGYEKRVKEETELTKEQLKTRYVGKLDPKKHLEDVGQELIEDNIVA
VSRQMIDKEATMPKKEGQAGHSNGDQMVTEEEL
>Mb|QLI63949.1
MHRASSFTSLSQSSAESNSLQHSIATGLMPFLRSVTIRQCTTRGTGIVLA
GRPSWQTRCTSSSSPQPTLNNLPASRLNPSPDDYASPNFADKAELTIYAG
RGGNGCVSFLREAFLPDGPPNGGDGGAGGNIYIQAAHGETSLHKLARRRF
IRAGRGKHGQGSAKGGSRGEDVIITVPVGTIVRELEREDPAGEEAMNIKA
YRALQKQRKKEELLLQEEKKRRESVEEAHEDEILDRKGRPKQAPEDEDED
LELDDPTRQKWLLYPGMSKSEMKSTSFPRLPRRHPLLQQPTAPIYLDLSR
PTPRPILLAAGGIGGLGNPHFTSREHPKPIFATKGEDAVSMKLTLELKLL
ADVGLVGLPNAGKSTLLRALTNSRTRVGSWAFTTLQPNIGTVVLDKYSGQ
PTARSHRKPSIEVDESSGAALEPRTRFTVADIPGLIEGAHLDRGLGIAFL
RHVERAGVLAFVIDLAAGNAVTALNALWREVGLYAQMRDEEDRDREAESR
VDWDFASDSSRGPINLMNAEYASPSAFEEGSAQSGLHIAGKPWFVVATKA
DLPNTQENFKELNVYLDKITNGEVAHPSGVEGAWTKNCAAIPVSAIHAQG
VDRIVHWTLGLLDE
>Mb|QLI63950.1

MATPSQMPPVPNREDIGATWHYLQSGITRIMNELERGIDMQMYMGVYTAV
HNFCTSQKAVGLTGPAMQSNHRGAHLLGEELYNNLITYLQKHLEDLVEAS
KSHTDEALLAYYIKEWSRYTNAAKYIHHLFRYLNRHWVKREIDEGKKNVY
DVYTLHLVQWRKVLFEQVSGKVMDAVLKLVEKQRNGETIEHNQIKQVVDS
FVSLGLDEADMSRSTLDVYRYYFERPFLEATAEFYTAESKQFVAENSVVE
YMKKAEVRLAEEEERVVMYLHQDIAVPLKKTCNTALIAEHSTLLREEFQV
LLDNEREEDMARMYNLLSRIPDGLDPLRTKFEKHVLKAGLAAVQKVQSSE
GDKLEPKVYVDALLEVHSQYQLLVKQAFNDEPEFTRSLDNACREFVNRNE
VCKDTSTKSPELLAKYTDVLLRKSSTSIEEGDLERTLTQIMTVFKYIEDK
DVFQKYYSRMLARRLVHSNSSSDDAETSMISKLKEACGFEYTNKLQRMFQ
DMQISKDLNKDFRGHLESVDSLKTVDSTFSILGTGFWPLQAPSTHFHPPV
EIATEIERFSRFYKHKHDGRKLTWLWHLCKGEVRAGYCKNSKTPFTFQVS
IYQMAILLLFNEKDTYTYDDMVTATQLSTEVLDQALAVILKAKVLLMDGG
SGERPKPGRSFSLNYEFKSKKIRVNLNLGGVKEAKQEETETNKTIEEDRK
LVLQSAIVRIMKARKKMKHTQLVSETINQIRSRFVPKVGDIKKCIEILLD
KEYLERLDDDELGYLA
>Mb|QLI63953.1
MGKKVFFDVTWEGPVMQGGKPTNTVKEQSGRISFNLFDDVVPKTAENFRA
LCTGEKGFGYSGSSFHRIIPDFMLQGGDFTRGNGTGGKSIYGDKFADENF
KLNHDRPGLLSMANAGPNTNGSQFFVTTVVTSWLNGRHVVFGEVADEESL
NIVKALEATGSQSGTVKYSKRPTIVKSGEL
>Mb|QLI63954.1
MERSRGIPDQELFLSKPAPEDCDSPTVEPLRIFKPKSPEPAGRTSASPAN
KPPFPLPPGASSSAAPLPFPDDDEVRRPTAPRPYGSAYNDTTPRLDTSSV
EKRPGLAERRGASPKVIHSPTTPEADQGLFAQPLKDQPRPNPAATNYPSY
QKTYYPPPGAATATTTTTTTASSSSTQAPNGDQGVNRFSSTASNSTTRAS
RGSPPPPETPVVEPGTIPGGGIEARYAASGISGTATLTSLQAHQAQSAAA
SQRLAQYGNQPPPQRPWTPTESPDQAPSGPPTVYQGLNPISSPEPRLGAA
SAPPQPAQAPNPNQYQQNAPVQVSVLEQDFQRLGTKTPPPAYSSVNPNGG
SAYPKEKQRPQQAAPPGAATAAAAAAAATTTRPNTTPPTATASTTPPTKP
VALAAGAGAAAATAAGMISPALQHPGHPAFANDPRPEQNGQSLQSPAPSI
SLQTQTPASPPPMPEGWIAHLDQNSGQYYYIHLATQATQWEFPKGPNPIS
HEQTPLSPTASTYGNPLTSPMFGKQSMASPMFPPHTPGYAESIMSVASQT
PTAVGFSGPPPGSGVEMYRIQPTNGVYFGPYLRYVNMDIENGVWRGSILI
VTDAHQPPTIHIHLSVDLSPNPKQLEPRNIYTHQRWTFYKYDIDLQMSES
GTERWTYAVTSHLGCTRYEFVVAGRYETGWRFIAHSGNDFASGTSQNERA
RLGGVGFMWKDVLQKNVDCGGFHVQLGLGDQIYGDRLWREVPLLKQWLAM
SGRDNKKNVQWTARHEEDVAHAYFHFYTSHFDQPFMREAFAQIPHVLQIN
DHDIFDGYGSYPDYMQSSPIFKNIGRIATEMYLLFQHHTTTEMMRNIRTD
NDIFTITGTGWHFVKYLGPAMAVVGPDCRSERTQARVMAGPTYQGIFPKV
ATLPPSVQHVVWMVSVPIVYPRLDAVESLANTVAAGKKAVNTTYNILGKV
TSSVAGVVGGKEVVSQGFSQVKKAVGKTGLMGNVLNQFGELDIQEVLKDL
WTHDTKDLERTYFIRTLQSIAQQKGIRMTFLSGDVAASGAGLVHDPTHPG
DHKTMYQIISSPIVSAPQGGYILKLLHNQKTLYVPKNGQKSTHEVSDTKE
DMMEIFHTDASGASRELKKLMGRRNYVTFLAYDQEAVNQVPFSPNPSISS
SQQGLSKLSLAVDFVVQGDGAYTATTKYGPVIIPHLEYGR
>Mb|QLI63955.1
MKSFLTRPKRKLSPEPKPVTDDDEPTEVKLVLLSSVHPEVDQETLLDLLL
AHDGSVSQASQALKSNKYQQGARKSGIIGHQQSLRQYTMGDSTSPTKKKP
KSKKGSTLHLYDPEDVAEHTPCTIIHNFLPPEDANSLLKELLKEADSFEK
ITFKLFDNVVSSPHTSGFFVESYEEMMAQRSEYYYNGSQLTDVRRITPEL
TKVKPIVQAAVNQEIQHRIKTRYPDGKKLQHQSPNPWIPNSAFVNCYNGA
QQSVGWHSDQLTYLGPRAVIGSISLGVAREFRVRRVLPKDGDTKSTDESD
AGGQISIHLPHNSLLVMHAEMQEEWKHCVTPALSIDPHPTAGNKRINITY
RDYRQRMHPKYTPRCVCKVPCVLRVVQKKKENFGKYFWMCHAGNVPGKEG
CSFFQWAEFDDDGNPVVSGNAVKYSER
>Mb|QLI63956.1
MAPAEDSATRHVSPSEARRLVQDILTGNGVPGENAALIAKCLVAADLRGV

DTHGMNRIPSYMERIRRGVLDAAAQPAIDQVTPAVAQVDGRNGFGFVSAH
QAMRAAISSARVYGIGMASVKRSNHFGMSAWLVQQALDADMMSLVFTNSS
PALPPFGGRSKLLGVSPIACGAPGEPDFILDMAPSVAARGKIYKAQRRGE
AIPAGWALDAEGRPTEDPAAALGGVMLPMGGPKGSALAVMMDVFSGVLSG
AAFAGHVTGPYDPSGPADVGHFLVAVKPDLFMGLDEFRGRMKYLYERVVG
ADRAAGVDRIYFPGEMEQRTQAERERTGIPLVQAEIDALNAEAARVSAKP
LVFMDE
>Mb|QLI63958.1
MRIFLLPLSTRRTFLYAQRLHATTSPPNTSLLDRGTAFAARKWSEWEKKP
SGWQKKVVEYGNHAFRRIPYEEWGLKSVPPLSTRRRQDEIEGREKVELVY
PGSVLRDEDVLQVTGQLAGEREGLHRRRLMWCFVGMPITLPFALVPIIPN
LPFFYLVYRAWSHWRAIAGGKHIQWLVEQKLLKPAPSSTLDKLYGQAPKL
AASVSPVDKEQVLLTQEQVTSFSESLDIPALEIELERAIWQVEQAIHKAD
EQQQQQQQTQKHGDGTNANHDVKSKDSKEKQ
>Mb|QLI63961.1
MPRGRRSLQAGGMPLLEVSESPDVNGTRRTRSVSSRPANGYEIVVSSDST
QPVRRPRGRPSRASLLSAITTSSSSSPSHYSGVKPRRGRAAAIGSTSVSS
AATPQELSSDAEYSTPATSKNPTPLTAEGSLSVEASKSLASLEVRLPARS
RADDSDSERALRNSIYSMKANRKLTEIVNSDEEDDDSSDTGWDARIARRL
QDEEFAKADEGALRSSSLSGSNRAVTATTSPLPTRGVAKRGRASTRLSMQ
LAPPAKKSRIIPDSDDPPTSDFDMDAEIAAAAALDSELSEPPELSEGEAR
FTDSDEVNEAAMDKDGGTSDAFSDDEPLSVKKKGKQPQKPRTLSSKAKMP
TRRGRPSTAATGSKPNLAIRRNRAQDVVQDSDDDDVAEEVVSIASDMSTV
SSVSAGLSSSNSSDAEEGVSELRRIAANRRAYRSGNTRRLRKERDRLEYH
HPEIVTMWKDLENMPILKAGMAPQPQSISRQLKPFQLEGLAWMKEMEKRE
WKGGLLGDEMGLGKTIQAVSLIMSDYPAKQPSLVLVPPVALMQWQSEIKS
YTDGTLKTFVFHGTNQKAKTITAKELKTYDVIMMSYNSLESMYRKQEKGF
KRKNGIHKEKSVIHSIHFHRAILDEAHSIKTRTTMTAKACFALRTTYRWC
LTGTPLQNRIGEFFSLIRFLNIRPFALYLCKQCPCSTPEWAMDENSRCSH
CNHAGMQHVSVFNQELLNPIQKFGNLGPGREAFRKLRLMTERIMLRRLKR
DHTDSMELPVKEIYVERQFFGEEENDFANSIMTNGQRNFDTYVAQGVLLN
NYANIFGLIMQMRQVADHPDLILKKNADGGQNVLICSICDEPAEDTIRSR
CKHDFCRACVSSYIGSTDAPDCPRCHIPLSIDLEQPEIEQDENLVKKNSI
INRIKMENWTSSSKIELLVHELHKLRSDNASHKSIIFSQFTTMLQLIEWR
LRRAGITTVMLDGSMTPAQRQASIEHFMNNVDVECFLVSLKAGGVALNLT
EASRVFIVDPWWNPAAEWQSADRCHRIGQTRPCTITRLCIEDSVESRMVL
IQEKKTNMIHSTVNADDKAMESLSPEDMQFLFRGS
>Mb|QLI63962.1
MGIAILNGILTSLAEINGPRPLQSPSSNASTPGEERPQSLPSRFIACVRS
AESAKRVKAALWEHSSIVKVVQNDNIAAVQQAQVVLLGCKPYMVNNVLGE
PGMADALKGKLLISVCAGITADHMEIALHGSVPTTNPEDDGRCRVVRAMC
NTAALIRESMTVIGISDPPLPPDMKTLVTWIFKRIGDVVYLPANNMDAST
ALCGSSPAMFALMLEAAIDGAVAMGLPRAEAQRMATQSMRGTTGLVQSGE
HPALLREKVCTPGGCTIGGLLVLEEGRARGTISRAIREATVVASQLGKGV
QGVNGTRFNTTG
>Mb|QLI63963.1
MAGGRIDCYLDLASFFSYICIADLRLNLDKLAAHGVDVEFHPIFLGGINH
LSGNRPPWTLPAKARYLIYDNKRAAARVGITEYQAPDDLFERAKTQSALR
ALLFIKANYPKEKLLSAMHFLLHKFWTPPNADVVDESSLRALLAQATETP
GGGAKLFTEEEVGRIMDGRAEMKKKLIEDTAKVVESGAFGCPWFLVTNSR
GETQPFFGSDRFNHIYMHLGIPFQDVAILPPSSAAKL
>Mb|QLI63965.1
MASGYSAANQPRRTSPPSSSPSPPPPPSSSSSPSPSPPSDSSSARASPEP
VRWSRRSFSPSRRLSNAMLRARSSSAPRTLLQRAWRKCLDWSRWLIQYYL
SLSLLYRVLAVLGCLLGWALIVLTIVYSHRFFSWLTPVAKSWRELPGGWL
IVFALIFVTAFPPLIGYSTANTIAGLVYGFPNGWPVAAVACTLGSLAAFL
ASRTVLSRYVDRMVGKDHRFIALGQVLRRDGILYLTAIRFCPLPFSLSNG
FLATIPSITPLSFAISTALSTPKLLIHVFIGSRLAVLAEEGDKMTAGDKA

VNYIGMAVGGIIGLAVGLAIYRRTMARAAELALEEGQDIGHVEQGSGRYD
DIDDTDATLLDSEDAAAIMSGDDVSLWGAQANAWGTFDDDDDDSSRKPNR
>Mb|QLI63966.1
MTNRSPDPTDDGDEQSCNPDVADTEAMACRPQSRSRSASRTGNDPGEFDW
KRSNTRNGHAKMPSPVTPPSLSRPFMPAATTMALRKSSPGLSGPKGSRRN
GTVSILVLFTAGIGIIILAAILKSLISYQIEPKGCRMSYMRPSYIHFTDF
DTEHTRFATKYSLFLYREQGVDDEAKLRGIPVLFIPGNAGSYKQVRPIAA
EAATYFYDTLQHDGGSVDSGVRNLDFFTVDFNEDITAFHGQTLLDQAEYL
NEAIRYILSLYSDPQRTTRDSHLPDPTSVVILGHSMGGVVARAMLVQSNY
QANSINTIITMSAPHSRPPVTFDGQIVQIYDEINAYWRDAYAQKWANNNP
LWHVTLISIAGGTLDTVVPSDYASVEPLVPETHGFTVFTTGIPTVWTSMD
HQAILWCDQFRKVVAKALYDVVDSNRASQTKPRAQRMRLFRRRFLSGLEA
ATEKTIASKDEIVQLALDDESSRIVPVGDRLILDRLGNQRDPVVHLLPIP
PQETSVPKRFSLLTDSPLVDAKENGPLEVLVCTVSPGEVDSATIHNSYSA
TIPGGSQARLACKSTSSDVILLPASTASTQYPFYLEGENPIRPFSYLQYD
LEDLSEYNFIAVVDRATSQTAGFVISEFSDRDDIQRDFPTSFSHIAMFGL
SFSLPSDRPMAAEVQFPALTSSLLAFHLDLSQHQCQSTRHLFTPMVRQYL
DKPYESKYFVNAKRAHISLHGIAPYVPPPLELKSTNSGLGFQIWADPSCG
ILEAKLRIDLLGSLGKLYMRYRTVFAAFPLLIVTLVLRKQFRAYDQTGIF
IPFSEALDLSLRRSIPLLLVSLTLLAVSPEGTSRAIPNLIWSKRHLNAFH
RNELLIGTENPSFCLLVPLIGLVCIGVCAVLHYIVLALTRSLGILYAFFS
NAPTTETASHKRIASQALVPSTPRRRMVSTVVLLFLVSTFIPYQFAYLVA
CLVQLFTTVRAHRISNIADSTSNANYYRYVHSILLLMIWVLPINLPILAV
WIRNLAVHWLTPFSSHHNVLSIMPFILLVENLTTGRMVPQVTSRLRHVTS
VLLFGTAFCAAVYGVSHAYMLHYLVNIIAAWLVILHSTADSWSFAGFGAM
FEGGTDESRNRRKTP
>Mb|QLI63967.1
MGGTDDVKDGKPAMEKQETSGSQSGFSGFMDDLKEKFSESKLHDAKVALI
HKKHQIGKFGNLFNKDHRHDEEHEQRCDAKRSAICEQHRFQSFFPERDGN
LIKWYVDGCDYFWAVSVALEQAQESIYICDWWLSPELFLRRPPYHKQDFR
LDQIIKRRAEAGVKIYVAVYKEVEAALTCNSRHTKHALDALCPEGTPGHG
NIRVMRHPDHNVFENAADMTMYWAHHEKFIVIDYSMAFIGGLDLCFGRWD
ARQHPLSDVHPEGVSEEIWPGQDFNNNRIMDFQKVADWEQNELSKAEYGR
MPWHDVSMALIGPCVYDIAEHFVLRWNFIKRDKYKRDARYDWIMLEGREG
EDEDLVGVQRPKHPVGDYLKHPISPLSTKNLDNRGTVRAQLVRSSADWSS
GILKDHSIQNAYCEVIRKAEHYVYIENQFFITATGDQQSPIHNTIGRAMV
DAVVRAAKDGRKFRIIVIIPAIPGFAGDLRENAASGTRAIMDYQYKSICR
GENSIFGQIKKEGVDPTQYIFFFNLRSYDRLNKTHAICEAEKKTGISYQQ
VQRAEAEEIMASGVHGYEDSSSDEGEGGSRFHDLNPKHKHDREKRKANTS
SSQQKAAEDAQRARKIFEQEKPEEEVISAASVAHHAMKTESSLQDEPWDN
HDDEESEIKNWIQEELYVHSKLLIADDRIVICGSSNLNDRSQLGDHDSEL
SIVMEDTRKIQTTMDGKPYEAGYHAATLRRYLWREHLGLLPPQDLDAKDD
INAQPPTVNPENDIYDNDESWNFVADPLADQLWDTWMGQAQKNTKIFRNV
FHADPDDHIKTFDDYDRYLPPKGVKQGHIYDQFMPAEEARQKLSQVRGHL
VSMPMMFLQDAEMAERGLQVNAWTESVYT
>Mb|QLI63968.1
MALPGTRPKLPVSVDKPTPYTFDLGLLLAEDPNAITLDRSSLEQSLAEVA
RDGAQSLINQLLTTCPISSTNDGVLLSLPAPSTALPREKPLPEAKAKTKW
ERFAEKKGIKAKTREQRRNLAYDAESGEWKRKWGYKGLNKKGEDYPIMEV
DMEKERERKEGTSIRGDGRRERKEKIKRNERKMRKNQRDALSAR
>Mb|QLI63969.1
MSSANKEDACPVDHKTRDAWLSQARAAEATKSQAAAAAQCPVDHTSQAQS
KSWTQAITSYMPWTSASTAPATPEVKPRNAPLTTDRVISTIPRSATGSEA
CPVDHGASANAPNAEIESGVDASGNWVYPSEKMFFDAMKRKGYDARVADM
KTVVPIHNAVNERAWQQIKQWEAPYLSKSSCGGPKLESFANKNDRMTPTA
RINTILGYTAPFDRHDWVIDRCGTRVEYVIDFYAGRPGAKDKAGPSFYLD
VRPKLNTWEGVKMRAMRWTGLA
>Mb|QLI63970.1

MPRSKSVADDSPLGSEKRSVRKRSVGTMDLDREATPSKRGRVSAQEIINV
KSSSVDDSETEDHDQEIQVGATDKASPSIDVAEVPKATPKRRGRPPKSQS
QTSTPSKTPAKSIFATPVKNHETESATPKRQAADRSARKKSARALIENVF
DDGDDDDEEDDDEVLVREIYEDSDAEDLGDNADILTDGLNDDTSGNVTPS
KTRKSRPKRTKSPTPPRDLPPHELYFAHNKPGRPKTSDNTLGSVSLLTHD
EYFNVLHQHKDRHADDVEYLENLHAESFPQWAFELSQGYSICLYGFGSKR
ALLQKLAKYLHTNHRGKPARQIVIVNGYSHTTTMREVLTCIGSAIDPSQK
IPTSTPAATVQIISSQLSNTNLGLTILINSIDAAPLRKPGTQAILSQLAA
HPQINLVCSADTPDFPLLWDIGVRSAFNFVFHDCTTFAPFTVELDVIDEV
HELLGRKARRVNGREGVAFVLKSLPENAKNLFQLLVGEVLIAMEEEGNMV
DEAAGVEYRMVYNKAVEEFICSSEMAFRTLLKEWVAFFTLIRICSWLLTG
YRFHDHQMITSRKDALGTELLSVPFQKDELEAVLEDLMS
>Mb|QLI63971.1
MPMDIVDRELADAEADASPGRYRRRASDEIERVLSASSASTSSESNDGDS
AFRRNMSRVSTQNDLERHPTELSRIATARSQHNITVGGGLRSRTVSRASK
KPLPAFGAGKSYPPPLPEREEYVVEFDGPTDPLHSQNWPMRKKVLTAAML
SYTTLTSAFTSSIFSAATTSVAAEYNVGSEVGLLGTTFYVLGFAFGPTLW
APMSELRGRRLPIVVSMFGFSLFSIACATGKDIQTILICRFFSGLFGACP
LAVVAAVFSDMFDNRTRGIAVTIFSMATFTGPLLAPFIGGFIVSSHLGWR
WTEYLASIMGFVAFILDLFFLEETYPPVILIDKAADLRRRTKNWGIHAKQ
EEIEVDFKELIQKNLSRPMRLLFTEPIILALSIYMSFIYGILYLFLTAYP
LVFVGVHGFNSGQSGLTFFGMITGQLLAGAVVIMQQPWYNRKLAGNNGIP
VPEWRLPSMMAGGISFTIGIFWFGWTGFSSSVHWIVPALSGIFTGFGLMS
IFLQALNYLVDAYLMFAASAIAGNTFLRSLAGAGFPLFARQMFQGMGIQW
AATLLGCVAVVLAPMPFIFYKYGSRIRAKSAYAPTFAAPAMKNSEDEPGD
SSTEKEGENHAALASAPRKESENGVLQNA
>Mb|QLI63973.1
MYTLTNIATHRFSGPEETYVLDVDRTEAGLVTISSDQHLSLLRPARLPDG
PVASWKPQHGNLTSLRVFDGSSSLVCTAGEDGTVAVWDLRLRGDQARAAQ
FNAAPSPILSVACSASTQTIAVGTELRDHTASIFAWDVRAGARPRAHYQE
VHSDDVTQLAFRPGQPAFLLSGSTDGLVNVYDTRVADEDDVTLQTFNHGA
SVHRAGFLTGTEVVALSHDEQFALYDVDEGAEGGDAAQNWGDLRSVLGCQ
YVADVMGKTDGSGAIIGAGAQERHGFELVFLARNQGHGPKWVFDRENSVG
LPGAHGEDIVRAFCFFDDAQVVFTAGEDGNVKAWRGG
>Mb|QLI63975.1
MDGLTRIQCRSLMKLSSHVPRAAAHAISRRHASTLATTNAPGTKERVVIL
GSGWAGYAAARTLSPSKTTRILISPRSHFAFTPLLASTSVGTLEFRAAIE
PVRRLGLDEFHQAWASSIDFKNKTIRVEANLRGDVSAATNRPEIKGEEFD
VKYDKLVIAVGCYSQTFGIEGVKEHANFLRDVGDARAIRLQVLQAFETAD
LPTTSDEERKKLLHFAVVGGGPTGIEFAAELHDLIKDDLSRMYPKLQKHC
AITVYDIAPKVLPMFDSKLAAYATETFKRQGIRVKTEHHLTRIRRQGHVL
MLQIKEEPEEVGAGIVVWSTGLMQNPLVKTLVESDIEGVGKI
>Mb|QLI63976.1
MDMGDMHMHDAGLGMDYNYAFARDYWYIIAVVVGGLTALRGIEIYTGKQR
LKACKETSIEHATRPGNHVSQAWATATAITREMSHPQLYVSRVGFRWATP
PPLGRIMVLLCYWAMIIYFMSWNVVKNDAYFWERIGYRNAWVSIMQLPFI
FLLSMKFNIVGFLIGSSHERLNWLHRWVARTMFVTATVHGWHFWTEWVRA
DFLEYELSIMPLVRYGLGAWGVLLFNVVVGFVPIRRMVYEIWLIQHVLSS
VAMLVLLGFHIPADARYLLWMSVSFLAFDRAARGALTAWQNTRWKPSTSA
CEGMRRLGHSMSSRAIGDSITVLTIKDVHFKWHAGQHIYLWIPRLGFLES
HPYTIACAHQIKGSCCCNSIQLIIRSHGGFSKRIHNFAVKNPAKTLTGYV
LGPFGVTTRWETFETLVLIGASTGASFTVPIMEYVAYSENTSCVRRLEMT
LVARTAEEMEYYVERARDAARSAQEKGIMVRLHIAITGGYSETDSGVPLV
QLRRDVKDGSTEGSIKEKAAASYSGKSVEDGLRSRSRNSCSSTSRNSTST
VDMVREYASRPDIEALLREPVEQAWGETAVVVCGGKEIVARTRNCVGKLS
DERAVHKGTGAQGIYLHVEEYAF
>Mb|QLI63978.1
MNSLRLARAAVRARPAAFRAVSQRRTYAEAVPDKIKLSLSLPHQSIYRSQ

DVVQVNIPAESGEMGVLANHVPSIEQLKPGIVEVVEESAGSKQFFLAGGF
ATVQPNSTMSINAIEGYPLEDFSAEAIRAGIAEAQKVASGSGSEQDIAEA
KIELEVLETLAAHVK
>Mb|QLI63983.1
MTPKLKPLLLPPLVEQRFGLRTDQQQVKGPPAMATPDYGDQSFVYYTTNS
SASDITSPVTPIFSPKGHQRCSSSTSSLELPMQLAQECPTSPSQASSTMS
SLRQLPDVEEEPLDRGEDTATLSGQHFGLYSCLCDQPCSHRNSSEAIFPS
DFVSECDIDYDMGFLSDTDFTTDPRYPRKKRTTTEAALSEFTSRLGARMP
TLKRWRSSKRAAPRASPTTGLTLENVLARESMSSRSSSMSSSTHYLPERA
AESMPPVPSNPSMSFYSLSDGAGTGLGIDITPEDHSNLERDRAMATTPLL
PPLLINPLASPPRESPLQSPKVETSPTPDAPLSPVFGAPAQFSRPPLSTR
PSMSSLRNVSSSTEPPLPLPAILQEHDEWSDKLGHANFTISPQPYELETI
NPETIAKFRNDWDLARINYTKHLVRTGENYGQTSKIYNLTERKWAEVELR
WQTTFGDIVKNTSHSTAGSVSESRNQSRGRGRGRSSSSNAAALARLPAHD
DLVAELEWRRLEDCLPSAVPQMLKSLDADGKFPGRGDEDIVGPMQRDAVM
VRARSEDAKGRFWKTLADRVGLRK
>Mb|QLI63987.1
MSLTNCRFYEEKFPEIDSFVMVNVKQIAEMGAYVKLLEYDNIDGMILLSE
LSRRRIRSIQKLIRVGRNEVVVVLRVDKEKGYIDLSKRRVSPEDMVKCEE
RYNKSKTVHLIMRHVAEKTQTPIETLYESIAWPLNRKYGHAIDAFKLSIT
NPEIWNDMTFPSKPVEDELKLYISKRLTPQRTKVRADIEVTCFGYEGIDA
VKTALRTAEAKNDSEDANSDTQVKVKLVSPPLYVLTSTSFDKQQGIILLQ
EAIESIRKSIEAAGGNLTVKMDPKAVTESDDAELQALMEKRERENAEVSG
DESMSESDDNIPETI
>Mb|QLI63988.1
MSAPVFWSTPLKYCRWAARERPALFWSVIIGAAGPIAMPIVPPIRKYFGD
ADPAPIPVTYPVPTGPRKQLTGYDD
>Mb|QLI63989.1
MNRLDDILRRYTVQGEDTKDRLLGAAFVVTDKEGTIYTGSSGRLDLDPKS
PLFTERSFTWVASLTKLATTISILQLVERGQLDLDADLRHLIPELRDASI
LHEFDENDNPVIEANAKPITMRQLLTHTSGFSYEFSDPVLLQWSRTQPNR
NVSRLQWSKLEVTTPLRFAPGQSWGYGVGLDWAGQVLEALTGKSLGQYMQ
ENILDPVGLNDTGFWPERIPHTAPRTAQNVQRTKDGKLAAALWPTPEKHE
IESGGGGWFTTASDYALFLRAFLAGRLVSEETMREMVKPQLNEAQAEFLR
TIAFHPMVHNTFAPEFKPGTNIDHGLGGMLNVEDVPGKRKAGSIAWSGIL
NSRWWVDPKTGIAGVLIVNVRPNGDPIVAKLYDELELALYGQLLGQGAVQ
SRI
>Mb|QLI63991.1
MALWPFRRKSTRKRSRSGAALSDNEAAPVRNQADAVPAKTASKKARTEPA
KLQRRPRTYSFSPGRHDSIRIDRARRNAEAANQPRPNPDNEQASGVAWER
TPTLHPTRRKSSKRRRDDHDREAEIKAMSAYMPARVAADGHARSNSKQAT
KRAKTGGLSHQQVPASQVSLPYPDSVPSAMSVDSDFISYKISALDSLAPR
PTLKYTPSMRYTTSRGTTSGAAESLKKPLGERQPIQENQFDSRKRMDDLA
NELDAKDLRELMERDNRRRERRRQQDQERMDRRLARRAERHRREKAEAQK
AGTPPPENLERGVMGRELVGLGIEPASAVVTSSKRRDSDTLPDAPADMED
DAEPPKPLESFHRPETSSRDDEPAPSQEQTLLTPPRIEEPQQPVSASPQG
SKLAGMLRSKKSRSKSTLGSDKDQPADGASGRKNSECSTKAGSRLSFSSL
LKWGSRNQRYSGPSSFSNTSREEMQAAGAATQNQAQAEALARLQGEDMSN
PANYLAAKAGAGIPKRTKSRFREDLPEFPLSPPDSRVQSPEADPPLPVLA
EKSSEIDSRPTPPSRHGTPPSIERPQGAQSAEPHVSISLASIDSEGSWLS
GRVGSMRMSVKRDSLLRAHRRENAPSESPTNSTREDLAIADDEYLSRLAP
DRSPGTVASGRRSGEGRPSSDAGESITITDDVKWGRVGAKPEVVQFHNHN
RDTIRSQHGLLNIDSGDEEDNAILIYREVGHKQ
>Mb|QLI63993.1
MHVLGNIGHSFSQRRKTHKPKDLHIRKVSFSGSSLSSSPSTSSTCSTSSA
STARALSSARTPTTGSTSPGFGPLSAHPTFHPPPRLHDRPLISPERCAHA
PASTFFDDSTDDEDFDGGDYTVVGKAQALHFSHTTETTLLPPSPPPEHDD
APVFGDAQDYFTLQLASRRPPMPRSRWSDSTIQTLDLDLDRDLSSVDDLD

LDETPGSDAPVPLDVPNFSHKRSVSTPKRPPMRSLDSLEDLMKRSGWKRR
GVVFNETEGEGEGEAETVADL
>Mb|QLI63994.1
MSSMRNAVARRPHRERAQPLERGRLGLLEKHKDYSLRAKDFNKKKTQLKS
LRQKAADRNEDEFYFGMMSRKGAGSKLKDGKSWTGKLEGDRGNRAMDVDT
VRLLKTQDIGYIRTMRQVVVKEIARLEQQVVLTRGLDRLEDEEDEEDKED
DLDSDDDETPLPAKPKAPRKIVFMDDEEEREATMLDRLEAEQNDDGDEEE
EEKGRNAAAADGEGDDEEADPVKSLRRLKRQLENAKKQLKAFNDAEAHLD
VQRAKMAKTATSGGNTRRGRKIMVRARKR
>Mb|QLI63996.1
MSAQALNKIAPNSPSRQNPSELEQNIAQALYDLETNTADLKVALRPLQFV
SAREIEVGHGKKAVVIFVPVPSLQGFHRVQQRLTRELEKKFSDRHVLILA
SRRILPRPKRSARSRNTQKQKRPRSRTLTAVHDAILADLCFPVEIVGKRI
RTKEDGSKLLKVLLDEKERVGVDYRLDTYAEVYRRLTGRNVNFEFPQVAS
TEP
>Mb|QLI63999.1
MASNGTQETFSPSDVLAAVRTMRTGEKETKTKAHEYLERFQKSKDSWGTI
MGILQSTAEPEATLFAAITLRGKITYDLTTQVPPSELPALRGQILLLLKH
FAAGPKPIRVQLCVCLAILAIQMKDWNDVLPSVVQSLGDSPESHACILDF
LRVLPEEVTEGRKITLSEEELAARTQVLLGDNIDQVVQLLISYSQSSPAA
PRNPQLIECITSWLREVPVMNIINSPLLDHIFAGVSSDESGAEAAECLCT
MLRETRDIDESQDAVHALYPRVVALKPQIEKAAEADDTDQLKALTKVFST
AAISWVVGIAREPSHFRPLVEAVLECAARDKERDVIEHTFDFWYELKQYL
VLERYIQGRLELVDIFSKLVDILLRHLEYPRPESGNESDLFDGDREQEEK
FREFRHRMGDTLKDSCDVMGVTECLTKVLHAIKLWTQKYAGQVNGSDVPH
WQELEAPLFAMRALGRMVHKDESIVLPQLMPLLVQIPSHEKLRFATIMVL
GRYTEWTAAHPEYLEPQFNYIVESFQTESREILRAAALSLKFFCTDCRNL
LSGQVLQLQTFYDQILDKLPDLSKEEITEGVANVVAVQPVDETYRLLKTY
ADPLVQRLMAKANNASNEEGKLALADHLQLITIFVQNVVPYVAPGGENPA
VKYWQEIFPILSTVLDNFLDFSPICERICRCWRNMVTSYRTAMAPLLPEM
ANKLASSFNTSREGCFLWVTSAILREFSEDREHLDQATTENIYTFFEAQT
TAFLRVMADLQPKDLPDVIDDFFRLLIDALLYYPQKLIPSQLLVPIFQAS
VYALTLEQRDPLSSTLHFLRDLLSYGGDNPASSEGLPEAQAAEIRGIIKN
LLMAHGADLVKQTMAGMMITFPGDCFADGSGVVLALFEIMPAQTTEWVAH
TIELLPQGTVSAPEAQRFVTKIKEKLSSGDVSGLRHVRTLLQDFTTNYRR
RNVAPRDGLGQLEAARFQFSG
>Mb|QLI64001.1
MPIPFVLVCDLLDECHRLCIANKPNTQAVVDWFARHRGRLDAHDTDLAAL
LSTLLPEKRTDRVYCIKAPTLEKIIGRALMLGSSRIVELALYKQPGQCTD
LADCAERILNTTPNPTCNEKLRISVEEIDEVLHSLASRIKWSSPSIRKSQ
AALTPRGRCDLEDVYRRLTACEAKWFTRLVLKDYQPLILDSQLVYRCCNS
TLPLILKAQDDFATAIQTLQSAKGNMLPNSTRSTSRRDQILAVVKPKLGV
KVGRQNWFKGRSIKHCLDMGHGRMSVEQKIDGEYCQIHVDLTSGKPRVQI
FSKSGKDSTEDRHKLHGVIIKSLGLGTSTCNVRKSCILEGELVVHNDVEN
KILPFHKIRNHVTRRGRLMDVDLDSPPRSYENLMIVYYDILLYDDQSLLG
VRHSERFKILQKTIDCERGRAELVKRTLVDFHRPTAASELRKAFASVITD
KGEGLVLKPDDPYFNFQANGGRNSGLCIKLKKEYIGNFGDVGDFAVVGAG
FNPTKAKSYRIPKLQWTIFYVGCLNNKEEVKRWNAKPEFTVVSAVELTEP
LLKTFISHCNPVSVPLHKNDATRLHIPKGIEANAALTAVFQNPAVFDLRC
FSFDKPGNMGFWTLRFPVVSKIHFDRDYSDAVTFDELQEMAKVARATPDL
EDSQENLQWIAKLEGADPRGRAVDAVSQLTATTIPTPSPCSSRPPLSPII
QRQKSRSPEVVRAYRMSQKSADMPTASLTTPPPSSPAEEEARTPKRGSKR
KCPTSFSPTAAPALQTHLLMTTRLSPIAATKKLRKPLDDVDANVLQQSAT
PNSSFAEDGGTDQCVGLTKSPPLIHETQENEALGTPDEGNNPTKSDIRCT
PITISSDETSQATDISPKVKNGCKFAAKECHLFKHTILLASRALGPSSER
TTLLDDHGASEFATSLDDWLQENNYGATLGNKSPRIIILVDTVDKASETK
LVLAEVERARKELPRKTRGWIAVYDWRMLEHLKVMEDENTKKKYYDGFHD
PWRRWYCGIV

>Mb|QLI64005.1
MSAPANLESLQSMARLSRLTRSPVAAFINRHFPNATWAVSLRHRQQPLLM
AAEHRPETASEEVSEERTEPFSQSEIDARFNRFGVEEASAGPNDENVSLD
DLHVDEDHHKQGIRILVRPLTKLNGTRSVSPGSDNCRWLALRAEVSSSDQ
PEHKVLAVTQTSKDIKFSVRIPGESQDDAPRPPLWCELYYDPASDRVIFL
NRSDVPISLHKLSQTTLRSLPPSEIYAIDPGLINALQPGTWRIKVRDIAV
LDFRVLEKRPIALYQARPALPHQSSSLTSLASTSSMKRALSPDESERRAK
RRISDDSNPADDGVIMFLRPAADPLVFPLPNGKESRELSATTAHALLDAE
KGETIAVPGVCELDEYCLTKRDPIASTAQSDVFTADHSKVPDNILTVKVL
KTRMANGNDKPYGHERNVIRQADMWLRESQSQEGLQHESIVRYYGGDARY
LSLYMENVDAQDLAGIPRWRNKQTNLFMGTRTDAMRILRGIAGALGYIHG
RQLVHNDIKPGNILYSPERGAVLCDFGLSTPAANPPSGGGTPYYVPPEFI
GTKMRGPASDVWALGITMLYLLRKISFPDSRGGRHLAKPLYWQIAAINNP
NMPHKYGNGQPAVHQMRTWLGEIFEARKHLNPGDHLERLVKDMLTPNPNN
RINMNRVLQELLIGIPAKAG
>Mb|QLI64006.1
MAPDKTPIFRAVATSTRPLYQLLRCINFAPRVHVQITEEGIRFAADHSRV
MQGVAFLDKALFSAYSLNLPLSDDDEAVDLPNFQISLASFLEVLQIFGAV
DVATRAQKAEQDPYRSNLRNYRPDAFSNQALGISGTCTLLYGEAGDAFKI
TIEEAGVKTTAGLTTYVPELPDDIPFNREEMSFKIIMSSRSLLDSLAEIS
PTAPDKLAIIASKTSPFLSLAGTGDLGSSAVDFARGRDLLETFTIADRWA
QTYKFDFIKNSTEAMRIANKVSLRGDAQGVLSLQFMVEMEGGKRSFLDFR
FVPFITYDEEDEGETDTGIEFEED
>Mb|QLI64009.1
MRVVLVSGGVISGVGKGIIASSSGLLLKTLGLRVTAQKLDPYLNTDAGLL
NPLEHGECFVLADGGETDLDLGNYERYLGIQLSNESNMTTGKIYKLVLDK
ERRGDYLGKTVQVVPHITDAMQEWIERVAKIPVDDSGEEPDVCIIELGGT
VGDIESAPFIEALVQLRHRLGRENFFSIAVSYVPIINGEEKTKPTQHAIK
QMRMAGLIPDMVACRCERELDDGTIQKIARSCQVEYEQVIGVRDMETIYQ
VPLLLEQEGLLKQLRKGLALDNTTLPPARVQKGQALWDIWTKTVSPKHHL
QPVNIALVGKYVTLDDAYLSVHKALEHSAMRCGRKLNLVSVDSEHLEHDM
QQKDPTKYHNAWKALCEAEGIIVPGGFGSRGVEGMIETTKWARERKVPFL
GICLGLQVAVMEYARNIMGLKDATSEEISAHAEHRVIIFMPEGSKEQMGG
TMRLGTRTSHFKPGTEWSKLRAMYGGADVVEERHRHRYEVNPEYIEDLEN
AGLSVTSLDDQGVRVETIELKDHPFFVGLQAHPEFTSKVLSPSPALLGFV
AASAGCFNEISKAAPKKELLVNGASDASF
>Mb|QLI64011.1
MNGTTASARGKRKTQISPENSPPAKRIINGKLSPGDNTPDVAEIVQYDGE
SESDDIYTSAPIYIGTPGSLSEWQDTIQNVVRNVVAIRFCQTCSFDTDPA
LTSEATGFVVDSERGYILTNRHVVGAGPFWGHCVFDNHEEVDCYPVYRDP
VHDFGILRYDPEALKYMIVDGLDLRPDQAKVGVEIRVVGNDAGEKLSILS
GVISRLDRNAPEYGEGYSDFNTCYYQANAAASGGSSGSPVVAKDGSAVAL
QAGGRSDGASTDYFLPLDRPLRALRCIQQGNPVTRGDIQCQFLLKPFDEC
RRLGLQPEWEAEVRKAFPDETNMLVAEIVLPSGPSDSKIEEGDVLVKVNG
ELITQFIRLDDILDSNVGESIKFQLQRGGEDIEVEIEVGDLHSITPDRFV
SVAGASFHNLSYQQARLYAVAVKGVYVCESAGSFRVDNTDNGWIVQTIDH
KKVPDLDTFIQVVKAIPDKARVVVTYKHLRDLHTLNTTVVYVDRHWSSKM
KQAVRNDKTGLWDFSDLGDPLPPVPPVRRSASFIELDHMPHPGIAELIKS
FVHVNCTMPVKLDGFPKNRRWGMGLVIDAEKGLVLISRAIVPYDLCDITV
TIADSIIVEGKVVFLHPLQNYVIIQYDPALVDAPVKSARLSSEHLSQGAK
TYFLGHNRIGRVVHGATTITEITAVAIPANTGAPRYRAINVDAITIDSNL
GSTCNSGVLVAPDGTVQALWLSYLGERSPCTQRDEEYYLGLGTPTLLPVI
STIQRGESPSLRLLSVEFRAIQMSQASVMGVTDDWIKKVTQANRSHHQLF
MVSKRTFERVNQPVSLLEGDIILTLNGKICTTISDFDVMYSNEVLDAVIV
RECEEMPLQLATVPADDIETDHAVSFCGAILHRPHQAVRQQIRKLHSEVY
VSSRIRGSPAYQYGVAPTNFITHVNKVATPDLDSFIAATRKIPDNTYFRL
KAVTFDCVPWVITMKKNDHYFPTMEWIKDSNEACGWRRVTYEGKEIFQGE
AVDGVLPIAEDTDME

>Mb|QLI64012.1
MSEADEVAEDYRHALEDLSSNMRFEISNLTVIARENTEHALAIAEVLQQH
ILKAPPTKKLPALYVLDSIVKNVGTPYTLYFGRNLFKTFMESYAVVDNNV
RRKMEEMLKTWKDPVPGSMDTRPVFSHELVRPIENALMKARAATMPQPGA
IPGRPRSAMIPHRNTPTPPGMRGQPGGPAGPYPAQQYGYPNGSRPGEAPY
PGQQQQFLPSSSTPQPHGSIPASFQPLLSGPYGSAGAGTLAGISVETLSN
DIQNLIVAMRAEFAQNPNDNSVQNRLKALLDLQGVVRNTSLPPDQLELIK
NKVTELAAVTMKATPSQNSTPVPGYVPPQGHPPSHPAPVTPVPASVPEQK
PQVTLDSLLGPGAMAALMARQAAASQNATPAAPFPNAPIRSPPPTHAETP
KPPPQNPMSLLERLRQAGMIPSATPPSSGVPVVATPPPPPPPQSILPLNI
SSILASAKAAASQQGLDGLNGPVLDSTSLKQQFRPSTVAALYDNLGPPCS
QCGRRFKTDEEGKRKKMAHMDWHFRVHQRTTEAEKRGTHRSWYVDYQDWL
RSRETVDSDHIPAPDDGAGQASEANKGPKYIPVPDPASGINNVCPICQER
FENKWLDTAQEWVWLDAVLVGNRAYHASCHAEATRDREGTPGPSRRTPEP
VLGKRKAENAISSPKIRTLKTSM
>Mb|QLI64013.1
MHVTSIVLALAAAGAYAAPAPAPAPEASPWCWRPGQPCWKVKRVAEAFSESI
KSSGALKERTPEAEYSNSPGGAAYKIKRSLIELAHVASLTAREPAEYYRD
LSLETRFAADEGLDKRGEVAEDKRQWCSSPGEPCWKDKRDAVAEDKRQWC
SAPGEPCWKDKRWCGRPGQPCWKAKRAAEAVINEIRDMSKRDEGGDAPPS
HQGGHFPAVCNGPNILCLKTKREASPEANPEANPQWCWRPGQPCWKAKRD
LHALDLAARNVVESLE
>Mb|QLI64017.1
MYIKQIIIQGFKSYKEQTVIEPFSPKTNVIVGRNGSGKSNFFAAMRFVLS
DAYTQMSREERQGLLHEGSGSAVMSAYVEIIFDNSDDRFPTGNKEVVLRR
TIGLKKDEYSVDRKVVTKTDVMNLLEAAGFSRSNPYYIVPQGRVTALTNM
KESDRLNLLKEVAGTHVYEARRAESLKIMNETNNKREKIDELLEYIKERL
SELEEEKEELRGFQDKDRERRCLEYAYHHREQVTIQSALEDIDNARQDGL
DTSDSSRSEFLKGEKAIAKLDAEIHNLQRELELLQIDRRQHEEDRRDGAK
ALAKVELKVKDLKDGQSALEQARSQHEAELQSVQAEMSEKESQLAKILPD
YNKKKQQESDVRRQLDTAEASRARLFSKQTRGSRFKNKSERDAWLKSEIE
ELDMTMSTQKANKLEAEEEVARVRKSIDQTEKEVADLRSRLANWSGDRVR
LAEEATQARAHLDKLNDERKLIRREEDKLNSVIANARQEKDQAEREMSHT
IDGATARGLATIRRLKAEQDIPGAYGTLADLLEVNEAYRLPVEQTAGASL
FHYVVDNADTATYLADILFKQRGGRVTFMPLAQLRPRQITFPRSNDAVPL
ISKIQYDHKFEKAFQQVFGKTVVCINLAVASQYARSHGVDGITAEGDTTN
KRGAMTGGYIDPRKSRLEAVHSANKWRQEYESLIAQSRDLRRQIELKDQE
ITGAMSELQKLEQRLRQADDGFEPLKHELRNKSAHVENERSRLDAALVRR
DLVDKNLNGFLEEIAAHEAEIGTEFKKNITPAEEQELEQLSTLSQQLQKQ
WNDLSNQRRKLERTKQFLEVDLRQNLQLKLDQLSSQAFENSTSGGSSGGM
KEAQRELKKAQKSLHAVEANLQETEGKMEQLVGRIDELSGEKSQREQRQN
ELSTRIEKQQKRMEKTMQRKALLTTQAAECAKNIRELGVLPEEAFDKYEN
MEANMITNKLKRVNEALKKYKHVNKKAFEQYNNFTTQQDQLMKRRKELDA
SQDSIEELVEHLDRRKDEAIERTFKQVSKEFATIFGKLVPAGHGRLVIQR
RADRRQEPEESEEEARGSVENYTGVGISVSFNSKHLDEQQRIQQLSGGQK
SLCALCLIFALQQTESSPMVIFDEVDANLDAQYRTAVAALLESISNEAGT
QFICTTFRPEIVHVADKCYGVTFHNKTSSINCYSEEDALNFVEGQAKPTG
TL
>Mb|QLI64021.1
MSSPSSDASPDSAVQYAYMFEKDKGPTKQLDALLRAIARHVMLEIGEKAD
NHLTPSKLAAFYKAVGGDYDSLFVDTPHSTISYVWQITGCQHSLQPTRND
FDPPSIPALTYRGFSRWESLEILLGPEEHVPFLQYAVKEWNLKHPETGAD
FPVDLPWTCFPEKPDQDIDRWHKSCADKLWTAASNDDGATPRPPPANEQP
PPPKFTYAHFQEPFSFTSSPRPRPNDGEYFGRPFAYTHVPRRYASQRKAE
RSPDRPRRGDTSPDDRGRRRSFSDYASGRPDQDPSLPRGYSPSYLNPNMK
RSGPGRRHSHPRHEMTDSSDEEPVGDPREKRRRHPYSPPPPSVRRFVRTG
AASQSQGSSNGSSHLRGLRSDGRPEEAKRRTGPSPLGSLRDKFTETVSSI
LPNGLTSDRPRPGPRQNSGTEPIRTRRSREQVRPSRLNRSYSDMDTDESG

DPSVTESDGRRRRRTREEREREQERERERYQRGRVRDHDRDWEEDRDMGL
RREKSYLRRPETQRRTSSHADIDRNRDPPEWDLRDRDRDRDRDRLRDERR
KWERRNSPEEDLTSPAAAMAARRMHPEAAYS
>Mb|QLI64022.1
MSTIPPSAQQSAYPPTIPLDFNSKQPDKVTLYPLSNYTFGVKETQPEEDP
SVIARLKRLEEHFSEHGMRRTCEGILVCHEHNHPHILMLQIANAFFKLPG
DYLRPEDDEEAGFKARLDERLAPVGRIGEGEEAGDWEVGDCLAQWWRPNF
ETFMYPFVPAHVTRPKECKKLYFIHLPKTKVLSVPKNMKLLAVPLFELYD
NTARYGPQLSAIPHLLSRYNFEFVDEDGNVVAMTPGAGPPEGYVPKTKVL
AGEDMEMKEENGTI
>Mb|QLI64023.1
MARSSAAARNLSLMEELEKLEQSITLTLQEIDHNFSKAHRIVTTSIIPVV
EQYGEHSRAVWEASKFWKQFFEASANVSLSGYEELANDDESTVMEDSTAM
RDEASADHTQTHDQDMTATPGGTRDSTMHGDESLLDDAELSGSTPRPPVT
EMIKAGVSGHSSPFDRMQREIRGDNDGTTVLQDNEDSTVLFAQRTAQLPD
ISMTPRGPREDQAARQQASARQAKDPLLHRVLDKNYRVQATPHRPGIKVS
PLKKEKEKAAWQYDDSIMSSPEIAAPTLRSEVFMSPYKLMARQRAAAQAP
RTPGVSVQTPATAKKSRDVFALAEEDKGAGGAAAGQAGRGRQKYEIDWDS
DEDGDDDLYAGMSPPKTIQFVLPPSKLLQTPAREASRRIVDDILMDAGAD
MESSEYSPTMVKMNEDIMDDSF
>Mb|QLI64025.1
MAQNMDRGLDEIIAEKVCFCARLAKTSSSLTKPSSEPMALETDAAVEIPA
VATVMTIPGTGLHHPPLLATVYPAGCLGPPLILSNRCIQRLDDADSNNPL
LDLSLTSSFASLQQSTRDDRRNIDSEWVHDRYDESESRRGPAPRRRRESP
VSDNKGSKIRAENIHYDLTEEDLDELFARIGRVSKLNLRYDRAGRSEGVA
YVTYEQREDAEEAIKQFDGANANGQPIRLTLLPSRNPFDTAVMPGRPLAE
RISAPGGRARSTSPRRRLEDEDAARKGIDRYIPGRRSRSPMPRRSGGRGG
RRPGARREGGNNSNEGGRGGRGNPRGKKTQEELDAEMADYFGGSGHDSAA
ADAPANGAAPATEAAPAAVTDDIDMIE
>Mb|QLI64028.1
MSEERSKISLRSGKKRRPSRPTISAPRQISSPMPQDSPLGGAPPLVPEAV
PRPRLRPPPIAGGKTSDLVKQRYSTRFNNLPTDFDPSAPPLPAVPPIPQY
DKREPQRPAPSRAGAAPVIDIKALRDPNLVPDQYVSAVLGDATDDQIRDF
EVALRKLKGRAATDLQQNLMQNRTQFIKISKEAEKLKGEMRALRNLMAEL
KTNTTALRAASIKSDEPATIPGSGPSGMSKRDKRSSVADRSALWNSQMQA
LYKNVEGSQKFLPNAVGRHVVQNAGSWVELDNATYKSRRAMQIFLLNDHL
LVASRKKRKVDAPADARGPMIKLVADRCWSLLDIEVVDMPGTAESSGGRN
KLADAIMIRGGGQESFIYRTEKHEGDEKKTLLLNVRKAVEELRKGLQSEM
EANNKARETINYFASRDPGLLQKTELLETLSDIKDMLIEVDGKQQNLRWV
EGQMDELDIDIALQKFEPAVARVEKLKSLARGLRSNLVAQDFINFKVEER
CGRLAVLIIRELESKHKSQIKTKERVGWLTILGFDDRAREAYLNARTGLI
RKRARQCIFQGDLHLYIWQLSFVYFTIIHNTVQTFQSCFPPPMMSACVKW
AKEEVEAFNVILSRQLSSTERGGQVWTQCMDRAKEHAQKLSDVGLDFRNL
VGHDVGPSAQTAAEVPGPSAVGLGLA
>Mb|QLI64029.1
MEVPPAAISPEKTRGQEDDSLSKICEEMKTWLSGSPAVKCVESADEDTKI
KSSKPATAVSSKASSSILVLPKAASDANAESESVTSADMRKLEAATILSN
GPSNLPAETEKAMSQLGTRGLTAQDDCGTPVGEQHEKIKSIGSSKSTPHA
STLLSDTPLPAPLHIIQAATAVEPGEDVKRRLSWSVSNVSPTEGGHEEGS
SSLSSVVDQEADSHIVHLSSVPNAQEKIAHEYNGIILYDSTAEGSNCLIG
AWLTKFRGLPSVTVPYENLCNESFVQCVIDTENGEFLAPIPQPETVRDMV
DGPCEGYNDIGWRQVNMTSNLYISQEIKSRENTARTLRMTLEQLDHKPEP
IVGTEEEHWPKAKCVIRPAAPQDFAQIAKIINAEAANTCNAQIFQDKLAM
VDDVCKIFDACVAESRPFIVAIPAQDDFLDQSKWPRDSDEVYQEFAKYMA
AKPKPAAPVAGFAFVSDYRMNLYGAPCLSSRYTGQVRLAVHPDHQRKLYG
SALMDRLLLSISPFRRSTLDYVWECDDPKDVYQFPVTRNRRQYTQLYFEH
FFSQEDSAAYQPGAEFLGKFGFQEIGYFKNAAVRFDDQHHNHWLNMVVWQ
TDNTPTSKIVARPQLSGSAAGNN

>Mb|QLI64031.1
MRPVTAWLLAVHCAASAAEQFRLNQTYNASNFFKSFDFRTTGGIPTQNDD
NWSWVKYQNLTNAQKKGLAKIKDGEVYLGIDHTRVLNKTSAEGRDSLRVV
SKKAFKYGWTLGVGEWPKAGEIDIYEGWNLDIHNKPVFHVGPASEFGSCT
IEQGDQYSGIISSNCDNTFADNKTQWLNQGCQSRETENTIWADPKGGIQA
LEWTTEYIKLFTWPHGQEPANLSDEELDTSSWGQPSVQLRASNCDINRIF
QEQQLMFTLPVCGIPPGSDQFWKELDGGSGRTCDKVTREPTCIDYVSKNP
QAFKNFYFQIKDIRYLTQKVIKPTNHTTIATSTSTRSMNRTSTATSLTTT
VSLNHTTIASSLTATVSKNQTRITTATGSTIQDGQTGIQEAVTATSTASK
ASVSCKHRHGARHI
>Mb|QLI64032.1
MTPIPHTYGSNCRLAPIPQRPSENGMPQIVSQFFYSSLIPINELLSNQRY
RSSSDNKSVKRQLRPFDLGDNNALEKAWMSLPSDQYRTEHEAIRAGRYES
LRPSPGLLRLRQKLVEALALKHLELHAGTLYGLPLNNNAASTSAEMGELP
ACCPVLRSDASEMLQQHFCALARKAYPELTIEATEREVIETMTAINKTKI
DTFDSEQNSVLGNLSARRREAVSLEVEASPVTSAGRIVARQGRPQSSSRI
TPSLLGQTALDVPAPPRLPGTSVGMAGDSFIQAGSKSGFTTPRASTSDGR
QQLSTSKHHGDDRIALRHHVPDSKKETESEASVLDVVVGISRLHKVSLPA
LLMEPIYWSPVNDVAVVMRATWFYRDTMMPVAPLVANQLEAGYQELKPWT
ETWRDELNCAVEVGSLGEEKISYCLWPQDSKYFNSEMQKREPGISAEAFC
SAHCCRGDAAAQGSIDPVVEGSSSNKEGISRAFSNYHVIYKDEKRAFLLK
PSLRPSAYYGRRPASKIVKGFAVGIPVVRGFDSQAWDRIYGHGHVPDRDK
ETQQIAEPTSHSHENDEDRPCDACEADKDNGQVTDLILVAHGIGQKFAER
VESFHFTHAINGFRRAVNDEMRNPIIQHITRQDQNGVMILPLNWRMGLSF
EDGGPGNRQTSEQQTNESFGLKDIEPNTIPAIRSMISDVMFDIPFYMSHH
KSKMVNALVFEANRVYRLWCRNNPGFADRGRVHLIAHSLGSVMAVDILSR
QPTEVPRLDLERVEPETQFFEFDTTNLFLLGSPAGFFLLLEHGALTPRRG
RRKPGADPRDIMDENIVSDAGRFGCLAVDNIYNILAKEDPIAYLLNGAVD
PAYASALKPAYVPSTSTSIFKSVGDVMRQIVPGLSLPTDPLATHPERPSI
IRLPSQLELEVHDFTREEIAEKRVFLLNDNGQIDWFLRSGSGPLEIQYLN
MLSAHTSYWINQDFVRMLCLEIGRRPGRNFSIPVMRAVKGTKRFSLER
>Mb|QLI64033.1
MAQLFWGAFAQSFFIPKPVLTEDNLPDQSGKVHIVTGGYAGIGKELSTIL
YQHNAVIYIAGRNEEKATTAIEEIKAQNPDSRGRLEFLSLDLSDLTTIKP
ALEEFSAREKRLDVLVNNAGVMMCSKDAKGTQGHELQLATNCLGPFLFTK
GLVPILKETALASPPGSVRVIWASSITANLLSPAGGVKLGEDDAPQTSSS
QSTNYGQSKAANNFYAAEFSRRHEADGILSLAFNPGNLATELCRHLGPVE
LLTAKIVSHPSRLGAYTELYAGWSPDIKMEDGGMFVIPWGRRGNDILRND
LREAIEESETNEKSIPRRLWEWSDRETAQYA
>Mb|QLI64040.1
MTSTIGIPIKLLNEAQGHIVTLEITSGQTYRGKLLDAEDNMNVQLKDITV
TARDGRVSHLEQVYIRGSHVRFFIVPDMLRNAPMFRSRNLRGRGVGLARG
RATVSRARAGGRR
>Mb|QLI64042.1
MPRQPRQSRSSRTTSSYFQRPSRALNTSSSTSSRRPPRDISEGGSSMTAT
PSIAGIVSSTAKSNITAGRSRPSTSVSGRKSRTGTLSTMLGASDQQTIIC
AVAESRGISPSIGLALVNITLGEAVLSQICDNQSYVKTIHKLQIAGPSRI
VLMSTACPPNKDSVLYTLIDELIPDADIEVLDRSAWSEVDGLEYIDNLAF
KSDIDPMKVAVQGKYYCISSLAAHTFTISFAPNSLRIKYRPSEDTMMVDI
SVIQSLEIIQNLDKPKSKDSLFGLLNQTKTPMGSRMLRNNILQPPTKYDT
FIRPRYEALEELTSEEEMFHAVRKALENFKDIERMITISTKSSITHAEEQ
INHVILIKTFLESVPELYQALAECNSALLLKIRDICHPNTTDPILSRIHQ
VVEADVTYMTSPLDMRNQRTFAVKAGINDMLDVARQAYKELTNDIHLHVN
DIQAQHGIQLTVKFDHGRKYWLRIKSGDLNKDTLPQVFINVVKKKDHIQC
QTLSLVKLNFRLSETSNEIIMRSDKVIQELILDLRESSPQLFRVCESVAL
VDMIASFAHLATIKDYVRPEILDTLALKAARHPILDNIMFEGFVPNDYYA
TPQHRFHIVTGCNMAGKTTYIRAIALLQIMAQIGCFVPAEYASFPVMHNL
FARVSLTDNLEANLSTYSLEMREMAFILRNVNGKSLVIIDELGRGTSPRD

GLAMAIAMSEALIQSGAFVWFATHFVDLTRVLEDRPGVLSLHLASERSIS
DEGEPRLVMLYKAKSGVVDTNQHYGIDLARAIGFPEPFTKRAEEVAKALR
QQREASLRDSESRRLVARRKLVLSLHEALKQAYEYGSDEALPGYLKHLQE
DFISRMNDLEIS
>Mb|QLI64043.1
MPEPTYNFPSVEETTNHPAYKGTVWKLKPHSSGHLPVAKGRGGPLKIYWE
VHGTGPTKLILIMGLAGTLTSWQIQTHHFGHVHGAQNSVLVLDNRGVGRS
DKPLGRYTTSGMAADVLEVADHVGWTQPRQLNVVGISLGGMVAQELACLA
PSRVRSLSLLSTSARVDSGKTLPQAALDRLALLRPKTDAQAIRDTALAVF
PPAFLLAPDEVPLPEPAAEPMCAPADTPDGEYPRFGSNFQRFQAQELHKR
RAPGAFTLVGFLGQLLAAAGHSKSPAQLRGMADGVGRDRILVMHGTGDKM
LVVRNGERLIHLVEPGVAMLVDDLGHAPIFERSVWFNGLLDERLHAWSKL
>Mb|QLI64048.1
MESCATCATILRLPGAPTSAKIPDDRIVSCCGRVICGSCIQKNPRFSQYC
PYCQISSTPSPLPQGLKDPPSYALVSQSRTMDSGPPPYTLVDAKPQPTRN
EGASAGVDEKSTADDTLHFLNHNHDSITSLSFRYGVPAAALRRANNITSD
YLLQGRKTILIPGDYYKGGVSLSPTPIDGEDEELRKSKIRRFMTSCKVSD
YNIAQLYLDKSGYDFGASVQAYFDDEAWEREQASNGANKAITRTKNLGFF
WRR
>Mb|QLI64050.1
MAMDALRNLANNIPDWQRRLDDLNGQIERRQAELAAIARTNATSTDSSRP
LRNKGSSESLKPKDDGPMHPDLDLAVTPSQGQRKRIAPRTPPTPSGEAKA
TPASPPSPDSESRVALHRQAREAVNAHARALAQTKKKQKSSSVLSADGVP
PTYRTRSMIMVYYDSYVQGFFDELVRFISSSRNLMRKAKMAAKVAQIKRM
AELEIANDAKRTSDERFAVDAIPSLRYMSTRKVGTTSQELTSGEPSRPAG
PEQPADVYDSLDKSLEFVQSTCEHGAHQFLRDADCHDEIGKIQSRLVEVL
IAAKQEIDRVQREDPELAKETGELGKVRLHRPISVRRDMTAECKDEVSHN
DGCLTWEISPEKDATLQTEMALEVDTNLEEEDMDNLPVLQYRSTRLMRSR
PV
>Mb|QLI64052.1
MYLPRSLISKLYTHLQTTRHPTSPPVLILVALEPDALCGCRILTRLLKHD
YIPHKIQPVAGYGDLEKAGKELVMPMMESQGGSGGVVVCLGVGGMVDLGP
LLGLEQDGAEAPYSGVEVWVIDARRPWNLGNVFGGFPLEATSEAQSSYQS
RTPVGISGGQVERAYKSGKGGIVVFDDGDIEEAMEKERDAYLALVDMPDI
DHDQEAVGLTDDDDNDDDDDNDGSLDPELPRAGQKRKSWSDGYEDESTDD
EDRPHQRRRSNSSSPIADSPRRPTYRGLVSLRGPEAGLSSDAIEPPSAAQ
EPPPPSARALRRRLLRLRQEKEAILHSYYRMGSSFAEPISSMMYSLASEL
GRDDNDLLWLTIVGVTSMELYGRSSAGTAAPIRSNDSSRPSGWMGVRGAR
LRQLLRDEVRRLNPPEVTNGRVAPENAGVIPTTARNPEDTGIRLSPEPRF
LLIRHWSLYDSMLHSPYLFSRLKTWSETGIKRLHKLLAKMGVSLAQCKQS
YTHMDMMLKRELRAKLLKYGSLYNLDEMVPAVDTDDKDRAGAKDGWGFVR
SWGWRATLSAQDVGVVIGALLEVGKHVQTSEVAMPTSQLTHDAEDEVEFA
AQAEEWVGRFWEAYDALEDIDSLKAGLPTAQFLHRAIYRTGTSLINKKQI
KHLRAFRMCVVKDGPDVALFTHPAALTKLSLWIGEALAEQEREAHGKLSH
GGRGTPLVVASLNEKRSVYVVVGTGGGGGPDTAFLNREAAKRRRAEREAK
AKRKEAARLAKEKIREEKRAAKRAAGQEDDEEDELETESEASDSGLDQDS
GDDDDADAEPDKGYGLNKFGSAFQEVVSETNARVRIDSFEHCAVEVRKEG
LGGFLESLSMKAVVG
>Mb|QLI64054.1
MLRRPPIDGGGSSQQQKLFLAFAVILLPWFQLADAQPRQAAHSIISPHVA
PAAGVAVDVDGSYKDRKKPSREHHPAAFAAAAAAAARNDPAHWGATPISA
ATIETPSGGDHKRSALQKQSDHQKEEQHQLQAGHGSENTPHKPRLDDNIH
NHNKKQTRNDASAFATTLAPDNSVRAPSPLRRQPSGLTSPQYARSLEDWE
VEDFVLLATVDGDLYAADRKTGKQLWSLLVDQPMVETIHYRGNSSELDDD
YSPVDHYVWAIEPNQDGGVYVWIPEPNARPRWTGFTMKELVEVLSPFAGN
EPEPAVVYVGDKRTTMVTLDAATGRVIKWFGTGGSHVNQAESCLKPNALY
DRDAEECSTTGTITLGRTEYTVGIERRDGKPIATLKYAEWTPNNRDKDLF
HQYRVSKDNRYISTQHDGKIYSFDYARSDSNSVPARLFSQKFEAPVARVF

DVCRPWDATPDSNPDLILLSQPIMPSPDDHTSNSIFLNRTTTGGWYALSG
RAYPLIDAAPVAPASRPDGWEKMASWDSMTETKLSRLIGTHSLGSQQKVQ
QFPTLPDRPATGNDREDPDNQSVPQHVPVEIEEPTIVDKVKSIPQSAATS
VYDFFSNPVLIIFLIGLLVYNRKTFARAYQLWLNGSSPKDVFSYLLPGAT
PDDKLATQGTDVSEPNGKQSPKPATAEDNSSEANPPELDAKAEDRSPRDD
DIAKPDNESTTDTDSTPKSASPSKPGAGKDKLADGPDNGSVAGGTGPEVK
KKRAHRGRRGGVKHRKGRPRDSSQSRGDEPPNATVEEAVSNAKKLGDRPI
WEPDVLTVANDMQAVSGPVVRMGNIEVNLEEQLGTGSNGTLVFAGKFDGR
DVAVKRMLIQFYDIASQETRLLRESDDHPNVIRYYSQQTQGSFLFIALER
CAASLAEIVERPHAFHELANAGKVDLPGVLYQITNGISHLHNLRIVHRDL
KPQNILVNMGKNGKPRLLVSDFGLCKKLESEQSSFGATTGRAAGTSGWRA
PELLLDDDGRDLNLMEASTHSGSGSVLVQDGTMPHHRRATRAIDIFSLGL
VFFYVLTNGSHPFDCGDRYMREVNIRKNNYSLQLLDVLGDFAFEAKDLIM
SMLNANPKQRPTATEIMCHPFFWSPKKRLSFLCDVSDHFEKEPRDPPSVA
LEELERHAPEITRGDFLRSLPREFVDSLGKQRKYTGSRLLDLLRALRNKR
NHYEDMSDSLKRQVGPLPDGYLSFWTTRFPQLLLVCWNVVYNVQWEESDR
FREYYEPAGL
>Mb|QLI64055.1
MLPKTARLWALLCLALIVQSAVSQSFDVVKRDDNPTPTTIPTSSSSAPKG
TNSARIDNDGKDPKERGGESLSSTLSGTITDAPTSTHRPTSTLPLTIATD
GPLDNATFFNATIPNGQLPLPPRLTPGWGVAGVIMLVTGVIYTLVGIKNR
WIHTFFSTAYMTALGVSVLIVYVMNVPVSNALQGGYVVAVVMSGCAIGAA
AMFFKELTEGFGCALGGFCISMWLLCLVPGGLLHAVASKAIFIAVFTVAG
FAFYFSRWTRDWALIFMISFGGATITILGIDCFSRAGLKEFWAYVWDIND
NLFPLGADTYPVTKGIRVETAAVIIIFLVGIISQIKLWRIVREKREKRAA
ERAEDQRNLENEEENVGRHIEDLNARERRQWERAYGDGMSHSITDSRATD
YGDVGSEKKLRPSYVESKQRTSGEVIEMADMSESDTSRNGMDGLMANEDT
DEGKITVRVAADDVPESTTDIEAERLDEKAPSSVEANVLTDGKWYSKETT
AGKRRPQAHSMTEVPEVIPLPFTVPEAADSASEGDRSSIATFADDVEATA
AEPLKHRSLVKRLSQGSVTLLRSFSHRSGRTGGVGGDVASDAGESTEELV
LEKTRRPENEGGSLAATIDDESVSGGNLHSITGDDELRNIHEGDETNRRS
IEIDAQLGGKGDTIRYSVKSTKPKTKSSPTSTATSGPVVGISEPNEDTTT
ELRDTLPTSEDKAMQLGGEPYADLTPPEASERAKSVATASSTPASLTKDR
LPRPLSKAAMSYRTNEWAKHLSNAECPEPDALQVTLGRAKKEVKNSTEKA
APVNVAELQKTAKDGTPAPAVRRSISQASDMGNNTHLAKRSSKQDLSPPT
RLVTPSSSTGLETSGEQRTPLSPVIAPAALLRKSSGPRHSTSNFMPIAEE
NSLQAVDSPIVRDEASEKQQPASNNSSAIILDGSPATVLGRNPTSGVVSY
SSPQTLIGQREMFLRSKSQGSLLSSTPEPIFAHQGPGSDGGSLFNYPTYA
AAFNVADPDDVPLSQRKEIMRQSSLMSLSRSPSQSIKAPSRPTSGFESSE
SIVFNSHQPRRVSTLPTQAVRESQLASFRQSVQQELRSGTPIITPSGRET
PFGPSTLLGGGREAEVQRNIEMQRSVLMGQKEAEAQRRESQKREREHADR
AFDERMRNGDMLDAHREAMRKMQKGAR
>Mb|QLI64056.1
MPPQTHFSEDPPQVINQKDLELANERLKQSGSGNGLAAAGEGSGISASDE
TSSPPGTDGNGGAIEKVGTYDRYEITEDDCYDELGYSFPSWKKWYILTVI
FWVQVSMNFNTSLYSNAIPGISEEFGVSDQAARCGAMIFLVLYAFGCELW
APWSEEFGRWPVLQLSLFLVNVWQLPVALAPNFASVMVGRALGGLSSAGG
SVTLGMIADMWEADNQQYAVAYVVFSSVGGSVLGPIVGGFTEQYLDWRWS
IWIQLILGGFVQLVHFFTVPETRTTIMMNRIAKRRRKETNQNVWGPDELV
PFSDRFSAREILVTWVRPFRMFLTEPIVLVLSLLSGFSDALIFMFIQSFA
LVYGQWHFTTVQIGLSFIAIGVGYLMAWGLFIPAIRRNIKERKQKPDDER
AQYESRLWFLLYTAPCLPIGLIGFAWTIQGPPIHWIGSMVFVAIVGIANY
AIYMATIDYMVCAYGPYSASATGGNGWARDFLAGVLTLPAIPFFKNIGAS
SGKNLEYASTILFCISFVLVAAVYVIYWKGPELRHRSPFAQKLADARQTQ
LNEGRRGSIPYDPDERRGSATSAVNVRPGNERRYSQQHRFFGESRVTPRG
TPRGTPSASRRPSMANLAAKK
>Mb|QLI64058.1
MESRVADHVPELVGVALVVTFIYSGLSNPLSKVPGPWYAKWTDLVGKYQY

YNGNKVFYVHNLHKKFGPYVRIGPNEVSVADLDATKIVYSTKETFRKTIF
YRHLTAQPMANIFSTADVDYHRKLRRLMSAQLSESSLKSLLPAITSRVEL
TIQRMKEDMQTRGFVDVFKWCLFMATDVIGELAFGESFQMLEKGEKNQYI
RDLEGVGVVSAVRITFPRVIALARKYPLPIFQKNVQQARRIIGYAEQSLQ
RYQSLIDQNPEVPKQIIVTKMFKAREDEKLSFSEIVINAQGLIVAGSDTT
SNSLTYLLWSVCRRPELRDALVKELRTLPDDFTEHDLRELPLLSQCVEET
LRLYSAAPALLPREVPQGGVELGGYWLPGGTTVATQAYTMHRHPDIFPNP
DEFNPYRWAAPTKAMKEAFMAFGRGPRRLHLAYIELRLAAARFFQEFPTA
FAKEFASRAPTLALPKSFQNQHSPLCCEVLDMASDYDFEIEVPDLAEDDP
MRAFLPASFGKKSKEANIAAQIDRSKRNADGKAPAGALDASVSEAKKPHS
KGSSSDSDDSDDDSDDSDDSDDSDDGDEYPVSHELVLKTHNRAVTTVSLD
PSGGRLLTASLDCVINFHDFASMTPTTLRAFKTVDPYETKKSAATAESHP
IHHVEFNELSGGVFLCVSAHPQAKIMSRDGGILTEFVKGDMYLRDMNNTK
GHISEITGGTWHPTDKNLCITSGTDSTLRIWDINNKRSQKEVVVFKSKAA
GSAGRTKMTAVAWGKPSQGGNNVLVSVALDGTLVMYGGNGPFTRPAGEIQ
NAHKPNTWTSGIDISSDGRMVVTRGGDDLIKLWDTRKFTKPLVSVSHTST
SDHFPTSNIQYSPNSTSIITGSETGHLHILNPGNLRAEHVTPITPGSALI
TVNWHPKINQIITGSANAETHVLYNPTTSNRGAVEVMSRAPKKRHIDDDP
NLTMDQGLGMSGDAIITPGALSGARKSGVTGTGRSKDPRRPHVPQQTPFQ
KNQPDEKHVAENIPLARMLHEDPREALLKYADKAQNDPIFTKAWSKTQPK
TQYAELSDEEEDGPDKKKVRR
>Mb|QLI64059.1
MQPLYGSHPHTPRTPGTPNTPNSATNVPPYQQTSQAAQRPGIYSMTQNPY
PPNPGYGTSAPMMPQTTTAVSHPQPIAPAPVGGRAPPVLRPMPPGGIMAQ
PGISSPYGPGSLMQPNPVMHDGEQPTHVVGSQGRRGILPSAPGRPAAPTA
GTGAKSTVIPVKDADGKFPCPHCTKTYLHAKHLKRHLLRHTGDRPYMCVL
CRDTFSRSDILKRHFQKCSIRRGNPTGASHLSHPQAHVKKNAQAQKAVGL
GNEADLNHLNGLNNMPAEGMVHPFGMVPVSDGMNNMAGDQSHLSRSSSAN
QDRNNMAPSMGAPQPYGANVSNSMNNQQMPSYSMPPGQNGMPMYGGSNGN
QQSGLDWSQMFQAGAHQPLNNNIFHPPNRGQTQIAPKTGPNHDSGATDSS
SSDSVCYSMWGIPTNIQNPYSQLSSQILNFLYPPNEAIDPTLTGMNLYFS
PDNTKDFVDQYTHFHEHVPILHPGTFRVMEAHPGLAACMCCIGACYSNRV
VLSDVQEMMDALWTAMERDCRIFSEESLHDAGVAEVTKSSIEELQAALLT
SILHFGNGTSQQRHRALQIIPMVAAQSRRLGLLGVSGDLSIFSVLHHPEV
DGQYDLKSDHFDWIAWTEQELRIRLMHAIFMLDAFLELFFNVHAQFDPSE
VHLPLPCDDAAWNASTADNCASALGLNGDEIAQSVNSHGTRRAAQPELHL
VRQVLLDDGWQMKLGSTNSLGKTILGVTMIAIIRLSQKVGSLNYLHTGDG
LPSLDWIVPYDGSSQPIDPAISDEAHHGMDTRTFVMLLSALDKLQKSWND
DEATQTALRENGTRVYREGMFLPVISRHILLNPGFADLEQDDEDQYVASY
KAMKCVGASPSQQPDEVQEAITQFGEEEYILDLARIFRPLPPP
>Mb|QLI64061.1
MIPNYAYRVSLLAMMAHAVGAEEAANAQKYTNYAVSGIKSLNDHWYNVKT
GIWDDAWWNSGNALTTLADFAALRLAEANKLNVGGYMRNTFVQAQKVNVK
TAKFVNKAGMVSSVYCLDGSSGCMAKREFLGKRGFDDFINDFYDDEGWWA
LGWIRAYDVSGDDDYLGAAIDLFNDMQTGLGGPCDGGIYWSKDRQYVNAI
ANELYLSVAASLARRVPQNGTYKAIAVQQWDWFERSGMINAQNLINDGLD
ASCKNNGLQTWTYNQGVVLGGLAELFRATGDLKYAEKAVSIAQAAMKALS
VDGILTETGGCDAAGDCGRDGAQFKGVFIRNLRYLNDVAPHQEFKDFIIQ
NAKSIWAKDRTSDNQFGVAWAGPYYPAFGATQSSALDALVAAVAVS
>Mb|QLI64063.1
MQPGLKAKSRGLQAFARGKSDSNANYNAATENPSQPQPEQRPATPSANEY
VPSRQELAEFARLPLPGAVRNPLGSPRPGGQLSNPQKPQSPLRRGLASPV
RQQVNQIQATHQLQHDIFTGSQLGENFMMSGLSTPVNESEDLAIRRTPDI
KIEDQRQDRNLIQRLPPRQVPVTTSQKHEFAFGADGRLSVVPGLHRYHPS
HMKDGFYNNDAQLGPRPFKSQKSAYSLPMQSAPAHTKLPMRGVRINRGQL
PGTNALIIQEDKVKDKFIERRDGQWQEQAIEEKFNESILVNDSDDDVDAL
GAFGGMHTALKPNMQNGHRTRREDGLSSKISRNAPAKDKKRRLPSLDYDD
KILSSMTYQDLQEEPFDVVPQIPGMNGHDMSTSKLTTRLEQFQQQGEHEQ

RQFFANMSIEDWEDSGDWFADQFSDIMKRLRNARREKREMIRSFETEASH
REEAIRLQTDAIDRKLTRMKQDGQRVVGDKDL
>Mb|QLI64065.1
MADSTEVTRSWASPLATLALSIPILILVSRRTGTKWQNFPPFFLLTLLSG
AWFMIVSETVSEPYLDEIFHIPQAQRYCEGKFLEWDDKITTPPGLYLFSI
LLQKTAATVKLPWVFSCDTSSLRITNVLGLIVLASLTLLCRHEIESRLYE
AHSSVRLKAISKYAVHTAFNIALFPLLFFFSGLYYTDVISTAVVVGGYLN
HLKRVGRDRSSFVSDLITVLLGTLSLFMRQTNVFWIVVWMGGLEAVHALK
TLRPQHAEQPFMTTLTDQVKFYAWRYSVGDVHDPPLNLAWPDDMLFTAIS
LGIAALCNPIRVIRQIWPYLSVLAAFASFVAWNGGVVLGDKSNHVATIHL
AQLLYIWPFFAFFSLPLLLPHVIPILDIAGNVSRSLSQSGQRAGSSQPSK
TSRSFESSRSVKSKRPEGSKARDTRSTVSQVALETPSLGSPSPALRTADF
IFNSKFIPWIIYTLLTFVVSTAIVKFNTIIHPFTLADNRHYMFYIFRYTI
RRGSLIRFMLIIPYTISRWMVWGTLAGCADWISSSNRELCSARYNVNEPA
PYTSHPLWIARGTKKTQTNVEYPQDMSLVPSSSASTDGELRGTVEDDPLL
VSAEPTSTSTGLIFLLATTLSLMTAPLVEPRYFILPWVMWRLLVPAWRVH
DHAHINSLFEGVAPRSIVGKTKEIFKRYDVRLVLETLWFVAINVATGYIF
LMKPYVWKDEQGRNLEDGRLQRFMW
>Mb|QLI64067.1
MVDDSMLVAVAEVVSSLAADPRPSSSLSSSIGSRVTLPGPETAAKRALEI
ELQKLSLRVGQLENRASASATAVFPETPNEVNDSIFGDDAGSSPAGNRGA
ISNKSKLAHAHQGSLDCPKLLPRQLTKEALEGLRDHVDDQSKLLDNQRQE
LAGVNAQLLEQKELQERALAMLEQERVATLERELWKHQKANEAFQKALRE
IGEIVTAVARGDLTMKVRMNTVEMDPEITTFKRTINAMMDQLQIFASEVS
RVAREVGTEGLLGGQARIGGVDGTWKELTDNVNVMAQNLTDQVREIASVT
TAVAHGDLTKKIERPARGEILQLQQTINTMVDQLRTFASEVTRVARDVGT
EGILGGQADVGGVQGMWNDLTVNVNAMANNLTTQVRDIIKVTTAVAKGDL
TQKVQADCRGEIFELKSTINSMVDQLSQFAREVTKIAREVGTEGRLGGQA
TVHDVEGTWRDLTENVNGMAMNLTTQVREIAKVTTAVAKGDLTKKIGVEV
KGEILELKNTINQMVDRLGTFAVEVSKVAREVGTDGTLGGQAQVANVEGK
WKDLTENVNTMASNLTVQVRSISAVTQAIANGDMSQTIDVQASGEIQVLK
ETINNMVSRLSSFCYEVQRVAKDVGVDGKMGAQADVAGLNGRWKEITTDV
NTMASNLTTQVRAFSDITNLATDGDFTKLVDVEASGEMDELKRKINQMIS
NLRDSIQRNTQAREAAELANKTKSEFLANMSHEIRTPMNGIIGMTQLTLD
TDLTQYQREMLNIVNNLANSLLTIIDDILDLSKIEARRMVVEEIPYTLRG
TVFNALKTLAVKANEKFLDLTYKVDSSVPDHVIGDSFRLRQIILNLVGNA
IKFTEHGEVSLTIKERVNQHEVGMGEYAIEFVVEDTGIGIAKDKLNLIFD
TFQQADGSMTRKFGGTGLGLSISKRLVNLMGGDLWVLSEAGKGSQFHFTC
KVRLAADDTEGTLKQLKPYRGHQVLFVDKAQSGSGADIKDMLEQIGLHPV
VVDSEKSSALTRLKAGGALPYDAILVDSIDTARRLRAVDDFKYLPIVLLA
PIVHVSLKSCLDLGITSYMTTPCTLVDLGNGMVPALENRATPSLADNTKS
LEILLAEDNTVNQRLAVKILEKYHHVVTVVGNGWEAVEAVKEKKFDVILM
DVQMPIMGGFEATGKIRDYERGMGTHRTPIIALTAHAMMGDREKCIQAQM
DEYLSKPLQQNHLIQTILKCATLGGPLLEKNRERELAIQADAKSRNSKTG
EQGLLRPGLENRSMTTREPVANKEGESPSIMSTEEDGPWARQRRDLSDMR
SISG
>Mb|QLI64068.1
MAPPHRPRITILGSLNIDLVSYVPHHPLPGETLTSTHFDTSPGGKGANQA
VACAKLSRASDLSSPSADVAMLGAVGADQYGTTLLDSLAEYGVDTSGVAV
RKAAKTGIAIIVVDEPSGQNRIILSPESNHTLLPRHFEELPAPKPDLLIM
QLEVPFDTVMQALRAAKAEGIPVLLNPAPAQTLPASAYDGLAHLIVNETE
ASILSGRPESELEDEAGLDGVANIFLERRVSNVVITLGGRGVYYATQSGN
KGLVPALKANVVDTTAAGDTFVGSYALSIVNAGEGELDIDAAVKAANRAS
AITVSRKGAQISIPWKDELQ
>Mb|QLI64071.1
MPPKAKAVTKPSNPEPKDTPGPGPETLNDRTSQRYYQTNPVEKRIEEAGF
TGLTPAEKKVYSYTKLIQPIADHKIPLSNKTEREYWKQVTKEGLPIRRLR
QGYCWGKDKTGHDIGAYQLDKFDQLLIKQAQLAALDIHHRQFLINRRKAI

ASGRDFLDEESAQEKSRRKDMATLNKDLYGEITGSLSNNLDWDDVIPIPH
SEPDDALSKIAYPDDYAEATSYLRAVMAADECSPRCLRLTEHVISMNPAH
YTVWLYRFKIIQTLNLPVPEEIEWLNQVALANLKNYQIWHHRQLLLDYYF
PSIDGDEETIRALGRTETQFINNMLKEDSKNYHVWSFRQYLVTKLGMWNI
TELAATQNLIEDDVRNNSAWAHRFFLVFSDPSVATPDLPATMHDPKIPRS
LIDREVDYAKEKIALAPQNQSSWNYLRGVLAKGGRDLSNVRDFSESFISN
LGADSEDVKSSHALDLLVDVYHQAGDISKAILCLQRLWEKWDPVREGYWK
YRASELEETKA
>Mb|QLI64072.1
MGFTDFLSDAGLTLLNNWVQTRSYIAGYSATQADVACFKALKEAPDAKKF
PNAARWYKHIATFEHEFASLSGDASKPYTVYGPDVAEVTLNPAKAPAAAD
DDDDVDLFGSDEEDDAEAERVREERLAEYRKKKEAKPKTIAKSVVTLDVK
PWDDETDMVGLESSVRGIEKDGLVWGASKLVPVGFGIKKLQINLVVEDEK
ISLDELQEEIQDFEDYVQSTDVAAMQKL
>Mb|QLI64073.1
MGGSQPFMYNAAWRNDARFPATTFDPKAVTRASWEPKPRKPPPTGPLISF
DRHPDAHMVVSHRNNPYLPLGQRMKKWIKGLRNVQFLIRVLEINAAIGSL
VLLILITNIDEATGWIMRISPGVAIAQCSYAIYHLSRNASGRTPGSSAAY
HVFAATSDLVFISLYAFGAFSTHKNAQTWATRLSDQEIMKYFVPAVYYMT
IGAGGLHFISLLISLWLGMMFRRISLMPPDMNPLEDHLTARPFHKRSKSS
ITTGSSLEGGNRLSTTISLPDGAASESCGNQRPAAVPFLHTRAGSSYSIS
SRGSQADLPNRMYQLAPGNHPRNSPYSSSEKQCSAPRPSQLGRYSSIPAS
DAQSLRMGSASSNHQNKGRRGKSSESWMPTDFFIPRTDQRNCSVTIVPTS
SRAVRDGKSYAALVPAFHEADSAESDHDDKNDVTYNSPEALVSPETHPNP
LASHPTALHKSNNFMATRSNQDRDNYPREDFTLLSEVSSNLRKVNNSQDI
TDREPEVGRPWKRQRDSSIQLDDGFYSKPYGQLRSSTPPIFIGSDRKLSS
GVDYGLRYSGGMSGRRNVSGKVTEEGRGFR
>Mb|QLI64074.1
MEVDTIDEPITKLRENGALAVGKPRMELMPRHEREKLRRIREAHKAYGRG
KKINTKNIKDKKLRSNMKRLEDKYQDAAIKAKDAEILLENTAGFLEAEGE
MERTFKVRQEDIVSDIGVATAQKRFDLKLDALGPYICDYTRNGRELLLAG
RKGHVATMDWREGKLGCELQLGETIRDIKWLHNNQYFAVAQKKHVYIYDR
NGVELHNLRKHNEVTHMEFLPYHFLLATINTGGVIKYQDTSTGQLVAEMP
SRLGQPVSMAHNPYNAVLHVGHQNGTVTLWSPNAQDPLVKLLAHRGPVRS
LAMDREGRYMVSTGQDLKMAVWDIRMFKEVNNYYTRQPASSVAISDTGLT
AVGWGTQTTVWKDLFNKHSAVQEKVNAPYMTWGGEGKRIERVRWCPFEDI
LGVSHDQGFSSIIIPGAGEANFDALEANPFETVKQRQESEVKGLLNKLSP
EMIALDPNFIGNLDLRSDAQRKADRDLDAPAVDIAEEIRNRARGKNGALK
KYLRKQRKKNVIDEKRMRVDEMWNEQQKLKDKKRKEVEADLGPALSRFAR
KD
>Mb|QLI64075.1
MGDSDQNKKPVAAVPAWQREASAENAIEPEPPAQQTDYHQQLQIARRFLD
EDPVKSASPAKKAEFLRSKGISEGDVEKLLGHSDVDPESSSSQNDTPESG
STQTTSVLHNSATLSAASDRPPIVTYPEFLVKPERPPPLVTKNGIFNTLY
AFAGLSTVLYATSKYLIAPMVENLTDARTELHDVTYKRLDNLVAQLEKTV
SVVPSTTSKPTSSEDSDGSDAEDPTEMFHRDIGTQTSLIDQNATPAPKES
EPTSKRHADTLTSLTKSLSILKDQYRAQSEGFEDVKTLLDLLRDDLDGMT
YGGYTEFVGGYDIYGSAKRNEPEDEIRKVRDNIRRVKGVLLSTRNFPAST
R
>Mb|QLI64076.1
MLPRAAAAFWRASTASSFIVRPNIVRRVPQSTRPLSLWPFKGSKVPHDLP
VYFPRPPPAPKSHAFRKRLGRVVFTTITLYVCFQIFATVVFDPLLDWADH
EWDNLSDKEKREMEEMAGEEDDPLLFLPFPFTTKEVKQPPYKGSDPEWLA
FLAVNKDQQVQKDIKFGLAEIIRRGVEKNPGYVRLLGGKDIKLKKLWLDI
IYPPAPPPKHYVSGIIIDDEGIFWGDRPIDSVAASHVNMAIYPKAVALTV
WTFVNSLCRQTAQDVAKALGFNTEPPQDTSWQTVAINRMKEQGALGSGGK
HAVKVPSTTDKPANFPFPTANGDLFGPSIGSDNTQLDPRIQGALHAASMT
FTKNWQPVKQPPSRGCIRVDGLVELQGKNAVMAVYVLGWYDPKQKNYMAI

QTGLKHLIQLKQRPAAG
>Mb|QLI64078.1
MEQDSKPLQADVRRNQQVDNAIRAIREKKPVPEIDFTIHTMEDGTQVSTL
ERVCKDVQAPAMTKPTEEQFFQDDTHSKPDIAFLKQHFYREGRLTEEQAL
WILRKGTEILQNEPNLLEMDAPITVCGDVHGQYYDLMKLFEVGGDPAETR
YLFLGDYVDRGYFSIECVLYLWSLKIHYPNTLWLLRGNHECRHLTDYFTF
KLECKHKYSEAVYDACMESFCSLPLAAVMNKQFLCIHGGLSPELHTLDDL
RNIDRFREPPTQGLMCDILWADPLEDFGQEKTTDFFLHNHVRGCSYFFSY
HAACAFLEKNNLLSIIRAHEAQDAGYRMYRKTRTTGFPSVMTIFSAPNYL
DVYNNKAAVLKYENNVMNIRQFNCTPHPYWLPNFMDVFTWSLPFVGEKIT
DMLIAILSTCSEEELKEETPSSTSPHSPTTSQVGDDPNSIEFKRRAIKNK
ILAIGRMSRVFQVLREESEKVTELKTVSGGRLPAGTLMLGAEGIKNAISS
FEDARKVDLQNERLPPSHEEVVKHQEEERATALQKAAQEADNDKKLHQLS
RRLSTDRKNRST
>Mb|QLI64079.1
MPSVFMQRTHRAFSTTTEDAPPPFLQKLKGDLKAAMRAKDAPRLSVLRSI
MSANLNASKTSSPIRTDVQLVALMRRIQKSAQDAAADAKTAGREDLVEKE
NVQIRILDEYLAGSGVQTLGEAELKALIQEAVDASKSAGTATKSLIGDVM
KRLSGALEGKDVDKKVVANMVKELASQ
>Mb|QLI64081.1
MSSAKATKKSAFSCEPCRRRKVKCGGEQPICQRCAARNDECIYKLNPTLS
YTQRLEGRIKELEDQVANLTKSPPSLAGSSHSSPPVSGVQDVSSQTSRHA
SDEQGISRSFRSLKIDDKGSITYHGATSFFNLPSDRTGPGPSDMPFLNPS
SSDTDLQRRERLIHNAWHQRAMENLNEVPEPFQYLLNVHWCWIQPLFNFI
YRPVFTRDMQSMGPYYSHTLLNAVISHSIRWGRSDPSTKRLLDDSYHGGL
VFGKHARSMLFEELNNGVCTIPTIQTLLLLSAQECSQGNSTQAWTYSGIA
FRLLDHLGICVDSQRFPGPVPLGDEELEIRRRIYWGCYFWDKLICLYLGR
SPSLQHTNISPSHIMFDDSAENDLWVPFGIPQTDPTWKYPPTTAHSASCF
MSMCRLAVIFNEILIHMYDPLSQNTDAEIQECLNSQEPLLHQWWDQLPVH
LKINPISLPNLAPPSHIVTLNCLYHAFKILLFRPMITGRGQRECEGPSPV
QRYLVESVTSATSIMAIFDLFCRTFTINYCVLSLAYCVYIASSIFLLQVQ
AAPDDQQAMRKLTYCIQCLQQVRQISPVIASALNNINKELTAVGITVGSS
MQQSPQAQPSLSTGSSAPLPSGTYQTSADIGRQIPIPSHPVYQPSFSHNF
GPDAMSMEPGVFEAMSTLEPLSIRVGTIPESENPPSFG
>Mb|QLI64084.1
MPVFSFYIFDRHTECIYSKSWLPAQRPSSRITTSASDDAKLIFGTVFSLR
NMVRKLGGDDDAFISYRTGQYKLHYYETPANLRLVLLTDTASPSMRNVLH
QIYINLWVEYGAYSAASFASFMALVPILSFSLLLTKCASIVVKNPLAPVE
HKGGEGVKNELFELGLDQFIRGLM
>Mb|QLI64087.1
MTGRSTPSSSSVLLPMPGHPGSSWATYRSRMASLLRHQDTKVLIAFWLFG
LINNVLYVIILSAAQDLVGSSIPKGVVLLADVMPSFFTKLIAPYFIHHVP
YRVRVLVFITLSAVGMLMVALTPPSKSVSVKMVGVVLASLSSGGGELSFL
GLSHYYGHISLVGWGSGTGAAGLVGAGLYVVLTEWWRFSVRDSLLFSACL
PAVMFVSFFFILPLDPLRKSSQQKDYETVPDQDLTEEDVEDMPQGVASSA
LLAPGPSNVHAAYSLHSLEGSSSLRNNLRRAKSLFIPYMAPLLLVYIAEY
IINQGVSPTLLFPIESSPFEEYRGFYPFYGFLYQLGVFISRSSTPFIRIH
TLYVPSVLQVGNMVLLILQSLFFFIPSVYIVFIIIFWEGLLGGAVYVNCF
AEIMENIPEEEREFSLSATTVSDSGGICVAAFVSILLEPSLCAYQLAHGR
DWCRRIEAQRS
>Mb|QLI64089.1
MALTAQQIATVKSTAPVVKQYGKTITTTFYKNMLSAHPELKNYFSLKNQQ
SGAQSAALANAVFAYAAYIDDLPKLSGAVERIAQKHASLFIKPDQYPIVG
KYLVGAFAEVLGNALTPEIAEAWIAAYGQLADIFIQREKQLYSDAGAWQG
WRPFKIAAKEVETDNIISVYLEPVDGKPLPAFRPGQYVSLQIPLPELDGL
LQNRQFSLSVAPEEGMSQYRVSVKKEAIEPYTTEDLAEGRVSGIVCNRLH
DRYQVGDVVNVSPPRGEFFFDDAAVSPTAPVVLLSAGVGATPLMSILDSI
MKSSNAERPVTWAHAARHAHSVCFGKHIRDLAASARNVRSALFVKNIKDV

DEEGRHYDFAGSMDIDKLAEEGALYLDDKTAEYYLCGPEEWMIKMRDELT
SKGVHVDKVHLEVFRTGDV
>Mb|QLI64092.1
MPLLELAARFMRPLLAWIEATPVHVLLVCFILLILLGLACLFVLLHLVAP
KPRPAIPSEKTYITSSPSGKPTTARQLPCWYDRWLAERRLQEQKHVRPQE
AYPAPDAGSIEPAELRLSVVFPAYNEEDRILPTLEEAVEYLDKHIGRSTT
AKGPFSPSSRRHQRGHSNAAKSNTELGGYEILIVNDGSKDKTVEVALDFA
HKHDLHDILRVVTLVKNRGKGGGVTHGLRHVRGEYVLFADADGASRFSDV
SKLIEGCEEVIDGSFRGVAIGSRAHLVGSEAVVKRSALRNFLMRSFHLVL
TILTPPATSRIRDTQCGFKLFSRESLPHIVPYMHAEGWIFDIEMLMLAES
APATPVLGSDGGVIGTSPGIKVAEVPIEWHEVGGSKLNVIQDSIKMAVGL
AVLRASWMMGVYRRRLT
>Mb|QLI64096.1
MAYPTPYTYIQCPCSDQSAADRGNALASEDLDDDERTFDPRAPRSNYSLY
PLEYLLYCEDCQQIRCPRCVTEETVTYYCPNCLFEVPSSNLRSEGNRCTR
SCYQCPICIGPLQVSAIQPMSDDNLLSPEGQSSSNGGPYALFCQYCNWSS
TDVGIKFDRPSGIHSQLGKLNNGGTPKITIRDVKERRKENPDEPPLPDDE
VDRDLQFASLRTFYQEQLANSNPNLGGMTLQDSIGFSSPAALSRIMSLYT
GRGHPGRMQQGPADIMREALNSEEGLKSAQLDESSMIKKLTNDGWQATTS
SEQRAHQVSAGRFQDELRPIPYLLRTKRSKRCPACRHIISKPESKVASTR
FKIRLVAKSYIPTITLRPMHPTAAPVPVTSRPAVNDEEPLTPLKPYQYIL
TFKNPLFESIKVTLATSNTTPGRFASKVTVLCPEFEVDANTDMWDDALKD
DGRDKGRKRDEGAGQGETAKIWERGRNWVSIILEVVPASLRPEHQTLLGK
DKDDVDDSPLKEDEDILEIPMFIRMEWEADAQHDMVSAHGKDKEAKEKRE
LAYWCVLGVGRISHE
>Mb|QLI64097.1
MASSTTPPTPFSVTGKTAIITGAGSGINHAFAELLLSRGCNVVIADLALR
PEAQQLVSQHGDKAKRPRAVFAKTDVTSWPDLNRMFEVAAAEFGGFDIVC
PGAGVYEPHWSNFWHPPGSPESRDAVDAGRYALLDINLTHPIRTTQLALQ
YWIHPPAGAPRSAPAPAPAPAPASVDNPKRVVLISSVAAQIPTFRAPLYG
ASKHAITGFTRCLADLEPRLGVRVTAVAPGLVKTPIWTEHPEKLVNLDPE
KDGWIAPEQVARVMVEAMEKETIAGGTVLEIGRNKTRRVQVYNDAGPDFS
PGGGIAASRSDEGNNMVWEWLGNESIWAVHDWGNE
>Mb|QLI64098.1
MALTKQVSPRNPNDFPTLQLFLLAVVRLAEPIALTSIFPYAWALVKKFQI
GNEQDASFYSGLLISSFSFAEALMGMYWGGLSDRIGRKPVLMIGCLGTMF
SMVMVGFASNIWIALLGRGIGGLLNGNIGVIQTMVGELVTKPEHEPRAFA
VMPFVWSIGTIIGPCIGGTFADPHESWPNAFPRGSMFERYPYLLPNLLCA
ALLLVSIALGFVLLEETHPDMQPRVLLPADTYVSEETPLIETSDAMKRPA
VDLRAETYGTLRTVNEDDEYDRGREVYTQEKSGLTKVWNKRVVGFIIALS
IFTYHSMTYDHLMPIFFEDDRIVNKNTSTLWAIFSSSGGLGLSLRDVGMI
MAVNGCIALFVQAVIFPIAAERVGVYKLFLLVTVLHPVVYAVVPFLLLVP
ESLTFPSIYICLAIRNIFSITLYPLLLILIKEATPTSSALGKVNGLAASA
GAACRMIAPPVAGFLYTYGSKINCTALAWYGSVVVAIVGSVQCFLVPRER
NLEISEECASRPPCNEYNALTAEVSDEEQERE
>Mb|QLI64099.1
MPPTESSPLLPAEPRTEAPFPRAYDSKSLETRSTSSHSSGGSGSPRFDYG
DVLRDVIIGFSDGVTVPFALTAGLSSLGSTKLVIMGGLAELFSGMISMGL
GAYLAAVTERDTYEAEQEERAACPPPEQRAETHAILERYGVSRAAAGPIV
DELCADERRWLRFKMDFDLRAERPAAHRAWVSGVTMGLSYFVGGLIPMVP
YFLLDNIRDALVVSVAVTVVILLGFGYVKNYVAIRNHRAGAWGALQTLVI
GVLAAGTSYLIVKSLDRGET
>Mb|QLI64103.1
MTASMLSVLSGGLPSNVACLAIAPLPRPFGRTRAFLVSRGRLVVLIEDTW
INNQAYSVDLSELEDCSIAVDATYYLSQLLDTSPAHEPLLSALGGLTGIQ
THINENLDLWNKNRVLPFFIFDGQSITGQDEVGLKRRRAANKKTDEAWDL
YSRSEAEKAVSTFGANAGAYIIQNLYPLLQGILKKRGLHFLVPPYNACAQ
LAYFEMIDSEQCAGVMGPQELLLYPVRDVIIRSLDFEANKVTAISKKKIM

RILSVTEPMFIDALLMTGTSFLPTFPPLLDTSMYTNQFTIMDAVNMLRTS
DKAVASACASFNDILQAQDANWLDKYRKARLAVHHFIYIAENGEVVVNDF
ERLTKDNHEYLGLQLPEELFHYLNTGLIGARNLNSITHGQIVVQPTLDGV
VSDEYKKLITTKLVPIKEQALGLIIPRVHRGIGHKDITLRVWFDPKFSHT
INHRAQQPPPSQQVGSWDVKESDIRSVFPAGFAGPIYLEVLALANSEFVA
KTIAKESKIRGIDSLEMVTSVSIWRFLHLRGYVSDSHTLTKWGNALATTL
LALRDAAENRPEEVPGLDEAALLAFELVRHGILGAKHQENIAGLPRRGTD
EDKTSLVLISQCATLLKLRHQVYGYTGPLNKSLLAFRSQSSAVREVDRDL
IEAIVASMFIYGQSRRDREDYLEISQRLPFLQEPDIGLGIAVRTFFDEDD
ASHSKEKRNERLEEFPKTFVPFAEALTDDFRICVDFFNALNQGMQTLGTD
ELPAADRTAWSNAQAYLDARPF
>Mb|QLI64104.1
MDRARRTAATTRRKTLGDSTSPQSTPPESISDHPSASPVMAHASAANSPQ
PATQSRRGSKSLHHTFTFSTPASGNRGQGVKRRSRTMDSYGIHDDEFDDD
SPRKGGHTLRKRARVDYTFEHVDDEVVVPSSTSSARAKKRKSDHSYDSEE
FYASGAKRRGASLGADASGNVRRNPARSSALGRADHGDDEDVKDTIEVGV
SFSDMDESEVRRDSHSSTSSAAQSLDGSWKSAPTVSNFGVLYPNDKSTNN
DERPNAETRDMDIDPNLRASPPPSSSKQPTATVEPIEAQSESTDTPKVNE
PANVSSNKAEEKPELSDTKIAEIPNAQTLPVATQDLPAIAQAETVAESEP
LVEEQTEDNVAPTSDVVVSDEMKDRTSENNDLVSSELPKESEVEQFVKEP
VVTEPSPLKSQPTTAEPIDISPAKQSPSVGAVDNVASEEATTLDANEDSN
TKGSPPAETPLVVPSISVEEVMDPKMEDLKDQEAPHQERKSSPESIVTEV
KDKVVEDSDTLSPEKKEIFKPKRTSQLGPSKPQPAPVGRWAYLTPYMDGE
YVSYPEKKARSDDEAGGDETTPDDKDGDKDGNDMEPMIEDNDDAPDPSGI
EAPTPALNTPTRGSPVPDSMDPTATNSPAPAGDDADDGDESDSQEVPERK
RYYKYRKTRDPEEYISAIENYEDMTTAELYEILEAINISLVQWQTEWLDL
GRLVDDHENSQRRRLADAKYESRTRNPTQHGVNHEEPDFAVKGYKAKERE
LMSETRYLQGQDRIMAATYGFEYDPHPSKIGRQNPETQQVGIMTRGRSLR
NQPRQTVKASEADEVTGKRQRKPVQLFDPATQDVSRSSTPVPTRGRRRRN
AGNADEGAQVAFTSSFNGDGNSEAENEAPKTRRRRGPRASNVGPGTAEEF
FTPRSSDNLAQEETGRSGRRRGRQPVRYDETYSEFMDEDSQPEPKQRKRH
MLTLKIPRSKNFSEPSSAITDNGDSRPSTASSESSSHTAESSYSFRPKRQ
KRFRDDPDETDEAAQAPPRKRGKRSVPQPFGGIESSFALDQPVQDAQPGA
GVRKVAKIKVVRPAGDARNGTPSSQPGDDKPKDYKNMTKSEKMSASMKSR
WANGNMAGAVEKRKATLAAKKAAQAAAEQKLGVIAPKPKVKPSKKEPPPP
GQSQGGYVPAMPGINYPFPSNQ
>Mb|QLI64105.1
MADVKDPSPAEEAAARAKEADEQAALPYKWQQTIAELNVTFTVPGNMKSK
DLIIDIKKQSLTAGIKGQEPVIKGDLPHAIHVDDSTWTLSTNADGTKTVE
IQLDKVNKMEWWAHVITSAPTIDVTKIQPDSSKLSDLDGETRGMVEKMMY
DQRQKEKGLPSSDEQKKMDILKKFQDQHPEMDFSKAKIQ
>Mb|QLI64107.1
MSALTIPRIATRLGPRTWTCSACQGQLAVANSHPFGGLRRFASARGSSQG
ANGNGRRGSRSRRVLLFASASAAGVASTALAFTDEIRNSYESMERTGRVV
AALAICINDYRTTLNAKATIQDEEEQTKVLQACHKRCAERTFKVLEKNGG
IFIKLGQHLSAMNYLLPPEWTTTFVPLQDKCPVSSFASIEDMFRKDTNGE
LWDIFSEFSSEPIGAASLAQVHLATLKESGRKVAVKVQHPDLEAFAPLDL
ALTKYTFSTLKRFFPEYDLEWLSSEMEVSLPKELDFQEEADNAMRMKAHF
AKMPELPLVIPEVIWAKKRIIVMACESGARPDDLEYLDKNGIDRDEVSAT
LARIFNEMIFGDGAPLHCDPHGGNIAIRKNNARRGIGRGPNFDVILYDHG
LYRDIPLPLRRSYAKMWLAVIDGDMDRMKKYSHEVAGIGDQDFPLFASAI
TGRDFSVVSNQGSILQTRSADEEQNMSSSLQEGLIVDLVQMLSRVPRIIL
LILKTNDLTRSLDESLQTRQGPIRSFLILARYCTRTVFHEQLEQIRNKGS
LLWPPNGLRVFAAWLGYLRVEVKLEAFELWLGVKRVLGLKVDFAGGSQL
>Mb|QLI64108.1
MPADRLLNTVLQYYQDVHDAAKTDQIIGSTTHLLTQLSNPLNLGVLTSQL
LTAPAIWHRNDGAKTAIRIISVYHTAAVRVRDNQLEGSKPEGRSDGGGLR
CDEWTRAVVKGADERSRRWQHLLVLTGVLMGMEGYDRHSLSRALRNTLEQ

AIVMAANLALEENVQDGPAAAVSVVTALNFAFPILSEFHRAQINCDALLP
IAVWAITGEEGFCDAQFLRTVSQDTTLQPGHVFSWPERSSSCQLLRQMEK
QPLMANMGPLSKLAAFAVQHATDTTVVMQAHDALLVFTTRVFDAWQKNRL
SEIDASFESSYLSPETAQTTWPLLWQVFRKLMFGTTAVLQAIVARSLLDP
HMLVPGLAASIASRSLQILRNLFFISSRNGNSSFQVYTFSYMTSIDVLSR
DGLATEAFLREIRPADAILNPSANLQMNLDLFYLNVAEHLPLSLPTEACE
LLIVKPAIAYLSQEGPMSASKTEIFESAHSAILSVLSCPQHSALTIKITP
FYIVKVFDSFPQHISPRQFRVAFKTVMEIVSPPFPIAAMEPHMSETLLDM
LRTNTKTASTTLLPASPDAVSRALEETREEPLSEQSALVMTLVDSLPFLP
LPLVEEWLAITAQAMNEIEDPRLRLPVKKRFWDVLVNGEMDVERSTIGVA
WWGTKGGRELVLFGGAPEPPLMSGALGKDTTSRL
>Mb|QLI64110.1
MAEHSPATSGPAPLRSALKSDGDGDRTPPASGTLKVTAHALNLHYDLVAL
FAHSLREKYQYHICRSQAYKRIAVQIAEPVPDFDDESQSIKTFRAGPSRR
LSGKPASGKSSISEGSPALRTDDDSAPSSSHHHSQRHHQYYAEKLLAQVA
DWLEHEKRKKNAAAKPKSRRKSKSPPDQDRSTPTTAARERSDSVDSQSSD
VSFEKLEHILQDSLASLGLSSIPQHPPKLSRRRRANSKPSLNRTASSDTD
YVDGDAIVPSCDVWLDNSKTMSYTGGAANESDSTSHQAEENDPWLIFKND
IIRIAHTLRLKGWRRIPLNSGDTITVERLSGALTNAVYVVTPPMDLPEIE
GKKNPPKLLLRVYGPQVEHLIDRENELKVLQRLARKKIGPRLLGTFQNGR
FEQFFNSITLTPVHLREPDTSKQIAKRMRELHDGIELLPLERDGGPGVWK
NWDQWVENVAKIMAYLDKQLETTSIPPAKSDSVVHAWKANGFVCGAPWEQ
FLAMVVKYRTHLVNCYKPKKIIKERLVFAHNDTQYGNILRIKPDDEKSPL
LQPANKHKQLVVIDFEYAGANLPGLEFANHFTEWTYNYHDPVTSHACNHE
RYPTPEEQRRFIKSYVDHRSQFAAAGSTPRVKPDSGPPTPSLNPTASSSS
IVDFMLDARVPPGGWGAAERAREEQSDLHVRQLLEETRLWRPACSVFWIA
WGIVQAKVPGLDANNEPVSEEEAAKAEQELGPDEFDYLSYAQNRVFFFWG
DCVQMGLVKPEELPEKLRSKLKLIDY
>Mb|QLI64112.1
MRSITSLETMRASALVIMATRSQDQDLVAGVASMHGVKGRAGLRGRGGGC
QSDGHGDAEAFSVSAGEEPRPAVQRSCQVIPSFGWHPWFSHQLYDDVAAE
PTYKPPPQLPAADETDETLLLATKKVHYQAVLKPSPQDDGFIASLPTPIL
LSSFISSTRTQLEAYPLALVGEIGLDRAFRLPQTWDPALAESRDGALTPG
GREGRLLSPYRVRAPHQEAIFRAQLRLASETGRPVSIHGVQAHGILYDIV
SHCWKGHEKHIPSRREKRMIAPGAEDDSDEEDADTVTGKPYPPRICLHSY
SGPVEGLKQWFNPTNPATIYVSLSTAVNFGTISSRNKFPDIVRAVPDDRI
LVESDLDSAGEKMDAALEDMYRRVCEVKGWTLEDGILRVAKNLEAFIFGK
EP
>Mb|QLI64113.1
MSPAASRCSSTTATITLIQPFQPDLHERAWASIPHPTLPLIATAHAKSVT
VFSLSTLSCHSNLTGGHTRSVRSVAWKPGLPPHKLCLVSGSFDATAGLWR
WDGDSTAATAGAPPDENEVEITASGRKVGSADSDETSHIGDKDWEFTLVL
EGHDSEIKGCAFAPSGAYLATCSRDKSVWIWEDIGASETDDEWETVAVLN
EHEGDVKAVAWCPDVPGRNSRRQYSSDVLASASYDNTVRIWREDGDSEWV
CVAVLEGHEGTVWGIEWEPRPKNDRFPRLLSYSADGTVRLWTLQQDPEGQ
EDNDGFGAGSRSSLGGIPNTMRRSLREEWTCAAVLPTAHDRDVYSVTWSK
DSGLVATTGRDGKVALYKEADTPSVSVTASEGQHDNTACIDGSVMPSVSN
AQLSTMAPSPTTWELLTTIPNSHGPFEVNHITWCRRYDAGSEKRGEEEML
VTTGDDGIVRSWQVRVDDAPRTGGPEQGI
>Mb|QLI64115.1
MTGNGLLGGHEKKHKVTIVGSGNWGSTIAKIVAENTKQHNDVFEEQVQMW
VYEEEVVVTSDSKHYDPSMGDKPQKLTSIINKYHENVKYLPGITLPSNIV
ANPSLVDAVKDSSILIFNLPHQFIAKVCDQLRGRILPFARGISCIKGVNV
TDDGVSLFSEWIGDGLNIYVGALSGANIASEIAAEKWSETTIAYDPPPMD
NSRNPSPRSGSPNPGITVTDLQDVSHRDCRGRASKTKLTGVPAEYPPLDH
DCFRTLFHRPYFHVQMVSDVAGVSLGGALKNIVALAAGFVDGRGWGDNAK
AAIMRIGLMEMVKFGKEFFGETVQSQTFTETSAGVADLITSSSGGRNFRC
AKKAVEKGITVDEVEQQDLNGQKLQGTTTAYEVNSFLKARGLEKEYPLFT

AVNDILLNKRQVDDIPNLVAQVTDGA
>Mb|QLI64118.1
MLLLHQVGSVKIGEVVRYTVTYTPSQDRILPSPEKLYVRIRNTSTIALRA
AFVHGPYTLSVSAYPSNYNPNEKYPNHRRYGVPEFEPMLKAGGSWECELV
VPDDIRQAAGAGRHGGFGKSCEHDDECASWIIEVASQVIFSTSAAVGFEV
VVARDYKSLNLSQTLPIVGNSSAVPQPGRVCDHQQSIGAKDGHHPAQPKG
VFSRAVHLKVEDTATLWNTPRLPGWDDVCEKRVEASGADKPVQSTVKTGD
SGTNKCQSCSIGERKKKKVHLVILTHGLHSNLGADMLYIKESIDVAAKQA
RIDAKARRAKEKAAKKEVTTESKTEDTKKGEDEGDKGDDDEDEDEDDEEV
IVRGYPGNATKTEKGIKYLGKRLSRYVLSMTYPDQPFLPIGKGAVEGLAE
AMGGGHSHQHQKSPNSKPAHKHSTIQADAAKTKHDSQCRHYKITSISFVG
HSLGGLIQTYAIAYIQKHSPDFFDLIQPINFISLASPFLGLSNENPLYVK
FALDFGLVGRTGQDLGLTWRAPTIARSGWGALVSNLGESAHKKVYGESQA
ESKPLLRILPTGPAHVALKKFRNRTVYSNVVNDGIVPLRTSCLLFLDWQG
LGRVEKARRDAGLVETVVGFGWAELTGTNILSPRQRQRLPSDEQVQQSDS
GTSTPRGSMRQVPQPSDDAMNEDDRASIRSVPRPFNGDDDVPSDTGGDAA
HLSPLAGFFNLFKSNEPPKPQVTSAKQKKILRRGQTMPTNDSDTTNSSIT
DISDRKPGKATTGHEMEEDVDGMTAPPKTTFFESAGDLLNPSLPTTEYLL
DPSKRPRTIFHDRVYHPSDIPPPPMKKRQSTLVRRRTSGKMSATSHQQSD
SSSPYPSINHADSMESAKDYDDTVHTNPDKHPAEVVDGSSMRVEEKIARG
YHRDLSWRKVLVKLEPDAHNNIIVRRMFANAHGWPVVKHLVDAHFSDSEA
ARMRDDEEPNIERAFASHRSPDADGREVKRLGQLGQDGAGSPDSSEEPGG
GETNEAREAREALDEVPELRKKPDGDVSSLPPRLGSSDRADSMTWSERDW
VDSENESEIETHEDGKVGKQRHQPTQPTKDGTQGRLSPSSWKWAEKIVGK
GSTRSRSKSPKPGEDK
>Mb|QLI64119.1
MVHSKPEEPTGSTAETPAMGAEEPKLGHPSSPSNAHERTSTTADLKGKAD
ESDEEEWWPRIKTGTEEPKQTAKSDETDEAGETEETEAETPKMTHDSMVT
VRLSEPPSLSLDTTAVTPGAEPVTPTSPIEEETSTDADNDSLEKKEHTET
RDSLMRIPRVSAPIQSPTDANRTLQDELGLYGVDGNETDSSEDNEEVNWE
QLEKTEDEQMKDEETDNSTALLLARLEQENAKLATNPKSVKVQTVEKLSS
TKPRPPSMAQLRKMVSGPTPAALRYSMLPPPPMTDLEFYAALVKDYQQTA
ARLPTLLSNKIRKGIPPPLRGVVWQSMSGARDSVLEEQYERFCGETSPYE
LLIGKDLGRSFPGVDMFRDPDGDGQRMLGRVLKSFSLYDTKIGYCQGLAF
LVGPLLMHMPDKQAFCVLVRLMEKYDLRECFLPDLSGLHVRIYQFRELLR
LNMPALADHLDELQVETAYVSQWFLSFFAVTCPLPMLFRIYDVIFAEGAS
ETLMRVALSLMRKNETRLLACTELEDVMQLLLSRGLWDCYHYNADEFVQD
FASLSGVVTRERLSQLAQAYRESLIATANAARMSDITTAASRFLGRIWAA
SSKNGSLAPALIAGPARPLSMLRRSTSKQSIASTLNSMEASSISVVSTAS
TDASTISRDSANGDDEPSSRESTPVGPKTMANTKTTEERYLHSQIEDLLT
ALSELQRNHALLSNQLQSEREEREEDRRTVRSLLDGLRKRAVNHDEAKGQ
EEIIPQLGTEEEEPPIIAMEDDDATPTIEDPVLEPKSTAEDGDSEDAPST
EDMKELLDRVESRFATDDGNRKHRSSMLQSKPQLREELLNTKGQLASAVS
HSQELSRRIHEMDQELSSLREQLRERHGHVRTLHQDKQRLEKQIHGMRAR
ISSSSVSEPNNRDADPDWVGKGKNGGLREFKLGRSKSTPQAPGYCKRISS
LPQNPESVIAAGSGVATPPNEHEALLLELVQAKTAEATAKQEAEEAKQKL
EALRKAHVVPSSESHASSPGSASLGVFGRLTGQATTPPAENNLKPTAATA
GTSPNTGGFWGWRR
>Mb|QLI64120.1
MDDGNRLPAGQPPSWYKVHLFRGMVNDVRRRAPYYGSDWLDAWDYRVVPA
TVYMYFANILPALAFSLDMFSKTGSNYGVNEVLLASVLGSVVFAVFAAQP
LVIVGVTVGLHDTGFGASGKLTNTPSTVYDIMKDTGVDYIGFMAWIGIWS
LILHVLLAVTNSCNWLRWVTRFPCDIFGFYVAFIYLQKGIQVLDRLGDGA
PFYLSIMAALLVFMVAYVCGELGTSSLFKQPVRVFLKDYGTPLTLIFFTG
FVYIGRMARVELERLPTGLAFEPTSGRPWLVRFWHLSVGEVFTALPFAIL
LTVLFWFDHNVSSLIAQGSEFPLRKPAGFHWDIFLLGITTGVAGILGLPF
PNGLIPQAPFHTESLCVSKPVKRLDEKGQDKGDFTLETTHVVEQRLSNLA
QGLLTLGTMTGPLLVVLHLIPHGVLAGLFFVMGVQALEANGITAKLVFLA

RDTNLTPPDAPLRSIKRRSAVWLFVAIELLGFGATFAITQTIAAVGFPVF
IFLLIPLRALVLPRLFRPDELDVLDAPTASDFTMEGIGGSWGGAAGPPPP
PADRSGDGTAVSSRSSGSRGPGDRVFASPGAARRSREDLAELGRLPSARS
TATTSMSVQR
>Mb|QLI64122.1
MPRPQSPSRSSTASGPGGGSAKTATKRLLKEMDTWRKEQSDEKGIERLGP
ISDENLLEWESVINGQGVGAGYDGGRWLVSISVPPTYPLQAPKMRFVTPI
VHPNIALQTGEICLDLLKDAWTPAYSVLECVRAVRMLLSCPETDSPLNVD
VAALLRGGDVVGTRRLVECWCADDGGRYDGP
>Mb|QLI64123.1
MSSEDYYVILGIERTASEAEIKKAYRKTALQNHPDKNPGNAEAEAKFKLA
TEAYEVLTDSQKRSIYDQYGKEGLNGAGGGSGMPAEDLFAQFFGGGGFGG
MGGMFGGGSSRGPPKARTIHHTHKVSLEDIYRGKVSKLALQRSIICPKCE
GLGGKKGAVSKCAGCDGHGMKTMMRQMGPMIQRFQTVCPDCNGEGEIIKE
KDRCKQCNGKKTIVDRKVLHVHVDKGVRSGTKVEFRGEGDQSPGVQAGDV
VFEIEQKPHARFTRKDDDLLYKCDIELVTALAGGTIYIEHLDDRWLAIDI
LPGEAIAPESIKMVRGQGMPSHRHHNFGNLFIHFNVKFPEKNWTQDQSAF
AALQKILPQPSVQNIPPTDAMTEPADLEDMDGQSQNRVFGSPGMDEDEDD
GHPGAERVQCASQ
>Mb|QLI64125.1
MSAVCEKTDPLQGVFGGLPGAKFDDIVELWHGHRTPIIICALVVLVVIRI
YIHKLEEAEPLRALPKQFEKRIEPLSANKLPSAVNSEKPVAKPNGPRRIK
GGITKKPATEETKEYDKSRPIKMMVFFSSVTTKTEKIAQQYLSTLEKSVG
QLSRGTERQFLKPELHDLAEIDFEDFFVSPAKTVEDAHLFYLFLIPSYNI
DTINDTLLQHLEETHHDFRIDTAPLATLLGYSVFGFGDKEGWPDEADGYC
FQAKELDRWMSKLTGRKRAYPVGMGDMKSDHAERFDEWSSGVQEVIAHVA
SSGTLGEGAPGSGDAMESDAEDVAEDDDGEVLIGEDISGDDRESSRPLDD
VEDLGRMLRTSDGKGTANGKKAPLAVDFTSYGASSAKKRTPTAPVIKEMV
AKNSPTYAALTKQGYSIVGSHSGVKICRWTKSALRGRGSCYKYSLYGINS
HQCMETTPSLSCSNKCVFCWRHGTNPVGTTWRWVVDPPEMIFEGVKENHY
KKIKMMRGVPGVRAERYAEALKIRHCALSLVGEPIFYPHINEFLAMLHAE
RISSFLVCNAQHPDQLAALKAVTQLYVSIDASNKDSLRKIDRPLHRDFWE
RFQRCLDILREKRFRHRTVFRLTLVKGFNVEDEAEGYAQLVERGLPCFVE
VKGVTYCGTSTSSNAGLSMSNVPFYWEVCEFVKALEGRLKAKGLKYGIAA
EHAHSCCILLASERFLVNGKWHTHIDYKRFFELLEERGADGDWTPEDYMG
EPTPEWALWGNGGFDPRDDRVDRKGRKIENIVVREAPPPDSW
>Mb|QLI64126.1
MARKFFVGGNFKMNGTVSSIKEIVKNLNEATLDAEVEVVVSPPALYLQLV
RDSIRSGIEVAAQNVFDKPCGAFTGEISVSQLKDSKINWVILGHSERRTI
LKESDEVVASKTKYATENGVGVIWCCGESLEEREAGKTIEVVSKQLEALK
AQINDWSKIVIAYEPIWAIGTGKVATTEQAQAVHKAIRDWLKNVSDKVAE
ETRILYGGSVNEKNCAELGKQPDIDGFLVGGASLKPAFIDIINCKRQ
>Mb|QLI64127.1
MGDRIPPPMPGHLPSSFDDNEEFFNERPMQKVVRKLKEEPLVPLGVGLTV
FAFVNAYRALRRGDSRQANKMFRARVAAQGFTVIAMVAGSMYYSQDRQKT
KELRKLKEERDAEEKRQKWIKELEARDEEEKMIKASIDKRRQKLEAADTA
ANSGEEGAGTGGILGKMGLWSKGQEAAEAEQGAKEETKKIENPKSSLGAI
SEVLVKQKGDEGKK
>Mb|QLI64130.1
MATRPPTLTEILLDVSPPPWTLSAFMAYLSQNHCMETLEFTLDSQRYAAF
YDQLAAENPPSQESNARVCGLWDKLMEVYIVPCAPREVNIPARVRDRLLS
LPSGPSAPHPAELEEAGRIIYELMNDSLLVPFLQSVAPLHFMGQNDDSSH
HHHQQRRRSQNTAQHPDQPRRSVASPHSADFDSLTDDSDSNSPSGPEPMT
PPTTPPTSEYTFNSSPGGFQRAMAAHNKGWKKVGEKLGFSRKASSRRSTP
TSSSSDYDAALGEPSHGHP
>Mb|QLI64133.1
MLEPHAISRPMDQTNAYGHSRSRTTSSNIFPIQQQPPQQQPASHYPQAMP
THMQNTRRSPSVNTFSTTSSMQPPAAYRTSSSTDLRRSISSRSGGTTAPP

SSYVALLRRQKATVWCDRAQYEDPRVAAQQKAAKMRANLEVVGGGRGTVA
ASRTNTGISATGKVAAKIRHHGKTPVVGYAPGDTNHVGVGGVPLRLSATE
VEGESSDDDDTQAASRAHHRRTGSSGRSSIGSGRRGLQYRASGSSVTVGG
QRRSPGGTPERTGSLIERPERTMDDSASGRAQSSRSASSGERADNIGELS
SNPKLAINSQRLSVLGREKSVKSADELKRRGSVDERTATLTSGRLFIANP
D
>Mb|QLI64134.1
MECPIGFDDCYVVGEPKSADEHVSTSTITRKCTLLCDEEEPMTIYSIVHT
KTVTFYGNRTDYTPPYSTIKTPVYCLEFEESPASSTAEGDFPEVTQVAAG
TTTDFFRSLTPFIRPKVTFITTDKNPSVVFPSDPPPNYSATWSDPYPNPI
IHKSVPPEGDQGDQGDQGDQGDQGSLGRQGGLGRTPEGNNQPRPTFNVTA
KGNEVIINSEIYSSLKPDQTTTVSVDGGKFTIYPTAIVGLGGTVAKPPPS
PTKFGVPSPTNGTVGGLDVSLSGSQVAIGGVTMTMPPHGTTTNIQGQSVT
ISANQIIVGTRSFKFNPVQAQPESEVVVAGGEMLTVAGKSVVVVHSKTIT
YGPDTPITTEVVEGDTVTIGPSGVRVHGLVLGGFQANETDTKYEIIGGAT
ITKIAPSVAIINGDTFTVGPGSHLTTTVIAGQEFTLGPMGVIVSTITLSY
PFGSTVVTTIAPTGTWLNTLPIETSRSPKDDDDDSMGPSIRPSLSCAGVA
LCIAIGVLVFA
>Mb|QLI64136.1
MSALVGYLVNFLVGYVAVTLFFYMASLAVPKAGFVARALAAYISLLICAV
FGVLASVFFTLIGKQGISQWATARAFHYVMRLTTGIVFQVEDPDKILATT
RPAVFIGNHQTELDVLMLGAMFPKWCSITAKSDLKHVPFLGWFMRLSGSI
FIDRKNSKDAREAMAGAAKEIQSRQQSVYMFPEGTRSYAKDPVLLPFKKG
AFHLAVQAGVPIVPVVVANYSHVLHIKSMVFRAGTVPIKVLNPIPTKGLT
SADVDELARTTRELMLTELLALTAKAQGRPVAVPASVENSSAASSAVHVN
SE
>Mb|QLI64137.1
MTSWYSNILTRTTSQISSLRSTLLASENDGDTDDDTHVCRVLRNYYGDKG
RPLPSWLPPDDPKAPPAPAAQQPLYVQPQVGARYGGLQTGGAGGAGASGGF
SSLWDSAPQQQQQQKQGRAPAAREDVVARPVASQGAAGAGGGGGSAQDRL
KQRLWGAGRQSPGQAGGQFQPPGASYEDRFAPGSSYGGQSGGGPGGQGRG
RQGLPSGPRGYR
>Mb|QLI64138.1
MSGGHKPIRLPPLKTLRVHNPKRQPENPCIAIMSSVLACWASAGYNASGC
AAIENQLRKCMDGPAPPPAPANTVNYHLSRMQKHVTSPRKQK
>Mb|QLI64140.1
MSSNSEIEAPTEQPPAQVSAETAAATVEAKITTPELDLPKLQALPAEQQE
LFLLSFVSDLKRHVLGLVADDCTAQQFYLKKEIFQIINLATPAPSRVIRN
SLGKCLAHIFSKGDRKLLFETINDLISVITGSKGKSDGETRSKQAAVSCL
GDVFAAAGDSAIGLHQLVCTTLIKLLKAASNNAGLRAAVLHALAKLIAMV
QGSMDESISRDIWKQGRNHAASDKGSLVVVSACRCLKSLVKHTAYFRNSA
DLDKLESAIFKSVESSSIQVRHAAADCFAEALVQGYSPTGNGEVSKSKKT
KPKLKRASTHPGIANINEDEDDLPPRSASPAPGGKRVQDLALSVPDMLKI
LSTQYVKQSASNKTRAAIGVCYGKLFRRLGERTVLAKYLQIVESLSTEIL
GHSTITNNRYRLLITRRIVDTVLYDVIGRQILGESGQISAAEALVNNILK
NYPQALAERPEPSKQMLIIALRAVASLITSLGSASNRFAESCRDGLLQVL
QHPSYSVQVYASYCMKAFVLACPQQLLPCLSVCMNSLARELSLLNGGRNS
PRRCLGFAHGLSATLSTSSARPLYGSVDINSRVLTMATNLLKSSSQSELR
ISSIQIQVAWILIGGLMSLGPNFVKIHLSQLLLLWKNALPKPLAKDNTSA
RSFLEASFLTHVRECALGSIMSFLEFNSRLLTVDVSKRIAAMLQSTTAFL
KTLPSKKTTEDISQRLTPALQLYDLDMMVQRRVLQCYIKLVNMSPAGGSE
ALLQSNLLTLAISLFADPENYIPSSLSASIANAAGTFDSIWDVGDNSGFG
ITGLVSGFDIKQLPGQHESSTGTEIEVDSPEDSINKLLKSPVCGGLEHDA
SMLYIGGVGDSSILPDPPATEVINNAIQLFAFTFPLTPAKVQESVLEQIK
TFASAGLLQRDAGRKAAINVNVSLAILSSLKVAVKETRSPAGNVSNLAVE
RLLQDIVRDIVLDQDQYVRSLGYESVARLCNIYGNAFTNQEIKYLVDTIV
GNREPSARAGCAMALGAIQTKVGGMAAGYHLKTILGILMSLCNDPHPVVH
YWALEALSLASDAAGLSFASYVPSTLGMLAQLYVSETHHAEISSAVTMNL

EMELPSVAAVSKCVDSLINVLGPDLQDANKSRDLIFTLVNQFQDEDDALV
EKAALSCLEHLSLYAPGQMRFAEYVKLLQKYLKSENATLRDVSVDGLHNI
MKREPRDVLREAEDGFEEQLWLVLDSVPSHDGVRNIIRNWMHQSCIFETN
EWLHRFQTVLKMTRAKNEARDEARPVGGVPDLQDEEVAGFAAAVPGAKES
RDNSAPSEAEPLRWQVRTFALGCITDIFAIVAKDVAANGESAAQLALQNK
IADVVRMAFSASTSNVLELRIWGLKIIGAVLKMFGRTPDPDFEEAMLLEQ
YQAQISSALTPAFAADSSPELASEAVNVCASFIAIGIVTDVDRMGRILKT
LVTALENFSSDHENAGIGELKGLSSNAQVMVKISVFSAWAELQVASTEQK
YLMDVLKPYIGTLTPLWLESLREFARLRFEPDISMTLGPPSLSGSLDTIY
AALNRETLLRFYQESWLKLVDAIASLIEQDSEFVFDALDGKESREAGPNG
DTKSPAINYRDEPVAFFFVLFGLAFEALAIRPGQADSLATREQALDILEA
LKKILHPSVSGHAIYRPDVFAETMDLLDRLVLTDGLDIQSVIVDIARALC
VTHPSARRQSEDEGDLSEDIDQLFELTRIIVLVLSGLLPNLSEGNQPVRH
QMNEEAIILVRNSLDALVDAAEVFPSIIKTDLHACIIHIFATILATPSCQ
ELIVPQSLAILKRFVASASSSTSSGDDGNLPPQAQIQGCLRRFLSIYVNA
QKREESTSLAGVRNCLLAITILFTGGENQLPANHPLVARYLDELLDCLTD
RMTAKIAANCIRSLLLQSSPTSADLSIARYLFPRLVTFVTNVEPEDPERA
RSLVSHTLCLYVGIIPKGRVPAAMGLVIPALMTRAVGEGEDMYQETSARL
LELVAIDQAGFRATVGAMNSGQRTFLEEVIRSGQQSGSEANASSAGEAGR
PSITLKMNFGG
>Mb|QLI64142.1
MAKSLIRTASLAVLGPLALYTVFWSLAVIPFFQRHFLYAHKFNTLFWSDV
NEPERWGFAKHQVAPFYLKTPDDNNLYAWHILPLPLYHKHEDQIVQGASS
ETVPLDITTTESFRLLKSDPKAKLILYFHGNAGHIAQMFRADSYHSLTDT
SSYHVVAIDYRGYGHSTGVPSEEGLIQDAETLVNWAMNVAGIPSHRIVLF
GHSLGTAVASGAAERFARRGVDFAGLVLVAGFSDLANLLTGYRISGVFPV
VGPLAAWPSAVKYLQTYVVDKWHSADRLASIVRNTKKRLRLELIHAYSDW
DIPWQHEEILFQAAANATTSGLTQTEFDQFKDKHMKLSPGGDGFSVTVKS
NPDTIIRQQLVLHGGHNEIVASSSVLRAVMRCFDEE
>Mb|QLI64145.1
MGSSQSAATSSDAGQEAKKSTSLYRKYQDKKGARPISDDDILKYTGKTRD
ELKSWADGQPGVGKNQLAGKLAVGSASGLGGVATGAGYGGWGPSAEPQGA
NRGMKFPPEHRESQPKVVDDDDD
>Mb|QLI64146.1
MSRPTPPLSRQESVESTRQIALSSFLQEKLQKERRAESEKLNQSQSSLPR
SNPDMSVSADLRRTPSSPFKTSMEGNRPQSSAGTDQGKKKGLGVKEMEKV
ISNLHKQNFDLKLELYHRRERQTTLEERVDAMESDRQRMEEVNDKLLIEL
EKRDKAVEEAVAMIVTLEAKVDQLFRERAMVHRIENEGFFCPRDYDNMGY
KTPVPHSTGPDTMRMEEDAKVVNRMPSFLSDHSETTQNLRNVYLGTQASM
LSLTRVTEGSPETDNVQALGSPTLSVLSESSFVSVYGRKDKVMDASQIAA
DVDETLVLDGADSSYTKERNGKQPSRNHCRSVSSSKTADSRASSTGHFQS
ITDVIAGSPLQRLEQLDTSYGPNRESGHHPRSQIKDYSKGQLLPDKKPFG
LGSIKQDKHDALRRVTTDGQGGVRLHDQALPPTPDTTSSSMLRRFKDSND
TLSQQDLNEENSNGVVLNHLGSQGGSQHNHQLAAVPNELPEEADSWASPS
KRAIDLLFEHRGPPIPRPRSADESTASHRRGKGWDSDDDDSDAHSLQSSL
DIWMRESAQPSRNRSRASPDLFSFPTGSSKGGWAMSSTFKQNSELAENAP
LPPGFDYMRDLFSVRQGLFAGTAPPPPNRRSSLHAQTGSTPEPATSQGAN
NQGVDQGPPSTARRRSCHSRQNSVDLGKRDDMRTPVQRDQFASPPQPNSD
QKPKHYPPIAGQQHGARTGLNRLFRRSTSAAPPGPSSEQSNPVETAVSEQ
SKSFPTIGIPSWASRHSAVDDDRSGATPPPIMLNPRQGRRHTLGAEGETE
KSPAQDLSRSMTPGPTLAAAATASAPSTDIENSPAAAAAASSSSGRRRWL
PGFARTNNLKNKAG
>Mb|QLI64149.1
MPSLPASVDLDECISRLYKKELLAESVIEAICAKTKELLMRESNVVHVRA
PVTVVGDIHGQFFDLIEIFKIGGFCPDTNYLFLGDYVDRGMFSVETISLL
VCLKLRYPNRVHLIRGNHESRGVTQSYGFYTECSRKYGNANVWHYFTDMF
DFLTLSVVIDNQIFCVHGGLSPSIHSIDQIKIIDRFREIPHEGPMADLVW
SDPDPERDEFSLSPRGAGYTFGAQVVKKFLAVNNMSHILRAHQLCQEGFQ

VLYDDRLSTVWSAPNYCYRCGNMASVLEVSDNGERFFNVFAAAPENDQHK
DIQPGNEKVADGNALPDYFL
>Mb|QLI64154.1
MSRLAPSKRWTSFEGWDYNGMKKRLEDLIATLNKKVLIDHAERIIGQKIS
VSEPFSAGQYWICFELVAEDETLVIARVRLPRHPNAAPTVSETDEQYSIA
CELSAMRFMRQRLSTVVVPRVYAYEGPGSQLATDAGAVYMLIEGFRGNTL
QDSVPDLCGLPSAKLEHVMAQWATIQTALATLTSPLIGSISGITKTGEPI
VNKLSSAASEGLMSPGPFSTAVEYFTALGEASLYRASRHESNQSMSHFHR
LGALVFLDIVQSTPFFQASPAQYPFNHMDLGTQNIIVDDDLNFLAVIDWE
FAQTAPWQVNYYPMPFPLSWSDESINTILDDPQHLAHKNVSRQTDARELY
CRKFREAEAKLKEEGMPLGNEFAKVLESAASRIYGCFSKLGDVPEQDEGL
VYEMVRLAFNFDVERANQYLGSMSDKLV
>Mb|QLI64155.1
MAAPSIPNLLSLRGSGRGGGRGGRGRRQAGPSASSTGVSPDATIQGTDTD
AAVSRLSAVELGYLEDPYARFFVTGPPTRRLPIINRGLYRGTKTAITSIR
TDTDGHTGTYMRTKSLDAMVDAFLSHNGPSLKQVISLGAGTDTRPLHMLQ
KPGAQNLIYHEVDFEPTCRRKLAITQSSPGLHRIFKDLSIHDGGSWSAEP
SKGGEYHCHALDLRSISESITELPSHIRSDVPTLLLSECCLCYLTQQDSE
NVLNFFSSKIHSLAALLYEPMPLHDAFGDMMVSNLKARHIYMPSLQTYKD
QQGQLARLRHSGFDSVGYATIENTWDKWVDEKERERVDCLEGLDEVEEWK
LLAAHYVVVWGARGATFGPLADRNQSAEIQGQK
>Mb|QLI64156.1
MTDTPAPPSTQAPLPSQTVPPAPVDTVVKDDPTIAPTVEQTEEEPYTIKC
ICNFSDDDGNTIYCETCDTWQHIDCFYPNNRDEAIREDFAHSCAECKPRP
LDRQKAIERTLRLRNANNILEAQKEPLDKKTKRPPTKGARKKPKPNDLQL
NGHLENGKLASPPPPSSKKAKSAHRPSHSVSSQPSKRSPSYGQNSRSNPA
HPPSPATTPPDLPNDFQIHHYSAGFYSLYNEQEVPDTHNNAFASLAIPTA
LSRWLRDPDTMKQEVGRTRAEVFQDEPPNVDQMRPKLEVNDTTRSVEPGT
TLRWRCIESTTAIEKDVALIELNGEIGFQKDYCANPVNLWADLSCPLPFV
FFHPSLPLYIDTRKEGSLARYVRRSCKPNAQLDTYLSGGSEYHFWLVSDR
YIPAKEQITLPWDFRLEKSVRDRWLHLLGLSDDDSAAQDEPELDSAEYTA
ISNWIDRILSEYGGCACDLENNCAFARFHRHYIFGRNQSKGGKKKSRKPR
NHTISPTSTGHATNSRAASEGNIDEGADDVKAEGGSRSKTPSRDRTPVGS
FDQLGILTEPTDRDKRKVAMVEDSFRRMEQQQPPRKKKRVSDGTTSSSKS
KSRNGSAGPPGQYVDAGTSRSKSGSPATGRSPSIGYPKASGKPCPSTSGS
RQTSAGPRAMYCDAAVQTDPVEGHWISESCNLTPIKKKIISLSKRLMNNR
RQSLITEEERKRSCLTSQSCSVSAMELDSPISEHRPSVASTGSDVTSPTS
HTTDTNLAEIPQSIVLNGTTIETSKMKVLDLRVQLPPVPAFDSTGLAAST
TATPLSAASSAALSPFGTTSASNSFAVGSINGIAAAPSPVKKKLSLSDYK
SRLNKAAGKVAGGSTLLKPGLASPEDVQVEMTMDPQPLDKTDDPTPAVNG
A
>Mb|QLI64157.1
MPVYSDAPGLRAFRDDKPTLLVCWWATSFCTLMILLRVLGRFIRTERLFI
EDKIAALAVIPLVLRMVCVHFILIYGTNNADFSGLDLTAVQLHHKSVASG
LVLLSRFFYAATLWTLKCCILEFLKRITDLTWEKHHHTALIAIRWTLLIT
FIGVVISDLTECQPFRMYWQVLPDPGGQCRQGYAQLITMATCNIFTDLLL
VIFPIPIILRSNMLVRRKVQLVLLFSLSLSVIVVTIYRVPRIIQEHGRQQ
YRSLLASVELLFATAAANALVLGSFVRDRGVKKQKFHRNSGADSFDRSSH
PRRPTLHRQWGSDEDLVRDVGLGVDAELREPLDSPDVEQFSPVGRMAPNL
DADINWQGSKRRSPTEQSDDSLLPHDPLTKVDSNKKRLSFLDVGGLLEES
VAGSSGSYRRGSYTSSTLDPNSPNAAPPPSVPASNNGVRRGSTALLQDLG
GLLGPLNTRAARAKSKPGTELQPIPQSREIQAHNPHGNPSPELKDPGGLL
LPR
>Mb|QLI64158.1
MTTPFLRTRMLGIARQARSFSCTTRQCAAEVKSLGVIGAGQMGLGIALVA
AQKAQVPVRLIDTSDAALSKGLAFADKLLAKDVSKSRLTQEQADQARALL
KPSIKMEDLSDVDFVIEAVPEIPKLKFDIFGSLAKICPKHAILATNTSSI
SITKIAAAATADPTDTSASSRVVSTHFMNPVPVQKGVEIISGLQTSKETL

ETAVEFCKKLGKVTSISADTPGFLANRILMPYINEAVICLETGVGDRESI
DAIMKNGTNVPMGPLQLADFIGLDTCLAIMRVLYNETGDSKYRPSVLLGK
MVDAGWLGKKSGKGFYDY
>Mb|QLI64159.1
MDKYIDGRFERLEKALSNLIDSVTKYHPSTIQAEELKAADNELSKGLEEV
QIHQNNHLRIQKLRQSSAALDTQIRDTLTSLANTHKDIVSTHTTTYPSEP
NYPIVYEELLRYARRISKTTLPPAATLEGAAASPETQSPNPESLAQSALT
PSARTPSQPQSPAVNGTPQLTAEQPTQPPAANLNTSLPEGMSQYLNPLSG
QLFFPWPLEDKIRSGSLASYQVLVEQDIDPKGYDPATEEERRLKEEEERR
AKEEQEKAEREAREKQLREERERARAERERQREKEQAEWRRASIVSGQPD
GPGLSRAGTALGEKKQFQFTNLDDLDDDDDDD
>Mb|QLI64161.1
MAVDCPICNKPVKSSEINSHIDSGCTSFIADKDPSPPTSQQNGTSHTPSQ
QKRSASSFFSTPAPKRIHSDGKALAPLVNGTFPPIIGKKRSFDEGPGAGN
EGSGLSAVDQKAKKNGVVNGQEEDGQVSKRAKTQRSAPLAERMRPTTLDD
VCGQDLVGPNGVLRGLIETNRVPSMILWGASGTGKTTIARCIAHMVGSRF
IELNATSTGVSECKKLFQEAANELALTGRRTIIFCDEIHRFNKAQQDVFL
KPVEAGTVTLIGATTENPSFKVANALLSRCRTFTLRSLTEQDITGILKRA
REAEEAVYPPTPLIDDEMMGYLARFSDGDARTALNLLEVALSLTTREGIT
KEDIKSSLTKTLVYDRGGDQHYDSISAFHKSVRGNDADAALYYLARMLQS
GEDPLFIARRMVVIASEDVGLADNTLLPLATAAYTATQQIGMPEARIPLA
HCAIALCLAPKSTRAYRALNSAYAALREPGVASLPVPLHLRNAPTRLMRD
MGYGAEYKYPPNYRDGRVRQTYLPGELVGRRFLEERDLGTEVDPDLVMGE
G
>Mb|QLI64162.1
MTQELERIFADLGLSQYLNAFIDQGFDAWDTILDIQESDLDALGVKLGHR
RKLQRRIANARGIAPSVSLSSSVKPGSDESKQDEVKRDSSRTVAESNGVT
KRKYRRHPKPDENSPERPPSAYVLFSNRMRETLKDHNLTFTEIAKLVGEN
WQSLQPEEKDVFESQANAAKEKYNRELTEYKKTPEYRKYSQYLHDFKERQ
AKQYKGQDGSKRAKVEPARLRHGSTSSSATPNTTNSSASGSNSERLRGSE
PPPTRRECVDSTTSLAESPHSSAAPTPMSAQNSYDDAGPSPRAMQFDNGS
PTDAHQPSRHQPTMRGRGRADSMQQHLPSLSDMLDARQKAIVHPVADGLS
YPSRPANSSTRTSLDSASLLSAGRTPTLRHESSSNGTNISGSSMGSFGRP
PGEGPLPIHALLSDRTLVGSSVHEQQPNPILNGATGPMNTQQSLVGFAHG
PSGYGFQSDSSPFHHMRVEQSSEGDVVMTTDETLAPQRQSRGTKDNLDGM
NALLRAGEIVGRGERR
>Mb|QLI64164.1
MAASPAASSRRSAASSSASLKRSDDHQPKSPTISVDTLVNHLLVAKRSLS
SMNHVLRANELATEARHSHEEMLLLAAQTAFVCNYLRDQNAILVRIRRSL
QTTYEWGKRDFKKLIKAMDDVDGALTGTMDMLRDTHVQTLLQPSDRERKS
LLDFVDESSVHGMREVMKKSIQDLQTIQQSFDGDLLRLETDIRNLGQRIN
ESYPQKPGDDAHLTPMVLLQSLDDHSSSMAQLLASLTKHFDMCVTAIRTT
EGAAALARRKAAEVTQSQSSDGVSISGVIAEQESHMSDLEPKTAEDRAEM
LKVVVQDADEVEDVVREIQERLAAMEQEYTSLQEHVDHSKESYFGIISAF
ALVGQIGDRIDDYVAAEEDFRLRWELEKEAVFGKVREMNEMRDFYEGYAS
AYGSLVLEVERRRAVDERVQGIWRKAQDSVDKILEADRVSREAFRQDVGE
YLPADLWAGMQGPAQKWTVVRINDDDSAAASMVGRPDSGQGSRMASTPRP
PAD
>Mb|QLI64165.1
MASYYHYNTQAVHGAPMSHNHGGHRNRRAPRLSVSQNAQRQFRGARSMKD
LNESAALSAFRMKFEAGRSFELEDDLEFCPNLLTETDLVSISSASERSSL
ASNSPESSPTQQPQTVAPGFSLNSTSAAFIPPSFQTQHSNLKIHQPSATR
GRNAIPIINPATGITMSSPSPSVSPAAMQQSLGRRW
>Mb|QLI64166.1
MAEHNIVVFAGDHCGPEVVAEGVKILQAIEQHRPSVGKFVLKEHLLGGCS
IDTTGTPLTDEALNAANSADAVLLGAIGGPKWGTGAVRPEQGLLKLRKEM
GTYGNLRPCFFASEALVDSSPLKAEICRGTDFVIVRELTGGIYFGDRKED
DGSGTAWDSEPYSRPEIERVARLAGFLARGRGDKTVWSLDKANVLATSRL

WRKVMTETFEREFPDLKVEHQLIDSAAMILIKNPRGLNGVVVTSNLFGDI
ISDEASVIPGSIGLLPSASLSGIPDGKGKCNGIYEPIHGSAPDISGKGIV
NPLGTILSVAMMLRYSLNLPEEAKAVETAVKAAIDGGLRTKDMGGNAGTK
EVGDEVAKQLAAILTRP
>Mb|QLI64167.1
MRIPYVPDPPQPSTAEETQILQRIQARRAPRPLQPLDLALLHSPPLADGW
NSFLGAVRTQTSLGDDLRELAISRVAVCNGAWYEWKHHAPLAAQAGVSKE
ALDVVKTEGPLGRLSRPDVLTERQWAVLVYTDEMTRNVHVRDETFGMLKA
LFSDREVVEITGTVAAYNCVSRFLVALDVGERNGTGPDDVMTH
>Mb|QLI64169.1
MYALAKCSLPRRVVGLLSPANAIHGTTTRSRLLSTLAILEQRDGQLNQGS
LNAFAAAKQLGGTVHGFVAGSNVSAAAQEAAKVEGVEQIIKVDSSAYEKG
LPETYAPLLVENIKKGKYTHVVAGHTAFGKNLLPRVAALLDCQQISDITA
IENDKTFVRPIYAGNAIATVESSDSVKVITIRGTAFAAVTPGSGSAAVVD
GVDPKADTSAEWVSEDLAKSDRPDLGTASKVVSGGRGLKSKDDFDRVMLP
LADALGAAVGASRAAVDSGYAENSLQVGQTGKVVAPELYMAVGISGAIQH
LAGMKDSKVIAAINKDADAPIFQVADVGLVGDLFEKVPELTEKLKSS
>Mb|QLI64171.1
MPNGPPPGYDQATAERNTGPRSAGPIRKPVQPLDLPIIKHLNTKRVVLAS
ASPRRKALLQQIGLNNLEITPSTKPEDVDKAVYGPYEYVAETARRKCLDV
YTTCLETHLKSIPDPDVVIAADTIIATRDGRILEKPRSEAEHIRMLKHMR
DTEMHRVLTAVTVLAPREDAQHPGYCITTHTEETKVYFFSEANGLPDDVI
EAYVKTREGADKAGGYAIQGIGGMILAEKIEGSVDNVVGLPVRRCLAMAE
KVVFQQDADEFDDGATDEDD
>Mb|QLI64172.1
MSPYAVQSQSRDSLELASLASSSMIDGGAVSEVSSRPSISSSRRLSLVEE
EDPLDDGNPAGRLGRSYSVSSTFDFAANLFPLSSTTGAGGYAPVGAPTSG
ARSGGALGGGSLEKHKTLTYLNGLSLIIGLIIGSGIFSSPSQISSKVGSP
GAAIVVWIVAGILAWTGAASYAELGGAIPLNGGSQVYLSKTFGELAGFLF
TWTAVLVLKPGSAAIIAIIMGEYFARAIIGADAEDVNPWVSKSVALLGIC
TVTCFNCISTRMGTRINDVLMFLKFVALLFVTVVGIIVAVKGYTFNGEKD
NAWKPQDWFKDTSFDLSNWAIALYGGLWAYDGWDNTNYVVGEFRNPSRDL
PRVIHSAMPLVIVSYVLANVAYFLVLPVEQVNGSNTVAVMFGSKVFGSIG
AVIIALMVSASCLGALNSSTFTAGRLVYVAGKEGYIPSIFGKIGVHSQDP
SLTTQRTRNWFSKKIHRFVGDDEMGLFFTPIYALILNGFITAGYCVVGEF
PTLLTFYGVAGYTFYFCTVLGLIIILRVREPQLERPYKTWITTPIIFCCVS
LFLLSRAVFARPVQTVAVVGFVVAGIPVYFWRIRGRDRFRKPGDDGGHRP
WWRFWKH
>Mb|QLI64173.1
MPRRAASPAASEPELDILDSLYPGEDADSNQNFPADRDVDLDDILNDPEG
AEDGGDEAFIALQQAASYRKASNLKGRTVKKGGGFQAMGLNSNLLKAIAR
KGFSVPTPIQRKTIPLVLDRKDVVGMARTGSGKTGAFVIPMIEKLRAHSG
RFGSRALILSPSRELAIQTLKVVKEFARGTDLKAILLVGGDSLEEQFGYM
AANPDIVIATPGRFLHLKVEMSLDLSSIKYIVFDEADRLFEMGFATQLTE
ILHALPPSRQTLLFSATLPASLVEFARAGLQDPSLVRLDAETKVSPDLES
AFFSVKGAEKEGALLHILHDVIKMPMGTPEIAKQDSEKASKKRKRGESGS
GKPTEHSTIIFTATKHHVEYLANVLQRAGFAVSHVYGSLDQTARRNQVED
FRRGRTNILVVTDVAARGIDIPVLANVINYDFPSQPKIFVHRVGRTARAG
QRGWSYSLVRDSDAPYLLDLQLFLGRRLVLGQEGEEPSFASDVVLGSLKR
DLVESNVEWMNKLLHEDVDVSTLRGVAAKAEKLYLKTRNSASSQSAKRSR
EMVSSTGWNQLHRLFGDDVDNMEQARADMLARISSFKPQETIFEISHTDK
SHSEAAEVMKQLRKKITPRNRQAQKQESDIEDDDIPTKSNDNKVDMDSDQ
EASDNGSQGADQTNGWDEDELEVTVSNTDSKRGKTDWRDSEVFMSYTPRT
HNAVEERGYGVHSGGFVEAARDAAMDLTNDETAKSFGQPSRSKMRWDTKS
KKYVSRDNDEDGSKGSKMITGESGVKIAATFQSGRYDRWKKAQRIRSLPR
VGEAEKPGTTQGLPSGVRYKHKLDKAPKEADKYRDDFMVRKKRVDEAREK
RQGRFRDGMGSKKELKGANDIRKERQEKERKRAKNARPSKKR
>Mb|QLI64175.1

MPRDRPPTTGYERLAQADQFSDDSDDDLLAASSASLQPASAPRFAAITQP
RHRSGMMSPKANQRPKFRRRGGSHGGVDLKAINARLERWADEIASRFKRG
KGRGISGEEERLEIHHSVFQPPEGVRPVTAEGLAEDQPGVMTKAEFEAIV
ESVRLAIRQEVHPSMISQGSSGSYFARNPDGKIVGVFKPKDEEPYAAGNP
KWNKWIHRNLFPCCFGRACLIPNLSYVSEAAAYVLDCQLRTHLVPYTDVV
WLSSKSFHYPFWDRRSFYRKKKPLPAKPGSFQVFLKGFKDANVFLREHPW
PDQYWSGFRANDTHRSRRKRWTESCRPSTSGSPEDADSSDEESPEDADGP
ASPPRFTWTEQIKQSFREELEKLVILDYIMRNTDRGLDNWMVKVDWETGT
VSVASDPVHLNMETPQDDHENAPRPVDLEQMPQSATRASYPYRSQRPMNA
SSNKLASKDPAMHVGAIDNSLSWPWKHPDAWRSFPFGWLFLPVDLIGRPF
SQRTRDHFLPLLTSTAWWAQTQMALKRVFQVDEDFQERMFAKQIAVMKGQ
AWNVVEALKTPDHGPLELTRRTKVCVWDDLVDVPVAVPMRVTSSELRRNS
NVRQSMDEADIASSVPVNQDSSADLLGLQSAPVDMPRSGRFDEISSPNDD
GARSPDERRSMNGPDRNPFGNQAGSRSLNVYEPARHGPYQQRRYSFASPA
AARRNSNTIAQQLYGDSHDSYDGDDPDGDLGYAAAEGQMGNQRKVIVERL
EAVKSRNPVFTWC
>Mb|QLI64176.1
MALKGSDIAQHNNADSCWVIIHGKAYDVTDFLPEHPGGSKIILKYAGKDA
TEEFEPIHPPDTLDKYLEKSKHLGPVDMGTVAKEEKAHDPHEAARLERVE
QKPHLSQCYNLFDFEAVARRVMKTTAWGYYSSAADDEITMRENHSAFHRI
WFRPQVLVDVEHVDFSTTMLGTRCSIPFYVTATALGKLGHHEGEVILTRA
AHKQNVIQMIPTLASCSFDEIIDARQGDQVQWLQLYVNKDREITRKIVQH
AEARGCKGLFITVDAPQLGRREKDMRTKFTDQGSNVQSGQDTDNSQGAAR
AISSFIDPSLSWKDIPWFKSITKMPVVLKGVQRVEDVIRAIEVQADGVVL
SNHGGRQLDTARSGIEILAETMPILRARGLQDKIEIFIDGGIRRATDIIK
ALCLGARGVGIGRPFLYAMSAYGQDGVEKAMQLLKDEMEMNMRLIGCARV
EDLNPSLVDTRGLFVHSNSTAADALSQTVYDPLVVPSQRAKAKL
>Mb|QLI64177.1
MSFTLSVRQSPLRLAQRLPELSKTALRSAAPVRSLHTPAKPTTNFFTSRV
SSIAARNAFARGSGRSYYQEATQRASSGTGMRKLLVGGAIFGGTLVAINA
VFNRETRDDGGMPLYEREYLNNTFLHTGLGIGIIGLTARQMVQSGFVYRL
MVTNPWVVGLGGLALSFATMIGTRSISPDNYVPKYALWTAFNATQAAFVA
PLLAFVPVPLLARAGLYTAAMMGALSIVGATAKQEKYLYIGGPLLAGAAI
VAVSGFAPLLIPATAVRTLAFTENIWLYGGLAVFGGFTLYDVQKVLYHAR
MAQAGLMKRDPVNESISLELDFLNIFVRMVQILMMNQNRRK
>Mb|QLI64178.1
MDSRKPSLDLEKELTCSICTELLYQPLTLLDCLHTFCGACLKEWFRFQAA
KAEKAPTPPAPGEAIFTCPSCRSPVRDTRHNATVVTLLDMYVAANPAKAR
SDVDKEEMQAKYKPGDQVLPKINTRERTAEERRADEEDRRLIDEVREMSL
REATPGGLSQRDRRRRQRQTQSVDGRSRSSRDQSSSSRRRGTEHTTRNSR
SDDSSRPGSDHSASDREQRRRRRRSVSRQRQVEHQTSLRSLIGSSEMSER
DIEREIEDFARQIQEEGLLDGLDLDNIDLSRDDDLSRRITEAYRRRQRER
SRNESSRQPTSSSNNAASRNSEGAQTDARLRAPEGGSRSRASSRSTNGGG
QAEDRSRPPPTGSTNLEVRDAARRPRRRTASGGRSATTPVFSSTAETRPA
ARSSTDLGLRSRTSDPTAPRHSFSEGRSSSTPSVPASSQAPIELPASASS
NSNIASFANRVPQWNPNIADPSPATTHELPAAQISRPHLPAELAIVHSAV
SSPMSSPSATSGHRRTKSQLFPEPSISCAKCNKQHIEYELHYNCGICTGG
QWNICLDCYRAGKGCQYWFGFGYGAWKKWENLRQKGDDSLAKPHMLTACR
YLPPPSTPGGADGRKTLTTDDPRGRLETGTFCARCFTWTNDCYWRCDVCN
EGDWGFCNNCVNQGNSCTHPLLPLTHEALQAASRPHSPRSTGRPPAATVY
TGPQAANIGPFKPLTFRTRCDICQDPISPSHVRYHCFSCKSALIPEAPPG
DYDICSSCYNSLVSTGQISMENGHSGWRRCLRGHKMAVIGFADGKIGQWR
FIERDIVGGRVLRSEAFESPERNGQELQKSSWKNGDEKLERLVTKDVSAT
APSTVGSLSFTQTFPLDGGTGMRANARWAWYPQAGSDDELLFPKGAEIRE
IEDVNGDWFFGTYMGAKGLFPAPYVRLGQRM
>Mb|QLI64179.1
MDYHSIHGHDISSFDIAGTHRLPTVQASQALNELQDDGNLYLSTGLEELD
QALLPPTPAASPGSTSVGGIKRGQITEIWGPSGTGKTAVSIQLAANSIHN

GNDVVWIDCFQEMDVSRLENVLASIAEDAVPDGSTRAEPPAAGRGKFAQF
SCLTLPHFMALVSRPNAKSIPEGTALMVVHNLSALINSSLPRSVDGKTGP
KSNKGPTASTKRLQGLQFIINALQKVAATKNCAVVVSSQCATRMQSEHGA
TLVPAVNASVWEQGISTRIVMFRDWAWKNGALVSIFVAGLQKLDGKSGYD
AIERAAAFNVTRTGIARVECDASNAPVEPADVVRGKRKLEQTDLEVPDSQ
DDEDYGWADDDEAALPAPPPQWQGSEDVLLGQELGRGDDGRDEEEDGCEG
SHTPSSEATY
>Mb|QLI64180.1
MAVTVAIVGTCDTKLAELLFLRDEIGKHPEVKTLLIDVGRKPVAHDAVDV
CVSDMLLKHAGGAEDTERTRGQFIETVSDCTTKLIRDMFTKGSIDGVISA
GGSGGTALASAVMRQALPIGFPKFIVSTIASGDTGPIIGETDITLMYSVV
DVAGLNQVLRDILANAAAAIAGAASSYSMRREQRPRESPAKKRVAITMFG
VTTPGVDAIRRFLESRFPIETYVFHATGHGGRAMERLIRNGQIDAVIDLT
TTEICDFIMGGQMSAGDERLDAAIEAGIPNIVSLGALDMANFGPKASVPA
KYQHRRQVEHNPIVTLVRSSESDCRSIAEFICDKLKASKNTALTQVWIPK
GGVSMLAVPGGPFYDEAADAALFDTIKANLAQTGIKVVEEPGHVNDLQFA
THVAEALVEMMQLAH
>Mb|QLI64181.1
MATDKGLEDVPEGQIESNYDETVDSFDDMSLKAELLRGVYAYGFERPSAI
QQRAIMPVIKGHDVIAQAQSGTGKTATFSISVLQKIDPTVKQCQALILAP
TRELAQQIQKVVVAIGDFMNVECHACIGGTSVREDMKALQDGPQVVVGTP
GRVHDMIQRRFLKTDAMKMFVLDEADEMLSRGFTDQIYDIFQLLPQSTQV
VLLSATMPQDVLEVTTRFMRDPVRILVKKDELTLEGIKQFYIAVEKEEWK
LDTLSDLYETVTITQAVIFCNTRRKVDWLTEKLTARDFTVSAMHGDMDQA
QRDLIMKEFRSGSSRVLIATDLLARGIDVQQVSLVINYDLPANRENYIHR
IGRGGRFGRKGVAINFVTAEDVRMMREIEQFYSTQIEEMPMNVADLI
>Mb|QLI64182.1
MAADSSSSSSATAADRDVEAQQAQRSRKHSHWSVLVDQAGVDDAVLNHKY
PGQGTHESPYLVDFLPVDPRNPMTFSSPFKWTVMIVAAISTLAVSFTSSA
YSGSVRSINEEFHASTEVTILGVSMFVLGFAIGPLFWAPFSELYGRQKLF
FLTYMALTAFNAAAAGAPNMIALIVLRFMAGAWGSSPLTNSGGIIADLFT
AKERGIATSIFAIAPFLGPALGPIAGGFLAEAEGWRWVEGLTAIFTGTLW
IIQSLVAPETYSPVLLRKRAEELSKRTGKVYISKVDAAMPRKTLFGQIKV
NLSRPWVLLFKEPIVFLTSIYMAIIYGTLYLCFAAFPIVFQSPYPQGWGW
KPGVGGLSFVGIAIGMIASATGSILDNGRYARTAAKHGGVAPPEARLPPA
VAGSVLLPVGLFWFSWTNGTNVHWVVPIIGSSIFAAGLVLVFLSLLNYLI
DSYVIYAASVLAASAVLRSLFGCIFPLFTGKMYEHLGVHWASSIPAFLAL
ACMPFPFLFYKYGEKIRLKCKYSAEASHILEKILAKQQPAQEDEGITECE
EGGREKGNQDTKTAPTTEVK
>Mb|QLI64184.1
MAAETGASTGTDIPKREPTPPTSLGSIPQKRAFDDDHSPAVPSPLNPDVS
ASELPPPDDASQLKSSKPARAKKDSFKKRETKGGGRDSSPATPDHRSVKD
QNNVDSPLRYKLAPPKPSDFELPRGPFMTLHHEVDGLGGRVEFFEASDHV
FNKKNFRYTRCIADPLFPSTLYYRQTEPEPFGPRLNYEDAATSVFFDKSG
RGVTTDKGFRMARANVAVREGRWYWECKITKGVREAAEAGAKPEGGKHVR
MGWARREASLDAPVGFDAYSYGIRDVAGEKVFMSRPKAFFPAGDGIREGD
VIGLEIQLPSENMHRKIMSSTYNPAIDAPDDESPPGAEAPNIVRDRVPIR
FKAHTYFEKIEYHTTRELEDLMNPSPIGSSSSSSSESPNPLHTSPALRTLP
NSSIKIYKNGQLIGTAFENLFGFLPPASKPQPQVGAREGLDDGMLGYYPA
VSIFRGGAAEVNFGPDFWYPPPEYASAPSGEEDTPESRRKRLRAVSERYK
EQIVEDIVYDVIDEVDFWMQDGGGATDTTGVQEKSEAVAAAPGREEIKEL
VQDD
>Mb|QLI64185.1
MHPPRTTAVSSSDHLTRGEIETITTLERIGGAISLVAVCFIFVAYALVRR
ARNVQNTFIVFASISNVGASIACIIALDGLVLGEATSLCQTQAFLFEMFM
QSDPWWSLAMAINVVLVFYFRTSPDSFRRWWWLYCLICYGGPFVIALSLL
LIRNSPKGLVYGDAVIWCWIGGKWAGVRIYSYYLLILICIAGTIISYALV
GYHVFRSRNRLRSFSVSKSREAGHSDHVSAADHSQSDGFYGTVTTEVLVT

HTSASNLVHPKSAHTARHREPASFPGATLRSSADAAAFTSSVSAPPRRQD
LRLLNPIHRTVSACKSVASKFSVEDPIKRAYLRTSLLFGLSVLVTWIPSS
MNRIHSWINGDSPYTYQVASAAVLPLQGFWNCVIFFVTSWRAVKDDVMYR
AGRMPPASHNDSRIGTLERTVNREESRASTDDEAESLHTRSDVELRAVEH
TATKGVAV
>Mb|QLI64188.1
MAPRVIVVGGGLSGLSAAHTIYLAGGNVVVLDKQGFFGGNSTKATSGING
ALTRTQVEHGIPDSVKQFYDDTLKSARDKARPDLIKVLTYKSAAAVEWLQ
DVFDLDLTLVSRLGGHSQPRTHRGHDAKFPGMAITYALMQRIEELSETEP
HRVTIIKKARVTNLNKDGNNITGVTYEADGQTASLDGPVVLATGGYAADF
GDTSLLQKHRPDTMGLATTNGTHATGDGQKMVMAIGGNGIDMDKVQVHPT
GLVDPKDPGSKWKFLAAEALRGEGGILLNGDGDRFCDELGHRDYVSGMIF
KEKDKGKYPVRLILNSKASKTLDFHTRHYSGRGLMRKMTGQELAKEIGCT
PDHLQKTFQTYNSIADGKQKDPWGKKFFHNMPVDINDDFHVALMEPVLHF
TMGGIEINDKAQVLNQEKKPFDGLFACGELAGGVHGANRLGGSSLLGCVV
YGRVAGDSASNYLFQQALSGSAAGAAAQRLGQISLHLDPSAPGKLTVEWA
NGGSGSGAVAVQQQAGAAPAAAAPAGESAAKAKPDPKAFKIPETEYTMEE
VAKHNKEGDLWVVVKGVVMDLSNWLDEHPGGPQAIKNFMGRDATEEFEML
HDDEVIPKYAPEQVIGRVKGQEPTLEF
>Mb|QLI64189.1
MSANPISAPGGSRRRREQYHEADVVVVGAGVFGCAIAFALANQGRSVLLL
ERWMTEPNRIVGELLQPGGMAALKQLGLGHCVEGIDAIPCKGYNVIFNHI
PSLIPYPTIDDSGNVIYAWGGSGKEGKRPEGRSFHHGKFITKLRESCLAH
ENITVVETEVVKTLRGEYTDQVLGVETRTKNRETGDKKPDYFFGQLTIIA
DGYDSNFRKDAIGDKKAVVKSKFYALELIDTILPQESYGHVIIGKDTFPT
LLYQIGTHETRALFDVPKDIPEASPAAGGVRGYIKNVAIPALPPSVQPAA
LRALEDGKIPRSMPNSWLPPTKQMPKGVLLLGDAFNMRHPLTGGGMTVAF
NDALLLSQLLDPSRVPTFEDSKAIGAAVNVFYWRRKNVSSNINVLAQALY
TLFAANDRQLYALQLGCFEYFQRGMTDGPCGLLAGIIQRPAVLAYHFFSV
AFLSLWLNALAICAGPLGVLKAPLALIDAVLILWKASCVFLPLVWREGFQ
>Mb|QLI64190.1
MQPKFSPGTDETSTASALAPLLASSGGRWTLSSEGKALERSFKFKTFAKT
WDFMTAVSLQCKLKNHHPEWSNVYNTTFIRWTTHNPLGLSEKDVRLAGIC
DALAKDFGELDPEPASREVKGLADKAASSSGDCCAPRKEK
>Mb|QLI64193.1
MRFDFTFLAAILLSSTAANVIEPRHAVRIEDVTQGDDLFKRKGGGGGGGR
GGSSGGSSGGSSGGSSGGSRGGSSGSSGSSGSGSRGSGSGGGSRGNTAPG
SNAGGTSRGGSGPPRAFGGGSYYGGGAARPYRSGGISPLGGAAIGLAAGA
LLFWPGRWLYGAYLYPYHNHYVFHNATTNKEESLPVVCACGTYEVCGCDE
NNSTMTELVGNGTYDALNKSIINVGDYKGQKTLLVNGTLPNGTTVDGPDE
GAGVGVGMKSLVEAAGLWPAVVCVIAAICLA
>Mb|QLI64196.1
MVSTRSSSRADSAGPEPTSTPSTPTPATRKRKPATSAPSTTTRRRAHRAS
AFEHTPTFWTLLWMGVSLPLVIWDTGYVLGRPHTMEGGAAHWPLWLPYKL
YGEVDHIYGWKAFHANNGFTAAQGLLNVVETLMYAVYLWLWYSRGEATAH
GRVVTGRPGALAVLIGFSSAVMTLSKTFLYWSNEYFSGFDNIGHNNPVDL
VFLWIIPNGAWLVGSSYMAWSIGAEILDGLEAASHVKKE
>Mb|QLI64197.1
MTPQILHLPDGKKFTVAPVFGGLGFRSHNHHNLLHPYPGGWMTVLHTEED
RVGFEGPNGGEGQKDHDENLPPQTKRRTKPFTRPTLQNDTLFISSISIPS
DAEFKPAASPTREIAMMLWITLYWYFHQPPPDRELHTAASKDTPAGAKPV
GEWRIRIKRDGVLRGRNLIPKLERMGLIASEDTAVGTCVDDSGDGWTNMF
VSKRMFWQIPANLFLFTLQPVRGLDMPGSPSTSRPGSPIKDDTGRLDVRP
MYSQSQSQSQSQTPALTPTGALTPSGGQLAADVPGAPMPTSVTAAPAFPI
GPYFSSSHLPTYFPPPPLQYVYTDGARHPLRPKPPRMGEVFYTRFIDSLG
QYLSFRVASASASPVPYLGPLGPNPPEQSHLSSMSDRDLLQSWFASQRVR
DFWGSYTPEFLETALCSRHSFPVIGLWDGVPFGYFELYWVKEDILGRHVG
DEAGDWDRGVHIMVGEEWSRGRVAAWLTSLVHYCFTADQRTMNVCLEPRV

DNQRVLRHLDACGFSKEKQVAFPHKQAWFVKLRREFWDGPAL
>Mb|QLI64200.1
MAAAATRLVVCGGSGFLGSRICKYAVARGWEVTSISRSGEPKWDSVTSSP
LPPSWSHKVSWERGDILRPVTYAPLLKGADYVVHSMGILLEADYKGIVSG
KESPLTGLQKMFSPVRERGTNPLDKKAGEDIKPPNPTDQFSYEVMNRDSA
VALAKHAAAEKASAFCFISAAGGAPIMPPRYISTKRQAEVAIATNFPEMR
GVFVRPPFMYDSSRKITMGMAAMAGAGTLFTRMTGNYLKDFMGAAGTKPL
QVDTVAEAVVEALSDETVKGPVEVPQIEELANKGWRKTML
>Mb|QLI64204.1
MVSRKDAQFDHLTLNTSGPIPCALQGQAVLNTPYFNKGSAFPAHERRVFN
LTGLLPQNVQTLQEQAKRAHEQYLSQTGDLAKNTFLASLKEQNEVLYYKL
LQDNIKAMFSVVYTPTEGDAIQNYSRLFRRPEGCFLNINDTERVFHDLAM
WGEADDIDYIVVTDGEEILGIGDQGVGGVLISVAKLVLATLCAGVHPNRC
LPVVLDCGTDNEHLLRDELYLGLREKRVRGERYDEFVDLFVRAARRLYPR
AYIHFEDFGLLNARRILDTYRPKMACFNDDVQGTGCVTLAAIMAGLHVSG
QKLADLKMVVFGAGSAGIGIADQVRDAIAAEGSTSPEEASQQIWLIDRPG
LLTTETKVSDAQRPFAKNPADWTGQRTDLLGVIQAVRPNVLIGTSTVPGA
FTQDIVTEMAKHTARPIIFPLSNPTRLHEQTPEHLLQWTRGKALVATGSP
FDPVRGPWGAGGEQVTMEIAECNNSVVFPGIGLGCILSRARLLTDKMLVA
AVQAVAAMSPALRDPTAPLLPAVGDVRDVSLQVAMGIVKAAVQEGVATED
GIPSNDHQLREWIRVQMWEPRYRDLEKVEMEGAGRTARGELRKAGTVDRM
SDMSAYGAMAQQ
>Mb|QLI64205.1
MEKYHDLQRKNDIYYRNTARRFYRGAIHNQIRYYNSPLDLHKVIDCDPPY
TNGYESTEGTNTEQIPKLKGRTHGCRTTTVTSWATTTATKTVTGIESVVT
TITVPQETTIHDTHTVVDSTTIWVPTTYTETKTVVSEHPVTLTVDNTKYT
TKSTTIVTTFVSTVITSFPVVTTLTLPGQTITAPGQTITAPGQTITAPGE
TVTAPGRDVTQTLAGPTVTAPGQTSTITLPGVTKTLPASTIVTTQIRTLP
PSTVHITDDQETKTITRPGYTVIETSTLTLPPTTIKKPATTVTVTPMFDV
SLCPTPTGVSTPLDAKSNRTFGCEPGYVCNPPKPNGCNLWPKPPSDDFLC
DPNDCIPSPPFAKVEWQECETGYYPPSYGYFNLDPEGFGLSYDIFEYKVI
NETINGHPSMITTGNWGSQSSLTAWPKPSPPASHGQMRKRINDHKACHKS
KRDIVVPSICYNPCNQAYLIAQGTGKTDGLCDRDSKFRAAEVSCSNCIKA
NANSNSTSTGEAQKEPFEQFINFCGGTKAEKPPTASPTTPEQLVTQQPTI
GTSTQVDVSHPAFTPILSSENIASSAPSTPSPTSSVGSTNSMGSTHSVGS
THSVGSTPASDASSSQTTHSAPPSSGQSSPISTPPGSSTSGSSGVQRPGT
STGGETYSPTTTHRTAVGTTGGSGSSSSRLSNTVVESSLGFPSASASTSA
SASTSAPASASAPASASASGPSSKIGHGSGTNPGSSESTSNANAGNGGGG
QPGATTEGAPGNPPTSTPPVVTAAASHLSSLFYSAGAILVSSVLIMMF
>Mb|QLI64206.1
MRTSSWLAQIIPLVEICSAASSKRGLVFTPNAEHAGDNKLWVQSGSDLTW
YYNYQSSPSPAYSSLSQDQFEFIPMMWGIGSDPNDTKFLTDVKKLIDDGT
KIKHVLGFNEPDTSSAYGGSNIKPADAALAWVANFEPLGKMGVKLGLPAC
TGGWDSLPWLKQFLGNCSELVSTGKDKKNCTWDFLPVHWYDNFAGLASHI
GEREATWPNKSIWITEYAFAHQDLKPTQEFYNQTIDYFDKLDSIGRYSYF
GAFRSTNSNVGANAAFLDKSGKLTDIGSWYLGFAATGVTPESSKAALLEP
TVGSLILGVIAGILVGSL
>Mb|QLI64207.1
MDRSRPIGRSQAEEYEPLATSDDAALESSAILRDTDLIPFSWIEYSIFGI
LGVAMLWAWNMFLAAAPYFSSRFAASPSIQSTFQSAILTVSTITNLSVLL
ILSNIQYSASYPFRINLALIISTVVFALLTCSTTLFLGASPSAYFAFLLV
MVALSSWATGLIQNGAFAFAASFGRPEYMQALMAGQGVSGVLPAVAQVSS
VLLFPPDRSAAGDAASGGETSAFFYFLAAVVISVVTFVALVPLVRRHNRR
VEDKMVQRMAESINSIEEAERAARKVVSLWTLFFKLRWLAVGVAVTFAVT
MFFPVFTAKIHSVQEGAGAIFRPAAFIPLGFVFWNLGDLGGRIATAMPFT
LKDRPFVLFLCSVARVAFLPLYLLCNIGGRGAVVSSDFFYLFVVQLTFGL
TNGWLGSSFMMASGEWVDEGEREATGGFMGLCLVIGLTAGSLLSFTISDI
>Mb|QLI64208.1

MSTQWSKTLSFEDRHTLSAPSPSSTPDEPATHTWSRPPLKNLTPGQPMSL
AGISLRAFALGLTASLGLVSTALVLALTSSPLWRVPLFLCALSTFHFLEF
WTTAAANTPEATVHAFLLTANWPAYPVAHAVACAECVLTEVVFPGRRWAP
PGLGTALTLVGLVMVVGGQVVRSVAMLTAGQSFNHTVQTRKADSHTLVTT
GVYGLWRHPSYFGFFYWGLGTQLVLGNVFCFVAYAYVLWKFFSSRVRVEE
KGLVKFFGDDYVQYRRRVGTMIPFVG
>Mb|QLI64209.1
MTSMAAESATTILCTAKQTRFHIESPNYRELDVQGLHILVTSGEQASSTA
KAKSKRSDGLEILSNAKLLLKEGQRYALVGRNGTGKSTLLRAIADKLIPG
IPEGTRIAILQQTKLTDDDGDENSRQGRKAEASVLQEVIDRATARDTIEQ
EIKALSDGVDASDPFGPVRALRSLKHHRLQKRLFRMDKEARLRSGARGMQ
ARKALVAFEKVVADSDVMINQSSDEISPETLQAETQEAADMLAELQIQVE
PSRIAQIETRARTILTGLGFTEERMGKPISSMSGGWHMRAALATALLQDA
DILILDEPTNFLDLLGIVWLQRHLQHLAEGDRPPTVIMVSHDRDFISVCT
DLMILKEKQLTYFHGDLPTYEASQAERKLWLKKMKAAQAKQRAHMEKTIA
QNIKAGKANDDQNKIRQAKSRQKKLDDRMGLQVSAKGHRFKLNRDMVGYF
HTAREDIDVPPEERPVVIALPEPPDLRFPGSLLSLDNASYRYGPRLPLVV
DGASLTVGMGDRIGILGLNGCGKTTLVRMLVDEARPTAGSVTTHPRLKLG
YYSQHAVEAIKAVGRADPALTALGLLTREVDGQMGEGEIRGLLGELGLPG
RVASDTPLCKLSGGQLVRCELARLFWRRPHCLILDEVTTHLDYETVTALR
VALDGWDGAVVLVSHDRWFVRGVVEGNLDDDGDDGEQSDEEGEAAPRRRI
VYRLKGGSMTPLEGGVEEFEKSMEKRVNKLTVQ
>Mb|QLI64210.1
MVLADVLKRWQQIKGKGTFFLTGTDEHGMKIQRAAAKEGIAPKEFCDNYS
NKFRELAAAGDISHDAFIRTTDLEHKEAVSEFLLQLKHTLPEHLGLYKGT
HEGWYAVSDECFYPEDLVRPDIVPQTGKKIMVSDETGSEVEWVKEETWFF
PLTKYKDALLKFYDENPNWITPAHRMNEVRNWVENHLEDLSITRPAARLS
WGIPDPEDRSQTIYVWVDALINYITQAGYGRKWHSSSDNMGLWPADLQII
GKDILRFHAVYWPALLMALNLPLPKQLLCHNHWTMSNRKMSKSLGNVVNP
FFAVQRWDIDPLRYFLMRNGSFTKDMSYSNQLIGSVYAKELQANIGNLLY
RIARPKVTSKWSTLEAVTAFRQGAFEEIRDHTSQQPFLSLDDHLEKITSA
YYEEMEKYNTGGAIREVFNLLRETNRYVSDTEPWNLVKNSNPQSRILLNL
VIYNCAEALRIAGILLQPIMPTKASLLLNELGVREDRRTLEFASKGKDPE
YGTEAQTGDSAARLKKWDTIFPPTPNANDSDAEIMEQLSSALHDKTKNKM
NQVAELLAMEARMGEEAVAKMLAEMHAARLKTAKKTSRL
>Mb|QLI64213.1
MAPSSVCSLCISTLGRRMAPRALIQPPLAQRFVSRQAKARPSRMVLSDRV
HQSPASADKHRSRRASIDGPFAGMNRRTANVDRSRGSREARGSSRRDSTD
TTSRRDERRKPDDRKALKMQRALATVSYGRRTSLKDKMSHYDTFDQFDLL
PIVKSAVTEELFKNMVDIKPTPVQRLAIPALLGQAGPGENKTPSTGMESF
LLAAETGSGKTLAYLLPAIDALKIAEAEDPDIKAYKERFEEEKARQQAAN
FKGRTFDEPHPTMARPKVIVLVPTAELAQQVMKVSKSLSHVAKFKTEMLS
SDLKAQQIQRNLYGPKGVDVIVATPHLLASIADSDPNILSRVSHLIVDEA
DSLLDRSFASVTTSIVERAMPSMKKFVCCSATIPKKLNNYLATNYPKMMR
ITTPNLHAIPRRVQLGVIDVSKDPYRNNKDLACADAIYTIGREASAHEGP
VKGEVDVRRVLVFVNEREKTEEVAEYLRSKGIDAEALHRDTPEKRHGEVL
SMFTSPEPLRISTPAAAAGRRRSLSNVKALVVTDLASRGIDTVAVRHVIL
YDVPHTTIDFIHRLGRAGRMGRRGRGIVLVGNNDRKDVVSEVKNSMFMGQ
ALI
>Mb|QLI64215.1
MRHLGANGYPSFATNNSGFFARHLRRISSNLPRYNSNNTIPRYGDKDKPF
RGQNIPLIGRIRSISGRMGRKLKIRLLIVLVLLLCLYIFYHSPLVYWYRR
ASWGGGGSKFVIILAANVGGGVMEWKGAREWAIERDSVRNKRKYATRWGY
DLEIVDMKTRKKYAHEWRESWEKVDYVRSAMRKYPNAEWFWWLDLNTYVM
EPSYSLQDHLFDNLQNHVYRDINEYNPLNITHPLTYPYLDKFASSPVGDG
NVNSVNLLLSQDCSGFNLGSFFVRRSAWTERLLDIWWDPVAYEQKHMAWE
HKEQDALETLYENHAWVREHTGFLPQRKINSFPPAACGDGSGKNNTRFHY
NQYERDFVVNMAGCEWGRDCWGEMYHYREFSYWLNRNPWERFKEDFIALI

WWKLTGKWVKL
>Mb|QLI64219.1
MGNITSRPDDGSTPYLRDQNRLSISSLVVTNPRRRSSVNIVPNAFPASRI
VVSRLSGETSPIEYVQDPESSNNLTAAPNFLLKLNGDDELIFTFTFVIRQ
SHVPAAADGRPATSDTNISGLTFVYASTPREVSNLVTREFHADPNLHKNA
NVELVGDYSTGGNSSVTFEWTWKWKPPKPVEDKGGGWRNCCSFVEYDPRA
HRLDTLASFSYFVSSPGLPLSNPSSPSPQFLLSSPPKIRVASTQSVESRL
TGVDVDEPVSPLLAPNESLSQTNTNQMQEPVKVDVPCPKYDDDVTVSDDG
PVFRATLKALEQKTGNMRVQMKKVLKRAEHAHLQQTEANDAFVSFMEALR
EASSTNANAFQPALEHYFDKIAREVVAYERQNTANLQKIIIEPLNKLYQL
DIKQAEYKKRDFEEESKDYYAYVSRYLGQRQDSGKAKKLADSDSKYQNKR
RNFELKRFDYSSFVQDLHGGRKEQEVLSHLTKYADAQTKGYLATAKRIED
FLPQLSALSSEVQEADKEYQYRRREREEKRRQLEKSNTPYLEPEQASLHS
SAPLSSSLNGNAGNTSDSELGRADSTGSQIKNAIAIGVGGQGSATQGAEL
SRSPGSQGQSSLSSSTPGAKFKGFRDLEERDTAQGVVSQRKEGLLWALNR
PGGHVDPRNLNKQGWHKFWIVLDQGKLSEYSNWKQKLDLHMDPIDLRMAS
VREARNAERRFCFEVITPNYKRVYQATSEEDMNSWILSINNALQSAMEGR
GVPEPSSSSRTPADSSLKRDIGSILTGKTQHSGHGPLHHHSTTNAGMPQR
RITVGARPGAVRTTSSGYDENPDKLLQMVRNADQGNCWCADCGSGSKVEW
VSINLGIILCIECSGIHRSLGTHISKVRSLTLDIKSFTIDIVEVLLLIGN
RVSNMIWETKLNPSQKPSPQATREQRLRFITAKYVDRAYVEPISTTLSRY
ATPDETLLAAIKKNEIQQVMYALALKANPNVVDKMRGTHAIWLALAAADP
AQPSLTSSVNASETDVKAVPFPVAELLTQNGAEIPGSMPAFPLGRFAQQY
YEQKMGRGNNPGHDVLGSLPLHLSSSDRPQREREARLQKRVSAGGRLAKS
PIPEK
>Mb|QLI64220.1
MDGRMGMSTWHRKRRRSNSPAAGPSKRAKTTNNQDTGGDEDSDSIIEVQM
DGSEDEVQDKYAVLDQRLAVKQESGADNLACDFCLKLPLNPDGSIPACDR
QIIDGLVHECLACANHRYKTRDLNYKCVRSGQHPELEVFKRYSDSHPVNY
PKPIACEACKDNKKVKDSRCDADPWLQLRCTNCLKTKDSESELCMVKAMT
GDANLGWGHEVERQMGPKPNLQRGRQKWFRRECDICKSRPDKERGVQVCS
WLDSRDMGGPGDTREPCLQCKEGNMSCFDGGMLVGHPVRLELPTEWSSKA
DLGGGWVELRGDKDIVTRPQCRQCHAQKRHCRASAKYAEYACNWCWQTGM
VCRDLNDENKIYPLFDLSRVGIGNFCPFARCARCEATGRNCDRQRPCDSC
FFSGEGHMCDEWQPGASRTLNCLNRRINHGENMMVKPGPLYYLSMGYGPG
GVDDEKDGAQIEHYIGPPFARYAFKTVAKRKGEVLKQNKQVLVAETQRMR
LALIARGAPPNGAPGGELYGVNVDDITLDQLRTWMVKRWPEWTPQCNRTG
YAKNRDMEIVSKAYMKSKFMNTGAGAPDTTTLVEELADGEEDLQIMPVTE
PTFNITGERDQTGSGAVDAYMVSTTPNVANNATFVTIPSNLLSGGTQLRL
TLSDSERYHEYHNNSLDVYDNQSSRWVELRCFQTRPPCEKAIWDDFWQNR
NGSTNFGLFSIMNGSLVRHSPINQVFRGIRGIRGTASSSVTMPPQRFSCR
EQTKMCTGLTGRVCQDIEHGNIPHSVCESCALHDNGLLSKSPSRMTKLDI
VAMRAYFCNSCHSRVIGGSLSDMYLLGAKDVWGCRFTNNTTFLGSLAVNS
RGMNFCPVEHPITGCTCGPNIFDSHLCLDHRVKYARYAMYQSNRMTEWRI
GEGEQDLCPVCLVHHPKDMANPSPSYHELLSADGTSTEETVSWACLVCGG
LVLNQPKKNAIIGGWFSWFPVASVSDWGLDHKASF
>Mb|QLI64221.1
MTLCRSALAVAAAALVSITQAWNIELPPCIEPFKPFVYSGCFQDGNPNAL
VFRSSLDQQDMTVEKCIADCKGNGFRYAGLEYYGVCFCGATVNGAQLDES
KCSFPCSGNKTETCGGDNTLSVWQDPTFPKSPSDVTVDDYKSLGCYTDDS
PKGRTLSYPIDLDASTFTTKKCLAACEKQGFPFAGVEFGKECWCGVVLAN
DTAKADTSQCNMPCQGDSSDTCGGRGRLNLWVAKDLESLEPCGHKPGTTT
TSLPPSNTTTTKPVDTTTTKSETTTTTKPETTSTTQPETTTTTKPETTTT
TKPQTTTTKPETTTTTTPQTTTTTKPQTTTTTKPQTTTKPTTTTTKDIPHTT
TNLCTATVTLPPKCEWQCGSWCAPPLPHWGDKNGCIIAQKTCHKQVSSCF
KNAGWPGSIECFKFKAWCGFIDAYCASWCPSDSCGKWDCWDKHHGGGGKP
PSNPPTTTTSVYPCPTTSKPATTTTKPATSCPPEPTNICTQPTNQKYGYG
PGKPVGGIPLPVVGCNDCKDEFDEKPFKYYTEAESRNCPGFPWPRWPSVC

SQACEEQYTHCVATYNKGCESLGWRDSWRKRAGESDEEFAKRWSGPDNGG
SGTCAAAGSVSTGAWSARGSDSLQCWGWGGNNPAWALERCKAQYQDCLRV
NQWVDPKDKCKRWPGC
>Mb|QLI64222.1
MKISSKAQLAICIGFASYSNAQFSNWLSDQINTSICTWSQPRAALIRDTV
YLDGGDIWWNSGLASGKFGPVLDQGNFQGYVLSYNFSESFSKDTNVTGIL
VNKRMSKAIGGEQSGISSEPGYDDGGMLANDAEFFLYGGAVTANTLASNP
EPAANAVLAYRRYAYGPEKPLWQSGFNSGHLDDGVTRYIAYGGPVNAPSE
NKAWYFSGLTSPSRGPISVLTYENKTTLAMIPSNTLIELDMTNQLFEKWT
NITLPDNIKPRANPEVVWVPVGKQGILVVLGGVVYPDWASGDLESANETE
SKKLKSPEFMRTIDIYDVANKKWYQQPTEGGPGARTRGCAVVAPASDKSS
FNIYYYGGYDGIHPKNDFSDEVWVLSLPSFSWTLLNKGVPVHGRSGHKCF
KPYPDQMMVFGGYTPTIGSSFTCLDQGPVVVFNLTTGEWLDGYSPTKYGN
YGVHEKIIAAVGGTASGGATVSSPVPSGWASKELGAVFSVAYDTKKVKQY
WPYDSASSPPPPPGATTSPPEQAVFPHDSHLLQIILPAVLVPIIISVGLG
IALWYCCARRKKSSAASSTDSTADESGLNIMTWARGPGAAKRSMMIQSLQ
KPETGTSPDLEENSTFEPIATDKTDTTPHEMEDTQVSELCDTSSPAELHD
TGFTPMSTFQKQFGPYGFETTTSQYNIQPGLGSPGADSHTHDGEREWTES
PLLGTWSQPRPRLEIPNFSSVSESSKNQDPPMVSPEERSRPDIPISPTST
SERFGGDYISATAVMSPLDRTMSGESEDGRFPSEC
>Mb|QLI64225.1
MGGKTANKAGYFDKLKGLLEEYRSVFIVSIDNVSSQQMHEVRHSLRGDGV
VLMGKNTMVRRALKTFINDTPEYERLLPFVKGNVGFVFTNGELKDIRDKL
LANRVAAPARAGAVAPVDVWIPAGNTGMEPGKTSFFQALGVPTKIARGTI
EITTDLKLVEAGTKVGPSEATLLNMMNISPFTYGLGIEQVYDQGQAFPAE
ILDIGEEQLLGTLAKAITTIATISLAINFPTLPSVMHSVVNSYKNILAVA
VETEISWPEIEDLKDRIANPEAYASAAPAAAAASGGAAAAAAEEEKKDES
EEEDEEGFGGLFD
>Mb|QLI64227.1
MLAIRAISRPAVSRQCLRAAPRASWAVARCYSTAGERVAKYEGTKDAQGN
YLVSLIEGDGIGPEIAVSVKDIFAAAKTPIAWESCDVTPILKDGKTAIPD
AAIENIKKNKIALKGPLATPVGKGHVSLNLTLRRTFNLFANLRPCRSVAG
FKTPYDNVDTVLIRENTEGEYSGIEHVVVDGVVQSIKLITREASERVLRF
AFQHAQSIGRTKVRVVHKATIMKMSDGLFLNVGKEVAKDFPGIEFDAELL
DNTCLKMVTDPLPYNDKVLVMPNLYGDILSDMCAGLIGGLGLTPSGNIGD
ECSIFEAVHGSAPDIAGKALANPTALLLSSIMMLRHMGLTEYAVRIEKAI
FDTLAEGKALTGDLGGKAKTHEYAAAIIEKL
>Mb|QLI64229.1
MAAAAKAPMSYGPPKSHVLDELVMGTNSSSIVSKRSVEKLYFPEEPHYFR
YFVKKFQRRAPLINRGYWLRLRAIDVVVRQFLFENRTTKKIVINLGCGSD
VLPWQSHARHALLCDDVLFVDIDYPDLMLKKRSIVLETPQLREILGENFV
VSQPDEDQILLQSDRYCQIGCDLRDISRLRHSLETLSPLADCHVLFVAEV
SITYMDTKFADALIHWSSSVGKAEFCLLEQLLPHGPDHPFAATMLSHFDK
LKTPPKSVSKYPTLAKQVSRFTGLGYTNINIWDLWEAWSSAKFVSSSERS
ALDNIEPFDEWEEFVLFGRHYFILHASATHQQEKIPFSPKYNHKEPCEKE
SIFPVSMVKHDVQTSKRRFGDVLAITDPTGAAYAVHIMGLGTAGREESHD
IYSLDGKMDMPIVPITGPPPRMCHSITDLGDYGMLLVGGRTSPANTLSDC
WVLHKGPLCKWVPTSSLPIPLFRHAAIRLRGSSLALIVGGKTGPSTISED
CYVFHATKGWSKCAILGEAPGPLFGAVLCNSPARLASDGIFTGLLAGGIG
QDGLISSKKYRWKLDNNDSQPTIRFTKHEEEFEQLDDLAVFGAKTVNLES
QTLVCGGVGACSRSQGQSILMVYMPDNDAYAVSKMGTCPEGKALPFMVGS
AIMEAKDSIVILGGGATCFSMGTFWETGVFQLSFKGKEDDGVGSCGFTRH
EPLKVRFLASQRIVSSTRQTLRQEANLEKATVATIPRVRLETPAQFEDIL
RTGLPVIISNANFGGCVQKWTPSYMIDRIGYNTKVVAHECKHDHERMDFN
SKNFRYVTEPFGKVMERAEAGERIYLRALSRDKPSERPANIQDDFSALAE
DFCLPRELDSANRCLFSSVLRVSGRINMWLHYDVMANVYAQVAGSKRMVL
FPPRDVEHLQFAPGASSSSLNVFSELQTSRMAATHPYEAVLAPGDMLFLP
PCWPHAAATITDWGIAVNVFFRDLESGYAIGRDVYGNRDLAAYEKGRLEV

ARVGKSFQHLPLETRRFYLKRLAEELESTAEGV
>Mb|QLI64230.1
MASTLPPLGAGNGGAHTQHSLPSLPAHLQSDTHITGHLASRFHVSHPTAK
LSSHALVCLNTYTSSSKGPDGGKPGSAMAGAEDMADRAWIRLGHRSENQA
VVFLGESGSGKSTIRSHLLTALLDKSSTPLSNKLSLAAYVFDSLTTTKTA
TTPTASKSGLFYELQYDTSATTNPILIGGKLLDHRLERSRIADVPTGERN
FHVLYYLLAGTSQAEKTHLGLDDGASGSKRWKYLGHPTQLKVGINDSEGF
QLFKTALKKLEFPRTDIAEICQILAAILHIGQLEFESTNNTSATGDDSGG
FSHEGGQTSTTVRNKDVLGIIAAFLGVSAADLQSTLGYKTKMIQKERVTV
MLDPAGARAHANELARTLYSLLVAWILESVNQRICAPEEDIANTISIVDF
PGFAQQTATGSTLDQLLNNAATEAIYNFTLHNFFDRKAEMLESEEVSVAP
TSYFDNSDAVKGLLKSGNGLLSILDDQTRRNKTDIQLLESLRKRFEGKNP
AIEVGSATAKLPGSNFYTENAAASFTVRHFAGEVEYPVKGLIEENGEVVS
VDLLNMFNSTKSEFVGRLFGQDVLQTVNHPIEKSTVMQATISSKPMRAPS
VMSRKGGRGRGIASQRRQQESNLFDSGNTHAESRSPKGGNKGGIDQGASG
QFLSSLDNVQKAVTDPGTNAYFVFCLKPNDRRIANQFDSKCVRTQVQTFG
IAEISQRLRSADFSLFLPFGEFLGMADAETILVGSERERAEMVIEEKRWP
SNEIQIGSTGVFLSERCWMEVARLVDVSAAQGRFALPSSDGYGNDGATPG
ERDAFGASKEQLLSGGNTPLMYGEKGRPGYFGSDDARSEAGASAIGGGDM
FKNFDTREQMAERGNEKNLEEIEEFKDSPSRKRWVFTVYFLTWFIPDFLI
RWMGRMPRKDVRMAWREKLAINMLIWFMCLVAAFFIVVFPMLICPKQNVF
SAAELSSHDGKDGNSAYVSVRGYVIDLGSFADRHYPSFVSRKTMLNYAGM
DVSSLFPVQVSALCQGTDGSVDQAVTLDYKNINMTGSPALINSQDLNAQY
HDFRYFTNDTRPDWYLEQLIMLKGNYGKGTIGYSPEYLAKLGAKRQVIAS
IGSRVYDLTAYTVGGRRLRVKPGESLPSDPKLADFMDQKVVALFQSQAGG
DITKFWNSLALAPDVKARMQTCLDHLFYVGDVDTRNSTRCKFAEYLVLAV
SILLVSVIAFKFFAALQFGGKNVPENLDKFVMCQIPAYTEDEDSLRRAID
SAARMRYDDKRKLLVIVCDGMIIGQGNDRPTPRIVLDILGVSETVDPEPL
SFESLGEGLKQHNMGKVYSGLYEVQGHIVPFLVIVKVGKPSEVARPGNRG
KRDSQMILMRFLNRVHYNLAMSPLELEMYHQIRNIIGVNPTFYEFMLQID
ADTVVAPDSATRMVSAFLDDTRLIAVCGETALTNAKSSFITMIQVYEYYI
SHNLSKAFESLFGSVTCLPGCFSMYRIRAAETGKPLFVSREVVDSYATIR
VDTLHMKNLLHLGEDRYLTTLLLKYHSKYKTKYLFSAHAWTIAPDSWKVF
LSQRRRWINSTVHNLMELIPMAQLCGFCCFSMRFVVFIDLLSTVVQPVTI
AYIAYLIVLVATKTTVVPITAFILLGAIYGLQAIIFILRRKWEMVGWMIL
YVMAVPVFSFGLPLYAFWHMDDFNWGTTRVVAGEKGKKVVISDEGKFDPS
SIPRKKWEEYQAELWETQTSRDDSRSEISGISYGTKAQAVVSEYGFPSRP
DSTTGFAAHALPYDSRNNSRMSLAHSDIGHHRMSQFGGSQFFNPDDMVGL
PSDDALLAEIRDILKTADLMTVTKKGIKQELERRFDVPLDAKRAYINSAT
EALLSGQL
>Mb|QLI64231.1
MASKLVPNDPRVREETASIRGKQYNYILGEPTSTPIETVFLIHGFPDMNF
GWRNQIPYLVSLGLRVVAPNMLGYAGTSRPDDLAQFSHKSISADIRELAI
KFVGEDGQIVLGGHDWGGALVWRTVLWYPEIIKAVFSVCTPFAPPNPVWV
PLEDHIAAGRLINFKYQLQLAGPDVERELQSEEKVRQFLNGMFGGWGEDG
KMGFSTSEGVLFDNLPGLGPSPLVSKEELDHYAARYMLQSAPPMRGPLNW
YRTRKINYEEELALTKDFKQISTPALFISATKDAALPPAMSAGMDRWFKD
LTRGEVEASHWALTQTGDEVNKLIGAWLNKVLDGAVRASL
>Mb|QLI64233.1
MTRRTPLPSPQSSVLPLRQPSAPAPAPAPAPVTTTITTASPKSKNFILAD
MDGRASRENRACASCISKLTPSEIGQVNWHLAQDQACRLCQVAQCKPEAF
TKPFCDFLQENPTVFHTVDYFKRKLSDSGFEELSARDSWNGKVHPGGKYW
VTRNGSTIIAFTVGAAYKPGNGVAMIGGHIDALTARLKPISNRPSRAGYV
QLGVAQYAGGLNQTWWDRDLSIGGRVVVRDPETGKTSTKLVKLDWPIAKI
PTLAPHFGVGMFGNNNKETQAVPVIGLQSSSQHDAEPLGPEGAFVNTQPP
RLVQLIAKQLGITSYASILNWELELYDSQPAQTLGMDREFITAGRIDDKL
CSWAALTGLLAADTNTDDSYIKLVALFDDEEIGSLLRQGARSNFLPIVIE
RTVEALNPSTYGPGLLGQTYAKSFLLSADVTHAGNPNFLENYLDDHVPQL

NVGIAICGDSNGNMTTDAVSTAILRRVGEIIGAKTQDFQIRNDSRSGGTI
GPSLSSAMGCRAADAGLAQLSMHSVRATTGALDPGLGVKFFKGFLDLWEQ
IDGEWS
>Mb|QLI64234.1
MPPAQASDPEVDVDMAHEEDDDQVERLINEEYKTWKKNSPFLYDMILGTA
LTWPTLTVQWFPDVKEPEGKNYRMHRLLLGTHTSDESANFLQIADVQIPK
AVAPNPANYDEERGEIGGYGNPGDVAAIKCDIVQKIEHPGEVNKARYQPQ
NPDIIATLCVDGKILIFDRTKHPLQPASLGKVNAQIELVGHKAEGFGLAW
NPHEAGCLASGSEDKSMCLWDLKTLEAESKILKPFRRYTHHTQVVNDVQY
HPISKYFIGSVSDDQTLQIVDVRHDNTTTAALVAKRGHLDAINALAFNPN
SEVLVATASADKTVGIWDLRNVKEKVHTLEGHNDAVTSLSWHPSEAGILG
SGSYDRRIIFWDLSRVGEEQLPDDQDDGPPELLFMHGGHTNHLADFSWNP
NEPWLVASAAEDNLLQIWKVAESIVGKDDGELPVDELDR
>Mb|QLI64235.1
MASEKSSSSLLQVGGRPRALSSADTITSASSNPFLTPTVATSRTASISDE
LSAALRPDPGTEEYFRVENNPFAFSPGQLSKLLDPKSLAAFQALGGLRGI
AKGLQTDTASGLNVDETSVPGAISFDQAVRSSALSSIGEDKSAPNPNHSS
EAFTDRIRVYKRNILPAKKPTPLWKLMWNAYNDKVLILLTVAAVISLALG
LYETFGVHKEPGAPPPVDWVEGVAICVAIIVVTAVASLNDWQKEKAFVKL
NAKKEDREIKVIRSGKSFMINVHDILVGDVLHLEPGDLIPVDGIFIDGHD
VKCDESSATGESDALKKTGGDHVMRALEAGHNPRKLDPFIISGAKVLEGM
GTFVCTSVGVNSSFGKIMMSVRTEVEDTPLQKKLQGLALAIAKLGSAAAA
LLFFILLVRFLVDLPNDDRSSAVKASAFMDILIVAITIIVVAVPEGLPLA
VTLALAFATTRLLKENNLVRMLRACETMGNATTICSDKTGTLTTNKMTVV
AGTFGTTSFSKSETESVSQWASQLPPDTKSLLTQSVAVNSTAFEGEENGQ
PAFIGSKTETALLQLARDHLGLQSLAETRANETVVRVFPFDSDKKCMGSV
VRLQDGSGYRLLVKGASEILLGYCSAIADPQSLAEDDLATAKRRQLLSTI
EQYASNSLRTIGLVYKDYESWPPAHAEIADGQAKFPSLLCDLVFLGVVGI
QDPVRPGVPEAVRKAQHAGVVVRMVTGDNIVTARAIAAECGIYTQGGVVM
EGPVFRKLNDPDMEAVLPKLQVLARSSPEDKRVLVTKLKELGETVAVTGD
GTNDAPALKAADVGFSMGISGTEVAKEASAIVLMDDNFTSIVTALKWGRA
VNDAVQKFLQFQITVNITAVVLAFTTAVYDPDMKSVLKAVQLLWVNLIMD
TFAALALATDPPTEKILDRPPQGKKAPLITTNMWKMIIGQSIFQLAATFT
LYFAGGSLLNYNTDDPQVRLQLDTLIFNTFVWMQIFNEFNSRRLDNKLNI
FEGIHRNYFFILINVLMVGLQVAIIFVGGSPFAISPSGLTSDQWAISVLV
ACICLPWAVLVRMFRDEWFGAAAGVVGNPFVAVYRASGRVWARLVGGLKR
SKRGDEAEREEGRVDGDAPEPRVNAPAIVVDEPLPAVVEPTHVERGRSP
>Mb|QLI64236.1
MPTAGLKTIISLSFVLAVGFLLVILSCALYQSYFPLLVVATYVVAPLPNW
ICSRCANPDDFVESSGAAVLDLGRFLTGFLVVMGMALPIVLAHSDLIRVE
AMVMSIVGGLLIYGTIISFSLFFHEEQEF
>Mb|QLI64237.1
MSSTLSNSSVASPPYWMNTHAHQRNTSSVSVESVLPAGAIILQDNDTSEH
QDRNNACWAKSVEVVNYTVVNGGATSVGAFIVWNVRVETLSGSYMNIRKR
YSEFDEFRSKLVKSFPGFEGAVPSLPPKSVISKFRPNFLEKRRTGLQYFL
K
>Mb|QLI64238.1
MVTDPLYQASPLCKPSPNGRLVAVLSSSTIIVRSTETLQTIHAVKLSPEL
PRPISNLTWAPSSSKILVSAGDYIQVFAACDGSFQATIHGPAAPAGGKLP
MIQFGSHDTEVLACAPFGIKFAVFDVAASKVVEISNPKFHHPTSASRGFS
LRPDTGHLLMLTRVGGKDMVSIHHPLTRRVTRSWYPDTLDAQAVQWTPDG
QWIVLWESPAQGSQLLVYTGDGQHFRTLDASSLRPDPTTEPESDMQPGIK
SCQLSSNAELCATGDHSRGITVLQVGIWRSVMRLMHPTAIAPRETLQVWQ
EQVSVGVDGRNIHSFSRSTHTVSPPTFANGSNSNSTKQSTDVRGGCSSIS
FDASSTLLATRLDDCPCTIWIWDLAAAELRAALIFHLPVIFTWHPSIRET
LLICSQDDREQCMSYVWDPLSHGPTPLYAEKYLPASKPASKMQATWLNNE
TESPELLITNSQHYVLLSLADADQEPSPWEAAVGGVELDGPSSGRDEAES
LGSARPIVIADDASNLDDTFSFRYT

>Mb|QLI64239.1
MHHHRRKSGHAHNTSMTDVRKSVTATDPTSKHKRPGMTRRHTPVNAQKLG
RSHREREREHQDAWEDERESFPQYCMTCEKQFVPHDERHLYCSETCRRVD
QQNSSSHSSQIRAYSVSSNPSYYSPGNQEPRDIIPRASPSRPNSMHFSQS
PPASPGTIPASHHISALSALRSLNIGPPSPPSPTGSNGGSLWPFSRSVAT
SPASSYRRPSGPYLSSTYDTGHHYAPGAYTYDSGSHGLDRPLPTRHPGAY
SRPKSIELVTPVLGR
>Mb|QLI64240.1
MATEPVHPLRISKNTPTPSPKKTMASPLPRPLSEISPTEKRRNSPSWQPY
SPKRSALPGNDSSPFQSSPQESSTSPRLFWQSRNSENMLHGACSGSPSPN
RRSSIERLQRASRVRNSNILALEQKQEYDPTRIPHIERPLAKVQGTVLTS
SSSSGGFRSSDSDLQEPDNRSSLSMMSPSKISPKLPEITQKSARSPSKDQ
ASPTKSSLSPTKFNKNTFDPDVGTWSGHSSLSEKELPDGRYLSRHHKSVT
FDAAPPQINEYEMATPDLSSIGTNSREGSYDSVEDEDDDDDLYDPAHIDV
QDESFDESLEDTDKTPVVGPDDWRGDSPVIHRVLDPSKFEGSPIPEPGPM
MAATGRTTPTNNKRSDSATSNGDHRPLPPLPGTGHSRSQSSGSNGASPGL
SATAERMLGGSHRSLPVPPPAFASKSDIQSFGSQKMTLEERLKLMMLSDD
NGDSKTAAEQQRERRMRRGAGRERGGTPHSEAESETALEAFEVDDTIGDI
SGIDFQIPPKISRESIMRRVNGNNAVDERSGHHISSPAASPQRSLLPHAE
ESVVDLDPDVPIPSTEDSLVESDYEQEASVIITRNSEDGEDEDVLDYYEE
ETLALDEDVAEGAQTEEEYVEQPQITQQIEEVAATAEKQDHSPHQHLKLE
PFTTGLGFDGVDDPFTSPKAVEEPVKHQPIHENETRSSEQQILEPPKRPA
TPTRRMSKPEYDGSGWGEPDDEFADEPGSPSSVIHHPVEEERTESPPAIP
ERIATIKASGAKLKTRISNTPSDLAAMREARRQVSREVPNVPPIPEKHRN
RMSRDMGAEGLLEQDEFIARHPSFKNRSLTLDLDMGLSLDQDFERVIEAQ
KRGYLMRQNTKVITASDKDGDDHSRARSAQSSPVKQGRPETWVVEPWNGE
TRPRSMRKRITGASDAIPPLPGRDSNATTINHGLEEDLQSEVATIDSGER
GRLFVKVMGVKDLDLPLPKNERTWFSLTLDNGVHCVTTAWLELARNAPIG
QEFELVVPNDLEFQLTLNVKLEKPAYLDAQIATKPVKTKTSTFSRVFASP
KKRREMELRQKEEERQQREALARQRNCAPSGYELLSPLTAEDGSFARSYV
CLKEHEHRCFGRPYMAEIACFNEWATEEESFACSVKSKRGTAAAAVVRRA
PYKIGKLELQLLFVPRPKNSTDDDMPKSMNSCVRELKAAEERLSRNWEGH
LSQQGGDCPYWRRRYFKLVGTKLTAYHEATRQPRATINLSNAKRLIDDRR
ALMEKETTGKGGRRRRSAFAEDEEGYMFVEEGFRIRFNNGEVIDFYADAA
EDKEGWMRVLSEILGRDSSMDEINGVRSRAKWCEIVLRREEQLRRKAEGR
RVHSRSKSTVS
>Mb|QLI64244.1
MAAMANGTANGVTNGDQLVRPVSTRFSDIPSAIDIPVQGDQEDEAVEIDL
EDLLDDPTELCTLFENERAAKTYWMTVALAYAKQKKIDHAIEMLIRGSGA
IQSSNPRDKVSMICCLCWMYLWKSREAPRVAPDGVRVSEAKTKEYYLQLA
TSSLNDAARLNPSFPPIFLARGVLLLLRASLQAPSKTAGGIGSEKNELLK
TAVKSFDDALRVSQGKNMLALMGKARAYFSMHKYPDALATYQDVLHKMPD
LVDPDPRIGIGCCFWQLGFKEDAKVAWERSLEINPDSKIANILLGLFYLD
ASGHVPVNSDDFLKLYRKAMTEYTQKSFKLDKEVPITCSTFAGYFLSRKA
WENAEKLAHKAIQYTDVNAIASDGWYLLARKAHYNDDTEKASDYYRRADD
ARGGADTGYLPAKFGVAQLSVLKNDLGEAKLRLEKMIQQSKNHEAMILLG
TLYAEEIFAGQKSDSKEDKSAEMKKAITLLESVRNAWKDPKRTIAPDASV
LLNLARLYELDQPEKALQCLQQVEQLELDQIPKSERPTDAENEAAAQVEM
RKFLPPQLLNNIGCFHSQAEKHELASEMFEAALSACMRIGEKEPGMDTDA
LVSSISFNLGRSYESRGLTDKAMEVYEGLLARHDDYTDARTRLAYIKLRK
NPNKEGPDAVAKLYQENTSDLEVRALYGWYLGKVHSRKRPANIVEDPEFR
HYKHTLQNYDKHDKYALIGMGNLYLLQAREMRRETESDKQKRSVIYGKAV
EFFEKALSLDPKNAYAAQGVAIALVEDKKDYKSALTIFNKVRETVREPHV
YVNLGHVFAELRQYSKAIENYEIALTKDGKANDPVILSCLGRTWLNRGRS
ERDIDAYGKALECAQQALEVAPDQVHYKFNVAFVQIQLVTTIQNLPENRR
TAEQLETAAAGLESAIESLDAIATLPQTPYPKHDIEQRANMARNTLRKQL
ERAIAKQKEWEEKNKEKIQAAKEQREAELKRREEQRQEILEKERERQEKI
RKEREAIAIRDRALAEQRAEEERQKQELEMTTDAETGEKVKRKSKAAGRT

GEGRPKKTPKKKKAARDEDDSEEESHTKKRRRLTKKESSKFKSAEIVVDS
DDEHEDDDDALERAEREMERDGMPASDDDDDLAADKMDVDEDDAPARSGA
EDEDEDTAARQAKRSRRGRIVEESEEEE
>Mb|QLI64245.1
MVSFFGLKLGSDRKKAQSKQAQGKAPPKWNRFDRNALGEGQYLEKDLSPP
QFPSSTSRPGTANSTRSMSNWRAVFKNPAMTSSLIDLAPPKRPPSTGSLR
HAASHVNLRPGMALPTALGGNGLRPGTPSRPSGAKKAGWVNPLDVHFCKN
PANTHPGTPLGRNPVVTGPVALGSPNNPTGQFDFGQHTDGQETSKNTNKH
VPSATAHSRKSDEEFVASNQYPSPPPSDTGSEGALSPMNLPALDNHSPTR
KRDAASSPGNVDMPEPTSPPSPMPLVQPTSPDRLGGPIVRNSPARRDTFA
FHQPRRQSFTKEFEEVHRATMMHPPKEGFSGNFADFDFGESVIRTTSTMS
ETEGDPSFEAARRPSTSPEATKESGRGLAPTASEPIPSSSLKQTEREESR
RPAPTYTPEGQATNVIDQLPRSPRDRALRTLGPPPAAAGAYRAHSNGTGM
GFPQGFKSRFDSEKWSRATPPRPLQPVLPPSVIHDVETAAMRSFGPAPDS
GIGYTSREPTPRQTEKLPSSPFSRPPMEGNFPMSKGLPRGRRPGPPPPRP
SSSDDDNALPFPMWSDFDMSEPRRSAMPAPLTPSRPSPSQDPRSGLSTST
SAASPRIPSPTFPSLAKSISNSSDSFGKSIDMDNNEPLGSTTLGGFPSLD
GRPSTSGGTSSIRVEAKKAPPRPAPVTLPPSSNKGIHAGLRSPVLTESLG
TFI
>Mb|QLI64246.1
MPTQHVKPGTEEHLGDVANALLKARKVVVVTGAGISTNSGIPDFRSENGL
YSLIQAQFDAASRQARQDEHDDHDEHEEAADNARPTKRRKTSMDAINEPD
WITVKVGPDDCQDALQDGPSLPQHTDRPELLEDSAVTAATAATQIVVGTK
FRTPQSKTETRSSPDLNSSPPSSPSAEFMISPSTVRSHTHRRLTDAALPL
SSSPLSSPPPVLFDPFDTVSPSEASSGRRSSTSLSEVDETDSPPNSLPSS
QNSAGSRSALPNMKGKDLFDANIWSDPIRTSVFYTFATSLRQKIRHAEPT
TSHRFISHLRDRGKLVRCYTQNIDQIEEKVGLSTRLQDGPGSRGRFSRRS
TANMTQLNKMVEEAKSASSDKLASETSSLSDAPADSSEQSQPSSSDENNI
PATDREPRSDNQANAIPKVRRELPRSGVECVFLHGSLDLLRCFLCGKVCS
WDHDNRESETLSGQQPECPHCVGATAARQERGKRALGVGKLRPDIVLYGE
EHPSSHLISPIITHDLSLYPDMLLILGTSLRVHGLKVMVREFAKAVHCKG
GKVVFVNFTKPPDSIWGDIIDYWVQWDCDAWVTDLQARIPKLWQELEPAR
PRRKRESSGTSEDSAKEEKKKPPPANPVALRDTKVTGAYWTFKVLSELHR
ITGNLQPENVPARRASLPAGITAAGIVAEVFPHAPTNKVQKETKARQKGS
RKSAPGALEKGQRRSSTLNPNHGRSRKKAQAMVSSQVPDHVPEPTTSPAL
TSVEPCSTNSILSSVKENARVRKRKKVDGEEIPAPKVGRRRGGSSRHKAK
KDDLKLPPLQPLGTNRPALLYGKPQPLEPKSPPSGPLTSLSPNLRSAKTF
QRHNAFFLDELLVGLHSAPHGHPFQNYSENVPPTQAGTKEEANAAFALAG
LRTSPVMARTMPRKQIEVTAPIYRRVLAALIFNALHTCDYKRLFRSQTTV
VMGHQDVLALRAKSAESPRPFGSEWKREFPFDPEWHNLNHGSFGTYPKHI
RDRLRAYQDQAEARPDPFIFYEQPKRIDAAREELARLVHAPLDTVVFVGN
ATDGVNTVLRNLAWAEDGKDVILSFSTIYEACGKAADYLAEYFEGKLEHR
GIAITYPLEDEEIIRAFRTTVKEIQDEGKRAKVCIFDVVSSRPGVAFPWM
DMVKACKELGIISLVDGAQGIGMVHLDLTAADPDFFVSNCHKWLHVPRGC
AVFYAPIRNQHLLRTTLATSHGFIPKLVQRTTPMPPSAKSAYVNNFEFVG
TKDNGPYMCVKDAIEWRRDVCGGEDKIVSYLWDLNKKGIKLVADALGTTY
LDNSKGTMTNCAMGNVSLPVWVGERGEGAKETDIVVPEEDRDTVFQWITE
TLVRDYRTFMLRFIMGNRYWIRISAQIYLDLADYEFAAKALGDICERIGK
REYLK
>Mb|QLI64248.1
MPWALIPLREIGFPAKQYITIVNLTPHRFVLDRSRTHSYQMDTFDWDDVP
QGRARQNTAEYTKNVGKNPVDDGGEASYTIQGTNKRFEIRARTHIPDSYP
RRTIIDLSGMGLGQREYLDPGKETAVTLVITGSDSYGFTASLRHGRGNWM
KGLYDVIKDRQMQHIVMPATHDSGMSRISGKIGSIGTEANTQTQGLNIYN
QLRAGARLFDLRVGSVHKVANQDSYSFWTMHVNDELAKVVIGNTGESLDD
VVKEVNQFTAENPGEVIFFRVRYLNGILDIPTAGPIYWSADIVNNFFSKL
KGINNRCGHLDLNTKFNKQKASYFMDQNGGKGCVLFLLNGNLKPDVPQDS
VPDGIYRNSMMDVWDHWSNLPDTEKMANDQVASWKTVGRSGDFKNDQFLI

GQWLVSADAITTTALSIQNIGILPTNPALYWMGVNNMSPESWPTAVLVDY
IGVVVADQWSWEQLSAEMYTFAIGMNLYMISENCDINQKRSPLLPGANSA
VNALDAGQVASAWNGIIYANGTVDDDPAPTLHPGRAQVLKKGTEFLNGTI
LQSDVENQSC
>Mb|QLI64250.1
MGLPGPKGASQSETHLPPHDRPSVVSVPVTPGIGRESYDQDYFERNALQS
PSTTPGTDSKVAQSPTISRKDARPGSSSERRRRPKQNSTLDGNIISVTFT
VPHVLEYRQDSDWELRRCYNRSALLDALHHLSDNGPHNHTVVAWTGEIRQ
AADSNDDAVSDDNLPITPSSTSISLGGNARMQIPPSKNVFVTRADQLRLE
QQLHEDDISIVPVWLADENDMTTEGIRLHDQSRWRRYAEHDLCALLHYRQ
HPPSEPYSEGGRWADYCQMNQRFAQRVIETYQPEDTVIIHDYYLMLLPLL
LRERCPDISVAFYLHTPFPSSELIRCLGRRQSILNGILGSDLVVFQSFHY
AQHFANTCARILGLEATSRAVQTATRRVRIEVIPMGISIATVHALAWKAT
AIEKCALLKQQHGGKKIIVAYDPKDRLGGVDKKLLAFDRFLTKYPEYRNR
VILIQVMSPTTMEADDAQEADYATGVGELVAEVNRKYGSLDYMPIQLQSQ
NLSVDEYFALLRSGDVALFTSVRDGMSTTSLEYVVCQRDGHGPIIISEFS
GTASSLKEAIHINPWDTDGVADQIYRALNMEEEERRDIHAAVYRRVAERD
VRHWATTLLRRLKRAVGARDSRYFGANGLADIGEK
>Mb|QLI64252.1
MSAQSSSYSRNPGPVRRPSIGSRLSFAVSTAEQGVASIPPPAEGQIEEEI
AEIKRYEDFTTIDWVQDASREQARRKTRQKRAAGLYDRGQQGWRYRLWAS
YDAAQGWIVVTIIGAAIGLNAAFLNIATEWLSDIKLGYCTTAFYLNENFC
CWGEDNGCPNWHRWTGFGPLNYSLYIIFGTCFAFAAAILVKSFAPYAAGS
GISEIKCIIAGFVMKGFLGLWTLVIKSVALPLTIASGLSVGKEGPSVHYA
VCTGNVISRCFAKYRRNASKTREILSACAAAGVAVAFGSPIGGVLFSLEE
MSNHFPLKTMWRSYFCALVATAVLSAMNPFRTGQLVMFQVKYDRDWHFFE
IIFYIILGIFGGLYGAFVMKWNLRAQAFRKKYLAKYAVLEATLLAAGTAI
ICYPNAFLQIDMTESMEILFLECEGAEDYHGLCEPDKRFRNIASLTIATV
LRIFLVVISYGCKVPAGIFVPSMAIGASFGRTVGIIVQAIHEANPKSIFF
AACKPDEPCITPGTYAFLGAGAALSGIMHITVSVVVIMFELTGALTYILP
TMIVVGVTKAVGDMFGKGGIADRMIWFNGFPYLDQKEEHNFGVPVSQVMR
TSVVSVPVNGMTLAELEALLSEDEYQGFPIVEDRSSRILVGYIGRTELRY
AVDRIRREGRIDPNAKCIFAPPPALNAVTPVTPSVTISTDFASSTCLDFS
RYIDATPVTAHPRLPLETVMELFRKIGPRVILIEYHGKLTGLVTVKDCLK
YQFKVEAAENPKDDHRINQGQEQLWDALQKAGYWISGHISTMSRGRIRLS
SDEDRARARGPILDGDEDVGDEGVELESRR
>Mb|QLI64253.1
MDQADIPALLARLSSDEDAARKMAVFKLQTSINDPSFADVFISSGGLVVL
RGLIMESGGNTLAYSLQSLTRLLEVDMGWDIFEGPSASAFVEHVVELIVI
NPLVNILRGAMSILVALVSHSQSSPPASPSPVGSTPGSFGFRALKPAVAV
YPQFFELVIQQLQSADHALCANALMLINALIRDSISNGSTYSSTNGSTGS
GGEDWAKLVKCLQDLGLIKAVHQLMQSSSIQDLAHPVLEFQSLTKVLLRK
WREVVVDLDRPDHRRALKGIHLASAPERAVNGHSKDDAAEGGKKDGRKHN
PEKWRRLGFETESPAHEFDTTGYLGMMDLTDYVRKHEDGFQKLLLEQATR
PLQERCPIARASLAVTIILYHHFEVDGVEMEDSKGYQGLDGKDHDRLFRP
LLLQWSRLHAAGLLGFLRIWKATGAESDDFEKVAELVRILIEQVVGRASR
TKDILEVEDDILEYDIAQLRELQMELLELSFDDTWGQHLYQVRETLKQEA
LQFVKEQRVRCLLQGSWFCKPASHNKGREDAEEAKAVSPWRFAKLSHNRR
YLHYADFEEQMAQDPGLDVLTNKMDLGTVSSVGSNVSATDDRRSHASGST
VQNLASHSKTTTKITMYAGEVNEHGEADEYPMLTLWPTSHSLASEWLDGL
LMLLNQAPITAETSKLINLVSEYGLKIRLLNVRMDSAFEGPPPGAGVVPS
RAGLDEDYFFEV
>Mb|QLI64255.1
MSSPARPSSKSAASASGASAPPIQFATKSSVASPKLNNEMEMGSLAADGQ
AGDDDAAVAVSPEKDIMQLARIGDIVAMEKLFESGEFDATYSDDEGITPL
HWAAINNQYAMCKFLIEHGAEINRKGGESVATPLQWAAQRCHYYTVNLLL
QHGADPLITDAQGYNTLHISTFNGNTLLIVLLLHQGIPVDVLDTFGHTSL
MWAAYKGFPSCVDVFLRWGASVHAADEQGFTALHWALVKGNPGCIQKIIE

YGADRFAKTESGKTPSVTATELNTQPAWHRALRECGYDREGNPVTPPWPG
ANYFLKDKRAFTSKFLFFWPWVMIWGVLIVISYAPVFLGLPLAAGVVYCA
HWCATQVLEYAPSDMRHFHKTPWMAGIFAGSLFLVGVNWLSTVVWTTIRG
ESSHMILNLVFGVLFGLTTFFYAASMRYDPGFVPKLNGIAEQRAVIDELL
ASWRFDEANFCVTCMIRTPLRSKHCRRCQRCVAKHDHHCPWVYNCVGINN
HRHFFLYLVNLTFGILAYDWLVYYYFGVVSTDAPASCNVLGPGFCKLINS
DAYTLLLSVWVSLQLIWVTMLVFTQLVQVSRAMTTFENMTGIHTREATTA
LTSTGTPLDPSLAAAATSTTSAGGHGGRHKGGMLKQWSRILGVDPFIETI
TGRGAATGNSRRKKKNPYSRGCVSNCKDFWCDPAPVFGQRENGTAVLGGG
KVDYTAMYESPTLMSLTGMRTRGGYESVETEEV
>Mb|QLI64256.1
MGIETLLRKGGRKEGASRARKDADEYGPSYDKQWRRRQAHKYLLEPLTAP
EPNQETGLGSSHVNPYITAAKERLEAQTAAATSAASATSAASPPPSPAAK
KKENRPRPWLLKGLQGIPAYWPSTSATPHQPYPTPPSSASPTRRSFDVFP
SSPFATSYQSRQSSESSHLSSHRNGSQQSTRASSESISTAALTTSSAATP
PTSPSQRNSTSSAGDNNNALCKNNTHSPPQCQPRYARLSANSSSTTWGRQ
SYPEYPRNQLDKARRIRQNASNSTDSNKKKNNSNRKSSSEARLAERFPGD
MSHRPLDMIKRDTRAAERPHRHRKRISETDTIDALDTIGGAYHHGGPYDA
TLRSRNLNKKYSPVAAVQDSNMEAIRATPRENIMDSLIKHVPLQGTASIP
SGAPDMRGRIMYYEEGADLMREPDAAGGAYKRWDGIQYHPDDLKGKGESS
FTLERDLKKGRRHRHHLSEADAFEMAPGTNRFSRWTASHQRSVSQHSAEA
LSSGVTYKNNPDGDLRRAHSTGNKLGDGLKRRFGSIRRKKDTPSEAVP
>Mb|QLI64257.1
MSHSHSHEGGASHSHSHDGFSAQEHGHSHEILDGPGSYVGREMPIVEGRN
WSERAFTIGIGGPVGSGKTALMLALCLALRQKYALAAVTNDIFTREDAEF
LTRHAALPPQRIRAIETGGCPHAAVREDISTNLAALEDLHGQFSTDLLLI
ESGGDNLAANYSRELADFIIYVIDVSGGDKVPRKGGPGITQSDLLVVNKT
DLAEAVGADLGVMERDARKMREGGPTVFAQVKKDVGVDHIVNLILSAWKA
SGAEEERVQQGGPRPTPGLDGLDGGKSG
>Mb|QLI64258.1
MLKPGLILILAAVASARQCQNITVPVSISSRNGVFDMAPPSTDIDVTNLF
LRMAWPGNNGTANLLKGYKTVSGNYTLAATYCEPDNGPGHALQILTHGVG
FDRSYWDFSFANYNYSYVNRALDAGYSTLTWDRLGIGHSSHGDDPVNEIQ
IFLEIAALKALTDTARGGQLRGVAHKFNKFVHLGHSFGSAMTYALTALHP
DITDAIVLTGFSQVPQFLAYFVLGANFAPVADNHLLAGHYAAGYVAPRDS
IGVHINFFGPGDFDPEVLRVATQTGQPAAVGELLTVGSVPKSSEFPGPVM
IITGDRDIPFCGGNCMNTMAINGSAPNLVEYSRDSFKKASAFQATVVPNA
GHGLNYNYNAPDVYRAITDFIKASV
>Mb|QLI64259.1
MLSFILIQNRQGKTRLAKWYAPYSDDQKIKLKGEVHRLVAPRDQKYQSNF
VEFRNNKIVYRRYAGLFFCACVDTNDNELAYLEAIHFFVEVLDAFFGNVC
ELDLVFNFYKVYAILDEVFLAGEIEETSKQVVLTRLEHLDKLE
>Mb|QLI64260.1
MKAIIAIPATLALILRAWSKNSLTPAGLFAATLTAIAHAYHPWNLPFALL
CVFFLAGTRVTHIKENVKATLTLHSKGSSGGEGPRTHVQGMVMRRVPMFA
NSLMASIFAVAHGYQLRARAAAYADPNTPNPKGSMCYSWGGDLLVVGIIA
NYAAVAADTFSSELGILSKSQPRLITSLTLRKVPRGTNGGVTLLGLVAGL
FGSMVIVAASMVFLPACTPGTAPTLGGGAPWTLAQRRVFMGGLVIWGALG
SVLDSFLGGMFQRSVRDVRSGKIVEGEGGNRVLVSATSRAAPEAAVDGGD
GGKAGADKQDGSAIDDQDEDDAKYNLRNKHRKPSFGDQRPSRVIESGWDL
LDNNDVNFLMAAIMSVGAMGVASWYWGVPAESILSP
>Mb|QLI64262.1
MVVLKAESEWGYAPPPQTPYSIISNIPGWDMLKGIRDGDMAPLAKVVHIY
PRLSPTHYVRTLAGGVAKAVGLEGKGCMVYLDPVMWKYTASHVSHQQRRQ
FAISPNKLIFKAVDVAGHRLYAVLFEPEHTKALMLSWGTPGIGLSIRGAE
QLLDKIDTLTEVPFENADAPPKPTWTPEGPSHELLRRRIVELLQHGAIDP
QKVQCRSNDVFLYPSGMGAVFTAKNLLQEYRPGFVNVELGIVFHNTHELL
HEESPGGWKHAPKVDKQGIDDLEAWLESQKSKGSGATFVIVEYPGNPTLD

TPDLPRLKQLSEKYGFVFIVDDTLSSFGNVDVLAQSDLLLTSLTKSFSGR
SDVLGGSIVLNPLSPHYAELSKRFAASHHNQLFAADAEVLLSNSQDYFER
TSRLNDNARAMAEFLGKTIGQPDSPVVNVQYPSLLPSKANYDAFKRRSTP
ELPTPGYGCLLTVEFDSVETARAFYDRCGFYPSPHLGGHVTIMLAYNMFM
FGKDKDEAAALRPCGVKEESVRISAGLEDAQDLLDTLQDALNVAIEVKKG
SKTQ
>Mb|QLI64264.1
MNPHQKNKVDVKSLSPEEQRLFRLYGKLPSRSDHFAKHLKDRKYFDSGDY
AMSKAGKGDGVDAGAVGSQHPVPENIPHLSSPINNGASIPKHGHHGSIAG
IQAGSPVKESSFLHRETSAEDASTGDDSIPSNNKENVSLTSAPLQSNEGI
PIRR
>Mb|QLI64265.1
MSSDYSYDDQAQFFPFFILTLTGLVTLPITYNLLQSSKDDSHLAPRIQTD
YKIQHGDVVASLRAAQKRKQRKIKRALVAVAGWGLMGLMAYLIMTTNPAE
QKLWNPYDILGISESASENQIKSHYKRLSVKFHPDKLQPDPAKNETVESL
NEHYVELTKAYQVLTDEDVRKNYIQYGHPDGKQSMSIGIALPKFIVSDGN
GKYLVVLYTGLLGVLLPYLVGSWWYGTKKRSKEGVLMESANNLFRHYDEE
MDESGIIAALSAGKEFESVLRGDQAESGLSKIESRISAEGEASPFASGFS
VKDKGRLEDLDSGVRRKVLGLLWAYLGRVELDDAALTKAKYDIAPIARAL
NQSFAAIALAFGNIGPISGSFLANQHIIQALSPKSSPLLQLPHFTSKIAM
AVEGDSKTHMTVQQFMDLPDAQRRQLSVGKGLLSEKQYKSAVSVAKQLPY
LRVAKAFFKVTGERCIIPSSLVTLVIKGRFIPPGTQNVPAVNDLDLEDVD
PAEDDLDALMGRKKKTITGPDGKPTSVEEQSILPPLAFAPHYARDHTPKW
YAFLSDSKQGKMAVPPFTFDRFDEPIFDEQGKPTFNMQTLKAQFAAPPQA
GHFTFVMHVICDSYVGFDTKMEVTLVVEEAAKAAEMDAEDDISEPEEDSI
AGQMHALKTGQAPQPKRRRVDEESDDESGTEEEEEETSDTNTDTEDES
>Mb|QLI64266.1
MASQLLPLELIDKCVGSRIWVVMKGEKEFSGTLVGFDDYVNMVLEDVTEF
DYSGTHTKLPKILLNGNNICMLIPGGEGPA
>Mb|QLI64268.1
MGKPVALVVGASRGMGRQVAVDLARNGYAVVVAAKSTSDASKNVPFPPDP
NSPDSTITTVAREITTAGGDALALQVDVRFEESVNKLVSDAVAAYGRLDV
LVYNSGAIYWSSVANTPLKRFQLMQRVNPEGLYAVVQAALPHLSPAARVV
VVSPPIYSRFFRGKTAYAMGKVGMSVLTKGLGMDFAREGKLAAGMAISSM
WPAVAIESAATQHFGGSSPEERRDLRKATIFSDAVLGVLRAPADKVNGEL
FLDEDFLRDFCGVADFQKYAVVPGAQPRRMLPAKLPDLSVAEQDDEGKRI
DLAKPKI
>Mb|QLI64269.1
MPEAITSTASPAAPHEPPFTYTANNGYVETSQEVPPEVRMPRGAPHPEEY
NEDDDNDFDDIFEDDDLDEEEWGGDARDLTKNYNRQRQLQDNTNGAAAAP
RSNQQKPTANTFASVDDQVSALSKYAAKIRLDTVKQSDEKDKDKADRATS
DQVLDQRTRMILLQMINRGFVSEVHGAISTGKEANVYGAMLVDDKSGDAV
HRAIKVYKTAILVFKDRERYITGEHRFKGGFDKGNNRKMVKLWAEKEYRN
LRRIHTAGIPCPEPITLKLHVLVMGFLGDKKGWAYPRLRDATLTGDDVDQ
QWRSLYIQLLGVMRKMYQVCRLVHADLSEYNILYHNGLLYIIDVSQSVEP
DHPRSLEFLRMDIKNVGDFFRRKGVDTLSDRAIFNFITVSTGPVEEPGLG
ETIDKLYETRESAANEDEAAALEIDNEVFRNQYIPQTLEQVYDIEKDAQM
LSKGEGGDLVYKNLLADQVVQPKQDEGGEGSEGSDDESGSDVSPSDSETD
DEALFDKGRPRGRKFEDKDEKKQHKQAVKEAKREKRKEKMPKHLKKKIVS
STSRRKK
>Mb|QLI64270.1
MSNPPPFQSALLAGALAGTTVDLSLFPLDTLKTRLQSSAGFFPSGGFSGI
YRGIGSAVVGSAPGAAFFFCTYETAKGFLRARAAVPDAVAHMVAASLGEV
AACAVRVPTEVVKQRAQAGLHGGSSRAALAAILSQRSARGLGAVWRELYR
GWGITVFREVPFTVIQFPLWEAMKSWRRKGRTAGEDVAAAESAVFGSVAG
GISAAATTPLDVLKTRVMLSKDRVSVAEVFGTMVKQEGLRPFFAGIAPRV
TWISIGGAIFLGSYQWAINTMRTIS
>Mb|QLI64272.1

MATPQSTTSYEDSGSKKLEQITFRFCSECSNMLYPKEDEDAHKLQFTCRT
CQYTEEAQSTCVFRNVLNNSAGETAGVTQDVGSDPTVSHVPRVICLYCGC
PVFCYICGDLAADMTAPRREEEKDNEAEMEWTSLSQFVPLDGYDDPFAEY
DEELMDEAEDEVYTPTSSDFNSVANSESGLSDYHVRTITC
>Mb|QLI64273.1
MSDNPRGVEHTDSIDSHDSAPKQPEKTKSRRPPNTAFRQQRLKAWQPILT
PKTVLPLFFTIGIIFAPIGGLLLYASSLVREIKLDYTHCINDAPDFTTFA
DMPGSAVESAFKNANSSVRAQWARKKDVNVTLHNGREIKGNQCVLQFTIP
EDMGAPVLFYYQLTNFYQNHRRYAESCDLQQLKGDARSYSDITGSKCTPL
YGIKPNDTGKPYYPCGLIANSMFNDSFSSPAWQNPPNDGKARTYNMTDKG
IAWDSDKDLYGPTKYKASDIVPPPNWAIAYPDGYTTDGMYRPPDLQNWEA
FQVWMRTAGLPTFSKLAMRNDQDTMVSGIYQITVDDHFPTIEYKGTKSIL
LTTRTVMGGRNNFLGIAYITVGGVCIILGAIFTATHLLKPRKLGDHTHLT
WNKVPASKSSGPSTAMASGREIRPGDA
>Mb|QLI64274.1
MPKSVCIVGAGPSGLLAAKNLLCNAPKGCFKVTVFDAHEGIGGLWPTSKT
DTKRQVHPLMVANQSKHTMQFSDHAWEDGAPQLPLAWQVGRYLEQYTSRY
LSSHPDFSLHLNTRVVRAERGDGDGSGWDVRLESGGREQTQHFEHLVVAS
GYFGKPITPEGVSIAPSAAIPVIHSSQYRDLKNLFGTRSSAKGKILVVGG
QMSGVEVAGTIASHLSSATNSPDDFSPKAAAAPFLPLDFSSYNRNNRPLP
LVNTQGHIDEKTAQVVNGIFLNALGTDQSAFSPLLYVDDATKTQPPYLAV
SDWYCDFVRSGLISLSNGKFESISGNTAKLDDGTKIDDVVAIVLATGFDP
SPCVSYLPSKILKTLNHSPQHTEQPLALAFHGTHHPDVTGLGFVGFYRSP
YWGVMQMQARFLAQYWSTPIDARPEALELKLKSDKSIQRTLDLRDDPRLS
QFPMGDYPFLMQEFSEALSIPRVDPSLSGIPTLSYNSLPLDMLTPARYPS
PSDDASAKADAEKLRQDTVHVARDGLTTPRFMPRAVFRSLLGTWKLEREL
TSKLPTHPSGHFSGTARFLLRDKTSDGLQCARQSLGSSDPDEEGGMEYLY
IEDGEFKTEQGFGFRASRRYVWRYNEKEESLSVWFAKPGDPKRADYLFHE
IEFLQPENGREKGWSAKAGHLCIDDYYDVKYNFAFQAVNLQNWSIEYTVK
GPKKDYSIKGRYTR
>Mb|QLI64275.1
MLLNIVLGFAFAAVVAAKPIATDDYGNTLETRGGNVQVSETDLTNMKFYS
QHSAAAYCNYNTAVGKPIVCKDTACPLVMQNKPVVIASMVGRLTGIGAYV
AIDSIRQEVIFSIRGSNNIRNYITDVIFAWRSCDLAHQCKLHTGFAEAWD
EIKDAASAAIKSAREKNPGYKVVITGHSLGAAVAIISTAYLRRDGIPIDL
YTYGAPRVGNDKFANWFSSQQGRHWRVTHENDPVPRLPPIFTGYRHVTPE
YWLSGGDAFQTVYDLSEIRVCKGIANIKCNASRFITDILAHLYYLGYTGG
CSASPLRLRDGQEEDPLPQDLKDRLTAWSQKDQQFVEENDI
>Mb|QLI64276.1
MAALPDLTDWLVDSNDALNVSLVSPSKSGLRLVGSFHPSFTYPIFGDEEK
IFGYKDLKISLRFRANDMRPHLETTYSKQLSPSAGVEEPTDINAVLQTGN
HLPKVAFVKSSDFESSSQQLGDNWTPPGTLQETIDGPDGQYEIWKGSLED
PAIKQLNSRIQILVPMLIEGGSYIGQNPESDSSDIDLSDANRWTVFFLYR
TQKSDGEPDKKSYVFVGYSTVYRFFYFGRPPTPPPESGDKWELPDGSFDL
SNLPCRTRLSQFIILPPFQGKGNGATLYKSIFQHYHKHDQTHEFTVENPN
EAFDDLRDVCDLTFLKTIPDFVKLKLDSSVTIPKKGPVPNLVVGGDKLEE
IRLNAKIAPRQFHRVLEMYLMSQLPSSVRPSMALDDDRPAPTQADKHLER
LWQLIVKQRLYRHNREALSQIEQAERIDKLQETLTGVELEYARILAAYER
AAGHGLGPSQSNGKRKLDDEAGDKTPSKKARVEDA
>Mb|QLI64278.1
MIRLGAARQAALGISRVRWTCPACLCRRQYSQKPEKREPPDHRKIGLQQE
LFITSAYSPGSPIFLPNGARIFNRLIDFIRKQYARYGFQEVITPTIYKKA
LWAKSGHLENYADDMYTVTGNTSTPDKDAGGCCGGGHGPAEPDLEADNEY
GLKPMNCPGHCLIFASRVHSYRELPVRYADFSPLHRNEISGALSGLTRVR
RFHQDDGHIFCRPSQVEEEIKKTLDFVKVVYGVLRLGVGYRLALSTRPKD
HFIGTEDEWNRAEDRLKMALDSSGMEWSINEGDGAFYGPKIDIVIKDSDG
KEHQTATIQLDFQLPKRFALEYQAPAPEYEARGETTNDPVLLAEYGPVRP
VMIHRAVLGSVERLMALLIESYDGRWPFWLNPRQAIILTVNTSRPVLDWA

DEVKEILLGLKKEAGQTSTEGPVNPTGLAVDVDYSARSLGSKIREARING
YSQILVVGEEDVKHGQVSLGKERMSPMEMRDKMCKLADAFE
>Mb|QLI64279.1
MKGRPVEDVVYDRLYAKPKASDPQNFQTFLQRSLIPEVRQETHAFYGHLE
TQEAKYPGLDYTHPTHRIRLCRWPWHRLLFQAFDALRLTKAEIRGLTKWE
GTKWAKEKFEKEQGITIRDTTADEVSDWVPPSEVQLPETSSITQAEPEQI
QSTTSIPSLGVLGDEDDDANQEDESDGEFESVGVYLNERLRDRATRREAG
DTSIVLDEEWEQWLKNAIDSGELALLTEQMTEQMIRRAPSTSVIPAALIP
PRMLTAARAGQWSEIPEVLHPMLLRTLEAENPSLRAESRRSRVTAAAAAP
HSSLPRATRPTIRLPNAQDAF
>Mb|QLI64280.1
MPSTHQQDKPWDTDDIDKWKVDTFTSKDNVGGTFAEESSFVTLFPKYREV
YLKEAWPLVTKSLEKHGIACTLDLVEGSMTVKTTRKTFDPAAILNARDLV
KLLARSVPAPQAVKILEDGMACDVIKIRNLVGSKDRFVKRRQRILGPNGS
TLKALELLTETYILVHGNTVCAMGGYKGLKDLRRIIEDCMANIHPIYHIK
ELMIKRELAKDPELVNESWDRFLPNFKKKTLSHRRVPHKVTDKTKKAYTP
FPPAPEQSKIDKQIESGEYFLAKGAKDRAAREERNEKQKLRKEEKTKERE
AEFVPPEENRPKKKRKKSSD
>Mb|QLI64281.1
MNVDDHVMPSGQHYSASNRVPNIQQFMDQLDAEKKERDAAIDSQLKANRL
HKEVEDHTGSEKPLRKDLRTVRDPVTGKDVQIQDAKTDFKEAVENPQLSI
PNENLGKEATIATSSKQSGEEYRYAQDVTAPPDPVQEGSTSDVPIRSEKT
SVLFYKTPSVSYEPMFATLERKGNVLCAGIFVSVIVVGKLFGGRLLGLVP
LGFCIASGVFLWIKDLIRQGRDLEWSSEQERGETATVNLIPESVEWMNTM
IGLMWGLINPEMFASVADTLEDVMQASVPGIVENVRVADISQGSNPIRIL
NMRALPDSHVKDIKDEIHKENEKKMDKDELAAVEHAGEFYNMEVSIAYHA
RPSGEDVASKAKNMGMQLVFYLGIKGLFGVPLPIWVELVGLVATARIRCQ
LTPDPPFLKTVSFTLMGVPHVQAGCTPMIEKGVNILNLPLISNFVNWAIG
AAASMYVAPKSMTLDMSKMLQGDDIKKETQALGVMFVRIHKATGLSKQDQ
RGSKGGGSDPYITVSWSKFGKPQFCTRVIQDDLNPVFEETCGLLVTSDLI
KADEQLSMELWDSDRSSADDVVGKVELSIQKLIQHPGKMFPQISKLRGVK
ADSTMPGELHWEVGFFGKTQFRKALRTHGKDLKLPKELVDKAEFQEEKGT
ITNEQEDAVVHTPPDPLWPSGILSVVVHQIVGLELANIKGSDGKRKGKEY
EPARPEAGEVKEEQSRKLPSSYCTILVNDDLVYKTRVKVVSSQPIFNAGT
EKFIRDWRSSVVTVSVRDSRNRQHDPLIGVVPLKLSDILQTSSESTRWYP
LDGGIGFGRIRISILFRSVELRLPPPQLGWDIGTFEFLSGAITTTGYAPV
SKVKLRLRTGGSSASVKRQFSTRDADGLKIDISGGQDGKIMRLPVRHRYR
SPIFIEFYPAGSRHADAYAALWLLELVDGEERDFDVPIFKCDNSVRLSQN
YITQENYREIPDTYIEQVGRVKFRGRFSAGTDSDHVRFVSDNDSRETIES
WEACYAEGVRQQEVRAEVPPLVQKLHDQSLTQGRDVLAQADDKEKERWMA
KDGTDWSGAFGQDPKQLMARRAEDRGDGEEYDDFDEDGDYDDNNDDGDDD
DDDNPNLGIHDAASEPSHPSEGSDAARQSMDTDITSGSGASQTGSGKNPY
KAYKDYKGHSRDLHRKHRGLMQWRPMRNVQFAKDEAKFALRKVTKLGALD
GREPDVETEV
>Mb|QLI64282.1
MATRRSQFHQEVLIDTTPLPDNVPAVKEIGASSAPLLSASFFIGARCRDY
NDDYMQCKTENPGRGEFDCMKEGRRVTRCAQSVLTDINTHCLAEFRKHWE
CLDDRNQQLWQCRPAEWKLNKCVFDNLKLEKKIPDQPKNVTPVELRPKQI
FSDVRIGPGDGKPFIAGQDAKQ
>Mb|QLI64284.1
MPSHGSPSPGHQQGTKRQRQSSYDAASDRNSQTSPSEQLAGDASQSAAGP
ADAANAGPAKGGQSSNFRNVSACNRCRLRKNRCDQKLPSCASCAKVGVAC
VGYDPITKKEIPRSYVFYLETRVELLEKLLVANNISFPPAENLELCSRPS
ADAASTLSATDSGYSGQSESGDRAHRGQHHALEALKAKKAPQSPALASII
SPPKARSLASASGVSFARVVFAAVQYSVSEQTGSAERSAGRKQSVSGASA
SMRDSFFGLHTRPTIQPAPFPKKEEGLRLATLYFEHANPQIPVLHRVEFM
QTFEKAYKNASKGLTARELYMINMVFAIGSGVILGEPVRTPAPTNPRLPL
DQAPGACQPEEYHASAIVHLEECLSTSGGYLEVLQAVLLLANFALLRPVP

PGLWYITGVAVRLAVDLGLHHEDGGDADAIPPEPTSDPHAAQAAEIRDGS
AQDRGRRHWIRDLRRRLWWCTYTFDRLVSTCVGRPFGVSDEIITTELPSL
LDDEYITRNGFLEPPEGDDHPSYKHVAHHYFRLRLLQSEILQVLQHNQAQ
IARASNANQAKLYPEMRSHLPSPFLVHFDSFRSWRVDIDRRLYLWKVSAP
TREETGVAFSTEFLELNYWQAIILLYRQSLSVPAMFEGEYNTSDEVNSPT
AFTAELREDEDGIYLKVAEAGQKILRIYRQLHLSGLVSYTYLSTHHLFMA
GISYLYAIWHSPVVRSRLSMEEVDFTVLAAKSVFSDMVDKCPPAETCRDA
FERTAKTTIKMASSSGGFGVPPQQPRRRRRETMPWTSTQDGSSMKPPARA
HRHRQSEAAPFPFDLSLSDGLSSPSMSNAGDMAAQHTPPLGKTSKTYDSD
AFMSGQGAARSGPSPSDAMSQDDGGSSIDPSLVPSTPTTRRSAPQHGASG
GSFLGQQFGLQGSMDYPDAQTMEFLNNLGATPDGDINGVDQAHMDLGFGM
NWDGMHTEYGDGQQINPFDTFFFGGQQGSGGVGNINEDDKNNNDSSNGNS
NNRNNN
>Mb|QLI64285.1
MRLALPPKPRFLSGVPPNAIGIVALVAAVNLVVWASIAVVLHHHPALISA
AALSYTLGLRHALDADHISAIDLMTRRLVSLGQRPATVGTFFSLGHSTIV
IVTCVVVAATSGALRDRFDGFTRVGNIVGTAVSATVLVVLCAANAWILYG
LVGALRRELRRRRGLAAGTLEPALPYDHDHVHGGGVLVRVFKFLFKAVDR
PWKMYPLGVVFGLGFDTSSEVAILGIASLQALKGTSIWLILIFPILFTSG
MCLIDTTDGAAMVTLYSSKAFSSDLVALLYYQIVLSAITVLVSAFIGIVQ
ILSLVQNVAEPEGSFWDGVGAIGDHFDIIGGSICGVFLLVGIASILLYGP
WRRRVDRQTQGLTAAEDESGRGSEEPASPFDQPEVPQPATAK
>Mb|QLI64287.1
MASSSATPPSTSTSTSAAAAASLQVAAADAPSDGKLPARPKQLVNDPART
GFDPETKWWMNYFKILSNQMTPEGKFHYREWRYKVHEERDCKRCEEYKDW
LFTYSPVVRFLSDKIQDLNGKLDRSNIHCRRCPSVLEEDGTVNRQAGGFD
PNHGILICANEVRDRKHMEDTVAHEMVHAWDHLRWKMDWVGDKDLKHAAC
TEIRASMLSGECRWTREAFTRGQWSVTQQFQNCVRRRAIQSVMARPRCKD
DVQATKVVNEVWDSCFSDTRPFDEVYR
>Mb|QLI64288.1
MAASGPPSPISEKQAFDLNLQHGHKDLVQAIAFNTYGDRCATGSVDGKIR
VFNRHKDGTWRLCDTWTAHGGEVLELQWLPATIYPNLVASLGIEGWFRLW
AEDPSAAPGRRFCAGRSVGGRPAFDTRSTRAPYRSFSMKHNEETRHTYLA
LLATDGRLTVYENDQPENLSEYTSIDEFSVCSKPNRGEEASFKVRFDPNP
EPCYAALRAGVGADSLGIVVAGMDTVKVYRSRDIVTASYGVAQTQKEFYL
AAEIHGHRGLVRDVAWAPGNIRGYDVIATACQDGYARVFRVDTPFSDDDG
KSWSAGDLLKSRGHTLSARDSPTKDEAAEKHPHPSTLSASLAKSGNAGDR
QWSGQPGQVHHTHREISRLDSHRTPVWRVGFDDDGQIFGSTGDDGRLLFY
RQTPDGVWAKSSELVMVKARMATP
>Mb|QLI64289.1
MAPLPPTAGKAPSARALRMQRITGQIHPLLPSKVFRTDAHVSDDFINSKR
DRRIMKHSSFVSRITSSKIAKNKRRRPKNKLKTTLEGLADALPELKSGEG
GEEVLAGRVKHKSLRSKKGALKKKEKIVKGEMERFGASMAKLSAMHRQDA
TRKTTQSRTDEDGYVEDGSDDDGDDENMATEARAQKSISQQMSTTTPAAT
SDRWAALRGYISATMEQNPAFARK
>Mb|QLI64290.1
MSSSSSCLQVENAVEILSNPTSNQSVKEQAFEYLNQLRSDPQGWQACTNL
FSRAPRSSEVVRMVCLEVVNYAVHTQGLDAESLSYLKYTLLQYVRQSYGP
DAQQEPDPASLQNKLTQTLTYLFVFLYQDGWQSFLDDFWGLTGLQSNRDS
VSGVLFYLRILSSIHDEIADMLLSRQSNDAKRNTELKDQLRAQDVHKVAE
SWKQILNKYGDNDAVIELVLKVIGKWVSWMDISLVVSQDMINLLLPLVGR
TTSGGGEDKVRDAAIDTLTEICGKKMRSADKMELVSFLNLQDIVGQLIAS
APLSTHKGTSKYDTDLAEAVAKLVNTVMSDIVKALDDNHIGEESRERAKQ
HLHGFLPFLLRLFSDEYDEVCSTIIPALTDLLTFLRKLGQLPQDYSSMLA
PILDAIIRKMRYDETSTWGNEDEQTDEAEFQELRRKLQNLQKTIAAIDQQ
LYMNMLSNLVASTFQTLDQRGSEMDWRDLDLALYEMYLFGELALPNQGLG
TKNQPSTEASERLVVMMQKMVGSGIANFSHPAILLQYMEICVRYCIVFES
HPDYIPQVLENFVRLVHHDHVRIKTRSWYLFHRFVKQLRSQVGNVAETVI

HSIGDLLPIKAEVPGEDADDDMSSDESDHSADALFNSQLYLFEAIGCISS
THSTPADKQAMYARSVMDPLFQDMEVHLPRAKSGDAQAVLQIHHIVMALG
TLAHGFSDWSPGSAATSHHPPPAKAVSDEFSRAAEAILIALNQLNSSAEI
RTACRSAFSKLLGVLGSAVLPQLPQWIEGLLSQSSSKDEMAMFLRLLDQV
VFGFKAEIYDVLNVLLTPLLQRIFGGLSEPISGTDDEIQLAELRREYLSF
LQIILNNGLDGVLISEANQGFFEPMISSITELAKTLDGNIGPSRLAFTLM
ARISAIWGGPDVATISQNPVAPTTGPAPAIPGFDRFMLDRFHSVCWEVMR
NPSFRPAQDAQTRQVLTEIAGLEQTIYTKTGDVFIRELQNGLFPTLGING
DEFLRSLTTSTDKKGFSSYLQGLLKNRR
>Mb|QLI64292.1
MKLYAIAAVAALVGTGFSAKNERTFAVLRFTNKQLTKGRMDPIVSPGQVS
THVHSIFGGSNFGLGSTGKDLMDSKCTTAMIKGDNSNYWVPSLYFKDPKT
GKLEDVELFYANAYYFFEPTNDDIKAFPVGLSIMSGDQTIRTPPKDGATS
NLDPSKGTVNPIKWTCPRSNFDSPSWPAGSNGLAAGIQDPNNKGEGVGFP
DVNCDGYASPLRADVHFPSCYNPAAGLTNYKENMAFPTDDGKGKQDCPKG
WIHVPHLFLEVYWNTPLFKDRWEQGKGQQPFVLSNGDATGYSNHADFMAA
WDEKLLQHIIDTCNAGSQGMDKCPGLNGLNKDDCTIKSQVDETVDGVLDA
LPGDNPITGWSYGGNGTEGGGNEPKPSQSQSTGGDNTAKPSSTNKAGGDN
TSTAKPTNSQPAQPTNSQPAQPTNSQPTQPTNSKPTGGDNTSKVSSAAPT
SDNKPTSTKNPDNATTKTQPTSEAGSSKPTLPSQPSACTPKIHTVYETIT
VTAQLPGATDRPVSNSTRTAGDFKYAGCFKDSRDRVLKDDVRPNLGAISN
AKCVAHCKAKGYNLAGTEYGGQCYCGNELVRSEKLDDSACNIACEDDKSD
TCGGGWALTVFSKDGEASLKDVKSRRHAHEHLQRHRSPHY
>Mb|QLI64295.1
MPVVKGGVWTNIEDEILKASVSKYGLNQWARVSSLLARKTPKQCKARWNE
WLDPSIKKIEWSKDEDEKLLHLAKIMPTQWRTIAPIVGRTANQCLERYQK
LLDEAEARESSSLGLMGPDGGETQAPSADDVRRLRPGELDPDPESKPARP
DTIDLDEDEKEMLSEARARLANTQGKKAKRKARERQQEESRRLATLQKRR
ELKTAGINIKVITRKKGEMDYNADIPFEKKEAPGFYNTSEEKTQNEAQLR
GFDPQKLNLATKRKGDDDDDDDGDRKRRKNEKEGLSESQKAAIKAGRMQK
IREAEQSSKRRPLNLPAPQVSEGELEDIIKMGKMGEAANAVARESDNDAT
RGFVSSYSTLNTGAPIRTPKAPEQEDHIANEIRNIRALNDTKSALLGGEN
TPLYEGAGSTGFEGIAPRKQAMATPNPLATPLRSGAGVGATPGRPGQTPM
RTPRDTFSLNQEDGMPIPSATPRDIKMHDLAMRNQLRSGLASLPKPKDTE
WEFEIPDEEQETARADDTAEEDAAERDRREQAIRAAEEELERRRRTQVMQ
KDLPRPSIVDLTMLLKKADSIQNAAEAMIARETALLMANDAAQYPVTDSQ
VKGIYKPIERFDDDALADARLLILSETKPRPNLEQIQDFFESRAKNSMLL
GLGCYNDDEEEQEAAMRAAFDAVQDSIMASAEQGAKLEKKLALHLGGYQK
RQKMLKDKISDATDALEKARNALSGFKTLAISEDVAIDRRLEALREEVMY
VTKREREAQEEYRKARDELSALREDGRNGYH
>Mb|QLI64296.1
MSLTPTMPKDDSKPPLDDGYYDLGTHHRHITTKHPVAQTWFNRGLVWCYG
FNHEEAAKCFQRAIDHDPSCAIAYWGLAYSLGPNYNKPWELFAKNELTTT
AQRTHRAVEQAKTHSANATPAERALIEALQFRYPSTDAPAKSDYCIWNRD
YADAMASVASQFPQDLDVATLYADAMMNLTPWQLWDIKTGEPAKEARTLE
IKRVLDKALAHQGGDQHPGLLHLYIHLMEMSQTPEAGMPAADKLRGLVPD
AGHLNHMPSHLDILVGDYARAVIANTDAILADEKFLKREGPMNFYTLYRS
HDYHFRIYGAMFSGQSRVAFETVDMLEASISEELLRVENPPMADWLEAFL
SMRVHALIRFGKWHEVLALKLPQDTELYCVTTALLHYAKGVASAALNRVD
DANAHRRLFRDSVPHVKPSRTLFNNKCVDILDVAEAMLNGEIEYRSGNYD
AAFAHLREGILRYDWLPFDEPWGWMQPVRHAYGALLLEQGRAEEAWGIYR
ADLGLDDTLPRVHRHPNNVWALHGYHECCVKLGRMAEARDVEPVLKRALE
VADVPVRSSCFCRTTVTARI
>Mb|QLI64298.1
MTTILFLIFAVEVLAQFITAVGAAQINNLIWTLINYLPVSTSKAAAEQRT
LQAEYLKVRRDLNATSSQDEFARWAKLRRQHDKLLEKLDASKKAMEASRA
RFDNYLTAIRMLITKVPQYFIPFWYGKEPMFWLPYGWFPYYAEWIISFPR
APMGSVSAPSWQLACSVVITLLAEILMFASRQISAQPKTKVAQPGEEAKK

PGKQTAEKSEEKKEL
>Mb|QLI64299.1
MGRRKIEIKAIKDDRNRSVTFLKRKGGLFKKAHELSVLCSVDVAVFIFGN
NKKLYEYSSSDMHQLIAKYQFHGVPNEHKGPSDFNGGDDDDDDDGDGTPP
RGHEGSEHQMMPPQFHGHGHSQPPFAHIRHHTPSASPPVPNGGPFQAHPG
HPGPRGSTPQPPMGSRPGSRNDMRRMAPNSMVPQPHGAHPGIAYMPTPPI
YQGPNQPGMMGPQPAGQYPYAPQHQHHPHHHHQQQQQQQQQQQQHQSYVD
DRRPSAPPAYAPQPPPHAIPVASRPEPSPPHTASQQQPLAPQHPVPHISP
PPQPDRRPQDPPPPPRVEPKTELLDRQPLGLNTDIAIKKLPQRKSHSIFT
PIEENRSILSQHLASFASESQGIKTESNRSQSVDASSRGGPSASPQLKRS
STQNSLDKARAASVSSGPESAFTPPSRSNSLKIGGPGGGSRPRGPRLTVQ
IPDGASEPGSAGGESNSPHNSTITPTQAPRHNSVVLPPPSPSASALLSAG
ATGPPNPFARPPPQQNVNGETPVSALPSRFLGHELLPSPGSFYQDWNFRG
NDSNTLPSPLNFATPVVGTGPSFLRDDHHGSGANNNSSTSNNSSGSTAST
AATTTNTTATSGTMSTGVPLRMTKRKSPEFGGGGHGESQEDATEAKRLKV
EYT
>Mb|QLI64300.1
MAPNPSPPQRNFEHPASSHAKKKRNGPYPIQPITAFYVFFAAGMIAALYA
PIQDCDETFNYWEPAHYLSHGYGLQTWEYSPDFAIRSWLYVGLHSIVGSV
SEFYFIRYGLAFVCALCQTVLFKVISLTMNGRIGMFFLISTVICPGNFHA
SASFLPSSFAMYMCMLGAASFMNWRGGIKTAHGIFWFAAAGILGWPFASA
LCAPYLLEEFVLVLFSDKDAFIESIIRFFRGVIAGLIVLFFDFLINLFFY
KKIAVVSWNIVKYNIFSSTGGPDLYGTEPWTFYFKNLALNFNIWFVLALL
ALPLFLLQKVISPSGQGFQSGLRTVVFVTPFYMWLAIFTAQPHKEERFMY
PVYPFLTLNASISLHIVLAAVGSTDPSTIAGKIPARLKLLAVSAVMLLAL
DVSLARLYGIYSAYSAPLRIYSRLWQSSHGQTPVGREEDIVCFGKEWYRF
PSSYFLPRDMHAKFIRSEFRGLLPGEFSEAKIGFGFWSGTWLPTSGLNDR
NEEDPGKYVDIRACSFLVDTQYPLRTEPLPPNEPDYIADKDTWEVVQCKP
FLDAANTHILARTLWVPELEIIPEKFRRKWGQHCLIQRKRV
>Mb|QLI64302.1
MASTLSPAAVLRTLQANRGLRQVQTARRFFSTPPRGVLSVSEPSLRTLQH
ASTASQQSLTPLGIQKSSRRHSSACACHGGSRKKKAYIALGSNLGDRVAE
IEKACSEMDRRGIRVTRTSSLWETEPMYVLDQDAFLNGACEVETDLEPLA
LLDQLQSIENEMGRRKLIDKGPRNIDLDILLYGDETVSHERLNVPHIGIP
EREFVLRPLSELAPSKPLDASKPWKLVQDYLNELPTGKPLSSVTPLRGAC
QSLMPLVHNRKTQVMAILNLTPDSFSDGGKNDLSNLRDTVLRHIQGGATI
IDVGGQSTAPGRPEVSAEEEASRVIPAIQLIRSIPEASNIAISVDTYRAS
VAERAVDGGADIINDVSAGLLDPDMLSTVARLGKTICLMHMRGTPQNMTQ
LTNYDDGLVPTVASELLQRISAAEEAGIRRWRIILDPGIGFAKTAHQNLE
LLRRLDELRAWPGLGGLPWLVGSSRKSFVGKVTKAEELSDRIWGTAATMA
AAVQGGADIVRVHDVSEMVKVTAMADAIWRPS
>Mb|QLI64303.1
MSPYWNNNSCSPFLGPEMPCTLGNLASYAIDIRCAEDAITGIRFAEDNNI
RLSIKNTGHDFLGRSSGGGSLALWMHNLKQVEFFDYASPLYTGPAVRVGA
GVEYSEVYMAASARGYRVVGGSCPTVGLAGGFSQGGGHGPLGAAYGLAAD
QVLEWEVVTASGEHLALAGHGEHADLFWALSGGGGGNFAVVLSMTVRAHA
DGPVAGAGFSFTNPGNDTVFWGGITAWLRHLLVLHTFHRGITTVFAITAK
SFALEFATLPDTNTTDTINAAMAPFVTQLSDLNISLAGSYQSNVHKDFAE
HYSHWATQTYASNITLGGRLIPRSVVQDEVSGLPALVNTFRDIAHRGAVI
LSVAANVTNVNHIHNAVLPSWRDSLFTTTFARSLPQESTWDAILADYRQL
NAWQEDLRVITPGAGTYMNEATWDNPHWKEDYFGSNYNELLAVKTKYDPR
HVFWANAAVGSDEYWKATKDRRLCRI
>Mb|QLI64308.1
MGRLAGKNAVVTGAAGGIGLETTILFAKEGANVLMADISEPALEKALAKV
KQLVPSAKVETKKCDVSKEADVKALVESVDPWGGLDIIFNNAGIMHAKDD
DAVNTPEEIWDLTQNINVKGVWFGCKHAVLSLRRHKKTKGSIINTASVVA
IVGAATPQLAYTASKGAVLALTRELAMVHAREGFRFNSLCPAPLNTPLLQ
DWLGDDQAKRHRREVHFPTGRFGEAIEQAHAVVFLASDEASFVNGHDFVV

DGGMTKAYVTPEGPAAAAPQNNASKNSLA
>Mb|QLI64309.1
MAPRDEVLSWYRAATETLISAPAKDDRDPESAAVNVLLQRQAKALLVIHD
ALGDSSPHQVNESLLTRRLSDVVSISASKFYAYRFDLLPCQWRQIHTDAL
ILSTHHDVLCSLGQNAWLADAVLDGVVEKLDRAVITAGGAGVLGTAWVEK
TLRLLEDLCDALPGEPPAAKRAKRAHAHFALGEPYGRPALSPDRTCPARS
GWSLAAFERYMNGDTSPRPVVFTDLVAAWPALTSRPWKSPCYLLSRTFGG
RRLVPVEIGRSYVDPGWGQELVPFGAFLSSYVSPEGGGEEVGYLAQHDLF
SQIPSLRGDICTPDFCWSSVPMHPADPARNKAPVDVPLVNAWFGPARTIT
PLHTDAYHNLLVQVVGTKYVRLYPPWSKAMRPRGEEDGVDMSNTSSLDVG
VLEGWDEDAQGMTEEEREAARSELESEEYWECVLEEGDTLLIPMGWWHYV
RSLSVSFSVSFWWN
>Mb|QLI64310.1
MSQLGRCWAVSRRGACNRSILAPISRNGALRFYAHQTAAAGPRAGHLNKE
ELLKAWNQFRGSNSLTQTDPDVITGFLGKRRNVGKHLSFADLTTSSGEVI
QICSHVDGGLESHEKFRQVPAFAPVVVCARPEPPAQAAGEEQNSSSSKRT
LYLRDIRSLNTVPKDLIVTSDVQFPQTKRHLQIRFHPELQARLKFRSWLK
GQLNQSLLEKGFTDVETPTLFKSTSEGAREFLVPTRQRGTAYALSQSPQQ
YKQVLMASGIGQYMQWARCYRDEDLRTDRQPEFSQLDMEWAFAGSAEVQA
DVNDILLAALSRLRPARSYSDIRGERIPILSDILNEPAPADESPDHKFTT
LTFAESIAAYGSDKPDLRIPNQIHALEDIEPYRNFVNMITHLSDPLIEAF
TFPLKDCSPSEARKFVIKFMDNLPPALANNPDGKPQILIHDSRQPLSGFS
SLGFEYESVLDKLSQGQEISDGDLVVFQAREKPQGQYCLGSTKIGDIRNA
LWKSLVEEGYMEKPKLGDPESLQFVWVTEFPMFKPVEEGEPGQGGAAGIS
AAHHPFTAPLSENDLEILFTDPLQARSAAYDLVLNGIEIGGGSERIHVAD
IQEFIMRDVLKMTDQRIKDFSHLFDALRAGCPPHAGFALGFDRLVALLTD
TSTVRDVIAFPKTMRGEDPFVRSPSKLNNEQLAPYGLQLMSKSK
>Mb|QLI64311.1
MDKRQSFQRVVDENNHPGTCWEFERTGTCKFGKRCRYQHPATSAKLAKEP
PTELTGFFDNNGLGVSTQRIDGKLREWKRLLGNGVTASRPPPAVVSRFFK
LGLELMEGDVGSSQEVIKRLANDQGLPFVKDVVERHLAVALQMDPSITFW
NTEVKPLFQLITHPRVIHSSVLEQEVMTIFNFILGVGGSRMNRLFGYLVL
LVTEWPDATDIVSKMSAVELSLAVLSKVLDCNTINIVNQNFATLATQFAE
IVATISEGQETFATLQSEKYIDYIRRRLQVGDDIPEMSGPTRDPVVREEF
VLRRDLPGTLSAEGPRHDNDFADMSKISILPTCEEIMSPREEYLPTSDSS
QWHIGGIRGRLDREFRLLREDTVGQLRDTVRDVLEHLRRPQEAQTAVKNT
LRTYTYENPVPMKVDFRRNGGLELQVRCSQLMAVAKLAKKQREEWWQRSR
RLQAGALVCVISASGLVQFFVVSQSTLRSQSEPRFQRPKDKMVSTDEGDQ
QQVYTLSDDPEYLFVTLQLVEDKVEDVKELLQWYGVSDQHRRRYLVEFPG
VLLDSFRHTLAALQQMYKHQDIPFSQILAPASDAGAEVVVQPPGYAREPG
FNFDLSCLQDGLLVSPRNLPDSSELAAGSTLDATQSAALLNTLSREISLI
QGPPGTGKSYTGEKIIQVLLGNKKRAKLGPILCVCYTNHALDQLLEHLLD
AGVKGVIRMGSQSKSDRMTNLNLQNIVKRFKKTKEEAAKLGQAEGGIKQV
IQAVGCTLQELADCKTWGTLKAFLLENYPHQYESIFPPEEDGWIEVTRHK
PEQKLERWLCTGDDVDAVPRDIPTLEQSDLWEMNQPERLVLFEYWMKRIR
DPIILLISELHEEYSAHIQERSKVSNEVSIRCLGQANVIGLTTTGLAKNI
KLLRSLSSKVLLCEEAGEVLEAHILTALLPSLEHAILIGDHLQLRPQINN
YELQSTNPCGVQYSLDMSLFERLVIPPHATDPRFPFSSLETQRRMHPSIS
ALIRPTLYPDLNDSEGVHMYPQVVGVKKRLFWMHHEQLEAGASVQDPLNT
SHSNDFEVEMTTALVSHLVRQGEYQQGQIAVLTPYLGQLHRLRHRMASIF
EISLNERDRDELAALEIDVDLSSTSSPQNASKSSLLKTIRIATVDNFQGE
EAEVIVISLVRSNTQNRCGFLSTPNRINVLLSRAKHGMYIIGNSNTYRNV
PMWNDVIKIMDAQGNIGTSIPLQCPRHPDTPILVSQPDDFLLSAMKGNHA
LPARSPARCVAAIRNAADCAISPVCHAQKENASLSVHTLSALCPVLYPAI
GYHVQDDVKRCLDVDIDCPDIKFCQSCAPEDIKSVCVDFLEMKEYQDIDL
DQDPCIFPDCGHFLTVASMDGQMDMMSHYDTDENRMPVNIFKKSEPFSMD
GSGVRVCATCRGSLRNIARYGRIVRRAILDEATKKFISWSHKQYGLLSSR
YLEEQEALGDAEVLGAATARAKLQRSKSSVLRFLYLDCLEEHALSERYGD

LLATWVDIRLFANDVKVDEQPFQRVADLVRHANRQHKTNKEFRFDETVIQ
MKGYILARTLLLKCEMTILNDFFARCNGRARLPVKFVLDFTQQFRDCEEV
IQIANKSVHPKEEVQALIFEAQLCALSCKVDACSVPPGFEGGAAPDKDDV
QAYGFALLERAREMAQMYPSTMIFAGEIDAVESSLHGSYQTLGKEEMRNI
VEAMAGDIGTGGRWYYCENGHPFTIGECGRPMEEARCSECGAPIGGRNHR
LVHGVGEATELNQITRGMGRIVL
>Mb|QLI64318.1
MTTQRDGVNPLRPYYRAPVIGETTDPASASSANPFAVGNVTGTARYASKA
RDVLADIDYKDYLNDSSPSVIQNAKELVEELIWKYTSVLMAQPFEVAKTI
LQARDQDENAVWATTEPGGLKRQGSSQAGSIYDYNDSDSEGDEAAFFTSN
TPVTPTPSYNRSSGNRRVTSPLQSQAPKRPIIPEHYITIRRPDSILDVIG
QLWQKDGAWGVWKGSNATFLYTVLQSLLENWSRSFLSAIFNVPDLGVKDD
IDRLIDIASPYPWASLFVAATAAVATGVLLSPLDIVRTRLIMTPISKGQR
RTLATLRALPSYLCPSAIAIPTVLNSLIHPLLTLSTPLVLRTRFLIDSQV
SPMTFSVAKFFASSAAILIKLPIETTLRRGQMAVLCSPDYIKALGGTEQK
LDTIVPIGRYNGTIGTMYHIASEEGTRENPAISNPAAKKGKAKTKSFQPT
HIKGQGLEGLWRGWKVNWWGLVGLWTANVVGNGGEGEF
>Mb|QLI64319.1
MAEGPDATASRPSRRQWTLPPWLNHFNIHDGKIVFRCWVAAWVASLLMLI
QPSLTNLGVAAFFALLILIAVPPASILLVYVLASFSLLLGMCFGWLWGLL
TMKAALAARPDSETQQSLLALQQAAVAQANQTGQPVSWEALVLVHNGFML
DARVTAVFYVMSCVFIYALCRLRMKNMKFVLLQLFGIISIDIFLIFGPSL
PQFEAQLGTILVKPAAVGVALGSACCLFFFPQSTSYAVLEQMEKLVLLQE
IALQATRRRLGDQDVSLGELIEAKAKTVGLYKAMQPAIAFLPLDFSRGRW
NAEDVKGLQTPVRVAMIASLSMIEFHISRINVIQKEDKLAKHHQDEDKNN
HNENGIGMHQLKESSELLHALQSPENGEVQDRTRAALLDTTADVLQACSQ
SIKVVAKCINTVNNRRWVGQPSQQSFDELEQELRTKLDALRSSRDSCVTN
TTEKILSTHQDLFDAKGDLKPSIESGSRLLRGIVVAMVIEERVIGIATAM
EKLIEYVLHLMTTRKTHRIWIPSRMRYMFRWLLDGRLSVPVSGTTTDAAD
DPDNTADPVLLQDQAEEAHRRLRISRGYYVRPRKKSAATRVIAATFRWLF
DPAGMYALRMVVVTVATAIPSAIPSSAGFFYREKGIWGVISAQTCLLPYM
ADFTFSVVSRGLGTVLGGVMAMVAWYIGSGSGIGNPYGLAAISALMILIL
IWWRIFLPPAFAQAAIMTGATYALIVGFSYDHFHIEQYGLPGVGYEAFWK
RLVTVLLGFVASCIVQLVPKPPSARSHVCKTLSNTVGTLADHYALLLSHW
GLGQKNSPLSAVAEQISIGVAETLLSLNGPITLLTLEPTFGHFDQYTLKE
TQKLCLAMNQSLERLMDVSGSLPKEYQDRFSNVVGFVDDHVIGDVMAVLG
VVRLSLKSGAPLPERLPAPLIRNFYAWWHDKHRTAILNTTLVRDENYRRY
CVAISSYLRFLSAVDDLVLVIKGAVGECHVVRQWENV
>Mb|QLI64321.1
MEEMDLDAPAGRKRKADDIPDDPRPARRIKALDPDVVNKIAAGEIIVAPV
HALKELVENAVDAGSTALEILVKDGGLKLLQITDNGCGIEKEDLAILCER
HTTSKITAFEDLSSIGTYGFRGEALASISHIAHLSVTTKTKDSPLAWRAH
YLDGKMVAPKPGQPAEPKGVAGRPGTQITVEDLFFSIPTRRRAFRSYSDE
FNKIIDMVGRYSIHCQGVGFTCKKAGESSNTLSIQSQATTLDRVRQIYGS
SVANELVDFSASDARWGFKAHVLATNANYHIKKTTFLLFINNRSVESANV
KKAIEQTYLNFLPKGGHPFVYLSLEIDPARVDVNVHPTKREVHFLNEDEI
LHAVCTELESRLAHVDTSRTFKTQTLLPGAKPVPESMDEDQSSPRIMVTG
KRRRNSNDLVRTDTYARKITTMLSHADSGEGSKAKTSKEEEPLAVPENIE
YETNDREMTACRLKSIKELRSEVRDNIHHDLTEIFASHTFVGIVDDSRRL
AAIQSGVRLYLVDYGHTCFEYFYQLGLTDFGNFGVIRFSPPLGLRDMLQM
AAETEKKVLGAADHDFPVEKIVDKVANQLIERREMLHEYFSLEVSPTGEL
ISIPLMLKGYTPPLSKLPRFLLRLGPNVNWNDEKECFDSFTRELATFYVP
EQLPTPPAPGGGEEEEEIPEEMRTRRQHVRWAVEHIFFPAFKTKLVATKA
LMDGGILEVASLKGLYKVFERC
>Mb|QLI64325.1
MVSTKLESALLVAAIATGVIGRPGSGASPANKASPEEFLHSRNLPDGYYV
PSYYPAPYGGWISDWQESYRKAREFVASMTLAEKTNITAGTGIFMGSAPR
VGFPQLCFNDGHNGIRQADNRAVAIGKEARGKGVNVWLGPTVGPIGRKPK

GGRNWEGFGADPSLQAIGARETIKGVQEQGVIATIKHFIGNEQEMYRMYN
PFQYAYSANIDDRTLHEVYMWPFAEGIHAGVGSVMMAYNAVNGTACSQHP
YLINALLKDELGFQGFVQSDWLAHMSGVASAIAGLDMDMPGDTQVPLFGF
SYWMYDLTRSALNGSVPMDRLNDMATRVVASWYKMGQDNGFPETNFHTLT
RDSVGMLYPAAFPDTPSVVVNNFVPVQDDHNEIARQVAQDAITLLKNNGN
LLPLRTSQSLKIFGTGAQTNPDGPNACADRNCNKGTLGQGWGSGTVDYMY
LDDPISGIKTRAQDVQFYDTDSFPDQIDQPKDTDVALVFITSDSGENTYT
VENNHGDRDASGLYAWHHGDQLVKDVASKYKNVIVVAHTVGPMILEKWID
LPAVKSVLVAHLPGQEAGQSLADVLFGDVSPNGHLPYSITKKEEDLPESV
TKLISAPNIHQHQDTYTEGLYIDYRWLNKNNIKPRYAFGHGLSYTNFTYS
NATITKVGQMTKTPPTRSPKEGILDYTQPIPEPSEAVMPDGFHKHWRYIY
SWLQASEAKNAAADVKKKYPYPSGYSTAQKPGPRAGGGQGGNPALWDVAY
KLSVVVTNAGLDHSGKASVQAYLQFPKNITYETPIIQLRDFEKTEVLRSG
ESTTVELQLTRKDLSVWDVVLQDWVIPVVDGGYTVWLGGASDDLRVSCHV
DGMKCQSAATSE
>Mb|QLI64326.1
MSRVVLCTRHLGLTATTKLSQPAPSFIQHTGSRPPRHLPVRTFRSTSHLP
EKSSHSPKANASWSSTSDTKSLSDIAFAFDIDGVLYRGGQGIPGAREMLR
SIRSKNMRYVFLTNGGGAHEDAKVASLSKRLGLSIDEDVIRDRVILSHTP
MRGWADEVKKQTVLITGSHPETARKIANEYGFERAVTPADLIHANGDLYP
FDNLKDTLHSEFRPLPDGKSASAIKDPYSKSLAGNALKIDQILVWNDPRD
WSLDIQVIHDLLVSHNGYLGTLSSRNGNRGLPNNGWQQDGQPELWISNLD
LVWKTEYPVNRFGTGAFLEALKGVWSAVTNGAELHYNALGKPSQFTYDYA
HTRLLQYYSTMKGGREAGGRKDATPLRRVYMIGDNPESDIRGANEYCPED
GTEWVSILVRTGVWTETETEREPRYKPGAIVDDVVDGIVWALRNEGVDVD
RKTLLAGGG
>Mb|QLI64327.1
MASSSALRPSVGCLSRIGSHFASSRAATFSTTAQQCKRKTKDNNKNRGVS
SLYGSGPREHLSMSDIPLPKPRDFKPKVAVDENHGLWGFFPESGKALWSP
KETEEHGRAWSVEELRKKSWEDLHSLWWVCCKERNMLATSKAELARGKFG
FGDREIEARDEEVMKTMRAIKHALTERYYTWQDAIDVAMSDPEIDMHAED
GQVYKPSAYEEEVAVEDAWAKEKGAEQEPVKADKELSR
>Mb|QLI64333.1
MPASKNNEQPQLLFVDGTFEELAKEMAEYLKAEEAAQLLSKEKVSKEDVL
AKLVAASAGLSTVPEKEYTAASNLVIHLVLQSADPKKYLQTLCANLAKVP
VNSSVHGPGLSLNALATVFNLLPPEDVIRARIFLEIVKFSRAHNMFDSMR
LYLDKLGEWLESWEASEEIERMVYENVAEAALEAGEESISYDFVLKALRT
FDADEKDEITSEDAQRLSLRAIKMAILSNTHFLFQDLRAIPSIQALSDSH
PIYSQLLDIFAEQDLEDYNDFTEEHEGWVEQQKLDHEKLHRKMRLLTFAS
LAAATPSREIEYAKIAKALQIPQEHIEMWAIDVIRAGLVEGKLSQQRNKF
LVHKVTYRVFGEKQYQELSTRVDHWRATLQNVLGVLRQEQTNAKAQKERE
IQELERKLNNAGVSGGNRRQQNPQSQQRERTDNDD
>Mb|QLI64334.1
MPSFNGTAGPSDDDEDDYMNMTFDDPVPQKETSLQRTQRLKKESLARGLI
KSKAQLAEEAAAAREKALSTSMLEAPGAKKSKGLAMMAKMGFSGGALGKK
DHDGESSSKPEPIKVSIKEDRGGIGLESEKKRKFDEAMEERGTKTVKIDP
DEYRERVRKEREDERLEKQFHAAQRVAERMDEEQVAHDESASDEESESAP
RPKPLSSRPLKSIPVLYRGLVRHREEKERDRRMRHDLEQSLSRLPTYDDA
DEDEDDKRALGKKKTVYVTSEDLDEEDEELDSFNALEPAERLTRLVKHLR
EKYWYCFWCKMGYESSEMDGCPGVAEEDHD
>Mb|QLI64335.1
MDDRNSALHVSLQPKKPPIWTKYGERFGVSNSSNSLPSRQATIEHYWSRE
ATRVAAQNEQDAASTGHSPSLGPRIAVLLPVSPSPSKAYPGPKQEPENEA
PLPNQLELPKRVKKNVGLLSQSPGQDTAQSSATPPPSSTSTPSSYSRGRP
KGWKRGMSYAAMRGNTPPTWKTVRSPRQSRTKAPQPGFGRRPGRPPRPRS
PPPKDLYHKANPHFVNFLCEWDGCKAELHNFDTLRRHLHIVHQQNGKWPC
KWAHCGLDACADARQWRSHLEDAHLTPFRWHVGDGPSNSSGMRAVLPDDA
QDELPDFLMGSDGQQVTPSVKNQQIEDFATWRENRRKLKELLIQRDQNLP

DESSESSGETD
>Mb|QLI64338.1
MSAASSASSLRRVAPLVIPAAGRHTATVVFIHGLGDTGHGWADAVGFWRT
RQSMNEIKFILPHAPHIPITMNGGMPMPGWFDIKTLVKGADEDGPGVLQS
RDYLHGLIQQEIKDGIPADRIVLGGFSQGGAMSIFAGLTAPVKIGGIVGL
SSWLLLNQKFKDYVPDGNINKDTPIFMGHGDRDPLVLYDLAKDSEKALSS
MGYSVTFKTYRGMEHQACAEELGDVEAFLSSRLPPKGH
>Mb|QLI64341.1
MRRTLQSALLLGLSSLAGAVFKDEVGDIDFHYSLVGLPQVETTFFHRPRK
EDKASLLYTLGDVGVIGAINPSNGELIWRHQISHGIANGGGHLRAPEGEN
WVTSAYGPKVQAWNALTGRNIWDMEFKGVVKDLEILELTESPRKDVLALF
DEDGVTVLRRLHGALGTVVWEFRETSKDLPVQVSTNIANIYVLSLHGSPG
SYNLKVTSLDMATGARVDHWSVGNKGDIHGAEDVMFVGANSAAPIAAWAS
RGLSKLSVNVLGTKTKQEFTLPDGTETLRIHAPHLAQSQPHFLLHIGTKT
GHKAIVYHTDLKSGQVSKAYELPHLSGPGAFSTSSDAANVYFTRITANDI
SIVSSESHGILASWPYLPKDGHAAVHAVSEVIKKAGGKEFAIRSAAVTDA
DDWVLIKNGQVDWTRHEGLSGAVAAVWAEIPEAERLAEVLAEEAHANPVA
AYIHRVNRHIADLEHLPAYLARLPQRIIDGISGADLTGHKDGLHRDTFGF
SKIVVVATRRGRFYGLDTGRQGKILWSQAVFNVDPGESLAIRGIVAKDAE
SEVTVVGSNGERATIASATGRILEAHQAGSFTKVASTAVIGDESSQWLLS
LGANGLPVPNMPIGTIPNDTIVLRDGSLGVKGVKFTAKDGEVIKDDIWQF
QVGKGQKIVDIVSPPSHDPISSIGRVLGDRKVAYKYFNPNTVVVAAIEEA
TSTLSVHLLDIISGQALASQVYNGVDSTKGLSCAMAENWYACTFFGDYTV
NDGTDRTIKGFQVAVSDLYESPEPNDRGPLGDAAEFSSLNPVDTPTGVPL
PHVVSQAYVFSQQLKSLSVTQTLQGITSRQLLAYLPDSNSILAVSRHIID
PRRPVDRDPTPAEVEAEALMRYTPQFEIDGRGIVSHELDVLGVQEILATP
AVIESTSLLFAYGVDIFGTRAAPSGVFDILGKGFNKVTLVGTVAALFVGV
LFLAPVVRRKQIDRRWEAFL
>Mb|QLI64342.1
MRTLASSPLRLCRSCPPPSYPQPRYIQRRALHASRLLRYAPQNVSDAEIA
ALARQHQHSLSLADLVKHGRPPLSEKSLLSSANFTLSLLPIRLARRIQAL
RNLPYIVVSNPNISRIYNNYLHSLSILLPYWHAASQGHPITTLQDEIEFT
NVLAELVATHTDTIPILAKGFLECRRYISPAEVTRFLDEHLRARIGTRLV
GEQHIALHFSSQPHFAPEDSPTPCPEHPSYIGVIDTALKPSLTIDSCAGF
VADICELRYGSRPQIYVDGEPDTTFAFVPMHLEYIVTELLKNAFRATVES
RAREPVVVTIAPEPPLKEQPGGAPLIKQPNTDRGQFRSDAIKPLDDNAPG
VTIRIRDRGGGISPDVLPNIWSYSFTTFSDDDGFPGSGGNTGGDGLSAIA
TASTGGSSIAGLGYGLPLSRAYAEYFGGGIAVQSLYGWGTDVYLRLKGVG
NLN
>Mb|QLI64344.1
MKFVKSVHTVAALSGIIAPKDLDVESQTSVRNVAATLAYETMTYYSGNTT
DPKSRDFGNLKQPYYWWVAGALWGALLDYYHYTSDPSYNDVVIQALLAPT
NLGPNKNYMPPEHADEEGNDDLFFWGSAVMSAAERNFPQPNADLPSWLDI
SSNVFNQLVGRWDTKHCGGGLFWQILASNPNGLKYKNSVSNGGFFQLAAR
LGRATGNNTYLEWAEKIWDWNAQVGFIDKESYRIYDGADISDECKSTNKK
SFSYTTGIFLYGSAIMAEQTGDDKWKQRTEKLLDEAGKYFFTGKDNNIMW
EAECEPNSKCNYDMVTFKGYLSRFMWQTSRITPSLSKKIEDLLVPTVKAA
VATCTGGKSGHQCGMRWYEGGFDGTVTLGPQMCALEVVQGLLIKDSPAPL
KGNDIKQNKDDQFKPIDTYSNNLAVSSKREISSTASSPPDPAATSSLNKG
KAPKKSESAAVASNKWALVAVAFGSIVAVWGLA
>Mb|QLI64345.1
MSSDPLLPIAPARVRALLLPLGKIKTQRFAEFVKLLQAEYVVHLRDISAD
GRPNRNMFSPLAYPDGAMLYDLITHVPPVSHLALSPFDLYREPLVLIALA
DGEELQDKTFSKRYSANRAATTVERNVHALYQELEELRDNYPKVLVHQVV
IFDYISPEGVDVHTPEEILFVPPKDKRKRTTIKTVMCDISSLVLAEMTTL
AKSFEGMSTIESPGLYSAKNMNGNTWGGNGDGSPASLSRRNSQFSLPQQL
SRSSSASSVADRTQTRLSMPPTTSRSPSNAAGRPSTPPRSGLSTTPMSPN
GGQSGSSTGPSSPDQKPQRSDVNGSRDLSRDRVSVQGFGPGGANDRWRLR

GKGRTSVLIGSMYLQAGRWGDSLRELSEGATAARSLNDHIWHGKALELII
MNLVLLGWSKLEFQIPTVCLPSHERSTSISGVLKSEADSANYSQPKHIRH
LQNLLPELLDRILGLFSRISSENLPPLPLAETTIRFCKLQSAMHFSGGML
DQKAFGAIVTGELPTQTLTTSPRFTVTPSRQQIVNMLFKAFPSSGTELLT
TADRTSILSGIANVLGALGYHRKKAMVIRELVSVLIAGLVEARTRGAAEA
GIHPAAGLVSLVPGSDRTSAAGVALDLGEGDIEHGIEPFLELLCRSYGVV
GFDMRKKRDGADDSDSATVARALIQSSTRFFGFTEIKLNILRACINFSEA
LPDFDGVLKFSSDLMRTAGSGIAPGPKREDAAPHIHKDEQVRLVTNIART
AALTERIGLGGLAAEYWDEFLVRGIALEPLPNTRTPIPHAKSVLPGANAS
RASQDVNPFIYNPFLKEPDEVAAENLVAGELATFKVTLQNTYDVEVDIES
MRLDTEGVDFEPVPENVILGPYRTQLVRLKGRPKASGSIKITGAVVKIRG
CRERRFPIFARHWTPSRAEKIKSKGPPTLEGGSRTAPVKPEMKVLNLNVV
QPQPLVVVKSTSLAQSSVMILEGERQVFTVTMQNTSTTTPVDFLLFSFKD
STQGPLQAALENREATPAELYEYELVLMKKQALRLPRNNQNRYIPPGGEA
TFEFEILGKPGLTHAAIQADYTCLGVPRDEVTEQFYTRQVLLDLTVTVNA
SVELSRIDAVSVQGQVPPALLKRVGGDERTASPDKYFLLAVDLRNAWPSQ
MSVQLEGEDGMMVGDSILPGKTSRVVIPMKRVYLEDPHESVPSLNPSRNR
QFVVSTSKISPDMERANREAFWYREKLLENLKATWQTTSVPKRNGVVELR
NIRLTPRMIEAIKFDEVDIAVSIDGAPDNVAYVDEFMQMRVQIANRTAKP
ILPLVRLMPALCHRPLNVALDYTRKFAWNGALQQQLPKLKGHQVTEFVIG
VTALCRGEFELAASVEEAQVLDGGLKEGRDLGHRRSDTQKLMDAALGVKE
RRVWYARQPCTLRHGVARTFRVRTEEQRLQDLEKIRKYRALEDEVRDKAA
SAAYTPELFQLTSKLLSINPEYYTIWNIRRRCLLSSLLSPATSHQPPDTR
DSAPDHVEQQASSDRDVLQSELSFTIPLLMQSPKCYWIWNFRQWTLSQAI
LRLPAPAARQIWQTELGLTSKMLDKDQRNFHAWSYRRLVVGRLESPELQG
RSMAEDEFSYTTRVIHRDLSNFSAWHNRSQLMARLLEERGAGAQQRAALL
AEELHLVREALNVGPEDQSLWYYHRFLVAQIVDRGGVGGTFVPDLTADEK
AAYLGREIDEIKELLEDYDDIKWIYESLLEYTLALGRLDQGRGRAGDLRG
WLAKLRVLDPMRIGRWDDIERQVE
>Mb|QLI64349.1
MADHHISSKFSKLNPFGSDDEDGGEGIAVDSVGGGGRSTRKAHHAKQQLN
VSNALRKYLAKQKVIAEHDAGIGSNETTPALRSLLGKSHFDVPESLLDRS
RPLPEYFISSSHNTYLMAHQLYGTSSASAYETALKTGSRCVEIDAWDNSA
NPEEPKVTHGFTLVSHILFRQVCETIRDVFDEEKAIAQKDPSYAVAPILL
SLENHCGEQGQKRLVEIMKEVLGDRLLAQPVIQKGHREQSGEDEHVCLAD
LGACIAVIVEYDLKGDVGAAEESESESPSDNEQEGEEAKAARKAYKEKTK
EAAPAGIIPELADLGVYAQSVKPVDNSWFNPGTLVNGPHHHLINVSESGL
SSHLPDESIAIAAHNSQHLMRVYPKGTRISSSNLKPLKFWAIGAQICALN
WQTFGTSNQLNDALFSGSEGYILKPAALRSGGDGRILTGKKKRLRLHVAG
ATDIPVHEDREADSLRPYLTCNLYSPGDIEGDTYKRKTTPYKHHKFSILH
RGDNPPVTEPIWDETLEWVYEDNELVFLRMLIKSDDAWARNPMIAVAAIR
LLYAVPGWSFIRMMDMKGGETKCTILVKIEIDDV
>Mb|QLI64350.1
MATVVPPPSKRQKREELERSRVQQDVNAAMAGPEGSFKARFLDGDGNQVC
DVIEVPLADASEKNLSLLLNTLLSRERERYLPYRFRIHIPDSDIIVDQYP
KDLLQLLRSHGIENPFETTVTLTAEPQSVFKVHAVTRMSHRIPGHGEAIL
AAQFSPETNTRLATGSGDKTARIWDTETGTPKYKLAGHTHWVLCVAWSPD
GKRLATGSMDKSVRLWDPATGKAVGSPLRGHSKWITNIAWEPYHLWRDGT
PRLASASKDMTVRIWVVNTGTTEHVLSGHKSSVSCIRWGGTGLIYTGSHD
KMVRVWDAAKGTLVHTLSSHAHWINHLALSTDFALRTGYFDHQPTPITED
EKRAKAKERFEKAARIQGRVSERLVSASDDFTMFLWEPSQGNKPVARMVG
HQKQINHVTFSPDNTLIASTGWDNHTKIWSARDGKFIDTLRGHVATVYQC
SFSADSRLLVTASKDTTLKVWSMASFKLVVDLPGHHDEVYAVDWAPDGKR
VASGGKDKAVRLWAN
>Mb|QLI64353.1
MAVSELTASAVARGAMAVDSSDVVQGAASTRPTALQNRAAFSKSPFVRAA
ENSLVKWQILDADSVDRAKRENKLLFLHIGYKACHFCRLMTQESFSNPEC
AAILNESFVPVIIDREERPDVDTIYMNYVQAVSNVGGWPLNVFVTPNLEP

VFGGTYWPGPGTSRQVTTESEDESPDCLTIFRKVRDIWHDQETRCRKEAS
EVLAQLREFAAEGTLGTRGLTGTHPIATPSWNIPSNPTTTIRARDKDAQV
SSELDLDQLEEAYTHIAGTFDPVYGGFGLAPKFLTPPKLAFLLHLNTFPS
AVQDVVGEAECKHATEMAVDTLRKIRDGALHDHIGATGFARCSVTPDWSI
PNFEKLVVDNALLLALYVDAWRIAGGKADSEFYDIVLELADYLSSPPIAL
PSGGLATSEAADSFMRRGDREMREGAYYLWTRREFDSVVDASGHDKQISQ
VAAAHWDVQEGGNVDEDHDPNDDFINHNILRVVKTQDELSRQFNISPDTV
RQHIQAARKELKARRERERVRPELDDKVITAWNGLAISALAQASSALKPV
DSARSDKYLHAAESAAAFIKASLWDESSKLLYRIYREGRETKGFADDYTY
LIHGLLDLFAATSDESHLAFADALQKTQNSLFHDSDSGAFFSTTASSPQA
ILRLKDGMDTSLPSVNAVAASNLFRLGALLDDERYSALARGTVNAFEAEM
LQHPWLFPGLLGGVVTARLGPRESVSDVKYKVGREKAV
>Mb|QLI64354.1
MGQDSTAITQSSGYNDGWATISPYSPSPYDGSPMTEYSGFGTFVPPGVHS
DSMPRMPQQGGHQHQHQMMHHSATPMGHHQLPMLNTTWPSQLTNPTPSSG
SFSAPATSIPPVPRIPRAIDTPKLPSQADKGRKTLTTEQKRAMCQYHEEN
PGTRQADIGLRFGVERSTVSKVLRHKDQYLRRDQEPDPAALKRSGKAKNP
DFDRTLSNYVRRQQQRGFDIKDEEIMEQARLFAHASGNQDAILGSLTSGW
LQKFKQKHGIGTSRLSRRASETNIADSTRMPEHNLVRNGATSNDISPASP
TQPLSPLSGSRSDEEVKREQNIDYDFAYRQQHSQPTTSLATDIRDNPGSS
FSGGTLSPTGTFNFSPDPNAGSFQPLGMRAEMPPEFHREKRSNTFPSIDV
SYGNQATPATEPMTPQLPAPTTAPSSALDSPTNDGQIAHFSINTGLTSPP
ALHRTSSSSSIAARSSSISLTNTGASMTPVDSSPVSPSQEDARRAANTLL
SYLHNMSSTGQLFQGDYQAMLQLTKKLEIHQHQSNRPSAGGLSRIPEGDN
EMNATGPAMMQAS
>Mb|QLI64360.1
MASGAQSFYELYRRSSIGLALTDTLDDLISEERINPQLAMKILGNFDQAI
TEALQKNVKSRLQFKGSLDTYRFCDEVWTFLIKNVTFKMDNGSQSVQADK
VKIVSCNAKKPGEGA
>Mb|QLI64361.1
MPLSHRLKSMADRLAVESEDGLTSSQRMLINHDLKPVEKARRQWGPWNYV
GFWIADSYNINTWMISSSMVVGGLSWWQAWICVWVGYSISACFIVMNGRV
GAMYHIGFPVANRASFGIWGSLWPVFNRAAMACIWYGVQAYIGGNCVYLM
IRSIWKSWDRTTMPGPFEPGVSSTPDFVSFFIFWLCSLPAIWFPVHKIRH
LFTAKACFVPPAGIAFLIWALVRAGGVGPIVKKSNTIHGGDLAWEIVKGI
MSSIANFATLIVNDCDFTRFARKPNDAVWSQLFAIPLGFAFTSLIGIFVS
SSSSVIYNEPIWNPLSLLGNFIDDGGAAQRFGVFVISLAFAVAQLGTNIS
ANSISAGSDMTALLPRYINIRRGGYICAAIGLAMCPYNLLTSANNFTTYL
SAYSVFLSSIAGVMSTDYYVVRKGYLSVKELYDARKTGPYFYTFGVNWRG
YTAYIAGILINVVGFAGAVGQQVPKGATYIYNLNFFCGFIVSSLTYWGLC
KISPVPETSDQWLEVGGAIDDLQVAYDATNDEYDVEKVTAMPGKDGNRAT
GF
>Mb|QLI64364.1
MATGRLSFLYPHLLRAARTSVTPSTVATRWVATTRSKSSFAARHGKAVEP
TWNKTQEAEAQSSRSTESKNDGDQAPAQEETKATEATKTTDTKQPRAQPR
AAADKIASPELEQSSSVQAETATMSSADSPTSKAGDSAATTKVADSSNAA
EEPSQSTSDVDSPAAPSLEAVLHMPSPDKVEHPHMAPPPYVHHFDSYSLV
KQLEEGGYSREQAVTSMKAIRKILGQNLDVAQKSLVSKSDVENETYLFQA
ACSELSTEIKNNRRLQEEEMRQQRTHLQHEVDILTQSLNQELLTLNDAVR
GLFNDRNMAVREEQKSVESAIQKINYKMSILLSSDSKSEIEGVRWVLIRR
SVVGLVFLAILTLGMIRYTTYLAQQKKKESEKRKRERERLKRDGGRNDNT
SAADAAMILSAS
>Mb|QLI64365.1
MPDDNPPTSPAPQPPKRPPAASNGINTTKSTGFATGINASNAPPGHVIRR
AKTTDDGPSLRRKSHTSRSEASFDGMSPGLPRRSSNFSDYSLSEARDILN
PRAQTSQELPSPESSSLASLSLAFALLPAIAGALFKNGHAVITDVLLLGL
SGVFLHWSVTQPWAWYHAAQQVRIQQEAHTQTAVEADDDRHYASADAASK
TTNLDNVPEEEEPPKPNGPSTAAQSKRGKDGGTTPQQQAALRELYMHEVL

ALISCFLLPPLSAYVLHYIRAQLSRPSEGLVSNYNLTIFLLISELRAFSH
ALKLVQSRTLHLQRVIHGNPFASPTQAGAQIEDMIERLSRLEARSLADEF
VRQHSDGLDPVQAEETANMARDVRNAIQPELDALNRAVRRYEKKATMLQY
QTDSRFQALDSRVDDAIALAAVAAKNSRSKNALARTIDSLVAIVLFPFNT
ILQVITLPLRSLIALINLNKRAHIAEKPGRTTRSAKQSSPRYNGDRVPPR
VMKR
>Mb|QLI64367.1
MDIDMSRRNKVPRSLSDAERARLDEYIDSIHYSARYSDSEYEYRHVQLPK
AMLKAIPKDYHDTSKGTLKLLWEEEWRAIGITQSLGWEHYEVHEPEPHIL
LFKRPLNYQAPQ
>Mb|QLI64369.1
MDTLLRWPDIPHPAVVATTAAVATASLIVVAKATLWPRRRSVLRSPLKTV
LPRASREELSELVYLPDAFPGARDVETPYGTIRVYEFGPEDGDKVLLVHG
ISTSCITLGRIAHALVERGCRVMLFDLFGRGFTDGVGDIPHDTRLYTTQI
LLVLASSRLSWTGTSGFRLIGYSLGGGIVIPFANAFPHMVSSLVLLAPAG
LIDAASFGAVSRFIFSSGFIPERILAVLTGQRLQRPIASSRAARSRAAGV
LAVAEAENLAGEKTPLEERVLDYVRWMVHNHEGFVPAFMSCIRYAPLTEQ
HEEWKGLAKRERGTTAVIFAETDELISADDYARDGLPLVGGEGHVFWRVV
PGSHDFVMTHVAAIMKELDEFWDMKT
>Mb|QLI64370.1
MASRIHGRADDPSVIVGLACRVPGANTPSKLWQSIAEQRDVQQKMPEDRL
KVDSFFHPEPTHKGTTNARFGYFLDQDIGNFDAGFFGISGKEAEAMDPQQ
RLLLEVVYEALENAGITLQEIRGSLTSVFCGSFTNDYNAMLTKDLEYYPK
YTVTGTGDAILSNRISYFYDLHGTSVTIDTACSSSLVCFHLGSQTLQNGE
ADISIVVGSSLHYDSNVFVTMTDLGMLSTNGRCAAFDASGSGYVRGEGIA
CAILKRKSDAIANGDSIRAVVRATGSNHDGKKNGITLPNSVAQEQLIRST
YERAGLNPSHTQYFEAHGTGTAAGDPIEARAIGAVFAPGRDEPLFVGSVK
TNIGHLEGASGLAGVIKTTMALENNYIPPNMHFKTPNPNIEFEKWKITVP
TKLVEWKVPDGVPRRASINSFGYGGTNAHVVLEEYTQDEQIPAPIPPEPV
ANGVHSRPYLVPLTSHSPKAGEMSEETLKAYLNNTEDATIADLAYSLSTR
RTLHQQRSFVVANDKAGLVDQLSSPRPAAPWAIAKNPVPRLGFIFTGQGA
QWFAMGRQLIEECPHFRQSLQRCDAILKSLPDAPEWSIVAELNKTKETTL
LGETLYSQTICTALQLAIVDLIACWGIKPSAVIGHSSGEMGAAYSAGILS
FESALIAAYYRGRYMSSSRSDGVPGGMMAVGLPENKCLEELKPYAGRLTI
AAVNSPSTMTVSGDQDAILELKEKLTEKKVFVRQIAVKQAFHSHHMFPLA
PAYENALKNNPAFKTNTPTCRMFSSVSCRRADHEKMGASYWAANMVQAVR
FSDALTGILLDEEDEQNVDILVEIGPHPALKGPARQTVQSLKLDLPYVAS
LTRGVPDFEGLLNMAGTLFSLGYPVDIVAANQNLSQTSKGALVSTPTGTK
LHDLPTYTWEHRRYWSETRYIKEHRQREFRHATLGHRVAGSVVRHPLFRN
YLRLSELPWLNEHVVENKVVFPGAGYISMAIEAAIRTDRVESVKMIHLKD
IVVKNALLIPSTDEGVEVLLQLKPVTLSAKSHSDTWYEFNVFSYDENSNC
TSHCHGLVSVERGDAAPINYTCRYPEGTNLNELRKKTYRSMPAGTFYKNM
AELGLAYGDKFRLLKGSIESGVGFAVSDLVFDPGHLPSEAGDETVLHPTL
LDSFFHVIFHAVENRLGRPLDEPYVPSFFRALRISGNFFDGRYSTAVKKF
QVASFTKLPSPRVAINDMIMQNDRGELMMEIAGLEVTSLGREDPNGQGPR
TLFYRQRWQPFFDMMTNVEGKPVSDVVDVFAHQYPNTKMLHITSDLDKTR
DFIQHLGCGKGERRRFKQLDVWSLQGNEFGEEAERLSKECRGLVNVVEPA
LDSYDLIIVSETGVNAVSFMNGTGCILFDGQKGVVTAELQELFSSPFCTA
LRKAVEPFTLNSELSVVMPTCKPSARTSLILKELESAHGGDLIFTSFPEL
AHQGTALLAKDVVVLAGLDESISDKAAFKGAQALLTSLEKNVIWPTEGAT
LEANRPDNAMFIGLVRAARSENDTLRAVNFDFGIDSPAQTVAANIVRLLD
GRITEDELTERNGIVYIPRVEADDERNCKLRNGPDQEPRLEPFGTAQTRT
PLALRIGKVGLLETLYFGEDTQVMDTKINDNEIEVETKASSINFRDVAAS
MGIIEDSNLGDECAGICTKVGANVRGFQVGDRVVALRPGQGAHRSLVRNP
ASWCYKLPDNMSYADAAAMPLILGTAWFALDHTARLAKGESVLIHAAAGG
VGQMAIQIAQRAGAHIYATVGSPAKRRLLKDVYGLKEEQMFSSRDASFAE
GVMEATGGKGVDVVLNSLAGPLLHASWACLAPFGRFLEIGKRDIHENTKI
AMDPFRRNVLFASIDLVTMFEKNEALGERLFKECFALLANGEIRTPATIK

QVPYSDVVKGFRLLQMGKHTGKIVLVPHAEDMVPVMRGGYRDVKLFSSSK
TYLLVGGLGGLGRTLSQWMVRKGARKLAFLSRSGADKAEAKATIDWLIER
GVDATVYRGDVSKFTDVRSCVDQIGSLLGGIFQAAMVLQDKPLETMTYEQ
WQHCCQPKVEGTKNLHEATLGNKLDFFVCFSSVAAVVGSKGQANYSAANC
YLDALMRHRRELGLSGTTMNVGAVTGVGVVAENEELQKIMLRMGMDTINE
EELLYQLEEAVLADKPGRPVTARGCNGHQIITGVGLISPDVYWASKPMMK
NLYTNHDFGAEGAGQSSKNLLALLGEEPDIEKKTEILLDGFLEKIASVLA
TPRDSILPSNPLSAYGLDSIVAVEFRKWFRKEVQADIALFDILGATSINA
LVSKTARMVTTISASKHTAGDKPEKKSAVESESTKEDSNVSAMATGQLIK
SQHSGVVPLSTFQSRLWFVHSFLEDKSFLNLPIVLRIRGKPDYPTLQKAV
QEMAVRNPALRTAYFEGDDFAVQEPLEDFELGVDFRDLTKEVDTEKALEE
FVKYNRKIEMNVEEGEVATYSLAKLSEEEWAVVGMIHHISIDRASLFPIM
SQFVGIYDALRTGNDLATVSAPEFNYIDFTLWHNARLASNLMKPDLDWWR
TTLKGIPQSSKLLPFSKGERPARSDPLRQKVRANLNAKLFARMKRIASQS
NGTPFHFVLAAFRAFLYRYTEDKDLVLLMVDGNRPHPDTDPLMGFFVNLA
PVRCNDDCDVPFDQLFQATKTRALDAMAHSGVPFDTIVDIMNVKKTSSHM
PVGQIAVNYQIHGPVPTYTTCDFVVENVESDDIPTAADIQLEAIETSEHS
MDLKIEYATALYDNPDMERFLDNFNTFLESCIKDHRQPVDEISMCGPLEI
EFLKKNYWNMETKENQWGEQSVLDVITEMARQHPQTAAIKTSDCRSMTYR
QLIESAESVASELLNAGAQPGDKIGLIALPGLEAVTGMLAALMTGSCYVA
LDTDFAHDRLSFMLSDSGAGILLTGPGQDGLVAELLSKMVMAPKVIRIED
AAAAGRTVAHRRARHPDDPFYMIYTSGSTGTPKGVVLRESNTQAMLATLN
KDYAFSHEDNFLAHTTTSFDLSIVQIFGGLTAGATVSVASWETRKDPVAL
ADFMMREGVSVTYFTPTQFALLMEFNGEALRKCSKYRVAYFAGERLPVRV
AKAFYDLGTPATLYNTWSPSELVVQTSISKIDYPGEGIVSLPIGYPMDNC
RHYLLDIKGNPVPAGQIGELVVGGVQVGAGYLNRPDVNAQSFVEDPFASE
DDRNRGWNRMFKTGDRGRFRPDGQLEFHGRIAGDKQIKLRGFRIDLGELE
QVIFKESQSLKKAGALVDIAVVARTVDSDEQQLIAYLVPKASITNEADKI
AIVSHLHRKIKPHLNYYMLPNGYQFLAKLPVTLGGKVDRRNLLERRLQLL
FPSSITTQPGNTVSGEPTEDLEGTILALFRTTLGGEIELNDSFFERGGNS
ILLVRLQAKVKKQFKIAPPLPALIREPTAAAVCAYLRRAKGGDGTDKGGL
ASVISWNVETNLPNTSQYIPRYGIPRIDRDELKSILLTGAESFVGIHLLA
EILRTKRDATVHVLGSMEKLEPQVLIDLFQKLDLLQDSLTLDDINSRVKF
VPGSLNQSNFGLSRSAFRELGQTVQSIYHLGGHVSLLKTYTSLKPSNVTP
IFDIIKLSGLGSHLSDINYLSTWSVAHLQTWSASKRTREEYATGEEDSSH
FSPPTEDESGYFKTRWVAENLLTKAARRGFPVTITRASGVTAAVRGPGVP
DPGDEFIMRIVVGMIESGMVPQIGRADQPSFAVDVVPVDWLASSLFALTS
EKQALSRVAPSEVCSAPRIYHITNPLPLRLKDLPRIIAELRSEQTRPRMV
SLDEWLDSMESAEGENAAGQVVRSTVLKQYLSTGGVMFSLDSRRTMDILD
GLHPGVAAGCPAVDAEFLRALLERLKGADDVFSG
>Mb|QLI64372.1
MPAASMLRVAARGSTTFFRAAARPAAQPLMVRAFSTSMPRRSEHAEETFE
EFTARFEKEFDGVQDVFELQRNLNNAFAYDLVPSTSVIAAALKAARRVND
FATAVRIFEGVKAKVENKGQYDQYLNELKSLREELGVPLKEDLYPEGK
>Mb|QLI64373.1
MNNSGSSRAPPPPPLNLASLPPVKFNLDKTKTVDSEVVSIPSSSKRSASY
SSSASTFSGQSSSQKTSSSFIKSSPTVNVHTTCGRHTDQYIFGGPSLRDL
AKSIKKRF
>Mb|QLI64375.1
MADSHPDIQSILAALAAQRPPPGASSQTPPGPPGQSYPPPSSSTQAPPHY
QAPPPTAAPAFGASGHGLPAPASSGNVDLSAIRPVTSGTMNFDAIVSKAR
AYAADNGATSYDRPLVYGADSRPNDRSYRRSRSRSPRGEYRDDRRGGAGR
EYGRDRSYSPPSRSRFSPRGGGGGGRDRSPLRGGDDNSETIQIESSLVGL
IIGRQGENLRRIEADTNCRVQFLAATDGGPFRQCRISGPRPRRAEVKDAI
NRIIEDSGMGALNRPAQDKNRDPNKGGATALRDGEDHMQIMVPDRTVGLI
IGRGGETIRDLQERSGCHINIVGESKSVNGLRPVNLIGTVEAAARAKDFI
LEIVDSDTRGDGPAAKKATAVVVPRSEPPPRDATGSAGPDKINDSVYVPS
DAVGMIIGKGGETIRDMQNTTGCKINVAQSSGPGETQREIALIGTRDSIA

RAKLAIDEKVDAVRQKSGSGGPPRGRGHQDVDRPSYSQGPDTSSTNPPPA
SSQDASDPYAQYGGYQNYVALWYQSLMYQQQQQNGDGSPAGAPAPGS
>Mb|QLI64376.1
MEVDDHPMGDADMTNGTSHDNSVRPEDDDDTASDEASYDDGVYPDLQDLA
LEQLRRRGLLPTGCCYDDRMKLHMNADFSPNTHHPEDPRRIHEIFKAFKR
AGLVYTGPEAELPKIIKECPTRYMWRIPARPATRQEICLAHSSEHMTWVE
HLDKLSTAELREMTKRYDQGRESLYVGSMSFPAALLSAGGAIDICKNVVS
GQVKNAFAVIRPPGHHAEYDAPMGFCFFNNVPVAVRVCQQDYADVCRKVL
VLDWDVHHGNGIQNIFYEDPNVLYISIHVYQNGHFYPGKPPSPETPDGGI
ENCGAGPGLGKNINIGWHDQGMGDGEYMAAFQKIIMPIAKEFNPDLVVIS
AGFDAADGDELGGCFVTPPCYAHMTHMLMSLADGKVAVCLEGGYNLSAIS
NSAVAVARTLMGEPPPKMTIPKLNREAARTLAKVQAYQAPYWECMRPGIV
DVPAVQSLNANRLHDVIRSAQRLVLQEKHNMVPLYVQRDQLYKSFENQVL
VTPNLHNSRRILVIIHDPPQILAQPDPIDSSLQPHNAWVVDGVTRYIDWA
ISQKFGIMDINVPAYITNDEDADSYIPGYRERILQEQVQQLMNYLWDNFL
QLYDADEIFLMGVGNAYLGVKVLLINRDCKSKLSGVVNFVTGNLRPVKSD
IDPDLSLWYKENSRVYVAGDHACWSDHDLTKKVQKRRFGTVVRSPKLGLN
KMMQEHADEVQGWILSRVSATSRGDTTEEEDEDDGNGIGGVRAG
>Mb|QLI64377.1
MPAPKAAPARHRLTLAQLSAYDDILTDALVDHVYYWTTVPKNRPSYHPSR
GLSENAIAKIIQDELVLWKDLAAAEEKLLAMDGLRKFMNKLKTDREKDDF
KRHLRRYLQIYLPDCPWEVSSTNRYTIVSHEASITARRYIKRNESIKYLS
GIQVVITPEEETEIAARKKDFSMVISSRSKSTSLFMGPARFANHDCNANA
KLMTTSNAGIEIVAIRPIESGEEITVTYGESYFGEDNCECLCGTCEKGLR
NGWEPEEGAVSIQTSAEEDRPETYALRRRRREDSVGGSRTPSVTPGIRPR
VPRTKAKSSRLSNAYGSSGAVSPAPGSPVGRKRKADTMATPPITPAKKPK
HTIEQELHPAASRGSSVSESILSSNGEAVETDVTSPEKDSPEPVPETPIR
LLANRLKGSADQKEDHTITAIAPFSPESIQSQQSPQSTKDGDTNESSATS
KPELSAMSISAILNAPSSSEPEVTAPISASIEAVDMSQIATNVDEEEQPR
PKSRRRSFSKQASPPAKVRRPGDYVLTPLLLSEPEMAWIQCTVCNAYFVQ
QNAYYTRSACPRCERHSKLYGYIWPKTEKAGPSDKEERILDHRIVHRFLG
TNDERKARGRKCLPEREETEEPSEPERGRRLLRAPIKKKEKSSDEVQDSV
TVKLIEESGLRRSGRPRKCRCA
>Mb|QLI64378.1
MAAKDAITCHVLDTAAGRPAKSIRVRLEGPRGSQPAKEFESITDDDGRIK
VWLPYSSATASGEVPVYTLDDVLGQAEASSTWTLRFDIEGYYGEGKTFFP
EAVVVFRVQKGQHYHVPLLLSPYSYTTYRGS
>Mb|QLI64379.1
MAGAQYQNPPQAPPLFTGTTESVVADINKNNELTKSILDKVVADVKPANA
IFDSVLEPGLVNENELGRSYRVAQFYQYVSASKELREASTKAQEISDEFN
IELKMREDIFKLVDAAFETRDSQKLDGESLHLLQKERQKYIRNGLLLPAG
PQRDRFKEIQKRLSLLCLQSQKNLNDEMGGVWFTPEQLEGVPPDDIDISG
LEKGTGENEGKVKVTFKYNHFFPMSKYAIHEDTRRTYVIAESNKVNNNVP
LFREIIALRDEAARMLGFPDHASLVISEKMAKTSGRVMEFLGDLRERLAP
GGVKEADRLLQYKKEDCEARGAPFDGNLYMWDVPFYSRIMKEKEYSVDEA
EISQYFPVDSTYQGMIKIFEQIFGFVFVELSKEDRARLSPTGKAEDMTWH
EDVIVYSVWDDEAAGSGFCGYLYLDLHPRDNKYGHNANFNIEPGYLNKDG
TRNYPATALVCNFSKPSATKPGLLKHHEVITLFHELGHGIHDLAARTKYS
YFHGTSVAGDFVEAPSQMLENWCWTPSVLKFLSRRWDTNEKIPDDMIEKL
VKTKHFNSATSNLTQLLYGLFDMTVHTPKSHEEAKNMPIARVWNEFRRDI
TGIKGPEAQGQGLEWGNRFATIGHYTGGYDAGYYGYLYSQVFSLDMFHSF
FKKDPMDGKEGRRYRKLVLGRGGSQEELQTLKDFLGREPNTEAFYQELGL
A
>Mb|QLI64380.1
MANEQISLPYLAGILLLSGLIIRYLFFSGTQTPRPTRSPEDVRRSRETAV
ERIQQMFPQAERRSILWDLQRNGGSIQNTTERILAGRLETPPVTFQPPPP
PGQATAGASTVSATRQADKPSQPDLITRYNLKSKITEAEFEEDAKSKGWS
SNRDERQASLQKRRDEMILAARRKMEAKIAAEKK

>Mb|QLI64382.1
MAATAISGAAFGAAMMAAGFHEPSVVISQLKFENWHMFQTFLAATATSAA
IYAVAERLGYAKISPRNSSPLGLLSSYDGNILGGGLLGAGMALSGSCPGT
LYAQLAAGVRSGFHALDGAIIGGVLWSGLVSRLVRRRREKAGARPEPAVV
GERLGLSNGTALLLLEAVCVAVIAATTLYTPASPTAKIPGALAGLLVGGA
QLVSILTRRTMMGISGSYEEVGNFFWWLVRGADPNAKPSSYQNILFASWV
AAGAWGLLRLVPGLASGPAYEISPLLAVSGGVLMAVGSRMAGGCTSGHGI
SGISLLSVSSVVTIMSAFAAGGVVAPLVH
>Mb|QLI64384.1
MAPLYLFLAALYTGLTLAAQCQSIQPKSDPQTAPGVTFKVLANDLSKPRG
VIADPKGNLLVVEAGAKGVRRIELDDGKGLDTCVTKSSSLVDDETLNHGI
ELSSDGKTLFVSSSTGVYAYDYDAEKGTVGKAKNIITGMDQGGHATRTLL
VPKHNSNLLLVSRGSDGNIDKDTVEIGSAKSQIRIFKIDHLLKIDSPVRY
SDGEVLGWGLRNSVGVAEDPVTGYVWSVENSIDDMQRNGVDVHNSNPGEE
LNFHGLPNDTTSDVYGKNFGYPSCVAIFDTSNVNGYPGGAKIGLQMVGDQ
MPNNYTDRWCQDDTVSPYITFGSHLAPLDIKFNFQGSAALISFHGSWNRK
PPNGYRLSRVAFSEGYPKADKSSPTAEQELMWNKDNSYLKMLLTNIFFAS
LATGELILNYDGSLEGRVEEFEVEEMVIDAVGGPSKLGALKNPLRQRDSD
DPMFGSAALSADGVLHVLNKRQSCEAGYWYCSGPCKLREQCCGNDRCIPS
SAECCKNGRYCEAGNHCYILSSQGNLPVCCTDSRCTARVENGRTTYATSR
TTTRTYTTTRKQYYYWTMTWYYYYYYWTYSTVIEASVVTSARTSTTSVLS
VSTTAAAAASSYFSSISSTLSFSTPASATSLASLTGSTRKGAEGTDGPTA
TDTPNSGGGSENTADNGGGNEKSSPTTAATGATGTRRPDSPGAAASLWSS
MDILTTMSVALGVSTGALAVML
>Mb|QLI64385.1
MADIGISGTEFIASYNWLDDCYPKIFIPGKPPLWTPPLIRRQLQEDKGIF
YRDRNAASYPKHPIEPAIVSVMQMHSTPRHVDVFACASTLRKLLGFLQGD
DRPFRMLVEVIAGTVHLIRRENRPKEQILGVRGYGHTFPEAYTTWEADVK
RSISHQRILGYRFCGLDMMVRYEGDGYIKSDKTPLKTSNKADLLELLGGL
QVDRAGKATSSDDSESILHVGNAGEEVKQEQVFDLKTRSAKRQDEDILAE
ELQRLWIAQIPNFILAFHDRGHFANVQIQNVRDKLKDWEAQNQSLLRGFF
HLLGHIIDTAGDYGDSQLEIVRPAGGGLEIRKRAPEAGSVLSGPVRQIWA
TWLSDGEDAGHDVRAASLDMHTAKETSSEDDTACDDQCGYCGKCA
>Mb|QLI64386.1
MLTPLRQVLLAALAATSVHAIPQAAPTTAAVPSPSSTSSSGQACNNSPTL
CGRQYNAVTHMGAHNSAFLRDSSTGNSLAGNQLKNATAALNAGLRLLQAQ
VHKPNSTLELCHTSCDLLDAGALESWLKDINAWVTKNPNDVVTLLLVNSD
NAPASDYGAVFESSGLAKVGYKPQSNLLTSTWPTLQSMISANARVVTFVT
NMDYSASTPYLLPEFDHVFETPFEVTAIGGFNCTVDRPSKANPASSSLSS
GFMSLVNHFKYQSLIGSIQVPDVDAISTVNSAGTSETGNLGKHLQQCKTE
WNKAPNFVLVDFWDKGDPIAALDSMNGVTDATGRSTSQSSGQSAGSNFAK
DRKFGIGALVAFVSAALLLV
>Mb|QLI64388.1
MQSRRLPPIPPGPTGRPPTLNHINDVNEPFPTRDALQAATKSHSQGNFTI
SARKLPISKAGAIDHLTEKIGIPMPEMIFGDNIVSILHRPSKWYFEFNTP
DALDAVDKTDKHMLKVAYARDWESTREGTTQNIKEVVKPYDWSYSTTYAG
TVGVAGQQQQQQQYDGPSSSEAASSPIVPQFQPTDKKIPVELLKRRDPIL
FFDEVVLYESELDDNGISMYSAKVRVHQQRMLILCRLFMRLDNVVVRLRD
TRIYVDFETDEVMREYTAKEAKFDDVKRKLAMSGRLPDDITVVLRNPNEL
DPLLDIVQHQTEALCLK
>Mb|QLI64389.1
MAGLGGSKAEPPPTSTGGWDFLLKPFGRPTDERSLPTSEPDQILNSKLEE
EPSKTVKSVGDEDSRRAVIGLPRAQTLKRRLSEQRVNLEPVLPTPDERRA
ASEDRRSAQSYMGQAILPHCSPRSTVPGTFSPSTSSLNGYGSDLSSQLPS
DPGHYELSNHIMDQEQLNLESRCYDGVPKDAASVADSIDEATLAEELTQK
WILNLSMHFRDGSKREKFFVTYREKEYLWRRVTISLDYRSVPPNSLEWKL
SHTSEQREKNAKIYEAIRGSIPVIRFFDTVTNLKLQTTDDRLHIHVVEDL
NEVIHFPTADQVKHLKCDFFRESDIVFDSHLSGFVYKVHVDGKALIKKEI

PGPETMEEFLYEVNALGNLRDSKDIVRLHGLVIDDFSDQIKGLLITYAEK
GSLLDVIYDNCKERELGISWPRRQRWARQIVRGLNDIHELGFVQGDFTLN
NIVVDAAGDAKIIDINRRGCPAGWEPPELKVLIEAHHRTSLYIGVKSDLF
QLGMVLWALAMLDDEPEAHGRPLMLGPEINIPDWYRQMTEICLDPDPRKR
LAPSDLLRMFPPDIEGRKGSTVTADESRSSDGYSVDYPPHTQQYIKTVSP
SNGWQCSGKTYVNTSPDMYGPQSPMRGRSPPSSPPSAFDGDIDEDSTTEA
GKVVDVGLRPETPLSLGQYDIDSAAYLPTDIRLEDISPKRSTIPVMDRKP
SLGDGLQTISTPKLERQDTAIHAPTRHDTAETQLPKSTPKIEDSHLAESL
SGHVDELTADMTTATEMVDMHTVSDAHEIKTMESDADGGVSLVLGHVPTA
HRLQSHSDLHSEAQQDVQQNKRPPDVNSCRSVAMQIDPSMNKSTMNTGRE
TSTDSAVSNKLHTRSAYNHREEARLGDFRIRSSIPDSVSGSHLHQRPISI
PVVLTGVGAGLSIEYDTIGLGNMVTVDDDFDALARPATV
>Mb|QLI64391.1
MEEQLVQLLANIQLPDQGPRQQAEIELRHARTNPVFPVSLAKIASHASVS
TGVRQAALTSLRQFIEGNWAIGDQDDEPIIPIDDDTRAILRQSLLDLALS
QEEDRKVKISASYAVGKIAIHDFPEQWPNLLPTVLSTIPSGTDAQLHGAL
RVLGDLVEESLSEDQFFSMARDIAKTLTEVALNENRKPMLRALAISVFRS
CFDLMNMVKDDHSQEVKAFAEELLAQWNPFFVSVLKSRLPEADVSTGTQP
DSWNHIVALKLQVVKTLLRIRRVFPNLLLPQSTTFFSAVWEELNLLQTPH
EELYIKTNAQGRLEDSDNLPYTLDFLILEELDFLNQCFRSPPVKAELDGH
LQAHASAQDVPWMVEIMRMLVSYSRVTQEEEELWDIDCSLYLAEETSVTA
NYTARTAAGDLLIKMGEWFNEKAVDGLFGFTKSLFPGDGSLWRSQESALF
LFTMLLSDFQDLSKTIPDAVASAYLELVDFTINKPDEPLLRARGYLVAGI
IGRSFQTPLALLDRMVHAITNEESEVVQVACIKAVEGLINSGRVGADRQV
PIITAIQSYMNEKDPAEMEEADELLVVLAETLRSTISLDTKIALSSDIQS
TDLLFMLAKLGASNFQVTMIISEAFEDIVASLSDSASFSALCARTLPTLT
GAFDVASVTEDNPLVTVATELLVVLAENGSEPLPAGFVATIFPKLNRLLM
ESEEGEVLRPGSEIVKWVLSHDHQQVFNWQDANGRSGLEVCLHIIDRLLG
PSIEDNSASEVGGLAAELVEKAGQERLGPFLPQLLQAVANRLASAQAAAF
IQSLILVFARLSLNGAQDVVEFLSQIQINGDSGLQVVMAKWLENSVSFAG
YDEIRQNVIALSKLYALNDSRLAQIQVKGDLIVGADDGKIKTRSRAKQNP
DQYTIVPAPLKIVKVLIEELASAAGAGAAASTAAAAVAAAEFDEDNDEDG
WEDDDDTLDLSLAATKADLMTFMEGGPQRRPDDETHSYLTEFFVRCGREN
IANFQEWYNMLSEDEKTRLNLVANSAG
>Mb|QLI64393.1
MDSEPFENAGTSFERRRMPYASSQPSERTETFGRSGPRLAGESTAAKEEA
PNPQAKPKGRRYGPRTCRICLDTEHPKFPTDIPRTFGVSTRSSRPVYESD
DPELGRLLSPCKCKGSSKYVHEGCLDAWRLTNPTATRNFWQCPTCKFTYR
LARLHWASRLSSKWAQVFLTIVVLIVSLFVLGFIADPIMDLWFDPVGSIA
DTVSSVVSDLDAVRPPVYEEPSTWAEHFTKGFFSLGLIGAIKSFVAMGPW
HWINFRAGGLFGSGRRGGTGRSRMDNFNLLFVIIGAFTFMMAVWRFVKAM
SARILTNVSNKVMDVGDEDDEYEEAIPDGGAAGPS
>Mb|QLI64394.1
MANDEYDFLFKVVLIGDSGVGKSNLLSRFTRNEFNLDSKSTIGVEFATRS
IQVDSKTIKAQIWDTAGQERYRAITSAYYRGAVGALLVYDISKHQTYENV
TRWLKELRDHADANIVIMLVGNKSDLRHLRAVPTEEAKSFASENHLSFIE
TSALDASNVELAFQNILTEIYRIVSSKALDSGDGAQATIGAGTNISLSKP
ADDESAKSGKCC
>Mb|QLI64395.1
MEQPADGPIQSIAEPGVKASGPLLYSMRKEVAELLGRHQTGFPGAQPVSF
ARQHLDELQKQDYYVCEKSDGIRYLLYSTSDEEACEAHYLIDRKNDYWFI
TNRNLHFPLENNTQAFHRDTLVDGELVWDSLPNGKKEPRFLVFDCLVMDG
NKLMDRTLDKRLAYFKERLYTPYKKLFKEYPDELRFQPFFVEMKPFQLAY
GIEMMFKQVLPSLKHGNDGLIFTCRNTAYKHGTDPHILKWKPPEENTIDF
RLKLTFPLVEPDEWERKEGITEPFIDYDSVPKSELLIFLGDSGDKYELFD
DLYLTEDEWETLKSLNDPLNERIIECNQDDQGRWRLLRFRDDKHEANHKS
TVVSVLESINDRVSEKDLYRAAGPIRDSWKARQARSTQVRDR
>Mb|QLI64396.1

MDSTIFHMEQKSRRNYRLIPPRIIFPTVILALAAAFASPAAADKDLSTSK
TWAPASLLPTLDHPEPSLAIPPPAAVSSTERISRRLGKRASNGEYSSIQT
AQSDKRSAATTTRSNQTSTADTAASPLPSPFDNPVPNAFQVPGSDDSCPK
FIAALLSDPTFKSCYPISMLIQTSTSFFNAQKELVSIVKVLDATCDVDVA
KCTAYMTTAAQNLTASTNCKRELDQNQTQVVQAWQGLRAYNVLYSATCLQ
NPTSSSYCFASAVTNISNPSDAFLYFMPYATPLPGTSVPSCGWCVQNTMF
IYHTASANRRQLVASVYQDAAIQVNTICGPNFVNSSLPAAENGAFGALVP
SHIVIAATTLFTLSLLVWFL
>Mb|QLI64398.1
MSQPGTGTTSMPGQDVDGKGVGRVISKVKNAFKSDKREAAASSTQPPSSP
RDDTRSDASQGSTPASAVPKLKILEERAKKMGEKFGLVIESSDFLSHSPK
ETVLRVDKPIRMRVRRNCHRCNTTFTTGNECTMCQHVRCEKCPRHPPKEG
EAGYLAHLAQLEAELKANKENPPIVPDYFWGDERIELKRPSKTGGQDLVH
RKPRQRVRRTCHECQTLFATGSRKCENCNHIRCTDCPRDPPKKDKYPFGY
PGDAFGPDSDARFECHSCEMVYPAGAEDGTPCIMCGLEKSVESPRALPRK
VQPAPDLEVLSRLQARLDGLRAKDST
>Mb|QLI64399.1
MALDLKDIDRYDFYAGFLKSSPKITIFPKPLKRSSLHSRALSNAVSPSRN
DSASPGQRDSAHRPPPEHPQVTTATWVCTICSFSNPIPVNFDPAAANAHT
PLPPCLACGIKPTLAHVLKAAINNASQRPTPVINTSSGFSSLSNRQHESD
ALLATSTNPLDSFQQSRGQALEQNRSGSSVDAGASFACPRCTFSNHPSLL
ACEICGAPLLSQNTPSIKTESPTVRTESPGPVHEAPSNAPSGIDVADSIK
ISFRGGGMQIFYERLKGAITQRKWLLQNAPPVPKNGRSADPNTLAGSSDT
STSRTKTAGIAGLEQLGLNMHKNNEMLIGSAFEDLEALMSSAKEVIALAE
RYARQTNGVTGGASAEENAILAESASQLGLVTTKDIVAGGSSEYLYISEL
SRNLAEFLADDSRGVLKRAGGIITLVDLWAMFNRARGGVELVSPMDFEKA
ARLWESLKLPVRLRTFRSGVMVVQGHDRTDDATIKSLLSWLQDLHEFPPE
REVTWDWREFGRGVTALETAERFGWSIGVAEEELLMAEEHGALCREEGLE
GLKFWKNYIDTGDAPPPKSKARLEDEAIIKALQDSGFM
>Mb|QLI64402.1
MARIQIPLDALTSRLNLGDRFSGFTSGPLTGRFANLRPISEFLDFKRLSK
PANFTEMQSRVNYNLSHFSSNYAVVFCMLSLYALLTNWLLLFDIILVVVG
MWFIGRLDGRDLEIGTFRATSSQLYTGLLIVAVPLGLIASPFSTLLWLIG
ATGVTILGHASLLDKPIDEAFSGEAV
>Mb|QLI64404.1
MSLPSATSKSLTLTKATKDERVYIWTRHQPIWGHRLTAQAYIDREEHLLA
YQLTKDGGLTSWILTDPTSTDAHGAPGGRPILSAVETYRKRAVVRDPAGA
VRDVTAHGVASVFTFDEFRRQGYAGRMLALLGEALAAQQAEDPGSAEFSI
LFSDIGRDFYANHQWVPFASAHLTFPTAPFTTPYDDRLTLVTGDNLQEVA
RLDEQTLRRKIAHPPGRGYTVRAAVLPDFATYEWQMARERFLCDYLLGEA
PTVHGAMYTPADAPGSRVWMLWSNILYGGKEKPQDNVMNILHYALEDENI
SDEDLSRALGAIMGLVHTEAQAWLCSRVDMWNPDERTQRLLENMTHLQGK
LIVRDQTNIPSLRWFGQAPVSEVEWVDNEKFEWC
>Mb|QLI64405.1
MTQTGIDHGRRRAVLYPLQITLLLLLPDVFEVASNLREAKSNNLIKKVSF
LDSLRKGLRNGNERAAYCLVSLLRAARHFEAESDSALVSYAMDVQDEIRE
SVFSLSQSNIATPQFDQDMITGAFVSLAHLGLDGSVETLTKACISPPAPN
SFKIAVIQACSYFARLARRSETAKYTALQTSVLVLIRSEFESEYMNFFKA
RHELSQDGSRKILEILRYFYASPGPLLRDLSGETAGMAFLKPFLFCVLSR
DQSIRQLATKVANKLFIMNKDSVGTSQLEYCGATEELGQNLWIRSSSILL
ELCGEIEKDISLSTITQLREFLGARSALLKGMPILSHLPMDVPDIHLAAS
KLETTLLISICSPNIAVCQLATICIGLLVDECTHVGEYTDSAKLLSSTYR
NQLVYQELSSPAFHFTGLVAFQKRMRGFLRQIRLPTQGIVDAWETAFRQW
IHLSKEVSLATADGVDDRMLSEWRNLSGFLASLGGICTYGYATKLDEAIG
DLNWIDRVSSEHHEEPLLSRFLRLSIQLLGCNNIKVREAMREVLSTEVSS
ILYAPLFKALESELEVLLAGVLASPDKGQENEVIFAEQAASLLKIMVEKL
ESPSDLGSISSVHPGVLTLSFAKFLDGALDGVATLRVKVKICHLCEAVTK
RKEHLNLRDDIRIRNQLLEYIFGWIARPHALKQEQLGNGPRQDEFRRVQK

DLDKACLRTLGDLTFRLPLQPADSQSDAGMSELKSQMFHTYFNRFLSLLN
YETQEMSRQELPLMGNREEGLSNADMVITILSNLLSANIDVGLKHSLNIG
YHENVDIRAAFVRVLCNILTQGTEFSSLTDAAVSDKYVELLNLLTTDLSL
SVSMSAICPASEVDELTICLLTVFEQRGLTFELFEALIRQEVEQTENETE
ILRRTCVATKMLSVYAKWKGASYLRTTLQKVLERLMLTSNDLDLELDPAR
VSTPGELQKNAAQLQIVAKVFMDDICASTSNVPSSFRRICSIISDAVLPR
FPNAKYTAVGAFVFLRFFCPAIVAPEAEGLVSVAPTKEMRRGLLLIAKII
QNLANNVLFGTKEPYMFPLNPFLVQNIHLVTGFLRDISVSTRQADVAPPA
IDMFDFGSCVSLHRFLYDHWDHLRQTLVSRERKEHLRTPVEIARTPPSVF
EPLRDLISNLGPPPLAISWNRPQISCNTPPLYSRFQNFMLRNAFRSAESF
LTSRAVYDGGESKDGLSIVCVILRHIENESIDYDTLLYCYLKIASRLWHE
PFGLFIDATCYNGRSEPSDEFFSKLDLLTPSELSRNLSRIYIYNMNSAFK
RCFRRLLRNSTRDDSSVFHPDNVEYYLIGSLQDLQVHFHLSQLHLPKETI
SVVTETRYMFQTITRLSKSKGKVEVVIKVGSQFLQITTVKKQEVLSGLRL
SSVINDIFRLSDIEEASTPQAEDDSNFGLRADGGKIVMFFTSSKRADILQ
TIRGAKTIHGKDSRMVKPVERLVRPQDVPGTMLNLALTNLSSPYHVLRLA
SYNLLGALCRSFDFGVSGRLVCNRDLAVPLEPTRFIVDISRELAQAEPQL
TSDFLTEFFVSWDSIPDEQKPLSLEYVAPWLPGLRTAVLVVEVDGDKGRD
KVASLLRKLIDISVLDQNLTYCLEKYIWPCIAQDELLLEIFTDELIKAAL
SYGNGADTLEILSSVMVGLGTVTLRARIISRLRKALNRSSLRPTRLLQDN
SVWEEICILLQFCLGLSFDSRVQSQMFLPEIFHVVTMLANTGGSEVRLLV
YKLLINSIHAICASFSIDDAKLVRLRSILDVFGDTRGDIFTAPTLLARDG
ASVSTSFDSGPALASTENLTSVLFEVCLIAAPSMDIANAWRARWMSLVAS
TAFQNNPAVQPRAFAVMGYLAREEVDDDLLYQVLVALRNSVSQFGEDGNS
EMLVSIITSLSKMMAKLPSSSRYGLQLFWLAMSLVRLVPAAIFNCAAQFL
GAILTNIGTIGNVRGDRMVPLLLQCRGQLEDAALPLDDAYGIRFEEDTFH
FAVCACLVRGLTDTATRPTAIQVLSSFLEMTSRSEDPAPGSPNHAIHGSP
YLALLLARGMQEDFMDSLWWSDLKPEAISDILDSRGVQGVDNMKDKDLLL
ISAMELVDFHSLEDAAQARSLHWLNSLAEARPFVFTSLCGALPSVLDDVL
LHSQESAALEAAHVLLRTLASDQKYAQAMGSMQSLNSQLTEMGFGGLWKI
PKQSTVEDAKGECFELTEKLIELIVM
>Mb|QLI64407.1
MAPSLEEPEPVSDVLANPIKQKPQLVAPEPEHCPGPESQQAGQADSCAGC
PNQAICASAPKGPDPDIPLISARLENVKHKILVLSGKGGVGKSTFTSLLA
HAFATNPDSNVGVMDTDICGPSIPKMMGVEDETIHVSGSGWSPVWVMDNL
GVMSIQFMLPNRDDAVIWRGPKKNGLIKQFLKDVEWGELDFLLVDTPPGT
SDEHLSVNSFLKESGIDGAVVVTTPQEVSLLDVRKEIDFCRKAGIRILGL
AENMSGFVCPNCKGESQIFRPTTGGGKGLAEEMGIPFLGAVPLDPRIRMA
CDYGESYFDSFPDSPACLAFKQVVKRVASQLGLDTTNVLPDE
>Mb|QLI64408.1
MLIPAAALSLIGLAAASPLGERQVVPNYPSKSTSKGFHLVVNVTDLSKDF
ESPVHNTYASSIHVGAGLALVGQSASTGYGRIFYQNGTVDEQRYSQSNIL
SDSGTPPFPSGLTLVKDPNSQTVSTAHLNGGAGQSGIGITRFPEPYAFLD
PETWVACNESQPYYGGKYFVILKQAETTIGDDGSINRNIPDGCAPVRLVP
ECTKLNDLPAGSTSSHEYALDSECYPDVKSLDWTQYGP
>Mb|QLI64413.1
MYELFLTALVEDKDFNAACAVLSGFCAMSPWETVNRVLYLQGPPRPSGIT
NQSSIDKPLRKDLALLWKELHQSLSRQSCILQVRYEIVKDRDMGPSAAPM
DLDSMPGVLRWADFPDPPHGRPLLTQRKMVEIWEQKKLLPIMRDNNYRFK
TEILEENYRFFREEIEFCLTRQYFLRSIGDYAPLESRGGQQPSPLTSLPA
WDAVTPVDMQNRWILLVKSHVVQDNKPDDIKLAQDQLLSIRGELEGAFDF
KIIDRKVHDTRIAQQQQGIQALPQKVMLGKA
>Mb|QLI64414.1
MALNLRNSTRAFGSLKPLTRAALIGARGYATAEPDLKATLKEVIPAKREL
LKKVKAHGSKTIGEVKVENTLGGMRGLKAMVWEGSVLDANEGIRFHGKTI
KDCQKILPKGKTGTEMLPEAMFWLLLTGQVPSTNQIRVFSRELAEKAQIP
EFVSKMLDNFPKDLHPMTQFAMAVSALNYESKFAKAYEAGMNKSDYWEPT
FDDCVSLLAKLPTIAAKIYQNAYRGGGALPAEVDLEQDWSYNFAAMLGKG

GKANENFQDLLRLYLALHGDHEGGNVSAHATHLVGSALSDPFLSYSAGLQ
GLAGPLHGLAAQEVLRWILQMKEAIPAGYTEQDVHDYLWSTLNSGRVVPG
YGHAVLRKPDPRFEALMDYAAARPDIAQDPVFQLVEKNSRIAPEVLKKHG
KTKNPYPNVDSSSGVLFHHYGFHETLYYTATFGVSRGLGPLAQLIWDRAL
GLPIERPKSINLEGLLKQVEGQ
>Mb|QLI64416.1
MAAGDEHSAETSGPAVERNASAANATPGNTTATESGDATPCQKPGDNHEG
KKRGEKGRAERSRQLKNDKKRKKQDDWREFKRRKLSDKAGESGNSEKKNP
FSQEEIASEERRPKRKVAVMIGYSGTGYKGMQVNGDEPTIERDLFRAFID
AKAISKANADDPRKSSLARCARTDKGVHAAGNVISLKLIIEDDDIVDKIN
AHLPEQIRIWGIQRTNNSFNCYQYCDSRFYEYLLPSYCLLPPHPQTYLAK
ECEKLNKEYGAEDEVAAVMADVQDFWAHVEEKEIKPILARLDPELRAVVM
ERVHAQEAEEYENNKKAEAEAEQDHAPDAGAADKPDEAGDAATRPEEPKR
VSLESHKPKHRELAPIDFALRDIKAAYIAAKRRYRVSPERLQRLQRVLDK
YTGTYNFHNFTIQKTFSDPSARRHIKSFTVDPKPVIIGGTEWLSLKVHGQ
SFMMHQIRKMVGLASLMVRCGTALDRVADSYQEQKMAIPKAPGLGLLLER
PVFENYNRRARDTLGKETIDFGKYEERLEAFKQKHIYSRIFEVEEKENTF
HGFYNQIDQFKANHFLWLSAGGTKVAELNKESDKTEVDKALGDEDENPEG
GEG
>Mb|QLI64418.1
MSRNGPSVVAKTSGGNSAYHNFHNDFLHVSDLNERRRLALAEVDKAPFGW
YHVRACVVAGVGFFTDSYDIFTASLLTTMLGIVYYPGVGAMPTSSDNAIK
LATSAGTVVGQLGFGMLADLVGRKKMYGLELIVIIFATIGQALTSGSPAC
DIIGLIIFWRVIMGIGIGGDYPLSSIITSEFATTKWRGAMMAAVFAMQGL
GQLCAAFVMLFVTLGFKGSLEGAKDKQHCTGDCQVAVDKMWRTLIGFGAV
PACIALYFRLTIPETPRYTFDVARDVEKAGDDVKAYMTGKHEGEPDEVTR
ATMAQQAKDNLEVPKASWGDFSRHYAKPKNFLLLLGTAGSWFCLDVAFYG
LSLNNAQILQTIGFATSGKSVANVYQFLYNTAIGNLIIVLAGAVPGYWVS
VATIDTLGRKTIQLGGFIILTILFVVMGFAYNHISSNGLLAIYVLAQFFF
NFGPNTTTFIVPGEVFPTRYRSTSHGISAASGKVGSIIGQGAISTLKTHN
GQNWMDHVLEIYALFMLIGCFTTLLIPETARKTLEELCGEDDYAVTHADR
GSNGTHDLNGEEPVKTS
>Mb|QLI64420.1
MKRSREPEEFLDSNFQDGDAERFEFRDSSIPTAKISQLDIAVQDEDDDTP
TMKCSLPPHKEPIPFRSYQEYESHYSMFHTNRCLECRKNFPSEHLLGVHL
EEYHDPLIRVRRDKGEHTFSCFVEGCERKCLTHQKRRLHMIDKHMYPKNF
FFGVTKEGIDGRRSLLVEGDHHRRRSSTASISQESPRRAGIIEEQEAGSK
RDGKNNKPLFSDTVKETPSKGQNVDADMADLTGAMSALQFVPPSIRFGRG
GRAGFSKS
>Mb|QLI64421.1
MDIDPYPASQPVHGQDQLAPSGHDSGTNSSQSQTQSITDSQTSNASSATD
QVATPPGSDSGSGVERPPVAEVTAMLKNRYANVGPEVTTGVSSQDSQLHQ
LSAIAAVQDRLGLDAAGLPRKRMADGEVKDRKGSMSPVKGHSRTTSTVSV
ASTAGSTIGELSAELRTRLSYAMVKVNNGWQARNLDEVESLASQATSPAS
STSTVHRRKGSSASPRLPSTVTPSHPQFTYESMNYKGKSNSPPSANFDKP
TLAPPASIQPSSSMPAPRSNPRRNSNPRYTPTMLSHSHSASANASAQTTP
ISNGIRRSHSHLDANMYSPHHKNVREQDAIETLLFMSSPGNSASLKHTFS
PSGSPGPQPSAPPRSVGERHALPSGPRRGLPGSRPPSSAKKVGFDKSPGV
PPPHSPMDLDSPQQQHYAAHSRSTPKRQATSLASRHRTTLSLPSGLGLGN
GTARKALRDEDIERMLDHAGAELADSSDDEEIQLPPGRNRLAGAMGI
>Mb|QLI64422.1
MGKKRSREEGKDAPPADAGVDKMDEDSSDDEDFDMVNVEFEWFNFDPEID
FHGIKSLLRQLFDVDASLFDMSALADLVLSQPTIGSTVKVDGKQTDAYAL
LTALNTHVHRDTAPMKDVLKYLVDKAQTNASLAAVPELVNAGKHIGLVLS
ERLINIPPEIGPPLYSMLIDEVEAAVEDKEPYEFSHYLIVSKTYQEVEST
LDVEGRKKKKAREESSTFFFHPEDEVLHKHALAYGSYSYTKENDSVADSK
RAFQEAGIKPAGHMILIEASRFPGAVKAIADYLQPQQ
>Mb|QLI64423.1

MGAIYYLLHPNQLRSIIQWKLWHNPVHRRDPSTESETLKECFRFLNLTSR
SFAAVIQELNHELLVPISLFYLVLRGLDTIEDDMTIPLSKKVPLLRNFHD
TMEVDGWQYHESQEKDRELLEKFDVVITELKKIKPTYYAIIKDITVKMGH
GMADYAQNDEMIKNGVNTIDDYELYCHYVAGLVGEGLTRLFVASELGNPK
LAERPSLTESMGQFLQKTNIIRDLREDWSDGRRWYPKEIWSRHVDKWEDL
FDPNYEDKALNCISDMVLDALKHCEECLFYMAGMREQSVFNFVAIPQGMA
IATLELVFRNPDVLKRNVKITKGDACQLMTECTQNLYTVCEVFRRYARRI
QKKNDPRDPNYLNISVRCAKIEQFIETLYPRQDPKQLNLENNQKQRNEPT
MDTSETLVMFGVVALCLLFVSGLMIGTAWLLGAQFPNIFKEASILLQKLN
DPAPAVTTAITTAREEL
>Mb|QLI64426.1
MLKAYKNLSPKTRAGVGIGLIAWSFAGLYLSDQAEEKFGYTPSEKDKEEL
WRWAPKVTTVDKSDTSK
>Mb|QLI64427.1
MGESRQELIQWLNSLLQLNMTKVEQCGTGAALCQVFDSIYMDVPMSKVKF
NANGEWAYVQNFKVLQNLFAKHQIDKPIPVEALIKCKMQDNLEFLQWTKK
FWDLNFPDHDYDAVSRRKGGALPSSNSAAPRAPVSVGAARRGGATAAAAP
RVAKAAGAGPNTVALQAENTTLKETVTGLERERDFYFSKLRDIELLIQQA
VEEDPEIEKAEDGLVKQIQLILYSTEEGFEIPEEGEPLDDQETF
>Mb|QLI64428.1
MSSSSYSSSTSSPTPSMSAVSPPKSSFEMDDDVPPRLAADKTEPPLKKTL
HTAVYISLWIFISNLTILFNKWLIDTQGFRFPILLTCWHLVFATIATQVL
ARTTTLLDSRKKLPLTPRLYARTILPIGIFYSGSLVCSNVVYLYLSVPFI
QMLKAAAPVAVLFTSWAWRVAEPNLASFLNVLWIVAGVALASVGEIHFSL
IGFMYQMGGIVFEAIRIIMIQVLLSGDGMKMDPLVGLYYFAPVCAVMNFL
VAIPSELPTFTWAAVSKVGVGMLFLNASIAFLLNVTSVFLIGRTSGLVMT
LTGIFKNILLILVSIIIWNTKISFMQTVGYAIALAGLTYYSLGYEQLSKL
FQSSATWASGVWGATSAASRGSGSSASRRCVLISFFIFGGLFVFVAALQH
YDPSTKSLKLPAWFGTE
>Mb|QLI64429.1
MNVQYNSALRQSKSIRSELDTLNAKGSPTPGEIGNVSASVSSFTKIIDEY
HRLARQEIVPKKQEEAFERVKRFREDLSDFRSQIDGLKKSRDEAQYQNNG
RELLGRRPYNATPENPYAGTPTNTNSSFHPRHTSTAGAPLTTGSGDETRE
AHAFREQAFFQQTHSALDDYIARGQAVLGNLGEQRERLKNTQKRLYSVGT
TLGISGDTIRMVEKRAREDKWLFLGGVVVFFAFCWLVLHYLR
>Mb|QLI64430.1
MAVAAAQVGSPQQQPSLLHLIALVDNVPIDFETNCTPYYRLQLAPDPRTH
GYIHPNTVNSMPWPASFTINHDTRQVTLSPPSPETSLSLSAHANAAFQAA
IDAAIDGNMFPMLNGMHSEHFLLMGAREFVQIERFAASLFGIATRGAHLT
CYVRAPDGLKIWVAKRSPKLFTYPGMLDSTVAGGVKADNSPLDCILAEAT
EEASLPADLVARLVRSVGVLTLANRNPRTELHHSEVLYVYDMELEEDIVP
TPQDGEVEEFVLMDCAELRHRMFNGEFKPNVCPIMIDFLVRHGEITPEGE
RHYVEICNRLRRRLPVPTVPDQY
>Mb|QLI64431.1
MSDSNNPSGRSNSLQRMLDLEKQYMFGRLHAHSQPSAPSNATSQPATPAV
IKNASPTRPGILTRRTQGNMQRNRPKSNMPPRDPSLPPQLPPLAINAGAP
ITMSGSDLLAGHGRDYSPRDEDSTIVPTPSDKYPKVVSFEFPPTESAPAP
CHSPSWEAYERRKTEKKTERREREESRNGKAKKLVKQPPASSPYSMQHAS
ASDVDANRGRRRERADSSTAVNNTSKPPRKARSRSGSFVSLLRAPFDFRR
SSVDHGVDSSFIGGIKLEMQRNATAQQALDHQAVEDESNIHPALRKGKQT
DRWSAPLKSPPPLHRAGVDGENQRRYPPITRNKTKYHHKTMSLVSPTAPA
VPDLNTIDKWRAKVGLRTLSRPGSHMGLDNDAHLNGPDTVAVDLKQTISE
PRDALLFDPTRPTRPTSAASPQKPLHVVFPASPPPREKMLEKKPKLDHSN
NVSISSCSTGDTFDTAPSSPPPEPPRRSSKRNSTLSLDGSIPPLPSPKHS
SSPQPSPVQDAPPRRSFPLCGDGGFQFKDTAKGKAIEAPVSISQAATYIP
SPGFQLPGSSSEDSGSEDFHSTSIVSTPATSRPQSEKSMALEPSKPEPLE
LQRGKEKSLPAPPTTTYPLHSTDNSEDEMDHIQAVAEKVLNVYNDIAVQP
PGLCRRSNSQSTLTTDASMPQPLQEKPLRLRSKYNQPGSSSQNPASPATY

LEEARRQPPTAPTQRAHKQRLGPPVSFVLPDSDSDSPVTEKSLPSVPDIV
GRSLRHKSTPLLSTSDREPVAKVFVECCNCKYYHDMPSNLYEAMANPESV
LSPVDKYGYAGALSTAVKCSWCRHEMSTRCCAGLAATVYIKERLH
>Mb|QLI64432.1
MKTTWKDIPPVPTQQEFLDIVLSRTQRKLPTQIRAGFKISRIRAFYTRKV
KFSQETFSEKFGAILESFPRLQDIHPFHKDLLNTLYDADHFRIALGQLST
AKHLIETISRDYVRLLKYGQSLFQCKQLKRAALGRMATICKRLKDPLLYL
DQVRQHLGRLPSIDPNTRTLLICGYPNVGKSSFLKSITRADVDVQPYAFT
TKSLFVGHFDYKYLRFQAIDTPGILDHPLEEMNTIEMQSITAIAHLRSAI
LYFMDLSEQCGYTVQAQIQLFKSIKPLFSNKLVFLVVNKIDVTRPEDLSP
ELQEELQGLMKAGEIEMLQLSCNTQEGVQEVKNAACERLIAERVNQKLKA
GTNNSGNIGGRLADVMSRIHVAQPMGGQSLETFIPEGIKNLKKYEKEDPE
RRKLARDVEAENGGAGVFNVDMRADYLLENPEWKYDKIPEIFDGKNVYDF
IDPDIEAKLAALEEEEEKLEQEGYYDSDEEIDDDEEAEVLRKAELIREKQ
QLIRNEARMKKRLKNQAIIPRSKTKKSFSQLEDALDQLGVDTRNLADRAQ
PKDVRRGRSMSRSRLGTEDADSMDVDSKPRDRLRSRARSMSRAPTTNRRE
DGVQDELTRSKAERIAKLNQKRMNRMARQGEADRHTTVSLEKHLYSGKRG
IGKTGRR
>Mb|QLI64434.1
MAAPAGRTLFKKKDGILTLTDDYQIVTWTPNSGGPPTISLPVSNITNLQQ
TPETAAKVMLKIFEKADNGADPATYLFHFNTAEAKAEAKAVKDVLSKILA
DMRSHDTGVPRPNVATPTAGGASTPAAAGSGSAAMAFASAVNSQASSSTR
WFDDNQLKNDIELQQSLMKKDKSLHQTYVEAITTKPESISGAAFNSQFWS
TRTNLLRAHAIEINQKKGAYNVLSTVKPRTVDGELKLNISVEQVQMIFAQ
HPLVKRIYNENVPKLSEADFWSRFFLSRLSKKLRGERITDNDTQDPLFDK
YDVTENTEGVQSKIMAQSVPHIIDIEGNEENQGGFKGGNAKDVEMRPRAN
IPIVKTLNSLSEKIMANVAPSDVYDDNGSRERDEFNELALRDLRGNTAEH
RIMLNVKEQNRFFQKQDSAPSENARVFATQKPKEVLSKIRNTLRKFETDS
SDGASLQAAIAFEDESDSDEESQKHIQVGSRAAIKVAEKEVMEGVLQQRA
QKYGHSTDATSPMGLPPSMTEKCTLTHATSIEFLHQFWTAFLSGDPDRAA
ELQYLAESLKRSAARVSAVAEEAEKERESVIRQRKQEIRNHFERTGKKIR
WKSDMVGGGRDAVIKLMQPVLDALDKAQAEYSRALAAEGIQISTEGS
>Mb|QLI64437.1
MHLSALLTLLPAVLAAPATIGRRAEPAPLFTPQAESIIADKYIVKFKDDI
ARIATDDTVSALTSKADFVYEHAFHGFAGSLTKEELKMLREHPGVDFIEK
DAVMRISGITEQSGAPWGLGRISHRQKGSTTYRYDDSAGEGTCVYIIDTG
IEASHPEFEGRATFLKSFISGQTSDGHGHGTHCAGTIGSKSYGVAKKAKL
YGVKVLDNQGSGSYSGIISGMDYVAQDSKTRGCPKGAIASMSLGGGYSAS
VNQGAAALVNSGVFLAVAAGNDNRDAQNTSPASEPSACTVGATDSNDNRS
SFSNYGKVVDIFAPGTGVLSTWIGGSTNTISGTSMATPHIAGLAAYLSAL
QGKTTPAALCKKIQDTATKNALKGVPSGTVNYLAYNGNGA
>Mb|QLI64438.1
MEDLPNDAKSKEVVRLWRAWRTVHEMVADREYELAEEEVNISLDRFRDEY
CNPDGSVNRSKLQFSARPSDAMLKKNTPPATESNPDPVPDCGPVWVEFLT
DKQFGVGQIRQFAKYTISNNYKTGIMVTHVPLSPAARKSLASVENLAKIE
CFLEDDLLVNITHHELVPRHVLLSRDEKSALLKRYRLKETQLPRILQKDP
VARYLGLKRGQVVKIIRVSETAGRYASYRLCV
>Mb|QLI64439.1
MSTTVEKIKEIESEMARTQKNKATSFHLGQLKAKLAKLKRELLTPSGGGG
GGGSGFDVARTGVASIGFIGFPSVGKSTLMSRLTGQHSEAAAYEFTTLTS
VPGQVVYNGAPLQIIDLPGIIEGAKDGRGRGRQVIAVAKTCHLIFIVLDV
NKPLTDKRVIEAELEGFGIRINKEPPNITFKKKDKGGLNITSTVPLTHID
NDEIKAVMSEYKINSADITIRCDATVDDLIDILEAKSRAYIPVVYCLNKI
DAISIEELDLLYRIPNAVPISSEHGWNIDELMELMWEKLNLKRVYTKPKG
RQPDYSAPVVLRSNRSTVEDFCNAIHRTIVEQFKTAIVYGKSVKHQPQRV
GLAHELEDEDVGKYTTHRFGLLMYTIYVNRNLFEGELRP
>Mb|QLI64440.1
MEPLESAADVVHPEVRAHISSLVSALGGTSAEDDGNYQLGDDALEVLRDL

KRWIRFYDEKTNRMDVARCIYEANLIEGDLLPILRTWPENAMDNKFRSRI
ALACFELMVPLTWPIDWDKERMTVNHHRHLPVLELAQVQYKRAIINFDGA
RILHAAVRAALPAMALPIGDRTARDQGVIKLVLFFLRNMASITPPPNDKY
EGDESQVSRSAIIDAFSYQDIFHVLLMLSSNMGEDFRTEDTTVMEIIYHL
VKRVDPEKLFMNEQQLNKAKATELSALMSKESAMLQSHNRKGATRHNRFG
TMIWVKRDDGKMSSLSGQDALADAAVRNQKMDEIKKFKPPRRARKDNQNE
RDLGPPVKLNARANDQLRKFVDEFLDSGFNPLFQHIRKTIDREAPHLLQH
HRRQFFYLVAWFLEAERVRRKVKKPNRKESADISGFQLVAGVLNQEMFIT
LSRALQESYDMKDWTELTAVMRCFTQILLTVQEMAESANEDDEQIAENVL
SRLFYEESAHDAIANIIKTYKDQGFDYLDASTELVHNFLRILEAYSKQNV
DMQVRSRKRTRRKKKQAAKEGGDEEEAVLDDSADDEEAAERTSKERKFDF
HKYSTRFAPQGVVDTFIRFTKYYRDLTDTQLKRAHRYFYRLAFKQDMSVM
LFRVDIIHLLYNMIKGPNALEKSSSMFRDWEELVKQILRKCTKKIQERPE
LIIEMLFSKLQGTAFFLEYGHERQTTSKSQRKPAAELEFKHTEERDEQVA
IAVGALLDKNGSEHISWVRQVLSDAESERRAWTAAADAKRDAEAVFGDDP
AITDTEPKTPPVFSVKPDGAERRTAMFKNAHLRLLMKLSGMQLMGPASEE
TPESLWIIPETISADQLKDTIHFINQAEFSPPTFEDGFLAEHQLKRKAAP
KKKAAFDDEEDADDDGFLDDETLFPAGGPTSRKLADEKKKPKKTRRRRKA
NNTVELDDSVVEEKVRKRREKELEKARKIKSALYVRDGDDEFDSDEDEAF
FAREREIATRAKKAAESAREPTLLPKKRKSTAVSDVSDEDEEEDEQDDLN
LARRILSSQEGGESDTDDTPVDLSDGEARKRRRVSVEERDGDNDADMDVD
IITKMAEMQDDDGEETTQVVVTRRPRVRGGFIMDSDDDE
>Mb|QLI64442.1
MEPLSDNEMAGPSTIRSLSDHQPEEEGAPRFRIPPRDLAAVEIPAVVENI
DRAVKAFGRVPSLEHLMDPLRNSVPLYLNPESPFCKPIMSHSAASHNVVL
KLTVPKRTGRRRRRGSNGPWQGDVNFSDAPGANGKEKVQSIARLDEPKVL
RRKLQDNAGRYQVEAVGTIKHTHRFRALADFYWDMNRSEFAQRYAEQVLP
GDVQKLKEFTLQSGTDRPPNVDIIPPPIFTHMSLPFNYFYSQNPYVRTTE
DGGTINLTAVKQVGYFISAEDPAPDGPQVPPDMTDPRMIEVLAQLEDAFQ
HRPLWTRRSLLNHLRGKLQSWNELKKYLNYAAYQFKGGPWRDSVVPYGTD
PRTDPKYRIYQTLMFKLKKHKRTIKHQPWQSLRKTQIGESQNTPSMEADS
HIFDGKSYHTDGKVWQVCDITDSLLKELLDNAAVRPQCDVVNSGWYHGGL
WAKVKAIMKTKLVAIRFGRHLTKEDFAQTLEFGDATPIRTVSNTFHLPLP
NLHLTTEEHTVLHGRGPLKKKSTGYNVRFRDIGKSPSVAHEISPAPFIDS
SVIGTDRMESDDEDSGSERDDDDDEDDDDDGSENEEYPDTGYYNHMEQYG
DHQYGDGDAEMDMGRSAYPELD
>Mb|QLI64443.1
MGWKEAVWSFCRDDRVHLVGIGLATFGLVRSIITVLTIIRDENKIPPKEP
KAQYITQETEDSLRLDTLEKLLVHPNYSVREVATKILCDRAINDPQTLLT
LLYGITRPDYEERMRCLRALALLTGQTMGLDGLAQLNSDKAYSALVRSLE
LSLEDAEPPDLTNSHWDEYYLRDMAERFNLMFIHELITKYPSQALIKARF
VEKWLAKQKWGDDPVARRDNFKAYMHLKDNRLVDIVDKLKRSRRGLRALE
QAGLIDDSRQQIRTPDMIMEIEEEIVGESPSVNRVLRPMRETSVEEQRLR
RQHREAMVLNDGTRPLGREDIIERDHGSPP
>Mb|QLI64445.1
MRPTIRVLARYLEPGTPTGLAGLWTHSTPRSTLLYLYGTTLNKLQSIPET
SLYRQSVEAVTKHRMGLVENIVPPGYNEWAAKAKELISKNPEQFRVASGR
VDGSEARTVKLGDRVFIIGAKHEAGDIRYEEWDGEADEGGELEGIRTPAE
RQDQVIWAERKPLQDHEKIEWEDEPQLTAEQVQELEHKIAAGLIEEVIQV
AEGELLIIDTMEKARVWEDLEERPVDGQWEYFDRKTMAQGSSLSTNLPNS
INPFSRSPSERRQLSLAGLKDTDEDPTRGIECFPHRGIDRKAPEVTVSEE
EEEEEDDADELDASETSQATAEEPVRMVRSGKDKRNQRRPSLAHPFSRLD
TLLRSIHQLLDQAEITKAARLFGIVLQLRPGSRPIDVRQHNIWAIGAEIT
IRGGEDVTLLHRGLQHVDIESHEYTDGMLSGSKSYTRIRIPRRRHPSENL
KKLKAYFGALIQKYPYDHKFPRKTSALHFQLAMLTCEVYSCHAMYASADS
PTTALQLDLEEVPPQISDRYSEDVYQNDQSEMTMNVKQPGSEVNFRIQRE
RNQIYAREALKDISKRMDSLINELPYSKNHHFLQLRATVSLLIAELLALD
NGSQQGITNEPTAAIKSEQEIASIMLQRIIDNGGHTARPLMDLMGQCSNL

KKELQQQVLYASLPIRST
>Mb|QLI64446.1
MIPASPDQLRTADKGRTSDGIIRRCQLSYAKKSDIVRVSLLTLLKLLRRR
QSCSTAINNRAASVNMRKPYRFYSLNRPKLRQSWNKYNLYNLYRNAGREP
QIRGTPTFFQQKWAAKSKTRAYHGEHIPEKKWVRLFSRRLLSAVDLPPEY
LAAHDGSEQAAGRGSGLTTSNITAESFSKVPKPSTYERSRRRPPPFGDVN
KLLSDQFTGMTPYMQMTFAPLERRLDTAVFRALFASSVRQARQFVIHGAV
KVNGKKMIHPSYQLNPGDMFQVDIDKVMYGTGQQKATQGNKRLAENLNSR
KQKADAFYQAAIDKAAANARTVASTSEAAAEAEKPEAEAVSAEESVEDQP
ESAGESVSGPGLESLTPEESWRLNNRALKFLLKDVKKILKNNPKDLSAKE
KKQLRLFRADAKRFLSQPEDSALDVQELIEELQLQMKSHELMRESFEKLT
IAPVSPDATAETPAAEASGQQTEYNRQRQVDKGLDGLSEEQKEKAVRIMG
ESQLSREEMRKLARLLQYDEENPIDDSKPYATPWRPRPFMSAFAFIPRYL
EVNPNICAAVYLRHPVARKGMGEVPTPFSYLTNQLTHNWYLERG
>Mb|QLI64447.1
MAESDIQTRFSPNSPLEPDIITEIQSILRLHDLSVDDLFFKWESYCIKMD
LDAQAGLSLSRIRNLKQSIQDELEKSHRATQVRNERKVLSATPKASSGGD
VFGMLDGLVPSTPATARLNRSGGVGGSALKKRMDGLKVGSSPARMDDQLK
AMNGLPCVSFVLDDWFRTDFDIRTTSFADRLNPGDVVEVLNGHLQPAEAP
IAPFAEPRIKLTAASDIKKMAYKPLAMKLSEASEILDDRIDEFITVVQDH
HKLEDSAFGSAASQSTTEVVAVGRIASDSMEGRLNTASLVLETSRRTGMG
LRVPLRMEKIRTWSFFPGQIVALRGHNATGNEFVVHEILEIPLLPNAAST
SSALEAHRAKFRGHADAMDSDAEPAPLTIMYASGPYTADDNLDYEPLHAL
CSRAADSYADALVLAGPFLDIDHPLIASGDFDLPDEAVQDPDTATMNTVF
KCLFAPALVRACSANPHLTVILVPAVRDVLDKHVSWPQDAVLRRELDLPK
SVKIVSNPMTLSMNEMVVGISTQDILYELRSEELAKTSGDLMARLVRYLV
EQRHYFPLFPAAERTRLPKTGTEEGLATGAVLDVGYLKLGEMVNVRPDVM
LVSSSLPPFAKVVESVLAINPGPLSKRKGAGTFARMTLHPPAVGEDEMQS
HKIFDRARVEIVRI
>Mb|QLI64448.1
MWPSTPSADSLTRPRIAVAVVSVVAAASVGYYAYRAHQNESSRETPTGGG
LHRSNAIRRPRRTTSIFQYGTPLDENTEGTNFRVPGDAETVVDVDVETSD
DWWNEPPATTVPLSPRAGHTIVSLLFRVSEDNARRNGCVHRGCQCNSCGM
VPIRGVRYRCANCADFDLCETCESQGVHNKTHIFYKIKVPAPPFGPRQMQ
PVWYTGDPDTCRRNIPKGLIARLSRDTGFERPELEAFWEQWTYMANTDWR
DDPEELCVAMDRKTFERCLVPTGGSRHAAPNLIHDRMFSFYDSNKDGLIG
FSEFLHGLSYRKRRDKLKRVFEGYDIDGDGYVNRRDFLRMFRAYYVLYKQ
MHKDILDGLDDQLLASTEAQQLVSSRQPLSSLFGREGRIPSADSGFRIEG
KTNRRDGSIEVADGFSSVFTENRGDTAPREDILASLFAYDTDTRQTRRLV
SRSDADTNWRTVRRVSGGDLDRAYWSSLLDPPATLDDIPHVFNNNEDEDV
DANVDEDEDEDEDDGDMQTGESGRTYSESRARAIARSRRLAPKLEKQRLD
MARKHLHDRWKRRQFYLDEEEGGIAPDDWDAEEDILARVNLITEDSKARD
PAPITTRSRSSSKVRFAEDTDDYEIRSNPSTSSRSVPERWGGMDIPDAER
DAGKEILYQVTQQAFNELLDTIFKKAEDMAVAAAESAKDREKYKAQIDAI
DLPESQSQAPPNQPRPESQPTSKAISEKSLDELLANTGYTVVPDEPRNAV
DLDGSVEIIHEEQVDGAGSNTDDERAEYRDPTLPQFRPNNIAEADELAAR
QGASELEHVPRSSSHQAAQEEPDAATLRSWKRLTIAEAEAVKRGGWGRLS
FEEFEDIYKGQEDLGYRLDYLGSWIDFCIP
>Mb|QLI64450.1
MAFNFNWSPLTADAEFYRRARDLLTTALNKSPKPPIIVDDILVSEFNLGT
VPPDLEILEIGDLAEDRFRGIFKMCYSGDAFLTLKTRVQANPLNTYLFSK
PSFTSPQPLAAASGLTIPLSITLSDIQLSAFIILVFSKQKGLTLVFRNDP
LESLKVSSTFDSIQFVRDYLQRTIEQQLRNLMMDELPAIIHRLSLQLWCP
DQANKGTETPTEDADVKGADPFASPPLDPVDANGNLLDYNTVSELTLNGG
GEVPYLFSQKNLLRLAALTDSHRTLSLFTPGINEVVFRAWSGFGDRSEAA
SPPFATPSLARTSSFPTGGSTTYTFSDSGSTAHGYLPSRPSFVGLNSAAN
GVAHGANHRSRPGRKKKTRVVNLRRAKSEAGSSDVTSSEDVSETASVNVP
SSEPVMSTSIPEEPASDGARVEGVPTGKVRFRPAHSEHVLPIRQALMSDI

YKKKLEQMASTQEEVKTPESLINTYGMITEKAPFDKNGNPIRDQQSQASN
SSDTSSVILEQAWIMKMAGEIARRVYDEKNRHQGIWEEGESAPPPAYEAA
TH
>Mb|QLI64451.1
MTEPAVEGKPFQLYDIVIVGAGPVGLMLSTCLARWGYRVKHVDDRPEPTK
TGRADGIQPRSLDLLRNMGLKTGLMAHKPARVYEVAFWNPDKSGNDIVRT
GTWASCPKFIDARYPFTTLLHQGHIENVFINDLEKNGVLIQRPWTITHFE
SDEQENPEHPVRVELKHVDGTTTETVRAKYLFGGEGSRSFVRRLLNIQVK
HIDPIQHVWGVMDGVVKTDFPDIKMKCTIHSHHGSIMVIPREDNMVRLYI
QIASSEEPDWNPRKTATSEEVQETAKKILHPYTIDWERVEWYSVYPIGQG
ISDRYSLDQRVFLGGDACHTHSPKAGQGMNTAFLDALNLAWKIHAVEGGF
AHRDLLKTYEIERKGVAESLLDFDNRYAKLFSQRPPAAKEVEAATEGCPA
NRGEAEEDDFVKMFKESCEFTSGYGVAYGPNEVNWSVNHQANSTLIHPEG
TKLRTGHIFINSDVTRVVDANVVHLEQEIPLNGSFRIFIFAGNPAVTHTA
LQDLANGLGAERSFYGAYARKDVDTVSHHERHNPHSLFFTICTIFAAKRS
SIEISKDVPRLLARYRDHIYADDIWDRRVPNSRASAHAKMGLDEERGAVV
VVRPDGYVGVIVSLVEGSGTVNALNEYFSAFCSKNLVAGSSQL
>Mb|QLI64452.1
MDANTSSSAFPSMLAGSKYGNINVPPQLDMILDAVTTASPWTIILTILAM
CVVYDQISYLMNKGSIVGPAWKMPFIGPFLQSMNPKFEEYYAKWASGPLS
CVSVFHKFVVIASTRDMARKVLNSPMYVKPCVVDVAHKLLGHDNWVFLDG
KAHVDFRKGLNGLFTRKALECYLPGQEEVYKTYFKKFLSVTKENNGKPVP
SMPEFRELMCAVSCRTFVGHYMSDAAVKKIADDYYLITAALELVNFPIII
PFTKTWYGKKAADMVLVEFAKCAAKSKVRMAAGGEVTCIMDGWILQMIQS
DRWREAQEKGENMEGVEKPSPLLRMFSDYEISQTVFTFLFASQDATSSAA
TWLFQVTAQRPDVLDRVREENMKVRNGDPDAEINMDQLESLTYTRAVVRE
LLRYRPPVLMVPYLVKKPFPITDSYTVPKGSMVVPTTYMALHDPEVYENP
DYFDPERYYSGDAEVKGAKNYLVFGTGPHYCLGQVYAQLNLALMVGKASV
QMKWTHHPTPLSEEIKVFATIFPKDDCPLTFEKREW
>Mb|QLI64454.1
MSNVEVPDNLKQQKPARGGPPSVYFPLGYKDAAYQWWTSVTPTMAERNVL
SLVPYLKEAVDSITSTPVSTTGTPDPYGTRTWRRSLVQLSGKNRALNEIC
IERQGEQVQETLVMVHGYGAGLGFFYKNFEPITRIPGLKLYALDMLGMGN
SSRPPFKIHAKDKEEKVKEAEDWFIDALEEWRQARKIERFTLLGHSLGGY
LAVSYAIKYPGHLKKLILASPVGIPEDPYAVNANLPEPEESTLQNEFTQD
QQSVTEGRGTPPTPPNGQAANAPKRPLPNWLVWLWDANISPFSIVRMAGP
FGPRFVSGWSSRRFNHLPPEEAQALHDYSFSIFKQKGSGEYALAYILAPG
AYARRPVINRIHEVGRQPIPQADGSTGALKETGIPIVFMYGENDWMDVAG
GLAAEEKLKEAKVRALLHGTDDEKRNENGSVKVVIIPKAGHHLYLDNPDD
FNRLLTEEIEETRAAGKKRPAL
>Mb|QLI64456.1
MTRLLSIASLGVLVASTLTTANSLPKGDVDYCSVYNRAPTPGCQPVPEGF
LRSRGDSLSTPETDPRVLEDAFSALSVLQNVYFEPVNGTWPTSIDWTGAV
VETVIAGMLTTLTQSLDSTERGRDWKEKENLISSIFAQVTHSFFGQNAVA
ILDQAYDDVLWVVLGWLEAIKFVRLHSSLHYPGTEQTCLNVPGRLSDALG
TISFHGYNWYCTFAERARAFWDFATRGWDTSLCHGGMVWNPRLLPYKNAV
TNELWISASISMYEYFPNDEFDDAWTASQGFPTNDPLYLAAAIVGYKWLK
DVNMTNSQGLYVDGYHVDMSKPNNVECDQRDEMVYTYNQGVILTGQRGLF
TVTGSPSYLEDGHTLIQNVIQATGWDLGENAAIDNVNERPRGKLPPWRGI
GRGGILEEQCDADGTCSQDSQTFKGIFFHHLTAFCAPISPVDIPGDANVN
QTEFSKVQSAHDNACRGYLHWVKYNMDAALATRDKAGRFGMWWGADIFDA
FDASRQTDGIDHTTPNATDYRNKGTPMDVIWGLNMTWHPGSKTSTGLCKN
LLLRPDPKLRIGVSPELSVDSQIVLGRQNSHGDPNDRGRGRTVETQAGGL
ALIRAYWELSQS
>Mb|QLI64457.1
MATQLATSEPAPSVRPTATSATSAPDGDEKDAVQGSSPAEIPVRTKESFN
KLMVERYTMRDAIASAALGEQLNQTRQSSQEMREKIHEYRMVRNEYKPWF
PPSRLYGEGYNGFGNGYTENQQPASRIIYPAQKPRPGRRTTPSFKFARKD

MLKQAEQHEELVPLRLEVEWDKVKLRDTFTWNLHERLLAVELFAAQLVED
MALKPPAAQPVFEQIVQQMREQLNDFYPFAFAEEDALDPELPYSAYKNDE
MRILVKLNITIGQHTLVDQFEWEINNPNNSPEEFAANMARDLSLSGEFTT
AIAHCIREQTQLFTKSLYSIGHPFDGRPVEDSDLVSAFLQTPLPAVFRPQ
QQAKEYAPYMYELSEADLERNEMVFSREQRRQKRSINRRGGPQLPDLRER
QRTIRTLIVSSVLPGAVEDVEDSRLYKRTAGTIRKRTARDGEISESEESD
DSSPDSPAPSQMAGTARTRGMRGAASAAQQRMANIGRSETPETGSLHHHE
TRTSRRGREETEEPSHLWVTLKVSREKLRRVIKGDYRDLKAPSQPQTPVV
AHARAPSASSSIPKPKVPFTPTSASSTPQPAAAPAPTGSGSSSFPAGQLG
RLPAPPPVPGQSPPPAVSISSPIVTDHVHTGQSLTCASRQPPPPEWLVNA
LKDLEKQYPNGDKFEAIMKYSYLDPDTELPLQPQTQPFPEGVKYAWLPRI
RCLDCATKLYTPGPDMTAQKFEAHLKFSGHRAKVNQRLAAQNGGASGS
>Mb|QLI64459.1
MGFAKILRGIVRNDAMRTDPDQIYNAKVLALVCSACFGGMLFGWDTGAIG
GVLTMDETRERFGYLHRSKTDKSNLDQNIVSTLQAGCFAACLVTSWFADR
FGRRWSLIGAGVITVIGVVFQAASAIDGTLAVMYVGRFIAGLGVGAASTL
TPLYVSECVPRAIRGGLTAFYQLFIVLGIMIAFWVNYGCLLHVSAPAVFI
VPLALQALPAFFLITGMLISPESPRWTARKDKWEETSQILVRLRGLPADS
EYVSGEIQEMVDQLENERRVMGDSSFKSLMKEMWLVPANRRRAVISIVLM
ICQQLTGVNAINYYAPQIFTNLGMNGTDSSLFATGVYGIVKTVAVCLFLL
FIADSLGRRKSLLWTSPMLVIVLFIIGIYGRVQPPVTGEPVTAFGYVAMA
CIYLWAAIFQFGWGPACWILVSEIPTARLRAANVAIGAGTQWLFNFIMAR
TVLTMQNTMGYKGYGMFFMFGSFDILMGLFVFFFVPETKGLSLEKMDELF
GMNETAKKLDAEPETGRPVSVHEDRVTKT
>Mb|QLI64462.1
MSAAQAHLMAAMPDASLFNFPPTNEPTPLSPASVAVSIMRPFNIPDNIYQ
AALDPKVPLTIAALYAVTAKALNKFNASRNKKPWAMSKTKPFFAFVVFHN
VLLAVYSAWTFWGMLSGMRRSIVSPFGPGGVAATADSLCRLHGPRGLGSS
LYFNDTSSHWDSADPNTLIGSLDGGQPSSTQMGRIWNEGLNYYGWIFYLS
KFYEVLDTFIILAKGKFSSTLQTYHHAGAMLCMWAGMRYMSAPIWVFVLV
NSFIHALMYTYYTLSAFSIRVPMVVKRTLTTMQITQFVVGASYAILHSFV
TYVAPITVTKIATEAIDLPAAPADAAMPTATGGFDSLKKFIFGSAALDSV
ASGVAKESALITETSYVAQPCISTANETFAIWLNVLYLAPLTYLFVSFFI
ASYVKRSTANNKIPGKTNRRLSNVTLAEKAGWDAARGLEREVYGGENMVN
GHANGHIEAIEEADEPASTKTNGRARRKA
>Mb|QLI64463.1
MSGEKLNVIALISGGKDSFYSLLHCIHHGHRIVALANLFPISADGGGDDS
TAVRVIEPGRHDVDSASATTIDGHDAAALPDSERDLNSFMYQTVGHEIIP
LYASATGLPLYRQPIVGSALRHERDYDYTSAHGQGSAAAADETESMLPLL
RAVLARHPEADALCSGAILSTYQRTRVESVALRLGLTPLSYLWKYPILPS
VPGALADEAQLLSDMAAAGVEARIIKVASAGLDERHLWEAVTSETGVRRV
KSSLRKFGAAEGASLGEGGEFETIVVDGPDILFKKRIAVPDGGRKVIAEG
GGSTWLMLRGAKLEDKAVHGQETPSSVREPALLEASFQSVVDALDLTTPV
QHAAGFRSSKLLPKSDVQFRADSDTLHWHVVADASATGPFIQNEAIHVVD
KIKSLLSTNGLDSSQITSVLILLRNMADFSIINSEYGKLFTKPNPPSRIT
ISSGDLLPQDRNIVMYITAPVANAKVARNGLHVQSRSYWAPANIGPYSQA
IEAAVSVDGEPTRLRSVTVAGQIPLIPASMALPTASKTLLRHQIVLSLQH
LWRIGAEMKVQCWSSAVAYFARQHGENVAQESAVLAGQAWKLAHGSPDCN
DDDDDNGPDPWDLKYNQRFQVLGATETKTSCPSIPDWSAYTLRQQNEPDS
SVPPLFTAEVESLPRGSLVEWHVHNGLKQIEAGSVELIHLPTAGTVGGDG
WQSWHLIVKAGENHIVYTTMAYSGASQDGPVVEFRVLERELGAMYRASIH
TLQPNVMSLAANPLPYLGYVDLRRTESVWGHNGSDDVEIPFAVVPCHSLW
DSAGQRLTAVALYKSILTDACAS
>Mb|QLI64467.1
MAPSFPGARAEVDYPIYSIDFDPEDANRLVVGGGGGAGCSGVGNKITVFD
VSHQDEIRAAGEIELSRDEDSVMSIAVGPHKGKTTYVYAGINSSPEEIAK
GTNQHLRLFSVDPSKARPLAAMTGSPSKPRAFPEAKIAEASRTALFANPD
ANAYQRLLRVSGSIGAAASALGKEPQIAVFETTTPKPKIRGLLELTKEAE

AIDIIQTGDDEFQLAYVDRYELYIVNIGSKGNGEPQMVFSMPDDHGERPQ
FRSIRYLTPEFVLAVSNLPKKNTGALIQGLRLPSSSHEKARLAATARISR
KMSAIALAVTNLSLPASPTAPVSNAQFAVAVAGNDSSISLYTLEHQVSPA
LNLLLNLYPFYTLKNVHGNDNISGLAFSTFITPKTHIRAQYVKLASTSLG
KTVAIQSIPLKKFVDVQAPRNRKGPPRPTRYVVAMQSRAPSARPLIITLS
IIVLILAIVAQSVKELYGVGKPVIFAQPFLPSWHGSLRQPKTQPSYSFEN
ELISSLVDGNMVLGSGEKFVLLEDDQPVVVEGDGEQATKKLRVNVRDSDA
HGSARAWDQLHVDEQDAWKERLRDAGAWTQNMGENVFKGILFGELAGVVG
RVVAG
>Mb|QLI64469.1
MAPQLDSYFKQVDDSANHFIERLRKAVAIPSISAEDARRPDVVRMGEWLA
GELKSLGAEVELRPLGKQPHKEHLDLPPVVLARYGNDKNKRTILVYGHYD
VQPAEKSDGWATEPFELSVDDKGRMFGRGSTDDKGPVLGWLNAIEAHQKA
GIDFPVNLLMCFEGMEEYGSEGLEEFIQAEAKKYFADTDAVCISDNYWLG
TEKPCLTYGLRGCNYYSVEVSGPGADLHSGVFGGTAQEPMTDLVRILGSL
VDTNGKIQIPGIMEQVAPITKEEEGLYDGISFTMDNIFESLGSKTTIHDD
KKNTLMARWRYPSLSVHGVEGAFSSPGAKTVIPAKVIGKFSIRTVPNMDI
DTTNNCVYKYVEEQFAKLNSKNTMKVYAQHTGKWWAASPKHWNFSAAAKA
TERVWGVQPDFTREGGSIPVTLTFEEATGKNVLLLPMGSSTDGAHSINEK
LDKRNYIEGIKLLGAYLHYVAEEPQTVLDEFVPDRLCASLTGCIIGRCAS
QIDIQNSTTPLSASGSRQNGPETQHYQHLSNTAGLPKFLAAKFELYLSPT
AVDAWLVVDGFIKSSRLGRLVDTDNTLQQPAGTWPGRQTPNFGAFLIAQR
PRYPSLHDLLDGSGPHIANHLERLYGPLWKGLPGRAATPAG
>Mb|QLI64470.1
MTDEPSEADVFAIPDFWKTSRWLDQLASETSTSLFGHGLDALYNVKSTSL
PLEPIAVDTDGFFKLPEVGESHADDPDELQDSDMTASTIQEHQEFEDKSD
IWTGVSEPQPRPSAFRTWETFKTGTLQPYIPMLLSEAGQGAYDALLSWPT
DSLNLQNTSTPAVETRAYFTSVLALCLGRESVFFARTTEDWTFKCTLPEL
RVSGYSRQVLQGVQRQAAKCGSTFLRLGAFCQSSYAPSSSRCAIALASAM
SQILQAVEHRVSVDGRFPESLLQLQTTIREASTILRPLDDLAARIPRDLP
DEEILSLVFQEASTFEFSERYIRDMLCEILQRISSPWVESVEEWLGTRRE
VGMPFARSNIGETKGFVTVDVEVFIDDFGREVEDIDFRLDIGRVPQFLPS
DIAESIFETGRNLRFIRTFHPDHILARSDVIMSNRPPAARWLFDWASILD
LEDRIAQYQDGILDAIQESRSATAYVAVHSKLGVDEPDSDLELTFFGVDE
STMEQRLIESMDHFSRPLIESSSDDSLSRIVQEKFSSEIEDDSIASDDTP
HWSLLPILSFGGIAAAQARIVNRESLRLLFEANNVQLHLKLQRDFHLLGN
GLFCSRLSHALFDPDLETTERQAGIAMQGGVMGLRLGGRDTWPPGSSELR
LALMGVLGEAYGSQGVTKASTSINRDSGASPLPGDLSFAVRDLSEEEINK
CMNPDALEALDFLRLSYTTPPELTWIITPVHLMHYDRIFKLLLRILRMLY
VVNRLYQDTNSRLNTWVEEDTSYRFVRESQHFVSSIASYFLDSGVAIAWQ
SFENKLNSIRVHLDKPDAAPDKCAYSPNQLRDMHSQVLERIMYALFLRKR
QLPVLKLLEEIFGIVLQYAKQSRLQALGEEGESAELANSAHMYAEFRKKV
QVFITVCRGLSEKARASSKNTADGASSEDSGFGDDSLIAQLLKLDMGDYY
CRR
>Mb|QLI64472.1
MQIPGSEESPFTSLFGLKLIPIGSESQAPSTTGSESPSTARSSPSSGRRR
SVSSMKDLIPLKLSPSSSSVWSDSQPTPADEGMADRAADCLPEVLARDLE
LSAAVFAEKKMTAGTPVISLSAEERARLRASRMALYSPYLAASARVHSLD
PIEERDSTASFVDQEVEFPVEKYELDSESSSTTSHCSSDGAEPPDFRHRG
SVATTATSFASVACRRKNSLLPDIQYECSWIEADSDGEDHVDSNTQDYNV
GSLSPRPQTPPCSEFSSSITETDPNLDISNPSIHTHNSPHRKSHSVSGGF
PSVPVQRQLSGRSLSVPVRPSTMNGPIIKPAENSFKNDQLQTMCPTQCQS
LKTMRRTQSLRTSATDSSFPTHRLQKPSLQPAICLETSLVTNACSVSDDE
GLSPVANRDSAKPHASFVRPPTPPQPLRSVQSWLNSSLQPYPWASHGDEA
ARAVPLPPDAIETLRVSVACFPETMLLTSSLTVETIRSYAKKMRHPVTEA
LIIAGDTSIQPPRKSLWRKVINYKKGPQPSDWKTTPMYSSSRQRSSTDST
SSEELDVPKSWTPIKNVFGHCSDYICDAIYAHIVSYNYVSALVARNPAPV
AGNGRTSSMSSRDQQQQQQQDDIPKKAASLLGLTASASAEAAASMGRFPR

RVSSPLGEWNKEGIMTSRNTTPSSQDNALRVIQSGLLQCIARLVATAKLM
AESDTGEERMVDMGAEDADMLFMRSLCEIVRMAEEAS
>Mb|QLI64473.1
MPRPGAFGSSLVDLASYLLTNGPRKVYPASRRVRVIHNNTTIVDSTKAVH
VWEHDGYPQFYFPLSELKNCSKRDIQLVRSDGVARAAVVEVTIPSRNGIK
ETKTDRVIRFTDDRSLGALSGLVRLEFGAMDQWLEEDIPVYVHPKDPFKR
IDILPSSRPIEVRVGGKTVAKAASSVHLHETGLPTRYYISPGAVDPAVLR
KSSLVTRCPYKGDAEYYGIVVDGKQHHDLVWYYRLPTHESAGIAGLLCFY
NEKVDILLDGQLLERPKTHFG
>Mb|QLI64474.1
MDNLTAAILVVSTTAAQDPSTDASASVLRNVLAEDGGGKWQVMAESIVSD
DVLAIQRRIIQWADGADAPHLIITTGGTGFAVSDGTPEAINPLLHKQAPG
LVHAMLSTSLAVTPFAMMSRPAAGVRNKSLIVTLPGSPKGAKENLQAIIK
TLPHACLQAAGANSRALHAGGVKKLEADAGISSSKSKQGHSHDHRHHHRH
HGHGHGHGNLVRHSNPEATALSNDPALGPTRRHRESPYPMISVGDALALI
SEHTPDPDIHEHPVTDKIIGSVLAEDVSARENVPAFRASIVDGYAVVAPK
DGTMKGVFPVTAVSHAAPGETKALTEGEIARITTGAPLPPGATSVIMVED
TILKTMTDDGKEEKEVEIQAEGVKEGENIREVGSDVSQGTVILNKGEQIS
GVGGEIGLLASVGVAAIRVYKKPVIGILSTGDEIIEHDRAGPLRLGEVRD
TNRITLISAVKEWGFEVVDLGISKDKPASLEETLRNGLRQADVLITTGGV
SMGELDLLKPTIERSLGGTIHFGRVAMKPGKPTTFATVPVKDNNGQRRSK
VIFSLPGNPASALVTFHLFVLPSLHKQAGVSPVGLPKVSVFLGHEFPMDP
RPEYHRAVVTVGQDGVLTASSTGGQRSSKVGSLRSANALVCMPSGTGKLK
KGSTVDALLIGALRTS
>Mb|QLI64475.1
MKSASRLFYLSVFALWASPGTCAAGSESNCAISPKSIVQDACASYSTLEN
LNDRVKPALDDLTRTTDFFSYYRLNLFNKKCPFWDDSNGFCGNIGCAVET
LDNEEDIPEVWRAKELSKLEGPLAKHPGKQERKQHPQRPLQGELGENVGE
SCVVEYDDECDERDYCVIEDESASSKGDYVSLLRNPERFTGYGGEGSKQV
WDAVYRENCFQKSSFSHSANLGMSYNYNPAALGFKQVMDAAGRQAQLEAQ
RLQHPNIPFVSSTGYEVDDECLEKRVFYRVMSGMHASISAHLCWDFLNQT
TGEWQPNVQCYKDRLHGFSDRISNLYFNYALVTRAVAKLGPELAGPDYTF
CTGDPTEDLVTRVKVSEVAKQAASIPQIFDESLMFVNGEGPSLKEDFRNR
FRNVSRLMDCVGCDKCRLWGKIQTNGYGTALKILFESDNHSNKIPVLKRT
ELVALFNTYARLSSSMRAVGKFRQMVDAESGHKPAAATESAAVEDEKVNE
EGAEEDDFDPSYLWRELEDMKRRGPKSSSFRDQWAHETALIRQSVKVVLH
GWLHGSILIYRIAVTEAHRLWLYFLGLQPGPGLVDYRWAPKDKKTEL
>Mb|QLI64479.1
MLEGLVAGLLNRFLGMYVQNFDPTQLKVGIWSGDVKLRNLELRREALDQL
KLPINVVEGHLGELTLVIPWSNLRGAPVKVFIEDVFLLASPKEEAEYDEE
EEERRRQRLKLEKLDSAEILKERNREGMSQEEQKKSQSFTQSLVTKVVDN
LQVTVKNIHVRYEDSISAPGHPFALGLTLEEFSAVSTDGQWKPTFIQDSS
TTTHKLATLGALAVYWNTDSDLFGSGREASSPLKEPLSHDEMVQKFKGMV
GKANALASNHQFILRPVNGQAKIEIDKSGDIHTPKFKANLLFEEIGLVLD
DDQYRDALMMVDLFHYFIRHQEYKSLKPKGVRPKEDPRAWLLFAGNAVLN
KIHERNRKWSWDYFRERRDDRQRYIELFMKQKQQQQFTTEESEEFRKLEC
KLEYEDLRFWRSLARNQLKKENAEALRKQPQQQQQQQGWLAWMWGSQPQG
TIEENEENTQMTEEQRKELYEMIEWDEKQALAEEVDVPREAVKLCLEASL
STGSFTLKKSPHDNPSDLLSLHFDVFKAKALQRKDSVLANISLGGLRVND
GTTPDSLYPEIVRVKDAPEVRKRKSLSLKELEIDKEEPFFQFEIEKNPIE
REGDFAIVGKLKPLEVVWNPNFVVGIVDFFRPPERHMESITALMESAGAT
VEGIREQTRAGLEFALEEHKTVNAELDLQAPLIIVPVSITTANSTCLIVD
AGHIHVNSQLVDNKTMNEIQSKQRQSYSEADFQRLESAMYDKFIVKLTST
QVLIGPSIEETKKQLVKKSADSQLHVVEKINVDFVVETSILPKAPNLTKL
RVSGHLPMLHATVSDSKYKNLMKVIDAAIPKLGQSQQPESSSENSQTLQQ
RPRLLSANSTRSRRRSQRERRQSSAFPFMQPQSAVILEDIDDDEDDFEDA
TDGVDGQQLKIQQRSFEFNFKVDTLKGSLYRSDPDRVKPDTLLVELVAER
FDLAFYTRTYDMAAEVSLGSVAVEDFVENPPGEFKSIVSSGDSEDLKAGR

SLVHVKFMKINRQSPEFMPVYEGVETNITAVISTINIVVTRKTLLTLLDF
ILITFTGGNNDQEAPSAPTIAAGEGSDEPQLEQSIVDAPKPNGNVGSIRV
KVDLKAIRLILNNDGIRLATLSFNHGDAGIYISGKAMRISARLGDLSLVD
DVNLGVSADSSLRKLVTIQGDDLADFRYQTFDPENQKAYPGYDSSVFLRA
GSVKVNFVEEPFRKIIDFLVKFGKMQALYNAARQAAVTRANQIQQSPSRI
RFDVVVKTPIVVFPRLYVSERDKGDAITAYLGEIYAQNKFVPLDDSKNSD
IAMKISAGIRNVRLTSDFHYSEDRSEELEMIDGVDLGFNITYVEHKEGVK
RPDTEIEGTLSDIKLRLTQYQLKTLMEISKSVPAALAGEGDDSDEEAARM
VDEGTLQRARGAPGNGNNSHDQSLIDLSPELSSLQNAWTKLDLIFQVNTV
ALELIMAEEDEPVGDLAKSSLSRFSLDDTRFKTRMLSDGSLEAELLIQSF
TIQDSRRQETKYRRIMTSSNKEAQQLMASVTMSGGKERSLIAMATIDSPR
IIFALDHLFAIQKYITEGLAVDDTSPMDDESIGETVDESDTDSMQVTYNA
PGTERTQSHASRQRNEVSPDKSQKQKEESTMSMAFRVNLVDAQVILIADP
LSSSSEAIVLSIRQVLLSKQHALTFQISETGMFWCRMDKFDTSRLRIIDD
FSIQLNMDNSQVGVTGIHIDVEPLILRLSLRDILLILQIISRASELSGNE
TKHPPETAAEQKARELRNAGLKYKSASGRGQSTIAKTRGTKTITGRTIHP
PTKPQPATVPDREELSATIDGVRIVLIGDLHELPILDIGVNNFTATAENW
SSNLKAEAAINMYSSVYNFAKSSWEPLLEPWHVGLGVAKDPTSGLISVDV
ASKKTFDITITTASIALASKSLTFLTSEQNVLDKPRGVEAPYRIRNYTGF
DVVLHSKSPSSEEPISLRLEDGAEQPWSFEHWEKMRENLLTESSQDGVGI
QLEGSGFDLIKSVKLNREGEFLYGLRPKTENILHRLCVEVKLGSDNVKYV
TLRSPLVVENATEIPVELGVYDAREGHLLKIEKIAPGDSRPAPVGAVFEK
RALIRPDGGFGYEWSRDQLWWRDLLQRPTKQLVCNGENGDPFYFQAHAQF
DRSNPITKIYPHMKIKLSAPVTLENLLPYDFKYRIYDKNTKKDWSNFLRK
GGVSPVHVVELSHLLLLSIDMQDTVFKPSDFSIINSGNVEDFRKESRIVV
KDDQGLPLNLALHYFKIPNSGGAFKITVYSPYVVLNKTGLDLRIRMKSFL
QQAKPAAGQFPLVDTSDRERPKALPFMFSYGNDDNRNRALLKVDESEWSK
PQSFDAVGSTSEVVLNSASKTKEIHVGITVKSGEGKYKMTKVVTLAPRFV
LENKLGEEILVRESSSSGYMTLKPGALQPLHFMQKSAVKQLCLCYAGVDN
HWTSPFNIADIGTTHVKIAKAGQRQRLLRVEILLEDSTIFLHLSMETKNW
PFSMRNESDTEFTFYQANPNVDEDDVEDGSGWRPIRYRLPPRSIMPYAWD
FPAAKFREVVITTNNKERHVKLAEIGNQIPMKFITASGDQKIIDINVAAD
GPTQTLILSNFRQSRSMYRPKALSRTTSGPEAFEVKDQDTGATFRAQLKL
SGIGISLINAQLKELAYITFRDVQLRFSDSPLIQTVSLAVQWMQIDNQLY
GGIFPMVLYPSVVPKRANEVDAHPSLHAMVSRVKDESYGVTYIKYASILL
QQMTVELDEDFAFALLDFSNVPGASWSKVEQEGKLCDEDLDIPEPSQQQG
GQDIYFEVLNIQPMQIDLSFMRTERINAEDKTSSRNPIMFFVNVLTMAIG
NVNDAPIRFNALILDNVRVSTPVLIQNISNHYSQEVMFQLHKILGSADFL
GNPVGLFTTISSGVTDIFYEPYQGLILSDKPEEFGYGIAKGAASFAKKTV
FGFSDSFSKFTGSLSKGLAAASLDKQFQDRRRITKARNRPKHALTGVTAG
ANSLLTSLASGVGGLARKPLEGAEQEGAFGFIKGVGKGVLGLATKPAVGV
LDMASNVSEGIRNTTTVFDGQELDRIRFPRFIPNDGIVRPYNSREAMGQY
WLKQVDNGRYFDEQYIGHLELPKEDMVVMVTFARILLIRSKRLTSEWDVP
LKDIQTIAKERTGVSLVLRGGANGPFIPVGGGSERAYLYKMIGVAVEEFN
RRFRGGE
>Mb|QLI64480.1
MDNTDLIKTVRGLDQNGPGDDGQNLQQLWNFLAAAADNQFHAAEESSLRW
LLKSMNGTSEAAETLRRYPLTWTILDRVFQRIPLFSLAKSLADRKFMAVL
QQTLKDVSKPVGSTEQTTPTKRKRSTLIAYSVDALRSINGCLETAQTLFI
AVKSLLNRLDNTLETFSRDKIGAEHIRSLFCTSAVDATAIAAPAFKICEN
LLSSDSHDQIKGCEDWIQTISSVWDLHLQGTDDVVNVAIYLFRPASVILA
NVGPFSSANRAQVRDSLKEKWSSDMQAFMHRNFALPGRGAFINHRTLEAF
TTALEICKGIIHLAAPALYFIASSTYKDVADSGLRKDNVAWIKETFQAVE
LAIARRQDRHILMESILEQALGNSAPVGVDDLRRVCRDYALQDDGTRWSL
VSKIAQCETDVFQISDDSIELRKEICDRIIKEDNDESNKKAIVEVIGAIQ
DGFRTHRDLPSFLRLWFQQLSDTERQNFRADSAWITFIRERSQKDSLTSI
IETGMSPQRLNEVVAWVKENSSKQNPQATVLFASTVVQSLHSEQYVDAIG
RQLFDLVEGLKASTPLTALRWRVVSMTVWCVAPDERQGIWGAVKKRLTKA

LEKSPILSAEAYEAFRCCYEIWDSVSPDGPWVEEPAGLTEAFTKRLAVEM
SSACVLKGTLFSTALGLASEAVFNEDYGYEQYIAWLLNGSSRFNKLYMSK
AGELPPALGEVIASRKLSTDGLAILWAALIRNETNLNETKLSHGLVDRLL
AAFDESGKEKNWPGEKGQMWMRALSSIPLDCFDRDQREKLTTILAKRQSM
LTEPATKDDVNSWKLLLSLICKIMKRPTFHEGMRFSDLVEASQTLSHLSM
TSEDSIETILEVIERFSHMASLVIKQMAGHVDERSAKYFREATTFVSICE
KQADGSSDHKLDLPALHMTLLKSLGTELTQSVNARSNTDFAPLLTTTRKA
LSKCITKVIKFCVSEKKVLELSSLPVDMSIMAAVDAAAATEFSDKNDLKS
SSIRKLEKRTKQAMQDGDLRAWKIQTFLRAYLSDKLEVSHPTTFDDLKNL
PHRMREVVLRELVQSVSANMDGLSKLAYLRELLRELKGGCNTDGQLLAIE
YLANQIIESSDFQLQTDDGFNIGMVHSDLTSLLLRKPSNSNIICRILRAL
LEKRPQLMSQWNIEITLSTVSDLASDRHAGESTTSYPWLCKLVEVIIKKH
RLRLEGHFHLLLSTLQALLQNLIKSQQHSTATDNLAQEAKAQLYARLVTL
ICEPTVGAVSRSQLHSSLDSATDAAKRSAGRHMYLILVHYVKLQLETSVS
TEVRDALEQAMNSIFDITPPEGRKILNDTMDSSGRAILREMFKRYVKFGK
WSGV
>Mb|QLI64481.1
MRLPFRKKDKKRDGPSSPVPAEFRSIAALDSLRSPPSQLSARILAQLPDE
ALQRIFISVCPQALDKSYESCEESANDEGCMLCDVRDLSHCTQVNKKWRR
VALQVLYHSVRIDPVHYCRMEAFLAEQRKKKSRFNKNGIPEDPALARLKL
LRRTIRENPTKIGSLVQFLKTPYMIRESCHIELAQVIAKLPMLQYVDLPE
GMFRDDPAYTTLRLEVQARCPFIKKATYASGAERSFATLVSGQIWPGLEV
LELTSLKIDPLILRAVLSCLRQLRALKVSETYSLSDDVLVADESLHPLPA
LEELILKDTPSLTSAGLVDYLSRNETSQALKVLTLKDTGIQPWKLQEVLA
VAPSLKTLAIQSRVSEQFPSNEPIQSLTHKGLRTLRYEITGTPSAGPFAT
QGYYSYLSNSVLAGSLPRLRRLYVLDEQFPEQLQNLPPPMASFAGGRART
LSNSSAHIIPSVHVSPPDISLSSPTHQGLGNLSSPTHQDFGGVLSPTRKN
FGNVLPSRQNFGNMASPVRQNFSNAVSPNRFSSNSLFAQAAGGFPPTQTL
EIFSKSDEFGQWNFARVDSFTGAAAGPPRRPTSSYGLDADVTGQGWDRGE
ARRSIMIGNGMSGFSPAPRDDGGEGFTFGPVPVPGGANPSTPRSSTGDVK
RARGMGSI
>Mb|QLI64482.1
MQPELTLIVAATRSMGIGLNGTMPWTGLRKEMQYFARVTTRVSPQAPPNS
VNAVIMGRKTWDSIPAKFRPLKNRLNIIVSRQHSATLPAEIAPSEPVRVS
SLEQAVEFARTHPLISRVFVMGGGQIYDAALRMDAAKRVLLTSIEREYEC
DTFFGLDLRGDAAGSLGWRRRQSDEWREWTGEIGDAKMEEGGVGYEWQMW
ERE
>Mb|QLI64483.1
MADEKTPRASNEPATEKKGAEDVEAGQSGLTRSLQGRHLQFIAIGATFGT
GLFLGTGAALAKAGPASLLIGFVFIGLVVYSVMVALGEMAAYMPVAGAFT
VYASRFVDPTLGFAMGWIYWFSWSITFGLELTAAGLIIQYWTQAVNIGVW
IAIFWVIFTAVNFFPVRYFGEMEMWFSSIKVVTVIGFIIFGMCINAGAGD
QGYIGFRYWTYPGAFAEYVVSGASGKFVGFWSVLITAGFSYQGSELVGIG
AGETENPRKAIPQAIRWTFWGNFVLFVGTVFFIGINIPYDNADLQSTAQN
ASASPLVIVAKLAGVQVLPDIINAVLLTAVLSAASSDVYSSTRILYALAD
EGLAPAVFKRTNRYGTPYWSVAFCSLLGLLAFLNLSQSGETVFTWLLNIT
AVAGFIVWALINACHLRFMKVLQSRGQPRSSLPYVAPFQPYLSWFGLFFN
ILILLTSGFTVFIKWNTSDFFVNYVSILLFIVLYLGHKVVFRTKMIKIED
IDLTMGRTEI
>Mb|QLI64484.1
MGIKGIYKELGPGKRISLSKLAADSLESANRPYRIAIDFAIWHFQTQAAQ
GGTNPALRTLFYRLVRLLGTPIQPIFVFDGSNKPMFKRNKRSGRGDGATT
AAAKHLIRLFGFPTHDAPGEGEAECALLQRHGIVDAVLSEDVDTIMFGCT
KTLRNWSSEGKTSTAPTHVSLYDVGDMALGDLGLDREGMVLVALMSGGDY
LPDGVPGCGVKVACEAAKAGFGKSVCRLKVSDKDGIQAWRNSLRHELQTN
EKGYFRTKHKALTIPEDFPNIEVLRYYTHPIVSSESSIEAIRQTMEQKHV
FHLTALREFTRENFDWDYRIGAVKFVRVLGQAMLVRDLANSQIAAQHVKR
ISGRRTHFSTDGTPELRLAFIPEEVVPIDLAKEVDETVNPGRTGLALNSD

DEFDTPEGVPTINAPKLFDISKPDLAWVLEGLVKNSASDVFQEWQLKEQA
KAVRRSPTKTKKKAGITKALKGSDMPRGSLDKYVTATKAALASNQGSSKA
LEPSSSLHKSLARPKATPSISKQSSSSPTRSPKTAATIEIPQEATRRDLY
FIKSPSAGIPFSSCISFPVRFSL
>Mb|QLI64485.1
MSEDHPAASSFGEPNAQPPTPKQTPTSAVFPSPVFETPKHYQGSFVEAGG
LTPRFAEEYSVFNATPGNLRGTQGPFPDLIPIAPVSSSASHKRLLSAEGL
AIEIAAHTNHFSPNPSATLPPVAPAHRLPSSPNPPTTDTASSTAGFKSPL
NPKLSETPTSSPKKARRCTTAEEPTQVISPPPTARKGERWLAPGLNMQND
QSFGRSDFHDLSQQEIAALMSSATDMFGYPMSAPAATHSNFWDPTMSMNM
DLDFTAAGTNMFQPPTPSSHRHTGSFDWNSEIQLFQDPNAPPSSNQENVQ
PTKEERPLAPKPTASDIVCTGAHDSTLLASFAPVLEDPFNMISAGDAVDP
GLLFSRPQSAPIAASFGTTVQSGTAEAAIQSRKTPVGEGRRSGSVRDRKN
STKDPERATASSPVKSLGRPGLGRSMSENRGRKTVRRGSLPMLAPAARTA
APDPGLRGSKSTGRLPGRISPLKTQQRLSSLASIPEMSPQARQHRSVRLS
INAHGRATVETLLGRGGTAMTRSQSSQDLSTHRSWSSAEDDSDTDDEPII
IPSRNNSFKASFALPDPRKPVGSMFSSRRSASDRSNSNSANEGESEAETV
INEKPGKYGDATSELRKVVEDRQKRSLRLGSATSSQRSLNTIIRNFPGGI
ISPTSLTESSYGPDSYNVRCVCNSSKADETDGFLVQWYVSPSPAGFTFQS
GSLT
>Mb|QLI64486.1
MDDAAAKHWYVQVENSEKESFIQKAAEVALICVATFDVGEECLLELLSAE
ENVAIFIECSIMIRENIHTVDPEDQLHAIWFLTWKRITYTGIEALLNTIT
TTKCLDTAIGHCRNTGTLHDPQPWLVTEFHWVFTKRRIPGSTEHRRDYFN
VLTAEFLVNGLPSGKLPRDIEEHEAYSTFFGHARLDVNECSEKGMQYQTV
HKIHGHDVSLGLLATPESFQESLGCEENPDFIIAATADSGCQYDLVPGRL
FHKALPNEFFEKHVHWLFRKGRNMVIEFRPKHLPWKHEGTNWVLHKVGDY
WQLAEPSGGLLVDTAREASKQVTDAFASLAEPEDIHIVYFPRTKHLEVHL
PRLQLEFYIEEKSSRILSRQYQGMWVDSCQDIDTLIGLHQKLVLRDERSH
RLLLIPEGELNLATPEMNDTHVFVTIDVPSCGKVYAYELDTYLGKLCGRD
FESILRLALLHGYTSGCVPDPFTGYTGTERALQIVRSAEINSPRAVSPER
AKGELLLQASWQEFQPQILRRRNEAKKEFEDHLLGIKKALLRGSLPQNSL
TELNTAAIQPNLPRVCTHFLVEQLASNDQLPLQWKRELAIFATRLSKWQN
WDRLLLLPGTAHKDALKQLHGLKPRTWDPMMHPEWLVFEVENNISIRDIQ
IEFANAMMNPPNRHNSIMQLNMGEGKSSVIVPIIATSLCRKGNLVRLIVT
RSQFAQMSQIMTARLGGLLGRRIYHLPISRNMTLTEASVGKLHKIIQDCV
ESKGILIVQPEQILSLNLMISDYSSKCSEKAREVLYDIKHLLDDSCRDIV
DESDDVFSPRYELVYPLGKEQPIDFARERCSIISQILTIACKKAEAIVEE
MPGGLVISPVGPGRYPKLSLVTREASELLERRIAEEICLTGLIGFSISQT
SSFSTYKEKILEFWTNRALKETTREFIEKDLCWAPQMTSFLYLIRGLIAC
GVLSAALRDRFRVNYGLDKTRMPKTDLAVPYRAKDTPSVGSEYSQPDTVI
TKTYLAYYYDGLNDRDLTETLMHLLQSDRSHIEYQEWVKAAPDLPKAWHD
VKGINLEDTQLCKRDLFPRLRSLRVCIDYYLSYIVFPKQLREYSEKISAS
GWDIGRSKELVTTGFSGTHDLKPLLPLTMRQQDLISQAHTDALVLEKMLG
LHNHTHFLDPPNSSRISEDFLQKITTQEPDIRVIIDVGAYLIDLTNEQVA
RTWLGILQGRNLMIDAAIYCDDNGDLTVLDRHGKTESLQLSPFARQMERC
VVFLDEAHSRGTDLKLPSTSCMRMRLLGDGQSVIYCVPNDIQSQIKASAN
RSTKQPPSIEDIIYWSIEQTAIETGQLLPIWAKQGGQYVQQSKTWAQIRE
SPDMSLRRASANAFKETGIRTIKSLYQPQNSFNIPTDDDNEMAVLIHERL
KEFQHLGAVDNGCSEEREQQRETEQEQQHELEAQVTEQRHLHRSRICSVA
THEVHADVEAFVKFGTLRDHGVGIIGAFTSLSDTTAALQFDLRNFPSQLK
VSRDFANVVRETAGTSQEHCDLFKRPIHHILTTAGEKGMITNMLIISPYE
AESLYKMIEKSKTTKLHIYAARQNSSYMPVDDLDLYTIPTSPIKRQVPLH
LKIELNLFAGQLYFASFKEYTDVCTYLGIAWNDTFTNTDSDGFIIRGKGG
DEVPDCLNLAASPIPFLKSFITNVRHYGNNISKTHVGKMLNGEFLTESDF
PKRQKRKRNEEAEKDGLRSVKREKVNEDQGSMFVKQEEDVEMEG
>Mb|QLI64488.1
MLRARPMLASSLRSIARIPTQPSSTTPTLAVSRPIIAVAARFASSCCSSA

PPSNALALRTSSLLSPPSSSSSSSAFFTPSASHLSPSPSSHSFTTLRMSP
DGSPPSAEEVSKYLRQSHDRLFEHNRAWAAEKAERDPGYFAKLAEGQSPE
YLWIGCSDSRIPAEQITGLEPGEAFIHRNIANLVCNTDLNVMSVINYAVR
HLGVKHIIVCGHYGCGGVKAAMTPKDLGLLNPWLRNIRDVYRLHEKELDA
VADQEQRYNRLVELNVEEQCRNIIKTAAVQQSYAKNQFPIVHGWVFGFHD
GLLKDLKIDFQAVLNDVQKIYNLADA
>Mb|QLI64490.1
MSLVAQFLTQINQYVRLQQGDNLRAWLQVEPNSAKSYYDMAAELRAKFNT
ASGLEAAIDTHLPVDDDVPDGQAAAWPSFQSFMKDYLTLWRDINYDDLLG
THELLTALVNSCGTAFTHPTYGAMLLQASMSFSVTLARFTLQLNRRPDLT
RRIRSVDEDSGGKSVAESSAEIIQKIFTTCLTDRSVGRVPEGKKVGVYMF
ANLVLKLLFACRRTNLAKMIFVNISTISPPLSLYPASQRVTFLYYLGRFN
LSNQHYQRAALCLEQAYLQTPTPLVSHRTNILTYLIPCNILLGRFPSNLL
MQRPEAATLRSVFVPLCEAVRSGNFIQFQHHLATHETWLFEKGLLLALTH
RLRPLLWRSLTRKTFVLTYVPPADASSRKAATLDISHLHAAAVYVQRRLE
GWIHYHPNARGRPSSQANPLFMRAVTNNVQESAETTLVPPPRGPTKLRPN
EGLIWGNAEITLDDVEMVVATLVQQGLMHGFVAHGQARFAIIGAKAKGPV
VAGWPVVWQAIQERRYEEDFDINEVPGWVKG
>Mb|QLI64491.1
MYLRTVHAESRIPALRDLIRSYPLGVLTTAIPSSTEPFIQATHIPWLLDV
QDESSETELGVLRGHLARQNPHSKVMIESVSSSSNNNNNNATNTNVLEQD
VLVLFTATPSHYVTPQFYTETKPSTGKVVPTWNYAAVQAYGKARIYYDSK
SDETSHFLTRQITDLTNHTEISIMDYTGQNGRPDQWNVTDAPDRYIELLK
KNIIGIEISIDKLQGKFKMSQEMSKGDRDGVVQGFATLESDVARAVSEVV
QERSEMREAAKR
>Mb|QLI64494.1
MDDVEELVIKPFRDVVEKGKLAIENAADSPDMLMEAQRLVKVGERGLHHI
ERSCKKLYNDYSSNFVAALKENDEITNIRCQLTNLLWDFEDFLEPDTFEA
SRFKELQKLDRDVAPKVYNILITMKLEPPNFYLSQPSPPLSPHPPSFSHI
QPAPQHPYDLGFHRGSGSVCGSQGDVRSVTDVPNVEDATAQFRTLMENRQ
RPSVHSHVRDPGLGVLKLEIPPEQQMPDPPRPPSPDPWDPQRAPYSGHGV
MENYSPADRRPTSIRAESPIDPAISPLGPDSRRRISSTDAQQSSGSATVR
PEPDTRVQDESRQSGSSTWSSSTNATSHGTRVRPYTFSSTIPEEEQTPRK
PSNPIHIPQLQVRPPLPPIPLEHRKPLESSSTKGVDDMGRQYLSRQPQGQ
PPNVTLPAPSSLQNSTHGTPRALSLRPPTPKSQAELEVAPGMTGRADYSD
LIPVGTESNTESLTELVEVSLSQKDCTIGPSSTFYLYKGFCDGAKEVRQG
GIGVKKTKRPGFAGTTTVARCTGCLFELDFSQIEIDVNKEDKGNFYKSGI
NYRLRFLQKSHLSAKRVDDVLYACVFCVAARRTVDECDSTVFTNTKALFT
HLSHHPRPLPEVPGIAVVEGLEMPARLRNDFDVWFRNPAEAHPAQLNMSE
IAGKATGITRDHSRRLYGQRLLFDRSPALELCHGAKVTGITWPEKHNGEW
VFAWHDGIHASTPADIVKLDAPPSEEIRMVCSSLIRAKSRWKFVYRDKEK
DKSLWLKFDKNEAITNISYASPDHWCWSGTNAKGKWGIFPKVFLDPSTIQ
ELTTEGADRALTLSREKNKSLSMLSRFPKRRPSGRPPSVAESTSSRETQG
GLSSLRNSRGSRGTEDAMSGLVT
>Mb|QLI64495.1
MEHPVQEIASVITTLTTGSPQEQQDTLAEYFSPNASFSHPFCHVPSFGKG
AVPLARNTDSLWVILCIYRWYRTLSPNIDITIDSAVFDQRTSTLYVNIRQ
TFAVWFIPLYSARVRLLTVLTLERRTSSSASASRAALSGPGQERAKYYIS
AQEDLYQMNDCVQFLLPRVGRWLWLLWQLFSTSLCVLGALAFLPVYYLLN
GGKTAKKVA
>Mb|QLI64496.1
MDQRPGSSHEDRLRSESADRAVKSEHAVSSPQIAGSDLASASFKPSPTPL
PSTEAPDSAAMAVASKKKGTATGVKKVPKRPGPGNSKRKKKKKTGSAAPG
VDDASGEEESDNGPYCLCRGPDDHRWMICCEKCEDWFHGECINMNKEIGE
NLIEKFICPNCTTADLATIYKKTCALRACRKAARLAQSQPSVFCSNEHAQ
IWWERMISRLPKGRAKSGLNGQLAQDEFMALLSSNIGSRDEDGTLKLVKT
PFSDDSTNGIEAEGDDALSKLLSDEEQQVLDEAAKARFELAEETLLCHKM
FTLIELAQERRRAAITDGRFGDDICGYDSRLDAVSARDVFSAFIKSPEGE

EVFKNSKLDDPLGEDDMVRGMCERKRCKTHSGWQKMLPLGVKHHIREMAG
QAAEVGEEEQIVRKAVLERWKRRQAESNWVESLEE
>Mb|QLI64499.1
MPVAVANHSHRPTTKVSHKPFKSKAASKHELRDRAKGRVPNEGSQRRTPH
QQVMSKFDRRNQAKQARLTKHREHLKETSIFSGKDGAPRIVAVVPLCADG
DAKVAIKQLNGSLDIETDAQDGNFRVSVDRFKQKLQYITLQRELTACLDA
ARVADFVVVILSANEEVDAVGELILRSIESQGMSTLFTLVQGLETIEPAK
QRQSTMASLKSFITHFHPEQEKLYSLDNRQECSNLMRSLCNTTPKGVRWR
DDRSWIFAEDIKFAANESESTVVTGVVRGRGMKADRLVQLGDWGTYQIEK
IVAAPLAKHAKKGDETAVEVEAENLLEVPSEDRDDLDELAPEDVVMDAED
DSEMAKEPAPKKGVLLDEHHYFSDDDAEERKIKKKVPKGTSNYQSAWYLD
DVSDSDSDMEDMEIDDHKGQDEEIGAEDGIEGYAQPEPTEAGPSEYPQSE
MMDVPDAADDAQQLAQFRARKRDEAEDDKEFPDEIELHPHVLARERLARY
RGLKSLRTSPWQEDEDRAHEPEEWRRLLQVPDYNSSRSRAVREALVGGVA
PGTRVYVYVKGVTTDVAKSHKPSAPVALFSLLRHENKKTSLNYLFNLSKD
YGKSIKSKEELIVQCGPRRMVIKPIFSNPGSTPNDVHKFCRYLHPGQSAI
ASFMGPMTWGAVPVMFFKRCAPGTTNADGEQDIGLSLVGTGTALPPSTSR
VVAKRVILTGHPYHIHKKIVTVRYMFFSREDVEWFKALPLWTRRGRTGFI
KEPLGTHGYFKATFDGKINPQDAVGVSLYKRMWPRASRPVREPLLEGSAA
AVGQGQNDDVMME
>Mb|QLI64500.1
MSSTSRKKDKVTESLSNLTLDTSKSNKKASKPKKKAAVADSWENDDESSG
SENELRPSVASPTVPSAPPPTPFAANYASIPGWDDAGAHASNSPSGADST
KRPEKTDAVARRMIAAGLGMKALKQTEEQKAYQKSIREQERKKKEQERQE
EHRKRMEADKAKAAVWDD
>Mb|QLI64501.1
MQAMEETQRQNPIKGPVFWTTPSEVDAWNIALTSDDVFKTDATQKPSTPP
NQAYILQYHDDIKCIDSPPVCVSCPSSADKLGPQSRRVILDNLPADVTIP
QVLRSIKCYGGIISTVLANSLKKANTRTKTAMIEFVYPEAAADVVSHFHG
NKTVFLDSKGGEHQPEVYVVPTPSYLHTEVNHCLLDRGCTRALFMPSFPE
EAIWYLICTIGMKHINNVQLSDGHLTIELTSLFEADRAGKEIRFGNTDME
YDATKHGMCYIPDSSQGEMQDNYDSHNGFIAFTDTNILQTTWDREPYNRY
RPQPLHETPTGKDAIRHTSGPTYEETLAEYFSVDPSEIWSYLEDRRSFQD
TTYNIIGSTIKLTRRKWSWSISAEDEVKLLMANTLHDPDWATEWDEYFKS
RGMANLRTWEEYGTLAKHRRERAAEQGLAEGIVPICDGCPCECEDLKKVP
VPKFIQDYSACKRVDAEAE
>Mb|QLI64505.1
MAVLVSSDNDPRQMDNSSASSSPGLPPLRHADDSDPDEADIEEHMGPQGF
HFRRSTRTDPDDRHHDPSVDPVLQRFQDMIQSFSQPRRSEGTSLFGRPDN
DSYSPPRFRRTTITSGTFGGGTASVTIFSGPVFGSRGAGAVGEDGDESTN
PNNLDPFQTLFSNVIRDLGPPDGAGEGGPQFGLARSLQEILNHFSPANAM
MGDAVYSQEALDRIVTQLMETTSQSNAAPRASNEAIANLDRKTVDKAFLG
PEGKAECSICIDAMKVGEVATYLPCKHWFHDECIVPWLKQHNTCPVCRTP
IEKGQQRQENNRGESGTGAAGAPGPSNVRFHVPSPTPPANFSRNNSDQSR
DIGNRSENGYDTPTGRAAADLVSSVNLDPQSRPSNPSQSRLNEALRSVAN
TQRERENDRRNRANMSEMSYDTSRMQRRTSHSPPSPRAFNLAEQGARMRQ
RSPSENDRRGNGDRETRRQSGALSWLRERFAGGNGSGGSQGSSRDEQQP
>Mb|QLI64507.1
MTTTVCPIEALPNEVLAASLACLSTRELIQVVPVSRCFYTLATRILYRRL
IDVSPLPDNRLILDCYHPSERLTTPPLSCRYLGLKRVGEQPITDESLKFS
DLGKLYSSYRPVMKEESRDRRRASRRTGSKALQGDETATQDVYLDHDLLF
SQLCAVTYVVKEASRPGFYISQVNTCDGVIRVWRPWLAEKANLNGAAEGL
HSKIVDFDKFLWVDVGKNVGLRFRVVLGPAERLPLLSGPGDDSPVSYTLI
YEELVVRTTTLLMAVEASTVQEASPSSKAIIISQI
>Mb|QLI64509.1
MTDSKKGSAYGAPAGDTDFRKTWDLGEYAAKAKEREAKEKEEAKARYEAK
LAGKKYHKPLTGDETYTTARRNVIDLTAQVGKTQLVPAGAGVGKRGRSAG
FYCESCDLTFKDNKQFIEHLNTTQHLLNTGQTTEVKRATVEEVHERISYY

IRKKEELEKEKATSLQERLHIREEEREKELEERRQRRRDEAEKKRKEKED
AAKVKTEYGEDVRIEGEHDEDDMMAQMGFTGFGSSKK
>Mb|QLI64510.1
MATGRERREGKLNERLRGAGRTNIDDNDTFNLDIPGIATGPAVQSSSSPG
SLPAHPAAQTSLPSRTTSNAPDTRQETESPYQPAGDADLPGPRSSSAPVS
APSERDDPDLDEPEPVQPPSSAVFRGGPRVARTITEEITESPAGKPGSGH
RQRINEAVNIGGAPTGRTPDISDEDDAQTQPSSPLANKTRTSNVSRPSIP
AVPEESEGEREEEPSDEARTRPPQQRPGIARKEAVVVEEEEEEEEEEEEP
EESDHSDEGAVEIDDRRAAQSIGVKRPRPNKTRSPELGSEQSEDSQPEEP
VQKRHRRSPAAQRQPAKRPKPQPKQKRHSSGSDRDDDAAIEITVQRFVNN
FAQGADDEDELQQEIPFANRGGETVVDVFSQVCEEMISSTLENLQKMLQS
SDDLGKKKELRVKIRAIGAYREELNSRLLQHAIHLNHWHSLRRRLRHVQK
EKLALRDEIIRLKGEREQVALRMDAVRIKHEADSKESTSRLNASSLMHDI
DLAVEQGREAPELSRKAEKEADLGNLDLVLAQISDQVSSTSSTGGLLQQV
QDFNAFLERAATALESG
>Mb|QLI64511.1
MSLVSDQVRRLDAYLDRLPVISEAASDDGRSDVEERAFVTTEALTSPRLD
ELLRIVQSLSATSASDSVLPLHRVKALLAQSGLPSLQPHGINQLDQTEQP
VVKSAYEDEIEWLLVTKATVHIYGAILNSLLDQIIPLNDDIWYWSDVLNS
YTYSSLYTVQTSPVRFWAWTLEVYSASKMRLQSLSVRDSPVEFVDSTAAG
VSQQWRQFYGIVRDTIRERSFANIQRKVLSPVAFCRSEARRKQAHLRKLR
EITASGLGVLMDEGLQFGHDDDKADVHDSHDLKGVVERSVALMDMVLKEV
CTLDVNIHDFEDKVFAGVEEDPELSVHLEESVTLDRPAVLSRRLLRIIDK
TLPDHFNNMQSLARENGRPSQLIRYWLPTAVGVLSSTTVLRILINRQDEI
INWITNLGGTIRDFWFNWVVQPTTKVIRTIRHDSTSDIAIMSRDSLKADR
ESLERMVVDFAKDKPHFAYEGNSSITDIQLAEIRNKVAEGDVTPVLRAYE
QDLRSPFVGAVRGDLVRSLLIQVQKTKVDLEVAMTGIDSLLKSQELVFGF
VGLTPGVLVSIGVLQYLRGIFGGRSGLRRDKKSGRAVRILRNIDRILSEA
RPTENNVLSYKDHGLLLCEVHILRSYARNLMPRDVEKDFLEDLDDLANIK
GIQVQTKALERIRWAYARWLK
>Mb|QLI64512.1
MDAAPDSAKPVAIIGMGFRLPSGISTDAQYWDLLINKKNGRCRVPATRYN
VDGFSGGKTQTQFLATEYGYFLQTELSNIDPSFFSMKPAEATLLDPQLRL
LLEVAWECMESAGQTHKLVGSDTGVFVGVFGEDWHNMLHRDDLMPNTCRV
LSAGDYALSNMLSHHMTIRTACSSSLSGLHIACQSLNSGDCSAAMVLGSS
LIMDPSMTLDMSAQGVLAPDGQCKIFDTLADGFARAEAVNAILLKPLEAA
IRDGDPIRAVIRSTAVNSDGKTQHVGTPSAEAQHSLILRAYQSAGIDDLS
KTPFVECHGTGTMKGDPIETTAVGKAFGTRGTYIGSVKPNLGHGEGAAAL
TSVIKCVLALENKTIPPNVNFVTPNPRIPFEEYNLQVPGEPVPWPEGRCQ
RISINSFGFGGANGHVILDSAASFAAYCGAARQRYSSSDSDSLEHGLSHS
DENFLSLSLPASVSSQSSDCDSTVPEEFPRIVVLSARSEESIKRRIAAFE
AYMTNSPPAINDIAYTLGMRTAHMSHRAFAIHHNDIHQALQFSVGKKIIT
GKEKETKVAFVFTGQGAQWSGMGRELVHSSATFREDIRNMDKVLQSLPTA
PKWTLEDVLCRDDEAETLSCHIAELAQPLCTAIQVALVNFLRKCGIKPSA
VVGHSSGEIAAAYAAGAIDYSEAILCSYFRGLVTTKLQSDGSMAAIGLGR
DHVTAHLVDGAQIACENSPKSVSISGNSEAVCKTMENIEAVNKDTFMRKL
KVQVAYHSPQMSPVATKYEEMMKPYHTGTKAWKCPFYSSLYGKLADPKMG
LGPLYWKDNMVSPVLFFSATRQLLDDLPEITAMVEIGPHPALQGPVRQII
ESKSPQPSVTYLGTLVRDKPAHDAVLTTLGRLYTMGHDIDFSFLNPGGSV
VADLPRYSWDHKRYTWTESRLSKHWRMRQFAHHELLGIRCTTPSEFDATW
RNVFRHYDIPWLKDHMLNSDIVFPAAGYVAMMGEAIRQLLGSEAYILEDV
LVKAALIVPEAEPVEIMTTMRPFRLSGLSNSSTWYELSICSLCADTWVEH
CVARGKARSEGGNGNSKQTTTAPLQRQVNEDCFYNRLQYLGFCYGPHFRR
LRELTADTDRQHAVGAVTDTTNKNEAFYALHPTTIDLALQLSALAACQGI
GRRLDILAVPVELKHIMVCPGGPELVLDAVTDTDNNTASILAMSEDTGKT
ALEIRDGRFLPFNNGGLQKQEPLHAAHVEWLPDLDLYDKIDFARTNGNSR
KVNKTLDHATAVVVVQMLHELDSLGIDAATTPGDLPKYIAWLREEKNKYT
QDEELASLFSLSSKTRIEMLDSLRRQVQDDDDETSLALANMYVRLATSEN

IAAIFTGSIKPLQICLDDKGLESFYVNGQSLVSPRDFLRLCTHSKPTLSV
LEIGAGTGATTEAMLQGLVAEDGRRVYSKYVFTDISSGFFVSARDKFAKW
DGVAYQVLDIESDPAAQGFELQSFDLIVASNVLHVTSNLHKSLLNVRSLL
KPGGRLILQELVAPPNWRCIPFLTGLLPGWWAEKEDSREAGPLVSVQRWN
TELLSAGFSGTDIVKLDDEHPYSITAHMTSTALAEVKEPLGLTFLYRQVR
HEFACKLAEKLHLLGHQVQWVQLGSEENDTKAESTGCDIISTIELEGSSF
MDDISEADYGHMMKLLARHKRGILWLTRPAQFACETPGYGATTGLMRCAR
TEVGLDCWTAEIEDLDDTALDSVFALSRKFCQRGPMDRGVDAEYAVRRDG
VVYVPRFHLESTENELQQLSGDDVAKQLVIGQLGSVDTLHWVTHTEHLDL
APDEVEVDIRFVGLNFKDLLTTMGILHGQKDSLGLECSGVVTRKGSAVDT
VNVGDRVCAMGSGLIRTRKCFPSKLIIQIPDDMTLEQAATLPVVYATVVH
AIVNIGRLRKGQSILIHSAAGGVGQAAIYMCRAIGAEVYVTAGTEEKVQF
LMETHQIPREHIFDSRSDRFLDGVMGITDGRGVDLVLNSLAGDLLQASWC
CVAKHGKMLEIGKRDILEHGRLAMDMFQGNRAYCGIDLSAMDEEQVTELM
GQCFNLRYKQHLKPIEPVQTFGPDQISEAFKLMKKGHHMGKLVIAIPENG
SKIPAKTSDKSKLFSDKPTYLLVGGLGGLGREVARWMIEKGAKSLCFLSP
SAGSNQHAAFIKELESQGCRITAIAGDVSEMQDVERAISASPSPIGGVMQ
LSMVLRDCALIETTYQDWKAVQNPKVKGTWNLHRALINTKLKFFILFSSI
AGLLGHPDQGSYASANTFLDSYCHYRHGLGLPCSVIDLGGVDGIGALAVK
SNKISHFNSLGLFLLQQDHVMEAIEIALHTSAPTSLTVLPRPVGGFTWKP
QIAIGLASARASSIPVNLQLFKSDVRFGEYVNLAVDEDQREVKKEAMLRE
VLDQVEADPSVLDTPGTLDRLSLAIGSTLFEYLSLSENEMDIHAPLESIG
VDSLVSIEIRNWWRRTIGLEITASEIVKAGSIEGLGKLLVTGLRKKYNKG
SDLPSDAANKASS
>Mb|QLI64513.1
MSGFDSVGTLLADIANRLLEGLRIFSLADKRHWRQDEYEQVKALEEALNE
AKKDFQELCPLVNGQSQYEHDRKYETMQELRLLRDKFRAHVQLLRDWSRS
GGPIDPVWESSQRCLGAFLVRRTQAKVAVQPSRQVSEADYQRRQLEELGA
CTKIGSFDRFGEEDMVFICDFCDGHLIWEDLENVPTERTAASLPTRQGNS
QHWQATGTSSSGQQEKLVIFPPIAVANHLAPLHGDWQARLLCPFCEDEAK
KPQDEDDEEELYKPNDEFEDVASLQEHMEWQHVEVAVPSGQQSGNCAVM
>Mb|QLI64514.1
MISLPISSTDDFHAQKHNWQQPSHLHNSHQYRDEEELLNDIPALDSDLER
QLVFQGNEDSLQPFHDCLTHPMQSDSWGGLAQLTPKIGRFSHQRESSLSS
LGSTGPASPYTQNTSNPQIAFTDSTFEGLAEMNSQDLTNNQPSSYYHLAK
SMGPTPYPTYHGMDGTIAEMAYPVTVSGPGKRPRMDKGLLSAPELAGAYN
RSHPASVASSVAGDSPATPAIGELDQKSSRRKNGKTPMTPDLSYPAASDL
ASNFLDYGHIPKLDRTMTDVYGDELYNPNFTITATSSPQPHSVTSVGSDV
FSQRINAANHQHLSAAHSPASTTSRARSPFRTGSPFATSSSHQFNCSQPP
SREYDQQIQQPLAHLENAGEPETPKTISPKDAVLEFNESEADGLFPLFPQ
NSSGFEIDTLSKTAPSSDHNESYIGQNMGSLHQNDQFGYLPSQVPTGIQV
PQQYPFIARPATSHDTPPRLSSSGASSVVSGGNTPAPRTRPTSRGAEGGT
YTCTYHGCTLRFETPTLLQKHKREGHRQTQGLGAPRVQDMGMASGLLNTQ
AGPHRCDRINPSTGKPCNTVFSRPYDLTRHEDTIHNARKQKVRCNLCTEE
KTFSRADALTRHYRVCHPDMELPGKSRRRG
>Mb|QLI64516.1
MHIHPLITFLALPLLAVAARPKNAILLSQVQSLTLRGNGAKTTNRRVSAI
PQLKCTSSRELCSLYSIDTMRCTNQGSSYGGEDIEWSCTATLPEELKLGS
TDVICEGYASADDPYVLKGSCGVEYSLLLTSKGQERYPHIANPYGGYFSD
GRGGTDLSAWLFTVVFVAVLGWIVYSACVAGNGGRQARGNTGRRWGGGGG
GGGGGGFGGGGWGPGNDPPPPYPGTKPSSSGSAWGPGFWSGLAGGAAAGY
LAGNRSRNDERRGYDRGWGSNTWGSGPASPRASPAASWGSSSSGGSSRHE
STGFGSTRRR
>Mb|QLI64517.1
MVLAVDLLNPSPAAEAKKHKLKTLVPAPRSFFMDVKCPGCFTITTVFSHA
QTVVICQGCTTVLCQPTGGKARLTEGCSFRRK
>Mb|QLI64520.1
MGNNLSSTFVPDTSKVVLSPPDAFGDILVVIIWGLVLYVCAMIFTTCALV

DRWKGPMDRIRVGGGSVLAAMLLSTAWPVVMAYIVISG
>Mb|QLI64525.1
MASSLSAAKQQLRSLVKQRLAAVPADSIVTQSRKICESLKEFRPYVEAKS
ISIYLSMPSGEVQTDAIVRHALDSGKQVFIPYLHKSPLDTPGTPARVMDM
VLLKNMQDYENLQPDRWGIPSVDPATVHERQRILGGLDAHRSDRAGLDLM
LLPGVAFDFDESGAIRRLGHGKGFYDFFMNRYLAKAASPANEAKPILFYG
LALTEQLLSASSEEQIPMGQYDRPLHGLVLGNGQIKESSRSITAK
>Mb|QLI64526.1
MGAGMSWLSNLLWAKKEIRILILGLDNAGKTTLLYRLKIGEVVTTIPTIG
FNVESVTYRNLNFNVWDLGGQTSIRPYWRCYYANTAAVIFVVDSTDIERL
QTAAEELAAMLNEEELKDAALLVFANKQDQPGAKGAGEISEALRLGELRD
RNWSIMACSAVDGSGINEGMDWLVQTVNQE
>Mb|QLI64527.1
MLASRIQRRAFSASARNLSKVAVLGAAGGIGQPLSLLLKQNPKVTELALY
DIRGGPGVAADLSHINTKSTVKGYEPTAAGLAECVKGSDIVLIPAGVPRK
PGMTRDDLFNTNASIVRDLAKAVAESAPNAKLLVIANPVNSTVPICAEVF
KARGVYNPKTLFGVTTLDVVRASRFVSEIKGTDPKDENITVIGGHSGVTI
VPLFSQSNHPDLSSNAELVKRVQFGGDEVVKAKDGAGSATLSMAMAGARM
ADSLLRASAGEKGVIEPTFVDSPLYKDQGIDFFSSKVELGPDGVKQILPV
GEIDAAEEKLVEACLGDLKKNIEKGVTFVAQNPGK
>Mb|QLI64529.1
MAAKSSNPLISNQKSSQVQAVLHPLVLLTISDYITRHTLRQQKGPIVGAL
LGQQNGREITIEHAFECHVREAPEVEGGYLLDASKFGARLEQMKTVHKER
QLDFVGWYTLLPLTGPTPTILPIQTQILEGWNESAVLLGFHPDEVLNHSV
GGKLPLTIYENNYEVDDQKAANPDGEDKRMEDGEAALKLKFSEVPYSVET
DETEMISMNYVAAGGANATAASAPASTVAKDEKQAWSVASTSKGKRRLVE
PKAYETDTIDETGVSHLTREEEEMVASLTTKANAIKMLHSRIRLLTAYLE
RLPPSDVSGEQPDSETMDTDHTIPSLPILRQIQALVNRFDLVIPSDEEAF
NKELLQETNNANLVNLLSSVMQGVHGARDVGKKFHVVESAKKVGRKGRAE
GYLASPGFNMPGTGDLLI
>Mb|QLI64531.1
MTRFRPCIDLHAGQVKQIVGGTLDSASSALQTNYVSQHPPSHFAELYRSN
SLEGAHAIMLGPGNTEPAKQALQAWPGHLQVGGGINDKNAKEWVDAGASK
VIITSYLFPGGKFSQSRLDAVLGALGGDKNRLVIDLSCRRRGEASWFVAM
DKWQTVTDMEVNQESIKALEPYCSEFLIHAADNEGLQKGIDEKLVERLAE
WCSIPVTYAGGGRYLEDLDLVKRLSGGKVDLTIGSALDCFGGNGVKFDDC
VVWNRRQEE
>Mb|QLI64533.1
MRLSTIGAGLIACLATNVAATALTYKLDANEKACFYTETKKDNEKIAFYF
AVQSGGSFDVDYIVEGPNAKMILQGEKERQGDFVFTAQHAGEYSFCFNNE
MSTFAEKYVDFEISVENEARTAQLPSKQGTSPEQTSVLEDSIFKISSQLS
TISRNQKYFRTRENRNFSTVNSTEKRIVNFSVIQIGLIICMGALQVFVVR
FFFQGARKGYV
>Mb|QLI64534.1
MSDYTLYAYFRSSCSARLRIILNLKNISYDLIPVNLLKDEQLSAEHKALN
PSASVPLLVSKRSPDAGFKIGQSVAALEYLEEVHPAVPALPPTSDAKGRA
TARVLCEIIAADTQPVTNLRIMRRVRALGGSAEEWNKELMGDGLRAYEAV
AKDSAGKYSVGDNVTMADACLMPAVWNAQRFGVDLSQFPTVSRIFANLSE
HPAVQKADYFKQPDCPEDLRRK
>Mb|QLI64535.1
MSMKRKVPAKLAAPVVKPSAKIPTKTTIDESRTVVATADALQSSQIEAIE
ISSDTDSEEDTSDLEDNAEEDQTNGEETVPKAAAAAVAKVNGTNPKANDH
ELEGSDGEEETSPSFGELLRGNETIDVPALLQQSVSASTTAQPARTAIVP
PSHQSLTTVLTQALKTDDTDLLESCLHTTDITTVRNTIERIDSSLAGILL
NKLAARLYRRPGRAGNLMTWVQWTLISHGGALASQPDVVHSLNGLQKVLA
ERAKGLNSLLALKGKLDMLDLQMDLRRKMQRNAGLLHQGEDADEEEEDAI
WVEGETSPVGRKDLANGARSRRRRGDDDDDDDQDNEDLPMTNGVGDSADE
EEDSDVAEDDDSEVAEESLDEDEVDHEDVDDSMGEEEESDVEAAPPSKMQ

KVVKSFSKRK
>Mb|QLI64543.1
MSPHAVLSGTPSNGKPTVDLSKFAVTQNAFLPATSPPTSLSDSYYEPWEL
IAQHLPALIESNRIRDSVRQLPVLSTDCLVSEAEWRRAYVILAFMAHAYI
WGGEKPEQILPPQITVPFLRVSSHLEVPPVLTYAGANLWNFSCSGSDFTN
LHNLSLPISFTGTESESWFLLISVAMEAKAAGILQTMMAALEAVKTRDYP
VIASALGQLRNCIDGVGALLERMYEKCDPMVFYHRIRPFLAGSMNMEAAG
LPRGVFYDEGHGKGRWRQYRGGSNGQSSLIQFFDVVLGVEHRTHGKPGQE
SFHAEVRGYMPGPHRRFLVHVARMGSIRELALAEPATPEQRGLREAYTAA
TEALSEFRNKHIRIVTRYIVLPSRQPWTGGPRTNLASSSSSRGGEQGLMG
TGGTQLIPFLRTSRDETAEAGKLAREKE
>Mb|QLI64544.1
MASSMALRKLASGSAFSRLARSQARAFSTTRPAARVIANHPLRAKEASPH
VSTKYNVIDHEYDALVVGAGGAGLRAAFGLAEAGFNTACISKLFPTRSHT
VAAQGGINAALGNMHEDDWRWHMYDTVKGSDWLGDQDAIHYMTREAPASI
IELEHYGCPFSRTEEGKIYQRAFGGQSQKYGKGGQAYRCCAAADRTGHAL
LHTLYGQSLAHNTNYFIEYFALDLIMQDGECRGVLAYNQEDGTLHRFLAN
NTVLATGGYGRAYFSCTSAHTCTGDGMAMVARAGLPNQDLEFVQFHPTGI
YGAGCLITEGARGEGGYLLNSEGERFMERYAPTAKDLASRDVVSRSMTME
IRDGRGVGAEKDHIFLQLSHLPAEILAERLPGISETAGIFAGVDVRKQPI
PVLPTVHYNMGGIPTRYTGEVLTVDEQGKDKVVPGLFACGEAACVSVHGA
NRLGANSLLDLVVFGRAVSHTIRDNFKPGEKHKPLAADAGSDHIEVLDQV
RTSDGPKSTAEIRLAMQKAMQTDVSVFRTQESLDEGVRKMNAIDPLFKEV
GIKDRSMIWNSDLVETLELRNLLTCATQTATAAAARKESRGAHAREDYPD
RDDKNWMKHTLSFQKTPHGKVDLSYRRVISTTLDENECKPVPPFKRVY
>Mb|QLI64545.1
MSSKDSGGDSKLFARGKVAELRLELNSGGKKDKNYAIKKVALKRIVANMT
MSNNDMVALFPDIIACMHIPSLEIKKMCFLYLVNYARVRPEIAVKAIPVL
ENDMEDQNPLVRALALRTMSYIHVKEFVEATVPIVKHMLRDNDPYVRKTA
AFCVAKLYDHDRQMVERSDLIDRLNSLLRDDNPTVVASALAGLMDIWERS
DAIKLTIDYSNASKMVAILPDCSEWGQTYILEALMSYVPQETGEAALLAE
RIAPRLSHSNSSVVLTCIRVILYLLNYIADQKQITTLCRKLSPPLVTLLA
KGPEVQYLALRNALLILQRRPEVLKNDIRVFFCKYNDPIYVKVTKLELIF
MLANENNIDEVLTELREYATEIDVHFVRKAVRAIGKLAIKIEPAAKRCID
LLLELVSTKVTYIVQEATVVIKNIFRKYPNQYESIIGTLCEHLDSLDEPE
AKAAMVWVIGQYADRIENSDALLEDFLYTFSEEPVEVQLALLTATVKLFI
QRPTRAQELVPRVLKWATEETDNPDLRDRAYMYWRLLSTDMNAAKQIVMG
DKPPITAEAERLEAPTLEEMCLNVGTLATIYLKPVQTVFRSARPRKLQES
PALQKQNLLVDADNQKSISMFGRGGQPTNIDLRSRNAAPANGEGNLSQAV
SDADAYFSSIGTQMAAMTLDQGEDMFGGGSGNMTGYVVSAHAPQAVLQPA
QGAGSNGDLLAL
>Mb|QLI64546.1
MKFFGGSKKQSSTSTSSSRKHTPSSSVDDRDFLQHQTHPHSEPASPTKGS
SNNKSPSKRSSRPVSYREPKDPAPEKSSSSSSSTPRHARLSRHSTEPSST
SSSRRSKFDPDTHPLNLPPEERRRLSKLSNSTMSDHMDIDREPVNGASSP
ASQPQSNPSAQANFSVPIPNGTHHNDAPAPPPHRSNPSSPVPTALDDAEA
YKAAGNRFFKDKNYTKAIEQYSKAVDLFPDSPTYLSNRAAARMSNGQYAA
ALEDCSRAADLDPQNSKILLRLARIYTFLGRPEEAMTTFGRITPAPSAKD
MAPTKEMMYHVDTAKHILKQGTGVTMALHAIDQAERGLGPGVLKPRKWQL
LRGDAHLLVGRENNLGEAQGIAMALLRNNAQDPEALVLRGRVFYGQGDNT
KAIQSFRMALTCDPDYRDAVKWLKTVQRLDRMKEEGNVEFKAGRFQAAIE
KYSEALQVDPNNHSINAKLLQNRAQCKIKLKQYNEAIADAEKAVSLDPSY
LKAKKTKANALGQAGNWEESVREWKAIQEADPEDRTIPKEIRRAELELKK
SLRKDYYKILGVEKDCGPDDVKKAYRKMAIKLHPDKNLDDPDAEAKFKDL
SEAYETLSDPQKKAAYDNGDDLMDPADMFGGGGMGGIDPEILFSMMGNQG
GFGGGGFRSAGGFPGAAGGFPGGAQFNFANAGGGRSRGESEEEEEEQEAV
QWDTDASDSRDANVPEDHATAKEELMHEYQQRRDARNNLVDTLRQYGMLV
LAAVVLLPPHLAVAAVFCMLLVYMLMRYVRPSSRTATKPTG

>Mb|QLI64548.1
MASHILASVDARSSFWLSSSLAEAISALSQPVAIFTRDDDSKTQQQQGLG
IVSFLTAIGTAVAIFAAQFSLFALLRNRLARIFKPKTYLVPERERTEPPP
GSLVSMIRALITYDEREVIKKCGLDAYFFLRYLKTLLVIFVPICFVVLPI
LIPMNYVGGLGREIDIKDSARNSSSSKAVPTGLDTLAWGNVTPQNTSRYT
AHLILAILVVIWVCTVFFLELRVYIKIRQDYLASPEHRLRASATTVLVSG
IPKKWLTEEALLGLFDVLPGGIRNLWINRDLTTLLDKISLRDNVHKRLES
AETDLIKAVKKAQLKQRNAEDKKRRRKLNLKALTREERVARDAEEDAEAN
RLARTEEGKDAGHDQVVPHTVDAGIRESMLHDQEKTSLQDTTRGPEIHHN
PLHHVGAGLKGVAGKAGRGVNDVLVTKNGFMDLSSTMEHSHQRVPSPAPA
ESLEAPRTSSLDGSVLTSSSSRAIKPPHGAGHRYGMENSVRKVDNVDDLY
LNAKPRFWQFWKAPQGGFAFPEPHGTSECASATEESGWQSFKHYIPFMGP
KEEPFKYEDAYECQYKSDMETDAEWAKYIKRRDRPTHHLPLFGLSWMFGM
PLVTKKVDTIYWCRQELARLNLEIEEDQKHPERFPSMGSAFIQFNHQIAA
HMACQSVAHHLPKHMSPRAIEISPRDVVWENMAINWWQQWVRSIIVVIIV
VAMFALWAIIVAWTAAIGQADALITSNGWLSDLERNKKLGTLIKAIAGVL
PATVLSILLLLAVVIFGFLAGLKGAKTGSQKTEFVQMYYFAFLFVQVFLV
VSIASFFFRSLGEIVNSVKELSSVDAVLNLLAQNLPSAAIYFFSYMVLQA
LSTSSGTLLQVMTLFMWYIMAPMSDSTARQKWARNTSLNQVNWGSFFPVY
TNFACIGLIYCIIAPLISIFAVITFGLLWLAQRYAMLYVNRSGTDTGGVL
YPRAINQTFTGIYFMELCLAGLFFIVNDEKRRNVCTPHGVIMIVVFILTL
VYQVALNKSFSPLFRYLPLTLEDDAVIRDEAFQRAQDVRFGLIQEDEEGG
RANLGEKVTQVPDTDENIELQNLDAGQTDGLNKRFRPVKQVGTWARAGGK
QVGTWAMGGGNQLRKLKNLTDNTRAAQYRRSQRKKDLDAQLTMGDALYGG
IPDDIEDLTPEERDALTRHAFLHYALRARRPTVWIPRDDLGVSDDEIRRT
NAFSKHIWISNEGTALDSKVRVVFGKNPPDFSEVDLITL
>Mb|QLI64550.1
MPRRCARTIERACCAIATYIPLIFVYGLTTWAIWMVIEIGSVAPKGSWLG
TASSTVGVVIYLLLNWSYTTAVFTDPGSTTNRDGYGLLPTTTQNHYPPAT
SVTVKSNGEIRFCKKCQARKPDRAHHCSTCRRCVLKMDHHCPWLATCLGL
RNYKAFLLFLIYTTILCVYAFAVSGTWVWSEVIEEDVEKLDALLPVNFIV
LSVLSGIIGIVMGVFTGWHVMLALKGQTTIECLEKTRYLSPLKQPYRAAH
NPANRLPQAAQQFVDFHTNALPGITRPEEGEERRPSNDFNYSPAAAWRAP
GPRDSPQPVQLSYEEHERLQSRRRYEEYLDEQDSTKLPSAFDLGWKKNLR
HLLGPNRWLWAIPICNTTGDGWSWEASPKWLEARDRIKREREEQRAREIS
AGWGGQDDDMGYPSNPNQYTPTSAADGNAPRRVLSKADRVLGRDPNLYAD
GSQGMRMQRLSTRGKSLEDELLDTDNDEDEGAGSPKANSRDKAERRALDV
VTNGKAWTRGGASGMLRNATHSGSPGDASPLDDQGVD
>Mb|QLI64551.1
MSRQINQPSNQIKLTNVSLVRLKKGKKRFEIACYKNKVMEWRSGIETDLD
NVLQIPNVFLNVSKGQTAPKEDLEKAFGKGKSTDDIVLEILKKGELQVGE
KERAAQLERVHNEVIGIVASKLVDPRTKRVYTSGMIEKALDMLSSQAHNA
DKDKSATASGAATPTTTPATGDDGEAKPRTKEHIWTGVVTTKSAKSQALE
AMKALIAYQPIPVARARMRLRISCPTSVLKQAVKAAKAAAKEEDGEQKAP
GTVKDKILGYVEQVENQDVMGSEWEVVGFVEPGAFKALSDFIGNETRGQG
RVEVLDMAVTHED
>Mb|QLI64552.1
MSSASARGNYSHPSLEDDDLIDPDDEADLNDFDDPLENRSARQPLSGNIG
STSSRPLNESYLTSSIPGEDRRAPQNTIDETVWETVRRDLLAVWAKLREV
LYPRYLLGGTMFDSEGGLRGAYSNIRGAGITGTREELTGLASRMMDAEAL
LQSNMSPGLRDWDLWGPLIFCLLLSLLLSIAARAEQKDTVFSGVFAMIWL
GEAVVTLQIKLLGGNISFAQSVCIIGYTLFPLVIAGLLSALGLHWIPRIP
VYIVLVAWSLAAGVSILGGSGVVKNRVAIAVYPLFIFYIGLGCLCFIS
>Mb|QLI64553.1
MTHQAKVLSSWGARPLSTEATTIKELPGDEPNDVVFESKYGLRSVVLNRP
KKLNSLNASMIRKIVPRLVEWEKSDMANIVVMKGAGEKALCAGGDVAALA
EYNQEGADGWKKSADYFGLEYKLDHYIATYGKPYIAFMDGITMGGGVGLS
IHAPFRIATEKTVFAMPETTIGFFPDVGASFFLPRMNGAVGTYLALTSER

LTGPNVFYSGIATHYLHSTSLPDLEARLAELRFRDDDPLPKRLAIINDTL
EEFCTGLPYDQPMQLGGAVRQAVDRCFSKNNVSDIIAALKAEKGETADWA
QKQLATLNKRSPTAVHVTLRQMRVGGKWDIAETFNKEHQIASKFMQHPDF
TEGVTALLVRKEKPKWQPESLEAIPASENVSQPFFNFEKTQSLELFADRT
YSEYPYQGLGVPTEKEIKAAISGGGYTPQELTKTIVASRNGRQGIADVVN
EIISRKTTVDAQGKVKWVNEDASAGSRL
>Mb|QLI64555.1
MSGSGYDAVVDVDDEGDLGHTDLQEDLEFHNSNFTDTSHSARKGPPSGLP
PPVTAPSASSSSKRFLWTISFYSQFFDVDTSAVLSRCWAALYPRANFLDV
LEGNPDLYGPFWIATTVVLILFLGGTISDWLSTRGTAQFAYDFRLLSGAA
GLVYGYTLFIPVALFLALRYFGSESANLLECWALYGYSNLIWIPVAIISW
SPIEILNWTFVAVGFGLSVVFLLRNLYPVLSATDRQVSKVLLIIVVILHA
GLSLTIKILFFAHNSPVAKKPVGDDVPADDKPAFLL
>Mb|QLI64556.1
MAVQTPAKPSIAANGASNHDGLPSLTFSVVTKEVEKQDAMRLVADSIAQQ
RQTASFSVIVHPACFVGLVAACTAIYRQNAHRDFGTALVMVCGLVIAYLA
GVRLLTANYIRIAESLKWKEWITSPEGKEDYVLAARYGDELIATTVLRLV
APAGSTKKTRLSSVKGGSGVIRAWTTKYRYRNKGIGGDMLRLAVLTTRSK
CGDNAQVTFDPDHANSARPLHDMFNRPFIKREERAKKALAHALEACDRGE
SSFQME
>Mb|QLI64557.1
MGPVKKNLKATKKFEKNHLKGVLDKRKAVAKIKQRQQVKDKKQTRRAKDA
EFFKGTDDGSTPDKRSNGGKGKQSGEMSVDDFFQGGFDEIIDSKENKAGK
LGKRKRSEPKDDEEMSDGSGSGVDISGPPAASESEDGSEDDEDGGMSKET
MDKLAEKDPEFYKFLKDNDPEALDFDDDADLAEVDELSAGEDEHEDEQPK
KKQKKAKAAAEQDRKDEEPSQSNELTKSTVASWRKAMEEKNSLRAARQVV
LAFRCAAHLNEDENDEGETQQRWVINSPEVYNDILVLALKQIPVVMNHHL
PIKESASGRAYAQIETKKFKTLSILLKSYTSSIMYLLGTLSDDKTLKLTL
SSLTPILPYLLSFKKLVKALSKAVVNFWAQPASSETTRITAFLVLRRLVV
IGDKGIREMVLKATYQGLVQGSRVTNHNTIEGINLMKNSAAELWGIDPAV
GYTTAFTFIRQLAIHLRNSIVNNKNDSFRMVYNWQFTHSLDFWSCVLAEH
CSPLKEAEAGKENQLKLLIYPLVQVTLGAMRLIPTAIYFPLRFHLIRSLL
RTSRATGTYIPLASPILEVLSSAEMKKAPKATTLKAFDFAVAYKAPKSYL
RTRIYQDNVGEQIAELLSEFFLLWSKSIAFPELALPVIIQLKRWLKQARS
RNQGNKNAKLVSQLVTLVQKLEANAKFIEERRAKVDFAPKDRAQVDAFLK
DLDVAKTPLGAYVVNQRKAREEKNRMLEEARKEEDRKRREDEKGVVEDES
DDEMEVDGEDETDEEDDNDAVELEDDDEDSE
>Mb|QLI64558.1
MPSAAARRLYEDQIPSFMSVFDLDADTIAEGETVTLVDTKMVDMPPKQSA
FADPSQPPALGQGGFPPDPTHRHHDSTSTQMSESTESSPTTTLSTTDSSP
LSDPSPSSSPDSPINLIPLNDYPSTTFGCRPATSQNNMLCIPESHALDRP
MTSPAPRRPRNMKGLSIQPPLTVSTTSKCIATEPSSPSFIKPKIPGMKRK
PSQLSLKTGASDLVKKTSLEVPASPAMPPILQRRALKHSTSSPHIFAAMK
SSTFGPLGGMTFPKVLERNESGLSEVLRPMKQKSVMDLDESIVEEDSPIK
TQLANRSEYEPFHETANNEDQKSPGYPNGPIAIYGDNIFLYLEPTAEEAS
RFDVVINVAREVQNPFLAAERMFENASTTGNESPIPDTAATIASFATAFE
YQPIEDSTIDTPTTPKANPWHVPEYIHIPWDHNTNIAPDLMHLCETIENR
SKNGKKVLVHCQQGASRSASLIIAYGLYQSPELSVNDAYYAAQAKSRWIS
PNMKLMYSLQDFYKELTKKRSPSSSTHRPRTGRSPTKHRLTLSADAIDIT
SKEPHTAPLPGEETPSDAIDSDCPTRPRGSSTPGSQAISPGPASAPLSYS
WKDHCEKTVAESSPSVPNQPFEPTLPSLQPLPVLQTREVLAPPCDAPIFA
KSENLPQRPKTGLGQPEPGFGLPNQSSTILGLRSFESPGAFPSFPLRKFQ
KLSPQDPIDGTRTERTVTLSTPYPDESALLSPRAETMTTNPLHDVHEVAG
MRFVESPPTPTESLFSPREASFPTDPFSFFGRMMGFNRPAQAADPRSPPT
RGEAPIIRSIDELL
>Mb|QLI64559.1
MAFPFNPNTVKRNARAYGFNTQEAAIFAHIALDQLRDLNASSSEPVTVEQ
IAEAIKQYATKEVERERAIKLEVRDRGISILDDMEADLLEMGTGACKSKH

SKIPEPRIAEEETDGQPVKRFKFDDDGLELYFGKDDPYGIQAEVVPLSLK
YYNQDIITAMCSRVELAVELGKQLAPEDLVNLYATSRTFHNAINEHMLSS
IRMWIAHRAPEAGKIFQFRLYRRHLVMDPAGRTWREQCQGTKAARTNPKL
MAQARSVPGLRYLQLVLRRDRCCRQIIGIMARNGFLMPGSMHGTLLRLWL
LMDIATSGQRQALLRNRDIWSDNHIYNAQMFFMKLLMAFNDPTYGPGSPE
LLHLFLGQKGLYPLWQLLMRKRFTTLPDLIDLQVRYQYEIPPELVANEAG
EQTTIHGVPLHEVGTGHYEGWGLGDSHLLRPDELIPLEATARTLDLEHHL
IYMMMWGYFDTRDGDNIVPTEEDMYISDEEDVLKHMDTSYHWKKKHSLKK
KFFHLSAEEKQKIVDDDEDDRLRALAWTGDVSEGDDDEEGEENPYPLDQE
INRGFIVRSPRKDEKKVSVPEMNDKQGWEEFVNTALMTVAPDFREDESLR
AQAWHNYSNDEMEGEWDWPAWLAQDEARRGVEGTEEGEHESEAETVIMED
EDLADGEDTGSEVEDGDDSDGDLHDPLLVELRDYLNGEGVQPSAELRQLL
GGYA
>Mb|QLI64560.1
MSSFFTIPGSQKKRKRAAAPEAPKKRIAGAKPSSAKSSTRTGAANSKRRV
DRDESISGSDSDDDESYDSASNPGDDEGVEDRQSSDDEGETAAEKRLRLA
ERYLENIKEHVDEAGFDAADIDRDLIAERLQEDVAETKGRVYRQLATQLA
FSKASQTFFRTNTNSVTSIAVCAPFVYTATKDLVLHKWRTQDLPQYQFPQ
TTKRKPKKPPAPPRKRPQLEAWIKGNKSKSKDKNYKRHTGQILAVAASPD
GKYVVTGGEDKKLIVYEADTLKPIKVLTHHRDAITGLAFRRGTNQLYSSS
RDRTVKVWSLDELAYIETLFGHQDHVVDIDALAQERCVSVGARDRTARLW
KVMEETQLVFRGGAVDKKNAPQDIDPRSLLHEGSMDRVAMIDEDLFVTGS
DNGAIALWSVTKKKPVYIEPLAHGLDPAINPLDASAEVNPDPKKVIPAPT
PRWITALKTIPYSDIILSGSWDGHIRVWKLTEDKKKIEQIGVLGDFADDY
DNESVKDAPSSNGTAESRRPIPGLINDIAVFERGERGKDGLCVVAAVAQE
HRFGRWKVFKGGVRNGGVVFEISRIPTAQVNGDHESSGSDDDESDS
>Mb|QLI64561.1
MALYRRPSQAKLIFAPAGSSPHGQAEQFTKSTLSRRDLHPTTPTPQFNSW
FSQAQASSIPQPEACTLSTAELPSGRVSSRIVYLKELDAAGGFVVYSNFG
TSRKAADLATNPHAALVFFWAPLQRQVRVEGVASRYSPGESQEYFDTRVR
GSRIGAWASRQSQVLEPDGAVDGDDGRAQLEGWVADAEKTFEGREDIPVP
DFWGGLRIVPTRVEFWQGRSCRLHDRFVYVREEGRDGEDDKWTLERLSP
>Mb|QLI64563.1
MTTTATTLKGQPLDKPVLDAMLRRRMFYTPSFEIYGGVGGLYDYGPPGCS
LQANIVDLWRKHFILEEDMLEVDCTVLTPHDVLKTSGHVDKFADWMCKDP
KNGEIMRADHFVEAILEARLNGDKEARGQKVEEKDDPKKKKKAKSEAVK
LDDAVVQEYEEVLARIDNYDGPQLGELIKKYDLKNPATGVLPSDPVSFNL
MFQTSIGPSSNLPGYLRPETAQGQFLNFAKLLEFNQSQMPFASASIGKSY
RNEISPRAGLLRVREFLMAEIEHFVDPHGGKKHHRFHEVEDVVLLLLDRD
TQLSGKTDTKRVTVGEAVKSGLVDNETLGYFLARIHLFLDKIGVDLSKMR
FRQHMANEMAHYACDCWDAELLTSSGWVECVGCADRSAYDLSVHAKKTGA
PLVVRERLEEPLVIEEWQVDIEKKKFGPLFKKDAKTVETALLATSQEERE
KLAKQLKDSGKISLDVAGVGDGKVEVGSDTLKIEFRKRIENTREFTPNVI
EPSFGIGRILYSLIEHNFWTRGSDGGDEARGVLSFPPTVAPTKVLIVPLS
NNVQFRPIIKKLSQQLRLAGISSRIDDSSASIGKRYSRNDELGTPLGITV
DFQTLQDDTITLRDRDSTAQVRAEESKILDAIKALVDGSKNWEKVASELP
KFEGQEVEVAVR
>Mb|QLI64564.1
MPRTKVTVKYGKRSTRTKAERLFAELPQSPVRKPVVVDIDETIASVTEKL
STVQIEGAPKQPRRSPRKSVKRIEIIDDATDSSDTPEDLGDSDYRNSKGD
SQDAASRSNDTSEEDGKENLLDEIAGDISLRILTWDEVCPPGDRIEKIAE
ASYAEVYRVTNERGTSVIKVIRLTSPIKPQTKAQVRSKLVDEEPHSEEDV
NGELQISEWLADIPGFVVYKERYIVQGKTSRALLETHQVFQRRMKRKDPG
RAQFYPSPSRYLDDTKFLVVELGDAGTALEDWELRNESELWDIFFLEAIA
LARAEELAMFEHRDLHEGNLCIRQARPRTQRKPGSQGYFGYSGVDITILD
YGLSRAEDLSIDLAKPIAYDLERDLGLFTSTHAAQCRVYRQMRSFLLRAD
RKCLPPEAHKTPYAKGVDGPLCWEAYVPYTNVLWLAYLYEYLLDNFRGDA
KELAHFEEQTADMWAYLNPDADDSVPCFGAAGDIVCFAVESGWIREEQLI

GSEASMIEREDSIIVSKEDGDVDVLSRRLSKRL
>Mb|QLI64566.1
MPSEEIFSSICGPPIAANTAVSKDVGIYGHSLTPSWAIKSTFKKSSAPPH
GIAVNDTHIFAAQDQKAHVHVYSRLRGNQETLVAFPERIRSLALAGDVLI
LGTSEGRLILWEISTGRQVTTPPCHVQAVTCLAVTPYHLLSASEDSNIHV
WSLSRLLELGADAGHEPDLTLSNHRGAVTDLVVGPSTNAETSLCVSASTD
KTCILWNYQTGQVLRTLLFPSPPLCVSLDASARALFACAEDGGLYLVELF
GDKPLVGSRSAELASIVVQVNAPLGVSDVADGPASCLALSHDGTSVLTGH
TKGKILHWSLIDNSHPTELANLNASVTNLVYLPLLSPKKPCKVASVVKPT
QSQRQYSITAQLKGDFGEGSRFSYMLNSTGLPVDVIESATASVLNSHSGT
KEDTRLLKENEELKAIIEEQKELQKATMQYRDGGKTS
>Mb|QLI64567.1
MHLSILGPLCLASAVSAWLPQDRDLQAFNQTARFEKLGKRFKPSLPNGVT
KIRGVNFGGWLICEPWMMQNEWRNVLGCGGSASEFDCMNDHYKGGNREAG
NQKFEKHWRDWINPDTVQSVHDVGLNTIRIPIGYWSYTDIVDKASEPFAD
GDRMLPYLDAVVQKAADLGIYVVIDLHGAPGGQQQDAFTGQNNQPAGFFN
DYNFGRAEKWLAWMTNRIHTNPAYASVGTIEVLNEPVSRHDAAGRYPAPG
EDPGLVQKYYPAALKAVRDAEAALGIADAKRLHVQFMSSKWDAGDARAAA
AVARDPATAFDDHNYIGFALGSSNGDQRRLMHSACTDSRVVNGQAFAVTG
EWSMTSGVDWKDAGFFKRFFTAQQQLYEKPGMDGWIYWTWKTELNDPRWT
YSYATYLNYVPTDAAALEKNVYQDVCSGFR
>Mb|QLI64568.1
MDPRHRQPAPVPRSNNANSNSISNAGSNSASPFPPASIVVPPPLHSAHHH
PFATHTFAPVSAYAADPRRASDTPYYPPAGRASLPAPELGHNHSQSQSHS
HSRAQSASSLPSLPSAREHSRVMPPPPTSPSQGQQGGQPPPPHHHHHQQS
QSSVSYGPMPTRPPSLSAGPSTSFPSGRELPALSSLSRSGGGAGSSSMSI
SSMLGGPPPASRDSQPPPHYSSHGPPPSSGPGYGPAMQASPRLPSASSEY
PPFRRPQTPDHPRPYDPRASAAPSPHGPFATTPDLQRYGTPQGYHQRHPS
APADISREPGRMSAGPPPTQNKPPPPYGGMPPRPLEVGRSDDPYARRDDS
RPPPAMEFNPERPGLRPYPYDERFHPDRDRQPVPEAREREGRERAYSGGA
DSRRQQQQQHMSPHELGHRDTHPGQSAYGRPPEHRDTRDQWGRPTTSDPN
YRPPLEHQRPQHSEYPPASGPYSHHGQPYQASPPDRYPPTSHPPHPSGNA
PAPPPQSYEADRARMDQIHPHPPQPQGPPRREEPPPPAPAGYGTSHGPSF
DSPRKESDDHHGPNGLQRNLLAVQDINRKGRMSPLPQAVQGAQPQQPGPA
AEPGIKSEFGRMFSGIGSGVSGIGMSSPVASSAATAFPSQTSHPAGGKRE
DIESVGVDSVVDNIKGPKPGRRRKLKDEDARDDDSSGRLTPAGRANKRAK
PHQHHHHHHHHHHHHHGQDSVPSPPTAAAQFKNLKGSTPTASPTEKSHHH
HHHHHGQKTGAPAPASQTKPAPPVIPPKTKTVVASKLVLDSVANRARHHL
GDFIYEPELKPGRLLPNTPTHRGFSSNPKPLPWDIIKDKENCLLTVKVPL
IHLSPVAREEITARAYLWGTDVYTDDSDVVAACIHGGWIKGEWTDDVDTA
MLDLEQGTAEGGKRKTKIPTPELPSRESEGAITTPPPLGPMDIPANRDLH
VNVLILPRLVKYAGSTRFGISSREFGGQYGSRHSVHDGISYMIKSIRWVE
NGAQPQARLRGKGRRERMRKAMKEVTASFGNIHGLDVEQSKESNASPQRG
DIIGNWRKKDQPDAPAEVVNDKPDQERAASEGNKENRVVTTQPGNSEEPA
KETKKTPDQDVEMTEADAGKKESDTVEEK
>Mb|QLI64569.1
MTVFVCSLFLPKTIQFNLPGTPPRRNTLQRRSKTTIPPPPLPTAPVIETQ
PSLFSPRPVITPPRTPTGETESPDPFANEDGLRIQFPHELGSPTDEVASR
WGTRANQPLSRANSPPPAEIFERDRTLEKARELGRRGLLQPKTHMRSDSH
DRVFSSAPWRVVDADQGNGGLRNAAEAAARDGKLGDYTWVGTLGMPTDAL
EGTQQKLDIEDTLTNEYDMMTVFCSDKDFDGHYSHFCKQILWPVFHYQIP
DNPKSKAYEDHSWKYYVNVNQAFADKIVQNWKRGDVIWVHDYHLLLVPGM
VRKKLPDAKIGFFLHVAFPSSEVFRCLAVRKELLEGMLGANLVGFQIQEY
TRHFLQTCSRILSVEATPEGIEMEDRFVDVVNTAIGIDPISLDKNRGERD
VLRWLNILQERYKGKKLIVARDKLDHVRGVRQKLLAYELFLNKNPQWREH
TVLIQVALSSSQNSDLDATVSDIVTRVNSSWANLAYQPVVYLKQDIDYAQ
YLALLTIADALMITSQREGMNLTSHEYIFCQDGKIYPNRKHGSLILSEFT
GTSSLFSKSELSVNPWDYRQCADAIKQALEMDDDEKTRRWENLHQSVAQH

TGSHWFAELLSHLDRAYEEQHKRAQTSVPRLSIQDVCKRYEASNRRLFML
DYEGTLVSWGPVNQIIPVSPQRTLDVLNDLLLDERNIVYVMSGRRPEEVD
RVFRRVPGLGLIAENGCFIKDCGSEKWVEMSDSAHIDNWKASVKDILRYY
LERTPGAEVEERHCSVLFHYDGAEDQEAAARLAADCASHVNDACESQRVH
AIVLDGTIAVEPLDWTKSTAAHMVFEKLLTTQACSEGKGEGKKPIDFLMV
VGDGREDEKVFKWANKLGKRRTIDNVVTVSLGNRSTEAAATLSQGVSGVL
SCLQRLASL
>Mb|QLI64571.1
MDMDHSTMHMGMTGTPTAMSMPTSTADSHSHGHDMEGMGNGCKISMLFNL
NTVGSCFLSSEWQITSTGMFAGSCIGVFLLGMVLEFLRRSIKEYDRFLVR
QHVSKFQSSASPAAAAATGSVGSKEGAAITSCAVVPPFRPNVLQQGIRAF
LHLLAFVVAYILMLLAMYYNGYIILCIFLGSFFGAFIFQWETLPLGGQQT
SAAQEATVCCG
>Mb|QLI64572.1
MSNMPSDTEAATTSVPLEPAQRVLAGKKSLWESIRSNPKVIFIAFFASLG
GFEYGYQQGVLGQSLVMTRFMKNFPSVVESSSATGWLTSVLQLGGIVGSL
SAGVLGELISRKYTMFIACCWVILGSYLYIGARDGLPSLLYAGRFFTGVG
VGLFSGVGPLYNAELSAPEMRGLLVSFYQFATILGIMLSFWVGYGSNYIG
GTGDSQSNLAWRIPSIVQGIPAVALAIGIWFMPFSPRWLVKVGRDDEAKS
TLAWMRKLPADHELVQVEFLEIKAEAVFERKAFARDFPRMAEQGSKSAFR
EQIAQYINCFRTKDNMKRIMTGFLTMTWQQWIIYYATNIFQSLGLTGGTI
ALLATGVTGVVFLISTVPAMLIIDRVGRKPMLLVGSVVMGVSMVIVGIIV
AKFRHDWPNHVAAGWTAVALIWVYIAGFGATWGPVSWTLISEIFPLSIRA
KGASIGAMSNWLNNFAIAFFVPPMLEAWAWGTYIFFAVFLTAGIFWVWFC
LPETKNATLEEMDRVFGSHTGERDAELLREAQREVGLTALLEGRVDRTAK
AGVEKHIEAL
>Mb|QLI64573.1
MWNDEDNNPYGTSFDRRDSQTSSSINPTSPSSRDYRSFDAPRTPTSGSED
DEPMFSQATAVPRHESHDHGAPQDEPVQKRKPGGYTSRIEQILYENPNMP
ILITDAGKSLESGGRYIVYTIRTGDLEVRRRYSEFASLRDALTRLHPTLI
IPPIPEKHTMADYAAKPTSAKQDQQIIDLRKRMLAVFLNRCRRMEAIRDD
GVWWRFLDPNASWSEVLHSHPVASIPKAILKAPPLDTANPTPAHNFLPVP
STSAKLKTTAGTTYDINPSVPQTGTHVIGRFPPDSTTQSEHDLDPYFISY
EASIKELEQLLMGPMEKVNRRTLSHLSSLAADLCELGSRYNAFALSEQAP
SLGPAIERVGQAADLSYIATEELAGSLGASFAEPMRENAQFAGVVKSVLR
YRVLKRVQQDLTTEELAKKKGLLEQLEQSEAEARRIEQYLSSSQQLSPPP
KRSTSLKEPSSQHRRDTGQDDTESIDSDFPPTHGDFSSSTPSASQGLPER
SASVSHKKAPSSNSITNKIFGPLRHAIQGVADVDPERTRRDTIGKTRESI
GQLEQAQVASELDVKEASASVLQDLKRFQRDKEDDLKRYMLAYAKSQIEW
AKKSKQQWEEARAEVDKIDEN
>Mb|QLI64574.1
MAPAVASNRDAAVCPTDDEAITPRNRTRSWDYVWRSGVAGGVAGCAAKTI
VAPLDRVKILFQASNPHFAKYTGSSFGVATAIRDIYHFEGGRGLFRGHSA
TLLRIFPYAGIKFLAYEQIRAMIIPGKEYETPLRRLLSGSLAGVTSVFFT
YPLELIRVRLAFETKKDGRSSLSSICRQIYNSSPVEKSATARLPSAPAPV
AAAVESTASVAGSIVPKTGLINFYRGFAPTLLGMLPYAGMSFLTHDTVSD
LMRHPSVAQYTTLPRRKNQPSDKPAPLRSWAELTSGGVAGMISQTSSYPL
EVIRRRMQVSGAVGDGHRMRLGETARLIFQERGFRGFFVGLTIGYVKVIP
MVAVSFYTYERMKLVFGI
>Mb|QLI64575.1
MADGGAAEVTTMFEPSKTNVHTMDHEKKHATEAQPENIQSPMSPTAPHVP
FPFPRRTSLEIDDYFTGPRDISKHSKWPIFLQMHGSILPKMILPLIFIAG
WSTAVTVISMKIHDLGVQSVLLTITGFVVGLGLSFRNSTAYERYAEGRKF
WAALVLASQVLGRVFWIHGLDHPEADPRESVLKKLSSMNLLVAFAVALKH
SLRFEPYTAYPDLQNLVGHLDTFAKEATNEDASSFTAPKKNFFKGLGEYL
GVSFAASNPRKTLKKATRPLGNLPLEILNHIAVTIDHMVRNEQLKVPMQQ
TLAYNNLSVLNDVMTGCERVLNTPLPIAYTIAISQITWLYVVLLPFQLVK
YVGWITIPATLAAAYIILGLLFIGREIENPFGQDVNDLPLDGFCDQIAAE

LDIIASYDMQNPYGFFMKSENMPLYPVSTASAGTWIHRSEEKLREAIKTK
PVTTFEWRRSRSGHHQGSSTQAGDHNV
>Mb|QLI64578.1
MANFLASIFGTELDKVNCSFYFKIGACRHGDRCSRKHVKPSYSQTILMPN
LYQNPAYDPKNRMNPSQLQNHFDAFYEDIWCELCKYGELEELVVCDNNND
HLIGNVYARFKYEESAQKACDELNSRWYAGRPIYCELSPVTDFREACCRL
NSGEGCVRGGFCNFIHRKNPSEELDRDLTLSTKKWLRLRGRDERSMSRSP
TPEASKRR
>Mb|QLI64580.1
MATIYIDEDIGKDEPTQQGTEQSPFKTLQFAYLQHPAQAQYLTRKSESAD
DEAAREWKPAAKSAMKKVVNYFEQQKKKAAREQELAIHRKKEEEERHKVL
DEAKKIVLEEDPSLPKPVTIKLDEAGDHIALRKADDADSKGTRVRVFGRV
HRERKQKDVLFVTLRDGYGFMQVVISGKLAKTYEALTLTRETSMEIFGEM
REVPAGARAPLNRELHADFYKIPKHWRAVGGDDAITNRVTASTEHSTLLD
LRHLTLRGENASSVLLVRDAMEYAFNRAYHEARIRKVSPPALVQTQVEGG
STLFKFDYYGADAFLTQSSQLYLETCLPSLGSVYCIEKSFRAEKSLTRRH
LSEFTHIEAELDFINFDDLLTHLETLICRVLELVLEDPMIAGYIKTLNPE
FKVPERPFMRMRYSDAIKWLIDHEIPNEEGNPHNFGDDIAEAAERKMTDI
INKPIFITHFPAHIKAFYMKRDPEDDRVTESVDCLMPGVGEIVGGSMRID
DYDELLAACKREGLDPEPYYWYIDQRKYGSSPHGGYGVGLERLLAWLCKQ
HTVRDCCLYPRYMGRCKP
>Mb|QLI64582.1
MSDSESGTKLVVPKFSSFKARDPARPTKADGGKGKQRERSQSRDRHRDKR
HRSHHSQDSRRREERSSRHRERQAAPPQDPAQGPKALLEKTTSSAFVIDT
KGDHLILKYGLDRAKVPAYYRYGGGRVLGTAGRLVIHREGPRDQFSLRFP
GDGAGFFDKDGLRSKSFRLTRDPIRLRARASESDEDDTFLAVGSSKKRKQ
DERDSESEDDEGLSYRSIEGKAKAKAALESDHDSSGSEDEVVDLEHDNPL
RWKSIQLSRQVKDHPGDIDAWMELVDHQDDLQRAGETIDERTTANTAHSF
SEIKVDMLESALSNAIVAEGREVVLVRLMREGMKIWPSKLAAKKWSEIEK
EVENNFELWRAHLDFAMSNISSFQYDDVKQMLLDRLRAVLSRAGAQEKHW
EEAIYVFLRTARLMHDSGYKELAAAAWQALLEFNFFRGGSEMPGTGMEAF
RDFWESEVPRLGEEDAKGWKYHMSTGGQVEAPEPRKDNQVADIATRDAYK
AWAHYELGQAQSAKLPARTMDDGTEDDPFRVVMISDIEPLLFMVPNVLLQ
GRPGELLLDAFLIFCGLPPLFRSTPLAELAFDDQFTSSSTVTSTALRQAT
LLDDPEEVRRKPPSFSRVLNAALSPSLLFAGADWFQYFGSSLTNLSVETA
FVGNVLKQLVHEAEVESLALYYLGFSFASERPLVKKSAKALLKKYPNNAE
LYNAYALAEFANGNADVGKKVLVSAMEAPSLSVGAPGFQLHKSLSWEELK
AGNRTQAITWLCSAFDNSLRQAQADHGSISPAVILKARQEILAHIHQCMY
EGRANDAAVFVEGLTLLSYLTDGGNTEPTSDAQGNISAAMNAIEGMSAEF
KSRGDANTRSHERMLQFGAQLLYLNATRGPFRRIYLREQLSKFLDFFPRN
SIFLSLAEWADSSLRVIDETRQLLYDKILIKQHDCLSSRVFSIEHEISRG
NPNTAKAAFEHALSSDVCKTNTRLWIAYLRFSYAHRELRPKVKDLFYRGL
RHCPWSKDIMMEAFCSLIRDMKSDELRSVYNTMTGKGLRLHLDMDEFVER
WRRDRHGRDGK
>Mb|QLI64584.1
MDSSPTQVKVIFTTDEQDLQLPESKRQLLVPADVKRYGLSRILNSESMLD
TSSPIPLDFLANGTFLRTSIEDYLKSNGLSSETTLTLQYVRSLIPPVYQA
SFQHDDWVGDVDVLSATAPASKGIDGVVNDRIASASYDGLVRVWNPSGEA
IATSSAGRAGGHTSRVNAVRWLSPSKLASAGLDRKVVIWDYSESEDNFSG
SLKHNMELWGHEKNINAIDVNCATKRILTASSDGRVGLWTSSKKAAPQAD
AESLPSSHSAKRAKLSAASNTAQRGPLAMMPMHDDQVTAAIFHPHDSTVA
YSASQDHTVKTIDLTTQKEVSRLTTLHPIMCAAALPGNSLVAAGSSARHI
TLLDPREAATATAAMTLRGHINMVVSLSPSPDNEYSLVSGSHDSTCRVWD
LRSVRPATSEEGSGSVSEPVYTISREWLSGKKLPPAGDGAKVLSVAWDKS
WGIVSAGEDKKVQINRGRGLLAS
>Mb|QLI64585.1
MVPAPQALPARFPCWCRAVYSWGGESKRDLGFIEGDLIECLNAGDGSWWV
GRLYRDRRTVGSFPSNFVEVLPEEFRPMTRSVSPLPGSNTPSPKNTPAKS

KTFRKPFEAYAKAPHYTTAKQPTSYRDPSKVNPAATPTPVSTRLAAGAPA
PKVQHQRAKDKSGGSFSRSVHDTIERVPSPPTVHGYGSRAPSPAPSPAPS
QTYRDRGSTPDRDSAPPPAPPPHRHVGGGGRPVDMSRHTSNASFHPYSPA
AMTRHESNASYHHPPQTPRHASSAPHNEMHQRELNGDYTPQGRSPRQYSP
DELHMTPSPLREAMDSVMEQLDQLDGLGSSWDGEQVQQAEVVEQPLDPWA
PESFDAIAHKSRRKGSVTTRPLTSTGIAHHDEGYETWSGGSSQETSFQNG
SRGQKHELPELSNYVERMEKQFQTMHHHSKSVGMAADIDLDAEEDIPPPP
PPKGQPYQRPRSSAGEPAGRPRQLRTQKSAYEIGRGKGAASGSVGRTLTT
RTNTTNASTGNQSHTSSSTQSSGRTLWSGVSAGGISSTSAGSMARQHMRT
QSAMDAREAELDRPDSPFTGVTYHSSHASHVAANGRPRAQTGLEDDLTAG
IGGLVQPKAPKRNIFKKIFDSAKTGVASNRGSLAAGSIAGSNSVRASPFN
RPRPSVAPSAMSSRMTGISGPDYGSNAARDMGLAGGSSVDWVQVRRDVNR
SNSLSKAERNERQDRCQMMEHPVLNPVDEMYEDIEGDEAADGMPVREAIN
YQSINLSQVDKNSRFIADLPPMTTAIQLATTYVCRPYRSDVQRLRAIFTW
VSEKICWEEDFEGDIDTRRVIQAKRACAEEYAVLVMEMCAAVGLECEIVR
GYLKSPGDVADLNMMPRSNHWWNAVIVDNEWRIIDCCLASPSNPKRGLYS
SANGATADSWWFLARPTEVCWTHVPEHHDQQHIVPPVAHEILLNLPCTCP
SFFRNEIEMVDYNTAVTRIEDLEMVHIKFNVPADIEVAAEVEARAYTRDA
DGDVFESGDVLKKRALAQAEWYDGVKRYTVKALLPGDEGQGTLKIYAGKR
GLMHSIKDIPHPLAFALPIVHTGENPPYDFVTRHPTPHAQRHDIYVVQPQ
CQRLALNNTFVFAIRQHPSSTLSGPNSALTPASNPGRTSPNPFARPSSAM
SMNTSSMSGSTPSSASGTVAGKKPAKLAIQTPGGKILRLMRKEDRKGIAA
GSKGLSGDEDVSDGGTWETIIKCSERGVWRGLVLADRTARWCVFAEWICV
G
>Mb|QLI64586.1
MADEAPPVHIIERDGKQFKTITEGKATILVPHETKAGQDTTEEQQVFYNP
IQQYNRDLSTLVVKVYGEMLLEKRVEAFNSKNVKQGKKRKRDEIEQDNEE
ATTQATEQQQLEKEKAENGHTYKPSFKILDALSASGLRALRYALEIPFVT
SVTANDLSASAAESIAVNADHNGVAGKFTVTNDDALALMYRSIASDLSRR
DKRGNPSKSHKFDVIDLDPYGTAAPFFDAAVQSVRDDGGLLVITCTDSAV
WAGHSYCEKTFSLYGGVPVKGMHSHEAALRLILNAVATSGARYGIAIEPM
LSLSIDFYTKVFIRVTKSPSAVKFLGAKTMLVYSCDQGCGSWETQPILKS
RPFPNKKGIGSFYKHTMAQGPSTDRLCQHCGFKMHVSGPMYGGRIHNQDF
IHRMLDEIPKASPETYGTLSRLEGMLRTALEEYIPGPELPTDVDPKDAEA
AVVDHTPFFFIPGKVSSILSCSTPTDDMFRGALMHLGYQAGRSHCRPGSI
KTDAPWSTIWWVLTEWIRQKSPVKVSKFKESMPGWKILYDAGIVGHENAK
SDGSQGPESIDIKMEGTEQQQQQQKDEKVLERTEAEDNAGEKKEPLTEEE
LRKTLVFDDALIQLGRQKGTRYMRYQVNPHKYWGPMTRAKG
>Mb|QLI64588.1
MLTRTIIPINQNWQFKQAEKDDASYLAVSQFPTHIHLDLLHHKLIPDPYI
GQNENEVQWIGETAWTYKTIFSSPGTNGRAKAVLAFDGLDTFATVVLNGK
IILEADNMFVSERVDVSDVLRKTGDNELVITFDAAYLRGWKEIEKHPDHK
WGCWNGDISRLGVRKSQYHWGWDWGPELMTCGPWRPIHLELYESRLADLH
AEIDVESSLKSARVVAHAIVEGTASHVRFNISFGNKSVSSETVRIKDGEA
VATFKVQNPELWYPIRYGSQPLYTIKSTLLDESAELDSSSKRIGLRKVEL
IQRPLLDQPGTTFFFQVNNIPIFCGGSNWIPADNFIPRISKERYYDWVKM
VANGNQFMLRVWGGGIYEEEPLYDACDELGILVWQDFMFGCGNYPAHTSF
LKSVEREARQNVKRLRHHPSIVIWAGNNEDYQYAESEKLGYDPKDEYPDN
WLHSNFPARYIYEKVLPDICRELIPSTYYHPGSPWGGKNTTDPTMGDIHQ
WNVWHGSQEKYQNFDKLVGRFVSEFGMEAFPSIKTIESYLPKGKNDPERF
AQSATVDFHNKAAGHERRLALYLVENIRYAPDPLEYYIYCTQLMQAECIA
SAYRLWKREWRGPGREYCAGALVWQINDCWPVTSWAICDYYLRPKHAYYT
VKREMAPISVGMTRREHTHAKDKYTRVHIEREIKIEIWGSNLNLEDLTAD
CVVKAWDVETGRETYSHTVSTRHFFPANASTEIISMNVPVTNKNVGEEGR
TVMAAYLYQEGKQIARYVNWPEPLKYLHLQKPKHLRAQLCTTRDAIELSA
EVPVKGVAVECDDDNVKFDDNLIDIVPGEIVSIGVKGVIKDTKFTTQYLN
MLG
>Mb|QLI64589.1

MLRNAANAARKAVTELSQYPKPGDKLHGFTLVRSKHVPELELTALHLQHD
KTGADYLHIAREDSNNVFSIGFKTNPPDDTGVPHILEHTTLCGSNKYPIR
DPFFKMLPRTLSNFMNAFTASDHTFYPFATTNAQDFKNLMSVYLDSTLHP
LLKQSDFTQEGWRIGPENPLGETAESKKLVFKGVVYNEMKGQMSDAGYLF
YIRFQDHIFPAINNSGGDPQKITDLTYDQLKNFHANHYHPSNSKLFTYGD
MPLFDHLQELDARLQAFAKKNIDEEIHQPIDLDGPYEVTLKGPMDPLVDP
DRQYKTSVSWITGDTTDVVESFSIALLSTLLMDGYGSPLYRGLIETGMGA
DWSPNAGYDSSAKRGILSIGLTGVQESDVPKLKETVQGILKQARDNGFDK
TKIDGALHQLELSLKHKTANFGFSMLNRLKPKWFNGTDPFDSLAWNDTIS
AFRAKLAEGGYLEGLIDRYLLNDNTMIFTMAPSITFGEDLAREEQQRLSS
RIDDAIQSAGGEEKARQKFEQQEQELLVEQNKTNSEDLSCLPTVHVKDIP
RAKEPVVVRDEVQNGTAIQWHEAPTNGLTYFRGINTLENLPDDLRELVPL
FTDSIMRLGTKDMAMEQLEDLIKLTTGGVSVGYHSTPSPTDYSASSEGIV
FTGMALDRNVPVMFDILHKLVLETNFDAPETALRIRQLLQASADGIVNDI
ASSGHRFAVGYAESSITRSAWLRQQVAGLSQVKLVTRLAGRPESDQFEDV
ISKLKQIQQFALSSDNMRTAITCGTESVTQNSNSLQSFMKALPQGVSDLK
NPEPRRLPTDRKTFFPLPYQVYYGGLSVPTVSYTAPDGAPLQILSQLLTH
KYLHHEIREKGGAYGGGAMYKPLDGLFGFYSYRDPNPQNTMSIMRNAGRW
AVNKEWSDRDLEEAKISVFQAVDAPKAVNQEGMGKFLSGITEDMRQKKRY
QLLDVSKDQVKEAAQKYLVDAIERGEERTAFLGERQSWVDDSWKTQEMNV
AVGE
>Mb|QLI64590.1
MIILSLCRRMSPSRRKLAHNLGHGVAFKSIALSRRTLHSQAASQQDAEVQ
SIRNIGIIAHVDAGKTTTTERMLYYSGVTQRVGDVDAGNTVTDFLELERE
RGITIQSAAITFNWPKAQNCTPGTRPQTVNLIDTPGHQDFRFEVDRCLPI
LDGAVCIIDSVKGVEAHTERVWGSAQEFKIPRIVYCNKLDREGASFKKAV
LEIGTRLKGWPLVCQIPWWEKEVFVGVIDIINSIGYRWKSEREKIHYGPD
ELQEELSASNPDLLAEMQLARQRLIEGLADFDEAIMDEFLAENPDIPNST
LKQAIRRAIRSGDGRVIPVFAGSSFRHIGVEPLMDAITDYLPNPAERPDA
EVRVGSAKHGLHSVLDSKGKKTAANIASVASIFKVVTHPKEGVISFVRVY
HGTLTRNASSFNTNINAQERPMGILQISASKTQDVQELTVGQIGALRGLK
KARTGDTLITTVGGKPVPEHLRHVQIRPPEIPPPVAFLQVEPYGNVAAQE
LQTALENASREDPSLRWGRNSKTDQFTIQGMGKLHLDVSLYNMRQKRRID
AEFGQIEVDYKESVTQPTKPQYTVFDRPVASKPGRAACTVILQPIDEENR
HALLESGIEIDGNIYEIEVPQSQESSTLAFDAEEARHQLLNGAIAGLARG
PRRASPVHSCHVKVSLDTSEGSLESPTGGHFSSVARAAVQNTLRDAFDKQ
QIGVLEPIMLTHITCPEATAGTVQHDITAGAGGQVLEVKDRSAESMGDDL
IDVTKIYAPPDPYDSVTSLRGKRSTDRMVEIVAKVPYKEMLDYDDHLRSK
TAGRHSMTMSFDSFARVVGHREKML
>Mb|QLI64591.1
MQFKDTARQALFAAALVAPQLTSAFYLPGVAPTSYKPDDQVPLYVNKISP
VAAMQDYRLHSVVSYDYYHPAFQFCQPNGGPKYVSESLGSILFGDRIMTS
PFDLRMLRNETCKPLCKVSYPEKMREFINDRIYQGYSLNWLVDGLPAGQK
IQDELTGTTFYSPGFSIGDYQFTENEEEDEKLSFNNHYEIWIEYHEVNGN
PNQLRVVGVVVQPSSLAYTGEPDCADNHPKLIFEEGDKNQDVYFSYSVYW
RKSDTAWATRWDKYLHVFDPKIHWFWLIDTAIIVIILVLTVMSILVRTLK
KDISRYNRLDAISLDDLNGTSAIEDGVQEDSGWKLVHGDVFRTPSHPLLL
SVLLGSGAQLFVMTGCTIAFALLGFLSPSNRGSLGTIMIILYTLLGFVGG
YTSARTYKAMQGEQWKVNIALTPVLVPAIVFAAFFLLDLFLWAKQSSGAV
PFTTMLVIVGIWFVISIPLSVAGSWLGFRSPQIEAPVRVNQIPRQIPPVT
TYLRPIPSMLIVGLLPFGAIFVELYFIMSSIWFSRIYYMFGFLFLCYGLM
VVVCAAVTILMTYFLLCAENYHWQWRSFLAAGMSGGYIFLNCLLYLITKV
KLGGLAGIVLYMGYSALISFLFFILAGSIGYFASWWFVTRIYSSIKID
>Mb|QLI64592.1
MASRLALRGAPLRSSTAVVSRAALPAGATSSTTSASFVAVARAPVARFAA
ARCFSQTSRAASSKAQLDSIRKDPAYLDMQKTSDEHPEPWMDFTARHIGP
RDEDIPAMLKQLGSGVESLEAFVAQVIPEDIMLPPQKKIQPKTYSESGVA
ELFKAMHAQNEIHTWMNGGGYYPVEIPGVIKRNILENPAWYTSYTPYQAE

ISQGRLQSLLNFQTMVSDLTGLPVANASLLDEGTAAAEAMTMSLSNLSTS
RAKRPGKTYVVSHLVHNATFEVMQGRAEGFGIRLELMDLSAPDAIQKIQA
LGDDLVGVLAQYPDTNGGVGDFRELVDVAHKQGTLFSVATDLSALTLLTP
PGEWGADVAFGNSQRFGVPLGFGGPHAAFMAVKEASKRRLPGRIIGVSKD
RTGGRALRLALQTREQHIRREKATSNVCTAQALLANMAAMYAIYHGPAQL
KEMAVNNLRHARMVQSAAQHYGLTVPTATLDPQGRVLSDTVTISFDDPKV
CQTLRKELLERGISGGKAWTSSEVVLAVPTTFTLDTYVRIVDAFQAVAKK
NHTDRNLDVANKFWTEGWKPTLDKIVKDIPQVVRRESSYLTHPVFNSYHS
ETEMLRYIYQLQSKDLSLVHSMIPLGSCTMKLNGTTQMELIGHDKSSNIH
PHAPVSSYKGYQRLFSATSAQLAALTGMDATSLQPNSGAQGEFAGLRAIR
KYHEQQPGPKRDICLIPVSAHGTNPASAAMAGMRVVPVKCDTVTGNLDLA
DLEAKCKKHEKELGAFMVTYPSTFGVFEPGVKKACEIVHAHGGQVYMDGA
NMNAQIGLTSPGALGADVCHLNLHKTFCIPHGGGGPGIGPICVKSHLEPF
LPHKDSQTPVSSASFGSASIVPISWSYIATMGDKGLRKATSMALLNANYL
LARLKDHYPILYTNDNGRCAHEFIIDARPFRETAGVEAIDIAKRLQDYGF
HAPTMSWPVPNTLMIEPTESESKQELDRFADALISIRSEIREIEDGKQPR
QGNVLRNSPHTQKDLLVGDGEGKWERPYSREKAAYPLPYLTEKKFWPTVT
RVDDTYGDTNLFCTCPPVEDTTQS
>Mb|QLI64594.1
MAGPGAMYLKFGPDIILCLCPLRVQNKDAQRYLQWLSIPAQTFNVGNYRR
NDAPQPTADFFDTNNAEGERKRRAAAEAAVADMLAWFRTGGVVAILDATN
STKERRKWVSDVCAQHGIEVLFVESKCDDQEIIMANIRDVKTTSPDYRGQ
DPEAAAQDFLNRISNYEKSYKTIDQDGDEDEYTYLKIMNIGKQVIINRIQ
DYLQSRIVYYLMNLHIRPRSVWLSRHGESMYNLDGRIGGDTLLSPRGEQY
ARKLPELVRESVGDDRPLTVWTSTLKRTIATARFLPLHYNQLQWKALDEL
DSGVCDGLTYQEIKDRFPEDFAARDEDKYNYRYRGGESYRDVVIRLEPII
MELERSEDILIVTHQAVLRCIYAYFMKKDQAKSPWMNVPLHTLIKLTPRA
YGTEEVRYEANIPAVSTWRGKGSTAKHENPTPESL
>Mb|QLI64595.1
MAAAKMTKNQMRRAKKKEQKKAQKPESTRDSPQEKVEEKHQAKDVADGAN
HVDSNTNEINSHTAEPPVTIDAPVLDDSLDEDPAFAVYKDIFTKFGASLG
EDEVAKEANAGNQGTVFLGDDDGIPDEEEEDEKPKLSKKKRKELNKLSVA
ELKALVSIPEVVEWHDVSSSDPRVLVQIKAQRNVVPVPTHWSLKREYLSS
KRGIEKPPFRLPQFISDTGITEMRDAVLEKQAEQTLKQKQRERVAPKMGR
LDIDYQKLYDAFFRFQTKPELTRFGEVYYEGKEAEVDYQHFRPGDLTDAT
KEALGMPAGAPPPWLINQQRFGPPPSYPTLKIPGLNAPPPPGGSWGFHPG
GWGKPPVDETNRPLYGGDVFGLAQNGAQPQATQAQLQTGEAVERALWGEL
QPREEESEEEESEEESEQEEDADAGGLETPSGLETPSGYSTNLNEYGSGV
ETSVAGEMDLRKERRGYDTEESSAPRSAYTVLPERQVRAEGFFGSDRAYD
VKAGQRAGMPVLGSEDDSRKRKKPGDVDVALDVDSLDQHDGINRDALRQQ
FEETRRQEGIGAKWAHDDDLTDLIAEESRKRQRTEKERQDKKKESRYRF
>Mb|QLI64596.1
MTRNAPNHTVWRDRWDVAIHHLFWVVCVSAIYLATELTIWGLSFVLASAQ
LQYFGSIVGMIVIFSVMAAAGQVCPSCGDFYHRHIKSKVDFINAQLGIGF
SVPIILLNHVLGIRDIGYIIATSAITNVASWTLVFLLSLSGLLLLTTLQS
YASKHVQGKENKVSAFASRYEVGGTIRRAAAQCYDRPWQYLTSDDSVEKT
EDRKSSRADVKDGAVVNQPVVEAKVQDRDRNLWHILRSNGYVILCLVGII
GIGIPLELVVHDGRALDGFSLWLCWLVAIRLQRTLKSSNLFGSNVRCRHS
LATMVNPVLVTTLLMLGFTRIRGLLKGSRGIAKVLGDFSSGTPLYTIWTA
VASDSLMPDGPTPWFGAGDFALSLLECGIVAWGFKLYECRKQLFSMSGIS
TLLFSTTAAAGNVFLSVMLARALGLQPEESLAFAARSATLALAKPSVKAL
GGNVALNATLVVSNGILGQLMYPVLLDKLSVPTVQPEPQDKAGEGKIDDD
GPVTVAAGTAIGINGAAMGVSYLYEVKSRAAPYAALSMTMFGVMTVVFTT
LEPFKNTALKLASR
>Mb|QLI64597.1
MSSSLKSLALRVPRATSCKSPTAIIRCCSKASMSTATRPAGLTNAAPSQP
SSYAMSMKLHSYSLAASSKSVLRLVPRARTTSPMTITSQARCFASAASSM
SSSGTSSGAGAAASKSTLDWDSFFKLRLRRRRIQLFFSVTTGLLGGAGGA

ILLSTGMAEPVVMQIPLDPFVTLGLMTLACAAMGWLVGPSIGNQVFYLLN
HRLKAQMMSKETEFFARVKKNRVDPTNSSAGNPVPDFYGEKIQSVSGYRQ
WLKDQRAFNKKKTRAFV
>Mb|QLI64599.1
MVRVTDDLALSPDALSLYYASDPLLGHLPVLIFHGPSTTANYTLNTSRVQ
IHILSSAGFQSFPRITISPSSPFYGVVDHLPREIQVDEVFRGLAFGLFKY
FSELPDGVKGYLKNLYPTRGKRPGTASALFGEQHAAALVASMVKSDNAAE
VIASLQDALQTQHINNVDLDFVLPPGAIVPLQPADLEDVPDDEDDILDPT
LRQYGGYTSLVKLFGEPVFLPTNRLRRAPSKPTSLNRGRLFTQDQKYELR
MKLAELVDTEERYVMKLRELVNTIAKDFRESARARPEGSLSPSEEELERL
FPQSADQILQLNSAFVDQLRKIMDETEDGALRDMDQAASTLGSMKSSGSA
RAKDPSGALSMARLFHEWFPKFTQSYQDYIKASQHFPILLNSFLDQQSSF
RQRVNQTGEQTVRSILIEPVQRLPRYSLLIDQIVGSLPMTHPALQPMLKA
RDIITSICSMDDPLPDQPHVANRLRNMVESWPRDLEPHGRLIVAADFIEL
MPPFQGGTSDLDSSGIFLLFADCIIILRKIGFTMTGRDLLREIDKPSAAE
LLISMTNAAGGPASYEFAFTGWHSLADVRFTESEDGSLVWMTSTRDMKDA
HTGEYKISKAPTSRCFLLQEAFEGKATKWGEDVVKARVEARFSETERENP
CWTLRSVRMQDNNLGLHVAVFQEGAHQLVEGRREPAAIRVVVDHEKGTKG
APPGHYGVEIVINVSSNNLKRVSMLTAGLNGKQFEDDIDLEDLLPTMSRR
IIQLLSTQFNVSNTHLTASMVSYYTKTIRGLNFSTRTEKGRSFLAASPVK
LLSSLWNGSASNSPDVVVSGVKSQQQPSLHRANSIHSMFGSIKGKDSRPD
TADGRPANPLLRLEQTFTGFTAALQSRKGTIIGRTLLNRSLVDELSVNDI
YNRFIETPFEYDVASDIGTEVVFVAFEKFLHIAWADQMGPVMTMQAMDTL
LERANKRVPGNFADFVNYLFRDMAPQNRRAFITVIKLLADLLDGCGNDSD
RGALTLAFAELLVTDGEAHNYINLLDRLVEDCDKISEDQSAHQSFILGSF
VSESLVSGYRQGKAHTGSITSNASSLRRKFGLNALLRQNSQDERPSVWRT
LSKHRNPATGDTSSLSRGAHGKLYRAHSIDDNSLPKQLHRRGMSRDRPPI
AGAFDETPQRPTSSRRYDFPLDTIGEPSPTERIVSNTKNPKKKRRSSLSD
LKSLMAAATLDEQPFQPLQSTKQTSGKMNNNPISPKSAPLSKIPVSPSAA
EALRASRQKEKENLADSSKKIVSHVSEHSGGNSSKSPETPSRIYRHSKAL
SVSSIPTLRPSKLASGADYPTRPVSPTRSNAQRLRLHSPQKLRERLQSEK
QFAQEVDSSLKSELSKIGDEMARVNKARSPNSQTDMSGFTASLKELEDRI
GVMMRELREQQATTQKELDTTMKSTEAKVRAIDQLHKEVVAENELLYEKF
NGELGKIIKAVRGKGRDDKEELVAKLKEQGEETARAKKENARLKREMVSL
KTMLRGAD
>Mb|QLI64600.1
MTPVDDGHISLRLKHGIHTIFMFADAQAPMSEITKELLELLSERYPEGLT
TVVSPLKTMAIPANPRLAYGVLNVPNDPSRGWKRIKTGEDESSTPTACGL
KNNSIVAFSFVSGLEDEDVLFEVEWPKDDDEMYDQAG
>Mb|QLI64601.1
MDALRTAIQPITHNLPPPIRDLGVSIIGDKCYKTLLLDVDLENTECLKLA
VSKGLGIGIIAASSIVKVPQILKLVNSKSAEGVSFLSYLLETSAYLITLA
YNVRNGFPFSTFGETALILGQNIIISVLVLNYSGKASMAAVLVAALAGSV
AALFAENVLDMKALSYLQAGAGVLGVASKVPQILAIWQEGGTGQLSAFAV
FNYLAGSLSRIFTTLQEVDDKLILYGFISGFTLNLVLALQMIYYWNAPSQ
KAKGKRKAVPIEAKAKSSSTGTPKKGPTTRRRG
>Mb|QLI64602.1
MDSDVMSDPAFSLGEESDGYVPQKKSKPKAAAKPPAKATGKKLTQTTLKT
TKAPAKRPKPDSDDEDSLGGSSSGFSHTPPSSKKQKKAPPPKKSGGVPLA
EVENDSVLIDVPPTKPTSKSSKTATEMYQKLTQLEHIIKRPDTYIGSVEK
TDQQMWVYNKETSQMVYKNISFVPGFYKIFDEIMVNAADNSQREGSGMTY
MKVNINRATGEISVENNGKGIPIVIHEKEKIYIPEMIFGHLLAGSNFDDN
EKKTVGGRNGYGAKLCNVFSQEFTVECQDSGNGKRYKQTWTDNMQKMQKA
KITSNKSADFVRITFKPDYDRFGMPEGIDDDLEALLYRRVYDMAGTVHGV
KVYLNGEQIKLKDFKAYCDLYAKSIAAERTAEEGGTPTCTVEIDKDKSHP
RWEVAFTVSDGTFQQVSFVNSIATTTGGTHVNYIADQITGSLLKALDKKK
KGHSLKQSHLRNHIFIFINCFIENPAFNSQTKEQMTTKVSQFGSKCVLGD
NFLKAIAKSDAIENIMDFAQKKADKMLSKSDGSKRARVSNAKLVEANLAG

TRRGHECTLILTEGDSAKGLAVSGRAILDPDKIGVFPLRGKLLNVRDASL
DQITKNQEIQNIKQFLGLKHKQVYTDTKSLRYGHLMIMTDQDHDGSHIKG
LLINFLQVQFPSLLKIPQFLQEFITPIVKVWQGPDPKKPLRLKSFFTQPQ
YDEWKESHKAELSRWQSKYFKGLGTSSNEDAQVYFTNLDNHLKEFEVMKS
EEAELFDLVFSKKKADARKEWLGNFIPGTFLDHSMKEITYTDFVSKELIL
FSMADNLRSIPSVIDGLKPGQRKVLYACFKRNLTKDKKVVELAGYVSEHT
AYHHGEASLQQTIIGLAQNFVGSNNINILEPSGNFGSRLAGGSDAASPRY
TFTRLAPFAKKIFSPLDEACLKHQYEDGKRIEPEVYSPILPMVLVNGADG
IGTGWSTTIPNYHPADIVKNLKRRMGRLDEDGAEEKPFETMTPWFRGWKG
NPEAAGPDRYKFNGIASQNDQNPNEILITELPIRMWTDDFKAKLEKVISG
NDGPTWIKDYKEFNDHKTVHFEIMVDEKHLPKILEEGLLERFKLTKQVAT
SNLVAFDTRGQIRKYEKVEDILEEYYVHRLQMYHDRKQYWLQVYDNDLEK
LKNQYRFIKEIIDGELIVGRKKKAVLVSELRDCKYKAFPPGGQKQKTADD
EPDESDNEDDDDDDKATAAGAHDYDYLLSMPIWSLTQERLDKLKDAINKK
KDDIEGLSAKSEKDLWSADLDDFMTEWDAQVALEQELQTKIRRMGRRESK
KIGAGRNRKGKGDDDYEPEKKSKGKGKGKAAAAKAAPKVKTETKSAQRFA
DMFGTKTKKEPKDDVVDLSDNFSDDDFAALSRNKPSSARVTELSQSASQS
QAEEPEVPRPTKRAAASKAKTLFEVSSDSEVEDVEMIGDIGSMVKGIGKP
AGDKSGRLSLYAMNRPESSQGNSGSSSLQKLKTKASKSSMDIDSHDETNY
EMLAKSSPHKIKDEEDAAQDSDDELAVTAASSTTDTGLAAVKKARAKPAT
SKAKAKPETKPRAKAAPKATTLSPAAKAYAAKKKTIGVKKIALEDSEDEV
DEPPSPVSKPKPAARGRPGRAAAAKRPVIVDEDSSLMQDESDDPFEMDDD
>Mb|QLI64603.1
MLRRQLTVFAAFIFFTVCIYSALRISHQETKLVVKHTGNTRSDQSPLKPS
LHEVQKLESTLTLAYEPTTINDARLKPTQAEELQEDWGDATTIHGGPRAK
DVLSSKRHPIQHLMTDARRELEGTTKRQSKDLESAVKEYRRRYEIPPPPH
FDKWFEFARSKNVQLIDEFDTIYNLITPFWGLKPKTIRDRAKEALGNDNA
LIGVAIRNHQISHMEGGMEWQRNATQGMLDGFLKYLPDMDLAFNIHDEPR
VIVPHDDLTRLVKKAKTENMPASNSVKRPTNDFSKTSPELSDKMIFEQVK
HSRFNSFSRQATWTHSRMSCPPESPARILEEDERWDDLSQYGVSELGFVY
NSTAMADICFSPSLSSTYGFFDRPNTYKVVHDLFPIFSQSKISSYNDIIY
PSPWYWYDKVKYKEEEDMPWDEKDSKLYWRGSTTGGFSRDGGWRRQHRQH
FVKKINAGGQAKIVKNQGSQSRPKWAVTEVSRADYSTLTDVSFSHIGQCD
EGDCKAQQAFFNVSSRVNQTDAWGYKYLLDMDGNAFSGRFLAFLQSMSLT
FKFALFREWHNEWLKPWVHYVPLSLQGDDWLETVRFFNESSLGAEDGKNM
AMESREWAAKTMRKEDMEVWFFRLLLEYARVIDDQRAIIGFDMFSSGKKL
>Mb|QLI64604.1
MVNWNVFRILGDLAHLASKGILIFAIHSNRSAEGVSLITQVLYALVFCTR
YLDIFRTTILWNLLFKIVYISSSFYTIGIMQWVYPRSRERELSWKLGGGV
LAAAAVLSPFMMMIFYDQWGFITWMWVFSEILESGCILPQLLLLRQTTVP
TVINSFYLLALGSYRALYILNWFVREFDTTGRKPEAVAVIFGMIQTALYV
DFAWVYYTRQRVKLRGGGIVDADDMRRGWLLRRIFGKHVESADDEESTPA
LGGEGNGARRAARPGWGARGISVSADEGVFDADRHHDQEEGLDGGHIDPD
AKMHDPDELAKALDDDEDEAHPSAGSSKGSNGGNTPSGMRSGDEWRE
>Mb|QLI64605.1
MPPGFSAAVAQLETFVLPERLSGSLSDRAMAKAMVRAWRRHGILQIAMSP
SQQQIYAQANAASRDFFKATPLQKQACVNECSYAGYVASGEEITAGVADY
SEIFTVTKDLHPEDPRVLEKWPCHGPCPWLDNRMKSPMLNYMADMGSSGD
KVLQLIELGLDVPQGSLTKYTDDGWHHMRVLRFPARHRTNGKGKAGRGIG
SHTDYGLLVIAAQDEVGGLFVRPPQQGESFANWKQSVAGMKEDEPGWLYV
PPTPGVFTAFPGDMMQYMTNSFLQSTPHKVGLNVRERFAFAYFHEPNFRA
MVKPLPGFNGSQEPADGIHYGTHFTNMALRNYPTRITTTKLLEEGRCEML
SKERLRDEMV
>Mb|QLI64607.1
MLKCSPVVRAFVRPRRQITHFSRLQRASYQFSRCYTKSAADDGSPGFKLH
YPNPPTTNHSDLDSFIAYARRKGLDANSTVYVGTHYEYKVSSALAKYGFF
LKRIGGSSDYGTDLVGTWTLPWSSPSAPFRVLVQCKAGTQRVGPQHIREL
EGAFVGAPVGWRGTGVLGLLASERAATKGVRDSLGRSKWPMVYMCCSKEG

IVSQMIWNQCAEELGLDGYGVSVRRGDDKDELVLMRNGAVIPLLQEK
>Mb|QLI64608.1
MMKNPLSAPFELESSSFQTPAAVVSDECATAFDLDNRIHYDLTPPGEKAE
FEQEVLPVTDPISVTTQPLVAIIGVGYVGQHLAQVFSRAYNVLGYDISEF
RIHHLKKTFESNDRVSFSSSPSAMSQATHFLISVPTLLLPDKRIDSSFLR
NALSTVAQIARPGSTVIIESSVAVGMTREYLGPLARNRGLFAGMSPERVD
PGRTEPPAHAIPKIISGLDDEVPGSLAAIHELYSRVFDQVTPVSSPEVAE
MTKLYENCQRMVCIAYANEMADACVSHGIDPFEVCGAAATKPFGYITLMP
GLGVGGHCIPVNPYYLFSNNSFPILRAACEKMTRRPGEIAERVVTRLCRR
LPFCRPRILVVGMGFKPGQAHLINSPGLELAKSLVLADKVEVSWVDPLVS
QESIPQIPRFDERDWSVASLEASFELIIVAFRQSGLNFELLGQLRNVEVD
MWCR
>Mb|QLI64609.1
MIPSTIYRCRQSDTVRQLSSPSASRAEKQALDRDVIVSVLEASATRRDAK
SYLQKYTSKKPLSSDLSRASAKDSEAVTAKGDGLEPVNVAIVKLRLPQEL
SPDALYGIARTLSQLRVLGLLALVVVDCGIAADRQTFDNEAFRLCEAIDS
FGSPGAKLADHVFLRRHTNDNPSVVPKFRCGMRVEDLGLLNRALQRGMIV
VVPSLSRQDEISSPIPAEANETTITLAKFLTGLQLDGMNGESIDGVAEYL
RPKRIASVERIILLDPVGGTPMALRPDVSHRFINLEQEYGELIGSLKSGE
RITRIANVQSHIANLSLAKDALSLLPPTSSALITSPHAAANTRSGIPSKL
PIRALGHPPFGFDNMVTTRNQKNPLLHNLLTDRPAFSPSLPLQRIQDETR
GCLQNAESGSATLVKRGMPVTVYPDPRVHPWQPPNPGLPRLRLTDNCIDL
PRLFHLIEDSFNRKLDAQHYLKRVNENLAGIIIAGEYEGCAILTWEHPET
LDEQTAYDSGRFVPYLDKFAVLKNRQGSSGVADIVFNSMHFMMDSFMLDD
EGVRDRIRQAEDFLDPSDPHARSYRSDIILMLQKNQRRLTVNLDHVRDHN
NKLAQSILDQPFDYLLAFNHALKKIVETVPQARPDQTAKDTVYYCAWAGS
FGLNSCNPRTLSSHHLNHMVSLEGIVTRCSLIRPKVVRSVHYNEKAGKFH
FREYQDQTMTNGVTTSSVYPREDDEGNPLVTEYGFCTYRDHQTVSIQEMP
ERAPAGQLPRGVDVILDDDLVDRVKPGDRVQIVGIFRTLGNRNANHNSAL
FKTMILANNVVLLSSKSGGGIATAAITDTDIRNINKVAKKRNLLELLSQS
LAPSIYGHDHIKKAILLMLLGGMEKNLENGTHLRGDINILMIGDPSTAKS
QLLRFVLNTAPLAIATTGRGSSGVGLTAAVTTDKETGERRLEAGAMVMAD
RGVVCIDEFDKMSDVDRVAIHEVMEQQTVTIAKAGIHTSLNARCSVVAAA
NPIFGQYDPHKDPHKNIALPDSLLSRFDLLFVVTDDIEDTRDRQVSEHVL
RMHRYRQAGTEEGAPVREQMSQNLGVSAESQVGPQQPTEVYEKFDAMLHA
GVTRTSGRGSAKKPEILSIPFMKKYIQYAKTRIKPILTQDASDRITEIYV
GLRNDEMEGNQRRTSPLTVRTLETIIRLATAHAKARLSSRVEDQDAAAAE
AILRFALFKEVVVDESRKKRRRTQAVEFASSSEDSSDDGDESEGEAANPR
GARSTRSATRAIHPTTRSRTEVSRAISGDGHGSVSNNGNAAVVDSLSTQH
TTPRRSGRSRGSALDTSQSQTSFASSASASALLSRTQEDSQIEQDLASEA
AGLVIDDAAPIDTRRVAVFRTALGQLLNSDLFEDDSAALDAVISAVNRKV
SSGDGGAFERAEAVKALKKLEEANHIMFADGELVYKI
>Mb|QLI64611.1
MSSMFKKKGGLAFKPKAPVARPRPGASAPTTKTAPPPQPTIDDGSETLPE
TAPEVIEKEPDTHEITHGVPQTAAQKLAKEPEKELPVEPSRRTTRRASQL
ESSSRPQVPKETPAASGTTSTTVPVTEPPKADTPSQSAPSARTRRQSAAK
PPPRTSIETPEQSTSSEEGSTARTTAPPRETQSSSATASEEPTSASRASI
RTRARRPSTTGADATTSAPEGEPSRPKKRQRKTSRQHDGTETPAPRKRKT
STPNATGSRSSSRRARSLTPEDAEDQVVDLQKLKMSDLTRDLRIGKKFSR
HDELRERERKARLKLKLDKDGDTASEAGVDGTQSPAPGSTNDTTTKANSS
TSTPSTAAAAAAAAAATAGPQFRIVDGQIIVDQSSLVMDRHARAAAARAN
EDMETIEENDFTRLITSSSFMNTSKLRGPNVWTDPETELFYRGLRMFGTE
FEMISKMFPGKQRRHIKLKFNREERHNPKLIDAAVMGEKTIRIDIDEYRA
FTGAEFESLETIEAEHRKIEETYEAERQRVADEQAEIMRKKKEELFADED
GEDGGLKKKGKGKKKGKQAIQYGLNGEPITGD
>Mb|QLI64613.1
MSSKRELSDVDEGEVSEPDPKRAKKQTGAMPRHQHQNSSIDPTWGQKYVF
SSLQDATTIPYGEESDFEDDADAMAYLLSVRKEAYDIPHLLVAPKVQIGP

QFPPELQQDDQGHVDRTIYTTGAVDDPRGYYDDGAYIAMPQHDDEDEKAG
QAAHSCGDDERELHEAYFSSVMARYTHLHQLLHTTPPPDAAKRLSSTKET
FAAPFGRDSTTNKTWANIMRNTDPHPLQLALMSKETVIRILRVLIGGTFL
RRGHTLSERTSQWLWGLLARLPDRGELNHTEIGWVRDLGRRAVLLSRSLA
EMAALRDELADGGLGVNEGVDASSSDEDVVGTDDDDAEGVDSPGENDSPA
EDSNQESVARDEADKGDPGADSTLEGNATGRRDEDEEGEIQESETGDVAM
DLGSDSDSDQGGAVDESLEAAKRELLSRLGASGAEQELDTQEDDATLRLR
MNMRATLNMILTVAGEFYGQRDLLEFREPFVGM
>Mb|QLI64618.1
MELDRDTSTSTPATSAIPFEPHQSQPQPQSASPAPDHTQSHSKSESHGAF
SPGQRPKKSSTTCSTCRFRKVRCNGLRPVCSNCQRLGFPCSYDDSDADNW
SLALPRRRVKQACLSCHSRKARCSGHMPACDRCRSQGIECVYRPGKRAKG
TGRGGYGEVRSPRSHDGDDKDRTKDRDQDKHDDSDTPVTDPANTPSTFNH
EIPSIDESFDSLTSRTFDQFFRHVHHIPMYSFLHRASLMEQYNSDKVDKA
LLLALIGITSCLTDMGAGVREYGDRCIDRAEHLIFSDYSRPSTFKVQALV
FMIKHRILSNKYPSAFMLLSMASRFAAALRLNHASPNLCFLAQESRRRLM
WSLYCIDTGISAGYRDFALWRADRIFVSLPCNERNFEFDLPQAAEMLDPA
SNGPDIPPPEDVGSLALHIRILHLRQKIIEFTKDVLVGRTITDTSSLQDP
VLALHKQLDDFAGRLPSSFQFSESSLRLRAYSPRICVFVMIHVWWHQCHC
DLYRMTMAGFREALPEATLQKLDPSFLAHCQRQCVDHSLAMASIFSSMHK
LGARPVADLDLALCAYQCARMLKYAFHGAAHNLGLTVEVVTEKAKVCLHT
IKQCCMGPAAGAIRSDLERLISQGLGSEVSSPQDTMVDSNRTNSAGTDAP
SRHPVLRNIEVTEEPSVISTTQTVACRTNSHPPEPFITTPVTAATAMPMQ
TSLSAQELATPNAWTTAPAFPSMEAAVQAHAEPEPMDLTNASNKSDIGAG
ELNSAYEGALEIFDQDNGLAHAMGVELSPWNWSSDLQWPDFLSSGVGV
>Mb|QLI64619.1
MAAEYATTRRQKKRLIVCCDGTWMNSDYGFSKSSPFSKKGTLQVPSNVTR
ISRCFKRRCADGTLQIISYESGVGSGSNTLDSITGGAFGTGLAERVREVY
AYLCANYMDGDEIFLVGFSRGAFTARSVSGMIANLGLLTREGVEHFYPIF
KDMQNWENEDYVDPFPSVPFRDKPKGAHADVEYRARLEKMGYTRVYQKNN
NLIKVKGVCVWDTVGSLGIPKIHWLEKLGWHDTALSDRIEHAFQALALDE
TRTPFSPAVWERPRREGLRTVDLRQVWFPGNHGNCGGGWDDQGIANATLA
WMMDQMTSIGVEFDTPSLKRVVEQTSDFYESPEAHNQKKDRSKLKQWAVD
PIYDSNKPLRPWALGSIQKVGGVFYALSGDTIRTPGMYKQVDPKTSACRE
SYLQDTNEKVHSSVRVRLACEGLGLNDESVWTCQALSDWKLKRAIFEYDE
EVHHYKPYDRYRDRPSREGWIWKYVGSEKNAPGQRIMPEEPLGPYERYVL
ELSGGSPNVYDYADRRANRSKRRSRSERSRKTLRGASHTY
>Mb|QLI64623.1
MGQDTDAQGHGDGHVALPVRGGFRSHDEQMHAPESADDDFNSLMASSMAT
LLNTASSPVSPGQSSPPLDQVKNSFFVPPARPSSTPFPLPDNRGTRVCCL
DDSDYGTDIEMFNESSSSATENTMAHASRATPFDELPTEVHEAILDHLFG
YCISPTSSSAMRISSLAKCWSGAQRHCRRRELADLALVNQVWRVLVQQRL
YRHVKLKATIESIENAMMHLADKPHLSCHIKHVEVWFPVFQPTFGPLALS
NTLVLPTVTSDGLTNATYTLPANNCTLDEVFRFVSLALPCVQVLSLEGGE
RRKAPKVVCFRHQEHLLSGRALEPVPTVRTLVTRGQWNLMRETGDFHAIL
GALPNLKEWQASYSKPKSKSYITMSEFVPSLPMRITSFNLCLENDYRREG
VMPAFYAKVAMKTHICARMAEVTTRLEHFSYTGRVCHRFFDMAGRLANPM
TTRLRSIDLTVKNCCRHSSSYHDSGSGIQELGFIEAFERLVLSAVRSLDK
FKEVEYLRIRFVDLDSILPPLNPYFLMANGECSGVWSDAIVAELSRVRPR
VQFTELCESFGNISYSKDGRMVIMPEYPRTRISSLKLSNYRSLATRITIQ
>Mb|QLI64624.1
MPQKRRARNAAATSNDTENGGKSKKRRVSLACDACRAAREKCDGARPECR
TCISQQRSCSYTPATKKRGVQTGYLRAIELSLAWIFEQMPECEERLLRFL
SRGDRDATKDHRALLGKGIAGHHLQRLWNQNRVRKAVDGLLSDAQINNSE
DSGNDLDTDGESQANALDATSRRSGESVTVPRMVQDPESAHERRLRLPTN
WKRLLDVYVSYTHCWLPIVNLDPLRALASSYPSQGIRICSNVHDGSYLRH
SELWAVLTIAAYQDGGYTEGAGSRSPNISEIFDVSHTLIPADDGGFDIHS
INAMIIHSVILIGKKKTLAASLLLGKSARLLQRLRLIEDVTTQEEGDFYL

IQHAVVSLACSFLDVLTSVFLHQHPMRGLSHIKGTRQVEAADFADFDQPW
VPVPEISATSTSSTIPPQIAQPIRTLTQLHAFASVLSDHLTSHMSGENLP
GVTGPEVLVKILDSRFNYCNSLISSGSTPMIPSAYLVKLLFLTATIELTS
NPRSSLLSGFLEIVESCFAVFGAGHTPPAVVLLLQIVQRRADADEMGESG
IRKWRSITERLQNIWREREPYAYTLDEPPIYSSEVTEPHNLRQIIFTPIP
TGTDPDAKGHAGSQPRDLGVKALTDHEPAQQFRFGYQDAIPNPAFAIDTP
ITTRLNRGSANTANTHTAMFPGNHRMSQSFDYDAILEDFGSIGYTDNIEM
DTRFMTNLGFAPGCDLAEIFQGDFGV
>Mb|QLI64626.1
MGGPKKRAFDAKNQRPSKKQKRQQTQQYDSGSGSDGDEDQDFDAVNLLDS
DDDIHNATVDDAAESDDADSSSSEDEQSRVKKAKKSSKPAKTAHKDDAES
AEGENEDDDDEESDVDEYDLDSDNATSRRKSKRNDPSAFATSISKILSTK
LSSSKRSDPVLSRSAAAHEASKAAVDSTLEARAKRHLRDQKKKALEKGRV
KDVLEASRDDTTGEVESSTAEILDTERKLRKVAQRGVVKLFNAVRAAQVQ
AAEAEKDARKQGVIGINNREEKVNEMSKKGFLDLISSGGGGLKKGALEEA
>Mb|QLI64627.1
MPNHPKDSPPPTNTMLSVATPIFGFNHHPSVSSPLSSSPIRASSPLSPVD
RNTLPQRQIQSSPIQQPSFKYASRPVRKNPLLRKREDAQESRRLNFLQSV
RQKQDDKAWQRRDIEGQFLKNSLLADIGRLSYDAPALSDTDLEDAMAFKE
DEKLEHNDRHGSERDDMTDEVCHEEEEFEAMIASYEQQNQLGQRPSSPLL
SDEDYDDVFAELIAQEQAYSQPQSSGEQMDMS
>Mb|QLI64630.1
MGKVHGSLARAGKVKSQTPKVEPQEKSKVPKGRALKRLKYTRRFVNVTLT
GGKRKMNPNPGS
>Mb|QLI64631.1
MFAVPGWSVSADALKPETASASGPSGVKSKKRKRNNVESQSITSTNVADL
YEAVVEGKGKLPPSNQGSKDDNNSAKRAKKQSMTTKAAAQDDQPKSSKKD
KKTKNKNRDQNTTTANDARLPEPLDNTSSTKPSKPNTTSTPSAAAPSKLT
PLQASMREKLISARFRHLNETLYTRPSEESFTLFQDSPDMFTEYHEGFRR
QVKVWPENPVDSFLSDIRSRARAKPPTKGRPGPPPSQRNKMALPRTTGTC
TIADLGCGDARLAESLQGDRSKLRLDIKSFDLQSPSALVTRADIANLPLE
DGSVNVAIFCLALMGTNWIDFVEEAYRILHWKGELWVAEIKSRFGPARKS
AVVEHSVGNRKKNSAALGKKAKAARDAEADALHGEDLAVEVDGLEDKRRE
TDVTAFIEALKKRGFVLQGERAEAVDLSNRMFVKMHFIKGASPTKGKGVK
AEDASKMGAAHKKRKGFAPKWDDDEHAENAEDEASILKPCVYKIR
>Mb|QLI64632.1
MASGTARAASRANSYADDRMRSSMPNGSFRSERPDPRAPEASHPEAQYPG
MAHKRTASGNPRPVNRSISTQERRYEERRVTERTFEAHVDRILKRNPSPE
KQHRRSATGERREGEVRKQKSTEFRPRDSQSDASQAPWDPEATLLPHTSA
PLASRISIPPAASTVPQAPQPKSLNELTLEIQEAAIVEDLLFVFMGYEGQ
YIRFAKGYDPTEERDRLTGPGFKISPGLDPSLADLTRTMLKMATYYSALE
AFVDVQSRVEFGAVNHALCASIRKFLQDYLIMIAQLETQFLTSESFTVHV
LNIHTMPTCQMMHQLYLLAHELLKKNGIVDDETEDEDDEIENDDAMIERL
REGGELIPGNMTSKKICKGGVVLGLITMRLERMSGDPAARALLTSLLRDA
SRPYMVMLNEWLHHGSIYDPHSEFVIEERKSINRERLYEDYTDEYWDRRY
TIREKDIPPQLQGVKDKVLLAGKYLNVVRECGGAAASKVVTDVPTTFDDP
RFLENVNNAYAHANKSLMQLLLTTHALPARLQSLKHYFFLDPSDYFSYFL
ELGSSELRKPVKNVNTSKLQSLLDLVLRQPGSIVSLDPFKEDVKVEMNDV
TLIKSLQRVVNIRGIEQGEPLQPVANQHIEKDKNATGFTSLQIDYSVPFP
VSLVISRRTIWRYQSLFRYLLSLRYLESQLSTTWQTQTRGVSWAHKGQNR
ALELWKRRAWTLRARMLVFVQQLLYFCTAEVIEPNWTKFMSRLSAKDGDP
TKPSAPTRTVDELMQDHVDFLDTCLKECMLTNSKLLRIHSKLMQTCTIFA
AYTKWLTQELEKSDPDLSCTTKPKTMTVDQWNRFQATKAASRPQPDSSSA
PTDPESRVNNLSEIIKKWENNFSRHLQILLDALNFYAATETVVLLSLCAR
LSTANQGTEFAGRGQDEDIG
>Mb|QLI64634.1
MTSGPSIIRKSGSNNRSNLELVNSLRDSFSEAKRDYAEGAHPNGYGTGNG
VADGIPNGIPNGISNGNGNGNGNGIRSGTSTPPTSVELWTERDGKVLYIP

RINWQAAGLREEPNQYEITVKMFLLPGTSVEQRVQYIGEALALVSKELGI
QTIDLLVASFPGISFEGTCEWEADKKNANQGNLDEELATWRIFEELHNQG
LAKRLGVAEFGSEKLSAFVKRASVPPVVDQINLHDCCNVPPPLKQLAAVH
GIELNVHRDCTDILPPGTLRELLGSGPRGANVLADAENGGVGLQGNIVPQ
WVVRYMAFVRDRGVIENKGYFAGAELAC
>Mb|QLI64635.1
METPPSPSLSNTPRPRLARTSSVSPQNTTTEPQMPVSIKREEEEDRHPVI
ETIDLFDQGYESSVDAPVTAAADLRLELEQEKRSYPGASTWADEEEKLFE
ILFQRADIPLLPAHWHVDFRGIPVVDSIFSAGDGVPSIIYSRSMEKEFQA
TMALIRLIDLTSSVRTTVQSGLRKKATQLIKRCLDKYIAWAAEDGGYSHL
KIVPNITTAVMDAELGEKDITSTMKNRMRALAMAQREFLRVDRDDSFWDV
SEVRGGEKGADLSTLIVEKYLKRVEELSESAKAPSRNGSRQGTPRPGSAS
RDDTTPTRRRSPPSPISNGLTPDEQQSTRGQIKYRYRPPVVYGLFILRTS
VFLLTVDSAKGDSAYVSFHVDMHFMDRHQSVWNALTAAIAVCLARDQLSE
RTDEFEELSASAEESESEA
>Mb|QLI64639.1
MASASEKPPTTPGSSASDTGEESGPEQCIPPKDDAHLDTWRGWVVVAAAS
MSLFMYMGVIYSWGILQAKMADAKGYSLTTLTFVGSLATSFMVSLCILVG
KLVRKFGYRRTALVGAVFLGLGEFLSSWVVGHLWALFITHGILFGVGGGL
TILPCSTAPLQWFRRYRGLATGIVFGGGSLGAAVMGVAANELVNKVGIEW
TFRILGFMLWAVCLPAAYCIKQPLLSKSAVPQLQWYRWREPKFVLLILGG
AIACFPLFVPPYFIPIFARSIGSSGSTAIIALTIWNLASTFGRVFAGYSA
DAFLGPLNSLNISLLFCGVSALAIWPFVTNMGLLVFFTIINGVGCGSFFS
LFPLAVGTTFGAPNTMGILPILWQAWIFGYFMGTPIAARLYSLAGTGTDV
SLYRPAAYYAGAASFVGIFFMVAIRLMNSKKIFTKV
>Mb|QLI64641.1
MKHEREPNGPVSDPDCQRAVAATDPVDAAPAAEPSLRAEPVSRAVSTPST
PSSPPPPQVVHQPDLHRVPTAILEKMATVPASQVLTGSHDVEAVSRVASA
PYSAFPRRTKIWITTMVTIASVISPMTANVYFPALDSVADDLNVSIALIN
LTLTTYMIFQGLAPTIFGDFGDTAGRRPAFTLAFTIYLFANIGLALQRDY
TALLVLRCLQSAGSSGTLALAYAVVADIASSEERGRYMGLVGAGVNFGPA
VGPVLGGVLSHELGWPSIFWFCAIFVFVWLIPWVLSVPETCRNVVGNGSV
PPPRWNMTVIDLLRPGGVNQRPASAPKVKIRVPNPLRTLSIVFQKEEGLI
LFISAIMYVNFILIGATLSILFKEIYEYDDLEVGLCYLPYGVGCCMVMLL
QGHILDRNFARIAKKLGMATSRKRGEDMLSFPIESARIQPLYPAITVGAA
ALIGWGWSLEAETTVAVPLVLLFIVGMLVPSSFGVLNTLLVDLNEDAPAT
AAAANNLTRCLSGAAATAIVDQMFDAMGRGWTFTLLALLMVACLPGLRVL
EKRGPRWRAERARQKADKQDN
>Mb|QLI64642.1
MMTTALPYMNRQNAMNNRSNGSLSNRRASLSSFPSSRSPSQMSQNSRQAK
SPQFPVAETDRTSDPSSKSKTPSLAPSSQHSSLSAPYRLNADARSSRRLR
SQYPRGSADNHVEYILVASFDIDRGPVMEHQYPVAITGDENMLAELMLPD
QAHARNQDWTIFFLHKDTSQEEEDAERQVKERRRQMRRRRRNRAAGVISE
DDEEEFDDEDDDDEDVDDESTDSEPEGGEGPPLIHVLNLVNTKHDKSVKR
GAVVKAMAICTRHPFLHIYKPLLLLALEEYFKSPVPETLSMLYDAVNAMD
LSLMPRLSILERHLLLASENRDLFVEKFEQMIQIRMAEDKADGLIDGQVE
DSRSPVKSGGISRAGTKAHVEGGSQALYSVPRDTHEFESKVMYKGIPIPI
KVPTAVMPETVGDFSLIKLIQNFSEPHAKSPQAFTLHPHLTTNGAGTHPI
IVLVNALLTQKRIIFLGHNMPSGEVAEAVLAACALASGGLLRGFTRHAFP
YTDLTKIDDLLNVPGFIAGVTNPTFEMHPEWWDILCDLPSGRIKISSKIE
PAAVTEGLLYFQQQNPPLATVASGNANNNQDLTGDGAFMIDILKSIAVRH
GERVIRAKWRDWVTKFTRIAAQFEESVYGASALYIGSEDQDISLPGASGH
GYVWPDDAAKNKELAGNVTRIEGWRNTRSYYSYIQDLAQVYTVRPLKGLD
LAHMHDRLRMQRLSPSQSKDIYLTLSKCIHSYDEICLLLNVAPESHAGLF
YLALGLFHQDREVRLKTSELLSRIAEHEAGQHWWKGLSRFEKLAYERIRR
EINLEMRAKLEKEGLSPSYERRIS
>Mb|QLI64643.1
MSDIEDELLALAGGDSEDEGSARSRGGSESPRRSRNDAGKSKKSKRRARQ

DDSEEEGEASSAPSSPNSLESAPMDESDSDAEPAPRVGGTDEDDKYPVDG
KFTSEAERAEIMAMREVDRERILAERMDEVERQRQRRRLRQMIEETERRN
VKKKRSADTAELEDGQRKTSRQRTGKGNESAMDSLRRARAEKAKRKEDKT
RGRAYSPARRDSDEEESGDDFSRARSRTPEKEQEKEAPPAELKDFERVRL
GRNEFAQVCFTPGFEPAITGCYIRIALGPHPETGVEQYRMAVIKGFTTSR
PYALTGPQGPFVTDQYVRAAHGKAVKEFPFIAASSGKFTDNELNRYQVTC
TNENVKLPTKAYLADKIDEINNLINHKWTSEEIKARLAKRNELKRRFDPA
ERERVGKLLDEAVARGNETKAQELQEELEQLGTQRLAFRTTLGSSKNLDG
PRAATEQDRLAERNRENRRMNAELVRKAQLKEKARAREIEMALKRGEQVA
DDPSRRLRTKAKFVHDVNDTPQKLSANGSEASTPANGTPKVAATKPEMLS
HLAKLQEKNYSESNGVPGIHKPLMDDDVIGALDLDIDVEI
>Mb|QLI64644.1
MASSQLRSIGAFARQLRLPRRAFTTTTRQLEAAVPATSRTETALAEESQP
KPIEQAPNRVDVWSRSQKPRSNAMTGPRFEQTDFDLQPQPLSAMEMIHRE
PVRWTHDRIVACDGGGGPAGHPRIFINTDKPEIATCNYCGVPYANEHHRK
HLESLPKTSYPLS
>Mb|QLI64646.1
MAAAAEPRQFGDAIVAFSLERVFPEDDTSLLPVSDIDLEPAIRALTGAKA
DLETEIHTINEETRDDVSSWVQNAKTLQEDIIRSKTIANDIIRQSEAPDV
SGEAIEDAEDKAQFLNRELQYSRQLHEVLNNIKTANQLLGQVDTARSERR
VVDSLHLLEQSWTAIDRIGVSKSCRILRRLDHRAFELKSSVHEVFNNVWK
ELIQFNVEERRVSIRESLPEGGMTLSDAVIGLKAYKETDERMEQLWRNLD
GSLISPRMDKTKQPAPSVAAKGDELSLTGSSDGSVAALLSDLELVLVFVA
KKVPMELLDSLVNFMMPDLIPRLIQQWLNPAVPSSLSEIPMFQSIIQVAR
TFCTALETNGYTGFDELKEWVSKSHMTWLGRCRESALDKIRTKLSNGIGK
PKQVEKIEKHMVTVAEGKELATTGAGAAADTNDWGAAWDDAWNDQTQEPE
PPTRAKEDDGADAWGWEDDDSPQQAAGDAKESEVEDDAGADAWGWGDEET
SAEPEVSKPSPKKQKMESSPEKTRELVLKETYYISCMPEPVLGLIMTVLE
DGAALTADGEKYQLVASTAPGLFSLPTFALALFRAISPYYYSLNVGGNMY
LYNDAMYIAEQLTKFSEAWKKREDLAPRARNMLRLENDIKTLQNFANRSY
ASEMSIQRTVLQDLVGSSQSIILQDEPEAAIESGMARIRYMATTWENILA
RSVWSQAIGSLVDALANRIITDVLDMPSIGQDEAYNIANLIATTTSLDDL
FLPSKLKGTEPTRNEAATTDQYAPNWPRLKYLGEVLQSDLNGIRALWCEQ
KLSYYFTVDEIVDLIKASFEENPRTRQTIKEIQNTQYPAVDL
>Mb|QLI64647.1
MRYSGRFAALAGTVLFSAGIAHAGYLLDTTYDSTNFFNSFDFFTLPDPTG
GFVQYVDGQTANQEGLAVYAKGGIYMGVDSKTKNPPNGRKSVRVSSKKSF
TRGLFIADIAHMPGSICGTWPAFWLLGPGWPSSGEIDILEGVNAQTKNSI
TLHTANGCTMSNQGALPSTQFSSADCGALGASKGCQQATVDASNYGDGFN
AIGGGVYATEWTSDHIAVWFFPRSNIPQDITSQNPNPASWGAPTARFVGG
NTCNINNFFKDHQIIFDTTFCGQWAGDVWKNDAKCSTLAPTCQEYVSGNP
EQFANAFWVVNSVKVYQQGGSPGPVQHFAAVNPSSGSVGPQDQNHPQPQT
QVQPQSTQPATQPQPTNGQVWNGQPQPNSDTNSQPPPPKQDNSWNGQVWN
GGGWNSKRRNRAVEKRFSA
>Mb|QLI64648.1
MDVEMTAGDAGQQQSLTQQSESSAKASPPAANNAGSNAANQMSFRRQRAS
RACETCHARKVRCDAASLGVPCTNCVAFQIECRIPTPKRKKAQNAGNQTT
KDSDSDREADDRPHQTTPGSASSYPRPPTVSHTSDGTPSSSMTEAQVRQE
EVDSGTYLNLVMKPKFTRAPITEAGRVAFLGESSNLTLLVHDRQGSSDVV
HYPLPENVRGSRARLTELDNVEIEILHQRGAFLLPPRSLCDELIEAYFKW
VHPIVPVINRSRFMRQYRDPKNPPSLLLLQAVLLAGSRVCTNAQLMDANG
STTPAALTFYKRAKALYDANYEDDRVTIVQSLLLMGWYWEGPEDVTKNVF
YWSRVATIVAQGSGMHRSVEQSQLSKSDKRLWKRIWWTLFTRDRSVAVAL
GRPVHINLDDSDVEMLTEDDFIEDEMDRASEFPPDPIHVQFFLQYVKLCE
IMGLVLSQQYSVASKGRQRNAIDLTHSDMALADWLQNCPKIVYWEMPRHH
FWSALLHSNYYTTLCLLHRAHMPPSGSRSFPDDSPYPSRNIAFQAAAMIT
SIIENLATHKELRYCPAFIVYSLFSALIMHVYQMRSPVPSIQQVTQDRMR
SCMQALKEVSRVWLVGKMVYALFESIIGNKMLEERLQKAGGKRHRKMQQS

LSQLEQQSKVQDVSKRKYDDMAIDFTANAPTPQESYERSRPQTPSAIKTE
NTSAMQPPAVASPNSRPSVADTFMGGTNSRPQTRPATPFNPSFSVPATPP
DLYLVTRNSPNLSQSLWENFQPDQLFPESSSMPAFPNLSPTQTHQEPNHN
LMAQMTPNSMSQGQASNQFQATGQGQGNGGVPTQGFHASAGMWHTNFDGG
VHDGQSPDSWSTSSVQGQPAPTTLNVEDWFQFFGINGDASNLNLDVSLS
>Mb|QLI64649.1
MALQSKDTTSLTASYKSPENEHFTITESIPTSPSTSVQDKTHFLEALRKA
VTDTQEQINKELTARMEEDKAREAASGATIAARLGADEDKEEENYGEEVQ
EEED
>Mb|QLI64651.1
MALSAQEISQLRSALQDAAVKCSERCLYQSAKWAAELLNALPETDTVDDA
TPADGSNYTSPILTPNADPEEAALETKELSKYLLAKSLFDCKEFDRCAAV
FLPESLLSSVLSSPSETTVSTPSQRSKGKAKAVEASPASGAAPLPRLSQK
SLFLALYAKFMSGEKRKNEESEMVMGPQDLGTVVNKQLLVVGRYLAAWFK
ERTTTDDEVLGSQGWLEYLYGMVLAKEKNDAQAMDYFVRSVHKYPMNWGC
WLEMTSIISRVEELNQIARHCPQNIVSFMFHLHTSLELYQQTPNLANSLA
QLLSIFPTSSFLLTCNALLAYHAKDLMAAEHHFSRLLSLHPHRLDSLDHY
SNILYVLNMRPKLAFVAHLCSSVDKFRPESCVVIGNYYSLLSMHEKAVQY
FRRALTLDRSCLSAWTLMGHEYVELKNTHAAIESYRRAVDVNRRDYRAWY
GLGQTYEMLEMHTYSLWYYKKAAGLRPWDGKMWMAVGSCLQKMGRERDGI
KALKRALLADAYYDVGSSFGSGGDLLGARGATGHMDPEILLQIAAMYDQL
GEEEEAKAYMELCVAQEEGGAADANNLGESSVMIHTDSPASEDYADREDG
SAGADGGGEGTGVTAATSKARMWLARFAMRTSDYTTANRLATELCQDGVE
VEEAKALVREIRSRMEASGMFGSDS
>Mb|QLI64652.1
MLRTTFSKTAAFRPLRATFTTTARAMSEGDTGAPPKTGGPGPLTDWGDWF
LYSDAFQRRERASEDWAIRQREKERLLELKKKLAEQQQHLQRLSDHIEEI
TRDQGGEKN
>Mb|QLI64654.1
MEDRRELRRMPRGERCPECGSQKWYLQDGLRFCARGHQIEGFIQFDVGDE
EDAGKMGAVARREKEKREVEKRQLTGQEGRSLFLEAVQLLLRKQVSFLIR
SKGHREELETVVRDLWDLRIRGYTSNSTETNLANTELEMFSSQSTRTEEG
TNVSWHSRSRTQNWDPERGTDWPMPRMTETIVLCYLGCLLLRIPTRLADL
YAWVSNGSMPYLSAFQELPREIQERMPSSYANMMKLCSRSGLNGEDLYRT
VIDTVLSYRLNYDMTFPELNYLPMAVQYAKHLCLPVEAIAVAIRLASVLD
LMFYFPVSKAKLYFLDNPEVQLISLLVVATKMCFPLDPSQTSLLDPAGCC
LPQFNWASWKTGFSSDGPKSGKETQEQVDFEHITPNQVTKLSDEKFDAYV
AHLSESIGHGSYNEIVQFFPAETVAVSSPPQFSTPEEKIEGRARRILAQA
VKPNNMDGRRREVTYEAFRTVEDISDISYAFYKEAGNAAGLSTSTMTRAI
YMLEQRIVSWQKKHRKEI
>Mb|QLI64655.1
MSAISSIVSAATATLSSPTATSTLRASNQGGIIDGANPTKYNPKDPIILF
IIQTVLIVTVCHLLHWPLSKIRQPRVIAEVVGGIILGPSVMGRIPGFRQA
IFPDASLPNLSNVANLGLILYLFLIGLETDVRFLISNWRIATSVAVAGLA
LPFGVGCALAYGVYHAFRSDEGLVPIKFEVYMLFIGIAVAITAFPVLCRI
LTELKLLDTNVGVITLSAGVANDVVGWILLALCVALVNAGSGLNSLWILL
SATGFMLLLLIVVKPSLLWLLRRNGSLEHGPSQSIISLILLIALVSAFFT
GIIGIHPIFGGFMAGLIIPRQDRFNIRVTEKLEDLIGALFLPLYFTLSGL
NTNLGLLDSGAAWGYVFATTFAAFFSKIIGASVAARFSGLVWRESFSIGV
LMSCKGLVELIVLNIGLQANILSTRTFTIFVVMALLTTFATTPIVTVLYP
PWYQRKIAAWRRGEIDWDTGAPISSASSSEGPKDDSSLGLERVNRLLVYL
RLDNMPSLLRLLSMFGDPSTPSSTPTPSDTGTDEIHDVSTTPPSRDRAVW
AHGIRLLQLTDRDSSVMTVAQVDDFSRHDPVVNTFRTVGQLHNLSASGEV
AIMPETRFAEALVAKSYSMASDLLLVPWTETGDMGDSQILSSTSVNDKIG
SSYGSFVKSVFDSHDRNVAVFFSRSDPVAGGKAKSEERAKLMRQYSFGAL
KEDLPIAPGSNEPYHILLLYFGDEDDRLALHLVLQLCEKSRATATILRAR
SSVGASNSVGDALFERASSQLPASVASRVNFKTETGPSTVEELVQYAITE
TQSGVTESKAGPELVVVGRRVAAKLDEGKLTHRSREDLVLCLGNLAGNLV

AGGVRADLLVVQAKAAENK
>Mb|QLI64656.1
MSIDLNWETLTSGPDGDALAERIRSFIHDKFQTVPLPRFIRSVTVHGFDF
GTIPPELELKDITDPLPDFYEQELGEDESDDDFGSDEEAAAHLAAERARH
ALAGNRKRPSGIRTALRSDFAGHDLGSPFLGTSTPGILGGPGLNYLQGHL
GTGTHTPLAAVAGAHLGNSWMAPGALDSSDFAHAASHHRNPSQSSISVDN
FSVNLEGTSPPLREKSSVSTLAPTAVSMSRPPTRDTHLVDSAVDEGDDGR
HAALDASAVDDGLAAHRREPRVEDVQAVFRICYAGDIRLNLTAEILLDYP
MPSFVGIPLKLNITGLSFDGVGVSAKIRKRVHFCFLSPEDALAAVGSVDG
GEGGESSRGEQGARKNTRFGGLLQEIKVESEIGEREGGKQSLKNVGKVER
FVLEQVRRIFEEEFVYPSFWTFLV
>Mb|QLI64657.1
MASHEARIHLQEEAIPRSSRPSGRRGGRGGRPEGREVQLSKALSRLLRHQ
AENAGIKLDEEGFAPLDRVLSWGPIKSLSPSLQEVKDVVETNAKKRFTLR
PADPLAPEAPSAAGYLIRANQGHSLKVDEAALFEPILLGAADLPGKVVHG
TYFAFWKAILESGGLKPMSRGHIHCSDRTPEEGAVSGMRRDAELLVEIDI
EASLKDGVTWWRSDNGVILTDGGEGGVLETKYFKMVTSRTADLGVLWEDG
VKIADLPADLKFRAPEGKRPGGRRGGGEGGGGRHRRS
>Mb|QLI64658.1
MSDEVSQFLEQVERLRGQQIEDDEARAREREEFLAAKRERQARREERARS
ISPQKSSPMNTPSPRSSHQSAQVSDATRLGSPSRTLDDREPPASPPAGHD
SMTSPGLPAKDSDASAELDTQPTESSTPPARTSTLSWQRRPASRGGARPL
SMVAAQNATQRSLAGSQEPASATEETVSKDQIAKALGSKDPSWFRQTADR
GQNSAAYRRSQVEDEDRLDMSSVRAQLPGMSLTNPVSSSRSSPSPQPSPE
PPSHPGLASPVTFNPPRFDTASAEKQPTQEPASLSGRASPTRSPSPTKGL
GGFVQSAMMKRSDSVKRWSVTSPPGLTRADSVAATRGARSSVHMGSRPQS
TVRRDSATPTSSRPTSQHGDRPSDLGDISKSSSLMSIDTGRAEEKDEDAG
IPSSPSKTMDPRRWSPTKSSWLENALNRPESPKPSPTTAQPTWRNNANAE
SSRPKSVSHKHQVSIGGLMRQSAVGNVAKSNPTGLGGIYSPPPNANRPAY
GQGSKPSTAQQSDPDLPRPDEMTDKEDTKPTDKETSSPSATKHTPVMSPP
PLKPKPQTPPTMDFRSNLRQRPAEKDTPKSGEPEFKNVFGNLRKTKTQNY
VAPDELKNNILRGKSALNVTDGPKKSERKDEFKDAILKKRADFKAVQSEG
RGVTRASSTASERPLPEGLAKRVELSKSVAAMAKKSNVPETPTPTPTKKE
DSPKPAPAPKRVPSGSAFSPRSPNTAIPPARSPSTEKLRRVSTEPEAAKT
TVEPQATPTLQKEMNAPSKLAAQSRVVGGKLADRFNPGLAGMLARGPPPM
ATNGGRSSEETGDDNGKPGSGDATEPSAPGPQLTHMTKGRARGPKRKAPT
GAAASTLSQTARQLPPAIVEAAKEDEPTVQEVAINKLGTQHVSTGPAGLD
EKPITPTIQQEVDTKTTLGNTPSLPPVTLVAKTSEPRESLEPAARPVSRG
RPLPLGPRASTPSSPAKPSPQPEEDASSATSPKKFDTKKMSRFLDESPSK
DVQRDTAAEPTWLRHKRTGSQSPTKLAEPPSPTKSDRDSLPSMREASPRS
PRFGARSIPISKPSFDEPVTPGGGQSRRVRPLSGLSGDGLKSPAAISSPT
RSPTKQINEVSALLTEFFGPPQTREAINVDPAQILMNRPDTGDKVTSLGL
QMFQILGDGKKLPVSPQNERVLFEQEMYVCAHNFINGSGKKVFEVYFWVG
DEVPELSAEDAQLFVQREARSLGGKLVRFQQGKETAEFVQALGGVIIVRR
GSSNKYDSLAPNILCGRRYLGQVAFDEVDFAPSSLCTGFTYLITKGGNCY
MWKGKGGDVAEVSCARLIGMDLTLTGELIEYEDGNEPTSFWDLFGDNSSK
PHSADHWRLKPNYDKYCSRLFCSDADSRQQIFELSPFNQVDLLPTSIYVL
DAFFEMYIIVGSRANAQYASFRNALDFAQEYAILASGMEDRPFVPVSTVV
LEGIPRDLKRVFRKWDDGRSPTVTNASVGLKRGRSLRIVTLTQALQALRE
>Mb|QLI64660.1
MARRRTRAKEPNPVEAEEHDPMQDDIPEPEPEPASASASEAEPDHVPAGD
DDQDQDDDVEQRSLEFDDELSWRPAKPIPSATLLDRLERLSKELADFDQG
GVQLDSLKHVATSLAHRNLLQHKDRGVKAYTACCLVDILRLFVPEAPFTD
DQLKMIFTLFIKDILPALFDPTNPYNSQHKYVLMSLSDVKSILLLSEIHG
ADDLLLRLFNSTFDGVSTSSRASNEEQVAKDVEIGLTEMLMEVIDESPGS
IPATVIDAIISQFLRAAPPGGGRSKEQNGKQATLLHKTEPAAYVMAKNIC
NGCADKMSRYVSQYFSDVILNASRFATKSNGFRHAEESDEEDGHSGPSEA
DLKSLRQAHMLIRELWRAAPTILQNVVPQLDAELSADNVHLRIIATEAFG

DMISGIGAAGPPAPPTLDPAAYPPLRLMDDAIGTPPVIDTETNALTRPYS
PQSFAQTHHATYRNFVGRKNDKAGTIRAAWVTAAGYILSTSAGGIGLSRE
DETELIKGLVDKLNDSEEKVRLAAVKAIELFTFRDVILKLGVTGGVEKEG
SIFASLADRCRDRKPAVRVDAMVLLGKLWAVGAGEISDGQEAVTACLSGV
PSRIINAFYANDPDLNVLLDRVLFECLVPLKYPLVKGKGGKSAAGSQGKG
GVTAADQDRIRAERILLMLKSLDVPAQKAFFAMQARQPQFAKGVGIFIQQ
CEAYNGGVIDGNEDKVKTSLSKTFQWLGGFFPEPLKVRGDLQKFAKLNDR
RSYQLVKYAIESESDFKTVRRAISELITKLQGTPAVVCLETLIPLLYRSS
CLMFNRSHLATIMDYSKSDKDGFAAVAHLVLNDISQRNPDIFKAHAEDLR
REIIEHVPSDKKINDPTVVDILKAYSSYAKKYPKDINYDRSFTQSLMSYA
QYASPPRAAKYAVNILLAKGDDKSKVTATNLLRNIMKGLEYGSSHFLARL
ASISQLERLVPSVTADSDEAIRDLTINQILRQVRTDATEKDPSWVDDADM
SEELQAKCLSLRILVNQALAAEREPDAEDRVKVVFKLLKTFVVTEGEFCK
TKDTPLHHKKRLRLLAGLLMLKLCTVKAYDEKLEPTSFNKMAELVQDSEL
QVRRHFMEKLQNYLTREKLRPRFHTILFLVAFEPASDLKNRVETWIRSRA
RYYEEKKQRVMEALMGRLIPLLAHHPDYSAEMDDLVDFASYFLFYLNAIA
TEDNISLIYRYTERVKQVQDGINPKASDNLYVLSDLASAVIRKYQERKGW
IFQAHPDKVGVPSGLYTALPSAEVAQQIAKKLYIPDEIDERLDDLLRAMD
RKKKRKSIHEHGDPPAKRPRTQVKTVIREKSKANSSKISSSKKKSAKPKK
APKPKKHKEEDEVAPSERRRSGRAHKVSDYKERADEDDEEEMLEGVAEWD
YGEENSDVEESGSDEGSELSDDDDDDAESADEEEEAEEEAEGEGEEEHEE
EAPAKRKGSKVASSPKGKASASVKASVLAKLDRTSRARGRGGRATRDDSD
MEVDE
>Mb|QLI64661.1
MSRTVDKLPPPKTRAQVKVIVATASRTGTRSVYEAMTILGYKTFHMKECL
YQGGVLHMEILAEAIRAQYSPSSGVKRYSRPDFDKWLAEYDCLVEIPYFL
GPGLLKAYADDPNIKFILSDRDPDKWVTSVNKTVGEVITKMNHFPLNVLQ
YFSRDLHKFLTLNKVIYGAIADGTVPGDRDNKAAMRRNYVS
>Mb|QLI64663.1
MYMGTIPEHFAQIVSRHGDRAAVITRSPTPAAFETTLTYYMLDLLSNRLA
SSLASLGVKKGDRVAISQGNTPEFAALTYACFKLGAVLVPLNPSFNAAQV
EAALKHLAAEVLIIGAVTDLAYKPGRGRSNEELLTSIAGDLRASAPQSEG
VPSLKRIVVVDNRVSHQDVRFKLDECRALTPYRALLDGSDRSVVPSAPLD
ATDTINIQFTSGTTSTPKAAMLSHRSILNNGIFIAHRMGLEPADRIVVPP
PLFHCFGCILGYMATATTGSAILFPSPAFDPVATLRMCADHDATGLYGVS
TMLVAVLEALDAGAVASPPAHLRKGIVAGSSVPEALMKTIRRRLGLRDLV
ICYGMTETSPVSCMTSPADPPDRRTASVGTPMPHTAVKIVDPRDRARLAP
LNTPGELAAAGYLVMKGYFGDDARTADVRVAEPRLDGSPGLTTWMYSGDE
AQMDEQGFVTITGRIKDLIIRGGENIHPLEVENCLFQLDGVMEVSVVGVP
DERLGETVAAFIVPHRGWKGDDGQHAGEGDRVLKKDAVREWVRSKLSSHL
VPKHVFWVDDYPKTASGKIQKFKLRDMAVKILQGK
>Mb|QLI64664.1
MAPGTRRANRSGYAEHDDFEGLPVRQWRQEWVNVAPPLPQEPMQQNDVWA
IELFHGMPKDSALLAPHSQELLRAARSGQLYKRPAPAEEEEAEAEIPQTA
EKNEKKEEETTVKGFSIKLWKQIPRNNEGSGLSHLAKRRKGTVTIASRTI
EDKATGPTVTRATVRRIDAAGNPYTEEVTLAEGQQVVGEIISTRVEAAPA
PAAEAFVAPVPPPRRRPPPPKRKSKAGPGRGKKKIKNPLPGEGQPAGVAP
IGGGTATVVKIEGQGENGIAQEGASTPNPDSEMADDDDDDDEGDDGEEGE
DEDQEDVDHQGDNADDSKIQDEEMTDVVEEAKLPTTSKPVVDAMDGITQT
EVVPPLTLAPALASLATSSPRPEGSPLKNVMMQSPTEVQAQEALPPAVEP
APVSGLEPAAIPGMTSAHSPTMEQSQTTDAESKVESTIVEPASTVAGDED
KAPAHVDVPAGDAPAGEAPEIQRLAEQEQTETRPNAPSPPKTASPDVALR
EEALLPPPPEEVGNISSPKADDGQDRVSDSEDKSRESHQSDFASVSDRPP
LHPQDSVMTEDTIKPEDSASVRFPLSESGAPSEAGTASMEDTKDAAVAAA
EPIEELPKEPSPPKDVSPVAESIVDTPLVNTQTEPEKPKPEEVEVSTEDA
PKEASSPRPDPIITDAPEPEVSRPVPEPEPIPEAKEPTPPAPVPEVSHEE
PVSAPEEPKEMSHEEAAAQEADVPMEEAKAEEPAVAQTEPIEEDARDEVK
DEPTKEPTVEPALEPTPVDIQAEAPKAPTPPAAEPPTADAIPEPQPADGE

ERKDNQPANE
>Mb|QLI64665.1
MKGYAEEEEMLEIGTFQRRDQYHPYEQQEPSIEEFPPRPARGTPRGPASN
AYSQPNSFYPDEFASPSPGWPPSIAGRSESQTLVDSAESRSSVRSWRTGQ
QTPVDSTGAPRYSVPFHNDSSPSTPMNQSHISLTGLLKQAQPPAGGKDWV
DRESIDIVHKRDDSDIWRGWRRHLFRLVPLLTFANTGFYIAYLALRIACV
VWAQNAFETTYSAAWIFIAVEIAVAIPSLMHNSWTMMSMKKRYRPKLRLR
GYDVPTVDVFVTCCGEDDDLVMDTVRGACDVDYPYDKFRVIVLDDGKSET
LEAAVTRLGMSYPNVYYMARVKVPGQPHHFKAGNLNYGLEQTHLLPGGAG
QYMAALDADMIPERDWLRAILPHLLIDNKMALACPPQLFYNTPPSDPLSQ
SLDFFVHVIEPIKDALGVAWCTGSGYVVRREALDEIGNFPLGSLAEDVAT
STLMLGKGWKTAFIHEPLQFGTVPEDFGSHLKQRTRWAIGTVDTAFKLKF
CLWGEKIRQMTLAQRFSGFLYAVLSLYTILVSISLFAIPIILVMGKPLVA
YASEEQLRWLIRACFASTISNRLCEFVLFIPAGYHTGQRGSRYQLWMSPY
IALCLIRSFMLPKWLGGQVQAFKPTGSLSSALNERDPALKKNMLRRVWTI
LINYMGIFHLLFVYFTLVGVVLTSYRCFVRTDSTRSLLLCLVTHAFWPPL
TFIFICSSLWTPVAYAIDPPQMPEREQLLDRDPKTMVAHPTPKSKKIAFG
GQAAWFEFELTTSTLYTTLIFVVSFIF
>Mb|QLI64670.1
MNKIRAIQELNRREIENGITPEGSWHVDYRDTAFIYFGGLPYDLTEGDII
TIFSQFGEPVFLKLARDKETGKSKGFGWLKYEDQRSTDLAVDNLGGADIS
GRMISVDHARYKPRDDEDPEEFNVSWEAMMRREGKLKDEDEESEEEEVAR
PMLPEERELAILMRDHDDDDPMKGFLVEEKKREVEEARSKADRKERHGDK
HRRRHRRRDESPEERDKRHRERSRERESKRPRDDSRDRERRRRRDHDEDR
DKRRSRRDRDDDDDEREWRRDRERDRPKHDRNRSQERTERHRDDEKRRRR
SRSRSPRRHED
>Mb|QLI64673.1
MDEPEQCIICLDPLPHPSSLSSPPSPSPSHVNGAGIKPAPETTAESDPVA
DEPNHLNIVAALDGCEHIIHDTCIRSWAQKTNTCPICRTPFHCVRVYNGV
DGTAISTYDVIDKKQVAEFDVQAWLGENIVDQEEEECNPCPICNSAERED
ILLLCDSCDAAYHTHCIGLDHIPEGDWYCMECAHLFQLTQDQPERTDAEP
ESPRPQLVQRPHPRDVRGYHVRTRQRLRRARRQARNAEWQGAWGQFSGRF
YEMSDLDLDNHDDEDEDLEQFRRFQQLDRRELDRWRQRMDIANRLGARET
FASNIPPQISERLQPPPPPVEETRDERRAWGAFDRAREAEVTASGTRKRK
SRSVTASPAEPVQEPERKLKRPRTRRLPTQAEASASASAPSPSTSGPSSA
RPTTSTAATTTGTSGINGTNGINGTNGSSLRNGLTRMESATPLVSSLLKE
LEPNPLSEDETPVLTPNRRVPPEASSPALSPSPSNHSSPRALSLTPPPLP
SLNGRPTSPTLSLSTHIEPRYPPANYSPTRSNSDHGDSDSRSAKVDPRPL
ELRQPRPRRAHQVPQTEEGSPTRWTMTQEEKKSINDIVKSALRPHWRAQK
LTTEQYATINRDISRKLYDEVKGAASLNEESRRTWEKRATQEVAQAVAEL
SA
>Mb|QLI64674.1
MQATQKGPKACTTCAKAKARCIPRADGGEKCERLKKDCFSRPPAPPRVKK
RPKRSRVAELEKRLNELSSQFEGAQPASGQSVSAASPPQQFTKASDKTDM
YSFEHLFPSPSPTGDDGYEATAWSPEVMKEFDSPWPLPAESEMLLMVYRE
MFAEYFPFVLIPRELSSADLRLQRPFLWKAVMVSATIFDATRQVKLGEEL
LADIGKASIVDGTRSLDMLQAVELLIGWWYFALKSSQVTNLLFLARSMCV
NLTAMSYGSQGEDAKYGPLDHLRAYAGAYYLNTIIFTTNKKTDVLMSTTQ
LDHCCKVIENSMEYPSDEYLIKLVKIQQLAQTISITVSSDGMAPMSLPLV
MVVQSFQDQLDTFRASLSPRLAQNPTLLCHIHVAEILLKDMAISDQRCSP
DNMQVSDRLQLLWSCVKSLSDFFEIRFAEPEIERPRFLCIIASDLAYTLI
TGIKLLTLQLPGWDVASITKELDMVVIFGKQIDHLMEIMAKRKSGLLSPD
RCGIEDPLERLVRLMRTAQELVSMHVNGGSPLQMVDDMGAVAWRDIMNEA
PWGMEDSQGLIDLAS
>Mb|QLI64675.1
MAPLILHNVPDEECYVGDDGIKRPYAMYFNQNDAHTGTSRSRRSVAETGS
FGKSTRRSRSRTGTPARSRENPTLAAADKLFGDWVSNQAAVAAPSNNQRK
SSGLMQDDAPPQRVVKSIPVEVILRGYRSSVQQYAAISHYEELAGAILED

YPRDPPASQRRYKSELRDPALTRRRKLSPEERALVNRADGGEHWVKVTFE
STEAANDAIYASPQRILGYLVFAEQYRGIPPAKDEACPDVDSIMDSEFER
AQSMPATMGTPRRKSTNNNSLPPMLHSRLIDMSPSESKSSSQTLDTATLT
PSHTSSATMQEAPGVSSGVEPMELQEEDGIFCRRIPTARRARLLPAEQAL
LPQQSVMQRFVNAVPFIKWFSGSMIGNEVPRTETGEFDWGRASLYWKIIW
WLDATFGLFRGDVYSVDKDD
>Mb|QLI64678.1
MGTAANHETFAVNSASGSRAVEHGTINRRSPRQAILIDAQRYSLAGQWDI
SRPTYTRNTRNSGNMEVLLGITGKDFTIIAASKAAMRGATILKADDDKTR
ALNKHTLMAFSGEAGDTVQFAEYIQRNAQLYSMRNETDLSPSGLAHFVRG
ELATSLRSRHPYNVNLLMGGVNPITGKPHLYWLDYLASLAEVPYAAHGYA
QYYCLSILDKHHHPDITLGQGIKLLTMCTDELKRRLPIDFKGMVVKAIKA
DGIVDIVLDDDKVVKSA
>Mb|QLI64680.1
MASSAQPHDHDMYQQGPEPFPDIELQHTNRNDRDSTHEKPPLPSLDTLNS
LQHQESTGIETVALSPSVQRADASRHIDDLELLRAERLISNQEHEATKTA
SKNRAHNVEPEDAFNPTVTKEEHVRKRNEDAALYKFWLFLKKFPRFVRYL
VYLFPGAALLLIPVLLGAFRYNDGSNLVGGDGGVELMWFGIWLEIVWASL
WVSRMLTSLLPHIFQGVATMLGSTTTKKWRDIGAQLELHTALFLWFLAIL
ISFKPTNNGHRRGTPDKDSSGNDWVDIVNKVIISLFVLFTLNFVEKILIQ
WIATSFHQRTYSTRIENNKGDIRQLVQLFEFAKAKLEDTDKFWQGRDGNS
NASGTQTPMANFHEGARQVLGKVGYVAGRVGNDLIGRKVNTNHPRKVVSE
LLRTTSSAHTLARLIYRSVVREDRDTVYPEDLKQVFATQEEIDAAFGVFD
KDLNGDISMDEFEAVCNEIRLEKKAIAASLKDLDSVIQKLDKVFLFIIVV
ITVIVFVSIFSSSTAAGLASASTSILGLAWVLQATAQEFLQSIIFVFVKH
PFDVGDRVTIYGSTGANMTGDDYYVTEISLLYTEFKKMQGHIVQAPNSVL
NTLFILNQRRSNGLSDVLPLQFKFGTPAWMIDELKARMLDFCLANKRDYQ
PTIITEMTGVDQIRSANMNMVFIHKSNFQNELLRLNRHNKFVTELVYQLE
QIGIQGPLRVDPGGSREYPMYYAGSHPPPYSNGKEQQHDMSASNVASPAP
APSRGAMPRHNSVRSTHISTTSEEAIVDFQDVFENRREESQARRMESIRE
KHIAEQAEAEKESRASTTGAALSPIHSVNSQQRSRFFGRHRSGTKSQQHQ
PQQSDIV
>Mb|QLI64682.1
MHSSFTRIQNVFGFFTTVACVLAGFIAATDLLAARNPSGTLIPTNIQVVK
GRPHYYSSKKEEYAIIKFSLDADLSSLFTWNTKQLFVYVTADWPSAEGQN
ATNSAVIWDSIITNPSADHLQNIGPIAMKKLKRSAEGKTIDPSRGLLKLN
NQRPKYQITHPSGKIASVNDVTLKLHYNVQPWVGLLTWNMDKVIGWWFPM
EKGVSEKFEFPAVKTKDAKKKAKKA
>Mb|QLI64683.1
MSATFSIQPIGRFTGQSHPVKRPKEFACFSYDENHEFRLDDSSMKYYYTP
QLGADLSKGFEKFQKLDDSGDEHLDSLLKTILAHERETGKKIDANVVTWR
GMMTKIMAALFEQQDGFEMNATLYQNCIFIEENNEYKRQSRAKENHRGPR
RGPPLEVMQYWGISLPPFRFLILLTQDYKNSPPPSPPGYKFETLSTLPKI
WAETSREYIENRENQIVNNKEQYCSVVRTGIGKTVLCLGGEVDAIWDSKP
QPGQPINWVELKTTAEIRSDHDMDNFHRKLMKFWIQSFLLGVPKIIVGFR
TRDGILMDVKDIETHNIPQTVNSRQGARWNADMCVNFAGEFLEFLQSTIN
DEGVWRIRRHPASPTIEVFKVEETGHGNILSDEFKNWRIKLSLGPSTEP
>Mb|QLI64684.1
MFGTIASPLYQQSNALQGNREKLTFFPSAQLANSYQQLLDEFSSSELKSV
GNYTLGRLIGKGSFGKVYLATHKLTNGSKVVLKSAHKDDANLAREIHHHR
QFVHPHIARLYEVIVTENLVWLVLEYCSGDELYNHLLQHGPLPVAKVQKI
FCQLVGAVSYVHLQSCVHRDLKLENILLDRNENVKLVDFGFTREYEGRTN
YLQTFCGTICYAAPEMLKGEKYAGEKVDVWSLGIILYALLCGELPFDDDD
DNITRTRILSEEPKYPDHLTSDAISLLKLLLSKRPLPRPAFPDILAHPFL
VEYAPAQQAILDVKAHSPFSTALEKDCLERMRSAGVDIDAVIESVLAQKC
DALAGWWTLLLEKEERKMLRRDRKRQEKELERSLRRLSAASSRLDRMTPI
LQDVDEDAGLTSQFIRLGEHGSRRGRPERRSTNYHGDFTVTDLPLLPESG
RPHNDIPPTPVDKDSIRSASTSRHRRPIPPPKEGVIRSARSRGSTLHLVT

TSDTLGIRSNGSAAQNTQQNGQQKTKKKPSQAIIAHWKNLSHWFIENATK
RRKGHERRTSRSTPNLHLKDSSGNSGGKDVSPRPQTSKYPTTSSVDSGHP
TASLPKGVVANGLTKGGPVNGQANVPTRTPSGSSFPPSAHIQPLRPRVAN
SSSYKRQSLSPAPVTPRSAMRRSSAGLRGRKSTSSSVSSIRSMHHHRHSH
SKASSTSSAGSVSTSKTPLGRGQSPHHSVKVLPATPTANSFPSNIRLVRG
SPAPPPLRIYDEGMPSGNQQPPPGSPNPFSSGVLFAKRKRNIFKGPSLNF
GSGGHHISRNANSGSHSRSGSGSALGRRSGEITIQEEDEEPVVEEDEEEE
DEFEEVESFQPIVGRPGEIIEEQILEDEPLQLADAESPSAENAPATAKVD
AAKS
>Mb|QLI64687.1
MQFHVKRTLEGGHDIRGYELPSWALLVFLVDILALIPVFLLFQYTFKLVY
PVFAMVEDENPPAYEPVPLDQSVSGGEEGVARNSFKPTNGRPRTVTSSFR
AINRLLMANGGVAANFRGFFCYLAQVLLTTLLMGIFTGTFGGYLFPVATL
LASLALVQYSTAWVHIVISEPSPLPFWSRLPPFRRTFEATWQAVTIFWFA
SEVTRFVPVIMALVLGITMPQGGFNEPLDMDALGPGFFAKSLVVSVVTVA
ASVFVIIPAYVVLVRVQASLLPVEQSTIIPFDRSFEGKVEPEVVGGKGYA
TVSDAWTTFSKTSWRRLLILYVKMFCLSLAAMALFVAVLVPQTIFIAKHS
KEVR
>Mb|QLI64689.1
MVPGDMPQTALAYKSTPISRDPWNTGHSALVDPYGNDGFGNGFYSPPSIM
NPSHSYAQNGNIPRSGGGYNTRADTFSSPSSGYALRTPATASSSMAPAFN
GYKSPGKTTGWDNHGRYSPTGSLEEMVASPSLSDYSVENVMGVTSPQQKP
RHSGKRGSQRRPSNRDEFDGPHQFLQRPPQEVIDMQERELPHLPTNLHVQ
EQDTVLNQVNDRLSQCAFDFVAKYQFPIPLTQDMRPVERPQDREWTEWVY
LLKRLATKRRIPARVLYNGQIKQFVTILENSLEMRHAAKHQSRPLKDDRN
ILQLISAGIQVAKILKDASAMDYLDHLYVETESRIQERTKSRFRS
>Mb|QLI64691.1
MALTRSSRSTRDPAVNRRNGNRSSRIAASRTQMRPKPNYAEASTESDDAS
DAQSTIVSEPAESGPESDLESSMSHLAKERPLRLAGRSERNKRLKSVSSN
EVVKKQPQATSSASPIKSRTRGERSRPSPPSSAKRRKLLAQDVATNSHVP
APFIPDWRDPRIPFACWADIFLYAAREGSTEVLSNSWLIQAATTCKLFTE
PALTAIYRCPIIKNTAKAKRLTLLLDRPSSETLYNYRVKVEALHINVQVV
PQSLLFQLISPLTRLKELIIYTPMDQPPYRELDQSLRWHYPEDIFRALDA
APTESLLASDKPFPIALKSWEWSGRFLGGHVPTIEAISCLHKTVPFSQLT
RLSLTNFQVPSLKHLHIKPGSEEKELQTYHEDGAMIQAVAQAIAPLPSLK
HLVFESSTVLNDRLLPLLPRNLVHFELINCWEVRSEDLSLFLCTHGSELR
ALTLMHNQSLDLGFLTDLADTCPKLRELHMNLSYYRHHHCVNDADPMYDQ
VLLPSQIPKWPSSLRVLNIEHIRDWSVDTAEMFFQSIINSAPLLPDLRFL
AIKTMLDIPWQARATMRREWRNKMERVFLRPVVPPNFHPTLRHPDEDEAP
LAVKKMRLPESTSPSRRSSRIASNSQVSEAQSRSGKSTRHRHGNVSYKEP
DTDDEEFNSSEGDDDEKDSIDSNKSTNVLSLDQDRPSEEFLHRKCTTVNI
LFDNQKIRELQYSMEDFGSEESDSEEEWDGDYEDDEHVVVF
>Mb|QLI64693.1
MPPHVPRKRLRGSPGREEFKSGAGAKQTATPSKRKFTLYDDLDAAATPAS
AKTSESLLQSFGDDSDSSLSSFSDEDFEDVPLAKRPKVNPNVSDDDDDDE
DFEFEDVEAPAPSNSTVPVVSGDLELTLNKDTRLSLTNVFGGKKGPSKRE
RKVRNATHCLHVLSLLWHNAVRNSWLCDPEVQGIMISHLPPKMWDEVERW
RQNSGLTKQPAKAGSSTGRAKNSAKLKRKERESRDWGNAARRLEEGAVDM
SHGDPLFRLMQSLTSWWKQRFKITAPGLRKWGYMALERLDRLTKAYKDEY
ADCNRFGERIASLDEFRQCAMKCEGSRDVGAQLFTGLLRGLGLEARMVAS
IQPLGFGWNRLEDAELEPELAELDMKAAKLAKTTAENSVKKPNKTKDKSA
ATRSKRQKSTRAAAAVETASHEMDDLQREYTDTDDESVVAMQVTPKKSKT
PVSKFDPDLEYPHYWAEVLSPVTNKYLSVDPIVKSLIATNREITEAFEPR
GQKAEKARQVTAYIVGYSPDGTAKDVTVRYLKRQVVPGRTKGVRMPIEKV
PIYNRHGKVKRYEEFDWFKSAMSGYRRGTKAHPITEIDDDEDSNDLKPAR
PEKKEVKEGEETLQYYKQSKEFVLERHLKREEALKRGAVPVKKFKNKAKG
GKAEEEDVYLRSDVLQVKSAETWHKQGRAPLAGEQPLKRVPYRAATTNRR
REILEAEAATGKKVLQGLYSYEQTDWIIPPPIKDGIIPKNEYGNIDLFVE

HMLPEGAAHVPFRGAMKVCKRLKIDYAEAVVDFEFGHRMAVPVIQGVVIA
EEYHDEVMAELEKDEAERRRKEDEKRRKAALGQWRKFIMGLRIVERIRQE
YGEVDENVSVFGHSKDVASKPHAHAAAVATQHEDMAGGFLPEGYEEEHEE
EHHHTSSFFPVGDDEDDAGDDAGLTIEHH
>Mb|QLI64696.1
MIFSDFYRSPRSPLSKLRLHHPQLPAQLPTAAPEWRADEYADLLSKDKTK
QKEAVKRYLTDKVRSDWTFAWPPVVSNENVASSRPEGPGASVELPNQANS
SKKRGGAQAPTSKEDGYRGGEGTELDEPESDTGDDNAIVDDDNDDAESVY
SVMSEDAIHYRPRTEWTSDLSDDEDMPPKPSPFRFDNPDSIQSAIQDIVR
ARKARRRRALREEIAWNEGLACFEARRNAWTGAKTVRVRSKPVTTPPASP
RSPRRFFFRRSMSGSPPNPAVAVLSQHGECCGAVSDSSSASKDAEKDLKK
HQSTDSTVSDSTRIQTYPIETLLPVGQPLLPPTNPLRASISPAVYLSLYD
KVILHNLQPACPINLSDMLRSCVAGWKRDGEWPPRCSAPDPMPATRKKKK
AAPASATDNTGNVTRRMSFGLLGRDKDDEGGAGKGIRRSLQRALGIGVMA
GMGDA
>Mb|QLI64698.1
MDAAFFLSLSAGVVSVFLMKYGLQALKWLASALYYSIPTRYKAAQRPEDD
HIQILVLGDIGRSPRMQYHAISVAKHGRKVDIVAYKETARHPDLIGNERV
SMYALAPQPEWIAWGTLPFFLNIPCKVIQQFWTLFYTMMWATPAAKWIII
QNPPSIPTFHVALIVSLIRGSKVVIDWHNYGHTILAQKSLYSIFVPFYKW
YEIILGKFLGNANLAVTDAMARELRGPKFNLKNPVHTLHDRPLDLFQPIT
STKARKEFLSRLPETKPHVGNILDGTMRLIVSSTSWTPDEDFNILLEALV
LYANPSEDDASSEPPSPVLAIITGKGPEKEKYLEMIKQIQDNGRLPGIQI
LTAWLSNRDYASLLGCADLGISLHKSSSGVDLPMKVVDMFGAGLPVAAYS
AFESFSELVKEGQNGCGFETAAQLTEILKRLFSEKGQGELAQLRKGAVEE
GSLRWDEEWDRVMAPIIGIDAKAGAVR
>Mb|QLI64700.1
MDRSKARPLEPLELKARPLAFHAIAWSCDAELAVATGDTIHIFLPEYPRA
GSNPDDPGDDGFLQPQFSLTLTACAIVRPDPAINGPLCAFAGVALPPSSS
TEPLVFKGVGNAEATKAGSALGQTLRVEWSPNGLGQNLRPIVTVMTTSGS
IVALGEYIDQKTAMSSRSRTRTFRNWKLLWGLGANLPIPDGESKSGFRNM
NERIVSFSWAKEIAPGMGLLAYLNDAREVAIMGIQFFYKPPESDESSSEV
AGWEIFEIDRFDGRGPHDATEAADPEFVPTGGPFSLKWSPWHITNDYRTA
TIAYLGKNHIGFRRVTIQGRWHRGQDPSIRVEEADTTSICTFLSSDAFVE
WEDTIWQDSNGQMARGIIATPFVVKPFQVDLCANPGPRLAPHSPRDCSTS
YPPPDEISTNPITGLIIHHPDPSMKPAEPLYTLIRLSATPTNQDWYQTNL
PPTTPFPQWAERIQRSVTRQVSRVEALGGIDSESDTDSEFDEPEVHDMTP
VSEEAVSSKVHPHRFRLWGLAASPGDGSIAALVSKHATQHAERRPRSTVL
FSWDTGERTDSTVPHVPKLTTEGTIWEAMYGRILDMPSFLSGTTDRSLVH
ETSLRKMFKGVIPKQKCVFCGTSLVMLGIESICEKGHSFATCTATGLAIM
APGVSRVCSVCDLRCLNLSELSRIAKEHLGPSTVVESAGEVCGGCGGKFL
F
>Mb|QLI64701.1
MPVNHVVLASGAVVAVSVAVAAALAMYENPELRRYADDIRRRIAVALHSL
GDGINPAERAPRFNRPEDAEGFLQSSRGVGAEPGVDADEATRRRQREELL
YWNSVLLKKKEEESAAQPPPAALPPAESSRPRAGTRGSSFDDFLRQDENA
EQGTYVFNTGADIRDANSGLRHRVQVAAPVAAPVAAASLYANPFADEHCV
ANDEVEDVSATYTFPQSQEHMSDIYSATTRENDDAQSRTLDGSPALIELP
SPNFGAPSPPPATLERQLAENEYMSAGQDDREDAYASIQAWAHDSGRNFY
SPLPVTPVAPVSEPEVISDGQLTPTDSVSIIGSGECVGNDNDTRSGRPFD
VISESEGMLTPASWSEVGSVVSETEGHGHLDVNTPLHG
>Mb|QLI64702.1
MAEKQFKPALIVVDFQEDFCPPNGALAVPAGRTIAPAVNTLLALPFPLKI
ATRDWHPRTHVSFASNHPSSEPFTSRATIRHPSDPSRSYTTTLWPVHCVQ
NTPGAQLVPELDLSQVHQVIDKGQDDRVEMYSAFHDPFHVSDSGLAKKLR
EEHVTHVYVVGLAADFCVKATAEDAVAEGYETYIVEEATKPVAPDAWETC
REGILAKGVKMISIHGDDVARVKAL
>Mb|QLI64703.1

MEQMHSSSWSPSQALPDNPQDQLKQSARPGARFDDETDHGPVWDQTIRPT
TFTTPSRHQPHSRESSGDKTIHSPSAVASPRGHTQRQGLGVAIERTIDHS
KTAVYGHHRQTSIVHGFQHSRNGSHSSMSTSPLSPQMIAAAGVALDRLDM
QSVTTRLEANMLPPRPGTSLAGPTLSPIGGIPMERSASAADISPPASAQR
KPERMQSKSRRDHPAHQSHSRTHRDDQKAVGDYALHVLFTSFITQAEEKL
TECITVPFDPEPNIDRICGPGVDPAFDQLIVALGHIASPKPKALIDLMML
WRKSKSDAANEARIQLQQLRGSQPGGLLLRRNTEPLQPGPGAGAADPTSP
MNVTLSGKQELVSQQERRSTVSIYILCRVLLEVICQSNLASITTEMEDKL
ESIIFGQLKIADPEQLMLSPLKLANWNLFAQLLGYMSGINFPGVSRRFID
DLDASLQERLLKSPTSSTGRDFEGKMELVLGGMKHLNIKISPESAWEKSC
EFLVSLGRLFAKSHGQRVKAAFCQVIDMLLLPIAAKASNSHLMHPKWAEV
VSAISFRLAQLFAKPRHWAVAFPLTATLLCVSSPDNFGSGWLQLVLSLQT
KIKDRFTKPLCLQVISRLVWTYLYRTNDNFQTTVRKLDDVIRLVVPPSKR
SIVSSDTAVVEPLIQIIRIIGFKYPEYCFRTVVFPLINAELFTTNKELRV
DQLDPDRIVVGIRAFLCIMSDLEKGEQGRPPFPQFYGVPNSADRLPTSPV
LSFPSKGPLSTPSSGSKEEIISRPVLTHVLSDGVREYYLQFCQILGKITI
VCDNTFGGQAALDEKFNSPGPKTPISESFTFSRRDEPPNASDQKQAFYEL
LHVAVQALPRCLSVDIPFNSLINLLCTGTAHVQSNIAESSANSLKAIARQ
SHAQQVTMGFARFIFNFDDRYATMSDGGMLGPGHIESTLRLYVELLQIWI
EEIRHKSREAASDQPDSNASEKRAMKLDLSSIWAEVDQAEAHGLFFLCSQ
SRRVRHFAVTVLRLITEFDKALGKDASEEKETLRLINILENDSNQVMNFN
DEHLSVAERSRLQRGLQNANSKGAVVELCTSDVSYDATLWFKIFPNLIRI
SFDKCPFAVTICRDLICNRILQMYKPIVYLSEPTRGLYYGPDAVGRMGNR
SPAAQPEVMIEQWKLYLIFACTTLADPGSLPTGQTPPDGQHARKTSKPSS
ADRIVTARTLFKYLIPLLSVSSASVRDAVVVAMGSINIHIYRTLLEELQG
QVSRCNDEARARIHQRTNSSPRRNRKMDLLRTEITHVFKLTSHFLKDPQV
YQSEFFLNNLTLYAKDLKLFLMDGDLQSDDEFHKLRRHYCGLMEALFEGI
NKTNDPSRWMTFESRKSAFSLMEYWCGFSPNQGQTQVDTRGQPLINQQSL
GERGAVSAAMEIEKRNLRTAALSAMAALCGGPISITTESGVTLQFDMRRM
LAWIEAIFNSGSDRMNVTGQRALRNLVIHNQEYPYLLEHCITRCYVTDVP
KVLESYFSVTIEVLQQQNNYPCAFYKLLGLCLFTLGNDRSEIRSKSSTVL
RSLEARQQRNSKIQDFDISISDKTQAVYKLAQFEISKRLSKQYTELAFHV
FSEFTYYFKDLQPVAQRNVIAVILPWIQTMELKLDPNGGPTAQSYVLLAN
LLELTIKSGGALHNEVQALWQALATGPHPGNVRLILDFIMHLCLERREQN
FVEYVKQIVVFLSTTNSTPGIKVVEFLLMQINPKAMVPNEKRESIPPPPD
INGFPYCADLNEALPVGTKQAGFSLGQLSLILLVDLMVSPVHLTAENVPL
LLQVVTVLWDHYTPLVQEQAREMLVHLIHELVISRLDDVSVDVAKEFIED
LVDAIRRHDRTVVWGYEDSNGKVDDQGNKVPPSMEYLTSQVVETFQVTFP
AIKEQWGRLSLTWATSCPVRHLACRSFQIFRCVLTSIDQYMLGDMLARLS
NTIADEDPEIQSFSMEILTTLKTLLVKLDSEKLLTFPQLFWTTCACLESI
NEREFLEAVEMLNELVSKLDFRLPNVRRIIADGQPERWDGQFDGIQPLLY
NGLRSSLCWQPTLDTIDKLVKLPADPLIGDDSRLFFALLANFPRFLNELE
NASPTEKTMKTAKLLRVEADRLGLESIAQALDDYTSGRQLDSGEYLTQLF
IALREHFLPQQDFKMITFLIGLLTNSLSWVKVQTMRILCVAIPEIDMRNP
EFAGHGSDLISPLLRLLQTEYCVGALKVLDSIMAMSGSHMDKHYLRMIMT
RSTSKAVRKEYERTPSLFGIPECSGWAIPVPASKTDATRANIHAAFYMCQ
STEGSSTEATPTAEVEFHEDDFPYGYFDSMERTETMLSDDGRAEVPVGDL
VTKLDSLDDFFDEPSSPTTDEDGRSSRTVTEFSPESFESGTELYDEQILP
ILHEASNTTAFQNGFIDRPLGISRDLATNTMNPGAFTAGMSPRPALHSRS
ITSPSAPASYQSHMTGEVGSDDEYFSAGFSDADDERPDARSRIKSPKPGP
RPTMRRVDNRSRDETLREPTLPQVTPRMPNKAFQANIASPGDNL
>Mb|QLI64704.1
MAGPYPQLVLFGDSLLQRSVDLQDGFSMQAELQSRFIRRLDVVNRGFSGW
NTANAVKFLSEIFPKPTETSPKIKYLVVLLGANDAAIPTSSQHVPIDEYK
ENLTSIVTHPHIQAHNPKILLVTPPPVDEIKLTKLGENGHAPAARLSAVT
ASYAEKAREVASENPNVSSVDLWKAIMDKAISMAAPNDYIQGGHLLGSPE
NGKQGGLDSLLPDGIHMNGEAYRVFYDELKCLIGEEWANLAADDRSGYVF
PDWRVLNPLKE

>Mb|QLI64705.1
MASMFEQPRNGTLFLGGQKISGSDIRDQNVLATQAIANVVKSSFGPSGLD
KMMVDDIGDVTVTNDGATILSLLDVEDPAGKILVDLAQQQDKEVGDGTTS
VVLIAAELLRRGNELMKNRIHPTTIITGYRLALREAVKYLNENVSIKVEN
LGRESLISIAKTSMSSKIIGADSDFFADMVVDAMQAVKTTNNRNETKYPV
KAVNILKAHGKGTRESMLIKGYALNCTVASQAMPTRIQDAKIAILDMNLQ
KERMKMGVQITVDDPQQLEQIRARESGMILERVELILKAGANVILTTKGI
DDLVLKMFVERGAMGVRRCKKEDLRRIAKATGGTLLSTLSDLNGDEKYEP
SYLGHADEVSQERISDDECILIKGTKAHSSSSIVLRGPNDYSLDEMERSV
HDSLCAVKRTLESGSIVPGGGAVETALHIYLEEFAGTVGSREQLAIGEFA
QSLLIIPKTLAVNAAKDGAELVAQLRSRHALSQRIQEGDGSEDEKTIARK
KGYKNYGLDLMRGKVVDELKQGVLEPSISKVRQLKSAVEACISIMRIDTL
IKLDPEQQAEDDGHGH
>Mb|QLI64706.1
MPSKDAVDVPSFASTQLALLDRELKSEVDETSQLISNHSPAGLQRAGLAI
TNLVVSSQRTGLGGKTVLELGPDSATSATGELPEHGVRTGDIVLVAEQPA
GSAKKREVRELERKGSRGVVTKVQRGFVNVALDDGKDEVSFPGRVWLVKL
ADEVTYRRMNQTMEKLQGLVEAEYSSFMRVLFGLSSPSPLPQDLSRDPQA
GDIEWTDPTLNDSQKDAIRFALASREIALIHGPPGTGKTHTLIELILQMV
KRKQRVLVCGPSNISVDNIVERLSPHKLPILRLGHPARLLPSVLHHSLDV
LTNTSEAGAIVKDVRAEMDAKQASIKKTKSGKERREIYSDLKELRKEYRE
RERKCVSNLISGSKVVLATLHGSGGFQLRNEEFDVVVIDEASQALEAQCW
VPLLSARKAVCAGDHLQLPPTIKSLNSKANPRPKDERLPIIKGMTLETTL
FDRLLALHGPSIKRMLTTQYRMHEEIMRFPSEELYGSKLVAADAVKARLL
KDLEYGVQDNEDTSAPLIFIDTQGGDFPERSEEDDKDNPKKGRGSLHSES
KSNEMEAALVLQHAGQLVGAGVRPEDIAIVTPYNAQLGVLAPLKEKFPGI
ELGSVDGFQGREKEAVIVSLVRSNPDGEVGFLGEKRRLNVAMTRPRRSLT
VIGDSETVKRGSNFLKKWMEFLEDNADLRYPDISSIVN
>Mb|QLI64707.1
MQATSGETTSLRSFAKFLPSHDTAVCLVPPKKLWPSINSVRRLYDKAFEK
WPPHVNLVYPFVGPEVLSDAVDMLCQIDLAALVPKTISLQEADYFTHRND
NTLILRPGKHGPSTFAGELVNGLRQQIGWLALDSYQPHMTVGQTEDAKSD
SHNFLLNKARLLTPVNWEIKQLAILVRDSSRSMRLWGYVDIQNQQCQQSP
SLLGFQLDGSSQETQSQTTFAFDSSTLSWKTALPSTPPPDSIRQLVVASY
NVLAEFEWPPDTSRYQGLISTILSERAAADVLVLQEVTDHFLPYLLQHQG
VCARYRFATHGPPGQPGHGPLPNLLNIVVLSKLAFRWNYVPFARKHKGAA
VVELAVSHQDPQMISNPQSIILAACHLTQGLADGAVVAKKNELHKLIQHL
STEFPNHPWVIAGDFNLATSVFTIDQARKRGQLSSCGYQSLCEIDGMFAN
YGLQDAWLLTRLRSGVSRDTVGSSQSPEDLFEGEQGATFDPTTNPLAEKA
VGGNLNNRPQRYDRIIFNGALSLAACGFNTFGWPVCDTGDGQSVASDHWG
IRCLFKYLERDANFAAQNASNSVLTKFRKSPSSLGDIGSLKHSLMSLGCL
PTQADQEDRSKAFQLLQSVLCEPNKNQTRHQLRTGPDMVLIPVGSYGLGV
WTPESDVDCLCVGNISAKIFFALAISRLRRAKSRGISILRIVRAKTGIML
ELSIFAVKFDLQYCAAALVAEKYPDIMNHPATDPAFNLPAQTLSKLKPAR
DLAYLKRSIPDMAKYRIAHLLLKAWAKSRGLYSSKFGLLGGINISVMLVP
ICKLLAYNGANVGTIDILTTFFHHYRNFDWRRNMVFDPLFHGTIKYHRTQ
RESMCLLGWHTPSLNTALNASDSTIHTLSAELIRASDLLSQEGVNWDTFL
RGTQPPLSFPELTACNAASFLDAYRSYVKIDASYWGSSPSKGRRFVGWLE
SRCVMVLVDMNRKLADLVARIWPARFTLNASHGTADIGNEEFNYTYLIGL
TWQHDDIENKTREDTQHAEKSLSLILRDFENRLRRDQKYYDPCSCWIMTS
LVNAHGVRQFQLDPQPWKDDNAGDTDSEEDLELVDNESDDGKEEPKAGAA
ISLATPSSDPVWRKASPSVLKAGGSRKLRTAADVMNRLRWDSAMDASNYL
VGYEDRFTGAKEKALEQWKSEQTDEEFIPQHRILYFKRKTDGFFVWERST
RIDHIFGSGM
>Mb|QLI64709.1
MLFQTVASIALLATGAVAVEARQPYRLAVMALPGQSLMRRDTSGYKPDQK
QCKAGNTCAESCGADYQQCAGGNPDVAHCFNPKAGESCCTDDSGNSCQDG
YYCTHDTKAQTWCCPKDMDLTACAAAYTISGLVAATSKPPPSTTSQAPTT

TSIPPTTTSSSIASSTTSSTPVITTSENTNTTTSISTASIGKSTAWTAVN
STISTHVPTQPSAGIPSVTGAPPPAPTNVNAATASGVSALLLVAAGLLAL
L
>Mb|QLI64711.1
MPQSDTVQADARRFLDERGSTAALAPNGLNPATIMEKAVKDRIVESYFYK
EQCFALNEADIVDRVVEHVRFVGGTHGDAQKPSPFLCLAFKLLELGPSEA
VVREYLSYGGEHFKYLRALACFYYRLTRRAADVHRALEPFLGDRRKLRTR
GRRGAALTYVDEFVDGLLTRERVCATSLWKMPPREVLEDLDELEPRVSPL
GDLEDILDEEDEGEGEGEEGPRDEDAEEGEVGGDMDVDREEGGLSRSGSR
SP
>Mb|QLI64712.1
MSLDKNKTSPKPASKPRRLPEGFSTMNKDSSFTYVHMSTASNADLFEDFC
KDKLPSDEIFVPPQHQPLNPEDEDDVVPDQHAAFGITRATKEVRGAAWKD
LGLGELMRKGPPKGVSGKRMPR
>Mb|QLI64714.1
MEESNGLGPELESFRQQWLSEVRTKKGEQSTQPQLRNSVPSASTAPPPPR
KSMARRPSPAKSRKAPPAAEEEDEYLHGRSFDDSPEPSGNTIDGSVRMPP
ERELVSALDHYEEAMEKEALGNMGDSLKLYRKAYRMDHGVHRRYREKHFP
QHKQPQTAPTASSTQITSTDATPAGGDAPVLLPIKDLIASFAGLQIETAP
PETDGTPPAPCPLSCLPSELLVHIMQDVAALDVGDFARLSLVCKPLAYLA
ATEERTWRHVCLGARFGFAAMHRRWSKTVEWEALDEDDGPAGTTTEDAAA
TASLVPVPYASWRAMFRGRPRIRFNGCYISTVNYVRSGQASTNQATWGGA
PIHIVTYYRYLRFFRDGTAISLLTTLEPTHVVHHLTGDLLRLHRDSDNAH
AHLPSAAMQRAYKGRWRLSLGPGGDTDAEQAGPPRGEEGDEQLREGDVTV
ETEGVDPKYMFRMDLSLRTAGKAARNNKLVWRSFFSYNRLTDDWAEFTLK
HDKAFFFSRVRSYGVGE
>Mb|QLI64715.1
MKSYEHIPYNSSKKDFFAKTGRECIEAFKYTFQIPRNPVVFTVMWDYNIG
LVRMTPFFKCMGYPKTRPSQMLDKNPGLRDICPSITGGAVFAQGYWMPYK
CAKAVCATFCYGFAAALIPLFGPGFPSECISPDSAHFSSMTINQELIKEV
SEQAQQLQQGPAKQSATSYIFPQRPPGRNVRTPRPTTIGGTPRMNTEPIS
RPQTYLNPVTRRQQQNDKSRADDQGFYPLPRTEPSAIRRPAQTSGVVLSS
HANEYFPAARAPETTSLRRSRQIPSTSHSYETSTRCRKRRKLSSDASCDG
VYDSTPRHSQSTRTSLPKSRLPRGTNLSEQRCMEEREAGSSGTTNTFVTG
AGEGVVDDGCRSQAGPFVWTPGRRKSI
>Mb|QLI64719.1
MKKTLLLCFIHGFKGGESTFGDDYEFTKHLRDIVAQKLPKVDVRVLVYPK
YETRGDLGECVSRFRDWLEDKVIDMEVASGTASPTVDPSVRTILVGHSMG
GIVAAELLIALASEKPIYSEDGIRKSDDDEGRPAFNALMFPYVQGVLAFD
TPYLGISPGVVAHGAESHYGTAASTISQMGGLRTALWGAGNTTAKAPAAP
PSAWQKWGKVAMYAGAAGAVAAGGAAAWMNRDQIAESWTWASSHLEFVGC
LARAEELKKRVAFVVRLHEELGVGYANLYTRLGKGASSKKISMAGTVLGK
DRTFCNLPKTMNAGDWMEAVNDKAGDETVAHMTMFEPKENPGYEKLSHDA
AGLIAQWSRNDWYDKSTETTTAEKVESVEVDEKGKGVEKEEEKENTEKIA
PSS
>Mb|QLI64721.1
MPNRVAIRAPVDFEELNSASDVDNAPEVESSSESDYLDSSGARKRRRTAS
ADDGGDSLDLDQAEDGDDSEDDEKHFQTVLSTIKAPSRIKRKTNHTQAEQ
EAMIQPSNPILAPTDPNTSFSSLNVRPWLVQSLANMAIKRPTGIQKGCIP
EILKGRDCIGGSRTGSGKTVAFAVPILQKWAEDPSAIFGVVLTPTRELAL
QIYEQFKAISSPQSLKAILVTGGSDMRAQAIAMAQRPHVIIATPGRLADH
IRTSGEDTICGLRRVRFVVLDEADRLLHAAGPGSMLPDVEECLSVLPPAA
ERQTLLFTATITPEVRALKSMPQKPGKQPVFVCEVDTQMLAIPTTLHQTH
IQIPVTHKEHYLHVFLLTEANKDKTIIIFCNRTATADYLHHLLRLLDHRV
TSLHSKLPQRQRIDNLARFRASAARILVATDVAARGLDIPEVSLVVNYDI
PRDPDDYIHRVGRTARAGRTGESATFVGQRDVELVLGIENRVGRQMDAWE
EEGVNLETRVLRDALKVVSEKKREALLEVEEGREVGGKRKRAKMKLRAE
>Mb|QLI64724.1

MPAGTKRDRDGNARSRSSQDAPRTRFQSLFENFRDELDEHYDRKERVVKA
SRDVTAQSKKIIFALQRVKHINQGLPPSTEKDVTTRMAEITALLQSISPD
VQSINRHRYSWSLRCLEELVEALSFAHYLRHQKLITPQEAAASTPGDIAL
TPHDYMFGVFDLFGEMMRFATVTTAQHGEMLGGEDGRNILGDIQELGCAF
EALREIPTRDYRNKMEAMRQSVSKVEKLGYGLAVRGSERPKGWVPDMKDD
VEAGSP
>Mb|QLI64725.1
MDGTTASSGGLRVGKLLPKGLSAKRRRRKDKNGRDSEASGADDDTRSTSA
HSSQRRFKRELTLESDDANSTNIADDDDEMDDRSFGSFESGADPETADRG
RPASSSAPARPATISAHPSLIGYLTTSSPVVQTQHLDSRSHFLSSSQARG
YSSGSSDKSLSPEKPSKAKGVPGFQNSIPEPPAPAKIASADRKGRASTPR
IETNPPRTPPQDDKPAPVIVNTPPTPTDRADHSQFATSPDGRPSTNGADP
KLRADGSAAASNMNIHRRSKSGSAAIGPSKLSHITAAPLTPTDETGGETT
PSATGFFSSMISAAQNAATSISNTIPSTGLGIGSNKTRNGTPKSLNSPAA
SQTTLNSQPAPEPEPRIEDTMDRKESAVRTLGSGDLSLSQLGLPESSITS
PASARFADVADTRTRSESAPTEPQTSATDVLPAESSSRPRSLAEPASGDQ
TPPQTDLSDLKGPVQRSSSLRSAMKPHRKRGSSATTGGTIAAAITSATNA
ALPTSGGIGAPKLTGFAIASKKRNRDFHNLFKSVPDDDYLIEDYSCALQR
EILAHGRLYVSEGHLCFSSNILGWTTTLVMSFDEIVSVEKRSTALVFKNG
LMISTLHAKHIFASFTSRDATYDLIVNIWKLGHPTLRSTLNGVQLEGTGG
DKTEKVEEQQGVMEEEPQEASGSEDDTDDDEGDDEEFYDEEEHDEVLETQ
STDMTAVEPDPERPATRKVSGMTAVNGVASEPSKDASPPPGGAPDFPGPS
AHAPTDCGDSTAHYEKVLGDEIIPAPLGKVYNLVFGPGSVTWMNKWLSTE
QKCTDIQMEDKKGLTADNKSRTFTYIKPLNGSIGPKQTKCVVTETIDQMD
LEKAVNLSVSTQNPDVPYGNLFTVKTKYCLSWAENNATRIQINCTVDWSG
KCWLKGTIEKNVNDGQGQYCKELFASLKSAVSSRARSGTATNGIVKGKKK
LKRSKALQTVVDLEASKGGKSDGGKQNWGPLEPVRSILEPCVDVLQPILT
GNVMYGLLVGLLVAMWFGFGSPPKNNAPFGPDVGFYSPNRQAAYEEMWRR
QDSELWEWLEERVGMERLSSDQPSARKRGMEHRTVEDKLREERMDEREIQ
EAIRVTEEKLRVLREVIGKGRPN
>Mb|QLI64730.1
MYPEAMKLTNTDSSQTKLKVQLKLAIARLRMVQQRDEQLGKSQQRAMAQL
LEAGKIDSATIRVENIIRSDITTELHEMLELYCELLLARAGLMESSVCDP
GLEEAIKSIIYAAPKTEIKELATVRQLLGEKYGKEFVLTAMDNLDGKVNE
KVVKKLSVEPPKQELVQGYLEEIARAYGVDWPKREKVTPPPELIDGFDEG
DDEDNQSGGQKILAEQLTTGRKLSKGEEELNKATPPQGFAGPRSPVTVTP
PRRTTENVHPKVTLGSVELKPNKRMEAAASRKEPEGAVPDISDLERRFAA
LKKR
>Mb|QLI64731.1
MTGPVTTNHDDTILHISPPATTSPPSEPSPPARPVANRAATPLGLFLDSS
GFSKPSSTKGPTTAGGAGAGMGPTGHRADLSPAEMPDLAAVSPAPAHTSA
VAGVVDSVPCAAQEQQQKRPDPSVEAVEAIIVSPGPASGSEFGSCFGSGS
GSGSGFALQSSMMSPSTTLGSSVHSVHSVHSPSAPARPHPIQAPSPADPR
DSASQMTKSTPPPDRDAELTSSADLVDARPLRTASVRADTRMVHPKPIAR
PPALSHGVPDKLIRSGSATGNIAQLEATAERLSMTSSIDDAIRDLHGELK
RSDSRRSAKLALATMASVDENTTSPASQLTRHLSTTSSIVSTNNAARHGG
YSPAGFVLSPNTSLTSRLRSGSGNSSGRQDATHDAILSRHGPGKSSLRSV
LSTKLSLAEISESEPISLNQKAFDEADAAPPLEEQSAESLQLPEETIVGT
SGTDAFHQLHEDTLGPQPDSQHAPRHTLPPAPLGPEEYQYEQRPGSVHSN
NTSEQARDAFIDFDGVHWEPQNEEDLYVPPEIEPDLPTPRPLPISMPRPT
SYIDPSSGQKMLYYPARVPAMLNLPPKLSSKPKAATRNQRRSQVLSVMMD
APTTQDTSASPKRQRQSTFSDLKGISSAAPEAPVRDSWLPDPIANHRDSF
AALSSFDPFDGGEAKSLHAAAEDSPPTPPPEEAAECSVVPDTLRRPERLS
KALKENRKSKASMLDTLPPQLRASAFFDLPSVAHEVEIKDGSAMATLDSI
LDASTSAPVSAFTDHVYAGKLGPEIYGKAKKNTARQSVATLNGLAPEPQA
KKRSSMMWLGKRNSSYNSDEQKRPHSSSIPVATPEADGSQDGQNAPGSID
IADGAIEADVDKGDNEGEDEEGDETAAPEDAYLGPPTTLLAELQLRKHQQ
KQRVRNMGKGLPNGMYATLLDMDTVAEAQRKNRQNKRVNLAWEDPDANLD

QNGSDDEDVPLAILAAMQRGAKNLADLDRPMGLMERREQEDNEPLSHRRA
RLHGLDAPPPVLHQRHSVMSLSAAQALQVPSSTPRVEVVPSPEPEVEELE
EETLGDRRRRLAAKDGELPMARPVSRAFSSELLSQFGDAEDATKESKSKK
GDGKETQAAGASGEDETLGQRRRRLQAEREARQREMNYGNSVGDQPAQEP
RRRLSLANVLAAHPKRESDRRTQEESLRAEEQRRAAQDRDAKMAAMRMQM
PSSLPQSGLERSGGFLGGAFNDGTGGHGLKAGLPSTAVNSQGLLPGKRSS
VMTSYGPGFGAPIGQPGYGVVNGYGQGYGGLNSMGSFNGRPATQMYGTGM
MSMPMNNGSMDRVEQWRYGVRP
>Mb|QLI64735.1
MFMARSEYDRGINTFSPEGRLFQVEYSLEAIKLGSTAIGVATSEGVILGV
EKRVTSTLLETSSVEKIVEIDRHIGCAMSGLQADARSMVEHARVECQSHA
FNYNEPLGVESCTQAICDLALRFGEGADGEETIMSRPFGVALLIAGFDEQ
GPQLFHAEPSGTFYRYDAKAIGSGSEGAQAELQNEYHKSLTITDAETLVL
KTLKQVMEEKLDAKNVQLASVTKEKGFRIYTDEEMAAVVERLPAN
>Mb|QLI64736.1
MDITSYVVQGRDQALLYGDYSTYHGQLAKRLLSSRKKLGIVTKNRGKFQK
KEEIAAEQVSSNNEYVHLLLLTSERAWAQAMSFKASNSATQKDITGRTKS
HIVSRLNKASRAAQRLVELLSDPASGSSTNDVLEAKSYAALIRGAMLFER
HDWEPCLQSYAVSRVIYSALASAGKSDLFKDLLSETIDPSIRYAAYRLKT
PRTVPIPTIAKKSFPRSDDALVKEINRLDPSILAEGDDATKLGNAAENAP
RTITWRSRQVQIEDAQISLAWASVETAKMNLTRNFAQSQDKQPYEIAAQY
DEILMTTQDAVDATKQAIDELKAEGVPQSDPRMQRLQITRTAVNYEMISW
RIGRNRVLTGLQDGGPEDYNASRRRKIKAPKDTEAGEQKKQRELPPSKKL
AKLKEKVALYDGTLQNVQSIKELPGVAADEALATKLNGYEKYFGSLKCFS
IARSHAITNNATNALALINHAHSLIQEASSNLSQGNDDVNQQQTTPVSID
VTAEAVSYLSSLLNGELQRYRAIVHMHNLRDAESVAAKGDFDSPLISKLH
TYPRRVNLRNIVEFPPKMELIPMKPIFLDVAWNYIDYPGKTPAPTAALGP
SSVGTKEQPKKKGWFGFGR
>Mb|QLI64739.1
MPTAPPSRPPTTYLDDEDLSVKCCTPITSLVAPVEVGNIGSGRAGTSAFF
AWPLLGPLLFENETSDARDHCANERTFLSYLRLSVFMAILSVAITLSFHI
NSKPTDLERRISKPLGAIFWVFSVVMLGLGLANYIKTVNKYSKKAAIVQT
GWRTQLILGTLAFCIIGSSIVLLVTTRIRHQGGG
>Mb|QLI64740.1
MAEFDTLDIVVLGVILLGTIAYFTKGTLWGVTKDPYANAFANANGAKAGR
SRNILEKMDETGKNCVIFYGSQTGTAEDYASRLAKEGKSRFGLETMVADL
EEYDFDNLDAMPSDKVAMFILATYGEGEPTDNAVEFYEFITGDDVSFSEG
SDPALQNLNYVAFGLGNNTYEHYNSMVRNVDKALQKLGASRIGEAGEGDD
GAGTMEEDFLAWKDPMWAALAEKMGLEEREAVYEPTFGIVDRENLTVESP
EVYLGEPNKMHLEGTAKGPFNSHNPYIAPIAESRELFSAKDRNCIHMDVD
INGSNLSYQTGDHIAIWPTNSGDEVDRFLDIIGLKEKRNNVISIKALEPT
AKVPFPTPTTYDAIVRYHLEICAPVSRQFVATLAAFAPNDEVKAEMARLG
SDKEYFHSKTGPHFYNIARLLDTVGKGEKWTKIPFSAFIEGLNKLQPRYY
SISSSSLVQPKKISITAIIESQAIPGRKDPFRGVATNYLFALKQKQNGDP
NPSPFGKTYALNGPRNNFDGIHVPVHVRHSNFKLPSDPAKPVIMVGPGTG
VAPFRGFIQERAKQAQNGATVGRTILFFGCRKRSEDFLYESEWEEYKKAL
GDSLEIVTAFSRESSKKVYVQHRLKERSKEIGELLSQKAYFYVCGDAAHM
AREVNTVLAQIIAESRGVSETKGEEIVKNMRAANQYQEDVWS
>Mb|QLI64741.1
MKYSAVAAIVSSAVAVSASPVEKRAIGGVLLCTGANSTGTCKYSVYELNK
CHQLTEPFYQNTNTFAVDGENFYCFPKLVNCTDICTSPTGCTFGAVDYSY
EHKYNLSAISWDKYITSFQCGMKQDPQTNNNGSSSKRFLS
>Mb|QLI64742.1
MANDFSLFTSLRFDVKLKHVPSMGLAYAGWNYRNESPLYMLDYHRDRILR
AAVHWDWDKVIEKLSGDAGLRNLAEAAEAEIGPSETGPFRLRIVVTKQGD
ITFHQYDTPGLDIGNLFPESLPAPGTSPSPSQPQAPPLLTVVVDSVDSSR
SEFTHFKTTNRALYDDARSRAGIGSVSAANSVEVLVINEEDNTIMEGSTT
TPYFWRGGRWITPPVSAKYSRQDGSGGNDGTSRRWVLERGLAVEQEIRAD

QLVDGEACYISNGVSGFRVGTISLKRGGQ
>Mb|QLI64743.1
MAAQTGIEAALAKIMDVVQKQNEEMSELRQECAQLRHSNTEISQLLRETV
MGRRDSTGLAVFTPGGDGSLRSFSPHPLPKLSPAMRYIPPPSPSPQLTVP
AMDREIPGFYVIIPAGGAGTRLWPLSRENHPKFLLDIDLSGRSLLQSTWH
RLVPLAGSSRMTVVAGPCHSESIQTQLPQLESDNLFTEPGPKDSMAAIGL
AAAILLHRDEDAVIGSFAADHMIAGEDAFLNSVTEAVKVAKEGFLVTIGI
APSHPATGFGYVKLGDKLDVPNAPSARLVSAFKEKPDAYTAAKYLSSGNY
RWNAGMFVTKASTLMDLVKEYEPELHSDLTRIAKAWEDKTQRETILNEVW
PTLEKVAIDNAIAEPAAAEGRVAVIPATFGWDDVGDFSSLAEMLPAEANS
PRILGDRNLVVAQQAPGGIVVPGSGRLVACLGVDDLVIVDVPDALMVTTR
ARAQEVKSLVASCRKAGFKNLM
>Mb|QLI64744.1
MDFLQRLARYLDRPLFPWKKLILGFSVGQYLFESFLTLRQYRILQQTKPP
AVLSKEISQETFDKSQAYGRAKAQFEIISGLWAQIQNVAFIQLDVLPKLW
SWTGDLLLKWAPSRFTGEISHSILFVLTFIVIQQFLSLPTRVYSTFVLEE
KFGFNKQTPKLFITDMIKTNLLTVALVPPILAAFLKIIQKTGNQFVFYLW
VFSAGLQLFTTTAYPIFIQPLFNKLSPLEEGELKTKVEGLALSHKFPLQE
LFVIDGSKRSAHSNAYFYGLPWKKHIVIYDTLIEKTKTDEIIAILAHELG
HWKLGHTTRLFGIAQAHLLYVFSLFSVFINNVSLYNAFGFHTTHPIIGF
ILFSDALSPMDTVIKLLLNILSRKFEFEADDFAKGLGFRSQLASSLIKLH
AQNLSTMDADWMYASYHFSHPHLSERLKALDWKSKDSLTSEKDREGVATA
TGRDEL
>Mb|QLI64746.1
MRNLRNVRFGRWSHPDITSACWDPEKDEVVCTIGPTADSTSIELVRVSEN
GLTSYKSVASWNAPSPSPDLAVDKVVNIQYLSSSGTTCLVLEGGDIITVQ
EDQFSDHGAHIEIMGSIDAGISAARWSPDEELLIVTTKANTAIFMGSSFD
PVAEVTMTADDLKASKHVSVGWGKKETQFQGRGAKALRDPTIPEKVDEGL
PSAHEDGSTTISWRGDGAYVAVNSVQEGIRRVVRVYSREGELDSASEPVD
GLEGALSWRPSGNLMAGIQRLADRIDVVFFERNGLRHGQFTLRSPEGSVM
FHEKIRLEWNSDSTVLAVVLNDSIQLWTMGNYHWYLKREITISAEYLCLS
WHPEKALRFASASSSETAIFEEAFHTARGSCLPPYDNGAVAVVDGETVKL
TPFRTANVPPPMSLFDITAESSVVDVAFGPQNMSFAVLHRKGIEIYDWPM
KNGRSIKPNLKSKLSFESTGVTDRVVPLRICSTSGSQFRCLVSSGDSNLV
HFAASGDSGTIDILNSQEILVSTSTYEDATSIESYGQDISGNLYKLSETS
TDLLPLQFPTHLPWFEIVKFDGELTAFGLSRNGHIYANSRLLAKNCTSFV
LTASHLIFTTSNHFVKYVHLVSIVEELEVPEDDPEKDERCRSVERGSRLV
VAIPTNMSIVLQMPRGNVETIFPRAMVVAGIRSLIEEKNYSRAFAYCRTQ
RVDMNILYDHKPTQFLSNVSLFLDELNSMTHIDLFLSSLREEDVTETMYK
DTRKSRTQLGIETEPVPTRPSSKVNIVCDAILKALQARKATNIQNIITAH
VCKSPPALDDGLMLVAELMREDEKVAEKAMEHICFLVDVNRVYENALGLY
NLDLALLVAQQSQRDPREYLPFIQNLHALPELRRKFEIDDHLARRSKALS
HLQALDNFDEFCVYTTKHSLYQDALRIYRYEPTRLQSITNLYAAYLESKS
SYREAGLAYESLKNYAKATSCYRASGATCWQECLYTAQQQSPPLSAEAMT
DLATSLADALWEAKDYTSAATIHLEHLSSLETAIKCLCKGYHFAEAMRLV
VQKSCPGLLESAVDVGLAEALGSTTEFLADCKGQLLAQVPRIAELRRKAI
EDPLSFYEGERPGGMDIPDDVSVAASSRISTSASLFTRYTGKAGSVGTVG
TGVSRATSKNRRREEKKRARGRKGTVYEEEYLVNSVRRLIERVSGTKPEV
ERLVFALVRRNMAERARAAESLMNDVVEACHTAISEVFAAPPDNDDQQQP
VEGVGELTWTAKGGDAVLQEWMEGRAKKQEAPVITALQKLTLLG
>Mb|QLI64747.1
MQRDASSETAPVTSSTIPTTSRPENPSSVPGTGPATQIFDNICTSTGQRE
GGGSSHLDPESPVVLLPGDGTDTYELADYGEIQESKKAHAVEKRRQEKRD
RKRQWIETQDEMKFSHSIQFNAVPDWSSHYIAYSNLKKLIYQLEKNAHQS
RAGGDSESRPLISSEEPTEVFSRALGIELEKICSFYVAKEGELLDEVNQL
VRDVMERPSLESPDLRRMSLGEYNRPHLRPLSSTGLASDDDMDDSGSDDD
ETTGLHKPKSSSGRRRTIANIGQHQVDLAASSEFTRSVRRHSTVEDFGDQ
SVMFASGLFSSSIMLKKRIVSLYVQLCELKSYAQLNKTGFSKVLKKFDKI

LDKELKGPFMRANVDTAYPFKNETKKIIEENIEKMEDAYTEVVTAGDREL
AKKDLRSHLREHVVWERNTVWRDLIGIERRGEAARFGQSLLGQDQTAAHK
RLQGDDEEIAAAKQFRTPLGRLSLPTWLTDSSMLTLLGCLGAFAALLAIP
IMDKPEEQNCLAMLVFVSLLWATETIPLFVTSLLIPFLSVVLQVVCDENG
SKRKRLGAKQATNAIFAAMWSPVIMLLLGGFTLAAALSKCKIDKRLATLI
LSKAGTQPRTVLVANMFVAAFASMLISNVAAPVLCYSIIEPMLRTLPSDS
DMSKAVIIGIALASNIGGMLSPIASPQNVVAMGIMKPEPTWLQWFFIVIP
VGAVSIFFIWMLLLVTFQPGRGTAIAPIRPVKERFTGVQWFVSVVTIVTI
ALWCASHQMDHIFGDMGVIAIIPIVLFFGIGILTKEDFNNFPWTIIIILAA
GGLSLGKAVRSSGLLHTVAELVSKNVEGMSLYGVLVVFASLILVIATFIS
HTVAALIFLPLVFDVGMSMDQPHPNLLVMGGVLMCSAAMGLPTSGFPNMT
AIMKEDATGQRYLRVRHFISRGIPSSIITLAVVITLGYGIMQVAGLD
>Mb|QLI64748.1
MASNGRRTALVTGATGLLGREITAAFRQSPKWNVKGTGYSRADGVDILKV
NLENEDLNELNTVLDETQPHVIVHSAAQRFPDRVDKDPEGARSLNIAASR
RLAQVALSRDILLIYISTDYVFPGIPGDAPYEADATPKPTNLYGQTKFDG
ERAILEEAAKVGKPGSAIVLRVPVLYGHAETPAESAVNVLMDSVWKAQTE
GANIKMDHWAVRYPTNTEDIGRVCRGMFQVLIIPTTHMARFKQYTANFNC
ALYDLVHDPKRVFVNMATKEFFPDMAAKYLDANGPDHRKALPSILQFSSE
DKMTKYEICQLFGKIMGLDTSNIEANTQGNDPNAAVQRPYDCHLSTKALQ
DLGIDISTCNFVDWWRREIRAFSK
>Mb|QLI64751.1
MPEPAHSPQRDRARDRDRDRDRDRDRERDRDPSQHSKRLSITDRILGSLN
QARKETSAAVQRRTSTSASTSASASASASARQSPIPRIREWLSTCNTQHG
RHCSGTDSRDAPTWRPIHLIDCDERCLVRSKPADRYSALSYVWGVSNPRD
TGSDAAAQLLTTNLDAYQLSLPDKGVPQTILDAMWLSKKLGIRYLWVDRM
CIVQDDEQDKSEHIEHMAYVFANACLTIISAYGDVHTGLLPLDPRRPSRA
PRQGNPDHNGLLLSSKWNTRGWTLQELVYSRRAVFFFEDAVTWECHCDLW
QGNAANMTKILRGKKPACTNRLSEAAFAFQHTEWPDMDEYARLAMDYSMR
RVTIVDDSIRAFAGVTHVLSRIFQGGFAYGMPLMFIDIALLWRPQATIRR
RAMSRPPFLPSWSWMGWWFDGIPVDLTLWRAAADYVEETQAVKRGQESKR
FKPTHPFRIKTVVAWSLSDRANSAPIDNTGLRMREFRSRKGAGQALPPGW
SKSGSHFKHDSDDLTLFKYPVPVEDVPEDGLYESRPTELSIPGPLLYFRT
TTAFFDVDYAISMAPKDMPNPPVAIGNIFSRSNRWIGELRSHDGWLGVQS
SNYGGEEKLEFIAISSAMERKGSYVFPMDRFSESMDDDGVVQFVNVLWIE
RISGVAYRRGIGHILQKAWDVEAKEEVDIYLG
>Mb|QLI64752.1
MSSHSEAVCLDPKSTINRQIERANIPTAGRLAGQPARERTMPHDAVYGFP
PEAFDASLLPDINRQFLEPHDIEAFVRALQAPDPLHSPADETGGLKSPRS
GASPATLTKRNSQAGLDDANDHKSADAIAAAAAAVGGDLPAPTDTPTQGT
FFTAQNDWAPVSPALYRRRRSSKKCKQKKGASKAVEGLLGTRTKDETREG
YLYQLSKWPLLIFVFAWLTGLAIAYLSTRWYIWVYEHFFTWRGRRETLRR
NMRKASTYEEWVSAARELDAFLGRQTWREENEFAYYDSKTVKRVWDQLKK
LRIKAEAQEIQSARQHGDHKTAMEDLKSMVEACVKNNFVGVENARLYSQT
YYGTKNLVQNFVDEVERSIKLLLRTKQLGTEEKRLLFKHVYANYGRTALC
LSGGAGFAYYHLGLVKALLDADLLPDVITGTSGGALIAGLVATRTNDELK
KLLVPALSQRINACRESATAWLPRWWKTGARFDSVDWAERCSWWTRGSMT
FREAYERTGRILNVTCVPADPHSPTILCNYLTSPDCVIWSAVLASAAVPG
ILNPVVLMMKLRDGTLAPYSFGHKWKDGSLRTDIPIKALNTHFNVNFTIV
SQVNPHINLFFFSSRGSVGHPVTHRKGRGWRGGYLMSAFEHYLKLDMNKW
LKFVRYAELLPRPLGQDWSQLWLQEFSGTITIWPKSVPSDFYYILSDPDP
SRLARMLHQGQQSAFPMLKFVTNRLKIERLVEQGRRETRPWARRGSIQAV
MSEEDLRSLLVGEMAGVTTEEDTDGDGEEAITLTAVEE
>Mb|QLI64755.1
MKRSFSDVDNDVESQPRPARANGFNPKRQKQYGTGKHKAKEGSLEYSRKR
ARNIERLLHRKKDLPANVQNDLQRELESHKSTVSDKSFLKRRSAMIAKYH
MVRFFERKKASRLVKQIKRKIEQNPGTEDADDLKRQLHIAEVDEAYTIYH
PHLDPYISLYGNSKSEGNDKGEDAAEDDSETKQIRAAAKAALDAKRPPMW

SVVEKTMEEGPEALKQLRERRSADDSAHSAPATKKHSPTTRTSSNKPDSR
QGHEQAHDQQSRAGAKDKASKQQKGEQSQLNRRERRRLMRETIAAEPDED
DDGGFFEEM
>Mb|QLI64756.1
MERGYVRELDLSNCGPASVATRVGVVPGLRSSAAFFTFLRANLALSPSAL
SSNYNTPYLEPFSPQPPQLQPPSPSPPAASSEPPLAPIDDIPPLSLDALK
SQNDKLDGLRLVADSVSEMRHRASKAVVLHPLCLAAVAAAHMAIYQFVYI
PSRDVSRALMLVSGITMIYMATIRFLTSGYDTLAERMGCEDWLRADFGSE
DDIILGARMGEDLVGAVVLRLEPKRAPSTNPKRKSRSRSASLRGGKGVIR
AWTTKPGYRGQGVGKKLLREAVRLARDRCGKDAQVGFALEHANSTMLLPA
MFNKAFRKDEVCAAKALQGAVAEWDLAKKKKR
>Mb|QLI64758.1
MGGGGDTRSKVSYPVLVRRPVGVPKTSILKDRIEPFYKPGQYDKVNLLAN
LYNARFSGKPHVKIYVWSAPGQDRPTFKEATSHEFKETHIGASFGPSWTT
HWFRLHLKVPKEILDQELLVLEWDANNEGLVWTECGIPLQGLTGGGDRIE
WNLPDSFRDGKEHVIYIEMACNGMFGNAPNGKTSIAPPDQNRYYGLSKAN
ITAVNVDARKLYFDMWELGDAARELPEDSAEQNHALAVAMRIIDTFQVNN
QESILKCREIAKEIIGPDVDSDKVYQHGKEPVVFGIGHCHIDTCWLWPFA
ETKRKIVRSWSSQCDLMDRYPEARFACSSAQQFKWLKQLYPSAFERVKRK
VNEGQFHPIGGSWVEHDTNLPSGESLVRQFLYGQRFFEVEFGSRSRTFWL
PDTFGYSSQIPQLCRLAGMDRFLTQKLSWNNINTFPHTTFMWVSPDGSQV
MCHMPPSETYTAEADFGDLRRSISRHKTMRVDSSSLLVFGKGDGGGGPTW
QHLEKLRRCAGISNTIGGIPKLKLGLSVDDFFERLAPKAKDFPTWYGELY
FELHRGTYTTQANNKMFNRKAEVLLRDIEQLATIASIKDSSYKYPTREID
SMWENVLLCQFHDCLPGSSIEMCYDESDKFYAEVFKTGDKLLADLYNSFG
VSSTLSNSLHESVVVNTLPWHRKEIVQLSETEVGVACGDAQLLPVRRFKI
QENKPAVALTSNGEIFVLQNEQLRIVVEKGCITSLYDLVNDREVVEQGCQ
ANKFVIFDDIPLYWQAWDVEIYHLNTRRELHYGKTKIHENKPHRVTLVTD
IKISDQSSMKSYISLGVALKGQQSQVDCSAEVDWHEDCKFLKVEFPVNVV
NTEASYETAFSITKRPTHYNTSWDMAKFEVCCHKFADLSEHNYGVSILND
SKYGFATAGKTMRLSLLRAPKAPDEHADMGQHSIRWAIFPHNGALSSVTV
KAAYAFNNPLKLLSAPETAIQSISEAPIKLINRDESSSLILDTIKRGHDD
EDVSLREGLRVNKGQSIILRVYESLGGHSRGTIETSYNVKRVNKANILED
ALEEVELGDGKFDIKLRPFEVATFKLQL
>Mb|QLI64759.1
MASLREEDARDTTAVPEPVKSPSTSGQPDTGDIEPGLTAPSLATRLNHAV
NENSPLLSSNESDPDDAARPTPALQHVDETQETKGLWYMIMLTISMGGLQ
LAWAVELSNGSPYLLSLGLSKSLMALVWIAGPLTGTLVVPYVGMISDNCR
MRWGKRRPFMVGGTIATVAGLMFLAWAREIVGGILGIFGADPKSEGVKVV
TIIAAVIGVYVLDIAINTVQAAIRAFFVDCAPAHQQEEANAMASRATGIG
NIIGFIAGYVNLPAHLWFLGDNQFKILCAVASIGLAITIALSTASIRERD
PRKDGSPIKKTPNIIAFFFNILKSIKRLPPQTKRVCEVQFFAWVGFFPLL
FYTSSYIGEIYVQPYLEENPNMTLEELEALYEQATRIGTFALLVNAIVSL
LTNIFLPFFIAPTFDGHPVSVNKHTGQPVATKTTGLPRRSYLDYLRIPGF
TLKRAWLGSLVLFAVSMFCTVLVRSVAGATVLIGLAGITWAMALWAPWAI
ISGEISRRDAIARVQRLQNMAPPQSNDPDAPSSNGNSQDMDAELGNEEEE
EEEEEVDQAGVILGIHNMAIAVPQVVATVGSSIVFKFLQKPRGTPGDHSF
AIVMALGGIFVLGSCIFAVRIKDDVDTTEEAVHDEARGALLADEDAGN
>Mb|QLI64764.1
MAAHKTSAVSDEPIEVLIALHNKFDLLDLAGPMEVFTTAQHDPKDETSKA
FEVTLAGAEPKVLSEQGAIVGSQISFKEAHERLEDFDILVILGGNSDEIL
KADTEPLGLINNFAELQKKDPARERTILSVCTGSLFLARENILSGLSATT
HPDYLTKFENVCSDAATRNMTERCDVVEDARYVVNNLRFDLGDEDENPYI
RRKSDAGRRPSNARKGSMSFKGSNSRRESIARRAAMRLGGLRVITAGGVS
AGVDAALYLVSALVDEECAVEAARLIQWTWNKGVVVDGLDV
>Mb|QLI64766.1
MKPAVGRFLILLPVILAVISFILSMLALYAGRQPGFMEDYAFVRLNTSMV
GHNLFDKGKKDDKSGDKKGGGGILGGILGGIVPDVEIPGANVLGDAKDWV

EDKTDGVKGKLNDITGTIADEIAKKIGIKQWYSLHIMDSCEGYFQPNATA
PGAALNVTNCTSSSPSHRLNLTDLLDKELRVGPAKLNLADIKWPKGIQDT
LDVLNNALLGLLIMYSLGIGCCAASALGGLAAFFKPDMRILALVNFTVGG
LAFLSIIIASIIVTVATNTGVEAVNKIGNPVGLYASRGEKFYNLSWVTTG
FMGFVTLFWMVRFCMVRRQRRLMTEKTWS
>Mb|QLI64769.1
MRVLCVAEKPSISKAVAGHLSGGSMQTHGTRNQYIKNYSFDFDFGQALGP
CSVTMTCVTGHLTNVEFTSEYKNWSYPPPETLFNAPIVTSIHDDKKSIAK
NLADQAQYARLLVIWTDCDREGEHIGQEIVDAARKGNASIQVKRAKFSNV
ERAHVLSAARRLVDLDKKQVDAVSTRIELDLRIGYAFTRFMTNSLRPLGG
PMESLTLSYGSCQFPTLGFVVDRYFRVKNFIPEPFWSISLMHNRDGKKVN
FSWERNRLFDRMTTIILYERCLLAKTAKVINVQEKATRKYKPLPLTTVEL
QKAATRLLRMSGQQAMTIAEGLYNKGFISYPRTETDRFDKGMNLKGLVQK
QSPDQRWGQFAQNLVNGAFKQPREGRHDDKAHPPIHPITYAAPSVLSPDE
GRLYEYVVRRFLACCSEDALGMATTVDLQYGDEIFGSHGVIVLERNYLDV
FVYEKWDNNELPRFVRGEVFQPTEAIMSEGKTSPPSHLTEADLIALMDAN
GIGTDATMAEHIQKIQDREYVATVGNSATIASEDDDGVQDRASSGRGRGR
ARARGGRAARGTSTSSSGRRGGVRVFIPTQLGVALILGYDRMGFETSLGK
PFLRKEMELKMKAICEGRTTKEVVLRESITQYRQVFMQTQEKLDVLRAAC
REFVFAQPAS
>Mb|QLI64772.1
MSSTNEEDPFLHVQQDVLSQLQSTRPLFASYLRIRSLTTTPSSPELASAR
TDLEAALSTLAEDLSDLVASVQAIESNPSQYGISSAEVSRRKRLVSEVGG
EIEDMREELHKKIDHQPAAREHLPDPDSFAVGDGDGDGDTYAEFEQQQQM
EMMREQDQHLDEVFQTVGNLRRQADDMGRELEEQRELLDVVDDTVDRVSG
TLQMGMRKLQHVVRKGEDRWSSCCIAVLIFVLILLLVLLLIL
>Mb|QLI64773.1
MTAVASPPTFPQISRPAWGAISGSNPMDPDEVRGMFAPRKSLSRTNSASS
LSSNASNTSVATNGSQSNGTSLSAASDLSQWSATAGPRKRPQPKTPWPPS
KPDVQPDYSRLPANRPNGMAAHHNGLQPGPGQNQMMQQQQMMRGPMGAEQ
FPPGQPVLYLLSLNGTFERKTISVPFAPDSLRIGRQTNQKTIPTATNGFF
DSKVLSRQHAEIYAERNGKIYIRDVKSSNGTFVNGTRLSQENRESEPHEL
QTADHLELGIDIVSEDQKTVVHHKVAAKVEHAGFLSNTNNVLDMNFGDLD
PANGTMMIPNWPLQMRGRANGHALIPGPGGRMMNNGGMNNQGNGMPQQRS
FFLAPVATDQILKRLTSEMRNARLQAQDLSRTNQFVNTLLTKEDVKDLEK
PEVPEPAKQQQSQPSVNGGIPFRSDPKTRFSEPPAPPPQQPLPEKPDVPS
LKRGPTERPKSGPPNSVAARQENMNQIIQLTEALNNAKRDIDTQTARMRE
LEEMLQKEREARELAEELAKRLEASTNLQMNGSVLTPADVTMTDISESAQ
EQEVEESGDSRSAAPEEAEPQKNDTSAEEVTASLQSRIDNMDEQMNDMRE
QLEQWKHRCETAESERDESRKSLAEMVVQLRAEEAKRSAAEERRRSRSRS
RATESKADVTEQEAPATAPPDPEKPDKTIAATNTSKTDTEDGPTLSRANT
ITPLSSQRGFASRDQSLQAGLPYASMLGVVLIGMGLMAYINGWQSPPPRI
EQ
>Mb|QLI64775.1
MAKPHHNQQNPASGTHASRSASASSTTATCRNTDGGQQRNPLRRALETLP
PPIFALPFFAFALAAIAIYFLSSRQVPAPNSVHSTPLDLLSIPASTYSNP
KSTMSSAEQTFIAIKPDGVQRGLVGPIISRFENRGYKLAAIKLVTPSKDH
LEAHYADLKGKPFFDGLIKYMLSGPICAMVWEGRDAVKTGRAILGATNPL
ASAPGTIRGDFAIDVGRNVCHGSDSVENAKKEIALWFKEGDVVSYKQSQF
DWIYEKA
>Mb|QLI64776.1
MSSALLRTTPVVRAGLRAAKPMAMASTSFVRGKATLPDLPYDYAALEPFI
SGQIMELHHSKHHQTYVNSFNAASEVLAEALSTNDTKAAAAQGPLLNFHG
GGHVNHSLFWENLAPNGKGGGGEPEGKLLTAINEDFGSFENLKKQTNATL
AGIQGSGWAWLVKDKTSGTLSLVTRANQDPVVGNFEPLLGIDAWEHAYYL
QYQNRKAEYFSAIWEVINWSTVSKRFEK
>Mb|QLI64777.1
MVIGGCPRQQQATSCDPIPARTVGSESNLQRHHVINEQLGANDAINRLKM

PFQRLVRSSAVRSSLAATRCLPVAQRRGFLPASLSDRKVFDAKYPDRQTM
SEAEDPGMNGGYINPPRIKRQFRDPYATWWDPQERRNFGEPIHEDNDVLG
IFSPYEYTWTKPGPGLIMIGSFVAVFLGLSGLVYLKYPDQPTYPREFEAG
LERELGGPGAVRARREGDEDP
>Mb|QLI64781.1
MSTAARRRLMRDFKRMQTDPPAGVSASPIPDNVMTWNAVIIGPADTPFED
GTFRLVMQFEEQYPNKPPQVKFISQMFHPNVYATGELCLDILQNRWSPTY
DVAAVLTSIQSLLNDPNTGSPANVEASNLYKDNRKEYTKRVRETVEKSWE
D
>Mb|QLI64782.1
MPGPERNPQTLYDKVLSNHIVDEKLDGTILLYIDRHLVHEVTSPQQAFEG
LKNAGRRVRRPDCTLATTDHNVPTTSRKGMKDIATFIEEDDSRTQCVTLE
ENVKDFGLTYFGLGDKRQGIVHVIGPEQGFTLPGTTVVCGDSHTSTHGAF
GSLAFGIGTSEVEHVLATQCLITKRSKNMRIQVDGELAPGVSSKDIILHA
IGKIGTAGGTGAVIEFCGSVIRSLSMEARMSICNMSIEGGARAGMVAPDE
TTFEYLKGRPLAPKFGSDGWNRATAYWKALQSDPGAKYDIDVFIDGKDII
PTVTWGTSPEDVIPITGVVPDPETFASEAKKATGRRMLEYMGLTAGTPME
EIVVDKVFIGSCTNSRIEDLRAAAHVVKGKKIASNIKRAMVVPGSGLVKT
QAEEEGIDQVFVDAGFEWREAGCSMCLGMNPDILSPQERCASTSNRNFEG
RQGAGGRTHLMSPVMAAAAAIVGKLADVRKLASYKASPHVEASVTPSTSQ
KAHVDERVATDAHEREVIGDQPEDSEPHTNTSVSTSNGGSSTAGLPEFLS
HKGIAAPLDMANVDTDAIIPKQFLKTIKRTGLGTALFHALRYSPDGSDNP
DFVLNQEPYRNAKILVCTGPNFGCGSSREHAPWALLDFGIKCVIAPSFAD
IFFNNTFKNGMLPIKIENKSDLDAIADEARAGREIEIDLPSQLIKNSVGD
TICAFDVEEFRKHCLVNGLDDIGLTMQMEDKIGEFEKKMTEDTPWLDGTA
YLKRPGQGGKLAAKAVPVPKTNRGEEKKEPLEW
>Mb|QLI64784.1
MGKPKRNVLAAAEETLTPPATLDPSQSVVRVVKPQGNNLYTCELPNRKTV
LLELAQRFRNTIWIKRGGYVLGERYEQGSGDTRADGEIVNVVRDEKLWRK
QPYWPEEFTKNNRDLSDEDDSTVGQMPSSDSEEE
>Mb|QLI64785.1
MVAGNARYVRYIIIAVFTLAVFYFVSNSKYDAVTIPTGLDHASTNTNAGK
SDDPRNEPQQPLEKPESSDNAATVPKKQNGNNAQSPLAMTPEDPNWDVEL
VSPAPGPRMNATFVTLARNSDVWDIARSIRQVEDRFNRRYNYDWVFLNDK
PFDETFKKITTSLVSGKTHYGEIAEEHWSFPQWIDQDKAKKVREDMAERK
IIYGDSVSYRHMCRFESGFFFRQPLMMNYDYYWRVEPSIELYCDIHYDPF
RVMHEGGKKYSFVLSLYEYAETIKTLWDSTKKFMKNHPEHIAADNVMGFL
SDDGGETYNNCHFWSNFEVGNLNWLRSKPYIDFFESLDRDGGFFYERWGD
APVHSIAAGLLLNKSDIHFFNDIAYWHVPFTHCPTGEKTRLALRCHCNPS
DNFDWKGYSCTSRFFDVTGKTKPKGWESEKD
>Mb|QLI64786.1
MGLSTFVSASLLTLQLFSSPSLAAPSPGFDVKFTNFTNVATNGSWWMADI
KRQGNAPFANGTYSVFRNVKDGEFGAKGDGITDDTDAINKAISDGGRCGD
GCDSRTTTPALVYFPPGTYLVSKPIIQYYYTQLVGDAASVPTLKASANFE
GIAVIDSNPYKSDGSNWHTNQNNFFRQIRNFVIDLTDLRQETGTGIHWQV
AQATSLQNIVFNMVQSKSSQNKQQGIFMENGSGGFMSDLTFNGGALGAFF
GNQQFTTRNLKFNNCGTAIFMNWNWVWTLHGISIDNCDVGINMANGGSVQ
TVGSVLLTDSTISNTRVGIITAYKPSQSGTNGTLVLDNVDTTKGVPVAVQ
NDRNQTILAGNSLIQSWVQGRSYVGATGKAGQDARSPVPKPAALTDSTGK
IVTKSKPQYETVPASKFISVKSQGAKGDGKTDDTAAFQQVFDNIRPGEIV
YIDHGVYVIKDTVRVPKDVKIVGESWPLILAGGCKNFADESKPKPVFQVG
QPGETGNIEMQDFILATKGPQPGAILMQVNVAGESKGSVGLWDVHFRVAG
ATGTKLQSDKCSKDPKTEAPPNPRCVGSFMLVHVTRDASAYFENTWFWVA
DHELDLADHNQINIYNGRGVLIESTKGLWLWGTASEHNQLYNYQLTNAEN
VYMSLIQTETAYMQGNPDARVPFKVNSQFSDPDFSTCTGPRCARTWGLRA
QNSSNVFVYGGGLYSFYDNYDQECVAANNCQDEMISIENSELHMFGISTK
ASVSMVTLNGQAAIQDSENRNNFCAAIAAFASS
>Mb|QLI64788.1

MLRTPASNLLRKGLVRSVPAAASRASSTHAISNPTLKNIEKRWEGMPLQE
QADLWMALRDRMKGNWNELTLQEKKAAYWIAFGPHGPRAVDPPGTNARVA
WGIAVGLAASFAIFAAIRSFAKPEPHTMSKEYQEASNELLIQQKADPLTG
ITSEGYSGKGMVQSPPKGN
>Mb|QLI64794.1
MDVHRCRFVPYRPSAINAVAFSHPKTRTAKQNAVARLAVGRANGDIEIWN
PSSGTWLQELVIRGGKDRSVDGLVWVNEPDQDLGDGHVAIGKSRLFSIGY
TSTVTEWDLEKGKPKRHASGQHGDIWCIAAQPQSDKPQVTGGFQEAQNSK
KLIAGTIDGELVMYSLEDDDLRFQRVLVKSPTKKAQMVSITFQSRKVVIV
GCSDSTIRAYDITKGHMLRRMTLGSDLLGGSKDIIVWSVKCLPNGNIVSG
DSTGQICIWDGKTYTQAQRIQSHKSDVLSLATSADGTAIISGGMDRRTML
YKQNSGAGQRWSKVWGRRYHDHDVKAMASFEHGRISVVVSGGPDASLMVV
PLKEMGRENHRMMSNLPQQPPVTSAPRARFIISWWEREVHVWVLRKSAAD
LLQSGEELVDINQNRKLLKTIVVKGDSNIASATINPDGTLLFVSTATDVK
AFRLQHEDPAKPSDVKLSSLELPVRLSQLGATQVKLSPDGRWLLVVQEGS
RVLMAQIQTEHTSTETAEAPIRLQRLSRLRRQIPRYILNGGLGSYDRSIT
QITFSADSKMVAVADLAGYIDTWALRNHDQDLQTGDEMETLDDAASASSD
NDSSDEEDEGASNMQDRWVRNPNAKLLPKLPSSPVVLSFSDYVPESESSL
GAEKDGVDDYILAVVTSSWNVLAFNPRHGALTSWSRRHPRKALPGPVRDL
LDLPKGILWQGSRMWVYGVSFLLMLDMGQDLPRVPEAEGGDSQLTQGTKR
KRVGQTTGAGGKMERENLAPHQVRKHGADEQWEDIDVVKAPEVGDSDSDE
DMVDADGELSKLRSTTISGKTALQSTGSTTVEAKKWWITYKYRPILGVVP
IDTPGQDVEVALVERPTWDVETPERYFAGEEWDRAN
>Mb|QLI64797.1
MSAELGQRRISAESTGFDAGAGPSSRLSFSHRRSASIEGLKEHMFQWGEG
HGSDDDDVSEHELLMDPNLPDDEHADAIEMGTRSVRKRREEEDSLYPEVR
AAVRNYDEDVPCNTVRAWVLGLSLVVVGASMNTLFSLRQPSISIGPLVAQ
IIAWPVGHAWARFMPKRSFTTWGFTWTLNPGPFNIKEHSIIGVMASVSFS
VAYSTDIILAQRIFYKQNFGVLFQLLLTISTQSLGYGIAGTMRKFLVYPA
SMIWPANLVAVTMMNTMYEKNDVRDPAVFGGNMPRLLWFFLVTVGAFVYY
FIPGFLAQCLSVFAFATWIAPQNAVVNQLFGGTTGLSLLPITFDWTQISG
WVGSPLIPPWYAIANTLIGVIAFYVIGCSVLHFSGAWYAEFLPMSDANTY
DNTGAPYNTSRVLSKDFTLDQEAYESYSPLFLSTTFAVSYGLSFAAISSL
IVYTYLHNGKQIWKQYQNSANEKPDIHLKLMKKYKEAPDWWYMGLFLVML
AMGLFTVLAYPTKLTWWGFLLAVAISFGFSLPIGIIEAVTNNRIGLNVLT
EFIFGYIQPGRPLALMIFKTFGYITMSQALSFVSDLKFGHYMKIPPRTMF
WAQVVATTFSCFIQIIVLNLALTNIPDVCDQHQRHHFTCPGGRVFFSASV
IWGLIGPDRMFSPGRIYSALFLFFVLGAIVPIVVYFGFKRYPKSPLQYVM
APLIFGGAGAIPPATPLNYFSWGIVGFIFQYWIKKRHFGWWGRLNFLTSC
GLDLGLALGTLFIFFAFTMHGIEAPKWWGNEIVDSTMDAQGTAVQGRVAS
GQVFGPKSW
>Mb|QLI64798.1
MAGSATIAFPWECRDKEDQAQASFNHPMRRTNQILMEENMRLKRILRENG
ISWSPVAQAHLIQQNPALRRRTRASMSAHELGCPSLPTEVILRILKFAMK
SPHPIIDPLSKLSHEHLSEKEKARGNQVAIHFLATCRALHTEGTRYFWES
NNFTFTTPEALRNFSELSSQYRNQITHINLRVIARYYDDQRRKHRLERGY
HAELKKDQLLKVRLRPKEPPLIRGGFRCYAWSQITDFLMALRAPYDPNYK
DKNNPRPRLLPGLLSLRLDLVNFSETLLPFSGSELHEVASHELGCTLNEL
QVTGMPFDDAGMKASAELSGMLKDEGLYLDGSASFVALVKQLQPLSGKRW
CARVVRAFKGREDDDDSDEYDEDDEMDGPHPHGHSHARIGTMPPAPVEEG
HPTSSRDPDKVIWKQVPLSRDDDARNWVQFSRRGGHEIADTDWETDDEGI
CPCCGEGHPGSSLLSFMMEQDDDDDDFMDFE
>Mb|QLI64799.1
MTRNGTSTVSVRHGAATGLGAPNDEDETCPVCKTTRYFNRDMEFLINPEC
YHRMCKTCVERIFKDGPNQCPYATCHKTLRLRGFKSAFFADLTVEREVDI
RRRVAQIFNKVEDDFESLDSYNKYLEYVEDLTFKLVNGPPEAKGEAEGEL
AAWEAQHKAEIERNKKLARESDDSRKRRLAAEQDAARQRRLQDLQQDAEE
KASAARFREEMLDSLQSAEVGHATETVDKIMLKKRGQQKRDSARDALSGP

AGGLSIRGLRDKTRGAADDNKPYDPFGGLDLAPTRVDMGHDRLQEYHSEW
IDVMRSKSEYLVGGYSADEYISRALYESFSGLGVFIAEDKVDRAVATAGA
SEAAVAGGKMDTDDVF
>Mb|QLI64801.1
MPWKPATGGNGQKPRAASSKSIRGHISGPIPIPDPMDEEFPMRDQTATVA
RNADEASSDSHLIPPEPSSNRSYKASHFRAVSAGNIDPLIAHQRQHPLSA
PRRASTLRGSSVSQHDRFTRISDQADGRDSKASKNSKPQRKKSTIRSALG
KLFGRKKKSETNEKAGLPDWKASDASTLLSNKQHRSDPTWERSNQMSIEP
KRSFSMPITEFDRALRSHSIGPEDVMAITSVRNSISADVRMSDKNVAAFE
PSTHLASPRWAGGSKLTGLSPRPASSQDRGTRMAGLGDDPSEIGRAISSE
AHGLKRRSRSLSVMPSLELASQGVVRRRSAEIRYWRESYAAPFMSPTSTT
ADEEIRQLLSGDTDEFTSSAEQRVVTPEQTVLMEAAEAKEVQTPTSTKTT
EPAGASERLSLDSRVGSLETRMSRLESIVLQLGQSIPALRQPSTTQLRSS
GGPRQAQLQTPDHIIHSMRIQPGDFSLADEHRQSTSRPSTRHSDASKMTF
GDISDSTPRATILSPVDGEARPNFLAAPRGLDATSKSRHGSLTFEHYTNL
MALFETERSAREALEAQVRSLGRQIQVMSKSMVYTNADQSESPSLDRSLG
EVSVFDYDDEDDERRHFAAPRFGFTSLGMEDSGTTAEDGSDKEYTESFVT
PVETSKSSFDMYGNENDPMLSNGRKLSLSHLTMKQPLTTMPQVATKAV
>Mb|QLI64803.1
MKPDSDLNDQQADDHHSHPDKKRKRSLNEQQSLCLVQHGKDVLGKSADQE
ENQPELGGGTKLTVKKAPSRCTSCVIEWPDAFKVLEETHKALNLVFTFCC
SRKHLATTFENIKPTVETHIKRPLMEEEVALVVALRPEGINFTYVDELML
QLDIKGSERDDTFKPSKSSRSQAPAHDASVGGWTGNESLDDHSRSKTGNG
SREVLFFEFVDGDLKREVQDKKTGEPVRPNRKLRDEQLKMPIYGQKQMAQ
LIERRNQKFKNAINLFLNKCVQDGLDPDVTLREQAQAFIPKPSPTNDVAA
ESRPSTIPGNIPKERKSIPEIVQELKESSWYTGQIVPDGHRVFEPQEAIY
GDLGFLLTQDLVNALYNAKGITRFYAHQAEALNHLHEGKSVVVATSTSSG
KSLIYQLPVLYALEKDYNSRAMYIFPTKALAQDQKRSLKEMMVYMPGLEA
TVVETFDGDTPMSERTAIREEARVIFTNPDMLHVTILPQEERWRTFLKHL
KYVVVDELHYYNGQLGSHVAFIMRRLRRICAAVGNRRVRFISCSATVANP
KEHFETIFGVKNVALIDYDGSPSGRKEFLCWNTPYKDPGDPASGRGNAKL
ECARLFCALLLRGVRIIAFCRVRAQCEILVNAIKQELESLGRPECINLVM
GYRGGYTAADRRRIESEMFEGKLLGIVSTTALELGIDIGTLDSVMSWGFP
YTIANLRQQSGRAGRRNKDSLSILVGDGFATDQHYMQNPDELFTKPNCEL
QVDLSNMLVREGHVQCAAYELPIRPEQDAQYFGADISDVCRTRLIQDEMG
YYHCHDRFRPQPSRYVAIRDTEDEHFAIIDITNGLNTVLEELEASRATFT
IYDGAIFLHQGNTYLVRDFLPDRQMAKVEKVKVDWTTTQRDFTDIDPTET
EAIRKIPCSLSQAYYGTIKIQQNVFGYFKVDRKGRVLDAVQVDNPPVIRY
SKGMWLDVPRTALDILQDRRLNSAAAIHAAEHAVMSLLPTFVISMPEDVR
TECKVALKEFSKKESQRKRPARLTFYDAKGGASGSGISTKAFDHIDHLLN
MALRRVEECQCQRGCVECVASVMCKESNEVMSKAGCRVVIRSLLNMEIDV
DQLPMGPEEFNPVGFETVIPAQPVPSRGQARTKIGTMKSERQDG
>Mb|QLI64804.1
MRFATSALALIASAAAASAASVTFWTLDDLVRTIYFTPNPGFPEVAPVTC
NNQQKTVVNFPDQWIGNYYAVQKGQKNAPGMLGEVNFGAWGGMTYFDVSA
IVDPNDQNNVKQMYPASGKSPMSGCPVFPCNNAYYLPDDVQTKVTHESDL
VTTLGEGFTGLNFS
>Mb|QLI64806.1
MAPSWIFGATIALVCRVAHGAQPGAVQPVAAPMRDLAWGQLNFLHTTDTH
GWLSGHLLDADGARPQYSADWGDYISFSQHMKKRADDTGSDLLLIDSGDR
VEGNGLYDASTPKGLFYYDAWAEHHVDLICVGNHELYQASTADYEANTTV
PKFGDKYVASNVDYVDRATGQRKPLAQRYRKFKTKNKGLEILAFGFLFDF
TGNANNTVVQPVAETIKEKWFQDAIREKPDLFVVVGHVGLRMTEFRTIFT
ALRKQNWHIPIVFFGGHAHVRDALSYDSQAFAMASGRYFETVGFMSIDGI
QKQSADDVSAAASLKFSRKYIDTNLLGMYYHTGLNQTTFPTDDGKRVSKQ
IARARKTLNLDHRYGCAPKDLWASRVQYPNNASIYSWIQDEVFPDVVVNT
KRKDKPRLAILNTGGIRFDIFKGPFTRDSTYIVSPFTSGFNYVPDVPYNI
ASKVLTLLNGADKILSTHGAETKFMAIPEVMFPSGHGQSPADAHQAEDER

LELRDAKPDLIRGYTTKDDIGTDGDDTVHEAIPFYNTPNCIQSEINFPEK
GDPETVDLVYIDFIQPWIIPALKFAGGDYTADDAKVYMDGTLTYKLSEWI
GKNWQGDC
>Mb|QLI64808.1
MGMEHVELSHEEVWDDSALIDSWNEALAEYKKYHSVHARGGSIRDLESKI
SQRAPESEPNKQANNPEPVSSTGNEDPDAIISSSDQAQSSQKGATAAASA
APPPQALLGSIRDDNLKRLLMSWYYAGYYTGLFEGQQHGQQGEQKPQS
>Mb|QLI64809.1
MAGELNVRPSSGSKPKTNHVPSPDPQWSAISTKLLLAELQRRKEDGERPA
CGSKETGWYDTAAHVFALLLILTLSTLACGFPLISRRATTGKRQKTIIFY
CQHVGTGVLLATAFVHLLPMAFESLTDPCLPYFFSQGYTPLPGFVAMVSA
IMVVGVESYLTARGAGHSHSHAHNYFDSDESDGGGEFQDVPLHDHPGMSA
GRTRNHRPRDISLGDDTEAAQGLVAGVSPVPESTPTATIHRRASAENEFN
EGDSDLELDMDELDPAPVNHNHNGAYASLTKPGTRTDPSEPTLHPSSPEE
TRRQLLQCLLLEAGILFHSVFIGMAISVATGPAFVVFLIAISFHQSFEGL
ALGSRIAAVPFPKNSIRPWLMVLAYGFTTPIGQAIGLIVHKMYDPASMGG
LLVVGFMNAISSGLLLYAGLVQLLAEDFLTEKSYRVLKGKKRLQAYLAVV
AGSLLMAIVGAFA
>Mb|QLI64811.1
MAHAGRRSRLDPGEASPSFAQTSAEFRLDDSDDEAVIDEFDLRHEAGGSV
EAYEMVERDAGRRRMSARSASTVASFQLYTPDEDLAVRRKFDRKLVLFVA
LLYLLSFLDRSNIGNARIAGMDEDLQTTPPRDDWYEWALTAFYISYIAFE
WMSLLFKLVPAHIVVSMTVLAWGLTASLQAIAPSYPVLIALRVLLGIGEA
GFTGIPFYLSFFFKREELAFRTAIFISAAPLATSFASTLAYIIVKFASLS
PIAPWRLLFLLEGLPSVLAAVAAFTTIPDSPSSAPYLTQREKKVARLRLR
RDHHHPSSHQPHEPASGLQPRDLLLVFADPVAWLTSAMFFLTNMAYSSLP
VFLPKIVADMGHDALTSQALSAPPYLVAFVAVLLTAHASDRLRARTVPIV
LHALASAAGYGALALAGPLRLPPFLRYLAVYPAAVGFFNVVTLIIAWSIN
NQASQTRQGGGFAVLQLIGQCGPLVGTRLYPDRDAPYYTSGMGACALAML
AVAVLALVLRWYLRYRNRKMDQKGVDSEHDEYAVEEEGLVASGKRKTEPA
HSFRYML
>Mb|QLI64814.1
MLARAFLRNGFGLLVPLAVACSIYLYLYPVFSRCAFPLPSRDPDAAFEET
KKLHWPYADAETNAQAHALPTKQAPFRLLALGDPQLEGDTSIPTVYLGIF
PHVKTIFQRLTFQIWHDSLRDRVRVILHDIIDIFFEDIPFIFESWRKRFD
LFGNDFYLAHIYRTLHWWTQPTHVTVLGDLLGSQWIEDDEFERRGRRFWN
RTFRGGERVPDDVAMWPNIEYNLSGILDGSAAEEVWRRRIMNVAGNHDIG
YAGDLTPERIERFERVFGKANYELRFELPVTDPETNATIQDPETNPASVR
LPPELRIIVLNDMNLDTPAKDQGLQDATYNFINAAIGTACAVEYKGQFTL
ILTHIPLYKPAGICVDSPFFDFHGDHDGGGVKEQYLLSSDASKGILEGIF
GVSRDPQAAGQGMGRPGLVLNGHDHEGCDTYHFVNQTNGTTPDERSWESR
KWQDVRVSNIQQLQQHPGRREITVRSMMGDFGGNAGLLSMWFNHDTWEWE
HEYVDCPLGRQHFWWLTHFIIFGVILFGSSYVAVAILESKGVDVDARLRQ
AFVWAQQQLKELEKKRQARKNAAVSNGKPGGKS
>Mb|QLI64819.1
MLYVSFSLGRKWFDCAECHREQEKHPLLQSFEMVFACKKCKKCFRKDTQE
FEESDEYCPHCDNHFVIDAKTPKAALSVESEDVRVNNKMLRDERVRQNDY
RTMFDPDDDADKLG
>Mb|QLI64820.1
MSTIRVLNNVPVHLPEGLSEKQLLSFRPFQQWTTTLDKSLTRQSAPGHPF
HKDPYSLRSITIQSFDLWGHERIGFIKVLASVSNSAGEALPAGAFLRGPS
VAMLVMLIPDDAAPDSDERYVVLTVQPRVSAGSLSFVELPAGMVDDAGSF
RGAAAKEIEEELGITIHEDELVSLNDLAAEAAGHREDDDEGLPTAMFPSA
GGCDEHITIFSCERRIPRSQLSEWNGRLTGLRSEGEKITLKLVPMKNLWM
EGARDAKCLGALALWEALRREKKLKHTRQEQEN
>Mb|QLI64822.1
MSAPFPNLYTHRKVAESAAKTCDVCYKPSTSVLITPDKKDFFYVCPSHLK
DTYFCTPKIDEAALKAKREKEIAEETEKLKKEYEERQRKKKEKESKDKDK

KDNKEKDKDTDKKDSKDEEKVADQSAQNSKDDGSGIPNEEPRVFELKSAF
YQQRLQRKRQVEAAKRDRERVSQPGYFPSVPSAPPAK
>Mb|QLI64823.1
MSFFGLGTGPRGHNTAAPGFSQAHDPFAGLSGRGNEDDALDFEDTYDGLG
DQLDETDDVFNDDTFGGDSAVAGNVGKDFDFFGQTAKVADAIEEEHLRFN
RQQPAPRATAAQPQSQAIPSQTTSNYSTQSTQRPARTGYEKYREPEPLPD
LHVDQSIWGIAPAKTTAHTSQGATQQMSTSASRKIMSLEEVEASMRAQAP
KQAQSQVPLSDPAIYQQGPATEYIAGQHVTQHQQQGHPGHGPPVTILQRP
QSMQARPTEQMPTPPPQQSRQQAPLVQPTQILQNPNRLSGESAPMSAPGH
YGHRSQGSIPGLAQLQAHPQMLQMSEDEKAAFLDQETKRAKRNHKIWLLS
KDNGLMTPQDKNFITRIQLQQLVSATGNPVEGSDSSISEDFYYQVYSHIR
AGQRQNPSQPLSNFAQTYLFQTGSRHGGMRRHGRPAENHIQRMEQQVQRA
VEAAKNKPKNPQLVIAGSLGKISFSNAKTPKPLLNIKRAETETPRPSNGK
RSPHQEAVLDRKRILRNVEKVYDILMKIEDHIRLIPPPMGNQPDIELQEQ
HRQWAVTLDAYNAKLWTELKVHEPIGATVPHPFIAFLSCAKGKKAIPRIF
PHLTFEQRTTILTMIIYHLDQLDVVQGAAVTSGETNLNSRMRENIELFIS
TVMPSLMQYFNETGLDIVDGVLNLIATKLNVDLIARTRIGVSMLTLILSR
AVLLKQTGAGNPEQWEKWDQTFEILFSKLEPSLPYIFPGSVNTGIDVYVW
QLLAAMGVSATHDQQTRLVLAVKDRVLDTVSMSKTLPPTMAAERLGSVNL
FMRSIGLDVELLQ
>Mb|QLI64824.1
MAHAPLLRNNTAPVFHPNQSGVSVLPHNNMNSVPRGSQLSGSTAYTSSST
SLNSLSSAATVVPGQNGGSVVATANIINQKADASRSLYQICVSLKQRLSQ
VPDFGPFLEQLDPTDPVDPLWNLFRSGYPLLTIYNSLQPTEPLKVEDPNA
SEAKKSKIAIFRFVQACMKELQVPPAQSFVITDLMGNDTSGFVKVTQVVN
YVLDLAEQRGLLLQLQPYPEDEPAVATGGAQMSYRDHIVKELVDTERKYV
QDLENLHDLKKTLEQKGLIPGDVVHQIFLNINAILDFQRRFLIRVETTNS
LPKAVQRWGAPFCFYEDAFDIYQPFIANQRKAAQIANQVFEKIQQSEHPV
AADFNTLDGFLLKPMQRLVKYPLLLKDLAKKSEDEEVKDDLNTGCEAAER
VLLKANEAVNRDLLDEALEELITRVDDWKSHKVESFGKLLLHGVYGVITG
KSDQEKDYEIYLFECILLCCKEASGKKDRTRSTGPKIRNKNAKLQLKGRI
FMTNVTDIVSLSKPGSHSVQIWWKGDPGVENFTIKFLNEETMRKWAGGLE
TQRKEHAPRLSTSQESINNDFAWTRDQAGGLENPYLLQQDEEDDEDYGPA
TAPAQFPMPSSMGTAVPRTASSSSLRQRSATGESTQSLAGIARAPPPRFP
LPMPPAPLNIQIQGNGAPSPGSRGGDSYFSPVAESPASSRTSTTSGIFPG
PAYQFPPKAGTPQAVWDDPRYSPSMPRAPSRDGSSPNPIGMGSRTARGPS
LPAMATHSQTAAQQQRSRSYSTPDVNVAGIPRHRQPSQGNIPAVPGIPQH
LHPAHDGSIPRSQTGSPRNDLPIRTSTQSPGAQRERMHQHSGSLGGTMSH
FPHQQPYPRGNTPTGGNGLRVDSATANSRNVSPSLGTATLPPTGNPVASP
DLPMPTQLKVRVNCDSGNYVTLVVAFNITYQSLIDRIDAKLARFMTSSIS
KGMLKLRYRDEDGDFVTIESDDDIQIAFMEWREGVRNMYSGGVGEIELFC
VGEGQ
>Mb|QLI64826.1
MPTELEELVGFIAHPNPQIRLAATENLVPYSTSEPVIFKTEEMKPIKNLK
LLIRDHPKIAEHVLTMLINLSGDPEILEDLANDDKFIGVVLDHIVGQGEP
NANLLAMLLANLAKLDSFESILERKQTAPSALGSDDSIMNQLMDLFVKGQ
DGAYNKKANFDYLAYVFADLSKHEKVRQYFLTEQKYDGVIPLTKIRVFTE
HESDVRRKGVASTIKNVAFDIDSHPVFLDEDGINLLPYVLLPITGNEEYE
VEDTMEMLPDLQLLSPDKKRDSDKKNIQTHIETLTLLTTTREGRELMRRV
KVYPIVRETHLRVDDEGVREACERLVQVLMRDEEGEEGGASSQVQEVDED
DEIVEV
>Mb|QLI64831.1
MASSKEMQPSSRPLTLQRPSVSSPSAAAIPSRSHARNNSHSLLSSSLNAN
HRVTRRKSVTNPGPNVAALTAAVANGEQIAAIPITNGGRRNTISKAALAK
TSLVGSLPSPPASLPTNGAILDIKQDVHDSAIDDGNDVSADEDDKSQQAR
IRRASDGSPLVKEPKKSNRVEVRCDKCGKSYKHSSCLTKHLWEHTPEWSF
TSKLLISKHQQVQLLEAASVLVAMNDKESTATPPESTKDSASEPDSSSPA
ASGYSDHVERQSSADTTPPPMMEESGLSGLSYRDGSGAGFARSYQSTVSG

SLPRGSAFGHFRQPSHERRPPSSGANHTGEDDRDLAAAVELLSCSFNSNN
GSHGTVTVPLDAPPVPPVPAQYLDQAASFSSAGFINSFPQRQPESFTRGE
MRRGGEDVKMEDSDDDFDMQSRARSDEDDDGVFGRMEE
>Mb|QLI64836.1
MASQTRDASSKPGVQEPRPSSSVLLLSPTNEILLLHRVKTSTSFASAHVF
PGGNLDPLHDVDDGDIPGPESAERHRDGPAYRMGAIRETFEETGILLATK
NGALVNLSAEQRDEARKNIHGNKVKFGDFLKSIGAVADTAGLIPFTRWIT
PTNVPKRFTTQMYLYLLPISKRSVPSEILLPTPDGGVEHTAAQFAPSQSW
LDKAADGSIILFPPQVYLMKILTRFLTGSTSALEEGPLHYTAQRKKLVSF
LRRRVTAETDRGKQHPTATISWADKVISPYHLLFREEDGRVVLALDKPGP
ELEGSDRGGDWERVALVKFGRGGPSEVEIRMREEVLAEQRAKPDTRL
>Mb|QLI64840.1
MSKRRPVEEAIEASPVKKLHIDCAPQYDDGDDDFTPSESPSSMADAESPA
TVATTPRAKFPSDLKTLACTWPGCTKAFNRPARLRDHLNSHTNSRPFKCP
YDDCDKDYIEDKHLKQHIKAAHTNERKYACQVPGCGKSFVTGTRLKRHQA
VHEGAERFRCKDCGQSFRKRETLTKHVRKEHLHIRAYACSEQDCTESFDS
KPALKRHREKIHGEIKYWCGECGLKAEEDGTQTRVGFTTELLLHAHLKKE
HQNCLFCDFKPSSRWELDQHVDMYHSGKTVKDRQTHACTHEGCNKKFTKK
SNLKAHVRSAHEGFRFVCGDVTPSGPEFAAWTNDQGCGAKFSTKVRLDDH
IRFIHLGHERPKLPKPGSPSESPTAFIDEISGLANQAKHTVFCPDCNEGF
KRYHDLQVHLSHGHQQPDPPSHSAPFLHLDDGDDELSLGREEFHASWPED
TAQDEIFAAQMDYGPPKDEWLEDEANIMLLARDSSHEEHIDPNLARL
>Mb|QLI64841.1
MASPLLAMGEKLSVTTAIIGLLAVGGKTIDALWDLNTPANKANSIFATAL
QEIKQCRSTVHVLYKTFSLVESAQLPFPERGTWIEADYLIATMTDTVLAV
SDLQAICATLNLERQGLATPPAEDLAVGHDYSSSYEQSIQALCSRIRWHN
LSMTMMMTILKCPGESDAQNSRVGLERRMTRLLAANTNLSGRMRQLEDVF
DGQVVDRELLPHYSPPARQQAFWVPPRLASSAASMITTSAGGDQRSDNQD
TLRVLSPFSGYTLADIPVLSIIPLPVTTAELVDGNEFYTFAYARRVSRDL
SELMQYQAGQGTSQSLGVILGRTNNTSIDNGGSTSSTGSNESGPVVGEVQ
PDTQRKKWRFRRFRVKQRWRFP
>Mb|QLI64842.1
MPRDPLIGLVGKPSAGKSTTLNSLTDASSKVGNFPFTTIDPQRAIGYLQI
DCACARHHVSDRCKPNYGACVDGRRSVPIELLDVAGLVPGAHQGRGLGNK
FLDDLRHADALIHVVDASGTVDAEGKETRGYDPSVDIAWLRSEIVAWILG
NLMQRWPSIRRRHVAVKATAVETLQGQFSGYGATSTVIARTLDRAAVKEP
LEEWPDETVAHIVNAFVDEKFPTVIALNKIDHPDADKNISKIAKMQDPKT
IVLCSAISEIFLRKMAKQGYIKYVEGSEFIDTREDLIEQGDPDGGGLKEL
DEKNRNRIENLKDMVLYRFGSTGVVQVLTRAAELLGLVPVFPVRNTATFS
SGASESKVVFRDCVLVKKGSTVGDVAKKVMGDAPIAYVEGFGGLRVAEDD
YVAVGKNDILSFKVGRG
>Mb|QLI64843.1
MLASRLSRALPRATPITARSAAFARPLAPRFARYESTSAAEGEKVQGSVI
GIDLGTTNSAVAIMEGKVPRIIENSEGARTTPSVVAFAEDGERLVGVAAK
RQAVVNPENTLFATKRLIGRKFTDAEVQRDIKEVPYKIVQHSNGDAWVSA
RDQKYSPSQIGGFVLNKMKETAEAYLSKPVKNAVVTVPAYFNDAQRQSTK
DAGQIAGLNVLRVVNEPTAAALAYGLEKEADRVVAVYDLGGGTFDISILE
IQNGVFEVKSTNGDTHLGGEDFDIHLVRHMVADFKKTSGIDLSSDRMAIQ
RIREAAEKAKIELSSSLQTDINLPFITADSSGPKHINMKLTRAQLEKMVD
PLITRTIEPVRKALKDSGLQAKEIQEVILVGGMTRMPKVAESVKSIFGRD
PAKSVNPDEAVAIGAAIQGAVLSGEVKDLLLLDVTPLSLGIETLGGVFTR
LINRNTTIPTKKSQVFSTAADFQTAVEIKVYQGERELVRDNKLLGNFQLV
GIPPAHRGVPQVEVTFDIDADSIVHVHAKDKSTNKDQSITIASGSGLSDS
EIQQMVEDSEKYAEADKGRKAAIEAANRADSVLNDTERALNEYADKLDKT
EADGIKEKIAALREFVAKSQSGEGSATAEEIKEKTDELQMASLNLFDKMH
KARNEASSGEQASGEQQQGEGEKKDENKP
>Mb|QLI64844.1
MGSVLNVSDAPTVRVFIAGGSYAGLSVATNLLDLGQGLSPRMFQQPYEHH

PNLPRVNWDITVADERDGFYHLIGSPLALADAEYAKKVWIKFQDLPGLQT
PQIKFIQASVSSVDCEAKTATTIDSTTKVASTHEYDYFVAATGLRRVWPV
VPQSLTRKQYLLEVEEHIHSVASAQHGVVVVGGGAVGIEMAAELKLVKPD
VKVTLIHSRDKLLSSENLSDECKDKALELLKEAGVDVLMSHRLASTTEAE
TANGSPKYEIEFTNGHKMSASVVIMAMSKSKSTATYLPSSAIDGDGYVKI
ESNCTFSPGTPNAEFHLCAGDLTKWSGIKRCGWAMHSGHYAAQNIHKFLL
KKHVGQEPKFAELQEVPPMIGLAVGKTAVASGPEGTVFGEDVMQAYFKHD
LGFTICWDWMGLAGRKEDKTD
>Mb|QLI64845.1
MAHLANPLATTSQLYQHTVLSSLPQDLQESIFIATQCLTQAAGQLLDLPQ
SVTARANVILARYWLVDSPMAHEFSDASAAAIYLVAKVGPIPRSPRDVSN
VYAYLLSSSSTLFKTGHVPKNDPKTYYQSEADYFSFQNRLLALESRILYA
LTFNTHAALPHALAITYLQTMDFLSQPKSSISRKTIQHLNTALLSPQMLY
LTHQPNALATAAIYNAARDVGAKMPDCDWWEVFDVDREELGFLVVGMRSV
EGWFRQRKEETPELFKGMLTRKLIEGEMRKRGLQVTNGEGKEDEEDEVMK
IMDNR
>Mb|QLI64846.1
MVGFRQLLTPTSSSMNSRQPHFNKFYDDRVGAHRHVQSPERILDRFNEPT
IIITAVVLFVACVAKAYNLELSLDAIFYTATTYLWDFVVFIVPTPFIVVL
DNWLSPSSMSESPPSTHAAKSKAMRRLMGLDRSGGMMESVFQARSRALSV
TGCVLGLKLDPERPAGLGNRDNSCYQNSILQGLASLPSFPEYLSTCVRAI
DTYRPTNDVAHTLRSLIADLNDTSNNGRTIWTPSLLKSMSTWTQQDAQEY
FSKVLDDIDKSVARAVKSMRRYSGLEADCNKDDSSTSEHSDDSGYQSLAS
SSDSAAKKSLRNPLEGLLAQRVACVQCGYSEGLSMIPFNCLTLSLGLDKN
QHDLYERLDSYSKVEAIEGVECPKCTLLKAQRLLGKLIEKLKENGSSSEQ
MEEPLRRLEAVELALEEDDFDDKTVSEKCKISAQGKVNTTKTKQIVIARP
PKSLAIHVNRSVFDPRTFDMIKNSAPVSFPLTLDLGPWCLGSAEDVEEKL
ASHSESSEEQWLLDPKSSMIAGDLSPSRLTGPIYELRAAVTHYGRHENGH
YICYRKYPDSEVSSDKDSSSADVDDTIDRDTTVGDADQKKGLTPPHRQNS
GWWRLSDHNVSKVDEDVIMSLSPGVFMLFYECVDPSMILQNETDNMEDAQ
EVSTPNSESLKTANRADAENREGISEAQVLPVDERETSEMPSMPSPASDT
STSETVNTTTDTMDAPTVIESATSSVSRWTHDEDDAYRKNRVEE
>Mb|QLI64847.1
MSDKDKEHFNARAGSSYAASIGITHRTTPLKCSWTGDMRVRFSDFQVNEI
NTDGTVLHLRTIGLGNERTQLEQPQNSESNARNEDSSTPQDEKPQPEQTI
EVPQEDVKVLEDLSTAQFAQDLVKLFNYGNGPSSNDANTATSPVIDDKPK
RAQLHQEVRRIFKSRLETTTGHDGVIVANYVSPRKGKKSRGRRGQPGDDG
PMGDYLHFTLYKDNRDTMDAANQIARLLRVKPQIIGYAGTKDRRASTTQR
CSVRHQRKRALAGLNGKLWGISTGDYEYQEQPIHLGQLLGNEFVITLKNC
KMAGETAAQSPSERLASMRNNVEPALKHMSSHGWINYFGHQRFGTHDVGT
HQVGKLILGDDFEAAVNALLQYDPEIAAKAEAGQVPDEPSKKDDALRHLA
CMLFQTGKDLNRAVKVIPRRFAAESCILRHLTRMGKNSARDFAGAITHIT
RGLRSMYLHAYQSHVWNHAASQRYALHGNKVAKGDLVISDGETQPAAAER
ERDQDGDEVINPVEDEEDAPLRARALTEEEAASGKYTIYDIVLPSPGYDV
IYPDNEIGQFYQEFMGREENGSLDPHKMRRLRREFSLPGRYRKLMQRFLV
EPTVEFKLYEDDTEQMHPTDLDLINGAKKNGIQGKRKRDEDDRGPAKKIK
ADDGEGQAEAKMETANSEQEATSSNGAEANTETELPAEPTKIAVVVKFQL
ASSAYATVTLRELMGDRLDEVTPSDEGVGNS
>Mb|QLI64848.1
MNWHFDMTNRGSERRDRSRPTNGREATRSLKAAPSFIIDTHRHHQRQPAP
RIAISALTAILALHFAPARGPNQQPHKIHHITSSLPQTYVKMSMRIVPAN
SNPESFTHTSHTSSAPSAPGVHDTLRHGVGQSPYDAAANKPSSAHPLEAR
LKNWEATQQAARMESLRRTFGIAEPVRRGMELKIVRDGEWRPLALGNGLA
SVHEDILKGREDTITWEDVFTGEEMKGVPGVHDEMEKKLKIQ
>Mb|QLI64849.1
MGLLGISLIVASVVYVIIRPPGWLAHILLQWRPSGGVIADTPTRQKRVQD
DTAPRIQEDALSIEEPLHSLNGLAKKATPAESKQLKDAAVMPPSPPQSVI
ELPTLMVDEPEGSTTPKAMANKPSDPVPSFSLSPEPLTSSKSSSSMMPPP

PLPARLSAGRMPTLPQFPAPNSAQRARGPVPNRGPPGLSVPSAGLTPPPT
HSSKPQKPSRKVLLDPGHSPLDWARISGPNADLRGVEPSTPYLRVTPSML
RNQTGRKGKDAWMALNGKVYNITPYAKFHPGGIPELMRGAARDGTKLFGE
IHPWVNYETMLSACLVGLLVEEPDDGSQESEMDKMD
>Mb|QLI64850.1
MLQMHGGQQQQHRTDDSTGEGDRGRGKLVPPLSLPKNRSTGNLALSADAT
VDRSRNRMSFGGTPTSAMEYDALTRPSHINDHEHGAGITGLRRIRQQHPP
SRAPTMPNSNASSRSPSVVALSRSTSMSAMLASTASMQHTAGPASPSFSE
DLSRFPSESLHSFSFAHQSEEFIHNRQTVLKRSMDFMKDRMGWPMSSTQA
GLASAHARVTGDVDTQNMLELLARAQLIGAGNLPNPDPAAVTAAPLTGPA
DVTGENIFEKQFMPPLSRTSTTASDSASPFTAQIQAPKSPLVRFQTSDGA
SVESLPAVPELEHEALTSIGDDERLDDEELDPRAKPTSRPISLKRTMTDT
LGIALQDKLMDTMAQPFLAGQPMYEEPITSPTTTTAYTGPPTKTFPSALS
PTPHGHTNRWVPAAQAIFTTEAKPPWTIIAANDLACLVFGVTKAEVRKMG
ILEVVQEERRAWLERKLLRTDEESEDDVKEGTVRESPASSVASTLLGGRS
GGITAQLLSKPNSRSQPSKYVQRRAQTVHSGDPNPPTTRGTGQHRSNLSR
GVLLCGDVVPIQKRNGATGSASLWVKEKRIGLIWVLEEIHEDVSYVTIDE
EGSVKSISGASDTIWGSQATGSDFDVARLIPRIPRQGIDPTIGEIDFAQI
ARRKYFTCPNAHQINIPCSVEQVRGKLELRVSTFPHMAGIIVVDPVSLKI
RSSNSVFCAALFGYEKPDGLPIDSLIPDFDKILDILTQEDGLQMLDGMVV
PEARFRRASAFLALKEHRPDAAARFLQPDGLPARHRDGSDLKIDVQMRVV
ESEKQTNIAEETVHEESDEDEANSGDESFPVSHKEMVFALWITYSRHLHA
HARLSVDSPATSGTTTPLHQPSPGQTPPVHTPMEIASDDDSPPKKEERVA
TTLSRQLRDVALNAAAKLTRQPKPTEVSTSGSSSPARAAEPARKTTIRDY
SILEDMGQGAYGQVKLARHKTSGKKVVLKYVTKRRILVDTWTRDRKLGTV
PLEIHVLDYLRKPEFRHPNIVEMEGFFEDDINYYIEMAPHGLPGMDLFDY
IELRANMQEPECRSIFVQVAKAVHFLHAKALVVHRDIKDENVILDGEGNI
KLIDFGSAAYIKSGPFDVFVGTIDYAAPEVLAGKPYGGKEQDVWALGILL
YTIVYKENPFYSIDEIMDRDLRVPFTMSEESIDLVRRMLNRDVEERYGIL
QVINHPWCKAID
>Mb|QLI64851.1
MEEPSTLSQTNEPISRPSPTSTLVSANLQQLPAISALAVSNAAQSPPQLR
ATAAPQNPTYTGATPPAATNGNGNGNLPTCQNCTTSTTPLWRRDEFGSVL
CNACGLFLKLHGRPRPISLKTDVIKSRNRVKTIRPDLAAKKKAQQQQAAN
FGLGADVSGAALSAQNAAAAAQAIRRTSQKPNGNEGSDSPISRTGTPSMY
NQGLPSFMVDDPYQGGSFNGTSENGDRDMPQTHEQLIAHNSSLKTRVSEL
EVINELFRGRLSQLEQQEAAARHGQEIAGVEQTQLRTQLDASQETVTQLQ
TQLEDSHRRENNLKRRLDELELELKAVQDALDGDVERPNKKPRIGNEVPA
AVPTPMAEEEQPVPQAEPQAEPQAEQPEEQPEESVVKEETMTEPAAAEEP
AAEETINEAAEETNETIDEASIEDAVPAASAS
>Mb|QLI64852.1
MSPKQIGPIAQAWYKWKALRLPWRKRFFIGYDLQGNTYWEFRLAKPSPGT
SPSDSRWRRIVHYPRSTHYSQVKVSPLWHQWLRHTRRDPPSLEEQAGDLV
RQERMKHLAAEADARWEAKPRVMDDVRPGAAAAAAVPQEQIPRPAETQRA
KGEETPGEQEQSPEQDAARQRRVEGKEDPWAKARPQGPGETWQPTAWNPA
AAAVAKKR
>Mb|QLI64853.1
MEGRAAQRSGVPQCRRRREHHQVDAASSNTLPAQGSARPKPSEMNWLRSR
PDTSWISAGPASSFPNLGEDAGSLIRSRLCDAQLTPGCKIFHVPKDQPSQ
ASEMHISPESLESPVVGDELKDQVLVFQYRGNFHAVDHSYPLSNGIPFDI
EDFGVVLSAGLSCPKHGWSFDLFTGTADRAAYKLAVWEVQLREKDGVTVV
DRRGGDDVGAQDRTVWVRRKPRMG
>Mb|QLI64857.1
MRGSRLIYDGLWRCLCPAVDGRALRRAVSSRAPGVVTTARAPSAIACTRL
AQARHVTTASASASIQNQMVTRDAAQQTKHEVPREERLLTPAPPSEETLR
QASVEEIVAALVTMRDAQGWSFHGQEIDRHDRVIRLVRHLLSARDQTPSP
FIYECMMDAMVDPQGSVKGIRKLLDDMASQNMKPTAAMCHSALAALTNHP
DYALRQEILDTMQEFWFTIDTPARQSVVLGLLRDEQYELAYSRLTEMIEQ

GAHIAIWVFDIFVMVFGKLGFVDEMMSLLYRRKSIQGEDRAISSILYYAL
DVCSSAFHYPGTLFAWNSAVRSSLFQPADGIVENVLATAARHGDASLATE
ALDMMSRRTRVLAYHYEAVAEAFAASGDMTGAFRILCIMKKNGIRIVRGN
TRVVSEALRRHPKLMSDAEAALRAVAHDEQLPVAMVGVVVEALAETQGSE
AAMDLYRDVACLCGEHANSTMMQTLIINSPDEETTRALVKDYTAHIAEDD
DPVRDSHVYGALVTRCAEAGELDLAFRFANQALVAMGGSKTRRLDWAKVL
VAKAAEVEDGRIWGVVDELRKDEETGALVERLLRQMRMTKRASELKM
>Mb|QLI64858.1
MSTDPDSQSNGYDGHTHPIERSDSSVSTATSENNEPDTSQPVIHQPVPTR
PRMPSRKSSGPLVVPRDSSEVGPVDPQFGPDDVRAMSPRRTSEDIDRLGK
EAREEMRRHAKALQESLITIFNRIEAVREEHDKLDNNNKFLQKYIGDLMS
TTKITSSGSRGKK
>Mb|QLI64860.1
MSSMNGSSFAAGSPAMTASAPDAFGRNLLSSSISSLQTSTIASRHTSQIS
KTYRQASTLFLTRRLPEALTTLTPLIAPPGSADDGEPAPVAGASRTTRIK
VWSLYLTILNAILEMDPEEGKDAFGNQEWRALCSKVRDGQVWEEVVQNGY
HGVEGDVDSDVVINLATILLAHARDQILNQRRLETYLATTTTPNLDLASK
LNGSPAPGSRMGSRYRSPAPGTSGANTPRDLNARVKILELYTLHVLPRNN
EWDYAREFISVSPVLDDERREAFVQALQSLQEEQQEQERLEREERRRQEE
ELRREIEDTKRQRAENEARERKRLEEERARREASEVDYGVEQSPSSSTSG
NKSRQSRASRPQHRPNKPSVSSRQNGKAVAPPTFTARATMVVTRLRSVVD
ELARSLNSNPALLMRFLAFVVGFLIMLGHKGIRQRIQRVLGASWNKVMAT
AGMGTKVSYI
>Mb|QLI64861.1
MLPPGSVAGLTALLAAVPSVHAMYTSNSPVLQVNAKTYHTLIAKSNHTSI
VEFYAPWCGHCKNLKPAYENAAKKLDGLAKVAAIDCDNEMNKQFCGGMGV
QGFPTLKIVRPGKKPGSKPVVEDYQGARTAKAIVEAVVSKINNHVTKVTD
KDLDAFLKPQGPKAILFTDKGTTSALLRSLAIDFLDVISVAQVRNKEAKI
VEKFGITKFPTFVLVPGGDKEPIIYDGDLNKKDTVAFLKQVGQPNPDPAP
AKPKVKGDKTKAPKSEKAQSKKTKASSSASPDTEDSATTPEASTQTTATE
PAASTPDVISITTVTTKDTLVEKCLSPKSHTCVLAFIPVDASENSAKVTD
SLSQLNTKYVHGHRQLFPFLAIPSNIEGLDSIRQALGLEKDVELIAINAR
RSWWRHYDGNFDVESVEAWLDAIRMGEGSKKKLPTEIIAVEEVVEEDSDK
KTDAEEQAPADEVKHEEL
>Mb|QLI64862.1
MGAARRALPTRLLCRSCLKHQQRRFASSAAPPKPPPSGLAALPSRQLLSV
SGPEATKFLQGIITANMANAEGLPRRDAFYSGFLNATGRVVHDIFIYPFR
QAKQDDGYLIEADAGEMARFAKLIKRYKLRAKVTVRNVPPDEASVWQAWD
DASPLDIAASESRVVLKDPRAPGLGHRIVQLNHKAPELDVDASTEDAYTI
RRYLQGVAEGQDEILREQALPLESNMELMNGIDFHKGCYVGQELTIRTRH
RGVVRKRILPCAIYSEDKAPPQTLSYDPECASPESLTADMIPAETSIGRF
GKKGRSAGKWLKGVGNIGLGLCRLEIMTDVVLPGEQAAATYKPGNEFVLE
WGDEDNKSDVKVKAFVPKWLREGLSAQQG
>Mb|QLI64864.1
MNVERWKRRSASVLSLNLPATNHYDAKPQSQSTESKAPRVLTRALRSLSS
SSMDSITATSTRSNSMRKLQKTPSSGSSSMIDRLQKRVSKDSPITDRPGS
PTEQLQPYSAMEVLQSGSLKTDVSLLKAKSEYLVLSDQVLVKFNNGDSAR
TAFPQLFGTSADGHNTSQCHHTTSKLSSGDVRLEISLRSVVAAFNDDSAG
SRSGIEIWWFSPWPKLAYSKAHFYFSLPKERDLWLASIHRAIRAKLRKSS
SSALISDNLRSRIDHIVRAAEGSADAGYQSIIFPVARRVFGGGMKGSATE
EAPDNSDISSFFLIIGPCMCHFVEVLKADHATPPGDLRVKAISHGTVTLT
RFKASVVSHEHRFVMCFRSPFGRETRIDLASSQYRRIIETLTKADRILKP
MWPQHFQQVIFDIRGLPPPLQLTSGNDLGGLKRSLEAYCAAYQVQVPNWT
IDWSPPPQPAFRLLPSNGRAYSPMQLLAVFRALRYNSFFKAISFRGVDLS
SLVGKNDKSKYGDSLAYKSLNGVTISEEHHEILLQSPILEQEIHALLFAS
ESIRSLDMSNVLGLGDKANRLSRYQYDLTSLSKTTSEILRPFLELWRRQL
CICHSLSLSGNPIALDDLDELADLLTLDQVHLRRIDLSECSLGDAGLSKL
WVSLAGQADSLEWINTSNNQGVVRFEIIQSTLSGLRRLSKLEIAGNTRIL

SEESLFDELTMQTWQLQELDLSGIMLNDATVDALSNYLQTANSGSVHTMR
LNNCGLTGVQLARLFYAMGRARPVELYINGSRLDEGVDDLCMAIAEGYGP
WCLFVQMVEFAMEANYIKLLKALTVNTSIECLSLAGTSTPDAASSAACQA
IADFFAKNCTVRFLDISGFDARLDEGRLGREFSKALISLRTNESIEHLRV
RSQMLNINIGDLAEAISENKTLHTLDCEGNDFHLSNYRHLVSSLDQNTTI
RYFSAFSAEELNKATSKSVDTAVIGAPVRRPSVISRFKSDKTANGSSKPL
VQRLKDEWDTAGSDLARILRRNQVLFDHENTAEPDESSSQEYHRNSITEV
AFSTAFGGLPLKDLQSQRAKGGIRSSQDPQQAVSGDLAVRRHSRYGSIGS
RELATRPVSMISSEIAASPSTEEDSNGSSGAPTPPEFESPIEQEFGLAAE
IFASTIFDEQNYTYSDGNDADDGLQMKRYRRYMGDPTSRIDEEEGANDTE
LP
>Mb|QLI64865.1
MSAPPPPNGPPQGGIPMPMPGQRPTPEQIAEIQRRVAEDAEKAGMTVPEF
IEHIKKQAMEQQRMRMMQQQQQPEAGEHVHGEHCNHDHGDGHHHHNHQQG
QPQPIVPGPPNPKALALAKFLKGQDLKPRTSILNGERKDMFRVKRALRAL
QTPAYEKARKKNPLLPEITDRASLENAFKLLPLSMLALRVSKIEPEEGPN
GKKPKRTKGQWNVRIEQQQDAGDDMYYVWLWEGSQVKRQLYAALALVLIF
IIVLYPLWPLKLRQGVYYLSWGLLGLLGLFFLMAIFRVILFCVTYFAVPP
GLWLYPNLWEDVSFMDSFRPVWAWHESAADKKKKKKAKSAARASGKVPNN
AFAASTGQIAPTTATTTGTDTQINVGNVQQRHYEAPKVEELADDE
>Mb|QLI64866.1
MASPDLVAAEAEWREQFAAMQTALANLKLPTLSKAEKSYPDDDDDLEGFS
SGNDDHDVWDFISDTEQDEYSSDLVDVDVDGVGAAEPESALHWFLDASAG
IAANNNLSPDVFQNQIMSVLGSGEPDYELQSHLTDLVGFDHLDFVIDVLS
KKDVLVAESKGRDEKHPTSRRLLNKSEREAALKRQDFEHKSASLSPASKK
EPHYPHVYRSYQAGNTLSFAGKKYGLPVGSERLQFEKYEEYFVPAGRKGT
LGPGEKLVAISDLDGLCRNTFKGYKALNRMQSLVFPVGYKTNENMLICAP
TGAGKTDAAMLTILHTIGQHVYPNPIENPEATEFAVDIDDFKIVYVAPMK
ALAAEVTEKLGKRLAWLGIKCREYTGDMQLTKSEIVQTQIIVTTPEKWDV
VTRKGTGDTELVQKVRLLIIDEVHMLHDERGAVLESLVARTERQVESTQS
LIRVIGLSATLPNYVDVADFLKVNKYAGLFYFDASFRPVPLEQHFIGVKG
KAGSKQSKENLDNIAFDKVKEMLERDHQVMVFVHSRRDTMVTARMLHQKA
IEQFCMDLFDPSGHPKYDQASRDMKSSRAKDIRDLLSKGIGIHHAGMARA
DRNLMERLFGEGVLKVLCCTATLAWGVNLPAAAVVIKGTQVYSASDGKFV
DLGILDVLQIFGRAGRPQFEDTGIGMICTTQDKLQHYLTAITEQQPIESK
FSTKLVDNLNAEIALGTVTSIQDAVQWIGYSYLFVRMQRSPTAYGIEWAE
IRDDPTLVQRRRQLAIQAARTLQQCQMIIFNERTEELRSKDIGRIASQYY
ILHTSIQVFNTMMQPHATEADILKMISMSGEFDNVQSRDTEAKELTHLKN
DVVPCDVDGGIDTPQSKTNILLQSYISRQQPEDFALSNDMNYVAQQSGRI
CRALFMLALNRRWGHQCLVLLTLSKSIEKRIWPFQHPLHQFDLPKPVLNQ
LDAKENLTMETMKEMEPAEIGGLVHNHSAGKTISRFLNHFPTVHVEAEIA
PLNRDVLRIKLYVIPDFSWKDQIHGTSESFYIWVENSDTSEIYHHEFFIL
NRRKLHDEHELNFTIPLSDPLPTQIYVRAVSDRWLGAETVTPVSFQHLIR
PDTESVYTDLLNLQPLPISALKNPGLEEIYAQRFQFFNPMQTQIFHTLYH
TPANVLLGSPTGSGKTVAAELAMWWAFRERPKSKVVYIAPMKALVRERVK
DWGKRLAQPLGLKIVELTGDNTPDTRTIKDADIIITTPEKWDGISRSWQT
RGYVRQVSLVIIDEIHLLAGDRGPILEIIVSRMNYISSSTKNKVRLLGMS
TACANATDLGSWLGVKEGLFNFKHSVRPVPLELYIDGFPEIRGFCPLMQS
MNRPTFLAIKNHSPDKPVIVFVASRRQTRLTAKDLINFCGMEDNPRRFLR
MDEEDLQLNLARVKDDALKEAINFGIGLHHAGLVESDRQLSEELFLNNKI
QILVATSTLAWGVNLPAHLVVVKGTQFYDAKIEAYRDMDLTDVLQMLGRA
GRPQFDNSGVARIFTQDSKKDFYKHFLHTGFPVESSLHTVLDNHLCAEVS
AETIVTKQDALDYLTWTFFFRRLHKNPSYYGLELSAEEHSTIAAQQLANE
YMIEMVNKSLGELAESKCVEVFPNGDVDPTALGKIMSYYYLSHKTIRHLV
KHAKAQASFLDVLSWMSRAIEYDELPVRHNEDLINDTLSANLPYPGHAFG
LPMWDPHVKAFLLLQAHMSRIELPITDYVGDQTSVLDQAIRIIQASIDVL
TELGYLSSCLRMISLLQCIKSARWPTDPVVSILPGVEPESTKDNTPLSKL
SVLKPNEVNQLSKKLGLKSAQQLSRVARAVSFLPNVSVSTSDVTALSVTV

NIKRINALVDRGARIYAPKFPKPQTEGWFVLVGDITRDEVIAVKRATWAA
PGAKSLGQSSTPSVKTVIKLPEAVGGQARKVDILVVSDGYVGLEYKIKGV
DIPGVPMVDDAIGSKKGNA
>Mb|QLI64867.1
MTSHISDAAQASSGSSNGSDSKSNTPAPSSATSNTSQHSSGDDNLTCRWN
SCNAKFVSPETLYEHICERHVGRKSTNNLNLTCQWNSCRTTTVKRDHITS
HIRVHVPLKPHKCEFCGKSFKRPQDLKKHVKTHADDSVLSRPGQDHQGLN
YRPQGAKPPSYYDHNGQIRSGGFSHHPGHGYYAPQPSTGYNLYFNQPPMS
TPRHEHIGYHASTSAGGYDRKRAFDMVDDFFGNAKRRQIDPTSYTQIGRS
LLPLHGSLSVPNGPMAATEQYMPQPAPAMVHGGPAPTQNPLAQQYYLPMP
NARTQKDLVQLDNLLGQMQDTIYENANHATAGVHIHSGGEGGFGGYRHSQ
SPTVLQRGSPTGLHVAAEGYHPVSAASMASPLTAISSTGTPAVTPPSSSM
SYTSGHSPSPSASSGLSPQSRHSTTTTSSVMYPSLPTSLPAVSQGFGSTT
ATLGPSFDTSERRRYSGGMLQKARTIPPIRPADETSGRTTPKAGDSALSV
GSPSSESDVSETTREREEQYDRWLEDMRVIETLREYVRGRLERKDFVNEQ
EAEASRRDGDAMDVDVRSPPRQPISLREGGSSLYPRLPVPGS
>Mb|QLI64869.1
MDLVNTTVGALNQTQDYFSVFEEVSKYNVNLNYFERLWAAWYLYMQNDTL
ATGIMSFVMHELVYFGRCLPFMIMDKIPYFHKYKIQSQKIPTLKEQWDCA
AIVLISHFTAELPQIWVFHPIATYFGMDYGVPFPSLLKMVIQISILFVME
DTWHYWFHRALHYGPLYKAIHKMHHTYSAPFGLAAEYASPIETMLLGMGV
VGSPIILLSVTGDLHLVTMYVWIILRLFQAIDAHSGYDFPWSLRHILPFW
AGADHHDMHHEKFIGNYASSFRWWDFFLDTEAGPEANKRRRERKLKAIKD
AKKEN
>Mb|QLI64873.1
MEPQQQQQQPQQQQSAQQQPTGGVPGPAGRRLHIAHRRSPSELTPLMSMF
ANPGMEQLAIQQQIELLQQQQQQIQATHQQYVNMGMIPPGQSLGHNGAFN
PLQPMGNAFQFPNQMGQQNMAPPSQPHSHRRNQSAMPNMGMGPPPAPSSG
ASGSNFGNFEAPPAHNRENTGGRGGRGGSGGGHQRRHSLALADAKKAAEI
AQQKRTTSGFQFPAAPAPASDKPDDENKPVAPSNLDISVAQGSNRGRGGH
GRSQSMAVNGRGGSGLRSSAFSLGGDGDGRRGGGHARSGSRNFEGNWRTQ
GQNLDQAGNTGQTSSFQPGHRPRGSINQSVSSIGAFQYNPNQPQLMQLPG
QMMIPQMYGQQLNPIQLSQLQALQAAQMSGQQFQGLQTSQHAGQLGGQQQ
QQQQRKTLFTPYLPQASLPALLGDGQLVAGILRVNKKNRSDAYVTTQDGL
LDADIFICGSKDRNRALEGDLVAIELLDVDEVWSQKKEKEEKKKRKDITD
TRSGSTNQGNRDSGNNEDNNAAEGGIRRRGSLRQRPTQKKNDDVEVEGQS
LLLVEEEEISDEQKPLYAGHVVAVIERVAGQMFSGTLGLLRPSSQATKEK
QEAERAARDGGNNRHNDSRQQEKPKIVWFKPTDKRVPLIAIPTEQAPRDF
VEKHQDYADRIFVACIKRWPITSLHPFGTLVEQLGRMGELKVETDALLRD
NNFSSDEFSDAVLRSVGLQDWSVAKEEESELAARRDYRNEKVFTVDFNGS
AELGNAIHVKSRPDGKIEVGIHVPDVTHFVKPNSLVDREAKKRGTSVQLI
NRFCALLPPKLSSEVCSLGPDQERYTTSVVFNVNPNNGSVAEGDSWVGKS
IIKSSGKLSLKDIDQALENASEFKNEVVPVNTVQILQAVAQKFREARLGA
GGEPIAPLRLFQQLDDENNPVQDNIFDSTQALELVEELMHKVNSYVAQRL
AEGLPEKALLRRQASPNPRRLQTFVDRMNALGYDIDASGSGALQNSLFKV
DDTDLRKGMETVVLKAMHRAKYFIASKTPKQIWPHYSLNLPLYTHFTSPT
RRYADIIVHRQLEAVLSEGKIEFADDVESLAKTVESCNTKKESAQNAQDQ
SIHIESCRTMDKKRQEANGDLISEGIVLCVYESAFDVLIPEWGFEKRVHC
DQLPLKKAEFRKEKRVLELYWEKGVPSSAYVPEDERPKAAASQRMTNAMA
AARQAEEAERVKKEREEAARKQTITGTISTDDVDALFDDDDEDNTSDLTE
AMAGASLAERPTQSVPGSPARSASNAGKLHRTRSDSKVPVTDAVETRLTN
KEKYLKLFKLREEGGDYIQDVTEMTRVPIILKTDLSKSPPCLTIRSLNPY
AL
>Mb|QLI64875.1
MPATKLTNGLVAILGLTSMVYSEKSSKGDLILCDCGIGDDKEHPDWSTSR
QMNWYKDIKWPASAANYPNAPDMAVQVPYKDGIYPWIPQGVTATMPNGEV
WTAYIEDGTPDGFKAGSAVSSKDGKNMLNCWAYRGRPVSAAINKTVSHDA
ICWTAFVCNHDNEPPSRPDDMDHPSTMTSSTATPATTYYTKPPKTVTATV

TTTSGQPVPTYNPNKGTLSIYAGVNPRFINWQDTWQSFINHFVWDKNSGR
CIGEPARGIGYNITIDCAGIQIDEDTHMTLLLIKALRDVGLNSLWFNQNP
VIPGGNGPNNTSPNWVVMPESFTLEATDLGTNNVVGRLAYKTHYDNFLTP
PCSICEIGRFDKNFFNPIIESMKGSYPEFNNFTIKGQCDPWIACV
>Mb|QLI64878.1
MSLNAGHPTPKSVRSLQVTDATLSWRDLDDAEESVSTHEIVFTIDFSTAQ
RPNYIVYVLREDADAQSHPFQLDLLQTDAIPAELRHGHLINGIPPHLQPT
PSNKVDVIVSTKSGTGRAQSFWQAALQPLLQSASSHPVVEQYNLVVTQDA
QSVRQYAKTIDGPPSAARTIILLSGDGGVADLLNGREPVPDAPLPLMALL
PLGTGNALFHSLHKPCATTPGPTPLVLALRTLFLGAAANLPVFEAKFTPG
SHMVAHADGSQAALQLPHRRQVSSLYGAIVASHGFHASIVYESDTPAYRA
HGAKRFGMVAQELLKLSHPYKAALDVRPPSSAAASEDTFERDAHETHGYI
LVSMVSNLERNFTISPASRPLDGKLHLVHFGAVGGDRAMEAMMKAYDGGK
HVDVKWDDGERVRYEEVGEVRIVSGEDDERWRVFCVDGTIVQVARGGGMS
VARASRELLRVLVDEGARA
>Mb|QLI64880.1
MSPPAAVSPTQKVAELVTPTSSSKLAVKAGSNNAQTIEEMMGQWDNFKFA
PIRESQVSRAMTRRYFKDLDTYAESDIVIIGAGSCGLSAAYVLGKQRPDL
KICIIEASVSPGGGAWLGGQLFSAMVMRKPADAFLREIGVPYEDEGNYVV
VKHAALFTSTIMSKVLSLPNIKMFNATCVEDLITRPSEEGVRIAGVVTNW
TLVSMHHDDQSCMDPNTINAPLIISTTGHDGPMGAFCVKRLVSMQRIEKL
GGMRGLDMNSAEDAIVKNTREIVPGLIVGGMELSEVDGANRMGPTFGAMA
LSGLKAAEEALKVFDLRAKQNAI
>Mb|QLI64884.1
MAQNRHWEQDKEATIYIGNIDERATTATIYEIMLQMGPIHNIHMPRDRVT
QNHQGFGFVEFRTPSDAEYAANVMNGIKLFGKSLRVNKASADKQKGADIG
AELFIGNLDPMADEKLLYDTFSRFGPLLSLPKVARDDSGMSKGFGFVSFG
DFESSDAAVANLDGQYMLSKEVSVQYAFKKDGKGERHGDEAERELAKQAK
KRNIVPEAQALPAFVTQQSQPQQPPPAPPQQQQQQQQQHQQHQPPQGGQ
QPPPVPAGFDPAMIPPPPAGLGAHAMPPMAAHHHAPPPPMPPNFHRNPPH
SQARSSVPLPPPPTGLPARPPQSQNYTNPADFHPGAFRPPSGSPNPPGYP
VAPPPGFPAAPPGAPGQQAPPGAGPPGFMPPPGFQPPPGFGRR
>Mb|QLI64885.1
MAPAEDRMARFKALQARAKTSSATNRDEATKESQRLGTDQSQLTALKRKQ
DIAAHKLVKAEIEESGKDFERKRAWDWTVDESEKWDKRMKKKAAHRDNNA
FSDPQQESNKIYKRQLKNITPDMEQYEKQKMAAIEKAAASGGLEIVETED
GELIAVDKDGSLYSTADTATLTQNKPDKAAVDRLVADLRRAEEQRLKKRK
ERMAKNGDDGDVTYINEKNKQFNQKLARFYDKYTADIRDSFERGTMI
>Mb|QLI64888.1
MTNWRELGEIPDSEDDEGFYSQPLASRPIATTTDAVVPVYDPDPGIWDFP
GSPNNNNNDNVVTNPRTPALSIPYEEQVLDLVASSPLSSALSEDGLPGVE
DLFPGDDTQQHVGKLEQGDAKLLGASQAESHCSSAVAQDLPLSPDPPLDN
HSFSRKPDGQSQTIGTGGISEAQRIAVRYERSLRPRKPIQEHPYLLENAQ
YSSFLKLHGVKPLRMAIENERRKRDAQPGGSQEDDFQEDSQESCKPDTLD
DSSTDEIGLPGDLDAVLLPSSTPLRQSPTARRIGISSPVSSHGDTDNTSV
VDEDLPALEELLTKPPKLVLTKANKRDATPLTSSIRKRMRRNIIQSDPVD
DDSVQAASDVQSPFIQFSPSLRVPPLLETGSDGIPNPQVCDRSPTLSAAS
ISGRAMGATNRATYIASSDEDDGITPANDNPTSPGSRGSDTENETVTTMS
RRIRGVLPASWLRLDRQAGRANAKNSLSRRTRLLRSPEIEHKRGVAQSRH
ATNGTAALPIFYDASDEEEVRSMLDVNENKCDNPAQLEIDHPESPIRERD
TNHEDDGDASAIEDNGVDFMLSGPSRKRQLKLTDSLDQGARKRPKRKDQI
TKQSYGRLRQVGIKAHLHESPMPRRPDSSLFEHSQKQDLRKRKSIYLSRK
PAGKQARTSPPKLSILDTIQPDAPQFLKIAARTARTRQLQGRSSPYKKTI
QLATRQDQLDVASVLIDWRSGSIKQRDSVTAATKSPQKKRGRVVLAEASG
NRHGNNITRAQSRPGVSKKLVKHVSNGGSIRYLPAQAHPQRQHLKSARKI
AAVKKTFSPSSIRPAQLERDEIVQMTKFEFNTRKRFLDRLYQNGHSGVRK
PSSEPSSLRGSTRNLGVSMQDTANKQPDKAKQILGGSRNPKKAAPRRIDI
DAPQYSHAKDPVPEVYSAEPSIAGTEPAQAKLVGLGPYGTQYTHHFEIFP

LHPDVFFHESTLIGSGELQSCATAVFSMHTSEAQPCMSISLNDHLFRWGA
WNDQTSSELGVVLDYIAEGIEAQLAGKPAPSPSELTPVLAASFVLGYIKD
SFGFFEQSQKLSFLSRARECLNNFNNRTDSILRTARFSIQGIGHVLQIYD
RLMLMAFLMLRFCNQDPLLITEKFSVETCLQSIGETAISILTELGIGSLQ
KFYTELHNRHFRESGIRNDYPVINSWVMVMKVMDLAHIPRASFWDVAQRW
IAPKEDLSSSNAQDHERIWENVFTLLPLMEFSNTGVLVAGQRHSVSSDGW
TIPQKLLRQVFQLYERNPRQATSFNYYCRALVGRCHYLVQEWGWKKCVSI
VGLIFDFFGSQQLSHLRNEEVFKSPRFLENLGGRPNLAIEPEDRCFHVFL
KLVAVAIRKLRAAGSMKDISNLVTRIVPNHNRQHRKDENVHARDLAALRN
HHDLLCTLFWAAPAECRPSPILIQNLVDPADSHKEACLINLRAWTQLARF
VISSGETMISWRPFHTWRNSFFEQVLKQYNSVASEVALQLSALGKELSHS
ITEEMIHTTVAVNKAALVDVIYASLTASLDVIRHAPDFPTATFPWNIPQL
RTIFTQFSVTLPDLAWRLLRVALATLETMLSKIDDFKANEESQQSESQIL
NADVADDALQHLDQNLSKTFFGMARRVLATPVNEQDPHTVKQSQVECTEQ
IVILAARMSTRFINGGLMRLAGAFKGSYQLFSESPFTLDLRQRGYVVLFI
STLLRYGIDDFGDAGFSLCDIWVSSLAKPRASLKYEIELGKELRRRGNMF
VPEAVLGISIQPDYNTNRDLLEYAISSMRKAVRDAGPTLRAAMATEYSAA
LKVMMDQIKDDLKNGTHTSNEHDGYVVFIQGVISLVKTYGSDICAVDNYF
CQISKEYSPSAEDPQLKVANLKSYGLRLQDGDARIGQSLFHLLFNNAKFS
IFNGKLTEEVHMLGKGMSNSGVKTFIVSKMLPSIIHASFKENDAFPLLDI
YVEALALCLSGKAIPLELSADDAPTICTLLQAMLNGINGLFSGGRSLNGS
QIHLANQAIGLGNLLWPSIHVLSMSSSQPPLWTKMWSLLCDFWAVVSKCR
ICLQSSNGEELSSEVAQFESLVIGTREARNEADEFGSDVANFAENIIQDN
AGPTVDLSSYRYSDASSLLNSFIQSNLAHRLPANDRPLQDIIAPLAALTF
TVIIESKVNLLPEEELLAPLLESFPGRGHQIRLLATLLYPSAAPSRNLVL
HGTEATGKSSITRRLLANLVDTRPCTETRSAGLKYAVINAAQCITARHLF
ETIVGSVADALKLDGESGETSSSTSTAALGRRRRCETMAQLSATMSTMLE
GPARDPAWRFILVLDEVDRQRDAPPTLIPALARLSELIPCLTTVFIVTAP
PAGFLRTTAAVYLHFPPYTKPEFVRILSLSPPPAISGATQLETTDLWTRF
CAAVHDAFVRSASRSLPSFRHSCQALWPRFTAPVVAGTYSPKEFSKLLVA
ARVHFQDESLLNPGIVSVRPEPSSATMTTTAVPPAASSRSFATTTTTTTT
TTAASYLTALLPIAARLILLAAYLASHNPTRHDLTLFSTYHHGRKRRRGG
GFFANRGTPRTKHRRIARKLLGAHAFVLERMMAIFEAVRSEWSPDGSSVG
STGLDGDIGMAIATLASLRLLVKVGAGDMMDRSGKWRINVGWESIRGIGR
SIGVEVEEWLVE
>Mb|QLI64889.1
MRLAPSRGFACLPVRRASTFPNTDSFSSTYALLATAVIRANHGHITASPK
GSFALMTQQRRGIHSSERKSSTVAELPGAVPPPANLEPSRAPLSVLPLSM
IIRSLATTVVSSSPLLLPPSLQIMTLLAHSTNPVFNPDRNPILRYFLKKS
FYAQFCAGENPAEVQATINRLKNIGFTGVILGYAREVVLTEKQAEEVELA
AGQETQKVIDNEIVPWAEGTLETVRLAQPGDFVALKFTGAGSLALHELKH
KLPASPYLSKSIDSICQLAHERGVRLLFDAEQDALQDGIDDWTMEFANKY
NTSPDTCTIYGTYQAYKKKTPAVLERHLAMAQEGQFSLGVKLVRGAYLGS
DPRQCFFDTKADTDACYDGVAASVLTRQWNTTLEGNGEYPAAHLVLATHN
AESVQKARAICDAGGAKSEIAFAQLQGMADEISCELVEASQSARAADASA
TPNLPAYKYLVWGTTGECMKYLLRRAQENKDAVQRTKSGRDAMWAEVVRR
VKNTLRLA
>Mb|QLI64893.1
MDIDNDVEDDDPITASYPVFLNPALPLGRRLLVLQQPNRTDDTPRPPPTE
LRLKAHSGMVEIDLPLDNTTAYDREKGLKWGRILQSSMASKNGGSHGLAG
GFGFGAVQQRGKKKGEMDDDEYMDWNEAVRQDKVLRTQTLGGQYPDTDEV
QYMVGVFQGKNLHLTPVSSLVHLRPQLHHLDAATQQERNASASASKEAAG
SSSTAARAIHMTIKNTADGDAVTTETMADRLRFVQTENWRKMRYTDENDE
AAWEVYNESLFLQLRNDTNKSEDEGETKEGEGAKKDMEELVPRFGAKWND
KQMLEAVSGIEKPDPEPEIKPEPKGKQDAEDTRTSKGKPRAGAAPPATRK
PSRAKAPSKAAITID
>Mb|QLI64896.1
MDRQRKRELRTLNIRAWDGEADILVVSNSLDSSLKKNTAFIKRLRTAISP

ATLATFLQEIRTLSLHKYLSEIIAACYEGLCRLKSPGEIEAGVEIVSALH
QRFGPTEFTEYLAWLLGKGMATPDKTSLKSLAIEVREKEEKERITRQRAL
LRVITELWLVGVVRTLDDATKPDDATKGAAAGKAVELKNRASSTKAGGAA
EPFPLEVLKDLLGYDREHANLPLLVIFVKSFSWDILGVKPTGSDGRKTVE
EDGATSGQNDIVQVVDEATELNQTLDQQPFTPPDMAERFRKVLKKYFDDV
AAHIVRDQKSIQSQARRNAEAYVKSGEVFEDRQANYEKQVKAQERLISNA
QVIADAIGAEMPDLKESYDALATSSGSIGLVKTSEYLRTMGEGAGIWTDE
DERRFYENLVDLKGKVPPILLEDGRRKKVDGEEQVGKKTDAASESSDAAK
AAAEAVEDQSMAIANKTIGAQVDALLARLPELTNKDVIDQTAIDFCFLNS
KASRNRLVKALTEVPKGRSDLLPSWSRLVATLGRYMPDVPKGLVDYLDAE
FRSLQRRKEKDFLGQVRLSNIRYLAELTKFGVVPEHVVFHCLKVSLDDFS
RMNIEILCNLIENCGRYLLKNPETAPRMASFLETLQRKKSVQHIGQPERM
LIDNAVYYVDPPERPAIEQKERTPMELFIAKLVYSDMTKRNYSKILKQIR
RLHWEETEVVSILEKVFSKPGKVKYGNIHLLGILLSAIYRYHPEFVVKVI
DNVIESVNLGLEQNDFRFFQRRIAEVKYLGELYNYRMLEHPVIFDTMYKI
LTFGYGGPPIPGRLNPFDPPDDFFRIRLISTVLETCGMFFNRGAAGKKLD
YFLSFFQYYIQTKSSLPMDIEFVVQDIFSLTRPQWKIATSIEEATKALQL
AIIQDQKTAGIDRAAEVDDATSGASSDDDNADVDDVEQDGEGDEESASED
DEVEDGDDESANDSDFNDEAIVVTRQEEVVDPEDEADFEREYAKMLAESL
ESRKFERKPLFDVPLPVRPKNRESSTGAPQDQEPSAPNHKMAFSLLTKKG
NRQQTRTVELPSDSTFAIAMKTQQQAEKEEQQRIKNLVLNYDLQQSEDQE
GSERATTFYHNRVDRSSKDRGQRVRKLQLSDVDW
>Mb|QLI64897.1
MKYSTTIFTALAPFAMARRVANVFPPPAGIVQRNAETESSESGIQVSRGK
QVEGITANSRAEVIVIWLNPGNGASKTSINEQVTVTKTVTAGAGGEATQA
PGGSATASAPGTGATHTITVGGANGLAFSPQELRVPMGDMLLFKFGSQNH
TVTQAEFATPCKPMAGGMDSGFLANPNNTVSPPPEVAMQVMTDKPLWFFC
AQKGHCGKGMVLSINPTAEKTHAMFQGMAIAQNGAGSATPITGGEQGSTP
PPPSAPGGVGGNSTGGAGGAGGSGSSGGNSGVTPGKATMGADGSYHCVVT
CNSGSFPDASQGVGAFGGMSGALPV
>Mb|QLI64898.1
MSQVASQNRFAFLGNDDSGEDKPIVPVKTVDKAPARTTKRNTEPQAPAAP
TRGTGNRRGGPRGNEGAFRDRGVGSDRNHARSTDEAPRDGPRGGYGARVR
GGRGSRRPRDNDDRHTSRTGVHPGSEKQAAQSWGANEGDAELKDEQAGEA
IAQSEKKEALAEDAAGEEAAEPEDKSVSYATYLAQQAEKKLNIDAVFKVR
EANEGSKPDKKWAGAKALEKNDDEDFISGSHGKKQRERERKVKQTIDFDP
RFVEPERTRGGGRGGRGGRGGRGGERGGDRGRGGNFRGGRGGAASAAPIN
TKDESAFPSLGGK
>Mb|QLI64899.1
MGGALDSSSNARSNSLYEPGQNAISTLLQPPIVRTGLQPHTSAPSSSSHK
PPNSRDIPPVALTNIPIIDPAEFSPYVAQVGALYEQLRRVKESEDESAKS
RHNSKPGEQPDTDGLLRPTTKSHRRGSTASVASLASIDAPSPSRRSNPGL
GRKAVQGPSPLSTIPTVYFDQDFHLENPRTFDVVSERSEVIRPSSLGKDS
SSVPAAPRKALATNAILQEKLSWYMDTIEVHLINSISTASTTFFTALGSL
KELHSEAADSVERIKTLREELEALDKEVATNGILIAQKRRRQENLKQLND
AVCQLRCIVDWVGRCESLVDAGEVDKALGSIDSLERLIAGEREETDGKEP
LGIELRDLRGASALQGVNQDLNTLRFRVGKAYELQFVNVLLADLRRHTES
VSSADVLLRWDNAASRGRGGHSRSPSIFPTYLTATEELKAQLMPNMKGLH
RAKYVAAAAVAYRDAVLREIRNLIRRPLPSSSDDDNESMLSVSTTSGSRH
LSSQEKSANLARNLRALDAEDAEELLVKIYVGVTETLRRLTTQVKVLLDI
TSSLSDASHLDGVKSPPARSPVTSPRPDRHISNPMAAADVELQEELHKSL
DINSLLTQAVDIANDKIVKVLKVRAEQATHLPLDLFLRYFALNLYFTTEC
EAISGRSGTSLKTVVNGHIKEFVQRNRDAEMQKLALGMESDQWNAKDFTE
SNTKLLDLILGCSMQDASVWTENNKIWIPYRELEKPEIVTEPSESNGTGK
DKTRSAVIESDKFILPNSAVLCLDGLGSFLHLIAGIPSMTTDVASSLIAY
LQLFNSRCTQLILGAGATRSAGLKNITTKHLALASQALSFIATLIPHVRE
FVRSHAGSGAGVSSLMGEFDKVRRLLQEHQDSIHQKLVDIMSSRAAIHAK
NMRAIDWDSARPGVVQPYMEILAKETTTLHRVLTKHLPETSIQMIMEPVF

ASYKDQLGAPLKEATPKTEAGTQSMLRDMEFFASRLGKINGFGDTGDFLA
NIVKSKEVKPKTPEPVEETEGSQIKDKSADDKKPDTEPATETKEEVVDTK
EESKDESPSKGSEKQPED
>Mb|QLI64902.1
MKLSQVSALVACVPAATARFVELMEADHVVLRPDEPTFLIETAPGKTQWV
TEEQKWELRRDGQRFMDITATKDLGSQGLRIKESVEFPKKCVNQEEVKSL
AKNLDTAPMKANLEKLSSFHTRYYNSEWGLKSSDWVLEKVNEMIKEAGAD
KTVTAQSFPHTWKQHSVIATIPGQTNSTIVIGAHQDSINLWLPILAAPGA
DDDGSGTVTIMEVFRALLKSKDVVQGKAINTIEFHWYSAEEGGLLGSQAI
FQEYEKQKRDVKAMLQQDMTGYVKGTLDAGLPESVGVIVDFVHAGLTKFI
KTVIEQYCAIPWVETKCGYACSDHASASKAGYPSAFVIESSFDKSDPNIH
TTSDLIEYLSFDHMLQHAHMTLGFVYELGFHNFAKAVEGEGEL
>Mb|QLI64905.1
MSGLATKQQSLKLFEKLKSKPANKICFDCGQKNPTWTSVPFGIYLCLDCS
SNHRNLGVHISFVRSTNLDQWQWDQLRLMKVGGNESATKFFQQNGGTAAL
NSKDPKTKYQSNAATKYKDELKRRAARDAKEYPGEVVMTDGADDGSSTPA
GEPDDDFFSSWDKPAIKRPTPPLSRTATPPVVGRTPSPFLNAGNGNGKDT
PRPASSLAKSDSTGESKPAASRLTTSAALRKTTTAGGPKKLNVLGAKKTQ
KLGAKKVAGDSIDFDEAEKKAKEEAERIAKLGYDPEAEDEPTKDAPGAAT
ILSPTPVSPPRSGTSSHTRQKSNAEVERLGMGVARLGFGQVGGGKSTAQP
AKKNAGGFGSVGPVKAVEDDSNSYARNKFGAQKGISSDEYFGKGSFDPNA
QAEAKSRLQGFEGASAISSNAYFGRPEEDIEEEYGDLESAAKDFVRKFGI
TASDDLENLTNVLGEGATRLQGAIRNYLGS
>Mb|QLI64906.1
MPEEGVPLSPNRELLTTPEIVMLSSIFVSQGVNKIRLTGGEPTVRRDILS
LMRQIGALRPRGLKQLCITTNGISLHRKLDSMVEAGLTGVNLSLDTLDPW
QFQIMTRRKGFDAVQKSIERILEMNKVGAGIKLKINCVVMRGVNDREVLP
FVELTREKDLEVRFIEYMPFDGNKWNKGKMFSYDEMLDLIRGQFPGLAKV
KDDKNDTSKTWHIPGFVGRIGFITSMTHNFCGTCNRLRITGDGNLKVCLF
GNAEVSLRDILRKSNRGEPIDEEAYEAMKQIEMDRKQGRVPPDQPLGVAP
NEAELLDVIGIAVKGKKAKHAGLGTLEHMKNRPMILIGG
>Mb|QLI64908.1
MEPPGNDGPDGSSIKPVSSLLARFENLSQTAPLVPPQNAPSRPISPAPKP
DRLRAFKLSQDPPPSPSPASALASSLAAAPPKTPVKSPAKPIQLHATEPN
TLRDGSPALTHTSTRPLVDGAKSPALTMPPPPALKVLPPQSPHKAHSSAV
MSANQSPFLDPTTATSQELPGASPIPIKLPSRPITPIHPLSAPRSPRLTS
TQPPSPPPPRRSVEIRREPKQPPPPPAPRLDKSRKTTPVSSSLPDTRKIS
TSSKGNIVDETSPFNSPPASPEKTDGDESAPELPMRPRAEPNKDLVSRPK
PVVGAAFELPPVHHSIAVRRTERDPIVNGTTKPSLITQHPIDDRFAHSPK
RSTSDLTTMTYGANSYSSDKPPPKPPRPTQQRTSATPDILSSSAPAGRSQ
QQQQQQQPQLTTHQFPAPPTRNHVPVKTVGRSADKEVPDPRALMLPPPSI
SASRPTPRSIEPEPAASTEPAAHVTAFPDCSNVNRKPPHAKKGCHEIQTK
YDPRIFDVCGDLACTSGQLTRVWNIRDGEQIMSLAHTEGVRATAVLFKPA
ADPDHEGGFIWIGTNMGELMEVEVATQRITEVRAGVHGKAEITKLYRHYN
QLWSLDEGGTLNLWPGDNDGVPNLKSHPIIQPSRLPKGATFSMVVGSELW
HATGKAIRVFEPSTDGSRPFQVLVRPLTADGCGDVSAGTLLSSMPGKVYF
GHMDGKVSIFSSSDYTCQMILNVSSWKINGLAGVGHNIWAAYNTGKICVY
DTTEQPWTLKKEWQAHDQSVLKMKMDPSSAYRIDQLQVASLGTDSKVRLW
DGMLQDTWLEDDIKSKDIDYCEFEEIKAMVFTWNAGASTPHSLRYSKGDG
DSTFFQDLLRSSGSPDILIFGFQELVDLEDKTATAKRLLKSKKKEGTEQE
RMSHQYRDWRDFLLKTLDDYMPGDDLYHLLQSSPLVGLFTCVFVKSTIRD
RIRDLSAAEVKRGMGGLHGNKGAIAIRFRVDDTSLCFINCHLAAGQSQAN
SRHNDIAAILEANLFPTELDPETRLDTFTGGGDGSMILDHELCILNGDLN
YRIDTMSRDTVVKAVKQQQLAKLLDRDQLLVARRRNAAFRLRAFEELPIT
FSPTYKYDVGTDTYDTSEKRRSPAWCDRLLFRGQGRVRQLDYKRHEVRAS
DHRPVTGSFRLWVKKVDGAGRARAWMESQGRFESVRAQVMANEKMAYLAH
YCGFDEATSRALIEEKSSRKVHRSPSRNRGGGY
>Mb|QLI64909.1

MDFNSSSPFPEGVISFLDTDLYKLTMQCAVFKFFKDVPVTYAFTNRTPDK
KLSRVAFKWLDEQIRKLGNISLSNGEYQFLKTHCTYLTDDYLNYLRDFQL
RPREQVTVTFTTVGKDSGADSDIGDIDIKIQGTWSDTILYEIPILALTSE
AYFRFMDTDWNYDGQEELAFHKGMQLLEAGCITSEFGTRRRRDYHTQALV
FRGLVKASKAAAEKGFPGKLSGTSNVHLAMRFGIPPVGTVAHEWFMGTAA
IFDDYKKATELALRHWVGCFGSNLAIALTDTFGTAEFLHSFSLPVQTVEG
GMSGDIFKNEDGSTKTYAELFTGVRQDSGDPAEYAKLMRNYYDKQGIKEK
KIIVFSDSLNIERCLEYKKVSEELGFQPTFGVGTFLTNDFTHLTTGTKSV
PLNIVIKLSSAAGRPAIKISDNIGKNTGDKDLVEKVKKELGYVEREWKEG
DETSRWGKEENAQA
>Mb|QLI64911.1
MATEQQRLPTRERRPSTSAPIVDITGGVGPAGISRPKHKRTFTGFGPGEI
KSVEASIPEPQREAWKRNQITSFTDKDGFEKEVVRHVETTLARSMFNCDE
IAAYSATSLAFRDRLITHWNRTQQRQTYRDTKRVYYLSLEFLMGRALDNA
MLNVGLKNVAQDGLAELGFRIEDVVKQEHDAALGNGGLGRLAACFLDSLA
TLNFPAWGYGLRYRYGIFKQEIIDGYQVEVPDYWLDFNPWEFPRHDVTVD
IQFFGQVNKKTVDGKTVSVWEAGEIVQAVAYDVPIPGYDTPTTNNLRLWS
SKASGGEFDFQKFNSGDYESSVADQQRAETISAVLYPNDNLERGKELRLK
QQYFWVAASLYDIVRRFKKSRRSWNEFPDQVAIQLNDTHPTLAIVELQRI
LVDIEGLEWDNAWEIVTSTFGYTNHTVLPEALEKWPVGLVQHLLPRHLQI
IYDINLFFLQSVEKAIPDDRDMLRRVSIIEESQPKMVRMAFLAIVGSHKV
NGVAELHSDLIKTTIFKDFVEIYGPDKFTNVTNGITPRRWLHQANPRLSE
LIASKCGGNDFLKDLTLLNQLEKHVQDKQFRKEWAEIKYANKVRLAKHIK
DTTGVVVNPASLFDVQVKRIHEYKRQQLNIFGVIHRYLSLKAMTPDQRKK
QLPRVSIFGGKAAPGYWMAKQIIHLINSVGAVVNKDEDIGDLLKVIFLED
YNVSKAEIICPASDISEHISTAGTEASGTSNMKFVLNGGLIIGTCDGANI
EITREIGESNIFLFGNLSEDVEDLRHAHTYGSHTIDSDLDKVFNEIEKGT
FGTPHDFSAMIAAVRQHGDYYLVSDDFHSYIETHQLVDEAYRNQDEWIAK
CITSVSRMGFFTSDRCINEYAEEIWNVEPLAVDRH
>Mb|QLI64914.1
MTADDDSSDLSSLSSLSPVPSDVELDDNSDDATTKSKSGILKFFPKLSEQ
PPKEPSPPPRKRSASPPHEYVLADNPDIAFIVMFRSRFSDAFPKSLAHFG
PQELERDVVESIPGDRVEHLLCAILGLLLNRKQDVKLGHYGRALEDAITS
HKNQWPASWEEKNPLSGGATFNSMTPTKRLTLLRTLILWAMASSDTVKGL
INQAYKQNRQEDDLNQPRSVQPWGSDGDKRRYFLVEGQDDTNFRVYRESN
PAGTNRTWFSVAGSIEEFKALAEKLETKDGGPKAKKLSQKMLQVIPRFEA
GEEKRKRREYRQMRKEQFKRPEPGFSMYEGRTRGKRIKYTYSDDEDAFFS
DSTGYRRSARNTGANTPAETGPVTTSSGRQIRAPPRLNMVVGDSAPGSIQ
GENSELEHDHSVGASGRPRRPAANQDTNGWSERNSRGDGTDEESEAEFGD
DEEDAHVPEESDDEDDFDEEEAMVEDDLDDQRQSLVVKLSVTPPKLRTVL
SPMDEGSNMLPTPDTQDWKVDATIPETRTVEMQDVPAPEAHSIPSEQVKK
EPTQTDSGDVTEKLDAIRAPTLSAIPSTPLAFRGSPEKPHAQLESPFGNF
NDTT
>Mb|QLI64915.1
MFETTISYVWGQLKAAKPAFISKKPHFSFITVHYFWIIGATIVGSIAMFV
GGKGELAYIDALMFASGANTQAGLNPVDVNRLNGFQQAMIYLFSVTSNPI
TLHGFVVFLRLYWFEKRFRSWAREIRSRRRTLTKSKSKARNDVHLAEQGV
NGRDITLMPQNGRKQRITNDGILLDDGHETQHNAEVANKSDESDTTTATD
DQPKLSEPNHPEKSDGSPQDHGAGENRTTSLPGHTAITFADTVKRSDGLG
DEETKFPQRRTNAEHIAILERQRNQDDDEVLRIPGPRDAERGLGPRRLEA
SDMDEDDNRLARSQTAETFPGGVAQPGRIRERQSTIVIAEPEKHHRREEI
EEKAKAIGGTMDALRYRKPRIFNPSQNKHHEDEEDGRRTRPVRTRTLDNI
RSVLSSSKTEDMPYLSYTPTMGRNSNFVGLTLEQREELGGIEYRSLRTLA
IVLLVYFWGFHLVILTCLLPFILHNDHYGDIVVADGVSRTWWGFFTSNVA
FMDVGFTLTPDSMISFNTSAFCLMVMWFFIIIGNTGFPVMLRFIIWVAAK
LAPVGSGIWEELRFLLDHPRRCFTLLFPSGPNWWLFWILVVLNAVDLLFF
MVLDLGGEPIAKLSVSNRVVIGFFQAASTRTAGFSAVAMSEIHPAIPVLY
LIMMYISVFPIAISIRRTNVYEEKSLGMYQPNHVEDDQDASALSYVGTHL

RRQLSFDLWYVFLGFFIITISEGAKIVAGRFSLFDILFEVVSAYGTVGLS
MGAAGVNASLCSQFSVVGKLVIVAMEIRGRHRGLPYGLDRAILLPSESRF
RKEAEEQEAALARTNTALTHATASGIQHQDINLTRTRSKSREHVRANSNI
VAQLLQPGPAVHREGPSHQRMSSKDSHGHPFGSMRTFSEPHPDDEQMDEL
APLPSSGSKPWNRRVDSAPVS
>Mb|QLI64916.1
MAINAAVSPLSLENGLIQCLRAANADVTPTGRKRSVDDIENDIIPDPIKR
RKLDDAAAGSPSTPKALKAIASAISGVFGAARQSRKEVNGTEPSLPATST
QPKPLLSTNKNGRLLSPAAPAAPAAAAHPASSPMKRPAIKIAALRGTIWD
NGDLPESKKCTVPKRPVKQAVMGNRYQGPLPPASVYQPKEATASAQKPGS
IAGNKALPPKGILTPTKKRGRPRKNVTFNGGTNGEVYFDDLPKTSTARKP
KAIKAQMAQQEDDGIVCEMCYKPDSIAPNEIILCDNCDFAVHQQCYDIKE
VPEGDWLCKSCSQEDVLKAPGEPTPKTNEPSYPVEVPDIPNLDHHLRGFQ
RVLLDRCSGRRRIRMFGQEEPYEKVRQLIEQTVVAGEGNSMLVIGPRGSG
KTTLVENIIVDLSKEHGHEFHVVRLNGFIHTDDKLALKEIWRQLGKEMEV
EDDLLNRINYADTMASLLALLSHPSEIMGTDENLTSQSVVFVIDEFDMFA
SHPRQTLLYNLYDIAQSRKAPIAVLGCTTRIGVVDMLEKRVKSRFSHRYV
YLAPPKSLPAFWQICRQGLMIDKPDVESEGVDVHTEGHIEFHKYWTQKIE
ELYKQRQFQHLLQYHYFTTKSAAAFFTEWIWPLSSLSSRNLTMNSPTALS
PTTSLAPPDSRMQLLSTLSYLELGLLIAAARLDIVAHTDTVNFAMAYDEY
SSLVGKQRVQSSTAGMLAMGGSARAWSRGVAGVAWERLIALGLLVPAGIG
GSRSLGHGGLDSKMWKVDVALEEIPTAVKLSTTLARWCREI
>Mb|QLI64919.1
MATTEALQQQKPLGDTLEDQKTRLDRHGYLFGQKITHSLSPFLHQIIYDH
LGLKWSQIRLDSADMSQFLQLAQHPSFYGASVTMPNKVAILAHLDEMTDE
CRDVGACNTLFLKQRNGHRILCGTNTDVVGIRDSFHHNVADPSHTFHGRP
ALVIGGGGAARSAIYALRKWMHVTDIYLVNRDKSEVDAMVADCSSRGYGH
GLRHVASVDEARAVDGPGAIVACVPDCDPRTREELVARQVAEVLMDKRHK
GAMLEMCYNPSPFTRLGALAGERGWQVILGTEALIWQGLEQDRYWTGLRI
DQLPVAKVQSAIGEKVAQRSQSRL
>Mb|QLI64927.1
MEKAKKAVSNFLSQDGKHKTTVDENVRKPVTEEHVFPEQHEQITTAVDRE
VHQDHHQTVIQPIKAKETLPEKHTHNTVPVEHKTVEHGNKEDVKAALKRD
AENYVNTSTTHDTKQTATAAPVVEGERTQHHVHHHIQPVIEKETIQPEYI
HTTVPIHETHHAAPIHHETTVLPAKTMEEYTAGRGDLQAHSTAKVKEFPG
CPTLKDKSLQSDTKGQQAIHGH
>Mb|QLI64931.1
MPSKNKKHAKENGGISAAVTKQAAAKLSSNHYSNITQGDGNAASNKSTAS
DTSSPPANTPEIKPSTSPDDGNPVVASPMLDALSTISASPNTAPSMTTEW
AKTIMAGSPGNLINLTSESPPTQPSSYEEAARLHNGWATTQRPFMSPSPA
SPSPPNHGRRPLSLQSDTHYHFAATPPRSSAAYRRSSLYSQYNPSQAASP
HPPLPHQPQPHFYGAQDLDLTVTPQSGMKAGDRGLFFGFDRLPSEPGLSS
QTQKVVLAGYEGGLQVYAVNKRGVELVASLKGLRGGVHHAKILPWTITGQ
NSSFFPLVAVVVHGPVLPTRPTDAGCRQETTSRETSDRSASPRPGYAPSE
GPSTRAAPPIQFYQTAVEVYSLKTNRLVDVLLQTQRVPINMEVSLSSPLF
QPPTPSGAFNIKADDGTIAVGSGVTGECWIYRQLLELQNGHMFACAGKLW
TTIQQGQRAEAAADTDKKGSSSSPRRSSPQAPIFAMNGRLIAYCPSNPSS
QASLRAHLPVPTLGRAPGISSMAPPHLPAVSSALDLPISDSMMNKFMRET
TQELISGMKWVGQQGLQAWNSYWNQPPTSQQQQPRSPPAQWTGSRAQQTD
PSQFPPTHGTSTPATVKDPGLVSIVDVEKLPLSATVHPLTTFATPLGCSF
VSFSPSSLALFTASIKGDVQTVWDLLRIQHTNSSPLQTTLAQNETVGPQV
RQIAQFSRMTVARIVEVAWSEPHGDRLAMVTERGTVHLLDMPFSSFMWPP
PRRRRIPQKGGPEASDGSSSAVSMASGALGAAYQAAKPFVSRSRRGSNNT
VSPSAGNILRDSAAQGGRVIAASITHSLGKTGTAINQLRHTGENRVCLPP
SAILPSPACVSWLRNRRSQSLFSIGGGLIRVFPCSTRTSSSVPGKRISRA
NKYKDMRVPLLPDDIVAPIVRQIIELGAADEYLELSEAEMEAGNTMTLKP
PHVATAPRHGMDSTIPQAEIESSAPYQPFHTDRRVALCEYRRIDSTAALL
ANVGLDGKPNKRKNKQNFDAMKGNASAWAFGQDIPCVKIDLGLATANEDD

YIGPDDHMALPQSAMERVMQYEGSESIVVTTRRRRGARQGDGDEDGFFED
DCEVLDFADQRV
>Mb|QLI64932.1
MAFPPQYREPPPSLPSILSSGPGRYNMHRDMQFSGSPAISIPGLDPKDDV
PPPLPPPRHLPFLEGSANPEDSKKESLDRFSSSTHSGYGSMHPGSFMDER
PSYKRRGTGSSTNGDEGYASYTATERFVAAASHDTRDSRPTEFLHHSKFH
FQTAADLHGDSMKKKLNPIRTFDRSPRSATSSALSEQVRRQPDRRAPTLS
MPVQLPIHTRSLLDSSARMTDTPIFSAVSPRSGPFRHFPGDHRFAKDGSD
YDQSPRGRSQRNNSDDASSTQGSYDFTGVEDMEIDEATSPKRLHPDEAYA
SVGHKRRAASPPAGDSLLHGVSSDIRRRELGSRGSPTPRLTVMPQASSLS
SMSSAAISRSNSYISTMSIPPSSVTTAMSFGRRSPGAASPGGISPTSCNS
PYTTPASLNHSPRNSVSGRGNTHGRTISGTSPRKLADIQKPAGSKVQPVL
MCECCPKKPKKFDTAEELSAHEAEKQTRSMFADIRGRVQHFPAMIVRFMI
RQPVPEKPILAAIVVKIFFAQVEDLAMGPCPEELSQNTPRTKTGMIVSVT
YKMFTNLASATPPRNFIERTIFGST
>Mb|QLI64934.1
MSSIASCALRSASRKATATAPLRALPRAALVAARTTPARRGYVTESKQDQ
ARVETAIKLDKKDFVDIPPPPMGAPADAKVSPMAEVLKQAAVMEEGQRPI
YLDMQATTPVDPRVLDAMIPFYVGVYGNPHSRTHAYGWESEKAVEAAREH
IASLIGADPKEIIFTSGATESNNMSIKGVARFFGRSGKKNHIITTQTEHK
CVLDSCRHLQDEGFEITYLPVQNNGLIKMDDLEAAMKPETALVSIMAVNN
EIGVIQPIEEIGKLCRSKKVFFHTDAAQAVGKIPLDVNAMNIDLMSISSH
KIYGPKGIGACYVRRRPRVRLDPLITGGGQERGLRSGTLAPPLVVGFGEA
CRIAKEEMAYDSKRIKTLSDRLLHGLLSLEHTQQNGDENAFYPGCVNVSF
AYVEGESLLMALKDIALSSGSACTSASLEPSYVLRALGNSDESAHSSIRF
GIGRFTTEAEIDYVLKAVQERVSFLRELSPLWELVQEGIDLNTIQWSQH
>Mb|QLI64935.1
MPLASQLSNFFFAGSSASAGDSSSDEHVVGLAEPPRNAGIHTKHSPGRSS
VIASDSGRTMKSEDIETEGRPPYLHAMIAGGIGGSTGDLLMHSLDTVKTR
QQGDPNIPSKYTSLGRSYYTIWRQEGVRRGLYGGWIPALGGSFPGTVMFF
GTYEWSKRFLIDHGLQHHLAYLSAGFLGDLAASVVYVPSEVLKTRLQLQG
RYNNPHFRSGYNYRGTLDAARTIVRTEGASALFYGYKATLYRDLPFSALQ
FMFWEQFHAWARVYKQSREIGVPLELLTGAAAGGLAGVITCPLDVVKTRL
QTQVNPSSDQVTKLKEVASSQKRHISTSSPSTHRPQPGAIALETSSVITG
LKVIYRTEGLAGWFRGVGPRGVWTFIQSGCMLFLYQRLLHQLEVMMPAEK
DDL
>Mb|QLI64936.1
MPATEGAPQSGRDEGSRGRGRGRGRGSGGRGRRGRGGNAQTAVNATPANP
AAQHVAAAAAAARVQLLKQQSQTADANAGVPTPATPADADDDADICFICA
NPVAHHSIAPCNHATCHICGLRMRALYKTKDCAHCRTPAPFVIFTDDTAK
RFEDYTDKDITTTDSNIGIKYTNEDIVGDTVLLLRYNCPDESCDFAGLGW
PDLHRHVKSVHRKRMCDLCTRNKKVFTHEHELFSDKELEKHMRRGDDKPG
AADQTGFKGHPLCGFCGERFYDDDKLYEHCRMKHERCFICDRRDSRQPHY
YLDYNALEEHFKKDHYICSNSECLEKKFVVFESEMDLQAHNLKEHAGKAV
GRDARLVDMSSFDIRQSYQTERRGGHRDGEGRGRGRRRGGRGRDPNEDTV
PPTSAQPLRRDELALQRQLAIHSAQSVSNRTFGGQLSAPAGQSLTTRSTP
SGTSTPARSNNTASPQNGLASAMDSLTVTDTSNMSPEERARLVRHSSVVE
RAANLLGNDANRLNTFRQHISSYRQGGFTGPQLIDAFFTLFADVSSNALG
TLVREVADLFDDKTKADNLRKAWQDWRAINEDYPSLPGLSGMHGATSSSS
GWANAATANPASPNATAPAQKHSNRVLRLKNSTRLGGPTPTSIKATPGWI
SSPAVQRPPASSSAFPLLPPASSSASVSTSASISRPTWTSSSSSSQPAQP
RSAISRNQDAFPSLPAAPKPTTTIFGYGSGRGVRRDYGHAESNFTWGASS
SAANTTPAEEDDDAATSKKGNKKGKKVLVQWG
>Mb|QLI64939.1
MYRATVRTASRQALRSLRSQPFQSAPRRFASTSTPAYKPRTWKGSAVRWG
LALGAVYYYNTGSVFADEATIQSIAAPSAFTESDVPTVDSVIEQKRKQLK
PTPEEQAKPIEKQNSTPETQPKTESQNTVVAAGSPEALEEEAGQEGAFNP
ETGEINWDCPCLGGMAHGPCGEEFKTAFSCFVYSTEEPKGMDCIEKFQGM

QDCFKKYPDIYGAELSDDGEEAADDLQQAPRGEPQESLAAIATKDSSPET
DNLPGVKPLENNGVPKKWEDATDADKEDAKLEQPSVLAEEEKKEKKSEEK
QD
>Mb|QLI64940.1
MTRIASLVALSAASAALQLPLQRPINDIAPHSAFHARPLVSSGTLQDSID
KDRLWERAEELYALARKSEDEFNHPTRVIGSAGHQATLEYIKSELASLGS
YYNVSEQPFDAYAGRVREFRLVIHDKVPASTTAFSLTPPTKNNEPVFGQL
ILVRGTGCKASDYPHKVKGNIALMQRGTCPFGDKSQLAGRAGAIAAIIYN
TVDEELHGTLGKPSPDHIATFGVSSKDAEDWVENMPRGEIFRGSAYIDAT
VQTIKTNNIIAQTTEGDADNCVMLGGHSDSVEEGPGINDDGSGSLSILEV
ASQLAAFRIQNCVRFAWWSAEEEGLLGSEHYVKSLSDKENAKIRLFMDYD
MMASPNFAYQVYNATDAENPLGSERLRDLYTDWYTSSGLNYTMIEFDGRS
DYDPFVRAGIPAGGIATGAEGVKTPLEAGFFGGEEGEWYDKNYHQIGDDL
NNLNMTAWEVNTKLIAHSVATYAASFEGFPERTREVGVASFRKPKCNALS
PQFCHS
>Mb|QLI64941.1
MADDTPRRSRSQSPARRGPRPKRGGFRWKDNIRRNDDRDDGHRRDFRDRS
RDRDRGRGYRDRNRDRDHDRTRDRPGDRDRNRDRNRDSNHRVPDRLRSPR
RNDRDSSEKADGRKEKTSKPAPASVTGGEEMIIVHVNDRLGSKSAVPCLP
SDTVGQFKIMVAARIGREPSQIMLRRQGERPFKDHITLEDYGISNGVQLD
LEIDTGD
>Mb|QLI64943.1
MANNDDGLCYPTEGIMSKNGRKRYSIGVRSQHWQLRSLISAEKKNIVYFP
GGNGSNHVQRLNTLTRECETIKLLTFAPRCLVAENGWLCCGSENGEFVAI
KLEDAIESNQQPLLGLHLDAETRHQLNLGGASRDEPLLALLAHARRSSKS
LIAKSMKLAKDRVNCVTLWFPPALTPLEAGSYTEPVAVLANNDRTVVLVS
LREFDQNEKIDPLDVVTYPDFVNRAILSPDGRILVAILDDPYLYVHERVE
TEGGISTPPASEDSGYRWEQRQRFLLKSQRKDDRSDSRGSFAACFSPSGA
YLAVGTQHGTISIFDATLLLDPSAEPLITTFKSSRPESGPGAIRDMAFCP
GPFDILAWTEDKGHIGFADMRSNFIIRQIVDINVEADFRHIDILDRNTVD
PRLLDGNSDRRSNRSPPSGSTRTRRQGEALETLNQPLTPNETVVLEAIQG
DRRRRERAALRSTEERARDGGPSDLALTYLSSLRPSAADGESPRPRQRSS
SVGRAMGDILGSYREQRERQQERLRNARQLTREGSEGQQPQSTSTRRTDQ
RWSEQRWVDRVEETVAAMRGQRERQDSSYLNVLEILQARERPSNDTDNED
SSLLVPLVNQVVNRWEESAIRGTLATDHGVFEVPPSPDNTAGLAWTEDGR
TLFVGAQTGIYELHINVQGRKFCPSITMS
>Mb|QLI64945.1
MNDPSSPPRRQDLFPSILDDRSKPAPQQTDAAPAICRICRGEGTSAEPLF
YPCKCSGSIKYVHQDCLMEWLSHSQKKYCELCKTSFRFTKLYAPDMPQSL
PVHIFLEHMAKYFVRNVLLWLRAVVTISVWVCWLPYFMRHVWTSMFWVSH
EGFGPGPFSLAFSEPTRSTRPTRPAPTCAASPPYPFNIAADPIDNTTIAG
KITFLNSTVGSLPQTLLSDVGFLRNLTRNATVNRSVIAVIEGQIITVLVI
VCFILVILVRDYVVQQQPEINMRAAFADQENNPPAPAQQPVDPEVHDDND
TPEFDGTDSDGETLDAGVEAPGPLGLDNGRDIDNGESALSYTDIATRNAT
EPDRMVLELAADSQESSAQDEAAAGSDNRASVIDYLRVYRRAGGNLEKIL
QIVEEEDLEDKLGYWVDVTRRSMRERETASQDISGPSTSQEPVQSLPPFS
PALDLNKPNDWGQPLPPGDEQEARQGNSKGKEREWLPISPDLSPGHMAPD
YDVASGPSRPRARSDGPEPSNMTNPLANNSWTFDSLPAANEVVPELMPEP
HTPEAQATAQAPSDNAGMRRRTVIPDSPPHSSHGDDVASIDHGSDITHVV
PDEPVAPVPAQRGFVGRIANFMWGDLDQRQDIEEAEEQEPAANGVAAGAA
GDDGADDPWVDVPIAGPAEANVDPNAGEVDAAAAAAGMDAEAMEDLEDFE
GVMELLGMRGPIAGLFQNAIFCVVLVTMTIFACVCIPYNIGRASVLTISN
PILLIRMLMGLSKVIQDAAMMIGGFGAWCALNIVDMFTSIIGGAMGAHIV
SARKASWGLWTGTGARVVEYGLMIEFPLFTPKVQHFSAVSHEALNALKEN
IGWVLGQVDSGLKALAEPDYTALIDGRFVAATSTTIQSFVSTLTGVASLL
SDPSSWIIDLGEVTTEAPLDPSLAYWSGLDRFWAILIGYLTVFAVGALYL
KRDSPFSRGAVMQAWEAGVIDSLHQASGIMKVILIISIEMLVFPLYCGLL
LDGALLPLFEDTTFNSRILFTCNYPLTSIFVHWFVGTGYMFHFALFVSMC

RKIMRPGVLYFIRDPDDPEFHPVRDVLERNLTTQLRKILFSAFVYGALVI
VCLGGVVWGLYYTMPGVLPIHYSSNEPVLEFPVDLLFYNFLMPLAVKFFK
PSDGLHAMYTWWFRKCARGLRLTYFLFGERRIDEEGILQLSPELKGQVPP
HKSLLLELDENNAVIPKTWRDTFEGGDAKPNPSISKGDMREMRHKKIHLV
KTGQLERSGRFVRAPASDRVKIPKGQQVFLKVSERNRRKDGKSDDDMYAS
TQYQLVYVPPSFRARLFLFILFIWLFAAVTGVGFTIAPLVLGRAMFKMLI
PAHIRTNDIYAFSIGIYVLGSLAYAVFRLGHLTARLRDWTSKIGNKLANG
NSGRRVISGLARGAKLFYAYFFLLIVFPLLVSTLMELYFSIPLHTYMNPP
WQQNETTTANQGGEAGRHTIRVIQSWTLGLLYLKLATRMITSLFPDSRAA
IAVSSVMRRGYLKPDVDIFTRAFVIPGVALSILAIFGPPTTAYLLIHFGM
LSPSQLTGAVADRANASLVFRYSYPVAAVSAVMVRYAVGLTRVFNRWTGG
VRDEAYLIGERLHNFGAATAGSRKARKAWGAAGGGRL
>Mb|QLI64946.1
MPLRLAAGRALAAQPLTLHGASATAHLRRFHASLTRASDFAKQNHYERLG
IHHDASPGEIKKLSSPHPPPLPQLQKTRRKRPTRSKTTTRRSFYLLSKSH
HPDVNPAPGAAQNFSLLSEAYNVLSDASRRAQYDRDVLRLHHPHHHHHHA
SPPAGSYHSTTAHQHQHQHQHHAGGRPPSGLSKRRGTFRGPPPSFYRSGG
WGAHADKRRQAHEESTATSTSSSARGDPWKHFHQAPGQAQGGMGPGSSPF
GAGRPDDDVPHFDRRAHARTHQRQDERRHERRRAMGDDDVEFEPQTSLAG
HFFIIMGILAATMVAPLVYLQFMRLGRRNRDGV
>Mb|QLI64949.1
MPLEKIYVVRHGFRSSWSVDPRTGVYLGIPSPTGIQGDPALTSYGIDQAK
ELGRHLETLDPAVEKVYSSPFYRCLQTMVPFMDLQREKPGAQNGQMPQVS
AGESDETAKSLLTGGTVVTKMLPEHGIREWFGSAPFDHPEPAAPGVLKAM
FPSYDESYTSAARPSRKGETLEQLQQRITVALRAIIQQCDADGTRAIVLC
SHAAVIILIGRILTGLIPASVDVDDFQAYTCGLSVYSRSSGGVAEGNNIS
DTRDARHSTSSSTPDTLQANRIGGWDCELNSDCSFLSGGAERGWKFTGDE
SFPDTGSLSQVEPGSKL
>Mb|QLI64951.1
MDDVDLREQLLDRRLQLTEGLNGLPYDLILYLQRAVVHSDLGYPDLAAGD
AYKALLLTDEVCNEGFEYHEKAMESLLMHTGVPIPEVMAFGDLPEEWSPA
VIESLGGGEEAARRLAGLASIRAYQILALSLLLCGCLKSASNFCQRGLKL
APENKELLNAQTHITTVARRRLRRDDFDTNDLPDYGLVRREVYPWNDHEP
DRFAEDSLKALNADLKTMAPKCAVQVATLPVLLEGASNTDHYDIIPTCKQ
LGVFAVEDIEPGEDVLREYSLLTANNRLKDPVCDACSSDLPPLGSNNEAV
NCDECYDTVFCSQFCHDVAQERYHPAVCEKDVDAIAKDPEASEADETLYL
LLLSRVLALASHQEVHPLDVREVRFIWGDFVPTATNDIDISPNAGPPPEW
TLPFSFKYNIETPLHILEKMDIDVYTSLPQYDLWILNTLYAKFRGTASAR
KNPRDGRPDVAAVHPYWCLANHDCDPNVTWDWGGRMVLWARKERVVGGRP
GGIKKGEEILNHYCDVSLPVQQRREWARGSLGGWCMCKRCRDEASQNGVE
PQA
>Mb|QLI64953.1
MVTTRRSAGSSMEPTATGSTARQPQNLSPPRNPPLLPTPTERLLLAVFPL
VLVFGTLFSVLSPETRSAHYDHMTQSHSQDPSAAPSYFARKSNIFNVVFV
KRGWGWTTFAFYFFLFTHPSGGAGGLDITPRRLRAVLRWAAVTAWWFLVT
QWCFGPPMIDRGFRWTGGKCDMAERDVLQGSADIGEAFTAVACKAAGGKW
NGGHDISGHVFLLVLGTCFLAQEIGWAVSRWSSRLAEERCIVLPDGAVKS
ANVEAETAAGQGGGYALGIGTRTALGVAGLNLWMLLMTAIYFHTWFEKLT
GLLTALVGVYLVYIVPRVVPAVRAVIGLPGV
>Mb|QLI64954.1
MDSSQAPLMADNREGDDVEAEEDNVHEGRLTKDVDANPGVFVVALTIAAG
ISGLLFGYDTGVISATLVSIGTSLSNRDLTSMDKSIITSSTSLFALLISP
FSSVLADRLGRKRIVLYADILFILGAVLQAICSTVPVMIAGRCIIGAGVG
AASFVVPLYIAELAPASHRGRLVTATVLFITLGQMVAYVVGWAFSAYGSK
ETGWRWMVGLGALPAGFQAAIVVFMPETPRWLVKAGKPAQAKTIIQRVNG
GAGTVEDASAIIKEIEIEIREEENLGRRRDQQSTRWKWLGAWRDLFGEGK
NQRALAIACLLQGLQQLCGFNSLMYFSATIFTAVGFSSPTLTSLTVAVTN
FVFTLVALGLVDRIGRRRVLLYSIPFMILGLVCTAVGFSFLPSGVTNSQT

QPQSESGSQWAAYVILISIMLYVASYGIGLGNVPWMQSELFPLSVRSLGS
GIATATNWSANFIIGLTFLPLMDALSPSWTFVLYALVCGIGYALVWRIYP
ETAGLSLEEATALLERGWGV
>Mb|QLI64955.1
MSTQPAVAEPQPQTRPATQKLSVLHGPLEPPLVDLTLGELLQLQTYQHGT
QECIVIPWTGARWTYNELNQQSSSLAQALLDMGIGVGDRVGIMAGNCEQY
AAVFFAVAKIGAILVILNNTYTPTEAMYGLKFSDCKVFFTTQTIGRLDNR
KLLAALDNKTDAPKVFILRGEAGKYTTYDSLIKSTSRKSYDTLYRAMQRV
LPHQVVNLQFTSGTTGLPKAAMLTHHNLVNNSRFIGDRMRITAEDILCCP
PPMFHCFGLVLGLLAIVTHGGKIVYPAEVFDIPSTLQAISDEGCTAVHGV
PAMFDSLFQAEFPPGFNCDKLRTGIIAGAPVPKYLMELLVNKFGMTEFTS
SYGLTEASPTCFNAFTDDALSTRLTTVGTLMPHAQAKIVDRDGNTVPVGS
RGELCMAGYQLQAGYWNNSEKTSEAMVRDAAGLLWLHTGDEAVFDENGYC
TITGRFKDIIIRGGENIYPLEIEERLMAHPSVSRAIVVGLRDKHYGEVVG
AFVERLEGSPQPTDAELRDWVRSTLGKHKSPAHVLWLGQDGVPCDVPLTG
SGKVRKFEMAALGNEILAKRGQTSKL
>Mb|QLI64956.1
MSTLQAVRYSRGKLEVLDQLRLPHEFHYDQVSTRQEAFDSIASMRTRGAP
AIAIVASLGLAVELFNGPVAADSPQQVIAHIDEALDYLKQSRPTAVDLTN
AINQLKARIRTSGETKASIIQGFIEEAENIFEKDLKTNLAIGDYGAEWLK
TQVGATSDNTVSVLTHCNTGSLATSGHGTALGIIRTLQANGQLRHAFCTE
TRPYNQGSRLTAFELVYEGIPSTLITDSMAASLFRTKKAEKNIVAVIVGA
DRVVRNGDTANKIGTYQLAVLAKHHGIKFLVAAPTTSIDLETETGNGIKI
EERKREELTQVTGAVIKADGKVDESTKVRVATADQRIEVWNPAFDVTPAE
LIDAVVTEKGAVEKGPDGKFDFSKIMPERWAKVAGV
>Mb|QLI64960.1
MATQALNRDEISNLSFILDKPYQVSYAERPKPTLSSPHDVIVAVNYTGIC
GSDVHYWSHGAIGHFVVKDPMVLGHESAGTVVEAGSQVTHLAPGDRVAIE
PGSACRRCPDCRAGRYNLCDAMVFAATPPHHGTLTGLWASPADMCYKLPA
AVSLREGALVEPLAVAVHMVRQAAVQPGQAVVVMGAGPVGLLCAAVARAY
GASKVVSVDIVPSKLDFARRFASTHTFLSRGGAPEDNARDIRELAGIPRG
ADAVIDASGAEPSIQTGLHAVRVGGTYVQGGMGKPDVNFPIMAMCLKEVT
ARGSFRYGPGDYELAVELVASGKVDVKQLVSEVVPFDRAEEAFKKVKEGE
VIKILIAGPNEKIDEV
>Mb|QLI64961.1
MSMFRAKKLDLGCFVKARTIRDHTKRKVFEQFETERQALRYIIRNTTLSP
RVRAEAQLQLTQMHAYTRPTQIRNRCIMGGQGRGVLSDFKMTRFNFRMQA
MAGNLPGVKRASW
>Mb|QLI64962.1
MTVVNGTGPGKPVLFFDIDNCLYSRSTKVQDLMAELIDKYFAKHLDLPWE
EAVRLHKEYYTSYGLAIEGLVRHHRINPLEYNAEVDDALPLQDIIKPDPE
LRKLLEGIDKSKVKIWLFTNAYVTHAKRVVRLLGIEDLFDGLTYCDYSQM
PLICKPHPDMYKKGMREAGVSDVKDCYFVDDSYLNCTKARDFGWTAAHLV
EEGLPVPEKTASQYQIRHLSELRAVYPQFFKPV
>Mb|QLI64964.1
MASATKYIQLARTLPEPLQRFFARWPPAAVQSPGSSPTQYQEQRPDPFRF
YKHPVTGKWHDPVYSYRRQAQLVQLAREHGVEDLLPETTKGTEYRLAHRV
EHGLRVKGTGVGQKVKGHIHERHMIAKMEERRKAMLEMPKLIKAWKRVGK
KNWTKWPK
>Mb|QLI64965.1
MAAANNMHNLTTLIKRLEAATSRLEDIATSTELPKDVPALKQAIASPAVE
SQAPTPPQTAAASAKTTSSQSEDPVPESIEEFQQFINSSVGKYVKISTEL
GGVVAKQAVEVLKGFQEQRKFLLITTKATKPDASTYQTILKPTNDALMAV
TELKESNRPDPMYTNLSAVADGIMMLAWITLDSRPHKHVEESLSSAQFFG
NRVLKEQKDKDPKQVEWVQSFYQVFSDLAEYVKQNFPSGVTWNAKGKPAG
DVAKSFLQTATALGTPDAGGASAPPPPPPPPGPAPMLDIKAEPVPEASVS
SGGFDAVFSELNKGSAVTKGLRKVDKSEMTHKNPSLRASSAADPDGPSRG
KSPAPGKKPKPESMRVKKPAKKGLEGNKWTIENYDRETLPIEIETTLTQS

VLISRCNNTTVILKGKANQVTVENSTRLSLVVDTLVSTVDVVKAQNFALQ
VIGTLPTVMLDQVDGGQVYFSKESIGTRVFTSKSSGINLNILSGPDDDYK
EVPLPSQICSYYDENKGDLINEIVAHAG
>Mb|QLI64968.1
MTKRTKKVGVTGKYGTRYGASLRKQVKKMEITQHAKYTCTFCGKVTVKRH
STGIWNCKGCNRTIAGGAYVVATPAAAAMRSTLRRLREIAEV
>Mb|QLI64969.1
MSDDEDFMQESDEEQYDFEYEEDEDDESGDVDIENKYYNAKQLKLTDPSD
AIGEFLGIPPLEEEKGEWGFKGLKQAIKLEFKLGKYDEAADHFAELLTYV
KSAVTRNYSEKSINNMLDYIEKGANGKEAAKSMEKFYSLTLQSFQSTNNE
RLWLKTNIKLAKLLLDRKEYSSVSKKLRELHKACQRPDGTDDPGKGTYSL
EIYALEIQMLAETKNNKQLKALYQRALKVKSAVPHPRIMGIIRECGGKMH
MSEENWKEAQSDFFESFRNYDEAGSLQRIQVLKYLLLSTMLMKSNINPFD
SQETKPYKSDPRISAMTELVDAYQRNDVHAYEKVLQGHQDILDDPFIAEN
IDEVTRNMRTKGVLKLIAPYTRMKLGWIAKQLKISQPEVQDILGFLIVDG
KINGTINQQDGILEIASDADIKRIQAMSSLSASISELFGAVFNEGDGFRS
KERDGSFEQSMDFQGLSMGRALRGAGQQRAKKGKIPSAMWA
>Mb|QLI64973.1
MNPRSERLHVGIIGGGIAGLALGIGLNYRHVSFTIYEAAPGLNAIGAGIS
LGPNTTRAMALIDPALREGYEKIAIKNRSPEKRHNFADFLLAEPGLGAAR
GFHGAAVGSKDFVRSGAHRKDLLEMMKSLLPGNDCIRFGFKAISVCQVGK
RVQVSFEDGQAAEFDAVVGCDGGKGITRQAVLGHQFPDQVQLTYSGRYVY
RGLVPPGKAQEILGKYADDSKIFIGQGRYFAAYPLSGGGFNFLGGRQKDE
PWTHSHSTQEVSRDDMRNDFDGCDPRLLELLEWAKPLRWGLFHHLSTPTY
THGRIVLLGDVAHASTPHQGAGAGQCFEDALILSHLLGQITDDQEIPAAM
EVYDSICRPRAQEIVRTSNDTGLLYTMTHPQCADDVAKIVASAKGRFQWI
WTHDLQADLAKATEDLDRLRKQA
>Mb|QLI64974.1
MGNEESALVAESVKPSTLTERSLAAVAAYLNDESQQRKVVVLTGAGISTA
AGIPDFRSPKTGLYNNLARLNLPYAEAVFDLTYFRTHPEPFYVLARELYP
GKFHPTVSHAFIKLLDDKGMLQMLFTQNIDCLERRAGVPADKIVEAHGSF
ATQRCIECRAAFPGDLMTEHVARGAVPRCREAGCAGTVKPDIVFFGEMLP
AAFGERAGHARAADLLLVMGTSLTVHPFAGLPELAAEGRPRVLLNLERVG
RLGTRPDDVVELGECDEGIRKLADALGWRDELETCWRAVVGHEEADRQLR
SAGGGGREVEGEVEDEVRKLAEGVEEALRLGESEGEDGAGPSAEQGHEAV
VSEEESAAKGGHSMARAESSPETPETRVEQHVPRAAAEAAGSETVDEKEK
EKEKEAEKPSL
>Mb|QLI64977.1
MLKSVALSAGLIAGANAFWRMECPGRVGLARMDPIINPNEASPHAHTIHG
SNGFSESATTADLLDGNCTSCRVTQDKSAYWTPTIYFQDAKTEKFEIVPQ
VGGMLAYYLLYGDNITAFPPGFQMISGDNDRRTYTLGDARQPDPDRSQWA
GLGQTTQEALAQRAIGFNCLNYAKNPEQTLYRHYLPPKAELDQDCTDGVR
FEIMFPSCWKGGNAIDSEDHKSHMAFPDQVMTGTCPEGYPVRTPSILYET
IWNTYAFKNRDGRFVVSNGDTTGYGYHGDFMTGWDPEFLQRAINKCTDLS
GLIQDCHIFDVVDKEKATSCKIKKPLLKALFAEDVVGPMANLPGGIKVGQ
VGSGDHGKKPPAASKPAASSSASSSAQSPVSKPSAAVSPLPGLAFKENAP
SSSSVPSPTTTSVTPPPPPPTTPSVQPSYYSTEYITNGNQVTQILWVEKW
VTVTDYGNSPAPTPARAHDHRRRHMRHGHVHGKF
>Mb|QLI64978.1
MAPSKSRLDDTFKALLARREHQGRLRRLTLREPATVDFSSNGYLSLSTNP
NIKTAYISHLQSSSDDFSLGSCGSRLLDGNIPPAENLESQIAQWHGAPAG
LLFNSGFEANTGLLSCAPQPGDYVLYDELIHASVHDGMKLGRARAIPFAH
NRVEDQPGEQETPGPKPLDAVLRALLRGQDGHKVRSGQTNVFVAVEGVYS
MDGDAAPLEQIVECVEKHLARGNGHVIVDEAHSTGWMGERGRGLVCQLGL
EERVWARVHTFGKAMGCAGAIVLCTPATRSYLINYARSLIYTTAMGYPML
TAIQTVYDYLQSGQADGLLANLMSLMQETHTLLLSVCQKHSPPPQLFRIN
TSPPESPIIPLFSAHSRSLARHCQQRGYMVRPIVAPTVPKGTDRVRLCLH
AANTVSEVRGLCTAIEEWLEQQKIKDLSPRADGVQRPSVGGLTSLPLAEP

SKL
>Mb|QLI64980.1
MDDLYDEFGNFIGEEAESEDASEAGVEAGADAGDYVYDDEPEETGGVTGT
ELMEIDDGPSNAVVLHEDKQYYPTAQQVYGEDVETRVEEEDAQPLSEPII
APVEQKKFNIEEADLPPVFFDRKFMTDLMNFPEQTRNVALAGHLHHGKTS
FMDMLVLETHDIAEKLDKRSGRKRDEKLRYTDVHILERERGISIKSSPMS
LVLQSAKGKSHLVNLIDTPGHVNFVDEVAVAFRLVDGICLVVDVVEGVQV
NTEQIIKHAVLEDIPLTLIINKMDRLILELKLPPKDAYFKLKHVVEEVNT
VITNAVPAKASEKRISPEKGNVLFSCTDLGWCFTLQSFAKMYTDTYGDVN
TDDFARRLWGDVYFNPRKRSFTRKPVEERAARSFVHFVLEPIYKLFTQSI
SAPPEDLKLVLASLNIQLKPAQYKADAKDILKAVCQQFFGPSTAFVDMIV
RHVPSPIEGAQRLLERAYSGPLDTKVAGSIKACDQDGPLVMHVTKLFNTS
DAKSFYSFGRVLSGTARPGMSVRVLGEGYSLDDEEDMTIATLGQVFIGET
RYNIPTDGVPAGNLLLISGVDNSIVKSATIVAPKFEDDEDAYIFKPVTHF
TESVLKVAVEPINPSELPKMLDGLRKVQKSYPLLDTKVEESGEHVILGTG
ELYMDCVLHDLRRLYADMDIKVSDPVTRFCETVVETSATKCYAITPNKKN
KITMVAEQLEKGISTDIETGAVKIRDPIRKTAKFFEENHGWDKLAARSIW
AFGPDDMGPNILQDDTLPAEVDKKLLNTVKESIRQGFSWATREGPLCEEP
IRNTKFKVTDVLLAGEAISRGGGQIIPTSRRACYSSFLMASPRLMEPVYA
VSVTGPEDSHTEVYNVLSRRRGHVLSDGPVAGTPLYRVNGLIPVIDSFGF
ETDLRIKTQGSSMVSLVFDNWSIVPGDPLDREQIIRPLQPASAQATARDF
VLKTRRRKGLSEDVSVKTFLEPEFYQSLMESGMLGDA
>Mb|QLI64982.1
MDVAYNQHSDRAKRKNRSTTNINHLSLAPLTVKLPINDSDAIPDSLAAQA
HATYLQGKSAPTTPRLLSRGSVTPRSRSHHRAPSAPSGPLSKSKSSTYLG
ATGYGKKSRSGASTPRRRDVFGNANGNENDSDWLVRAGALLSSEAREYKG
QAWLVSKQSSTSLAGMRDADEEAFERELAREREVASQRASRRGSSVMADD
DATPNGSRFNSRFNSRKHSMAETRSHLGTPFERAAAEGDSYFPSSPEAAI
AGPDFVNLDEKLEELERDTTQDDEATVRRLVRHGTVGRGSWISNVIGWSL
FRVDENDEDSEDDDEDYIDDEELARTGRGGGSHRHFEGILHAPVERLPPP
MSDEGGWKDAAWLLSVASKVMF
>Mb|QLI64983.1
MSPTWRIVMGCDEAGVSYKDAIKRDFESDSRVTEVIDVGSKESSDKTAYP
LRAAAAARIVAEGKADRALLICGTGLGVAIAANKVKGIRAVTAHDSFSVE
RSVLSNNAQVLCMGERVVGLELARRLAKEWLAYEFDTTSASAKKVDEITS
LEA
>Mb|QLI64984.1
MDSNSNAGPSHSRSQRSLRAPSDRNRASTLSAASLAGPATPAESDAPADE
YFEPLSTAASRRSRISTASHGWASSAGVPESNATERPRRPSIRIRRSSLG
VPQSTSSTQDAATAPQQQQEHDGGNEALRGNRPRSASQPSPAPPPVGDAA
ARGARNSRRMQPQIALPRLTEEGARPTMAELGLEGTSPLSPSVSLPPAPA
LERSHSDGEAGVDEAEGKKPRRSIVGRLWKPSFRRTSVTAPTQGVDPMDT
DEYNDELVDWLDIIDPEVQTLSTLTNVQNSLFVPDLGSWVNRRPTYVLSQ
SDTLLPRPVSAREPIQQPEQVPTIREDEEEAPRPEEGEADQPPYIRRTDT
ITSRLSDSHYAALPHGRKLEGWSAAEKWELDDRVRHMMHSRRSRFKRQMK
GFGQYVRRPLGFFVTLYATLITLFGLAWVLFLIGWIYVGEQQVYAIHIID
SVLVALFAIMGDGLAPFRAVDTYHMIFIARYARKIAKAKKQAAKELARTD
SSPETQNTDASEERRGTPTPPPPPQEEQRKPELPNGSPTLHQPAAADGIP
PITLEGDNNPGVDLEDAKSETCSLEYDYLTPKQRKSLEHHQKKLSKSHSF
YKPHETFTHHAFPLGHLIAIIVLLDCHSCLQISLGATTWGIDYHHRPFAI
TTVILCVSITCNLTAGLVIMFGDRKTRKKDVVELLERQALTGDAIKKVER
KREKEATPEESERDKESVRQQEVDVGSEEKNGDKGKDKEIEVPTSRTGQV
LGLAGHEVGSLR
>Mb|QLI64985.1
MAAAGPAMESASCVGLGLNLAQDADISHFSLRSDAVAPETKPAAPRNRFQ
ATVDDDDSDQDTVVASPARNKVADSVSSSSPSPSSSSRTSTQRPNGDFPD
HHQPASPTPASRYGKARPSPSPSPSSSYSHASPSSSKSHASSSSTAYGTP
NATLTPPSSAESSPKQRPAVHFSTRPPVVLHHRSNSYPVATEPSSPRYTT

SSRGDGGPHMSVVDRQWGQLFTEHGEPTRRLASVLRGLANFMVREYEPRN
SLVIPPVKMFALYRRYKLDKEQFPFQGIFDYQSRHCLRSLELLYQDLSCE
YHLVQDHHSTRPYIPALTPVGLQTWLTAFIQAAPDAESYRLQSILMDIPL
EADHQGPAPAERLPKQLSRHLFPAHRNDRTYRDIVCALDDWSKRMGSSSE
PPSPSWGNLLFEAFRGASHMSSGPASMVRREKPYQTDDYKRHSSRKSPPE
DVTISPANTSRRRQKSTSTASRTPQEAKYYYSSSSSSTSSSQREKAYNPE
PRYFPSSRSEPSRRRYSEPSPERIKYDRHTSRRRGKSPKAARAASIDSER
RYRHPPRSYSSSSYSRGAEDVDRGRMAPDSSSRTPDALPGRVDSYRFFQG
RQQGPTYDDFLRGKARG
>Mb|QLI64986.1
MRFSVAALSTLLAPSAALSLFNSESQRAITSNDDLKIPGESPLEHCSDRK
AEGYVEIKSVDLAPNPPSAYVLSPSRLGEMVVGYPVANDYADWFVLFDSS
GQDLVIKAEGTVKKTIKEGAYIDLTVKYGLIRLLKTKADLCEQMGEVDLK
CPVEPGDRVITKTVQLPKEIPPGTYNVEADVFAANGERITCLTATVKFNM
PGMDFLGGEL
>Mb|QLI64987.1
MAVRRALSPCALTRPLSASIIPILSKTHIHPSTIQRLQKTQSFASRSPPK
APSYADVFTLSDIPLSSFESVISRNGSSFTDLSPEEYYRVAQKFSDAINR
GSSPWTSSFTGKDAIPAETLHKVACIMRQITGPRSADAFATALWSTASEM
GYRPSTLSLARQLVRSGAYGRVPQLRRVEARFKGLVSSGKDADALTAEGE
LLLEQGRLDAAAAVLTKALKLSPRFEWRPYCRLCLGRAYLKMGKVDDARG
LFEKLAQEGLVEADMELAEMLRGSDADEARQRMYTAACNGRRDMFTRLSE
MELELEAVGGEKDGEERRLWAMEWSKLADGRVEY
>Mb|QLI64992.1
MRRLVCLANPPKTPFCFLPTSPIWASHINKDRAPRHAMPLYRPNVPTELP
GKALPDHLTQSGIRNQQRLVPELPRSHALSYIALLSGIAETPFSQSSQDT
GKFARGFRGSETADPIIEEKNQECCFAVSMLSTSRSRDTSSSLLSPHPSL
LLLDCGSDHTLAWDTKSAKAEISARAPAYPSPPMSGSPSLPPKDPRNFYD
RSEGFGAYSTTTSSQDVYRTNSGQQRPVDLRLQLPPPRRPPPQYHPRISV
QYRPESNMEPPYGYQGSDGRAAQAALYSSREPPDLNKGQHLHQYGSMVVG
TSSTSQQSVESGPNFTENQSMGSPKSQRKTKGHVASACVPCKRAHLRCDA
QRPCSRCMSNGKEDSCIDVQHKKRGRPRLRDDRDTRFDPLRPPHSQDVSP
RRPLSIYPSSGTGPPLFDDPYERHPSFRQPEVSTGNSFSARPGERASSSD
SNSYNTSLSATSGSPEPVAYLTMALEFVKGSVSFWDAAGLPNMAGRNLGD
VVLPAELEKVSQIQSHFNNEQKRREPNYLPPILGTGSQSIQGLGFTIEDF
GRFPLNFHDHMAFSKVQIKQDNNTKGQWRGNLTEDDHTQLPNKNPLGKII
RAFDKAFSFPLYDLEQSNQRFVNQQPGPVTNGQAVLT
>Mb|QLI64994.1
MGLLVWLGLRRSNQWESLTVMDHLIDSPLTFFTVNFYHLVLFLRGTPFRP
PRHQPSIRVVCISDTHDQIVDVPPGDILVHAGDLTNAGTAADIQKQLDWL
KTLPHPVKIVIAGNHDSYFDIRSRCDDDRRRGARLNLDGLIYLQGEISAH
KIRGREISIFGAPDIPECGPKSFAFQYSPTRPPWLSKVPPQTDILVTHSP
PKHHLDLDLGCPHLLREVWRVKPRLHVFGHCHCAYGKESVYFDDVQFAYE
RLLSRPRRGFFWDFIPNQSWVDMFEIVYHGIHSVLWKWLMSGPGSNQGSL
MVNAAQMYKNSGKAVSRAVVVDI
>Mb|QLI64995.1
MGVASAILIVLITILFPPLGVWAVAGCGMDLFINVCLTILGYFPGHIHAF
YLEYIYYDRREQAHQGRFPTSRAPGVYSDNVQTGGQGYGTIVQPTR
>Mb|QLI64997.1
MQPFGGPMGRVAWAGSRRIFRQDAIVFARKTAQRQSLLSRRTATDLQCST
QGIFSQSRLFSSHDPPKQTEPVSSPKNDSETNPRDPAKSANGPNSSAAEL
PSVTDSRRSDLSQKFGQTMDNLQARALTASQTLNDITGYSSIEGIKREND
ALEVQLAEAHARVRNARQAYKTSNTKRATTQREVTTLLARKDTWSPADLE
RFTELYRTDHVLEGEVSGAQEALTEAEAEEQSLSQRLNAGILKRYHEEQI
WSDRIRRASTWGTWGLMGMNFVLFVVLQFFAEPWRRRRLVKGVVEEEKRV
LEEVRGELEAVKLSLAKKEEEAVPRPMEDSLAAAPAEPEVTTTPSAEVSW
KEFITDPARWRAAAADLVSERRVDLRMKDASALALQGAAMGAAVAGSAVM
LLVRRT

>Mb|QLI65000.1
MESEPPSVDEGRSSRRGRFMGKLFGKDKKSQDNSPAALDAFLHSSADNLE
VTHPPPPPPPSLPKLAKLDTTISRYPQALAVNQQAQQNRPMAPGKMESKS
PRISPRPNKKGMTVRFADSYPEIIGEGGDESDIPTAEIARNKRARPPPPP
VHRSPRLGPAAPPAGGLRGPAGGPVGFPGAGSDDFQPKPLQRTQTGYSSI
YGPEKEDRNTSLVPPGANDQGTGNTLVPGGTASARFLETSARRDDQRRSF
IEIHQAQMREAEGKAFALAARTASSTSQNDWEDGPRPAVSNVDDSPDSSR
RYPSSPSAQMKKKPALDYSPAASIASNTSSTYQLSNPSQTSLPVRQPSAA
SQNGALPGGSSPGPPLGHPPPMAARSASIRLQDAVVSAGEDDALNAFVTG
TKHLFELFRLHAETIQPLLTCAPKDCARAAIWWFLKGRMGLESAVRERPN
SPQSQMQNELDRQQAYSNLAKGYWLCREAIPEIMDGHRLAPDAEITGVSQ
SLVSALRKLSQSMKRNGFLPPEEPFLPQTIDKSIWLEYPSLSQDLNALLS
GNWSSGLTATQHPVSTLQLLEAFPLGDTVEHFSYGRVMADAFLMEQGRES
QRIYLPCILSMVRPQKSAGLVFIIASQNGCVQLAIQENKNVGPVWEDLRW
RNNTCAVEVRMPRGFLLVVQLNQSDYRTLWNMYDFGSKVQSTLRPRSDEL
VVFRNNLRSFQYLDADPQSRVFPREAVPQCDVALFEKIYKENGPAGPRSW
HCGFRIAVVTGPRTRATSGVQHNFPPPLPVQFGFFRAEGDAPALSVRFVN
GRQKGRMVFVFGDEKERVKFHSLLTGTALNHDERIMSDAPLKSFMISQSL
REPTGIMSFSRMPWKAARVVNDEFSSNGDLPPTVLADRLKVILEYQNGTV
ADRVNVAPGELRMRLEVSNTCLLRVLRQPQQDITMSVSEAQVSKELPTNL
ADALQLLKANQTIRSYEFNNLKDLHEFQTSMTGFEVIFDAIAATFAISRR
RMVVPIHKKWEAGLSRIQVVKLEDRQLQLLAFFEDFQHGHCMNFVLKGTD
VYETFQRGSKAGLRFVDAKFPLPQLSADKDSDYDDMAFVCLDLPDLPGEH
DDISIMFENEADRDLLCKCLPATPKGSSRISSRLR
>Mb|QLI65001.1
MDTLVSRYSRPAYEQNEPFENEAQEMMDATSSLTTKFAMPPISQPSSWLR
AATDDRSNPDCPIKIAHGTTTLAFRFQGGIIVATDSRATAGNWIASQTVK
KVIEINSVLLGTMAGGAADCQYWLAWLGMQCRLHELRHKRRISVAAASKI
LANLVYSYKGMGLSMGTMCAGVTKEEGPALYYVDSDGTRLAGNLFCVGSG
QTFAYGVLDAEYNYDLSVEDALELGRRSILAATHRDAYSGGFINLYHVKE
EGWVKHGFTDTNPVFWKTKLEKGEFSNVTSELD
>Mb|QLI65002.1
MSVVGVDFGTLQTVVAVARNRGVDVITNEVSNRATPSMVGFGSKNRYIGE
AAKTQEITNLKNTVSCLKRLAGRSFNDPDIKIEQQYITAPLVDINGQVGV
EVSYLGKTEKFTATQLVATYLSKIKQIAASELKLPVSDLCMSVPPWFSDV
QRRALLDAAEIAGLKLLRLINDNTAAALGWGITKLDLPAPEEKPRRVCFV
DIGHSNFTCSVVEFKKGELAVKGAAWDRNFGGRDFDKALVDHLAKEFKGK
YKVDIYTHGRAMARTIAAAEKTKKILSANQQAPVNIESLMNDIDVAAMIT
RQEFEAMVEPLLNRTQAPLEQALAQAKLTKDDIDVVEIVGGGSRVPALKE
RIQAFFGKPLSYTMNADEAVARGCAFSCAILSPVFRVRDFSVQDIISYPI
EFAWEKAADIPDEDTSLTVFNKGNVLPSTKILTFYRKQPFDLEARYAKPE
DLPGKQNPWIGRFSVKGVKAEGQDEFMICKLKARVNIHGILNVENGYYVE
DQEVEEEIKEDEKEDSEKKDPDAMDTDKKEDAPKKTRKVKKQVRKGDLPI
SSGTSSLDAAQKTALSEKESAMVMEDKLVADTEEKKNELETFIYDLRAKL
DEQYAEFASDDEKTSIKAKLEIAEEWLYDEGDDASKGVYIAKLDELRALA
GPIVQRHFEKIEAERRAVQERNEAEQAAKRAVEEEARKAAEAEKAAQQGS
NDQEMKDADASQVQSEEPTESK
>Mb|QLI65003.1
MADFLASFNFGTDNEVAPNDGACASLDHLQGRLRALIEAKTTATRAEHVS
TTVDVAGTAEFAVDLTQGDKDNMNNVDPLLSGGAEDTDRRHEGQTHVYTA
MDVLRDQPSQAPVVQKAVAEQIARAAGVEDKSVWVLHSFESAAHGWDFTF
ICEQSVQHWKAQNEGKTKDIVGEYTKKEPDPVLMSRPAFDCRGRLLITFS
RKARTISVKYNHTIMHKSVADLTELFKPPPMIGPQKPDKKTLAKAAKLAQ
EEAAKVRRKKNTEHQRGQDGEPKGQRRRRNKKAAGETQQAHLSLVEQAAQ
ALDHADMSRLGEAVAAMNNDDDGHGSAVGWATNPSAGSAIKPLSLNVTPE
EAARRKDVATKLLTEAGVSPDSLSTDQLNIFANQAPELQKESLNMLVTYG
AQRLQIIHPSNKESSAPVPQSDVSPSGEGAASGARTTTTELVLEDSTPKG
KRKRSSRPLGKSRLSCFTCKKRRVRCPRERPTCSECQDSQVECHYPPQPS

QKPKQKSDALATDDADADTEADDDNVEGEADETVMTEANANDTSMTEANA
NAGQQEAPQDDNDFSSDSGAPYSYPQIPGPDLVRDSAHDLAHSFHQQPTQ
APYFQSASGLALPQPDPLDDAPRSQLNNGMTLSESSAYYPSYPPPEPAPV
TEPQREPEERQPVEPNIRTSNHRRQASNNTRTNTNTQKSSWAQQHGNDMF
IPTVSQASPLLQHGHPVSQSPDLAPAAAHINMEDAALLTHAATHNQSYAP
NSSMRIHRPTPPQPKSPFLPPYHSRSASQQSHRSSGMMPHSRSSAFQAPP
PPPPPAAAAAAAADTHSNIRYNNVQSRSSIGGLPTARYGSSDGVDSSNKI
GYKPYSYQKSGSSAGTSNHGYQSQSFHSPMTSSGMHGSLAAYAGLRPGQG
TPGDRQDHRQGQHGQHGQHAQNWQGESQGNGFGQSQRHGYNWGMGEGWNQ
GHNQGH
>Mb|QLI65004.1
MSIDNKPNPPRDESDDSADSTPEREQNNSATVSGNSNNAVQDAQQPKRKG
GRKPIYATSEERKQRNRQAQAAFRERRTEYIKQLEETIRIHESNLHNLQS
AHRNAADECLMLRYKNSLLERILLEKGIDVQAELQAKAESPNLGPAHVPQ
NIVQPPPIQRPIMNRQHHNRKSNSSIAPKLEPGSGAVGSAMQAPKSATSP
KNRPTPSSHSNSPNNAASTFSPAPSDSISMRGSISGLTRQHLSLGGTTQP
SRSTVLQVATPRSVAGTGASYYPTPAFQNHVEQADHDYDSRADMVDDSEI
ETPSGHGSFGGAFNSDTTQPMLLSPTSTGPAHHEQGGHQASLSNHHFPSM
TQLLDHQNLDWDPFGLSASMAFPNQQFPFDQSSLR
>Mb|QLI65006.1
MSTTTETTTRRESSFDSPRSPGATIFDQRSSFSGRSLTEVVSGNTTKPSP
ANELCLNAVRDWKTCLETLCEAFKVSLADTYKSYERDATPEMVDLLFTSR
KFRREAVHRMRNASVTRLLSADPQFFPRYEIRFRNYERVKKEATEVRQLL
QSGESGISPSREVHEFLIAQRGDAVLEFANIGNEASHNDPVLRFRVSSYM
LSETSPIFARMFSGPANSMLLHEAEDITPHIPPSPVPYVCKDGSEAKLYR
MPQHELNRHQSLEILLHAAHMHNELIPREVGFDQFVAIADCSMRYKSTSP
LEMVVEHRWLPRWMHRGADDMPDGVLVISYAFGCRQLFTRMSKSAILNLL
DEKDLQSKPWPQKIKDKIWAVRCAKLAQIYSCCTNAIQEHIRQPNRDSPR
ETYAHPQAEAVTSLQTATAAPPAYAATLTSPPRCPKGSHSCDASNLGWMM
LVFNQMNLLPQILQPSVLSHMPESEEPPRSLTQLVETLRMMPSPASPVHR
GGVCDPSPAFRAAIADIYNSVTGLTLHDISGKSHGWALSKHRMLDPQTIP
ATGLSRMAASNDNYSVVNEFPDSIRFQILLEIGDLSDLHAAAQINKGFYE
TYQTHELTLMRNILRADRSRTGFALVPQRAGNSEDKIPKEVSDMIKREGP
IEGADAVTIGTDDYDYSDEDDEDDLEVIEGTEAPPRNTTLSSPPLTIDPP
GYTEDERRSSEEAASPTTPRQITLDIPAQSQRSPRKPDETVTVVIEEPLM
TDEEAHRILWPDPIVSESPTPINPSSPGVEAMHEKFRVGDPSFVVALEDK
TLVITGDKQLRSELDRRIGLSKKGQDSRPNSSGNASGGCGSKS
>Mb|QLI65007.1
MARTKQTARKSTGGKAPRKQLASKAARKSAPSTGGVKKPHRYKPGTVALR
EIRRYQKSTELLIRKLPFQRLVREIAQDFKSDLRFQSSAIGALQESVESY
LVSLFEDTNLCAIHAKRVTIQSKDIQLARRLRGERN
>Mb|QLI65008.1
MKRLGSLAKGLPALNPSLRAGPRRTSPSLHKKFQHGFSTLSKRTTAIGAS
IPATIVAGTLFIWWLYPTDEFAKLSPPHPRRRRHYYDDEEVDEQEDEDED
EYDDQPETAWDSFAKRIENIAIVTETEWSAFSERVAGMILPDWSKLVPGY
LRKLQRELSMAPGSLADEIWEDAHDPAINPEITYTAQVRVSSQLCDDEKE
FLARRKRVIKVALAKYLGLDEHEIHPDDIPTIAMCGSGGGLRALVAGTGS
LIAADEDGLFDCVTYTAGVSGSCWLQTLLNSSITGGNLKRLLEHLKNRTS
VHIAYPPVAFQSLVSMPTSKYLLSGLVEKLRGDSKADFGLVDVYGLLLGA
RFLVPKGELGVNEKDFKLSNQRDAIKFGDRPLPIYTVVRHEIPQTEETNR
GPTEREKKIAKEESWFQWYEITPYEFFCEEFGAGIPTWAIGRKFNNGKDV
PPETRFHLPEIRVPLLMGIFGSAFCATLSHYYKEIKPLVLSISGLSGVDQ
FVSGRDDDLSKVHPIDPASIPNFAYQMHGKLPDTAPKSIYEQEHIQLMDA
GMSNNLPIYPLLRPGRDVDILIAFDASADIKTDNWLSVADGYARQRGIRG
WPVGIGWPKAGESVEETTMQLVDAEASSSQEAARRVQQAKQDQESLRQTA
GSIAKDPQADDKEAKYSPGNEEAGDLGYCTIWVGTTQERSPHPSPPAKAI
NDTNKWQLTEPNAGLAVVYLPFLANKKGPNISPGTTDFLSTWNFVYTPDQ
IDSVVDLARANYNEGRNQIRSTVRAVYERKKLLREHVEKKRVNGLEILSM

>Mb|QLI65009.1
MSATKLVSDAERRFGESPNERDARVERLWSRLDPTGKGELDLKGLQKGFR
RIDHPLKNANELLKQIMKEVDTNHDGKIQYEEFRKFVEKTERQLFLLFQA
IDKDGNGKLDSAELQTAFRTAGLSVSNRRVADFFHDLDRNNDGFVSFEEW
RNFLLFMPACEHDSRLQAVLSYYDSVVNVTPEGDSLVSDETLEGLGTDEL
SSSSLFYSLFGSLVRVASPLPPATIPPGSPAAPAQDNDPTSIDADLKTDA
VEVLSQPGANAVASADEDGDSIPHQTTQRRRLVATKPTRLEPSKPTQSIQ
RVGDGTSEQLMEEPPQSSAGVESRPQKKFKLTHFAPDPGYFLAGAIAGGV
SRTATAPLDRLKVYLLVNTSTSSETAVAAIKQGRPIAAVKNALRPISNAV
KDLFRNGGIRSFFAGNGLNVLKIMPETAIKFGSYEAAKRALANFEGHGDP
RHINSYSKFTAGGVAGMIAQFCVYPLDTLKFRLQCETVKGGLTGSALVRQ
TAVKMYADGGIRACYRGVTMGLVGMFPYSAIDMAMFELLKNSYRTYYARH
SGCHEEDANPGNIATGMIGATSGAIGATVVYPLNVVRTRLQTQGTVMHRA
TYTGIWDVTQKTIQKEGYRGLYKGLTPNLLKVAPALSITWVVYENSKSVL
GLH
>Mb|QLI65010.1
MAAFTITRKQRLMATIAISAAFFVAELIAGCYTHSLALIADAFHYLSDLV
GFVVAFVALTVSEGSQPPPKEFTFGWQRATLLGAFFNGVFLVALGVSILV
QAIERFTHTLHIDEPKVVLVVGCVGLGLNLLVMSFLHEHDDHDHGHGDGH
SHSHGSHDDAAHFDDEAFMMVDDPENRAESRKVSVTSSHHEHKHSFVAPS
KPGRDLGIFGVFIHVVGDAINNVGVIVSAVIIWKLDSPARYYADPAVGIF
IAIMIFLTAIPLTKKSGSILLQIAPGGIDVEDVKHDIEMIPGIESVHELH
VWRLDQRKTVASAHIVVDDRTVKHFTEKAKIVMECLHAYGIHSATLQPEI
LPPITEQHVEDVSNARETTVHRRRLNRSAKEYVVYAYMAEFAPFPKLPAE
IRLAVWELVQPPPRIIGQVPCRACWAECFAWRRGRDANCSRRRQCEQRNH
PDWRVRYIVHPRRDAVFPLLHACQESRAVWLPRFHRPAARHLVCETGVAS
ADEGDQTAGVRFDVPFINYEADILTVFDDWAFAGALNTVDRRPQTEQGEL
DPFVGLRRESIRHAGFCESAEELWSALLSLDARTLPRLRSLSLVRMGPLP
PGQETERSAPGEMMCPALSQGVDCVVRDVAFPGDAYHAFFHHWRSRDELF
EPRSELPLFKAFHRFWKAFLWHLLNREARRGFGRDYSGWWTFMEYVGRSG
VDAAGSVCVLSKVDGCGAGGHGHEDMLDWTPAFQLRCRLLCEEGEAHGLK
ALREANAGKVGGISERASLERLLPGEYVSGW
>Mb|QLI65011.1
MASESRLYTFSGETKEHLRKFRLTTSRAKDPQAVIYMIDKNTHEIRQALD
DYDEARVYKSLKEVAEDLPDHTPRFVLLSYPITTSDGRLSVPYVLLYYLP
ITCNAEVRMLYAGAKELMRSTSEAGSVIDIESAEDLEEVPEKLASK
>Mb|QLI65012.1
METPVIPLLQQVHIGLGPNGYAPVTSAVTADGTACARQYGQLMDNLLRLC
PGHVWPKDSYKANCPSPILVNQRHQGRLERLHEALTSAITDIVQRWWSDT
EANFPQRMPLAKDEEELLRWLDDQVSRGKAMPFSSCRGSWRPDFLIKDSP
PVFREAAANGEPGEGEVFCITEINARFSFNGFMHEALGQEALDDMGLHQL
GLASATNSAQLLDGLMQLFQPELPLHLLRGEEQGMDIHMFVNVMRRRRPG
LTMRPITPADLRLVPNPEGKTGFKLCCVVGKGDESAASSTTEAGELVEEI
HQLGLELHQRELSGLEPEMLRQISLRCFNDMRTVLLVHDKRMLGVVKQEL
DRLVSRGVLSPAQARVLDAGIADTMIPGSREMNELLRLAQKDPDSRNQYL
LKPVRGGKGAGIVFGDEMNPHEWVAALERLQSPVLVPGVSCVVQRRINPR
LYDVVVKESDSRARYPLVGTYHVVHGKLLGLGVWRTGPDRICAISTGGAW
MCSVIRQD
>Mb|QLI65013.1
MFALFKSLVVCALVAVAQADTIKITARNDNTFNPNSVTAKKGDILEFHFQ
PKNHSVVAGDYKYPCSPLPIGSGFFSGYMDVESGEGDKVFRVTWNNTDPM
AFYSSQGDECPKGMVGMVNPNGTETLDDYKKRAGGLARGVTPGNSPYGGD
VTGNTSPSSSGSKNGGKDKGDESGAGALRASLLALATAWGAIILKSSMMR
GGVASSRGAARQLRAGLHHSRNQRFTGQYSSCIGRIGFRPTAIKHFPTCQ
TTLPAPTALPPYTIGLRTAATIVESQLHGEGPLAEYDRRVENGLLRDDDH
QRGIIESLQNLYNELRNYHAPEVKHPSLDLLKPARKSVFSSLFGSNGKAQ
SAISDIPDNLPRGLYLYGDVGSGKTMLMDLFYDTLPSSVKTKTRIHFHNF
MQDVHKRLHKLKMQHGTDVDAVPFVAADIAEHGNVLCFDEFQCTDVADAM

ILRRLLECLMSHGVVLVTTSNRHPDDLYKNGIQRESFIPAIKLLKNRLHV
INLDSPTDYRKIPRPPSGVYHTPLDAHANSHAEKWFRFLGDSSDNPPHSE
TQKVWGREIYVPRVSGRCAWFTFDELIRQPKSAADYLELVRAYDSFIVTE
VPGMTIRERDLARRFITFIDAVYEGNAKLVLTTEKPLTELFISKDEIAES
LLKNNPQSTEQGEKAVSTVKELMEDVDRQAEELKNSNLFAGEEEAFAFMR
ALSRLKHMESKEWVERGMGLESQGGKEDKDNWSKTRSRQMEDSM
>Mb|QLI65014.1
MPIFQDDLEPDRHRHPGRPSPLQREPTSSVLRRLSEESLPTELCEGPVPN
DPTRPTTPRIDEEAAISCRAELIERLKRGESPTWIPNRHLESILQRRDSP
THIRTSPESPKLLPPVQITPEKVDPGTENAVQDRLQDGLNIERPRSALHS
GDFTHDQAEQDRPSSILNRQRVESQLASESSWIATSPPRDFTPFNFERRI
PFQSDAGSFRSVTASPLSASLSSSFAYHPPTSPLVQSESNDELDFSMAPR
SLDVPSRELNRRLSALNSAGSSPFTSPSPLGGSRLSFGRRDGSLPWQAHQ
PRRSLTSTPTFALHGTPPQTSAVFRARRPSFGPDSSPIQHASMVGSYEES
ILRGRMSTTPSKPFDFTAQIGVLGKGRCKSCLRCPPHVSLPFAAVYYSYS
NTAHGRSRSDDGPSPYVGQIDLENGLPNSEEESRSKRKAHGRYLDRQPIN
NQSVDTLMPDFELDHDRPGTSRPRRRTGSSKAPPGGSYRIPEKGQIQIII
KNQNKTAVKLFLVPYDLTGMEPGTKTFVRQRSYSAGPIIDHIPNLSETDA
QSRPMLRYLVHLHICCPSKSRFYLYKSIRVVFANRVPEGKEKLRNETTWP
EPRFTPYKPIRVMHPPLNMSSGPGSMLVADKAFRRRSLGITFGGASSRAF
DMVDGFDSSGGGNTLPIEPIPFRLPSQGRGDSDTSNSASNPAAGPRSPES
SQASRPSTKDSAGLGVWDASGDDTGSPYEKLSRGDFGYGGSPFLGFFGNG
SPGGTEGLLSQRLRSLGVNRPQPPESAEDLA
>Mb|QLI65016.1
MDSELPPTSQHDSGPARAEAQPGFSPADIFDNLLATAGRASPLTPFFLFI
YQILGLRLGLDLSVIMALLGVFWVTIKIGGQGYTLVNAFIENHLMHSVTV
PQDDEIYDHVKAWLSKQERVIKTSRLVAQTVKNDDCDDKDLENLDVTDDG
DETPDQQRLNVLKLISRVRHQYFPAIGKTRFWHKNRYFVVHRSKNVVMET
SEFPLKVNADEEIKISCLWSSREPIDKLLLEAKEMYYCEMKKRTIIYRPE
VVDHTNRPSSWRLAAKRPKRSKETVILPGAITDFLLPDIREFLMTKTERW
YTARDIPWRRGYLFFGPPGTGKTSFVAVIAAYFLLDIYTVNLSEPNMTDA
NLLRLFRDLPRRCMVLIEDIDVSGIQRDRDSKGVERNQGTANRIGMITKT
FSFGGLLNAIDGVAAQEGRILIMTTNKPESLDEALIRPGRVDVKIGFHNA
TKQQSAALFCRMYGDSTPLEPNKPASKGIKDQNSDKVSLSQEEIKSLSEE
FSEKIPDRLCSPAEIQEFMLLRKEDPRKAVDEVESWVEKMKEQKNSGSN
>Mb|QLI65017.1
MNVVIHRFRDYEFSPMRNRVVLGDNGPQTFPSRISSAASQVPWVVEASSA
ARSGIPQATVLATSCLLIVLHLIRRRSKRSLSHHAVKSRASGLRWGYEVL
SQMARAAALAFLAAAFFQGHAHVLNVVLVAYIFALGLTRLVTEAPWRQEA
LYHINLIIVAELAILITAQLLPCIEIDVECTEDASVVGAAGALSFGLAVA
MCTPRQWIQPQLAYEIPGFHVPDEPALEEETSWVNYYCTYQWLTPTIWKG
TWGKLDMSGIPKLAWYDEPLYLLRQVQLARAASKSTLWTTLRFQRTELAL
MSILIGLAYIVENVSPFGMFQLLAYLDDPGSAVYRPWIWLLLTFFGPMSR
SILFQGYVFTSTRLIVRIKSAMTQELYQRALESMELEDDPFAGKFDDDKE
KKGKENSTKATSAGRLANLMAADVEAIFKARDVIIILVGVPAGTIVSFVG
LYRMLGWVSIVGVVILVLATPISFLLGKWMYRTQKKVRKAQDSRISLVTE
YLASIKAIKYFAWEKPITEKIVESRALEQKALWGVSVLQVFINQITQIFP
YLSLLVMFGLHVIIDKQRLTASTAFTTVYLVKNIRRNIMMASYFARSFAG
AIVAFGRLDKYFASTVPLVQYPEGPLRIEKGYFRRNKKAIFRLEDISLDF
VQGGLNVVTGQSGSGKSTLMLAILGETYLESGSISVPADIAYASQTSWLQ
NETIKDNILFGSPMEKVRYDRVLTACCLPEDLRELPDGDRTSVGENGTSL
SGGQKARVALARALYSKAPLLLLDDIFAALDAKTSAGVWKHCFCGDLLKG
RTVVLVTQVPWIAAQADLSILLEKGQVKAAEPNIGVVRRPIKIAEVLGGD
ADDSSETEVETPPEPDLQAISDALNDPDKVVQDVSVKDIVDQEAKASGKV
NRWTVIGYLGYFGHPSFAIACILGLFLANIFYFGANFWLSIWVEAYEKGG
PVDVAYYLGIFAAFIFLEIIAFGFIVIMFEWGGWRAARRLHNDFIRSIMS
VPLSWFKTIPVGRITNRFSGDMASIDGNLSGMVRQIVDIFMMLFFRLGAV
SSIMPIFMIPGLITCFIGVLIGEMYTRTAVVIKRLTSSSQSPVFSQFADT

LSGLAIIRARKDKAQGFRFDLADKLRIWSATAEANYNCNRWVGVRIDFVT
SLVSLFAGIIAISKAGIVGAGLVGFSLTNANDLSNTVLYMVRAMNDLEVE
MQSFHRVKEYVKLESEDKNDRPYPDEGKGQYADDETHVIPKNWPRSGEIE
FRNVTIRYDPDGPDILSNINLKFNAGERVAVVGRTGSGKSTLVLSLLRFT
EIVSGEILFDGVDITKLPRTRLRESLTIIPQEAVLFNGSVESNLDPTGRV
PREQLEAALDNCKGIASFSGDVTEEDDNDANAAIRGHAQAITLSTEVDAR
GENFSHGQRQVLSLCRALIRKSKLMLLDEATASMDYETDRGIQQILRDDL
EAADDDRTLVTIAHRLRTIIDYDKVVVMSAGRVLEYGSPKELYDAKGQFY
DMVYHSGEMEELERLLEEK
>Mb|QLI65018.1
MALIQALRRSIKGDKEKQHHTSIATKSAVAIIPPKKVIRALYDYEAQSSQ
ELSFSKGDFFHVIGRENDQDWYEACNPALPDARGLVPVAFFQALGRTERD
SAQSDNSRPPTAKSPDHDSGYSENAPHPITTAVPITHRSTKSTDIDNKIF
ATVMYDFEAQRPDELGATKNEQIIIIAQSTPEWFVAKPIARLGGPGLIPV
SYVEIRYVRTKEVVPNAVEAVKRAGVPSVEEWKRMAANYKNSSITLGKFA
GPDAGGQRAVEQGMESMSLQEGRQSAQNGSQHRAGQHSISQSQQYAHNQE
ITPVYAPVRAWIPRYCFAEDKYWFVIVAVLEDGRHWELSRYYEDFYDFQI
ALLNQFPSEAGHDGTNKRTLPYMPGPVSYVTDAITEARLHNLDAYVKNLL
DQPDRVSKCDLVKKFFAPREGDYEIAPNSADEDMRLSQGSQPSAEGFGNG
GSRTNLNGNAYAVLPATSRQLSNSQQGTSNQPQQAVMKVKMYYNGDLIAI
RVPSEVDYQQLCDKIRDRLKLSPDNQLQLFYKDEPTGNKPNLMSDSDLNF
ALERHEKLVLYVEVA
>Mb|QLI65020.1
MSGPPSRIRSILGHLLGPAQPPPHIHHLSPTFFLERAALVEPNADAIFHI
TPNGVALRRSYQTFADRARGLAYYLKKHKLSRIGILAPNTPAFLESIYGI
VAGGGVMVPANYRLKEDDIAYIFEHADVDCIIVDKEFEGLLGVFTKKHPN
VPLIVDLDTDATEGPSCGQFNEAILEGLAHDRELGGHSWAGLQSKATNED
DMLAIPFTSGTTSRPKGVVYTHRGAYLAAMGNIIESGLNQGHCRYLWTLP
MFHAIGWTFPWSVVAVRGLNVCLRKIDYPLIWKLLKEEGITHFNAAPTVN
TLLVAAKEAVKLPNPVQVTVAASPPSAHLFELMTNLNLMPVHVYGMTETY
GPITKCYHLPEWDNLPPQQKYAKMARQGHGFVTSLPIRIIKPDQPEGVLI
DVAKNGQEIGEIVFIGNICAKEYYKDAEASRKLFAGGVLHSGDLAVWHPD
GSAQILDRAKDIIISGGENISSVALESMLVQHPDILEAGVVAVPNDHWGE
VPKAYVTVKEGKNIRGEDVISWAKNQSNISRFMVPKEVEVVKELPKTSTG
KIKKNDLREWAKKGAKR
>Mb|QLI65021.1
MVVDTAYYDTLGVQPTATELQIKKAYRKLAIVHHPDKNPNDPAAHEKFQE
IGEAYQVLSDTELRKAYDKFGKDRAKPQEGFTDPAEFFTSIFGGEAFEDW
IGEISLMKDLTATMDIAMAEGEDQPETPANVEFPGTEEAKKQSAQEFAEK
ETAAAAAGAQGAPGAPPPPTVVVDEEKPSGTAHPATAAGPTPPPRSDATS
PAPSSSSRNRTQIPLRPALSDRPHDEASIQAAEEELRQKEKKKGGLTKEQ
REQLAAYEKERAAIRQKRVDTLAEKLLDRVSVWTETDKGDDVTRAFQEKM
RLEVENLKMESFGIDILHAIGQTYVSKASGLLRSQKFLGIGGFFSRLKDK
GTLVKDTWNTISSALDAQQTVEDMAKMEAKGGEDWTEEKRVEYERRVTGK
ILTAAWRGSKFEIQSVLREVCDSVLYNKKVSLSKRLERAQALVLIGDIFL
KAQRSPEEEGDYLVFEQLVAEAAIKKDKDEDKRKRDKKDRHHRHAEEVAG
DAPNVPKA
>Mb|QLI65022.1
MSFTFGTQGSSSAPSNNATTTAPASGGLFGATSSGNTPSFSFGSLGSTTG
NNSTPTNSGGSIFGQKPAGDAQKPAGGLFGGASSSSNTASAPSGALFGGT
STTPAAGSPFGGTSNTSSTPTSAAPASTGMFGGGSGALGGSNTSSSKPAS
GTLFGGLGSSAGGGLFGAQTSSGQSQSTTTTAAAPSSGLFASSGTSLFGN
KASTTPSSNTLSTTPTTATAATPAKPMFSLPSTTPAGAPPTDSSKPASTA
GTAAGGLFGSAAQPTTSSASTSAVPAAPGLFAGAQSGGGSSAGSLFGNKP
AATAGAPAGGLFGAPASSTPSTSATSGAATATAGSASSLFGAKAAGTVGS
TAPASTTTSNSLFGGTAASSATTTSSAPATAGGTTGGLFGNKPATTMTSS
ATPAVTSAAGGLFGGGQAGSTAAAKPASSLFGATAAASAPAQPAAAAPAS
TAAPTAAPTSTMGGLFGSKPATDATNKDNASKEEPKPTTQNGTNSALGAS

TTGPTSQMARLKNKTMEDIVTRWASDLTKYQKEFKEQATTVSTWDRSLVE
NGEKIQKLYLDTFEAERASHEIERQLAAVESQQEELEAWLNRYESEVQDM
FAKQIGPGEQLGGPDQERERTYKLAEKLTQQLDEKSRDLSKMVKEINDIS
GTLSKGTKTEDPLSQIVRVLNGHLTQLQWIDANASALQAKVSAAQKSSGS
LNNHYTGPESDAAEGFYRSYYGRR
>Mb|QLI65023.1
MTSSDRTVLVLYGSETGNAQDMAEELGKLCQRLRFTSRVEELDNVNLNEL
LQHQFVLFVISTTGQGDMPHNSLLFWKKLLRKKLPHDCLGQMKYSCVGLG
DSTYLKFNWAARKLIRRLDQLGASTFIEPCEADEQFPEGIDGSFVRWADG
LRKHLLKYYPDPKGRGAVPENEMLPPKWSLAPAIDKRAEVSRKSHFNGDT
KKPPSTPPLNQLPLPYGLQATLVGNKRLTPEEHWQDVRLVSFDIPSSLQV
NPGDCLTIYPKNFPEDVQKVITLMEWDAIADDPLDLSLCSLPPTLCPPSP
STLRDLLLHNIDINAVPRRSFLKSLSYFSDNEDHKERLLEFTTTEFLDEY
FDYATRSRRTIIEVLEEFYSVRVPPSHLLEVFPLIRGRDFSIANGGKHLT
PASPSPATRIELLVALVKYRTILRKPREGLCSRYITHLPPSTPLTVTLKP
VLSPIHGAVNAQRPLIAMATGTGVAPVRSLIHERMTHKSSAPMILFFGNR
NKDADYFFAEEWNALAGDKLTVFTAFSRDQREKIYVQDLIRKEAKLLEGL
ISQKAIFAVCGGSTKMADACKHAVFDPYIEGGDEVERKRILDGITWWQEI
W
>Mb|QLI65024.1
MADTSGSQSQSTDPAAQLPTFAAAPPTSAPSNVVPQLSSPAPILRPAIPG
GRSGGRPPRLGISIPASPSVKPVGSQQQVQSTPSRPPLPTLHLATPMGTR
ATPYEQPPGSQGIQPGQSADGGSESSAAHSRSGSFGPLDGRASNPTSAGS
QFSALSFASQYIIPVTRTQGTPDPVSAVGSMYSERSEGGVSMERDGSHQD
SATFAGWTLEKLKEADVEDLDDEGWRIASMNNFIEELGHLGEGAGGAVTR
CRLKERKTVFALKIITTNPDPDVKKQIVRELGFNKECASEHICRYYGAFV
DPSTATISIAMEFCEGGSLDSIYKEVKLQDGRTGEKVLGKIAEGVLRGLT
YLHTRRIIHRDIKPSNILLCRDGAVKLCDFGVSGDFGTKGEANTFIGTSY
YMAPERITGQSYTITSDVWSTGVTLLEVAQNRFPFPADGTEMQPRAGLID
LLTYIVRQPVPTLKDEPDVNVYWSDNFKYFIECCLEKRPDRRASPWKMLE
HPWMLDMRPKRVNMVKYLSSVWKWEK
>Mb|QLI65026.1
MSSAATKMIRRKKNVKKGIQFCLMVCGASGTGRTTFVNTLCNKDVLTHKN
ADDPTDAHVEDGVKIKPVTVELELDEEGTRISLTIVDTPGFGDQIDNEAS
FSEIVGYLERQYDDILAEESRIKRNPRFRDNRVHAMLYFITPTGHGLREL
DIELMKRLAPRCNVIPVIGRADSLTPAELAESKKLVMEDIEHYRIPVYNF
PYDIEEDDEDTVEENAELRGLMPFAIVGSEDVVEIGGRKVRARQYPWGVV
EVDNPRHSDFLAIRSALLHSHLADLKEITHDFLYENYRTEKLSKSVDGAA
GNIDSSMNPEDLASQSVRLKEEQLRREEEKLREIELKVQREINEKRQELL
ARESQLREIEARMAREASATSVSAPTEANGDNEA
>Mb|QLI65029.1
MADQDSHYDFLEDVDDEQSIISTRGLEAFGRKVTTTASHLMAPNAEATAH
HYQTAMKQVHRQLTRPSVQQSVFALARRTPTEIVRSRLSTAEIQHRAVSF
LPDDLLSNIPEDDNAYSLFQGFKASFPEMTDEGKKHRRRVSRGRTLLDDR
QRTGSPGPVASLSHVQKEKAAMMHEFELLGVRKSMASSEIREIDNKIANL
QGMRKIILERLAGLEQEEALLEHDIIDVEGRLEVAQALADEAEVIARNTP
TKDEVDLVGDADDHDPEFMSQSVYEKLPGSKNSTPRRPKKVHRRKSMPIL
HEHFEAGSNIRQLRAHKDTVTAMDFDAPFGTMVTAALDDTVKVWDLNAGR
CIGILNGHTASVRTLQVEDNILATGSADATVRLWDLSRSSYDPHGNSYGK
EDEEDEDAIAFENPNDQPIDPPEDSMNDCELYTLQAHVDEVTALHFRGDV
LVSGSADKTIRHWDLEKGRCVQTLDVMWAAAQASATANIDGGWRPTGRAS
SSSADFVGALQVFESALACGTADGMVRLWDLRSGQVHRSLVGHTGAVTCL
QFDDVHLVTGSLDRSVRIWDLRTGAIFDAYAYDNPITSMMFDTRRIVSAA
GEDVVKVYDKVEGRQWDCGAGITEEGKNPGVVEHVRVRDGYLVEGRRDGI
VGVWTC
>Mb|QLI65033.1
MKNLSQIKDGVAASFRTDPTINRVRGWAGPASRRLPSATAEYLAEKLPVA
QWLPHYDPRWILRDLIAGVTVGVMLIPQGLAYAKIATVPIANGLYASWFP

PLLYFFLGTSRELSAGPTSILGLLTAEAVEDLSRQGYRPADISAAMAFMV
GVYALAVGLLKLGFLLDFVSAPVLTGWISAVAIVIGLGQVGSLVGLDLPP
NVPGIIHDFFAHIRGVKPLTLAIGLTGLAFLLVLEQVGKRNKRGKYVKFV
CTSRAVILLIVYTLISYLCNRGRGNDLLWAVTKVDTHGLPAPRPHDPALL
KKVAVRAFAPLIAMSVEHLGVGKAFGLRGDYSIDKSQELVFLGVNNMVNS
LFGAQATGGAMSRTAVNSDCNVHSPVNFLFTGGLIVLTLYELAPALYWIP
KATLSAIIIMAVAHLVARPSQFYRFWKMSFVDFVGSQLALWVTLFTSTEI
GLAVAVGFSIVYTLLRLAFPRWIGLSHLETENNHVSLPCAGAASTSVDVP
AEAYLVQYTDDILFPNAERVKASIIQSIKVHFDPASDANMVVDKSRRTWN
PATKKQIIKIRKRKGITAFSGDETPLRRVVLDFGRVSFIDTTGVFSIIEL
KMELRRYIGQDLEFRFVGMVDAVKERFDRSGWEFASPGRQRAEAADVVYS
SIDMALWHPGKSDDKDDLSKEKALDA
>Mb|QLI65034.1
MVKKSIDAKVRPLANPALEKASLLGAARLYVSKESLIALTNGLENGRPCV
VEKLEDQAQGPVRREASLWILPEKNLSPNVVMMTRAFQDATGFRIGDQVR
ISLLESMPDVDEVVVQDVSEEQQQAKDGRGARHAACWEFSLSLSMERADL
IFPGMVFEGVNINKLRRTFKVVSVNSHTNNVAKFSISSSTIRILAEGDDS
AAADESEAFTGGELAVTGVPGLASQISTINRFLKGFTRQFWVKDERESCA
FVIHGGHGTGKTFILQKIADTRWGRPFWIKPPDKLAAIRETFKQAHTLQP
SVILIDGLEDLISKDRSNRDSVIDVIGEELDALSAEARARNALPQVVVIA
TCLDYMTDVPAKLQKRSRFRENIALPIPRASERLEILNFFDPPVHPAEKR
ACLDSVAQKTHAYNGDDLANLVLNAKKILGNRLDEEGLDPCTAGEQFLSK
DDMEQALRITRPTAMHDINLKPPTIHWQDVGGQESLKKVLNRMIKNTKNT
NPSSRHVLRNPPKGLLLYGPPGCSKTLSAQAMATESGFNFFAVKGAELLN
MYVGESERAVRTLFERARAASPSIIFFDEIDSIGGQRGPGSSPSSGGSRS
TGAVNMLTTLLTEMDGFEALTGVLVLAATNRPEAMDPALMRPGRFDQVLY
VGPPDHAAREAVFGVHLRGLALADDVDVREAARLADGYSGAEIKAICNEA
GLAVLDRYDDDETGTAKLEIGMDDLVAAMERTPRNITSFMISGYENWSRQ
FRRK
>Mb|QLI65035.1
MLSILRKARLKDKEMRILMLGLDNAGKTTIVKKIMGEDVNTVSPTLGFII
KTIDYDGYKLNIWDVGGQKTLRSYWRNYFEKTDALIWVVDATDRLRIDDC
RDELHGLLLEERLSGASLLILANKTDVEGCMDEQEIGKALRLDEIRTHQW
NILRCSAMTGENLQEGLAWVVADAKKRLFLY
>Mb|QLI65036.1
MALLRLLAVLAMATSVATAANPTTSDDMGPAAFMWPEDRVWSAAADNTAP
CGSVARVGNRTNFPLTNGQVALVDQKEAHSVQLSISYDDDPKEDNDFTTL
IKPEAMAELDKGHTCVNVQNPPPFIKAGANATFQIKYVASFDKPDKETFY
ACADITYVEFANFKEKVPCFNATVPEDSKKDTPTPTSDASKNSGSASSGL
SGGGTAGVVIGVVGGVALIAVAALLIYRRRQQRLRMLRQKFSPRNIKPDG
QHRDSPSVRSESA
>Mb|QLI65039.1
MDKYSDQRSSTANDSDVHSVSLEKEGLLDGASIHESKPPRMRSQSRVYLL
LAALMAVANVVLGALLVAQFRSSQQKAHSHRHDHHEKKPTPIPIGGLPQV
NSHPDWLPPEEWRTEIFRLHEIYGEEPVGTAKEAWLSLIPKGKGFIVVKN
DTELPNMPGLRHPEHEQHACVAIFHQLHCLYITYAAYWNARAGNFDDIPP
EHLIHCWDYLRQSIMCAGDTSLEWVHRLDSPPGETSGWGFQHTCKNFDAI
FDWAESHRYTETVEID
>Mb|QLI65041.1
MNRIFGSKQSGPKPTLSGAITNIDTRIASIDTKLKALNGELSAYQEKLSK
LREGPGKQAIKQKALKVLQRRKAYEAEKEKLEGQVWNMEQASSMQDNLKN
VMTQVDAMKTTKKELQKQYGKIDIDKIEQLQDEMADLMDVGNEIQESLAR
SYDIPDEVDETELDAELEALGMEQELEMEMGGAVPGFLQDEVMPEFVDEP
PQTEDKVKEAAG
>Mb|QLI65042.1
MSASLPGSRDLPVSQYDLATYLGRVKHAVGLTDPSTLLAGTSGLERAKQL
VTDYKTGKIASMTPELWHAKKVVDSTLHPDTGEPVFLPFRMSSFVITNLI
VTAGMLQPGLQTPGIIAWQVANQSLNVAINSANANKSSPMSAATLAKSYA

VAVSASCSVALGLNALVPRLRVAPATRNILSRLVPFAAVATAGALNAYLM
RRGEIAAGIDVRPVLSADQKRGLQEQGLSERDVPSLGRSRRAARLAVYET
AASRVFNSSPIMIIPPMVLYHVQTRQAWYRNLMEGAYVSSRPRLAAGIPI
GLNLVLIAATSFAVLPLALAVFPQQQEISADSLEDEFRGKGGLNGKVWFN
RGL
>Mb|QLI65044.1
MSASAYDRHITIFSDNGRLYQVEYAFKAITAANIMSVGVRGKDCAVVLSQ
KKVPDKLIDPTSVSHIFQISPSVGCVITGSIADARAFAQRAQGEAAEFRY
KFGYEMPADVLAKRLANISQVYTQRAYMRPYGVATTIISLDSEFGPQLYK
CDPAGYYIGYKGTAAGPKQQEALNHLEKKLRNKDCAEGTWEDVVELAITT
LSTVLSMDFKKGEIEIGICGGPRTDGKEGTNAAFRMLTEDEIDERLQAIA
EKD
>Mb|QLI65045.1
MAGSILSVDNVGNAHPETPGYNLNLYMRDHRHGGLSPAVVESVAGLSAGV
ISTLVVHPLDIVKTRMQIHRSAGPPASPPTTVTILRSLTSNSRPVLSLYR
GLTPNLVGNATSWASFFFFKSRFERAIAHANRRVRPSAADYFLASALAGA
STSVLTNPIWVLKTRMLSSDKGSAGAYPSMLAGARTILRTEGVRGFYRGL
AVSLLGVSHGAVQFAVYEPAKRVYFNNRVAEGDVNPRLTNEATVVISSVA
KLVAGAVTYPYQVLRSRMQNYRADERFGRGIRGVARRIWMEEGVVGFYRG
LVPGVVRVMPATWVTFLVYENVRYYLPAWMA
>Mb|QLI65046.1
MTGAEMAQNAPATPASKKLHGRAFYESIGSPKYIVAPMVDQSEFAWRMLT
RSFLTDEEKKKMLAYTPMFHARLFSQEGKYRKAHFQSTKPDSQEPWLDGN
PSIDRPLFVQFCANDPEALLSAAKQVSPYCDAVDLNLGCPQGIARKGHYG
AFLQEDQDLIFRLINTLHKELSIPVTAKIRILEDKEKTLAYAQNVLKAGA
SILTVHGRRREQKGHLTGVADWKTLRFLRDSLPPETVIFANGNILQGGDI
DECLAATGADGVMSAEGNLSDPALFAKAPPVGEEGREYWRGKDGRGGWRV
DAITRRYLDILHRYALGVEPPARRPLFVPGDNTAWMEEQPQEAQQDEERP
HKRRKKDTDGGKRDEPSPNLVAMQPHLFHLLRHFVSKHTDIRDLLARSRR
DGIQGYERVLEAVERRVAKELLDYEATEGKSFPDELESLGDGKDDEVEGE
SSKATVRRCKRPVWVAQPIVRPLPAEALAKGAIRLSKKELEAKAKGESVV
TDVTGGHKPEEGKAEVKNGDKAVEEGRKEEIKARDELVSG
>Mb|QLI65047.1
MAPAALDNANPTILSASQLPTRQKKAAHRHGPDSRYSDIILSKPAFLSQP
CCDNGVVWPRGDDAVNHDQYAPEPIDEQEIYDLIATISDPEHPVSLGQLS
IVNIDDIHITPSPALGVPDANTIVQVTVEITPTVTHCSLATVLGLGVRVR
LEQCLPPNYRVDVLCRENTHSQDDQVNKQLADKERVAAALENDSLKGVLD
KMLETCV
>Mb|QLI65048.1
MTLSSTSNETSLQDSTAAADAARTAKVHALTQNLLSKSPIYGLILADIQL
AAVAAGTVTLRLTLSATHLNSKGGLHGAVSATVVDFATGLAICAHDLRER
TGASVDMHLTFLSTAAAGDTVLIRSTAERVGGSLAFVSVAINKLGEDGSE
TPVTRARHTKYVRGTKEP
>Mb|QLI65049.1
MARPLRVAGEKKLWCPKLGIRFSPSSNSVPLYPPLDMSRFVQTASEDNDI
RNNFTNEPFGYCANAVMEQDLATLATTRHDAIPQPSKNSTSTNPQIPLQR
PTTTINQESENSQGKEVSKPNPEGDPEIVEPPLTPLSFNISPSLFYAARA
AKAGSSESFWSHTMYQRTSDQGAVEKVKVHYCTSKHTTEQVCRKHFLGEA
VLGFDLEWFPYASRSSGTRENISLIQIASPGRIGLFHVAMFAKGEDDLVA
PALRTIMEDPNVSKVGVHIQGDCTRMKNYLGVQVQGVFELSHLYKQVKYT
AAKTPKLINKVAVALSTQVHDILKLPLFKGDVVRSSNWMKRLDYKQILYA
ASDAYAGIQLYHVLDSKRKRLNPCPPRPHHAELGLPIPVVESESKPEEEA
GHLSDGSFSSFGSELEAELLALPLIPAKPPVRDARILIAEKKAAEYRKSK
TSGVSAPPTSLRAYFVWHSNLDLNPEAVAQLLRDPPLKTNTVVSYILEAI
VSEKMTYDKGRLKSEVLPLLDQRAMKVGSRYGALVRSCERTDEET
>Mb|QLI65050.1
MATNGDGSSLARAESPTGSPRSASVSLQAAATMNAGLQREPSRRSSTSSL
ARNPLSPNTSRRRSTVLMNLQLNDPSVPAPGEMAQEHSGYHRSSPQPLSG

SPLMADPHHHRAPSLGELHQELEAEQEGHVNRLLQMIRQQQLELQRLQTS
RPGHSADPAAEDSAIAETVPRPIYGAGGASQSNIPGSQPVGSYSRSPVFP
RGSMEMARAEMHRRSRTPSRGASPRLRATSISQESGDWVLGGRDESAFYQ
AETQMLTRENQMLRHRIRDLEKQLAEFHPGSGTGTEPSIPSHLTQSSTSG
EPAGEVGPKES
>Mb|QLI65051.1
MLYTRFQSLLRERRRARRDIEISAPFDLKLEPATLPGVSQEELSILREKA
AASRIGVLELRPESPSEYHKRSRPSPRLRPVQPATMEIETRPLWY
>Mb|QLI65053.1
MEPPEQSVSLDDPSSPRSSISSTQREIWPGPSATANLGQGHQRCPSGPSL
HSQQQELQHQHQERRPTASPSPSPAGAHSHKISASSIAASNASRQSRYVD
SVPSETNAEPFSFPIASQPTPIPVKGAHSPSWHSRPQSGISDAGSDVLVF
GEPHPSPTHSDILRIRAGLNAENAAHVWLPEPSLTARSSLSDLQSRRLSS
TSIYSLASARGIINYSSSAHGSDSGTLSRSVSGLMSSSKGASAGTPPEPG
LSTAAAATSSPNGSNPNGQHNLATRDATSQPLDLMRRNQRTDSNMRAQPD
RSRSRAKRRFSGSTATSTHSQGSDRGAHREKEEAKPAPWGTIGICALDIK
ARSKPSRNILNRLIANRDFDVVVFGDKVILDEEVENWPMCDYLISFYSDG
FPLEKAIAYVKARKPFCVNDVPMQKVLWDRRVCLRLLDIIQVRTPKRLEV
NRDGGPQVLTPEMVKHIKDISGITIEPADATTAIRPSKVELLDDGDTLSV
DGRVLKKPFVEKPTSGEDHNIIIYFAKSSGGGARKLFRKIGNKSSDYVAD
LTIPRAITEPEGSFVYESFMQVDNAEDVKAYTVGPNYCHAETRKSPVVDG
IVRRNTHGKELRYVTSLSNEERDVASRISTTFGQRVCGFDLLRAMGKSYV
IDVNGWSFVKDNDDYYEHCANILRDLFVKDKLRRGGATPPLPSPETTDLD
PMTKIVQQGREREQQSGPASSSTSRSTRGHATTTEPQNEDSGQKLSGETT
SPRPESTFNSTVPSTSASPLLPPPSEIALPGSSTAASSVQSAPISAQPTQ
APSEETPTQPPPPPKHSWKLKGMVCVIRHADRTPKQKYKFTFHTDPFIAL
LKGHQEEVLLIGEAALASVIQAVDVAYEAGIEDRGKLRALRNVLVKKGSW
AGTKVQIKPMFRKKRLDEVSTSIEVPAVLDMAESPPERKGRAGECDGSEC
TPRSSSKRHDSLSGVTMSKFTAAEESLVLDKLQLIVKWGGEPTHSARYQA
QELGENMRNDLMLLNRDVFDEIHVFSSSERRVSASAQIWACSFLDRNDLP
EDFITTRKDLLDDSNAAKDEMDKVKKKLKGLLRKGNERPAQFTWPENMPE
PSEVQTRVVQLMNFHRRVMQYNYGKLYNGAASSLGANSNASSERLSMEGS
SASISSLSHANAVNGIQSRWCSGEDAELFRERWDKLFSEFCDGDKVDPSK
ISELYDTMKFDALHNRQFLEWVFTPPKSMLEDEYSQMEGKQKDIDEAKAS
EDAKGEKGQNGSFDGSDKTDAANRSVKKLFRRRSFLNSLRHTNDEALPEQ
YFRLYKGANQTAMKSDPRTEPLQELYRLAKVLFDFICPQEYGISDSEKLE
IGLLTSLPLLKEIVQDLEEMQASTDAKAFFYFTKESHIYTLLNSIIEGGI
ETKINRSTIPELDYLSQICFELYESETKAPAESALGDESTFTYSIRITIS
PGCHVFDPLHVQLDSRHCIGCAPRRSLTPHIDWLQVIKTLRAKFNQ
>Mb|QLI65054.1
MPSTTCFLCGFAILEGHQVRAVYKKAPSDDINRNTPTADVLLSNLLPYDD
GFLWIQNDLEHPMLIACVTTNERPEYHGLLYGFRPFHEGPIRPGYPIHEP
CYKMLCQVQRGLDMTADVQAIFDVCTSFPLSNEGAFWGHNHEMLQPLAPP
EGEDGFFNLYSGESFSLGRDCHYAENPMDPNDLPELHEMMARLEHHPNPV
EDAQNSISICTKETSREERKSVRNLQLADPIFTDSQKFLGFWESRFAPDS
DLWYVYETMPRGLGDRWNLWYQLCIKYHNCRAAINRRRILRLTHRMCHLI
HQRHENSKLLRGSPSDITNDEAFWQNDGHDNKIALCMVPNITGIVDQNIE
LHDDLRHVAFSFTTVSNQRYISGLRLYGANNEERTLGYFHSKDYSTPSCG
YQSRAGEFITGFDIALDNLGVRGLRFLTESGKPLEWVGEHTGLPKRRLTA
AFDDGNPVPRCGFKLVGIAICSTSDPWPSVSQDDEGFAREVRREDQQVDL
RIVPGKETWYPDLPPLGLKFVRLNKADRPLQQPYHYCIFGGSRGERLRHL
NKVVIRADKHVRSVAFHFDQDGERMEQVEFTRPRYGLKLGEDLAMVSLAI
DGAGGERITRVESVLDPWTKILVSLQIFTNKGQTICSRTTSNPENTSSSE
DTPSKDKVDIYDITQSPCETGRIMVGFFGTMTAHTSQGGISSIGVVPHEL
STLYEDRKTAGEIRYQPCSVYRQQEQQLEIRKMASKWIACGLVLQAGLAA
ADGTGMVGLGKTMYNPPCAFACRASISSCPLLCTPKGEDAETPPECFTTD
QAFMRTLALCISMYCGQSDHPSLAKIEDYWESHLASSTVAKFEWKPAMSY
GEALRLAKIDEQSAVGRNQTSAGGGDHHGHKRQLLRRHGGSHGSEDDHDE

MDHVDSPLPVIKPRAPLNVTSVVAQKDWTKSYNGAKGFEINEAGHAKYTL
VLLLVALFAPAVLSLVRFVPGLSRSRTWQWISSMLVEPPAWGKSHREPVS
PYTGGGLMPTRGQALYILLISVLNTVFLVAPYTALFPQTSFPSIESMEIS
TIGNRAGVLAMGNMVALFVFSARNNVLLWLTDWPYDTFILLHRWLGYWTI
LLAVIHSLMLFGYYVAEGMYEDEAAKLYWTWGIVATVCAAALWPSSLLVV
RQKAYELFLCTHQVLVVFFVVGYYYHIWERYRHNWGYEIWVFVAIAVWLL
ERSARLLRVGLAGLKTAVVSPVEGSDGDYVRIDIEDAFAEGVAYLYFPTL
SWRFWENHPFSIASSFSAHATDAGGSLENLHVTDVEKTARTTTRSAEVEV
EAKADSPSTTASTVATRPSGKTRVTIAVRVRSSMTARLAARVSAAGGPIR
IPVFLEGSYHSSAGSQLSRCTRILCIAGGVGLSAVLPILRQHGNRPARLC
WGIRNDSLRSVFRDEMSSLSATVDIETHVGSRMDVGSILREELTAPCGSE
DRGPIGVVVCGPPGMADDVRNTICTLGRSATVRGGAQCIQLTLVNRHSAA
RSTDEMMPTARLASALCWCLVGRLAAAKPPRRSADLCLQSCQNALAPVHF
SDAEVLPAKSRVEMCRSQRAVTSMYLCLDMFCDQDARAAEVAALNETCVG
SDMDALPPISIISNYTPGDIERLRHIRLGETFAPNATVKEVVVPAFELYK
AWFDTLDAYRYVTHHHFLYGFSMILFWLAVLAIGVGNRIILGCIRFLKTY
RLYRSRRSETATWVKSRIVIPATFGYRCATEVWCGTIPPRIQSLTIAAFA
IMNLAFSIFGFSTPTRQWLRYVSDRTGIISFANFPIIWLFGMRNNFAIWL
TGWDFGTYNNFHRWVARIATLQAVVHSLGYTVLILKEGGWTYFAWWWTQM
FWLAGEVATVLMCALMACSIYWLRRQKYELFLVLHIGMSIFILVTMLGHV
SIFNGHYDAFFWVPVFIWVFDRVLRILRIIFFNPTIKPTVATATYNLSTN
IVRLHVPCHFRAYKVRPGTYYYLSVIDDKRFWESHPFTVASVSDSHLPGK
PFGEQAPLLESDVIGAEEQEMAKPNPKFLTFLIRPYDSFTARLKDLASED
SPQPASIRVLVDGPYGHSQPLHLFDHVLFVVGGSGVVVPMSYLKVLTGRN
KQTTSTHIHWAIREPEFAAEVLSNDMAEALGDASFAIDLYFSAETERNIG
ANMPPKASRHYRRPNAAAIILAAAEDAGERTLAVVACGPAKMADDARRAV
TEALDIFPSQIEYFEESFRW
>Mb|QLI65055.1
MPDTSGANGTNGRTVPVGNGKATYAEKHNIAEHFIGGSRLENVSPSKVKD
FVAQHDGHTVITNVLIANNGIAAVKEIRSVRKWAYETFGDERAIHFTVMA
TPEDLQANADYIRMADHYVEVPGGTNNHNYANVELIVDIAERMNVHAVWA
GWGHASENPKLPESLAASPNKIVFIGPPGSAMRSLGDKISSTIVAQHASV
PCIPWSGTGVDAVEIDKKGIVTVADDIYAKGCVRSWQEGLEKAKEIGFPV
MIKASEGGGGKGIRKAVSEEGFEALYKAAASEIPGSPIFIMKLAGNARHL
EVQLLADQYGNNISLFGRDCSVQRRHQKIIEEAPVTIAKPDTFKAMEDAA
VRLGKLVGYVSAGTVEYLYSHADDKFYFLELNPRLQVEHPTTEMVSGVNL
PAAQLQIAMGLPLHRIRDIRVLYGVDPRTSGEIDFEFKSEGTAQAQRRPT
PTGHTTACRITSEDPGEGFKPSNGVMHELNFRSSSNVWGYFSVGTQGGIH
SYSDSQFGHIFAYGENRQASRKHMVVALKELSIRGDFKTTVEYLIKLLET
EAFEDNTISTGWLDELISKRLTAERPDTMLAVVCGAITKAHIASEACIAE
YRAGLEKGQVSSKEILKTVFNIDFIYEGFRYKFTATRASADSYHLFINGS
KCTVGVRALSDGGLLILLDGHSHNVYWKEEVGATRLSVDSKTCLLEQEND
PTQLRTPSPGKLVQYAVENGSHVKAGQTYAEVEVMKMYMPLVAQEDGIVQ
LIKQPGATLEAGDILGILALDDPSRVKQAQAFVDKLPPYGDSVVVGSKPS
QRFSVLRSIMFNILNGYDNSVIMASALKELIEVLRNPELPYSEWNAQFSA
LHARMPQKLDAQFAQIVERAKSRHADFPAKALQKAFQKFLEEGLPANDAE
TLKTTLAPLSEVLDAYSEGSKVHELNFIQSLIEAYVDVEKLFLSQAQEDS
VILRLRDQNKDNLAKVVQTVLSHSRVSAKSSLILAILDEYRPNKPNVGNI
SKYLREPLRKLTELSSRSTSKVSLKAREIMIQCSLPSLEERTAQMEHILR
SSVIESRYGESGWDHREPNLDVIKEVVDSKYTVFDVLPLFFAHEDPWVGL
AALEVYVRRAYRAYILKQIDYHNDETDSPQFVSWDFQLRKIGQSEFGLPL
QSAAPSTPGTPGVPDMNVKRIYSISDMSYLTSKWDEEPTRKGIIIPCKYL
DDAEELLQKALETLAFHNKQNRQHNATAILKDLSGKRKPFNSVQKDVKTD
DELSAVLNVAVRDAESNDDKELLTRIKPIVEQFRSELLVRGVRRLTFICG
HGDGSYPGYFTFRGPEYLEDDSIRHSEPALAFQLELGRLAKFHIKPVFTE
NKNIHVYEAIGKAVDTDKRYFTRAVIRPGRLRDEIPTAEYLISEADRVIN
DIFDALEIIGNNSSDLNHIFMNFTPVFQLDPKAVEQSLQGFLDRFGARGW
RLRVAQVEIRIICTDPQTSTPYPLRVVITNTSGYVVDVDMYAERKSEKGE

WVFHSIGGTSEKGPLHLLPVNTAYGTKNALQPKRYKAHLMGTQYVYDFPE
LFRQAIQNTWAKAVKDQPALASQQPKVGDCASYTELVLDDKDTLQEVNRE
PGTNACGMVGWIFKARTPEYPTGRRFIVVANDITYNIGSFGPKEDNFFNK
CTELARKLGIPRIYLSANSGARLGLANELMPFFKVAWNDPAKQDAGFRYL
YLDEKTKENFKDDVITEEVTEDGEKRHKIVTIVGREDGLGVECLRGSGLI
AGATSRAYNDIFTVTLVTCRSVGIGAYLVRLGQRAVQIEGQPIILTGAPA
LNNLLGREVYTSNLQLGGTQIMYRNGVSHMTANDDFAGVSRIVEWMSFVP
EKRNGPIPVSPSSDSWDRDVVYCPPQKQAFDVRWMISGKRDEDGSFQSGL
FDKDSFVETLGGWARTVVVGRARLGGIPMGVIAVETRSVENITPADPANP
DSIEQVTNEAGGVWYPNSAFKTAQAINDFNYGEQLPLMILANWRGFSGGQ
RDMYNEVLKYGSFIVDALVKYEQPIFVYIPPFGELRGGSWVVVDPTINPV
AMEMYADVEARGGVLEPEGIIGIKYRKEKQLETMARMDTTYANLKKQLEN
SDLTPEEAAEIKKKVVVREKELLPVYAQIAVQFADLHDRAGRMKAKGTIR
DSLEWVNSRRYFYWRLRRRLNEEYILRRMASSVLSSSRDSSPSKGTDLRA
GHLHLLQSWSGIVNFDTSDREVAEWYEKERKTIGEKVDALKAEHLSAEVA
EVVRTQKAAGWKGVREVLRVMPVEEREAILKYLKE
>Mb|QLI65056.1
MSAIDSHPSVQKFKPTALRLAKQIRHRGPDWSGNITSNNTILCHERLSIV
GVESGAQPLTNDDESLVLAVNGEIYNHRLIRKHLRHKYHFKTTSDCEVII
PLYMEYGLDAPNHLDGMFSFVLYDKNQDRTIAARDPIGITTLYQGWSWKE
PGTVYFASELKCLHTVCDKIVAFPPGHIFDSKTGETTRYFKPTWWDPTKV
PSTPVDLKVLRHALEKAVRKRLMAEVPYGVLLSGGLDSSLVAAIAQRETL
RLKQKAIAANAAVDPVGNPDLGEGLVGIDDDDNLSTLTYLPQLNSFSIGL
PGSPDNKAALEVAKFLGTKHHVMTFTIEDGLNALSDVIYHLETYDVTTIR
ASTPMYLLSRKIKAMGIKMVLSGEGSDEIFGGYLYFHAAPDKKAFHEETV
RRINNLHYADCLRANKSTSAWGLEARVPFLDKEFLETAMNIDPQDKMITK
DRMEKHILRKAFDTSDEPDTAPYLPENILWRQKEQFSDGVGYGWIDALKD
HAELHVTDEMMKNPKTEWGTDIPDTKEAYWYRLMFDEHFPPSCASTVLRW
TPTWSKQTDPSGRAISTHNEKYENAA
>Mb|QLI65058.1
MESSDDGSTLSTNHINTLITIERTGAGLSMAAILLTVAPFLAFKKLRTTP
NTFLVFASMANAGASVASMIGYDGLRMGEESTLCQAQGFIFEWFMQADPW
WSFAMAFNVFLVFFNNANPSKFQEYKWIYCVVCFGGPLLPAIVLVSIRDE
ERGPVFGDAALWCWIRPKWSIVRLYAYYIPIWICILLSILIYIAVGYHVF
RSRNQLRNVTFQNPTRNWRNGSLTDSENKISGEENPTRQNVCYGLAITEV
QVTTNFPGFIDDDIPVLPTAVHRIPSRMCGRSWLDSHNDSDSERVIPTQL
FETTCSSGGRYTNSNNALVGKFGALKSTASMKLKRLDPVKMAYLRTSGIF
GISVLITWIPSSINRIYSLWNGGQVSYPLSIASGCVLPLQGVWNAIIFFT
TSWSDVQAEAKTLKARLGYGGVCEYPRGTRLESRLGISSVETYDSFNESQ
CHGTKRCGCGKCVGEVVRLEDWNGRTSPSRI
>Mb|QLI65059.1
MRTSFLSSALVVASFAAAKEAPSSTTHAPACTATASSGTGGFFDLRPDTA
HPSEKGKQHKTALAKDYHARGYDYGKNFTLNICGAVVDPVTEVVGISKSQ
WTNVSAYYMSHGSIYSIGSESMDLVSRGRKLVLQYTGGSPCGTTKSNKST
RTPSSPSANYNYANKESALTNQPHQVETLKDDKEEKSSRRKSTTISFLCD
RDPSSSQASISFVGVDEDECSYFFEGRSIHACAQAEPHKPGSVGPGSVFG
IILVVAFLVYVLGGVFYNRTISNARGWRQLPNYSLWAGIWSFFSDMFVIA
FSSCARCLPGRRGYSHLSSSPRNRNSDAENRLIDQLDEEWDD
>Mb|QLI65060.1
MAALTNAAELDSKSLSPRFRVGGQLSIGGITFRPNLRMSNTDASILITLS
VISVATFSLYVFRNRLQRLLPRWLQPFAREEGHASNKRKAWTHWTLILAL
ISISGFILSVVPICLAPNERRGILDIVPWMSILFMTALDRPTSTPRILLL
QYFLVLSSGMGIYSAQFLDHHLRSIDPFRVARLALSLFGIVSIGSMPLRN
PAWDAEDIANPKLPPSHHVRSPEDNLTLFTFWAMTWVYPLARLCRKKEIT
VEDVWQLPHDFQHTRLYLAFRELQGKLLPCLIEANGLDLSIATTLAIVEK
LAEVSNIRLTSRLYSALDNGNPTEAYFWCAVMLCVDFMRQISKTTSSWYS
RKAYERSRGETFIALFGKLLTRAVPGSDVTEKGPEEDSVGTLTRPNNTLR
RGWLSCCCGRKHRVKSTPTQATPASNAKVVNLVRGDTYEISQRFWDFPKL

MSQPIKVIVTIYYLIDIMGWPSCVGLGLMILFLTSNSLLVRKVVQLERSR
TAHSDKRAQAVAHFVEASRPLKLNGWTSSWSARIFKFRTMEMLKRLQIAR
VIAAISTTNVAGGSAYPFASICLYTLILRQGLPNDVIWPSLQLFNQLEAS
LKEAFDLITAYWKAVIPVERVNKYMEEPDRDEDSLGITDASDIEFHGASF
AWPSTNHLVLSQLNLKFGKGLTIIRGKVGSGKSSLLLAALNEMELHGGDL
LRPDEPVGYAQQLPWLQNKTIRENIVFHHGFDAARYRQVLHACALGPDLA
AFPDGDLTKLEEGGVGLSGGQKARVALARAVYSPCRILLLDDPLAPLDHD
TASNIVRRFLQGPLARERTIVMVTHRDDLVLRIANQVIDMDDGTARRLSQ
SEIKDELEHPYHATSQDTIVEELEPTHETHEEEEEDLQEAPEEPSETGSV
PLTVYLNYMRAGGLYMWAFLALFYGISRYCDVSEASLLESWGNETAKDRV
ISTRGYWSLPNPQKFPQIWLAVFGGLALGQTISYGIAQLLLAKISIDAAQ
GIFKAAIGRVSKATFRYHDTTPTGQLKNRLVSDMGMVDGGILAPLEGFVY
NFIALVLSIVGIATHQPMLLIILGAVAVLFVYFFRIYVPISRSLRRMEMR
YLTPIIANIGVMQDGLVTIRALRVEGRFQDRHLDAVDDFQKQDHFFWSMA
FWLDFRLSISSACTRAALIIFMIWRGTPASAIGFVLTQTSISMASIQQLC
EKFAQLQLDAVSLERVGMLDKIPEEPNGDEEPPADWPRPCDDVKFESMSF
KYAEGLPNVLKKVTFEIPGGSTCAVLGRTGSGKSTIANALLVTETASEGT
VKIGGMDLAKVNRTALRNRVTFIQQDPTLFPGTLRDNIDPEGKFSDEECE
NAIQNVLGKEWGLTSQIDAGGKNLSQGQRQLVGIGRAVLRKSGLVILDEA
TASIDRGTAATVQKVLREGLSKSTVITIAHRLEAVEDANWCLRLEKGQVL
DCGPAKDLGKKAKEEES
>Mb|QLI65063.1
MVPTRLASGPRKFHVLLGTFIHSKSRTELEYIHNAGVAVDHGGTIVAIAR
DCESVNVAKDRVLEETGWTHSDVDVVACREGQFFFPGFIDTHVHASQYPN
VGIFGKSTLLDWLEKYTFPLESSLSDLAKARKVYTACVRRTLSHGTTTAT
YYATVDVPATNLLADLCLSIGQRAFVGRVCMDRKELCPSYYRDESAEQSL
HNAMESIAHIKEIDPGYELISPILTPRFAPSCSSEGMKGLSAIQKREDLP
VQTHISENKSEIQLVADMYPDSGSYAKVYDDHGLLTPKTVLAHAVHITED
EARLISQRESKVSHCPCSNSSLTSGEARVRWMWEQGIEVGLGTDMSGGYS
PSILEAARQATLTSRHLAMQIGDEVLREKTKLTVEEALYLATRGGAHCLG
LGDRVGGFEVGKQLDAQLIALAQVQDDGSRAGEDDDGYVDAGNVDIFGWE
TWEERIAKWLYNGDDRNTKKVWVKGRLVHHRR
>Mb|QLI65065.1
MGRRPNPLILEYFERGPKLNDNSNRYPHTCKQCGENFPKGRIDSLTTHIT
KRCPAISDSERMRACLELHGITQARTAAERHQQVQAAQAAQAAAQANGQQ
ISPSALPQGWSALETLAEASRQVDLNENSRGNKPSTAQPQANGVQNAERF
ELQEQYTLDNPPTNYETTRAQATGKKGTAAFQTPPPTATELSPEERLQAL
LPASESSPDATNLSVAVAAAARLNPSFLDPQLVNGDATPTSPSVADSAGA
AAADSTPTTAVDTTAIQPWGEMTYLTVTSPAPIPTDNPPVAPLPLIRGGV
RMDTSAGVLNGRSRNFRSKFTAARRQEVKEVRKVGACIRCRILRKICSKG
TPCDTCRKVLSPRVWMTGCVRSRLHEQLDLYSAGVQVVLSQNRINMLKEQ
LNLVNNGAVVEVSHFAETNKNIVLAAAAAVIEPLQQEGVTPEAQESLHQV
IMIDQDKDDVPGKVESYMRDVLDLFIEREPSKFMRVTLETAKSQLAIKTD
EPLKKALELWGLVESIDRERQWSIAEKPSADSEHGRPIKEAENESDADIY
TMICMQLNAAAERKANSTSKALLNLMDRQLADSKTKVGFNIYLAALIFLN
CVEKSTWAFKAWEQDHLRPGWPLERDPSVFTQQGGNLAGLIKMLLGIRKA
LPQTCRGADGKLATQDQDAVVVKFFQDLDMEYDTIEARQRGSQFSPADSR
SLELMFCSHLLLPNAPA
>Mb|QLI65066.1
MESLLSYRNSECEALKDPSYLELVISILILVGLLISYLPQHYRIISRGTA
EGISPYFVLLGTTSATSGFANILTVPQSRAAIGCCKELEAFQCTAGLLGV
AQLGTQWVCFTLILVLFLIFFRYRDATIPSEELAGESPKWQTAVGVGLAC
VVHGLLVVIITGIFTIALPNHLTAWANFLGVMATVLAAIQYFPQIWTTYH
LKHVGSLSIPMMCIQTPGGLLFAASLYARLGPKGWSTWAIYLLTAIMQGC
VLVLGIYYEMAARHHERQQSNQNADGTYSSLQNSAPPRPSLASRTYSEGW
ERGLPGPFTGHPERYAETEEDLDDIQEREQRAIERENQPLLRPGGIGNPR
KNYRTTKKR
>Mb|QLI65068.1

MKIAYLSLGLLSLLTPLTAAWSKEDREIFRIRDEISAHEPNPAATFYDIL
GVPPSASFDDINRAHRKKTIALHPDKVAQRLRAERAKKAGGKGKKSAIKP
PSSAEVKAAVKQASERQTRLSLIANILKGPSRDRYDHFLAHGFPLWKGTD
YYYDRYRPGLGTVMVGLFIVGGGAIHYLILYMSWKRQREFVGRYVKFARD
TAWGGNLGIPNIEPRSSTPATASDEEAPPPVPQNRKERRMQEKEAKKEGG
RGRARKVTKKTPSASRDASSGPTGVRKRVVAENGKILVVDSLGDVYLEEQ
DEEGNVNEYLLDPNELLQPTFRDTAVVRLPMHIFNLTVGRFTGSRDAVSD
SETVDAEEVVDVSQQTPTSESAGDDFELLDKSTDSLGKAKSSGMEKSGKS
SKRKNKKR
>Mb|QLI65069.1
MSSTAAGEPTQAINSASKKKNNRKKKNANKNKDTTPLSNGDATHEDVQND
NDEDDDDLDTAASTDKLKPEGNGRAVEPSKATDGTSDSDPTAKLEAMGKE
REALRAEVEQLRKQLESIQETHQQEVSQLKTDLEESNSAKEHAEEQYQTL
LDRVEKIKESLSGRLKRDKAELEEARERIEELEAQNEQLQGSAQSTGEDV
EKLKQELQDANRELSTLRSRNNLSAHNWHKEKEELTRTVQHLKEEMETTA
NAMGEWEVLAMEERSIKENLADKVGDLEEQIQTLKKNYETAAADRDSQAT
LVDNLQNALREIQDARKKELRDMVETTESQLQAQKLVAQQAEARATEAEA
AKDEMSKELERTAPFEKEVREKTLLIGKLRHEAIVLNDHLTKALRYLKKT
KPEDNVDRQVVTNHLLHFLTLDRGDAKRFQVLQVMAGYLNWTDEQREQAG
LARPGGSVGSLRLPTSPFHRTPSSPSLNADVFSEPTSAKDRESLAELWAN
FLERSAQEGAGDGLSKDSGSRKASVSSITTGTAVAKPDSGP
>Mb|QLI65071.1
MSSLQRTRSLRKPTIPPPAEKPVRPTATDATRSGSPSRLPLKPFARPPTT
ILPGSSATNTTTAASSRSSRPAPTSLARSSSLSTRANGASAQETAKKETS
RYPPASGANRAAARTRPTSAGSGSAEPARRPPTHVRAKSTATSLPPATSL
PPPSSGRVGSQTTAARGGHGRHVSMSVDNKGPSTPAKPSVAPPAAVAKAQ
AQPRLRPAFSTLQQHYSPAKSQAPKPLTSAIIAPPSPSKLPANIAASAET
SRLQAELLQLHLLHRDSGAVQAQWHASAEAKLGARFEELGRESGRVSERE
RFAAESDNILALRRWARGGRYGIEDKIQSLDEILTGLWTLSEPAGRYARV
VKRFERWADHMCDAEEARHDIGRMLVHGHDSLFVGELDATWREECAGLTR
RLETWRARLAEIDDLPTQEGVATAEASSLGRMLAGAGNLVHDMLTELHAM
QEIEQQALAREDEWIERVNREGDDDIDTPRAGAVWRVI
>Mb|QLI65073.1
MNKLANMRHWSERMSPNFGMGRPNMPMNGLNPHQKNAAQTLGPINSPQAA
DASIHYSFNVPFASDLAGPNTEDIVHATTDAVLRWTHPADAPDDVQVHEL
PVHAQNLANLGRLCRDLSVGPLPIEAHVISSSPKNGKGQVTTVCLSGSPE
LVHKSRETILNEIPISLRCTTIDIDGNLVCDINAGVLKKPVVETLDHISR
FCGVDIFLLGPKLTPIVDGMTGDSQLRMDQRWRVAIYGDILSSEHAKARV
LIHIDTLLGRTFDVTKIELSFHQLVCGRHRKNIKLIESSTGTAIYFPPLF
SQMYRYCPQNATRRDPTDIFITGETIQAIELAKQKLHETVSRIRVYMKDV
TIPAAKIDSILLGRLDKVRKILEANGTYIMFPPLACQRTMVRVQGSESLP
VERTVREIMSLAGQFYGAGWYMQQPEVRQQLSPVEIRTMLSDICANAEAD
LSYDRMSFTVTGSDDAVKSALTVISDLKFVTQSQYQIRVKIELANEHKEF
VSGKKNGKINKIMGQSNVQIIFDGFNEYNFNIDVMAASYDSMKQGLTLVE
QEMPASISFHVPDQYHKRIIGIGGQHIQRIMKKHSVFVKFSNAMDRGGMG
REDDDIKVDNVICRTPARNAQNLDAVKNEILEMVDRADSEYTTQIASVDR
LYHRELIARLPEIDELEQKYNCKINFPSTEEASDEVTVTGPQWQVPHCVD
ELLGMVPDKHELVLARTADLVKFLESPQFATDLISKLKAQYEVEVTYHQN
PDELTEEGSPTVTLVWGFTRNNAGGLRDAIDFLEAQMAAATVEASVVKGS
IPRPKSDSFEDSLQYFDSKLLQHAPASSSSDSPVKSGFGAEVARERSSIL
DRLRKPGSMTSISSFLDRRKNSSHSMNSNFFKGSSNVSKSSLISIESTRS
FNADRNPWNDSGVNLPEDENPWGPRPFSKHMDNKLAIPLPGDVTPRHNTR
ASGDSGRPSTSHSMNSGYPAHIGPFR
>Mb|QLI65074.1
MSTEIGVVFCSGGGLFLIGGVMLFFDRAMLAMGNILFLIGLTIIIGPQKT
LLFFARKQKAKGTAAFFAGLTMILMRWTFIGFLVEAYGIVILFGDFLGTI
AGFARGIPVVGPYIGMVVDRIGLGRRNAELPV
>Mb|QLI65075.1

MGHEDAVYLAKLAEQAERYEEMVENMKIVASEDRDLTVEERNLLSVAYKN
VIGARRASWRIVTSIEQKEESKGNSSQVTLIKEYRQKIEAELAKICDDIL
DVLDQHLIPSAKSGESKVFYHKMKGDYHRYLAEFAIGDRRKDSADKSLEA
YKAATEVAQTELPPTHPIRLGLALNFSVFYYEILNAPDQACHLAKQAFDD
AIAELDTLSEESYKDSTLIMQLLRDNLTLWTSSEAETSGAAGQADAPAKE
ESGAAAEAPAATAEQTKASE
>Mb|QLI65076.1
MKSMQGLLHKTSLQTAHEQQQRFFAPQSVAGQKRPWSSNDGFVTSHQHAT
TATNSPSHSIVHFQKAISLPSAPKRLSASKITQVSGHHTTGEYSQRRLIS
ATPLSSQDSELDLAHQTYGLPRQIVSNFSLLGINHIYPWQKNCLKGPGLL
TGERNLVYCAPTGGGKSLVADVLMLKRVIEEAGTKALLVLPYVALVQEKV
GWLRKVVQHVKILDDSMPQGAENGPWRRRADYGTIRVVGFFGGGKVRATW
DDFDIGVCTLEKANALVNTAIADGSISKLRSVVLDELHIIDDDHRGYILE
LIAAKLVCLGQQVQLIGMSATLPNLNLLAAWLHGHTYETRYRPVPIEEHL
VYDGKVFRAGPNDGLGHTPSQLSSQDVSAGKTAPVRRIENSMHKEFTDSV
LNAVVSLAHETACMGYGVLVFAGSRGVCESDARWISRVMPQLHELAADVL
KKRLDLLGDLRSLSTGADPVLEETVLYGVAFHPNQLFPDAGLTSEERDLI
AAAYDAGTILVCIATCSLAAGINLPARRVILHNVKMGGEYIGPAMLRQMR
GRAGRQGKTQLGETYLCCRQPDLEHVLELMHADIPKVSSCLSTENRRIQR
ALLEVISIRLATSRESISDYFSNSLLQWSQSPDFVHKGIELSLEELQSMG
LIMPDSSTGYAPTQLGKAIVASAIDPDDGVFVHKELVKALRAFVMDGEMH
ILYVFTPVHSFGTTVNWQVFRNEMEYLDESGMRVLQFLGIKPTSIIRLAQ
GAILKETTVEEKRMARVYRRFYLAMQLRDLCNEVPIHAVARKYDMPRGSV
QTLSQTCQGFAAGMVKFCEHMGWGPMAAALDHFSDRLMAGARSELLALSK
VPFIKSRTARVFFENGYRSVAALANANPKDLVPILMQAQPNKLRIKSQND
ELMEEKMLAKANVISSAANRLWNIQMQAEMEEE
>Mb|QLI65078.1
MGFLDPFTDGLSIGPGGMSRPSKHRRDASRSRRKHRSRSRSSSRTRGGGG
GANSIAGALLGGLTGDSHYNKHNSSRGSFFGVGNNSRSSFFNFGGGGGSR
SSYYKRSPRQGFMQRAYKRLKRLIRDLIHYAKRHPWKVFFLVVMPLVTTG
ALAGLLARFGLRIPPSLERMMGMASRAASGDSFGLVSDAVRMAGDFGGNT
RGASISRGRDGGMQWERRSSYGSYGGYDDYGYGNSTGRGGYGGSGGSGDS
WSNTIADVARRFF
>Mb|QLI65079.1
MRPLTEQETKVLFTKLANYTGNSLKNLIAPLSDGDRFCFRLNKDRVYYVR
LSIANLATSIARDKLLSLGTCIGKFTKTGKFRLHITALEILSEHARWKVW
VRPNGEMPFLYGGNIVKAHVGRWSDDCPEHQGVVVYNMNDTPLGFGITAR
STAEARRLDPTGVTCFRQADCGEYLRDEDNLFATT
>Mb|QLI65080.1
MPPTFLTRDALSPFVEGSSISARSAMPSTDGHFATQPWRSLLVGSKRLAI
RSFSTISRRDDDGQNHLIDLMLVILGLVFAALILASLLYLFHRRRRLQHQ
RNGTLPTYEDVKQTNPRGLTIETTHNGRSSVFYIGRDGQPMLQNPHSPPH
SPDNVPQIHITFPEEPDENGRPKSGRVLVVRVGDNAAVGLEPMHDEQLPA
YEKESKGQFQSIDMDNIGGLKEKDRNLFQ
>Mb|QLI65082.1
MADPALHDCPMQKIPVEVLLRITYHLGTPDLGSVRLTCRSMEQALYTPFV
IEFFTRKQFMIAEDSLQALIDISKSRLAHHLRFVHIGLDRFSEGIQRPLP
DDDKERKFRERYANNFTLWNTGHHRDMMAEAFRNLPYLEGVVIRDANSHR
RSRDGPNAEWHSYGYTTAFHDTGISLSQGMTGIWNSGFPYQYCSQVFTAV
MFALGAAKASIKSIEILSRNANHLRDFAFNIPKFMEPSVVPVLQGLEKLH
LCIDLSWRTPHVGLPAIVGSSRYSPDLFIRNFLAHATNLTNLRINEHRTN
SLGLASLIDWMVDGGSDGTATSMPKLQELSLGTMNIDAPRLLKLISKFAL
SLESLELWKVTMLRHLPSDAPSPAPKEIFWAHFLKKLIEIPCLELRHFKM
GMLQQHWIEKPVPAHVAFKGRGPTRQYTGPDWRQFVTEIMPELEVQWPYE
AADETDEDNDITDEVLEGLELGLFDDDEMEIPNPAELLIQELLESNDHVH
FTTHAPWD
>Mb|QLI65085.1
MPRKPLTDRYWARVDSGLTNDDLSLPKATVQKIVGEILPPHGGVAFAKEA

RDLLIECCVEFITLISSEANEISEKEAKKTIACDHITKALEQLGFSDYVP
AVLEAAAEHKEVQKGREKKADKFANSGMSMEELARLQEEQFAQARERHG
>Mb|QLI65086.1
MAQSTAHRRLLQEYRALTNSPPEGITAGPVSEDDLLHWECLIQGPEGTPF
EGGVFPAELKFPKDYPLAPPTMKFLADMWHPNVYPSGLVCISILHPPGDD
PNHYEHASERWSPIQSVEKILISVMSMLAEPNDESPANVEAAKMWRERRA
DYENKVRDGVRRMLGL
>Mb|QLI65088.1
MACESCRLQTRTLLRAAIRASAVRAAPARTLNLSRPTSSSQPAPTRWFSN
TPQRRILGMGTLGESYRVLGASEKLYKICAQPADYHISEEARNNDQVERL
EDGEELGTPIDAGNVWHKTFGLQPSFSTWSHVTMLHLYLLQARVRCFERD
TFRNWQQQLVDHFFFDCEKKMHLDHNITSSALRQRYLKDIFVQWRGLLLA
YDEGLIKGDAILASAVWRNLFKGAPEVDPRSLCAIVGWMRSSLASLEAAS
DVDFPQKAGDILARPVDVFWSRLEEPFKKAFEGEAKKKNGE
>Mb|QLI65089.1
MPPKRKLADIEPEPTAVPPSLPPSVEEAYRRKCVQLKNRTNEVEDANDAA
RLRLARIKRQVEKLRIERAFLLEQLAKRTSTNVEDSEGSPSPPPTPKDKP
LRIKRGHRKSALLDLDSKATPNSSFKGPASPSESQAKRGSADPDDTPTNG
ISKPSKLPKNAFELYCEDARPILEAKSKDGDGDGDGDGDGDADADLNVDE
ELTRAWEDLPDADKEEYETKFEELSKKADEKDDEDEDKDGTEDTPEKEEK
AGDADKPETQDEDVEMTNYDTEDQDQDQDADQDGETRMEKDGDD
>Mb|QLI65091.1
MSRECAACDRNLPQSSYKANQYAKGPGVSRCKQCVHGHYSDNPSTRHSDA
GRFNNSFRAHIPNSALEQPFAEGAFRRVARGTYTSGPRQGQACVVKWFKR
GAVFSKDYFHLDIKAVDKALEFVNGFNDLGIINKTVKINVPSVWQFDDDN
DNEWAGQSVLCEPFIQNYQKFNSNSGWTDETRVWGEVMQALSHFSYHVSG
GYYVLCDLQGGIYRHQMVLSDPVILSRHRQYGVTDLGPKGISSFFAQHVC
NDFCRPHWTQPANPQQHFRPIPGTSMMRRTVPTEHSRPLRTRFPDR
>Mb|QLI65094.1
MHLPYPPRKRSNPPPFRPMSSNISLLRRSRLKNIAIGALLFLGALYLLFG
GKKPDPYHEHVPSGHPSVVLVTVIDPSEWDTAYLDTIKENRERYAARHGY
QAMVVNALDYDTRGAPRSWAKIFAMRHALSKYPDCKFIWYLDQDAYIMDP
TKSLEDQVTRPSRLESLMIKDYPVVPPDSIIKTFSHLGGESIDFIVTQDK
EGLVHKSVVIRNGEFAKFFVETWFDPLYRAYNFQKAERHALEHIVQWHPT
ILSKLALVPQRTLASYSKSTIGEQYQEGDFVVLFPDCTKTGDQSCQTVSS
HYWEKMKKAFGIA
>Mb|QLI65095.1
MRTSSPPNSPTSLSRSETLTLLILSGASLSVLLNTFQGDGEPLIASLALS
IIAFSLCYAMIRWLGPTFMRAGFRGRDLSKSTRIEIPECMGAVCAAVYLL
TVIVFIPFPFYKDIVAATSGGGNRDVVLEQEHVDHGRFLHRFPHNKLASY
LGAIISLQTIALLGIGDDLFDIRWRHKWWIPGLASIPLLVVYFVDFNVTS
IVIPVQLQPYIGELFDLGPLYYVYMACIAMFCPQSINMLAGINGIEVSQC
LVIAALIAFNDCLYLFTPYPHPATDSHLFSLYFLLPWIGVSAALLHHNWY
PAKVFVGDTYCYFSGMVFAVVGIQGHFSKTLGLLLIPQFFNFAYSVPQIF
GLVPCPRHRLPKFSARTGLMEPSVTPWSPDRQPHPLVAQGLRLLGKLHLL
KVTVDEKGSFVETSNFTILNLWLVWRGPLREDRLATEITLLQLLMGLFGL
FVRHKLALLVFKEDNWGTTAY
>Mb|QLI65096.1
MASTADRSRLRRERTFVGSECAVCEEPLEHTLQGERILQFSCSHVSHEAC
FYEYIRELDGQYCPTCEAPLHLDTSRGGNVVDISSSPKIANDLELGTNSE
VDKISNLVRTAGSDTRSTPTPTPAWDSQTVRPPSVDSSQRKPHSQQPSSL
GRDSGTRGSHRDSRDAALSDRYSSSRHARSDSEVTGVASSGGYPETMQST
PWRHDYDVQAMETTPGSPRTISRNPIPSPTVTVRSEFPTINRSRQQQTLT
CLVTVEVADNKWKPDPEDIGSSPHLAQQVSSKIDEIATRVVTPTPSVPRF
IPYESPEVLEEMTENLRNRVDNWHGLDFSRFGQLRLYGTLRVGKDKVSWQ
ELECYLFAEMLICVKEKKLPPQAVTQWDDDGAPRKATRCTLKGSILIKKH
LNEVSETGTIDENVLTLSLSVAELPQFHLRFDNRNQLKLWNQALLDLNAV
ENSLFRSPEFDRGEVSEGDEEVEWQRSRPQRVSSTASSWGPKSVTTAPTE

YTNFAVKTPYHPYSLVHVPIDVVVVVPISSSMQGVKINLVRDALKFMVNT
LGERDRMGLVTFGSGGGGVPIVGMTTKAWPGWTNILSSIKPVGQKSHRAD
VVEGANVAMDLLMQRKHNNPIASIMLISDASTADADSVDFVVSRAEAAKI
TIHSFGLGMTHKPDTMIELSTKTKASYTYVKDWMMLRECLAGCLGSMQSL
SHQNVKLKLKLPEGSPAKFHKISGALQMTKRASGRDAEAALGDLRFGDKR
DILVQLVIMPDSTSQEQLPQDPWDNIVSGLEALGGSVENDAERTLSVEEV
PLVQADLSWGDILREGTLQHMPRPSLLAITMLPPSSSQKKAWHNSPPIPP
HPSIVQRRMELLTSDMLTRALTLVSRGQHDRAHTLLSETRSILKGLGKGG
LPPVPSPPNKPSPTTTHPGNESSPNPAGTPDRKRSPSPPTSATSSGGNGY
PATQIARSRSNDGLGLGSGIDPGTVAALDIELESSLEWINHPAVFGRDSR
KAVLQAIGVISSQRAFTFRTPIETLWSGRVSGIRRLADKSREWREEGGGE
GGIMEEA
>Mb|QLI65098.1
MADAGLVQASLIWTTYAIAVVLCFLAAIITTFTWQTPRDRSAVVSIVAII
TLTSLLATVLLLPVDIALVSATNSVSQGAKKDWATPERVNSILLTLKIVY
YCLYSADALLCLVVIPFAYFWYEEYDEIEFEEQGRTWHSRFWAACKYTLF
FVALVVVLFLLGFFMPSPGDSSKAHWDLDYFKDLIARNHAEKALTFALGL
LITLGTLLYVVYTGSGLAMMPIAFIKSAPSMSAPQLSATTASQLEQNRER
QRQLEMRNSGRQDGMSRKDRRELDALIREEQTLVRRERLAAEAQGEGKSR
VYQAWLRLCALLRPLKMLCGILLLATSLLIWVSMLITGIDKAKNSVCKQR
CGYILGNIHIFQPLNWVFVKLARVFPIDYVLMALLILFLFSSTISGIATV
GIRFLWIRIFQIRRGRTAPQALLIATVMLTLAVLAINYAVGMFMAPQYSI
YGTQTFCSNKPEYPGAQPSCKGQPDLVRPCSEQLDFKHAKDICTPSVMST
FLNRITMTWPFFGIVDFWAQFAFLAVFLIVFATAIVRSPRVNIAELDEDA
EDAEEEGLLAATGRRLNATWEDVRRSPAYGSTRNGRSDNSRA
>Mb|QLI65101.1
MGLLESLDSSITDLFGQWNGYSTALATTLVVLITYRIMSATEPDAHPLLL
ARQSMPSSVRKEGESAVYRSQSAPHGMALNAGLNVKDPGAPKFSRGRDGD
LRDVWRKAVSGDAGARGRLLTVLGSENVIEHKMDDINRQINIIGRYIAEQ
GSIKVAIYLPNSIEHLITLLACSFYPNLTAVLIPFDVSESELISMLRRSA
VDTVVTATGSFPLDAVVNAYPSLRQLIWVVDEGSRHMDWNDVPEGMGSAV
NVTTWQDLLNDTPAAAGTELPASEDSTGAPGDIVTFWQGKQGELEEMVRF
TQANLASGISGQIAAIPTRERLTQADLFLPAESLSNMHTLTLTLAALYSN
SSVAFNSVAGRSPDLVLATQGVAPTVIVASPATLLRTHEESTRRLGGGLA
KLSHSLATSTLTQRGVHGGTNFLSAFAAGTRPRIGTAPGKLRLVYVAERV
GGDTPLLSTQILSDLRVYTGARIVYALTAARVAGAVAQTALFDYRLTPGI
EAHFGVPPSSVEIYLKDTEKHNTTDDIIEGEIVARGPCVSGGEARLGVAG
KINDDNTLSYV
>Mb|QLI65104.1
MALAPSSPRLPSPPPPAEIQISPNSPSGGAPASPHATQMEQSVLDANSRR
RIHPGTKAADMAAGPPLVPLSELDSAFQLQEHLAALHYDRTASGTQAITR
ETAHQLTQPPVGIDRTIWLYELCRFLISKCNQLVVGFLFDTPPCSAQTCP
EMRASEWQFLCAVHEQPKSCCAIDYCCHTLDWAANVVSDQKLFPSRFAVL
SDAHSKNAGVKNLVNVFRRLHRIFAHAWFQHRSVFWSVESQTGLYVFFKT
VCDQYDLLPAENYKLPPEAEGLEAPPIEPEADKPPPAILKPASQQRPAGT
EDDGLHSGGRTNTRRHIRSSPSTGSAVTTVIEADEEDTDNVSNKMESLHI
SDTRPTGQEPESAEVPVIVERSVIEGASKSEEQHPPEPVEPPAPTEAETE
AEAEAEAEAEEANQTIVEPELASNQDEDQNTESNIALPSAETDTRPSEPH
VEESCSETKTDDEESKQPTDETAPDPSESTEEEKEPAKETKSEDMAGESG
HVPDPE
>Mb|QLI65105.1
MSAYAGYSKTSYGAQGGDDSGGFFAGGSQPGSQGGKSYQDESLRPVTVKQ
ILEADEAFSGADFKIDNTAITQITFVGQVRNINPQPTNVTLKIDDGTGQI
EVKKWIDADKADDGNADQYELDSYVRVWGRLKSFSNKRHVGAHVIRPVTD
FNEVNYHLLEATYVHLYLTKGPLGQDGAANGGGESMFVDGGGYAGNANGG
GSGQMLSKVSGCSPLAKRMFNFMNGTPGMNEGVHLNVISSSTGMSVRDVL
AAADELLGQGLIYTTVDDETWAILEC
>Mb|QLI65106.1

MSSVSHAGPDAMVTIKVTFEGVTRRAKMPLREMVPRVLEEHIRIFLQIPT
DTKIMIERYSDSAAAFVMLDASNMPVYKQLYRAAKAKSKLKLRVSVLPQS
DASSPKPVTIEDASESAQTTPIEKPELACANPTPLDAASSARTTLTKQYD
SNLLQEAAKIIQDHQAEFHNHIRQVMKSTDELASLTSQMASCQPFTTTSN
APEPSEPLSPVCPATGAMFAVCCNSCEQSIPDAHYHCSTCDDGDFDLCQS
CVDQGITCYSDDHWLIKRIMNNGQIVNSTTETIAPKLKLVANAAPKDDAA
KVDDSEKEIFLPGLFEKQTLPPAEYFCTRLAKPVTQRAIARFAAVGNMRT
CNQCVRELPEREFLHCTDCEDYDLCQPCFAKDAHGHHPKHAFTAAVPGTE
MPTHIRVKMNSGRNQVHHAICDGCDAYITGVRHKCLDCPDWDYCAECAEN
AHFVHPNHRFAAIYEPLADLHTSFLSQPVHMGICCDGPLCSASNAITSYI
RGIRYKCAVCHDLDFCANCEASPANDHNKTHPLIKFKTPVRHVSVTTTGE
HQDGKRMPAMGDRSSTISKATETVGSSAANAVNAVQTVVDVKPTEADIPP
PVPSEKPLAKSELDSVAAPPAPTTEVKDEELRAVFLRDTVADGTIFPPNH
VFEQTWILRNEGATSWPAGCCVKYVGGDYMGHVDSSHPAGISELVSASES
TICYAPLAPGQEYSFTALLRTPIHSGKMISYWRLTTPDGMRFGHRLWCEV
NVRAVAAPEAKPQAQSSPTSVKNEPVGEPSQSSSVMIFPKLEKESPSASV
HEDAETESPLASAEDGFGQCDEDDEWDASEDGFLTDEEYDILDASDEEFL
EEQEKKLLKK
>Mb|QLI65107.1
MEFVTALRGTFDDNKPSLFEVLSEQQLNGLLPPTLRYLLTVATQRHPRYL
LRILNSFDELYALCMLAVERHYLRTRGGSFTENFYGLKREKALRGEIPRA
SISAPNLVRETLKLSTSDVWKNLAVLVGVPYLKRKLDESYEINAPRALLG
SAYTRMPDNPTVKDRILHYYRWFLMNIYPSVNAAYYFAILVFNLGYLFDR
TKYHNPLMWLIGTRLRRMTGADYQAIDALSQPKPGNRPGSRSIFSPSELG
SKVLSSLSLLLPMSIFALKFLEWWHQSDFAKQLSRKATENIDLPPPIVNG
VAKKSKSSEGQEDKAEKTEEDGHDKIMSAEDAPIATPSMLPIYVVPTPKD
TSHCPICQDDIVTPTACQTGVVYCYTCIHKWLEGTHDKQESFMEKHKGKW
ESGIGRCAVTGKRVLGGTEGLRRIII
>Mb|QLI65108.1
MSDDSPHKRKRSLGDTGDRDRDQKKMHLGDSRLGIEDLHLDVGEKYLLCR
TPHPEPLTRIAQDLYEMCGLTSLAAEVAREKPNGEKNALRKTYKGHIKRL
GVAGHFDVQKKKEDAPSEFMAILQVPELEWNVHQVKGREITDGLSATTLS
NLGRAMNMSKGPIPKPVWDSSVLGDLAPSSGNASKPISAKPSAPGTPLAS
TPNTIGRPKPPILAGQDPNRPRRNVKKRSYGDSSFEGYGEGYPDDDGGMD
TGYSTGEGEGGQKRRKKVGYDSTNGNESLSSDMALKNTAASPPNALMRQQ
SYGPGMVGA
>Mb|QLI65109.1
MAEQLRFDGQVVVVTGAGGGLGKAYALFFASRGASVVVNDLGGSFQGEGN
STKAADVVVDEIKKAGGKAAANYNSVEDGDKIIETAIQAFGRVDVVLNNA
GILRDISFKNMTDQDWDLIMKVHVKGAYKVARAAWPHFRKQKYGRVINTA
SAAGLFGNFGQTNYSAAKLAMVGFTETLAKEGAKYNILSNVIAPIAASRM
TSTVMPPDVLEQLKPEWVVPLVAALVHSGNTTENGGIFEVGGGHVAKLRW
ERANGLLLKADDSYTPSAILKKWDQVVDFSNKPQYPSGPNDFLTLLEDSM
KMGPSEQGDKIDFTGKVALVTGGGAGIGRAYCLAFAKYGATIVVNDLMNP
DDVVNEIKKAGGKAVGVKASAEDGDTVVKAAIDNFGRIDIIVNNAGILRD
KAFTNMDDSLWDPVFNVHLRGTYKVTKAAWPYFLKQKYGRVINTTSTSGI
YGNFGQANYAAAKCGILGFSRALAIEGQKYNIFVNTIAPNAGTAMTATVM
PPEMVQAFKPDYIAPLILALCGDSCPDPTGGLYEVGSGWCGKTRWQRTGG
HGFPVNVKLVPEEVVKHWKDIVNFDDDRVDNPEKTQDSMMKIMGNMGNVV
EKVDEPAASNEYLDAIKAVIGKEGPPVEFKFEERDSILYNLGLGAKHTEL
KYVFEGAEDFQVLPTFGVIPIFTAEMPFDFGSIIPNFSPMMLLHGEQYLE
IRKFPLPTSGTLESRGKLVEVVDKGNAAVVKTALTTVNKETGEDVFYSEM
TAFVRGSGGFDGPKKGQDRGAATAPHAIPKRAPDAVVESKTDENQAAIYR
LSGDYNPLHIDPSFAKMGGFKRPILHGLCFFGVAGKAIYETFGAFKNIKV
RFAGTVDPGQTLITEMWRDGNKVIFQTKIKETGKLAIGGAGVELLDGGNS
KI
>Mb|QLI65110.1
MSSFTRVLRPALRGSRGLAQTVCTSSRIASRPAAAARNMNLARPLSTTSA

LRSETIDITDIPPTPISHLSEVEAAMAESVSKFASEVILPKARDMDEAED
MDASVVQQLFEQGLMGVEIPEEYGGAGMNFTSAIIGIEELARADPSVSVL
VDVHNTLCNTAVIKYGSEALKKKWLPRLATNTVASFCLSEPVSGSDAFAL
ATRAEETADGFKINGGKMWITNSKEAEFFIVFANLDPSKGYRGITAFVVE
KGTPGFSIAKKEKKLGIRASSTCVLSFDDVVVPKENILGERGQGYKYAIS
LLNEGRIGIAAQMTGLALGSFENAVKYVWNDRKQFGSLVGEFQGMQHQIA
QSYTEIAAARALVYNAARKKEAGEDFVKDAAMAKLYASQVAGRVSGLAVE
WMGGMGFVREGLAEKFWRDSKIGAIYEGTSNIQLNTIAKLLQKEYTN
>Mb|QLI65113.1
MSSPRGGSSPVHNDHHPRRPESTSTTSHASGGPRYCRWKTAEFHDQECSR
YFDCPSHVVEREISESDHVEQSQRADSPEAGPSAGIQISDTEDAPRRPST
DDESNTRPESGYSHVQTQTALESEASAAHRQSAIQSASLSTTQSADEHQN
EPTVVLPIRSSSIPIPPPSQSRESAVREEAARAQMHNMPDPRVNWADLES
RYRGAYLQTAHEGEHQAMETLPRTMIRPRDVHRPPAPSRRRESEGRRASQ
DHRVNRPRGDGSVTPEFVLPRWQPDAEVTYCPICHTQFSFFVRKHHCRKC
GRVVCATCSPHRIIIPHQYIVRQPGSDISMPPSLLVDAMGAGYLDVNGLS
GGERVRLCNPCVPDPNTAPPESPTPSIGSPRSPHQRSRSSFGPSHGAGHP
SNRYGAVFTGGDNRDPLQYHYARARSITMNPSVQSSPNLAPSSHRMSAPH
VHPSHLERFLTAGPSNIYPSSSRHRHSSYGYGEISSSSSRQRALPHPPQI
AEEDECPVCHRELPLRLLPDSEALREAHINKCIRDHSTYGSLRPPEGQES
SAPPMLSRRTGMYIYPATEKDCVDDAECTICLEEFTVGIPMARLECLCRF
HKSCISAWFVNHPGRCPVHQHDGFGF
>Mb|QLI65114.1
MAFQTSILRDGEWVTETVNLQAALKASTTPKSAARAHPEPPSCGILSRTI
VESPVVHRILPVRLRSETHNDIAFVGDHFVQISELRRDGQVHEVVRKSDF
GSRIRGAAVIGDSLQHGLDDDDPADMVKSEDQDILMRDSLDTGLDSRHLL
PPQLLVLILESGDTMFTFLRERQDSTLEFVFNKRESPKNLSYLGYHISVD
PSSRYMAAASPDGVLVIYEFESMAGLNQQYRQQGFFDPVKSIRIRAIQGV
IHKLEFLYPRPEDDYHIILLLIVTRRERRSAEPVSRMVTYEWEVGDNLKE
IFAEEKAGNRLPKEHRMPSLLIPLRFNTAFFAVSQPDIGIVKNCLSGSPV
FEVLDTDTPGRTPLHHGADKPLWTAWSRPFRRKKYFEKTDIIYLAREDGA
IIHVEIDAPELVPSVTNVGCLNTNINTAFTIAYDVFSDVLIIGGDSGPGG
IWKLAPRTDLEQVSVLPNWSPVVDLVTSSGRPLKASSALEPRNTNLRSAE
MNNLPSKPESLFSASGRGIRGNLTQWRWGIQGRIGLDIETGEPIRCSWGF
SMSGPEGNGLFGLLALPDSSILLHFSSDFSQVDAVPPDSTAFDLTSRTLH
ACQAQSGLILQITESSVALLSGSRTSLHPLQSILGIVGACAENAFCADDI
LVLSTHNDGNSQLHILQIEEMSIRAVKSWDIAGEVTCVSLFKTAGNYFVI
SGSVIDSTSWVFAYALDGTIIVAEAVDKKRVGPCGFEAPSEAQPFEALTS
ICVVRESSNSAELVAGTRCGHLLNLRISDQTSERVTWNSEAMGVAPVDVF
PTHGLFDGDFAAMACCDNNITMMSNFSMSSLKFQKKHFIWLTDSNDASMP
SPPIHSVFGLGLSLSGYSGHMSLMLLAGSRLLIAEIWPHYSLVPRSLPLN
GSPTRVIFSQAWNCLVVALLKDGKSTLAFIDPDSGMHLASACDKDRNALE
FISGLGHPGDRIYGLNEWLYVKDGKTFAFLLVTTKEGRLLIVSVNKSESR
TGDGGGKRLQYWTRYKKVMAKPIYSIVGDNDGIIFCVDKTIHWDVLDLTE
KKLRPMKQYDVDSPVISLRVFNGKIFALTTMHSLEVIDHRAGEDNPMSLI
HTDAISRTTTHMTDIGTDGRESDVVGRWPITMLSDHRGGLVGVWIPWGQR
DKEFQTIFEAILPNSVRRFTHARSRPLWLGSETRNRYGVVASSKEGSEVF
GVSLDGSLQHFTLVNLELWRILSLVQILAQRNRLFNAMGGPPSDSDSAGS
EDDVDIEPRLHPKLMHIDGDMLYRCLENRLLEKLVGTADGLDLFCEYLDG
LDGGRWTDRFRDGIGNSEEREAAYFNLGYDILSYILAPAL
>Mb|QLI65115.1
MSAPSSPERAAAEETRSKSPARPASAQDVVEPVSQVAAPANPDPNVEDAN
SPDEEEEEEEEEEEEEEGEIDESSTSHATEEPGHDGPPLPQEPPPSGDDDG
WDCQWDPNTQAWFFHNRFTGKTQWDNPRIVSSSSSANDRAATSQAAAPQP
PGELPPVSGYNPAIHGDYDPNAWYAQKYNQADEPSRPLEVPSLDDGYTSA
AAFNRFTGQFQHEDMGPGRHTDEAKSRRQMNAFFDVDAAANADDGRSLKA
ERANKRISKSELKAFKEKRRARKEEKRRAWLRD
>Mb|QLI65116.1

MATKQPLKTTFDVEHVIRPIFTGGSIAIDNEAQILATTLGEDVVLTNPAN
GKHLAQIEGDGEMVSTLTLTPSGSHLILCSRSLSMRIFSLKRSPSDDTVQ
ATLSQTLKPHGTPVVVLAVDRTSTLLATGGTDGTIKVWDIAGGYVTHSFR
GPSVLVSALHFFEIAARSKDNKSSRKGKKGVQAEDDSDNEVEAASSVNFR
LASGSQDGKVRIWDLNKRGCVANLDSHVSDVQKIDYSPEQHALVSASRDK
TLIWWDTKSWKIRKIVPCLELVETAGFIDNGRLTFSAGANGCLRIWDTDN
GREVTPAQSERSEEEGIVAGIYLPGLSFILCVQVDHTLAFYKSPTKESEA
SLGIPEPFRRISGTHDEIIDLGYLLPDRSIMALATNSEDVRLISVAQAST
EGASAPWDSHATPYFGQDVALLRGHEDIVISLDIDWSGHWIVTGAKDNTA
RVWCVDSARNSYTCWATFSGHAESLGAVALPKSVPAEGSAARNNPLDHPP
ACLITGSQDQTVKKWEIPRQRQQQGSKPRAVFTRKAHEKDINAINIHHSG
SLFASASQDKTVKIWSMEEGEVQGILRGHKRGVWSVQFSPAQLPTIQGND
GPVTGKGVILTGSGDKSVKLWSLADYTCIRTFEGHSNSVLKVAWLNLPSQ
REQSRKPVQFVSAAGDGLVKVWDANSGEAECTLDNHEDRVWAVAVHPETN
TIASGSGDSTVTFWKDTTVETQAVASQAALKMIEQEQELENHIFAGSYRE
AITLALQLNHPGRLLNLFTSVVTNGNPDEGSLCGLKAVDEVLGSLSDEQI
FLLLLRLRDWNTNARTAPVAQRILWTLVRSYPASKFSDLSVKGARGQKSL
SDVLQGLQVYTERHYRRMEELVDESYLVEYTLQEMDSLAPMEDSNASLKG
LDDGQGDAVMVG
>Mb|QLI65117.1
MAHITSSAAPVGSIIFSIFALLVISIIVLLILRYYLPLRTTPGFYLVPIF
FALWLPSIVVLLVPIDLASSAATDDETTRGIWLPERVVLVSWRITYWLTF
VLTWAILPILGEYSDAGYHEPSDKLKYSLRQNAQFYAIVLGASAIGLVYV
FIAYQPSILSLKGLVMTLAYCWGLVLAIYLMGHGLVAIPRRLIRNASISG
RLKRLQSQAPKVHEHMEDTLVILEEVEAQVAELCRRKTGSALSFQEWIEE
LQDMGNILPSQVPSGSGIFATNGRSVPPVITEKYLAELTRSFVRARHARS
RYVDEWSRLVHEAGELQAILNSAASKKLDFGEPSPHTGVWGRTKILSPYT
RYIFYYHVIPYTQVAFGLFLALASACVVWSELVRYTFPRLSVIRLSVVHH
WVGDKPEVGFAGQVVSAFWICYMCAAALLSMTEVKVWRGRALVKRNTSYE
SAFWYSMQVAKLTIPLSYNFVTFLSKEVYEKTIFFKFLGQLLEKTAPGRW
FDDLFPIVVLFPVVATLFGLYGKVKRVFVGIDVIDEGDENGPTYGSGSWR
EGRGLIERELGGTATYRRRGDTASRVALGEAGSRAVPILSIPAVRDPPPL
PSQTPVPSANTGRPGQISRAPIYDDAAEDDNMLVIIGNRMKNTVEGIEAP
KWFQQIKKPKWMGGNNENAGNSQNNSDIRRWFGGEGHIRL
>Mb|QLI65119.1
MGFSATQEHHDAILQEIQNRTQADWKYLIVDEASKKLIYNSVKEDDILSS
SIATIERIEERREPNPGMEAIYFLTPEPHIVDCLLADFDRRLYGRAFLIW
TSLLDPKLSRKINEFPGVGSLMVQPQQQRPGSRPVANPKTLLIDFYPRES
HLVSFKDPWSFPILYHPACNNLIPKHMRILAQRIAGVCITLGEYPKVRYY
RPTAAFHEASVLSSHLARFVQEELDVYAEWHKNEYPPPSNRPPSTLIITD
RSMDLMAPLLHEFTYQAMAHDLLPIKDGDKVTFHTTVNAGTKDEKEEDVE
LCDDDKIWVDSRHRHMKDTIDKLMGDFQKFIDENPHFTKDTENGGAPTLN
TMREMVAGLPQFQQMKSAYALHLNMAQECMNAFQNHKLPDLASAEQTMST
GLDEDYRKPKNILETVVGLLDDEAVLPADRLRLIVIYILYRGGVITEDVK
KLLLHAALPPQDGEVITNMEFLGGKTSYTLKEPRQQPPPLFPKDPKAFQP
NEDYALSRFDPVLKPVLDELTKGTLDQTIFPYVKPPSDPNEDLLAAQGGS
LRAGRPNWAAAGRRPPENRQRIIVFMAGGATYSESRVCYEVGREKSRDIV
LATSHMLTPKLFIRQVADLSRDKRQLDIPMERPKPRAPAHLFERPPPPPS
QQLNPGAGPRMAGNPGRAPPPPTGGLPGRPVVPPGGAPLPPVPPPAPPVS
SMSNLNLNSNGAPPAKLQGKPPKEKKKRNFLGMKK
>Mb|QLI65120.1
MVSQPDQPSSGSNTASNVSSRRGHQYSSASVVSRPSLSRTKRYNRSHTGG
RSYAPQNEFPVFAHSGDVEILIRLGAGADTTTQRYLLHRHTLTRCSGFFE
ASTSMEWSKARPLLAGGNELARIGEEPPSTLDSSDSDRFAVSGVPGSGGS
APRKRWRYELDSGVGEGDIPMLVQKEETTPSEDSHPSFPSLFGSASGSNT
KSASRSKHGPPTTNHVSFSRSVANLAVTPSMSQEDEDLLRDYDNLFRIFY
NYSPVLDGVNVADAYVQCKSLLALADQYDALAVVGPRVDHHLLQFQSRLW
KQIAKYPISYLRLGYLARSKVIFQEALIHVVGQWPVGERSIRAALPDVVL

DIIEDKVDELEEIISRVEGRLFRLTLTGAKGERVTPGNNYLDWLVVSLFR
QWLADNTSPQPSPERHGNSRSSNNPPAPPPPVPPLTSIGRAYRTLGSGNP
STFLNHEDCKRFLKLTPELYRRDNLRRFEKRMDEMKAMARDLVRPLLGSG
LELDLAGPSRTSEGISYLTCTAVGNRDLPWPVEG
>Mb|QLI65121.1
MDSSHQFNHRPGQNDVPPLLPMEMMGTLQYLDPNMTNIKPDISGVIDKGP
FLSSDREWTCYRRNYLACICSFSLSPSYPGPGIQFTPTPMTGTSTTQTFQ
VYGFAMCISAVVADNEQHAIELVQHTPKRDKGPISKPSKTLIAPKTSSTP
HNPSLSIYGDGSGLSSSRSLYADGFGGQTNVGQQVPTEHTFERIQFKQAT
QNNGKRRAAQQYYHLVVELWADVGTQASDQFVKVAFRKSAKMIVRGRSPG
HYQNERRNSQGNGTGGAAGSGGYRSMGPMGDFGNGSGMTGPGLGGYNSGY
DGRGTNYNNVRHQEMSPESIFSSGDDKPSNKTYQYYASGAGSSSSYESHS
DRVDLFGHRNGTDTLHSSLPNHATNERKVKPEFDFNMLPSPFSGGARTDD
HHRERFDGRPSPGGFYPSIVPPTGLGAL
>Mb|QLI65123.1
MHSTTLPRLPIFEAIVKHDPQSTAVIHSSSGRTFTYGELLGDIRKARERF
YEASGKKDLNGERIAFLVGNSYDYVVTLLAVLAARSIAVPMSPAFPAPEL
QYILDHSEAALLVSSPRFASKAAEVLATDLTTKPAHLELKKHEGGGMCEV
VSLEEGSDPAGAGLMLYTSGTTNRPKGVLLPQSALTAQSRSLIEAWKYTP
ADHLLHVLPLHHIHGTVNAVITPLFAGSSVEFMFPFNADAVWKRLAAPFL
PTNGTNRPHGTTKPPNSKVTFLTVVPTVYSRLLASHKLLPHPIQAAAREA
ISPANMRVNISGSAALPTPIKTAWSDLSHGNVLLERYGMTEVGMALSCGL
SFTDRIDASVGWPLPSVQARLMDVDTGEIIPLGGEVDSQGRERSGEIQLR
GPTVFKEYWRNPSATAKEFIPDSDGRGSWFKTGDVAIRRAVPAASHSQPW
TKGPLYFIQGRLTADIIKTGGEKVSALEVERELLSLPEVSEAAVVAVPSG
NWGQKVGAVLIINRDVVQKWSPLDMRRALKSRLANYKIPQVMKIVEHIPR
NAMGKINKKQLVKEIFADEHSGDEL
>Mb|QLI65125.1
MANWNTWNPFGRRESHSSGSILTYKLLTLLTWLLSVIVTIYYAIHEPKDG
FTLRRRIWDQNNLIRTAFTMSPVLGDIYWVILFILQFGYITHLFSRTPET
VNYAASVGSHFIVNNLFHFAFIMLFVRSHFIWAEVMLVINFFNLSSLYFR
HNTSPRFIHTPVVSGPLAWTFVALYWNGAMMVYHPHNLVARIFGNIFIWA
ILAYGGFFIVAYKDYTMGFWLSLLAAAIGVAQFERQIIALQWIFAFVIMA
VLFLATVTVAVPAWTGRDYLWRREAQPAGEAERAPLLHD
>Mb|QLI65127.1
MFRRHWSGLPKDATFPSDLKGLGYFVNDDDEIRSVESPEQYFKFFINKNE
RINQCQRFEFDKALEDIIHQRLVAEGLDKVALPLGSTTSEKHIVVFATPD
LPSKSRIIVFFGEPTKVLGMLAGRVANGPGGINKGSLVSVVQELQKQVAS
NKDSSPPGVFIANPGQLYWWPEGRRMLTATDSTAIPLPSLVHAGRRHIPE
INTVIAHETPEKHVESVFSTLLQVMSERTKVDLIAIGQSCELVTKFLDDA
TNWHAWKDHLDAMLLMGTVYPADLTNQALRDFTAKRARAYIVSTEPLDTP
LAPPSGNEEEQIPAFGCPCYSSSEPFYAEMVLIRALKPALQYLETVALTP
GYENDDILVAEKPKQEFTDKDWEKVADSEKPLIRVVDADLMKQEVKNRKR
WRKFLENGEACDTDSSDDEEV
>Mb|QLI65129.1
MFSRLVMPKPLKDGRPRTTSQAVLAWICGPGRRMATTATLRVAHQIRRNL
TLRRLLSFPHFLFLFWVVILLWGERWVFDSKVAKCDWENWEKWPKEADPH
HLIFVADPQLIDPHSYPGRPWPLSPLTVLVTDNYMRRGYTALQRQLKPDS
VFFLGDLFDGGREWKTRQGAKFVDPKWGGERSATEKKWVKTWHRKYDDDY
WIREYQRFSNIFFAPFNEAGGVPGPYQRGRKLVASLPGNHDLGFGAQIQV
PVRDRFSAFFGETNRVDVVGNHTIVSVDTVSLSADTSRYKDEHDLKPIYG
PVHEFLDQVQATKRKAAQQELAVWHGVDRGLKLRHKVEDVNEADLSRSPM
DPGEGAPDFPTILLTHVPLYREPGTPCGPHREHWPPSKSTLRKDGTVDPT
ARDDRNAISVSAGYQYQNVLNDEDSVKLIKKIGNVVHAFSGDDHDYCELV
HSSAQENVPEITVKSISMAMGVPTPGFVMVSLFNPIDAHGKPIPNSPEKT
IQTHLCLLPNQYHTYMKYITFIIVSVVLLLIRAYLVPALHLTPFALEPET
HAAPMLPIYKDKVKMEPPEYGALRSPAAATSGARSQANGGNARWASKKSK
NGQRWGWSNGNGGPRITLDDHFYDGGKANRGRRELRLLGRELWTTTWRLA

WLTVLYWAYLARKG
>Mb|QLI65131.1
MSTLRPLRGGCLCGRNRYIIALPEDGINEAQVLFSTDRSHQEPLATPLAA
YIRVPLSWLHSTTFAFFDDETHTMIRRVYTHPSQDHTKRQFCGFCGTPLS
YWSEEPRSEAEYINLTLGSLLQEDLQDLEDLGLIPEEGESEPEEKRAASP
NRGIALRHSFGVPWFDGMVDGTRLGRMRRTHGIRRSDDGTVKVEWEIIEE
SDGLENDVEMETGSASGKRKLQDRDG
>Mb|QLI65133.1
MPSRSDITYFGAGPALLPTDVLEDAAKALLNYNDTGLGVAEHSHRSQLAT
DIINQAKADLASCLDIPDDYEILFMQGGGSGEFSATAYNFAGAWVARKHK
AMVDAAGGDAADPKLIQQLKDTVDKHLKMDYLVTGGWSQKAAAEAVRLFG
AEHVNIAADSRESNGGKFGTIPDESTWKLSEDAAMVYYCANETVDGVEFP
EFPKSLAPGPDGKGPIVVADMSSNILTKKIPVGNFSAIFFGAQKNLGVTG
VTVVILKKSFLPPVTPQPSAALMRQLGLPIPPIVFQYETIAKNNSLYNTL
SIFDVYVAGRVLKKLLQTYPDKVQGQQAVSEKKAQLIYNALEAFPDIYKV
IPNKAVRSRVNICFRVTKGDNVDAAEKAFLEQGVAQGLTGLKGHRSLGGI
RASNYNSVPLEGAEKLAKFITAFATS
>Mb|QLI65134.1
MSDQSRLELINENPIRNGLDAFRALFTSICTSRNLPSTIDTLRQLGAEDL
RNLALTLFTSLRNLPISGSLHSTSGSATLRSVLLRLITAAASDSFDFDRI
KPLLEIAISDDTKDVQIWDFVSTAAAESTPPPRSIASSLQQTPWSQNTSS
FVNSSEYRQNVDPVLKLELEHLYVGLPNFHKSFFGDIPELDMVSEAVFRK
CIEGDSPLFKEGWSGWPAGAKESDVLTWIGGLISQLEAFADNRIPTSVAR
RKLLAQPKTPLEGSTGKRSMDIGFVDSDIIYKPDTTDSRYRWAHILVVGE
LKNNPKADIASVAWIDLARYAREVLAAQDTRRFVLGFTLCGSLMRIWEFD
RLGGIASEQFDINKEEGGLQFVATILGFLRMNEEMLGFDPTIVTTSGQKY
IEIERNGQTECLVIDEVIKRAPCIAGRATTCWKAHRNNDPHNPLVIKDSW
QYVDRDEEGELLQEATEKDVTNVARYYHHETVRIRRTDDDVRNNVRKGLD
VTKATNFQPKRTLPSNVGGTVSRISRKGRSSSSGVKRPSSETDAALPPSK
RSYSASPTKPSLDVLSNRIHRRVILRDYGEPIYKASSRAALLDALEGCIE
GHESLRKAGFLHRDISVNNLRVNEDKANPSWRAFLIDLDLAIREHRDEAS
GAMGKTGTRAFMAIGALLGEQHSFMHDLESFFWVLFWICIHYNGPDDSRT
VQRFEKWNYVEMAELAELKKGQVSHEQDFIKTTSEYFTTLYTPLIPWVNK
LRKVVFPNGRRWEMEDARLYLRMKNVLRDAITDPTVAPA
>Mb|QLI65135.1
MEQQVHKPHRKSKEKKKHTGDHNPKAFAFSNPGKLQRSAARSQDIKEKRL
HVPLVDRLPDEAPPRLVAIVGPPGVGKTTLLKSMVRRYTKETLSDPQGPI
TVVTSKKQRLTFIECPNQMEAMVDIAKVADIVLLMIDGNYGFEMETMEFL
NVLAATGMPGNVFGILTHLDLFRKPQALKDAKKRLKRRLWTELYQGAHLF
YLSGVMNGRYPDREIHNLSRFVSVMKNPRPLVWRNNHPYSIVDSFRDITH
PTKIEEDPNCDRSIVLSGYLRGTNFAAQGQRIHVAGLGDFTVANMEVLPD
PCPTPSMEQALAKITGKTGRRRLDEKEKKLHAPMSDRSGLKIDGDAIWIT
REKGFSFDKDAEGVERGEGEEMIVGLQAERRLLGQMEDGVQLFSGGEKLK
EVAEEEDTGRKTQRRARIAERSESDVEDEGMDDDDEEEDEGFVSGSGDED
SGAEVEFSESKTGKMFRKDADKSKGEDDDVAFADSDSDLGSISGDDMGMD
DEDEDFGSDEEAAAMRWKDDLADRARKMHGKRRTYHTGDLARYMYDDSMT
PGEALKRWRGEGGEPEEENIDDSDDDDFFKKKSSQEKEDSTEDRSIPDYD
YDDLAAKWAERDNIEALRRRFTTSGLGGDDDGEEGEVDGGDDDDFGGFGD
DDDEGDGVFEDLEAEPAEKEQETAEDIAAERERNAKRKEELKQRFEEEDR
EGFLNDKANARREGGDIQEYGEDDWYDAQKAMIRKQLDINKEEFETLDER
QRAAVEGYRAGKYAKIVLEKVPAEFVTKFNARLPIVVGGLTATEDRWGFV
QVRIKRHRWHKKILKTNDPLIVSLGWRRFQTMPIYSTTDSRTRNRMLKYT
PEHMHCFGTMYAPLIAPNTGFVCFNSFSASNAGFRIAATGTVLSVDESTE
IVKKLKLTGVPYKIFKNTAFIKDMFNSSLEIAKFEGASIKTVSGVRGQIK
RALSKPEGHFRATFEDKILLSDIVFLRAWYPIKPHRFYNPATNLIGWQPM
RLTGEVRRDQGEAAPQLKNSQYRKIERETRHFNPLRVPRALAADLPYKSQ
ITTTKKQKRDTYMQKRAIVLPKNSEEKKARAVMQQLLTVRNDAAAKRRAK
KAENHAAFKKKLADNEEKKEAREKRETKEFWRKNGRKRGAEDGGSGGGKR

RR
>Mb|QLI65136.1
MGKSQSKLSQEQLTELQNSTHFDKKELQQWYKGFLKDCPSGMLSKEEFQK
IYRQFFPFGDPTSFADYVFNVFDSDKSGSIDFKEFICALSVTSRGKMEDK
LDWAFQLYDIDGDGKISYDEMLQIVEAIYKMVGSMVKLPEDEDTPEKRVR
KIFRMMDKDENGSLDMQEFKEGSKRDETIVSALSLYDGLV
>Mb|QLI65137.1
MVNNRNYDFLIKLLLIGDSGVGKSCCLLRFSEDSFTPSFITTIGIDFKIR
TIELDGKRVKLQIWDTAGQERFRTITTAYYRGAMGILLVYDVTDERSFNN
IRTWFQNVEQHATEGVNRILIGNKCDWEDKRVVSTEQGQALADELGIPFL
EVSAKSNINIDKAFYSLAADIKKRLIDSSKNDQATGTGVNVGDKTDAGGS
KCC
>Mb|QLI65138.1
MDGKQQLAQGISSESLRHSSSMSSISRLRRFLLPMLAIILLLRGVYDISY
RYKFAGPGITHLVPLEAHIISKCPDTRDALRELILPVMQRVYDKVDFKLN
YIGTPTADDGVECKHGPSECMGNIIELCTRELYPDPKINLGFIMCLTKDY
PHIPERALVEDCALEHAIDIRAINECAARDDGAYGMGLLRNSIQRTTDYS
KVAREQLTDLRRPMLP
>Mb|QLI65140.1
MPLCGGSKTVQRKLVLLGDGACGKTSLLNVFTRGYFPTVYEPTVFENYVH
DIFVDNVHIELSLWDTAGQEEFDRLRSLSYDDTDLIMLCYSVDSKDSLEN
VESKWVGEITDNCPGVKLVLVALKCDLREGGGDDEADDAAASAAAPADGN
ANSERPRGPLIEYDKGLEVAKRIGASRYLECSAMKNRGVNEAFTEAARVA
LSVKKEREENKCTVM
>Mb|QLI65142.1
MVKPELPAEVTDLLASPVELVKWLEHEDAELGRSHANLLFEKLLKDAAQP
KSMSGQACLRLCGLVEQSSVSKSDDLRLWAFSQEVMLKLFNFYIEWNESD
KHRSMKLVLDLVARCILKNPDRDAALQAKKYVSDSLISIVIGRSTKPVVK
SAIKTLDHFLSKGVFNLCDIRLTYVSFRTDIRTQDEVEIWRNFTVELFHW
MHLQLVRPTVGKLIVSVYRLWRRGQDVGAVPSIEIWHQWLLDSLTEEPTL
LESIKNYIFLPLFKADRSEALAFLGRMNEDQAVSAASGMSLDVPALLQLA
ALETGKKVGLIEEPALGSANSQAEHNSSITLHEKVLESVLAHPSHEVRSL
ALSLLVTSPSTTRPYSPVALDLLRKYMGTFFADPDAKFRVEVASKARDMF
RRVRGAIHVLKRSIPRARAKAQKANAPPSVDKDAVSQPILYQSNLISLPE
AQLANCLDYHVEFLRWYLGFLCEELSPTASYQRHIAALKAFMYIIRLEGD
SNKTWETSDDQELFFNCLDAKWARALFDLVMNPFEDVRDLSATALAKCYA
DGRYRRLTLTGVEITKVPVEEITQLSQRAHELVRRTARADHSDGASKSSQ
LLYRFLESGDERIKLLAAMVNELNRKVSMAEKDLGQAVVNAPLHGDFASL
CHTWQVVSEIKLSEVELLEANKLQRDIVSCCERAWRAVRDVLCDDSPEGH
LPQELEEVDGLDTKDLLSYSFRSVHESSNLMRTIILTIQNRSRNGLIIPS
KEVFERIGNLTFTQLATLRHRGAFTTVSSTFATCCQQTKYLQLEQNERTL
LDIWYEGTLNAIDSQASTTRRSAGIPSMITGILAANASHPSFAQVMDTLM
AIAAREAKVSETDGSRLPQVHAYNCLKDIFKNSLLTALGNKSESYLPQCL
ELAASGLRSEVWAIRNCGLIFLRSLIDSLFGTHESKAVIEAGWDGKANRI
HYHRYPNLPGVLKNLLQSGHSILSESSTSATAAAESVFPALDIIRRAGPP
DLLRDEIQVDVTAYLSSPVWHVRDMAARTLCSCLLHEKWLADTRGIFDAA
MTSKSRNKPNHVHGILLTLKFVIDRLSEVARERLLVDLPELHSFLVQTKV
DTCFPDCPDIIASYLEVINMIWFFQTTNKHPLSPFSITMPRNRGSALLKI
QRAIHDVYVLSTLDEPVSQLQSLLQSQQVGLDGLVAALEIIPKVWDPSLC
SQDTLASLSNLYVNVCLQTDYSKAQVLAVENLADTIDKLLQQKAIDKIPR
DALVKLWLGLPARPMNPALSNAVIRASGSVIAALIRPEQSTPVNIHSWGQ
IMAEAALDNKTFDTRFAAALSLASFFTASPPTSPEFLPALLSLYDLLNDD
DDEVRDVAALAANSVIGAALVPIEATNRLLQFLGTQFGTAPEFKAIIADR
LVGHFGVQATKLESWESAEVELARALEFDDSLFVVEENNLFIDEVRETKR
WVEVFESLEWDEKDEHLGKLGSWVQGGIAQVARLAELEDGPLGWASNPTA
FAICTRIIRCSVALGRKFKHSELEQGVALAKEALRSRSTTVSKLLAEAWI
DF
>Mb|QLI65143.1

MNTVRFVRPIAARVPFQRSTLPRVVRAYSSKSFEYIQTSTPKPGVGQVTL
NRPKALNALCTPLINELNEALNEFNASDETSVIVLTGSERAFAAGADIKE
MAPLTFSEAYTKSFIESWSLLTTQIKKPIIAAVSGHALGGGCELAMMCDL
IYCTEKANFGQPEIKLGTIPGAGGSQRLTRAIGKSKAMELILTGKSFSGA
EAERWGVAARTFPTYEALMEETLKTAETIAGYSKVAVQACKEVVNKSQDL
GLRDGVEFERRVFHSLFGSQDQKIGMKAFAEKTKAEWTHS
>Mb|QLI65145.1
MKSFALLSALCGLGSAHFLLNYPKSIGFDDSNEGNAPCGGFTPDLSKDLV
DFHVGGDAISVKLTHPQGNWLFRVTTDEKAESGWEQIFPIVQQSGLGDFC
EPQVTVPSKYAGKKGVLSVVSSATDGLLYQCAVVNFVDGKGDKPSACVNA
SSVKASFTDDSKLTALVGSGSSSTPTSGSGSSTASKTSASSTATHSGAAS
SLHAWSLTSAGWGGDCENKLSG
>Mb|QLI65148.1
MQDDKPPVINYVVSFILVGLAWGLTTPFIRRAARAHHPPPHPTLHLDSVK
NHWLRSRLYGAFFAVADLLRNPRYALPLLLNLTGSIWFFLLIGHAELSLT
VPIVNTMAFLFTVLGEWYVEGKVISRDTGVGMVLSLVGIALCVQSKS
>Mb|QLI65149.1
MHFPIVSLLSLVALVSAAPANLQARDATNCGGVQYTAGAVQAAADAACNY
VQSGSHAGSSTYPHRYNNYEGFDFPVAGPYNEFPILKSGQIYSGGSPGPD
RVVISNSCNLAGVITHTGASGNNFVGCSGTS
>Mb|QLI65151.1
MARKSSKRKPSSPDHTNGNSAKRSRNANGLREPHPLAAEAERHGIVLRKY
YPPEMSNARARAYNNNEIPRPMEDLVTALYNTTEQRQRVEAKDSVIHWFK
TDLRCNDNRALALASEKAKEAAVPLICMYIVSPQDFEAHLTSPVRVDFIL
RTLEIVKRDLAKLDIPLYIETVERRKQIPDRILELMEQWGSRHLFANMEY
EVDELRREANMVHDLAENGKSFEVVHDTCVVPPGQLQSGSGNQYAVYSPW
YRSWVAHIHADLDLLELFDPPNKNPGTARKRFKDLFDCAVPEAPSNKQLT
KEEKRRFHSLWPAGENAAKERLDKFCEEKIGGYSDNRNIPAQDGTSSLSV
HLASGTISSRTCVRTARDRNKTKKLDGGNEGIRVWISEVAWRDFYKHVLV
HWPYICMNKPYKPEYSNIAWSYNEEHFKAWCEGRTGFPIVDAAMRQLNHN
GYMHNRCRMIVASFLSKDLLIDWRKGERYFMEHLIDGDLASNNGGWGFSA
SVGVDPQPYFRIFNPLLQSEKFDPEGVYIRKWVPELQDLDSKEIHDPYGR
GAGAKAKKAGYPKPIVNHKDCRDRALNAYKEGIANGM
>Mb|QLI65156.1
MATPELTIAKAALSASLFRADPASISRPSVDTFFQQVTSTLTQCSRPNVQ
VCTKPPFEKFFVPALVHAKIVSLEIAIQTCKDYILSNIIHSSGRSTALAK
YLVALSNAQVDDAAHPRPSTKRRRLHVLYIVNDVLYHAARQGNQDFRTTV
EAYLPPLISAAASFEKCPKHIKKLQNLVSIWKSKQYTSDAVIPKLEEALA
GSTVSATPEPVNTSLKLAKEAPFILPSFHGDTSTAWYDLPAATWLPHLVP
NSTKPMLPDLIRPIQLAPGPADNVVTAAVKALLSDAERIFSRDRRPDDAH
IDVNEMGERIVLDEITGEVIGGETQTKRSIRVSIFSAQLFAQPPLFITIL
QTQAHVTFQKPQPQPQPQQKLHTPISLALALTLAITVTNPQPPQILQQQS
PSRLSETISPANRQPQYAIQHAHTSPTGRIHRSLAATASSPPSTRLGARS
CLCSSDDGCVERASSPATSTNSPSPSTI
>Mb|QLI65159.1
MPTIYSSLYSNFIRQGSTVAKSITTHGYAQSFVAATHPHVLNSQNRPVFG
RRHTNRLGLSALQFHSTFHKDLNGASDHKNAHTHLPLDAYFEALQKHQAS
GELDKEWTQFEFPKQIEWKPSAATVLQGEDAVVAEAEALAASETHSAISA
EDEAALAHIDAALERELEARKLLDAAENAPAVGQSSSVPLSRTGTPSGRR
SKSPAIARTSSPDVDRQSQSYADHLFKLSAAGRYAEIPAVFEAMLATGVK
PIASAYNALLVAAIHIPSKKIEIVSKALDVYADMVRRRISPDSDTYDILV
GLLASRSVEVSEMKVALEDKRQRFGGMDEPGKFMLASHELEHAILCEDDR
LDLAMKLFNSSVDVDAAMYSSETYHQLISACAESGRVSDMLRLFEHMEST
KTVPFASIFPTMISAFAERGDLVSAVECYNEYRSLAVAQDGGKATLHDRV
DSEVYAAVINAYVVSDKIEGAMKFFEKIVKEYGVRAKDIKDALVSTGFVK
GFISRGIYQEAFQWAQSVEAESQSEAMIRIATVAADHNDNITATEAYANI
VAEPQALVTPTTALLAMSIRGGDVAAATKYWHALNNPEVKATVDFIEPTV
MYAVALIGSGQVAEGLAQSEMMFKRIRASEAELAEEIDEGVDFIRRYMER

RGLTDPREIASQTHNGTPFMPTPSVASFEDTFDPYAHNTDFKGSSLIAED
LEGGHGRKGSRLNDALGRFRNIRRAGRHPRYITYAKLISAAARDGKMDLC
HELFAMARTDMPLLAQYAVVRYGWFSILDAMVAACLTLGNRGRAEQYHQE
LLEMGAAPSANTYGLYITTLKDSTRTFDEASAAVQIFHRAKSEGVEPSSF
LYNAVIGKLGKARRIDDCLFYFAEMRASGIKPTSVTYGTIVNALCRVSDE
KFAEELFDEMEAMPNYKARPAPYNSMMQFFLTTKRDKTKVLAYYERMKAK
GIAPTSHTFKLLIDTHATLEPVDMPAAEKVLEVIRASGQPEPVHYSSLIH
ARGCVLHDIDGAKKLFDSVVRESLVPVNASLYQALFESMVANHQVADTEP
LLAQMRQKRVELTPYIANTLIHGWAAQNNISKAREIYDAVAREKREPSTY
EAMTRAYLAVEQRDQAETVVSEMRTRGYPSAVVNKVLELLGGGREAPVL
>Mb|QLI65160.1
MNHARVKALKGNGKTTSKKAIKSGRASGNITPRSSPLPSLLTSPTHSATH
SRVTSDVSDSEDEGDFEFDDTMSSVHSGGSGAATPDEGGTSTFDAQALIE
GLQDKKHNNNEVREQFLETYLRTLRNRYSADTHLWLDPAAPELVEVFLKD
ADRAPTARERLLNLQAFCLTVGTTDDLEIFEGAERTLKQILVDDDDDQCK
TYAIYALGMSVLYAGGLEESALEVMQYLLDIVHSDGESIESHDNAAVVSA
AIVGWSFVAGHVSDFSDYADTAMDAFVDQLDSSDTEIQCNAGQAIALIFE
SSRHHEQETGEPFQLPYDPQRLIGRISELAKLSSKSVSRKARRDLREGLI
SVVTSLERGVGPYYSTALYLPEKDSYVPPSQRTEDGQAEYGYRCRLRLGN
HSTRIDTWSLLSRVNMMKILFRGGLQHHVFTNPVVMECLEDAHWGEEQAA
AADYGGGGKPSKGRKR
>Mb|QLI65166.1
MAPPSSSSSSSSSSASPSASPSSSSSLARINNIANHISPAMASATSFPAD
TVPQAPEDPLFGLARAYKADQSSLKVDLGIGAYRDDNAKPWVLPVVKKAD
EILRNNPELNHEYAPIAGIPDFTSKAAELILGADSPALQEKRATSIQTIS
GTGAVHLGALFLAKFYKGNRTVYLSNPTWANHKQIFGNVGLQVADYPYFS
KKTNGLDFEGMKAAIQAAPDRSVILLHPCAHNPTGVDPTLDQWKELAVII
AQKKHFPFFDCAYQGFASGNLAQDAAAIRYFVEQGFETVVCQSFAKNFGL
YGERAGCFHVVTSPGPDASTTISRIGSQLAILQRSEISNPPLYGARIAAT
VLNDPKLFAEWEDNLKTMSGRIITMRNELRSRLEKLGTPGTWNHITDQIG
MFSFTGLSEAQVLKLREDYHIYMTKNGRISMAGLNSKNIDHVASAIDKVV
REGQ
>Mb|QLI65168.1
MSTTPQNATPSSAEFLSRVQLPPSPPIRLQPVSTDPTESYENTKPNTEAS
DYWNGGQQHKDVSPTANGHEVAPGQVDQKRSQPSPGEAEHSVEIPAASSG
DHDGSAAAGRRSVQFARQDPQLLSAGGHSRQGSWDGPETPGRHKSLSIMS
KIKALASAPSTPSLKSPIAPPFAESASQSATNSPTSTRFGTRLPNILTEE
QSDAEADVDADADADVDETADESAIPENGTRKKKKRRMRRFAKGLPSTPS
TPSRFASDADTWGAYNRLLRRRASMPDTSMASENPEQVLSEGDTRSGPKR
RPFRRGHSFMGTTTQPPGEGDDLNDMESSAVVGRRNGHLRRITVFGGGGV
SDGDAMTPRRPFFNSERASNFGAQKWKQVKSTLKLLRQKKEDRFDYFKSA
ELMAELRAGAPAVLMLASMIQRDEHGNKRIPVLLEQLKLKIKDSSPIPDD
DKDRHWIFTMELEYGSGPSRMCWTVIRTIRDIYNLHFRYRFALNNDKYML
GRDLGGRPKQPKFPYSAFPYLRRARNKDDDSDEDERASVRGDETAGEGTG
GEGTAPEDHGDGTHDEQHQPTRKKSRANLWGMRRRSTGFTDPGELSNAEG
TAQAIDIATRRQRYVEKQRRILEKYLSEMIRWLMFRADSNRLCRFLELSA
LGVRLAAEGSYHGKEGYLHIQSSKGLDFRRVLTPAKVIARHSRKWFLVRQ
SYIVCVESPENMNIYDVYLVDSKFSISSKKNALQQIGSKDKAKEIDLTVE
ALPQKHHTLTLHTSERKVRLFSRYQSVMKQFEDSINEMLKQTAWYEKKRF
GSFAPVRNGVFAQWLVDGRDYMWNVSRAINMARDVIYIHDWWLSPELYMR
RPACISQKWRLDRLLQKKAREGVKIFIIIYRNVEAAIPIDSEYTKFSLLN
LHPNIFVQRSPNQFKKNQFFFAHHEKICIVDHDVAFLGGIDLCFGRWDSP
QHPIVDDKPTGFEMTEQPKDAEHCQLFPGKDYSNPRVQDFFRLNEPYEEM
YDRSKVPRMPWHDVSMQVVGQPARDLTRHFVQRWNYLRRGRKPTRPLPFL
LPPPDAKLDELEALGLTGTCEVQILRSATTWSLGTDETEHSIQNAYIKMI
EDSEHFVYMENQFFITSTEAYNTKIVNRIGDALVERIIRAHKNEEDWRCA
IMIPLMPGFQNTVDEQEGTSVRLILMCQYKSICRGEQSIFGRLRAVDIEP
EDYIGFYCLRQWGVMSNDALVTEQLYIHAKTIIVDDRVALIGSANINERS

MLGTRDSECAAIVRDTDMIESTMAGKPYQVGRFAHTLRLRLMREHLGLDV
DEILEEERQQELDRDVFEKEMEEIYREDNADIGVGSSKTRQDSLLTPRQR
SFNYELDMEHAKALQDTSLPSSESESESESEAGSKKGKEADVGLSPQQKKQQ
RLDVSGYGEDQWKLAEQSGLDEGRDSVIVGGREVLVHGMNTEGRGTICQP
KPPHELVIMSDNRHLGNEGPGTGDLPPMPAMNRRTTEQLGLLRPAQLPPL
PVADDTDIGGPAVHVDPTTGRPKSGAFHPMAADITPTDISKDCMRDPLLT
SFIDDIWNRAALNNTKIYRRVFRCMPDSEVRTWQEYREYVDYGTRFRASM
EGKPNGEESESKAETNNQVESTAGGAGIGAPGPEAIVAAAKEKTMKQLPF
LEQDMGKLDITVAQNGDGELNEKSMEPAQQPRGPLDSNPVQRSEKIADAS
PTTVPNGHVGVPVPEEAPAKLLEPQASRNTDRKTAFATLDKPPSRETPNI
PSQAQFGSVKKRRRANTKGSRRGFSLEDMPSRADAEELLKLVQGNIVQFP
YDWLLTEEQNGNWGYQVDGVAPLSI
>Mb|QLI65170.1
MSFQPTQIFEEGTTEEKGENARLSAFVGAIAVGDLVKSTLGPKGMDKILQ
SASTGDIMVTNDGATILKSIALDNAAAKVLVNISKVQDDEVGDGTTSVAV
LAAELLREAEKLVGQKIHPQTIIEGYRIASQAALKALEESAVDHSKSPEA
FRKDLVSIARTTLSSKVLAQDRDHFATLACDAVLRLKKSSDLSHIQIIKK
AGGKLSESYLDEGFILDKKIGVNQPKRLEKAKILVANTSMDTDKVKIFGA
RMKVGATSKLAELEKAEKDKMKAKVDKIKSHGINCFINRQLIYNWPEQLF
TDAGIMSIEHADFDGIERLALVTGGEIASTFDHPEQVKLGTCDLIEEVII
GEDTLIKFSGVSAGEACTIVLRGATEQLLDEAERSLHDALAVLSQTVIEP
RTTLGGGCAEMVMAKAVDGAATRVDGKKQLAVSSFAVALRQLPTILADNA
GLDSGDLVSRLRKTIYDGLTTYGLDLMKPGGGIEDMRDLGVIESYKLKRA
VVSSASEAAELLLRVDDIIRAAPRRRERM
>Mb|QLI65171.1
MAVPPIIHSEKAWVTLITNESYLPGLLTLNHSLRTVKSAYPLIALHTPSL
PSSCIAALSRRGIPSIPVPYIAPRSGKKYLEDARFNDCWTKLIAFSLTQF
SRVVQLDSDMLVLKNMDELMDLALDPVSLSESGSETSKRVFASGHACVCN
PLKKPHYPSTWIPANCAFTHQHDNPDLAQVEGADPARSLGDLNSGLLVIN
PSEVLFEQIIEHMDAHGETYAFPDQDLLADLYRGRWVPLPYVYNALKTMR
TADVHGAIWRDTEVKNVHYILSPKPWGELDEQGRWKGESEINGWWAEANR
RRLEREKEEGI
>Mb|QLI65173.1
MAEPTSAPAHADYETRFPRVTIQFCTQCKWMLRAAYFAQELLSTFSTSLG
EVSLQPSTGGTFVVTIHHQPRDQAPRAVSTVLWDRKTDGGFPETKELKRR
VRDVIEPGRDLGHVDRHRSAHAASKTAGESSGGADGAGGPAAAAAASGAA
MTDTPCEDCK
>Mb|QLI65174.1
MSQPGIVLAFNPKQHSHLTPYIAAIHASCITQDRTMATFLPPLSHEKLLT
WWKERIAEVNDGKRLIWILVKEIDASGRPKGPDVMGVVMLSTPYSETGAF
RGYVEKLFVHKNFRGRGGARALMEALENGAANMGRSILLLDAETNSAAEA
VYRKFGYVELGRVPGYGMSPTGELKDATFFYKQLQ
>Mb|QLI65175.1
MATPPASTGTIVFYDIAHRPPVAETCCSPNPWKTRFALNFKGVSYSTSWV
RMPDIAKVRRDLGQSAGRKFADGTDFYTLPVIQDAASGATVGDSFDIAVY
LQRTYPDSGGGDLLPAQTLDYTFNQSVAFPVPLSERPDGGFAEYLRFNTN
VDAAFTTHTPLMVEGLPLDPATADETKAEFVRRAGVGSWNDFTLTGEARE
KLTESFRGMLGDLSKLFLENTDGPFLLGQRASYADFIVGAWLRMAQATLP
KPEWEDVRRWHGGVFGRLHDALEAYAQVK
>Mb|QLI65177.1
MDQPSEQYQVQGMSSNVQDLPNISTPSPSTDLGLDSTDGFLLALNCDNAD
PSSHLWGVDITPSMNTFGNSPASQSDSAVDSPASMDTPKDTTTTNRQKPR
SIPSSFASPPSDSTESGETEKPDEPYAKLIYKAFMTRPNYSMTLQEIYQW
FREHTTKAVTETGGWQNSIRHNLSMNAAFRKRDKVVSKASRNLSSATEAS
KRVNEWVLEDWAVRDGVQSTTRYRQKTYAKRAGSAGRNGVETWAVGHSVK
RALSGRKGGCATRAARVGRTSPYGQLMQFPHHSQQVLDFRPVSPTYASVA
DIYDMRPALPGQYTAMPQYTSLDAYREAIMGRCGGNATSEPQQLRPADSN
SQQAMANGGSARLANHASPCDEHTAQSSRMPALDMAFGPGEVQRHASVKY

EDAEACVSTLPTCRPYQPLQGSSYLWWNQTGQ
>Mb|QLI65180.1
MPSPQQSQPGSFSLFPSPNASKPPPASRSLQSYRGRRSESQERTPQTPQV
QRVATPDHTQARGQPSYTPPVQFEPARAETPSNWPLSSETPQNSEAPARH
HGLETTADTAAVQQPRNIHDVPGRAETAFSEANTLVRSNSGRSRNSIAKN
PLRDHPSPASASQPLRSIFPTYNPDVSLNEQEYAPTQMGPAHIPRAIISR
QSVYEEPESPGTQSAPGRSPPSSPRRPAARGRWPLRIQTQPPPETPTPCT
TEDLKSLWKVANGWKASVSESRVFCLKLSRQKDAPVYTLSSRSSQPFWSL
RLDPTSAAAYISLTRHDPAKPCKAPIPEAGTSPGAPASSSGSRTDAKHRH
EALSSTLEEESRKHPPNDGLVALLMPAAAARMAVDKADDAASVAAAENEC
ARLVWDEDTATHFLVHNALARPFRVAVDRSPAYSRVEYTLEHDESPRHLA
KLTRDGTGGGWVELDTGVAAQIDSFYILDVVVAALLLVAAGEDRSSPTPM
ETFEPPPRAPPAAALGARRGSARWSKLSIRRDGGKKKRHMEAFEMDVESQ
NDSLGKGPHGRRSAGDKPPFLIRVVVKLAKGVFKLAIWVLAALFKAVGGV
FKCLYSCVGSKY
>Mb|QLI65182.1
MSTAAVAQPQHPIFSSTSSHIRSAPKKNMSITQTYYLAHKARAKLSREAA
QPDHDLRLLVGHANLLDSLMLELADAEREQERWFNQSVRGASNPKPADRR
VQWADRIVEQPEEEYDDDSSDSESDSDSDFDEDDVEMTNAVSARVPVAAQ
RAQLVNVDDEEEMGDDLEEDYAQLELVRTPSHSTSPPELVDHDSESSDDE
SMPPSPADASLPYNEKEAAKEEGEEIFNDEYYTSQQTSAGLVTAISVY
>Mb|QLI65183.1
MPSLLGHAEPNGVFQETNGHRRPETPAGRMALTDYSINPSPSSEEKRAKL
RQAVPDDLLLPNGYPDYLRMIACATSRVYEACKVTPLSPAVNLSNRLECN
VLLKREDTQPVFSFKLRGAYNKMAHLDPALSWKGVVCCSAGNHAQGVAYS
ARKLKIPATIIMPEGTPSIKHMNVARLGGHVVLHGADFDAAKEECARREK
QDGLINIPPFDDPYVIAGQGTIGNELFGQVNMSKVEAIFCCAGGGGLIAG
IGFYVKRMAPHVKIIAVEAYDANALAQSLRKGERVVLNEVGLFADGAAVK
TLGEETFRICQDVVDEVVEVTTDEICAAIKDMYDDTRSGLEPAGALSIAG
LKKYVSQHPSQDPNRTLVAVTSGANMNFDRLRFVAERAAVGEGKEVLLAI
TIPERPGAFAKLVDTIMPYAVTEFNYRFADQNVANVMIGLSLTAPPAKRN
EELSMLIERIRAEDMVVTDLSGDELAKSHLRYLVGGRSGVANERLYMFSF
PERPGALERFLKALRPRFTISLFHYRNYGGDIAKVLAGISCPEEDSGELA
QFLKDVDYPFEECTNSEVFKTFLRS
>Mb|QLI65184.1
MARPALVCRAVGPYAGRYLTVFEPSSPHTITLATCIADELIHAFDQTFLL
STKGRITSWADALEEGRKDYQERRDHFLKFIRHPEALTELTVDPLADDPD
SPWNTVRQDEIIRTEIQQDVQRLPDEVNYHEDAVQGMILDILFIYCKVNP
DRGGYRQGMHELLAPVVYALEQDSIDSEASGNDARLDAKMLHVLDSAFIE
HDAYILFSKLMEQAQSFYEVVNGSTPSNHDSQPVIMQEQLSAIVERSRFI
HEICLQKVDPELAVHLTNIEILPQIFLIRWIRLLFSREFPFSQLLVLWDT
IFAVDPSLELIDLICVAMLVRIRWQLLAADYSVCLQLLLKYPPPEQPHGP
HTFVDDAVYLRDHLDFTGGSSLIMKYTGRIPENPNDIKPTQPSRGAESEA
NSVRQLGGRPPISPSRLTQQPPSMEAFFQGAARGANRVLERGEKLGINQA
VRDAMGEIRRNVQSFNEARQAQRSPRSILSDEGASKVLAAMERRNKELAG
LLNDTVTNLKTISLSGFEDEAKSLELIEVAAAKIQFVQIYLEDSTMDVPV
FSTPKNKLVSSPEGKEKVVEEATETRATTANQLHVAPATSSDAPAPDVSK
LEINEEKHSSKKDAADPEKMVASQDDQPGEAPPSDPLGNNTVKVRPAPVP
TRSTLAQSSFSWMLEPDETTPSRAPAASKSPPSQHGKRHSNNLSRERNAF
LFGEMTAEATDRSAPPKTDDIFGMEPIAKPKGKTQGLFHDK
>Mb|QLI65185.1
MPSLGFLKKKRTRDSNPDPSTSTPTSPVAATSPSKPFDSSTLQQRTSGSQ
AKTAPGTAPAPSQGSAATAAPASQQQQMNPANAQGGTFVTPLPMQTNQAD
SQSLPSINNLIHQPQNGASTNNYSSFNPAALGAQISHPPSASPGTDPGHM
QQQQQQAYPQPAMQPQQQAGHLPDQQQQQYQQQQHEQRVHHQHQGSVTRV
TKGKYSLGDFDILRTLGTGSFGRVHLVQSKHNQRFYAIKVLKKAQVVKMK
QVEHTNDERRMLSDVKHPFLITLWGTFQDWKNLYMVMDFVEGGELFSLLR
KSGRFPNPVAKFYAAEATLALEYLHSKNIIYRDLKPENLLLDRHGHLKIT

DFGFAKRVPDKTWTLCGTPDYLAPEVVSNKGYNKSVDWWSLGILIYEMLC
GYTPFWDSGSPMRIYENILKGKVKYPAYINTDAQNLLERLITSDLTKRLG
NLYGGSQDVKNHPWFSEVTWDRLARKDIDAPYTPPVKAGTGDASQFDRYP
EDPEKYGGIGGSDEYGHMFTDF
>Mb|QLI65186.1
MDPDEDVEEYSVDEENFASYESEAANAHLRGIASSYSAVNHSDSDSSYAL
SEANHVVSEDDDHLASRSLLKRSAEQAFNNLPFKRQKGVLNPEYLDLLNR
EIDDAAHQVCLEDIPEGKSDLRVDLPGSQVGLTFWSTVEKAHFFESVSRL
GKYDLPGIATRIGSKSEIEVKQYVELLQTANDSRQSCLRRRSCLEPAEYP
AAVEISQQCCHFQEEAADTVSVLQERREEQREKLKWGQKWDVTPSLARRL
GHRGEESDDTSDMTLKFSELFHLSNWLQLSERVFMNSSVPGNNWNYIDHQ
RPSMWATAFDDFYSIAVSVTRRLVQTTLFISMSRIRAKKELVPTTRDIVR
RKDVEAAVASLAMPHDSKSRWIKSARRLRLDVFNEPPDKDDEEAEEEPMT
YEEVETILSGNNDGIEIQAPKDVILTDEASLDGDLKESDESRDNDTGIDS
ETEDDDDEKHEITREANEILLYSTPELKDIQAAKQALKLRITTERQQEAQ
AEMHDEYASYQAEIEMWDILKRKPLMELPKVEDPGPVHRSILSVDNVYPL
GRDWASKLEYYGEWETLDTARHDE
>Mb|QLI65188.1
MAQFVLDLFYSLGNCLNCFPGSPTLKINSRSFKILRLLGEGGFSYVYLVE
DTATHELFALKKIRCPFGAESVQQAMREVDAYRLFAHIPTIISAVDHAVA
TERGSDEATKTVYVLLPYYRRGNLQDMINANLVNRGSFPERHLMTLFLGV
CKALRSMHEYQAPPAERMEMGNEEDAPGANSRRGTGSKMGTRGKRTEADE
EEDQERPLMESENRVSAGGKVRSYAHRDIKPGNIMIDDSGSTPILMDLGS
VAPSPIPVTSQSVALQIQDTAAEHSTMPYRAPELFDVHTGSVIDTKTDIW
SLGCTLYACLVGKSPFEMRSDETGGTLSLCVLGGDWRFPDETPGGAKRVN
SISQAKARAASGDHSAASASENTNPTISEPVRNIVRQCLKVEPAERPGIG
ELITMVEGVIEDLPDEGGL
>Mb|QLI65190.1
MTAVDVILEARDARLGFNFEFLVFFIICGLLTVFFLLYFNRLFASVVSYA
IRTYTWHRYRVYIDIKAIQISLLAGRIFFTGLRYHGSNETFLVQHGDITW
RYWLRRVRDADINVSNFSHGSSVSDSQKNSNLPCRIHARLVGVEWFVYNR
SPAYNDLLNGLLSDDPSASKSSGISNATQEAGLTSRRGYQNGSAEKNKLA
VDATAQAQFSEKPSQPPPSSPPERSSTASQSVSETVDGNDASHSLPAMLQ
LLPIHVEFQKPAVVVGNDNTKAILIVKANSASTIVDAGPTNTVDPYRQLF
KIEFDHPVIEMRENDEFKEDQVARATRRREDTSSLETPAQRSFFRNRRRK
VFNSLRNMVPYLRRSVESFSADSRAAMSTGVSQIPGSGHWQGLSRYLDDR
YQDDKARWSSVEYGAVNTILDSPSAALTVYWDSVSKITANARRHSSSSFH
ASINGTEPPAWGMSLSVKGGTVNYGPWADRQRADLQRVFYPTLSKNSTAA
VPLAPGSWRVATQFNLFVELEDAVTLRIPTREESKNWRWRGKEPPVGRQA
VTGKKRRNRNKKSSKGNAQPARPAGWLEIKVPSNSTVSYSMDMLGRSTGY
QNRLDIDLPSTELRSSVNHDLMWKSGPQRISCDLSNPLTWNTLRNWSFNI
TCEEMKLYLLRDHIFLLIDLVDDWATGPPSEYLVFTPYIYHLKLNLNNLQ
LYLNVNDENIIDKATAHDDNTYLILSSPILKAETSIPLDKFRPSRNTIPF
DIRTDTFDLTLHAPQSNTQAAFLSSNELGHGESLAVTGSYEYNATTSVGN
TDTLILDVHGQSPYAYLYGFIIRYLILLKDNYFGDFVHFRTLDEYQEQLQ
LEAQKSNTDAVHRPPNKKSNDLDVILSVRVDDPRILLPVNLYSASRYVQC
ELANLSVDLRFTNYYMDLELLLSPLSLSLGCASLSPGSPNSSYTNTQLFV
DGVRVFGHRTFGLPPSEPTYLCNWDVSVGMVDGECTADFLTSLAKGGVAF
GFMFDDVENALVPYSSLVFHDVTFVRVDIASVRLWLHVDEAAFLLSTGSI
QVRSNDWGGTHYSKRANISIPKIEISCVNAESASRHTSRRNHPVDTAAYL
KTDVQLASIGRKFHFQQELKIQQDLIRREDQRTGRTPFLLRKPVDPNFVP
EPFDPPAQCVPPPAFPLTEVDDDDDASYRSIGTSRFSGRLRRQSSFLSSS
SSNTSLRGPRSQPRSRARSRQTALSTQPSGTEKERRVSGQRASVLSQDLR
LAAIQQPSQDWQQKEHIEHSSVAFSSQYFAPHFPLGGVRPDGREATFEEL
EDEPDNDFFGTDASLSDLDPNSLSDNHAQASTMIDFTTGITAFISPEAVR
YVNSLLSALQPSEPDDILDSLQTSTIADIFSEKKQSHITGSMQDILIKLP
KANIRFLNSSMLDSPDASQEEQDQYDIRISKTLLITRTVKDRTGDNVTSR
TSLHFRLKSAEMSASERLSSIETPQAAIMVNIDDISVSLGAKDITYFDAD

IGSIVGSTASGKVEYLASLIHRTGSIASELGNLLSETASRNNNRTKYLVS
QLLEDGHATNDPPFLIRPSAVLRSTHEHMRTVDSWKLMMRLRQIWATLSQ
SQRRELIDKCRDNPPEAHPDVADHVISSFQKWRSWDLDNPRNAVLMKKIF
GKAINEKEEPPQEYPLLGACQLGELQFVLDPGPKQNNIGFLDIALRVDKR
PPGQPNVFSEMKDFDGSLTILNLCCAEAAIHLNWELCELAEDVLRLYNKA
QPQPSEKTMSSKQPKRKVAQSSAAYHVVFEVVKGSIEAETVNLTAKSIGD
GLKTSILLCNSGEELSVANAMLNSNAVMSKLQSHGKILGVLQIQKPSIFV
AHELHKTTDIDAHVVKVSASSPNLTLRVKDDPIGLLEVLDLLIRDEAAQV
YRLKELIPSPSEGQNKRDKIKQRISTFVLNVAMFLDGYTISIPLLQSLTY
KITGSVARASCAANFEKDIVFDFDIKENSHEIQINVKNEPRSISLLEIPP
ANGRITSNMQSTENVISVLASMEVIRLDASAIYSLLAALNRPQISGAIEE
AQQQVKIIQGHVSETFGTHREMKAENASLPTSTPNSAYNIHLTLAGLRIL
ARTHLKTVEEPMAQILFALDKVTLQASTQQDSNSSPLKYPDLYVNLRQIS
LDISRGKENALRSCGNLTTGINISASSQRGDDGREDWSLHFRNDDLTLNL
SPETVSTIVDVVGYMGSKIRDLDTSRELEYLRKLRQSRPKIRINDDEQDW
GEETDIFESVLSSITYRFELRNIRFCWDVADGTIDLDYTKEDLVLSIKLI
EFGTRTRKSARLTIEDFLLQTVPPGQDKAIRSLHSALLPEVMFNVAYVST
ADARRMAFQAVGDSLDLRLTSAFIVPAANLIQSISLSMKNAQAASSQWTI
EGPAAVESLTSKKTEDLTPPRQSQRRMFGNKRLENLLVDADFAGAVVYVS
TKRNAASAVSESKYSQPSLAGKYGQFSADDSGSGAVLRSPGLAWKAEYRD
NGKEDPSLYGEIKINASSNILYPSVVPLVLDILSSVKKVVSDNDANADTE
QTPEQLEQPKLKQTKSGDEDNFLTADPSTVLGRLRLNLGLRICRQEFSLS
CQPIARVSATACFDSMYFTLITVTSQEQGNFFAISGVIAKPQASVQHVYS
RDSTASFELDSVAISFMNSKHFSGTSGVSAILNVGPMKVSVNAKQVQDFL
LFREIWYPEELRQKSAVQVPKMPTETSQAHLVQRYQEVAATAAFPWTATI
SIAALDVCVDLGQSIGKSVFQINEFWVSSKKTSDWEQNLCLGFKKVGVDC
TGRLSGFVALQDFRLRSSIQWPQREEALNETPLVQASLAFNALRVKAAFD
YQAFLVADITSLEFLMYNVRDGRDGNGDRLVAIFDGEAVQVFGTTTSIAQ
AVALYQALQKLIEERRENFKSSLREIEKFMKRKSISSRTMLQTPPAVPKL
PVDDTLSKSPISLDTDVVVTLKALNLGVFPSTFSDHQVFKMEALNAYARF
AASIEQRKIHSILRMTLGQLRIGLAGVRDAEAPKMLNEMTVEDVVQRATG
SRGGTILKVPRVEAVMETWQKPSSNHIDYIFKSAFEGKVEVGWNYSRISY
IRGMWANHSKSLEQTWGRQLPMTAVKITGVPETEGEQQKITAEVNVPQSK
YDYLALEPPVIETPQLRDMGEATPPLEWIGLHRDRLPNLTHQIVIVSLLE
LAGEVEDAYSRILGST
>Mb|QLI65192.1
MPVPSTPAPLAPSPPSHSNPGAVTEVSNQRQIASRPLSKASQRPAAGSSA
GTAGSLAPASGAPGPPSRAPLPTKQPKANTSAKTTGGPVPLPRLSAPLVD
KMAMAAVISPSPTAEAPKISMTSKEWVIPPRPKPGRKPATDTPPTKRKAQ
NRAAQRAFRERRAARVGELEVQLDEQKDAHQQEEVKLKEKIRNLELDLQS
FRSRCMLLENMLDRERQERIRAETQAETLKSRHGDDFFRSQSISGPRHSH
SQPHSPVHHHTRHSLSGGRNEHLSQGRNFSISNIITPPESLDISSNNPEA
DAAITCGNCSPNSRCACAEEVLASTSAGCGKCGFGTACQCLDGMANNVSA
GQDMKRPLSPSAGSSDNKRHRPDVDIHALTETDFTAMFSRKPAADATPQS
PYDQPPSVTQMSSEESMAFKESCGFCKDGTYCVCAEDTAGMATPAMTPTE
STQPISHQTQTPPPSEPDILPPPLAMEMTADGAVKLPRRIQGKKPVHKAS
PSSKGGCGANGPGTCAQCQADPKSGLFCRLMNAKYSREQGSGGGCCGGKG
AGGGCCKSKSKAQPVPEKITLPSLPSLGLSCAEAYQTLSSHRNFSKAADD
ISSWLPKLKTTQHGGPVTPGRQAIEVEAASIMSVLKEFDVRFGREC
>Mb|QLI65195.1
MSSGASTTLAHRSVATIRSGTSQAPASTSTPHTPPRTVTSSFGSPSTIRA
EDDFVLLEIGSRYIRAGFAGDSLPKACLSCGPGQQRRVGDFRAWQEPASG
PSRKWAADHEIWRYDVRDVDLGLFRDKLDRLLREAFTRFLLIDSRPRRLG
LVLDSAVPLPLLSSVLDTLFTNFQTPIISLMSTPTMVTVGAGVRSALIVD
MGWNETAVTSVYEYREVKSSRTVRGGRSLNDALYKLLHALIPSAGDDESE
DRAVAFEECEEILCRLMWCRPSAFKSSQRQSTQLDTVEEQDENEGESNQP
SGIAEVPLRSTNPPSTIHIPFNKLADVCDDSFFDPSAARTTFDDHELPLH
LLVYQHLLQLPVDVRAVCMSRIIFTGGCSNILGVKERILDEITSIVERRG

WEPVFGQSVEALRNGRRAQRKASLQRSSTSSVGTSESGGDDSDGLRSESG
TTDSQEDQVEAKITRNRPIRPQVQGRLRAIHSLGAWAGASLMCQLKIPAL
ATIDRELWLQQGANGASRPCDVDFKNQQRQSMGTIRGTGGHHTNWTLGVW
GAL
>Mb|QLI65197.1
MHSNRPHPLLEQVPLTVSPFVSLPTATTLSYNYKTMPSNIPPSAMGIPTA
SEDPSQQPKARFVVSGSGHAAHPDDIIDSCRALLDHVTKVQESAERELQE
FEDQIKQRDLAEKRRVAPGWLDSESRLLQPERMSAQAGPTAGATAQHQQQ
QDPPPMGAGTAQPVVDAGAELDRAFGGMALK
>Mb|QLI65200.1
MATDNKAHLLPPSYKTQITAWLAEDTPTFDYAGFVVGEVPRRATLLAKSP
GILAGVPFFTEVFAQTGCSVEWHHAEGAPLDPSSSPGGKVQVATVAGPAR
GILLGERVALNTLARCSGVATQSRRVVSLVRGAGYAGVLAGTRKTTPGFR
LVEKYGMLVGGADAHRMDLSAMVMLKDNHVWSRGSITDAVRAAKAVAGFS
VKVEVEVQSEEEADEAIAAGADVVMLDNFTGDGVRAAARSLKERWAGRSF
LLEVSGGLTGDNVEAYVCNDVDIISTSSIHQGVPHVDFSLKIEH
>Mb|QLI65202.1
MERKRKLPPRAAARQEQAAKKRNVTTRQSSTPAVAEPPPREPTPSPPPPP
PPPLPKSIEVGKPLPTVEEPQPTNLSCKEYQSVSESGVMSESLSRSRHKW
ISEGLFEKFWSKPHKRKGVLQEDPNNPPKDSMMKVGLVTLTIEPHIVEAT
MYAVKTSKLLPHQQQRFKPNPQPPGQATPTGQPQQPTVVQPQPQQQQQQP
HPNPSAPAQQPVLARPVLQYGPPNGAMRPPPIPTSPVTTTPTSMATPAAS
TPGATPAGAVPVSTYSTSAAPTSAAPVSPKSSSPALPAVPPASSPAEMHV
KATSSPNPGPPSVAQPAQPSAPQQGQPQAQSQGVVASTPTPTPAPKASQH
SSPPPNTAPLASGAHPPSSTIQTPLQPAGASQLITRPVANAPPRQAVVSS
PAAGPSSSTASRPAGTPNQAQPPAPSNRPPPAAPRAAPAPAAKPAANDPV
IALLAQKASADPELRDLMKRVAVGQAKDGELAKFQKIIDQLNAEYRQRGG
QQGPSADRLLVDGRTVKYFADEVRTILDIVLASNPNQKSSELRPPPRSDP
LVVLLVKTALENQRIKDMIRRIAEGKPGFTDATDLKQTLDRLHRDAKITP
KATTTPGPSPLRQPPVNGSPAASQPKTQAPQGAQQANQQIKSKASSHVNR
PDLSAVVFDFGSGDRCLFPRYSILEFLPSQSGQHVVASFLIVRKGSASEY
GGDPKLDYYQPVTIRLQTTTGRHLENLARVVAPQDEVRRYMDDVMDNMTR
AEYVLLAMRLPRVDFDPQEAVPVVEDSKASSPTPKSEPETDNNTTPKPGV
LWTTGSSNKATVSALPVKLQDPEQEAQSKYERLIRSVAEKEVDGL
>Mb|QLI65203.1
MTICQLPPSVALITRRSLLIPATTMGSQITASSLPQLLEHDTCVKLAGID
VDGILRGKLVSKKKFLSIAEAGFGFCSVIFGWDMHDQTYLKELKVSNAEN
GYRDILAIPDLSTFRRVPWEDNVPFFLVNFFDPETKKPIAPCSRGLLKTQ
LDKVHAKGYGAMAGAEYEFYTFRNPNSDSASPAGFLLQNPPHQLPSLTEG
MFGYSLNRPVHNKDWYYDVYNTCARFSCDIEGWHTESGPGVYEAALEFGE
VADMADRASLFKYVVKSVGINHGITPCFMAKPKQGLPGNSGHMHISIVDG
DGKNLLARDTTDPDAKWNDIAALSDLGRHFLAGLLEGLPDIMPMLAPTVN
SYKRLVENFWAPVTVSWGLEHRAASIRLISPPTSKPGATRFEVRTPGADS
NPHFVLAAILGCGWRGVEKKLEIPCPPLALGQDTGGAGDVGERLAKSLKE
ATARFMRKDSIAREIFGDDFVDHFGGTRENEVRLFDEAVTDWEMKRYIET
V
>Mb|QLI65204.1
MPRDSQPPPSPTLELASLSPTSSISPDSPLLTPTSTAPPPRWALPSRTRL
QHLLDGTRTASRAILLRTRRLAPLPRRLSWTSGVLCTLLAYTIACLALSI
PPFASRLPAYTGPHAVGAIDMEVPLPQARLIADPVFKHSHTPAFKLETVL
LTLYYPASPSASSSSPPLPWIPRPISLTAQGYARFARADNFLTRPLFTFL
LWLAAGSVTIPAATDVPLLADGGAKLPVVVFSHGMASSRTDYTHYLGSLA
SRGAVVAALEHRDGSCPGTLIRLPDRPPLQRPHFRASDLLASPTNRTPVD
TPSMKRHQLAFRDAEIFETIRLLRLLDANASVPSTRDPPKSLGSFASRLD
LDTLVTAGHSYGATAALQALRHAPSPSDKGPNPSVAGIALDPGKESGPLN
AALNVSLLVVHSDSWSRQRTLFFGRPHFDTVRDLVADVLERSGRAAWFLT
SLGTSHPSVSDAPLIEPLLLSWTTGATLNVREALGEYVRVSHDFIQSLRT
AKVDGILQEKVTHTEYNKWVSDERKASFPKDMARLWEIHVSPASSNDLKG

PDGQT
>Mb|QLI65206.1
MTVAADLTHRSSAQSQVALEDRFEVLKEIGDGSFGSVVLARVRTAGANVA
RRGSVVAIKSMKKTFDSLTPCLELREVVFLRTIPNHPHLVPALDIFLDPY
SKKLHICMEYMEGNLYQLMKARDHKCLDNASVKSVLFQIMRGLEHIHSHN
FFHRDIKPENILVSTSSHQEASNSFRRYSALVTPPSTPPNYTVKLADFGL
ARETHSKLPYTTYVSTRWYRAPEVLLRAGEYSAPVDIWAVGAMAVEVATL
KPLFPGGNEVDQVWRVCEIMGSPGNWYNKAGVRVGGGDWREGTRLASKLG
FSFPKMAPHAMDTILRAPQWPASLSAFVTWCLMWDPKNRPTSSQALMHEY
FNDAVDPTRPKSSASKILGRKQSDLGRTVKESTSTTQIASLPSWFRKSLI
GRSDVTEPAVVQAPSKDAPIPRPSPVSAAPPVEAPAAKARAPPAAKRSTW
TNGLSNVAPMPILPTIRPISPLSDAVTARANNGQAEIKTKKLGRQLSVAS
STNGYTEMHRQQAERALNGETGLASPPSGHKESFFSHLRKRARRFSGRHQ
TPVSPSYEDMEAQIGCGPWASNRSSMVVDQQGGPIPKAEVYDSLDKALRD
VQGNLDSRSALPASHQPTSNGKLKRHHSLPHQQPRSVDNLIGAARAGPIS
SRTRRAQAAHGVQQYEVPDEEDELLDEALTSARRAMRHMDGSAKPLRQSA
SNLGLATPHPYPSPSPSASGSQVLYGDGQEALTPKPLDLGKKSYNQYKWP
TPPYEGSEWGASASASIWAAGNRF
>Mb|QLI65208.1
MASDLSNAAQVASSVASSLAAPWDPSAAQPLTDAMITASAKVAAAHEPTG
WLGFFGWLLYVVFNLTSTVLYWVVRLATISIPRLLYALFSSSWTVTMNAT
TLMFILVGVVSAVSWVVRYRILNMYSRLPPEPQRKEPEIDLFPDSHEEGN
KGGLASYLDEFLSAIKIFGYLERPVFHELTRSMQTRKLIAGETINLEEEK
GFCIVVDGLVEIFVKSGRHKTAREPLAAHDLDTSSDEEVAPDQQRYQLLT
EVRNGAPMSSLFSIMSLFTEDVRLRLNDEASPVPPNGESDYRRHLSVGES
ETQSFTPAPASMPNTPHLDGKGGQPDDKSVSAEGKSQLLPVGDLHESGSN
IPPISLDPSSPTQPQRPKLPGRVASHSAHPDIIARATVDTTIAIIPASAF
RRLIRIYPKATSHIVHVILSRFQRVTLAHAFDYLGLTGEVLQTEKSMTKY
TVCQLPNILRGDALSRLKEKFKHERARIAGDDERNGIALHNAAATRRRRS
SSTTLRREAMLSSMTKHRPSSVLGSNLSVEESPQNRQHTSSHPGDLANIH
ASRNAGRSPITPGASAKDVTSPLAHRSFNPFDSQRHARISVDSRESLDED
NLFRSSILECMFKSLGLNGSGSSSREPESVDGSPRLLSADHRRKTFGTNN
AFGFMGPFETSGDGETESITSSGLTVHTPPSAHILSHEIKDEVEIVFFPK
GSVLVEQGERNPGLYYVVDGFLDIGTYTTEQRPHNILQTSGTASFNIDAR
NNATRRTDSNKDNGRTRRSVSLVKPGGLAGYIGSVSSYRSFIDVVAKTDV
YVGFLPGAALERIVDKYPIVLLTMAKRLTHVLPRLILHIDFALEWVQVNA
GQVIFHRGDESEAIYIVLNGRLRLVADRKDGGMSALAEFGQGESVGELEV
LTETVRPGTLHAIRDTELVKFPRTLFNSLAQEHPNITIKISKIIASRMRA
AVEDPPKNGAEESGSGAINNQRKSTMNLRTVAILPVTAGVPVVEFGNRLM
NALAQVGPPNGATSLNQSAILNHLGKHAFNKMGKLRLTQYLADLEEKYGL
VVYVADTSVNSPWTQTCITQADSILLVGLADGSSATGEYERFMLGMKSTA
RKMLVLVHNERFSRPGLTRSWLRNRMWINGGHYHIQMPYSTNTIPVHLPP
SKRLGKALKERVQVLQAEIQKYTSRKVHHTPYYSPDTPYKSDFHRLARRL
CGKSIGLVLGGGGARGMSQIGVIRAMEEAGIPIDVIGGTSIGAFVGALYA
RHADIVPMFGFAKKFFGRMASLWRFALDLTYPSASYTTGHEFNRGIFKTF
GNTQIEDFWLEYYCNTTNISKSRVEFHTSGYAWRYIRASMSLAGLLPPLC
DEGSMLLDGGYVDNLTVSHMKALGVDIIFAVDVGALDDDTPQTYGDSLSG
MWAFFNRWNPLSAHPNPPTLAEIQGRLAYVSSVDALERAKTMPGCIYMRP
PVDDYGTLDFIKFDEVYQTGYKFGKEFLQKLKDEGGFPWMEEAEAKEAMR
RTKAPRRASI
>Mb|QLI65211.1
MIRSAARLFQADLSVAPHLLRPKTFVKKIGKNNEIYYTLVKKGLAHNLDP
EKEYKFFEMPNLLLDLDAEGTRHVRSVEDTDEKLMTARWAEVPRRRAVQT
RSRAFEEQLYESRGKAREIMSQPTNLWRITSHDIVSAALHGGPGPQRKSA
RELTPADVISGSSSAPTPSRNSGIIEKLLLENGIPQHAVNEDQLLLRWMT
LRYNSLTQSSPYHRRTAPSPEQLKRALQTQTTILGARRLVFQALSAGTSI
NSFQNETKPAISLPHQIRSTCAYILSQDPRDRDLYLQALTFLGNLSERLS
SLDAQLGPILCGLALKLSAEIGSLEALPVWLYRSHHTCSNGKLLMKDVLS

ALTSLQRTLRSKRPVNLQTIEQRQLLFQLLTGIDENDTMAKDSFRTFGTT
FLEGDSSSTARRAYSAYIALLGQLGAARTLWREWRELPSSVSRDEAVVLG
FCNALKQAVRVMPGSDGEYTPDSSLAACAALDYHAIGMQDTAAWRARISS
NMAGQANVNVAACRAILDLPLADCIKGFGAL
>Mb|QLI65213.1
MLYLSRITSLFDYGAIFLMVVTIIVFLVPILILFPPVPVDHSDALRQTHS
KLGRPQRESHLRSQYDARHKPQAGKTPKIESLYIYPIKSCRGIELDRAKV
LPTGLEHDRLYTFAQLKPKSTPAVTEPGAQTDSSSAWEFLTLRQLPLLAN
VKVDIWLPDAGKKSRLLGSLQGGFVAVRFPWQERGFLGLVQLLVAKLSRG
WSAVPEKEFILPLEFPSKQEIDARGYKFANIKVWVDAPYALDMSNELPAE
LATYLGVKHSLGIFRSDPSARRKVFRTAPRNEMSGHEAEIDFHDAFPVHL
LNLTSIRALESKIQKDRFIDRIDVRRFRGNIIVSGLKEYDEDDWKSVKFK
HPGRSETMSRFDVSCRTVRCKLPNVDPATGIRHIVEPDRALRKYRDIDAG
APKMGCLGMQLLPTFPTSSNLERLESVLEVGMEVDVLERGSHLYVKQSAR
>Mb|QLI65214.1
MATTTVTVTETRTEPMTADTILRLFPDIDTSSEALSGHDEEQIRLMDEVC
IVLDDNDMPVGKASKKLCHLMSNIDKGLLHRAFSVFLFNDKNELLLQQRA
SEKITFPDMWTNTCCSHPLAVSGETGSNLPDAIEGAKRAAQRKLEHELGI
KKEQVPIEKFRFLTRIHYKAPSDGKWGEHEIDYILFIKTNVDLKPNPNEV
QATQYVSADKLKKLFEDPSLKFTPWFKLICNSMLFEWWASLDSGLEKYTN
EQEIRRML
>Mb|QLI65215.1
MANLPSQHPTLSIHLSDITLTPLITSSSSPSHLDSLTALTSSALSSQTAA
QRAHLGRPQRIMIEYPDSGAVVLQTYLDPRESSSSVPSSRSTTHKDIDTM
TSGTTDEDTETQQSPDDAAPMLVGVVVAGSSEEAREARRAAARLERVGRE
FQKEWTAQLERGAGAD
>Mb|QLI65216.1
MASRSSYRGGYNSMYSHRHHYGIDSRGYSQSPQHGGSFHNSQSTSPYGAG
RSGWNGQYTPEHQHPPPYHHGPGQYGPPSGPADHYHSQPPRSPPYGAPSG
PMNSHSSGNYRGGPGSQAGSQRGIPFQGRGGFRGPNTKAWGPASSESTAR
SHPQHADEQGARRYSTAAGSREDSGSKPTEMDIDRESRTSKPSTGVHVPP
KGDVEETPAAPSRPTPTGPGSAQSGASGKFSFAFKASSKPTPTAPKPEIS
QKFNAPPKKDISTKDSTTEREPPRSAPTEPASARQRLDFRNPPPDGPKIA
PRTRKVMKTMRRARERPQLEPDLVGSESVFYRKPGNESVVGSGTYGKVFK
GVNVYTKGLVALKRIRMEGERDGFPVTAVREIKLLQSLRHANIVNLQEVM
VEKNECFMVFEYLSHDLTGLLNHPTFKLAPAQKKDLAKQLFEGLDYLHTR
GVLHRDIKAANILVSNTGVLKLADFGLARFYAKRHQLDYTNRVITIWYRS
PELLLGETQYTAAVDVWSAACVMVEIFNRTAIFPGDGTELSQLEKIYSVL
GTPNRQEWPGLVDMAWFELLRPTVKRKSVFFDKYISKLTPAAFELLSAML
QYDPAKRPTAAEALQHAYFTTEEPLPKQATELAEIEGDWHEFESKALRKE
NERREREARKAVAKEPVREKEKKRAPEDGSDGEREVKRAHTESKEA
>Mb|QLI65218.1
MWLDRLAGGPSGASGQSTPQAGNRSYSPLPRRTSSNLSPYLTSQRAGHSP
RSSSLSLASNDSSTSLLPASRRLNGSSLRHSSTIPNLPDPVETLERLLQQ
PGHVENDAETQKARTSSIVEADLDLDFDFGGLGLKELASSHPPEHAMTAS
RQPQTVEEYHQQCAKFEDLHRSITACDDVLNSVEINLADFRNDLAAVSAD
IESLQERSAALNRRLENRKEVEKALGPLVEELSLSPEVISKISTGHIDES
WAKMLSELDRRTAAHKMKTSSALQHSKASEELGPLLEKLTSKAIERIRDF
LVAQIKALRSPHINAQIIQQQNFIRFRDLFTFLHKHHATLADEISLAYMN
TMRWYYHNQFSRYERALAKIKIHILDKTDTLGHEDTTRMTAVLSSARATG
PPHDVFNLGRRIDLLTTTNQAAMSSYLAEEDQATHYLEVQFRNYNLALID
NATAEYTFLATFFSPALSYSQISKTFNYIFESTFELGQTLSKTLVAETFD
ALGILLCIRLNQRFAFELQRRKVPVVDGYINGTNMLLWPRLQVIMDRHCE
SVRQLTNSLPSKPTRSAADVAKMTTAPHVVTQRFGQLLHGFLELSADAGD
DEPVVASLRRLRSEIEAFLAKQSLSYGADRRKSDRFLYNNYSLIMTIIGD
TSGKLATEQQGHFAKLKLAFEVDA
>Mb|QLI65219.1
MVHQLPYALDAETPLSPSELQVLRAQYEKEGDMVGVQTKFNYAWGLVKSN

NRNDQQLGVRLLSDIFRLSPERRRECLYYLALGNYKLGNYGEARRYNDLL
LDKEPANLQASDLRQLIDNKVAKEGLMGVAILSGVSIAAGVVGAFLFKNA
RKR
>Mb|QLI65220.1
MFKSGISSFARASRPTFSAAASRRALKPAALRFPLNSRFASSASVGVGKI
HQVIGAVVDVKFDTAKLPAILNSLETDNNGQKLVLEVSQHLGENVVRCIA
MDGTEGLVRGAKATDTGAPITIPVGPATLGRIINVTGDPIDERGPIKTDK
FLPIHSEPPEFIDQSTSAEILVTGIKVVDLLAPYARGGKIGLFGGAGVGK
TVFIQELINNIAKAHGGYSVFTGVGERTREGNDLYHEMQETSVIQLDGES
KVALVFGQMNEPPGARARVALTGLTIAEYFRDQEGQDVLLFIDNIFRFTQ
AGSEVSALLGRIPSAVGYQPTLAVDMGGMQERITTTKKGSITSVQAVYVP
ADDLTDPAPATTFAHLDATTVLSRGISELGIYPAVDPLDSTSRMLDPRIV
GEEHYNTATRVQQILQEYKSLQDIIAILGMDELSEADKLTVERARKIQRF
LSQPFTVAQVFTGIEGTLVDLKETIASFKAILSGEGDGLPENAFYMVGDL
ASAKEKGAKILADLEKN
>Mb|QLI65221.1
MNPKAANYIEQLNSARCEGNWDAVPELVRKVRKHAPERQCLTLAAETECA
ISQVTAAGARPSAAQTAQDLDVSSRFPKLAAAVNEEHHHHEDRFQAKVCM
GWLHWVVGEYNDTMANLPGSLEAEGISQERMDTVSEWTNLCALKSSYLKA
NCLMRNDQRQEALSALRAGTPSLNGVWCGHGVRKQLRYWSELFLTEYCML
SSQAVENEELNLDDPNSLASFRAWARYWDVMGAPITGGFGFKGSVPRRRI
WAEYYEALSRILETDLPYTGGHVDTISADLPARAQLRLEIKQTEAAYRAL
ILTETTFPRADEDREEVEDFVKRVMMNWAILCGRGWREQDLGAGGRAALS
RNVLETLYSAATRTYHSTSILRSLFSVHLALAEFDLAFKAFDSYLEIVKK
GKARVEKTGHEEVSLDDDGIVLETMAQAVMALCRYGHRRAGEKARQLGAE
LEDQLSKLSHTKSAENGVPPTDDNEPRNEWRAHVSPSIIALAWQAIGLSH
AHWSRITHEAASITEIQSKAIRCLRKSLAAEFGRSKDIRSFFSLALLLAE
RRELTAAIELVRSALLCNKGHEESYSLYHGPYWQERTLIPVWHLLALLLS
ARQDYAMAARACEGALDQFKDSTILFGKPDANCRSDHLKDLDAHRTLEGP
VRGLVDDMDDSEKESILEVKMTQLALVEVVEGPDAAVNASYELLSLFSRL
FGTLSAAPTAALPAPSLPPKTSGTIRSIRGSIFGTKVDRPRSPARQQSIS
TVSEKSEVATSRPATSHSSQIAAPAIQITDEKDTVNGSRRGSTGPGRRSD
SRQRNSLKKRDRSGSRTRPVTAIAVTHQPTVVDGETFFTPTAEGDSPDFF
NLNKMSAARPTSSSRVKPISSMHSIMTATSKSSDVTELTIDVTHASPNVL
PLVQFPKEKEKAQKISLLIKIWLTIAGFYRRAGMLDDGKAAVLEAQKLLT
GIESEAARDPSSTGATIGASGWAERRSIDDLWGDIWSELGLLGVARGLPY
EAKSDFEVALTHSPDHPTATVGLCNILLDTFSEKVLPPSTMPSLDGLYQN
ERGSLQSPSQGYHERFADTLPSSPLGLGPPITDRVPPLKMDDTAARNEPD
QLPAPYKATHLPLSDRLAARERAFTLLSGLTRLGTGWDNSDAWFALARAH
EESGQPEKAKEVLWWCVELEEAMGVREWRCLGNGGYIL
>Mb|QLI65222.1
MDNLWNRRTNSGKLSLSTSTNGPNGSSAGDASRNSSVPRRHGGDISAHGK
ASPFNTPGGNLASPTSGASSAFGLGSGAFASFGSAKTPKSAGNPFDTAMG
SAVAKTTAVKDSGKAAIGKAPSMASIAENKVSSLTVASKAHPLKDAWSFW
YRPPISKAHGFIEYENTLHGIATVQTVEEFWEIYSHLKRPSTLPVVSDYH
LFKKDIRPIWEDEVNRKGGKWVVRMKKGVADRYWEDLLLSLIGDQFGDAG
EDVCGAVLSMRNGEDILSIWTRTAGGRVLKIRETMKSVLNFPPNTRVEFK
THDSSIQQRTAIEEQRREKANQNQGGDRRHTQRQSHE
>Mb|QLI65224.1
MSEAKNNGEPNRHEEAHGLLASSDFDDHEDDIVVYAANGDGKREQGSHSD
TSPSPPKVKTPRTPNRVQFDLNPAVIGDANGSAHGGRRSSSEESFEFDDD
VEIQGHHSHRVPLLTDMEAPSVAVANTLGNDANDDGNRLEQAMRRPKSGL
KSAFMNMANSIIGAGIIGQPYALRQAGLLSGILLLVGLTVVVDWTICLIV
INSKLSGTSSFQGTVEHCFGRPGLVAISLAQWVFAFGGMVAFGVIVGDTI
PHVLVAIWPNLGTVPLLGLLTDRRVAIAVFVMGVSYPLTLYRDIAKLAKA
STFALIGMLVIVVTVVIQGFFVPSESKGSFSTPLLTVNGGIFQAIGVISF
AFVCHHNSLLIYGSLKTPTIDNFSRVTHYSTGISMMACLFMALAGFLTFG
DKTLGNVLNNFPSDNSMVNVARLCFGLNMLTTFPLEAFVCREVMLTYWYP

DEDFNLRRHIISSTALVASATAISLLTCDLGVVFELVGATSAVAMAYILP
PMCYIKLTTKSWRTYMAYAVVVFGVAVMVISVVQAVDKMVHGSDEATQCV
>Mb|QLI65226.1
MASPQQIRTPITDLFKIQHPILLAGMNVAAGPKLAAAVTNAGGMGVIGGV
GYTPDMLREQISELKEYLTDKNAPFGVDLLLPQVGGNARKTNYDYTKGKL
DELVDIVIESGAKLFVSAVGVPPKHVVEKLQKAGILYMNMIGHVKHVKKC
LDLGVDIICAQGGEGGGHTGDIPTTVLIPAVVEACKGHKSPVSGGPVQVV
AAGGIHNGQLLASALMMGASGVWVGTRFILTDEAGAPKAHKDAVRTAGHD
DNIRTIIFTGRPMRVRNNPYINDWETNRTQEMKELTAKGTIPYEVDLERF
MGGEAKEHSGVESYSSNATSEAEEDDDEGDDPLEQFRPYLMGKCAAVVNE
QKSAKAVVDEFVNDAVAWIKKGNQMIAKL
>Mb|QLI65227.1
MKLTTGLITAFAGYVAAAQQSAQVFIVPAGDASSPPAITPSLARLLLLQR
LAPSGRGLSLHDIPNGVGPEHAVSLISRFGKNIPPLFSDGQATEPSQLVL
MLEGMDDKQMKDLQKALSMSPSFTIADPPSDKAHRDLMELDFYRAGVADG
SKCSIQQLVNPLENCWAGKRSAAAKFSIKKNPELLDEVPKQIKQLIRLAK
SGELQTTIVALPSTKSSSNWLDEQQQELRRRQAEQVISSFDRPAANAEPT
STPKPDPIFDSSERIKACYETKEACMASTRNCSSHGECQDKYAKVDGSKN
TAACFSCHCQRTRSTSGSLTTWAGPTCAKKDVSVAFWLFAGFTLALVGIL
YLAISILFNVGEEQLPGVIGAGVSRSK
>Mb|QLI65228.1
MADKLTDAQVADLLTILRSDSSLDAKVQFVTVIKSAIKQHNVPESCIAQL
FDGLRTASSAQHAALVNAGFTALNHLLTRLSRQDPKLLAREAARTLPLVI
EKLGDQKDKYRSLAAQALNTLYVASPADAERFVRSSALAGKNPRAKEGGM
HWLLQTHQEHGLPFRGYVPILMELLEDADGMVRDTAKATVIELFRTAPNT
AKSDLKRQLKNFKVRPAIEQAIVKALAPAGGRPETPADSLPSQLSQTSRS
TLVASVSSLGSERPITPMPDAADAVDPQYVNTNRELDDIFKDMAWFFEGK
ETEQNWLKREQSINTLRRLNAGNAATDFQDVFVSGLRAMLDGIIKTINSL
RTSLSKEGCGLIQEIAISLGPAMDPMVELLMQALVKLSAGTKKISSQMAN
VTVDTIISRVTYNQRLMQHIWGASQDKNIQPRTYATGWLKTVLKKEAGHK
SHMEHTGGVDLMEKCIRKGLGDANPGVREKTRSAFWAFWGVFPARADAIM
ADLDSTAQKLLNKDPNNPNSPKKTDAPARPGLGLSRSTMASSKPSLREAM
MAQKKALAAKNLPARPGSAMAHISSPVRTTSNTSTHSTASTKPTATRTRP
ESTLSVNAGGMSVAPMRPTRRRPEMAARPATAGPYSVRDRPGLDADSPES
VKSKRVTPKAPKAVTPKKTLPKTRPGHTSHASESSIPSPTFPSAHKRIAS
PRGSPVLLKHSQTSPASVSRAVPARSNSESMPVPNFASLHVSAPEQAEKL
QESPGMLRSSPAAAVTRESNLGTQVQSPSCTLKVYEDPFTDGAPSATKPT
FNVPVLEDKPVNEAAGNLPKPNGESLMPDTLDSPEKTRQNSRLLDSGITK
IKNKNLEVHGFRKLQSLIRDSKTVLTDGKFEDLLLGLFQYLEDPLPSLPA
EKVQDVKAQILSTIKLLLKKERDNFQPHVSKGLESLLETRGACDTRAHVV
SGLELLADELVTIGDGSEIVVVLTKRLQTCTDATTEGCRTLSMGLHVLKE
MLDKRAEFVPVDWELVQLTDLSSRCLESADSGVRMDAVKFCVSLHDRVGD
AGFWGAFKDIKEDPKSLITYYIVKKQREQGSVS
>Mb|QLI65229.1
MTTNAVPDTQLGLSQEEIQLLRQGQAALGGGSTSSRTASRASSQGLLLLD
SSSLAALGRYFDRLMANIEQNIRYLSEQAQMFTQVQYDRAGNIIDGADAE
IERYRQILVQLDELETDFDRIAHIKEIVKGYRSRVEELERDLETSGTSSR
HHKHSSHKHSHSHSHKQGSSHRSRH
>Mb|QLI65231.1
MASITTFNSASENYDRLLEADTVNMQTRGSGVGSSNGGDGLGPVPDITAP
QKMISAMSGSLLTSLLVTPLDVVRVRLQSQRTPTSAVDFSKLALRTTTLT
PAQTAELGVTACCREVFFQGNSAELCIAVPRGEGIVEPAASSVASGADCA
VHEVQKKTYNSTFDGLRKIARNEGFTTLWRGLSPTLLMTIPANIIYFTGY
DWLRYNPVSPLSRLSSDNAPLVAGSTARILAATAVGPIELFRTRMQAAHG
TSTTNHLVETFQGVRDMVATHGYVSLWRGLTLTLWRDVPFSALYWWGYET
IRSRLTDMREDSQGRPFNRAESLQSARRRSQSQENHMETFVDSFTAGALS
GTLASIVTTPFDVGKTRTQVFRDGPSGLAGKAHAAEEKNMMRLLWHIFKT
EGAPGLWKGWIPRVLKVAPACAIMISSYEVGKRVFRGVNERSMGEREEDN

>Mb|QLI65232.1
MSSLGPSGSNAHGLNFSQAGLRRSAAFPPTHANQHQHQPNKQSPAPHAAS
PYQPQGYTPQQPPQGTYPPHLFASPVNPNIPNGHPPSRTPNSHVPPNALG
VQRQRAAPPRQQMPPQPNPPPMMFPTNILPIRATLAIQATSSTHLIISSK
GITNSIQATNLINSSSSNNNNSSSSSNNNISNINSTSSTSSTNNINNTNS
IRHTRHTRHILRIRLIRLIQAIQAIRSTPQQQQQQQQQPSQQPQQQQQP
QPPQAQVTPQPTPQQSTPQPPQQQIPQQQMPQQQQQPPQQQQVQQVQQVN
QVQASPQQPAAQVPQQQANHAGSEMDVDGNQDGDASEVNGLDSKLLNDPS
YVPSQPMGEMMSPPPEGGSYPTLEAVQKAVLRYCTSVGYAIVIGRSKKTV
PGLKKVLFVCDRAGKPPSRVSPEFRKRKTSSRKCDCPFGFFAIEQRTQWT
IRYRPDPAHLQHNHGPSESPSNHPAARKLDSKMVAAVKQLKENGAGVSET
LQILQTENPDCHLLPRDIYNARAAINRNPQKVATGLAENRPAIYTKPQQS
PEDRIRADLRRELAKAREDLQKIEEEKQKEIDALKSQLEEKEKLIKRFEM
FIDICNERVMVQRERLAGPDGNLAIGGNSVM
>Mb|QLI65233.1
MPPAHSSGLATSRHAPVVLLTDRLEKPSLDDRDYRVVRLENGLEVLLVHD
PKADKASAALDVNVGNFSDSKEMPGLAHGVEHVTPQLLFMGTKKYPGENE
YNQYLAANSGSCNAYTAATSTNFFFEVAAKPANDEEPSDANPSPLFGALD
RFAQFFIEPLFLENTLDRELNAVNDENRKNLQNDTWRLNQLNKSLANPEH
PYCHFSTGNLEVLKTKPESQGINVRDKFVEFHDKHYSANRMKLVVLGREP
LDVLQKWVVELFSGIENKNLSPNRWTQEPLYRDADLGTQCFAKPVLDSRT
LSLLFPFIDEEGMFETQPSRYISHLVGHEGRGSLFSYLKNKGWANSLSAG
AYPVCPGSPSIFEMEGLDHYLEIPTIFFQYVAMLRESPPQEWIFEEQKVM
AEQDFKFKQKTLASKFTSSISSVMQKPLPREWLLSGQKRLRTFDASLITK
ALERLRPENMRLVIVSQEYPGNWDKREYWYGTEYRHEKIPQSLMAELQKA
LTMSKNKRLPELHLPHKNNFIPNKFDVEKREVSKPALAPRVLRHDQGART
WWKKDDTFWVPKANVFVSLQNPIISTSVKNCVEATLFTQLVEDALEEYSY
DAALAGLQYSASLDTRGLCIKLSGYNEKLPVMLEQVVNTMRDLDIQEDRF
HIVHERLVRAYENSQLQSSFQQIGGYLSWLNSETRYNVEEMAAELKHATA
GAVRLFQKQILSQLYIEVYAHGNLSRGDAVRLTDMVESMLRPRPLPRSQW
PIIRSLILPRGSNFVYKKELKDPQTINHCIETWFYVGDEADRQLRAKTLL
TAQMIHEPAFDQLRTKEQLGYVVFSGTRSFSTTSGLRFLIQSTQKPKYID
RRIEAFLVQFGKKLEQMSDSEFENHKRSLIVRRLEKLRNLDQESSRQWGQ
IDGEYYDFELNQHDAAHVKPLTKMEMVQFYKTYFHPCSSTRSRISVHLKA
RGLDTKVMEILKEAGVEDVPKKKRGSVDVLREYLESGEILESEKLNAVIS
KIQKCGLPPAAETDATNGSTKDTGAVETAQKITDVRQFKASLQASSGVRP
VKQVNYLSLPASSPAFVHELLGQLTVKTSSSKKRRLARPPPSTKQVLDAP
ASPSPDSALPSCEVEPDDDPVLSFSAELSSVIASREPEAPRYLLRSEALP
VATASSLPPRLQHTHAASLVSTDYASSTASSPCASAYAELSLESDRGGDE
TGSARNPGRSQSPFVVPRRAMMNGDADLPQRSSSPLKRRASSMDPDAAPS
KNGTTMDTDASQPSQQIDPSSTEFPRAMSVDAPDVNGHEASLSQLAPPPL
LEQVKIIEMLLKAFAEAPPQEGSVAYLVSRTWVDKALSLRTGSKPAAAGA
ESISLGPVDNTDIIQETIKDSSGRDFVRLIPGLGNESFELFPEDAWKMVM
DWYGIKEGQQPIARIAVNTADSKQAPPNILYEFHPPIFHVHRLWSDVSTV
PIEQSLKAKNPPPLIVARSSTTHAQSFLREIKTLAGVGLERKVRLFTIPA
SAQVTRQETDAASALTPPDSPGRSRGGNENQSVWPTLLVDVVSFSKVRDI
RVSVPLIDHTADDKFNGQSTLQHYELTTDQTLVLDEAIGNSYVSNYTGRM
KAADKPTPSRLLGSTISSKTSSNRSSPVFDGPMTRGRVQKKRHGRSIGAV
GLHNLGNTCYMNSALQCVRSVEELTKYFLTESYFTEINKTNVLGFEGRVA
IAYGNLLREVYEEGRGSVSPRDFKSTVGRCRPTFSGWSQQDSQEFLGFLL
DALQEDLSRIKKKPYIEKPDSTDDMINNPEAIKEMADKVWDITCRRDDSV
IADLFTGLYKSTLKCPECGKISITFDPFNNLTLPLPLENMWSKSVKFFPL
NDAPVKIEVELSRHSSMESLKQFISVRTGVPVERLMGAEEFKDRFFKIYD
NTQDVSEEIQSSDTPTIHELEAVPTNWPSKKPHKKYRSMLDIDTPPESAE
WSEEECHTMVVPVLHRRPQIPGRGPEGISPPHFITLTREQASDYDVIKRK
ILQKVATFSTWSKFKDTQESDASDGVDADLVITTASDADSSGDSKIISNS
VEGEDDMVDITMRDAVDNISNRATISPITQPTILKQFNTKAPKFADPANF
LNPELQNLFDLCYFKNDTDGPVPTGWSNVDNTRTLPRLADRIPEPRVKDG

DASSPGSSNSTGSGNEESGDEDEATPESTQTRMIEESSEEDAHQAAKING
RSGRHNSKYNQGSRKKFKGHKGYGKKGNKRRDKQTRASKLSQRANIVAPQ
PMPPAVSDGGPLIRLFEGIVVDWTEDAWDAAFGNNTNKPDPARGSRTFVD
VQTLHDPALKITQRRRVTRKTRGITLEECLDEFERAEILSEQDMWYCPRC
KEHRRASKKFDLWKTPDILVAHLKRFSSSGWRRDKLDVLVDFPIEGLDLT
SRVIQKEDGKAEIYDLIGVDDHYGGLGGGHYTAYAKNFVDGRWYNYNDSS
VHVVSDPTSVITSAAYLLFYRRRSSGPLGGPRFKEIFDKFDKESVHSDNE
ATESGEEVMTQRSTKTTVTPLTGMDDDDDELPIYDGNTIRRSIEDEGNYH
SGSKGALDMTQSWSFTSLNGNGVNGNRDTDCASDDAQFDSSGDDRSKALS
EQDTDMASAAPFEEGASWDEQGVISVPADGAGEGASEEVTEIHLEGDKMT
RSA
>Mb|QLI65237.1
MASFFSDTLSSHRFHLFATAVLSGATVALILGYQGREREQRLSELKRSIP
SPGDGGHHTNRLNNFGASSESAVDKEDARNQALSRRALAGDFDEELILEQ
LARNRVFLGPEGLTKLRGSFVIVVGCGGVGSHCAASLARSGVSKIRLVDF
DQVTLSSLNRHAVATLADVGIPKVHCLQRRLIAIAPWVKFDLQQEKFDGS
VASRMLGPWEDGRQPDFIVDAIDNIDTKVELLKYCYDHKLPVISAMGAGC
KSDPTRIVIGDIGASRDDGLSRATRRRLKLLGITSGIPAVYSTEQSGEGK
AELLPLPEEEFQKGVVGDLSVMPNFRVRILPVLGTMPAIFGLTAANHVIL
SITGYPLNYVPAKGREKMYEGMMTFVQGSEDRLARMLGLDTVGLKTPLTS
GDVAFLAEELYHGRSAVSGISTKLVLTRWQRPEDTNMSVIGEGKDTQKCS
TVRLRDLVCMTKEEATRHEKEIFKARKALGEVYDTEMIDRIEAKLAVAAK
YESYR
>Mb|QLI65239.1
MATRARLLRGVGNGRCSHVSSLVAPAASRGLPKATARLASACRSSVLPPT
RCLGSRSFSGTSASRAAKLTTDSYPGLTRDSRFSQLTPEHVAYFKQLIGQ
DSAVIDGINADAAADIEPFNEDWMHKYKGQCRLVLKPGSTEEVSKILRYC
NEKMLAVVPQGGNTGLVGGSIPVFDEIVISMGRMNTIHSFDEMSGSLVLD
AGCILEVADQHVAERGYIFPLDLGAKGSCHVGGNVSTNAGGLRLLRYGSL
HGSVLGMEAVLPDGTVMNDMCTLRKNNTGYDLKQLFIGAEGTLGIITKLV
VQCAQRSPAVNVALFGIESYEKAQRAFREAKLQLSEILSAFELMDGTSQR
LVHQVRGGTRPLENDYPFYCLIETSGSNSEHDYEKLEKFLEDVMSNEVIA
DGVVAQDATQIKTLWSWREGITECIGHWGGTYKYDVSIPLSEMYLIVHDV
KARLSEAGLLGETDDHPVVDVLGYGHMGDSNLHLNIAVRRYDKEVEKVLE
PYVYEWIEKRQGSISAEHGLGLAKKNYIGYSRNETMIGLMKQIKTLYDPN
GIMNPYKYI
>Mb|QLI65240.1
MFSRLAINLAKPAFKPAAAMAPRMAIQLRFISQATAGSTGRKMPSKPTKS
TAPVESVPATFTIRDGPVFNGTAFGANANISGEAVFTTSLVGYPESMTDP
SYRGQILVFTQPLIGNYGVPSNERDQYNLLKHFESPHIQCAGVVVSDVAL
NYSHWTAVESLGEWCAREGVPAISGVDTREIVTFLREQGSSLARISIGEE
YDADEDESFIDPGQINLVKRVSTKAPFVVESPGASLHIALIDCGVKENIL
RSLVSRGASVTVFPYNYPIHKVAQHYDGVFISNGPGDPTHCQETVYNLSR
LMETSSIPIMGICLGHQLLALAVGARTIKLKYGNRAHNIPALDLTTGQCH
ITSQNHGYAIDASTLPSDFKEYFVNLNDGSNEGMLHKTRPIFSTQFHPEA
KGGPMDSSYLFEKYLDNVRLAKSKALEYRDNRPSQFMLDVLSRERVGVEP
VPLASAA
>Mb|QLI65242.1
MAGKGSDGKKRPVALNLAPKRSNSSNSSSTNNSLKIPRTPRFAEATSVHS
PVDMKKSPFADPEQPTEGTSQPGDVGFGYIGNDHDSAAGPKSPLKSAMKV
PGTPARQLSNPLSPTFREEEMLEKREVSTDKEQVKDIKIKTRVRMAKFAL
RGVNFSCSLIILSMLSASFAIFNSTKSLAAASKFTPWAPNTQQTAWPQKL
TLAMACVSLFACILVFFAYCRGGHRRATKVNTYYTMFAIGWFILSMLLWV
VTAVIFQHSKNNSNNKDMWGWACTNNQRAEIYHEKVDYALVCRLQNWTLI
CIIIEVVVEVISIALYSIVFYRFYSKRRLMKSMDMRDRARSDLYLAQLRT
QSAPNTPGFGPKSPALSSYAMSPRHPPAAYRNLSDIDETSPFTPGGKVVV
PQSQFGTPSPDFKLQPPPTKAPSATPKTVQAAFAPPATRTPSPPVQPQHA
PAPADEPTYEAVPIPGAYAGQAVKSPPPGQMTFGVAR

>Mb|QLI65245.1
MSPTMDANASRLPSGLVSPRHVRSRTQSISSDRPSTIGYGVGLPPVFISP
PAAFIASSAASQIITNDHDSHADAWYDQNGIDPAAETATVSASALQLVNS
FLDQLLFNVLQVSKSTALSALRPAVIEVLKPKLGRDVVSNADEELREYLG
GANEEDYEQFQGPSSSRNWDGELVWKRTRLRCMVYSSLGDMEEEDEDAYM
EEENLDIGAHEQISDTISAPVAIFLTSVIEYMGELTLTVAGQAAYQRVRS
KIEKELKDGARNTSDPADRIVVTDLDMERVALDRTLGRLWRGWKKRMRTP
ANDIAGSLFPKGPPTPCKPGQMEIAHDALSPPKSATSDSGSEFRDSQEHF
EDPIMEDVQPMDIPLPLGDNDVDEIEVPGLTHYSDDEGDDEGDDEMDDDE
VQVRKRPKSLLISSSMFANGLPTPTKSQPHTPTVRSRQRSTSLPTPRIPH
FPPKTQRHIPPSTSESEADQDTVAEIGVAMSTDGHGSLSEANAEPIATSD
VPAKKDVDARSQSSGSDYDGTEEVAFEKAEIMTTSRISMSSSAQSTDSDS
NGKATPMKRSSSVHSARIIDVPAPRSPARSRPTSLDATEQLRPVRVAGVA
SGFARTAASDDRSKATSNETPIKAAINALPPRSSHDKRKPSASNTAPISE
LEEEASQNFTRAVNQPDSSADATEMEAAGSTSSAMSQRRKGMLVTLNTKI
QSSSKYEQSPVSARTVSLPATPASPDIAKGNAPDLPKKASVHSGWSSPKP
ETKSRLSIERTRTHESDELSLPAQQQNTSGSRQVSQAHTSVSSVSSGASR
LKPVRTSEDSSSRSESVARNFEELIQSNQTITYTLTPENMRDIDAKRSLD
SPVVTKFARRKSEDARAHQYSPKASPILTPSAAGNLPSPRLPPSPASPKI
VESKAIINPAGPVPPAPTTITAPTARTNGATAREARVPSDSVSDFAEFIK
STGPAGETKSYTVPKLGSPPVSSDKGYRESRRASATSLANRLRYQPRDAT
VDSRVDNSDLIDFIRQGPPIAASNHRIPRHVAPFRTTMDSDQMSGAVGGK
AIDANIPDIRSSRTSTNVTESSMPSMQSSVNSKSALIKHKTPKIPNKLFD
DDDNMMPKPKTRRVRDPYAIDFSDEEGGVGQDDHILDTPKLPVKKEESLA
EFLRNYEPPPEPVSAPAPRLPRKKASAPSLIGRFTRGGKDKEKEKDSASI
KGMPESRSVSRASSGRGYIPIQVNIPPGYDKYNPMNGNIGAKRARGPSTT
SSTAGRVPMKKFEPREAASKRSETADLAAFFRDSGPPLNNSPMVIDRRSS
QQEDSGGLTKMFGRKKKPLAF
>Mb|QLI65246.1
MYARYTTTLLCNTLIQKSISSCSLTGDATRPLCADTCADFAQSEAYITAD
KNLCSNPGPDLSKLIRADFATCSLPEDALTSRCIAGFDNESDNCGFGNST
IGLCSYCAAGGTNSTDTCCYNANAEKRCEGVKLPPITATMTFTPPTATST
PSSTGTPGDSNGSDGGNRLSGGAIAGIVIGSLIAIALLALAIFFCFRVMR
RRRGSQQGSIFNQPSPARKGPLMAQAKQTAPEGYEVLPGGRVARMAALEG
HSGGSPTQRSGNNGVTAFVGDAPRKGTDTQSSSDEYAESPERGGVLRPPP
TSRRQGSLSSGSALGSDGPQSPGSAGYSSPLGMASQQSEQLPFFKDYYSQ
DDIHPSDKVAVLWAYQPRAPDEFSLERGDMIKVVGIWDDGWATGIMLDER
ADEWEVRRQAQRDSGVSNTSGIRDESPEAQGEMKAFPLVCVCLPEHWRKT
IEGDGSTESASSPGPTVPTSIS
>Mb|QLI65250.1
MASLDKPRHVKYWQRCFTSYLPSAYTTNDSTRLTFAFFIVSALDLLSVPF
TSKERSSIRSWVLSLQHPDGGFCGSPTHVMSGQDATKGSANLATTFFALL
LLGVAAGTDDEARAAFTGVQRRKLLRWVTKLQRPDGSFGQVLWEGEAVGG
RDTRHSYLASAIRWMLRGEGDEEDIDVERMVDHIRHTQTYDGGAAELSQH
ESHAGYAYCAIAALYMLDRPASSTAPHKSEAVGKGVPDRDALIHFLVHRQ
FNYLVEQEQDESDEGDANYVQAKLGDLSLSHHCAHVGYNGRWNKKADTCY
CWWVAGTLAMVDSFHAVNIPPSRQYLLDVTQHRIGGFSKHAGGPPDLYHS
YLGLAALALMGDTDLKEFDVGLCCSKETVRKIELARAGLFGNRKGDFDGD
GFWESIKKAD
>Mb|QLI65252.1
MTITVILGAQWGDEGKGKLVDGQCQEAQLCARAAGGANAGHLVRVNGVSY
SFHLLPSGLINPKCMNFIGSGVVFHVPSFFSELRELEEKGLTAVHDRILV
SDRVNVLLDLHIAVDGLEERELGDAAIGTTRRGIGPCYQTSRARTGIKLT
DVFHPDLFEQKVRRLADGYQKRFGELFEYDIKAELARFDEYRETLKKYVV
DGVSFMRSAQQSDTKIVIEGANFVTSSSTTLAGIIGGLTLNPKNITETVG
VVKAYTTRVGAGAFKTEDTEEIGTKLQEIGREWGTSTGRRRRCGWLGAHN
LKTLNLVVVNYSNSINYYTSLNLTKLDVLDTFETIKIAIAYKIDGEELDS
YPADLDILNRAEVVYHEMPGWQKPTTNARTYYDLPKAARDYVEYIEKFVG

VKIKYIGTGPDREAMIQRS
>Mb|QLI65253.1
MIDKLVGLAMLVASSVIFLYYTIWTLLMPFVDKDHPLQDFFLHRKWAIRI
PVILILLGSAVVGSFLGLVMIRSNRKKAAKAKAAAKKKN
>Mb|QLI65256.1
MDYQPSPRRLSSSTCSIASNSSWTSHDECPPLATSRRCSVRFFTYQHDRK
PSTSPSSASSRSCEGMSQKETRELWRCMLHLQQRYGCYNSTRIDLAVNAG
EAGIDLMPNPFIIDTLNDSLIDLPHEGWEMLDRCLRGANKEPQTKPKSHK
KPWRRTRVGIPVHHTS
>Mb|QLI65264.1
MPRKLPWKTEPQGSPPTTTPKKQSPRPEPSTSTRDASTRPLVSRRRSPEH
GASRIRSPSTSPPPEPPAQQFMIAGPAGDDRYRMVEDELLATAGLFTAHL
HRAEYTRLKRLAAAQNAAAIREMERPVVPGPATATATRRRGRAARTAARR
GLVRGEGTRGGSSLQGLMETPRGEGARLTGVGLGAASRLREAVVSARLPE
EVGGSRGDGEAPSFAGAVDDDDGDDDPFGVRQRRVRREKSREQMVRRTGE
RETRSPKRPERDTIPGFF
>Mb|QLI65267.1
MSRAAAAAAGRNPHPDFKTVESSRPPAPRASFNVTQTTAPDWTFGSGANT
PEPPSAHVSIDPYEPGRPAVFNYKLLISGIVPRPIAFLSTRSGDGSTNLA
PFSYFNMVNHDPPLFVVGFASSVASAKDSLRNLLESKECVINVISDSFVE
AANSTSIDAPYGTSEWDVSGLTAEECDVVKCPRVREAVFSVEAKLDMVKE
WESRVSGQKTGVMVVLEGVRFWVREDAVNEDRNMIDPAVLRPIGRMGGIT
YSRTNEAFELPRPKFEEDLGGHQGLERIKTQK
>Mb|QLI65268.1
MAKSLELKEQGNRHFQSGDYLAAESLYSKAIIADPKNPTLYTNRAMSRLK
LSLWESVISDCQTCLSITPDSMKAHYYLSQAFLHSRDYESAVTHAVRAHG
ICAKTNDKSLSQITAAVLRCKKARWEHREKLRAKEAQALETTVLDLLAKD
ADDMLADPATSEYEKGLIREDNEQRVQQMRAVFERARAQADRRREVPEWV
IDDISFAIMVDPVVTKTGKSYERSAIMEHLQRRPTDPLTREPLVPSELRP
NLALRQACEDFIEENGWAVDW
>Mb|QLI65271.1
MEGAFTHVGNHLISDSAAAIKAGADDLSALDPDESLLYGKYEGGRGGRRR
ADDDDNQTEAFEEDENDSLNSVPVEGMSGLKLKAVDEEKELPPHACAYCG
IHSPACVVKCLTCNKWFCSAKGNGTSTHIVNHLVRARHKEVQLHPDSTLG
DTVLECYNCGTKNAFLLGFIPAKSDTVVVLLCRQPCASSTSTKDMNWDIS
RWEPLIEERAFLTWLVNAPSDVEQLRARHLSPNTIAKLEEMWKVQPSATV
ASLSIASNIDDDPDPVLLRYDDPYHYQNIFGPLVKMESDYDKKLKEAQSE
DGLIVRWDYGLNNKHLVSFNLHKIESGDVKLAVGDEMRLRYNGELREPWE
GVGYVIKIPNSQSDEVGLELRKTGNDKLVPTDLSHNFSADYVWKATSYDR
MQLAMKTFAVDDMSVSGYIFHTLLGHEVQLQPVQSRLPRKWSVPGLPELN
QSQIDAIKSVLQKPLSLIQGPPGTGKTVTSATIIYHLAKMSGNQVLVCAP
SNVAVDQLCERIHNTNLKVVRLTAKSREDVESSVSFLALHEQVRLSEHNS
ELVKLSQLKNDVGELSSQDEKKFKQLTKAAEREILNNADVVCCTCVGAGD
PRLSKMKFRNVLIDESTQSAEPECMIPLVLGCKQVVLVGDHKQLGPVIMN
KKAAKAGLNQSLFERLIKLQLAPIRLTTQYRMHPCLSEFPSNMFYDGSLQ
NGITHEQRLRRDVEFPWPIAETPMMFWSNLGNEEISTSGTSYLNRTEASN
VEKTVTRFFKAGVKPSEIGVITPYEGQRSYIVTTMQNSGSFKKEYYKEVE
VASVDAFQGREKDFIVLSCVRSNENQGIGFLSDPRRLNVALTRAKYGLVI
LGNPKVLSKHELWHNLLVHFKDRKCFVEGPLTNLQACLLQFSRPKVSFRQ
KNNYQSQYGAGGYTNGRFNGSATPGRDFDSGSMMSYIPDDVSSIQGSAFG
GAALNSAFPPMFSSFTPEQWPGLPGVPGRGNKGRSRATESIAGESVANSE
LTDTTTSVIGGKGVSQGGVSLGSGLHDAVTGMRPVSYTQSDRLKQYVESN
GRIAPGSGFGRRYDDDEKSVSTAFHSQIGGGYD
>Mb|QLI65272.1
MSEDDVDMLGPDGQGSSKVDRDRRAPRFSWTPAYETTFFRSLCESVQLGL
RENSSFKAEAWDRAALALQDSHGAYPAKSHLINKSDNARKRFRLWRGLRE
DPEFMYNPVSKTVTATEEAWRAHIEREPLSRALRGRPFDHEEYMEILYPD
VIGSGGAPKRIMKPRRRTDGQPGDESDMPGTGVLNLQPESASGQNTLESP

GPARSSLSQTPIGSATASGTQLKPSSTTIPPRTTPTQPSALTPPDESANH
AKKRVMPAPQTEGAFGATPPPSAAKSGNAMADAPSPEKKRRTSIHGDPVP
VVTSTSSNCLHHASSSDSPTAVQLPPPVTLATPQTTAADSQPNGLSTCLS
SSRWCELALEQFFKDFAGEDMDLQIRVAETVLVDESRALVYCKMPCGPKQ
SPEDAAAPRPAEVVTTESASTASLIDVDVPSVRTVPSDFLEQDVQTETQA
GRLERDVEAAKAKAEQARKKAGKKARDTDSWLVRQFSSLSDGSAGALVIT
NVAAVVGVSSYLGYRAWGLYEKGNLTWKNAGVGVGILAGVGAVEAVFGRY
LYKGKKNGSS
>Mb|QLI65273.1
MDITTSPSQIPAWVSLTSEETEDVDEPPERHQKFERTCIRRPQDQSSLLT
KAILAEPEEVPSHPAHSILLGTQRRRSMTSNISVASTADLTSDTGMTSPC
RTNTPSPPIPEMAALRLHHEIGTAEKNHLAVAAAPQPPKKRCIQFACAAK
PPVAQAEAMVKAPSADQAPRRPSIKFAPHVGSASTQNTPPRQISDPGTRP
HLATNSESPSTPKKTSALSTALHTSPRPSAVHRLSNKITLARPKFLRANS
KDLAKDSSQFHEFASGTAREEDWIRQPSSAATRLTIYDTLTKENHFRRLG
AEAEEEAELEEEEAEAGSDEDEEEEEDDDDGENDDDLEEDGEEDFQEDSD
GYHTDSETGFADSDEEDEEEDENMVLWTPSHATSRQIQVVAPIRRPSVNE
PQSDSSAASYGSRSSTRRTRPRRIKAESDAPDLPDSTDFVCGTLDEDRPL
EEAYLSCLAARRNEKLRVIPQDIDPSFPASDPDNDDEEEIYNPVHHDSDD
DVWVQGRMEDLHHGQDRSRRRRKSEQASPRKYRSPPPRRHHSPAPKNRGR
SPKPLFGRHSPKRIRSPAPKAMNTPLQTPRQGPHVQFHLAGRPGMTQTKS
LPRAPAMFPPMKGSKTARHQHHHDSDCHLRGAIDIVKGLERKRQRRKEKF
FQKYCNRARRGQIPERKPQPGRGAERMKELGLLMAGKKDQGNYVMSI
>Mb|QLI65275.1
MPKSLIIGISGPSSSGKTTLARLLRDIFPNTFILHQDDFYKPEPECVALP
PHSPQCSHEGRLPVKNGLLDWDSAGALDISAMTEALSYIRQHASFPVLAP
HRLTFCSQILTSSKPTLDSKEDQNSVGKCPVPESTITALRSRVSDALPSS
HPLRGENLRLCLLDGFLLYSPSMQAVHSHLDLKLFVRASYAKAKARREAR
DGYVTIEGFWADPPGYVDKIVWPNYVEEHAWMFDGGDVEGTFNADVLESQ
GIKAPKEAPVDADLEYVLGWMVDVVIDELRKHL
>Mb|QLI65277.1
MAPRKRAASGIETNGEPVPKRRSARQATASVSNSSSGPVSTQNIQVPRNA
GTKASSKQSKTDTEAAAKKPTKQSTGGAKASKAASPIASKKDAGAKSNER
AASEDVDVDSIPTTNPEAPRHEGEWYWLMKAEPETRYENGIDVRFSIDDL
RAKTKPEGWDGIRAYAARNHMRNMNAGDKAFFYHSNCKEPGIAGTMEVVK
EFSEDVSARRPGTPYYDSQSTKDKPRWSLVHVEFRKKFAVPIPLKELREM
GKPGGPLENMQMLKQSRLSVSRVSSEEWAALCKVADEKAKEAGLEHETGK
LKK
>Mb|QLI65280.1
MSFRALASKPPKGSVSMDPNCAICHAPATLACECEARGLEMAIRQAEDHM
MRSVYNDIRSWVRGHAQDYVLEYFRLLTERRKNAHTTHLDQITAHSFYHY
NAPPHPNQIAEAQGALKRGIDEDWQASVQRYPEVLEYFYGLVELSLPADD
EPAVKDPPLSALNGHRKATRRSLEGSTSTSSEGRRAPPALSGAPFLPTSY
SGRDLALDRRGSQPPARRPSTTAHLPPVPSSYYGGFR
>Mb|QLI65281.1
MSTGSLPQSLADDGHDHDHDHGQPHSISRQPSHARQTPRSSNTSVALHRE
KPPPASLNSLDGWTKETTAGGKSILTPGPDAATTPYSPPIQHGHNHSHHS
HHHDHSPDHHHNHEVGHKHDHAHIDEHRSDPGKAPRSLFTRTLLKYTARF
PILHAILVEKDSRRIFYFMVLNFGFMTVQAFYGYVTDSLGLLSDSIHMFF
DCVALMVGLLAAVMSKWPPSQKFPYGFGKIETLSGFANGILLMLLSVEIA
FEAFERLWEGTKTKRLGELFVVSSLGLAVNLVGMMAFGHHHHHGHSHGHS
HSHSHSHSHSHSHSHSHSHSHSHGHEHDHSHNHDNHKHGHSHGCGGSDHG
HDHSHDNENMHGIYLHVLADTLGSVSVIVSTALTSIWGWAGWDPLASCFI
AVLIFLSSMPLVTSSAKRLLLSVPEATEYNLRNTLGGILEQRGVVNYWAP
KFWLDDRTGSADGEKLVGVVHVVVARGYGMDEARDRVRNFLKSQGVEAMV
QVEREGDNACWCTRGRGSVASPSASKML
>Mb|QLI65282.1
MSYIAPVHKPTSIRHALRIRFLSPDVEDLVVAKANRLEIWRLTDEDMTCL

HTKLIYGTISMLQRLKPKDSETDLLFIGTDRFQYFNVAWNPETNQLDTVE
QTIEDVAEQYMRHSQSQNKCLVDPTGKFMAMHLWEGVLNVFRLPMRKGMT
TKLEALDQVRLTELWMKASTFLHSQTGHPKIAFLYKTQTDQEEARIAVYK
LTKDDSRGNVARFDPHRERELDQIISDPYASMLIPVPFREEKRYHVRNNE
GAKAHLGGLLVVGETLITYFDSLTYSRVSSTLQDPKIYVAWTEYDDVHYF
LADDYGRLDLLTIETTTESTGIVVTGMAISPMRFPDSSGCTSRASSLVYM
GNDMLFLASHHGDSQLLRIDIDAQVMVVAKTLSNNAPILDFAIMDMGNRE
GDSQFGNAFSSGQARIVAGCGAYHDGSLRSIRSGVGLEDQGILDEIQDTK
GLFTLRSHQSSHVDTLVLSSVADTRVLRFDSAGGIEEVYAFQGLTLDMET
LLAVNISDGQLLQVTPKSAVLLDSESGVNLCSWDAPSGKAITAASANKGW
ALLSIDGSLLVSLNLHDNLAAVLRDTSGDESSTQPDQISCLHAARDSPDI
GVVGWWASGTISIVDLATLQPLHGEPLRQTEDSASVPRDIALVQLHPPKV
SGPTLLVALEDGNVVTFDMSIQGYTISGRKSVTLGSSPARLHVLPQEDGT
CNVFATTEHASLIYSAEGRIIYSATTADDATYVAPFDSEAFPNSIVLSTD
SHIRLSHIDKERLTHVKTLSVKETVRRVAYSPTLKVFGLGCIKKELIHNE
EVITSSFRIVDEIIFQELGKPFIFNTSTSLEMVETVIRAELPDSMGNLAE
RFIIGTSFITDDDAMEENDTRGRILVLGVDENRQVYQIVSHNLKGACRCL
GTLGEHIVAGLSKTVVVYHYVEETTVFGSLQKLAAYRPASFPLSLDISGN
IIGVVDLMQSLTLVEFIPSEDGSRAKLEETARHYQPGWATSVAHLDGERW
LEADAQGNIIVLQRNPEAPTEQDRSKLEVTSEINIGEQINQIRKLHVASN
ENAVVSPKAFLGSIEGTLYLFGEIAPNYQDLLLTFQSRLQDYIYAPGNVS
FNLWRAFRNKAREGDGPFRFVDGEMVERFLDLDEAKQELVCEGLGPSVED
MRNMIEELRRMH
>Mb|QLI65284.1
MHSRKALHEKQINEIKAAATPRKASLRLDDIENASPGSALGTPRGGRIGE
ITEEVMVGGVSVTPIKRVPILANFEEWMKMATDNKINATNSWNFALIDYF
HDMSLLKEGDGVNFQKASCTLDGCVKIYTNRVDSVATETGKLLSGLADSS
SKKKGRDGEDAEGDESEDELDEDGNPKKKSKKKTQRSSEATLAPSFSALQ
LKKFELEFTVDPLFKKASADFDEGGAKGLLLNHLMIDSQGRIVFDSSDDS
GDVAPMEKNDEMEDADPDTDEADITAQLEERLGEESSKEDEEPTVEIDLV
SLGQKFLPSLSQLDGLDVCPSLKNFDLGDSSGSMDIPFLRAPDDWRDQDK
NHAAGDHSGMLIDDDAPIEFDDDDLGLGSFDIGGDVAFGEGGEAWARDAA
LAPQMRIYDAEMGDDGVDGDGFDDNGEYIVSMVKPRNADKMHEDILGFFD
QALQKNWSSAEHWRIRKIKDANKPAGETKKRKEKEPFEIDFASPLESTMA
NIIHTPASSNSVISMPKKDWKSKSRNLLPDDKHFNSKSLLSLFLKPKARL
SARRIVGGFGPRMGYGQDQKDDNQQIEMDEAFWAKQKGPEDTALPEGDYD
ANFFQDDGLPFPGGDDADDDDLEFADAREHLSPDGDPGMTEAGGITAMLG
GETFTNAAFGHTLVTSTRRIRPEYVNYARKATKVDVRRLMKEIWKGMAFD
QIDIPPEERTSMTASSEAEEKPLPPEDDPTLNFTEVMNGLKSVYPKSAMN
DISTSYCFISLLHLANEKGLVIDKSENLEELKIRKDWTAKVSEGGE
>Mb|QLI65285.1
MSQDYFTGKGKGPLRSDPDPDAPKNSSPSSSSRPHYMTVGSGSTSEHAAR
LQNMLDVDSGYGGSVAGEDHHMAMTSATNSTSDSSQHPRGRASAGHQMWY
YRQRAILGRSINKVLDLLQSLQQMNTEWPAHYPTIRPADATSSERPGMGR
TYSVSGDAASSSSTTAQPPLLRRSLTAADASTAESSRAAKNRPAPEPRLV
SPQIAQELSILKLDLKLGALHQAELVHSLEKNSIAALLDGKISSSIRHLL
SLRERIEDTSSKVLITGDLNAGKSTFCNALLRRKVLPEDQQPCTAIFCEV
LDARENAGIEEVHAVHKDCTYSRHDETTYDVYALTELEKIVTDNETYTQC
KVYVKDIRTVDESLLNNGVVDIALIDAPGLNSDTTKTTAIFARQEEIDVV
VFVVSAANHFTQSAKEFIYAAAAEKAYLFIVVNGYDTIRDKERCQKLILD
QVSGLSPDTRKESSDLVHFVSSNAIPAGPAPPGGPGGNGSGSSSGGGGGD
DPGDDGAKGKGKDLDKIRDFEALEASLRRFVLEKRARSKLAPARTYLLNI
LNDIHSLAQVNEEVAQSEIDRVSAELREIEPQLASSRKARSEVSEQIDRN
IEETCQDVYDHSRSELNTAITQAGRTNYDVPYPGVFGAFQYADDIKEAML
SHITDAVTRCEEYGREKSVQGVNTIKQLGLLHVGDEFQNLQFRPDVMFRR
KRDALARQVHIPTELWDFVDWSTLLQRQEKFAGTGMALTVAGAVVPRMLG
MSNWMDHAMLTTRVLSNGNLRQLIVPGIVIAAIAGVAYVLQQIPNSLPPR
LATKISSQLAELDYIHANSSRISSSVRKVLRFPADSLRVGLDQSVKDLGN

KRDEADKVRHESEEASKFFHELVGRSQVQRSMVEAVDLDAPQELH
>Mb|QLI65286.1
MASNSARGGPVGANEGAETPKPAAQTTVPDIGAPRAADGAPKEKSEKDLE
KERKKAEKAAKIEAKKAKAAQMAANAANKEKKAKEKKAEEAVPPYVEDTP
AGEKKRIRPFEDPNFKAYDPIAVESAWYDWWEKEGFFKPEFTPDGKVKEA
GSFVIVHPPPNVTGSLHMGHALGDSLQDLMIRWNRMNGKTTLWLPGCDHA
GISTQSVVEKMLWRKEGKTRHDLGRTKFVETVWEWKEEYHKRINKALTHM
GSSFDWSREAFTMDANLSAAVAETFVRLHEEGIIYRANRLVNWCTHLNTA
LSNLEVENKELTGRTLLDVPGYEKKVEFGVIVHFKYPIEGSNETVEVATT
RIETMLGDTGIAVHPNDARYKHLVGKNAVHPFIPDRKLPIVADEYVDMEF
GTGAVKLTPAHDPNDFNLGQKHHLEFINILTDDGLMNENAGLYKGQKRFD
VRYSIQDALKEKGLYVDKKDNAMKVPLCEKSKDVIEPRMKPQWWVRMKEL
TGPALEVVREGKIKIRPDTAERSYFRWLEDIQDWCISRQLWWGHRCPVYF
ANIEGVAKDTADDKLWFSGRTREEAEKKAAAALPGKKFTLEQDEDVLDTW
FSSGLWPFSTLGWPNNTHDLDTLYPTTVLETGWDILFFWIARMITLGLKM
TGKIPFSEVYCHSLVRDSEGRKMSKSLGNVIDPLDVISGIKLEDLHQKLY
QGNLHPSEVEKATKYQKTAFPDGIPQCGADALRFTMINASTGGGDINLDV
KVIHGYRKFCNKIFQATKYVLGSLPKDFAPAADGVIRGKTLAERWILHKM
NSAARDLNVAIADREFQKSTNIIYKYWYAELCDVYIENSKAIIRDGTEEE
RQSALQTLYTALEWALTLIHPFMPYITEEMWQRMPRRPGDNTKSIMVSKY
PQYSPALDDPESESAYELVLAATKATRSLMSEYSLKENAEVFVQAYNEAA
LKTCNAEVASIKSLSGKSVKTMHVIGPDAPRPAGCVAYPVSTSVAVFLHV
KGRVDMDAEISKAQKKLEKAKMSIQKQEKILFDPGYIEKVSAAVREGDEQ
RLADAKQEAISFEETIKQCEQLKLE
>Mb|QLI65288.1
MAVDQGTQTGIILTTSLAAFISGWLFGVFTTRGYLISPALAEERHRNLHD
PVESDESDIDEDDTILDHAPNWANGAAADRKQGLRANARVGGMTERQTLD
ADSNEECKLVLVVRTDLGMTKGKMAAQCSHATLACYKALARSAPDSAERK
LLQRWEKRGQAKIAVQVKSEAELRDLRRKALDAGLMAEVIQDAGRTQIDP
GSVTVLGVGPAPKSAVDKITGHLKLL
>Mb|QLI65289.1
MTLQQYESWYKANGIEALLVERMKAKQKKEAAKTSRTRDSTPSVILKSRR
KSKRGSNKTKVKAEQDDMAQDYPLFPGFFPSEIDAEVDEEFASGDMLSLK
GQVWPGMGKMDLANDDMKRTRNQRKPNSVIEKMKHISEGIEPTQVVMTPD
LEVERVKGVYDSSSPIPGQEEETPKKTVRPKRKRSQALAEISVNVPRRAS
RRPGRRNGQSKVKASPEESNGDSILPIAASTTSLRHGNDVFRDNDDDGVS
GKPP
>Mb|QLI65290.1
MLFPEEDAQLLKAWIVKRIENTSDADSDVLAEYVIALLKHDGDKESVRKL
CEQEIPDFLTEDPKAFLDDVFQAITYRSYLPGAHPPPKPADPLPSLQNKS
PESSRKRRLEESGPSDDPEQNGPQGGRAFKQPRKGRRGREDRADRNSNGY
GMMMMPPFDPNNPMEAMMQMQAMGLPWPAMPGQQGWGFGGGAVGRGGQRK
KGRGRCRDFDTKGFCARGSTCVYDHGNGGGDGGDEYDPSNAMIKPLNPET
PSRGRKGKRGGRKGGSRAPFSAEGAVNDRSKSTIVVENIPEEHFSEGKVK
EFFAQFGNIVDVSMQPYKHLAIVKYDKWAAANDAYRSPKVVFDNRFVKIF
WYKDEKDKLPASVSNNPSFKPGSGLDEMDAEPELDMEEFQRKQEEAQKQH
QEREDKRSELEKKRLELEKQQQDLLAKHQAESERLRAKLAGKDGGSETTG
SSGTDMLRAKLAALEQEAKILGIDPNAEVDSSEYPSRGGYRGRGYRGRGF
APRGRGAMRGTAGRHAAYAQFSIDNRPKKLAITGVDFTPSEKDEQLRHFL
LNMGEFESVDSNPSVTHVSFRDRKTAERFYYSLHGNELPGVEGKLELSWV
NSPLAPVNLKKSAHEEGGTVMPGDAMAGMDDAREEGEVREKERDANATPV
NMDYEVGDYGWE
>Mb|QLI65293.1
MARLSIAQVTRPSTTSIPKFLAPAFVQTRQASVVRIKKVKKKRAIPKDFK
RHNLEKRQFPQFSLCEAMRVLRAVEVGQPPASIKYEVHINLKTARNGPVV
KNSIRLPHPVQSDWQIAVICPEGSDIATAATAAGAVAVGEETLFEAIRKE
KIDFDRLICHEASEKALNKAGLGKILGPKGLMPSKRMKTIVSDVTKSIRD
SAGAADYRERQGVIRMAIGQLGYTPDQLKVNIQALLKKVKSECAEISEEV

SKEVHEVILSTTNGPSLSLNGKFNDVEGETQPEALAGVM
>Mb|QLI65300.1
MRPEVEQELAHTLLVELLAYQFASPVRWIETQDVFLAERTAERIVEVGPA
DTLGVMAKRTLASKYESYDAAKSVQRQILCYNKDAKEIYYDVDPVEEEPE
PVAGSSAPAADASSPAAAPAPVAAAAAPPSSGPAAQVPDEPVQAIDIVRT
LIAQKLKKPFLEVPLSKAIKDLVGGKSTLQNEILGDLGKEFGSTPEKPED
TPLDELGASMQATFDGNLGKQSQSLIARLISSKMPGGFNITAARKYLETR
WGLGAGRQDGALLLALTMEPAARLGSEGDAKAFLDDVTQKYAANAGISLT
STSSSGPAGGAGAGMMMDPAAIDALTKDQRALFKQQLELFARYLKMDLRS
GDKAAINSQKSEKVLQAQLDLWTAEHGDFYAAGIEPVFSPLKARTYDSSW
NWARQDALSMYFDIIFGRLQVIDREIVSQCIRIMNRSNPKLLDFMQYHID
NCPTDRGETYKLAKELGQMLIENCKEVLDVSPVYKDVAVPTGPRTTVDAR
GNLNYEEVPRASCRKLEHYVQQMAEGGKISEYGNRTKVQNDLQRIYKLIK
QQHKMSKTSQLEIKSLYGDVLRSLAMNESQILPKETNKGKKSGLRGTSQN
KGKVETIPFLHLKRKTLHGWDYSKKLTAVYLNCLEDSAKDGVTYHGKHVL
MTGAGAGSIGAEVLQGLISGGAKVVVTTSRFSREVTEYYQSMYSRYGSRG
SQIVVVPFNQGSKQDVEALVEYIYDPKNGLGWDLDYIVPFAAISEAGRQI
DGIDSRSELAHRIMLTNLIRLLGCVKTQKAERGFETRPAQVVLPLSPNHG
TFGNDGLYSESKLALETLFNRWYSESWANYLTVCGAVIGWTRGTGLMSGN
NIVAEGVEAFGVRTFSQQEMAFNLLGLMSPTIVDLCQAEPVFADLNGGLQ
FIPNLNEAMTKLRKDIMETSEIRRAVSKESAIENSIVNGADSEVLYKKKT
IAPRANIKFDFPPLPDWKNDVSPLNDKLRGMVDLDKVVVVTGFAEVGPWG
NSRTRWEMEAYGEFSLEGCVEMAWIMGLIRNHNGAIKGKPYSGWVDAKSG
EPVDDKDIKQKYEKHILEHSGIRLIEPELFEGYDPNQKQLLHEVVIEEDL
EPFEASKETAEEFKREHGDKVEIFEIPDSGEYIVRMRKGASLWIPKALRF
DRLVAGQIPTGWDPKRYGIPEDIISQVDPVTLFLLVSTAEALLSAGITDP
YEFYKYVHVSEVGNCVGSGMGGSAALRGMHKDRFLDKPLQNDILQESFIN
TMAAWVNMLLISSSGPIKTPVGACATAVESVDVGYETIMEGKARVCFVGG
FDDFGEEGSYEFANMKATSNTVDEFNHGRTPKEMSRPTTTTRNGFMESQG
CGIQVIMTAQLALDMGVPIYGVLALTTTASDKIGRSVPAPGQGVLTTARE
HAGKFPSPLLDINYRRRQIERRKKQIKQWHESELEYIHDEIDAMKAQDAS
FDEKLYAQERFSHIEKEATRQEKELLRSMGNNFWKSDPSIAPLRGALATW
GLTIDDLDVASFHGTSTKANDKNESSVICQQLRHLGRSKGNAVMGIFQKY
LTGHPKGAAGAWMMNGCLQVLNTGLVPGNRNADNVDPVMEEFDLIVYPSR
SIQTDGIKAFSVTSFGFGQKGAQAIGIHPKYLFATLDENTYQQYCAKVEA
RQKKAYRYFHNGLINNSLFVAKSKAPYTDEQLSKVLLNPDARVSEDKKSS
ELRYSDNFMKQSEKVMTSDRAAETEKVMKTLAQKVANKNSNIGVDVEDIS
AINIENETFLERNFTAQEISYCKDAPSPQSSFAGRWSAKEAVFKSLGVAS
KGAGASMKEIEIVKDDNGAPTVKLHGEAASAAKKVGVKDIALSISHSDNQ
AIAVAVANF
>Mb|QLI65301.1
MHTFVAAVALSVVGVAQAGFSIETIHKESAPILTHVDAEVVPDSYIIKFK
DHVNEAAVNDHHSWIQSIHKDGEEERLELRKRSLGSSPVEAFAGLKHTYN
IASGFKGYSGHFHESVIEKVRNHGDVEFIEKDTIARILRPVTPDESVQET
CTPETEKQAPWGLARVSHRKGLSFGTYNKYLYAADGGEGVDAYVIDTGTN
TDHVDFEGRAKWGKTIPAGDADEDGNGHGTHCSGTIAGKKYGVAKKANVY
AVKVLRSNGSGTMADVVKGVEFAATRHVEQVLLAKDGKRKGFKGSVANMS
LGGGKTQALDAAVNAAVKAGIHFAVAAGNDNADACNYSPAAAELPVTVGA
SAFDDSRAYFSNYGKCTDIFAPGLNILSTWIGSRTAVNTISGTSMASPHI
CGLLAYYLSLQPAGDSEFSVASITPKQLKETLIDISTQGVLTDIPNDTPN
KLAWNGGGCSNYSKIVAAGGYIGRPRTSEPKPVGNAVKEEMKVLSHKVSQ
GVKDLGDKTEKFVEKIHDVVDKEIEHFISEISV
>Mb|QLI65302.1
MSWKSSERLMDTIRHYAKFPATGVSLRQMVQFGDKPSIGTLFRASQFLAE
ELPIRLAHRVQELDDLPDGLNEMPSVMKVKDWYAQSFEEITQLPRPNLSK
EIRDRLMKPTKGNGRHIQRLSDATPNPSIDEGDSSGWGGLTNSSNGNGKA
KSLSRRYFATVDDTGDWPADLQLYNQRFAQTLHTIKRRHDGVVTTMAQGI
LEYKRRRQRMQIDGTIQSFLDRFYMSRIGIRMLIGQHIALTDQSHHRDPT

YVGVICTKTNVKDLAQEAIENARFVCEDHYGLFEAPRIQLVCNPNLDFMY
VPGHLSHMLFETLKNSLRAVVETHGMDKQEFPVTKVIVAEGKEDITIKIS
DEGGGIPRSAIPLVWTYMYTTVDRTPNLDPDFDKSDFKAPMAGFGYGLPI
SRLYARYFGGDLKLISMEGYGTDVYLHLNRLSSSSEPLQ
>Mb|QLI65303.1
MALTEYVFSPSAALAIVGLILSSYIFDYFVTNGKLRGIPAPFGAQFSNLW
LLMVCRRGDRFKTVDEYHAKLGKVVRIQPNHVSIADDAAIPTIYGHGNGF
LKSEFYDAFVSIRRGLFNTRVRHEHTRKRKMVSHTFSTKSVVQFEPYIHE
NLDLFVRQLDKLVEQSSHRTQGGKPEAHLDALLWFNFLAFDVIGDLAFGA
PFGMLEKGADVAEIKSSPDSPPIYAPAIEILNRRGEVSATLGCYPQLKPY
ASMLPDPFFSQGLKAVQNLAGIAIARVESRVENPPDVDRKDLLARLMEGK
DDNGQPLGREELTAEALTQLIAGSDTTSNSSCALMNYLIRDPRVLQKLQA
ELDASIPKHVDVPTYDMVRDLQYLGWVIDEALRHHSTSGIGLPRQIPPES
PGVVIQGYFFPSGTVLSVPTYTVHHSKDIWGEDADEYKPERWENVTTRQK
NAFIPFSHGPRSCVGRNVAEMEMKLILATWARRYTVTLRQGPMETREGFL
RKPLGLEVGLSRR
>Mb|QLI65304.1
MASAKEYGLLCLENPLLDIQAVGDQALLDKYGLKANDAILAEEKHLPLYE
DLLNNYDAKLIAGGAAQNSARGAQYILPPNSVVYLGGAGDDKYSAIMHDA
VTAAGLRVEYRVDPEKKTGRCGVVITGHNRSLCTDLGAANHYDLDHLKKP
EIWSLVQNAEVYYIGGYHFTVCPPAIMELAKQAAEKNKIFVLSLSAPFIP
QFFKDPVDASAPYWDYVIGNETEAAAYAESHNLPSKEPKDVVKHLANLPK
ENGKRKRVAIITQGTEPTLVAVQGEDQIKEFPVHAIGKEQINDTNGAGDA
FAGGLLAGIMQGKDLATSIDMGQWLARLSIQELGPS
>Mb|QLI65305.1
MARPAVLWLLLALTACALAAPQNEVSKHALESLSLDDLDHELQTCPIVQE
LSAIKHAHHAAAPSSLTTRLFAVLFPGSPAVNALLATLYISGPPNFLLAL
CPTNIDPSSLSVMVAFAVGGLLGDTLFHLLPEIFLGEDEPDRARFVLVEP
NRNLVLGLAILVGFMTFVAMDKGLRIATGGAGHDHHSHAHAHEADAQTTT
AAASGADTGTGNGQVKSRRKGKGKKEHADEDHHKEVNPSVKLGGYLNLIA
DFTHNITDGLAMSASFYASPTIGATTTVAVFFHEIPHEVGDFALLVQSGF
SKRAAMASQFVTAIGALMGTLIGIAIQEFGGGNTDGEASMPRNAGLWGTS
LTWGDMLLPFTAGTFLYVGTVAVIPELLETGPNKMQELRKTLVQFSAVAV
GAGIMLYISWHD
>Mb|QLI65307.1
MTNAIVVVGAGVSGLTTALLLSKSNANVVTIIAKHMPGDYDIEYASPWAG
ANFLPMSTRENSRWERRTWPEIKRITEEVPEAGIHFQTSRIFRRDKDMEG
PSCPSDPMFDHDPWFQSVLPDYKELDAASLPAGHDSGCEFTSVCINTAMY
LQWLVGQCIKNGVVLKRAVISHIREAKNYSHTGKPADIIVNATGLGSLKL
GGVEDQAMAPSRGQIILVRNELHPMITTSGTEEGLPEILYTMQRPAGGGT
ILGGTYDIGNWESVPDPNIALRIMRRVVEVCPEIAGGKGIDGLSVIRHGA
GLRPYRKTGVRVERGKLDDGTHIVHNYGHAGWGYQGSYGCAEGVVELVNA
IREENGESLVTEPKLFSWDN
>Mb|QLI65310.1
MVSKASAPKKKSRIASRTAPAETSPAPAAANGTHSQRHQQTLVNIFADAF
ANVLSSEHFPALLQEIKQALYSRDFGRAFGREDYLEAYAARWSPTRALCY
ASVFVGIDAHLEVLASDAAGSVESEASHDGAADSPGEARPRRGGIKMLCV
GGCAAEHVAFASYLDQTERAGTLALFDSAPWEHVASMLQMQLTTAPALSK
YASAAARASNAALVDPSRLAFSFTQADVLSLTAEALLEQVGSAPLIVTLM
FTLNELYTEGGIGKTTKFLRSLGQVLPGGSLLLVVDSPGSYSEAAVGKEK
KRYPMQWLLNHTLLEGESSGYTWDKLESEESMWFRMPEGLSYPIQLENMR
YQMHLYRISKKS
>Mb|QLI65311.1
MAGRETMPGKRRPSEVLEHQELQNLVRIASDLREKQGLAIPNSSDLSDND
DDPRLDPTSHTFDHYRWAQRALMSLNKSGISLERQGVIFSNLSISGSGSP
LRFQETVLSSLLLPFRSLALAVLGRAKSSRPRRILDSFDGLLRSGELLLV
LGRPGSGCSTLLKALCGHLEGLTLEPGSRIHYQGISFKKMTRQYRGEVAY
NQEVDEHFPHLTVGQTLSFAAAARVPRQRPPDLTRQEYIDTMVSVVMALF

GLSHTFDTKVGDSFVHGVSGGERKRVSIAEMFLSRARVGAWDNSTRGLDA
ATALQFIKSLRLSADLGRACHAVAAYQSSQSMYDLFDKVVVLYEGREIFS
GPCADAVAYFEDMGWHRDSRQVASDFLTAVTNPGERTPRPGMQDKVPRTA
AEFADYWRRSKEAAKLKADMEAYERAHPLGGMAWQRFQESHEKQQARHTR
ASSPYLLSVPMQIRLCLRRAFQRMRNDVPTTMSTVVVQLVLSFIIGSIFY
NSPNTSDAFFQKGAVIFFAVLMNALITINEIMQLYSQRPVVEKHARYAFV
HPFTEALASSIIDLPIKLLRCSLFSIVLYFLVGLRAEPGPFFVFYLFLIT
TVLVMSGIFRSAAAATRTVGQAMGIAGILILALVVYSGFMIPQSYMHPWF
AWIRWINPIFYAFEGLLSNEFHGREFGCAQLVPPYGTGSSFICAAVGAVP
GQRSIAGDAFLKANYGYQYSHLWRNYGILVAFLVFFHVTYLTATEFSKGR
PSKAEALVFRPGHAPRRLYQGDVEAPEKDRVSVSTAPGDDKMGHLPRHRD
VLTWRALNYDIPVQEGTRRLLNDVNGWVKPGTLTALMGVSGAGKTTLLDV
LAQRVSIGVVSGDILVNGQVTTSGFPRRAGYVQQQDLHLGTTTVREALRF
SAVLRQPPSVSKADKYQYVEEVMQMLGMHEFAEAVVGSPGEGLNVEQRKL
LSIGVELAAKPSLLIFLDEPTSGLDSQSSWTICAFLRRLADHGQAVLATI
HQPSALLFQTFDRLLFLAQGGKTVYFGDLGLKSSTLLDYFARAGVRRCGE
RENPAEYILEMVSGRDDSGIDWAEQWSKSPEHDEVLEEVEALNRRQAVAR
TESTADQDVSREFAQPFGTQFVHVAGRAFRQYFRQPEYIFTKFALGIASG
LFIGFSFWKADSTLQGFQNALFGVFLLATIFPTLVNQIMPKFVAQRALYE
VRERPSRVFSWKVFILSQMLVEVPWQVLLGICAWACFYFPVFGTSGSPDT
QGLILLFVIQFYMYAATIAQMVVAAIPDPALGAMLAVLMFGMSFIFNGVM
QPPDALPGFWIFMWRVSPFTYYVSGLAGAALHARSVVCSASEMSVFDPPP
GQSCGAYLAMFLDKAPGTLYNPGASSGCEYCALSSADQYLAARRIYWGER
WRNYGIFWCYMVFNVFGTATLYYLFRVRTWKRRKVTRP
>Mb|QLI65312.1
MRGFVSAAALSLVLVGTPVSGLDVQIQDEASIKSTAGTIAYGLMKYYTGN
NTGDTPGNLPDPYYWWEAGAMFGTIIDYWHLTGDSSYNDATMQALLHQAG
DTKDFMPKNQTRTEGNDDQGFWAMAAMSAAENKFPDPPSDQAQWLALAQA
VFNQYVLRWDPADCKGGMRWQIFQFNNGWNYKNSISNGCFFNIASRLHRY
TGNNTYGEWATKIFEWQQSINLITSDYGVHDGISIDPDGTCSRIDMLEWS
YNAGIFLHGAAAMYNATSDDKWKKAVDGILKHASEKFFKDGVVYEQFCEF
NKVCNNDQQSFKGYLLRWLAATSQLVPYTYDTIKPLLEKAGTVAASTCTG
STAPPNFKGQPGTACGFSWIPQGRFDGMVGVGEQMNALDAVMYNLVRKST
PPVTQNNGGTSKGNPSAGSGGTEDPTVLKPLTTADKAGAGVITMLFIVGA
LGGTAFMLLEFR
>Mb|QLI65316.1
MTNSFNFNNIGQRQDGVDNTNMPSPTSPTQDAGQASSSSHHQSGNVTVYP
TTGPDSYTSPILAPGSSGQPALNPRSCVTCRRRKVRCDKQMPCSNCRRAQ
IPCVFPAPGRAPRQPRPKDPNAPPKTSSHREAELVKRLRKLEGIVEELSG
QIDEPAARGPSTTASPDMQSGMEGLSLRHMPGTLDHIAGQEGSPRGTDSS
AGHSGEAGRKEKQPNFGRLVSDDQRGTSRYVSSGFWSKLNDELDAIREET
QLLTGEEDAEDSDFEGTPTDSPSTGFGNITDHQGFILGYRSADVDLQKCH
PLPSHASFLWSVYQENVEPLIKLLHVPSVELIISDARRNHGKLTPGNEAL
VFAIYFAAITSLEPDEVQANFDANKDDMLAQYRFAVEQSLAKANFLDTSD
LAVLQSFTLFLIVVRRHDESRFSWALTGLVIRIAQGMGLHRDGTHLRLSP
FETEIRRRIWWAILVLDLRSAEELGTDMTISERSYDTLKPSNINDSDISP
GSTEFPVPREGRSDCAVSIVRCEICGLSRRLVAAASAMSSLCPGVDQSSI
AERERMLIEVYQRVEHKFLQHVLDETDPLYWVAAMIARVIVAKMCLVIYH
PMLFPGSEYELTDEIRQRIYVAAIEVIEYGHKLNTDPRCKQYRWLFKTYT
NWHAIAYTLIETCRRPWTALVERGWDAINGYDIDPLELAKRADHAAVFLP
LRKLFAKARKHRESELARLKANQDEARRLDFAERMNPAQARFGPVPGAEN
VMEQMREKWWSLVRPDGSSPMPSYSIARQPPPPQEPASATAASGNDTLKP
PGNIPVQLNLSSAAMEYMNELMAQPNPNVAEFWRIDNIANNGGNLGSSGS
APSAPMPLQNEMSEDILRQQALMLQAQPSKDDGLTPYLWSDPFTVMNTQF
DVTEDADMLGDDFDWQDWSQSIRGLEMGSTQKHEGW
>Mb|QLI65317.1
MEDPTWAWPAWKFGMKRDDLFTTLHDQYNTFSFTLQDPEAFHHDVYEISR
DAESTAEFHRMMADRRQMRLRELNESLETLAVEIIANPTLMGTDQWQYAL

QLFRTKSYDSILRYFASYLPEDYLDRHDTHSTSSASFSETDTISTVASSV
NDAPSPFMTDDDLFPNGPVITAEPDAIKEKPHHSSHPQGPLSAPHSEDAQ
SEPSVSSPPSDAESNDYLTNPPSRSMSFSGSESGHLVSELIRRRCLEEDL
NEKSRSDESETAFTFVCDSFEPIDVIEGQSHTPGGVVDFADEDEDIPTAQ
YPDDVFNSLDLTFSLTHPPSAHDTLDSDTPTPRPEAAAASYMEFRSVLSH
SRKAPSYQRSPSPKCCLANRGAGSPVREVRRSPEESLSKIQKSVQDFSRR
HPKTRRRMD
>Mb|QLI65319.1
MSRYAGPMPPVPRRGPRWSHLHHPGTVSFDLGSTESPSSPGSTSTHSTIR
EDTRRKTERHCTISVNDGYSKDEVLLNLDHLGGDIKPGALMALIPLKGDS
KNATCGYGSVSKQPHEHLDSARAASGTYSDHDHGAGHTYIFVAKDMSKEM
KARHPDAEVHVAKHIADAFFMRKGCHVLLRPVNQHHPAVEATHVEMSFKD
QYLSRADMWRLAAGELTQRTVYKGQSLFFMGTIKAQVTAVYVNGHTVHSA
FFGKDTRPIFRSESAKYVLFIQMAREMWDFDSEASGEIMFNKVVNGFLPA
LFKRWAVLKVKHLVSIVLFARVEYDTGISADLATSSLLDDYYTGMQPYGN
RRPYKDFYRVVVSEMASGEWTTILYQLKREFNFFRRDITLHHQKISTFGH
RPSDEDGAKDGISSPAKAESSLSIHGNVLEAINMAASQFSHDYIDRDLTR
TGISIAVITPGPGVFEVDYETLRRTTESLVGSGIGIDLICMASMPLHSVP
LFKYRNPQFSEDARHSQGHALSRSFHSRDSTPSHPTPVIGSYQSMSGSFS
PTKAYSLAGRIETLALTGANEEYCYALPQWLHVSFWTGAAEESLSYAGIA
LTVSNKVEQEDKDWFKVRCRMYDLQMRSVLDANEIETAPLQSDSSYPSHL
VQASAIAKKRPDRPGDTIYIPNKQAPETLFDHVYGFQKFVPDKLVRPGEK
SVCKHLQEYDDAKARLPRARPYHQHQPSRNKPEFEDVARKQLNDDTSAMG
TSLPERKASTYLQPSHMQASRKFSLTTTEVEKPILSAPKPSPEPPPSTAT
FKKAPSIKQPKFMRQISLGQRGFGIAAPKVVAAELKIETVGAAAVSSNLR
PPSSAHSFSDLRPSTPQTITSRSSSASAQRQKYETAASTTDGVPSTPSIP
ISRDSFNRPGTAVSQVKSPSVLPVAASTRRERRPEDNDVRFSNALRAEDA
QKVYTSKLRAGVVPELPSTLSPTTAITPWLTLLNPSNPERHKIDDTILYS
RWQHVFPRTSDMKVQKWKTLCCPAAVPLTTEYFPAKAQFDAEYQRHPYNI
DQNLDDDVVDEPKSRKDFIKELISMRFSQGFQVIVGPSVARAFGQKVIKV
ADVFARDQPLDDGTSVFMSVGNTIHQLSCVNGSEVEVNIYLRKPTDVTAE
SQRYPSAYRPAIRTIFDTEYETRSFDILTPKMDRNWNTIDSYLAGHYDQM
MDSLRFWRARFVLIPLGPKHPLIQRVQTSDNPEELRIEGIKRLGQLWQKH
RYVAPSDRRFQPTAKRRSPLDIVYKTEDPSVVIAAEVETLPILESLEGPH
RKGGLVTRKERFQKGNLNLAALAEAMQQPVENGGVPLRNRRWHLRLHNAA
FIGSDMTTWILDNFEDLDSREEAEELGNALMVPNEPKHKDKDKSKDKEKD
KEKEHAEPKSEKDRGLFVHVERRHNFRDGNYFYQIAPEFAKPQPGWFSSR
RRDVTSPAAPGSDGGSMDSPRPNMPRPLVPNDPKSPASLGPAIGSSQLLK
NRPKVVLSKMIKYDIDPRKRSYRPERIDLHYDRLHNPDNCYHIRIGWMNT
TSKLVEDTIEGWEREASQYGLRLVEVPINEACRITETNPFRKPYRIRLAI
RPPLERPETYYDPNSLDLQAGPSKNFYQTVILKNFDFVLDMEAASNFPSD
VDVRYSWGKPDFSYTQYIHRSGAVLAQITDEGDFLVLANRLYNKRTSMAR
DKELRSQVHSEQQQQQQQQQQQPQPQPLPPLNTNTRAMAQIAPYVSGHP
TTNLPESAAPLASPQVRPTFYHFSPASRPIDASIYGSKISYPAGRSEPEV
LKTELEAFCLNEVALTAFYKDILDKGQRPHGTPASTATTQSIAGLEPVPE
ASIPSLGLGPGILGGDGAGGVGNGHSAAMVSMRMSSPMAFLRRGSVQYDG
MGLGSKGK
>Mb|QLI65320.1
MRLSSVPAALLAAIVGTAQADHASDLKSISLRTHSIQQPYLDSDMQSRWF
DFGGDTIVRTDSYIRLTSDLPSQSGWLFSRVPLTATNWEVEVEFKISGKH
QLYGDGFAMWITKQRGQMGPVFGSVDRFEGLGIFVDTYKNNRPGVVFPYV
MAMYGDGQRSYNKNDDGKETELAGCSARGIRHASVPTKMRLTYIQDKQLK
LDLQYREEDEWTSCFEVDNPPQIPNIAYLGFSAETGELSDNHDIISVSTK
NLYNSPGSTTDRAGANKGRPIQSNVVKEGSSWTWFFTKIILFFVAVGGMY
VGYTAYRAKSKSHRF
>Mb|QLI65321.1
MAAAGQSSITVAVRVRPFTIREAAQLQKTDDSPVFLGDGSLAGPAPKLHQ
RGLRSVIKVVDDRCLVFDPPEDNPVQRFSRSVVPTSKKVKDQVFAFDRVF

DENTTQSDVYEGTTKNLLDSVLDGYNATVFAYGATGCGKTHTITGTAQHP
GIIFLTMQELFEKIDERSQEKSTELSLSYLEIYNETIRDLLVPGGSQGGL
MLREDSNQAVTVFGLTSHHPKNVQEVMDMIIQGNEYRTVSPTEANATSSR
SHAVLQINISQKDRNADVNEPHTMATLSIIDLAGSERASVTKNRGVRLTE
GANINKSLLALGSCINALCDKRQRAHVPYRNSKLTRLLKFSLGGNCKTVM
IVCVSPSSAHFDETQNTLRYANRAKNIQTKVTRNVFNVNRHVKDFLVKID
EQMALINELKAQQKDAEQLFFAKFRKQCDKRDGVAREGVLRLRAAYDHSA
AERQERIATMKKLKAFERRIAMLTGWIAAFDTVCEQRADNDSMPQNLISI
RKTAQGIQVELENSRQHIHQKLEKATWERAIDTALSHSLQQLEGLEGIDS
GEHATLTREAELLKANFGREAYREVLEQEKLGDAAMMQMLLTAQFEMLAS
LSDTLAMDEESAVAQAKQIIHKLLDTGYTAAGQVVKPDGSLPVLEIFPPT
RKGTPKRKKSLGAYVKPIAPLALAAVQSEHVFASPMKSSPRRRKVLGTAK
KGISFTPVKKSKPRSVRWRDDESEDGTLADFANTPQQMDATPDQSSAERT
PIPPPQPTYLVEDEQNRETSSPRIEAPEEEQAATPASKPGRFQAGFLSKT
RMSVQPQGSPLLQAPMLSRSVSSSSPNSGRLSPLRTLDVSKTGNLSPPSL
PAAHRSRLSPPQPTSLRGIVDENNPPIQGTPSSGSDSESSMIDVRKLRSA
IQSAKARRPSALGGVVAANGRRVSSISTIPERAMPRPSMPSALASSTNGI
SRARRGSLERRKSPPLTCSPPEFKGDRNLTAGQARRMNLGGSVRLESTAS
SPRDGLKEAPRRVTISVGSTPASHRRQETRGATAWR
>Mb|QLI65322.1
MSKSTGVDAEASELRQRQTRDSPIVTNQPQAVAVESLSTEDGPDHDKPKS
NSKTFGRTPDGTIFIVPTTHDMVSQLLDPRQPKNLSDAVVLGILAFMILV
AYSLPAGWKRPVFAVTFLFWRGCYNVGIGVLLHIQSRHRRLITWAKRWKL
FEDPKTGNNPRPWLYNLLKRELEAKIPEDYEFEKAPLEYNTWLVFRRVVD
LILMCDFVSYCLFAIVCGHTPEGENIIVGAARWALGIALVGFNLWVKLDA
HRVVKDFAWYWGDFFYLIDQDLTFDGVFEMAPHPMYSIGYAGYYGISMMA
ASYEVLFISIIAHAAQFAFLVTVENPHIEKTYNPPLPRKRDLSRLQSDSN
VSPDMSDHNSTDRPASTPKMATQEPPVPVHNMVGFRNMDLFRVPDSMVVL
LPMYVAILTLATPTTPAWQAFFVAHALLWRVWYHLGLSIILDKQSKTKMW
TRHFLKYGESAGEAWRQWKGLHHISMIMCNASFVAACYKMYSMPSDWGYG
LVLLKHVLGGGLIALQLWTAFSVYDSLGEFGWFCGDFFWDQEAKLNYTSI
YRFLNNPERIFGTAGAWGGALITWSRAIFIMALVAQLLTLYYISYVERPH
MQKIYGRSMRGEAGLTKFIKRALPPPVKGWQESVDKVLDDTTHFVEDFID
SARPKFASGVKTIVRDTSALFNIAPARITLTKIPADLAGLDPKLYSLSVE
GTPSALSNIHDRATGKESFTGRFPKHVKTMAYEYGAPLRVKWRAPANHSD
KDWIGLYMVTDNRSRESTEVPSLGRWAPTNEGVYDALTADSSIVTPEHPS
PKSDSSEPDMVEGEVEFNGDKLWWTQGVFEFRYHHNGRHMAMAISEPFEI
RIAQFAEDDVDLDSKGMYAEAVEAAILPVVRNCFDRDPDIAPCTVDEPFG
SYVERDGKYAKRIVYAIREMFGIEFAPAVVLADGNVQKLAWRICNAKEVL
APYSMSHSKGSATPAAQEFDNKT
>Mb|QLI65326.1
MDSTNPPTKPCHNCRRQRLRCDQSYPHCNKCTAAGKECLGYGKLFRWTGA
VASRGKLAGRTSSAPVDAAARTAARATRVAGKKADLDFLTPSSSPPSLCS
DAGTPVDMSMSEYQGDAKLVLRSPSPEGVVTSPWALADPLYQDMQYDHRY
YLSYFTNRVCKDLVSQDIPERNPFRGLLPLTRAHPLLQHIIVAASAAHMS
NLVRAPLAHHPKGVDDGVTVGIEQASRRALQDALVAKSKALTLMRGAVEN
IKTTGGDVVLAAALFFINVELIESGKHGWKAHLEGAGRIMSLLQPDSVAD
TALRDYMLSDCFIYFILASAFMPAASFDTQSYFQPSQIPFILQRAAANSY
LCCPPEILTILHSASQLSNVSPDIASEEDVRAAGLVLIQEAQAFDINGWA
NDVRNMSYLQDAPIQSRIHAGSAHRLAACLYILQAIPSMSQMNDHGQVAE
ALSRDIFKHLSSIPDNDPNFKATTWPTFIAGAEAGGRARREWVMDRLQRL
VRSCPWGFIYTAMETLQVIWDLDNKGQGTKSWVQTLKDPQMNFLMQRSKN
FQDIWGIAMDQITSDRMLPSSQQVRCLDRLSILDDDAEGEDSLMPFWLLL
QNILSGKRNSSSGVLLKCRIQYPTAFEDPLVLPGTIACSNKLSTILGPSS
PPPYAEYRADCIGTTRTLPTRKHQVPQFWRSLAFIQNGNCLLGGATVSLY
VIRAVVA
>Mb|QLI65329.1
MRRVVVTGLGAVTPLGVGIRQIWNRLLAGESGIVSVASRQPQARWKELTS

TIAGVVPVGSEPGQWRASDWLSASEQRRMSTFTQYAIAASDMALKDAGWD
VTTPEDMEATGVCLGSGIGNLDEIYETSLAHHTDGYKKVSPLFVPKILIN
LAAGHIAMKYGFQGPNHAATTACTTGAHSIGDASRFIAVGDADVMLAGGS
EACIHPLTFAGFGRARSLSTAYNDNPSGSCRPFDAGRNGFVVAEGAAVCV
LEELEHAKARGARIYAEVKGYGCSGDAYHMTAPREDGRGAFLAMKRALKN
AGVKPLQVDYINAHATGTQVGDVAEAAAIRRLMLGQEGVSAESQVTVSST
KGAMGHLLGAAGAIEALFAILSIYEGVVPATQNLHKPDVGINFNFVPLTA
QEKTVRVAISNSFGFGGTNSSLVFSKM
>Mb|QLI65331.1
MSAMAGTKLSKGSRISNATSIRSIRALAPLLDRVLVQRIKAETKTASGIF
LPESSVEKLNEAKVLAVGPGAMDKEGNRLPMGVTVGDRVLIPSFGGSPVK
AGEEEYQLFRDSEILAKINE
>Mb|QLI65332.1
MLPGWSASQQAPGVSQRTQPVAYSYAASPAFVPVQVRQGFSQYAPPTPPY
PGYVPPSVGAQSPAQAEQPKPKTEWPESVRKYVQRSFLPENDDPTVSRAE
VEAKLKETIGTAKENGTLYTIDWDAVPLPQALVKADRDALYNSGNHTPSS
SESVAKSRKRKSSDSVDGGSNQPPWHNANSRSSLETRISYPSPDKRSTFD
DNLAKSSKFQKEAASKRKRRFESEYKAVYRSPSPTPPSSGPIIGTSEVLE
KRYLRLTAPPIPSNVRPVHILRQTLDLLKKKWRKEGNYSYICDQFKSMRQ
DLTVQRIKNDFTVSVYEIHARIALEKGDIGEYNQCQTQLRSLYSLGLKGN
PIEFKAYRILYFIHTANRTGLSDTMADLTAAEKEEKPIKHALQVRSALAL
GNYHKFFQLYLDTPNMGAYLLDMFVARERLAALCNICKSYKPDVKLRFIT
EELGFESDADAAQFIIDHSGQHLLEDRSDNIVFLSGKAGQLFESSRSEAF
RKVDIKGQI
>Mb|QLI65335.1
MGPENNGCSREELIAPRSQTINDLKLQLVLSLVLGVSAFITFCILRPRWP
SLYAARERRLDPKINLPALSNSFFGWIPQLYRISENQILAAAGLDAFVFL
AFFKMAIRLFSIMAFFAIVVLEPINISYRGNETWLNPNKPEHDGRDRDLF
GSSQILYGNGLDVLKDNDEDKSNEKPYLWAYVIFTYFFVAVTLYSINWET
FRIVDLRQRYLGSQSTVTDRTFRLTGIPIKHRSEEKLKDLIEKLDICLVD
SITLCRDWKYLDQLVRHRDLLLRKLEASWAKYLKIQESSTQHSDATQTQN
VDDDTVGQVRGINRDEESAENARLLSSQQDQPYMFAGDRPQVSIRYGPLL
LRSQNVDAIDYYEEKLRRLDEQIVQARKKEYEPTDMALVTVDSVASCQMV
IQARIDPRPGRFLTKPTPSPSDLVWKNTYALRGIRRLKAWAITLFITVLT
LVWIFPTAFLASLLSICTIDRVLPKFAAWLSDHTVIRSFIQNSAPTLIVS
LLNVSVPYLYDWLSNHQGMVSQGDVELSVISKNFFFTFFNTFFVFAVSRT
GFEFFNVLRRFLKDTSLIPRIIARDVEDLSLFYTSFIILQGIGLMPFRIL
EVGSVFLFPISRWLSSTPRDFAELQKPPKFQYGFYLPTALLVFNLCIIYS
VLLNGEIILIFGIIYFGLGYFTFKYMLLYAMDQPQHATGGAWRIICYRIV
IGLLVFETVMVGQIASSRAFVQSVAVLPLIPFTIWYSYYFNRRFEPLTRV
IALRAIRNGREQNGEGEQAVEDEASDDDGPHFQRQILRRGSTLDEFKERG
LTFVNPSLVTPLQLPWIYHDPPALADDDVTTEGDRSSQGRPTLILPNADS
SLGIGEDNVWVDSTQRS
>Mb|QLI65338.1
MSHIENVDGVVFDPNLTKPDVLVLQNLVADIREYQERDEQCQRRRDKEGS
DKEKGQSHRDAATITALAALNDPKHALFEPTVFSQVDTGDIRLPLSIDAW
IFRPYVRVARSIVRVETDVVMLTHLLLYFSTSVPSAMFLFWKFTWVHGLL
HFIMQVSYMGTYTLMMHQHIHMRGILNKRLSILDSLFPYITDPLMGHTWN
SYYYHHVKHHHVEGNGPDDLSSTIRYQRDNVLHFLHYTARFFFLIWLDLP
LYFIRKGWPAFAIKAAFWELSWYTTLYTLYQYNPKATFTVFLLPLLLLRS
GLMLGNWGQHAFVDHDEPSSDYRSSITLIDVPSNRYCFNDGYHTSHHLNP
RRHWREHPVSFMKSKELYAVQKALVFHNIDYLMITVRLLMKDYRTLARCM
IPIGEEQMSMSIEDRMALLRSHTQKFTEKEIQDKFKAK
>Mb|QLI65340.1
MQTPEKRRGGGGHALRTGSRHNHSTVSTPSTSGTAASSNPNSTGSGSGSG
SNSMPAMPPRAAMVNNNAAVPEDAHETASSNASSGGASVTDKDKDGSSAP
ARSASIRYTASSSKERQKERDAVLVREKDERIAYLEREMGIMEREFQREL
DKLSQNESETATFWQAKHSALNQQYLRTDTELRLLRAEVDVREAEREELR

QGWEVLRRELKERDDEMRGLRSQIRGLKEFVSTSTRTDGQTSDEVFGDGM
ARLGNGLQNWVITNFRRTRLVDFAKVDEATLAELSELVPMYEALAQTVKV
HLLQSVVSKILVEMVFDTYFVGLSDDQTRCFRQMEGLLSSFSTDESVNQW
RASTLALIRRDAPQLLQSSTTEYVEKVISRINRILDSITDSSTSSAPSRS
EARDSALRVLVNNSVELARLLVAQKAVLRVYMPEVLPHQQVMFEPETMED
LGDEDEEALARREIWCVVFPGVIKHGDENGGRMQFRNVIAKARVLCSPEN
>Mb|QLI65341.1
MRRLDFASGCQNTQPETKTTGAASSTRIGPSKSSKSSKPSRSSIASAPAG
TPTPAAAPPARNTATTTRRIVSAPSSYSPSPRQTPTLKPRVTATVSPTVA
PRGAPTQPHLPLLSRHSARHLPRLPTPGLAANTTNSSTTPAPAVAAVIAT
TAGASHASLRSSSQHHKQHVISLAVKTRSPPASLTNMPPGERPRQPQLSA
AAARSINRTPLTPKIAAKGPPVTPLARRPHSSTPTLAGSQNPRDDVSTPV
TAFLTHNITPRSGTRQSRVDSANSTPNGTPNPERTSDSFENNNNNNASRL
GLGVSPPANDLPRQTASQLDIPSDPNDSSKFFYASDARCIQQGGLQRPGS
VSQKSANTFFYANGSTADGGRNISPPSNAASISLASPNPESATTKFFYAN
GKPDVSLRPGILQSTSESVVSSNSRIATSRPPTGNPNAAFNIPPPQRPAS
PIKAASTAMSQTLRNNVASPPSPKRTSLVSSPPSLPQQASSASRRKVSID
TAPKEMGGHRRAGSVPGFEPLTLPRFNLSTKCSETASVSPPLSPGFSQPA
MTMASIFQVADELQDETESREGDAQSDVSQVPRSPTKSAHFGDSVSELVA
NARRERKVQDLEITNASLEAINRTLERQLRKQTAELRRYRRLSRSGALSA
ASSRVTSLALTEPPTDMSDVDEEDEMELEGDMDSFDDTDLSSNDTMSTED
IMLSGAKLDARRRRDERRLHLDLTKHQELLIDSQRINQSLKRCLDFTETL
IKEGQKALQYNVRVSDVKLGGRVLAPSDDYDDEVSYQEEATIQSGSHGLE
PPWPKTSQDRDSGIELPTDGG
>Mb|QLI65342.1
MASKAMWEVDPETRSKLAALQKESKNNICCDCSAPSPQWASPKFGVFICL
SCAGVHRGLGVHISFVRSISMDAFKASEIERMRLGGNERWRTFFEEHEDT
QMRGITWEDATIAERYSGDVGEEWKERLSCKVENREYVPGEKKPAAPAVK
PASSPALGGQRAGSRTGTPLSNSTSRSESPGNRGAGGKVKVDDRYFSRLG
AENASRPDDVPPSQGGKYAGFGNTPAPAKAGQAGIPNFDELQKDPMAALS
KGFGWFTSTVSKTAKTVNDGYIQPTAKQLAEGDFAKQAQLTASQFAKQAQ
MAGKSAQEGFNRFVEGNDSNHRRDAPLDASRKDFWDDFSSLAEQKKQTSS
SIGTSAMGMGKKGSSVAQPTKKADDWDDW
>Mb|QLI65343.1
MPAASGDSSKPSSAQSSKPSSIKSHDGSRHSPGLIAQDFDSASESSHLRP
HSERSSVKERFTRMFASKDLPKLNNGNGEHAGRPRGPSLNGASSPASSRK
DSSERLSEKPPSNASKVIPGKDLGSRFACNSEVQGGHEHHLKSSRRQEKL
SDMWRSLLGKKPEATPDSDLSLVSNWVDSLRQEKEEAGDRKGGPNVSSTL
VEKYGKCQEVVGRGAFGIVRISHKKLGGSEKLFAVKEFRRRPEETEKKYS
KRLTAEFCISSSLRHPNVIHTLDLLKDAKGDYCEVMEFCSGGDLYTLILS
SGKLEVQEADCFFKQMMRGVEYLHEMGVAHRDLKPENLLLTTRGALKITD
FGNGECFRMAWETDAHMVSGLCGSAPYISPEEYTDKEFDARAVDVWACGV
IYMAMRTGRHLWRVAKKDEDEFYARYLEGRRDEEGYGPIESLHRARCRNV
IYSVLDPHPTRRLTAAQVLKSEWVREIKLCKAGEEGL
>Mb|QLI65346.1
MWRRTYLLLVLIRLWFALSPSYLHPDENFQGPEIIAGQIFSYPVRRTWEF
TSEHPIRSVFPLWPVYGLPMLLLRWLWIGNGHDGEIPPIAVFWTLRVLMF
LISFVLEDWALHELIPSPRHRRVAVLLVASSYVTWTYQTHTFSNSVETLV
VAWSLVLIQRIVDTRQQSSLLSSTVLGIVAVFGVFNRITFPAFLLIPGLR
LIPHFLNKPFSFVALASAALITTVIAIALDTAFYSSEPVTWANLISRPTI
TPLNFFLYNSDTANLAQHGIHPWYQHVAANLPQLLGPAAALVFAKPHLSL
RLYSAISGLFVLSIFPHQEARFLLPTVPLILSSVELPKNKILLRTWAGAW
IIFNLFLGVLMGIYHQGGVVPGQVFMSKQPDATQAIWWKTYTPPIWLLNG
KNEVLQTRDVMGLKGDALLTQLTELATCDTPADRRNQEYLKEKNGTYLIA
PASATWLDPYLSNKGLHGLRFREVWRYRKHLNLDDLDFGDDGIWNTLSRV
IGRRGLIAWRVTKSCQKLSNSS
>Mb|QLI65348.1
MACLIPLSLNAKCQERRYNGGMLKRLEAGSQLYQPKSTNQPQSEQEDKET

LGIMRPIPRTRMEWKAALGDIRRDYMNRRFRECSTRCHDILHHKDELETT
HPVHLVYLRFYAATALEMQAHAIHHSSPFRTALLKQAYDHLSAASKLAKQ
ADEDTSRPTSRFPSRFSSLHSSAGSNSSGSTVSTRRSSPAPSLSSIDEGA
MKPSQPPRPKKRVAFCDEPMMEPIIRPDSPTLGFDEWLGRSSPEPVYPES
ILKNARPLLPTIISAPLESASPDPSEAEDIDPFFHTRSIHRFCTILSSIR
RQVTAHMTTLEIEIAACHIPSVPAHTNHELKALDARARIERLRACGWKRA
RFDAERYERLRENALADMAG
>Mb|QLI65350.1
MAEGDEKHGIGSDDAEIGVATPVPNEEPPIDGKLEKRLLLKQDLRILPLC
ASIYFLSFLDRSNIGNAKILNSSTHDDLQTSTGMTDYQFNIALMVFVVAY
AVFEVPSNILLKRLRPSRWLAFLMFCWGALTICLSAGRNFGGVTALRFLL
GAFEAGLFPGLVYYLTFWYRHNERSVRVAFILASATLAGAFGGAIAYGIG
HINKAHNLRGWQWLFIIEGIPSCVCAVLVFFLLPDYPETASWLGEAERDL
AQRRLLHEGSKGTQPAMTWADAKATLTDWRLYGHYAIYFAISPGFASLSL
FSPSIVSGLGYQDLQAQLFTVTPWAVAYVCQLAVAYSADRFNARGLHTAG
AAAVAAVGFIVSAALPPTAYASRYGGLILATSGAFSAIPPLLGWLSSNMY
STAATGLAIAINVSCGGGLGQIPGVWIYKAGEKSRGYPTGHWTNAGFQIF
VAAASVGLWAFYRVKNRRLTEEARRNGVEPTLFKL
>Mb|QLI65351.1
MLNSSSHRLWICTRCVRRVTWPGTTATSILQQRRWETTAAAEATRSSAPS
DHHVSSRHDDAVLRDLFDAPSGGLSFPSFSLKKSQGLFKNRYLTSPDGFF
VFAQKNLEKATRLVQRVLSASTTEDYQGVVKDLDRLSDLLCRVLDMSDFV
RTTHPDVRFQQAAAGAWSMVYQYMNQLNTMTGLNDQLGKALSCPEVTSAW
SDEEKTVAELLKLDFMKSAVNLPKRARDRFVELSSRISDVGSAFVQGMEP
EIKEVMLPSHRFYGLYPGLAATLKIRSQISIATTGPESAAALQSVYDEET
RKEIYLAQRTASHCTIERLESMLKLRAELAELAGFESHGHMALKDRMMAK
SPASVMKFLLALRNHNAPIVQQELLELTEKKRERLAMPEVDLQAWDRDFY
MEKVRAEMRSRVRHEDQLTAFFSVGTVMQGLSRLFDRLYGVRFVPREALP
GEVWHPDVKRLDVMSDDGEQLAVLYCDLFYRPQKSPNPAHFTVRCSREIL
ADELNEATEDMVSATPIFDTPESAANDGMETSQRDGKLNQLPTIALVCDF
PKNNSAREPAFLSYYSVETLFHEMGHAIHSILARTSFQNVSGTRCATDFA
ELPSTLMEHFAADPTVLSLFARHWKTDRPLSYDLVQERITLSKRFEGIDT
EHQIILAMLDQAYHSPQVKSLSFDSTRIFHEIQHTFAHGPKDPPGTSWQG
FFGHLHSYGSTYYSYLFDRVLAERVWRVVFKGGDDQAAISRENGERLKQK
LLKWGGGRDPWRCLSDTLNDERLAAGDEESMALVGSWGIKDHHQISYRNA
DSPRSARVAIRNSVS
>Mb|QLI65352.1
MTPSSPHRSSQQTSVSVSQPLASGNALSQLQPAQVRTLRDGFQILDRDCD
GMVNREDVADMLSQLGLPNSASDVSQFFPPSKSQTIALAAFLNSLAETLC
QLSPSAELLSAFSAFDDDDSGQVDWAELRDALLNTAPEPGERPLAAAEVE
RVVNGFTGRRAFNRNINAQLGAKKGEVFKYNEFVNSIMGSNGASEGSSQE
NSEEQ
>Mb|QLI65353.1
MDTNMEDVGRAPADVSPVANEPATIPTLDGWIESLMNCKQLAEADVQRLC
DKVSNIEEFKEVLAMVFGFVQCLASILPDPLFMLGAILLLLSWQEKFYRR
SRMSSQWYKCPVTVCGDIHGQFHDLMELFKIGGPNPDTNYLFMGDYVDRG
YYSVETVTLLVALKIRYPQRITILRGNHESRQITQVYGFYDECLRKYGNA
NVWKYFTDLFDYLPLTALIDNQIFCLHGGLSPSIDTLDNIRALDRIQEVP
HEGPMCDLLWSDPDDRCGWGISPRGAGYTFGQDISEAFNHNNGLTLIARA
HQLVMEGYNWSQDRNVVTIFSAPNYCYRCGNQAAIMEIDEHLKYTFLQFD
PCPRAGEPMVSRRTPDYFL
>Mb|QLI65354.1
MPKSKRAKVFHLTQVTKKTREQKDKLFQNIRDAVPEYQHCFVFSVDNMRN
SYLKNVRHELNDSRLFFGKTKLMAKALGQSPSEAIAPGIEDLTQYLSGTV
GLLLTNRPTESILEYFNNFAPVDFARAGVASPRDFTIPAGVVYATAGEVP
VEHDVPLEHTIEPELRKLNVPTRMVKGRVVLGDESGQGEGYVVCKEGDVL
DSRQTRLLKLFGVCVSEFKVKILAYVPTGTVVAVNGMITLLLTFLLPPPR
YWSSASGEVTEVDPSAMEGVGEN

>Mb|QLI65357.1
MSEIEKALVELKALMKYRTEQLGYEEDFRGLGLTSRKNLCLHPSVKREKS
GSVVDARCRSLTAGFVKEKKDRGENVAVCVYHDNLDLLEPHNLIPNGVWT
FDGILRYGEEHKQCPYFTARRMMQYCNVVIFSYHYLLDPKIAERVSRDFS
KDCIVVFDEAHNIDNVCIEALSTDITEDSLRRASRGAQNLEHKITEMRDT
DQEQLQNEYQKLVQGLREADEARQEDAFMANPALPEDLLREAVPGNIRRA
EHFVAFLKRFIEYLKTRMKVRQVISETPPSFLAHLKEHTFIEKKPLRFCA
ERLTSLVRTLELTNIEDYQPLQEVATFATLVATYEKGFLLILEPFESDTA
EVPNPILHFTCLDAAIAIRPVFERFYSVIITSGTISPLEMYPKMLDFSTV
IQESYSMTLARRSFLPMIVTRGSDQASISTSFQVRNEPSVVRNYGTLLIE
FAKITPDGLVVFFPSYLYMESIISMWQGMGILDEVWKYKLILVETPDAQE
TSLALETYRTACCNGRGAILLCVARGKVSEGIDFDHQYGRTVLCIGVPFQ
YTESRILKARLEFLRETYRIKENDFLSFDAMRHAAQCLGRVLRGKDDYGI
MVLADRRFQKKRVQLPKWINQGLLDVDTNLSTDMAVSSARRFLRTMAQPF
HAKDQDGISTWGYEDLQKYKEKMDMEKIRELEDAAKIPGDEDNAPINPYE
FDDEELDQDMMELDDF
>Mb|QLI65360.1
MSRQRPRLRNGVDIQLQSAFQDGNWSVAMRLASQRARTFNDQYFDIVKVC
AESQLDDPNAKFAAVAAVDKFVRDGTVVKDVDGIDLLEWATLDLIPEDAF
PETIGPLRVRAVKAAPKDKIAATRCLQSCLLHWDLVSAQQIAAIIDRSFS
QERDFMFWNIVITHMLATSSQSPPEKKKLYGMLAQRQIQRAAQLAEQAHA
SESKEAPARGIKTEEEILLLYDIVETHGTADDVKTLIESPLFSPIAQFRL
GRKELFQRTAANYRKQQDWEALCNLCESCLSHTDENGELSLLACDWLVWK
NFIEGASYLKSCNTEIVERLKSLLLRLARSSGLKPIYKRNILLARVSSAF
KLQTHDDDDMKDDQPFSLRLRELLNYIQDQKSSAACFDDVKEFLEQLDIS
GLKHVAYTYVAQLIQSVDDPIKRARVSLLSLKTQYLLSTCPIGRVPIAGE
KPSSKCLVCNAKFESESCPTCLTKISEEALAAYKSASKDFGENPVIQNEI
LPELSVAVAFCNVKLTFRSRPGYIPSSPPVSQHLLRALFILEHQAFLTPK
HSQISLLLVQLHLLLGSAHRSREAWHGLAVKRTIVDSLAPIFYDRLSTVS
PIILDSSDSWGWELVETLRSHYVNSLKLKMPRRLIDAFEEASYGSIIDMP
KYIENLRSGCTRAMSLVEEVRADRLLGEPCGEFLNDARFYEVPDDLVLRN
VVDHGSFPSWDCYSSRPVYERLRLGPGPSNTRCHLALLAEAFQDILSYRP
PTVYKATAAVMGIDNTFVIEMMCRIGNSMSKFVHKASGNCTSSEMLYYEA
VNVLATLIPLCVGIDKSAPLSDIFSQITEAVKAAITTQLDELPKLDGTVA
QTVSALRSFHEVTMLRDTAMAAKLTAQWILSFNEREKERDRSGSSNLPKE
VVSQVKGLQAAAEAGLKDGKSLIAQLKQEVGTGSEFVGKLRPWVFEDGAG
ELNDLVEDGTLKEVVESWRQNLAGWGQQQPSVSIYHELPTGQVRVTRCTH
PGWKKLLAMAQGKPPTSHEGGDDDFQTLAIKCVVADLGIL
>Mb|QLI65364.1
MDTSAGAPPSPFLEPTSRTAPNTLAIISLSKEHYQPITLQESIGAVDGYA
EGVTLNTRFGSFPHSTLLDIPWGSQVRASVVDTGSRGRKRRRDQVEDEET
PTSNQDDGNDTPTSAPAKKAIAASSGFIYVLRPTPELWTSSLPHRTQVVY
TPDYSYVLQRIRARPGTRIIEAGAGSGSFTHASARAVYSGYPGSDADARG
KVFSFEFHEPRFEKMDAEIKQHSLQGIVQVTHRDVYDEGFLINGKSPKAS
AVFLDLPAPWEALHHLARRKTLKGGKLDENPGDWDSPLDPNKSAHICTFS
PCIEQVTRTVTELRTLGWTDIDMVEIAHRRFNVIRDRAGVNIPSEKGFSA
QPADVAEAVKKLKSDLKRTQDFHKAQTSQPVSDTSESKMDVDESTPESNT
GDSNKTEDPLWLQGRLVHRTENDLKTHTSYLVFAILPREWDEAAEEAALA
KWPCGGESKTIGNLDKQARKQEKREMMAGKKQKRTRTNDTQTA
>Mb|QLI65368.1
MDTRFPISRDDLYNLQMEVKQVQYTQSNHAERLLLRLEKRSADDSALKSVW
NSPFPGILGGTPHQGPVQTPHNDVFDDLDEQGEQLLGSLHLGPAEEEPVR
RGAASRANSVRFDESALHGSSWGGQSNRHSGDFGPLRPGSGLMMERTLSH
KSDGRHSSAGHSVHSHHSLASGRASSLGLDTNYAGGDDDSSSFEIPGPPV
SLYVLGTVPSIVRCWLTTNFAHGTLLYADVCTGSQKSVVDSSLLKELDLS
REVERDIDGVDRIRLNVYLAEAVVTRHESHNGSSTGGVPSMAVAFEVTGN
EQSSTIGDRKGIRIYIGSDALRAHSADVLFSQNTMVLYGNEQERLRVPFV
RPEDEDAFRYIYTTSMVPEKPKLNATATPFVLVDSRASNNGRNGAPSPGT

LHEKAAPAQPISPHESDMESHKSEERQATSEHGGDSDNHGREANYSETSG
KDDTSASEVSRRESSSTSTGIWGSWRQGAGAGPDGTQRETGPLSGYQPAG
RSGRNMKVLKPLKSNSNSARTGASYEPPLPPKFSTEGRRKSQVSLNGDGG
GGGISVANRWDVKRSVSSGAELKMESSNRENQKGATLPRSANPVGVASAF
SWMTPATKASKTSPAGG
>Mb|QLI65369.1
MAPEKRNEFLDAPESDDDQIDNYNSDDEITKGRRSAKRRRVDSDDEDAGS
DVDRSASDDQGSIEQDGQKGDSSKQVKESKSTSRKELPKLETGLPDITKP
LTKKNLVASEEAIKKSGVVYMSRIPPGMKPSALRSLLSPYGRLNRIFLAP
EDPTVRARRKRAGGNKRVLFTEGWVEFVKKKEAKATCELLNGHNVGGKKG
SFFRDDIWNLVYLKGFKWHNLTEQITTENAERTSRMRAEISKSTKENKEF
VRNVERAKMLDGMQAKAKKRKAADAEEEPVQQGERSFKQVKQVKRDDKMA
QDQPESVTRVLSKIF
>Mb|QLI65372.1
MGSSPQQAMNGDMVDDHAAKADTNDIKQLQQWNGKSVVRDDALRVHDLIQ
AHFDGQPSAPSVCSWDGSLTYHELDQLSSGLAAELVARGVGCEMFVPLYF
QKSKWTAVAILAVVRAGAAFVLLDDSHPLPRLREICEEAHATIVLAASEF
RDNAQQLVDESLVILVGETQSQQWPRDSNESWRASPVTGTSALYAVFTSG
STGKPKGAVNEHASLCAAAEASGRALAMSSQTRAFQFASYAFDPTIMDFL
RTWIYGGCVCIPSPAQAKNDIAAAMAQLGANLASLTPSVARLLDADALPA
LRALEFAGEPMLQSDLEKWADRVLLVNAYGTAECSVYSVICSPVRRDGNP
HDIGFAAGCVAWIVDPADHHTLLPIGQAGELVLEGAGIGRGYLSNKELTA
ASFVLGPAWLHQFRGEKSRHIRLYKTGDLAQYQPDGSLRCLGRKDTQAKV
RGQRIELGEVEWHIGDLLRQRHQVVVDVVQVHRGVGTQAVLVAFVRLRDA
VIDQESPATGLFATPTDVFRLQMEKVEMELKGRVPAYMVPGAFVALNYIP
LSISGKTDRRRLREETAQRPWAGIEVYMLHQKSRHYSPKTDIERILQGSF
AHILGLQAEELALHDSFFRRGGDSISAMQLSAHCRRRGLSITTQEIFQYP
TIAELNARILTQVDIQAPSTALPFPLSPVQHMLLQQKSLPGRESQGFQVP
SRTLLLRLARPISLVELESAIQSVIHRQPSLSMRFTTGKDGQLMQTVTSL
IRGSYQCCHIQTSAASVATLDPRPERLNLETGPLLLADLITTQSDDSQQL
LLVASQIVVDVRSWKIILDELSSCLTGGDFSSSVATGSFQSWCEAIKPHI
NGQLNGQPAGWTKSSFWGSVDSSSTKSSRTISTEFILDPSRTDLLLQRGQ
HALRAEPPEILLGVFLHAFRISFPGRDGLQIFMECDGRTAGVSQVVGQFT
TYTPIMLPSGQDGDVVDVVRRAKHAHRKACKEHSMHSKHTFPMEAIFHWK
QEKEHRALGSGSGVGELFLQEISSLGGSEPHQLRKPALFELNLLQSSSSL
TASLVYGEHMLYKDAISQWFQLSQDTLDELLRRLENLPFRPTLDDFPQLS
ITPAGLETLISDALAQQDILSPSDISDIYPASFIQQGMLLSQARDPQAYW
SRTSWEARSRDGSVVVLHRLQEAWQQVAMSHSILRTLFVQSQDGFHYQVL
LTSPPLPVKILPSNAPDQIQVTANEAPGVLESRTLLPWSLTLRPLNSSSV
TCEIQISHALMDGESMQLLIRDVQRAYDAEQPKRAPPLYSQYVGYLNEVS
RDPVLEYWKDYLEGTEPCHFPAFPSSQPTTAVGKLVDLDIVIQDTHKLFS
FCREEGITLFNVVQLAWAIVLQCYTGNETVAFGYLTSGRDVPIDGVEDMV
GPLINMLIARIDLQGDLSVRTHLHECRDRYMQSLPNQHCSLAAIYRALGH
SGTPMFNTIITYQKLLPINTSATIEFIEILDQQPTEFDIVLNVRAGREDA
AVSFTYWENKIPPELARSIKSTFSAVLGEITTRPDVSVSEVNMVSAADLV
QIVDWNSPPAATVSACIHDMVREECIAHPDRLAIDAWDGSFTYQQLWDHS
SSLAACLIDQGVGPEMPVPICLERSAWVPVSKLAVLRAGGICVPLDPSAP
VERIQAMLEEATPEIIIVSSTTTNIDFQSSARKIVISLDMFAHSAAQLLD
ASSPVQPSNGAYILYTSGSTGRPKGIILQHDGLSTSIREQSRRLSINGET
RALQFASYTFDVSVWEIFAVLTSGGCVCIPSQMARMNELATFISTVKVNW
VAFTPSFVRLFQPKDFPSVRTMILTGENLPQDIADTWCSKVELVNAYGPT
ECTICTTQPVEAGRWTMGTIGWPVGGSGWVVDPSNINRPMPIGAIGELIF
EGPVVAREYFRRPDATEISFVSARPSWLPTSSYNGCRLYRTGDLVHYNPD
GSLRYLGRKDSQIKLRGHRIELEEAEFLIRRHLPPGYNVVVDVIHPTDQA
RSSILIAFLCNEADTSQNLPPQSLLIPASDSFRSLSQDIQTKLPLSIPNH
MIPQLMIQISMIPRTKSGKTNRRIIRVAASRLSRTDLEVLVDARGGGGKM
QPTTPNEKLMQSLWATALDLPTESVGVSDNLFQLGGDSITAMKIVGLTRA
AGYTLVVSDIFSRPSLSRLAEGLQRRDLSSVQNSAPFSLIPSEEKPEALI

EIAMNECQASCDDIEDIYPCTALQEGLVSLTIKQQGSYVAQYQHRINPGV
DIPRLAAAWDMVVAANPILRTRIIQSQSARTFQVVLKHGPTCVIESSSFE
ETMKATKEARIALGASLLQCVIISQESETFLVLTIHHAVYDGWSMTLLLE
QLESAYAGQSLKLRPFTGFVEYLSRVDISASNKFWRSQFTDLNCAVFPTP
SVQLNRANMATTLQLVEHRLETPSVGSKFTLSTIVRLAWAMVLSKHTDSR
DVVFGATVAGRSAPVADIEQMTGPTMATVPFRVSINPAESVLESLENVQA
RTIDMIPYEATGLHNIKALGADAAVACQFQNLLVIQQQSFYQPKLMHEV
QRFTENIAAINTYPLTLLCNLNGDHILIQASFDSEVLSKAETETLCYHLG
QAILQLVEKQSLNVSDIDLVGPETIQQLQAWNQNIPPQVDGLVHEVIDGH
CKSQPQALAVCSWDGDLSYSELDSLSSQLAEVLIERGVGPEVLIPVYLEK
CKWTPIAVLAIMLAGGAFVLMDPAHPLDRLKLIHSQLDAPVLLTSTSLSS
AALNIASTVILLDSDEWQEETDNKSTTPKVWRASGVQPNNRVYAVFTSGT
TGTPKGVVIEHAACLTSMQALIGPMGIDRNTRALQVASYAFDVSVSDHLA
TLLAGGCICIPQASQRENNLALAVEGLQANYMHITPSLAELLRPEDVPGL
RTLVLSGEPMTAGLVTTWAGAVKLVNAYGPAECSVDCFVNASITSMTDHS
NIGYATGSVGWIVDQEDHDKLAPIGTVGELVVEGAILSRGYLNDEQKTSA
AFIEDPAWVRRFPLKSGPRRLYKTGDLVHYHPNNGSMCYVGRKDTQVKVR
GQRLELGEIEHYLKECFAEATRLMVDLIVPVSDDERPALMAFIEIPSEYH
DLRGEASAHGLFNQPSDTFRAKVQTVVAQLRKKLPPAMVPATFIPVVKIP
LSSSGKTERKTVRQAASRLAREEKRHYMAASKVKRAPTTDTERLVQSISG
RVLKVPVEDIGMNDTFFEHGGDSISAIRFVGDARQAGWTVQVVDIFSEPT
LAALAGKMHRSEGDLTARLIPPFLLLPDEAEISRLCSLVASSCSISTDRI
QDIYPATALQAGLLALSLKQQGAYQGYFPIRLPSGVDLDSMKKAWESTVK
ANVILRTRIVQDADGQLYQAVLDSEVEWHEAANLDEYFKVEASIPIRMGG
PLARAAIVQDSKQGGWYLVVTLHHALYDGWSIPMLVQDLQTAYMGQMLSP
RPFNGFVDYICRADDVASSEFWRNEFEGLSCEQFPPLPSAHHVPSIDGFL
EHTLALRGLSSDFTISTIVRLAWAAVISSYSSSDDVVFGTTVAGRSAPVP
GIENMTGPTIATVPIRIRVDPNMCTKDALGDIRTQGTQMIRFEQTGLQSI
RSLSPEAALACQFQSLLVIQWMNEESTESSDFSVAYEYAHDEKAFSSYAI
NLTCDLASSAIKFRVAFDSHITEPAVMQMMLFQLSNAISRIMDNAMFVRD
LKEISPQGMQSLLKRNAHLPPRVQACVHDLILSYSENTPDAIAVDAWDGT
LTYRELDSSSSSLSAHLLSSAQVKPETIVPILSEKSMNTAIALLGVMRAG
LAFLLLDPSQPAPRLKSLCEAVEAKIIVASAKHADLASTLVPEARVVVCS
RQDLASSGFPNGVALPVPVSDPRQIAYCVFTSGSTGTPKCILIEHSSFCT
SSLAVGRLIGMDQSTRVFQFASYAFDVSITDHLLPLMHGGSIFIPHSKNV
KNNLPASLRESRANFAELTPSVARMLDVGDVSSLQILVMSGEAMTQADVE
KWQDKVRLINLYGPAECSCAATGQNPVSLSHPPQVLGKGMGGVCWVVDPA
DDEQLVPIGAIGELIIEGPIVGRGYLNAPELTASVFVPSMRWLQRVRGEE
DRVYKTGDLVQLLPTGDLKYIGRKDLQVKIHAEVLHPNEQQGKSILAAFL
EVPHLQKLPNGHSSSTEMSILPATASFLADVQRTESELRDTIPPYMIPTL
FLPVNRIALSLSGKVNRGVLQNLGRNLNRKDIKAFINPRRTKRAPETEMQ
RALGMLCAKVLNLPLGEVSIDDSFLALGGDSISAMSLVARANALQIAMSV
GDVLTQSTIEKLASRAKNTTALLLPQPEELDVSFALSPIQEMFVDVVPQE
SHNHYNQSFFLSLARNVSVEDLEDAVREVVRHHVMLRARFSQDAEGLWTQ
SITSKPESSSPYLFKSHEVADVASIEPHALKTQQSLNIVSGPLLAVDLYQ
VHGQGQYILLVAHHLVVDHVSWRIILSDLEESLEASSASNARGCRNIESS
LSFQSWCHLQAQHVREQLPPQKVYPTPTENHVPLSTTFTCMDYWGLTSRS
NTCSDIVSIGFTLDAEVTESLLGHANDALRTRPVEIIQAALVYSFMKQFP
DRDAPIIYSEGHGREVWDPSIDLSRTVGWFTTIWPTLITVHPTHSLIEVV
KRTKDGRRQVRNNGWAYFASRFLHPAGRSQLPLEVPLEIVFNYAGSFQQL
EQRQGLFHMAETQYTRFDVAATATRFELFDVSAVVNRGQLQVEISHHQAM
PQARIRQWGSSFEAALREASVKLQEIQRQFTLADFPLLQIGYSDLAHLVD
TVATVAEVHDITEIEDAYPCSSIQLGMLLSQAKDAAQYITSTTWEVQTME
GIDVDRFIRSWHKVVNHNPILRTIFVESHSGQLWDQVVLRHSAGDVILVD
ADGINSPVSQEFGSVRASRPWQLIVTRAGVALLCEFRILHALVDGFSTSL
LEQQLSRAYGDQVFSEPVATLKDYISYVQSLPSGVATQFWATYLEGSDHC
ILPTLHHSQDPNYPGSVSYVTRKLDDLRPLQVFCGENGVTLFSLVQLAWG
LVLRAYTASDAVCFGYLVTGRNMPIAGIDEVIGPLINILVSKMHLDGSNA

IADLVQSIHSDFLRGLGHQHTSLAEIYHSLGISGRGLFNTVVSFQTKSEQ
QQVNHTSGLSIRTVDSDDPTEYDLTLNVSVSKDDMEYALWYYEHSFTPSQ
VHDLAAAFERALLSSVALTNAKIRDVDLLSEDHLHWLQLRNEVLPETRQV
CIHEAIEPFFLSTPSAQAVCAWDGDFSYAELDQLSFSLSRVLTHRGVEPE
TYVTIHCEKSRWTSVAMLAVLRAGGAFTLLDPSLPLAHKQTICSDLGCQI
MISSKTAEDASSLVREMIMIGDGEDESWVVPPSEPIRSSQAKPTHAAYAV
FTSGSTGKPKGVVIDHSSFCTSAMAQHQSLSIDSSTRVLQFSAYTWDVSI
MDQLATFMAGACVCIPSESQRVNALAEAIQQYGATWIQTTPPVVRLLEPT
QVPSLQTIILIGETPSYDDIKMWQGRVSLRETFGPTECSVLALVNPDLSS
DPRNIGRESGCICWIVDPDDHNKLVAVGAIGELLIEGPILGRGYLNDPLK
TSAAFIENPGWAERFRNNQSVRLYKTGDLVHYCPDGSIRFFRRKDTQVKL
RGQRIEPGEVEHHISNSFDGAERVVVDVLALESEASRSFLVAFVKPASLN
DNDVDIDADLFAPPSTAFSVQVALTQARLQNQLPKHMVPTIFLPLNFVPL
TSSCKTDRRMLKRRAAALTRSELETYMHAGASVKQTPTTEMEQKLHPLWA
KVLNLATEMIGTNDDFFHLGGDSISAMQLVFQCRRVGISIGMEDVFRYRT
LAAIATHARNEKNSADALAVEKIDEPFPLSSSQQLFFHFSPHGFDDFNYC
SVMKITRKIEAERLRRAIRTIVERHSMLRSRFSQDCDGSWMQRITSQTEG
SYRFQHSIISGENEITQVLQSAQECVNIQHGPLLVADHVQPDGQKDDYLI
LVVHRLVADLASWHIILGDLETVLSQKNPIPAFAPVPTSFQAWCSLETDS
KARHHLTGNRLLPHSGEQVPMVCPLEFWGMTGRPNQVKHSNTTIFTIDHS
TTNLLLGRANQAFQTQPAEILHAALVHSFIQTFVERNAPIMYNEGYSRQP
ADKSIDTSNTVGLFATLHPTLVNVDNCQDLVEMVRRTKDAHRSATPNDWA
RLSSLCKDSRYSESILPTPVEIVFNFEGVKEQLENNNSILQIIRSPYTEH
QETNRASEAPRFALITISAVVVQGELMAYFTFNEQMKHKVLLKDWVDEYQ
KSIVRAVMGLSHASPAYTLSDFPLLTTTYEDLDAFVARVRDQIGLQEGEN
SAIIENAYPCSPIQRGILLSKSKNNDLYSPRFTWKLQVAGQNPIDLSRLR
EAWKSVLDRHPVFRTVFVQSSSHMEYYNQAVMVSLPDNITTLQYDDEDVM
ELVRDYRVTRLHLDQNALWHLILCQSKRGDTFCDLKVSHALIDGTSMAVL
TREVQLAYDEMLSAGAGPPYSDYIRYLQSTPVDTVIDYWKKKLSAISPCM
FPRISSQSRPPSTSNIKRELCSVELEIEDASIIYQFCRQHGFTISNILQV
AWALILRCYTTSKSVCFAYVTSGRDAPVSNIEGAVGPFINVLINHMPLDS
DMPLLDVLRVNQDDNVDNLKYQNCSLAEIFHAMSIGAAELFNSSMSVQNR
TSDYDSSGLSFEFSVVNGEDRTEYDITIHALVDRQGISLSLNYWKDEHLS
AEQAGQILRTMKEAVISIATHPNVKVGQVSLLSPEDQLQLHRWNEEVPIT
LNSCVHDLIERHFQCSPSSMAVHSWDGDLSYQDLDRLSSRLATRLVDLGV
QAETFIPLLFERSMWVIVSILAVMKSGAAFVLLDPTSSVSRLEKVCADAK
VKIVLSSMANKEMLRHLPVDLVVVNREAEYLWPSVVVLPSVAKPQNALSA
VFTPESSGKLNGLIIEHASFCSAVGANKGPMGIDSNTRMLQVASFASDTS
IGDYLMALTQGGCVCIPSASQLGDVEKAIHDLNANWVNLTPSVARRINPS
RVPSLKTVYLGGEPTTSWDVARWQGFVRLVKGYSPTEFLVRGTIRPNTLN
TSDPHDIGVGTGAVCWIVDPANHQRLLPPGAIGELLLQGPILPRGDLGGQ
KQNNTSFIKPPIWLQHLQSSVFSQLYKTGDLVRYQSDGSILYMGHKDTQA
KLQGQRLELEEIAYHIRQYFPQDRDVDVVVDVIRPDNGNAKPMLAAFISH
SFSPVIGGPDNVVFVGPSVPFQMAILAAQTQLAEILSPSMVPAAFLPVEG
IPLSVALKVNRKLLREAASNLSLKQIDEYVDFSPLSDEGRIPASELEKSL
QVCIARVLNKAPGHIGLDDNFFRFGGDSISAMTLVSQCRRQGMAVTVADI
FRHKTIRRLASHIQSAKATAAEIQESVGTVFDLSPIQQAFFDRTSEGLNN
FSQSFLVKLRQPRESIETAIATIVKRHTMLRARFRKEAGVWVQLVTDDIE
HSYRYRNLTLSVAEMTKKCFVESQAALDFMQGPLFVADHVTVGANQYLFL
AAHHLVIDLVSWRIILNDLEMLLGGESLPPLCSLPFQVWCHLQAEHAKKH
LIPSKTLPLLANIPDFELNVEEYWGVPKCSNIHADVMSIKFNIDARVTKL
LLGPANEALHTKPVELLHAAILLSFVKSFPDRPPPIIYQEGHGREPWNSS
VDVSGTVGWFTTIWPALIRAKVDSSFMDVAQLTKDIIRQTQANGWSYFTA
RYLHPEGSKRLTFREPMEILMNYHGSYQSLESKDSVLQVTSDILDIPAGV
DPRMPRGEIFDIEIWVSGDTLEFDFTYNKHSLRQTSIQNWVSSCRSLLES
AAEELSDASCLSTLSKPPQLSLTGPELCQPSQHPCYIGIDPASVQGAYPC
SPVQQGILLSQERNNALYTTRTVWKVSTPDSRLPISISQIQQAWAQIVMR
HSALRTIITESTSRDRLHDQLVIDHGAGKVIVLDSIDSPMEPLGTFPWQF

IIKPTCNAEILCELAISHALVDGISISILGTELSGMIGGKAIDSPAMPYK
DYISYLTSVPRQQIADYWGEYLSDVNPTLFPILNDASEGELHFVTRYIDA
QRIGAFCKTYGFTLSNVIQVAWALVLRCYTGTDDVCFGYLTSGRDIPLSG
IENSVGLFINMLVCRLQLDSNHQMLSILQRNESDFIRSLDHQHCSLAEIQ
HDLGLNREPLFNSIVSYQSASPVREQNRHRAHDKHALALVETVGGEDPTE
YGLVINVNASSDHVSLSLSFWDAFLSPEQGSRLCDSLVQTISSIIDGPST
IVGDIDLVSQEELNVIHSWNAQLPKTEEACVHDLIFDQCRVHPGAPAVCA
WDGTFNYKDLAQLSEDLAIQLQTLGVGPDVFVPLLFEKSKWVSVAMFAVM
RAGGAFILLDDSYPEARLREICETSRCPLVISSSSTYGKAAPLTERVVMV
SQSEAAQWKSNIGTLSLQQNVLPHHALYCVFTSGSTGKSKGVVIDHSCYA
TSGLSLQKRLRVTNESRVFQFASHAFDVSVSDYLTTFIAGGCICVPSEIE
RINNIPHAVQTLDANWMHITPSVAQILHPSQVEGIKVLVLSGEAIKPENI
TTWGHSAHLINAYGPAECSVDCVVNDQVVSSPLSIGYASGATCWVTDRNN
PARILPIGVVGELLVEGPIVGRGYLHNEEQTRRAFIERPPWLQSFRGHDA
YQRLIYRTGDLVHYLPDGSLKYVGRRDSQVKIHGQRVELEEIESHLRRFF
PGSQDVAVEILQRPDVASQKVLTAFIRRQALDSTRQEFSIFESSQEFVTD
VHIAELRLVQNVPRHMVPVMFLAVNHIPLTRSGKIDRSKLRNQATKISWP
DGGSSVEHTSRRSLTETERKIQLIVSRVLEVRPDVINIDGDFFQMGGDSI
TAMQLVSLARHEGLSVTVRQIFILPVLADLAREIQQSTSVSDLASYVVEP
SSNTDHELLRDLPWKDLPFRKDDVVDVIPATIVQAFSAGRPQNYWFLELT
GPLDTARLKRACSELVEKHSILKTVFIPCAGQILQVILRSLPLDVIECNA
NQKDLRSFAEEYSRQDFPSQTLYGSVPLALTLVRGSDERHLLIMRLSHAQ
YDGLSIPKLMANLLDLYHEKHISVSADFATYVRHRNRSQNATTFAFWRSL
LDGSSMTSLFDQPVATGALNDSRSSGLVESIANVPLPTPPRGITLATVVK
TAWALAQSKLLRRSDLVFGQLVSGRNTGVLDLEDVIGPCINMIPVRVQLH
PGQTISELLKQVQAQHVETMPFETTGLQEIVKNSTSWPANTDFGSIVHHR
FSQDQSALESNELSCRIDAWSPLSLPAMNVWVSTMAEEDGLSINIFSRSE
IATQAQIDRLMEEMSAMLAICREGVCVPGEDSYDDWVHV
>Mb|QLI65373.1
MARPRGSSGASDESTGTSHDQELGSMYDYLAKIILLGPSGTGKSCLLHRF
VKNEWRVLSSQTIGVEFASKIIKVGTGARRKRIKLQLWDTAGTERFRSVS
RSYYRGAAGAILVYDITSHASFRGIQPFLNDARALASPNLSLMLVGNKLD
LTSESLIDTSLPPPTPSSVGSTSTITAGGASSLSISTTSTTGTITGRDYG
GSIGAGTQQRATIAPDGREISTSEATRWASTAGIPVVTEVSALNGEGVEE
VFARLARMILTKIELGDIDPDDPMSGIQYGDGGGWNTASDGGSIKSSMTG
ATIDDNISGLRRRKRGRNRNQNWGLREWEEVFTLSSRNRRGGGCC
>Mb|QLI65375.1
MPWWGKPKEPKEPKAEPSPDANANADQQKQAFDPDKLPAREKLPRRLQNI
VEKSDQDSRAPPTTDTKLRYAAYASRFRTILLSAHRYVAYTSDIGESFRP
VAHPRVVRAAYGISWLYILGDVSHEGYRAYWHNQRVLNPQLRLSPHQEKV
TGLRAEDNVNLQPGVVSPLEDWRTVMVQRAIFQSIASMGLPAFTIHSVVR
YSGRALKNAKNATIRTWGPIGLGLSVVPFLPRLFDEPVENAVEWVFHKGF
EVVGGQKYVGSAPSTGREDMLNKPIKPKTE
>Mb|QLI65376.1
MATSEGLEPPPEMGKTDPGAHQLSEPESSDSDDQFTDAQSGTATPHATSP
MPKPRAENADSSCSPAKIPAAERKEKGEADAQSEEIAQQDDPKTEIQTEI
SKQEVPVTVLAPVDSADAPERFSSTDAKPDVVLEIAADDEHAEHGGDENA
DEEEGDGFGDDFDDFEEGGQDNDFDDFEDGFQQPQATAAATQAPPTVQPS
TLPFSIPDFDGLGPDEVMSTAEPCLDSLFPPQELVFPPPQTLPQESSIFL
TPRSASLWSQLVAPPPLAPPDWIRSRTRRLFLVSLGVPVDLDEILPASKQ
KKLVLPSLKIRASSPRRSSDSRSASRLQQSEGNASSTSVDTQGKPRGSKK
RKGPPPQPELDLVAARYLCMTTDEALDGMTNEELKAHVSKLEAMEGTANE
ILEYWKKRTDEKIGDREAFEGVIENLVKHARKVRK
>Mb|QLI65379.1
MYNRESKIMRKLLGKAAPDSATGDLASITGTTTPIPTSYRPSKSQNAVYV
AGAPISCFDVSADRRAVVLGGAHVLKTILLDDLTASSFNFYDGIDVRTAI
TTRQSTVSRQNVVADQLNIRDVKWHGNSHVYTACASGKVFSYDLTRLGAG
DNEPLEYILTQEDSRQMNTLDISPHLKTWLLTGGQDGTARVFDCSNTTPA

RHGLSFRQRFAPLRCIDPIRKVRWSPANGHEMACCTESGVVMKWDVRQPS
RPVLRINAHEKSCSDIAWHPDGIHLISAGMDTKIHTWDLGNTADKRQKPK
WTISAPAPVAAVAWRPGLWSASAHARRVAQIAVTYDETSSRRYGTSAVHI
WDLARPSMPYKEIERFETSPSALAWQDQDMLWTVGQDGTFNQCDVAFAPK
VIDRQSTSAMAFSPRGDVLMFLDERPLSHRPRLPVVHQSDAMHRATYSSS
PSNQGLSISRSDSEDDVIGTFLGSRRRIPRKRRPSGRGTIPLSTTPPSGG
APFPDDNKQNMGLEQSINITGVFRSQQAMSFGPIPAAKPVQIYRFLSNTY
LETLKRELPYSDGGKPLVERVGVIMEQYAKAAEVANLYRLAQTWRILAYA
TNLLLKRRAQYHFEVRVGQFQKLQVDDVVKGADKLKASEMYANGEDTPRR
PSAQRSSIDGRFHSIRSLLAEEIESTSNVPTPVARPADSATSTATSGHDD
AVHQHGKRLSPIVEPESLTIGPAAHGSFGESQSPRKRHDSEPISVGSEQT
DDSATSYTEGYDFYDAEVLAKAIDVPMPRRKNSSRGVPYRGRTVRQDSDE
SFVQMFSISNGTKRSGRMGSSGDAFAKPLNREQSDLERESGTSSADSPAM
ANTLTGRNDRSPRSNESQEEVFMISQTTKTDDSYPSQPSFTSQDTDGIGL
GESLHNSVPDIRTSRSVSPKKKESIQPRHDPRPHVVETDYLPWSNDPPYP
YPVNAHNSKASPASISPLNPYSLLLSALDFESKTSALNASAMILLLRPLV
PTSVIDSHRASAILRQHHQRLMRMSLFVEAALLRNLCIQGWPDGLPDWGD
NYISIFGGAQQGVKVGLFCSSCRKPREVDPAGGRDAVWTCERCRSVMAPC
PVCGHREAERPSFIPSETTPSASDVESEMWLSEWWYCLGCAHGGHASCLQ
LWHGASEPVAAADPSAKYSDGCCPSDGCGHACLPGKYRGETTTARSDELG
RAAVDSSRAREGPASRGGSRRSSPGAPYERSVKSDINDVPQSKAVGMARE
ALNKGGGGILSSSPGRSSGVGDRERRKSVKFARTDR
>Mb|QLI65380.1
MSDEKDAVDYSLNNPDTLTKYKTAGQISEKVLAEVSKLCVPGAKIVDICQ
KGDKLLEEEVGKVYRGKKITKGFSHPTTVSPASYVTPYTPLTSDESEATL
EIQPNEPIKVQLGAQIDGFGAIVCDTVLAGENKDEVLTGRPADLLLATHY
ANELLLRLMVPPGLLAQGTDEEKAKAASQKPPTQAKMTSLLEKVCEAYGC
NLVESTTSWLFDRNEIESTKKIVLAPQDGGKGDGVPAISEVWGVEMGVSL
GSGKCKALDGRATLHRRTTQTYGLKRPTSRKILSEVQKKFGTFPFSLRQL
EDERDAKSGVVECVRGNVFRQYELVGDKDNAPVARLLTTIAITKNGITKL
GGPPALDLSKVKSDKKITDEEILKILEQPLARNTGKKSKSKKKKKPAKKA
AAAAAEDSDDDSDDSDE
>Mb|QLI65381.1
MSSSQAKWREEQILIICPGSRTTMAQLGCVELTPPMHRIPTRMFKDGDEW
RPYHTFQRSKVVNGVEQQEWVEDVDEDKGATYPIQGGRIVQMDAFLAFLD
HVHSLLTTTYHNTPIMLMASPQWTRPDCEAIAQYIFEKTRTPALCMIHSG
IATQYGLKWPNMTVIDIGYEKVDVTAIHDGRVVNHIDVGSSGADSPISGG
EVFTQRLMKLLKGKSFTHGMAEQLKKSTICEVLPYAANELGLVELPEDSA
PAGALPGGSAAAAVQAADASKPAEPVKPSATGGGDNGDGEYANNNNGDDD
GVLDVAAIVTSGQTKEFLAKKEKEKGKPGRKPKVQDADVAPAKPARLPNS
KRKMNTFFYDEIVQELVPPEPKQNGTNGATDTPGDKPAPGHQPPKEGESN
SAPPVDGAAPASNGGETEAKPAEFTSDGALPAAVPAPAVAESKPDITQAE
HTVSVSVSDTPEYRPKRVRRDIEVGLERFTFADRHEIDRIVTAIYRTVQG
IDDMYMRPQCWDNLVFVGNGSRLRGLKENILQTLNARHLISPSTATMFTS
ELPSNMATPSGTGSQTPTGSFTGVPHQLSTSGVNPLLQAATTASTLGIGA
AGGTGAAATPAAGSDAAGPAGHHFHSQTPTSIKTANLPGYLAEWTKNGFE
EAMFLGAQVAARIAFSLHSNMDAQSIEAQRLMSLSRVDYNELGPKGIRTH
SMLS
>Mb|QLI65384.1
MGPNFTGHPAAMGHPGVAGHPMGPGMPPNSAQGAPGGGMPQQFAGAHMAV
GPGGQVNPALMGAMPQGANPHAHAIQHLTPAQQQMFHQQQQQQLQNQFNN
PSAMAAMRQQQILQHQQQQARQAFMAQQAIQANMPVGVNGMPMGMQLGQL
NPQQLQQLRQSGRMGPGQHPQAQALMAHHMALQQQQQQQQQQQQQQQQQQ
QQQQQQQQQQQQQQQQQQQQQQQQAAQGQQMPGNAGQPMGMNAQGLANVQQ
QQAQMGAAGQSQMGQPQQTGQPGQGQPQPQQQQQQQPQPQSQPQPQSQPP
QPQPQQSSQPQSQHTPQQGSQAGTPAPSGQHTPAQTPAPPTPSQPTPQQL
QNPQQQQQQQQQPQNQSQQPGQPQPQQGAGPAQPQMNAATAAAAQHMALS
NNLFHQQQRRENLKGQCLLKLMQFSEHLSGFPGPRAKDDLTYWNGFVSRF

FSNNGVLRHSVLINDADESTDKQYEIAFPAIARYFHTHFSSGVRSMQLIM
DKGLTDRPLPGDGHCIENQRASLVYWFEAGSHLVATGTLRVQFDNEQRIE
LFEFVTTGHEEYISRKQVIEAAKPAHMWIKDWHKVNSQDGKTSPEMSKKS
KAKQLKSPQTQPPEVLVDLPDSAVNSKGVTEAVHQFLEIVEVMGQMNPLF
GFCQGNPGVGPYAALEQYVGTYINGNAQAVNGQPIPQGGPRTPSFGQFPM
GASPAAAHMNLPGSPHIGSPATGQMQAPGMQLQPSQQGTSSSGPSANTSP
ASNKRRRPSGVKTEDDGSGGVTTSGAQVNGVGRGKPPTPRMPKRVKGNPS
>Mb|QLI65386.1
MGKKSFRGKGRRGGGGGRGGAQANGGWRDYAPFDKNNDKLEEYYNALLQL
PEEEKQQYWEALRRELPNSFRFTGSKGHALAVKRLLQTRYIPEITKIEHH
DGRPVEPPKPVPWYPDELAWWMTTPKNVVRKFPPFSAFQKFLVSETTVGN
ISRQEVVSMIPPCLMDLRPGMVVLDMCAAPGSKSAQLLEMIHRGEEARVR
KVLRKFAKEDGLDLGNETQDEIDADLEADPSDNGRATGLLIANDSDYKRG
HMLVHQLKRLSSPNLIVTNHDATQFPSLKLPSTDPAAKPTYLKFDRILAD
VPCSGDGTLRKNANLWKDWQPGNALGLHTTQIRILVRALQLLKVGGRVVY
STCSMNPVENESVIASAIERCGGPGNVEILDCSEQLPALVRRPGMRKWKI
MDKSCRMWDTWEQVETFAREENDGVVPGRVSQTMFPKLEGTECYDLPLER
CMRVYPHLQDTGGFFITVLEKKTDFKARNENEPKTAATNGKSEASDEKKE
VKPAETAAQAPAATKTTESKPEGDAVMDDAGTKGTKRTHEEDDSEQTAKK
AKTDSSATTPAPLVQPAEPVTKPKKAGPPEEPFKYLDPSHPVIQNIKQFY
NLSTRFPDNRYMVRNELGEPAKAIYYTTALIRDILTENEGRGVRFVHGGV
RMYMKQDAPSAEVCRWRIQSEGMPIVQGYVGEPRVIHLHKKETLRKLLIE
MFPRIADEEWKNFDEIGERVRDVSMGCCVLRIEPEAGDLDFTERMALPLW
KSIHSLNLMLPKEDRAAMLLRIFNDTTPLINNTLNKHKDQKNQKDQKDQK
DESKHTESETKQEQALDSADLEDMPSPE
>Mb|QLI65387.1
MSLTPPEYLFVVRHGSRLDAADKSWHLTSPTPYDPPLTYGGFLQARRVGN
QIASILEQAKAADETRLTLNGSRKRKRFKVVIHSSPFLRCVQTSIGISSG
LAQFLPNSMYNPSDLILPRAVPQTEPQFQFKSTLLRLDSFLGEWLSPEYF
EMITPPPGPALMLGGAKAELLRREDYSIYDDVPELEAPQPSSGSLWQSPT
LSPAQSPRSSSTDSEESINMSALGAALPTRPDVKRGYIPPRPIYAASSSG
SIPEGFVAHARDASVAVDYQWDSMRAPLDFGDGGKFGEEWASMHKRFRRG
VRKLVNWYASTESPADLVPAKSAMDDHSGAEHESHDGDVETVVIMVSHGA
GCNALIGAITHQPVLMDIGIASITMASRKQNLDYKELLASTPAPPDSSEK
TVVAVDKMYDIRLSASTEHLRRSSLSTPISAKSNLMSNIWNTNNGSRGRT
STLSSIGGPVMSPFTYSDPFSSTGSRSTSASAALPPIVRRDSASQRPLPR
GSALASVGFGHGAGSPNTGNRSPPATSLGFGLWSAGPPSAGPSSLRLMDD
GNDDVDDFGSILPDFDQKRFKMSSPTKEEKEPDLPAESTSSSAIIDDAPT
FPELPPTRADKVKGAPTPLMFAGPIKLNTNLGPNKPVEEMKVRQLGAGLG
GLWGIPPPPDEAERFRDMSHTKRRWTVNERA
>Mb|QLI65389.1
MPASAAPVAAGGSESDGAKLAVWKEALYERCKEAGPGNDLFSQDDLLRLD
VIPNKDLLLLARVVQSLSDDKLFITMREASGQVLWKWRDAQEAHKYKQCS
TDEQVMVYSLIDDSGGDGIWSQTLQKRLNMHDSVLKNAIKQLQTKGLIAP
FKNVEHPNKKMYIKASIRPSDRATGGPWYTDQNLDEAFIEELQRVVFDFV
KRQSGYRSTHGGAGASASARSQVPTKGVIKGTAADKGKKRDASEMSKPPA
KAVKASAGATPRREAALLPLPAGYTAYPTVRDIARLLSSSGITNNTILSE
EDVKKLVDVLVWDNLIEPIKIAGRMGYRVSRVAKQSPQNWAGREDPTGRS
GDPELYLSPYTEAPCGKCPVFELCEEGGPVGPSNCEYFKRWLGSDVI
>Mb|QLI65392.1
MADLDLQAVHDEMVAIAYEAGRMILSANPADLDTGTKLNSVDIVTEADQA
VEKMVSGRLAASFPSVSFMGEETYKPGMRLGPEPTFVVDPIDGTTNFVHS
FPNACISLGLAVQRSPVVGVIYNPWQDLLFTAIKGKGAYMTRSKGTTPQK
LPLAKSPRPLQGLGTSLIAVEWGSDREGSNFELKTDVFKKLAASRESGGS
MVHSLRSLGSAALNIAAVAAGQMDAYWEGGCWAWDVCAGWCLLAEAGGRM
VSGNPGNWDPELESRVYLAVRGAPTGQKDLVEEFWKVIGDRKMGYSV
>Mb|QLI65394.1
MAPRKNAQSTTAELSLVHLKNCLVNLPPSLVSLLLNLNTPVQNVVVELAY

KPASSGGQPSSPQSIFVGWTGMPSKRRAAPLVSRDGISGSRGSRDQEIQV
VEMDATLANTLDLNDGQKVTATVHIDPPVAHTINIEPLTPEDWEIIELHA
TFLELNLQSQIRALPNPAFNNGTTTVPPHPLTLHLSPTSTASIKVVSLDP
APSADVAFVKLSPNAEVIVAPKVRAKTSQSSGDHRSVASTSKSRRSGGST
MRRRSTREESKPALFCRAVERSACREWFDDGLLDPDISIWVDRDRLLSSS
FRGVKYVLVDVIKPAGLQKPQAEDGTAPPASIASSKVVARLKPWDDPPDG
QTAALSSPLCAVLGCPGIVGGVVKLQAAPPPLPKGAVQSVRVFPFAAGSK
PNEGLRFGSDSKAEKEESSKRFRHMYSGANNTEGLLQGPITDGMVVGVYS
ELEAPQGWEGGFVKFEYGAEYAAQEHSKDAIFWAAGLDEKTQIEFQQPQA
RPPWIAHSDVFDLLPKNDSLLVGIDSLLENLQKQLAHLSSVLLTGGMGSG
KTSVAKYMAEKLRRDMLFYTIYFSCRKLVNDETRISTIKETLTRIFMSAS
WGARLGGKAVVVLDDLDKLCPVETELQVGNDNGRSRQVSETIRSIVRQFC
TRDGGIVLLATAEGKDSLNNVVVSGHVVREIVELKAPDKEARRRVMESIA
RQDSIPDKDPDELDSNLSRPPTSDGGAMGDDTEAWLHGSDADKPKSKSTG
FIIDSDLDFLDMAGLTDGYMPGDLSVLVSRARNEAIIRSIAESPSDATDG
AVRLSRVDFDHALKGFTPASLRNVPLQSSTTTFKSIGGLHETRRVLLETL
EYPTKYAPIFAQCPLRLRSGLLLYGYPGCGKTLLASAVAGECGLNFISVK
GPEILNKYIGASEKSVRDLFDRAQAAKPCVLFFDEFDSIAPKRGHDSTGV
TDRVVNQLLTQMDGAEGLSGVYVLAATSRPDLIDPALLRPGRLDKSLLCD
LPSLEDRLDIIKALFQKVRLSSELTDSDDGLTEIARRTDGFSGADLQALV
SNAQLEAIHDVIDVNGPAASSRHGGGNKGNPAGRNSTPSFVQFRYGTDPA
LGKDNMPQSKSASLIESAAIMSKLEQIKLARKKAKQAKKGYGALESSENK
ESASTDHREVVIEWDHLIKALDNTRASISNEEKARLGRIYHEFVVGRSGQ
MKDGQGSMEIGGRSSLM
>Mb|QLI65395.1
MATIAPSPSATPDTNGVNGVNGAGQTKSDIANLPDPVDQANGADQSPQAK
PDAANSPKAANDADNETDDNAGQGTSNTKHSQPRENETGPTLKPKSLQAN
GSGLKKAIKGKKPPGGFDTTPLPDAPQGYTIRFTFRYAANLPPADFSTAS
SDPFLTATLTASNPKRHKEDPDLVHRTRTIRKTTEPEWNEEWVVANVPPS
GFKLKCRLYDEDAADHDDRLGNVTIEVPQVFEQWEGIPPPGKEYAAKKRV
ISKRALFVQGIASLLRSDTHLTPKLCVSIEVLGKSDPPFAQMCTLGPTRW
TKHFSPMIGRITGTKVNKDEEHDQEGGSRQATSTGQPEEKKSQKYDFQAN
EMQLQGPVPPELYHRYVEFRPIISSMFSSKGIRGKILNMALHKQHNRVYN
YDQSTEWGSFEPCSEEATQAFLRLAHFDEGGRTFTYVLTLDGLFRFTETG
KEFGIDLLSKHTMHSNVETYIACSGEFFIRRLERPVASDDPEPAEKTHPK
DPISGGPPDESPPANPAYYQLFIDNDSGTYRPDKSILPKLKEFFEKNFPN
LGVVTLHCEDEELQKLKEDQRSAKKKEGHMVNLVMNRSLSSISSAESELN
DRDETFESGKKSKREAALALLENPHTIKDALKGSEPSNKGKAVEA
>Mb|QLI65396.1
MNKQDSPESSTEPSECQSALTDFLASHNISARQIRDDAELRRQQAAAQAR
QNGEQLTEEVEVAVAESSAAGSRQRASRNEAGTQKSKRKTQEQKAIEKIK
ASKAFKKRKKQADDSGDDDEIARAIFESSHAPLPGQMENCAICEKRFTVT
PYSVAGPDGGLLCAPCGRETAKERQGGKPKKKPRKQTGGIGSRRSIQSRI
LDGDVGTKSLATLCVQTLAKNVDMADSLGDLPEHLIDKIARIFSKRRLLR
PETLPLFAQPTTETIHIYDGARLGDQDYISIFQVSPKLRRFKARSAIQFK
DGVMDYLLSRDIALESFYLHGANLLSEQKWHEFFSTKGQTLKGVQVYYTD
RHFGDDTIVTMENHCPNLLRLKIENNQKVTDKGVEAIGNLASLEHIGLQL
QNSPSPGAINAAVSKIGHNLKTLSLKIVPDADDTVLQTIHQQCRSLAKLR
ITDSEKMTDEGFANLFTNWNNPPLQFVDLQKCRYIDASQPRTNHNNVGLC
DEGFKALMAHSGQGLEDLNVHACRHITKQAFEDVFSETSTYPELKTLEIS
FCEEVTDFILGCIFRACPKIKEVNVFGCMKVKEVKVPRGVILVGVPNAQG
MMTEGVD
>Mb|QLI65398.1
MDQLHLTHSLADAFNALADEVQILADRKTVLEHKLRFAHEQFQYLADKYA
AGTPEIAETLAKLQLPPHTSVDDTSPVPLPQRRSTPQPLHQIAVIIRDGR
RMASKLVALSSSSKTTGSSRETLSRTSQDETSMSTALEQDFTVEGKKGNL
QCPFSKRVSVSGTNDGESRPSVDHTPHSSADPICAAMFEEATSQPAPNGT
SSKCPIRFMDKHSPEEIAHYVETHKHELPRSHEVCLRRYQRNETQIKKLD

SKYGNIVSMIEGLSQLHQPMLPESEEQLQQRDAGDGDSNVRVETWAQAVS
GTAESDASEELPPLDEDRQSHFDRPLKEVRVGESPSRPWGISVPIYELSG
HEDEHPLSPPPAPVRMSTAVHDHVPPAATNGAGKCPFDHTKFSAGFAGPP
PSTPKPEEKPSEPVQGLGLGDTPFTNLKEAPPTTTGPPPQPTFINPASTH
AEDSRGPQMVFTGPVFIGYPMDQAIEFMNRYRGPQ
>Mb|QLI65399.1
MGQQNQQMRRARLVASVAATIISLACGTNYVYSAWAPQFAERLKLSATQS
NLIGQFGNLGMYSLGVPVGMFVDRRGPRPFVLIGAFLLVAGYFPLHMAYD
KAYGSVVALCFFSFLTGLGSCMAFAAAVKTSALNWPGHRGTATAFPLAAF
GLSAFFFSFIGSVLFPGDPSAFLKLLSFGTVGLTFVGFFFLKVYPHSNYR
AICHEEGRRSPSLSAHLRQSYSQSTPHGIRHSEEPGTSNTDSTNTHMASS
PALSGPNTPSNNPSSSSKPVNNDIEEDAVDETSSLMSSTTPELVENVVAS
SVDTDRSHRIDIRGFSLLRSQSFWLLFTIMAILSGVGLMTIK
>Mb|QLI65400.1
MSAPVIPPGGTIVQTFRRSFVDVPIDAEKGNAISTTEFLDAAESLTTMFD
VMGSVAFSPVKSDMLGNVKKLRERQLAAPAESENIQDLCRNELKTKKHTA
TEGLLWLVRYSFPLMPDQELVQKLMNTSGLEFTCIALSSNVAKPSEELAD
SFRGAYGQTLKPHHSFLVKPVFSAAMSACPYRKDFYAKLGQDSGKVQDDL
RVYLAALEKVVGILKGFLDSKDAKW
>Mb|QLI65401.1
MGRRPTPQPLPLGSTKANTSANSIRSTHNRGHRLGATGATAAAATGFGLG
ATAQATRTGTAACIESTAETAVQTANVSPIHPRPPVSLQPSLPLSLQSQP
SQHSLQSELSQSQSHSRSQPSVSLTSRFAATKRPQTSQASQTGRGHMLTL
GHETNQPRPPQLQLQHEQSPPYHDQISGSASPAFRQVTSDAEKRPSTKSG
FFHFNKSSKSSNQLPQNPCPQLSDSQEYLATRGPEEPRAPKSENFPYTQE
YLQKPPLPSRSELSLSSAGDCDGTSAHSPGGKRSKTKSFGLLSRTRSHRD
KDRNNNLKAAPVLAPATLGRNENYDNAMPLRTAPVAQDRAFRDMMNSSAR
NRSEDRAARRDSSRDKEKGALPSSLKENGSGFFGGLRSSSTRAADIISKG
LFGKSSRSGSTTEREHAVDDEHYILKVLNLPLVEQTRITRIAKRLETSKD
KTEFWMPAFPWRAIDYLNHKGCDVEGLYRVPGSGPQIKKWQRKFDERYDV
DLFKDDDLYDINIIGSMLKAWLRELPDELFPKAAQDRIARECSGATKVPQ
LLVEELSNLSPFNYYLLFAITCHLSLLLSRSDKNKMDFRNLCICFQPCMK
IDAFCFKFLVCDWSDCWIGCKNEAKFIEEEYRLFDQPPPRGITVPNGPSH
IDEHNDRQLSSTDSNKPSDYDNSNRVLQRQFSGSNASIGSRISTISTNVT
TNSEREPQETTRQLRPLSPIKPLSPIGF
>Mb|QLI65402.1
MNFPGGSSGAGAPTPGFGPQDPGVKAVQSAMESCYGKSVMSGVMGFGMGG
LFGMFMASMSYDTPFGTPVTNSAGQNISSLPLRQQLKHGFKDMGTRSFSM
AKNFGKVGALFSGIECGIEGLRAKNDLANGVAAGCLTGGILAKNAGPQAM
AGGCVAFAAFSAAIDAWMRQPKEE
>Mb|QLI65405.1
MELAASRSRVRRSLLTDANRAIPDCPSRRMVKPLSFKGDKKPKKRKRTAD
ADRDDGEAGPSRVQKLNNDDDAAADDDSWVSADVATDVVGPVMIVLPTEK
PSALACDPNGKVFAMPIENVVDGNPTSAEPHDVRMVWVANKVSGTDNFRF
KGHHGRYLACDKIGFLSATSEAISPLECFNVIATADTPSTFQLQTLRETF
VTIKPNTSSKSTSPAEIRGDEDKITFNTTMRIRMQARFKPKLKASKEEKA
LSKISRQELEEAVGRRLDENEVKVLKRARREGDYHERLLDLKVKNRHDKF
G
>Mb|QLI65408.1
MATTSNMFLYSLTVQPPNNVVQAVLGQFAGTKEQLIITGAGSQLTLLRPD
PSLGKVTTVLSHNVFGIIRSLASFRLAGSNKDYLIIASDSGRITVVEYLP
AQNRFSRLHLETYGKSGVRRVIPGEYLACDPKGRACLIASTEKNKLVYVL
NRSSQAELTISSPLEAHKPGVLVISMVALDVGYANPVFAALEMDYSEVDQ
DSSGQALEEVETQLVYYELDLGLNHVVRKWSEPVDPTASLLFQVPGGNDG
PSGVLVCGEESITYRHSNQDAFRVPIPRRKGATEDPSRKRTIVSGVMHKL
KGSAGAFFFLLQTEDGDLFKVTIDMVEDEEGSATGEVKRLKIKYFDTVPV
ATSLCILKSGFLYIASQFGNFSFYQFEKLGDDDTELEFSSDDFPVDPQAS
YDPVYFHPRPAENLALVESIPSMNPLLDCQVANLTGEDAPQIYTVCGNGA

RSTFRMLRHGLEVNEIVASELPGIPSAVWTLKLNRGERYDAYIVLSFTNG
TLVLSIGETVEEVSDSGFSTSVPTLAAQLLGDDGLIQVHPKGIRHIRNGK
VNEWDAPQHRSIVAASTNAHQVAIALSSGEIVYFEMDSDGSLAEYDEKKE
MFGTVTCLSLGEVPEGRVRSSFLAVGCDDCTVRVLSLDPESTLESKSVQA
LTAAPSSLAIIAMDDSSSGGSTLYLHIGLHSGVYLRTVLDEVTGELTDTR
QKFLGPKQVRLFQVTVQGTTCVLGLSSRPWLGYSDPITKGFVVTPLNYVD
LEWGWNFSSEQCEEGIVGIQGQSLRIFSIDRLGDTLTQKAVSLTYTPKKL
IKHPEQPLFYTIESDNNTLPPDLRAQLMADPAVVNGDATVLPPEDFGYPK
GNRRWASCINVIDPVSEEPQVVQTVDLENNEAAVSAAIVPFASQDNESFL
IVGTGKDVVVNPRSFSEAYIYVYRFQEEGRELEFIHKTKIEEPALALIPF
QGKLLAGVGKTLRVYDLGMRQMLRKAQAEVAPQQIVSLNTQGSRIIVGDI
QQGVTYVTYKPTTNKLIPFADDTIARWTTCTTMVDYESVAGGDKFGNMFI
VRCPPKASEEADEEQSGLHLMNARDYLHGTSQRLDLMCHFYTQDIPTSMA
KTSLVVGGQDVLLWSGLMGTIGVFIPLISREDADFFQSLESHLRTEDPPL
AGRDHLMYRSYYAPVKGIIDGDLCERYTLLPNDKKQMIAGELDRSVREIE
RKISDIRTRSAF
>Mb|QLI65410.1
MTPFPSAARAEMAALEGRNESQDSQAILDAMKAEFGVGTRPGLVPSRAPQ
EKKATTHKAANNACVNAAAAEAGVDGTRVLDDIPDTAVAEDVVRNSTSDT
IAIQFDKPRASLDLDKKPVTVTERAQGTTKMDVSQQTPTQVNADRDYAEF
CEPLPSSPTYDRTQITTNDEPRTLNPDDTGAVDFGNLGEFARPSSQVSED
GGFENTRGEWRGPDNTSQLQANHASTPYKPPDLPPETPALPKNPFATKNN
GAVPFGGTQLFGQTQMLSSAAKIASPTSSRPSPNIFLNSISPNVMETSPL
KNRANVSSPTDIRTSSPTRLHEVPATVLKNNTLSIVVEETPVSLRSQKDE
LIPESPTLKTPRPSASHQPMAHYEPMKESQERKGSRGARDSRPASDSDSD
DAFQKLDRKKRVERKRAQAAAEMDKVSFMRTQRRMSDEQPGKKRRKLGTE
DTDAQGAKNEGQDQPLVKDSQKAITQSAEQPSEESTKATPGVAVETMDAQ
KELADAQPAPELLDEEMIPATSPVRSSPAIYQRGTAPSASEPELPMLRGS
GTEQGQDAVDSSALPPIRRKSQRTYGKRGRGTRTRLAVVSSAEEKEQEPA
EPTKEPVDRVEGSEDTAKNKNKNDALPSSYSEPPVPMGTRSRRTEQRTRT
PRRSINVDRHVLTSSSLTNLSGTPVPSSKTTPGTQDSHCPSLPGSVHLVS
PENSVRPLRRHPQNGLKSESPQPVTKAMRLSRRSLRLDSDSADELCHSPS
ATALDRSTHSKSVRSFRTSLTSTSRTRRLFDGMVFALSFSGSQAQRTKLE
TKISQAGGMILHEGFQELFEQWTVAHASSIQEDGSPPLRLTKAGLEHGFA
AVIADSHSRKAKYMQALALGLPCLAPQWATACLKRGEIIDWAPYLLCAGA
SQVLGNAIRSRILAPYSAVDATLPETMEARDKLLDGEKLLIVMDHKKSRK
ETKQQYLFLALALSPSAVSRVTTVQLAGDAVRHAELSGSPFEWMYVDSST
ATVEDVLAAAQESGKRKRRVASEPVGLGMRVLTDELMIQSLILGRMVELG
EMDE
>Mb|QLI65412.1
MSSSGARSGERMVHQDFIARIRFSNALPPPPNPPKLLDIPNMGLASGQYT
TPGFASRLAREQPLNIEADAELGMPLDLVGMPGVFDGDERSIQAPAQAPA
VHPHDRALLRPIAALGKPKVAEANVSFLRRTEYISSSTPKRHEGGNPRAL
LAKARKPQRHAPEAAADSPQVIKRKIDHGFDIAQQDLKDPKRIKHPTKKH
LKVVQAIPLLPDLYAFPDSGAYVTVKFQTNPVGKSNKYDTRLLSGLLRPI
ERTAAEDAAYEAALEAHEQDPENNPKPQNLMNYDFYLGQSSSVADNFRRK
FDVDDPDHDGEELYTHQGESGGYFQFNRVRAYETSHETELDHPTKYEDEV
ILAYNDEDTYPKQKAMYYYPVMQKSTIRPQRTKNIARTIGISEEEEQIVD
QLDITVDDPTEEMLDAMRKYEHHPLGWEQEAEEEPAQDQDAGEADGEDAE
ANGSEGAERRSPSGERDAEADEDED
>Mb|QLI65417.1
MAPATQFELSPPPGDAVSAVAFAPSDSSKLLVSSWDKKVYSYNVASGGSE
GSLTNTYEHRAPVLDVCFGANDNEAFTAGMDWTVSRLDLETGDITPLSKH
AAPVRRVVFSKDHSKPHLPYQTVPLLNNIQDILVSASWDSTLTLHDLSST
SAPIRIPLPAKPHAISSSPTKIVVAMTGRIIHIYDLNAITKLFASGGTEL
KPWQTRESSLRYLTRAVACMPNDAGYATSSIEGRVAVEWFEDTAESQARK
YAFKCHRQPAPDGDGDVVYPVNALTFHPVHGTFASGGGDGTVALWDAEAK
RRLKQYQKFSNGVAALAFSNDGKYLAVGVCPGFETGQEDYTGAGATSVLI

RELGENEAKGKGAK
>Mb|QLI65418.1
MSSHPRRCRSSTSASVPVTSKSFDILRNLLDGFATAASLTGDNGYPDIDK
LVKPLRDIHQHVSAFGSPALQDDFRHARGFDILLDVLRAFSGFYDPQKRS
QADMMSLFRLLGASLNVLSSVLRNHSGNKRFFRYRVAGGGWEALEQVIAS
IGLGGAEPDPWVSCHVFGKLLAFALDDEALDLLCQSIAKLLRPEAETAPP
STSNGDDDKEMPPSDDGDDQWDLVLARSAENIGPNVRDIVKSTSVIQFPE
ILRPVVSFWMAMPREANSPATPSSIFVLETILCAISDSIFNRAAVHSTGV
LSQFLHAAFGQEVSLAMSERERLLAICKLLMFLGVNEPADTQFLLAASRP
DAADFCLEMMSRYAGPSFFQFDLSLHGHSSLELSTLGRPFPPQSTAGYTF
TAWIRIENFDSTSHTTLFGVFDASQTCFVLMYLERDTRNFILQTSVYSNK
PSVRFKSVAFSEGKWYHIAIVHRRPKTMTASKASLYVNGEFAEQVRCNYP
LTPPLSNDNNESFASFNSNHNKTNPVQAFLGTPLELSGKVGPGRVVSRWS
LASAHLFEDVLSDDYLAVHHGLGPRYQGNYQDSLGGFQTYETSATLGLRN
EITHPGKDENSDILRAVRDKASTLLPECRIMLSILPSATFPENVQYLDTG
LLRSLPRISTRNLFRISSQEGAPLAINCAVPSLPDALLRAQGMASFRGTP
IVAVPSYLDENLWRLAGFTPLALKLLERATTADETVRSIEILFHCIRKSW
RNSEAMERDNGYGILGMLLRIKVGYGTQIAGEPAAERLLISNEDRDSLLF
RILSLILGFVGYNHAEPIDSFIVNPLAYRILLIDLDIWRKSTSRIQELYY
KQFLTFAVNSKHHEFNSRRLTRMRIVKRLLDAMKGEAVSEDVVSHFMLAF
EILVKSNLSQEVMRALSLFITYAFHSPTNSNPRTPKPLSSIPRPGTPGTP
KRIAVDFIRMATPPSGTKFLSRRQLGVRVLGMYSRILCEKGNTNHIKKFA
RTVTNKWLLYLLADDDAEVVMHGCRILARLLVSHGSNYTSKFAGKSGGFI
IMASKLKRFWANAKLWPICFSILFGYDAANIGLEQAIDPSSLLTLFSKRK
VAYPESLVIITSMLQHGLRDVMNRLDESDSQTKGSDVSDSRAEAANLFPV
EHKMTNPEKADSSLFDENVLLGVIRFLLDLQERSADFRDFAISSEWMRML
LTAVYPCIVSTDAVTPEVELNSRDSALTFEGSDVIIRPIGRGSAPAPIVR
TTSVDVLSSPQSTPPKGTPLRRASSFVLLTAQKASSADQAHSELRSRPSN
DFSGQISGGPVFDGMLELVLRVFLDQVWNRKDFSGFSLFAKVPPGFQEHQ
AYFESYLWRKILGQLTNRIHANQKSLCEPRLLTNIFRFCSQMSEAIFEGW
FIDGAEVAIDFIGSILEYLQRPDISNLKSVRLCSQAVSTVRNCLLRIVLL
KLSDLENAQVTEKEAKGFMNMLGYWQMSILACFDHDDDYFRLFWYQLYMK
LIDGKISIRIAAANLLRIIMVQKPDESTALIRSCITPDQRQLSRDFQKLT
EVDDETFVLWVDKHRPSLDRLFFGSIAKTWEDFVIVENQRTAESAKSRLS
KRKDKLKVWYSDGVNASKTLLNHDVGNTAWMKSIFNSEYFKYQRLLQDQQ
DDMAYLGAAYRKMEKDLRRPGAVFSEPTPLKWKLDRTEGRNRMRLRLLPD
ASGIDEKYQSKGKAIEAASVPLRVNTAINSTPPTNTPSRSLSSTNLADGN
DAAATVEPDSVSDKQEGGFVAEDDFELVDDPNEPIEGEDTFEDKNRKVMR
RLEHGDQVQAAYNMSRITGLEACEGILIVGKDALYMMDNVFQCASGDIVN
VWQAPPEERDPFTMVVTGTKTLEKRQTAGMRDQDSRHWKWHDVISFSKRR
FLFRDVAIEIFFTDGRSYLLTMINSAVRDTVYGKLLNKAPHTNTANALPN
PEDAWRLEALKTVDESPQGFGAGLGSRIGTLFNSSPSNPIVKRWQRGEVS
NFHYLMMVNTMAGRTFNDLTQYPVFPWILADYTSEDLDLEDPATFRDLSK
PMGAQSQARVPGFVETYSALREIGQTPFHYGTHYSSAMIVSSYLIRLPPF
VQSYLLVQGDSFDHADRLFQSVADAWASASSKNKTDVRELIPEFFCLPEF
LTNINGYDFGRKQSTGARVDHVRLPPWAKGDPKIFIAKHREALESPYVSE
NLHLWIDLVFGCKQRGEAAVENLNVFHHLSYAGASDLDRITDANERAITA
GVIHNFGQTPHQVFNKPHPAREYTQCSTRRLDTSIFSLTSLPRPLLESHE
RVSSLIYAPKLDRLLCASAFRLNFAPYDKFLEWGYADNSIRFFFSDNRKP
AGLFENLHTGQISCACFADSKTLITAGEDCVVSVYMVHTSVSKPVELVPR
SSVFGHKTLVTAMAVSKAFSTFVTASADGQAFLWDLNQLTFIRKLPLVRL
VECAAMNNVSGEVLLCSGPNVLLYTLNGTLILDQNVCGEQDDYVHSCAFY
EGAGDEWLENCIIFTGHSKGRVNVWRKCVLAGKWTLELLRRLDHIDFKGD
TQDNTDAGITCITPMPTCVYTGDEDGRVYEWNFGRGDR
>Mb|QLI65419.1
MAPKVRTGCKTCKIKCDEGRPACERCCKTGRICDGYDGPPATGVTTPSLT
LTRIASSSGTTQEVRALRFFVERTAVQFGTFAPDDLWSSRVLQLAHSNTC
IRHALVALSSYHERYWSHDVGGETPYGLRQYNLAITELVKSGADRPSYLH

IQLVSCTIFICIEILRRNISNAMYLFKYGCRMIEGAMKQVRKGGYVGGAT
GISIEPIIRLVEAFFARISTQVFLAIGGDIDHKLADIISPMLKLRMVTMT
PKPTLSSIQEARDSLLKLALQYRRDMTMAQSKALGIRFDAWTEAFDEFRR
AVDRNSLNSTDKRALALLELHRRYLYINIAALNQPDREDPSMWDLWTDQF
REMVEFATEAGGLDAADVPADNQPQFYMEVGILPALFFLSSKCRDPEVRR
RAIDIMETNHIQEGIWNSKMAAKVAKRVIALEEGEFIVKSSTDIDGLARV
RRVAVHAGPEVAYLNVGYELHCGWVQEELD
>Mb|QLI65420.1
MFWISRYIGLGTLLNLALLCHVNAFYIPGWSIKSYKEDQQIPLMVNKVYS
DNTQLQYAYYDLPFVCPPTGQHKSAAGLLSGQSIPLNLGEVLRGDRIMAS
DMELAMKKDTSCNVLCTREISRSDLRRAKELVHDGYVTEWIVDNLPGATS
FVTVDKTKKYYAAGFKLGYTEFSPSTGKARYYLHNHHTIVIRYRQAAGRA
GARGEKIVVGFEVYPKSIGNGNRRDTKGCPVDLQNINQPLELYMAPNKTL
DAAAPIYTDSSYQPAATEVDDDSAGGTISIPYTYSVYFREDDTIEWSHRW
DLYFVNQEEGSRIHWLAIVNSLIICGLLTGIVMIILAKTIRTDIQGYKDA
KAEDGKLRSKRKSRSGNRTPKEKPGLLDQGDDAENDADISSDDEALEEVT
GWKLLHADVFRTPRSGNLLAPLVGSGMQLMFMAMGLVLLSAIGVLNPSFR
GGFVSVGVGLFIFAGLFSGYFSARVFKSFDGRDYRANALVTAFLFPGLAF
ALVFVLNLFVWAQASSTAIPFGTLIAIVFLWLCVQVPLVLAGSYYGYLKA
GAWAHPTRTTAIPRQVPRQAWYIKSLQSILLAGLIPFAVIFIELLFVFQS
LWQDKSGYYYVFGFLAVVSMILVVTIAEVTIVTIYIQLCSENYHWWWQSF
FVGGGSALWVFAYSIWYYFFKLHITGLVSSLLFFSYSFMACCVYGLLTGT
IGFLSAYAFVRRIYSAIKVD
>Mb|QLI65421.1
MLSSRFIGQLASTARANIVTSVRPPVASRAFHLSAARRIVHNVKTAEEFQ
KAVTENDRVIVDCFATWCGPCKAIAPILEKHSEDAVFKDRVHFVKFDVDE
LPAVTQALGVRAMPTFFFFKDGKKVDEMVGANPPALLEALKKIAA
>Mb|QLI65423.1
MAGSIFSSPAPYHIISYGTLLGTSFFHSFINGPVMFKTVERPAFSAIQTK
LFPIYFGLQTILPAILALTFPGNTLAGVSSGISGLLEASNRWHSLAPIAA
MLVTGLVNLAILLPATTKTMKDRHGQAKRDGKEWYEPGPHSDEMRALSKK
FGMLHGVSSLLNLATFVSALAYGFTLGSRLQSVVDKI
>Mb|QLI65424.1
MVPAPILLGLCALSHLALSNQDEHLRPSAELARLRAPQIFNAVHNALRQW
GSSVHHNGMSLFLATVPPGVLFHHGNLRPQSPSEPDWLAYEVEHAEGFTR
SFGPPPKQNPGSSSAHRQNVLGGGAERGWLHVYRTTRPLRFLYVDGMSGG
KTDMGTLDTQDYLLRSRYSHEDKRQGNTPIRERERAEFLCRLCEEWDLQG
IIRMEAGFEIIKCDFTDGLEQVQAFRRSSRELRSETRQFEYLRGIAERYF
DIGSSRTVIDYSSMVSAFFFSVNLTNPDAGRPDLPRLSEATGAELAAINK
YFNATVEKRRDETPPSIDWQDVSDIIVSRYADRLEYMVQHVPSVDVLALE
VAFLLDVYIDYPDEDEPSLENAAQHCRDFYLQAISPASESDHLIHAAFRQ
VTTEICTALFAVRELLESEQLTGEQTLAAARKRLQSLVDYLDWARFKRCS
GCGVDEVCVIPMWPFGNEEDYYHPRCRNSSDTRGGKSYWGRFTP
>Mb|QLI65425.1
MPPSDNNRKPPTAAVNLIAGGGAGMMEALVCHPLDTIKVRMQLSRRARQP
GAPKRGFIKTGVEIVRRETPLGLYKGLGAVLTGIVPKMAIRFTSFEAYKQ
MLADPATGRVSGQATFAAGLAAGVTEAVAVVTPMEVIKIRLQAQHHSMAD
PLDVPKYRNAAHALYTVVREEGFGALYRGVSLTALRQGTNQAVNFTAYSY
FKVWLKEWQPQYADPGTNLPSWQTTLIGLVSGAMGPLSNAPIDTIKTRLQ
KTPAQPGVGAWARIAAISADMFKQEGFHAFYKGITPRIMRVAPGQAVTFT
VYEFLRQRIERSNMSFVGGNYEE
>Mb|QLI65427.1
MVSELCPVYAPFFGAMGCTCAIVFTCLGASYGTAKSGVGIAAMGVLRPDL
IVKNIVPVIMAGIIGIYGLVVSVLISDGLKQELPLYTGFIQFGAGLAVGL
AGLAAGFAIGIVGDAGVRGTAQQPRLFVGMILILIFAEVLGLYGLIVALL
MNSKATQDTVCQ
>Mb|QLI65430.1
MTASSRGRRALQGALSASPNVCSRCATSTRIATRFYSSTTAATTNLSPDA

AAPPPTRATPPYDVRSGLILTRPPLLTRKLHPFENAFFFYQKRLEERLNT
PFITSIYFKPDTARRLDWSLKVGERQGTVAKDIGVYNGKGSMAWDDELKV
GDQLSSQDSVLNSLLKDAEARVSDDAEVIAAEDVVPVERPVERETEADKK
GDVKRLDRQLERTLYLVVKGKDGWMFPADVVSRDENLHEAAQRVLDQAAG
VNMNTWIVGRVPVAHVVKQPVQTADGTVQTRGQKTFFLKGRIMAGQADLK
GNPFGYTDFKWLTREELEKELSPEYFRGVRNMMSDR
>Mb|QLI65433.1
MHSAKYHERVTSGEIAPQYEDIRYARNKGLGQPVATSRPWWNPRYWTRKI
WIAVVAVVVIVVVVIIAVAVTVTKKNAYPDYSPLTYNLQDTYGGQSFFDQ
FNYFTGYDPAHGLVHYVPQADAVNRNLTYATQNSAVLRVDTAVGPGSNPD
ASTGRFSVRLESKKTYNGGLFIFDVKHTPYACGAWPALWLTDPSNWPDHG
EIDIMEAINQGAAGNAMTLHTTGGCSMGVKRKQTGETDQENCDHGANNNA
GCAVKESPSTFGTKLNDAGGTVMAVEWRSAGIRMWQFARSAVPSDITGKK
PNPSTWGTAAADFPSTDCDIGSHFKNNSIIVNIDLCGDLVYGSWDKSGCS
GTCKEVVANQPDSFKTAFWEFGSFEVYQST
>Mb|QLI65435.1
MLSTLVLALAAVHGATAHFGLTYPPWRADTLAAENEAKYSQWSYPCAGVD
YKAGNVTDWPIGGGLLALDLHHPWTYVFVNLGLGENATNFNVSLTPQFWN
VTGKGTLCVDKLPVPVDVQDGALASLQVVTSGASGSALYNCADIRFKKDA
KTPGNCTTSKGITVQNVTGQSSAGNASGNGTSDGKQSAGVAAGANVVALV
TVAALASGFAMGL
>Mb|QLI65436.1
MSSFAAKRKARVIKVDDEEGSDGSSSSGLDAAGSKEDVHKPLFGSKSGRK
PFRQSGLRKTFNPGEGDTSAATGSTGNEDAEDDGPVVVRPNRPGAGKQKK
KGPKTRLSFGGGPESGESEDLATPKQVPLGQRALENSAVRRGIAVRTLPT
RSTQEEEEDRPRYSKEYLDELQSSTPSTPQDISNLHISDADEMELDPSEL
DGALVVEYPGASSPKQGTQILSEAEIRERKERRARLAQEQGFLSVEDDDT
DAFGRKKKDDGRLLADDEDLGEGFDDYVEDGGLSLGKRAEKERRKKERKQ
MAELITAAEGHSSDDSSDSDAERRMAYEASQSKAGLDGLKKPRKNPAGDL
LQIPPKITPLPSLSECLTRLQATLREMENDLKVKNAHLDQLRKEREDIVK
RETEVQALLDETGKKYQEAMAQGKIDADAASAAGPNIELVGERGLDSLGT
TPRRPDQSELDAVS
>Mb|QLI65437.1
MLRRPPTTLQITAEDVAAYEDRRANEALAAAQQARTEAQAYTQAQAQAQA
QMEGVQESPDAAGRRAREERLGITRRR
>Mb|QLI65438.1
MNTSPDGLKVNQTLGASRDKAHNPMSRSRLLFQPPDTAPATPLQLANSDG
LLSSLKRKLSDCTVDYPRRRATIACEVCRSRKSRCDGSKPKCKLCAELGA
ECVYREPGVKLDAGDKLILEQLNRIENLLQINLAANQGSGLHMSQSSPSI
SNGTASGGDQALLSNSSNTASTIPNGGLGTWVSVPTGTNISTMPRCHTNA
TMHLLQWPLIRDLVSTPYDPQILLQLEMDREPLHTLTKTPCVDLSNTQAY
IQAYFDRVNIWYACVNPYTWRSQYRTALSNGFREGPESCIVLLVLALGDA
SLRGSISRIVPHQEPPGLQYFTAAWSLLPGMITTNNVLAAQCHLLAAAYL
FYLVRPLEAWNLLCTASTKLQLLLMAPNRIPQKQRELVERIYWNTLLFES
DLLAELDLPHSGVVQFEENVGLPGGFEGEEQGQARIGHDDLWYFLAEIAL
RRLLNRASQLIYSKDSVSSTTSLEPVVAELDYQLTQWYEGLPPPLQFPFS
RTMLHDPIQAVLRLRFYACKTIIYRPYILAVLENEQAVLDPAVRECCHKC
LEASVRQLEHVTEQ
>Mb|QLI65443.1
MWSKIVKIFCPAAANAGDNAVGIIVLQILRLCTVITLATMGVAYWVFILN
VDKSRTYFVFECASLCCNSIICLILLFSEFPVFKAVKRYFVSSWPVLSDV
HGVAWLGSIMLLLGCNIFGNLNTPAMDSEKVAAPFLKLTLAAGVLAFIFG
VTNIVCSFVWRNGKEGITSRDVRANGALAKGRRQSPPAYASTYTDQYGKQ
GAKAGCFTRFTDKATTLFSKKKESARPEISAPVIDVERGHSEKDRFSPVM
PGLRRPPTVMHPMHNNTDSGSSSMYSDLYSDPDPNRI
>Mb|QLI65444.1
MDFVKKAAEGIKGNSGEKKDQAAQGQGGEQQDYVDKAFGMAASKSGHNID
RNTQEKITDAGRSAYEKATGSKVNPKISN

>Mb|QLI65445.1
MSPGTMTTTQVYLLPLHDDGSPDVEKQYIYIAPLSNDPVTLRFAIEGTSS
ICRHGSLWVNIPEKGREFRRDSFREFKLTPDFNRTIEISIQIHQPGAYAF
YTTYAELPSLEPRISEAASQASNQLKRTPTYYIDVAPRLRLDGRPLPLPA
LSVFSVISKFMGKYPTDWDSHLRGISDRGYNMIHFTPLQVRGSSNSPYSL
YDQLGWDSGCFPNGESDVGKLIQSLEKDNSLLSLTDIVLNHTAHNSKWLQ
EHPEAGYNLSTAPWLESAYELDSKLLELSSKLQSLGLPTDPKSPEDLLLI
MEAIKTEVIAKIRLWEYYALDVERDADAAVDTWAGTGTYTPQDTESIREA
VEQLRDSSVTQQADFLRKFALLGKDRLGERFRRRVDPNIAASLLAFSLGR
YEGESSNSADKAAARLKMVEILHALNVPLYEEYDGDIKEILEQLYNRIKY
VRLDDHGPKLGPINDENPLIETYFTRLPKNGTTSKHDPRDLMVVNNGWVW
GGNALIDNAGPDSRVYLRREVIVWGDCVKLRYGSKPDDSPWLWKHMTEYA
RKLATYFAGLRIDNCHSTPIHVAEHILDEARRVRPDLYVVAELFSGSEDT
DYVFVKRLGLSSLIREAMQAWSTAELSRLVHRHGGRPIGSFEVDEIAHGD
PSPSPDSESGRDLTREVIRRIKPTPVHALFMDCTHDNETPAQKRDARDTL
PNAALVSMCSSATGSVMGYDEVYPRLIDLVSETRLYTSESSRSGPIKVGR
GKNGIAGVKKLLNQIHTLMGKDGYDETHIHHEEEYITVHRVHPESRKGYF
LIAHTAFPGYGNGNGALTPVHLAGTRAQHLGSWTLEVDASAEATAKAVND
SHFLVGLPSRVIDIAGVDVDSQGEDTVLTVRDNFPPGSIALFETWIPAAE
HSTGLDTYVTSGAKAAWSQLNLIDLNFLMYRCEAEELDSNGGRDGTYNVP
GHGKLVYAGLQGWWSLMKSIIRENNLAHPLCQNLRQGTWSLDYIVGRVQK
MSETEGNEQLARPATWLSERFDAIRTIPSFLLPRYFGLVLRTAYTASRDR
ALELMGENIEKAQWFIQNLALVSVQQTGYVKSASLWPNKAVPSIAAGLPH
FAVEWARCWGRDVFISIRGLYLGTGRFDEAKEHILAFASVLKHGMIPNLL
SSGDAPRYNSRDSIWFFLQTIQDFIRYAPEGVDLLRSIVKRRFLPYDDTW
FPTQDPRAYSKESTIEEIIQEALQRHATGMKYREANAGSQIDSQMKDEGF
NQDIHVDWETGIVFGGNQSNCGTWMDKMGESERAGSKGVPGTPRDGAAIE
ITGLLYSTLSWLSELNEQGKYAYSSVKTAAGTSVSFKDWAGRIKANFERC
YFVPLSSKDDYKYDVNPAIINRRGIYKDLYKSGKEYEDYQFRANFPIAMA
VAPSLFDPGHAMHALCLADSVLRGPTGMATLDPADLNYRPYYRNSEDSED
FATSKGRNYHQGPEWLWPTGFFLRALLKFDLMRRTTPEGRTEAFQQVTRR
LAGCKKMIEESPWAGLTELTQKNGEYCPDSSPTQAWSAGCLIDLYMDGRE
EQQKQQ
>Mb|QLI65449.1
MAAQQQRWGHFPDSQGSQGLRDEYDDYTYPTHEHRRSLDAQPPRRSKSNR
ASVQTANTDALTESTFSPHSPTASSFAGAAQGLAPRPPSYQRPVFVANAA
PDVGAGMGAGMGNVGANVNVGVAVSNIPQEHVSHMPEETSRGPHRRSSEQ
FQDSLSESTRQALQALPNTPKVPRGPPVSYHQPYETSEPPYTRLNSNSNN
VSPTSFQSLSLPPGRFAVDDGMEPEGYYTTRREDYPDFPRSQIHDVTCGK
GVVAPPMMPQDIAAGGTRRPSVTLPSERRKKFANDRSPLQRLELTLDSMT
KEEKRARVQAAEQRARERAAKRAAEASTAQSGESQGRRIPIEPPRYHPEA
IVPTVPVDEPQMNLAETGNRGHPYLQHQPVQHQHPIPSRYRAEAADHGHV
AGPPPPLHVPEQREEHGESRSGLPRRNLSFRERAARNEKEHILKQASPVD
QPTAFFSGGPSGGTNIARNGSNKLRKEPPTDQWTSTKPNMERKVPGAAIP
EPLSATRAHGAAGRLPRAIRDKELPPVPTAQELAQGIQRRATEPIYRKEL
RPDEEYIPPATSSNANTSSIQSPGTEQRDLAVVGRRPERQESIHSVEDLP
QHRVSNMIFKDPENLRPGEGLYQPPVWLNEFQNATVGFLGGTLLDLTENH
QAISDKNKAWWETTGGRKNSTYSSRPRKAEAFDGEYDDTNAPTRFKPSLY
LKCGPLLRYCGIRQEKVPTRSQNSALSNREIWRGSVMIVTHDLESSYDIA
PMLRLFVQDIQLLPPPPLQVNGDLPPEYVDPIAGHPKLGRRGETIYVRPV
EHLEEARDLSRTETDEGLYEKSRSPPDIPPPNGTTELPQSFAGRQKRILT
DGEKLQKYKDVRGFRLHAERGYTFWRFNIEVELREKQQRIAYRINRGPSM
AFWVPPRGQAMNIMFHSCNGFSASANPDDLSGPDPMWRDVLNTHQSQPFH
VMIGGGDQIYNDSVAHECSLFDEWLGIRHPQHKHAAAFTASMQDEMEEFY
LQRYCMWFSQGLFSLAASQIPMVNMYDDHDIFDGYGSYPHHYMKSPVFAG
LGAVAFKYYMLFQHQSVLTETENTEPSWILGQEPGPYIKELSRSVYVSMG
GKVALLAVDCRTERTEHDVMNNQTWETIINRMYAEVRRGHVEHLLVLLGV
PIAYPRLVWLENILTSRLMDPVKALGRTGMFGKALNNIDGGVEVLDDLND

HWTAKNHKHERSIIMEDLQDLAIDKSLRITILSGDVHLAAIGQFYSNPKL
GLPKHKDPRYMINVVSSAIANTPPSDILADVLNKRNKVHHFDKQTDESMV
PLFHHGVDGKPRNNKHLLPHRNWCSIRSWSPGRTPPPTPPLSTYDRSPSP
PSVVNSNGGGAGGLFRRLSLGGGTRPTSNRFDGSRTSVRGDRPPVSGGVG
GLFRSLSRRNSSDGPRPAKLTRTMSLGSGEGKKKGFFGLGRRGSKSRPDD
GGINGQWGVESDDEDGYFVDNHPSHAVRPSGLRGGGTYEHSEFSDDDEMH
FTARPPQHTQTTGNQLAGPPRGDDEPDSMVRPFHRTPTGLSVKQLRKAER
FTVDLEGGLDICLNVEVNSRDPTGITAPYRLLVPRLQYEYNPADDELQQP
AKESQQPTGFKRFLSFRKKPEKPKLQQEHGHDGEESEDENEDYMSDESSD
VPPRR
>Mb|QLI65450.1
MSPTEDKTGADGSAELNFGDAIDMNTFDQILEMDDPGNDDFSSSIVFGFF
TQAQETFDNMDTALTQKDLKTLSELGHFLKGSSATLGLVKVRDGCEKIQR
YGKNENLDGSSEPDSELCLKRIAEALVTVKADYQDVEGKLKAYYGKDDEA
>Mb|QLI65453.1
MKASHVIGACLGMAAFAAAHMEMKDPPPIRSGFNPYTGWDIDYDMISPLN
SDGSDFPCKGYARYLGTPKGRPVATWTAGGTYSMTISGRIPHDGGSCQAS
VSYDRGNSWKVIRSWVGNCPVLGDSSYSFTLPSDLPAGDMLFGWSWFNNE
GNREMYMNCAAITVKAGNKWAQRGAADSYQSRPAMFVANVGNGVCTYEGT
DVEFPQPGPDPVRTSRRPHAPGHNGCGNWGGDY
>Mb|QLI65454.1
MATHMREKRVAKERQKIERSGLPPGIELVDGDSLKEWTFDIRVLDTNPLY
QNQTYRLRFAFPQSYPIEPPEVTFDMKPDRPVPIHPHIYSNGFICLDLLD
TQGWSPVQSAESVCMSIQSMLTSNSKDERPPGDEEFVRGNRLRPRDIEFL
YHDNTV
>Mb|QLI65455.1
MPFEDLLPFLEEEIKDADEETFLLYSNAIPSQNLGFIDPKATSLEITLAD
RDLTIHQSPTVLGSTRAGGTTGAVLWKVAPLFAEWLSAPANPLFTSGILA
PSSTVLELGCGISPLNALAVAPRIARYVLSDQGYVRKLAERNLSANRAPV
GRSRSGAKSVQAGGDVHFRPLDWEQDEVTPGLAAPGSSFDVVLAADCVYN
YALAGPFVQTLASSPRTQALSVLPPPSTRTATLPARPTSTPASTLSETTD
TSGAEPVSAPSASSTAVGTLTGTAPSANGTAGATGPPVVTSGAGRKGAVI
LGALGAVVLAI
>Mb|QLI65456.1
MVQTSTVVTATVATAAAAIVTYAVYFDYRRRSQAEFRRDLRRNERRQVRA
EKEEAEASTRAQRDAIKAQVDEAKEEGFPTGVEEREAYFNEQVMSGEMLS
SDPTKAVESALAFYKGLKVYPAPGDLIKIYDSTVPKPILDILAEMIAYDS
SLDIRTRSPGGINLGDIPNVGLD
>Mb|QLI65457.1
MDPSEVSVRDDGQPTAPSTPPLRRRYSMEEDMYYNRVMVAPPRRLSQPPA
YEPNPNDSDEGEDPGPGRRKGSQAVFDQSNEAEEGSESLPNYQSSLYIEG
VFSRKHEIENTTKRAEDRHWHTNFVTLNGTALNVYTVKKDWGWGRSRDGP
TICPDNPPWVRKSKLEKSYSLIHADAGIAADYTKRRYVIRIRAETDQFLL
SCIELATFVKWLEALFAAIDVAAPIDDRDFPRDMSIPRIQRIRWYQGQSP
ALPGYAVSTEQSRNNETSVEGPELSSTTTQTDSNVGLSSSTRPPPTLQPL
QEASANPPPRIEPLSRRISTSSYPNPSIDPHTGKWFPEHQWSSAHDLLYA
KLCYSNLLFRSPRKSNYIISKGKQWFVDWGTGKMVRVLPPTYGEIDFFGP
WQVIHTENPRI
>Mb|QLI65460.1
MAGLGPRRDASARIQKPDTSLDRLAALKARVAAATGASKAKGDINSLLED
PKSRNSSLVTRDLPIKTAQPRAQQHAGQSPYRTPAANSRKFGQAMQSDAI
DNPYLDENPAHQSSGARQREPRRLVFNQKGKYIQQANALRRQAALEAMKK
RIAEQTRKAGINEDLDVEKNFVIEQPPDVEWWDEGLIDGSSYEQIGNPSS
LKIISPDSIVTEYIQHPVALEPPQDRHVLENKAMFLTSKEQAKIRRQRRM
AELKEMQAKIRLGLIPAPPPKVKKGNLMRVLGDVAVKDPTAVEARVNREI
AERHQKHVKLNEDRKLSKDQKNEKLSKNQERDADKGIYLLVFKISSLENG
QHRYKIGMNATQLALSGTCIMHPKFNLVIVEGGAWSINKYKKLMLNRIDW
TENSPSRRGDDRPNSERDWLQAEDQDGALKDMSLNECRLIFEGEQKARVF

RRWSSKVCETDAEAREALARVKLETFWSLAKGTLGIS
>Mb|QLI65463.1
MANRSMASALRSLRHQAGPSRLDRISRRQFSSTAPNAVRAIFSETDNTEL
NNTLNSIQEKIILPAHLPEKQRKMVFDPKKRSHLEQNPIIIEVDGLEHKF
SSIDRFKGIENSKKALSKALDNMHSAEDWANLATLLAGYKKAGIKLKANH
WGKIVRVAGTKGHIFAVIECAKQSDKTGFVFRTPEMVVRVLAFVNDKITE
SAWDASETKQALKWAEAVLDLLQRPEHAVSSQRTRERLHFSRLVRGMTVF
ARASAVKVGQAAERDINLLRDEVALLSSLWEDAVNKDLSEVPEFANLNPL
VERNASDNGNHIPEALNGSAYIQVLTQNIRGIELAQEVLGGDAESLSPIL
NSLKSHLQDFAAKSETRTEAWAEEYEKVMGSKPNWPAVAAPAAGA
>Mb|QLI65464.1
METWLEESGAVGLDGLEVADFPVTGRGVKARRPFKQGERILTIPSGLHWT
VKRAQNDSLLGPALCSARPPLSVEVTLAVHILFVRSRESGYDGLRRHVER
LPASYSSSIFFTDDELEVCAGASLYTITKQLLQRIEDDYRDLVVRVLVQY
PDLFPLDKFTLHHYKWALCTVWSRAMDFKLSDGSSIRLLAPFADMLNHSS
ESKQCHVYDASSGDLSVLAGKDYEAGDQVYIQYGSIPNHRLLRLYGFIIP
GNPNDNYDLVLATHPLAPFFKQKQKLWALAGLDSTCTISLTLTDPLPKNV
IRYLRIQRLDESDLASIALRQAADEKISNSNEVQVLQSLVESIASLLGSF
GTRLEKLEEQLATGVYPVGGNAWAAAHVSLGEQRVLKLAKKKAENLLATV
ESESSKDGWLPAPVQCANCEKGPRQLMLCGRCKAVMYCGRACQVAHFKDH
KATCRATVSQKG
>Mb|QLI65465.1
MSSSVLKKPVKLACIQLASGTDKAANLKHAASQVAHAASRGSNIVVLPEC
FNSPYGCQYFPDYAETLLPSPPAPEQAPSYHALSAMAADNKVYLVGGSIP
EFSPDTKKYYNTSLVFGPDGALLGSHRKVHLFDIDIPGKITFRESDVLSP
GDKVTLVDLPEYGTVAVAICYDVRFPELAMIAARRGAFALIYPGAFNLTT
GPLHWKLLAQGRAVDNQIYVAMCSPARDMSATYNAWGHSMIVDPMATVVA
EAEETETIVEAELDGGVLGEARKNIPLGSQRRFDVYADVSASGGGV
>Mb|QLI65471.1
MPPEESKMAEPIVDKGKQVDQGESESEDDEPAATTSAADTPAGTSTASKK
KKSKRKKAKQLLTGKSEEQKQADEVTKAIGGLTPQQMKELVSLNPGLMQE
LAAASGSSNPSPDQVANMLKNMNLADIMTGLAASGKNVKDMGAYKFWQTQ
PVPKFGEEGAKVEDGPLKMQKVEDIDKEPQPLVAGFEWVTVDLMDDGEIK
EVYELLNGHYVEDDEAMFRFNYIPEVLRWAMMAPGWQRKYHIGVRASQSR
KLVAFISAIPVQIRVRDKTFTSSEVNFLCVHKKLRGKRLAPVLIKEVTRI
SNLDGVWQGLYTAGVVLPRPVSTSRYYHRALNWKKLHACGFSPLPAGSKP
EYQVRKYALPETTATKGLREMQDKDVDAVVGLLKRYLGRYNMAPEFAAEE
ARHWFLPKKDSKQVIWSYVVEDNGKITDFFSFFCVESSIIKNNDVLRVAY
LFYYASEAGLSEPFDKPALKTRLNALINDALILAKRAKLDVFNALSLMDN
ALFLEQQKFGAGDGQLHYYLFNYRASPISGGVNAKNQLDEDHLSGIGLVM
P
>Mb|QLI65472.1
MCNYIYKELSCQHHYHLVESWCSKYIETERRCPPTIVSKQYWGDDICCAY
YEVI
>Mb|QLI65473.1
MALTLLMTACAFARLGAGAICLEKATTIPDGWQMLDEIPDPSTPMRFSIA
LRQPEMHGLASKILANGLLGMGDTRSLRTPAQDDVDHVMEWLSSNGVSEA
KVDTDWIRVATTVGEANKLLDMQLRRYSYDGKKPILRAPEYNIPDSLDTA
IDFIHPIANFMTPEHEVSVAPPLAEDAGLHRRDVACSPATTPDCINKLYG
INYTTPDGKSCIRFGIAGFLEQWANYDDTRRSFEHSRPDLVKAGYNFTVE
LINGGENQQDLGTAGSEANLDIQFGMAVGYPTNVVYYSTGGRGAKIGDNG
EPLTGELDDNEPYLDFFHHLSSKKNEELPHVLSISYADDELSVPKAYAIR
VCNEIGMLTSRGVSVLSGSGDGGAKGGRNATCRSNDGSNKDMTISTFPAS
CPWVTSVGAVNNGQEPPNGSVFSSGGFSAYFERPDWQDAAVSEYVDALDG
HLEGYYNSSMRAVPDISAIGTRFQTVINGSPFMLDGTSASTPVLAGMIAL
VNDARVRQGKPVLGWLNERLYSDEVREVLQDITAGQSFSCKFSGGKEPGG
WPATEGYDAITGLGVPNDFNKFMDVLVNMD
>Mb|QLI65475.1

MRRTNLFFFAGIQLCSARSASFSIHDDLLAYPQFEVVFASDYISEKDAQA
LVNRNNQQHPTYSAEFSKTTGNHAHATTTTDAAAPSSNESDEIASYSYEI
LNLSPHRYLCSIPVIEPPGPVNETENALAKVEEEREHQRAAVKGWELVSK
LEGSCLYYVSGWWSYSFCNNREIVQYHAISVSSNGQIPRRDPNGQEYILG
RVPTLPATTGDRKKKRQQRGFDDPPRPPAELQVKGDQRYLVQKLEGGTIC
DLTGKDRKIEVQYQCVPGIKTDKIGWIKEVVTCSYVMMINTPRLCSDVAF
QPPVEKIANPISCKLISESDASTLLLDQQPRAAQPDNDGTKENIRQKETN
DEVNEKKDAAQVTVGGVLVGGKRALSTGDDDGKPLKLQGLGHLFAPQPKI
LQIIAEAASKENGGKVKGLTEEELEKLNIDPKAVQEMREKLKKLAGEKGW
KMQLFQMNEDDEKELFGYVDDTEEEEGEGKPGDGKVDSGKTKSDGDAGGE
KGPNHKEPGKQRMKSREKKKDEGKGSEEKFFNRDEL
>Mb|QLI65477.1
MTGKNTVPAAPWRAAFLSHIEQVQQPTFTLSSLHPASPPSSSSSCPPQFV
PRARTVVYRGMWASLTPNPKNPAQLNPASYETDLPTITTDARMEKVSEIF
SASNGGDSSGIGGAVEAVFWFPDSMTQWRLRGHACLLGPDIDSLEASSTR
EALAPYMRKAGDAEWSWSRELTAQFGNLSPFMRGTFRNPPPGTPITQVPG
EGLGLGQVVEDLEDEVARKNFRVLVIVPEEVDRVDLSNPERGQRWNYTLE
LRGHGAEWRETELWP
>Mb|QLI65479.1
MATPSQRSLPTRHNPFLLENVPEYLELVQRRRLGQTKLTPKMVGGDDAES
TALGVFDYAHLRAPLPKGIVSGIFKSSPTSYFLMRRSFDGYVSATGMFKA
TFPYAEASDEEAERRYIKSLPTTSPEETAGNIWIPPEQALALAEEYSIGI
WIRALLDPAKIPVSSYSNPDSPPKNISAPPKFDFVKASAKLAPPSTSTLA
KSSRSRRSVSPTKGSRRGPASPRKRKIQSKASSTEPTADKAEVNGDSKNG
KQEDIVLKTSEFEPAVVLEPRTEDATMKIRVDEVSKDAAGHETKHTVTEV
DVPLPVAGEPPSAEEVARMMEAAKEMVQKDREADEKHAEQQKKYDDDKGT
DTTTTTVKKGKRKAGDISMSEKDDEKAHSMAEEAKEEQPRAKKVKTEAEL
RKDRVKNRALFGIGATVAVGALVPWLVNFL
>Mb|QLI65481.1
MAPSFDHLREADLDDDEFNEEDIDISDLREKFEVSLEQGYDAFVVIDGLP
AVTEDQKPKLVKFLLKKLNSVGKTREDLIHMPMGDDGKSLSFAFVEYSSP
AEAAAATRQLDLVPLDKKHTMRVNKLTDVERYGGEGRIDEKYTPPHIDEF
AEKEHLRWFMKDPSGRGRDQFVMYRGDTVGVFWNNEKEQPENIVDRQHWT
ETFLQWSPLGTYLTSIHAQGVQLWGGQSWSRQARFAHPYVNLVAFSPTEK
YLVTWSNRPISIPETGHPALSIDDDGKNYVIWDIATGIPLRSFANLDLPK
AEEGKPAPKLQWPAFKWSSDDKYVARLTQGQSISVYELPRMNLLDKTSIK
IEGVMDFDWAPATVQREGVKNYEQLFCFWTPEIGSNPAKVGLMSIPSKEI
VRSLNLFSVSDAKLHWQSEAAYLCVKVDRHSKSKKSQATTLEIFRIKEKG
VPVEVVDTIKDTVINFAWEPKGDRFAIITTTEPVGVTAVPPKTAVSFFCP
EKVKGPIAGNFKHLRTLDKKNSNAIYWSPRGRFVVIATIHNQQSSDLDFF
DLDFEGDKPESDKDMTANLQLMNTADHYGVTDVEWDPSGRFVATWASVWK
HAMENGYHIYDFKGETLREEPIDKFKQFQWRPRPPTLLTKDEQKQVRKNL
REYSRVFEQEDADRGASADLAVVEARRRMLEEWYAWRADVEEEIYEERVA
LGLPQDPHVALLESKTKAMGVDSADQEQVIEEIVEDVLEESEEVLA
>Mb|QLI65483.1
MSNPLPSMPPVRPTAIPVDAPSSTWDRISSWVSENKAVVYTIAGVAVVVT
GAGVVYYLNSDSQKSRPESSPKLSKKERRKRKEAERKAAETNEPTPAPSQ
AKSVAVESEDEIPEIDESTVTKLSPEQREQYAAKLKQAGNRAYGDKAYNK
AIGLYSQAILCKPDPVFYSNRAACYSAMSEWDKVVEDTTAAIAMDPEYIK
AINRRATAYEHQKKYSEALLDFTASCIIDNFKSESTAQAVERLLKVFAEH
RAKEMMASRPPKLPSPIFVGNYLQSFRQKGRPAGLEDSVELDAETGKGQL
QIGLQGLEKKTGEGYEDARQAFEKALELGDLGEYEALAYNLRGTMRTLLG
NHAEATEDFDKSIELDPGMIQSYIKRASISLELGEPDKADAEFAKALEQD
KDDPDVYYHRAQASFIKGDLAEAQKDYQKSIDLDKDFIFSHIQLGVTQYK
MGSIASSMATFRRCIKNFPKVPDVYNYYGELLLDQSNFSEAVEKFDTAME
MEKQTKPMSMNVLPLINKALALFQWKQDFKEAEQLCQKALIIDPECDIAV
ATMAQLLLQQNKVTEALKYFERAAELARTEGEIVNALSYAEATRTQVQVT
EKYPKLAAKLAGGGAPPGLGMGR

>Mb|QLI65484.1
MVNHRALLSLLAFAHSTTAWGAPGYGGFTLRWQDSFPGAAGTSPNTGNWN
LVDGYLNVNNELQRYSPSTRNLQISGGQTVQLVPWRDGSTRMGWTSGRME
SRYVFTPDAGRVTRVEAALRFGGAPQPAKQGIWPAFWLLGDSIRHGVQWP
TGGELDIMERVNGLWTGYGTVHCGYSASGGPCNEPNGFGGAVGIPDDGWH
VWRLEFDRRSGDWAQQTITWFMDGRQFHQISGARIGDYNLWVALCQRPQF
FILNLAVGGNWPGYPNGNTQDGYGSMMEVAYVAHYST
>Mb|QLI65488.1
MIHEQVVSNIIPSPGSPPGMTTSKSSKSSSFQSLNSDDGSVLADVSHFED
IGLDDDNVTLKVNVRPGPTPLVTPVARNLAGGKRPPGPKPRRSFPNLHTS
VYASNPRSTSLAGLTDPRPLSLNRGHSTVSLPVARRNRSTSPNLSLNPRD
INLPPKPQPRPRRGSWQATRRKSLQDLEHECDEDDGDDIPDGIVLDNVPI
SPRPANERPPSRAPSISPSPDRAPKERVRSIGNGTPAVAQAQGSLRSPSW
RSDASDKPPPSPVKSRANSWNLALADLNAESKALTEKLEEHADELHERSS
RRPSGAARPNTWNSSHMPYDHVYDKKARVKSSTPELPPLRRTDIMIDPLP
ISKEKEAVLSRTRPSWLPPKDPAEERRHLKEYQKMMAASAKADERREAAR
RSRTESRDNTADNLMHIWEGDIIPRWNDAIRERRTRDMWWRGVAPRSRGV
VWARAIGNELGLTEATFRTALARARDLEDRVRTDRADAEDARRAKWFEQI
RKDASDKTWKNLKIFEITGPLHSSLVDVLSAYAMYRSDIGYVFGCNTIAA
LLLLNLPNAETVFIALANLLNRPLPLSFYTADETAQASAYNLVLQTLGHK
SLPLRDHLTKTMRDVTPELYLGGMFLSLFTGHLAIDEAARLWDVYVFEGD
ALLVRAAVALLLSREMALLGSKTSDEVLAVITRANSSTGAARAVAAVGAE
DRFMAAVREAGKA
>Mb|QLI65490.1
MSTIQHIKNLIRHGKQARNVNVDDEPSRKNDVPPAPAANQEKMNISDPVL
HQPLPHKEPLEAYSETPGDTQNRAAQAHNAAAHHVEPAQDVNAKASGTSK
KRVDDASLAKLVAEENASRSKFPRYPGLERWELIEKMGDGAFSNVYRARD
LQGDAGEVAIKVVRKYEMNSMQRSNILKEVSIMRQLDHPNIIKLIDFSES
KQYYYIVLELAPGGELFHQIVRLTYFSEELSRHVIIRVARALEYLHEEKG
VVHRDIKPENILFNPIPFIPSKVPKPKQPGDEDKVDEGEFLPGKGAGGIG
EIKIADFGLSKIVWESQTMTPCGTVGYTAPEIVKDERYSKSVDMWALGCV
LYTLLCGFPPFYDESIEVLTEKVAKGQYTFLSPWWDDISQSAKDLITHLL
TVDPDKRFTIREFLAHPWIQGTGPTPSEERRKSEAVIRGFDASKLDDVGK
RYDFRSPGAVNLREVFDVSYAVHRQEEEGKRRAQVGARAGGMGKPLGGLD
EEDEDADEMQVDQQAQSQAPNNGTHALEQSMRNTNIRDQEALARGRERER
APAPAPAPERGYGQHSAAVTAAARQQVRDRNRQKGAFELTLENATLLGKR
GKKISAMGA
>Mb|QLI65491.1
MDAPDSDFDIKLQKISGDLIADLDRALTTSIRKPDGHGGTQVRSFVRARE
IFKSTTMFKVLDMYQELPQLLDPHLPKWIPLLAESYLEYSQMRRRIKKAA
DQSGLLVPVDYAICRILYSFCKVRGEKVIIRFLNAETRYLEVLLSAIEEA
EEKSPTTGESQTHGWEWEQRYVVLLWLSHLLLAPFDLSTISTLDMEESAV
APIPGFEWPENLPGITIRVIPLAIKYLSSPGKEKDAAKALLVRVAMRRDM
QQLGVLDALVRWALASLRPRGENPAQSTYFYLGVLSFLAGVLQSSAETSD
MDKYLPSVFQCVHDIPLGNNEVSKSIIKLALVRKMILKVIRSVIASLLRQ
NRQSMANTEMTETAIGYLLDSVSDNDTPVRLSASKALSIITLKLDPGMAS
QVIEAVLDSLNRNILWTKPVGGQAGKPTRNLSAVNPLEWHGLMLTLSHLL
YRRSPPAKQLSDIIHALLLGLSFEQRSTSGGSVGANVRDAACFGIWALAR
RYTTQELLNVPTTSVFAAKAHPDSSSILQVLGTELVVTASLDSAGNIRRG
ASAALQELIGRHPDTVEKGIEVVQTVDYHAVARRSRAVEEVAIKVTKLSS
QYGEALIDAVLGWRGVGDIDAQSRRVAGAAFGALTSELAQLDATPSSSRF
LLAIHLVIARIQALVKRQVEERHALLLCYASVLDMVSQTPTAGQGDAQID
SALVQTILSSVHDIIEDTRVTEYRKPELVAEGASRLVVSALPIIQAAVLG
NQPGPAWMSGHALPSSSRSREYIASTSKLLKNAPCHEQVGKLLSALKSVT
PTWLARNEPEAVEPASEAALVLLLFSTSQDQEKIIQEWAITVATKPTTRT
VTTGQGYFHALAMAQPLAEQLRPSDPDVACQALLDRWAKDDDIDMRVTLL
QSLVRSRLLRAQANVFLSLLVDGLNDYTTNARGDVGSHTRVQALRAVWKL
WGDEASPDAKSDVMNTSARELFPCILRLAAEKLDRVRPEAQAVVALMMKE

SDSESFRSLTFSSKAYFESLLKLMLSDAPSPVLEPCQVDRSAWMAELMSG
LVTSADAGNDDLVIASRAALTDFCETSQRHLDMVCDALALNLRAHYADDR
IAVPTLEIIAFLFRVGVFHRSSGVNLRHLCFYTQKAGYKSGNVRKLWACV
KVYSGVASVPTGNQGDGQEAAQAGVKEARKRLGALMHHPWPRVRSVVVDE
IWGLSGFAEDDGARLTSVDWSAAGKTQIDATVKALGLG
>Mb|QLI65492.1
MASGTLGTWRFLPLPVSSSIPVLLVSFETGPSSYVIRVSDMANIWMESLD
RKAICMRGWSEDTSIDPSDTAENMTTFLSCLRKALDSKQAGHDETSLTLA
PEDNSGAADDGITLKITCPLPGFHALKWPIHLKKQSPSAVATNLVLPLIQ
AQFGTKHEIQSLIQRIAEKDAVVTKLSDKLEAMGTGLEHVFTALSGRKKV
TRAAAEDRVKGLAPFNEQRWKKELDSDDVGPSSVNDLIQHVFGDTGLEFR
STLEVDKSPELDKWWHDFLPTSQRFQRKEPKGSASKRQAVTPPQQPSNCD
DDDDFQVQSTPPHLRSAKKSATVSNVLPADDASTDGEEDSILDSNPPPAL
SDRTRQPETSRPGGSRLGAIGGKKQPAPPRAPSPPPRRKTANPPVDDEET
ASEASDDDATASVPASSPIPSPVQRPSKTAGLGRIGGGAMNTHPLDSASR
PEASPPPVARDTGPRKLGTIGGKKSKVERQDEDTERGRLATRRANSPKDE
KPRETSEERADRKREELKRELERKAAAGPAKKKRRF
>Mb|QLI65493.1
MPQANKRSRPSNHADPSSPHIKRPKFARGESIDSEDELYRDPSHVSKKRT
NFSALPSPKRRLFSRGDIPSTQFTKPAHAHIMTSTTTHSSIPDLNVRRAV
SGKHIYNADDDQRGQLILQQDGNSATLVPKFPAEESPPLQWMRVPLESIT
TIEHAMSSSHYVRILRPRGTDFEANLWLEFTDHRDVFSLVQAACHVKTST
CTSETLRRRWQKAFDSASTYTTQNTRQTPNSTTSRLEISPPHATTKVHGQ
TEAQKEPTRGPRRERIIDKLENSVSAEQIQATGDAKDDDVTVSLAPTTRR
TRRSSPVHITRDPRPDRWTDRNPLWRASWHKSLIFPPTGKNRATVDDGDI
LRLDEGEFLNDNLINFYVRYLQFKLESERPELLSKVYIFSTFFFEKLRST
KGKVNYDGVRAWTAKFDLLSYDYIVVPVNENAHWYLAIICNTPNAVSGMP
KDDATPAKEEATPPGIGLVARDMPDVSIHSDDASTPISKEPVDLEPPTSS
RTLQESSPAPKIATSNNRLAAASHVDPRLPRIVTLDSLGNPHAATCRVLK
EYLIAEVKDKKGIDLAMVPTGMTAKKIPEQDNFCDCGVFILGYMEEFLKD
PAETVRKLFQKEPVNWDIRPSLLRNRVRDLLFKLQKEQHEWLERERAEKR
LLSAKKKKKVAATTRVANQLAFPPTTVREKLLRKQSDATAVLAHVPRLLV
GDGSGKAEPDATRSPPKKQLPGDDNQVKLVNPPNGNPGTSSTSPDDVFYS
APSSPSRKPAAKNADKAKPKEPATKPTGSSQQKLDGYLSHRLPSSSEVEA
QSTMTTPKRQNLASIELVDVEDVVLISPQVITRQKSTQLESSPYFVQPLR
SSQSSSPKRLRARYDGVERAVDLT
>Mb|QLI65495.1
MPSQRPYFLSSFIAAFRQQGPTLQANQQPNKHNNQSAAVGSSTSSSSYPQ
TSTAPRTIATSAAAAAAASSAQANSRSPPPSVSIHSPRGNSAASIPIPNP
SASGRRRGSDSSSEGFRDAIGADKWYIGGRTAGGEEKFFKLGVVRRVRSN
DGLSLDRLSL
>Mb|QLI65496.1
MATETIPPAVGGSPKLEEDKSRREEAPLKRKASLGDDDDSPYTKRQRQDD
DDGSPPRRRPSIQSEAQDRRASATQEEKKRGKRLFGGLLSTLSQTSTNSQ
HKRRQEIERRQQERMQKQRDEDDKKRADKVARIKETRIVEHIDFEEQVMR
NKHSKLLALAEFLKTKSQPSIYYLPWKRTSDQEDAIDDQVRSAKDTIAKE
EAEFKSKKELHLSRYQPRRPSSDKEKDGIGRLETAHVGKHGEERREQEPD
TKDQDKSVHHHDPHDESGDVVVEADEDTVIY
>Mb|QLI65498.1
MDLNDLKSTVSNLTLYDLKAGFRKAQNAVMNYTEMEAKVREATNNEPWGA
SSTLMQEIANGTFNYQTLNEIMPMIYRRFTEKAAEEWRQIYKALQLLEFL
IKHGSERVVDDARGHITLLKMLRQFHFIDQNGKDQGLNVRNRAKELAELL
GDVERIRSERKKARATKNKYTGVEGGMTFGGGFSGGSGSRYGGFGSESGN
YGGYSGGVYGDGGGFGGQHDGYRESGGGGSSGAGRSDGFEEYDEFNEDER
PSRASRKSDHTSTKKPAAEPAKKKEPEVDLFSFDDPVQSSSSATPAATQS
SGLAVLAGPSSGNVGADDDDDFDDFQSATPATQPIVQPLTTPLTSTATST
AQFAAPQPVSAPQTANLSQMVNTSSISPGPNSSATHGANYSAFAVPMTQN
QPPKPTGFQPAGPNYFSGVPSQAPNPAPTNMSAMGNMASTGPTTMANMKP

VTSPGSKPAGASGGDAFGALWGKASSSIKKTDKSASGPAMGQLAKEKSSA
GIWGAPAVSSQPGSAAGNSGSASGDLLG
>Mb|QLI65499.1
MSTSSRRSFTPNRRVKSSSRDIARSRASSRAALRPQHFVVEHEEARSFAL
RTAYLHYLLQPRAKRKQYVPAPKPPARSHSSVGQLVQEYISGNSTSLKLP
SGFPTQLLDRVGGVVRGSEPLPGFSDAAVKRSFAEAYTAFSEKNFRRTIE
KERKFEPLVLMFYSSATKAAQKGKAPDDDSWKLLPDRHLAMFVRLATNIL
REHGHDRDRADLLSRLTSLEKKLLTNDQNLVDSAQDGKGKTIEVVVPLSY
DVKDMPMVQVVAAIFGMSLSEVQSEINQHRQTWTEEAALKDLKSYQHRLN
TTMAGALRSQDFDVEDAFVEWKKSESPHLSQMMLDILTARPELAKTSSSS
YEKQLPSRPQSMIGTDQAYADLSRVISAADGGINAFDPAVSLANLSIGCE
PGSIRAVEETIYTFIPPEPREFYKYILQHAVTFDQLHADPALEYKPLSKQ
SVDLLTELCVRWRIPQFSRLIALLEVAARKFLDQEIVPEELDAIFDFVKS
PMPEVRKPPHITHYATPLSDIETSRWTMHDFAVYQQTFHSLHDALLRELY
ELMEQCYDSKPPNIAAVMFLLENHIGGDPMFSPRPNALAGFSEHLAQGLC
QRAADVYRDYMQKEIPAHKEEWDFAHVIQLGKSVTKLCDRIRKRYKNNME
IMGVNPLSILVQEVFPSFEKDAVAIIETIMRGADEVGEELPIRDGFDLYK
ELVAIRRIHVESLDGVPFEFDIEDILVEFVWRFIRAAEEKVNGYVDEAIK
KDEFRVRAQGAEQIPLDSERHSVSVIDLFRILNQTVDQVVSLGWNNDEHY
ARFMTSLARTVATAVGRYCEVVEQRFAKEMDRPSAEEVAAQSRTTQEKWM
QYAKDAWSNKEKIEPFQFYAESFVKLNNVEFAMQELDKLEKSMNSEACAT
LLEKIDGPKKKVRKPSKYTFTIKVVEAEDLKACDANGLSDPYVVFGDEYQ
KRLHKTRIIYKNLNPRWDESFDITVQGPVNVIATIWDYDTFGDHDYVGRT
SLKLDPVHFGDYLPREFWLDLDSQGRLLIRVSMEGERDDIEFHFGKAFRH
LKRTERDMLTQQINDTLSLETLRNLLGIGGIGASMTSLWKKRASTAAAVT
PSQIEGALARLFTYFDENFAIMKQTLTDATMIAVMTRLWKEVLMTIENLL
VPPMSDKPSAKKPLSRRELDVVYYWLEMLFNFFNARDEQSGEQLGVPAEV
LKSPKWHELASLNFFYFEDSGNLIRESERMASATAQRAQQALLQQAPQQN
RMSAPASFGASFGGAGAFASMGTIRRGKSIMMNRNLGTMRKAKEAKRREA
QADPSDDMILRILRMRPEAAKYLKERHRQRERQAATVAAALIVRNSVSQG
WQTGGAFGAGQFGRSNLPRR
>Mb|QLI65501.1
MALISRYLPFAPSATTAQAVTYLLGISLFSISFLVFLNSSVSFVITDLIG
QKKNVGDVVGTLGFADEIVALVACPAWGLISDRLGVRWVAVMGYAVIALA
LVLFVQAKNVYPQLLLARIFFAVGASAAATMVTAILPSLTDEGDLSDGRS
PSRYKPNQRLSVAFSVESEATITPERYTRSLSNDSRDSRQQQDAVAKSAK
PSALAGLVGLFTGCGALVALSLFLPLPARFGDIDGVTPGQAVSYSFYVVG
AVSLVVALFVFIGLRNLKGEEGKGWTVLFGISERDAYARLGENGEDRTQR
QKTIPYLHLMKDSVLLGFADSRIALGYIGGFVARASTVAISLFIPLYINT
FFISNGFCQGSPNDPSPELKKECRAAYVLSSILTGVAQLMGLVCAPVFGY
LSGRAGRVNYPVVVATVFGMIGYLMLPQLPSPEFKDVDDRGGTPFIFLVV
ALIGVSQIGAIVCSLGSLGQGVIAVELPRSVRREPSIAPGEDESAESEPL
MRRSTNEDSASRVRLKGSIAGVYSLCGGAAILLLTKLGGVLFDSVSRGAP
FYMMAIFNGALLVASLCMDASQAFATANQGRGH
>Mb|QLI65502.1
MPESGWLQWTIILVLTALFLSLVVVAQVVATYWAAYLAAPQEIVTFIDTV
DFSIQENESYDRDVAKVQRLEDKIRLGQLLREIQKGGDDLREQLNSLVVS
DGATTLRTSTRLLWGAKRAQLEDRVRRLDLLRMRFLVVYMGIMTSVVDRN
PPPPPPLPRDPEKIMSFSSPTKPSLKKSLTETVVKRPPLRRLTTQAIGHQ
ENVITPHRKGWAGVVQELQTSPRMQQRHASIELAMAQPTP
>Mb|QLI65503.1
MAATANINPTPQSVTSLVSKLGDADPDFRFMSLNDLLHLLNIAKPDFLQH
DYNTAARAVDSIIKTLDDQNGEVQNMAIKCVAPLVGKVPMAIIAPMIDKL
SSMVLKNSVDNAVTSLALRSVIIALPRPVPGISTTPDVQQSYDAIRRVLI
PRLIGPNAATPRRQTTDISLPSVPAGMLQGDDASPETVDVLIEVVRCFGP
LLQSVEVEAMQHVVMQLLESNRHSSVVKKRAVVAISMLAVYLADEHLRDV
IQRLAFRLSDPGTGDVSRRLYISIIGSMARSIPIRFGPHIASTVPLMLKA
LSEEELQDHLERLSDGEDIGIEFNEVREAALVSVEAFLASCPQEMRPFTD

DIIAATLRYLKYDPNYAVDEEDDDVDMDEDEDQEDEDDGFDADDGFEDDD
DDASWKVRRCAAKALYTLISTRGSGDLLDNGVLYSQAAPSLIKRIDEREE
NVRLEVISALALLIRKTGEGLHVANFSLDDLDPPIAAPAPLNRKRRRQSS
AGTATTVSQFMAGSGLTSPVLERVPTTGPRADLAQLTPGIVKASIKQLKG
KTVPTKQSIINLLDDIVLVQRGGLASFFPDLVGSVLDAAKSTGHGATVSS
LASAGGSASATLSTLRIATLRLISDISKTHSSSVLQPYLTSIVAGVINAV
HDRFYKISSEAIRTAEELVKTITPPRSRNSGSKFKAELESLYEVLMDKGS
ANDADAEVRQRAIHALGALISRTFGDGSNLLSADKRMKALNILRERLKNE
TTRLAAVRAVDNVAAFASSPGELERGWIQDVALELSAQLRKANRSLRGSS
VNALKHLILSKASQGQLEKQTVDGVVSALMPAVTNSDTHLLGPALVILAS
LTPSHADVTVSKDMVAALCQLLKSHFAGIALDQLLEFVARIGESGASQTL
MQGLLKDVSISGDPSVVGKVIGTLLVTGDTSAGVSVDSFISELENSSKTG
DEARVSLALAVLGETGMRLGSSSPMRPDLFLRQFHSEPDKVSLAAAIALG
RAGSGNASEFLPVILENMQAGGNTQYLLIQSIKEILLSIAVQSVDIREYA
VPIWDQLRKASETADNRVVCAECVGRLVILDPSSFMTKLQVFLLIETEIF
FVANSYQALLKDTSSGIRGMAVQAVRYALPESDEAFDNELKNYLIDMLLV
MLQDTDTEIRRLAMSTLNSAAHNKPDLILPHLGELMPFVLGESIIKPELV
KEVMLGPFKHTVDDGLEVRKSAYETLYALMETAFTRINNIDFYDRVVAGL
KDDNDIRQLCNLMVSKLIVIDPDETARRLDSIAEAYRGVLSIKLKDNAVK
QDVEKQEEANKSVLRVTLLLGDKMKAMTGNAGAVTSNAGAAGIWTSYWEW
ANKEFEKQLKGLREESKELQSRMV
>Mb|QLI65506.1
MAATIKPIEGRSIHLIQSGQVIVDLCSVVKELVENSIDAGATIIDVRFKN
QGLDLVEVQDNGSGICPANYPSIALKHHTSKLSSYSDIASLRTFGFRGEA
LASLCALSSVTITTCLESEVPRGCKLGFEISGKLASTTMVASQRGTTVSV
EKLFHNLPVRRRELERNIKREWHKVIALLNQYACVQTNLKFSVSQQPTKG
KRIILFSTKSNPTTRENIINIFGSKAMSTLVSLDLLLEMHPSNVGPDLRT
DQESKQVRILGHVSRPVHGDGRQAPDRQMFFVNGRPCGLPQFAKTFNEVY
KAYNISQSPFIFADVQLDTKMYDVNVSPDKRSILLHDQSLLLDRLRSSLI
RLFDSHDYQLPTTQVLITETPSCPASVDANRRSIPLIRENPPYTSESELS
ATSHGEPDGGSPSNTGFVKGKSSQLAMREEKIASGGREGAKIAPESLTKW
FGCDANRPPISNSASQNTHVKQNRPSLSLFASSRTIALTDSDTEQTPQSQ
SLRRVDPIADTAPRNTTVVDHGTNSHKPSEAVSDSRSPTAFSWNAISSGP
STSLVQTIGHGDTVLLIQKKKPHKGAVPQTSENSHSSHLPNSGHTGKVCS
FSEFQERNAQHSCSGCSSDPESTGDTILDDKLSSSNTKAELSEASTLHPK
GMAKDNTENLGGARCVTPHSNQPQIEEDEHIDRSISDSNDEDNVAQGDTN
GRPHANSSAARRTRALGDGLRKKFGTAQHSQIIRATEATLASLSSSWLAY
SVRNISGPVEERVADITASDAESKLPLIIAKDDFSEMRVVGQFNLGFIIA
VRPKSHRHTCHEANDADELFIIDQHASDEKFNFERLQLNTVIQSQRLVYP
KALQLTALEEEIVLGNLSALEANGFKIQVDSTGGSPVGARCQLLALPLSR
EVAFTLNDLEELITLLAEESSGSRHIPRPSRVRKMFAMRACRSSIMIGKP
LTTSQMYSLVRHMGELDKPWNCPHGRPTMRHLCSLLAWDKAKWVADLHGN
STTESWLAYSMDN
>Mb|QLI65507.1
MQKLVKRTAQAQRQATRRARQQMEQDNIDNRMRNRQALRSAVYEIRQNLK
DARQARREDWEMGPLAPKRDLGFNNYGAFKETVRQDWTNYGLHQARPQII
EHRCAWAGGVRQLNLAPQDRVVIMDGPDKGKIDRIKDVQAENGTVTLENR
HRALSVGMFDNPARSQAMPISVGSIRLVYPLRNPETGVTKDVIISQLKAV
PPNMQSSNMSLDRWQYGKKWDRLVAGLNVVIPWPEVQVPEFEATKADTVR
EQAEERTFYYGLLSPPMPDQVLDELRNKYSRFRTRHEPWYIQQKEMEEAG
KKGRLEAIESMQTPLEEFHERQRELRDTQGEPELSEEMLEKIGEYMAKKK
DAALHQAGVSEVSSPQAPVN
>Mb|QLI65508.1
MASSATSSLRSLVRPSIPLAPRIHPVILSRVAFLSTTSARAAAPPPTIKS
RRDLPKKVKRTFKKKTNVVAVKKPNPGERKAFRKRIQLSNNSALPVRNID
ELTPETMANLESKGKMFALPDKVVDQLRALEAFKTTQTWNLFRRPHFLVR
EETAQLVIKMEKSIEKKEALKCVLTGSKLSGKSLALLQSMSYALMNDWVV
VHIPEGQDLTNANTEYSPVEGTEPMQFAQPVYTLKLLQNIYKANKAVLEK

LALQKDWSRFTHLKQGATVADLVLSAKESENAWPTLEALWTELTLPGRPP
VLLALDGLAHINKMSEYRDSSFNQVHAHELSLVRLFVDALSGKTKLPNGG
AIVAATSDNNTHYHPSQELVLSQLEAGQAGREIPKPDPYERKYDERVYES
LKNSYVLRIGGVSKDEGRALMEYWGASGMLRSVLNTSTVTEKWALGGHGI
VGEMERVSLMTMRM
>Mb|QLI65509.1
MTDSKRKANGHSATEADDRSAKRRKLAEFDLSKGESRESTTAYGLAFLEQ
IRRTADKSGRLVAIYFENLLPREGNAEYYKRTRMPICLTSIEEKLNKGGF
KNLAELESYFKRMIANAKEFYPRSSTVFEDAERVRKALSNYMTKTNPAYQ
TRNYQAVPTPLPPEDGEEVEEEEEEGDEDAEGEEEPAQEQEDEDGEEEEE
EEEAEEAEEEGEEGDDEEPASRRRSIILKRSRGRPSLDLASHTNSPRRAR
PAGRPDHEYKNVPFKGLNFQQAQEKIVEELLRYKEPEYEGYFEPFVNLPP
RALRDYYRVISDPLSLKKLQKEVLGIQGRGDPTGISDFKSWNALAERAKL
LWNNAYFYNEEGSEIYELAQELEKAFNEQLKKAKAVVQEPAQPKIKLKVG
QSIDTPSSSKKITILVGGRGSSVDSPTPQTAQSIEPPAGGSHVNGTARQP
ALDAARSASASMPSPSPSTHVGLKAEESARMSPAVLAQTPAEAPPGPPTP
AVEAPPPAPPPPAGPPQIQSTNPMANGYVEPKRLRAPGKGIKDALISRLR
IQLHPSMQPGDPVLLDVLPLPHEMQQSGTVNVPPIINRIFIVPMLPDLLQ
DRQYSLWVLIDKQPLKPCHQPIPNQLPQERAFDVMLHPGVNVIEAHLIAA
IPRHERVPGGPEVELEVFTAYVNVLRN
>Mb|QLI65510.1
MNLNLSASLNAARLLFKPSMCLPHHTVSTFNDLPIPLDKGLRSNGYKSDI
RAVVLDKDDCFAYPDAKEVYEPYKKRFDSLKEAYPGRKLLVVSNTSGATS
WDKDLKQAAEVERGTGVHVLPHAVKKPGCGSEIMEYFRQHPETGVTDPSH
IAVVGDRLTTDMMLANMMGSWGFWIKDGVVPMQQKSIFSKMERNLAVFLA
DYRGLQPPRPHGRVSEF
>Mb|QLI65511.1
MAMIDVSRQRRSLLADATVLHHLPPELLDIIRQQSTTKLLDAVTQAALCP
PLTEQVFVHFENVFPDICARWILNATNESLHIRTAASFARLLPFAPYLAS
FLRCTDDISSITTQPSSLQLLLPKFDQSSLSRLDGQSLLESLITAWRLLN
FDFRTYSPLASASCMQALFQHESLPVRYLAIRVFCLLLHASDFKLEALIH
EHVGKEEAVTGSLDGALVDFGFLSLYEQNRAQRVLDLQALLKCEEDTCEE
KECLQTLTPYVVSYGNVLLPRPHGPVGDPSSLVMTNTTTRNLERLATLLR
DSDPIMLYGMAGVGKTSLIHEIATQLGMYSNMVTLHLNDQTDAKMLVGLY
ATDSKPGSFQWRPGVLTTAVREGRWVLVEDLDRAPTEVLSTLLPLIERKE
LLIPSRGERIKAASSFRLFATVRTSRGLNGRENLPSLVGMRFWQTLYTDP
LTQSELEEVVLQTYPILRKFVPGILAVHGRLAQLGSNVSLMSRGRSVMDR
QVNLRDLLKWCRRLKECLLTAGSTNGDEPISETTRDWMFMEALDCFVGSC
PDVELGRVLTYAIAEEMHMSKDWADHYLSANIPPLEENDRAFCIGRAQLR
KKRLHSRLQKSKRPFASTAHAKRLLEQIAVAVKLEEPVLLVGETGIGKTT
VVQQLADSLGHKLIAVNLSQQSEVGDLLGGFKPVNVRSLAVPLKEEFEDL
FAATGISASKNQKYLEQVGKCFAKSQWSKLSKLWREAPKMFTKIVTELER
SQGDQAEARDGDAQPAKRRKTLSKLQTLQELRPRWDSFSRNLEQFDVQIS
GGAGAFAFSFVEGNLVKAVRNGDWVLLDEINLASPDTLESIADLLTGPNE
RPSILLSETGEIEKIVAHPDFRIFGAMNPATDVGKRDLPIGIRSRFTELY
VKSPDTDLKDLLTIIRTYMGNTSTKSDQAADDIARLYLNTKRMAEERRLV
DGANEVPHFSLRTLTRVLSYVSNIAPLYGVRRALYEGFSMGFLTLLDRDS
EKMLLPLIYHHLLDKHGNPQSLLSQPPKHPNDGRQYVKFQNRNHDRHYWL
FQGDQTPIERDDYIITPYVERNLLNLVRATSTRRFPILIQGPTSAGKTSM
IEYLANFTGNKFVRINNHEHTDLQEYLGTYVSGPDGKLRFQEGLLVQAMR
QGHWIVLDELNLAPTDVLEALNRLLDDNRELLIPETQEIVKPHENFILFA
TQNPPGLYGGRKVLSRAFRNRFLELHFDDIPEDELEYILQQRSRNTSPPD
CRRIVTVYKELSRLRQTSRLFEQKDSFATLRDLFRWALREADTREDIAAH
GFMLLAERVRNDEERIAVKEVIEKVFKVKLDPQELYSASKAPELKNLTQK
ANAQGVVWTHAMRRLYVLVSRAIRNNEPVLLVGETGCGKTTVVQLLAEAL
NQELHIVNAHQNTETGDLIGSQRPVRNRGAILEAIRTDLKVVFESLGMEI
PTSVEENVEQYKALPQTVKDTVPQSIRDRLAGNESRSKALFEWSDGALVE
AMRNGHFFLLDEISLADDSVLERLNSVLEPQRTLLLAEKGIDNSFVVGAD

GFQFFATMNPGGDFGKKELSPALRNRFTEIWVPPLSESEDIYDIVKTKLV
DGSKHLVDAMVDFASWFARKFRSMATSAFSVREILVWVQFINSFKADDPV
ISFVHGASAIFVDSIGANPSAILATDLKTVNAQRQECLNKLGELIGQDVS
KIYPTEPMLSITDTLLSIGDFSISRDPTGSADPTFAFHAPTTRLNAMRVV
RALQMRKPILLEGSPGTDLMDLFGTDVPIEGEEAGNFAWRDAPFLQAMQK
GEWVLLDEMNLASQSVLEGLNACLDHRGEVYISELDQVFKRHPDFRLFAA
QNPHHQGGGRKGLPSSFVNRFIVVYADVFKDEDLKLIASHNFPQLSEEVI
AHLIEFVSRLEHQIVVEKTFGSQGGPWEFNLRDALRWLHLLRSSDPFLKT
AQMDDFLDIVVRQRFRTSRDRAEVDKLFARVIGWEPRAHDHVHNLNSQFG
QIGFAVLPRNLNVQPEGLPNINIVPRLAELESIMICVKQNIPCILGGPSG
YGKSSLLSYVAALSGKQLVVFPMNADIDTMDLVGGFEQADPLREVNAALA
DLREYLQDSVMTLVPSAAPGEVLHLLHLLDRHNGDHESLTAILISMKSLL
EQVSSDTEVGVALTRMKQLLEGPLVLANPRFEWLDGVIIKALVTGQWLVL
DNANMCNASVLDRLNSLLEPNGFLSINEHCDSDGKPRIIRPHRDFRIFLT
MDPKYGELSRAMRNRAIEIHINSPPPESVPCLDKVSAIESGLQRYHESRL
PSNASAGDISIDTFQVAVDNLSLGDGALLGRFVNRGTTDETLRSVFTDLL
EFLKTTQGSHILRNIMQLYATLSSDTIADFPQALPIHPLNNQIAIKLLEN
ETSAHWMGTCYETSRRLHCIRQIIKKHHSQAQSAKLGSLSRLQRSLISDR
VAAVSKDSTVKLAGFLSSIMVSIERYLDQEVNSTENFLARRHLLEILGLY
LELTICLSSSHEFDEAYFQAHIGLGLRKLDEFSQMMTGETDSTFLSQALG
QLRSDFNSGFQLSTGLSMEVLWNAFRPIPIANKAVFDRSVEVDQLASRFD
ALRWKASACIFDLSKAQQTLATTYKIVRDDSVPTEQLVADLTTAIQDLET
RIGQEAQETVPFFASDFENLRQLSILRSLASDSLTPELDNQLVILSNLPT
KAIMTLASASDRAFNLQSLSYLAHQGIFSWDGKLTTSLWKKLHGLRNVNL
SSLALLETELPVVGKHLTGLSPEISADPLVNLNDLLRKLMLDTFEAHDSS
LQNTLGAIFSEAEANISSISALAVNGLGSSTAFNDLLQDIDSVHLRQTTT
DHFIPSVVGIIASKANPNKAAYFSALAWLQFSIGLVKLYVPDRIFDPQLQ
PKVELKFFEQLEASLQGKLAALKAFQTSFTGQETSARIQLIHQQLDKLGP
APKEIQPVFRPERSELHQLHAEFSNILKTITSSSLSGVMKDLNSDSDNGV
AGALHLIRENISRLVDRISSRFEAYQDMGRPTVNILRCLLVGLSLCDTSK
AANLEPSTLRLVESTPFFGLKTQDVSLRRVGTKSFEFLSLMSANVAVGGL
QGLSSADREAVFECFHSFYDDWNKKLDADRKAEAARTSMYRFRGTLEDEE
EFDAMEFNELFPTFDDEDSTARKDIKPDQVRDTSLKVADSHRRIFFEQQD
PQDALRESCMAAGQRVVGELQDYATIDRSINSKMLASTLLLLGEKMEELQ
SSSSTKTYNFYTDANLAEARQLVSLAHQIKARFRELQLVDEIGHMQPLTD
VIQSCDKLLEQVHGEPLAKLLPKVEQLHAHVYEWQFGGWASKTYAVLSLH
NLLTDTVIRWRRLELSTWANLFDMEQKKCQDDAYSWWFVAYQVVVAVPLT
MVDSRSEMQAYAKSLVENLELYFSSSIVGQFQTRVALLRQLLSHLNMLAQ
DYPALDAICVAVDNFVQYYTRYEKTAEEAIQKGRIPIERKMKDVLLMASW
KDTNINALRESARKSHQKLFRLIRKFRGVLGQEMKMFMAQGLPDESLTIG
TSTESEPVASDLPMVKIEALDALLPGWLPDHKRLANLPKTISVMRKITYS
PEMSSNMSNLVLDFVTELNTSMAELRKETPSILNDENKDQIKHLKTRKRK
LFADTLRDLRTMGLQHNLSQDKLAEQKSLAVVLSSLAAVKFLESAAAKEA
EYYLHKTLDLAPRAREASREHSDDLTGAEVARSAGFVEGLISFNLTQRQS
LGAATQLRESLQQAVQEFKNLGNAAKNGTLVHQRFDGDWTNLVQWLGPAI
QFAIRLTEMHGKIGNIDNKAVIQELQEWATRLAARSAALKTLVQLPVGIS
SVAHDKMREAVLKDLDEIDHRIGQLAEIREDLAFILSHLKAWTTTESGSY
ASSIGDTTLDTFANAVSSLADTVLVAIENAKKKGVVVPREPGEPGWLTKH
CEAFFAMIGQLRMNNVERTVRRCTDMIRQIQLDDEQTSTAAACVIHIASP
ILDQFLTLCSRSIGQGLEMHRASAHMAYNLTKAFNQIVSQGFCTPQEKSD
ETSGDAGQLEAGTGLGDGEGAQDISKDIQPDEDLTELAQEANKEKNEDIQ
DEKDAVDMADEDLEGELGSVAGEDEDEGSQKGDEKEEEEDDIDEEAGDVD
DLDPTAVDEKMWDGNDDDDNADKDQQGNEAKGQQQDDEQMAADEDTKQKD
AEKEPEVREEKEQPREGEEEEEEAEVEEEDVHAQEELNRQDQNVQENDAL
ELPEDMELDLDDKEEVSDNDDLDDLSDPEGEADQPEEQPAEKDEIDEDME
DAAGQQLKEEAGEVEDEIGEDEEEVPTGADEVKDEVESDVDDEVEEESKE
ETAQDVTQPPQDNENTDVDNAAPSDVKSTGLDQNQQLMDIDDKFQAQAAE
QEQGDMGDGAADQDTSAGSKGATSRSNETVDKSQQEDDDENSARSDPFRK

LGDALEKWHRQNADIMDANQEDTNNQKADERENAEQGRQEYQHLQNDEAA
ADTQAMGNAEEDEVQPIDEAMAIDDEGEKDDPTSNRMEEDADDEAKEAPD
KMDLGDSEEAREAANSKEADDGRTGVQTRQGNYNQEETPSEADDGVAEDG
EPDDGPIEETSEQLSMTHIGDEARVPRDYGECLQQWSEFQTKSHALSLNL
TSQLRLILTPSQSTKLSGSFRTGKRLNIKRIIPYIASSYKRDKIWMRRAI
PTKRTYQILLCVDDSKSMGESSSGSLAMESLVMVSRSLAMLEAGQVGVVG
FGGDVFTAHELTEPFASDAGAKVLQKFNFSQDRTDIALLIRQTIDKFRAA
RQQSGGNGDLWQLALILSDGLTPSPAHDAIRRLLREAMEERIMIVFIIMD
DTGKKKGDSVLELKEAKFVKDDNGESRVVLKPYLDTFPFQYYLIVHNLEE
LPTALAGLLRTWFAEVTS
>Mb|QLI65513.1
MRWLSPNLFYAFTVVVIAFGSIPKGYDEGGFASASGLRSFLTDFGLTRGR
WTGDPSQLATRRAVVSSLGVLGAAIGAGMAIYVTDVIGRLRTWQLFTLLW
MTGFFTVTFASGNVGVLLFARIWGGIGAGGLTVVAPLYLSEIAKAKNRGM
IVSVYMVVLLTTLMLGFFISYAARRTMASNREQYRVVLAVPQIPVGIALF
CSLFLHDTPRWLASKNRHDEALIVLARLRNKSMEDPEIQSEYREMQQEVV
SKNLILADTSTWTIIKEVATVPSYRNRFLLGLAMQTFAQWSGGNGITYYI
PEIFRLAGITTNRALINAGGYGATKLVFTMVFTWGLIDYFGRRRCFMTGL
AMQCATHIYMAIYMAIWRRSDNQSASTAAIASVFVYAIGWSIGLCTVQYL
YGTEILPTRVRGVCYSVNMMVHWLFQFAVVRTTPPMFRNLDVWGAYVFWA
LVCFIGLIVLGIWAPETKGVPLERMGELFEGPWYKTWKAKVNMNGDGESL
HLQTSRDSVVHQHENRNKAPVD
>Mb|QLI65516.1
MAAPNSVHSRSHSLLLLQKLLNLRDSASPLTLVLDGLEQPAQPVLDEFMT
RAKISKTKVILLSFATVKKPQTVDVMVKAAGRDLRLVAKELAAHYPVFDP
TQPREKPSQRAIILIDSLNSLASTAPSSLSHFLSSILTPAVSIVSVYHTD
VPILLPPSFNEYEPHPFTILNHLATAILRLSSLPQEIDRQKARNRAVQEP
EWGLKEDREGVIIGLKPRAPDQDRGVVVDMELRRKSGRTVTEKFILQRHL
LAAGKSGTGRITLLTDHAMFRPAEADRDDAEEPESTFSLGLTEKQRRDRE
GIVLPYFDAQTDIGAGEGGRILYEMGREDDFDEEEDEI
>Mb|QLI65522.1
MTRDPSRPQLVIHSKHHVSDRTASSGSFHAGAPIPTLGSFRRQSSTHKYQ
TFPVQPPKTPLGPPPVPSQDAVSESTESSASGEDDETPLPVRQLLLLAFL
SLSEQTALNSIGPYLPEMVASMPGVPGGENGLYVGLLASAFALAQLSTNF
LWGYASDIVGRKPVLVAGTFSLMGCFCVFGFCKEYWQMVVIHALMGMLNG
NAACVPTVLGEVTDRSNQSRAFTYLPVIYSLGSITGPALGGLLVNKMGSE
YPYLGPNVLGAAMLAASVVVVGIWFEETLDESGKSPWRPAWISRLVAWVD
GSGKPGDRRASWSSRLAEASASQQPLLSSSSSTSSSVDQDDADTDGDDVK
DGGVSGRPVWKDLLNRTTIILLATYLVFQLSNISFNSLYPIFASAPAPAG
RDLSPKKIGFSLSAAGLATIAFQAFLFQSIKSKIGNLGSYRYALLGLGVS
MILMPWVGYLDDDPPLNIGSGRLWLFAELAMVLILKNICAVGGLSSVMLL
ITNSAPSHASLGTLNGVAQTLSALGRSFGPFVSGGLFTLSVNIRPKGEAL
SWSLFGGLALVGWGVSMFIRGEGLESEDWSDDDDEAETA
>Mb|QLI65526.1
MAPGSIWSRLRIPPSGLARAVTLNLMGFATWIPVIAWFNMHVAELTFVDG
SSMYPFLNEDKDSTLRRDFFLNYKWSPQEGLERGMVVTLRSPYNPEVVAV
KRVVALEGDVVRTKKPYPIPTVRIPQGHVWVEGDGPAGSSLDSNTYGPVS
KRLLTGRVTHIVYPLRKFGQVRWWEHESKLVE
>Mb|QLI65527.1
MRSILGAAAVLFAAAVSAVSTAGNRLLVVLDDVAEKENYKQFFGDLTERG
YHITYETPKSEHVKLFHLGERTYDHLVFLPAKVKALGPNLTPNILVDFVN
ANGNILVALSSTTPASSSLTSLLAQIDIGLPAERTGTVVDHFNYDALSAP
ESHDVLVLDAPTNVRPGLKNYFEVPGVLSFPHGAGHTLGPGALLTPVIRA
PSTAYSYNPKEQAEAVDPEDLFAAGKQLTLVSVFQARNSARVAVVGSAEM
LQDKWLDAKVSRPEGSKVKTENREFAKRLSGWAFQEIGVLRVNNIEHQLK
GDNETNPEIYRIKNDVSYSISMSEYSWNKWEPYTLPATDALQLEFSMLSP
FHRLDLQPLSVSDSATVYGTSFTLPDQHGIFNFKINYKRPFLTYIEEKNT
VSVRHIAHDEWPRSYVISGAWPWISGIGATVGGFVGFCAIWMYSKPVGGK

PKTK
>Mb|QLI65534.1
MSSSDVFTMRGSLAAIPMELDKAHFVPLVTLFASTFVAFHIAQAIYRLWF
HPLSKFPGPVLYSVFYLPYLYQAYIKGRWIFKTTELHRKYGPIVRVGPNH
IIVDGEIGWPQVFGRRKADQSEYEKMPAVHKAKAYSIIGATRDIHRRQRR
QLNHAFSDASLTQQEDIIQAYVTLLLERLQTRADEAEVVDAVKWMNCTTF
DIIGDLAFSESFSSLQNNAVHPWVKLIFDSIRFLAVRRFFVNFPLLRFAL
SMFSSKFGTKQHPSRDHAKNKARRRMALGQDPPGGRKDFMSYMLRKTRDG
QVGMSEEEILETCPTLVIAGSETTASALSAFCFYMDKNPRAYAQLTEEIR
TAFGAEEEITMQNASHLEYLQACVNEILRIYPPAAETPARVSPGDFIEGV
YIPANTLVSVYQWATFRNPKHFKDPESFIPERWLSPSHPLYDEGFKNDNH
AIFKPFSYGSRDCIGKNLAINELRVIISRMLYRFDFNVLEGQDDWHTKQR
SFIIWDKSPLMVRFQTRQMAN
>Mb|QLI65535.1
MPVQDTISTLVEWASSHGATLHPSIEVYQDPQTGLSFRVKPSAKSPVQPY
EPIVQLPTSLTLSYFNAVSEQGTASPDAFRGEFLARAAPHVVGRLFLIKE
YLKRDKSFWWPYIRALPQPGQGNKSQWALAPFWDADEAELLEGTNVEVGI
DKIRNDVRRDLQEAQELLRLHGDASGAFGRALTTELYQWAYCIFSSRSFR
PSLVLSDEQRRSLPRGVTMDDFSVLLPLFDIGNHDMTTEIRWDLDDERQT
CELRVGRTHMPGQQVFNNYSMKTNAELLLGYGFMLPATKELHNDYTHVRK
RTAAAPRASEEYLISLRPLSHPSSVLARAKQSLTLDPATAVLGSFRHVQP
EMVWDIFCTLTTPEQRAVLIPVPEGKPKDTYQRDRFFSGKVEGEARMYLQ
QTVAIIQHKVLQELERLNETDVEAVGGQEADLNRNQRLALDYRERCRKVL
EGVLESMSGDDILDDEF
>Mb|QLI65536.1
MTSMSGAAPQARSSSSGVRTPLFTRSSFIDDDFATSLCRNRAFQPQWPSS
NGGSRIVVLGSVRLGLAAVNMSFLGNPPPNYHTPPFPSLNVNTLQDRTPN
RTYTLYRITDVWKFTVLWTLITYIFFHLGAVLVAVFSHGLNKGSWRFLWA
VPIIYLLIAGIEAIIAGSIVGLV
>Mb|QLI65538.1
MEWFGHAKVEFTHAPAPRAIREKDGKETDLLKIVEKSTPPCHLNPLLFNG
HIQTMWTATKPSGPPVYYRRKTFDADHKTYKGTFAVDFAVQPYEGGDETL
PRRTTYYTDEEFDNIGSDDSKPMLVVLHGLSGGSHEIYLRHTITPLLGDG
GWEVCVVNSRGCAGSKITSGVLYNARATWDCRQTVKWLKKTFPNRPLFGL
GFSLGANMLTNYCGEEGEDCLLKGAVVCSNPFNLEISSKMLQNRFIGKEV
YLRVMGFAMKNLVSTHKEALKKYTDLDLPSLEKVTYLYDFDREVQCPTWG
YPTEDAYYRDASSTDAVLNIRIPFIAVQATDDPIAVKEALPYAEFRQNPN
TIMITTSMGGHLCWFEMGGSRWHPKPICNFLNHLAFKADLDSITPSRVPT
PENPKHGADYNPMRRKLQILED
>Mb|QLI65539.1
MTAFLLSICALDRCLALFDEYHRRLSQRQLVLALASRAGSVRRRRAVAGH
HRAASPPFSIQIPTQRRRPHARPSTRHSFHSISKISKRHLAMASHSEIRR
RSSAADRTPIEASVLSSTIGAATTSTFASAPESASSSSPLTAANALTSTT
PGPPRTSKANKHRSLDSSSMPPPPLPGGNMAAPSSPKRHSRTESTSRIPN
RLSLTLPIALPTSDPSRPTPAATASTPSSVPPTPQDHSALPSPSNVNEFI
TAIAAKERRVLELREALAREESELTALKRQFSLMDSLHKLDTSLYDSTSR
VFTPTADGGVQSPRRSVDMDRRNLLLQNQGTPTQSRRTAFRGRHTRTLSL
LSPARTNSEFSALEDKIGPDARPPPIDQRPTQAANPMLAKRASWQPRTQQ
SPPVSSQFMQDVRLGFKAFVEDIRQITIGDEPINGQGQSPGQHHTGSMNH
SGPSDQARSRNGQTGQRIASRLDASSPVTAPPTPNSTGRDVSPEKSRPAK
SKHFSWTPLGFDSMDDTDWANWESPATAKSTRWSGSTVHGGPMDDIQSIP
EYEEHTTPVKPKNLQTPILSPNKLEEMLPNMVNRLSPSNIKRTANNLLDE
WEKSLMAPTASADEPQANKENTTATETR
>Mb|QLI65541.1
MGLRAQSASQDPAEQQNVVGRLCDRYDVRKALLPASGDPATRKLEVDTEP
KILASDEGQDSTKAPRYQVTYHMTHRKPSPLHEIIEQCQICLANSKRFFG
DHIDVGQLLNSTPRHPRHHTTDKMENDRGEIVDLYVPRKCSATNRIIKAK
DHGSVQISIAKVDENGRAISGESHVYALCGFVRAMGESDDSMNRLAQRDG

LLKNVWSAQR
>Mb|QLI65542.1
MDPGRRVMIDDRRPPSRRDATSARADAYNRISKSTKPGPSKTAQNAAKYL
TEEEQSRKFVADEDKFVLKQSKKKADIRVREGRAKPIDRLAFNLRYIDEE
RDVFDDDEGDMDIQLDAPEVIVQGLTLAQLKELESDISSYHILETDRRNR
QYWEALRAICVDLKSKVDTNTRDGRVVNSVVDDIDRILSPKSYDQLEALE
KQIKTKLQTDENIDTDYWEQLLRHLLVWKSKATLTRIYNEIKEKRAEMLK
TRGPVARPKQDAKAISNAAVPSEPQPSPQPVANEDASNVTNALYDREAAR
GVSENEEIFNAEEQVPTGSKPQWADTYTARKPRYFNRVQMGYEWNKYNQT
HYDHDNPPPKVVQGYKFNIFYPDLIDKTKAPTFKIIREHGRRKGESFAAA
GEADTCLIRFIAGPPYEDIAFRIVDREWDYSAKRERGFKSSFEKGILQLH
FQFKKIFYRK
>Mb|QLI65543.1
MQQPSSSGNQEQHLRAQLELLQSHDAETSTASGSCDSRSALSHSPPARTA
NGYEELAAASHEAARALSKTQAESHIHPDLRATPGHPSNMMPMGPPSGHS
PGGSPSGPPNASIAPAPSSSAPEPGAAGDGRKAKRELSQSKRAAQNRAAQ
RAFRQRKEGYIKKLEQQVRDYAEMEQTYKVVQAENYALREYVIHLQSRLI
DTQGEYPPPPPNVNLSQSSAAQPPPASAPEPAPNPGVGTPLEAVAQAVAG
LAAQEQLAESQGRYPSPEYKPEPRDEDSRTVDEINRQLHQQSEENPGRPA
DV
>Mb|QLI65544.1
MAPTRRSTKPDATPTSKTEEKAAEANAAVIAEEEEEEEAAAEVVESTEGK
LPFAVQFPLIIALNFALSSLGRMVISQVSNGELELLTRTQDTRGEVALVA
GWRMFELAVGWFGKLDSLEAGAASILAHGPTFYLLAAFYNLTPTTALSAL
AVDVLSVALPFYLVRPLSSTYGSPTRADRDVLDIPMQLLTTILSTGIYTV
TLVLSLRFLMPRILVVYFSGLPSLEPAYSASYTDVLPVTVLFGLAASTFI
FAPFAKTGKAEEDAKIEQFDPVSASLGQTVCWNFWGYTAKTKVVIRRTAA
AVLLSTVGTYLACTKTMYGIESTGAAAYASLWAVAALFAGVGLGLVGGD
>Mb|QLI65546.1
MALHLVPPQPSSEESASNQGDAAEPCFDDNSSDLAQTPVSNHGSVVDAIE
DHESGRDHDGGWLTPQDMAILKVLGSEPCPMNCLGQHHHQCLKRLPSGTK
PVKLVGEGSANAVFEIKVPPRDRAGRDLKGYLLRVAKVPSLGAAPTYNYV
YQQQFLQTTIKPILGDHVVNQELVVLHKTGIIQELNNLLRTIDHTRKDKF
KGTYVGETDLGFLVEDMRPQDPETCTLVEFKPKWLSQSPSAPKNAIRCRQ
CAMELRNLVKDLSRNKARPEQKPCPLALISSDSPWPVRSPFRMAPHLENQ
GSKEFYLEALGAIPTHPAIRDLKAQQDIHDTVGPLYAASSDPFFPLAMTL
RDCTCFAQIHKCTPSVRIRFGDFDWKDPQVKFERWRGVEEELIEKGFYTA
EWILCDGTFYRPPTLCLLEHMPTVSKKDMAIIEIRDSKEDRSTDPQQHAF
PLQAPPGTKIHRCQTNTAVLKRLLEPYKKEAPDFTRPARKRSAS
>Mb|QLI65547.1
MSCPNDPETADILAQAKEEGWKRCYRCKALVELKEGCNHMTCRCGAEFCM
ICGIKWKNCDCPWFNDDDRRADFLNDMNISIPSIRGDLGDIFRGNGPPAP
PELRGHTEPPPMMMPVRRRPRTYQEEMHMRRQQESFDADFARQLQYTGDY
DEPHSYSMMGGLGDIHGIGNASEHYMNENYRRGGHYRPQTQTTAVHDRPE
YGDTRRSRGRLETSQERRLADRLSETRSGFGSPPAAGPIYPVGMSVPPPP
VPAAPVPIPPSLALRHHTVEAEMYNISPYTPRTERVVGRRMSRDYEDEAR
VHSPPRSHWRRGRETRPKSSELAGLNGTGQGMNRVSQWRAFVEPGIPDGE
STVGHA
>Mb|QLI65548.1
MASASVTVIKPNGPLPGAQASDTTNELPRPYKCPLCDKAFHRLEHQTRHI
RTHTGEKPHACQFPGCSKKFSRSDELTRHSRIHNNPNSRRGNKGQHHHHQ
HHHHHHHQGLPPHMHHEGMMAPPPAPKTIRSAPTSTLASPNVSPPHSFAS
FAQHPHPPPMHPYNRGGDISMLAKAATQVERETLTAPPSHSARHYYGHSM
HSSRGHPNGLSSYHMARSHSSEDHDDHYNGMRHAKRSRPNSPNSTAPSSP
TFSHDSLSPTPDHTPIATPAHSPRLRPFSGYELPSIRNLSLHHNTTPALA
PMEPHLDAPQFPPQLNAPRSNGMSLTDIISRPDGAQRKLPVPQVPKVAVQ
DLLSDSGYSNSGRSSTAGSLAGGDLMDRV
>Mb|QLI65551.1

MVLRKHELEGKLGEESEMIKSGELRDENPLDQSDEFNEFLLACRHGDLRK
CQEFISQGVNINGKDRFDYTPLIIASLCGHYELVQLLLDSGALAERNTFQ
GERCIYNALNDRIRNLLLQYDFSKSSDPYVYWSTHISGLLNRSTPTTSDI
SLTSDSRTFNLHKFLLASRTPYFRKKLEAQPETPSWHLANTVPVEAIQIV
LRYLYLGDLPKDLVPAGSPISEEDVVKGLDKISKQLEVDHLWEAILAGND
RRLARQRYQDEEERALKQIGDFFSAKVLGGKMLVDTERVNDVRWKHDNPA
FADVLLRADETHDDDEEAQEAPVLNGQGIPIGTGRRPRKSALYPVHKAML
IRSGYFDRMFSGDFVESHRSENLHIVTVDCTPAVLELVLTFLYTENAACP
LEHALDLLYTADMLFLDVLKTKAAQAISTLGSGTSSALVDRTHKHARGLA
NGAGHSEQQAELVEMEPINIYDVIHAAWDLRVQRLEEFAARYLATRLEDY
IDESDFQELIRESAERIQRREETDTIELLDDIRYYLSERFRLRFEDAGLD
DMMGEDGEIDAEAAVSLAEQGDVMVNGHGVQNGVGGNGVVKTLDGEMAED
EFASDAINYQILLGKIDAMLERLKLDA
>Mb|QLI65552.1
MTSHRTMLLLNPVYAFVVPFLFVVTVPLAIFAGITTTLAFGVLIFRVVIV
YLDVALSLIPQSLASFKLRRRYIRHDTGMPPSFSTWYGSSSGDSSAASST
QQAAPLHLRRRRRPSSSLSMTSTGGSTTPVSELGLGLMPSVGPERDFEGI
GGWRSGDDDEAWTTINSRMELPDRQFTRHHCRSASGGGATTPGDGGVLMM
KSRRRSPESKPVMRTATSPNSSRARTPSASRVQNLTTLGNGNSDSYFPLT
MSPKASKKMSIL
>Mb|QLI65558.1
MGRRTTSASSLTESATTSTASTTSTTIPSVFKSRPSSSLDVAARVHESQL
HAQGVPLQTLAPRSTASSSSFPPQPSEAESLSYTGPRESTSVVRPASRSS
SSSPSIPGHDPASPSLSEAIAEPTPGCLDHPLDEADMASKKTPMLRAMDG
SRSTSSPYGSVTLSHDDSPVSSDSEDDHNNHRLRRFGSQNRSQLSLSSRR
SMTSWRSEDLDGAALMSGLEGRFGIAESAFPSEILDHDKDGQDIGSEDGL
LLEDASSTASTIEPEDNSPHELVRASVPPTDNTTLSINTPRMWCLSVIFS
ILGSSTNLFFSLRYPSVAITPVIALLLVHPLGHLWDMILKRPDDPDYVFV
DGVRTQAVSEGAGQTHQKHATSWRQWLAQGRWNEKEHTCVYVSSNVAFGF
AFATDVIVEQTKFYNQPASIGYQLLLTISTQILGYGFAGMARRFLVRPSG
MIWPGTLMSAAMFSTLHKQDNKPANGWTISRWKFFYIVWSGAFAFYFLPG
LLMPALSYFSVVTWFAPKNVVVANLFGVASGLGLFPLTFDWAQITYVGSP
LLVPFWAAMNVIGGLAIVMWIIAPVFYYANVLYTSYLPILSTGVFDNRGK
IYDVSKILTSEFLFDRDAYKQYSRVFLPVTYMLSYGMQFAGLAALLTHTF
CWHGKDIWRTWKRSIEEARQNGKPTYRPVPESQSHPISSSTGSLGSENYS
RFSASTSNVDNLISREDIHCRLMSRYKDAPLSWYLLTFVSMTAIGMFIVE
FYPVHLPWYGLLLSLAIGAIFFIPNGIIMAVTNQHSSIYLICQLICGVVF
PGRPIANMVFVTYGYISSAQGIKFASDLKLGHYMKIPPRLMFLVQVVATL
VSSVTQIGVLNWMFANVKGICTADALNGFTCPIARVHFNGSILWGVVGPG
EFFGPGATYRALVWCFPLGALLPIPLWLYARNKRGSIIRKVNLPVIFGAM
SWIPPATGLNFSVWAVVCYVFNYLIKRRANAWWGKYTMTLSAALDSGLAF
GIVVVFFGFLYPGWMKDFKWWGTEVYKQGCDWQACSYRTVPEGETFGPDA
W
>Mb|QLI65559.1
MFSSLLRRPKRGSRRLDIHDLSVSPSSGPPRQRQFTGRAHATADFTEADD
DDEDTNEEDLIGFDEEADGVHDEVEEEEDADDADHGADEDNRHRALPVLP
LFSATHLDALPIYSITHSIRIIVSARTETTLSWDQLRSPQVSQFLVKPMQ
QQIRTQHFSRATIYALMANCLQFAKEGQRYAANAGISNTRAKVCELLALK
LLKEYSTRELIDALAYDFYPLQGLPGAQPPLPEGKSYSKSKQAMTASRTS
TLEVAIRASAKHFLAHPVVIQQLEAIWNGAITFYSSADSLHRELPPSPWG
GMNGEIAKPDSRTPLLGSLPAKPGQYHVPSERRTVMLYDPRQASMFKLSR
LRVPRYRSFLSTLSLAVLIGLFLAVLSQRSVKITGLELVFWFWSAGFMLD
ELVGFNEQGFALYIMSFWNIFDLGILVLLIAYYCMRAYGVFLVDPHHWNA
MAYDVLAANAILLLPRIFSILDHYQYFSQLLIAFRLMAVDLAAVFVLVLI
MCSGFFVFFTLSQNASNGGEVAYKIFQILMGFSPAAWDVWPTYNWLARIL
MMIFLIICHFLVVTILITVLTNSFMAIASNATEEHQFVFAINTISMVKND
ALFSYIAPSNIFAWLLMPLRYCMPLKHFVWLNRTIIKVTHFPVLFMIFLY
ERFWLAPSMFEPTDLVENHGRSRTRAISFGDAAQRTAMFSPNVRVREESV

AGFHKDRALEEVFRRVPDATTLRTQRRNERRKTQTAIRNWMEQHDEGGMP
PTNWPTLDSRAVPDWQRRMSVGWDKPSSHLRQVSEVRSTASDPADFMSNP
GASFRNARGRGSRVELILPEYKDHTDADGDDELVTNDEDEDEATNAGRSQ
QFEKHGVEDGEDYFTTSLGARSAKLASSHESFARAATTIRQAHPRRGAHA
RTLSTNTILYNPEDQPRPQPAPPVSPPKAIPSRRPNTSGRTTGTDTAAHT
TRHNSPRRAPQLSSSKPRPILAPRGMTDSGGVSKSAMLSLAPRDRLRQAR
RLSSVDLSIMSDNMGIEDNNAGLAGSFQTQMAMALMKDSRMRAAGGLEAA
DRDRMGRLVLARMKTLEESFADVIKEMRDLKNSSTAPQSQRNSSGDDFKA
GIASSEHERRKKSKGDVTPRKVYGKRPVSRRSMKESKVGLSMKREPRGKG
KGVAASSSDNGDDGFAKKGSSM
>Mb|QLI65561.1
MDITINCHPERNEGVINDTFINHDNVYSSRSLRSQPISHLTHNISTREAT
QRDVTGSSPGRLSGTGQGDLVDSIRERDSIEVCVDEGGVEEQVSMDGSVG
VSKTDGCKILQVADNDAAVDSFGSSMSAVTEQSEASSSFVSSKATTPVEI
EFSKLQRGDKVSQRLVPKTYHRSHERLSLTVRPKSSIPADIPVHQHAAEC
LDAAESSRLKPYALNSEEYHLLRHHISHSQVTTYLNIRNAILRIWLQKPW
AIVTRREAVGCANARWFDAANVCYDWLVRRGYINYGCVKLPDVPTMKSGT
FNDRKRRKIAVIGAGISGLACARQLEGLFKQYAERFYDMGEDIPKVVLLE
GRSRVGGRVYSREFKTKLNDQRPEFEGKRHTAEMGGMIITGFERGNPINV
LVRGQLGLPYHALTAETTIYDSNGKPVDPIRDELVEKLYNDCLDRVSEYK
FKAQPSKVIEGNRDLLDEGRDSPGDGSKTIMQAEEATAALPHAPSVSEQS
VPEKINLVPVSADKLTGRVHNQPGVPASEKVAEKAGMMGWSLKPGANEDF
DLNLEDAVSRRGSTLGSVLDHGVTRYKDLVDLTPQDHRLINWHIANLEYS
NATNLHNLSLELWDIDAGNEWEGNHTMVIGGYQSIARGLLQCPTPLDLST
KFAVKSIKYQSTSFEGPATIESEDGVSVSADNIVCTVPLGVLKQGSINFE
PALPAWKLGAIERLGFGILNKVVLVYDEIFWDPQRHIFGVLRNSPNRYST
SQEDYALNRGRFFQWFNVTHTTGLPCLIALMAGDAGFETERSNNESLVEE
ATEILRGVFGNKVPYPVESVITRWGSDRFARGSYSSAAPGMQPEDYNSMA
RSVGNLVFAGEHTIGTHPATVHGAYLSGLRAASEVLESILGPIEVPTPLI
LPKDSLLLQKRKEAAKDPRQARLEAYELEVWDHIRGQIGERPLRPNKVAG
NAYLLYSKEHSEEARKRCEENRKPGKARSFPNEVRMMTSKMWKETPPEQR
QPYEEQAAEQKKAYNTALQVYNATVHEWDQKAIALRAEYERAHPSVPGPE
ELREEQGTPNKYRRAKQVSYAEGRDADMDV
>Mb|QLI65562.1
MASSKPINASATKSVTGPAKTDLDGHNLPPSPAPSSPRNGRRRYALATEL
VYTEGKDQYGASSIPIYQSATFKQTSAAGGQQDYDYTRSGNPTRTHLERH
LAKIMNANRALAVSSGMGALDVITRLLRPGDEVITGDDLYGGTHRLLTYM
ATNQGIIVHHVDTTKVDAVQARISDKTAMVLLETPTNPLIKIVDLASIAQ
MAHDANSKALVVVDNTMLSPMLCNPLDLGADIVYESGTKYLSGHHDIMAG
VIACNEASIGDKLFFTINSTGCGLSPNDSFLLMRGVKTLAIRMEKQQASA
QAIAEFLESHGFRVRYPGLQSHPQYDLHWSIARGAGAVLSFETGDAAMSE
RIVEAARLWAISVSFGCVNSLISMPCQMSHASIDAKTRRERQMPEDIIRL
CVGIEDVNDLIEDLSRALVQAGAVTVTLDGFHATGAAAELGKTPLTQE
>Mb|QLI65563.1
MAIQYLILLSRQGKVRLAKWFTTLSPRDKAKIVKDVSQLVLARRTRMCNF
LEYKETKIVYRRYASLFFITGCSSDDNELITLEIIHRYVEQMDKYYGNVC
ELDIIFSFTKAYYILDELLLAGELQESSKKNVLRCIGQQDSLEDMEYLPV
QDFEAVTSALNFNTPDCNVTGGCDLYTTKSTGSDKKLYKNIDKDLDSQHA
ALLKLGASLSPPDREHMLATSPSMQLFSTSSAFGPLSDLSSRRTFAYLIA
TLNASHPHYDFSHVLRPNDFKRERNLRRVMANLDSILQNVRPGFEATSFD
SSLGSDLNSVWGTQCWSLIDKEMRLNECTIFSYQPDPDPFEEDESAIWAV
HYFFFNRALKRVAYLYVRVVPVVSSTSPTLRPMRVGQAKRNIDSDAEGAS
KRANYWLGDRHVELVNSLDDEEEQMDDGLFWNRGEDGDLVLYSEDEYLEE
ADEDDDMDDEDEHNTHMRHTERLMSEDIAGRMEI
>Mb|QLI65566.1
MKRILILDGGLGTSLEQKYNTKFNPSTPLWSSDLLVSDPATLLQCQSDFG
DVPVDILLTATYQVSIAGFAGTKTPKFPHGISPLDIPPFLETAVAVAENA
TRAHHGTVALSLGPYGACMIPSQEYSGEYDDAHDSQEALCQWHRERMQLF

GRVRGLASRIGYMSMETIPRADEIAAMRAALDQVPELAGVPFWMSCLYPG
DSPCLPSGEAPETALRAMFDSRVAKSVPWGVGINCTKVWKLTALLKQYES
VMDMLVQDGTLLEWPALVLYPDGTNGEVYNTVTQVWEVPGDGRDVSRVPW
EEQLAEVVRGTEGRGKWRQIVVGGCCMASWQDIARLREILLTA
>Mb|QLI65569.1
MASRLDRLVTILETGSTRLIRETAVNQLADWQKQHPDELFNLLSRVVPYL
RHKDWETRSTAAKAIGKICENAPLYDPNEDDGPSEPKKEENAGENGHIKK
EETDKENSLTGHDHDLLKFESLDVNSILTYGRELLRGGGIEYSLASLDPQ
ARLEHQKKTLLGRLGLLGRRFEDEEMPVVAQSQTLPVTPIDGSNGNGLAR
SDSISQSQGGEDSQLSSRQLNVLKRKRKREAMKASQGKGGFGDLSLRRST
TAGSESLIGDETPMAEGESKKNGKMNDYFNLDRPTDVDEDTKVVSEFKGP
VIPIKSELEVEDVLEGAEWPYERLCDFLKIDLFDPSWETRHGAAMGLREV
IRVHGGGAGRVRGKGRYDNESLNKRWLDDLACRLCCVLMLDRFTDYSSDT
SVAPIRETIGQTLGSALKHIPSKSVHDIYRILYRMVMQEDLELERPVWAV
CHGGMVGLRYVVAVRKDLLLQDGDMIDGIIRAVMKGLGDMDDDVRSVSAA
TLIPMAKEFVTMRPDQLDGLTSIVWESLSNLGDDLSASTGRIMDLLATLC
GFPEVLDAMKASAAQDEERSFTLLVPRLYPFLRHTITSVRLAVLKALLTF
ANLGDDTSHGWLNGRILRLIFQNILVERDQETLNMSLELWNALVGSLAKN
PAILADEFAPHIDSLMELTLHPIGISRNPIPMNATLFQKPSGGTYSAQGM
APPAGRRLSSPDGADRAPKRRRKSTKTEEPISTGMTHDVDGHMMQGDVDL
VGMDVLIRSRVSAAIAMGKIMSLVPSSNLDDFDALLVPGLTSAFSSTQLS
ACLVIDEFAKNCTSPNEPSRYTEQLQRMVELDRPSSYRDLVNFVQRARTQ
CQQLIHLFRDHGKVSHGKLPVLPVVVQGEPEAGPNAFSIATAEKCVGDDF
ERLKKIMPPGQRLIASQQLSEARNVAEAAIEEAKAFKDARDTRIRAGAAC
ALVAMRLLPKKPSPLIKGVMDSVKTEENQLLQNRSSATIARLVQLFAEKG
RKGPADKVVANLVKFSCVEVAETPEFPVHATKTSCILSMQKEEDRVDHPD
AAKWAKEAKAARITRRGAKEALEILSKAYGSTLLETVPSLRKFMEEPLVK
AFTGDLPAEAKDPESVFGQEIVDAMSVIRTMTPTLDVALHTFVMKMLPLV
IKALHSELSVFRYMAAKCLATICSVITVEGMTALVEKVLPSISNPLDLNF
RQGVTEAIYHLIAVMGDAILPYVIFLIVPVLGRMSDSDNEIRLIATTSFA
TLVKLVPLEAGIPDPPGLSEELLKGRDRERTFIAQLLDPKKVEQFKIPVA
IKAELRSYQQEGVNWLHFLNKYHLHGILCDDMGLGKTLQTICIVASDHHQ
RQEEFAKTGAADVRRLPSLIVCPPTLSGHWQQEIRTYAPFLTVTAYVGPP
AERKAIRDKLGETDIVVTSYDVCRNDTDILGKHSWNYVVLDEGHLIKNPK
AKITQAVKRLASNHRLILTGTPIQNNVLELWSLFDFLMPGFLGAEKVFLD
RFAKPIAASRFSKASSKEQEAGALAIEALHKQVLPFLLRRLKEEVLNDLP
PKILQNYYCDLSDLQKKLFEDFTKKQGKKLQSEAGRDDKEAKQHIFQALQ
YMRKLCNSPAMVMKPGTPVYDDTQKILQRQGTSVEDTIHAPKLTALKDLL
VDCGIGSDDGDTNDPLYQPIKPHRALIFCQMKEMLDMVEKKVLKELLPSV
SHLRLDGSVEANKRQDIVNKFNSDPSYDVLLLTTSVGGLGLNLTGADTVI
FVEHDWNPQKDLQAMDRAHRIGQKKVVNVYRLITRGTLEEKILSLQRFKI
DVASTVVNQQNAGLSTMDTDQILDLFNLGDSGPNLISEKAQKEVDGREED
MVDIETGDVLRQPGKKAWLDDLGELWDNKQYEESFDLDDFMKTMS
>Mb|QLI65571.1
MSPNVQETKASGRKRCHGEFAADMMKVDDGGDAKTPQPTAIRRSGDSLLP
LRAQIEPHSSPGGSSPLTEEGSRTPTRDSPSPTTQTDTTLDSNSCDQKNL
DTMVTSSKGAASKRKRLTAEEKVAKEKEQAEKKKEREEQAAKRAAEKARQ
EEEKAARVKEREEKRKQKEEEDRLKAEQRDEKKRKKEEEQRRIQEEKDKK
ARSQPKLNAFFKIPDTPKKSANGSNSAVGSPIMTNGGDAPAKPMETEYER
LFKPFFMRENTRLAPSATQMDEETREAKSRILDKFVASQRDTDNQELPRF
DPVEALRLPARTPRRGRIHHPVKHIMETAYRDADNSNIVGASGATSIFPE
ARQKLASIPQKVIAFSHDVRPPYYGTITLKSYALGRDAMVKLARKPTGRR
LPLDYDYDSEAEWQEEEGEDVDMDDDEEELDDEDDMDGFLDDADDAGLSR
RVFGNTMEPECTGVCYENYHRLGPNRTVYEHKMEFIHEGLEQTWGIDPFS
SQYWEPEVKAQPAKVSQSAPESTTEMPPPPAPTNAFAALHGGAANVSAPT
KLVKAELLNDFKRAILDNKALSKVGIIDFIFHQFRNSVSRAEVKNTLEHV
AEKKGAGRSKEWDLKPGHEIVL
>Mb|QLI65576.1

MSLSRVSSDAELPSSSLAQRQVPQDLRSSEMNAWGISPPGFTATDELVTA
FQEGGQRVIDICGENMERWALYRSFLDALTYLDQVKVQFHDQPDVYNRFL
DIMKDFKSQAIDTPGVINRVSELFAGHPNLIQGFNTFLPPGYRIECGAGN
DPNTIRVTTPMGTTVQSIAGRGQGEHGGPGASQPLFPERGTQWQQQQQQR
SQHSIESPEANFSTPLQNGASLFVQAAAQSGAFDAPGSAQRNASAQGQSG
AGTVPGARQTPGPASGPGGAGTGPGNQANMERRGPVEFNHAISYVNKIKN
RFQDKPEIYKQFLEILQTYQREQKPIQDVYAQVTTLFNAAPDLLEDFKQF
LPESAGQAKATPGRADESAPTGSSQTPQPGGQKMPPLGSFAPPPSASKDN
KKRPRAEKQAAAAAETAPPETNATNRLQQPVVNGTGKRAKLSHARATGDA
STIEPTLTPVMPEPYPPRPSATSNQEEIAFFERVKKFLSNRSSMNEFLKL
CNLFSQGIIDRNTLFHKGALFIGANPDLMTFWKTFVGVNSQDVVIENRPA
APTEKLSLSNCRGYGPSYRLLPKRERLKPCSGRDELCNSVLNDQWASHPT
WASEDSGFVAHRKNQFEEGLHRIEEERHDYDFNIEANLKCIQLLEPIAQQ
MLAMSPAEREAFHMPAALAGQSTSIFKRITKKIYGERGIDVVNDMYSHPF
DVVPVLLARMRQKDEEWRFSQREWEKVWHAQTENMHLKSLDHMGILVKST
DKRNLTAKHLVDVIKTKHEEQRRERALRGKAPRHQLAWDFSNKQVILELL
RLMMLYSMHNGQHSTQEKERILDFFETFVPAFFDLPEEMYQDKLPKMQPD
SGEDDVEDSTPAELTNGRSRRNGRKGDLLRGVLDPGRNGSKPRSQKEDSA
ASGSKETTPDVTSANEEEMADAPEESAVPEVSNERWMPTVPKPVVVGGDN
ALLDEGGELKADGFFTRPWYNFFCNQTIFVFFNIFQTLYNRLLDIQQSQE
LVAQAVERLNRPKPARDIGFTENHMTFFEPTDDPETYWPKTVELIEEYIT
GDVDENRYQDVLRHYYLQNGWKLYTIQDLLKTLCRLALTCSSTDAKEKTP
DLIHQYLTSREKEETSYQTEISARKFAEKCVKDGDIFVICWFPQKAEATV
RWLQRDETTFYMDEMQVRERWQYYISSFIRVEPTEGIPRSRLQKVVLTRN
LPSGDADSDSDEVPKPLSYSENLTTSICLKSSKMVWGPGTSEYFIYDRSP
RTQEERERREKFTKALSMHRESKLLEKMVHNPSWTKDMGPEEVQEQNKNF
RKWMDDGVAPPNGDVEMD
>Mb|QLI65579.1
MASRSDFLPRSTRRLLRQGRHVTASPCPICARSSLAARPRSSPRLQNRSQ
AGWPRLLSTSTNPRTELQNTLLELQKQSPNLINISKLQLALQGLRQDAGN
EIIRIAILGLANGTEADATAARVLHALLADPLQEVQPWEKELENRDGSTP
LIVRVGPPRNHAVSLEIEKEVGLDEMHISCPELDGYNFEFLLMGVDVPYG
APGETTIQTLEDAVLVPTVDIPSANDRVSPISTPVHQALLVADGLIGAVN
VSALPVSETDESISAVVQMEGVTKEQLQASFDVVDVSLAEKGLGLFRQGP
QNAMDYERLWFSSNVPTLLKWLKDGAKSGAADETKPAVRRLIASTLQNTS
SQIQLVEARKLSKALTLHSDDPAVAALNKRLADWAQNAHAELQDELDLAF
KGRRWRKLGWWKLFWRVDDVAMLTNEMLGQRFMPTAEQELVYLTGRIAQL
GGSSPEYPQPKSSLDATESRKLGSGEHAPPLAATSSYSLPKWPGHIAFTR
RYLQNETVPALQSLAQKLVIQSLGTSGITTSLAALLYMSSFASTIYEAGA
VAALGIVYSLGRLQKRWDAARTFWEGEVREEGRKAVRGAEESVATVLESN
GSNGTNGVEEMKELQNTKELIAKAEDALARMK
>Mb|QLI65584.1
MVTTQQLVLSSLAGLAAWQSGSSSTVAASTTAGNGTITNDAYFYGQSPPI
YPSPEMTGGSQWAAAYQKAKALVSQMTLEEKVNVTGGVDLRTGCSGSIYP
NKRLNFPGMCVSDAGNGLRNTDFVNAYPAGIHVGTSWNKDLARRRGAAMG
GEFRRKGVNVLLGPMVGPAWRVVRGGRNWEGFSADPYLSGSLVAQTIQGV
QSRGVQTSLKHYIANEQELNRNPSGEVEAVSSNVDDKTMHEVYLWPFQDG
VRAGTGNIMCSYQRINNSYGCANSKTLNGLLKTELGFQGFVMSDWGAQHA
GVATALAGMDMVMPSGDGFWGDHLVKAVKNGSVPESRVTDMATRILTTWY
QFGQDAIFDKPGIGMPLDVRQPHETIEGRDHNDRPVLLDGAIEGHVLVKN
TKNTLPLKSPRMLSLFGYSARSADSLSPGPGDLLINLWRFGLTSVSIDDV
LSSQLGGQQAKYPNIAVNGTIMGGGGSGAGTPAVFVAPYDAISMRAAQDD
TAIFHDFGMPEPVVVPSTDTCIVFGNAWASEGYDRPSLHDNFTDSLVKTV
ADQCNKTVVVLHNAGPRIVESFVDHPNVTAIIFAHLPGRDSGTSLVRLLY
GEAGFSGKLSYTVARKESDYSHLLSPDEPAGKFQKFPQSNFTEGVYLDYK
YFEMHNITPRYEFGFGLSYTTFSLANLVIQRVNGGNSGEWAEGAIIPGGQ
ADLFDNIATVTVDVSNTGNMPGAEVPQLYVGIPGGPAKQLRGFEKRFLAP
GTTVPVEFPLTRRDLSVWDTTAQKWRLQRGQYNIYVGTSSRDVPLKSSLT

IN
>Mb|QLI65588.1
MSDKAPSTLQSYVDSATGAVQSALGSLTGNTGDQAKGDAKKDDAKVEHEA
SQASVKVPGATVSSDGGVSKDHPDRAAGNWNQTVGSAKEAVGGLVGSENL
KSAGREQNLEGQQQEAKGQLNDLGSGMAARAQGTVGGAISNITGDKEGQA
HYDQMRADGKAQQRGVEHDLEKRAEAEQKKE
>Mb|QLI65589.1
MGQGFSLATPSAGSAGIDIAQLQDVQYERSIGNARFMKSIRGRHEHGVVL
VKVLVKPYAGVNLDQYKQKIIKERKALAHVPNALGFQRIIETDTNGYLVR
QFQYSSLYDRLSTRPFLEDIEKKWLAFQLLCALRDCHAHDVYHGDIKAQN
VLVTSWNWLYLTDFSSAYKPVLLPDDNPGDFSYFFDTSGRRTCYIAPERF
YATGDSPPPKAKMTWAMDIFSAGCVIAQMFLESEIFSLAQLYKYRRGEYD
PVITHLSVISDKDVRDMIAHMIQIDPEKRYSAEQYLEFWKGKVFPSYFYN
FLHQYMELITDPSSGNNPMSGSQRNMGESDDRIDRVFYDFDKISYFLGYQ
IEKRTPKATLYRSRLSLSHFPVRLSIPNHEHTVSADLEPPEDDGTLIFLT
LIVSSMRTTARASSRVRACDILLAFSERLTDEAKLDRVLPYLMTLLKKEE
TDSVIVAAIRTITQLLQLVRSLASTAQRFLEIASSLRADGSMPITDPEVE
PGAEAEANFESVFDNAGRELFEILESHTKQLVEDPDVHVRRAFLASVAEL
CMFFQEHSNDILLTHLNTYLNDHDWTLKCSFFDTIVGIAAFIGSTSLEEF
MLPLMVQALADTEEYVVQAALHSLAQLAGLGLLSRPKTWELVDLVGRFTM
HPNIWIREAAAEFLSQSAKFLNVADVRCILLPLVVPYMKIDTMADLGELS
ILDSLKRPLTRAVFEQAITWATQSERGVFWKPLQKIQSRAFGSSSARPFS
DLSANSMTKIVRNDEDEQWLSRLRNLGLKPDDEPKLLALREFIWRLSRSK
LRDSAGEPSLHTGLVSLKELGITPQTVFFNDTPLRDSSITPDLDTPAGPY
TIADALLDASMTIDDTLSKRRKAVLHTRVNRVQSVGPRPSSKRRFLSPEG
STRTSSQHSRRESSDGRPGGSRDDDVFIRDGPYASRRAMRHQSSALSLLD
RRDGNKSAAETSTSDANAFGEVEGPFAQGSASTSPTPTRQHEGGKLVLSQ
ARHTYEGTDPNIQRMLDQMYIDNFPRDVLEFGPMVQPISRTKARAVGIQT
GDDYWKPEGHLVATFGEHKGPINRIVASPDHVFFITGGNDGCVRVWDAAR
LERNITHRSRQTHKHADGAHVLAMCFVENSHCFVSCASDGSMHVVKVDTI
SASGVIRYGKLRVVREYQLPEGEFAVWCEHFRQESNSVLVLATNRSRILG
IDLRTMSVLYVLENPVHHGAPTCFCVDRKRNWLCLGTSHGVVDLWDLRFK
MRLRGWGVPGKGSIYRICIHPTKGRGKWVCISGGTSQGEITVWDLEKTLC
REIYRTSGTKDGPKGYSAWDVDEDKPEGMLDRFATNLENSEAASADRGVR
AMIAGTGAAEDSRDIRHAFLLTGGSDKKLRFWDISRIENSCIYSGHPSDE
QQPTFIATHPATAVTLNIEKAHRHGASGNTTAAGERPRGKSSSKRPARST
VISMQQQQLLQSHLDAILDVMLLEYPYTMSVSVDRSGVVFVFQ
>Mb|QLI65591.1
MVTDMKNGAFEHDNMDIPKGDRVLPMFSLKDRTAIVSGAGAGIGLGVAQA
FAEAGANVAIWYNSNKQAVTEAENIQKMYSVKCKAYQVNVISADAVDKAI
GEIIREFNGRLDIFVANSGIAWEDGPFLDGHVETAKKVMAVNVDGVMWCA
KSAGIHFRRQKKEGKTIDGKPLENYICGSFIATASMSGKIVNVPKLQAVY
NGSKAAVIHFCKSLAVEWTGFARVNTVSPGYIKTEVSSFAEAETEEQRKN
KIPMGREGQVSELKGSYLYLASDAASYITGLDMIVDGGYSLL
>Mb|QLI65593.1
MEHDSGMKLRHNLEAQEALISKICRIAGAPGLSISVLHRGTEVYSHHVGH
ASVKDGKLPDGDTIYFIGSMTKAMVSALVGILVQDGTLDWKAPVSHILPE
LAHAFDGRGSEITLVDLMSHRIGMAQADALWLQCAGNILLSKSEALDAWI
SQPTVRAFRADFLYSNFAFETVGRIIEESTGKILGANLQEKIFGPLGMTR
TSLNDEFSKDPNAAKAYFPLKDGTPVEVPVPTISDKTIMAASGGVRSCTN
DLARFYKNLMHAANDQFNHQRTSTPGSPFKQLSTIWSPHNQLPVHSMRET
SYALAWARTQLPSPLGSFNYNKHLVPAMPVIGRGLPSRLVVYHGGSMQGF
TSAVYLLPETETAVFALQNSSALCDACDWVPQMLVQTILSGLPQCDIDFV
QLATVSAARGVKLADEVEEELQRSRERGTNPRPLKEYVGRFWHESKSFRI
DVTVESDETLSMCFQGMPSETYCLRHLHHDVFVWNEPHDVPARRGRFQTR
PVESYRIRFGSTSQDGAIDRLYWLYDETHAEPGMFRTEGGLLAADVDMPN
>Mb|QLI65594.1
MDHSQLENALLQVPGQQLQTRVDALKRTVTYTGTTSAPATLTNTSTISTR

APSPAVSDIGPDYENPLEASSRWYLQAKAQTVRYAASLGFSIHNRSDPPA
PYHSREIWLDSTLSQWKGRKKIKVEVWNPPRISVGPRAAVINLHGGGWIL
GQGTDDARWAGAVVCDVDAVVFTVNYRLAPSYPFPTPMEDCVDAVLQIAS
RASEFGIDPDRIILSGFSAGATNALTSWIVLQDPSPWNYQFPSPPPSILG
LVLFYPTLDVTITRPGKRQTCNRPERTLSPGMTDLIDASYFFPPIPREKW
TDPRMSPGLMSDDLVKKLPHFHMILCEYDMLLAEGIRFAERLDQHDMPFT
LRIVEGEGHAWDKPPPMAPKESVFVEYAKATESIARWLGRDYETDKESTS
SMRTRRPRFRRPRHLSIRSRSVR
>Mb|QLI65596.1
MELAAGGFDNDTRGWIMSVVSGIACIFGASIVCVDFIVRQFPGKRSFRIQ
ESNIFLACSLSLSFGVMMFSALYSMLPESRDYLRKDAWADQPAGFVVMAC
FIGGFIGIQAVSRLLHRHMPSHVVDCDHTHDNATPQDSFSCNHSRRQSRV
SRARRRLSRPRSSHSHAKLDAGHDAENGVALAGETTPLLPSSIETDSNGH
RNPLKRHASTRADGMSQRPTSPLLASRGHATTTDLEAAARRPSMYEVQKR
VMSFVRDTKCNCEEEGSCYGYTDPCGQECFKHLSTRSQSGPKHAASLRTT
TGPFYLHSGSIFHAGHGHDDPDASISPLYRISRATSREPLFYPTDEETPD
QEEHGSYCSSTEEGDLEISQHHHHVPTNAFLSIGLQTSIAIALHKFPEGF
ITYATNHANPTLGFNVFMALFVHNISEGFAMCLPLYMALGSRWRAIAWSA
VLGGLSQPMGAGAAALWFKLAQRSNITPNALVYACLFAITSGIMVSVALQ
LFVEALSLNHNRNLCIFFGFLGMAILGLSNALFAAH
>Mb|QLI65597.1
MDDYMFRFYVDECRTVEEATSYLEILNYSDPFYNSCEEHALTEDEFDNFL
YRRGAFAPPDKMRDGTRLLSGIRLIVQKNAKDKDTFTPKVISLPKDSYED
MVRALKLPFRGIETTSVVGPFFWSSFDQDDDDPHLQIIHRKSDVRKKGRT
RGWEMMLSHSFKTNMTTGYLKGTPSSDVVFALQHLRACSAQIGHPMLLPI
IILSYDLSTANDQKQRDARDWLRRLENAVSLRNEVEEHEQYFQDGLLEVD
GLNRDLVECHGHVMWKRPQAYWAVAEEMEKAMGRYHDKMATVREEKLRLV
NGETASAEDAERRHFKEVDKLHRSMTARLEFYKVKLKGLENYIHTTLERL
KVQREALYNIMSQREARLNLEIAGEQRRIAHASKRDSTAMKTISLMGTLF
LPGTYLASVFSMTFFNFQESAHPVSLDLWIYFAVTIPITAAIVAIWVWFD
RRREAQYARDDEDLEQNIDKMEKEIMFHLRKRTMSKTHTWNSLSSPPRP
>Mb|QLI65599.1
MSTFADDKAPICIPFITQLLQTHRTKSPDRPLIIGLNGMQGVGKTTLVAP
LAAALNARGIHTLVFSIDDFYLPHDEQVRLAASHPENALVQHRGEPGTHD
VPLAKAVFASLLNNLPTSIPQYDKALFSGQGDRLPPSQWRPANHPGQPPV
QVVIFEGWCVGFRSVPAAQVEAKWKGPSRTLHKHKLEHLQFVNEELSNYD
ALTDSFDAFIHIDSEDAEYVYAWRQEQEDSLRATRGDPTAGMTPEQVVRF
VDGYYPAYELYTDGMRRGIFKDRPECQLRMIVGRDRKVKEVVRI
>Mb|QLI65601.1
MSPIPDSLSGEDGLHLAQRRQHHRRGVKPQYRSLNRLVRAGAKHLVESGQ
TDIFPDCHQDHSQLDKASLRSHIQQDIRQYYFNIGKSAYRGDQIPNYNGP
TPRKLDGQDDGRDLAYSDIELPSDRSRLVRRPTLEREDAFWYASTSKIHI
RRRVETANEDQQVADLYRMGLLYDNDEQDRSGGDSFNLNSIRHDEPLYPI
RPARRTRKHKAAKDGFADQALHLNLSFSDLGDDDVLAQYLMAWTRPHADE
AIQHAPQELAESQPPLRVIYELATSQPSFDLDTSQPPELVLDALSDYDCF
SDGELDDTPSQREVQEHADNPPADSWVMLGDDS
>Mb|QLI65604.1
MSEKLTFPAPAAAALECHLPRPDTQTSNRPFVTLTFATSLDSSLSLAPGV
RTHLSGPGSKAMTHYLRSRHAAILVGVSTVLADNPRLSCRIEGSASQPRP
IIIDPHMRWTPRSSDQVFQICRSGAGLAPFILTGLLEAQIPRESVELLQQ
HGGKYIRIAPATSSKDRVRFEWNDVFQTLLSEGLTSVMVEGGGQIINSLL
SPTNHNLIDSVIVTIAPTWLGKGGVVVSPDRVCDAEGTPQAAARLSNVSW
HPFGEDVVLCGTLAPQ
>Mb|QLI65607.1
MADIKIDGKLFQERISHFATAWKNDLRAKDGLFGGAASILIMMGKMEEIP
EFHKNNAMHFWLLGYEFPTTLMLLTVDTLYILTTAKKAKHLDQLKGGRFP
IEVLVRGKNAEENEKLFVTIAEKIKAAGDKVGTIAKDTSKGPFVDEWKKV
FSEQCKDVEEVDISAALSTHAFSVKDENELRAMRTASKACVALMTPYFLD

EMSNILDSEKKVTHASLAEKVDRKLDDDKFWKTVELPNKGKLPSDFDSAQ
LDWILGPAIQSGGKYDLRFAAEANNDNLHAGIIIAGLGLRYKSYCSTIAR
TYLVDPNKAQESNYKLLHMVHNSIIKDVRDGMSAKDVYNKALSLLKIKKP
EMEKHFLKNVGWGVGLENRDPTLVLNAKNNRTLKDGMTLIIHTGFQDIDN
PQPQDKHSKVYSLVLTDTIRVTTGEPVVFTAESPTSADANSFFFKDDEEV
EPAPRKEKKDHKVGAVATKNITSTRLRSERTAQVDEDAENKRKEHQKELA
AKKQKEGLARFAEATGDKNGAEVKKFKRFESYKRDNQFPLKIKNLEVVVD
SRNSTVILPIMGRPVPFHINTIKNASKSDENDFSFLRINFLSPGQGVGRK
DDQPFEDASAHFVRSLTFRSSDGERYSEIATQISNMKRDAVKKEQEKKDM
EDVIEQDKLIEIRNRRPAVLDNVYIRPAMEGKRVPGKVEIHQNGIRYQSP
LNAQHRVDVLFSNVRHLFFQPCAHELIVIIHIHLKDPIIVGNKKKTKDVQ
FYREATDIQFDETGNRKRKYRYGDEDEFEAEQEERRRRADLDRLFQGFAQ
KIAEAGRNENIEVDMPIRELGFNGVPYRSNVFIQPTTDCLMQVVEPPFMV
VTIEDVEIAHLERVQFGLKNFDMVFVFKDFTRAPFHINTIPVEFLDQVKE
FLDSSDIAYSEGPLNLNWPTIMKTVTADTHQFFADGGWGFLQENSDDEQE
EESEEESAFEIDDDEIDEVSESSEEGSDFGSNASDDDDDDAELDSEEEGE
DWDELEKKAKKRDREGGLDDDDRSGKKQKRKR
>Mb|QLI65610.1
MAVVGSGPAGFYTAYRVMTKLPDAHVDMYEALPVPFGLVRHGVAPDHPEV
KNCQEKFEDVASSPRFRFIGNVSVGLCSRAKEQGMVRLRSLMQHYDAVLL
AYGASEDKRLDIPGESTLNGIYSARQFVGWYNGLPECSSLDPALVNAQEA
VIIGQGNVALDVARILLEDINVLRNTDITEHALAILSRSRIKRVHVVGRR
GPVQAAFTIKEVRELMKLASVAFHPVDRSLIPVDLKSLPRASKRLMEVIL
KGSEALVESAPKSWSLDNCLAPSRFLGGIQSPEDVAGTEFNVMKLTDAFN
PLSKTMATGQTLQLPSDVVFRSVGYKSVALPEFAAMGIQFDESRGVVNND
GLGRATQVLAIDQQTSTCQQVPGVYCAGWVKRGPTGVIASTMQDAFITAE
AIVQDWISGAEFLRPKGDTPAQGWNGVRQDEGSHTERSVSWDQWLQIDRA
EKQGGKELGKEREKFTNTRDMLAVIQ
>Mb|QLI65611.1
MLSASSPITFPKSSPAPKRPTPHEEPTKSKPRVDGPFILDGDDSEDESQE
PQNGSHIAKRRMLQHPPEHDDTAPIMQHEENHKGDDTQTSVETSDIRAST
EPPNWSAKPSLFSGLILETISPSTYSAKTCCGKSATIKQRKTSAAVSYET
MVAARSKTKEGRAKRSYYGIEIHDLINKAAKEIRNKPLTVSTKPDTIIPS
LEAPISGEKPKKTLLWTEKYRARNFMDLCGDDGTNRQVLRWLKRWDPVVF
PGQAKAKPVMTRRPGAKHQLEEEKPHRKILMLTGPPGLGKTTLAHVCAKQ
AGYEVLEVNASDDRSRDVVRDRIRTSLGTESVKNVTNNKASDGSQKIAKP
VCVIVDEVDGVTSGAGASGEGGFIKALIDLVLTDQRNSSAPTSEYSHKKK
KGDDFRQMRPLVLICNDVYHPSLRPLRQSNLAEIVHVGRPSLDAVVGRVK
SIFEKEGIPCDKDAARKLCEAAWGMTSGIDAKRGAESTVEGDLRGVMVVG
EWVAGRFRSCLGHANQRLTRPWIENNILRDLSSSAAGARGLGRGNVRDII
QRVFQEGGGFPKQAMDLSEPKTLHEQPKAELGFGEQQKKYAMGRLGQMVD
TSGEISTILTEIFAEYPNREFNDDIYLAKPNQAYDWLHFHDSCQSRLYAS
QEWELAPYLSQPILACHHLFASPKKHYTMASGFEKRWGGVEKEEDNSPPV
PFSGPRADFQAREAEKQIRSQLQALQSQLSPTLMRSFRSAEDVATEFLPY
LIRLVSPDVKPVVVGGSQGTMASVRKESEKVMVQRASQVLAEVGIALHKG
KVESEMTGGRGPQYVYRMEPDLDSLSTFETAVALLHSQAPTRYAVRQVLD
QELRRTIALRDNSARQARFQAGAASQVQTPLPVHDDKENKALPEDKPDVV
VMRDFFGRIIESKPLAELDGNCDRRRRHEERNVWVTYHEGLNNAVRKPIS
LKEFLGVL
>Mb|QLI65612.1
MASRPRRSAATKANESISVHAKWADASERSERSTMTSRRSGRTSGAGASR
DLPSSPDESVTVKTTSRSTRGANRRSDRFDGGEIVHGTRNRGGRKNYVID
SSPDEEEEEDAEGDDVEDADEDAEGDMEVDAEGDDIDAEGEEDAEGDIDM
DAPVVSRTIRVSQPAPRSAAKVAASKMAIGEDEEDEDEDDDDELSDPVDS
DEGEQTMGFGDETMADEDAEGEEIEVAGEDEEEEEEEEEDEAEEGDEDAE
GEEVGNAEAESDGGTREGTPDLTKMTKRQRARFEEEPQEYMKLSDEVQVK
KHFTAEELSMRRQEMARRRRNLSEKRNEEVKMETINKLLKKQAPKINRKA
AAAAARADSPTEELYRPDPTLIRWVNSKNGSRVSVPADIMTGPAGQVFSK

GGGLASGKMVEEVA
>Mb|QLI65613.1
MSSPNNTSTSSRENAAAGADEKPRLTEEEKKQNHIASEQKRRQAIREGFD
RLTELVPGLEGQGRSEGLVLKRTVEYMRQQMQERRALIERIEQSGGHVDD
ELKKVLNEQTHGRSKG
>Mb|QLI65614.1
MSQNVTSASLAPSDPEQHDALRALNRSPHPYHHQSSELPHSSERFTTHDV
SRGSGNDGAHLSPTRLPAFSKESSPGSESGTEADDEHFLKGLPAPKTKLH
KGLRDYDEPTSGFTTPAPSPAALDDDHIQAISQRLRPKVGQRKKRWLDVL
RRNRALVRRLTEVGLVGSLGFMVVTSSHVSPLFRVWRKDFELLGLLYLTL
LGLYPLRVLAWGFGTRRPSKWIPLRIPTNFDPAPLLYPPAITTFVCLLVA
RSNPGTILPSIILSISSLPQALIPKLDVDTSYDTLHWALSCLPLVWGPAL
DSSRFDPTYAFLSAEALVLLYPVHQTLCTVLHYLTTTSLLTAELQLLSIA
LISVLILSASPQAKILKGLLWGGGLAVLVFCGPVIRWGIALARVPKWRFR
RPSSSQKHRFWKELRKLLSWHRIKSDMVGSVYEDSPSETTSSADEEDDIP
IFRGPLRVKTLGPNLDIRDTTESVGTSGVTSPNDISGGISRRHTMPHPDK
LRKSAVHTPSGRRKRAISLSLRPYIRLTQEQALVRKWAYAAYVYTAIIGI
ILVGVRTYVEQFALNGHEPIGWALGYMFGNLPWFRFQVVSANLERWVCLP
AISGAKECHSGWVQHLRHDAFGEANTRLILSAYWLTILVFGLVVVFKLKE
TYEVDTRRKVFHFMMVGMFLPAVYVDPAYAGLALSIILAIFLILDLLRAS
QLPPLSKPIASFLAPYVDGRDFRGPVVISHIFLLIGCAIPLWLALASLSR
TGSGCLSGWEVPTRDVSMVSGVICVGLGDAAASLIGRRYGHRKWLWGGGK
SLEGSLAFAVAVFAGLGAAGVWLRVGGWPMTDQPVGWYASARNAGICATM
GSLTEAVLTGGNDNVIVPVVLWTCVKSLGI
>Mb|QLI65615.1
MAADDSKDDAAALSALELEAKEFDKDAEIDRILRAFRLDAYAVLDLQPGV
AESDIKSTYRKKSMLIHPDKTKNPQAPDAFDRLKKAQTELMDEKHRERLD
EAIADARMLLIRENKWTVDSPELKTDDFAKKWREKSKEVLIDNEHRRRRQ
MRAQMQEEGREQRKQDAELEERKRKRQHEQDWESTRDERISSWRTFQKGK
TGGDGEKKKKKKLKPIG
>Mb|QLI65616.1
MGKNDKKGAEKSAGKGKGADKESGGKGKGAQAINVRHILCEKHAKKEEAL
AKLNDGVKFDEVARNYSEDKARQGGALGWKTKGSLDPKFEEAAFALEPST
TNNPKIAEAKTEFGYHIIMVEGRK
>Mb|QLI65617.1
MSRTNISSGSAFEAEIGYSRAVVVDDWVMVSGTTGYNYQTGEISDNVAEQ
AEQTLCNVDKALMEAGSCMADVVRVQYILPDRDDFPKTWPVLRKWFGDVK
PAAMMIQSALMKEEMKIEIEVTAKKGCGLEK
>Mb|QLI65618.1
MRRGRSPSSGSPYRALAHHQQANSPSTSTNPSSVIRSFDVDSNPARPVRP
SPLTASTIRDMPLDLVERIRSFPLFVSAPGEFLVAIGNHLKPQINGPNDH
IITEGDDAKAMYWLVRGVVAVTSRDGEAVYAELKPGAFFGEIGVLMDMPR
TATIVARTKCLLLVLKKEDLQLIMPKFPDMEKAIREEGQERLNLLKKQRQ
ERGASLKTPKADFAPRTTVPGEVSTGESGAIKDGTVINSKKRKSPSPGII
EDPAAGSAIASGYVNIRKTLKELPLFSTLPPDILHFLGLSVQPKTYHPFT
DIICEGTPGNEIYFIVQGEAEVFHEQRKEQDASEEATISIQHRPRLKAGQ
YFGEVASLGLSPGRTATVRSITTVECLMVPGEVLEELWRRCPPGIKYQVE
KTAKLRLKTQDEDVDMTGHDGKIKALTTSSPSTPSTPTRGILPNLTFSTP
SSQGSPSKDDSEIIELKDPDPFLSVNMENLRNRRRHSLAPPTPQAESLAA
GRQNGYRPSIIDLTPIKFSSSETSSESGVPAKRARTATSRSLSPPAIPPL
SDEILVRIFKHLDIRELIRLRIVSSHWRQLLATSPEVCNDVDLSHYNRHV
TDHSIIHILAPFIGNRALSMDLNNCFHVTDEGFTILWRQCGKNIKRWRMR
SVWDVSAHQILEMSENAKLLEEVDWSNCRKVGDNLLGRVVGWVVPESPTV
KKNVVVSTPTQKRQQQEAKAKPPTANNPPPGTVIGCPKLNRLDLSYCKHI
TDRSMAHLAAHASDRLRSLTLTRCTSITDVGFQSWAQYRFEKLTSLCLAD
CTYLSDNAIVALVNAAKNLTHLDLSFCCALSDTSTEVVALGLPLLRELRM
AFCGSAVSDASLESIALHLNELEGLSVRGCVRVTGKGLENILRGCTRLKW
TDVSQCRNLEHWLRAGSIAKWGFDDRTTKPVVPTGLWAAAAHEGIGEIPP

PKTMSVAMLTTPSFLPRSGLGFLGRRARQPVRFIIEKGVQGLR
>Mb|QLI65620.1
MPPPTSRREILARLQSQIQQGKPIVGAGAGIGLSAKFIEAGGGDLIIIYN
SGRFRMAGRGSLAGLMPYGNANDVVLDMANEVLPIVKSTPVIAGVCGTDP
LRNMAFFLKQLHDMGFSGVQNFPTVGLIDGQFRANLEETGMSYDLEVDMI
AMAHKMGLLTTPYVFNPRESEKMTRAGADVLVAHMGLTTSGSIGATSGKT
MDECVTLIQEIRDAAVAINPDVIVLCHGGPIASPDDAQYVLSRTTGIHGF
YGASSMERLPVETAIADITRDFKKIRT
>Mb|QLI65621.1
MATNTAPSSHGQQVTGLCSPRLIVARVDDNEDTMDRSRPSLPSSFSEPPT
LHGYSSPMRQHKRTPSAHREIKETLDARVEFTSDEADGRTYHRINQYVIV
EEIGRGSYGAVHRATDQFGTEFAVKEFSKSRLRKRAQSHILRLGPQSSPG
QYLPRPRRACGPLSPQLTGLRAGEENDALFLIREEIAIMKKLNHPNLVQL
IEVLDDPEEDSLYMVMEMCKKGVIMTVGLDENADPYNDESCRHWFRDLLL
GIEYLHAQGVVHRDIKPDNLLLSDDEVLKIVDFGVSEMFDKPENMRISKS
AGSPAFMPPELCGKHQEVSGKAADIWSMGVTLYCLKYGKIPFNRPTVLEI
YEAIKSDDTCIPDGEDPAFVDLMTVILEKDPEKRITMSQLREHPWVTKGG
CDPLLSAEDNCEHIIEPPNELELNRAFTRKMNHLLSMMKAIHKFRTLLAR
IRARNNTGVVGSSTSLQRLMSEDESFNAADERAKAEEIEALLARRRHLLS
QQAEDADKGHAREIGEQEPLLLGIGTGARDAFAWDEATPDVVADSPTAVD
FDVYDRAYEEAIKRGMEARPSKKPTMYLTKLVKETDYFKKIENLVGGTVP
SPPSPKTEVKKATHVLRDRLPSEPRAKLAEIIDKVALSTPETEKDMEQPG
LERQT
>Mb|QLI65623.1
MPPKHRDSSSQRLSSDHDSRARQRHDGSASPGLRTSPSDDGGKSSRGPSP
VLTAAESAAKVKSTGGNLPGHGGQVCSNCGTTRTPLWRRSPQGATICNAC
GLYQKARNTARPTSLKKPPNVVAAGSSRSTPPKPTPSGPKSASNVNSGNY
VSAEQMPTGTCPGGGRCNGTGGAEGCNGCPAFNNRVSKTAQLNMMQRSKG
CGSRTDLSNSEPVPIDVNALQTQNQDASMVIACQNCGTTITPLWRRDESG
HTICNACGLYYKLHGVHRPVTMKKPTIKRRKRVIPAMQDEEMEDMAESPE
APKMEVTPERGTENEDGSINLGTRRRPDAHPLTIEPQPAVMRSSGQVSPL
PSTSDLAAYHQRSGYSQSVSSHANEDNRLPPMSSMAAVSERQSSMSPASF
LSPRRKRSFSTTDTEVGSNADLVPDSAKRISSIKSILNPTGSGGMNRPGS
ADMDDYTLPPLRSASGLLNPHVHDVSPSAGSRNDGQGHADKIENERLKAE
RRLALQREADQMREMLAAKERELMEL
>Mb|QLI65624.1
MPSEVDTKEAREVNRQSLVIDEEKSASVAPAPANEQREVVTPALQEGDAP
ATSNWEIRSWYLYYIGANGLALFNFGPTAFQNLLDQAAGDSELLYFAGRA
RDVNSIVLLANGMSFAIQAALFLVIGAYADFGTGRRWVLLVWSVIAYGIG
FGWLGVHDAARWKVAAGLYIIGLVAYQLTLTYWTAAFPSLARNTAHLRAS
RAAYEAGEITQRELDGRDEMERSRLSNVAFWIQSCGEIAILAVIVGIMFG
LRVDDNPSNNNWGLSVLVAFATACWLALSIPWFVLEKKRPGMRIPPGRNI
VTVGLWQLYEALGQIWRLKQSLVYLAGYFLLGDSLNTTVTVIATLQNQVV
SYNTLTLTYLLIVGIGAQALGIGGFWLIQKRFNLSAKTMFNAVMVFIVLL
DGWGMVGNWTDRFGFKNVWEVWLYQAYYGFVVCPWYSYSQIMISSVTPRG
HEFLFFSVFNIIGKASSFIGPLISSAIIDATPGGQNNSAPFYFLFALSLL
SALGIWAFLDLEKSAREQEAFLVEEKERVYGEDTAAAKEKLAA
>Mb|QLI65635.1
MSSADHVLQQDRELWISNGNCLVYLHGSEATKDKDATKAPVFKLPFSFLL
MTRCYPLIQRFLVINNPSERGVRTPRTPGEINKWHRSNPKRTVELFIPAP
LNADKKKVERHQLLMRNFFAWVLRRPLVGETLGEAMIGLLNCMHEYRSGG
KVDNAADIMGYLDEKGYLCLAGQPAHALAVLQLAETFRMKDLYLQALAHC
VGMRDMLLCRREFKHTSPATRNLIHESENELKSRFNRASAMLENFLDQEL
SEAHLGIPLGLRAHLERFRSFLLSFYSVRLGYYPPSSFDAAVYRAMAGDF
KALYRLLVDEAYSSSEAMPSVAVGGICTLQLIQSLDMHSHLEPMKHPLPK
LPQLDPRPRPRRLAWLPRGPKHRADERQLGHAALLNASNWEEDVLDNSLV
RAYRQFEEDCALERKRRDRRERVSLVDGRKVRWILIYAVHQTLRYATQRP
HGVQDDPSARYLVTIAKDTLPPWQDKNTRGGLIRSQTEPALNRLGRLHDA

DLATAMERFEIKPDIDYFALTHKQDAPPPPRSRRASLPPLATDNPSALSR
ASSFKQVLNRSSTIRKSVRRLTQSAAPPSLPPSPVTAGKPLYHEIVVHGY
GNGTNDVRLGSRAGPPLPSSDVGRSASTASETASSNASTVPSSASMVDTA
ESSLGSPVPPDSPIEILLPKTRRWSTHDAAVPYGNNSPPVSKSNSLRRRP
ISSAFEGYTHAAKAFGHFVDNERRSIFANSRQSGADPKRSSSIAHSRSIA
LPIREDDIAEETCLLPRDSGDWTAMQAFLDGKGVDTDAGHAWEQYADLGG
LTEVW
>Mb|QLI65641.1
MPPASADIPIIDLSGPQAEVSKQLVDAAADHGFIYIQNLGHDIPAASIDD
AFALSEKLFSAPLDEKQACSIQTNNRGWTSMHAETLDPQNQKVGDFKEAF
NFGEFINNKAQQPIPPTVAADEARFSAFADLCRALCLKILTLLGQGLQVN
DFLSSAHFKSPAGSGTILRFLRYPPPSSTSHTVDDVRAGAHSDYGSITLL
FRLRGQAGLEILKKDGTWAPVPVSPPGTENDPSPPILINIGDLLSYWTNG
LFRSTVHRVVFPANRAEGVEGESSTDPRYSIAFFCHPMNDVLLEPVPSER
VRGFVEEGVVKNPYAERKVLTAGEHLLMRLRETYGTLYEDKKD
>Mb|QLI65642.1
MSGSRPRHNIDRFLEEEELADRMLKVAGSTQDTSTAKVAEDAPAHQAEDK
TGKGKTDGDWVDVQAPGASGGTAAEGSWVDVGSTKKVENQEGGTASVHET
KKPRGAPGGKLKMTLGLPVGAVMNCADNSGARNLYIISVKGIGARLNRLP
AGGVGDMVMATVKKGKPELRKKVHPAVIVRQSKPWKRFDGVFLYFEDNAG
VIVNPKGEMKGSAITGPVGKEAAELWPRIASNSGVVM
>Mb|QLI65643.1
MAVERIGSIIKHLAPGSAINQIQSKNPDDIVITLAVRTPLTKARKGGFKD
TSLEYMIYALLKQVRERSNLDPALVEDICFGNVSDGKAAYKLRAAALAAG
FPNTAGASTVSRFCSSGLKSIADIAHSISCNSIEIGIAGGAELMSAPGDR
LEQPFDEAILKKSQDAADCMQPMGWTSENVGRDFNISREELDKYSAESFQ
RAEKAQKAGWFDDEIVPISTKVKGPDGEVKEVTLTKDEGIRPGTTAEGLG
KVRAAFPQWGPLTTGGNASQVTDGASAVILMKRSTAVKLGQPILAKYVGS
TVAGLAPRIMGIGPSIAIPKLLAIHNIALADIDVVEINEAFATMAVYCRD
KLSVDWTKMNPRGGAIALGHPLGTTGARQVVTGLSELRRQKKKILLTSMC
IGTGQGMAGLFVNEVV
>Mb|QLI65644.1
MVVNHFRNKREAHIVNRSSVHQWNPFRHLWDTPADRQRRRETWGGPELEA
QTGQDGRDYGGRLAHAPTAPNPSSGGIPRDPSNADTITSSAKESDEATRT
RSVYNGEEKVALNQFLGHDKSGSLRNRRPEDDAANLEGDPSLAQPTADDN
GKMKKKVKSGLINWHLKPKEPFTVANQLQRTFLSSWINILLLAAPVGIAL
NYVHSVNRIAVFVINFLAIIPLAAILGFATEEVALRTGETLGGLINATFG
NAVELIVAIIALKDDQIAIVQTSLIGSILSNLLLVMGFCFFFGGLRRQEQ
HFNHTVAQTSASLLALAAASVIVPSVFNVAASDISQREIAKLSRGTSVIL
LIVYIAYLFFQLHTHHEVFNEQSQKVPAKPWSRSGGPKGIKQGLAMPSSM
ISHAIPDQEENQRLSKMLGNPKSLDEGEDEDDDPQLSLAVAIATLTISTV
IIAFCAESMVGSIDSITKSGGLTQEFVGLILLPIVGNAAEHATAVTVAIK
DKMDLAIGVAVGSSMQVALFLIPLLVVIGWGMDKEYMDLSFDIFQVAVMF
VAVLLTNYLIGDGKSHWLEGFLLICLWGIIAVCSFWYPNTSLEQANGTGT
TH
>Mb|QLI65645.1
MSTATTTSVSPANGTVSKGNATRRRNRNASPGPVEGESEDATAAEKPRAA
VAQKKYRHVAAVHSKSRPSCLSHDSDATPSFIGFRNLMVIVLVVGNLRLM
IENLKKYGVLICLRCHSYKNEDVLIGGLLYFLIPCHLLVAYLIELAAAKQ
ARGSRKRLKPGSTGPSEQDKSKFHSTWVLVAWAHGINMTLALTLTTFVVY
FYIHHPLVGTLTEMHAVIVSLKTASYAFTNRDLRHAYLHPVKGEHIPELY
SKCPYPNNITFGNLAYFWWAPTLVYQPVYPRTNKIRWVFVFKRLGEVCCL
SAFIWFASFQYAAPVLQNSLDKIASLDFLMILERLLKLSTISLVIWLAGF
FALFQSFLNALAEVLRFGDRSFYDDWWNSESLGAYWRTWNRPVYTYFKRH
VYVPMIGRGWSPWAASCAVFFVSAVLHEVLVGVPTHNIIGVAFLGMFLQL
PLIAITAPLEKMKWGHTGKVMGNVIFWVSFTIFGQPFAALMYFYAWQAKY
GSVSRQPMLALQT
>Mb|QLI65646.1

MASTGVDRPAPPSYSDQEKAAVLGGKLNDGSHAEPVTSRRLSLSSGSKHD
ATHRQLKSRHIQLIGIGGTIGTALYVQIGKGLLNGGPGSLFIAFSFWCTF
VLAITLCMAEMVTYLPLSSPFIRFAGRYVDEAFGFAAGWNFFVLEAALVP
FEVVACNFILHFWTEAIPTAAVIVIVIVLYALINLLAVKWYGESEFWAAI
GKVVLIVGLIIFTFIVMVGGNPKGDRFGFRFWNDPGAFAPLYYEGSLGQW
LGFLQCLIQAAFTIAGPDYVSMAAGEAENPRKVMPRAYNAVFYRLTAFFV
LGSLCVGILVPYNDKELTAAFKNSAPGAAGSPYVIAMNRLGITVLPHIVN
AMVLTAAFSAGNSYVYCASRSLYGLALEGKAPKFMARCTRKGVPIYCVGF
VLLFALLAFLQLSENAAVVLDWLVSLVTASQLINFSCVCVSYLCFYRALQ
VQGISRDSLPYKAWLMPYGAYYALVATVIMVFVGGYPVFLPGRWSVADFL
FSYTMLAVFPALYIGYKVIHKTKILKPEEIDLVKDLDTIEDYERNYIPQP
PANRFEKMLDLLFG
>Mb|QLI65649.1
MPSPTDVEPAASVASAALDKAGVSARQPSRGSYPSISSILTTSSERLTKA
AQDVHPISDSPGAETVLYLAYGSNMSAKTFLGVRGIRPLSQVNVAVPSLR
LTFDLAGVPYMEPCFANVGFRNVPDQKKNTQAELIHQDMEWDGGLIGVVY
EVTQEDYRTILRTEGAGSSYKEIVVPCYPIAPKEIFPKGPTPFLARTLYA
AYTSNANDDRSWWQHLLDGRQRPDPDYAQPSLRYLSLLRDGGREHDLPTS
YQQYLASLHPYFATTWAQKVGQYLFIALWAPLLIFYLITSRFLADETGKL
PPWLAGGVTSIFNMMWMSYDAVFKSIFGDGERTEEDESDERKSLLGNRKA
>Mb|QLI65655.1
MAPSPAPHERPPFSATDVVASEASGSTKVTGPTSPTHRTPTTASGPNGKS
TSAGPQKNGDRGSPKGSGASSGAKSSPNGPNRNEIGRAKVIVKKEPGSPT
LPNMKPRPRRLDLSKNTLSTPNNPASAVPMSARDSLGIQEVGIACLSPGF
VTQDPVMKEQLQRSMSVREQQRSIIEQRLQQQSAKGEGPSDRDKDALSNF
AAKTPGMSRRGKAPPGLSIVAPSHEHFANERVIQSAPLGRSFTGPQHHAS
MSRQVVNHPSHLSSNSHIHHVPANQTNNRLPPISDVFGHSATAHPDNAPP
PFQRGAPLASPSLHSAHQQPPSSGRPREYKSAEEAQHELAGGRPELLPKI
VHYGGHQPPTPPSPAAGSRQLDGSRTSTKRRTRAEYEEGASPPLGYGPSA
TKRGPFGEGRDSPETQRAKKDEFLRLCSRAWDLFHS
>Mb|QLI65656.1
MADTAPNGGSILETTTNNAAAAYDSVTNGPVAQNVKDQNAKTQAEFSNLA
ASRKTPTTPAATGQPLTHYHSLFSELLSWKNPRASAIAYASIVSFIFAVR
YLDVIRWGLKLSFMALAITVAAEVTGKVVLSHGLATQMRPRHYWTVSRAT
VDGMIGDVHELINFFVIEAQRILFVENVGASAAAGVAAFISYHLVKLVPY
WGLAIIATTVAFFVPLVYTSNQELIDQHLKNASDAISAQTAQVRSVAQKQ
ADQLTTMGKQYAGDYTGKVQEMLRGRGAPAPSGPPKQPTLVAPAKKPEFP
TPPTEEPKSTKTAEPEGEVPEEPVIPETEPLVAS
>Mb|QLI65657.1
MPPFNLETCARPNILALQPYRCARDDYKDDGTNVLLDANENAYGPSLDPA
AVEHARSSLGGLDPLGLHRYPDPHQPELKQLLCRLRNTHAHTPKAIGPEN
LFVGVGSDEAIDALLRCFCVPGKDRILTCPPTYGMYAVSAQVNDVDLVKV
PLTPAPEFALDVPAVTAALSSEPGIKLAYFCSPGNPTGSLLAKEHVVQVL
NHPTWNGVVILDEAYIDFAPRDSSLAELVAEYPNLVVMQTLSKAFGMAGI
RLGAAFTSPPIARMLNSMKAPYNIASPTSALAQSALGDRGLAVMRANRAK
IVAQRERLIQELPKIEGVGRQRGGTASNFLLYEILNTLGKPDNSTAVAVY
EKLAEHKGVVVRFRGKEHDCLGCLRITVGTEDEVTKFLDSLRNALIEVRG
VSGK
>Mb|QLI65659.1
MARPMGSVRLKKANPLTLLLGLVLCIFILFFLVSPSTKTTPSKLPSVTAQ
HHLSPPTSPYKKSSSKSGLQTGPPPVIRYNLNNVTITANPIENQEHVLIL
SPMARFYQEYWDNLLRLSYPHELITLGFILPKTKEGNAATAALQQQIQKT
QKRGPGKDRFKSIIILRQDFDPAVESQDESERHKMANQKPRRAVMAKARN
SLLFTTLGPATSWVLWLDSDVVETPASLIQDLAQHNKDVIVPNCFQRFYN
DDKKMDERPYDYNSWQDSDIAMQMASKMGPDDILLEGYPEMATYRTLMA
HLATDGGDQSLAIPLDGVGGTALLVKADVHRDGAMFPPFSFYHLIETEGF
AKMAKRLGWQPYGLPNYKVYHYNE
>Mb|QLI65660.1

MASTTTTTTTATSTRALRSRPSLSRLRGPSSFASTPNLNTLFTAQSKLLP
PTLGLPRKASLAALTPSSLASIPDVSESYALDTVLSDSSPAMAPATPRRQ
SAEDLLVGDTVDVPGGMYGTVRFVGTVQGKKGTFAGVELHQDFAARGKNS
GDVDGISYFATSTPGAGIFVPVVKASRRGSSTSSFPMTPTASANGRANGV
NYTPPGLPKFSASVGPGARAPSPGPKRPPRTSLPRPDSPVRRTQMTPGPR
PSIGTPAPRPPARYGSPTNNSRFTQSVRGTAGDPNKKPSRLDRKPSIGGP
RSASVLGSAPGFDEESTPSLSGSPRRLGPNGLGSSVSVLNLRRPASRANA
ANEEEMERLRSQLEDRDRQLKDQATTLAEMESSLVELQGLIESTDMPGMR
RNSLDDKDSAQLRAMLREKNDKIAMLTSEFDAHRADFRSTIDTLEMASTE
TERVYEKRIEELMGDIRELEARNLDVDSVASQLRQLEELVQELEEGLEDA
RRGEAEARGEAEFLRGEVERTKTELRREREQSGPGGGAPHDDKGALLKEL
EQKEDEIRGLKAIIHSLSRDSIGDAEDRTPTQRRGSFMNGGEDGETARAS
LARQVAELQSLLDKKNGREEELEQELEALRGGNLATQKNSGHRSSLRDSR
DTVIPVRFYEPISPEVNHTREPNHRRGNTLDTMHESDAYSTATENSTLWC
EICETGGHDILTCTNMFGPEGAAKASSDIKTGKDVVREGLKLQNSLGVKD
AHPAPLSPTKPKAAFPTAPAIKILPNPMESGPVAGKESGVVDTGKWCAVC
ERDGHDSVDCPFEDAF
>Mb|QLI65661.1
MADPKIQELLNKPRNELTEYEIAELEEHEFTSGPLSILQTAVKSHTQVLI
SIRNNRKLLARVKAFDRHCNMVLENVKEMWDETPRLADGKKGRPVNKDRF
ISKMFLRGDSVILVLLS
>Mb|QLI65662.1
MDKEAPMNETLRAISKARQEGKYSDLLLACEDEKMPVHKIIVCPQSPVID
KACNGPFKERETGIYEIKDASIDTVRSMIDYMYTRQYSPTTQPDSVEPSG
ASYPIVFHARMFELADKYMVVGLQTLAASEFNKAIEQETDVCKFLRSVPE
IYSLASTASDKLREAVVWEFRCWIACQEFDATVKEVLREVIGKVPQFTEE
LLDSFLDSTLLDECSCSEGRTKRIRRALDMPKCDVCGHYCPRVRVNAS
>Mb|QLI65663.1
MTVKALRAVASGKNGVGSFILQCKKMDFHYCDWAGSSKGMNGFIKSLLPK
FAAANPQVEFAVSPRPGKHPVIIGHYINGRTKPICVRNLSPYEILQKVEL
LRDASGEKLKRTNKAVTSTNPSVRGIWSPYHGKGMVV
>Mb|QLI65665.1
MANPSLNFITFNQDHSCLAVGTSKGFRIYHTDPFSRIFSSDDGNIAIIEM
LFSTSLVALILSPRHLIIQNTKRASVICELTFPSAVLAVRLNRKRLAVIL
EEEIYLYDISNMSLLHTITTSPNPSAICALSPSSENCFIAYPLPKPREDS
DSRRPAHAPPQSTYVSPTSGEVLVFDTLTLKAVNVIEAHRSPLSCICLNS
DGTLLATASETGTIIRVFSIPRGQKLYQFRRGTYPSTIYSMSFNLSSTLL
CVSSTSDTVHIFRLGTPPGHTTPGGVPIETPSPPRQDRWSRARSYDDGDS
PGNSATDSPRSDAAESGPGNAFPSGGQQGHRRQSGSFSNMLRRSSQIMGR
GVAGVVGSYLPQTVTEMWEPLRDFAFIKIPKSGVSGLTPRATGSSIVGPL
RSVVAMSSSSPQVMVVTSDGGFYVYNIDMENGGEGYLVKQFS
>Mb|QLI65666.1
MANRMSMFSVGSEGPGNQRAGGPQTAQVSTTTLLNAIHNIYLASQPHQLE
SSTSLVVNTWLTAAQGSPTVDSTLASKAWEHARRRAEDGCVILGSLHQST
PSVLTPFLTSFPFAIPSTLYKALDAVQPFLRCVTPYNPSTPRQAALSVAL
TLNLTGNLTAASLALSQGGIDTVNGLLNIPSEAGYRAFDVFYYLLTSAST
PAEREFLGLKTPSSYALLSRSGTYGPPSYLPTADDGASADDFRQALKDIG
IKGSAHRNFISTLAGLLKLGNTLDYGVESEALEEICEDVSGLLGIEPDTL
LRQCSTEDRWTFIGGLYESLVDWVISKANEAIAAQMIRIRNGDESPDGRG
VRTPTSDQASGDTVSITVMEVPQITLGKALSMRSIFDDSQGINAEMIEDG
VNVSPAGSSVLREMQQAVSDVGPDLGIMTGPEGRERQHELEKREVVLEKI
AYAAEEGGFLRKLLFPIDGEGINLGRAGRFDLPAVLGSNRLWYHLSLHPT
DDSPTQLAALPSVTSAWSAGTVSRQLRSWRLPEWANRRNRNLDFTADFDL
DEFASRYAVLGCRDGKDGIESWVLERGWSNGEVFVGKDRVWMREGAWWEA
ESMLDLKPEHNDMNTPAMNINPFGSGFDTGYSANGSGYFPPQGIEPSFNG
SNDQLMHSRNFSQSQVTVGQQNPHAPPSIAPTAMRNVSNGDYGLGNKGDT
YKDDIYYNPEGDFTGTMDPELAKNKKLQTKEVSFGRRVWVGFVWALTFWI
PSPLLRYIGRMRRPDVRMAWREKLVLVFLIFLINAIIVFWIIWFGRLLCP

NFDKAWSQNEVKTHQGEDDFWVSHYGKVYDITKFWRQQHSDTDVKTTADN
MQPLAGFNLDNYIFPPLNEVCQGLGIAETTRLVFNETAEFSIAVHTSGFY
GNPTSKLHDPLWYFNVFEPKIKEYYKGDLVWGEQDVQSQGNNMQRPWAIY
GNQIFDLTDYFKTKDVYKDAAMYNFLDESVSDIFKNNPGQNVKSLLDQVI
QNSIGNETQHAKVMNSWLCVQRNFYKGITDFRDSPRCTVNNWILLAFTII
ICAVILIKFVAALRFSSKRRPSPQDKFVICQVPAYTEGEESLRKALDSLT
ALQYDNKRKLICVICDGVIVGQGNDRPTPKIVLDILGVDPKVDPPALPFK
SVGPGSEQLNYGKVYSGLYEYEGNVVPYLVVVKVGKESEQGKTKPGNRGK
RDSQILLMSFLNRVHHRAPMNPLELEMFHQINNIIGVDPELYEYMLMVDA
DTSVSEDSLNRLVSACANNAKIAGICGETSLENDEKSWTTMIQVYEYFIS
HHLAKAFESLFGSVTCLPGCFTMYRLRTVDKGKPLLISDAVIKEYAVCDV
DTLHQKNLLSLGEDRYLTTLMTKHFPYMSYKFIGDAQCKTAAPESWSVLL
SQRRRWINSTIHNLVELMRLKEMCGFCCFSMRFVVFIDLFGTIILPATCV
YLGYLIYRVASHTGQFPIISIAMLAAVYGLQALVFILKRQWQHIGWMIIY
IIAFPIYSFILPIYSFWNQDNFSWGNTRVVIGEKGNKQVVAVDDEGFDPR
SVPLQRWDDYALANNLPGRRGGYQEKHDYGYPDQYEMDEIKSVYSAAPQG
SVLTGMGGRNTYMPPQSPAPFSNMNRAPTMQGQYHDAPASIRRQSMMSLG
TQMQDMHRSQSPYQDFPANRGSVMNLRGQGSMTPSLGMAGNRASSAMGYT
GGHRPPMGPDAMQSTTSFDFQRGHVGADDTAIVDAIQSVLHEIDLDTVTK
KQVRALVEQRLQTELTGERRTFMDRQIDRELENM
>Mb|QLI65667.1
MATENINSPATSVEKEPNGVNGVQQRHYHHHHDISQVDSGFAAASGGSLN
PGLWKPYEHRKFANPAPLGLSAFALTTFVLSAINTHTRGVKEPNIVVPLA
FGYGGLVQLLAGMWEIACGNTFGATALSSYGGFWISYAILLTPNWGITAP
TGPYEGHVSSPLGFFLTGWFIFTTMLLLCTLRSTVMFFLLFFTLDLAFLF
LACEQYANDMGNATAAVALQKTGGVFGFLAAFLAWYCALAGIQDSSNSFF
QVPVFHFPWSEKAREDRRKSERQQV
>Mb|QLI65668.1
MKFAKELERDAVPEWRIKYLNYKAGKKYVKAVSRAINRTPKTSANRTQSL
FRSSNHGRTAILPTSSSGPPPRAPNRSNGSATPGSNPISIAAHADEDEAL
RGDGNDLHYGSFVPTPPKHSPLRQSEDKASRDFALPAPAISASDSLGLLK
PTAAIKGRNTVPTQRRASTAEPVTATRNDIVEQSSAARRQSITGGALAES
PSQLRRILSNAYGPMKRDMPWMDGGINEFDLVREREREFYAFLDSELEKV
ESFYKLKEDQAGQRLALLREQLHEMRNRRIHEIASNRDNDPDNGQYTDAE
NSKDNPNGWVQPLKAKIFPLGPNSKALRNMPNTPYLPASASAGDATRDYS
RRRQKEDVSYRTAKRKLKLALQEFYRGLELLKSYALLNRTAFRKLNKKFD
KAVNARPTLRYMNEKVNTAGFVNSDTLDGHIKAVEDLYARYFERGNHKLA
AGKLRSLSKRSRSESGSSFINGFLIGTGLIFTVEGLVSGSQLLFDSDADL
RIQTSYLLQLYAGYFLMLFMFSFFCINCFIWTKNKINYRFIFEFDQRSVL
DWRRLAEFPSFFLLLLGIFMWMNFSRYGPDWLYIYYPVILISITAAIIFF
PGPTLSHKSRSWFVYAHWRLLLAGFYPVEFRDFFLGDMYCSLTYSMANIE
LFFCLYANKWDNPSQCNSNHSRLLGFFMALPPLWRLFQCVRRYKDTCNVF
PHLVNGGKYIMTILSTVMLSLYRINGTRSNLALYIAFSTINGIYVSIWDL
FMDFSLLQTDARHFALRDILAFKRRWPYYFIMVADPVLRFAWIFYAIFTH
DLQHSTLVAFAVSFVEIVRRGMWALFRVENEHCANVSQYKASRDVPLPYR
IEPLMDRASEEMSPVLMSDEPRHDLAQSGSSTAVASTLSALRRRADTVSA
RSFSKILAEAHKQDFVKRRKPGEVVEDEAIEQSDEEEDEDDEDDEAEVAS
LLEAGEARG
>Mb|QLI65669.1
MQRQPSESSEQICQSNNTPAPSSSPQHGFLHHGPSDIRQVRSASSSCGSH
SASPHSVSDLDREPRDAVDADKLDEKLRGLSLHGKPKTSRNRPITAGQRV
SDYENALTPPTPRQALGFKVIRRSDSSDGPQMTDFPNEILTHILSHLHPD
SHAAVALVSKRFYALVTTPHAWRMAFMRYFPGHTCLDTTSAKRTADLWAG
STSDVVRSDIRYFGRLTPLATWRSEYLFRTRLMRSLARGKPGSSSGGIGA
SGGANKSSKRRSAVLTYNSKLPWLVTNLHAVFSSGKRPPRAIQGAGDLGV
ATMGDPTSGKIEKWGLEDPFSAAQLEEVVPNLVPYGLGDGPVAVPNVMDV
SQPYGVIAGEGFPGGRAHFRGINESCGRYLGGDTGVVDTYPDVPKIPEMS
DAICSLWIAKSPAVPAATQSMCGMLTGSALGVVTAYSLGSDPNGPRYVNG

DMTARWVLSPGVPIISLKVDDNYSVKRKLSFRVWAVALNALGEVYYLTES
PVTTLSRGKGNDVIRHAWLAGRTAYWHLVESTRRTARPDELDKNVVRGAY
TPRSPSNDMILSKEQLAAEAREIEKYLRYKPSHFRKVCEGWDMRRRLEVD
FAGDDGKGAGEGIFVIDCGLAEQTPARVLRYCRSLASPVVQHEQNVPKRG
DSTATAQIRPRPSLFGSTEMTTAMDCATPEPQSPHPPPPTPMSPLRESIA
NLHDWDCTAFALKGGQQAVNITASSLDCSSLSLLTLGEDPLHVAGESTAA
PGVFTATTTTPTKTSVEKAEQAVTEIPGRRARLLAIGTDTGIITVWNARD
EVGHQAAQPLRVLHTDSPKISCLAASALYLVHGGSDGLVQAWDPLASTFE
PIRTLNARSNGRVPRHMMTMNPTLREGDYSAVGAIYLDPDPTVLRGVVSF
GAFLRYWTYSSASHPTGRKRRARHSDIHGRIASRRLGGTVSGYIAAEERE
LRRENEARAREQAHLRKRFGVGALGDLTEEEALRYAEMVSEEAYLQEEQR
RASDSAADASLDTASSISETTVDIITPDPSVTDVSPPMASVTDNDESEFE
QQIQQAIRLSLLEGVNDLGQSPRGNSSSDAHFPIQIRVKTKKAKGSRSTS
TSPGPTSHTPVNGSRSNLNTEDEDLALAMSLSMQEQQEVGLGVHVGSEFP
PLDTEGVGKGKGISRQ
>Mb|QLI65670.1
MTLTPSAPEHQDSALPASPSASFYAMSDDEEGGYNTITHAETGRGVKLLF
SKSKVYVHPTPSAKDNIPGYIALLQQKPPTSGRPTSSSSHESIAPGSSDL
LLAWVPESALGDSTSIYVKVDLCDGDSPPKQSYLVPPPPTVTSHSGSVGG
YSFAVPVSAVYSLLVRPPSIGWWYGSIIINSRAGDSFPALFFHDNECQST
ILQKKKLTRDNFDPFGESGQMFWGADEVLRWLKRYVKIERSGAEPNIYLV
EPTKDDLEGFGSKTAGVSKQITPGDKAAGSSREAQMDPFVKFVKETGWNI
MNQFSKVTTFTRRAAQDFAENNNLPPQVKRLLRNPEVQTLQDEFDSARIY
LARWAMGIAEQSERDRRGRIWTVKDVVDLEDTDVGEFELLEGASALSLEE
RRRPVTMAEWETFFDPETGRLSLTVDEVKERIFHGGLDAEDGVRKEAWLF
LLGVYEWYGTADERKAQIASLRDQYYRLKHSWWERLEGDGGEGEAGEWWR
EQRGRIEKDVHRTDRNVPIFQGEDAPHPDPNSPFADVGTNVHLEQMKEML
LTYNEYNKDLGYVQGMSDLLSPIYAVIQDDAIAFWGFQKFMERMERNFLR
DQSGMRGQLLTLDQLVNFMDPKLWNHLQSADSTNFFFFFRMILVWYKREF
GWVDILRLWEGLWTDYLSAEFHIFVALAILEKHRDVIMGHLKAFDEVLKY
VNELSNTMDLESTLIRAEALFRKFQRLVEAVDKKQNFPGPRASCSSEAPS
PQPQSQTQTPPKGKETETQQKIISPELRNLLNRKVEVLSRKTVAQKGDGM
PGK
>Mb|QLI65671.1
MSLLDGVEQHPLLHTNDNLPPIKDDPVQEFFVIKQEARLDVNFRERRIDG
TVDIYIVIFSDNVEEIHLDAAHCEIDTNNVTITEMREAHGEAIEGQKQKV
PAEYRDPYQRLSHSKSWNWRAEHHDLRRRRARNVFHNRKTDVPAENREIE
GCTPVYGSLKVSLRGGKDSERPKLIIRKSALNVDGANKGHKQFKVTVPFT
NKNPRDGLHFVGEDALDNRYTHMYTRHSIQPGTASCIFPCVDDHGARCDW
RISIRFPRTLGDALEQALATQKDTTVNGVDKYHDHDDRKTMLAEEDKLRE
MTVVCSGFLMEEMIDPQNDHKKIMTFEPEKKVSVQKLGFAIGPFEHIDLS
SEFRTEEDEVKLGVSALKVHAYCLPGRADWVRSTGAALTMAADFLTYTFA
KYPFGNFKICFLEDMVEDTVPLHSLAFISTRLLFPEEIIDTEIETTRKIV
FTLTYQWIGINMIPNTRNDLWLVLGIAHYMTDLFMKKLCGNNEHRFRMKI
LSDKLVNVDIDRPSLFDLGPNLHLGEFEMDFMSLKVPVVFFILDKRMIKA
SGGHGLTRILQKFLTKAQIESSDRSTILDTEKFRAVCEKGSRYRLEAFWN
QWVYGSGCPRFEVKAKFNKKRLCVELTLNQIQHQAAKRTGGLEKDDFLRV
VKERRAGLKLGEVQPLFTGPMTVRIHEADGTPYEHILEIREDATRSTKFE
IPYNTKYKRLKRTRRMKEKHSGGGSMEITENADDALLYCLGDVLQTPDDQ
RDWELIEWDPETERKMDQESYEWIRVDADFEWACDMKRTLEPYMYVSQLQ
QDRDVVAQQDAMLYLTHGPLHPIASGFLVRTLIDRRYFHGIRTMAAEALP
RQSNIKGIPLLGLRQLMRAFREMFCYEQTNQPIPNDFTDKKQYNVRRAII
KAISEVRDHTYRCPLEARQFILNQLLFNNNEDNPYSDHFYIATLVEALAT
SLIPSKKDDWLSRQSKAPDEEERLFLEKAMEQIERVLRRDEWTNSYQNVW
TIAGLDAKQRLMKAEVIPKSYPDFAQYLLDGSRDLIRIKAFEALIDLGAM
MEATFFTFFLFALMTDKSPFVRNKLIQAMASGLAAIAFGEHTKIVKNEPT
KDDIEETLLLVQDSAQEIEARKERIARKENLDAALKALRKEMDEMYVKDE
RFYSNAMRKALDHPGLGRNEVESLLDLAAMMFEETQGWVLTLNLPKAWKV

ERHAQRLADRLIVNFKSHYKTQPKHPITFPDVSEVPRPAPVPRTSSIKIS
TTKVSTPQRPIPAPGKSETDTIVASLVSNTNGTATKATNLGNGATPASAA
PKRSRPENDNGTPVPKRPKTESSITNGDKASKKRRMVTLKTGNPKRLAVI
LGLPLGLAPVARTALPTGPPKEAVLGSSKDSITAKPRKPLPSGEVARKPL
PGTASPVPQSTPKVSIDTKVATPSAGKASSPPASATPVSARPKIKIIRKP
QPPPPTS
>Mb|QLI65673.1
MTGCNPKHFNLQDDPFELFLLSEGEKRIEEKVYSGMSNTSDFILNKEDHT
LGNLLSEHIKAHPNVYMAGYKLGHPNVPQLFIRVQTDGTKTPREVFTAVC
EKLINQLESLHQEFTREWELRRITNTGEQGNMQGTGF
>Mb|QLI65674.1
MNIAQPISSEVDAVDFTFLSPEEIQAISVKRIENDSTFDSLLNPVHGGLY
DPALGSWGDEPCATCSLISSSCPGHPGHIQLPAPVYHPVFMDQAYHLLRA
TCVYCRGFRLPPKDLHKYMCKLRLLQHGLIREAHVIDALGETDSSEMDEA
LGLGESEADEENSSFDNVTRKREQYVQVCLRSSALRRGDTRKGKHEGMAQ
ARREVIKQFLAEINKKRQCASCDGISPPYRKDRYVKIFEKALSPKEQVKM
IKKNLSRKDAMTRHYDAKLDPKADVEMSEPDAIVESLSDQDEEEPDVDGA
STQRYISPMEVRARLSELFKKEQDLVFLLYNSKPRTRRSPDVTPDMFFIT
TILVPPNRYRPEARMGDSQVTEAEQNSLYKSILRGCSKIARVYSVLKSEE
RVDITQLHQAWTELQEGVNALIDKSKNPVQGAAAKRNEDGIKQKLEKKEG
LFRKNMMGKRVNYAARSVISPDPNIETNEIGVPPVFAQKLTFPEPVTSHN
FRDMQQAVINGANTWPGAVAIENENGQVQMLKNKSVDERISLANQLLAPT
NPAAVRLKNKKVYRHITNGDVVLMNRQPTLHKPSIMGHRVRVLPGEKTIR
MHYANCNTYNADFDGDEMNMHFPQNEVARAEALHIADTDHQYLSGTQGKP
LRGLIQDHISISIALCTRDTFFSRGDYHSLVYSAIRPESGHIFGERIQLV
PPAIIKPVPRWTGKQVISTILKNTQPINYGALNLRGESQIKAEQWGTNSE
EGTVLIRDGEFVTGILDKSQIGQSSGGLIHAIHEIHGPAIAGKLLSAFGR
LLTRYLNMRAFSCGVDDLKLTAKGEEARRQELQAINRVGLEVASNYVSLK
DVSDSQNPVLLERLEEVLRDDTKLEGLDLLMNQSTKDITDKVQKVCIPNG
LEKPFPRNQMQAMTTSGAKGSRVNASLISCNLGQQVLESRRVPIMVSGKS
LPCFKPFETNITANGYIGSRFLTGIRPQEYYFHHMAGREGLIDTAVKTSR
SGYLQRCVIKGLEGLMVGYDTTVRDADGSIIQFLYGEDGLDVTKQTYLKD
FGFILRNLTSEVAQLRYDPQVVKRLGADRELFTKYMKSAVKHSKPGDPKG
RDPLISAFNPATNAFVLSEAFYNAMSSYLKANADGLIREKRDKSAPDSSN
ALNKKNAEVLFAMKYLQSLVEPGEAVGIVAGQSVGEPSTQMTLNTFHLAG
HAAKNVTAGIPRLREILMTASKDISTPAMSLYPRDSLSKQEAEAFTNSIS
ALPLSHILDSVIVEEKVGRGKIYPSAKIYHIKLKFFPPQEYFEMHHASIS
DVLDAVEWKLLHHLIKLSKAELKKRALDATAATPDIGAKVGVVETAAASG
ETVGDDDDGDDAGDDDFTSAKERANRLEAVSYGPNDDADDEIQQAMDEDA
AADELEVESLDGNPQKETTSEDEDESAERKVADSAKPRAQRVMCAFPEVT
GFEYDEAVGDGCNVTIEFDAKVPKFLMLNIVQRAIKKTMVREIRGIGACR
LDEEGGGGKERVIHVTGANIGAIQKYSDQIDPDRIQTNDIVAVLSMYGVE
ACRSNIVRELAGVFGSHGIKVDNRHLNLIADYMTRNGDFTAFSRLGLQGN
VSPFTKMSFETTLAFLKDAVLDGDWDELATPSGRLVMGRLGKLGTGSFDV
LTNLPTVHF
>Mb|QLI65675.1
MASRSAVNTRHIYKALTRPTSARLIGLARIQNAALSSSPRAKSGAASNPA
MAFPCLDALETKSATLQASRSSSGPEPSYTSGPTETFHCKDPLLLDWGGI
LPEFTIAYESWGAVNADKSNVILLHTGLSASSHAHSTEANPQPGWWEKFI
GPGAPLDTNKYHVICTNVIGGCHGSTGPSSIDPGNGERYATRFPILTMQD
MVRAQFRLLDHLGVDRLHASVGSSMGGMQSLAAGVLFPQRVGRIVSISGC
ARCHPYSIAMRHTQRQVLMMDPNWNRGFYYDAVPPHAGMKLAREIATVTY
RSGPEWEQRFGRRRADPARPPALCPDFLIETYLDHAGEKWCLGYDPNSLL
YVSKAMDLFDLGRDSQLAARDRRQARRDGTYAADDAACSLTLPPRPYEEQ
PASEVDTSAPLEPASSRPPEDLVLGLAALRDTPTLVLGVASDILFPAWQQ
REIAQSLRLAGNRGVTHVELSEEMSLFGHDTFLLDLEHVGGNVRMFLN
>Mb|QLI65676.1
MAGPSAPTELRFKNPAVLFVITARPVLHARAASPASAAVCDLQEKFRNAI

YEFDSIVATTTKLLTFANAVNIPVHATTQTAAKLGPTVPALASLLPSPPH
DKTKFSMLIPPLAAALPPASRVALVGIESHICITQTALDLRDAGHFPYVI
ADAVSSCNRTEVIIALDRLRAEHGVTVTSSESWMYECMGDASNPAFKTLI
GVVKGAVADTKKVLETLPPTSKI
>Mb|QLI65677.1
MHAQSLVVYICALFAFVSVSAADIQKRSFSVSRVANPNFTGRNGPRALAK
VYRKFLVPLPKGLEETMHAQDKRQVAAAQARKRSPEAFRPMRLRQPGRRG
LLDDLLQGLGLEDGNQNGNKGEQNGNQGENGNKNGNKNGNQEGNQNGNKN
GNQNGNQEGNQNGNQNGNQEGNQNGNQNGNQEGNQNGNQNGNQNGNQNEN
GNNNGNKNGTTGAPPQGTAPGTNQTGTVEAKPEANDAEFISPVKIGGQTV
NLDFDTGSSDLWVFNTQLGTGSGAGHRVFDPQRSQTFRLMPGASFNISYG
DGSGAVGNVGTDVVDVGGASFPNQTVQLATAVSQSFVQDQSNDGLMGLAF
SKINTVKPVKQATFFDNVKDSLAAPVFTADLRKAASGSYTFGTIDESKFK
GPMAWIPVNTTQGFWQFTSERFAVNGGQPKTSTPGGQAIADTGTSLMIAD
SVMVQAYYDQVQGARVNAQDGSVVFPCNAKLPDLDVDIGGVYMARVAGSD
MVFADLGNGECFGGLQVSPIRNLAIYGDIFFKSQFVAFNAGNTSLGMAPH
A
>Mb|QLI65680.1
MDSVLRQSKAMCPFLKAASPATLRALSTSARPKPSPCGGTMSKLQVFAHR
CPVMGKAMAVQSARNGVAVAAGLRAFSGQAKVNKARIHTSRTQEARAVDT
PILEKRENGYLAHGHTGSAGKFNYEGFYTSELDKKHKDKSYRYFNNINRL
AKEFPRAHMANKEDRVTVWCANDYLGMGRNQRVLSKMKETLDEYGAGAGG
TRNISGHNRHAVELEGTLSKLHAKESALVFSSCYVANDATLATLGSKMPD
CVILSDSLNHASIIQGIRHSGTKKIVFKHNDVADLEAKLASLPLHVPKII
AFESVYSMCGSIGPIEQICDLADKYGAITFLDEVHAVGMYGPHGAGVAEH
LDWEAHQKGSPRGTIMDRVDIITGTLGKAYGCVGGYIAGSSKLVDMIRSL
APGFIFTTSLPPATMAGAKTSIEYQMEYAGDRKLQQLHTRAVKEELNNRD
IPVIPNPSHIIPILVGNAELAKAASDMLLQDYQIYVQSINYPTVPVGQER
LRITPTPGHTKEYRDELVEAVDQIWGRLGIKRTSEWAAAGGFIGVGEQGK
VEEPLWTNKQLGVEQAAKEMMATGHGNNGGGFTEALLEREMSNTARTAGV
AAH
>Mb|QLI65681.1
MPYNTSAIPPRKEPTGQTQLPLSRVKKIINQDSDIAMCSNNAAFVITLAA
EMFIQHLAEEANTQAKLERKPRRNIQYKDVANAVSTHDRLEFLEDVVPKT
APYKKVKVTALATQARLRGDLKIPEERPESSPQVSATTNGGGGNPIVNGS
VSSGGAFTVPLRNDDRQSGDDPNEQLEMEMRQATGRDGDVRMTG
>Mb|QLI65684.1
MKIENRTFLISGGASGLGKACALELIKNGANVAILDMNEDGHELVKEVGS
SAKFFVCDVLNTESITKAVQGTAEWAKASNKPIGGVIPAAGVSTPATILD
RDGAAFSLDDVDFVLGVNLRGTLDLVRQGVAEIAKTEEGPDGERGIVIMV
ASSAAFDGQKGQISYSASKGAVAAMTLPMARDLARFGIRVVTIAPSLFET
RMTSMMSGKVRKSLEATFEFPKRSGQPEEFAGLVKHSIENVMLNGTVIRL
DGGSRPSKI
>Mb|QLI65686.1
MSAANPPRPSRSLKGKVAIVTGAGSRGETIGNGRAIALLLAEDGAKVVCV
DREEALAQRTAELVLAENEGASALAVAADVSSAEECARVVDAALRVFGRV
DVLVNGVGVLGPPGTAVDVDAARWARGLEVNVTSMMLMAKYAVPAMRDNE
PEAGVRGSIVNLGSVAGLRGGTPSLLYPTSKGAVVNMTRAMAAHHAKDGV
RVNCVCPGMLYTPMVYDAEGGMSAEVREARRRRSLLQTEGNAWDCAGPVR
FLAGGEARWITGTVLTVDAGATCALAVSMPDGE
>Mb|QLI65691.1
MADSRDNKAYKYRQEISQMMYVSGETGEPSVETTSIIEDIVRQQVIELLR
NCTELASRRGSKSISTNDLIFQIRHDQAKVSRLRTFLSWKDVRKNVKDSD
DKGADADLAGGDDAVVPGGPVDEAAKKNKKAKVGLPWEPASFYSVEVPER
DDEEDEEEEEMNYITLQRLRKADERTKAMTKEEYVTWSEYRQASFTWRKG
KRFREWAGFGIVTDSKPSDDIVDILGFLTFEMVATLTEVALKVKEQEDTA
RAQSGADNAGTKKRKLQLGGLFDPPSEGKTPVEPRHVQEAFRRFQQWPKK
TRAMLNGTRIVQKTPLNII

>Mb|QLI65692.1
MANREVLSSRTETLNKYLKLDQKGSIMAEYVWVDANGETRSKSRTLDEKE
YKPEDLPVWNFDGSSTDQAPGENSDVYLRPCAIFPDPFRGSPNIIVLAEC
WNADGTPNKFNFRYECNKAMEAYADHDPWFGLEQEYTLLDHDDRPFGWPI
GGFPAPQGPYYCGVGSGKVVMRDIVDSHYKACLYAGIHISGTNAEVMPGQ
WEYQVGPCVGINMGDELWVSRFMLARVAEDYGVKVSFHPKPMKGNWNGAG
MHSNFSTKEMRAEGGMKHIEDALKKLEPHHAECIKEYGEDNEQRLTGSHE
TGSIDQFSWGVANRGCSIRIPRETAAKGYGYFEDRRPASNADPYRVTRVL
MTSIFGKL
>Mb|QLI65693.1
MDRIWGDGYVPNDQDLLRSRLRTTGITESVFDLGQLTYRMFDVGGQRSER
KKWIHCFENVHCLLFLVAISGYDQCLVEDKDGNQMNEALMLWESIANSHW
FTKSAMILFLNKMDLFKEKLPKSPISKYGFTDYHGPEDDYKAASKYFLDK
FRALSRNPEKEIYGHFTNATDTNLLKITMGSVQDMIIQRNLKQLIL
>Mb|QLI65697.1
MKATIAAIALAILADGAVARERVDTAPQAPAPRTNGRSFTLNQVQNKDFQ
GHDGPMSFLRAHFKYAKKLPDYLSKLVEIDPNFRIKFGLGSQADGQIGTV
LADPTPLVDTQYAIEIGIGTPPQHIRLNLDTGSSDFWVFSTDTNPSMVDR
QVLYDADKSMTSHFLAGESWKIKYGDNASASGYVYTDRVQIGETFVDTQA
VQVAVNLTKDLSSDNFVSGILGMANSAANTVRPTPQRTYIHNIKDNLAEP
LFTANLQRQAPGSYNFGYIDDSQYKGSITYTPVEPSFPLWMVSTTGYRIA
KTSHNEQIKSIVDTGTSLLLLPETVVSSYYAKVKGSSVHPQLGMRLVPCN
ADLPDFYLTIGSYRGRVPGSYMNYGRISGNSCFGGMQSSGDLPFSVLGDV
FLKAQFVVFDYGNARVGFANKQLYE
>Mb|QLI65698.1
MAGKKGENSKKVAGNARKAEAAAQKAAAADARLEAEESENWQKGSKSNAK
KEAEAAKKAEQAAKKAEKDALLKEEEASLGGRAEPKKSKAPAKKSRGLDL
SQLDGGAPSTLNASGIDNALDALSLATGSDDAKIDKHPEKRFAAAYAKYE
ERRLTEMKADGSGVGLRLEQRKQRIRKEFDKSPENPFNQVTIAYNATRDD
LDQVKAHEKSKIEKRLGPYAAAVLRFQISFPDTYPKLPPLILFSTDIFHP
LITPLTTYMYAKDVQDKGTSSATDQERLPPGAFSLRHGFPQWFGRGQSAE
PGSRLASGERLDSSGSDGTGAPPEGVVMSPEPSHASSPDYSQTCKPGMSV
YTILKYIRSTFDTPEVLDMVPLEASGNPGAWHAWRAHRRKHGKLADQKPS
SGDDSDSDGVRNSYSGKAEEPSLSPTTATRQPGQWNWDGVWEDRVQKGVA
ASLSEPVLYGGSGVPDEMIHFLAMEETDLESVKDDIRRTLGSAA
>Mb|QLI65700.1
MPQFNYEYRASRTIEYAVLTAAACLSIYWYLQPVLSPGDPVKPTRVICSK
ARQAAKLASADSTRASSLAPRKLAPSYAPGVFPGGRDVETAYGTIKVFEW
GPEDGEKVLLLHGIGTPCVALGDMAREFVRKGCRVMLFDLFGRGYSDAPS
DLPYDDRLYTSQILLVLASSRIPWTGSSSFHIVGYSLGGALAAAFAAYHA
NLLRSATLICPGGLVRPSHVSLKSRILYSEGLLPSWLVNGLARRRLEPRP
GTASADIPDPSVNTVDGDDSQDVDFDQVPVCAGDASGPKVGDVVRWQLRN
NAGFVPAYMSTIRNAPIYGQHEGVWRRLSEQLALRREDEQVPGLEGGRIC
LILAERDPIVVKEEWIADSFAVLGEEAVDIRVLKGGHEIAISKGKEVADV
AMAAWRR
>Mb|QLI65702.1
MAPIPVYSSSPINAAKPAGVTPKTADSNKGGQAQLEPPQTSGEQAFSPAQ
PGARPMLPKATGAPQPGGHVLATPTRTSSVGNNPPPPQPGAVPVPPGGGS
GTGSNIPPPPRAGESLQNHQSPPVTTMPPQMGYQPPRAPLPVQARSSTTA
STVGPPPHSGPPPHSGPAGVSAPLQGNYPTAGISLPAGGYQQDVNAAGYN
SYQRAERAYEDESEASVWDTARKWAASAGESLAAAESEVWKRINKD
>Mb|QLI65703.1
MSERQNTLQRINTETSSIPQIEVMSPPRDKKPKRTPRDTGFPEPFHSGPN
TTLPHPDADLSPNAMLSYEDVCEERMIKRHRLSFLKKHKRTVSYGVINPQ
SEALSSIVSLSMLDFDGKNNNQLRSTSTTSLRLSKDGGDSIRSSSKKDED
TPPTSPDVAEHRRKSGLFSRFKRS
>Mb|QLI65704.1
MVSTAGAIVITIIVLLTAAVGGWVVFARMRARRLGLPPPTLSSYLPWKRN

ETPYGPPQPAPGGVVGWFNDQVRKVKNRNNRSAAGAYERPLQGGSGGRRG
FGPLDPDEAWDARVGNEADGYGYYEEQELGRHSNTEYPGGSSYSMNLATN
PRADYDDEERGRKPGRPDVSQNKNPFDDDAVSSLRGVSPRPIDTAAAHTH
RSRPSQPESDGSSPTERRSAFRENV
>Mb|QLI65705.1
MEPIFRRAASSLCGRCTASLRRQNVIGRVGQLAIRPVGAIASMSTKPTTP
SNSASRTFTTTAESKSEAAAPEKDTPTPSLRLSQDDLFHPFSSSPVPEFR
HRAAFMRQHAQCPHPDHKPTKLPTVAPKPDNSGEPTGTMSPAHVDFECPD
CGFPVYCSKEHWMDHYEEHLKICDTLRQINEDDHDLRSGRVFFEGNLPDL
QMDEAAVNMTNWDTFMYTREFEAVNSDRSMRQITRLLTYPVTIGSVLHEL
SPYSLKKGERLTPEGLKSFSALRYNLHPPRTGRGAGVNQLRPEPPPVRVF
VLGARAESSLPRAAWTQLAHLFPDSRLHLVFIGPESMANRDDEFPLPERT
PSNPFGAIVEDRVWYKMKISTIVDYYHTIHKTGHFAPYDPYFDCFVLFHP
GLGHPASSHEWEETLPLLLETKVPIIATGYTQFDLERDVEWVHKKSKGEF
DILLEPGENTFRSLRWDLNDMDPQDISCGNWGVWAFRGKRYEATTKDI
>Mb|QLI65708.1
MDTHLPTGAPLYFGPRRPKRLKIDVACDTCRSRKVKCDGARPACGNCSKR
FGQKDHCRYSNSDGTPAAPHKDTSPSTYTDWPPAKNEPQQQPHPAQVSNL
TNHHVPPGGDPYPSRNCQEPHSSSQVSTFRPVLPGPSPGKGSADVKHAIP
RDKRTAAISDAGSVRAPDASPSAIDSMTAVVDDDVSTGEFFGSSSAGSFT
SQIKAAVASRLGQAQPKPARPGLSLGVGNLNAPRRAGMHNSANEVLPPRR
QADHLMNVYWFYVDPLYPFLDRRKWEQNYANLFAGTPIDTDEVIFVATLN
IIFALATQLVESMEPENRDETSGVYFKRAKDLLDLTFWDSGSLELVQYLL
LMSQYLQSTNLPHQTWMIVGSAVRVAQSLGLHLPETSALQPTTSQRELLR
RIWHGCVLMDRMVSLTHGRPAMISRNLASAVPLPLTSSNAKPNDYGRLTE
GSFFVKSIELYEITHRVLLDLYSEPGSRPRSTIHDDRKDEDLAAVMQLDS
AMMKWEESLPKFLVLSDADVANNEVSHRQAVILHIRFLHARMLLLRPVVA
RVCLPPPGFGVAAARPPDSLQSRVMQQCTIYCVEIARSIISLLLKYQAND
GTVGLLPAWWYRVYYLFSAATVLIAAKLRTDIMNLQDINQAWSEVMKVLQ
AHEHVNTSARRCVAALQILSSKILNGGASTMDSSAHFDTTVSPMDSSVPH
VGGIQPAVQQDQSFSQFPELDIRDLTFTVDDFSWLNDMNTWNVLNDT
>Mb|QLI65709.1
MEDSTTYGSPANGASPDGNPILAQPPLQAEAIPGDSQDVPMSEGVTSEAN
IKQDSATPGPGATAAEGTEALQADATANADTEMGDMPAPGSKGITAAEGD
GATDNQETKSKENVESAAREHLVSQTHAIVLPSYSTWFDMNSVHDIERKA
MAEFFNNRNRSKTPAVYKDYRDFMINTYRLNPVEYLTVTACRRNLAGDVC
AIMRVHAFLEQWGLINYQVDAEQRPSHVGPPFTGHFKIICDTPRGLQPWQ
PSADPIVLEGKKNLDTDKKSASSAGAKNDLNLEIGRNIYEANSKGLSVNK
SETKANGETPTTNGVSGTEDATMSAISKVNCHQCGNDCTRVYYHSSQTDA
SSKAKYDLCPNCFTEGRLPANHTSSMYSKTENPTYTSIVDRDAPWTDAEI
LRLLEGLERFDDDWGEIADHVGTRTREDCVLQFLQLDIEEKYLDSEMPMS
APTGLSMLGAQHGHLPFSQVDNPVMSVVGFLASLADPASTAAAANKSADE
LKKGLQKQLNGSKRNQESNGASNGGEKASGVSREQDQTRYGGESMDVDVR
HEVTTTTTTTTTTTKNRSIASIPLASIGARAAGFASHEEREMTRLVSAAT
NVTLQKLELKLKYFNEIEGILRAERRELERGRQQLLLDRLAFKRRVGKAM
ESLKTATTIGGDQGIRMAQEALLPDGEKLGFQPVTGEPSVQAPSSNGQMK
SYEA
>Mb|QLI65711.1
MSFAIEVPGEANPLSLETLCHALQAATSNDFAQRQTAGQQLTSWEQTPGY
YSSLQAVFLDKSIPTNVRFLAVIQLKNGIDKYWRLYAHVRNGIKPDEKTL
IRSRLFQGTIDEEERNLALHNALAVAKVVRIDYPANWPDALSHTIGLLRS
GKDGNQKHLYGTLQILLRVVKELGTARLRKSQTALQSVTPEIVYVLNDIY
AEKTTLWLSFLSSNQGSGEDANVAMHNSLLSLKIIRRLIILGYERPHTDK
SVEQFWTISQNQFGQFLNFVGQEPTIPPIYQDLVGKHLLQFTKLHIDMAE
QHAASFSILPNSLHLVHAYWDLVSKFAEVFDSSGGIRQGSGDAGSSKAKV
EGPLQERLALKGLLLLRACVRIAFQPVQTFKYRTPETKAEQEQSRNFIKT
ELLKDDLVIQMVNSIITHLFVFRRSDLEAWEEDPEEWEQQEQSEGNAFEW
EVRPCAEKLFLDLLTNFKQLIIPPLLSYFQSAQSSQADIATKEAVYTAMG

LAAAHVVNVFDFDSVLSSTIVNDAQQQGGLHKVLRRRIAILISQWAPVKL
ADTSRPLVYQIFQHFLNPNDETNDLVVQITAARQLRWIADELDFSVEAFL
PYTSDVLSQLIQLIRNVAVDETKLAILESVRILVTRMEEQVSQFGDQLMS
ALPGVWENSGTEEYMIKQAVIAIFAALVMSMGDESQRYQNFMIPLLSEAA
RPGSDIHVHLIDESLELWNAILMQSKAPLAPEVISLAEMALPLLEYQPDT
ASQALTAVESYILIAPSAMLEDKLRRPTLAALSGTLNSKSREQVRLGTVC
IEYLIRAATQLGGSNGISVIVQDMLETGFMNQIMTNIHDAWEAHQTTGPN
RKISKLNTITEGDYLAILARIALAEPNMFVQMLTAFGSLEQVWSWLSAEW
FSHLSSMDHQERQKLYLLGLTRLIELPSPMQELTLSKLQDYFDMWTNVTS
EVQEGELGGHDTLVWGELEPSEYDTPKMIIERQTMAKDPVHSVPTFSFVS
ARLQDLVARVGGEAAFEEQWAVNVDKDVLERFGQLANAAR
>Mb|QLI65712.1
MPPSDYTAVGGGALKLKGAKVQKKKKKKDKNDLERNLSAGEGALAKSDGA
PAADEQLDHRDSKGDQSDDNGAVARKTESERKYDEVRKKRLQKMAESSSS
RPELLKTHKERVEELNTYLSKLSEHHDMPKIGPG
>Mb|QLI65713.1
MGDVIKEENGTKRKASEDPTSPAALKRIKHDESVDAAEEKKPVAMKPIPF
PEKPAVIEERNGEIEFRVVNNDNERESLIILTGLKCIFQKQLPKMPKDYI
ARLVYDRTHLSIAIVKKPLEVVGGITYRPFKGRQFAEIVFCAISSDQQVK
GYGAHLMSHLKDYVKATSDVMHFLTYADNYAIGYFKKQGFTKEITLDKSV
WMGYIKDYEGGTIMQCSMLPRIRYLEMGRMLLKQKECVQAKIRAYSKSHT
VHAPPKEWKNGVREIDPLDIPAIRASGWSPDMDELARQPRHGPNYNQLLH
LLNDLQNHQSAWPFLVPVNRDDVADYYEVIKEPMDLSTMENKLEADQYAT
PEDFTRDAKLIFDNCRKYNNENTPYAKSANKLEKFMWQQIKAIPEWSHLE
PEK
>Mb|QLI65716.1
MSGFPSFIARLARPFTQSTRLSVAPESSSSAPGAVPEGAQRCTVAAGCFW
GTEHLYRRQFAGKGLLDAKVGYIGGDLSDPTYRAVCGGKTGHAEAAQIIF
DPEKVTYRQLLEFFYKMHDPTTLNAQGPDTGPQYRSAIYFHDAEQEKVAR
EVTRQVNEQWWKGGVVTELAPAGRWWTAEEYHQEYLDKNRGGYECPSHFL
RPFPPLQ
>Mb|QLI65717.1
MPTDKVPVTVSYRKRGLAPPVFVAGSFSDPQWELQEMHGVIDESGEHHFT
IQIPVEPGKEYYYKFKAANQDGWVLDGHATVVKDDQGRPCNLLKVPMTSN
GADRSHTPLVEATERKDDGPVAPGQHTIVASGPKSLLPNVFRDKTHEADQ
RSGTPIKQVAEVAAEVADTAAKLDEFDTAVSSPAPIQLRTDNDMAVEEEI
ATPLFAHESFGAYEFVDDGLDHDYFGKNPKSPNSMVSDSGCSLGDVDIDD
PTLEKFPSDRPSVIDTLRKIQSSTDEGRSSFEDGQHSTPATSRRTSLDSL
DGNGGQLSPTSVRKQDNRASRSSFGRPRSAVSLECIDEEPKASGDVSPGT
KHDPETQASGTQGDGSTTDEDSGLVMKIAKV
>Mb|QLI65718.1
MSSSSSAVQAKAFPDGTTDYVPLRGKKADASKVHISDTPMTWSNWHKHVN
WLNTTFIIFVPLAGFISAYWVPLHLYTAIFSVIYYFNTGLGITAGYHRLW
AHTSYRAGTPLKIYLAAVGAGAVEGSIRWWSRDHRAHHRYTDTEKDPYSV
RKGLLYSHLGWMVFKQNPKRAGRTDITDLNEDPVVVWQHRNYLKCVIFMG
LVFPTCFCGLMFNDWLGGFVYAGILRICFVQQATFCVNSLAHWLGDQPFD
DRNSPRDHVITALVTLGEGYHNFHHEFPSDYRNAIEWWQYDPTKWAIYAW
KMLGLAYDLKQFRANEIEKGRVQQLQKKLDQKRATLDWGTPLEQLPVISW
DDFVAESKNGKALVAIAGVIHDVTDFIKDHPGGKALISSAIGKDATSIFN
GGVYTHSNAAHNLLSTMRVGVLRGGCEVEIWKRAQFENKDVTYMNDSAGQ
RIHRAGTQVTKVSQPVASADAA
>Mb|QLI65719.1
MEQVHPQPASHVLETPIWMMIVRGLQFLISLIILGLAGALMHDLYLDEFG
LSVATAILTWCILFYAVFTEKIPPWRTAYHVLAVMVLDGFLVVMWLATFA
AVAARRATFKFNVQVGGCIDDGSIIGSKTCFTRRQLERRDILFKSGGEMM
SSIAGLGALLWLMFIATFVWTIVMFLRGRKEGRFPIAPGGGGGSNNYQME
PKAEQGASVTQPQQQQQYTPYQQPIQPQQTGQHEQSPLGQNPTPAPYQPA
QSPYQQQPPYQPPQDAQQYAQYGQTYPSYQQHVQQGSELSATPLPQQPYS

PSPVTSSPPPQQEQQYYHQPQQHPQ
>Mb|QLI65720.1
MENIERLRKEIAVRESELADIRASLATAEAQQRQASPKLNWKWPLEEHEY
QRYGRQMIVPNFGLDGQLRLKHAKVLLVGSGGLGCPAAAYLAGSGVGVLG
LVDGDEVEVSNLHRQIAHSTARVGMAKVESAITFLRELNPTVSYKAHVAH
LTPQNAEDIVSQYDVVLDCTDHPTSRYLISDICVLLKKPLVSASAFQTSG
QLIVLNSPAGKGPCYRCVFPKPPPPESVVGCGEGGIVGPVVGVMGVLQAL
EAIKLISRGGLEAYDSAVPEIQQTMMLFSGMADSSPFRSVRMRGRRKECF
ACGENAQLTLDYLRSSMDYVQFCGVTRPVQLLREEDRITAEEYQGLARSG
ADHVLVDVRGKEHYSLSHIPGSINVPISRFMSHRAGETLPEGIPADLPAD
VPIYIVCRVGNDSQIAAAKLKELGLDRNGQRFVGDISGGLRSWKDAVDDS
LPFL
>Mb|QLI65722.1
MASNTKDSLLSRHYIDYVYVPAVLLVAGTLIVKREWVPYSAALALVLGAY
NFWNFQIKKVLKPDVFQEFELQEKTVISHNVAIYRFKLPSSKSILGLPIG
QHISIGAPLKQQDGTTKEIVRSYTPISGDHQPGYFDLLIKSYPQGNISKH
MASLVVGQAIRVRGPKGAFVYTPNMVRHFGMIAGGTGITPMLQIIRAIIR
GRADGDKTQVDLIFANVTPQDILLREDLDALVKEDSGIRIHYVLDNPPEG
WTGGVGYVTSDMITKWLPKPADDVKILLCGPPPMVSGLKKTTESLGYKKA
RPVSKLEDQVFAF
>Mb|QLI65723.1
MATRSLESASSSPLSFYTANDSEIEESTTASSPTQTLPCPYRDARQLPRE
LKGHCHIFLEEQLYTCAINLLNSTLGSGTSRRSSSPKTVPVPPPSHLALL
NTLIIHPIHTTRAEKSEYRDVAALALDYLRNLLTVVGPVNAGFRSAFQFH
ASPRWTRRSGFTSPNADSDMSDGDSDRDHDRLQGRMANEGSLWSRGQDLW
ATVGWAFNTSILYPQRWRYWRVWLEFMLDVLETDWAERQRQDEANHEANG
GEGAAPLTARSESMMAMYMEQSMDGHVALKRIIKALFADGGKLSSSSFPE
VFEKEPRGPKKATRKRKREQVLDLENGKFGDYFDDDSISSGVSEPPTPQK
PRDGRNSDTMGVLSPGLVESISIRLRFFKMLSAATAALRKQRELGNLYED
YVLAIKKLPLPLYSLYVAQRENPLPPEIHVPLIQELFRLLLPQSGKSTAK
VDPEGDANGILTLPMLEYCYVPMAANTVSLEDNAKLSLLVENAIQLLWIS
DMIEYTDSFGDACGRGIRAREAKAKKKRTGKAHAESSDVSAKDVLTRSGE
RIRMLVQVLKETALDLSD
>Mb|QLI65725.1
MLEYFTYKKVKKHKAEKAAKEEARQAAEAATRTDTSAAESSSNNKHAHGH
DHDTRHRRHDGHEAVIRPDDESFLEELLANDDVPAPPLPPRLYLRDLDWP
SDTDDATPSTSQQAPPPPGKPADSESDKDKDKDKEPKKPNRISALFTRSK
PKDDALKPLPTTAPAEQEKEKKDLSKVLDRLNLSVKNNKVISTDSSAALQ
SFTNVFKDLVNGVPTAYDDLMGIVDDKDGALAKGFDKLPNSLKKLVTQLP
DKLTSSLGPELLAAAAASQGIKADSSSGLKGAAKSVFVPANLLQLVTKPG
AIVGMLRAIVEVLKTRWPAFIGLNVMWGVALSLLLFVLWYCHKRGREVRL
EGERTVDGSDRIEELADDPALPAPEPPGRVSTLRDEDAEVVEAPGRRNLS
R
>Mb|QLI65726.1
MSELEKPSPVDQGKGDALSNSDLGNIVDPDAGLSEAEKKEAERKLLWKLD
LTLIPWLCLLYLICFLDRTNIGNAKIAHLEKDLGMDPKSLKYAATLIIFF
VSYAMFEALANFLLKKWKPSRFIPSIMILWGCCMLGMGFVKDWSGLMAAR
WFLGVAEAGLFPGVNYYLSCWYKRNEFGLRAAIFFSAAALAGSFGGLLAA
AIQKMDGLRGIPGWAWIFIIEGLLTIAVGFLSFFMVHDFPDEAKFLAPED
RARVLLRLKNDNQHSARHEDFKWDYMWAAVKDWKTYNGMLIYMGPLMPLY
SFSVFLPTIIQSMSFTSADQIIRNQLLSVPPYAVAAVLTVIIGFWSDRVQ
KRGVFIMGCAVIGTVGFVMLISTTNPAVQYAGTFLGAMGIYPPISLTIAW
VANNIEGVYKRGIVLGFVIGWGNLNGVVSSVIYRWPPRYFQGHGTIIAYL
VVCMFGGSLLFATMLKFENRKRSKGERDHWIEGKTEEEINALGDKRPDFL
YTL
>Mb|QLI65728.1
MKPLVAGLISAAAASAAFNPLAQQQPIMPGGDRLPAQPDNKSYAQETPPP
PAIKFKTGSSPPPPPPKDQDAHENKYEPQAQPQVSLSDTIGPLRSISSFS

SFTRLCESTSSLLADLSARTTVLAPLNSAVDSLPRKPWESPADYRALGSQ
AYDGTGGQDRANKNLLRFVEAHLVAQSPWPRGEKAKTLAGREIWWEEESD
GQRVLMPDRVAVDRVASRVSNGELWIIRGVINYT
>Mb|QLI65729.1
MASKHPVPSRAALNALRGVVLTTSCSVILLAEERRRRLQLARAAIENARK
LHMVQSNRGPLALAETNTPWESRFAEVDGEVLSMAPLPRPRTSTRRRGRS
HLIGTRSESEVHYDDDGRTQSTSRADTRSKVSKSPVDVDVLGRAFDMVNL
ENMRLSPLKSHCYTSLDWKAPRAFTQSGASKAPLSSTRQSTPDTKTSPSQ
PAEDSPEAEYELILDSNEGLHVDSIGSARLYLEKSGPEGLTVRPFYDEAI
VVLEQLLQDMELTGAKKADVSERINLATMIFQRVAAFGPPLPRATRPLRS
QGIRLLQLVSTASPDKLATTLAILLPLSKDPLKFLQPFIECTQHGSNRKA
VRDALRFLSQNARSCSWARGVLVCRLLAQLAKTQANYDKTKRVYHVLQDA
GLYKEFGIPQSTEYKIRRLMAILALERGDDSLANTELEFLGKLDSEACRS
DIRLQTRLITSNAKMGKWAEVCSDIEALGQISNTQCIGFQRLLIRTTDIF
DQSHNAYELQMFLRKLATEYKLSLKHRWIYAVLDYYASRRQVEPVFSWLQ
FCSSHGLQIDSAFNKRFFARCRKFWSFSDKTIQSLEESLRTRARTVPQTA
SEMGQNELQRQMASLVSNGEWEQVTEIYETACFSGTDHSVQCLRLAVLAY
TKCRNPDIERASNLIQSAYAKGGDISEVLTPLLLAKLERGDDPSSLINGA
LRMGVRIHDSAYNKAAQALSGRGDHRAAADMCELAARENGNGELLYNEYN
FANLVFAYTGSANYRALQSLLSEFTSDVQWWHGSRTCKETIKLAMKTAAM
RSVAHSQNSAPHRQALDELDEALIHVKKCRSTKDDRRAVSEAYVRLAAMP
SSKIHSKSNGRFSNKASKLRREREVAQIAEPVLGVASSSG
>Mb|QLI65730.1
MALRLPPGLVPSEVAFLCEMELVTVVPRQRLESIHLLAGQTPQLRPPRRS
NLPLWLALLLKKQRRANIVPPPWMHPDSLRDVIHHETKVDTKGWAPPPPP
RSRADSLGNATRINAFSGKETILSPPFLPSCTADAPSGALPYHWFELAEM
LLAHAGDDIASASEVRSLLRDLQEVRAAKMRSSTAQLESGVDGVMSLRGV
GAMELAESRGFVTGVVEGVRTIGASAEAMRREEEEQGRDDGEESDDDMGL
>Mb|QLI65731.1
MALYAQRYMLRGFASRCYITSRRRISTLKVPDQTRFSYFLSPKDGTRDLL
TNLSGHAVLENSRGHDAAVVLATPDLTKQLDDQEFISGLVKLLSASASIS
DFHLLCAVVDNIAPALGDVVPLQGISVLRGNVDSLLPQLWHPNPPREKAD
KDSVAALTFSVGKSTVTLPLTRTTFHNNRASTLLSNRYSLSEGQPELKDR
KEKQWQHVQITLNQPVQSTTDLGLWAPLSPVTRARKITESFGNIVRGIEI
GGKSTPASTELEDSVNTMLANRKATGSDQGPMGVWAVITPETSESESLVL
ADAPNPADTLEGRIVQRKDVETTARYLEQQHQLGSRTYQVLSGGGGWGPK
KGLLSLDPQQTHFALSEAEEMERFMKTMDSSGFAPPGAHIQFFVPAMASP
EIITCPVTGVVFGVPLGIETSGEAGTAPKGYLVADHFGALSSQGVYLSGR
TDADSASCYESKISVPGCRIFVGNPEAKPGGFFNFLGSGSLADAGTIALM
>Mb|QLI65732.1
MADDTEFSSFDENAWKAQDAADDREIARLLEQSQEGGNSSLKLDDTPFDQ
SNKADDAQDFEDISDDDLPDEEPSASTSLELPALTDDGGTSNDADDLFGE
GPSSPTDPILGPPSPGPRVRDTDTADDSHPIDSSLSFAAINFDPDPHLNG
AANQDPDIPAVVESHEDLLKAAWPAFKKGRVLTWSELLPPKKATWKEKKP
LKKPKHLVTSKLNLDLAADQEKMFRIPGTATSGPNHRALESSLAGFVRCE
NNGSNQEEDRSHFDLDQESDSETVVGFTLQDIDLACQDWGDQINAVETEF
KKRVAAEKELLKPKKLVEDHDDEWDAEFLMDTMDEQPQQPKKRRAVKFGL
PEIPRYAASTFDNFEDVTRRGAKRVHLDMNDPHLLLDTDMHLRAKRPRLD
SKVKRMANGNMGRDISQRFNLSNDEAYELLKENHQSKVRATLGNISVEHS
MPAIKLSWPYYKVKLGGTTDEYHRPRFRYKKFSGHVIKFDKPQHHKRKMM
KGKAHEVYRLSKDLSLSDNSTAVLFEYCEPRPRVLNNFGMGSKLINYYRR
KDNNEDDQLPKQELGEYRMLLPEDRSPFSLFGTVDSGETVPTLHNEMYRA
PVFKHMPRGTDFLVVRSTTGVEGSKWHLHKIDHLHVVGQTFPSVDVPGPH
SRRVTNASKNRMKMLAFRMIRHSQTDNCQLSDITKHIADSTDTQNRQKLK
EFLQYDRESGEKGMWRLKPNEMLPDENAIRAMIKPEEVSLLDAMQLGIKE
LEDAGYDPRNANIDDDVMDVDAEDEDDDEGGSKAPKGAKKPGEKQEETLA
DKMAPWKTTKAFIDACAQKAMLQLHGEGDPTGHGLGFSFIRTSMKGGYIE
AVQGPLATSADAMEREKRANGGHAYNVKKQQAMYEEGIKEIWEKQKSTLS

DAQEHDDKDVAVTEDEDDRFNVHAAATPAQFDDGTSQISGFTSASRHVKK
AICITREVRLQDGTTQTRTEVVHDPVVISQYMKRRTEADLELKDIYSSRP
TGNADHDRLAGIRIKKELERLEKNKARRQAREQQKELHQKASAGEAGSPG
AVDKVPTVSPPLYPSFDWWGAASHKEGQAVPTKKQARRKSVLHRLMKSSN
REAREREKRRQIEERATARKVRKEENMAEKLKVQREREQKKIEDTRLREE
ARRQEDQAREGKKRRQEELRADKQRQENEQRRKAQEEREERRRLRLEREE
ENQRKKEEKAKARPKFNYGSCRP
>Mb|QLI65736.1
MGHVIRGTTLAAKKMAWPPAVSLGNPQAGCWRASPWPMIAPSALKLQFSS
SSTRHTNYTNSRDEARGRGSTQRKVDDRPWHRESSGSLPKSTSPDPTGGD
ATKGRLLTTPTRLLKLILPIPFHPEQEYINSNETKTNKPKQEAVEPLALL
VHPQQPLSYLERLIQAEIPPLRVKDHEKLPEIIFRAEADYTGGNKSDEKR
GESNGNGSNVASYSGLGREGPSKGDTHWVRWSGSTEIGDFIRDAARGREF
SVTIEGHGEELRVAVPSFKDRTYYMRMRLRQMSQEIDQMATVKRECDLLA
HKGAHALAKGGFAALAGWWGIVYYVTFHTDMGWDLVEPITYLAGLASIMG
GYLWFLFISRDLSYKAAMNVTVSRRQNALYQERGFDPAKWDQLVHDANGL
RREIKFAATEYGVEWDEMKDLGGEEVKEALEEEKSDRERKREQEEEDDDG
DDDDQGQGHDQGQERKRKRSESSKTS
>Mb|QLI65737.1
MMRNVATRASRRVLPTIRPRTTQYRSTAIRAIFTTAVMSAPVGNPFSHAI
PAADSFQLLPESQKTGEAEDALYEAQIKEVEAWWSSPRYSGIKRPYSAAD
VVTKRGSQKISYPSSVMAAKLFNLIQERQAKGEPVHTMGAIDPIQMTQQA
AHQEVLYISGWACSSLLTTTNEVSPDFGDYPYNTVPNQVQRLAKAQSMHD
RKQWDARRKMTSEERAKTPYIDYLRPIIADGDTGHGGLSAVLKLAKLFAE
NGAAAVHFEDQLHGGKKCGHLAGKVIVPIGEHINRLNAARFQWDVMGCEN
LVIARTDSESGKLLSSAIDVRDHEFILGVADPSIEPLAETIQSMEAKGAS
GSQIDIFEAKWVKDTKLVTFDEAAVAHMQKEGVSREKINEYLNATAKDRD
MGITRRRALASHYSKTPVYFNWDVPRTREGFYHFRAGMEAATKRALAFAP
YSDLLWVETGDPNVEVAAKLGRAVRQQYPNKGLVYNLSPSFNWMAHGFTP
ETLKSFIWDIAKEGFVLQLVSLAGLHSTATISCELARNFKTDGMKAYVEL
VQRREKDLGCDVLTHQKWSGASYIDGMLGAIQSGSSSSRSMGEGNTEGQF
N
>Mb|QLI65738.1
MESSPIVKAHDHAAAAAVATRQSADTTVAINEHTLAAGEFANAARNTTSV
EALRTLKLLEEHHRRLAELLNLQPDPVSQTSEEDVNEKTSLDEKGASTTH
QSGKKLPSLTQKGFTAREMSSSIASNLASARGIRSKYRGQPLSPSVSNDQ
APGNVDPHPRRDGSRTKMQSIIDTQPGKPTWVPPMAPHKTEPRTNKSAAS
SPRIDQTTASAMASEDAGYARFYNSFGSLINKISAPLAFAGLPLIQEEPT
PEQPTTEQPVASSPPKRRASRQPPTSLALDPDLSKIYSKATMRALAREGH
STNDSFYVVPTTGHTMSYANILTYAEKEKRRLGASLHSNDPEDEDDDFVD
ARETPVPVSPGARRRIGLAHKTERDLRNTIEELSLQNRSLKDTLDVVSKR
LHAFEASSQNSAMALAESYRILRPGSPSAAPHAPNAAGDEALRKKTLNME
EQLAAATKQMERLEKDNRKMQKTLDKYREKWEVLKAGAKARREGKSGEGE
DV
>Mb|QLI65741.1
MDETMADDNLRARLDKLQNILGKKITLDLSGRSDRVIPGNRETQVPSWED
EHFDLKMRQYETSNDHDAETATYMANPIKSPGTYKDIDSATLKIGQSVDE
EISFCPWHAVVHYPARFIGKANKPRAQPFFDRVLEGKTWDFFYLHNPAEP
DQKPCLLVPTAQFVGFLKGVNQILATKLVIPGGPNKRLFDLHFGVGGTPR
PRYLLRSRDQKKLHIDEWPAANEEDLRVYKDAADVHRELWLSEWEKTKQH
SFPDNRGGSYRAEKNREARLQMLSQTQLFLGLQGARDPPGKPVTFICVDI
EAIEVAPNPVSEIGIAVVNSERLKGIAAGPSGANWWDFIEAHHIRVKEYA
GLVNYRFVQGCPNNFDFGTSVFPRRAELVDTLNTTFAKYANGAHDVVLVG
HDIKSDIKYLSTIGFPIDQLPGLIGNVDTQAIYRAWKDGTKSPSLGSVLV
DLDIRYKNLHNAGNDAVYTLRAMVAIAVVGLHSAQDAQLLEPS
>Mb|QLI65744.1
MDSAVDMNDASKVLDLSRIRFQLIRLEDTITFHLIERVQFALNKTIYTPG
AIKIPNSNLSFFDWYFSEQEKLQSLIRRYESPDEYPFFPDAVQTPILKPL

NYPKILHPNDVNVNDKIKKFYIEKFLPAVCPDFGRRDRGESQENYGSAAT
SDFACLQALSRRIHFGKFVAESKYRSDPETYKKLILANDRAGIAESITNK
AVEKSVLARLRLKALTYGKDPSVSDDSEEHGKIDVDAVVAMYEDFVIPLT
KEVEVEYLMQRLEGKD
>Mb|QLI65745.1
MASKKTKAAASAADDNLDELFQGIGDDAKSKKPTNPKSTKTASSASKTQG
DDDILADLESQLASEKSASRPHTPRGVRRPAGTPPVGDDKATAAAAAAPS
VRKSTDSARSLRANLTPSATSSEPHEAEKKGAAEQSQQQQSGGGWWGGIL
STATGVMKQAENAYKEIQQNEEAKKWADQVRGLSGGIDVGALRNYGDELR
TRALPTFTNILHTLAPPISSHERLMIHITHDLVGYPSLDPLVHGVFARVM
SQVEGGDLLVVQRGRESSSGRYSDGSAGWRDGPWWRQTDSTRELGLVKGM
IEGTKLCRAGAESYANEYFASHGGIEQAKTRATEDLSESNPVRTSDIFLS
VQAISTDGDKALFAGSASAVKEKETSAVKEQDDTDELVCFAVYVLDPVHE
IEFSTVSQSIPSKWVRWLDAASPLTPRTSDENQDNPFDAGIPEDIKDIIE
SGGVDPREWVAEWIEETLCLSVGVVAQRYVARRMGVGEGGLGRGKKRVDE
LIEANAGEVARAGII
>Mb|QLI65746.1
MAVTDQDHDQDNFSSISWTEHDTAVNSVAGQHGHHDGEASGARRSGRELD
PHGLGSEKLECTVGSPLKENDGSKDAFISYLITTHSTFQSFQKETTTVRR
RFTDFVFLYKQLMRDYPATAVPPLPDKQRMEYVRGDRFGPDFTLRRAHSF
QRFLTRLSLHPILRRAPILHSFLESPDWNATMRSRSARTSLSTDPSNSSG
VFDNFADTFINAFTKLHKPDRRFIEVKDKSDKLDEDLGNIEKIVARVVRR
EADLETDLKDLAEQFQKLITLEPGVEPEVHAFAASIQDTAAHLHTLRDVT
DQDYLGSLRDMQAYSLSLKNLLRAREQKQLDYEQLTEYLNKSTAERDSLQ
SGYASSGAGSFIRAKIEDVRGVDHEQARRERQRKLELRVEELTTEVERAR
ATSDMFDDEVVREVADFERIKRAEFKTQLGGLADAHVGFYGEVVDLWERY
IKEMDQESVLAA
>Mb|QLI65747.1
MSSASRTLASRLVTVPAAACRRVQKGRAIRMESFTSKFPTRSRSAPRVIP
LSVPAHRSFSTTTPTRHGHIDPPKPGEELYVTFVEKDGEEHKFAVSEGDN
LLDIAQANDLEMEGACGGSCACSTCHVIVTNEEFFDKMPEPEDDENDMLD
LAFGLTETSRLGCQVKMTKELDGLVVKLPSMTRNLQASDFQ
>Mb|QLI65748.1
MGRPSHHREDSSSSKSHRPAQPSSLRQSHAASSRDSSQDSGPAFRQAVPS
RDDAARGDATVGGNTDESTPLISDGGSTERRRSSAVHPGVCNHGTFSPRP
MTPDEVFPIARDDIVTTGPDGLFDNSSSQESIDEGWKKWFSGRIRTKRMG
ETRELAHRAGFRDTPLMYLSYYIPCLNWMQQYKWSYLQGDLVSAITIASF
YLPMALSLASNLAHVPPIHGLYAFVFQPFIYALLGSSSQMVVGPEAAGSL
LVGTVVQQSVDSGGSAEDNDLVHAQICGVVAGMAGATVLIAGIARLGFLD
SVLSRPFLRGFISAIGFVIAVDQLIPELGLNDLASDDGASHGSTVEKIAF
IVENISEAHHLTFAVAGISFLVIMIFREFKKRLEPRFPSVVFVPDRFLVV
VVSAILCWHFRWDKQGVEVLGAVKAANGGVFAFRWPFQMAHMKHIRSAMS
TSFLIALLGFFESSVAAKSLGGNDAIQGIELSANREMIALGTANLVGACF
MSLPAFGGYGRSKVNKTTGGKSPVSSILLSLISLLSILFLLPYFYFLPKP
VLCSMISVVAYSLIEEAPHDIAFFVTIRGWTELGLMAVIFLSTIFYSLTL
GMAMGVGISILLVIKHSTRPRIQILGRIPGTNRFENAESGRACLEFIEGC
LIVKIPEPLTFANTGELKARLRRLELYGTSKVHPALPRLRSQESNRNVIF
DIHGVTSMDGSGTQVLEEIVRNYRERGVRIFFSRGPSRRDHAVWKLLERS
GIVALSGGESHFVNDVEEALRWTEYEDSIGVRSGGSG
>Mb|QLI65749.1
MTTTVNLDNDAIVQPGLVDGENKGHPYSPSKGSMDAHVRKASLQHHDVML
DDDEYEGKPTDEEMQTLRRVSGKIMWSMWTIAFVELCERFSYYGSAVLYT
NFVNKPLPVGSTTGAPLDPNGQPGALGMGTKAAQGISLFNQFFAYLMPLL
GAWIADARMGRFWTLHLAIGISTIAHAILVAASAPGVIVNSKSSFAAFII
GLICLCVGTGFFKANVSPLLAEQNDDVRPRIEVRKGERVIVDPAVTNTRI
FLYFYLCINIGSLAGQIGMVYVEKYVGFWLAFLIPTGLFLLAPLVLWSQK
KSYKLKPPTGSLLSKFIRMANFARKRSGLKNFSWEVAKPSRVPLEERPSW
MTYDDAWVDEVRRGLMACKVFLFLPIFFLSYNQMTANLTTQASTMERHGV

PNDIIQNLNPISIVIMIPIIDHLLYPGLRKLGVAFTPIKRMTTGFLIASL
SMVASAVMQYYIYEMSPCGWHANTEDCPPAPINVWAQSLPYILIGIAEIF
ANVTSYEYAFSKAPENMKSLVMSINLFMSAVSAAIGQAFVPLSGDPLLVW
NYTVIAVIAVIGGIVFWFCFRHLDSEEDKWNMLKKSEFIGKQQPGVGKAH
EAGEAA
>Mb|QLI65750.1
MGKSRRNRAGTSQRRDPLAKPVKAPSDPELAALREAKIIPVINDLQNPDP
KSRSAAAVAVANIIQDTKCRKLLLREQIVHIILTQTLTDAALQSRAAGWC
ILQGLVLEEEDDFCVHLFRSDILPAIAHAAKNVSDKLSSLGAGSSEFSPP
EMPLVFGISASLITLLTTLAEAADDILEAISSNTTIIDFLFVLVAQDVLV
AQTNTDDAESDGISSLRDVALACLMLLTEENGNLAKKIVTNSKCYQAMSS
LKNHVTGEGVLACATLHNVFSALEGLKDAPNVTSADDSLLIPTLTKAIAT
IQPDQQAANGSQGRSNPLKMQRLALETLASIGTTLNSANVGTPVPAKPDA
EEPKDDEDMNDADAGGSNAQDEVEEEEEDDDDDEMDQDAMEADMEMVTGV
DDDDDKANIDDMPVLKALLQTALPELIRISSFQPTNNDSLQLHGLALSAL
NNVAWSVSLVDFSVDQNAGIQKAWAPVGRTLWEQVITPILSSDTADVNLA
TQVTGLAWAVSRSLGGSTPLKGDEHRKFISLYQATKSMDPQDSEDPFQRL
GVKCVGVLGQLAADPAPASLNREIGTFLITLLAGLPDTPAADAVEALNQV
FDIYSSEGFAYDGEVFWKGNFLNQLEAVLPKARAMVKSVDKKTQPELRAR
ADEAVMNLGRFLAYKKKKKPDDVNGS
>Mb|QLI65752.1
MAYEPRGEHGGGGQDGTSMKARGRRPVTDYSSTVTHWLRNRVPNYKGSYN
GEAERPSASYIVDMLPPAARPTNAADTIPIKHLHSSLNKIKHPINVVRWT
PEGRRLLTASTSGEFTLWNGTGFNFETIMQAHDSAIRALEYSHSDDWLIS
GDHDGLIKYWQPNFNNVQSINAHSDPIRDLAFSPNDSKFVSASDDSTLKI
FDFALGQMESKLEGHGWDAKSVDWHPTKGLLVSGSKDHLVKLWDPRTTRC
LTTLHGHKSTITKVMFERVRGACLATSARDQTARVFDLRMMRDICLLKGH
EKDISTLTFHPVHANLLTTGGMDGALFHYLLDSPNPPAGQPLTVAPYDSM
DPSTTPAQSVWPMHRVPFAHDYAVWSLDWHPLGHILASGSNDRITRFWTR
ARPGDADVSQDRYHIGEAAAEAQGTWDRRGNRRQRQEEEQQEMEDEMDAL
VDQDATKLPAPSSLPGIPGLPLGGGSALPGLTAGLPPPPPIPGVGSSGPP
PPPFPLPGLNGAPPPPLPGLDPNHPPDPAQLLEMMKKAGVPLPPPGALPP
GLIPPMGAIPPPGNFPFPVPPPPMHTEENDTRRRAPLPSQEESLRQEQRQ
GKYTRAR
>Mb|QLI65753.1
MPLPGSDTEVKASSEAAENFASHYYQAINNRSTLLPFYINSSPRYSIAAD
ISINGSVVATPADYTTLLEAQGQGVRYEIESLDAHVINPSSKYGAPDNIH
DNNKVEKNGSRMTIVVTTMGRVQFGKGREAPRKMFNETFVLVPNWDSMAR
NPPRGVKRWLIMSQNFRAL
>Mb|QLI65754.1
MAANMQQMAGAGQMMPQQLRRPTTNPHQIQQLVYQKLLQHSQPPNGLTWQ
GNVSLNDRMGKTMDLISNIALGVANLDHVQAAEVGCEFERKTFMDSSSKE
GYDQAMANKTLDFFKKRQANEPNIQNNLNANAQAQAQAMMNMQAAQMGRG
MGQVPQGFQHLQHQVPPGQLPQQAQQHSQLQQQQIGMGRAMGPTQQAMAM
QNRQPQQFPNDMSRLAQSDRAKVMDLAVKMMAQASEQQKASTRMSLQQRL
SPQQLAEFQAQGKDPLVLFFQNQAFQVLKNNMNRLQAQQQAGAQNQNGAQ
AATAVMMQQQHSQQSFQQRQNMMNAGQPNDFSQFTPNMESIKDQQMTGLM
AQQAGQMVVPASSGGAQHATPQPGNQNMPGAQGQNQTPRQGQQQQPPQGS
VSLQQMKMNQAAQQSQNQLQMKMQQQQNPQNGMTGGMPPSQSPAMNTLNT
PVSRPPGGMNPMGGQGMGQGNVAFGDQRFNQGTQRPNNQAFNNMLANMSP
EQRQAIQGFPPDKLNEFLRQWQSQRQEQVPMNAGQMSQQHRPQNQFAGQM
NLNMGAVQGAQNLQQPNGGVPINQPQQPQQQMQMPRIPIQNNQAQAMMDA
MDLPPHVQNIVGQLPMDVKKWRDLKIWLSQNSTLAPTTRNQLGALQQRQF
QILMQRRASMQQQAGQTPNVNAATMPTAGGQMPNHQQMGMQRVVGNVNLP
NHVLQVTPQELMQVRNQRSNFSHLPDDQLRAMVLHLKRQAFLQQQQQQQQ
QQQQQQQQQQQQQQQQQQAQQMRNQQPQGQTPNQTPNQAQQQAGGAQNRL
GVAPVPVQQTPQPPNRTSQSQTQPSPATMAQTASMQPTGQRQQGQVVNAQ
ARMNSQTQPTPKSLKRPSTDDAVDTATTSNTSTPAPTRPISQPNQSTQKA

PHTLSPQQIAGLSPEQRARYEQFMKLQLTQGANVGSQPTADALARLRAIS
QEEQRQFSQESMSEMSMNPQELGETGVKLQRIAQDMNKVSRGLTKWYSIT
RDDARAKMFFRTRLRIMKQFSDGEAMKVLKDKFSINATEIDKARAMLESM
AKDLAASMYGRGMMKPGQQAPQGQGAGQNQIAQSQQPQSQQQAQQQNQQS
QPAPLNAANLEKNSQALKNQQKSASKGGASVPPAPTATQPPFSFGASSPH
GNPSYLGKPKDVMNLQLPASRKKQKVSGQQPGQTPQGATPSPKTAKNASP
EMRRQEPPKPVFLCKEPECETGSFGFPSEQALQQHVEEEHTKPREDPVKF
VKENLALALGLEPDGSLKKNQKPADGATAMSLSTSKQGQTPKNVAATPIS
QDHAMKRTGSALSKSADGKSKSDAPGSKQATPSAWAGCTIDPQALLSNLG
FENGLPTIISDANLYRSLTPKDTPESSKDSGSSEPNSDISEGAALEIDMT
WQTFDTDLLLDLNNASLEGDLSGLDPTLLLDPPSRAPPDWEDIDIDFSKP
FQLDMSMYSMST
>Mb|QLI65758.1
MNPPPEYHNGPLAPTNTPKAVEEWEDDDVITPIDATEQVHICPPTSAPAP
LPSRNTKSVNLRASRLSTPKVRRIRSRQRQKAQNAKAGIRLITDMSVFRR
NNHVTNNMRSPPGRKGKFVDADALRALEGEPSSASVGNWNWLKKGSGKTP
EPATEMRKGRNKDQQLSPEDRPIVIGISLESSDVEGREISPQNPPVDDSR
SPFPVPELSNPHAPKAAKADVQGHKSVWSPDTPDTSYSFGSNNVASSVYS
QFIMPNQIPKEQACPPVPELPPNYKKAAHQRVLSLELGKSRQGDDESGTP
CTLFEEDGVPSPQRHPKSREFDRSPDSAASRSHGWWDHVVTPFVDKKMSF
SSQKYKAESPKSPKSPKFHKGMSRDELWENLDVKEESRSRLSPNVAPARV
QAPIVRAPAPQYDTKHGKNPQSLFTKTQTSAAEPSMRQTPQIVISRDSPS
PSDIPPPYSPPKREQEGKPIRYRAVFPPGHPLHSQFPPTPRPASPGLAGT
MTSQRGQQKGAPNHASAVPTTQTQPITTQPLPVRAVGTFVPQEHAYSAAG
SRHKVERQRRRHEKEDIIARRAGGFWRGRGCMSSGGCFGRTGPEGRKRRR
VWMAIWAGIIVLILLIIILLAVLLTRNHSPAETPSIWVNLTDFPPMPTGVL
TVVGPDNTAARSVCTEPSTLWSCSLPKDQQSSVLPYKANQPTLIMQIQWD
NGTRRSWNVANADAPAPLSRRAFGAASHALSALRRDPVPTEFFPDPAAPK
FREMWFLGETTDNIKSDQKGGEPAPFYISLLRSTNDTVPTPNLSRRGVSP
NIGNTTFKDLIPPPDLEKDGTSAPAVMIPNPVQQPIRLFDRGLPTEHYGF
YTYFKRTIFLKSVNIQNNTDNNIPLDEDGGCRKTEASHLVTWGDTRLHVQ
IWTRALSSNTSSLLKADGSKGIGGTGELVRPGTMPYPVTITQDTHGGDPD
KKLVWDRPIDDRLQVTTTEAQALVNDMGIGGTWVNKRGTGDAKFGGFDGG
SGGCKCEWVNWV
>Mb|QLI65759.1
MQTPILKALPATIVVTARASRAPSLRAFHSTPHRRSGNGSVPGGKPSDDS
ITEHDNEPELQPATETTGGRARNQTPGALKNRLARRRAASALPPVELPSS
FLNNNVSLYSVERLPRLPVALVEDATREKTSRLFQGHTSLSRQKHQTLQS
YFDTALTTLLLRDCELAMDFERSKAHDKGRHWNTSASIQSVVRIWDMILD
SAWHIVDCIYPARQPEYTYKVRPFWWWDIYKHLDQRTQNSRDQLLALTLS
LDERLGHAEQLAYPLSHAVADLPVDTLLSIHHAVKRDLQALPPTVFDAKS
SKRPITILSMSGYGGKSISQAVGEHLAFWNDADLIHLDAQDLSVLVGDYL
GLNWAYSRGAVSMMGFRAAELNGKLAKEPGDNSRLSEEDDHDAEAGIIGL
RASTGSLDDDLQKIRHGEYDCFSKWENLKIDKILDHIIRAPELKAGSQTP
QRPILIHLHDIVELSMTLEGSLLLGRLRTLVDTAWHQGMKIAIVGTSSSE
KSSEEYQNAVSQFAATDWVITRHLEADRVERRTVAKASSGQARFSLQKAD
YFTENICNINRMLRALSPNDSPVVVELSNDNVIAYLHSGNPKFSLLRDSI
LPIPEVYHLACAFRALDDQNPGTGPIGFLERCVMGPLRQQPGQRFLDKDD
SEMDIEADTNHQSREEPKSDGRIGMKLNEYEKRISSGQINRENLRTTFED
VHAPKDTISALKLLTSLALVRPDAFAYGVLSQDRIPGCLLYGPPGTGKTM
LAKAVAKESGANMLEISGATINDKWVGESEKLIRAVFTLAKKLEPCVVFI
DEADSLLANRSMFSNRASHREHINQFLKEWDGMEETNAFIMVATNRPFDL
DDAVLRRLPRKILMDLPLNADRAAILRLLLRDESLDGSVSLDDLARKTPY
YSGSDLKNVCVAAAMAAVEEENEMAAKHEGPEPYHYPERRVLRRDHFEIA
LKQIPASISEDMTSLKLIRRFDEEYGNGRRQKKKASMGFGVLEDKGHANG
SEARIRSTISAE
>Mb|QLI65760.1
MATSLASQLAQIAANSKSTLNFKTQKAAHSKSLIWEPRVAATQSYQTLYT

ICHPGFEELCQLDNRFARFQSTIFSEESQTQDRTQLSASENEELDRQIEA
FLRLAGSKLRLMPAIKSIEWLIRRFRIHEENTALLLMTFLPYHSIPTFAT
LLSILPAKVPHEFRFLDPYIRSLTSPPRSVIVHQTVHHPEFLSSVSEYTL
ESCRKQYHYPALVMFWGGLMTEAVNGMLDRTRSGRASVQSQNTQDLLHRL
GPIFAESLVMKKVPGLQIASYMAMTVFVSKGNLEDNAVTAFMQQTVYGWT
NETIRPGLVCLVIMAQFRSAKQMSNKVTKALMKVQDIGPMLVEIGKEHRV
DKLANGLCLALIERFSKKGDSRGLPTIMAILGSTILKEKQIAVIFKSLLL
AALKLDDNNDEDGALRRELGSALISLSQTTGKSGEIIQSVIQDVEFDIEE
LEMKLDLSFRTRNPPALDENTTTDQLESVKKPRDLSGAVDELATRKESLS
PCLLPEPNELFEEFSHLFFSIVSETSQQSDMMAKFDGIPQLGREAAFKDS
TYFSFFIRIWSGPYPALARMAALNMVKNRLKSSDASKMDVQALIPYAVTA
LSDPSKRVRQAAAELIAVVTDIYTPPISAKSMMTWDSGLLYGEAKDKKPL
PVDVTAKMLHLQLLPSIEECVMDPEHITSILKSVIEQGKYQVAPQPALDK
KDHMGQSARLEILSFFASHAQLTPLILVKNRLLKSLNEVKGVGNTPRTRM
LLPLLNWWTSLSESEIRRRCTAEKQDILTLDSRFVDVVVPNDTEGLEFLF
KLLKEPAQREKADLTRAIFGRLRKMWPSMKDPVKFMVAEQLLEISQESPS
SSDDESSIVSIEAAELLRAVPLTTDILSFFLDSIQTGTKMITEPPPNKRR
RTSSADVSRSLITHVTPELSQALRKVTFVLQLVDNSDPVSHPELLDGLFT
ALSELQHFRTVVGSELGYLQNLILRSLLAMMPTYKADKALTIDSSGGYGD
LLVNCIQKSSSPVVQNAALLLVANLATTAPNLVLHSVMPIFTFMGTSVLR
QSDDYSAHVVSQTIKEVVPPLIASLRHGKNGPVAGASDILVSFTTAYEHI
PSHRRQGLFVALVETLGPRDFLFALISMLVDRYDTSEALLSFIVDLLNNF
DIKTQLETFVKVVELVSDLFKPKPGMSFVLLGITDDGDKDVEKVALRQLS
ALPSFLANKKLRSQIGKLADREDMDTAEVREFYATLLENILILADTVKTN
RTLHGRCGSALSNLLNLLSIGEFIKAVENLLDRPDLGLRQKVLRALEVRV
ETESNTDPASRNALLSFLPQLTAAIRESSDIRYKHTAVTCVDKIAEKYGK
KDVEAVVAAAETIAGQHCLGQEEQRLRVMALLCLTSLVDVLQDAVVPVLP
IAIPEAVKYLEASLEGENADGELHSACYGFISSLAEHLPYMLSTYLDRIL
EVSSRSAEAGLDGEVNESRTSCLEFLAKKLEAKEVFTALERNWEKATDAG
FSAIHEFFTVLSTAIDKHNKSSITKNISVLSNILVKAFDLRRQVDAKGEK
SEASLREVADIETSLNEAALKMIYKLNDAAFRPLFVQIVEWTSSNLPKSD
KSGRNLRQYSVYGFLNAFFGNLKSIVTNYATYVIDDAAKILGAVNPKSVE
DRQLWSRVLQVLATCFEHDQDDFWQAPSHFGAIAPVLMDQFLQAGSVDVS
ERLIPAVVELAAAADSQAHQKEINTALLKHLKSEQTTVRLAAVKCQQALT
DRLGEEWLLMLHEMLPRISELQEDDDEVVERETHRWIVKIEAVMGESLDS
MLQ
>Mb|QLI65761.1
MRAALFAGLVGLASLSYAGHAPRDYKDNDYYVIQIDSSVPPEEIASRLHL
RHEGTVGALSDHHVFRSRKTEHDVVRRELLESKRKKRTLEEYDVLDNIRF
SAKQELRRHLHKRVIPPPPPGAHIPRAVADPALSAVKKQQTIMDTLSIKD
PIFTAQWHLFNSVEVGNDVNVTGVWMEGITGKNATVAIVDDGLDMHSEDL
RENYFAEGSYDFNDHDPEPAPVLSDDHHGTRCAGEVAAVRNDVCGIGVAY
ESKVAGIRILSAVISDEDEAEALMYKNDKNQIYSCSWGPSDDGRTMEAPS
VLIRRAMLKSIQEGRNKLGSIFVFASGNGAKSGDNCNFDGYTNSIFSITV
GAVSRDNQQTYYSEPCSAQLAVTYSSGGSTSDGFIHTTDVGSNRCTDRHG
GTSAAAPLAAGIFALVLEVDPELSWRDMQYLVMDTAKPFSAPGVVWNQTG
IGKQFSHAFGYGKIDTYDLVQKAKTWKKVKPQAWFFSPRLEVNGAIPEGP
TGISANFTVTKDMLKEANLERLEHVTVFMNVNHTRRGDISVDLISPSNMV
SQIATTRSGDEHYAGYVNWTFMSVAHWGESGVGKWTLVVRDTEQNKHNGS
FVDWRLKLWGESIDAKKATELGMPTEDEYVDLPVTQSPTPTASPTPTATA
SPTTTATATSTVPPSWFPGFGSKTAMLWVIGAAVLIVLFCIGLCIYLFIA
RKRRNTPRDDYEFELLDEEEADGLNSNEKGGEGRRTRGGELYDAFAGASD
DEEEFDEYRDPSTERLAGNGYEEDQYVVGEESDDEGSSGAAETMPLGGGS
RS
>Mb|QLI65763.1
MAPRSEKSQLKRQRGSLPEDATESKKPRRSDRLSSQTVGAVGAAAAAAAD
KTPIINKQHLPSPVTHVAAEESSDANKEATPTPSAGKHEDVTPRKTDEAF
PQSQALSSPPQDTQPLSQFVDRHPALSDDVEDEVKEGVWGYLVPLDPKYG

DKPLVLKRRNACPQPDSVDAAVNGEKTPLNTNKPAPIRAEEIFERKNAKG
AASGGYLIGRHPECDIVVNEGIVSNRHCLLFTENNGTDTVAIVEDLSSNG
TFVNEAIVGRNQRRELEDQDEIAVHGKARFIFRYPQSRRTSAFSQQYALL
EKLGKGHFAEVYLCIERSTGQRYAVKIFTKPPGMEDKSKTEGLQQEIGVL
MGVSHPNVLCLKDTFNERDRVYLVLELASEGELFNFIVMKQKLSEDETRK
LFLQLFQGIKYLHERNIVHRDIKPENILLVDKNLHVKLADFGLAKIIGEE
SFTTTLCGTPSYVAPEILADSKQRKYTKAVDIWSLGVVLYICLCGFPPFS
DELYSRDFPFTLSQQIRSGRFDYPSPYWDSVGDPALDLIDSMLIVDPERR
FTVEQCLAHPWMTQNSPGVNDSTGGLVGGIAGLEVNRRAPARERTLLATL
NSVQVTAQVAVGDDKAPVKVFSKNQQRITNASKEKAPDHHRAAGEFMEMG
GKGDQPLFGDDSGSIYATATRTGQKGKAHGR
>Mb|QLI65764.1
MAASTKFLREYKLVVVGGGGVGKSCLTIQLIQSHFVDEYDPTIEDSYRKQ
CVIDDEVALLDVLDTAGQEEYSAMREQYMRTGEGFLLVYSITSRQSFEEI
TTFQQQILRVKDKDYFPMVVVGNKCDLEGEREVTRQEGEALARSFNCKFI
ETSAKSRINVDKAFYDIVREIRRYNREMQGYSTGSGGGSGVNGPPKPMDM
EDGEKEAGCCGKCVIM
>Mb|QLI65765.1
MPPPPHQKPENVLKRAHELIGVNQAPAALNLLHEHVTSKRSRNVPIASLE
PVMLLLVELSVEQKKGKLAKDALYQYKNISQNTNIGTIELVLKKFIELAV
EKVTAAQQKADEVQSSIDATTTTNVDDLEATETPESILLATVSGEQSRDR
TDRAIVTPWLKFLWEAYRTVLDILRNNARLEVLYQSTAMQAFDFCLKYTR
KTEFRRLCELLRNHVQTAAKYSSQMHAINLSDPDTLQRHLETRFQQLNVA
VELELWQEAFRSVEDIHTLLSLSKRPPKNVMMANYYEKLTRIFLVGENYL
FHAAAWSRYYTLLRQSSFMVASGQGKKADNPPASDADLQKAASFVLLSAL
AIPVISTSRSRGAMVDFDEARKNKNSRLTHLLGMTQAPTRNRLFRDALSK
SLLQRARPEIRDLFNILEVDFHPLSICQKISPILTKIGEDAEMERYIVPL
QQVILTRLFQQLSQVYETVDLSFVETLAQFPEPYQVTRGTIEKFIMNGNK
KGDLAIRMDHATGVLSFDNDVFSSAKAGSAGSAESETGTVQRLQSTPSDI
VRSQLTRLARSLYTTCHYVDPNFNKERVAARQAALQRAKAGTEQERQDIL
ARKETIQKRKEEASELQARREKENARQKRLREQALQEAEDKRLAAEQKDR
EAKRLQAERDRVRKDELKKQIAELKMSDKVLDIDLDDLDNLDTNRLRAMK
LAQLEREKNDVNEKLRITGKRFDHLERAFRKEEVKKLSEDYAEQVEVDRA
IYEKVKVKTLKEAEEKHKESVELKHRLTRLVSYYEDFRSSLHERRRDEFE
KRRRDAERELEKQIAARKKEFRDRKLREKREREEKERELREAEERAAREK
EEQRQREEARKAELAKLKEQRDKERQEMLEKAALQQRREEEALARRKAEK
GAAAAPSAPSAPLQTETPEGRKPPVIGSGSWREREASKNAAAAGAASGSE
SRPGPAMERTDSNDRSSGPPRLQLAGSGNKPSWRDRQAAKGDAPSSSDNT
APPATSRGFGGPRGAPPPMDRGGSNRGDSDRTSSPAPQSESLPPRTAGKW
VPPHMRNKA
>Mb|QLI65766.1
MASSRALALALLALLKACNANDRGSTPTPTVTTAPIFIPYYSEEQWSAVR
GSIVSMNQTATQTTYTIFCPTATQVACDLSLEFPFIIVEGPSTLKFHGTL
TSTYIADLECNMKGTTAATCSGYSSYCSGYTNGKYTGPTEVSWTSTLTGS
NIHWGTLTLADKPSETDDSLEVTATNIAVPTVPSSQTYNSTPTQTGGGDN
LHSSRSWAPTTALLSIFASVVIM
>Mb|QLI65767.1
MSLTAIPYNRVQTIGNILFATRGGKIHSFNLSDHKHISTWQHPDLERHSI
AGDSQNEPKSETKTPTNAENVPETEQSEPPAKRQKVAEGDLNISAGDATA
TKEEIVQKKVKGGKGKGKSDNHGDKSRMGRVVDRPLITLLTCTDDGKHLI
AVSGHDKSVWVFEHDGEGQITQLSQRVMPKRPSAITTGPGSQIIVADKFG
DVYSLPLIMTPLSPSSLALRAAPAPKPSSTEPAANTFTVHSKRNLEALEH
QRKQLEFERSKGGEAKLEGPIFESHLLLGHVSMLTSLIVTETQGRKYILT
ADRDEHIRVSRYIPQAHIIEGFCLGHKEFISEMTIPLRREDILVSGGGDD
ELFIWDWKAGRVLSKTNLLALAREIAPQTTRIAVSGLTSLVYPEEGGVTY
VLAVCEDIPAIFTWQLTPDNELKNPGIIQLPCNPLHVAIASASNETALKL
VVAMDPGQNVQAKSLHAYTLTRTEHRLSSDVEIPIHGQRSDGPEMQVSES
EIRNMFYAIENLRKQGGGGEDAGTDAVAEGNGEEEASPAEG

>Mb|QLI65768.1
MATNQVSLPPSLEDARIHGLPSAAYYIPDFISEEEEHFILGKIAGAPKPR
WKQLTHRRLQTWPSDLVQNRLLDSPLPEWLENPVVSRILSLSTVKSDGGS
KPGPDLEAEHIFAQSPHRRPNHVLINEYPPGDGAAYWPVVCTVSLGASLC
LNLHRNRDDGALDPVPAWRILQEPRSLLITTDDLYTDYLHGIADIEEDLD
LSADTIVNWSTLRCSQQFADGRNVRQTRTSLTYRDVLKVSRMGNKLGMFS
RR
>Mb|QLI65771.1
MADYERKVLEFYQLPTSYPEMWPPEKDAGEESAEDDHGAKQGRRKSRYEA
LGSAFGGRKSFVGESGQGGIGNLVQKDEADPLGTSDSVVRSLKQLGLPVQ
DDARLRNRFMLSSTTFSPALFISQMHSNADTNTLLQGLDVLSQSIDQKSA
SLKVLVESNFERFVKAKATIDNVYKEMKYRGIDPVQQRQSGRHSRHSSRT
SFGRSSASGATLGLNNPLALPISDNRKKNALIKESEYGVMGIKAPLLDVS
AKAEDVWGPALGGREKEKHLRNVSTYLDEFRDFVELSATVADSIKRKDYD
TLVESYNRARKFADEAKQLSRNLNGQSPSDKQLYMLVLAGRMWNDVDQQV
QAFKKDIWKKLVSLHFSRSESMGGRQQDQHMELIGLLFELGVEDNPIWVW
LLSRHDHLRSKIQGSADRSKVEIEVLRRRLANNEKPAPQILASYLRTLGR
QSIEGKISSSDSTDVIELWDKMLAFLAGLISSQGILGEVVEFWQTAQGFV
SGKTQQSLPVGYNGESRSQHRLSPQAVSDLEKGMVELVELLREHVHAFFV
EIPPEDISLLFSPPLLSPMSPASAGGVNNPLSPTSLRDPRFNFDSNNPPP
PSPRRGESWEKLAFWPPWSNSISGVEYLSKMLSLVGSAAAEMASLSPISS
ADGQVVELLKTLVGMSRDRCVTALCAAWNRDAENIKYVEDWRRPAESGDV
TRMPAIFAAFEGAVLSGMQKILYIPEAMEKPGAGNIVLPPPTKLLQMVRS
QYVTTLYKALSGMVENAERALKKNDDEWEVSQDASPAYVPNSIPSKSILD
AGDRNIRMLLTLSNLQALRSQVVPSLNSQFENAFSVKLTDESKTIRDVLG
QIDARLFQSYTKQSIETLHAIISAGLSLADWEPPVNTRPSTAKPYIYEAL
LALVLVHSQVSTTAPSLTSQVLSFLLEQTSLQLLDAFRKRPRYSLEALMQ
ATLDVEFVAQTLSQYTTDKASELQSQIYQELDGRTDNDARARLQSELPEM
RSILKKLREASKNEFACFKKQKRSGPKVDPGAASSKEQA
>Mb|QLI65772.1
MSYYENAQWPTPSQNNWEHQQGTSTPTRAGASGPQPQDDFAFSYQFDEVD
RAFENLQKSGKGYAMGARRKPTPKASLRSGAPIFTDGRVSHGSGPRSHPM
NNTFEGPQGHNLHNFYASQRHQPSRGSNEAEQVMQAKRRMAAQRERELRN
LHTEQQYQRNALTDAPQHTNTTPVNNNAKHMSEEETRELIARQRSALYGE
GPFADKTSYVDETGNIRPGVPGQSGPSSIRGPSPLAFEAIGRVPSGAEVV
TPGSASDHNATIADPGPRPQSTSSPHNSGPTNRAFDNAVGSQSRTSTSSP
TGGSPPRDFVPGSKPGQSGATVAPIGTRPSGTPSTTTSGKRSTTPLASSS
GGWGRGNGVWGQSSGLGAQASVWG
>Mb|QLI65775.1
MPGYIVTCKDDATEDQIKAAKQHALDQGGKITHEYTLIKGFAVSFPNDAI
TTLESNEHVKAVELDGEVKAF
>Mb|QLI65776.1
MASKVKPGVTSTGIIPVIPPIPLSVPAQGSRGSNPRKDPSEGKNRESVPT
KLDHSLPITHDPNAHQKIVRHRFLSSAEWTVIANGIGGLKDGESHQPTHP
THWLWPSLGMPRGLYREVVAHKYRFYLLFHFTSVLRWMLMVVQLLIGAAL
TALGAMSLVNGTPITILAAVNTAMAGFLALLHNSGLPDRYRNDMAEFEEI
EDHIKEVLNSSIVPAGQTAEQVLAEFFDRYREAKSTVAVNMPANYASKQM
LSPEKQINKGSFNPGPGTGPVPANRNVKGSYANVHSGTDNGAQA
>Mb|QLI65777.1
MSDAQASEAERNIEIWKIKKLIKSLEAARGNGTSMISLIIPPKDQVSRAA
KMLAEEYGTASNIKSRVNRQSVLSAITSTQNRLKLYNKVPPNGLVIYCGE
ILTQEGKERKVNIDFEPFKPINTSLYLCDNKFHTEALAELLESDQKFGFI
IMDGNGALFGTLSGNTRDIVHKFSVDLPKKHGRGGQSALRFARLREEKRH
NYVRKVAELAVQNFITNDKVNVAGIVLAGSADFKNDLNASDMFDNRLQTK
VIKVVDVSYGGENGFNQAIELSSETLGNVKFIQEKRLIGKYFEEISQDTG
KVCYGIDDTLKALELGAVETLIIFENLEITRWVLKDSNGEEVILHLSKQQ
EQANRDKFLDSATGQEKEIIAQESFLEWIAEHYKEFGANLEFVSDRSTEG
NQFVKGFGGIGGILRYKVNFEQLADLSDDDEYYDD

>Mb|QLI65778.1
MASRTLETRFERMTVQDENDHGEGTRLYAKGKTVVASSVAAQQLSLGSSR
TNLFKVALQSQSHNAVAAVTLPSQAAQRKINNPASPTRKPLPSAGSARTS
EEGPEAAVGKASSLAEQPGLPKQFHLGMFEIGRPLGKGKFGRVYLARERT
TGFICALKVLHKSEIQGGGVERQVRREIEIQSNLRHPNILQMFGHFHDSK
RIFLILEFAGKGELYKHLRRENRFPEWKSAQYIAQMASALRYLHRKHVIH
RDIKPENILVGIHGEIKISDFGWSVHAPNNRRKTMCGTLDYLPPEMIKPG
SSDNYYNEKVDLWSLGVLTYEFLVGEAPFEDTPVMTQRRIQRADMSIPSF
VSPEASDLIKRLLVLDPEKRISLEQVQVHPWIVKHCVKGERATNREKGR
>Mb|QLI65779.1
MASDEIIWQIINQQFCSFKLKTDKKQNFCRNEYNVTGLCNRQSCPLANSR
YATVRQHPTKQTLYLYMKTVERAHLPSKMWEKIKLSDNYTKALEQIDERL
IYWPKFLLHKCKQRLTRLTQVQIRMRRIAAEEERLGEKLVPKMAPKIKHR
EEARERKAEAAAKLERTIERELVERLRQGAYGDQPLNVSEDIWKKVLNAM
ERDGQGERDEDLDEGVDSEAEDEEVDSEAEDGDKEIQYVSDIDESEAELD
ELEDWLESEEEDEDEQEDDSEAEEDKVGAKRKRGRVVKMNNKKAKQEKEK
LTLTHDLAW
>Mb|QLI65780.1
MPTSDSAISFGASTSASISTCPSVPKTKEYMDSLVLPSQAKLEHIAQVQE
EKEVEARRRARDEWRRRSASGASDGIHHAASTGHHKHERDEAAKVIQKTF
RGYRARRELQGYSLNASTRWVTAVREAQFRQGIKPHATAASPSAADGDVL
ALEDPVDYRPASARQKWKKVSLVARRAGHDASDSESDSDEGDLEGMTSEE
KAAAKERRLKATAKRRQAARMLGLQYFLEMVDSKHRYGSNLRMYHEEWKK
SDTKENFFYWLDYGEGRFIELDTCPRDRLEREQVRYLSREERQYYLVRVD
AEGRLCWVKNGARIDTTEQFKDSIHGVVPLDDPTPAFAPTASQNLHSQDN
DDDDNDDDSDSSDESKREADRAAKYADPGFDDSKGLKKVTHISASTIFNQ
LLRKSVKKNTWIFVADTSFRLYVGIKDSGAFQHSSFLQGGRISAAGLIKI
KKGRLSSLSPLSGHYRPPASSFRAFVKNLKEEGVDMSHVSISKSYTILVG
LETYLKTRKKGKELVGKLMHGKDKLVAPEEVQRREEEEKDKSESAEKERR
VLEREREEADKVKREMSKSDRVMEKIKSLPVHTK
>Mb|QLI65781.1
MAAQLIMPTLPGDQPQSRHFLPNQHHRPQHARSHSYQVPQGPQISPLDTS
AMPQHQPYSTPPSPKGGSQHFRQGGQPMYMPAVLRPCHEFTSRKVTRCKT
AGSTSSTDSDSTLRRSNTAIMSIPGLSLLGQRLSRRSTDEPSKTLDGEWK
LDAFPEVTGLPSRMHWKPDTESTVCDDPTCKRTFNYFVRRHHCRKCGNIF
CDWHSSAVLPLDQNGSFNPRAGPSRTCNHCFQEVKALVHTRNNSQSSSST
ASDAMPPTPINAPTAPGVTPPHKAEVAASVPRDWNWSTF
>Mb|QLI65785.1
MARNSEKAQSMLFRFREAQAADLGIIDAGRARRPKGITEVDSVPLCEKWR
GQVLKEISRKVSRIHDPVLSDYQIRDLNDEINKLMREKHMWEIQIRNLGG
PNYMRTAGRIYDEEGREIPGGGKGYKYFGRAKELPGVKELFDAAKAKAAR
KEDKPLEDRRDLRKNVDAAYYGFAPGEEDDALLAYEAEKERLAVANLLKN
GPQEPPEGWEALPGDTGDGQVWVLPTMEEVQQELIERRRQKLLDQL
>Mb|QLI65787.1
MFWRFGGYANISTIDTILDKPDFTLEELLDESDLIQELKQHNSKLIEYLR
EDSILEKLLEYVVAPRLEVFATPDDPADDESKGKSRLLPFSRPRASSRAT
DVTDNDEEEQEKRRNRYALVASEVLSSDTWSIYEALTENRDLIRKFWKFL
ERPAPLDPLQASYFTKVNESLFEKKTDDMMELLRSIPNVVAELLRHVECP
MIMDLLLKIIALDRNEGGQGIVEWLFSKDIIPALLSCLNPKHSWVVQTAA
GDFIKAIITISANASQNEQQCIGPNELTRQLVSEPCVEQLIGYMLGGGNP
LTVGVGIVIEVIRKNNSDYDPDMGTESNQVPSSRDPIYLGYLLRLFARNV
PKFMSLIMDGPAKKEGSESTFGEKLEPLGFDRFKTCELMAELLHCSNMGL
LNEVGAQELISTRDAVRQRLRHEGRLGPLREDDQSADDLTMRMSHGAVHD
EGRRFEVTNADDDGFEEVESSKEMSEDTSHEFVKAEDDITAAPPSSFIDK
DEDDFVDEPLSSPRLSVGSDKINEQRFEDPDLVVAPLSPSKSKPAELAES
AKFANPDSIQRTEFDSATREIDEHLTSTQQPTESSLSTSALPEPTPSGSS
LNPSDLGDQHRGLSPHPEDTPAPLFSSASAPQSVALDQTAQKTEIIVHPP
AASHPVHGMESSEQIADVDDPSAALGQQQLPQNINIPLEHVVGDFLKMQF

VEHRVVPAILTFFFSYPWNNFLHNVVYDIVQQVFNGPMDRGYNPTLAVSL
FEAADITTAIINGQQASDESQAKTKTRMGYMGHLTLIAEEVVKFTERHPP
ELLSEMVLDKVMSQEWINYVEGALAETRERDNAILGGVRPEVALGHRASI
GGGNGLGGVGLSGLGGASATGSNALAEAGLNGGIEISEGSNNGIGPFAIS
AGTLMSGFGSSSDEEDDEGDVEEEDVNSEVSGDSFDDRSTGSPEVAANAN
TDSPVDVISISAEPFGAISGDSSVDIRPRLGSPVRFTSTRDYEEDEIMED
GSPQDKWSGNHYDEHGTERSLPS
>Mb|QLI65790.1
MASRALLAMATAFFGLANALGDTDTITWGGDNSRAGYQTNHNMDPSIVGS
SQFGQLFRTKLPGVWVGQPEQIFSQPLVYTPSGGDKQFVYFATTQNNVYK
LDAKTGQIVASRNLHIPFLTADLDGCVDINPTIGITATGVIDPATDTLYL
TAKTYVDQNSGEKAQGRPAGRYYIHALDVNDLSERPNFPVDLEGIIARNT
AERMFQGGIHHQRPGLLHTGDFIYAGFASHCVQYNFTGWIIGWHKTTGKI
VEKWATEGEGVPNTIKGGGIWMSGGGIASDDKGSIFLATGNGYASQLADV
PVNGFNPPTALEEAALHMTINADGTLRLVDFMMPHEKRELDGADKDLGTS
PLEILPSEFSCGAIKRIGVVTGKSGKTYFLNLDNLGGYKNGKDGLDDVIQ
VYENLNSVYAGAGVYPLEGGYIYINVIQHPSIVFKFSCNNGSPSFSKVAE
TPTSNAYILGVSHGTTTSLNGQPGTGLLWITDVQGLNLRVYDAVPQGQTM
NLIQSFNIPAVTKFTRPVFGNGIVYVGTTQGYVYGFGSPVKVPLNCTSPV
DFGGVDMKNSSIAKPVTCKALIGVTVTGIELNDAKNKDFSLSGLPDLPLE
LAVDQTFTVNAQFSPLKVGLQSNDVVVNTTNSVTGFSTGTHARLTGTGHS
AGALLDLTPVILTFKNAVTGEDPNGISESIIASNLGNGILTVQTVLYSTT
GTDGPFQAWNGQGDLVIGKFRFQKLPPTIAPNSAATVNIQFDTSESGTYS
CYVKFVSDGGNRTISVAASSGPPPKALLEFQTPDETGWVKYKPGTPFSFG
NITENNSRSLKFRVTNSAARGAAQLSLTVSKPPFGVAGLIRALNQVDLAE
GTSLGAGESANATLICNVPYAQWNTAPYNGTVQWTMNTNDATLGKQFIQF
FCNAVAEQAPPLLPNGLGQFKYVGCYRDNTPGRQLPNQILASDTMTSADC
IKACHEKGYTFCATQYHRECWGGNTIPLQKVDDANCNYYCMGALKQICGG
NGNNNDGTYMSTFGDTLKWDGNTTQPPSASGPVVNPGVSGYTSIGCYTEA
TDGRALTNGVGTEKKTVKDCVDGCKAVNYIYAGVEYGGECWCGNEFTAGA
VPAPDKDCSMTCNDNSTEYCGGPSRLNVYKLDAGKTASATSALPSVTVKT
TASVPASASSTTTLAGSSTVATSSASTTTSIAEISTVTSTSSTPTPTGPV
KKAKVGNWNFQGCWTEATHGRALTSRAYADDNMTLESCAKFCHDLAYFGV
EYGRECYCGDKLQAGSVRADNQDDCNFLCPGDKTEYCGAGVRLELYKYGA
SSSLLPTSTSASNPLSPGSSTKTQTPVTSVPTTRITTTKLAAATSSLGSS
TSADTAVSSLSTTSLDSTSIVSSSTETSTTSATPTPTGPVISEGNANFTY
YACVSEPSGGRLLKSQVLNTDNMTIGMCLGKCNKYQYAGVEYGQECWCGD
KLNLGGDGNGSPGKNVTDKECSFLCPGNSTEYCGAGLRLSLYSRKQITKR
WNWSIL
>Mb|QLI65792.1
MTSGHDAEPSRQYPPATTPEELDHLVEVIKDWAIGNGLAVRTPPAVISSE
ADPTRITAVPAPVTLFPTAFPRQAFLQGQKAQNAYNELVIDGDDFVRDLW
AVHETVKSEGYTQPLSLGLFRSDYMVHEHKSSESPTAQAKQVEFNTIAAS
FGGLSVHASRLHNFLAKTEYPLLGQPLAQGTLDLPANNTIEGLAGGIEAA
YHAYGDSELGHDKCVIFLVQGGERNVFDQRHLEYQISKSSPTIPVFRLAF
TDIMKHTSIAKSPKRQLLYTLPRNGNRVFEVAVVYMRGGYGPDDYPNQQT
WDARCHLERSHAIKCPTVLTQVAGIKKVQQVLATPRPSSEPSILSKFIKD
DTPSAIALWNTFTNIYPMDTSDVGLEARKKALDPEQCVDYVLKPQREGGG
NNIYGSAIPGFLKTVPEAHWNSYILMELIKAPAENNFILRNGAIEKGRVI
SELGVYGTCLWNQETGEVIRNEQAGWLLRTKGEESQEGGVAAGYGCMDSV
SLT
>Mb|QLI65793.1
MITGAVLVSTSLLGSGFSTKFPHFFVTQGIFFGTGTALLYYPATGAITEW
FNEKRPLALGLAASGSAVGGALWSTYIPKLNNMLPHGAIFLVIGGLAAPL
LFLGCLLIRERKGAAGHDESGDEVRPSQRSIRAAVLEWRFLALTWSLVIL
NTGVLIPFNFLVVYAREKEVSENVAYNLVAYTYLGSIIGRIGSGLLADFF
GCFNVLFWESIIVGVMTLWWIQVHTAAELTAFAVLFGIFSGGLVPMGSAC
VAQTTPDMGHIGLRIGVMMACCAVGALGGAPLSVALLNLAG

>Mb|QLI65794.1
MVSGPQTPASTRTQWIVYAVASGACAAFNGVFAKLTTTELTSTLAHKLAT
VLSLSSHENIVEVAVRAIFFGLNLAFNGIMWTLFTQALAKGTSTTQVSII
NTSTNFMLTALLGLFIFAEALPPLWWAGASLLVAGNVIVGRKDEDRGEDT
PGEVGPEERSDEAIPLLKEDGDEDEDVADLGDLSR
>Mb|QLI65795.1
MASHEEDSMPEETQGYKLSQPKQSLAQYEQMDAGDESLQRYKKSLGLGGG
KDVSDPNDPRVCIIRALTLDSPGRDPVTIDLSTPGSEKSLKDKPFKIKEG
SKFTMSAEFKVQHEILSGLHYVQVVKRKGIKVSKDSEMIGSYAPNTENQP
AYTKKFQEEEAPSGMLARGHYNTFSSFVDDDKKKHLEFEWSFDIAKDW
>Mb|QLI65797.1
MPTATQIVEALLTPSIPVILLGFLIMIGGPILLHFVLASSATYTIAPTVL
LIGPENAGKTSLLTLLERGTNPADTHTTQRTQSVELNATTDAKTKGSFRN
HDDSSGTYTKFLLVDTPGHGKLRNVAMAKLADADKLKAVVFMVDAAALGE
QETLAPTAAYLHDVLLHLQRRAGDKGKNKAAVPVLIAANKMDLFTALPAN
LVKSQLENELTRIRASKSKGLLDSGVGVDEIGSEDQDSWLGEYGSEKFSF
QQMGEFDIDVEVVPGSVTTKEADVDKWWWWMAQRV
>Mb|QLI65798.1
MGLVDYSSSSDDEAPAPPKKRLKGQEHVSDSKMPPLPEAFYDLYASTVRQ
SVVDDPSLHQGRTRLNPHVAGNWPTHLYIEWHPTEVQHETLDALIKKTQQ
ELGDDIQLHSFLNSDLGTDLPLHISLSRPLSLPTSIKDDYLTKVKHSIRS
CGTGVFSVKPAGLAWYKSPDSDRTFFVVRIAPTDANDDSKHLRASTHPEL
MMLLTRCNTVAAHFDQPPLYEQTQGESAATAFHISIGWTLGAPDEETRLR
ALKLFQDKEFEDIHAWNVKVDGVKAKIGNVVTHIPLLGNVKTKEEDDFAD
SLYGS
>Mb|QLI65800.1
MPSLKVAALALLAATGSALPAARRGNLVVRQAQNGTAPTTEVSETELNDF
DILQIALTLEHLEEAYYREGFAKFPDSDFAALGLKPEQIEDLKRIGQTEE
EHVSFLQSTLAQAGVQPVQPCQYEFNVTDAKGMATLGALFENVGVSGYLG
LAKRIKDPAILSAAATIVTIESRHQSSLRVLLGQTAVPQAFDAPLSLKSV
FSIAAPFIKSCPQGSNLAVTPFPALTMEAAQSGEASAMTVGSTVRVAAAS
GAAAATHCAFTSGGVVPGGTAFTPFSESAGCEIPQGVAGVTYLSLASSAP
LDGALTDDITVAGPMILAL
>Mb|QLI65802.1
MAPASPSPRRTPSGRPRGRPLGSTNAARAARQALTVTSATEPPPKRRRYV
PGGPGGGGRFVETDDLEIQVTTSAPRSRTTAARSAINGKSPSVMPRRERS
TRSRASASEELEEMRWGSAAAVAASVKQAEDYKPREERSWEEFHPNLDIE
KTFMVLSADEVDGVARDTPSTPALQTPGTPLANGSMTPSRQINAASTGTT
PNPQGKPDANASDTQAGTPSRRSRRPTREVVSFYNARTSDLAPTPRTPKI
LPIHNQTPKERLDLKLPSYRRTNRIELFESKTFGQARYVDKAMSNVGYQE
SDQFIRPDSTLIKAIDTNAEEDLDLTPVNSNEDHPHHTKLGKVEYDMDEQ
DDMWLEHLNTLRKQNELETITREVFEITITKIEKEWHALEKRIPKPNPKP
PQTHRPRSSSAAAVNGETQSGEEPDSKCAICDDGDCENTNAIVFCDGCNL
AVHQECYGVPFIPEGQWLCRKCQLCGRGIPTCIFCPNTDGAFKQTNSSKW
AHLLCAMWVPEVSLGNHTFMEPVMDVEKVPKTRWKLSCYICRQKMGACIQ
CSNKNCYQAFHVTCARRARLYLKMKTSHGALAVLDGSMVLKAFCDKHCPL
EYSQESNVHQATRSAKKFYKRNMRGRIWADNLAIANVIAAQQRNLLAETP
ESAQGDKEKAASEKKKNEQPPKNMWKLPSGAPIIPQAVFDIVEASIQRFP
FRKRKDFLSEACRYWTLKREARRGAGLLKRLQLQMETFSSMELTRRDFST
MGPNGKARLARRIEFAGSILKDLEQLKDLSEKIVQREQMKVDAAEMEDEF
VNECYFPVAKHLPLAIEKAISLDKDLFTSGLENLRSRVDQRFYVTALTFA
QDLGDVIGTGILNAPSSSSSNSSESRFDATDASPAKSNFSDIRERRKLGK
RILKAVQPLLETALRVESEISNKSYETVQKELENLIDASVDTSRVAITSA
TTKQEEDTIMVDAPDSSEITVKSGFNDEDVDAEGDPMDTAEDAEGQNGGN
IEVNTSGLGITVKSEEPPVSSRKSRRTKSNNSLQPSETPPESEAYVNLAA
PTASTTGVPGPPTPPHSNGSFGKEPSDPLTEGGVLWYFKAMQPRGTSILG
EHWAAGRDAVRMLSEELTDLDDEELKGLRAEVDESVAAGLVNDDAGDGGK
VKGRSRKKRASGRRR

>Mb|QLI65803.1
MAALQQAGGGASQAQRAPPALTNTVISYLKTIFESHADADGRWTLGQIAR
FIQQVQKNSDKTTAAAKLLQSTEIDLSAFLQYMTSEDSNITEPWNQSDLS
WPLSSYFISSSHNTYLSGNQLYSDSTTDAYTNVLLRGCRCVEIDVWDGDE
SDLSASSTESSADEKADDEAVPTKSKRQGTFDRLRQRIPDSLTSKLEKTS
LAKTRELKHTGKHNTDTANGAPPDGDSKIVPEVALVEPRVLHGYTLTKEV
SFRDVCNAIRDSAFTVSDLPLIISLEVHCGAEQQLMMANIMKEVWSGMLV
TEDEIKPGLLPSPEDLKRKILIKVKYAPPDTPAAEPDSSAEDDSSPAEAA
PPKKPSKVVQALSKLGIYTRAVSFKTWTQPEASMPTHIFSLAEKKFIEHR
EKHAQALFDHNRDYLLRAYPSGLRITSSNMMPTVFWGSGAQVVALNWQQT
DEGMMLNEGMFAGTCGYVLKPEGYRPNLKSKPTLNKITYKTLNLSLTFLA
AQSIPLPHGDKSDKKFHPYVKTELHVDACNAPAHGRPGSSESLDTDVRHK
ARTKTHKGTDVDLGRQQVSFNGIDGLVEELTFVRFTVRDDELGRDDLAAW
ACVRLDRLGQGYRFIHLIDSKGHLTNGTILVKVEKTLVEEKPDGKEGA
>Mb|QLI65806.1
MTSEDIKYKHNGANSPDGADVEPALTPISLVNTDSPAGSGFSPLVTVVGF
HHARGPEVETWFGADEGVDPAIKYNWPLLPFMALSDGAHASEEDFSYFTL
LKPKTNTEPATSLFGISLQKAVVVIADSPQSFGMLRERLSIVTQAWFAQR
EFTDTEILRRFQESLADEKARGLVEEDTERDQHLGMSLREFIHEFKWQAL
VLLKCCLLQPKMLFFGSRCDRLCMVQFSLISLIPGLIRNLQDCADPELDS
YEKKLAKPTTLQSSNRNSLLTFMGLPLQIFGKGGFFGPYTPLQQLDILAD
FGTKSYIVGSTNSLLLQQKDRYSDVLINLDEDTVSITSPSLKAALALTPA
DRRWIDFLTHEVNETWDEANPSRPKNMQYIGSEEFIRSQFEAYMLGLISS
VKFRNFLISQGEDPSTLGADSDPAVDFGSEWVEAWKKTENYRIWDAHTDT
NLFAVSEPKHPCAGGLTIDDIQRRVADQIKDLHLDERLAQGRDILGRNLA
AGREKASTMLNKLYSDVEYLRESQRRRAEDSRTQTTNPKSSTEKSQYPVD
LTKAQQTMNSVGAKASAYMSSWATWAGDKRKTGGWASGWGGKKSPSPSTH
NNPPISTSPIDKDYQMVSPPDSRSTSTDDPKRHATHASFSESILSKTSER
PVSGSSEMDDSFQDVMGSPEKVSHKQKDEDVTPKLNNDGFQQEDVMGAKM
SEKGGMDDTSEGKGTEVKASE
>Mb|QLI65807.1
MSGFFEQLPLPTIDRPFGVHLWPIFSKAWEAVVGYPAEEFKFVPFETPMS
TLKSTSIFIVVYYMIIFGGRELMRSREPFKLKALFLIHNFYLTAISGILL
VLFIEQLVPELYHNGVFHAICHYDGGWTQPMVVLYYLNYLTKYLELLDTV
FLFLKKKPLTFLHCYHHGATALLCYTQLIGSTSVSWVPITLNLTVHVVMY
WYYFQSARGIRIWWKEWVTRLQIIQFIIDLGFVYFASYTYFTSTYFPWLP
NAGKCAGEEFAAFSGIAILSSYLVLFISFYFATYKKGGKSTTRKSLRRMS
QAPLPDPLHVQAAAANAKSNGHSNGATTTGAKANGAATRSRKA
>Mb|QLI65808.1
MVSKRKRVLEDFDPNKSDSEDENFDPSEARAPRNPKKVRPSRGPRPAGGR
KRSHRYRGSDIDEDDEILSDSLGEDSFGEDESEDQDAPVNAAGRRTRKAA
RTHHSYRESSADEEAEDVVNDSEDAGQDAQELDEPSARRLVKLKVPKGPS
AGATRRATRASTADVDDFVELSNSGRHAVASRNSRSRSPETYSRTRRSSR
SMKGLHAAPETIDEATQESSNRAADDADEPDELSHDYKPVEKKEDEDEDM
ADTTVVIADDVAEPVKEAEAVPETAAGAGAGAGAGAGDGAGTGAGAGADD
NDDDEDDDVPITRRTRSSRAAADASNEDKAEETHETSTNSRRLRRGRSKK
STQEPSSDFEPGDDSEEEAMSETKGEDIESPTPRGRKDRRSARNSRRGAD
SGDEELDVDLDELNEEARELRQSSRPRRRPRDESPPIVYQESRRARTRVN
YYMPPLTAANIEEEEVADQAPTPARRRRGGASIGWERNLNTTFGPFGGGG
GGGSLLNGPWGTGAAGGVDSDSSDDEMVHRSSVGGNIGMTPTAGAPAGGL
LPGGLGLNPEATPNVGKIKDRKALADADPLGVDMNVDFSKVGGLQGHIDQ
LKEMVQLPLLYPELFAKFHVTPPRGVLFHGPPGTGKTLLARALANSVGSG
GRKISFYMRKGADALSKWVGEAEKQLRLLFEEARKTQPSIIFFDEIDGLA
PVRSSKQEQIHASIVSTLLALMDGMDGRGQVIVIGATNRPDNIDPALRRP
GRFDREFYFPLPDIEARRSILSIHTKDWGLSDPFMASLAEKTKGYGGADL
RALCTEAALNSIQRTYPQIYSSTEKLVVDPNKINIHASDFMLSIKKLIPS
SERSATSGAKPLPRAVEPLLRAQFSEAKRLLDELLPRKKKITALEEAMYE
QYNDEDHGFAREAMQQEFERSRVFRPRFLIYGPHGMGQAYISSAILHHFE

GVHVQNFDLPALLADGRSMEQVIVGLFTEVRRHKPSVIYIPNVDAFYTTL
SGTVAYITFKTMLRSIPPTDPVLLLATAECAKYELPSQLIQDLFGFSRRN
RMEIPRPERDNRMEYFKATLEYIKKKPAEFPDPENRKKRVLEELPVAAPI
AAKPPTKEEVKALKRKDHQWLNALKIQLQPIMDQINRKYKKFRQPVIPQS
HIDYLFAESDPNYVRPDLAGGTIRPYEIVKDKYGNDVLRDTATGKCYYNL
ETTTIEERLSNGFYARPQDFLFDIKALAKDARNIGDKERTLKANELLSNV
EVDIASIEAGNASVDWEGLHRRQVQRAAEAAEKERKRKTMQSILERVQSD
LGGGNDSDSQGPLTLGETVPGSRTTARFQLRSPLSNGHEEAAGQDQSNLS
NGTEGASKEGDVRMSGVDDDTQHATQASVMGPPAKSENDSTKHTGMTQIS
QRSAVTALPPGVSPSAVVNDASTTKTSDPSTHRSSNFSTQLTNGNHGAQQ
SSGDGDQDVELPDTLVVPSQATPSDEVWPHSQAHGLARGQVAPGTTNMSG
SQVSTPSQARHSGDQAGDGLIQPSQEPQSQEPHGLRYSGSSASQPPAVEG
AIENLLHLLTDGTSGCTIEQLEQINRELMDEIWKSRHEWNRHAVVVHLGG
VFNDVISDIEQIQAVGSSSQRDSHLDR
>Mb|QLI65809.1
MAVLGKRKAPASSISEPDANELLRRHFEARFQPLEERLSNTKSEEQADEG
DSDEDSEWGGLSNDDAAGSDEEEGDDAEDLLEGESGDDDTPAIEIIDHST
PQLPKESMSKRELKAFMSSRPPDQSATPNPIQPAVSSNPSTLPEDAPSLL
KQDLELRRLLAESHLLAPHAAAKSLSSASSFAAQPKSFAAGRTRQKATDL
RVQALGSKLSIHKQDKMPMHMRKGMAVAADAREAKRRREAKENGIILERE
TGKQRRTRGRRSDVDRPGVGRLRGAELRISERDVKRIEGNRDVFGRNGRR
>Mb|QLI65810.1
MTETTEAEEAIPIEELTPEEANRIIHSHRKVRYGKQRKVKCDNKQPCENC
VKREHPQLCSYKPNRSSATRNASAAQNGQAKKRARSFSDAHEESRKSERG
DSWPRTIGISTSLDDTESVGDRYLGQNSIPALLREQSSPIEKNVSVDIRG
DMRSILGLDTSAPFPLMSSHHLHRLAQDISAELPSDREVMKLFRTYKEIP
QPFWGFVCDIDDFESKLMVYLEDRSRNASTAMKSSRPVAASWLAILFAVL
AVGSQYHESPYHIRTRDSQRYIQISFHFLRLGNFLLRPSFDSIQALLLTS
FVLLNDMKAEASWALTGLTCRLAQSLGLHRTSSLEGRDSVPPDAKVKEMT
RRKLWWTCVWHDTLTSLSFDRTPMTNFPSCDIPISSDTAPGKFTYLEMMY
HLIEIISRRLNPDAIATSTYAQIVDNCEAVENLRRRSLPHMQDKEQCKTA
LHRLQYYAIRLHTSFVISVACRPALKRTCDFDPKQKKELADRCKDNLTQT
VRMFLAMHQLSVIPTRTWAFTYHGLSSALLLGILCETKADPEIRQLQGDL
IAALSATAAKEASSPEPHIAVSDKDIELSGPLWRALTALKNIYEHGTVIG
TLLKREGDSSGGGSGTRTPLPPFMLSSASAASLGAQLRAAQAAGDPREDA
ALAMAEMQNGASLQDFAGITFPQPLPVSMNLDGLPGLSVDQAQLAPMDLY
DSIWGGVDAMSFDFMAQPPPGQPQQQFYF
>Mb|QLI65811.1
MMSARSAAGLGAMRAASRTTCVVGRGFRISTSTRLGLKESSSQTDADYDR
HKRDSLAKQKKGAGHWKPELASDSEEAVKADRASSGREDISRLQERTKRS
AEESSRSGTSVRDGM
>Mb|QLI65812.1
MVGKVSERVLLREGLERTDNGMKLTTWPDVAPINQKNYYTDYMKRDDQVL
ALRLQNDATRDRLVQGARDRDRLLSKPANGELPLPVPDLTEEDGAATPSG
GVDPSRIIVIHPGSQNLRIGFAGDALPRTIPTTLATKFPQTEAEMYEALP
RRQFEAKTTDQQYGEEWSKKYSKMCSELKSEMRANKRKVLPNSKELVQTF
NRRTEPEIIEKHNDPLEIEWTDVKTLEDPDSLASCFIGNDALRVPDDSDP
KFKLWWPLRYGWWNEDEYSSQEHLYDDFETLLDKALRQELGLTTNSEWQQ
YSCVFVIPDLYDKKYVEQILRSCMTWFEFNKVCFVQEGIAATFGAGYTQA
CVVDVGAQKTSVTCVEDGLCIEDSRINMKYGGYDITETFIKMMLYDNFPY
QDINLQRRYDFLLAEELKIKHCTMSQADISVQLYNFHVRAPNQSTRKYTF
KTYDEVILSAMGVFDPSIFDNSAKLRGRRKLVERSYNAYDVEIPDDPTSA
AQLAVLALVKPSIMSAANGPSHSQSQSQLEVSTPSKERSQPFNFLGKGEN
GVNGTPAGSHAGSPAPEGNGTPAPYIFGAGKEALNVDSPAPSGSRATGTP
VPGQQSQYSQAPSGIFVDSNARSATDIAVERDGVLPMAPLDIAILTSIQN
AAKGDDKKLRDLLGSIMVIGGGSKIPQFTVVLEEKIKARRPDLYDRILVS
RSARDMDEQVVAWKGASVFAKLSTNDSWITPFEFERLGARTLHHKVLWAW
>Mb|QLI65813.1

MADTGLPPNWEVRHSNSKNLPYYFNSVEKVSRWEPPQGTDTEKLKHYMGA
HHSAGSRPGAVPGVPDGKIRAAHLLVKHKDSRRPSSWRESEITRSKDDAM
EVIKAHEAKIKSGAISLGDLAPTESDCSSARKRGDLGYFGRGDMQKEFED
AAFALKPGEMSGVVETASGLHLIERLE
>Mb|QLI65815.1
MSKRNLLLCFDAFGTLFSPKASVAQQYAQVAHQCGVTSFTQDELQSRLSA
AINQERERNPNYGKDTGLGATRWWTNVIQATFKPFIPHNQALPPALVPKL
IHRFASNEGYAAESSLLSTLRAFRQRKARQGFEQVIVGVVTNSDDRVPSI
LSSFGLNVSPLRYGTPGGPPVSPEEEYDIDFHCMSYDAGVEKPDRRIFQA
AELMLSRVIASRAGQTPTPAELASWQKIYVGDEYAKDVVGATNAGWNPVL
LDVEGRAMEIPKLEDCPAKSVNALFTEHEVVRVRSLRDLAAWMTGVE
>Mb|QLI65817.1
MAAVYKSLSKTDGQKPESANNGVKKNKQRVLILSSRGVTYRHRHLLNDIA
SMLPHGRKDVKFDSKSKLNELNELAELYNCNNVLFFEARKGKDLYMWLSK
IPNGPAIKLHLQNLHTMEELHFTGNCLKGSRPILSFDAAFDSQAHLRVIK
ELFLHTFGVPQGARKSKPFVDHVMGFSFADGKIWVRNYQTSEEEGSMVLS
GDDDKGKKPKGGATSRAGNTEIKLIEIGPRFVLTPIVIQEGSFGGPIIYE
NREFISPNQLRSDLRKAKASRHNARVEQSMDRLSRKGELGLRSSSGQKRT
KDELSNKSLFA
>Mb|QLI65819.1
MSKAKGATGAVDQVVKLIVGAGQASPSPPVGPALGSKGVKSMDFCKEFNA
RTAHITPGTPMPCRVTVRPDRSFHFDLRTPQTSWLLLNAVEAPRNKKGNR
KGASKPGHETVGTISLKHVYEIAKIKQSELRLSGLSLEGLCRSIIYQARS
IGINVVA
>Mb|QLI65820.1
MSFFGQPKPMGSGGFFGQSQATQQAQPAQQTGSVFGQTMGSQQPGALGNS
MMAQQQQVQQMPALAQSQAQLSSSLWQPGKETPHQKPILDQIKLVTEKWD
PANPSCVFKHYFYNKVDEAHIPFYKPQPHEDPREWEEALQNKPAPGFMPV
LCSGYTGVADRLKTQKRAISEFNTRLHQVNGSLDAILQRHELETEVRALA
ARRRQTAISERCLALAAKVQILRNRGYALSGDEDDLKNRLQTLEREVQDP
ALGAREEELWSRLIVLRGYSDRLNKELDKPAGQDGEGLDPETEAKAKRVL
EDYEKQLQHLKKELEALGVDFEEWEKSRNASLKSR
>Mb|QLI65821.1
MDLASFVDRLRNGSAAKFPADANTLAFAQKLDSQDQLKHLRDEFVLPTKG
SLKKRALDGSLPGQSASNGINGAGGQPADADKPCLYFVGNSLGAQPRAVR
EYLDAQLETWASIGVNGHFTDMDNSPLTQWQDMAEDCAKKSSDLVGASPH
EIVIMNTLTINLHVMMASFYKPTAKRHKVILEWKPFPSDHYAIESQVIWH
GHDPDKSMVKIEPDDNSLIPTDKILRTIDEHAEETALLLLPGIQYYSGQL
FDMASITAYARARGIVVGWDLAHAAGNVELRLHDWDVDFACWCTYKYINA
GPGSIAGAYVHERHGRVDWAAGGDAVGGGRPSYRPRLAGWYGGDKSVRFN
MDSTFVPTPGAAGYQLSNPSAIDLAALSGALSVFNKTRMRDLRSKALVLT
AYAELLLDQMLDEAGGDDPGAAPLFTVLTPRDPLQRGTQLSVLLREGLLD
RVSRALEDNGAICDKRKPNVIRVAPVPLYTRFEDVWAYMQILRGALGL
>Mb|QLI65822.1
MVRGQPYATAGRNGKNGNAMPPFPTRQMFVLACCRICEPIAFMSIFPYIY
YMIQDFHVTDDPSKISVYAGMVTSAFTLAEFATGVLWGRLSDKIGRKPVL
LLGLLGTALSVLVFGFAPNLPVALFARALGGLLNGNIGVLQTTVAELVTV
KEQQPRAYTIMPMVWCIGSIIGPMIGGALARPCISYPDIFPPGTIWDRYP
YLLPNLFSAATVFVGVIIGFLFLEETHTAKKLQRDAGRELGDRLVDLIAT
VPLCGRLGRPAEKKSLLVDNGVMGYNTTSTGSSGLSEADEPLPVYQSRES
SPNLGPQSDTDPEATSDSGRNELENRPLVIFTKPVIMNIMSYGILAFHTM
TFDQLFPVFLSTEPPINGPTVELPFKFVNGFGLDTKTIGVIMSVQGIYSL
FSNYMIVAPVTTRLGSLRLFRMLAFSYFALYLVTPYLVLLPQNLRMTAIY
ILVIWKCTYATMAYPNNAILLANSAPTKEVLGTINGIAASTASLCRALGP
TVSGFLYAAGLHTGYSGLAWWFSGVVTLVGAYLSSQISENGSQATGVGND
DPLLDERLLDDYDDNDVV
>Mb|QLI65823.1
MASGELASGRAKKLIERFKNNIEGKSTISTNQDANHFLEATDILCRCKTP

AFYVESIISSAKGVETLGSVVRSDLSTGFISSVMTMLMKHLSNPMVKTIN
SGHFLQRILFAVLCPPTFWSAVLKHQQSTGFSVGEVETFAWLCLEIVSMK
SPELDTLYGDVGALMDSQSLIRAASHPVRKLAYRIEKILKLHSIAATATT
GHGPGGRHDNDFLDFRAISIYPTADEVRSMDEPFLQRLDDVFDVPRESRS
ATYLSWLYRLLREDMLADMRDNLAIAWGQRKGRRKPPRLGGLSLVGFGDG
SKTRVNPLALQLQCEDGIVFPRSIDQRDRKTFLEGSGKSFMKNNSIGVLC
RNNDIIAFGCLIRNVDLLLKKTPTVVIKFINVEALKNSLEALLHPMKGEL
QFHVVDTATFAYEPILQRLKEIYEIPLEDMVLDPKSATSLYQPPEKLSQL
LRQLRAALDKQQEVNLSSFGHIGGSIRVSGAQLESLINGLENCVGQIQGP
PGTGKSFIGALISRIILKLTNYRILVLSYTNHALDQFLEDLLDIGVSERD
MVRIGSKSTTRTEPLRLETLCKQPAFRFSFEVNATIRKYKSEASDVNERL
VDVGEKLATAYVDPDDILTLLEFSEDATPFWPAFQVPKQDDGFELMGKNN
KPIRAGEILELWVQGQDSGVVRALLASMDPASRSVWSIPISSRTEFLNQW
CSKVRHDQTQEFASLAEMSSRIQREIDSLYNESKRKVLRSKRVIGCTTTA
AAMYQSIIESAQPDMVLVEEAGEILEAHIITALSSSVKQLVLIGDHKQLR
PKVNNYTLTVEKGEGFNLNMSMFERLIHQGHSYTTLREQHRSHPEISHFV
RRLAYEHLENNPKTLEREKVRGLKGRVIFVHHEHQEEKLADVADRQLGES
KKNAFEAEMVLKMVRYLSQQGYKSKHMVVLTPYLGQLSLLRQKLSRDHDP
WLNDLDSYELTRAGLMTQAAAKVNKQPLRLSTIDNYQGEESDIVIASLTR
SNSSGDIGFMAAPERLVVLMSRARNCAVLFGNMHTFTKSKKGGELWGDFF
QALKEKECLFDGVPVYCERHPDREALLKSANDFDQHCPDGGCSEPCGKPL
SCGKHACDLRCHRAIDHSQFPCNKRVSHTCVRGHKSKILCSTKVEGCKSC
AREDEEIRRRAAQDLKLEIMRQEKQAAYASELQKIGDEIDHYERVLAYKR
EEQTQQAQLEEKKAKLKGLKAAQERREAVESSKKAQARKDQPRTGNSKNS
GSKLKSQAQQEWEWMKEHGEPSNEALNKLMDLIGLETVKEEFLAVKSNID
TKIRQELSLSEERLSCSLLGNPGTGKTTVARLWAEFLACTGALPGDGFKE
TSGSKLANMGVSGCEKLLEDLKDNGGGVLFIDEAYQLSSGNSPGGKAVLD
YLLAEVENLRGKVSFVIAGYDKQMETFFAHNPGFPSRFPIQMRFNDYSDD
ELLQILQRQVDRKYKGRMAIEDGPDGLYFRIAARRIGRGRGKEGFGNARA
VENVLAQMGKRQSTRLRQARRSGAKPDDHFLTKEDIIGPEPSATLVNCKA
WQRLNEMIGLAEVKQEVRVLLDSLTTNYQRELEEEPLMSFSLNRVFIGSP
GTGKTTVAKLYGKILASLGMLSNGEVVVKTPADFIGSVLGQSEAQTKAIL
ASTIGKVLVIDEAYGLQGAGGADGSHVADPYRTAVVDTIVAEVQNVPGED
RCVILLGYKEQMEEMFQNVNPGLSRRFAIDSPFIFQDFDDDALSRILDLK
LKDGGFQATAQAKRVALDILSRERNRPNFGNAGAIDNLLSKAKGGYQKRI
SAGKAKRSQLEAVDFDDNFDRRSATDVEELFAGDIGREKVISLLKNIQDR
FRQLKSLGMDVDEEIPFNFLFRGPPGTGKTMTARKFGKLYYDMGFLSKAE
VVECSATDLIGQYVGHTGPKVHKVLNNALGKVLFIDEAYRLAGHGFAKEA
VDELVDSVTKEKYKGKLIIIILAGYVKDINNLLSANPGMTSRFPETIDFDP
LSAEDCLKLLTSSLAKRKADIERKGKLLDLSCLQYPSEDFERNILDLFRS
LSGVEGWASARDVKQLAKDIFRNVDLSSPSLKVEQDEVSEALNTMLLKRS
KMEQDAAATDLSLDLANLQLREGSRPPLPSQSNISTKSQQEERTNKTDDV
VSTEEPDQDKSACDGLGIRDAGVRDEVWEQLQQDWAEEQRREAEYRELKE
AQKSATDENRERIVRQLIEEEKRRKEEAAMKARLMALGRCPVGYHWIKQS
GGYRCAGGSHYMSDADINAL
>Mb|QLI65825.1
MVFLNMGGPSKTDEVGDFLSRLFSDADLIPLGRLQGYLGPLLSKRRTPMI
QKQYAAIGGGSPIRKWSEYQSAEMCKILDKTSPETAPHKPYVAFRYADPL
TEEMYQQLLADGFGNGKGGRAVAFTQYPQYSCSTTGSSLNELWKWRQRLE
GKRVDESGEGTITWSVIDRWPAHSGLVEAFAQNIKAKLAEYPESRRKDVV
LLFSAHSLPMSVVNRGDPYPGEVAATVYAVMERLRFSNPYRLCWQSQVGP
SAWLGPQTATTVEQYVAKGQKDLVLIPIAFTSDHIETLYELDHEVIGESG
HTDTIKRADSLNGSPVFIEALADIAKAHLRSGEACSKQMNLRCPSCKSER
CTETKKFFARQQANL
>Mb|QLI65826.1
MFRRSAVRLAVAAAKAAEPSAHTLAVSKAQGISKGLTGAIGNTPLIRLNR
LSEETGCEILGKAEFMNPGGSVKDRAALYVVKDAEERGLLGPGGTVVEGT
AGNTGIGLAHVCRSRGYRLVIYMPDTQSQGKIDLLRLLGAEVHPVPAVAF

DNPDNYNHRARRHAEALDNAVWTNQFDNTANRRAHIETTGPEIWAQTAGG
VDAFTCATGTAGTLAGITRYLKDVSGGRVRSYLADPPGSVLHSYISSGGK
LVERSGSSITEGIGQGRITDNLKPDIDLLDGSLHVSDDKSIEMVYRCLDE
EGIYLGASSALNVAAAKEVAEKLGKGHTVVTMLCDGAYRYADRLFSRKWL
AEKKLLGAIPKHLEKYIVLP
>Mb|QLI65827.1
MIPRSRLCGRLILERSAAHASRTSTSSISPRWALSPACHSRHICRSTLPG
IRPGLVGAAFSTTARLRKKDDKGKKDDFFDSSAETLTEPLSEEEVKANLG
NKKKDAERAAADAKSDSSTAKADAGSKSQDGKSSGAAGSSAEGGSGSGGD
SSGGDGGKKGRKSSEKALQKPVVPEVYPQVLAIPIARRPLFPGFYKAITI
KDPNVASAITESIKRGQPYVGAFLFKNENEDEDIIRNVEDVHDVGVFAQI
TSAFPIHGQEGALTAILYPHRRIKLSSLVPPSAAEQIDGKQEPDTPEVIP
QQNSGEEDAQQEKKGDVVASFEEGAVEKKPDQVAEKYEPTSWLKRWPVSL
VNVDNLADEPYDPKSPVIRAVTNEIVNVFKEVATMNNLFRDQISTFSMSQ
STGNVTSEPGKLADFAAAVSSGEQNELQDVLSCMNVEERMQKALIVLKKE
LMNAQLQSKITKDVESKISKRQREYWLMEQMKGIRRELGLESDGKDKLVE
KFKEKASKLAMPDPVRKVFDEEINKLAHLETAASEFNVTRNYLDWLTQIP
WGRRSPENFGIPNAKKVLDEDHYGLQDVKDRILEFIAVGKLRGTVEGKIL
CFVGPPGVGKTSIGKSIARALNREYYRFSVGGLTDVAEIKGHRRTYVGAL
PGRIIQALKKCQTENPLILIDEVDKIGRGYQGDPSSALLELLDPEQNSSF
LDHYMDVPVDLSKVLFVCTANMTDTIPRPLLDRMELITLSGYVADEKKAI
ANTYLAPAAKDAAGLKDANVNLTDEAVEELIKSYCRESGVRNLKKQIEKV
YRKSALKIVQELGEDVLSEEKALTDEGKAALEESQKKKTEEEAAANDEAK
SEEARAATEATEAKTAEKPRKALRVPDSVDVQIGKDNLVDYIGPPVFTSD
RLYDVSPAGVSMGLAWTQMGGAAMYIESILQAPLRASTRPYLEITGNLKS
VMKESTTIAYSFAKAFMARQFPENNFFDKAKMHLHVPDGAVPKDGPSAGI
TMATSLLSLALDTPVDPTVAMTGELTLTGKVLRIGGLREKTVAARRAGCK
TIIFPKDNMSDWLELPQNIKEGIEGHAVGWYTEVFDLVFQDLDREQANRS
KVQDVKKEVERSEEDEEKQAERQA
>Mb|QLI65828.1
MIVPYYGLQAQSEYCTRHVTQKFLSPEKSYLSYYDVLLTVEDIKSLKNDW
LTDNNIAFWEEYLERETLPRYPQARIVLLRPSMSFLLMKEPDIRHVRSAL
PDFSKVTHVFLPINDNRNVSVAEGGSHWSLLLVSLLDGIAFHYDSLGGAN
FAEANLATRRMGEVVGRQMRFINLEDSPQQENGSDCGVFVCLLMRHLLVK
RLLSANAREKVSMSMAGKMVDSNGGRKEMLRIIENLRKEGERRRSRSASP
FTNKTPPRIE
>Mb|QLI65829.1
MAEIDKEDPLPCPDWIVSTLSNVHIARDRRWFGDDYVTFPSHTCSLGQRS
EVVGIGTVCLPVKASLTTKGPGSHGILRLCNVLHVPAGAVNIIGNVISDD
FYLEVCAPTARVAAKLISRKKGKTAALLNSGPGQASHMYSVKLSGYPVGP
RVGIPWVWSHEVMMEDMCSNWPLGEQERYLECEYRKERVSTAKPDSRSLG
VEKSNTSKIDPTRETLVRHRERRQAASLYLAPEELDYVNNHWGDSASFME
NLCLNEHMEKDCLRAKHLASTFADLYHEEDEYWEAREDHRKPPVVDRLFT
PRQLYYMNRDHGNSSIFMTHMDLDVNNEDDCIEAQDLAEDLCFDSDDEDD
EDADYLDENGLCEDEMEEYEPGMDGYELADPGIPARRSRDQVLGYLNVGT
YEAGPRQLAEHFLSEEELNFVNQTYGDSMSFMTCFNLRFYKEVDCLEAKY
IAAAEVRGRNKGEPRN
>Mb|QLI65831.1
MSLYHEASDILSTSSQEGGSLKSRVYQRKGIKSTPKQLYALVFESCKWSA
ILKEVIEGADILKIERKLTPTLALLLVHDLLLAKGGITLPQSHGLRSSVE
RHKGRLNSEFTRARLRRKASSLELLKAEVDKAAAGEEAAHPRWVRINAIK
STLEEQLETTFRGYARAGTIQDVVAKEGKHILIDPHVPNLVAITPGCDLS
KTEAYNSGKIILQDKASCFPAYLLDPRSEDGDVIDACAAPGNKTTHLAGI
IRSHEPEFESEPQTIFAFEKDSRRAKTLEKMVKIAGSNKMTRIALGQDFL
QVDPESERFRNVGALLLDPSCSGSGIVGRDSMPELHLPDPPQQNPQRESK
SESTNKRKRKYEEIRNSDQTVIRDDDGNEIVVESEKDLAARLNALAGFQL
TILLHAFRFPSARKVTYSTCSIHSQENEHVVMAALNSDVAKERGWRILRR
KDQVTGMKDWPVRGSTEACNGDEEVAQGCIRCYKDDGHGIMGFFVAGFVR

DHANASEDGDGDGPYMRDERGAIVRDMLGMPVLKSTGTAVSLVGRDDKAG
DNVSMTNSNESHDDDDGSTSASDDSDAEESGWEGLGD
>Mb|QLI65833.1
MAADTKDFDVVRIARSDGADTGPGYWPSTNTAPKRTGKDVAAAAVEKTPR
VKPQMTRLAEDDPRFIEWRIKLGILLKQELSPNPDEGNPWYVQFPRGYWL
YEKSKHLWVSGYPIKTKLYKSPQEFGVHLIWLLSASRDYKDCCCVHCNLP
NPAKLAASVEETLIITPGETPIPVSAATASGTGVKSSSNTPMPAPVSAAA
QRVTPVPLPAIPGQPGRTPPTTSATPATKPPTSKSTSTKSTNHLIVAIPS
RSNSNTPQPTPQQQQQQQQPVPKPTPPPSQQQQQPQPVRWSLSGSLLFRA
GELTWYQNGNTWRLGIVASPSPNPSSPTHTMLPLGHALVPQQTVTKPQSD
LRPFHAFSVPPVAVAELKDKPFDQIPWDALFHSAANEPPKRELLALDASK
LAASRIDASYSLWCPLSDDPNADTLPYYGCFFGAERIEIGDCLRIKPVPS
EPSVGDASIMGLRYIFTKKEYPGAVFFRGNVYRLAKPDSPPASVVPEDQL
PVALRDETSWRNQLNPANPRRWVLVKENLTLNEQLVKGRFYPSHRLMPIL
NPAGFNAALAQGKVDEQVPYLNNRMDGGVGGHIGRKANRMDSLGASVPQG
SMISLEPLIKEDGLRVQG
>Mb|QLI65834.1
MEASTNAAAVRVPGHYRTINSHATDDAPDIDMLSRSLPTSGPLLEPEPAD
LAMLRPVASNQSCTGRSRDHAEVGVPNGAAPSERSPLLRPRASVSGLSTS
SILTDSEPALFLNGTSPSHFWYIFSQILTVHFIGCFDGTIMASSHPVITS
YFGAANSASWLSTAFLLTSTAFQPLLGRLSDAVGRKPLFLGSLGVFGLAT
LWCALADSIESFVAARAFCGLGAGGSMTVGSIITSDLVPIERRGAYQSYM
NIIYGVGSALGAALGGAMAEALGWRWVFGIQIPPLVTCLCISAVAIPVDL
GIMGERKTVMQAIREFDGKGSLLLTVAISFLILGLNLGGNVMPWSHPFVI
ASLITFAVCFPAFIWIESRVSKPIMPLRLIRESPRSNLVFSNAIAALLSN
AIFFNIPLYFQAVLLMSATDSGLRLVAPTLVSSFIGALTGFAITWTRRLK
WPLVCGAISYLVGTICLFSLRRSLPSVAYFLTLLPSSIGGGFQFPGTFMA
ILASSPQSEQAVVSSTLILWRSLGMVLGIAVSSLLLQNALVFYLEDYVSG
ELKDAVIRRVRASVEAVAQLDEPYREQVIRAYEASLRLTFGFCIFMAAIS
VAFVMPMKLKRLPARKYVKKSRVN
>Mb|QLI65835.1
MKSSLLLAMIPAAIAHTPDFKTSEAGNPFVDGFYADPDNEFYNNQYWVYP
TSSYEYDKQTYLDAFSSPDLVQWTKHANILVASDFTWARRAVWAPAPISR
NGKYYLYFGANDIQTNSELGGIGVGISDQPEGPYKDALGKPLIGQYYNGA
QPIDQDVFIDDDGQAYIYYGGHGHANVAKLNEDMVTIGTFDDGTSYREIT
PENYVEGPQMMKRNGTYYLFWSEGGWGGPDYAVSYAMSTSPTGPFKRLEK
ILQQDPAVASGSGHNGVIHVPNTDIYYIVYHRHPLGENIDANDRHLAYDR
LYFNADGTIQPVRMLVHDNFNDGNMIGWTTYGGSWDAKTNALRAGASAGG
KALLNTNFGDVDFSADVKMPSANGGDAGLVFRASTPALGTDAYDGYYAGI
GTSGKLVLGRAEGGSWTQLASVKADVVPGKTYRVRVTAKKNAISVYAGDS
TSAQLHVLEGTFGSGMTGVRVYQTDAVFDNVRVEHL
>Mb|QLI65836.1
MQLRRAARVILVGAPGVGKGTQSERLLGRFPQLASISTGDLLRSNVKNRT
PLGIKAESTMKAGGLVADDLMLRLISSELRNRGWLQSHGPPEVMTLSSEA
TTTAEMQSRDSMAFGAFAAGRPPSPAQASEDPAASFILDGYPRNAAQAGS
LEGIVPINLAVSLKTPFEVILERIAGRWVHEPSGRVYNTSFNAPRVPGRD
DITGDPLTQRPDDTEQVYRARFQKFQETSEPLLEHYASKGVLVEVEGMSS
DEISPKLYQVFEQRFVM
>Mb|QLI65841.1
MSGFQIPGLGQAKPNETLPPLPADVLAAAASVDATEAPPASSEPHGTSQP
KQSDNKTEGLNTTVATAALQATEEPDAMIIDQPDSPSSLTGALEAAIGGL
DSTDNAPEQPQPQTNAVALQNDQSEHPEWEVDSSPYESSSESSSSDSSDE
DSDNEGYELLGVEETARLLMEAEGGSDDEGDHGKGSSAARLRTKNEIAEE
IVPKPDITITSEMKIEELGSIEHIVENTVLVKAITPGEYQVLDTGSALCT
AERAVIGAVAETIGKVLQPMYTVYFSSEQEIKDLALEVGTKVFYPVDHAS
YVFTQPLRNAKGSDASNFHDEEVGDDEMEFSDDEKEAEYKRALKQKKKDK
WRSKNEDKAGGAARGPHPLSQEVSADAGLNYDDDDGPYKPLARPPGFGDG
LAPSESYEPAPRPGFRRGRGGDVRGRGSRGRGGAGRGGGRGGYGNTSRDG

YSLPPQGGQQNSQQIPSQPATQQAAAPPVMPNFGFQLPAWPQSQAAPGQY
PVPPPPPPPGWPAQGQGQAGAPNTIINPAFVAALMSQMQQQNGQHGWGAQ
QQPPPNGGQAQ
>Mb|QLI65842.1
MLSRAARPALRAAVTAPARATAASPNGAATYATLREIEGRLKSIRNIQKI
TSTMKIVASTKLTRAQRAMTESRKYGETANEVFEAAETVAPESEQKKSLI
IVCSSDKGLCGGVHSGLSRRIRALANEVTEPFDLVIIGEKCKAQLQRTNA
ESIQLSFAGVGKDIPTFADAQAIVDQIVMLPTEYTDVKILYNKFINAQSH
EPTFVEAFSEEAISQSPNISAFEVDEEALVNLREYSFANNLYWALAEGHA
CEISARRNAMDNASKNAGEMIGKYQILYNRTRQAVITGELVEIITGATAS
EDM
>Mb|QLI65843.1
MPIEPIAIVGASCRLPGSSNSLDSLWELLADGGEAWSSVPADRFNEAAFH
HPNASDPNGTNNHQGGHFIDGDIRDFDHAFFHLSPQHAAAMDPQQRILLE
LVYEAFESAGWAREACAGSRTAVFAAIFGTDYDRNLCKDVLDLPVYHSVG
TGIAILANRISHAFDLRGPSLTLDTGCSGGLVALHEACQSLRNGESDAAL
VAAANLQLMPDHYIGMSSQHMVSSTGRCYPFDLRGDGYGRGEGFVVVALK
RLSDALRDQDPIRSVILNSGINQDGYTASGITHPNRVAQADLIRDTYDRL
HLHPQDTAYVEAHGTGTVAGDSEELAAIAEVFAGRNRSLPLHVGSNKGSI
GHTESTSGLASLLKAILMLDRQVIPPVAGFTTPKPGLPLDRITIPTQRLP
WPLTEGMVPRISINSFGYGGTNAHAIVERGPRTYDASCSTTSTNSPRLFV
LSANNQGSLKSLLESYVNWIQQHPKIPLADISYTLCHRRSALPWRFSCVA
DNESSLLDTLERGVNLTSTRPPPSKRQVIYVFTGQGAQWAGMGHELLLET
TPSSVFRDSIRISRDILYELGATWDLEAELLAQDGGGRINKAELAQPATT
AVQIGIVMLLRSQGVRPWAVVGHSSGEIAAAYAAGRLSHHMALRVAFHRG
LMPGVSKDRGLPPGAMLSIGLGQGDAAPYLHDLTHGEAAISCINSPNSVT
VSGDAAAVDEVMARIIARGDGTFCRKLRVDTAYHSHHMRAIADEYRTRLG
DLDLGIDEPISPPGGLRGNEEVKYFSSISGLSWVSDFAASYWVDNLISPV
RFSDAIQTLAREHHKHNGGLALFVEIGPHAALAGPIRQCLAASNVPKLEY
NYLSALKRGTGAVETMLQVVGHLFERGVRVNFDEITALTPGFHTAAVRPD
LPSYCWDYSSKHWHESRLSREYRMRREPYHHLLGVRMTELASMEPRWRHM
VGLATLPWLAHHIIDGLIIFPGACYVCMVVEAVRQLAKEQYSEQALEAVS
LRDVSFLRALVVPDAPSRVEMQLSLKHRADASPLSFTFSVTALSDGKWHV
FCTGVVEGVMALDKPETESVMLDPHPKQDWLNGIVVIPEELYNEMAADGN
TYGPSFRGLRSLTTSTDGLRATAVIEVPDIAASMPAQYQASHILHPTTLD
SMFHVGIPMMKRWHGSGSVMPVHIGELLISTQTPALNSQGSKLEVSAKLT
SSHFRASNIDMTVVSGGFPVIYASAIESRSLAYHADRAHGTQDEHGICYE
LAWRCDLNFLRTSDLTRNSTLADLVSFICFKTANLSIAELGESPGVLAPA
FLAAVGVHRGTVTSYDVVERGNKPIDEGCKRLIGYPIRRRALDPDNSLES
QGFAHNTYDVVLVSDLGSLCYVSTLVKNDGVVVIVLKPGSDDNWQSTLRK
TWPSHDVQLTVVNSVNGNRIILARNDKTKHSHSSSRVHIITHSALKTTPS
WATALIGKLHETGYEISSGSWSQDVIQRSSDARNTCIMVIDDLAQPILSD
RACFNSAITLLRQDHRIIWLSLVSPSSMHQITGVARTAHAENDNLRLTVV
HAALEAIGSPVLVDLLCHCLTYAPTRDSLAHCEREYRVSRDAAVLVPRLR
RSDCLNRAIRASEKASGPSSNIETEPYHYINSTRRLALGPVQSSKGDGDI
VFIDSHTVELAEGQVEVETEAFVLSKSQNLTSYQLGEYSGIVRRVGKGVR
DFSPGDAVVALTLDGVVGANHARVPSSHVSRRPDSLSPAVAAALFLPTIA
ASYAIHHLAQISKGKSKVLIHGVLTDIGRASVAVARVLGATITATAISQQ
EALEITRQLNIQLENIIVSRPSLLRPRLDELLQLDAIIRISEDSVPAAAW
SCLKPFGQVVVFNSSSSIAFPSPPPENATIHYCHITNLVQAQPDRLADLV
SLATPALQRIPFKGIDLYIQDVAQVREAIHLVRQGGKVILQASPSSLVRT
VISSSVSMDWEAADASYVVAGGMGDLGRRLLLLMARRGAMHLVTLSRRSI
EPEDHRSFQEQLKLVQPGCRLYCLVCDITSESSVQNAADTLIRTGVPPVR
GVIQSAVTLHDRTLETMTFDDFFAVAHAKVDGTLVLERVFASPHLHFFLM
LSSAVNITGASGQANYNAGNAVQDAIAHDRGPGFLSMNIGWIEDAINTSN
NKTILQGLWRTGLRPILGQELSRYFDHLLGAASSHSRMRQAIIGFNAASL
SHTSASNSNVHSAMFCHIRGSLAAEESSSSTNSVRSFDEVVEGGDLDTII
DFIGSAITRRLMTLISMDDDQIKDRNGSILDLGLDSLVAIELRNWITREF

KSPLQSSEILTDQPIRDLAEKVASRSSLLASGLDKEATADSLENGDAEDH
HSAGAVRPSTSAHSTGKYVSEKLPPLPLPPLSDTLRLFEDSRRAIDTANH
RRNTSDAVHNFLRGPGPRLYKSLQKTDSDVIAETYDRQVYLDRREPLPEQ
GPFIFIHSIQAPVHSQARRAAILTIAAFDFIRLLARGDIAPDTLHGEPIT
TEGRNWLFYATRRPGLGVDHMERHAPNHTVAVLRRGHVFQLRLPDVEQTL
DMLAVTRVYDDILTASCDTIPPVCTLTADERDSWALFRLDLERRPQNAAV
LACIDTAAFVMCLDDESPTNSGERYTQFMINGAHRPFANRWLDKTLQFAV
TANGISAEIYEHSKLDGIDTNRLHAHIARAILAHPPSETADISSVSPPYA
VQQLMWEPSSATLQRIEHVRAQCQVYGPLDHQVLDVASLGFRSLRGFRLQ
PNATAHLTVLLALYLVDGEIRPAWEKVSLGTFARGRVEWVQTVSPATRAF
VEAAAASYTDSNNRTRVRALLHQATSTHSRAVVAAGRGLGAVRALYALRG
AAQEQDKELPELFRTHSWDATRRGGPGQDVKLGFMRLAPNDDANGNIGAT
PGDDDINGHWDEAGFLVCGERGVYVHCNVWEKHARFAVSGRPAYVTKLCK
AMERASYIITSLLDDPSKRPT
>Mb|QLI65846.1
MADNKVYRASTTAPVNIAVVKYWGKRDAKLNLPTNSSLSVTLSQADLRTL
TTASCSASFSDGDSLMLNGEPSDISGARTQACFRELRARRAALEASNASL
PKLSTLPLKIVSENNFPTAAGLASSAAGFAALVQAIANLYELPESPSELS
LIARQGSGSACRSLFGGYVAWRMGDKEDGSDSMADLVAPASHWPSMRALI
LVVSAAKKGVSSTSGMQQTVATSGLFQQRIAQVVPANMDLMEKAIKAKDF
AKFAEVTMRDSNSFHACCADTYPPIFYMNDVSRAAIRAVESINAKAGKTI
AAYTFDAGPNCVVYYLEEDAPTVLGAFAGVLNGVSGWKDDSSSVKSGAKL
EDGIASTLKDGVSRVIMTGVGEGPLKTEEYLVGEDGQAVKR
>Mb|QLI65851.1
MAGGVKKPVNIFRLGNLGEPDGVFNWRLWFAVVSFGLLGAARGIDEGLIS
GAFNSPDFQSTIDYKSFSSVEQANIKANVSAMVQIGSVAGALIAFLVCDR
IGRIMATRFLCVLWVVGIIIFMIGGVNGNLGAIYAGRFIAGLGVGQTPVV
GPIYIAEVAPASVRGLCTCFFTGAVYIGIVLAYFTNYGCRLNIPSSSHNQ
WLVPTSLHIMMAGIIFILTFMQYESPRFLVKQGKNERATHVMARLRKQAP
DGEYVTREIASIQAALDHELEATKGVGWAGKLKEMFLDKSNLYRVYLATM
VQLLSQWSGAGSITLYAPDLFEILGITGSEEGLLVTAVFGIVKLVSAVLC
ALFLVDVIGRKRALLTGITLQAISMIYVASFLTAVPELGTVKHYVLPASK
VPASRGAIAMIYVSGCGWALGWNSMQYLLTAELFPLRIRALATSWAMTLH
FANQYGNSRAVPNMLLPTSDGGISPMGTFWCFAAVTILGGVWVWFSVPET
SGRTLESMDRLFDLPWYKIGLHGNRDAEQQDMAYNEKQRIAEETHGAGQH
IEERKTTTVA
>Mb|QLI65852.1
MSLSQLSQLLPLPEEELQQVLQYASTLSKAEVATHFNNLLGDSPLAVDFI
ASFNSHRKDPSGARAPSLETENTALNSDSADAVPKAKRFQKKKKPAIHVP
EARRVDGYAGPSSKAYKKDLDFEYIPQRASAPSSNHASRSATPPAQAQAK
QHASSAGYLISDGPPKVKAKSNPGSRTSTPKPASGNTTKVSIAGGTPMAG
QSTALADLDAAIRALEITTNPTLENGKRKACNCVATRHPLQGAAPNCLSC
GKVICMKEGLGPCTFCGSPLLSADEVQAMVRELKDERGREKMAVNASAHR
RAEVSKKPAPFTQTRDDSASGAGSSLSEAAVKARQHRDKLLNFQAQNAKR
TTVRDEAADFDVTGAMNGTGSMWASPEERAKELKRQQKLLREMEWNAQPD
YNKRRQVISIDLVGGRVVRKMAAVERPVTPDDGVDSEPDEGVLGETSGNT
GGQNRTNGGAFSGNPLLGSLMRPVFEVKGKDKDFGAARSREERKKGWRRV
QDDLDDNEDVILDGGAYGYKDGGDEPACG
>Mb|QLI65853.1
MAAMRGPRLQVGSAGWITEERNAALQITQSEVDEFSYSARNELDWLNEHM
ANIFNENEINFAETFKTPAKLRGKTPRTARKANPLETRVPLSEVFSATPN
GASSPISRLQSPGVHDDAARGSPSPLRPSPTRVPVFSQKSAMQGTQDSGY
FGSQDVVPVNIHMDSDSYDGQDSLQQGAVQTTTIPLKNSPANASTQPSPE
KTFQTAREDQTARVSADTSKKSIPKAQLNLHRESEYEDDVSPERQLSLEA
KQGAHYPDDDARSASDGSSPIRPIVRKSSLNFASLPAREPLAAGKSNGAR
ASRTSHLDHIRTSYYNRTTGGKSLGGNLTKPDRDENQENEMEVDDELPSH
PITNQDNKTYTQRLQDQINMLGQSQAKNVQTQPQSKAPALKAVSPIPLNT
LQSTPGAFPEDDDDDWIEPPATLAAANEERPSLPKSHSADVMEGIQGKKT

IGETDFVMSDDDNAEVDKQFARPGKGNGAKHTYGHGKSASVPVVPNMRPA
MEDTHYLKKVVSVSNPSLLTVAEAGSLGTFKSPSRALRESPLKQVKNKLS
SILKSSRGLLASSAAISAEGKSLMSPTSTRLGLHSFMSSESVVSQQGTGS
QASQSAPANQDASPTRLTRRTRASVEKEKEEKRREKEAKRMEEQNEKLEK
ARQKEREKARVFSKEQEKVAAMEMQIASKKEDERSAVKETPKSARTGHRN
AKNSGEETTSLNRDVDMEDAPSMPPPSAPRSVGPTHATRNREIKRPFTRE
TQTRPKQAPTVIRVNTGSQHSQYRLSTAQPDVSSLSASQTQQQLTSKASK
ASLQLKQSTQSLRGAAPSSRAKAQEVAARKKEQEEKETQKRRDAKAEMER
KKAAAQEEQRRQEQLKRQEAERQRKQQQQEQASLQSQSQTKGNAQRKAAI
EKAKQTRAPPPAVRSQPNGPPDSAMGQDSQPARPQSRMTSTANWGQDELG
RPVNAVLSNTGKAGSKRNFVQEKGDDGHEKRPQSRGGPSYQTNDAKRRRT
SDAFDDEADASGQKNIMGPPVRPSAGFKKELPSKTVFQSGYTNAPHGATR
DLFKATITAQHANQMKASHPLEMAQISKGAIPFAPNPNAAGPAYKTPARP
GPANTAKSAAKSVTRSSPRFQNGEAIELPEIQTDDDSEDDEANGVVVAPW
ADSPDLRKALVNQETMDPSLIFGPPRPLNMEEVFSKSKDRWHKFRARTSS
ANWTGADRLTEDDIRKDMAARDKLRRDGGWSYEMSKDML
>Mb|QLI65854.1
MDYSASINESEDPAASPWGSSPVQSPQHSRTGFAPIPGDNGSSPFPYSPP
PGILEASGDTDGFQRSDTATTESGTEGGDLDQSATLASSAAEFAGESHPH
ASQPPQQAGEPQPRVPGSNQQAEQAQKIPPAQQRPQFKLQAKITGLERTG
KKDPVLRFDVHTNLPAFRTTQYRDVRRLHSEFVKLGQHLISANPEALVPA
VPPPVTAAGAGTDEDENRVKAMIQRWFNYVCSNETLMRDDEMVLFVESDF
GYSPLVKMKQPATGVRRKILKQFAPPPDDTPELADARPTVKLFYLGTMDA
GHKVDKMVKARRGLGLAESDYGVKLSSMHVQEPHPGLANAYKKLGRVIQT
MGDYHAAQATAQAAAIGDPFQYHSQDAFIVKESLTNRQILIREFLQSQEA
TRGKLNAADRLKASSSVRREKVDEAITALDDARQFEANLYNKTNRVTHNL
VQERRKWFARTSADLRTSIRDYVLREIDAERRTLALLETVRPDIRSIDAS
GGLSRLGRESHPTVRRSSLASSQGPKGDAWSGVPRRSDATRSTSSSFMGK
VAEEAEGKEDDVRESQRVPGLAGVNEEDDDDRIDARNAASRLATSTF
>Mb|QLI65856.1
MAVDYSVYLVTDSTPAILGDKDLISVVEASLRGGVTVVQYRDKHSAKEDV
VAVAKKLHQLTQSYGVPLLINDRVDVAVEIGCEGVHIGQDDTEIQQARKL
LGPNKIIGVTASSKEEAISACQAGADYLGLGTVYSTATKKDTKSIIGPSG
VADILAALDAAGHSSMPAVCIGGVNASNASAVLAGSASPHKALDGLAVVS
AIIAAADPAAASRDLLGKVITAKIPDVVAAVGRKTPLSHNMTNLVVQNFA
ANVALCVGASPIMSNYAEEAADLARLGGALVLNMGTVTPDGLNNYAQALK
AYNQARRPVVLDPVGAGATSVRREAVQTLLASGTFAVIKGNQSEIQTVHG
ASVTQRGVDSSSSLTVPQRARLVRSLARQRRCVVLLTGPTDLISDGRRTL
RVDNGHGYLGMVTGTGCTLGTTVSAMAAAYEQDALVATVAGTVMFGLAAE
MAAQRDHVRGPGSFVPAFLDELFGIRKATMEGDSTWLALAKVAAVDVADD
E
>Mb|QLI65857.1
MAPPPEIAIPSTSVSEDVGSKPYTLYNITLRLPLRSFVVQKRYSDFARLH
NTLVQQVGVPPPEPLPGKHWLKSTVRSPDLARNRQIALEKYLRTIAETPD
RRWRDTSAWRAFLNLPSTSTTSSAVSASGMARPAATGAADPQTWLDMYRE
LKQNLNEARQCLSRRDAASDSGNSTAAAEAGSAASKVLFKAGILVTSLSD
GLRKMQESNRLGEGELRRRRDLVSNARMERDGLEKLSTALFTASASGSNP
RNGGNSSVSAAAADEKSTLLKSARPTTGRVLGAPLPETAQTRELDNEGVL
QLQKQEMASQDQAIDQLADVIRRQKAMGIQMKNEVDAQTEMLAALNGDVD
RVNDKLGVAQNRIKKL
>Mb|QLI65858.1
MAPSAIDDPKQPRPDGKAYPPANIFPVKETRFEKYIEPQADGYKKAFEQH
KGNAAIVIDNGSSAIRAGWSFESAPRFSIPPIMSKYRDRKLGKTFSFAGS
DCYADTTARGHIRGAFEAGTGIVSNWDVMEHVLDYVFLKLGLNESSDGVD
VPIVMTEAVANLPYSRKSMTEIIFECYGAPSLAYGIDSLFSYRQNKGNTG
LVVSSSYTSTHIIPVYNAKAMLGQAIRLNWGGYHGAEYLLKLIRLKYPAF
NAKLNVSQAEHMIRDHFYVSKNYDEEITSYLDWTGLEDRDVVIQYPYTEE
VVVQKTEEELARIAERKKESGRRLQQQAAKMRLEKLMKKEQDLEYYKNLQ

AKLVDETKKEIRRQLDSHEIKDENQLDKIVKELEKSIKKARTKDVGGDPE
EDQEQPNFDLLDVPDDQLDEAQIKQKRQQRLMKSNHDARARAKAEKEAEK
ARVAEEERLDKERRENDLDNWLEEKRQARLRTLQKIKERDRLKADLGNRK
SLASQIRMKSIANLASDNPTKKRRRGGDDDDFGANDDDWGVYRQIAVGEN
SEDEQEEEDLATNLKNLEQELLQYDPDFDYEQTQEAQSDWSKSLLHAFAR
GPRPFDPASQAQLSQIHLNVERIRVPEIVFRPSIAGVDQAGIIEIAGDML
NQRLSGMPDRDALLKDIFLTGGNTVFQNFDERVREGLRALLPADSELKVR
RAQDALLDAWKGAAGWAGTPAWKAATITREEYREKGPEYIKEHDMGNSYA
>Mb|QLI65859.1
MATSAEKPSKTGDVVASHSAPRKFKASDLPLSSATRAAIESLKHSFKKGG
AYDSIRKQVWDKFGASDCETIVKQAILEVAEKEVDRNPQQLLTLDPRKAS
ALIDGALERSGVYQKAEGVIAKLIDVDAIEERIREIRRAEIGEEAAESER
IRGAKTDEEYAADTEARQAERERVREELRLKEAAIEEEKRRIKQEEQKKE
ERERAQAELKRQAERDERRRKREEERAERERLRDLEREKRHKEREARDKE
REQRDRERSRDRSRHRSRNRSRDRSRDRSRDRSRDRSRDQDKDKDKDRGR
DRSRDRRRDRSKTKETASDQKMSKEDNERLEQEALADLLRESSRSDQAKP
ELEVDAALAPPPRKSAPASAINPIKRDSRKASENGSRSERRDSRTAADSK
LSGSRDEKRSSRSASRAGDRDREKEADLVTGTGATEVDQNCDGIGPGLGR
GETSAVSEVVHGIVTTGVGVGRDEENAPDPPHDLNGVNGVGPAQGQTVGT
EAVSETDPARRPGQKDAGVGLEDASGVDQGTAAAAGERARDRDRDRDRDA
DRDKPRDRDRDRRSRSRSRPGTSVVRTASAAREEHEEWKLEEVRKREKEA
KAYLAAQKDAKAKGLPIPGVDDKKPVRDDRRRSRSRSHSRDRTRYRERER
ERDRDRDTDRDRDRDRDRGRDRDRDRERSRERERERERHDRDRDRDRDRD
RDRDRDRDRDRDRDRDRDRDRDRDRDRDRNRDRDRDRDRDRDDRRRRDRS
LSPRRERRRSRSTSRRRRSRSR
>Mb|QLI65860.1
MTDSNTKRQSTLRQQEARPFSNGNTSSDIENQLAAAQKNDGPPYSVFTPW
QKRAIVLGAAVTAFFSPLTAQIYLPALTQLSRDLGVTNTQINLTITTYMI
FQGITPMFIGSLADSGGRRPAYVVCFIIYVAANIGLALAPGYSALLGLRC
LQSAGSSSTVALCTAVVADVVTSAERGQYVGFTVIPTVLAPSLGPIIGGL
LSQYLGWRAIFWFLAIFSGITLVLIIFFFPETCRRIVGDGSLTPPPMYRS
VWQSLHLRREKKARRRNALSKSTSNSSSASKSGNRFKFKPPNVLGSLFLL
FERETGLLLWTSSIVFAGFYCIATAMPSLFSKRYGYNEVQVGLMYLPLAL
GSIGAASIVGPSINWNFKRHCIRSGIPYDQSRQNDLSNFPIEQARLEIGL
PLLCLGAVSIIGWGWAMHAATHVAVPCVISFFLGIGMIGYNNTTNILLVD
VHPGRAGTATAANNLTRCLVGAGASAAIIPMIDAIGAGPAFTLIGGLYFV
GIILLLLILQFGMKWRAEAKEKTQTSKLKTKERHQQGLLVVDEPRPAEDT
RNRDLRNA
>Mb|QLI65861.1
MSLFAPISMRCVFRNRSYATQSGHGGSESLSPKRRSVTPFNDDGYVPWSE
LSAGEKTARATQQTFNFGFVLVGLALTGAVGYFLWTDVFAPDSKISQFNR
AVDKIKKDPRCIEVLGDAKKIIAHGEETTNKWRRARPVASSERVDQQGNE
HLLMHFHVDGPNQNGVAQLHMVRRRGESDYEYKYLFLDVKGHERIYLENA
DSTSSVGKKQLSFFGVKW
>Mb|QLI65862.1
MPREIITIQAGQCGNSIGSQFWQQLCLEHGISQDGNLEDFATEGGDRKDV
FYYQSDDTRYIPRAILIDLEPRVINGIQTGPYKNIYNPENFYVGKNGVGA
ANNWGDGYQSGEAVCEDIMEMIDREADGSDSLEGFMMLHSIAGGTGSGLG
SFLLERLNDRFPKKIIQTYSVFPDTTNSGDVVVHPYNSILSMRRLTQNAD
SVVVLDNGALSHIAADRLHVQEPSFGQTNQLVATVMSASTTTLRYPGYMH
NDLVSILASLIPTPRCHFLMTAYTPFTGDQVEQAKTVRKTTVLDVMRRLL
QPKNRMVSTVPGKKSCYISILNVIQGEVDPTDVHKSLLRIRERRLATFIP
WGPASIQVALTKRSPYIPMSHRVSGLMLANHTSIATLFKRIVRQYDGMRK
RNAFMEGYKKTAPFSENLNEFDEAREVVADLIAEYEAAEDADYLSPDNGE
KATSAETDRRMG
>Mb|QLI65863.1
MDEIAKEYDVIVLGTGLTECILSGVLSVKGKKVLHIDRNDHYGGEAASVN
LETLFKKYGNFKAGEEPWTKYGRLNDWNIDLVPKFLMSSGELTNILVSTD

VTRYLEFKQVAGSYVQQGAANKATVAKVPSDAGEALKSPLMGIFEKRRMK
SFIEWVGTYNPKDPATHKGLDFNTCTMKEVYDKFGLETTTKDFIGHSMAL
YLTDDYITAKGQAPEAIERIRLYGNSVARYGKSPYIYPLYGLGELPQGFA
RLSAIYGGTYMLNTNVDELLYDGDKASGIKATMKTGAEDMKFETKAKLIL
GDPSYFPNKTKIVGHVLRAICILKHPLAGTNDSDSAQLIIPQSQVGRKND
IYIACVSSAHNVCPKGYWIAIVSTIAETSANHHLELQPGLERLGKIEEQF
MGPPIPIYEPLEDGKKDNLFISKSYDATSHFETTTDDVKDIYRRCAGEEL
KVEGLREGLSVAAEE
>Mb|QLI65865.1
MLRNPLCRASSQVLRGARCSAALSRSSIPTVSARTSSWKLAAVRRPLAAT
AARSYATDALHSPPDPSDNFLSGGAANYIDEMYMQWKQDPKSVHVSWQVY
FKNMESGDMPISQAFQPPPNLVPNMTGGVPRLAGNLALDDGSDVTNHLKV
QLLVRAYQARGHHKANIDPLGIRNTAEGFGNIKPKELTLEHYGFTEADLD
TEYTLGPGILPRFKREGRDKMTLREIVAACERIYAGSWGVEFIHIPDREK
CDWLRERLEVPQPFKYSIDEKRRVLDRLIWSSSFESFLATKYPNDKRFGL
EGCETLVPGMKALIDRSVDYGVKDIVIGMPHRGRLNVLSNVVRKPNESIF
SEFAGTLGAEDEGSGDVKYHLGMNFERPTPSGKRVQLSLVANPSHLEAED
PVVLGKTRAIQHYNNDEKTHRTAMGVLLHGDAAFAAQGIVYECLGFHSLP
AFSTGGTIHLVVNNQIGFTTDPRFARSTAYCTDIAKAIDAPVFHVNADDV
EAVNFVCQLAADWRAEFQHDVVIDLICYRKHGHNETDQPSFTQPLMYKRI
QQKEPQIDVYVNKLLREGTFTKEDIEEHKQWVWGMLEESFDKSKDYTPTS
KEWTTSAWNGFKSPKELATEILPHHATGVDSKTLDHVGEVIGSAPEGFQI
HRNLKRILTNRTKSVVEGKNIDFPTAEALAFGTLVTEGYHVRVSGQDVER
GTFSQRHAVFHEQENEKTYTPLQHISKDQGKFVISNSSLSEFGALGFEYG
YSLQSPNALVMWEAQFGDFANNAQCIIDQFIASGEVKWMQRTGLVMSLPH
GYDGQGPEHSSGRLERYLQLSNEDPRIFPTEEKLARQHQDCNMQIAYMTT
PANLFHVLRRQMHRQFRKPLVIFFSKSLLRHPLARSNIEDFSGEDAGFQW
IIPDPEHQTGAIKAPEEIDRVILCTGQVWAALHKYRADNKVDNVAFTRIE
QLNPFPWQQLKENLDMYPNAKTIVWAQEEPLNAGAWSFTQPRIETLLNQT
QYHDRKHVMYAGRNPSASVATGAKHLHKKEEEDFLEMAFTVKQDKLKGE
>Mb|QLI65866.1
MTPKLGLLLAVYFQSLLAFRFNRELVANEKPMCGPGEGSCEMGSCCSEIG
FCGTTAEYCSGSQCQLDYSHTCDTLVPPDGSDTSEIPRPRVGKVPYGPMI
TSCKNPGMVALTFDDGPYIYTTELLDLLAAHEVKATFFITGDNRAKGHID
DPATAWPSILRRMYNAGHQVASHTWTHRDLTEVNETVRRAEIIHNEMALR
NILGRIPTYIRPPFLECSAGSGCEETLGDLAYHSISANLDTKDYMYDDPA
LIQRSKDRYSSTLSPDSKENSYIVLAHDVHEQTVHNLTEYMISLARERDY
KLVTVGECLGDPEENWYRSAETRTVGKRNLCSGPAKPQVTSVSPPLNTTT
KISPNQRCGGSTGYICPGSGFGDCCSHWGYCGSTPEFCGTGCDNDFGDCD
PSPQGVLDTTNGLCGARYSATCLHFAGKTCCSKYGYWYVPFLVAKFIGVL
TIQVAGVRLSIVVRGARTSTVIVTERRYFGFKTLARATPRAPGSQTPRSI
ESATSVDSQFHTSNVSLWGDVRQEEAKLRWDAEEELKRVSKELLRLQKWA
LITGLVVLNSALITISLLLPQAVYLFLALLSCNTILQAGMVVCIVANVIW
IKIICGCRKKKNTAPATPERMVLLLPCYNETKEEIARSLDSLVSQKGIDE
HARLVFIVVDGNVRGPGMEKTTQDYFLEDILEPGTSKYFENGYRARDGLF
MPVKTRVGRYKGIPFVFVGKRYNQGKRDSLCFARSFLYHFKKRSVNAVTI
FNKELFEYLGNCLLENGLPNVDYLVGMDADTVFDDFCVVEMVNTIRKNPK
LVGVCGHVCVDFDGNNFGLWSLYQSVEYSQTQGLRRMFQSRITGKVNCLP
GCCQLIKVDEATFGDTVLRERFGYCPKPNDVMTQQIMGNYSEDSIHASII
FSLFPKKQTAQALRSKAFTIVPQTWKVFLSQRKRWALGSISNEFVMIFRP
GIILVERLQSIVAVMTWAITPFIISAVAQLIALFAKKGAKVFEDEIFVGL
ISVLWVRYLYSFCIGFWLPRNNLQRLQYFVGFIIHFLSSPFINIIILIYS
LLYADDFKWGDEALFV
>Mb|QLI65867.1
MSASATVARRAATRTPLLRNAQALSRGVTPSLMARHIGRQSQVSALALLI
PMRQVSTEHHRPDSQHSGLPPGFNVDQAKKPLPKENPAVEAKDVDAQSPT
ATAGQPDTTQAEAAMTDLAAKKDDLAQQKEVTKAEKEEKKLTIWQKVKKE
AQHYWDGSKLLATEVKISWRLALKMAAGYDLTRREHKQLRRTVQDLGRLV

PFSVFIIVPLGEALLPLALKLFPNMLPSTFEGQKSKEAKATVLRSTRKEV
SSFLRQTLKETGLPLSQATAQKEEFANFFRKVRSTGEAPTHQDVIKVCKV
FRDDMTLDNLSRPQLVSMCRYMNLNTFGTDMMLRYQVRHRMRQIKRDDKA
ISFEGVDSLTVLELQMACAARGIRTHSVSPARMRTDLQSWLDLRLKEGVP
STLLVLSNAYMYGQGSGEGSSQIDALIGVLSSIPEELFHEIELEVHNAEG
AATNKQRLEVVKEQQELIEEENEQNEESQSTGMATPRDVEDIDEKEERIM
HAEAEGADKKAASEMVDAEREQAQVTERQAKTQSNAEQK
>Mb|QLI65869.1
MASRVPLSWRLASRSVAAGRRACSTTPLSWTPRTAFRGGKRQQRFYSSDS
GPTGNKDRVKFWPFIAVIAIGSAGYVGLVNRRKDMTRTSDSKTEGQLPPP
AKSSPTFSPSDVTVLFVLGGPGAGKGTQCANLVKQYGFTHLSAGDLLRAE
QERPGSQFGDLIRDYIKNGLIVPMEVTIQLLENAMTEALQSNGNATKGRF
LIDGFPRKLDQAHKFEESVCPARMVLFFDCPEKVMEERLLERGKTSGRAD
DNAESIRKRFRTFIETSMPVVDYYEKQAKVVKIDATPTPDQVFANTKARL
TEKFGKEF
>Mb|QLI65870.1
MDALQPLIHRLTTTPPATYCQTFFLLAAASVLSLQLLPNEARSTLTDYGA
RRPRGSSAPTSSKLASMLGKLTAMGQLPHSYFWHFYLLSTCLSAFWAWQY
LTHGSVVSFIAERQVSLSGEPSVELDRVLLAWLMMAAQGGRRLFECFWVV
KPGRTPMWWVHWVLGLGFYGLMGISVWVEGSGAILESWQAPTKAVVLTSR
ALFTLTVFFGAWFAQYKCHGYLASLKKYTLPDEGMFKYIICPHYTSECVI
YLAISIMAAPPGRAFNVSVLCGLAFVVANLGSTAHGTKKWYAGKFGADTV
ANKWMMMPGVF
>Mb|QLI65871.1
MRVNQATGISTNKVLLVPYEAHHVGKYHEWMGDPAIREATASELLTLEEE
YENQQSWRTAHEKLTFIICEALPTADRASPSIKAKVVDADDRMIGDINFF
IYQNDDPEETSPNRKANAALRGEIDVMIANTKHRRQGFGSASVQALLMYL
RENMKHILKEYACSLKLGQVDLAELMVKIQEGNIGSRRLFENMGFKQAGG
VNYFGEVTLLVSWAKVEKLVDEWLSSGEVYREVRYEIS
>Mb|QLI65873.1
MDFKSFGKNLSDFGAQITPFASRTFQFTKEQFGQAEDKTQLPPDYIDLEK
KVDALKQAHQKMLAVTSQYSNEAYDYPPNIKETFQDLGRTVSEKVSLLSS
ATTTAEAQAALVAPPSAKPQPKTFSHAVARASLTSSQLLHQNHTGAGEDQ
LATALEKYAITMERVGEARLAQDSQIQSSFLAGWNTTLNTNLSFATRARK
NVERSRLSLDAVKAHAKGTTFKLGHGGSRPEQHEEQGLSPEAQEEIEKAE
DEFVTQTEEAVGVMKNVLNSPEPLRNLSELIAAQIEYHKKAYEVLSELAP
VVDGLQTEQEASYRKSREEGS
>Mb|QLI65874.1
MTGNTPRTSVHPGQQNDLNSPSDGSAHAAIAMNSNTNATSVAAAKRRSRR
RAASSSQKRRCISTACVACRKRKSKCDGALPSCAACASVYGTECIYDPNS
DHRRKGVYREKVDTMKARSATLQVLVEAILNASEDDVASVVDRIRSCDDL
DSVAQSIVNQDSDSGATGPDDIDEDSVDNPAVKGEEELARNMGELRFENG
SVRFIGGTSHLLHLGGSQNDAIESDLEPEQQLTSDDPITSWTRVTSDSNL
IAHLMRMYFNYHYSYFTTLSKKLFIRDFTAGQANVASRNCTAYCSPLLVN
AMLALGCHFTDVAGAFVIPGDARTKGDHFFAEAKRLILEDDEFARPRLVT
VQALALMSVREAGCGREAKGWVYSGMSFRMAQDIGLNLEVTGSDREHVSQ
EEIDARKITYWGCFVFDKCWSNYMGRLPQLVKTTCNVTKFDVDNDEDLSI
WSPLSDKGLDKSFEQPSRTRSAALQLTSLCEISSDLMVFFYHPNHIRRSR
GKSAELKKLSEIHQRLEHWRKVLPKEFEPADGQLPNVILMHMFFHLQYIH
LFRPFLKYTPSASPLPAHVSPRRICTANAGAISKLMRLYKKMYGLRQICN
IAVYMVHSACIIHLLNLPEKTARRDIIHGVKHLEEIADDWLCARRTLSIL
SVLARKWGCELPEDAAFVLKRADEKYGYYSTSDVPSPASTHATLGVTSDD
AMSKSSGEQSPLGQFSQAQITPGVGSGYVGDGMSMLQPPQVVEGVSDRLS
HREQTAQPEGMSLGFSGNVMERWLGDGNPTTESPSVFGSPFFPIDHQVPE
PQNSHPVMPRRMAGSAGNEPPNGQDWMLNDGARWQQSFESWGVDGGVPSN
GMFIFNQDRNNRFNQPAAGYHDDQGQLTFENLNASLNGSSWLPGLD
>Mb|QLI65875.1
MPSPKRRIDAETAEKLAAEEGFITEEESEQDDGSEDESGSSEEESSSEEE

QEAPRRLMIRPKFVPKSQRGKEALNKEGSEEEARLAEAAAKQKAADELVE
EQIRKDLAARAAGKKHWDDDENSDSDVDTTDGLDPEAEEAAWRVRELKRL
KRARLAIEEREREĽAELERRRNLTEEERKAEDEAHLAKQREEKENKGKMS
YMQKYYHKGAFFREEAEAAGLLQRDIMGTRIADDVRNREALPEYLQRRDM
TKLGKKGATKYRDMRSEDTGRWGEFNDRRGGKFGGDERFRPDDERFKPDE
RDAKGANAVPLGDKERRRDDGRGDSYRPRDRSRSRSPRREGDRRRKRSTS
RHTHDGDKRRRVSPL
>Mb|QLI65877.1
MDARSATSEPQHPYPLSPWTATSTSTTLSTESTSNGAAHSPSSESPPDAA
AHPVPAGAAAPFAQLEFLPFLFPQQTIYHAQLLHYNKLISPSRGGGLQTP
PLPPVVSSPHVQAGPRSRSSSRVSLKDASASKGAASGGNGNRSHHNPDKI
NRPVITSKDAPSKMPPKHSVETSRSIPQRSNAAATLAHGQSHSSSVPSTP
HQHARQFSFESREPSPTAANNHSPRSAYSETNSTLPSLRPLPPRLGGCKY
ETAQINSRRRIPYSVGSDRLEKLDLRTVKNKLSEDEERKLATDMREIYNR
LLPTEEVEERRKKLVQKLEKIFNDEWPGHDIRVNLFGSSGNLLCSDDSDV
DICITTSWQELEGVCMIADLLARRGMEKVVCISAAKVPIVKIWDPELGLA
CDMNVNNTLALENTRMVRIYVEADPRVRQLAMIIKYWTRRRIINDAAFGG
TLSSYTWICLIIAFLQLRNPAVLPALHQMPYKMPKSDGTPSDFADNLKKI
KGYGSKNKSSVAELLFQFFRFYAHEFDYDKHVLTIRQGKLLTKQEKKWNY
ALNNQLCVEEPFNVSRNLGNTADEYAFRGLHLELRRAFDLLSEAKLDEAC
EQYVFPKEEERVWTRPAPQPRPVLLRSSSQTHSGRGGRGHHRGGRHNNNF
HRGGGGSNRRASSSIPAYEQNMFVPLSMQQDMSWLQTPQYPFQYTQQDLM
TQIAYQEQMRHFHNLYAQGPFLPHQVISQQQQQQQRLSINCGSGQQSSDR
SRTNSFDNVPVTAPLRPEIYALYGMNIGHAFLPQNGYGSYPSSPATTNNN
AAQDFRRSLQRSTISTEGGASMSSSSVRSQSQPAQRSPSANPGAGYQLSS
QTPASAKLSAPRQTNGITIPNFMPDDADFDETPKAASESPESVDAKSSGF
FQARSLSPTRPAQSQQQQQQQQQQQHLPNGIAFGDLATKSSSPERRRLSI
EQLPQTLLDRRMRRASRSPSPLGHARAFSVGVTSSAPLTSAPFPSSQGSK
ASSRPLVVNGSGLKTAVTPPGPSPSSRAESLGSEDSPVAGMENALHISTV
PVWPMQPPANTTLQPSAPSRIQQAPSPLALDRPPIVVNGSSAPLSMPHCA
EDASFKDRIALMNSIYLTSQPNQQEAPNGATARLSPSARQRLMSRQPQNG
VIAPLDLAIGDNRVGKPTAIDAAHLSPVYETRTPSPTTLRKQEPAASMPT
WLPNKPPYKAEASKPTRVDLSKEALHSPDHRRPPFVDADKGQKSQKPHVA
AAKSAPQRENGHVRGAKSEGDGGWQKAAGKGKKKAHNTVSTQPSQAELPP
KNEFERKGG
>Mb|QLI65878.1
MSGYPGQGRYGAAQHHGGGGGFSQGQGPGFGYGYGNYGGPPQGPPQGQYG
PPQGNYGPPPGQYNSPPPGNYGPPQGNYGPPPPGPRPPSGPQGYGHSPQP
QYARPPPANPPPPGYDAYGYPMQGGTYAYRAQAGPPPPSAPQQFGHGAPQ
GYTFQYSNCTGRRKALLIGINYFGQKGELRGCINDVHNVSNFLMERYGYK
REDMVILTDDQQNPVMRPTKDNMIRAMGWLVKDARPNDALFFHYSGHGGQ
TEDLDGDEDDGFDEVIYPVDHQQVGHIVDDEIHARMVKPLQPGVRLTAIF
DSCHSATAMDLPYVYSTKGVLKEPNLAKEAGQGLLSALGSYARGDMAGVA
STVFGFAKTAFKGDDAYNKTMETRTSPADVIMWSGSKDDQTSADATIANQ
ATGAMSWAFITALKQNPKQSYVELLNSVRDILASKYTQKPQLSCSHPLDT
NLLFVM
>Mb|QLI65879.1
MSGSQQGWLSSLSSLPSLIFPPVSWFINGSPDGSSGAPANLLPLVYSEST
VDDAEASETAADTPSSSFANSDASRPWLPRFPKLKRHYPPVERIRTERSP
KANANPPHSYRNPRSFPRQRFLADTIYPRLEPSDPRQKDICFALSSLHRQ
RAPQEPSPAPRALRPPEHPNTVFVEINGIRTNKRRAEDLEVDLVAAIQNS
THTNPPSSISISRTGFFPESESKFQEEIQAHMSHAKPLPELITGMMTNIL
STFSDLTGIFSRLLYNQAYEVSESRSYIQIKDPSGLDVAKKRVKLTSEKS
DFQWLDRDGLHSLLSRYSHFRHCFDKASSILLAHPSRAEMNKLLKPLLPV
LADNADDLNALSSGCDGVDLCRFWFYSWRSMMTYLNTVYEIGSFIKMKNK
HHRAPTVWPNSPSSETLETAARIRRFLSDPSLFVVLKSILELSDELVNYP
MPADMIDRVVLDMAAVQHNIVLPSYVQSEQFHERLLEYFPPPQRVDPDYA
CPVLPGAFPEADSEFHDVGLVEPEVPPLPSLSVEPPPRISKRGSTKTDRL

RAKFFAAEISRLTPEEFQTKYCGPSDDHRNIENDYSTDFIGKKPLTTAHV
GVVRSILKDRKAPKRLTMTRAPKAVRFTEGTITPRQRTHLGLDIRRLLPS
SDRATDPVFIGNIKLGQSGPADDKLDVDEWEYLRAQQTNRHVNSIFHGAK
ISKHKPPANDAIDPSLAIERMLSVSSIESLAISDDTRAGIAIKKEQVAQQ
AAEDARKYAEERARQELEEKLARTGGLRAPEQPLVSPLSDHWQERAQETL
RKSPRTTLAKTGDGIDLRRHDFAKVVKATEWLNDEIVNGCLNWLDQSINS
AAGIKDVKKSTRKCLAMSSFFFKFLRENGFTKSERTLRRHGVEKKNFLHV
DTLLLPICESSHWTLLVIRPSKKTIAHMDSLNPRGSQTYISLGLGWLKYI
LAETFVEDEWKVTQHEAPVQTNGYDCGVHTITNGMCVALGLNPIDTYTTE
DMPQQRLRLASMLLNGGFTGEFDLRVY
>Mb|QLI65880.1
MATTRRLITTAAPVLLRNTAWPAPAAACRAICSSASSHCARNIPGHRQAV
SARFAAGRRWFSAGREDAVPGSRIWSFDEVKQLVDGKRASDNIVIVDVRE
PAELQTTGKIPGAINIPVTSAVQSFHISDADFEDVHGFGRPSRDKTLLFY
CKAGVRAKSAAGLAKHAGWENVGEYSGSWLDWEAQKGPVERVKSGKGLGP
N
>Mb|QLI65883.1
MRRDNDEVAAAMADDNGTYFTYRPLSNLPTPPPTYKESKSPSTIASSSLE
DGEPLKAKYRGPAIHLVNLIPSSASFAIASVPIVQAILSRADLPIETVAL
AVCILDSLDKKFARAWRLSCPLLPGTWATSSTNKRHTLPPTPLLYQHQQH
QMLHIDSVPPELIIILAALVIAVKFIEDPQQPTQYYCDAWGRGTWSHDQLN
ATERCIMESLNYRIMPLCDEDCLSDAMVDMQLAGRQLDWDTPPLSPPDSL
ASSGSNMDRNFVLGHCRSKTMMPSTAALGLGLSLTPSESPCTTSCEAVTT
PRSETLGTTYF
>Mb|QLI65884.1
MASADVSIPRRERERERGPQREADSYRPPPRERTPPPVRTEEEKQAAAKA
EYEKLLNMRSGGTYIPPARLRALQAQITDKTSKEYQRMAWEALKKSINGL
INKVNTANIKHIVPELFAENLVRGRGLFCRSIMKAQAASLPFTPIYAAMA
AIVNTKLPQVGELLIKRLIMQFRKGFKRNDKSVCLSSTTFLAHLINQQVQ
HEMLAGQILLLLLHKPTDDSVEIAVGLCREVGQYLEDMQPSISVAVFDQF
RNILHEADIDKRTQYMIEVLFQVRKDKFKDHPAVKEELDLVEEEDQITHR
AELDGEIDVQDGLNIFKFDPNWEENEEAYKKLKAEILGEGSDYEDDDEGD
DESSEDEEEEETKAMEIKDQSNADLVNLRRTIYLTIMSSADPEEAVHKLM
KINLPAGQEPELPSMIVECCSQEKTYTKFFGLIGERFAKINRLWCDLFEQ
SFAKYYDTIHRYENNKLRNIAQLFGHMLGVDALGWHCLSVIHLNEDETTS
SSRIFIKILFQSIVEEIGLPKLRTRMTDETLRPNLEGIFPKDNPRNIRFS
INYFTSIGLGALTEEMREYLQNMPKPALPAPAAAADESDSDSVSSYSSYT
GSSYSSRSRSRTPARIRGQPVPFTAAFTFSVIRFKVTVSQARTQRLLLGL
ASTTIRLRKQIALAGAKTKRKATQRLVQLVQPLSVARSPPPFSVRQSASR
TGPFAEGRVVPSQQLFRKQRATQ
>Mb|QLI65885.1
MGVISSTPAAEQVPAHGVEKTSGAGRRILFLDAYDSFTNNIVSLVKDALG
SNTTVHVLHMDLRTIDSDSTPHWTRQQFLDRLADFDAVVCGPGPGSPLHD
ADVGAFKLLWDLPREQSVPVLGICLGFQSLVEHFGGGIRRLRRGLHGMVR
EIDHVQVPPTDIFDRVPRFKATLYHSLCADIGQDEVPEQDWLTARWLPSR
RAPELQPLAWILEDYDGENRHDPERILMAVRHTTRPLWGVQYHPESVCTD
AAAKGVIRNWFRQALKWNEAARREVPRNPSVGYVDSLNPSNHSLPGLDSG
RKLSAFPWWNDLQSDLARRGIRPGYACRQIKLPGGVDTADIAEVLGLGCK
DTIILDSSSTVNGDPLARSSVIALGVDEALRLEYHVGDSYLVLRQPAASG
RPEKSQRIYLGEDVDPWSPWHVMSEYWRWRKVSEDAHMPTFKGGFMGFVT
YEMGLSTLSPGSVSKARGHHRPDLCMAWVSKSVVLDHQAGVAYVQALTSE
DCDGLWIDDVVQAIETSPAWQQPGRGRRPDAKSRLSNEALLDHVRKGGGR
LHVSVPDSNGYESDVSKCQDYIRDGESYELCLTAQTTMTRPHGNDQPHHT
RTSPAPRDQSPSKLPATDEACRHGTPWQIYRALRARQPAPFGSFTRLGGA
TIISSSPERFLMHDPKGLCSMRPMKGTVRKSSAVSTLAQAEEILHVPKEE
AENLMIVDLVRHDLYGVCGAQSVTVPDLLRVEEYASVFQMVTAVNGQLPA
EKLEAGTDPTRSEFPFTGMDVLASCLPPGSMTGAPKKRSCEILQAIEPTE
RSVYSGVIGYLDVQGKGDWSVTIRTMFRWDDEQAPAQAGELEPREVWRIG

AGGAVTTLSTPEGEREEMFTKLCGPLGTFSDVA
>Mb|QLI65887.1
MPSFRTIFLAVAASFSAVANADYTIDPDSVPIGTRKAWCDQEMTTCPLIC
QQVEPRTTIDNTCDPESLTYGCLCGNNLQPNVSQYSLSLPYFVCTQWVIQ
CKDACPTDACKSDCQQKHPCGAQDPVRPNATKTAGASATSTGGSNTIYTN
APGGGSDNGKKGAGAALEVGRTYGLAFVLTSLFAGFALL
>Mb|QLI65888.1
MRLSTSALVLGAASSAVGLQDQKVLSNPKSTLGFDFNKLGNVDFDVWTKP
LREAFGEATSEAKALWQEVSMLAPEAVDAFKQHVINAKPKKHSRIPNSKW
DHVVHGADVQNIWVKNEKGESHRKVGGKLDNFSLRAKKVDPAKLGVDKVK
QYSGYLDDNEQDKHLFYWFFESRNDPATDPVVLWLNGGPGCSSLTGLFME
LGPASINKKVEVVHNPYSWNANASVIFLDQPVNVGYSYGSGTVSNTVAAG
KDIYALLTLFFHQFPEYAKQDFHIAGESYAGHYIPVFTSEILSHKDRNIN
LKSVLIGNGLTDGYTQYEYYRPMACGEGGYPAVLDESQCLSLDNALPRCQ
SLIKSCYDSESAWTCVPASIYCNNAMIGPYQRTGRNPYDVRRDCKGGNLC
YDELGYISDWLNKADVMEALGAEVDSYDSCNFDINRNFLLQGDWMKPYFK
LVPKILDEIPVLIYAGDADFICNWLGNQAWTNKLEWSGHKGFSEAKSKGV
KVSSGNGAQEYGKLKSHGNLSFLQIYKAGHMTPFDQPEASLDFLNRWVAG
RLDA
>Mb|QLI65889.1
MANMAISPIRSTATSTYTGSTISLPIARQQSNNMDGMGGVAVKKEGWASV
KESKNFIQPWKQKYLILRKESLDFHKTEGGKVSYTLYLKDVVNVGRVEAA
GTIFEIKRKPDGASNSPGDDDGQTKTLHIKVKGDDELYEWIDFIYGACPG
MGGVSNPTNFSHAVHVGFDPQTGEFVGLPPEWSKLLNSSAITKEDYERNP
QAVFEVLDFYTDLAKRAENPNQYSSMTPTPPGGMQQKQIGAAAGAGIAPP
RPAPPTPTQRNMSYGTTPPQSSNGQQRRPTLGDQQQQRQQMQNMAPNYVS
QDALREEQRRKQMEAQRQREAEDRERRDIEAYNASLPKNKVPLAQQEIGG
YGGTSGGSPHGDRFNPTRAAPPAPKLSGSPANGMRAQRPAPPPPSSTSSP
RPPQAAQASSSRDPTGQSSRIPRNDGSPASRNPNGTQSQPRQPQPAQGVS
RLPAPVKPLNVAPKPSQAHPAEGIKAAEAALTAKPSPSERKQDVRMSTMS
ENEVMAKLKEAVSKDDPNMSYSKQKKIGQGASGSVYVAKIKETAQGVARE
VLRQQGARAQVAIKQMDLAHQPRKELIVNEIMVMKDSRHRNIVNFLDAFL
RNSNQELWVVMEYMEGGALTDVIDNNPSISEEQISTICNETCHGLQHLHS
QSIIHRDIKSDNVLLDARGNVKITDFGFCAKLTETKSKRATMVGTPYWMA
PEVVKQKEYGPKVDIWSLGIMAIEMIESEPPYLNEEPLKALYLIATNGTP
RLKKPEKLSKELKAFLSVCLCVDVKSRASADELLAHDFLKYGCPLGSLAE
LLAFKKHAK
>Mb|QLI65890.1
MSPFLPPKASILSPTTPVQPDRQSKPTTGRKPSVDAHRSYSISSWVKNIL
PSHRPERRGTLRRRGYWEQQELTHSNQHEAHFAALGRRSRRSATAGSNHI
TEWNLARDLSDGGKTRRDCEENMVLDADRKWSWAPGEDAMKSRTDIPDME
QVQGASRTDQNRANSAAVNSLGPQPPSAAELQNIEAKKETRQLRRNLKES
GDYLGVQGFNPETSQLDVVTSTESDRSSSLSQETQQKLLILRNTLRDARH
SYKSAKERNDSQVKKIPWKNEKEKLCRLEKEKEAAKEINKTIKWKRHARQ
WSSAQEPDLSPIAQSIVGIAETSRKESHNQNSKLEHHDSMSNLIDLDSSC
KTSFPTSAMHDDQPLAQSPDSAATVVKTSQGQSLADPTALGSSAWELFVN
GISFDSSDEPVPAKVPEASLAGSERTHIRQLENSEQPVLGRKSDKCQETH
YSVLREELKQNNANIEPTNHGSFLDLRRQRQEDPRGETAIINSLATAMSK
YTKPSIRPRRLHLPHNVVNLSSQCKKDKGGPTTINCNKKGEESQSSSMMT
MDQVLRKRQSEGIQPTQEKPVIATGSRGKLMQTWGHWIPRRRGSRSFQSS
QDNTASHTAAGLETIPQIPDSWATDLTSRLGQQIFAHRPETNQRVNTNST
IRQQTGTEVEELHRLSSITETSKHIDKDKDILARDNRESLAWKEVETKQP
VPVSYACIPITTTTGCGQQDLQVSSRANTQPDTKNPSTLRLERHLGGMAS
DLPLAARSERNLGAAVMKPLRNQWRSVGQANHGIGTDTMLKKRHIQIDER
MERSTRPVAKTGNTATPDVVVSKPEERVGASKAIKSLKKPVVHGVDKRSR
VVSKISTELKDASIRTECKLAMPEQTRPMAQQDEPDGLSDASQGPGSFPD
LNVIHTGGDGCQSSSSGDTATDTVSHMAITEYLETVRCHLLAFFLLYWEI
VGPVFTSRSEYWARNRRHESTLGDCFALLLALPGAILAVMGLV

>Mb|QLI65892.1
MPHEILVGSSCYTATSAQEIKARANKTSQPKIQQIRGQWVYFVDLKSSNK
AVLDNVKTLLQDVDSEPLLASESTSNTITVYVTPRYLSPWSSQATNIAHV
CGLKDQVNRIEKGRLIVIDFEEPYQSGQDATFRDVLYDRMTEIFSFEKPE
LDVMFAAGVPAPLVVVDIFADGKDPLSVLRDYNKEKGLSLDESEMAYLVD
VFKKLGRSPHDVELFMFAQVNSEHCRHKVFNANWTIDGVKKNKSLFEMIR
NTHKKTPDYTVSAYSDNAAVLQGESANFWAPDYSTGTWTMTPEVAHILAK
VETHNHPTAISPFPGAATGSGGEIRDEAAVGRGSVTKGGLAGYWVSDLLI
PDHRAPWEVDIGRPAHYASSLDIMLEAPIGSARFNNEFGRPCLTGCFRTL
LTPETPAEGAKAEASAWRGYHKPIMLAGGVGTVRPSNALKEERFVREGAH
VIVLGGPAMLIGLGGGAASSNASGESNADLDFDSVQRGNPEMERRAQMVI
NTCTAFGEQNPIAMIHDVGAGGLSNALPELVKDAGFGGNFELRQVESADR
SMSPLQIWCNEAQERYVLLINPENMNRFTSICRRERCGFSDVGAVATKDA
NGDAKLILTDRESKEYPRPIDLPMDALFPPKHQQERNVNTKKANLTPFDA
LASLRKAFGADLDNATLVKKTVERVFLMPAVGSKSFLITIGDRTVGGLTA
RDQFVGPWQTPVADVAVTATSFSLGGKQRTGEAMAMGEKPTLALINPGAS
ARMAVAESLMNIGAADVMGDLRRVKLSANWMAAVSHPGEGAALYEAVEAI
GMEMCPELSVSIPVGKDSTSMKAVWKEQNEVKSVTAPVSVAISAFAVVED
ILSTWTPQLRRVEEVGESILVFVDLAEGRRAMGGSALAQSLGAIGNEAPD
MKNFNLITDYFDALSQLHKSGVVLAYHDRSDGGLITTIAEMMFAGRCGVD
MMMDGISKSGSAGDMIEALFNEELGAVFQVKASDEINFKRCFATCGPPPG
LIRKFGVVKASPNQSLTIRHGATTFANLDRTEMQQWWSKTSHQMQRLRDN
PACADSEYAAISDVSDPGLSYHLTYSPSENILPLTSSITGFFNKTPRVAV
LREQGVNGYAELAFAFRAAGFEPVDVHMTDVLGGRSLADFTGIAAPGGFS
YGDVLGAGQGWARSVLMHESTRREFEHFFRRPDTFALGVCNGCQFLTRIQ
ELIPGTAHWPTFVHNESAQFEGRYSMVTIQEDESKPSVFFHGMNGSSLPV
VVSHGEGRAKFSSPNSLQELTNSGMIPMRYVDNRLKVTEKYPYNPNGSPG
GVAGVSSRDGRFVAMMPHPERTIVAGVNSYIPPKATEEWGDFGPWVRIFK
SVRRWVG
>Mb|QLI65895.1
MDAIDIGAPTPTPLPDPPVKDFMTDAQWETLYALLDGVLPCITSTTSSDV
KDKNSGILLSDTDFEALIDDCTAALSNPPSRHKIKEYLEFRPSQDENFRD
DCLRSLAIVPQRNQLIKILNLLGGHAGSMLLTGYWTPITQQPTKVREAIL
KSWATSRLTPLRMLAKALAQMAQKANAIHSPYFREISGYSDAPKDWKPVE
GYEYNFIRVPAGTGVHEITTDVVIVGSGCGGAVSAKNIAEAGHKVLVVDK
GYHFPPSQLPMTQASGSHHLFDAGGVFFSDSSSMGVACGGCWGGGGTVNW
SVCFRLQDFVREEWAAAGLPLFTSSDFDDSMDRVWDFIGASKDAIRQNPR
NQALLDGTQKLGWKGGVVEQNTGGKEHYCGQCHLGCGSAEKRGPTVAWLP
AAGDAGAEFMEGFAVDRVVFAEDGVTAVGVEGTWTARDENGGVHTPQNTR
TQRRVVVKAKKVIISGGSMWSPLILTKSGIKNPNVGKHLHLHPVNLVSAI
FGKKDLASWEGGIITSYVDEFENLDGKGHGVKLEPVVNVPYATYSLQTWR
DGIDAKLLALKYRHIGTFIVLTRDRDTGRVYPDPRKGTPRIQYDTSDFDR
EHTIEGIIALAKICYVTGATEIRPHLHHLEPFVASDGGKRQAEHQAGKDP
EFTDPEFAAWIKKLRVADNQGPTAMWMSAHQMGSCRMSADEDTGVVDMKG
KVWGANNLYVADASVFPSASGVNPMVTAMALADWISRGVASELAGE
>Mb|QLI65896.1
MSDNGLIGTIAPGTRLMSGPPQDDAATASSVLFQSSDKTVVLLDVPRSLE
EAQVLPGCRLLRRVYSDPPPTKPFPTPDPKRSRRRAGHRGRCDHASGHDG
GAAHDWPAQPPAAQIADLMTAAAVQSALQVMCDGYNGPFHLPRKPLPADI
EDVNAPSPLHVQDARPLYGSIQDLRQVFADTAPVFKLVVLDPPWPNRSAR
RRSNKYATATNLDDIRALLTSIPISAHLASDGLVAVWITNKASIPELLTS
PAGVFASWGVELVAEWTWLKITAAGEPLYDVESVWRKPWEKILIAKPIGA
PAPPGLKAKVIVAAPDVHSRKPNLRLLFQDVLGQQDYAGLEIFARNLTAG
WWSWGDQVLHFQGQQYWEQSC
>Mb|QLI65898.1
MRFNLGTSSSGHRPLPQYKTCLNDLPSTRRSKAGRLAKCIVGGTALLAAA
GLAAHYLVPSLDSLGYKTGQGPPPDKVLTTHQLQALQPGNRSISVGGDRR
LRIFMPTDSPHINLCKSVMSAVALGYPAPILLNWGGEFNRPEWHLAGSHI

AKLESLLSVIENMLSLAEGEDGDVHQDDLAVLVDAHDIWFQLPPSVLIQR
YHQLNREADERVRQQWDDAQGFAADFPIQPPKQSILVTAAKDCHPKSDSG
SDPHYAHWPESPMPSDMYGHGTDKVLTMPLDPARKFKKIRPRCVNSGMIM
GTMGSLRNALTRAKAKVETVAHRGRQLWSDQALFGEVIGDQEMWREWVRE
LGSTWNGTASENYLSRLPADVRGIAKAALKGERFEYGIGLDYSFGTIPPT
CSAEEDGYFVKMDDRQALKRESARAGVPNGEVRVNGVPAELDQAGVGSAH
LSGIKWGDASLYTDFFFGVTPVGIHHNAYVDNLKSIRLKEWWRKMWFYPH
LRNLVARALLPMDAGDTIRPLARIPAQEGGQVTRYWTPKSDLRNRMVKVF
EPASLDAKSGGSFASIEWEGVCQRSGQKPWHEELFGDKKVPVVAPGTSLP
PPVDPALIATLLTFLSLPPATSIAPLEANAAFHRIYLIHYASPPPPELRD
LRRSPGGGTSLVLRVSGPQAPPYLKTANEVAVMAWVRGHTRIPVPRVVRH
DCTPRNVLGCEFTLLERMPGRSVDGLYARLPEPARRRLVNRLVDVLVELH
AHAFPHVGGLQLVGGEGGEVVPGPLLEDTCWFLPDAEAEFGTDVPVGAPG
AVGPYASHAEFVKGFVGAFVHAICVRERLGWVRDLVPRLAALAGALPRLR
LDTRMVLAHKDLHFGNVMVDEDGELTAVLDWEFAGVVPVVRWDPVRAFLW
NGVYSQEGHDEKYRMRAVFERELERRGVDKWWEATNADIDAVWDVVRYVR
AIVELCPRGDNMDLCRVWRAKAEEALAKLGV
>Mb|QLI65899.1
MGLLALGTALEWPEAKKRADQVREWGIKQLLEIWNKAKGKERDAMLWGDE
VEYLVVTYSKEEEKVLLSLRQAQILEALAADKELAKEGCVPALLDGPVEK
PKSHPLPVFHPEFGRFMLEATPGKPWGIGFKELLDVEPDMKLRRKIAKEH
MLSKEHPITLTTFPQIGVPGQFTDPYYPPSGSKLRSQFVPDEIANPHIRF
PTLAANIRWRRGRKVQVNVPVFHDKNTPTPWKDPTANYDLHNWPEDDDVR
NGAAPDDFIHMDAMAFGMGSCCLQITFQAKNITEGRQMYDQLSPLGPIML
ALTAATPIYKGFLANTDVRWNQISRAVDCRTPEELGEKPLQNDRWRIPKS
RYASNSTYISTDPRLRPEYMDPDLVIDPKIKAKLMEGGMDDLLATHFAHL
FIRDPIVVFEEDLQELDLNKTDHFENIQSTNWQHMRFKPPPAENNIGWRV
EFRPMEIQVTDFENAAFSVFMVLVTRAMLSFDLNFYIPIKKVDENMERAH
GVDAVLKERFYFRRNLFPLRPSRANTAFGEESRPGSAMPSRSGSPGLPVD
DEYAEMSIDEIINGSADGGFPGLIPIVESYLDSVNVDVETRCQLATYLSL
ISKRASGELDTTARWIRNFVAAHPRYKHDSVIDDAITHDLIGAVIAIGER
ESAGKNFAGLGIHGLPRLLGRFRGGCGQDPMTNGEDGNGDLVAQWAAVKE
NELGNGSRKRKSEWIDGENEAPGRE
>Mb|QLI65901.1
MRSSVVTSGLNSLRSVGSRPTAVFQGPWMATAIPRPALPSMEQARTGATL
VDSPTAAHEQPEVVENTARQANAKKILQDAVAATGPRQNWTRREISAIYY
QPLLELAHQASLVHRRFHSPGEVQLCTLMNIKTGGCTEDCSYCAQATRYQ
KGTGLEAKRVESVESVLAAARTAKDNGSTRFCMGAAWRDMRGRKNSLKNI
TEMVKGVKAMGMEVCVTLGMIDAEQAKQLKEAGLTAYNHNVDTSREFYPS
IITTRSYDERLKTLGNVRDAGINVCSGGILGLGESSEDRVGLLHTVSTLP
SHPESFPVNALVPIKGTPLGDTTPIAFTSMLRTIATARIVMPATIIRIAA
GRKTMSEEKQALCFMAGANAIFTGEKMLTTDCNGWDEDSAMFGRWGLEPM
KSFDKKPVQGAGDIEAGKI
>Mb|QLI65903.1
MSLTYGVGGVASFLTVVGAYMLFTGSGEAFNVGAFLESVSPYAWADLGIA
LCIGLSVVGAAWGIFITGSSILGAGVKAPRIRTKNLISIIFCEVVAIYGV
IMAIVFSSKVDKTDVVAAASAESYYTGFALFWSGLTVGLCNLVCGIAVGI
NGSGAALADAADPSLFVKILVIEIFSSVLGLFGLIIGLLVSGKAPNFGAT
S
>Mb|QLI65905.1
MSKLHPPGVKTVGPDTNCTLDNCPAEWSIYGFRPSLAANATFLGLFILVG
LAHGYLGFRWRSWGFMGGMILGCLSEAIGYAGRIMLYNNPFSFVGFMVQI
VCLTIAPVFYTASIYVTLSKTIMYFAPDLSRFNPQLFYWIFIPFDVICLI
LQAAGGAMSSNSNGDDRLGVNISMAGLALQVVVLTAFVVCFADYMVRYVR
SGRTRSFGWRLNTFFLGLASSVLLILARCAYRVAELKDGYNGSLIREEIP
FIILEGVVIVIAAVALCFGHPGLVFGRDEARKNSHDFEESGTSTPERK
>Mb|QLI65906.1
MATEPEPEKTAQPQKEDEEEAEGGGGAAKPGPRALRLQEIYAASLARTLD

KLSYDNVAPCYPTIARRASPVLRQVQAQMVERLRDKCEREFDAILGARRV
VRKMNELEGLVADAEARRKLHGEEDLPTPAHLLSPEEVLRAHLGPRLAEQ
RGLLNARLQTTQAQNGLLADHVKAQREEIDALLGKLDAAVEDVRSANGVL
GGVVGELAGEARGIDADMGDASVS
>Mb|QLI65910.1
MARKSIPVMEWAQAHHSVVFDDDYEPRHIAPARRGGPEQRFRSLSMSLSW
KRPKHSLFGHDDMRDPTHHGNDAAIDDSHDGPEHPQGPTHVKQRRASGSL
KGIIRRASMSLKGIMHRRPSVAAADVIYEQGASNERPTTSHGPWNRLGQA
ANFHRSPSFYDLDLYRDPNKHVPIPGLGDEPPVIPHHTGAAAKASAAMQN
EYFARQRGLNPPSSDDGNDRESGIGIAVTRLSVDLDSVEQESVLEQDSRI
SRIDFVSELPAELAIHILACLDASALAKASAVSRQWHHTVSNQHIWRESC
LRETTGTYATSRPVQPGAGMGVPKIHPQNDWKQIYKVKLELSQRWKEGKA
RPVYLHGHTDSIYCLQFDEFKIVSGSRDKTIRVWDMHTMECKLVIGPPEV
VDGPHLLVDEEGNPSHYAAGSDNEQTALSLPNTVSFPEHHKASILCLQYD
DEILVTGSSDSTCIVYDVKAGYRPIRRLRHHTAAVLDLAFDKKHIVTCSK
DISICVWDRETGALLRQLRGHTGPVNAVQMRGNTIVSCSGDFRVKLWNID
TGKNIREFVGHTKGLACSQFSEDGRFVASAGNDKVIRIWDANTGECVREM
KAHENLVRSLHIDSVSGRLVSGSYDTDIKVWDMETGNQLLDFPRWHSSWV
LSAKSDYRRIVSTGQDPKILIMDFGAGVDGIGQLETSAAQATD
>Mb|QLI65914.1
MSLSEEPTYGAASIPAQQSTEENAATKENQTQRLYRNSLPSSSDSPSSQL
PLKGQPHLPRTESNLPVAAESETGARKQKDQDSSSDESNDTSSSNSSANG
KDDDDMLSVRDLSSSSTSSLAMPTGDVPALPAKSSLRASRLLASIPQKRA
PNDDQPMLPHAAPHQVYLSSEEDASSSADDFSDFDDEMESDGEGGSQKPS
TRNMAREDTARVVAVVFHGKPSIVTLSPRRSISPSSSEVGQPGTGLLRTA
TEPSLQHQRPRSVSSATSVMKFNHPPRSSSMTTGFEKKRPHFLHIDPYAK
VRDEAAEHLRGPMTPTSMFRRTLSLVKKRSKPNLHQSESQLTPMEKVGEV
EEEEDPRESLCLEPNSVGPVSNQRHGSVSSPRSEASSPISPKSRFRTGLS
LGRQRSVKA
>Mb|QLI65915.1
MSIIDTTRDLAALFEQQAQATPQAIALEDEKRTLTYAELDSETWALADRL
RQYGVGRDCLVGVLMNRSADNVVASLAALRAGGAYLVLELAYPAALLRDV
IDDAMPTVIVTQKAHASNLNVDIPVIIIDQPDMTVGANEPILVEPKPPLP
AEDDLDRLAFVSYSSGTTGKPKGIANPHRASVCSYNLRFGLSDLKPGDRV
ACNVFFTWEMLRPLLRGATTVAVPDSVSYDPEGLVEFLAARKITDTLMTP
TLLATVLTRHSSLADTLPHLKSLWLNGEVVTTDLCRRAFTALPAVRLLNV
YSASETHEVAAGDIKSFIDYEARVCPVGPPMDPSTIYILDDAGNRVDIGT
SGELYVGGDMLARGYLNLPETTAKAFQIDPFSTGLDARMYRTGDMARMLP
SGLLEITGRVGGMIKTRGYTVQPGAVETTIVKHLAVRACAVVARGDGLDK
QLVAYVVPDKGEIRNRTVLVVDESGYSPVARRALSDHLAHYMIPTLWVEL
ETLPTNGVSGKTDLKALPPPPSPKTPNINAKKEHDTRIKMDTIIKMWALS
LNIPATAISQSHTFFDLGGHSLTLADLAGRLTRTFGFPIPLAPLAGTPTL
EGHMEAVQAARDGHTAAVQADLPAVLLADSALPDDIKSNGKPMCRLGDAE
TILLTGATGYLGAFLLKSLLESTSAQILCLIRFTDPNIDCRPAGIARIRK
NLIDQGIWNDAMLERMEVVPGNLARNRLGLSPEAFQDLASRVDVIVHAAA
TVNLVYPYAALRNANVGGTREVLRLASRGGATVHHVSTNGVLPPSKTAWT
EEAMLDIDDVPTKLPDGYGQTKWVAEMLVYEAGRRGMHVKVYRPGTISGH
SLTGATNTYDLLNALIVESLHLGYAPKVDGWLAEMTPVDFVSQAIVTLAN
HPCTKQKLYHLGDPDPVTAENVFQDLEALGYPTKRLPWDDWVALWKEKRG
SGRGGDDPFTVDILRGGMPTVDTLELVTILSDGATQPALDLYGVSRPKID
RNLWETYARHFFARGWLPRPPLRLKLNGVHPHPKKGRLSGCVAVITGASS
GIGAAVAAKLAGEGAHIALAARRIDELEKVKTKLSTCSGKILVHKTDVTS
KAQVESLMKTATEKLGPIDILVSCAGVMYFTMMANCQTEEWERTVDINCK
GLLHCLSSTVPGFLERKRGHIVAISSDAGRKVFPGLGVYSASKFFVEATL
QSLRLETVGSGLRVTAIQPGNVDTGLLNISTDAEALKKYGEPTGAKVLDA
EDVAGAILYAVTQPEHVAVNEVLIEPRDEPI
>Mb|QLI65916.1
MDRDRDSRGRRYDDGEVVRYGAGESWRPTPRGGGPGGDRSPRRPRSPIRD

RPRSPRPRSPRPRSPMMPGSDSYVPGRYQPRRRSRSGDRFRRDRSRDRDS
PPRRRERTRSPIRRSPPPRRSPSRRGSPIRDNRYDRPRSPRRDWDRDRDH
VRDRDRDWDRDRMRDRERDFDRRDERRRSRSPFVRDRRERSPPVKMAPSG
PRGGSYRPRSRSQSRVGERYQTYRRASPVRESGISSAITSQPPSERASPH
PGSTRARSPLQSREQSPHRTVGSGSGSVRDTPRSGPCEPPSASRSPPRGP
AALRAPPTGPSAGRTLSTSVSSPLPSSSRHPQTPGAGPHRSDAPSPTNPP
SGPRGYVAQRGYPSRAGRGGWSQTPSRQMSGLSPSPTTPGGSTSIPTGPR
GAPSAGSSTPTQGRPFNPPTGPASQHGNGPRQSLAQSLLATMPPLIPGGK
LDSSMTPFILGVTRELEPHYRKLRDEEEKMREELRAKQERLRKSLYMWDR
LERESRAWELRSDLSEKSMKNLAGEGMGGAAF
>Mb|QLI65917.1
MIQLKTMLNCIDNSGAALVECALVVGQKRHARIGDRIVVVVQEQRGASSS
GMAGISAAAKVKRGDIRHAVVVRTRYPTQRRDGSVVRFDDNACVLLNKSG
DPVGSRINGVVGAELKRKKWSKILSMAPMQA
>Mb|QLI65919.1
MAAQKDYYADLGMPRDADIEAIKKQYRKLALKYHPDRNSGNEDEAIAKFQ
IIQEAHEILSDPTSKAKYDATLGRGRYPGASGVRGNPWADVGQQFPTPPR
RTPAARNATSGAQRWQTRFSSGVPPTAKQTFGSDPNAKKNAAKAFENMRK
NTRGEPRPSEPPPPPPRQPPRTESARQRQEASFGSRKSGYYPRSTMPGDE
PPVSNANYTNSERRNVPQPPPRKPVPSPMPDPLGQFREKGASSDSRHGRA
KNARDGNLADEGSASDDNQRSSNVPRRPTQEERDANLRVPTDGGQHSQSA
DEGTGTYTNTSPESRADARFDADPTNETAKSPMEDNSMGNKDEPTMYETP
LNHHRHKVSIFSTYPGSTKPGKAWTSPTAGLDSSGTVYQQRSPSGKKGGS
VTLLSFERQQTNILNRLINNKEGLPWLTEEKECILSTDCYTETSKHAQAA
NRTRSHSFSFPINDDTFAETPNVPGGHSFAKSSLDDINTSFVKDDNSNTY
QFNAGTTEEDANVGFMPSSCTQRARHSTIKRPTMRHSDSANTTSHAAQVE
GGFNPDGWSDKFNAQTFVPQPQPGPSASPTRASRTNSRKQKIRPMAGTAA
TVEESSSEDETYEWRGRNAQAQEQAPATDSPQAMDIDSPMSSGASATQTA
RNIPVEPSRPEWRAGKVAGGMADDEKPQRPAKVPLDVNAAGSEDSEEFRA
NFADLKNVPPFSQQKVGLESFDGMKDNLPFESKASDGPRAKVPRPVPLEF
PSVPLAPRLPPTVAIDGMKPNTASWTKYVGDFESYLRQWDTFNGLVVDHF
ATRKLLIENTRASKGYGFLAARGDADLQAYYSWVQQDNDVRRQWVSVCEE
HESRMREFMAFRDKMKDSRA
>Mb|QLI65921.1
MAHYNTNGPKNGVKSVEGPREQNGVSRPFTLQESLPYSPQTSTVPFLPDI
IPDPSVGSGSPSLRISDLFSTQDYDRVNQEATSQPHGAKNLKQTVDHVLH
DLKPSKRTQYKFPTVPNGAPSSSAASTHNRIISQGLSPIAKAVYERVGSF
FKATKPNISSPKLVNGEAEQPLKLAADSVSQSPTIHVQPQSTAKNNAGSG
KTKIEVAISTKQSFDRSQYAEISYDHPDQNIITPHDQLIVEAQQYSQSNP
LTAKSSIVVNNAGLLIELPTSGIKREDYQEFKVAPDAPENLSFRRKERQE
FEDGQDIIGASLDQRQRGEAALDSLDALMRQVFSAVGGALVMEPGLEHIV
TLTADQEASMTATTQQKMHTAIQKVIGLRSFNAVPLENLLRVMKLSEASL
KQAEGLDIRVDESWDEAAVESWVQQLGELQTALKSARTCLRIMSGCREDK
QLYSEAVITKCVNIYKAVTEDIVMPLVELRNSGSSGGLFKLIQKHKKTIA
SVFVCCQKLSALLAELITKIELSETVINTLEFTSSKLIFVENAYLEKDSA
IGLQKFDGIRSVAMDMLCQIFLIKPEQRQGIIDDILTSLEKLPVGKLSSR
QFKLADGGSIQPVSALIMRLVQASSGRVGGGNGAGQGSNIRAMDIDGLGE
DDEDDRPLKRNQATATILNEEQGAQQPAVAIQELEGVAAPLIDTAARNAS
YVINFIVKRAMGSTKSGDTPYRNLLDLFVDDFTTCLDLPDWPSAELLLRL
LMVMMVQLFEAPKTAAPAKNMALELLGTMSAAISRLRSHVKKSAGAFISS
DAGKYSKYLSDLATHCLEQRCPMENIIAWAGPYRGVLEYLQERSSEDPHL
SSAVSFLISDWATRVHTTYDNIQDNDSEGDKELGRSAFRLRMMIEDRQWL
ASEYTFKAVTTSQARFAFSVILLRSPFCESFGKILNILLGSMASDQATVR
SKSLKSVNQVLETDPSILDGDSTVIQLILDCASDSSTQVRDSALGLLGSC
ISMRPVLEASLTPKIIDRFQDAGVGVRKRAMKLARDIYLRNRGKNLRSAI
ANGLLRRIQDPDEGVRDLARQMIEEVWFAPFYTNENTASFETSLAEHVSL
VIQTVKGGTVTEILDKVLQTILKPGSKSLDGPFGVCSKLVGIMFGLIDNP
ESEDPTVPSGRDALQVLTIFAKADPNLFNFEQIKLLKTQLASFTGADELA

AFRAVTVIYKRVLPQLPNVHTEFLADVRLQLLKGIGKISSRGALDDLIAC
AHTVCELLKDFAPLGNLVASSLVGIQKLSKVPLDAKRLNLVAAYSIIVGS
VGKHCDLDKQARIFREKFPGWKGDSVSKLIVDILSPFSSPQQPLDARKAS
IEAIGLVCQSWPRNYDLVKVYTAFQQVFQDKIPILETMILRSFKEFLVTE
ERRSEAAAEAPKEKNKELTVMGGTNFDDVASGATQRFLKEITRIALSSQD
EHAFLAMEVLGSINRQGLTHPKETGVTLITLETSANRKIAELAYLEHRSL
HEKHETVLEREYVKAVQSAYNYQRDIVKDSHGATMEPFQSKLHLLMEVLK
ISKMKNRQRFLEKFVGQADFELGKLNVKAEMPHQVDFARFIAENLAFFEY
HSIGELQTTVNMIEKMVASTGASVAQAIESEVFNVRMDVDQEEEQRKTLL
EGDVPVGVPVGGTQPDVPPPAVPVPTLSVEPQRLRQLTAGSIILLSLWEV
RTHLRRLFGMGTNRHDNRAKALAKDLNKTPLKVQGVHGERVWDEIVSHMN
GLSSQESMVQKCKAFVELMNVDKEFKVAEEDDEMGMGGGPSTPSEGEDDD
DGGDRGRKRKAAGTPGGRKKRARSGSQTRKRGRPRKQSVEQSDDGDFDGD
WI
>Mb|QLI65922.1
MASLRDLYGKQGNGKDSHNFGVDYVLHYKIPPEERDEAEAGFVQLIKSLT
KVGLAAEVRNGDPGSLLVFVKMASTELLGQQVYRGRLHDWLQGVRTSGPN
SDITKALEDEPVMEAERLRLVYQLIARPENEGGTGISQASSKWKYVVDVF
PIHDQPFNKAWIQKWSKKYLLDDSDLEDIRSKFGESVAFYFAFLGCYFRF
LAFPAALGLGAWVLLGQFSFVYGLGCGLWTVVFLEYWKKKEVDLAVRWGV
RGVSALQLPRTQFEWEYEAEDAVTGEPVKVYPYMKRLKTQLLQIPFAIAC
VLVLGSLVVIANSLEIFINQVYDGPGKQYLGFLPTMILVIFTPTFSAVLM
SAANALTDKENYDTVDAHKAALIQKQFVLNFMTSYMALIFTGFVYIPFGN
ILLPFLDFWRRTAQTLTFSDKPLPTQLFRIDPGRISSQMFYCTVTAQVVN
FATEVIVPYVKHKAFAKAKEFKSNGLLQAQDHPEEAEFLRRIRDECELEV
YDVTDDYREMVMQFGYLSLFSVAWPLAACCFLVNNWVELRSDGVKIAISC
KRPIPWRSDSIGPWLNAIGFLAWLGSITSAAIVFLCSGSQDQKRGAASQI
TAWGGLLSILLAEHFYLLTQLAVRFVMNKVESPGIQQVRKERYLMKKKLL
AENLGQPAAEKASIPGVEGGEKITRQALEEEARQASIRGHGSPEEIFWQR
QRSMEETILIGRKMIEQQMAAENKKQQPTPGPSPRA
>Mb|QLI65923.1
MSDVEENNAPEVSEEVEVSADAPKGQMSVLDALKGVLKLALMHDGLARGL
REASKALDRRQAHMCVLNENCEEDAYKKLVAALCDEHKIPLIKVQDGKQL
GEWAGLCVLDREGNARKVVNCSCVVVKDWGEESQERSILLNYFQTSQPS
>Mb|QLI65924.1
MPRVLHVQKIDGKPGKVYYPIKIREAPLPEVGPSQLLVRMDAAALNHKDL
FIRRHLYPAISFENPLLADGYGTVVEAGAAVKQRNLLNKPVLLNPMLMWE
SDPVGPERGVVLITGASSATKTGCAQDYALVEEDDVVPAPAHLTPAEGAA
LPLAGLTGWRALVTKSGNAEAGRNILVTGIGGGVAIQVLQFAVAMGINVY
VTSGDQAKIDKAKRMGAKGGVVYKNDGWERELKSLLPESRPYIDAVIDGA
GGNIVSKATKFLKPGGIISQYGMTVAPKMEWTMQAVLMNIELRGSTMGSK
REFKDMVAFVNEKKIRPVVSKTVKGLANLEGIDGLFKDMDAGTQFGKLVI
EINDRPSSKL
>Mb|QLI65927.1
MRLRDDLILGLSAIVTAGAAAPEFDWDSISPSTDLRYHDCYAAFKCARLK
LPLDWKNSTDPRTVAIAVIKLPAKIPDHDPAFGGPIFTNPGGPGGSGVSF
VVDNGHFLRDYVDSPGKKHYDIVSFDPRGTGNSRPLANCFPVAALARDAW
KLETRGNGGPDKSLAAISYGLALYDGYGRRCEKADAEGLNGGEIFKYLGT
PSVARDMVAMVDSIDELRKTEAARDGPVELLKRNEGDVPRLQYMGFSYGT
ILGNYFASLFPGRIGRIVLDGVSNADDYATGPGWLTNSVDSDAIVDKFFD
GCFHAGPQVCRLARPQDKSASDLRTRFWPWVKQIDEAPVAGVGPLGSSVV
LTGTDIRLVLVGAAYTPLKSFIQTAEVLDNAMVHGTYDTILAMIESGLGA
PLQDACPVANQTAKPDLGTDPLTAVLCGDGEDIRGKKPSWWARYVDRQLA
QSAVFGGLMSTVRFPCSGWRFKPNWSFRGPFTSPEPARDGEQPQRGQPAA
PIMFLTNRLDPVTPLASARAMARKHPHARVVVQEAMGHCAAISAYSECTR
GIVARYFDTGKLPDGEAVCEAECGPWDAGCARTMPKSSGEDAGEASLARR
FPLGV
>Mb|QLI65928.1

MAKKARQRISYVLENAKSSEGGHRLGVNGLAVDTDNGILYSGGRDGIVCA
WDLDLDLRRPSTDTHDSADKTRSRSKFRAQAQPHTHWINDITLAQNNTAL
VSASSDLTVKVWRPHSEEDCTRTVAIGEHADYVKCVATPPASINANWVAS
GGLDRKICLWDLNGAGKTLEVDVKGEEILEKGSVYALAVSRDIMASGGPE
KTIRLYDPRTGDKVSKLVGHLDNIRSILIDDSGDIILSASADKTIKMWSV
KGGRCMYTFTMHDESIWSLFSEDPSLGIFYSSDRSGLVAKTDVRGSMEDL
DNGLSLAVAQEHCGVSKVVAAGGHVWTATNRSSVNRWGDIDTDTDACLPE
PFRHQRAASAASNKLRQATVAGAQATSNKEIPSESILRISNTAVFPARGV
VDAEPNNLNESLTRRGSEVVVEQPDPEVKPIHQTPEETIEGQFGLLKHKM
LNDRRRVLTLDTAGDVLLWDLLACKPVQSFGKQHLEDVEALVNTREAVAP
WCSVDLSSGNLTVVLEPFNCFDAEIYADELKLDEPVEFREDQRISLGRWI
LRYLFSNLIDEEIKRDERYRQKLNEEVSRNQAAGRANAPTSIDIPPSNVS
NWDKPDQLATPRANGSQLHQGTPGLAIGLATPAPGAALPGVPEEVVSSPL
SPTEKGNTADKDDYFAGGSAVGPGAPATESSEMKTSTDNGNEKGKDKASD
TAKLPGSTFGKKFRISFSSKKLGRSSSQATQDKPVVVDEKAEESESSSTN
EKEVDDSFWGVIQKIRNDYDKQLADAPEKLIETKVTPSLPSETPVLKLPP
GTKIIIQEEMSGGSANVYQGTVENVGKDADIIEQKAPMWLGDVLLQNLVP
FKEPVKISFVLHPMGGLIALSPADGNNRLNANRMLRVKKILAYVAERIEE
VPEEPQPNALPPEKYLELYCNDQLLDPLMSLATIRTHVWKSGNDVVLYYK
ANGRKEIIPPKPASSIIPADTPGAPSPLGAPTAASESTTSTTTAVT
>Mb|QLI65929.1
MPRARRARQAPPTTDSNSPEPAAVTDDHDEQRGRTRGRTRSTRSTGARLR
LDEEDAIRAANQRRDAALDSLANEDPTSTPGPDDTRSSIEIGRRAMATPA
MRRDTTGLDLADDDVFGDLGDSFADGDIPDAPHSAASSSGTLGHFKTRPR
SRQSSIIGRNDPPIRPSSRGTNTPGVSSSFNIGIFRRRAREPSILATSRR
ARSQTGSIAGSAASSRAGSVARDSELESEGEFAPEAESTPLDLRRRSRRS
QAHVARDPAPSPSPLPEGRRRSLRKRKSEGNAQSESPRPEKVARVEANGG
IDISSDSELSELASASPPAAHFARATTPINMDEINAPPASSDSEADDSVW
PDIHTLAKRHRRPSINTPLRNNRDDLSDVSSPPSLTHSPNYGTRTRGSKH
QRSQQPTTADLTNLLPKRRYKHNREESDIDTEGEDGEDTRPRRGKGSRPP
SRTTSRQGRNILKPKQTPVSVRRSARTASKTHHHEEDKENESEEEQDNSQ
FQPLPDDTFDVGASVTEDIQNADELKRASTKFKEVDRWELSFEEVTEPSS
PLGAR
>Mb|QLI65931.1
MTLPHRTWNFDIDRFLNPFVPSPPWRFLPYPVARWFGYRKAKPKDAGNLM
PVFWAFIGVFAAILTIQAVSKRVPSFQARGAPIIVGSFGAAAVLEFYAIE
SPLAQPRNAIIGQLISAFVGVAIGKLFQLSDSFKDIQWVGGALACACATS
LMALTKTVHPPAGATALLAVVDDTLIHIGWFMLPVMLLGCALMLGVALLV
NNLERRFPMYWWTPEDLSKGRKSILRRRHSAELKKDDMDEEKTVDSPTAP
APAVTSKKSTIDGSENDAESSDRDQTGGMCVKFTHAHQHAGEVVIRKGEV
VIPEHMFLTQEEQQFLETLTYRL
>Mb|QLI65932.1
MRFTAAAAALMAGSAIAGDVSTDYTTQLVTITACPESVTDCPARSTQVQT
SVVPLTTSTVYSTKVHTITSCAPQVTNCPAHSTIVSTETVAVSTTVCPVG
PTSVPVPVVPVPTVPGHGNNSTGVVVPPPASSHVVPPPASSGPATQGPPP
AVSTNSPVTQAPAPPACPAPSVTAITKSYTTVLTSVEYSTIEVPCPPTGT
VPQGTGIPPVPTGPANPPSGGNPPAGGNGTTPGGNPPPVTAGAASFAGSA
VFAAVAGIAAFVLA
>Mb|QLI65934.1
MADRYSFSLTTFSPSGKLVQIEYALNAVNQGITALGIKATNGIVLATEKK
SSSPLADQSSVSKISNITPNIGAVYSGMGPDYRVLIDRARKVSHTGYKRI
YNEYPPTRILVQDVARVMQEATQSAGVRPYGVSMLVAGWDDGIEPEDEAP
AAEGDEKKPTSKTGGIQKGGPMLYQVDPTGSYFPWKATAIGKSATKAKTF
LEKRYSEELELEDAIHIALLTLKDNIEGEMNGDSIEIGIVGPPADHLLGV
EGVEGATGPRFRKLTPQEIEDYLTSL
>Mb|QLI65938.1
MSTDPALMPSPPADLTQTAEHTIKERHSLTLDQRRALRRWAEAQTIRPPH
KACIAWFHSQFGQQISQSTVSHSLSAKYSRLDGNNPQLSGSRLRFGNWPD

VEKLVLLWYRQVQAAGRPPTNDELGEKAKSIFLQLPRYRGQNVPEFSPGW
IHRFKKRYSLLVRRQRRSGDGAINLAEDIDYLADCVPRVMAVTSDTSPAA
IREQVLRVVGVEASLNTCALVRDEVLRRMSTSSNHSLLPQHAVPQPEPIP
PPPPPSEQPMYADDDPEVVLQNALRQLQQEEQAAEEQAAAVREERERVER
AGIHVAGMVATPGPQDSSSDPRYTMPTQEMGGHDLTLTPIHSDGPVSSND
RPIRCPFCVNQRMLRTIKEAVEHMSTHVVV
>Mb|QLI65942.1
MSIFAHIKKSRQQAKEHNAKLVEQKKKESDQVPYKHVPTHAATDAFASAP
PSWREADRSRIIEQNRRRSAMAASGHHMSMPGVPRVGSSLSRVSYPGEGV
AATIRMPRPYSYAGISPFPGVLRDSREMVYTAPDMTYVHPGSLKGKEVPR
VYDSQRISPASSKGDPSPVESSSDSTSSQDDLEMKPSNAARARAGDGAKA
HRLHPSRARRISDVSANQYTLSNIGRGNTSNYTRDSRPPPSMRGFGSIPT
VPVMPPMNIGVLPGTIAAHGYNGTSPSNTLTPTSRRSSSTSLPGLSGVST
RQTASASMAPAVSSAHKSEYPLNRLSISSFESGVPTPSSRMTVGQSAGAG
IAMAYSSTSQASSNGRHSQDVDSMRQDTWQTQAFVPDHQHMDRSRPPSTL
AHGDLVNVFPEQAGLEAAPSKGGKSKKLSKTGGGKLFKKNRWSSSKVSVV
AV
>Mb|QLI65943.1
MPDGLSYLSENSTCAMSTHDGADHNPSEMEDGVPAGVSPLEETPCQKKAR
EVQERGWRRIIRNFTPSWFAVNMGTGIVSILLHNLPYNGQWLQFISYIFF
GLNILLFVAFSCISLVRYIAYPGIWRAMISHPGQSLFLGCFPMGFATIIN
MMIFSCAQWGDWLVYLAWAFWWIDVLLSMATAITMPFIVMHRHKPGLNNT
TAALLLPIVPTVVAAATGGIVAEALPNHHHAFTTLIASYILWGIGECLSA
CVLALYFHRLTIHSLPPKEVIVSVFLPVGPLGQGGFGIQQLGKVAVKLLP
VTPAFKTVGVDVARGAEILYVLGVFLGMVLWGFALVWVCFALISLATIRD
FPFNMGWWGFTFPLGVWATCTSMLAINLDSEFFKVATTIISLSVLLLWMM
VASRTLRLAVRGDLFFAPCLKELKEKEEREGDAGEKV
>Mb|QLI65944.1
MRGIFKAATAVAAAATCFQPGRAQTWSRCNPVQGGQCPPDTALGMAVNVD
FSKGAVNSFAASGTPSYGRDGVSFTVSRSGDAPQLASLFYIMFGRVEITM
KAAPGAGIVSSLVLESDALDEIDIEWLGTNPDEIQSNYFGKGQTTTYNRG
QFHSVSGTQAGFITYTIDWTQDRIIWMAGGKVVRELKASEAQANQYPQTP
MQVKFGAWAGGDPSTNAAGTVEWARGPTDYSKGPFSMLVQRVVISDYSTG
KQYRYKDSSGSWQSIEAVDGSVNGNFGKGNSLTVTATAVGTAPTTTGIVP
IPVGGIAKDGSPATATQTGWPWVASASPSGGTVPSGWYINSNGKIMRSNA
AALQKSASFSVAILGPLTLGVVVFFYRLW
>Mb|QLI65945.1
MILSTQISRLDGLMLCASVDDSPEPPAVSSLKSQVRQILRTLTRNSTPQA
SIESPPHAIHYLIDSDIVFLCICDSSYPRKLAFTYLADLSREFLTTHSLQ
QLQSPSLRPYAYMDFDSFITKTMATYADARAAQNLDKVNDELRDVTKVMT
KNIEDLLYRGDSLDRMGEISSRLRDDSKRYRRAAVRINWDLLLKQYGPFA
GLGFFVLLFIWMRFF
>Mb|QLI65947.1
MALREYKAPVNYDEQQSAFEEFLNDFKTASPEHTITTAMGDITIDDDDLS
DGYDFMDEDDESGDRRRQEKANNRIPQHKYKDLLQQLADRTIDEITIDLD
DLYAWESQGQEELHLVDSIELNTKHYVDVLSKAVDKIMPQPSTDVSFKDD
VLDVLMARRQARNRELQEAAERDPTAEDDQFPAELTRRYTLVFKPRSRTA
EEPTKALAVRQVRGDHIGHLITVRAIATRVSDVKPIVQVSAYTCDSCGCE
IFQPITDKQYGPLTMCPSQDCKANQAKGQLNPSTRASKFLPFQEVKVQEM
AEQVPIGQIPRSLTVLCYGSLVRKINPGDVVDISGIFLPTPYTGFKAMKA
GLLTDTYLEAHHIHQHKKAYSEMIVDPRLVRRIDKYRQTGQVYELLAKSI
APEIYGHLDVKKALLLLLIGGVSKEMGDGMKIRGDINICLMGDPGVAKSQ
LLKYISKVAPRGVYTSGRGSSGVGLTAAVMRDPVTDEMVLEGGALVLADN
GICCIDEFDKMDDTDRTAIHEVMEQQTISISKAGISTTLNARTSILAAAN
PIYGRYNPRISPVENINLPAALLSRFDVLFLLLDTPSRETDEQLAKHVAF
VHMNNRHPDIGTDNVVFTPHEVRSYVAQARTYRPVVPESVSDYMIKTYVR
LRDQQQRAEKKGKQFTHTTPRTLLGVVRLAQALARLRFSNQVSQDDVDEA
LRLVEASKESLNAEVNTGRRGLNASSRIYNLVKALADSGACRADDAEDDD

LGVELSMRKVKERVIAKGFTEDQWLNALDEYTTLDVWQTTGSGARLVFVT
AGNDDEDNMDD
>Mb|QLI65950.1
MAAKLAALPVLQSIEDCSDFSKTVEPFVDQFFALPARLVASIGSIENLQR
LYVETNPLITGFAASIFLAGVVLIVSEVNRNYSQVDRMWSILPNLYIIHL
AGWARMAGLPHSRIDLVATFGTLWSIRLTYNYWRRGGYNVGSEDYRWEIV
KSKVPAFVFFILNATFISLIQSVLLFAFSCVPAYAILLSTRFEPNVTAAD
FAYFAVQLLLVISEWISDGQQWEYQTAKHQYNKNAKLPKGWDQADLDRGF
ITAGLWGYSRHPNFFAEQTIWFVLYQWSCYATNSLYSWTFVGSGSLFLLF
QGSTWLTEAISAGKYPEYVEYQRQVGMFIPTSIWPYKAPSYKPRVIRTSE
LAKRHEQKKKQKQT
>Mb|QLI65951.1
MAAPGDQRIAVPIDDPNADTEWNDILRKHGVIPEKPPSPTPMIEEAILQG
RRLAHENRLEGKDLDELDELEDFEDDEFLDQYRQKRMAELNNLQKKSIHG
SVYPISKPDYQREVTEASQKGPVFVNLTSGTGTNVESRVLSELWRQAANE
YGDVKFCEIRANQAIENYPDRNCPTILVYNNGEIVKQVVTLVTLGGVRTN
MRHIDNLLVEVGAVSESDMRIIKRRQAAEDAEEERLAGKSIKTGTAGRSR
NNDEDDDDWD
>Mb|QLI65956.1
MAPSHSASKRKAGEAPISPPPIKRSLQSTTTKSAVANFFTPTSQKPKDRT
TWSERGPNDDVPATLLVGRYVPEAHNSGQKPRRKIAAFDLDSTLITTSSG
KKHASDATDWKWWHPQVPGILQKLYKEKDYQVVILSNQGGMTLHFDPSYK
GPKANVQKRVTEFKQKCSAILNSLDIPISVYAATERDIYRKPNTGMWKEV
CEDYDIPENEVDLEHSFFVGDAGGRVAGLSGSGSEGLVAKAKDFSCSDRN
FAHNAGITYKTPEEYFLGEKPRDFARDFDVAQFPFDEAVSSSEGPSKVFE
RKNEKDVVLFCGPPGAGKSTFFWRYLRPLGYERVNQDSLKSRDKCLQAAR
ELLSEGSSVAVDNTNADPETRALWVDIASKAKVPIRCVWFKTPLHICEHN
DAVRAHNQSLNPEARQRLPKLAFTGFASRYKAPQIKEGFQDIVELEFKFL
GTKKEHDIWARYWA
>Mb|QLI65958.1
MRQVDEVGFCPHPLPHHFSIHALIATHQTRDTTHPSVNSVLSSIPQPPST
MDRSQASSSNLMDNHNRLVADILTRYRTLMMLATVQAEGERSNATPETMA
VSGISMKMEFDGLNSSIKDLLSLSRKIKELWVFGPLGQGDPDRKAKDAQI
EDDVALVSALLNGLDGRRMRDLAQRCGGTWEVLAKEDATTAK
>Mb|QLI65962.1
MPPLIDSPQIKQAELLRPLSFHFHTYVWPFAIIWPIFLRYYLTPDLYEEY
IGAPEWTFVWCGTIITLQSLVWLSTHWSVSLEARFKATRVDTVEDAQLIK
VLPIANAGSGEICKLVRDKVGGKLITSFLFQKRRFLYNTETKSFSTLQYD
IDSEPKPTIGHFQSFRGLEKQSELTRVEQHYGTNTFDIPVPTFTELFKEH
AVAPFFVFQIFCVGLWLLDEYWYYSLFTLFMLVAFESTVVWQRQRTLNEF
RGMSIKPYDIWVFRLGKWIEIQTDALLPGDLVSVDRTKEDSGVACDMLLV
EGTAIVNEAMLSGESTPLLKDSIQLRPSDAVLDPEGLDKNAFLWGGTKVL
QVTHGNPEQEKPKLSSHVPTPPNNGAMAIITKTGFETSQGSLVRTMIYST
ERVSANNFEALLFILFLLIFAIAASWYVWDEGVRKDRKRSKLLLDCILIV
TSVVPPELPMELSLAVNTSLAALSKLAIFCTEPFRIPYGGRIDVACFDKT
GTLTGEDLVVEGIAGLGLADSNIVDTKEADGAHSTMVRVTEASVETQLVL
ATAHALVKLDEGDIVGDPMEKATLTSLDWGLGRNDILASKNKYSGAQGTV
QIKRRFQFSSALKRQSSVAMVNGISRTGKKVKGTFVGVKGAPETIMKMLV
NVPADYEETYKYFTRKGSRVLALAYKQLTVDSELGSSKINDLKREKVEAD
LTFAGFLVLHCPLKEDAKEAVQMLNESSHRVVMITGDNPLTAVHVAREVE
IVDRDVLILDAPEDNVDGKKLVWKSVDDKISIPVDATKPIDAEILKTKDL
CVTGYALAQFKDEAGWNSLLRHTWVYARVSPKQKEDILLGLKDMGYYTLM
AGDGTNDVGALKQAHIGVALLNGTKEDLTRIAEHSRNTKMKEVYQKQCDL
MKRFNQPAPPVPVMIAHLYPPGPSNPNYMKAAEREAKNKNMPIEDYIRAQ
GHPTETIVSPGAQSLANAQDARQAEVQKKAAGFADKLASGMLEAEIGDDE
PPTLKLGDASVAAPFTSKLRDVIAIPNIIRQGRCTLVATIQMYKILALNC
LISAYSLSVLYLEGIKFGDTQYTISGMLMSVCFLSISRAKVVEGLSKERP
QPNIFNIYIIGSILGQFAIHIVTLIYVARLCEKIEPRSGIVDLEAEFEPS

LLNSAIYLLQLIQQVSTFAINYQGRPFRESLSENKAMFYGILGVSGLAFV
CALELMPEINEQMKLVKFTEEFKIKMAVVMALDYVVCFVIEKGLKAGFSD
YHARDIAERRPEQLERENARKKIEAEKKAADEEAKRMEKVAEFERQVAER
RRKLEEWRAGRRAQ
>Mb|QLI65964.1
MTSIKNMVLGAVLLWAQLIAAFPTPIGGDGGLLSLLGLGSSRNGDKNASP
TPAKSNVKAKSRIVPNSYIVVYKNTTSAADVKAMTASVSSQLKKRNLNKR
GFEGQPLSTNVRSFQINNWHAMNFEAEESMALEVGEYDGVDYVENNTWFS
TQELVEQTNAPVGLQRLSEAAPVGEQAKKGSYVYDSSAGNGTTAYVVDSG
CRTTHRDFQGRATTIANFVKGERATDANGHGTHVACTIAGARFGVAKLAT
VKCVKVMNAKGQGTNADIIAGLQSVVEDVNKTKPQAATMNMSLGGGRSKA
LDMAINNVFKAGVLPVVAAGNENQNAKNVSPAAAPNAVTVGAVNATTDQK
AGFSNFGPSVDINAPGVDVQSCGIESDSDVSTKSGTSMASPHVAGLANYL
MRLENVSDPAKVTALLKNLSKDTDATVEGGRRDTTPLIANNGNQKDKNTF
LDENGPVKGGTQGNGAASN
>Mb|QLI65965.1
MAPQKRKPVDDDFITTLSDNDEDPFKEEDAPVRPPKKKTKTSKKPKKAGK
DDEASDEEEPQGIWGPKDDDDDAMDSDFEFVADDAGEFGDEEFEGWGFEG
AKKGMTEKKGVDLEEIIRRRREKKNGKAGETLENADQEAETEDNLEMALD
DGDDEVLADDAFGMNADSEDEENEEEAHGSGDGEDTDASDKSDADEDDHD
DDRASDDDSVATPEAHPEDDASDATDDEEDAEEQAKRDEFFAPEERQKSD
KIVDLSSFQGMSLSRPILRGLAAVGFTKPTPIQAKTIPIALMGKDLVGGA
VTGSGKTGAFFVPILERLLFRPKKIPTTRVVVLTPTRELAMQCHDVGTKL
ARYTDIKFSLAVGGLSLKAQEVELRLRPDIIVATPGRFIDHMRNSASFNV
DTVEILVLDEADRMLEDGFADELNEILTTLPKSRQTMLFSATMTSTVDSL
IRVGLNKPVRLMVDSQKKTVTTLVQEFVRLRPGREDKRMGYLAHLCKTFY
HERVIIFFRQKKEAHRARIIFGLLGLSCSELHGSMNQSQRISSVEAFRDG
KVSYLLATDLASRGLDIKGVDTVINYEAPQSLEIYVHRVGRTARAGRKGT
AVTLAAEGDRKVVKAAVKAGKAQGAKITSRVIAPADADKLQEEIDGMEDE
IDEIMQEEKEERQLAHAEMQVKKGENLIEHEAEIKSRPKRTWFETEHDKK
KAKQAGKDELNGLRESLKKKGSGKLSNKDKKTLDAKSMRSEGRLWKKGKG
DAEAHKGKGPGKAKAKVKAKGKGKGKR
>Mb|QLI65966.1
MDALVKYKSKSDAIARRGHSHDRIMHTSFKDLIPLRQRVDAEKIDLSRPD
KESVAATTERTKNALAALISGAVAAQRPKNVNVGNRNDPTFVRYTPASQM
GDDSQKETRIMKIVERQRDPMEPPKFKHKKIPRGPPSPPPPVMHSPPRKL
TAQDQEMWRIPPAISNWKNPKGFTVPLDKRLAADGRGLQDISINDKHAQF
AEAVKMAERHAREEVQQRALMQQRLAEKEKAQKDENLRALAQKAREERAG
GGRGSGRSPDSRDSRSRSGSYSGSDSASDSEDSEIREREKARREKRKEEE
RKLRQNRMGAERRIQVMAREQNRDISEKIALGLAKPTQSKETMYDSRLFN
QSSGFDSGFNEDNHYDKPLFAAQDAISSIYRPRANMDDDDPEAGDKEMAK
IQKTSRFGEALGKGTFKGAADVEAREGPVQFEKEIADPFNVDKFLSEVDQ
NSSAKRGYGLNDEDRQPKRPRVEDEDD
>Mb|QLI65967.1
MSWLTISPESHFSIANIPFGIISTSSDATKRPAVAIGDYALDLKAFASGG
GFSASSEIQKHQDVFAAESLNNFAALGRSFHRSAREYLQSIFSADTKHPQ
ILKDNEQLKKTALLKRADVQNHLPLNIGDYTDFYAGKNHAYNVGVLFRGP
DNALQPNYTHLPVAYHGRASSVVVSGTPLHRPWGQILKDPKAEPKIPTLS
PCQRLDLELEMGMFICRENKLGSPIPIDEAENYIFGYVLMNDWSARDIQA
WEYIPLGPFTAKNMGTAISAWVVLADALNNSRTAGIPNDKTLLPYLQEKK
KDNCLNIHLEVDLTTAKGNKTTISRSNSKNLLWSWPQMIAHHTITGCNLR
PGDLLGSGTITGDKPGSEGSILEQTQGGKVAVKLQGGEERKFFEDGDTVT
IRGWAGEQGALIGFGEVSGTILPAQPLF
>Mb|QLI65968.1
MESSGPDTPVLDPKPHDDAPEKQKNEQKSELNGHADNSAPQSEPQSKPKA
ESPREQTDKSLPNGNSPPTQEAQTTEPAKTSQLTDEPTPRSDSEPKPMSE
APGTLESQEGLATQSKEPEKSPPAIDEEEKKSTELSDTTKPEQSDVAAPT
VEESDVKPPTEEPAKKTASDTAQDTEMTDVPSEKDNKTVEPSASGEATVT

SQDPTALPTSQVDLGPSSMSQLAIDTTEKVISPTEPTSDLPMHDAPGVKV
AREREEDASEEPAPKRARTEPMEDEAAPAPSTTDVPSDAQAEVVVSGTES
PGIAPAALNKLSNWLDEETNKREISQFQRREMRKVIGRVKKTKAGGHFRD
SVQKLWPGLWDSYVAKVDRPMDLAELERGLRDLNGPYTTYGKFQSDLRLI
FDNTLLFNGPHHDITASAVTAVRAVWEEVLPIPSEEPAKPKAVPKAKPIR
ESRTVANADAARRQSTGPTASPVAETPANKPAAAAATQDHSADRRSSTAT
EGDRPKRTVRAPKPKDIDYTTKPSRKKLKPELQFAEEVLAEIMSGKNHQL
NAWFMDPVDAEGFNIPDYYSVIKKPMDLNRVSRMLSEGEISNLKEFDKTV
RLIFDNCYKFNGPVEQGNPVSAIAKQLEELYLAQMKGKDAWLAKYAKANA
PASASNASDDDEEDEEEDGEDAVDAPVDTRAVEELQAKLDEETKKLNSMF
LTANQSMIDIQKNIVEMIQKTLIEKAREIQSARSKTKNDKPKKAGKPAKS
KTAGSGGRKSTGGAAQSKKAGGSKKAAPKKSLTAADKDQIANAINDLDGP
HLDRAIDIIKRDTGQNENNDGELELDIDQLSSDALLKLWELCKKALPNFA
KDSVPVASAEISRPPAKQAANANKSAGGKSKKNKPMSAQEQEARIAQLKE
LRGLYKDGQEPGDETRGPQLTHGAETSDDSDSEEE
>Mb|QLI65970.1
MSGQLASYKQEFLKAAIEGGVLKFGSFELKSKRISPYFFNAGEFHTARLA
GAISSAFAKTIIDAQQNTGLDFDVIFGPAYKGIPLCSAITIKLGELSPQN
LDTVSYSFDRKEAKDHGEGGNIVGASLKGKKILIVDDVITAGTAKREAID
KIRKEGGIVAGIVVALDRKEKLPAADGDDSKPGPSAIGELRKEYGIPIFA
ILTLDDIIAGMKSFASDDDIKRTEEYRQKYKATD
>Mb|QLI65975.1
MSSANNHKTVGLPADNTRLQQPSDAVPMENMAQGTTPAEDLKPAARVDAS
EESEIVATEPLPASKAIPTDPEQRSTENEDNTEAVSKGKEKEEVAPVPPT
KERSDSIMAIGPAQDDIHTTPGQSDGPVCNITLLLTSGSRHPYKIDAKYL
TRRNVAMPDETESGQPDPFSISIYTLKELILREWRSDWEAKPASPSSIRL
IHFGKLLDDKEQLKKYQFSTESPNVVHMSIRPQDLDEEEPKSGSKNLPGG
SGDGQRSRPGGMCCVIL
>Mb|QLI65977.1
MDASKPLGGIQPRLFKRIATIILAFALSAGIIFGLFTYAPEKYLQQMSNA
VGISSGTRPIPRCGDFTGSNEIWKESQTKYRNLRDDKFTIAMQTYRRPKE
LNDTLHALLSEKIPSLTEVVVVWNDVENAPPPNYQSKHGVPVRYRPSREN
SLNQKLWPDPAYKTQAIFLSDDDIYYKPKDLEFVFQTWRKFGRRRMTGGF
TRCADRDADGGWKYTGCSTEEGQNYYNMILSGLAFTHISFMDYYSSQDEV
PKKIRAYVDEHFNCEDIALNFITSLLSGEGPLLVKGKEPYVSFVPKNGIS
TKPGHGEARTKCVNDYYDMFKCMPLINETARIELGVLIS
>Mb|QLI65978.1
MARATPFSGPNEPLHIFQDDVFDRSAPMTSHAPMPSVTKPARRPLSSSNS
NIVLNPPTTNGMSLSPHKMRSSSPRSPLKASSQGNKLHMVSMAPPMGRAP
TTDSLQKRPHLSKFKTGPQRPHIDMLSFGKENIHPQIFPAPPTINLSVEN
YFQKPHGKRVLMDAPQIKDSRPAKKTKTEEALPPHDSFPPITDDGTKPQH
SYAQLIGMAILRSPMRRLTLAQIYKWISDNYSFYNPNDAGWQNSIRHNLS
LHKNFIKIERPKDDPGKGNYWGIEPGTEFQFLKEKPTRKSAPTAENLPVM
STRLEPSRPATAPFQEPTLPPPAPMQHSTLPPLPTSQATQPMQQELSSDA
TIPISDNIGPEDVGDKTADNDLPLESSLYSPLPANMHSSPPVSRHVEARS
GTPPPMSRNPASSISRSHKRKFASMDDSGYISSLESSVMRPNQKALLLTS
EADRPRLTRGRAEEEIARIRNSSPLSPTKSRSLSLYGPVSSSPLRQSNEH
HQTLPPLTPVVTMKPPVRPPPSASPNTNLRIHRDKVRHMLQSPLRRVTGI
GEESIPWSPAFNLDDSVYAFDDMGLPSTEFDIFQDFTSMDDHLFPGIGSV
DAGSPVKRSAKRARLDRSVSTSALGEISNSAVRKSITSAPLLKVSEHSPS
RFLETPSKAFEGMGSPSKLFQQSPSRLASPSKFSAMLELSVDHDWPTLGI
DPSDFGSHSNNGNTDFTGLDILQGFEKIGSGSQSSRPQQQQKGSKPSLSR
SYSTAF
>Mb|QLI65980.1
MAGSQLKRLKASLREQGITGPQQSKKQRRKNAEQGKARSDKRLQRGVVLE
GIREQFNPFDLKHAARGPKFDVTTNRVATGDAAKGIRGRPGVARAVGEER
RRETLLVDMQRRNKVGGIMDRRFGEYDPTMAPEDKMLERFAREKQRSHKK
TSMFDLEDDIPMEGGLTHGGKALEFDDDNLVDDFQEDDLDMYDSDGSER

EMQRLKRLRSMAAADNDEGEQDEPERKKTKKEVMEEIIAKSKMHKYERQA
AKEVDEDMRVELDKELQSIQTLLTSTKGSRRENGANSTHASSIAGVDRDA
FDRNFDLEVKKLAQDKRAQPTERTKTEEEKAEEESKRLKELEDKRQRRMK
GEDVTDSEDEEEDKDKAADLGGMEIDDDDDDFGLGSGIRARPTATEMGFD
DEDDFIIDDDLVASGSDLEPVDSDEDSEEDDDMSQQNEVDEEDEFTKGLL
NEEEARNPIFRIVDSDARAAAAEQSDEHGLPFTFPCPQSCDEFQAITTAY
SHKSLPTIVQRIRALHHPKLDSKNKEKLGNFAMALVDFVALPWNEKTSPP
FAVLESLIRHIHSLSKMFPTEVGRRFRHHISDIGQDRPLALEPSDLVVLT
AIGTVFPTSDHFHQVVTPSLLTMGRFLGQKVPHKLCDYATGAYLAILSLN
YQQLSKRFVPEVPGFCLNTLCALSPSAPAKRLGNFPRHEPPAGIRAQKLD
AIAVRKLTLGDCAAVSSKSDSAKAAIIATMLDILDVAADLWSGKEAFLET
FGQVVKVLEHLTSKTCRPQLPSQIVDRAEKLIAKLGRMLSLAQISRRPLE
LHHHRPLAIKTYIPKFEETFDPDKHYDPDRERAELAKLKAEHKKERKGAM
RELRKDANFMAREKLRIKKAKDEAYEKKFKRIVSEIQSEEGKEANAYERE
KSARKRSRNR
>Mb|QLI65981.1
MSLVSGEKTNFQFILRLLNTNVDGKQKVMYALTKIKGVGRRYSNLVCKKA
DVDLNKRAGELTSEELERIVTIIQNPTQYKIPTWFLNRQRDIVDGKDSQI
LANGVDSKLRDDLERLKKIRAHRGLRHYWGLRVRGQHTKTTGRRGRTVGV
SKKKGG
>Mb|QLI65983.1
MASPVPILPRGGDPWNGQYAQQQRQYQQQHLPAQAPQAPPAEVDENGVQI
RPMGLKVHYTFDREAKVNCLARYPQPLRVQTVPIDETNSIGVVDLRSCIH
AVTECSPELAGQENDYTIYAVDFSEPDFPLVGQGMLSWALEAMQGESMMQ
QPKMVTGRVTKNLLGVFGGGNRETLEVRLRLSVAPRSNKPPPHNNSNDMQ
QYSRPMESAMTPTGASEWNTFIQSNPQIGQTGHVSRVASPALSQGQGPNH
HHQQPSMMGNRESFAPVAAQQQQMPTQDVHRIAPVPVDPSEQRADGGAPS
SRPSSRASNRASRRKPPTGRPRGRPRKNPVEGNTSGYEDGTEGEDGPAKK
RVKTTQVERTAPNLFANGPESLRVAASTSGSLRAFRPILANPEGASGNSN
HLQEVPRAPTPVPDGPLHTGPGRGPGPVKLRRESTLSQQTFSNNPSFAES
RQPLSPSQEDDRSPESIAPTPNYSVDSPADIGSSPPIQRATPFLRSSPPP
SSPVLPPMPPTELPRDASFATDDLDELFGDEANTPIPAPTEAVAAAAARK
PTESKNASGVPVQVFQMQDGPAGQDLVHICSYNTPQPVPATAKECASDTQ
SLPPLKKAPSRTASRKKKTKPASPLGMAPTPPPTTDAVENAASPLLTAEP
GMDETARRTSNLVSIAPKPTAEQLQPQNTQRDVEKEELPTQGLAVAKAPR
QLSRSQSAGPLALPTIPASEPAGPSSLSQSIVAENEFPTPPAPSALKRAA
STGPLTLPVPASDPVVPIALPTSMEHTNSTFAKSDAVPAPSSPPIVSSRA
NKNIVKKHAIKQRLEEAIMNGEMPPYCSNCGAIETPTWRKIWVQENDGIP
EYCEYSEKPGKITAIEILQRDADGKPTVHRLVKKSLGSSDDRAKWQERLL
CNPCGIWLTKCKSHRPQDRWDKDLSRTGQERRKRGTGKGKKSRGKDDGGV
NPTSEAYLPTDALGPIEPSSPKHSDLHKSQPDRDIGTHEASASLDMQEVQ
SNPGSTHSRGSDTAKSPVDVEFDQAVGSTKRLLFPSPRKDGIPKTLEEID
INIVQTSDEGLSKGSGAGKENMTSTRQDGESNDHDNLEAVFKNPTVARPS
TPTPISKAGTPNEPFKTPTRPTPSHRPITRSISRSIRSIRSIASPSQVLV
QETPTKTSRSAEGGRPRRSPRNHHEGFEVFDTPISRTISQMFSEGNGFGG
DDLDLGSLPTLDNNAGSMIDFGNLLSTDAVLPSSPPKDGSMSFDYHASSS
VWAQWDIDTTGGMDTGGT
>Mb|QLI65984.1
MDRGCFTLARFGDATSPKPLVLSGHTRHVDLPPWGMVLTLLRRMMAAEVK
KVPIPRRGVDYRGKIVLAPMVRSGELPSRLLALHYGADLVWGPETIDRAM
IGTTRRFNQDTRTIEWTRMSSQGHKEPPPDAKESIIYRVFPEKEANKLIF
QIGTADPDRAVQAARLVAADVAGIDVNAGCPKPFSTSGGMGAALLRTPDK
LVAILEALVKNITPEFDIGISVKIRILETPADTEALVRRLVATGITGLTV
HCRTTPMRPRERAIRDQLRMVVNICHEAGIACIMNGDVENRDQGIKLAEE
YGADGAMIAVAAEKNPSCFRSEADGGLLGWEPVVEQYIKYAMEISNRFSN
TKYLLAQMIPGKAKPYKTVQQSKSYTEVCNALGLEHLLEKAQDCDVQRGL
DKPKEKLTKKARKNASALAAGGQMVEARAQSHHRKTLSERNVGSKAAPEP
APVATAV

>Mb|QLI65987.1
MADDIDSIVKGAPVSAANIKTTSGPTPIAPAGGKQGDPLNHAPSSPSMVY
LNLLILEASLRAQFLELRARRRHHTFFLMLLTLWVAFFGYKLYFAPREDG
RGVGGSIYWALEGAQKVCFMGGIITAILVWGTGIWDRGIRWPRRWFAISN
RGLRGFNCKLVIIRRPWWAEALSTIGFFLTYGLFSHTASSSYRHVEPWLL
RETERELQLSAEGHPMLVLPHDDEEKGGHEEDLAPGGDYVKLLLLPKPFS
PTFRENWELYRTEYWERENERRALLRKKVKEHDRSLAKQTYGWLWWLPWR
QTPQRKPQSHKTLHRHSSSEKVRRNRGGSVRRPSTSSSRSQTPTLDLDDG
QLGRRPSSASEKRRKKQSSISMSKSKRSGTDSRSVTPEAPSPLTKEATIS
TEGSSTEGSGRVLRSAGQKSGRSSPEPEKTGS
>Mb|QLI65988.1
METDAIHRYRSRILREMNANRANPFNSPPSSTGSHGTVTMSSLFSDPDGE
STRRLNEDIARVTAPRKLPVNWEAAHRKWPEYFSKPDTNKAPIFDDANDV
RPWKPQSKENKPPVSNVKFAVDDSTQDIWPSTTKTRAEMQPRVDNESDIS
SILDKSPARPVSNYGATKSQKNPSPLSKPHTRAPSEPVGNEQRRSSITDA
FNRLQRASLSPRNADQDSPNVSSAKSSLTAVPPSPASIASPSHNASNVRS
FFMPDVSHLGDFVTGTLKFSGSMKNGVPIFVKHGKVHDRQERPSAAAHAQ
VDGIEVPKDEEKIFVSMDMIRDEIVSLQAAHDKVQEYAEGLQLQVERLEA
QLRHGRFSGSGYDQSDKIMAQKKKLELEVATLQARLDSATRKISTNEIAN
DAVAHERDRAISRLQDACEDISKLTRKLSVRERELSTSREQLETSDHIRR
DNDTLRRDLLSLKHNRDAMELDNASLRTDNENLRKEQKRLREEIDSLRAK
KSNRNDSILAENRSLHTNNHRLAEENDDLRENLDAAQHELDVAREEIDTL
RMAVETITQEKSALQGDNDSLVRHNEKYFNENKILRRENSGFERSVHDLH
EQNLKMKEEVDFLKQQLDHCRPSHKNEISGRLDEETEENMTSAYFLPDIT
VKTNESHPSEPTATKDMPTLPEVTTQGYTSELETRDISQRTSNNRQPVKT
SESHPAEPTATKDAPTLPEVITQGYTNEQETRDTSRRTSSNRQRSKSRPR
ASSKVVEFSIPEKSVIRNKNSGNTANRGSKRRHARAPSTTELDLTNEDVD
ITGDLQSEDFTNQDAAISLRVRVCLKGLMNRTK
>Mb|QLI65989.1
MARTKQVPVRRESSSEFFNKQTASWVDTDDRGDKLQVTNGYTTAANGPPS
SSAKPEAGVVQLFISVAGIYASFLTWAYLQEKLTTTPHGSAAAPERWHFP
VFLNTIQSLFAVTVGSIYLLFSTPKGSSIPPIIPSRRILGPLALVAITSS
LASPFGYASLAHIDYITFLLAKSCKLLPVMFLHITIFRKRYPLYKYLVVA
AVTAGVAVFTLHSGKKSKKSTKSEEANVVWGLLLLSINLLFDGLTNSTQD
YIFQTFRPYSGPQMMCANNMMSTLVTGLYLLASPYLVATGIGEWLGMDVA
GGAGELPAALDFMRRYPAVWKDVLGFAACGAIGQVFIFYTLSTFSSVLLV
TVTVTRKMFTMILSVVAFGHRLTHMQWLGVGLVFGGIGVEAGIARQEKMA
KEAAKKTIAKKDS
>Mb|QLI65991.1
MADEENRPAATHNSPRTWLLTAALSPLAVRLIRQLLSHGDYVVACLPPQE
IDHEQRSAEFRELIAECKSSRKDREGWKDRIRGIRCDGNMSSCISAVTDA
VAVFGRIDILLCCRSEAVVATVEELSTNPHTQSLARQQFESNFFSQVNFI
KATLPQLRKQHTGHIIALTSTCGHLGTPGLSMFSAATWGFEGYCDSLAYE
VAPFNIKVTVVQPNMEIQSLTGRLTFAPQIPAYVDAYPAAPNVRDMLSNV
LNSDPDTAVPEPLEESGATPSTPGSTSLEPEPGRGGIFDRYPRLTPQAAD
ALVNETVHALTAIGGHENPPSRHIVGSEAALAVKEKLKTVTEEMEDFVDS
SLAVDIFESELTEEAKKGKRPRDKSPEAGGSNQPSQ
>Mb|QLI65993.1
MVGDNSSNDTLPRYEQDSLLPRADNMAPNTKKAAAECISDDALIHLKSYK
YSSVDKSPVSKYILGPWWNAFVNILPLWIAPNMVTLLGFFFILGNIGLLV
IFMPDLVGPGPTWLYFSFAFGLFMYQTMDNVDGKQARRTGTSSGLGELFD
HGIDSLNCTLASLLETAAMGLGTSNAGIFTALCPCLAMFFSTWETYHTHT
LYLGVINGPTEGLLIACGIMVASGIWGPHIWTLPLANALGDSLPGLAQML
GQTSFRDIWVGLIISSLLVGHIPFCLYNVALARQKQGLPVTPVFAEWTPM
AVFTLGVGAWVFSPYSTLMTDNHLVLFCLTMSFVFGRLTTKMILAHLTRQ
PFPYWTVMLAPLVGGAFLGNLPRFGLPRISATAELYYLWAYFFFSLVVYF
RWAYLVINAICDYLGINALTIPKEKQIANKQARDTTKLH
>Mb|QLI65996.1

MSLVGSLKGDMPQQVRSLYRQLLRQGEQFSAYNFREYAKRRTRDAFRENI
SVQDPRKVQELVQKGLKELQVMKRQTVIGQFYQLDRLVVEGGISGKDTAK
DGEVVRQKEQGWN
>Mb|QLI65997.1
MQRLRPLRAILTRGTRTRTYSSFQTRPAPPKLPASQQAEFERLQREAAVS
SAFQPANTGAPAESLSTQGQTQAQANTLTEEESVNPALFRGAPPEFDGDK
NPKTGEVGGPKNEPLRWGGDGDWSYNGRVTDF
>Mb|QLI65999.1
MYKKLVSVLAVAGLWLDGAVARSRISCRDDLNAFITKQNHISLDGVLANI
GSDGSRAQGAAAGAVVASPSKSDPDYWYTWSRDSALTFKVLIELFIGGNK
SLQPKIEQYMTAQAHLQGVSNPSGGPDTGGLGEPKFHVNLTAFTGPWGRP
QRDGPPLRATALTIYANWLIANGGQAEAANTVWPIIAKDLAYTVQYWNRT
GFDLWEEINGSSFFTLSASFRALVEGATLAKALGKQCPDCETNAPRILCF
LQSFWASGYIDSNINVNDGRTGKDVNSIISSIHTFDPAAACTDATFQPCS
SRALANHKAVVDSFRTIYAVNKGRTPGRAAAVGRYSEDVYYNGNPWYLAT
MAAAEQMYAAVYQWRKIGSITVDATSLPFFIDLMPNIAAGTYAKDSDTFT
SIIKAVTAYGDDFINVVKQYTPADGSLAEQFDRETGSPKSAVHLTWSYAS
FVGAVERRSGVVPPSWGEPNANTVPKICEAPPSCDSTMTFNVKVTTVPGE
NIYVVGSITELKNWSPADAIPLDASQYTPSNPLWSAKVTMPAGTNFEYKY
IKKTSEGSVVWESDPNRSATSSTGCRSTGTLNDEWR
>Mb|QLI66001.1
MASADHDPLVLLRKAISSSQPFIPSASDDPGAKECPLSEASHLQFSAQGI
ALAIETPTRFISNDKPVDLRSIYFAWLNRELAIPEYNASATTLNEQLAAA
GSTGKVQNLGFIERLDLITWLEAASEESEYIKPVAGDADAAAAAAAAGT
APATKSGAVSTAAQARAGKGTMDPRLASVYDGERRMGDRNTVLRGIKPTD
FSHVRKLAAPFIQKKAQAVSAPGAASSLSLNQKGPTRRPDPIILLSPSAS
SLLRMSNARSFLEDGKFVPPDASASTASMLHVQRVIRAIDPNRPLRFILV
EGSEQFKPEYWNRVVAVFTTGQTWQFKNYKWSSPNELFKHTLGVFVGWRG
EQAPESIRSWGHRVLSTGVDRWRGGDGADASRFRDKEVVEQIWKSIEMNM
RSKGWRFDAAPTAI
>Mb|QLI66002.1
MAHIVPNLLLTRFRQMPFPPQNRETPEPRPSDKTDADDGTYHPTPIDIVV
VRIMLIRSVKLPPDLVDAIFDYAEYWAHSTNAIDYRAEHQELLRISGACK
NEDQFLLRSYPVGLTGIDGQDTLSEELAYDTQEATPLPRGTDCDAKYFAK
LAKYPTPRLLNPARKVVFKIKSHDQGWASGNEEKGTYKGSWTWFEAGLEK
FDAAQTSDAQSTRDAPQETRESDSSALPVRALRAVYPKIEQLPKSDDDEN
KEKEDENESEAEAKYKYVHHLEAQPEWEIHRNLRAVGALQEHVVTWSYLD
DIKAESDAGKVLDDQGRGRATGDGSFVRSLVLGDVITVWGKARFPGWVNC
GDSVKVEVYWAV
>Mb|QLI66005.1
MPNIRITKPCHNCRRRRLRCDRSWPTCNKCTLSGQECLGYGKVFVWTQAI
DSQGNPRPSPASGRRPGETGAFQPQAPPDSGGTRGDVAVGWALAGTGGLD
PDQIHDVRVIYAEHAHDERVFRGHHQQQPLALRGAMSGKDIVSPGEMAVT
DKDVCIANTPNPDHQVDMPIRAGLLGGRSLTDPLFQDLDRTSRAYITHFN
DRVCRDLVARDSDSNPFRELIPLTNRHPLLLQVLIATSAIHCCNLFRPTN
SIPTSLTNPGDYLAQLRLRDDSSRQILIHALTAKEKAIAYLRQVMGSLNL
ARSEVGSVVALAAMHFFVKFDLIDFEPRVVVEKEGEEVKKIQGWRSHLEA
ASIVLELLALDPTRHASTRILRDCVIADCFIYHILGSTLAETAMASRIAR
CGSELLEVMKRVEVNSYLSCPPEILKIILSASQLSCQGPYTDFDLAAADQ
ARALIVEAHAFDIDGWVDQLRQLPDVTDIKSRFHVASAHRSAVCLYIIRA
LPVVRVVYPVDANIFVNDILDHLGQIHDEDPYFKATSWPTFIAGAETRDL
GHRKSTLNRMFAIWQICPWGYIFAAIRMLKHAWQIQDTNPESNVNWLQEL
KKDGYLIV
>Mb|QLI66008.1
MGGLNLEVFKFGMYVMFPIGIMYYFGTNLDDRFSVPNFWPRPEECNKLPR
DRDEVKAEYERIVARQRFRQAQLQEEQRQRALLQGNRNNGSDS
>Mb|QLI66009.1
MAEQLNMGGLSLGDGQQPQRSYIPPHMRNRPSAAPPPPAAAPMNGGAPNG

APNGLSNSSWAGNNTNYAGRQQNNFAPDGGAPPTFQSNRRGGWGGRGGGY
SGGHDGHSGGGGYTARGSGDGQWRDGKHIPGPANPRLERDLFGTIDDPSK
QHTGINFEKYDDIPVEASGHDVPEAVLQFTTPPLDEHLCRNIELAHYKVP
TPVQKYSIPIVMGGRDLMACAQTGSGKTGGFLFPILSQAFINGPSAVPAN
AAGQFGRQRKAYPTSLILAPTRELVSQIYDEARKFAYRSWVRPCVVYGGA
DIGSQLRQIERGCDLLVATPGRLVDLIERGRISLCNIKYLVLDEADRMLD
MGFEPQIRRIVEGEDMPPVADRQTLMFSATFPRDIQMLARDFLKDYVFLS
VGRVGSTSENITQKVEFVEDIDKRSVLLDILHTHAGGLTLIFVETKRMAD
SLSDFLINQSFPATSIHGDRTQRERERALEFFRNGRCPILVATAVAARGL
DIPNVTHVINYDLPTDVDDYVHRIGRTGRAGNTGIATAFFNRGNRGIVRE
LMDLLKEANQEVPAFLEAIARESSFGGGGRGGRSRGGGGGGRGSANRDFR
KFGGGGGGGGGGFGGNSGGGFGGPQQTSGYGGGFGGPQGGYGGGGGGGYS
GGGGSYGNPGGPGAQSWW
>Mb|QLI66010.1
MADTDHQPAATDHAQIQRDASDAAARAAIPPSPPMPSSDPGLAIVDHDGS
AADQDDLFKLPAAEALRLLSASVELLVLMTGDIPPTPPPKTPTDPQMTGM
QVEKDNMVRSRSQQSLANARQQAERAAAHRQHGDAGEATSGIDRLQAYSA
SMSAQLAAQQHASAMPSTSAEPIDGVRLRQHPDADAEPYIIVGADSRPVN
LQHGAITRKFYSKKEPPIPISQYLLRLHRFCPMSTAVYLATSLYIHRLAV
EERAIPVTKRNAHRLVLAGLRVAMKALEDLSYPHAKVARVGGVSEVELAR
LEISFCFLAGFELVVGEEPLRRHWEELRDGRAQQALRGMDVPTLKLSKRP
REMAQASG
>Mb|QLI66011.1
MAGETNTLANAGDKVAAPLQNQHHTPIAGMDYNPDPALNPANQHTHSHHH
HSARIDAAGGHDDAVYTSGTTMDPSLIPAQQHGDNHHAHSVDKKDPLSLP
EYSDNEKAEAGMVSTGRGSGSTDHRHRESGGGFKGFYRRYRPVFHLVYFL
FFTGWWIASLILHRDDKNWVVPFLLWLAISLRLFFYYVPSRYLSTAIRWV
WSHTVLVVYELIPSAFRIPLGAFGALAVIIVGSFASEEVADNTRANRAVS
LFGLAVMLFVFWATSAHRSRINWRTVIAGMLAQYIIGLFVLRTGVGYDIF
KFIADRAGDLLGFARQGVAFLTSEETSQTPWFFFSVIPAIIFFISLVQVL
YYIGFLQWFIKKFATFVFWALGVSGAEAIVAAATPFIGQGESAMLVRPFV
PHMTKAELHQIMTCGFATISGSVLVGYIKLGLNAEALVSSCIMSIPASLA
ISKLRYPETEETLTAGRVVIPDDDEHKAENALHAFANGSWLGIKIGATII
ASLLCIIAFVGLINALMTWWGRYLNINDPTLTLQTVLGYLLYPVAFLLGV
PRDNNDILLVARLIAQKIITNEYNGFLDLTKDKTYAGLSPRSRLIATYAL
CGFGNIGSLGIQIGILGQLAPSRGGDVSKLAVSALISGVVATLTSATVAG
LVITTQITNFSSAPQS
>Mb|QLI66012.1
MGLFSGNKMPVAGKTVLITGGSEGMGLSVAQQLSAKGASIILVSRSTAKL
EEALKTVRASAKDPSQRFHHIAADVSSPSYAAPLIAAATAWNNNQTPDIV
WCCAGTSAPQLFTEMEMDSLRHHMDVNFYGTAEMSHAILRAWLEPSAPVT
LEPKHLVMTTSAVALYTIPGYAPYAPSKWALRGLADTLSQEVMLYPQNVK
VHVVYPGTILSPGFVREEQVKPEITKILEASDPRQTPDQVAAASIKGLEN
GDYFVTVNWLGDLMRLGMLGGSFRNNWVVDTLGAWLVAILWIFVQPDLHG
KIRAYRKKHGHPSARTKDHVGLDGGRSN
>Mb|QLI66013.1
MASGDIPSDQFAVLPIRMPPMPSFPHSAVHEVRIRRNTPKIPTANDSRSL
FLKNIPADSTEPHFRAVFTNLVGAGRFETIFFHDEATTALPVGPAQATKM
QGFARKRKMKDVEAEERELEEKASQLPQIWTRQLHRSSCTAVVLLADERS
VRLVLKAIAKVQKSKKYPVWGENMADEVPELGSSWVSEHLQLSRVDKSAT
QAAVHAFFNAYNRKEKEAIELAKRLRNEPDEDGFVTVTKGGRAAPASRGE
AEEAKQRMLDKAAKKKSELKNFYRFQLREERKKQQAALLRRFEEDRRKVN
AMREKRGKFKPET
>Mb|QLI66014.1
MTESAPKRLKTGPKIGTHNGHFHADEALAVHMLRMLPTYHDSPLIRTRDP
DLLAACHTVVDVGGEYDAQRNRYDHHQRGFTTTFPGRRTKLSSAGLVFKH
FGKAIIAQKLGAGVPQDAPEVDLLHDKLYESFVEALDAHDNGISVYDPAG
ISAAGLEKRYSEGGFTLGAVVGRLNPNWNDERPSDPDEAQEAEDARFYKA

SLRIGEEFERDLDYYATAWLPARSVVQKAFDKRSEFDPEGRVLVFEGQSV
PWKDHLYTLEDGKPTVLYVLYPESTAPGAKWRIQCVPESKDSFVSRKPLP
EAWRGFRDQELDGISGIEGCVFVHAAGFIGGNKTFEGVKKMAVKALA
>Mb|QLI66015.1
MADGEPVRDVKNHLLFEIATEVAHRVGGIYSVIKSKAPVTTAEYGDRYTL
IGPLNHTSAAVEVEEMEPTNPEIAKTMQAMKDRGIGMLYGRWLIEGAPRV
LLIDTKTAYGYMNEWKTDLWNIASIPSPASDDETNEAIVFGYLVAWFLGE
FVCHEKKRAVIAHFHEWLAGVALPLTKKRRIDVTTIFTTHATLLGRYLCA
GSVDFYNNLQHFDVDAEAGKRGIYHRYCIERAATHSCDVFTTVSHITAFE
SEHLLKRKPDGVLPNGLNVTKFSAVHEFQNLHQQSKEKIHDFVRGHFYGH
YDFDPDNTLYVFTAGRYEFRNKGVDMFIESLARLNHRLKNAGSKMTVVAF
IIMPAQTTSLTVEALKGQAVIKSLRDTTTMIEQSIGRRIFERSLKWHEGD
SMPDEKELISGQDRVLLRRRLFAMKRSGLPPIVTHNMVNDQEDPVLNQIR
RVQLFNHPSDRVKVVFHPEFLNSANPVLPLDYDDFVRGCHLGIFASYYEP
WGYTPAECTVMGVPSITTNLSGFGCYMEELIENSSDYGIYIVDRRTKGVD
DSVNQLTSYMYEFCGKSRRQRINQRNRTERLSDLLDWKRMGMEYVKARQL
ALRRAYPNSFAGDEEPEDFIPGVEQKISRPFSVPGSPRDKSGMMTPGDFA
SLQEGREGLSTEDYVAWKLPAEEDPEEYPFPLTLHTKQPGGESPLPPAAL
NGN
>Mb|QLI66016.1
MADEGVAEHYQVLEELGRGSFGVVYKGIEKATGETVAIKHIDLESNDDDI
QDIQAEIAVLSTCSSPYVTQYRGSFLRGHKLWIVMEYLGGGSCLDLLKPA
NFDETHIAIICRELLLGIQYLHTEGKIHRDIKAANVLLSETGKVKLADFG
VAAQLTNIKSQRNTFVGTPFWMAPEVIQQDGYSFKADIWSLGITAMEMAN
GEPPLCHIHPMKVLFHIPKNAPPRLEGDFSRDFKDFVAQCLTKDYERRPS
ARDLLKHRFLRSAGKVEALQELIARRQMWEANQNRQKHPIYYQETLQTIS
PKDEDQEWVFDTVKSVAPAPPKRPTIRHRKPSSMLASEEGLRKLDIAEGP
LGPSSPATGTMRKCTVRRTPSLAHSNSIRSSGSPRPTVAPKKPLQPDMSF
GNSGSTMRLFRRVPSDSSTNGQIGRPSSPDDVFCDENLPPRIASPVEPYG
KEAILGRRLYNKAVEPTLAELHAQTSAMQKREALAKLSDAFASLDAVDPE
GAYHLMSNLVASMSQDNKLNSSFLRQPIQKAPDDGTPLGTVIVKSSAPSL
ASPTKLILSSNNPHLKSHRRRQAESPRTSLTEKELEREKSSLLEEKYPGR
DALSGMEHCKQLSDVLYQRWADNLRIRWPAV
>Mb|QLI66020.1
MGVISNTVASLKTLAGPNHKYRAIPAPVTGRNHAQQIFDERPPRSRFFKL
SMSVMLLAVFVFLGFAFARDRSDSESCVNNGSCTHDFRKHWGQYSPYFSA
HKGSIKPDIPSGCEVTFASILSRHGSRNPTAGKSKAYKDLVERIQKDVKD
YGKGFEFLKDYKYTLGSDDLTPFGEQEMVKSGKKFFKRYQKLAEDSTHPF
VRASGSERVVMSAQDFVHGFYKAKGKNGSKYLEDILVIPEDNGSNNTLDH
GTCGAFETGPNAELAHDKQAPWKNIWATPIMERLNAKLPGARITLEETVY
FMDLCPFNTVASEKSTVSDFCRLFSKEEWRGYEYYESLEKWYGYGPANPL
GPTQGVGYVNELIARLSHSPVQDHTSTNETLDSNPSTFPLNRLLYADFSH
DNTMMTVYAALGIYTNATEIPTDRRVSPKTADGYSASQAVPFGARMYVEK
MHCGGDDYEMVRVLVNDRVIPLKGCKADKLGRCEVGDFLKGLDFAKKGGL
WDQC
>Mb|QLI66021.1
MSMKKPLLADYLHNNVFIDPKQLIGEALHNRAESVDHELCEPGDEDTFFV
ADLGEVYRQHLRWKKNLPRIKPFYAVKCNPDPKVLRLLAELGAGFDCASK
GEIEQVLAMGTTPERIIYAQPCKTNSYVRFVKNAGVKQMTFDNADELYKI
AKLYPEAELFLRIMTDDASSLCRLSMKFGAAMDTTESLLAVAKNLGLNVV
GVSFHVGSGASDPLAFYKAVRDAHAVFQQAAEFGFTMRTLDVGGGFCGDT
FEQMSSVLRDALDEFFPAGNGIDIIAEPGRYYVSTAFTIACNIIARRTVE
DPTLDGKGYMVYINDGVYGNFSSIMFDHQHPVAKILRSNGRTLYNTASAN
ACSAGEGVEYSVWGPTCDGIDRISESIRFETILNVGDWLYFEDMGAYTKC
SATQFNGFSNSHDVIYVCSEPGAKFLLNM
>Mb|QLI66022.1
MTVHSNPAVAAGPHPLTQLSADEFTKARDIIVKQYGSSQSLYFRQINLEE
PSKESLIPYLEAEHAGSLTADTPRPARQAHVEYDLIKVDRHELVRAVVDL

DAAKVVRSDTAPPHSYPYFTTDEFTQFFDHCVASEMFKEAMTEFVLPEGF
ELSIDPWPHGPSDKDDDPRLMQGLVFAHDARKNHPDTNHYSYPVPIIPVM
DWVTKKVIRVDRLATGGSDDGLDPVPRREKPRKLFEKSKPAEWIPELLEH
PMRTDLKPLNVVQPEGASFTIQDDGLIEWQKWRFRVGFTPREGAVLHDVC
YQNRPVFYRLSFSELTVPYADPRPPFHRKQAFDFGDGGVGRMANNLTLGC
DCLGAIHYIDSLLSGPDGSPMPAESVVCIHEADEGILWKHTNFRTNNAVV
ARARELVVQFICTLANYEYIFAFKLDLAGGITLETRATGMLSVVAIDEDK
ASGYGNVVAPGVLAQNHQHIFAARIDPAIDSYRGTQVVVEESHGVRKNPT
TNPHGNLYEVRRRVVDGPTHVDAEPRLNRTLKMENPDRKNAVSGRNLAYK
LVAPATQMMLADEESVLSQRAQFARHNAWVTGYRDGEFWVAGEFTNQSRA
EAGGIGDMVRRGDWFTEEGKPSGANGHGQRSSPVVWPVYGLTHNPRVEDW
PVMPIEMYQIHLRPVDFFEANPALDVPSKKNESSVLVPCCGTNRVANGNG
CASA
>Mb|QLI66023.1
MAEFPKHPFLLTVEETAQALNTDIDKGLTTAQVTELQQKYPKNELDIGDT
IPWYSILTKQILNAMIIVLAFAMALSFGIKDYIEGGVLAFVICLNVTIGF
WQEYRAEKRMDALRALSSPSAMVLRDGKTQVISNPDVVPGDIILLKMGDT
VPADMRMFEAMNLACEEGQLTGESIPVEKITENSILAPGTEKAATSEAEV
GIGDRVNMTYATTVVRKGRGRGIVTAIGMTTEVGKIAASTAKKRRKAGRS
MNWRKYGKRQPLVGLAKRTYDLIGKFLGLTEGTPLQRKLSALAYVLFGCA
IILAMVVFGVNHFNLRNEVIIYATSLGIAIIPESLVAVLTITMVVAVTVM
RKANVVVRDLSALEALGGVTNICSDKTGTLTEGAMIVRKAWIPSSHNYTV
RESQNPSDPTKGRVTYSQSKDEPAVEKEEPKRDYDRERSAAVLKFDVPDE
KLNPKPAKQAPEAEPETTMTDELQAFLLSAALCNLATVRYDDEEEKWQTT
GEPTEIALQVFAHRFNKGKKSLETKGWKQLAEFPFDSSIKRMSVIYDAPA
GEAGSIIETEQSMVFTKGAVERILDLCSYIGTGAEQQPISDQLKEDVLNQ
MNVLASQGQRVLAIAYRPWGGRFTAKQSSSAAEDEKLRGDVEKDLVLLGL
AGIYDPPRRETKPSIGECSQAGIKVHMLTGDHPETAKAIAKEVGIIPKNL
GVLPEKVAQSIVQKATDFDKMTDEQIDALEELPLVIARCAPDTKTRMIEA
LRRRGAFMAMTGDGVNDAPSLSRADVGIAMGSGSDVAKSASKIVLTDDKF
NSIVAAIREGRRMFDNIQKFVLHLLTSNVGEVILLIAGLGFVDHTGFSVF
PVSPLQIIWINMVTSSFPAFGLGREAAAVDVMRKPPQDKRRGVFTNQIIV
DMIVYGVIMGACTMCTFCIIIYGANNGDLGENCNTEYTDVCDAVFRARAA
TFAELTWLILISAWEFKSLRRSMFHLNPDDDSRFPVFKDLYSNRFLFWSV
ILGGLSVFPVVYIPFLNTKFFKHKGISWEWALSVGFTITFVAGIEAWKFI
KRRFHLLEDRPVQRGAWGQGGPDDQGPKFRKTLSVSSFKTWASFSRKDTG
DSVSRYSSSRGNQGIVQGTEHV
>Mb|QLI66024.1
MNGNGNGVIADEEQPLLGRKPSPPSLLSKCRKQMTAEVSRSWADLVLLLC
YVVTGLLDSASTQVWGAFVSMQTGNTVFVGLGIATVFNPPVSARLYKSGV
SLLSFCVGSFFFARFHRYFSPKRRWVLCASFAAQALLIVAAAVVVTVWPA
PGDERAVGWTVLVPLALVAFQSCGQAVTSRALRYNALTSVVLTSIYCDLF
SDQDLFKVRNVERNRRAAAPALLLVGAVAGGLFSHSSFGIQGALWAAAGI
KSIMVVLWFFWPTEDEES
>Mb|QLI66025.1
MSVAQLRSPRLANQLARHVKAARHFSTSAALRKELQDAYILSASRTPTAK
FNGSFLTVPAPKLGAAAIKSAVEKSKVPVEKITDVYMGNVLQGSVGQAPA
RQAAIFAGLPESIEATTINKVCASGLKAVAMAAQNIQMGLTEAQIAGGME
NMSQVPLYLPRASGLPAFGHVKMEDGLIKDGLTDVYQQFHMGNCAENTVK
NHNITREEQDAYAIQSYKNAQKAWADKAFADEIVPVTVPGRKGDKIIDTD
EGFLDVKIEKIPTLKPAFVRDGSGTVTAANSSTLNDGASALVLGSKTIAQ
EYGTGSRVLAKICSYADAAMAPIDFPIAPAKAVPIALERAGITKDQVAVW
EFNEAFAAVILANQKILGLEGARVNPLGGAISLGHALGSSGSRILTTLLH
QLKPGEYGVAAICNGGGAATAMVVQRIESV
>Mb|QLI66026.1
MEPQVISENTPLIPTTPPTSDPRPRNARTVTFCPDPVTKTFEPESYSSRS
SAASASSNLPPGPPRVSPPVLTALNNKLKRRNSHGSVTIPTTTTTAQPYL
GSKIGPQRSTKKTEKLKLLPNPDIEDGEDEESGRDVYSQYTRIKDPTARR

DAARLGKSDRDRLPRVTAYCTADKYQMDSLMRFLKGRGKARGANPKIIDE
CIYTPYSYTKPTRIHQDPVRSYLRRHSTGSEIMEDEVQRHQTLGLQNHDS
GYADESSANNIPDPSHLGNESLLGPAVTEEAPAADDILDFDTQVHTPEVF
LFDYGVVVIWGMTASQEAKFLKELAKFELEKLAPDDIETELFNFYYTREY
QARIYNDFITLRDRNNYMIKLAISHALAQSVKTSLYEELIATTVDTCKDI
PAHIALTGKINLSRKQINMQIGDLFILRIAIHLNGSVLDTPELFWVEPQL
EPVYQAVRSYLEMDQRVGLLNERLDVIADLLAVLKDQLSHGHGEKLEWIV
IILIAMEIFVACINIAVDVWAGDM
>Mb|QLI66027.1
MADATVPSLRESLCSEATPLPVRFRALFSLKHVARTGEPAAAVAAIEAIA
AAFASPSALLKHELAYCLGQTANDAAIQPLRDVLSDLKEDPMCRHEAAEA
LGALGKPDNLELLKYFRDREGEDVVITETCEIAIDRIEWENSETRKQEKL
RQSDFASIDPAPPLPESEKNVEQLGKQLMDTTQPLFMRYRAMFALRDLAS
PPDCATAVPAVLALAKGFADSSALFRHEIAFVFGQLSHPASIPALTAALS
NSEEASMVRHEAAEALGSLGEEEGVEETLKRFLHDKEKVVRESVIVALDM
AEYEQSGNAEYALIPEAASASA
>Mb|QLI66028.1
MVRTSVLHDALNSINNAEKTGKRQVLIRPSSKVIVKFLQVMQRHGYIGEF
EEVDDHRSGKIVVQLNGRLNKTGVISPRYNVRLADLEKWVVKLLPARQFG
YVILTTSAGIMDHEEARRKHVAGKIIGFFY
>Mb|QLI66029.1
MAAPTTLPALLASLTQSLSLTQEAAPKIAAIEPPENGISLLDVKNELLLS
YLQNLVFLILLKLRNSKPDPENISQLDESVRAKLVELRLYLEKGARPLEE
KLKFSIERFLRTAQDAQREQKAKEQQSKSKSKKNADDDESASESASEDLE
SDEESEADEQPQARKGNMGAAPNLGAMIDEVSTSRGGREDGPVGVYRPPR
RDRVVMETTRSREKSQRRATKSNTMEEFVNDELATAPMAEPSIGTTIVQG
GRKMKTASQRMEEAERREYEETNFTRLPKESKKDRAKKAQAAGRSGRMQF
GGEEWHNLGEGVDRIDRLTKRKGPPGGGVRAMLEKSRKRGFDTTDGPRGS
GRGVEMGERFQKRAKMLEAGRRDRGKKR
>Mb|QLI66030.1
MAMAPSRRNQRHRQSSLSLSIAPNSAVPEQLPPVSALFLIDFDVKAGYTI
TWKRSVPHVELDGQVEYKSLPSGLHTVRHDLIYFVDGAHAGLSAFVNEPC
EEEEARNARMIAVGVLVPLSYGRLGRAWRHAENLKELAAILAKDRSQTGV
LEKYWNSHKASENEGQPNDLGPANLPDSPTQERRTHKRKGSASDGTSLPP
AEHRLSPFHPAWSLVSLLDKFGPLIFPIHRAALLRKRILISCHAPVHEIC
DFVYDISILSNIPFSVADRLPPSSPTPRLRPLFTIGVHDIPFLMEDFEAS
KRRQDGTADSGGEAGSGWIACTTDSILAMKDTLWDMLITMPPEHSTNAVD
KVWPTVEYPRGTPVKATQRDLRRFNALRSGLIRLASSPQQQEPDSPPFNS
SAVRLSTSHSHRLVKDEEDPHIDELIEPLSWAALAYNGYMWWASAGEQLR
SEEHEESSRDAALLADLAPAPASSPRPLSESLASLTSRRASNPDEARTEL
AIIAYFHRLTTQMLSIIVDLVESADEAYPSPYRDEQGSELDDQEGDEAEV
LLNSGGLGLDANVITVDSHCIENMGLDVWSASDAAFVQDLVDAYFGRASR
IEGKGVEVCGVRVC
>Mb|QLI66031.1
MPSFAGLTASGYGPGTSDASQDVGERGERGYRRRKLAAMAGNLYRSGQQA
VTELRDSYVQSRGRGIDDFAMDQSTHIPGAFPDVAIASQGNEQMVLFPSY
AKRHIKQDWTRMPRKQPVDQPELRNEEWWRQEWEKHEDEKAVVDVDVRGW
IYSPHVGPMTRRNRILIGLARQLSGISAPKTDQGYGPASGEPKTQHQIHA
EIHDEEKIAQKAAMIEKRGQEEKRVAYAGGYSEPSQDPNTLGVEAASRFG
RRGSYTPDSAPPSPSMPARQPSGLGELTDAELAVANANLMARVAPFLTNP
LVAMPITIFFYNDTKSQSKTILTDDAGHFNIRAALEFIPTHVRVLANEKL
STTQEVKITEPAGVSLISDIDDTIKRSNISAGAREIFRNTFVRDLDGMTI
EGVREWYTDMHRLGVSMHYCSNSPWQLFPVLASFFKIAGLPAGSLHLKQY
SGMLQGIFEPVAERKKSTLNRLVKDFPHRKFLLVGDSGEADLEVYTELAL
ANPGRVLAIFIRDVTTPEAAGYFDAAFDFTHGKISSMTLDYSRSGSVKSA
SRQNSAPGAVGETQAASGPVMGTLIDFSDEPEEVKLDEAAALAQVKMANL
GRPAGAATSALAGRKPPPPPRPAKPAALRSAPSHVNASNGGAKADMPPPK
PKRPQGLGAAGLQPLTTILSAGQDGAQTTEDSSTPAPLEQEPAPPPLPRR

RSGLKSISPRLFGRGASSSNTDVDFEPLPPSAAAPPPSFGFSYYRSGSRS
GSSTPSGSPTLGAQGANKKLELWRRRLARAHEQLDHQGVALYTWRTGDDV
VAEAVGIVTSAQEDMKRRQGR
>Mb|QLI66032.1
MAQSLASSKFLRGSFTKCQRCFSSRAFQPTPLRPTLPRAPVQSRQPILQK
RTKYKTVEQAKSRHSTGPFSWKAGILFVATCGALVWYFEFEKGRMQRKRI
ADASKGVGRPKVGGSFELLDQNGKPFTSEMMKGKYSLVYFGFTRCPDICP
EELDKMARMLDVVEERAPGALLPIFITCDPERDDPAALKSYLAEFHDKFI
GLTGTYDQIKDLCKKYRVYFSTPQNVKPGQDYLVDHSIYFYLMDPEGDFV
EALGRQHSPDQGAQLILDHMKDWRK
>Mb|QLI66035.1
MPGNDGMLPGGTMWMSPPTSEPRKRPAAIYTNIAYTSGDEMNPISAWSGM
HLQQVTDPSTSRSSPITQEMLPLAMSGHDEWNQPSCGEVIDFHGLDSEGA
IGQAYTTDEAVPIIDLRYSNQSQEAANLNMEDGIKQRRLSNSSLTVSTSE
PMSDIPSSYEDYPAGLSEAPSYASDYLSTASNRTSLMSSIQLSPVASPRA
TPQSRPEQVRTQSRGRASPSPRPSVRSAPYTLETSKNQRWSTGSYAPTQN
RRQSPMIFHGAQDGYATNQRMSLQNYPPLMPAQSFHPGGIQGHPPHQPPF
VVSGQPMFARHGMIPPAHMPHQGMFEQPPPLPSHGIFKMLQSNGDPHMLH
GHYTDLSDPPDLFGPLAEEQVPPPEEDMNHSDPNMIPYEQDLRFDGDLYT
PRWVRGHGNKREGWCGICKPGRWLVLKNSAFWYDKSFTHGISAATGHPFE
EPLDTRRMDGNPDVWEGLCTSCNDWVPLVSSKKKGTTWFRHAYKCHTHQK
VKDMPKRRREMGSSSRKLAAQTPKTKLDATVQQPMTPQSVTTPVSGLHTP
CSVPPLMPQLHLQVLHHQQQMPPHTEPPASVSGPDSMCLSGGPPPISMPP
STSIMGNMI
>Mb|QLI66036.1
MATDKPDVPDQRAGVPSRNPLPLSASQESQVRDIYYARVRSQCAEEIKAF
AACALGRTLTVSFACRAEHRVMNNCMKLHATPEEHDAAREEWFAMRMERH
RQRERKAKMAVAQEEFLREWWGLPEEVRASRQRELEKLGQAERVGGMTAK
DRPHGPDGGR
>Mb|QLI66037.1
METTLVRGSSDEEPQSSASTTPSPEPTSTKPTFVIEIKAAPKYTPGCGPA
LQSLRLLPPASSSAYIVERILLPSPGLAADGKPLPKRMTYIVGWHDLPAA
SLLVPAMDVLDYVSPRTLEEWEETMEEELDQERIRLAGEKKVGFEGLKQR
HKARPPTHTAIEPAAAIEPETEADDVVRPKMGAISLSTPQKRKLADFEGL
SDNDNSPSNQLAREMFGEDGREDSWDVPSAGLGNEDMRLEFVSDTVDSDG
ARKPKRPYINTPVPLPSYINGILEYATSSIEPKPSPTPTFKADDASQHPG
HTDLVASIEGTTSSSAAGFNAVTGSSIFPNMSNTRESTRDNAAWTPAAGQ
ANSSAKKSTRPKSAKKRHKESPLIEKMAEDGEPTWEVERLEDVAVYDVEG
QGLVRYFFVRWAGDWPPDQQTSWEPEGNIPRNLIRNYFKMDKKRRAKLAA
QNQRQRGPPERVGWGNSNATSTGSVNEMYQTEDQGDDDLLGAPHTINDKG
SLFVGDDLDYDTFYEDESETRDLREKASAALPPLFGVY
>Mb|QLI66038.1
MSSSAIRSRLAARGIRSSPASRVHADASRTSIPGSRRQASSSANGSVQAG
RAKAILYGGALAATLYVGYLYTTDTRAFVHRYLVPPALRALFPDAEEAHH
AGTSALKTLYGLGLHPRERGNSLRTSELCTSVFDTQLSNPIGISAGLDKD
GEIPDALFALGAGVVEIGGITPLPQAGNPKPRVFRIPATDGMVNRYGLNS
RGADDVARRLRDRLRRFARSLGLTEQEVLDGAASVPPGSLQSGRLLAVQI
AKNKETDERDEKAIADDHVYCVRRLARYADVVVVNVSSPNTPGLRDLQAT
EPLTRLLSAVVDEAAKTSRKSRPKVMVKVSPDEDEDTQMEGIVEAVRRSG
VDGIIVGNTTKRRTGLVPEGIKLKNKEQKALMETGGYSGPAMFDRTLDLV
GRYRKMLDAQPASSDKQKILFATGGITNGEQALKVLNAGASVAMVYTGMV
YGGAGTVTRIKSEMKNQLKN
>Mb|QLI66039.1
MASMLGTSAVLQGMADALPTHQQADDSSDLASSYEAIALLVHAYLAALGF
RLRGFDQDKVIPDCESLAPRLPPKWNSGFGSLSFVYKHKQSSMDFVFRID
RMGAKVEVRGLAVGDENIHRFERTVRDIVNSAALPVRIAMTDGQEDRSDL
VEKLRNVFVSGQAISDLLNDLKVKIVQKLIPKLQSEGYVETEDAEETARS
ERRGQERRNPQRPFEPSVEPNIPYHPPGPLPAMARPRPGAPVGDFPPPGF

EDEYEINRPPRGNPIAMPGGRHPFNIGHDDLNPPGLGPHDPLRGSFVPGG
LPHPGAPGGMHPTFDDPLFTGEGGNGGYGGYDPQAPPGARWDPVGPGGTP
RFPGPGQGPGNGPFGGSGGFGGSGGFGGGHII
>Mb|QLI66042.1
MKMAWDIKRPGDIISRPLPPRIPNSPDSFDTNNVEYFKDGDIPPRSMTSS
SFSNCQKPIKYGRGKYAHVELVPQPSDDPDDPLNWPRWRKDLNLFSLLVM
VGLIGGMKTVFITTAGSLATHYRVSNMAIGALTAGPLMVSTLTSLVGSIV
AKLYGKRPVYLISTLFLFLGTIWNMTAGDNYGSCLGARVFQGLGWGSFET
LVMGSIQDTYFEHERNLPVSIYNLLTITTTWGSPLLGGLASSNAKSFTSQ
FRIINCLYFLALPLLAFGAPETVFDRSRATITPLPILGLDGRRPRRFRHR
RNMETAVEYIKKMKPLSFKASITMSTVLQVPRAIIAPTTSLLFVLTFIPY
GALWGLTSSVSILTTPAPISLDVSLTGTMMTGPWIITSLVVGGFCFYRGL
YERFTKRVSYLIISTGGILSLTGLLSYGLGIHNFMTADPGPPAPFFTSNT
AGQISLPLLSLQLGILAGGTYTLDTITRPLLARSASFTSSSIAVAQRSIG
DMHSGVIILRNLAAGVFVLSMPNAFTVYGALKSVVIGLGTTQVILTALTM
TLWWYLDESIWRADGRVMRLVDLKLLKQSLNFFDAD
>Mb|QLI66043.1
MLYLVGLGLSDETDITVKGLEVVKKVSRVYLEAYTSILLVDQSVLEEYYG
RSITIADREMVESNSDEILRNARNEDVAFLVVGDPFGATTHTDLVIRARE
LSIPVRTVPNASIMSGIGACGLQLYNFGQTVSMVFFTDSWKPASFYDRIK
ENRNIGLHTLVLVDIKVKEQSLENMARGRLVYEPPRYMTVGQCAQQMIEI
EDEKREGAYTRDSLAIGAARVGGKTEKFIAGTLEELCSTDDLLGPPLHSL
VLLGRRTHELELDYVREFAVDKEKWDKIWKEDYGKQL
>Mb|QLI66046.1
MGTILLPHLVTGWHVDQAILSEDNRLVVIRFGDPTNNPDLDIMDEVLSKV
APLVQKWAVIYVCDISKVPDFNHMYELNTNEEPFAVMFFFRNKHIMCDFG
TGNNNKMNFVERNRQDVVNILEAVYRGATKGKGQVNSPKDYSTRHRY
>Mb|QLI66048.1
MDSNMMPQTAQQNAVFSKANFAQQKLGQYQVVAPASPVCSRPGSSCSQPP
TLFSNGPAVLTPTGSPPPNGFRPAVMLESEFGDSSYFPSTPPLSTSGSAV
GSPKNYEVLQTPLNPMFSGLDGFVDVKSGYESTESSILDWASCDSPPMTP
VFIHSQPSRVPSLSSTTSDLSNISCPSLSPSPAPYARSVSSENDVDFCCD
PRNLTFPATSNSSLAPEFTLDCLGEEEDSPSEQSALDTVLSQTAFDFSPA
IASGLPAFEELSDFESEDDLSNLVKPGEGSRPRACTGSSVVSLGHGSFIT
DEDFSFDENDTFQFPSPSPPSSVEAVDDGHKDKRQKKTDTKDSQSVEPVM
DTAAADNQTAEVEEQSEQASPEPSESHSSAEADTPSAPMPAPTNRRGRKQ
SLTEDPSKTFVCDLCNRRFRRQEHLKRHYRSLHTQEKPFECNECGKKFSR
SDNLAQHARTHASGGAIVMNIIDNADPSAYEAGMVAPPPVDDYANYGKVL
FQIAADVPGSASELSSEEASDNGKKKRKRSE
>Mb|QLI66049.1
MSTVDMGSPQKISILGQDSIVADYGLWPEFIATDLVNHVQSSTYVLITDT
NLHDIYVPPFQAWFQSQQLADNARLLTYAIPPGEASKSRETKAEIEDWML
SQKCTRDTVIIALGGGVIGDMIGYVAATFMRGVRFVQVPTTLLAMVDSSI
GGKTAIDTPMGKNLVGAFWQPKRIYIDLAFMKTLPVREFINGMAEVIKTA
AIWNEDEFTILEQSAADILACVRSKGLDRLLPIKDTLTRIVVGSARVKAE
VVSADEREGGLRNLLNFGHSIGHAIEAILTPQLLHGEAVAIGMVKEAELA
RFLGILRPHAVSRLSKCIASYGLPTSLKDKRVMKLTAGKECRIDTLLEKM
SVDKKNDGDRKKIVLLSQIGRTFEPKASVVADSDIRTILSASIRVTPGMP
NGLKVTVTPPGSKSISNRALILAALGSGPCKIKNLLHSDDTEFMLSAIKQ
LGGASYSWHDAGEILEVTGNGGKLSASREDLYIGNAGTASRFLTTVLTLC
SSTQGSNSTVLTGNARMKVRPIGPLVDALRQNGAQIEYLEQEKSLPIRVH
STGGFQGGMIELAATVSSQYVSSILMAAPYAKSPVTLRLVGGKPISQPYI
DMTISMMSSFGISVTKSTTDMDTYHIPLGAYKNPSDYIIESDASSATYPL
AVAAITGTTCTIPNIGSVSLQGDARFAIDVLRPMGCSVQQDEHSTTVTGP
ITGQLKPLPQVDMEPMTDAFLTASVLAAVANGETQITGIANQRVKECNRI
AAMKEQLAKFGITCTELDDGIKISGKSLSDIQTPNVGIHCYDDHRVAMSL
SVLSVVAPGSTIITERECVGKTWPGWWDTLAQSFKVKLDGSDITDSTSGV
KHASTRRLHAKRSVFVIGMRGAGKTTAGRWMAKLLDWKFIDLDEELEHRS

GKTIPEIINSPKGWDGFRQDELDLLQDVAENMPECHVLSCGGGIVEMPKA
RELLQNYCNSGGMVVLVHRNIDQVIEYLMRDTTRPAYTTEIRQVYERRKA
WYEVCCNHIYYSPHSAPNTESNSIPDDFKRFITSITVGNSCVKNIAKKDE
SFFVSLTVPKLNENLHAISSASVGSDAIEIRVDLLDNWSLEAVTEQLSLL
RFVSKLPIIFTVRTVSQGGRFPDAANDHRHDLYRLALRLGVEYLDVEVTS
SDEILQELTETRRNTLIVSSHHDPAGSLSWKNASWIPFYNRALQYGNVIK
LVGNAKSIEDNFDLANFKSRMMADHKTPIIAINMGAAGQLSRILNGFLTP
VSHPDLPYKAAPGQLSAAEIRQGRALLGQIKKREFYLFGSPISQSRSPAL
HNTLFGITGLPHEYHLHETSSTENLDEVLHSESFGGASVTIPLKRDVIAL
VDELTPAAQTIGAVNTIIPLNHDIPSNKRRLLGDNTDWKGMVYTLSEAGV
INPIKNESAAVIGSGGTTRAAIYALHFMGFETIYVVGRDAHKVNTLASEF
PRDYNVNAITEPAQAADFTHSPSVIISTIPADQPIDNGVKDVVFTLLSLS
AVSRVKRVLLEMAYKPHHTQIVQMAESIGTWAVIPGLEVLASQGWYQFEA
WTGIQPLYHRARDAVVGSTS
>Mb|QLI66050.1
MKPFNQPDRSRLSPLTNKRLNRNILLTAALFFLTSLFLWSRREPAPVPEL
PPNKLWLEPDEINKTYSFPTTSDFQPVSLSPGNRSTQELCASFPRHKLSR
IQPVLKMGYSENRDKIESQLDSVSACFSADDLLIFSDMEETLRGHKVIDI
LAYLPDTYRKGSDTSKFQQYLLQKEMKENGTLETDKEAAKKIDGWALDKF
KFLPAVERAWAMRPNKEFYVFYETDTYILWDNMFRFLDTLDPDTPLYMGS
ASPGRRDGRRLQGTLFANGGPGYVLSRGAMKKLLHRRAGPNGAFIDPPFS
ERFRYLAHDGECCGDSILGFAAWELGIVLQGFYPLFTPYLLTALPFDGDH
WCHPLVTMHKVATQDMVKLWRWEFEHRKHGQPLMYADLWHFYRPGQHSTR
DNWNNEARGSFVPLGERQADSSQACQKLCRADGECMQWVWKGRDQNTCHL
LHAIHYGGPQEAHEVEGKWIDYKSGWETERIDQWRLSRKCDAASWVGPSL
KRVF
>Mb|QLI66051.1
MASQPAEKGTLPSQFDSERSSSRARDSLDGKGTPQNEKADLKRALTKADS
KVVAAPPKATDPDEMFRHLPPDEAEVLRRQLVTPELKQGVAVLYRYASRN
DVIIIAISSICAIASGAALPLMTVIFGNLQRTFQTYFYSAGQMSYNSFVD
ELSKYVLYFVYLAIGEFVVTYICTVGFIYTGEHISAKIREHYLESCMRQN
IGFFDKLGAGEVTTRITADTNLIQEGISEKVSLTLAAIATFITAFVIGFI
NYWKLTLILSSTVFALLLNIGIGSSFMLKHNKNSLEAYAQGGSLADEVVS
SIRNAIAFGTQDRLAKQYDKHLAKAEYFGFRVKSSMAVMVAGMMLILFLN
YGLAFWQGSQFLVDGIIPLNKILIIMMSVMIGAFNLGNVAPNIQAFTTAV
AAAAKIFNTIDRVSPLDPSDDKGNKIENLQGNILLENVKHIYPSRPEVVV
MDGVSLEIPAGKTTALVGASGSGKSTIVGLVERFYDPVQGAVYLDGQDIS
KLNLRWLRQQMALVSQEPTLFGTTIFKNISHGLIGTQYEHEGEEKQREMV
IQAAIKANAHDFISALPEGYETNVGERGFLLSGGQKQRIAIARAVVSDPK
ILLLDEATSALDTKSEGVVQAALEVAAAGRTTITIAHRLSTIKDAHNIVV
MTSGRIIEQGTHDELLEKKGAYFKLVSAQNIADAEDLTAEKEEEDINEHQ
EELIRKMTTNKEGQFAVDPDDDIAAKLGRSSTRKSVSSIALQKNKPEGEK
RYGLWTLLKLITSFNAPEWHLMLFGLVFAAICGGGNPTSAVFFAKQIVIL
SQPVTPANRDQIKKDSDFWSAMYLMLAFVQFLAFSAQGIAFAMCSERLVR
RVRDKAFRAMLRQDVAFFDKDENTAGALTSFLSTETTHVAGLSGVTLGTL
LMMSTTLIAACAVGLAIGWKLSLVCIATMPILLGCGFFRFWMLAHFQRRS
KAAYSSSATFASEAISAIRTVAALTREHDVLKQYHDSLAEQQRRSLMSVL
KSSALYAASQSLLFLCFALGFWYGGTLIGKGEYDQFQFFLCFMAVIFGAQ
SAGTIFSFAPDMGKAHHAAGELKTLFDRKPTIDSWSEEGERLPQVDGTLE
FRNVHFRYPTRPDVPVLRGLNLTVHPGQYIALVGASGCGKSTTIALLERF
YDPLFGGVFIDGKEVSSLNINDYRSHIALVSQEPTLYQGTIKENILLGSA
KEVVPDEAIEFACREANIYDFIISLPEGFNTVVGSKGTLLSGGQKQRIAI
ARALIRDPKILLLDEATSALDSESEHVVQAALDKAAKGRTTIAVAHRLST
IQKADIIYVFDQGRIVEEGTHSELMKKNGRYAELVNLQSLAKHG
>Mb|QLI66052.1
MSTPRTKRQFAGSSVDPSQRQITSFFASRAPGDAAGEPQPQPPRQPLLPS
SVQANLLNVGMRVRKSVPEGYKTSGTSAFKLWTDNTVPTAPASRPATQTT
LRAASKELLPFCGINKVGGLDSQPAFASDYSDSDSDSSVPEADDVPELTM

SQESVDSNAPELSSRKRCYSDDERADCPTLLALPSKGWDDQVSPRSFAPS
QWGDSRVMAVPRTRVKKRPSLKHVGQENAAVDGDFGEADFLVFGDTRDMD
LS
>Mb|QLI66054.1
MAPNPNPPVILQPPPSAPDTSTRATSPNPPSTTRTGIVVFSGGSAANSLV
DVFERVREANKTSLSYVIPISDNGGSTSEIIRVFGGPGIGDVRSRLVRLI
PDNDVPETVAIKHLFNHRLPKTYEAARAEWFEFLDATHALWTDVSSPKRE
LIRAYLNTFNLEVIKRMRPSSRFDFSKASIGNLFLTGARLFTGSFEAAIY
LLSSICAVPDRISVLPALNTNFAHHIAAGLQDGTVITGQNDISHPSYPTA
AVPGAGASSTGMQTPALSVGHDTEEHDKIEDANLPGSLPALRRPAIVFSK
AEDEDLASRIDRIWYINPYGQEIRIPANPRVLDAINTAGTVVYCIGSLFT
SLVPNLVLKGVGEAIANPAIKNKILILNGTADRETGPSDNPLTALDFVAA
VANACADSRGWQKPSEEEYNLYVTHLVHLESPASPRVDKHKFGQLGIDTV
RLYGPKDESGRGGRYDATALGQTLETVVGRKDIRVDRSRRNTLVG
>Mb|QLI66056.1
MSETQHPRPMPAHADEYMEKGRDGVVHDVSNAFDKPEHGAGATSDREAQA
GVLEVEAIATVWSKTSLIVAYVLIWIVYFIMLTQQAAEAALNPFVTSAFQ
QHSLTPTVGILSSIFGGACNLTVAKVLDVFGRPQGYMASLVVVTVGLVMM
AATTSVEMYAAAQVFWTVGTAALLYSVNIFVADTTALRNRALMTALTASP
NIVTTWLGGPISQGFLEGPGWRWCFGALAIIVPVLCLPLFVLLMMNYFKA
KRQGVIKPRAGSRAPWQSLLYYCREFDAVGLLLITAGLALFLLPFNLYTQ
QALGWRSPLIICLLVFGVVLLALFAVWERFFAPVTFIPYALLLDRNMVGA
CVLGSVLFISFWCWNSFFSSYLQVVQDLSVTEASYVVQIYGLGSSLFSIA
AGVAIRYTGRFKAMTLYVAIPVYTLFMGLMIYFRDPSMSVGYLIMCQIFI
SFAAGIIIMTPQIAAMSAADHQHIAVVMAILSMFSSIGGAIGLTVAGAIW
QAVFPVKLAEYLPPEDLPNLVSIYAALDVQLSYPIGTPTRTAIQHAYSDA
QSMMLTAGTAIWAVGFAAVAIWRNTDVKNLKQVKGQVI
>Mb|QLI66059.1
MSSVFAIGAGAAVAAFLGRAGLVAWRRSRGGVGAMGKAFYKGGFEPKMTK
KEASLILSLNERTITKDKVRKAHRTIMLLNHPDRGGSPYLATKVNEAKEL
LDKQT
>Mb|QLI66060.1
MPATRGRRTAAAAAVPANPLQAHRLAFSGKFPGFSHKDLNTLFTSLGASV
TPSVNGRNTHLVCDEKDFLRKAPKVRDAEAAGVSLVKPEWVAEVAKLRTD
VDPSNYLWPEEQHDQGDHETPDDCSADGSGKKRPIAVANSNGSDLDAGAD
DGHVAKKPRGKAAQVKDEAEEEREEVVDQGNSVKEETIETKLKELSKGQF
LKKKDIVIPVDERCPLQTYAVYIEPRSGMIYDASLNQSSTSNNHNKFYRL
QILFQGLTYKTWTRWGRVGEMGQNAILGDGSLDDAKKHFEKKFKDKSGLA
WKDRTKDPKPNKYAFVERSYDEESEDGEQGTAPDAKKENEQDYKPPECTL
PAQVREVMELIFNQNYFDATMSSLNYDANKLPLGKLSKATILRGFQQLKD
LASLMDDPALATSQWDMTVAAATEHLSNTYYSLIPHAFGRNRPPIINNEA
LLKKEIELLESLSDMKDASNIMKIDRSKARTVHPMDSHFQGLGMREMTPL
DHSSSEFDYLQRYLNGSKGSTHGINYKVRNIFRIRRAGEYERFNDSVFTK
IPSDRRLLWHGSRCTNFGGILSQGLRIAPPEAPVSGYMFGKGIYLADMST
KSANYCCSYISNSHALLLLCEAELGDPLQKLTDASYSAGEDAQKGGMYST
WGMGSTGPSSWVDAGAVHHSLEGIKMPDTKVAPGPTNVDGAYLAYNEYIC
YDVAQVKLRYLFHVEM
>Mb|QLI66061.1
MATDAAVAKEGLVIPLIDFSKFLTGTPDEQKTAAEAILHGFQTAGFIYLK
NHPIPVDVLSRTFARSADFFALDTDAKMKLGWTTPEANRGYSSPGREKVT
QLEDIAEVEKVRSAAPDLKESLEIGRDTDPKHPNQWPAEDGKLKGFRTDM
NDFFSQCQKLHVEVMRAIALGMDLQEDFFDRFVHVGDNTLRLLHYPSVKA
DVFRVNPGQVRAGEHSDYGSITLLFQDSRGGLQVKSPTGYFVDATPIPGT
IVVNAGDLLARWSNDTIKSTIHRVVEPPRKEDSSYPPRYSIAYFCNPNTE
SFIQTLPGTYATEKDKKYKGVNSGDYLVQRLTATY
>Mb|QLI66062.1
MSSNQYDSQASTNYKEAFALFDKRGNGRVTVDSLGDLLRACGQNPTLSEI
KDLEQNAGSEFDFETFQRILNRPGGFRDPGEPEEYCRGFQVFDKDMTGFI

GVGQIKYILTNLGEKMSEDEVDELLKAVDTSSGQVNYTDLVRTILAN
>Mb|QLI66064.1
MAEDEKRAAVAEATEATEDAARRPSASPTEKSEPNSEPFSYDEDDSFARQ
GDQPRTPTAIEHDLNVTEDDLLEAKELASQYTLEEVRDIMIRVRRIHEKD
PNFPTTVIEKVKTFLDNDDIFENPDQHQHLIQEMKLEAALVTSNSPYSEV
RAVVSNKDDPKTPCSTIRSWAIGLIFSVLLAFINQLFDIRQPAIRVMANV
AQLLAYPIGKAAERWLPDKGFTMFGIRHTLNPGPFSKKEHMLITIMANVA
YNTPYTNYIIWVQYLPQYFDQPYASHFAYQLLIALATNFIGYGIAGICRR
FLVYPSYCVWPASLVTIALNSAFHTEMNPPVGGPFRSIWRISRIKFFYLM
FAAMFVWFWFPNYIWVSLSKFNWISWIAPGHRDLNIVTGINNGLGLNPFP
TWDWNVLLWDSADPLMVPFFSTLNRFIGVFASFWVVLIFWYKNVYNTGYL
PINTNRVFDRFAQLYNITKATDDHGLFNAEKYESYSPPYLGAGNVVIYMF
FFAIYTSTLTYAALYHRHEIAMGFKALFYSLRLKKKPVKEERVLDVHNRL
MRAYPEVPEWWYMACLAIAIAFGISGIVGWDTHTSPGVVFWGLALCVIFV
IPVGIIKAMTGIEVTLNVLAEFVGGSWVDGNALAMNYFKSFGYVTCAHAI
WFCNDLKLAHYVKIPPRQTFVAQMVATFLSTIVCIGVLNFQMNNINNVCT
PEAQYKLTCPGVNTFFTASVLWGTVGPQKIFGHRGQYSQTLIGFPLGVVI
VVLIWALNKKFPKWTWARQLHPVVILYGGIVWAPYNMSYVWPSVPIAYFS
WIYLKTRFLGLWSKYNFVLSAAWSCGIAISGIIIFFSLQYTETDFDWWGN
NVSEMGCEGNACALKTLGPGEWFGPGPGKFN
>Mb|QLI66068.1
MAGVKRSLAAMMADERSLSLGDTTEPITRSRSAKTSVNSSEYTSEAPSPT
LPPLNGHMPRFTPDASIVIVGVRGAGKTTLAIMAASALKKRIVDLEVAFQ
RATNFSSPTYSKVHGTTQCQIKQGDVLQSILETNTTGCIIVCSWMERRVQ
GLLRQFAASNPVVHVMRSKEAVQEHLRVESGTKWETFWSTSNAFFRTCSN
LEFFNVSESAAVEKSLSSIRNSNGVDLPPYLALKQAERHLLKFFSQIYPA
GTIPFFESAYPLASVATEQRQYTYAVDISVEEIIHNRVDIEDCSAGADAM
QITLEDIQTDWADCRYLDEHTKMANRVTEAVGLVRRSSVLPIIVHIGFPN
PSAATTSTYFYLDLLSHVLTLAPEMMTVDLRLDDNAIAKLASLKRSCKLI
GHYAPIANFDSWTSPQWISHYQRAVRLDCDLVRFVRPAEAVTDNFEVARF
RSTVESLPDSHAPIIAYNSGNLGKHSAFLNPILTSVSPRLESQNNEKQRQ
TGLLADSATRALYASFLFDPMKLYVFGANVGYSMSPAMHNSALEVCGVPH
RYEPYSTNSLQQVRHLIQDPNFAGASIGLPFKVEFITLTDSLSPHAQAIG
AINTLIPIRHLNDDGTVPTGAASFFRGVNRAGPVLALYGENTDWIGIRSC
IRRGLSPANAVRPATCGLVIGAGGMARAAVYALLQVGVSNIAIYNRTVEN
GKKLADHFTQLLQKSEFEGLGAGVKTKFEVFPALHHDWRSAFRLPSIIIS
CIPTHPIGDVPSPELRLPESWLENQTGGVIVELGYKTLNTPLLQQAMEHA
SRRWIAMDGLDLLPDQGFAQFELFTGKRAPRKTMKKAIFENYPDQYGRSN
PEELRRRLQTMLE
>Mb|QLI66071.1
MPVQTTIAKVTSVALRISAILFFRFFPAHFPSITFALFAIYIPAYFASYR
AGPALEIVDDEVDVTVKQTTLTSETPLLSETGQPPEPEVVAQEVDVEETI
ILGEKTERPWKVLLYGYPSATQPKWSIITLLINVVLALLTADALFRARWY
YSSEDLSFVRLGYVSPSEANFLVREPDQAKLPVTLQIRAKTSEASVDAPA
WSNAADIQQTTNETDFTQALTVSLRYSEQRWYEWRTSNNHSGEFLSPPKP
GSMPTVNDGKFTFLSTSCILPRFPYNPMDHALAIPGLRHLARKLPDLGAQ
FMLFLGDFIYVDVPERFGKSSEEYRMQYRQVYASPDWAPVAQNLSWIHVL
DDHEISNDWSSNTTGIYSAAVGPWHTYQANVNPPKAVQAGTRSTPRQDAT
WYEFVQGPVSFFMLDTRSYRSSNNAPFEEESKTMLGQDQLADFLAWLGRP
EPKGVKWKFVASSVPFTKNWPVNVKDTWGGFLFERRKILEAMWEAGARGT
PVVILSGDRHEFAATKFPPPPESNWPESAAAHEFSTSPLNQFASPFPTYK
QVDSEDVKLHYIPSGSSKFGSFTIENIDGQSILQYTLYIDGEERWTTQLS
APIVEEAAKTSGSFWDRFKFV
>Mb|QLI66072.1
MDAKPVAPEEIKEGDVEVKATDSNAAAAGAPKPAATTEDVPDPDEDDLDD
LDDMLDEFSTVKLEDGANKPTESSSAGPSTSTPKQGDAAPKESPAAEEEP
FSEEDFAKQLQAGMADLLGELEQSPEMQQQFEDMFKQMAAASGDAAPGTG
AAGSSSAKAGAPGTASEAPFQETIRRTMERMQASGDQATAAAATSSGDDF

MSELLKQMSSGDLNGEGSEEEFSKMLMGMMEQLTNKDILFEPMKELDDKF
PEWFEKNKSSTPAEDLKRYEEQRVLVREIVAKFEESSYSDSNASDREYIV
DRMQKMQAAGSPPSDLVGDMASAQEAFGGADDQCNPQ
>Mb|QLI66074.1
MASLNLAKRSFAPVPIETTFQSNRKGTQPGGQPGGPNPELTPEPSPRSAS
PPMPQPRQRRRFKPQLIETSRRTRRVGDPGPATRPTDKTDITPYTNHIYV
AKPKPRRKHGEALDDESIPWIPPTRRESEDEGVKEYLLEIAAREAARQME
EAALAAFPNSRAREGGVAHFYFRESSESDNSAENTPQNKDNGMQPRVRRK
SSNLGLNWWHKHMQSHAEQLAYERGDDEMAFGGVDEDTIMRTDSDLDKMD
LPVPPDPLWTTTNRIPPEDRRDSMAELQDLRDSLTPLSRPDAAFTIGNRF
QPGDAAYGRPSPDQRDVGYSPFLRPVDRGPDVAFGRPFGAFGLKPEDPKL
HLMRQAASPPMLGKDLTFRRCPSPKQTKLETDHPFANHDVYKEQNRDVTG
QGGLWRGFCCRSESNTEYVVPAEFHGANMLVTPYPPASPRDGAGFDSPSM
SDEPESMFSSSNSSESLNNISPTRAEHRSRTSQSKGLHMLHGLDDRLRRE
KAQVELDEKIASEFDDEFVTQVYNYLSLGYPATARYFDGELSKISRVSVE
ELCRDDERQMAKGHMLEMSLQATSETDRCPRWKALRTYVTEWARQHPDLD
NLDPLAWGVRERRGSWAI
>Mb|QLI66076.1
MTLETLRESVHADTNIPASSLHIYHNGRLLTDDTKTIEQLEIPNGGMLAV
HVRHLRGNTGASERAAQTQPPAQPPRPQGSGDNDPELIRLQILGNPAARE
QLQRHNPELAAAVDDPVRFSQILQNSQDRERREREERQREIERLNQDPFN
IENQRKIEDMIRQERVMENLQSAMEHNPEVFGRVHLLYIDVEVNGTKVKA
LVDSGAQATIMSPAYAEACGIMRLIDTRFAGVARGVGTAKILGRVHSAQI
RVGNLFLPCSFTVMEGKTTHLLLGLDMLKRYQATIDLVKDRLIIQGEEVP
FLGEADIPKDEEPVEQEATIPGPAGTTIGQETGAVLPPQQPSTQASSASA
QSTQPQRSNPPASAPSAAASASTPNVTAAHVESLIAMGATREQAIQALQA
AEDNVDVAAGLIFF
>Mb|QLI66077.1
MDANGTFQSPQPPPWPSSETTVPLRGSRPGAEPAGQSATVQQSLNPNLAV
AQGPVLQPSGPMPAWNPAALLQPSRRGFSATDLRNLPSGRPQPMYNHVAN
GHSRASPSVNDQMVFQFTSASDTSSNDHTSGSSTPTPPNGETSVSNANGV
GLWIERMNNVQRRSNVPHPKRRKTEDPQDFAARASNMSIRSSSGMLGDYV
RDRRQETDAGRLVPQTPTVDLTDGTDDDVMVLDDPKDEEVCYGMIKTSLN
CSKVPSPKPGMQSLWGPGYNPGIKILLKRQSGERSLKIQACDHTREMIGL
VELSCAAALTPLLDTNIRLRTDCRIPPHQKKPGEEPGQAISRAYNLDVVL
YGPQRYARSIGALLSKHNLKLIAPHMVQKGIKVFNPHVSEWRPPPPKVYP
TNAQGTYTSVANRTVEEIRSEVMGVFDSLKRNDDLPTMDPDARILTPLLK
HQKQGLYFMATREKSLQEQSTEKGMVSFWRTKVNARGEKLYSNVITGQEQ
RAPPPETCGGILADMMGLGKTLSILSLVSSTIDESHKWEQVPIVQRPKMD
PRLSSNGHSAMQTNFDPAAVTRHVKSTLVVCPLSTVTNWEEQMKQHVAPG
GLSYHIYHGSNRIKDVDKLAEFDLVITTYGSVSNELGSRRKGKDGIYPLE
EIGWFRIVLDEAHMIRESSTLQFKAMCRLQAERRWAVTGTPVQNRLDDLA
ALLSFLRLHPFDDRSKFNRYIVEPFKACDPEIVPKLRVLVDTITLRRLKD
KIDLPKREDLVIRLNFSPEERSIYELFARNAQDRVKVLAGVKDGKALGGN
TYIHILKAILRLRLLCAHGKDLLNEEDLAALRGMSAEMAIDIDDDDEDGP
TLSHQKAHEMFSLMQDTNNDACIECSKKLNAGEGQNLDAEQQDDILGYMT
PCFHVVCRNCCRSYRERAQQFLAPGQNTGPCLICGSHVRLEFVELRRDDV
DAEHDGPAKTKAKDTKKRFDKYDGPHTKTKALVEDLLKSKAASQACPNEP
PFKSVVFSGWTSHLDLIELALKPAGITFVRLDGSMTRMARTSAMEKFRED
NTVEVILVSIMAGGLGLNLTAGNTVYVMEPQYNPAAEAQAIDRVHRLGQK
RPVRTVRYIMLDSFEEKMLELQEKKMKLASLSMDGQSKALDKAEAARQKL
MDLRSLFK
>Mb|QLI66079.1
MRTSLCSHLLLLLLGSSFIAVASASRDTPRDSQVRLTGPIRHHQVDPVILD
ALKKHSDPVDALVSLHPEAADQLAQPRLLRVSGEPTARWMTEGDKLRLKR
KGHKFVDITDHEDFYKQNVNALAGEAHLPNLSHQHIVKPLLPQVSTKRMR
HVLEHMTSYYTRYFGSVTGEESAQWLHDHIAEIIKESPFHTHISLEVFTH
SFPQPSIIARFEPKVRNFSLPLTILGAHQDSMNYLFPLLPAPGADDDCSG

TVSILEAFRVLATSGFIPINGPVEFHWYAAEEGGLLGSQAIARYKKESGA
RIGAMMEFDMTAFVAKNATESIGFIETEADAPLTKWAADLASEYVSIPAK
VYKLPSGAGSDYMSFTKLGYPSAFASEGNPSAGAFPGEFDPYVHTAKDTM
DVDDDTGIFSLEHMARFSELAIAFVVEQAGWDNKWR
>Mb|QLI66080.1
MAQDSDVTPSAEVAASAATTTTTTDAVEAKELPIRQTSFGTAAPGQTLT
GKQEHYLKRELISKQAKWEIDELNSPTALQRFGAPFRSERGEVPPEESEL
PILRFVFVHHVRDFPFLDKAKEKEFWQDKLQVFLESFAVKRVSSSEDRLE
ETKRRKLAIKAQKLIELMMVSGIPTSSGFEERIRFSEIEIVDSNAIETGV
MHSIPEGNYINGWDVNVAGVRVMTVKGRIRQHKHAEFLLRIKQKGELEHF
VGRRYGDFSRLHKRIKTELPGRILPPLPKKNKSSTTASGLWTGWSNADDS
DASSVSSVSTRLTNAPVPQSGGISSLLSPTAPSQRKPGSAQSIRSARESP
RPSVDGKQSPEKQDADDQSVVLWRESSRVSLRAFLRSLLHNPQIANTKAM
QEFLSGDPITPTDDDVEDIMRRKAIDEKRIEEQKQFYEIARKRAAELDEY
MEHFRRDIVERNGLTMLFKEIKEKETIQDLSLRYQKFAEWLRIEIAAVIY
HLFLAEDNSPEIFAQAKRIHSLIPYSVLKNVIRIANPAAVMSGVLDIFLA
QPFGTRSLMQRIFSLTLNDGIKSFQKSIDALTNKIADPVFTDKLKRYTDA
EEDLKAAIRLEAEEEQIDLIVAIMRSDLIEPELTGEQIQRLFNAYVAFNN
AVENVDEELKQGAQLFSYLKQLLKLCTRQRDKAMMLRLIEEPVTLQLFRD
LFTIFYEPLVRVYKSANVYNSVTDFAVFIDDMIQVVDKCREQDASADPNQ
TVQAFIDLCQRHEHNFYKFVHEVHTHDNGLFTQLMGWIEGILEFLRHGPK
NGKLNVNALFEGGVSAGILDKEKAIQEINSLISWQEARKKWHHDKTRQKM
AAEGGPLGSVGAIGTGFDSTDFGLDGQDLQEMAYDFESESEKEAEAEEED
ELDPISAERRRRAKKQDRLRRSAGEPAKPVISEVHKLHDNFLVMLREVLA
S
>Mb|QLI66081.1
MSTPDSNVPTWVSQLRSPPGAKARVPGVPDPIGFSSGPSGSKKPNKDGKS
QPPRKAPTGEEMETLKLKKAWEVALGPVKALPMTVIMMYMSGNSLQIFSI
MMVFMAFKNPIAGLMATNQAFERFNTEKNAAQMLQTKLVYVAMQLVALGV
GIWKVNSMGLLPTTRSDWLMWESLRESVDKAITAL
>Mb|QLI66082.1
MAFTTNATIASFNGRILKLTHQSSSTSTPMNLNLFLPNTASSSKPAPLLI
YLSGLTCTPDNVTEKGFLHAHASPLNLALLYPDTSPRGTNLPGEHDAYDF
GSAASFYIDATKDPWDTNYRMESYLTTELPELLFANFKELDAQRVSITGH
SMGGHGALTLFLKNPGKYKSVSAFAPICNPSQCPWGQKAFAGYLGDDKAE
WAKHDATELVRRWKGNLNCLIDVGTGDNFYNSGQLLPENFVEAVKGAGVD
GVNVRYHKAYDHSYFFISTFAEDHVRHAAKALGLL
>Mb|QLI66083.1
MNPASRIPSLIFHLQKHCTPSVLILSATPLSRFHSVPASKLRLRRRPVRD
GPLKDDAGHIVIVKMADEKDTLQLGGDISWDIPEAPWTTARPSKRDPARF
LPQAIAHRGYKALWPENTMGAFKGAVEVGAHAIETDVHLSRDGVAVLSHD
ASLTRCFGIDKKLADCDWEYLSTVETLREPKQGLPRLQDLLEWLVEADKL
QGDEHSHRPWVLLDIKLDDDAKDLVNAISSAIAQVPASTPWQGRIVLGCW
NASVLKASRRTLPDYPVAHIGFSLSYARHFLPIPNMGFNMAFHTLVRPYQ
GRRFMRDVRAARRPLYAWTVNHPRWMRWCLERNVRSGGGSVVDGVVTDDP
RLYLDVCRRYEDDVDAGAPGRDDRGSLGQTVGLAVDIVKRHVLAKLFFWY
RRHVQGKLDDLDGKTKRQIKRD
>Mb|QLI66086.1
MTRALVVVGALVALPSSIAIGFDDFYDFADTQILLQLDARAELATSPPHY
PSPWMNPQAPGWEDAYKQARDFVSRMTLAEKVNLTTGTGWMSDNCVGNSG
AVPRLGLRALCLQDGPLGIRLSDYNSAFPAGITAGATWSEHLWRDRGKAL
GAESRDKGIDIALGPASGPLGRAPTGGRNAEGFGSDPYLQGKGLANTVIG
IQESGVIACAKHFIANEQEHFRQAGESVPRGFNISESLSSNVDDKTLHED
YAWPFADAIRAGVGSIMCSYNQINNSYACQNSKLLNGILKDEMGFQGFVM
SDWQAQHGGAATAVAGLDMSMPGDTEFNTGYSFWGGNLTLAVINGTVPAY
RIDDMAMRIMASFFKVGMKPGKQVPTSFSSWTRDTFGYRQAAAKENWEQV
NFQVDVRGNHAAHIRESAAKGTVILKNTGSLPLNKPKFLAVVGEDAGQNS
RGPNGCDDRGCDDGTLAMLWGSGTSQFPYLITPDSALQRQAIQDGSRYES

VLSNYQWAATQRVVAQPNVTAVVFVNADSGEGYIEVDGNAGDRKNLTLWK
NGDDLIKNVSSICHNTIVVIHSVGPVLVTDWYQNPNISAIVWAGLPGQES
GNSITDILYGKTSPGRSPFTWGPTRESYGTDVLYKPNNGNDAPQQDFTEG
SFIDYRHFDKVAPGKNSTGAPIYEFGYGLSWSTFEYSNLKVEKRDVRPMS
PPNGKTIAAPTFGNFSTNLKDYAFPPSIRYVYQFIYPWLNTTASGKEASG
DPHYGQTAEQFLPPGATDGSPQPRLASSGEPGGNRQLWDVVYTVSATITN
TGKRVSDEIPQLYISLGGKNEPVRVLRGFNRIEGIAPGQSVTFRHEITRR
EISNWDTASQNWVITKAPKKVFVGSSSRHLPLSADLS
>Mb|QLI66087.1
MTSSDSETQQMTGTSSVDQTMYTLSAQSRRSRPGLVQTNFNSNSQTVHVP
GSAISASSAASSSQQGSPIMSPSTAVLTTNSSIQASPEPLRPRDDDQFSA
SFQLPDSVLTRKTSTNSLGRSWVGSTSPIAEAVVPESRSNSVSATSTGTT
PTTTSKGNLMRRLSSRASRASRTLSGRRRQSSATPASRDGSIGPCFLRRR
SDSNNTAPDYVPTPSTDSESDLDERDDLLLIKSSFLDHIPKGSSANSTTG
SVTGSANNMAGPVIPLQLQRGTWLRKISKKNRSKRICLIYERETNKLVWD
RARPHKFLHVDDIREIRFDSDIQQYARDFNIPESERLTWFTILYTMPDKS
KSKLMHLMADSIETTNLWINFLDAMLRHRQDVMTALMTFNDKAIQQYWQS
EMTKKFGDNPRSEDQEELDIIGVMRVCQNLHIYSSQSTLQANFRLADARH
REALRFEEFLDFVRLMRQRPDVQRIIRSIAAKPDKGLTFVEFLSFLKDYQ
GEDIEANVTTWERHFNKFVRKHRADDLGGLKVLQDDMYMSEAALVGFLSS
RQNGPLAEEPQEYTLDRPMNEYFISSSHNTYLLGRQVAGQSSVEGYIRAL
AQGCRCVEVDCWDGSDGQPQVVHGRTLTSAISFKEVMTTINKYAFVKSSF
PLWISLEVHCSPSQQALMVDIMKEAFGARLMTEPLDPSSDKLPSPSELME
RILIKVKKPRGGRDESLPAEPVGRRRGSSLSSPVSKPTVPDNGAFSPSPS
LPQSPLLSASNQARRLVSKSRVNTIAEGEVHDVPSSSTSDNESGSEIGGS
VRRSQNKTTRYLGALGVYCAGVKFFGFEDPAAKTFNHIFSFMETSFAKHS
RTKDAKMALDIHNMRYLMRVYPDGLRLASSNFDPLIYWRRGVQMAALNWQ
TFDLGMQLNNAMFQGGKDESGYVLKPDELRDIQIQPYNSAIAEGKKERSV
VTFAIDVISAQQLMRPANLAANKSMDTYVEVEVFHANDKRDKKEVGSGLT
HALDSPLKFQTDVVRENGFNPMFADGQFKFTVTTKHPELIFVRWSVKLAS
YGESYNSKEGSAIATYTAKLNNLKGGYRTIPLHNHAGDQYLFSTLFCRIN
VEPIQTKLFDIAAPVQDGGKLNRFGGKVFGRTSTSPRSTFEKSSMEKNSF
DSFA
>Mb|QLI66088.1
MASAPATVDHVGHVPVDDVASPEDTNPSKQDINPWSVSGEVGDDGKVKAI
DYTKLIDEFGTSKIDDALLQRWEKVTGQKPHRFMRRGIFFSHRDLHMILD
RYEKNDPFFLYTGRGPSSDSMHIGHTQVFDFVKWLQETLDVPLIVMLTDD
EKYLFSEKRTVEEVMGYSRTNAMDIIAAGFDPKKTFIFSDFAYVGGAFYK
NIVRFAKRVTYNTAKAIFGFDGSSNIGKIHFASIQGATSFASSFPHIFGH
DEKKTSSIPCLIPCAIDQDPYFRLTRDCAQGLKFAKPALIHMRFLDALQG
PGSKMSASDDNSAIFLSDTAKQIKNKINKYAFSGGRETLEEHREKGGNAD
VDVAYQYLTFFLEDDDELKRIKDEYNSGKLLTGELKAICIEHLQKYVGTF
QERRSKVSDAVVDEFMSVRPLEWKGNPRVPRADLVVPVTKTDGATPTGDA
AGTGELSKNAMKKLLKEQQIAAKKAQKAKEKEAAAEGGSEA
>Mb|QLI66089.1
MVLSDSEPDFDDVEAIGMPSSQELAGKAVASKRSRDRTANSTTRVTKPFQ
RTKSAKSGTGHLKLPAMARQILMEKSKNNTIEQAGAHGKHMDEPDKIALG
KSYPNLSSSHEQGRLAGLSRNTGSSGKLYTQNIEKEHEPTFENGHEEPKS
VEQLGQQETEPVIDAEEQFPTEYTNNMDEVSTQGRLGELMKKYDSLKTRH
NELREVGMIAAERNFERLKQQSEQNTAASNKLIQSLKDELNVQSALAKQG
EQARQQLERSETNVCDLESKICGLEASLSSARSEIKSLSAKLSASRAAEA
CVKNPGSALKPGVIGHKSAPSEVVRAAQAREDLYGDLTGLIVRGMKRGDG
GSVFDCIQTGRNGTLHFKLSLDAGDASDSYDDVQFTYRPQLDTDRDGDGL
RADKAFQSFPEPYPQNEKDLIPRPLAPFLRSRVLQEGTFGVSHTRVPTES
RPGHVALIAGLYEDVSAVATGWKLNPVNFDSVFNRSRHTWSWGSPDILPM
FEQGAVPGRVDTYMYEPEFEDFSQDALRLDYWVFDHVKHFFAAAATNQTL
NKALRQDKIIFFLHLLGLDTTGHSYRPYSKEYLNNIKVVDQGVKEITELI
QKFYADDRTAFVFTADHGMSDWGSHGDGHPDNTRTPFITWGSGVAAPEVH

PGAIAPGHDMYSSDWGLDHVRRHDINQADIAALMSYLIGAEFPANSVGEL
PLSYLAADNSEKANASLVNAKGILEMYRVKESNKKTNELRFKAYHAFDGE
GSSPENRTAAIANLIANGQYEEAIEESNTLIQVTLQGLRYLQTYDWLFLR
ALITMGYLGWMAYAITTVVDMFVVHEVIAAQRTPLGTATFLGVLFALYAS
FIISKSPLTYYLYAFFPVVFWEEVYAHRQSLYRGRLILFGHIQSAGGAAS
LFLHAVFYIAVIQSLAVGYIYREVLTGLFVLAAAWPFMYGLSFIQYHALL
SMTWAASCLTMSTFTLLPAMKVESIGLVLAGGFAMFLVGFLYLILEDIGL
ADFTWAVNSNPSLKKTNKNIQRTLTGIQVGLILLSMLVTRSSALSLQAKR
GLPVGNQVLGWAILIVSLLMPLLYRLQPNTHYLHRLVVIFLTCAPTFVIL
TISYEGLFYVAFSATLLVWVRLEYAAEIFYRKHQQSNGSLVQPNTLDQDL
IQHRALRLSDARVALFFMVLLQSAFFSTGNVASVSSFSLDSVYRLIPVFD
PFSQGALLILKLMIPFALISANLGVLNKRLGVAPSALFMVVMAISDILTL
YFFWVVKDEGSWLEIGSTISHFAIASLLCVFVAGLEGVSTLFVSGIDVGD
GQSELDPTTNQSGSTMTENGTPNGHSHSTKAKRQ
>Mb|QLI66090.1
MQPGQPPPPPPPPTSGRPQTRAPGSFSPLPGATSPPVDPLRQQPSAPTRP
PPLRIDSSGSDDSLEAPSMPIAASRQRPDSPATPIYAQFATAHANASSSN
VHQNFSRPGGGPSRPAAVGLSSPVLKGHSRKHSATQGYFDSTLPSTATSN
LSHVSMSAAAGQAAAAAAAAVTSTGSLSASQIAAQAAVMSHQNTSHVRQR
SQTVPFPGEGADGVRRGSGSKVPLSPPILSLTEASAPRDSAFVAHGGQHY
DKAAGSHSSAATAAANVVFPRSGHNSPREKSISPQPFPPFPPVPPLPATI
PEKSKAEKSKHKLFSRPGKSSSSKGEAKDKNLQSPGKIGSALSALQRGNF
STSSLVDPPTQSLYSLNNSSSATIRPIEVFSEERVKEKEKKHHHFLSRQK
QKLKDEYHLPLSSASSNSKPTDPNAPSSLYNFNVPASPGPGTSTFSKGKK
DKKLGEKSDGRLDSESTFNLAGDWSTSSGLHPLSQQSTLYDVAEPGKAVS
QNNLALDEAWPYLKSKLLVVFEGEDLRLPVEDFNRVVQTHIQWCIHKRSP
SSMVDDLRDLLTTGFSILDRTLRLTPEDQFIPTVVELWMFTFTSILPYMQ
SVFLPLDLEVSGCGVIMTPEQARDFWGGVVASPSSSNKPAGDAPAAAVLD
VRRLVLTAYRDIVVLPRYDTLKTIFSRLSLEFLPSSFAHMALASPPLERM
LSTSPAESQSFIIRPGTATSVDPLVGSFNSTSTTLLGEGSERGRSRAVSN
VSFGSHGSDSAGAIRPFTPSGLPALSSVREQNVEDSKQLTDMVGRMLQCM
SVLSSIGGDDADDEGSKKIEELCKMLKLNWLGRGRTGRNRRGIVGGRVRR
DEGREELRVA
>Mb|QLI66094.1
MADEHDLEFESADAGASTTYPRQCSALRKGGFVVIKGRPCKIVEMSTSKT
GKHGHAKVHLVATDIFTGKKYEDLSPSTHNMDVPVVNRKEYQLVDISDDG
YLSLMDDSGNLKNDVKKPDDLDLVKKLDALFPTPETETNKQKDTNVIILT
AMGEEACIDAKEAPKGT
>Mb|QLI66096.1
MIPRQGYAPTPHSYVPNSSFSATINLDEEVKLTGSRAERDLQDSLAELFS
IIVTLDELEKAFLKDAIPEAEYTEICERSLRQYKALLADETIAAEFQGLE
EFKAKWDLQAPRATERLRVGMPSTTVTASSSAPSQQASSAANNTSGVLIL
EATQEFITFLDAVKLGMLSKDQLHPLLSDVIQSVNRVTDQDFDNRGKIVQ
WLITLNQMKASDELSEQQARELELDIQQAYQGFRRTLT
>Mb|QLI66097.1
MTESAVLTTAAASHHHQHQRQTSAKTNANASKHRATVSAALSVGASLSTG
TRSTEPANTAVARGSLYGRGGRCTGPGTGTSLSSPNVPGASMPSVQVPAT
ALEDSTPTLNAHPTCRASTMAPSAATATSTSTRTTSSVTSIPHSGPNEQN
RPPSSPPRAAATVAAGGHHLDNVHADDSPPLTRHSHRQYNTSSKLPPPRP
ADSRRGPPAASSFPSQPHRPHHSHPGQVPPSLHGAPSSALPNPVPEQRPA
PSIPDNIANTGAAADANADANADAGREAESTTHHHPSSPFAVSPSATSPP
RPAVHSRASSYHASPQPATSSSSDSPPAQPKRPASCPESGPDHADQTTSP
SSLSPTGTTKPLSWRGPASRSSNGVSTSDGPPPSLNTQRLHGVEAVAQTT
AQPQPLPETPRTKSTQGQRELLLLNRLSGSSSSDDNRYPSPQRPPVSYRP
TQKRHSIQPATLTPGRVPPIRSFRSSDSRKSLTQDMNPMSRPHDLGELYA
TSPEEQSPHSFEGRHAQGGVSLNDGESGRQDDGGDVFLRIARDEQMRRLR
GDENLDDAQSSVSGIRRSSHRRPLSTVVPSYHHQASPPRHRRRLSDQQDR
PRPLHLEDDQGSEISRTTTFRSLLREKAASVHPGEEIPRTRSGGANLRPS

PLSARSTVGYDNSQEASIYARRRASITDSNSTVGRTPGYRPSGLIQARNH
GSSPLARTIDLQNKTATEAPHGLEGTESTASTTAPSTVWDELDDLKSRIN
RLELTGKLPSTSGAAVSRMSEERPATATTTVTTLSGSPKRQVNGQIAEVS
STTSSQREAHPILHEALVKSRPYLSLEVYQALQSAANDAMALSSMMGTPA
LGEAVGPANIAGPASPPALAQHDGPATPTAPKSYSSLPTPRRVAEHGIPK
SISSPRPLSKFEERRSSILNGTALPTPRATGSAPTTPIEGNSQRRSSLMI
SRTRRAGTEEPEVARSPTMLRSRRAGTEEPEEGRQTSFLRNRRGTVGDDG
GEGRFRPPSRADTDVNMLRDPGREYPSDSPSIAPDSISQIPSALPRRRFL
STSVYSSRPAGSQGSNIASPRRYNDRQAGEQDFGDSVGESRAQRQGPPLS
KGIIHSRASSLSTRRNRDSLISTSSGATIGPYR
>Mb|QLI66098.1
MAALPLGASGDDAAGQLLERSPSPPPGNLEGAGQGSQFCLHNSSCSHLDG
CTCVLDDDHLVRQFRDSISASTSRSSRSSTLSTASTSLTAADCDCTQSTA
TGPGCRSQTRSSLPGKRSRRDREQLQAARLQRSGRDGPPLGSKDKPSKHS
KANWSRRRSGNTSGTADALAAVEPANEHSLVKMSTLEQQRWINVQQKTFT
KWLNTKLVSRGLEVKDLVKDFGDGVMLIHLLESLSNESLGRYASKPKLRV
QKFENANLALDFIKCRGIQMTNIGAEDIVDGNRKIVLGLIWTLILRFTIS
DINEEGMSAKEGLLLWCQRKTACYDEVEVRDFSASWNDGLAFCALLDIHR
PDLIDFDSLDKHDHRGNMQLAFDIAHEEIGIPRLLDVEDVCDVPKPDERS
LMTYIAYWFHAFSQMEKVENAGRRVEKFVSNMQGAWEMQTAYEKRMKELL
AALRAQVKEWQLAQFEGTYADAKAQAKDFAAYKRGQKRDWVAEKSDLATL
LGNIKTKLGTYRLRPYDPPAELSLDALERDWGTLAKAEMRRAQLINETIR
DIKNALRKSFADKANDFAMALNTMQLAISGLDGDIEDQWHHVKKLSDNLA
PLDRYLETIEAVDAKCYEANIEENDFTTYTYDELAYELGLVKSSVQKKLA
FLENQMVARNMTNLTPIQLEEFESVFRHFDRDDSNSLHEIEFGAALASLG
LVFSEEEMHDYFLETSGGRDEVTFEQFIRFMVDVTEDQNTAEQVFQSFRE
VADGKPYVTEMDLRHSLVPDEVIDKLVEMMPPHKGPDMSQDRGQPQFDYI
SFMEKMIDAEGTQDDVPSGVLQDRTNLGDTNPRKSPKANGFPG
>Mb|QLI66099.1
MTAPGLTAEQLASFQQNGYLIIRDALAPSTVKSLLDETHALLTSFSLKDH
PLTRFSTGETTDHVGDEYFLTSGDKIRFFFEEDAFDAAGTLTKPPAKAIN
KIGHGLHALSRPFARLIDDGPALRHAGELSPADVARSLGYRDPRCLQSMV
ICKQPEIGAAVPPHQDSTFLYTDPPSATGFWYALEDATLENGCLSFLPGS
QRWAPVGKRLVRKAGGVGTEMVDNEGTRFPDGGEHGREPSAGAEAGEYVP
GEVRAGDLVLIHGNLLHKSERNTSLKGRIIYTFHIIEAEGTEYDERNWLQ
PPEAGFTKLYS
>Mb|QLI66102.1
MLRWLVSAVFLALPIATTIGVLLGMQAYNHAKGRPPPFSGGDDGNGLPGI
GTPKGKDTVEIKCDQAFGFDPKSKGQNFILNPNPWGWKKGEEGALCMLVN
FNGNRTYATEFSAPVFNTTWQYPRVTGGGNNVHAFPNAKVDSKNFPVQIG
SVSKFEFDVEWSLSLKNDTHEEVTDADVTANQINANVAIDMFMDKDSTKA
EDSEKAGFEVMVWFADFGTDAWPLGKKNTPDKGLVKSQTLEGVEFELYSG
LNSQNQMVLSWVATKPTTTFKGDLKPLIDTIFAMNNANYPQKTDYLGYLA
FGQEAYSSTANVTFSVPSLAVDIETST
>Mb|QLI66104.1
MPGAFPPPPVNTIDWSNVGFKIREVNGHIEATYSKTTGKWSPLRFVTDPF
MRIHGMAPALNYGQQAYEGLKAFRTPGDETITVFRPDRNAARLQHSADVA
SMPEVPIDVFLQAVRSAVALNAGFVPPHETGAALYIRPQLYGSSAQLGLD
PPEEYTFCVFVIPTGVYHGTHPVKALILDEFDRTAPNGTGHAKVGGNYAP
VLKWSGKAKREGFGITLHLDSARHEEIDEFSTSGFIGVVAASDDDVTLVV
PDSKCVIDSVTSDSVQHIARSWGWKVERRSVQYAELPRFSEVFAAGTAAA
LVPIRSITRRKGPVGGSLPAGPRVSSTADADVVTYIPEGQADAGPICLKL
LTHLQAIQLGKAADEFGWRCIVSDKDLDVQGAAKEANGTATPQTVDQMD
>Mb|QLI66107.1
MSRTCLARLRLHTSSPGSAKSAARLALPRAPYSSAASMPHRHKVLVVGAG
AAGLSVSHQLLRSGKFAKGDIAVVDPADWHHYQPGWTLVGAGLKDKNKLK
MRTEDLIHAKLKLYNDSVGAFTPQDHCVTLETGTRIQYDQLVVCPGINIK
YDSIKGLPEALANPDAPISTTYGYHTCDKVYQNVQKMKSGVAIFTQPTGI

VKCAGAPQKAMWLALDYWKRAGLYRPGSEQDSSIKVSFATGLPVMFGVAK
YSATLDQLRRERGVEGLFQHDLVSIDGNQATFVLPDGKQVARRFDFLHVV
PKMGPHAFVANSPLADAAGLVDVDSSTMRHRAFSNVWALGDASNLPTSKT
AAAITAQAPVLVSNLLCALEGAKTNAVYDGYTSCPLTTEYGKVLLAEFKY
GGEPKETFGKLVNQAVPGRGFYYLKKDFFPWVYKKFMVKGAWGGPKGFIR
QR
>Mb|QLI66108.1
MAALSHRPPIEDDTPSQASKTHHRSHDNHHAHHTPPLKHYTSAPRSSTSD
RLTADLTTLPASSHHQRHSSTPSASSFSRSQRSATSLLSRAAAALDRTQN
AFAGISEPVIRPRHSNSALARLSLQSLSAPSPEPSSPGRTAGSKAFSVNG
PFAPAPRAQIKVVSPAAQANHPPSQPYSETDPSLPAPIRVAPSDKKMHQT
SSRLLRMTDDDRPFTRDFKDLFATLIVSLLPLSAHRVRLSKVEYTFLSED
AINNLGSLKFSQSNRMPDPKDPSRIVTTTTTTTFSMAKDMARSICQRFVE
ARFIESADGKYQQVYNMKGSVWQLTPKGITVLDRFCSRNGIQQKQVGELS
NMSSAQLVILERDQQTDKLLHDWGTIEVIFRRFVGADRRNVKTSVTAADS
DSLHDYRDGLTGVKMAAERKVNGKAYRDTFTGKATTDWLMDCSTIVDRRE
TVEIASLFVEYDLMEPVVQDRAFMSQNTGYNLFQPTKHAIYQLTTRGKEL
ISGSGSRGRASESENGTGSQRNGITRDSNTQRLEKILNDAALRLLFRENL
RETHCEENLSFYKDVGEFVRSCKDAIRQAQKAPNASSMDTIKEIMAQAYG
IYNAFLAPGSPCELNIDHQLRNNLATRMTKAVGQDNTMIETLQEVTALFE
DAQNAVFKLMASDSVPKFLRNAKYEQQLRNYDLDMSARAPERSQSRSNRK
>Mb|QLI66109.1
MNPYTTNPKHIPESDLYADIPLYGRYSPRPDDFRVDVQHINSSSQESIQY
WAAVIGLCAESNMIFPAEEGRDVFALGSVIVKSSHRHQHEKIDYSYADAN
EVQAITIAKSVLDGVNVPDIYFSGKINGRAVLIQERLPGVCLNVALPYLS
EVQKNSFKLQAREILRQLHAIKAPSSRETRDHIVPDPNILTSGRLNPREA
HILFSANVDQDMSFMHNDFTPCNCIVNDDKIVGLIDWEMAGYLGWKTAGE
IHRKIRSPRREQYFHANLDEGELEDMMWWNDLYDVLP
>Mb|QLI66110.1
MPSPNPSRPPSPTPSQLPPVPGSPVYSIASTANPLSSFNLPLPPAPRPSH
AVLTKQDLESSHLAYADLVASAKSYRLALATLSTAASSFAAALESCARLK
ESRAEPIGHHAAGAAMTASFTTAKGACTADTLLSASGVHHLIANHHQILS
ETVYRSFEVPLLHDLDKWQTVIDDEEETYQARIKAQSREVKRLEKEGLKL
HRQRRRDVARFRAHLVDLTRKLDGLTTLHADHARALLRESQDTSARIVDA
SCSLVRAEVDIFESLARKGWTGGGLDDLLDKGVDLFAAEDAGGHAAAPGA
ADASATLFSILPPRSILADSASDAAARPAHGRGDSLLMESERYQSLAALA
TEQSQRHNGVGAGAGDADSVFSADFNRPRGARPFSPQPIRRVPADVTFES
LGAVEEPPADAPRDGEHERQPDEQRGEHDDDDATRGRTRSSSDLTRERHP
SQEEEEETAADTWEDS
>Mb|QLI66111.1
MASATALPKHPVLRRTATSATASATDSESSAAVTPADSPRHSASSTSLSS
LSSLDVDASKHEYAGLLDTYGNPFTPPDFTIKDIRDAIPKHCFERSALKG
YAYILRDVVCLASTFYLFHNFVTPENVPSTPLRFVLWGIYTALQGLFGTG
LWVIAHECGHGAFSTSTFINDLTGWVLHSSLLVPYFSWKFSHSAHHKATG
HMERDMVFLPRTREQQATRVGRMVHELGELCEETPVYTLLHLVAQQLIGW
PNYLLTNVTGHNFHERQREGRGKGRKNGFGGRVNHFDPSSPIFDQKHAKY
IVLSDIGLAIAFSVLAYLGNRFGWANMAVWYFIPYLWVNHWLVAITFLQH
TDPTLPHYTAEEWNFVRGAAATIDREMGFIGRHLLHGIIETHVLHHYVSS
IPFYNADEASEAIKPVMGKHYRSDTRDGPWGFIRALWLSARWCQWVEPSA
DAQGAAKGVLFFRNSNGLGTKPISMKTQ
>Mb|QLI66114.1
MASLAGACRASARLVGRASSRGLTTSARCLAAQNFVMPALSPTMTEGNIA
TWKVKEGENFSAGDVLLEIETDKATMDVEAQDDGIMMKIMAQDGAKAVQV
GTRIAVLAEAGDDIKTLEIPKDEQQQSSSDSAAAPKQEEAIPEKKTKSTP
RASTGETHEQKYPLMPSVEHLVKQNGLSKDDVSKITPTGPGGRLLKGDVL
AYLGSINADSPAAVSSRLEKLSHLDLSNIKVAEKKAAPAAPQEPKPSRHE
ARPLEINVPVSLSKVVEVQKRIQKTLGVFMPLSTFISRAAEVANDDLPPA
NRAPTASELFDQVLGLDKIKAAKGSRGVYLPQISAVPPSTLFEPPTRPAK

KADIIDVLTAKPKKAAREHGIATVPGFSNGLNVFSLVVPREEEHRAHMFL
ERCKAILEEEPGRLVL
>Mb|QLI66115.1
MADQEEDFSSLPLPDRFTHKVWKVRKAAYEDAAKQFSASPDEADPCFRPF
LNDPNLWKSAVTDSNVAAQQEAIIALCAFLKFGGRDCALRTRGLTITPMV
EKCLSSTRAATKQNAQEALLLYIELDAAGPVVEDVLPGLTNKVPKNVAAT
LNALTTIIHNYGCKLVDPKPVLKALPKVFGAADKNVRAEATNLAVELYRW
LREAMKPMFWGDLKPTQQTDLEAQFEKIKAEPPPKQERLLRSQQAAAESA
PAGADDGEDGVEDDEEAVEVDAFSLAEPEDITKKIPANFTDMLASSKWKD
RKEAVEALYQALNVPRIKDSDFGEVNRGLAKCMKDANVAVVTQAAQCIEQ
LARGLRQSYGKYRAVVMQPIMDRLKEKKATVSDALGSALDAVFVSTSLTD
CLEDITAYLSNKNPQVKEGTMKFLIRCLRNTRTVPTKPEIATICESGKKL
LSEGSPALRDGGAEILGTVMKIIGERAMTPYLDGLDDIRKTKVKEFFDTA
EVKAKEKPKPAPAAKAPPPSAKKPLGAKRQPVKKAGAPVMPAETPPAAAP
AAPKLAAGGSKLGAPKPSGLAGLKAPQKRTLGGPGTASPRRPATGPPVMP
PEDEPAAPPPPQLRAGLSRGLAGRSLAKPAAAPIMPTADSPPPSSSLTAV
ERAELEELRAANERLSRQVDDMRQERNKFMSEIQELKNQNAGLIEDHTRD
VLSIKAKETQLVRARSDAEATAQANERLRRELERLKRALSRAEGLGAQSG
VGSPVTSPTHDDVGIYRDSGSTSTSRNRMSFTSTMSEEKENGELVYPRSK
LSPELRRSGSGASSGRGSPARGFRTAVPPTYESLEPTAPATRERPVSALP
QPTNGGGVESWRRAAEVTSQLKARIEQMKAKQGLTRP
>Mb|QLI66118.1
MGVPKFFRWLSERYPAISQLIAENRIPEFDCLYLDMNGIIHNCTHKDAGE
DVSFRLSEEEMFIRIFNYIEHLFGKIKPKQLFFMAIDGVAPRAKMNQQRA
RRFRTALDAEKAREKAIKEGVEMPKEEPFDSNCITPGTEFMAKLSQQLRY
FVNKKVSEDADWQGCEIVLSGHEVPGEGEHKIMEYIRNAKAQPNYNPNVR
HCLYGLDADLIMLGLLSHDPHFCLLREEVTFGRASKAKSKELEHQNFYLL
HLCIVREYLEMEFQELHQEGTLSFPLDLERIIDDFILMAFFVGNDFLPNL
PGLHINEGALANMFKIYKAVLPTCDGYINENGVINMARLDKLLTELSKLE
LVSFENDISDEKWFASKQMEKKLEAKNGGSNKNAKTGHLVITSFQRDLWK
QKIRPFVSKRSADALDLGITLKAADRKFAQDIADAMHLDWSTKENENGDR
HLVLSFPARRGGGKDEDEDEEEEEGNLAAFRVIKSYDKALVVDVTSEEAQ
QQYEKLYNEKYAGWKTKYYLQKFPEWAPEKYEKEQIDLCQNYVQGLQWVL
FYYYKGIASWPWFYGYHYAPLISDVAKGLNADLGFKKGQPFKPYEQLMGV
LPDRSKKIVPKVYHDLMTNPNSPIIDFYPRDFELDMNGKKMEWEAVVKIP
FIDEKRLLDAMAPKNDLLEDDEKSRNGFGVPIKFTYSADVNFKYPSPLPG
IFPEIQSCHCIENIFDLPDMEGLEYVSGLATGALVKVEALAGFPTLHTLP
YTAQIIEGYGINVFQQDSRNPSIVVTLTDTNLRTSIETFKAKLGQRCYVG
YPFLQEAKIMKVQDELFSYELAEDGQDVVAKDHSNREASDFSKEAEYLES
WHAKRLGVIVGQVECLVHVHMLKGLLKTEEGALIKEYAENPSVRSVYASQ
TVVDEVVNEDERFIEKEALPIEEEFPLGTRAFFLGEYAYGRPLEVTGHVK
GKTEVVVSIPRNKEPEFSKRIIHQAERTNPYTPSFAVAKQLGVHPLVLSK
ITSSYQVSTAAGLRLNLGLNLKFEARKLKVLGYSRKAHTGWEFSSLAIKL
IADYMVAFPDFFAAIQKGAQRSEICETDLWNDPVVASQRVKDVVAWLKKQ
ETSKFERVPLEAEQLDSEVVTALANAGEQVYQASQDIDIKKLKGVPRAAI
LKPSDAELVLGNQNFSLGDRVTFAASAGKVPIATPGTVAGISRTATALLL
DVVWDTSFMSGTTLGERAPMFRGQTVPSSSVLNTTDKQVISASSKSQQKR
ATSTISTGFYGSVGVPQYKDAPAPAPLRGGWRGALNGSGHSTPGRNSPSR
GSPGRGRGSPSMGPQRGGSNGTAPNLLHSTLVYRDGSSRTPPEQTANGTT
WRGGGRGRGRGSHHETPSPQPGKYSSVPPPASLDGNGGGRGRGRGRGRGG
ANRGRGGNGRGGTTPPARS
>Mb|QLI66119.1
MPRPRRPQPRKSKSRRTVDTSDDNDWYTIRNILDERLVKGRVEYLVDWDD
NQKTGEAYSPTWSREVTDAARNEWEQRKRSRVTEATEVRTQDSQDSQPPR
PSNWRHLQRPQTAGPSGASRRRRSPSDGVDTHRPAKVAKVAHSATPSEEP
VPSIVSISSPDSLDDPDGLDRLTQQSLNAPRSQQLVVELEIKSDFDTSEY
ASQHNTQSSSQSSQPIAELEEQDERSLFASQLSRQTIPDSQEPSRQTWTQ
DKQVTSLAPSLQASQGGISEHEPQIYSWAQASSQRAGHNEEPPTSSHWDS

SNYRDEEKRDRDQSNSNIPSHQPAQEEAQSLVASQDFDPRAPATKPQSVQ
SPQGNKNLAAIPTISPVFLSQVVAAELNLPSSSISSQESPHKSVVVETPS
DTKQNSASTHPLTTDLAAEFRDQSQGESQDAQVLQIDPFVSHVRAFSVSH
DTAHASPTLSPPKIDSQSSPEPPFAIAMENHSSANPTPQPSAVDELSQIL
NLDKVMAEGPSHPLEEHSAEHSTPAEIPATNPSIQDHDQQPSSAEHESAG
PQSEQLALDTPLVSRISAVVSMKSIVDAIFAHPDLPASSTMMLDALHNPQ
PELSTVSPADISKQPDLAAPLPLMPSLLTHDNLSSNATRSSGVSVPMGQP
QQDQESSDDSGDESQEPITLRHIITLPFQASLRPLYDDTLLESKREVTQF
GAIFNSEDYVEPDETLVQKIDQVFSRLHNICDYPPDAVGSTLEDLPSDQL
IKYCYDANPKFNFIYELLQGLTQDTEVLIVARSTDLLRLLYRLTETLQIN
CVCEEIGKSRSEFTSIARVTLILPGKNVEEDDFDIVIGYDHSFGGSEIQK
KLEPEIPEARSPMVLILVTTHSIEHIDLYISDDLTPLERKNALMSGIVRS
RHLVSDPDRGYSEPHEIASLFLDYLNGQTEEITWKPIPVPEDVMDIYLNS
QSRSQMPVEGTPELENARKRKLDDSDDEDVKRMRILPHRQSTVQTNEAPV
PDDVKALLDSANIENTLLKASHVHISVPISVLQVLAEKNNELKRQLEAAD
RDAEYKSLISGLEARIKEYQRTNARVYSSQREALEDRIRFERETRKAESN
LVQANETAQRDAERAQKKIADLEATVARLTADPSHPDEPSPLSKTQTLLQ
ESLGKIAMLEKRLENAHKDADYARSLYQDATATSSALRGENADLSEQVAE
LSKKTPETLGKVHEIQADATIKTYLHQIQGLRTQLRERELELDYTKEELR
TLRNGRRETRQVSVPRSPRMGVMSPRTGRATYGSAGGSTSRGTSPAPTNV
EGAGVLAGMQFLGQPANGRWQHLRD
>Mb|QLI66120.1
MPETASPDNDVSIADPQAHETGTDANNLHEGDVITAGLKAKDVSHEAGTN
VLANTDAPAYTNGHPPRHVNGNGGHDEEDSRPAKRRRTHNSTPPPTPSSA
KKLKPISPPWKRISAEGPTSFTENGRRKSGRINTLPLELQPQGKKRLTRQ
SLGSKVGSQKKLLGMPKSTSRKPEAPPSRISSRSLRRRSPSPPAHPTRQS
TRTRRAFRAANASDAQDELSTPTHDPTYQFHDGDGRSPRIKLRVGRSGFI
PLVHADQVRKRPKVGTSFEDFWARAGTIPVEDGGLFASEDGPIYTDEAAM
KDAFSMTRIEQEVQPGGILSLGRCSAFIPEEEEEPSRQYARQDHLIKAMA
NFRKLLLLEQQRHRAIAKKLAEACRDAWVDRQPKSAEQLEAEARQKWIGH
YRYVIRAMLGTWENVRAQVNRRRLEQWEAEEQKRVKAALNEAVNLSEQKL
QARRGLDSEQVSGDDDGFDDLSDDLSTMDDDDIDESNMASGDSDGDDEDG
LDSDNMSTSEEEEEGVDGGIKDTGDEGLTQEELRAKYANLPDLKESDKQS
SIDATASDALADTDVGGETSDESVDMDDDLGSSDVGSDDDDNDGDEDESD
EEEGDEDAGGLLGLLFGKSELKQMKQDATEQRDDGTDLTVASPELGRDAT
EGDDPTLKQQVDEIASLVSLPSADYQPFTSAHDHPVVSLVEDSANAENSK
AASQDQEGSKIEPSDRNTTEMVPPLQEMQAGSPNEDVTMADAHDEDAVQP
QVSTQAADLGKPSSPETEPVTNPTSPRRSQSPPTSDTKPSEVEASSAELA
VAKPSGSRSVTPHTPSTHKSDIPFLLRGTLREYQHDGLDWLAGLYANNTN
GILADEMGLGKTIQTIALLAHLACYHEVWGPHLVIVPTSVMLNWEMEFKK
WCPGFKILAYYGSQDERKRKRQGWNNDDVWNVCITSYQLVLQDQQVFKRR
RWHYLVLDEAHNIKNFKSQRWQTLLGFNTQARLLLTGTPLQNNLTELWSL
LFFLMPAENGVGGFADLQEFQDWFHKPESQILESGRDQMDEEARAIISKL
HKVLRPYLLRRLKADVEKQMPAKYEHVEFCRLSKRQRELYDGFLSRSDTK
ETLSSGNYLSIINCLMQLRKVCNHPDLFIDRPIMTSFRMSKSVPAEYTFT
ERKVRSLLADAPMNTVNLGFLNLVPTQHEKLSSVNADRIAQLSSHRALMD
LREAQKVRAQAAHTIIDPSTVGSSISYLESGARWGRFEELQHCVYLNALR
RQQRPVYGKNVVDLVSINAYKRPCRPKPKIPQKIMSWFEGDLAFVRALTP
TLDQRAETLKSTISKFSCVTPPVVTRDLEQVLLGPKAVQAFTDEDLRLSA
PVRWAPFLPKQAPRDPWHESRMRHSIQFPDKRLLQYDCGKLQVLDKLLRK
LQAGGHRALIFTQMTKVLDILEQFLNIHGHKYLRLDGATKVEQRQILTDR
FNNDPRILCFILSTRSGGLGINLTGADTVIFYDQDWNPAMDKQCQDRCHR
IGQTRDVHIYRLVSEHTIEANILRKASQKQMLDDVVIQEGEFTTDYFNKI
SVREVLEDKVDFTSEGVAAADAALERVLGGPDTSRDQRTVGRALEQAEDR
EDVAAARVAEREIQADDADFTEKTNGTASGTSTTRQGTPAGKSVMDSGLD
LEEMTPIGEGEIEYNAYGESMGNIDQYMLKVMTEELKHTKLELPKGMKKN
KKKGRDTRKR
>Mb|QLI66123.1

MASGVPHVGRKPATVLSATELIGNTPLVRLNKVPQSLGVECDVYAKTELF
NAGGSVKDRIALRMVEEAEKSGRIKPGDTLIEPTSGNTGIGLALVGAIKG
YKTIITMPEKMSAEKVSVLRALGATIIRTPTQAAWDAPESHIGVARRLEK
EIPNAHILDQYGNADNPLAHEHGTAEEIWEQTGGKITAVVAGAGTGGTIT
GIARGLKKHSKDIKVIAADPQGSVLALPESLNQEHVNEPYKVEGIGYDFI
PDVLDREIVDKWYKTDDEESFVLARRLIAEEGLLVGGSSGSAMAAMLKAV
KDYGFKKGDVVVVVLPDSIRSYLSKFADDDWLAANGLLSTGSAETSLAKN
ATGGDASDPYAGATIASLRLKPVTSVVDTAPCSEAIETMRDKGFDQLPVL
SATSKLVGIVTLGNLLSYISRGRATPQNPVKDVMFNFSRLDEIVTDPRQS
ASVAKTTGPKRKFVEITLDTPLSTLSKFFEWNSAAVVTARSDEKGSLSKP
IGVVTKVDLLTWMVNKK
>Mb|QLI66125.1
MKTLIFQVILGTAAVFIAGCDATSHHHRMHQAYQRLAHPHSHVHSRAESN
VLVKRGTCAFPTDDSNLVAVTPDQKNAGWAMSPDQECKPGSYCPIACKPG
MVMNQWDPDSTYTYPASMNGGLHCDENGNVKKPFPNKPNCVEGTGSVKAI
NMCKKHMSWCQTVLPGNEAMLIPTLVKSEAVIAVPGSSYWQSTAAHFYIN
PPGTGTDGCIWGTEDKAIGNWSPYVAGANTDSNGQTFVKIGWNPIWESSG
LKGTKPGFGVKIECPDGGCSGLPCQIDPSAAEGSVASKDAAVGAGGSSFC
VVTVAKGSSAHIVAFDGSGGNKEPPKQSSSAPPPPPPPSTSQQPTTTQIP
TTSSTPPKPTTTSVAPTTTSQPTTTLAPTTSSSTLTTSSSSSLSSSSSSS
SSTTSYAPPTLIPGIFHENGTSLSNSTTTLASSTAPTTSIAETTVPNVPI
PTEKKGEAGRQQGSAAVAGLVVALVAAACLF
>Mb|QLI66127.1
MSHPEEMDDDLLDVEDAAEEFENSGDDIAMDSDAEDEEEEVLLQNDSIAY
FDLPQDSLFTIAQHPLHPSLVAVGGSAGPEDDAPGAGWLFDTSAAASRPV
LPASYASNPDVTPKSTQLDSLFALEGHTDSINTLAWTLPRGEFLVSGGLD
GRMKAWKADVRPGTVDMKLVGEAQEVEEVNWISACPSPNHPNTIALGASD
GSVWVYTIDAGDASNPLQIVQSYFLHMAPCTAGAWTPDGHLLATVSEDSS
LYVWDVWGEAAARNIMSENGMTVVSLTGEDQRFEVEGGLYSIAIDPRGSF
VAAGGAGGAVKVVSLPRLSPASSQQRGKSPADATAGGQILASLHTQGDSV
ESLAVSLTGATPPTTLLAAGSVDGSICVYDATRRFAVRRHIAGAHEDHSV
VRLEFVKNTWLLTSCGMDGVVRRWDLRSGGATGTTAAGATDRGLAKEWKG
HRGDGEGGGVLGFVQGETGERVVTAGDDGVALVFEA
>Mb|QLI66129.1
MVLVRFFLYIAGARLAVTAPNVPVQPPSTSEWDLSKPLNGFTFQLAKARS
AAPTIDKPALLYSRITSIKGSSSNQNAVSSLVQILQTPPSHGQHTYQNIS
TTGNLSTQYAIQCGWDGAPVWLLLDTGSADTWAVQTGFECHDSHGTKHNQ
AACAFGNPHVKKFQNGPIDGLHFYLKYGSGEKVFGPMGYADISCGGVSVP
RQQAGLANYTYWHGNNLTVGILGLAYPSITSAFYGEIGQEAAWNAIQYVP
FLTNAIMQGSIDPVFSVALAKNSSDGIIAWGGLPRVQRASDVYAETDLII
ANLVGSAETSWRYSFYTIIPDGIIWGQRMDKSKFPYIVDTGTTMNYLPPP
LAEAVANAFLPRAVYLYQWGSYFAPCDAVPPRFAVVISGIEFWLNPADMI
YRDLVDPMTGYCAVAITSGGSGPYILGDVFLQNVVAVFDIGGAQMRFYRR
A
>Mb|QLI66130.1
MEKFPDHVDAPRFPNGISRSPSPLKPASGYGLPNGAISHSDRRSTPAARK
ENGHTQAAVNGHGQNLSPWSEDSKRGHGRQKSLSDAFRTIRTRKGSVSQN
AHEIADALRAPVSPRLVILCLMWYTSSALTNTSSKSILNAFDKPATLTLV
QFAFVSSLCILLAWLATLFPILREKVSALRQPIRQPSRDVLMATLPLAAF
QIAGHLLSSTATAKIPVSLVHTIKGLSPLFTVLAYRIFYDIRYPQATYWS
LIPLTAGVMLACSGKHSYGGQMLGVVQALLATLIFVTQNIVSKKIFNEAA
KAEAEGPGVQSKKLDKLNLLCYSSGMAFVVTMPIWFWSEGITLLKDFLHD
GSLDLSNKKDAMDHGRLTLEFIFNGVFHFAQNILAFILLSMVSPVTYSVA
SLIKRVFVIVMAILWFRSPTTPIQAVGIALTFVGLYLYDRTSESNRADRK
ARLMTESRIASPLLPLEERPSRPASGMNGVTTQPYDRGYLSSFSGGNSSK
RSIE
>Mb|QLI66131.1
MNTEQGKRAESLLLKSSRTYGIQTRKDDVQAAINHPHHGPLFAEWALTHL

TSDTLLTADELDVYMALDRNGRVDQLADLHDLSEVPAVTEDELTAAVEEL
KRCTESISKQTETLRQQQDALARMVKTQAENTTRREQLERVQRKKRELER
DQLTKEVERMSQEITLRVTELNQHGPALNDSIATLLQSDDKLLSSLQKLG
WELDQPDLGETQDIEKLRETCMRLIKATVETVRTKLDTIYLETLVTAERS
GTAKPATNEQVKSLQDEVESLYSEILPVAQMSIDQQYLEPALQTLSMRNS
QSIGKTTTSLTYINECLTYLVDRMDRLHAHVKSHKSHQAATAAITAAAKA
EIAVVIPPKKPRQAIPPSPVRTRSNTSEARQRRRSSGAQEESPIETLLQD
LAVTLPPDCIKPQDQIGFLESVLAERTLKGNDVAKGAQEGFEIAARAHVE
DARQAIQLVRDSLLAESSFGTLKLVDPDMDGSISVLAQEVNKAKEKLRSL
QGQSIVARSEKRDEFIQRWGS
>Mb|QLI66133.1
MSRAAHPHPQPSASPAATDASLSPSQASTLLASFTNFLTVALHSILYHRQ
LYPPATFLTARAYNLPVHQSRHPGVCAWLRDAVAAVAAQVRAGSARHVAL
VIHAPASFDVVERWIFDLHSFPAAWGDEDAPLTHFNNASRHPPAPDAPVN
WTDVNEALRGALSRISQVAQSRPHLPEACTFTIGIELRDEALPPIQHPQL
WIPAQTEPQIPSETRLEPGRAVRGTATAPIRSVQAGPLYFECWVEQGKLP
PGTTSPKAPSPSFSPTFDSSGGSPDATTS
>Mb|QLI66134.1
MTSTKTEGMSIHFGPFEVTKQVFLTTPHSYALVNLKPLIPGHILVCPLKS
HLRLTDLSPTETADLFGTVQLTQRMLAQKYLPEPGNLLSGSFTVAVQDGP
DSGQTVPHLHVHVIPRRKGDVGDSPDAIYVKMSTEDGNIGGAMWDRERRP
APAGRMPRIEDADRSPRTQEQMEAEADEYKTILRNLGIA
>Mb|QLI66135.1
MKATQSSVMDLPASTDVIAPTSTTTPSRSSSVFHGHDAFPSDPYPRLSIS
TPARILLGTVSSGVIGFTLGAMQGGQMAQLRFRAEHAHKMPDTTTGWYLY
HKSKNYHAMQGGIREGFRMGAKTSFWSFVALGLESTVDRYRGSSDMFSTT
MATLTVAGAFSLCRKWNLDGIAHSLSITSSHRASSSP
>Mb|QLI66136.1
MLGRLLHLGSGGPATPTVESSSNNASRPILSLESVQEDIHTRNLLFPDAQ
ALYQHRNDQVFPLSTTPTTPGISTANAFDYSEDVDLDVKDVRVVVMQDAL
GPANASLLFDSHPPPDAMATSDRSPALQEARRDPISSRKGSLTQSARPLV
IPSESPQPRQGAFDRRASIHSRSQSYHETESQRAAREYREELATFSSCIF
GNSELMAYKGTSTKVHVVPSEARANESTTSSVFSDGKSSIGRSSGRSSKL
SQSYSSQVVSPTIGASHPLGGVPPRQSDRRKVLITRLFPVSLAADDADHS
VTPQNHNSDDNGGFPFPAQSDESSHKKKKPQPKQRRTPMYAVVLVVQIPK
PSSRMSSAVPTKSVFRESGSYQDQELFSSSYNSAKAGWNMMSTSGMYGEA
ADSLDIEDRIDSLTRHWDIIMRTLTHLQSLVATTIHSMLKQADSTSPGPY
SGSSGSNFVSRTPSMTERRGNELPRAKPPKSTTKLVSLLPNCLADDVNIA
SEVGLARSRIVMGLSAARVITGQGRWGIWRDEAIWTSKWAASTERTAFFY
NLLTGFLATHTDWLQALCGPSYRKRATLARRNRNEEDLSLPARTIIISDD
KMAARRLIFLLSAFLPANQQIPTARAHRPSASAPVGGYSNSPPTFIIPVL
REESLRRKINRRSGLRRTSHSRTTSQSARNSGHVPQLAQIGLDRNHQRRA
SDAASIRTTNLPMLGTDLVTRKSSAATTTTIMPETTTPHFTSVQQRDCQR
RSRPGSSGSVAADDLKRSLKRGEHSGQAGGVSRPPSSGLKWGNLINGLWS
PRRRDSMDSGTYSQGSADLRSPIKTSFGRTDKLSEIVKESPEERFGAAIV
DSAQEVDAPRDNYSGVRGRGSFAQADRTPDPTGAFESPVKTSINAEDGVI
DVDVPFPEYITSFESAISSPSSSGYLSTPGLPSGLEAFEQTRFAIEGDLP
LNAAGWLNRFHPDFALQALPPQDNLLDKVKAALRAEPSPAPTTQSNDLSE
RWVDVSSVMIADATNNSVSRIVYRRLVKPKAQHDPPSGNLGAGGYTMHTP
SILPYETQLEEEWIEEPIFTCDEALRDAVERAIHFNSDASKGSSTTSSSQ
TPSDFQGSMESDSTMPDTLSEVTQLANPPLDAPRVQCKTVILSALEDMFR
EAVDSRDTGHTGRANNRLNRHRHSILYDAVRAWVASMDAVE
>Mb|QLI66137.1
MKVTNESELATATPTLERATESTQRWNTKNLGWRLAADAAGAACAGVLVA
PVISIIDRSIMENASGRSSLLDSIRSSVRNFILRPGPALFSRPCALIFIL
YGGTYLTANALDTATSTATNKPATAISSGPGKFAASSAANVGLCMYKDRS
FVRMFGPVGAPRAVPLPSYALFTLRDCLTIFASFNIPPLLAPYIDSRLST
EFKKHLSGQTAAQFVAPAAVQFLSTPLHLLGLDLYNRPGQSPAVSWRDRF

QIVRSNWLISSAARVCRIVPAFGVGGVVNFKVRHSLMTKLE
>Mb|QLI66142.1
MGDNPSSQRSADGGASRPTARSRSVSFRRQSTTERINEILESGRDRADTM
STDPAQFSRQLSPQAVAASEDEPAPDELTGIVSRGSGQNYQALYMVGTTR
SRPSIYSRQSRPAAEDGPAEDAPNQSPESERESRSWLRRKLGALQSVELE
NKGSVARDHLALERTFLAWLRTSLAFASIGVAVTQLFRLNTTISDGNGNS
GSATLRRLGKPLGSAFLGISILTLLLGARRYFRGQEWVIKGKFPASRGTI
ILIALVALAIMLASLVVVIVIHPSDEEL
>Mb|QLI66143.1
MASRDEDTYCCAQKADENNTITQIDFVTLGMFIIDDIDFQPPTPPVRDVP
GGAGTYSALGARLFSPPPLSRSVGWVVDKGFDFPNKLSTLIDNWDTGAVI
RDDFDRLTTRGWNGYVGPADRRAFKYTTPKKRITSEDLTPNLLFSKSFHL
ICSPSRCQELITDIVSCRKALAAPEDSYTKPIFVWEPVPDLCTPDELLNC
TNTLRLVDICSPNHAELAGFMGDDGLDPETGEVSTLSVERACEQLLASMP
LQSYALVVRAGDKGCYIAKNGGRKRRSGPGKTSNSQKALIHGALQPDIDM
EALFAGLLQDDDGIIAREEIEVDPGLERWIPAYHQDGRKVVDPTGGGNAF
LGGLSVALARGERLENAVAWGSVSASFAIEQVGMPSLVRNDNGVETWNGE
EVQQRLQEFDRRL
>Mb|QLI66144.1
MSHRSWEDYNVQDLGFIGREGRDVEGRSSDEIVFDSDDDEECEDIFAGTW
DAATVAKRRILRAKAATMSSPVVMGPVNGHLSPDDAMTSTVDAEVGQSDT
DIPDARESPMDRSSPSASEAQPHETPDHIAEDRASSDDDGSSLDNASEDG
DFDMQESAASPQDGDAEVNRESSPASSRPSKRKTTVAEEDYMRENPELYG
LRRSTRARETRKMVDSDDSDSEDDEPVNRRQSKRRRVNTSQPSSKRNTPL
LQPSTNESDSDSDTYGGARAKSQLKKARLQRESQPALALAEKRWSSRRAA
QQVQQGMYEESDFEDDEDEADMASAYYVADYVDDSPYVEKVVRHRLRDGL
ELQYDAGRHDFEYFIKWQGKSHLHDTWETIESLRNMRGFRKVENYFRKVV
EQELDIRFGDDIPPETKEQFFLDRERDEDAFEDYTKVERVVNVRDGEDDT
EYYVKWKGLTYEECTWELASEISPEFQDKIDQYLDRSSRSWQSDRRETNP
DTRGRMIKLDSQPSYIQGGELRSFQLKGLNFLCLNWTRGNNVILADEMGL
GKTVQTVSFLSWLRNDRRQEGPSLVVAPLSVIPAWCDTFNHWAPDINYVV
YLGPEDARNIIRENELIVDGNPKKPKFNVLVTSYEFILQDWQFLQTIKWQ
TLAVDEAHRLKNRESQLYARLVGFGVPCKILITGTPIQNNLAELSALLDF
LNPGKVNIDEDLDSLSAVDAQEKLEDLHKSIAPYILRRTKETVESDLPPK
TEKIIRVELSDVQLDYYKNILTRNYSALCDATGGHKNSLLNIMMELKKIS
NHPYMFPGAEERVLAGSVRREDQIKGLITSSGKMMLLDQLLSKLKKDGHR
VLIFSQMVKMLDILGDYLSLRGYKFQRLDGTIAAGPRRMAINHFNADDSE
DFCFLLSTRAGGLGINLMTADTVVIFDSDWNPQADLQAMARAHRIGQKRP
VNIYRLVSKETVEEEVLERARNKLLLEYLTIQAGVTDDGKAAFKERLNKK
GLKTDGPSSSEDIQMVLKMRSSKMFEQSGNQERLEQLDIDSILENAEVTK
TKVDDKMNLSSGGIDWDNFMQITDVKVDDINLDWDQIIPADKLAEIKADE
EKRQNEEYVAKLAAENAPRRATIKNRNKENDRADRLKKRQREQQQEEEEQ
RALLADPKRPLSDKEQRNLIKAYFRFGSMNDRESDIIQEAKLVERDHEFV
KSVLDDFITAAQRAVDDNNVKLVEEEKKTGKSLTKKDRKAVLMDFGELKK
VNAETAIERPKQLQLLRKAIRSQSDWKTYRLPDATKGANYTCAWGAREDA
MLLVGIDKHGFGAWVQIRDDPELDMQDKLFLEEHRVGKKEERNKANDKLK
APGAVHLVRRSEYLLSVLQSKYSGDRAAQRIVDNHHRNNKKNSVPNGHRG
SGTASPAPHAIKKHRDRDRDDQRARSNTDDRGSARPDFKRKHALSYDDDR
SPKHRRVEDGRRVSKHGIDRDRDRDRDRLDHRRYRDDDDRRHEKHRHRHE
DERRRDERRRDDDRYRDRDRDRDRDRDRDRDRDRDRDRDRDRDRDRDRER
DRDRDRDHYRDRHRDRDREHARDSHRDRDRDRDRDRDHDRDHDSRTQDER
RTKALRRLDELRRIGDNKDERNRDNDAMIWYLLKPVRENFERILSTTKDN
VKSSKERAAIFGVELVAIGTFLNERLPVTAADESLKNNFWDFLAALWPVD
DTSKIVTGERLSNMYRILTRNKDSGSSSTRMNGA
>Mb|QLI66147.1
MEDRKRPAIGGSDDLAAPPSKRVAVNGSKTKDDAIEMKEEGWIEAYTKGA
IYRQMQEYSRKAATAESRLEELHKRCVHHDDHLRIIDGWWRQLLEELEVA
AEPTLSGNAPTTDPPYLSAIGFKDLSEFQSHLSDKGKTIKTKAESLLSRI

ASQRGSLSPDAASLEAKVNNLLAAQKEYMLKLDRLNTEKDQLSEQLNTAT
LRYFKAEKKLDRAKSSQVQKLEQQAFANATRPSGATAAAAAASGEVGSEQ
HEVNGSSDELLLKYEETTASAAKQKEQLDSILSELKTAQDENSTLKARQN
TWSDEDFIRTDVFKQFKAQNEDLIKRINNLEATNKQLREEAEKLQAERTS
YKTQLETDASQVTQELEADVITRDQDLARVRSARDEILAENTQRKASMEQ
EKASLGHVKELISAKDDRIAALESRLSRLEPAEDQEMVNADDLTELSEDE
LRQRYKKLQQDFQSINQELPSIEKAYKKMKDQAQKKVLDFVALEEKVSLL
IAEKSKADQKYFAARKDADTRNNEIRSLRHQNSKSTEIITQLKDLEAQNR
LLLGNLEKQVADLKQSNALLTAENKKMESTSLEAVRRSDSLNRQISDLTS
LVKSRDNASAAVRERNTVQEAEVEKLKVRIEHTQKDRDNWKNKALSNSSE
EEEMLRTFALCTICRNNFKNTALKTCGHLFCNKCVDDRISNRMRKCPTCS
RAFDKMDVMPVHH
>Mb|QLI66148.1
MRPACVRCSALHPHIHVRAPRTPLRSRRAAPAACLDPSWQSMPQCRSQST
SCNHFVDSERIHPASPPPQLLPSAKPFRLAVKDNIATAEFPTQCASRILR
GHKSPYEATIVTQLRARGGIVVGKTNMDEFGMGSHATNSMHGAVSNHLSA
DEPISAGGSSGGSAVAVMLGEADIALGTDTGGSIRLPASYTGAVGYRPSY
GMISRYGVFPYANSLDTVGMLAREVKPILHLLVETKLDEEHDPNDPTSLS
PASRRRCAKACPPLLEDMSKLTVGIPLEYNVAELDPLVRQAWVEAATALE
SAGVNIVPVSLPSTKEALCVYYVLAPAEASSNLAKYDGVRYGARGEHGDA
GGDTLYSETRGAGFGPEVRRRILLGTYSLSSEAMDNYFVQAQKVRRLIRQ
DFDKVFKRDNPLHEAAQFDLSDMDAETALQDKQGPPQVDFLLSPTATSFA
PTLNDVLTKSSLEAYMGDVFTVPASLAGLPAVSVPSGRLGEGRLPLGLQL
IGQYWDDKRVLAMAERLKALAA
>Mb|QLI66150.1
MSNIQPAAQGTKETGLRVPSNGKTIYHRPLNRTKTAELSQASFAYLFSEM
VTYAQRKVKGIQELEQRLNEQGHPIGLKLLDLLLYREPPRSQLRPLSIIS
LLHFIKQNVWQHLFGRQADRLEKSNNPDTPDEYMIIDNEPLVNQYISVPR
EMSQLNCAAFVAGVVEGVCDGADFPARVTAHTVGEGDMWPGKTVFLVKFR
SEVVERDGLLAKS
>Mb|QLI66153.1
MTSTQLPPLNFGAGGLRISLFSPTLPEPFVETEAPHPDQDKSEKGDELEQ
TELTQPPLTPHNIPIHPIPALQEAFSESLDEATNGVPGDKPKLRELDARG
RREALITQDRGDDPFDAKWRLRPGQNQHELVKLIGQISFGVYLLLNGMAN
SNAQVVNILQGHIDEVDEFLEVALEDLAQAIDDLNERIEHLKLPLANVQV
FEELLEDRNFRLEIIDGNEKIDHVLSRTNAALKQWDEDIEAGLQSTAAFT
SWLHDEEDATWRMERPDVADIFDAMKGNTEGWLSAFDDMSDRVQEASNVI
VRLSTIISEMEKKAGEVSRRTWANIQPFSAPTSSSQAPNDTTKSVSSSRQ
SRNLPLRQHQHQSSVQSVATSTRSASTIGLDLGEEFPLPGGLPLLPPHRS
PLRRTPGNHSRTQTPTQSAPQSKAASKRADAPSPSPSASEEPLYVLQPRT
YTPQPQEPSPSPALKSAGTSSPKPQDPTQSQDNALGIDGQGAPQARSSIV
PQVSQKTNSPDTMQIPPRPVRPPSPGLGTPRTTHSNPLDSAYGSDSESIT
QRAASRAGSDVGRSPARPHITHSPRSDHQQYYRPVRASPHSALQQRPHTS
VGSRGSYMHLRSQPSAIGGMSTLSNVTNAVYDDDDRTQSSGSNTTGPTLR
KKKSAFGWFKKAFSLDEEERAAYEARKATEYRDRYYDPNSPKFIDGRRIR
>Mb|QLI66154.1
MPVQLLPASAAAFAPRAPSVNVVLASKVEPWLTQTLKRVNKVKRPLNSVP
QHQRCLTETLSSPNAIWTLASLMLSKLPEAEMPREPLEELFSYQLVHVEA
YIVHVDMVLRNEVAYKLTTDTIDALVEYHDKIHCADAKASTYDWSEKEQQ
CKKLHQDFVQAINKFVYRTHVSALEGLEEEGAGELLCGRSEEVRNSLIGL
MKPLLPPPPRVVDVVRQPTLLPSSPVNNMWSSQPTMPSSLPAVEPWRVLP
SSPSVPSTSAEPNNPPMWATMGINELQMPSPAPAFTQPHATAALYFTSPP
ITAPMPAALPLPSMLSSSQCGVSVGMGGFGWDRYQEYATIM
>Mb|QLI66156.1
MKKPVTSLLFALLCAETTSLSITPGDDASVKSAASTIAYGLMKYYTGNNT
GDTPGNLPSPYYWWEAGAMFGTMIDYWYMTGDASYNAATSQAMIHQASPT
RDFMPINQTRTEGNDDQGFWAMASMSAAENKYPDPPPDKPGWLELTQAVF
NHYVSRWDEDNCGGGLRWQVFTFNAGYNYKNSISNGCFFNIAARLARYTG

NSTYADWATAIFEWEQKAGLITKEFAVLDGIHVDHGGGGTCVNRTDTQWS
YNAGIFLHGAAVMYNLTGGEVWKARTDGLVRHSLGEFARDGVIFEPACEP
VRGCDYNGLSFKGYYIRWLAATIKMAPHTYEAVYPVMRKTAEAAARACSG
SDASFRGVPGTACGFAWTDGGKFDGLVGVGQQMCALDAVMYTLVDGVGAA
VTHDTGGTSRGNPAAGGESDDSHLPLPKAITAGDRAGAAIITIILVGIGI
GGSAFMLWDEDL
>Mb|QLI66158.1
MGEENRDLVRLAGEYADRDIDLYNLLGVDALTAKEDIHRAWRKRSIKYHP
DKARENFDPEKWELFEKARDILSDASARAVYDGASKAKLLRRQEREAMDK
ERKKFADDLEAREDAARRAREEKQQMEREMLQKERERLAEEQQMRAEETR
RQAEAAQEVEDLAEARRRLKEKKDEKARKKQAKESMRATLGSVGKPSGPA
NGVVNVPGDYVADLALNKQYWELVCDKLRAIQAVRNLQKEDTPAEILQEA
ERVVQEVRGKIHEAEARYQRETATT
>Mb|QLI66161.1
MFLHHAKFALRLLLATAALAPAVNGQTQCRSRDGPKLGAVASENSICSRI
GTRLLEDGGNAVDALVGTVFCVGVTSMYHSGIGGGGFLLLRSPNGTYEFV
DFRETAPAAAFEDMYKGNVNASMYGGLASGVPGELRGLEYIHRKYGALKW
ADVVAPSIKLARFGFPVTEDLVRYMDSTSPNAFLTQDPSWAMDFAPRGFR
VRLGETMTRKRYANTLETIASEGADAFYTGAIADATIRALTAANGTMTLE
DLGNYTVALREPVQLQYRGYKLTSTNAPSSGVVALSALNILSGYDAFLDP
SRSNLSTHLMDEAMKFAYGQRTKLGDPSFVAGLDKYTKDMISPQAGAEVR
SKITDQKTFDVSYYDPEGLESLETPGTSHIVAADAAGMSVSMTTTVNLLF
GSGLMVPETGVIMNNEMNDFSIPGESNAFGYIPSAANFARPGKRPLSSIS
PIIAETADGKLYFSIGSAGGSRIITANIQNAIHLLDGNMTVEEALGQPRL
HDQLVPSRVSFEYTYNNRTVAYMKSLGYNATWVAPGQSTAQGLRLLPNGT
FEAAGEPRQKNSGGFAV
>Mb|QLI66163.1
MAMMASLLRPTPLLLIAALLRAALLVYGTYQDAHSAIKYTDIDYLVFTDA
SRYVWHAQSPYARDTYRYTPLLAWLLLPTVSFFSFGKVVFALADLLAGWL
ILRILRRRGIPPEQAGAFAALWLWNPMVATISTRGSAEGLLGVLATALVW
AAEGHRVNLAAALLGLGVHFKIYPCIWAPAIAWWMDDDRPRRKPARRRAR
FLSRDRIQLAAVSLATFTGLNVLMYAVYVSPPFSSPRRAAVTQTDFRSYG
RPFLVHTFFHHLTRIDHRHNFSPYNVLLYLASSRPGIPLHAESLAFLPQL
SLSCVLIPLVLAKKDLATTMMAQTFAFVTFNKVCTSQYFLWYMIFLPLYL
PTSTFLRKPRLGMTALVLWLVSQGAWLHQGYELEFLGRSTFFPGLWASSV
AFFLVNCWILGVIISDGGAHTAPKARTE
>Mb|QLI66165.1
MSAPPPPPPHGEAPRSSGLPPGKYDIFVIPEHSAGAGFLYLPSLKPSVNS
FFAGFASALIMVALFQSMAPAFRTWWETFQGLGNMGITLLILGVGFGAWA
LGRTQKDTPGSNYHYQSSYSSGESGPGTGPGGFASSPGASPNGGAHARGH
GGAPPPPPPHGSPPPPPPRGGGGPSPQSRAQSPPPPPEPEKPKTSWERPP
PQPDPPQPSTPQPSTPQPSTSKPGSPKPQTPKGTWEKAREETRKKEEERK
VKEAELKRREEAAKRLAELRAKEARERQEREKERERKEKEIKDKKLKEEA
EQRRKELVEKLKREREEKEKQKEKEEQKKKEATATEGGTRVASTYAFSGA
GEKTNMWPNGRPSVPPQATNPPSPERKPPAPTARTYVSTPEDDAFSYRPY
DQPNRKSRRPSLSSFAGSDASWAQSHTTARTSPPPSVRGPYSTKDPDKIV
IQAVYLFMSQYSKTPASQLISGVGSVTDGLILRITTEGLFIDDDVRGVPQ
REWDVKAWTLKLVEVWCPPHCLKLPASATSNTSSGPKSAPHLLYKMATAR
RNADRDSQKVLNGDEADAYLTEMLRACKDCCRLGLCERTFKNTNIPSLTG
QTGVWKSKGLHVLRASVRDQEGKRYVFVMSEAESWKLAFGLQRLRGGTQV
RHLGVAGMTPSEARGTLEMLGWLD
>Mb|QLI66166.1
MAIDVISSALSINSKSAATFPQADKFEELVLAMKKVLGPSSGLTSDDVDV
GSLTDLMINYDSNPLEWSKYAYGDASRGYTRNLVDEGNGKSNLLVLVWSP
GKGSPIHDHGNAHCLMKILHGSLTETRYAFPEDDDDEGPMQVISEKTYKE
NGVTYMADELGLHRVSNRGSDFAVSLHLYTPPNVAKEGCHIFDEKTGKSS
HVPGCHYYSAYGRLLK
>Mb|QLI66169.1

MSSEANAGSVAATADKAEKPKFSWLRPHPIFVIILVGSEEQPFGIQKDFL
CHRSDFYRKYFAGNQTEDTVEHVVKLADATPEVFGLAQNFLFTGKVMSGD
EGVPSYDSLVGLWNLGHKLGVNGLCDKTLEAMTECRRLTERIPSTPLLVQ
VWRDTPEGSSIRMLLLSWAAEYMRSSDSRAEFAKSLPQEVLSELVVAMSS
FELAAPTPAPAPAPASAPSHSPVVSAPELPRKNVHYLEDQSEDDLNNIRK
NRRLSGPPTIIARTDQSLGRKASRTPLPKPQKRRTSAAYVDSRNFSTGQK
LEFCADLLTRMLSGPGFWTRLVGPFRDPVEPAEDGVPDYFDKVKRPMDLT
TIKAKMDRREYATEDAFVTDMRQIFENCYTYWKKGDPMWLAAEKLQKTFE
DKFSHMNKWIAKMGGEEGE
>Mb|QLI66170.1
MTLRERFLHRLPNYNGPYNVGYMDVEVPARNPRPISNLKRGGKPVLRLDT
VLMGIYYPCDVEKTIQASGGAHNLRRVNWMPAPRIATSKGYAKFLNIPAA
PVTGYLACTSLFTKLPAFRNAQLADSWAENGPKEDVACDKEDGGKDGCDS
EKPTFPVIIFSHGLGGSRLCYSTICGELASFGFIVVAVEHRDGSGARTLV
NLPENVSATEIESSTAEIVSGNDEEHGSEAGAGARKKKVERKTKRGVNPY
YIMDYILPKDNAQDTSPHNPRGVDRHLRNAQIELRLEEIKEAYHVLSLIN
GGRGHDVARLNLRKKGNIGSSSRGLTGITWDSWKGSMFLDKVTAMGHSFG
GATTIQLCRDQSLAWLGQGVILDAWGQGTPARGDGPGNIVAKPIIAISSE
AFMHWKENFERVVGFCQEARESKTLCWMLTIVGSTHLAMTDFAVLYPHWM
SFFMKSMVNPLRACSLTVATSLEFLSLTLPPGHIKHKTWADEDLLLSAPA
PSEPEEALIEDHAPEYKWVAVRLKIPNEFSKRFKAWCRRLWRTLMCNAVE
GDALGNGLHDYTEQDELWTHISPRCADVASYRSALG
>Mb|QLI66173.1
MADGGSMQPDSKQSQPSSAPAPAQQQNSNTEDEAKGKKPAVKKRTKTGCL
TCRKRRIKCDETKPRCKNCIKSKRPCEGYTQRVVFKDPANLGTAHGHFGP
TVYPPGTRQQGASIGLHPNPANSQASLQPIAPRPATFNGSHRQPMSFTQV
HSSHGSLSNYPPIPNPLLGPDAAHLAQNYYMGSSHGEVPPNVLGQEQDSR
EEQLGTSFERDMPSNRGYGWPDRTQYGHGGIEDEVAMDESDDESPDPQSL
TVRDYRDVLKPYTRGGTEVRTFSAFAQNYALSEYMDYVHSSELRRHDMQT
IFRHFVSVTGPTMSLYERDPTSLASGARFDGDATLDNNLWSHTFPLLSFH
HPGLFHAIMAIASLQIANLQNTPATAALRHYHISLRRNARNVSSLQRNTK
PTTVATTLLLAYFEVWSSDHTKWCNHLLGGRLLFRQIPLRQMSRICLPVK
RMRHFPSPLSAPPSIGPRRDPADLDYGLLSTITGHHVTAEDYGLQEGQLL
DEECLWTTDKDIETYDMLRDLFWWYAKMDVYQSMLGGTKLFMEYEHWTQC
PPRAPLSSINSIFGTYDHLILLLGRLASFTSKDLSRKRKAFRAKGGGSFR
GGSSPSQFPGIVPTLGKFHAPIGFSAQSCYSDESDSPGDTATPESNELAE
QEWESIRQAFETFENRLDARFKPLNSEYADHKDTPFGTALQYRTYSVAGI
WMNYYMGLIHLYRSRPNMPPAAMQAAGMAAPSTAGYAIQIGRIAAGLTDN
CSTKKDISTTLAAALIESSFCLFVAAVQLQDNNQRCWVIQRVFDITRLTG
WQTARKIAIGCESAWIKVAQLGHGPEYKRPQHLEEVPTSVWMNPRKLDQR
IQDLQNTTETRLVLHRSEQTNLALGLLGVETDLEVLDLREDR
>Mb|QLI66175.1
MTAASSTESPATHQFPLPKILEYPASTPPCLITQGAEGRLYKTTYLRPDV
PCALKYRPPKPWRHPILDQRLTKHRILSEARILAKCRRDGVRVPAVYAID
ESAGWLMLEWIQGTPVRVNINERLGSRTVGIENDARLKDLMRRIGVAIGN
IHRIGIVHGDLTTSNMMLRPPSDSAEQVDALSGEIVVIDLGLASGSIHEE
DRAVDLYVLERAFGSTHPRAECLFGELLDAYKGCFKQAATVLRKLEDVRM
RGRKRSMLG
>Mb|QLI66177.1
MGAYPGGLIYLNATLPSRSAHWYSVMAHEGPSSGFAPYVDHPEEYQVFLS
AKANKSESIQVAIDSAAGLNHNAAQERHGYWVSSMPRVVYVPPGIYTFEN
PLVMNTDTILMGDARDPPVIRAAPNFAHDVLLNGMAPSYMPNAGELANTV
ALKNIILDTTAVNRSKGLTALYWGVAQGSHLQNVQIIMPQSIDRKDGHTG
IRLGVGSILSLGDVRIENGLNGIWHDGHQSALFKNISFYRNTVGLRVSNG
SAVTLLAPTFENVDVCVNHTSGSVFVGIVDGTSINSGITFLSGKTNNDMT
RPSLLIDNLDKRRDPEYEPENTVQVKGRTVLGPVAHVNSFSYGNTVGRNH
IYGVTTNNSITRSRIAAPGGRIPIHPPPNYALSAWSDFINVKDSKQNGGH
TVRGDGMTDDVQALTKVLQHAVKEHKMVYFPFGDYRVSSTLVIPIGSRIH

GEAWPSISGAGPRFLNAENPKPVVQVGNVGDVGTIHIQDMRFTVADVSPG
AIVLQFHAHGKKPGDVAIWNSIVNVGGLRGAPDMVRRCADDSHPCQAAYL
GMHFARGSSVYAENVWNWVSSRNAEDFFAGVDVAAKGGVLVESTNGTWLH
GLGSEHWWQYQINLRSASNVVLSMPRTEANPGQGGTARPMLPWPGTSDPG
GWGDPDYSRCRVSDPACRTGVGNYINGGSDIYYYGAASWVSRAGAEHPRC
YAGYHCQEHMHYIAKTPENLQAYGLCSNDTMYALRLGDGTRVVEHGTFYG
PGWPKGSVVGRYTT
>Mb|QLI66179.1
MSDDEAPTLVPAEGQERASKPLGMRKNGKQWHEQKKAFRPSKGLTTFEKR
AKERAAMAQMKAKEKEMKEEKETMRKEKMEKIREKRAKKEEKERYEKMAE
TMHRKRVERLKRKEKRNKLINS
>Mb|QLI66180.1
MPASRGWHPRHLWPPWRHANEISKKDDDLPLPRHSKHGRSYGAQWQSARM
PRRTWVKRLAAYALVIVFLVYLFNRAHSPSSSALPGSRDDPDAVAAAYKE
RFDPARTAHLDGAPKKPSPPHQPPPAEVDGQQKGISSPNESIDEESKEGH
KPDNGRAKSPPKYDGPIRYPALASSLGAIASTGGDSPHNRNVLFAAASLK
SVSTLLPMACEMGIERQSYVHFAFIGTADIAMQDLLKVNGIDKTCPLILH
DARPDHSQMSTESRMSLAIVKALYLLNFHMHPQAVFVDSTNAEESYFLRA
VRDQVSSTQSALIELPEQSRTSLAWISKLDSAALSAWNDVRFDILIHAAP
TGTANLERLLRSIARADFAGIQTPHITVELPYVVDAPLESYLANFKWPRP
TPSDGQRPQMISLRRRITRQLMEEEDSSVRFVESFWPTDPRRSHVLVLAS
HTELTPQFFQYVKYSLLYQLHSKFARMEDYHASLLGISLSVPTTLIDGSQ
PFTPPKPVHGDKDEPGETAFLWQRPNSEAMVFLGGKWVELHHFVAQTLYK
KRSMSSTPALLAKKQASKEYPAWLEYALRLSRVRGYFTLYPSRETAKAII
GVHSDVPDAPEEYSKDESRPKSPKDYSDKVDEDFDPVSPVDMLETLPHNG
ELQSPGPLPLLSWDGQAKTKDSIAEDAGEYTTLFRREVGECGEDYLKNHP
IANNMAEDLFCKPKA
>Mb|QLI66181.1
MSDFGDDDVGVGGDEPMLEEEEVTEYYDPEVLDNEDGERGENGDDADNVV
VSGDPSAAANAVKAGEKSLKDKKIPEADRTTTPYMTKYERARILGTRALQ
ISMNAPVLVDLEGETDPLQIAIKELREKKIPLIVRRYMPDGYYEDWTCEE
LLQ
>Mb|QLI66182.1
MSTTCSLSQTPSEPSSDTKKRKPLTREQRDFLSSALRVNQAGELAATLIY
KAQTPLVVRQHPHLRPLMAHMYDQEAGHFATFNDLIHKHRVRPTALYPLW
SVLATGLGWSTALMGREAAMACTEAVETEIGDHYNNQIREILEMITQWEA
EGYQVSTEIGELVQTLRRIRDEELEHLDHAVEHDAKKAEPHWLLTGVIRA
GCRGAIWVSERV
>Mb|QLI66183.1
MPSTTRQSLFRKVQSFKTDYAPCTITQYVSERSGMQVVVADRQGPKINGY
FTLATEIFDDSGAPHTLEHLVFMGSKSYQFKGLLDKLSSRAYSGTNAWTA
TDHTAYTLETAGWEGFAQVLPVYLEHIILPTITDEAITTEVWHIDGQGND
AGVVYSEMQAVQFRSPEIMDLKARRLLYPENVGFRYETGGMTEALRVLTP
ERIRQFHRDMYQPRNLCLVIVGETDHVDLLQILDEFEESIKDDIPPLDAK
FDRQVPPWLDTAQPPALKESIVTTAEFPEEDESVGEILIGFFGPNCVDLI
ETSALNILLTYLCGSSVSVLENVLVEKEELASSVTQWWEARPNSVIWLQP
TGVATEKLEFVEKRLMELLKEVASKPLDMEYMLECIKREKRQVKFHAETS
ESFYATNIITDYLFGKRDGSTLQELGTLSEYDVLEKWTDEQWRAFLSKWM
AHAHHISILGKPSHELAKKMKADEEARIAKRKEELGAEGLEKLAKRLENA
KKKNDEPIPAEVIDRWSVPGTESIHFIESDTARSGRAKSIGLGIGQAQKV
IDSATDGKLPLFIQFEDVPTNFVHITIHLGTSQVPFELKPLLSIFSDNFF
NTHILRDGKQVDFEQVVMELERDSIGYNIGSARSLGDADGFMIGFQVEPD
KYSAAVHWLRTMMFDSVFDPQRLGAAITKALADIPEAKRDGRGMAAEIDT
AIHMDKTSLAVARRTLVKAVYLKRLKKLLKGEPEKVIEWFNTIRKSLFTF
QNMRFLVTANVSKLQNPITTWNALAETLTTQEAMVPIPKLVNLLNDEGRK
PGSVGAIIVPMTTLDSSYSVSTAPSITSLSDPKFPAIMVAIGYLETVEGP
LWNAVRGAGYAYGSYFSRNVESGLLSYRVYRSPDAYKAISASRDAIRKIA
VGEVPIDKHLLEGTISQIVVMFADEQATMPSAAQQNFVRSVVRGLPKDWS

KDVLRQVRDVTVDEMKSVMQEFILPCFEPGKSNVVVTCAKLMSENMETAF
KDMGYKVQTHELSYFHDDYGLKAGEEEEEEEEEEEEDEEDEGSEGSYDDD
DESDED
>Mb|QLI66185.1
MTDRGNPAKAQTYLTSSSLSTLSHESASNAARPRNRRVETIDETYNPPTT
TGITKSSGTSYPIQGATHGNKTVPLTLERSQEDQIPNQGSTGGDLAQFLG
DSWTQSWTSVQAFASTLISGVDNSPKAIRSTHLYPRTPRGSRSETWGPSP
PSGGQRNNDVGVGSLAKRRDALKAAKTASILESHEGVNGGLDVIGKHKRR
TSDEIRPLDPEPEEHLVYVHNVEPSDTYAGIILRYKCREDIFRKSNGLWS
RDSVQMRKWLIIPVDACEIKGRPCDPPSWSDEREADLLTRNSSIARESSF
ATDGSYRGTLSLSADSSHIDAKQERAEDLPWSHVRWVHIDSFRQPVEIVR
VARQALGYFPPRRKKSIRTASPISTPRQSSDLSSLAPNSTERPSTHRLSS
VSGHPLIPGTPMSSQSRAGSEATNSRPAWMRRPGGVGSMSRTVRAPGPDT
DYFNSWTRKHIPGLDMEALPSMSVMGSETANFGFGQSASGIVESPFENCR
NASSTSRQGTGLDRAAAAVEHWLRGALAKRPSTPLLGSRLRPTGLSADPD
ATDLIELTDTASDDGKAARPVSTSILGSGHSVTTGRTDGGLRIKGRNKFG
VDDRATHKKDD
>Mb|QLI66186.1
MLPLSLLNAAQGHPMLVELKNGETLNGHLVQCDTWMNLTLREVVQTSPRG
DKFVRLKEAYVKGNNIKYLRVDDDIINQVSESQRGQQGSFRGGRGGHGQP
RGDHGHGGRGGGDRGRGGRGGGRGGRGRGRGQ
>Mb|QLI66187.1
MLSAFTARPVIELRQRDKSKIETILAYGDRILVGLNSGALRVYRLNDPSS
HQQGESPSAVDQGASNTSPSQNGGPPVSRPSGKPTDLLREVEKFSTRAID
QLAIIKEANIIISLSNYYVSLHDLQNYDHIETLSRTKNASCFAVTSNIIK
DKDTGIPEIISRLAVAVKRRLLLWNWHESELSGDVVEIVLSEAIRSITWA
NATKLVCGMNSGYVVVDVVTREIVDIVSPGAVGINGQGSRFGAVSTAGMG
YVGLGGYTPKPLATKLSEGQILLAKDVNTMFVDDSGKPLEKRQIPWTSTP
ETIGYSYPYILALQPPAKGSLEVRNPDTLSLMQTVFLPGAAQLHFPPSTV
SLAHAGKGFHISSDRCVWKMDATDYDSQVAELIQSGNFDEAISVLNMLED
ALLKNKFDTLREVKMLKAEMLFKKRKFRQAMDLMNEDDVHAPPERVLRLF
PPKISGNLSQWEESQHDDDADGDAAKTTNGERPGSREGTGDSAAAANVGG
FAKLFLSGHKKAAASDAVSVPSKKDGADVEDTHSVKDVHGTEDKPLEGKD
LKNAVLELNSYLAGTRARLQRYIDPITGKLKDQEDKKRSIEEAAERFLRT
TQTESEKKLEEELRNTFRLVDTTLFRAYMFSQPSLAGSLFRIPNFCDPEV
VNEALLEHNRYTELIDFFYGKKLHKQALGLLHRFGSPMKSDEAAPSLHGP
DRTIQYLKNLPPSEMDLIVEHAGWALKSNPTYGMEIFTGDTENAETLPRD
RVMVFLRTVDAQLERQYLEHIINELDDTTHDFHNRLVELYVKSLGGMKRG
EEWDETMTKFVAFLRDSRQVYSLTKALGMIPKDDPSFYEAQAVILSNMGQ
HRKSLEIYVFKMRDYAKAEEYCNRVYKSQDTTQSSPLNSKDNASIDTDDS
TQSIYHILLSLYLKPPSPHAVQLEPALDLLSKHGSRLPATSTLSLIPDDL
PVNSLESYFRGRIRSANSLVNESRIVAGLRKAEQIAIAARLNIGDSEING
QGGRNRHVTITDERHCFVCHKKLGGGMRFGGSVVAVLPDNTVVHYGCLNR
ALGQKKADG
>Mb|QLI66188.1
MGRDLGPIEAVPVPPTQTYSRYRSLRGKSVSSPRDFGVFRDDDIPQSDSI
CAGLEQSDSLNSLRSRSKSVSYHGNATGSKDPPPVAPAPTRGILSPRSIN
ISAPSGRKILENIKSPIFSSAIPPWKSFSKINKPANKDVGGTDGDCTAAG
PQSQNQADNPEGQEKGQEKGQEEEGQEDGQQHGHLSNDRGDSVQLPKAES
TNEPAVEDEPKEPRDDSAVSDIKQESGKGTDHLADEVARLEAETDRILAE
QKKLDIARLQAHLVAPPPKLKRQLLDKLIFFSRGKRSAGASQPGTPGTIV
SAIFSPATSRSSRDSIGEPATPSLPGLSAFKMSFIAPGGKGVVPQTDAPV
SASNGGERRITVRCFSSTLSLLVTADTTPVDILGATADQTRHDIDPAKCV
TIECYSALGLERRLRRYERVRDVMNSWDKDKQHSLLVIKSESPARDKDLD
LQAAPVTTEPPQGFSMQMHHSSKTGKWTQRWVTLLENGQIYAAKSPNANA
SSKDSIVLCHMTDFDIYSPRESQTRRHLRPPHKFCYAIKSQQKTVVFANT
ENFVHYFSTDDAQQAARFFEKVHAWRSWYLVNRMVDLQEKSRVPQIALGP
RSNSSESPKKSIRSGNTSVRASTVSLQKDDEGEPLMNVNAFRMSKIVITP

EEAKTRRSMSVKSKSSVNRSGTVKKKMHADAEFSKTAKGAESEFAAGGLL
GNAYDAKLKQSDAAPAVSKTAPDGPFTVAPSLLNGGISNSSSDASEKAKK
QSEEEKKRPEVTSWFPSAAEHSARTRSQSFHPPSSSYQQRRPMTAHTGAM
AARSRSEKHPAPLLSFSKDFPEPPRFREGPAGVRQAPGQPLVSYATGGTM
REPRDGIPRRNMSRRGLPASHDGGFTGQPPPSPHRPSPSPGSLARSKTSS
SPKRFNNGDVHSPPMPATSSRRRLNNGEPHNPQLNRPEPLVNRAK
>Mb|QLI66190.1
MAVPPKFAGHKLEFAPSPSTSSAPHSKHTFELYVDYCCPFSAKLFRTLSN
DVIPAIRDNKSWASNLTLIFRQQVQPWHPSSTLMHEAGLAVLRLAPGKFW
AFSSQLFEEQKDFFDANVVGETRNATYKRLAKIAGKVGVDEAQVYRLLEI
SDKPAENGSLNSGNQVTNDLKVVTKMNRLVGVHVTPTVVFDGVVQDTSSG
WTLDQWKEWLSKNVV
>Mb|QLI66191.1
MASSTTSKATRPASPAASIGLSSPVSFRKRRGSTNSISSTIDKEQLAQAL
DQIHTSASRCESLTTFNDFAPPPEGIPALENRPGAGELVQNGLSGLYNRF
REAVGGGSPTKTIPKEAKEIKDGSGQTSEVASKRSSIAPGHSSRPSVSSL
TRVDTNATITTNSISTIALTDVSSPTTGSGNVETRPQIQSTKSNSINLMT
GPRSSSTSRQSLPTNATSSVVADPTIAPPAPLKRDASRSTIRTEDSGGQG
SSRRSLTIAELQAESTSLYDSKDGRLPPRAGRDDESSADGSLDAPFSPIA
GAPSTTSTTNSRHHARNPSVTSSMLHPDDMRRTPAVIDRISRSRSPRYPG
SRDSSLDRGTAAASAINTSAHDSVYHDSFGQEIQSQHLHPDFGRIPGTTS
GQERASDQMNAQLDKMRRQVLSKEFWMKDDTVKECFLCQTPFTAFRRKHH
CRTCGCIFDSKCTTVVSGEKFGVQGSLRVCKNCLEVISRRFDGSGSDDSG
DERSFLPKIFGPNNPKESSETTESKPRPMSSSVAVGSEGLEDSRPLTTPM
MAIPATRRVRESANRASAVLEIGAPQLSRPGSSRSLKSLTTSGRPQSSAG
HKRHHSKHGFLGRFKPAPEQQAPFRKGIDEESSKKPKFPAFHDDNIIDPD
LADYMSEDSSGDEQMGIFATMAASDVQSTSYEHDRSAFPSYLNQTRKYRH
RPGEKSISGMSYVSRGVLDETNGPISLLNHRRSTRRRNLSISGSAHHHGS
PRPKSAMYKGPSASSEALFGLEHPSQSGTQLTRSDSLRRKRISKQELNRS
SLKHVDRLLYQLLDDAQIPNPGVWQKSLVPILLQLTDDVTPDVAKGEDMD
LRHYVKLKKIPGGRPGDTSYISGVVFTKNLALKRMPRRITNPRIVLVTFP
IEYQRHQQHFMSLQPVIEQEKEFLRVVVQRITNLRPHVLLAQKGVSGVAL
QYLSEANISVAYNVKDTVIEAVARCAEAEIIESLDMLALPVRVGRCSAFE
VRTFNNDYPGRKKSYIFLSGCRPDLGCTIALRGASGALLRQVKHIMEFMV
YVVYNLKLESSLLRDESVEPPEDSETSLSNSLQGLNESFRSISSAGECCK
QGPTVVVNQPSSESEPPSQNTVDSVSTIPEDAELVGQAGGPAPEPAKMVS
LHAYHTHASPESQVPDDIPMPTFYSDMVAKYETKILSASPYVKFKQPYLL
MKAREQERRLLYLRQLRDQDAVEEDPEKAGRHRFQLIKPEMVEKIGQKAP
RQVMEVLHAVHDAEYDKALFNYQTQTRHWEAYYIQGNLDLFDPYSHQNIV
VLYSVICTDTKIPCIEPGLLAINFYDEQHVDTGMDADCTLGQYIEDVAYS
KNDICNSNGCEKKLVDHHRTYVHDEYRITVFVEHVPNPSSRRPELGNGIT
MWTYCKLCKKDSEETVMSDATFKYSFGKYLELLYWGRGLKLKSIEDCPHD
QHRDHVRYFSLRDSRVRIHWDPIDLLEIVVPRARITWKVANDLKLKNEIY
NKMEERWSKFMSSVRARLKSIRTESLLPEKAESCKAEVERLMKKTQEEQP
AIIRQMQRTYVESKYYEVVPFNKIIREMLEKAGEWDQAFSKFEADFLGDK
DMRQITMMQLKKIFTDNESKESLASNDGTGSVGDVDDRPSQTSTEAAEKS
TQPTEYTDTTMETSVASSKALDAKHESEDEKVYIPAEGAIERVEGLDLAA
SPEAIATPAPSSQSPASTEIASRIPIAQTPVTSRPPATPTGAGRHPTDSA
ISSQSLSERIDQMRREQAMQGGEGNMVASKAVPERGSSRKSGPNSSPPIV
RTTSHPVRTLPRPQPAAAKANGPKDSKSSTTADSATETPPEGSIKVDKKL
SDRLGLTALKNRSKTATSGIPRLSHKKKESKVSTLARHFEQLSREFEKER
IRDRKERAASLRQPRARLPRTSTKAIVQVYGDVSEAFEEPLSTNEQIPSR
DGDQNQTSVLVKPEASVPKSEPAGELPTQVEPNTTKVDDQTGKSETDHAP
DGETSQATSDDEAMTSDIDSSIADEFLPDLQELAEALEPSTDIPDELPKH
QKTSLMRYLANFWAETSASGWPPLEYPINSTDHIFVDSDIIVREDEPSSV
IALALNSDDYQAKLAGIMRDNQDVTELEVDGISDGEPKSSPISEIGDGIM
YEADLEKSLHRVTGTHLKYQFKEGAAIMTCKIFYAEQFDALRRKCGVAER
IIESLSRCLKWDSRGGKTKSVFLKTLDDRLVLKSLSPIETSAFLRFAPSY

FNIMAEALFHELPSVIAKMLGFFQVIIKNPTTGTDVKLDLLITENLFYDR
SPTRIFDLKGSMRNRKIQSTGEQNEVLLDENMVEYIYESPLFAREHSKKV
LRASVWNDTLFLARQNVMDYSLMIAVDEERKELVVGIIDCIRTYTWDKKL
ESWIKDRGFAGGGRNRPTVTSPKEYKSRFREAMARYILQAPNCWHLFNNP
QLSTNYAQARFEEPEAGAN
>Mb|QLI66192.1
MTTADANLIIPVPWDKLLLWVQQQIALEMKTGQPVAMTRPQLEALSAFVP
FNVEPDVSDQGYVSKLIEKVQSLRLANPVWTDQGPTSIPINGHFQPLWRC
VCTLQHHGSFPREGFGFSVGEQAPFFQSKKNAKLFAAKHALSYLTQARPA
AITPGKLLRMDRPAISPLQLPKPHIPPPLNVQPLGKRQRLSGNSPVPEAT
PSKSLRTSVPSTRSPSNPSVFEEVTSLSHRLALGSPTYKIEPDPAGDSLF
CGRPVFKNDMRIPPDLGIVSGIAGESQARLKVAESVLEWLQAELQRRQDT
FESLWSAANSPKPATPNTQ
>Mb|QLI66195.1
MISSSARASRSASRYSSPAQPNGGTVSKPLEVSRSSANEASRSFMQRWLE
PSVQSKPSFEEAGLLRGGVLENMAPLGALPKAKKTSGENGMPTRKIILKT
STTHPPQKPPGRETPAHDDSHVVAAASPAVSPPVSPLFPPAPASKRKSLV
ARTKDDEADDEEYDPRGDARRRRSRRVSLPAQRTMAAAVPPTTTITTAAQ
APSPARERRSSTSIVLKPATPVKRHSRSEAGKHGLTDKVVEAAVDEALKH
YRYPTAWALKTLYEEKSGDRDFVAMIQDVFSQTADEETMDEFYRQIEERK
RQGKKDNRGCYFFVPPATNSRFTPHKAMAAPYAHLIHEPEGLPRESMTRA
AKKAKTSHGGAGPGTSNAATPGRKGTAAAGAAAGAGAVGTGQTDKATTKI
IRVKTPASRKRARRDSGSSDSSLSTAISMSSPEVRVSSLNSPSAFRGAAA
AAAAAGRGAVRGGGDEAAGEQAVEGGGRGARRGSVAVASTAMPTTTPTTT
PTTKGTTKGTTKGTGAKSQTAPKSQPITTRGKSVASSKQLVSNSSRPNSP
TLPNTSRTRPHISSSCLADDASMPGRVSAAELFPNLKVKSAEKPAVSPAK
DAEIDALDAPAPMPEEEEESFWDRRRTARRVTNNLSPHESSIRAGPAADR
ETTPVKTTRRTRQSLAASVTTRATRSASKRPNDDLERTVSPIAFSLPGDG
SSAVGSRAATPTSLRPAKKQKTGLRVKTSPVKKKGGAAGVPRPSHELNSS
SLNGIAPKDPNADNDEYCSACGNTGDVVCCDGCPRSFHFECVDMVQSDDL
PDEWYCNECLIRRYPSRVPIHKGIFGSALNNLEKSIPRAFSLPKRVQNRF
EGVKAGADGDYEEVVSNKTARKRNGTDEPDFFKQREDGQAVLCHSCQKPA
TQIRSIIPCSVCSFYWHIDCLDPPLAVPPVLKTWRCPAHVDDILIDVPSL
APAHRFRRLKGNQTVTPAFGRGLKNNGHIEIDWTDEPDEPDASGWPDHRS
FGRTYKLPAKGVILDFIQQLRNQGAGYGSRQNESKLIPYPTPPSHGDSTS
PLAGSALDRKMQEMQVSLNLLGLQQHRSDGVDKLTAALMTAADDSMLALM
AKSNADNLASGQLTAGDRLGLRAMLAQMDAVGTRIRQLLAEDSSTPAPQP
IKAEASSPVIASTTGLNDPHAQKMDHCVPVSEPTPPSTVDHGEGTMDLD
>Mb|QLI66196.1
MPRLVRRRPLWDRITSTLNPMDFLLWLSEEIETRDWDSGLVGTQLGLGLN
FLFLVVRANSGSKQASDDIFGDDGSSRWLSMLIYPLSWGLIAFSIINAIY
TVSRSRKYRLFEANIDVKPSTSSVRRVKVQSSPVASSPLRFIADMITSDS
AESRAHPDKSRDVWELSVWDPLPVCVRLACLFSPGHVLVYLIFLPLAPLD
PRPSVTVFNSLIMQVVLSAQMLLLSSRYLQQAKDSAIVQKEVFHEYDAKF
VQPRIHPIVRDVGTQMSDDQPVISREFVQVGTPTTLIRRSFISHGNPHVD
SEEATPIKRFNSPSLRAGNVMKPQIFTPATASRRTELFTPALSSHRSSGI
RHSLPAGYTPSSNVPAAYTPAPPSTSTVGTSTGMNTNFGGSLGVYAHQKS
PLKKAISMGDMNSDNFSPRGSREAAAYEQSGQEPPSSPTKYSEKRRFTST
NINSSPHPFANMGKHSASERFPKRWRG
>Mb|QLI66198.1
MGDYDRTQSLDAGVADGPSKSQFRPAPSPSGSKVPLYMQSSDGKHVLVRR
VRRKEDGSWKHFARWFVENQISLSFNLLALLFLCHNFIPKARAHTRKYFQ
LAYYNAKTGQYASGFDDAYFITFCIVLFTLLRAGFMEYILAPFARMQGVT
KKKDQIRFTEQAWLLVYYSVFWAMGVYIYCKSPYYLNLREMWTDWPNREM
DGLMKGYVLAQWAFWLQQIIVLNIEERRKDHWQMFSHHIITTVLISSCYF
YHHTRVGNVILVIMDVVDLFLPAAKCLKYAGYTTLCDIMFGVFMLSWLMA
RHFVYVMVCWSVYAHTPEIMPSGCFRGSNDHLIGPEDPPAGLSWLIEPFL
SSTGRVCYNETVKWSFLTPLLALQGITIFWFTMIVRVAMKVLRGDGAEDS

RSDDEAVEDEDEYVYEEAEPLEEEVDADEIDLRSWERRTGVKRQASSSGV
SLPGHSDRKELLGRIGCEKQVD
>Mb|QLI66199.1
MLRAGFLLIVAGTLAVLATSVTICLEIISCVHAAKNSKLLIASSTAAALE
SVVLLILGALGIAQAVPTKVPLSWKCSLPAFITQLFAWLAALVASVVALV
YLQEWWTQLELDDEAAEGRWKVFVGSATVVAFSTALQLGSIILHFATSRD
VTAGTASSFHSNEEGRTMNIKSVRYSRTTVGAALQEMTSVRSNESSPPSP
ESSSMGPIDYLKASMSQAIRPAASRTKFLKSGRRRATSLDSKNPRKSTDT
SFDSWDTSSVDMYNRQVVIEMSSPATLNRGLETIPASPSGSRSPTRPGTP
LDMELEPPRMLYRAESYSSSVLSQQDPHRLTPDSSVNEAHIHPLFRSDSP
TPPPAASPGTVVLAAPNAGQVISCKSSMQSMRRLRSGSLPATPSPLIHQA
SFDLLKQSKMGDDGDVGRKSSGTRSERKMTPPVPEWLLSPSMKTSLESFQ
EQKQENDEET
>Mb|QLI66200.1
MPPSKGSNGSGAQVSHRLRNFFRMNSAASSGSEKEKAAANNASRTSASNN
AAASDMPGPKPSRHTKFFTNTVGRLRAHTSASEGNQLEEAMSPTAHANPY
FAHQGQPGLRHHNEGSVPPSPPDTPELKVSGPDGIPADQATSETKEELAR
RLRRVASAPNAQGLFASAESSSVPALPIGIPAAQRPATAELGKDPLVADS
ESGSIGLAQSRSESSKSDSHAIAGEDVLSALRAPSTSLAFRRTYSSNSIK
VRNVEVSPASFDKIKLIGKGDVGKVYLVREKKSSRLYAMKVLSKKEMIKR
NKIKRALAEQEILATSNHPFIVTLYHSFQSDEHLYLCMEYCSGGEFFRAL
QTRPGKCIPEDDARFYAAEVTAALEYLHLMGFIYRDLKPENILLHQSGHI
MLSDFDLSKQSDPGGKPTMIIGKNGARTDALPTIDTRSCIANFRTNSFVG
TEEYIAPEVIKGSGHTSAVDWWTLGILIYEMLYGTTPFKGKNRNATFANI
LREDIPFPDHAGAPQISNLCKSLIRKLLIKDENRRLGARAGASDIKAHPF
FRSTQWALIRHMKPPIVPNPTKGIDTVNFRNVKESESVDMSGSRLKGVPL
DSGLATPGAEIEDPFSEFNSVTLHHDGDDHHQHIVHHPSVNQQVSASS
>Mb|QLI66202.1
MDRLSCNVIYVNRVVGEDRLLRAAPDTNSVANEDAQPEWNRDDAKELVQP
LLDTFGDVHVCGTGAACLSTLFQLQDGSMTNITPTLVLLDTPFNDHIQDQ
HARSRSSSPTPPLVAHSSEIHTPNEELYGLGLLQKIITEAHLRNMSKLVV
PVAIISHPETTVPNGGHQMTDGASEAVQIPQATLAANRQLMRTCLDLGAV
DVLISPLNSKCITTLEICAYKAHRDATKEQQALMEVAKGRKRSWVGVNEQ
KPFAYLREAMVSGLMNGICRLDSNDGQLAGAHIAVSSSRQVEIADAVGRW
HFCAHSFSDDELLVAALVMFKHALADPELEPWRIPADQLISYLVACRAAY
NSFVPYHNFRHVVDVLQATFNFLVHIGALAPYPAGRQQGTAADKSPIAAL
LSPFEALTLLITAIGHDVGHPGVNNGFLATLNAPLAQLYNDRSVLESFHC
AAYSQILRRYWPSAFEDRKMRSLMISSILATDMGLHFDYMKKLGDVQERL
QDNNSTDGWNGRQLEDHKALACSLLIKCADISNVARRHDTALKWMHILSE
EFSRQASMEDELDIKSSLMAQPKKDILSLSNAQLGFMNMFAIPLFQGVAD
IMPAMRYTVDELEVNKRLFEQKVQQEKARQSPDEQRLRRGVREGTFSPRT
RSVVGERDTPEGQSVVKIEQVKNLTDGAAEEKGEAPQVVADVVARVEQQA
PIAPEEPADVAAQVPPQGTAAPRQDLNGTTSAFDAVRELAESDPFNCRSR
GDSGTTESKTSAQFGRQRCSETTEGSASGATAGDWHSQATTATTGKMALS
PSTQGTSIVSNESIERTPSATGLNISPPSTSSHGSPGTVLRDTGAGDDEP
SHGGSIVKAEAKSLRKKSSRFRMKDFPFFRRNKASSPPLPASDATSMGRD
GKF
>Mb|QLI66204.1
MSKRTVFTTVTPLPLGLTRDAAVAFLHDHLQMIDLNPLIKDRHRIPPPRH
APDEELSCVWYSLTDKVSYLPGGLASSDVTYTAAFHDLPRGIQTHCYAPM
GVETRSKWSIGGSMPGEPLEPMELGLGAPTTGLYLREDVDLRCNIFMASF
VKKTIKKSHSSLVDRLHRVAKGQTSSESSTTTLQQQQQQPRNAQTTLPG
FSWQRDNRFHGHSRNVSTPALASGTNPFNDRPLPPTPPAELYDSKPLPSY
DPLAQREPQAAELQ
>Mb|QLI66205.1
MAGRNERVSTYTSGSGETDGRNGEQKKNLWTSMLESVASGKRLPEKNLLV
LGGTPETQREFIESLSGTEARRNFDRQKTPPVANNFALGYTYYDVLDADQ
DDTLARVSLYLLSQPSSEFATLVSPLLTAETVSHTAIVILLDWSQPHKWL

RQIWSWIQVLEEVMGGVNTEARNEMEDVMTSWKERGRVGASTNLDGTPTA
TATSGDGDGLLPLGPGEWSEPLGLPLCVVCQNAQKMEFLEKNKSWKEPDF
DTVLQYLRTILLRHGASLIYTSQNTPSQLPLLIHSTLGITSLLKRQPLKH
NVIDRDKIVVAPNWDSWGKIRILGGTFDTELISQGWEADIKLPPGSDPGA
GNTDDESPTPPQEPSAIAQYEQWCRDPNSGGLAVVESAMSDGQAVGVESD
DPQDFLERQLKILEAFKAKAPEKGGSPLPAGRRVEFPEEKSVHDHIGPVQ
FNMGGIQVDADDMLQRLKDRNAHAAPDDEEEQEELPVGNMAKEFDNEQLQ
NFFSGLMNRTAGAADSPRS
>Mb|QLI66206.1
MPGDVENVNGETESLLGRSRHRVRRFFDGFIDFAFQGNILQIAFGLIIAN
IFTDLVKSFVSAILMPPLAILFPVNKNIEEKFAVLRPGDGYNSTTGYNTL
RQALDDGAVVMAYGSFIYQVVSFIMVGFALYGLAHIYTLISHDPIIKHTK
KCKYCRNRINEKCLRCFNCTSWQDGREERNGC
>Mb|QLI66207.1
MEPGDSGSRPSVHPNPAIITEQHQRHHQPSPSAPPTSYQSPREHLERRYQ
EQHAMATATVLAPSSHYPQSSSYSSYPQHQTGTAPPPPSNSGSNMISSEH
RRSSDDKEQPGRQSLPSISEVISGTRPGQYPPPSQPNLQPGSGLPSPFTP
APRSYSEPEKHSPRQLHTSSTYPPRQENLPAFTDSPRPPFNGRHSLPPVS
DRRQTPPSKPDFPQHRLAESQQPSDAHASNGAYGHPPPPTSHPYPAGHLP
PGQVPLPTYPISPRHAVPPPPGQYDARGQSVRHEEAEYPSRPRFEPTPTR
HFEPWNYQEALSHTGSASRTLLCFAEAYGRIAQEQHGSHPLPERLPTERE
VNDMLGNIDIIKRCLEQVRDAVQVSIQNERAREGTKIKGAFEEHQDVSMY
DNGMKSQYTMTEVKKRRGRAAPPGRCHSCNRIDTPEWRRGPDGARTLCNA
CGLHYAKLERKRQLEARSLRPKPEDRN
>Mb|QLI66210.1
MAIAMGWQKPDNVAGSSAPAIMVGLFVASGGLLFGYDTGAINGILAMDAF
KTQFGANCHSTTTSDSSLSICPKDASLIVAILSAGTAIGALLAAPAGDSL
GRRISLLISVGIFCIGAICQVCASETALLLVGRALAGVGVGAVSVLVPLY
QSEMAPKWIRGTLVCAYQLSITVGLLSASVINIITSKLDDSSAYRIPLGL
QIIPALILTAGLLVLPETPRFLVKQGKNEAAGLSLSRLRRLDITHPALIE
ELQEIIANHQYELSLGPSTYRDAFFGTPHLGRRTFTGCGLQMLQQLTGIN
FIMYYSTTFFNGAGVNSPYTKSTIINVINVVSTIPGLLIIESWGRRSLLM
TGALGMATCQLFMASFTTAAGEGFKNIKETILVVFCCFNIFFFAASWGPV
VWVVTSEIYPLKVRAKSMSLSTASNWILNFGIAYATPFMVESGPGSASFG
PKIFFIWGAFCIVAFIFVWCMVYETSKISLEQIDEMYERVNHAWNSKTFQ
PSWSFQQILDQGWSPSGIPEHELQTTASQSSADTTLGDSGSSTITIHHSE
EGQQARQSNENKAIPMATVDFSY
>Mb|QLI66213.1
MPGGYHHRNMIPATSPVGSYSSNESEVPGILPETVWVEGDQNDTPTDFPN
IRETISADALQASPQEQHEDASHQGIACDNETSGTPSATAMSPRAYQLEM
LDQSLKQNAIVVMDTGSGKTQVAVLRIKAELDRSAPDKIIWFLAPTVALC
GQQFDVIRLQAASVAMKLLTGNDQVDTWSADTWDTILDGVRVVVSTYQVL
LDALCHAFISIDRLSLIIFDEGKHPGSKIMTDFYHHNKHAAKSIPNILGI
TATPSMKEDLANMEILEAVLDAKCISPTRHREELLKCVKRPQIYSIVYDA
PRSLQYTESMYRLRKEFLNLDITKDPYILRLQEDPSDRNRRALVRAVEKY
DTFVQNQIKGLWGRSAEILQQLGTWAADMYIWKSSAAFLARINTDAIFND
WVDAEKKYLADFIRRVSPSCPASMPQSGSEVSEKATALVRELVSVEESTV
CLIFVKERATAGALREFLVSCPRIVEKHRIGSMVGTSNYQSRKLALYELV
GQSSDPMAALQSFRSGKINILIATSVLEEGIDVPACNMVICFDHPQTPKS
FLQRRGRARMKESKLILLSEHSSSAVAKWEALEENMKAVYQDTQREIRAI
QSLENSEESSSTFMEVESTGARLDFENAKSHLEHFCRVLSQGEFVDSRPD
YIIHSHEDCDSSSKPRLSATVLLPSFVPSQLRQIDSKFSWRSEKNATKDA
AFQAYQTLYEAGLVNKNLLPFKYEDIPGVETRAPEVEVDALLNPWFGVAK
AWTEGDQSWLYSLKCCDHMHGESEYNVLIPAELSQLRNIQLYLNSSDLCE
LRFDSRRPISAADVAAMPDHTSALLALPFAHRWPIEEKHHIIRVWTQEHH
VSMGGIGGKTFDPNSSDVIEGKYLVRNESNSPYHYVGLLPSKPPVESVQS
SIFDYENAPADEPYLVVKKWTKRSDFLHPVLSSDATPTTTKPYPRVIPLP
WATVDSIPLKFAQFGMLIPSLIHELEVTLIAKELSTTILERINITDLRLV

REAISSRSAAEPVNYERLEFLGDSILKYCTAIQVSAIHPEWPEGYLSFFK
DRLVSNSRLSRAAIDVGLAKFILTKTFTGQKWRPLYLENYTGNPGKVSPR
KLSTKTLADVVEALIGASYVNGGITKALNCISLFVNEIQWQDVSANRALF
FKMARDQDKLPAELKPLEHLVGYQFNKKSLLIEAMTHQSCMFDYQRRSYE
HLEFIGDAILDFIIVRKMYTAEPLLAPHQMHSLKTAMVNADFLAFLSLDH
QMRRTETAVTSNLQIVDKETTPLALWTFMRHSSEAIGIEQATTLKRYEAL
RGEILATLRQGSHYPWALLAGLQAKKFYSDIFESLLGAVWVDSGSIELCE
CMLQKFGVLSILDRLLKDRVQVQHPKEVLGKMAVTQTVTYNVDVQEAADG
DRAFACRVLVGQRVITEVVDGVTKEEVKTKAAEAAIRIMSAERDKAAEVK
H
>Mb|QLI66215.1
MANSSPLRRSQLLSSPDRPYLSNFLLDSRNNELSHRDALAAAQAEHDRVR
EAAIRVYELHELKEEHNRILEQGRREQERLKAEAAIAAEEKRLQELKAKS
IPKPPPPPPEPAKPVEKPAGEKKPVSVVSEPREVKGSEPLAQTQAKTQPS
APAPQQNGLFSNLGKPVPSSPFAAAGQQTKSQQPSVLDQNRTSPPEAKTS
QSQQPVAPSQTPQLPKSSVDPLSDQYIRIHQALKQLRKDIVAASKVAGSP
LKGKVGTIRREIRVSIGQLTGGKGANVSSTTKIMSLLRQSLDGQLPSPPV
VVGQFVAIPREPSTQDIPNNGAELPSLFIYLMNICAKGIISQFINEGGAN
PKAADPIGVFTAQVFSHKDFSWRGQSLIDILVAKFRVVCPVLFGSRGNEK
TEKGRQALGWKKDGPSWIPEQAHNNRMAGLGAGFASISLRDFSKASKTNP
YPPTHYWKAFASIVNCPTAQISNTQLVVLRSMIDGHESRFINFYGNAAIA
ALQLALVEFPKRAPANSPAAGSLRALADILRSDSGLALA
>Mb|QLI66216.1
MPPRKRARGQAVATPSATRDDDAMDIDTPAASEAGDPSKHDVSNYNNMWT
DDQVASLFKGVIRWKPAGMHKHFRMIAISEHLRNHGFDPDIYQHTRIPNI
WDKLRTYYNLDLIDEREYFDDDETEDKYVDFTLPHSHFLDSMMQRAVADS
SEAPTSPPQLELSPELSHARKRKRSGTASKARATDAEDTEDGTDAPSPAA
RPARGSRGRTRAASQQAKPEKNDKSDKGKDKAEKDKAETTEEEEDAENQE
EESGSGEDEDEDEDEDEDEDEDEDEGEGEGEGEEEEEDEEDEADESAEEG
GTPASRSTRAARKRTVAPKPTTRRTRSRK
>Mb|QLI66217.1
MPPVTAVPTGGQHGPSNFDKLKMGAMMGGTVGTIMGFIFGTVNIFRYGAG
TQGIMRTLGQYMLVSGTTFGFFMSIGSVIRSDADPKLHELYMRAQRRPIV
NMASPAWRKSP
>Mb|QLI66218.1
MAGLPPGWEWDYDGQRWFYKYKPTGQIQYHFPAEGDEFPDFVEAGVPAPD
LAPEERLESQQQVKRQTSSAGSGADARSKRAHMTGAGNGSDNKYAMSATA
KPVSAIWDGDDDAEGDGVFQPENFMFLGPGTYTDVSPLAEEEEEAAKRSV
VGTIGERVQGGETTGTTAAASAKGVSPLGSSKTTPMMAKSEPSTSSPGPV
DASIRQGPAVESAPVTQSAHIVQSEPIPSQPPVEIESTAVVAEASDSSVI
HMIDSREMPVEMMGDTMHRFDPVGFVAEMPTEHTAAAHIELYPEPVEIAD
NTVLAPVETAAAAVSATAIAELPAKNSPVAGKEAPVVDTPKGPEGNQSTS
GESIPSEDGTTSTEQQAPSDHVQSHGQRVVPSEQPTAPLTQNAGASYANQ
VLRQDPGQYGRHSNEHGQVESSMPTELKTFKIARKQPGSTPGTPIAQQSQ
QPDYQAYMPGQMATTAHLPIEKRHSSIGLQREASLMMNMKRSSVSIDSNS
VPRVLSPAQHPVPNASCEPLSTQFTKPTTDENKTDNTDLSLSHAPSVLKP
ARNRKSVQDSLPQKQVPTPAALATPPAQPKPESRRSEGISKFPSVLRPAR
GRSSSHPGQVINSSVRQPDQHAAISQDPLPLRGRMYQVNNNQTTIPPRQP
PFQHPLWATVYADHPARPASVMPIINHVPYGSQYSAAGFGLGLIPRGNQS
SQPSLSGCRPPELQDQPSPLQRMQEAIASQPPRPQTVVGAIEADSANTTY
TTTIVPPQQLVSVQPVIKDLVTEQLDRPQNVPAVVQPKSVDSQLQVQPAQ
KVRDEDAEQPQPQPQPQLQQKPSISQSQLTPTQLGQNVAGEQPTLSQNLQ
TAASIGTAVETQSLPTQQEPQKKLPPSFFQVQPLQQIQNAEAGQPSQLQS
LLAPLGIAQRAGGHPPSIQQESSTTSRLQDTQNAVAGQPSQMQNDTSRQP
PGPQSVIETIQHSSPPKPPKNAFSPDPSQPGSSQPTYPGMRRASMFTSSE
VSPARPRGDSQVSALGSPLSTDAARRGSSNTTINDPNYTPSPLTDTTGPL
AQNGWTQSIALGQQNESPPVPAKIREHAEGSFFPVQNTSDTRASQVSPPT
LDVARQQQENTISKPPTQFQFAQRAVQQKAKYLSQPAGQQEQEIRLPQES

AQDTEHPIGYQVKPLAGRSGQTPNDQQTVLSPGSGHILGRIEERDESEPG
PRINQTQQIELPSSMASSIQHSIPGTSASFHRSTLHHPQPSAPTQVQRSV
QTIGQLPMASTPLGQPPSGQLPPIQKPQDLVPPVHMQITMQTQITPGQRA
SNLNPQGQWNPPVNYNAPTQGSQEKERKWSKWFKGGHSKPKSAPQQMMSP
QPGPQPWPGGPYSPNHFWQQPGQQPGQQRPFGHGQSPIKIVNMYLPGKSR
LLYMLMQLVS
>Mb|QLI66219.1
MSYYDVDAILTDAEKVPCQFELDVPYLGHLDNSSGLKPGTPLSLPLWLAE
MLALASAGEDSKAPLTLNLPPCLSDQVINALKADPRAVALRDQSPHFYGV
GVRMLDLFDERELGAVLRTTFVVRANDVGLHARKAEDSVGGQGEEFLRGL
DEWERNLFRRGHEGVRGAKEWTEKVKRL
>Mb|QLI66220.1
MRYEDWDILLFPQNCKVPVKEFKVACHVVHDPESPHLGGTFGLPTVCCFV
PSLPTGTPFRVSIHSWNAPVVSKFTNSYSKFPENVKFEARLFIDGRLVAS
TALDRESLWPHIVAHSFDFSKSGDLEPLSFPGFREELLQQNFWSPADDLG
RIKLVLSEGFPRDSITTPFERVKNVVAFSFQHAPLEVLESSSIAWPNPSM
WRRAPFAASMQVPTLQPDDSESHAHSPQRRGSSSDKLSYATNSFQSTGTN
SVLMPGGRRVTHPVYGPCFYPTQNGLDPFGNAGSYFEWLNCMDIGMTAAA
GDNHFNPNHSSVAQRSTRRTSTDVSMPDYVPAKSDSQCVEQQLAPEPFPG
EDGRQSEQSKAPINAPSADVEHKREGAAFPVPTDNTSFPPELADSLTNSL
LNQPLPMNLHHSAFHTPAAEVISRKEHRNQQHSGPSPTTAMDGSVDTQER
LETRHVSQQLYTPQGPSIAIGKVGMAENSLNHSDYCASAMTRGNALQACC
PGHKPSSEVQDEEAKHGGGKGAKRMRNFISVSSRAIDEEDETRRLSPRVR
LTSFLEGQASADTTP
>Mb|QLI66224.1
MASVTSHTSPHTSPYDFDNTEDESWQYLEYSSGASAPGSVGFLPSPASGS
LNGYAIVGHTHAHLTPPQASVSPLSLVEMDQTMFLPAETSFAAQEGFGAS
DGPAGFVQGSENESAGGSGSGSFLTPQEYLFADGGLSQREMNDMAPFMSN
FLTSEQTTTTTDFTPSLFHMDSTITSWTAISPAAANNHVVSFEEFISSSN
TTTNSITSSSPTGSSPKSPSIKSEFSKSPIALRKVKDAKIEKKTKKPADS
QSGKFVIVTPNSITAHAGRPNPFECFEAMRATQRGRKGPLAYETKENALQ
VRRVGACFCCHSRKVKCDKERPCKHCKRLMLHVPQVVCWQFQDFIPALFP
EFIRAHFKKDEIVKFLKDNVDGFVVRGVSQPCEIGLFCGLRFSAVLSVKA
KFFTPKTCDVLQHWHTNPQGGSQNLQANGSAPIGLELNTTAQRDDLRRKV
KAYVADIINEPFYAEQVTDSVRSTQLPVKILRIVQTYAKQSDSPMVKRAL
SIYAMHYIMTRHLCITPKSILALQPGLVSQNTPFITPRVLARQIKSLIDG
LILREMQVIFELFSKSLKPKHRREWAPCTAAFLVLCLFMEAVETTAENFV
IYQNEINKRNMSPPEYKRGFALDICKELENMPFKQFAYQFHNIYQTHARD
VNTKSFNPLFDESFEARGELDGPAVEMVRGMRELFAGEDWQDMQFLADDE
IILSRGESTHPIDSSFLYTGRLVAKFLLSFTDENVIFGGKI
>Mb|QLI66225.1
MVGSIVRQLFSPATRRPLTSPLASALLKPTRFTPIFRSAAPAAPAPFSTT
PARGATLNQVLRGIRKGKRARHAVSPALSNTNCPALKGVCLRVGVVRPKK
PNSGERKTARVKLSSGAVVTAYIPGEGHNIQQHSVVLVRGGRAQDCPGVR
YHLVRGALDLGGVANRTTSRSKYGTKKPKKATVG
>Mb|QLI66226.1
MDKMGSPEHSSLEQALELMSRVPLIEDGHNDWMHMIRAYYDFHVDERFEP
QRDLAGHVDLKRLVEGRAGAVFWSVYVECPKEEDDFSDALHLCSLRDTVQ
QIDLLYRIVDLYPDKLEIVQKSQDIMRIFKSGKCASLMGAEGLHQIGNSS
SALRIFHRLGVRYVTLAHGKNNLYVDSATSKAPVHHGLSSHGRDMVREMN
RIGMIVDLSHVSEAAMVDALNVSQAPVIFSHSSAYALVPHARNVPDHVLD
LLKENGGIIMISFIPWLTNKEPEKSTVDDVVDHIVYVGDRIGYHHLGLGS
DFDGMPTHVQGLEDVSKYPNVVARMLERGISPQDVERVIGLNLIRVLRDV
EEFAASHCRSISVLEDKVPQLWDDGIRTHVKRLYPNAEHDKPHGNIG
>Mb|QLI66227.1
MAFKNHGRQYDLVVFGATGYTGQLTAEHVAKFLPTNLKWAVAGRSESKLQ
SVVEECKKLNPDRLPPSIEIANTNDESQLEALIKKTFIFITTVGPYCVYG
EPIFRLCAETGTHYLDCTGEAPWVARMIKKYESTAKDSGAIMIPQSGIES

APPDLISWAMAQHVRTELDAPTKDVVVTIHKLNSAPSGGTLATVLVLFEK
FSLKEVIESTKPFATSPIPYTSGAKPQKGLLQSILGVTSRPNLGLLTTSI
AGTTDQTVVTRTWGLLHEIPSRKKEFYGPRFTWSEYFKARNWLHGIGIHF
ALAIGSFFLVFVPPVRSLVKRFIYQPGEGVSREDMEKEEVEFRGTATPDI
ESNPSQKQAFCRAWFHGSLYMLTGLFLAEAALTILEDDLDLGGGVFTPAC
LGQKYLDRVNDAGFKIEVRTVEG
>Mb|QLI66228.1
MGNIISSPMHKLPHGESDDGPPAKRRRISSPDSLDLDHLVASPRLSESGS
TLRIEVLKILHKDSKKVKKYQGTPVPHDILTTKARCKVSMFDMSSGPPQI
LHCQSQLCDLITFKNPNGPYRIARVDLPAPFFVPRESILINRPDDGTFDL
SDSYQLLIELEAANAAHWPPLDEQDFGIPTQSLYPPWGTTRHWILSSRFD
SVFGRMRNPLSLSARYPSDQSSYQTNYVMDVDLRWTAGFKALRRLEKGSL
PCITAIHPDVDMFSDNAMSHPGEYGLNGVNGHTDSASPQDQDDELAGDQT
PSRSLRAREKNKIYNLKVLSDQALGRERKHRGRNSNTEINGEGRVQYILP
SDQPISLEFYCCITCGACHDSMDELQLHLQTSHPTYKYVLETTSQGPLFR
VAALRESTLSPRKALQLARAANPFSIQALISGDQNWAMSRLGPDGDEVFK
SPTKNFLGRIQSGSPVSKFSKPPLRRPVKAASASKVLVPNIPQPLFHPIS
KAKLKPGQDVPQNTPDITWLIQKHRESIADFSDVTAAEKEYIWEWDGYIL
RQNITSAAYFPRAWLNFVEEKAGWLARAQHRMLEFGKHSSVLLARDVLDD
EDMQQAFRYINEARAKSAKGQSPHGEMLAADDRAKGMASTKQSPKVSQIR
KSANGCTICQLPVIGPRLLLCSNTVRSPGV
>Mb|QLI66229.1
MAPDPTSIDNSNDNSNTNTNTASTPNRKTKKRVVWHSSSQDYGKAEAEGT
SPDSPADGGGTTSTNTSLAPASTNAAAAADSTLHSTVATSTSNSGNTSGN
GSGTPIPILGVKDLRSEPGSPGLFSTPPGAPDAVELRMALNLVLTEEESR
REEQSFLSVATPRAPRPALRRNTSYDDPEERERADQAESRVTERQLRSMN
DARQRADRLAVSVGSYSASGSRRNSDVEGSFQPLIRDSDDHGSSFADDAT
VTSDDDLDKERFRYRTPYSRRQRFIEDHDTAESLVRSHTQKAGKSNLFHE
EPGSSGTNTPVSRQTYGNDYVPRPSKYRGGILSSLLKLYQDDRRAPGNNT
SGQLTPLSSSTPVMSPRPSPPTSRPPSEAGGSRPSSRAGGSRQRFTSGLF
TNNRARHSTTSLTELVKSSSMFAAPGSSEISDHWSEKFKKEKADKSPKPK
KEQIRITVHIAGIIARHRYLLKLCRALMLYGAPTHRLEEYMTMSSRVLEI
EGQFLYIPSCMIISFDDSTTHTAEVKVVRVPQGIDLGKLCDVHNIYKDVV
HDKLGVEEATDGLHKVMERKPKFHTWFRVLMYGIASACVAPFAFEGRFID
LPVAFILGCIVGMLQLVFAPSNELYAHVFEVSASVITSFLARGFGSINNG
QLFCFSTLAQSSIALILPGYMVLCSSLELQSHNIVAGSVRMVYAMIYTLF
LGYGITIGASLYGMMDSNATSETHCKDPLDRNWYFLFVPGFTLCLCIINQ
AKWRQTPVMTIMALAGFCVNSYCSKYFEGNSQISNMLGALTVGILANLYS
RLGRHMENAWLDVVEWWQYRVKPRFSRHRCRDPDAWSLPSMSDPESNTAS
QKKQWRQPRKVGYSLAAAAMLPAIFVQVPSGLAAGGSLLAGITSANQLTG
NSTSTVDTSAKTIGNLDGTAFNVLFSVIQVAIGISVGLFMSALIVYPLGK
RRSGLFSF
>Mb|QLI66230.1
MTGTKLPEVDPMTRMQVDESVVGHTEIDESLYSRQLYVLGHEAMKRMGAS
NVLVVGLKGLGVEIAKNIALAGVKSLTLYDPAPVHISDLSSQFFLTPADV
GIPRHDVTAPRVAELNAYTPVKIHESTGLDADLSQFDKYQVVVLTNTPLQ
SQKTIGNYCHSKGIYVIVADTFGLFGSIFCDFGENFTVIDPTGETPLSGI
VAGIDEEGLVSALDETRHGLEDGDYVTFSEIEGMEGLNGAEPRKITVKGP
YTFSIGDVTGLGQYQRGGMYQQVKMPKPINFKDFTASLKEPEFVVSDFAK
FDRPQQLHVGFQALHAFQLSKGRLPNPMDDDDATVLLGAARLFIKEEKLE
VELDEKLLKELSYQARGDLSPMAAFFGGITAQEILKAVSGKFQPIKQWMY
FDSLESLPTSTKRSPELCKPLGSRYDGQIAVFGAEYQEKIANLTQFLVGA
GAIGCEMLKNWAMMGLGTGPKGKIIVTDMDSIEKSNLNRQFLFRAGDVGN
MKSDCAAKAVQRMNPDLVGHIQTFKDRVGPDTEGTFDEAFWESLDGVTNA
LDNVEARTYVDRRCVFFRKPLLESGTLGTKGNTQVVLPHLTESYSSSQDP
PEKEFPMCTIRSFPNRIEHTIAWAKEYMFEKCFVKAPQTVNLYLTQPNFM
ETTLKQGGNQKETLETIRNYLTTERPRTFEDCIAWARMQFETEFTNKIQQ
LLYNFPKDSETSSGTPFWSGPKRAPDALKFDPNNPTHFGFVVAAANLHAF

NFNIKSPGADKNIYLRELENVIVPDFTPDANVKIQADDKEPDPSAGGSDD
EDELQKIISSIPSPSTLSGFQLQPVEFEKDDDSNHHIDFITACSNLRAEN
YKIEAADRHKTKFIAGKIIPAIATTTALVTGLVVMELYKVVDGKNDIEQY
KNGFINLALPFFGFSEPIASPKVVYKGPEGKVTLDKIWDRFEIGDVTLQE
LLDHFKAKGLTIVMLSSGVSLLYASFHAPAKMKERLGWKLSQLVENISKK
PIPEHQKEVIFEMVAEDMDEEDAEVPYIKVRVR
>Mb|QLI66231.1
MSSQSQGIKLIHNPDSSGYRLLELPPDLANLLESDTAPVLTLESSDSSTV
LRTPSKTYNLRQKNTSNGLYLLAPHSPSDLPDQELAVISIIHETVELDVV
PEMPVRRDKLADTGSRGKWHEMFGKGR
>Mb|QLI66235.1
MEGRGTPFDSNPSSSDGQYGEEWARDLRLKFEGLLRDKRMNDLRSCTRSH
SPRHQSPGLSGRASSANLAAHASFTDNRPATAHGSETPSTPPSYSCIRHL
PKIPTAPAAGDRDSQKFRNLMISLSLTPTKYENPGLLDEALQTIPLDRIY
SEAEEECQVLQAEAESMGDGRRPEWGYQDCVIRALLRWFKRSFFTWVNNP
PCPVCLSPTIAQGMTAPTPEESACGALRVELYRCSAQNCGAYERFPRYGD
VWRLLQTRRGRVGEWANCFSMLCRAVGGRVRWVWNAEDHVWTEVYSEHQR
RWVHVDACEEAWDSPRLYTEGWGKKMSYCIAFSIDGATDVTRRYVRNNES
AAERNRCPEEVMLYITQEIKNIRRSNMNKDERFRLEKEDQREDEELRGYV
VASIAQAVTDLVPGASSSSSASTGRIVPSHGQDVKVPAEQLVQQTGSAAW
LMAQQQARNQQFQPPRDPRQQ
>Mb|QLI66236.1
MPPPKSSRTAADDHKTDTTGGPKEKGSGPASAKMRRGASQSSASQLREAH
AANTPTSAPLQSASEAQAPTINWSSFERDALHTYRREHQLNTPASFASKY
HQLLLSRSGSIGLYSPTMTRKQQTRRQSKDNLAKAVRKHFNGLGIQENDV
VVDFIYKIRNEKMARAGGPNRQPGIISEH
>Mb|QLI66237.1
MASTTSPAASLLKRQLKEIQTGKDLPGISCGLVNDSNIFEWEVMLMINDD
CKYYGGGNFRARMVFPQTYPHMPPSLTFQTPIPFHPNIYENGNLCISILH
PPEDDEYGYETAAERWSPVQSPETILLSTISLFHSPNDESPANVEAARLL
REEREGKHKEFRKRCRKCVRESLGED
>Mb|QLI66238.1
MASTDEPQSLRTAFEEAESKRRLLESSPDATSPTYASDLNSVLALYARVL
DKISSVSLFSPNEGLEDMATSSLPYILVNFTIAELVQRTPFTVPARRKLV
LRAARDAYERFLSLADGYGLVSGPYAKLLERYRDDEEQFAVVSKTGDMAS
KRDAKIASFKAEKGLREKLALLRSDGRYVEKDEEAAREVYLADVAWKIHQ
TFQGLEGLNREAEMLAMAPDPDPETSARHAREEADDASVRLDQPFGRSGR
AGGPLLSREGRPLQPFTLVGSRADLARGVFRPGHNLPTMSIDEYLAEEKR
RGNILEGGTDPPRMEVDEDDMDAVDRETYKAREWDDFKDDNRRGAGNTLN
MG
>Mb|QLI66239.1
MDDLFRNPFGDDSSANGNAQRDTGGGNGPTLIRTVYKTMAPTFEGPIAGY
STLHDDPNPEPTQDSSPTSKPDPKPTPSTTSTTKSKPKETLSMPTAIFKP
DSSSSAPETLLIKATGSPALSPSIDYGAQPTSTPTLNFVSSTPTSDATPA
KSGDEGTSAGAKAGIAFGVLGGVFLVGLVAFLLFSRRRRQAGERRGAYNE
KSYANANANAFDAMTVRSDPHAPRISLRPVTQFLPNWGLDKRTSKSAGMA
LNSAAAVGANSKINAMRERPSTSQSTHPANPFGNQAERVSESNSYEQGAV
PRSGPFTANGPAIAAGAAAGAGVVGLTRKASMRNNTERNIDLTLPPTLGS
IPPSPAGTEFSMTSASSGAAMPPSNGAAAIAAAGGPPNSNVHRVQLDFKP
SLDDEMELKAGDLVRLLHEYDDGWALVIRLDRSQQGVVPRTCLSTRPVKP
RVPQGAQRLGPPVNPNGQYPRSPNQPQGPGQRPMTPQGRPVTPQGRPMTP
QGTAPQARPRMGPGGPGTSSPRPMSPAGPPPSPAPRFNGPPPLPVHPGSQ
PASPVGMNRPTGPVGPGRVNPAQSPPPGPPTGPPTGPIGRKPVPGQAY
>Mb|QLI66241.1
MATSTLVIRRMTRGQHLLDQRDSPSSTSFSSAYRKPALCVDRAVAENSPK
NKCRKPNLRVTFSDRVDMISSSFLPTRFRGEPDRSQSSAAPSRIGKKLAP
LLQLLSRVACSHPIHTVVIVAVLASTSYVGLIQDSLFEGPVSLGKADWSS
LVEGSRDLIAGPDTQWKWQGIEQDVASVADANHLALLTFVFPDTSSTDSS

SSAPRSHVVPTPQNLSITPLPVTENSFNTYTLDSILAYSVPKKQAPEFVS
AAKEIPDEQAEEIVTHHGREKKMWIMKAAKVNTRNTIVQWLSNAWVEFID
LLKNAETLDIVIMVLGYLSMHLTFVSLFLSMRRMGSNFWLGMSTLFSSVF
AFLFGLAVTTKLGVPISVILLSEGLPFLVVTIGFEKNIVLTRAVLSHAVE
HRRTQARDAKPGRKSDERTQSQNVISYAIQAAIKDKGYEILRDYAIEIAI
LTIGAASGVQGGLQQFCFLAAWILFFDCILLFTFYTAILSIKLEINRIKR
HYDMRMALEADGVSRRIAENVAQSNDDWAQSNSSGSKDTTLFGRMRSSTV
PKFKVLMVSGFFLINVINICTIPFRSSASLSTLRSWAGGLGGVVSTLPVD
PYKVAKNGLDAILSAAQASNTPTRVTILTPIKYELEYPSVHYALSSSFGE
SGLGSNDSSVQFGDYGVGGRMVGSLLKSLEDPVLSKWIVVALALSVGLNG
YLFNVARWSIKDPNLPDHSIDRKELARAQRFNDTESATLPLGEYVPPTPM
RTEPATPALTDDEGDGLQMRKVKPARSESQSEHRSIEELQKLIAEKRTHE
LNDEEVVTMSMRGKIPGYALEKTLRDFTRAVKIRRTIISRTKATSGLTNG
LDSSLLPYEHYNWERVFGACCENVIGYLPLPVGVAGPLVIDGQSYFIPMA
TTEGVLVASTSRGCKAINSGGGAITVLTSDGMTRGPCVSFETLERAGAAK
LWLDSEIGQKTMKDAFDSTSGYARLQHMKTALAGTNLYIRFKTTTGDAMG
MNMISKGVEHSLNVMSTEGGFEDMNIVTVSGNFCIDKKPAAMNWIDGRGK
GIVAEAIIPADIVKSVLKSDVDALVELNVAKNLIGSAMAGSIGGFNAHAA
NIVAAIFLATGQDPAQVVESANCITTMKNLHGSLQIAVSMPSLEVGTLGG
GTILEPQSAMLDMLGVRGPHPTNPGDNARRLARIIGAAVLAGELSLCSAL
QAGHLVKAHMQHNRSAAPSRTGTPAPPPMTPVSLAMTNAQDKSKSAAAQQ
RAKR
>Mb|QLI66243.1
MCTTNCYTYVYPDGHRETIKQPSLCPSSRHGQPCSQNIVFKHDAQSVSYG
HPVAPPFVSPATYAPQYYSNYPPTPTYSPRASTPTYRSGDESDRSYHSAS
RSRRRSVYTSPRMESSSRRYEQNGRTVLVHNPPTPSTPPPAFTFPRTAPS
SPSFANAAPYIVDTSPRGASSRRPMIVDERPRHGQDAARIHIEFHDGGRK
KKHRRQTPSTSSYDSRHSYTSASENDEVRRRRHEDEAARSETRRMTQEEE
IRQQRLRARIAKANAEIARREAVPLPPTLKRSSTAAKATHHLGDREKELI
ERIQTLEIKEQARQDRGRSSKRDDDAQKERLMERMLPRRRATVGPGSRRP
RVEYTDGVYRWE
>Mb|QLI66244.1
MTHHTVDEEARAEVDVLNSRLEKTTQLTKKIQASLGRLEVSGQSVRDVAG
PLSSETRRLQLLGNNVDSVLSAIDRLRQPADSKDNEEQIIRAGPDKAGLS
SYLASLKRLNKSHREMQASNLRANQHTMTDLTRLIKSGNSQLESYFDKLL
RGETPRSIEPLHYITKDKPFPVLPQDKIARLGLVYAYLAGGQKPVGHDSP
ASKIYAEVRGPYLSSSLANLAAASVNTAKKKNLGAIYRVGTNGIGTYAQA
MEAIFLSEYDNICSIFKREDWGPVFQGTCQAALSELARTVRELNAHIKGH
LGTDCYLAYEITEILSGLLGNLETRTGELKASLAAALKPVRETAKASLSE
LLEDTKRKAGGLQTLPSDGASIPLVADTMQRLQAMVEFLRPISSIMISLG
DGGWKPSSASAGRSADVIPSLASFDIGADGREIFGHYCLDTIDTLLSTLD
QKAKILLRGKSVAGVFLANSVVTVERMIRDSDLGPLLQSRLEVLDQWRKK
ATAAYTDVCRDLSVYLFDTIHTNRTQRPTSGQAADSASVIKSLSSKDKDK
IKEKFSQFNAAFDEMVARHKSYSMEPDVRSMFGKDIRQKLQPLYDRFWDR
YHDIDKGKGKYVKYDKSSIASVFLSLAS
>Mb|QLI66245.1
MAITWDSIRSLLLFFGPMLLPKAISWYRSIRTSPHGLPIQPMPPRVRLAL
GLLFALSAIYLVKTLPAFAPENLFTRTQSRLQIPVDVLFNRVAVLRPGNI
LTASDHALRAKFVNMESRLLYAQFGPQVLSDCPFCNADEPKSYLYYALPS
LLWPHLANLIALAAITSPSFTGKHGYQWRTLATIASLTLAGLEAYIAGSY
SYKANAKAPRLSDVDFFYWSMRSYRLVALAALNAVLGWVMYLSSTNRAFV
QLPSTSERVEDVAKGLLTVKSKLNAIGIINNTAMRDEELRSRCQTYWAHE
VRLMRDVMEEREVVEGVNDALSNRINIDSISKDAETYTDSVLEPLRQLQQ
SE
>Mb|QLI66248.1
MAPGDGSDLFWYDGTIDADRRSPEPKDGPTFARAAQTFSSKRKASQFSLR
SLTKSLSKRPRLVAFRQWAVNIYHDSSRRLTEAYRRLKYQRRFGEFRAWK
GIGRRRQRPQPADDVKDKSAQPYGVFKYESGPGNQEWWKDGVTRYRAPSW

MFRAK
>Mb|QLI66250.1
MQAPLEQGGSALAGNSTKMGIKAITHVESYTPRKLSDDLPSIMLPKPMVH
PHTPPESTTSSPRLKLEGPFPSISRDCSPLRSSISSLGSPRNHSLVRLPS
LEEFDLGVEALARSYGPARPYTPPSPLRSLRRTSILPQPLPSLQAYVSQD
HYAPYHMNDAYIHGVSALDGYPSPPPDGENRHINQKYTTEEGDFIIYAWH
DKKLKWQRIKQDFAAMFGRTPERTVQGLQAWYYRMNQRIPLWDQDGWLIF
DNEDDLEPKHISIKCRERDSQDKPMEPLGLAQRYPERAIHYSWVDPDLKR
KCQDWAAKRAMQYRDRRERRKRKEQRRLKL
>Mb|QLI66251.1
MRKALLSSSEVSALVSIIVVILFTLALFLSGYTLQQRTLQDLRVAIRPGH
TKPSPKAHLPDYFTRETVRLQDGTVVVRESLADKDEKARIAKKYELREDE
AKGKTKTRRKQKQKQRQGDDVRLAPEQREHDGEAQGVGGTKRQATEEQLA
MLETIKERAAGQAWGVDHPDPLAKNPLPVTRAERRKLIKDEIQRLAQADK
PVYYQRRLW
>Mb|QLI66252.1
MHKGVHGNGTNGPSNGKPHVNGDGPLVHRIKADRKQSSPMAPAFMVSAPG
KVIVFGEHSVVYGKAAIAAAISLRSYLHVTTLSKSKRTVSLRFTDIDLVH
TWNIDELPWDTFQQPSKKKSYYSLVTELDPDLVAALEPHVDVSPDSPEDI
RRVHRNGAFAFLYLFLSLGSPSFPGCLYTLRSTIPIGAGLGSSASIAVCV
SAALLIQLRTLSGPHPDQPPEEARLQIERINRWAFVYEMCIHGNPSGVDN
TVATQGKAVVFQRLDYNKPPAVKPLWDFPELPLLLVDTKQPKSTAYEVAK
VGKLNKMHPKLVGSILDAMDKVASSASELIADDDFDEEDEESLMKVGELM
TINHGLLVSLGVSHPRLERVRELVDHEGIGWTKLTGAGGGGCSITLLRPD
VPRAKLDELEERLDKENYAKFEATLGGDGVGVLWPAILKNGTEEDEEGGM
EIDLEKFLNAEGTEGVEKLVGVRDGGEREGWKFWRVESR
>Mb|QLI66255.1
MASLTSASRLCLRSAAKPAMPAVRALSTTAMRSDNAASSYQSPFKMGSDK
GSSIPDFGKYMSKKSEGSNKLFSYFMVGTIGALSAAGAKSTVQEFLVNMS
ASADVLAMAKVEVDLNAIPEGKNVIIKWRGKPVFIRHRTQDEIDQANKVN
VASLRDPQTDDERVKQPEWLVMLGVCTHLGCVPIGEAGDYGGWFCPCHGS
HYDISGRIRKGPAPLNLEIPEYDFPEEGKLVVG
>Mb|QLI66258.1
MASVQAPPAVQPPGAPMPAGATKQQAEEVFRKLKQMKEQGVPPTDPEYIK
ASHFLMSFQQQHNMRRNQQFIQQQQMQNGVQTGGRPQPGTPQSTQPNAA
SQGGVASASASAQTPAAASTASPASGSGSTQFSQQQLALLRQQIHAFKLLGK
NAGVSVQLQQAIFNQRQRRQTTAIDNASNSAKNAQPSQDAEKAPSAGPEM
ATEDESPIPKAHTYKTVKSPYGTSMIRPSIKYLDHAQRKNRWFIPGVFPT
GIDFDHLRYEREVVIFNRMSQRFSQLKDLPANLAHWDSTKEILEADDTLK
RKAIIEMKSLALYAKQRALREKIGRQMMHYDNLAMTTNRSHYRRMKKQNV
REARITEKLEKQQRDARENREKKKHSDFLRAICHHRAEIQESANSQKTKS
HKLSRLMYAQHFNIEKEEQKRIERTAKQRLQALKANDEEAYLKLLDQAKD
TRITHLLKQTDGFLHQLASSVKAQQRHAAEAYGDDAEPFVEEESDDEEEE
SGKKIDYYAVAHRIREEVTEQASMLVGGTLKEYQIKGLQWMISLYNNNLN
GILADEMGLGKTIQTISLITYLIERKLQSGPYLVIVPLSTLTNWNLEFEK
WAPSISRIVYKGPPNARKLQQEKIRQGRFQVLLTTYEYIIKDRPILSKIK
WFHMIIDEGHRMKNSNSKLSATIQQYYVTRFRLILTGTPLQNNLAELWSM
LNFVLPNIFKSVKTFDEWFNTPFANTGGQDKMELTEEEQILVIRRLHKVL
RPFLLRRLKKDVEKDLPDKTEKVIKCKFSALQAKLYKQMVTHNRLVVSDG
KGGKTNARGLSNMIMQLRKLCNHPFVFDEVENVMNPMSISNDLLWRTAGK
FELLDRILPKYQATGHRVLMFFQMTAIMDIMEDYLRYRKFEYLRLDGTTK
SDERSDLLKDFNAPDSKYFMFLLSTRAGGLGLNLQTADTVIIYDSDWNPH
QDLQAQDRAHRIGQKNEVRILRLISSNSVEEKILERARFKLDMDGKVIQA
GRFDNKSSETDRDAMLRTLLESADMAESGEQDDMEDEELNMMLARSDDEI
TVFQKIDEERARDPVYGMSAGTKAKPRLMGDDELPDIYLNEGNVVEEETE
DLVLGRGARERTKVRYDDGLTEEQWLLAVDDDEDSPEAAAARKQARKDKR
EANRLKKVAILNSIDNSPSASRASTEEIETPKKRGRKPGSKNEKRKAEDG
DEEPPAKKRRGPQGRQSKGGSAAADSRVSPQQREVLQKSLRGLYDALMNL

EVDDIEPPAEDDESDAGKRLIIGPFIKLPPKRDYADYYVIIQNPICMNHI
QTRIKKEEYSSLSDLRKDFQLMIRNCQTYNEDGSILYQDAKTMEEFFNKK
FQEELEAHPELQELEEGGSKAGSAAPSGQGASTPQPSGGTRIKIISSSAR
ASEAVNGAKSSAQSDEE
>Mb|QLI66261.1
MALSRIEGQDNLYVGGIWALRRSDTLTEKGITHVLSVVGFSPDSLKNFKD
EPWSEYGKQFHHLLIDIDDVDESNLLAELPKAVKFIDEGLRGPRNRNTSS
ASDHAESQGGTVRDGSGESGIVDEGLERLKISSPNSTAESTGAVSQAGGV
FVHCAAGKSRSVSIVVAYLLWRYPNRFDANVVPASAYGNVSSKKNSGASA
AHSSKQSAGRSRSRKETAEEAVELALGLIRRTRPMAEPNNGFMQQLALWW
EMGCPDDVETHPLYQRWAYRREVEEHVAVGQAPSRLRFEDEEPSATAARA
AAAAGGGGGGDTQLTLRCKKCRRTLATAPFINKHVASGSSKTFDPRQPCP
HYFVEPLSWMRRELEKGELNGRLSCPNERCGAAVGRYDWKGLRCACGGWV
TPGLSLQRARVDEEMRRNHGAVGQGQGPGLSEFAKNMGIRMPPGSGRGGG
NL
>Mb|QLI66264.1
MAPQKPETFMLSTEAQQALPHDAQVALQQVDNLKYFLISAPVDWQPDQYI
RRFLLPTGEYVSCVLWNNLFHISGTDIVRCLSFRFQAFGRPVKNSKKFEE
GIFSDLRNLKSGTDASLEEPKSAFLDFLYKNNCIRTQKKQKVFYWYSVPH
DRLFLDALERDLKREKMGQEATTVAVSEPALSFQYDSSQSLYEQLTKTQQ
ANSSSFNAQQATSFSQGQSTSPVMRAMDSMPPPSMMPQTMAAPMGEPMDH
HMVSYEAMPMAAAVPQQVAHVKREPDFTRVQYNQNGVPITHGHQRHASMP
AYGLEYSPAPSFVSSQYEDYSNRGISFEPITPPQQALGMTGEPAYIANEE
TGLYSAIPDHLSGVNGLNGMVQLPPSNLAGSQFTRSYGTNNVYSVIEGSP
TYKQRRRRSSIPPSMSAIPGAPVPNTAAHRPSDLRRSVSASVGPVAEGEE
SADNSPPGLSYSASGMSMGSQQHKDVVDLSRHGTPLSNVDGSPALNPMTL
QPAFGDELSHDGVIKDHRRTLSGPSGVIRRARSATVMELGPYPHKSHSCP
IPTCGRLFKRLEHLKRHVRTHTQEKPYICPHCSKAFSRSDNLAQHKRTHS
REDGGEGSFNLSAEEEEEYSGEDQLGSVEEASPTSEAAYVPTSLNAAANE
SVSSNSISNANNSAMHQPQTFNSLQTLSMPMTISQPATINASGMM
>Mb|QLI66267.1
MKASPWVSIAAPLLLFGLCQGHLKPGHSFEPTLERRGVDTESGGFDDEFP
RHVYRGEIQRTPKEVQKDGGFYSRGMQRILSGDKLSWEELEDGSSLFRHA
AGDTAPFTRYVSTSADPATSLTFAVNDEDPVEKGYIYKIHADKRMVDVNR
SLGKYSPYPAQTEHAAIGFIPFEQIEGWWEVTYKHDFADPKVGKKTQDKL
RRGQLKHFHRNPHFSPNFQKLRGNGVAPQLAGFPRLSTAWDEDTWKQFKT
LPVSKSLDDMIEAVCTGNGNDNCIKQLGQPQSKPTKPKTSPSGPIDPAKP
KSLATSFRVYAKAGTLVGFNVLAPYLRNVLNQLRQWDHPIGWAVKELDDS
INGFQEYIGGPRRNDISGNDNQAALINFFKRVFWILEGPRRRPQDLQLLS
YGKRNKMRLISVNDVLRTCERVDETPPDEQQLKTNLKDACTKVRKKAMEL
EMPTAAELEIGRAACGVCGLAWSPQDGQCKDKTGAILWPREPPAESTCTH
HDSTKCGGEQTAAQLQTGQAVCGICGSNWYPDEGKCRDAAGVLLWPFKTC
SAGAGRIQCEGGQTTAQRETGQAVCKACGKYWDPEGSKCRDEAGMLIWPP
QHLPSIKPLHPSEKCRGGNDMTCQRRQIAAEAERGRIVCGTCRFSWDLRA
RKCRDNSGAVIWPPKE
>Mb|QLI66270.1
MAGIKALPPSSAHFSSANDDGLVCLELEDGSAYAGYSFGAQKSIAGELVF
QTGMVGYPESLTDPSYRGQILVITFPLVGNYGVPSRETVDELLGDLPAHF
ESSQIHVAGLVTDSYCGEDFSHFLATSSLGTWLKEQGVPAMYGVDTRALT
KKIREKGSMLGRMRLQTRLTQINGVTNGIAHDTPLENFAEIEWVNPNEKN
LVAEVSVKAPKLYQPPASVAARRHPSGRAIRVLCVDVGMKYNQLRCFLKR
GVEVMVCPWDHDLAKEAGDQYDGLFLSNGPGDPEVLDVTVKNIQAVMEKN
RTPVFGICLGHQLLARASGASTSKMKFGNRGHNIPCTSMVTGKCHITSQN
HGFAVDASTLTDGWKELFVNANDGSNEGIMHVDKPYFSVQFHPESTPGPR
DTEFLFDVFINTIANCTEDPKLLQQPVSFPGGTIDENERLHPRVSVKKVL
VLGSGGLSIGQAGEFDYSGSQAIKALKEEGIYTVLINPNIATIQTSKGLA
DKVYFLPVNAEFVRKVIHYERPDAIYVTFGGQTALQVGIELKDEFEGLGV
KVLGTPIDTIITTEDRELFARSMDSIGEKCAKSASASNVDEAMRVVKDIG

FPVIVRAAYALGGLGSGFANNEAELLDLCNKAFAASPQVLIERSMKGWKE
IEYEVVRDAQDNCITVCNMENFDPLGIHTGDSIVVAPSQTLSDEDYNMLR
TTAVNVIRHLGVVGECNIQYALNPFSKEYCIIEVNARLSRSSALASKATG
YPLAFIAAKLGLGIPLKEIKNSVTKVTCACFEPSLDYVVVKMPRWDLKKF
TRVSTQLGSSMKSVGEVMSIGRSFEEAIQKAIRSIDFHNLGFNETKALMS
IDDELQTPSDQRLFAIANAMHEGYSVDKIWEMTKIDKWFLRKLKGLSDFA
KDMSRYTTKDIARSPNLLLQAKRLGFCDRQLAKFWDSNEIAVRRMRLEAG
IRPFVKQIDTVAAEFPAFTNYLYLTYNASEHDVSFDDHGVMVLGSGVYRI
GSSVEFDWCSVRAIRTLRQTGFKTVMVNYNPETVSTDYDEADKLYFENIN
LETILDIYQLEASSGVLGAMGGQTPNNIALPLHRAGVKILGTSPEMIDMA
ENRYKFSRMLDRIEVDQPTWRELTSFSEAREFCNAVNYPVLVRPSYVLSG
AAMNTVYSEKDLESYLAQAAEVSREHPVVISKYIENAKEIEMDAVAKDGV
VVGHFVSEHVENAGVHSGDATLILPPQDLEQTTIERIEEATRKIGAALNV
TGPFNIQFIAKDNDIKVIECNVRASRSFPFVSKVMGVDLIEMATKAIMHQ
PFQEYPPTSLQPDCVGVKVPQFSFSRLSGADPVLGVEMASTGEVACFGVD
KYEAYLKALLSTGFKIPKANILLSIGTYKDKKELLPSIQKLQRIGYKLFA
TAGTADFLQEHGVQVQYLEVLGRDEDINSEFSLTQHLAKNMIDLYINLPS
NNKYRRPANYMSKGYKTRRMAVDYQIPLVTNVKNAKILVEAIARHVYLDV
SKRDFRLSELPPPPPVVSAKTPEMKASLPSIGQVTESPRRRGTDAVLASP
IRGAGHIDELALPAAIQTSAISASVLKLLSQPSEFKNANVLSVKQYTRAD
LHLLFTVAQEMRLGVQRAGVLDVLRGRVLCSVFYEPSTRTSASFDAAMQR
LGGRTVSVPATNSSVQKGETLQDTLRTLACYGDAIVLRHPDEKSVDIAEK
YSDVPVINAGNGSKEHPTQAFLDLFTIREELGTVQGLTITFLGDLRYGRT
VHSLVQLLRHYHVKVQLVAPKGLELPKDVKDQLVASSQLLGESDTLTPEI
IAHSDVLYCTRVQQERFETAEQYERVKNSYRVDNTTLKHAKSHMVVMHPL
PRNEEVAEEVDFDQRAAYFRQMRYGLYCRMALLALVMAS
>Mb|QLI66271.1
MELSDIFRIVNLAVAVITILGGIASIFGFQLQSIILGAYMIVFGLAIALL
EFQIPPQVSRYANFLFSFIGRGIFYILLGGLLLGGHVLSNIAGSIVGIVG
VGYIALEFIPSIEPPSNMREADAGWGAEQV
>Mb|QLI66274.1
MSMDEEKGQGIVPKTPGEKDALDVDSSDKADQGAAGSTRSGILAQLRNLE
ARMDAKFGIESEAIVRKRAEDKKDVPWTEELTMALLWASGTMNTSCFATG
FLGWQFGLSLKQTILITIFASILGGIVTGYCATFGAATGLRQMSVSRYSF
GWWPNKLIAILNCIQQMGWAAVSCITGGLALTAVSDGHVSLILGIVILAV
VALLISFVGLNAILVYERYAWIVFFVIFLIIFGETGKYADNSTPASITGA
TLSGAVLSLIAIVYGSSAAWCTTASDYYVHYRADVSRTKVFLMTSFGIAI
PTSIGMLAGCVVASSLNTQAEWRDTYEHDGLGYLIQDMLYPRGFAKFLLT
LLVLSGINTNVISIYSASLSFQQLARPLALIPRFVWTLVSFACILALAVG
GREQLNTYLQNFLSLLGYWCTSYAIILLEEHYIFRKGKFENYDFEGWNDP
ARLPLGIGAATAFGLGVVSWCMGMNQTWFVAPLAKVIGDNGGDVANELTF
VVTALGYLPARYLELRILGR
>Mb|QLI66275.1
MGEADSYANGGNERGPPQQWGQQQYGQPYGQPQQSYGQQPYQQQQYQQQY
QQPPPQGYYNQSQDQNQNGYHQPPPPDQYGAPPPYSFNPPKGNDPNYSFD
QAFKVEKPKWNDLWAGILLILVFAGFVAVSIISIRGYVTGSRGNGIYGGA
NNFSLNNNTIILFALGLVVAFVLSWGYVAVARIFPKQFIWITGILNICWA
IGTAIYYLYKKYWSAGIVFLIFGLFTAFAFYTWISRIPFSALMLKTSITV
SKKYGHVYLVSLIGGLLATAFAAWFSVTLTAVYVTYEPSANNPKCGADGS
GCSKATVIGLVVFITFAMYWISEWLKNTIHTIISGVYGTWYFCVHNFPKG
ATRGSAKRALTYSFGSISLGSLLVAIIQFLRQLCSVARQQAGDQGGVGGM
IGYVIFCILGCLIALLEWALQFLNRYAFCHIALYGKAYIPAAKDTWNMIK
DRGIDALINECLIGPVLSFGAMFIAFATSLFAYLYLEFTNPAYNRDGGFT
AVIMAFAFLIGFQIALIFTTPISSGIDTIFVASGWDPQVMINDHPELYQE
MVRLYPKVQQAIQVR
>Mb|QLI66278.1
MSSSRDVEGETALADMVVSGRQFYEAQQYKRALEQFTRAMKYCPCARGVK
RERCTCKNFERVAAEEGCIFKEAMYNCKCDVGRTFNKCDNRNHIQALDYR

AATFEALGKLDRAKKDAEWILELAPRLPDTMKGYLRLGKVARLQKNHEFA
WKIYNAGISINKDVEVASSPKLQQLYKARTPLHRHFSRKDPLRLPTEIAM
LIFSYMEFTELPPCLGVCKLWRRTLTSPLYDRLWRTMIFPGRGMKRAPRY
DALKKMLSWAGKGGARKIVIPLPKAFLLNQQKLTLLLKASPCLEYLEIGP
QFDILMFPPKENMWTKLRHVSINGTGDPAKPAWSTPVIPIGGFPLAFLNN
AAASLEHLVLTGIPEPWYMTQSVPFLPNLKTLRMCNISTLRQSFPIFFLS
DAFPRLEQLWIGPNIPNLDSNSLSGWREKWHTMWNHLKVLIFEESSVVGP
ISQVENSLLTLRLLTCLNYGNSLQHIRFDIPSDNEDRHGRHRVFSNSRTL
HGDIDIPQYPEFRNLHSLASKSLCISPDILRLLFSDALNMGKIQSFDIVF
PIESLNDRVGDRSITHLKGYEWLRGAASITSMGCYRFRFRSYPRNDDDLP
LPQFLASFPNLETLSIFSENYEEAEFAGVVAAILKATTLKTIYTTSVKGA
VMDQLRTVAESQGVKLIWGHQPQAWPVPLDES
>Mb|QLI66279.1
MAITPTQFAKKTAQSANWSDAKRRVLSSYREWIRAAPEIQTMYNMPLPIS
AIRTRVRQEFERNRYAAKLPVVDVLLFKSHAEYQEMMNFWKQTTHVMAYF
KEENFRGDKRLPSSFMDGFLEGRN
>Mb|QLI66280.1
MYSSSNAFLGGNSQRPGGQQYGAGSFNTGMGPGGPQQQQQQPSPFAPQPT
GFAQQPLQQQYTGFPMQAQPTGMPQSSQQPLQQQYTGFPGQTQPQQSYQT
GAVPPMPSIPSQYQQQFQQQKQQQQQQLQQPQPQPQPQSTLPVPLPTGFA
STSPVQSSAPAPPPMKPQATGFTEMAASFHTGGTAKPQGRRAEKTNKIPN
IRLSFITAQDQSKFETLFKSAVGDSSTTMSGDKARDLLLRSRLDGDTLSH
IWTLADTTRAGQLYFPEFALAMYLCNLKLTGKSLPASLPDNIKNEVSSMV
DIIGFSVVDDVPASSSATNAPSFQTDTTSPPVIQQPQPQPSNSQLLQSQM
TGFPGQQTGFGVQPQGLQTQQTGYPGMQNPQPTGYQGPRPPMPPMPPMPT
GFGQGPNDGSMAVPLNAQPTGRPGQWGLVNAPASGLPNIDALQARMMPQQ
GREAGGYTTQGLQGNAVIPWAITKEEKTRYDALFRAWDGFGKGYIGGAQA
IEIFGQSGLEKPDLERIWTLSDNGNKGRLDLDEFAVAMHLIYRKLNGYPL
PNNLPPELVPPSTRNFSQTIGTLKSMLNQESDFRKNSGAALLPQKTGVSY
MKNRSFRGTAVGAQGGRKDATVFRNDDEDFGYRSSARRRLGNSSPRSESP
SSVASSDDLSLDQLRKKIKEKQVLLDAMDFADERNNEEDDILDRRDRRES
EELYRRIRRIQGDIDAHPDAALATGDSDAERRALKRQLQNLTDKVPELAS
QVRRTEKDIMEARLELFRLKDAKAHPNSGPQIVGTGPGGTVTESDRLKAR
AKAMMQQRTAALTGKKIDIGTEDADAPKRLEEETIKVKTEKESNESMVRD
VEDSVRDFSRGIEDSLKEGGKDDSSEHEKRRWEDALGVEDEVRDFIFDLQ
RESRAARLRSQDKGSARAPPAVEQSRPERVASPRVESPISTSRTATPPTA
GGSYNSYKTPEERAAYIKQQAEQRMAERLAALGIKAPVKPGETAAQRAER
ERAERSAKLKQAEEEDARREAERQARLADEQGVPPPVPASETPKATARAP
PPPPTRKAARVDTSEEDVATLVEEERAAKAAEEARINKEREEQQRETRAM
EESVKEEEDEFAKEQEEAAARLKALEEQVRQGKLRKEEEKKRKKAALAEA
KEKEAKMAARRVEIEAAKQRELELQRQLEALDDDDSSSDDDDGPEQITPQ
ESTPSGSQIVHQETEKWHSPPPAVLPTAPPVPTVVTSPPADIESKNPYDS
VIPQPAESSSISQAHDYTPAPPLPRPGASTNPFNRMGQPPAAAPGPVSRK
RADSDDWGSDREADEDSDDDRPGGGNAAQLASILFGTMGPPRPLSATGRD
STPASPAPANDTPVASPPPPPPPAPPVPVSNGPTSPPPPPPMPSSDAPSA
PPPPPPPLPPTGAAPPPPPMPGAGAPPPPPPPPGDAPAAPPSGGRPAGFLS
EIQLGKSLKKTKTNDKSASAVAGRVLD
>Mb|QLI66281.1
MPSGQVEPALTNGHDGHVHSAPVPGKKGKGKKAIDSSEASRLLQARISQL
EQDAAGEKDQELEIEREVKRANRDLMQQVAKMDDMQKIEHLTKRSSELLA
DMRRLEKENQRNKKRGDTLQKERDSGRTELGKTVGLKEKLEKLCRELQRD
NNKMKNENKELQTTQKRNNVSWDEKFSTVLSKLEGYQEEKDTPRKQVVDM
EVDELFRVRFKSFIEQYELRELHFHSLMRTKELEVQYHQSRYEREKKTAE
AEASKARHLQAQVQAFTKTETELRNQLNTYVEKFKQGLKVEDTLNNSNDL
FLSFRKEMEDMSKKGKRLEKENEALKRQKDATAANIIRMAEERQGWKKQV
DSAEKKVNKLMSIIQQMQHQGRKGPPGLASVMENAYSENQGGAEGDDESD
YSNEGEDEELSEFDDDTEEEPQPVQKQAAPVPYGPERPPAPTQQQATAAT
NGH

>Mb|QLI66282.1
MPLSQIEKSVTHLLVATKQLLETLTQWSRGQATDTQVSDVYVRLGYEFNM
ACRAFTAINVDTSDLGNVPDLLRHILEATLSQEASTDSLEKYLPRIRDII
INLLHGLKRKQQRLRQRQGRERDASLPERTTSVSTAASGTSGLTNLLDEG
LENGYRPDPSQRGDPNQQTNTSTPPGRRYNHQRDQARAENIPDQSTITQN
IPSAPPYPAEDTTMPPSSSASDLNIDAFPPPPPPPPDTQSSALAALQKGG
DLERRASRRYSQYQISKHLGGAANGMPILPSQTTPIPNRGRREARESLRA
VQSRESVRHSRTLSSQSKSAAVEASPSRMPGVPEASSSAQQQPSGPAELS
DGPAVQTPEEKYAPSATLNGPLQDLSSFMRESAPPEPDVKQTRLQSKTDE
PASLPHPTESKPGESFFQVSPPLTPTKDLTLFLQYKSKVKKIVLAEGRDE
LSIGRLQLAFIEKFSWNTQHNGTDLPEIYIQDPVSGVRHELEDLSDIKDR
TVLVLNVEPLDEVKRHFDDGMLALTKIVQSMKQNVDDQGTTLQRVSDRQQ
ETATELARLATASPVPPAAEGGVRMLPGAGRRLNAGQAGELQSLRRDLAV
LRQTYSSFQSDVQNSMTSIRQKAANVKSAASTATIPDIEGNAGYSYVTNG
RKQLNSDSDRLVGKVDDLQDLIEDLRKDVVHRGVRPLPRQLEEVAKDITN
LTKELKKMEEYMANEKPIWTKIWEKELEDVCQGRDELRLMEDLMVDLNDD
LDKASETFTLVEQATKEQMKDNGTGASASTARQFSRGLSHLGNSLDQSAA
KEDMLGEVRALQPNHENRLEAIERAEKLRQKELEGRRGNAMLREIATYVQ
DGKLKKSGGFEEVERSRKAKDERIRREVWERQNGIIPEDPIGEEELQSEE
QAEQSADGEEFHDAAPTPVTEDAPKEAAETAA
>Mb|QLI66283.1
MAAGNDIASPVAASAQESATTDNDSTGVMVATSPAKNNFFSFFQLLFYAS
PTWLDYLLLVFGTLCACCAGVPFPLMGILFGQVLDNLNDASCDAESSQST
GDIQSEVNKKVLILVYIAIANFIVIYLYIVSWSIFSRRLEARIRDRYFQT
LLRQDATFYDSRQAGELTSRLNSDIQIIQAGTSEKVGICIAVSSFFISSY
VVAFVRDTKLAAILISLIPAFLLMAAITSIFTQKYAARMSDAIASASSIA
QEALSHIAVVQAFGAGPRLEAKFASTMMTAQKEGMKKAVTAAIQAGTLFF
ISYSANALAFWQGSRQIADAVENGGNNVSVGRTYTVIFLLVDACVILGSI
APLLPIIGAATASFQKLKADIDAPSGIDSQSGDGDKLPDTIDGSVEFHDV
SFAYVSRPDRPVLRGVSFSCPAGKHTALVGLSGSGKSTVAGLTARIYDPT
DGIVTLDGHDLKNLNVKNLRSFMSLVQQEPSLLDRSILENIALGILNSPW
PEHQRFQPVILGSGLAEIAAKLAAGKDLASLAGAHGPDMVDLVQLVREAA
SLADASGFIGRFEFGYGTQVGIGGKLVSGGQRQRIALARALIRDPKILIL
DEATASLDSASEHRIQMAIESIAKGRTVIAIAHRLSTIKSADNIIVMNGG
EIIEQGSHLELMALNGSYAGMVRLQNIESLQNDDTPSMASTARGDVDTII
TEKDSVLEEKTADTFQVQDKEQPALNSEGIIPTTSSLDSSKTGWEIIKDL
GRMMRPHLSWFLLAIAAGIIVGASFTGSGLIFGNTVGRLSPCNSPGDIRW
AGKFFGGLFFMLAVIELLANSISWSSFGNISEKLLYKVRVLCFRSLYEQG
LDWHQADGRTPASLLSVITADAAAIGGFSGSIIGNCFSIIVNFVIAIIVS
HILAWKIAIVCLVTVPILLGSGILQLRALTRFERKHADAFSDAIGITVEA
VNSFKTISSLSLENEILGNYRRTLRPPYKAMAAGTAYANIWLAISYGTSN
LIYAFAYWWGSTRITKGEYTSTEFFVILVAMLVSAQLWGNMFTLAPEVSR
AREAGSRILSLIEQGSSKDLAVKEMALSSTQTTAREDIEAAAEGPPKSAS
PNRGATVTFKDVSFAYPARPHIPIVQNMSFTIQAGQFCGLVGPSGAGKST
ILSLVQRMYRPTGGAIEINGSNICAREGTEFRNDIAVVPQDCALFDGTIK
FNVGLGATPNHEATDEEIKEACRLANIHDTIMALPDGYNTECGPNGSRLS
GGQRQRLAIARALVRKPQLLLLDESTSALDAESEKALQEGLERAARGITV
IAITHRLRTVRKADVIFVVEGGTIVEKGRHEELVEKSETYRINALQQMLG
S
>Mb|QLI66284.1
MAARVLMQRRSLAPLATIALGGLAFAPATVLAEGPVDKKKPIYDDLDAPS
ANDAPAPAAPIKALQFSKPPAEEEPKKEKKPRGPSPTDRLAVHIGKARMA
LYRFAVCTENKVNETMDSAFNLEQSFTNTIASLAPSRESGEKLMPGTIYV
LVAAMAGSILTRNRNILLRSTVPLAFGVGAGWTVLPVTMRNISDLSWKYE
QRFPAVAETHVNIREGLRRGISFAKVHSQVGVQYVDDKVTDAREAVEGWV
KQGK
>Mb|QLI66285.1
MALPSGPTIRALRLFGSRGPRTTRRAPRVQHDFRSYATTLESNSKGLVLK

VSEGGKSSPKRLSQLWLRDNCRCVKCVNQDTMQRSFNTFQISDKIAAKSI
HQNQDGVEITWSDSHQSSYSWAWLRSAVYGVENKKLKNLGDQTLWSASIA
SNPPEVSFDSVMAKGDVSGMADLTEKIRGYGICFVTNTPKTPEATNELLE
AIGPIRNTHYGGFYDFVPDLAKADTAYTNLALAAHTDTTYFTEPAGLQAF
HMLSHTPPPNKANDDSSLGGQSLLVDGFYAAYRLKKEYPEAYETLREVRL
PWHASGNDGVAIAPDRAYPVIEASPYKFHRVRWNNDDRGVVPLNVNVDKW
YSAARRWDSILRRKQNEYWFQLEPGRVLIFDNWRVLHGRSAFEGLRRICG
GYINRDDFISRWKTSNFPRSEVIAANMQLK
>Mb|QLI66286.1
MRSIPSRLPIIRIANGTFHRHHPNSQVSHANPSLFNGLSFELPSSSLQPH
NWCVVGPSLSGKTTFLQILRGRLLCEPPTARSYPYLSGEDVSSRLRNPQN
AIRYVGFDAEAIGGGLGGSVTTSAYLSARYESRREITDFPLRDFLLGNTE
LNPLKAPLGEDNEGAVSKELLNRVVTDLRLGHLLDLPVTFLSNGQGRRAR
IARALLTRPEVLLLDEPFMGLDPTTVSGLSPLLQSLAEKATPRLVMSARP
QDPLPEWITHLIYLRNDCQVSSMGPKEVVLDGLRRYVQGVKLGGLLEDDK
LPIHTLSEVGRVLGSNVPCTPTNGSQGSEHELHPANPDAEPLVEMDGCRV
QYGDKIALGNWSQETPQGPKDGLVWTVRRGERWGVFGPNGSGKTTIVSLL
GSDHPQAYSMPIKLFGRSRLPEPGSSTKPLTFWDIQSRIGHSSPEVHQHM
PRGHTIRKVIENAWAATFGARPKLDASARAKVDAALRWFTPELRPNSESN
LRGDDLSWADEYMFGEVSFSAQRVALFIRSMIKSPDVVVLDEAFSGMDDA
VRDKCALFLTDGENKIHAGGEVVRSEVGKRGEVVVEGLTDQQALICIAHV
KEEVPDCVREWMCLPEANTGRRARFGRLDGPLGHDERRWFDVWGFSA
>Mb|QLI66288.1
MSSNTPMEDAIRAKITAAFDPQTLEIYNNSHLHSHHKAMQGVTSKETHFR
LVITSEAFKSKVQPARHRMVYALLRDEMALEGGIHALELRTLTPEEEERQ
NKKKAAEAAAKEEESKEA
>Mb|QLI66289.1
MDRPEPGLVKWSPNSSFDSFVHINLQHRVVQLYEPTGHANKRQFDFSKLS
RHDDFPPLTTYDWSPADTSLLAVGTSSGIINLLKIDDNSNAYAEIGLKMA
RTCQAVAFNTTGLLAVGLDRVRMDQSLHVWDVSRLSAQELPKPGFPNAAS
TFVDSYTRVEPSVSVSSIKFFEDSPLTLVAGIKGQGVRICDLRDPSGNIT
FQTKCNNNLAIDYVDQHFFASSALDHPGVMIWDRRATSRPLASHSYMQAV
EEDELPWGGALRLDKVIETDSDPFLTEGKHSLVRSLRYCRDRRGLLAALS
PTGQLKVMETNKEIMSIGMAAHEGPELLQVQRSHEMDVSYRDNSRRNDRI
VSFDWVTLDSPALRPRLLVLRANGAFDILEQPSRTSDHVYKLVPWQAPHR
GLEENSPYLDLMKFEPAQTSEILGPLINEQVLSDVPIFGPEKADIADRIS
DALKDSVSVSATIEHVDEISRPLPDSFFNATSITQKLKSIRAYVRDEQAD
QKAEDGQQEQGNGPKHLDNRPNEISLASNSLVSCREIHEALLGTLATVKG
LPREAQCVVDHSMLLRAKEKYLFDAATNRTVLSDDSWRRFLWDWVADAEM
AADDGGMLLGNMDLSYLGVHSIWTNDLGKNSMARLAPGAAFPEPAQWERV
IGQFCKKRRLPKFDAVPTKKPFHRQLCLEICSWGDPQRHDPNGVDREADP
EYPTAIHTMVASRALFRGDTEQAISILKKASAVHPELLFVSLSLQLLGRG
GKNLPEEHLELDDAVASKTDPYLRAISSLIATGDWSAIANQRSLPLTDRA
IVAVRNFDDSQLTEWLEQNVSLAIEEGDIEGIALTGITDKLVDIFARYVQ
KFHDVQTATLVLAIAYPRYIDDIRCRAWRNAYRAHLQRHRLFFQRTKFEV
ESTKRSKRDGVPTLKPPSRQIALRCVYCDAEASLSNQGVAPPTSNPTLET
RNPLLATSINSGISCPNCGRHLPRCVVCLEVVGVPRSDKPEEKESTRMAG
RFPTFCLRCEHVLHLDHARQWFARHVECPVPECRCTCNFRANPELSYH
>Mb|QLI66291.1
MASVTSLDKDLRKLRLDKYTPEAANEARSWIEEALGEKLPSQDLLEGLKD
GVALCKLVNLATPPPGTKFKKSPMPFVQMENISHFLRACQSPPLNLQQHD
TFLTVDLYEQKDPAQVLQCLGAFSRAAHDAHPGRFPDPIGSRARAAGVIS
PQISGYASPTGLRGRGTSITSNSSSAYGSKPVLPRRTAGGDTTPTGRRSP
TKSPTMTNPASPGPVSSWSKREHEGSTAPAWNIAQYGYMGGASQGNLGIS
FGGMRQITSAGPHVPSLADKERKRREEAERQEEEERQRRAQQETEEEEAR
REEERRWEAETQRLREEERRKAAEEKRRWEEQERQWRLTEEMRRKEEQEA
EAKLEEERKRARGRGDTRLQGQFLSQYQAEQAEADRHNNRSQKLEEELER
ARQREAEYERERQTRSTSRPAGEIVPNKARSRSRHRGTSTAPSTPSAVGP

QRSQNRYESWTKDDERDYLQSQWSEHQKGSRESSLEPQRQESVDVEVPPP
LTTTPRPLPEPNVTPKPKPKPKPQSTQSPNPISSSSRPLPDPKKYAAPAS
ASSQNRTDRFLATNTPPSQTKPHQTYSRELGATEERDGEDHRRVQSQAQT
KAAGWASKSLLEREMEMERQRQQEWEDAQKETAKAVRSEGGVDGIGGGIG
GRWDVGQWSGYTGGDSQNRGAQGIGSGRRQIVGPRPLPGSGR
>Mb|QLI66294.1
MRKFDNCVLLTRVGSFYELYFEHADEYAPLLNLKLASKKTNAGPVPMAGF
PFFQLDRFLKILVQDLNRHVAIAEEFPNDAADKVKSGGLMHDRRVARIVT
PGTLIDENFIDPYANNYVLAIHLAGSKEAPELKSAMVEDHGIRVSPPASP
MLLGLAWLDLSTGHFFTQSADLASLPSILSRIGPREVVLDENIESQKDHD
IFSILAEDRHLITYSPKGGLTDIGDWTPLLESPVPESARKTFTSEEVLAG
GLVLNYVKGRLQGMTVRLLPPTRHESMQIMNIDKNSLRALEIKQTIRDGL
FRGSLLHGIRRTVTKSGARLLNEWLSSPSTSLHVINTRQNLVARFIAAEH
LRDSIIMLLRRSQDSQRLVQKFSLGRGDADDLLALAHTIRATEDIVILLQ
EAAASGSDAAQNECLSGLLSRIDLTQPLKLARKIKGAIDEEGIVHQHEVE
DSEASQMLALAQDIVATEGSQEDASSLHKSKKKRPTSIKEYYAEDNDAWI
MKPGASTNLKRLHAQLSSLLDEKIELSDSLREKLGASSLTLRWSTSLGHI
AHIKGKDTKNLTNIRALSSSRSTTSFHLSEWTTLGQRLDQARFHIRREEQ
KVFQSLREQVVRNLVKLRGNAAVLDELDILTSFARLAVEQNLVRPKLNNS
TTHTIIGGRHPTVEGGLVEQGRSFVRNDCLVGSQPDGLLWLITGPNMAGK
STFLRQNALITILAQIGCYVPASYAELGIVDAIFSRVGSADNLFRDQSTF
MVEMLETAHILKQATPRSFVIMDEIGRGTTPEDGTAVSYACLHHLVTVNR
CRTLFATHFHGVADLAASDGLCTVDGGGPVQLYCTDVEEDDQGGFVYVHK
LRKGINRRSHALKVARLAGLPERAMDTATQVLEAQGAPSTFSTCAASGAI
>Mb|QLI66295.1
MAIRDRFRRALRKSDSSDTISPTNSNTTTATTSETSSLHKSTSSKFARTF
AFSRRSKDESHEESKRNKKNKKTHPSQRPLTLQNLKHQEMLSHFSMTFGA
SDPSQIESASFIGVSPCCTRAPSLEIDRAEISHSLTGSPNPDSVRD
>Mb|QLI66297.1
MPIRLRMRPQPVRDLSPMSARYDFDMPDIAEDDSFRDVVKKLSLYFTDVI
ELPSTFEQLRTTEAGNCVRVLVDHLGQNCTNPAIVNALLALKWHYAAANE
HLSLRETRSFACEITAWRFLTRLSERQAVDFCLYEIPVAPEHEHRSQQAS
HDQEAGETSPLLIRRNSSDEGLPTIVVGSARERTKMLSSLSRLTHLGDES
DDEADPTSAFKSLNALEIAAIANAKRFLSQHVVQKIITGIWNGDIVFWDT
LSVNSVKKPRYYNPHTSDPFSRLRVPKYLKTWEVLFFVVFLCLYYSVLIT
RDDTRITLTEVFLFIWIAAFFYDELSEWIDAGSIFYATDIWNAFDMIMIL
IGFLFAGLRVIGLAKHNAAMNEMAFNVLSLEALFMMPRICSILSLSPYWG
TLIPCLKEMGKDFVKFMVLVVIVYFGFLTTFSLIGRDAFSFGRMTGILTK
IFFGSSYVGFDIMDQIDPIFGPPLMIIFITLTSILLQGSLTGMLSNSFSR
VITQAREEYLYVYSVYVLEASTSNRLTHFYPPFNLIALVIFRPLRFILPR
VGKFRAGRIVLLRATHLPIVGAIRLYEYIRRNSQDNDEYAGFRGPRESKK
SSRPAVHKRSSSGYIRQHVSLSQESTLLKADGKRRDGDGDDMEEPKGVEG
QVAELHQKIDQLTSLVIAMQTKSAGDDTPPAGT
>Mb|QLI66299.1
MPLDTSTYSLALLRVDGRRWNELRRLHAQIRTQDAADGSSYFEMGHTKVM
CVVTGPSEQQQAQAQAQRRGGQAPGRDAASIIVNVVIAGFSSVDRKKRAR
SDKRTQEIEITIAKALSSTVHTHLFPHSSITVSLHVLSQDGSLLAALINA
ATLAVIDAGIPMTDYIAACTAGSTSSYAAGDDSADPLLDLNNQEEQELPF
LTVASLGDSDRVVALVCESRVQVSRLEGMLVVGLDGCKQVKMLLDKAIKD
RGVKMIREGAVQRSDTVAMELDE
>Mb|QLI66300.1
MLYNSIVAATALLGLAAAQVHTDCNPMERDCPANPAFGTDHNFNFNSTPS
SDLWETTAGTVSYDAKTGAAFTINKQGDSPTLRTKFYFFFGRTEIHLKVA
PGRGIVSSMMWLSDDLDEVDWEFLGSNKTFATTNYFGKGRQDFKNGGSHP
MTGMQDDYHNYTTVWTKDSIEWFIDGNHVRTLNSKDANNTHNYPQTPMRM
SVGIWAGGDPSLPEGTRLWAGGDTDYANGPYTMYLKSAQVTDYSSGKEYT
YGDRSGSWESIKIESGNSTAVDALNKQPDKSTSEKWNDLPTGAKAGVYAG
GGAVGALALGALLWYYIRQRRIGAAEAKAAALRDEEDHRENARFLKDGIN

PDGFTDHGQEYNARELRAGGMASENSYHVPESSPFDGPFDEKSRLGSSAS
NVSTLVGAAGAGAVAGGAMGAAGAMRGQPGRKASPASSNHGFDFGVPPSP
GHPSRSPSPGMPPGSPRQHQMRSGSAPDPYSRMGSPGPHQQGYGLQRMQS
PGAMGPQRSFTDNQQPGYGYRGPASSGGNNGW
>Mb|QLI66302.1
MRRACLVLGLASLAEVAVAGLYPGITPDNHTCALVEPVLSCSCKAKLDKV
EDTCCVETFGGLLVATQLWSTFTGLEDKGQIYPKNDWSIHGLWPDFCNGS
YTQYCDLSRQYDPKPSPNTTNGQPGGIPVPAYKGEPIETWFGPYGKLDLL
AYMNKYWVSQLDPNWVFWAHEFSKHATCFSTFQKECYGPKAAEHDDLFDF
FETVVTWQRRLPSFRWLSDAGIRPSNRTSYTLSDIRHALTKEFGQQPFIG
CGGPKYNETEAGKGSKDNGRTEINELWYFYHVNGTPQRGDAEKLDAGKAG
GRLTTCAQAKGAIKYYERTKGSEE
>Mb|QLI66303.1
MSSLEAKIVVLGAQGVGKTSLVMRYCKGAFNPSQITSTVGASFLTKRVVD
SDTDTMVRLQIWDTAGQERFRSISRLYYRGANACILCYSITDAQSFAEMG
VWLTELRRNLPNDVVLHVVGTKADIVARDPSARQVPFERCIAYVAENLAP
GLGSTPPPTATPLNAPGSMPSVEPRTPSSKRSSGFWAQEVGWDACHEISA
ESGEGVEEVFRVVARKLVEQNRKMQQALLLATATPGTPGYEAGMDGGYFD
GMNVRGSFRVGRDRRSWLFSPAFSPAVTVDRTGATAQEQNEPDGASKGSR
RCC
>Mb|QLI66305.1
MGVFSFFRKVYDIDTLDTRFMSSSSVPYQTVIEARSDPDAVKEAADKARA
RAPPPKWQTPEFYLYYFVFLWAVPYMFWVAYSVSRPSDPRYHKYERFLSD
GWIPGRKVDISDAQYHTFRGNLPYLAALLIFHPLLRKVWESVYTGANGNR
GPKGSSRLEQRALFDFTFAILFLVILHGISAFKVLTILYINFQIGRKLPR
KYVPIATWVFNISTLFANELCEGYHLRNVASHISPPVKSASTGQLTDSPL
MQTGAWLDGFGGIMPRWEVLFNITILRLISYNMDYYWSIDKKASSSLEKK
QLDPANLSERDRITISAEPADYSFRNYIAYAIYAPLYLAGPILTFNDYIS
QLKFKAASIEKPRTIRYGVRFLLVLLAMELVLHFDYVGAISLANPVWGEY
SAAQLSLLSFFNLHIIWLKLLLPWRLFRLWALVDGIDPPENMVRCVSNNY
STQLFWRAWHRSYNRWLIRYLYVPLGGASFRNWAAAAQSTVTYLCVFTFV
ALWHDIQLRLLIWGWLIVLFMVPEWTAAFLFPKRKWESRPTEYRMLCCVG
AVANVLMMMAANLVGFAVGLDGLQSIVAGILHEWSGVIFLTVACACLFVG
IQVMFEIRESEKRKGATLKC
>Mb|QLI66306.1
MASLASAARDIPQPSVNRDNLTVVAARSDSATVARSHTLPTPPNSISPAL
PAHGLKAQLQKAKLEPIDSDLDLHDNASSHGRSLSPAALESAGAITAPML
AKYHLPEILLNHGPLAIRHIMGYLTTSVPGFSGIPPTKARRLVVGALEGR
GGEGGGVEGDVEYEKVGWGRWDAKRRGMASRERQGTPPSSYGSAIPISKA
GGRGARGSMADEAFHFSHDDNDMHIMMETEADKMSMDGSASASCSEAPDD
DVVMNDDPEDVTDDEDWAAMGAAALRAESYPNMAAAQSNGSMRSPNFPPI
GGLRTFSSSSGMARPPQPTHLDLSALACASDAQEREAVEALLRLGSV
>Mb|QLI66307.1
MALDSAPHPTDAAISHLWHFIETRGLDLDVGSSQDPRQGSARNDSSGGGT
LSQTAGSKGSSLGKLGTTFIPVSVFTAVCLVIFILLRRFCKRVYAPRTIP
ELRSPDVPSPPLPGGWLNWIVPFFKTPDTIVLNHGSLDGFFFLRFLKVLR
NICLAGCLITFPVLFPIHATGGSGLTQLEMLTIGNVKDPQKLLAHVFVAW
AFFGFVLYMIVRECIYYVNLRQAYLSSPYYADRISSRTILLTCVPKEYLD
ERRLRKLYGDSVRRVFIPRTSKALVKMVKEREQTAERLEKAEIALIRIAN
LARQKQLAKDSKLAEPSTAAASITESTGSKQDLVVVPSDSTANDTCRGDL
DEAESPGSIIRLESLRRNPDKDNDDTSQHEGIKLNSIMADENEKLDQDDG
EYEHPYGLSMDLPDVRGSVAAQWIPVQRRPYHRPIGNFGRRVDTIRWTRT
RLRDLNLQIFKMRRQVRRGDGITLPSVFIEFYTQEAAQAAHQVLTHHRPL
QMSSRLLGIRPDEIIWSCLRMPWWELIIRRFGVLTLVTAAIIFWAVPSAF
IGTISNIEGLTQKLTFLSFLNKLPSVILNFIQSFMPAVVLSLWMAAVPWM
LRFCGAQSGIPTITRVELFVQNVYFAFQVVQVFLITTLTSAASSALGKIL
SNPLGAKDLLAENIPKASNFYLSYIMIQCLMSGGMRLIQVFGLIRHYIVS
RVSAVPRTRYKRWCKLDSPYWGGVYPIYTNMGVIALSYSCIAPLVLLFAA

GGLFATQVVWKYNLIYVLDSDMDTKGLFYPRALIHLTIGLYLAEICLIGL
FALKGAFAPLALMVLFFIFTGLVHFSLSDAIAPLLLNLPQTLPLEGEIQE
EEKAKALREKELATARLEGEDGATGAANDYYDSEQVFGDEEQLEQSDTEE
EEEEEDSGDEHGPITGTRAVEGAASIGSTLKEWAKATTTAKVNKEINSPG
VQEFLAKIQFWKHDKHANGEPPGFLARWLHPEEYEDFVALRNTIPGDQLR
HVEYPSEFRKRCYLPPEMWAPKPTLWVPRDEARVSRQEVAHTKKYTPISD
KGAELDEKGGVVVYFDKAPIKKERMLL
>Mb|QLI66309.1
MLLSEDPATLISHTVNNFNIGPDKLAISRIGESLSTLQQARELRMRDAES
SLKKLARQLNTLSSQHAELTSSHSATDHASKIATLDTRKFRTAKAASDAE
MEAERLAQQAADLSARLQELDLQGVEGDENARRRDAVDDEVLLRLKVYRS
LGIDIERDEKDGEWTRAVVRNDGRGDVHVVNMDKKFSRYFYANYFWQTL
>Mb|QLI66311.1
MATSNPTVVDIHTHMYPPQYIKILESRTTIPLVRKFPQAPEPRLILLEAE
LGDLEKAINDPSAKPPGRPLTSHFASLDQKIHFMDTHRIDISVISLANPW
LDFVHASHAGDIAESVNVEFSDMCGKHHGRLFFFGTLPLTAPLETLLASI
SHLAALKYCRGVILGTSGLGKGLDDPHLIPIFEALAAANLTIFLHPHYGL
PNDVWGPRAGEYGHVLPLALGFPMETTIAVARMYLAGVFDQVRDLRMVLA
HSGGTLPFLAGRIESCIFHDGQLVREGKVGENRRTVWDVLKEQVYLDAVI
YSEVGLKAAIDASGSDRLMFGTDHPFFPPITTDEQGQWESVSLNARAVAE
AVGEGSAEAKAIMGSNAIRVLRLEEKA
>Mb|QLI66315.1
MGITFSKRDAATYSAAKVGGKLLQTRTNGKSVLGTLVAPLLPMFLENNPL
PNGSPWSLLNHTTDYYEHFPRTGVIRSYDFTVSRGVIAPDGYQRSVLLVN
GAFPGPLIEANWGDTIQVTLHNNISSAEEGVALHWHGFLQHGKPWEDGVP
GVTQCPIAPGKSYTYSFEAELYGTTWYHSHYSAQYAGGIVGPMIIYGPRT
ARYDVDLGPVMLSDWYHRDYYALVEETMKPNGRPVRSDTNMINGKANFDC
SKLPAGDKTPCHSNAGIARFKFTRGKTHRLRLINSGCEALQRFTIDGHTM
TVIANDFVPVEPYDTKVVTLGIGQRTDVLVAADAPPDAYWMRSNISEACS
LAGERHAYAAIYYDGADEARQPQSQPWDVPDPLTCANDDLALTKPVMRRR
PIAPDLTYDMEVGLFKNASNITLWSFGGVDFRGDYNRPTLLLSALGNHTF
EKQWNVKNTQGAKSVRVHVINNTPVAHPMHLHGFNMYVLHEGEGPWDGTI
VNRDNPQRRDVVQVRKHGHLVIQFDAADNPGVWPFHCHIAWHVSAGFFAQ
FLTNPDQVARYRHTMPHVVAETCRQWGEWTNTNIPDQIDSGL
>Mb|QLI66316.1
MRSALGLPTRWAAILCLLAALFCLIQPSASVKEHDFKKCDQSGFCKRNRA
YADNANTHSSTWSSPYEVLPETAKFKDGQWQAVILKTINNGEKAALPITV
SLLKSGVARISIDEEKRQKKEIELRHNSKARKERYNEAEDWVIVGGLELD
KQAQVAFQDKSQANIKYGGDDKFEVVIKFSPFEVDFKRDDESHIKFNDRG
LFNMEHWRPKIEKEKTEGDDSKAEENAKTDDGEDESTWWDESFGGNTDSK
PRGPESVAMDISFVGYEQVFGIPEHTGSISLKQTRGGDGNHHEPYRLYNT
DVFEYILDSPMTLYGAIPFMQAHRKDSSVGVFWLNAAETWIDIVKGKDHK
NPLSLGKGARTSTHTHWISEAGILDVFVFLGPTPRDLTKKYGELTGYTAM
PQEFSVGYHQCRWNYISDDDVKDVDRRMDKYKIPYDVIWLDIEYTEEKKY
FTWDPHSFADPVGMGQQLDTHGRKLVVIIDPHIKKVDNYAVSSDMLSKDF
AVHNKDDETYEGWCWPGSSNWVDCFNPKANEWWKTLFKYDSFKGTLENTF
LWNDMNEPSVFNGPETTMPKDNIHFGNWEHRDIHNLNGLTFHNASWEAMA
SRKKGEMRRPFILTRSFYSGSQRLGAMWTGDNQANWEHLAASIPMVLNQG
ISGFPFAGADVGGFFGNPDKDLMARWYQAGIFYPFMRAHAHIDSRRREPY
MLGEPYTEILTKALRLRYALLPSWYTAFFHANRDGSPIVRPMVWTHPSEE
SGFAIDDQLFLGTTGLLAKPIVEKDKFSTDIWIPDDEIYFDYTTYQILKT
QKNKRVTVDAAIDSVPLLMRGGHIFPRRDIPRRSSAAMRFDDYTLVVTVS
KDGSAEGDLYADDGDTFDHEKGQYIYRKFSLADGTLSSVDAEGRSAKSVK
AGGWLKAMSQVHVDRIIIVGAPASLNVKEVQIASEGKTWAVKVDYHSAEK
GRAAFAVLGRVGAKISEDWSIKI
>Mb|QLI66317.1
MTYRGTLPTGQPGFPGQQQQQDHHQMYGSPMGGYQHQGHFQQPQSQPPHQ
QNHQGLYVNGIGDQFYGQQHQNQFVQQQSQYAVHQQEMLRMQMARQQQQQ

QQQQQQQQQSAARFMGGGGGGSGGGYHDYQAMMRQTQHMHQQHSWPQPVS
QQQQYAGHPSPRPSLTGMAQTPQHAQHMMQSQQSQASDHMLQRMYQQQQM
QQQQATQQILRSPQIHQQRVSPQPRGSPLLPSQMQRKHSLQGQTGQQAMQ
SPIQQQLQHQFQSPPPPRMKSSTPVQNQMLLQQPPTQSQTPPQPHHQRLQ
PTPTPPPPPQQQPAPEPVQEVQEPPPIPVPEPEPEPEPEPEPAPAPILDV
PVDEPPLPPAIQEPINFVNPLDIFNAPPPPPLPPLPMGDPNSWGKKEPDP
EPVEDSITVKPSELTTTVKSSPVQESITVHTAVKPEMKPEIKQEPKYEPM
RIKSSPHSSVTNSPALSTKSPSITKRSPAPKPRVNTIPIMVAIAEECLEK
ARSSVHDVAMSLDPEKVDEYQKLIATSLSCLEAVMQNNKLAPREEARLRM
RYAAILHEETENLMEAETALGKGISLCDKHRLIDLKNCMQYLMLKVLFQR
NHKAALKAVDGHISDCEAFKHVQWYYAFRLLKSTFYMAIGSASDAAALEN
IRAVQNVATSRGDNAMTVFASILEGLALLKTSKDSNIERVQSCIAQVAKF
QFDPTVKIMQLEMLTLVLDFASSLYRQSPDTTAQKLRLLQQKLDACEEWN
NVKADFLIPVKKQPSNTRPVSEETSGIIRLAGEGDGDVDFIVMSFMTKVE
LRSLVFTFSGLANMHKPSSQGRRSTEFWLEGAKILDTWDSSTSGIPYGPS
VSLSTAIRQRSWRTEAQAYLNVLLGLLAASHCQWGKVKQFMEKLETLITP
STQLTIGLLSTYLTGVYHQGTGNLEAALAIFLEGRFDIAQTSGSSKAGQQ
EIALLAGLNRLWIMQHPSCRNDYQTHELIEQLQPFCSNHWNIDLRTAWHN
VVAALETDPPQQLNQQKQHIQAAMSGSKVTNNILGAAVTLCIMRSRFFEN
VIGEQALKSARAASKQAQRSGNMLWQSVADGMLAQSYEVQGHRDESIQEW
EKATREAKDAFAGSW
>Mb|QLI66321.1
MSSAIDGSLRRGSQNEDPKEIYDAISHELSQIYHPYIDIELLPKGFETEL
KNTYFVKEEHCLAIPKLRLVQAFTAARQILISHLNKPGSISTDDVRKATS
VMLLMDSEHLTAANTRKRLLLRQTLAACERKAELGREMYFVNSLLSSHLH
RHTKSPVLWSHKHWLLRQYLEAKVPIDLMHDLESVVFVAAERHPKNYYAW
TYARDLFVSRAKVKPGWDAEQDEELMRMTAKWCRLHHDDISGWSFLLHLA
LGSPQHAQEIFRQTARLVQTYTWRGESVWNFLRTLVLVPAMRDSSEVRQS
FVDLWHHVRQDIRDAQSLDAKVLDRAAAWAEIPRP
>Mb|QLI66322.1
MSFAEVLKQVKSLSYETIIFDTAPTGHTLRFLQFPSVLEKALAKVSQLSS
QYGPLLNGVLGSGGALPNGQNLNEMMEKLESLRETISEVNTQFKDADLTT
FVCVCIAEFLSLYETERMIQELSSYGIDTHCIVVNQLLFPKKASECEQCN
ARRKMQKKYLDQYEELYAEDFNVVKMPLLVEEVRGKDKLEKFSELLMKQY
IPPE
>Mb|QLI66323.1
MHLSTTLLASAMTMGVSAATNNILGFNSGATFADRSAKFKKDFEAEMKGA
QGLKGAPGTFNTVRLYTNIQAYSQDDPIQAFEAAMETKTQLLLGIWASGT
DNIDKEISALQKAVKTYGKEFTDLVVGISVGSEDLYRDSQTGSKNKAGVG
NSPDSILKFIGDYKKAFKDSALSKVPVGHVDTWDAWTNSSVKPVIDAVDW
VGVDEYPFYENGKGNSIENAPYLFEKAYGATVNAINGKPIWVTETGWPTT
GPDWDEAKASVENAKYYWDEVGCRSLFNKVPTFWYTLRDSNPDNKVKFAI
AKDLTTPLFDLTCPKTFKTKPKSSSVVSSATGTATGTATGTATATGAVNG
TATATGASRPTSTGGSGNPGSDGSNGSNGSGNNGGSNNGGSNTGGGSGTT
SGATASSTVVKGASSTVEKFSGAAYFVLAVLAGSLTLL
>Mb|QLI66324.1
MSANSIKVVARFRPQNRIELESGGKPIVTFSSEDSCSLDSKEAQGGFTFD
RVFDMACKQQDIFDFSIRPTVDDILNGYNGTVFAYGQTGAGKSYTMMGTN
IDDDDGRGVIPRIVEQIFASIMSSPSTIEYTVRVSYMEIYMERIRDLLAP
QNDNLPVHEEKNRGVYVKGLLEIYVSSVQEVYEVMRRGGNARAVAATNMN
QESSRSHSIFVITITQKNVETGSAKSGQLFLVDLAGSEKVGKTGASGQTL
EEAKKINKSLSALGMVINALTDGKSSHIPYRDSKLTRILQESLGGNSRTT
LIINCSPSSYNDAETLSTLRFGTRAKSIKNKAKVNAELSPAELKLLLKKA
QGQVTNFENYISTLEGEVQLWRSGESVPKDKWAQPGDGIVGARTEPRTPR
PSTPSRLTAESRAETPAATDRASTPSLTLEKDEREEFLRRENELQDQLAE
KESQVTLAEKTLRDTKEEMAYLKEHDSRLGKENERLTTETNEFKMQLERL
TFESKEAQITMDALKEANSELTTELDDVKQQLLDIKMNAKETGAAFDEKE
KRKAEKMAKMMAGFDLGSDVFSDNERSISEAIQHIEKLHEISSVGNNIAP

DEFRDLRTKLVETQGIVRQAELSMFGSSSGEMDSRRRHELEERLEAMQQQ
YEDLLTKNLSESDVEEVKARLEHAYANRQTTQVELIDELKADAAQKAAEN
SRMKTLIEDLQQQVKAGSGATPVTNGKTIQQQIAEFDVMKKSLMRDLQNR
CERVVELEISLDETREQYNNVLRSSNNRAQQKKMAFLERNLEQLTQVQRQ
LVEQNSALKKEVAIAERKLIARNERIQSLESLLQDSQEKMAAANHKFEVQ
LASVKERLEAAKAGSTKGLNATTGGFSFSAAGSRIAKPLRGGGGDGPNVP
TIQSLQNEGATNKRSSWFFNKS
>Mb|QLI66326.1
MPESAPAAAPASAISRPVSEALLNEKWDRCLSNLLVKSTLGLGFGVVFSV
LLFRRRAWPAFVGAGFGAGRAYEECNINLKLAARDLKKQQQP
>Mb|QLI66327.1
MPFAQLVLGSPGSGKSTYCDGMHQFMGAIGRACSVVNLDPANDHTNYPCA
LDIRSLIKLEDIMKEDRLGPNGGILYALEELEHNFEWLEEGLKELGDDYI
LFDCPGQVELYTHHTSLRNIFYKLQKSKFRFVCVHLSDSICVTQPSLYVS
NVLLSLRAMIQMDMPHVNVLSKIDKVSAYDELPFNLEFYTDVDDLNYLTP
YLESESPALRSERFAGLNEAIANMIESYGLVRYEVLAVENKKSMMHLLRV
IDRAGGYVFGGAEGANDTIWAVTMRSESSMLDVQNIQERWIDQKEEYDKM
ERDAEEEQARLAEEQAME
>Mb|QLI66330.1
MPGQSRKVVCVVQACISSGGNIRCSPRSTHVRCLSTTAPHQTSRRTPDFN
SGFTGSYDPTAELGRGPMFNKANFGVPQFYPRDLKRRVDEYVVGQERAKR
TICSTLFNHYQKLRRRHQHEHEQRNFQEKLMRQRFARDRELHQKRRDIHP
VEDEFPGHNESVRQLHDATEFEENPLDHLYVPEDPSAPEHVKIDKSNLLL
IGPTGVGKTYILETLSKQIHVPFSICDCNSLTQAGYIGQDVETCIERLLI
EANYDIKATEYGIVVLDEFDKLARRESPTGRDVGGEGVQQALLKLVEGTK
ITINVKDNRSSRSTPPITTNYSSGGSSSSAPQAVPPGGKVDQYTIDTSNI
LFVFCGAFVGLDKAVLQRVAKPSMGFGGELRGRSTLSGSKHILPSSVYAH
IAHHDAMPEASFTPLDLATPADLQSFGFIPELIGRLHNICALSHLSTSDL
LRVLTEPRNSLVAQYTALFETYPSRLRFTEKALYAIAERAAAIGTGARGL
KMEMERILAEPMFDAPMPYVLITEDCVKGSGKATYWGKDGRFEIDRRLEE
ESMSRQDTIPQVTFERMREAGQSGG
>Mb|QLI66331.1
MADFHPELQAKLDDLQRELQEGDITEKGYQKKRTLLLSQYGHGESSAPAL
APTIHEPPPRSGLRTKQPDDNPPHSSYDGHRASAYNAVRENGSDPGSPIY
RPSSGYGTPSTTSQAAETTPAALLRPGGPVAERSVPLQRDSLFITAATAS
SDDPSRSGTMVSRDYAFNPDQQGAYMDAGHQQQHHHLHPQQQFDGRTRTL
QDSQGYFSDFAGQQHYDQTPSGGEYGAPHRYSSGEAFSPTAAMAPPMLTA
NDLPPPEILEYLQPLEPREVPFAIHDPHDANITMSRFDNLAAVLRHRART
TAKVPAYWVLDSKGKEIASITWDKLASRAEKVAQVIRDKSSLYRGDRVAL
VYRDSEIIDFAIALMGCFIAGVVAVPINDLQDYQRLNYILTSTQAHLALT
TDNNLKTFQRDITTQKLTWPKGVEWWKTNEFGSYHPKKKEDVPALSVPDL
AYIEFSRAPTGDLRGVVMSHRTIMHQMACLSAILSTIPGSGPGDTFNQSL
RDKNGLLIGNRKAGSEILLSYLEPRQGIGMIVGVLLTVYGGHTTVWFDNK
AVDVPGLYAHLISRYKATVMVSDYPGLKRAAYNYQQDPMTTRNFKKGMEP
NFQAVKLCLIDTLTVDGEFHEVLADRWLRPLRNARARQVVAPMLCLPEHG
GMVVSVRDWLGGEERMGVPLKLEHETTSESDSDDGGETAKPVPPNGFGSL
LGGGTTTTTEKNAGNTEMNEVLLDREALKTNEVVVMAYGDEVGKKAPNEP
GMVRVGTFGYPIPDATLAVVDPETGLLASPHSVGEIWVDSPSLSGGFWAQ
PKNTELIFHARPYKFEPGEPTPTPVEPEFLRTGLLGTVIEGKIFILGLYE
DRIRQRVEWVEHGHEIAEYRYFFVQHMVVSIVKNVPKIYDCSAFDVFVND
EHLPVVVVESAAASTAPLTSGGPPRQPDTALLDSLAERCMEVLMEEHHLR
LYCVMITAPNSLPRVIKNGRREIGNMLCRREYDLGNLPCVHVKFGVEHAV
LNLPIGVDPIGGIWSQIASESRANILAPADKQYSGIDRREVVIDDRTSTP
LNNFSCITDLIQWRVARQPEELSYCTIDGRGREGKGITWRKFDQRVAAVA
MYLKNKVKVRPGDHAVLMYTHSEEFVFAVHACINLGAVVIPMAPLDQNRL
NEDVPAFLHIVADYNVKAVLVNHEVDHLIKLKPVSSHIKQSAQILKIAIP
NVYNTTKPPKQNNGLRDLGLTIDPAWIRPGYPVVIWTYWTPDQRRIAVQL
GHDTLMGMCKVQKETCQMTSSRPVLGCVRSTTGLGFVHTCLMGIYIGTPT

YLLSPVEFAQNPMSLFVTLSRYKIKDTYATPQMLDHAMAAMHAKGFTLHE
LKNMMITSDSRPRVDVFQKVRMHFAPSGLDRTAINTVYSHVLNPMIASRS
YMCIEPIEIWLDTKALRRGLVVPVDPEAEPKALLVQDSGMVPVSTQIAIV
NPENRMHCYDGEYGEIWVDSEACVKSFYGSKDAFDAERFDGRTVDGDPGV
AYVRTGDLGFLHNVSRPIGPGGALVDMQVLFVLGSIGETFEINGLSHFPM
DIEYSVERSHRNIVPGGCAVFQAGGLVVVLVEVSRKPYLASIVPVIVNAI
LNEHQIIVDIVAFVSKGDFPRSRLGEKQRGKILAGWVTRKLRTLAQFAIR
DLDVGAIGESGSGSGPGDLADAHRMSTGSIRSSGVHAGASSLRNVEHAPQ
ILEQEELDVQMNRMAHMPPASAPGTASTVTHDDQVTPTGFAQGVSSDTLS
QGTLSRNSASQPGYFDDAQRNSEAAYNDDQAPAVGPKPDSSRSQDHPNAA
KKRMVLPGVDGREGFQFWDSPPGEDDETDWTAEAIMHMNLAGDMSSR
>Mb|QLI66333.1
MGKKKRGHPDIEEVLVRPWCYYCERDFEDLKLLISHQKAKHFKCDRCGRR
LNTAGGLSVHLNQVHKETLSQVENALPNRQGLEVEIFGMEGIPQDALEQH
RSRIIQNFYQAQEDRRIATGNPASGQAMPRKKVRVEASEDLLKRLAEFRA
QKKAGTLPPAAEERTNTPPTEQGNQQQTAAQPTYPGQQAYAYPADSLPAR
PPSHALGGTVLPQRPPQAGYWQPQGGAAGGVGDDVDQLIRMAEAGVKPQQ
AQEADDAGDRKAKKEKKGRMIYDDVEFSPEERMAALPRYAFVPQVGA
>Mb|QLI66336.1
MNSNLFTKAAAPARVIAHAKFGGQPKIRQCLNKGAKLHTAAFRTSPREKC
TRVKRNIIPSTKRFFHSTDSLSQKDPYKALGVSKSASASEIKKAYYGLAK
KFHPDTNKDPNAKEKFADVQSAYEILSDPKKKEQYDQFGAAGFDPSGAPG
GDPFGGAGNPFSGFGGQGGFGSQGGFGGGFNFEDIFSAFTGQQGPFSGRR
GARSNPFQQEILVGDNIEVQASITFMEAAKGTSKTISITPLTACGTCTGS
GLKAGTQRSPCKSCNGTGTRLHFMQGGFQMASTCGTCEGTGTTIPKGSEC
RTCSGNGVVRERKTITIDIPAGIEDGMRLRVDGAGDAPPTGKTADPNART
QRGDLYVFVRVAKDPKFSREGSNILYTANIPLTTALLGGQVNIPTLDGSV
NVKVATGTNTGDKMTLTGMGMKRLGARRGGAGDLRVEFRVNMPKYLSANQ
RTIVEMLADEMGDKTARRVMNVSSTSNNDASNPDSHKNEGFLKSMWHTLT
NHPAHQKEGDEKSSADKPDAKKDSKPDNKDNKPKDEPKKSDSGPA
>Mb|QLI66338.1
MSGSFEKSVKGATKIKNAPPKTKYIEHILVATHSGDAGVGEVFRSLQYRL
RDSTWTVVLKSLLTTHIMMREGEKNATLSFLAKHRNILTVGHFADAQTQG
RNIRHYANYLTERARAFRDTKIDWVRENDSRLEKLAVDKGLLRETEVVEN
QLSALVKCDVLETEPENEITIAIFRLLVLDLLSLFQVLNQGLINVLGHFF
EMSKTDAQRALEIYRTFTRVTDHVVQYLSAARMHEHHTRVEVPKLKHAPV
TLARQLEDYLKDPDFEVHRRQYLAEQDAKKTGGSSKAAKVDFPKSPTKPA
SPNLNNPFPSVPDAKFDGKPQANKGPDPDLIDFFDSIEQKQTTLQVNPQP
TGIMPAAAPFQAQPTGMPFPQNGFAPQQTGFVPNNMFQQQQNMSGFMPQQ
QPQPQLQQPQPLQPNFTGAGFGGFTPQPSFQPGTLGSIPQNLEATFLNPN
QGFQYGQQQQQPQQPQSLQAGQANPLQLNPLQPAPTGTTNPFRQSMMPLA
SMPGSSSPFSSPGPMPQVQRQSTNPFARSPTQSTSPFQSAIPIQAQPTGT
NPFAKMQQQPQQVPPPDQSLNPGPLGPQPTGTTNPFRHSAFVNHNTGMGW
QHNQASIGGGLDQLPTVPVFPRPAQQTPWQQ
>Mb|QLI66340.1
MADDVQGAGFSPKNEPTHVETTEDAETRATRRELKQSSISDQPGKTTATD
DNRPETPSVGISDEKIDELKEQVSSPKKKRAHDQLDGEQDSQDNDATSVT
STDSAKDRAMRLEPQKKRHRDEEPVDIVSHPPSGTEEVDQSTQNNSKMPT
QPTKESSNSTFAESAFGKLASSSSGFAALGSSQGGGFASNKPTLSSFASV
KPVSSSETSGTSVNKGQAGNAPKLSFASNSGLSPFAGLGSGTNGVGSSKF
GSGLSGIKPLGSFGAAGTKPLQSEKAAKPFGAPESDADDDDNDNEEGDD
ESQPDDQERGASPEKDADEKKRPKLHKIAVDDGEAGEVTVVSVRAKMFCL
DKKEGWKERGAGMLKINVPHASVEFDDDGAVIPGSFDASGLETNGDSADS
DSAGPKVARLILRQDQTHRVILNTAILPAMEFQEKASLKSVGILFTAFEG
AETKPVSITIRVRSFQLYTFSVGAEGTD
>Mb|QLI66341.1
MAQKTTLDEFEAVYPKLEEAILEHARKYKLPQGELDWLKANLETNPLGGK
CNRGMSVPDSVSLLLGSALNEEQYFQAATLGWMTELLQAFFLVSDDMMDG

SITRRGKPCWYRQEGVGMIAINDAFILESSIYVLLRKYFRSHPAYIDIVE
LFQETTFQTEMGQLCDLLTAPEDKVNLDNFSMTKYQFIVIYKTAYYSFYL
PVALALHQLNLATPKNLKQAEDILIPLGEYFQIQDDYLDNFGLPEHIGKI
GTDIKDNKCSWLVNQALEIATPEQRKILEGNYGQKDDAKEAVIKKLYDDM
KLKERYEAFEEKRAGEIRAMIERVDESEGLKKGVFEVFLDKIYKRTK
>Mb|QLI66342.1
MSAIAQDEVQDSSPPHHDVPQHAATTAATPQDLQLIWKWNEAVPETVDGR
VHDLITQRAQLQPDEQAVCAWDGNWSYRELDERSTNLAHRLVHMGIGPDI
VVPLCFPKSKWTPIAMLAVMKAGGASVVLETSLPSDRLYSIVQQVEPILI
LSSSMTTELAAQLTTRPVIIVDEASTTPDVTSILPEVQPWKSLYLVFTSG
STGTPKGTIITHSNFCSAIHHQRAILGYSSSSRVYNFTKHSFDVTWSDFI
FTMTAGGCLCIPLQQDAMSNLAGSILAYNANFINITPSVGSMIRPSDLKG
TLKQVVFAGEALPSHQALEWAQHTRVINLYGPAECTVGITLAVISDIANI
DSSSATIGHGIGSCSWIVDQSCHDSLVPVGAVGELLTEGPVVGAGYYRNA
EKTAAVFIENPKWLLAGPPGGSGRPGRLYKTGDLVRYEANGQLAYLGRKD
LQVKINGQRVELGDIEHHVRAHLAESTDVGVVAELGTPRDSDRPLLTAFL
QISTPAARQGRDGLEGELRRVIVGLNNRLATQIPAYMIPSVYIPLEVFPK
NANGKMDRRKLREICETSSMQELMAWGSPLPEKKKPTTRMECRLQDLWSS
VLGLDIGNIGVNDSFLRLGGDSIAAMRLAGAARGHGLSLTVADIFNHSVL
HDMAGVVGELSATSSFIQPYELLNDHTEVGVIRSQVAGACGVNAAEIEDV
FPCTPLQEGLIALTAKNPGDYVSRHVFQLQPTVDIKRLKRGWGEVVAMTP
ILRTRIVNLNQQGLVQVVLNQPIRWAPKGGEMDLAKYQKEDGGLTTGLGT
PLARFALLEESYGEEIRRFFVWTIHHALYDGWSKPLLLERLAQAYAGEGT
LQTSPQFQGFVRHILGMRQDEATKFWLTQFEGSEAQTFPNLPSPRYQPRS
DKMLTHAIGELQWPKMMGVTASTMLRAAWAIITSCYTNANEAVFGVTVSG
RQAALPGVEQMTGPTIATVPVRIVLDKRKTIQELLLQIQTQVVDMTAFEQ
TGLQRIRRISPEAERACDFQTLLVIQPAADQKEKATHDSLFAPQSDNDHW
SLDDEMAEFRTYPLSLECYLEDQGVVLLTRFDSAVLNADQVAILVRQLEH
VVRQLCSAESAEAKLEDVLLVGQEDLQQMWHWNSVVPDTADGVVHDLITM
TAKQQPEAQAVCAWDGDWTYRELDGLSTRLAHHLVTLGVGPEVIVPLCIE
KSKWMPVAMMAVMKAGGASVAVDYAQPEDRLSSIVKQDLARRLVPHQPVV
VVGEQLVAGAKLPTTQTKTPTPTTKTQTIPSPTSTAAAETPLPVVNQSNT
LYVVFTSGSTGAPKGVVVTHANITSAIRHQRETLGFTRQSRVYDFASYMF
DVVWCNLLQGLSAGGCICIPSNDDRRNDPLGAITRLGANTAIFTPSTIRG
LGLQSLKGLRHVHFIGEALSADDVGGLHPDSAVTNLYGPTECTTFSTAQQ
VTANKASNSDKRQRIGIGLGLGLRTWLVQPFDHSKLVPLGCVGELLLEGP
LVAAGYLGEEEKTAAAFINDPAWLLDGTPNHPGRNGRLYKTGDLARYDAD
GTLTFLGRKDSQVKINGQRVELGDVEHHIQSSIAHRQDVNVVAEVMKPRD
TSNSILVAFIQVDIASEPRDEETMQNELAKITAGLADRLLARVPAYMVPS
AYIPINEIPVTPTGKIDRRRLRAMGQQWTLESLVRTNLPLSSERPQPSTV
TEKRLQSLWAAVLGLKADSIAADDSFLRIGGDSIAAMRLVAAAREQNLTL
SVADVFQHPLLSNLATVVGEEAVGTSGKNAHQNEPFTLLNSGEDLESLKK
QIAAKCNVEARQVADAFPCTPLQEGLLALTAKRPGDYVARFIYPLLPSVS
PEQFFKAWEAVVSATPILRTRITDLGERGLVQVIVDGPIGWSRKDELMSL
KDYEMVDRQLTTGLGTPMTRFAVLQDPVDGNRFFVWTIHHALYDGWSISL
MLNRLEAAYSLNEGSVLPFSPPLQTFVNYIMDIDQSIVGTYWGEQFQGSE
AQTFPSLPSVTYQPMSNAFIKHRIQDLKWPKSDVTPSMVIRAAWSLLATK
YTDSSDTVFGVTVSGRQARVLDVERIIGPTIATVPVRVAFEWTEMTVERL
LRQVQAQAVQMTEFEQTGLQRIRRVSAEAERACGFQTLLVVHPVEDEEEK
NTSSRWFISRRDENGEDDTNVTEYDTHALTIECSLERQGLRLRIAYDANI
LDAQHIKRLATQLEHIIRQLCIPENASKSLSSIDMVSAQDMRDIWEWNDT
CPEALDSCLHDMISKTAQILPDALAVNAWDGDFTYRELNDLSTRLAYYLV
CIGVGPETIIPLYFEKSKWTPIAMLAVMKAGGASVLMDSRQPQDRLRAII
DQVKPVVVVSSSSNAELASKLTNTPIIIVSSKHLERICGEKLLERLPQVQ
PWNKAYLIFTSGSTGIPKGAILTHANVCSAVRYQQLAQGYTPDARVYDFT
SYAFDTAWNNFMHTFTIGACLCIPSESERRDDLAGSIERFKPTILDITPS
AATTLQHSTIQSLRTLILGGERLSAEYSERWRALVDLKLCYGPCECTPTA
TVATIDCSIHGEPTIGRGIGMVCWIADAKNHNILVPIGATGELVLEGPLV

GNGYLGDEAKTSAVFIHNPPWLLRGRAGKLGRHGRLYKTGDLVRYNSDGT
LSFIGRKDAQVKINGQRLELGEVENHMARHPLTRQSASLYPYSGPCAKKL
VGVFSLEIIHRADNSPPIELVGADGATAVQDHIHALQGLLGEALPSYMIP
SIWVALKDIPLNPSGKLNRRAVEDWLLRMDSETFARINNTGLSPAARKPK
TDSERILCDACSVILNTPLSEINLQRSFVANGGDSISAMRLSSYCRVAGL
IFSVSVLLKSKSLADVAQLSTIATDATVNRRKEYREDFDKAFTLSPIQKW
FFAQTPSEDVTKEDYYCNQGFYVKVLRPVLTKQASLAIDMIVKRHSMLRA
RFERSKSGWMQRVLKPIDAVYHIGSSDAQSIHEIKAITAKSHKSLDIERG
LVFAADLYNLPSGDQYLTLIAHHLVVDLVSWRIILDDLEALLTGKKILDC
FPFQVWSELQTSEARSSKFAPDRVLSTKNVQNNHTFWNFTEHISNTATDH
IEKKLEVDKATTALLLKDANTAFNTEPIDLILSSIWDAFFYVFPEREGLT
IFNEGHGREPWTTDVDLSRTVGWFTTMSPIHLSRSDGDNRANITRLVKDA
RRRLPANGWAYWVSRHLNDQGIAAFESHASTMELQFNYHGQFQQLERSDS
LFEVVNFDDSVSQVGPLLPTSVLFDINVAIEAGITKFSFSWNRHLAHQDL
IQAWIEQINTSLQAICFELSNRRPERTLCDYEFLNLDYRGLDELHGNVLS
RIEALNDTQVENIYPSSPMVDGILLSQMKGTGSYETSQTWEIRPRESHVI
NVGALADAWQAVVARHPSLRTVFIHSMDASAAFNSVVLKSCRGDVVLVES
NSLESALEIFKELPSIDYTQDKPAHRLVLCTISGDNRVICKIEMSHAISD
GASTAITMQDWAKAYSGELNVEELRSISHGFSQTLTSVSKTDRMAYWTKK
LCAMEPCHFPRLAEAMPADDKHATGVATLELGDETFHHIQRFCEAQSITP
ASLFQSAWALTLSAYTGSDSVCFGYLSSGRDFPVKGIMESIGAFANVMIC
RTDISREWSGQDLISHVHNQVLDDLGFQHCSIADIQHELGLPFGQNLFNS
IISFQRDNDSLSEGMVTEDLVFADLEWEDPTEYDITISIRHTDTLVYFTV
DHRLSCVSNDQARSVVSLLEKVVKFLVGDGRASSAAVNKPQNQLSAMENI
SERDLQDIWRWNKTVPEAVHGLVHDIIANVAQENPYASAICAWDGDLSYT
ELNGIATKLAHHLVTLGIGPDVIVPLYFEKSKWMPVAMLAVMKAGGASVA
LDRTLPEERLRSIIQQVNPVVILTSAANEEMAKRLADCVTVPVSDAHLAK
LSTSSTSLPDVKPINRFAVRYQQQALGFKSTSRVYDFVKYAFDVTWSNFL
HTMTSGACLCIPSEDETSNNITGSLISYRANFADLTPSVASTLRPADLTT
LDHLLFSGEALTTHLAAQWAEQATVLNTYGPAECSVKATFAMVGKADASA
ASIGSGFGMCTWVVHPTNHNMLVPIGVVGELLLEGPLVGAGYLDDAAKTQ
DAFIENPAWLVDGARGRRGRLYKTGDLVRYNIDGSMTFIGRKDAQVKING
QRLELGDVEHHVCSNIAYHAPVRVFAEVIKPQQSDKSILMAFIHVEDGPA
MTTPQRAKQTGKVTAGLNERLSTQIPSYMIPSAYLLLDEAPMTATGKTDR
RRLREIGQQLTFEEIMALNYKSGEYKRPSSTMERRLQSLWASVLGVREDS
ISADDSFLRIGGDSIGAMKLVGIAREEGLALTVTNIFKQPKLCDLAKVVR
QVSRADCEIIQPFSLLKFGTDSEAARDQVAKLCQIDISQIEDIFPCTPLQ
EGLLALTAKRAGDYVAQYVLPLQKTTDVTRFKRAWEAVLQMTPILRTRIV
DLLGQGLVQVVLKENASWSECSSVSEYQQADQKLVMGMGKRLVRYAVTDA
PDCQQRFVFVWTVHHALYDGWSMPRIMERLEQAYKGDVVLRQAPPFQRFV
KHIINIDEERAKQFWQAQFEESEAQVFPVLPSPTYQPTANSSTAHYIDSL
HWPKTDITASTAIRTAWSILAARYTNSNDVVFGVTVNGRHAAVHEVDEMT
GPTLATVPVRVAFDWEQTVESCLCQIQEQLVEMAAFEQTGLQRIRQMGIK
AKQACNFQTLLLIHPPEEAVQLETEIFACKDQDNDDDEENQLAQFDTHAL
TIECDLAHHGMTLRFEFDNSIMSKRQIEKLAGQIEHVLRQLCDPEQGKIR
LTDIETVSKEDLKDIWGWNATVPETVKTPVHEIVTAMCQKQPEAPAICAW
DGDWNYGQLDDLSSRLAHYLQHFGVGPEVIIPLCFEKSRWSPIAILGVMK
AGGVCVTMDSNLPEERLASIVQQVKPTLILSSLAKKDLATQLSTGKPVLV
IDEKLFLSLDTPALEPLPKVQPSNTLYIVFTSGSTGTPKGVRITHSNFSS
AMLHQHSAHNFDSIANPRVYDFASCAFDTSWQNMLATFDCGGCLCIPSEA
ERRDDLAGSIERFGITHSELTPSAAMVLPLSTLKNLDTLVLGGERLQEEY
AKRWASLVTVKNSYGPSECTPTSTVADVDPVHFNGASIGRGRGVNTWVVD
TTTGDSLVPVGGVGELMLEGPLVGPGYLGDPEKTASVFINNPPWLIRGAP
EHAGREGRLYKTGDLVCYHEDGTLTFIGRKDAQVKIHGQRVELGDIENHI
LRHHLTRQSACLLPKTGFWANTLVSVFSIQKIQQSFDEVDTTSGQQTPSL
ASVSSSTSPDTDSTSSSWSSMEDSATPSSQPSTDIVLEDSTTAIQDYIAI
MQSILETSLPSYMVPTRWIALKEIPLNPSGKLNRKQVENWLTEMDQDTYA
KLSSIDNSLVCRGPETEAERVLRDACSLILNVPASEINLQRSFVTNGGDS

ISAMRLSPHCRSNNVVFSVATLLKARSLAEVAKLSSAVTSLILPRTEDFD
KPFHLSPIQQWFFNQSPDYLVDKAGHYCNQSFYVRINRVVDIKEVRVAIS
KIVELHSMLRARFQKQDHVWTQSIPQATEAVYHFEAVKLKSISEMQSLAA
QRQQSLNFERGLVFSADLCELDAGEQYLVLIAHHLVIDLVSWRIILEDLE
TLLTGGTLLESLPFQIWNDLQYQEAKSSRYDPDRVLSTEGISNDLAFWNF
TSDTPNGTQDHTDKTFIINRHITSLTLKEANNAFNTEPVDLLLSAVWDAF
FRIFPSRDGLTAFNEGHGREPWNAEIDLSRTVGWFTTMSPIHVSRSIGNN
PVNIVRLIKDARRKLPANGWAYFVSRYLNSKGVEVFGPHDSVMEMVFNYH
GQFQQLERDHSLFDSIALDDVSDVGPNLPASSLFNINMSIEGRQTHVSFA
WNRHISNQDLIHEWIDQIGPSLGAICESLIGRSRERTLVDYEFLSLDYTA
LDGLQSRIIPEIEGLNGTEVEDIYPCSPMVEGILLTQLKDVGFYETSQLY
EIKPRGSHVINIRQLSTAWKDVIARHPSLRSIFIESLDPDMAFNQVVLKE
IRGDVVLLESDDLDSARALMQNLTKVKYHKLKPPHRVSLCQVHATNSLLC
QIEMSHTITDGASTGILIEDWAKAYKGLLGKDNLIETSRGFARALTSSIS
ESKRSYWKKKLAGLQPCLFPHLDKICEPSEVVSWSVVEISGAEFAAVQKF
CESYSITPASLFHAAWALTLTSYTGTHSVCFGYIASGRDLPVPDIGEAIG
AYANMMVCRADISPDWSKQRFVQYLHHQVLEDMGYQHCSLADIQHDLGII
SGQQLFNTIMSFQKDVDEVQEGPISQELDFIDEDGDDPTEYDVAISITVG
AKLAKLFINCLPSRFSNEQAHRIVSLFKTAVTRLIGDDIPQSYESTLRSI
NLVSEDDLKDIWQWNSIVREAADSCLHNLIRTTAQRRPEAYAVNAWDGNW
TYGQLEDNATQLAYHLATLGVGPNVVVAIYVEKSRWTSVAMLAVMKLGGA
SIMMDSSQSENILRTIMQQSKPVLLLSSSSTARFAAKLTDRPILVVNNDL
VEILARAAAVPLPVVQPSNMACLVLSSGSSGPSKVAILTHANLSSAVKYQ
QDALGYKPNSRVYDFMPYSSGITWEIFTHVFTIGGCLCVPSESGHKGVLA
DSISLWQPTIMSITPSAAAMLLDSTVQSLETVRPEDADEPRIGRGVGINT
WITDTQDGTTLVPIGSIGELVLEGPLVGAGYLGDAETTAAALIHNPQWLN
RGSGDPKYPGRTGQLFRTGDLVRYNKDGSLTFIARKEAQVKIHGQHVELE
DIENHMVRLPSIRQAACLVPKSGPFADKLVGVFSLDGSYEGDSNADNTEL
HLMARDKAEEVQDNISSLQALLDNSIPVYMVPDVWVALKNIPVDHSGKLD
RKLVDNWLSSLDAEMSARISNAISSASSPVTATERVIQDACSHVLNIPAS
HINMQRSFIANGGDSISAMRISPYCRASGILVSVASLLRSKKLVDVAESA
TVAKGPSLSREEDFDKPFSLSPIQQWFFAQSPPDKVNTTSYYCNQAFYVQ
IKRPVSPDGVSLALRKIVQEHSMLRARFHLDETKGSWTQLIPKPSDGLYH
FASLQADTLPAVQSIISQRHQELDIERGLVFAADLFTLPSSEQYLALIAH
HLVVDLVSWRVILEDLESLLNGGTVLEELPFQVWHSLQKQEAQSPNFAPE
RVLSSDVHNELEFWNYTPTTPNTFDDHIEESFEVDCATTAILLKEANEAF
NTEPVDIILASVWDAFFKTFKMRSGLTIFNEGHGREPWGAEIDLSRTVGW
FTTMTPMHVSRDHTRNNSARVVKDIRRQLPSNGWAYFTSRYLNDEGIKAF
ESHDCVMEVLFNYHGQFQQLESDESLFENVAFDIFDVGNDLPASALFSIN
ASIEGGLTKFTFSWNRHINHQDLIRDWIAQVAPSMQALCNSLLACQPSLT
ISDYEFLNLDYKGLDELQNRILPALQSETGTTVTDIYPCSPMVDGMLLSQ
IKDPEAYKTLLTYEMKHHNSLQPLNIDVLAKAWQAVVARHPALRSVFIEG
VDKKTAFVQAVLETYQGEIIICEAENRTAAVTMIRKLPSVDYQRLKPAHR
LVLCTARDANTIICQIEMSHTIVDGASTAIMLNDWSMAYSGSLGTETLAE
TNRNFVRALKSSPLANKMAYWSKKLAGLEPCHFPTLSDAPSPGNSIAVVS
TDLDGDDFTRIQRFCESESVTPASIFQSAWALLLSTYTGNDSVCFGYIAS
GRDLPIDGIAESVNAYANMMICRADVSRNWTPRQFVKRLHDQVLEDLDFQ
HCSLADIQHDLRLSPGYPLFSTIVSFQKDDADAADDVAVGDLVFVDMDHE
DPTEYDISFDISYGKSQVAFSLDYRLSCLTNEQAERVISLLRVITMSIIN
EKTVSATLASIDMVSEEDLGQIWKWNSVVPEKVEALVHDLISDTARKFPN
APAICAWDGDWTYQQLDTLSTNLAYELLKLGVGPEHMVPLCFEKTRWTPI
AMLAVMKAGGSSVALDTSLPEERLRIIMQQLKPTIILTSSIGRDLVGRLT
TGLVFTVDEANVARLETPPNPAMILSDVTPSNTLYVVFTSGSTGTPKGVT
INHSNFASSLRHQRGAHNFKAGARVYDFASYTFDVSWQNVLSALECGACL
CIPSNSERQNDLAGSLERFQVSHAEMTPSAAQLLPASAIKRLDTLILGGE
VLSPELAKHWASMANVKNSYGPCECTPTTTVATIDPDNVQVASIGRGFGV
NTWVVSATGDSLAPVGSTGELFLEGPLVGPGYLDDAERTAASFIENPSWL
LRGSATHPGRKGRLYRTGDLVRYQPNGSLTFIGRKDSQVKINGQRLELGD

VEYYISSNITYKGDVRVVVDVVKPQDSERRALVAFLGVEETSADLDKIIA
GLADRLAAQVPAYMIPSAYMLLESIPMTASGKTDRRKLRAMAEKLTRVEL
MAHGPTQATRRAPTTPKEIQLQGLWSTVLGIPREMISSEDSFLRIGGDSI
GAMKLAGLARDAGLSMTVTDIFRHPCLSDLAKTIRSSFGNEHEVVLPFSL
LSSKRDALEWQAQIAQLCDVSAPLVEDAFPCTPLQEGLLALTLRRSGDYV
AQNIFELKPDISISRFQRAWEHVIATTPILRTRIVDLHTQGLVQVIIAEE
PTMWPEPTSTILYQEADRKLGMGLGTPLMRYAIAKDSHDRHCFIWTVHHA
LYDGWSMPLVLERLEMAYNGTVLQPPPPFQDFVKFIGGVDDGGVEGFWTS
QFNQIQAQAFPSLPSPVYQPRSDAGISHQIKDLAWPKTDTTASTAVRAAL
SILIAVHSDSCDSLFGATVTGRQAPVPGIENMTGPTIATVPIRVPVDRES
TVKQLLQQVQAQVIDMTAFEQTGLQRIRRVNASAKQACDFQTLLLIHPIE
EPGKSTSSLFDDAEDEEDVDGDEIVDESFAGFDTHAITIECDLENSGALL
RFRFDSQVIKKSEVEKLAARFDLILRQLCDPDMAQLKISDIDTVSPGELK
EIWDWNSVVPETTNYAVHDLIAENASKYPDSPAVCAWDGNWTYHQLDSIA
TRIARRLVSLGIGPDVIVPLCFEKSRWMPVAMLAVMKAGGASVALDTTLP
PERLRSILQQVSPSLVLASSSSQRLAEGLTDQPILVLGEGSQEEFDEPAL
QTLPKVEPWNKLYLVFTSGSTGVPKGAIVTHSNFSSAIRHQQAITGFGKS
SRVFDLAKYAFDISWSNFIHTMAAGGCLCIPSQQEALDNITGSILAYNAN
FIDITPSVASTLRPSDLRCLKTVVFAGEALTNHQASQWSKHARVLNMYGP
AECTVKATLAELDGTCSSSAASIGRGVGLCTWVVDPSNHDKLSPVGTVGE
LVLEGPLVGSGYLGDATKTAGAFIESPAWLLRGGPGHQGRRGRVYKTGDL
VQYNVDGSLTFVGRKDDQVKINGQRLELGDVEHNLASSLNATSKFQLAAE
VITPRDGENAMLAAFIQSSGQLGDFESEMRTLTAGLNDRLASKVPGYMIP
SAYIMVDKLPLSATGKLDRKQLRAMGAKLTLLELMSSTGPQPERRPPSTP
MELCLQKLWAHVLDLNAEEISADDSFLHLGGDSIQAMKLAQRARSQGFHL
AVADILIGPKLSQMASRLECLDGKPAAHALEYRRFSLLPPESRLEIEQIL
VEHGISKDNVQDILPVTDQQARYLLTTYTTARSAVYYHTLDHDGDFDLGQ
MQHACASLIRRLDMMRTVFIAYKDSFLQAVLMNLDLDVGVFETATSSLDG
YTLELKKQDLLCNLQFGKSLTKICVIRQTREQKYRIVIRLSHAQYDGTAL
AKMWAAFEAAYKSAPPGDAKTSFSHYMHTLSLVDKEKATEYWRTMLRGST
RTPIKHQTTHRLGYGLGPNIVKTLPASSLQSEDFTFATVLKAAWSYVLAK
YSATEDVVFGNLVHGRNQPGTQDIFGPCVNIVPARVVFQQDWSARDLIAT
VNSQQAASMPFETMGSRDIIHNCTSWPKWAFFSSVITHQNYEDRQTEESS
VDFDSADLSTGDIDSVEVYITSTPGADGTDIDFTFTDNVISHSLAQRLAS
DLADTIIQFYRAMDAKLLAPRDIRSLPALLPLPADPEVALAPPTDEQMAA
LRGCPAKLRNALGTAWKDALGLRTVPGNESTESFFELGGDGDNAGQLAAH
MQRQGYRLRIEDVFEHPTWFELLLHMARQ
>Mb|QLI66343.1
MAVSQHELYKLSATQAAGLIRTNQLSAQDYAKALLDRVRERDPQVRAWAY
LDENAILKQARKLDALPPEKRGPLHGIAVGIKDIFLTRDMPTKYYSRLHA
QDGNAAADSACVSVLRAAGAIIFGKTATTEFASMTEGSPCANPHNSRHTP
GGSSSGSAAAVADFQVPLSIGTQTGGSVVRPASFNGVYGFKPTWGTVSAE
GTGIVSVSADTPGFYARSVDDLEFLAHLFRLDSLLTEPLHPLSIIGARIA
FVKTHIWPEAQSGTRAAWNLAHRLLAETGAKVEHVELPERFGSCPEWREI
VVAEETRAAYLPKYLQDRTKLHADIVAFVESTNVPSRKKLLEAHDGIARL
RREWDLIASQYDIIITPSAIDEAPEGLESTGTAIFCEMWTILHAPAMNVP
GFFGKCGLPIGLQVVGARYNDMQVLQGGKLIGQIFSRP
>Mb|QLI66344.1
MAPELRKRKSVSEVQKSNGATTKTSDKPTPKGKRKAAGDVSPVSLKKQKP
SKKAAAPEKPVAKSPREPKPETAEKEAEEKDDVLEIDDSDDSEGEDNENV
KTLAAEIDSGDEAPVDKTLSFKPGQDVGKIPHVTKELAKAAKKATGEPGV
LYIGRIPHGFYEHEMRQYLSQFGPVTRLRLSRNKKTGASKHFAFVEFEEA
TTAEIVAKTMDNYLLFGHILKCKTIPKQQVHEDLFKGANRRFKKVPWNKM
AGLNLQKPLAESAWKNKLDKEKDKRAKRAAKFKHMGYEFDAPDLKDVPPP
QAAENNVEEPKTIEAAPVEEPAEEETKATKTPKAKGSAAEPESIPTPKAK
AGKGTKGKAKKVKA
>Mb|QLI66346.1
MAVAMLATTGLSEHTSNWAVLVCTSRFWFNYRHLANVLSIYRTVKRLGIP

DSQIILMLPDDMACNPRNAFPGTVYSNSDRAVDLYGDNIEVDYRGYEVTV
ENFIRLLTDRVGDEMPRSKRLLTDDRSNILVYMTGHGGNEFLKFQDAEEI
GSFDLADAFQQMWEKKRYHEILFMIDTCQANTMYSRLYSPNIIATGSSEL
DQSSYSHHADNDVGVAVIDRYTYYNLEFLENQVKDLNSPRTVGELFDSYD
YEKIHSHAGVRYDLFPGGAEGARSRLITDFFGNIQNVEVDRDSSSSLDEQ
LLALSKTIAILREKEAEEDAAPRNTSSVPASVSTEPVTKLQGATVLTNDK
WWNKKVVGATALAGCGLLWAIGSFLEV
>Mb|QLI66347.1
MNTVKSFWLGWGSLCVAGAGAYYFAKREINADRQAKLQAARKKREDLRSL
EYSGPGSTSSSVTNGNGAPAITDTAGSPSQEAGNDPAATRHAAVTESQQT
REKSKYESSTPYRSPKGDRFS
>Mb|QLI66350.1
MAEFVRAQIFGTTFEITSRYSDLQPVGMGAFGLVCSARDQLTNQNVAVKK
IMKPFSTPVLAKRTYRELKLLKHLKHENVISLSDIFISPLEDIYFVTELL
GTDLHRLLTSRPLEKQFIQYFLYQIMRGLKYVHSAGVVHRDLKPSNILVN
ENCDLKICDFGLARIQDPQMTGYVSTRYYRAPEIMLTWQKYDVEVDIWSA
GCIFAEMLEGKPLFPGKDHVNQFSIITELLGTPPDDVINTIASENTLRFV
KSLPKRERQPLKNKFKNADAPAIDLLEKMLVFDPKKRITATEALAHEYLA
PYHDPTDEPVAEEKFDWSFNDADLPVDTWKIMMYSEILDYHNVEAGVAAM
ESQEFNGQ
>Mb|QLI66352.1
MATATPTEAQIQHQQLIEQLDIHSIHKTFRNQHWKPNQRRNKNLKAIVGD
ASRREASALATPQEGSGNATPARDDGLSTSGTSTPATTNGRDQAPNLAQA
SRSLSKLVLEKSLKPPGAIASAPTATYTNIESAPSLAHSKHYCDVTGLPA
PYIDPKTRMRYHNKEIFALIRSLPQTSAEQFLEARGAHTVLK
>Mb|QLI66354.1
MADKPKPAAKQASPLKDAYLILYNFASAVAWSVVLGRTLAVLYLRGPAAV
YAVVGEWTKWTQTAAVMEILHSLLGVVRAPVSTTVMQVFSRLLLVWPVVN
TWPFLALSPFYSSMLVAWSVTEVVRYSYFALSLAGALPSFLTWLRYSMFY
ILYPMGITSECMLVYAATGPAAERSSFAPWALYAILAIYVPGSYVLFTHM
MKQRSKVLRSSKTKKGDKAQ
>Mb|QLI66355.1
MASGYDRALSVFSPDGHVFQVEYAGEAVKRGTCAVGVKGKDVVVLGCEKR
SAMKLQDTRITPSKIQLLDTHTALAFAGLNADARILVDKARLEAQSHRLS
VEDPVTIDYITKYVAGVQQRYTQAGGVRPFGISTLVVGFDPGSKVPRLYQ
TEPSGIYSAWKANAIGRSSKTVREFLERNYKDDMDREATIRLAIKSLLEV
VQTGAKNIEIALMAPGATMEILPTDEIEGYVKEIEQEKQEEAAKKKTGRT
PGTGSAAILTRGQDDSAAE
>Mb|QLI66356.1
MTLKRKRSEPELCSSPSSCSSIFSSPPSNSAIDVNPQAFYNMPPAHLNSR
TMKRFRNNRPSEEIVHQHTLNLLFSAQQQQSQTVRETGFQPLPQTGSASE
PRKQQSLHRFWSINSAPSPVSESTVEQPALAPTSCEDCGAGLGPGGNAHE
MAVDDLTADNTTCGACGKHVCFSCSVSNLGEEKRCLCCAGRKAFGNIGWP
NMGLSLC
>Mb|QLI66357.1
MATNTKYQPAPQQDPDDYTQAPPAYAEGSSSRDESQGLFGPPRSSEDNLP
DDFKFGGSVAEATVDIRNQFVRKVYTILTVQLAVTAAVSGLTFFSDAYRT
WIQSHPGVVWVSLIGALVFMGLTYWKRKSYPTNLLFLSLFTLAEAYSISV
IVSFYKTSIVLNAVILTAGIFAFLTLFACQTKYDFTSWMPYLFGALWALV
LFGFMAMFFPYNSTAELIYGGVAALIFSGYILVDTQLVMRHHHVEEEIAA
AISLYLDIINLFLAILRILNSQSNN
>Mb|QLI66359.1
MAKPDQLPAMDPFSPAHQPDEGYSEDPLNPTVHHGLPASLATLRSPAELP
SWLASNASALPVSVKKELTLALLDDLPTSAIAEIVQRLNPRLYIDFVQYL
PAEICLKILGYLDPVSLVGVAQTCRAWYELALDRKLWERLYHLEGWKAVA
SEIELCEGRVNVGLTSSIGHLHRIQNADGQTNKIRAILNEDEDQEMTGTS
RSQDMVPEPVGIEGSSIFGTPGSSFSSNRQLMSLTGGDVDMDISFASSSS
AYKGKGVDRSCGSSDVVRAKRKEPGGTPEPSLPPILPPDHPSASIRSTLW

AWDIGSSRYRINWKYLYNMRRRLESNWELGKYTTFQLPHPQYPEEGHQEC
VYTLQFDADYLVSGSRDQTMRIWNVRTRRLVRPPLIGHNGSVLCLQFDAH
PSEDIIVSGSSDSNVFIWKFSTGELIQQITKAHRESVLNVRFDKRILVTS
SKDKTIKIFNRRPLRFGELGYGAGESAFSAVGKTIKRYGYEPDLAQELPV
KEPYTMIGRLEGHGAAVNAIQVRDRTIVSVSGDRHIKVWDWPDQVCTQTI
PAHDKGIACVEFDGRRIVSGSSDYEVCIFDAPTGLKVAQLRGHAHLVRTV
QAGFGDLPYSKAEDEVDAKIVDAEYFKAVEAGEVARDGERRNRRTRRVNA
GSSRPADVQAFGAKVPPGGGGGRKYGRIVSGSYDQSIIIWRRDKEGVWKP
AHHLRQEEAAAAAQRNMALAIPPQPILQPTFSNQHQGGLPMHPSAGPPAM
RIPASRSRLPPLGEMSQASVDPSVGAQGTSGLYMNLIDQVVPLGARALEQ
TLANYPAMLSHHNYLQSVIEREPSPLARTQLRQAITAALVAFQTGQQSTR
QRPASTPNPASTAPPAAAAGPSVAGPSNPPGMPATYQAADIPTPRPTQAI
AGPSLAAIHVAPDVNMATAATPMPNPLPTPTPAPAQPAQVPAQHNPPPPA
HHHPHIAGAENAPARVFKLQFDARKIVCCSQAPVIVGWDFCNGDSELEET
ARFFATVD
>Mb|QLI66360.1
MTLLALKEDRPTPSAVYNWRVYACAATASFASCMIGYDSAFIGTTLALPS
FVDEFNFASYSPSSLALLKQNIVSVYQAGAFFGSLGAYASSYFLGRRKSL
VLFTLVFIVGAAMMLGANGDRGTGLIIGGRVLAGFGVGGCSNMTPIYISE
LSPPAVRGRLVGIYELGWQIGGLVGFWINYGVNTTMAPSHSQWLIPFAVQ
LIPAGLLLIGCFFIPESPRWLFSKGKRDQAMKVLCWMRQLQPTDIYIVEE
VSYIDEETERYRREVGAGFWKPFLALKQKRTQWRFLLGALLFVFQNGSGI
NAINYYSPTVFRSIGITGTNTSFLTTGIFGVVKTALTVVWILVLIDHMGR
RNLLMVGALGGSICMWFIGAYIKIAGADPKSSSGNLSSGGIAAIFFFYLW
TAFYTPSWNGTPWVINSEMFDQNTRSLGQANAAANNWFWNFIIARFTEQM
FNAWGYGVYFFFASLMLLSVVFVFFCVPETKALPLEAMDRLFRIKPTWRA
NTILMEELRAEEENFRHNADGVGLEVEKIETEQVESSSGSVPKV
>Mb|QLI66362.1
MKDKNDVIHEFNDLVNMTAAELEKWLKSGDSQAVGWPKEDDGGGESVGHD
SGRKIVQILKDNPQKSPDKYTDDQIEHMRKVVSYCKRHLAQESKSNDNKS
AEEVKKTKSYASLKNWGHDPLKEREGDGDEDEDEDQDEDEDEDEDEDEDE
DAGQDDEQDVGTKRGTKDDQDGENKRQKTDKAASKNQKNGHSGAAKSANK
QDTKDDDATEQGSQPSSDEDDGQESKKSDKSDKNGSSGKKEPKKGDTVSW
EWGQGQPEGTVKQVKHEKASVTTKKGNTVSRDGTEEDPAVVIDAGSSKAV
KLNHELN
>Mb|QLI66370.1
MSVILCTAGYDHTIRFWEALSGICSRTIQHPDSQVNRLCISPDKRFLAAA
GHHTVKLYDIKSTNPNPLLTFEGHTGNITGVAFHCEGKWMVTSSEDGTVK
IWETRTGTIQRSYNHGHPANDVVIHPNQGEIISCDRAGAVRIWDLAENTC
AHELVPEEDVPVSSVTVASDGTLLCAANTLGNVFVWTLKQTYDQTQIMPV
THFSAHKEYITRILLSPDVKKLATCSADHTAKIWEVNNTKPCDEGEKDPK
PFPLEATLTGHQRWVWDCAFSADSAYLVTACSDHYARLWEVHSQQIIRQY
NGHHRGAVCVALNDYSETR
>Mb|QLI66374.1
MSTAQFEWHPAKLLEDNAGLQDFALLVLNQPLKNGAILRRLWKNSSVRVA
ADGGANRLHQLSSFQGKFSNLQAIIGDLDSLCSSVRDFYSSQPTPAQVIH
DTDQESSDFGKAITWIRKTQPSALDIVALGGIGGRVDQGLSQLHHLYLYQ
TDPAYANGRIYLLSGSSLTFLLKSGTHMIHVKEDGEDEVFGKHVGIIPLQ
EPSRVTTKGLEWDVTDWESKIGGRLSTSNHVLPHTKCVEVHTTKDVLFTV
ALRPVEGEEEA
>Mb|QLI66376.1
MSSQLPIDTFPAWAHLNDVQFTHVNLQDVGEGKGFGLVAHEDLESAEADG
TSKGLVTIPHDLVLSAEAVEDFAKVDHNFKQLLEAVGRQSTRGDIMLYLV
SQFAQSSRPKGLSPTPWTEYIRLLPRPIPVPTMWTEPERLLLNGTSLEAA
LEAKLLSLGKEFDTLREVSEDFPFWNEFLWSGEEVSLEDWVLVDAWYRSR
CLELPRSGAAMVPGLDMVNHSSKATAYYEEDDHDNVVLLIRPGCPVRSGE
EVTISYGDAKPASEMLFSYGFIDPNNIVDKLTLRLDPFPDDPLARAKLRI
SNSGPTLTISRKETEDSQGASSIVWRSPFVYLMCLNEEDGLSFQLLQDTA

GDRQLRLFWQGEDVTDQADDFETLIQGHDLCPVFKLRAITVLHEKVGEQL
ARISRGPSDDELEPLQAAGLLRAESIAAARILKDVESKLLGHALEALDNE
RTNLLANNHVVAYLGSKEDYPIGQEAQATAANDDADDFS
>Mb|QLI66377.1
MSPTIIHLRAETKPLERRSPLSPEGAKALLDAGYVVRVEECPNRIYKTDE
FKAAGAEIVPTGSWVNAPTDHIILGLKELEADGSPLPHTYIHFAHVFKKQ
FGYATELSRFSKAGGLLYDLEFLEEGGRRVAAFGRTAGFAGTALALLSWS
HQILHPGTPMGPAPVLDSAADLVALIRSKVQEALPANNGEYPRVIIIGAL
GRCGTGALDCLKAVGIPEASILKWDLPETSRGGPFPEIATSDIFVNAVYL
GAAPAPPFVTIESLSGPERRLRVISDVSCDPNSDNNPVRVYSRHTSFENP
TTPAEGKLDGPELRIIAIDHLPTLIAREASDEYSSLLLPSLLTLNRRDTE
GVWVRAEKTYRDRVAELP
>Mb|QLI66378.1
MLPSGLRQVSRRGLRQFSSTPATQADFTHAIIGGGVVGLSIAHRLSQQSP
SSSSVLIERHSSLGTETSSRNSEVIHAGIYYGRDTLKTQLCLRGKQLLYE
FCEKHGVSHRRTGKWIVAQNDAQRRELEKLHAHCADIGVPVRWVPEREVR
DEGEGVRAAGGALESPTTGIVDVHGLMVSLAGLFEDAGGVVALNSTVAGV
APLGARPGSAGWRVDVRDSLTGEASSVTAETVVNAAGLGCVDVYNMVVPA
ALRKRLYFAKGNYFSYSASRPRVSRLVYPAPNPGAGGLGTHLTLDMGGRI
RFGPDIEWVDSPDNLAVGRERLEQAVEEIKLYLPGVDETCLEPDYAGIRP
KLSYKGAGQAGKNFNDFVVRMEDGYHGWVNCLGIESPGLTSSLAIGERVG
ELFYGRK
>Mb|QLI66381.1
MPVDYSKWDALELSDDSDIEVHPNVDKRSFIRAKQSQIHQERQQRKLQIE
AYKYERLIHDGLLKRISSLLSSLRTHASEAAGRNPGEIAFKAVMETAGDP
KDDQVPPRPEGLHSDGKEPKTYSQMMAALLDEVNKALDAKKPDDRYQAMI
TELQGHVDKINEMQRELTPKLEKLLKEEGKKITSADIHTGFDSSHVAKAK
PSEKKGASTEVELLNPNALSSDSQSPSDKAKIDEEAEVEASPAAKKFAQI
DSNDYTASLQFLSQNPQILTEKETDGLLVLAFDAALEKKDGYCRQYVHQA
LLLQYCRLLGKDGVRLFFQRVTTKGHQAQTVFYDDVRDTYTRIEKRAQEI
IAQRAEEPEEGVEQIQLHAVEPGTVIQIKVPSAESQDTDEQHAREIFDGF
KPEMKKALESGNLDEVNKVLGQMKVEAAEELVALFGEANILSLEEQIIDA
TTEEGQKQLKELEAAAKAEAEAEAEAAGPVGDPE
>Mb|QLI66382.1
MSVPAFSDIAKPANDLLNKDFYHLSATTFEFKDTAPNGVAFKVTGKSSHE
KATSAAIEGKYTDKPSGKTPRPKSHRRPGIRRDGSLPVVSVSRILGSAAG
WPLEFAATIQPGYAQLLTRYTRTGLTLTQTWNTANALDTKLEVNDTLAKG
LKLEGLFNFLPATAGKGAKFNLFFKQPGFHGRAFFDLLKGPVANVDAVVG
HEGFLAGASAGYDVNKAAVTAYSAAVGYAAPQYSAAVTATDNLSVFAASY
YHKVNSQVEAGAKATWNSKTGNTVGLEVASKYRIDPVSFAKVKINDRGIA
ALAYNVLLREGVTLGLGGSFDTQKLDQATHKLGASFTFEG
>Mb|QLI66384.1
MWLLESKDAFDGRRLWLRPGKTYLFGRTASEPGQLAISHTTISRKHLTIT
VETVKAGFAQHLTGRSKLTIEDLGTKIGTVVNGRKIRGEKYVAAEAELEV
TMGKCPSKFRITWFPVAFTFSFTSKELQTDPLTTLQERFEQLDVKLLTDY
NIEHTTHVVSRKRNTAKGLQALINGKYVVTETFLDAITQAAEANDASEPS
SLEQDFDTYWPSAMEHLPPRGGEPVQHPDSIYAPDRDRKDVFEGYTFIFY
DKTQYNNLMAPITNGGGKAIFESITTGETHTDEFVRFVKQVAGEKGLGSF
DDGSEGKGVVLVRYLPAKGGHIEWYTEFITAVSLRLDHRPIEQNEFLEAI
LTKDAKILRRPLEVESAPASQTAENGEPEPTLTENVTSVQTEPLIEEQPQ
PRRTRTTRPVKRRFAGFDDDADIDMDDLVVGSLPRDSAPGRAEKQDEGGL
FVSQEPDMAGSQPPPGIYDSQARKRTASIEDDLMDGMAPAVARFKRQRLE
RGEDFAPPSPLPEPEPATVTAKKTKEKLNVLAMAARHREEEEARARAERE
DLANLPDDIDLAEIRRLNIVEEMEIRVSTRDARTREKDVVDGRWNPKWNG
MKNFKKFRSRGEATGRLPVRTIVALQEVKNKEFGIGDNYWLEDEGVERKR
SPTKVRSAQSTQNSASVAEPSPRHRATHVVVSESEEEDDAPDSVRPGKQT
GSVGTSSTKVTATPSQTTSTSSSRQARATQEKRSAAESLSGQRAKRQRPA
LRAIQADSDDSEDELKFRFGKRR

>Mb|QLI66385.1
MAVIDRGVVTSVPMSNGDATPTSPGTPVNGKGRKRLLRGLQRISSSPSLN
SLRRSRSASSPYSTRGTLSCVSLAAVGPPQTPSSARSSTPQPSPLGLPSA
SISLSPTSTHEFPLYDNIEGHLSVRKVDNIDQNGPPATATVPAGLGFAKQ
DGLAKALAAAKAPPKPAFPFWEKMPHELRVQIFSFLRPKELVQASRVSKS
FYRFCFDGQLWTSLDASEFYQDIPAESLARIIVAAGPFIKDLNLRGCVQV
EHYKRTEVIVKACRNLMNATLEGCRNFQKTTLHTLLRTNDKLVHLNLTGL
SAVSNTSCRIIAESCPQLETFNVSWCDKVEAKGIKAIIQSCPKLRDLRAG
EVRGFDDISTAETIYTTKNLERLVLCGCVELTDDALKVMMQGIDPEIDIL
TERPIVPPRKLRHLDLSRCNRLSSAGVKAIGYAVPELEGLQLSGCKTLTD
AALEPILASTPRLTHLELEDLEELTNALMSEHLAKAPCAETLQHLSISYC
ENISDVGMLPVLQKCLRLTSIDMDNTRISDLSLAEAAAMVNKRSKRSLKA
SDRPQVTLQMVVYDCQNVTWAGIREVLFRNAQIRPAAGEPGKVTYPTEII
GLKCFYGFQMTVDEHQTRVLRGDLLSAARLERKWADYMQANEEAGMGGAG
HRRRRRRAREAHAQHMNEEDGGIVGRRRARTMGACSVM
>Mb|QLI66386.1
MFNWAKQQLANVAGTQEPIYGASAIRSVAEEAKETPFTELSRDDLKWVAM
ESTCVETQSFYLVSDEGHIGLAQVIYSNVAGMRTTCQFNSKIFSKDPSKP
HLWCSTPLNHFEFNEDKTSCYADDCALELSKDGNEYTIKSMNDPRSIVNL
KITRTAPGFKAGKSGTTKYGTDLENPWGTMRHAFWPRCAAEGTITTPDGP
IDFKGQAMYSFALQGMKPHHAAGKWNFANFQGPNYSAIMMEFTTPPSYGS
TLVNVGGIVKDGQIITAGPGHEAIHTDVKNDSENEWPEPSSVKFEWTGNK
TDAKPVSAVLEGPLGERTDRVDVMAEVPGFVKTIVAAAAGTKPYIYQYAP
KMTLKLKIGDEEVTEEGQLFMEATFITE
>Mb|QLI66387.1
MPKTQPNLYAFGNVASLATALRGYVVQSQNAGFSRHDSFKVAISGGSLPK
TLAQALLAPSSGPEDVIDFTKWEIFFADERAVPLDHADSNYGLLKSELLE
KIPADQQPTVHPIDTTYLNDTQELADQYEKTLVSSFASRDSVRLPIFDLV
LLGCGPDGHTCSLFPGHPLLRETDAWVAPIEDSPKPPPKRITLTLPVVTH
AVRVAFVATGGGKKDIMKEIFDSLEPTLPCSLVNEASGDRCSWFVDEPAV
EGVGFPRKAFL
>Mb|QLI66388.1
MNFAPIRRPMGCLKSVFRQARQQRTAQRSLATAAVAPTGNKAISERQRAA
HGKVEKFEIYPQIPSIRSTNPDPMPILRQQQLAQLDPAGARTRLFSKDHA
DSAKVGDVLMVTTQGGEPFSGAFIQIRRRGVDTAILLRGQMMKTGVEMWF
KVYSPSVTGIDIIWRRPKRARRARLTYMRKPKHDMGSVDNLVSAWKRERY
ALRSKSKQAGGKKARA
>Mb|QLI66390.1
MARALSAPSNLPSLVKTTFAKALSGGDLHYFPTKVTVLPVNSVPFQLRFS
PALASKPKPPPQQDASPSPNQPSDPFANPPPALYIADVGPSHYLVLNKFA
IVPEHFILATKEFKQQTDALEEADLEATLACIEAFARADGEGLFAFFNSG
EHSGASQPHRHIQLLPIARMRDGLVSGDDDGHAPWDVLAHDGGRLEQAPF
AVFSEPIAPGMSGADVHGAYVRLCGRARRAVGERAPREGEMSYNMAVTRN
RLVICPRLAEGAAVCGRGGQDVGRLALNGTVLAGTALVRSEDEWEALRAN
PGCLADVLGKIGLPGQV
>Mb|QLI66392.1
MAAANSVVQLSALSPNETFIKLPHPYLTEYTVQKTTAPPNKTAGDVPFYQ
LCERPGSSTQKLPAPLHNTKLYFSEPADLKSSELPADSNNGAWARARRSP
FSVVVWDGAETPSLAQAWLLLYVLFTARPGMESVRLELVGANAATLGAQL
RDVLLAVDHPPPAREKRASASSNADEYLVLALRSTFWQGAGSPFGPRPVW
LPAESPPSLHGAGSLASYPLTPLQHTVTITSAGDPQDADRCQQSWHPIRP
AKPAPGAVVYSRWIPHLGETFSMVSLDYQDEEHLRLFHEWQNDPRVSQGW
NETGTLDQHREYLRNIHEDPHQVAVLAKWDDAYFAYFEVYWAKEDRLGGY
YDAGDFDRGRHSLVGDVRFRGPHRVTAWWSSLMHYLFLDDPRTMHVVGEP
KNTNATVVMYDLIHGFGIEHFVDLPHKRSAFVRCPRTRFFQLCPLGENDK
VVGGMRIGLVPKL
>Mb|QLI66393.1
MEQQSSTSKVTVEYFDPHHVYKLLAPGLVPRLPLRNLHWQSHAGPLRSID

TLHVDLVPGDASQPPEPAAPKSRRSTNASRDDGFQTQQVGGEASSTDTVD
KQAAATKTSPSGQRRHQIPGLRSTPYLKVLLVRCDDNDSYKATVRAEVRE
WIKEHTPPSNSSKKASNQEKHDAFEWLILHVVIPNTAAATQPRNTGSKGE
GGSAEKASTTSRWRPGSTPLMEKFRSDFNSSSKGAPDRVAQIRIGINDVP
YDLLPRVVPAVPSGYSETEQDAENAWNELMSKFKSLILSSFDMRVTQYEE
DIKEKDGQRSLPGWNFCTFFILKEGLARGFENVGLVEDALVGYDELSIGL
DSVIQEQVESGAPEKHGGVMLTYTEELQRMARQVLTELSGDAGDEVAVDL
QRQETASNDADDIPISSSNKAYRDMILANQVSVFDFRCYIFSRQISLLLR
LGNAFATREELLAKLMDQRNSILHGVAPLLPPMNNDDGPENLGMLSEVCR
RTLEFIPVISQVMRQDITASLLNDTNPNTADNAQVTTLDPFLLETMDNMV
ASFAFTIAQQILVQTSTKALPIPLPTLVMEDGAEPKSSIPEPKTMLHPAR
SSSLQAQPSSRPPSNPNFPGPGRKPSLAEPEPQASSFLKVGLEELAARRA
ELYMLSRRILEGLGKKRGWSNGWDEAPLVGEPGLDGMEEISLDDGGAGGG
CGRGSSIGGKSSTESTAPSIAGVDSQILRTATDNTVDFYRLYEILTGKAS
HHFAVANHVHSVTTCKADLAMLKFHTKEYKAATKHFEEATPFFAENGWSL
LELSLLVMYCQCLSELGSDDHYVNVAMTLLIKSCAAERERRERKAAVVAL
PKATILDSSPTKQVAAKLFALTSNLSSEVKVPLSKFFMDVELEGTPVYHD
RKDGFSLKIQLRSLLPEKLTLDSAKLRITCTDGGPRRDINLEVDEEIVLS
PGKNSISVACNSVIPGSYRVSRLALVSSKLFLHHEQDVNSLPPRTSSIFQ
DPDVTLFQCPNALDVQLTASRHISLGKNNALDLIIRSGWNTLKGCEVKIR
PTTGGLRLLTTEAKLVDESIQFAKRPESGALFFGPMEEATSITVRLPYSI
EQDLGDVSVKVEVTYVTTSDESFQLAKSIMIPVSLAVGVNVQDVFKHNAL
FSRFNVDTASSSPLRLFKSELVESELFEPSFGIPPANTVMVFPKQHATLL
YKVKRKEGAKVAARSGRILHLKLYYSVLQTEIEERIKESILEGLKDTPLE
LYSKVTAARVLEETRNGLEAHDLERAALVGEVTTSYLEKARWAHHLRGLG
CVPGTRDDATTKLAEFLDDWQRKHPRMTLPTGTPDEPWSIVIPVEVPSLS
VVHTADIRIEQSLLDHLEGPSAGAPAASVNQVLPATLHLKWTRIWDTDAL
AREDEEFSYEVTAPSDAWVIGGRRRGHFVIPGGKAPSTMSSTAETEAEIP
LVLIPQREGYLPYPTVEIKDVPPAEGHRGAASTTAPCEVDWRNLGETVRV
VSERRSVTVSLDASGPGGGPLVCESEGMNRQKGRIVA
>Mb|QLI66394.1
MISNHFFTQLPPPSTALFVFSTLCSLVAGNVLPQETKTVEAHAFNVRPWP
LATLAPASPLELLRRESNTICGYIGGDPNLPATCLAGSHCAADVENGVIG
CCPDKGPCNAGIFTGCVDRNSGPQTVADPYIYTCRGKNVCYKNMFEGGYF
QYGCGSASALATTVVLTASGRLPLDLTTISVKLTATVTPLSSPATLSSEP
SRTSDSSTVLESPVASGKPPEPSETHGGAAPSSNGSSSTNTGAIIGGAVG
GVAAVFAIATLAFLLWRRKSSNVRRGPGNEKDSRYIRSMAPAAHREFEPL
PSSHDASEARFHAGYGNSTPGNSTPGNRTPGISTLAASTSDSVSAVSGRS
GSPRVQPYSHDTIDAVATRSGHTNYDSDRVPLTRELDDFSHGFNSALEAI
ESDSDVEANRNHMATYPGPRRGGGGGGVLWQQNRRRSRNLAWM
>Mb|QLI66395.1
MANSRRAVAAAVLVFLIFFLTLRSHSSPAQSRGSAIRAHAASSGNAHDDS
MRAVPQKPMIDMSRMSTYDKLAYAYPYDIETKFPNYIWQTWKTTPADSNF
AFREQEASWTLQHPTFVHEVITDSVAESLLQLFYAAVPEVLETYNALPLP
VLKADFFRYLILFARGGIYSDIDTYAIKSAIEWVPSHIPRETIGLVVGIE
ADPDRPDWAEWYSRRIQFCQWTIQSKPGHPVLRDIITRITNSTLALKRDG
KLSSFQGKNVVNLTGPAVWTDTIMDYLNDGHFFDMRKSQGKIDWHNFTGM
ETSKRVGDVIVLPITSFSPGVEQMGSKDYDDPMAFVKHDFEGTWKPESER
HIGEQNQDS
>Mb|QLI66396.1
MSAEPLPPPLKIPEVSRFINRANQLRTIKPAIAYWCEYHAVNQIVTKSLH
TTDDDCFAYTKGLIERLEATKTERADDEAIVDNTAGQAYVEQFAQETFSR
AERTLRANKVSRQTADTFDAAATFFDLTHEWGTPEPEIIKKIKFAKWNAA
RILKAIREGKDPNETNPQVQEDEGSEPVLSPTDPAVQQIMSPPVASVEDA
PDDGEPPIAPVQEGYFPPTTAQPEPFVPSPLTQSPGPPASVMHPPPAQVS
PAPSPDVHPPQQSPQIPTKLPPPEPSAPTPSAWNNVPSAPPPSAPTPQYV
PPTVAPAPAPAPVRQAGPPAPSAPTGFAPNHKSIEQAQKHAKWAISALNFED
VPTAVQELRNALGMLGAQ

>Mb|QLI66397.1
MGGGDLNLKKSFHPTLRRNQQAVYEEEQKALAERKRTQQRINEIKEERAK
EELQRQLEDAGGKKRVDRVDWMYQGPTDGQVGTSEETEAYLLGKRRIDNL
IKGTEHKKLEKSAGEESFMALQNANSARDTASKIRDDPLLAIKRQEQAAY
EAMMNDPIRRRQLLASMGMEDEKNKERSRDKEHRHRRKHRRHRSRSRDAE
DDERRHKRRHRSYSRSRSPRRQRDDGDDEERRRRHGRRRDSGVDRSPRRA
RDDSAGRERRYESSHKRSSYQDSDGRRRHDSPNSGDGGLRRRSPSPDRHD
RRRSDHYARRDGHDNRHRDDNRDDNWRGSRNGTGPHHSGNGHGHGNGRAR
HQARGKDEDGETEKARKLAAMQSAASDMDLDREKRLAALEQEERAAQKAD
ERARERGGERGFVNGLHKQAGKLGLGERMGRNRQGYQRDDD
>Mb|QLI66398.1
MASTTSLKELVKTINTFLVDPSLPLPDTFVDTLTTFTQKQEEDDNSAADL
LQDSLLSVFDKRVKGNAKAVGPWIAILRRLLPVLQTPERILPWFDACKGI
LDRADLDKYVVDETVASLMDLITLIDEFQISFAANSAANRIIDRLFQVWM
DRFYPALLDGNTSSEYNERLIRHALCNFGKKRPKELFTAMDGYFVQSKYR
KSALRFLCDFIQIQPPHLHQVLHTSLFGNLMTCLQHDTSTTIVSAALTTL
IMLLPHMPSSLVPHLPALFNIYGRLLFWDREHSRPAEWPDSDGAGPEPAP
GWEVATFDPDVDDQSIAHLPNYYTILYGLYPINFMDYIRKPQRYLRHANA
ANADEVEVQPTEMRHRSETFRRNHLLHPNFYSLTIDSEKTDFGRWIKSEA
AEVVAECMGLCLETDLYNFADLSSAEIPGSSPGVVNTTAALGNDVLEKSK
ADAALLSGPGGKHDSWRNSHLTASDSASSNQTPVTAMRRGSQSSVPSHRD
STGDGRSRTGGTDSPTLTLSPSHPQLQDLIQSNKVIKSGLHQSLANDSVA
SLALSHQDSVTEKAPPGAPTASAATAPLPLTSHSPFPTSEMSTQVVHLQK
QILLLQNDLSFERYLKQQHMAHIGDLRRKQMAEAATEAETQNLIMMNRNL
KSRFEDAKNSEMQVKKESEKSRAMAKKWEADLANKLKDLRHKSKKMQAEM
EALQRELEESKQEGDKLRKLLCAAEVKELNWKQNMQSIEIHGAEIDRLKA
EVERLTRSERDHQAKELERQQMINSATKTESRAEALKMRLDAQENEVQRV
KKLFQSQVAALQTQLSEAQEERERPGSNSNLAVESALAASRAKQAELQKQ
HALLMRKYTALQSSLLDMQSEQPPEQTRATASQHQPTDADYLSLSTSPVV
VKARSHRALSNAEATGHNMKSAGGSQSGGTTTGSTLTKEGSGSQAEASGS
PISPDQRTFSGLQSRLRRDSRDKSKDDGSSASGKPKKEKKSSGLRGIRGF
V
>Mb|QLI66399.1
MGDRLTQLQDAVDQLAQQFVACLHFVQRRHDLETLGPNDKVRDIKQEPHQ
REVDPLPADEFAAGLKELSRDLIIKEQQIEVLISNLPGLDNSERDQERNI
KDLEEDLKAAEAQRQDALKERDHILAELDSVIRSVRRP
>Mb|QLI66400.1
MKLSYIGIISYDTREGIDPKNREPSQELAVVKDVSSYGLLTRNNYAQFMT
FFAKQLAEKAKPNQRQSVAQDAQDFVFHVYSHKLGVCGVAISDAQYPSLA
AHKLLGNVVNEFLAKFPASSWKDLTASSPRANIKDQLAEYQKELQQQLAQ
YQDPAQIDTIGQIQRELDDTKQVLHQTIESVLGRQEKLDELVHKSNDLSD
MSKGFYKQARNQNRCCVLM
>Mb|QLI66401.1
MASAKRSNSSLPPGYSEDKSKGAMLRFQDSLPRLPVPTLEETAKRYLKSL
HPLLSATEYSNSKAAVKDFIKPGGIGNKLQEKLIAKREDPATKNWIYEWW
NDAAYLAYRDPVVPYVSYFYSHRDDRMRRDPAKRAAAITSAVLEFKKLVD
NGSLEPEYMKKLPICMDSYKWMFNATRMAARPADYPAKFSSSENKHIIAI
RKNQFFLISHEIGGKQLNSTELERQFNKVYQAAQRVPAVGALTSENRDVW
ADARDLLAQTSAKNKDALRAIDSASFVVCLDDASPVTLEERAHQYWHGDG
ANRWYDKPLQFIVNDNGTSGFMGEHSMMDGTPTHRLNDYINDLIFGNKID
FSDPSIRSDLPGPQLIQFDITAPIQAEIDRAVTDFHTVINQHQLAVQAYQ
GYGKGLIKKFKCSPDAYVQMIIQLAYFKMYGKNRPTYESAATRRFQLGRT
ETCRTVSDESVAWCKSMSDASQDDQARINLFRSAIDAHLEYITAASDGKG
ADRHLFGLKKLLAPDEEVPAIYKDPAFGYSSSWYLSTSQLSSEFFNGYGW
SQVIDGGFGIAYMINENSINFNVVSKSLGSDRMSYYLNEAAGEMRDLLMP
TVASPKAKL
>Mb|QLI66403.1
MVSLRPLSRSLPIASRAALFSTCSAASARPASLRAAGFANKLSTSPLSSA

KPRFLPSSMRALTTAREKVKVLLCTYDGGQHAKDVPELLGTTENELGLRK
WLEDQGHTLVTTSDKEGVDSKFEQELVDAEVIITTPFHPGYLKKDRLPKA
KKLKLAITAGIGSDHVDLDLANKTNGGITVAEVTGSNVVSVAEHVVMTIL
VLIRNFVPAHEQVERGDWDVAAVAKQEYDLENKVVGTLGVGRIGERVLRR
LKPFNCKLLYHDYQPLSTEKAEELGCELVSFDDLLERCDVLTINCPLHSE
TEDLFNKDNIAKMKPGSYLINTARGGIIVAKDVAAALESGHLAGYGGDVW
YPQPAAPNHPLRTAKNPFGGGNAMVPHMSGTSLDAQKRYAEGTKAILQSY
LSGKHDYRPEDLIVHKGSYATRAYGNDKKTS
>Mb|QLI66405.1
MTFEDEERRGGTPSGVVEREAEDYFGHVSTEESPLLNTDPPPDVVPDKAF
QHLVLSMCVLFAFIVEVSVFIMQPPLQQVMEDRICGEIYPDHPVGIMSKT
DGRCKDNRVQTELAMLRSWEISAEMFVPFFVQIPYGIIADKYGRRPVLFL
ALFGAVIQTAWILLVLGMPNSFPVWSMLYGNIAYLIGGGGQMAAAMIWTL
LADAIPVAKRTSVFYLLYAMILIIGVIVNPIAALLLKINPWIALWLGFAM
LVVGLFASLLVPETLALRLQADRKRPRGASVDGPMHISNNPRKSWLEHAV
FSMKNDMGHIWRYIFASKSVMILIFAYAINYPIKLNQTFNLLQYMTRRFN
WEWSTATYISTVSNITAAVVLLVILPAGSWVLVNKRRVGPLNRDLILARI
SLIFVIAGSFLTAAAPTVWLFITALIATSLGAGFPTLCRALLNAVVEPHT
VATLNTTVSMMETVMGLVSSPVLGWLLSKGMELGGVWMGLPYLVCAGLAV
LTGVMILAFRLPTGFAQSSS
>Mb|QLI66410.1
MDGPITEALGAHHPYYPLGVAIPTYVANTMNAGVLVSIFAAGCGVIFVPT
YLFAVKTRPAISFGEVLVAFWWVLCGSIHLTFEGYFSYNHFDMASKTDLY
GQLWKEYSLSDSRYMTQDPFVVCMETVTAFFWGPLSFFIAYATITNHPWR
HPLQLLVSMGQLYGDVLYYVICWYNEYVNGVAYSRPERYYYWAYFVLCNA
FWIVIPLALMGYSAKTIAKVFEKTKVMNGSAKKHL
>Mb|QLI66411.1
MVLNVFRIAADLSHLASILILLHKMTQLNSCSGISFKSQALYLFVYVTRY
LDLFSTESLYNLAFKIMFIGSQGYIIYLMTNAYKPTNDPNVDTFRVQYLL
AGAAVLAVLFPYKYTFSEIMWAFSIWLESVAILPQLFMLQRTGEAETITT
HYLFALGSYRALYIPNWIYRYFVDTHYKTDWIAIIAGIIQTVLYSDFFYV
YYTKVLKGKKFKLPV
>Mb|QLI66417.1
MDRHELSVYNPNPQRLAGPQLLHHLVTSPNEATAIEFLSGNEQTSISYKE
LHDASDRLCHSMEALIAESRDIETDTVIPVLIPQSLHLYISLLAILKAGG
AFCPLNADAPPERVKFILKDVSAKVVLVTRELAHRIPAECNVKVIQVDTQ
SFESEHEREPSFKVDPERLAYVMYTSGSTGTPKGVGLTHSAATQALLAHD
RHIPEFRRFLQFAAPTFDVSVFEIFFPLFRGSTLISITREEMLDDLPGVM
RRMDVDACELTPTVAGSLLKKREAVPKLKLLLTIGEMLKMPVIQEFGGSS
THESLLWAMYGPTEATIHCTLQTSLPSESSPGSIGVPLDTVSCFIIKPAE
SADDAQDFTLLPQGEAGELAVGGFQLARGYINRPEQTSSVFIDSPYGRVY
RTGDRAVMKPDGTLECLGRLSDGQVKLRGQRIELGEIEHAALRTAGCHGA
SAAVIDSQLVLFCSVEQGVGEDDILFSCKSWLPRYMVPSELVIMTEFPRL
PSGKVDAKKLKHNFSEKNTTKNMAVDTLNEPTEQEKAALNIISETLHRSV
NIGSTLTSVGLDSLSAIRLVSAFRQKGYEISVTSLLKCRTVADLCTSLQK
APETSSQRNETHAPLLDSRSISEQHPLLAGLSQLVENCMECTPLQRAMLA
QTEHNPELYCNEMLFEASFNVTPQALFKAFEAVVQSNQILRTGFLHREGQ
YVSVLFSEPLDGQITVQTKCCEGSNVRSPVDILRPFLVQIIDNGEEMSPN
ILIRAHHAIYDGWSMDMIIADVSCIVKGGVPASRPQFKQVLDFQSQVTIE
DEDDSRAFWTSNLLGWNKISFPKLVGQAPSDQILSEEAFLRISPDIVQSM
SLSHGISSQVIFQAALAMTWQGVVGNSDILLGSVVSGRTIPVPGIERIIG
PCIAAMPLRIDTSNMNANIDALKAIQAQNRAIMEHCSVPLAEICKLARLA
PSESIYDVLFVYQQSLYEWELQNSVMKQIRHIDRLDTKLLVEVEPRKDGY
ALQMTYHSSVLSGDFVQLLVEQMKEHCEKILAQPTGPLMLSKHLEGLEMS
IFREKTRVDNEPADVAALFNASLMRNPLAEAIRFVARTAEGELKESTLSY
SQLSTASNQIAHYIRGSGAEVGDVIAIMMNKSTTLYTSILGIINAGCAYL
PILPTTPMERVREILRQSHTKYCLVEDEVACCAHEFPNSTTVLNMSTASF
RTMPSNPPFVQPDPDRLAYVIFTSGTTGVPKGVAVSQRNLASNITYLDTI

YPKSAQPHLLQACSHAFDVSVFEIFYAWYAGMCLCAAPNDVIFGDLEYWI
RELQITHLSLTPTVASLIDPKNVPQVEFLVTAGEPMTTSVLEKWGSLLFQ
GYGPSETTNICSVKRMSRGDNIEHLGWVFPNTSVVVMVPDGLDAVPRGWV
GEFCFGGSQVANGYLNDTTLTTQKFIDHPSFGRLYRSGDLGRMLPDGSLV
IMGRLDDQLKLRGQRIEAQEINSILTSTELVTTAVTILVQSKSGRSNQLA
TFYKPSSAVSSSNPLDVTKSSNQMLFATLKTKLPAYMVPSYLVPVPYVPM
TSSGKVDRRHLLHWFEGLSSAYLEDASPALNQPNVAESWSDIESRIATAI
SESMHVSLNEIGRWTPFLTLGVDSISAIGLAKSLRTHLDSQVAISAILQN
PSVAQLGHFLARDDGKGLAELSSKMKTSFETFEREVGGSLSDRIQDLEAI
LPCMPLQEAMLIQGQESYYNRILLRLHVSPEEMKTYWRQLSNRHSILRTC
FVTTTNSRYPIAQVVLRNWDLPWREFEVTVPSLEGASREHLNSLPDPLDS
LVPPCSLGLIRYKGSNFLSFICHHALYDGIAMENLWREVEALAQGCQLPD
AVSYLPFLQQALSLPVGVESFWQEQFRGFQSSTAFARSARSGINQSTNSV
SIDMSFKDIQQRVRSSGVSLLSLCQASWAAVLACVFDDPDVAFGNVVSGR
TIGVDGVHKLVAPCFNTVPVRLDTSRSAQNIDLVKAFQELNSKLLPYQFS
PLKLVQKVVGGRRRHLFDTLFLLQQPLKEMDKNVWTLQEDVGNMDVPIVC
EVIPCPNLNSIVVNLHYDMDMITPDLASVVTDMFKFIIRSVVSLPFGPVP
SRSTIPSHFSQGLLGLVLKRERSDEDSIETTSSQWSSLEMKIRKVISNIS
GVSEAAIRRETTIFQVGLDSINAVQVASALRSQNLPVSSSDVIECSSCSK
LASRISQNSEELKQENVWIDFSAFQNQVKPYALEKLRPTTEIEAILPCTA
VQNAMLMAFMHSDEANYLNSLSFKIKETVDLNSLFVAWQLLHGQHPMLRT
GFVPVSHTGSTFAMVRQKSTSLTHPITLFDRLPFVLEDWKAECRSFMLAN
LHEPAWRVALVNTGSQMLMHIVIHHALYDAQSLDGLFQGLSCFLLGESCT
FSNIEPALADVLSRAKSELSEGKKFWEEQAVRVVVNKFPIMTSLREPVGR
LTVNEHISSMSVGELQEATQAVGVSLQAALQASWSRILASYLGEQSVTFG
VVLAGRTSDETADSPFPCLVTLPIVAEAEDSNMGLLQNMMAYNSNLYKHQ
FNPLAEVKKWLGHPASPIFDTVLVYQKTSGPHLNTDQWKLMEDLPSVEYS
VSLEVEPLEDEQLHLRLTTRSDIVPHEQADLMLKQFDAMLTHLLKHPNAT
QNEIYKYHQSLFSITPPRIPVIDAPVEFVHQFVERNAELQPNFPALEFVS
DFNGTPSSKMTWTYKELDDLGNQVAHLLSKVVRVGNIVAVHFPKCPEAYF
SILGILKAGCSFVALDPDAPKARKEFILEDSKAACLLITNNSDLLFSVKI
PAITINEESLRSHPSDPIMHGEAFTPQNTCYCLYTSGTTGTPKGCEITHE
NTVQAMMAFQHLFQGHWQKDSRWLQFAALHFDVSVLEQYWSWSVGITVVA
APKDFILDDLTGSISKMEITHIDLTPSLARLIHPDQLPNLCKGVFITGGE
QLNQEILDSWGPKAVIYNAYGPTEATIGVTMYQQVPINGRPSNIGRQFPN
VGSYVFQQGTDIPVLRGGVGELCVSGKLVGKGYLHRPELTNERFPKLSEF
NERIYRTGDLVRILHDGCFDFLGRADDQVKLRGQRLEIGEINHVIRNNTL
GVKDAATLVMRHGSKDVLVAFLVGEGEKASDLCMVSDEDALGAKARASCL
DSLPGYMVPTYFIRLSRIPLSPNNKVEAKELKMLFHSLLPDELVKLTGRD
TTSSSSEIDAAVMKRLIEALACFTYLPTSAIHETTSIFDIGVDSISALQL
STALKRGGFPKSTPAMILRSPIVSDLARLLSRKLTSTKERDETKEIEQVL
RAYQQRYRALVCRELRVKSADIEYIAPCSPLQEGMISAAVAEEGSHMYFN
SFDFHVKDDVPMDMVRHAWETTIRDHSILRSVFVKTIDGYLQVALHGIDN
FWRDLVASDDGDVDNILEQARLSWTRDNTLHILSPLQLIQVKGPGKRALR
LYIFHGLYDGNSFNLMNEYASPFWAKHLEHWQRCEMPKISPLASNNAEIV
SLSRRLTIKRLDELRRQQSVTLQTIVLSIWTIVLQRHVSKPFTTGIIVAG
RSLDVPNVENTMGPLFNTVPFFNLTLNGLSWEALVQKCHKFNTDILSFQH
VPLRDIQKWCSDGQSLFNNLFAFQLEKPDPNPDAIPWTIIDNKATSLDYP
LAFEATRTKDGHLSLYLVAHPDIAEEDTLEQMLDHFEQVVLTVEPGTVIA
LPPHSNFSFPKNIKPEPFSPKTFVPERLHWTPVGLALRDEFCTFANIPAE
DASPGTSILDLGIDSIDAIKISARLAKRHIKLSASQIMRHQTISAIAATA
SNLNHDALLKSSIDTGKAEIQSRLHSYIQGQGVDPNNVELVLPSTALQES
MISAMVQSDFQLYFNHDILELDKTVSLSKLRDAWLEVIKSSPILRTAFFE
IDDNNPDMVYCQVVYKQATAPVAMHKLAGASSLQDLMKTATETAIRGEAR
NNLFQVSLVSTDSQNYMVLSVAHALYDGWSLALLYNDLAKAYRSELEPRR
YSEVLMSQPIIYEYSDVNGFWGTYLADASPTVFRSGRPSAPLNPSNIIRN
EHLSSKSLTEINIFCKKTSISLQSLCMACWTAVAAHFTRSLDTVFGIVLS
GRDFEGADELVFPCMNTVAVRSVIHANVSSFLRYMEANLADIRDHQGFPL

RKAQAAAKLGGKELFNSLFILQKTPNIEASGSMWRSIGGASAVEYPVCVE
AEPIQDRLCWRIACKSDVLSQLDSEDVLVKLDQILNFFLDFPESEILSFT
DGLVSICALPQIAIEEQPANSHTTATADDDMDDAYSWNHTALLIRQILSD
VSSLPIESILPTSTLYHLGLDSISAIKVSMMLRKVQIDLKPRELIGADSI
KEIVRLAELDHKPQKEESFNAAQLKLPASVSQESLLNLHNILQQDVEAAL
PATPLQIYMLSAWQNSNASVFFPEFCYEIIGDYTYEQLVEAWDQVWRHIP
ILRVRFMSTGDIEMPWIQVILKPSSNSSGHMSEPLWRFSVLKVEDGGSWL
LRLSIHHSLYDGFSLPKLVQLYYEAIQNTAESAITPNVDLISWQNYTIRP
TLKASIDSRNRFWSEYLVGCSTSPGSPIPYASERVSYIRRHAFLKTKELQ
QVASSHGISLQSLFLAAYAKTLCVNVGAQDNTQSVIFGIYLANRDTETDN
LDETYPTMNIVPLRVQIAPGDNLIKVAAAILKDLQLIQSNGRATVGLWEI
YEWTGIQIQNFVNFLSLLDDDANSGKPGLVRRRTEDKLSTKGLDLLKQPW
LQNNAVRKAYPASLDIEASVHEDSLDIGVFGSTDSLSHDDSSRIVDNIIQ
HLASVKLA
>Mb|QLI66418.1
MGVFLSLNWFFLLAGLALTSTGTAAQNLVQVQLTSGTKITGRLNSGVETY
NGIPYADPPIGPLRLRPPQKISRHLDSVDGTGIAAACPQLLLSPADKNII
SSIGSQILDLPFFKDVKGQEDCLTVSVQRPANIKPGDKLPVLFWMYGGGF
ALGSTNTYDGASLVREGVANKQPFIFVAVNYRVGGFGFMPGIEMLNEGSA
NAGLLDQRMGLEWVADNIDQFGGNPDRVTLWGESAGSISVFYQMALYGGN
SSYKGKPLFRGGIMNSGTTIPAEPMDGFKGQATYNQVLKEAGCAGAVNNT
LSCLRELDYDTFYNAVTQTFSGIFSFSGTKISFPPRPDGKVLPLSPDELA
ATGQYYAVPIIAGDQEDEGTIFALYQQSVKTNDDLVNYFSQFVFPNATKE
QLSELVEAYTKDDEGSPFRTGPINSLYPMYKRIAAIIGDMTFTLSRRSFL
EATAKANPNMPAWSFLSSYAHPLPILGTFHASDLIQIFYGIPPTHATSST
RQYYFNFLYNQDPNKGNKGQLNWPVWKENNTLMWFKTQLDNGYLTDSFRN
NQYIVLAKLAKAKVLRQ
>Mb|QLI66419.1
MAISWKSFDFFEVSQVSLPDDETRSLFDANEISSICSGSDSLFLGGSDGS
VSIISKSWKAVRTFQAHDAGRVTHMRQVEGTSLLITVAEDLSSEPVLKAW
ALDKLVKKTNMPTCLSTLTINNGRRQFPISAFAASDDLSQIAVGFGNGAV
TVIRGDLIHDLGTKQRIVFESEEPVTGVQLATDEKLTTLFISTTTRIVKL
GLSRKGQGLPPKTVEDSGCAWGCMTLDKETGDVVVARDDAIYTYSLDGRG
PPKAYEAPKTLIAMYENYVALKCLPTGVNGRDPDSMRRRFGGGGNDDLFN
ATMFVLLEPDLRVIAHSETIMSPVRFIFDVWGDLYTMSEEGKIYRYHEKP
LQQRLEMLYQRNMFPLAIELAQGSGMDSQQQSIIYRKFGDHLYQKADYDG
AMMQYIRAIDTTEPSQVIRKFLDTQRIHNLIQYLEQLHEHRKATADHTTL
LLNCYAKLKDIAKLEKFIRSPGDLKFDLDTAIVMCRQGGYYEQAAYLAKR
HGETELVADILIEDSKSYGEALGYIWRQDPEVAYPCMQKYARVLIENCPK
EATKLFIDYYTGKYRPRKSTVAIPGETSSTGGLTSGTSSAVQNLTNYLPF
PYMGTSSGPTATPGTPSNIKQPASNGTLELGEDDHPPPKYTVPAPRTAFS
SFIDHPDEFITFLEACLEEGDLKVSDRTDLYTTLFEMYLYKASEKKGHHR
EEWEGKAKKLIEGEHVPMESSNVLLLSDLSNFRDGTVLVKEQAGLLFDIF
RSYTSAKDTRGAIKALRKYGPEEPQLYPAALAYLTSDPKVLEEAGPDELA
NVLSKIDKDGLMAPLQVVQTLVGQSSAGGVATMGMIKPYLHDTITRERKE
IQDNRRQIDTLRVDTEKRRAEIADLGSKPAVFQATRCSDCGQGLDLPAVH
FLCKHSFHQRCLRGGEGDEVECPQCAGGNDLIRKMRQEQRKAAEKHDLFK
IELEGSEDRFGTVARWFGRGVMDGQNKDDDI
>Mb|QLI66423.1
MCGTCNGDATGAAPPAANSTTDNAAPSASTSNGVYPAQGNPASSPYQSVQ
DYISNVGRFKIIESTLREGEQFANAFFDIDTKIKIAKALDNFGVDYIELT
SPVASQASYDDCKAICQLGLKAKILTHVRCNMEDAKKAVECGVDGVDLVI
GTSSFLREYSHGKSMDMIRSIALEVIDYVKSKGVEVRFSSEDSFRSNLVD
ILHLYQACDKAGVDRVGVADTVGGATPRMVYDLIRTLRGCVGCDIETHFH
DDTGCAVANAHSALEAGATHVDTSVLGIGERNGITPLGALMARMVVTAPD
YVKSKYNLKALKSLETLVADAVQVNIPFNNPVTGFCAGIHAKAILANPST
YEIIDPHDFGMTRYVSINSRITGWNAVKARIEQLGLGSEFSDDQVKEVYG
SPLFSAVFHYTGDIDTMRRTAKIKQMADIRPLAIDDTDSIIRSYHLDLKV

>Mb|QLI66424.1
MSAANTTGPSPTFYSSSSSPETPSAPSPNPNGRALPAPGDSATTLDVSGD
GTTVKLDHLGPLVVNVDGTLSRISNWSEMAEVERQNTLRVLGRRNKQRLE
ALRKAGKE
>Mb|QLI66425.1
MTADLAPAASATSGTSVPATRVKSSASTTSQFGYPRGHLGHLSGHEAEAL
DQFKNLLEERGAWKREPASHDDQTLLRYLRARRWVVEDAYKQFKDTEDWR
SANHIDTLYRTIELDAYEQSRRLYPQWTGRRDRRGIPLYVFEIKNLDSKT
VSEYERVGAKSTFSDAQTDGKTSPGLLRLFALYENLTRFSQPFCTQLTDR
EFPDVPITMSTNIVDVSGVGLKQFWNLKGHMQAASQLATAHYPETLDRIF
IIGAPFFFSTVWGWIKRWFDPITVSKIFVLSPHEVRPTLEAFIEPRNIPK
KYGGELDFSFGQLSVPDPSWEGVVAWENGYSSFPSGPLLWEDVDDGKRAA
CVALGKDKGAPRNVRICTVPKTWPAEEDAQTQTDGQGVTAEGTQTNGEVS
EGTQTADETRDDGAQTEEADGITNGEIVEKLKLTEDKTAAAQVEVAPAAA
A
>Mb|QLI66431.1
MASDELSAGLNYLTDAAHLLRQTAPETSAHLMRQRDDLMLHNNLTQHEVQ
RQHVCGACGHIMIPGDKTLLKLELREIHRLRRQAASQKKKRQEKPNTASS
GPTKSISCGHCGRDTTISLSAPEAATRRKISRASTAKKTVPAEPQNQKMT
ANASSKKRAKNRKGGLRALLSAQQQKSATSLSLADFMG
>Mb|QLI66434.1
MSSGIRDKLYLPVVGTQLVGMLILDLVPFYPKFLWQSPSSLLHPLVSLRK
WWTIQSGDPYFASCTHEPWFEAFLYVELLVQLPLTAYLAYKLGSMKPTSG
ATELAALAYACVTFMGATACAYDIWHMGADKVSVEHKPRLFWGTYLPFAV
IPALMAVDMYLRLLARVQDPRPTKKI
>Mb|QLI66437.1
MTSYDSDSSDGGEFTETNVLLGYASKDSQDETISKLGGIPEWLEADKPPS
AALARCKVCKDLMALILQLNAELPERFPGHDRRLYVFACKRESCRRKQGS
IRALRGVRIWGDETPAASAAKAEEEPKKQETGRNNDGLALGDALFGGKGI
GGGSGANPFSSNANPFSTSSSAGSSGNPFSSGQQKKDTPKEAEKDAKPAT
LSKSFAETLSLGTTPAGPPPPPEPWPETSAQPKPYPTLYLAEADYETLEP
TSTKIPDNARIEEADAPETVSVLDREAFESSMDAAFQKFADRLAQNPDQV
IRYEFAGTPLLYSRTDAVGEKLSKGSIPGCPNCGGKRTFEVQLTPNAIAE
LEADDLSLEGMEWGTIIVGVCERDCVPRHVGKDEAGYLEEWAGVQWEELT
KQK
>Mb|QLI66439.1
MDIDKKYDNYDFPVTAAERQDGHAGHLNEQQIAQVHQLRMMLEAEGYTDR
LDTLTLLRFLRARKWDVKLSKAMFVDTEKWRKETKLDETVPIWDYPEKPE
IAKYYKQFYHKTDKDGRPIYIETLGGIDLTAMYKITTADRMLSNLAVEYE
RLADPRLPACSRKVGNLLETCCTVMDLKGVTVTKVPSVYSYVRQASVISQ
NYYPERLGKLFLINAPWGFSTVWSVVKGWLDPVTVKKIHILGSGYQSELL
KHIEKESLPVEFGGTCTCEGGCENSDAGPWHDPQWARPAWWEKKADEKTI
QNNGSKIETAQDGPAIEPAPIAETAKDETVQTAAA
>Mb|QLI66441.1
MPEPGRKPEPDKRAAAQQAVDILHEISTLLNCHLDRRAISICVSMIEKGV
NPEALAGVIKELRQEAQGVERQVAGRRA
>Mb|QLI66444.1
MTDNGIPLTTVRSNASSTGARKANTGNTTSYDSSPENTFEKNESEMHHRH
AGRRRKQGDDKLSRRGTAGSDEVSVNALGRLYAKIVGFSVVTRYMVYIVP
VGLILAVPVLIFRFTDFGDGTFVGGRDGPTLFYLFLWILIAWCALWVAKI
AAWVLPKVFMFFCGIVSMGVRKYATVLKNLSIPLSLFFWALAAWLSFKGL
FNDALSKNVVWVRNMERVLGALFVSLVVFLVEKALVQLIGVSYHQRSFAN
RIKASKREIHLLGLLYDASRTLFPMYCEEFAEEDYIINDSIEMMLRGKKG
KMMGGNGAATPMKIIGDVGRFGDKVTSVVGHVAKEITGKQVFNPNSAHSI
VLEALEKKLPSEALGRRIWMSFVLEGKDALYLEDFEEVLGPAYKQDAEEA
FNMIDSDSNGDISLDEMVRKTVEMGTERKAIGEGMKDIGQALRVFDKVLM
FVVLLITILIFLVFFQSSVVTNLATAGTALLSLSFVFAVTTQEFLGSCIF
LFVKHPYDVGDRVEVGGTQMLVERISLLYTVFTKTAQNQSTQVPNIVLNN

LWIDNVSRSKAMTESFQVDVSYDTSFEDIELLRAEMEKFVRHPDNSRDFK
PDFTIGVGGVGELDKLTLEISIQHKSNWHNGVVRATRRSKFMCALALALK
KVPIYGPGGGAEALGGPTNPTYSVAVTDEWASKSRDAVAKDKDAGRMVPV
QTGQTDAEHRSAEEHAAAELNARSLAVETSGNWGEFRDDRSQRSPSEDPR
RSRDIESVRNELLKKESQRGRRKAGEGLQTLSPTETHGGYSAHSPRAGPF
DEEAQTGIPQRFYSVSRSHEPTVPEEEEGLHPTGPYSVGGLSHKPSRGPS
SGHR
>Mb|QLI66445.1
MEDDARGSESAVAPLKAANGDHQPKKLPKGIVLGEDGKPCRSCTSFAAWA
AMTKENARQNNPAGSNKTLATNMSQSSNPPSDCPPDVEALGRGTWTLLHS
IAASYPESPSMSQQSDLLSFVKLFSKLYPCWVCAEDFRTYIGREVPKVES
RDNFGKWLCGAHNDVNRKLGKPEFDCSRWQERWRTGWKDGRCD
>Mb|QLI66447.1
MASGNNDPKLLYAVDGIKAYHIANGNEHSLTPSGPQTLSLLMVPTASGFA
DPSGIGSGEEDFYLHLHLPPELDLPMPATTQIYHQPPTSYLIPRWDLGPD
AGAFTRIEFPPAGSRPGIQEDVDTFETILAQCTAFLERAPPPKPKRAKTA
PVFGASAKAREAAGEELPAYNPADYTRGEGYVQGSLSSSGKGGRIVLVDE
EDGSVIGELSEGFQVVEDTGIKPGSKDPVEIALPTDGSQAINIQPAPAYY
EEDRIHPAYKKSTIVSMGMRASRLLITTSDYVSHTLQGQADNFTKSTQPV
AKPVTFTPTTHAHIRKINQFSNKAAGMSAATVGSIAKVAQNFGANLSRRK
DGKARGFDKDGNMIETYKPGMLNKSLMAFNTVVDGMEHAGLSLLTSTSSS
VSKVVEHRWGPEAGEVSRNLGGGFKNVGLVYIDVTGVSRRAILKSVAKGM
VVGTVKGGGQVIVGGDEKSKTIPVAAGSGNGNGEKASYGDSSSVSGGYQG
NGKKPA
>Mb|QLI66448.1
MASKLTLDSTVKLNSGYTLPVLGFGVYQTPKEIATDVCAQALRLGYRHID
SASAYRNQGPSAKAIAASGIPRSEVFFTTKVPMSGKPLSYDNTHALVDTA
LRDTGLDYLDLVLIHSPYGGTENRLGAWKALVELVEAGKIRSIGVSNYGV
HHLDELEAYIKKLEQERGGPGKGGVISVGQWEVHPWLTRSDIVAWCEERS
IAVQAYCPIVRGERWGEPGVKALADKYGKTEAQVLLRWSLQRGHVPLVKS
VTPSRIAENAAVFDFELTDDEVEGLATRDYSPCAWDPTVEPIDK
>Mb|QLI66449.1
MRATPMRPTLLPIARLVTRYTCFTRASFHSHEHPPSAKSFGDVENAILSA
AYQHVPEHGFSHSALSLGARDAGYLDISPSALSDGVFSLIQFHLVRQRLA
LTEKSRALFDASNTNISGNGIGFKIATLAWARLMANKGIIHRWQEALAVM
AQPSYVPASLKELAMLSDEMWYLAGDKAVDSSWYTKRASLSMVYSTSELF
MTNDKSPGFVDTRKFLDRRLEEVTTVGGFVGTLGAWGSFTMNAGVNVLRS
KGMRV
>Mb|QLI66450.1
MSSGTELEHIDAVDAFARTLFVRVEKYPSPLFTEVAAAVRRLHLALRHLR
VEAADPDSLLRSADASVYARQLRPIVEDCDFALKQLKTTLEKFDGAGGRE
TDGLVDRIAAVRSRLANETTSVDMFLDTVQLNNPANNAPEVVPQGCEPNL
ENIKDKVDNIAARLFRRWNTNSGIINDEDSMWQEFKSELEMEGFSPHILH
KHKDVLRAYIRGLQAMSPADGGSPPSVRGLLEHEAAKRPPPLPPKEPLAP
KELYSSYVENEKCFPIIKKEWRMPDHAPVAGGFDNDNHSFSSDYGSGAVE
GSLALISTKDLINMDSISAGMAVMHLKPSSSHLGAPGVTSNPGLTTPEMS
GSWPPPHITNGQSNLPNSVGGSPNMGYPQPGYPSASGAQPGTFSSSPGIV
SRLAPDRYGKEIPMDASWTKIRRSLVSPEVLQRAGVRYEARPEYVAVLGR
LSREQIQDYARQSADCRAARSGRYPTAQRQNRYTQRDRAESKSSREYNDD
ESVLWDESDATDHEDATSTRGRKTYPYIVSPPSKDKTSPSSTVMPRPILK
NKNENHVRFDPEPHEVDIKSSKSYDDGYNRRRENEPRRNRDHRDSGKREE
RPRGDDNRHRHPNRSEHGRRRDRRDERQMRRKTWGETIGVVSIGGAAASL
LGVLTEAAVGF
>Mb|QLI66453.1
MPDTSGYGYDIRDDDTLVGDDNSSVSSLGSASTLVSEGANPGRRLPNIRP
HIDHRVDVVIIGSGITAVGVALSILPLMASRGMAPNVFVLEAMGLCHEGA
TQHDALMSFLPCRVFRPDGGLLPEKVLDFQVKNAEAVKELESKYLDAGAR
EFEMEELYDNDEDLEFGKRTQRPLDELYPQYGATILEGDEVPHYGPLRAR

GARTHTARLIRLHKLVTSIWADLRTQYANLCISTNTCVRHITLTTGSSHP
YIVTSSWGSIRARHVVHATASSAPRLLPEIGLTAGWKHRLLLRPGARFPK
REGKHIWALHLESHPFTVIRLPGGNDEIDELLIDWEWVSGRDHGDMLSTV
DQIYSYMESVFGPEWKDAEIMPQNRSSTIGITGDSLPFVGRIPQDYTNRG
VSSSEQAAVEAPFSDDGGLATPGEWISAGYNGQDTAFALLSGYAVGIQIA
GMENEELEMVPGRPGGRLNDWFPKEELSLDDDRMMRANLGPLG
>Mb|QLI66454.1
MTPSLLGQVGLMALVACLGLLLCQRLLKRKAGYRLPPGPKPLPIVGNILD
LPPKGEPEFLHWFKHKDTYGPISSITVMGLNLIIFHDKEAAHAVMGKKAQ
KTSARPQLNFATLCGFENFLITHQYNNTYRKHRKMVHQEIGTKSLSAGFQ
GTQEKEALHFTLQTLHDPDNILKHLKRLAAAVVLRITYGYSVERNGPDPL
VDVIEHAMENLSRAFVPLSWAVDAVPAIKHLPDWFPGTSYRKTAKEWKGI
NEAAAELPYDFVKRQMSQKAHRPSYVSNLLEKNMIKTGNETSMTLDDEEA
IKWTAVSLYAAGSDSTVAIITSVICALVIFPEVQQRAQEEIDRVVGNDRL
PTFDDRQNLPYIDGIVKEAWRWNPVGPMGLTHKSEEDIVYGEYMIPEGSY
LLPSLWWFLHDPKDYSDPHTFKPDRYMAPLNEPDPGDLAFGYGRRSCAGR
FFADASVYITLVQLLAVFRIRKERDANGNEIPVKLEAIPGMVNRPKEFEF
KVEPRSPRHVELLRRIEGEQEPEFSDAGRLSTKAG
>Mb|QLI66455.1
MAGLNPQITNLVIMLGMMQVSKRIPFDDPNVLNTVRAVYLGSNVLIALLY
LYIGSQINKKKDLTTLKYVEPPPMGSTEEGKLVTTTIQAYDKDQLRALFR
GQMMGIAIMGFMHLYMKYTNPLLIQSILPVKSALESNLVKIHIYGQPASG
DLKRPFKQAAGFMSAMQQGQAQSDKKAVEAAEKSGRGGAKEE
>Mb|QLI66456.1
MPSTQDPSEQVKFLVSCIGHTNNGRPDFAAVAEELGIVTKAAAQKRYERM
LKANGVSGTKSPTKTADDGAIPTPQTTPVKRKAAAARTPGSAKKTRGKAG
KDVKKEELDEEKTKKGVKKEEDSESDLSDPPTSDDIEI
>Mb|QLI66457.1
MAEADETPPSPSTFVPPSVHREALLAGSSFTHFSPVPPALLTPARASDPS
SLPPLCLGVDEAGRGPVLGPMVYGIFYLPQPQSDPLLRDAHGFDDSKALT
AAARSSLMRALCTAGTDLHAQCGWATAALSARDISAGMLRPSGAYNLNAQ
AMDATVALVRGALERGLNVREVYVDTVGPPATYQARLQRVFPTVKITVAK
KADSLYPCVSAASVCAKVTRDAALEVLYEARRDENGAGPGMEWGSGYPSD
ARCVGWLRRNMHPVFGWGPECRFSWGTAKDMLETKGGVRVDWPVEEEAGD
SMRVTDFFAAGRDDETDELGGWYGSPAGLEAF
>Mb|QLI66458.1
MSLPNFPDMPGAMGAVHGMTMHQMPQHNQPSPQPAKAFLCATCSKSFARR
SDLARHERIHTGLRPHVCDYPECGKQFIQRSALTVHKRVHTGEKPHHCEI
CAKRFSDSSSLARHRRTHTGNRPYKCPYADCQKTFTRRTTLTRHQNHHSG
TIEEAAAATAAALAASKAQKATRARSEGDHLSSHGSPLTTPSPGHRTMSM
SPSMDMTTATNVNRHPGEFQYLQQNGLPPHLRVGSPNSAASNGYTSGGMR
PTSHPTSYGPPPTLEPSVEQHQPGSSGGSPHMSSVGWQSPSHVPSPSQGA
GSYVYPEPDTYPQNAAMGQMYYNASAQMRRPQSAEPGLVHMA
>Mb|QLI66460.1
MADTGDDTGHVSEPLDLVRLLLNEVVFVKLRGDRELKGKLHAYDSHCNLV
LGDVEETIYAVDEDEETINRKSEMLFVRGDSVVLISPQVPF
>Mb|QLI66461.1
MHSLDSPPLDINYYISPSTCVDSTTPDSTRATAEASTVAAVNPASPPSPS
HTSPTTRKLAAGSGITRDGVIVGKFVLVEEAEAEDAAHISTGHARTRMAQ
RRSSYPFSSASELSGRGLREWQSSKERRFHGDEAIAPHTAAEGNCSPAGT
DLGNHPPAAVLATSQQQLNEYRARLARDQEIRELSLKGLLMTQSEKTRVS
PILEEAAGMSSPPMLTAALTTTSTESGNTVRGTMTPAAVAQTPSYPFPRM
ARFPSHQTSSKWVPYQPTASPLSRQQFQSPLEYRGLLDKVSSESSTPASN
VAFEPPGAASENPHGFDFPSPNLYDLSLMLSAEPGLDAWWNTVVQIMRDI
YKAERVTLAIPADTTDIENVPWGQKATYNEHQEDELSLGYLAKASSASDG
AAAFDDRTADSPSTAAPRPGLRTRHSYTAFEENKHKSLDQNSIMPPPKRP
TNLSRSKTQYPSPRVAAELHDDYTKLNKDALDEHDATAETQQAIPSWEAP
FMARYEGQGQVLPVLQALDYEADPLIDHTGVMRVLERGRPVALTRTYPYL

PRTKPDPKQTVEQKSASKGSSADSKRIKRPRTESVSKLSTIFAGTTQSKG
MGNNHVGDKLKTTTISSSLDDEDPRPPTPKYEEYEQAPPSPWSQSPAPSP
AVRADPKENPFFTDAMVDEDSFNPGSPPTSYTGMRPPEAIGVDNSWTVLH
IPLTHVLLSKPTRSFKLDTAALEQKSHLRSRDERHVSAEADQVSPEQVKR
NRSSPIAVLSILSPIIPYPSNLRHSLENLAPHMATSFSLCRHYSNLETEL
AGLRRRRPSTTGFGAVAPFGSHVDGGAFLASAQALVHQNSLGGSITSPSD
YSAISKSTTASPLVTPGWEHGSLNHLVDRRQPLGSPSGSQALESYFATKQ
RLVVKEASTSATQRPRTVSKDSSPSEKRHSSLAGVRFGNEAASSPQQQVV
ERGYFESEDSMTKPQKEEEEDEEDAGAPSSSGGGASELVSDKTEDGGKAD
LNTNNNDASHYGHSVLHSYGADFSSTFQSLPPSSSLSARLPPTPAVPPRS
GSLTSMAGDMPPPSDRLKGLILDSLPAHVFVSLPQTGETVWVNSRFLSYR
GQTVADLSADPWGSIHPDDRAGYLKAWGHSLRTGEQFSRSVRIKRFDGAY
RWFYARAVASKDKRGVIMQFLGSYMDIHDQHIAELKAARQEEIEASEAKH
RLLANLIPQIIFTATEDDAITFANDQWLSYTGQSFEDSLGLGFMDHVHPD
DLARCRIPIEHGGQPLNDAQSPPEEKGHGGASSSGTQTTPTARNVQQQPA
VGKSGMRNVQPALSRASSSGSESIYSLPSAELTELARKGIIKVTTDTSGR
LSYTTEVRLRSKTGEYRWHLIRCVEIDTFDFGRGASSYFGSATDINDHKL
LEAKLKEAMESKGRFLSNMSHEIRTPLIGISGMVSFLQDTTLNEEQRDYT
NTIQTSANSLIMIINDILDLSKVDAGMMKLKYEWFHTRSLIEDVNELVST
MAIAKRLELNYLVEADVPAWVKGDKVRIRQVLLNVIGNAIKFTAEGEVFS
RCRVYTEEDPAVCNENEIMLQFAITDTGRGFTKEEAELIFKPFSQIDGSS
TRQHGGSGLGLVISRQLVELHGGKMEGTAIPGKGSTFTFTARFTLPSPED
HPNIPISPGSTASTGSRQGSSENVNHQTTGLSAVEELRGSSSSPREGEEE
GANPKGIHSASPVSACASGSLEPPSRPPRYLTGPPSTASGHSGLARFSEA
AKASGQDLSQMKLEMAVDRGSPPRPSTSEVGSPPPKSDLRPPLYSILIIC
AQHHSREATTQHIEMTLPKDVPHRITAIASIEDAEPFLGGEDAMRFTHIV
LNLASAEAIIDVMDRITKSQKIEGTTIVILCDSVQRQAVQKLAADTKHER
FLSENLVTFIYKPVKPSRFAVIFDPEKVRDLSTDRNRSTAQQMVENQKAS
YQEIEKRMGNKGYRVLLVEDNPVNQKVLNKYLKKIGVEVEVAVDGVECTE
IVFSKPHSYYSLILCDLHMPRKDGYQACREIREWEKLSSLPKLPIIALSA
NVMSDVQEKCVAAGFSDYVTKPVDFIDLSRAMAKFF
>Mb|QLI66462.1
MEIAEASTTSGQTAQADQQQPQETTTTKTTTPAADQTTTAGAENAEPAAP
MSKNALKRLRRAQEWEDGREDRKRRRREKRVAGKARRREERAELLAQGAD
PAVLFAKRAPARLVPVSLILDCDFEAYMTDKERVSLSSQVTRCYSENRNF
KYKAHLWISGWRGKLRERFETAMESQHAHWKGVSLEEGDFLAAAARAREG
MRSAEGGEMADSLQRSADAAAANRWERDEREPFPLPGPVPELDAEHADVV
YLTSESPYTLERLEPNTSYVIGGLVDKNREKGLCYRRAVERGIRTARLPI
GQYMKLRTRQILATNHVVEIMLRWLECEDWAEAFMRVIPKRKGFEMIADG
DEDKTGNGGEDGDQGDQGEDGEENKEGGEKNGEEDKEDGKKSGEDKEKNG
EDGEKDKEDEEDEEKNKEDGKKNKEDGEQNGEDETKGSNSQDPK
>Mb|QLI66464.1
MSKGRVCLAYSGGLDTSTILVWLLQQGYQVVCYLGDVGQDEDFAAVEAKA
LKLGAEAFVCENLQRELVDDVVSRAIMCNAIFEDQYLLGTALARPIIARS
MIRVAEKHNCAYLSHGATGKGVRFELAFIALNPSIQILAPWRMPEFIEKF
QGRNDLLKFAAENNIPVSSTPKAPWSMDENIIHTSYEAGILEDPDHTPPK
ELWMRTKDPQDAPNEPTEFSVHFEKGLISKVVVGDKEVTDSVESLKLLNK
IGYENGVGRVDIVENRFIGLKSRGCYDTPGLTLARLAHLSLEGLVMDGRV
RELRDQFSSITWSRLIYNGMYFSPEREFVENSVVFCQHNVTGVVRLSAYK
GHAYVLGRSSNASNLYSEQDASMDSLEGFSPMDTTGFIAIQAIRLKKYGE
QKIKDGQPLSKS
>Mb|QLI66465.1
MQLKTTQRLELPPTADMHVHLRQAELMELVAPTVRQGGVDTVFVMPNLVP
PITTVERALEYKAQLQKIDPNVHYLMSLFLHPSVTPDVIAKAAAAGITGV
KMYPQGVTTNSENGVANVEAFYPTFAAMEENDMVLNLHGEVLESLAPENT
TLEEAFLPTLKKLHERFPRLRIVLEHCTTSAAVEAVKACGPTVGATITAH
HLYLTGADACCDPFAFCKPIPKKPTDRDALLKALVSGNPKFFFGSDSAPH
QIISKTSAEQGKAPAGVFTQPVATQLVLMALEEGVVRGDISENDITQEKL

EGFLSRHGRRFYKLPETSDKKIILERKGEKIGTSVKSADGKFEVGISRAG
TEIFSLAWESV
>Mb|QLI66466.1
MAPPKIITTSFLVGNLHCPSCVAVIKKTLEDEYAGRVFWVSPNVVTSVVT
VEHEDASADFIRDMGKTLEDVGFEICAVDTTGTMPDELNRESDAEQARTS
QDSRRRGSGALDLWWRLWGARTPPATPHQGKAAAHLDNCDSCKTTAAAAA
HENGRVLEEQLTRHVVAPFSPQNLIKATSKATTTPLDEVVIDDTDAVPDL
WRATLSIGGMTCASCSNTITDEIQKFPWVAKIVVNLVANSATVDIKEQDR
IQDVVNAIEDLGYDAALDQVINLKEQKRVSADARDVEIQIDGIFCPKCPQ
RICKTLNNLSFSRIEILQAPTLDKPFLKIRYTPRAPDFTIRQILRAIEAT
DPSLRASIYHPPTLEERSKAIRAKHQRELFWRMMLTLILAIPTFVLGIVY
MSLVPDSNPTKQYLMEPWKSGLSRNAIILCVLSTPVYFFAADIFHVRAIK
EIRTMWRPGSRTPIIERFYKFGSMNMLMSLGTTIAYVSSVAEMIAAAAKK
DDYVPENRFYFDSVVFLTLFLLAGRLIEAYSKSKTGDAVGALAKLRPSTA
VLVEKDPIRGQITTTTSIDQLEYGDVIRVPHGASPATDGIIITGETNFDE
SSLTGESRPIKKAPGDEVFAGTVNKGDAVTISVTGTSGKSMLDQIVDVVR
EGQTKRAPVEQIADLMTSYFVPVITLIAVITWIVWFSLALTGHISKEELD
SAGGPAAFAFQFSIAVFVVACPCGLALAAPTAIFVGGGIAAKHGVLAKGG
GEAFEKASKIDCVVFDKTGTLTEGGEPKITDSELFPHQEVKDLDKRALLA
GLKAVEEQSSHPIAKAIVNFCGDEVASAELGKIEELPGRGMKASYKDKQF
DIAVGNEHLMRDLSVTLSQSVVSRLQSWKSEAKSVAVVAVKPHEQQSPWI
LAAILSISDPVRREAVAVVKALRARGTQVWMLSGDNVTTAKAVAQRVGIA
DTNVLAEVLPSEKAEKVQYLQATLHSHRTVRRRHGYSTRRAMVAMVGDGI
NDSPALTTADVGIAIGSGSDVAISSADFVLASSSLGAVLTLLDLSRTVFR
RIKINFGWAVVYNLLAVPIAAGCLYAIKTSSGNHVRLDPVWAALAMALSS
ISVVLSSLSLRARVPGLGFRHSPVVVE
>Mb|QLI66467.1
MSKYKDKDGGIVLSFGGQWVSWAHTIVAYTAFLSALIVGVSLHYHKIVQN
EFYGYPQEWFPSVSATIGDRYPERSFFMIFIAITSGPRFALVGLFYLLTR
KPGTKLPGFIASMGVLRTLTCGGWTYITSTDDHDWHDILMISYIVATLPW
TLGCIALSPPNPQAIKYRKYLATAFFGTLVPLIYFFIQHKVHRVAGAYTI
YAFFEWALILFDVGFDAVTALDYSTFEVVIRDVKGLSKGDNLSSVPSAVM
EKEKEKATGGLYSLRFTWSEALDTAADVYHGFVFWSMLTSLGLVVWYFPL
WHMGISGYEAFVLVSISPLLLVGPLRSAVISNQRIIHLLSLSGVAAYLVL
DPARRLFTVGFGVAMSTLGWVATLHAESLHEARFESRILGLLVGLILSST
AKFAWQTNNPIWPIMHEANGGWNLTGLVLGVLAALRFTRKAPLTSGTPDG
AQRGSTVLAACGVGGVFFGMHSLLSDTSTMILWVWEGFPIRGPYFSTHGW
CTLAAMSAGLFIGICKPSLAGSWPQYAVGTAGAMVLTFFSHWFGYYGGLV
IAAYLMAVAVPLLSNASKKSPAVTFGLGFFIYVFLVLFHVWVVAYAFVPG
GPLVREHTDWIMYSMMGLIGAGVYDFNASQPRKQQPRRTSASQHKKYFGF
ATIIVNILFLCAAFMRFPTNDYKPYHAKDRVLTAGIWTIHFSLDNDMWSS
EYRMRDLIKEMELDVVGLLESDLQRIIMGNRDTTQFLAEDLGMYVDYGPG
PNKHTWGAALLSKFPIVESKHHLLPSPVGELAPAIHATLDVYGELVDVFV
FHSGQEEDPEDRRLQSEYLAQLMGSTPRPAFLLSYLVTKPLEGNYNTYVS
EKSGMHDVDSTDWDRWCEYILFKKLKRVGYARVSRSTITDTELQVAKFVI
PNSAAEAQQLDSVSAEERNRRVQESEVPEGWRFPAMFRGQGVRDHRYHVF
DEPRYFN
>Mb|QLI66468.1
MEQQSKRVPRQKHDRVILQFDYDCFYAQVFENKNPALKSRALGVKQKNIL
ATCNYNARGLGVKKLMLISEAKKICKDLVLVDGEDLTPFRDVSKILFNFL
RSHSWNNRVERLGFDEVFMDVTDVVDYNIECLNAGCLSHSFFHLSKTDPQ
DGFKCNFTAIAGCVEGTVPDEAPLENKTYLRLLLGSHLANHLRTKMEEDY
GYTSTCGISTNKLLSKLVGSRNKPRNQTTLMALNEQDVIAFMDSHKTRQI
PGLGFKTSMQLESQVLGEEVDADSHSFTSAVYARDVRLHPSISPGFIETL
LAGPGAERGVGVRVWGLLHGIDQTQVKEAGDIPSQISIEDTYKGLETMTQ
ITEELHKLSCSLIRRMRIDLLALEHNAEKPGTQRWIARPKTLRLAIRSWP
TRQGRSSASNFSRISRSGPLPGLVFDASADIDALAQKLVAEALLPLLRRL
ETAKGHKWNLQLINICVANMVVGAGDDKQGSGRDISVMFRRQDEVLRPFK

LEPLSDDEAPSEVSTGDAEGLGSGVDWNGSDNSVCQDCGHSLPSFAMPAH
LRYHEMGE
>Mb|QLI66469.1
MPRTRSTKPAASESTDTPAATLASKYTLTPRDGSSPGIFILPKTATPEAR
IVTLPHPRNGKPSRYLICPKTGIHEFTKISAPQSAPRSWLIETRADSGDD
DTDSKEADPHVDIVSSSDMFVATTIDALFLVLPSLVDATVQKGSDEKKRL
FLSSDDHFDKLPEESSHLSEILQWEATRALIESRMEIICDTVEAGDEKMF
RLSEKKLVATILEKAKRMSDGGLPATLEEKFVKRALEAPILLHRSTLAAK
VSENQAAAEVATESEVSTPVTDSAQSQSTTVTTDSNSSFVSQPSTAATSF
VEDGQAEDAAMTAIQASAEVTALQRLRVAFDFICSRYVPTATAEQLKLHL
SQQDMCSVDFSPLDKYRSKLAELRAEAVAATSTADYSRKRMGDEEEDALR
QEKKRKLEEEKKRKANESRGVRELKKVNTSGMMKLSHFFKAK
>Mb|QLI66470.1
MVVFDGHEYLTEEERRLKQDRERTKYWKKWGPYVAERQWATVREDYSADG
DAWSHFTHEHARSRAFRWGEDGIAGVSDTHGLQNIAFSFWNEEDDFLKER
LFGLSNPQGNHGESVKEAHFHVDNTPHSYMKFLYKYPQKKFPYQDLIQEN
GRRGKEDKEYQIIDTGVFDENRYWDIFIETAKESDDPDELLFRVTAWNRG
PDPAPLHIIPQVWFRNTWSWGHESEDKRPSIAAESELSAKSKHYSLGDRY
VYYSPSPGVGSSGEDVVPLWMFTENDTNYELLYEQKNKQPYVKDAFHRYV
VGGEKAAINPSKTGTKSAAWFNFNEDGGVNPGECAVVRFRFSKKNMTYLD
EEEFDDVIEKRREEADEFYYRISPLPISDDLRNIQRQAFSGMMWTKQHYH
FIWDQWAKGDPSSPPPPPERMGIRNSQWRHMHCDDILSMPDSWEYPFFAA
WDSAFHCIPLAMIDPDFAKKQLDLFTREWYCHPNGQLPAYEWNFGDVNPP
VHAWATFRTFKIERKMYGRQDLDFLERVFQKLLLNFTWWVNRKDTDGKNV
FEGGFLGLDNIGLFNRSEPLPTGGVLEQADSTGWMAFYCLSMLNIALELA
KHRRIYEDIASKFFEHFILISDAMTFRTGHKDEKSLWNEEDGFYYDAISW
GGPYIQQLPVRSLVGLIPLYATATLEPELLNKLPSFRKRVEWFVQHREDM
AERNMASIRKRGKGDRLLLSIVSKDRLEKILKRMLDEKEFFSDHGIRSLS
KFHKESPFSMEVNGQQFKVGYVPGDSDTGLFGGNSNWRGPIWLCVNFLLV
ESLQRFYLFYGPEFQVECPTGSGDYMHLGKVAEEIQHRLQHLFARTDDGR
RSINAGDDRLDFDEHWKDYLWFYEFFDGDCGRGLGATHQCGWTGLIARMI
HDTGTNCRLPQTPRTPSAAMAHYFDDMLQRGLDLGTPRSPSMRRIRRSST
ARSIGARSDFEASANGVDDDAHSVTNSVIHDDSERAREREEADSHLHRYI
SDQLERYKDESVNGGHADEYETKA
>Mb|QLI66471.1
MASKSFNVGVIGYGMSAKIFHIPFIATTPQLKLHSIVQRTPRDGNSAPQD
HPSARHYTDAKQLLADGEVDVVVVTTPPDTHFALTKAALEAGKHVLTEKP
FVPTAAEAEQLMALARQRGRLVCVYQNRRWDTDFLVVKHLVAHDVVGRVV
ELDSHFDRYRAAPPTNWKGALGIPSGGSALFDLGAHLVDQAYVLFGMPRA
VHGRLLAQRSGAADLFNPDAVAAELTYPDGKLVHVRISSLSAETDQPRFW
LRGARGSFRKRGLDPQEDRLKAGVAPSAPGFGREDPDSMRLVLVGEDGAP
REAKVPDLEPETYAAFYAAFGRALETGRDEDLPVKPSEARDVLRILEAVV
ESAKTGRDVVFA
>Mb|QLI66472.1
MIESIGHLEEWWLSSGFKHLTDLVNVDSLGLGPGQVGFVEAIQKKLKAFD
NDDLHRQLYKDESSPLPPSAMPNSRKKLGTIKGVFERTKDGRLDHIQTFE
VLAMLEQIEIHRKRLAASAKQVIDGGLLKDHISLLIKKHGAASETYSSLQ
LYFRAHCMVDSLPQNNIDRDQAAKIMARLNALFPPNFKACGKDEDEVEPS
SDIVNLRGEIRLCIYEHLMRPNKMLQDNEQSFKVNFTSPCSIPSYEICRN
SMVKYAEEFQKFEELCFIIFRQIEEARDDSPPTWLKVPLSNGSEIHGIVQ
TAYRSWNTIAVKTSTVARTSSNSRHTSSPTLTGLIASQNTNPLLEDMPGH
ELSAAKKSIAAFSLDSPTPDDLDCSEFHIHPLFQDQKPASDEMKRPGVIL
VKKRSSRNF
>Mb|QLI66474.1
MAPGAKSSRVKPSEVAADTKRHFIPMVNANYAEMFPPYSILYRQPAELAI
IRRNLSTNPPVFRIEAGDPVMIAINHAAADSQTTEGSRVRIPFICAANER
RPGGDWETGCSGYEEKLCRRSNLSATLSSPWPNTQVPSNYPIPSTGGILS
DSVVVYRGPHDHYERFGRLYDLPVVSVPPTRWPKLKENGTMYSFAEERDM

MREKIRGALRVCLYNNYDRVVVGDFGLGNSCRNPPQEVAELWRDVLLFDP
DLRGQFKYVVFAFEDPSQSTTRFIWDEMARKDKKGGSSKVKSRGNAPTPA
GFSQGSSTNLATDMAIFQMAFGHAEIVRVLSARDPRYGLDMITS
>Mb|QLI66476.1
MEDDRAQVDLGIAADIEEAQLTQAHQPEPTSDDAPQSTTKQPKKRFVGRR
AAAEAAAKNGTTSADGESGAIQAAKPRRAPRLLNRVPKDILDDPNLKEAI
ALLPANYSFEIPKTIHRIRTSGAKKVALQMPEGLLLFATTISDILTQFCP
GIETLIMGDVTYGACCIDDYTARALGCDLLVHYAHSCLIPVDVTKIKTLY
VFVDISIDTAHLLASLERNFATGKTMAVVGTIQFNATIHGVRSNLEAAGF
KVLVPQIAPLSKGEILGCTSPRLKDEDAVDMILYLGDGRFHLESIMIHNP
SIPAYRYDPYSRKLTRETYGHDEMQSLRRSAIHTARTAKRWGLILGSLGR
QGNPHTMALIEKRLKEKGIPWVNLLLSEIFPGKLAMMSDVECWVQVACPR
LSIDWGYAFPRPLLTPYEALVALGVKEEWDKGSVYPMDYYGKEGLGRARP
LEAS
>Mb|QLI66479.1
MEGSSKIKPAARVSGQKQDVWSIINEAAAASPKQPIVNMGQGFFGYNPPD
FILNAAKDALDRVECNQYSPTKGRPRLKKAIADAYSPFWGRKIDPETEVT
ITTGANEGMLSAFMAFIEPGDEVIIFEPFFDQYISNIEMPGGKIVYVPMH
PPETGATKTSSAADWTIDFAELEKAITPRTKMIVLNTPHNPVGKVYSRDE
LQKIGDLCVKNEIIILSDEVYDRLYYVPFTRMATLSPEIEKLTITVGSAG
KNFYATGWRVGTFRSTLPFSPVSPFNSIFFRLSSLTFVLLSPLTGWLMGP
ANLIKYVSAAHTRICYSSVSPLQEACAVGFEQAESEGFWDKTITDMKSKM
DRFNEIWAELDLPYSEPEGGYFVLVNMKKVQLPADYPFPEHVASRPRDFK
LAWFLIQEVGVAAIPPTEFYTPANAHLAEDYVRFAVCKDDEVLEEAKRRL
RGLKKYMQ
>Mb|QLI66480.1
MAQASTKVQTLIDEYPVVVFSKSYCPYCRATKEILKKLGAEFKALELDQI
PDGAALQDALEDITGQRTVPNVHIRQKHIGGNSDVQSLNNSGKLEGLLKD
SGALKA
>Mb|QLI66482.1
MADETSGNSCPVSGDISGDDSQSQPLPTPGLKPNPIVDDLKKNHLNKGVL
IPLKTTQEVRQDHTTVYAYITRAPTKSANDVITALRTLRPEGGANPLPHL
RTCAKPTDLPAHLKTQFMNDDPVGRQIHTSKSTWIYIIAGEAREISKSDL
IKTLSTVDALENDPFIATITIPLLAPTSQVQAALWSSQYWPTVYRKNNPL
GPHPSMVGRGTDEIKEDASLWMALAHQVALQAKEAGIGEAMGAVIVQRGE
NGTELVGLAGDARKHQECNGAILNNTVTHCVLRAISMVAQKLVRHEHREA
GITAEAANLYYDCFQDQPLIELERRCFEQEHPNKDGYLCHGLELTQPCAT
PANCAKLASKALLLLLSSGYGSRFTQTVGRNHYFSKEARIRVYVPTRLLS
TTATRSVFGFLNRIIKTALPDSEMNKRAAPGVNATASPPKKRKTDNVQKF
YAVQAGFRPGVYMTYAECSAQTAGFRGAVFKSFTSRSDAEAFAEGKKVAA
SSDEPERFYAVAVGNPTGIYTDWNEAALAIKGVKGPKYKRFGTRMEAVVY
IKQHGSREAIEALGETYAAAPVELPVSPTPVVASTKKTASKTAAKKAEPG
VQRPAEDVLQIYTDGSSLANGKAGSRAGVGVYFGDGDPRNVSERLVGDPQ
TNQRAELMAMLRALEIAPLEQTVQIISDSQYSINCVTQWAIGWKHKGWKT
ATGENVKNQDIIRAVLDKMDERTKTGANTYFHWVKGHASDRGNVAADRLA
VRGAKMAC
>Mb|QLI66486.1
MTFATEIHTFHSGRPQPQTSSSSSTFKSIDPTTGEPLATIYTTTPAQLND
AVAAARAAFPVWSQTPAPKRAAILLRAAALLRERNDALALTETLDTGKAW
CETSTVDVVTGADVLEYYAQFIAGGLPGQHTRLRQDAYILTTHEPLGVCA
GIGAWNYPIQIALWKSAACLAAGNCMVYKPSEVTPLHGNTLAQIYVEAGL
PAGVFNVVYGDGPAVGAPLVAHPDIAKVSFTGQVSTGSKVASEAVKSMKS
VTMELGGKSPVVILPDADVDEAADVAMAANFFSTGQVCTNGTRVFVPDTL
LAAVEKALVQRCREGIRMGLPRDEKTNFGPVVSAAQRDKINMYIKHGKDV
DRAKVLYDGSVDSQKDKPTPGGFWVPPVIFTNCTDNMRVAREEIFGPVMC
VLPYKVAGKSREDWVAEVVRRANDTPMGLAAGVVSSDVGLAQDVLRQLDA
GITWINTWGESPAEMPVGGWKMSGIGLENGHEGIQAYMRVKSTLVQLGKG
ACKGMFAKL

>Mb|QLI66487.1
MRTGFILGRTCFRPLLRSQSSSVPLIRRVAFRLHHTRQIPWTGPGTKGYS
ARRVILQASTGVAALSAGAFIEISQTDNNGTEQTGEHRMLEVSREEMRKA
VGDHETGLLRAAHKAVLFFDLYVWEPVCTGFRFLHLLAIFVPVIITVPAI
WIGKRRPERDNERSGTLWWYQFLVKAMELAGPAFIKLGQWAASRTDIFPN
EMCDTMSKLHSNAPAHSMHATRRTVSAAFGGREFDDIFDEFDEKPLGVGA
IAQVYKAKLKPELAMSKGGMNGSEGYGGLSENVRRNVETVLKSSPKRVPS
SYVAVKVLHPRVDRIVRRDLKIMHFFASILNAIPTIEWLSLPDEVAQFGE
MMKLQLDLRIEAANLERFRRNFKDRTTASFPYPYTDFTTRNVLIEEFAQG
IPLADFMENGGGVFQHDIADEGLDAFLRMLLLDNFVHADLHPGNIMVRFY
QAAQPELRLRKKPDRNAHDQEADVTEKVLERLRPFRRRKNKAAWEAELAK
IDAEGYRPQLIFIDTGLVTELNATNRANFLDLFRAVAEFDGYKAGHLMCE
RCRQPEAVLEKEVFALKMQHLVLSVKSRTLALGNVKIGDILQQVLSMVRN
HHVRMEGDFVNVVISILLLEGIGRSLNPNVDLLSSSLPILRQLGAQSGAT
MAKSGDFSVILVWAGLEARKFLQASIEDVERCVKYDLLSPNV
>Mb|QLI66497.1
MARPYEPGPSTSLSTNTPSQTPSSPVPEEDDDSGAASPELPLTMTASVML
ADLPRDATAALASVGGFPQEKVVVKFKPVGAAPSLAQELCKISATRKFEE
VVRYLRRKLRCKDTDSVFLYVNSAFAPSLDEVVGNLHQCFKNGQDQLIVA
YSMTPAFG
>Mb|QLI66498.1
MAPSAAHTADGILPPIDLKAIRPFWKRRNGILLYFLLTSSLFTSAALGID
GSMTNGMQVLPTWQERFGHPTGSKLGFFGASNAIGGVIPFLTLSWMGDKF
GRRIPTALGSLVIIIGVLVEFFATSLNMYIGGKMVLGFGSGLVQMTAAVL
VTELSHPKERVQVTTFYNTSIVLGYVIGAWATYGCFRIPNQWSWRLPTLI
QIVPSAYQLALIFFSPESPRWLVAKGRKEEAREILVKYHGECDPGSPVVA
FEFAEIQEVIAKEAEQNMTWREFFSSVPNLKRIGLCFATAVFSQSSGNLL
VSNYLTQILKDTGVNADKDITLVNGMVTLWQYMVALTVTVIIDKFKRRTF
FLVGSGGVVVTFVVWTIAAQQYLEKNSLAAGRVVLACIFIFQAFYTFAWT
NLVVTYPLEVVTYQMRAKTWAFVLLTIQVASIFGGYVNPIGLENIGWKFY
IYYCVWVTIIFLVVYFFFVETAGPTLEELAYLFDGAEAKMNIMHATKGIE
DSAAHMEEKPGQKLV
>Mb|QLI66499.1
MDDGKHGKAIEHHASDLDNNLSKQMSRQQLQHKRSRETRFQNDLAHLIPS
RWWFASSAFPMIAATLGPVASAFSICALGSQWRQKLVPGQNVDDAVFIQD
PAWLTIVNAIQLAVALVSNAFLLLNMTKRVRFSIAQPITIIGWYISAICL
LSLNATAAGPLQYNLDPPQDYIWSQAFYYGIWAAILYFVDASLMAVTFWG
ASSGHYPKDFNLTRSQRTLMLQTILFLMYLLLGALIFSKIENWRYLDAVY
WADVTLFTVGFGDFAANTTLGCALLIPYALIGVISLGLVISSIRSMILER
GRRRLDARMEEKNRRRFIRTMTKSGHDEILSPIDDDTVSNWGPENAGLPQ
NEFDRRKTEFLLMRKIQQQASIRRKWMAMAISTGVWILLWLVGAVIFVSA
EEPYQQWNYFDAFYFCFISLMTIGYGDRTPNSNAGKSFFVFWSLLALPTM
TVLISNAGDTVVKFVTDATIRIGNITILPGEDGFTGNAKYVANRVSFGRL
FRSATITIREHEREKRIRKRAEDLTGFDPKDVKDTEKQQNESSHSGGSAS
HGHREDDEERQGKHKLCQKTEIPAGRPSLAQVQDRLDDLPIGTELHLLLV
KEIQTVSQHLREPEPRRYTFEEWAWYLALIGEDERDPLNHRKVLPKAKHR
RTRRHKHKHHNRHRDKAEQGRDPPQDDVAETIRAPRPSMQLAAPIPVQLP
DTDAHLKWSWVGNRSPLMGGQEESEWILDKLMERLEESLWETKIDEDDAN
SP
>Mb|QLI66500.1
MRPTQALSVGRYRHLKLTTKDVGKGFYKGNRTGSMGRHTKHGGYVIEWNK
VRTYAVPPNLKDFKLTPFVSRQVREVPGDYKGLEKGPQDPYFYVEQWKRY
NGVD
>Mb|QLI66502.1
MSYKRSRSTFEADLHAPYALFGTPLPDETDSRDDGSYLPLWKQDVRDEKG
RRRLHGAFTGGWSAGYFNTVGSKEGWTPSTFVSSRSNRRKDDTASTTIQQ
RAEEYMDEEDLEDAAEAQQLQTSQAFAGLGSSTPDEMRAGGLMGLFKSSG
DTKGLQLLRRMGWKDGQGIGPKIRRGARLDVGMSGVVSDNNKMYLFAPDD

AQMIQFVRKTDRMGLGHQGGARLQSLGKTGDSDQDNDDDDALDKNGRPSL
FSATKKEAKSQRGAFGVGVLNDTGSDEEDPYEMGPKIRYNRVMGGEKKKK
KKATAAVNPSLKKAPVFLSKTARAGHNLRRCHDGRLPLDGFVLAKVVEDL
AAALSQYAPPPVPEGWKSSKDPLNMGDSSKYTSTADAAKVSTLDPRSRAA
LLGETALPGKSVFDFISSSSRDKLAALSGNKHLPPGLGQLPEGHTPLSED
DRRQALWNEVPKLDQSTAAAALSRSSAGPYADDEAKRGRYRKYLMSQANP
GQDLPDKAPRATDEDYIREMNEFYNCARIFKPMTGFMASRFTTSKTVLNP
SSNNGNGSDDKTRTDLLSKPEPKSKDAAEEAAVLGMFGHMTRSVGDFYPT
RLLCKRFNVRAPAHVAPDRESDNTSSTPKRSDSRAMPTQWSTSDLNVSVS
APPAVEAPTSSGAVTPVQQPVEVNPEKNDAVEGEAANADVLKAIFGDSDS
E
>Mb|QLI66503.1
MGLSADVMANGHEHEHILRPRARKGIRTQATRTLSGGSDGQLGGVGESAP
ASGLTSGQSTPVPENAPPSAVSLSCARKLARAEQKRRLFPTIEYASRVSH
FDPESDYRDFHGFFNLFWIGLAIMGITTMLRNMRDTGYPLRIQIWSLFTV
KLWHLAIADFLMVATSLAGLPLHAVFRRAPAGAVLTWERGGMAIQSIYQV
LWLALWIAVPFVLEWTWTAHVFLLLHTMVMLMKMHSYAFYNGHLSETEKR
LRSLDAPGDEADTSPAYVYPTANLRRGSISGIPSSQNDKPLSLPELTKQQ
QQQHLHHPSSSSSSSPSAAARLDIQTLREALARELTSPMGNTTYPRNLTW
ANYIDYLFCPTLCYELEYPRTPRINWTSLVAKTVATFGCIFLLTVTSEEF
ILPVLVDAQRRLTSSPVSAPEALLILAESISWLLFPFMLTFLLVFLVIFE
YVLGAFAEITRFADRHFYADWWNSTDWLEFSREWNVPVYSFLRRHVYSAS
RPHTGKLVATLITFLISAAMHEVVMACITKKIRGYGFICQMMQLPIVMLQ
RTRCIRGRKTLNNVCFWCSMILGLSLICALYVLV
>Mb|QLI66505.1
MSTPNSRKRAAPGASPMVNYPQRMQQPFPADSAVPDNMLRWNGDSADFVD
GSAHAANPYGMMPTPPQYTQVPAPSNSLTRRDMNQALIPTHTRGFDGSVE
PWAGFGDGNNLLPQNDEEGLIEQDNVEALEEMAQKAKRDAQGRRKGIPPF
VQKLSSFLEERKNEDLIRWSEKGDSFIVLDEDEFAKTLIPELFKHNNYAS
FVRQLNMYGFHKRVGLSDNSMRASERKNKSPSEYHNPFFRRGHPNLLWLI
NKPKTRATSKKSSNKNEIGELDSDEDQIQDEVTAQNTAAGPNVPTGRSLP
PASASSSAEPQALPKKEMTLIREELTKVREQQRLIHKAINRLQHTNNDLY
NQALMFQSQHDRHQSSINAILNFLANVFRKTLEDQGNTQSVGDILSSMIT
NQGQPSHQGNVVDLGDFFQPKMDSASNMGGAHKRTRGLLPPIPNHQADAP
TATQSPSTSSTPYYPVGNHNPEMGHVTELVDGSDTPPSLRQELESNPHER
MMKIINDHNATNKSGMDLPEATELVTNAPNTLNKDQRSKFVDFMAGQTTS
PPSQPPSAPTTAKPTPVPPRSAKASVSPSPTPADAAVPSLSPIMRSPQMA
PPSLNQISTNQIDLDQLQRLQSDQDAKIHELSELLGPLSPSGRIPGLEDG
AEGYFDQPHVDLDQFFDSNAFLNDASFGDGNDFNFSIDGAADDVKNGEPS
PTGTEEIQRDDFDTTQKNGNKRRRVA
>Mb|QLI66506.1
MVLLVLESQNKTESLLSMYKGVSPEDSAGILSRTFWWWLMDLLAVGKQRI
LRVSDTPRLSSSLQPSLTRKAVLQAWNDRGSQPVIISRAISFITNDQGGV
AQSCRAYEIFFAALIVYFGLAISSSMYKNTLNKLDILIRESLVHLVYDKT
LNLSHHGSDAGRVVTLMSTDIDSVSEMGRLLHASWGQAVELVIGMILLAR
EVGWVFPVPLVIIVFASRVSRFVAQNVRSRTKSWNDATQARISTLSTVLN
SMRSVKAMGLSEAIAKYINELRDKEIDRSKHVRWMQVLYNASANANGLFT
PVITVVLYAMLAASRGERLDTKTAFTTVAILSLVTHPANMMMTIFPKFVG
IMANVDRIQSFLLEPSRVDCRKIIDGSQFTETENCTGIRLDDVTVKFKST
TAATLKHINLNLPKHSITACAGPTGCGKTTLAKTILGEVSPVQGIVTVSS
RRIAYCDQRVWIPIGTIRDIICTFATKVDESLYQEALRVCCLDYDLQRLP
DGDRTVVGSQGVNLSGGQRQRIALARMVYSGASIAVLDDPFSALDGVTEN
KVVDNLLGKNGWFRRAKVTAFIITYAAQHFQHSDGIILLQDGHISAQGDW
RAIKSRASEMLKFNFEETQSSTVEPTGKSQYSTAKQAETDADKDLHRSVG
DMTLYSYYIRSVGVWNFLLLATCTALYATFNTFTTYWFKLWTESDGDRVV
FYIVGYVLLAFTAWAATSANLWSTFILLAPRSGRLLHQSLLTTTMAAPLL
YFSSTDVGIVLNRFSKDISLVDRQLPLALSNLCSQIFKMLAQAVILMQVQ
RLLLVTLPICIVIVYLVQRIYLRTSRQLRVLELESYSSLNSWFLETTEGL

VSIRAFNWIKPATGKNLENLETSIRPSYSLVSLQCWLNLVLDLLVSCIAI
GIILIAALWRSETSAANVGVSLNLILVANTTLVRLVESFTQLEVSLGAIA
RLNEVETQTPSEDRPWEDQVPPSSWPEKGSLTLSGFSAGYNDDHNVLRDI
NLSVGRGEKLVICGRTGSGKSTILLGILRLIESSGEVEIDRRLLSQVPRN
IIRKRAFITVPQEPLFLPSASLSFNLDPDAQASEQMLQEALQSVGIWDVV
CEIARGKDSDPLSQPASSLQALSAGQLQLISMARAIVKMKIISQGVEYKD
GGVNVDSPKPILVLDEATASLDAETEGRIYDIIESEFVADGHTVVIVAHR
ISVLAKTMRPGKDKVAWLQDGRVVKVGDYEEIVKFASSTAIGEGQ
>Mb|QLI66508.1
MATLTPGRHQKTQRHAKPDSRQQKQKLHQEDLQKLERAVNDLDAKSEDIK
TFSDLPLSQPTIGGIKASHFQTLTEVQQQAIPLALKDNDVLGAAKTGSGK
TLAFLIPVLEKLYRAQWTEFDGLGALIISPTRELAVQIFEVLRKIGRYHV
LSAGLVIGGKNLKEEAERLARMNILVCTPGRMLQHLDQTAGFDANNLQIL
VLDEADRIMDMGFQSAVDALVEHLPKSRQTLMFSATQSKKVSDLARLSLK
EPEYVSVHEAATSATPTNLQQHYITTPLPEKLDTLYGFIKSNLKSKIIVF
LSSGKQVRFVYESFRHLQPGIPLLHLHGRQRQVARLEITSRFTAAKHSCL
FATDVVARGIDFPAVDWVIQVDCPEDVDTYIHRVGRTARYQSKGRAVLFL
DPSEEPGMVKRLEQKKIPIEKVNVKEKKKKSIKNDLQNMCFQNPDLKYLG
QKAFISYSRAIHLQRDKEVFKLDKLDLDAFASGLGLPGTPQIKFHKGGDI
KKIKNAPRDGMSSGSESDMDLDGEYGKTKKKDKKKPEVRTKYDKMFERTN
QDVLSSHYAKLVVDGGNESGSDDDFLSVKRRLSDDDLDAEIQKGHNSNVK
ALYSLGGDEPYVIDSKRREKALQSKTKMLKFRGNPTKLVFDDDGESHEIY
ELQNEEDFKRAGPADEQRQTFVESESARVKAADVDDKQLAKQKKREKREK
RKAREREELEGDEAPMLVDAPDGEDPLALLRSLPTAGEQSDGEPPKKKAK
KWFEDEDDDDADNKKKSRRKGKVLQVTEEPETLEDLEALATGLLDG
>Mb|QLI66509.1
MPAPTYIISKIADPIFAVFIGISAATMRINREEKAKGYTTQQTIDNGLRR
IGLSKKTE
>Mb|QLI66510.1
MEAPQDFKSQQEGIQKSLVSTVKSVNRLAAEDLSFLRTVSPDVAEQLDDR
SARILHLSTRLLQSAAKACGVKAPRLEDVEDIDMSWQSIEDVVDSVLEKA
DTAVDEYTGLIKRKEPPSADTGPKAKRPRPTGKLVRNANITKPQAMFERK
PDNFPTGPWKPILANKPHATVSLEQSLFTAPGEDGTAQYKHPYETEISTM
KYPEWVFQKHDPIPSQPIDSTKATWVDTYEGVLEMLEELRKAKEIAVDLE
HHDFRTYTGLVCLMQVSTRDRDWIVDTLQPWRHKLEVLNDVFADPSVVKV
FHGAYMDMVWLQRDLGLYVNGLFDTYFACNLLNYPGRSLAFLLSKFVGFD
ADKQYQLADWRIRPIPEDMLYYARSDTHYLLYIYDNVRNELIEASDKSDP
EKDYINQALERSRELALSRHENPDYNETTGEGARGWYNYVFKHSHLALNG
EQFSIFKALWKWRDETARQEDESPNFVLGPTNVTEIARVNPPDVKALHSL
LPLTARLAKARLNDIWARVQEAKTRGGPSLMQFFTTLAPQSATKSKLPKF
PRERVELPILEGPNVSMGIMAQSKLFGSMPISSRWEDSKGSPTHMEGNIP
FPWQRFVQDSATVDGAQEENTAHQVVEEAPSTMETPKLKAQMEELDEEFT
LKTGRKRKSESEPDQDDETSSSGESESESESESESESEPEPEPPATEKDM
PIADSDGVISIVDNPPKKTKKQRQLERRQKKKQEDLEIAKKREAKLARKA
RKQEKSRQGKEEQKKKYNAVPFDYTKAPSVMQANRGQAQQGGKKEQKKVF
DPYSKTGDNEIKGARKMPPIRGERSATFKK
>Mb|QLI66511.1
MDNLATRMVNTDGLPPDYYIGVDVGTGSARACIIDETGDIKALASENIKL
WQPETGYYEQSTTDIWQCICECVRRVVIDSNVDPSLIKGIGFDATCSLAV
FYADTDEPVPVTGPEFTNDGQDRNVILWLDHRPVDETELINSTKHKLLKY
VGGKMSIEMEIPKILWLKNNMPAEQFARCKFYDLGDALTHLATGNEARSF
CSTVCKQGYVPIGVDNSEKGWQEDFFETIGLEDLSKNNFERMGGVHGVNG
TYFSAGESVGTLSRQAAYQLGLPMGIAVGSGVIDAYAGWIGTVGAKVDLG
DDELNANVPHNDLSQAFTRLAAVAGTSTCHLAMSKDPVFVPGVWGPYRDV
LLPGYWLAEGGQSATGELLRHMLDIHPAYNETCALAKAEDKHIYDFLNAH
LEYMASKHNAPGIPYLGRHHFFYGDLWGNRSPIADPNMKGSMIGLDSDKS
TDNMALWYYATMEFIAMQTRQIIEQMNTAGHEISSIFMSGSQCQNPVLMN
LLATTCGMPVLIPRYVHAAVVHGAAMLGAKAASHNKEDGSEPETLWDIMD

RMSKRGRLVEPGTDAGEKALLNAKYEVFLEMCQSQQLYRKKIDKAVEKWS
AEFGGD
>Mb|QLI66512.1
MSSPSKKRKLNTGGKQHGPSTRGLEFFFAKQRQSSVATSSADAADVPDLA
STELTDEELARKLQDEWNKEVADESKNETENTRSTSPASNLAIDELSPAA
QPELEPRVAKEAELPASKGNTLTLQSTGMAEDAITTNIPLDEPPLIFQPS
KYLDLLREHWAAEGGNASYSLLTRCFVLVSSTASRIKIVDTLVNCLRLLI
EGDPSSLLPAVWLATNSISPAYISLELGLGGSAISKALKQVCGLDNRSLK
AIYDKYGDAGDVAFEAKKKQSFTLRKPKPLTIKCVYQDLVKIASSQGQGS
AQAKQRIVDRLLQDARGGEESRFIVRTLCQHLRIGAVKTTMLIALSRAFL
LSRPPGADYSPLSIPELYKLKKEELAEIWGKAEETVKACFARRPNYNDLI
PVLLEIGISDELLIRCGLALHIPLRPMLGSITRDLSEMLTKLQGRDFACE
YKYDGQRAQVHCDDGGKVSIFSRHLELMTDKYPDLVELVPRIRGEGVSSF
IMEGEVVAVDRETGELKNFQTLTNRARKDVEIGSIKIDVCLFAFDLMFLN
GQPLLDRPFRERRELLRSLFTEVPHNFTWVKSLDATSSDSETVLEFFKSA
TESKCEGVMVKILDNLVDIPYLGDQEGPAAEETEKLSTPKSKSKAKKKGN
GATAAGEGDKKKTGSRRKPLLATYEPDKRLDSWLKVKKDYNSSFDTLDLI
PIAGWHGQGRKAKWWSPVLLAVRNEETGMLEAVCKCMSGFTDTFYKAMKQ
FYDNGEESGERKNTRSQMPSFIEYSGPMPDVWFEPQEVWEMAFADVTLSP
TYTAAIGLVSDERGLSLRFPRFLKKRDDKSLEEASTNEFLAGLWRKQEAK
AVPREAPDGRGDGELEQMEDE
>Mb|QLI66514.1
MASPALETKTTNRELTQAHRFVVTAFLTGSRILGRSFMAAYKQAQAASQY
QRAQAKAGGGAASGRASLASGMTLDEACKILNVKPPAGGQANVEEVLDRY
KRLFDANDPQKGGSFYLQSKIVRAKERFERELGPIREQAEQEAQVKEGWK
PKVYKDK
>Mb|QLI66515.1
MSDYAPPSGPPPPKAPEVPAGWVARWNDQYKEWFYVNVYTKKSQWDKPTA
PVFPVDENAPDDPPPGYEPGNGPAPTDTKKNPYEDRSGQNKHAGSSSAQD
EDAKLAAKLQAEEDAKARGGPTPPAGYATGGAADSYAQGQTGQSQGSFPT
ELPPRDDARGKDRGFLGKLFGKGKTPQSGQANYGGGYYPQQQQNYYQSPP
PQQYGGYAQQGPPMGGYGGGYGQPPYGGYPPQGGYGGGYPQGGYQQQPGR
RPGGGMGGMGMGLAGGALGLGAGVLGGALIADAIHDHDQSEYNQGYQDGM
DNDYGGGGDFDGGDF
>Mb|QLI66517.1
MSGLDVEALLEATAQGSAQQNSNTLNDDSSRSDKNGRPQNGTDDSRDRDR
DAGQDRRRRDRRDTSPRHRKGTPGETTPRSDTGSHRSRRRSESRDRDRFN
SRRHRDGDYYRGRRGRSRSRSRSPRRRYRPRDDRDRKDRRDRSNRRDQGR
ARDRSSGRRSRSPQLNEDERDSRTVFVQQLAARLRSDKLKRFFEENAGPV
NEAQIVKDRISGRSKGVGYVEFKDEETVQKALQLTGKPLAGIPIIVKLTE
AEKNRQARNTESTSGNPNSVPFHRLYVGNIHFNVTEQDLQAVFEPFGELE
YVQLQKDDNGRSRGYGFVQFRDATQAREALEKMNGFDLAGRPIRVGLGND
KFTPESTANLMHRFSGQNNFQGSAFSGAGGRGGQASTFDRAGGRDSDKAG
GASALDDTDVAGVNFNNYSRDALMRKLARTDEASNGREDQVVVKPKTEAK
PLPVNVNMASRCVVLHNMFDPEEEEGDDWVKELEDDIRAEAERQYGHVVH
IAVDPNSKGDVYLKFDKVQGGENAIKGLNGRYFGGRMINASPVVDAVYSS
LFSRTRAI
>Mb|QLI66519.1
MWTGLIRRFSTEDTRLGSEADTNDDQDKDGVNGVFTPSGRRALNASPFRP
PPLEPLTLHGYRDSTPSSARLLTSAIAEEIRTMVPERLRIVDDWHLVYSL
EQDGASLSTLYQKCRHYEGLRVGFVLVVKDQEGGTFGAYLSEYLHPSPSY
FGNGECFLWKASILASLPLPPSADTTNLTRSTTLAPIPSSESGTSTPSES
VRFKAFPYSGLNDCYINCETGFVSVGSGGGHYGLWLDDSLNIGHSSQCET
FGNEPLSDAGEKFGVWGVELWVLGA
>Mb|QLI66522.1
MGNDDAVSDKHPKGPMPVLIRASNGKSKRNRSDKIKMSTIVEPQDLDSFY
TRFADICKSGMVALKPRDRSKKKAKAKKKKAAS
>Mb|QLI66523.1

MLDQMANEIKLISGSSHPNLSAKVASRLGINVANTMSLNYSNQETSVSIG
ESVRDEDVFILQSTAPGDVNDGLMELLIMIHACRTASARRITAVIPNFPY
ARQDKKDKSRAPISAKLIANMLQVSGCNHVITMDLHASQIQGFFNVPVDN
LYAEPSVLRWIRENLESENCVIVSPDAGGAKRATAIADRLNVGFALIHKE
RPRPNVVGRMVLVGDVQDKVAILVDDMADTCGTLVKAAETVNQHGAKEVY
AIVTHGILSGKAIENINASCLAGLVVTNTVPLGDKTERCPKLKVIDVSAT
LAEAIRRTHNGESVSYLFTNAPV
>Mb|QLI66525.1
MGPTVGNGVGGMDMGMGMGMSMHPNGTNQPQATEYTLQGVMRFLQTEWHR
HERDRNAWEIEKQEMRGRIAHLEGQARRADATQKALKKYVTILERKVKDQ
AAKLKGTAATEAAEAAKNDRAAKIQEKLRTSSDKYSVPGVEGIEVEKPDD
DTQRIELKTFLDQCQSEFLYLMITPANPLPPRDSPPLPMIEDLREVEQFG
MPPPQHMDRQYQMQRQNVNHVQDFNVMNARQQPPQNAGPQMQQPPANFAP
KQELQPQPMLRSAGDNQQAMYGTPSDWSAPVSVTTRPVEENLPGPNHAAE
ALGGIQDATESRDKIPPNADPDGWDFPEGTFPEPGAVPQNPPSATNRPDT
DVFPNAEYLPKSPNRVPSGGHRRKGSMSRRRSGEHELGLGSSQKSDSGNF
KLRFGLRGHLDTVRTVIFSGGGSPGEPEICTAGDDGMIKRFHIPRLDHPG
PFAGAASDLDVIANFTHRGHSGSVLSLTSWSPAPNFSTGGRAQGDGWIFS
GGQDATIRVWERGRVDPKATLEGHTDAVWALCVLPTTLGAIFGTSSPYGN
ADRILLVSGAADGSVRIWSVSAPPQLSSPQPGSNRGMTGRGGRVRGNSMS
SGSGFSNSPQASVASSSPFHYTLVHVVSRGDGSKGSPTCIAPLGTSGETF
VVSYSDAAVIVYDTKTGEQVGSMDSMETYDGSVKTGVNAIVATTIGLDQG
NQHHGSSLGGEEENAAGGPTGGRSMAGSGVEGTIISGHEDRFVRFFDANS
GQCTYNMLAHPDAISSLSLSPDGRELVSAGHDASLRFWSLEKRSCTQEIT
SHRVMRGEGICSCVWSQDGRWVVSGGGDGVVKVFAR
>Mb|QLI66526.1
MQRALSSRARATALSSAASRYRAGAGLGQQLRFAHKELKFGVEGRAALLV
GVETLAKAVATTLGPKGRNVLIESSFGSPKITKDGVTVAKAVSLKDKFEN
LGARLLQDVASKTNDVAGDGTTTATVLARAIFSETVKNVAAGCNPMDLRR
GIQAAVDSVVEYLHKHKRDITTSAEIAQVATISANGDHHVGQMIANAMEK
VGKEGVITVKEGKTMQDELEVTEGMRFDRGFVSPYFITDAKAQKVEFENP
LILLSEKKISAVQDIIPALEISTQTRRPLVIIAEDIDGEALAVCILNKLR
GQLQVAAVKAPGFGDNRKSILGDIGVLTKGTVFTDELDIKLEKATIDMLG
STGSITITKEDTIVLNGEGSKDAISQRCEQIRGVAADPTTSEYEKEKLQE
RLAKLSGGVAVIKVGGSSEVEVGEKKDRFVDALNATRAAVEEGILPGGGT
ALIKASAQALGDVKAANFDQRLGVNIVKNAITRPARTIIENAGLEGSVVI
GKLTDEHAADFNRGFDSAKGEYVDMIESGILDPLKVVRTGLIDASGVASL
LGTTEVAIVESPDEKGPAGPPMGGMGGMGGMGMIATVSQECITAYNDLKL
SKKYKYIIFKLSDDFKEIVIEEASDDKDWDNFREKLIKSTTKNKSGVVGK
GCRYAVYDFEYSLATGDGVRNKITFIAWSPDDAGVQPKMIYASSKEALKR
SLTGIATELQANDADDIEHDTIVKTVSKGLAG
>Mb|QLI66528.1
MSTDKITFLANWHATPYHAPLYLAQAKGYFKDEGIKVALLEPNDPSDVTE
IIGTGKVDLGFKAMIHTLAAKARNFPVLSIGSLLDEPFTGVVYLKDSGIT
TDFRSLKGKRIGYVGEFGKIQIDELTSHYGLTPADYTAVRCGMNVSKAII
NGDIDAGIGLENVQMVELEEWLASQGRDKSDVQMLRIDELAELGCCCFCS
ILYIGNEKFISENPEKVRAFMRAVKKATDFVLADPHQAWKEYVDFKPVMN
SPLNWKIFERSYAYFSQDLKNVERDWDKVTKYGQRLGVLSPDFKPNYTND
FLGWGLEPDSADPTGDQKRMVELQRNVACCGGFHRLPLTAKA
>Mb|QLI66530.1
MPALDLQQPLAADDTRILGQDPLIPPALLTSELPMTDSAVRTVIKGRNDA
ADIVTGHSDRLLVVVGPCSIHDPAAAHEYAARLKALSEKLADDLCIVMRA
YLEKPRTTVGWKGLINDPDIDSSFQINKGLRVSRQLFVDLTSNGMPIASE
MLDTISPQFLADCISVGAIGARTTESQLHRELASGLSFPVGFKNGTDGNL
GVAIDAIGAAAAKHHFMGVTKQGLAAITRTKGNEHGFVILRGGSRGPNFD
KENVQAAKKSLSDKSQKLAIMIDCSHGNSQKNHNNQPKVAKVVADQLREG
EKAIIGVMIESNINEGNQKVPPEGPSGLKKGVSITDACINWEDTITVLED
LAAAVRTRREINGRSTNGHAKVSLVEEA

>Mb|QLI66531.1
MPTMLQTPSRASTASSSSFQPLSRQNTMSSYDGTRSARQSKRYSMSALYM
SMSANEGEMQIEDDLAKAQKVLRDLKSKISSQSKKNFVLEKDVRYLDSRI
ALLIQNRMALEEQNEVASHLEDATEVQEGVFPNDDKTQKYGNLLFLLQSE
PRHIAHLCRLVSMAEIDSLLQTVMFTIYGNQYESREEHLLLTMFQYYNQS
VLTYQFDNTPDYSSLLRANTPVSRMMTTYTRRGPGQSFLKTVLADRINSL
IELKDLDLEINPLKVYERMLEQIEEDTGTLPANLPKGITGEQAAENPKVQ
AIIEPRLTMLTEIANGFLSTIIEGLEEAPYGIRWICKQIRSLTKRKYPDA
NDQVICTLIGGFFFLRFINPAIVTPKSYMLIDGTPAERPRRTLTLIAKML
QNLANKPSYAKEPYMAKLQPFIHQNKDRINKFMLDLCEVSDFYESLEMDN
YVALSKKDLELDITLNEIYAMHGLIDKHYQELCKDENSHLAVIMSELGPS
PAQVPRKENRVINLPLFSRWETAIDDLTAALDITQEEVFFMEAKSIFVQI
LRTIPQSSAVLRRPLRLERVADAAATSRNDAVMVRKGIRAMELLSQLQEL
GVIDKSDQFDLLRDEVEQELQHLGSLKEGVIAESAKLEEVYKTIRDHNTY
LVGQLETYKSYLHNVRSQSEGTKRKQQKQQVLGPYKFTHQQLEREGVIQK
SNVPDNRRANIYFNFTSPLPGTFVISLHYKGRNRGLLELDLKLDDLLEMQ
KDNQDELDLEYVQFNVPKVLALLNKRFARKKGW
>Mb|QLI66532.1
MASHYSNSYYAPASNEQPRRQERTRSSNGTVSTTVSSSSGRESAATHMTD
VPAYSKKIVVVGDGGCGKTCLLISYSQGYFPEKYVPTVFENYITYPTHSP
TGKTVELALWDTAGQEEYDRLRPLSYPETDLIFVCFAIDCPNSLDNVLDK
WYPEVLHFCPYTPLILVGLKSDLRYKKSCIDMLKTQGLTPVTTEQGLAVS
EKMGAQYMECSSKEMLGVDEIFERAILTVVANDRRNYDAMGSGSGNEKML
PIPGMKPPKKKKRRCQIL
>Mb|QLI66534.1
MSWAGFKKNVNRATTQVMMKTGHVEKTNDRDYELEERRFKTMEAASLRLQ
KEAKGYLDSLRAMTASQMRIAETIDAFYGDSGARDGVSRSYKQAVEDLDS
ETIKALDGPYRTTVLDPINRFCSYFPDVNECIKKRSHKLLDYDALRAKVK
RLVEKPDKDATKLPRTEKELEMAKLAYEQLNEQLFSELPQLIDLRVPYLD
PTFEALVKIQLRFCAEAYSRMAQVQQYLDADTRDLYAEGHLDARVEQVLQ
EIRELSISGTV
>Mb|QLI66536.1
MSSPPSPPAAYEQAHVHAVYEAIAPHFSATRHKPWPFVQQFLTSLAPGSV
GLDVGCGNGKYIPVNPTLHMLASDRSPSLARLARAERAADVVTADGLSLP
YRPRSVDFVICIAVVHHLSTRQRRRDALAQMLGCLAPRGRALVYVWALEQ
RASRRGWDESSRQDTLVPWVTLRKGAQPDETHHRYYHLFREGELEEDVVA
AGGEVCDRGYERDNWWVIFGHTR
>Mb|QLI66540.1
MSAWMNDAVPNHNGNGFPHMNDPNSAGAMMDHSAFLANPGQFNPGQFPNQ
QQMAGMQNGPMRHGSPTYQNQNQTANPNQNQNQNAVYQTNPVIPSKRPRP
REDSVTGSPGQNPGMQPPSRSETPQQQNFGGFQPAGGAMPQQPPGQFPHL
QPNGSANASPSPIMGNQMRPGNVPQRVATASPHPFSPGPQQQFTGSQASP
IPSEHGTPQPNQYMQNIPPGYNPGYAQSPSNPRPSPNPNVMAGGPAVGGQ
MLPQQMGQMPQHMGQMPANMYQQMQQQMQHQVPQPGQQQPPGQQQPPQQG
QPRPQGMMDQQKMAAYQMRLQQQLQGNMQMQAQMQAQNMGRGMMPKQQVP
GMPNGQMPQGAVRQQPRPMGNINPEQFMKNLTTLMNAKGLPLDPNPIVGD
RPVNLVMLFQLVQSKGGYKPVTTGNGWGLIAQSLGLPAQIPTVPPTLRQV
YERNLAKFEEAWIAQQKHRLMQQNPNMAGPGTPQKQMQPGQQMNQGQMPQ
LQQQQQQQQQQQQQPQQPHLQQQTPMKPGQTPVNGFSTPQPPQQQPLPQQ
PQLPPHVQQQQQHPHLHQQQQQQQPPPPQQQPQHPPISNAAPGHRNSMSR
GVDPSTPNDFSMQSPSHARPGSVSIDGRHATPLPAVGAEQHMPRMPPKPE
DYSPCARELSTHGGVDLHAANLLGAELERWEPTVPSVNELGNIDISALTR
SLQCGIHGEVRLALDTLATISSSQNQAHFLHLRACDDLVEALVDCAESQV
DLLVEHTTEVADEIQLTSYEDVVRGCRIERWSVRDVPAFGSIEYQLDGAV
DRLICITTILRNVSFPGENNDNHIMLCEESVIKMLCSIIRYLGTRTMLLR
THANTLDFMKDIVVLLSNISSQLEIPGREQALSLLQFLLAFAPVPGPTMA
GDTLNFTPYDPSIHSYLPHAVDALAKLLARDEPNRGFYKTLFTIDSSSPL
ANELLVRAFALTVAPLPDKAKEQLRPPTFPPLFETRKPFLMQGLLGAEII

ASLVPGSEPGMARALLASGNCLAQNLVQLIQDLCFLYEKPPPSTRGGSRA
VAARKDPELVYIVVVAVSLLRRLAEKARDSSGTGSAGIGDLLPQPQMLMD
ALMMQSSEWTKEGLLPQLTAVLNLDG
>Mb|QLI66541.1
MAPSAVSPQQRPVDSGVVQPQAGNFTGYDHVTWWVGNAKQAAAYYVNLFG
FKHIAYRGLETGSRYFASYVVGNNDVRFVFTSPLRSAVHLGPEEPISPED
KTLLLDMYAHLERHGDAVKDVAFEVDNVEGVYHKAVKEGAVAVQGPQRLH
DAEHGSVTTAVIRTYGDTTHTLISRGSYSGPFLPGFREGKKSTASVALPD
VRLARVDHCVGNQDWDEMVSACAFYEQCLSFHRFWSVDDKQICTEFSALS
SIVMASPNNLVKMPINEPAAGKKKSQIEEYVIFNSGAGVQHIALLTPNII
DAVTALRARGVEFINVPTTYYDTMRLRLKTEKRNWELKEDLDTIQRLNIL
IDYDEGGYLLQLFTKPLMDRPTVFIEIIQRNEFEGFGAGNFKSLFEAIER
EQAERGNL
>Mb|QLI66542.1
MAFANEPAGAKTPSPPSTIRTPPAPQLGFHDSWEPYSPPRKSARLSSQRA
ANRTPSPRASHRQQPGSPRTAKKSLARTGAPMVSPVTSPRKRSHPALDSR
RQVSSSLTAEGTANAAVALGLNVDKGVQRSTTASVSQSAGMLPTPSKTPQ
KPPNEKTAASIQSFARNLFSSEEEVLPSPRKRRSKKYSGVTMESFTAEEE
EDPITIFTDSQDRIPEKDNSEANPFYGSAATAAEPSKRQSRRKVVSIPGE
GAQSIDEAARREDGIVYVFRGKKFFRKFSEQDQSEEIAASAEDDELRLNR
PLTRASVKPRLLFPTQKTTAEDDEEALTDVEDMNLIDEHVPQTPMKAHSI
PAKTPEAPKYAPVSPPDTRRTTRSTNKLADTPMKGTGRKSPFDSWPRTKE
HKSQSPAPKRAGESLASETTKRTRA
>Mb|QLI66543.1
MAPVGDYADRPTQRPARDDDMDRRDSHRSSRRAPDGGRQERQRSRDRRRR
SRTPDSPGRHRRRRHRSHDGERERGSGGNLDIRSGRQERARNDERSDRRR
RRRDDGESDEDRRRDRRNPVKQGGPLPSQEDSFAIVKGEEPEKPKEKPNF
GSSGALAAASNSIAQADGSSIVLKYHEPPEARKPPTRDQWKLFVFKGGDI
VDTIDLSLRSCWLVGREMAVVDLPAEHPSISKQHAVIQFRYTEKRNEYGD
KIGRVKPYLIDLESANGTVLNDSKVPDSRYLELRDKDMIQFGNSTREYVV
MLAPRD
>Mb|QLI66544.1
MSDVDDYSITGSDNAADVGELPGVDEDFGAIEMFDSNAAPSTFSSQAPVA
VMAEEAPLEASPRKSKRDKKGKRDRKRKSSQEDDVATSPIPEEEDSSRKQ
RKPKRKSTKGAEILDSQPTQHEPIEPSEDTPWGQLQEEEAAALSKTRRKR
KLSDTVDGKGRKKKRSHDQEPESQDDEAGSSNLLRKRKEQRGDLESAIYE
DDEPESDTQESPTVAHLRRRSQSREARSRENSVPSESQMDVDPGAEDRTK
TAVAALAEADGDVERLAREALDEHHIGQQALEEGQNVDQDPDMSNQYPQE
PLNASLESMVEAELPIAKSKKSRSTRKKAKPTYFEQDPIPDIVSEDEGNR
NAWEESPSPSAAMPKARRVKRAAKKESRGRKPKRAKLSQSMRGGSADADD
AFAQASGQPRNRLTGFKQGRFTDAELARIARAIESYRADHDITQQEVNEL
IHAPGGTTAGDTHAQLWSRIFAECPDRHRQKVINITRKKFHNFVARGTWT
PDQETELAELIQRHGTKWSYLGSLINRHPEDLRDRYRNYIVCGQNQRKDT
WDEEEEARLTQHIIESMIVIDQLRASQPSKTLLQKSYEELIDWQNISELM
GRTRSRLQCITKWKSLNIRINGKDHLVSSQPDSQISFRLEKARRQISAMP
VEERYRLVMAVHATAVGADVKIPWQRLVDKPFRNQWHRYTQMLLWRRLKQ
TVPNWETTSVRDCAQYLIDQYNQTGDLPEVPDELFDDADEMQYMQAMAPV
PSAPGTQDDERGQVSTEFITESDAESDHAEPNGETTQLEGEEQPGEQAHE
DVEMEIDPALADVAQPPKSTPTKKTSGKSPGSRKRTRKSAAAVEDSTEDT
RQESQEPRQTEEDNADADVEQFHEKTTPRKLKSYRGKKAVRHSSPPASDA
DSVMDDMQDLPARVAVA
>Mb|QLI66545.1
MDPLHAPPPFLKSSASTERGIPCALSVPSLPSMISGVLIFNQKGENLIFR
AFRNDCRPRLADVFRIQVISNAQVRSPILTLGSTTFSHVKHENIYLVAIT
KSNANAALVFEFLYRLIQLGKGYFGKFDEEAVKNNFVLVYELLDEIIDFG
YPQNTETDTLKMYITTEGVKSETRPEETSKITMQATGALSWRKADVKYRK
NEAFVDVIEDVNLLMSATGAVLRADVSGQIVMRAYLSGTPECKFGLNDRL
LLDNDGLLTLPSGNRMGTKATKAAAGSVSLEDCQFHQCVKLGKFDTDRII

SFVPPDGEFELMRYRATENVNLPFKVHAIVNEVGRTKVEYSIGVKANFGS
KLFATNVVVRIPTPLNTAKIVERVTQGKAKYEPSENCIVWKIGRFTGQSE
YVLSAEAILTSMTNQRAWSRPPLSMNFSLLMFTSSGLLVRYLKVFEKSNY
SSVKWVRYMTRAGSYEIR
>Mb|QLI66547.1
MSAKLHPPSKPSQPKSNPLPPLIQTPSGLALLELQGTINLPQSKDGQALS
GIEIGRIDFPDFVPDAEGSAWMKRVHMYVGQHQRLTGEVKKLPNAIAVIS
RRENRVLENSAGTYLEEGENLEVVEIVKYKIMFSTRPEPVGATNAS
>Mb|QLI66549.1
MSTDVVSDVAPLDPGAPASQSQGSGPSYDPEPTAHAAGPTPTPASNQHAT
DSEDQIECAKPTAAVFLNSPPDSNNATKSDASDSELSELEEEPTLDDAPS
APTPQPASASAPAAPEATTTPVPAHSPTEKPEGAKDEDEEDIGEVLPDDW
SGSVPIFRPTCHQFKDFQKFMKKVDHYGMKSGIIKIIPPREWKDAQPPLD
ELVKHIRVREPIKQDIMGSNGTYRQVNILHGRSYNVPQWRQLCEQSEHQP
PARRGERRANAEKPKPARSRNVVSKETKLSTPKKRGRGRPAKNKGKKRPD
DGDERPMTPVSPKPEPEEEKDKAVDSIEQELGEEVQEDDESTIGRMSGQM
GGAKPAKPKTQSVSARRKYSRREGSTMIDEAAFKDFDYRMDISDFTPERC
EELERAYWKTLTYAAPLYGADMMGTLFDDRTDTWNLNKLPNLLDVLGTKI
PGVNTAYLYLGMWKATFAWHLEDVDLYSINYLHFGAPKQWYSISQADARR
FEGAMKSVWPADAKACNQFLRHKGFLISPHHLLQHYGIKVNKVVSYPGEF
VVTYPYGYHSGYNLGYNCAEAVNFALDTWLEMGKIAKRCECAQAQDSVWI
NVYEIERKLRGEETEYEETEDEDEEDDDDVSGLPTPPATNGVKFKDASRK
RKRGQGEKKTRVKRVRLRLKTKVEPPCCLCPNDIRHFDILPTADGRKAHK
LCALYIPETYIDSVDDKEVICNVEAVHKDRLNLKCLLCRSRRGACFQCSH
QKCARAYHATCAAAAGVFVEDQDIPVFGEDGTEYKEQAFEFSCRFHRVRR
DKKTDGDALEMDTNIRSAAEKLKTGEICQLQYLKGEIFAGVIVENRLQDE
TLLIDLLPQGEQLEVEWKWLVLPDPADYHLPKASAKALPMPASRKAKDKL
NTKRIHADKPRKDDLFAEGYRWAEFELYEASNKGQAKVDFGKSDQIWHYL
GKTSTEARAQYTEDPTQQRHNPKSNFLDTIPKPPRPVPTSKPTYNHQSYL
AKAMAYSYSLAGNYTAANEQVSFEKPYIYKPRRPAEANFTKPTTFANQGC
AATHTSSVGSAPVFHTYDHQTANFRPQPTFTHSMPQNHNAAPVQIMPHVQ
MPQAAGNTLPQSRQTAYQKPANTPSAQNSKPVWQVHSSVYQKYPFFQVNY
NRDFSRYRTPYSPWGGFTNGYEGNLRAHLMATQQNSIQHPTFGGTPGPQG
HVQQVQQSLVGQPVHQVHQMHQMHQAQPIQQVQQSSLPQAPKPKPAPAAK
PFKVGLPAAFHIPLQTTKSAMQVPAKVSPVPLPPNVLAAMAKASEGQQAP
RPEPRPLKDQSQPTQTHSVEPTPPSNQPSVKPPGSQPAWANQPQLGFSAI
PVTQQQHPARENSLTPVGLTPQEFTDVPGSESMQFVERMMQNLRRASMNG
RDGQDGHIRG
>Mb|QLI66550.1
MAPNASTAAGQASGLIDKLQSSTDEVLNAAKTSPEFRNAEHEAKERRIAQ
MMADQMSMPMTVNEISVHGAKNIRRGFLGPIFSPLLRDGSSAPSTVGDVM
AKLQMASSKLSGLQILREPPEIYLSQASQVDPSTSPSDVNISIGLKELPL
FKLQTGTDVGNGEGSAYGSLLWRNMFGGAEMLTLNAKAGTRTRSAYSANL
SAPVLSDPDRRISLEGLASAAEKPWASHEEVVKGASLRFSWLNAERDTHS
VEYSGAWRQITGLGQGASPTVRQDAGDTIKSAIKHTFHRERRDNPQLPQS
GYMLRSGLELAGIGPLGGDVSFSKGEVEMSGAIPIPLPGAQERSGASVGA
GFRMGMLYPLPLGFDLGGKSAPSRLNDRFQLGGPTDVRGFKLGGLGPHDG
VDAVGGDLFAAASVNMLLPLPYKGPESGLRFQMFANGGRLVALKNRSKST
EAGLQGLSGSAVRDGMLGAVGDLFNGAPSMAAGVGLVYAHPVARFELNFS
LPLILRRGEVGTKGFQMGVGINFL
>Mb|QLI66552.1
MVSVAEKDGPALQDNKPPTKLPMDFPIWTRAEDDEKDRNAGLSILQRRSR
KWNRDRGVAHAADPAQRPSRLDSRGLFKDIAQPVVSGIFDALSPQDGPLS
SDDEKNPKSEGLKQPAPIQTQPLPRLPSLKSPESPKSPKSPNSPNSPKSP
TLPANPTPPPPPAYPTPSPLPPPPPPVSNPGNPKPTEAVVNPIQPPPGNT
WPDSSYGPSMGFSTLTPSASTTRRTTTTNAPPPSLAPIVTIPVPLSTGPR
KGVNPLPTLDAVSTFSMSMSPTSPASPASPASPTRSSSVYPTPISDSDKA
YHPGHDKGNIHPKVEMALVALGSIGSSIIVAFIGWLIWRCHKRRAVDSS

RTWKPHFPKNMPTDKPKLLVTNALARIPIFKNRMDDRRRRWTNLDQADLG
PFSEKAYVAEAQSMAIQQPPLSGISVQTDLVRTSTHADVPRNGATLHVPK
VSISSIEPQAGSTLRSGAQLPNTRMSDISSLSSGFGDGQFIIGTMSSGTN
SANNTASETTVKAPLAVARRESIGALSQRRDTVYTEASEDTPARFRSVNS
WVRQQTGRVTRATQRNQASDAPPVPALPPEQDFDLMMPDGEEPRRADSVT
GYGVAR
>Mb|QLI66553.1
MSISNGQPAWPPPVLHHANGSRSLNHLDDGTARTQTPVDNTVELVPEAPV
DVEEESRRALFADLYRKTEDKIALLFSEDGSYNLDAIAGLKRRAPTPTVT
LPPTTDHEPIKEPPYKKAKRAIDEDDYDDDDEDEDEVATAPAPVLKAQGS
AASIAANSLLSPSKSGSSPVHSLNSPGRLGEKLKSSQEEPQSRSKAGESD
DNAIKNLEEARLATEEAARRSFHTIFYTLENDRTAMLEQKRLEDSEKQLQ
AEMDSNQANGAGGTQASNQGSLSSANLGASSLTLKHLIARIDMKRDQVRA
SDAELRLLMNEVRKNRSKWASEENVNQEELYEAFEKVLTELKAHTEYSHP
FLTRVSKKDAPDYYSLIKNPMDLGSMTKKLKSLTYKSKTDFVADLDLIWD
NCLKYNQDMNSQIRRMANGMRKEAEKLIPLIPDLVVRSRAEVEAEERRKQ
NGGDDDGGDDSDDEPIMSSRGRKATTKGAKSRTAPTDQKEDTPVVDQKPV
LQLNGLLGKAGREGSEVDGSNGFSTPPIAGSITPSGALNGHSGIASNADA
MDIDGPSLSTMTAFGEATEQAYEDEEYKIWKQTTKKDRALTVKERHFLFK
GNSLNTDAPALLRNRAGMRWFLKHQREAETMGIAAHPHLASAAGAGKDLA
PSKQPETLAEGIDEETEQVVPDYYNPLTSIPDIKPNLQWVEDGEGQVINQ
HEAFLRLVPPGSFIAPKSRLTKKMDDNIRQIQETRKLATKISVIKQMQVQ
SQVYTNQFPKSSIETFLEQDIEPHFISDDGPVMASETCQDALKRSVAKIL
YHTGFEELQPSAIDTLTGIAADYFQKLVRTFNIYREAEKTSVQTPQGPRT
KSRFTPEEVVLHTLEESGHDVASLESYAKDEVDRLSSRLGILHERMKLHL
TDLLRPALSADAGVDGVGAFKDGSDQFMSGDFADDLGEDFFGFKALGLDK
EMGLDSFSVPFHLLHSRVRNQYQMQTQTAGAISADMFEALSPSEPVSNEG
IQEEIGLVKNFFLAKLHANGDQPLVEDEDLPVKQRKPRPRLGASGKIISA
QKRPPREQLALAKKKKKMELAAAAAEAKANASPEKGANAGTGSTNTTPAK
KKSITITPVPPPNPALLALTASMERTDSMQSQAGTSQTEKDENIAMMSPD
SIAQ
>Mb|QLI66555.1
MTVDDTLSLPRILCLHGGGVNGTVFRLQCRAIIAQLGHRFRFVFMDAPYT
SRPHRDIVTVYGDFAPFYRWLAWSREEPGLPPWARAGDVMDACLAAMERD
AGDGPWAAVLGFSQGAKVAASLLWAQQRADEVLGPGRARTAFRFAVLMAG
SAPVLHLDLRLPPPPPHVASPDAFSTDFTDYPDPGAPPSGLHVLRLPTLH
VHGLQDPGIQRHRCLLERYCMPATARLVEWDAGHRLPVKTADVKAVVTEV
LRLARETGAIPPDVDAL
>Mb|QLI66556.1
MAPELSPTKSDKVSKALVPDENGYTRLHITPLDQELVKIVIPASVLPSAR
NISFHTLETFPEKRYGFVDLPQMEAEKLKRKLHGATLKGSKMRIEKARPE
DRVEPTGDVDTEIKSKKRKSKDESESSKRRKRDHKVVEGVVLEDRKVKRG
WTESADTKRKNKKTKDKSTKDKDQEKRRRQKSKYTDQDECLLKTKLPPNA
VGNLPAADAHKKKKKKGNVREVTVHEFEKTTKFPSFLKNTAPETNGKPAT
EFVEGKGWVDEDGNVVEVVKKQDLPVTTPKRKASSKKEAAVQEESDDDDT
SGSGTSSEEEEDGDSDPDESEAVNVTKVEAEEKDSGSSSEGDDTSSDDSS
SEEEEDPASSPTNKADNTRPMSSSSSRSLKIKIPPPPTTPSASKVHPLEA
LYKRAKPSETAATQTAAQESQPFSFFGGGDADDDDIEDEPAAPSIPMTPY
TRQDFEWRNVRSAAPTPDTAHPSRTSFPQADDVDMDDAAEEEEDEDQDGE
EGAPARGQSGSSDFQTWFWENRRDLNKSWMDRRKGAAKEKRHRENKARAS
KAI
>Mb|QLI66563.1
MASPISPASTTLPSRATTGLSGHTEEAVPDFDPNTTAGLLAERLQAWKHA
CGYLEEYVTAIEKIHGRQAKEYEKALKTISNPLREGHHFDQSLGGIAGFF
ENIRSNTQAQINTNIETEKSLKGSVLPILERLHKEIKHKAKELHGGAQSS
AKEVEKLRNVTQKQIELLGQQTASYDSAGGKLHAGDDPYVIHRGVMHRLS
NQVVAENNHHNDLIAVQNNFQTFEAHVIEVFQQAVEAFVQLAGGQGEKTR
ALYNDMLGTIQCVPVNFEWQNFTQRCSDRLANPNDPPRAVDAIQFPNMDH

AATKPLIEGSLERKSRNKLSWGYSTGYYVVTPAKWLHEFKDSDDSRIDPK
PELSLFLPDAVIGTPSGDKFNVKGKDRSGTMSSKLTGSTELAFKAHSAAD
AQKWFHVIQQVCGATGPAEPTSPTPSSPATASPATIPPSVGKAGEKANDK
VETSPAVASPQESKPDALAATTTVEGGHKAQEAGVTGGEAVSSPSDEKKP
TVA
>Mb|QLI66564.1
MAVNRIRGAFAAPRKGETFELRAGLVSQYAYERKESIQKTIMAMTLGKDV
SALFPDVLKNIATADLDQKKLVYLYLMNYAKSHPDLCILAVNTFVQDSED
PNPLIRALAIRTMGCIRVDKMVDYMEEPLRKTLRDESPYVRKTAATCVAK
LFDLNPAMCIENGFLETLQEMIGDPNPMVVANSVQALSEITETAPETRAL
VVTSATLKKLLMALNECTEWGRITILTTLADYPASDVKESEHICERVVPQ
FQHVNPAVVLAAVKVVFIHMKAVNPELVRSLLKKMGPPLVTLVASAPEVQ
YVALRNIDLLLQAKPDILSKELRVFFCKYNDPPYVKLQKLEIMVRIANEK
NYEQLLAELKEYALEVDMDFVRRAVKAIGQVAIKIEEASAKCVQALEDLL
ATKVNYVVQEVIVVVKDILRKYPGYEGVIPTLCEHIDELDEPEARGSLIW
IVGEYAEKISNADQILESFVEGFMEEFTQTQLQILTAAVKLFLKKPSNTQ
GLVQKVLQAATAENDNPDIRDRAYVYWRLLSGDLDVAKNIVLSQKPTIST
TMTTLPPSLLEQLLSELSTLASVYHKPPESFVGKGRFGADEIQRAAIQEQ
RQNAADNPIAASVAAAANGAGPGASSNNIENLLDIDFDGAAPASHEQQSA
SATPDRVASPAGGAASGGMADMMSMFDAPPIDATNNGGAAGPSSGSGIND
LMGGFDGLSFGDSNSSQPLPAAMQLNNAQGGSSTQTQKKDSDDLLGLL
>Mb|QLI66570.1
MASSTDTGQLLPAAVRQSNFEQAVAYSLHLWPALTLAVQNNWGGPDSADK
RDWFAGAIVDLFPEFSDAPQTKPIEEPDMEDVETVLLQVMIDEFEVNVDD
DSGTEVAGNIVRARAECAIGQFDEVNKLNQRFQNLKGKKVDQMFTKAEDA
HQDTDWESDDDDDDDDDDDASDVDMDDAPAPRSAPKEKPAPQIDEDGFTKV
THKKK
>Mb|QLI66573.1
MTSQQPPSASRRPRLSLQIKTTVCSPPSKGLRGIPLNPSDPTTFNTMSNI
YVTAIERSSAIQSEPVTAIKTLQNFSLQSPIERNGLKSRVATSFVPTYPE
TPLTAQPVSPQQMDFVYPSTMTATPPLSAGPVDNSSKVFTFPPSADSSRT
NRPPEPPADDRIPRRRRTPHSLPGITSQLPYTHPRSLHSILRNSPLPPRT
AIPPPSPRRQSLRLQEKAAKKVEYDSPLEEEITTSKYVRSHIDLLSEDAS
PISPVYMHASSPGITAGEITRLPDVALAFTANEIQDGGQTPGPVEEMGRR
MAGLAASPVRRKREKKRRWVWTIGQDDEDDSMGGAVAASRAESAHKKARG
EDDVPQLTYEELPTPSIESISSVAGSDADVSDTCSVVSADHTARRCLSVP
SDTEVEAKTPIAPTGGQKKRDTPIPELADNGDGSTRSVLAN
>Mb|QLI66575.1
MHLPLLKLVALATAAAAAARDATNHPRRVKEVKNVAIIGAGAAGTSAAYH
LQKYAEEEGLAVNITVFEKTDHIGGRTVTVNAFNDPGQPVELGASIFIKD
NHIMYNATRDFDLSLTGLTEPQPADYTAIWDGKTILFRSEAGASKWWDAA
KLFLKYGLAPYRAVQLVKSAVGTFLRLYETPYFPFRSLTQRAFELGLLRL
TGVTGEQYLRDNNINPDFVRDLMQSATRVNYASNMAYIHGLEAMVSFSTE
GAVAVAGGNWQIFEKLLQHSGAVYYPNTSVATLAFQKGADKPGSAPKYLI
STKSSGSTSKAAKLPTAFDNVIIASPWQFSGIEAAKGVIKHRIDTIPYMK
LHVTLFTSPFLLQPSFFGLEAGTKAPSNVYTTLGKDEEAHEGPEGVGRTG
FYSISTLRTVTNPKTQGKEFLYKIFSAEPVNSTFLSDILGTPVPSCFICN
ETTGEVEPISWYYPHWFYSYPIESPRVTFQDPIVGRGLYYTSGVESFIST
METSALMGMNVARLMADDFAGVTRSRGGNKDTASQDTSPRPPREDFWDSM
DSEMGASMNFPGADEL
>Mb|QLI66576.1
MKSRVIRNLPIRRATTSIPKTAFATPVRAASSVSQRPNSDHVSFPGALKS
AFTTQLSFEHPESYKALPTYRVVDQHGAVVDQSFQPDISDETVVKLYKDM
LFISIMDLIMFDAQRQGRLSFYMVSAGEEAVSVGSSSVLDPEDPVYCQYR
EQGFFKERGMTTKEFMSQLFANKNDPGKGRNMPVHYGSKRLNIHTVSSPL
ATQIPQASGAGYALKMQKLQDPNSKAKVAVCFFGEGAASEGDFHAAMNIA
ATRSCPVIFICRNNGYAISTPTLDQYRGDGIASRGIGYGIDTIRIDGNDI
WAVREAVKKAREMALQDGGKPILIECMTYRVSHHSTSDDSFAYRARVEVE

DWKRRDNPITRLRKWMEAKGCWDETKEKEARDSLRKEILKGFSEAEKEKK
PALRTMFEDVYEELTPDLKAQIKELRDMLDKYPDEYDFSEYDGGKESLKV
>Mb|QLI66579.1
MSAGTVLVTGGTGYIGSFTSLTLLENGYDVVIVDNLYNSSEVALDRIEKL
CGRRPVFYKVDITDEKALDHVFAKHPSIDSVIHFAALKAVGESGEIPLEY
YRVNVGGSIALLRSMERNNVTNIVFSSSATVYGDATRFPNMIPIPENCPT
GPTNTYGRTKSMIEDVIRDCVNAQRNKLKKADQPFEQWNGALLRYFNPCG
AHPSGIMGEDPQGVPYNLLPLLGQVATGKREKLLVFGNDYKSRDGTAIRD
YIHVVDLARGHLAALKYLRENKPGVKEWNLGSGRGSTVFEMIKAFSAVVF
GPENPEQLKYEIVARRQGDVLDLTANPALANKELNWKTELGMEKACEDLW
RWVKNNPEGYRQEPPAELLAAIKTPSS
>Mb|QLI66580.1
MPQKRRRLEEPAQPALAEQSSSLRRTTRQTPVLPPLPPTSAAVAKVAQAP
AVADPTAMATTTVTATATATTTGANGPGSAVSGRPQLQPTGPATTASTAT
ATTVSNPIANLQTPVPIPTPLGSGHQQPNRSRERPQSTIPPPPIPPTYSP
PVKTPVPVPVVTPISPPSIPRPPLPSLRSNPPAGRSMSGISIKDNARPSD
LVAPEISYHVPQLAVPGRVQSSLLRPHGPSALPPRLDRRPAPPVVTPVLP
PKPPTLPVNDRGPPRADRNIDKVVLGDLCFRTWYPSYYGKEVLGDTSGNS
RNGGGSKDGSNEASGKASRKDKDNQPLLERLYVCPSCFKYAKELVAWREH
VRVCERKAFIPGKKIYRHPRGRRKVLVALEGKGPVPKRRRGDSGIRYTEE
IVQDEGEWSIWEVDGEKHGLFCQNLSLFAKLFLDNKSVFFDVTGFNYFLL
LYTPPARPIAPETEPIQTRPPQVCGFFSKEKMSWDSNNLACILVFPPWQR
KGLGALLMGASYEISRREGILGGPEKPISDLGKKGYKRFWSGEIARWLLS
LENTVSQPQPPQVAGAPVMTQELIVDVNDCSRGTWIAVEDCLGVLRDMGV
LEDAGMGYGKPEKKADAAEEDESREPPEGGDGAAHKPQDLVSRSAEEKRS
LAVKVVPRVRIDKEAVRRYVATHRISLERTCDPDGFVQGYAMKVDSSGND
GEKTGVETA
>Mb|QLI66581.1
MRFGKTLREAVYTPWKDKYIDYGKLKTLLHEDKFDDDTVPWTEEDENRFC
DEIFNVQLEKVARFQQERFDALKQRVDAAFEKLKELSPAEGDKPATRTEE
ETSELKELEKELDNITNEVKELKKYSSINYTGFLKIVKKHDRKRGDRYKV
RPMMQLSLSQRPFNSEQGYSPLLNKLSIMYFAIRQQLEGASAEQTVPNLD
TQGETHNGERYTAHKFWVHPDNLLEVKTLILRRLPALVYSDQTSKEVDGN
DSPAITSLYFDNKKFELYSEKVDRQSETSSLRLRWYGQLNTRPEIFVEQK
AADASGLSEEHKFTIKDKYVKPFIDGEYAMEKAVQKMERQGQPEEKIESY
KATVAKIQDFVKQKQLSPVLRANYSRTAFQKPLDDRVRISIDTNLAFIRE
DTLDSERPCRDPKDWHRADIDNSSMTYPFRNINQSEVSKFPFAVLDIKLK
EDSNRRRPVWIEDLMGSHLVHPSPRFSKFVHGVASLFEDHVNNLPFWLSD
LETDIRKDPQTAFEQEEQRRAQRADDAIAVGSLIGTVPSSYRAAQSSPVG
KSYLADRMAADSKAALSTSLRRKSGIANGEEEGESSNSPQQLEEQRQRRG
GYGTLSSVLPGLSLSKYAKAKRAQKARLPEGVVEPKQWIKNMGELKVEPK
VWLANERTFLKWQNIAILQGSLALALYTAAGESLLAEMMGIVYVIIAAFA
GLWGYYIHQARREMIMERSGKDFDNMLGPMIISIALMVALVVNFVLQYQK
AFSKLNRGNNGTGVISEELR
>Mb|QLI66584.1
MAIGNKLTTGLKLCLAHYCDIHGPTPLMVTEGLRSPCSVCFEEVDPISTA
TGKTKCDVRTRSGGPTSAPQSQSSVALNNTFRDLSIGRSGSAPASEHDAH
SQRAALRRSTTASGNTNYTVSSSALDTPPESPRPVFQQPRRDSSFRRTYD
DTVTKKQGPCDNCAMTLPRRQTSGATPEDYATGAHGPTLRTKAPVERVFG
PAGQTSPPNSQTSSDTDGDRETSTRRVRSRRTAPTASSSRNTSCSSTSTS
SERTPYHFHYVNYTSTHEPILADSFSLIRASCLRALSFETLPRPPSTSTP
TSSPQLNTSPSFVTTQSPGSAATGGPIFFGDALAGYTTAYIFRIPDVHAR
GHKRVYAFLALSTHKERLAMKTFAMISAAFRDLATWIQQLAEAEAERTTA
NSSPIGSVMYGGGAQPQPQASAFDPPAAVDRSGSSFLTGGSAFTRRMGGG
PGASSLKARGLAELVGQPDFFIELHGKFVQLLFEVGVALNS
>Mb|QLI66585.1
MSTAQGGGPDPQAMQAMLAPALTVHTPEEAAAESSSRATVRETTRPTSVD
LSREGASESSDEGDRGVRMSPAAARRFARRNLRRTAATPDLVTSLVRPPS

PEAFYIAQDAHGHPRQRAISRPAAMLSQPYGRRHSNAGPDRRTGPLLPPH
PSSLDIPNKYGGNCIFDMHMLTYVSDPDPNLLCPICHDPLVDPVTTPCDH
TFCYRCLRRSIASSPSGTACPIDRELLLWVDCFSAARLVRTQLNCLMVKC
PNQGRGCESELRRENVEKHATTECRFREFTCPDTACDKKLRDKPKDDKCR
HREVGCALCHDQVEEADKDMHLLSCSQAKTRCQSCWQLVCRSQMKSHHDL
ECDGVEVGCPYKDLGCPVRTIRGEVGAHSFTCAFHPDTPSGIVIRNQREI
IQSYGDLGSQLRDMQLRQDETAKRIDDMLANRRINGEGESRNDAVYSDNR
TMQDLDAGFEEVHQNLTHLEARQSMWTLNQVMPIREEVTELRNNINMIRM
HVNWLLNRSREEGRIRAANNTGPTAGIRRDSSGDAPPILSERRRSSSADM
DVPRL
>Mb|QLI66586.1
MASAPASVRVSGPPNSSFLIGYPGISATLPRIEGKVEIRPGQGYSMPVPV
SLVRICLQRRETIHPDADSLTKRHLGTPRKETTDVVGKEVLLFRCATGKE
AESVIAMDLPFVLFIPFGRGGEETNRRIPPASLQLPSRTAETYYELVVTV
QQGHSNQYKYNFPVPLQRYDTLSTFGMYNKPETKLVTSDNVVHLGINLPR
WSYGPGDPITVYIKLSPNPDWLTKAKRVSIEKITLAIEEEVTYNHEGDEP
VKKVNKLCKQLQTVKMKVPEAGYATNIGLVFPCKDLRDPDGIVRRGKPAF
PLYEVSSFTTTASLYKIEFYLSIKVQLSGARDVNLRQPIVICPLDQQACK
EEMDAIEQAAKDAAHVDPNNPMLPARTIILANDRDSLRALGLCLVGGQKK
PFID
>Mb|QLI66590.1
MSFPQTTPLRPVPGAFLNTPAVASRFQSGSDPVRRQLFPASSNNQPGGQV
GGNLASGGLGTTATQGGGLASSRSSTALTAVNTGDAGLVAANPLPTPRLE
NVPPVLKAAKAINAFLQMDESFPDLDSYCRQGASSDYELSRSDSSWAPFH
KTHMYPIPNQVFDHYNAGELQTLMGLFAEINHAWVVIDNSLYLWDYTHAD
PELIGFEEQPHTIHAVALVPPKPGIFVKTITHILVVATSSEMILLGLSAT
DTPAGTKSVALYQTKMNLPLRGNDVRVISGSADGRIFFGGSSDIDINELY
YQSEEKWFSNRCGRINHTNPGWASVVSLQSTFWSHKEPEHLVDIVIDDSR
KLVYTLSSKSTIRTYHMDAPDRLNKVIEKEKIHCLRDIAHMITQSKLLTD
KLRIVSISPITKQEASKLHLMALTDSGCRLFFSATNASSYLYGSQSNLAP
QSMQVQFIKFPPSQASRRSGLFSQTGQPGESAVDLESTILTASRQGIRFA
PGFFLDFVSSGNNASIDTLFVSGPETGRIKRVSPTAPLRYFEQGSWINIG
SRAEAVGLITKPFAAAAQPLGFGNELAVQFDEPPSEFAVLTNTGIHVIRR
RRFVDTFAAAIRDAPGDEVLDSITRRLIQLYGRVETISTALAIACGHGGD
GRPGAARAIDQATEDRARALFVDFGGQPTMAETDGTALTTESVKLSSRHD
ALALYLSRLIRKLWKSPVIRTGVSPTGGITVDSAVPLSKLNITQENLERL
RRFIETNRGLIQGMSGPSDLQRVSTRQEEVALQAEHQALHALQKIMESIS
EGISFVLMLFDERVADIFTRLDEVSRQQLKELSYEKLFSQADGKDLAKLL
VKAIVNRNIESGSNVETVADALRRRCGSFCSPDDVVIFKAQEQLKRASEQ
PLNTNQFRNLLHESLKLFEKVAGSLTFANLQAAVTQYVEMKYYAGGIQLC
LVVAREKDRGNTALVWVNDGKPSGDPRANAFNDRKRCYDMIHDVLRHLDA
ASSTEPEMVDGKLTLIATKRLEAYDVVNGSDDEVFHFDLYEWYIQQGWTD
RILAIDSPHVITFLERLAGTNVEHADLLCRFYTNRSRFFDAAEVQAQLAN
SDFPIGIKDRIKLLSLAKANANVATVGVSRQQQQQVNHEVTDLLDVANIQ
DDLLQRLKADDRIDPERKAEIELALDGKIQGLSELFNDYADQAGYYDLCL
LIYHTANYRNPTTIAGTWSNLIQQTHDEVMARQENPEPGMPTPPLPYEAV
TSKIQNIAHHTSLDSFVFPIQTLIPELCRYAVAYQQDATIGADPTWPVQL
FLTLGVSHDMIVRVLENIFDTQDYGFSGMARNRIIEIIAYVVNDWVADVR
RRGGAGKGGSIGPSVADLVSRCESALPPPGQGNNNGGMDLADIRRVLRAL
RREISGLIERVPTGSLRFM
>Mb|QLI66591.1
MSSNQQNGAVLGREFTQNVIDAMGPKTSPRLRQVMGALISHIHDFAREVQ
LTTDEWMAGVELINEAGRMSNDRRNEGQLLCDVIGLESLVDDITFTTAIH
SANGLTASAILGPFWRHDTPHRDNGTTITFDTPPDGQVVYMHGKVTDVTT
GKPLQNASVDVWQASTNGLYEQQDDDQKEHNLRGVFHTDAEGCYSFYCLR
PTPYPVPMDGPAGKLLGMMDRQVFRPAHIHLMVQQEGYKSLTTQIFDHKC
KYLDKDSVFAVKDELKVEFVPRKGDPQAPLELEYNITLAKTS
>Mb|QLI66592.1

MSAPPADGYGQYPGQPEQPEQPAQAGFAPQDASAAAPSKKKKRGYAAQAF
EVGSGANAAVGGQIQGGGQQYGMPAAQQPVAYGNYPQPDAQHQAPGPGYQ
YSQAPVQPQQQVPYGYQAPDQGYASAGPQPSGPGVAGITQGMGGMQLGGQ
PQQYQQPGQAFTQQPARVGPLNQLYPSDLMNNPFSVSELDLPPPPIVLPA
NSSVTPSPDANCSPRYIRSTLNAVPTTNSLLKKSKLPFALVIQPYGALHD
DEDQIPVVQDQVIARCRRCRTYINPFVTFLDHGHRWRCNMCNLSNDVPQA
FDWDAAAQQTVDRMQRHELNHSVVEFVAPQEYMVRPPQPLVYLFVFDVSY
AAVSNGMLATSARTILDSLSTIPNADRRTRLGFLAVDTSLHYFSIPKDED
ENGETNMLVVSDLEEPFLPVPHDLLVSLTESRQSIEKFLQKLPDMFQNNH
SNGSCMGSALRAGHKLISSLGGKIVILTASLPNIGDGKLDMREDKKLLGT
SREGSLLQTANSFYKSFAVECSKNQVSIDMFLFSSQYQDVASLSNLPRYT
GGQTWFYPGWHASRPEDALKFASEFSDYLSSEIGLEAVLRVRATTGLRMN
AFYGNFFNRSSDLCAFPAFPRDQCYVVEVAIDENLNKNFVCLQAAVLHTT
CNGERRIRVLTLSLPTTTNLSDVYASADQCAITAYFSHKAVERTLDSGLE
AARDALQAKLTEVLQTFKKELAGGSMGGGGLQFPANLRGLPVLFLGLIKN
VGLRKSTQIPSDIRSAALCLLSTLPVPLLMRYIYPRMYSLHDMPDNAGMP
DEETGQIVLPPAINLSSERLVPYGLYLIDDGQTQFLWVGRDAIPQLIADV
FGVEERAQVHVGKGRVPELDNDFNERVRAVIQKSKDHKSLGVGSITVPHL
YIVREDGEPSLKLWAQTLLVEDRADQGVSAAQWLGVLREKVSMR
>Mb|QLI66593.1
MSPQVDEGPLISDATGVTDQADSHYNRGDSDSQQIHDQDDTISMNPGHQA
ASVKKKKGKSRGNKSTAQRGPTALPRNRGTGFEEYFADPPMTPDEAAEEK
LEIYADCLPFEERIQSCIRRFRARRRIQGDQIRYFDEYLFLGGIDTSGNA
FSGLDTSDLKDLTPAQRREATVTDTVNGACGADDRFYNGDDEHWSVDFAG
VAAGLFSTSLGQLTGFEPKKTESLISLVENFLRYVLLHEVCPEYKDNIES
ALQVCNQARQEWPALNQLTAELPGYFNLAATELFSSVAVGDWSFLSFSKP
SWYDPKTVFLAVCALCDEDLTLDEFHQDRVEASKECTYTLEVTRVEGPCS
LTLDKFASLLIEGSDCGVSPVGKVFFKPAIIEDEWETPSMHISPQDANIW
VYLERSLLTNVMPKMKMDITLVELNTGIRFIKTIHQIVPSFYTFLPQQMM
RHYKQPRDDDRPAPSVHDTGTEEGQSTE
>Mb|QLI66594.1
MGREKRKVKPPIRASGPKERRRRNQKFLSDHPAPAPAPQGLVAKPTLPKS
RHHTYFEFVENTDKKEKILQVKETEDKIPPPGFEFVPIGNPGLTKACKDL
SREKDAMIFVVSPVTTNNSLSQQVNRLGHHVRQTIVEEAKATIADLPESG
AATPDGNPEPIPEIQAEYHAQADAALRDLFPRIPNTDREIIIQHAFTRVS
FSNNRLPLCSLTNRNTSQRATTKGEPPVGLCGEIPLARRVQLAVLAHIRH
NHTRYEELLKEAGWQVARKTVETLCLDILVKWRGDEETGRDQLDEILREV
VVISDSEDDSSDEEMVDDSSIDSDGDVSPSSVLISTKSTTATVLPQMAAV
EQPGSPGPTTLAEGRTARAKEKAPARSMDHVQKTNRKNQRGFKRYQAAWQ
QAILRNRGADDDRSGFIAGGVGEYNPRHPQMPTRQLEAHQSTTYNGSQVE
RATPSIGFTVNSGLPPMESQPYTRPVATPPLYAYQSTEIQASRDALSSSV
YEFSGESPSTLRMMSPIANRLGDMAVRSIEPASSETVMQPAFVRAVPPRQ
QDHLDPLPSRPPLFHTIRSMSPMDSEIRNSPDRGGRRIISDYPAEGSFQA
NSFALDAPTYVPQERVDYSFISGAPSYARPALQGWSRPYEHPIWADPPRG
DRIIVNASRPGTRTNPILMEDRGGFFERVALPLEPTVFTHQKEDSLTLHS
EPYRATGSYLVSSERDARILRNNRGHDSLEVMPINRPGALHNSHHSERFY
GHQPQLYQSCVPEESNIRDVESRDRARHCVASRYWPAQEVRRYPQTDAPL
QNHQPSYRSHQGKRPAADDHYQNASRGRRRVRPSDDVIVLE
>Mb|QLI66595.1
MTSPGEAAAAKDTVSGDLPIRRPSMASSNFDIVSTYHKFLESDPNLTKPV
AAIQSLIELLNAVPSKTVYETLDRIKAHSDRLKASVENPVPLSAGTDLFL
QYLVSSLKEQEDSFDAVRQHLLRNGRLFAQRAVDARNGVAKQGWRFVRED
KCVLTHGASRCVTALLERAARNLNGKFRVVYVRDETRVAESDRVVRELRQ
MGIPVAEIPVAAVAHVMGLLRQVHMVMVGAEAVTNNGGIISRIGTFQIAQ
LASKVKMPFYVAAETHKFTKKLPMDQRDLGLRKHGYWTQHVLDFSTDSAS
KQLENAVDYTPHTLITNLITENGIHLPTYVCEQLLDIYGNLHES
>Mb|QLI66596.1
MATTAAQDEYNSLVANNTARGTLDLDHRDDPELSAHDDDDDDDLDEETIY

RNAKIEAAMRTTTGASELKLPPASFDSGRSTGVKGVIADARSFETARKTT
WIDKAKSARRSILGIAGINQNGGAKSESETDDDTMSAPGSDEESFLQQWR
ESRRRELEREANRAVRTRRTSPSVRVYGRLDEVDASGYLDAIEKVGRETK
VVVFVYDHECDVSATIESALAPLVKSHSTIHFVKIHYDEIELDNAAVPAL
LAYHNQGDLFANLTGIIEMMPDEESFGTRSLQRLLERHQVL
>Mb|QLI66597.1
MLTSRLVSRVAVRPSSWQHSSLPTVISSPGLSRWTRGYASSSEEKDLVII
GGGVAGYVAAIKAGQEGMKVACIEKRGTLGGTCLNVGCIPSKSLLNNSHL
YHQILHDTKNRGIEVGDVKLNLANFMKAKDTAVTGLTKGVEFLLKKNGVE
YIKGAGSFVNENDIKVALNDGGETTVRGKNILIATGSEATPFPGLEIDEK
RVITSTGAIALEKIPETMVVIGGGIIGLEMASVWSRLGTKVTVVEFLSQI
GGPGMDTEIAKSTQKLLKKQGMDFKLNTKVVSGDKSGDMVTLDVDAAKGG
KPETLSADVVLVAIGRRPYTQGLGLENIGMELDERGRVIIDSEYRTKIPH
IRCVGDVTFGPMLAHKAEEEAVAVVEYIKKGYGHVNYGCIPSVMYTYPEV
AWVGQSEQELKNQKIPYRVGTFPFSANSRAKTNLDTDGLVKMLADPETDR
LLGVHIIGPNAGEMIAEGTLALEYGASTEDIARTCHAHPTLAEAFKEAAM
ATSSKAIHF
>Mb|QLI66598.1
MFDLFAMLLSSVASFLFPIFASYKALKTSDPAQLTPWLMYWVVFSICLLV
ESWLSFILFWIPFYGYLRLLFFLYLILPQTQGARVLYEEHVHPFLADNET
SIDEFIASAHERLKAAGMTYFRRAIEYLKTNILNLPPSEPEPSPSPSSAG
PQGYTQSLLARFSVPTTRWAGTANTGNEFYNLLASAVSAAASNAGGFAGS
PGGSSGRSMTDSGTLIPPNLRGSAEKMNFIAAQRERLNIVLGALDREAQQ
IQRDGTGASHTVPPGGFAPTGTHDMDDDQVTQRPPSGLSTLSALSKSRSE
TDFEKVEAESGAEDDVALRRRNVPSGTAGGSWMPWGWGGTGDATPGPGGP
KED
>Mb|QLI66599.1
MGKSSKDKRDAYYRLAKEQGWRARSAFKLLQLDEEFNLFANVSRVVDLCA
APGSWSQVLSRVLIKNEQFGLKSWQDGEAHLRRSMLSVLPASTTPSGKSA
VEAAIAAQHAASRPRDDVKIVAIDLQPISPLAGITTLQADITHPATVPLL
LGALDPGGDDAQRAAEGAAEGKASHPVDLVLSDGAPDVTGLHDLDIYVQS
QLLFAALNLALCVLKPGGKFVAKIFRGRNVDVLYAQLKIFFEKVVVAKPR
SSRASSMEAFIVCLNFRPPTGFHASLEEPLGVGERLDKMVKERELQLPVV
ARAAMQNPQTGAWDCTPASVMGVNALDSSSITELEVYDETKDGDGSGNYN
NNNQGSDRSTRWIAPFVACGDLSAFDSDASYLLPEDHVSLDPVQPPIAPP
YKRAVELRAAQSKAVR
>Mb|QLI66600.1
MEYDTKTPKSVTSDPTSFAHDSVRNRWPVILTGAIDDVHRTAHNADPEKQ
AEGNKIIQQLVDLKYGVLHDHKLTPIIVDDGYPDEIARYNEEIKQLGEPT
WLDVPWLFSECYMYRRISTFFSLTKHWKNYDIFSRQKMDTFRTSRNAVVE
LASRYKEFVTELRSNKDATHDEEAEKLLFTEMFEICLWGNATDLSLLTNL
TYEDIQKLQGSQARKAAEKNILINHLPEAYAILKKARDEGKKERRVDFVL
DNSGFELYVDLVLAGFLLTSGLATHIILHPKSIPWFVSDVLPGDYGLLLN
AIADPKKFFETQSDEEKAQDKTPAPLTDKEAEDLLFVAQEWTNFYAEDRI
ILRPNRYWTAGGSFWRLPAEAHDLHEDMKHAELTIFKGDLNYRKLTGDAH
WDPTTPWTTALGPMGPGSGINVLSLRTCKADVVVGLPAGKDEELRATEGG
GGDTGARRWAWHGKWAVVCLSKGS
>Mb|QLI66602.1
MPAAVETTSAPNNNGSIPAAVQKQAIGASSYLQRCLDWAAPPPSRQRAYE
TTSAFASARPVLFSFLAAQALLCFFPLLLFATFSLCTVVFGLGAAIIFAL
FWIGIALLVLIPTLLVTSSVAVLVWAWSAGSFLVARWLYQRASLRTSGEA
RVDTGGKQVAVVKDEDGFSGSVEHVKAEQ
>Mb|QLI66603.1
MGAGRRMKRKQGAPEPLSEAHYAKLKRKAGLPVDTRTEAPDNKRRRTGKN
GQPKANGASVKKAAAAPKKSNGDKPAKPKPGSKSKAKPVMPGLDDSDVEL
EDDEFDAAELDIEKLSDLEEEEGEGATLGDDFLGSDSSVLDSDDENDAEK
ATFSEDEDESDAEEKLTAANIDGLSRKLDRKLAEEAAANAAEMAESALQT
NIDGDKPHIIDDEDDELSAKTSALLAPDLQLLRTRITENIRVLDDFANLA

EDGRSRSEYTAQLIKDVCAYYGYSEYLAEKLYNLFTPREAFAFFEANETA
RPVVIRTNTLRTHRRDLAQALINRGVTLEPVGKWSKVGLQVFESSVPLGA
TPEYLAGHYILQAASSFLPCMALDPQENERVLDMAAAPGGKTTYMAAMMK
NTGVIIANDPNKARAKGLIGNIHRLGARNVIVSNYDAREFPKPMGGFDRV
LLDAPCSGTGVIAKDPSVKTNKTELDFMQLPHTQKQLLLAAIDSVNHSSK
SGGYIVYSTCSVTIEENEQVVQYALRRRPNVKLVDTGLAFGKEGFTSFMG
KKFDPSLRLTRRYYPHAYNVDGFYVAKFKKIGPTPAQAPRDNTRAARDAE
DVVDRTPIPADDEDAGKDDDFGGWDEDEDNEYMEKGRRNAMRRRGLDPRS
NAKSNKGSK
>Mb|QLI66606.1
MPFVANTPESLLDRADSKNPNSTCRGITSSGRPCRRPISRSSAGISLAPG
TRGLRVDLSDESLYCWQHKEQATHSAGSSPGPRRSTKPILEARSSLDTLA
DRLGVVELGEKNTHKHSRDHGRPLNLPAKASRPRTPPKLQFCFCFSIPLE
EVNEPARPKPRPQLEPIQEPASASMPNLPSRHHHNSKTGGQQRIATPSTS
PGKASYRSGRSTVSQTARFKDLIPDSLDMATASTLMTELAKPHADSEEAG
YIYMFWLTPTSKQSPPPVDAARSLLVPPSTSSSARSRRPSDIVSTFADPS
GKGDKKTMLLKIGRAANVQRRMNQWQRQCGYGIEMLRYYPYVAAVNDSPG
TSRTPRMTPHCRRVERLVHIELAGMGLRADMSTCDACGREHREWFEVKAT
REGIRKVDDVIRRWVDWDEASLD
>Mb|QLI66607.1
MSVVSLLGVKVLNNPAKFTDKYEFEITFECLEALEKDLEWKLTYVGSATS
DQYDQELDSLLVGPVPVGVNKFLFEADAPKTTRIPDADVLGVTVILLTCA
YDGREFVRVGYYVNNEYDSEELNTDLPAKPIIERVRRNVLAEKPRVTRFA
IKWDSEASAPPEFPPEQPEADLVADGDEYGAEEAEEEAAAEAAEKTELQP
GEDAEMAGVEQENGNAEENRPEEEEMSEDGSVDIEGESEDELDEEEEAEA
EAEAGEDDAMEVDDAEKQTDAPAAKPVTVSS
>Mb|QLI66609.1
MHIIKPSWLSHSGEQKDFEVYSCHISPDGKRLATAGGDGHVRVWSTDAIF
NAKDASYTKPRQLCHMSHHLGTIHSVRFSPNGRYLASGADDKLICVYHLD
KTPAIAFGNNDPPPAENWKTYKRLIGHENDVQDLAWSPDSSLLVSVGLDS
KVVVWSGYTFEKLKAIPAHQSHVKGITFDPANKFFATASDDRTIKIFRYT
PPAPNSTQHDMINNFVLETTISAPFKSSPLTTYFRRCSWSPDGNHIAAAN
AVNGPVSSVAIIERTRWDSEINLIGHEAPTEVCMFSPRLFHTVKPDENAT
SNGTSNPLLVTVAATAGQDKTLCIWNTNTSRPVVVLQDLSSKSISDLSWA
PDGQTLFAASLDGGVVVVKFEEGELGWVAQTEENVRALQKYGASRKGMGM
PEDVDGLLLETHSKAGESKAVESRMGALMGDLPSDTPKESTPAPNGTKAG
SEKHATATNGGSEPAKSPGEEQEDKAADKAADKIADRVKELKSRVTIGKD
GKKRVAPLLVSSSGTGQSSLPQTQLVGSTATKTTQNDAPQTTLDLSKPFD
GLPKGGIASMLLGNKRKSVTGEADEDEEEPAARRVAIGPLPIVTNGIDGV
EPAALTLVQNGVVPTPEFLRPAVLNPSISFSQVRLAVPRIRSHILRPMDR
GVLQPDGTSEDASKAPESIIMEAKNDTNPRDPSHVMVTKRGKLLWQEFLP
RAVILVTANKNFWAAACEDGSVHVWTPAGRRLLNPIILESQPVILECREH
WLLCITAVGLAHVWDLKMQASPHPPVSLGPILDVATTSLNQHTASPGPGV
TSAHLNTNGYIVVTLTNGDGFYYAREMYTWQRLSEAWWAVGSQYWNSNDS
SISALQSTAVGATSTETGDETTTATVSSGIIPFLERHTTNEFLLKGRAYA
LQRMIKTVMQRKESEDLESTISIAHLENRIAGALQLGAKEEFRLYLFMYA
KRLGAEGTRPKVEELLNSLMGGILREKHIENQQGKGWYGVDDEICGWNRK
ELIRGVVLILGKYRELQRLTSQYARVLDLSLEESAVDVDNMDVEA
>Mb|QLI66611.1
MSIRPLPKDVVDRIKSSSAITSLNQVVCGLLTNSLDACCTKVNISLDYVL
GNCTVQDNGLGIEPSEFEQDGGLCKLHHTSKSPPNPNIHGSHGSFIASVA
TLSLLTATSHHRHHASQASLSVHNGKVLARHVPALPDQRFELFSHGTRIS
IRNLFGSMPVRVKQRGLLFSDRVRLAREWESLMRDIAGLLLAWPTEVSIS
LREANTQWEQRLRSGNNEFVSRISRIFTQSGLAGSEDAGSWVPVSASCRR
VRIKGAISTNPVATRRSQIMSLGIRPIPNSFGTNVLYEEANKLFQGSNFG
VVDTEEHIGGIQGKPRKGIERWPMFYLEIHLLGTDEDLAMEDVLGDSKHS
LQAIIDLLKVVCYGFLKKHLLQPKQIQRIAEVPVTARTRHPPRRLTGQSI
SEGQKCSRLPSAATPEPEPRRPCSPFDAWNRLKVGRAVAEPKVAYQSVES

PATADKPTTRRLVGDRGVLLQRPFDDAAEIEPSVQSPHMNEPEVLVEATT
EKNNTKSLEVDSKAETSSPKTRLTRQVEQISEGRPKAQPQEWLQNIIRSW
KNPVFELTEPRIPSLDQAIPVERPVNANYDGPCSKFYSQEYHVQFESAAM
ALDGRLSRTSLAQAEVIGQVDKKFLLLRLPLHTPNVAQGTEASSTLIMLD
QHAADERCRLEELMSQYFQLNPLQAVTELLEKPIVFEVSAREYELLGRFH
SHFRTWGIMYVMCKTSSMNEHQIEIKSLPPSILERCRTEPKLVIDLVRKE
IWKLEDRTIPPQPVYEPGKPWVSSFHNCPQGILELLHSRSCRSKVTTNTI
IFVQPANNSQVPSCLMMSSARTNASEWSNVYRDVPFHFSVRTGARAWYRW
SISD
>Mb|QLI66614.1
MDNYQKLEKVGEGTYGVVYKARDLANSGRIVALKKIRLEAEDEGVPSTAI
REISLLKELRDPNIVRLFNIVHSDGHKLYLVFEFVDLDLKRYMEALPVSD
GGRGKALPEGSSATIMQLGLGEVVVRKFMMQLCEGIKYCHSRRVLHRDLK
PQNLLIDKEGNLKLADFGLARAFGVPLRTYTHEVVTLWYRAPEILLGGRQ
YSTGVDMWSVGCIFAEMCTRKPLFPGDSEIDEIFKIFRQVPLLLLHSTPE
IYSQNMMDEEVWPGVTSYPDFKSSFPKWKRDYRQPLCQNLDQKGLELLEM
MLVYDPAGRISAKQACNHPYFEEYLAEQQEEVGRMNGYYN
>Mb|QLI66615.1
MEDILTAVSADGSSIMPKLAPHLSRHLLFPLIQFEGDQAEEKGEDEKAKR
ILSGKIKLLEDTNMTDYVATLYCELHGTSDPPAEYTKKRQDVLAQLEKYE
QATAKIADLLTQDEVVNGLRSDKVANLEFLKNQHGVTMEMVNALYDFGQF
QFRCGQYGPAADMLYQFRVLSTDNDKVSASTWGKLASEILSTNWESAVDE
LKNVKEGIDSKLFNNPRAQLDHRTMLVHWSLFPLFNWEGAREPILDMFFS
AAYINTIQTSCPWILRYLIAAVITGRTRARNSSTQQKQLKDIVRYVRQEA
YEYADPITQFVNALYIAHDFNAAREALRQAEDVCRSDFFLASSADAFVNA
ARHLICESYCKIFSRMNIRDLSAKLGLNPDEGEKWIVNLIRETRLDAKID
SQDGTVIMNHPPNNVYQQVIEKTKGGFFRTQVLNAAVSK
>Mb|QLI66616.1
MAKRQRTSETDPVREKSYFDQQREALLSDIALSFEHVLANINKLNRSLES
VIEVGNEFSSVEALWSQFENVMAKDEAKHQGEKTEPPGDEGRE
>Mb|QLI66619.1
MARNGVHIFLDMSNINISFQCALRGKFSIKEHARFVPLPQLNLQFLTEIL
VRDRKIMTLNAGCSVLPHRQEPRFVQELRDLGYRVDLRERKRENHDFKAG
EASSSDEFVFSRNGGRYVEELVDETLQTRIAESVMEFFKDQGTLVIATGD
ARPAKYSDGFFAYAERALRMGWNVEVVSWRASLSSHWNRPNWVEQWSGRF
RVIELDDYIDHLLV
>Mb|QLI66620.1
MSRRQARTSVRFSTYSMTPSFTPTVQTSDTAQAEISDIALNLDRMENKAL
SSQRVMLSDEKTDNMSKLALGAKLDRALGRRMSGQDAVMRPRRNVSVSVT
MSEKKALSERA
>Mb|QLI66621.1
MTRRRYNIAMVSDFFFPQPGGIESHIYQLSSKLIDRGHKVIIITHSHDDR
KGVRYLTNGLKVYHVPFLVIYRQATFPTVFSFFPILRYIFIRERIEIVHG
HGSLSCLCHEGILHARTMGLRTVFTDHSLFGFADAASILTNKLLKFILSD
VDHSICVSHTCKENTVLRASLDPLMVSVIPNAVVAENFRPRDSPSSPGAA
FAPDPSPQRLGPNDMITIVVISRLFYNKGTDLLTAAIPRILETHPNTRFI
IAGSGPKAIDLEQMIEQNVLQDRVEMLGPVLHEDVRDVMIRGHIYLHPSL
TEAFGTVIVEAASCGLYVVCTQVGGIPEVLPSHMTTFAKPEEDDLVVATC
KAITAVRAGKVRTEKFHEQVKKMYSWSNVAMRTERVYDGITGTISEEEFY
GFDTGGYNGSRVRNFALIDRLKRYYGCGIWAGKLFCLCVVIDYLFFLFLE
WWFPRENIDICPDWPRKMLADNGKKEGQSNRSSTSQGRAGHD
>Mb|QLI66623.1
MESVKQAANYVSETVQGAVSGASHEANKEVAKDSNANVGTRLSAAKDAVG
DKIDQNAHDGKAEVHKQAAKN
>Mb|QLI66624.1
MMRGSSPLVRSTRGLRLQRPASFVCWQCRSIHISTAPSTESPRVPGDAFG
APIDGARDMADARFEVLGSPYSLLSVTLSASQKLYTRRGTLVAVAGKPDN
AQSTLSLLNPVTRAAFGVPFVYQRISATTPITALISTKSPTTTFTVLHLD

GTTDWMVSQRNALLAWTGHTLTLSSRIQRRLALAHWGSTQLTGRGLAALS
APGQIYQLTLGEGEEFVAHPGSVVAYSVSRNAPQPFRFKSTSLRLQVPSL
TSWIPEMEFVKTVRNSDVYKFLAGALHNFRTMARRTIWGDRLFLQFKGPA
TILMSSRGVRVADVLSREQVNEIADAPAGVLPSAVELANKPKQGGQVLDK
SQADEAASNLQVGSPPKDSKVVLEEGRELKEFVR
>Mb|QLI66625.1
MADKSATEQRKADWAIMKEMSRYLWPKDSFGTKIRVGLAVSLLIGAKLLN
VQVPFYFKSIVDSMNIDFAAVGGTAAAVAGSMIVAYGAARIGATVFQEVR
NAVFASVAQKAIRSVARNVFDHLLRLDLSFHLSKQTGGLTRAIDRGTKGI
SFLLTSMVFHLIPTALEISLVCGILTWQYGAKFAAITALTMVGYTAFTIW
TTAWRTKFRRQANAADNKASTVAVDSLINYEAVKYFNNEKFEVARYDKAL
GEYEKSSIKVATSLAFLNSGQNIIFSSALTAMMYFAADGVASGSLTVGDL
VMVNQLVFQLSVPLNFLGSVYRELRQSLLDMETLFNLQKVNVSIAEKPDA
KDLVLARGGEIKFQDVTFGYHPDRPILKNLSVTIPAGKKVAVVGPSGCGK
STLLRLLFRSYDVQDGRILIDDQDIRDVKLDSLRKSIGVVPQDTPLFNDT
VEHNIRYGSIDASHEEVVAAAKRARIHDIIEKFPDGYSTKVGERGMMISG
GEKQRLAMSRLLLKDPPLLFFDEATSALDTHTEQALMININGILREKGRT
SVFVAHRLRTIFDSDLIIVLKDGKVAEMGTHRELIDRAGVYSELWSAQEM
LFNEDGSEVEETKEQREQVAALKQSSTRK
>Mb|QLI66627.1
MVASATLPAPIGEPQKLEKKPIKFSNLLLGAGLNMFEVTTLGQPLEVVKT
TMAANRGDSFATALGRIWARGGPFGFYQGLIPWAWIEASTKGAVLLFVAS
EAEYYARAAGASEFGGGILGGITGGVAQAYATMGFCTCMKTVEITKHKMA
AAGVKPQSTFQTFMDIYRKEGIRGINKGVNAVAIRQMTNWGSRFGLSRLA
ETWIRSATGKSEKDKLSAMEKVIASGLGGGLSAWNQPIEVIRVEMQSKKE
DPNRPKKMTVGNTFRYIYSNNGIKGLYRGVTPRIGLGVWQTICMVAFGDM
AKTYVEKVTGDSVSAKH
>Mb|QLI66628.1
MAWPYGFPSLTHEEKLLRRQTLDLYACIAHYSALVPALLFLLYRLLKRVA
SQVKSGSSSEQTRYSVVPNSPTVKAQLQRPSARFASQWRKVVWWFSDDVY
FLGLHWGQKDEWVLGTIWTAWLLVLSIKGTGNDYFHLTKRFGAIAVSQLP
IQYLLALKALNPIAWIFASSHEHINRYHRVLGRIIYGLLWLHLIFYNAYF
IMQAIWLKRIFDSVVFCGVLASFGYHGIATTAMRKAREYSYRLFFITHLV
VALLTPVLLFFHASSARFYIAEAFAIFILDLAVRRITTINAPSTVENIPG
TTLVKVMSSIPVHKLTPFRATPGSHIYLSLPPQGRSAAVPSSKSAIFDYL
YNPFTVAAVDAEEGTISLIARVRNGPMTNVLSAYSSATPRPPSESRKITL
AVEGPYGTMATKYNDLLKWGATKVLLVSGGVGATFTLPIYQALQSELSSA
KLQFIWAIRGAGDATWAVSNKTTGQVVLEDPNIQLYLTGDLGLREQDNGD
SAGGSVELSSMRRTNGRLTANHNKKRPNIEKIVDDMFRGSPDESVAVLVC
GPIEMNREVQRRVRPWVMKGRRVWWHNETFGW
>Mb|QLI66629.1
MNPHATPRPFPGRSIGPSSLQHQFQQNQQNQQPQPQQQQRQPLAPQVSAN
EEYASIADEDREHIDEVFDLMDMKKKGWLNSYEFKHSLAALGFDLSKPEY
FRELQSYGSVPPDWHDQQSCPVNRYYVYLDQFRLCAAKLIANRDPREEAA
KVFAMFDYDQDGFISVEDLRHLAQEIKDERTLSDEEIQSMIDHLDHDGKG
GVSLDEFMQMMEEAG
>Mb|QLI66631.1
MEADIRAVLPNIDPVVSDYSVGYLTHASTAWTDDEETSGLSPLSEAAAAI
TELLLSASGDFNPSVEEKVKQLVEKWVDKYAEANGGQRIGPSIARRLDQT
IQVSTQRNMSSTLAVATGGVDLEQANARKVESKVDRKKLEKAERKIAAKQ
QKKTFKVVEYEASRLLDQPENTQSYEEFYMAVNPLQIGSSGANKTKDVKL
DNIDVSIGGNRILTDTTLTLAYGHRYGLVGNNGVGKSTLLRALSRREVAI
PTHISILHVEQEITGDDTPALQAVLDADVWRKVLLREQEELTASLAELEA
RRAPLADTSADAAKLDREKEAKDSKLGDIQSKLAEMESDKAESRAASILA
GLGFSPERQKYATKTFSGGWRMRLALARALFCEPDLLLLDEPSNMLDVPS
ITFLSNYLQGYPSTVLVVSHDRAFLNEVATDIIHQHSERLDYYRGANFDS
FYATREERKKVAKKEYENQMAQRAHLQAFIDKFRYNAAKSSEAQSRIKKL
EKMPVLTPPESEYSVKFTFPDVEKLSPPIIQMSEVDFGYTKDNPLLRNVD

LDVQLDSRIGIVGPNGAGKTTVLKLLIGKLEVSKGIVTSHPRLRIGFFAQ
HHVDALDLSLSAVSFMSKTYPGKTDEEYRRQLGAFGITGTTGLQKMALLS
GGQKSRVAFACLALTNPHILVLDEPSNHLDIEAMDALAEALQAFQGGVLM
VSHDVTMLQMVCTSLWVCDKGTVEKFDGDVQAYKKRISAQADAAGVVKAH
>Mb|QLI66635.1
MPKRKRSGQSSVHAELEKYQDEVFRALKASKGFERQRLSKRLRDPGITQD
KVHRLEREIAVLKSLDLHQTARAHLHLSLLKVKSIASSADLPQELLRGVS
KPDLPEDEKAALHNVTSGLYNREPVKQVVGRAVEAVCGVLNVPVPENDRR
TRKNKKEDGEEQPSARAEKLPDETKFATPSENPADEWDEESEFEGFSTDV
DDPGSVLEEQELDSDAEVKEVTEFDKLDGLLGSSSDEEDDWSSDKYAQFR
GRETVNLDDISVSGSGEESEAESVSQASSQSLSPPPEKKQKKDKKASAKT
GPVRDSTFLPSLMGGYISGSESASDIEEAKPKNRRGQRARQAIWEQKYGS
GAKHLQKPQKKSGRDSGWDMRRGAVDGEDRGRGTPWKKGIQNPVARNGMR
GDGAPAPVAKRDDEGKLHPSWEARKKAKESQKAVAFAGSKVVFD
>Mb|QLI66636.1
MPPRLLPSSSSICCRTTSPAPVGYLTACLAGLTIQQTRNASILGSLANNK
GAVHIKKRVGRGPSSGHGKTSGRGHKGQKQHGKVNAWFQGGQTPLVVKHG
RMGFTNLRAPKMSEVNLDQIQEWINQGRLDATKQITPKELIQSKLVGSVK
DGVKILSRGSECLKQPIDVMVSRASAGAIAAVEAAGGKVTTRYYTKLAIQ
RLLRGESVNTDMPLPVGKEHVDTVLAEARNAPFRYRLPDPTSRRDIEYYR
DPAHRGYLSYQLTAGQSPSLYFKVPGERKIKSAAKAEKKATEETLW
>Mb|QLI66641.1
MSAADKPSSSGVDTSVDAAVPPSLPPPSNRKAPITAVLAAAPSRADALLA
HLFRCMQTRSGAETVLLFACYASRLTGSVLEAAAGAAIRQSARRLVALAF
KLPPATTVVMSSTAPRPSLAALALQLGGRFKALAAMISETRTMGRLWGLL
GLYFAAKRLVLKSRAASSSSNEKNKLDHSSAEDASEALFDTLVAYAQVAS
LIVYQASENVAFLSSKKVLPFAPATQGRLGLLSVRAWGLYVAMEGARLLV
ERGRRTVRDAEWAAAWDKSFYRNLAWAPLTVHWGSTTGGFLPDMAVSLLA
FYPASGAMVDLWRSTA
>Mb|QLI66642.1
MDNSFRSTVLPRAQGDGKGEGDMDRHGRGHDRHQHHAAARDVMNAHAGSA
AHRHLDSSSTTASAASFSLRSPTVPTGSAHAHARAHTSASANANASSASA
SASASMSASTFHAPPPFASPGRNTNASITTSASGHHHHHHSHGSAASPPL
QSPPRLSPPLHHSSVAHHHAIGTGHRHHGHGSAPAAAGRVASSSPYMASS
SSSPAGAAGPPPLPPPSTSSNPSTSSSRDHHHQHGAPPVSPRHPPASSAS
NYYPPPASSPDSHHQSLHHTRDKPASGRVYDPTTDTTKERRVSDSWHNAP
QNSTPKTARDPHPYERTPADQHHSSSYYSNNNGHYTSPRSSYNRPRSPLS
HSHPSLPVGSHSPPGRTAQLPSPSERHVPAATLNSTTNGSSTMSTIIKSE
PRAVSPSKPVSASSTNTPGRSADPMSFSNILSSAEPAPKPAPEPRGPSPE
PLEPEQDGDTEVEPERDVEREPEPTRARKAESVVRKKPGRRPTKGRASDI
RDAEAPKGKRRSSMKRESPQPRSTSKRLANGQPKATKTWSSEMEKKISNS
EIQIENDVPSLDPTEFDEQEYKQRAQKRRRIMIDFDADQGRLRRDEYTKS
AAKKLLLHSELGKRRYDDVFYDQALHEVREQELFAEKERKKDMQRKRRRE
KSMAVTIELKEAALARANAAADENERQKHLRDAERASKKAQQTKLILQKG
IKGPARNLELNLEGGTMSSFLASDADTPKKGRGKGGRLKKSKEQKKAEKD
SAEAAQALLDAGKELPPKEESKVRIKIKGRSNKEKEKEKDTEKTKESKEP
VKEKKDEVSELEKKFSSKGYNQIYEQIWRDMARKDVNKTFKMAVDSYATK
GSNLKKTAILASKEAKRWQLRTNKGTKDLQARAKRVMRDMMGFWKRNERE
ERDLRKAAEKQEIENARREEADREAARQKRKLNFLISQTELYSHFIGKKI
KTDEVERSTDNPDIAQPSNGIPQKKLDIAEPSGPVGDRVTDFENLDFDNE
DETQLQAAAMANAQNAIAEAQKKARAFNNEGDLDMDEEGEMNFQNPTGLG
EMEVEQPKLINAQLKEYQLKGLNWLVNLYEQGINGILADEMGLGKTVQSI
SVMAYLAEKHDIWGPFLVVAPASTLHNWQQEIAKFVPEFKILPYWGSAPD
RKVLRKFWDRKHSTYRKDAAFHVCVTSYQLVVSDVAYFQKMRWQYMILDE
AQAIKSSQSSRWKSLLGFHCRNRLLLTGTPIQNNMQELWALLHFIMPSLF
DSHDEFSEWFSKDIESHAQSNTKLNEDQLKRLHMILKPFMLRRVKKHVQK
ELGDKIELDVFCNLTYRQRAYYTNLRNQINIMDLVEKATLGDDQDSGTLM
NLVMQFRKVCNHPDLFERAEVTAPYSFGYFAETASFVREGSTVVAGYSTR

SLVNFELPSLVWTEGGRLNKASRDNAKAGWRNKALNHMMNIWSPDHVREN
AQESKAFSWLRFADVSAGDVYRATHDCLFTRAVNELQKSDRIGQMSIIYD
EEGEDKTFTPAHALFNICARENRRPLAEITTEGILDNLMNVARGSYNDSG
LSRLEIAGRSRASAPPVEVSCNSMSAVRERQDMMFNPAIRRALYGPTFAE
EKSLVEQKVPLELYPSRSLLPKPDSERKRFTEFVVPSMRKFVTDSGKLAK
LDELLFKLKNEGHRVLLYFQMTRMIDMMEEYLTYRNYKYCRLDGSTKLED
RRDTVHDFQTRPEIFIFLLSTRAGGLGINLTSADTVIFYDSDWNPTIDSQ
AMDRAHRLGQTKQVTVYRLITRGTIEERIRKRALQKEEVQRVVIQGGGAS
VDFSGRRAPENRNRDIAMWLADDEQAEMIERREKELLESGEYDKQKKRGG
KRKKAEAPASLDDMYHEGEGHFDDGSRAGASGTATPAESDTKGKRGRGKA
AGKRAKTAKQRLAIADGMVDA
>Mb|QLI66645.1
MPETPKKKEPTASEAMFFFAIVKHTRNKADIDWSAVAAEQGFKNAEVAKV
RFGQVKRKLGITTNTDTPTASRTPVKKGTFAADTPTRVTKSSGRAGSKGR
ARGKDVKVEGEDDDDETVVGMKNEHLDAELEADVGSFLTEEASFMDGEEH
TDPF
>Mb|QLI66647.1
MAAALFNVKEHIIDASHIREFAYATANSQDATLKLHVKQYTPKDNPSPRR
GDVTLIGAHANGFPKELYEPFLNDLYQELNKCGVRIRDVWFADCAWQGQS
GILNQERGLLGNDPSWYDYSRDILHMINTFRMPRPLVAVGHSFGANVLSY
TALLHPRLFHSMVLLDPAIAYYHTSLPMDFRASPAAASVGRRDRWPSREA
AAALFKKAPFYQAWDPRVLEQWIKHGLHNTTADPNGEVVLATTKHQEVFT
FLRPSWPAFDAEGKNAIDPKWMPDAGTSIKVEYPMYPFYRPEAINTFIRM
PSLRPGIVFILGGQSNVVSDADVKTRTETTGTGLGGSGGVASGRVKEVVH
EDYGHLIPMEVPRFCAQHAADFLKKELDIWAVEEAEYEEWAKKSNTDKVT
ISQEFKKHLGVTDRPRAKM
>Mb|QLI66650.1
MANDEDYMAFLDKANQDLDDGKALAAKQKEQSSAAFKAVDEGSQAPRVIR
DACQDAVYVTDADEPFEEVSLKWSGDGLPDETEFAKLIKHWDADKADVSI
MDPVDWDSQGQYTKLIEAVREATKGNDVRVYSVVRDKTRTEYWVVSREEG
RIVGVKALGVES
>Mb|QLI66653.1
MEQVQSVATEARTEPTEDRTQDPHAASEASTNESSQLHAQPTAQQAAKGV
TICGVCEKAASKYKCPRCYLPCSVACNKIHKENHPPDPEPQLIAKPENPE
PLISSSPVTLSDPANPFRALDTSEHLQRLFRKYPGLRQQLLDIHAATQPP
QEAPDKRIPASLMQGVPKKSNWNHDVGIKNGKEALRRARMADGEAGEGIR
EYTELILHLINKRDDKDEVTAMLQQQVSQEDTKLIEQLMAQERR
>Mb|QLI66654.1
MPGKTPKSNGNEPIENGFRNNGDIDMKDASKTKGKKGAKDGDEEMTVVVP
PSKGSKQASKQPHDADGDVSMSGEDTADNGEAKVDPVVQTVADIKSNFAL
LDRAVALFDARFSLRALRSISLIRKHLTADIIGQAIVDTFPATITSGNIA
KTLLVAIGRESMPLGRQSGSEMDVDSVDTKASKNGKKENKEIIPEIDIFL
GILIQVYLFDAKKYQQGADFSKYLSDRIQSLNRRTLDSLSAKVYFYYSLF
CEHIGPLPPSPESPIVSIRPTLLAALRTAVLRKDIDTQASVIVLLLRNYL
LTSHISQADLLVSHTQFPENAVNNQVARFLYYLGRIRAIQLRYTEAHEHL
TAATRKAPSSNCAVGFSQTATKLLMVVELLMGDIPERATFRQPALEDALH
PYFLLVQAVRVGNLEDFETTIADHADTFRRDGTYSLILRLRQNVIKTGIR
MMSLSYSRISLRDICIRLHLGSEESAEYIVAKAIRDGVIEATLDRERGFM
KSKEVGDVYATREPGEAFHDRIRACLALHDESVKAMRFPMNQHRLELKNA
QEAREREREMVKEIQEGDLDEDDLGGDFEGI
>Mb|QLI66655.1
MLTTLPDFTPRDSHSLWYTSSRGGTAQPQGLHDTHHGDSPTHNHGSGQSR
SRGHIIERTALARLAADEQFMHRRRMQVQNFGSGWLRPPGVAKTLHQMRE
EKREQEEHQEAMRREQLAQELAEAEGGGMPEEGMMDDVQLDGAHDLDDEI
PDADDHFGMGDNDEDDDGDEEGLEAEDPEELDDEALREERQNDLMAARMR
MTDDAFREALVRGDPDGDDMYGGVEELEEEHQGHMLDEDDFAHDGMEANL
DDDIPEAESGLYEHTDSEAELSSSDDEERDEEQEDEDIGFVPRTAQMAPP
QSPTLRGRRVSGRESLDLSNILSQDESSFMDSSPVQRRRHG

>Mb|QLI66657.1
MAIIRHSVAPLRAVRCARQPIATRPFSVTSRRQGGGHGESQFEPPTGWLW
GIKPGEKPEPEGWEWPMYIFCGSLLAAGVALAFKPDTSVSTWALEEARRR
LEAEGVLPDPSKDKKE
>Mb|QLI66658.1
MKAAVVLSFVAAAVAGAIEPREGHCGGDNCARQVTGTRDGLTAITSRKND
CSNFMKTTVVPEATTVTVTITVDADEPASVTKRDIEYRAATEAPTAVPAY
ASSCNNPGKYSSACSCWGITAVTVTAPVPTKTATVTSTADSCEDL
>Mb|QLI66659.1
MTKLGHPLGEVNLTKAAGVYGTVQAIFDAGKESQAVFCHVCPHCATLELI
FHKIRTSTSVEPAAFLRKSFQMVERLGAKAIIFTVDVGWESKRTLEARTN
GEIHKSSLGAFMATGGFRDRNLFWDDISWIRRYTKLPIIVKGVQSTEDVE
LCVKHGAEGVMIRLQVQIETYRPDLFRKFDIMIDGGVRSGADVDLARLQQ
METAPSSFLIVGSGVFGLSTAWAITKRSQFAKTSITIVDDAANGQFPPDD
CASVDSSRIIRADYADPHYTAFASEAQKEWRKQGDDDLGGQGRYTESGFV
LTAYEPKELKVGTKSGMDYTKESWKNCVDVAARDGYPADRIKVLENTKTL
NECLGTDTYPGDWGYLNTLSGWADAGRGMEWLYERVKATERVNFVNAKVD
YLANEGDRVIGAKLTDGRLVTSDVVLVAAGAWTGSLVDLRGRVEATGHVL
GYIDISDRELALLSKQPVALNLSSGLFIIPPQENTLKVARHSFGYLNPVM
VHNALPLFPSPERPSFVTSRPSTSRDGGLARLPDEADADLRRGLAKLSPI
KGLETRRWKGTRLCWYSDTKDGDWLVDWHPGWKGLFIATGDSGHGYKFLP
ILGDKVLDCMQGQGGALGDKWKWKNIDDETVGKETNGKFKGLVTMDGSRG
GDPGMVLAEELGKKSAAKVAKL
>Mb|QLI66660.1
MRTTILLSALCAALTTHANPQTAEISIQPISSSHPQAPLPFASISYDLAA
LDSSTITSYDAPDIPDSSSLVRIGVYDPKVKSWLSGTTVASTENFAKGYS
PTITVSVNSRGDVLSAALKGVQIDAGQTRDFGPKVVVLTEGRGPQPVLNK
PVVVSKDGKKVEEEVEKTFLQKYWWMIGIAVFIALSGGGGEQGK
>Mb|QLI66662.1
MSRPSQPMFQRTSSGTNLADIGPSSVKRSSFQPKNVLQTPGMTRQFSDAE
RRSSIYRPRQSTVGGVNNHQGFFQTTLQPAGVPKDPRPLKDRSFQMRMGQ
ELMEYMAQNDFEMQMSHTLSPNVMKSPTQKDFNYMFQWLYHRIDPGHRFL
KNIDQEVPPILKQLRYPFERSITKSQLAAVGGQNWSTFLGLLYWMMQLVQ
MLEGYAANKYADACLEAGVDVSDDHIIFDFLSTAYKDWLAMDDDTGDDDA
ERILAPHIDAMARAFEDSNAKYVSELEMLEAENARLHKEIEDLEKATPDP
AVLDDHFKIMEEDKTKFEEYNNLALQRSEKYEHRIQVLHEELDKVVDELK
EVEDERRSLQRAVDAQGISMQDIDRMNSERERLQKGIETASQRLDEIKKK
VAEKESEAGQKLEELERTVDKYNTLAYQIAVIPATAANARGRDYELQLTI
NDSSDFTSTNLNASRNMAPSAERLLADATTGYQAGHILNLDLRGQIRSNF
LMLRKEISDRRSAAMEDMMKDHDLLDGIKEAIEDKRNEVEALQHRVRAAG
EEYEKTREVTTAQKLSSDAQIEKMEKELSRMRAGLSESVQLLEQREINTT
IEYEQLVLRASSLREELHTEIDRMLNDVIKFKIHVQKNLDDYEGFVADEL
EKELGVDDHKDDTRGMDL
>Mb|QLI66663.1
MTFGYRNRLTENDLWELAESENTRAAAKTFQDSWSVELVGKRAPSIWKTL
FHGFGRAYVQAILFKVWADIFSLMQPQLLHFLISFVQSRGNLTNGVTLAL
AMFLVSVVQSLCLHQCFQRLSYTGVKVKAALISTIYVKSLRLSNGARASK
STGGIVNLMAVDSQRLQDCIQFSQHTWSAPLQIVLSVHAGRSSLVLVMIP
INKTTTVAIKALQKQQMKNKDARSKIITEVVQMMKSIKLLAWGPAFMDKI
SHIRNDKELATLCRVGKLQALSGFIGAAAPFILACAAFSAYVLVQASPLT
TDVVFPAFALFHLLTPPLAILPTAISSVTEASVAVTRLSAFITADEVQEN
AAVHNGTDTQQTNPAIEEVNFTARAGELCCVVGRIGSGKSALIHAILGSL
HKVKGTVAVHGSVAYMTQDTWLLNATIRDDITFGHEWDAGFYENTVKACE
LVSDFVQFPNGDLTEVGDRAAVDQHVARHLVDNVIGRHDLLQGRTRIVVT
NSEAALAESDAVVLLQSGRIAERGTGTQTMEAMRDFNDVEGKFGTLHDED
KVNMSADVSSTGKLQVISNIQGLTTMRQVASQEADPSTAHRADDERRRGL
PRRRIDVESEPIIRTERSKQGLVGWKVYGDYAKSNGLVFVCLYVVALIGS
QAAEIAEYRGYSVKCRYCWIVLLHKPDIRRDALSSVADNQTWSNIWLKHW

SETNNDIGENPQVAKFIGVYVAFGRGSAVLVFVQSMILWLACSIKASRTL
HERMVFAVFRSPMSFFEQTPAGQILSRFTRTWKPRPDIYRIDESLARQFN
ALFVSAARGFFILGVIALGSPLFVVFIIPLVLLYTYMQRYYLGAKRELKR
LDSTSRSPVFAHFQETLGGVATICAYGAQERFIRENERRVDANMNAYIPS
IKANRWLGFRLEFVGSVVILLAAGFAVAGVAFRSGQSEGMAGLTISYALQ
ITQCLGSLVRATGEVETNIVSVERVLEFPGLPSEAPDVLESHRPPGSWPA
QGAVKFVNYSTRYRPELDPVLRNINLDKRAKEKIGIVGRTGAGKSSLVLS
LFRIVEPALGSVHIDDLNASSVGLLGLRRRVSIIPQDSALFEGTIRDDLD
PDKAHDDTELWVALELAQLKGHVAGMCGDLDAKVYEGGSNLSQSQKQLIS
LARALLKPSTILVLDEATAAVDVGTDMIIQKTLRDNESGHRTIITVAHRI
HTVIDSDRVVVLDDGEIIEADTPAALIEQKGAFYQLGREAGLAE
>Mb|QLI66665.1
MSVIYIRSSRPHDLEAEGQANAVKKRKWRVSIRVNNNAPTSADIEDPFLQ
VDYKGVFEQYLRSSDRPRWSSQNPIPEEEDAIELAEKRIEEYGRLLFDQL
DNQTGLFNSGESEAQIYVVEHHENRAVGNGGIHCLAWELLESVEIARLPK
LRLRVTRVSDFPAPRFLEPPRPDGPRLAAIQADRNATFNILLVIARDFSR
TGAERDPEPDLAQWPLMNIQKKLRSRLTLEIVRPGSLEELDRHLDIRAKQ
NVDFNLVHFDLHGRIMRNESGVPVPWLLFARQHRPSASEYSFPQTQLAKA
EAVAEVLSRHRVENVVLNACLSAYNRSGAATNLAHIFLRHGISNVSAMWY
YVHWKTVKTYVEAFYDKLLIECLDFHVAAQRARESLRRQPTSLAERTFQD
FFLCVNYARDVHRTDSMLREVSPSPSAKSHELTITLSNTSTRSSRSGIRI
KSPSRFADSFGFDDEPILRLRLHLLELEYKLMTFRIIYASDLDQAGSDLN
GTLGKMVNMWLNTNLIDEVHYYKAKDFSKRNLISGAVSVSPRTKHTRLSH
GGPLQRLFPRTVDSLRQTLHIVKEVDSVVDPGMQADKLENQRREERRFLA
QEGLQQFASKLHEEGCSYMIFVGSQNAQWWRTYLQALNGEWWLNMPWSFA
VHTRFTKEM
>Mb|QLI66668.1
MSDVEDSVDNEPPVIDPYEVLGLPREATADQIKSAYRKAALRNHPDKVTR
EQKEEAHAKFQEIAFAYAVLSDPARRKRYDTTGSTSESIVDADGFNWSDY
YREQFRDSISADAIKKFAEKYKGSDEERDDVLIAYEECKGDMDQIYERVM
LSDATEDDERFRSIIDEAIAKEDVPAFDSYAKESKKKRAARVKAAKAEAE
EAENHAKEIGVHDMLFAKKGAKSKGSSEDALAALIQKRQQDRSADFLEHL
TEKYGANSSKGKKGKKRAMDDVEPSEEAFQAAAAKLKGGKAAKKSKK
>Mb|QLI66671.1
MGPNSFALGALCLASLAFARTDLVGCTYFDTVSAYPGGQPFATRIWYVPG
SGELCEFLDCGGGRAPPKTTVPGCAAYEGTETYSPSFINPKTLGHAPATV
SPAKTTSSSAAGTPTAASSPGITGTEVPATTEASAWSETGSKTAMVTSTP
SGSGSRGAAMTNTTKPTTISSTGAAAGLPTAGPVVLGSWMAAGVVAGLGL
F
>Mb|QLI66672.1
MSVNPAPSLQLADYLEKLPGTTFRKLYQQPSTAFAIFRRMLPHLAKTLVM
RILYMPKPMPLSDLDLWVKPDAKRQRDHAISTLKGLHIIQISMPSKEKPQ
EMQLTTNFKNSLRLALTGGGEHNSFGVPSTLLVPPEIDTPFLDRYARKRW
EDILHFVVSSVGYKSTGDVSGPNKSVKELLIAGRLVDRRPNGTIGITQAG
FTFLLQEPNAQVWTLLLLWLEASETNKAAGLETVDMLSFLFVLASLELGR
AYDTNALTEQRKNMLPSLLDFGLIYIPQHKRSMFFPTRLATTLTSGGSSL
RTISEGVAAATAAATTTAGGSSSQSGSGGAGGPLGPSADQKGSVIVETNY
RIYAYTQSTLQIAVLALFCKLNMRFPDMVAGRVTRTSIHQAINFGISADQ
IISYLAAHAHEQMHRSAALANKPILPPTVVDQIRLWQLENERMKTTGGFL
FRDFEDHKEFLDTARFAEEIGVLVWRSDKSGMFFANKYEQIRDYLKSRKR
IE
>Mb|QLI66673.1
MATQQILFAETIAGMKKAFKRKAYESDSDSEIDSFTNRGHKLQKRARFVH
KGHLAPAAGPSAYKEAVDYAGVRRNILYRNPPLVDDEGYEITSDDDDDRV
GVAELAAAELNPYSNIRLEHILAPLTASTELPHHPTLSKAFTSKTLNELI
NQSCTLMRKENQSLWEVRHLWTSLCGDGVWMPCEMMIGSNDIDLYSDEHV
ARFLQSLPNSGGSARSSVAAVNGDTNPYNSKSSVYGRQGQLVGTTADADV
SMMDETAANNEEEKGKDKEAAIKSEPVGVKVDRGPNETEANNGIANINGN

GDAQSKQNGNNGGVTSDAPKEQSAGQFSSESKSPSFIHPIFLPPSGAKPD
RNMGIPENEAEDIRRLLALYVQKQEEVARGAMRLHQGLLRAERLRSNVLR
WSKAEAHCGPNRDMSDGEDWYDKEEWGLTEDLKKGQDEEEEDTTTTGKKT
RNRRA
>Mb|QLI66674.1
MTPEPIAIVGSGCKFPGSSSSPSRLWELISKPKNVATKPPPDRFNMDGFY
DPNPANPLTTNAKESYFISENIRAFDASFFNIAANEATSLDPQQRLLLET
VYESLEAAGLRMEALRGSSTGVFCGVMCADWEAVVGLDKAVPEYAISGLA
RNNLANRISYFFDWNGPSMSIDTACSSSMVALHQGVCALQNGECATVAVI
GTNLILSPNLYFAASNVKMLSPESRGYMWDHRANGYVRGEGIASLILKRL
SDAVADGDPIECVIRASGVNQDGRTLGLTMPSGKAQAQLIRSTYALAGLD
PTRPEDRPQYFEAHGTGTQAGDYQEATGIYSTFFGASPKTSTDDVLHVGS
IKTVIGHGEGCAGLAGIIKASLSIQHGTIPPNLHFERLNPKLEPYSSCLK
VPTEPVPWPQLPPGVPRRVSVNSFGFGGTNSHAILESYEPNLHRNPKSHL
NGTSKHLPSLLPFVFSAASERSLGAVLEKYARYLKDNPTVKAADFAWSLI
EKRSALKYRLTLYAPTIEELQTEIDKEMALRKADNPSTVISRPDTGKKSI
LGIFTGQGAQWPQMGLDLLSTFPVTRGWFEELQESLDQLPEEYQPDFSLF
EELAAPKSSSRIQEAAVAQPLCTAVQIVLTRLLATLGISFDTVVGHSSGE
VAAAYAAGVLNAHDAIRIAYLRGRVAHLAGANNRAGSMLAAGLSVQEATD
FCEKPEFAGRISIAACNSPSSVTLSGDADAIEEADLLLKSQDKFARRLLV
DTAYHSHHMQPCSDPYLRAMTGCKIQLGQPTATTWFSTVHAGKTINASSH
GAALVAEYWKDNMRNPVLFYQALMAAIAASPPGLIIEVGPHPALKGPALQ
SLSEVSQSASSTPYIGTLSRGSTGVQALAVTIGSLWAQLGAEGVNVDQYM
SLYDQSRKLQFVQDLPSYPFDHSQSYWTETRRSKAYLGRGRRHELLGDLS
EEVTEGEWRWRNFLFQRNLEYLEGHQIQSQTIFPATGYVAMALEAAALMA
EGRTMRLVEINDLVINQAIAFLDDNKGIETVFRAYQVRSEGGVTKAFFSC
HADIGGTLKTCASGQIVVTWGKVDENLLPTKPPSVSGMSAVETDEFYASL
GKLGYGYTDLFRGITQLTRKLNTSRGLLNNVESDSLLLHPATMDCGLQCL
LGAIGAPGDGSLSRLQIPTRIQSTIINPMFCGKCSVSAGKSLPFEAAVTG
LSADGASGDVSLFTPDGHGLIQFEGVHVSPLMQPTAKDDRPMFSQITWGS
IEPNAEPVDGPPPPLQFCPGDKDDPQHMCFSVIQEILSKLTAQDRKNLEG
FRRDVVEWFDHVVEMTREGKHPLCNKEWADEDPGEVLDVLAKKAQPIIVE
MTEKTRKHFLGFLRGETPMIEVYRQDNLLTRFYDQEQELKYMSLRVGDLA
GQLAFRYPRMKILEIGAGTGSATRAVLRRIGQYFHSYTFTDISAGFFEDA
EATFTEHADKMVYRVLDIEKSPVEQGFDANSYDLVIAANVLHATKFLQPT
MTNVRTLLKPGGHLIALEITNENILQDAMFFCAFEGWWLGKKDNRPWGPK
ISVPKWEDLLRKTGFGGVETIIPSPEKPEYAYWGYSTFVTKAVEDGVESL
REPLAVAPAASDKLESIMLIGGATEKTSRLVPELQRLLAPYFHQVYDALT
IDSVECSDAPLAAILCLADMDSPCFKDLTAQKLSCLKRLLEVGRRLLWVT
AGSESENPYLSMSKGFLSCIGYEYKGSLHQYFNIVDPDAVNATLLGNTLM
RMVQSEFTNDYSLSKGVGSVELELRFEDNVMKIPRIMNATSLNQRYAAAQ
RAVYNQMNLEQSTVELRSTSGNFEFVETADGVKDASFDEHQSMVQIRVRY
SVSLALRVKGAGFLSLILGTHEVSNARFVAFATKNASRISSPSAWCWELP
NHIAESQEPQFLKIVASAVMARNIIEQAETNTGLLVHEASDYLKRAILTA
AVAKGVQPHFSTSVAAVAESSSSILFFHDASSSRTLSRLLPTNLSVIANF
SNTSNGVFSKVESLLSEDVQRHHNGTLCRLSPLPSKGLNVAAVSETFKMA
RVVAGDVMLELAKHFESSEKTNVIDVEQVPRRGLRASEVEIIDWAQASEL
PVRVSSASSQVRLSGSKTYLLVGMSGDLGQSVCHWMITRGARNIVLASRN
PKVDPQWIDEMSKLEANVRVESMDVTDRESILNVDRTIRQNLPPVGGVVN
GAMVLQDQMFSDSPLQSILGTFKPKVQGSRLLEEIYGGDGLDFFILFGSA
TAILGNMGQSSYAAATNFMRSLIRRRREKNLVGSIIHPAEVCGVGYISRM
GSDLSRLMNKLVGRHIVSERDLHETFAEAILAGDPASGRDPEVMSGFTQH
DPEETPDLIWYSNPQTWPLVNYRLHSTSSQSTNTLMPIKQQLASATNVAE
AEEVVLAALKAKIIQKLHLSEDISVTPDTRLAELGADSLVAVDLRTWFIR
ELEVEIPILHIQSGASIGDLAATATSKLPSGFIPNVGKD
>Mb|QLI66675.1
MVTLTEVEDEHFQSSQAGPDVDDDDFTDTDSEISNDSDYDPSNETLAERL
YALRDIVPPTTRGWISSRVNSISSKAWSVLSFSGKGAWVITTSALFFGVP

FALSFAEDQQLTAMEQEYNMRQTGGELLTAGSEQNTADKVGAALGDGQAK
PSL
>Mb|QLI66679.1
MKLSGGLALLFASTIPTVAGESDSSSSLTSVITKLPACAAKCFGGALTQT
KCSLTDQKCICSDQVLLETATACIMESCTAKEALTVKKLTSEGCNAPIRD
KNAAYIRVSDVLGVISGLFIFQRFAFKLWAKQTFWWDDWFALATVICGSP
STIINAYGVGVNGIGQDAWTLNFDQLYNFGKFFYIMEVLYFFEIALVKLS
LLLFFLRIFPAPAVRRLLWGTAAFTVVYGIVFVFVGIFTCSPISYFWTKW
DQEHSGKCMDINVIAWSNAGIGIAIDVWMLAIPMWQLKGLKMHWKKKISV
AAMFMLGTFVTVVSILRLRSLVKFGTDSLNPTWDFFEVGLWSTIEVNVGI
WCVCLPSFRLLLVRLFPTLGGSTARSYAGYDSSNNRPSEKNNRSAGLGPS
ATATSGVDDWQNRSSPVGNQIAYHKSYAVEVSDLDEVALMTLRDHESRTA
ASSSRGSA
>Mb|QLI66682.1
MPSSLQKTRKHIAKKRNGEVNALHAKSRDSLRLHKAGVRDQRLEKLAAAR
SKKEQPIVDRVNFFQESLAEKDNQSLDIETVQSFIKRFIHQYDEEYDDLK
KARRLGRPATAREDLLKLKIASLEKEYQTGFVIPDVMSAEHAKNLGKWEG
SWAYLTTLPWIRVSSSGQVRSAEFPSKGIN
>Mb|QLI66683.1
MKIRNFYIALGLGASQVAALYPGGPRLPGSVRPVPVPDEDEEPAAAQISS
NIKAYNMSVPIDHFHNETKYQPHSNGSFNLRYWADVSHYKKGGPVIILHS
GEFSSEGRLPFLDHGIASILTQATGGVGIVLEHRYYGTSWPTDSATTENY
RFLTTDQALADTAFFSKNLKIPGHEQLNLTAPETPHILYGGSYAGGFVAI
ARKLYPDVFWGAISSSGVTVAIDDYWQYHESTRYFAPGECSPTIQELTAI
IDHALLKGSPRDQLEIKEIFGLRDLFDDEFASYLSDQLPSLQGTNWDPDL
DDLGFGTFCAIITSDSLLFKSTEYLLERVRYHVEAAGHAHDSSKPLTMRM
LNYIGYIKDNVKRDTRRCRGKSLRECLSVRYEKSTTEINENTWQRSWLYQ
TCTEWGYFMGGASTPKDRLPMISRALTAKFASYRCESFFNIKSRPNVGII
NKHGGFNFSYPRVALIDGKQDPWRSAGVHAIGLPSRQSTPSEPFELIDWG
VHHWDENGLDVKSLGPDRWSVRPKAIQDIQRDEVEIVKQWLREFEKPRHA
RSGLEL
>Mb|QLI66684.1
MAILETAASKAAGPATYRSTKGNGFAKNVSLIFLTFQNSALILVMHYSRV
MPPEGDHRYFTSTAVFLNEVIKLAISLTLALYETSKTLAPSTPATVLFEQ
IYNGVFSSDGWMLAVPAALYTFQNLLQYVAVENLDPVHFQVLYQVKILTT
AIFSVFLLRRQLGFKGWASLVILTLGVCIVSLPSSEKTTNSLLLHGVPDH
FFPRSKHEIGQTVAGADVPEAALHLSRRSATYEGIAKDLPPADPIMNYSV
GVTAALVSAVVSGLAGVHFEKLLKESSTNASVWMRNVQLSFYSLIAAFLG
GCMYQDGAGIREHGFFEGYNAVVWAAILLQAAGGLLASLVIRDADNIVKN
FATSISIVISFVVSVWIFDFAVTLTFLAGTSLVLLATYIYSVPERRLHRP
PAMRIVSFEKPAIEQTVTPLQTPRTADVRRLNTDPLEGRGVGKTTSRPSS
PMLPRVASRTHVHKEV
>Mb|QLI66685.1
MPEIAEVARIVHFLRLHLVGRKIASASAIDDSNVFGKVGTTGAEVEAALK
GKQVVSAGNQGKYFWITLDKPPHLVMHFGMTGWVHIRGEKTAYTNYYKKM
KDSELTTWPPKFWKFHLKTEGKPEVEVAFTDARRFGRVRLVDCPGADIRK
HTPLKENGPDPVIDTDRFTEEYLRGKMQARHVPVKALLLDQAMISGIGNW
VADETLYQAKLHPEQYSDSFSDSEIKKLYECIRYVCQTAVDKLGDSDQFP
EHWLFNHRWGKGGKGSSSSKLPNGEKLAFITVGGRTSCYAPEVQKKTGQVV
PGAKAQPIKDESDEEMLPAKNPRKKKLEQDGVEGGEPFTKKQKTRVVKKE
EDVVKSEPGFPSGRRRSTRLRN
>Mb|QLI66686.1
MSDSSTALEQPRPQTPKRPGASALFWLGLSYTPLIARVSLSHSLNLADSS
PYQDLRSAVTVAFLRAFIAPKEGNPRPFSQTQRRTVTKLPIKGRIWVSNY
TVPIPPEPESITEAVGKVIESLNNPDVPVPKTYLPHVAPVSGEWTGYRAG
AKQDEPAPSISDREKYVEMMKEVKKPTTILYLHGGGHAFLDPASHRPTVK
KLAKITGGRAFSVRYRLVPQSPFPTSLLDCLIAYLMLLYPPPGAFHDPVK
PEHIVISGDSAGGNLTMALIQVIVELNRLGQRITWHGEPRDIPIPAATAC

NSPWLDISHSSRPYYGTIPGAFDYLGPLDDMGRKGLTPCDIWPAQSPRKF
MYAADDIMTHPLVSPVMRRDWAGFPPVYFCTGWERLAYENKFLAQKLERD
GVTMVFEEYEAMAHCFALVLGSLAESRRCLDGWAGFIRQAVENPAGLRSS
AMTIHAKTLRETPLDFGDLFDVSEGEVRKRVVDKVAETQAWIPSESKL
>Mb|QLI66690.1
MTDQSDRAAGRLGPEPQDTPDTAPDDEAVNDPETPVADGRGELVSLEGNE
PCAASAGQGRGDDDSEGEQPEEEEEENDEEDEEDEDDEDDEDEEPRLKYA
RLTQSLSGLYRNGDATSAFLVGGDKMIIGTHNGNINVIQLPTLQPLRIYH
AHSASVTSISISPYPPPLPHDRIETPARPQQPQPQPSPRPDASPAGASRR
PKEPPQLPRIPSNDIYISTSSMDGNVCVQSLLDMKDVHLRNFARPVQAVA
LSPEFKSDRTYLSGGLAGQLILTVGGGQGRSTSTTTGTAVAAASGWLGTM
GLGTNTGKDTVLHSGEGTISSIKWSLSGKYVAWLNEFGIKFMRSKLHLES
AEAEGAWHRIGHCDRPQTEEWDTMASVWKGRLEWVDEQAIETDESRFSGD
GAVNLVTPTPKKTVERLLVGWGSTIWIIHVHHGATSGGRHGRDKPFGRAE
VAKILRMDCIISGISLYTQSTLLVLAFCPTDDDDDDDETPEKAIRGHKSN
ISAGSAGSEPSGGKRRRQNNQPPELRLIDLDSQMEVDKDRLGVSRFERLG
PADYHLGLLPAQAAATAVASKGALEALAGFGNDMWNAAINPKSLFSSGAS
IISKDSGEDTASVSKTLSATGTLRRGQVKGSGPVTIHPSLTKPGIKIFIH
SPYDCILGTKRDLADHLAWLVEHEQFHLAWELLDENPEIIATTPERPGDV
PPATPSKGQAAVDEFFDGDSVVESTQQDIHSHSVREKRRIGELWIQQLIE
NGNWRRAGEVCAKVLATPGRWEKWVWTFVGAKHFDEITPHIPSQTMHPPL
PTTIYEVVLGHYIQNDKPRFRELLDQWSTDLFDVRAITTALENQLKFRDV
QEDSIEDGEKGRDWLIVMESLARLYEAGGRHREALKFYIKLHDADSVFRL
IRDRHLAEAVADDIPGFIGLRVSRERMKQMTEQDLVEATSEAITLLVDEA
QHGLVRPDVVVEQLEAKALYLYLFFYLKGLWRGQGIREHSAENIDRLVMD
SQSLVDSYADLAVHLFATYDRPLLMEFLKSSTSYTFEKAVQECEACSYHD
ELVYLYSKTGQMKRALYLIIDRLKNVQKAIEFAKEQDDPDLWNDLLDYSM
DKPSFIRALLEQVGTAINPITLVRRIPEGLEIQGLREGLTHMMKEHELQH
SISSGVAQVLRSELAAAQNDLRSGQQKGIKFEVVQPGTAKSPNMSSKGQV
GTDKDVGDKSDNGTLQHRHTAPQPGRCASCHEPFTEFEMETILGFACGHV
FHVSHLLELLHKGKKADIDLGGAGSENGRYSIGMKVMRARLLRDKGATGN
TL
>Mb|QLI66691.1
MGRGRGSGHQPARRPADLTGIISIAEKNAFVSLVNTITDNMQKDVSNPFD
SLPVRPVLGDNGQGQWLSLPLLRHQDENKENTLTGKTFRGNIPDTYSTTH
TKSHHAVETEESARFQPLGELKKETLVFFRKWKSSVLQRFTEINVNDMTA
ETSGPYGRGRSDRGVYRAARGGRGGRGGSTGPHCVPTRHKDSKLAARHPP
VPNTLWSLHPDRRELLLHIALLILLSLQDYKANSRLLLLKLTSSLNLSFE
TYQSGEAQVAQGLVRAALDALLEDAAKPDEETGGPKFKMVLGGPVNLTAV
LKYVRIGTRQRGLGLSTITAASLLGTMAENAVLMGSLFGINPAKLPSKML
VTFARDIQDFAFVRLCKDPYNYDDVTMLEVSHRRLHVLVALSGFLTDSKD
VAETWKFLNSQSELYAIQWEHAALTNLGSALETVIKSTAWESAKKEIASK
SIFKTLMTSAWPQPLLRISKIVDNAWSVGMVRAEKAGAMLADALMRHKFQ
GERPVSLIGYSLGARAIYACLMVLAERRQFGVVDSVVMIGAPFPAESRVW
LTMKSVVSGRLVNVYSENDYILGFLYRSCNLQFGIAGLQEIQGADGVENH
CVEALPKGHLSYSSLTDHILQSIGWEDFHVQVLNKENPPRGIPRTTSQNP
PNPPRRGRYRR
>Mb|QLI66692.1
MLSRQVFRAARAAAPQRAVTLRAAPVRSFAAAAAADSQPPIAVFGLDGTY
ASALYTAAAKTSTLDPTAKALSNLNAIVEKDPKLTQILAAPTLTAEDKTA
IVAELAKQAGAGGETVKNFLSTLAENNRLGLLKGVCEKFGAIMSAARGEV
ELKVTSAQPLDNKTLSRLETAVSKSQYVGQGKKLKVTNEVNSDIIGGLVV
EVGDRTIDLSVSSRIAKMNKLLTDTL
>Mb|QLI66693.1
MSWRNQGITGSNNIPLGKRRFAEEDGVTDEEPSRGRDPEPRTEADGPRRR
KKRNRWGDASENKAAGLMGLPTAIMSNMTSEQLEAYTLHLRIEEISQKLR
IDDVVPADGDRSPSPAPQYDNHGRRINTREYRYRKKLEDERHKLIERAMK
TIPNYHPPQDYRRPTKTQEKVYVPVNDYPEINFIGLLIGPRGNTLKKMEG

DSGAKIAIRGKGSVKEGKGRSDAAHSSNQEEDLHCLIMADTEEKINKAKQ
LIHNVIETAASIPEGQNELKRNQLRELAALNGTLRDDENQACQNCGKIGH
RKYDCPERQNYTASIICRVCGNAGHMARDCPDRQRGASWRNDGRPAGRIN
GGDAVDREMEQLMQELGGGSGPPARIEAGPGGGADQNGADAKPWQRGPTG
GPAPWRSRNHDSNDGESRGSGAAPPWARERNRGGHDHHGDRDRDRDRDRD
RDRDRENGYGQGYGSSGSAAPPPWQQQAPGTQGGYPGYAGYGGYAPPPGM
GAPPGLSQAGAGLAAPPGLAGVNALIQQYAGGATQAPPPPPPSGDAPPPP
PPPPGNQPPPPPPGA
>Mb|QLI66696.1
MDAPTPKATPKRKRCGDISLSPVKFSFNPSTAESADDGSNSPRSKVAHRF
RGLALGSGGGVVNDHGHEADVGDGPDFARKRQRQDEVVANASEEDFQPVT
DSPTPRSSQAFGPGLNAFAEHLASQTPTLVAAAPEPENKPPRRRRAGTPP
LNMHKSPKKAKKYDVEASESPFADEDDIIVDPVRAALTWHDDEITVYDPY
DKDDDGTGINGVGFKPTPAMAEARAVRRRHQMAEYRKREESEARARRSQR
RRGEEPPSARLKKKSPSRKVRFADSERQSAVVIT
>Mb|QLI66698.1
MTAPATGLIASANIKRKILAFVVSFTNKLRIRASRYSRYDPERRSRSPRD
RSPDRFDRASQYGDNDRRRSSAEARSNSSGFPPNRDGFGDSLRREPPRGP
KALVDAPSGPRGGAFGADFRGRGRGRGRAWPPRDESRDRGRDRDIDYRDR
YREDRSRERERERDRERDRDRDRDRDRDWRESRDFRSRRSPPPGRGRTPP
RDFRDRDRDGPLVPDADRSRRGSRDGGPPSAGSSNSDPPFGMLPFGRGGG
FMRGGRGRGRGRGDWPSDRGRGRATYEDRGDRYPRSRSQEARWGRDRDRDDR
DRGDRYPPEADIIRRDPRDDPRERNDRDLIRPKKEGRPSISQEPSAQGRE
VSPPPVAPSAPAFGSVPSRTGADGVPVTTSGSGKLPPSAPRALTDRPGSS
GNSAPMESSAPVSGPIRAGSHDGPSIPVGPRAHQQRPSSKQWINPDLKKA
PVSPKMMRAQTFAQSGSISRRDSRESDHHHFEQRRPRSSDADTDPRGSGN
FDNRARSNHSAEPGEITVKSESERGSVTLESTKTPETGQSFKDDHRPSTA
SNAAIKPVQAIKRVRKRPAVGVVRFSLPPKTALKDQDSESDDDEDMADYF
NMEIEKTEAELARLPKPSLPTEVVAQYAALSHGSMARILNESEGLTDVLG
TVTETLDVELSKSHDVVTAPTKDVDATAALIETPVEVTAKVETEEIAKPD
VVDTVPEPITKTEEMDLDAPRAPSVPASDVQSKDAVASKPPSRPVSRSGA
STSGTSILAHEGTKQPSQPPESIPEATEAASKPPSTPSQIPDDDDETESE
DEAYLDLKTVRRFMTTPPIDSLPDYSCDSWVKDKDLLATFDTDSIIDEYV
MEHLDKIHLEESTQQEQARKAYADKYIDYLEFTLSNDPAAVKSRDKFSVA
VPMIEPAGTITPEPKPEGRSSGRRFASERDLERVLQASMREDEERKEREL
RAQKEKYRSEKEAVIPEMYWDAEERAKAQYTDRSGYTPQDRLVSTWHVLP
PLNNFTEEENELFEKRYLENPKQWGKIAEMIPHRDFGTCIQYYYLMKKDL
NLKEKLKKQPKRRKKGGRGKQRSSALVSELGNGDEGTEESQETGENGERR
RPRRAAAPTWGFEQPATDSENATPAGTPGRRGASAAARGEQGEKVDGRRG
RRRAKDKDKDKDKDKDKDKEKEKDKDTKASKANQSLSVTPGGGSGKGRSR
SSSRVPADIPTTAMPPDAGRAPTAGFDQTVPSGIHPPFPVQQQQQQQQSQ
QQQQLQPQQAIPGMERVKTIPPPSISDVMAAPSLRPEPPPPPQPAMTTFN
LAQPQTERKAPAQASSYWSVSESNDFPLLLRSFGSDWTAIAAHMGSKTAV
MVKNYFVRQKDQGKTEWDAIVQEADGKRTRGEKRPDPPLPTTGGRGRRYD
NSSAVGSTRPLAVAPGMEMHNEPAQQKMEATNQPPRSQPYPGGYGVPIAQ
APTQQQQQQQQPAQQQHQHQHPHQQQQQQPPPPPPQQQQQPLLAQPVPGQ
QQQQQQQQQQQQPPPQPPPPPPPLAIQQHAVPQPASQAMSPGPRPALRAP
AQAFGFPEHAREREVVAQAHQRVPLPPKGGGGANQVPELRDQRPLAAAQP
IPSAHPDSMLERQQRERERERQRDRERELAARQAERQSMRINAEAELAHQQ
RHYEQQPPPYGHRHQSSIGQGPRGEPLSLSRPSSQEPSRSAAAQPYPPPT
MHQQPPPPSHAVRGMMDHPSVAQSPPLGPSSRPMSSLQQRPPPGQAQEPY
GHAPPPGPPAPTPSGASRPPTEPRKTSNIMSLLNDDPPPASKRVSEVSNV
PSGPSGTPPPPTMGRPPPPGPAPPSQMRREPEPQYSPYGRAATNVSAMPS
LKPTAYGGSPGQPPHMGAPRSTMGMPNEPVPADRDPYYRGHAAYQAGHHS
GNNSPQTAHRYPPAQAQPQYPPQGGYASYGPGGQPTHAASPPPPQYGGHG
SAARSREVPPSGRDNAWPQQPTSSGAWPSQPSKPSQGPPPQQQWAPPHPS
STPKPSTPAQAWASAPPPQHVGMRDDRGAPVYASSSQPPQHSMQSRYPPP
GSRGPEPVPPPPAQGYPRYASTPGPGPRDPREHPRSYTPGYDSRGPPPGS

GYPAPDPREMQMRDARDPRDPRDPRDVMGRGLRPHEYERHPDQYRR
>Mb|QLI66699.1
MSSFEPVVVIDGKGHLLGRLASIVAKQLLNGQKIVVVRCEALNISGEFFR
AKLKYHAYLRKMTRYNPTRGGPFHFRAPSRIFYKAVRGMIPHKTARGAAA
MERLKVFEGVPPPYDTKKKMVVPQALRVLRLQPGRKYCTVGRLSHEVGWK
YQDVVARLEERRKAKGAAYYERKKVAARQLSDAKKNATVKAKTAKALAAY
GY
>Mb|QLI66701.1
MNHPTAYTPLESLFLFQSLLTHGADTGAFVRISELLRNNALVKSDKSFDA
ARLAPDALQQLFLLLLREEVKSGTGSTTTTTNATTATAGPSTNTADTSAS
PASRKRKLGSPPLPSLKDAYEHIEKVPALLDRLYARYRDTIVKEIREDEE
KFATVQKHIQFLEKNEKERLAKAAASQNGASMLAPRDGRPIPGQPPTPTP
GQAGGIKRPVSTTPVYPPRPPSTSTTQPTVAAPSHLQSGSTTPVRPPQPS
PKPPTTAGSGLQAPAGMPQPGSRVLQPPGQPPMPTLSPRPDATVALKPKE
ATPAAAAGQATTAGTLKWEKPYQPPIPQTPGTAGQTQPLAPANAAAGASK
ESQQQQRLPQQQGQYAGQVLAPTVAAQGRPIPGKQVLVAPQNAGQLSTPL
QPAPPRPGGVSASQGQPPQQHPRPISTTPVIPPSRPLPPQQAQQAQQAPR
PIAAASQTPQKGVTGPVAAPQKRTPAQAAQKQQTQRPSPSASPAPLAQKD
RPYASYNREPPRPAIPEHIIRQAAVAQAAAAAATPNAKKISPVTAAPSTP
SVTTPIAALTRGFGTKWASHSTPSTPRPITAEPESPAYEPVSPPPRDTSI
LSDTPRSSSKRDLTKGGLKADAVKARGRPPRISHGGRRMSTTPSVSGTRR
SQSVVSQADELAMDHPISSTKIKHEVLTPRHIEETGDTTADESVHGRTNM
ATPGSVSSRLAKRKRQDSPSVPTAPPTQVLWTRGFTKVSSSALDQISSHR
DANMFATGVRERDAPNYRQIVLQPQDITSIRSAIKQGNKAALQAASSLPG
GDPGTASVWLSISEDLIPPKGIINSAQLERELVHMFCNAIMYNPDPDRGP
GPAFLKRSQDEEEEVVGYRVDENGVVKNTRSMFVEVEKLLGDLRSAEKDR
GVPPPSATRPASVATPADDTAEDEDELAGDGNTATSVVKRRRVAARN
>Mb|QLI66702.1
MLGNTAFKFTPDTASISSRGTSISVATSNEAAIIRCIENGEYVDGQEIDD
DTRSLTSSVRQHIVDGGLRYHAYHAGQYAFPNDENEQYRDNLKHVVTLHL
CGGIYFFAPIHELLQRGAYVLDLGTGTGAWCVEMADLYPNSTFDGMDLSP
IQPDWVPQNVSFMVDDIEHEAGWTYGENELDYIHVRHLIHSIKDRREMWR
RIYSHLKPGGYVEVQEFKYAAACDDNSCDGPYAWRDFLNYLAAGLEALGS
DLYGMQYVERELLEAGFQELHYQDLKCPLGPWAKLLRLQECGHLLRDIVN
SGLEGLTRRPFRDGLKWTPLQIQMFLIDVRKDVNRVENGIPPFHSYFPFR
SIYARKPMVT
>Mb|QLI66704.1
MDAGSGMRPTRPQSVKELVAQAENFTFNANIPFKHWTRAAETLYQEASFA
LSDGDYGRAYMMLYRHSVLVLRCLQTHPQFKDPESRKAFRPLSKRIDRVI
ADLEEIKPIINDEYAEWERVRPAGSKLEEQGPLKSPSSYGEFAARDPSLS
GNATVLDASENQELAVELAQKELARRDTVRQANRRTRVYGDTILTSRLGD
GQKLDFSLQSQMEAVRRATESSQQDLYESQTHKPTTTPHREAVPQSYVYP
SISKSRPLQLDEAPQTPPRPPSLSPPSRPPKEYIHAPPRDPSYISRRELP
VVPNKVPVADSLSASYPSYEVARKNQPPLPPKTLDGPSLPKKERLAFKPG
AYLENGDPIRSVFLPSKLRAAFLDVAAPNTNKGLEMCGILCGTPVNNALF
VRSLLIPDQKCTSDTCETENESAIFDYCAGEDLMVLGWIHTHPTQTCFMS
SRDLHTHAGYQVMMPESIAIVCAPRFTPSYGIFRLTHPPGLDHILDCRQS
ETFHQHSVDNLYRETEYPNGHVYESEKMPFEVKDLRRK
>Mb|QLI66705.1
MLDDNLPTFRLRQSSDNPQNTLLYFTHNGSEPSPEYLLKRPLPSESRNQY
ALGLLDVNHPSVIYAEVLLQPEWTQPSLSAAELRAQNGVSTPLPMTPEAF
NVLLYNPDQSIAVKRNHGSWSKSNTWDFEVPERSFKVPSSSQIDRDTTPA
IPELAPKVVFRWKKDGRLSKDMTCYMSGRSVGGKKSKEPDITIAMFQAGK
NESAVTIYEPNMARVEVEDRKGLEVALILSAEVIRDLYLVPRQDPFNTAG
GPGPSAPKTSRPSASPPQQMPMASGALGPGVSPAGPGASPSALQSSVDAE
TRRLQAMVAEETRQTREREKRDEEEQKRIRKMLEQEEKERRRREAEVEKE
TERLRKQYGVPAPPNGGPPGSSPALPPRPGQNQYYASGGLGSGQPPMPPR
PNSVGPPPQPESSNAGGRRKHSGPFGGSVSGLFHRSDEDRRNKVEKKRSV

HF
>Mb|QLI66707.1
MAQDQGKLSSMTEPVDDDEPDEWDKRIFSTGCADENMKMTDCYFEKKDWR
ACAAELPSSPPPPPATHLQTASTSTPPGHSPAQHRRRRRRLSPTPPDPVT
CAQTSPHETMMLSRRAASAAARALATGAPSLPLAARFHATEASAPAAQPS
PTPASPTPAAASKPAPAASKWAKAKTAAKPARSASTSSSTSSALTEADVI
YEPRTIAYRAEGSQPPLDAASIPAVDWANSFHGISSRPVTEEQFRALMAP
LAIKDIEVKPDGVVYLPEIKYRRRLNEAFGPMGWGMIPKGDAVVGNSIVT
REYALIVDGRFVSQAQGENAYFSPDQLPSSVEGCKSNALMRCCKDLGIGS
ELWDPHFLRWFKRTHMEQAWVEHATTKKRKAFWFKKGEVQVSYPYSLAK
>Mb|QLI66709.1
MSPPTGNAGLEKESNLARLLGSGSAGIAELAIFHPVDTIAKRLMSNQTRV
TGASQLNQVIFKDKASAPLGRKFISLFPGLGYAAGYKVLQRIYKYGGQPV
ARDYLAKHYGKDFESAFGKKTGKAIMHSTAGSLIGIGEIVLLPLDVLKIK
RQTNPEAFKGRGVLKIVADEGFGLYRGWGWTAARNAPGSFALFGGSAFAK
EYMFHLQDYNKATWFQNFVASIAGASASLVVSAPLDVIKTRIQNRNFDNP
ESGFRILANMARKEGFSSFFKGLVPKLLMTGPKLVFSFWLAQTLIPAFDV
AFSKK
>Mb|QLI66710.1
MICHQLSRRAVFITRPLQRTLQPLLLQRAKRYGAFDANLDQEALAEARTW
YQSFDASQLPKGSTTYARSSGPGGQHVNKTETKATTVYSVRDLLSTLPRN
LHAAVRSSKYYVASNDSLTFQDQTHRSRTANADENRNKLLEEVIRIYKET
TPTETSGEKKKKYEAIEKRFHEARIKQKKFASAKKQSRKGPQG
>Mb|QLI66711.1
MKCPCRAAPWRIFLQSLAQVHNFPVSTSTARPWRPAPVPRPVSSQRRGLA
ASSYWRRETTRSAVAAENAVDKRENVTGTAAETKPLRKNRARDKKTENEA
PSAKEFQTRTIEKSSPPSPPPKRRANKAKPHDQSPDLNIAQPKTPEWQSQ
KAALKEKFPQGWKPRKRLSPDALAGIRALNAQFPDTYTTRALADKFEVSV
ESIRRILKSKWTPSVAEEQERQERWFRRGMQVWERKAAVGMKPPRKWRRE
GIVRDSWWHERRKEAARREREWEEEEKEAERARRNYGARSVGGEDEGRGE
AGSGRW
>Mb|QLI66712.1
MAPAPIPTIIIIGAGVIGLSTAVNLQQTLKETMGPDLPQILLVAREWPGS
IPGAPHQHSVDYASMWAGAHVRPIPATTSQLQEEAKWLKRTVAEFDRLAA
EEPASGVTRTLGVEYLEAPDQAYQQQEAASFKKETGLPGFKKYAKPELPE
GVALGYEYETFCINSPVYCETLLRKFVLRGGKTMQKDLRSEWEAFSLADN
VAFVINASGAGFGDPTCFPTRGQTVVTDLVDVQKTVTRQYKDGSWSFLIP
RFFNGGTIVGGTKEPDDWRHAPCVPTRKRLLEEGLKLEPLSHGDDKITPR
KTMKEVGVISDIVGRRPTRHGGMRIEIEERRIPHPNKDMNCSVIHAYGAG
GRGYEISWGVANEVLTLASPLLARL
>Mb|QLI66714.1
MSQTLGLTRLAYSRVWHQISASKPHQTLTTQPDVTPPSLGRLASRIAVLL
MGKHKPIWDPSTDCGDYVVVTNCAALHTTGRKMWRKSYYRHTTRPGSLKE
LTMDALMEKHGGGEVLRKAVRGMLPKNRLRDKRLARLKAFEGDAHPYKDN
LVRFGGVVVGSEGWQQAVEKIRESDKKRL
>Mb|QLI66715.1
MAFLILVIGDLHIPDRALDIPAKFKKLLAPGKIGQTLCLGNLTDKHTYEY
LRSITPDLKIVKGRNDVEATSLPLTQVVTHGSIRIGFLEGFTLVSSEPDL
LLAEANRLDVDVLCWGGTHKFDAFEYMDKFFVNPGSATGAFLNSWGGVGE
DPTPSFCLMDVQGISLTLYVYQLRKDDKGNENVAVEKVTYTKPVEPAANS
S
>Mb|QLI66719.1
MGAAPANQRKKVTLGTLRSLHRKGEPITMMTAHDFPSAHVADHAGMDVIL
VGDSLAMVALGMEDTSEVLVEEMLLHCRSVARATKSAFTVGDLPMGSYEI
APEQALATAIRFIKEGRVQGIKLEGGKEMVPTIEKITTAGIPVLGHVGLT
PQRQNSLGGFRVQGKTSSGAMSLLEDALAVQAAGCFAVVLEAVPAEVAAL
VTQKLAIPTIGIGAGAGCSGQVLVQTDMTGNFPPGRYLPKFVKKYGDVWG
EAMKAIEAYRDDVKAREYPAKEHTYPMPQEEFAAFEKGVKALENVADQA

>Mb|QLI66721.1
MRSALVSLLAAAAGASATSADGVFEFTPNLVPLQENAGTDKLFPMGDCFG
FKLEEATIDGMQEAMRKGTLTSVKLVTCYMTRTFQTQQYINSVMQINPDA
LAIAAQLDDERRQGKVRGPLHGIPFTVKDNIGTKDNLETTAGSWALLGSR
VPRDAYVVAKLRKAGAVLFGKATLSEWADMRSNNYSEGYSGRGGQCRSAY
NFTVNPGGSSSGSGVGVAANCIAFSLGTETDGSVINPAMRNSIVGFKPTV
GLTSRGGVIPETEHQDSVGTFGRTVRDAVYALDAIYGVDCHDNYTTAQRG
KTPKRGYAQFLTTKETLKNATFGIPWNSFWVHASAEHQRTLVSLVKLIED
AGATIVNNTEITNYETIVSPDGWNWDYGTTRGFPNESEYTYIKVDFYNNI
NKYLAKLRNTNIRTIDDIVKYNLDNDGSQGGNPWPLGNPAWFSGQDGFLA
SLETKGIQDETYWQALNFCQSTTRGGIDDALRHENKMLSGLLVPSNVAQS
YQISAQAGYPAITVPAGIDDESGMGYGLAILHTAWAEEELVRWGSAIEDL
TKGTQYARTLPKWRGYMQRNLPVPL
>Mb|QLI66722.1
MPQVRRGSRSLDWHTRPLATILSSDNASPNASEPLNDIIPDSRPPAGSSG
SNNNNHNRNITISPPEPPSLDSHPRSVPGSPISSSSALHEPPRRSLSYAL
RPPAPAYYDSRAATRTEYARRGRTLQEYYDANPLLLPQLPFTWHHGRRRW
RLFLFAFFAFVDASVVPIALYYALNYAGHVEGWIIFAVVTTIWGGPTYLE
FAVRTFRLIKKERFYRPLGTDSRWCFDMLTWASVITITAVTALFIVGSAP
HIVWLRVLCMPAPAILYCLGGVMALIMLWHVMGWKAPFRISSTAKGEPVL
PAAYYFIEDVIAVNAKAGRPYREALAARYKASPRFQRMLYVQSIFWSIPA
LVLAAALTVIAVVHQVPATVAYGICWAVPFIWCAVWGGISVWWCKRDMIR
ERLEWEQSCGEVEKQPPPRTESDSGSGVMVGRPADAVAV
>Mb|QLI66724.1
MASQAELAARQSNADVASPIEPSTPEEVGPLPVSSQVTLPSTEIPGSPID
PDQSFKTEANDEQAAAMVIPSSLTPPPSTQVAAHSNARRTFSQSQQSAPY
SPPATILNNVRDREVGTDYAPPSPDQVLEAPADELRSMLQTCIAEQQKLK
METAHHKLQYNLLRLQAEDDAKRAAVEHEMIRREVDALRMAEHSRQARRE
LSTASDSTQAKYLQMKMWYEAAMEENETLTRRLKVAKKVIQQKEEETMCL
TEERDLLLTRIRENREHFHMLCSPGGIFHSALTPKQQYISTPQQQHRTPH
GGPHQTPRSSQRGGDTEGREHGLSALLQAMSQSQDNNSAPSTPMTSHRAV
QKHSGRHNRNAQSLSSLPTTPVSRSRDGGLLPSADLVPQTEPRHRHVEQQ
VVPTTPNPKLDRRRKSRESTISADDNEELARQALESVEAMHSFTSQGSKS
APQGSRQRRRADEADEEEEVFDSQASQAATEMLRRHPGRSFGVAPSAGSR
DASPGPGQGPVEQSAKLQARLLSSSRGGGGAKDKRKFSGNALAVDDSRRE
HGSPPKKLRAGSMTEEQRVGLGIQYSR
>Mb|QLI66725.1
MATMTASTEPGRLSTDKTEVDSGITSPPENTRPRDDHHVTEGKAQQSEDG
GPDYPHGIKLVLIISALCLAVFLVALDQTIIAPALGAITTQFQSVKDIGW
YGSAYLLTTTALQPMYGAVYKLFNVKLVYLGAVFIFEVGSLLTAVAPNST
AFIVGRAIAGIGTAGLFSGSIVILSLSMPLQKRPIVFGLIGGMWGIASVA
GPLLGGVFTENVSWRWCFYINLPIGGIAMVIVALFVHINRNTSDTENMTF
MERLLQLDLLGTFVFIPAIVCLLLALQWGGADYPWNDSKVIGLFVGFALM
IAVFVGIQFWQGDRGTLPPRFFKNRNTLCAMMFAFFFGAGFFPLVYFISL
YFQAIQGVSAVQAGIKILPLLLATVLCSVITGGLISVIGYYNFVIIPCMI
LYTVGSGMITTFDVDTPLRQWFGYQVIAGLGIGAGFQIGVLVVQTVLPQE
MVPVGTACVQFFQALGGAIFVAVAQTLFQNGLIDTLAKDNIGIDGKIFIN
SGASQIKHVLEGMGRLDALDQVLEAYMIGLRHTYYISVACAACAFLAVLG
LEWRSVKHGPDGRRTKKKKQETAASATV
>Mb|QLI66726.1
MAEQEPHSLASTFPNPPPFWKDFTPDRVARIDELRSAFAGGASDHASSPV
VRIPGLPEELTNLQPPPEPSDGRWRVFGDQYMLDDKLPTLEEQGITNLPT
TGLSDSKDAKHYDRAFELKKLVKSLLLNFLELAGTLSRNPAHAEAKIQDL
RTLFINIHHILNEYRPHQARESAIAMMQDHLDKTRSETDAIRMQVEKAKS
VLEGLGKLGQGDVELAGEEGAGEAGDETETMRIRREMDLWAAADAEFAL
>Mb|QLI66731.1
MSEAARWKATVFVGGLSSVVTTTHLLDAFIPFGEIVEVQLPKPDSRKSTD
AHRGFAYVEYEDPEDAKEAIDNMDQSEFFGKILKVSHAKAPKSADEGLGS

RKAVWEQEGWLAEHAAEEQTGALGGDELAANSSAADPMQGLEGLDVAGPK
PV
>Mb|QLI66734.1
MSSSARDQALVATFAFGAVVCASLGTVLFHLRGGALSLFRGDLRLVLAGF
LLFASLWAVDGFVATFIDQTSASGCQIAVAFAAAFDQLARVVLEEFLFWA
MKSDARATCGVLFPQAVFFLRFIIGGVFVGVQRPQFDPVCVATNLLWPLG
VAVLCTDAFIVLTLVTRASSVGVFRDMRVKGPVGSRSRGIALIMMALGLW
IPLSVPMILGISSFGIATRTALPAVGVLLVIGVIAFYFKDVVLPLKAISQ
KTASFLNKFPGLSEAAPPPTREPRNQEASAVESSVPPDRFGRPREYSREV
TTSATARALPSGDGNNQLPIISRPSPGQAAAGVGGMPVKGELFPPIRTGS
LSTKREESLMQPTKKAVIKGGKLVISYPVVQEDKLSEASPLKRIATVDLA
TAARQDRERRVNPAAPRQLSTKRYSRQATKELQRKQIGGDSATGQTRAST
LEPIEERASASQAASSAVLSAPATPSGDVRQRSPKHISQCFQGTFPSVVP
AKNASGSGSKSPTSRNSSSGVPLKPPPRSPLRPFPENTTIATALKGPDKP
LAETLDRTEDHVEEFKNSAAAPSSQQQTSQTSQTPPSRSESVSRRKSIVS
PGKTIDVLVPPVPPLSPRDVDSSSYYQLERRSSKQHRRSGPSSSIPRSGS
VKNNIRPSRKRPKTPPPTDEVKSSKTPVQLRETTGLPSHPRARAAEASAS
KSQDPTVMFVNSIDYNDPGRVRDIIDEIATDLAAKADRGNSDNKVSVVHR
PRPIPRKSPITPSGSAKESPTVGLTSDLLPPKKEGEARLKASVLTATAGE
TPSQPPPPPPPPPKSKPLEPARTKTSVSLSKTYNPEVKAYITPVAQTGIT
PPPTILQTQASHDRPKPEKNVNDRVTVAKVTPQEYDEPPPSGKRMEDATR
YLSKFSIATTMSPEDPPSAYLPSPAFKLLRSSQEGLRRRSSPVLPVNDAR
ESVTPTSIVIDQPGAQSATPSYAKQYIKEQDLEAGYVSKSLGADLVAPGM
PARVSYADSALPNDEDDDREMVTFMLDRNTYYPANVASTPSPQNSMYGGT
EGKKGSWHCRVGENPPTFSNRESMQSRRFPKPPPLELHKIFKRIKGSPPQ
PPPLETAKQAIDEIQEQLKKFEDSNMDAAELEKRRMSLLKDIEMEMGMEE
NRWQKMRDDLTRASVLTVGSHPGSPLPHVASPLASQNMQSPAMNVAESSP
KNNSPYLSGWEKDTGRPRSSVLYTADQVQMSRPRRVPDRFQVTTANNTSE
ISEPVAREGVSITTGNTSPVAPSSSPSNNETYSRLGNSNREQRPNQKEEVA
PAKGVLWKSDSINQTPEITKSQIALWNSASDSAIQSTRSLSPGPRPETRK
PREFSPVEESGLWDNSPESFIPQAKSPSLWRAAPQSPKSPPPSQEPATRP
RTLRPPRKSKRISSLPDILENPEPLENNRDALGIFRFPWGETSDAATLYT
PFQNQMFVLPGNMGAGVPPIYPTLELQMQILRSQQQPSSYFEHYDDEEEA
VENISEDSDNDSFYDDDEYEDDSFEERTLWEIASLLKSDRVPSRESLFPN
NSNTLPGSLTTLGPTVPVRPSSVPAAWQVAEEEEPEHTESEPSPMPLLRR
ESSSPTLPPPEVSLWVNNVHMPPSTVNQGLFQDGKLWDTYVEIYKSTARA
PPRQTKASDITSTSLWSQPAINTGSPPIAYLWSGESKSQSTVEATVEITE
ISCPNTPKPEFLWSIPEPILDEDVFGLPQPKPEHWDHYNDLGEVILRPRP
RQSGHLNIESQSLWTQGTPQEEQTNNKVWLEGITPASPSDGTNEGDTSLA
TDMTLLIETRINSQESEEDAISIEEQPTYDTGIIIAPHDDIVISSPRGND
LWVPISTLPLDEEEHQGLFKIQQTSYQHDKGYRRSSKSPAALETRPTPRS
SRQPLQKLESHALWAPATPQTFAQDWVTLSSVRPSTPPGQLSYESGSESP
VSDISSVYSNVTGESTVASFLTGQASVQLKPHLVTDADWMATPKGTTGPA
IRPRNEIARSVPSWAAALHDSPEGAAQPPTASASQPEQEALQSTSEETQP
AVAQVDAGEEISAPEKEPNPLSHRRETQIGGFDPARHHPVFNVYILDTSN
GDYHPAAEGYIHTLVNKNPDLIQR
>Mb|QLI66735.1
MTRALQTISLGLLVSSLYLALYLELIPLPALVQQQIVPVLPFWALISFGA
LLLFRLGWGIMTFNDVPEAHQELMKEIDMAKGDLRKMGVDVD
>Mb|QLI66738.1
MAENGTTLPTNSSTEETGFAQGKGKGKAAAEGAPRTEDAAMDEEDEEDDD
DDDEDEDENGEGGDEEDGMEEIDLNNIVEGGRRTRGTVIDFAKAAEQHPV
EEDEDEDDDDFQPPTEDTEMSG
>Mb|QLI66739.1
MLLYTRLAAPKVPRKRSRAGCNYCKDKKKKCDELRPTCSRCQEHGEECRY
EPVKPRQRHKIEVSNKKVSNEESPLLPGDSKSCVSVQTTPDHQLALTGTS
TSHETDHPVPWDSSESISKDCGRDSLGPLGHDSLPLLRNGTQHLPGSLLV
HSKSSHNPSLPLTAPAATPSPLFEFCAPAFEEFSNDYHHRVLVNHFCNTL

SHLIVLREDEGNPFQQLVLPLAHNSPAVKSAIYALASAHLEGNRAQMMEN
DQKSIQFHNEAVRNLANLIGKGNNGDKNELLATIILLVYYEVLVQRRRSN
LVEGHLKGAMAIMRNGPTRNDQTSLFLDRAFRFYDVIAALSSGTAPVSDH
PSCDSFTGLLEQTDSQGSSSPPVSVDALLGLATSLWPVIHRLSGLLTLKN
CMEESVSRGDFSGTADLRARFGAECAAVESSLEEWQPMLPVVCDPQTNLE
QSTASFGARMSQFQSIFNTALAYRHSALVYLHRTIYDSPRGDPTVQRHTS
ISLVHCEATVRHEGPMGALLWPLFVASCEAVEPTDRDLASKAFRGIGQRQ
GMANIDRAWEVVQEVWNRADKIDDHSPEAIITPGQGKKDLWRRVSEDMGM
AIVFG
>Mb|QLI66741.1
MSTRQSALVTGASRGGLGDALAQELHARGFRVFATARTLPKVQHLKDLGM
DVVKMDVADSTSIRNAAAQVASLAGNRLDILINNAGTGYQSTLLDADIET
ARQLFNVNVFGVLDVTQAFGPLLIASKGIIVNAGSVLGRVPMPFCGVYNA
SKAALDSLSKQMRIELAPFDIRVIHVNAGGIQSDFLSHAAGPKGLPDKSP
YYAGHGVLNPWISGEITRSKYQPTRRQDYAKSVVDAIVKHNPPSCLWTGY
SAWVIWFTTSFLWRDATDLMMWAMGAPDIKVAISFNE
>Mb|QLI66743.1
MSFGKTVKLNSGHTIPRVGYGTWQAAPGEVGNGVYEALKTGYRHLDLAKI
YGNQKEVGEGIKRAYAEVPGLKREDIFITSKLWNNKHRPEDVEKALDDTL
AELQLDYLDLYLIHWPVAFKPGEDKFPKVANGEVDIDRGVTISQTWKAVT
ELPKTKTRSVGVSNFSIEHLEGVIAATGVVPAVNQIERHPRLPNAPLIEY
CAKKNIIITAYSAFGNNSWNVPLLVNTPEVKTIADRLGAAQGKTVTAAQV
IIAWSVLGNHTVIPKSVTPSRIRENFQEVELDEQAIQDLKKLGETPQRFN
VPTTYDPKWNINVFDDEKDKNAALQVLIK
>Mb|QLI66744.1
MGVIRKKTVTRGEGGVKYVCDVCSSDITSTVRIRCADQSCSDFDLCVSCF
AKGESRNNHDPGTHEFRVIEQNSFPIFEREWGADEELLLLEGAEIYGLGS
WADIADHIGGFREKDEVRDHYLETYVNSPNFPLPKRCRPHDCELANQVPR
EEFQAQKKRRIEERREKSKSAPALQPKTKPTASVPSCHEIQGFMPGRLEF
ETEYANEAEEAVQHMQFDPGDGMNPRTGELEPEMELKLTVMDIYNCRLTQ
RVDRKKVIFEHELLEYRENTKIEKKRSKDEKDMLQKAKPFARIMNHKDFE
DFNQGIIDEQNLRQAITQLQEWRSLKIGDLRSGEKYEAEKAARIQKAIPM
GSMDRERLASSQRSKQQAAPEPPSGASLLVAPELAIRPAQTNGEAVNGDS
KPLVNGQANGVNGINGVNGHASARPKYTPQPISGVQPLQLNQDTAADLHL
LTPDEAKLCEIIRLQPKPYLMIKEQILKEALKTNGTLKKKQAKEICRLDS
QKGARIFDFFINAGWLGKA
>Mb|QLI66745.1
MGKRNNHRRRRLTPSLKLSREPPRINHSSGATGPTSPPETPTGSPHMARK
PPQHSAVYFGYSGRPLEQDPNIKPVGHGHQFSYRRVIGPEQLSPRSSSSD
ESEGEEEQAGEELDDDSSVSDIARPQSIINSTAASSEDASSGPDDETSGD
GDDEDDEDADDEDADDEDADDEDRGVEEEDYEEEAEEEEEHVEVNARPFF
RLPSEPETVSISAAGSEAGDEAARQSRVAAVPSSAASGCEFTVEDIDAMD
SGCDGLEVLLPTEIESNRSRSRSRHKYVDKGMVRDFKNLNCSNDVSENEV
DAEGELGMDTESVFLKRQKEIRRNRRVSMSSTGKRTHSEFSEFTDSDNSD
TGGLDVNEVGSSARKLRKRLHRGSLLFQDPPAPRIDELDEPDSSENEYDA
ADPLARELPYWTMETMEIMEVESP
>Mb|QLI66746.1
MSAAAPTRPSRRAAMRRAAIIDSDDEDEIGNTTKIRADDEAEYTPEASRT
TRRQTRGGRLSVAPGSAPTRRRSRVKKQSIASTEPDELEGGPDSSHAVDA
DTTIQQTAQTPPKKPSPRKRKSAAPRASIAPTEDLAPPVPKQPEPFEGPQ
AHGSPLADITAVSVNAAQNVEETQANVKPIKPFDSIMDKPLDIILKSRTT
TIPFVEEPSTSPKSRIVLTHLILNNFKSYAGRQEVGPFHASFSSVVGPNG
SGKSNVIDSLLFVFGFRASKMRQGKISALIHNSAQHPNLEYCEVEVHFQE
VIDKPTGHEIIPNSKLIISRKAFRNNTSKYYINGKESNFTTVTTLLKNHG
VDLDHKRFLILQGEVESIAQMKSKAANEHEDGLLEYLEDIIGTSKYKTPI
EESAAEVETLNDVCMEKSGRVQHVEKERNSLEDKKDKALAYIRDENELTM
KQSALYQLFIHECNDNITVTEEAVNQMQAQLDAELEKHQGSEQFIKDLEK
QYTKGTKEVETQEKQTQSLVKELAKFEQERVKFDEKRKFLVEKQKKLEKT

INNAQNATNEADETIEETKQDIETRTQEVADLESQVKIEEAELANIRESL
KGKTQAFSDKIAAKQKTLEPWMEKINQKQSAIAVAESEMNILKEKANAGA
VAIEELEGKITTIETDKAAKAKELKACEKEKSELQKEAEKMNSELAILAQ
QEPKIQAKISNSRQKADEARSSLASTQARGNVLTALMRMKESGRIDGFHG
RLGNLGTIDQKYDVAVSTACSALDNFVTESVESGQQCIEYLRKNNLGRGN
FICLDKLRSRDMSPIQTPENAPRLFDLVKSKDKFRPAFYHAMQDTLVAND
LAQANRIAYGAKRWRVVTLAGELIDKSGTMSGGGTTVKKGLMSSKIVADI
SKEHVAKLEEDRDQWETKFQEFQEYQRECEGKLKDLNEKIPQLETKMQKL
GLEIESATRNLSDVKKRIKEVSQEHQPSTSDSSRVAALEREVAKLNGEIE
QLRGETASVEEEIKALQEKIMEAGGEKLRSQRAKVDDLKEQINSHNEETS
NAEVRRVKAEKQKVKLGKDMAKANKELENAVRELENLEADVNNQGEKSDD
LKAQVKEAEADLASKREELRAFKKELDAKTAELNTSRAVEIEMRNKLEEN
QKTLAENEKRHRYWEDKLSKLVLQNVEDLVGAPSPTEQAAKAEQPEKEED
SSTSNEDIEMAESEAEEAEDASMADASTTEKSTEGGSNNPPYELPRYTPD
ELADMSKETLKGEIAALEEKTQNASVDLGVLAEYRRRVEEHAARSSDLQA
AINQRDAAKKRCDDLRRRRLEGFMEGFSAISLRLKEMYQMITMGGNAELE
LVDSLDPFSEGILFSVMPPKKSWKNIGNLSGGEKTLSSLALVFALHHYKP
TPLYVMDEIDAALDFRNVSIVANYIKERTKNAQFIVISLRNNMFELAARL
VGVYKVNHMTKSVTIENKDYIDRPQPQPQQRPQPGTGFTTTLALR
>Mb|QLI66747.1
MSWWPFSSSSRSDEIRSGTAIPTRQERAVCWAARDAYFACLDAHDILDAN
KDAAAARRACPRQSDDFERDCAAAWVKYFKQWRVADIQKKRRLDELRKQG
AQEMEVSTTFAPDGGAGAKGTGKEDIQDMLERKKVK
>Mb|QLI66749.1
MGADMASQNSETTPAKLSAPKDKKCPYCKQAFTSSSLGRHLDLYIKEKNP
KPPDGLHDVEAIRKLRGGITRRQARGSLGARKDSTPAGTPRPVPKRETPN
QEIEPMGADPMPKDGTYAADSSFGKFPFVPRWEATGVINDIPPRGSWDGD
AGSDPAKRPPMQRTVSKQAVQKAQFDLKQKLSDATDNARAAELALRELLG
SWRAAKQHIDNSSMPFGFDPLSLDFPALTLQCLHPPPTLFSSTQHPTSTS
WSVQSPGQREFKALRSYFNEEFRAWKVTCAAATTAASEELAYPPTGHLHK
DTRDAVKKAEKTAGNLEKQVEEHLQSAYAVWDSLPGQRQQELWILELARG
VGRKHKEMEIIKEQQHRLKQENAHLKMQIDQLNRLQQPREFKLLSPSTIP
IDRDVISYAYEQGVRGGKIVGFDMEDRHVDIATVVAKSIERWKNVIVSTR
VTSSGMSAQRPLDQPAQTPVNLNSPSSSQSQGLSQTTQQQQQQQQNNQS
PAQTNNPSEKRLSTASTSGRISENTAPSTSTTGPPSIAEEEEGDQNDDVS
DQDADAEMEDDDSFAIMNASPAKHAHAPMQQTAALEVPRTRGLVQQQGAS
PDMRFMMQNGAVSPVGRSAMTMTRPMPNMNMPMQGNHMHGSDMPMMQQVR
GDMYMEQ
>Mb|QLI66750.1
MPQDPNLYGQRPAKKQKKNTALSSSLDFTAQLTSLMSNTTSSTSTSGRTR
PSKEPKDDIFRSSKPKQQSSRKDDGKLVLKEVAGTEEETQNLARARRKME
DKARLYAAMKRGDYVPKENEAAPLVDFDRKWAENVEGKDYSSSSSDDDGD
DGNDVIEYEDEFGRLRRGTKAERERMERRARRGLLGAEELERMSARPSAP
TNLIYGDAVQAMAFNPDDPEKMEELARKRDRSATPPDQKHYDADSEIRTK
GVGFFKFSKDEETRTKEMKELAEEREQTEKQRQAREDQKEARRREIEKRR
RNMGTRRAQRQADNFLEGLAE
>Mb|QLI66752.1
MPIALSPPTRPKNGLTKFTNCRLVKGDALVTEDLWVSSITGKIINSQATF
FDELNLPDKTIDLGGRIISPGMIECQLNGAFGFNFSTLLDDMSQYGKKVN
EVNKLLVQTGVTSYIPTITSQRPELYQKALPFLGPSGANRNAHEGAESLG
AHCEGPFLNPTKNGVHNIDVLIEAQSFTDIEACYGAENLRPQSEGEPIPI
KMITAAPERGSMMKLIPEIASRGIIYSVGHSEATYEEASEAVGRGATMIT
HLFNAMRPLHHRNPGIFGVLGKAENLARPYFGIISDGIHLHPTTIKIAFN
AHPDGFILVTDAMHLVGLPDGAYPWTNGEQTCNIIKKGSKLLLENSDTIA
GSSITLLECVNNFLEWSGTGIPQALKAVTATPAAMLGFQGIKGSLEAGAD
ADLVIFSEEESTTGSGSKQLVLDEVWKFGGLLHTANRTKAL
>Mb|QLI66753.1
MATATMTVTAVPMPKKKGADLAPESERFLRCCADVANALIEDHESTKDGK

AGRDINLNSLRSKLAKKHKLANIPPLTAIIAAIPEHYKKYILPKLIAKPI
RTSSGIAVVAVMCKPHRCPHIAYTGNICVYCPGGPDSDFEYSTQSYTGYE
PTSMRAIRARYDPFEQARGRVDQLKSLGHSVDKVEYIIMGGTFMSLPESY
REDFISQLHNALSGYQTSKVDEAVEAGEMSNVKCVGITIETRPDYCLQPH
LSDMLRYGCTRLEVGVQSLYEDVARDTNRGHTVAAVAETFCLAKDAGFKV
VSHMMPDLPNVGMERDIDQFREYFENPAFRTDGLKIYPTLVIRGTGLYEL
WRTGRYQNYTPNGLIDLVARILALIPPWTRIYRVQRDIPMPLVTSGVENG
NLRELALARMKDFGTTCRDVRTREVGINEVKNKIRPNQIELVRRDYTANG
GWETFLAYEDPKQDILVALLRLRKCTEKYTYREELTGQPSSMVRELHVYG
TAVPVHARDPRKFQHQGFGTLLMEEAERIAREEHGSDKISIISGVGVRSY
YKKLGYWLDGPYMSKWLDGRDENGNQ
>Mb|QLI66754.1
MFSFKTLASLLVAALPLGNATPQAGSAANLVPDKYIVTLKDGISANDFNF
HLNWVRDVQVARAGRRRGLNFRGVEKTYGVGNFNAYAGHFDEHTLEAIRR
NADVESVEQQQVYHLHELTTQKDSTHGLATVSHREPGATEYVYDSSAGEG
STVYVLDSGIQVDHPEFEGRAFRGYNAVKDATDEDVQGHGTHVAGIVGSK
TYGVAKKTKLVDVKMFHDAGSTNAIILDGIEWTIKDITAKQIQNRTVVNM
SFGGGNSTALNKIIKTAYDAGILCVISSGNMGVDASDWSPASSPDGITVG
AIDANWRLWDHSNHGPVVHILAPGVDVLSLAPGNRLREGAELLRRLLMLL
GWPPIWQLLKTSTLQRS
>Mb|QLI66755.1
MAIPTSGVLLFLLLATPVYVLSLVVYRLYLHPLAKIPGPKLAAATSWYEF
YYDVVGRGVFCWEIKRMHDEYGPIVRINPNEVHILDTGFYDEIYAPASKK
RDKYADYVVFLGVPTGAFATCPHDHHRLRRKALNVSFSKKAIYEIEPLIQ
AKVDLLCGRFGDVAGGDRVVRLDAAFFALANDIITEYSFGEGYDCLGKDD
FQESFKGVLLTAVEAGAFIRQFPWVVGVVKALPLLVLRRVSAAFASLLEW
EAIVNKRVETLVAAARAGKPLADRSVFSTLLRGDLPPGEKSFQRLIDEGK
SLMGAGSETTSWTLTLLMWYVARDARILSRLREDLRDMPDGVAASSLLKW
LEQRRYLSAVVTEALRFDHGAIGRLPRIANEPLRYKDHVIPAGTPLSSTN
YWVHMDETIFPDPATFSPERWLQAEREGFPLQKYMVSFSKGSRQCVGQNL
ATAELYLTAAALVTRFDFEFAGTTEQDILPARDCFIPRPVVGSPGVYAKI
RGLDREKKMASNS
>Mb|QLI66756.1
MDSEDEYVSGMSTDAEGMQEYSGDEMSAADDFEDDFDEPDPDFGITSKDI
TKTKQPAHVVSFKVLKPSDIQHQQDDMINEVNMILDMRKEDAAIMLRYFR
WNKERLLEDYMDRPEKVLEAAGLSSNTAALPKLEAVPGFVCDICCEDEDG
LQTFAMKCGHRYCVDCYRQYLTQKIKGEGEAARIQCPADGCGRILDSRSL
DLLVTPELTGRYRELLNRTYVEDKDIFKWCPAPDCPNVVECGIKRKDLDK
IVPSVECLCGYRFCFGCPNADHQPAPCELVKRWLKKCADDSETANWISAN
TKECPKCSSTIEKNGGCNHMTCRKCKYEFCWMCMGLWSEHGTSWYNCNRY
EEKSGSEARDAQAKSRTSLERYLHYYNRYANHEQSARLDKDIAQKTEKKM
VQLQTTSGMSWIEVQYLNSASQALQTCRQTLKWTYAFAFYLARNNLTEIF
EDNQKDLEMAVENLSEMFEKPIVELSDSKLKVDIMDKTSYCNKRRVILLE
DTAENLAKSKWTFNADLTAPSVASARR
>Mb|QLI66758.1
MTTATPRRHRGGGGSRRIKQDPDTSLTEPFALDDINDVDVRSYVTNYGPT
NHIARQGRSDAPAPSERSLDTGPMLREIRTRPNATNGVSVESPISNHDDS
FFHTSFQDIGKKLKACNDTLGELQQLGVSHDVQLPELVLVGDQSAGKSSL
MSGLANLDLPRSEGTCTRCPLHIRVSRNSDWSCRVWLRKEYSFQPPTDRP
IVESDVTDEDPFFPWRKQPVSAVHEFKTMQDKSQIEEVLRWAQIAILNDD
KHHGLFIPGSGAIAVNRRIEDAAAETVAKFSPNVVALEIKGPDLPDLSFY
DMPGIFQNPADAKDDYLVNVVRNLSKAYIQHPSAIIICSMPMNSDAENSS
TFGLTRKLGATSRTIGVLTKADLLPEAGNHDQWLAIMKGCTHQTGLGYFI
TSRPQGKDLDELKKWEELVFESHSLGNWPSEFHGFSERCGVEKLKSFLSE
RLGEEFAKSLPNIKQKVKHHLDKITKQLANLPELPDNVELEIKTGLMAFA
DSARLKLEGFMSHFSVLPRNFRNCLLETKPKFTLKDKSDFPVLEISDDES
DAASVATTTPTAKRRTAPTNITPSKRPRLETPLHGTPSNGHVKPEEQHGL
TLRGTSIMAPRATIPKKTVLPEPFAGFTNVGRGFRTLRQVREEIEAKTKA

GMPDRTSDQVYEDLVVEAVRPWNGPMDAFIKQTMRELQSELESTLNKALE
GLKKRFIYLEAKRHLRRFLEEHRKETEHDLALLYHDEKERLLTFNDKTFE
QYRDDEYMELTRFRHFMRLKAVGPDPGNYISSDKLTEDKRVQEAKRRDQE
MLRLGRDPFARELEVIAYVRGYYRLAALRFADAVSQRIICRMIPAIRRQL
PHYLEDKLGIRGPDAASVYERLMEEDETTAGRRETLKSEKDKFVRALASI
ELLENGAAAAANMSMSSMAGVSMTETETATQRVEPMELDSRTEEDEV
>Mb|QLI66759.1
MLRPYWMVCLAALTAASLKDLANCDPLRKDMRNDPIAQKSCPKKTRTEPS
KLTVASDCPPDAAFAGKASFDFTTANWDNVLDFWAVDEATANDKKRLDFD
TDGNGVAMGMWKPGDAPTLVSSKYLLFGKVSVTLRAAKGNGLITAVVLKS
DSGDEIDWELLGAYDNQAQTNYFYDGQALFNTYNDTYDLAASSFDALQKY
SLEWTDQFLSFSVNDTIRKVWYVGEIPAAKWPQTPMQVKLGVWSVRNDSD
RGEIAWAGGVPDWGSAPYRGYFQSIEVEDYTGFCNQTDGYVEYQYDERTA
GWQKIRIAGCQSRPGPELPAPSPVHTGDATATETGETGGPETTAADDDNG
GAGLSAGPSSALAAVLCLVWFLVL
>Mb|QLI66760.1
MPVVTITSKDQFDELIKKTPFVAVQASASWCGPCKAISPMFVKHSDALGI
DDTYAFAKFDTDDVPDLAMELGVRSLPTFYFFENGDKTDSNVTGANPPAL
KKTVEEVSEKAKAKGAAGQGGFVTCGQLACYGDARGFWGGTKADA
>Mb|QLI66761.1
MPRLLNRSRPSGVDSSPARTSSSTSLPRYQPPSCPLNDRARRALGELSDN
RGTQHYESHLKDSARHLGLSVGDLHERLRAQHDRLQLLQERREQKGTNKT
PEEERLEAHLVELEREVDELTHESEQSIRDVIDRRAGIEDQSALLGDLYT
TAATAIAPNTAETRARRGVKQEPARDEEQDPEQQQGEEPVASTLEAFRKG
RATKQAEYEQLTPYQRYGVNNDYAGFKKIWHDAASGEDGPPLPDASRWFR
SDGRPVMTRPGATTRRSTIGPAGDDDDIAVAREVLSLNCPLTLRQMEEPY
SNVKCKHTFEKSGILGYLPARGEVQCPQTGCSQKFSKSRFQQEFYLDEAI
LRRIQRVKQTQRTRDDMDVDDDEDAAGGDASLVFEEERRVPGRIPKKERT
>Mb|QLI66762.1
MSKQTPRKARHSRQPSNLIPAVSDYESDAAAIHAAYAPPPPRTNTELNLS
VLQRYLPSIQAILSIAANAVVYTFDSETASWDKSGIEGTMFVCAQSPLPE
SHQPRACVFVLNRRALDNLVVDLARVSHVEIAGELVILRVEGNWEEGDKV
IGLWIHNDKDETREISGATIQESWKVARSAASAEEQGPEAGPAMQAIGKR
LSLSDLFGKQHEHQTANY
>Mb|QLI66765.1
MADSDGEFVADMSDDELVDHQVTDDEGPSARSKSSRTKRKQKRNGEQAWE
TSKRSWETNLPEEDQDGILNLTVLEAEKRRRLLRDTTPLQRGIIRHMMLV
LDMSFAMAEKDLLPTRYRLTISYALAFVREFFEQNPISQLGIIGMRDGIA
VRISDLGGNPAEHIERLKAVEGQDPQGNPSLQNALEMCRGALFHTPSHGT
REVLIIYGALLSSDPGDIHETISNLIADKIRVSIVGLSAQVAICADLSSR
TNAGDDSQYNIAMDEVHFRDLFLAGTTPPVTRTAEQNTASLLMMGFPSRT
LAPGGAISFCACHSKPHREGYNSCGLTLILSTHLARSYHHLFPLRNWAEV
SWAEATRSTVCFSCLCPFPEAPKDKVDGVEKSKDDTRRPKAKGVSESGRY
ACEVCGNHFCIDCDVFAHQVIHNCPGCQSTVHHDVSATVNGNGYSHQTNG
AMEVDT
>Mb|QLI66766.1
MAPTRILKFSQSENESSYVLVQVSSKGSKPLDLKLVGTEGEAPYVCSLRH
DRVASLRVKNCPVSEAEWQTILQSVFEQQPLPDIQASATVQSGSSVSLTI
RKQVQGITQRLGAITLTHDGGEAIELFEWCATSVDAVTQTNAAASASDAR
VRDLQTAVGELKSQLDELVAAKQEDEVVLLQKFRDLLNEKKVKIREQQKI
ITDLSANDAPREEVDEVDEVDEVQTAKRGRKAPKRKAQAVENESPEEDVD
MTAEKGESEDEDAESTTEATASVANDEDEDEDEDEDMDMDRTADEAAREP
LPPQKTQGAPPPPRALPFQKKKQAVAADDSDDDEL
>Mb|QLI66768.1
MSRITRVRRLASLQLAIATPSSAPRAPFVACRAVSTTRAARSKNTEWVRT
KLWKGEAPGLEDPYTQRPEPEDPSNLPEEALEFQPRTDKTPAAVLESRLT
LPPKRTEAASEKELTSSDPTYVPATEAEGLEEIGTLNTWWDQEGHWGEES
EFRGFGSAEKVVDKDVVEVYLRQAVVEALALRETGAFSQWATKKWREGPR

SELDQVLAAEIQVQGSQASLKGDVAAIAQNLTAESDEMEATERIRPEEAR
EMLKTWDASWKGLVLDDQLKFALRKRLYQLTGTLIADAKLGAAHTVRHVL
TLAAKQPQPKKLAELLETRSDVQELANVKVHSRKIGPIDRETALGRWKVI
EEELTKRGLPVTGTAGMSKNKERDWISGKI
>Mb|QLI66771.1
MASEESAAQKLLQKHAEEPHTVTVEDVPDEELPVKPTSDASGSWAAPITS
KAAGKQKESSNPKAPLDTQSHELFPELGAPKVKTANVAPIWGAKSNANGK
TNGAASNGASRSSTPTSGTATPSKAAAPSMFIPGRNVETVTLEPQFVLPR
GQLKRPIPDIIKDINRKSRANITMANGANGRYKFEATGPQEIAQQALKDL
VQQIGTRTSIKVSIPYSARAHVIGKGGSMIRALQEKTGAKIQLPKVEDNA
LLADDDEATIDVSVEGNALSAASARNELLKIAGERSANVQTKVRGVPTAF
YPFIAGPGNSLAQALEENHGIQMRVPPHQAVCRAQAPVASEPGQPPVFHP
AGAEDDHIQLAGDRAAIQQVRTEIERQVAELHKQLQLEQLAIQRGRHQFI
IGDRGLPADDFFAETGCAILLPSEEEDDMVTVIGPADRVQAGLEKAMDLA
MGMQMSNIDIGRFHRNAPGGAAAHAGNVTRYLRQRKEIDRLEKLYNTHIN
TPFSQDGALPWELYSREGKNAIRAQSEITGIINGQPPARMHSVSIDPFFH
QHLRTDITPKVKADYGVHVVIPEASEAAAPVLLVFEGPDPADGPYQLPRT
KPSDAEIREFKKALDDAEKHILGLIGKQEQLSSTTLDVPAKFHERLRRFI
KKEQESRASDQIPVRVTKVGTTITLRGPKSAVDALAAKANAFVEQEKEDE
KERGFTLSFDFPQKFANHLIGKGGSNIKELRDRFDVEIQVQDGQVELKGP
KAKAEAARHHIQNLSRALADETTHILKIDPKYHRELIGAQGGQINRLQTR
YKVLIFFPRSAKTGPEDQPNADAASDTGKPRRQQAPDEVIIRGPKKGADE
ARDEIFSLHKYLEEHSVTATVPVQQKQVGSLIGQGGAALDELRQLTGARI
DVPADRDADMVEISIKGTASQVAKARKVLEEKRTIFDDTIVQTIDVDKKH
HKALIGSGGSNLKDIVVGAGGSDDRRELARTIQFPKQEADGNTIKVEGRT
DVVQKIIKRIQDIVAERESQISDVIEVPIEHHRSLIGRGGDTKRQMESKF
TVSIDVPRQGDGKTGVKVTGRPENVVQAKEHIAGIIKQQQGETIQIPRNV
HHAVSNNGQIFRQLRNNHQVTVDHSGHGIPSKPDNSARANTGSLPLITDD
ADAAADAHSWKVVKTISTEEGDIPWVLRGTPENVQKAKDVIQKALEQAKK
TDATGYLILPDPKTYRHVIGPNGSKVNAIRQQSNCRIQVPRDQARDEAIE
IVGSQAGVEKAKELILVAVREGQKPRE
>Mb|QLI66772.1
MPPDISSNIRVFVRWRDQTVFAGEDVKCTVTFKNVAPTPSQQQRQQQQSQ
QQQKPHGERQRLAVPLQARAKANVGLAAPPPTNATRGHRRSALSLSVSST
NSHSRSGSVQWSQATGSGGDWRPGHAHKRSLSIVSIGSANNAEDHVQRNE
SPSRPHRPHRGHSRAASLQISAQGATSPGPGPQSAPFFPRHMSSPLFNAS
YPPDRFGRLSGASTAPNTPGLGGQRRAPKSPARREMPNFRFPGGPSTRNA
EAASPNLKSPDGLLSPRQVSPHESNGLAVRLKEQPQPPSAVDRPAARILA
SNTIPGGTPRSSGEFYSMSNNSSETLVSEYVTQPLARGNGRSPHTRRSSG
MSPQQQKMPESLMMGYAQIQGSFILDGSLISLGPFEQVKKKGAVGGRGGG
VVGLEQTKRDTGFLGSFGWGNISNSLGFLGGEELSTIKEIRGAVNSKSIP
LLSTPQSILFVDLQLAPGESRSYEYTFRLPKGLPPSHKGKTIKISYSLVI
GTQRAGGAKEQQVRSVEIPFRVLGSVNSHGEILGHDLMSPYILLRDAAEV
KSLGKHVRMHKSRASFGPPATMNEFLNYVDDLASRRRDSTGIALLSPTES
TAQRRPSTYEEVGTAKEAIHLAIMRSNMAGDGQQSANRFEIARNGQRVGV
VMLTRPAYRLGEVVTMAIDFTDADIPCYAVHTTLETSEKVDSSLAIRSES
SLQRVTRKVYVASSEAAMYSRRVVFTPTIPISATPEFVTTGVNLEWKIRV
DFVVPYQGSDTTHLGELQVPHPLLEQISQDEKGGLVLVAIENLACESFDI
SVPLRVYGAVGSGLERLERDEASEEGLVV
>Mb|QLI66773.1
MSTQPPQVLSRKANGPSSSATSQPSDQIKVRQRVRASQAKDNDTGGNTLK
PAKGKGPADRRGGASHKSANDGEVAPKAPRSKPQNEGEKLVIRRLPPGMT
MEECISILGPEWEISKGKVDWFSYVPGKISIDPSKPSRPGRAYVHLVRKD
DIMPLSEVVRNATWEDAKSTFTNPSLIGPPVLEFSIYKKVPGTKKRTDTR
QGTIDQDPEFMAFLEGLANPAPMRESIDVEDADESAKVEVKVITTPLVEF
LKEKKANRGKDGSGKNSKSGKGKSGAKDDDSFGRKKGKESRTEKQDKTPK
ENVKILTKKAATEQAAEGAKKVASEIATANAAATTAAAATDAPKSRRAGI
AAAARILQRDLGLSPGSAHRRARHDAAKAEADAKAASSSTKDDNGTTADV

ANAASSTASAASPAEVIVQAPKSRPDSPVAPKPQAGRRNRGGKGGAEKGK
PAANEISGPQPTVANPPVILRKKNDSETNKKTAEPQVQASGPVTNSNGNG
RQAGKEKANSKLASQKKSPAVSANATRAFIKHVNASQGVNDAKLREALGV
FGTITLVEIDKRKGFAYVDFSEHEALVKAVAASPVQVGQGNVQVVERKDK
KLTAAAAAPGGSAKDDQTPNGEKEKEKEKAPGGRGRRGRGGGKAAAGTAN
GQATAGPTLASTGG
>Mb|QLI66774.1
MADQAQKRITAIGQQLEPSPGGLPRIAKVAGKSNGPRAEGKVVIITGANS
ALGIGRATAHQFAENGAKAVYICDYADSNLAAHKQEMTGLYPAVDVHIWQ
FDAADEAQVKAVVDDALDRYGRLDVFFANAGITGLNVVFTEFKDSDFMEV
LKTNTLSVFLAAKYAAPAMMKTSPSKPTSGGSIIGTASVAGIRSNAGSTP
YSASKAAVVSLAQTIAYQLAGTGVRVNAICPGLIETGMTAPVYQAARARG
NEKKIGHLNPTRRGGHADEIARVALFLGTDESSYVNGQAWAVCGGLSAGH
PYVPGRLS
>Mb|QLI66775.1
MSSSGLTRRRGAGGGGIANTEAEASNAASRTNSSHNVRDSGPETSYENSE
NGHKIAFDPRDISESAERSKQPKLTMMEEVLLLGLKDKQGYLSFWNDNIS
YALRGCIVLELAFRGRISMEKDPSRRRFPPADRNIEVIDDTLTGEVLLDE
ALKMMKQSEKMSVSSWIDLMSGETWNLMKIGYQLKQVRERLAKGLVDKGI
LRTEKRNFLLFDMATHPVADGGAKEELRRRVRNVLTQRTVVLSGNQFLPE
NLEFRYLRTVCMVCAAYAANVLENALSTLGHEARERAFAQTDELLADYSQ
WPFGKKATSNGIGANLPQAISEEISKAKDKELQLEVVAACLSVFTRLDSL
L
>Mb|QLI66777.1
MSTNFRDRAIAEVQKAIAADHAKEYQKAFDLYMSSMELWVKALKWEKNKA
LKATMQEKMATYLDRAEKLKQFLQSEADTNANGGKALMGANGASTGKGKG
APEDDDSKKLRNALSGAILQERPNVRWEDIAGLEGAKETLKEAVVLPIKF
PSLFQGKRQAWKGILLYGPPGTGKSYLAKAVATEANSTFFSISSSDLVSK
WMGESERLVKALFSMARENKPSVIFIDEIDALCGPRGEGESEASRRIKTE
ILVQMDGVGNDSKGILVLGATNIPWQLDAAIRRRFQRRVHIGLPDLNGRA
RMFKLAVGDTDTALQAGDYNTLANTSDGFSGSDIANVVQHALMRPVRKIL
QATHFKPVMKDGKRMLTPCSPGDPEKIEMTYDGVNSDELLAPDVSLKDFE
MALEDSHPTVSKEDIEKQIDWTNEFGSEGA
>Mb|QLI66781.1
MRNPSLHLPQLLLLPWPLASLATQVDPLTYVNPLIGTTNGGNVFAGATLP
YGLAKVVADVDGQNTGGFAMDMSNVTGISSIHDSGTGGNPSLGNFPLFPQ
VCRSDELNNCQFRIGDRKVHYRASSVVAEPGLFGIELASGIRVSMTISEH
ASLMKFTFPKSKGNHPLIMLDLTDLWQSRQNASILVDEKSGRMVGNGTFL
PSFGAGSYQLHFCVDFAGAKIHDTGVWVNNRAGTEPKQMYITRGFNLFYL
EGGGFARFSPGPDNTVTARVGLSFKSSEQACGSAEREIPNPLQDFDRLVK
TAKDAWREKLSPISVKPGGADKNLLTSFWSGAYRSFISPQNYSGENPHWD
TGRPYFDSFYCIWDSFRAQHPLLTILDPIAQTQMVQSLLDMYEHEGWLPD
CHMSMCKGWTQGGSNADVVLVDAYAKNLSSDIDWELALEAIVTDAEDEPL
EWSYHGRGGLQSWRKYKYIPYLDFDPLGFGTNSRSVSRTLEYSYNDFCLA
TLAGGLGKHDMREKYMHRSMNWQNLWKEDQTSVIKGKDTGFKGFFQPKYM
NGTWGFQDPIACSALGSFCSLTSNPSETFEASIWQYLFYVPHATSSLISL
LGGDDAFISRLNFFHESGLADISNEPVFLTVFLYHYAGRPGLSAKRAHQY
IPSSFNNTNGGLPGNDDSGAMGSFLFFSVMGLFPVAGQNVYLINAPFLEE
VSVKSPVTGKRATIRALNFDSAYKNVYVQKVTVNGKPWTRSWIGHELFTE
GWTLELTLGNTESDWGKKAADRPPSWTS
>Mb|QLI66784.1
MPVPFETLLPYAIMIGMFGVTGTGLAVVKTWRNEGKRPRYSLDQWDRVLM
DRDRRLTGTLRGQTDKPEAPPGFEFSHGWKLEKRFT
>Mb|QLI66787.1
MPNTPPPALLPPPEGIFRSFEDLMASVQRVAKDQGYGIVKLRASNYRDGK
PTRYDLVCDRGGVKYNSTAKKRNPSTRKIDCPFRAKAVCEVQLGNQWRFA
LQEPRHNHEPRVPSGTPGQENAPLATTIRSFTNKLDRLSHDIAQGFIRIE
QRLDNIEKRMENLEARAGGYEPRFQTIESRLQNMEGPRMDGMGMDDVESR

LLASTVM
>Mb|QLI66788.1
MSIPGLGQIPVQTVHSSTRIIVLKPSWEWRFEVPAGRKITVKALSGTAEK
DGVELALRNAYSFSAIKSKILTWHGCELEVDGRTDDEFVAEYASPAANPA
NAHVNLHARLNEMRTAAVYERREGPRVLIAGPPTTGKTTLVRTLASYATR
QGFEPIVVNADPKEGMLSLPGTLSASVFATAMDPEAVDGWGTTPTSGPST
VPVKLPLVYYYGRNLPDDDPEFYRELTSKIAGTVSGRLSEDEAVRSSGVI
VDSMGVSEKSKIGEDLLAHIVDELSINIIVVLGSNRMTAELSKRFSTERT
SLGEPIQIVGLDRSEGVVERDEGFLEYSREQAIKEYFFGDARRALSPQIQ
QTDFDALVIYKASDYSAYEKATLSRDEPSSVMQHWTLAVMHASPKDPPEV
KED
>Mb|QLI66790.1
MSLKLEIETWVAALARYDNNEFDDALGEFDRIADTSKILFNMGVIHATLG
EHEKAVECYQRAIRLDQYLAVAYFQQGVSNFLLGDFEEALANFNDTLLYL
RGNTMIDYAQLGLLFKLYSCEVLFNRGLCYIYLQQREAGMQDLSYAVKEK
VVEDHNVIDEAIREEAEGYTVFSIPVGVVYRPNEAKVRNLKTKDYLGKAR
LVAASDRSNAFTGFTGSEIKNAGQNEVKDDRPADNISFAATNLVKPGIQS
RRQQSEPPTSRNVFPPTPPPENDRPNRGSVRNGSRPTPAKLTIQTQDIGR
KYEKAPSPEEIRATRSASSSVSRGFSRRDLPPMQRRPTRPIEEEDEADPA
DLYDMYQGRGGGSSMSRDSRPSTRSRQQPRYPEEDDGSDYDGSLGGAEFE
MVSNNRRRQGSRSDSRAASRRPEIRKIRVKVHADDVRYLMIGTAIEFQDL
VDRIKDKFGLRRRFKIKIKDEDMPDGDMITIGDEDDLEMAIQSATNQAKR
QRLEVAKMEIWIFDL
>Mb|QLI66793.1
MPPKAADKKPASKAPATASKAPEKKDAGKKTAASGEKKKRSKTRKETYSS
YIYKVLKQVHPDTGISNRAMSILNSFVNDIFERVATEASKLAAYNKKSTI
SSREIQTSVRLILPGELAKHAVSEGTKAVTKYSSSTK
>Mb|QLI66794.1
MPLVDDGFEALLEPFYNGKKLTDPVNTRDDKFQLLPAFLKVKGLVKQHID
SYNFFVEQEIKDIVRANRTIRSDVDSNFWLEFTDIRVDRPRRQDHNDAKS
RTEVTPMECRLRDMTYAAPIVVDIAYIRDKQRIIRRNVPLGRMPVMLKSS
RCRLSGANNAQMEAMNECPLDPGGYFVINGTEKVILIQEQLSKNRVIVEA
DEKNNIISASVTSSTHERKSKTYVTLKKDRIVMTHNVLVEGIPIVIILKA
LGGLSDYEIMQLVAGSDGRYQDEFVINFDEATRAGVFTQHQALEYIGARV
KMGSRKAMFGPQVRRNNVEEGLDALANLIIAHVPIEGLDFYPKAIYVAMM
TRRVLMASQDSKLVDDRDFVGNKRLELAGQLLSLLFEDLFKRFMGEVKMS
IDKFLKKNNRAVPLDAVHMISNHANNIGMGINRAIQTGNWSVKRFNMNRA
GVTHVLSRLSYISALGMMTRISSQFEKTRKVSGPRALQPSQWGMLCPSDT
PEGEACGLVKNLALMTHITTNVDEAPVKRWIFTLDTGVEPIRNFSGAEMH
REGSWVIHVNGTPFALTRYPKRFASRFRTMRRKGWISPFVSINLNSHFNA
VHIATDEGRICRPYIIVKNGKQKLRHEHLKLLHLGKVTFDDFLTKGIVEY
LDVNEEQDALITLYENQITQSTTHLEIEPFTLLGAVAGLIPFPHHNQSPR
NTYQCAMGKQAIGAIAYNQFNRIDTLLYTLVYPQRPMVITKTIQLVHYDK
LPAGQNATVVVMSYSGYDIEDALVLNKASIDRGFGRCQVFRKYTTELQKY
PNGRRERIGDPVNQVNGGTTRREVKYEALDDDGLATVGYRVRSGEVMVKK
ETPLDLTTTGIGLDRGPSEYRDSAISYRIPDPAYIDKVMISQTEKDTTVI
KVQTRQTRRPELGDKFSSRHGQKGVVGIIVDQEDMPFADNGLCPDIIMNP
HGFPSRMTVGKLLECLTGKASIIDGRPDYGFGDAFRSHPVKDMSQVLLDY
GFSWEGKDYFTSGLTGEPLEAYIFNGPIYYQRLKHMVQDKMHSRSRGPRA
ILTRQPTEGRSRDGGLRLGEMERDCLIAYGASQLLLERLMLSSDGVTLDI
CQQCGLFGYKGYCNTCKSTRQVTQMQMPYAAKLLVQELISMNVGVRLQLE
DEFPHPD
>Mb|QLI66796.1
MRLSNKSILLLELAGAALAMPARRAVTAPVADHDKAHDIKEAFEISWNGY
YKHAFPHDTLHPVTNSYADDRAGWGVTVVDALDTAIVMNSLKIVQPMLDH
IAKIDFTTTAEANDAISLFETNIRYLGGLLSAYDLLKGPYKHLGADAGKV
DALLKQAQSLGDSLSVAFDTPSGIPDPTIFLNPNKKNSGTDRNNIAEIGT
LVLEWTRLSDLSGNKTYAELAQKAENYLLHPKGAPEAWPGLIGTWVSTED

GSFLDSNGGWSAYDDSFYEYLIKMYVYDPKAFGEYKDRWVAAVDSTMKHL
VSHPTTRKDLSFLSAYRGQQTIPNSGHLASFAGGNFILGGIVLGEEKYKQ
LGLEITDSYYETYVQEAAGIGPEGFNWVDAGLSAGNPNNRPPPANQTEFY
EKAGFYNTSPYYILRPETVESVYYAYRLTGDKKYQDMAWDAFQRIRRLCR
VNDAYAELTDVSKKNGGSFVDEMQSFWMAETLKYLYLTFAADGPVHVQGQ
GATNQFVYNTEAHPVAVRGR
>Mb|QLI66798.1
MPMADIHHSWSSTPWQRQHHLAVPMLHKSRVPYTRPSNSLQHVHVWVPTK
YAPDEPRAPSPSWLPPPDTTLWIVYIHGGAWRDPLDTADNLAPALKSLSP
AWLASREAPVAFASVSYSLSPYPQHATHPSAPSDPSRNATHPRHILDVLS
AIAYLQSAAGFGSNYILAGHSCGATLAFQAVVNPARWGVDPDRAPVQAPR
VVVGLNGLYDMPKMVARPGHGYRQHREVYEQFTRGAFGDDERVWRAVSPV
YIEDWAREWPGGRHALLVQSREDTLVPFSEAEALEASLLKSKGERLSVEL
VEGRGDHYDIWKQGTLLAEMLTRAIDVSGL
>Mb|QLI66801.1
MALSEDQIRIIAENPLDDALKNIRIKLRQCDDVPSESIVASLLGALVTSS
AALDLPAPDGTTDVAEKLFIIRRNVRGSIPRLETFKPLIDVVVTHATDVE
IWAAVIDLINTLHPPTPPPSSVAPTFKGTPVKTSSSRLADSETRDIVEGE
LFEEIKKCTFRNVKGFCDKFFNPKSWHRERKAMLRTMMTAHDGKTWTEFP
STPEEKPVWDWLCSLEERFLAEAPHKLHTTRTANQFKERKGQMDLFWQTP
AANADDTFEYKHVLVVGEQKKSNDTGRFKATLLQLARYVRGVFADQPTRR
FVHAFSLCASTMELWVFDRSGPYSSGPFDIHDEPDKFARALVGYATMDDN
AMGLDTFIERENGRRHVTLDDASGNAMRARLDTAMVRQRAIVCRGTTCYE
TQNSQVAKFSWASDKRKPEAEQLKLAEERGVRGVARAVAHRQITTIAELR
SGLEFPTHHKFRHEAIYSEDPPSATASTNTTDHKRKSSSDNTSDNASGSK
RRRSNSQKSKLATEFNDQLSVGKAKPSLYMPGEDLWENRIYSCLVVSPAG
RVISDFKTIKELLESMRDAIRAHQSLYITGNILHRDISSNNIIITEPERA
DGFKGMLIDLDLAKVRDSGPSGARHQTGTMQFMAIEVLRTADHTYRHDLE
SFFYVLLWMCARCSWINRFGRRDEKPPRESILRKWEVGGFRDIARTKAYH
MSVDGLEEVTDEFPEAFQIIKPLCLRIRKILFPLDKDERMSVGTPAGSPD
QLYNAILDAYDEIIHKLSKD
>Mb|QLI66805.1
MANHHPSPTMMQLQQQQQQQHHQAPVAPPQSVPPGHFAPSNQIAAMNEA
VWLQIGSVSELLRVPDEAMHAYERALQANTNSTAAMNAIGMLLKGREAFD
KALEFFRAIVQLEQNNGEAWGNLGHCYLMTENLQKAYDAYQQALVNLRDP
KDPMLWYGIGILYDRYGSYDYAEEAFSQVMQIQPDFEKANEIYFRLGIIY
KQQSKFNQSLDCFKYIVNSPPGPLTQEDIWFQIGHVHEQQKDFDSAKAAY
QRVLDHSPNHAKVLQQLGWLHHQQNTSYETQDRAIQYLEKSVSADNQDAQ
SWYLLGRCYMAQQKYPKAYEAYQQAVYRDGKNPTFWCSIGVLYYQINQYR
DALDAYSRAIRLNPYISEVWYDLGTLYESCNNQIADALDAYQRAAELDPG
NPHIKARLQLLRSGGGNGSGQAPAPQPADVHPQAYQAAGPSGPVGPQWGG
SAQQPPAGGPPPAPAGAGDNWAGRLSNVNPPPQPPNPYENRGEPFRGPPP
PPQRQPSPQQDQQMRQGGYSEPRGPAPPGPSRRGPSPAPGGHQYGQPPPP
PSQGPNPADRRVANPNWGGPPPSSVGPPSNSANGANAPSNGAAPFRPTSS
PRGEVRPHDNRMPSPKTAYPQHPGASSYAPHHPEGPPPSASENGPPPPGA
GPDAAVQRPDDRPPSVGPKRMREWEEESVAKKQANEENRARLDDMRHRRP
STPPRDAYRRNSNEVRRSDEQRRPDEPKKEEARPQSDSYHPSEAAHHPQN
HSISSNQLPPMQPGNVPPGGPQDKTQPGPPPKEERPASEQAPTAVAPISA
EPERAARKMDVDEDYDDSPEEDKKTGIVANGSGPSSGSSDAKATSPASAG
VNGTSGAATKTE
>Mb|QLI66806.1
MVSLKSLVAAVASVALLAHSSLAHSVKRNPVSRISLVQEPVIQAPSHRVH
AFSKFDLTFSLQHGQQNIRLALEPNHDVLHEDFSITHLNEDGSVREIVKV
PRSEDKVYRGDAFIERPGIQGWSKAGWARVTVHEDGEKPIFQGAFRIDGD
NHHIVPRSDYQSMKDEGDPAVDSADDDVMVVWRDSDVVDYYDGHGELKRD
LADSSKCNADTLEFNPRFWQDVDETGNSLQSTSLPSLFGRQIDSGGGGTG
MNLLNSIGSSAGCPTTKKVALVGIATDCNYFSAFNDTKVIKRRIIGVVNS
ASEVYESTFKISLAIKNLTIFDRGCTGTTPANTQWNVKCSDGVSINDRLN

TFSKWRGESKDENAYWTLFTTCATDTAVGLAWRGMLCRAGASDQGSSAQN
ETVAATNVVVKSETEWQIFAHETGHTFGAVHDCTSQTCPIKGPTQDCCQL
SREQCDAGGKYIMNPSTGSGITSFSPCSIGNICSGLKSNQIKGNCLTDNK
NVQTDVDIGSQCGNGIVEAGEDCDCGGEAGCNGNKCCDHKTCKFTSGSVC
DASNEDCCTDQCQFASSGTVCRPSTGECDVLETCPGDKAACPVDKHKNNG
DSCGNGLECASGQCTSRDLQCQHMANSLTGRNNTKACPETECMLACTSPD
LPSNQCVTYNQFFVDGTSCGAGGHCSSGKCDGSSTIKEIGQWIDNHKAIF
IPIVSVVGLLIIIAIISCITGSIRRRMHKRRVRKQAVPGMNGSSWPPSSF
SGQQQWGGPRPWNGPRQTESSGALFPPEQQHQQQQSGFYDPPPYPPPPAA
NADGRWGNQRSLRYM
>Mb|QLI66807.1
MAEEDGQELVTKPFKFVTTGKLQPDEILVRDSANSTDARFPNVNQTKHCW
QNYVDYHKCIIAKGEDFAPCRQFWLAYRSLCPSGWYQRWDEQREASNFPV
KLDA
>Mb|QLI66809.1
MEPRARAGKNVVAQLLYAHGDVKNPLPETVRVLDEILTDFMQSIAFEATR
AAHYSGRQKIKYEDFEFAFRKNPAFLGKVQEVFEKQKEIKKAREILRDGE
DEIMKDAAEEERRREKEDVTGAGGAGKVNRAEEELGEADDDAEAEMDALG
KKK
>Mb|QLI66811.1
MPASARTATSRLRLVPRIAAPARRSLATSTASAKDEQWPQRTPLGAYYTS
ILRDPIPYPFAHKPEEPPSTADPSVLPPHRRPTTATTTPPARGPEPTPPP
QTAQEKARIIFGSRLLGPAEQAGRLALKQAKSTYLAGVLVPPQPEEPDNC
CMSGCVNCVWERYREDMEEWTASKKEAERRLRAGGRTVGAADGGGSETSW
SAPAVGGGKIAKDMWDDEVYRGVPVGIREFMKQEKRLKERHAREGTVGG
>Mb|QLI66812.1
MVESSKMKCLGADCENDAGTLQCPTCLKVGLNDSYFCTQDCFKRNWNQHK
TVHKIAQGKTQNGTLHNIIPPSPKVVSKCNEVARFYNPFPSYAFTGSVRP
VYPLSPMRTVPKSIPHPDWSVTGIPKAERRLNRSKIDLLDAKGQEAMRKV
CRLAREVLDIVAAELKPGITTDYLDEVCHNACVERKSYPSPLNYNHFPKS
LCTSPNEVVCHGIPDQRILLDGDILNLDISLYHEGYHADLNETYYVGDRA
KTDPDSVRVVETTRECLDMAIALVKPGTPIRDFGAVIEKHAKSRNCTVVA
TWGGHGINTEFHPPPWIPHYAKNKAVGVCKPGMTFTIEPILALGKSREVY
WPDNWTNVTVDGKRTAQFEHTMLVTETGVEVLTARKENSPGGPIPMPATP
NDTAKTNGATHA
>Mb|QLI66813.1
MTGSEGQSVYESADLVALLRKQLENEQPAPYQGGSMLSVDFSSPLGGAAR
AERIRQMSHNHVRFAPNLSHETAAHVSQQPAFEKSRFAQEILVKIQHSLR
RMPGVSSSESGEIVGQLQEVGQVISQETSQLVDALMNVTLDQEDTDKAVA
RLSLDATILNAEMDDKAQTIRDLRTELDAERKKREQAELDVKKVMGDLQR
LREDFKKLQEKATRKDEGADADVRDDIVKSLEDSIRYLEDQVNNRRSLWL
MKHSDPQSVARAIETLADSVQDNQAAHQSILSMKSRTARGANNYQDTRHQ
ENADARPYGEMAPPPRPPSVFQAPSTSCAVAPPKFGPLQAGPPRAPSAFS
HERRYQGSASSWSSAQGRFAQAGNPFFSNAASSFTRPPTRRPYQAPTRYQ
PTAAAEFQPGYAPEHAPARPGTAFGHHQPPREYYPQTPTTSRSRYGRPAE
PSMSEYGSAPSSAGNAAAARGILSGPLVHMNEQAVAAWNESIMELYAMIR
AFVERHANVPDPAMAMKMSTLPLWPVLLATYHPLSAKEAASYLDIHLRAD
NPKCCLVTRVIIDYIVNRVWVPNAWIGSNDDCTYALLELEKDLEKTQGQP
SAQRQPLLDRQAHILEAIIKLEPLAFHREKVGDITTALMATIQPLLNRLA
NPADAYRDLELVADLAWDLSSKILASRLTFDFRFPEVGARFSSQSMLPIW
PALEPTELQAKHWRVALVTTPVVTCRNDTGSNISAHSVALADVYCMQ
>Mb|QLI66814.1
MSADLGADSAPPNLNLSPEEKRVYAQLFRQADAEGAGFVTGDAVVALAEK
TRLRNDQCQTIWQIADSEDRGFLTPTGFSVFLRLIGHAQAGREPTAELAL
QQGPIPRFEGLWTSPALGSPTSSSPAPLQAQASGGAVRIPYLTPDKVSEY
AAIFERLPLQAGRLHVEQARLIFEKFGLPKETIRDFWTLTGIEERGFFVL
PEFAIAMHLFTCIKAGSLRSMPKALPPALYEAATRRGAAPRQSPSNTGMG
PIPRQLSGSAQMRVGSPLGRPPIAPAAAGGDWAVSAADKAKFDQIYATLD

KTNKGYISGEEAGPFLRQSNLPEEKLAQIWDLANFNNQGQLTREGFAIAM
YFIRQQRSGVGGDLPETLPENLIPPSVRNQRQPPPFPAPVARSAPPQPKS
AMDDLFGLESTPSPVPAPVQTTMSTGGSNANDPFAGGSAILPPSSPIRPT
TTGTTFKPFVPSSTFGKGLTGPPSQGDVPKQSEDLLEDNDPEASKNITGE
TTELANLSNQIGTLSKQMQDVQSKRTTTQGELNQTNSQKQNFEQRLAQLR
ALYEKEAEDTQSLEKQLRKSRADTQKLQSECMTLEGTFRDVQSQHQQLAA
ALQADQQENATLRERIRVVNGEIAKLKPQIEKLKSDSRQQKGLVAINKKQ
LSTTEGERDKLKNEAAELAKGGEDAARQMDSNSPTSASAQVASPALSTVS
DNNPFFKRTASTDIMGAFASPPTKPYADKSLDDVFGPSFGPGASTSTPPP
AFQQQQHTGTSAVSGASFHTGRSSPSISRQGTMNMEPPAPPASRQINSSF
LPFPDHSESLSSSRQVSPPASRAEGLSADSNGESAAEEGEKSETQGATPT
ATEAPTVSVTEAALKAKAEEATDSAPKPDPFSSTDQAKAKADFDNAFAAF
AASGKGKAAGESNVDKSTDAFNTEFPPISELERADESESESERGGFDDDF
TAASPPNKGVNKKDAVATEATEAKKTAAETPVNVGPEIPAKEPVEDTNKS
SEQPSSPATVTNTSQPTLTADDIFGAAAPNAVPQNTTGKTVFDDLDDDFE
GLEDAKEGSADDDFANISRDDFNPVFDSSPPASQAKSESTAFGNESTFDF
VSHSSAAGSTTQQKTADSHDWDAIFAGLDSPSNLAPPGPGGEGGGDKHAE
RPEPPGRALTEQGVHDDPILKSLTDMGYSRSDAVAALEKYDYNLEKV
>Mb|QLI66815.1
MDKDQLAQLLQASQVPNTEQVKAATADLQKNYYSKPESLILLIEIALTHK
DGTVRQLAAVQALRLAPRHWASTAEDKKPLARSHLLEGALKEDSATTRHA
LSRLVAGIVGLDLENGDAEEFLKQLLPLNNSDNVQSREVGSFILFSMLED
SPEHFEEHTHQLLQLFQSRIEDPESKEVRINIVQAIGVILMNIEPEEDPQ
SLKAVQGFIPNIVNILKAAVEAGDEESYQTIFEVFHSYLACDPSFLAAHL
RDLLQFMINLGANTNAEDDARTQALSLLIQCVRYRRMKIQGMKEVAAELM
IKAMQIITELDDDDEDEDMTPARTAISLVNTLASELPPRLVAVPMLDQFP
AFAAHSESGYRMSAMLSLGNAAEGAPEFISTQLQPLLPTVINLLCDSDIR
VRHAALVGLIHLAEEMADEMSSHHEQIISAVLKNLESASQGTTDKKNISI
VRCACGALDTFGDGIDTKIMAQYGPNLIGPMIKLLDHEDYGVKAAAASAV
GAIASSMDKEFQPFFDGAMKALGRFVMLKDSDEAMDLRSATCDSLGRIAI
AVGPQAFQPYVVDLMKASEEALHLDNPRLKETSFILWSNLSKVYGTDFDH
FLEGVFKGLFASLELEEEEIDLPGVDPSQLAEGAIIGGKRVKVKAPSSEE
DHIIATGGEDDWEDLDDFAGLEDFGAVTAVALEQEIAIDTLGDVISNSCN
TNHLESYVEKSIEQIIPFADHTYEGCRKNAISTLWRIYSRVFQVWEESSG
SKWQPGMPPTPAPPASIAKIGEALHKATMDIWTSDSDRSVITEINRNVAA
TLKACGPAVLVAKPDMLQELVSVVSSIVTRSHPCQQDLGAEDEEQEIDAG
SSEYDWLVVDTALDVVSGLAAALGTQFGELWKIFEKPVLRLVSSTEDVHR
ATAVGTIAEVAKYSGEAITEFTESLTQALVRRLSDHDQLTKSNAAYALGL
LILNSNDTVKTIPLYPQLYEKLEPMLSVHEMRITDNVAGCLCRMMMKHPD
NGFVAQALPAIVQVLPLTEDYEENEPIFQCIYKLYEQSNPTVQQLTPQLV
GIFEKVLGEPEEQLEQDTRQMVQRMVQTLRQ
>Mb|QLI66817.1
MVTTRGFARATRLPRRFASTASKAARKQPPAPAPSPPPPPPPRLSKSYKP
SPRSAPSQPRRAPNPSRPGDTLQSLWRRSWLPLSGAALLSGFLGFYIIGT
AAASLKTRPCQASCEHAAPTGRPPALTGDNAEQFDKELDLPERWMGITKL
RKKLAGHAKGHVLELAVGTGRNLQHYNWDALGGTHPAPGKQTAAGGISSF
TGLDISADMLDVARKRLVAAVPPVADSEPSAGRSALADHMGGRLSLLDDR
LRLICSDAHRPLPRAAGAGATYDTIIQTFGLCSVSDPVAVLCNLASAVKP
DTGRIILLEHGKGWYGLVNGLLDKNAEKHFEKYGCWWNRDIEALVDEAVQ
KTPGLEIVKLDRPNIMQMGTLVWVELRMRGKR
>Mb|QLI66819.1
MTDGGELGPRPPGAAPRRQRAPTINIDTAAVSSGGNVVEMPQESTESQPQ
PQPQTMATPISPQDDTVSPSTIVEQPPFGFNSQRSELQADTSFDSRENRP
TSPHNVSSPVLSRTHDGGAGFLSVPNQLRSRQNSVDSDDVARSVTSHGDT
TVVASSYTQTDKLKASGNEKIMKDTRALNPDTGTDDDFNVDNNPFAFSPG
QLNKMFNPKSLSAFFALGGMRGLEKGLRSDRKSGLSSDETFLDGEVTFDQ
ATAENDKKATEGAITTTAAVARMQTNKSRGNDSFADRYRVFRDNRLPVKK
GKSLLELMWITYNDKVLILLSIAAVISLGVGLYQTFGQSHDDEPAVEWVE

GVAIIVAIAIVVIVGSLNDYSKERQFAKLNKKKQDRNIKVVRSGQISEIS
VFDIMVGDVVHLEPGDLVPVDGVLIDGFNVKCDESQTTGESDIIRKRPAD
EVYQAIQNNESLKKMDPFIQSGARIMEGVGTYMATSTGIYSSYGRTLMAL
NEDPEMTPLQAKLNVIATYIAKLGGAAGLLLFLVLFIEFLVRLPKLPDSV
TPAQKGQNFLEIFIVVVTIIVVAVPEGLPLAVTLALAFATTRMLKDANLV
RHLKACEVMGNATTICSDKTGTLTQNKMQVVAGTIGTAQRFGAARPDSQD
SSDESVEMDTATEVSAAELTSMLSAPVKDLLLKSIALNSTAFEGEVDGEQ
TFIGSKTETALLLLARAHLGMGPVSRERDNATTLQIIPFDSGRKCMGIVV
QLPTGGARLYVKGASEILLAKCTRTLSDPSTDDSVTTLSAQDGKIITGLI
ETYASRSLRTIGICYRDFEVWPPKSARRGEGGGSDIEFNDLFQEMSFIAM
VGIQDPLREGVYESVKLCQKAGVVVRMVTGDNKLTAQAIAKECGILQPNS
IVMEGPDFRNLSKREQEKIIPQLHVLARSSPEDKRILVKRLKDKGETVAV
TGDGTNDAPALKMADVGFSMGIAGTEVAKEASAIILMDDNFASIVKALKW
GRAVNDAVKRFLQFQLTVNITAVVLTFVTAVSSETEKSVLTAVQLLWVNL
IMDTLAALALATDPPQDSVLDRKPEPKGASIISITMWKMIIGQALYQLAI
TFLLYYGSPKGILPLPGPDDIPEPAQINTLVFNTFVWMQIFNQWNNRRLD
NKFNIFEGLSKNWFFIGISIIMCAGQVIIIFFGGAAFHIADQPEDKAIWG
TLWAIAIVLGFISIPVGVIIRLVPDWIILALVPEFLKKRSHSVPGLTISD
EEMEMYPEPLAELRDELNFLRRVKGGRLNNLKFAIQHPRETILQRSRSPS
HSRSNSLHAPQTPTGEENFGSPFATPNSGAKSGSNRSRSNSAVRATALMA
GVVAAGVGAGWSPAGRVSAEEMQEGSNNSNNKTVEKRNEGGDTSESRKRE
>Mb|QLI66820.1
MQSLWARAGQAHRCGCRACSTAIGAAGRRATGATGRRKATFAEIFTACYS
SVFATAAIVDAVRKDDRRRELDRQLEAARRELSELQERSSSTSSQVEANA
PDLSMQQMDTLWKSLKAIYRNRPFMKEIDKPAVIDASELIANLKREYYNT
PGEPSLHSSRQINYDQLERAIMAEERDSRIGSRESLSQAQLVRESLSVEY
LVEQLLCRAEILDNSSVECPSFDEARGLAAKGYPNFSFRSVDPGKAAKNT
ITLNNQIRSLVNTHDLSWKEKIGRVCFNLLVSAHPPDMHTYNTLIVAFDR
SGHHVFSDALVYSFFHRRLLKPTPSTFTAILNHYRVTRNHGQFLRTLACL
TGLDVRSGGKMGRRHIKDIESWGMEKWAANTKLRTSTGNWVWEHVPLNVP
LVETIINGLLHFKLFDEAASFFVSCMRSGVALSTSVVKQLFDECVAALDW
RAAVRLIRGLSDSQKRWEILLSQEDADTAAHLVGRAFALIDICGLSSRRE
PEGRLISLDISGPKLNRLLESLSKANLLLPDVNVHPGSFQDARTREGAEM
TLSKSRLLQLESMWKEYEFVRKTTNSIESKLLYPEFSSTFRTSMAIHIGK
SAVQRSVILAREFQDVVPASGSLSLQSPREAHSDQPSEEERLSEEMEAMA
VNDEWTEQEGGPKLREAQIATRGWSRKQDGLVSEHVPEWAASYINYKGLK
KLVKALAQKAGNGETVDPAELFFALDRNLEDVDSFYNRKFAEACRRLNIL
HNRYGRVPDVVATLDQDEVEEVMGALLELRTQLRNLQWFGEINRRGFVKI
TKKLDKRVPQISYQHRYISTKVDPKQFAKDGNISRLLAEINRWLSVLTDA
QQIDDSMSDRSTRSLGRASAKAMLNLPQSQLDILDHAVRNDDVSSLKWGL
TDANVGSDSFAHSLLLNLLQRSISARSRQCITYLLTQVNDLDEPDDINGR
NCIHRLVIHIGRTKSSTQDDESSAYPVPLGTQFTNRHLQPAVSAAVPVKA
INTRESNLLSKDDEAVQILIYLLDQLSESQRAALKGRDSFGRLPLHYAAQ
FGFVVVCQIIMKYMQDWNQFDAKDGIDAPEWHDNDGFAPLHLSVIGGHPL
TTQALLQGENWQGSSEAKVQIRKTIPKSGASLALATKSNYEPIVKMLVDA
GVDINWTDKTGETALHIAARFGHEDCARIILRGSGSQKANMELAENSYSW
TPLHVAAVDGALSVIRLLVDAGADVSKPDASGWTAKEHAALRGHMDIARF
LAGHTNEEEAAARIAQGMQQSNPPENSSIDGRRSNIPNSNTPRPAEPVKT
FGHRYLTDESLVLVSLGSMDTRKNTEAVHLDSVPLTEAHSTQLDTALSVV
VSANGAHGDPTTIDLPVHEGISTEPIVFKTKDATKVKLLFDIIPTYSGSE
KNKIGRAVALLPSVKKTIGSRRMNLQGDVCVPIMSSNLEVIGTVNFNFLV
ITPFYHPNIEITSRQTYWRKVSSTMLIGHRGLGKNLTSNKSLQLGENTLP
SFIAAANLGAQYVEFDVQLTKDHVPVIYHDFLVSETGIDAPVHTLTLEQF
LHINPDKARDRGYKNGANTDSLDDLTIRPRSNSFAPPKRSLSMGYAGTGN
EEMEERMKHTRDFKAKGYKANSRGNFIQAPFATLEDLFRQLPEEIGFNME
LKYPMLHESEEHEMDTYAVELNSFCDTVLSKVYDLAENRHIIFSSFNPDI
CLCLSFKQPSIPILFLTDAGCSPVGDIRASSLQEAIRFASRWNLLGIVSA
AEPLINSPRLVKVVKEIGLVCVSYGVLNNDPLMVQRQVKEGIDAVIVDSV

LAIRKGLTSGESTVDGSNGSPTSEDSLKLDIEEK
>Mb|QLI66822.1
MDDYYKTNALPPQEGQRQMPQQHEGQQQQQQQPSGNVIDMINRSSVSGSQ
MSLQPPPSAMPQLIQNQPMISGDSLDDIIRNNHTEMHRRKSMPHAYGSPL
MQDENPRRMSSIAASEDVMAFDPSQHDLGNFQFSQPVGSSFHGINPSLEM
TDQMGGFVTDATDYSGISADLIGPMNPPPFGNVELSQMPNEPSLSLFPPS
PVPGAGSGARPMNSGFVSGQMPNLSATFGTEPAPSSSTLTATSNTLPGSI
DDHPDGMMHISTSQLNAVNPPASRVPETMVANPVSSNLGQPLPHSLSRHI
SSESGITQPSSTSGPTTPATTSTPRESKEKTIYSKSGFDMLKALWLVATR
KDPKIHLGAVDMSCAFVVCDVTMNDCPIIYVSDNFQNLTGYSRHEIVGQN
CRFLQAPDGKVEAGSKREFVDDGAVYNLKNMIREGREVQQSLINYRKGGK
PFLNLLTMIPIPWDTDDIRYFIGFQIDLVECPDAISGQELGGVKVNYKHN
DIGQYIWTPPASSQWQPENGQTLGVDDVSTLLQQFSPKGLVSDWHKQSWD
KMLLENTDDVVHVLSLKGLFLYLSPSSKRVLEYDAIDLVGNSLSSVCHPS
DIVPVTRELKDATASAQVNIVFRIRRKQSGYTWFESHGSLFVEQGKGRKC
LILVGRKRPVFALSRKNLESNGGIGDSELWTKLSTSGMFLFVSSNVRSLL
DLQPESLVGTSIQELMRKESRLDFGRQIEAARRGKIVSCEHEVQNRRGQG
LRALTILYPGDAPEGQKPSFLLAQTKLVKANRVVLATATSNSQAASTSSQ
SNAVPELHSGAGQVSQPPGGGLPLGTQDAALAALAAEDNIFDELRTTKCS
SWQFELRQMEKVNRILAEELGGLLSNKKKRKRRKGVGNMTGRVSPRNSFR
GGNNGNGDSQSKKSPSPIHSSPLHKEFASENPTSQAVVAKGDTDETDMLN
PSQLGNHVSSEASESNLVQRQMMAMPPPSQPSLSSGAAGSGMTSIREEHE
ASQS
>Mb|QLI66823.1
MDKILENPYVAGIKSHVEQLGPIPQPVLGAFAGVGALWLTYKLFGYLQLV
LSAFVFSGHNLRKYGRAGSWAVVTGASDGLGKEYASQLAAKGFNLVLVSR
TKSKLESLAKELQEKYHGKDLDIKIHAMDFAKDDDADYEKLAEVVRGLDV
AILINNVGQSHSMPVSFLETPKDELQNIVTINCLGTLKVTQIVAPILKQR
KHGLILTMGSFGGWTPTPYLATYSGSKAFLQQWSNALSSELADDHVDVYL
VLSHLVTTAMSKVRRPSLLIPNARNFVKAALGKIGLGGYQTAPNTYTPWW
SHSFMLWLIENVPGVNSPVTIWYNKKMHLDIRRRALRKQAREAKKQ
>Mb|QLI66826.1
MKSRLTSTLGGSSIVYERRAVSQIRTYNWPPLQLNFWIFVMLLSASSIVG
VFSSFIQIQTQLDLPIPWYFPYFITVGSVAILFMGFIFWLIATRRLLPAI
VMIGAFMLFVLWLVGLVIVSVQLWGPSGSIQSTCNLQVFNRSPHGLNQET
LAWMQQKNICQCWHLVFAMSLTGIVFLVWVMIMAYQVFVNS
>Mb|QLI66827.1
MFTFCPLQGALSESTASQSLLELDGGVKVLVGLGWDETFDLGKLEELEKQ
VPTLSLILLTHATASHLAAYVHCCKNFPLFTRIPAYATRPVIDLGRSLIQ
DLYSSTPAASTTIRQTSLSEIAYAYTQTAATAQNLLLQSPTPDQIARYFS
LIQPLKYSQPHQPLPSPFSPPLNGLTITAYNSGHTLGGTIWHIQHGLESI
VYAVDWNQARENVFAGAAWLGGAGGGGAEVIEQLRKPTALICSSRGAQKS
AQTAGRAKRDEQLLETIKTCVTKGGTVLIPVDSSARVLELSYLLEHAWRA
DAASDNGVLTSAKLYLAGRNMSSTMRYARSMLEWMDDNIVQEFEAFAEGQ
RKANGAVEKKEGGPFDFKYLRLLERKAQVSKLLDQVASAQGEVAKGRVIL
ASDTSMEWGFSKDVLKGLAKDPNNLVILTDRPTIAKDDRPSIARTLWEWW
RERKDGVSVEQTSTGDNIETVQAGGRELEIREASRQALEGDELAVYQQWL
ATQRQLQATQQTGTAGSLDGPTDVVDDASSESSSDSEDEDGQQQGKALTV
SATLGQAGRKNVILKDEDLGINILIKRKDVYDFDSRGKKGRERCFPVAIR
RKRNDDFGELIRPEDYLRAEEKDEDNADGSHLALDDDKLGKKRKWDDVIK
GANGPNKRPQPGKGVAEDGDDGISADGHAADDLDDVEDTEPEEPAGPCKL
VYTTETIQAKLRIGFVDFSGLHDRRSLDMLIPLIQPRKLILVGGNHEETM
SLAEDCRAALGMDGDKAVDVFTPSVGVWVDASVDTNAWVVKLADPLVKKL
KWQNVRGLSIVTISGQLLATNTTAEATEPSDEDSSNKRQKTEPSTAVALT
STALANSSGVLPVLDVIPSNLVSAARTAAQPLHVGDLRLADLRRAMQGSG
HGAEFRGEGILVIDGSVSVRKTAEGRIEVESVGLPVVGRRNTLYEVKRAI
YDNLAVVAGA
>Mb|QLI66828.1

MGSERWERDRFEDDRYYMRGGRGRERSEDRYDRSRYYEDDFVRDRRYHDD
EPRYEPRREPARQPEFDRRMVIEKERDREYYRDSSPRRPAFLRRQSSLDT
YDRRPLRNIFDQREEYPPPARREDIFRDEYRAPPYTPIPLPKARGLPPPG
RRDDRYYDYRPESEYYPEDEYRPMPERVREREIIRERDRRDRSRESRGTR
THTHRSSSRSSSSSSSSSGGGATVRSEYPKKGKTKIPARLVSKRALIDLGY
PYVEEENTIIVQKALGQDNIDELLKLSEEYIKVDQEVIAARSAAGDIVEE
RIEVRKQKVIETKAPPPPPPVVVPAPPPAPIVVPPPAPVPVPVPPPPPAP
VIVDAGPRVDIVDKTVIREISPSRSSSSWDSGHTHHHRHHHHHDDGALVV
VPRSRSRSRSRTRRDIRAEIKALERELVHRPRAEVEREVVRTERLPDGQL
VVYEEEVERTVARPKPPRIEKDKKGPPPAIIRAMLGTLT
>Mb|QLI66829.1
MPGRQSHGRSLMAAPKAKAGGKKGSKARSQKNALNAFGIAQDMYPTKYKR
TPRARELDAEIERKHGRDEAGEDEDEDEDEEEQPRRKKAKGPSRADGDGE
DGSDSEGNEWRLGGLRDDDEDSEIESDDAFGDSDNEKFEEYSFRGSKSKP
QDDDSEDDSNDDEGETLGADAIDLATALDQWEEESDDEPEADDESGSGDS
EEDGDESEEDAESDDDESEDDDDTEADPEKLNALKGLISQYGGENDDDKT
KSSAGQKLSLKDLGLSGLNDPLMKKSLKLMNKETKEKRPGVAQKLAVPLS
RREQGRLDRSVAYEKTNETLDKWTETVKQNRRAEHIVFPLPQNSATAGLD
TSEIQPLSSAKPSNELESAIMSIMEQSGLSMNKEPKPKPQEYDDEGNLLS
RKEVLARKRMERELNSREAKRAKRIKKIKSKAYHRVHRKQRERDEMAAKE
AMEEAGEIDSEEEREAQDRRRALERVGQRHKESKWAKIGSKAKRAVWDDD
FRTGLTEMARKDEELRRRKEGKAGVDGDSDSDATSSSGSDDEDDIRRQLR
ELEEDDDEPQSKLMSMKFMQKAEAAKKQANDDMIRQIRRDLDGDSADDSD
DMQADDEVGRRSYGTADSSAKPALDTSTRAPKRSRVPGPQDTDVDIIINN
TTATTKQPPEPSSSTTGAWSRGETRRKKSAPLRGEDLDLSANVLVASQPK
SRPTNSNSNRDSDSRKNDSDEDGSEAEQHMPLAIRDQALVARAFAGDEVV
AEFAREKGEIAEADDDKVIDNTLPGWGSWVGDGVSNREKKRHQGRFLTKV
EGIKKKDRKDAKLDRVIINEKRIKKNDRYLATQLPHPFESRAQYERSLRL
PVGPEWMTKETFQDSTKPRVLMKQGIIAPMSKPTA
>Mb|QLI66830.1
MGSTVSQGVDVPVAAASPSGEQQKDDASTTSSNSSPERQSCSAQSPGSVS
TDHKSTHSRSTSNRENNPSCSRMLFSSRSRAIFHPRSHEEIYAEQAYLSL
TLHAHIAQHCDLMNRYSLTEAETFQGESRKCKRRARKELGLLRGQLIHAA
EQEQIIFARLGELFVEAKSRESRNVAQNQRLQGLRGLRRYSHPQNQQEIR
RSMSSLNGATPAFIPQKDLFSHPEYDEDVASIADTGKDDREVSENCLGEL
LEPNDEQFKEQRLLLRRMSGDDYTSLLKKDRRLSLPTSLGLT
>Mb|QLI66831.1
MPAQKSTGRLADLERDGFVVVRGIVAGQKLEELREASLRVEKLGRSGQWP
HIRTVGKQFPPWTFSPEKGIWGVQHLMNPDLPGHEIFTRQYFSEEILSIV
RELLQCDDDELVMELFNMLVRPDGDFELRWHRDDIPADASAEEEMERLGR
PAFHAQYNFALWEDESLVLIPGSHKRPRTETERNADPFEKVLPEQLVVKL
GPGDIAFYNNNILHRGVYDSKKDRVTLHGSVGHSGGSKLRARNVLQHGIG
SYVDKVDFSKLSDGERQRAEEMRARLVKLGSESGDVGYSLQG
>Mb|QLI66832.1
MNSQGAHDVSPEAMQARIQQARREAETLKDRIKRKKDELSDTSLRVVAQQ
AHEPIPRNQLMKAKRTLKGHLAKIYAMHWSTDRRHLVSASQDGKLIIWDA
YTTNKVHAIPLRSSWVMTCAYAPSGNFVACGGLDNICSIYNLNQQRDGPT
RVARELSGHAGYLSCCRFINDRSILTSSGDMTCMKWDIETGQKVTEFADH
LGDVMSISLNPTNQNTFISGACDAFAKLWDIRAGKAVQTFAGHESDINAI
QFFPDGHSFVTGSDDATCRLFDIRADRELNVYGSESILCGITSVATSVSG
RLLFAGYDDFECKVWDVTRGEKVGSLVGHENRVSCLGVSNDGISLCTGSW
DSLLKIWAY
>Mb|QLI66833.1
MAPNFRIVGSLAELYPASKQKVSESSTALWKERRVQQRWLLNSHPEVFVG
GATDLGDYLKEDMERFEKSEDGDNDGPLLAVGEMNDVRAPPRLKGTPLLA
VATGESGARLRLTRMDESQWRWGDNKDAILNLSVVDSIHQEEETIWPGDG
LPIMKIKFATAYWQQGSVRWLLVQSQTSVTILCPEYHRNPQPDSGRSSSR
SQPPSFIAPNPLVTLHHHQTGGNAFSDVCFNPPSVGSPPQIAIIDECGYW

TVWSLQGTWQVAKKTLRLSLYKCGHICEGVLGTIPRVCSYQAQRHGMLQI
GRTDGDDSPHVTTRRAEYIRQSLGHSQHVLVWNSERISLVDLESDSVSPH
SDILSAQSRKKPICILDVQKSPSNENHVFVLTTRQVIWLDVLPEGTEPRT
IAQPKTLLACSHVGFWNTDCKMSVCQVSENKLDGSMVFVFSPFADQLTVY
WFNFSSDTELPQWHRHITSLSARSKERSLSKAQSIRVQPAELELSAAGDE
PGPGTEYAHADTKFYQITVLDRDLGVRYCICTSSIDPSLEVSLPTFRIGW
SKSANSRRWKRRRKRFLHHMTKAFVLPDSVTDNDLDSVLQKQKSGPNDDD
TSGEVTLNEARPVLLKFGSIAQTITSHLQAAISEGESGLPMQLIDAVQRI
IEHGIEDNSLPLTSWIQIVSTLDKPLVYGDPGEGMEAEIEGLLGSADEHV
VVTQLRRRYADEPPGSLLGYPLLMEELSELWLTAVADSLPNEMQTIRKIW
VAEIARDIFLSSYGVMVQNVPLMGPSGYDLVEESQVDSFTPRSSNQVPYS
SQASASSSQASVLADDVPDAAFQRLKLLAPTIKQGTLGSLKPPKVLSYWP
TERGIDTENYVSSIAVATEEKFSHAKRRLQRIEAKRKAQSEKFKRPAFMR
QGFPASDGLGEDASSMDMRPPPVQVMSSQQRMPDSSQSFGVPGPSVTMSQ
PVSGAFGDRKKVKKGKRKSGFR
>Mb|QLI66834.1
MLFKTALGFAAIAAAGAPIAADDSAKALETRWGARISNNDFANIRFYAQH
SAAAYCNIGVPAGQQIACGNNACPLVARNRVKVVASVTGDLTGTGAYVAI
DTFRREIVVSIRGSNNIRNYITNLIFSWTDCNFTKQCQVHAGFAQAWDEI
KVAVNRAITNARRRYPQYAIVFTGHSLGGAVATIGAANLRRSGLWVNLYT
YGSPRVGNDWFASWFSNVQGGQWRVTHEDDPVPRLPPIFSGYRHITPEYW
LSGGNGGNTYKTDYTIANIKVCEGIASTQCNAGRDVTDINAHLYYFGAIA
SCAPSSLQLRDADAQDDPLPKDLNERLTDWSRKDQEFVKSENV
>Mb|QLI66835.1
MKDPIARTASPYTDPSVQGPRFKIQESRTDLRKMISSNVNKTALHPGGVQ
PHHNHTELEEELHETAHIDYDRVAIIPNPSVAALYEDALVYETGSAITSS
GALTAYSGKKTGRSPLDKRIVQEPSSENDIWWGPVNKPMSTEVWKINRER
AVDYLNTRNRIYVIDGYAGWDEKYRIRVRVICARAYHALFMRNMLIRPPR
EELEHFHPDYTIYNAGSFPANRYTEGMTSPTSVAINFAEKEMVILGTEYA
GEMKKGVFTVLFYEMPIKHNVLTLHSSANEGKNGDVTLFFGLSGTGKTTL
SADPNRALIGDDEHCWSDRGVFNIEGGCYAKCIGLSAEKEPDIFGAIRYG
SVLENVVFDPDTREVDYDDSTLTENTRCAYPIEYINNAKIPCLSESMPSN
IILLTCDARGVLPPISKLDRAQTMFHFISGYTSKMAGTEDGVTEPQATFS
SCFAQPFLALHPMRYAKMLADKIDQHKANAWLLNTGWVGAGHSQGGKRCP
LKYTRAILDAIHSGDLAKVEYENYGVFNLQVPKSCPNVPADLLNPEKAWT
AGSDSFQKEVVKLGKLFRENFTKYESEATEDVVKAGPSV
>Mb|QLI66839.1
MSFNVAFLNTAGMTPVPMPGHHQTSRQPGQASRRSGSASPSSRHKSNANR
RLGHARTSSGSRASCSSHETALIADRPAVAAGHNSAAAQLNSNSASSANS
QTHRSFHPPSAFISSSTSPVGAPTKASSLNPPRHRHRHAADGPHGVDMNF
SALSAESVSSASGTASLSARTTMTLDNQGPGDNLLLSRPFIQRNGRTYLN
DTSLPYPLPSDLTELHRQSLRTLLLIQLFGAPVCSPALTKKPPQRVLEVG
CGSGFWSMMCHRYFKERGHGSICFTGIDIAPLSPGSADLTEDATQPDPDM
RWKFVNHDLRQLPWPVPSEEYDLVMVKDMSLATTNVQNQHFIDEYLRLLR
PGGTLEIWESDHLIRMLRPHVPGSAATADDAEEQEAASSLGAYVMSSNTP
LSAPLNNFLVEYNTWLTRALENRDLSAVPCTLIRPILLQESETVTDVKSR
RLAIPLSEVRWEREGVGGVVTRDGKQTGEGRGKDQKPERTLTAGQIALRK
TALLTVVQQVQALEPILREVSGKSQDEWDVWLGKMMSDLMSESGASWGEC
LEVGAWWARKRKRGHDV
>Mb|QLI66840.1
MPARTLPTFTRAEVEAHSSASSCYVTIGSKVYDVTDFVDDHPGGGSLIID
YAGKDVEEILKDPTSHPHSEAAYEVLDDSLVGFVVNEKSPATNGSAKPAN
GTATSKDANYVHPRTGMSCEEDLSKDTDYTHDFKKHKFLDLSRPLFLQVW
NGGFSKAFYLDQVHRPRHYKGGESAPLFGNFLEPLTKTPWWIVPSLWLPF
VAYGTYIASQGLHNPFVLGGYWVFGVFFWTFVEYCLHRFLFHLDDYLPDN
RVFITLHFLLHGIHHYLPMDKYRLVMPPALFVILASPFWKLAHAIFFYNW
HAGTAVYCGGIFGYICYDLTHYFLHHTNLPLWYKQLKKYHLAHHFLDYEL
GFGVTSKFWDQIFGTELQTSIKTK

>Mb|QLI66845.1
MSTTLRAARGVLGLSRPRAAAAPLHTHNQRRAKPSSKPFRPDSRQWHSTF
SSVNPEEVSHFNSLASEWWDPHGSSRLLHLMNPVRQDFIRACLQSQPDTK
ASRLRDASLSYLDIGCGGGIFAESAARLATTKRVTAIDPTPSVLAVAKAH
AKKDPSLSGKLFYQQSSIEDLQVPREPCDAYDIVSLFEVIEHVDEPGPFL
ERVRPFVKPGGWLVMSTIARTWVSWFTTNLVAEHLLRIVPPGTHDWNKYI
NEDELQRFFLDKGWDSPRVMGVVYVPGLGWKEVRGGEKVGNYFFAVRRNG
>Mb|QLI66846.1
MSKRSSVASSSANGAMGKTSFNHNQPAIDKQKTLLSADVGHFSLVRAMHL
ADLITLMNGFCGVMSIFSSLRYCLGDPDSFDNVWLALAFLPFGLFFDFLD
GRVARWRNKSSLMGQELDSLADLISFGVAPAMVAFTIGFRSTLDTVGLAF
FVLCGLTRLARFNVTVSVLPKDATGKSKFFEGTPIPTSLFLDGLMAYWVY
CGQVLDDVPFGAWLKDSALEFHPAVLLFMLHGCLMTSKTIRIPKP
>Mb|QLI66850.1
MAVNHSTAVGATLQDLPKSWHFTESLPPDSAFPTPADSHKTPRDQILPRQ
VRNALFTWVRPEQQKDPELLAVSPAALRDIGIKAGEDKTDDFRQLAAGNK
LYGWDEDKLQGGYPWAQCYGGFQFGQWAGQLGDGRAISLFESRNPDTGKR
YELQLKGAGLTPYSRFADGKAVLRSSIREFVVSEALNALGIPSTRALSLT
LLPHSKVLRETVEPGAIVLRFAESWLRLGNFDILRARGDRDLIRRLATYV
AEDVFGGWEKLPARLEDPEKPGESPVPGRGVPEKELQGPAETAENRFTRL
YREIARRSAKTVAAWQAYGFMNGVLNTDNTSVYGLSIDFGPFAFMDNFDP
SYTPNHDDHMLRYSYRNQPTIIWWNLVRFGEAVGELMGAAGLADDAAFIS
EGVKEDQQEEVISRAEKLIMQTGDEYKEVFLGEYKRLMTLRLGLRERKPA
DFDELLSPALDTLEALELDFNHFFRRLSNVKLAEVSSPEGRREKAAVFFH
TEGPPSTVSEDEARDRIAKWLDKWHARVVEDWKDGELVSDEKDQERIEAM
KRVNPNFVPRGWVLDEVIRRVEKEGERDVLNRIMHMALNPFEDGWHGKTV
DGVAYKGDAEEEQRWVGEVPRPGRLMQCSCSS
>Mb|QLI66851.1
MASLALQFRVADAADAPQIQHLVESAFRASDPRQDWTGNTELASHFCLAM
DEVQSKLINPDVVTLVAHDTSDPAALVASIEVSKRDGAGRLSMIAVDTSH
QQGGVGRRVLEHAEDYCRRAWGVDKFSLNALSTRRALIEWYMRRGYEKTG
ETSRFPAERFPELRLPEDICFVELEKSLKRRNSLPLHRQ
>Mb|QLI66852.1
MPRKSIDAGKSNGSTMNDTVARKTERSHEENQERAYIAASRRADRSIEAR
VQSARQASEIHKKRTGKGFKITEDIVMKEEMYEEEDDDMPRSLHLLTAHM
ATQSPQMNSRLESYLANKVAFSHALAQSNMAWRNHGVNRAFAESFPNAFP
LHPPNISQPPNGSEHVNLPNQGIHQHPECYSPPPAYSPPAYSPTNYSPAP
FSPGLPASMPPTAPATGSVSLAQSTHPFVPSFPYQPARNDNRRRSRTSIS
PNRKRRYAHTKSPPALGRQAKFSPPATPTPATTPSLDSNLTADTPSDSFE
PITTPGDASAFTTMLPAEARMILQGVGADDVLCQTLAGQDLFNGQGDFTQ
QWMDPSVVYDSSSMPRVPSMNGVDPGLVPNLYGNGATDSMASKCAMPSPT
DEESWNAFINENIWTADQNQ
>Mb|QLI66853.1
MDPLVASLVNAFVKSWPSWHRLRLGNNLRDEEAKLQLPVDMATIMAEAHK
KSQSSGIAAFAGAWAEKSLITLIYGSNVIETAGSTLGITTRLCQDIFRGR
PVDAYIDKKDPAYREHLENLAETHRKSDMANVVRSRREVIGHANALRFMI
DHVILNNEAWSEELILQTHRILYEGIDDDVVPGKYRTHEVAVCYSSPGDK
KTKKTSLCMRAAAVPRYMTAMVEHLNHDITQAEASGQRDPYTLAARYHHQ
FVMIHPFADGNGRMSRIILNTLLLKYAGHVAPFGCGNDKDDYLAIVRRAG
VVFHREDMEVDFGQQTCQFEFAKFVLAKSKPGLEQMWAWASAEPEDEFLF
RAVDRQVDGGCCYTSERHRTL
>Mb|QLI66856.1
MSFAARNALRLSRAASPRIAVPRPTARLFSASRINAVSDTPQKKSVVREK
EVPVTVYGAGQGQGDKHTVHVPEDAAQVPYDSPVPPVEYDLVQPLGKKVY
EAMPHTMRNMSVYGKVIIITGGARGLGNHMARACAEAGAKAIVIFDANQD
LGDEAAAELHDKSGLPVSFFKVDVRDGAAINAAVESVVELYGAPDVLVNS
AGIADSNIKAETYDPAMFRRLIDINLTGSFLMSQAVGRAMMAAGKPGSIV
LVASMSGSIVNYPQEQSCYNASKAGVIQFAKSIAAEWAKYKIRVNCISPG

YMDTALNRVPALEAQKKIWKSLTPQDRLGGVDELNGLCVFLASDASSFMT
GSNVIIDGGYSCY
>Mb|QLI66857.1
MTLTVELKTPITGPYQQPIGLFINNEWVEGVDKKRFEVINPATEEVITSV
CEATEKDVDIAVAAARKAFETTWKQVTPQQRSVLMIKLADLVEKNVDLLA
SVESLDNGKSITMARGDVGAVAGCIRYYGGWADKIEGRTIDIAPDMFHYT
RPEPLGVCGQIIPWNFPLLMLSWKIGPALATGNTIVMKSAEQTPLSALVF
ANLIKEAGFPPGVFNLISGFGKVAGAAISSHMDVDKVAFTGSTVIGRTIM
KAAASSNLKKVTLELGGKSPNIIFNDADIEQAISWVNFGIYFNHGQCCCA
GSRIFVQEGIYDKFLEAFKKRTQQNKVGDPFAKDTFQGPQVSQLQYDRIM
SYIKSGKDEGATVEVGGERHGDKGYFIQPTIFSNVHADMKIMQEEIFGPV
CSISKFKDEEEVIKLGNQTAYGLAAAIHTKDLNTSIRVSNALKAGTVWVN
CYNMLSHQLPFGGFKESGIGRELGEAALANYTQNKSVAVRLGGAIF
>Mb|QLI66859.1
MDTDREQIVVGSDQSPNQRQLTFQSYQQPSPSGTTASGRAPAQKRYELID
GQWVQLTAAGLPRKKPGRKPGSTVKPKTMDGADPSKVRKPRKPRDPSALP
IQRKRKIAPASDADPDSDVPRTASGQHGGISSPSQQHQLLGASDLRGSPK
MPKREGYPGSMQSILNADPPSASIPVRSSGLSYDPIRGNYDPVRETITSQ
NANPYSSTSGSPRGPAQAANRSPSIASIIEPQARTATATATSPSMSQHGH
PTASAHSRLHGKDSSSMPPSPSYRPTSTLGAPPTAVDSKKESTVAAIQRP
VVNQANFTTISNGPIRRSSPKLKPATGVSTPRTDTLDDFQDSEGRSILDF
GKARPGEEVQAPTIVLTIPIGHGENNKYVNFMRLAEEQYGWDALHPRQAA
NRDRKARIEAATASLEKAELGRESGDEMSLDNSDGEASNPENGGTSGPDA
QVKPKKKRNFKEDQYDVEDDFVDDSELLWEAQAAASRDGFFVYSGPLVPE
VEKPAINSTHERPKRGRGSRGARGSRGSTRGGTGTGRGGGPGSRGGAVTR
KPRITKAERLQREREKAERESMAQINKTPTNGYVLQPSTPAFSVSELGA
>Mb|QLI66860.1
MSKMLFTGPFVRRRSNFLRYAVLAFILLVAFWKFHQRAEDLVSFDLTVNP
PPGGSKGTTADDDKTDKSQSTDSDSVKPPVRPDSTRRPEGTVPTVDSEGR
HPIDKLIYDAQHQFAAVASGESKTLEQAAQAYRKARGRHPPPHFDKWFEY
AQSHNALIVEAFFDQIYEDLEPFWGIDPAPMRREASQFEMTINVRNGTAT
AGSDWMWTRIWLDMAKTIEALLPDMDIALNAMDEPRLVAPWEDIDAYMKK
ASHTRKLPKAAKTVNKFHSWPEPKTGFLAGDIVDKNWEKDEFYWKIVRRG
CPPNSLARTTELQTSFQDPPTISNAYAKPHFYKGYVSNYTKSIQVCHQPD
LQGLEGLLIRPLSTKSTKVMFPMFGGSKLTVNNEILLPAPMYYNGEERFV
GNGDHGVEWPEKTDKAIWRGVATGGRNTEDNWRGFQRHRFVAMNNGTKVA
RVESGEDRAENFVLPEKEYGVQARADQNLGPWVDEFADVGFIDLMCNTPD
QGNYCNYTDFYFKAMDGIKLTEQFNYKFLPDIDGNSFSGRYLGFLRSTSL
PIKSTIFREWHDSRLVPWKHFVPMDNRFTDYYGIMEFFRGYQGRNSHDEA
AKRIAMEGKEWAERVLRREDMQIYMLRLLLEYARVMDDDRESMGWTDDVA
MNPSLAKSWSWWW
>Mb|QLI66861.1
MKFTAVAAISMAALVSAQSISDIPSCALSCIQRAVLSTHCEGTDLKCICK
KENFSTIQGAATPCVIEQCGTETAVNKVLPATTNLCAGNADGGSSEAASS
AADPTEMATQMTEAHPTEMAEPSESEAAGDTECHATMASQPCPATPSSPA
SNGTAPPAPSTPVTAGAAGLAPIGGLAMLAIGALAL
>Mb|QLI66862.1
MRDSKESDSAPEDTGHDANAQPDDSILKLLPPPGGASASGSTTGPSAMES
SYVDAVTIDHSRAPDVTITEEAVREHFKDQESSFLPTLSPIATRTTAEDA
TGADDTIDTSGRKKDQNAEKRDDDHTSDVDVGNTTSSLENLDSSPTTAAA
ARTISRAVSMASSATRDGRTAPAAADEDDYRDETEHSFLSGADDPFLQSQ
ISDKLDTGSTPSNSLRIGKRSKYLRSRYGSHRSSTSSLITNPESQDGSEA
TIGHGVDYALQSGGAVPALGMLRSTSNPFQRSISVGSMVSGFGGDDLNDL
PNHHLDTLPEVSSPDRPNHNDPLRTPKASRENLKATAPTDTVIAQHVKNV
QVPESLVKEYRFKNSLQTPRRPSNLTTSISTDTITKTGRNLTLKEQSSTI
ERLSKENFDLKLKVMFLSDRLDRLSEEGIKEMISENVELKTSLAVLQRDN
KILRKRIKELERQARDEDNRPSTASSTDQTSKTNDEDAYEREQELIYLRE
RVEEYAIEIERLRNESLSRESEKLQLVQMVKTLGERSGSNAGVPDESAVW

SDLLEQETVRREQADEDNRKLRDEIFRLKKELSAQGNMQHTTNIYNISKK
PRDGAFSPTRATSGLSGETNVPDTNLGGVNVLVDELRRESEQLRHENAEL
RREVGAQTSMLTSRNREKERLYQEIEDLKMAQRRGGPAPSTIDSILDRSA
SRAGGHERSQSRGSGRTRQTLAEEESEREGLENKLAEVRDKLSEVRLQNQ
ELQRELESCMVDFEAAVEGKRQAEDNAMSLQEDLDTAMNDLVALQAERDE
AVREQGEMENEFEALRHEAQEEIDAMEAEADQRNEEIQRAYTDLKDRSEN
FDALQEEMRKMSEALVRLEDEQTGKLRRIQQLEEELDSSNKELEDLEQKL
MEANEKNQRFSIQQESSQGEIAFLREEQEVDKIRIGDLEAAIANAEQNLR
DEKDRARELDNRLQQERMHREIVADKEKEEVQQVVNELNREASNAKDEVR
RLRKSLSSREVEATEWKERLIELENNLREALGDLNGTRSSLLKSIAKMQR
ELENTIRELDTTKASLVEKDRIIKQRDALLESHALESRKVTELLERERVA
HRNTKAQYDTFQRTHQHLTQTASTQDVRIAELESTRGQDRRRLAQLEQTA
RDQLTERNELLLMLWHKLSALCGREWLNNNTLVDRQVVPSVEVIATRLPG
FSKNLVGAIKAIEAMFGGLHSKIKSVERDLHREYQTLENNLEVRTKKLDR
LETMLRNSVASGSLAPQDMHSRMARLEDAYRQLKVENATLRTANDVRSRA
AHDGTTTPLGRGTTVKGSPSPGIPRGPGDRDRSRTSQLSQDRNSRSGGSS
RSNTASGIPRPSSGNFTSMEVALPSEGEPTNNDNRWLLRLRDMEYKLKME
REGRNQDRAAARQRLGGLELENRDLRERVRRTNTDTE
>Mb|QLI66863.1
MGQSLIDRIQAKLELFRLEQRYTRRRNRRSTFQSNAIYVDGEYVYQTPNT
TGSSTDSSNDHVDALHGEMVSPGKNMPVTAFPPESRPEISMPERKKLSRF
SSIPSLGSVFGSKERPRVVERRTSMIR
>Mb|QLI66866.1
MPPKKAEGSAAPKAKSTHATYQDMITDAIVNLKDRNGSSRQQLKKYVKAN
NNLVNVSDNMFDSLFNKALKAGVDKGAFAQPKGPSGGTKLAKKKAEPKKP
AAKKEATTTKKAAPKKPAAAPKAAGEKKATATTAAAATTKKAAPKKAATA
KKPAAKSDKDAALTKTKTGRVAKTTKPAAKKAAAPKKAAPKKAAAKA
>Mb|QLI66868.1
MSVALTATAVPVVIDLTLDTPTTVTPLTSPYNHGDPTQQFQLASQQPPAK
KRRVENDGPGAEHVEMRMREYLLPYVQKAVDELARDACLVDTIAIEAVTT
LLKSARFREKVQLSNGLLTAEDRADIEGLATQAVQQLAGLTQYRRPQPQP
IKEEAPSSALVRPKLTLRAEQRAFQRLESAEPTSKPDTGPTRVPRPRVRR
QYVVPKSSDGLPTASTWLQLQRRPYLHYSARQDITLGVNRPVNVARHRVQ
LPAVYHADFSSLEIAEIMKIVGQHLSQHIPRTREGLEQILRHYNVPRIIG
DSISGRTQQDIYNLCSDLCAGKASQPDNSRVLSLEEVSPERRARNRRANR
LPALLLAREMEGNVGFGRMRRYENFQNEFQKCHEDGLSLVAEFTNCAGDI
SAMSWVPDGNIICGTTAHSDAHNQQYNKPGNLLLCSTERGTLRAFPDHRI
PRPFVEKGENSTEAMRQSQDPWLYSSVVSTDYDEMNGRAFTSSFDKTVKV
WSVAQDGTSMQATATWHHQGNVNFVVAAKDGSGRVATAADVPTQAIRIYT
VDPNNVQDSYYYTISCTRTDADGSDKWAYCPATMQWGQTPGTHHLLAIGY
SPRSFTGDDADIPVDKLHSGEIVLWDAERRCRVPVLTVTTANVFEIVWHP
ILPVFIVATSPSGLIVEHGVRTHVHIFQLDRDREDGAYSEYQKLDCYASD
INELTIMPNSLCHSYISAACTDGRVYIWDTAQGDRPAHILKHGEPLGAVI
CDREKEDEGVKFTAWGTTPDRFYTGSSDGVVKVWDVRRKRRPFVRNLLEG
PGPITCGVFSPNHNKLVVGDATGRVFLFSVDDRDEMDSHFLTIPGTNRRV
RRPKPFIPHPEPEPPTTSSPFPASTPSDASIAQYSRDRYLSSNSLILHPN
PVIGAVQGPAYASTGLFRLDAHAYFDPQGPLLPEFERNQRAAREASLGPE
TRSIRRLKGPPVQTWVEKSLHDENVAKDFDSRSLDNWSFCQLVNLGVRFN
MDGDEGWEFMYEETPVDTD
>Mb|QLI66869.1
MEKYSQFRDRATGISPFLPVTTPLSVVSLATHTVLFIFRLPFFIAYAASY
FVLFHHLPLPVVARKMALWGLMAIPGIWWVDLQLDGVKRGTLSGQPLHRF
PHPGSIIAANFTSPIDAVYLAAIFDPIFTVSYPGERRVQHVSLFGAIMQA
LSPVKMTPPANVKLVQIEDLLKAYPDRVIAVFPECGTSNGKAILPLSPSL
VDAPSDVAIFPVSLRYTPADVTTPLPGLWLGFLWNLLSRPTICIRVRIAE
SQMNTAATKNSNANASNGTNELRHRKDGSSDISAEEQRVLDRVGEALARL
SRVKRVGLTMKDKDAFVTVYNSKKKS
>Mb|QLI66871.1

MAGLVRLTAALAASLSLASAATINHDFNVTWVRANPDAAFERPVIGINGK
WPIPRIECNIGDRLIINVNNQLGNQSTSLHFHGLFQNGTNNMDGPSGVTQ
CSIPPGSSFTYNFTVNQPGTYWYHSHNDGQYPDGLRGPVIVHDPEFPYKK
EVDEEIVLTLSDWYHDEISTLIPKFISKTNPTGAEPVPNAALMNDTQNVT
ISVQPGKTYMFRVVNIGAFAGQYLWFEGHKMRIVEVDGVYTKDAEAEMIY
VSAAQRVSFLLTTKNDTSANFPIVASMDTTLFDVLPPELNYNSTGWLSYD
KSKDYPAAALVDELNPFDDMTLEAYDGMELLPEPDRTVELNVIMDNLGDG
ANYAFFNNITYALPKVPTLYTALSAGEMANDPRVYGEYTHPFVLKKNEIV
QIVVNNLDSGRHPFHLHGHNFQAIYRSNESAGTFADEGGETDKTFPRVPM
RRDTMVIWPEGNMVLRFKADNPGVWLFHCHIEWHVVSGLIATFVEAPTEL
QKTLSIPPTHLQACQKGNVATEGNAAANTRDLLDLSGQNEPPAPLPAGFT
PRGIVALVFSCVAGLLGVAVVAWYGFSQAVEEAPGAVTSIIRNADISESD
NSQIVSSSTNAGRTTT
>Mb|QLI66872.1
MAMLHCENILIHGYKFNLEKLKGPHSVVTSKYDSVTHTHHNTTYTLDVCQ
PLKKSGTSKSTEECPNGTRVCAISRFIQGDVDKVETVTAIAGSLENVGGS
QFEYEATRLNGSDSNSDSKKQGLRLVLKGGKHPLSGPVKDRREQKAVVEF
LCDPDKEGNEGEWESEDKYSKLRRREDKKEDDKDDKDGKDGKDGDGDKDE
SFGEHQLKSDKAALLWESYGPEKDADVLRLTWHTKYACEKRDGDNDNKNG
DGGDSSASWGFFTWLVIVAFLGIAAYLIFGSWLNYNRYGARGWDLLPHGD
TIRDIPYLLKDWTRRVLNTVQGTGSRGGYSAV
>Mb|QLI66873.1
MPDKILDDISHRRYNPLTDSWLLVSPHRTKRPWQGAQEAAVANTLPEYDP
KCYLCPGNARAAGDTNPKYDKTFAFVNDYSAVKEQQPDYEPDTKPNGITK
RNSSDTTIVDFSADVESMLLRAQSVKGVCYVLTFSPKHHLTLADMPAPEI
LPVIEHWTRIYANHLSSSNPLDKLAENLDISMPKDEAPIPKDEYRYMQIF
ENKGSAMGCSNPHPHCQVWTTSTMPEEPGKELSQMTKYRANNGGRHLLED
YVQLELAKGERLLWQNKSFVVVCPWWAVWPFEVLVLPKRHIRSLVDFKPE
ERLQFAEAIQEVTRRYDNLFECSFPYSSGIHQAPLRGTKEEIENSYFHMH
FYPPLLRSATVKKFLVGYELMAEPQRDITPEQATARLRDCGGELYRNNLK
>Mb|QLI66875.1
MASRFVPAKGKALGHALRMNPQVALLGFLLPAWQTSAQRHFSTTTKRPSK
LGRTPISIPPGVELSMSDLKTSKVATSYKPTVKKTIIVKGPLGSLELDVP
EFVTLQQDLENKTVLLGVEDANVKHQNEMWGTSWSYLTNYVMGVSEGHTA
ILRLVGVGYRASVERRGAKEQYPGQKFLCLKLGFTHPVEEGIPQGVTVTT
PSPTRILLEGTDREVIMSFAGRVRQWRPPEPYKGKGVFINDQTIKLKQKK
IK
>Mb|QLI66876.1
MATQRVTYRRRNGYNTRSNRTRVVKTPGGELRLLHIKKRGTVPKCGDCGS
KLSGIPALRPREYSQISKPQKTVQRAYGGSRCGGCVRDRIVRAFLIEEQK
IVKKVLKEQEQSQKKK
>Mb|QLI66877.1
MEESAPKRRRVSPDPEAPTAASRPAAQAPTNLPTTTTSQSPNRRPRPSFA
SPTRASLSRYNPQILERRRSAFRSPPKVTTTAPAPSNPAPGAARPPPEAA
GNIGASESNQPEDTVEQTGGAPRPATSSQAGPAGPPLRTPTRPSPRPFPP
PAPEGDDELNPFLGRMQRREPITGVPIPPPPEPELPPAVSDPVSSTPPRG
IHSSPRWRDRDRQKKKSPMKSSPLKPRGVSTPARRVGVSMSPAGSAVQRE
LPLQDRSLNANTNAARQVPASDKNMHKLKERNRLRREIAVLRREIELAAR
ENERIRVMQRAGRIVAPENEEDVLNLVQKTLVATRTAPTPGLAHQMTQAA
LNPMGILPFGPPRLVSNQPSPETDEANIKSHHPVPMSAADDLPFLQLFTP
FSATSQVSVLPSAQNQPLRQRRQITLRSRDVPGLFTARVDMVVNAMNLNI
LALKVPALEPCARPELGPFVEKICSGDCNRTMQHNVGILSWAMGEWYRVA
VQRARFWFQLEQSLAAKGDYLDAISRMRSEPGLGEDNSQDDDLDTCSKSD
LFRFLGQQSYDVPVPTITGEGPESAVRLEWKIKLDWTGEAQSKMGLFLGI
PGKWRQADERGVFGQFAKLFEVLIEGGQPPRKAVQTIVALLTGA
>Mb|QLI66879.1
MVKGRVLVIAGSDCSGGAGLEADQKVLAAHGCYAMTATTALTAQNTTGVK
GIHVIPAAFVEQQIEACIEDVGVDVIKTGMLAAAETIDMIARQVAKHGIT

SLVVDPVMVSTSGAQLLPREAIRELSMHLLPRATVLTPNIPEAILILSEN
GDSALAEKKICSVSDVEFIGRRIQALGPEWVLVKGGHLPFRADMSVAETE
HEKHVVVDLLVGPEGKVFRVQSPYQPSTSTHGTGCSLASAIAAGLAKGID
VPAAVHSACRYIEAGIRSAPKLGKGNGPLDHFHSIQSLPFAPGYFIEYLL
QRPDVKDVWETFVYHPFVMALGDGTLPLESFKGYIVQDYLYLVHFSRANA
LAAYKSKDIESIRRANKIVGYIIHEMQLHVNYCKQFGISEEEIQATEEKQ
ACTAYTRYVLDVGQSEDWLALQMALAPCLLGYGAVAQMLHSHRLTRTKDN
IYWPWIQNYVAEDYTSAVRLGSELLEKHMRLQSPSRVEELIKIFIHGTKM
ETGFWEMFPYK
>Mb|QLI66880.1
MHPAVSASEAALQKDNEVACTLLIELLAHQFCYPVQWIKTQDLILGDQET
NRVVELGPGNTLVKMAERTIAQKYAQQDTAANISRQLLSFQQNYDQISYE
QRTVNPSSHEKTEDASGGSEPKPSPLVLSASPQQSAPATVVQQSTVMAVE
LEDEPARVIDITTTIVCLGLKKPLDGIDTTKSIRALSGGRSTLQNEIVGD
LGAEFGAIPDGAEDMSIDDFCSLVQGTFAGKLGAKCCSAIEKMTSHKFSG
SFQGSGIRKYLKKQWGLGPGRQDSVLLAALVSQPQARLQNHDEAKVFIDG
IVQQYIQKHGLVLPAESSFAGPSAAQTMVGVEVLDCLEERQKAVLREQLQ
LYARLLGVDLDAPEAANAALRQRISELEGQLDEWNTEHGEAYASGLKPVF
TPLKARVYDSWWNWAMQDLMILISTATSGSLSDIEPTMDRLKRNLVQKAH
PRLVCAIRHFSSTTTKAHVAEILRDVLSRCQTSVNEMPMVYWHDQSMAPV
TTVDQSGKIVYSERPRGIRIDDDVLPRIGRKVCNTWEYLDSLSCTLHRVL
AGARTTGLSFAGRNVLVTGAGEGSIGCSLVSKLLSAGAHVIVTSSSYSPR
VTRFYQRLFAEHGGKGSKLILVPFNQASVRDVEALVTYIYDDGGLNLDLD
YVIPFAAISENGRNMDNIDSRSELAHRVMLTNTIRLVGRVKHHKESKRII
TRPAQVILPLSPNHGTFGGDGLYAESKLGLESLFDKFYSEDWSDYLLICG
ALIGWTRGTSLMSDNDIVSEGIEKHGMRTYSQDEMALQIMALMDPAIVSL
CTDEPLLADLSGGMAQIADLKGVVTGIRDAINQLSEERRMMRLEALSDAH
AVGMEEGIETQANIDFNFPALPDWKTEIMPMADQLKGMVDLDHVVVVAGF
AEIGPWGNSRTRWDMEVNGSLSLESCVELAWIMGLIKAHSGIVDGRPFSG
WIDKSTNKPIADREIKEKYHDFIRKNCGLRYAESKPDSPSWTGMESLHEV
EISKDHEPVEVSKETATQLKNIHGENLIVISNEETGQSKIVLKKGAKIMI
PKLLRTQHTVAGQVPAGWNPSTYGIPEEIIGQVDPVTLFALVASAEALQS
AGISDAYELYEHLHVSEVANCTGSGFGGSASIRKMFKGRYQDQPAAKDIL
AESFISSTSAWINMLLMSSAGPNKTPVGACATALESLDTAFDLIQAGKAK
MCLVGGFDDMLKEISDEFANLKATVDADDDAARGREPSEMSRPATSTRNG
FVESEGAGVQIVTSANVALEMGLPIHAVIALTRTAMDGASRSIPAPGQGL
LGSAAQTQSKYDSPLMSMAYRKRSLQKRLQQAAEMRELQISDLGEEIAHL
KRQGCAFDEANYVQHRMASIEQDAKRDRAEALKLYGNHFWIHDGRISPIR
GALAVWGLTVDDIDVVSFHGTSTKANEKNESSVINEQFKYLGRTEGNVVP
VVCQKFLTGHPKGAAGAWMLNGCMQLMADGRIPGNRNADNIDAALQQYDY
LVFPNKTIEKPEVKAFVVQSFGFGQKGAMAIGVNPKYLYATLDEDDFMAY
KQKRHVRERRSNRVSSERFCNNSIFQAKDETPYAAADRSRVLLRSEA
>Mb|QLI66881.1
MPRIKLVSRDSHGNDFIDSVLMPTPSQITPLNSIPSSPEAMDWSSYYPAY
VADTPPVESTHPQKLTKDVEVVDIGCGFGGLLVALAPLLPESLLLGLEIR
TSVTQFVQEKIWALREQNEGKLYQNAACIRANTMKFLPNFFKKGQLSKIF
ICFPDPHFKARKHKARIVSTTLNSEYAYALRPGGIVYTITDVEALHHWMV
QHLDAHPSFERVSEEEEEKDECVKVMQNETEEGKKVARNKGQKFVALFRR
IEDPPWE
>Mb|QLI66882.1
MAGDEAATSDAAAAQTRALPPLTDHDFKIYNRMADRMEQFHNYFRQTWTL
LWTAATTSRRPQNLTQKQFLQEAISFARHLSAHHGIEEAHVFPLLATRMP
EFDARRGSLVKQHEQIHKGLQEFERYVKECHAGRDEFEMGVLKEKMEGWG
GVLWEHLDDEVRMLGAERMRAVWSKEEMMRMPM
>Mb|QLI66884.1
MAPTAEKDGHKAKDDSTHVKELYPMVNWKYDLFLYMMGNLVDLFFREVVP
RGSWKVPQSGPVLFVAAPHANQFVDGLVLLRTLRQEAGRRVSLLIAQKSV
HGFIGWASRQAGAVPVGRAQDATKSVPGTIYLPDPVNDPTLVRGVGTNFG

TGDGQIHGMVFLPPGKKTASVSSDIAQILGPEEIRLKRPFKGKLAMQQLT
GRDDIDEDGKFTNKSIKGPAPGYEGTKFKAAPNIDQTKVYEAVFSRLRHG
GCVGIFPEGGSHDRTELLPLKAGVAIMALGTLAEAPNCGLKIVPVGMNYF
HAHKFRSRAVVEFGTPFEIPPHLVELYRNNQRREAIGQVIDTVYQSLSAV
TVSAPDYDTLMVIQAARRLYNPTGKKLPLPVVVELNRRLAMGYERYKNDE
RITSLSASVKNYNLQLRYLSLKDHQVQYARMSILKVLFLLVYRSIKLLLL
FFCTVPGLLLFAPVFVATKIISIQKANTALAGSTVKVRGRDVMATWKILV
AMGLAPTLYHFYSIIIVFKVWQDRLWGYVPMWVPLWLAYIGIWPAMIGLT
FAALRFGEVGMDIFKSLRPLILCLVPSSDFNIHTLRLRRAELSAQVTDLI
NTLGPEMFSDFDKTRLVPDVSRLESGGATPERAHKRKDSDQSNTSADVET
PPMLSRRSTTQSSRALPRNDSFSNIGTVGIFSTRPPSRSRSRSRSSSAGG
GFGSGGFPISGFTTLDSAEGFNEATRKIREAMKNRRKKSDKLHAADDDDD
EDSEEEGYDEARKKNI
>Mb|QLI66889.1
MAAPLVTVSESKDLRGLNLIAAHSHIRGLGVDATSLEPRAASQGLVGQEK
ARKAAAVILQMIKDGKIAGRAVLIAGPPSTGKTAIAMGMAQSLGPDVPFT
SLASSEIFSLEMSKTEALTQAFRKSIGVRIKEESEVMEGEVVEIQIDRSV
MGSTKQGKLTIKTTDMEAVYDMGSKMIDAMTKERVMAGDIISIDKSSGKI
TKLGRSYARSRDYDAMGVDTKFLSCPDGELQKRKEAVHTVTLHEIDVINS
RTQGFLALFSGDTGEIRSEIRDQINTKVGEWKEEGKAEIVPGVLFIDEVH
MLDIECFSYINRALEDDLSPIVIMASNRGNSRIRGTNYRSPHGLPIDFLD
RVVIINTHPYASEEIKQILSIRAQEEEVDVSPDALALLTKIGHEAGLRYA
SNLISTSQLVSAKRRAKQVSVEDVQRSFQLFYDPARSVKFVADSEKRLIG
NDGAVDLSVTNGSAAATERMDLS
>Mb|QLI66894.1
MALIVDKHRPRSLDALTYHPELSHRLQSLAQSGDFPHLLVYGPSGAGKKT
RIVATLKELYGPGVEKIKIDSRVFQTTSNRKLEFNIVTSVYHLEITPSDV
GNYDRVVVQDLLKEVAQTQQVDQSAKQRFKVVVINEADQLSRDAQAALRR
TMEKYSPNLRLILLANSTANIIAPIRSRTLLVRVAAPTHEEICSVLAMSA
KKEGWGVVKGLHQRIAMESGRNLRRALLMYEAVHAQNEKVSEDTPIPPAD
WEALIGQIAKEIMDEHTPARILQVRAKFYDLLTHCIPPTTILKTLTFKLL
NLIDDGLKGEVIQWSAFYEHRIKTGTKVIFHLEAFVAKFMRILEMYLMSM
EM
>Mb|QLI66895.1
MLIPKADRKKIHEYLFREGVLVAQKDFNLPKHPDIDTKNLFVIKACQSLN
SRGYVKTQFSWQYYYYTLTPEGLDYLREWLHLPAEIVPATHIKQQRSHAP
PRGMLGEEERQRRPFGRGRGGDRGDREGGYRRRDAGEGKEGGAPGEFAPQ
L
>Mb|QLI66896.1
MTEYWKASPNYWCKHCAVFVRDTKLERQNHEATAKHQNAIKRSLRDLHRN
HEREERDKERARREIDRLNGVVTTSSTGPLRLGRASAPGSSSNESSMKKQ
REQLAEMGVAVPEDFRGEMAIPGDWTVTSTRVIQSAGDGGANDEKKADKV
ATGVRKRDEKEEDKEEEEAVKGLFKKPRRWGRDSRKMPEGEDEELDALLS
GTVKLKKAEGGDVKSEDQDEVKAEEDAVKMEPETRASVLGGEVKEEESEA
RVKEEGDGDAGITPVVFKKRKPKSLRTK
>Mb|QLI66899.1
MREIVHLQTGQCGNQIGAAFWQTISGEHGLDSNGVYNGTSELQLERMSVY
FNEASGNKYVPRAVLVDLEPGTMDAVRAGPFGQLFRPDNFVFGQSGAGNN
WAKGHYTEGAELVDNVLDVVRREAEGCDCLQGFQITHSLGGGTGAGMGTL
LISKIREEFPDRMMATFSVVPSPKVSDTVVEPYNATLSVHQLVENSDETF
CIDNEALYDICMRTLKLSNPSYGDLNYLVSAVMSGVTTCLRFPGQLNSDL
RKLAVNMVPFPRLHFFMVGFAPLTSRGAHSFRAVSVPELTQQMFDPKNMM
AASDFRNGRYLTCSAIFRGKVAMKEVEDQMRNVQNKNSSYFVEWIPNNIQ
TALCAIPPRGLKMSSTFIGNSTSIQELFKRVGEQFTAMFRRKAFLHWYTG
EGMDEMEFTEAESNMNDLVSEYQQYQDAGVDEEEEEYDEEAPVEEPLE
>Mb|QLI66900.1
MAEYNMDESAIMDSGSPGGGPSERLGSRHRDSAYDRSPLANKTRSLSDGG
GTPSKLSPSPLIVEASRRTSKDDTHIKDAQPPSTPRHSDFRGLSLQMAPR

QFTPPPASNAYVKPPPLSPKLDHSQIYASPTNILPRRSRGLDFSRAATSL
HHSTLAESSPDSSPTIGDRAINIPDRGNEYGGPEQTSSSLWSIMGNHEKM
NISSSLGSTNQVAVGSDSSSSSDDDDLMDEDMDEPYLTTPQVSKTTNIGS
TGPFGSPAMSSLMNFQQRQRHRKHPKKKVRGPLGLGFNLAAAALSKSPPS
NGAKARRESISWQANQLHISAADGYESGKSQSDADGNGGGEGSQRGVIKR
AVTRRGNLLPKTKTFARIRAALFEEVAPAEAETRREAEVVKQVRESDVAL
EPRVSQAPAESGPAASVLSSPNLTNNEPVDDQEDDVNMLSDLAPGLSSSF
KQQAIKNSKGKTFWDTFSESSSMGGTRTTPPPAAFLPRGSSSGMSEDNGI
DSPVPGLASAHSASQSSNAQGPSAAEITRRINNKRRRDDDFDPVSFKRRA
VSPGMSAHNSPILQSPMQRDSAPWGSRPGSTGGDKAGSSAQSETGSVSGG
TPANPAAGTTGRLNSKGRIGFQGMVDTNDGIMRMSIE
>Mb|QLI66901.1
MARRTEQRMEFPYGTPQSGITPQQQQQHHPTPVQPPMNVPQPPNASAAQ
QQAATPTPAQSRQRKRPAPNTAPSPATVTPAAPVAGAGSQVQPPAPTPPT
PQTAPSTLQPQAATEDAKTTPVQPPPAKKSRTNTPWTPQEELRLKQMRDS
GNSWAEIAKTFPTRTEGSVKKHWYKDMHYAEFAEDESQALLNAIKEYENN
KWKVIGQKVGKPAKACEQYAKEHFPDLFANGKGR
>Mb|QLI66902.1
MASVAAAAPAGTGTSASHPYTCNTCQVAYRNIDLQKGHMKSDWHRYNLKR
RVASLPPISSEVFTEKVLQARATSSAEAEKAYFEAKCEPCNKTYYSENAY
QNHLLSQKHKSNETAAGGPRRRHDDEATSVISSTFSLGEPTSVAKDELDT
DAEDEFNQVIESLQNAKVSAEQRPSPVSRPSNPKPTAPGASKNGDGEEDS
ESTTPSHSVAEPTWTLNSCIFCNFESPSLPLSVQHMERFHGMFIPERPYL
ADLQGLVKQLQRKVSEYHECLSCGKVKSTVFGVQTHMRDKGHCKIPFSTE
EEQLAIGDFYDFRSTYSDNEEEDEDDGSDTEEERRGGAKLGSRRKVRLVG
EDGEELDGEEDGEGWETDSSASSLDSADLTAVPAEGHIHQYERLNKHPHH
SSRDPRNHHQADGWHSRAHKHTHAAFYDDYELHLPSGKSVGHRSLNKYFR
QNLNNYPTPEERAERLAIEGPESGEESHKKDRSIILRNGQRFKRDVVPRG
TVGLANVSDEKRRAVRKSEHRGRDLEQFTTKRTDWMYGKRNNNQKHYYYR
FDGGG
>Mb|QLI66903.1
MPALPSSAAPRIISRALRSSRHSPRWTRQLSTHTARRSPSLLRPGFHINV
PSRPVVPRRSALPSLGIGGTRSIFIQTENTPNPDALKFLPNHRVVPEEIN
SPFIEYLNPRSTISPPYPSPLAAKLMNIDGITSVFYGADFITVTKAGDAN
WAHVRPEIFALITEAITSGETIVSIAERKEGDEDIEEDSLAYNENDSEVV
GMIKELLETRIRPAIQEDGGDIEFRGFDDGYVKLKLRGACRTCDSSTVTL
KNGIEGMLMHYIEEVKGVHQILDQEEEIAMEEFAKFEEKLKHQKAAQ
>Mb|QLI66905.1
MCLLLQQQQQQQQQQQQQQQQQQQQQQQQQQQQQQQQVQQQQSHFNRMTGNH
SAGGTGGNMGGAHAQDSGHENISEDNRRTMAYIADLLNENTREAALLELS
KKREQVPELALILWHSFGEHLSKAEMTDARVMTSLLQEIISVYTLLNPSQ
LTAAASNRVCNALALLQCVASHNDTRTLFLNAHIPLFLYPFLNTTSKSRP
FEYLRLTSLGVIGALVKNDSSDVINFLLTTEIIPLCLRIMETGSELSKTV
AIFIVQKILLDDNGLNYICATYERFYAVGTVLSNMVAQLVESQTARLLKH
VVRCFLRLSDNARAREALRQCLPEPLRDATFSSVLRDDAATKRCLAQLLI
NLSDNVVDNSQGVSNM
>Mb|QLI66907.1
MFTSALKSFSATNITANYSTSEHPTSSAGPWKIYDATKKSTGNAYSVFVF
DKRTLSNGNSLGRSGAAGFKRTVDEVVERLKKDASNLAKLRHPSILELVE
PVEELRNGVLQFVTEPVSASLSSVLQEKDDQERSGGRSSRFVTEDASGVK
KRREIEIDELEIQKGLLQVSQALEFLHENAGLVHCNLTPDAVLINAKSDW
KISGLSFCSPADNSTKPTSIQGISLGEVLNIDPRLPRHVQLNLDYTSPDF
VMDNNLTPSADMYSLGLLAIALYNSPHKSPLDSHGSVSSYKRLFSSGSTT
PSASNGFLSSRPLPKDLSSHVLPRLIARRPAQRMTAREFQESEYFDNVLV
STIRFLDSFPAKTPNEKSQFMKGLNKVLPSFPKSVMEKKILPALLEELKD
RDLLSLILQNVFKIIDLLPSARRAFGDKVRPALKEIFVVNAKQGQEKDPA
RDAGLMIFLENLSLAADNSSGKEFKDDILPVILAAIECPTPSIIDAALRT
LPSVLPVLDFSTIKNELFPVVATVFSKTNSLAIKVRGLQAFVILCGGSTD

AAADDGLNGLVENKKTSSSSALDKYTMQEKIVPLIKAIKTKEPAVMMATL
NALRIVGENADADFVAMDILPILWSMSLGPLLDLRQFQTFMELIKTLSRR
VEDEQTKKLQELSGTANTGTARPNEDFLAFGGVTGTTFDSGTGGNEDDFE
ALVKGKGGMSASSNGNNGFPSWDKTPSVMSPSTTTIGSSSSTPKTPSFSW
STQNPTSPSTTTTTTMTQPAMGSGQPGFRTVTPDLARFSTLAPSSTQYSR
PLQPTQPSTQQPSFQQAPQQTPSSINWSATAAATSNPWASSTSNYSNYSS
HTPTQSGSSFGGMNTSMSNMSLGQQARPSLGGSDSRNSSFSLPPPPGSTS
SPNTSFSIKPPPAPGSWGAPTTQKPMAPMGNSGQQMNNNAGGKKSSGLDK
YESLI
>Mb|QLI66909.1
MSAPGSGFLGRSSSSNANMRGLVQFIADLRNARARELEEKRINKELANIR
QKFKDGNLSGYHKKKYVCKLLYIYILGWNVDFGHLEAVNLISAQKYSEKQ
IGYLAMTLFLHEKHELLHLVVNSIRKDLLDHNELFNCLALHAIANVGGRE
MGEALSSEVHRLLISPTSKSFVKKKAALTLLRLYRKYPDIVSPQWAERII
HLMDDVDLGVALSVTSLVMALAQDNLDGYKGAYAKAAARLKRIIIDGEYT
IDYLYYKVPCPWLQIKLLRLLQYFPPSEDTHVRNMIRESLQRILNLAMEA
NKNVQQNNAQNAVLFEAINLIIHLDTEHALMKQISSRLGRFIQSRETNVR
YLGLEAMTHLAARTETLIPIKQHQDIILGSLKDRDISVRRKGLDLLYSMC
DATNARIIVGELLHFLQNADFAIREEMVLKIAILTEKYATDVQWYVDISL
RLIAMAGDHVSDEVWQRVIQIVTNNEELQVYAAQTALQYVKSDHCHETLV
KIGAYILGEFGHLVADQPRCSPIEQFMALQGKLSGCSPSTRAMILSCFIK
FVNLFPEIKPQLLHTFEVYSHTLDSEMQQRACEYLTLASMPTDDLLRTVC
DEMPPFPERESALLSRLHQKHANTSDRRTWVVGGKDANSDGTELGMAKPG
GLKRTFSSAAAINGGRANGSANGANDLAGLNMNAPSPSEPKMLKAPNLAS
AAHLSPGWEKGFDRLMVRSDGILYEDGQVQIGVRSECRGQAVCIIAYFSN
KSPSPMTSFTTTLDLDESEKSKLTWDVRELPDSTIAPGGQTKQVTVFESK
KIFDKSPTVRISYLAGALQAVTVKLPVAAHKFMDPADLSAEDFFKRWKQI
GAGSREAQAIFGLTSGKSNTRELTEKFVVDTVEGLRWRVLDMVDPNPKNI
VGASVLHTSEGGKFGCLLRLEPNYSSQMIRLTIRATDDSVPAIMLKLTQE
RLAQGVSTIPEHREPPTRQEISEAFANTMVT
>Mb|QLI66910.1
MAPSTNQLEEALINGTYHVFTAEPDATTVNKVRKHVEESMGLEEGFFTSD
DWKQKSKALIKQYVDKLLDGWLPESKPERQSENRTKRDASEGSPSSPKRQ
KHAAQNGQHTTQDEEPHGSDKEAKSKSKNVTSRRQSRTVDSAKDSSDNLE
PPLPKKRKQKGNGASGKAQKGSPSIGEEDDSKDDAMSSIEVEVSNSVVNE
EEEYSDVIDEGPKPKRKKNQRRDSSPKPAKPKVKKTTESTPDDPDEAEIK
KLQSQLVKCGIRKLWHNELKKYGDDARAKIRHLKKMLTEVGMDGRFSEAK
AREIRETRELLAEAEAAQEMNRLWGMGTGGRASRSKNKSTKVEESGGSEA
EDAGGNGSTDDKDEEEGTSFAARRRRAQADLAFLGDDSGSDE
>Mb|QLI66911.1
MFGGGSNSPGLKTESESKAETKPPSPGSSPKANPPAPIKTAITSPASGEK
RSGNGSPRYNDTGGSTDKKRRSSGVGSKASSFLASAKNTLNFSQVGRGNS
DLSAQTPLQKLGKQDPALAVPQGQHNNSAGESLPGPKSTFRVGVWEDRNK
KCRRTMEDTHAFLYNFLATPQPNSDAPSKAKSEKNDSEGTGNEMLESDNG
YFAIFDGHAGTFAADWCGKKLHLILEDVIRKNPNVAVPELLDQTFTTVDT
QLEKLPLKNSGCTAAIAVLRWEDRVSNEKPAAGATKQTPKTNEANNPDKD
KPSSSPRGRTASASSTDTQLKPKLPTTRQRILYTANVGDARIILCRGGKA
LRLSYDHKGSDENEGKRIANAGGLILNNRVNGVLAVTRALGDAYMKELVT
SHPYTTETVIQSDTDEFIIIACDGLWDVCSDQEAVDLVRDVQDPIAASKQ
LVDHALSRFSTDNLSCMIVRFDKTSTVAQGVSTSESEGGSDRIAAARVSE
AEKIVSDTKQAIAEGTVPAVGVSASNSGRGYDTIPREGGEFIVTTLSEPV
AEETESAVAEDDSPEVSTKGKDAPEAAEAATKPDEGPEK
>Mb|QLI66915.1
MGESSPTRPKKFNTELTDYSLPRHQQDGPYADNLDLDVIVIGAGFAGIFM
LKTLRDRGFKSVIFEAGSDLGGTWRWNSYPGAGVDSEVPEYEFSWPEVWK
TWNWSSNYPDYRDLRAYFDHVDKTIGVKKDCSFNTVVVGASFDTAAGKWS
VRTEDGRTAKAKFLVLGTGFAARRYIPDWPGMDKFKGVIHHTSFWPEEDV
DVKNERCAVIGTGASGVQVVQAWGPVAGEVKVFQRTPNLALPMRRRKMTP

EEQERKRALYPELFTLRETSFSGFYFDWYEKNTFDDTPEGREAVYEKAWR
DGGFKFWLGIYKDNLFDGEANKESYRFWAKKVRARIDDARKRDLLAPETM
PHYFGIKRPCLENDYYEQFNRPSVDVVDISNNGIKEFTETGITLDDGTHH
EFDVIAVATGFDIVSGAMTQLGLQSINGTNLQQEWASGISTYLGISVSGY
PNMFHLYGPHGPTLLSNGPTTVEVQGRWIADMLDKIQRQDIKYVNPKREA
AREWTRHVVELNDKTLIPTTKSTYMGGNVPGKVYEPMCYAAGVASYRAEI
RAALDSMQGFEVVSNRIAVNPRL
>Mb|QLI66916.1
MTEPLSKVDSAVQGLSSSPPKEKGHRRASSSAAGVMNINDLEKDGIELKI
AIETQKTGWKINKSPTTVEDSSVLKVMLTKPPVKKIDLHFPLGMEVTARN
MKGVTIKDAMDAIYKAYKKRADDELDNPYLAGFEWDKEESWTRLVVHLQK
DAGTSGHGGGKKKKGKKDDE
>Mb|QLI66917.1
MASAAHCLMCFEALDAHLEDREPLSLSEIESSWALYTSSVSDSEPGPSRN
DQAPKTPALRRLAADADSSASSSSASPAWSSSTPATSMSSLSLGTSSAPL
FVTWNTVSGDDVSLRGCIGTFESQPLGVGLPEYATISALHDTRFSPIVKE
ELPELQAAVTLLTDFEEADDAYDWEVGTHGIRLSFTDRGHRYGATYLPDV
ASEQGWTQDETLYSLIRKAGWMGGRSRWKSLELKVTRYQGKKASLNYPEY
KKWKDWVVSKQ
>Mb|QLI66919.1
METYHGFVRTPGDAIKLFEACRLGLLPRVQRRLSEKERQSIRSGSVFVWD
EREAGMRRWTDGKSWSASRVSGSFLTYREMEGKRGGGFGSGRRGAGKTPD
SGRGSDEDHDDGEPEGYRYKADGLMKQSFSIITSTGQHLHLISYYSRPQP
GQPELPQPSSDPSLRGIAPPKGMYPESSMGETNQTPALTRAPMQQPYMIA
PHHAHAHQQAYVHYAQQGYGWPPSPAATPPYAHYAPAPFPPGTHLHSLHP
PHMTQVSSQLPIAYAASHYTHAPPPPHYDRPSLPPLPQSKPASIPAYQRG
ASPPRLQDSPGHRERLGSHGPLPALNSVTAPIPHPGMTPPTRNHSVSPPR
SSHESGLPSGFPSGHRSSLSALLHPASDSSSSKPASNGSSSSSPRISNAA
LEKCGVSEDARALRMLDKKFCI
>Mb|QLI66920.1
MAKKLSPTAFTKAVMRSFMADSGLEPRLLGPRLRIVSAQPGRVDFELDIH
KDHTNRLSTLHGGTLASLVDLGGSLAVASTGRFATGVSTDLNVTYLAPGG
KPGDLLRGTATCDKIGKTLAFTTVTFTNGKGQLAARGSHTKYVAGTMGPD
GPYVAPPEFSEDVD
>Mb|QLI66921.1
MPFLTPTDRQRRIIQLSQEWGVDLPPAPAPLTRPHNPPSFWTPKDDQVAE
DLMQRRASELSQSRRQSSLSKAFSSNNLKKGKTWDPREVLEVLSSWIANS
GSPGVAEALILKLSAAGVDFSGNQTKQKSGILSRRKSVDTFVDRSKFLKL
AVQGNQYEMLQVLLPHADGFSIDQALAPAIRTGNTRVAELLLRYGACASQ
TPEGQDAFRQACVVQSQSHIVGLLLRSESRPNAAWISACMCDAARSACLE
TVLYLSRSTADGNHNHAEALKLAIALGRQDIALAIIMGNHPPHGPGLDEA
FHNLYEHPSLDPATKLAMAELLLCAGAQGNALSQALESACETQFYDMASL
LARYGASIEYNDATMLKRAIARGELELVSSLLTDSAKLSPALASSCVPLI
AKQAPFETRVMVLSLLLRKGAKGVALDDMLIDAAEAGDIESVDLLLNPVY
PESTPITLKDSNHTRLSNGIPRHEVASVDHRSGEALRTAVLRADAAMTDK
ILSAQPTAETLSIVFPMTKKLSPKDRYRMAQVFLKRSLSGPCLHAALHDA
INEDASQRDNALIKLLLEHDADINFSHGPGLAAVIKQKDIKLLGSLLHKA
SPQTAAARVMDAMEVSDHRERFEMMSMLINAGAVIGAQEIAAALSQSLSE
KPVDMSLLRLLLQQGGADVNLLEGAIAKKAVSNPDPKVLDMVFANSRPSS
TTVTCAFNEFAPLPSTDSKAWKLRAILSKSKHKEDLNWVLVHEVQSLLKT
NTSKASLSTLKILLDSGADPNAYKAAALCHAVIGANVKITDMLLESKTPL
SAAALGTALPHVLRITDPMERLTLTKKLVDAGAHPLEINRALVHAITAYP
EDVSLQNALAAAADTSDGEALSLSISKESPETMDLLLSKSQSSLETRSTM
VVKAMEVKNRTARHHMCKNLLRLGVSTETASSALLVAARDGDVNLGDLLM
AHGASIATNNGQAIIEACRGGSAEVLSVLLKQDGQMNKSIMEAGFQAATE
VRDLSKRAVVFEQLLKRGVAGDLVDAQLQSAARYGEDGEAVLRVLLAAGA
DPNFNNGECVVAATRSAFIGSLELLLGLWGEGGHQKKVSQPTLSRSLKAC
WNLSRDSRFQVVNDLIKAGLRPTDDMHIALNEAINEDDPEERLVRLLLNH

GASPAANGCKTLVDAVNNSASSSLALLLEKNLPQEDISRAFNQAFTTETF
AKWFTDRGYETANLLLDKGAHGASLSGALVLAMRNSAVETEMLADKFVTL
LIKHGADVNYNNGEPLQQAASKANLSWTRQLLECRPTIPTLSLAFQCIFD
TALSQDEVLDLFKMFAEYRDGDVRIDVLSSQQGLEPVLVRAISQYPRSTT
ILETLLDAGLYHDQATTYKIHADVDKEEAMTLLIWAISQPQKRVSTSVIE
LLLARGAKVNAETSLSRTTPLMLAIQTRRPDLVKILLLEGAEVDILDYLG
RTPLSMATDIGGDISVQIMSLLLAADPSRDDGSLHNAARDLNLPVVKVLV
QSGHDPDFPSPLHEGRSALAEVCLHGSSKGEMSADRERTMQKVMTFLIDN
KSDLSLKANDKTLLQLCFAASDPVATTRCFLKSGMWKLINKPFNFYTSEG
YTYSPTMYIKKILPQTDASDALLKILKANRASDVYYAVSGAQPPDAIGLP
EDMAVQERARKARLERLAEETQDFSIVMARKREIASVEQQILAQKAEMED
MRRRKLHNEDIAAVRSKAQVEESLASAAHTRRLQEHHSLAESSISRSRAL
AASELETEEARQRKALEWETRLNTERVDNARALSSIRISERQEVERIDKG
AETRIKGRLEAQRKLVESQEKLAKRLADGPSAGINDPRRQIGYVTELS
>Mb|QLI66923.1
MSLSHAEASQSLDLRVEDYLDDKLQSIADLDTLDELLVNVETQRNQLQSQ
LDDAVRELEKARRTTEDRSDSLQQQIDEFNELQASIDTRVKIAAASDAPS
EAIARLQRPMKKLQHVELAQKYLILLQDVERLRAEARSYLPDSPKAALEP
YAKLKGLALKLRSLSGNEELHLVDHVQAVTESLWNEMKKTMSAELEGVLE
KRHWPKVDPHAEMDDEWIACVEKLIDLQMPEITRSSSTLPLLPFNVMTAI
FVAEFRFHFMSDKPTSNPQSIGTHCFPWFLTMIEKWEDFFRNNLGHLLAT
KFHETPVAENTVYLDPVSALITAMLPVMREKVHAVANEAIKNPPFLSSFI
SQLMTFDENIRQRFSYDDGDDDKGWNGLTGEILDHHFDAWFRVEREFALE
RFEKIIESADGRKIDYDYSVTGKMKPTFAAVRITDLLRVVTGKYERLRKL
KHKTKFLTDIQLDILDGYHDRLRGSLEAYQSITSTLGRTLHGASKEQLAA
LEGIGALETLCKVIGSSDHIANTLTEWSDEEFFVVLWEDLQTRDAQRSKR
NSAASGIAPDSILGRVPSHLSDNGSSDGGIFDETVSAYSSRRKAAEQLLV
NALVDSHAKAFRAYASKVQWTTVGDAAVLDDPSQFSITPELDEPLRILQR
NFDYLTKALSTASFRRVWHEALDKLQDLLWTSVLLKQSFTTLGAAQFAHD
GGAVFSLVERYIPGGSGALDSLREGMQLLNLATAAAADSAEGSAPGLTLK
EASDRVFTDNDEARKVLEELGLHVLTPVNARYILQRRVENNENIGW
>Mb|QLI66925.1
MDRPVHDYSQTGLPSPYPSSFGDNHSEGSAADHASGAQYPVKQEVSYPTS
ATPTSEYGVYPQSSRSGTFQEHIQRSYHPATGTGSGGMAQQQNSPSFTQQ
DGRNHQAHTVHSDTGVPIDPSIAAPSPTYPYGQHSPYAANPDMTHNYSHA
NSGIYAQPRPDWTGYSQHSQIASGHPVYSPSQASQQAQQRPNQVYSFVPI
PGAQQHKRPRRRYEEIERMYKCGWNGCEKAYGTLNHLNAHVTMQSHGQKR
TPEEFKEIRKEWKQRKKEEEAQRKAEEERHRAAAAAAQAHNGGPDQGPDV
PPTSTYPGSRPVQLPPIGYQPNQYPAPPSGAVPQQQLADYGAGHMYPNYQ
PHSPYGQPTQSMYNQSNGGPPGSH
>Mb|QLI66928.1
MSSSTAPSATSSPATDAPDEPQTDGSKLRTFLGILKKFIGVSDLASVRFS
LPSQLLEPTPNLEYWTYLDAPNAFIAIGTSEEPLDRMLEVIRFWLTKDLK
YAKGKPCKPYNSCLGEFFRCNWETEDNAPRIETAELNGCVSGSEKGSLRS
LKVPPKSDKNESTVSLSVPKHGASTPDKKPIRISYLTEQTSHHPPVSAFH
ITCPERGLTARGFDQISAKFTGTSVKVLPGEHNMGIFITLDKHGGETYQL
THPAAHLGGLLRGALSVSVSEMAYITCPETKLKCILHYVEEGWLGRTTNK
IDGVVFRYDPENDTKTRVQDVPDEDILIRLSGPWREKVVFSLGPKPMKSV
SPEHQYTIIDIAPLHVASKILPPEDQQLPNESLTLWGGVTKAIHAKQFSK
ATDLKVELEEKQREKAREREKNKETWQPVFFEHVVGNGGKPDLTDKGKQV
LERAQQGDWSMEGIL
>Mb|QLI66932.1
MTQLSDYAGPTPGKGEDNGESIEERCFSAALICLEGHFTQSPPMGPQTAA
RVNSMISKTLEKTHTLRTAREALSRNVAIWIWLTRIFAAAIPSLTTRSVG
PLSSLNDPDRGVSPQESTALIVMNHASIKEDLGTLIKLMHIARNLLVNAE
PEVPQDICAAVHFDQMVYQTIILCVNVTSKGYDGEILDETQRLKLADITE
LYKKLLVTSLQQAHNWTAKHDRNKMSFWFDVLFDEDEGAFGGNEDGLGDG
SGFRPDVAKQQVQHWLDRNSRMCDAARKLLKDYAQNHADKPPGNLAPIRP

LAWNWLPEDSVNMPGDLEDSNEKMNPVWKPDETDNFEQDRVYGRVSREID
TWWLRARDPNYEQWVVSMPSVELAQSRTEHCKTNLIHRYAHSYRGDHSPP
PEAADDELSDHEHCHHCPECHECHHHHDDEEDEEHEHEHDDEHGCEHSDH
DHDHDHGHEHDHDPECEHHHHHHHHHHHHHHDHDHEHEHDHDHDYVHDYA
DDMIDDEDADDDESYGDGPLTGLLTEVPNILDPKQIEALHMIVKSCILDN
AGSGLTRAGENLQKTRCRMFLALDCGKCLLRELLVFIAVWNKDEQSLIFQ
VTTQIVEALHHSALIPYAWNWLRIPKDIISPAQTVLLRLVNHMFRARVNN
NTTANNSNNNPAAANTSASTTTARDVPSHDKDNSRDIKLVHFFFSYFRSR
IVPECAALMHLQAQIREDKCDAAEFPVDTWDMERAKDGLGQYLDFLTTVA
EMMDTRAKLIEWEAIYDLITILSGLEAGVAKKPLIELPKRSPSKPGSSSP
STVSYPTPNTTTNPMVERPYATEAGSLPPSPPPPDMEEQAHKYPWPCVKG
QIFTILATLLQPPPGQSSPGNSQVQSQMVKYNGIVPLLNSCAYDDHNPFA
KERVTICLKWLLDGCEAANNFFRELVSMAPQPNLKPPPAGTAVSTLRVDG
IQGDIKVQVRSRTAPPVGRAGVVEEVEPYEKDLLQRVAGMSLNSAPATSR
GSIEDDFMA
>Mb|QLI66933.1
MAAAQGDTPTAPAAPSQSKSVVAHLQDWGSSSLPPALLATLVTALHARPL
QKLPLFLFTPPLLFSSYLNLSGYPTGSAGLTAAWSGLYALLALRRRQPLR
SKFSARGLVRGTAIGLGAANAVAGGWVYFGGDFAKDEEERTRRNRWAPKD
D
>Mb|QLI66935.1
MSSPWKKQPEDVVDSVLKRAEVSKIARRLQNRLALAQFKTKHGWEDLTLD
SIEPKLEEEMRRRRLCDGDVLSDSSSSASDLPYPTKALMSSPLKAPLFSD
AIGSSNGSSGHRKRTYFASFDHTASSPSKRFRASPTAHKSFSGHSTWKDS
HELAQSSPIKPRRQQHFTTSGGPDVSFFQQRRMTDALTSPNFAAPSDDDD
DLLPAHSFTVNNVRSSPPRTPPLQTRGLASKRSKETLDAGNGGKTGEEGA
DLLLYLAASPSPAVKSNRGRMERLSTPPPKNSKLDLPSSMMTTPGGGNLF
PNTPGQGFDFADFVNITPSPAQKPWRTPGPMSARTPLSVTRRRLTFEEPL
A
>Mb|QLI66939.1
MESSSTPLADYFWIAGVESVSYDDPAATNTFTNSLPVESTIAEDGEPDDT
ATNGYKPTTARHSRQNSASRLSKISVDGRYSIASTLDELDGNTRSNRSSA
TIKPVKPAGLNGNAAAMNGISPEVLIQGPTPGPGLMGMPDFDFDKALLKF
AAERENFLEDLSFTAGAKLQSRPPMINPRAERIRADEGLPSGRMSPLRSL
KGSIRRKISFREMNSVRKGPTVVRQGASSRAASVRTTKRLSNYNSVIPPP
EPLNTDPDMHPLKRRFEPVLLDRYPPKNANDELAKRGKFPDYVPMFAFPN
DIQIVSSDDRPRSTWHGFTMTGEDNAKLYGITVIIWTALTAERAEEVERR
CEQWRQNHMSNEERELAASLGVRLAGERSHLSQLLSKLPTIPSGSPARDR
LEDEISTVEEKIALMTDMLRPLRHGAASKIEGLTAGESGLWIPRAYGVLG
RDAACMGFWKEWLRSIVVPMTDGGVLRVPPSSPKVGRWQPLERYVVNLCT
EAFSPLGSKTQVELGVRELRLYARKEGANELPGCRTIDLYALFRCLSLEN
VVALFEYAMSESRIIFLSSHTSMLHLACHALVNLLYPLQWASIFIPVLPA
RLISALEAPCPYIVGVERRYERIDLPDDDYVLVDLDKDTIDATSQPHRLP
RQHRRKLMSLLQVAAPHTLRYGVATGPPPYAIESFPYDSFSSENGSVFTS
TAEPTTLGKWVSQNSSSFADRTPPEDVHPPVFNAFTVSSVDTGKLDHRPS
TSKSSKTSPQSSVSPASANFPPMPTTPISRSDSGFALTATLREKRSGHFG
EEKGRRSSSFGLDKFQPMHKPSLPLLNGHQPNASISGFSVVSESSFGGGY
APSAYAQSTLAASTIMPCMQIQPVRNTETTVWVEGHCFSLNTKDSSSSCT
ICDDKSEGDGFYECTSCKTVAHGRCLGQVSLVCPEAFHPDRVRAAFVRCL
ASLLYTYRKHLGRPNKQQKSNGQLYAFDMDGFIRSLPYDQQEYATMMRDT
QAFNEFIHSRETTPATDPSIRLFDEILMAKKARGRPGLSSGLSRLSTIRQ
SHGASVSAFGLAAASRTSSSKAPSYLTNTSDHIWRTASVPLPKGNFPGEY
RSVITRIPSCLDRTLMREPRAIQGVPRMEQRGTRAFVRKQVPSMLGTTPP
S
>Mb|QLI66940.1
MPESAPKSSKFRPLLPAPPSDDGSIASSSRRLAKQSSPVTSACIECRKRK
TKCSGSRPTCTACIRRHVECIYDRGPDETQAKVVKRKYEELKSHSTPYER
IFDAIRSRSEADVGAIVRKIRRGENVDDIARQVEHGDLLLQSFLVPEARY

RYEFPLSSNLPPHLLLQSNVYLKSLIYKWAPPLSLGDAAESGPSSSHQQP
ENAFSEAEAPYLMPYHAAKIINTRLSSIAPSKWTSVSSDDLLMRRLLSAF
FLQDHDWWTAFQKDYFLEDMSKMHDELCSSLLVNALLALSCNYYRGLDGR
TEFWNPQTLGYRFLAEAKRLWEYEKGLDKLTTIQAALVFHIIYSGTGADK
IGYAYTVQACAIAHKIDLFNPTSVTENTRLRDARHYTAWALFSWQSLMSF
HYFRPPLFNRPPASLPDPKHEPEWYGEIWLKYPLGRTLFPSSSGDVFKAR
AEFAVIQNEAAQRLFAGSTPAPQLSAAEVAVFYFRFKGWYDSLPEALSPR
RAVLPSHLKLHMHYFQILLRLFQSPSLINEPGSEADAAVVNLFGQPPQEL
LSYAKVSLETLLRAYYLRHGFEWLDTYLLSLMLDVAFIAIEGMKVQDSDA
DLSALRSTIILLAKGIYEQGQNLFLGKLVFNVVRGKMRSEDLDTLGQLAR
IEKIGEKEAIGLQQAQMEWPVQITSMADDPGSKKIGDLSKQLMETSLELP
GEDITL
>Mb|QLI66941.1
MSSPDPLNDSTMAELASVPPSSVARRVTRSQSSQRFLALGSSPRKQMFEL
QVGDRMSPQRLLVTVETDGQQEDISIGSNAKRKLFQSPISTLSTPARRLR
GRTTTTTVPLRESIEEDGDDATTPKARGRQRKSNGTPMPSAGTKRRAGTP
VRRTPRRLRTLASTDGEEPSSEASEEANVQTTPTPRRRNLRPRKTSRPAP
SSELGTDITPRPSTARRGRRRQALAPDELLELADEVGDINLDTTQLPPV
TSEDEVDLVRAPSESTDDGSLNAPDPAPAPPPAPAVPSSPSPNFAPAVAE
PDSDIWIGSASNEATQRASARTRKDVIPESSPRFPLTTYTEPQLDRFETS
QEGYYGDYGYADAGAAASDRSSIDEPLEDEDPAATAAPAGYDTIAQGEDF
SMIFMDSIPSLQAEFNSSVHPVVHEELGDETNLIINNTLESLRQADRDDP
EEDNDVVDTIEPAEPQPGEELAMEEPLVERELVGEEVPERSATPILQSPS
PNRPSPRWSRSPRKSTNSSPLRHRVLKQNALQTAESPAQTKQTDKLLSSP
WSDRRLRSADRLEEEVSKSYEDSFSEIPEAVLTAATPRRPQASVSYDDLE
DEIDQVRMVDEDDDAVAPDEDQQQVDEPVKMTEAAIGQGEEHIRSFEPPQ
QIMEQQVDESQEVDEPEGEGVEEFGEDDQTEEIGVELDHDHDEEVTSRQD
MLQESESEEEVGEYDLVLDEKLKLHPESVPEQVQALPLKANTNERRDGEY
EYDEDELDDAELAELETEQGSAVMDQTADADEDEELDRTEDMVASSQLDE
EDQEDADEEMADEVNEDEDYEVVDEPALVAAAIASAALQSSPSRLPTPDD
TPPQAELQTADDVAGKSARGSRPPSSLRSSSISPGHFRETLRATVEQPQE
IEDLGEEEVPEVEEVSEEEEMPEEEEMPEEEEMPEEEEDQEMEEDQQMED
EVPVYEKNPEGELFVPEAQQEPSLDESTRRSVDATPPQQISSPVQEPASL
QHETSSDKMVRPHLSTIIRAGRVLQSITSDPPSPEGREKQLGSPFRSSGS
KDSWSGSRDSQTSRRMSGSPLQGRSAVPTRIEIDNQAQQETSDSALRSAR
YPSFLSPVRRSKEPSVERNRSDGSPSRHSSASSMRITPPSDGVMSWVERE
GPISPSLRGDNSLREAARLPELSAKIEEPRSAGQVDAPLVQATVAEPPRE
STPVVEEPEAEQDASDDETDIWELEAQRETPRSVRQRPFGKRVPTSSNRR
GAIPSPWTKRSVHRPAISRMISQAVPDLSHLTEEPSAIPDQPPQSSSPDE
YSRLAQSQKEEAEAKRAANASESAAKASKFDLSSFFSSPAAIPGMLAQKL
MPARTQSTVQPANPPMSMAGQAPPVVPTSSMFPQIPQKDFRPGSSNRSSI
FSPARRKQPSSPLELQEESPEQQQQQQQPKADAERPSSPTTPEQVPMPTI
AQKQNFTPRPGQASNSFFHSSSNSTAAVTPPRMQLSHADIHRWTQQTSNA
SEASSDFQRPLLRPLPPKNASPTKSSLRSPLKPRTPGRVVEFTSSVLSPA
EQAKARLDNRSAQSNSILSQDFFAPPPPQSIVQVDKENASMDDISMDDAP
PLTKPPPPEPLSQTTWSRRHWLLLDQLLQYRRSSPFPVNYTRGADNYLGK
TVKSHGEAMTLDRWHLDVVDAFKAEVGGWDADILAKRLFALILGEARRME
ARDRRVMFH
>Mb|QLI66942.1
MPHCYLQLVSSLTFLSLSFLFPFAHSGPTATEQVFHAIKRDNNPLGIDWS
PAPAPEDGPAFSARALRDTKYLPAQIGGIVAAYGVSLVLVAITLLSLAKK
RRQHIQAGNDEADFDGSKAVYDSDSPSASFPLNQFNPQSHLRSSVVPNFS
YPSPINTQFNDNTLPPLKPYIYPSPISSVGHPGVNPSVDQSVVAQDRKMA
QNQLEEMYKHVMEHEDAKQRGIVLDTPIYPNQQRTSTSDKSATTLSGRDR
AKPASLNLSAEHEGKTQSRTSSFFSSLRSPRKKPIKGVHISPPIMTPQSA
TFPRYESREMNTIPHRNYAPPPPPPIPTDQTAFGAQVRSSGAPPTPSMSP
GSVQSIDERINSQLGPSNYPNTARSEVEPESATSQTPLVGLPSSPKAGAT
FPSLPSSPKPGVRFQRANAPSAVRTGGNLPLRAYEGALASPSATPRTTKQ

TVFERRGPLSPTTGKTPMTAGAVPYSPYQPFTPLVPITPSLVTKEDRKRM
RRMVPKTPTTELVKSSEDMW
>Mb|QLI66943.1
MVAPVRVIPNAAKRATSLLRTIQYTHPPSCPCHSNPGYHKAPPSIVPRAR
EAGRRQYATPTSVNQGLKEYAFEMAASSIRFGPGVTQEVGMDFKNMGAKK
VCVVTDPTVDKLDAMRQVREGLTREGISFEVFSNVRIEPKDSSIKEAIEW
VRPYQPDAFLAVGGGSVMDTAKLMNLYLNYPDADFLDFVNAPLGKGRPID
KPLCPLIAVPTTAGTGSETTGTAIFDLVSKRAKTGVAHRNLKPTLGICDP
INTRTMPAAVKASSGLDVLCHSLESWTAIPYNERTPRPPNPIMRPAYQGA
NPISDVFSFHALRRTVKYLPRAVKDPDDFEAQSEMLLAATLAGVGFGNAG
VHLCHGMSYPISGQNPGYKHAGYDVPAPLIPHGVSVAVSAPAVFRFTAAS
NPQRHLEAAEAFGVDTSNVKLESAGEVLAEALTKFLADLGDQPAGLKELG
FGSQHIDALVEGTIPQARVLMLAPGLDKELQTEKEQLRRLFEDAMNH
>Mb|QLI66945.1
MRYALLALGAGNLAMAMFTNTTDGQETAGKAVSNTLPAVVGDYELLGCSA
ANDDFKSFVQVASTDHMDLDFCSASCHSKFMAVSGKDCFCGEKADAAQKL
DAGMCNSPCPGNQAQSCGGNGGGNGRRDGAAPMKAVSMYVRSQAAEAGGA
ITKTITTTKVATVTKCSSTVTNCPVGKPTYVVTKVTETCPRPTEAIEWHK
KKITCYGGYCAPEIPRQSEKQRVLCDGAKCHAESCYNKDWSSLVLCKGDD
CKWSTSDDDRWFEKKIVCFDSKCAWENCHGDECHKKFVCRGEECKHESCS
GDDCHKKFICDHKGDNCKLAPPCNGKDCPKPPPPCHDKCRKAQPPPPCNG
DSCRAVPCQGNDCVKPVRPTGISRGTGTGTSRNTGTGRSTGTGTIYPHPI
PTGPPIVAGAGNVVANVIGAAAGLLLVL
>Mb|QLI66947.1
MAFHGVPAMVSAAAEAWPSIANSLMGPWRSATLPTRRLKPTPISDSQAVG
PTASKSSLTQLPDFIRAANPSIYTLPPIPKGAVPMKIHNAVSNLSQIGLR
AANPVLRQPQFQPKGYVPRAQWRPLVSIDMPRFTFQGVTMQRRGFGTGFG
SSNGISRNQLATVEESANRNAGNANMQNAFYQLLLRANMPGILVERYQSG
RFATNAATEEAYKKALTALNAGTNTTSGPSAPSGYARGQWATQEQAIANA
AVAGSSYGSGAKGGKGEPIHVVVQESTRSLIFRWVKFFATFVVVTYLCFA
AVTILIETLSTFRRGPGAKTDSEVKAEKQTTRFSDVHGCDEAKEELQEVV
EFLKNPENFSDLGAKLPKGVLLVGPPGTGKTLLARAVAGEAGVPFFYMSG
SEFDEIFVGVGAKRVRELFAAAKAKSPAIIFIDELDAIGGKRNPRDQAHA
KQTLNQLLTELDGFDQDTKIIIMGATNLPNLLDKALTRPGRFDRHINVDL
PDVRGRLAILKHHAKKIKAAPDVDLDAIAARSPGRSGAELENMLNLAALR
ASRARATVVSKEDLDWAFDRVTMGAERKSMVVTEKEKEMTAYHEAGHALV
QLFEKDSSSRLYKVTILPKGPSLGHTAHVPQMDKYSYTAAEYMSNIRVLL
GGKMAEEMRYGDDKVTSGVSNDLEKATDLSFMMITNFGMSNVLGPVEYGR
RYENLSSETRAMIEGEVQKTLKKLYEDVRKLLTEKRKELDLLAQALVQYE
TLDKKEVELVIRGEKLPGRSIVPRGPLVLPIPSDTPQPPGLGNVPHPQPS
GSEAPPPAQADSSGVDS
>Mb|QLI66948.1
MYTNKISALLGAIAAIEQASALSLHRHGHKLQKKQDVVDWVTVFTTVYVT
EGAPAPEPTVQTNDGIVTSQHVVVTPTPKIDAPANFQALLSSSAPAPAPS
PTSLVTQVKPAPSSVSQVKPAPSSGSGDGSGSSTGAPFSGKRGLAYNDPN
LANLFGKECKSGACGWAYNWGDSPGSLDNSYSYIPMLWGNKMDFFDRWGT
SCSKANGAKAVFSFNEPDNVGQADMSAADAATNHVKYMNPCDGKALVGAP
AITNSNVPGEGRDWLKQFVSECETKGCKYDFCNVHWYSPAAQLDQFFEHI
EEAHVICGKKPIFVTEFAPTGSDSEIDDFMTKAIPKLDSLDYVYGYSYFM
IQPNSLLSSATSLSSVGKIFASA
>Mb|QLI66951.1
MDRTLGLLATLVVVIPVLYMYTVSVVQSRFPAVRNKRICLLIAHPDDEAM
FFAPTILALTRPETGNHVKILCLSTGNADGLGETRKKELVKSGMHLGLRD
EDDVFVVDNPADFPDSMTAKWDESRIATLLCSAFAPQLGRQRADDSAKPT
ANIDVLITFDGSGVSSHPNHISLFHGARAFVGALMRGKSGWACPVDLYTL
RSVNILRKYSAFLDVFATMASWAVGVRHEDKAHPGGLVFMNQLVGEGGLP
TAWGAMTQAHRSQMVWFRYFYIAFSRYMLINDLKLEKIKGR
>Mb|QLI66952.1

MPEFAENDSPESNAPTPEIAAGFKNDEKQPANSSRFVHPSGKSGHGLGMQ
ILRGIAFTVYFTFCCFAIVVTQTIGAPLYFINREWFYAYMAMTKQSFALT
ITVMTHIWGPALVRISGDESVAGQILATPGGGVEFKFPERMVMIANHQIY
TDWLYLWWVGYANRPGMHGHIYIILKESLKYIPFIGTGMMFYGFIYMSRK
MATDQPRLAYRLNKLKQKKIDPSGRAYFDPMWLLLFPEGTNLSRNGRKKS
SQWAEKNGLKDPDHVMLPRSTGIFFCLNELKDTVDYVYDCTVAYEGIPRG
KYGEEIFGLASTYFQGRPPKSVNLYWRRFRLADIPLNDQKEFDIWLRDQW
YKKDALMEEYLKKGRFPALTGGKTEYVETEVRTRHPWEILQVFTVMGTAG
LLWHNVRKFLSTIAAKVGTMV
>Mb|QLI66955.1
MAEQITSTPAPATATAAVKASADSGKPSAATPPAPSPSHPLGLADTDIFG
ETSEEMDQDRLKQIAAAGNAHFHRLGWKRLAVVTIVEAVALGALSLPAAY
HTLGMFPGVFLTITLGSIAIFTSWLVGQVKLKYPETASYADAGRLLLGRF
GYEVFGAALVLELVMVVGSHALTGSIALSDLNDGRTCSIVFSAVSAIILL
ILAIPPSFTEVAILGYIDFGSIVAAVGITIIATGIQARDGPGGLSGVGWS
AWPKEGVTFSQAFVAVSNIIFAFSFAIGQFSFMDEMHTPTDYMKSIWASG
LIQIAIYTLTGALCYAFIGPSVQSPALLSAGPLISKIAFGVALPVIFISG
SINSTVALRYIHGRMFKNSVLRYIKTPRGWASWIGLVTVFTIVAWVIAEA
IPIFSDLLSLASALFVSGFSFWIPAIMWFKLLCQGKWYSGRNLLISLGSI
VAFVIGAVTLVAGTYSTIVDIIKETNDGSAHSPFACRSS
>Mb|QLI66956.1
MSQGSHPTLINLPPPGSNPETPSEMPGTPTSTTTSLSALSTTAIKDGHRG
HFASGPLGRGHQHNPSTTSLEAERADRISRLAGLERVSTLRAGPVAAGEG
GGGGARGRASSISPQTTPTSTTGGGFPPNFPTTHNLTPSYFDSNGQPVAV
TKMSTVGSASATESHVGDDTRTTAGERDEDMFSTDTNYREADSVASMGAE
PDAMDEDMDMMATRSVGGYEDRMSDDGSASLVGFGEGAGSTVSGPIYHRR
APPGASAAHQAWNLERTNSGMSDYRRDRDTMMSGGDTPMSASAIQERRDA
RMMDGVAADAPHHGMLTEQDNFVDTTFRGPLPTGRHPTTRETAERIVQQL
DRGEARAGSSGLGSPGVGGQSLGKLYFEGDKRDD
>Mb|QLI66958.1
MASSAQESAPARKLTKEERAEQRKKKIGPPIRYPFWFGGSASSMAACVTH
PLDLSDAPKSMSGTFVHIVKTDGPLALYSGISASLLRQLTYSTVRFGVYE
EIKSRYTKSGREPTFPVLTAIAMTSGFLGGIAGNFADVLNVRMQHDAALP
VNERRNYKHAIDGMARMAREEGALSWFRGWLPNSSRAAVMTAGQLATYDT
FKRLLIDYTPLGDTLTTHFSASFLAGLAAATATSPIDVIKTRVMSSTQKQ
GILRIVGDIYKTDGLRWMFKGWVPSFLRLGPHTICTFVFLEMHRKTYRKV
KGLDEKNL
>Mb|QLI66959.1
MGRVRTKTVKKSAKVIIERYYPKLTLDFETNKRICDEIAVIASKRLRNKI
AGYTTHLMKRIQRGPVRGISFKLQEEERERKDQYVPEVSALDYTQNESGQ
LEIDSETKDLLKHLGFDSIPVNVTQVTQNNPNERGPRRFGDRPRRD
>Mb|QLI66960.1
MRNKKARRNDDRDRPRDGIRRPSPPRFAPSSSSYDRPPPRSDTWRPGTRG
GDDRNGQYNHRSNDYRSSDSYRPPVPQGDFTFRFNKPAGIPDLPPNGHQY
NNKGPRRDRRGPPRKPGRRWQPPHPSERALISGATLNLPEERVDDGEGGA
KFRDLDELSDDDELEMDISSASDSEGPSRKRARTNDASEEANSVPKWSNP
DPYTAMPCPDDSTRKKRDVVKLIRKARVEGAAKAKVSTEAEDFLSFDTTE
DEEEEEDDDDDSVAEIPPPPSEPPPPLPPPAPASFNHKHPDDRNRRNGLP
VPDHSGPLGSRKRTADDEIKPPDYPDYGQLKKASTKPSKGSVTPTWLPKK
NEDPCPWQTIDHSATRNMAFRLHKEILDFYDYVRPRAFEQRIRDNLVENL
CKAMRRDARNFASAQVHAFGSFMSGLYLPTADMDLVVCSASYMRGGPPTY
LSAKNWLYKFQKFLVMQKVADSDSIEVIAHARIPLVKFVDRLTGLKVDVS
FENLGGVTAVDTFLSWKKQYPAMPILVTVIKHFLLMRGLNEPVNGGIGGF
SVICLVVSMLQMMPHVQSRSLIPEHHLGEMLLEFFELYGRQFQYETNAIS
LTKPVGYIRKSDVATLNYRNRDRLSIIDPNNSSNDISGGSSNTNSIVARF
ADAYSTLRDRMKEIALNPEKGGVLEAILKGDYSSFRRQREYIKHVHEQNI
GPCSD
>Mb|QLI66961.1

MSVKFERDTLRQTTQNVASNALPDGKIPGTSGKHPLAEKVGTALTGGIQN
AGTKGYLAAYIKQLESNPLRTKMLTAGSLAGAQELIASWLAKDRNKHGHY
FTSRVPKMAAYGALVSAPLGHFLIWLLQKVFRGRTSLKAKIMQIIFSNLV
IAPIQNSVYLVAMALIAGARTYHQVRATVKVGFWRVMRVSWITSPICLAF
AQKFLPDQLWVPFFNLVSFVIGTYINTVTKKKRLAALRKKHFGDGRPGAS
MGRPDDYPPMGGPNPPY
>Mb|QLI66962.1
MGNFISWINENVPSGEDNGNGQSDSTNKPQSVWGMIDTLIPVLIVSAVYL
IFFLIFRRSQRRYYAPRTYIGGKSERSPEMPSGWFNWFGAFWKIPDTYTL
THQTLDAYLFIRYLKICTVICFVSLCITWPILFPVNATGKGGQSQLEILS
YSNVNVDESPNYFFAHAFVAWAVYGFVMYMITRECIFYINLRQAYLLTPH
YAKRISARTVLFTCVPKEYLNEAKIRQMFNNAVNNVWIAGNTKELDEKVE
ERDKTAMKLEGAEVKLIQAVNVARTKALKKSGNNNESEQDTETADIISRW
VPDKKRPSHRLGPLGLVGKKVDTIEWCRSELEKSIPEVEKTQLGWKQEGN
FEKVGSLFVEFHTQADAQAAFQVITHHHALHMSPKAIGVKPVDVIWKNLS
IPWWQLILRRYAVYAIVVALIVFWAIPVGIVGIISQVSTLVKLPGLTWLN
DIPEKILGVISGLLPAVALSILMSLVPVIMRALSGVAGAKTNTEAELFTQ
NAYFCFQVLQVFLIRSITDAASTAIVQIAENPGSVFSILGSALPTTSNFY
ISYFIVQGFTIAIGVVTQVVGLFIFRLLYKFLASTPRAKYTKWTTLSAIL
WGSLLPVYTNIVVISIVYSVIAPLILFWSTLALFLFYLAYRYNLLFVSDT
AVDTQGLIYPRALKQLFTGLYLGEIVMIGMFSVVKSPGPAVLMAIFLVFT
ILFHITMTRSLNPLLYGLPRSLETEEAIFQTRGTEIDAEEADRETSNEGD
VTNGAKAGSSTKKILSGGNDYGVEKKGNMFTRFLKPWVFADYATLRHIVP
QEQSINFAQLQQAGEENERNAYFPPSVNSQPPQLWVPRDPLGVSKHEIAL
TSRVIPISDKGAYLDEKNNVVWSEDLTNEEELPPIHDEKVYY
>Mb|QLI66965.1
MSTPTLAEKLDKIKSPGLEGQKRNVVVLQAVESTIKEQNAAPTPTGYFAA
LLALLQQAISNGSVNTELATPVVYLLDTVTPYTPQPLLRSRFTQILTLLA
PVLLMQDAEAILIRTSTGCLESLLLAQDAPSWELSVSQIGPRRAVAGLLN
LSLDHRPKIRKRAQDALKKVLKNPPPSPSLDHPAADMCAQTALKNLEDLA
GKAADARKGKKSSDTTHDPALIHALHLVKTVATASGGWPSTKIESLCELL
LGIAKTGNEYMTMAGFEIFELIFESMSAEVSSSKLPRLMEIISELRPAAN
DTQLVPPWLAILSRGFDVSAQVEPDETFQKLPELFAMIAQFLESHSENIR
ISASECLVSFMANCIPQQVILGPSIYEEKILDKITKTVESLLTVQYQAAW
LQTFNVLGAMFTSLRWQSYPRMMNITKTIGEIRENSSFRNKKEADEIIGQ
AVQAMGPEAVLTALPLNIIKPVKGQAGRAWMFPILRDYVSNTELDHFKTE
MVPLSELMFQKVLDHGSADKTMEIKIYETLVQQIWATLPGYCDLPMDLME
AFDQGFAEMLANLLYKQVELRLDICRALKTLVESNQAIASIGDEEEDFML
QSRVSCDTAQKNLTYLGQFAGNMLAVLFNVYTQTLPQSRGPILQTVNAFL
SITPPQEVVETFDRVSKMLAAELENTQKPGEKGKGPKAKDHMPSTAQTLM
DLVITMSTYLPRDSFAALFEIAAVIIYREDEPQLQKKAYKLIPRLATSEL
GKAALKERNTDLQQLILSSSEKVSAPARRDRLAAISALLPFISDDSLHFI
PSVLSEVVICCKENNERARETAYDLLVQMGQRMIAANGAKIDNSKVRHMP
DDTPAGTANIEEYFTMVSAGLAGSTPHMISASITAISRLLYEFRSSLTEQ
TLSDLVQTMDLFLTSNNREIVKSCLGFVKICVISLPLELMIPRLATLVPN
LIIWSREHKGHFKAKVKHILERMVRRFGYDLIHKNCPEADRKLIVNIRKT
KERTKKKKEATRTGQGDDASDESDEEQSGKRQYENEYDQALYSSDSDDSA
ASDDETTAPRQKKKSQKGGKTYIIEDDDEPLDLLDKKALANISSTKPLKN
RNPTKSKAKFDLDGKLILGADSDDEPAAEDGMDIDKPETSGVGAYVAALK
GKDAAKRGRGGKLKFSNRRSKDDEDMDEMDGDDVAMIKSKMSPGRAERGN
KFRGRGGMRGGRGGQGGRSGKGGISAGRRGLGIEKRHGAPAQGIGKGRRG
RN
>Mb|QLI66967.1
MKFARDFKETLANQDFPPHWVTRAIPYGQLKKCLKKVQRELRDLGLDPET
LRALLDPTTTSPVALQYHLKTATNSNFVQPKLTVYVHLQDGVAVDASLTP
TSRGFFERVAAEIRSDPMGLAGTHADTQSTETSPVLASGHDADRQADGND
HNMYETVEVPLVFDAEFFDMLQSDVNNLDALQTEEEGKMTAEVVELGKEV
SFVCRTSRFSKSDLARWRRIFELYLDAEVFFGTHERDHGTRSSQMALKQL

QWFQAEVEKGHLARDFKLRESRMAFSRFVNLNLSLLKNLQFQELNKLAIV
KILKKFDKRTSLGVSQTFRSAIQSESFLSRDMAKDICAQMSQQLVTVVPQ
LNDYLCPVCFSVAYRPVRLDCGHVFCIRCVVKIQRRNERHCPLCRADVVM
NASADNLDRVLEKYMKQYFAKEVKEKQRANEIERGIEDYGPGYTHHDCVV
L
>Mb|QLI66968.1
MSSPRRRIETDVMNVRCLPWLRAFSHADEFFLTRTTNRQEFFVRFKGPAE
TPFEGGLWKVHVELPDTYPYKSPSIGFVNRIFHPNIDELSGSVCLDVINQ
TWSPMFDMINIFEVFLPQLLRYPNPTDPLNGEAAALLIREPKSYDAKVKE
YVQKYATKDAADEAGAESEDEDDMSSVASFDDEDEEPAGQMDDV
>Mb|QLI66970.1
MASDVIDLISSSPVRTTTSRPSACVQQPAKRRDDIDDFDDLDDDVFDVLD
DRQAKRKRTTGPGDAQTNCDKLTTAISSKPPLPLKEQTSRYQNPTVWDPI
EFTSSLEPVSPAKEQIDKGLPSQTSVAKKAIGNSTCEDAISCFSSDPFAS
SPHPLPGRPLASKRAVSLDPFCSSPPPSLTTSRSRNTATEYGKGRDGLRW
QAGISRQSAIEVHEDVDTSLFADPQLPRWANEPVVLEDLITIDDSDSLSD
DANDALPAISDMDVSKRRARSPLRRSQSDLTWSKSRTCYSATSTKRVKPQ
KTAHEREVEKTKKRLEREEAKAAKAAEKQRAAALVEVNKLRTDKKVSTPE
MIVDLPSGLSSELRIQVQEMLQGLGVDHATWDCPKHSIIKWRRKVTSKYN
DDIGLWEPVPAKIVDEEIAMIIVTAEEVVAMALGDLLASHVADIKTHHGS
MKFVYLVQGMTPWLRQNRNNRNRHFASGVRSTGAAASSSSRTNNAEYISE
DTVEDAMLALQVEHGMLIHHTAVAIDTARWVINFTQHVSTIPYRMQRDQA
TSVAGFCMESGQVRTGDSAHDTYVRTLQQVVRVTAPIAYGIAAEFDSITK
LVQGLERGGPERLDSVRKSANKDGAVSDRTLGQAISRRMYKVFTGKDEDS
TDV
>Mb|QLI66971.1
MEAAAKTVEVLSKRILPEKPHHLAFHPTWRYRPPPEDDAAESRGAKRRFE
EWHNTRLQYLTFLSEADRGTLFTRPYYDMREEPVKPVPREVSTLAKGTGT
GEKKKLSLSDYKNKKTGLVASASPPEPAITKKKESERAAAATSSATSTPA
PAVADGTNKPPRQEPKRPDGPRIREPESTGAKPKPTRDHNIADTRLPPKP
PSLPPRPPSPAGKRRMPDADDERPQKRPKPEDRPQSNRDEAPRRKEKPLP
ASRDRERERDRDRDRDRGRDRDGSAPASRDDRGPSSSSLLNGRSILKSSI
NSGRSTSPASRPRGDSLNGARGNNANRTSPMKPDTSTKAHVPPLLSPLHL
GFEDRPSRGDNDDPSSRKDRKRQDGPSEAAAATPKQKKTDIKKPRPAVTI
PPLLSPTLPPAIEAELKRRKKAASDSSDERCRDGRDALGIKRDGDNDRKP
KNKLGHRRRLIVSLNVPKALRASFADIVGRAPARRKDSQSQSERENERTR
AGSEEAGQHVPARKRPIGAVTGASDGVAMKRPRSSDVSTHPKLATPATPS
KKPAAMSRVSSTNSVAQTPTEAVNSTPSASAAAPADRRPNGSDAAPKATS
AEAKAMSDKVERLKEVGKKLKHEADQTLKRFRGDTGSMRGKPGEAKVKSG
FVLSLESIIVFMMAFYGADIWRGMDHKIGDHRAWLSMLPMIEFLKNEMRR
CDVSNSQPLYAMVLMLHAMSLDELIRCYIRFDSLNDALAKEIVGRLREKN
KIWPRVHEVSSTIQNPRLRIDVHPWSTLDDVADASLRVLRLWCAEEKIDW
APTQSLKDLWTAPPGPGRR
>Mb|QLI66973.1
MPDIRSFFTPKGGAAPKPAPTKSEDAPKSKRTKNRKVVEDSDEDEVVETK
PAVKAAPRKKKTDPEPQGVVISADDYFASSKKRANSKQSTPKRGSSKTPV
KEEIPVRASPRHKKPDAAVQNGSPAKKKPTTSYKKHHVDDDDDAYIDDGD
NDGDDIYAADVKGQNKRKNDDYAEEESEEEVLPKPKRVASRGQAATAKDK
KTNSRATPAASRKRKSPEQESEEEEEELPRKKPTATKPRAPRVKKADEPE
DSEIQNILDSVATVRPPTPPPKDPDAKFDWRKAAAGGGNASIQPVASADL
PEGEEECLSGLSFVFTGVLQTIGRDEAQALVKRYGGKVTGQPSGKTSFVV
LGDDAGPSKLAKIKSHGIKTIDENGLFDLIRKLPAYGGSGKGAQKAQEKK
KAEEEKVKQQIAEMEAEEKKRKSEAVKAAQEAASARGAAGPPPQPAPAQA
AQLLTSKYAPTQLSHICGNKAQVEKIQTWLRNWPKAKKYNFQRRGADGLG
GERAIIISGPPGIGKTTAAHLAAKLEGYDVLESNASDVRSKKLVENGVSD
VMNNTSLLGYFAGDGKSVDAAKKKIVLVMDEVDGMSAGDRGGVGALAKFC
KKTEVPLILICNERRLPKMKPFDHVAFDIRFNRPTVDQVRSRIMTICHRE
GLKLPPPVVDALIEGSNKDIRQIINMISTAKLDQTTMNFDEGKAMTKAWE

KHVILKPWDICQKMLGSGLFAPASKATLNDKIELYFNDHEFSFLMIQENY
LRTKPMALNGKGYNKREEQLKALELFDQAAESISDGDLVDRMIHGPQQHW
SLMPTHAVFSTVRPASFISGQLLGSNFTSWLGNFSKQGKLGRYAREIHSH
MRLKSSGDHNEVRQQYLPVLWKQLVNRLENEGTNAVPEVIDLMDSYFLTR
EDFDAIQELGVGPMTEEKVSIESKTKAAFTRTYNSMNHPVPFMKASNVIA
PKKQTKEVPDLEEAIEEEDDGQAAEVPDVDEDEIDLKKDKYIKQPKKKPA
KKSATKAAADNDGDDPPKSKGKTKGKTKK
>Mb|QLI66975.1
MASPSRSRSRPPRHDLVTRIHADALETPTILLGSIDISNSQAQEDSSSSE
SDFQRTKPSRLPPRHARSLSNPFPSFLGAARKRQGSTSQPQADSDRGDVD
NAMAGPGVGNRHRRGGPMGSKDFATGNCMTCASLMRWPKELKVFKCTICT
TINDLAAPGSEALKPGILGRRRDGNAHSSSDNSTCQPISLRHCQRLVRQC
IHSYISMKLAPKPAPEHAGWRSPTASQSSIPRSDAHRNQQDHSHGRPRGA
SYGEDHDGCNSKYVFEEQPTLRPSPLRPNAALRSYSSSHSERPPPPGQPP
LNPSPMARRRASSMNGPCEPKRIFKPLEEYIAKAFGSFDSVNSSFSTENR
GRERQQGDQIRRKPLPSRGRSLSQSADAVPRGESQPGMVMDELDPKLLLL
GDFAENGSWWTGGRRQTGPTKQTSTRRPESPPKKPVTIKSPNLNWDELDS
WYSSTINAAEDWFSVYEDVCREPTVRPQTQRDLEVLEQELLQGQEHVQRV
LLKATESLLKRPGRPITNAGDLRFLLIIMENPLLHAELKGFGGILQPDGV
APPPPQVIPPRRNLLPSSGLLSGQHSGIIKRIIGLISNTPPECHNQLITW
FCKYDNRRFVRTKDLVSGFLSYRLLRQIDKKQPTEVDITAGLIPEMRAGR
SGAGFYLHDEIRNGGTSKKRESEHKIAYTEDWQIKAASRVLALLFAANSV
PGIRHGYSTTAVGVNVAAGSTREYGHSSGQLVPTTDFYNSMIDYADLVSD
FESWESKRSKFSFCQYPFLMSIWAKTHILEHDAKRQMQMKARDAFLDSIM
TNRNVKQYLSLDVRRECLVEDSLAAVSEVIGSGSEDVKKGLRITFRGEEG
IDGGGLRKEWFLLLIREVFNPDHGMFLYDEDSQYCYFNPATFETSDQFFL
VGVVMGLAIYNSTILDVALPPFAFRKLLAAAPAHGLGMSSRPRPSMQYTL
EDLAEYRPRVARGLRQLLDFEGDVESAFSLDFVIDTEKYGTTVQVPLCHG
GERKAVTNSNRREYVDLYIRYVLDTAVTRQFEPFKRGFYTVCGGNAFSLF
RPEEIELLIRGSDEALDIASLRAVAEYDNWESRQPDGSSPVVGWFWETFQ
EATPGNQRKLLLFITGSDRIPATGAAMLPIKISCLGEDVGRYPIARTCFN
MISLWQYGSKQKLEGMLWRAVFESEGFGLK
>Mb|QLI66976.1
MSGQPLAGAPAANYAAQPATSPAGPAPAPGGNAGATGGAMSNQNLNQIVT
DYLLKRGFNRTEEVFRQESKHLGPDGKPIQQLANIGPKKYQKAFKLLKEW
VDNNLELYKFELSKLLWPMFVYAFLELAGHGYTEDAKTFLKDLSSYFQPV
HADDLKTFSTIALPQHVAENPTAKLYKENKYRIPLNQHATGDLFNFLERD
SEQGGSVIRQLLVTYCQIDSTARGPITPFSFEAVFRRSKNADIEEIDAKE
GIPGVNIGLSNKDILDPAQPLKLGPLPMDPDLRDDVRAEIEDEEKRNPPA
EGTNSMLEDFDQKIKREEDVDAPSRADLPLPPSRPRDIMLEMQKVRENRD
RFRIDGRTGGIGTSVSACMFTFHNTFGTVSCMDFSDDGQLVAAGTSESYI
RVWSLDGKGLPTMNAHEKDAKFNSRKLIGHAAPVYDVSFSESASGPAQRL
FGDEGRHNPVMNGRPKLLLSCSADGHVRLWSLESWACLCLYKSHDGPIYR
TLWGPHGHYFISGGYDKALRVWMQDHASPQRLLVGHDTAISAIAWHPNGM
YVLSASDETDKSIRMWSVVTGACVRVFAGHTDHITALECAPNGKILASAD
LAGNIFFWDLAKGTRIKRSRGHGRGGIWSLSFSVESNVLASGGQDGTVRL
WDVEEPVDPHKAAAHAGLEAAAASAAADGAAATGAATDASRANAAAGQST
AVPTSTGTQKKKGKEVMVTHDQISAFPTKKTPVMRVKFTRMNLVMVGGCY
DPER
>Mb|QLI66981.1
MAAAGVDVGYVAGHLGLDQPVLTTLTTEPTAELVATLLQAVAAKAHEFDT
LYADKLQTDIELENVVRSSENRSQASKSTTEKALKDVEEARQKLKEEETK
RQSLENELQVLKAKNSDHAAEVKSLTDKIETLQSSNRTNLSIIESHNKRD
ETITEELTKQHQRNVELAREVTALQQSEQNAKGLLNSAKYREESLKQQLQ
LARRNSEWLETELKTKSEESLKYRKEKGARIAELQRENEDVKSQVDALKR
SEQQLRERLDAMQAKADEALVKLQKQEGAFATTIESYKQELEDQRRLVDM
SDQLSKKHSERVRDLEAEKERLRENYENEIRRVRMELEQERQNVTEMEER
ISQLEGELDEAQARIEQGPPPGSAPQTPRPNGSLAGRAASPFGTPVSIRK

SAMTATQAIDQLYQVKGQLASEKRRNHQLSEELDEMMTALEAKAPEIQEL
QAESDTLRSEIARMSELSQQSFEERDLARKAARKAESALATAQSETNILR
TQLRDLGTQIQMLVFNVYAIEKGMDQLSEEEKYRLQQLERGEITEEALSD
LSDTHQFITQKLVVFKDVKSLQQKNQDLMRITRELAEQLESEEALAAKHQ
AKEDHDRVEKLQRELENMTEESRSIKNTMESYKTERDMFRRILQQRGAGV
DEASMMRSSLDGSQRAPLASIEGSEQAESLIEALRKLQSEYDSFRDAQDG
VRKDLRTQIDHLSTEKNSLQTERVKLQGEVKLESERREMLQSNYIALQSE
NSELQKRVQALSETAAKQDIRTQQVAEELIETKGLLDSMRNETANLKAEK
NLWKDIQDRMSKDNENLIEEKNRLNHLLATQQSLENERNMSESEARRKAQ
AKIDTLERELSDVQRRLSNETDESKKLQLRKEFESKEAQKRIDELMTSLG
QIREEHVGVKTTRDHLQARVDELTVELRNAEERVGRLQPRPTPRPGLDDA
SQRQQELEAELHDLNNDISDLKRDLDMANTQLENAKAQAEQYRELSQSNE
ELLADLRGSQEQYRQEMEAMIEEKDNKIKELSQRIEDMSEELSRSNTELS
QLRDSQGDVARKYEDEKTILEDELKRLKEESARHAEATRYHQQDLRAQAE
IASKAQQDYEKELVKHAEAAKLVQQLRTDYNALKSESASLRAEAESAKVT
LAQSESSWEDRRQHLEQEMAELTARREDVNAQNKLLHQQLEGLTAQVAAL
QQRRSNGDEEGDEGMSPVHVGDALEGLRELNNYLRREKDILEIQYDLKSQ
ESKRLQQQVEYTQSQLDEARLKLDQERTQSAQSGRSSMAHQDLMEKLHEL
NLYRESSAALRNENSQLKDQIVEKNKKIEEMEGKVQPLEAEIDNLNMQKS
YLEEEIKQIQEDRDRWQKRTEGILTKYGRVDPAEMEQLKKTISDLEAERD
CLKQGEQPLREKITELDSTLESERQAWQTTRAKLTEQFKERSRKLTGEKN
EAAQRVNHLQEQLDKASGDLQGAQQLAENVGKEKSELEQQVQSFKQQVEE
LRQQIQQHQPEQQAPAADTTSVDVVSQLEKQLADARKELEAVNAQKLGAE
HQLEQLRAELQNAIAERDEATQKLQDEVAVRTNGTTSEDAAPGGTGDSTI
AGLSDEERKALEERVIAAESKAAELEQRANEAENKIQQTIKERSDRMRDT
LNGKLKDSRAKMEEEFKKKEDDLRLFFQQEKLIWQAENAGEKPVVSSQPP
ATPAKPEEQQQPAAAPSTPNVAAGGAPDLSQLDDASIRQFLSTNATVKNI
IAANIKKKLETESTRIKSEFEAKIASAREQAQLMESKKSTLRINMAENKL
KSATAKLGVVETAANETPQKPVVEVWELAKSAQPPPATAAPGTPAKGAKA
PMTPASGAATPAAAATAVAQHPVQTATPASKPASGAVPSPAPATNNPFAV
PADATKSDAPNPFAVKTEPAAAAETRAQQPAQAKQHQSQQQGQQAQPVRS
NIPMPRGGGRGRGGPYVPPSQRGASGNQDGGAVGQGGRGRGGHRGGRGGM
NPGVADFQPGTKRPRGDSEAGGGAKRARGAH
>Mb|QLI66982.1
MPAPTVEAPAVSLSFANNFWGKEDAGVGPLLERMQSAKTTSDELKSFYSG
TLARASIEDEYARKLLHLSRKSLGSHEMGSLKTSLDTVRVEVESMAKQHQ
GIAAQMKSELEEPLAAFAGGMKERRKIVQNTVEKLLKTKIQQTQQVNKTR
DKYEQECLKIKGYLAQGHMVMGQEERRNKAKLEKTQISLATANTEYESAV
KILEETTARWNREWKAAADKFQDLEEERLDFSKSSLWTFANVSSTVCVSD
DASCEKIRLSLENMEVEKDIIHFITERGTGQEIPDPPKYINFCRGDVNDG
QSEVSEDDNYSVAQFPRSINPAFRSSSPQPSTFESHHDPNSMLANNLAHR
EPREPPQPTSREAIVTPQKAPPPMRNSVDEQRRGQGQQKPPQYDVNQHGP
LAAVPHDPYPMDGMTMLCRTGPPGPQSDRSSQPHSARPSSRESHSDYSIP
TSLSSVEPPSGQASPVKQEPVETRSPEKRVLKKKSGFFQNHSPFRRKSTK
EVQAPSQNRSTWHVAPGRAELERTASPEPIDANASLALGVGQNVLPVTTP
DTRRRPGQGREQDVDQSDPIAMALAELKDVNLGKQSSLRMSADHYHGIAT
PAPGADPRSGRPSGREAPPSYNTQASVSRLGVPPPAVTSRAMKEATKKAT
DQSRAVYGNAGNRGASPASRPATRGSDMPRAASPAPTRSASPQPRMSGDS
RYRSASPNPYSGHHRNASQVSVSQQRGSSQGYYGSGSPHGSTRGSVRGAS
PASFRGDYDRPRSSYGGGSDMAVQLAPAGDDRYGSQRGRGAVDLYDGGSR
PRSKSVADPSRQYTRDGRPILHFARALYMYQAAIPEELGFAKGDYLAVLR
HQDDGWWEAEVHGGNGRVGLVPSNYLQPC
>Mb|QLI66984.1
MSIIQQHTSATLSDAWRTTNIDAYNEDSSVNFDTSTLHPPQPEISEAEAR
QLSGQVRQLLRGGDAEGALRGCLETPVYNGTEGAKDAHLQTIIEVLQSIK
ASDMSPLLRGIYESPGGSECLDVLMKYIYKGMAMGQPKSASRVTPQSTGG
FGSRSGVSNEPASAAMSVLLSWHEKLVEVAGLGCIGRTMTDWRRV
>Mb|QLI66985.1

MVEHDGLMRVFWPTDIRKSNRPGVVVGWRNSTLDVFVIAILDDVDASEVE
HHLKGGSFFRSAPHSPGRIYELCGHSSMHVLGVSNSTGTATVDPSWVCAI
NHPSSRTPTITCAKASSIQLILYDRPQPKRMQYISLEPIALALGDKSSMV
DDAKVDATEDLVEQQERSAKEARQRLVEKLKQHTISNRVPSARDKALRKI
INQVNWSWELEQTLQKNAWRLGPRPKRSLSVSEQVMESASTMRNYVLRQL
WTFFAVYLFPTIRKTFILFLLGHRMVAEMLLLILEFRIKPGYAALKDISA
TAQQIEIRLQQFCYWPMQYSKLRQRKANWASITTSHPDYIRFYNSLWLVA
NDVIIGIALGSYVIEHADWVAAQIGDLLRTYTVEALQSSISWLMDYPAGL
KLNGELAVFLGDLFLWVIDYWSSCIETLNPALPNIVWFIGFTSFAGASMP
TALFSDLLSLLTVHIYSFYLASARIYHWQLTILQSLFHLFRGKKQNVLRN
RIDSCDYDLDQLLVGTILFTLLFFLLPTVVVFYLNFAIARMVIISMKAGF
DTLLSCLNHFPLFALMLRIKDVKRLPGGIRFELRDTQDYRVTDTNTINET
PTSIIYLKSIPLSFSAMFHQYAQMAHRIRKHYLSPRVLLCLLTGQFVPPI
NRKNLYSLQYSMLPARRATIWDMWRAVNTEIPQKKTFQLPIIPPLPNGAK
RMAGNGRARAHY
>Mb|QLI66986.1
MAGDRGISAELKETWGDDTVYSASFCQPEDVLYEGSEDEDYDNSAARRRR
YEEAGQRFLDGAVPLLFSAALRGPFDRRSGFINPWISHRHSGTARSSTGS
SRPIEKVASSQSKNSVRKPKPDPTLPARSADCHLPSPESLKQAPYTQEHP
YLAADGIEKVNTWREQVDPFASNKNGFWVSDDAKVSTSIRKRSAPGSEWL
KKVSAKRHQSTTTQKRPPRQEQEDDELDELMADLPSSSFENAAPVMSPSK
RRSPRNAMRRTSCKVLVESDDELSPNKTAAATLSSPVSLRNGPVISASSK
RSQPPIYITSSIGTQTTPSRLRHVKVLDTWSKNRSPSQEDPPGEEPRVKV
EAASGQDIETTIENISKSRSVTESEPETIADEDDNGSAMLVEDGCIIVNT
TCEDATSQATNGQHENQRYEPESPPSNCKAEMDGNSEPIAQRADSDIEMD
NLPGIKSEPQGVAECTVPDSTRNSTGPDNNRNPDDSPVKETDIPVAQSHG
NWVMKMESVGEVVAHQGGADVKQEDNLSDMDSGDDSGNSCAATQKTFSIR
NIEPNRSQDTSHASTSDDIAVHDEKTASMESEFSTTSTRDYHTQSDSESV
KHETPTTPRQAERVKEDASEFIFKTILNRFVPSSPWNKLTRLALSPSSSP
SATFSKKCAVANESADQVEEADSQPSQTHKEELPSPRRNELIGDFDQLHK
PVQIQQTVSYSHSDRRLKDSEADDQRVPESQQSPWVNTDHAILSRSPLGI
LSLNISGAKPSSNEAENTAAVSPSTNIQNPWMQNPSSNAASLDQSSSSPI
NENIEQIPFAKPSLPRPRTPEPQFCVKSFSSFMSPSPDRNRGEGVSLSAS
GQVFRSTQGSLPSSVKSQWSQKRPGLRVSWAASLEEFEGTPSREDARIAR
HMPKRQTSPPPENLSGDFRQAHDTKFAKHFEAVANRRDGRSQTLIPTESQ
QLQSPGPFAMAETFMTADNNTAGLVSKDRESAEHTEPRACSRASEEPMDV
VEDMVREMGDFWDPWNIDAELDQARKGASGGQTSSIET
>Mb|QLI66988.1
MESGASQEPPLKKRRFFRDPGENSSDPILLSPGHSSSPVSAKEYVKDHEG
VKRDSDSRIALAELSENGSANIKSESPQPTSVQLDESTTLNFDKDMFESF
IGDKVSNEVLNIIRNNCGDNLERAVNMFFDGTYKKLQTKQQPLLESVGQR
PASSRTAQSAPIEESTTSPSLSLGTLPRKRYLGAFGAEGWATRSGSNMLK
HGDVVKIERQKIKPPPPPKIKNKLGVVSSPSPKPKTAASRTVDVIVRFTN
QSGTEIGRLTKETANWVSTLIDQDVCHFEGICVYAPERLRTNDTVYLQLR
CSLLRSAFEDRSFRFADDQPNNIFERGETTDEKELRLRQVALVRLFQEIN
LQPTRANSAAKDKRRELLQAAEMDEQKQRDAKKAAVPGKESESVPNSSDT
EEGEELEQDQLDALYKKAQCFDFNTPEAEPADSFAMTLRPYQKQSLHWMM
AKEKDEMNNREPSMHPLWEEYDWPTKDVDDNQLPDVEGVSKFYVNPYSGD
LSLDFPVQEQHCLGGILADEMGLGKTIQMLSLVHSHRSETALQARLTNGG
ISSVNQLARLGANSSSSLAAPCTTLVVAPMSLLSQWKSEAEKASKEGTMK
IELYYGNEKTTNLQALCSESNASQAPDLVITSYGVVLSEFSSLVAKNGDK
SFHNGLFSLKFFRVILDEAHHIKNRSSKTARACYEISADHRWVLTGTPIV
NKLEDLFSLVRFLGVEPWNNFSFWKTFITVPFEAGDFMRALDVVQTVLEP
LVMRRTKDMKTPDGQPLVPLPSKQIDIVDVELSKPERDVYDHVFNQARRT
FSRNVEAGTVMKAFTTIYTQILRLRQSCCHPILVRNRDIVADEVEAGAAA
DAATGLADDMDLETLVAHFTATTDEAVKDNFTYGANALEEIRSEAEKECP
LCFDEPMNDQTVTGCWHSACRKCLLDFMKHESDRGAVPRCFNCREPLNQR
DLFEVVRHDDEINMVSKPRMSLQRLGVNQSSAKVTALISELRVLRKERPQ

MKSVVFSQFTSFLSLIEPALTRINVKFLRLDGSMAQKARAAVLEEFTERK
GFTVLLISLRAGGVGLNLTSAGRVFMMDPWWSFAVEAQAIDRVHRLGQED
EVVVKRFIVKQSVEERMLRVQDRKKFIATSLGMMSDEEKKLQRIEDIKEL
LS
>Mb|QLI66991.1
MPRPKVLPSQRRRAAEACNFCRDAKKKCSGTAPCSYCLRRGVGSQCIIAL
RPRGSRNQAVSDGPRLPCDTQPHRVGLEAQSGMLDLETGGPRCTPSAQLP
ILNVDGKGRRKGPDTSSFRPLSPSDSRPSLEGPESPERRRHGASDASSVS
GNPHSRMLFNLRGERVYIGGAASLSFLQLVRSIVADQIGPSQFSHNEKSD
TMLEKESPSSMRNLPPSTLPELDVETKLLYSQCYRAVTEGFIDIFAPTEI
EEYFMLADDSSFSPQKRAALGMVISIGAQCKSAASARDIGQGYFRQAQSQ
AFQGLLEDPDIDVVRSFLLMAFYLLGECRRNAAFMYLGIATRAAVALGLH
SQESYQDLNDLEDNKRIRIWLSLRIVDMLVNAILGRPAATAGLSSDINTI
LDHASETEQTDEMARLIASHQIVSIINGIVDILYERKEISTSVVENLLNE
IESWSRSLPSCLRSPAKGGALSGSNYTKGAIGSVHVSCLYYFAVTLVTRP
ILISTLTAPPASGGLVQSQLASACLDAAVYLIQTCMDARKVDILYGNMCI
MKALVFAAGLVLGFEIFAKRSIEFDIDAAFAGARDILNFLATQSPQAGHY
YEILTLLSNAISKQRQNVASTGRSRYVSKIFTLQEAKEPVSDSPRDDQGR
LTPLAGGLIPPFAENAGNWLSDNRTPADPGQEVFLGWDSLDISQWDNFPF
VP
>Mb|QLI66993.1
MSSGKGLPNFPDLEAKLQKPTKQSAFERQKAEAEAKRKREAAETAAVYAD
FVKSFDRDDDDDDRGDATAPSSFGGPSRGRPGFGGQPPGHGVSRRHFVSS
GLKSGPGSLGPAPSSFTKKRSFNDFKGGGSRDRSGLLGFEEPDSGPAATS
VAKAFDASEDEDMGDVKDRAEEKATSKPTLRLANLPPGTSQAVVKALIPD
SLNVENVRIQPPAPAGPGGSERKCMVAIVTLSQETPANEMDTAVSTLQNR
YLGYGYYLSLHRHLSSAVAASANLPSLGSSSSGAHPFGAKPVEQPGGRDK
YSHHQQGFHRGFAPPSSYGQVGAGVNRAQLLHVPVRPPQDVRTIQLINKT
IEGVLEHGPEFEALLMSRPDVQREERWAWIWDARSEGGIWYRWRLWQVVT
GSEFSQVKGKYVPLFDGGHAWKSPDKSLVFEYTTSLDELVSDPEYNSSDD
EDMEEGPGDNNNGVEAEKTFLNPLDKAKLVHLLARLPTTLSKLRKGDIAR
VTTFAITHASRGADEVVDMIVANVEKPLSLTAANAEKKQDARPIHPGTAT
EEGSTNEGPDTSSASLVALYVVSDILSSSSTSGVRHAWRFRQLFETALRE
RKLFESLGLLAEKLGWGRLRAEKWKRSINLVLNLWEGWCVFPAESQELFV
KTFESPPSLKTEDTIDESSARTKWKTVEALPAAKRERSAASPPSVVPASM
AQDDIDGEPLDEDDVMGEPIDDEDVEGEPIDEDDVAGDPIDDDELEAEAM
DQDGGGGQAAGAPSQAESQDENGGVGGTQVSAGRTQQRKRLRAVDMFADS
DGSNEEK
>Mb|QLI66994.1
MPHSITGAKSSPGAVKDEPMDDSGVASRMGPAASDGDDVDMDHDADDVAL
VKKEIKLTDMVDTDDSDDEEFPSSAPVKQPPSSPPARIPSPTDIGALKAS
DPEVMRSFYQRLFPWRQLFQWLNHSPTPTNDFGNREFAFTLQNDAYLRYQ
SFATADLLRKDVLRLMPSRFEIGPVYSTNPRDRKMLRNSSAFKPLAKELC
FDIDLTDYDDIRTCCDKANICNKCWKFMTMAIKVVDAALREDFGFKHIMW
VYSGRRGAHAWVCDKKVRSLDDQKRKAIAGYLEVVKGGAQSGKKVNVRRP
LHPHLARSLEILKTHFQEDVLEVQDPWGTEERYEKLLQLLPDRNLNESLR
RKWDAAPGRASVSKWADIDALAKTGASKNLDARALLEAKQDIVLEYTYPR
LDIEVSKKLNHLLKSPFVVHPGTGRVCVPIDTRELEDFDPLAVPTVQELL
GEIDSWKGDDGAASQDDNGKGISDWEKTSLKPYVEYFRTFVNGIMKDERD
VRTKREREDLMDF
>Mb|QLI66995.1
MTFSSGFHMPGGFHTDGIHQGLFRPPVSPASSSGYLTQGRPCGEGPTPKR
KRIRDDARQFDHAQQQEGIHDDITTNASTVLITPAGRHAAHNGRAYTLAG
QLDTPASGPGESGILGDSTYSESDYRKALGSKRSRHDNDFTDPTGHTPLF
NLPTQPYQSSGWGTLAFSTIGGVVGKVWEFCKAGAFKGFYAGGGRGFEMQ
PGDGIIPDGSILDPAWLQNDEDDEQHHRIPGHFPQAYAFCDQEATEDTLI
SSGASTPSAPAAKRRQTAPTDELGRNWVMIKDHASGTDSTPRRTSAAYRQ
SPRNRNQGPSLATGRRISTPQNRRVSSKVASPAPPRGTPRNGFNLSPPAT

LEPARPASSASYASTRSPSPTKVTNIAPSIAASPTPHSSRGHGRRRSAIP
APSHAPFTHSRTHSSASTASSRGAVDDLDNSPRLDAEAKHLAARRHREER
DTDVRIAAFNKQLQDMIRQGKEALGTTIEVDGEDGGWEDY
>Mb|QLI67004.1
MTLLVFRQLLGRSVAGSCTIAAEDDFGPVVEGCLDNFDFTLLFQEAILSI
TPMGVTILLLLHRLLHLHKQPQKVYSGGLLQRAKLVAFAVFTTVQIALVA
IAALPGTPTTAASLACAVVNLLGAAIMWVSSHLEHVRSVRPSLLLNAYFF
FSLLFDIVRARTLWAISQNAAFASGFCASIGLKVVCLVLEAVEKQSILLP
EYATYPSEATAGFFNRNFFLWLCPTLIRGFSKELVVTELEANDRSLEPNT
HQSKLQALWNKWRRWNKKMSHSLLVTYAVHFRWKLAAAVIPRLCLIGFKF
AQPFLVQSAVVYLSELNGQHSRNSGYGLIAAYIIVYVGIAISGTKAQHKA
FRLITMIRGTLISQIFDHTLKLSTSSLNESKAVTLMSSDIERIGTGLRNV
HEIWACIIEIALAMWLLQGQLGISIIATGLVTLFCTGGAVWVAKGAGDRQ
SVWLEAIQRRVHATANMLGSMRGVKMSGLTNDLSANIRKLRLEEISSSYE
FRRLLVRVVNLSFMSTALTPVITFLVFSVLAKVNNSQTLTPEILFPALTL
FELLASPVSVLIETLGGVMSAVGSFQRIGEYLATEVHVDSRNNVTYEKHG
SFGLEMPPIIPRSIISMGRRGLENCVEVSKLSVGYGTNEAPVLKELNFEI
KTSQIAMIIGPVGCGKSTLIHALLGEIPKTSGSIHIAFQEAAYCSQTAWI
TNGTVRQNILGEREMDEAWYNTVIHVCALDTDLEQFHGSDLSMVGSKGVR
LSGGQQMRLALARALYSRKAVIFLDDIFSGLDSTTEEKIFARLFGHDGLF
RKQKTTVIMATNAVHRLSSADHIIALSSDGRVLEQGSFEELQATGAYTRA
LAPSKRKTASAGDPMTTQINTQLSHAIAPEVPKGLQRRTGDGTVYNYYIA
TFGWYKWSILMFFAACYGLLTVFPQVWVNWWATANVKQPSSQLAYYVGIY
AALSILSVVALFASCWHLIINLVPKTALKVHDTLLRTVLNAPMSFFAKTD
TGVTLNRFSQDLELIDMELPLSVINTVIIFAVIIAQSVLICVTGRYVAAV
LPLCVGIVYAVQKYYLRTSRQLRLLDIEAKSPLFSHFLEALSGLATIRAF
GWQNGYLEESKRILELSQRPYYMLFCVQRWLGLVLDLIVTGIAVVLVSIG
VEAKGVVDTGLMGLALVNIVSFSTNLKFLVTNWTLLETSIGAVSRVRSFE
QGTESENNDDGRQRQPPSDWPSAGAITFEGVTARFDESLEKNVLRDVSLT
IKGGQRIGICGRSGSGKSSLVSTLFRLLDPHAGRITIDSIDISTLARHEV
RSRLTAIPQEPYLLTGTIRVNVDPFGRASDEQIIRVLETVGLWKLVQEKG
GLDAVMTEGSFSHGQRQLVCLARAMTRESCVLVMDEASSSLDVKTDALFQ
QVIRSEFAKHTIIAVAHRLDTVVDFDYVAVLDDGVVVEFDAPEELLKRPS
AFRELYEAQKGETIEEEDEEISDSTTANEKWGWMWE
>Mb|QLI67006.1
MAAPESPQSTHSGSFEPVDLLTEPQSLARAVHARRPEYLRPHKIRVKIGT
WNVAACTGTDKDLERWFVGGQGIDKHFATLDLSRNPAVAHPDSAGDEDDP
DAVRLLGGNDIGLYVLGLQEVVDLNATREYMNRAVNVDSSPVGKWQAALE
AAMPPGYQRIMSEQMTGLLLLIYASPEVAPTITNVSTKQVGTGLLGYFGN
KGAVTSRLVLGETTRMVFVNCHLASGAGSSYLDRRCWDVDQILAKTKFDP
VVHAGVSEDDGETIGDEDFAFWFGDLNFRLDGLPGHDIRRLLTLHTRGEY
ALGDDHTPRPPEGEGIIVMRDSESDDDTTMRSSLHSRDQSFDSETTLPDP
GDFPEDPSQDPTSLQATLDSLLPHDQLRRMIAHRKVFHDGWREGPITFLP
SYKYDVGTVGLFDSSEKQRAPSWCDRILYRTRRDKQAFDAKLAEEIAAKK
KDEEMKSRGIDEDDDVLFSYDPDTDGDEQAEVAAYDYDEYDEAQDDIPRE
VVTKEGYVDRIALDIYTSHQRITSSDHKPIISVFSLEYDAVVPELKAKVH
AEVAWELDRAENEGRPAITIAMEGGKSGNENTVDFGDIGFLERKSSVFTV
ANTGSVPASLLFIESPTTDEVDSTSLTWLTTSFLHPDDDEASDLGKEVTL
EPGETVLAHVQAQVSDISYLRALNDGHAKMEDVLVLRVEDGRDHFIPVKG
VWLPTCFGRSIDELIRVPDGGIRKFLAEKDIKGAIPYDSDIRCSAPKEIF
KLTEAIQTLVERCVADEAMLESMTLPREPGWPFDSSTWTPGLKEQDETKA
DLVAALDNDSSITEVLPVELSSPHKLELLSSILLLFLASLTDGLVPPILW
AKLSTSFPNLTSLAPTAWADTKMHILDIFSSAPNHNIAFVFLTSTLARVA
TELSPATSEPLLPGLSRRFNFRRGEDDSEKKRKLRERKYAEIIAPLVFRT
NDRDKTVKEKERIVVEIFLSREAGG
>Mb|QLI67007.1
MALPRKEESSSQLRFIQYQHSLESEYLPAIRSLISKDLSEPYSIYVYRYF
LCQWAHLCFMAINPSDSSLVGVIICKLEVHSSHSPPTRRGYIAMLAVESS

FRGHGIATALVQQAIDAMTKRNADEIVLETEESNTPAMRLYERLGFLRSK
KLHRYYLNGNSAYRLVLPLRSVDFDVTQDYLIGEVS
>Mb|QLI67010.1
MLSSRAAPSKAVSLSRNAARGLATVQDGTPKRTYGGLKDQDRIFQNLYGR
FPPDLKSAKKMGDWHKTKEIILKGHDWIINEVKASGLRGRGGAGFPSGLK
WSFMNSKDWDKDTKPRYLVVNADEGEPGTCKDREIMRKDPHKLVEGCLVA
GRAMNATAAYIYIRGEFVYEAAVLQNAINEAYKDGLIGKNACGSGYDFDV
FIHRGGGAYVCGEETSLIESLEGKPGKPRLKPPFPAAVGLFGCPSTVANV
ETVAVAPTICRRGGSWFAGFGRERNQGTKLYCISGHVNNPCTVEEEMSIP
LRELIDKHCGGVRGGWDNLLAIIPGGSSTPILPKNICDDQLMDFDALKDS
QSGLGTAAVIVMDKSTDVVRAISRLSHFYRHESCGQCTPCREGSKWTEQI
MSRFERGQGREREIDMLQELTKQVEGHTICALGEAFAWPIQGLIRHFRPE
LEARMQKFAQENGGAALAGGWEANTRSQGKLVSPGQ
>Mb|QLI67011.1
MAAQVIANSGHDDMIHDAVLDYYGRKLATCSSDRTIKIFEIDGESQRLIE
TLKGHEGAVWCVSWAHPKYGNILASAGYDGKVFIWKEQGQNNQWQRIYDF
PLHKASVNVVSWSPHEAGCLLATASSDGNVSVLEFKDTAVDHATFPAHGL
GVNSVSWAPATSPGSIVSSSPGPGSVGNRRFVTGGSDNVLKIWSFDPASQ
SYKQEGEPLTGHTDWVRDVAWSPTVLQKSYIASASQDKTVRIWTSDPSSN
GQWDSKVLNFDAPVWRVSWSLSGNVLAVSGADNKVSLWKENLRGEWECVK
SIEE
>Mb|QLI67013.1
MTTNIPRPGPANLGPNAGLDEWLEEAKQCHYLPERAMKELCEKVKEILME
ESNIQPVCTPVTVCGDIHGQFYDLLELFRVSGGMPGETNVQAPKTATTVI
TSDDIEPPSEITNPKLRKKLRSPVGDAGAGGTEGEDDADAVAGSRPGSAL
SGANVTSAQSAETRFVFLGDFVDRGYFSLETFTLLMCLKAKYPDRIVLVR
GNHESRQITQVYGFYEECQQKYGNASVWKACCHVFDFLVLAAIVDGEVLC
VHGGLSPEIRTIDQIRVVARAQEIPHEGAFCDLVWSDPEDVETWAISPRG
AGWLFGDKVATEFNHVNGLKLIARAHQLVNEGYKYHFAESSVVTVWSAPN
YCYRCGNVASIMTVDKDLNPRFSIFSAVPDDQRHVPASRRGPSDYFL
>Mb|QLI67015.1
MSFNLSSCVPAPVCSDKTTKFTNLDSIVDISKYYGDSTKKDWVAQGEPAS
RDGSVLLTMPKNSVGTVLASTSYMWYGNVKAKFKTSRGRGVVTAFILLSD
VKDEIDYEFVGVDLETAQTNYYFQGIPDYHNSGNISLSDTFHNFHDYEIR
WTPDKIEWLVDGQVGRTKERKDTWNETSQSWMFPQTPARVQLSIWPGGAS
TNAKGTVDWAGGPMDWEAEDIKNVGYFYAAFSEVTMECYNAKSAPGTNNG
KSYTFNSVTGTNDTVVDGKGDTVLGSFEGTGFNMMKDKASASQSADKPKN
TAATIPGGSTGGTGQDHSGKSGSDSGSNSGGNSGGNSGGNSGGNSGSGNS
GGSSSSGCETNTFNQDCASGSGSGSGNSDKGKNSGTRASASALAMIIAGC
ALFWL
>Mb|QLI67016.1
MDDETRSRYESKAKDLRTELKAWESEWAQAHGGSKPGRQDIKDNPDIAQK
YKDYNKARDILSGKQPPPAKSEPKPKKRQPDPILTETPLKRTKYAQTPSK
QRTHDEDLMNTPAISRKLFSPAPVTSLGPTPQRDGRVLGLFDLLVEKELG
TPSKKDTSKPSGTPGRSRGNVQSTPSKRTGSYDHESSTRLGRTPMSSSKR
NVLNTFATPMKNRRGSLDAKTPSSVSKLQFDTPAFLKRHSLPAVDENAMF
ADPSPLRLPRKPLVRGLSEIVASLRKVEDDVLDDDDDLDALRAAENDDTD
IRRPTSPSLGTRQASDPAAVSTPAIMEDSQRPALLGGFDDENMYDSPVED
AVDHNGNPLPVYKKKGQKRTTRKVNIKPTRIKRPENTPKDGNGDEDGEDV
IPETQLINGPDADFADVASGSDFENEEPAKSTDKPAKATKKEGAVKKVAR
KVNELAHANFHRLKLRNNGAKGGPGYNSRFRRRR
>Mb|QLI67017.1
MAIGSQLSLLLAAATALAPAQVAAAVVQDLPRQHDASGSADVVVRQTSLI
QRATDQSPKGYAPSPVDCPTSRPKVRDGGGLSSQEREWLPKRRNETVAPI
RELLKRIAIPDFDSDAYLKNAASDPTALPNIGLAVSGGGYRAMLNGAGAV
AAWDSRSTGSQTKGNLGGLLQSATYISGLSGGSWLVGSMYTNNFTSVQDA
VNAPQIWQFDDSILKGPEQYSLLQYYSEILDDVDAKDKAGFDRSITDYWG
RMLAYQLINATNGGPGFTYSSIANDPDFSSGKNPLPLIVADGRAPGQKII

ASNSTIYEFTPWEFGSFDPSLQGFVPLQYVGSNFTNGSIPGNQKCIVGFD
NAGFIMGTSSSLFNQIIMYIKDGNSRYVPEDIPKFVVDALTTFLNALGDE
SNDIADWTPNPFKGWNTAKNPSANETRLTLVDGGEDLQNVPYHPHLFRER
RVDVVFSIDSSADTDSSWPDGASAIATYERSLQPSVANGTGFPAVPGKDT
FVNLGLNSRPAFFGCDSSNLTAPSPLIVYIPNYPYIYESNISTFQMAIKS
DERDAIVQNGWAVATQLNSTRDPDWAVCVGCAMLARSFERTRTAVPDKCR
QCFANYCWNGTLNETKPAPYVPSLYGKPILSKNSGAAGQELSAMMTTVGL
VVMAVTAFNL
>Mb|QLI67021.1
MNYPSPRTPGSTPIHEWDYSRMSSATPTPSPRPNWFDSSTPRRPATRAAH
SRASSYSQHGGLTPRTHMESPRYNSYGDYCTVDVSAKDFSSSRRHTAHYP
APPRRDRRHSYTYVRASTPYGESDEDEIIEVLGGGTYVLLAEPRARSRRA
PHLSKHDGGWYTNQGVRPQSSSYNYADPAYARASPYDSPQPQPPQARPPT
SHGHSRRSSASVPQRPSTVRPAASSQRSKPPPSTPKATEGDARRHKIPQG
YSLKNWDPEEEPILLLGSVFDANSLGKWIYDWTVYHEGPDKPIADMAGDL
WLLLIKLYGKIKRAEEIVGQIRSAENREIMNDFIDAGERLTDKLRALLKA
CESPMLKAAKKKGSGLGKNSGVEFVLTLFGRDRELAKTEKLMQGIRLFDL
RFDANCQDIIDNPTL
>Mb|QLI67023.1
MNQRQRQGEATDGSSRKSRVAIIGTGLAGLTTAYLVHHDERKRYGVTLFE
QADTLSLDAASITLSNAKTGKNERVDVPPRSFCKGYYNNLCRMYEHLGIG
FQRIRLVWVYTKASATSQYEPPPEPRDAETIPDSYFVYNRRLHELLLLSP
NFWHSSLRQILQTFYLAICHMWFFVACFLLQPWTSQSEPCSDVDADTKSS
SDSETFHHYLQRIRLPRQYAVYYLLPVLSIICSCSHAEMMDFPASDVVDF
VKGSFFRKTFVPQGGVNRVQSTLSKGIEDVRLGARVTQVTRVDQGMLVRW
EQAKDGRSSSSEEIFDRVVLAVSPNVAATIFSPSRSTLGVIPTAPVTSSI
LAPPSGGVSLARGEGQTPSGECSYRGGDAQIMEFRTAFHGGTAQSESFHF
LPSGVVIRTSPSACIAESKGSLHTSKFTRTLRTIRSRSMVERVMGKDTSA
HGSEDVWVNGKDNVWIVGSWCWDGLVLLEGCVVSAMKVAEDFGVSIPWSE
KQ
>Mb|QLI67025.1
MKIKALNRSLVDHQPPGSDAAKLPRNLDSALHPFERAREYQRALNAVKLE
RMHAKPFIGQLGRGHVDGVYSIAKDPNSLQRFASGSGDGVVKVWDLTDRD
NSIWRTTAHENIVKGMEWTRDQKLLTCAADRTIKLFDPYNTRADSKPISS
WLGAGAFTSLSHHRSRNAFAAASSVISIYDLERHTAAPEVLKWPTSTDTI
TNVSFNYVETSILASCSNDRSLVIYDLRTSTPVTKTILSFASNQIAWSPM
EAFNFATASEDHNIYLFDMRKMNRALNILKDHVAAVMDVEFSPTGEELVS
ASWDRTVRLWNRDRGHSRDIYHTKRMQRVTAAKWTPDAKYILSGSDDGNI
RLWRANASQSEGIKSARQRQALEYNDALTSRFAHMPEIRRIKRHRHIPNV
VKKAGEIKSQELKSIKRKEENERRHSKKQFERRRSEREKMVLAKEK
>Mb|QLI67026.1
MTLIRSAVCRACRRSQFQLRRNFATANGQQATSNTKVKLVEVGPRDGLQN
EKKTIPLATKIDLIERLARTGVSTIEAGSFVSPKWVPQMANSSEILEHLL
QNKVQAPVPMTYSFLAPNTKGLQNCADILKANPETFSTQLDPRADSNGEP
KPAMEVAVFAAATESFSQKNLNCDIQTSLERFKAVIQDSKALGLRVRAYI
SVVLGCPFEGFDVDPHKVAEIATDLLESGADEISLGDTTGMGTAPRTSAL
LRCMSAAGIRTEDIAMHFHDTYGQALVNTAVSLEHGIRTFDSSVGGLGGC
PYSPGATGNVSTENMVYFMETLGMDTGIDLDAMTDIGSWISTELGKSNGS
TVGKAVLGARARAAAEAAKA
>Mb|QLI67028.1
MFMPVATQASLKGITPQQLEETGCRLCLNNTYHLGLKPGQEVLDAIGGAH
KFQGWKHNLLTDSGGFQMVSLLKLAKITEEGVRFLSPHDGSPMLLTPEHS
ISLQNTIGSDIIMQLDDVLVTTSPDKVRMREAMERSVRWLDRCITAHKKP
DSQNLFCIIQGGLDLEMRRECCKEMLARNTPGIAIGGLSGGEAKADYCRV
VEACTALLPDLKPRYVMGIGYPEDLVVSVALGADMFDCVWPTRTARFGNA
ITKHGVLNIRNAKYANDFGPIEPGCECMCCRQGEGGMGITRAYVHHNTSK
ETVSAHLLTMHNVWYQLNLMREVRQAIIEDRFPAYIRQFFADLYESKTDY
PDWAVEALKQVSVDLTVEG

>Mb|QLI67029.1
MEDVFAIGSNGSGQLGIGHEEDVSVPKQVHFQPAPLGSRIMKIAAGGNHT
LLLDESGGLFWSGDPSAGACGISSNDSKIAVFRPVRLVHSGHDDIRGTPV
LIAATWEASFIVSRDDTGRSTKLFSFGTGMKGELGAGELIVRTPTATQLQ
DFPPPGTEIIDLAACMGHVVAVLDNGDAYGWGNCRKSQVGAPAGVIYKPR
RVADIGFGVHRAVCTKEATCFFGAPNSGQVRILGSDKWGIVSKSPRVTSS
WSDIGAGWGAVYILNRDGTLQAWGRDDHGQLPPPNLPRLEQIAIGSEHVV
ALSQDGDVLSWGWGEHGNCGPRVENNDVKGRWNVIASSKFIPADSRITRI
GAGCATSWICIGRV
>Mb|QLI67033.1
MAAPQPRSPVAADSESRESSLARDEGHVGLAPSIPGLFTSEPPIRDALAT
DSSQIQDETVEECMPFLTGYGHDNCNAHGIPPLLRDRHVKFLQKQLGLLP
SMFKGADPSRPWIFYWCLAGLSLLGEDVAGYRSRLIETVRPMQNETGGFA
GGFGQTSHLATTYAAVLSLALVGGDEAYELVDRRSMWKWLCSLKQPDGGF
QMAVGGEEDVR
>Mb|QLI67038.1
MHRVSTARLLSRALGIGERAASVPVFLCPAVGLRSSPAPRAPGHLARSSR
RFNSTGVSARAVEDGPPARVDQLVRQLPVTCSGCGAFAQTGDAQQLGYFD
LQSRRVRNWLHPRRSERPGSAAAAEDKIVDDVLKGLDEERLEALGLRAES
MMEDASARDLERLVTEKPPVCDRCHDLMHHNMATDTAKLTMHHPTVESLR
DTIEESPYKYNHIYHVIDAADFPMSLVPRLNVLLGDIPLRSRNRRSRSSK
FHNDRKTEMSFIITRGDLLGPTKEIVDSMMPYLRDVLRDALGRLGGRVRL
GNLKCVSAKRGWWTKDVKEDIWNRGGAGWMVGKVNVGKSQLFEAVFPKGR
MGDSNKRDTGRPAPTPAQAADDAYGQQEAWEDLNVGELLPPPQPTRNYPS
MPVVSSLPGTTASPIRVPFGNGKGELIDLPGLARSDLELFVKEPHRQSLI
MKKRIVPEQVAVKYGKSLLLGGGLIRITPRTPDMVFLMYNFTPIDEHLTM
TEKAIGFQEQTRESIGLENITLAGVGGRMKHAGTFKLCHDVTKKRAGPLT
RKNAVGLQVDRLPFRVVSVDILIEGVGYVEVVAQVRAREFERWERIQADI
ERAAAVEGEDAEYVAAAPGEEPSSQDPFKEMMRNRRDVLGEDGPAVKARS
RLPEPDWPAVDVFSPEGKFVDSRKPINGWLNNKPRVLAEHKKMRPRRSMR
GAKKRAKMERRKGDPDAADPLHATTTMTDRQRQQAAPAPSTSQTQTQTGP
QTETQRILRLRGGHNASGRSVQWAEDVVDNEGLGRKSSKVCCIYHKPKGV
DESSDESSSSSSDSDTDSEPESARGDGKKAPACGGHSHGRARRPKDGRRQK
RPPSPNAYEKVPKQKPKEESK
>Mb|QLI67039.1
MDASTPNPPTLGTDAVGSTIVFFHPDLGIGGAERLVVDAAVGLQTRGHKV
VIFTNHCDPTHCFDECRDGNPPPPFLTLEVCLLLKKIPPQGTLDVRVRGN
SIVPPSIFSRLTILCAILRHIHLLLTIHLTGELAALSPRAFIVDQLSAGL
PLMRFLAPDVPVLFYCHFPDLLLAQGRQSLVKRLYRVPFDRLEEWSMGFA
HAVAVNSKFTRGIVGNTWPALQNKVPINVVYPCVDTHTTHETAPDEAKLA
AGKKLILSINRFERKKDIGLAIRAFAQIPEEQRRGARLVLAGGYDARVSE
NVLYHAELQALATSLSLAHHTLTPAELGSAAAPPDAQVLFLLSVPGALKA
ALLRAARLLVYTPANEHFGIVPLEAMLARVPVLAANTGGPVETVADRETG
WLRDPADAPAWTNAMARCLALPDDQLAAMGDAGRRRVRELFGRDKMAQTL
DESLVQIAGLAEERRVSGAAGFGVLAAFIAVCAVLAAWFAF
>Mb|QLI67040.1
MALAPSRTTISHAKSSTTHPTSLAIQIRHATFIPRPRRPYTFTQLVTLSD
GSTYTMRTTSPNPIYRASKDTRNTLLWQPSEKSLRNVELDEAGKLAAFRE
RFGRAFDATAQSDESETPAAASEASDDVFADLITGYAPQDTSTMKDSGPK
KAPTRRK
>Mb|QLI67045.1
MASPRDPARLRPGLNLLTTNSLAPNTYGNHVNSPMSAVSMASPFMQSAQT
PSSAIQPYNPQEWVPSPAQMPDRSRQFPVDGGQASAVPPPPYSPPRSRTQ
RPISTAFEHTVALGSTPPPPSRLTISPLHRPSPEPPVNPLFPPPPGAGGR
GSSRDRRFGLPSLGRRRDHEQPQASSPDPQTAAMQMRRSTGPAMSQPEPE
HIVAPIPSTPFTGPPAARRAASTGAIDTPTSQRSRSTSQTRWEPGMPLPP
PPPGPPPSSSRSQSVQSIDRASVPIISPPTRRPPPSGVTSLGPVPPTPAD
WVDADAEPSQPSQPSQSGRGRSPGLVIDTTTAANATDVPDPLGSSNSAGG

LNRARAVRHDKTILQRRAESRSRHTSHGSIDAIAQPHNISDIVVPSGSVG
VMQRLMAGKSTPRSAGRRDSELPRTADSLPLQDSRNSTPRAPRSGQLEAA
TPPFSPYHGKGVQMPTPSKAVAPKALPTPPPQTRSASGSRLRDSSRPSIS
ALTPISKQTVISQTAEQFATATIERFRSFAANESAAASDADRVRLFADFI
VNESRIRRERYSSAIGAMGSEIFDLSRDLFRPMVSSSRRESGASQDEWTP
ASTDPSYCQRDSTSSIVHGEGLSTSAPASAGLSASPGSAQPQGNWTNNYM
PSLSPILSMSVSDNYENGSSRGRPPSRWWETDSQGEPRMFERSKRESKYM
GVPKDQWVEEEQGTGAYIGTSEYESSTEYPREKTGWHDQEESSSWTPQPL
RLSSGTPPDSSSSQTSHHRPDAVDVSRLVTLPPPYPRHYPALNNNHPELT
ATRTAVRALNELAEINNVKERFTIASSKRREEFSKAASERRQSLMANLQK
EISSGNIGYADAAVIESDSQQQEKNNKKELEKTEYEQFQNEVIRPLNDLL
TAQIAKADALYDNLSKHLFDNGQMDADMPQEEGDDRPELLEKLTLLKWIF
ETRESLHRAIYDILSDRNTRYREVVLTPYRLSGNNEKLRSAEAFFAEDAA
KREYSLATEVLDRARQFRTIVEEAVERGVALQMSAFWDIAPPLRQLLDSI
PEDLEGFNIQIPASEFEENPAYHDHPLQYLYSLLMHAEKSTRQFIESHTN
LLCLLHEVKGAVVNAKARVLETQVTEADGSAISTEDREQRANSMRQMEDR
RLTEDLQEKVRLVEEQWNSALGEGIRTVKDRAGVLLLSTGGWDETLEEEQ
SFLGLDQMTNDIHT
>Mb|QLI67046.1
MPVHHLMVGTWTPPGAIFTFAFDDQKLTVELIKRTPIPEDEPISWMTFDH
SKKNIYGAAMKKWSSFAVESPTTITHQASHPMLHQPEASSPSTNTRAIFL
LAAKQPPYAVYCNPFYSHAGHGTVFASSPSGALDTAVQHYAYQPDSGIHG
MVFDPSETYLYSADLKANKLWVHRRLAEAPSPPQSATLELVGSVECPDAR
DHPRWVAMHPSGNYLYALMEKGNRLCEYLIDPATHMPVYTHRQYPLIPPG
IPDPWTMYRADVCSLSHSGDYLFATSRANAFDLTGYIAAFKLAKNGAIER
QICLNPTPTSGGHSNAVAPSDWCDDWVAITDDQEGWLEIYRWQDEFLARV
ARVRVPEPGFGMNAIWYD
>Mb|QLI67049.1
MSTFDGIVAEFPEIRMIEGQGKAILYTGDIRAEPWFLNALARNPGILEYT
CGFKTLDKIYLDTSFLADIPFQTKSEGIADLLAKVQRYPEDTVFHFQAWT
FGYEDVWIALSKALNSRIHVDDYKLRIFNSLKSRSSNQPFSPEFHLAAGA
AVLTGHMCGNTQHLGCLTSDTSVRIHSCEKANMCSVAKHPSVVSIRPVVC
RLPGGESMMEKGVGGGGTDLSREAEFDCITHEDVYALLESLPAWNQVDSE
TRKDIRAMLASMSSSGRDFALNMSIEEFSENLTADMSSLIQSLANRLSSK
TGVAKTPVNERSDSLPRVILFPFARHSPYGELCELVKVFSPKDIWPCTVD
KQWIEKITVESLFGKYCSKQIFEHDKQMAPLIAQEHEKRSRELRTPMVDS
QCDTSFEVTPQSKRIQEQAVLSRPQLPNALVEGAQAQTCHPAHASSSEEP
SSVNSIYSNLHSKARTGEDHVSPSQHEAERGEKSSTAARKRPYQELDDPS
ECSQDSARTDSQMAGIAIDSVSSLGGSALRRDAYKAMLSNTNEGGAWVPI
SLLSTDANHHEEEVEL
>Mb|QLI67055.1
MDPKQKFYRHFQDSVAELQEQIDELEAVSAVAGERQEAIDHVLAGISKLQ
HEVSDAAEFTPSYDRKQYSDNIKNLSDKLNETVARVTPRSRFQFKRSSIG
AHVDMGAPENDPRLHPGSLSRNTQSSSGERHQEARESDDTVGDLPSVAAI
RDYNAELSQPSTQFVRKPSFSMARNIGIANQSHLHIILPSSAARATASGS
LTDLKWCIVDMSVPTAQGKPFPGLALRNISNSLIVAGHVNGPVHITGVAD
SIIVVTARQVRIHECKNVDIYLHCTSHPIIEDCTGMRFAQLPKCYSIEGE
SSKENQWDQVDDFKWLKAGQSPNWTTLSDAEALDEALWTNVVPGQPGVSV
EETLKKVGIPRQ
>Mb|QLI67057.1
MRESPAKLGLVTEFENDSPPALARPARINALSLISPTRKAKGCSEVLRKG
PLVPLSTEERERKNSGSRSAGIRENLDSPNQTMSWTLLSSSDQELCHHGS
HQQISAENSAHSSSINSTLAVGGSNFCCDDDPKTGSFSSLNPGSASVSFP
PGIGKFTYNSLDDERSSVSTDYNDTRQTGSEGPLKPIRENVPSPNRVSSI
KLDNESALPGKLSAIRSITPSDHEDLLSLFEQMQIDSHELSTDDGSKPIH
NLPVFITPPSSPPETLSSSHKHQAIPRKPRGSTPNASLAETLVDDWNWEP
TRQKKIKPECTVNQSKGVLRKGKKMEFEATKQAIAENFLRELDLQIANSQ
ISKLTNSTNGVKIVWTKSLHTTAGRANWRRETVRTKQSDGSIVDTMHKHH

ASIELADKVIDDENRLLNVLAHEFCHLTTFMINGLTTNPHGKEFKSWASK
CSRIFGHRGIHVTTKHAYEINFKYKWECTNCGLGYKRHSKSIDTQRHRCG
TCKGELKQVKPAPRAKVANNSGASGQSEYQLFVKDQMKLVKRENPDVPQR
EILKIIAEKWAIEKKKKQENASSHTKEDMVIRKRPDDTQAHFTTKLMVDL
TLGD
>Mb|QLI67065.1
MSHKGKNSAAKGKKPAKSGAESKGEDALQAVILADSFQDRFRPFTIDKPR
CLLPLGNTPIIEYTLEFLAMNGVNEVYIYCGAFTDQVEDYISRSRWAPTS
RSCPFSVVQFVRLSDARSVGDILRDLDKRSLVDGDFLVVHGDLVSNFMLD
GVLAAHRKRRETSAANIMTVVLRSGGDEDHRTKTNGITPIFAVDAKNQRC
LQYDEMTPLQSDHYLALDPAIPDELSTEFEVRSDLIDAHIDICTPEVLAL
WSESFDYELPRRNFLHGVLKDWELNGKMIYAEILEDGYAARASNLQMYDA
ISRDILGRWTFPFIPENNLVPKQTYQRHANGLVMEHNVSHTHDARMANTV
IGRDTTIGPGSKISNCFIGTGCKIGANVVLEDSAVWDNTTIAEGTRISRS
IIGDWVSIGKSCTLSPGSLVGSGVRIDDNIALSKDSILSALTLSGEPISR
DTKLLGASTNAAAFADLDEDDLDERDASRLQRSLIYSLADFNISTSSIST
LSSDVDSDADEDGRFLSPGAASSSRSRLSSFASDDSTGKLSFHADAVHGL
LDTLRGETRDFDGAKLEFMGLRLANDASDGMMRKAVATAFARRAAELLTL
EHGSLEPTKAAEKALTSKKGATAFIDEVGVGGEETEQAEYILATQKALRQ
IKNPEQAKLSTLLAAMLQQLYTLDILEEDGILGWWQDERAAEGDGMAALK
ENCKVLIEWLENAEEEDDEDDDSDDE
>Mb|QLI67068.1
MSRSTSSSSVITSCSASPVVVAPPALAFMPFVAPPTEQHPALRSPLPANA
YAYALGNQDGKRDSGHAGTTASDSVIHEEDCEDDSGRDETGQKQEEQRPC
SDKTRELGRDGAVPEAAKHIESRPEGRALVVEVQTDRASAAGAALPGSPA
STTANSPSLPQADKSSRVFKQPISPRKLIKKRSLRHSGHKGRRSSESSER
AKEEPPMETDPEPLVRGSMATPASPMHKPGQNRDASLQSVLIPPGGLDGA
SNSISSATTDLDAEFAPLAISIPTDNFLDDAFMTGFSFSKRGSLMFGGKR
AAAVDNGGGAMDLSPSTTAPANNHNRDQAEMAPPSIDQTMSGDASNERNS
SSFSTAMAQPVPDIRILSPDVEKESQKVRSLYQTDDAINWEDGARFSFCE
HLEPTPEVPAEEDSHDPDLVPIANLGLASAGPRSVSSFSHYPDKTRRETE
LAGGLEDWDDIDGADVDRYGFIAPKRPSSRVGTPTELKSAQFSPRRRNVL
LKRDSVGLSSSLGGRTPSRKVSARSLNTVHSGASVTSLRSLRSSRSVMRQ
ASNLLPQNRNRRWMDEAGDMLTVSPTLQDRAEEAQVEKISEGLKRKEWER
SEKWRKMARVVKQGPDGQGMEFEFDVKNSKLIERTWKGIPDRWRGAAWWS
FLAAAAKEHNAPSSEFIINSFHNLQQRSSPDDVQIDLDVPRTINMHIMFR
RRYRGGQRLLFRVLHALSIYFPETGYVQGMASLAATLLCYFDEEKCFVML
VRMWSLRGLDRLYRPGFEGLMTALREFDGTWLAKPVAEKLTELDIDSTAY
GTRWYLTLFNLSIPFGAQLRVWDVFLLLGDGVEESSPSSSARSTATPRST
MEKNSEAPAQASGLDILHATSAALIQALREVLLDSDFENAMKALTSWIPI
KDEDLLMKVAKAEWKTHQGKKKT
>Mb|QLI67069.1
MARFSFSFTGRKKQNVPPPPTGPRMSKAHKILGSTPLNIDAPGSWDDVSS
LTTSDDRSTTTGASYSESELRGHEYEHRVAIARSDEDWGEESDIIPHPLK
LDAISFGNPTESVRTDTTGALRKSQSSSTIRSWYDKSKLPLSISQQTSSS
AIAKGLPPNFQPGLNPAGVEEGFRSKKKPAKLMFSRTARKGSDSTCKDSV
AESGLDRMLRSPSIISSFSVMSTRSRESRRFRKKHTKEDPPSPIPEPPRP
VTSGSNRIGRGNTREPPSLYSHYEQMSLRNVMRQKSTPSLTMTENGILTL
QNVTEVPYDGHEEEVAVHQRQKPASQNPKLTIFPKADLQSSPERSVTRRH
TKTPKASKSLDRSLEGADLLQTSVLMLSSDSEEDDTEPRPTVRPSAPAPL
RKPSKFETRAPPLEECRPKMVSDPKPSEERASQSTKLGKRRSVSASNTYV
ARPSPENRLRAPTAMSSRSGTPLGSFCPAISSPRTSVMSYSSVNSGMTWQ
GKSGYGVQEARAMTVLPAWRSPGAELEEKQDEASNTTSRMSRQPILNQTS
STDQLTPPLSPTSVDFYIGSAHSSIDGPGSHSRLIAVSHQEEVLLSALRQ
KQQAMRRASMSQVSEESEVDSVAGIKGQPENCRREQQGMESDMSSDDAQT
LAKEGHRKEHQAKESQTTITGSTFEFGFPAPPSFKEDTNTHNEQSPRRLE
SCGVHPPIHTEGIVLRSDSIPTSPVGPPPTLALPKLPKHARNSKSLPSRD
NDSTQSQRDVTLYFDDSEPSPDLSDIQGWESATAPTANAVSPDAPLATSW

PEHEGGQASGKQVLPSSKLESQSFLYCDGFSELNVRNQGPNQEKEATPGG
DKDIPRPDSPISPEAFPAVPARRVTLGNMARLSAVGPSLFTTAGAPGRRG
DDD
>Mb|QLI67070.1
MRFPALAVPALAAPALTASITLYLPPVPNPFTLPASTHATLSTLGSAFSA
PISALNTFVFHNVTPGSYLADIHCKTDGFRPLRIDVARDADGKETFQAWD
TFRGNEWGNKGEALPVKDGSAGWGVEAKSLGKKMYFVDRPQFSVLSILKN
PMILMGLVSMLIFFGMPKLMENMDPDLKAEFEARQREGPMAAMSGQQQNP
LGNFDMAAFLAGSGKKEGGAGAGSVSDGRNEPVRR
>Mb|QLI67071.1
MSRSTLQAFLTTARNALRAPPAQRAKPLTFVVGNESADLDSLCCAIVYAY
IRSHTAPHALHIPLSNLPRADLGLRPEMAAVLQHAGLAPADLLTLSELPD
HLQPDETRWLLVDHNCLTGPLKRFRDRVVGCVDHHVDEGALPGGISPRVI
EPCGSCMSLVVDECKPAWAQLLQGADDDIPAGEQDRLVKLALAPILLDTI
NLTAEAKVRPKDPQAVRFLEAQIREPGFTTTSYYEQISAVKEDISGLSFR
DILRKDYKQWEEGGLELGISCVVQGFEYLLEKADTKQAFLEELAAWAAER
RLDVAAVMTTSHPGGSFHRQLLVWGTTDRGKDAVGRFRDIGRPLGLEQWE
DGGLDGDDGDGGNTRFAWRQANLAASRKQVAPLLREALKEGK
>Mb|QLI67072.1
MISSRVALRRAGHIRWSCQPRFTARRQWARYNSDSSKGPAHRQGGNPFAR
AAVYAGVFGLAAAGAYYYPELKESFSTEEEAIEPSKAQPRFEKSRRQPIS
KEDNRDIISSQHLQVKNSWEHPGVYAWGSNSGKVIDPESNDKYVKLPRRI
SFFNDQILRDLKLTHSFGAAVNEKGDLIQWGLGFSSSDPKPVTTLRGKDL
AKIEVSTDRIIALSRKGDVYSVPSSRDDLEGGVKETRQKSSWSLWSSTGK
EAINFRNLTPSGLGWGERVIDISSGLEHCIMLTSKGRVFTAASSAIAYPS
KGQMGIAGLTWETRPKGPYDQPHELLSLKGFEATAIASGDYHSVILDKLG
RIFSFGDNTFGQLGFTMDSGLPYATTPSMIPVDKLYAASGMVPKATSIKA
GGTNTFFTVDASAPTTAVESRSMVPAKRMPGTISDLWVCGQGVAGTLGTG
KWTHVSIAPTKIKALSSLFEFDEETNKNVPIKLKALSVGSTHCAAVMDNI
TKTNTSSSGSENETNWGADVLFWGGNEYYQLGTGKRTNMNTPTYIGPLDG
GEGDANKRRKGEMHRLCLTPKQTVRIGEGGKGRKVTLEQKVECGRLVTGV
YSAV
>Mb|QLI67074.1
MAAPAPADQGLQDRILSHMNTSHTRELTHYLRHYCGLTPSQARGASLRDV
TLQGMRIRAGGADYVVHFKPALQSWNDVRPRVIEMDAIARKSLGISDIYM
TAYRAPRVADALVMASVAFYFVSFLALPWIVPGSSIWSILTAYFPGGPEM
FRWSVKLIFYPVLAIHLIEPMVLDATRLQKHGVDRWSGQWWMWVVSCFFE
GLMVFKRFDSVIAEKRAQKEAKKH
>Mb|QLI67081.1
MEDSDNIVTAVSSEDEHRLWTEMSECLAADCTSHESIDDTLREWLEITAR
LRYCTVDSHDDFLACAHMLLESDIFRNNKDYVRTQIIHSLLQEDETAPLH
AITSFLFLDGCSDEATFPRMIEESCFPRLLELITAQQEDADPRLHRLLLQ
LMYEMSRVERLNFEDLTLVDDSCVHYLFQLIEGVSDDVRDPYHYPTIRVL
LVLNEQYMLASTDSVTEGSSTISNPLTNRIVKCLSLHGPLYRTFGENVIL
LLNRETETSLQLLILKLLYLLFTTKATYEYFYTNDLRVLLDVIIRNLMDL
PDEKISLRHTYLRVMYPLLAHTQLNQPPHYKRDEVLRVLRILRGSPNAHF
APADETTLRLVARVTKVTWIEEKEEEKEGPGQAEVARKFLGISLSPSQYA
SSISVGDVAGVMERPGVQTPSRKASTASDTHGKTDTEGNGAELSARAKKP
LPIVPKHRHGIPFTQTAKVCGSVNGEGKKIPPKAPPPRRWGRRKSLTEQS
PVEPIGEANPD
>Mb|QLI67082.1
MSEQMDVGNNTPAASAKGSNSLATKVTAVLSTSFSDTEFREALALIDQQE
FANDAKNRRQIRINLHKDVIDSNGTIIDNFGRVSEQLHRVRLILEKLKTE
YESIKTQVALAQSETSPTLREAASLLEKREEVFVKQRVLSAFKEYFLLTE
PELVALTSTAEPVDERFFDALSKARKISKDCEILLGLQEQTFGLDLMEQT
SKHLNFGFQKLYKWTQREFKTLNLENPHMNTSIRQALRVLAERPSLFQNC
LSFFAEARERILSEAFHTALTGTTSSGIDDASVKPIDVAAHDMLRYVGDM
LAWVHSATVSEREALEILFISEGEELAKGLKEGRDAEVWRLVTDDEGKDS

EFNALAALNDLVDRDVAGVTRLIRQRVEQVVQANEEILPAYKLATLLSFY
GLTFEKLLGPASFLSECIHSLEMQAFRQFRSLLKDNIAIMQTDSENIPVD
LAPPLFFSRALDQLDAIMHTYDSSLSSTDFRGNEVESVLADALEPFMSAC
DIMAKSMISLKAAIFAVNFNLAAYKCLAKFEFTKKRAERIRIDIREASTG
IVQSQYKFFRVQSGLESLFNNINDSQDAGGCCLDGDTLTKASQQLDEFLP
SALMDAIDRVKCVQDTVLARQMTEEAADLFCKDFEKLEQEIDNRDANRAA
NDAGFLRLQFPRTSAEIRVLLS
>Mb|QLI67083.1
MTTDKKRVVVVGLGMVGIAFIEKLLKYDAHERQYTVTVLGEESHVAYNRV
GLTSFFEHRKVENLYMNPLQWYDEASVSLGYHINTKVTYINSASKTVTCE
NGDILPYDILVLATGSHASLPRHTPGCDAKGVFVYRTIDDLINLIDFAAE
RKESTGCVVGGGLLGLEAAKAMMDLESFSSVKLIERNGWVLSRQLDQEAG
GMVVQQVGDLGLDVMLSRRVREIKVDGANHVVGVVFEDGEDLDCSTVCFA
IGITPRDELARASGLDCVPRCGGIAVGDDLRTSQPDIYAIGECASWQSQT
FGLIAPGVEMAEVLAFNLTQAKLHAPRVFKRPDLSTKLKLLGVNVASFGD
FFADRDGPAHLPAKYKKLVNGAAKQAQTVKTLTYKDPFQNIYKKYIFTSD
GKYLLGGMMIGDTNDYIKVVPMVKTMKELDVPPSQFILGAKKDGDGDDGD
DLPDDTQICSCHNVTKADVVNCVKDGSCKDLGAVKSCTKAGTGCGGCMPL
VTTIFNKTMAAMGNEVKNHLCPHFSYSRAELYHIISVKQLKSLREVMREA
GNDDTSSGCEMCKPAVASILASLWNQHVMAKTTHGLQDTNDRYLGNIQRN
GTYSVVPRVPGGEITPDKLLAIGEVAKQYNLYTKITGGQRIDMFGAKKQD
LVDIWSKLVAAGMESGHAYAKSLRTVKSCVGTTWCRFGIGDSVGMAVRLE
ERYKSIRAPHKIKGGVSGCVRECAEAQNKDFGLIATEKGFNIFVAGNGGA
KPKHSELLAKDVPPADVVPILDRYLMFYIRTADKLQRTARWLESLPGGIK
YLREVILDDKLGICASLEAQMKGLVASYFDEWAEAIRNPEMVARFKQFQN
TPDTVDSMETEQDRGQSRPVYWPKEPAAQDFGSMGGRWSSTAWEPVIEAV
YFDGADELPNGISATVKRGDTQLAVWRFRGRYYATQQMCPHKRAFVLSDG
LVGHNPAKSCSNDNDNDNGGGQQSAAPWISCPHHKRNFDMRDGSCSNDGS
LSIATFSVEPREDGLLYLKLPPVEELDAALGTTKWRVRKGESGDSPFAEL
DRKMQLVGRKSRRVNGIKPTVNGTLDGSADGTGSESARPALMAGGGGCGS
APDW
>Mb|QLI67086.1
MSPKTPVVGLLGGGQLGRMLCESGAPLDAQIAVLDAENCPAKQINNNRHH
VTGSFKDPVKIKELAANCDILTVEIEHIETEVLEEIDTNGVQVRNADGTT
TNKKVAIHPSWRTLRLVQNKYEQKEYLKNQGIPIAEQMAIESGDAMLASM
GEASEKFGFPWMLKARKDSYDGRGNFKISNQKDLEQAVKEFGNLSCYAEK
WVPFELELAVMVIRTEDDSGKMKRVVPYPVVETVHEDNICSKVFMPPRNV
SENVCKKAQKVATSVVEKLWGRGVFAVEMFLTKEGDIIFNECAPRPHNSG
HASIESVPYMSQFKAQLTAILDEPLPEVLEPHVSSSIMINILGGADPDSH
LPLVEKAKSMYGSRTAVYVHLYGKESKPSRKIGHITVTGFGSIKDLEAFA
QPLLKMVDAIRQERLQASSKALRVQAAPTPGNPKVAKGNPLVLVTMGSDS
DLPVLKAGIDILNQFGVPWEVDITSAHRTPAKMAQVAIDAADRGIKVIIA
AAGGAAHLPGMLAAYTPLPVIGVPVKATHLDGLDSLLSIVQMPRGVPTAT
VAINNSTNAALLAIRFLGAFIPDLLEKMKKYQLEMEHQVKDKATTLRDID
VDAYLAKMGKK
>Mb|QLI67087.1
MPSTDRNSTSERSERRASTRRSRRVSAENALGDYEYDLATHAVSDGFRPA
VNNPPAVPNLNSPPSPPASRFHLPRLPRSPPPVAPSPMKGFADFNRPSST
SKPPRFHDSLTLRNDGLESARVGQSSSSSSPVQGDILYQGSTPPSHPYQM
YPQRTYSNATSSTAPSSETLDGTRGPTHPYALYTQSTTTSEDPTQQRIPV
GFNGMGNGYRRQIGPDGEDAGDLIGPLGHMEELPPYSRYPEEPFQHKPAA
DCDNTVSAANATDGAGARTNGLTTSTPAQPIPGAGGIGLATRNPEFSSTE
DLAGMPRGAGSSIRSRTSLESYHEINGAARDFAEKPTLSKWQRRAKKKLW
GVVPCWTICLLLAGIVIMGVVMGTVVGTIVTRHNTSPKGKGQSDKNQPNT
NNVVFLEHLPQDLPPLATGCFSLPPMDKYNVPKACIKGFEQNAAWNCDMP
FRWYSMNVTELAGAADVSNYAMKFRPFDPKASKFIYGTQPPNVPDFEPMR
LVKDTEEVNRGPAWFLEVKYNKTVMVREDQLALPSPSGPQRRWDHMKGPF
PGLDNSRFSRKGLAATEGDKPWICTWPNVTLQVFVYPNQTFTPPKPTSTP

SSISSMPSPFSPAAPTPTPDEPPLPKFKEPYPKLVKFVESRSYNNGDYAP
ATCTQYRITSDGQDKEPVLENGMPVAFIVEEVGKPAKGPITQRVSDPVPR
DIFSLEGRGADVTPCSCISFSWSV
>Mb|QLI67088.1
MDAPSKDGASSGRRQTPLTSPTDARRNTDDNTTPSRPGDGFGESYQSTET
VRHRPEGAGYGSINNPPTASSSQTAAESSQSQDRPQSKGTPQRSGFKPPH
RVKSFPRLRKPPLSRRTSSNTPHRGEIFSADDEPHEVVADAAERQGYSTR
RRSQQASTPLSRLQSYRAGGDGEDEEETRAGGVPTTTTEEDEPEHSDELP
LEEHVDCDSDGEISEAESFTLKDRQQAINQTHPFGIRVWKPALYKKDRSI
QKFAQADIHSSPGGRVSNWLLLFNLVWTLVFGWWMAFFAALGAIVCLLFA
AAPSGKEYGRVLWGLAGYLIYPFGKFVRLEKDEAYSHEDGDEGRSISEYE
QWQSGDLEYGRLFFGPDGNRSIVGRSRQSIDSEHSETDSLLGRGRRGASR
DMHPRMKRRRRLFGRGEWNIGRVIFFIFFYCLISPALIVTSAICWFLVFW
IPMGKVTILLFDHLRRHPLALSFETHMGYMRNDDAPDSSILVCTYRAVGS
KYWKYTIDGTNIFLINLMAVVCFVIFDWAVLEGVLHVESFITSPAFLFTA
GLLSIIPLAYFIGQAVASISAQSSMGLGAAINAFFATVVEVFLYCVALSQ
GKGQLVEGSIVGSIFAGILFLPGISMCFGALKRKTQRFNARSAGVTSTML
LFAVVGAFGPTLFYQIYGTHELSCTDCEDLEPGGKDGPGNVRDCRRCYFS
QAPALDDRFYLEAVRPYCYLAAAMLFFSYLIGLWFTLRTHAAVIWNAEIE
EKRHEEQLQASLLRASQPSAAETTGTDIRDSHLYKRILGQSLKHVGLQPR
PEEMARQASAPDAAANGTAATPHVVPPKGSDTIRSTVNVPGLSQADNTAL
AREVAHIAATAATLASRDRERLRKLSSTPGQHGTVRTHQNQADEATAAGE
TATVHAGGHGGGGHDAPNWGRAKSSIILLGATVLYAVIAEILVDTVDVVL
ESFSIDQKFLGITLFALVPNTTEFLNAISFAMNGNIALSMEIGSAYALQV
CLLQIPALVLFSAVYAHIPSGSDPSLYTFSLLFPQWDMVTVILCVFLLSY
VYGEGKSNYFKGSILVLTYFVVVIGFYFSGFTSEVMGIERFDVMGEDGNY
KSFKTIGRGSSGRAYGL
>Mb|QLI67089.1
MSGFGDFTSICENAPLPLCASVGPTLPATNRVGIEPDCYARNIALANTII
FEGAASAMHIVALIMTIVMILHVRSKFTAVGRKEILSFFYIYMILTFISL
VVDAGVVPPASGPFPYFVSIQNGLSNALVTCLLINGFVGFQLYEDGTPLS
VWMMRICSLVAFAISFLVSLATFKGWAGLNPTNTVGLFVVLYLLNAIELF
IYVGMQVILVTRTLHDRWPLGDIAFGVFFFVAGQVILYAFSSKICTAVSH
YLDGLFFATVCNLLAVMMVYKYWDSITKEDLEFSVGTRMNNWEVKELLPE
DERRATVYADDPYGHTSSYDPPYSPTAHRYSTKY
>Mb|QLI67092.1
MSSKIFEPLRIGRITLNHRVVMAPLTRLRANDSHVPEPMSAEYYAQRASV
PGTFLIAEATLISARGGGGSHAPGLYTDEQVRGWKQITDAVHAKGSYIFC
QLVALGRAADPETLKRETGLEVRAPSSIAMENGGNTPECLTDNEIWNLVQ
DFASAAKRAMEAGFDGVEIHGANGYLVDQFLHENSNDRTDQWGGSIAKRA
KFGLEVAKAVVQAVGPDRVGFRLSPWNTWQGMDATDPAPLFCYLIKHVKE
IGLAYLHLIESRVLNNVDCESAGNIMPFMEEWGSAKPVLVAGGYNQDNFG
QALERDYKDYNVGVVFGRYFLANPDLPFRLRHGIPLQKYNRSTFYAVMES
RGYTDYSFSPEFEQNRRDNGNRAPLL
>Mb|QLI67093.1
MPSVKNPNGPSKNRLAARASSAKKVRRKRSESARNKISKQDVARGARPGI
LPTSGPRAKVSAKKARKLEKKMGYALKRRMEAEGEKQMLDAPDVDAEVEK
QLNAENDMDIQ
>Mb|QLI67099.1
MTTVRICVCGDESTGKSSLIASLVKDQFMSNKIQPVLPQITIPPSIGTPE
NVTTTIVDTSARPQDRTTLRKEIRKCNVILLVYADHYSYERVALFWMPYF
RSLGVNVPVVLCANKSDLAGQGNTPQIIEEEMLPVMAEFREIDSCIRTSA
REHKNVNEVFFLCQKAVTHPIAPLFDYKEGRLKPACVAALKRVFFLCDKD
QDGFLNDQEMRDFQARCFDKPLTSDDLDNIKLSISKTMPDANLERGIDQS
GFLQLNKIYAEKGRHETIWIILRKYHYTDSLSLEDKFLHPRFDVPEYCSA
ELSPIGYRFFVDLFLLFDKDNDGGLNDQELEALFSPTPGLPASWVDSSFP
SSTVRNEGGHITLQGWLAQWSMTTFLEPKTTIEYLAYLGFESPNVKDDIT
AALKITKSRKRRRRPGRVERNVVLCYLIGAPGAGKSSLLDSFLNRPFEGL

YHPTIKPRRAVNSVELPGGKQVYLIFEELGELEPAILENRFKLDACDLIC
YAYDSSDPDSFSHIVDVRSKYPHLDELPSVYTALKADKDKTTQRSELQPD
QYTSSLSMNTPLHVSVTWNSIAELFVALAETATNPSMGFPKTEEEAPDRT
SLYLALGATACAAVAAVMIWRRSTNAA
>Mb|QLI67101.1
MTAKVPRNFRLLEELEKGEKGLGAEACSYGLEDSEDLLMSNWNGTILGPP
HSVHENRIYSVKMHCGDKYPDAPPTIQFVSQVNLPCVNPRNGMVDPNQLP
CLAQWKRENTMETVLIELRRYMAAPMNKKTPQPPEGSTYS
>Mb|QLI67102.1
MTSFTTPRIRRTGQNTQFTHNLSRRVNDLQTYPIQSPQGATILLYGHENG
VTIIWRGGRRLKRQKKSPEPEKKQNGTSDDAVMVIDSDDEHPPAKAQTVH
KFEDKPEFESENEEVPYPEIVQTLDLTLGTAVLHVAVMSMAPYNPQDRQN
ATGALLSDRMVFAVSCVTNDVYVITLPLTPPSFESKAREELRAGLLAGQA
GSGAWGESLVLLSGQRRYSDGLAISLVQPKSSDPDSKAPRAVVASCSRQA
SGTLALWDVPLDPKARPERAIEPFQTEYLPQPLSSISFNPSHPTQLLTVS
PYQAARVYDFAVSSVPPDPEAIGPFPPQGSWLLSLYQPFARPTASRKPIL
DAAWISHGRAIFALLADGMWGVWDVDGVRSSSGSAAMSSKLASGLKGAAL
TTFSISGYIEGTGSLRSVATQQKENHTGEFAPMTPHTRRQATASLSSTTT
FDRLAAVRGGIRTIALPPKGKALQEESLILWVGGLEHVCVIPILSKFWDS
QLRKGSGGGVNLFSGAQPTRMVKLADLSTGLLGERCCGVGLVVASLKSGT
SPEEDGGLPVDVLIQGESRLVIVRQGEDGPGKKIAKMADSRRRRLFSEGE
RSDAIIVHGNPERTTSLSFNLSTSKPGTLRRKSSQSKNDDSTGQFGVSDD
TLQLPNRSRVGFDFMNDLSAAADVKTDVTARNVEAEMLDIMEIDQVLDNM
RDSRGSRRKKVFFEED
>Mb|QLI67103.1
MSRWGPSMRRFTSALKTTCHQTRLFSVEAAAHHANKTQVYISRSNDPLLN
LSVEHRLLQTTPPESTILLLYVNSPCVVFGRNQNPWLEVNLSRLAQIVNR
PDEIGWKDSAVKLVRRRSGGGTVFHDLGNVNFSVICPPQAFDRDKHAEMI
VRALKSLGRPTTSVNKRHDIVMDVQLDGKPAHSTFKISGSAYKLTRLRSL
HHGTCLLRSPNLSNISGMLRSPAEPFVKARGVDSVRSPVRNVDLDVEVFK
DAVVGEFAKMYGDFDVQTEFDSRASQDQRIASGYEELRSKDWIYGQTPRF
TFCTHPFEEDPRHRPELSFDHKLHFEARHGIIERFSIGGQQNFSEQQLIN
RSLHDISNWDTQLFQAGLGRKDAYDVGSWMNRILGTEFTQISSPAAI
>Mb|QLI67104.1
MAESALAEASAFSMATGIKQEHEPTGLEGLDFALPPELSSKRPFEEDIGA
TTETPEAKRAKTETETEEASLEDGLALLVQNALSNVGDLVDQFTSGHETA
PEQPASDPMEVDTTIPLPEPPAVRATFVSDPRKFIRDTNVHALGNLALSL
LLVLVQSPFEDTIKLVRGSDSQHEKTFRGLKCSFEHVHEMYSPGPVLSAN
ELEFRDPDSQHFIELANLAQLGLRLVDGTPKALSEADENFLAMFQCQLSE
LPPGMTELYLGIKTQRAIEFLVEKEPEKPSEEVIGEVLTHGLEHTLKEQH
GGGELTSADQNFVSSVQTRKETLQGEVKEESEPAALREKHPAEDLLRSFL
EYTAGRLKSLTDIGNKLGITIEISDSEPPPEPIPDATGDADMDLDDLSSF
FEKTASGLVQNALAGLKDEPTTTDPAEGGEEAAAQTASTENKSEAASAQT
NGKIDLMTDYKELEALVAESTSNYVKTTLHGLSPIPYQPTVPTSTTDSMA
AQPPYQAQVQHHQVQPPYYTYTQPPPEPQLPPLPGENLPPNQTFPSSILY
DKARQAALSKSSAHTRREGLHSTRRPWTQEEEKALMAGLDMVKGPHWSQI
LTLFGQNGTISDILKDRTQVQLKDKARNLKLFFLKTNSEMPYYLQAVTGE
LKTRAPTQAARKEAEERARLNSEEDQAKLQGIMALAGGLQHPPQGRTPAS
PVNAVGSGVVTPAQAAQAASSAQHHAGHQANAAGQGAQATSVPRPTATPA
SQTRPGGQANTQPSSHSVAQANGRSQAQLPPQVPRPQGQPQHHQQGHQPQ
PQQTQPAEQAEQAQQAQQPTRTPAMPQPTPNTQAQAQPQPAAAQQHHAAT
TQPATTSIPTSTPLAAAPPQPITSPARAHSPQVSPPAAQPVPQTQPAPTP
SPAPQQHPGPPPEAPKPDSPTQQEQDDNAAEAALLEGLQAVVAQSLTS
>Mb|QLI67105.1
MAPGIVTKDMASTSMVVFPIEEDMPPTLYQNIDEKALVAKSKKHLLDYGT
KFEPDVITGCRGLYIYTALGHKVLDWTSGQMSCLLGHGHPEIVKVITDHA
MQLDHLFSGMVSPPVISLADRLCSALPAGLDKAFFLSTGGESNEAAIKMA
KTYTGKFEIVGIGASWHGVTAQALGAQYHFGRRGHGPLMPGMHMLPAPNA

YRSIFRKPDGSYDWETELEYGWRMIDLQSCGSLAACIVECIQSSGGMHVL
PPGYLKALKKQCEMRGMLLIVDEAQTGVGRCGDFMAINHENVVPDILTLS
KTLGNGLPLSAVVASDEVASVAAEKGFFFYTTHVNDPLPAAVGDKVLEIL
FRDNLIEHSRKCGEILHSGLQTLHSRYGCIGQVRGRGLMAGVEIVTDRES
KRPALELAKRVGNRAYELGLWANLSSHPSFGGTFRIAPPITVTESQVEDG
LAILERAFAETEGTLPLY
>Mb|QLI67106.1
MGSSNHPALSSLNINTLTQDELSVRTNLDRIFASKQTGFQGQPSWILLTN
LTENQRYELRVCWSALEPTRFDMEAYTLDTVLENDKLLQSLNAYAAFRPK
IQTTKQSTIIPNSSSLLIEIHSAADYFTDNAELMANPPPVLVDLILDPFL
LNVLPKSLLPTVGYLSLLGVVTWFVARWVASSLQSVADSMETPSKKQN
>Mb|QLI67108.1
MHSHSGQFCPGHAVDQLEDIIQHAIALGFKTIGLSEHMPRYEEQDLYPEE
RDCPRESLQALPPRHEAYLVEAQRLQAKYARQIHILIGFEAEFIRPSFAP
LVRQLALPACVDYFIGSVHHVHGVPIDYDAATYAAAVRAAGGSEERLYQD
YYDLQHEMLLALRPRIVGHFDLIRLKSADPGRDVRQWKGVWDRVLRNLKV
VKDQGGWLELNTAALRKGLQEPYPCRAISEEWIKMGGEFTMSDDSHGIAQ
VGTNYGRGLSYLEQLGVRNLWTFERQPHPGVAGSTKATLSDKAVPIDVFR
QHLTS
>Mb|QLI67110.1
MAAPSSERKGSSSGSQSTMTVAQREGHDMPDANNLYPGDKSDEEKAPPTP
EEPVVADGGTVAWLVVLGAWCVSFCSFGWINSVGAFQEYYQHNMLSSYSA
STISWIPSLQIFFMNALGPAVGMLYDRYGPRQLLLAGSVLHVFGLMMCSL
GTQYYQILLAQGVCSAIGVSAIFQPSINVIHGWFNKKRGAAFGILATGSS
LGGVIFPIMVSRLIRQVGFPWAMRTCAFLILALLIVANLTIRAAHPPRPQ
KLTKAQLAKPFSEFDFVFLCAGFFCFTFGIFVPIDYLPVQALDAGVDPNL
VQYLLSILNAASLFGRLFSGIVGDKIGRYNIFVTVCALTGIWVLALWIPS
SSTGGIIAFAALFGFCSGAYVSLIGPLVAQISPLREIGFRTGIIFFIASI
GGLTTNPISGAILGDDKNWVGVKVFAGVFCFAGTFLVLIARIRRTGWKLA
VVF
>Mb|QLI67113.1
MGSEFGITPDTKPEVRLDGVGIFWIVFAVTWTVILISGMAFLWTKRDMPI
LRIRGLGLSFTAIAFLHCYWVAVTTGYVYGPLMPEVAEFWIMSIWFPFGI
ALFHASNSRFLYVANAQKQYVKKTGDLGWDGQRPRIRKTLVARWKMLPYT
QRVLIFVGLGMVAHLSLTIFMFLISRKFHSGWGISGTEVTGTAMEQKAEQ
GRGWEWWPSIFWQLFWAWIVAPVILWRSRNLRDTQGWRTQTIACCLSGLH
ASPMWLIALYVPGMAPVNNYFIPPQWIAIAMMMLEIFTVFIPVWEVRRQQ
SLCQETLDSIARWESRQKSVQHSGKSLHSGTSSSSWMGRTKAGSVSTTGG
SILTMDALEHTLAKNPEPLQEFSALRDFSGENIAFLTRVREWRAQYIVNP
SLREPVRDEKDSEKTLTLSRECFESALRIYIDFISSNSAEFQVNLSSHDF
KNLKAIFEGAARVVCGECSTPDPATPFADVPKPQSESGETARYWGDIPEA
FNEGVFDDSEMSIKYLVLTNTWPKFIRERRSFDGASSAEAGK
>Mb|QLI67114.1
MASRPLLSEATQVGHIALTNLPPQLSLPMLIKKLSATPTLAAQNDILRQA
GSVDSQASLQANKATFFFQLERELDKVNAFYLQKEAELKIRLKTLLDKKK
VLQTRDGISRRSSKFTTLEEGFQQFATDLNKLQQFVEINGTAFSKILKKW
DKTSKSKTKELYLSRAVEVQPFFNAPVISELSDQATTSLQELGAWSDGIQ
VNFQTPGHVVTSQHFMGTDEGDADTLLLDTVITGNLETLRELLTKMQSAS
SSTDGGSSLPDRVTRTFLTAIHDAPEESLRVLLDTGLVDLQSYDDINERN
CLHQATIYGKQYVLEWGLKAGVSVDRTDVYGRVPLHYASLHGRLDMLEAL
LNVNSSTINLIDHDNFTPLIHSIIHGHIDCIQRLLARSARIDPVSDSDHV
PLNLACEHGSEAVVELLLRHGANILPDAEGLYPQHLVARSGKTSELLLLL
KKFGADLDQVDKLYGWTPLVHAASEGNVDCLQALLKVGVDVNIVDEKDLP
AMYYAAWEGHLECMKLLTPFNTHIRASPLVLQSSPGPTDSSNAPGPMSLD
PDAIPILELPPPIIPLRRYGHNFLDTKTVVQLSFDDLHEQPLIFFQDGKY
PAARLTISSKLSDLIPKNIILPFQEDTRMVSFQVDNLDTFSLDFDVFPTY
GAKIIAKTVVLPSAFKTQQGGISKCCLPLFDPRLRAIGQITFSTQVIKPF
NGQPLEITDFETYWKATSQFNQPTSAVVTGSSLSGDYVRLFVQYTADGVP

VIWSQWTISCGGLDLPLCRLSLQQFLTITAENTCRAALPSLSSKTVDNIA
EVYHILATAGITLKDALAILPRGINVNLQVLYPTQEEEKALALGPALDVN
VFVDAILTVVFDHARAQRAQYQSPEAARSMVFSSYNARLCTALNWKQPNF
PVFLCNDLGREDNILEENMLRPSGRRGTSIKEVVRIAQSNNFMGLICYSK
LLDMVPALVDAIKSHGLALVMDKSTDSPSTSPMTDPFPRPPKGVDGVLKN
HGILRFNDSIDM
>Mb|QLI67115.1
MASVGCANGASAARSFIQIAKRGYSTAAARSSAPLEFLLPRTASTTHCHA
AFLLRSTKTTRTGRASLRGAPFSGGLRQSQKRTFTTTVSRQATVCVLNPQ
KDEDGNEMMLEITPRAAKRLSQIMKKDDNPNLALRIQVESGGCHGFQYLM
SLVTIPPKDTAEWSTVVNEDDTIFQFSDEADTTSSSEHGPRIIMDEPSLD
LLKGSKVDFTMELIGSQFKIVDNPYATSSCGCGTSFDIKM
>Mb|QLI67116.1
MPKYTEKDLQEAVREVHQGTSQRSAAKRWHIPRATLQDRLNGGISHAEAH
ECRQRFSRQQEKLLSRWIFHLVALGVPPTQGQFEEFASRVLVVHGDSQPL
GKHWVQGFLRRNSEVKLVKGYLFSSHSSEPGDAVCDAPSHQSRRVRLQEP
TCTSTHHLEGQLSTENDFREEAAATWDSYSYMDHDPEMLFDGEALEIPDW
IEPPGPYLEEYTFPLQD
>Mb|QLI67121.1
MLHRTNTLTASPNLPLDPNVVTIATGVVFPCALEADGFIPPVAYVHLRAQ
KIIAQLHWLPRLSPLAAMARSVKRPRPEPHAFHQLGKQGRKTGITLQDRG
ERDEHGMQPIDSIFSPHEAKPASNDDVSGDSEGADMSIASSSGPGPQTLL
KNRHSITYPIPKSRSPIKTKLNSPAKRNPHLEFPSSPSRSTLLDDNTVTR
KIDFAAHSSKPRLSTGKDVGNGLKTSRPSLLRSEDDESNPPSDPILGHQN
DPTMSDMVEQSMQLVNAMHSDNFTAAGFDQGPDDLPPAVGNDDFPEEEPR
LPSAKPSPANPSPIQQPQRKPPPQPQPIPSIFGTRKRPSLEIVAEEDESE
KDEREDEEEERREKRQRTSMPPPPLPPPPPLSRPKTAASRGTTAPLISKP
KPPIPKTEVKPVKRGRGRPRKTERRTKPADEDDEGDETIMEIQRAPPMPR
SRGLVSVRNEASDTQTRSGRRSFRPLEYWRGERVVRGEEEEAQDADGVET
GDFLLPSTKQVIRIPEDAPPSRRAPRSKTRTKAKSVAVGEAVQEDEELED
WEMDEGTISGGIVLWEPEHEFNPPTEADEVQMVEDRLAISADAIQTQDIP
LATFRFAKTLTLPFMGAGVVDLPPGAEKRLKNSRKMHMVFFVHYGKVLVT
INKEQFRISAGGTWFVPRGNYYSITNDYEVPARIFFAQACEVAPPLEEEL
PEGSYEEEQKT
>Mb|QLI67124.1
MDDVRFRSQHSPRNEASMAGFVSPPRNNGRISQHTSHDQRGNMPRRFTTD
SGRVPTLSSMTAALTSPPRGLGLDSAPDYNNALHKVQLIEQKKLEYERIR
EQRRRFELEMQRLDQQQRREAQELAQMEEEIRIAGHQSEPTTPPEYRDNS
GFPSIFSRPNRYSTSSLASPPGFNRPARSGSQLTSPPSGLTQQRYAFDER
AMPSRSDPNTRRNSDDEEKEEAVRQDPTSHRSTNALHRYSMPVTRSRNGL
YDMNLDQTNTTRFLFGDDEPNSLPRGRTPDDNFPTLVRREDQMLSASSAA
LDLALSPSPNPEKSNGWNRVNRHRHQQSMSAINGSSSSKSNEVITIGSRP
QSLRHSLDLKYISESPAEPSSVAMSPQSSHMASPPRLQSSFSANDVPTVK
NTSGSSILSGNANNHAQQHFHNHNASMGRIPAGAMPSRHNRELSGDNTMN
NGREQSNGFQSIQSALQASAAPFGPSLTTAVQVSSATAVSGPSTTATASS
FNNFYTPNGYGSNAAGSSGSFGMPMLTAGMQQMSMNGVNGANLYPAQNYT
GYGSLSYNQGAGQPRDSQARVIQHRRQLDNEAMSRYQNMPLESFRGQIYE
LCKDQHGCRYLQKKLEERNPEQVHMIWLETNQHVIELMTDPFGNYLCQKL
LEFCNDDERTVLIQNASQDMVRIALNQHGTRALQKMIEYVSTPQQVHLII
EALRFRVVELIQDLNGNHVIQKCLNKLTSLDAQFIFDAVGNNCVEVGTHR
HGCCVLQRCIDHASGEQKPWLVGRITEHARILVQDPFGNYVVQYIIDLNE
PSFTEPMVAMFQGCISQLSRHKFSSNVIEKCLRCSQAPSKDMIVEELLAP
QEMERLLRDSFANYVIQTALEFATPHQKYRLVESIRPILPQIRTTPYGRR
IQAKVSAYDNRGSAASSGQVTPAENTQGQIPLRQGHNRGMSGNASMLPSN
GITNGGLAGLGANQGVRQGVASYPNSSVLTVPPPSGSAPIQQPQSQQQQQ
SQFNQRAPGSFTPSHSANASVDASGETRWV
>Mb|QLI67125.1
MGHSKQHDKGSPAPPSEPPHLTQQHEDPASLGHLRAASSASSSRDTQTAE

TSSVPDERASSVGGHGAFSPWSAGSERQLGHHSAASDISGDRVFPIRSVI
SVEPTPRQSIMDENLNQSSDADSRRISGHWTSYRGDSSSDTRRLEFAPMS
PTAGQPSGSCADLRRKRAMSGPVSSIHADAVRQGSVAPLQLEISASDHGS
QESGPLGVGDVAAGPDSASITSGNQSLGEVPHITTRFTHVETDEGHAIIT
GRDGILQQCEDEPIHTPGAVQGFGVLLALREDGSGGFSVRYASENSGKII
GYTPKQLFRLKSFLDILTEEQQDNLLDHIDFIRDEDADPAINGPEVLSLS
IKPPKTGKSIKLWCAIHINNAHPDLVICEFELDDDREYPLRPADELTPDT
PHDTLHSNPTLEEIEESTEILSKPLRILRSARKRRGEQGAMQVFDIMSQV
QEQLASAPNLDTFLKILVGIVKELTGFHRVMIYQFDASFNGKVVTELVDT
SQTVDLYKGLHFPASDIPRQARELYKINKVRLLYDRDLDTARMVCRTKED
LDVPLDMTHAYLRAMSPIHLKYLANMAVRSSMSISINAFNELWGLISCHA
YGNHGMRVSFPIRKMCRLVGDTASRNIERLSYASRLQARKLINTAPTDKN
PSGYIIASSDDLLKLFDADFGLLSIQGETKMLGTIEHSQEALAMLEYLRM
RKLTAVASSQDVREDFPDLKYPPGFQVIAGLLYVPLSVGGNDFIVFFRRG
QIKEVKWAGNPYEKVVRDGTVGYLEPRKSFKIWNETVLGKCREWNEEQVE
TAAVLCLVYGKFIEVWRQKEAALENSKLTRLLLANSAHEVRTPLNAIINY
LEIALEGSLDHETRENLARSHSASKSLIYVINDLLDLTKAEEGQSLIKDE
VFDLSACIEEATEPFHVDAKRKGIDYQVTKDPGLPQFVHGDSRRVRQALS
NITANAVAHTHQGSVTVEMFVSEIRDGMALVDFVVADSGIGMNPRQLDAL
FRDLEQVSSEELESPSPEPVDKQRETRTLGLGLAVVARIVRNMDGQLRLK
SEEGQGSRFVVQLPFHLPDDSLSPESGGHSQDSSSATHTGKVIASASPNL
KATPAGEIMLIERGSHSNMAADMDKRSTGTGSAGSRRSVESLVSHGSQKS
DAERLIDAIQTPLSLNENQTGYFAHGQSSKGNNIQPASLTSTGTVRPPSP
GPHKQDAPFHPHPRHEPGTAEIRDSKTRVKAVKMPDEFVDLPPRIPKGDS
SGVLLQLPSQKPQQSATVDKGASSNKEPSLNILVAEDDPINMKILQKRLE
RVGHTVQHTVNGEDCAMVYKDKSPDFDVILMDMQMPIVDGLTSTKMVRST
EQSSEHKGHSAMADANRRIPIFAVSASLVEGNRQVYVDAGFDGWILKPID
FKRLSTLLAGIHESETRNACLYLPGEWERGGWFSSVEGNGSEDTPCSQTT
PTADPESHQLEGSVKTES
>Mb|QLI67127.1
MPSRKRSAPDQSTDRRRSGRLSASAQKSSYFEDSDTGSNEDEAPVKKQRR
LSEKRKSASKQVEEDQYVQDTAVEHHDDEGEEEGDDDEGDDAPRRVEIIP
LEKMRDTGGVEYEDHKIHRNTLLFLKDLKANNKRSWLKSHDDEYRRALKD
WQSFVEAATLTIIDVDETIPELPVKDVIFRIYRDVRFSKVKTPYKPHFSA
AWSRTGRKGPYACYYIHCEPGSSFIGGGLWHPEAALITKLRRSIDRRPDR
WRRTFNEPLLKKVFFPNAKANASPEAVIKAFVQKNQENALKKRPMGYEVT
HRDIELLKLRNYTIGVKIDADMLAKDTAQQKIQEIMRGLSGFVSFLNSII
MPDPAIDGDDSSDDEDEDEEEEEGDGDEDEE
>Mb|QLI67128.1
MTVSLAELDAHEQPSDEMRAEWKHFYRLDPSVVVQDPRIDDPRLPLSENG
FQASGKIDKTQAADAFAALGDHLRSFAQEDIPVISHPLLPGLLIMPNLVP
PAVQKDLLAKILHRDLSNPKHQTNLHLHYTLPYSSATCSPPDSDSVPDSP
LPSRPQSFFTLSPEVPATFTPKDPTVHKPLSAGQVLNRRLHWVTLGGQYD
WTNRVYPDSEPPRFPTDLAQFLEKLFPETAAQAAIVNFYSPKDTMMMHRD
VSEEVDKGLVSLSFGCDCLFMIAPNSRAGRDDDKDDKTDGHVQGEKKYLL
LRLRSGDAIYMTKESRYAWHGVPKIISGTCPDYLESWPAENGEYAEWEGW
MKNKRINLNVRQMRD
>Mb|QLI67129.1
MHANGDMSAKVATTDVCIIGGGPTGLFTGLLLHRLGVSVCIIDGKPSTLE
LGRADALNARTQQYLEVAKLLDELLPIGLKCNTSSTFADGEFKSRQNTWW
VGLENTLHRNFLMVGQPVVEKLIASKLGEGLVRFGEQVVSVTEDDEGVET
VTDSGFRVRSKYAVGSDGARSMVRQAIGATFTGTKPEMLWAVLDTFIDTD
FPLCPEIITFQLNGQSRVSWIPRERGMCRFYVLLDGEVTQARAEESIVEH
MAPHRVEFKKTEWYSTFDVKERIASTFISKDGSGRIILAGDAAHVHSVNG
GQGLNTGMSDAFNLAWRLAQVVKDQNLQPGAAQKIIGSYDTERRSTARDV
IDVAAALVRDTVHTAKQYVGTIEKNAGYITGMGVTYRESGSPLVQASEHG
IWTAGNRCPDMILNKADGAETRLYTETTYGKYLVLHIGGAPAPDFGFSDI
VTHYTIRPASESAAGGDDSGRTYTADWASKDDPFVVVVRPDMYLGFVGGS

SDEAKAYLTELYL
>Mb|QLI67130.1
MSWRVDGHFVAQHRRNIAAVNNEPCKCSLLVPNFTHDAPMTANDRQKPGA
SFAFKPRNNIASWGKPDFPNSSSRPPAQTSAARASASKDSFESDPALRDI
GAMVNERQPPKEGQPSHTKREQSPDDFDAVFDQLHRGFGSVKSQHTQGMD
PLSLASNASTGHPTFGHPTKMVSSRKQKADVFIHQKSRPEHHLGGTKMSG
PRPPVYQDRKPPAMPIFESNGPSKSRATYSDEIFYTDPKKANEDLKALLE
GGMEDDDEEEPEDAAASQPSQPQPKGSQSQHQTQGEGIPSPTQKVKKGSP
IGKDGTVNGLKVKLLPHQVEGVEWMRGRELGPVKRGKVPKGGLLADDMGL
GKTLQSVSLIITNQKPRKDSTGWKKHFGGLEKTTLVVAPLALIRQWEHEI
KDKVEKSHGLKVLVHHGPQRTKDFKQLALYDVVVTTFQILVSEHGASSDA
ENGVKAGCFGLRWWRVILDEAHSIKNRNAKATKACCALRSEYRWCLSGTP
MQNNLDELQSLIKFLRISPYDDLKQWREHIDQPMKNGRGHIAIRRLHSLL
RCFMKRRTKEILKKEGALNPGGKPSAAGEASTTGFKHTERKVVTVSAKMP
PAERKFYDRLEARADKSMEVMIQNKLSYANAFTLLLRLRQACNHPKLVEG
KLEKDKDALSTGSSQKSQDIDLNAVVDMFAGMGIVTKTCSICGRELTLDD
TTLGRENCAECHDDLEYFKNNEELHTKKPKRSTRKRSKKSKGKKAAKAKG
DEEERPRLRTRRPRNRNAIIDSDDEEEGDGSWLVPDGERGNVHLGKAGGE
EDENAEGGGDWIGTEDSEDDADDKDQSNLSSFVVEDETAKKEDGYQSVDE
AAQDDSLLSVDALTQAMASQTLEDKKPKPEASTSNAGSNSEEDDDSESDV
SESEPYSSEDDEDNGGWLRSGKQTQVLASAKIREVTKILHAEAKEHKFIV
FSQFTSMLDLVEPFLDKDGFEYARYDGSMKNDDREESLRRLREDQSTRVL
LCSLKCGSLGLNLTAATRVIIVEPFWNPFVEEQAIDRVHRLTQTVDVVVY
KLTVADTVEERIIDLQNKKRELAEQTIEGGAKKEAMKLGINEIIDLFKPG
GHHQDVQPGPLEDRRKATGERAGRGLLGRKPVREESAVFGRRW
>Mb|QLI67132.1
MVSSESRGSPPQRTGSIPVITFQEEPMSTSGDRPSPVSQAGDNTPFTTGQ
KNITVLATSKLINQAVPKQSTTEGSSNMSEAYPMDKLLARLSEQQAVLSQ
QNEALKSGDDEGNMCQRLPGHTSSSNSLPITPATDGFSSTAPSTRPASAT
LEEARSDSDEVLRLKLQLAQAQNKISKLDQELSIRTIKGESEMQAINAAR
GMDTTARDSVWMATDDDHSDTSEAMSTTTFTRTKGIWGTTKGSFAGNVLQ
GAAAEPQPGTWLGGRGFNQSFTDGSSSYQAMEGYRGDRLSPDPDLVMRQS
ATRRPNRYDHRASNSHHFASVGYNPVNGPMAQFEAIGGPMASANMHPPPG
LGAMGMGAYAGYQQQPVGTPLSPHASEFTSKTHWKNEGLPTEGPTYLPPT
EPLNYRRLLDRNVTCNWKYIVDKIVCNNDQQASIFLQQKLKVGTPDQKYD
IVEAIVAQAYPLMVNRFGNFLVQRCFEHGTPEQVINIARAIRGNTLNLSM
DPFGCHVVQKAFDSVPEEYKAIMVHELLRRIPETVIHRYACHVWQKLFEL
RWTESPPQIMKYVNEALRGMWHEVALGETGSLVVQNIFENCLEEDKRPCI
EEVLANIDIVAHGQFGNWCIQHICEHGAPPDRGRAVDHVIRYAAEYSTDQ
FASKVVEKCLKIGNSEFLGRYLDRVCEGRLDRTRIPLIDIASDQYGNYLV
QWILNNATPQHREMVAAHIRKHMVSLRGSKFGSRVGMLCTNHAVTTRPGP
GVGPGMGSRMGPGPRFAGAYR
>Mb|QLI67135.1
MASKRKASAMNAAAAAAEEPVDPSDELMFLCLGGGNEVGRSCHIIQYKGK
TVMLDAGQHPAYDGLAALPFYDDFDLSTVDVLLISHFHVDHAASLPYVLA
KTNFRGRVFMTHPTKAIYKWLIQDSVRVGNTSSNSTTQPVYTEQDHLNTF
SQIEAIDYHTTHTISSIRITPYPAGHVLGAAMFLIEIAGLNIFFTGDYSR
EQDRHLVSAEVPKDVKIDVLITESTYGIASHVPRLEREQALMKSITGILN
RGGRALLPVFALGRAQELLLILDEYWGKHPEFQKYPIYYASNLARKCMVV
YQTYVGAMNDNIKRLFRERMAEAEASGDGAGQGGPWDFKYIRSLKNLDRF
DDVGGCVMLASPGMLQSGVSRELFERWAPSEKNGVIITGYSVEGTMARQI
MQEPDQIPAVMSRHLAAGRRAPGGESERVLIPRRCSVAEYSFAAHVDGVE
NREFIEEVAAPVVILVHGEQHNMMRLKSKLLSLNASKTTKVKVYSPRNCE
ELRIPFKGDKMAKVVGKLASIPPPQDVNVDSASAPLVTGVLVQNDFKLSL
MAPEDLREYAGLNTTTITCKQRLTLSAAGIELIKWALEGTFGTVEELPEM
RRAQTANSNGVKNGTASEAAEDDEEEEYKPEEPADEEVAALVAAYLVMGC
VTVRYRNNGEVELEWEGNMLNDGIADSVMAVLFSVESSPAAVKRSSKQHS
HSHELVTNGTNPHAHPSAEDRLERLFWFLEAQFGADNVSPVETPRLPESE

SQDESMDVDGASTDVEERKRKELERLHKIGIPVPGVSIKVDKMLATVWLE
DLEVECTNKVFADRVRAVVERGVEVSAPLWG
>Mb|QLI67136.1
MSKTEQKACLIVIDGWGIPSEESPKDGDAIAAAKTPVMDELSNNAEGFTE
LDASSLAVGLPDGLMGNSEVGHLNIGAGRVVWQDVVRIDQTIKKGELDDN
QAIKKTFQGAAAGNGRLHLCGLVSHGGVHSKQEHLYALLRAAKKYQVPKV
FIHFFGDGRDTDPKSGAGYMQELVDYLKEVGIGQIATVVGRYFAMDRDKR
WERVEIALKGMCHGEGEASEDPVATVKARYERGGNKDKDEFLTPIIVGGD
EARIKDDDTVFFFNYRSDRVRQITQLLGDVDRSVLEDFKYPKINKLVTMT
TYKTDYPFKVAFEPQRMGNVLAEWLGKQNVEQVHVAETEKYAHVTFFFNG
GVEKVFPLETRDQDQDLVPSNKSVATYDLAPEMSADGVADQVVKRLGEQK
FPFVMNNFAPPDMVGHTGVYEAAIIGCEATDKAIGKILEGCKKEGYILFI
TADHGNAEEMKFADGKPKTSHTTNKVPFIMANAPEGWSLKKQGGVLGDVA
PTILAAMGLPQPDEMTGESLLDKNLKRGTQ
>Mb|QLI67137.1
MTPSALLNLTPDSLPARARILVPEKVSSDGLALLTPHYDVDVKLGLSAEE
VISLIPSYQGLIVRSETKVTPEVLQAGKKLKVVARAGVGVDNINVPAATT
QGIIVINSPAGNILAAAEHTIALLLATARNVGRADGTIKEGKWERSKLVG
VEVGRKTLGIVGLGKVGLNVARMAKGLGMQVKAVDPYASADLARQAGVEL
VPSLKDMLPQVDFLTIHTPLLATTMDLIGEAELKTMKKTARILNVARGGV
YNEAALLKGLDEGWIAGAGLDVFTTEPLAPDSVAAQLARHPKTVATPHLG
ASTVEAQENVSMDVCTQMLEILRGGLPTSAVNAPIILPEEYRKLQPAVQL
VEKMGRLYTQHFVKDKGGMIGDRKFELIYHGGLAGMPNTKPLFAALVKGL
VASFSDSYVNIVNAALIAKEKGIIINETHVHQSKDMTFSNLVTLKSIADG
PTGGKTEQIIEGYASDKRVYISKLDCFNATFSPEGTLIILHNYDEPGKIG
GVGMVLGSHGINIRYMQVASLDAEARQGHNTPPTQKDNEALMILGVDGEV
DAKVMEGLRKSEGVLDVSLIQL
>Mb|QLI67139.1
MATDAKSTGSAKVDAIVQNVKAAEPAQLSGLALYSRFALAGAVCCSVTHG
GLTPVDVVKTRIQLDPKTYNRGMIGGFRQVIQNEGAGALLTGIGPTFAGY
FLQGAFKFGGYEFFKQQWINTLGYETASQNRTAVYLASSAAAEFFADIAL
CPLEATRIRLVSEPTYANGLIGGFSKMLKNEGIGAFYAGFGPILFKQIPY
TMAKFVVYEKAAETIFRQYPKETLSDGMQTVANLAAGLTAGFAAAIVSQP
ADTMLSKINKTKGLPGEGTTSRLIKIAKELGFRGSYTGIGARLFMVGTLT
AGQFAIYGDLKKALGATGGVEIAK
>Mb|QLI67140.1
MFLHSGASLHGHEYSIKRSTSAPVQPPRRLPILKSTLRPLVSPSESSETG
YFASPYSEGLHAPLRASTDEANQRPSLLSQQRAASNSYTRKDLAVRFREP
GMDHIYTPSASEDEESIAGPEPLDGLSDSPARRKKRRRLLRKSTRYALAQ
PAPQLRTKQRRLLQIRPRLLLQLQEIGDRRAIPAFDLIPSQLVAGTLIIP
KLSKTFPRMFHANAELSQNDVLLVRSEDYASTSGMAPERSDDDRRSLHGR
DICAVISALPEDSENSAEIVLEDGVPWYASPMANGSYEFTRTDDGGEITT
ARWVRRFNGSTRNSIGSFEPSSSARSSVHDSLPDSRWTFSIIDPSSRRHP
IMGSLTSETVEVYDHYTTLSTSSGRFPPTRSFVPEAEGVDRSPSPAGFNP
ESRATLEVLPEQKALMIATASWIRLHQQGWPASANPKFARSIPSHCRSAS
ANMCSTSRRRTFPSYDGDSSRATSPINLSQPSDSLHDPSLSKHPHGKSMP
ARAMSTGRAFMKRRSDRMEGLATVYSQEPPRPVYDAEKLDTLPEEKVGCL
SRIRQWTHKLFHRRRDSIR
>Mb|QLI67141.1
MAQTPVVPPRPSRSSGKEAPTHLMPQVPPRPVNKRLDRSISPGRDRFAQS
PLTAGIPSEPVDNRLSKHYVQKEQAEDPIERASSVGMPSVGEEGMEYSAV
AAELEEEERRPSSPEQTRSVAEDLQLHAPKPSLPAQSAKQQVMAVTRTDS
DRAASFGIGQPSNGEDRAVSRASTRKRPESSISAYSDLGNHTDDDRGIPE
IGQRVPMNKHLGDVQAPSPGPEISKKHHSRKLSGRALPPGSYGLHGHGVV
PQDKLEKAYFQKHPEALEREQHTPRHERHNDFAMSSSDLNKLVRDTANHH
ARAASVELRGTPTDDVAFEASEKYASRISSSRPASTVFADGNAPISSQHA
ANEEDNAIHVDDSKNPEFYSYGEGKVDEESEEYTAPILASDELNKDPSPH
VHQPAIRPHLERRGSSFDGEDLPSRPPSRPRVQRYQSHQQEVKHTPLEDV

EEYDPLFPEDGKEEKSGEKEEPVDENCDRRHFPSKDIWEDAPNSVHYTAE
VSTPDVAEPERRRSSAQPGDRPITPAQAFALYQEELAEKEANKRGNTFLP
LQDSKPSWVSHQPHLQTEVRPSSGSSRRFPSRDIWEDAPDSHIHQAEVSA
SPVDASPVEKTKPDIPTKAVKEAELSPERPAVPDRPKGRQNSGDDAVKAR
PPVSDKPKPSIPPRPAKSSPGESKDGISSKTKPPVPNRPAGGKIAALQAG
FMSDLNKRLQLGPQGVKKEEPPKEEEAVDDKEKIPLSDARKGRARGPQRR
APAKSPAAATSSEAKAAAPVLSFSITQTVWSVDPEHGGFSFSNKDDSPAK
EDTKPEELSTALKPARMSAVEPEEAKGAPEQLESAKEVADSEPISKTEKL
KLLDDESAGGPDIEENIEKHTKDERKKDITLASNTAGEGILEATIKNDHD
EVEPIEVQEDVKS
>Mb|QLI67142.1
MSKPTYTLAAVRLDDVPEIGQLTADAFVDDRQTQMKNMGKEPYDMNKMTL
ESLPSMLHNPRCIAIKVVDKDSGDIMGVCNWGFRGFKPEEMPKMEGKPLP
LEKPEETKAGPEEKKEQGATDGNGKERLDREEETDPITRLQARTGADLEA
WMEEVMPEGTRCLFVIGLSVSPKHQGRGVGSALLRWGTDICDDQGVFAWV
HSSEPAWPMYEKCGFRTVRSLDIDLDEYAPAPPPNEGPNAKWGHYVFRYM
KYFGNKGEP
>Mb|QLI67148.1
MLSRLVQRRLAASLRNCKTTPSPSTSSRSVQRRWITPAPKPGDGPLMSRR
ADRELPEISQVRFRWGRTLPIFLAIVSVCSISIFNYQKTSSPIVSSTLYA
LRTNARARAILGDDIYFKHQIPWIRGQMNQMQGRINIYFTVKGTRGWATM
RFASHRPSSRSLFETTEWSLTTADGEWVDLLEGGDPFRGLLGDDGLAAME
EDDLHATRGYRQQGALNK
>Mb|QLI67152.1
MPYEHLTNPSMPPFPPSSLRSHDIQTFDSPAPLTIPEPGPPRAPTKINTY
GIPGEIEDMLPVFDACIRVGKLDRAALVLKRFNAVRVLSGEERILLNNQY
LRASLKQMRNSPDRRQAEELHKWYEMQIRSKGLPHTAETIACMLKASLLS
ERGSRLDRLVNRYMGMAPGESGLRVLSMDDILSDQDLAVITEICPKYNFT
VEPAEDMEMSEFTEEHPELRSTEASVDEAVDTISRGAATELIPTPQRGTG
LASLKEGLHLLRDLQDVDISKMSDADRRDIQLQLERDSIGCAISRWREAN
RNLAHMGISTVVSASSAEESLSCRMAGWLECLEEKLKEELALIKISENKP
TKSDQDLERCLYGPLISQADPSRLAALTILSVLSLGAAQGVDKGIVVSRL
VTNIAKLAQEDIETQRMEKAKADAKRAAKKLGNGIKPNRQVHFTGTTTDT
ETRTISQPNNENTTIIDDASETAKGLENATREPWSISIRAQVGSFLLKTL
IDNAFIKVTMEHPTTKQPISQKQPAFTHMQQPRRGKKVGVLHYNSNLVQQ
LKKEPIGDYIAKHLPMIVEPKPWRQLNEGGFLDSRTTFVRVKSGDVEQKL
YTEAAIKTGDMAQVFKGLDVLGKTAWRINKNVLNVMMEAWNSGEEIANMP
PLNPEFHIPEEPDSSADPLLRRSWMQAVKAIENERSGMHSQRCFMNLQLE
IARAFRNQTMYFPHNIDYRGRAYPMPTYLNHMGADHARAVLKFARGKELG
ERGLRWLKIHLANLYGLDKSSFDEREAFTNDNIPNIIDSASNPLNGSRWW
LKAEDAWQCLAACFELKAALELPDPTKFVSQLPVHQDGTCNGLQHYAALG
GDTWGAKQVNLEPGERPADVYSAVADLVKQAIDKDAEAENEFALVLQGKI
TRKVVKQTVMTNVYGVTFAGAKKQVCKQIDALYPDLGKECGLPNLVLSTY
IARLIFNALATMFRGAHDIQYWLGEIGGRVCRALTPAQLKEIRDSYEKEG
RKKKDELDDLMKQFRSSVVWTTPLRMPVVQPYRKSTAREIRTCLQSVVYP
VNEQTDPVNKRKQLQGFPPNFIHSLDASHMLLSALQCHEQGLDFAAVHDS
FWTHAADIDTMNGILREAFIQIHEDDVVGRLAAEFEARHKGSLYLAHLDP
SSPVAKKIKQLRKNSKLCPKEELVLEYKRNSLRLSGNPWDLEAAKKIVTP
ASVYEEMAGAEIDVSISEDAKNMGLGEIPQDEVDNDEGMTAIEMSDDMAA
DEDGITNSDDPHDAAVLSKRLMENMKISHFESQLIGAKKPNNSTKRVTKR
GTAVPVWLPLTIPAIPKKGDFDVTRLRESKYFFS
>Mb|QLI67156.1
MSHQRYPSHDPVKEPHSVSVKVLRLSRPALVPQYPSSPLPATKEAFLPSS
LSYKTPSTNPAPFLLSPILNLPVSFGSAYVGETFSCTLCANNDLTTTSSS
SSSPSPSPPPAKHIRDVRIDAEMKTPGPGPAHRLPLASGAPADLAAGETL
QRVVSFDLKEEGNHVLAVTVSYYEASETSGRTRTFRKLYQFMCKAGLVVR
TKVGLLGGGSSSSSRKRWVLEAQLENCSQDVMQLEEVGMEAERGLRCEGC
NWAEGERPVLHPGEVEQVCFVVVEEDEEDEDEDGSGADGDADGRVVFGVL

GIGWRGEMGNRGFLSTGKLGTRVG
>Mb|QLI67157.1
MAAADSSRLQEAQKLAKTDPRKSEAIYKEITSKAPAATSDAATREYESAL
ISLGELYRDEKKTQELVDLVRESRSVFSSFAKAKSSKLVRQLLDLFKEIP
NSTDIEVHITKDCIEWATAERRAFQRQDLEVRLVALYMTKQSYYDALTLI
NSLLRELKRLDDKLRLVEVQLLESRVYHALGNIPKARAALTTARTSAASV
YTPPLLQANLDMQSGMLHAEDKDFNTAFSYFIEALDGYDSLDESAKAQAA
LQYMLLCKIMLNLADDVNQLMSSKQAVKYAGKNLEAMKAIARAHSNRSLE
EYERALSAYRYELGSDAFIRNHLRRLYDAMLEQNLIKVIEPFSRVEIDHI
AKMVGLDTQQVERKLSQMILDKVIIGVLDQGAGCLIIYDETQRDESYDAA
LATIDKLSNVVDVLYTNQASMLE
>Mb|QLI67158.1
MSSRHSAARPNRAGEDFARTHHGEAGGDSGNAKFDVRNPSILAPDSREED
AILDADVIGSRHSTKRGAVNLDGYDSDSDNETFNTRAASRKKGKVDINEQ
LDNYDAAGSAAAASGGNVNGADEDEDDQDMFAMGGEADAPKEEEEEEDAD
FDKSGKKKKDVRFLDAAQIMGQEHGSKSGGHVRLDDQDSEDDEAEAQLAA
LEEGVDEEVGAGGLKRNAPKVEAFNLQAEMEEGQFDQSGNYVRKAADPDA
VHDNWLDGVSKKDMKKAAAAHEKREAEARQQRREEADVLVSDMLKTLILQ
LEPSESPLEALARLGRGQTKTKKIPKWKLKKMNKGAEDMEVDKEVDKEDS
AQDAEQTRIKESINAITDAADKLLSRDYEEVYDQDRELLIRAYRRETGED
WVEPASRHQDVEHEATATAEPPTQQWEFRWTDGRDGGAKQGPFDKATMKA
WQDAGYFAQGIEFRSAGSGDDGWTATATFAS
>Mb|QLI67159.1
MAARSGVFNECAVAFVPSNELTPKLIGDLSRIIEDNGGTICEPRRNGSIP
IEKATHIVSNTINFEQYVESQAIMIPVVSEHWISTSIMRRKAAQVRPFSP
DPRMIFAEIVVTCADLPLMDKESIIGATMALGGQESKDVTRLTTHICALS
MDAPKVMGALEKGFKGKIVLPHWFDACFKLGKRIDEEPYLLPDPEVMKKS
SDDAISIPANKNLVGATSVSPEFLPIAADSQGARPPVTVFQDRAVMLAAD
LHITARLAKAIGDIVLDGGGKLVRNVDDCDMYICQYRDGDEYIHAAQSCK
EVGSLSWLYSLIVNNQWQNPLHRLLHYPVPLNGIPGFKDLRITISNYGGE
ARIYLENLIKACGAEFTKTMKADNTHLITARDSSEKCKAAPEWGVAVINH
LWIEESYAKCELRAINISKYNHFPPRTNLGEIIGQTFLDESRIRDLFYPG
GEEKVSPSARRKRKILEAAADNAYHRGPAEGVVIGRDKDFDVMRDDEEDQ
NPSSLKSKKSTAVETPARSRRAHSGKENDTPPAMSTGSRSAKAKARDILQ
CIAPDIALYEKEKKRHSKAGAPWGGKRAANLAEKEKDSKTATSPKHKQED
EDEDTKRPSKKQRPSLPDVHMRLVVTGYTGWVGDSKKEDHDRRKMRALGI
LIVQEGQACDYLVAPQVVRTVKFLYALARGAVVLSSDFIDKVLETGDVPD
VDDFILQDQEAERKYKFKIERSMARAKANRGRLLQGIPIYCTEGVRKGPE
SYRSIAEANGAIFKLYRARSGTTIKPTTAEEDGFAKPEPVYLLSGNSPDE
KQMWVRFRTMAEKGNMESRIVDPDWLLDVAMAQEVRFDENFLIEKRNQDK
SS
>Mb|QLI67160.1
MDRDWEQDQSADGEGGEHLVEKMIAAAQGAMQTEQTADRPESEVMTEEDA
LKNLTGTVRDQNDLERDITIQASAALMEAEDKKDQNRMAKLDATRQRLQS
QLDKEKRRLERVAGNPYQSRNVQKEIAKLEEELGQITGDIADFQARIDKR
HQENQLDATTHSKSKRLAGESHREYLIRTGKITPFAKVGGARPEGIAGQL
ADTILEAEEEAAAEQYNQEAEGPTSHQLLRRPGFVDDVVVKDLKPSEYNV
ESEFSLRPRKKRRTERERSSSADFEPDHPSGSESADSIVWQQGNEDDLIR
RENRKQKAKLRAQEQEEVDLSKLDDGNENLYKRRLKDWILRRSRARRARR
QMADNEHTSAESDEDEDEWLKPSPDYPDHYINDELKLPGDIHPSLFGYQK
TGVQWLAELYKQNVGGIIGDEMGLGKTVQLIAFIAALHHSKMLERPVIVV
APATLLRQWVSEFHRWWPPLRVSILHSSGSGMMNPQFEDDYDVEHYRPVA
NRSLNAARSIVRRVVDKGHVLVTTYTGLQTYADELLPVEWGYAVLDEGHK
IRNPNAEITVTCKELNTPNRVILSGTPVQNNLTELWSLFDFIYPMRLGTL
VNFRTQFEIPIRQGGYANASNLQVMTAEKCAEALKETISEYLLQRLKIDV
AADLPEKTEQVLFCKLTEGQRKAYETFLGSDEVSAILNRRRQSLYGIDIL
RKICNHPDLLDKSLGSKTGYNFGSPKLSAKLELTKDLLQKVMIPNDHKTL
LFSQGKLMLNIIEKCMRECNISYLRLDGETPVDQRQPMIDRFNTDLSIHV

FLMTTRTGGLGTNLTGADRIIIFDPDWNPSTDLQARERAWRLGQNKPVKI
YRLMTEGTIEEKIYHRQIFKQFMTNKVLKDPKQRSSYDLSDLYDLFSYNA
GDQAAAQRSDVFKGAEVDISANTDGEAVARQRLAPITSVGHKKGDVEQEE
LSKMRIVAAMEDFQEDDSAHDERRMLEGIFSRSVNSAYDHEQIVNGPQKA
KADIRVLRQEANQVAREAAAHLRHAAQEARRVPIGTVTWTGEVGSGGRPG
GNRRRGGPSSAGIMSNLANRQGLDGGSGSSSRSGTPGLDRNLKAKDFVTM
IKAFINRQGGRVPSKMLVDHFNPYCPGKKQSDEFKTALDRVAILNKTGGT
GRGMWSLRPGVE
>Mb|QLI67161.1
MATENEPELLQNLVDSFHTLLDEALIVAIASDYNLKSPSAYKQAQATLQD
LAKSVPSEEASGFNPSGIPQGPEGEAQLVQDEPTATSTTSASRRTSQAHI
TDKSSTDNSSCVPDSPSLMPRLTTFNQDSEEDKLLLLQSMFADLKQFDIQ
YALKKANGEFQTALDDLLNVQYLQSTGQQIKGIDGFFELENGRSKGRKKK
KGKKKVASDADQSSDGTSSPSIASETQSQDEIEYVAERFGIRSDQVSPIY
YKLQCSKGATVVELLNQYISHGIETQDESGKESAERLAQNYRHVPEKYMP
TIVHVAGSIPQFADDIAALLNKYFSRQPKAKKMDLSYRLTPLPQEDIEGG
DVIVSPRPGAKPAVGLVAKPSPISDMNYEEAMSRANTFHQARRDAAASAA
QMHRRGASSPLFRQAASYYSDRAREQGRYAQSATSTAADILVDQRSTASS
IDLHGVLVQDGVRISRQRVQEWWGGLGEMRAKKLREDGGFTVITGLGRHS
ASGVSQMRQAVAAALLQDGWKLSVETGRFVVTGRR
>Mb|QLI67162.1
MPDALMSTKQTAARFANALRSFSASGDAFRVLSTVPPRTETPPPPPDSLI
VLDSSFNPPTRAHAAMASSAVRQAGPRARLVLLLAVTNADKALAPAPLEV
RLGMMEALARELAAAPVQVDVAVTTRPYFHDKAAAMEGAAGYGRAEQVFL
CGFDTVVRIFEPRYYGGSGGMGSALGPFFRRARLRVTMRPDGAWGTAGEQ
AAYVAGLEGTLGRSGGDAAWVRRIELVDGVEGAVSSSRVRELVRTGERGP
AVEGLVGAEVARWIDEEGLYRE
>Mb|QLI67170.1
MMSFDGGTAYAESDADDEYERSMGDHSPVGDSEASPIDSELSTSAEHTPT
TYAHRSSADRLPETIITEWNADECADFISTIGLPQYADAFVENEIVGEAL
VALQHEDLKSMGIASVGHRLTILKSVYDVKKAQDVPIESDHYFPLSADAE
AQYATATLKDIKHLVEQLRLRDERMNLLEQDLRRMTDDFRRLREDMLPAL
RLVKDQQTPLPNLSGGALGQGFPYEATISPPAPTPPPGSGPGGVKRQYSQ
RKILIGATPKNASPTQVAHDRSIAEQTLDPSSAAERAVLSSSHLAAMNGQ
TSSAHPSPNMPSPTSPQNYSSSTTLGARSYRGETSAPSNRSTMNDADHGM
YMSRDKGVIPPGPPRRRETPVPDTPTPNSNGSSVEIFKSFRVSMDDPCYK
VLPAALKKYQINAPWDQYALYIVYGDQERCLGMEEKPLILFKQLDKEGKK
PMFMLRKTTNAQVDNEPGSAGIANAARGASTGYDPPGGII
>Mb|QLI67171.1
MASRLEDAEKHMVEHRDLASASNEKLESQLAEAGDADSDYGFTKVEQRNI
IRRIDRRLVLTVGFMYCVSLMDRTNMSAANIAGMSVELKLINNRYNIANL
VFFVTYIIFQPPSTILVRAIGPRVHLAAITLLWGAVMIGMGFVQNFSQLA
ALRAVLGLFEAGFFPSCVYLLSTWYTRYEIGKRYSVFYVVGCLASACSGI
LAYGLMQLNGREGLTGWRWIFIIEGALTCGLAIGSYWLLVDFPDSTRKTW
SFLNLRERQWIVSRVQRDRGDAKVEPFNLGKFLRGGTDWKVWVYAMIFFN
TTTISYALAYTLPIILVSNMGFSVGAAQCLVAPPYAFAGIVMFTMGWLGD
KHRIRGLIIIGNMVLCLIGLPIMGWHSKAAVRYFGVFLVTAGANSNVPAT
MSFQANNVRGQWKRAFCSATLVGFGGIGGICGSLVFREQDKLTGYKPGMY
ACIACALLNVILVCVCDLEFFRQNKKADRGEKLLESDDSETSATDFRYTY
>Mb|QLI67173.1
MGSDPQYAKWPLLPLSQHVFTLTNAYASKPAQEAAVKALQDAIAEDKMAP
FYRYLAHPIDGILNAVGEGGALGPGKPLSRKSSLVGMVATKASTTSVHLP
WDEALYSKLKEDNDKELAEFQKEEDEAVEQAGETEVMAAKGKRADFWARV
GDKDKAIAAFEDVFEKTGILGTKIDLVLAIIRMGLFYGDKALVQKHIERA
TTLVESGGDWDRRNRLKAYEGLHLLTVRSYNLAAPLLLDSLSTFTSYELC
TYSNLVVYSVLAGSVSLKRVDFKSKVVDAPEIKAIMGDGEDKLMALSGAL
SAGPGADDTTGDKAPQAASTTAVNLTTLGSSTEQSDAEMAIDFSPLALLV
SSLYNGNYKTFFQSLASVEEQFLNQDRYLHEHKSWFIREMRLRAYQQLLQ

SYRVVGLESMANDFGVTVDFLDRDLARFIAAGRIPCTIDRVTGKGVIETN
RPDDKNKQYQDVVRQGDQLITKLQKYGQAVRLRGSERA
>Mb|QLI67175.1
MSYPDQRSDTEWQAVLNKEQFRILREKGTEPPGSGKFDKHYPEEGVYTCA
GCHKPLYKANHKFKSGCGWPAYFDSIPGAVTRHEDRALGMTRTEIVCSNC
GGHLGHVFKGEGFATPTDERHCVNSVSLNFSPDDKTVESGSRQSKA
>Mb|QLI67178.1
MSSFLRSKQAGIQNDLSASIRPELFMPDEQARYGINSQIGCLGYDPVQSL
LAIGTKESRFGPGKIYVFGQRRVQKILQPPRATSFHSVQFSANRLVSLDS
KNELGVWDLDTGARVAAQVIAGTAVALITDPMLDWAFIGLQNGDILAYDL
DRRTMTRSFRLPNFWHQKDPAAARAATLVCLSMHPRDVGKLMIGYSHGAV
IYSFKQNKPINFLEYTLPAGAPGGNGVGVDTMRRPRLTHAVWHPSGTFVA
TAHEDGSLVLWDYREQRVITARNLKDTGVEKPVPNSTAPSSSGRYTKIAW
CCKENCDDTGLLIAGGESPDTSPRNFTFIELGITPMYATSSWQVLADYFK
GKRQISISLPPGAQAVDFLLIPRSSPHFAGAQDPIAIMALVSSGELITMS
FPSGYQISPTNQLHPSTFFVHPFVTKVNVSSLERPRWLTMEEKRDQGELL
VTGGAKGPRPKKRFEERTIIQAAHGDSTIHIWDSGHADEIENDRQLQIDV
ARSLDRYDDIDITAMNMSSATGEFAVGTRTGEAIVYRWGGNRFYGRDNPK
QLDPNPKGLTDISSRAEPTLKEGLQPSILYEMMQGPITAIQVSNVGFVAV
GSELGFLTLIDLRGPQIFYQAPMTDFAKREKRSSFFKRDHHHQARDEPQK
EWPVVIEFGVMTLDEDKYSSICCFVGTNLGKVITFKLLPAAGGAYTAQLA
GVVAFDGPIVSLSPINAQTGKPALASAATVGSLREGKRVDGALVAVTQKE
VRVFRPANSKGASRDFDDVVCHAASVAELELQGFAVVAFFDDQTARAYSI
PGLKDLGQAQLSMIDPTKPAFVAQTGDVFGWTGPSEMTVIHVWGTGKPLQ
QSPDTLINPELQCPPRPTISNMQWISGTQYVSPLDLDLLVGGPDRPPSKR
MQDAAAAERRAGAGGLASQESWGAYLTRQVNERTERLGVVNEGMDSLQQQ
SQGWADDVTKFVNQQKRNLVMGSIKSKFF
>Mb|QLI67179.1
MHLVHSVVVSFWLAAIFVGYRLFLKWYQYRRPRQLGCEAANKYPHRLPWI
LDPSGKDLQRKRFEALVLGKYNQFYQEKFAQYGRTIEERSPAGKTIATMD
SHNFRAMLAANFEDYDKQTIRITPILRLIGHGIFTKDGPAWKHSRDALKP
LFLRAELSDVHRFKKHVDRMLSLIPRDGSTVDLKPLICRLFLDSGTEFIF
GESFNSLDRNATQGDELMEAFSQALIGTTKRRHAFAALGSLFNDTVDENI
DKIHAFVDRQVSRALAAASQPNPLDEKKRGGQSNSRYVLLDEMAKRIRKP
MTLRYEMMNIFLPAFESTAVVLSNTLFHLARNPDLWTELRKQAIALGDQN
LSFELLRSLSLFRYTVLEALRLHGSTGRLSRTAIRDTVLPRGGGPQGRSP
VFVPKGTVVSLDLYAHLHDAEVWGDDTDVFRPSRFEGRAPKWEFVPFSGG
PRICPARQQIITQSVYLLVRLAQEFAEVENRDPCMEFVERVKIFTESAGG
VKVALHETQSIHIAAK
>Mb|QLI67180.1
MAEFSLDATFIPALHKPPALLPIAKHRDSLLYVIETYPVTIVIGQTGSGK
TTQIPQFLEKAGWCENGKVIGVTQPRRVAATTVALRVADEIGCEVGKEVG
YSIRFEDVTSSATRIKFLTDGLLIREALVDPLLSRYSVIMVDEAHERSIS
TDVLLGLLKKIRRKRPDLRIIVSSATLQAEDFLKFFAANSEDPSSTSDHS
NSPEIARIVNLEGRTYPIDMLYLESPAENYVEKAIETVFDIHTEEGDGDI
LVFLTGREEIDNAIQAMAERIGQTRDRYGDLQPLPLYAGLSSEEQMYVFD
KTPEGKRKVIFSTNIAEASVTIDGIVYVIDCGFVKLRAYNPKTGIETLTA
TPTSKASASQRAGRAGRTKPGKCFRLYTEQSYQSLPEANPPEIQRSNLAP
IVLQLKALGIDNVVRFDYLSPPPSELMSKALELLYSLGALDEYAKLTRPL
GFRMAELAVEPMMAKTLLTAQSFNCLSEMLTIAAMTSLGTSVWFYHDGEQ
KAMESSRRKFAAEEGDHLTLLNAYTAFVTKGKKEARFCHENHLNYKSMTR
AVSIRAQLKRYMERFNIQVSESLASNRPSETDLKSKAEQIRRCLTSGYFA
HAARMQPDGTFRNVEGGTVMHAHPSSLLFNRKADWVIFHEIMETGEKTFI
RDMTKIEKGWLLEYAPGFYTVTNSTRMGE
>Mb|QLI67181.1
MKTSSAVIAAIIGFASYGSAHMQMSDPPPFLSKFNKFTEQGKQDDSMTSP
LLASGSNFPCKGYHSLVGTPQGQSVATWTPGQSYKMTIAGTATHNGGSCQ
ASLSFDSGKTWKVIHSYVGSCPLQDGAAFSFTVPSDTPAGDALFAWTWFN

QLGNREMYMNCAAVTIGGGAKIRKARGDTTPFSSRPAMFTANIGNGCSTE
ESKDVVFPDPGSELDTKSDKPAPPAGSCGTKGSGSGSGSGSASAPAPAAS
GALVNNSNNNAGNPSAPPASTPSPTPVSTAAAASTPKPVGSGGSCRAK
>Mb|QLI67182.1
MPIVTELTKRLGIRVPVVQGGMMHIGTADLASAVSNAGGLGIITALIFPT
PAALRDEIRRCRTLTKKPFAVNITLLPAMVPPDYGAYAQAIIDEGIKIVE
TAGNSPGPVIKQLKKAGIIVLHKCTTIRHAQSAVKLGVDFLSIDGFECAG
HVGESDITNFILLGRARQTLKVPFIASGGFADGHGLAAALLLGASGINMG
TRFMATVEAPVHQNIKEAIVKSDEHDTTLLLRRWRNTSRLFKNKVALDAL
KIEKESQSGEFSEIAPLVSGKRGKEVFVNGDPEHGVWTTGPVMGLIHDVP
TCDVLVKRIEKEAEQAFKERSALIVPESKL
>Mb|QLI67183.1
MSPPAYTYPPPPASTRHYSAHGTSSAFSSSALPDEDWTKISDLAERRRIQ
NRIAQRNYRKKLKRRLEDLERRAGSSDDGETEKKPQSNNPSKSKRQSTSK
TTKPSPPAPTAKPIQVSQGQFTPPMENNEEMVVFSAGFEKRERSNTPPLF
AYPSYPAPDELLLDPYASAQPYPSISAADPYPHYLAATTMPATLPAMTNY
PDIKQCDDGLSPYLNYSFVSGVDINMSSHYDSSSAHTPPLSHSYETSTSC
SEAGYEYPTTPLSMPGSPGMMPSQQ
>Mb|QLI67185.1
MPKPRGFIMRYGAILGIVVLSLSGMTSAMGWRSPEQKYVDKPLEEALRSP
PPSPYASGANPSIFAAALSELQELESEPFCHRIAARLLVNNCQLLDGRND
ATVMTNTGRAARDFVDFFAASLAICDLERANFAIPSTCHKFREPVLANLP
APSKPELHVITREIDDCLEGLARSDSAWSTWVAYRHKALGFCEAARADGE
KDHHIFLHRKLANILERLTTDAEVQVQNRLNELDRMFRESSDNAKTLTAH
VAGLNASLLYFEQVITHSILSKSKETEMVVQKGLNEARSLQQLMEEVLGM
MSLREEKHARTLETALEVAVTQINRDAHEVMKMLTAVAMSSLSLQEQLKK
SESQLSVVMRKQERVQEGMEELSLLADLVADKHHSHQEMLQSAQNETAYL
LASLEAASFSMGNLRASFSDLGSPLAPTDNFARAEAYLRDAASQGANMAV
LPEYHLASWVPDAVEFASIAAQSAPYLEKYRALAKELNMAIVPGTLLEPE
TSSGGGLANVAYFIGPDGAVLGRYQKKNLWHPERPHLAADIESPHTAFDT
PWGRMGMLICWDIAFPEAYKALVADGARVIISPSFWLADDGGEGSDLNPN
CEKMFLDNVCIARAFENTAAIVYVNSGAPKGSTDGKDGRGNTFCGVSQVA
VPIVGRLGGGESMGPAEEMRIFEVDMGLLDMAESVYKVRGDMAREGWHYG
PYRSA
>Mb|QLI67186.1
MAQHPRPLSQVLPPLILGTATFNYQYNDDPSQMPYTDIVRRALQHNITAF
DTSPYYGPSEVLLGDALRTLTPPPDRGGYFLITKAGRIANNEFDYSPPWI
RYSVCRSLERLNTPYLDLVYTHDVEFVSPTEVLEAVKELRRLRDQGLVRY
VGISGYPVEILVSLAEMVLEQTGEPLDAVMSYANYCIQNTQLGRPDILDR
FKKAGVDCIPNASMLGMGLITTRGADSSPMASWHPAPPRLRDACHSLATV
AARSGEHLEEIAIRWALENWAHVGSQFGSVAYPPELLSEARVDESGSPAR
IGVSVMGVSNVEELEETWALWKSVVGLASRADEDSAERRDRINRIVTQQM
WPRLGPWKDYSWQSGGEAFTNMRQAKGVVPIDAVAQRWGLIPKKFLENAK
I
>Mb|QLI67187.1
MASFRLTIEDGQFRDAHGRQVVLRGINVAGDAKLPSEPDQPSHIAHDFFD
GDNVTFHERPFSRGEAATHFARIKRYGFNTIRYLFTWEAIEAAGPGIYDE
QFIQHTIDVLRIAKSYGFYVFMDPHQDVWSRFTGGSGAPMWTLYACGLNP
QSFAATEAAIVQNTYPDPDTFPKMIWSTNYHRLAAGTMFTLFFAGKDFAP
NCIINGVNIQDYLQGHFVNACAHLAKRIHEAGDLENEVVMGWESMNEPNR
GMTGYVDLTVIPKDNPLKKGTCPTMWQTFLTGMGRACEIDTWDMGGLGPY
KTGTTLVDPHGEIAWLPEGYDDSKYGWKRHPGWKLGECLWAQHGVWDIAT
DTMLKKDYFAKNPRTGKAIDYPEFTNTYFMDFWRKYSKACRAHHRDCLML
MQYPTLELPPEIKGTEDDDPLMAFTPHFYDGITLMTKHWNSTWNVDVVGV
LRGKYWHPAFAIKIGETAIRNCLKDQLATLRQEGLDRTGNHPCLLTEFGI
PYDMDDKKAYKTGDYSSQSAALDANYFGVEGSQIEGHCLWVYTVINDHER
GDQWNGEDLSIHSVDDKLPPLSPVPRSAVAGQSATSLLKLSTTNNRDGSG
DDESVTPGNLQRTLTTPSISSSRLAKDPELTNAPGYRAAEAFIRPTATVV

AGNVLSTGFDLRKCTYSLKISAPKPASDEAPTVVFLPEYHFPKNQCEVAV
STGKWELSSDDSEGPVLQRLKWWHPEGEQTLDVQGIVRKHNVPEGSAEEA
GYLEQCQQGYGFNFGNCTVM
>Mb|QLI67188.1
MARPQNSPILAQLRNAKSLPEQTAALQALKNEIVGHVQKKEAWIGLGVLE
PIVRTLYSNRSPAKLNGKDATVQPPSRPLSDEDSVKLQALQLVASFANGG
PAFLHPLHAARVVPAILGNISPYTSPPQVVVSSLRALLDITSAARLAVSP
SPLDVQTLADNVFSSHHIESLHAMLSMTSSKYILQSQVVLASTLISRLCR
EDRHQHALTVGGILDSLAAQLARFAVADGLVVPGAEEHAYSDGLHEVFPE
AANPGAQIGPILEAISCIIGDSKYRANRLICSPSLLAIFPSIKFEPYGDP
RDSQQALPPRHNGLTAMEFILPSLPITTSRTPSAAASSFPTPDRSDSRTS
SRTSLSKFGSSAVSAVWDSPRFQALGNTVDVSNEDIESPFIPWLIHLVRS
LNDYDRLMAAAVLAALFKAGLGRKGVRETSIGLLVVPILVGMISKNDKDG
VELLDSFETSQLQILEKAPAILARTITDSEYLQKAAFDCDAVKVLTRLLR
RAYQPVEDMRHPKYWSPNPDTGMDVESTLPLAQLGENGQNPLLAHRIRIR
ESALKAIGALSAGKEDYRKALIAEDFIPFVVESLSEFPRKPKQAKDKSLN
ETARTGPVSTYGTNPQSVIIAGCHVVRTLARSVSILRTALVDHAVSIPIF
RFMSHPDITIQIAATATVINLVVEVSPVRELLTESGVMKVLCDHAHSDNH
SLRLNALWALKHFVDAVGPDLKKACLEQLQPGWLMQLIRDDDQETSFFAL
KAKESSADDLDEDMDMQQSDEPLRWFFGTNGTIRELDASRSSRLRQVEDR
LASVREAELNPTRKARNDKLAIQEQGLDFIRNFIGRPESGVSSEAPSETT
DMIDYLFSQLGQDRLFEILASKLRSKVLHPFSRRTPVSAGGTRVLHPQAK
IIVAVICILVHMAASIHRHRQLVIAQTELLKLLAQQVNSKDRGVRVSLCH
LVINLTWHDDETEAQACSHRAQELKRLGFYSKMETLKQHDRDLDVRERAK
TATFQIDQATY
>Mb|QLI67189.1
MSGAMGEEEIDLYGLLSIERDATQDQVKKAYRQAALKYHPDKVPVEQREE
SEAKFKEITRAYEILSDEQKRHLYDAHGMAAFDPSRGGGPGGPEVDLNDI
LSQMFGFNMGGPGGPKRPRRGPDEEQEYKVTLEELYKGKTVKFAANKQVV
CSQCKGSGGKEKAKSTTCERCKGNGMVEAIRQIGPGMMRRETVLCDHCQG
AGQVFKEKDRCRKCKGKRTMQEKKALEIYIPRGSMQGERIVLEGEADQYP
DQIPGDIVFTLVEEHHDTFSRLGNDLSAELTVTLAEALTGFSRVVLKHLD
GRGIHIERPRDKILRPGDCLKIAGEGMPMKRGEVKGDLYLLVTVEFPEDG
WLKDDASYEALQKMLPPPPPPIEAEEVDELEYEDGADIEKMGENSGDPRF
ASEWGDEDEDDGQPQCQTQ
>Mb|QLI67190.1
MNRFRTKKKGRDDNSIVGRPSIDAEPSGPFRMFSKKKMQDEEPKPEIDLS
TALPSHNDFRTSLLMTGLSARFSMLREQDDPNSKLGKASDDSVLFSKRQS
RLADFGVFTGGLDDIAEIESLKAPPLGRFDSYQSDDASSTTGSIMGRSKP
TEGNNLFGGRQKIYKLSAGSKAGMTGRALYDDDVAQSSFQKWRQAEKERK
SLEESQKNDTFDAEATLNYSRRRETSSTTSSVPSGARNSTAATSIASQPS
SIKESSSTSHLAGLERSVTRTRRLYEQGLTQDLQDQQTSALSRMDTLSRQ
RPFGSRTPDPASIVPSPTIATFGDRVSDRRPIVSKASAPNLRSFSPSTMS
SSQMSPVDSSSKFPRQETKPIFGGNPPLSPPISETEEHPPLAIQPNDRGK
ATAMGVFTRPAMQYDESRYTQRQRQLQQGRDSSTSRHTTASAGSSVAGRS
RSSSAQRPSVEKSAPKAASSETSTLNENCGSTFFDDSDDGSMNIRASAAP
QLKIDRPSDHDHPAFRKSALPTPLSLTSPKDSEHGGSLSPAPKTEPPEDS
PTLGPNAGLSGMVRQHLRHDSTASSVYGSTSHEVEPSVTETKAVSVRDDD
GVSDVANAVEQDEFAQNLAERAQRVRERLTSYVESDNERSAPPTPPQPEH
NKDLGASKSGSLGMLRSKSSRGSLLDRSDKERGRSKTLKSPSPQPSRVAT
SPSPRKASSSRGEPRNGEVSVQSNEPALPGKEENVHVGLKAFRQARRELQ
KMKELETQQRHSQKPSPNPERPPPNSRITSYENGPPPAIFNRMPRDPRDG
ESYRSRSRAGSRAASERDRSGSETSNSGRAYSRGPGLRNGAATYEDHYGR
RGLSGSPGPEIRRQPMETSGMSPYMSTGPSPVMSPSHSVGGFEAPGRQHF
PVMQSPQQPDVHAFTRSRNGSLLGAAASTPNLHGGPGAPPLPPINPRRKN
IFPRPSDENLGHHVPNRVFGVGEKDIIAEPYRGRMPMNPHQSRQSPPRVV
RPAIPHSAMPSAGLPGGMI
>Mb|QLI67191.1

MASNGTQESLSSSVANALETPEATKATPKLMHSLEHNSTVLAVAVSAKHD
SIYAGTQDGEIVVWSLDTFHQLRQVQAHKRCILSLFLSPDSSLLFSSAGD
AVINVWCPKTLRRLYEIYGFHDVGDIFCTAYSAQHDTLYIGAQNTTIQWV
RLGDDSKSASPDSQNHPDRRSHRFFDSKAVGGGATPRRSEDRWALIPRAQ
SVLEIDSGCIRHFAHNGYVYCMLMAQGPTVEVDADDDVLISGGGDGTIKL
WSLTGSSRNGRGHADGDDGGIEEIMTLGADDAESVLSIALDGSFLYAGKL
DGIVELWDLDTTQRLRVIKAPGGDVMSLQMGWGCLWAGSTNGWASKYSTA
HYCAHGHVSSGHVSQKYQCLGKWKAHQGKILASAITTHKSEPYYITGAND
NDIAIWSVKDLTSLKPNLTEKSNDLLLSTLSDFVSYKTVSSRSEYAEDCR
RGATFLGSLFKRLGGHVEMLSTEKAHNPVVYAKFSGKKEALEQRKRILFY
GHYDVVPADGRKGKWTTNPFTATGTNGFLYGRGVSDNKGPIIAALYAVTD
LMQAQRLENDVIFLIEGEEEFGSRGFEEAVQRNKELIGHVDYILLANSYW
LNDEVPCLTYGLRGVLHATICVDSPRPDIHSGVDGSHLSDEPLTDLTCIL
SKLKGPGNRIQIPGFYDGIPPITPEEEARYDDIASIMVRHQSQAVSEEKL
KQSLMARWREPNLTIHRYKVSGPDGSLVSSHASSHISLRLVPGQEVEDVS
KAMSWFLRREFGLLESQNRLSININIDNKAEPWLGDPTNEIFRTLGQAIIEA
WPDCFDRSDSPSGNVQASSVAKSSKPRQPLYIREGGSIPAIRFLEKEFGA
PAAHLPCGRASDSAHLDNERISVVNLLKAREIFGKVFARL
>Mb|QLI67192.1
MVGRKRTVPSRSSKRGEGSGNNDQPDIYQQMLTEAGVSAPEQSSPERPLK
RRRPAPEQPQRPLSDHPSGLTKTDAQLDTQESSGEDEDVEFQDVILPVPT
VQTVELESDDEDDEEEDIQFEDVDFSAPLADTNTSAEKSNSLELNLTAHI
SAASPSKRPGERRKPISKEERERRVDIHKTHLVCLLSHVARRNHWCNDER
VQESLRPHLTDKTVNYLNPGSHLPQFGQAESLKNGLRQASDVWKAKFEIT
ERGLRRALWAEDAGQLQDYEPAEDLDSCLDRADFREAAKKLQGSRDVGAQ
LYCALLRSIGVRARLVCSLQPLSFAPGAPTLPKPKVAKSAAKTTQTERTK
EALVKYEALAASAGTTTGSAGGSSARRRLGHPNATAYNFQPRPAAPKERR
AFEAPIRIRESAHPVYWVEVLDAGHQKWQPADPVVTRTFWKTKAFEPPVT
DKENCLAYVVAFEADGTAKDVTRRYAKAYTAKTRRLRVETPLDDGGQWWR
MALESFRRRHPTDLDQIEENELTGVEAREPMPRNVQDFKDHPVFALERHM
RRHEVLVPEAKPSGTVGAGSRGPLEKIYRRRDVRTARSAEKWYRMGREVK
PNEIPAKWLQKTARRKGFRVDDRDEADMDDDAGTPIYTIEQTELYEAPAV
RNGRVPKNKFGNIDAYVPSMIPKGAVHIIHEHAARAAFIVGVDYAPALTG
FQFKGRQGTAVLNGIVVAKEFEAAIRSVIDGLTDVEQEMEDERKRLAALK
MWRRLLMGLRIRERIWSRVDEDERKKADKEAEMEAELANAESDVTDEFNM
VIDDDSEGGGFIIE
>Mb|QLI67193.1
MTVTATTTNGHVNGNAANGSTARLNRAEELDDLLSAVRDLILPFIKAADD
AAAHRETGRLPLDATGTPHNVLVDAVQPHSLAAHLKFILPEEGQGKQGLL
EGIARVLKYSVNTWDQGFLDKLYASNNPVGVISDIVLSVLNTNLHVYQVS
PALTIIEKTTGRALANKFGFTGPRAGGVTCQGGSSSNLTSLVVARNTLYP
DCKTEGNAKHDFIVFTSAHGHYSVEKSAMICGMGSSNVWKVPVDDGGSMK
ADALRELVVRANEQGKTPLYVNTTAGSTVRGSYDPFEDISKICKEFGLWM
HIDASWGGPVVFSAQQRWKVKGSHLADSITINPHKMLNAPTTCSFLLGPD
MSLFNKANTTDAGYLFHGSTEDDVWDLADLTLQCGRRGDSLKVALAWLYY
GANGFERQVDHAFDMASYLWKLVQQTGNFAMVSENPPPCLQVCFYYAPNG
DLSSDAATNTNRTQALVEKLIGRGFMVDYAPGDVGSFLRIVVNVQTLPST
VEGLTRALEEAGKEVA
>Mb|QLI67196.1
MPSAALRMATLRAFARHGCNPSPALTRSFSVSLRRAEINKVFPSASAALK
DMKPNSTLLCGGFGLCGVPDTLIDEILQKPDITGLTAVSNNAGTDNSGLG
KLLKTKQVKKMVASYIGENKTFETMYLTGEVELELTPQGTLAERCAAGGK
GIPAFYTPAAFGTVVQTGDLPLKNKADGTPDEYSYPKDVKVFNGKSYLLE
HAIDGDYAFVKAYKADKLGNCQFRLAAHNFNGAMGRNAKMTIVEAEHIVE
PGEIPPEAVHLPGIYVKRVIQSTSEKNIEKFTFAKDESDADAKAALGTGD
TAAKRERIVKRAAKEFKNGMYANLGIGMPMLAPGFVGPEVEVQLQSENGI
LGLGPYPKKGDEDADLINAGKETVTLNRGAAVFGSEESFGMIRSGRINLT
ILGAMQVSGTGDLANWMLPGKVKGFGGAMDLVSNPSKTKVVVTMEHTDKK

GNPKIVKQCAFPLTGRACVSRIITELGVFDVDFAHGLNLVEIADGVTVDE
IKSKTEAPFTVSKDLKPML
>Mb|QLI67200.1
MSKRPADDNASQESKRKRYSGSTDENVAPNIRIPGPIPAMFGLTPWLPSD
IPSSLPCIPKILDPELEATVFLHPGLSDPLRGYERLEWLGDAYLELIASS
LIYQTFTSTPVGRCSQLREILIRNKTLAQYFREYGLESKAQLPGDLKKDI
SRGRGSSSDKDIIKTQADMFEAYVAAAIISDPENGMANTASWLKLLWART
IKDQIVANDRKRKMATQPNSTEQGSTKDMSSHSNLSAKEKLRIEIGVKGV
LVRYEDMPRMGRDRDLGLPLYTVGAYLDGWGERNKLLGTGTALGKKEAAQ
KAAQQALENKKLLKPYVAKKAAFMEAMEATKASEEGDTIS
>Mb|QLI67201.1
MSRLSIATKSRLPSSLRVESTNTAAIKAISRLSREALILLALDWLDGPSL
AYTAPYLSAPRADDDETPEDSADLYPPCKSIEELHDLYTDLQQQRGSRRD
VVSRILEGDWRHGLTLYQLAMIDFAYLDEHPTSQKWTAYKIQPLNLPTKD
ADDEILKPDEKSLNLPRFHPSTFLQSLQEQVLPDIKAHYHFSRPKKFPLL
LLRIFAIESPYNTDLALSGLNGSGNSTSFHSSRTIYLAFPDGSPALYITR
AQATGPISHGESKSLHSLIANGVPQALSRPRERYTLKPTSLVSRNLEALL
DKRGPGRSNAAGGGWSIYANEKDKKSPLDTLLPTPPLSRESSSNAETAQK
RSRPLSQHERAAKRAKTVAEARFGNSGIVSDGKGIERFEIIMQDPYPAID
GLFVQDSEDEDDRRNRQTDRRRSKIDAALRQALGDGEEEEDEIDPTRWTP
IVRINFQGPHVFAGIRQLVEAGIVDGERMPGWMTGEDGVTTGVVKNGLIR
GHKGSGL
>Mb|QLI67202.1
MARALSPSDTASRSSFSSVRENDDGLAQTFTRSKVSSYIEAPEDVFDESP
ISELPQPSFQAPLTQPQSKLHGFWYPPDSFKGWREIPIKGKKASRSFDDL
RKLGMSWESPPASPAPVKKHHTGSYAIGDAPLERLPSEVLASIIDLLVVE
IPPNGLTTRNTDLMALLLTSRSIHATTLTTLYRHVTIPHSRIFRKFLNTI
SEYPSLATIVRRLDFSHFNPSMLFSTASERAKTQNLTTDTLAQCLELTPY
LQEFLAQEYIDTDLGPPVLRKLFFDMPNLRALDFCGCSSTVFKNSFNAIL
ENSWPDELFITRLSFHKCLGLPSTVFETILPRLGKLTHLDVAGTRITDEA
LERIPLTAELTHLNLAKCKELSADCVVKFVTSHPSTKDSLVFLSLATDPT
NHLLLGKDDIDGLLPRLPKTLKSLSLRGSRMHASHIPELTRLAQTLEELA
VGRGLEMSDIHKLFCHGQEWLQHNLRFLDICDIETIIGSASTLLAPASAP
LHVIEIAERAYERAAKVNKNLQRVGWVAKEFGSRYWLVRMNADGSTRDNG
GRWWKMGAESWGMRKVPVAIAEVGGMYGSFMFGRRV
>Mb|QLI67203.1
MAYTHSPKEPIAIIGSGCRFPGDATSPSKLWDLLRSPRDLTKPIPAESRF
NAEGFYHPKGKRHGASNVTKSYFIEEDPRFFDSGFFSIAPREAEAIDPQQ
RLLLETVYEAMENAGLTLQGLRGSQTSAYVGAMSADYTDTQMRDLENLSQ
YMITGTSRALLSNRLSYFFDWKGPSISVDTACSSSLAAVHLGVQSLRNGE
CTTSCVAGSNLILGPDAYLAATSLHLLSPSGRSQMWDKGADGYARGEGVC
AFFMKTLSQALRDGDRIDALIRESCINQDGRTKGITLPSAEAQAALIRTT
YRNAGLDILRNEDRPQYVEAHGTGTQAGDPREASAISQTFFPPDQELGNK
PNIVVGSVKTIIGHTEGCAGIAGLLKVLLAMRHKTIPPNQHLRHLNPSVE
PFYKNLGIPTTLLDWPHVPFDHPLRASINGFGSGGTNCHAIMESYVPEIH
DHGPWGRPEALRNVQASPLPDVDFTPTPLIFSAASESALVAMLEKYARYL
ENTDVPIQRLAKTLYSHRSTLGVRVIFPTTSRLELLEEIKKKLSKVRDSP
GTEIGTRSSVIEFDENRRPRILGIFTGQGAQWPGMGQTLMKRCALFRTTI
ENMEQALALLPDPPEWSLKKEIMAPPAESRLGEAEISLPVCAAVQVGLVR
LLSQAGISFHSVVGHSGGEIGAAYAAGKISENDAVIIAYYRGVYAKLAIG
ADGKKGAMIAVGFGYEEGLNFCSNPKMKDRLTIAASNSPKSITLSGNEDA
VIEAKEMLDNEGLFNRVLKLDTAYHSSHMYPCAAPYLAALQRCDIKVGKS
SATTAWVSSVYEDNRTITDDQDADMKAIYWKDNLIGRVLFSQALECALDD
DRGAFDLALEVGPHPALRGPSLETIRSKLGSEIPYAGVLDRKSDDVTALS
RALGFVWTHLGSDCVNFDDYSSAFDERNSHIDKTPLANLPTYPWDHKSIL
WRESRLNRQVRHRSDRPHELLGNRTPDDTEYEPRWRNFFKLEEMPWLRDH
CIQNQIIVPAATYCVMALEAARFLGRGKHVVNIELSNIAILRPIVLDESS
GGTETLLSLRSDLDSNKGKSDVIHAEFSLSAGTVEDGHLRTAASGEIRVF

LANDGADYSTFFPLRSEKTQSELLPVNVNRFYESLSQMGLGYTGPFRAMT
SMRRQMDTASSVVEIDEDVGRSIPVHPTWLDVCFQTFLAAFAAPRDASLW
TAFMPTTIGRMAFSPATRATPNGPASMTVDAQLTELTPAFQAKLPTITGD
MSIYNSKTGRLEVHIEDFTMSSFLPATEKDDRRLYLKTVWRPDILSRATF
EAQQQIDSLHELKVIDACEKVVRFYLSKLRREKSFPEIVKTIPGLLGVVE
AATASTVSEPTEPELASILQEFGHHIDMTLVTTIGEQLLQNSGLSPVPAE
APSSLGDLVSRWHHEGLGFTQVHQHIGSAAKQISHRYTNLRVLQVGPSSA
SLVRALCQGLEHALDSYTIVDGSEHIVEEIRAGLVSNKLRVDAKVLDVEA
GLGEGNDAFAAGSFDLIITHKAFRKQAAALRTVRDLLRPGGFLVMMAATG
PQLRFPFFMLSAPAPVNDDQGSIQPQLLNSTRDEIHSILQKSGFSGVDSM
ALDNVEAKHTFSVVVSQAIDDDVNFLRTPLAWTSPITTSGKILVLGGSSL
KVTNIIRIVQSHLSRVWSGEIVTVESLSGLLSQEVEEAEAVLSITELDQP
ILERLNAPNFQNLQMLLDKSKALLWITQGASESPYQSATIGLGRSFMSEN
PQKALQFLDLDTVTGNEAVLAESLLRLIASAVKTTDASKGGSRLWMIEPE
LSLDKGTLLIPRLVPDKKRNDRLNSLKRKVETQALVGTRAVTLVPSLHNA
GQVAYTAEETLNHRSEPASDQVSLRVEYCSADPVLPNHHGDNLFCCVGRT
QDEERFLALSASNSSAITVPRAWTIRLSDDKLKESAILSVLFHALHRIKA
RVIEASMPLGYITLLYETDAHLASSIDGPENLSGKEYALISYKGHSNGDC
PRNGIYIEPHMSRKAMASLIPSKSRLLIHPGHRSDRRQLQAIQQALPKNT
AVVAFQDLDSDRLITHDVLLGALDSLPPSPTATFDSTQVVKASSLVMDGI
NSHSSVVVVDWTGDQVITLTQRPVNPQHLLSPNKTYLLVGLSGQIGQSMC
RWLVTNGARHVVVTSRNPDKEGLWKDELQRQGANIAIEAANVTNKQELVD
LRTRILSTMPPIGGVANGAMVLSDKLFADMSYDSFQKVLSPKVEGSKNLD
EIFSGDDLDFFILFSSISAVTGQRSQANYAAANNFMVGLASQRRARQLPA
SVIDIGMVIGIGVIQRTEDDEGISAMETALRKLDYMPVSERDLHHLLAEA
ILVGKSDECPEIVTGLETFNTASGNQPFWHRNPRFSHLKTDVGSTQAGQG
STNSVQRTLKEKLADARGPNDALQTMEEALVAYLGSSLKLSLESIFTDVP
LIDLGIDSLVAVEIRNWIFSEAGHDVPVLKILGGSSIKQICTDVVSSLSF
EAQPAKAPESQAQAVPLPSSRDWNKPSTETNSSDDTSSSSKSSDLTPQPG
SYAASRLSSASPDITLDAVEGKSSRPIPFRTESLSLGQSRLYFLSQYLDD
NITLNCTASYALSGKLDVSKFEKSILAVIQSHEMLRTTFYLDEQDAKPVQ
GIIEKSPFKMTIISGNSDMEYVKSELDRTRNYQYNLEMGDTFIATLLSHS
ADSHTVIFGYHHIIMDGVSWQIFQQDLAKFYNHPSLISSPKSLPAQYVDF
TVKQQRDIAGGAYAHRLKFFQDCFREPVSPLPLFPFAKVSTRRSLTRHAV
RDVALHINPETLNAVKKASQASRATSFHFFLSAFQVLLHRLLSTEQMCIG
VVDANRSEQAFSGTIGFFLETIPVLFQVDGDQRFSNVLQTTRSKAYAALA
QTGVPTEEILRACNIPASTTETPLFQVVFNYRMGASRTSQMQGLDMKFSH
YTDAKSPFDVVVSVDELNDGTAMVTFSLQDYLYDEEGAELLAKTYKHLLE
ALANDPHRPVGSIPVFDTTLIEQAVALGTGPKIELAPSSAGTLSKMINTW
TSKDPNSLAVKDVNGNTKTYFQLSERANGISAELISDETVSLSPICVLLE
PGTDTIATILGILRAGAAYVPLDVRSTNERLSDILEESGSTILLHHRATE
QRAGELQRLSKNSQRIRLFSIDSIPQNTTERVEDLSTTDGLAMILYTSGS
TGKPKGIPLTNDNIRTTILGVSKRVPLGREVVLQQSGQGFDAAIFQIFIA
LANGGTVIMGDNRAEPAELAALMAREGVTCSVFIVSEMQSMLKYGYDELR
TCSSWRIAMVAGEVFTINLLDQFRNLNRPDLRIINAYGPTEASICSSMHE
VLPSDIRTDYSSIPIGKPIANYGTYIVDEECRPVPIGWPGEIAISGPGVA
DGYLHLPQLTERKFKRRAFHKQTSGYDRLYLTGDKGRMLCDGSIVMSGRV
DGDDQVKIRGMRVQLDDVSRVLVQASGGNLVDAAVLLQGNDATTQRLIAY
VVFSGTSHIDDKPTYLHKLNQELPIPPYMRPVMTIPLDILPVTERGKLDS
RKLAALPLPEVSPNTCHQMTEYEARLRDVWTTVLGDISLSVPIQRDSDFF
SVGGNSLLLLRLKSEIRRVFAVDIPLSDLFQYSTLELFAARLTGNSQLDH
INWDRETEPDDAICQSTPSAKTPRAPSHKTEGISVLLTGSTGFLGTAILR
QLVTLPQVVRVHCAAIRDDGRDKPRQLGVDSPKIVRYSGDLALPHLGLSP
EQMAQICNDIDVIIHNGAEVSHMKNYRSLRAANFLSTIELAHMAVRLNIP
MHYVSTGGVARLSGAAVQTESSLAAFYPPSDGSDGYVASKWASEVFLEKL
HSRMQGQTWIHRPSSITGNDMPTLDIVHNLLRFSASMKAVPDLAGSTGSF
DFIHVDTVSKNIANCAVSSTGGDGREPPNHLTFVHQSGEEIVPVNQLKEH
MEAAEASPFRVLPLREWVVGAMEEGLDEVVGSFLLGSGDRIRVPLLQRGD

CP
>Mb|QLI67204.1
MVQISEVKGNKRDNRTAAHTHIRGLGLKSDGFAEKNAAGFVGQVPARESC
GVVVDLIRSHKMAGRGVLLAGGPGTGKTALALAVSQELGTKIPFCPIVGS
EIYSTEVKKTEVLMENFRRAIGLKVRETKEVYEGEVTELTPEEAENPLGG
YGKTISTLLIGLKSAKGQKKLRLDPSIYEAIQKERVTVGDVIYIEANTGA
CKRVGRSDAYATEFDLEAEEYVPIPKGEVHKKKEIVQDVTLHDLDVANAR
PQGGQDIMSMMGQLMKPKMTEITEKLRAEINKVVSKYIDQGVAELVPGVL
FIDEAHMLDVECFTYLNRALESPIAPIVVLASNRGMCTIRGTDDIVAAHG
IPTDFLARLLIIPTAPYQADEIKQIVRIRASTEGVAITDAAIDKISDHGV
RISLRYCLQLLTPASILAKANGRSQIDVQDVGECEDLFLDARRSASLLSS
EAGRDYIS
>Mb|QLI67206.1
MDNPHDYLITSPSRPRRPTDPDSSASWVMLNQGGFVKLGNERILLKLDSR
ISCDLSVPQELKARCTPFQCKSDKGTLFLTNKRIVYLPSKPTQEPKFESF
SAPILKFQDSSTSSSMWWGWVWKSDCIPVSGGNIPPDLPRIEVKFTFSDG
GMMEFNETYIRLRERLFQYQEMRREMGPGADIPDEPLPAYEGPGTNPPES
TTTLTVPRTNRSDSSSSRRAPDEPPPGYDEAQAQQISMRLEDHIRGEVDR
GATDE
>Mb|QLI67207.1
MATNGRPSIVGPDSGPSAPYPYKMEGKVISGFGRGSKELGIPTANLPVDS
ALTPWISSIPSGVYFGYASLNLPAGHPQRPASSSSPGPASFSVYPMVMSI
GYNPFYKNTVRSAEVHVLHGFTADFYDAHMRLLILGFIREEKDYKSLEAL
IDDINFDCEVAKSSLARQAWKAESVGDGAEWLLKEL
>Mb|QLI67208.1
MKAVFVVGALAASAAAHTYPNCEHDNCYRALIKDGLQDKAKAFCFDWLAG
TTTAASAIPTDFSNCNVQAASSACSCVTYTATHTTGPTTTSTPPPVTTTT
SEKPPTTTPTTSEKPPTTIPTTTKTEQPPTTTPTTQAPPTTTATTEKPHT
TSQSTTKWTTSTIYTTKTNTITSCPPEVTKCPGGGHTTVVTETIPVSTTV
CPVTETEAPPTTTATTKWTTSTIYTTKTNTITSCPPEVTKCPGGGHTTVV
TETIPVSTTVCPVTETHAPPPPPPAGNQTSTLYTTQSYTITSCPPSVPSC
PVGKVTTTVYPTGTTTIKGWTSIPAVPTQPTGVPPVPTQPTGVPPVPTQA
TQPTGVSPSKTNQNPPPVVTAAAGQLVGSLEFVAAAAGLAALFL
>Mb|QLI67210.1
MDAATIEETNRIRVSLGMKPLPVPGAAIQSDPSSRSRDEEAASTLESRQA
ESYENYKKHQEAAAAKKKREDRAAAIRKARDEAQRLAVMEGKGLGEEDAK
KGDVDAKAWLMGQKKRQKKIDKARKLEAELAAAEAEAAAAVQYTSKDLAG
IKVAHDTSDFLGGEDQVLTLKDTTIEENEEEGDELENLGVREQEKLGERL
DLKKRRPGYDPNDDEEGQGGILAQYDDEIYGKKGKKFTLDGSGTIAELAD
ILGQPAPKAGKATISLDDIIGDAPMSSDYLAPSEIKVKKPKKKKSKGTRQ
KQYDDDDIFPLETQQDSNAMDVDTKESNSKKRKAESDTFVDDDDLQASLA
LQRKSALKKRKKARPEDIAKQLKEEDDEAEQQTNGTGESTGLVIGEISEF
VSGLSKRDDEEEKKPRRHKTQTQSPGLPEDHPMENGEHEEQPAIKEESAP
PQAPEEETGVEEEKAIGAGMGVALSLLRERGLIEESRDSDYEALRAREEF
LARKRILEEELDEQTRQQRERDRASGRLDRMTVREREDWARQQNMWRDQQ
QSRRMAELFSDTYKPNVELKYTDEHGRSLDQKEAFKHLSHQFHGKGSGKG
KTEKRLKKIEDEKRREAQSLFDAGRDGGMSAATAQQLKKRREAGVRLG
>Mb|QLI67216.1
MSSIELYPTSPKHILAQFIGKSIRDVPTPAAVINVAAVRRNCERMLQTCV
KLGLDWRAHTIEITEFQVGTDAARPVNLIVSTLAEAEFLLPRLKEYKSQG
RKVNVLYGLPIPQNALSRLAAVAQVLGEGSVSVLLDDPAQIPIVSKFQGL
SGVVPHAHIKIDMGGKRAGVEAQSARFLELADAALEAHGQGKIRLSGLYS
HAGHSYGGDSRAAAIKMMRAELSAMLEGADRLTSRAAEKSMAGLPPLVLS
AGASPTALAVQNLITTAEEGQDNVPVQLKAEVEALSDMFATVRSKGHLVE
IHAGVYPTLDLQQLAAHSISASRLSWGDIALTVLAEVHSNYPGRGANGTP
EALIGSGGIALARESCKAYEGMAMLSPWGRKGVGLPTCDVEDFRGWMVGR
FSQEHGILTWRSGNTKSEPVQPDQELPGVGDKVRLWPNHACITSSHFGWY
FVVDEDEAGKEDEIVDIWVRCRGW

>Mb|QLI67217.1
MAVPQVNGDVSPRPNSAFLQHLLAYPLISDSIHTVKANEYAQRSIKLGDS
AYQTFAAPVLPWLAKPYEYVSPYVARADSLGDKTLDRIDERFPVVKKPTS
DLYNDTRSLILLPYNKSIEGRDHIFDVYASEAKKIEQKGLVGQGKAAVST
AFVVSNETLGWLSSFLAAKKAEATTVVKEKVKQ
>Mb|QLI67219.1
MPEGTQSPPPERQTGKQLHEPPGKGSGIDEVDNKGDKMKSELENLSSNPK
GPMDDHLEAKFSKEPGNPVGAHIQK
>Mb|QLI67220.1
MKWTLQFLAFAALAVLPSTCVADANDNTNNNEPKPDKDGKYWIYGDGISA
AFVAYGASVSNFMVKDQYGIERDIVAGFDNASYYGIDKQHPHFGGVPGRY
ANRIKNSTFEIDGKKYHVKANENPTKGHPDGVDTLHGGPDGWDWRNFTVV
SHSNHSVTFSLVDPDGKEGFPGEVVTVVTYSLNGRDWDLKMVAEATTKKT
PIMLSSHTYWNLDGFANNQTSKVFNHTLHMPYAGFRVGVDNILIPTGELL
PNKKGGVNDFWSEPKQIGASFGDEGMVNNCGFNCTGYDNCFINNRCAIGP
YDWRKQGPVTTLSSPWSGIQLDVFTDQDAFQIYSCNGQNGTMALKKTQGV
TGGNKDKFPRTIPKYGCVVLEVQDYIDGINNPEWMRGKKQIYEPGGDPYV
LQAKYRFSLVSDHEEKKGRGPSS
>Mb|QLI67222.1
MVSQELPKHDTAAPSADSAQPLHPQQQYPQQPSQTPWPDDPSEAIWEHVG
VERGSSGTPACPPPSHPVANTPPPGNSLTSQGDADENVWDEAKRQQPSQA
ETNSPGQIPDILRPGGNRAATNPFLKRKPVPQTPEAMPTEAFSKLSVDDA
SQDANPWQQVVDSQRTHMGHPTPQPRPVTADGIESDPWAANIDEPSISQP
SIPPKSPALISLKSDNDDQPAWKDELVNNGDQTVIESPPTAVADELLHQN
VWDDLGAFDKGKAKVQQLSAPGHAAQIDDWSLIDAESCSTPSKHAKEDES
ADERPPLPARSTGLQQKWVPSRPPVDAKAETYQVKNIRWHDAKSQQNPRT
SPILIQNENGPCPLVALVNALTLTTPADNPDTTFVQVLRSREQISLNLLL
DAVLDELMSPRRTNSEDALPDVSDLYAFLQSLHTGMNVNPRFVPTPAMVD
AYKRTSLTHLHPTERGNLIPGTFENTMEMSLYAAFSIPLIHGWLPSQTDP
AYSALERQAGSYEDVQNLLFREEELEEKLASPTGLTEDEQQLYQDIVTIK
MFLSASATQLTAWGIEVIGKAMRPGTFAILFRNDHFSTLYCHPQTLQLLS
LVTDAGYRTHDEVVWESLVDVNGERTEYLSGDYRLIGLTHEAGPSTNLNS
STTDHGGQWTTVQNKRGKGRQQDEETGSAVSPHEQEDRDLALALQLQEEE
DQKHREEQARRQRERALSEQYIEQQGHRPAPVNRDRRRTSTPGHGGIAPE
RRSSNTYSVPVTTSTSHPQQSGISQPLQPQQQQAPVQQQVVRPLVPPRRP
GVTRRAESNGDDAPPSYEQAAQAAAYIPPVGHPSHSQSSTALSRQTSASV
EPSAAASQVQGVAGQPLISVDPPTNFVPGSHSQRASMSSSSRRQAVGHGG
RDKDCVVM
>Mb|QLI67224.1
MEWLERMPAGNPGTSSSSVRGLPDNPAQGRRPVPNSAATSNGPPPPPPPI
PGLRNTSSNATLSASSPITSAAQIIGLAREAMQTALESEGQAAEAGAVGT
ALRAGVTVDLSRKGIQKLPEEVIDIVKDQLERLALSHNQLSSLPTRFSEC
TSLRYLNIRGNQIKEFPIALCDLTSLEILDLGRNQLRILPQDIARLSSLK
VLSVPKNQIRELPLCLADMGSLQVLKFEGNPISFPPKDAIRPQSGSPPHD
GIMRETEVTEVAVTAIIKKFLRHFAMTGRMEGDATGDESSEGGTDVPRFP
LRRAASGRFPIKVNGADVPDMRSPNGAPRPPPIPTRSHNRGLSQQSTAMR
RPGVMPLTIGNVNERLRSNSETLLRSDRGPDGRSRRMGIVSRKPSELGTL
DETQANNRFSHYRGLSHGSAMQGTPQGVKSPATPSDPYLQRPIYVRRLSV
LPERRRESKIFDPIIEAAKGILYSVFQIHPMIQMLMTLTSDGSAKRSSLE
IVFYNTNSHVEELEQEVQRHDTTLLENGQYSAIDNENVHRACHTLVSAYG
HVCTLLAENIDTFVNNGDARYIRTLLMLLYNSIMELRVTLATVGASRPGS
QSSNRSMPDASETIKPFQREPFATPTADRTGLLRSRNGTVVHNPAGLRVA
TDLPLHYANGSTRTAKLALATPRSGGSFSSTGSRDLANDYTGEDAQFDKV
FLSLQKSTDIVLRTLPSFHGQLQSDLRTAMQRRAPMALVQDWKHLIAMCG
YTIQQTEILRNRLSLIKLNDPSVRSHPGFWTLCNNFVNSWWSLITKIKKS
IDGLPLPPDTRMRLRPIQQCMKETGNIIVQSPWHNLMRSSGPNGSLGGHY
SQSASPTTQVPITPQSAALGPAMQATVSTTPHSASFAAAFHGNVFDRADA
LMANPGISMSRSGTMSRGHSGFNSLSSISSMSSDGIPSISAPFSPPNGGL

GLSRLHGGRLAL
>Mb|QLI67227.1
MGMSDGIDEANGCTAMSRKRSNSRARNSRPTTPLRPSSRSSFRDSARNVR
GGGAEFPLNAFEPAFAELSDAMADLEANMMHFQLMHESLARFSESFASFL
YGLNMNAFCVDFPEGPIAESFARDRHEETSAEGTTRLETDVEATFMYVCP
NHFSYNIFLFYVYRC
>Mb|QLI67230.1
MDVSAAITEPPTPFWSSTDTATGSENTDSSPSSLIVVDPSKASQHITLEH
GDFSYRWNVPKPHYSYVLRSKEQFLATISAGDEEETSFSSNVELVSRFLL
HLIEQDELDGCKTSMKAREECIEALLWRLETDFLRGADIHTLACQLPGNA
KQRQVVIQAYTAAMVCTKREDNRAELETALFPSSTEKTTRTYAIFGGQGN
TLDYFGELRTVYKTYRPLIERFLIVAEQTFQDILANASAARTRQYCRGMA
ILQWLREPQSTPDQDYLLSAPVSCPLIGLLQLVNFAVICRCLRESPGDIL
SHLSGMTGHSQGVAVAAVLATVTTWDSFDAALVKALTILFHVGSAAQGAT
SQVTISSNISAETVKNGMGLPSPMLNVVGCRLEQLETYINDLNIRLEPHQ
RVFIGLKNGLLNFVVSGPVLSLYSLSCMLRKVKATPGTDQSKIRFSARKP
DFSYRFLPVSAPFHTSHLHCAQEAALRELDTLQLATSELKLPVHHSSNGI
DMRTLKKQNIVPDLVQMILCELNDWPLSTAFTNATHVLDFGPGGLSGVGM
MLHRNQEGTGVRVIMAGLAEGKSIECGYRSEIFAKKVTFSENWNRVYSPS
LIQPTTTDMHIENRMTRLLGLPPVMVAGMTPTTSSWEFVSAVMNSGYHVE
LAAGGFHNAEQLITAINSLIHSIPAGRGICVNIIYASPKQVRWQIPLLQR
LRSEGFPIDGLTFGAGVPSVEVAREYMAMGFRYLSFKPGSAFDIDQVIAI
ARLDETYPILMQWTGGRGGGHHSFEDFHDPILKLYGRIRRCSNIILLAGS
GFGGAADTYPYLSGTWSTSFGRPPMPFDGILLGSRMMVAKEAKTSQGCKE
AIVAAPGVKDESSWEQSYNQPTGGIVTVKSEMGQPIHKIATRGVLFWKEM
DEKIFSIADKSKRREKLQEMRSYIIQKLNNDFQKVWFGRDDMGNCIDLED
MTYAEVLRRLVQLLYVAKESRWIDMSFMRLTADFTRRMYSRLLPSSPSIE
TYFGLRDPQKAVPNIISQCPTAATQVISYSDARYLVLLCKLPGQKPPPFL
LDMDDDFEVWFKKDSLWQSEDLAAVVGEDAGRTCILQGPVAAKYSTRVDE
PVGEILDSINRGLISNIMKDRYSDNVACVPQSQASSTVPWTAPFTPRHCF
VQYEGKKTRYHLAKSIESEQLPDTEKWLQMIAGCSGTWLHTFLTSSVVSQ
GSKIVANPIRRALSPIKGMLVEVTETGSPEEAAIAVFDLKSETSLSSFSR
PSLVIRKEKDILATFYTHETVDRATVSVTFKFTYHPEMPLHPIHEIMEGR
NERLRSLYYQLWFGADVVDRFPSPRGKKRPSISHVQPCDSIPENSEPEMP
QSIQAGFPFQLDRIERNTFSELSDESLGVASLENFIFNNRGLTISSSGIQ
AFLNNVGHEGHMTSPRGSDEKTAPLDFAIVVAWQAMIKAIFPTAINGDFM
KLVHLSNEFKMLGDGAPLRSHDRVSTHAEILAIRNEPAGRVVEVGAIIER
DSIPVLEVISQFLFRGSYTDLGVCFEKKVQPKTYILLDTPAKVTVLKSKQ
WFQLVDSEMDLLGETVIFELKSLTRPGQDSNYLNVQTVGQVYRQHAVTKT
RHVIANVHYQCEKTIGNPVIDYLQRHGSSVDVVKQKDAPQPLGDQESLRF
TSPATNHGYASASGDFNPIHVSEPFSKYANLPGTITHGMFTSAAVRQLVD
KAAGINKNVRMRSYKCNFVDMVLPGTQLEVKVNHTATKHGLRVLSFHATN
IITGNKVLIGEAEVDQQPSAYIFTGQGSQTPGMGMDLYTTSEVAKEVWDS
ADRFFFETYGFMISDIVKRNPKRLTIHFGGRRGRKIRQNYMDMTTDCNGQ
PRPVFKSITATTTKYTFQHSAGLLYSTEFAQPALTILGNAQFLHLKSHGL
VDPNALFAGHSLGEYTALSAIGAIMPFEQILSVVFYRGLVMQSSVTRDAN
GCSHFSLMAINPSRIRKGLTTETLQTLVSAIAAATGNLLELVNFNVAGQQ
YVAAGTLPALDCLTYVANYMTTHPTELEPSHLAKGTKLPAVIEMWAEQAS
KKPEPTQLKRGLATIPLDGIDVPFHSSFLRSEIDAFRSVLHSYIKPDAID
ARRLVGKYISNVTGKPFDISYRAVREVYEQTGSEILRSLLTRLCTLQYEG
>Mb|QLI67231.1
MTSPPSSPPPVESPKSDVLNHITENSLLKEERDARDENRKAEEQRAIAAR
KPRRKKKAETKSEREAKARELDELLMKSAAFSDILTKKTQVLGRVGSSLD
GKTLGEHNLQMAKQPKCMVGGTMRDYQLEGLTWMFEICSQGMSGILADEM
GLGKTVQTIALIALLREQENYLGPHLVVAPLSTLSNWMDEFHKWTPSIPV
IMYHGNQAQRQEIFRTNMLKNLKGGRPTAKFPVVCTSYEMVLRDQHNLSR
IQWEFIIIDEGHRMKNAEAKLFQQLRQFSSATRLLITGTPLQNNLKELWS
LLHFLLPNIFTDWEAFESWFDFSDLEDERGTEEFIADQMKQDLVKKIHLI

LQPLLLRRIKQDVAAYLPRKREYVLFAPMTKEQTDLYNVFTNKEIDTRQY
LEQKVTEKVNSAMASGSSTRASSRSSSHTAAVAKKNGSSNKSTISLPVRQ
SPRGKKAENKPISSIPNAFSLMMRKRSASQPLKGDAQKTPATPAKQATKR
KSPPTSVRSETKSARSSRHSTPASTRGRSQRGKTYAEADSDEEDALSDDA
FEAKLAGEVESEEEEEVDELQSPEEVERAQTLELAKKQLAQKKLGNPLAQ
LRLVCNSPHNFYNPWATSTHIPVDESIITCSGKMLLLDRLLQKLFDRGHK
VLLFSQFKTQLDILEDYSRELRGWNVCRIDGSVPQDSRRQQIHDFNNDAD
YNLFLLSTRAGGQGINLASADTVILFDSDFNPQQDLQAQDRCHRIGQTRP
VIVYRLATKDTVEESLLMSADAKRRLEKLVIKKGGFKTMGQKMENKEQLD
AETLRALLLKDGQVYEASGGDQILSDEDLDTLCDRSDEAYERAASGLDDA
DGYRVVETGADSIKMARRE
>Mb|QLI67232.1
MVLSAKAPTAHDGSAAIFSPSMARIAASTAKDWSYVDSWLASKFPDSYTL
PRFERNPSTLKALLAVASISERADDSRHLLAGAEKIAQGEGSHIDGYFED
LADDPPRSRMTSGIREQILSAISAHLDQDGKAALDAMGDAAVQHGISLPK
LEDLSNQLIALQTTLFETSQASLRADFWRSFLYREAKRTTDWLATAQSQN
YNLPPELAKQNLDLQRKTKAFIAHLSDTQERIATCIPSGKGSDPTIQLVT
EEEEQFLAVLSHKKDLDAQLMVFQGLPSDTNKARDEVDTLRQKLRGFTSR
RDQAFESLVEQASPVKRRP
>Mb|QLI67238.1
MHRPSPDLSEPSRNLEESNCPTSDDATAMDLDLAMPPSDDDVLEHDNRIY
TPPPRVAARFYRPSQARRRDSAASSRRNSISSAHSRCSSLHHGPSRNDND
QSKYVAQHLRRASFLEDRRARLADRAAHAEKVRHRAALAKAAPRGPTASE
LRTLAAQQARERNLAEIVANCAEEVRKAKQVAELTKERREQEIAKMRAQM
EERMAEAERRREELLNKSATKRVRGQSVMARKANETLAQVKEDTKPRLSQ
DDAAACIQGWWRGRTRKRIIQEFNSLGLSVDGIRDTSFDTVVELLAQEKV
LIVTARLLRICGLKEGEPRSVDEMAAVRTFLSAFLILGHPTQVLINKNGG
EPDALGSHRLPSTDLANPQLQDLVGKAKDLLVNFENIFVRLTASNRYTMP
PALESTLPEAYAVFYNAFIAWKSRDSNALIEVMLMQFVELDAIFQTVKDT
TGDAAASLYRQSIEDSQLMLIVRIKKLAGAEKGKKLIFKAVSEARKSRKA
KKQTGDMKPRVAEGSAGDVTATASSLVSSEAQTLTPPATPSGRSESKLAV
AKTGLNGLLPDNRIIVHELAINKEYQVPSEEYVEQQAVLARPLYMQMRAA
MAENDAESNFRYFILMAKNIKEKLQRLLKPGNSMYNFIGEIIDPELAERQ
FVVGSFSYEKFFTAMGSLIPKLCAPFRDDEAKDLVQNKLSDGDLIDRVEA
LNSFIDVMLCDYINYLLRIAAPQLIDCAAPYEAKRFSTDVEEGDMDLHIA
EAAWRLATTKVMNEVQKRDPEGINHPKSKPGAARFYAQMLVDVFTQPTPV
KLEQIPEMLRLDHRRINEVSCMIQRIITAGAVLLQCKNLLKRDVRSAWKT
EANRIMTVLEANHSSLDATVQGTMAALEAGRSMPAVTKSHLRALVTKVLK
ASQEMSQQNIEPREPVLRLLLTRLRGNILARLAPGSASEKVKAANTAGER
LASLGLSEFVERVRGISDLLDKVGIVDRAAHGAWWDEIATKVQQEETA
>Mb|QLI67242.1
MEASSLQDRLRDYSKSFDGLLSLIDAKTYYGEDTADQWKRKKQTKKEAAA
ARRGKLDPDSELNRNAKEVMDERAKNKRKLRQMEEQADDDDDEDEDEDEN
KNESDSDDDSFQAINGIMTEKPGEGLKRKPEESNKRPKLAKDDEHGTDTL
ETAEGTPKLSKKDAKREAKREKRAEKKAEKKSPKQNAVETSIQNANATES
TQHKNKAQFEGPLSEAKDDVVEVASRSESEPQSPTFDTTDPSNSLNDASA
EQASTTTSVSSTIPPSEKPKHIKLPADTTALRARLAAKIEALRAARKADG
PDGKPIRTRQELIESRRAKQEQRKQRKLELRKQAKLEDARKREEALASNS
PSVMSPAVELDEGAGSFSFGRVAFGDGSQMSHDLSYVLSQGKKKGPSDAK
TALLKVQNQKKRLQELGAEKQADVAEKEAWLTARRRVEGEKIRDDEAILK
KTVKRKEVAKKKSEKAWTERAKGVQQAQKERQKKREENIKQRREEKLLGK
AGKKKGGAKKKKGGRPGFEGSLGVGGRRK
>Mb|QLI67245.1
MDLPPAPTPPVNSGGMPSNKDRTASLLNLLKFSSGSGSSNAQHQHQHAQQ
SHHQQQQQHQHQSREPSYSQPEYSSPPTRILQPAPAGADPTGLLAALMRG
AHDAHDTDDEARQPAPAPAPAPAGHEPTQQTQPAVTNPFANGSPSNDTRS
YLLALLNRPKPSQNDQPMLTETSRSKGAASQSPEITTDSNRFFNQYQHQQ
PQPQPQPHAYQQQAHHQHAQPQFHQHQPPNSMREAYHQVSNDSSYAYQQL

QLASQDPPADPADMSALFQQLMGSLTQSSPQSHTSQPASHLSQPPFQILK
KEQSSPAGSQPRAGSERSPLASPPDQSRHATNRKSSLHVHKSTAQSPKQA
LPVEFESSISAEKSRETVAEAVNDLADQADKDAQEALDRAEHDLVPAGFV
EDEGSKEASGQEWPSKIAHTEIVKSVEEQIVQNIKNGLEKNEVYRATEMS
YMNDASDSGNATGENVRGVADSWESADLEETIEVEEEKELPQVRVYKFPQ
EPWNSISLKEETDEIRPEFRDESIMDIARLKKEFDQIDRNLFTASQTYMT
YGMSKQGGLRVIRQDDGKDAKVFTDTKDRIFNVAMSVTPPESGNVHREAI
IGTGVGGAVYWVQIKDGEKDHIEDPHLEQYGFALPPISSHDGDAPGGLLK
TRARASTAHPEFFAVGRGKSINFIWPAYILQHKLFKAGHDRVVDTEALLA
QCSLKINTGKAGKDFTFSQDDSVVVSLDKSGRVKFWDVRALTKVKEDSDY
RYPLPAESSLEVKEPLMTLATTPEGEKAWPTSVLLLDRKKAYEQRGPLRY
MVVGMKQNHTLQVWDLALGKAVQEFNLPHSKESDAVCSVMYHPASGMIVI
GHPTRNSVYFAHFSAPKYSLENVSQVDYIEKLLQPLKPNEACLIRKPSST
AIISGFREYSFANRGVLRSLDILYTPIAKQGMGHMAGQDPKDKDRLRFEL
YAMHSKGVACVMVKKSDLGGSDNTLVLDAVKEGLVTVSDFNKPQAVDPSH
GGNQQAKNTAHRAVPKQSPSQQSTSAAQSDAAQRGSDAATPSKPSHEAKE
AETPAQSSKEGQAEKPERKSRKKKGNAAAKEAEANGATNPPRGTQAGRAE
SAAKSNSTAAVASGVTFEVLDSTINTMQTRLTAVVSDTLKSSLKNLHVKI
DESARLRDENFNQHQLKLLDMVSEVLNENTQKVLESLIHHQFTELLVPAI
GEKATIAVSDVLQHSLQPNVASAVQKEIQSALPQALNRSLRSVDFVTAIA
ERVGTAVNSTVQQEIVATLSQRLTPTFRNMATQSAQQAAAEVHQQYNDKF
EQMQSQHAADSSKIDQLLSYVTTLTDMVSTMAASQSSLQAEFLKLKQQPV
HELPGASGLQSQARDGGPGFRGFGPQGGYPNNGFMAGPNPHSSFTGYQTN
QPPTYQQQQQQQQYMAGPDYLRSGQHVGSPRDSTSQTDVTSASKVASVG
GPFANHGSNVDSEADRDVHDVEVLIQDEKLQEALLKWIKGGHQREVFQRS
LSLQSPSRFEHLPPVLLLAAIGAISKDLDVNNLLKQRIDWIEMAVRSIDR
RAAERDWRELGREVVKQAYDTICWFIGKLNTLMEKYETGYALERRRLEWI
VGSSEHILQTLDRSQS
>Mb|QLI67247.1
MNATKRKFKDLLQGLSSPGPTSPDKEADKMSSSPRYGTGTASNDTILQKR
RRLGFPESMTPTLYSPSHTTTFSNVVLRKSSSQLNSKATSHGPAKYCPGD
RDELLKRLATFQEITDWTPKPDKVNEIEWAKRGWICQGKEKVRCVLCHKE
LVVKLNRKEEEGKEVPVLVSSEIAEALVDKYSDLVVLSHQQDCLWRKRGC
EDSLIRLSFSNASTTLAQLRQRYDELCARSPFLPYEFNLRLPDEVDIDNV
LSQLPPDFFSEPPPVVDKSGAVATDKPPNRTALALALMGWQGLTNSRIGP
VPNSASCHTCLRRLGLWMFKSKEVDEVGQVVVPAPMDHLDPLREHRFFCP
WKNPQAQSRGGVMSNGKDSMAGWRTLVQTIKNESDLRNLYSGKSKRTGQL
QEQRLASPSPVRGMPGTPGSPSPITPGPKMNIDASSTGTPQTDAGDDEEK
TREAKDKERWARLRKVKSLFDTKGSRKFRRELGRPGTGHSNKSTTGT
>Mb|QLI67248.1
MAKKAKARLINARLISMAMTGFFYTFKRPRTAPLMGMIKYDPIVRKKVLF
LETKKRSK
>Mb|QLI67249.1
MSCNCFIVPPHLLKAIANSTHNSEEIRKSAQNGLELHAKLTDARKNRLAS
LSQPRGARGEVPAPRPAPFIPETVLHHLSIAEDVDEAVRQRAKRDLEHFQ
SLMAPKAEPQEESQSMVKPKKDPKDAPYRAVYDAEHVTEPTELPGKLKRA
EGQPKTKDNAVNEAYDNVGAVLDFYRKKFHWKSIDNKNCDVISSVHFGES
YENAFWDPEQMMMVFGDGGEFLSNFTNCIDVIGHELTHAVTEHTSPLVYA
DQPGALNEHISDVFGIMIKQKKENETAEKADWLVGEGCLLPDVKGLALRS
MKAPGSAYDDPRFGKDPQVDNMKDYKKTYEDGGGVHIFSGIPNKAFYLAS
VAFGGYSWVKAGQIWWKAMNSGKIPARCSFTQFADVTVDCAEEEFGKGAA
EIVRKAWTDVGVTRVI
>Mb|QLI67250.1
MTLTVLTDSQVNDILESLTIDELDDFRHVLSSALHEFSTGTDAGDGDVYQ
QPHRITTLHPETKATSLYMPSCGPEGMGCKIVSLTSSAATQDPTVKPISP
TGVVNLFHPDGSPMGLVHASTLTAFRTALGSACLLTRRNHVKTLTVFGSG
SQAYWHVRLALLMRGSTIKHVHIINRRFSDSAGLILKKFTIIPADIKERE
GWAHTKFSIITPTFHDFERLQKEQIRSADVIYCCTPSRQELFDASILTSH

EGRKKGRLIVAVGSYTPEMRELPEGLLHLVTKPHEKHHRHFHKHADEGGV
IVVDTLEGVLKEAGEIISAKISPHQMVELGELVMLHRLAIEESETDTTGS
QTPASSEYEVPLDGDDKTPSMSSVYSDPVPRPASPSSPSLSNHSRKPSFP
LPFRTRSGGSADTEKKKEDSLARWLRDGTVVYKSVGLGLMDLVVGMHVIK
VASEKKIGTQIEGF
>Mb|QLI67251.1
MTPRRLNVLVYTGTGTTSESVRQCMYSLRRLLSPNYAIIPVSESVILKEP
WAPTCALLVIPGGADLGYCRVLNGEGNRRIGEFVRRGGAYLGLCAGGYYG
SSRCEFEVGNKPLEVIGSRELGFYPGTCRGGAFKGFEYRSEKGARAAVLK
VATGAFKDGLPETFSSYYNGGGVFVDVGKVTGRKVEVLASYAEDLDVDGG
DGKAALVLCHIGDGKALLSGPHPEFAPVNLSPQPTIPGYDNLINKLQEDD
DKRFSFLKACLEKLGLEVSGENATLPTLSRIHLSAVDNSQVSELLYAWAE
VIDKENGEEFIAGEADRFCIQSDQDGLTLEDLQRSLPEADEHLARETGIV
DYSTITKRVIVHEKALPTTEMTPRFDHKLYFSSLRRFQVKEDGAEDWGNI
LMYGDVVTSTNSLLEKNPKLISKLPTGFTFSAATQVAGRGRGTNVWVAPP
GGLMFSTIINHPAHLAASRPVVFIQYIAAVAIVEAIQSYGVGYENLPIKL
KWPNDIYALDPTKPASSKTYVKIGGILSQCGYCDGSYQIVLGIGINAINP
RPTTSISDLLPANASPLHLESLLARIVTRLESIHAQFRRQGFSENLEQRY
YRHWLHTGQAISLEAEGGVRARVLGITRDWGMLRAEETDAEGRGTGKIWT
LQSDENSFDFWKGLVRRKQ
>Mb|QLI67253.1
MDELFDVFDGQAGAQSASDSEREPQTSRARDKSKKRKADEAMNGTAAVET
PKEGDDGDMGDATEVSKAEESEEEDSDTPSQQDKKRRKKEDGTGPVMTDT
FQTATSREVAGAATLIPQDSSLVLSHNIQHQVALPPDLDYEYVPLSEHKS
PDEPARTWNFKLDPFQSLSVASIERDESILVSAHTSAGKTVVAEYAVAQC
LKRNQRVIYTSPIKALSNQKYRDFEAIFGDVGLMTGDVTINPTASCLVMT
TEILRSMLYRGSEIMREVAWVVFDEIHYMRDKTRGVVWEETIIMLPDKVR
YVFLSATIPNAFQFAEWIAKIHHQACHVVYTDFRPTPLQNYFYPSGGKGA
RIVVDEKGNFNEHNFNLVMKEIEDKKGAGSNDINAKQRGKGKNKKINKGG
VDEGSDIHKIIRMTIKKKFNPVIVFNFSKAECENMALNISSLSFNDDSEK
AMVRKVFHSAIESLSEQDRELPQIKNLLPLLERGVGVHHSGLLPILKETI
EILFQESLLKVLVATETFSIGLNMPAKTVVFTQVTKWDGVKRRPITSSEY
IQMAGRAGRRGLDARGVVIMMIDDKLEPDTAKGIVTGHQDKLNSAFYLGY
NMILNLLRIEAISPEFMLERCFHQFQNAASVPSLEKELMSLQQERDGLSI
PDEATIKDYYQIRQQLNSYTEDMRAVIQQPTHCIEFLQPGRLVQIYNPKE
AHDNVTGGLDFGWGVIVNHYPRRAPRLGEPEWEPQESHIIDVMLPISASS
ADITPGQPTGAMPPGLKPAGADTADTATINVVIPCLLTCMKAISQIRIFM
PKDGLKADADKDQARKSLAEVKRRFTDGIPILDPLENMEIVDDSFKKLLR
KIEVLESRLLANPLHMSPMLPSLWDQYSEKVQLIDKVKEKKKAISKAHSI
AQMDELKSRKRVLRRLGFINESEVVQLKARVACEISSTEGHELLLSELLF
DRFFNELSPETCAAVLSCFIFDEKVETQALKEDLQKPYREIQAKARIIAK
VSQECKLDVNEEEYVTSLKWQLMETVYAWAQGRPFIEICKMTKTYEGSLI
RLFRRLEELLRQMGEAAKVMGNEDLVKKFDESLQKIRRDIVAAQSLYL
>Mb|QLI67256.1
MSNPTTASAAAGTSNPPAAVATAATATATATAAAAAADDSASTITVNTKA
PSANFPPPKTDKPRPHVCATCQRSFARLEHLKRHERSHTKEKPFECPECS
RCFARRDLLLRHQQKLHQTSTPSSRPRNRRESASGAAPAQSRRKNSVAGV
NAAGSGAAAAPMRPRANTISHVDGAAMQMLASANVSVARGMAHSRHPSLV
GLPVHNLDHVFGGMSAALGQRGLQHGLPKLETSQVGSNDFDASGLRTAPP
MAVFNPEFDFEGLFFGPGSTINPNALHYNDSPQSMALDQTSPFGQGLPDV
AATQHFDDWVTGFEHQMSFNTNEHVIDGSSPSAISTTSQSGISDVMVDGS
NHPAPAGTSTMWQPSVMGPPQMPNPFAMDLNGSVFPDLLSGAPLSPQPAT
QKINDPYFSAPPTSLSSLSTSVVSGINSQPLNPPLGFNAAQETPSSLNGG
NHAASPVTTITDATRTAIVTILFHCLPFGSRKNPSLSSLGSPQIQSGGNS
SSNPAINVPSTQDLQRYVRAYLTCFHPHLPFLHLPTLSFDVSPDSIGRSN
GAGGNGCLLLSMAAFGAAYEMEHAQSRDLFDMAKKIVFFYLDERHKSDVR
RADTRLSTISTDSKVQQNQAQSSTPLWLVQAMLLNVIYGHTCSDKVTSDI
ASTHCASLVSLAQSSGLISQGKGNVPTKQDVDMADTGDWTRISEQEEQRE

WLQWKSMEEHKRTVYVIFILSSMAVASFNHSPALTNSEITLDLPCDEEFF
AAESGAAFASRGGVEAANHNRLTFFDSLGDLLRTNERQHSMGNSQPQFGS
PMNTTDTPLTDLKPSTFGCLVLIYALHNYIWETRQRHQNRTWTNIETEKM
HMHIEPALKAWQIAWSSNPQHSIERPNPFGLGPMSADAIPLLDLAYVKLF
VDFGPAKAKIWQRDWDGMIEELSRGFETQDEARSPTCSSETGTDPSSNSV
INPGFMDSPATQSSLNGMHADKPFGTGQDANGTGRSISRRERYLRTASFY
AVDSLSMSDKLGVTFADKTSHELPMQTALCTAECAQVVAGWITALQERVG
PYLGIVGQDHIDLSQVPAIILLEEEDIKLLDKVRDILSTAELKINMRLGP
NATGTIGGHESVLSPDGFTGYACQILRLAAWMVNKSAVWPVAHIAAHGLE
TYANHLRVRAEKSVTGSMPLTL
>Mb|QLI67257.1
MALKRINKELTDLGRDPPSSCSAGPVGEDLFHWQATIMGPGDSPYSGGVF
FLAIHFPTDYPFKPPKVNFTTRIYHPNINSNGSICLDILRDQWSPALTIS
KVLLSICSMLTDPNPDDPLVPEIAHVYKTDRPRYEATAREWTRKYAV
>Mb|QLI67258.1
MAGAHEPPPTAGEYVDTGPYTTDPPLLRDHSQLKTYKTSRFEYPDIRVFF
RPHPKAAELPRTPAPLPLVVCIPGLGGSVAQFYPLLNSLVDLASCLSVDF
PGGGRSRYAVTSWDAYTPEALGELLELVIEDHRDNEHGQGIILIGHSMGT
MLAAQLASKSASHQTSLSKYVVAVVAVCPTAGPPDQQRSSLLRRLLWMPG
WIFSLWRAWDGIGGPYSPSVSRFVGKEADLELRVMQYRFNQQSRTPVWRR
MAYGALPVHENGKPIGGLPSLDTWAGVKVPVYLIAGEDDHLTPPGEVDKI
LQVLNHRTNSTQSNDEHTNQEELAISSQSPTQVPSQTSDTIYDPTEESFA
TDKIGADSIDNADDEPSTPLESPAFVPPQPAHPTPVVQSFVMPSPANHAL
LYMPRCSRVLAGLISDFLACHVTQRLSLAWQLQYLSREGKWDVKNLIKWK
SVAPVSEEIGPEGRPIFRALKTLREADDVHSPSEFVGTWSHVVKDVIDIS
RDQPVYDPRGLERGGVHYHKFPTVSKIPPQPKEVELFIKLVDKLRNAQME
RADKEGWEHPEKCVVGVHCHYGFNRTGYFIVCYLVERCGFSVQEAIEKFA
QARPNGIRHSHFLDRLYVRYNVEEGPGQQPTP
>Mb|QLI67261.1
MLPTSPVVQQNGAAKIKRPVPPGIQTNGIASSNSSPSPSMSAKKPPTSAK
QAPNSASDRSITASTVRPVNRARRENSSQAAGRNSRNSAGMRTGSFSVDA
GAKDYGPPPYVVTSQYILSKYSGKPPSLVVHLHATHFRFDGQDGMFPYKS
PMGKFIEHLRARTLPHDILEYLIEAKVPFYDGCLIVQIHDHKSLAQTTKA
VTKASRTSDAVLPSSIHNYNQCLTPSPYVPFPKEEQVTENGIASKDTDNS
KQSPEEGKDNESVTAPTQPSESTTTKEAPKPKIFTAVLHPTPESLQSDLK
IKATTPKGDGKGVNDTPSLAPPSTPLSLVPPTPTASSMPPPANKRQKKER
MELDSSTIYAAEGQILLATTAPLILEPTRNPEETILLLEAMAHPKHSEPP
PQPKTRKRTVAEMAADEAAAADQERYMLVLDDRLSGNTSGAQGSSATDGE
AQTNAAAFEPRFERFKVIEDIKRDHAEKKEQEKIKQQENDRRLQLQRQEQ
LLQAQRQQAEAERQRREAVLKENQMRQEAQRQQLAARSTAAAAAQSQQQQ
QNQQTAQKQQQQQQQQQQQQQQQQQQQQQQQQQQQQQKPQSQPQPQPQQQPP
QQQQQPQPQQTTQHAHPAQNGITGNISNGVPAGGQSAMANNMQGAAQPRF
PAQMSQAAAASSPVIRQGTPLSSPMVGSVPMQQTNSNIATSPPRPPSAVQ
NGQMSVPMAHNMSARGSQQSHHSGTPRMPNSTPNMPHGTPINRQTVAATP
RMTHASPPPNMVAQNSQMGQAMMMNQQGINQHNPQIFAQMAAQQRAIAQQ
QQQQMAAMQNGGANAGLNPQGMTPQQQQVMQMMQRQMMLQQQQQQAQQQQ
HQQQQQGGMMTPQQQQFAQQYAQLQQNMQGNQTRQMSAQVQAQLQQMARM
GQINNPMQRQVSGQMMNGSNANMQAFAAMQMQQAQQQQQQQHHPQQQGQP
QPQPIGANPIQAQIQINARQIYSRLMQGAVAKFGSAENVPQDTLDKMKQS
SLLQAQQMLQQSMVQRRAQQQQMMLQQQQQQAAMQGMGGMMGHPGM
>Mb|QLI67262.1
MISRSSLAKSAQQAVRRPCSVQSLQRRGFAAAAATGAFETTDVSGLKVAS
RDAHGPTTKLAIVAKAGTRYQPLPGLTVGLEEFAFKNTQRRSALRITRES
ELLGGQLNASHTREALVLEASFLREDLPYFAELLAEVVSMTKYTTHEFHE
DVERVLHLKQAALNANVAALALDNAHAVAFHNGLGASVYPSSLSPVQKYL
NEEYIASYADVVYSKPNIAIVADGASTDALTNWVGQFFKDVPAKSLNGQT
LKAEASKYHGGEQRTSHPGGNAMVIAFPGSDASGSKPEIAVLAALLGGQP
TIKWSPGFSLLSKATAGTSGLSVSASNLAYSDAGLLTVQLSGTAASVRKG

AEETAKALKSIADGTVSKEDITKAVANAKFDILEKAQLRSPSLLQAGSGL
VNSGKASELAALAKGIDGVTADKLKTAAKSLLDGKATVSTVGDLFVLPYA
EEIGLQV
>Mb|QLI67265.1
MLVNTILVALSAASLALAGTPVESGNWVEDHPDYSQQQCAGGRVNGNTFS
IPKSPNGDTSGSGCSNGHLRAERRYKDDYSSGVHQFGGEFKINSMSGSRI
SIKQTFNGNSGPYFIMGVEQGGRLYNVEGGKTIADGVAKVGATVRINTVH
NANNHRFSVYVNGKEMLRDENAPGGSFYDKIGAYATNSGTGDLSITWNDV
QFWHK
>Mb|QLI67266.1
MAGGRKAKPAPPARPTNTLIVDNGAYTLKAGIVANGHVGEPRIIPNCVVR
DRSRKVFLGSDIAKCRDFGELQFRRPVEKGSIVNWEAQKEIWDQELFDNA
ATKCDPTEARLILSEPPNGLPALQTNCDQVVFEEYGFASYYRGIGSTFNA
YHDIQSLFRTPKDAPTAANVPAEVMLVVDSGYSHTTITPLLRGQPLHSAV
RRLDVGGNLLTNYLARLLSLRHFDMRNETYIVNEMKESACYVTLDFKSDL
EKTWKGTRGEKRPSYISGDGIVKDYVLPDFHSNTKGSLREYDPTRHTKAR
KLAAAGQTDEDVLTLRNERFAVPELIFNPLDMGMRQPGLPDLIQQSLQQL
PMGLWPGLLANIVVVGGNTLFDGFIQRLQKEVVQRVPDDCVVRVARPVDP
VTSTWFGAANLASHPNIEKLVVTKKEYEELGSALVARKFAAGLNLT
>Mb|QLI67268.1
MSGAKATLKAINDAIRGQDYDGAIEKAKELIKKDPKSYHGLIFLAFALDK
KNQLVEAENFYKKATELRPQDAQAFQGLIKVYEKQGNQRLGEYQTAVVSL
AQIFHEAEDLYKAQDVVDKFVDFARVNGDKLQYADALWIQLPESPLYPIL
EGRFPQPARTYETIAHILEDFEKKRINTLVGERRTRLGARLSEVKVEVKR
EVYAQSKLEPIYRQLINWTSNDEVRRDFEEKLLQYCYDRLLVTAAGPVKT
QEQAKVFGLANDMVTIKHPFKLAWDIAIDWQDGKDIKDWDAGLLRDYCTF
FPDSDMYKIITAFLTSNFSPFPPEAKAAEDTRAAANSASDDSEDDEDGGV
PIAYVPLTDEDRLMMISEGITGSESVFAYRLVGVYFLQIGEYENTVELMR
KARAFLDKERARVGISYENTGDTYALCLATSLVYFQSPRHHQEAKELFEK
VLERDSTSTSALIGVGLIFEEEKEYDQAIDFLERALKRDTSNLRVKSEAA
WVKALKGDWKTAKHELSECLPILEEKIALYKELMADTQYRIGCCIWNLDT
SRQARKQRKGDCAYAYWLSALNNNMNHAPAYTSLGIFYSDYAKDKKRARR
CFQKALELSAAEVVSAERLARSFAEDGDWERVELVAQRIIDSGIVKPPPG
SKRKGISWPFAAMGVAELNKQDFHKAIVSFQAALRLFPNDYHSWVGLGES
YHSSGRYIAATKAIVNAQRLEEHLGDGISADTWFTKYMLANIKRELGEYD
ESIALYNTVIETHPEEQGVVIALMQTMVDNALTCIEKGLFGKAVQLATYT
IEFGSKTPADVKETFNFWKSIADAASVFGSVQSRVVNFPASEIKELLEAS
YQEAFELLANVDKVGTDVVYAKGIYPKDEQLGVDMTRCIHATILCYKEAI
HVSSGDIHAQAVAHYNLGWAEYRAHACLPAQMRKKPSNYIKAAVRALKRA
IELESGNADFWNALGVVTSEINPAVSQHAFVRSLHLNERSPVGWTNLGVL
ALLSGDTKLANEAFTRGQSADPEYAHAWLGQAFLALLYGDVKEARGLFTH
AMEIAEASSVPTRKHYSSSLFDHILTAPANMTVASLIQPLFALNQIRSML
PQDLASGHLSCLFNERTRDSASAVKTLEKLCDVIEADYEATESRESLARF
TLARTDLARAYLAAGEYAKAVECGEMALGLSSDDADSELTGEQRKKARLS
SHLTVGLGQYYLGQHQEAETYFEEALGESDNNPDATCLLAQVLWAQGTEK
SRDKAREVLFEVIERQPDHVQSILSLGVVALLDQDESSLEAVVEELQGLR
TNDKVSTTEQSQIGEVLRAVAGMAEGHTQQEVVQQIQEDIMLYPHLPHGW
SSLAEATGDKYAAQMALKVAARGIPPRGILEAQDLAKAYAGTGKAADAQK
AAFLSPWDGCGWTALKVVTDGI
>Mb|QLI67269.1
MAASSVQIPTHPSRDSVQCIRRVTRENRTLWYQLTVLQQPERARACGSGM
KANSDRRPVDPHPVVELRIIEGPSVGEGKDITFDYNANFFLYASLEQART
MAHGRVQSAAATNPPILTGVPASGMAYLDRPTEAGYFIFPDLSVRHEGYF
CLSFSLFETTKEEKDFDMEPANSDLPPGVDWRMEIKTNPFNVFSAKKFPG
LMESTQLSKTVADQGCRVRIRRDVRMRKRDGKSSAFERREEDFRRRTVTP
APEDPHGIRNRSLSNSSEHRVPYAPEPHRRPSAVDAYHAPPPPPGYDSGP
PPSRSCHLSFGEHPTAQYATPRHYPAPPPLNGHVQLPVAAPYSSPGQATN
YVKPEQSNYGYPTHTRNPSSTCPSPVPSVKHELSYDRAPGNYVSPSPSRY

SETSRRDSHQSYPPTPLLPQPPRRTSQTSLAPLKIAALVSPSPLPPIEAQ
TEPVPVSPIIPTGGKRKHQDVFMHGLQNGQRQQDTQYGRFKGLSPETLEG
RYSRADGKVDVIQFNRYQ
>Mb|QLI67270.1
MSLFTVPIPPCGAHPGGSITATQPQPQVYLLTFVSPPDNRLTTAFCRALL
QALDILEFGGYTPGVVVTTSGIPKFYSNGLDLEHAINTDGFWQLFFDLWT
RLLTYPMPTVALLNGHTFAGGLMLAQAHDYRLAPDPKGFLCLNEVLFGAP
LKPPMAAIFRHRLAPQAYRTVALEGRRFTGQQAVEYGLADATAASLDDAL
RFIAERQLTEKAKAGVYGVIKTELHKDLLKELRKEGVDREEARFNEDQRL
DGERKEFGKVWFEQWNKENKSKL
>Mb|QLI67271.1
MKSAAILGLTGLAANVLAHPQRHHANEASLGKRGVDVSQFRMPGSSEYTV
STQAESDPAISSIQKRGDYVSSATQLVKTVLPDAEFRVVDDHYVDTDGLA
HVNFKQTVHGIDIDNADFNVNVNPDSTIFSYGNSFYNGEIPPESPIQKRG
FTDPVNALKAVVDILALPVNPTDATSEAMEGAETYTFKGTSGAVSDPAAK
LVYLTKDDGSLALAWRVETDVMDNWLLTYIDADNSKDVHGVVDFVSEFAT
LQVYPWTINDPTEGARSVQTDPWNVDASPFTWFGDGSQDYTTLWGNNAVA
QTNRDGHNNTNDFANSYRPTSADRKFEYNYSPTQSNSTEYQDASITQLFY
TANTYHDLLYTLGFNEAAGNFQTNNNGKGGKGNDFVVLNSQDGSGTNNAN
FATPPDGSRARMRMYMWTLASPQRDCAFDSDVVLHEYTHGLSTRLTGGPA
NSGCLNGLESGGMGEGWSDFMAVAVLAKANDTRTKDFPLGVWVSNKPKGI
RSFPYSTNIQTNPYTYASANEKNEVHAMGEIWANTLYEVFWNLVDEHGIT
ADKYPTFDDKRVPKDGRFLTMKLVMGGMAIQPCNPSMVSARDAILDADKK
LTGSANKCLLWKAFAKRGLGENARYNGGRNRTEDFTVPAGC
>Mb|QLI67276.1
MSATPRLRSGFPATPATAPRRQNPQETPSTVGTVSSNGNSAKSPTLPLAP
ENNTARRVASDPLIPLTFLDAPQQRLYALAVYVLLWAWKLYDWLQVIEDG
DSSWWLFIKWIFIDFAFLFGLPELRIPWLELSQFIVTTLFAGHVVFNYML
MFNIPLPWQAWLLGFAKVLYDREISISEHNVKISSILNNHSLIMGKQIIN
ILPEGSAVLNPDMLPYCLNRKDKIGASLPIYFNATVPAEIELIRIDLDTN
KEETIKIPTREVNKVARAIRDHNADPTADGFSWLYVVKKPGVYRLGKVLD
QYKLEVQRAAKETYVVPCPQAKIRASESSQRCIRDLSDLSLEVMGTPPLK
IKYSRSVNGINHDFRFQSLQPDGFASPLITGANAAAVSAADDFSWARPAT
VTVGLNESLTNPGEWEYSVDEVHDAFGNEVKYAKEGEDVDVANSKGLTQK
FTVRERPKIRMDGCDLRKPIKVAKGRAASLPISFSLSGSIKDTSHEISWE
FSPIDTLTNSGDHGSEATIFRHSAKNSRDRPFVSEAGLYTLKSVTSGSCE
GEVDEPSSCLLLNPLEPHLSIRSEDIPDKCAGNSIGLRVDIDLVGTPPFD
IHYDIISDGPPEHKTLRVPGLRSQLELIPKKAGRHRYVFTSISDAVYIGL
PLSGQDTILEQVVKPAASARIVHPRDTINACLDSEVEVDVALNGDAPFNL
EWEIVHDGKRKTERASNLEDNMFKIKTPVLAKGGDYILALSSVQDERGCR
TFLQEQIKITVRRQRPRGGFGLIEQKSSILAVEDTSLQLPLRLQGESPWT
IRYRNLNESGSELFKTAIAPNDHLVVKSRGVYKILEVHDSQCRGTVDKTA
STFQVDWFPRPEISLIQTESITSAKDAFIKQDVCEGDIDGFEVKLQGSPP
YHVSYELEHRSQQGSHSKSPRNFDAALSKASISMDTSKAGDYAYKFVGLA
DNLYNSDKKLRPLVVQQRVNARPTAVFSKPGQSFKYCVEEQEHEDKIPIA
LTGVSPFSVEVEIKHQSGSATDTYRIASINSNSYSMPIPREHLRLGAQQL
RIRRVRDAKGCQQKYDVNGPSIQVQLFDAPSIYPLESREDFCVGERIAYT
LSGTPPFDITYDFNGRWNAKSQTTNFRRVAEKPGEFIITSISDKASECRA
AVNMHKKIHPLPSVQISRGKQSRVDIHEGNEVDILFEFWGTPPFEFTYTR
STNAKKGHKSVVLDRRHDISYEHSKVIKASQEGTYEVVAIKDKYCSFSTE
EVSEKQDG
>Mb|QLI67277.1
MRRLSSARDKLFRSSSRTSSQSKKSESSRGLSSSTSGPSSYPGPNNFSDA
APPAYTPAYQPAPAPVFLSPPSHAPVPVPSPVPAPAPAPAGVYGGVSDGD
RYANLANFDTILLIDDSGSMAGSLWREAQDALKSIAEIVTMYDEDGIDMY
FLNHKSSQTPSDRYKAKGGYYNVARKDVIETIFGNVSPYGATYTGRRLEQ
ILLPYMRKLASANDVDDVKPINLIVITDGAPSDNPEQAILQVAKALDQQA
APSYQVGIQFFQIGSDSSATSALQQLDDGLGRYGVRDIVDTVSCQWEDAG

RHRAKRLTGDAILKVVLGAVDKRLDEHRLTNKK
>Mb|QLI67281.1
MKPAVSHASTMEPRRPSASDAADAALASLGYKGELPRNLSMMSILGLSFA
IMAVPFGLSTTLYITLYNGQSVAVLWGWVLVSLISICIAASLAEICAVFP
TAGGVYYWSAMLSTRRWAPMVSFVDGWLTLVGNWTVTLSINFSGAQLILS
AISIFNPDYVANQWQTVLCFWAVMLVCAAVNAFGSRYLDLINKVCIYWTA
ASVLIIMVTLLVMAPSRHSGAYVFGHYDASASGWPAGWSFFIGLLQPAYT
LTGYGMVAAMCEEVQNPEREVPKAIVLSVVAAGITGVIYIIPLLFVLPDV
AILLQQTQPIGLLFKTVTGSAAGGFGLLFLILGILMFAGIGALTAASRCT
YAFARDGAIPGYHLWSRVNKKLGMPLWALALSTVVDCILGCIYFGSSAAF
NSFTGVATICLSMSYGVPVLVLLVRRRELVKSSPFSLGKFGTIVNIICIV
WIVFAVVIFCMPVSLPVDSSTMNYASVVFAGFAAIAIAWYFAYARRNFHG
PPVLGNGEFVPGVTGVQHHGAEAIVGTDSETPDPASEKN
>Mb|QLI67282.1
MNGYPTGRAPYDYGAPNLLSHNVNPTDKPEGYAQDGNANGFAQDAPRHQA
SGPNSTPRSARVDLKDPIQVHLLTETALSDSKGYEILSQEEVDELKKHAA
LLTQRVETTRSNLAIQSKYRDATASMARLNAPGLNDEQRNREAERERIEN
ERKCDELAAELLNLEKRLLIPHRKIVEHTAAILQLTHKASRKKAAPQNGQ
LINGIPGSPESLYTYSHSRNSLDQVGDETYFDDPSAYQLDGMDRPRKNVI
EIPLKSPVREQNQLRVEMDRMREENIHLRSQTDGLLKRLQSLNASLRDTI
VRFNPEINQTYDEPPQVSAAPDIKLADLLKTQIEYLESGLVAVQAEQDSF
AGGIQMGERLEAMNLQLRDLLMTSDPHYTPTPLPLDSDVNGQMAYLEDSI
RAVDSHMARANSSPKANDGTGPVLTTLWDSMQKGIVEAKQRQDERKRSQI
ERGITEDDMDMSDDEDFDTTKPYSLDTFADRVQRIHARTITLRDQKYVLK
RQIKQQRELNKSDGEKDKELERKQRELEESRQQFNRSEKDRKDAQEQLSN
IMADFEKAREAADRAGTVTLELEERKAKITQLEKSLKATQASLAAAESGT
KGSESKLSAINAQIEELTEGKNVAETTIQSLRDELSAKKQEIDDTKKKID
DLESEMVDLKLDLTVAKAELDGAYGSRAERAERAADIAALREDGKIKKLK
EEIKRLQGEMKELGGKEKELRKELQGMTDECQRITSESVGLTGELDDALR
AKDKLKEELHSTQEKMAKLQEDLDNERLKIPNDGSRPGAGASVLSERFRA
TMREERKRFQDDLKDERLRYRKLEEELSKLKRTLGPGKSPLSPR
>Mb|QLI67284.1
MRLIIRDDATSASTYVANYIVDRIKAFNPTAENPFVLGLPTGSSPLGVYK
ILVEKFKAGEISFEHVVTFNMDEYVGIPRDHAESYHSFMWKNFFSHVNVH
PSNVHILNGNAENLEAECVAYEDAIKAVGGIDLFLAGIGEDGHVAFNEPG
SSLASRTRVKTLAYDTILANSRFFGNDINKVPRMALTVGVQTVLEAREVV
VIILGQRKSLALQRCIEQGVNHMWTLSSLQLHPHPMIVVDEDATLELQVK
TVKYFKSIEKVAKEAGFEQILPSKVRTGNGVVPTTRVKEVDSPTILAPQP
TTSRLLRATPATEYPVRSVSPELVPDRMASRIPEPALNKRLTPNPEQQQA
ALPQNAISA
>Mb|QLI67285.1
MVAPRALRSCTISSFRPSRCLNRTASRPFCTTHLRREQQRTPTAHRETHF
GYETVTESEKQDRVAGVFSSVAESYDKMNDFMSLGIHRLWKDHFVSSLNP
GATTPLGKPQRMLDVAGGTGDIAFRMLQHAHVDNANPNIHITISDINPDM
LAVGKQRSLDLPASYQSNLSFLEANAEVLPAQIKDNSLDLYTVSFGIRNF
SNIPAALREAHRVLKPGGVFACLEFSKVDKYPLLNAIYKRWSFSAIPLIG
QLVAADRDSYQYLVESIDRFPSQEAFRDMIVDAGFAVSGDGYENLTGGIA
AIHKGIKPL
>Mb|QLI67286.1
MDFFHQVKQALDAGQEAEQPTPTSNGTGIPGAPSPQFALLNYLFPGYSML
MSAAHAYVGVDFNSYVPVLLGVIAASVAWNYVKEEFWVFFEDYFMSSVTI
RTDDEIYNMVMLWLSKQKFSHNSRHFVANTNINSRNRFMCSYNASDSEDE
DDEAADSPVNTTTGLSNDLKQALHYTPSVGTHFFWYRLRPLTFERVQNRE
QILGMTASEKEELRISCLGRNPRILKELLLEARQLHMKKDDRKTVIYRAN
LADIYWQRCMSRLNRPFSTVILNEHVKQDLIDDAADYLNPITRRWYANRG
IPYRRGYLLHGPPGTGKSSLSLALAGYFRMKIYIVSLSSAAATEENLTSL
FHELPTRCVVLLEDIDSAGLTHTREDSAAPPAVPGQVPSQAITSANGTKA
ATPLPVPPGRVSLSGLLNILDGVASQEGRILIMTTNHIEKLDKALIRPGR

IDMVIPFGLADSRMTASIFRSIYAPYESEIASKVNAKDSDSEARRARLAK
KHAQISKRVDEQARQFGEKIPEFEFSPAEIQGLLLKHKHDPEGALAASGI
WVEQMRRDKKKEKAKEAEKKRQEEQNRRQEEKNVNEDTKDKTGQEDKETV
SSQGDAEKAFEKNKLDKDSLTPVKSDNPKKSPSDSGYETT
>Mb|QLI67288.1
MAGQFVRNNPGALGELQQNHQDVAPRSPRKFRDEPRAGAQSPTKSALGKL
TFRSNTDKETARMAKSRDPSPDKKPKKTKSGTNLVGLLSRPKSIKNLYKL
ATEEESRTKDKENMTPVVTPVNERPPPIFAQFTSDRAMRQGSSSENTPIQ
ATKDRPRSFQPPKPHHDLSQSSQSSSPSKTGGRATRPVSLHPGQPPRGKV
PFGHGRAKSTVTTPTNTSSELQIDPKDIDQQLEALLDRRNIPENQRYKMR
NLADTIKLEFIRQDYAEMQAARADGPGTTGTTGSTSSSEQALVSDSNSPN
EDEERPKRSRGRSFTFSRGKKDSSSPAKKSKAESTLGRHFRSRSTDSVAS
DARPSSSSSFKGSGFLSKIKVQQGPSDYVAYLRKVQKPELVEVGKLHKLR
LLLRNETVAWIEDFIQQGGMHEIVGLLNRIMEVEWREEHEDALLHENLLC
LKGLATTAKAMEYLNAVQADLFPKLLHMLFDPEKKGPSEFTTRNIITSVL
LTYIESAPLVERVIRAKNVLGHLRDKDPEEGQRPLPFVLEMRQERPYRVW
CKEVVSVTKEVFWIFLHNLNVVALASDKAARSTAQEGYFASNEDRDGPFG
YMLRNFPQERPPVPAAPYVGGVEWDATNYLASHLDLMNAIIACTPSARER
NILRAEFRISGWERCLGGSLRICKEKFYGSVHVALRTWVAAAAEDGWDVK
DVRFGPPPESRSPKKMGGGQKPKIIEAPPKLEMPKLDFGANQPSKESWL
>Mb|QLI67290.1
MTTGEKVRGGEDDNGAPAMSESSLSHTLSRTSHTRPLDRIRSQNGYGVDD
DSDPAPGDGGEAHKETASPGGSKDPYEVGWDGGDNDPLCPRSFSKGRKWL
ITSIVSHVSLCVTCASSIYTSTYTKMEPEFHNSRIVSVLGLSTFVLGIAL
GPMFLSPLSEFYGRRPIYLVSWTTYVIFLIPQAVAKNIATILVFRFFDGF
TGSAFLAVSGGTVGDLFARHELQAPMALFSVSPFVGPSLGPLLGGFINYN
ADWRWTYYVLLIWSGLVWGAVVFFVPETYPILHPAVMFVSDKEYEALTIS
LTLTDPILLRNKAKKLRQGTGDDRWLAPMEKVHKSVVQAVGRSLRRPFEL
LIFEPMCLNLCLFSALLLGILYLFFGAFPLVFGTNYGFNLWQVGTSFLGI
FVGMILGVLTDPVWHRVRTRLVQKLETETGIPGASEPEFRLPPAIMGAFL
VPAGIFMFGWSTYPWVHWIVPIIGSCIFAIGNVLLFTGIFTFLVDAYPLY
AASALAANAFVRCIFAAAFPLFGVQMYEKLGFQWASSLLAFLMVAMVPFP
YIFFRYGRRIRSNSRYAKS
>Mb|QLI67292.1
MGNSNTKESRPDHHDGSSDPRESGRSRNRMSRGDLGGFLSLQPPRDRGRD
RQDAPFEHRETRQEREARRLEKERLTRAKDRERSTREEHVDGGYLVTMGT
YTGTEDFSKPVVRQLQIERKLAPFWRGLSDWSDNWAEHQLVAAARGLPVP
AADAPPDPDLIPRPSPPQPEGANTHSLQNLTVPMGPRTLSAASDRSGSGA
GSAIHSPTTAAPPKTSSIKPRAKALAAALSVGSRNASSTDLSPKEVPLPH
DPFVNGQPIEVYLYKDASECPICFLSYPPYLNRTRCCDQPICSECFVQIK
RADPHLPEHHPNGETRDPNEEPNPEDPPEMLISEPSACPYCQQPEFGVTY
EPPSFRRGLAYSTYASNVASSTAMSSQSSLNSTLSPSSPLHVSRRRTHSL
SATAPNVITTDRVRPDWATKLASARAHQARRAAAATALHTAAFLVGAQES
RSILRPGRFGRRSTGTSTPGNEQTHNNPGEAEADPDERASSRNADGSRRT
RMEELEDMMFMEAVRLSLASEEERKRKEEKALRKEAKKRQQAEKKAQKKA
NKDPYGGNISGASGSSLSLGLGRRRGNSGASALRVEATSQGASQNTKGEE
ESSTAEPETTGELPGKGKAVDRGPEDATAPGSFAAASLPIPPSRSRGGSH
LRQMSNASSLGSSLVDTPSGSYTGPGFTGADGTGSRSEGDRDTGSEPMFN
FRSLAELVGVNIDDGSAHRDEGEASSGHPKDATSRPLSHVKEEEKEGAEA
EHVEKMCAEKAGQEEKRSLPALSSESTAVETIGGSELSPKVTITPGTPMP
DDNVHSSEAKQLHHPSVRVQPSEAMH
>Mb|QLI67294.1
MAPNVENAQPGANQKTLHVWSKPIEQPDESQSLGGDKTTNNARTTIADAV
GMIKTEDFTNVHNTPCARQGFLAGIAAGAGVGGLRFVLRGNAVKAANWAV
GMFILGSTASYEYCQYLRRAERIQMKRHIEVVSENKRDQARKAAEGKKER
LQLEQERMAAAQKPWYKFW
>Mb|QLI67295.1
MKNQKKSSSSSGRITDARFASFETDPRFRLPSKKRTKTTLDKRFAGMIDD

EDFTAVAKVDRYGRKLKSDAKKKALQRLYQQEEDEDKPKDEGEGEGEEDN
DDEVDDDELVQRELRAADEKERRKAYDPARHGGFSASESDSDSDEDSDGD
EDEDESGVALASEGDMQRLQDEQADVETGEVTNRIAVVNLDWDHVKSTDL
MALFSSFLPADEKGRILNISVYPSEFGKGRMQQEELEGPPKALFKNKGKD
SDNESDDGSDDGSDSEDEKIKNELMQEGDDEDFDSDALRSYQLDRLRYYY
AVMVCSSPETAQKIYEATDGTEYQSSSNVIDLRFVPDDVTFDDEPRDECD
KVPDSYKPVEFVTNALQSSKVKLTWDMHPEETSRKESINRAFTGSRANIE
ENDLQAYLASDSEDDEDGSEEDNGEDGEDAADEPKLSKKELVRKKMREAL
GLANEPIPKEAKNGPVGDMQITFTSALSESKPKDVDQEETTIAKYKRKEK
ERKEKKRQAHKAKREADSGEPRDNEEEHPAAAADDELGFDDPFFTTEGTS
APSKTSIRKEERLKKRQAREAAEAESAAAKARLTKVMAQDALDNQAEHLD
HFDMNEIMRAEKKKNKKGKKSKKEAGNSEDRGGLQEDFTMDVEDDRFKAV
FDSHEFAIDPSNPKFKATAGMKQLLEVGRRKRQVGDGEDEPSKKKVKKGR
R
>Mb|QLI67296.1
MATAGSDYLPDTMSTTSDAEKAEKPASVNTASIHKKDEIGVDERRFSVSG
AGNLSLDDVAAVDVRIRDLSVSVNTAPSWLEPATYQDLFDAKFNTTPRIK
HLLESVAADLQPGTLTAIIGGSGSGKTTLLNTMAERIISPRLNQKGHVVF
NSQMGVHNIRHAYVMQQDTLLPTLTVRETLHYSAALRLPSTTSAQDRRRV
VEEVIRELGLKECANTRIGNSQYRGCSGGEKRRVSIGVQLLANPSVLFLD
EPTTGLDATSAFQLVRTLKALAQKGRTVITTIHQPRSEIWNLFDNLVVLS
KGGPVYAGPILDCLSWFTSQGFPLPPFVNPAEFIIDIAAVDNRTPELEAD
TIARVRGLKAAWAEESAQRYPPLLQVVDNRNRKQSSLAKTHASFLRQLQV
LTDRTLKVTYRDPLGMAAAIIEAVLMGIVTGYIFFDLARDQAGIRSREGG
LYTAAGLQGYLILLFEVYRMTIDIPIFDRESSDNCVDALPYVLSRRLARL
PTEDLPVPFLFSVLVYFLAGFERTVSKFFIFFAITLANHYVSVCCAMTCV
VAVRHFAGASLIANLAYTIQSIACGMFVQVNTIPVYVRWIKWTAYNFYAF
SAYAGNEFEGNFYDCPLPGGQSNPACNQYSGDYVMASLGFPGHWVARPII
ILTSFIVFFISLSVFGLHFLKVEMTIARARSSDTDLSAGKEKMTAQSISE
VRAIDLRLDDFALGLDKRNIHGKKLPRKTILNPVSTTFKAGVLNVIMGPS
GSGKTSLLNAMALRLRNSVGTKYRPSGRLTFNGAVPSDSVIRSVCSYVCQ
DDDALLPSLTVRETLRFAAGLRLPSFMSTEEKYRRAEDVLMKMGLKDCAN
NVIGSDMIKGISGGEKRRVSIAVQVLTDPRILLLDEPTSGLDAFTASSIM
EVLEGLANEGRTLILTIHQARSDLFKHFGNVLLLARGGFPAYAGSAKQML
GYFGRHGYECPQHTNPADFALDMITIDLQQDDREAESRARVQTLIECWSA
GGSDELDNTVNRRLSEIQEADEPSEKLHGFPIEDVNGEEKKPTTKGILAT
AGEGQASSGSQAPPTRRSFNKAKVSTPAELGALVRKRASIFIALPLLLER
AVINTRRQPRLIMARTMQVVGLAAVFTLFFAPVGNDYYAIQNRIGFVQQI
GAFYFVGMLQNVAVYPNERDVFYREDDDGVYSVEAFLASYTLIEVPFEII
SCLLFGVLGTFAIGLPRTVTMYFACVFACFGVVSCGESLGIMFNTLFGHT
GFAVNIMGILLSVANSMSGLLSINMPDLFKAVNYLSPIRYATRSVAPYSF
RHIKFTCSSEQRLPGGSCPIETGEEVLDLYNFNVDPVVNVACMAACIVAY
RLIAWALLKVMRARWADRRRREKH
>Mb|QLI67297.1
MSGGITYNGVESATGGNIFKDMVFWVAQRVPMRNTILDHIKNNGGKVVPL
EKDAHILIADDARKDAHPDSYSWKYITDSVQYGAAQLTDRYRISGHTETP
RVRVGVSGPVKRTRTRFSDADDAALANWVLAHTEGRTGNKIFKIFAETHS
SHTWHSWRTRFTKFLANLPIADLEKLAMSAADLALQTTNPVSRSASSQAD
SGQQEEQLPNAAQALGKTQKKQAARTPHRPASVSPSTDPNNAQITAASIT
RSEQKSEEAPISSTESKGSEGGTQDAMEGEFYDQLLIFMEETGAKINLEC
CIGGKTVDLWHLSQVVDAQKMPLEEVDWMKVAEDLKYNLVENKSVTAELQ
RYYEDNLAEFFEMVETFSLNGDGEFEDEKHSGPVSPESDIPSSPPRQLSN
RKRPIDDHTPSTVEKSSKRTRLSRNAEIPSTPEEIVGVLTTQSPSVIKAK
QLRQAYSSSTKTSTSVEPSHAIDDDDDNNNETDDRDEVMAGTQSKRVPRL
EAQHSTFDVTPSQQLHSEALDVTPIPLNLRKGRDDPKEQQAGNSEPAQEP
EALIPEWKAAERATRRSLPASFQPAPGPLPSQQAEARARRPSAPIRVNLD
DEGNSQDIRECTEFYEALGYPRPIVIESLMRTTMTPGWPMTSLLEKLHNK
EGVPSNYEGIWTDRDDKSLRYADAIEARKSSASTRELNKAKRELDRIVRK

HTQEAVDLRRRFLRAQAIMDRH
>Mb|QLI67300.1
MDAAEYDTNILVVNNIASISFRVYEPSSDQSSSFGLAAIDIENSLREKGH
IVYYDASRRGIWHFRIVGKEGSDEVSSQELDSSLSLRGYSLIVGEEGSFE
PSSLPRPRSQQPQTGHTPTSSTSSASALDQSQRYAPTATSHLGPPGLQEQ
DSKGHTSVTDAKLVQGGSSSPKAYESFVVATLLTIQSAFCRHTDSIPLNY
RTVLLSQDALENDGSFDVVTEKTPTIGTFSAYLTTTGVLVVCMGISICRG
LLSYGDMLNQASDIPGQQILAAPFGVIASNQPAAQFDGGTASMAQTPNTQ
ALSLRGAPDIHNSLWKQACLKFLQLRGVAQSKLTDCFWINLLISKPKLQD
AKSDVKRSPMPNPTIMIPWPSPLCFRKRPVEVSSTSRVGETILRGHEESH
DPLGNAQSWFSSTPERDEKISKRKAERVATSSEGFGGDSRPTRLNGQSPL
TLRRPSTAAAGSMYPTPPDALQNANGVTPSLDGTLSSPGNPLSAAAVPEI
DNNGSVSMAMGDSFEPVRDFSESKRQRSDSNLLGEADHMFGDMGQDMFGD
NDITEADFNFFDEEPEDIDLEAALDEMGDATTAAIIGAEEHTADEEISNS
REPIKEEPETEKDAGGFTKPELRHAISVQNDHAMPRGRESRPVSMKRESS
PFDPHTVFKRVCASLAAPEKDGTIATDTNRMTKIFEKMDFDPTLPMINKK
YEQGGQFDYSMSTAVGNSRLDHGVLPETDYLKRHGKLHRGSQDHKLYTKS
LMGPLSGLERPISGRSPTKLDTQMSDDEQSSTESDQDDSSYTSDEPTSPP
KSSVKQAPVEEDAVSQVASLKEQDVIEEPDHQLAIELPRLSKPETPELPL
SMLFSDPEPLALELSLGDEDLIQAAQILTEQAATGSLDLCNTDHPANTSI
ISQGRATLLSDARTALQQLREALSVFLRGVEPVRLKAFLDIQDVLLQGQP
RMQPRPIPGRDANAEQIRPSNLYQIPGPHLEVRRSDAKLSVLPSAVAFWE
SLGLAPSSGSKDITAVCVFPGWKGMVDNAKTFLGRIKSVYEVLRLGSFEN
MALASEMDSGLLPYEMDRISTSPDATLTGHGSALIGTMETLRASLSNLTV
SDTNVVVYFVYSPNNPGTIVEACAAFQRFFDSYQKDLAAKKETATNELVL
QLVSSDLLSSPTSVVVTPSVDLVRLCMETYDRCTLFGGPMPAPAIRLEQP
LPRIIDFKLNTQPSVSLIRENSCIHVAYAQTVDGRWVTTAWTDDRGNQQA
TASYCLGRKGRPLSTSMSEVAYEIWQSTLDLISAWTVHWRVIITKCGPMD
QQEIDSWVNLAHTETRLQVTMILMTVDTDPWLQLVPPVVKLPHASVPFYT
TPVSTPQANIVSPEQSATPATPANVTAATPGGGEAGADTETDAILADVTD
QTWGAIVGHRLNNSLGVTDLYPALVSGFLIKRTGARAEDVPIAMEVNLIH
TEAFPRSYEPLLREMLSYFRGLATLSRARGMVSRETDVRPWHIAAAEKAA
RAMYLLM
>Mb|QLI67304.1
MSCIFCRIIKGEIPCMKLFESEKTLAFLDVGPLSKGHALVIPKYHGAKLA
DIPDDQLTEILPTLKKLVSATGAVDYNILQNNGTIAHQQVHHVHFHMIPK
PNDAEGLGISWPSTPGDMDKLKALCEDIKSKM
>Mb|QLI67305.1
MNDSRKPHTMVLPTRDFTHGEASYCPFPTVTAAFLHHAAAIPSAKAVRDL
SANIERVLTYRELAIHAQSLASRLRRLGVGPHQTVPLVVKRGAEMVIGIL
AILSCGAQYVPLDGGVVPDSTIKLVAEQCGGQNVVCITSTQDRIKKLLPT
HSPVVIEQTPIEDQEYDAPESWVDHASADGGCYVIYTSGTTGKPKGVDVT
HKNVTNLVCLSPGGLGVQPGMSVGQVLNISFDMAAWEIFSCLCNGGTLVL
RGSKWEPTVRQLDVFICTPTILSKYHPKLYPNIKVVATAGEPTSQDLADL
WATYATYWNCCGPTETTIVNTMSKHVAGDPISIGRPTPNNSVYILDNDGR
PAPVGVAGVMWAGGHGVTRGYVGLESKTKETYIPDPFANDGSNMYCTGDL
GRWREDGNIDILGRVDDQVKVKGFRVELDGISSSLASAPGVTRATALLIE
GDIHGFTTPSGQDEQSILAHTKKLQPYYAIPSKLHQLQTFPTTANGKIDK
NALRLMALAPEPKHETQNYDEEAAIGSDQSTLAEVRSISTSSTLTELEKP
KLDISKEVPDKKLPKHLRGFRHRIAIVYRFLFSLIGLLNVGALIALLLVN
PGAEWLATLTAANLVLAVLVRQDVVINTLYTITCSLPKSAPLWMRKRFAK
IYHLGGVHSGAGVCATAWLLGSTFRSTVAYTQNKTNDSIATIMVSWMLCV
LCCGLVGFAYPTFRKKYHDQFEGIHRFVGWTALGLFWVRTMFSVRDVTPA
GEDFGLALVKTPGFWMLGVATCSVASSWFWLRKVPVNTVPLSDHAVVLSF
DYTVPVNGSFTRISKRPLFEWHSFATIPAPKPSAIAEKPGYSLVVSNAGD
WTRDCIQNPPTQLWVRGLPTCGVMRIATLFNRIVVIATGSGIGPLLGHIG
QPSCPTQLIWSTPNPEQTFGKKVLDTIYDSIPDAVIHDTKKLGRPDLVKM
GYNLAKDFGAEAVIIIANEKITKKVVYGLETRGLPAYGAIWDS

>Mb|QLI67307.1
MDPNDRPPPSGPDDAPDRDHMRARRIAMLGNIARKPSEDKTDSSSNQPAS
SSTSTPKPQTSEASTPKPKINVTPAATTQSNPNPFARLGVQSSKIAGDES
AATGSSRPACKRPAPDVDDTDSAPPAQKPTPQLVQQQQQQKSETDEDYAH
RVLSQVFRVSVDPHHMANPQGQRLVFLPNLNQELNDSGDSLKLSIEYLLP
CWKRAVRASSTAKNVSAPRQEILDEAKRLCMSNCLFALTMPALYGRDPNP
QHDTLVPYLLKGIQDDGGLCFDFIREAIKRFDEDEAFPALFNDAMIKISS
QLSTLSLGDEYKPHVQALLTYTRFPVLIANLAQHPSFNMAQSAPGIERHT
ILGPFFRISPLQPEAIKSYFPGARSLDRVRISNAQESLRIVLRAHQDDLF
VIANAFIRAGPDTRSRTLNWFAYIVNMNHKRRAMQVDPKEVASDGFMLNV
TTIMDRFCEPFMDNDFSKVDKIDVRYFRRQPRVDIKDETKLNADQATADE
YYSKKVEGDSNFISEAFFLTLAAHHYGSEALNSQLKNLDREIKYLDKHIK
AMEAERPKLANSPHQLRLFEETLKRHTNVLEKTIALKHAIEGALLDERMQ
STSLRFMRYVAVWLLRLVTGSDYKPGQETQMIRLPLSSDNAEAFACLPEY
TLQNIVDNFKFIFRFLPKIIPSAVGDEMIALCITFLRSSEFIKNPYLKSS
LVSLLYSGTWPFMHLKKGVFGDQLIALPFANEYLLNALMKFYIECESTGA
NTAFYDKFNIRYEIFQVIKCVWSNDVYKQQLTRESKTNRDFFVQFVNMLL
NDATYVLDEAFTKFPKIRSLERELEDTSLSAEDRQKKEEELQTLGSQATS
YMQLANETLEMMKLFTKALSESFIMPEIVSRLASMLNYNLETLAGKRAAA
ELSVSNKDKYHFRPIQLISDFVDIYLHLGYSPVFVDAVAADGRSYKPEVL
DRVTRILSSKNAKDPADLAQWEKVKAKFEVAKHELDQAELDLGDIPAEFE
DPIMGDLMKDPVLLPSRHIVDRSTIVQHLLSDAKDPFTRQPMTIDDAIPQ
TELKERIGKWREERVKIAKEQAVGLRGEQMDTTEG
>Mb|QLI67308.1
MAAGAFAGIAEHTVMYPIDAIKTRMQVLSPSTTTAYSGVLRNTYQIASGE
GFFSLWRGMSSVIVGAGPAHAVYFATYEAVKHAMGGNQAGVHHPLAAATS
GAAATIASDAFMNPFDVIKQRMQIQNSSKMYRSMIDCAKYVYRNEGIGAF
YISYPTTLSMTVPFTALQFLAYESISTAMNPEKNYDPFTHCLAGAVAGGF
AAGLTTPMDVIKTILQTRGTSSDPQVRNVNGFLGGCQLLYQREGFRGFFK
GVRPRVVTTMPSTAICWSAYEFSKAYFIKRNDSS
>Mb|QLI67315.1
MAPKKHFKVHSLNKHIEDTKKGLSAEAQYNKRPIPKSKVSIKKEPGAIDK
AIFGNESEDDESGSSSSESSDEDGPDFLQKLQVSKTSNTATPKKPAKRDE
IADSDDERRSTAKNVTSAKSKRPVQSGAPSSDDTSSESESESGSGAELKS
VKGEQKVRANGGPLKTEAPSSSGSTSGSDEEEEEGDESSSDDEPDKKATA
TKVKGSTSSDQESSDSASDDESDDEGEPAASKKPKAAITSTNGTSTATTS
ETSSSDEGTDDEDQTAVQAKPVATRKSVPEEPSDDASEDSSEEDADVSMH
IADRQHDPRATVPDFIAPDFILRKSDGGTNGQDVALVQNMEIPMNQSQSG
DSVFSHDGEDYGISMDSMTPKSSIQILIPSSDGTRYQSASRPIDQVMQVK
KITQLGQGSGATSSVGPTPKPPARQQPEGLKARYQPYGVTSPMGQIGVDA
SFHIEDDTEMADAPAPAPAPAILAGSAQNKSDKKEKKRKEKGESETPRKG
KRKHVSSEDDAVAATEQLMGENLSAETKTKKQKTERVGSPDLGSDAPPSS
AVSKKQTLLVPPAIPGSTPSSKSKRSKSKPETPASKPRETAVPVPQVPGS
SQHKVSPVPIPQPGVASTPVADRDSKKSKKKKTKTPDATAKTQQSPPPSA
QPAGRNSKIKLTPVPMPKLKTTG
>Mb|QLI67316.1
MTDQLVDQLRSTQISDGIPVQTDDWKKNLKLPAKDNRQQTEDVTKTKGLE
FENFALKRDLLMGIFEAGFEKPSPIQEEAIPVALTGRDILARAKNGTGKT
AAFVIPALERINPKVSKIQCLILVPTRELAMQTSQVCKTLGKHLGINVMV
TTGGTGLRDDILRLQEPVHIVVGTPGRILDLAGKSVADLSECPMFIMDEA
DKLLSIEFTPVIEQLLQFHPKDRQVMLFSATFPLSVKDFSDKNMVSPYEI
NLMDELTLRGITQYYAFVEEKQKVHCLNTLFSKLQINQSIIFCNSTNRVE
LLAKKITELGYSCFYSHARMQQQARNRVFHDFRNGVCRNLVCSDLLTRGI
DIQAVNVVINFDFPKNAETYLHRIGRSGRYGHLGLAINLINWDDRFNLYN
IERDLGTEIQPIPPSIDKSLYVYENPESIPRPISNLPRNAQQQQPGSSQH
PVAHQPQSNWQNQNGQHNNHYGRGRGRGRGYRGRGSAAAGGRGRGPPREV
QS
>Mb|QLI67318.1

MPPQPDPKLNSAHEEHQHSIDQDHVKSAVLQDQLRHADRALALIGDGQVE
LTDEDSRRIRRKTDKVILAILVWVYFLQILDKSVLGYGATYGLKEDTHLT
GNEYSLVGSIAPIAQLAWQPFSSFLIVKVPHRILMPTLCLGWGIAQASMA
ACHNFGSLMAARFFLGLFEAGCLPLFSIITSQWYRRAEQPIRVAAWYGTN
GAATIIAAALSYGLGHIKSDLLKEWQIIFLFVGLVTIVSAPFVYWKLDND
VSSARFLTEHERAQAIERLRANQTGTGSREFKWQHLLEAALEPKTYLWIG
MALLLNVGASVTNTFGPLILNGLVSDKYMTSLLNMPFGAVQIIVILLASF
LAQKARLKGAVLVVFMLPVVAGLAVLYSVPRGQSAKPALLVGYYLLAFLF
GGNPLIVTWIVGNTAGTTKKSAIMSVYNAASSAGNIIGPLLFNEKDAPAY
KPGLRACLAIFVALVAVVLIQWANLILLNKLQEKKRVRNGKKAKMVDHSM
QSTYHAMGEESEDAVETAAGTVEFAGEARTGEKAFMDLTDRQNDEFIYIY
>Mb|QLI67321.1
MADDYIYPTHFMDNSRGLKNLIMAWTLRFNDVLDGDQLSAALWRLFEIGD
WRKLGGRLRVGKGGKLEMHVPRTFTKERPPVAYTKETFDVAIGEHSLAKD
LPSPTEKPSLQRPASAFRSLSVRPDAPSTIGQLASRDVPQVSLHVVTFTD
ATLVSISWPHTLMDGMSLQGLLKAWSLVLAGKEDQVPALLGAKDDVLYAA
AQDAAPGDEWLLRRTMLTGFKFFLFVVLFLWGVLTERTIESRTICLPKAA
MAKLRRQALDEAAVSGSGQEKAPWVSEGDVIMAWAVRMVALAQPSPRPVN
ALGALDLRSRIPELAESDGVYIQNVVIGSSVQLSKDDARQPLGVIASRCR
EALVTQATAPQLRTFLFALCKILDAGGDPTLLPGVWNGELFTISNWTKAD
FVKLVDFSPAVVTTGQAEEQRINPPGTMIYHQSFVLGHDATLRNVMAVLG
KDHSENYWMMGYFPPRTWAVIEKEMQKMGET
>Mb|QLI67323.1
MPQRNHHQDLDFEIHVDPSSASEPADEKLPTMEHEDVACAKEVTHDEQGS
RASVGQDDTITNNEDIMQSVEEADADDTPAEDSLLSTENLQPTVEEVVDD
PEDDSVLDRNVWAEEDDPDSRRESASSNMEDSDGDLERRHSMRTEALIHA
AAKNIVEDVADTPRDSIQSAASAPEEEEEASFVSESYTESHRQSLGAESE
AGQSQVSEEDHAAHTAHADEGGDSSSHHEHEDDVFSDNSPRSSMGSMSEA
DREGAKQISPPKTRTARISDIPQSDADEEFVPTIRGTRRPTFRSPSSVMA
MQMSSPPASATGTPRSSRRTPRSTVSRLGSPSVSAQYSPRKTKTPPRFKR
NTPPLVLLHVTVLPPRWVWGHVLDNAQPSELSPEGKTLWESWNQLQDRTA
DTISDRGILLPHPQNDYEVLEERLLETLDLPLRRRARILECGHYLGPSNK
MSLSEDMESEDEDYYDEHGSPRQSMVEQTHWCKTCSSQIRYDSLGDGKVY
RVKVYASNGLMRAGAWEACWKDMERVDVELEPIIDSKLQEELTCLAAEQE
RALEMEEEAAAAAAAAVDEEIQRHEEAEAAERSYMPEDMDTSIHVEEPPQ
PEIHVESSPPCENDCEMGDEERLREVYGSSPPPQPESSSIPQRRCSDFAP
KETPVSPSVEALERREERRESRQQASKGSSLPELIFEAVKVLMQDQRNIL
IGLLSLVVLMLAVRSASTPTDVGAKGVLPEPVAQVVVEKPVESASGIVDP
PKVDAEETTAREAINVQITESVQASSTTQVSETSLNDVPMSTIEITRTVT
QERTETRTETMIETTTATKFETITEMVPQTITETMAETITQTMSETITET
MVETVTVQALPTETPKDVPDIEEPEVVGEEAMPEEVAAGNEDEGGDGSGD
GSSDEGGDEGGDEYGEEL
>Mb|QLI67326.1
MTSEAPENPSSQPVTQNGQAEQQEIPDGPNPPPELPSGQDRLPTRKDTSL
REFLNKIDDCAPIIPDAVTSYYMTKAGLPPPPQTDARLARLLALATQKFI
ADIAADAYQYSRIRASSNTNNPMGTLGAAAGFPIPGQQAGQPGNKDQGKG
APLGIQRPGYGGGGQGGSQNRTVLTMEDLGMAVGEYGVNVKRSEFYR
>Mb|QLI67327.1
MATTTHPFPLPTYANPGYDGTKGGDIGNGGDSSTSSMGISTGGLVAIVVI
VFVVAIIGVCTAILFFIAKKREWKASEVLRHSVKKVVAAITPRRTEFPNS
VKRSMKTSKRDRSTLSDDDVPPTPRLRPEDLEKGRVSGKVQSKCPK
>Mb|QLI67328.1
MSALQARESIEQRIKARDSIPKAPPAYLPTAGSPLSVDKEVYATIQKAPR
VLIDEFTLPIRAGRAWEAPAGSIVKISTPEGPQVGDLNIWNRHNPRERFW
ASRTRQLHASHVSTHDRLWSVLPYMRPLATIIHDSLDWYGVDPHGGRVHD
LLGTRCDPYINTVLAGEQYDFHCHSNLTRAVKPYGLHESDVHDVINIFQA
TGLDEQGRYFMNPSPAQAGDSIEFLAEQDLLMALSTCPGGDLSAWGFGSD
SEKEMIKCCRPLKVQVYQLQDRELLNKCGWKPAEVSKYTGMHGVVSPEGE

KQA
>Mb|QLI67330.1
MFGLGAFVAALDPVEAYGNKFFTRNGTQFFVKGVAYQLVPDDPLIDTEQC
KRDFSLMKELGVNTIRVYHVDAKAKHDGCMRALDDAGIYLLVDMDTFGTY
IESKDLYWNSTQYERYSQVMDAFHSYDNVLGFFVGNENIATKDDSPAAPF
LKAAARDMKVYRDNKGYRNIPIGYSAADIVQLRPMLQDYLTCGGNSSEIV
DFFALNSYSWCDPSTFEASSYDKLQEYAKNFPVPIFFSETGCNVPGPRKW
DDQDAIFAKPMVDDWSGAIIYEWIEEQNHYGLISYGPKGDASQPGENVFD
GFTRKGTPTPISPDFDNLKTKWASMQPTGVVKNDYDPKQVSTRPCPTSTA
DGWWQVNGNVELPTLGETLTGTFTSQASTTASPSEKPKPSNSGNRGQGGG
NSGGSGSGSGSGSGSDNDGDGDRNGSGGGKKDEDNAASPDRHLAAAGASIAA
AVLVLALAL
>Mb|QLI67331.1
MSRAGSVSLRALQGILAVANLGLSAYVVNWYRTSTSQSPDGSVSFLLAAS
VISILSILYLELVPRFVSRLAHPYAALAVQGLNTALYFAGFIALAVYVGN
LTFCEGSVCRASRADAAVAAGAFCAWIASTILTAKQMIVGGAAARQKAAP
MGKV
>Mb|QLI67332.1
MSRQIEQALLSMMPTYGSDLPPSLVELAGSLLAQSRHRASTLKAEEEIAR
QYACANIACDRLKITLDLPPIEPRPPIPPRIYKRLYTHLDNILPNPTATP
RPTRNRAPSSKQRDKDTPSKSETRRIPSRGTPTKEAALAPFRTTPKNGGT
PAKGHILDSQNSNGHLHPWIQPVIRFMCAESDHKRLAPTVFAGMEHILTQ
EGRPTQDQWVIHHVTDLLAAVYFFVIMRVRSITSGAKIDREGYVPLRKEI
LALVAKANQEVAVPDVDESDAWEGWSDLKSRDFDAAVAKVNENDWLAGDW
YDGIVDVISSIVRTDAGLSDDDVENSEALLPTRRADTMLQERYDLLSEAR
KSDFIAWRDIMLDKIAQATPVQVVMEADI
>Mb|QLI67333.1
MQRGAPYTKRRFYQLVKLYHPDTHDKDAPPSSSPQQIPHATRLERYHLVV
AANNLLSDPNKRRLYDHHGIGWTADHRAPDLREVDKAWRHQPGNASRNAT
WEDWEQWRNERDGKPSEPMYMSNGTFAALVVCMCMIGALAQMNRAEAAGE
QYVDFVQQKDWAIGQKMRQNNMASAGRTKDERVDTFLRDRENLSFDFQPA
KYDDPSHANRSG
>Mb|QLI67336.1
MTSRCRLWINGREVDGHGERIPIEDPATGEIIAQSDAANQQDVDSAVENA
HQVFKSGVWSNASRHDRARVLGNIAEIMWRDLPKLIRLEVKQTGRAIREM
KAQIPSLLRWFKYYASVLLTEELPVLPTAGKLHNWLERVPLGVVVQITPF
NHPMLIAVKKLAPALAAGNSIVLKPSELTPMTSLLLGQMFKAAGVPDGVV
NILPGYGAVTGKALVSHALVRKVDVTGGTSAGQAIGSIVGGNLARYTAEL
GGKAPLVVFEQANIDAAVNGIAFASFIATGQTCVAATRIIVQNSVLPTLL
EKLQRKCDYILRNMGSPLNERSTMGPLISLRQLQKVEALVDECLSEGTGQ
VLCGGQRMTGTSELDGIDFAKGYFYPPTVITCAAGKSVTEARIWREEAFG
PVIVVVGFDNEEEGVLLANDSEFGLGAALWTQDLSQAFRVSKQVSSGIVW
VNTHHRNDPSSPWGGASKSSGVGSENGIDAYHDYTTTKSIIMNFASANEM
LAQDDWFREGAGEVRYG
>Mb|QLI67338.1
MDDGPVPKLSELLRHPDDLDKIPALKLEFSRKKGAVDGQLRNGLREQLET
TQSGMTGLSDGQKTVQLIKEEMMKIDRLCSESQNMIKDFASINLVSQAHR
NFVAVETMRRDLETFNDRLTVVENMLRQDDADQENMPNLLPCHYELTQLR
NIRDDAMEQIHRAEDPGLESTLEDYFARLDDTIDWFDEHVGLLAMDLISL
VINENNGLVVRFAVVIEAEEKSDQRVLALQEALKDHEEMAARFQSITDGA
KKARGYKDKFLQAIKLGAEQQLEQSREEFLDDASKLEGIMKWYFNDLNAV
RVGMTPLMPKKWKIGKTYAEIYHQLMHDFLIGMIEDPETTSAHNLEIISF
PEKYYRKMAKMGYSQEDLTPHVIDNREAELVRDFRQLIIKFLDEWMDRIF
NQEKKDFAERNVEGSNLDQDEYGYFRTKNLIALWRMLREQIDAGANSKRS
DVIEGVIDAMFLRLRTRQQSWQRMLEDEASRYEAGKVPELEGFQALQDWL
VATANDQIACIDDNDEESRLGYLSSFRTNFEPHVTPQYLERGESELNALR
DSYVDFSTWCITKFAHLIFAVDFRAVMPDFFTPKWYANTAMKQMVVTFEE
YVSDYRQVLHHSLVDIFIEIFADELLVRYLLSVRNRGAKFRRADPFRDKL

FNDISTAFEYFSNLPNPDVGNSIKQTWRVTEPFLGLLTCDKDAVADEFAA
FKAAYWDLHISWVESVLKSRDDFERSMLNAVKARAAQIGVVRGPETIMSK
VK
>Mb|QLI67339.1
MFSTFTGSSRRPRNVNLSGQAGNPFANTSWSPAVVSNTTKTVSDAQAERE
KRHLERQRLKAAGRIQRTWRGHLARRNLADERRVVFDNLYRNSSGISVAE
RLPVAFNLLLSFFSRRSDVDIQRSCLFARDCESAHTEHIAPRGVHRSRLG
QLASILVSTLHFVASEKALTSDVSILLKLITRLTVYDSHAMRNSLDQYYT
ALASLYSKREAEKWNELLFASLATPLQAKSGSRAAYKALAFNFFTRGDLY
LFENNNLLLSRAINAGKLAESICSVYGSSPPTLPSRGSLLWLLAHYIGLG
RASLDSSARLAYLRALLVQLSFLSSEISIRMSFKPQVDDSDDEIERSSLR
ALAPYISSQLLSLVDHDGISNVLSDFSSNLARSATEESYGTSLLAGYILT
LLHCFPAKADDTRMRLFLEQIPTVTGHVPTVKFLWQIMRQTSVFTKLRTE
SENPLEVIRRYLQSSSQLSQDSTEEQEWRVILLFLELYTFILRLSDDEDF
LSGMKTQIIDSGASDSRLRQCSLSLVDVEALTTFLKNAAFVLLYQARDLS
QSQKTTDTATKSRLDSYFGAEKHRSVSAEIATSPASTKVDLDGLRAIITT
AIKMLYERDSRKHFLPRDHWLMNAKLQQEDFINAVITEEERQSHEEAETE
DSDEEEVTVSEGFNQESILYSTMAGQRLSRHARLERLKRQQIRAQRERRL
AEMGPKLEILKHMPFAVPFETRVKIFRQFINLDRIRQDGNSPFGPAHPFS
RHYAKIRRGRLFEDAFNQFYQIGDGLKDTIQITFVDQFDTPEAGIDGGGV
TKEFLIGVTSEAFGNTEGGAGMFSSNEQGLLFPNPTAVDTLRESLWRRGL
KQDDDEYRDKVNALLRRFEFLGRIIGKCLYEGILVDLAFAGFFLLKWPSA
GPKDENTYKGNINDLRDMDEELYKGLLRLKNYPGDVSELGFDFTVTDQVS
PPGAPVKTITRKLIQNGDEILVTNDNRLLYISYAARHRLVVQPALQTTAF
LRGLRSIIPPSWLSMFNQSELQRLVGGDSSEIDIEDLRRNTVYSGLYEIG
DDNEEHPTIKLFWKVMGGFTDTQRRDVLKYVSSTPRAPLLGFAQLRPKFS
IRDGGTDEERLPSTSTCVNLLKLPIYTSEEALREKLLYAVTSGAGFDLS
>Mb|QLI67341.1
MKPSLALVAPSLLPRLCLSAVVTITAPPAIPSDEPQWKTADTFTSAILNS
TNFYRGEHNATAVSWNETLASFAADHLARSGCRFEHSGGPYGENLAEGYP
NATASVEAWGDERDRFDFGDPGFAHETGHFTQLVWKSTTAVGCGRRLCGE
SGWYLMCEYWPRGNVIGQFGDEVGREISAAALLRPSIVSVVALGICAVIL
VAA
>Mb|QLI67343.1
MAANTTSGAVDQLATDLNNTSLNNGSGDAKPTVDTTVGAAGSEDASAPTP
SSAAPHPQNSASLYVGELDPSVTEAMLFELFSQIGAVASIRVCRDAVTRR
SLGYAYVNYNSTADGEKALEELNYTLIKGRPCRIMWSQRDPALRKTGQGN
VFIKNLDVAIDNKALHDTFAAFGNILSCKVAQDENGNSKGYGFVHYETDE
AAHQAIKHVNGMLLNEKKVYVGHHIPKKDRQSKFEEMKANFTNIYIKNVS
TEASDDEFRELFEKYGDITSSSLARDQEGKSRGFGFVNFTTHESAAKAVE
ELHGKDFRGQDLYVGRAQKKHEREEELRKSYEAARLEKANKYQGVNLYIK
NLDDDVDDEKLRQMFAEFGPITSAKVMRDAPSDGSDDDKEKEEDNEKEPE
AKEEAKETEEEDADKKADKKGDKKLGKSKGFGFVCFSNPDDATKAVAEMN
QRMINGKPLYVALAQRKDVRKSQLEASIQARNQLRMQQAAAAAGMPQQYM
QPPVFYGQQPGFIPQGGRGMPFPQPGIGMGGVQGGRPGQFPGYPQQGGRG
GVPQQMPPNMYGMPGQFPPQYGQPGTPQFMAAMQAQQAAMGGRGAPQGGR
GVPPPNAGPGGMPGYPPNNRQGAGRGNGRNNAANGPNQAGRGEGNNSSVL
QSQLAGAQPAQQKQILGEIIFPKIQAINSELAGKITGMLLEMENSELVNL
IEDDGALKAKVDEALAVYDEYVKSQGNEGAEAGEAKKEEETKA
>Mb|QLI67345.1
MSFGGQTPTIIVLKEGTDTSQGKGQIVSNINACLAVQATIKSTLGPYGGD
LLMVDENGKQTITNDGATVMKLLDIVHPAARILVDIARSQDAEVGDGTTS
VVVLAGEILKEVKDHVEQGVSSQIIIKGLRRASQLAVNKIKEIAISTNEG
NRRDTLKKLAATAMTSKLIKQNTGFFTEMVVDAVLSLDQDDLNEKLIGIK
KIPGGSLTDSRFVNGVAFKKTFSYAGFEQQPKSFKKPKIVCLNVELELKA
EKDNAEVRVEQVSEYQAIVDAEWQIIYKKLEAIYKTGAKVVLSKLPIGDL
ATQYFADRDIFCAGRVSSEDMERVVQATGAVIQSTCSDILPEHLGTCGSF
EERQIGGERFNFFEECPEAKTCTLVLRGGAEQFIAEVERSLHDAIMIVKR

AIKNHMVVGGGGAVEMEISAYLHRFADKNISHKQQAIIKSFAKALEIIPR
QLCDNAGFDATDILNKLRVEHRKGSTWAGVDFKNEGVADMMERFVWEPAL
VKINALQAATEASCLILGVDETIRNEESAKPQAPGQLPPGAAQRAVRGRG
RGMPRR
>Mb|QLI67347.1
MSRFFRGGEDSSSDSSSDEEELYSEEEQEQELGQNDSEGSDDMDDSDMSD
EETSSDDEAGGANRFLRDAASDSESEEEEVRAKVKSAKDKQRDELDASIK
QIENGQKNGDWTLISAEFDKLSRQVVRLQDGGKTPKPYIRIVAELEDFMN
EALAKAKVTPKKMNAIQARNLNAVKQKIKKASKEYQTEVNAYREDKDGFM
ESEDEEPEPVKPKKVARFQEETAQPEELGDDGFATVGKGGRTLQYTPESI
FKHLRGVMETRGKKNTDRVEQIKVMEKLGEIANTPYQQIRVLLAIVSARF
DLSAGTSNVMPLEHWKAAEKELSTLITVLENNKDYVVVENVEEWDDDEKA
PALQPGEKYIKVAGSVVSYVERLDDELVRSLQSIDPHTSEYIERLQDEGA
LYNIIVRGQLYYEYLRKDPALEIPQDSVNRIVMRRLEHVYFKPAQVIKTF
EENCWKAAGDKVDSVITPRSQSSDANNLVIVLCNYLFSNSDGIIRARAML
CQVYFLALHGEYYNARDMMLMSHLQDTIPNFDVLTQILYNRTLVQVGLCA
FRKGLVYDAQNTLQEICGSGRQKELLAQGVMMQRYSQVSPEQERLEKQRQ
LPFHMHINLELLECVYLTCSMLLEIPLLAQTGSSPDVKKRVISKTYRRML
EYHERQIFTGPPENTRDHVMQASKALAAGEWKKSTTFIHSIKIWELMPNA
EDIKTMLSKQIQEEGLRTYLFTYAPFYDTLALDTVSAMFELDIAKVAAIV
SKMISHEELAASLDQVTNNVIFRKGVELSRLQSLALTLSDKASALIETNE
RTLEQRTQGTSNAFERQGGRGRGGQRGGQRGGRGGARTGGHTQRQAGGTQ
FTGGALGAAVRG
>Mb|QLI67348.1
MPSFLNDDNSPIFSAEADLLNLDPNNQFGLEIDKKKANGVSIAHTQPEPI
NESFDESSTTSTHDSETSDTNHLPMNGNSTRPTSMSSLANGINDSSANDA
YEKSTIKALPNGTLALPDRTAPKPPASGVNGNSASETTSSYLHQNSTALG
SPVVAYQHQFHDHKPAPWANGATLTQDSAQASTPESDLNDNDGQTSPVAT
QVLPVSELVPQKFSPHRFSSPPAYQSGGSALTTSPSGHLQPPHAGSLKQR
HTLEISKPPPGRGSKDGLDVAQASGRFSPTVAPIGHRRASLNLARRTTRS
MQSDAPRDEILPDEDAIRWAEVYRQKRASKRKRKEEEDDDRVLVGTKVDE
SHANWVTAYNMLTGIRVSVSRTNAKLDRELTDADFEAKQKSTFDITGNEL
VPSAKYDFKFKDYAPWVFRHLRALFRLDPADYLMSLTGKYILSELGSPGK
SGSFFYFSRDYKYIIKTIHHSEHKFLRKILRDYFNHIQNNPNTLLSQFYG
LHRVKMPYGKKIHFVVMNNLFPPHRDIHTTFDLKGSTIGRDYREDDLEKN
PRATLKDLNWLRRQQHLELGIRKKRLFLEQLQKDVILLKRLQIMDYSLLI
GIHDISRGNEENLRDKTLQVFNPGGEKTTDDDPPSVLMRTPSKMENVRKA
RELREKIRAERPIPIGQALDKMPDELRDFHTRPGFVFNQDDGGFRATHEN
NQPADEIYYLGVIDCLTHYGMIKKIEHFWKGLSHDRSQISALPPEQYGDR
FYNFVEGITMSAEEARREALRREQEANEGRVSADKSAKRHSAQRYSHGVP
PMPDHLPPAPPTSGSSAEVRETMQKASKEAKRTESSGVLETDIPDRVLTT
ASSPDKRESFPHEPILPVVEEAAEGSRNDGGSVWDRPKNLRNGNTIPPPP
TGPPPPTPPKERPPPPPLKSDGHEPDGQVLRPRLSKESLNKNLPPLPKDE
ETQDTGVRMV
>Mb|QLI67349.1
MNEQPALRWSGSFGVLPTTAASVKNLSGDKIILPQSALEQLLNAAPSQPT
TASSFTAWDPHNPYRSNGFGFEQRQQLPQPLTFRLVNSQNGNVVFAGIRE
FSAEEGTVGLSSFLIEALGIEPQQLNATNGNRTSDADAIDLTGDHVPSTT
ASIKLTVDATNIPKGTYVRLRPLEAGYNPDDWKSLLERHLREHYTCLTKN
SILSVNGVKGETFKFLVDRLSPEGDGICVVDTDLEVDIEALNEEQARETM
RQIMTKAQTEVVGGVSKGGDLDIWKDVSGHVSPGGYVDYILPSWDRSQPL
TITLSGIEQEDGVDLFVTPKSSRQRVLPRDSAHVFGDFSPAEDGVKSITM
SPTNVEMEGAEAILVSVHGFSSENQRTSQPIPFTVRAKVGSPTSKAGQDR
MEVDGEEHSSDEEQCSNCKQWVPKRTMALHENFCRRNNIPCPYCSAVFKK
DSNEWHSHWHCEHDSAKGNSAASKSKHDMIFHQHHPCQDCEFTTNSLADL
ARHRTSVCPGKLILCRFCHLEVPQEGDPFNPSPEVVLSGLTAHELADGAR
TTECHLCDKIVRLRDMETHMKHHELDKVSRAKPEICRNVNCGRTLHGVGP
RGQIGAGTSQGQGPGNDIGLCSLCFGPLYVSMHDPEGKALRRRIERKYLG

QMMTGCGKSHCANEWCRTGRKNTNAEAKGSSASAVLPLVKPLLAMVGTPD
GHMYFCVDETNQKRRKIAEMLAAEKLWDLEWCVAAAEAEKGNIDRMRDWL
QAWAPRS
>Mb|QLI67351.1
MSTIASPRDPANPLRRLPSGTPTSSGRPSLETARSGVTSPAPDAPSQPLP
KRSNRAALREYYKLRAPRIEVAGSEVPASDLDAPDFNAAEYVARVVGNSG
LEDMLRLYTQVVAEVRALDAEKKALVYDNYSKLITATETIRKMRTNMDPL
NPMASTLDPAIAQIYSQASSIREKLRESVPAPDSEEGWRRAAELRRQRTR
ELAVEVLATPQKLRDLVKEGKVQEAKRQWEMPKRLLVAWKETGVGGDHVQ
DVIDEGDAVFKPVPDLPQRTSRDDR
>Mb|QLI67353.1
MAASAAAPLRRSARNVKKEAAVETKIRSGDKQAASHAIKRSASPNLPKRP
NAKKVKAEPVSPSAKHISTISARDLGSIKTETAKADILKERKLKSFSAFS
KKSPYPDFARPTAEECRAAHKILAKRHGERLRPEEVVAPTNAAGCGDSPS
VLDALVRTILSQNTSNKNSTRAKLSMDEEYGGSDKWEEIANGGQARLEKS
IQTGGLAATKSKVIIGILQHTKAKYGVYSLDHLFEASDEDAMKEMISFQG
VGPKTASCVLLFCLQRPSFAVDTHVHRITGLLGWRPAAAGREETQAHLDA
VVPDEEKYPLHVLFVTHGRQCEECKAGGGNAKTCELRRVFKNGRFTPPRK
TIKDEDDGNIKKEED
>Mb|QLI67354.1
MSDRGRSPGPSGQGSSGSRLPSRAPQGSAPGSRTGSPSRSGQAAAGWVSG
PGYDPAKPATQPDKGNTRMELPPDAYVTEGQKDLFALRGNKFNTEGSNAV
IEVNQYRMKKFDFAQKIYQYDVIISPDNERKSVLMKKIWTNTATKAALQK
YQYNMWIFDGRKLAWAPGKVDRGELRFTVDLDEGKRPPGAQAREGARFHV
TLRATTEVHIGAIQGYLDRKIQFNTNVQVALNFMDHLLRQWPSQHMTAIK
RNFYSASGSGRPLLDGNVLEVHKGTYASIRMSHNITRGGTGLALNVDVAN
TVFWTGPQPVDQLMCNFLVLCDRKWRGLNPSTIGQVLRPVRNNEGRYQSS
DAFKHLRKLRKLKFTVTHANRPASDKVYTIMDFAFDEKYGGEGATARTVT
FECNGTQISVADYYKQKYKVHLKNPSLPLIFAGKGGFIPMEFAIIEPMQR
YAFKLTPDQTAAMIKIAVTKPQQRRADTENNYKGLQLSTDPYLKMYGVEF
EPTFTKTDARILPPPQVNFGQGNADPKFAGRWDLRGKKFWKQNVAPLQNW
AFIVMDACVGYQQLQNFAKTFRNAFLAHGGKCPADALLLNVPGDVKNDVA
QAIAWAHKQICLQRGYTQLLFVVVQHKNSPHYERLKKSADCRFGILSQVV
NGSAVANNNGQYHSNVCMKVNAKLGGATSRTLPPWKSSPTYFPKDRPTMI
IGVDISHGAPGGGSPSTGAMTMSIDRDANRYAAVVESNGYRVEMLTAANI
HVMFGQLVKYWMAGHEGAFPKHIIYFRDGVAEGQFAQVLDQEIREIKTYL
REKAPNAQPPKFTVIVATKRHHIRFYPQKGDRNANALPGTLVEREVTHPF
MWDFYMCSHVAIQGTARPVHYHVILDEMGIPVNDLQKMIYHQCYSYARST
TPVSLHPAVYYAHLACARARAHENVASSEGFRAGAKGHEMIRDQVAMGQT
MEGPSRGIDAPPLLQLGGKPEGDKPPADGEMRQRDFIRGTMWYI
>Mb|QLI67355.1
MAAEQRKLLEQLMGHQSTSRAAQLSLTDPKVCRSYLVGTCPHDLFTNTKQ
DLGQCPKVHSEPLKVEYEALSDREKQRYGFDYDYMRDLQKYIDDCNRRID
AAQKRLEKTPDEIRQTNVLLKTISELAASIANGQLEVEVLASMNEVSRAV
DENFRIKQTMQLKVEKEKELKALSDTSGPSGHQKLQVCDVCGAYLSRLDN
DRRLADHFYGKMHLGYASMRKTYDAFPRELKSRQRQGMMDGDDGPGGGMG
GPRGPRAGGYRSGRGGRRW
>Mb|QLI67358.1
MLIDGEKYACEACVRGHRVSNCQHSDRPLQHINKKGRPVSQCHHCRTMRK
SRSAHVKCDCGEKTSKCAHLQQPVDGHKETCCCNHGGRCTCAHKKEPTLD
TVPEAESDPDPLATAARPKPPIRRRRANTVHSDGVLSFDQNGNHKPASKH
NRASQKCGPYQLNRVNSTNSASSLGASPESMLYQDGRAPDSFGNRPRAAT
ALEQRRVKSETASPLMSSGSFPHLQSGLPPLDLSGIEYPSYVNNGSFEMF
SSGVPSEVDGPIFSAGLSAASVDWSHYDLGEAKPENFAPSSYSQAGTRSF
NGLFEFGSGSEQLPRLANTTSTSGEVSEVEDFLGNGDGDMDGGFGTSSFI
RQANIVGSSTDLTSIDYDSFYNKNSENVAVGTISMVEEDPAFWMPNYNEG
VATAVGESPDQMAATSMGTFWEL
>Mb|QLI67360.1

MSLDQVPDEIIQHLLHHVSPEDTLQCFQLLCHRSHRLANEGLLWRYHCRS
TFRYWHFDHGFGQKLHAAVASVDWKRLFLLRTRRNRTVSQLLDAILRTKV
GRLRKIERICRLGYDAKDFLLVQCHAPESAPDVLARRYFSNAILDSIHRS
IAIEEWHRLGLDRDSPPAHVAAQRLERALGAFDMFVLHDQPGDLDDISQM
LDHLVIQFRASCANLAELTTRETALALNGWMRAKNLTGLQNPERNYRNLR
NCLIGQALRHEEHESIPLISSALFCCLAGRLGLNAQCCAFPSHVHAIVFA
QSGQTLDGHVVDSCHEQLERMYLDPYGLDSEVPASDLQNLLAHYGWQTST
DAFLAPVPPISMVLRTAQNIKATFARILELQEDAHPELSQLLRGNNAMNV
DASLYSAMWASLMLTPPNTFEWYDRLASFLRRFAGSWPEDAWLVEKYLAP
MYNSFTPLRDGFAHHVNRGLGDPWDHWRLVRDEDDVIPSVSRRDSDANQS
VPFKIGQVFQHRRYGWIGVITGWSDQGTRPLPVAISRDTDVEGGAGDPSG
QPRFPIRPPNQFYFICL
>Mb|QLI67361.1
MGWLWSSSSKPPTEEKAAPTPPTEQSAAPNEPVDPEIQKFFDLFKHDPEQ
PSTNQRATPSSENESSSSSITSWLALKASAKSTDSDVPDAPVGDALSESL
LPTDMSCRQAFDLAWSCNGLGGQFNSVYRYGNMRSCSEHWDDFWFCMRTK
SYTGELKANMVRTHYRNKAYRRYGDGKPNSEDVWERRTERMPPGSAFNVP
VDAPTVGDDEWRRMEEERRKGVRKDMGYES
>Mb|QLI67365.1
MHRAALRAFRTTSTCFSAVRNLSRTSRASHRALFSAASQAAHRQPVANEP
LRSSMYFRRLPVALVSSIVVGYGAWYSYNSTGGVPGGPSVARSFTSTQGS
SDALPTRTVLVVGADELRQGTIVGEGPILKPTADGRRIVEMLTPEQATEK
LRALEQSFSVNRGQGVTRYDLVQLPSNDPIEDDHAEKIVQVPSRSAGAEG
QSSDWMFWGVFDGHSGWTTSATLRESLISYVARELNETYKSAKSNSPTDE
AIDSAIKTGFTRLDHEIVHKSVEKVFKASSKAVAAELLQPALSGSCALLS
FYDTRSNLLRVACTGDSRAVLGRRSESGKWTATALSEDQTGGNPSEAARM
RKEHPGEDGVVRNGRVLGGLEPTRAFGDAVYKWSRDVAGKLRASFFGRSP
SPLLKTPPYVTAEPVVTTTKVNPEKGDFLVLATDGLWEMLTNEEVVGLVG
QWIESQASSSGSSTSQFEAAWSKIFGSQNKPLPVEQSKTGANDGQRTPIR
LQQWGINPDAKDRFTVKDKNVATHLVRNALGGHNDEQVRALLTLPSPFSR
RYRDDLTVQVIFFGDGLKTGEVTINLDATAETAEPKPKL
>Mb|QLI67366.1
MSNLNSWEDDPAAQDENLSRRAQQQLNVNAGQGQGGFRPGAASFQPGAQS
FQPGQPYGGGFSNQYQQQQQQQYYQQGYYPQYGQQQGFNQYNQGGYGGNF
SQGYNQGYGGGYPQYGQPGQAQQQPTQAPASAAPAAAPTAPKVLTKEGGP
KVLSIGGDASAPKPKAKVLSIGGSGTSTPTKDKEEAKPEPSNKPEAGQKA
AAVKAIEKTGEKAGKAASSSKSSGRTSPTPSSGRSSPSGPGEKKAQREVD
AVTKEQAADVDDETLKEVYGKEHVNIIFIGHVDAGKSTLGGSILWTTGMV
DERTMDKYKREAKDLGRESWYLSWVMDLTKEERSKGKTVEVGRGFFETEK
RRYSILDAPGHKTYVPNMIGGASQADVGILVISARKGEYETGFERGGQTR
EHAMLAKTQGVNKLIVVINKMDDPTVEWSQERYTECTTKLSQFLKGTGYN
LKTDVFFMPVAAQSSLNIKDRLPKGIAPWWEGPSLLEYLDGMSALERKIN
APFMMPINAKYRDLGTMVDGKIEAGVVKKGMSLVMMPRKQTVEVSAQYGE
QEEEVPILQCGDQVRMRLKGVEEEDILPGFVLCSPKRLVHCVAEFEAQIR
ILDLKSILTSGFNCVLHVHSAIEEVTFAALLHKLQKNTNRKSKIPPTHAK
KGDSIIARMQVIGGAGSVCVEKFEDYPQMGRFTLRDQGQTIAIGKITKLI
TNSD
>Mb|QLI67367.1
MKISGRVDGSVRGAVLNRPSAGFRNVHTPRRTICRPIDIAAESTRIKPAS
CMKSSLWILSTFMPESRRPSAVQPRLACRCYSTAPETLAATAKSLPFSRL
LSNDQEKRHLMSFVGERGEGSVQEHFDFHRDPYRRGYAQPDGPNLQVSDK
KHDVQYPSREETFKISDELQAHISKLYAAIGQRLRHPNRITLETIYKLYS
QLPEPRMLYLTWQWRSRLLKVMGTPPKRDMESMLRYFALVADVKNAGLTL
RRTQWNFALAFATKYASRPTAQEMESALRLWREMEREANVMGNDVTFNVL
FDVAAKAGNFTLADMIYKEMESRGIEFNRFHHVSLIHYFGLRLDSGGIRA
AYKEMVDSGEMIDTIVLNCVISGLLRCGEESAAEDTYEQMKKSHNLALSM
PQRDYMMNKVVTRVLMMFSKVGKKHPQLKESLQTNIQLAPDLHTYKLLIQ
HYAIRVGNLAKVAQYLDEMKHLNISVHPTIFLVLFKGFYLHGGFLNSDWS

EQRLNGVLTSLHQAKNSYGKAFRIEQWLVIWALRATKKCSSNEAVLETFD
ALAPRWDIEGERQQFLHAMMENILQDKDMKSMPR
>Mb|QLI67368.1
MASSLLKSKFLADVQSEESSRKPSPQPTHVSLPYSSLPNGNGNRTLRSAT
VGYVAPEFAGKLEQMNSVKNILQQAGYIPETLIDEQIKWFYEQLGIDDVY
FRIETPDAIANHISSLYAAKVAAYSREDKKEEIRLDMEANDHAIYIDTSE
PGESNVSGPRYESRLEAKYLDHQGKTKYRVETFRSPAVIGASPSSKATLR
CYFVYQCQFAQSPKETDPKETNIERISDAGFWQKATENTKQIYQEIIELA
VNRTGPVIEVFDIEDVAEKRLVVAFRTRTARGMFSALSDLYHYYGVTSSR
KYVEQFSNGITVMSIYLKPSSHADGYFPPLDESIHQITKEISLLYCLPQN
KFHNLFAIGALSLQEAVYAHSAWVFVQHFLNRLGPEYASLSDMLDMDDPA
QAALLSKLKRRLRTETFTPDYILEIIQTYPELVRLLYASFANVHLGAKNE
QVAVTPAVDVLSDEKLKDKISKTVANEHDEMVMTAFRVFNNAILKTNYFT
PTKVALSFRLDPAFLPEVEYPRRLYGMFLVIGAESRGFHLRFRDISRGGI
RIVKSRSKEAYGINARNLFDENYGLASTQQRKNKDIPEGGSKGVILLDPK
QQDRAQEAFEKYIDSILDLLLPAQTPGIKNKLVDLYGKEEILFMGPDENT
ADLVNWATEHARARGAPWWKSFFTGKSQKLGGIPHDKYGMTTLSVREYVK
GIYRKLELDPATVRKMQTGGPDGDLGSNEILLGNEKWTAIVDGSGVIADP
NGLDKDELVRLAKKRAMISEYDMSKVSKDGYRVLCEDTNITLPTGEVITN
GTSFRNTYHLRDTGMTDAFVPCGGRPESIDLISVSRLIKDGKSTIPYIVE
GANLFITQDAKLRLEAAGCILYKDASANKGGVTSSSLEVLASLSFDDAGF
VENMCVDAKTGQAPQFYNDYVCEVQAKIRENARLEFEAIWREHEATGVPR
SILSDKLSNAITTLDEELQQSDLWGNEKIRHAVLQDALPNLLLQKIGLKT
IISRVPESYLRAIFGSFLASRFVYEFGSEPSQFAFFDFMSKRMAQITGQP
NGAEQTQIQNFVLLPCTSKPRLLLTSLCSLSHPTDTLDLQDHVAPSAKDH
HSNHSHLSDTLPGAPGLSIAPHQAETLRDVALETAAAEAHSPRVSWANGK
ADDDTSDLLQQYADDHPAAAKHDVAEGSLDIEMYHHEQQDALAIAQNGGV
SSADEDDLDADADDLDDDMVDKISSSPSIEDGALNPVEMPAAWPRRESSL
TSLTRQLDIANMPLEMVEPVTVGCVNPEQALPVPETSVSQCSKSESVAEW
QHRFDSRRQYTWSNVGRRWDCEEECEGLTEPEIGHLNRASCQEEDCFPCV
ITDRPENSARLTKA
>Mb|QLI67370.1
MKRQAVCQLQRVAASSRPIRLPLQIARAYSTHPPQARLNKPIDYSETQLL
AHSSKGPALGNHNEIPPEVRNGATRKMNLFQAINDALGIALAEDDSVVVF
GEDVAFGGVFRCTMKLAETYGAERIFNTPLTEQGIMGFGIGLAAQGMRPV
AEIQFADYVFPAFDQIVNEGAKLRYREGATGVHAGSLTVRMPCGGVGHGG
LYHSQSPESLFTHVPGFRVVMPRSPIQAKGLLLSAIRSNDPVLFMEPKIL
YRAAVEQVPEAAYELPLSKAEVVKGGEDITVISYGQPMYTCLSAIQKAEE
DLGISCELIDLRTVYPWDKETVLASVRKTGRVLVVHEAMVNAGIGAEVAA
AIQEDPETFVRLEAPVARVAGWSIHSALMFEKFNIPDVARVYENIKKSLN
Y
>Mb|QLI67371.1
MDHGRDPCPWVIFNDFGGAFCMGAIGGTIWHGVKGFRNSPYGERRIGAIT
AIKMRAPVLGGNFGVWGGLFSTYDCAIKGIRKKEDPYNAIIAGFFVGGSL
AFRGGFKAARNNAIGCAVLLAVIEGVGIAFSKMLSGGTKLEMPQPPPSME
NASL
>Mb|QLI67372.1
MEDSERRTAKRSRFDQTEPEPKRASRFDRRSRSPPSRRPEGRDRSPLGHD
SSSDAKGSPVDAAAAAAAAAARINASLQARKGIQHVDVPPIQSADANDGQ
SGQAKPHSVSKEMYTADGDYIQDIEVNDLRNRYLVTKSSTQKKIGEETGA
DITTRGNYYPNKAMSTAANPPLYLHITSTTKSGLEAAVAKINELIKQELP
QLVDERRFRRRDQEQPEPERERERERHGGRRKWPEERIPIDLEPIPGFNLR
AQIVGHGGSYVKHIQQETGCRVQIKGRGSGYLEGATNRESDEDMYLHVAG
PDAGMVEKAKELCEDLVANVKEQYDEFKSRPPRYNDRGGQHGGDRYRGGN
DRHHDRGNQSYGGYGQDRYGQDQNASTNSPAHGANSPATNAADYAAQYAQ
YYGGADPYAAYGGYANYVALYQQYYGAQATQAQAAGAPGQAVASTSASPP
PPPPSEAAPPPPPPPSAAPPPPPPSGSPPGGSYSAVPPPPGL
>Mb|QLI67374.1

MSGRLSSHASGTDGDGLMPIAVVGMSCRLSGIATSPEGLWQMLARGLTGW
SSNGSSRFQMNSFWHPQSDLNASFNARGFHLIKQDPALFDNLFFGVTNIE
ARAIDPQQRMLLEVAYEAFETAGITMQNVKGSDTGVYCAVSHHDYDKILG
RDPELSPGYRFTGTGPALVSNRISYVLDLHGPSITLDTACSGGLVAVHEA
CKAIRAGDVAQALVGGTNLILDPDQVNIISSMDFLSPHGRCYAFDSRADG
FGRGEGVAAVVLKRLDMALGDGDPIRAVIRGSNVCSDGRTPGVTMPSSDI
QRRMIQRAYQQAGLDPRDTTYVEAHGTGTKAGDKAEATALHETFCKARQE
GNRLFVGSVKGNVGHTESVAGLAGLIKTVLMLEKKMIPPNATFMKPNSDI
PLESWGMEVPKRMLQWPDNATRRASVNSFGYGGTNAHVILDAADDYLDNM
KHMCPPVAIRACEGKVNGDLAHEVAEVTETSSLAMTIIVSNPTEAEPTTT
HVHGHSTLQHRRSLKASPTNAMTRPRLFPLSHDQEGGVAKLAANFKRFIL
DKLPDTNESLDSLAFTLSDRRSVHRFRWCVAASSRDELVDGLEHIVEGTD
RALQQAEERKICFAFTGQGAQWAGMGRELLAAYPVFAKSMERSEKYLTHL
GADWRLLAELDKPWETSRINEAALSQPCCTAIQIGLVDLLETWGIRPALV
CGHSSGEIAAAYAAGIMTAQDCLKVAYFRGHLVKRLKEKKPELSGGMLAV
GLSVGEAQKYLDNDATCEKVVVACVNSPASITLSGDEAVLSSIQETLAAR
GVFNRKLAVDVAYHSHHMNMILEEYLHAIQDIKPLHCGQHVQMVSSVTGR
AVQGEELNATYWGRNLTSPVLFADALAEIFDTARKNEKRSNMLAVVEVGP
HSALQGPIKQTIKASKTAGSISYHSVLSRKQDASRTAVALAASLFVNGAT
VDFGRVNDPHGDAKKNVLTNLPTYNWHHNTTHWVESRRSAQYRHRKFPKH
DLVGVPSSDSIPSEPTWRNYIRLRELPWLKGHCIGGNTIFPAAGYVTMVL
EALKQQILGGGNPWKKMRIKFRQVHFGRALLVADDSVGVETFVTIRPFTY
TARESSASWNEFRIFSVSVNGESTEHSRGLVTAISPLPNQDHENMTDAES
LAFIEQVSEKARIVLPPKQLYKELRDVGIEYSHPFNGQEHIRASESASTC
RIKIPDIEALMPLHYQQPHCIHPATLDVCFQAAFSAMKVGDNLRGTFVLG
GFDDLEISSEIPSQPGKHMSVAASLRGLGHSNFASDSVISSSDDGKSEVF
IKIKGLILARTSGPSRQTSSQHLAGESLCHRLEWSLDPTCAEPHSIIKRC
LLDPDVLVAGRTSMCEQYCQAVVAGALTELSPDDEAKITGHFSQWLLWMK
SIRAGSYQPLQMGNALNDKIKSLGIYGEVLVDMAPRLVGILRGDVDPQAL
LLEKQRLYRLYTRDNIKRCHMQLAEYVKLLRFKTPNLRILEIGAATADGS
MPVLEALYSNKGVAGEARSESYTLACAPTAFFQDAETGWDKAQESLEWKR
LDMEISPKEQGFELGSYDLVIAANVLHATREINVALGNMRSLLKSNGKLA
VMETTAPKIHDGVIFGMLPGWWLGATDGRVHSPLLDASGWRDRLGHCGFS
GIDFEMHDFDSAEDHQVSVMVSSATSHPEPWCLGMQDPTRGVSTLESELP
ISKYPTNLTGSEGGPRQTILVISDGKESRLADHVAELLTSVESSVHAEKT
MLAEAQVSPGQLAVVLLEAVHPFLATCSQADWGKVRHILSNADGVLWVSC
GGAVEGTNPLQSLIVGLTRCLRSEDQHRKVVTLDLEPNHGSGLETAEQIR
RVYHHVFGPHGPPASALPEFEYAIRNGEILIPRLMCNSPVNEYVQDSVSS
YHPRHEQGMKPGRALSLKIHEPGLLNTLYWADSERHARSVGAEEVRVDLQ
YVSLNFKDIMIAMGQLEGHTAMLLEGSGKVVEVGEALRHQYSVGDLVFAT
DPDGVATTSVINKCNVHHIPRKLSMEVATAISLAYATALYSLRNVANLQE
GESILIHSGAGAVGQAAISLAQYLNAGEIYVTVGSADKRALIRERFDIPD
DKIFSSRGLSFYHQILRRTNGDGVDVILNSLSGEALQKSCSLLAPFGRFV
EIGKKDVISNARLEMASLESNATFTTVDLTLLAKRKPLVHQELYRTIFEL
VGQDRIKVLSPITVSPVSELETSFRLMQAGKHVGKLLLQLDPDMAISVQP
PRPPTPRLKGDCSYLVVGGTGGLGRATMKHFASLGAKHIFTLSRSGSDSP
IMRELVEEMRLAGVCVVVFKGSVTDTVAIESIKEQAREFPIRGVVQGAMV
LQDSRVDNMSYEQWRAAMEPKVVGTMNLHRVFGDTLDFFILLSSSAGIIG
SYAQGNYSAGNTFQDSLARHRASLGLPARSIDVGSVEGEGYTANNEAAAE
FVSRQGLQPYKVKEFLATINEAIWNPFPSGPSAAQLICGTRRADPSSQAK
EAALQRPDPKFSHIWTKPHRQASAKADSSHFNIQAMFESAKTADEAEKAM
QIAIKQKLAHLLGLPEKDVSTNRSVVSYGVDSLIAVELRNWILSQLESHV
QIFELMSPMSFAELANTVARRSRLVATGLFGHVKV
>Mb|QLI67375.1
MNAHVGNTDCDPLTQSVTVFRFASARHRVLVELPPPSSFTISITTSLLTM
QHPRSLVLLGAASSLAAVAAGSLPRGVGPEFASHYQGKTEFSCISDASIK
LSFDRVNDNTCDCPDGSDEPGTAACAYIDPLSPEQPLPGSGSALAKAQPA
LPGFWCQNKGHIGSYVPFVYVNDGMCDYDLCCDGSEEFGHVGGVKCENRC

VEIGKEYKRLADEKRQKMQRAANQKNAMLSEAQQLRQKAEAKIAQLNTEI
KSLEVKKADLQKKYAAAQLQDRGRVVKSEGAGGKLGVLIGVAKPRVNELR
NTLNKVMEQRNDLRSRVEELETILGKFKDEYNPNFNDEGVKSAVKSWEDY
AAREADIVHEQVIDADVEEILKEDSESSGINWAEFESGEEGSDTDILYTF
DAYLPGFLRVFMHDKVTAVRLWLIQNGILADSGASGSESQVVKAAREAAE
AAEKDLEDKVRDRDNETEDLKKDYGPFDIFRGIKGKCVSIDAGEYEYELC
YLDKTMQKSKKGHGHTNMGNFVRIDRQLADDEERLDGKSLGKGERMVLKY
EDGQQCWNGPRRSTEVWLGCADKEELWRVSEAEKCVYKMEVGTPAACDDP
ALREEKKDEL
>Mb|QLI67377.1
MAKPRLIILIRHGQSEGNKNREIHQTVPDHRVKLTPEGWSQAHEAGRRLR
NLLRPDDTLQVFTSPYRRTRETTEGLLATLTSDDPEPSPFRLSNIKVYEE
PRLREQDFGNFQPCSAEMERMWQERADYGHFFYRIPNGESAADAYDRVSG
FNESLWRQFGDDDFASVFTHGLMSRVFLMKWYHFTVEYFEDLRNVDHCEF
LIMRKQENGKFLLETKLRTWSELRRERALANSSSSSSIKEKEKDDTSKLD
RTRTFVVTRKWGGCPNGCNHTDHYKRRHDLEALRQKDNELSGHAAAASAA
ANGGMISARRHRHKSTVSMGDGDDEYSDQHVLGDAPLEKTRSLDEVMSSP
DGTPSYITAEDRLRSLKSPPLHLGRDFGGTYSGHASLAGSDSDVSSDLGR
RCRRRKTSHNGEEQAPRSNGDTSHCMGAHANRLGDAPPAAGSDMEDLDAA
EKEDRSIRGSVY
>Mb|QLI67379.1
MAQPTPVEPNGASTPVNDENRHLAVPGKRKRDSEDGAAADEMTGVEEQKP
AAPHRWAAGEQKDLIKSYFDVLKRYFAHFLLSGATPIRRDVAIVNIDVEP
AILKRPLEDRQIEDEPDTKRQKSSEPPALKSISDKMATDSYIHVEDVASD
VVSAADAALSEFRAKLPDGSGSVESTKAVVRIAEFKAQAIAILEREKAYP
KVAPLEAIDGGDDSKAASRKSQAILSVVGYAPQERRLFSSLPLSHDIDLT
EFNLPPGVSFSRVVPANPQEQTQTLGDLFGPPRPLPPLQPPKQPKTQAKG
NTLDFYHPDLADTAKYRGTCYFGTKLTSGYYLDYSNATPSYQTKTKQRER
AQSLAGKKPSTSELEMSEMESLFRGAFSSFAPCKDDAGAVIPSGIAGKMW
WQKNGSKNFQRMIEVEYYPDIADVDAFHPEIKSEDIDEDAVQEAIDNWDD
GAVDPSLEDALGTKRDPNDKEVDEILEEVSDLIGTLASYQRIRNLTLPNS
QNRQSSDPVNGDMLANSGPQPSEEEQATYEMLKTQLALIIKTLPPYAVAK
LNGDQLDDLLISTKVQVQTDQYRGVMEEDDAGVQARIRAQQQQQQQQQHA
AAQAAQAAQAAQAAQANARPPSQRSASISTPYANQYQHTPQYGTPTRTPS
HPQQYFRSMPTPSYHQPRAMGSPAQQHPRPPQPNQYTRPNGYPNQYATQL
AKAQTPYGHQNMPQTNQQRPPQYGQLQQQGPPNAARFQYQQQFQQQPQPQ
PPPPGPPGQPNYGAYMNGAGVPPRNMSPQVPPRHAYSPSPNMQPAQQRYG
TPGQGNPQMIRHGSNPGQHAQYSQSPGPTGYHTVIPEAQQQRILEQAKAR
VAAQERTAMFGDKMQPGFPGMPMGYGGNMEANRIAAARANIMNQQKPQTP
VGQRQGMNGTPQSQYVPPSKVTPVPVPPIPGAGQPQPQPGQPQQQQPVPG
PES
>Mb|QLI67381.1
MPGGKGKSSGGKSSGGKTSVEGHKKQQSHSARAGLQFPCGRVKRFLKQNT
QNKMRVGAKAAVYVTAVLEYLTAEVLELAGNAAKDLKVKRITPRHLQLAI
RGDEELDTLIRATIAFGGVLPHINRALLLKVEQKKKKAQAIEA
>Mb|QLI67382.1
MGRYELEYPKDATNLVNRKGDRGIYALEKIHSLINSSQLIHVSFNVPNSP
FPVTLPMIGQMGSFDRPSADLGDPLDLYIHGYVSSRLFNLGRGAGEDGMP
VSCAVSHVDGLILALSAFNHSYNYRSALLFGHASLVEDQEEKLYAMELIT
NSVVPDRWKNSRLPPTNAEMQSTSILKVKIASGSAKYRDGGVSDDKHDLE
NEDALNTVWTGVVPIYSTMGEPIPGPYNRVDLPPYAKEFFDEFSAENKKQ
SLDAANTKTG
>Mb|QLI67386.1
MQSSPGMLTKFESKSSRAKGIAFHPKRPWILVALHSSTIQLWDYRMGTLI
DRFEEHDGPVRGVDFHKTQPLFVSGGDDYKIKVWSYQTRRCLFTLNGHLD
YVRTVFFHHELPWILSSSDDQTIRIWNWQNRSLICTMTGHNHYTMCAQFH
PKEDLVVSASLDQSVRVWDISGLRKKHSAPTSMSFEDQMARANQNQADMF
GNTDAVVKFVLEGHDRGVNWVAFHPTMPLIVSAGDDRLVKLWRMSETKAW

EVDTCRGHFHNASGCLFHPHQDLILSAGEDKTIRVWDLNKRTAVQSFKRE
NDRFWVIAAHPEINLFAAGHDNGVMVFKLERERPASATHQNMLFYITKEK
HIKSYDFQKNVESPTLLSLKKLGSPWITPRTLSYNPAERSVLVTSPAEGG
SYELVSLPKDGSGVIEPTEAKRGLGNSAIFVARNRFAVLDVSNQTIDIKD
LSNNATRSFKPPVGTSDIYFGGTGHLLIITPTAVHLYDIQQKKSTAELSV
NGVKYVVWSNDGLYAALLSKHNVTIVTKALEQVSTLHETIRIKSATWDDA
GVLLYSTLNHVKYTLLNGDNGIVRTLDQTVYLVRVKGRNVYCLDRAAKPR
ILQIDPTEYRFKLALIKRNYEEMLHIIRTSSLVGQSIISYLQKKGYPEIA
LQFVQDPTTRFDLAIECGNLDVAVEMAKELDKPKFWARLSAEALAHGNHQ
IVEMCYQKLKQFDKLSFLYLATGDHSKLARMAKIAEHRGDFTSRFHNALY
LGDVEDRIQMFKEIDLYPLAYMTAKSNGLEDECQAILEATGATEDQLTMP
KLGEPLSTPKPRVPTFKANWPTKASSQSFFEKALLGQVEGLSLEDEPATA
DRLVLDEEGDGAPALKRNGGLAEEDDEDVAGWDMGDDDVPEVDSDFVNVD
STEAGGAGSSEADMWARNSPLAVDHAAGGSFESAMQLLNRQVGAVHFAPL
KSRFLEVYQASRSFLPASSGLPALVNYVRRTVDETDPRQVLPIIPRDLEH
LATNDLQRGYDSMKANKLEDGIKVFKGILHAILVNAVSSEAEVAEAKKLI
TSASEYAVAMDIELARRSLGAADVVAQDPAKLKRSLELSAYFTIPKIEVP
HRQLALLSAMQLAVKSKNYNSALSFANRIIANGGSTKIVENARRTKAQCE
RNPNDAIDIEFDQFAEFEVCAASLTPIYSGTSYEECAFDGSKYHSKYKGT
VCRVCEICEVGKHGSGLKLFA
>Mb|QLI67387.1
MSSTTQSVQCFGKKKTATAVAHCKAGRGLVKVNGRPLSLVQPEILRFKVY
EPLLVVGLDKFANVDIRVRVTGGGHTSQVYAIRQAIAKSLIAYYQKYVDE
HSKNMLKQALVQFDRTLLVADNRRCEPKKFGGPGARARFQKSYR
>Mb|QLI67388.1
MVVGNIYVIAAVSVVGGGLFGFDISSLSAQLGEQSYLCYFNQGPHGPPYT
NEKCSGPHELVQGGITAAMAAGSWLGALISGPLSDRLGRKYSIMVGCIIW
LVGSTICCAAQNIGMLIVGRIINGLCVGIESAQVPVYIAEISPPSKRGRF
IGMQQWAITWGILIMYYISYGTSFIGEQEYFGWKASSFRIPWGLQMIPAV
FLFFMMIILPESPRWLARKDRWEDCRSVLALVHGKGDPDHPFVAMELQDI
KDMCEFERRHSNVTYLDLFKPDMINRTLIGLFTQIWSQLTGMNVMMYYIA
NVFSMAGYEGNANLLASSISYIINVFMTIPALLWVDRWGRRPTLLVGSVL
MATWMYANAGILANYAEVVPGGINGVAAQSMRLTGAPAKGLIACTYLFVA
SFAPTWGPVSWIYPPELYPLRLRGKGVAMSTSGNWAFNTALGLFVPPALA
NIRWKTYLIFAIFNTVAFFHVLFVFPETAGKTLEETEAMFEDPNGIKYIG
TPAWKTKVVTKLVDRAEHGDVDAKRAAEYKLSDAHEHDVEKAATPPAAA
>Mb|QLI67389.1
MNLTCNTTLEDMKISPGSERLIAGRLNFHDLARVISASSTLIATALKPFS
ADFNFNHEPFSIIRILFMVPVYAISSYLQLEWYWHAIYFQVIADCYEAFA
IASFFALLCHYVAPDIRSQKSFFRELYPIKPWVMPVNWFAKCCGGERGIW
RLPKSGLTWFNIIWIGVYHYCFIRVAMTISAVVSQYFKRYCESSNSPVFG
HIWIIVINAIAVTIAMYCLIQFYVQLKEPLAEHKLFVKIVAIKLVVFLSF
WQASAISVGTSTLRIVHPNEVIAYPDLKVGIPALLLCVEMAMFAILHLWA
FPYAPYIVGAPRTYYPNPIADKASPAVENERQAPSGGSMGLMALYDALNL
WDFIKAFGRGMRWLCCGVKRRKEDISYRRNRGDGLDMDNLPDNKEGGGSH
EPGRSGAGHPGQHDEYGSVTQAYGQPSQQANPYYSSRVEERAGLMDNAQP
NPEARDYSTAPPQLQRDASPYQAFGANQSREFQHHDTRQQTGVMQGRYNP
YDGTGSGLAAPTRAQAAVSHSLWGPGQQPQP
>Mb|QLI67390.1
MSAIYNLEPQPTASAIIHTTLGEILVELFAKQAPLTCRNFLQHSLDGYYD
GTVFHRLVPNFILQGGDPTGTGNGGESIYDGGAFSGDLDPWPMDERRGQN
AGPTGINFKDEFHSRLKFNRRGLLGMANESRADTNGSQFFFTLDKTEELN
GKNTLFGRVAGDTIYNLAKIGESEVDEASERPLYPVKIEKVEILVNPFED
MRKRSRVATQAVEKSLSADKSKKKKRKGGKQLLSFGDEEVGEEEEPVFKK
AKFDTRLIAQEEEPAEEPKKDKVGKKNSAKEDKKKKKPTLQEHSDEERPA
SNAENIKQTSQPKEDVREEAEELSDVPEPPKKTALQKANEELAALKASMR
RTIHSEQPAEDTKRKSALESMIPESSMRARKRRPGGNNTSGSDDQAAMRL
LRAFQSRLEKAPPEKEITESAEKRDEGSKDATGAGDEAELCDLHFIANCQ

SCTAWDKQGKEESDDEGWMSHSLSFAADRLGKDLSNRKKAEEELVVIDPR
EKARTLKEDKRADREAKMGGSRAWDQARDQARNAKLARVSSLAGRGAK
>Mb|QLI67391.1
MSNDGYRTVAYFVNWAIYARKHRPQDLPVENLTHVLYAFANVRTDTGEVH
LTDTWADTDIHWEGDSWNDTGNNIYGCLKQLNLLKKRNRNLKVLLSIGGW
TYSANFKNPASTPQGRETFAKSCVELLKNLGFDGIDIDWEYPQNPEEARN
YVELLAAVRQELDAYAAKCVDNYHFELTVACPAGPNNYEKLEIAEMDKYL
DFWNLMAYDYAGSWDSVSGHQANIYPGNNASTPFSTNAAVDYYTSHGVAA
NKIVLGMPLYGRAFENTDGPGKPFQGVGEGTWENGVFDYKKLPLEGSEEF
SDDQAQASYCYSAANRKLVSYDTAPMAKAKAEYAKQRALGGAMWWESSGD
KSGSDSLISTVVETFGGPGCLQKLDNCITYPETKYDNLRDGFPNN
>Mb|QLI67393.1
MASPSAAPKGAPPAPVDAVLRNTLRYTISAREYATLHRYIISRSRTLRRA
APTPASVDKALQPQRKGGDDYNAAAVRHALRVFVATMVGMKGWEAISRRM
DPSARDKGPKQPLLKSPALRLSVSLSSILLLYRFLFRFLSRLRAHLLDPQ
VEPFRRRNPRTAAALTSPYAPAIGASLAGVALGVYPSQQLRVSIAIYAMF
RALEFGWNVCESEGLVWGVRGGKKRERPWWFGSWMLQPLAFGQLLHAVVF
DRDCFPAAYGKFIFKNSDGYLHARPEGYSGTAKWPSTTDVVESLSRMARL
NWPAYISPTMFPTKEVLPPGLEAISPLTSQAHPLITSLSCATLHPSDPSC
TRTLLTFWLGSFPPMARFFLVVCSALTIVPRFKALYHFPVTTIRKIVASA
LRMSAFATGAISTAWASICFFQTYLPRRVLATQRFFLGGFFAGLWAWVER
RHGRGVFLYSARASVDSLWKVGVKRRWWKAMKGGDVWVFVLALMVTGVVY
ERDARALREKEWRKGVSWIRGEGWRDWAIDDDEDADEANDKKME
>Mb|QLI67395.1
MAEPSHNPYARSPNPSTRSYDSSSVSSATSPRPPSRYLGGLLGASSRPNN
AASSPQPLGMTPLPPVNQGFPYGPVMSRESLPSAEAVMAGQGPNHGHLPG
TPGGQAQKRAYRQRRKDPSCDACRERKVKCDATETTSCSECSSRNVKCQF
TKETNRRMSSIKQVQDLEKQIEKIKRENSTLRRLLQEREGHMDIDLDALP
SIASAPKLRKRLPPNPELTRARLHVRNFSKGIWQPPSQHRIYATSVLECP
APELPPKTVTDRLLHSFCNSAHTMFPIIHMPSFQSAVDDLYRVHPPRATS
AWISMFFAVLATGSLFSPEAPTTATFYRPAEFLESARKAMDPWNNTHVLD
NARALVLITICLNEMNLKSAAWNWLGNAVRVGQDLGLYSECGSWSAIDTE
MRRRTWWATYILDRTMATEMGHPFLIDDADCDVSLPVAVDDQYLREGGTR
IPNGAEPLTHSLLAVIHVVRSYTALVKALDQPFLSATQLTTFDNHFNKCL
STFPPACEPGSTVPLAPHFLAPLAYLFHARLLLHRHNLSPSCSPETRLAA
VENCTHVALETASLIERTKSPADGATALLTVHIFRSALFLLLTNYLDQAI
ICIRALAAIDARRDVSMSCGRFLAFFITTLGVKRTDYASYVSRRSSIDQT
ELTLSLARDEELLVYVSTDIQASPNRSWLWSFQDFDTASPKTGMGSQSHP
GTSEKVLFSSEQRTGLNEEERRDWVGWTRLESMARGIGSVNVPYPPPLPP
PQVKSESPIANVELPRLSETSRYSVGHRGDANPPGPKRGAERISIANII
>Mb|QLI67396.1
MPYVSAARYGKDNVRVLKVTRDQTTGVQTVTEMTVCCLLEGDIDTSYTEA
DNSVVVATDSIKNTTFIIAKQHPVNPPELYASILGNHFIQKYSHIHVANV
KVVTHRWIRMDVDGKPHPHSFIKDAGETRNVEVRVSRKDGISITSSIVGL
TVLKSTGSAFHGFIRDEYTTLPETWDRILATDVDASWKWAKFADLKAVEE
SAPKFDKAWDSARNITLKLFAEDNSASVQNTMYKMCEQILDAVPETETVA
YSLPNKHNFELDLSWHNGLKNTGKDAEVYVPQTCPNGLIKCEVSRS
>Mb|QLI67398.1
MMSWWSSSANTALDEQIEKATSSSLEDIALNLEISDIIRSKTVAPKEAMR
SLKKRIGNKNPNTQLSALNLTDTCVKNGGSHFLVEVASREFMDNLVSLLQ
AVGAVAVNADVKTKILELVQQWAAATEGRHDLSYINEVYRTLQREGYQFP
PRVTVASSMIDSSAPPEWADSDVCMRCRTAFTFTNRKHHCRNCGNCFDQQ
CSSKTIPLPHLGIHAPVRVDDGCYAKLTGKGYRELGPPDRSPTYPHKTRS
TSAMQPRNARVDDGFDEDLKKALAMSLEDVKNNSRGYVEPAENRARATKD
SSKLEEEEDTDLKAAIAASLADMEEQKKRHAAALKEQTSNRPTSSSSTPF
VLPKNDYELTPVEAENINLFATLVDRLQTQPPGTILREPQIQELYDSIGT
LRPKLARTYGETMSKHDTLLDLHAKLSTVVRYYDRMLEERLSKAYSQQNL
GGYSLPPPRQPTSAYPSVPSQAHANPGVAESFYTGQVPPPSFQVPPAQSY

QQQQVPPQATPQPQFASYSQPSEPHSGQYALPQRTSSWQTRAPSVPQDPY
HPQQAPVVEPAQGIQTPQQAPASPSTERRGSYYFGTQQQHSVQVPSGPSA
PGPAPGSTPSPYPSLQHSMQYQQPASTTAPSQATPIQSHAQPAQAGRRPS
QTQTQAQPQSQQVLVPQQQQQQQQQQPTAQQQPAQQQFWHQPGAHQSTEQ
TPQQQPEAPQSWAYTGYAQPTFPSVPQHEPMKKPVQEEALIEL
>Mb|QLI67400.1
MRQRPVGLVSLARASFAPSRSRGRFLSTTISRQADDKLNRISSSITQPKS
QGASQAMLYATGLSEDDMNKAQVGISSVWYEGNPCNMHLMDLSKIVKESV
AKAGLIPYRFNTIGVSDGISMGTTGMRYSLQSREIIADSIETVMNGQWYD
ANISLPGCDKNMPGVAIAFGRVNRPSIMVYGGTIKPGCGQSGESLDIVSA
FQAYGQYITGEITEEQRFDIIRHACPGSGACGGMYTANTMATALETIGLS
LPGSSSNPAEDPSKIAECQSVGEAIKTLLKEDIRPRDIMTRQAFENAMTV
VSILGGSTNAVLHLIAIADSVGIKLTIDDFQSVSDRVPLLADLRPSGKFV
MEDVHKIGGTPSLLKFLLKEGLLDGSGITVTGKTMKQNMENVPDFPSDQQ
IIRPLSNPIKPTGHIQILRGSLAPGGCVGKITGKEGLQFTGKAKVYDSES
DMIASLERGEIKKGEKSVVIIRYDGPKGGPGMPEMLKPSSAIMGAGLGKD
VALLTDGRYSGGSHGFIIGHIVPEAIEGGPIALVENGDEIVIDAEKQVID
LNVPEAEMEARRKAWKAPAPRYTKGTLKKYAALVSDASAGCVTDGSIH
>Mb|QLI67401.1
MLRQAFSTSSRALRSAPRLAASQPLLRTQFQRAPAAWSSRAAQPAVARWY
SDAKEAPAEGEKAEKSEAKEEAGETDAVAELKKSLEAKDAEARDWKDKCM
RTVADFRNLQDRTQREVKTAREFAIQKFAKDLVESVDNLDRALTMVPSEK
LVVKDEASQDLVNLYDGLKMTEDILMQTLAKHGLERLNPEGEKFNPNEHE
ATFMAPQPDKDNNHVFHVQQKGFKLNGRVLRAAKVGVVKNA
>Mb|QLI67402.1
MLAVQHAPRMGSTQPESDPLLMFGYSVDSTQPDIFDAAPDPAPGAPLLTE
TDSKFLSSFFEDMTSNQYNMPSFGEGLNFSDAWLDLPPQFMGSSTSLGPQ
ETPADFGFSSDHQNDLSRMLSADSSMMPPPPPPAPSHTQNFSHQHSDDVL
NAAATLLQNGNRAGTPKSISSPGNSSNSGSTANRPVVYPVGHLRHQPMEE
FKEENRRVSRRSSQGNELDHTFTRWMWGSREKTPASKPVLGDFQWGSDAS
FSDAQGPANGQAISSNGDGPAYIKREEDPNAPPRKRRKSKNVRGTAVDED
GDDDDDDATSSKSGRKRKTKSERSASGSEPPSDTTAGSRRRKSGVNGAKP
ARENLSEEQKRENHIRSEQKRRTLIKEGFDDLGELVPGLKGGGFSKSTTL
AMAAEWLDELLLGNKALAMQVSALEQR
>Mb|QLI67403.1
MATQYEVEHNVQFAETQPRSRRVNMDSFFSLLSRVSDSGTATPHNNPHAT
PTLPDMANIFRLLQDQMQTLASTAPTAENRTFLMDLVDTLDGDILDPPDR
LRGVGQEFLDALDRVGRKTLRADDDCAICKIPYLEDPYCLVVELPCKGAH
RFDLECVGPWLRSKGTCPMCREEMGKKKVAAVQEGDDEDEDDMDMIYA
>Mb|QLI67404.1
MSVGTYSLPKLPYAYNALEPSISAQIMELHHSKHHQTYVTNLNNALKTYA
TATSSNDIAGQIALQNAIKFNGGGHINHSLFWDNLTPASSAEASPQTAAT
LMAAITKTWSSFDEFKAAFTKALLGLQGSGWGWLVKDGGSLRIVTTKDQD
PVVGGEVPIFGVDMWEHAYYLQYLNGKAAYIENIWKVINWKTAETRFNGG
RDDVFKVLKAAI
>Mb|QLI67406.1
MAKVKGRAEQFQDPDEAIIKDHDPEANGSSSEDSEDENLGTEHYVRVGKS
KLRQKESVSLGPEYRGSRVSRSALDNETEDDEDAEESEDNDDDEDDYDDP
DVADLEADEAESNDSEIDSDNALGESDAERFKDYSFRGSAKPAKPKGRGK
RATAADFMSVDDENRESDRDEDMDDGLDALVNGGDDSEEVDDEDEEDSEE
DDGDDDDDEEDEEDDEDGDDDETEEEETSGKKIAARPHLSALSNRADIDK
GVAIQKQRKIYDGLLNLRIRLQKALVAANSFPTVEAGTDAESEPYEAAEE
AAIKLLNTISSLRESIKAPTKTGEKRKRDFVASMSNDDIWEQLQDEDKDA
TGFRQERLEKWSKKVQSVNMARPSGISEKNKTLISALQEQLIVPNNRLVK
RTRVPRSCAPAQLAKKVATDEYVYDDADFYQLLLKELVDQRTVEGVADYA
TAVPTVMVTATKEAKVRKQVDRKASKGRKMRFTVHEKLQNFMAPEDRRGW
EQEAIDRFFGTLFGRKLELNEDESDHEEMEGVDIEEEGLKLFRN
>Mb|QLI67407.1

MEPSSPDAASFTLCPIIQDLVRTRFAVPGSIFLVEDIQTIALTESRRWQV
IRLLLGDGELCIQALLADSMHRFVHTRDITVGSYVCVQDFQLRQRTTAAD
AGKPQQQMVYLVVHDVKTLGCNDDVRRMHDEQQLADEAASPAGSGASTPT
KPEPAGQAPGDGDEFEDDDDDDDMEGAFDEFEALAFPASKTPAPAHPSKP
ALPVALPRDWHDVQTPLKLTTLRAIPNLPYRQNWSCNVLAIVASLSAVES
CHLPPYRQRTARITDPSTAKQVHLAVFLDPDEFNPRVGSAVLLTGVKNHR
FDGGSLKKYASDRGRGRWWFQDPWELAWCDVRGIKDWWAEMEAYFASQLS
EEVVGGDV
>Mb|QLI67408.1
MSLNLDPNNATFLKDEQGRPFIVVRDQGRKKRQFGNEAVKSHILAARTVA
NLVKSSLGPRGLDKILISPDGDITVTNDGATILQQMEISNHIAKLLVELS
KSQDDEIGDGTTGVVVLAGALLEQAADLIDKGIHPIRIADGYDQACDIAV
AELDRISDTIDFTKTETANLVKVARTSLGSKIVSKAHDQFANIAVDAVLS
VADLERKDVDFELIKVDGKVGGSLEDTLLVKGVIVDKDFSHPQMPSEVRD
AKIAILTCAFEPPKPKTKHKLEITSVEEFKKLQNYEREKFIEMIQQVKDT
GANLAICQWGFDDEANHLLLQNGLPAVRWVGGPEIELIAIATNGRIVPRF
EDLKPEKLGSAGVVREMTFGTTREKMLVIEECANTRAVTVFCRGSNKMII
DEAKRSLHDALCVVRNLVRDNRVVYGGGAAEIACSLAVEDAAVKTPGLEQ
YAMRAFSEALDTVPMTLAENSGLNPISTLAEVKSQQVKAGPDNRGKLGVD
CMGRGNNDMKEAFVIDPLIGKKQQLQLATQLCRMVLKVNNVIVSGSGDEE
F
>Mb|QLI67411.1
MAPRRVSGGPVAFLGRAWQLALFVTPFHRMFFINDLHIAYPHAVHERVSV
PWLFIYAFFIPLGVLILYNLTARSPIVKHEVTYLSFFISIVLTLFLTDVV
KNAVGRPRPDLLDRCHPSPGTKENVLVTIEVCTAGESHKLQDGWRSFPSG
HSSFSFAGLGFLSLFLAGQLHIFHHPIGGRDLGRALICLSPLVAATLIAI
SRCEDYRHDVYDVCVGSVLGMSVAYWSYRRHWPRLSIARCHEPYPRPGAD
LQSAAWQRVRDEEEGGRAMDDEYELQPQRPSRG
>Mb|QLI67412.1
MAEQKHARRRTSSNAATLPVRNGQHARKQSKAAVQPRSAHTADDFLHDFL
DASFDPAAYLNATLPPLASRQASSQPAGAVPLAELSAQAQTLLSQLNAQT
TRLSNTLTQLTDDILRSGSRLAYEVELLRGETLGLSEMLHETLKEDIEVF
VPGGLQPNHETRKEATRRGEADEDKDGVEASPPPADAAAATGANDSSDPE
YIKQLQTLTLVRSRLESVIQTFGDAMDFTFPPSEVSVSSGFLSVSAPEPG
SEQQSSEEKGQQVLRKLRDEIAGFLQQSDDPIQGIERAAARIEELKELSV
VWKGTAEEKGRSKFIESLAKMVEDRHRDLVREMDQAARREGRAAGSQGKK
PGGQEGAGEESKVPGGFGLISQLQKLRSGL
>Mb|QLI67416.1
MSTRQTRRKSAKLREAAAAAASSDDDQKPIIMNGNGSAHHTPEATDIEED
ENIFLFWPNIIGYIRIVLAIASLYYMPVHPRTCSVLYSISCLLDAFDGYA
ARIFEQSTRFGAVLDMVTDRCTTSCLLVFLSSAFPRWAILFQGLIALDFS
SHYMHMYATLVVGGSDSSHKNIDKSQNWLLNLYYTNKNVLFTLCALNELF
FIGLYLLAFSSPWLSPHLIKSVEETSGGFINPGAPVNTSLLRQMFPDPFS
ASALEIARANKMDSLIPWAIAGISLPFMVIKQAINGVQLYNASKWLADVD
IKMRKEKGLPRKNKAKRV
>Mb|QLI67418.1
MAPSLNNHSSFTRPRTGDRDGRPSTRDQAGADMLIPSRTSSLHSRITQPI
PSTFNVKPQQRTPKTLTHAYMVCGVGREPSQWVKAPAPAQGKIGHMKGAV
GQFWLPEILGSSPRLEQDNEIARALHSAMRACFPHDVEICTGRSQPHCVH
HAFVLQQDSSHTLYGICLRVWSRADEKRAETIRDLRKRTESDFYDNPDET
YWIPYCLSFLSRYPLYNLLGDYLRGMWIHWNKATNLFHAEEVSRILSFPA
PRLNDLVRIDMKDYALCYQFPSSPTGFQNFSMWPLFNCLSIPNIVGVIEA
AISPTRRIIFVSHYAAMLTIAAETVRYCVRVYEWSGLYVPVVHARHAQEL
VQEPGPYILGVTAECRSLFTAPTDALVVDLDRNFVLTSSPPTALTPGQRN
KFVTRLTQSLNGDVTPAGVPHHLRSAYGGGKLVPAGQIIVMRGEVESIQD
PEWWNQDAIMAVMDHVCEKTGRNTGIKAVFGGSVKKPLMTKVSMRHLNEI
VRERNQYSRDALEAWQDFINLKGRMDTELSKVNKRNNYLVEELESWKQQF
LKFQAFAEQLTKETQDLKVKIETHKRENRRLTGLLDQQKDDNARMSVRLT

GTEKQRDDALMALVLQQEMAEDLERERQRNKKELAQLSHTNSTLLRQRDE
ARRVVMHLQSLIGGQSHHMEHLIHSLTKPDELVAEVEAGYESDERGSSDG
ASGDDNTSQLKSPRSKRLSAASFADVADRHLKDKTDAIAHIVRNIADQCQ
AAVESLQLAHDAEYRSSRRHSGLSNTQSEDGHDDRSVATSDNGEERRLQP
GRTSSIPPTPDLIPNRSSTAMSFASATTTPERSSQQYNLKDEIPTKIVED
DEEDYEENRSEGGGGATNENGVVAKHTESLLHRPSGARISALGASGTR
>Mb|QLI67419.1
MPNPWDYTYASPLAGFEDAPPLSTDTNDDGKSLVNPPRDGLSEAYRSFAW
PLKTDRRGGFDIHVYYLQQNPEQAKYARELWERIRREFPELRVYKFWDRP
IGPHPVAMFEVNVFTPAQFGAFVAWLAIWRGPLSALIHPNTTVHQGEAAN
EVELRDHTERAIWMGERLPLDWAPFRPQSHE
>Mb|QLI67420.1
MSRSVVQPNSWVALRLPNDALRVLQVAPNTTISLGKYGSFPSNLLMERPF
HLTYEVQEKRPGENFSRLRIVPGAELNADILAETNENEQTDGDAAIVVGE
GEQLALVDESGKVVARSNREIIDDSARQTLTADEIEVLKREGASAGKELI
AKLMLSHTAIDQKTSYSLAKYKLLKERKFLRRFSVLPLDVVLLGQWLLEN
RDAWKVLELRPEMMGLLGCWADVHFGGEPAQGVREPHGGRWLAVDDTGGL
LVAALAERMGLLHHEPGEQAASPGPGEEEEESRPAAEAAAGQNGANGGPE
QQQEPPSSPQAQRTSKKRPRCRDFDDHFALTNTITLIHSNSQPNLSVLRY
FDYDAGDPNPTYPYHPLFTNLLPISWLQLVDPAQDVVCSEKPADATPEEL
ASWKPNRRGNYHRKRRRWARTNEVIENTRAGGFSGLAVASTMDPVSVLRE
ALPLLAGGAPIAIYSPTIEPLEQLVDCFSIGRRAAWVSSLPAEAEGKTQA
ELDRWRGTREFPINPTLVLGAAVQSSRARRWQVLPGRTHPFMTGRGGAEG
FLFTGWRAIPAQGRVSARGKFQKKRGEAA
>Mb|QLI67422.1
MSGHRPMSIDAMLDMERQEVLALLENKNLNAPTSPSRMRSSSPYTAAPRS
PVRSMLDIEEEPEGSSHGVTTQAPVRSMLDIDAPVITTQAPVRSMLDIAV
PSPPGSVHGRSSSPATPNEPIFKTHSASSQGSHHPRSPSDAAFKPAEFGP
RAGLRVDKTSEYQFSGILPQYSGHVGSKRLSQSGSSKRLPGAGVLGEALR
GADLSSLQLPGDTGRNSSAGGRLGHPKSKSPHERWAARSRSPATFSPLLS
PNRVVLDHGQVVDINNAYRRLSDANLAFSSGSLAQLPMRKRSDDAGEGRL
IKDYLGPDGEHLESSEEEEAFSSDDEDRGRKTAPRSLNPDAHEEGENSKS
RSRSGQGDRQTLSLLAAADEERSKIAQKQSQTYTYRSLLEPEIKITNSSG
DTVKPVKGGIHPNTSYDQNPTSTTPSMFDSDEEADMADIKRAQNLGFSMT
NILTAPDSHRSIRIIYRGDYHKTVQSADEEKQRVRKYLVATDLSDESTHA
LEWAIGTVLRDGDTLIAIYCVDEETGIVTGEGALVPDDPKAMKEQAAAIN
TVTNAKGIPAPVTPVVELKRASALHTRADSAGGNRTPGASPAPSQRGDNQ
RAVEERSRAIQEMTDKVLRLLRKTRLQVRVIVEVLHCKNPRHLITEVIDI
INPTLVVIGSRGRSALKGVILGSFSNYLVTKSSVPVMVARKRLRKQSKYK
QKAVKQVNNISNPAARSLANAKID
>Mb|QLI67424.1
MASEHKIHRPYVLATLPRPLDHTGGRIVAREVYGLRPGQKKKKRMELAVG
VDGETASIYDVPASRLITSYPIPPQESFTCAPYSIRLRRSGSEDVYRYTY
IATRDAKSYKVTLFRDVVHPDGKTTTSTTSQTLRASPIHITGSSSTTSSL
SGNTVGDVVAICEDGQVIYLTGEDLTLQWSSPFKPAIQDVIAGAIESLRV
EYVSSGSVTDFREGVFKSRLEIFSALPRALDVDPSLIFVVVKSASEGKST
RHLLVLATLPGGAAAAPGVQNLIPLDISPLPANSSREESPVYEVDIHSGF
LTELANDVLSVYDITSAVPKKKSMIQMAGTQSFIRLSRPFLLTTSTDSMV
LYNHQYSSVHGRADLDLSELPAEDQQARNCQLITYFRSQDLAIALIDNVL
VSIHVEPPHYHGKRRKEGLLIDSIGRGAPFEVQTKPSKGESVSAEFSRLI
PGSMTEEYLVAYHKEILAANEMLANNALGKWEDLLRQKFGLKNQNDPALA
NGEATVEAQELPEWDWQTETGSYPPVDRRWVIYAISQVFSVETTSSDEPK
PELRLALPDSNVTTYLVVAGHLTISNLISAFRDVLPLEASGIKAVARDLI
HSLTDADPSMTLLLNYLQATKLGEVELLLAIRSLMLSMDLLPDTNKLNKL
KLLANEAHEEGDTQEVDLDDLEREIAVTEHYLGDESSSRSRGLTLAFTKL
WRIPAIATVKALRDTVRTEEVLAFIYILRVELVRGAWTTLYIDPTSLDSE
GNDPPPDGVITLIADLLGRCLDAVGAGGWLFNDAMTWADKTETGDFLTAL
KLEVAAALEGLEEAVLLNGIVGEAVRYGLTAEKNWAARQVWTPNKPISLH

LEGKESRLLPLGLKMKLLPTREKVVSGGEVVNRTSRETGHMISQKVEAYS
LEKLAI
>Mb|QLI67425.1
MSQTSSASSRHRQCNKSQTFRPARNSLFDLLRRHARIPLFRPPVHWTDMH
WQLLGAHFQELPSCDTPLPQNIPGTPPSKGHLLPSKAITTLSHALTDILH
PDDACADLYSNATRKRSSAVQTVLSTLWPAIFGQPILFPQLHIFFGGRPC
PNPVHMQLMWHYPDDNVQSSQSSFRSISTRPAESNGLFSGSAPAAHNPTG
FPMICYIGQDQLATMRRSVFRIRPGSERCRNEPVSRLFFLRSKAVMPANL
HHDAYLVAVFLAMAQRHFYDAPVPSSLGDSRWSTWVGKSHRRAFEDLKLR
ILTHDSDMEHFIIYTGHITSKFLDRFHDPSTTPTSDEIKGEGENDPLGIR
IEYTKVPIWPILGLKERLGKGLGEEVVGQFNPSEMETWDTDSEGDAPRNT
KRKREALGDMFRFEEETEDDEQESSLKPKRQRLQEGPPIGVGA
>Mb|QLI67426.1
MTTEVSNTRLYLGNLPRNATKADIEAHFATHGTGDITEVKLMNGFGFIEY
KDPMDARDVVPDGSDFMGERLTVQFARGPRQRESGFSGHERAPPRPRRTP
HRMQITGLPNETSWQDLKDFARQPGLDVVYSETGRDSNGRGFVEYETAAD
LRTAVDKLDGREFKGNRVQCIADTQPDMPPRDGRGRSRSPGGRRPYNNVP
PRFDEYDRRGPPTRGYNRDPGPYGYRDRSPRREYHEDRARYRSPPRGPAP
VEDYPPARGRYEDPYRRDYPPPPPDPYANGRQYDRPPRDFPPREPAYPPR
DGGYAREYDRGGRYW
>Mb|QLI67427.1
MILRNAAIRHGRALAPPISRSALRLLSTEANSQNAVNSSPNASAQAWSAG
QSSSAQPKILSGSSSKKRKQKTVQEFIMSLGSDSAQEHSGASWTKPHQIG
SEGQEVTPAQQFADFQRIQKNTRSLGSHVERRYVPTELISNPPGPEHVSL
ELLMASQAHMGHNTSLWNPANSRYIYGVRQGIHIISLETIAAHLRRAARV
VEEVSYRGGVILFVGTRKGQMEIVTKAAELAGACHLFTKWTPGAITNRDV
ILKTQATKVVDHLDSELDGFDMYKGMARPLLPDLVVCLNPLENYTMLYEC
GLKNIPTIGVIDTNVDPSWVTYTIPANDDSLRAMAVVAGALGRAGQKGKE
RRLQDSSRGNVSWNTSPELERHMKKEVQAAVTKRKEVMGRIQSNVQGFTE
EEQKLLRVRNNEVDLEVSEDEMVNMLGETALKDAGEAVAEAGHVDSSKTS
SWKVGEGLADIEAQLQRVQQQTRHIELAVGSKQE
>Mb|QLI67430.1
MKSALIAAAALVGTAHAGVHKMKLQKISLEEQLAGASIEQHVRALGQKYL
GARPASRSDVMFNTKAPQVAEGHPVPVSNFMNAQYFSEITVGTPPQTFKV
VLDTGSSNLWVPSQSCNSIACYLHSTYDSSSSSTYKKNGSSFEIRYGSGS
LSGFVSQDVVTIGNLKIKDQDFAEATSEPGLAFAFGKFDGILGLGYDTLS
VNKIVPPFYQMINQKLLDEPVFAFYLGSGEEGSEAVFGGIDKDHYTGKLE
YIPLRRKAYWEVDINSIAFGDDTAELDNTGAILDTGTSLNVLPSTLAELL
NKEIGAKKGWNGQYTVECAKIASLPDIVFNLAGSNYSLPASDYILELQGT
CISTFQGMDIPEPAGPLIILGDAFLRRYYSVYDLGKNAVGLARSK
>Mb|QLI67431.1
MCSADIFLALLAVLFPPLPVWVKCGICSADSIINILLCCLGFIPGLIHAW
YIIAKYPEPAYEYESLPQEGGHITYVYVQTPHGPQCRPPAPNGGMNYGTQ
QNGSNRNQQQQQQQPQPQGSQQPRAQTGAASAESAPNQQGGVPPSYAEVV
AGDHKVQSQD
>Mb|QLI67432.1
MSQITDASIRDAITQRLGATHVEVTDMSGGCGQAFTCLIVAPQFQGLNSL
KRHRLANAALKEEIAAIHAWTAKCQTPDEWERDKAKAGPPMDGTVGGHVE
GTSA
>Mb|QLI67433.1
MANYTLQENKGIWAVPLEITDFNILVALLGGFISVFGLVSYLFKETFCLS
EALISLLVGVAFGPYAADWIRPWSYAACHRDGLTDAQCQDRLNAITLNFS
RLVLGVQLVLAGVQLPSKYLWKELKPILLLVGPGMACMWMATSLLVWGLV
GTPSFLHALAVGACVTPTDPVLSAVIVKGKFADHNIPKELQDLIVAESGT
NDGLGYPFLFFALYLIKYIGSGATEGGAKDAMGLWFAITWGYTIILSIIY
GAVVGWIGKELLYFAKKRNYVDHESFLVFAVALALFVLGTCGLIGTDDVL
ACFIAGNAFTWDDWFRLQTKDDSLQPTIDMLLNVTIFLWYGAYLPWNDFA
HNSIIPLSRLIPLGLLVLLIRRLPWIFGMHKAIHQISQVRQAIFVGFFGP

IGVSAVFYLYIGIEFIQQHLSDENSVPRSDVKDLAETITVVVWFLVVCSI
VVHGLSIPLGKLGYLAPRLHRVLSESLSETISTDRFSVQARRRIPFFGRF
VREPEDVEANLAAQVRQGTAPIRRTGQGVVPSKIKQPEQDSRGAPAAGQS
SHEDAESSGTRIDVEAEARVSGEWTPAKGRNQSSDEPEARKSEASTPGRS
ARREIQFSDEVPQSPPAPAK
>Mb|QLI67434.1
MSSLVSDFIINPVLRQARRFSEISRTTVTGEDHEPTPPSDPDTVSDSGTA
GGVLREALEQLETPGEEPIARRPETPRSPAVSTSTQETRVADAPETPPSV
DNTSHDHLGFPLSPRRGRAIPEDDGMHELRRRIQAINSEDAPSAEKARRM
HELLLEGYRASQVANRRKPTPPMEEESLGHQWGPSTSPGPLESLKSWYNH
ATDQPSTADKFILSDGDIAPTFAPIRPRKGSDVELQGQGSTTPPESQAPL
GCQHYERNVKLQCFTCKKWYTCRFCHDAAEDHSLVRRETRNMLCMLCGTP
QKASDMCINCGEVSAHYYCNICKLWENRKSKPIYHCNDCGICRRGLGLGK
DFFHCKTCRACITTSIQSSHKCIERSTDCDCPICGEYMFTSPKPVVFMTC
GHSIHKKCYDQHMKVSYKCPICNKSLANMGTQFRNLDVAIINQPMPPEFR
DTKATVLCNDCSGKCTVPYHWLGLKCSICQSYNTVELQIHGQGSQELQTA
AERAEAAASAQQPSPDIQRPAMTPPATSSRAATWIPNRRRHSSHGIELQH
HAPDRFARSLSPLESGIDSHLAQIAANIDSDDDILGLWRGNRSQDQAEDE
DEDDLSEEESDSDLDSDGINSADEDEDEDDDDDDDILLIGHR
>Mb|QLI67435.1
MASLKTMTLHSHAGGPNPWKVAIILEELGLPYEHKFLDFKQVKEEPFISL
NPNGRVPVLEDPNTGISLWESGAIIDYLIDTYDTANKLQYTSTPEKYQTR
VWEHFQMSGQGPYFGQLIWFTRYHPEKLQSAIDRYAKEVRRVTGVIDAHL
KKQKTEYLVGDKLTYADLMFVPWAVAVSSMAGDLLDLSGYDAYDAWFAKL
KSRPSAAKIIKEREAALAASH
>Mb|QLI67438.1
MSAYPGYGGGGGYGPPQSSQYQGNYYRPQQGYGGYPPQGQPSPQPQPGYG
YHQPHPAQPQYAYQHHGPPPPQQYAGHYSPAPPQPQPGYASRPGQSPGIP
PPQHGAYSPRMSGPPVPPSGLQHFGAGAPQGYAFQYSNCTGRRKALLIGI
NYFGQRGQLRGCINDVRNMTAYLAEHFGYRREDMVILTDDQQNPMSQPTK
QNILRAMHWLVKDARPNDALFFHYSGHGGQTKDLDGDEADGYDEVIYPVD
FRQTGHITDDEMHRIMVRPLQAGVRLTAIFDSCHSGTALDLPYIYSTQGI
LKEPNLAKEAGQGLLGVISSYSQGDLGGVASNIMSFFKKATSGEDAYART
LATKTSPADVIMLSGSKDDQTSADATIASQATGAMSWAFMTTLKKNPQQS
YVQLLNSIRDELATKYTQKPQLSCSHPLSTYHRIQAPSA
>Mb|QLI67439.1
MADIRVQSLRKPIDVSEYLFRRLYEVGVRSLHGVPGDYNLVALDYLPKSG
LKWVGSVNELNAAYAADGYARVKQIGALLTTFGVGELSAINGVAGAFSER
IPVVHIVGYPSTLSQRDGMLLHHTLGNGDFNVFANMSSQISCQVAKLNSP
SDIADQIDHALRECWVQSRPVYITLPTDMAETKVEGVRLDRPIDLTEPQN
DPEKEDYVVDVIMKYLNAAKNPVLLVDACAVRHRVLPEVHDLLAKSNLPV
FVTPMGKSAVNEQHDNFGGVYAGDGSHPPEVKDIVESSDLVVSIGALKSD
FNTAGFSYRTSQLNSIDLHSDHCVVKYSTYPGVRMKGVLQKVVKRIDPKQ
LNIRASPAVRNEAREHHDGSATITQAWFWPRLGEFLTDNDIVVTETGTAN
FGIWDTKFPSGVTALSQVLWGSIGWSVGACQGAALAAKDMGEKRRTILFV
GDGSFQLTAQELSTMIRHELNPIIFVICNEGFTIERFIHGMDAGYNDIAK
WDNRGLVDVFGGQGKAQKFAVKTKDELNELLAKPSFKSANELQLVEVYMP
KKDAPRSLVMTAEASARTNARKE
>Mb|QLI67441.1
MSVARPVHSLLGRCIASTRSRAGSPTATSVVQCQQFHSSAPRSKRRSRFR
NVKAEEMGLLNPSVLSKYRQEKFPDYTKEELELLKQKYTPEQLEALRAGE
EAINTDDVIIQGRLRDDPYRPNYIEDFTVLDPRYDVKPKVDVTPKQPEWL
SHNEWVDQYGSKMSQMTDTKTSHQLTRAMVRALRRVKESSGTELIDLTED
ELKDLENDPELLKKYLIEEDGKQEPDANASGPDLLTRAQAMKLDEAVDAE
WKKELDKLSSMTDESEVEPSNLELLADGPAGVNRAYSAEAAELGKVPGVE
GLYKSAADPEDEGQDDGGHYQEIKRLTGMTLKDIQSIYRKVLVTRWVSNQ
TRLGKVRSTSVVAIAGNGNGRLGLGIAKSTEAGLAAETAQLLAIRNMKPI
RRYENRTIFGNVKAKVSGTVVELFSRPPGFGLRVPHRLFEMCRAAGIHDI

HGRMPRSKNPMNSVKATYEALTSQADPEEIAIGRGKKMVDVRKVYYGGAV
Y
>Mb|QLI67442.1
MAPETRSRGVPPSSRVYNSSPSLQQVQFPARRKKIRRYEGPERPSLKQQT
LTQIDFVSGFGEDVIILSDSDDDIQDEDPGSEDKENMKPSLPNGQDQNAQ
EEEEKGQEEAGDDEEPVSIRRKRAAPSIKQSKKRRRTMGDELKKAITPRK
EDKSRRKTLDDMPTRSNYHTQTLTQFLGHQTSFVADSDDDLDLDKDDGDD
GFLSWLGEPGSPSAGRGKRDFSSPAMKRRHEATAAITASTADVSMSREDS
IIPQTPAKKNTAIRFDLPSGGLQSPSQRMVERYGAPDQQDSPLKNHSSPM
KPPLVKLTGEPRRVSPGVTRQSSLVIQDSYTTDGCITPSRNQAKVSQSKT
PTPLRVLFDAPSILNPVGDDDTPTKRRRPRRKSPSPKKLAAKGVLYEIPD
SDGEDDGFGEEDEDDGKNLDERYGVGVETQLVLNELVSTEESQHSCHDAV
PCSPPTSRMHTQAEPVSATVQYEQQSTENQPIHASSTSSRPTTPLRPKPI
RKPLPHLSVHTQIQSQPFESQRVHVSILQSLPAPSARSDILLPISTASLE
GLITGTTLHVTTSFKIPSQVVRFWLFENGLLRYMASVEPGQQVPPAGFSS
SSQGRWQFHTSQVYELNNPVCEDDMRQEGWLRGQIARYKYLPPAVIGQLL
WNLRHALFADEPCEQQVASSPVHPGSQPKLPRGDDEDRARNCPPSSAPPG
SMTVSQQITAQIHSDIASSTQFPTSDDMVPSTPDFNEPLKLRSSSRTTTL
PTPSAPKQPSRTVRPSQATTLSQPSTPEKQTQQPASMPPPPPPPLNPSQG
SVQFAESSYLSSLPIPSSLGSTSQLLTKSQMLPDSLIQDHAPPVQPEIWD
SDEEDASL
>Mb|QLI67446.1
MSSTVELTYQDVAEHNTKKDIYVVIHDKIYDCTQFVDEHPGGEEVLLDCA
GQDATEAFEDVGHSDEARETLDKIQVGVLKRQPGDPAPKAAPPTSSGANS
GNSDSTGVGIGVYFIIILIGGALGYLGFQYMAKQQQAQQAGAA
>Mb|QLI67449.1
MKPSTLRTARLALNGLQKRAYSQSSGPRHLMSIADLTPAEFSSIVRNAAV
RKQAVKAGQTPPSLSTSLSGQTIAMMFSKRSTRTRVSTEAAVTMLGGHPM
FLGKDDIQLGVNESLYDTSVVISSMTSCMVARVGPHSDVANLAKHSSVPV
INALSEDFHPLQTIADFLTIHEAFPSSKPSGPGLGLNGLKVAWVGDSNNV
LFDLAIGCVKMGVDIAVASPAGYGIPDKMRQLIISSAEGVSSPGKLSETM
VPEEAIKGADVLVTDTWISMGQESEQQKRLNSFAGYQITNDLAKRGGAKE
GWKFMHCLPRHPEEVADEVFYSPRSLVFPEAENRLWAAVAALEGFVVNKG
SLLQ
>Mb|QLI67450.1
MAQQQQVALERLEHITRGLRSRVGDDVRKRAAIQLRDLVVVCHRDLSQEQ
FLAFYNAVNNKITQLITHGSDSAERLGGIYALDALVEFDGVDVAAKYTRF
TQNIKTILRGKDINPMKPAAIALGKLCRPGGSLISELVDSEVNTALEWLQ
NDRVEERRYSAVLVLRELARNAPTLMYQYIPTIFDWIWVGLRDPRQLIRE
TSADTVGACFRIIRERDQEMKQIWMSKIFNEARQGLKVNTVESIHASLLV
LKELLEQGGMYMQEHYQEACEIVFKHKDHRDPTTRRTVVSLIPDLASYSP
ADFAHTWLHKFMVYLSGMLKRDKERNDAFLAIGNIANSVKSAIAPYLDGV
LIYVREGLSVQSRKRGSVDPVFDCISRLAVAVGQTLSKYMEALLDPIFAC
DLTPKLTQALVDMAFYIPPVKPTIQERLLDMLSVVLCGEPFKPLGAPQPN
TLSSVPAITKDVKDPQAYENRRSEVKLALNTLGSFDFSGHILNEFVRDVA
IKYVEDEDPEIREAAALTCCQLYVRDPIVNQTSYHALQVVGDVIEKLLTV
GISDPDSHIRRTVLAALDERFDRHLAKAENIRILFFALNDEVFAIREVAI
SIIGRLARFNPAYVIPSLRKTLIQMLTELEFSDVARNKEESAKLLSLLVQ
NAQSLIKPYVEPMISVLLPKAKDSSPSVAATILKALGDLATVGGEDMLPY
KDRLMPLIIDALQDQSSNIKREAALNALGQLASNSGYVIEPYLDYPQLLE
ILQGIIRTEDQRGPLRQQTIKLMGILGALDPYKHQQVEEHTPEIQRRVDS
NQMTDISLMMTGLTPSNKEYFPTVVINALLQILKDTSLAQHHAAVIEAIM
NIFRTLGLECVSFLDRIIPAFLQVIRSSASTRLESYFNQLATLVSIVRQH
IRNYLPDIIQILQDYWDKSPSLQTTILSLVEAISRSLEGEFKVYLAGILP
NMLGVLDKDSSAKRVPSERVLHAFLVFGASAEEYMHLIIPVIVRTFEKQG
QPVFIRKHAIDTIGKISRQVNLNDYAAKIIHPLTRVLDSGDPALRTTALD
TLCALIQQLGKDYVHFMRTVNKVITQHQIQHQNYDLLVSKLQKGEVLPQD
LTSESRFADIADETQFADLGTKKLEMNAIHLKAAWDTRGKSTKEDWQEWL

RRFSTTLLTESPNHALRACASLASVYLPLARELFNSAFVSCWSELYEQFQ
DELIQNIESAIKSENVPPDLLGLLLNLAEFMEHDDKALPIDIRVLGREAA
RCHAYAKALHYKELEFLQDQSSGAVEALIVINNQLQQSDAAIGILRKAQL
YKEGIQLRETWFEKLERWEEALAFYNKREREIPADQPTPIEIVMGKMRCL
HALGEWDALANLTGSTWVNSAPEVQRMIAPLATAAAWGQNKWDHMDNYLS
SLKRHSPDRSFFGAILALHRNQFREAVACIEQAREGLDTELSALVSESYN
RAYQVVVRVQMLAELEELIVYKQCDEKKQATMRKTWETRLQGCQRNVEVW
HRMLRLRALVISPAENMRMWIKFANLCRKSGRMGLAEKSLKQLIGSDAPL
ETMIPYWPDNRSDRSGSTPPRNIPAQVTYAMLKYEWELGQQPAGRRQGIS
ERTLYCLQRFTNDTAHRLDVAKAHLAAQAGSTDGNLPTDYGIQGQIDPSL
MSPQTQRALYDQTVLLAKCYLRQGEWLIALNKNDWQHSKVQEILNSYSQA
TKYNPRWYKAWHAWALANFEIVQTLSARAESQLSRADQTLLIEHVVPAIQ
GFFKSIALSVGSSLQDTLRLLTLWFSHGGSADVNAAVMEGISNVSVDTWL
EVIPQLIARINQPNKRVQQAVHNLLADVGRAHPQALVYPLTVAMKSWQNS
RRSRSAAQIMDSMRQHSANLVAQADIVSHELIRVAVLWHELWHEGLEEAS
RLYFGDHNIEGMFETLGPLHDLLERGPETLREISFAQAFGRDLKEAQEWC
HQYESSKDVNDLNQAWDLYYQVFRRITRQLPQVTSLELTYCSPKLLNSKD
LDLAVPGTYRSGQPVVRIMSFDTTLSVINSKQRPRKLILSGSDGVSYAFL
LKGHEDIRQDERVMQLFGLCNTLLANDSECSKRHLNIHRYPAIPLSQNSG
LFGWLPNTDTLHVLIREYRESRKILLNIEHRIMLQMAPDYDNLTLMQKVE
VFGYALDNTTGQDLYRVLWLKSKSSEAWLERRTNYTRSLGVMSMVGYILG
LGDRHPSNLMLDRITGKIIHIDFGDCFEVAMKREKYPERVPFRLTRMLTY
AMEVSNIEGSFRITCEHVMRVLRENKESVLAVLEAFIHDPLLTWRLTSTV
SPAGPNFQSEREVALAGPTAGRARRHSILNADVAPSELLAANGEPSGPPT
SRARARTNSSAAPDGNGINGAQETESQNARAVEVLDRVQQKLTGRDFKNN
EELDVINQVNKLIVEATKLENLCQHYIGWCSFW
>Mb|QLI67451.1
MQAPVLVMNTNSGERQTGRKAQLSNIAAAKTVADIIRSCLGPKAMLKMLL
DPMGGIVLTNDGHAILREIEVSHPAAKSMIELSRTQDEEVGDGTTTVIIL
AGEILAQALPQLERNIHPVVIISAFKRALEDAIKIIDEISLPIDIDDDKA
MNQLISSSIGTKFVSRWMDQMCELALQAVRTVTWETGNGKTEVDIKRYAR
IEKVPGGEIEDSRVLDGLMLNKDITHPKMRRRIEKPRIVLLDCPLEYKKG
ESQTNIEITKEEDWNRILQIEEEQVKAMCEAIIAVKPDLVITEKGVSDLA
QHYFMKANITALRRVRKTDNNRIARATGATIVNRVDDLQDSDVGTRCGLF
EIEKIGDEYFTFLTKCQDPKACTILLRGPSKDVLNEIERNLQDAMGVARN
VMFHPRLSPGGGATEMAVSVRLAQLAKGIEGVQQWPYKAVADALEVIPRT
LVQNAGKSPVRVLTDLRAKQAEGKSSWGVNGDTGTIVDMKEYGVWEPEAI
KLQSLKTAIEAACLLLRVDDICSAKKAQLGGAPGVGGGDD
>Mb|QLI67452.1
MRLPPLAAFPKASWQQQLPPQEWDSLVQAWVALCRAYLDLSDDDFKRQTE
SHASLVTFSSTFVHEVVAAPLMPKSTLLLRPVFHLISRLLRLLAPPVLLE
YSFLADFARIYPKKRTSPLIAGLFSTHSSSTLIEASLTTLKKLLIPILDS
GIKGDLKLVEAKLVSLSPLLHVSPHSCIQLLAGSDFFEGLVTCFKVMNPP
LRKAIVTTTYLCVVGLTEAEPPKWSMLSDQLFTLKTVAEEHRMGPLNAND
SLVAELVTATPLLKVLLRRAEERGAATESLKKRITALEPFKKGPMARPKR
PSRRKVDKGKGRQTNEDVRAEMHVHHMSQITHVQDLFPDLGAGFVSKCLD
EYKDDVELVVANLLSQSLPPHLATADRSEPLSPHQDIPHHSNLAPRSTPP
QVPTRRNVFDDDEFDRLSADESKISFGKKPQKNADELLKDKSTAPNKAAI
LSALASFDSDDDERDDTYDAADVGGTVDTMNQEADGVDDGNEEILFRAYH
SDEKVFGRDVGTRRGNARTKLREETGMTDEAIEGWAVMLARNPQQKKRLD
AKYAFAGQQVRLERTAWRASPAEPATEDSDIDGGGGRGGRGRGGGRGRGN
RGGNVAGPTGVSSES
>Mb|QLI67453.1
MADVPLRVISENSASERRITPSWSITTLKAKLEPITGIPPSAQKLSLKAS
GGSDAIHIEAADEDNTRLSSFPLAPYAELHVGDTRPPGARPNYTDTSGVD
KYEMPEEEYEKKTDSVLAWKKAQKLGRFDPNAPSHEQARLEALEQEVRQR
GIKVGKRCRIGGEDTRRGVVKYVGEVKEIPNGIGQWVGVQLDEPVGKNDG
SIGGCRYWGEPSTMKHGVFVRPERVEVGEFPPMDDLEDMEEI

>Mb|QLI67455.1
MSIPSTSQLFEAGLRNSPSQSGFLDSLVKQQLEIASRDMEMNTPPPLSSD
SEDLVSDLGEYFTRTATIWAQLTQRLIKGPHIIQASFRAHERSRAESRMS
DLAELAKIDMGLSKLRQIGDLSDRFSREVQEVWWPHVSMMNGGGQEVLQT
SISPFCDNKISSISESVSLAHEPDSAHFTSRKLLPDKSQLSHAGIGDDGS
GSEDSSDDDEQFQSIDMEALRQRGKGVYFCPKGMKCDKGGVDKDGNLVVF
DRNSSFAYVSLLVRCSTREASPIP
>Mb|QLI67456.1
MASVSRLSSSALRASLKSQAFNGRTVAFNGARYYSAKTQTLKERFAELLP
EKIEQIKALRKEHGSKVVDKVTLDQVYGGARGIKALVWEGSVLDSEEGIR
FRGKTIPECQELLPKAPGGKEPLPEGLFWLLLTGEVPTEQQVRDLSAEWA
ARSDIPKFVEELIDHCPTDLHPMAQFSLAVTALEHTSSFAKAYAKGVNKK
EYWGHTFEDSMDLIAKLPNIASRIYQNVFKGGKVAPIQKDKDYSFNFANQ
LGFGNNKDFVELLRLYLTIHTDHEGGNVSAHTTHLVGSALSSPFLSLAAG
LNGLAGPLHGLANQEVLLWLTEMKKVVGDDLSDKNITDYLWSTLNSGRVV
PGYGHAVLRKTDPRYMAQREFGLAKMPEDPMFKLVSQVYKIAPGVLTEHG
KTKNPYPNVDAHSGVLLQHYGLTEASYYTVLFGVSRAIGVLPQLIIDRAV
GSPIERPKSFSTDKWVEIVKKL
>Mb|QLI67457.1
MSLQGGDVSSPDDQEERKPRPSSAASSSPDTGGDRDSFEDAVDNADTTAD
TVDAGSVRSLTKRSVSLSKRASRRDGEDEENEGGEKSDEDEKDEKDEKEE
KDEEDEEDKRTSRDELVDSDKAEVKPAPGRTSSPLSRRISNTSNLDNVNL
DDETPAKPEAPPIPPEKDHASKTLSLSSITNALPSMAWSPSAESPSKSPS
PSNNAVAAPPPQPAPSPPARKRSAPFSWFSTNKDTSTSPPAQPSGRRNTA
SSIATLTSNPEMMLSTLTEENQVTNGANGNHRKSLKDRFKVLRMREEAGI
QLIPGDGDGQEDALGLVSTDGKTVPGDKSPLPPVPSPNHELAPGTASGVN
AGPSAMQEAQVDWDLWQSVVYEGPAAVARTSPEELNRAIATGIPNAIRGV
IWQVLAQSKNEHLETLYKDLVTQGTDKDKQNIRSTATSPTPSASGIASYT
NGDGVKSSASSIHSNKSAGNGSTSPRSEKSQETIAKAQAALVAEKQKKQK
ERAAKIQTLEKTIRRDLGARTSFSKHAAAAGLQEGLFGVCKAYALYDDGV
GYAQGMNFLIMPLLFNMPEDEAFCLLVRLMHQYKLRDLFIKDMPGLHMHC
YLFERLLEDLEPALYCHLHRRDVSPHLYATQWFLTLFAYRFPLQLVLRIY
DLILSEGLSAVLRFGIVLMQKNASTLLGLSDMQQLTTYLKDRLFDVYIDK
DPSQGSILENGFFGSSSSSIDKEVYRADQLVRDACEVKITPEILKIYTTE
WEEKTQARKKQETELQDLKMAKENLAGQVRKLEERVQAHDAEQKDLCNKL
VHCTVESDQLREEMETLRTDNEELRTDNEKLRKENEKLRDENDTLPGEND
RLRDENEGLQGQVKELRNVIQLQPEEIERKWKIERSEMVKKNADVYQENV
DLQNELHRLAKELADCKLRYAQINDEHETLKTKWTDMGRQFRT
>Mb|QLI67458.1
MANKGPEPDMKTSSQKPGDDLLANFRQQFPEVAAVTSEASAPTIAQAASA
VDHPIINLPPQDQRNPNVETDVFRDQDPTPRASHEPWRFTPSLLDPNSYA
FNAFASTQPGYYVPTPGGNNPLLHSQAGDLHTPTIAAGGLSTPLSLPNSG
DALHAGATAMDLSGFQALPPHQFHHFNPFIHGAAFATTSFVHQDTGYETM
DQDGVVLEAAPPEHRVPSMVPAVLQQPQAVGHMMQMGHALPPNAEKFRFH
CTLNAPTAMIKQADEIPITYLNKGQAYSLSVVDTSGTFPIPPGTKYRTFV
RVSFEDEEQRQKSSVCWGLWKEGRGTNEAHHRGGKLQAVEYVEAGQPAEN
DDKRARIEVESASFDGFCVTWTPGASGSHDVNLAVRFNFLSTDFSHSKGV
KGIPVRLCAKTSLVPADGSLQDAESSSEVCFCKVKLFRDHGAERKLSNDI
AHVKKSIDKLKQQLAQIESGMKDMGKRKRISSATKGSDPQRPGKVQKHKR
TWSMSSTSSNAGCGGSRMTLEEDLQIKLQTLQDMFTSTRPVSILYLRGED
LDDPDLHPVSLPGDMSPPNKLEVSREGPNWQARSTKSSVAGSMVSPSPSS
LSLVSQASGMWQNIDNTTASGKAFEQISRIPRMDDAGTLSGWIEALGVDP
SYRPPAEQAEKPVACFYVQYRDKSEQGKPTYYRAIYLSKRTVEEFNGRIS
SKWGVEPAKVLRSLRVIQGGLEVEMDDDVIRELREGQDMRLEIEEVIEQS
SQTKREWEMLLDETGADGDDVAPFAKGFVLRLTF
>Mb|QLI67459.1
MSQQPHDMGYLDYTGHASRSPTSRQNYGAGFASGLTLPRQPQRPFDVPLG
SSALYPSDRIGSGYNPRAMDSMSGHGGMPGGYMLDNGQSWNYNSVGVATV

NGAMNGPNRQRSVNRRAALPQTWTDQGAMGMPNNGLPSFPGSLNGGQMSN
GGLRVDHGQAHTSPDLRSTASDGDQLIPTAIVIKNIPFAVRKETLASIML
DLNLPQPYAFNYHFDNGVFRGLAFANFQSAEDTRVVIEAMNGMDVHGRKL
RVEYKKMLPEAERERIEREKRERRGQLEEQHRAPILHQQSSIQSLSSMSQ
TQQRTPSSHLGDVDLNDPQSLEFYTELVMFKRDDSREILVFPAGISPEHR
RSIHILAHHMGLEHQSIGEADSRQITVLKRQQPSPTANIQNVPANSLDVH
KRGLSRAATFDFAADRESRAAANNYSHVMGRQGPTLELPGSPDGSGVPNN
LRAAKSFADLRSFTPSPSQASSSYLAPGSGLNNMVPGSTARFGDYQPAHP
GNPGTPGPKGDSLIAGLSGLNLSPYDSNPLQSQGRNAPGAIGSQRPGVTS
HKGAPERQPRGPEWESTGGFGGRGRSNGHMQRGSDSSDSGARVAANPTGA
SRYH
>Mb|QLI67460.1
MARKATARQASAPGASSEDKATGFTPERFEKELQDLADKARSDTFSNRFL
GQLNIYIRIVLLLAFLALYSHVSQLNLSPVYGSIPSSIWHTKLVMGGCFL
GWAGNTFLRDSLPITPAKALPMVAVCIPGLQHFLAAYSQTFGAQWGPVVT
ESLTLLPLALFTSASVADYWEGARLGMLPNFVADAGPGIGSWALFKFFEG
QVGVYLPRIMGRLLVFTRLGFEIILSVAYMAFSPSKYLIYAAFPFLHTIF
LNTHLQSRAATQSLMSSLMTDNWLFIERRESVTGYISVVQSIEEGFRVMR
CDHSLLGGEWISHRGGPVSEPVYGVFAMLEAVRLVESAAPVDDKDAKALV
IGLGVGTTPSALVSHGINTTVVEMDPVVYEFASKYFDLKENNPPVLQDAV
SYTGELATTAPLTYDYIVHDVFTGGAEPIDLFTLEFFQGLHSLLKPEGTV
AINYAGDLTLPPPKVIYRTIKQVFPTCRIFREMPPDAESLKTKGMDFTNM
VIFCKKSDSSPLSFRRPTTKDFLQSPARREFLGLPNEIPETAMLIGGDEG
ILRKNETAKLAKWHETSALGHWSLMRTSIPALVWEKW
>Mb|QLI67462.1
MVSAKKHVPIVKKRTYNVHRCLAAKDDPCFSRFETTRLLGTKGMKELMST
TRFNRHQSDRFKCLSASWRKPKGIDNRVRRRFRGTMAMPSIGFGSNKKTR
YMMPSGHKSFLVNNVKDVELLMMHNRTYAAEIASAVSSRKRIDIIARAKQ
LGVKVTNAKAKVTTEV
>Mb|QLI67464.1
MAPQFGATFVPGGFDDYYLPEVVAPSPQRVTPQVPQNMQDDLQRLELEAT
DSEKRETREAPSQYSRQPSLSTITPRHSYPQTDQSGVPNTVTTQVDSDIT
AYKDTEGRAQRLDTMGRDAPSFSPFPKVAGDNIPPADDSKEEILWNARKH
VLHSQDVRMQISWAGDVLIWAEIAMDAVARESAEKPRPATPRIEHELRLD
ALNIVNYLAGQEHPQALYMRSKWLEFGKFGHRVDKREAYSGYKRAAELGN
ARSEYRMGMLYEQSNDMSKAKEHYYKGMSLKDSAALYRMGMMSLLGQHGE
TKDYAVGLERIRAAADGSDEDAPQGSYVYGMLVGRDLPDINVPDGLLPYN
LETAKMYVEKAAYLGFAKAQLKMGQAYELCQLGCEFNPSYSLHYYGLAAK
QGLPEAALGVSRWFLFGYEGVFKKNEELAFKYAQEAAAARLPTGEFAMGY
YYEIGIHVNQSIPDARKWYQLAADHGNKDAIGRLESLSHDKSLSKQDHET
TTLTRIKSQHGSQRGKRPDRFKQPQHMPTLSETTPLSPTDNKPHLSQPAY
TKSHSATASPRLSPQPSPGIKPTIVSEQANLPDPSRASLVGTDRPQAFNI
RLEGNAPQPMRSHSAAPYPDDDRPAPLNVNRPHSTAPYPEDDVPAPSRRR
YGHGLQPAHGPQADRPSSAFGIKPQSAGPRPIPISHSTGSLQPPAPAGPP
QSRVASAGWEPQGHPGAYRQSSPGPGLSGQRPQPGDPMYGRSDSGQPPIS
GKMAGGKLQKQPGQQPYSSVSPTSADFGRDYGPRTSSRPASEMPPQHDLR
NNSHGYERFSRVPGATGRPERLSSLPPQDTHQPPRVSTGFGQTTSSQHRP
GPVSGKPASAPPNATGKRPSEANHPSGPATQSAASAPAKPAKGPATFEDM
GIPQGKQDGDCVVM
>Mb|QLI67465.1
MLLRYHLPCPHLLLRLTRIVAEYLEERTQSQPENPFASLIPDQQIAIVPE
FTLESGITLQNVPVAYTTRGKLNDAGDNAMVICHALTGSADVSDWWGPLL
GGPGRVFDTSRFFIVCMNSLGSPYGTASPVTAKHGDVSQGNYGPEFPLTT
IRDDVNLHKLLLGELGVNQVAAVIGGSLGGMFVLEWAYFGKDYIRCIVPI
ATSSRHSAWGISWGEAQRQSIYADPKYDDGYYPFDDPPATGLGAARMAAL
LTYRSRNSFESRFGRNIPDPSKVQTIRERPRPSTPSEAHFHIHNDGHNIK
RKSTSLQGSGSPGSNKATTPPADAPDPQFHGPRKHEGVSSESSLTGGETL
PQSQYFSAQSYLRYQGTKFVKRFDSNCYIAMTRKLDTHDVSRGRAETIGD

ALAMIEQPTLVLGIESDGLFTFAEQEEIAEHVKNARLERIESPEGHDAFL
LQFEQVNNYILDFLKEVLPDIMSKEGQAPEETGVGKLTKSSTFGEAEVED
ITAW
>Mb|QLI67467.1
MKLHVDMLAGLVDNTSLLGILLATLALYLGYFTLRPVPIPGIPYNEDAVM
RAFGDLKAMKSAKYRRRWIWSQPREHGTVIYQLFLFPFRRPTVIVSDYRE
AVDICSRRVKEFDRGTRNRECVGITAPNFHFTMESRDPRFRHHRELLRDL
MTPWFLETVATPRVYENAVALVHLWGIKELKALERPFSANHDLYLSTLDT
ICAVAFGMDREMSAVGHQTRHIEGFKPVEHWGLGVVARFPTAPTDAYLES
ILDIPEMVAIAQKSFFPTLSQRLALLSPKHARAHWNRRRLIRKQTEQALR
RISSLGDQYTPKSALDHLLHREMMASSRAGRAPDFFSPAIRDEVLGYLLG
GHDSIATVLSWWTKHMQCFQDVQSRLRQALRQAFPRAVEEGRWPTAEEIS
STSVPYFDAVVEESLRIASVATLISRTATCDTQILGYSIPKGTDILLSLT
GPSLTEPAVPVSEPSRTAACRQAKDRIPAWGDDIGGYKPERWLKVESRDG
SDGVRVFNPYAGPNLAFSAGPRQCFGKKLALLQLKTTMALLLWNFELQEV
EASLSGWDITERLFNLPTNCYVKLGRTGHDKVAALP
>Mb|QLI67468.1
MRHERQKGRHLPQRPGVKWVPELPEPRQCDVRDSFKGSRPQLPQPPPMRQ
RQHWRTGSFDDKPGASACAKCLGRTFPNTRQRLARHQTCFFTTRHPPQPF
ASRASPLRPTRPAQYPEMRHQVATLLGAAPAIIAALASANASPLTPKHEA
GRCAFRGQCGKQSFFGKELPCVDNDVAKDPDAELRKELVDLCGAEWNQGP
VCCTLEQVQSLKSELGTPNTLIGSCPACKHNFFNMFCKFTCSPDQSLFVN
VTDAAPKNGKLLVTELDQLISEEYGTGLYDSCKEVKFGGANSRAMDLIGG
GAKDYHQMLKFLGDKKPLVGSPFQINYPESYEQPNMGPLDMTSKKCNDED
PDYRCVCVDCPAVCPELPAVRKSGSCHVGALPCLSFASIFTYSVLLFTFA
ASVFGHVAWRRYAQHRVERTRLLHESSHSDDEDEGGPVLTEAMRDRPTKR
YWINDRCDDLFYRLGHTASRFPGLTIGTSLLVVAILSAGWFKFDLEKEPA
RLWVSPSSPAAQEKAYFDSHFGPFYRAEKIFLVNDTQPSGPGPVLSYETL
KWWAEVEKSIAQLESKTYAKFLHDVCFKPMNDACVVQSVTGYWFAKGGVI
NPKTWKDDLRQCAKSPVDCRPEFGQPIEPSMVFGGYEDDVTEAQAITATW
VVRNAEEGSFAQLAAVDWENALRDRLLEAQKEAQDRGLRLSFNTEISLEE
ELNKSTNTDAKIVVVSYIVMFIYACMALGTPFKHIFRNPALLLVESKVTL
GLLGIIIVLMSITASIGFFSWVGLKATLIIVEVIPFIVLAVGVDNIFLIV
HELERVNMSCPDQMVEERVARALGRMGPSILFSALTETVAFALGAAVGMP
AVRNFAAYAAGAVLVNAVLQMTMFVSFLSLNQMRVEDHRCELWPWWQIKK
ARIHLNGSNGYVGGGRVSEIEEESFLQVFIKNTYAPSLLTKRVKIGVVTV
FLGLFAAALALLPTIEIGLDQRVAIPDGSYLIPYFNDLYDYMETGPPVYF
VTRNVDASHRKEQQEVCSRFTTCHELSLTNTLELERQRPNVSYISSPTAS
WMDDFFLWLNPIYEQCCVENHKTCFAGRNPAWNTSLYGMPEDEEFIRYLH
KFLATPADDDCPLGGQAAYGDAVVISDDDKSVRASHFRTAHTPLRSQADF
INAYSSARRIASDISRRTGTEVFPYSVFYIFFDQYLSIVPLTAGLLSALV
GIIFVVASVLLGSALTSAVLTVTVIMSVVDIMGAMAVFGVSLNAVSLVNL
IICVGISVEFCAHIARAFMFPSRTVMESNNTTLRGRDARSWTALVNVGGS
VFSGITVTKLLGVCVLAFTRSKIFEIYYFRVWLALVVFAALHALVFLPVA
LSVAGGSGYVDPESEGTAAQDLTDRRWRAIRVHDNSDSEDEDY
>Mb|QLI67469.1
MAKRLSRPYENKLAIVTGGSRGIGEAVARRLAAKGANLVLAYTSESSKAR
TEALVAELSTSHGVRCASVQADLSDPSAAAPQIISAARALFAAYSPSGAF
QIDTLINNAGVASNQHLNDAALGPITADEFGRVYAVNVLAPLLLTQAAAP
FLPTDRSGRIVNVSSVSSSIGYQGQSVYAGSKAAVEAMTRTWSRELAERA
TVNAVNPGPAWGDMYAQAGGAFWSINQPYVDAAPLAAYHGDRDVLDAVGE
DAARFDKTVREGMAGRRPGLTGEIAGTIDMLCSEEAGWTTGSVVCANGGM
KMSIA
>Mb|QLI67471.1
MANTTLKLTNRSPKQPIKRLPTTVDLPADATVDDLKAVIAKEAKISDLNR
IGIYDPTTKKTLKNRKARLADEPNVSAANEVLVKDLGPQIGWRTVFVIEY
FGPILFHALVVAARPYIYKNGDSDMSTTQWLAFGMIVAHFIKREIETLFV
HKFSANTMPVSNVFKNSFFYWALSGLLCAVSIYNPNSLAARADKPAFDLV

GLALYLFGETGNALVHLYLSSLRSTGGTERKIPSGYGFAIVTCPNYMYEV
LSWVGIIIASRDWTVALFISIGAAQMFTWAKGKERAYRKEFGDKYKKKRF
VMLPGLL
>Mb|QLI67472.1
MTDYTTGIMPLHPVHKPPYTIEAPDYPKVPGETIPRRHPRAKNGLLSRPA
DDVHNVFDIVRRSARIYPNHQALGHRKLVKLHRETKKIKKNVDGEVREVD
KEWQFFELSGYSFLTYKEYETRILQLGSGLRKLGLTSEDKVHLFAATSLP
WISMSHACASQSISIVTAYDTLGESGVEHSLVQTDATAMFVDPHLLKTAI
NPIKKSSVKTVIINEDCIFATGGEVEDFKKNNPDLKVLTFEELRKLGEEN
MVDPVPPKPQDLYCIMYTSGSTGLPKGACLSHEAVIAAVTGELTCVDECV
SDKEFVLAYLPLAHIFEMVLENLVLFIGGTLGYGNPRTLSDVSVKNCAGD
MREFRPTALVGVPQVWETVKKGVMNKLDSSSPLLRNLFWGAYGYKSFMSR
NKLPLAGIFDGIVFGKVRELTGGRLRFTMNGASGISDNTKQFLSLVLAPM
LAGYGLTETCANGALGCPLEYSPNSIGPISAAIDVKLVSVPELGYFTDDV
KVPQGEILMKGLPILKQYYNNPDETAKAITPDGWFKTGDIGEFDTHGHLR
VIDRVKNLVKMQGGEYIALEKVESIYRGAQTVSNIMIHADAEHSRPIAVI
MPNEKILVDKAKELGVDEHDMHTNPKVRDAVLKDLQTVGRRSGLSSMEIV
SGVVLTDEEWTPPSGLVTATQKLNRRVIRDRFKKEIEECLKNAS
>Mb|QLI67475.1
MASISFRARPLRAVSRSVQAQRWASYDKSALTKDALNHKVESGLGAGNSD
ANLLLNTEEKTISTATGDLPLSPLFDSDWIKARRRSRKHSPSKATGRFRK
KLANNPYAQALATPIRRCPNTAISLPRYFFQDFELVKHPQDDSPWWAPGT
LAFEGLIYEKIPSKDKIQGAYSETAAPQAQELGADTHASTAASSTRPRRS
PVTSYALSRKSVIDRIGGPNRRHAALLLATRTGMATGPNTKNPVWRQDMG
DVLLGMMRRQAVDALVCQSGMKGDGQEKLIQPCTKWDEVKEVQPRGCVLW
LPSEENSSTQYATLDLDGAKYGSKLTVHNLHWLLGENELARLIGASELFH
DNEILVLKQWKRKSVMKLHLLLWRLQGYLAQPPCL
>Mb|QLI67479.1
MVNPHLSLGIAQAVFYLPMLPLTIWLMIRNGKIRPRMAWWPLIPFTLMRL
AGGPVIIAMVANMEQHNGEPDIGLAVAATILLNVGVIPLIVADLGLTRII
LMDNFGDGPFTHRITVGLRLTFVAAIALLGAGGGIGSQPDPDLVHTGHNL
VLAGYIVFAAELVALTLMQVYYHARKSSLRPSGHQVLRGALLASPFIMVR
TVYGLIESAHAQETETLWNPVFGYPAASSTVLFALMALVMEYIALLIYLW
SGFSIAPDRGLGGSRDAGPVPKP
>Mb|QLI67480.1
MAMDKLHDHLSNTSTIKKLVGRLRGNSVSTPSTTQRDDGYTSSSSRAPSF
RIRTPQISRSPDSHCSSSTKKRGKNGHCARDAQLALELWNDAYDALRDDP
SCTGLVIAYENIISQELPDHLKMGGLNSSFHGKPADQRLELLTAITAAGL
EKGRSSKNSQAENSAKVVLDAVRDRVASVVAEYHAASVAWAGFCTLTPLL
LDPIVNQKDFRKGLVHVVGRISWYMHLAQLLRANSWGAEHQFREQQYAVR
ERLVNLYQKVLELEMNCVCATASAWNTAAKNVVGWNTLDKVVKNLLDMDA
QVVEIVEKHCAERTRETLLQKYLDLDMP
>Mb|QLI67483.1
MSGVAAIEEERSQYQEQLDIILQQLRDDPDSAELQALQEELTNVIGLLDE
NIAELRPNQPSQPTPKEPSPPPQPEKWSRENHPAFKKAPPPPEEKEEPPV
TYHVNDTVMAKWVSGDKNFYQARITSITGSSAAPIYTVKFKSYDNTETLR
SKDLKPISNKRKADDPPPQPSAPGVVSSAGATMYPGAQHEAQQDAEAAQA
PKPKKIKAKKELEAGKAKWQAFNAKSKFGKTNKKDSMFRTPEGIHGRVGF
TGSGQAMRKDPTRSRHVYQVNEDLD
>Mb|QLI67486.1
MARSRSTFALGLGLLCWITLLFSPLAFVKPVQADVSEYGTVIGIDLGTTY
SCVGVMQKGHVEIILNDQGNRITPSYVAFTDEERLVGDAAKYQAAGNPER
TIFDVKRLIGRKFSDKNVQADLKHFPYKVVAEDGKPVVQVDVQGTPKTFT
PEEVSAMILGKMKDVAESYLGKKVTHAVVTVPAYFNDAQRQATKDAGTIA
GLNVLRVVNEPTAAAIAYGLDKTEGERHIIVYDLGGGTFDVSLLSIDQGV
FEVLATAGDTHLGGEDFDQRVIDYLAKNFNKKHDVNITKNKKTMGKLKRE
VEKAKRDLSSQMLTRIEIDAFFEGKDFSEPLTRAKFEELNKELFKKTLKP
VEQVLKDAKMKKSEIDDIVLVGGSTRIPKVQALVEEYFGKKASKKVNPDE

AVAFGASVQAGILSGEEGTEELLLMDVNPLTLGIETTGGVMTKLIPRNTA
IPTRKSQIFSTAADNQPVVLIQVYEGERSLTKDNNLLGKFDLTGIPPSPR
GVPQIEVSFELDANGILKVSAHDKGTGKQESITITNDKGRLTQEEIDRMV
AEAEKYAEEDKATRERIEARNSLETYAFSLKNQVNDAEGLGGKIDEEDKE
TIIDAVKEATEWLDEHGADASAEDFEEQKEKLSNVAYPITSKMYGGDAPG
SQEEESDFHDEL
>Mb|QLI67489.1
MSPLALGESIPQDTAHAVSVSLPTWKSNIGYEEGEKWVVEKMATGYPRFF
IHKSIQALASDIVSDYGLSDQQAMLFPTPAAAQRCINFVTNAEPSYLQKI
NLVELALDMSKTLSPLLKKLAPTVSAILLPKAAFPIAKQYWQHTGDGVSS
RRAEFCHSLLRDGLLIPTNSTQISSEASPWAIRGPKRYQRTGNNGNSKDT
GSYNYEESLESSRFLEERFGRNLDLAFVERAKSAIRRRIAGCLVQDMDSL
PVMASNSRGLQNLHEDDVYLFPAGMNAIYNAHRALLYARGQMKSVNFGFP
YVDTLKILQKFGPGCEFFGHASSADLDKFEELLRTGHRFLALFCEFPGNP
LLICPDLQRIRRLADEFDFAIVVDETIGTFANINVLPAADIVVSSLTKIF
SGDSNVMGGSAILNPNSRFYGQIKKAMNYIYEDTYWPEDAIFMERNSRDF
FNRVDRINTNAEAICATLQQHASVKDIFYPKCNPSRHYYDEYRLPNRGYG
GLLSIVFHSRQHAEAFYDSVEIAKGPSLGTNFTLGSPYVLLAHYQELPWA
AQFGVAEDLVRISVGLEDTEMLQNIFNRALNATETMPKTDSDFNT
>Mb|QLI67490.1
MSDAVSLLRRLILESPPGEWALNRLRELLIGALRQGPVPQHVAFEMDGNR
RYAKSHRMETVEGHHRGFEALARIMEICYKCGVKVITVYAFSVENYNRPK
HEVEGLMQLAKVKLEQLTTYGDILDRYGACVRVLGQREMIREDVLKVVDK
AVARTKHNNKTVLNICFPYTSRAEITTAIRNTVQDFLNPPPPKNTPFSPS
RIRQKILSSQLDGKEPLPTILDADDDDDVPEETSMDNDEDAGDSSSTTLP
PDSPSPSLRTRSSSNSHLPNPESITTDTLNDHMYTASDPPLELFVRTSGV
ERLSDFMLWQCHQDTQIVFIDCLWPDFDLQHFIWVMLEWQWRQKQKERDE
TSTLVKAKSRRLVE
>Mb|QLI67491.1
MPPRMSLSSPRATATFIRRLYSTETEPLIRVTNIPSASSGHIRVLELNRP
KARNAISKALLTALRAEVDDVHAQYGPNGEETGVARPGVSANLGPTRALV
LASAVDACFCAGADLKERRGFTQEETAAFLANLRGTFTTLSNLQIPTISA
ISSLALGGGLELALSTHFRVLSSNATIGLPETRLGIIPGAGGTHRLPAII
GLSRARDLILTGRRVAAPEAYFMGIADRLVEVVPQDERDASDILPAARKT
TLAEAVRLAQEICEGGPVAIRAGLQAVAWARQEKENEMYERVVATEDRNE
ALKAFQEKRKPVFKGR
>Mb|QLI67492.1
MVKTRKERAGNAHVKLRQPDRSGPTEKTLLDFAHEQNLFQQVQQREKEMA
KERRKQSIADGDDDDGEVPTLSPGAERALEAALWTATIAMLHFTFDVLVQ
HQYGQEISWAHVCTRTGRAWLVFLFLFYFLHPFESDAVLLPGLPSRYQRP
LRQSIFFIMGVASGCYLIYISNTYGYLATMKQAPPLGCLWLWAVVELDLV
WGCLSLLLAAVFLKQGGYEIK
>Mb|QLI67494.1
MAARFTTARTAPLGARTSAPRTLTNGFQSRPYNSQGYLQNGGSSLPPGAA
PLLPNQGRVIQTGPIRVLCIADVRGNLRSLNELAKQARADHIIHTGDFGF
YDDTSLERIAEKTLKHVAQYSPLIPDPVKKAIQQGGNGPVKTRFPPSDLP
LSELPLLLSGEVKLDVPVYTVWGACEDVRVLEKFRSSEYKVPNLHIIDEA
RSMLLEIGGVKLRLLGLGGAVVMHKLFDNGEGRTTIAGGQGTMWTTLLQM
GELVDTAHRVYDPTETRIFITHASPAREGILNQLSVALKADFSVSAGLHF
RYGSSYNEFSVNPTLDHYRGKLAASKASFNDVWETVKGEVEPAIAQNEAQ
QNLLKNALQIVEKMPTTAAGGNPFGGPVTGQAALGQVDESAFKNMWNFNL
ADAAFGYLVLEIQDGRIGTEMRAQGFNFSHRGAKQQPGAAPPTTAASAPP
PSVSTAAPTVSTPAPPVAAAQPAAPASRQPSSTQQPRSASATTPAPGPAK
PATPQPSQTSNKSGGSKDNEKTSAAPNGSANGPEPTSSPAPKTLASDIIG
LFIMNVSSEDQCRDLFDEADKGKIVKIEKWGNQNKVVQFKTTEDRDAAMA
RLPEEIKTRTQEDRTRPLVKIFQHREGKTFSNRGGAGTWGANARGGATAS
GYRSAGGNSDSESNRRGGRGGRGGRGSDRGRGGRGRGGMKGEGGASSPAA
TPSTPAE

>Mb|QLI67495.1
MFNPRFLVVSLGNPLPKYESLHSAGHFALNGLAKALRHPPFREVKFGRQT
CLVSQGPKYTLVQSPTLMNISGPFVAKVWQEIVRQHDPSSTSLVIVHDEL
EKALGDVRLTPWDRSHRGHNGLKSVKKSLVQDKFPESPFARIAIGIGRPA
ERDAATVSKYVLDKITVEQRRVLEEAVPLDVAKRLVELEDEWRTEMEP
>Mb|QLI67496.1
MSTYNGRHAPNVSQYLRDLNTISPQDTTGPTEDAFNMEDDLALFTNTQFF
DFDSGQNTDFQAQPVKVDVEDATRHGSTTSGDVTPAQTTINDMPNLDFMS
GDFNFTEFNSAYAAAPQMNAFPDSAQGFQPLQPNHQVQIPHQQPQFRQGN
ARAGDKRKPDAMVSAPTPAPQQVNFEDASRVAAEEDKRRRNTAASARFRI
KKKQREQALEKSAKEMTEKVTALENKVAQLETENKWLKNLLVEKNEGSDD
ITALWKEFTKHASEKSKSAESSTTEDIKEER
>Mb|QLI67497.1
MPRKLSKQPSLTSASPTLTASPAPQIPPSLTDPTLPLPKLIVFDLDYTLW
PFWVDTHVTPPLKPNAAHSLATDRHGEEYTFYPDVPQILQLLPRAGSPVK
LGVASRTHAPALARDLLKMLHLPPAQEGDARPRRALDAFDAGLEIYPGSK
IRHIELLQRRAGVDFEDILFFDDESRNQETERLGITMRLVRDGVCWAEIE
KGVDDWRRRRSVRKGSV
>Mb|QLI67498.1
MSFNEFLKRRSLKADDTRRTRAAELTLRQSIYPICLVTILFFLWGFSYGL
LDTLNKHFQVVLGINRSRSAGLQAAYFGAYPLASVGHAAWILRHYSYRAV
FIWGLCLYALGALLAIPALKNHSFGGFCACIFIIGNGLGSLETAANPYIT
VCGPPRYSEIRINISQAFNGVGTVIAPVLGSYVFFNFSDERALDNVQWVY
LAIAIFVLLLATLFFMSDIPEITDADMEFQAKETHAGATEKPFIKQYKLF
HAAFAQFCYTGAQVAVASFFINYATATRPGTSDSLGSKFFAGAQAAFAIG
RFTGVGIMKYVRPRWVFLAFISCCIIFLCPSITQRGNTGISMLYVVLFFE
SICFPTIVALGMRGLGRHTKRGSGYIVGGVVGGAVVPPATGAAGDTFGDG
YAMFVPLIFMVMAWTYAACVNFVPAYRNVVDSLGEAEIGIVHRTEDEENS
KSTSNDEKSREEHAHIGHVTKENTVETDAALASAPKDL
>Mb|QLI67501.1
MPHAMSMPSTGLQALILCGPGSSFPTFTSNPDENPKAILPIANRPMVWYP
IDFCHRMGITNITLICPPSASKAMNSAFSINPFLTGLPLPRPDILAPADL
DYNTGTAEILRLSEVRNLIKSDFVVLPCDLVCELGGDKLLQAWMVKSASL
TDLLGDGRCTMGHQSHHSGGLGVWYNTKSGPVVKGEETDFIATSPLPSGQ
SATQKGSISPHLSKLVYSMPTDSLKDIMEDEKSLPIRHALLRAHPRIRML
TTHRDAHIYIFPRWILDFVKENERLDSIGEDVVGWWAKAGWQVGLADKLH
ITTVCDAAKSEDDNGSTSGSVTSPPPGPEEAEKQSTERLGETKSIGKDGS
LKSQANQHTPPVVAYVHSGEGQEKPAIVRRVDTAQLLLAISLQLAKLESI
EEVGLEAASPFAHSRKVAYPEGVKSRTTITKQDSLIAENVTVEEKTAIKE
SVVGANCQINEGAKLSQCLLMDGVVVGKGCKLTKCILGKRCVIGDYSVLT
DCEVQENLMVEPRTEDKDNKLMSSEGLEATEAEMNEVLQDVDREVAAATD
EAVED
>Mb|QLI67502.1
MSADDRKEETAQNPTEFFESIGNKVAGFSATTNDATQEDEEYRPVEEIES
LCMNCGENGVTRLLLTAIPYFREVVIMSFSCEHCNVQNNEIQAAGTVQPK
GTHYELRLTSLEDFSRQVVKSDTATVKFIEMDLEIPPGRGQLTNVEGLLT
TVMDDLEMNQEARKEQSPEVAEKIAEIITKGRSMLAGDAFPFRFYVDDPA
GNSFIAPDLKDGVGKWEKREFVRTPEQNALLGLGDTTEAPADFTNPGLTA
DGEIIPNEVYSFPASCPGCMHSCTTHMKMVDIPHFKQVVLMSTVCDHCGY
RSNDVKTGGEIPELGEKITLTVQDATDLARDILKSETCGLECPELQLQVN
PGTLGGRFTTVEGLLTQVRNDLHSQIFEVNGPGQGGDSLSSSEKTQWDAF
FAGLDAAIKGEKEFTVILTDPFASSFVQPLVDPPAPDPKIKRENYQRTEE
EEEELGLKDMKVEGYEEPENTGGQDE
>Mb|QLI67503.1
MAIKHNQKIVNNHFRKDWQRRVRTHFDQPGKKASRRVARQAKAAALAPRP
VDKLRPIVRCPTIKYNRKVRAGRGFTLTELKEAGIARPLARTIGISVDHR
RQNLSEESLAVNVARLKAYKERLILLPRKSNAPKKGDTKTDLSKVNTATS
ISSVLPIAPTDIAVKEIKKSEMPKPIDGGAYAALRMARSNKRYQGAREKR

IRDKAEAETAKK
>Mb|QLI67506.1
MLALPAALLGAIAAVSVAATKEHLDGLPAYHYNAPIKVECMNRSSETGEH
IEKNDEIQWIPFPVCNETGKPLEFHYGYEGELNCTLDFISDPFFHLLEFY
IHSDAPMSCRLPARPPAHVEIVGEKPPAQEYVPLVFALAGTLQMSHMHIS
THMNVLLHSMPKHHQRPHDSGVLDSAIAYSTSPLSHMEGTETARIIPGEP
LPLTFSVRWFPTPALPKTEGRVEWRGLGGHIYATTVFYSIVSFGAGVVVA
GMYTLGVVLPRRLKGRSMGGATPLGYGLSSGGVGNGWGYTKKD
>Mb|QLI67507.1
MESSQSADDERYSIDPSEPLKDVIVCCTSIPPEQRTDIAQKVSELGGIHK
YDLTPDVTHLIVGDYDTPKYRHVARERPDIKAMDAMWIEAVTELWKRDEE
IDFIALEREHRLKALEKCGTDTASPHGGAAAGRQSLLICLTGFGDQRDEI
AAKITANGGQYTADLTRRCTHLVVSKPEGKKFTAAKSWGVYTVTLGWLDQ
SIQRGLILEEAKFDPLLPVEEQGVGAWKKEDPRRASLGKRSRSASSNTNG
TADRGARKLRKTASMKLNSQRNNLWGDILGRSGSREYSFGNEQADSDQQP
RQQATPEPTIQQPHQHHQLPSQQNNGVFANCVFAIDGFSEKRQTVLEDTI
ATLGGSVASSLREAAAHRPTEHSHRFLVVPQTSQPESHPNVPYDNIEIVT
EFYIERCLHNKQFFSPGEHVLGRPFPLFPIPSFSGLVVCSAAFTGLELNQ
VARSITQLGAKFEEEFRRTTNVLVCRSLKAMRKEKLKFALEWGVPVVSVD
WLWECISTGFKVPPSLYIFSEIRDRYSAQTAPAPTEIKRQSQPPKPKGND
TTQSPPAARTVTTRPTSSRPKIMGGFDASAFEQDSDEKQSTACQISAPVR
GIPRLESITSADFFTARSQTVDTSAKDHDAPLAEVSCARLNKSPSPTKHT
VPLPRTKSDPWPSGLAEKPNISARRPSAPPPDDPLYRRDGHETTAHQDQE
RASKQQARAAERQALSTKLSSLIDSAAVPSPVDGNCHAQGPPRPRRRQLL
GRAISNASNGSSAASIDGSGPPPRQLTDSLKAAVAAVTDDDDDMDKQDTQ
PPSTQLQYADPEAQQVKAALMNKMMGRDEPTLSTGRASVAVGERSLRKR
>Mb|QLI67508.1
MGADINVDVLVIGMGPTGLGAAKRLNQINGPSWLIVDSSDKAGGLAGTDT
TDEGFLYDVGGHVIFSHYKYFDDCLDEALPKDDDWYTHQRISYVRYKGLW
VPYPFQNNISILPKEDQVKAIDGLIDAAMECRVANTKPKDFDQWILRMQG
EGIADMFMRPYNYKVWAVPTTKMQCEWLGERVAAPDIKTVTKNVILNKVA
GNWGPNATFRFPARDGTGGIWIAVADTLPKEKKRFGKQGEVTRIDAENKV
VHFADGTTIGYGKLVNTMAVDHLAEKMGNQELMSLTKQLFYSTTHVVGVG
IRGERPERIGDKCWLYFPEDNCPFYRATIFSNYSPYNQPQKDAKLPTLYL
ADGTKTSSEAKEGPYWSIMLEVSQSSMKPVDVENLLKDSIQGLINTEMLK
PEDEIVSTYHRAFDHGYPTPSLEREGVLKQVLPKLQDMDILSRGRFGSWR
YEVGNQDHSFMLGVEAVDHIVNGAVELTLNYPDFVNTRRNEERRLAQSFN
FASSALTNGTKVTTNGVTNGTDLPSRERASSKVSVNGGHSRKSSKQVN
>Mb|QLI67509.1
MEIPGYYYDAAKKKYFKVESGSTALSTSYSSDAVKRRKIHDAARQEARLQ
AELVKRHIKRHGLQRNVISGGLLSREVECTRVAEDGRGRRFDEDIGAAAW
AGGLQSKGRVRFMPSFGRGREVNMSCFWVGSTGVVYATLDEESLLGTHTS
TDENDVLTFGTDPVTLERRRTPQFRTEMIRCPQMSSIKYHEPSHRMLLTS
REPDHSCGLYLFSPLVSEEPRTRGQWLLGESDHYQRLAIHGRIRDEWLVH
QSTPAPPSSDLLCVMGTNAGVVLVRSNDSMSRAAPAQPPKGTQLPQEIFD
QDFQFSNHNVLLAGGRQPRLWTTDLRTPVTEWSCSRQPSAIAHLRSVNEH
HVLTAGLQDKMGLYDMRFFKNGPNGTAPLLSFPGYKNAAHFHTGWDVSPE
LGVVAAAQDDGTVKLFSLHTGRVLKSRALGAVSTDTPIRALMFHSMAGDR
FPSLWVGQGQSLRKFSFGARAFNDQA
>Mb|QLI67510.1
MAQFRTQTVPHRQRISTDDNFQHPDSLATIMLPNSPTGVHARQRQHRRQN
STPSALEGVNISPLSNANNRRQAAAHRRGLSLDMRRHQTTPQTARQDFNQ
VRMATNNTGLANTSQHHVLPEAQQQRTQARPGPNQMQYASMVSNSNESFL
ISPHGTPQTQRFAPSSCFDTNAGRFSYPAHVDMMMRKNQENYYNNMAESA
SFDLFSNNSALSTPTFINFPDSPAGQVWPADDASSRRNSRRISDGIMDRV
NKFEGMGIEEAQRPTTPPNQNGTNYYPPTPVETPQGRIFKQEARPGRFSD
DYDESMEETIKPNRNSRSGLRTQSVFAHMRQQAEQNAGSLSPPVTNKISD
AECLSGLQMQNSDYMNMNALRQEFVKIEDQNDAARLELDTTNSLSDLSHH

TSPDTQNHGQFVGAAFEGKLDLQHFARLDSAAQSRKSSPHRRTESVASMA
SAASIADINIDETRTDTGVTLEEISQYIHSPETTDGKWMCLFEDCGKKFG
RKENIKSHVQTHLNDRQYQCPTCKKCFVRQHDLKRHAKIHTGIKPYPCEC
GNSFARHDALTRHRQRGMCIGAFDGAVRKSAKRGRPRKRPDLETRREKSA
RTRKKNMSISSMSSFSGYSDSSAINSPDNEYNMLDDMVDLDIVSARTHGM
PSMSTAPMPTLIPRVMNQDMAQSPSVVSAHSYVSPEAIMDTAPSHPASPA
KSGVSHYNTPPPPELSQSSSPPPCHFFDLAPNTSSGADLAVMSSTNSMMG
SSGMSGTLPMGMGDQDDDLLLQFSQDDSGLVQLDRDSNMLMLSKFDDEFE
AAVSMFTNNDDMFFSNS
>Mb|QLI67511.1
MRLSWGLVLGLALGASAGRIAPRQNDASTTGPSPAASSIGGGSEPTTPTS
TSTSSSSSSSSISTSSSSAGGGDTTVFVTATVTSAGGGSTVSETTTVTAS
SVTTVTITTTDIATTTITMTDGETSTRTVFVTSTQTVNMKRRSVGNVLQF
YGNGPAHVDAQPTAATTATGIPSRDLSPERIGIEKRAVVTSTVTVTVGGG
GSATTVTQTVTKNVRSTTTTVVHTTSLITETFQANAKTTVTVTSTIVTKL
TVVSTGVVESTSTPTSSPNPPNNNDNNGGLSTGAKAGIGVGAGVAGLAII
GALLWFFTRRRRSPKPDPDDLLGPSSEVPVGVGGGGSRPMSEGLASTPGS
VPRRSPVLPNVQPEGYRGTAKGDGRAGYAKPEPYGAAYAPTRSTTNSSYP
PRSGTLSPGDQLPRHPTPDTTTMASVSPLSARPQTAELGNDGAGARWHTS
EAAEMASDNPAAAKWHSDNAHEIDSHAVMSHQSGPVYEMPTEHFK
>Mb|QLI67512.1
MPPNLGVSQMKLSATSKSAFPWTRDSYLPVLTPSIEHKPCQLLATSTHDG
PTSPQCRAYIARLRSPSIATPQLRLYTSTVPPRQQAFHSQLESIPDAHIL
QSFQKAAAPTNPQNLTEKIFQRHSIGLAPGKTVKAGDFISIKPHRCMTHD
NSYPVATKAMNLGLTKIHDGSQVVMALDHDVQNKSESNKRKYALIEEFAR
KHGTFFYPAGFGIGHQVMIDEGHAWPGTLCVASDSHSPIYGGIGCLGTPV
VRTDAAAIWATGSTWLQHPPVAKVTFTGLLPFGVTGKDVILSLCSLFGSD
DVLNHCVEFAGSEQTMASIPIDDRLSISNMCGLFPVDETLISWYRAKATA
AAVCDSPAKERFNHERVKDMAENRLVADPGASYAKEFYINLSTLSPWVSG
PNSPKIANPLKNLEAQNIKIDKAYLVSCTNSRASDIAAAARVFREAAKDG
QPAKIASGVRFYLAAASLSEQQIAEEAGDWKVLLDAGATQLPASCGPCIG
LGTGLLEEGETGISASNRNYPGRMGSKDSKAYLASPEVVAASALKGRIAG
PGWYQQPDGVEKVIIGEGSGDFAADQARSIESAFDKMINEVDSMIAIAEN
GQDETTTTAAKDEALTDILPGFPEKIEGEIVFLDNDNINTDGIYPGKYTY
QDNLPKEKMAAVCMENYDTEFTKLTKPGDVLVTGFNFGCGSSREQAATAI
LARDIPLVIAGSLGNIFSRNGINNALVCLEMPRLVEHLRKSFKDDAERPL
TRRTGWKLLWDVRRSKAVITEKDGTVWEEKVSELPPSVQEIISFKGLENW
VKSKIQA
>Mb|QLI67513.1
MGDIAVESQVNHAPPHKKAAPSTIPTIDNFEGLPTEGGDDYASLKKLQRQ
LEYIQLQEEYIKDEQRSLKRELVRAQEEIKRIQSVPLVIGQFMEAIDQNT
GIVQSSTGSNYVVRILSTLDRELLKPSSSVALHRHSNALVDILPPEADSS
IAMLGADEKPDVTYADVGGLDMQKQEIREAVELPLTHFDLYKQIGIDPPR
GVLLYGPPGTGKTMLVKAVANSTTASFIRVVGSEFVQKYLGEGPRMVRDV
FRMARENSPAIIFIDEIDAIATKRFDAQTGADREVQRILLELLNQMDGFD
QTSNVKVIMATNRADTLDPALLRPGRLDRKIEFPSLRDRRERRLIFTTIA
SKMSLAPEVDLDSLIVRNDPLSGAIIAAIMQEAGLRAVRKNRYNIIQSDL
EDAYSSQVKGTSDDNKFDFYK
>Mb|QLI67514.1
MGSTDFGNFNDFCKDSTLPVCNLFSGPRHDQNGPWGGCQLTGIPLSNNRH
LANLGSIILCGAAIATSLVLLLRSERKRAAVGRREMQVFLAGYIIISICE
IFSVGGFPLNPTARVVFSAIHIGMIIATTWILMLNAVIGYQLIDDGTPLS
LGLLIVSAGVLFIGTGYIALDTGLKWTGYWNSSYDTPNRNIALYVLYQLI
PLIFLVAFFVLEAILVLRVLGEIRPMIYLTAAALLFAIGQVFNYAISRFI
CDGTSGGIDGALFETLFTLLSVVAVWIFWSSITEDDWPMQTGASYD
>Mb|QLI67516.1
MATAFEPSFSASGSRPTMAPVGPSVVDSLPSINFGFDDLRDRMAKFTTKF
DEFIEQGRKRVLEERNKFRINVVELQEDQRMKKRDIEIVQVKTVSYQQTL

EKEDAETREMEAAIASLSAQRDAHLATRDSLKQQIAQTQAEIESRLAAQR
AHAAQQEAQQRFNVPELDFWITNLCLRIEGAGQADRLKFVYTHIDEKNWE
REAWFELVTSSRDYDVKHCRPKLERDDVERVLEKVNESRELVVLLKGMRE
LFVEAMKS
>Mb|QLI67519.1
MASQSLDIESGDRAASKVPILLLKTRSTPSDAYEEILTTSKPKNESLGRK
FDPQFVPVLRHRFDEGGMQRTRSLLLANEIGDRPGCRYGGVIFTSQRAVE
AFTKVVNDERGGHGESPFLQNTPIYSVGPATTRALRAIQVEPPLQIFGEH
TGNGEALAAYIQHHYGQWYRDREVRPPLLFLVGEQRRDVIPRVLMDPSLL
QGERIIVDEEVIYGTGVMKSFSEEFESILNDTRHEPERWVVVFSPTGCDT
MLRGLGLLDESSGKVNLEHRDGRTFIATIGPTTRAHLVNRFGFEPDVCAE
SPSPDGILHGITKFTTSRT
>Mb|QLI67521.1
MLHEILLSLSGHPSPLLHTPPSPEADALAGITPPERQLLSSARHISDLNT
NLIAYSTEISTTHSSTICRAVATAISSVHLAAFQRKVLQVEESILRNDAD
WVGAYNIVPLTAIMGEFQQWTRRLEWLWDTVQFILAKNTDGSSCRGSTLI
DRLRDELQSGYQDVAATAESLVKVAETAWLKQVSAWVLYGRLPSFGADDF
FVQKLHGSPEDYVCVENCLPSFVTGATAASMIFIGKSLNHVRAMNNTSSS
SGGVAHVSAKLQELSTLTLPLENTEFSKAVKAIRTSLSQNTLQKMLPLAR
VSEMIQLLRDFFLLGRGEFAMALTHEADERVRNRWRRAGNLAYEKDDGLK
NITVKDGEVAAVLNRTWAVLVSMQGQHAEEDDQLELARSLIQLHLTKSKP
PAALVTGRGLDVQAANVLAASPFRNMLFSVPAMLSLDLPSPLDMVISPSD
LQLYSSINAYLLSLRRAHMRLTGLWKITSLRRHYPAPRGASDDAVTLRER
WSDRSWLLRSTWTTASATIFFLAETEAYFQTEIVKTLWDDFHAWLIPSQP
QRSGHATPIDHHHSQPPSQHSHDPQTLATAHTLYLRTLTHRLLLTQASFT
DQLYALLQHIDHLVTHIHRLHSLFTSLDLEHDAGVVDAFVDLEREQSQVF
SSLHNIQLKVKQGINDVVSTLRDLESDATFLAEWDGQGLVDDEGEDRGYV
PCRVGGVDRLLMKLDFGSWLGGTAWEG
>Mb|QLI67524.1
MAVDSIPDFLAEQRDKAPEELQPLILDFETFWERKLWHQLTDSLVQFFNH
PKSAPQRLAFHKVFILKFADKINQLKLVDLALKAATQCKDDQERLSFLLS
VVKKVDNDTSQDALVFASVAVSRVKLNLNRLDDARRDLDAAEKILDTFDS
VETVVHAAFYDANASYYQRKMDFANYYRTSLLYLACIDLASLSSEERHRR
AYYLSVAALVSSSIYNFGELLLHPILDSLAESEDDSWLRDLLFAFNRGDL
IAFDALSDNVASNKLLNEHSTHLRQKIYLAALTEAVFRRPPHDRTMTFST
ISHETKVKQKEIEHLVMKALSLGLLRGTIDQVDEVAHITWVQPKVLDMKQ
IGNMRQRLLDWDSNVNQLGNWIETAGKDVWAA
>Mb|QLI67525.1
MALRRPLWTKASWNRPPAVSPSRTESPATCAHGKLLSQAHPRQRQRQRHL
ASHAPTPAAAAPLRHHFADFLPPRGRTELDLVLDAVQTRDPELLYKAFLQ
WTDLLSNPSSPFNDGAVRQAQELPGPSFSEILRSIDPLLATRKHDVTHGL
NLTQGQTQFTDVGKLVDQFGVRNHHRQVLRGMQILMEVRAGSRYGLAPAD
FDVLLRCAGAAVDYQAAKRFWAMMPSQGLQDVRTCKTWTEFIKSRFVVEP
AYYQWDRSRVAVMARDMYSNRSPLPMATLKRLDSVRHCINAFKRRPWNRR
PDELDEDMRRLVRRRIDFRGYKNHWIRGLFYGHEMDEELLCASLVAFARS
SSLHSIKTLIFKNYYGIMIHQDAESNPENPQISGGVDLPPRSPIKPTGNL
LNAIVEAFGSMSHVPLGMKLLDFVSRRYDVPIPRETWSNLLNWTYLCASK
PFKPTRKLLEHSQYAVISGADVREVWNVMTSPPYNVSPSFDDYDIYIKTL
LSQRSFVRVLAIVRNDILPFYEAVVDDYELAVQDEILQNDLGPSPEATHR
RHQAEVYKDYVRHRISSWFDKLLKTASANKGHRDGVVMKTMIPNLLAEFA
DFFPHQVRYRTAQGAVQITRPDAVRRFQWVRCWRSTLPQKKAGFHVRDLD
GSDEPDFAYPEAPVLNVRGWHRRPRRRIAQLGRAPRQEDAESRTWWHRLE
EELIM
>Mb|QLI67528.1
MSSPSSQPENAIPPAPLGQEAQPAVGAVPDAASTVLVPASSSADEVSGQP
PVFSQVIQSSSRTQPIRSRDADQASFKDDIKSRRPSQHGPRQPGPSLLTQ
ALASARGIPQGQKQSSNNNTPSNNIPSTHDNSGSSSASSGRSSRFQLNGH
LPPPDRETESTTSHSHTAGRPTPTKHGEHQASVGRDPSLSSSAMATPVMV

PVASVAIPTRDSTSIPRQFNPSNLSQAREMLMEHRDFLDRARGRASTSLD
LDRSTTDILKSRAFSLSASPEEASTPTRPSYLSNDPLRLPPTKALNNSTL
DVDSRLSHRSSLESKVTVSPEKTGKIWSIGSGGGDEEDGLVEQSVAEAMA
GVEPNARSRKASYSLRFFKEGLPPEDKPRRRDTKTTPRDKLSPTSEEGIQ
DDSTIATVTPPGHRIEEPSTSQSMRSGSSWVSDDYFNLESTDAVAQVEKI
SETLRSRLRPEGHEAKSKPISPPKEVGPTPASQPDVVEGQRRSADDQQEG
KRPSSSGIAGAGSKARDKHNGIGHDADVDADAEDADESGEEKISSAVFLP
HQEIQDTRTAAPDLSGSAQVQRPRSISRSSAHPWLVKADEPEPEPEVHGK
GETIAATDNSRSEHRDSHASPGHELPEKPTEAANVVEPILKSQKAPKPQP
TVTHYDDHVHHHQHHPRQPLEAIELIPYKHQVGGHTTIWRFSRRAVCKQL
NNRENEFYETIERYHRDLLPFLPRYIGVLNVTFQKQPRRKSTVRKDEASL
AGRKKALDDNNDAEKMLNGTDHLATDVPGNELTDPAPTRIVSQSLANTAL
QIPTVTFDDNKHILPRNLLQPTPPPEFFRRRSTSSAKIYQSISKPQTPRP
HLEERPNSWGATTINKRLRNEVFNDAFLKEPVEVQKHRRPHQRAIPRPTM
QRLLRPTTSDPNLAASDKQIELEQRPPIKVQPPSPHIHHNGHTQSDLGAE
TASLPGQSEDVGAPTKDLTGTSAPEPEILKATLTTGRRRRRYSAGGLRRR
PEDVRESRGDLKYFEEADDAEYKTHNERLAAEKSAKDAEANVTGHQAAYD
DAPVHGPALISPLDLTHSAVPSTLPSALPSPTDEFKKIPRPINPKEAKTR
GNRVEYFLLMEDLTAGMKRPCMMDLKMGTRQYGVDATPKKQKSQQEKCRT
TTSAELGVRICGLQVWNARTQSYEFQDKYFGRKVKAGPEFQNALQKFLYN
GVDLHSVLRHIPVILKKLAQLEQIIHGLRGYRFYAASLLMFYDGDTSEEG
GYETMYDSATDVATDTEETTRRRRRNLREIDFKMADFANSLTPMDDIRDK
ACPPRHPNEPDSGFLKGLRSLRQYFLQIQADIRAELDLGPRSGRGKSQWL
SGPMDDLQLDFEDDLDSGSLSL
>Mb|QLI67531.1
MNGHFAAVGDKPAGGSFEHGVQVIDEDKEFNENLNDYLQTTNVADSGFNY
HLISVFGSQSTGKSTLLNSLFGTEFSVMSETERRQTTKGIWMSKNKREES
GGAKMADNILVMDVEGTDGRERGEDQDFERKSALFALATSEVLIVNIWEH
QVGLYQGANMGLLKTVFEVNLQLFLKDKQSTPRSLLFFVIRDHLGTTPLA
NLRTTLIQDLTKIWSTISKPQGLENARIEDYFDFGFAALPHKILQADKFT
SEVDKLGSRFTAGHKGHKGSDHELEGGVFLAEYHRRIPADGFSVYAEGIW
DQIVNNKDLDLPTQQELLAQFRCDEISREVLVAFDLIVAPLEEQQGEATK
LGKILVLPDLGGVGSDARGALLRAFETQASRYHKGVYTRKRLELESKVDA
RLKALYMGQLAAAHKAGVGAFSDAVSNKVKAGQKAGGGYEFAEIVASEKK
KTLDIFKSEAQGLAIEDLPWTNFKPQYQLFEAELDEVSGKLRKEEMRRLA
TRVERWVKSRLGEAIGLEFNKLGSGRAGMGAPETGDKPSEKDLWDRVWNV
FAGIVKEAEGRFADRARSFDASDDEAEVGMWRLRRKSWVGLREKIEEEVM
EGNILLKLRENFEDKFRYDEAGVPRIWRPTDDIEGIYTKARESTLTLIPL
LSRFRLSTTYAPPDLVSFIGGQPAGVEVGDEEDLSPIGGVDEDEGKSLEE
EMTVLSEGKRQDLVVRFKKTADGVYVEAKRSAIGGVAQIPWPFYALLLAL
GWNEIFMVLRNPFLFIFLIMLAGGTYVAYTLNLLGPMMQMSNAAVNQGVE
IAKQQLRDFIANSEAARSALNVPSAPAYSERESISMDTLDGQGRKASPSE
EVDDI
>Mb|QLI67533.1
MAVSQLDRLDLPQTATALGKPVSMPSPNRAERLLVPIELFSDILCPWCWI
EKRSLEAAMKKFESKHPQVEFEVVWRSFCLNPLFKTKCGKIALYDEMAGG
PGKFQPHLDRVSAVGAHYGLKFSVTGDTGPTRASQILIANILRRRGPAAQ
ARVVEALFRSHFLDGKDISDEDVLVSLGSETAGLPADVVRSDLRDDDNGR
FVDDEAEAAVEEKGVEAVPCVTVLGKYKVGGYQEQEVFEKLFERIWAENA
RVLSKPWDND
>Mb|QLI67535.1
MRYYKYRQMADEAAHADSTLPPNRLALCTRIMTGHKLPFHADHIGSLIRP
PALSSLQEQADAGTVTAAQLREAQRDAIADIVDKQRAHGVRALSSGEFDR
KYYFSGFFEKLGGFRQVSPVPWELARPSAPPIAALKKAGRQYMMAAVCED
KIAYDASPYLDDWKLLRGSVPEELRAACKFTMPPPCYFHLRLAPGKSYRA
SVYPDDEAFFADLASAYRREIRTLYDAGLRNLQIDDPTLAYFCSDDMVAS
LRAEGDDPDRLLDLYLRAHNDCIAGRPADMHVGLHVCRGNFSKSVHFSEG
SYEKIAQKFFTTLDYDTFFLEYDDPRSGGFEPLRFLPRGKNVVLGVVTTK

DARLEDAETVKSRVREAAGIIARGQGRSVAEAMADIGISPQCGFASVSVG
ADGMTEQKMFDKLRLVGDIARELWPEASPGVSGSM
>Mb|QLI67536.1
MDGPRDRPSGIPRLSRLPVPRSSVPKTAPLASASTSTTTPVRPKASRDSL
GGGELNPPKLRPVASRDQLRNSVGVVGNRGSPLRSVSSRDQLRSSTLQPI
KTKPQLNQTQRKPRSPSSQQRASSTTPQPLAIDVSSNAANEDEWDHSPTQ
NPHASSPSTLSGNAAELGGEAIFQGDSLDCDQTAFHTFQTPSTRRSKPGP
SLAERTMETLAQIPPSPALSKTSSSFFDQGRPRSRADGGNSRPGSSYNSD
GFGRASSRQGSRPGSSAGQDDSSALFRASVNPFKTTLSAISGTPRRTSAI
SVANTPQTRAASNRALLVSTTKLGNTGISYIQDAVRSTSPEKKMFEKGSI
RSGSKSVASRSTKPRASTAGLFKKPSLPSMPKAAGAPSESWDGAILPSSS
VSGPTDSAPLTNRKSSAALREQIAKAKAAKRAAVRQASENHGSNGLHTTS
STVTNGFDLEHDDPFNLSKGEKPSVKVLQQRVTSARTTGRLNIAALGLKE
IPEEVMKMYDLESISSYGGTWAESVDLTRLVAADNDIERLDDNIFPDTSP
EAFDEQEEGQGSIFGGLETLDMHGNLLADVPVGFRRLANLTSLNLSSNKL
ENSSLDIISQIGSLRDLKLSKNQLSGPLNSALMKLGSLEMLDVHGNSISA
LPPDIENMQHLRILNMNENRLESLPFDSLSKLPLTELSVRKNKLSGVLIQ
QPVESLPCLQTLDASANQLTHLIPVDTVISLPVIHAISLSVNRLQCLPDM
STWTNLLTLAVDENHIANIPDSFIGLQKLRHADFASNDVRIVPPEIARMQ
SLSMIRLTGNPLRDRKLISAATDELKEILAGRLEPPPPYQEPGHPTTITG
LMGSLSEIDDRLKTVGDMDTFPPSNDGDVRSDVEDDFTTPPTSAPHTPNR
SRSQTVVKDVWFVKSGGLLDLSRSDMSSLNPNTCSTVASQNQVRQIQLHH
NPIASIPIALGAFGSTLSFLSLAYAQIAGDSYLTETLDLPALRELSLLSN
QITSLNPVTKFLKAPSLEKMDVTLNRVTSLPTSLKQAFPQLSVLLMASNQ
LSELDPEWIRGLKIVDASSNNIGQLTPRLGLLGGVDGLQRLEVAGNRFRV
PRWTILERGTEATLRWLRERLPTEEMAAWRAANGEDSGEDID
>Mb|QLI67537.1
MDSEHLSNNGEPKPRAMPADLPTSLDDRRHAPMEDYIPETEMYDGWQGQS
QFLTTPALAKPLNFGNLSLNDDADYERDIPKPPKDSDTRLMEMLKAQAAA
HQAGHGLEDEQTILNDDKLSESEKKDMLQKAMIMAASNGDVGKVKRLLGG
NGQPLIDVNAADEDGTPPLIYASCFGHESVVKVLIDAGADVNRQDRNQWS
ALMWAMTNRHKGIAKLLLDNGASSDQKTSSGRTAFDFVPPDSDMSFYLHD
NGYNIGNAGMGDDFYNPGFSQDRFEEEMAENELRRRLMMDSARDLEVDLG
NVGIDDQPEPADEFEEEQQEFDWSRCLHDQMFVFQEHELDRILDIIITKM
TPQRSPTQKPVPANMIFLSARYAHYHASPELLEKLLVTAMDLINDVVERC
QWDMTILAFWISNATLLLHYLKKDAGLVEATAEFQAHLAELINEIFILIV
RDAERRLDKVLDAAMLDHETIPGFEDITFQNEWKLFKRKQTVKEEPLEKR
FRPPSPKQRAKPAPRNVTSLLSSTLFVLDLYDIHSVITAQIVSQLLYWLG
AELFNRIMSNRKYLARTKAMQIRMNVSILEDWARTNNRQAEHYEGGDTRA
SGETVMDAARRFLAPVIQLLQWLQCFSSLDADDLEALVGTLQQLKRLTPQ
QLIHAATHYRPEVGEKGLPKSAMKYLVAIQKEAALKRDRRRSRAASPLPK
SGPDASSSPTTPVKGRSNSGNLLDTPGSVASARNPDANDASDEEEDAPEH
LLMDPALMLPFTLPSVTDMLVSYGAGFGGVNRERERKYIPSVPAEFLEKL
EVSGVRKEPMFEEKDWENEEV
>Mb|QLI67539.1
MTQLPTTFDKPVHIAVIGGTGLGHLEGYEPIAAVNPITPWGPPASPIKIL
SHNGTYIAFLARHGLHHQYAPHEVPNRANIAALRHLGVRCVIAFSAVGSL
QEEIKPMDFVVPDQVIDRTKGIRPFTFFEGGVVGHVGFADPFDAGLAAIV
KRCAESMEGDGVVLHEKGTVIVMEGPQFSTRAESHMYRSWGGSVINMSTL
PEAKLAREAELSYQVIAMATDYDCWHSFEDVNVDMVMKYMHQNGKNAKRL
VGAVLDQLSNIEHSDLVMAKKWEGYSQGGLKFMTKPEGRDPDAAKKIEYL
FPGVWES
>Mb|QLI67540.1
MDHQSSPSHQAPLESDKPYAPHDVLDETAKTAVVGLGGGFFLAAIRNAMS
KRNVGALGVFTRGAPIIGICAAGPAAYAFFSRTMMNLREKEDAWSAAFGG
FMCGGVLGLPSRRMPVVVGLGSAVGAIQGALYFLGGRIDSFKKESDEFER
KERIRRTTRLPVEQTVAEIGEGRGIQPPGYEERRRERIKETYGFEIDPVK
ATVEGSQ

>Mb|QLI67541.1
MPPQGSDLPVKKQSKWSPEEDALIIELRGSGMKWEDVSKRLPGRSAISCR
LHYQNYLERRSEWDEERKNKLARLYERFKSEMWAKVAEELAVPWRAAEAM
HWQLGEADMARRAGVVPFSLAAVNVEGGHRAAPPRANIHFQPPDTVGRHL
TPPSPRAMYARNPPVSAMSTGHIPRGENVGAPVPPPLAGAGPAQGSMTEQ
HGEFYYSPGPVSVGPGLAPIQHQGQPRNPGPLPSLAELTTGVSPYAQQLD
RPRSGRLTPGPKHGMPMQTPPSYHQQLDARVKRRASPETMKREGSHRRRL
G
>Mb|QLI67542.1
MHLSIAIIALAIAAPVVIAEELDANDVPPACAVICRPIVSLTNMCDINPN
EKYDDDHMGKRQLLDARDADDPDDRIEAECICKNKSFNVPTVMALCASCI
AQNGRKTEDINEIMSQCSFSSVSYAPLSTGIVSGIQVQATKPAGGNTPAQ
TTSAPAATKTSGAGGVGKLANSAMAVVGVVVAVVAAGLL
>Mb|QLI67543.1
MGIWDALSEIVEAVTPWSVVEAEAPAEEPKEEQAAEDESKDEETSEEATE
EATEAAEDAEEGEEEEEEEEEEEEEEDEEEIVDPKETLEEECKNSAQCAPA
KHHFDECVERVQQQESEGEAKEDCVEEFFHLAHCATACAAPKLWSKLR
>Mb|QLI67544.1
MRSQPQESTGGAVPPQKTNSSDFEILSSALPFGQPVPLDAISGPTYVTAQ
LLVQQIAYKLSDKIFSYSPETFDLDVAAKNWAAKQNTNIHGYAPQVLPLQ
TRTGAGALALGYIFSPDFDVSRRHIPQTLLAPSGSLQQLRGTLDQLSLLY
SVSSPFVAHVAAADYTDADGLVSNYDTALRLAEDLGLGLVSSSSTYEAQH
MSLFSTLLATVLPTLHVYDGIRVARETLRVVDALSESGVADVYNKLVSEA
GKLNSHLDTAGKVVELLKLFNDELGTVYQPFEYHGHETPDVVLVAFGSVE
TQVAKQTLNKIAADGAKVGVINVRVYRPFIEEEFLKSIPASARTIAVLGQ
VRDHVAVADDATQSALYGDVLAAVTFSAKFSEEPRVLDIKYTPAQSLTPQ
GLVNTLNKVFGNEDEAKALPSLVNAEQYTFWDVDNSAAVNSPAVIGHILS
KQSTTNVYVHATYDNLTQGGVVRTDVRASRKALEAPYDVYEADVAVIGDD
CVLKDVNVVESVAHGGKIILKLPNFKVEEVEKRLSAAFRKAVQEKDAQLF
VLDTSFSPAFEKDAQSKLLLELAFLKVAQPELSPEIISKLVLVEGHPPTL
EECAEAVNQCLSKLEVPATWAEVADNFEAPRLPISLQSNSFLPFRKEDVE
EALELRDWQTAAKGLVFKEAYETRTGLRPDLPVKTATIRVKENRRLTPSD
YDRNIFHIEFDLGDSGLTYKIGEALGIHAENDEEQVSAFIQAYGLNPAEL
ARVPAREDPEALEIRTVHQALIQNVDILGKPPKRFYEALAEFATDETERK
KLEALGSSAGAEDFKRRSEVDMLTYVDVLDEFKSARPSFNDLARMVSPLK
RREYSIASAQAVTPNSVALMIVVVDWVDTKGRTRYGHATRYLSRLQPGAV
VTASVKPSVMKLPTRDTAPLIMAGLGTGLAPFRAFVQYRAMQKAQGKEIG
AILLYLGSRHQREEYLYGEEWEAYLAAGVVTLVGAAFSRDQPQKIYIQDR
MRQTLGDIAKAYVQDQGSFYLCGPTWPVPDVTKVLEEAIAADAKASGKKV
DPKKEIEKLKEEGRYVLEVY
>Mb|QLI67546.1
MAICKFYQQGYCKFGSTCRFEHPDAGNNRNQSQNRFGTLASGAGGGSRGA
LQDALDKYSIVTDTMVKDLTSEAPQWILSAYAPGRNAPEQLFGGYPREQS
FEEMRLHFMMGKAAGNEQQALNQAQELYQNAQQQMQNAVRNIDEAARFVV
EAESKHPNRLDVCREGSQGAPFGEFQVGKRPSGIAQPAQANPFSTDSTTT
ASPFGQAAAQPTPSAFGQPAALGQRPNPFGTPAFGQPAQPSSAFGQPSQP
SSAFGQQSSQPAAPAFGQPSQPTSAFGQASTLGAKPSPFGTPSFGQSAQP
TNSQPGGFGQPSQLGAKPNPFASGGAANQGSSPFGSLGTANNAQAPVAVN
PFASSNADQINATPNPFASNNSNQNNTGQPSPFGQQQPTQNQSPFGQASR
PNPFASTTTTANQQVNNPFAQSTQNQTNGVFGKPSPAPFGQGASQQPSSF
GSASQPQQAVASATGGNNPYPPESTKQHPSLESYASRNMDGTLAAFKGKP
VTYKDGKPGIRAFDGTWTRIWFPDGAPGYYKDTELSVEAFDEQSLAQWAA
FAQTGQFAEGIMPELPPPRECTRWDF
>Mb|QLI67547.1
MGESAATTFASIFVLGVGFAGAGYLYHKSYKMLVLKKMTRAFEPGDPVLE
LAAVGKDVPHSHPPGEEHWVRRPEQEHIDRIITGQEVGHYYFILGEKGSG
KSSMLIEAMRKIDGDGVAMFEAHADPEIVRIRLGKALDYEFHEDYIGGYF
SEKGPRDMGALLDIERAMNKLEKVAMKLFRIRKKPLVVIVNQMHLLRNDD

VGRNLIELLQQRAEQWAAAGLVTMVFNSDDYWVYERLKLLSTRMEVLPVT
DLPKKQAIQALQRYRIRYFGEKMTQEQLDDVYDRVGGRLSFLNRVAKSHD
MLATCEGIKDIEKKWFLNQCWIMGTEMDDDVMDQQKWAAAAMVLAHALVE
KEESMTSTYDPVVGHILPTYPFHIAQEIMTRCDFIRDLDSLNLFTITNQA
DVRASSVPMHRAFREICSQPGFKEHLDNTIERIAAIESLGRTRELVAKDL
VLGGQYDIQQERNKVTVRLKMPEEEEKDN
>Mb|QLI67549.1
MAILRGPVEPRRMGLMLTKRLARTMVLVLALLMTVGIMLTRHMYDRAESV
LPFHRQHTGPAAYSFKSASRFSPVTFDDPAGMSRHDLCASFPRYLLNHVQ
PVLKIGHSENKERLDASFSSVSSCFNTDDLLVISDLDEVVHGHQAIDVLV
DLPADYYNLDSNPSFRNYTAQKEMRNNGTLNEETQTAIDGWILDKYKFLP
MVERAWLAKPDKAFYFFYEADTYIFWDNVFRFLQSFDPDTPIYMGSPSPG
RHDDEQDVETWFANGGPGLVLSRGAIKALLHRETDANGHYVDSHVTEKWQ
DLVAGECCGDSVLGWALWNVTIQLQGYWPMFNPHPLHGIPFSDSYWCQPV
MTLHKTTPKDMIDLWHWEFGQRQLHRPMLYSDLWRLRHPGEPSTLENWDN
GDWDSWQPPGEASVSGSGACEQYCRSQDDCLQWSWRGGDEKKCILMRSVR
YGAPRNPEYFLDDGKRETLPEYELDTQAQGKWVQYQSGWVEDRIKSWRNE
RTCEQVHWVGPSVSRVF
>Mb|QLI67551.1
MASQDIFAKPVDTKAPPSSIPVRNDHPVRRKGIQSQAPLQTNKFYSNFFL
GDQRCPSFTFPYSVAWAGGKGVSASWGMSCSHIEENQRVFGEVKFDGASS
FYLNPIGIQSMVISARELGEETTLSMDSVTAFSARVHLSKNETAQPAISF
PLVQGMAYITARFDGSVPLIQSGVFFKAVSRVTTNPKEHVTKYNFHLEDG
TTWRVYAWRTKGEELDLKVINNNRAESKSPFYGILQVCKDPGSKGSEDLF
DDGAGVYPVTLELSGSVFGQEGSYSFKFQKNGHQLGHLYMFALPHHLSSF
NGETMKRVQEVRLQSTTKGVATLVKGTEWAMVERHMPTEMDFAPWHPENG
SMKELSSEAKGIIRAAAAKELSQNIAAQTNLDSMYFSGKALVKFATILYV
VHELLGDEALAKAGLGQLKAAFGTFAANKQKYPLVHETAWGGIVSSASYK
TGDDAVDFGNTYYNDHHFHYGYHILAAATIGYLDPEWVNANRDYVNLLVR
DVANPSEDDKFFPLWRSFDWYHGHSWAHGLYAAMDGKDQESSSEDVMCAY
ALKMWGRVSKNSDLEMRGNLQLSIISRSLQSYYLYKKDNTVQPQQFIGNK
VAGILFENKIDHTTYFDPNIEAIQGIHMIPILPATPFVRDQGFVREEWEA
FFDKGRVDSIRNAWKGIIYGNYATIEPKKAWEFFTSRDFDPQWLDGGASM
TWFMAYSAGLS
>Mb|QLI67554.1
MPYRLDTHVLTVDANVIHKVDTANPANLYSMWTVFSRCADSVEQGRRLEN
LSWRLWQREQLVENANKNTASSAAFTTTTIPPKNVPSETRLQEVPQLSGS
VESLADDETVDFTSVSAPLEIRPRIRRLDSSTTRRDRRISSDDFEKMIVS
IVKDKAPLSAPSQTSPLMASTKQKLLVPPAPPALTRSGSTTTESQSSEQI
SEVSHPSPFPPPEGLRVQALPFPEPAAAASSQDVLPEPSSSPAPKPVQPK
KQPARFALGGSCSSSEHSQSIENIKATSAAVGKKPMFQIGGSSGEDSLRS
SGHSALPNTRISNLTAQALQHSESAVDSDTEAEYVDESAIDDDDSSDWED
STEDSGKSSVDDKFFQRVESKANLASRPSLITLMLAQNERARTLGNQSSQ
STPAIHRTRATPNGPSLGVSPNDSDEAPLMMKGMRHSALKPISEIPRSSA
QPITTHATHVNFPAAFSPRTTRRNMLATELTESLRLNLVWERQQKKLGSK
ALLKRRHTSQDVANLKQYPQKACMKASEDAEAHSWNQYFSKEALNGYHSK
GW
>Mb|QLI67556.1
MPMQRTFTIIPNAPAPQPAQHVRPMTTKQVRKAYKASTKTPPMSRAERLK
QDRAEQERIRKEFEREKAAAKAKIAREKKREKELAEREHKKKLGMPLVDV
RPSQSTIARFVRGNGSGKKRDSGGGTLEEAGKGGDDVEDKETGQEQAHRL
NHDRPELDLIPEEDDSELAEILDTMTNSTSKGTAQKGPDMEENAEPPENH
ALEQDNPPTTPPPHKQPSRKPKTPPGPLGEMKPPPRPYQAKPQRPVHSPP
PVIQQPPMSTQAILGNLDDFFPSSSQQALELQDETLDAMPGDWLSDMETP
APQQQDQAPPPPKRFFTPSGSDELVSLAIQRSKRTAAVEQLKDQEIAQAH
HTKEMATCKRPGYSTTNTTLPQKRVDSITKSSPSFGEDKENICICDMSGS
QETEYGGDWMDDLALDFII
>Mb|QLI67557.1

MSKVMRSVKNVTKGYSSTQVKVREATSNDPWGPTGTQMSEIAQLTFNTST
EFYDIMDMLDRRLNDRGKNWRHVLKALKVLDYCLHEGSELVVTWARQNDY
LVRTLREFQYIDEEGRDVGQNVRVAAKDLSSLLADDARLRDERSNRRNWK
SRVTGVEEYEPQHAEPSASRRPRDRRVLANDENDAEYRLAIEASKVQEEE
DRKKRESKRTDDDDNDLAKAIKLSQEEEERRRRELEDSNANSLFDDYPPQ
PSTSNQPQYTGFNQGYQQGNQVDFFANPVDQNAVMNQPTGFMNNAYTGYQ
QSQPTGFQPNYNQAFGMQPTGMGLYPYGQQQNNMTGFQPQATGFNPYAQQ
MPQLSQQSSESALQPGSNNPWATNKSQQQPSLMPTPTGSNNPFAQNNRPL
TSNPISTLGALPEQKTLSTFNNQLMMQHQQPQQQPQHTLNPPPQKEMNDY
QSKLNNLLATGDGMDTYGNTGELRIPAQHTAPGMFANSAGSGVQRLGANA
TGNPYMRQQFTGMPSVTYGSQPTNGQGSNNPFGAGPQQQQGQGHDLIQF
>Mb|QLI67558.1
MTDAHESCASGSESGWVTITVSHRKHNYTFELAEDATVTDLFDDIAATLD
IPVAHQKILVPKGPLLKSPFKQPDTPLASLQGKTLTLLGCGAAEAQAVQD
MSAKVAQRNAARAAQKAKMKHHAQRRSRPRSPDDARYTFLQVRPLQGLPH
PETSQKLLLRLKEDPGVRAAMRKHRFTVSLLTEMEPLAHTQATHEGTSRT
LGLNRNQGEVIELRLRTDAHDGYRDYKTIRKTLCHELAHNVHGPHDRQFW
DLCRQIEREVDAADWKSGGHTVGETSRYAVAGLADGEEDEHEDDGGWTGG
EFVLGGAGNIGAGMSRREILAQAALERQRKEDEAERKACDAAEKGWRPCQ
RSQQPSDKRE
>Mb|QLI67559.1
MTTQFLPQGSESSYDYIIVGGGTAGCVIASRLSSYLPERKVLLIEAGPSD
FNLNNVLDLRQWLSLLGGDLDYDYGTVEQPMGNSHIRHSRAKVLGGCSSH
NTLISFRPFRHDMDRWVEQGCKGWTFENMMRHADNLRNTFQPVHARHRNQ
LCKDWVKACSDAYDIPIIPDFNEEIRTKGQLTQGAGFFSISYNPDNGYRS
SASVAYIHPILRGEERRPNLTVLTKAWVNRIVVENDTATGVSIISNGQKL
LLKPRKEIILSAGAVDTPRLMLHSGLGPRAQLESLGLDVVKDIPGVGENL
IDHPETIIMWELKKPVPPNQTTMDSDAGVFLRREATNAAGNDGDAADIMM
HCYQIPFCLNTERLGYPIIKDGYAFCMTPNIPRARSRGRVFLTSADPAVK
PALDFRYFTDPEGYDAATLVAGIKAARKIAQQSPFKEWLKEEVAPGPKVQ
TDEEISEYARRVAHTVYHPAGTTKMGDITKDDLAVVDPELKVRGIKNLRI
ADAGIFPEMPTINPMITVLSIGERAAELIASEGGWKPGNKPARL
>Mb|QLI67560.1
MPRDRDLDNASSGGQSKRPPLNHRDSDSHSDTTNTSGAPSHPHRTKHHQK
QHHVGHAGRLHARVPSSKAVHKQHGNAHPHPQPHAHPSKLNRRPASRPTS
PTEFDQVALRPPPHRRATSEVKLSRQSFPANLPKSLSHTSLKRNRSHVEV
GKRTRSSDKLKRSSSGTGIQRQPAKPSKIQVHFDLGSDDPEDEWVDASGS
NSPYLSRKGSLNSSAQSSLRHGPSTENSRPVTPSTTSKSREPDRPSPDRE
TSHHKEYLTSRLLQRTPSHGAPPQMTVDLANAAPAQLSRSVSSTGRNVTP
SAGSNQDELVSRFVDAASSGLASQGSFYHPAHATGRGPNDVPRRPDSMTH
IDLAPKNDDAEAVPVPSAEIDNSALVPKAGRRTVGPAAETSRTQQKLNLQ
RASSVLEPGQALTGAVGVVGASPLIGVGGPGYDGGNSRDPRIGKLLERTG
MEYLVVRRYQNPVARSLNRLSRLPGMEKKRRIPRTAAGSLNGKRTVDVNA
RHTRNVSMPDPRQTALKSGASIRTNGAGSSFDGDDVAKLNERLSGASLVA
AEEEDGTNALLRNLWDKSMDLSASTD
>Mb|QLI67562.1
MIALKTQTDGPPRTRKQAVGEAPSSRVVSKPVPTSQADDPRGYQVEQLRR
RFSPKESTAADGTTSLVFKLKPSDPDFPFELAHLECHVRVPANYPDERPE
LRVKNSNIPRGFGINIERGWDRLAMERKGATILSIVNALDKNLEKFLSEE
KVETVKLVAFKDTRHLDDAVSVPTPELSGAPTEPVKSKPAARPYIPEESY
SKEQIAEAKARRAQEVRQLEARMGRLPRFRRSADGIVFTLPIEPKRRNEL
PPGLHSVNSLHLIVPLLYPLQDLRIQLNEADGVDAEPVEALFAVKAAQQK
SMSLMSHMNYLVQNIHALAKQTQSMAQKAAAKKQEDTDKANTVDTFVAGP
SGVSAEERSHVKVIPRPPEWDFVEDDEDSDESYSSASDGEGGVVLLHKAD
AESGAEPEPAPSQVTEVQERGTMMSFPSIELHGIEVIQVSILSISVKCER
CKTVNDMTGLKPGTEKMASCKKCATQLTAKFSQTLVHENSSRAGFIDLSG
CKVADMLPSTFIPTCARCSTASQGLVSVRGETMTNICRECHGKFTFKIPE
VKFLFVSPGSLPPATVGPRRRTEKLGLHAGEALPDRGTCAHYRKSFRWFR

FSCCQRVHACDKCHDGAEDHVNEWANRMICGWCSREQNYAVEACGFCGRS
VIGKKGRGFWEGGKGTRDQRTMSRKDPRKYKRLGDVNKKEG
>Mb|QLI67564.1
MSPHSEEVVLTSNGAADVALHGNALTPTSNGSNRAAEAPAPTLAPDAPTT
SDAPQPLTSASTATLPTNKPNAEGTGASSNRASTNGNGVATHHAKANSNG
QAVQEPLGSNGREFLNGAAQSSPYLVPTALDSEFDLICVGFGPASLSVAI
AMHDAIAAGRKLLPDGSYPKVLFIEKQNQFAWHSGMLLPGAKMQISFIKD
LATLRDPRSEFTFLNYLHRQDRLVDFTNLSTFLPARVEYEDYLRWCSSFF
TRLARFGQQVVSVSPNDNSKGPVRSFTVQSRDTATGQAHTYRGRHVLVAA
GGQPSMPKNFPLKHPRVVHSSQYANVIGELIPKTATSCRVVVVGAGQSAA
EIFNNVASRFPNSKTYLVMRPEFLRPSDDSPFVNTVFNPEFVGSLYTKSA
KYRNNFLAEARATNYGVVRLELIEALYERMYDQRRELGADEKAWPHRIMG
GKQIISIEPHGETLELKVQSVQDGALDGFIDDADVEVIEADLVIAATGYQ
RNAHVGMLKDAWNMLPKAAPGGLQFSKGISGWNVETEQGERKIAVGRDYK
VKFAPGTVADEAGVWLQGCCEGTHGLSDTLLSVLATRSGEIVDSIFGSQ
>Mb|QLI67565.1
MEQHVGRVEEASSIPINAASSTEAGLHSRSGSVPHINLNSNIEAKIKNPL
IGIPRRVLLGNVDEFCREKDLGEYRDLIRRGALVAQDPTGYEDITGSQAL
SQEEIESLRAEVLHKWRIPKVLYLTVITCSIGAAVQGWDQTGSNGANLIF
PHVFGIESPSVHDKLIVGLVNAAPYIGTALAGCWLSDPINSYIGRRGTIF
VAANFCLWPVLASAFCNTWQQLFGCRLLLGVGMGTKASTVPIYAAENSPA
PIRGALVMSWQLWTAFGIFLGTCANLAVRGVTADAWRFQLGSAFIPAVPL
AILIYFCPESPRWYIKKGRYRDAMRSLMRLRNTPIQAARDLYYIHVQLQV
EKEFMGHGDYLNRFIDLFRIPRIRRATLASFVVMIAQQMCGINIIAFYSA
DVFRSAGADQFQSLLATWGFGLVNFLFAFPAIWTIDTFGRRSLLLFTFPQ
MAWTLLAAGLCNLIPGQGGLHMGLVALFVYLFAVFYSPGEGPVPFTYSAE
VFPLSHREVGMAWAVATNLFWAAVLSISFPLMLDRMGVFGAFGFYAFLNM
AALVLIFLFVPETKQRTLEELDYIFAVPSRVFMRYQTTKTLPFWFKRWVL
LRKNETLKPLYTLDVAQHQEEEDEEEEYDGSDAAFETDKVRVEMKDIAGY
TTHTSATTDSIRRSNSS
>Mb|QLI67571.1
MADDRIGAPALNTNIESGQFDEKSQQHRQQAPAEQVPRKVAAAEDDDEDE
DIDALIEDLESQDGHEGFDEEEEEGSPGGGRVVPEEMLQTDSRVGLTESE
VVTRRRKYGLNQMKEEKENLILKFFSYFVGPIQFVMEAAAVLAAGLEDWV
DFGVICALLLLNAGVGFIQEYQAGSIVDELKKTLALKAVVLRDGTLKEIE
APEVVPGDILQVEEGTIIPADGRIVTEDAFLQVDQSAITGESLAVDKHKG
DNCYASSAVKRGEAFLVVSATGDNTFVGRAAALVSQSAGGTGHFTEVLNG
IGTILLILVIVTLLVVWISSFYRSNPIVEILRFTLAITIVGVPVGLPAVV
TTTMAVGAAYLAKKQAIVQKLSAIESLAGVEILCSDKTGTLTKNKLSLSE
PFTVPGVDPDDLMLTACLAASRKKKGIDAIDKAFLKALKFYPRAKSVLSK
YKVIDFHPFDPVSKKVQALVESPQGERITCVKGAPLFVLKTVEEDHPIPD
HIDKAYKNCVAEFATRGFRSLGVARKRGEGAWEILGIMPCSDPPRHDTAR
TINEAKVLGLSIKMLTGDAVGIARETSRQLGLGTNVYNAERLGLGGGGDM
PGSEVYDFVEAADGFAEVFPQHKYSVVEILQQRGYLVAMTGDGVNDAPSL
KKADTGIAVEGASDAARSAADIVFLAPGLGAIIDALKTSRQIFHRMYAYV
VYRIALSLHMEIFLGLWIAILNQSLNIELVVFIAIFADIATLAIAYDNAP
YSQTPVKWNLPKLWGMSVLLGVVLAIGTWIALTTMFAGTENGGIVQNFGK
LDEVLFLEISLTENWLIFITRANGPFWSSIPSWQLTGAILVVDILATFFC
LFGWFVGGQTSIVAVVRIWIFSFGIFAVMGGLYYFMQGSAGFDNLMHGKS
PKKDQKQRSLEDFVVSLQRVSTQHEKSS
>Mb|QLI67574.1
MAMRLPVRQLALTTRTWAAAASPSTRRFTSTASNLSDAAKKTINRDAELP
NPDPGADSPSAALVSEHAPYMVATYARPPPVFVQGEGSWLWDVENRKYLD
FTAGIAVTSLGHSDAEFTRLLTQQAKTLIHASNLYYNPWTGALSKLLVEK
TLSSGGMHNASRVFVCNSGSEANEAAIKFARKTGKVVDPSGGKVEVVSFG
NAFHGRTMGALSATHNPKYQKPFAPMVPGFVQGVYNDVAGIKDLVTERTC
GVIVEPIQGEGGVMAASEEFLVALAKRCREVGAVLIYDEIQCGMGRTGSL
WAHGSLPRDAHPDIITTAKALGNGFPIGAVVVNDFVSDKIKVGDHGTTFG

GNPLACRLAHYLVERLSDPALQRGVLEKSKLFQDGFAKLKERFPELVTEV
RGRGLILGLQLTEDPSAIVKAARERGLLVITAGTNTLRFVPSLVVTDEEI
AQGLQILEDAIAATR
>Mb|QLI67575.1
MVKRKVAALEKLDADFASLQYKIRRDPKSYKDDFLKQWEQYESQREIFLA
NPAGGTADSIESFHNMIDLIAHVADCYPEETRTYPDDLKLILNEHHVVLH
PDLRDKVVGSLVLLRRKDVIDSASLLTTLFPILVSSPSKTLRELLFQKIL
SDMRNSNSKSINHPLNRTVQTVLYNLVTADRSSPRAIWAIKLTRELWKRQ
IWTDARPVDVMKEACLSDSEKVVIGATRFFLGGDKEREELEDDSSDDEID
LSKIRHQIGINKKTKKQKKTYDKAVNKVRRDQQKKAKPHPLNFSALHLLH
DPQGFVEQLFSKHLQNTKSKLSLDSKLLVLQLVTRLVGLHKLTVVALYSW
FIKYLTPRQQSVTSILASLAQGTHNLVPPDVLEPLIQKIANEFVSEAAAA
EVAAAGLNSIREICARQPLAMTDTLLQDLVQYRKSKDKGVMMAAKGLLSL
YREVGAELLQRKDRGKDATIGLRHGLQHQQKFGEEAAGSIEGLDLLAKWK
DDEKKRRRLEKGLPETAGSDEEKDNEEDGLDSDGWEVGSDDSSDSGEWIN
VEHSDDEAPALKKQKQGDGDASDGESSAAAQLERMQLLATTIILTPADLA
KLQELRAAAAIESAASGRRRRNDPDSRRHTDDGLTAENIEAPAKLRKLTK
EEKVALAKEGKPDREEHKSTQAIKKSKKEAAGKSTSNREKARKKNFLMTL
GKAKSKQKRSLVETRKVLRGHVERSTRGGRRRNFA
>Mb|QLI67577.1
MQPSLQPRPKTATSSPHRIPTPSHTLILSAFAVLFLALALLIPHSLTNSA
VPRLRQLSSHLLRRIHLPTATMVTAPSYSHELEVAQLAVQRATILTKRVF
HEKAKGTVDKNDKSPVTIGDFGAQALIIAALKHNFPGDAIVAEEEAAQLK
EDANLRQTIWDLVKDTKLDDEKAEQTLGGAIKSVDSMLELIDLGNSPGGS
KGRIWTIDPIDGTKGFLRGGQYAVCLGLMVDGEVRVGVLGCPNLPVDDSA
RLTTDIGSNATDEGRGVIFSAVEYKGASSRPLTTGSLSPDSRHIGMRPIE
DLAKATFCESVEAGHSSHDDQAIISQKLGITEPSVRMDSQAKYGSIARGA
GDIYLRLPVKASYQEKIWDHAAGDLIVRESGGQVTDIYGKRLDFSVGRTL
ANNKGVVAAPLGVHDRVLRVVQEVLKIGQ
>Mb|QLI67579.1
MYARYVPPSKGAKATAPAEPSAPKSSPAPKPDAIESRLVEEFSYTRYVPG
AKAAAKSSAPSVSAPKSIQYFNDEADSTPKRKREPATEEQQGSESKKIKR
VKDATAPRDDTAAADSQPGAKKKNRRKRKQRTQANDSSDDSDDEPKHTTA
MSSVKIQDNVEEAPPVDIVENGADSRDRESDVEMAEGALEAEEQTKEKKK
KREKKKKQAKGVTEEQDKDDENDMARHKAVLERKTKSIKLATKIAQTKHD
ESDEESVESHGLEPLPQPAPVPEDESKPTYDTLPPWLSNPIRVAQDTKAP
FTDLGILPRPSRILAEKGYNEAFAVQTAAIPLLTPTSKRRPGDLLVSAAT
GSGKTLAYALPIVRDISNGVVTRLRALVVLPTRELVKQAQDVFEVCAKAY
EGEDRKRVRIGVAVGSQSIKSEQEAFIERESRYDPEAYSKIQEEQRQQRA
ITFSADVDDTHDLDSANIDARLGPWKGEVVDFSSKVDVLICTPGRLVEHI
EQTPGFSLDYIRWLVVDEADKLLAQSFQGWLDLVLEKFQVNKFSARDFPD
MAYSGVRKVILSATLTRDLSLLNQLALRRPRLVVLENDGAVQVAEHALPV
GLREFAVRVHEANLKPLYLLDLLRQSHMTGPVGNGDSSSTLKEDEDTSDS
SDTSSDSDSSGSDSDTTSESDTSDDESTPPAPKTQIPISLIFTKSNESAL
RLSRLLTLLDASLTSQISTLTSTTPTHIRRKVLRAFTTPSSPVRLIIASD
LVARGIDIPKLGHVINYDLPASVAGYVHRVGRTARAGRSGCAWTLIGDDE
SGWFWGKIAKNQAIRRTQKVERTRIEEMTEERVQGYEAALAILGKEALEL
RRSK
>Mb|QLI67580.1
MFTLTIKAEQTGVTNLRPDDTQDNPFWYMFKVQCTSCRETHDNYVGVNRF
ETNEMSGSRGEANFVWRCKNCKRESSASIKAAPLAYEQSEPPKAHRIIEF
DCRGLEFTEFKPEGEWLAEGIDSGTKFTAIDLADGEWFDYDEKAGEEVSI
KDIVWEIRRA
>Mb|QLI67582.1
MFGGRKFSFNRHTGAPKLRARRFSNAEPGANEVQSKVHRQFRNAHEGHLP
HAGLDASRASTGVIWCTERAAELGFFEDPDSWANLGQGAPEVEDDIAGCF
KRPETIDISVAAREYGPTAGIRPLREAVAKLYNEMHRKGKDSQYTWENVA
IVPGGRAGLIRIAAVLGNSYLSFFLPDYTAYNEMLSLFKNFAAIPVPLSE

EDGYHIHPDKIAEEIARGTSVILTSNPRNPTGRVVANPELAEIQDICRGR
ATLISDEFYSGYNYTSNCDGTTISAAENVEDVDEDDVLIIDGLTKRFRLP
GWRIAWILGPKEYISAIGSCGSYLDGGACHAFQDAAIPMLDPTLVHNEMV
HLQRHFRDKRDYVVRRLREMGFVIKYVPDSTFYLWLNLDGLPESIADGLN
FFQACLEEKVIVVPGIFFDLNPSRRRDLFDSPCHHFVRFSYGPRMDVLRM
GLDGIERVVSKHKKAAE
>Mb|QLI67583.1
MTADTSVADGLAPEIHGRLLLLSNRLPITIKRSDDGSYTFSMSSGGLVTG
LSGLSKTTSFQWYGWPGLEVPDNEVADMKQRLKDEYGAHPVFIDDELADR
HYNGFSNSILWPLFHYHPGEITFDESAWAAYQEVNHLFAQTVIKDVQDGD
LIWVHDYHLMLLPQMLREEIAKTNKKVKIGFFLHTPFPSSEIYRILPVRE
PLLRGLLDCDLIGFHTYDYARHFLSSCSRILGTHTTPNGVDWNGRFVTIG
AFPIGIDPDKFVEGLKKPSVQERIATLNRKFEGVKLIVGVDRLDYIKGVP
QKLHALEVFLTEHPEWIGKIVLVQVAVPSRQDVEEYQNLRAVVNELVGRI
NGKFGTIEFMPIHFLHQSVSFDELTALYAVSDVCLVSSTRDGMNLVSYEY
IATQRERHGVMILSEFTGAAQSLNGSLIVNPWNTEELANAIHDAVTMSPE
QRAANFKKLERYVFKYTSAWWGTSFVSEMTRLDSNNVQPKTLRNVSGAVV
GPGQDEDEAVSPTQNNP
>Mb|QLI67584.1
MALFGQPTTGASSSPFGQPAQNQQQTQGGGGIFGAAMQNKPTGGGLFGQS
TQQSTTPTTGTTGGIFGAAQQNKPTTGGVFGGGQQQTGITGGGLFGQTPQ
SQTMGTAGGGIFGSQPQAQQQQQQQQQQQKPSLFGGTTGSSLFGGASAAP
QTGAFGGSTLVGSSAQPAAFGSTTTASAMQQPQQNTGLLGGSLWGASTQT
AQPQASQPAGAYFDSLFAKTQKEGGAKTNMEDLPSLELGLGDLRHRLRKL
QSKQNEKPLDGKAHYLLAASGVDPSAAAKDLGLLDVQGGRVERTHGYAPS
EIDVETYLSNLQTKTTLDMISDGLERSIRDFDTFLEDNVAMEWDAQRKRI
YQHFGIRPREDSAAAARESQGGFGRSRRKSQAAGARTGRNSVLGNTMLQR
SVIGTPSRIGTHQSEFSDMDRSADASGSLKSRGTTEDRVLRERQAKLAEK
VRTLNVARVRKHPFPILTELGEVEQKSHEPHASHVVEAYRAMIEIVGEDP
EAETTINSATAKERQFAGMYLDENPNSAASVAMKKRILHGSTAFLEKQFL
REVESLIAKHPHEAKLGGLPDITSKIKAYIRLRSARKDLVPDNTELQQIQ
GEFVWAVVFYLLRSGHVNEAAQYVNDNSNHFMGIDRTFATYLNNYAASED
RRITSRKLLDRCTNEYIQRSRNAPENSIDPFRMACYKVIGRIELSNRNLD
GLNTDINDWIWLQFNLAREGDKTVEMAGESYGLAELQSSIKEIGLKHFPK
STSEDTTNGSFGMFFYLQILAGMFEDAIAYLYPFSYVDAVHFALALVYYG
LLRPSDPMSASNDLRSYSVKNLPQINFGRMIGYYTRDFRAADVVSAVDYL
TLICLNKDLGGEAGERQSNLCHEALRELVLETREFSKLIGDIRPDGRAIR
GIIEERGALIGLTEETDFVNTVTLQAASFADESGRTTDSVLLYHLAGEYD
TVVAIVSRALSEAISLEIGEDPMRLMPVKPRAGGAEADSQQGSSLSLASI
DDPVELARTMMTMYEREVMFYRKIQDQNKTACRVLLEMSSIKKLVEAGQW
AQALDKIRGLEILPLHAAGDASTIRAYASKFSGLSQPVSINVPNLLMWTI
ICCVRQREQLSNGQFSGNEGTRRMMVEQLKQMTLDLTTYTSQLRYRFPPH
LHEALARASAE
>Mb|QLI67585.1
MSAAQLLNPKAESRRRGEALKVNINAGIGLQEVLRSNLGPLGTIKMLVDG
AGQIKLTKDGNVLLREMQIQNPTAVMIARAATAQDDICGDGTTSVVLLVG
ELLKQADRYISEGLHPRVICDGFEIAKAEALKFLDDFKLPKEVDRELLLN
VARTSLATKLNSTLAKKLTPAIVDAVLAIYQEPAKPDLHMVEIMKMQHRT
AADTQLIRGLALDHGARHPDMPKRLENCYILTMNVSLEYEKTEINSGFFY
SSADQRDKLVESERRFIDAKLKKIVDLKKELCGTDGKKNFVIINQKGIDP
LSLDVLAKNGILALRRAKRRNMERLQLICGGVAQNTVDDLSADVLGWAGL
VYEQTLGEEKYTFIEEVKDPKSVTLMIKGPNQHTIAQVTDAVRDGLRSVY
NMIVDKSVVPGAGAFQLACALHLKSDTFSKTVKGKAKSGVQAFADALLII
PKTLAANAGHDVQDALAAMEDEFNDGDVVGLNLESGEPMSPELEGVFDSF
RVLRNCIASSSSIASNLLLCDELLKARQMGRAAGPGPGMDGPDDHM
>Mb|QLI67586.1
MPSATASGAKPGGDSSARSRDHNQGNQGRAYTEDQKAAVIRIRKCKGTAF
YDILALESVKTTCTEADIKKAYRKQSLLTHPDKNGHEHADEAFKMVSRAF

GILGDKEKREKYDRFGTDPDSRFAAAQAQNPFAGFASRQASGGMGRGGAG
MWEEELSPEEMFARFFGGGGGFGGGGPFGAFDTGPQFVFNLGGGPGIRVH
QFGGARPRRRPREAQEAEQQQGGLQNLMTLLPILIFFILPMLSSLFTGGS
SGSSVPMRYDDPQPPYTERRYTPNHKVKYFVNPKDVQSFSKSKLAQLDHT
AEANFIRFLDNKCEHENIAQRRLREDAMGWFYEDVEKMEQANRYPKPNCD
RLRSLGYRRT
>Mb|QLI67588.1
MDQDSAASQAQRKIELQSPQDLAYLVAKVRGAAAARINEAFPHVPGQGED
ELRNQIESLVNEYIDKTFTLAAPNLSINGLPVSSTEYLSPSPATRDTHEP
FDARKRQRVAELISQEEKLLEEVAALKRSVPGKAADEQAARVRDAIRRDE
ETVEARRAAVAVESGKEGGSLRVDRLERQDGVEAGFRGAVEALGRLKRDM
PSAVAKMERARVAGEYVLDAK
>Mb|QLI67589.1
MSDVDQAIADGRVPPDVTRAILLESKDRAAVVGISIVTILTFFVVCGRVV
SRTFIVRRFGYDDGLAVVSLALLIAYVGLCIELICLGSGRHFAYIQYVLP
VDIVMRTQVLDFVAHIIYTVALLVCRISGLAFYYRICSVHHGFLIAIQAV
FAVIVAGFMPQLFLLIFHCRPVTGLWPYGWEPGWERYTCLQWGLVYSVNS
SVSLVCDFLLFGIPVAMLKILEMPRKRKLQLACILLPGLGVVAISITRLV
LVIKGQWEADMSWSYNPMLAVETSEIGATLIALSIPGIKPIFDKYVLNTT
VYTESGNSKYNRSATSKRSRGTALSTFRLRSQHSMLSSQENTAAYGNEVH
AISQPDGHSQTEADGIFVRVDFDVKEGQLETGHPGHPGQQSDGKYVQY
>Mb|QLI67590.1
MFSEYASRFLAQSQSRLSGFAGQGDNDNNPRSNEWPPSRPGRSQRDAGRV
GTTHRSFLSRGYGGNPYQQNGSGSRFGNIGFASRISAAQDAPLFHSTLDE
FREDDDEEEREREAADLFALQRSRRVAAASKLAESTESANSRGSIEDSTG
DVDHPYQDRIFSRGIRSSWNGTRSSHKPPFAGDTLFEENEELSKSGEEQH
QDAGSHPQAKSKIMVDVGLDSQEEGDEDPPESLLGGEAPTDSSPPPFQRF
KSQSEREPFILRRTSTTESDVSSIRRNDPLEYEVAQPGNLLTEGELFKHD
PFFAWIYLISFAGMLSTFVLVWLHTSPQKSPVGDTIYATLQKSFDMLAVD
TVVAIFVSFIWLAALRSFVRPLVSVILIAVPVIMLSFSLYSFISSFQGRT
HGSSFQDSVMRWVSLVPAASCVLWLWLVVKGRRAIQQAIEILQFSSKILA
QNSGLLLIGFGCLALIVLWTWAWLAMFTRVFMGGYFSKSLVRFVIQMSSW
WLGAAFIFMYMWTLSVINAVHRATTAATVSQWYFHRNAGPATPSREIVTA
ALGHATTIIFGSICQSTLLSLLIRAPLLFLPRRIGGAITNLAGFWIPTPI
VALTNPLTITYGAIHSQSLAVSARGLDQMEIVSPTIPTTTLTPRALRLRG
QANGLLPYRLAKLLLVATRLIMATGLGLAGWVITAKQLRIQLPDGMGVRG
SAYAYVVGIMASFIGYSVMGAMEGILSGIVDGVLICYGSEKRMERGHGRY
CLEAAYLFGERRIGEDLGYV
>Mb|QLI67592.1
MMDKETKTAERAKADKADKSDAKDRDKDQGTTTTNSKSPKKRRKERPCTR
CIKRNIGHLCHDEPRDADAKKTKNGKTSAPVEESDTQSQAPSDVGRSSIS
SNMGPPTFDGMRQRSASGFSAGGVLGQGNAMPLVTPNAGSGLQGNGLNNS
GTGNANQFSGISDAWLTAQNFNDMNSYNPNYMIAPHVTHEFNLLNDFLHN
GLLDDNNLTGDETRQLTALGRLGQSDMLSGFGSGAGLSTGGSAPSTNLRG
NLMPPPPSEGKGGRRSASSTADKTREYYLQAADPSGNDTAEDRMARVLRA
KIDAGLLKPFNYINGYARLGKYLDGHIAPSSKQKILRTINQFRPKFREKA
QGLTDMQLIIVEMWFEKQLMDYDRVFASMAVPACCWRRTGEIFRGNKEMA
ELINVPVDQLRDGKIALHEILTEDSMVRYWEEFGTIAFDPAHDTLLTACS
LKNPSDASDHPVVKCCFSFTIRRDDHKLPALIVGNFLPHDPPPN
>Mb|QLI67594.1
MSRSNPPNAGGSRKISFNVSEQYDIQDVVGEGAYGVVCSAIHKPSGQKVA
IKKITPFDHSMFCLRTLREMKLLRYFNHENIISILDIQKPRGYDTFNEVY
LIQELMETDMHRVIRTQDLSDDHCQYFIYQTLRALKAMHSANVLHRDLKP
SNLLLNANCDLKVCDFGLARSAASQEDNSGFMTEYVATRWYRAPEIMLTF
KEYTKAIDVWSVGCILAEMLSGKPLFPGKDYHHQLTLILDVLGTPTMEDY
YGIKSRRAREYIRSLPFKKKVPFRTLFPKTSDLALDLLEKLLAFNPVKRI
TVEEALKHPYLEPYHDPEDEPTAPPTPEEFFDFDKHKDTLSKEQLKQLIY
QEIMR

>Mb|QLI67595.1
MTVPFRVKALYEYSSPHEDDLNFDVGQIITVNEEEDDDWYGGEYVDDHGV
KKEGIFPRNFVEKFEPTAPPRPTRSRPKKEPEAHMSEDVTAPAPVPPAPV
HEPEPEPEKEEELQHGPEPVEAERSVPVVPVPVTAKHSEPALVKSHDATT
APPAGPKQAEASAPKPKSGPPPVSEKPASNSFKDRIAAFNKPAAPPVAPF
KPTGLGASGGTSGFVKKPFVAPPPSRNAYVPPPREKPTTNIYRREEDPEI
KERQAEALGQAEVAGLVPGEQSQEAGAEDDQPKPTSLKERIALLQKQQME
QAQRHADAAAKKEKPKKPVKKKPEAPQSEADVEAEEGSLSASLERVESRE
TEVRTRTSTDDTHARAPLPRRRSSKGPTADESHDGNEADMSGAGDTTEGH
DDTTEREDNEEVLRHVQRVPATSTATAPGLHRETGEANEEGVEAGEEEYE
EEEVDPEVRRKEELRARMAKMSGGMGFHGMFGAPMPPMAKAPPKKKTVK
TGEASGADEDIQETAPRPAPPVPTPMALPGMGSSVAPSRGHPADEEEEDA
DGDEATPVQSRIMPPPVPSREQEVDSGDEEEDEATTPAPISEVSSPRRDI
SGPPPIPGGRPAPPPLPTESRAQPPPPPADIKSPTEGSESDDELSGGARE
KDLEQTSTQNRRLPPMPPAPTSLPPRSPPQSPRGDDTLGQSPTTVAGKRS
SRPPPPIPGSAPAPPPALSRPPPPPPPGGLSRQSTADIPPPTPTRPPQAG
EEEDEEVTEYDGDYDTDIASSVPHKDALKSHARDSSAEDNTLTSPIRDTA
PNIPPPIPSATAPRAVPPPIPSQPPPDHKRRSVDVPRGAPPPPPKEAAPT
AVHDAGDYDPYSYSSPPPPPPMGSSYGHKTPKIDEENVYFSGPSSPTERR
ALPSAQPPRPSNKQSMDIPRTSTSNRRSMDMHRPSVESDFIANDVDLAVQ
SGWWKQANQVPPVFQGRKDIHFESEETTTTNQGSKTVITRDVFVLFQDYS
QTVVTVRYNPYDASDVELEQRHEPPPRSLRQDQMEEFYERYGRQISEAAT
AKKDTVVSDGTPQGFVMELLRPLSGALPPVGTRSYGALVYANMANASTQQ
QDVIRAGDVISFRNAKFQGKHGPMHAKYSTEVGKPDHVAVVAEWDGTKKK
VRAWEQGRENKKVKMESYKLDDLRSGEVKIWRVVPRTWVGWNIQP
>Mb|QLI67596.1
MEVVDLLPAEEQRSVWDETERNFWLKFDHENDANYGKSEQRYKMREAETR
ANLDKLTVKRTQLTEKQDRLLKELTQVEEELARVVEECDDKATKLNILEQ
KYRQSEHDRLEQRNKIAKSMERFFRVKRGEDPDAPSERDKEQWHEVLRNS
TRSGDEGREAARAEALEQANEAARSTQALTPVKSALVPPVTRRSSNVLVD
IIDADGHVIGPIERTDPWNQWVEAIQDLPIKQQVRIRRGRKFTSDHLATI
YDRSEAKGVKWLACMIQATGEIQPRRCHSCDKNQGAFEDCVVLGGPLFQK
CGNCEWNRQGCHMPAPYKSANRGPRHEQMQYHDVGKEPEQLAFRMPPANQ
SRQTLRPYLSREAGIARSSSRLNGSPVLPEPIQMPTPKESREHPFSSLKS
HQALSGFTPANYRGRPPSRDIPTPSVQSVENSPQPTRQHLPSSDEITRSN
LILKHNGTVYTFPECVEGVPLVKIDEDHPYWDPRWPPVREIIQPQLDLWQ
EKYDESVERKAKGEGGSSKFQNGRQVNRGRKILEFLEDGDISPYQLLSKQ
YTHTGKGTITAYDTLFRMCETLGELAKYRLDISPVDWLRHRLHELIVEKG
ANFNYSKIMHDFYHDPKLAALRSKNGFKSIGRPSGYRAGQIGGNDNNSTP
QGGMKKRKSMHSQTDTPRQTPSSSHSPSLAQDDYSHSPLASNAAPYSTNS
SFNAPPIQKRLKPLSPVSTHPNDEFYTDGISDTDSWSGAPIQTLDWRIYQ
VKTRLFTSSTEVTQYWHWKEGRRLFEHQVLKDTDPISWGILRSPIDFDIP
LDDITRVDWNIDALQVHFVMSQFSSRIAKQDGKPTGDVMAAFKRETTMRR
FLNYCRQRRLKLVKVTADEIEQRWGNMQSEKLPDTDEEASPAFRE
>Mb|QLI67597.1
MAPLKRARIAVDSDDMSGDLDSTEGGSSLANDSQTTKRLRMSLEATHDST
PRTTTREDGSSSEEEEDDDDVDNGADIPNSPPQTQYEIMRDAGFKHLQNT
DKDDFQATQKLRRRPTNVVQNMAAESGIIESITCYNFMCHERLHVELGPL
INFIVGENGSGKSAVLTALTLCLGGKASDTNRGGSLKSFVKEGQDHGSLV
VKIKNAGSDAYQPDIYGDSILVERHFSKSGSSGFKIKNEQGRIISTKKQE
VDEISEWYALQMGNPLTVLSQDNARQFLNSATPAQKYKYFVSGVQLEQLD
NDYKMSQDTLDRTIILRDDLSEKIAHVKKEMEDAQRLAETVQRNNTLRER
ARHYRNQLVWSQVVEREQELELQNAELETRKQKIIQLEKNCDELSRALDE
ITEKLERAEATRNGLNEEYRAIGESIASAEGIYTGAKKDLTELHLEERDA
FARLKAVKADIETYETRIREEEAKLVASSGFARVEKEAEHKRAQAREAEL
NDQIEDCTQQLPSLQSKIAEIEHAVKRHQQLKEQKRKDIVAAEQGVRELE
KSTGSIYDGFDRDIIQLVKAVAADSGFGSKPLGPLGAHIKLLKPEWSGIL
EKTLGDALNAFVVRSKRDQSRLSSLIRQSGMKRPPPVYIAYGGRIDTASQ

EPADEYDTILRVLEFDDELVRSQLIINHQIEKVILIKDRVQAEAVMIDSG
PPRNVAACICFHDGKGKRGQGLRITNRAGTIGTAPTIPSGMRPRMQSDAA
RQLAMQKDNLKQLGLELRDLMAEEGQALQALQKAKQELEKHKKNMKALEN
DLRRTQADIEKAAEELDTFEGVDDRLDLLRSELSAKRTEETQLGNQYGSL
KLSKRELGAKAEEAKKKLDAARDEQKDFQNRVSKSDTKIQSYNSMRRITV
SKKNHEFEQVDMGRAALNRAESKRDEKAEQVQDFIRQAEEVAPGRVHIPD
GEDHQSIEKKYEKIGEQLAQREARIGATDQEIYDRANEASIKYESVLRQT
QDVDETIMLMKRAIEHRLHLWRQFQRQISARIRIQFNYLLSERGFRGKID
LDHRGRKVTIHIEPDETKKSSAGRNTKTLSGGEKSFSSICMLLSVWEAIG
SPIRCLDEFDVFMDNVNRAISTNMLVDAARRSVSRQYILITPNAIEGRAR
LDKDVKIIRLTDPRQRTLV
>Mb|QLI67598.1
MTTQLLANELANLIHESKRKHNDLRQAAEKSLEELKQLGSVSEQAAPELL
SQKPNFVNPFIIACGTKNAKFTAIAIVCLQRLIVAKALPKSKLNQVLEAL
MQASSAGLDVQLKILQALPSLLQNYSAELNGNLLVTALNICFILQSSKNA
IVNNTSAATLQQLVVSVFDKVVSEDKNGPDAPVVGEAASADGTVQLRAAA
LDAYRIFNDLCLMTENQRPEFLRFSSLPQTFGLELIEPVITNHASVFTTH
AEQAHILRVRVMPLIVSALKGRPSFATTVRLVRILYTMLRRHIDILPKEC
GDALEILTHLLDQDSALWKRALCMEVFRSIFSDYVLVRKIFSLYDDKKGE
RNIIKTLAATFVRLSTEKPAVIGLGHQSTMPMSDPSTNLAQSTDHAMLEA
GMSGIISSSINSEASNTGISTQWSSIRVPCIDQLDKTDPPSIPESYVYSL
ILSCLSSLSDGLAKFILPLTVPSESRSRRKASKHDSGRDSPASSDLPEKK
LAGLERTASFKKNPVPVNPLQLKDHPMYPDIKICAAIVDECWPAILATFS
TFLYASLDSEYYHSLVRSFQRFAHVSGLLQLTTPRDAFLSTLGKSAVPPN
LLTACMNSGQPKSVAPSTPSDTANTLFSNARGLLSVETLTPASPSAEKQR
NASFDAAAVSLNTRNLLCLRALLNLGIALGPTLGSAWGIIFETLQQADFV
LFATGKAPGRTPSIGRGAEHGGDSDSLMANFNNEIRSVETAASRLIESTV
DFPNAPFVEVVEAVCNLLEQNLPEKPEAGDSASPSPIESSQKQLGHGHRR
VLSFSTTVSASPNQEFLFALAKLGEIANVNLERLLMNNPAASGWNILTNE
IIHALDSPVLGAHVRVKAAETLSRFILEAATATTSIPKDSRGPVQLRLLS
SLRDALTTLNQNARSASVASVATDIEVHRIILEGLKSLIEGCGESLVSGW
DIAFDIIGSGFVTSNAIQERRRGPEAGLLSTRSPKLTRSAFGSLQLICSD
FLSSLPNSCFLILVDTLYKFCSQDDDLNIALTTVTFFWVLSDFLSAKENS
LEIRADLLNGSDESELERKAADHTQKGSDAALWMLLLLRLATVASDERLD
LRNSAIQTLLRIFDANGGRLNPEAWSICIKSVIFKLLCSLEEELDAASND
DVNERDKVDWHGTAVVVLDGISGLLANHIDVLTAHPSFNQLWRELLGHFA
TLLDFKVLEINTAAFKALSHVLSQTGDNKKPIFNKMTVEFAWDLWSRGIP
TSKAVGDKAEDNQNCLVAYVAALRDVYNLIQEDLTVETVRRMLTLLRQTV
EEASVSTHVSDIEHATSLQTQTLAAVQMIRTDVEGVISAVIKQVSEFVTL
AYKRELYVSGSKRTYVAMSKASMKLVETLILNHASEEDIYNSGAFSDALS
ALHKPIALKYDFPISTKSVQPWRLATSSVLSVLEATLPKIRALGIPSNTV
REIWVAVVGVADGILSAECDESADGASFDEDETFDISSFRSLRALIIPSL
GADDIPDKTRKSYAESLFKNSIIHNFTHTEQEFPDKEDKYGLHSLYAPRP
GRTVAIPPTQRRRMAYVAFDELFSLVSAQDPLESTASEPMKQKSRKNKQV
EQEEMVSTLRNRIASIAAPFLILRCALTIRAYVADQPLRGQMPQPLSQRK
ELVWTLTKLVELESRGEAIPSLEKARGERRKHLLRLYPLLVRALGVGGDD
TILRLLGEALEAVGSELGII
>Mb|QLI67599.1
MFQSKKPYSAVTVTIENLTSESYAEEDLSGIPDLVEVIKLQASGPPEAAR
AIRKKLKYGTVHRQIRALVLLDGLIQNAGSQFQRALADEPLLERLRVCGS
SNLSDPAVKKKCRELFQEWTQYANVPGLERVARLYRELPKRKVGMKREES
KVIQETENPFGDDEEEEAERASEQKKQDMPGPSGGYHPATALDMQDGGGG
GSSGLKKSKAKSKSKSKSKSKSGRKFDLEAEMKKMKSVIADASMASTALMNS
LQTINREKERISDNRAATQHFEACKQLRRRILRYIHQVENEQWLGALLHA
NDELVDALMAYEQLDHSMEADSDSDDELAEQTHMYRMITEKAQEGRAAAK
DVPDLSGLTLDTASKHAPARPPRPVAPTRRLSSATSEDAEEEEQDDDDDN
PFGDKNVISTPSAEQGEPRW
>Mb|QLI67601.1

MPICIECRHPVKTLWTKYSGAGDQSSGHNIRLSVCRNCGFFCDKYVEHDF
VVIFIDLVLIKPQVTMALGACSVCRVEEEPHLLHNTLMRNGDRFDVCTTN
CLSHWPCIHETILSPTPCWHSWRANADTEHVLTNAPAFHSEQPSIIRLGI
LLLLFDVYLTWERIEKRAGPAMFPGGGNLGNLAQQPIAVQYLFFLVLCTL
STLAFHLIIRFLTCSSYSPFDILGIMPRYARPNSVSTALLLSSYTKLFPI
LMVIWKYDIPAAARPLGWAVVANNIEALRILLDCKYYTACILALAGATSR
WAAGRFILIAAGLGDVDSISESSVAADGRALWALFLLIRDWAGRLAVG
>Mb|QLI67602.1
MERGTTPPPSTTAPVASPPTPEVTRRIEENRLRAKAIRDQREAEQRAAGK
TSNTPKSAAGFVPTEHVHVSKPVNGKRLYSEISSQASSAMASGTNRDGRN
KGDEEALRPARKFTKFVDYNMSSMTDTKGGFLTTEDDPHNYALGAKKPGQ
EDEQRPKDMTVQEWERMQLIRKLQRQKAGPFEPGISVLDDEEKRKKCREC
KSLEIDFVWEEVFHICVCNKCKEKYPEKYSLLTKTECKEDYLLTDPELRD
PELLPHLSKPNPHKSHWHDMMLFLRFQVEEYAVNTKWGSAEKLDAEYERR
EAQKKARKEAKFKEKLMDLKRKTRTDAFRRQAGTLGKSGANKFGDSIRDT
KHVHQWGRTVENEEGMTIKTCVDCGMEVEELEL
>Mb|QLI67603.1
MVAAPARKTNASKSRSPAKDRRAVSRTQSAEASLVSSSAPSSPAKTETPR
TARYSHHLPTDQQRKGNGRVAGRNLIMWNRPRMAEKLLLHIHYECSRHKL
QLPWDAIAHRFHPGSSGAAIIQHINRLRREVLVEGHLVPPLTQRSTATPN
DPNLRGYVRRGTEGDYVETTRPVYFDERLDDPKFSIPGDFPFNEESMEPE
VGLGTSSAGSEEGDYRQDSPTPMTRNSLKSSNGSMEFYETLAPQFVAPIE
VVRDTRPPHLQQHVCHEAVEMEPTHSRHFSHESDSGTNPFKGTASPNESF
ETDISAVTPGHVAFPSVPHQHTNHGYRMAALQSWSHPFMYQMSLPPKMHT
SAFSFDHQDCLQASFHMGMPFAMTSPYEPYMAVDPGLMKQGTAQPVAVAA
AAAKEGAMQAEPATAHGARIGDPCIGVADADETKVADISTPLT
>Mb|QLI67607.1
MAMLQAPSKYVTLVSGDGFEFVVLREAAMVSPIIKGMLDVRSQFAEAKDA
RCVFQEMSGMVLDKVVEYFHYWYRYRNSDDVPDMDIPVELCLELLAAADY
LGLDQ
>Mb|QLI67608.1
MLRARAWKGRGHPFACTYCRAQLSSATSQTHQALWSAQGPCTTTTATRHN
SNTGRSAPSNGGFPGGFAGSVAGSWGAFAANKPARTLDSKVSSGGSLLPH
ELAARSRMKGPRTKSNTSNTEANRGLDKVGSPLTRSKSRSRTHDARQAQA
TKSKSVLADVFKTMREPKTPPTPKSWSAAGQQGSSGQTTRFAPETPTVTF
SPNPSATTSQIAHRRAPPPEPSMHPAGGAPRQIGWGSFSHRKQQVPTHGH
SHGNDSTTWSEITRSARAKPRSHQRQKIANPSSITATSKGGPEVIAPEQD
FWGELDTRVAGFREVKGKGRRESAEADGLGKRPIDKAPSSDGDGSGHNQS
VASSHQVHDKARERERRKSRFDIEDEPASDDRRTKKSRKSARGRRVELDE
DWDDESSRRWEHKQRRKAEKEAQKERELHEENPAMPIFLPEYISVSNLAQ
ALKQRPDQFLVDMEEMGFENVTPDTIMTGETAALISMEYGFDPTVDTGSQ
RDLKPRPIPDDVSSLPSRPPVVTIMGHVDHGKTTLLDWLRKSSVAAQEHG
GITQHIGAFVVKMSTGKHITFLDTPGHAAFLSMRQRGANVTDVVVLVVAA
DDSVMPQTIEALKHATSAKVPIIVAINKVDKEDARIDQVKADLARHGVEI
EDYGGDVQVVCVSGKTGQGMPDLEESIITLSEILDVRAETDDMAEGWILE
SSVKQNGKAATVLVKRGTLRPGDFIVAGKTWARVRVLRNEAGAELVEAAP
GTPVEVLGWRELPDAGEQVLQAPDEGKAKTAVQYRLEMAEREQSSVQLAE
QEQRQRDKAAAEDKAEEDGDAHAASEPGILYQNFTVRADVVGSVEAVCGS
VLELGNNEVRPKILRSAAGQISEYDIDHAAASKSVIVNFNVPILPHIKQR
AEEAKVRILDHNVIYHVVDDAKAVLSELLPINITYRVSGEADILQVFPIN
LKKRVFKNIAGCRVRNGSIKKSSLVKVLRKGKVIFDGNIDTLKHVKKDVM
EMGKGTECGIGLEGFEDFEVDDQIQTYEEVRERRIL
>Mb|QLI67610.1
MASGTPYTVKWGIMATGGIAETFCKDLLTNPATRSVHDIKHQIVAVSSSS
SADRAANFIKKIDGPSTAKAYGSYAELVADANVDIVYVATPHSHHFQNAM
LALEAGKNVLCEKALTVTASQARKLVAKAKEKKLFFMEAVWTRYFPLSIK
IREMISSGEIGTVYRTLADLSFNENTPQTDGIVSFPDTHRMVNKDLAGGA
LLDLGVYPLTWVFQTLYHVQPSATKEAPRVLAAMNQYRTGADENTSIICH

FPGTRAMGVATTSMLVGTSPHGDATAAPAIRIQGSKGEIQVPHPAYRPLS
YSVIKKDGDGTVEVVECPIPTDPARGNWGHGMFWEADECARCLRDGKQQS
ATMPWDESIVIMQTMDDALRQGGVAYPDLITTDVFDAKSPLNTGNK
>Mb|QLI67612.1
MRYPPHRITLNVVARHIHSAVTSRRPRRSFHLTTPASLPRKRHFFTSNPF
LSNNSADQVKSASQPLRNESPTLSAAAAKRKPTRATTAKNSLRRVAIIAQ
QPKRVGDKSSPDDTETEIETTISATCIAESFNMPMVVEILSSHGFAIDPD
NTGFDASEVVHAKGVNGGDIFVFPSGTIVTWSLPPDVVTKQILRAAEEPH
PAQLCEVEDLDFITDTTRAESTMKGDMVVLGTRREHKDGDTLDTTLAKIA
FSSGLARSTKLAVLESALTAYFESTRNIPAILSQGSGVPLGRKFILQKTG
ELLSLRARLNHYSELTDSLPDIFWDSKSELGLEGYYDQVGRALDVNVRIR
TLNQKMDYAQEIASVLREMSSEQHGTRLELIIILLIAVEVVFELRRIILE
WGQEREAARAAKAANEAVV
>Mb|QLI67613.1
MVFSRSRERTKSVGLTDLADSKPVDDDDAKVEAPIDLQEAPPSAEAAAEA
SSSSSPGPSAGVRATQTPVIEPATESDDEFDLDSGLQGDDTTASLASSIY
TSFAYERGRRYQTFGDGRYPIPNDDLEQNREDMKHAMLMMLTEGKPFFSP
VGTHPQKILDIGTGTGIWAIEVGDRYPSAHVCGIDLTPIQPEWVPANVSF
LVDDCTLDWIERDVDLAHFRFMVMILKDIPTVLGHAYESLRPGGWVELQE
LHGVPLCDDGTMPDEDPVKSLYDTAGAAYKKFGMSTTLPAQLEPYLREAG
FENVHCRVMKVPIGTWAKGKIMRAIGLYQKMAILDFIPTLAGRPFQALQI
SEADAEIRVALARKGLEDTSTHRYFNYYFWYAQKPGSSRREEHDV
>Mb|QLI67614.1
MALRQLTLDELNRAALDQFVYQPVSREMISFLADAAHNVITCDSTMMPSA
SSKHPQQQQHLPPSPPRSPESQPAQPADNTLPTLEEFITQLVVSSNVQVP
TLMSTLVYLTRLKSKLQPMARGLRCTTHRIFLASLILAAKYLNDSSPKNK
HWANYTHINTDMYSFGFNRTEVNLMEKQLLFLLEWELRISEQDLYRELDS
FLEPLRARIAERHIRKMRHREEKRRQQEANARYPTPPASRGQSRCRHASP
ETVRSASGGSITPPGLTYSSSASSYASSITSSRQQSRSATPLESNPSPYM
YEVSQPQGSLYDSPIHVVLEADPAKSVPDLAASGNRMLPYEISVEQYQQL
QDGSAKKRHKRGMWGRLLGGAVAVR
>Mb|QLI67615.1
MAGVAEKARFYLERSVPQLREWEEKEIFSKEEIRTIVQKRNEHEHRVLSP
GNAPSEWSAYVKWEQSLESLRSKRCRRLKIHHLNSAHAGQGRVLSIYERG
VNRHPGSRKLWKGYLSYAASIKAAKRWRKTMAKALRMMPTDAEFWVIAGR
RSARNGDMAAARGFFMRGCRFCTQDCKLWLEYARSEMEWLVKVDQRKSEK
KGVDALRPDRVDDDDELCIADSEDEDEAEDGNMLPEPSRAQAKVIDKQAS
EQLKSNPAMDGAIPMAIYDISRKQAFFTPEVAESFFTMFASFKALSVEPK
ISQHVLDSLSEAYPNHPSTCNCYIRQPILGVQTNTAEFPRGLRDVLARLG
EKLDVTTDKLELSRKTTAWIDGYLARGGLDDDIRQVLEYTKEKLELS
>Mb|QLI67616.1
MAPGAADPEPQWPPRSPHEALLSTPGGRQRYREMMNRTSPSPSPMRSARA
LAAITSSPGAGLGIQSSGGDDEDDDEDEETLQLKLQEIQAKLKLKRLQNA
RAREKAASHRDGEADAQSDPVLPSPNRRARARTPGADENVRPASQNQVTV
PASPVRKLQFLQQQTSPSRVFLGIDKGLKAKDVSLKRAPSQKGNKPGTSS
QTGGYLNRSRSVHSSALSPETARPLSFNERLASARTEEAARAERIDRIQR
ARTNAFSIGQDEMEQFKKSAVEIPDEPLPPPTFTREDILSQHAPAASKLP
RSKTAPSLHQPRPDSHNSNLSSSTLGPSATDESSGTDEASFEVYSCFHLS
KRILPHTVVTRHVSKKKILNIKDLLRDVKSPDYALPDVEQDIVVFAVVAR
KSEPRAHKPTANQKQEDRGKYLVMTLTDLDFELDLFLFNSGFTRFWKLTE
GTVVAILNPNIMPPPPGRQDTGRFSLVINSDEDTILEIGSARDLGFCQSV
KKDGDLCGAWVNKKKTHFCEFHSNEAIRKQRSSRVEVNSSGFGGWEKRSK
FGFGQKKDEKKKGSSNYDWETRTQWFAARSLSAADLIDGKDRGAADRKEK
AESVKRSLEAKEKEREMMKKLGKIGNAAGREYMERAGLKGTAGQSPATTN
QTNGSVPEQEAQSTKRDALSLGLTSKDRTIHLSPIKRKRPDSSQASSVVG
STNSSSTAAAFGWGSNLRNKLSKMKEGEKLHKGGDPPPVRKKTRFVTEKG
IREAGRESLGMDFSERQISLDDDDDELIIV
>Mb|QLI67618.1

MAQPQFGAGTSASSPSVSATPGARQQSHQTGPSSGAGDTPGQLQHQHQQK
HQSSSQAADHQSSAQNPQHKRVYQACIPCRRRKVRCDLGSVDNPHDPPCV
RCRRESKECFFSATRRKRKTDDDEDSDVDEYIIRNGRKRLHAGESPPPRL
DRRFYSEVPLTPGGSHGRSHPLRRPDGSRPRQRRDSELDGDGDANLENLE
AQTAMRRAVYNPHDALDLLYKAATDRSVVESSGKMVNSADWTSPAANNHG
REGSIASATTAPTHQPTPQDTANRDVPRPRSMSAAKPEAELHIPQRQFQD
TAPQQTQQQPADPVLAQQPTLRTQPGYAEALRAWSRFRFVRAGWFTAQEA
IQYIDYYYKYLSPMTPISPPTFSDPASHLTLLTEEPILTVTLLTISSRYR
QMPGTGGHCRSHAIPEQLWTYLRGMIERCLWGQEAFGGGFCGSGGPGSSS
MIMEETETSSTAPWRGMRKGSLRTLGTIESLLILTEWHPRALHFPPQEAT
DELMLPDYNIGPTASSDDSSRNVGAGFGGKRIESWLEPAWRSDRMCWMLL
STAMGLGYELGVFDDIDEMLKDDTITRPEYMDETYRTRANRIKRLLLIYT
SQLAGRLGWTSMTPEHLRKADPAVARRRTATNEGNTPSTSSMVNGFNYVP
DLELDDQIIHCWAGISNAMHVGNEKLFRSRKYTTEIIQSGRYVELLREYS
PLLKDWYREFELFRLPQFIRHILTIEYEYVRIYVNSLSLQAVVERCTNNA
GQNGNGVDGQATNGPTQLSPQTMINYGKLPLGQLGGFTINDQEYVREVVG
GCRNLLRTVVEGLLPGGYLKHAPVRTYFRIISGAMFLLKTFALGAPRSDV
KLSIELMDATVEALRNCVVDDVHLGIRFADLLETLTSRLRNRFIQAPTMQ
QGSGKDPTPLPEGTMPGVAAAGGQGEHTASWVGKHAQKLREGLTGQYRPA
SPSVEGNNISATPFDLSTGNFPYPSASSIGPSTPAAHVENNGAAPSNGVE
MQLFEGWDNPGNEMWYLPPGPAFFQNMGDSSVAMTAEGVNVGGLDLLEYM
AMDPVQFSSIDGPGSTSGNAGTQG
>Mb|QLI67619.1
MKLGNPFVFRPGPVSFWTTIVYLAIIIPLIYVQETVPPAPSEKELPQGVN
LTEAWLDLEVITRSYHPFNSHSNDIVREYLMRRSRDILERNGIDYTTDLT
GGVPWESRYLSSAEHPVQAAEVSARPRGATLFDDRISNVTMTNPQPNNTM
GRYFEGNNFYVYIHGSEDPEGDWWTSDNVQRVARNGAGVLVNCHFDSVST
GYGATDDGMACISLLQLLSHFTSEGHQPKNGIVLLFNNAEEDGLLGAQAF
GYSPLVQFCNTFVNLEGAGAGGRAMLFRTTDLEAAEAYSKSPHPFGSVVA
SNAFERGVIKSGTDYSVFVDNYGQRGLDIAFYSPRSRYHTEEDDARHTSV
DSIWHMLSAALATTESLARTTSTQFNGPRSDGRKDLVQSGRPTAGVWFDW
YGSSWSAFALRGLFAWTLTLLITTPLVLFVVTYLLVRDDKWYFFATKVDS
TVGDGEETVSFGGWKGFVRFPFALVVATALTIGSVFLLAKVNPLIIYSSE
YSVWAMMISLFYFVSWLLLRGAHFVRPSALQRGFTLIWLFIITWVLSVFA
AVAEDRMNMGAVYPLAFLHTFAFAAVLISLLEQYALPAKQDFARQVSGEN
EEEEEQEQEQENLLGDGGNDANQQNNEERGDTDIAATPTETTPLRAGEEG
HGSSEQTTTFANTYRRPVPETRVETRSGNNRKRSFPPYENEQAWSGRLPT
WTWFIQLLLLVPLYVTVLGNLALVQTTSIGKTGTDGSSLLVPLMGVGILA
ILLLLPLTPFIHRVSHHVPLFLLLVFIGTLIYNLTAFPFSDNNRFKFYFK
QVVDLDKGSNVVTLNGLEEFVRPVISSIPTPAGQRIHCDEDPFLSNLRNC
QYDASLLPPDVADGEELESLISIEASKSKNGKTILVSLDALNTRVCYLDT
SFPIFGFSVHGGAKRDDRFGSFPPEGLQQIQLWRRDWEKGWNVTLHLGGH
VLPMNQDSVEATEVDEVDDDTSGGELKRRAAKELIVTARCAWSDANSANT
IPAFHEVKQFMPRWAVVAKKTVGLVEITKKIKVA
>Mb|QLI67620.1
MSTAYQPAMPTFEGRTLSGYPSALSHSSHIKPVVDDARSSHQYRYASQQV
QLRNGPYLSTEPAADPMLRQPPSSLTPVTESSVAAKRSHSTTPRRGSETL
IYHSLQMPKCISHTGGNLSDFAAQMTCLFWFESIDELKKAESIRSRQPNA
SIQRLPALAKPHEQFRKWVYNVLSTTQVTQNVILLALLFIYRLKMSTPQI
KGRAGSEYRLLTVALMLGNKFLDDNTYTNKTWAEVSCFAVQEIHVMEVEF
LSNMRYNLLASKEEWEQWIVKLSCFHDYYERASNLPVSPVHIPSPSHKAL
YSPLPSPTGTGIPPFTPSTKFNLSPSSRHCQNVSAYQSNATSPLASKISI
PVPSSRKRSPDEDLTEHPAKRMVHSAAAQIVQGASSTRLNGHVDSTRLPV
PHLTVVTSHSPDQFATGQNFSSQGSVPSASQQVVSLPPLQSGVRAMSTVY
QTGTPSAVALQPQSVSASSSSAITGSTYPITGLPIHPQVPYGTPTKHHSP
RNMAPAYGSSPLVEAFGPGSAVHTPIAHTPISNSPSVYLQHRASPYKPIR
HVNRLLCPPPSASLDQYHLSVPVQPNQMHYQPLGRRHDLRTGIVPEFLVY
NRGQQQHLIPHGPHQGHFTS

>Mb|QLI67625.1
MEIEALHNQLSRVLQQTTSAFQKVPGSAVLVRYIRSSYQNDPVRSAIELV
LILFFIRYLLSPSYSTHKQNFVKLREDEIDELIDDWAPEPLVAEQTVLEE
AESDKLPIIVGPTGPKVKLLNGRTVTNLASYNFYNFNANEQIKEKAIQTL
RTYGVGPCGPPQFYGTQDVHVKAEADIANYLGTEGCIVYAQSFSTVSSVI
PAFCKRGDVIIADEAVNYSIRKGLQASRSNIKWFKHGQMDDLERVMKAVA
LEQAKGKRLTRRFIVTEGLFETCGDVTDLPSLVELKERYKFRIILDETWS
FGVLGRTGRGLTESQNVDPQQVDMIVGSLSGPLCAGGGFCAGAKDVVEHQ
RITSSAYTYSAALPAMLAMTASETVNLLQSNPDILSQCRENIKVMKLQLD
PRSDWVVCTSALENPIMLLVLKPEVIKAKRLSIEDQERIFLECADECLTN
GVLVARLKTRPYAHAIKARDSSWVAQPALKICITSALSKKDIEKSGATIR
HAITKIMTRKISGKTG
>Mb|QLI67626.1
MPSAVALPRQDVSVRLRQAKQSELAKLVMQNQFGVASSRSSTSPTSSEPE
QHFAVQPPSHSSDVASYSYSPLQSHGTTQCYSMSAPVAIPTMASAVRMMK
DLTDTPPLSQSVNSTAPSSPRIPPLRQNSGSQTPRVRPHATTLNIPGMTR
SRVSPDGRIPQRDVAAKLVIVMVGLPARGKSYITKKLQRYLSWQQHESRI
FNVGNRRRNAAGIKTSARANSGQARRALDPPVEAATILLNGVPATNNDIP
PDQAEPTTLNLNSTNGEPQEEVDQSARFFDPNNEKAAAMRNQVAMETLDE
LLDYLLNQGGAVGILDATNSTIKRRQQVVDRIREREPKLGILFIESICRD
PHLLEANMRLKLSGPDYRNKDPIKSLEDFKARVAAYASAYVPLGEYEEDN
DLQYIQMVDVGRKLIQHRLKGFLSGGISTYLSSFNLSPRQIWITRHGQSV
DNELGKLGGDSILTERGHCYGLALYKFMTQKRKEWLMEQKSKLAQATFPP
HPGDNTPPYPDMHKDLDEKNFCVWTSMLQRSVETAEYFDADDDYDVKNWE
MLNELNTGQFEGMTYDEIARKYPEEFHKRAADKLNYIYPGVGGEGYLQVI
SRLRDMVREIERITDHVLIIGHRSVCRVLMAYFMDLTREDITDMDVPLGM
LYSIEPKPYGIAFHAYKYNEANGWFDEMPNYRPQKAARGSV
>Mb|QLI67628.1
MRIRLPFAGVFLFLLLVAGYAGLSKLQLGQYVNDKVLHAGTFFLLTVVFY
WIVDTNRRRTLNMTLVVCTLCLGVGSEFLQSFLPNDRDFDLFDIVANIVG
SLVGLGLCSWYHKRMLERKRQQKSYNPVPGEDDEDVELGQGQESGIIDAG
PSRVRTLEEEVDNWDENAPDDWDEDDVPETSAPPPVRSQEPDTVDIGDAK
KRTD
>Mb|QLI67629.1
MSHLVWKFYWENDVEKFRRLLAPAGPNTHALTKSPGIGNSGGGLLGTSPG
GPSSSPRVTPKARKTSGYTYGLGKFKDGGAMLSRHEINSRDHAGLTVLLR
AASSTHPNARDFVQALLEHPSLDLYAQDSESGWNALHRSLYAGNISIARM
LLAKERTDLTDRTNISSAVKVGQLIKTKDHEGNSPFDLYNSTIAMRSLKQ
DPSSMKSNDGLDDEDSDGETAQMVANAAFGIGQPVMGDEFYVFGSNKNLS
LGVGDEDDRQYPERIVLERPDELLLKFHKEYLDSQDIESPPSQPDLNDIP
TLVRNQRLAIRDIVMSKFHTAVLTDDPVSNLYICGVGRRGRLGLGDENTQ
FKFVPVQGPFIDRKIRQVALGQDHSMAVTETGELWTWGLNSDSQLGYALP
PVMRSDEEPMSLSPRQVFGPLKKEIVQGIAASAIHSVAHTGSSLYCWGRN
FGQLALMDADSRSLELQQIPRKVAASLLSAPIEMVSAIEKATTCLLSNYT
VWIFTNYGYNLVKFPYPDVFSNQNLSASSYTNRYEMGRRNIRYIASGGET
MAAITTCGDLFTMQLNSKMDTNQLAGSTTNPVKIKSAVTKPQCVWDSRKD
GVASVSVGEHGSVIICTDSGAVWKRVKRAKGKITAFDSALDVKKKDFKFE
RVPYITNCVGVRSSTYGAFSAIRNDSKVMSTDINITSQSIWDDVGSLLCL
NDFRASTLDADEKRSRKKWDAAIAREKPGSVPHEILRSANIEQDLLQWIQ
VNSFQYDDLNMEIRTSLNPDLRLPVHSWLLAGRSSVLREALSTYQSNDDK
GTLTDVITIEQNDDRALLTFIGIDIYTLLNLIVYAYLDVVIPVWKYTKEA
PTLAYRFRQVRTELMKLGTRLNMPKLEAAARLQVGLEPSLDQNLREAIAD
PIFFNDADMIIEVDGDEVLAHSHLICQRCPFFEGMFHGRSQGHWLAARRS
TSLEAELIRVDLKHINPETFRYVMEYLYADVGEGVFNNVCMDTLDEFSEL
VLDVMGVANELMLDRLSQICQSLIGKFVTTRNIANLLNEISPCSVTEFKD
VGLEYICLQLESMLENHLLDGLDEDLLLHLDEAARNNQLARLPCARSGRA
ELLLHEQYPDLALDIEEERRRRVREMAFKLAQKDEEKKLSASYKARVGSL
DESVLAAQTLERSHHRSRAAQNEPFSPHLRPKDSIADLIFDMDDEDGPKI

SSPMSPAVLAALDAKNEYNLDNIPKLPEAWRGTKGKSLADADSGSSPSLD
RISPSQLPGPSIEPVRRAGSSHQPISLPARPANAPWASPILPASKLDLKD
IMQEATSKSALTAGLASQAEKAISSNKPQARMSQKERKRQQVQQAEAEAA
AEKEKNNPVPWQAVHSAARPAPWKTTPSTPKTSLKDAMQSESAQGGGPPT
LSKKPLVAAESNPQLSKRRTASPDTRFPGQCRTAGSPAAPAVSYGPSSEK
NLPVTQSKPYIKPAVKAEPTLGMSMADIIGQQRLEQQRVKEAVAKRSLQE
IQEEQAFQEWWDQESRRTQEEEARRQARDKEKDQGGGNRRGRKGRGGKQK
PSGNLGGAEKTDEVSTKRTGNNTGPSKGRGGRARGSKHDTGI
>Mb|QLI67632.1
MGWFWAEPAPAAAHTPSGHPPVIGNTAPPAGCPMHQKTLDALNPNEKPRE
QAPGPSASGCPVPHEARTTQDKPKSLLSQLNPLNYMFPDLSQERAPNQAI
ALPTSREESTIPKGSGDGTWEYPSPQQMYNALLRKGYEDTDITAVESMVS
VHNFLNEGAWAEIIGWEQRFGKGLYNGWQVCRRGEAHAPEEMERNWDVND
AEPTLIRFQGRPKDLTPKATMLQVLGWIYPSKFGTEPPFDRHDWYVSRDV
NGERQQIRYVIDYYSGEPEPTGEPVFYLDVRPAATPRGAAERIIRWSTDV
WWKAIGGDKREQDPQPFFRHNASKPWS
>Mb|QLI67634.1
MSRTQVVQVQSVYTPIKDAMQRHENGVTKNRKAPLTGSGRAWREDEEAYL
LQTRLQKMPYKHIAAHLNKTELACRLHYHQLSHGSTRRKRTASCSSGSSD
PSPNQPAAYSSPVYRGETRSLSPSVAATSYLPKPLNSDMQLSRIMSVERS
PRLPAILPKPEGVAYSQGGEVVPRHPMVTPEQHHEPLPPVVGMHHHGSPA
YQSPVPPLRLDTGRLSPPSTSVHTPAHVDLGRLRSIYETHKSAFWAVIAD
EYGMNASPVALEQAWKTGACCQYSESNPITPIASPDKDRRTGYRMTGQDK
TRISSILDDDNEQRLR
>Mb|QLI67639.1
MPSFKPFASVTGRNSYSLFDIRLDQDFIVFRGNDHESSGQLLKGVVVLCL
SSPLRIEDIHLRLVGTLRLSWTDHRSTAPGVSGQKVDKATIILDHRWQPF
VGTHGKSMTLPAGNYEYPFEFMLPGDTAESVEGIREASITYRLKATVGRG
KLAYDLHAYKHLRIIRTLEPGALEFLHAMSVENIWPNKVDYSIVIPQKAV
VFGGTINMEMRFTPLLKGLELGDVTAKMIEIRDCWIQGATGLSMREHRTE
REVATWRFEVNREEHWQDMIQDTGQEGWALAKSLPLPKRLRQCIQDLNHH
GIKVRHKIKLTVALKNPDGHISELRATLPVSIFISPNIPFDEQGNLLSQS
QGSSAPSTENALVAPPGYGEHVLDQLYEDVDVSGFQTPAVQSGVSSPFYS
QSRSGSAENLASLAHHPSPIAPAALSSRLADVSLNPSQRNRSYTSVHSHS
PSGRVSPTSGHHGSAPRSEPPSQNLTRSNSEEDRSGRNSTEHVDLDPAEF
AELNRVPSYATAVRTPAWSRTPPAAGLVPDYQTALHAPRTPPATDITPEP
LSTISEDRSGENRGLTSAPSNLSSVVANSGSS
>Mb|QLI67641.1
MAPQDSFIEEEEDVCPLCIEEFDLSDRNFRPCPCGYQVCQFCFNNIKNNM
NGLCPACRRPYDEKTIEWKVVTQEEVAEFRANIQKNQKKRAQEQRQKEVQ
KREAEKENRKNLVGVRVVQKNLVYITGLAPTVREDELLKTLRKPEFFGQY
GNIQKISISNRKSPDGQHHSLGIYVTFEKPEEATKCIIAVNGSQNGDRIL
KAQHGTTKYCSAWLKNEKCNNPGCMFLHEQGDEEDSYTRQDLSSMNSIHT
QRPLPGGGGGSSRAVSRQHLPHPTPPPPASQPMVRTISRDGSDNGIDSSA
LPSSANWARNPQRSRRGSYATSGAASSPAISMSLPATTEATQEAVDDADV
SPAQSVANKGKQPYGTQPQASTSAQEEKEQRSPKTTDSAVAALLKTLGDC
KLPVFTTANASDDTHPPMFDIRGGEKRRAMREDEESRIDQEDQTETHTPS
EGEPETGGSLALGGEPEDRELGRDVHGFDQRQSSTQPPIQRGSTDGLYGA
PSSGSSFPQGSGNISRVMTPQQLASLRLQAGYTDQMPPGISAQNPFQNSG
HNRQSSRFNFANDAGNSTTNIKLAANPRIMAQQSSMMPSSFQSQSSNQFY
ATSMPGPPPGLKSTGTPPSMFGQGFGTGSGFGGAHKDSSNDLLQTLIGRN
RGGSTQPQDVGKREYMISSFSNQYPPPSTSTPAPASGLLASLYGNHPGAF
QDFGSKQKKKGKKHRHANTSSSGGSGLVDLADPSILQARMQQHQSQSNAG
VAGQQGLFGGGQSQEDELLSLDEATSSVDALVSDEPQGLYAGSGFGFDMG
MTRSVPPGFAILPEMVNSPISAMASPALSLHRQAEAVSTLFKAPPPPGLE
NGTVTPVQTPRKVIAPGLEATKNSRPSTSEARVLKHMPSQPQFEQQSVSS
PPGDEDFPALGSSQSIKARTQSPASPVVPTPKPTPAAKKRANRIVDKAAS
SHDQPASTVSDSIKKRPSTISIQPPSDKSVPSHDFQGTDKPVEDSPTFPP

LPTATASFPSPVHRSGPRTLRLMPTPRAPESPALPSPVQSVASKAVSISH
RPDTPGSEMISDTASIVSASISASRAGSPPPSKVGSAAVRTTTKSQQRKQ
RKEALKQETKIIAEASKPEPEEHAPVIGRKKKQKKERPAKAVVAREKPDP
PTTSASEPSKTEPEMPVQHESVNDKVVEETDFKSRTIQKRAAKNKDKEMD
MEKGQAKSPPTPASSPTVPSKLAELGNSVPAEPIESSDPADRQQFGPASV
LQEIKSSLLTSALDKLLLLRLVSSSSLRADLSQAANGASKSVYCKDYACK
CGEIQDDDLTALRAGKPVRKQFHVDGSRMLITPNGDCIRGLTPEQEDAFL
DLQAAIASTADNPGAFVAPRHQPGSGAFSLIKGRAVPNGRPNIFPTTAQP
QFQDPIGKLQREDALSYINQYVLPRLNLDAANMGFPKGASPLRDAAAASL
NALAPYFYGPDAAAGVGIYSAPDGARAMQDFSSSGGAPAADASKIGPANG
LGGISLMSVEDAEVRLAASRKETEKLERGLNAVIKRNRRLLIGGGN
>Mb|QLI67643.1
MNRLNTTSVTLAVTPTVVSTLFSHYLNRKPLKQRPTAHLSYDEGLHLMRS
FLEFASHHTVEELQAFTSQWVPHPQWIRVDAVEISDEFLSRSAELIQAQL
GPDGIAQVGGKQWWQWRKPKSTFEAEWIEMRSHYNARKAIDDPGRRVMLF
VHGGAFYFGSVDEHRYQLQRHARKLKARVFAPRYRLAPQFPFPCGLQDCL
AAYLYLISVQDSSTILLAGDSAGGGMVLSMLCVLRDRGIPLPAGGILISP
WADLTHSFLSAAADAPFDYIPPSGFHHKPSRAWPPPNDDDIAILRGEAVK
RANKTVASNSTRVSSARASSSKPTNSESANTDSTRDSAASATRPTKSLTV
SMDGKSVTIKDQIQIYTTNALMDHPLVSPIMQTTLGGLPPLLIMVGGGEI
LRDEQVYLAHKCANPRKYTRPVETLSEEEIELLEKYKPTYVQLQVWDDLC
HAAPILSFTRPAKFMYRSIAQFGAWALARAQKRGIDILDDDDISVISKSG
SDTDVAQNAQHREGEERQAERQEPGQVGKAGDPLPHFINHMVRQRVTRHG
AILPLAAESELPGCCMNASNIGVATEGTVKRWQETRGQWDSRYMSSKMKV
HKKIIKDMATGFQDFGAGENPPPTALAGRRLIESDTIEKKKTRSMGLALW
SFWGSKHDELTVEREKEADKTPESQDVDVDEGRGARPYQDIESQQLESSR
LDIATRIRSRRRTVIDEHQTEYSGVDENTPVAQLIEQRKEQEATHPGRLS
PDYAPETGVAGKRPYIDGIALPFSLKKDAETASMVTLTSAMTPAGEGSRP
MSPQESFVNLSGPPESEDQARAEQMLAARTTGPAIENSERDQVPQVREPE
QSKEADASK
>Mb|QLI67645.1
MATHHNRSAASPSVLSSSSSRNANGVNGLTPSRSQSTRSTSGRSQHSARS
YSSSPAATASASATPLASPARSSRPPLSASSGASAFAAAAAGPRLPAACS
LPKSASSSDSTAANTPVVSRSSSRSRSNSNSNSNANSRSHVQNHSQDLGH
GHSQSQAKNQSPRHSLQRVPTNGTSHQGAATATTAATAAVAAAVAARSTG
SQQHLSHHHPQQHLQHQRHRHHRHASTSSTASSRPVQDILPHHDYETSHL
ASYSHSKRSSSRDRTSLPSTTRGAVAAPESPSVSSSQAAARSIARQASTR
SSSQRHHHQPYPSSDMAPSAVATNNGSPSAPPSSHGASDPHHQSHSASKQ
TRSRTTIPAQSGKWILGKTIGAGSMGKVKLARKEDGSEQVACKIVPRGST
EEHQNRADKERADQSKEIRTAREASIVSLLNHPHICGMRDFVRTNYHWYM
LFEYVNGGQMLDYIISHGKLKEKQARKFSRQIASALDYCHRNSIVHRDLK
IENILISKTGDIKIIDFGLSNLFSPRGHLKTFCGSLYFAAPELLQARAYT
GPEVDVWSFGIVLYVLVCGKVPFDDQSMPALHAKIKKGLVDYPSWLSSEC
KHLMSRMLVTDPKQRATMQEVMNHPWMTKGFNGPPDNYLPVREPLVHPLD
PEVIHGMQGFSFGSPETIRAQLSKMIDSEDYQHAVKMYQREREAPPPAKD
AEKKRGFGFDFYKRRNSSNSRDTLTGPSSDTLQLGHDPLYAFSPLLSIYY
LVKEKHDREAAQQVPITSSGSHEKEKERERERTRERERERERENERDR
DHRDRDYREREKGADTMPELAPPQAAHTNATTYEMPGERPTGGRTRPRAR
THGEDDAPESARPHGHPPPPPAPETKVEQLQKKEGTAAGLLRRFSTRKRR
EPERLDKDRSHPPMVQVHSPSDPAPSPATPKKSFSIRRGRRDRDEPPVPP
IRSGSSQPQHSALLSPPLSAHGTRDSRRSGLGRSTSVNSAEMRRRQGGRL
PKEAAATSGSEQSVSNDQSGTSQRAHAHSRSASMRTKSLGHARRESIQRR
RLRREGLQEADVPEETDYEPDQSGVSTDRLDSAELAKPVFLKGLFSVSTT
SGKSVPAIRTDIKRVLKQLNVEFTEIKGGFSCRHTPSIDLNKVHDHPTSP
GQTPGHRRRFSFGGLMRNDDREEIRELDREQRQPPTPRTPGRPDRDRPER
ERDRSDRDRSDRDRSYSNSETSVDSIPRRNGGTTRRAPGETSTQVQSDLG
ESMVLEFEIFIVKVPLLSLHGIQFKRMTGNTWQFKNMADQILRDLRL
>Mb|QLI67646.1

MSVTLHTSHGDIKIEVFCESVPKTAEARHLLPPPFLFLYGKNFLALCASG
YYDGSPFHRLIPKFILQTGAPAHPGPDDPKGGRSIWDAAFEDEIRPSLRH
AQRGTVSMANKGPGTNGSQFFVTLDKAPHLDGLNTVFGRVIGDEAMATLA
RLEAEEVDRKARPLARGDVRIDRVTVHANPLAG
>Mb|QLI67647.1
MFMLRNVGKLIFGSGTQEALIELPQGQLYLVRPLSPKGYSELIFKDSAIR
IRRTNQDFHYQLVVQRVFEEGEAELIADEEGEDAEIDALAAERDEKTFLL
DEALRFRVESREGGETVFAWRDLSGDNGDLYEFVCDTQVPTAQVEQFVQV
AQQCQYERKYRKPHTTASDEDYQQFEFHDENPIPPASPLHSPVLARSIES
VDDMYAKKTAANTGAKREPLAAAAAASLPEPEPEAESEPELEPELEPETK
GSGQSKAPEPDSAPEPLEIYASLPAELHLFDPQPGHFELIDDSVIATVSE
VGQWQYWLQIESKERSWVGTSVVPEFNPVFDFEYLSFVFNHFSGDGTARS
WLLRFKDQPTLEKFQEAIMQAIWERLNETKWAKVQEKEREYVLDAFGDLN
MEDAPPLEEEEEEEEEEHEEEEDAGIRSDDYESEEDEEADEEKTGGDINS
QLAVGYKHDRSFVVRGSKIGVFSHTADNRLKFNTNISRVTTPGGKLMSPK
KVMLHSEDRDLIIQNGVDANKLYRMDIEYGKVVDEWKVHDDIPVVTFAPE
NKFAQMSSEQTFLGVSSNALYRVDPRLSGNKLVDTDMKQYASKNDFSALA
TTEKGYIAVASNKGDIRLFDRLGIRAKTQLPALGDPIIGMDVSADGRWIL
GTTKNYILLVDAQQKGGKNDGKLGFEKGFSADSKPRPRRLALTPEHVAQV
YHETGKPVSFTPAKFNAGEGAEEKSIITATGPYIVEWDLKKLLRGVKTPY
KIKRYQDEVKADDFKFGSDKNVIVALPNEVNMVAKQSLRKPTRESIMGDL
RLSGGRRSSGRISNSQSGRYKLGRDDIVNSPY
>Mb|QLI67648.1
MASRPQNIGIKAIEIYFPSQYVEQSELEKFDGVSAGKYTIGLGQTKMSFC
DDREDIYSLALTVTSNLLKKYNIDPNSIGRLEVGTETILDKSKSVKSVLM
QLFGDNANLEGVDTVNACYGGTNALFNSVNWVESSAWDGRDAIVVAGDIA
LYAKGNARPTGGAGAVAMLIGPDAPVVMEPGLRGTYMQHAYDFYKPDLTS
EYPYVDGHYSINCYTKALDAAYRDYCKRESKLANGNANGTDASKTPLDRF
DYLAFHAPTCKLVQKSYARLLYHDYLANADAPAFAEVAPELRDMDYEKSL
TDKVVEKTFMTLTKKKFQERVQPATQVATMCGNMYCASVWGGVASLLSFI
DPKALEGKRIGVFSYGSGLASSFMSFRVNGNVEAIAKNLNIPSRLEARRA
VPPETFDAMCELRHQAHLKKDYTPKGEPSTIIPGTYYLEKVDDMFKREYK
VQA
>Mb|QLI67651.1
MPPRPVRRRPTRFSVREPETPSNANPLTFQKPNLPPLQGTPSSRRQYSYG
AEVEPMPSRPGHGLSRSQARDIGSAVRNVLTAPGVNDEDEEQDEDEREQT
QKPQKPKNAQPEPDELAGGQLSRPPHLPSYLSGPSNTALATRSLLSLRNA
RPDGSDGDDARSFGIESEFYGDATIISTPRALPPQRPELRDELSGDSVST
NETPSRSTMNKPPAIIGENEEVDLVRQPQNAYKGPAKSTVGPSPLRAPPR
RPPQQSARQPRFEQQEEEEEEEEEEEEEEEEEEHEDEEEENELDTNATDK
EREGSEEDDLIRRQEPRPILQTNTPVPGRSQPAPRENVQRTRIGGPSPAP
PHPRPIPRLLPPNTTRGTLGPTESTGPDKMRRTQLLTGAPLEPRQRSNRE
HRGPNFEENSSEDGRWLDRATATTTSLFAQAKFLVNSSPFGLGRRYPTDE
SERDAAIQRDITEAEAEVARERLREEEEAAYQRSYQRWLWLKSLVPFIGR
EQTHTELDGAAARANNRWSTFALIRFLNPMTYVRAIGWLIHAVLDWIMEL
VYFIIPAGVWDQLSSVFGFLPHILAGALAFVAAFALATQLAGSTDSSWAP
NAIEATLRTFDDLRYRISDLIPTVTWPKRDRWVDLDDLWEDDDSSRDKLQ
HFLNRMEEEFLTLKRSGKIHDASLRKLENILPSVVHMELKDGRPVISEEF
WHALRDLIHGDGGFLAFDKVGSEYVVSSDRQWSAITSRLLRDPAFTTKLN
LTALGLEDRLSRKMATFWDKWVKDNDDKIAHLLGTAVDQIKSAGSQREFE
DRLNKIVSEQLSQREEKEGQTGKFVSREEFLRHLRNEIASLSSQLRAELD
DLRPQMESLVQQTVDLATKDLTHSMSRADITALVNNLIGKALADVNLEAM
ALGKIHNHWETELRNQVNYFAVGSGAKVDPRLTSATWDPQGAAIAPHQYK
HGWRGSEAFPPIAALEPWQEEGDCWCAARSVNHRGNPHGATLSVQLHHSI
IPQHIVVEHILPGATTQPGARPKSIEVWAEILDPEARERILDFGSVYLPD
DKNDWNYTPPDFPAQFAKIAQFVYEGTELHEGVHVHRLNSELLALGAHTD
RVIVRAVSNHGAENHTCFYRVRLYGFNVELDRDEGGNSYY
>Mb|QLI67652.1

MAHVNLSTRDVNVLEKIKDPESNPAAGVITDESLPRDPHVTNSADYDKVV
QQERNIIAKIQALETEFSESQSQDANEAAIQGYQHCLAELDTLISEFPKY
ASARNNRAQITRRLYGDAMLLEITTGIPLPLIEQPDATEKKKAAVRALQD
LETSISLLSPATLSTPISPQAARTLSMAYTQRAAIYLRTSKLLSDRLLDM
DESRPEAKWNRIEFEQAASHDLAYGGRYGNQIAKGLAVSVNPTAKLCGQI
VREAMKKEYGPSFDA
>Mb|QLI67653.1
MASRVFASRLATQMATKAARPAIRAPVAAASKRTITGSASPLQTLKRQQA
TTLLLATSRNAFQVQRRAYSSEIAQAMVEVSKNLGMGSAAIGLTGAGIGI
GLVFAALLNGVARNPALRGQLFSYAILGFAFVEAIGLFDLMVALMAKFT
>Mb|QLI67654.1
MDDEKATTKKKKGFFSGILRTKSLASRGLKAFRGRGASASDGDKLKQDRA
TTTTTTTTTATRRAPPDQEPAKARKAQRREYGNGGETPRRVNGDEAPRRP
KIDAAQRNAGDVPRRVTIDDTSRNGGEHTSRKLNGNSSIPRKNTRASKPT
AAPLADEPTPSSRRRPRRPKPEPEGDDGHADPKDRQSSKTFGEGRVDTPP
PPTLTEWPPPGLSNDDELPPQHAMELITRGAPLHKGHRRDIPHAPDNYYA
LYSTTSSSRSDDTDAMSLHDSMTPLMTLRLQGPGRPATPWESLEQPSCAF
LYGQRPGTITLNQWVSMSSSLPPTIALRDSGVLPRNMGLSHILERLKELQ
AGLEDDDEGLLYRILYKRILRDPERILNPHKTLDKQITDLLLVLSRPDWI
DFTEPKNQVVTRFIFNPEEINPDVYRKFFHQLLLSLELELRIHSRSHGEW
ARERLLGQIPPTIRWNLALARRWREHVRVDGFGSMPEQTNLRYKLKKRQV
KVLKRFAQAMKWPNLDPTMESLRVHDEEFRLDSISSDAFAFFSGLVLPGP
TFPFLIMNTLIDLDPDSGTDELALLSHINPQCGFQYCNSHTYWSASCIVG
KVIAPTCCCVAGWVGPGRPTLDLGRSQIARIRTRRPKQMDRRIGPEDVES
MKERSDPLGPAAQSYPVDEYTLVVPDEDSFAVDTVRIELLGFKPVVAMAE
DTIANPGPRVFDATVQFAIDGMSWPLRLMYDVSFVTAWPCSLPPHPLFFD
YVYRVVKADEVVRVRDWGGLYYKDLRSGSASARSSPAPPFYNMGYDDDCD
FIDDDGDDEKVLVVEAYGVADNEVLARAWCSHWGLSAVVADIRKTCMACA
IREAYAATITVVIMVDSRYDGDAEA
>Mb|QLI67655.1
MNGEDPPERPDYITNIINGLERYNPEAVGSLETYLQEQCEQKFCDSNANR
TLLKLYQLNPDRLKDEIVTNILVKAMTLFPSAQFSLALHLINPSAAATGE
LHEALTKLRSLNSQLEGAQYAQFWASVDGDDLCADLIADISGFEDMVRLR
IAQLVSQAFREVKLAHLESWLGLSEDATRKFVTDVCGWTVDGESSIKIPS
NPDNEAKKAEIREDVNVEQFARVIRRSWEETV
>Mb|QLI67656.1
MMHDNVIREQSVSSMEQDLSSSRQTSQPAQDTSSWGTSAASAGMGTFDFT
SAPPDVSIPSFDPAIAAFADLSFTQPPHGSPYQTGKNTMTSPQSDALLQG
RDFHDKKRMKVESDTPALDSIDYWISFDDDLDKMGSFEIDYSRRPDLLSQ
NRPGMASDSIPGLGSGLYSASTAPFREEDFFDDSAFEQVLSDDEEMFEAT
AKPGEKMTQLENTSTSQQPLDNNAALFNSAQGTDKFQPGSRLESTFKDVS
KQMRRTGPQDAEPDIDEQRRFLEEALNSGRIPGALIPPNGFGVGFGAGMG
CRPPQEFITRPLSPLGKPTESQSTSKDTSRAKTGEMLDETESKKPTTKRP
TTSRAGSSKKATTAGAPKARSADRIAHNDVERKYRTNLKDKIAELRAAVP
ALQTSGGDGDSEGGSAGGQQSAAKISKGTVLTKATEYIHQLEARNRTIMQ
EHQQLARRLQAFETLLNSTGRQTDMMMPNHSMTLFDPRGFC
>Mb|QLI67658.1
MASSPWDYIAKLVCIGDSGCGKSSLTIRLCEGRFSPHHDVTIGVEFGSRI
VPVGPPHTKPSSFLSSSVNPPAGAAASPGAADGLPEPPRDTSKTPQKHMK
LSLWDTAGQETYKSVTRSYFRGASGALLVFDLSRRQTFQHVTDWLNDLRQ
IAEPDIVVVLVGNKADLAQQEENKREVTREEAEEWARRNGVLEYVETSAK
SGENVEQAFMRVAERIFHNIQAGKYDLNDRRSGVKGPSAGGNRQVKLGGD
SKSAGGGCC
>Mb|QLI67659.1
MYEPVAIVGTACRFPGAANSPSRLWDLLKEPRDVVKDFPLERLNMVNFYN
ENGETHGRTNVHHKSYLLEEDVRHFDAAFFRINPKEAADMDPQQRILLET
VYESFESAGWSLNHVDGSPTSVHVGVMTDDYTLISSRDPDMLGSHAATGL
SRAILSNRISYAFNLQGPSLTLDTACSSSLVALHLAIQGLHSGEAVQAVV

AGTNLLMDTIWYIAESSMHMLSSDSHSCMWDKDASGYARGEGCAAVVLKP
LSKAIADGDNIECVIRATGVNSDGRSNGITMPTPTAQAALIRETYGVAGL
DPITDRCQYFECHGTGTQAGDPVEAQAIRDAFFPNGQHKEHDILYCGSIK
TIIGHLEGCAGLAGLIKASLAIQNKAIPPNLHFHQLNPRVEPFYNNLEIP
TSLLPWPETHSQPRRASINSFGFGGTNAHAILESYEPSDAASPSPLSSSV
HTTMVGGRLVGPFVFSARTPSSLLKSLKKLLGHLRANSSLDLDSLSNSLH
SKRSVFPYRVTIPSALDRDDLIQRLEDQVNTVATSTLSPLGGGTGDEPRI
LGVFTGQGAQAPRMGYALLQHCKLFQDSIRQCDLALKLIPDPPEWSLAEE
LAADAATSRASQAKFSQPLCTAVQIGLVDLLRASGIRFCAVVGHSSGEIG
AAYAAGLLTRRDAMGISYYRGHVAHLAQGNGGRSGGMMAVATSLDDAMAL
CSEQQFKGRITIAASNSPSSTTLSGDADAVVEAKRVLDERNIQARVLQVD
TAYHSHQMLACVDAYAAHLKQLNVRVLTPASDDDCPWYSSVKTNTDILAN
PPQFGLEGQYWLDNMVQPVYFSEAVKLAVQDASTKFSAAIEVGPHPALRG
PLKQTLTQSIDYTPHYAPLLSRDGDSIETFAEALAALWTISPSSIDFGGW
RRAFGLVAQPQTIKNLPPYSWDHTQIHWRESRVVQNYRLGKQLPHDLLGR
LWNDSQYEQVWRNIFLLHEMPWLKGHVFQNQVLFPATGYISLAVDAAKAF
VKGRAIKLIQVEDLLITTALAIGERDEVEVLFIIRSHVSPQNVEAGCVLE
AEFACYSYPDQRDADRTCHGRLRIHLGEFESADLAPVCISEVNLTPFNID
RFYGAASEMGFQYQGPFRGLTLLNRCWGHAKAGASWSIEDLNIDCTLHPA
ILDVALQAGLSTFLSAAENSMASSYLPVGIKRVVVNPNERFRGPNRSTNV
DIEAYMVSRGIGKLMEIDINLRDQMSPHGDVCGIQMEGVSFKAISDPQPS
DDRNILAKTVWGLDVAYGLIHPLADAVPLGPPLYTLENYERVALFYLQSL
AHSVNKQELERLKPQHQQLIYYINAIMTKVRTGDQSHLRREWLEDDSATI
EDVLSRYPGDVDMAMLKVNGEKSISLLRGDYESTNEVLEMSSKSSYQDTQ
SFVLCIQFISQLVRQVSHKFPRTNVLEICTGTGSAAASVFDAIGDAYESY
TCAGSSETVLSRLKESLVPTDLNKIGSFRHVDFETDLTFQEDDLGLYDVV
IVTGLLRASPTLAQVVQNLRRVLRPGGFLISMALTGASLRPIALMGGLEN
WSRGSNSRATDGYAVKTGDLDKLLHRSGFSGIDCILHDQPDSRMHGVSVF
SAQAVDDRFEILRDPLLAMGMIPPNPVTIIGGETSRVSSLIRGIDKLLRA
WAPEIQAYGRFDEVDMSRIQPASSVIVLQELDRPLFSSFPTENEMENLKE
VLGTCKNTLWVTSGRLVDDPYANIMIGIGRSLRLELTHVSLQFLDFDAQD
EWDVHALVRQFLRLVFSSSSPDTTEGMLWKEEPEVVIKDSHMIVDRIVVN
DESNEIYNSGRRRVVKLVGPDEPIETAQDADSPESQLVCSRGIDILEGDM
PVHVKLSVALHSESKFPCFLSYGQLKYAQETVLTLSRKDASIASIGNKSI
ARSSLAQNCDATTLVNLASCLIASQIVSRMLSDGTTLVCGASQELARVTS
SLAARSGRKVLFVKVTSKQQSERAEWISIHPHQSTRSIHKLIPSDTAALF
DLSQTDTKVVLPYLPASCTIQGFDTRSVTQQSVEEAIDIYQMQQDVFRTA
STPTIVDIKDVPQRRNANPGHLSAVTRWDREAQIGVTRQPLDPSTLISPN
KTYFLVGMASDLGQSLTYFLVRGGARYIVLSSRNPKEQPWVKDLRRDGID
IRVVKMDVTDRSQVRETVSLLRRTMPQIGGVTNAALVLEPGVFANLSAES
IGKQLQPKVSGTAHLDDEFRTDKLDFFMAFGSLLTVCGNAGQPMYHAGNT
FMMSMVRNRRRRGLAASILNFGMLVDVGYVARSDRSGSSNVEEWLRSFVP
TALSEADFHHVILQGIVEGNPATGSGEVIMGLETFTDRGQAVRPSWVDSA
FLSHMVRVSKSIGKETIDKAPASTQQWELDLENSSSVDEAIPPITELLSA
KIESMIHVSLHSIHPDEPLSHLGIDSINAIAIRKWFREKMAVDISMLKIF
GRDSSSSIIRTVAEQYLDKKSTQNAEPANVKTQPTPQGEVRSESATLMPK
ATLTGETQLNTSAMSTPTTNSIKNEASPTSYQQSTTESSIVTPEPELEYL
QSESLSYAQSGWFYLNTISEMPSSFNLTVKINIKGQLSINRFSRAFDATM
LHYDALQTCFLASAESSEPRQHVIKRKRQSRLTLLHSTAETGAADVKKAF
EETAKHEYDLSIGDTLHATLISHGEQSHTLVLGFHNIAIDAFSMAHLMGD
IGRAYQFAPLSPNPTSYLDFARQQFDDIRSGRFVDSFEYWKKLLNPAPEP
LPLLPLAKVKTRRRQRAYGHHYIETVLDGDLVQRIDKVIQYHHVTPMQFY
LSALHVLLCRLLNVDDLCIGVVFNGRDPTSKFGGTVGHLADVLPLRSKGV
LGKSFSSVLKDTAQTLLDCLDNANIPFAAILEKMRPETSEGNMPMFQIAY
DYRGVEESRLSPMGGCTLTLGELNYTTLYDMTVDVLHTASGDHVLNMRLS
DDFYSIEATQFMMETFINVLETISQEPTASVKDYKLFSNAQIQQAATAAQ
GSELQYSWPNSLCERFYQVATSFPKSVAVKHGSETITYCQLRAKAGLYAR
ILIEANITKGSRVAVHCEPSIEFYAVMLAVFHVGAVFVPLDVSLPAARRQ

TILRACNPDVLVFHRATAASAIAETADHVIKSFDITKLARDRQQDSPIPK
RVPCEAESDSCILFTSGSTGAPKGIRLHQRALMMYAEFASRTHGFGQITV
LQQTSFGFDLSLAQIYSAFTNGGMLVVAPVEARGDPDMLSRLMVDEKIEY
TMCTPSEYGLLLTYADAALRQCHSWRFAGTAGEALPQLVVDGIRGLKLPN
LTLTDCYGPTEAFIVTCRDIPVRAAATYDKSEDQNGCVGHALPNTSIYIT
SEQDGSLLPQGMAGEICIGGAGVANGYLSPEMSDAKFVKNPFATAKQVDA
RFSTMYKSGDRGILHPDGSVIFLGRTRGGNAVVKLRGLRVDLGEVAGAVL
EAAPNDLINAAVTVRGDAQLLVCHVVFRHGKTLSNEQLTQLVRTMTLPRY
MIPSAIVPLERLPMTPNGKLDTAALEALPLPLLSRQEQGEADMTETEAIL
RNLWINVIGEVAESADIGPSSSFFLIGGNSLHLVNLQYAIRARIGVKVHL
RILEQAVDLRAMANIVNKTSRVRFAAI
>Mb|QLI67660.1
MDTYLSSASALSLRRDPRMLKLPPYLSLVCILVGAIWLVLLPLDNYSRRT
YISENALLPGQVHTYFGGSEQNIFRAYRRELDTLVNESNYDINNHLEVIL
STAGIKVGRQNYTYNSAGKTYNGENVYGILQAPRGDATEAIVLVAAWSSV
NETFNGNGVALAITLARYFKRWSLWSKDIIILIPPDSRTGTQAWVDAYHD
AHDSNNVASLPLKSGALQGAIAIDYAMDQRFDGIHIIYDGTNGQLPNLDL
INSIVNIGGGQMGMQTAIQGMQHHNDSYRDRLMTMLRGMLKQGLGICAGP
HSSFIPYHVDAVTLQPYGEGWHDEMAMGRVIEGTFRSLNNLLEHLHQSFF
FYLLMQRDRFVSIGTYLPSAMLLAANFTIMAIFLWVKSGQPAAQVQMPVV
ATFEKPAPAKPTFSKAPGIPPAINSTERDLSLPLGIVAGCHAASVIPLFV
LSNVPARLLPSSLAMFCIFSAVLPFIISYLLPVCFTQRTQHFQLTKSLSL
LVLGMALATLATLNFSLAFLVGMLASPLTFITPCKNTISKYFVVALLSAV
APPVVVYPATHLAGSSVAEFLQQASFGWNVWAVYTPVVIWCIWWPAWLVA
MTNVLSGTSDGAEMFTTEKK
>Mb|QLI67661.1
MLPALNRRIYRIPVVRTGHPLGTLFLRSFTASPLRRRFAATFAHDEDDMA
TPDGKVPKASAVTLKTPKGTRDWSGQDMLLREEVFKMITDVFKKHGGTPL
DTPVFELKEILSGKYGEDSKLIYDLQDQGGELCSLRYDLTVPFARWLAMN
NIQAVKRYHIAKVYRRDQPAIARGRYREFHQCDFDIAGVYDPMIPDAEVL
RIIVEVFEALELGVTIKLNHRQVLDGMFAVAGVTPEKIRTISSAVDKLDK
MSWEDVKKEMVEEKGLASEVADRIGEYVRHAGTISETLQLLKSREELVAN
EQVKRGLDDMTLLASYVEAFGIADKISFDLSLARGLDYYTGLIYEVINLP
PKAEEGQASGKKSKKDDPASQVGSIAAGGRYDNLVGMYGKRQIPCVGISF
GVDRIFTILEARRRKDGLKQRRDVDVYVMAFGDKSSNGLLLERMSVAQRL
WSAGIRAEFSPKVKPKLPQQFKAAEGVPLGVILGQTELDAGQVRLKLLGQ
GENKGDDKPAEKDDGKLVSLDELADEVKKLLV
>Mb|QLI67662.1
MGSKHTPYSSIAETSRILDFLLPLFDHVSLPAQARNKRANVRFTGTRDQP
YFPIPFKETELAAALKAVEGCVASALADEKFGQEQRDRRIVVDQEKTTAF
LFQTYLARVGGLGKLDKGVRALLKDTDLLQAQSNPYRRMSANLYETAERG
EYYHIHGSLEASTTLKMIGLEPHRPDLESHQDIVNTIEPAVRKHTVEQLE
AMNARHRQAGVKAFRHDEFLQTPHGRENWSLPPWSVESLETSTPRVPLPS
TGDRRILSGIKVLDLCRIIAGPTIARILGEYGAEVLKVTSPNLSDVPFFQ
VDGNMGKRTAALDLKTPGGRRAFEELLRDVDVVVDGYRPGAMERLGYGPK
VLADMAARRGRGVVYVNENCFGYKGEWAGRPGWQQIADCVTGVAWEQGRF
MGLDEPVVPPFPISDYGAGCIAAIAALTGLYHRATRGGSWHGMASLLHYD
LLLFKVGLLPRHVQEELRRQAGPEFLGLRHAHSVEQISGTALRRMRQLFP
EFVDDARYLDHWYAEGYKANVSVVLPVVEIEGLDISFQRASRPNGSNEAS
WDGGDGGEEGDCRIAAS
>Mb|QLI67664.1
MTSKSSNTFSSRKNRRTSDDSPASLPKRSSDATAPTRSTPSTTTPTTTNT
SRSSKRRQSKTVTRDSTQTIASSSVFSRSSNSTGLTAISSSASALSPVET
AGAGDRDSFVSIVDDPFFQRFDPANTAHAEREAFDEGTPAEPHNDSDDNN
KTQPWPPPRRESLTIGPSQYWPHHPISAMESYNIAIIGADAVGKSTFVQR
VLRLSRPPVTNTSSARQVVDSIAHMVTLVELDLEHFELNPSQPMQWPKQI
NGQIVPRMDAALILYDVTTEESIRQLPQTVAALTNSGLPAMLGACKCDSP
EDDWEVNADELANYHLFKSCVGQYKISIDRPDISQACLQAVIRAAVTHRR

DENNETVLRRRAQSAANLEAPDPSAGRPLSEHSKHSRASSDFSILRTFPN
QQSGENYRTQASRSPRPGFRTDKSGSDSFLDIDESDSESRRYSDDIPILQ
RNDDVIIEKQPKLTGVTFDELVDRLLAPRLSRVDNNFSDIFLCLYRKFAA
PGELFSAILVRLDKARDDKAAHYLTRTATQLRIIEVVAKWVSLYPGDFAR
PTTRRNLEDFIKHLSTEPIFAIAAQQMRRNLYFNVIEDDDTGWANSDNSS
DEIASKILSKDMDELSAGIHALQFNGESDSLGGSENPSTDRGSRTGIQVQ
FNSYEEYEREATLLEPSDHLPMNKFRYHIFMDISDDDFADELTRIDWIMF
SSIRIRDFVRHVSLSAAQKDKCKSLQNVNRMINHFNHIAKWVANMILLRD
KAKHRAQMLEKFMNIALGLRRLNNYNGLAAVLAGINGTAIHRLAQTRALV
PADVQKRFARLVILMGTQKSHFAYRLAWENSPLPRIPFIPLHRRDLVSAE
EGSKTFVGPNDDRINWKKFEVLGEVLLPIMKSQGTAYPTLAKHETARDLI
LGCRMPTDDEDISARKDFLFTIATSLERMKGAESPSLRRKVEGLKLDSYI
QSEENSSEDTYQKDLVRNGIERSLWNLMTSNIPTSRR
>Mb|QLI67665.1
MASTIGKIIPLVIAILLVSAIAWVVYQVYESATKFRDTASERMGKKNVVF
TKDGVRVGVKHVQNEKYVDKTQSWVVKAWNMGQPQSQPKEELAKRRK
>Mb|QLI67667.1
MQPARDVPRFDQLQDYPHAVAGHAGTLCDSDGQVFVKPCTQAEIDFYELT
KSKYPDFADLMPLHIGNLVLSGPGEMDIGDIGDGLQTDPEQVLATIQEQV
ANAARESQTEDTITWVPSQGKKIQTNMSVVLENQTNGFKRPNILDVKLGT
RLYADDAPKQKQQRFQQISKETTHHNLGFRIAGMRVFRGSQDPSELDDRE
YKNYDKDYGRFTVNDDNVVNELKRFVFNEAAGIDEDLGKAVCATFARELG
TIIDVMSDHNIRMYSSSLLFVYEGDGEALKDAIRLNTEYAKRSTGPPATK
RIDSGIGLDDEDQALEQLPPPVLKLKLIDFAHATWTPGSGPDENTIKGVS
SLEGFFKEMAE
>Mb|QLI67668.1
MPAVMECVQTITTPFPVKQPAGKAASRPRGDLDIIDIRSNRVDFNLKDEV
ARMVIPKDGRARKLPTLLLYDERGLQLFEDITYLDEYYLTNDEIELLKKH
SVEIAATIPDGAMVIELGSGNLRKVCLLLQAFEDARKSIDYYALDLSKKE
LERTLSHVPDFKYVCCHGLLGTYDDGTVWLKQPSIVDRPKCILHLGSSIG
NFHRNEAAEFLQSYADILKPQDSMVVGLDSCGNPDRIYHAYNDSKGITHE
FILNGLINANRIYGQEIFHLPDWKVIGEYVYDDEGGRHQAFLSPLQEVNV
LGSIIKPNERIQIEQSLKYSKIGAENLWKHAGLREIGIWTRGDEYGLHML
QKAQIPFSKAPSLYAAQTLPTLSDWQALWSTWDIVTKQMLPRQELTEQPI
KLRNACIFYLGHIPTFLDIQLTKTTKLPPTDPASYATIFERGIDPDVDNP
EHCHVHSEIPDEWPPVEDITAYQGRVRARLSRLYEEGVEKLPRQVARAIW
VGFEHELMHIETLLYMMLQSDRTLPPPHMGIPDFERMAKRAFEARVDNEW
FDVPAQEVILGMNDPESGTDNVHFGWDNEKPARKVQVHAFQARGRPITNE
EYAQYLYTTNAAKIPASWSSNGLTNGTHKPNSANGTNGYKDVPTTLPDSF
LSDKSVRTVYGLVPLKSALDWPVFASYDELAGCASWMGGRIPTFEEARSI
YAFVESQRELLMGGRLSRKVPAVNGHLSNDGVEETPPCSSPSAVENSSAL
FVDLAGANVGFKHWHPVPVTSQGNTLAGQSGMGGVWEWTSSPLRRHEGFE
PMELYPLYTADFFDDKHNIVLGGSWATHPRIAGRKSFVNWYQRNYPYAWV
GARVVRDVE
>Mb|QLI67669.1
MTGTGAPSGGSSAGYGGQAPHRSYEERAAAREQMMTNIRETSQQDRRVYV
GNLSYDVKWHHLKDFMRQAGEVLFADVLLLPNGMSKGCGIVEYATREQAQ
QAVAQLSNQNLMGRLVYVREDREAEPRFIGATGGNRGGFSGGMSGGFNNQ
GGYGGPPGAGGRQIYVANLPFNIGWQDLKDLFRQAARVGGVIRADVHIGP
DGRPKGSGIVVFENPDDARNAIQQFNGYDWQGRMLEVREDRYAGSSGMGF
SGRGGYGGGMRGGFGGGFGRGGYGGGRGGFGFSGRGGFSGGGANFDASAA
ATAVAPNPFTDGATAGTEKGETIYVRNLPWSTSNDDLVDLFTTIGKVEQA
EIQYEPSGRSRGTGVVRFDTAETAETAIAKFQGYQYGGRPLNLSFVKYLN
QSSGESMETDTHGGLTQDQIM
>Mb|QLI67670.1
MGSRGTVAQPEGTPRSWCRDNFFLTTDKTFLDPKAVNEVFESDLMWWNDP
LELSQMCKMLDNCLTLAVFYVPDTEEEMMSNGGMPRYMNNGPDFRMIGLA
RIVTDYVSLAYLTDVFIIEEFQRRGLASWMMRALKELVDEWPNLRGLMLM

ASDKAAARMYQRTLGAVDFDKGPSAGMVMLEMGGKGQKDVPEEH
>Mb|QLI67671.1
MSSSLQGSPPRPLSPILDKPRRQAITRACDRCRRRKAKCDFDNAVGTCSH
CRDTNARCTFDLPLAKRGPKSKKSTDSSIPPESVSTFSGRRGIHHAPLVG
HPSSSTAAATFKSPPSSSTTTPWESSAQLAAGAGLSPASTRDFASVASPP
AAHGVSAVRRWRTLSRALSLRNKVLEQMVERCFGLFFEYLYPLTPLVHEP
SLRDGLSIFTSQSSNSALQNAPRNGNDGLADTLPALNPPAWPGISPDVGS
GPGGETLGCWHDATFTLITAVCAEAAFLLPKDLFPEGETVADLFLQASRD
CLNSYLEADLENPNANSITIRYFHSNCVHAAGKPKYSWHIFGEATRLAQV
MRLYDEAALEGLPPVESELRRRAFWIVYMGDKSAAILNNRPITMHKYSFE
TGVTTAYPTGMDNRSGSMASVSDVATPPDSHGSNFIAGFNANLKLWQAAS
DLLIQVRLFQDQKSIEFGAANRPSQALTESERQRLDSLFVQFITCMDDLP
PYLQSYTFAAVGGAGGPIAEAKQFVIQCANLQVSFHCLRMVITQKFEDIA
FFTPGAEQADLRKTEIVRDMLRVMHEAPFWSLQVNGEPYVEKIRLIGATL
LSIIHRNQASPLAARARSDFSVLLDILTRLDSKASDALKSTSTWAM
>Mb|QLI67672.1
MTSSLGNLIDYVDFDKRSLLEYHNKILTKLFGGNAKAANYGLAVAIFSLG
LFRDWLYKVALLEQPSHPLLETIYSQAAAYMLFAAGNTLVISSTYRLGIR
GTFLGDYFGFLLDEMVTGFPFNVTGAPMYWGSTMSFLSTALFFGKPAGLL
LTLWVYLVYVVALRFEDPFTAGIYAKRDERAAAKSGKKQK
>Mb|QLI67673.1
MFLQRSAIAAARRAAVSPVIARSFTTSFVRREAAKGPTTPSEQAAALAGA
TQKKVGDYKVLNDIKTEEDLFGPGARPGTVPTDLEQATGLERLEILGKME
GVDIFDMRPLDASRKGTMENPIMVRSAGDEQHAGCTGSPADSHVVTWLGL
SKERPIERCPECGSVYKMEYVGPEDDHHHHHHAPEFEEPKTFADFVKPEY
RYR
>Mb|QLI67674.1
MAEAAFAKTFLSTLDSRPIKLSADHVEDAKSFPPRPPYILPRMPSPMSKP
KTLLPGQERSLTVTLRSLRNPPLDLKLTSQPLNTSILDIKTKVAQQTRIP
VEKMKILHNKRPLTDSKVLKDVAGAEELRVDFSVMVMGGAAAIQPESREE
APAKASGWDALTTDAFWDDLKGFLQQRLKDEAAAAELGTLFQRSWESSRS
GP
>Mb|QLI67677.1
MATEDAAWEGEGSGVEYDPLAQPSAGLDQPEETSEAVSEAAEPIDENEED
QDDGGEYDPESVGFDSVSHIPDKSASGTPSQRPANKPKMSGGFLVEVSDD
EEDDNQDAPSGARNSAAANANQTSNGTTPLPIAGALPNAPLGLAGVNPVV
LLEARIQEDPRGDMDAWINLMADHKRRGRLDELRLLYNRFLEVFPQAADI
WVEWIEMELNLDNFVDAEQLFGRCLMSVPNVKLWTLYLNYIRRRNDLTND
PTGQARRTVTQSYEFVIDTIGFDRDSGNIWQDYVQFIKNGPGQVGGTGWQ
DQQKMDQLRKAYHRAIAVPMSSVNTLWKEYDQFEMGLNKVTGRKFIQERS
PGYMSAKSANIALDNITRNLKRENLPRLPPAQGFDGHEEFHAQVEMWKKW
IAWELEDPLVLKDDEPKAYKQRILYCYKQALMALRFWPEVWVNAAAWCFE
ADIRENEKVQGTEMLLQGISANPESVLLAFKHADHIEANYPEKEEEKSEY
AKAVRKPYDAVLDTLYKMGDKVKEREKLEVNTLKQAAANEPAVQESIEED
DDDDDDEEKKVRKSPTEERIAAIQQAYAAESHLLSKTISYVWIAMARAMR
RIQGKGNQTEGGLRKVFTDARQKGRLTSDVYVAVALLESVVYKDPVGAKI
FERGARLFPNDAGFMIEYLKYLHSKDDTTNARVVFETCVNRLVSKPETLQ
KAKQLYAYFHKYESQYGELSQISKLEARMAELFPEDPKLSYFTSRYSTET
FDPVAAPIIISKAVQMRPKQLIPAIEQPISTRESIPPSRQEQSPRPQFIR
ATASPKRPFGIEDEDLNPPKRIARGVSPLKGAAGRRLDQQRRNQTSALPR
DVTFLLSLLPPAPTYDETRLNPTIMVSLLRDTQLPDYASWKAKTGGQYRI
TSGNHSRQASGDFGNRPTSPYGRMAAASGAYRNSPLRTESGNAFAANPYA
PPPDASGATASSWQPPSAGAYGIPPPAGQYGGYRY
>Mb|QLI67678.1
MSLNGLDDPKVVEAYEAATAEPGGWFLLKYESRDEIEVLSRGNGGIVEVR
NTIAEYDETSPLYGFLKYRRRSVIIKYLPEDCSRIIQARVAVHFNAVCER
FSPYDTTFEITTAAELKDSKLSAACSLHTASCSTSSSTSSLRRRRLMEIT
EEEEEEKRATKRQSVQDADGSRPESPTGRRSTSEPVTLNSELASSPENSK

FSAATTSEVPQFVGVDERPTSPESDVYTYGSYPYSKPKVKLGPRPSVDAN
GRPQTAGNFRPVSAIPAGFKMFGKGGKKGKNRDRQNSFSSPHEELASPDF
SVAVNSTFDDGQERPVTSSGLSIDSLPLPGSTPKKPTMSREKARLMKAKQ
LRDQKKKMSMLPSTEAAPAHSEELKNVIDNATQGQDEAISCSDPLPETDR
RLSLNKDDSGIMLETPTSVAPNDQNSDLAPSDSHPASPLVGSSDAGHSTK
ASSISESTDETVHAKEEEQSPETKEKHPRDSVGLNLEEATHAIDGPADGE
PKSDVGVAVDEHTVQKPLDAMENLGRGTDNTATSIAEASRDSEDLSAQDV
DKNHDEQNSLEDNALAFSKFSANGTRNLEEDGQTLTENDHIIDTRKKAND
NDEVQLVREEKQAKREMQILPIQTTNLKMEENFEDEIQSAVVEEATSATV
IKTPLTPSFPPSPSKAQNSYTVRTVSNPVRGDLVLPSDASQSSARSMSSS
AAYLNHIAQKQQGGNLAKKSNIGSVISERIKELEQRSAPSTDALAAPTRE
RPSSTFFAVKKREPSRSPSVLDRANSFRHQTPSSPERTLESSPEGGRRNR
LERSGSVTSRLSMFEPAPGPRNLVVPTAAPSRGRPESISVTARIIRDHNQ
PVQMGFEPSRDPSEYNPLELKQSPLLVDHQAASPGKDYRTTDPASEHTTE
NPEHGKPGKPRQSSLSIVKGFRKERRKSVTSDGSNPAYSPTRPDTTHGSA
TFSPRLSISSHRSSFSRDRDVVLSPADSGSGDDAKSTNGDKKLSRASRFM
HRLSQLSGSRSKNNLSATSPPTTKEETREPVQSRPSTTGSPTIVSYMGDV
NVQFPDNLLWKRRNMCLDSQGYLILSALPAQNGRPAQGTKRYHLSDFRAP
YTPDVEVQELPNSVVLDLIEGSGIQVACEDRAGQLRVLQILQEAHATRGT
TYAL
>Mb|QLI67679.1
MERHKQAVAKIATAVRGFFDKKESYRIFHGSTNSTRPRPRPGARIVDISA
LSNVVGVDRSSRTALVEPNVPMDRLVESTLRHGLVPPVVMEFPGITAGGG
YAGTAGESSSFKHGFFNETINYVEMVLGNGDVVRASNEEKPDLFQGAAGS
VGTLGITTLMELKLIEAKKYVRTTYHRTASVAEAVEKVRAETQNPDNDYV
DGILFSKDHGVIITGTMTDEKPADARVQTFSRARDPWFYLHVKDRTKSTA
SSAPPSVVDYIPLAEYLFRYDRAGFWVGAQGWTYFKYVPFNRWTRWLLDD
FMHTRMLYRALHASGESARFVVQDLALPYDKADEFVNYTTDAFGIWPLWL
CPLRQTLPPTFHPYTGETETAADGSTAPKDILNIGLWGWGPADHDEFVTK
NRALEDKLVQLGGRKWLYAHTYYDQDEFWKVYDKNRGWYQSLRDKYHATT
LPTVHDKVKIDVDAAREERKRTKQLLKSRWPVGGLYGIWQAIKSKDYHLH
RRAEWKYKGDN
>Mb|QLI67683.1
MHQHVSRALCRGQNYLSQSNIYRTTVIQPSNLPTCRFASWSQSSRSLSTT
AATKPARSYTPVSRAPTPPPTKSPEELAGASYIVRRTPSIQLPVYRRFAS
GGNREVVLIKKVDGDRKKLLEDLVETLGVSKQDIRLNPTTQHIELKGDYY
EKTKSWLLERGF
>Mb|QLI67684.1
MADDTEYTESDIWASPSHDAPLAARPKTPKTPKTPKTPTGPPASETVSRD
DLLRRELEGVRSVNEGIEGLLGTLNRAGGNMQVVAKTVANASTLLNTWTR
ILSQTEHNQRLILHPGWKGVTQDLAEQEAEALEKQRAAERKAAEEEQRRE
ELRRRREEEETARRLAPSTSREVARREAVCPNHLHQAHQEVAELQG
>Mb|QLI67686.1
MAKTVIAFDLYGTLLSTESIANELARVYGEDEAKLIATQARRYQLEYTWR
INSMDDVAFDTGDIHSSMLRSTSGDATLFTPRPVTDDQVGLYRSFSELTR
HSFRQATAEIGLELSRDQEERILDAYNGLDTFPDADKGLALVSQTPSLKP
YIFSNGTMAMITSSLKTSPSLAKSGTTFSESNIVSVDPLGVFKPDPRTYR
HMVEMAGLTHQLDKVWLVSSNPFDAAGAVAAGLRSVWVGRQGTGWIDGLG
GALGVSPTAVVGSVEEAVREIIRLDGLD
>Mb|QLI67687.1
MAQDAPPSQATGAVLMKSEAMPDGSQPVEELDFNKLKRPITAEDLFLGMR
HMGFQASSMAEAIRIINEMREWKDPDTSEKTTIFLGYTSNLISSGLRGTL
RWLAQHKHVSAIVTTAGGIEEDFIKCLGSGTTYMSSFSVPGADLRKRGLN
RIGNLIVPNANYCAFEDWVVPILDKMLEEQEASKGTDEEINWTPSKIIHR
LGKEINDERSVYYWAWKNDIPVFCPALTDGSLGDMLYFHTFKASPLQLKI
DIVEDIRRINTISVRAKRAGMIILGGGIVKHHIANACLMRNGAESAVYIN
TAQEFDGSDAGARPDEAVSWGKIKIGADSVKVYMEATACFPFIVANTFAK
DL

>Mb|QLI67688.1
MSTPLEFPPSFVRPWKELLLSRQREQLGDLPNHHARDPESLITTSSPQTL
YVGTGHSIFTRSILPAILGARSSVHFVTCYWAASPSLDAIRDTLLQLAAL
RRADGGAARSTLQITIGFSSWGLFQKLFHTSSRDGQLYPPSQWGKLGLPD
QRTLASGGIQLTVKSLFFTPFSVMHPKYVIIDGERAFVPSCNVSWERWFE
GCVELQGDLVQILMAFHGRVWGTGVDQSSSIETADGRESGPYISEGDSRT
VDTENSETMFSPEDDISAIKSLRITGNDNVPTILLPSSHHRNPRFSFFPF
LSRSNPPMTPLNAALLTLFNNARRQIIILTPNVTSWPVLKALLDALSRGV
DVQIRTNKGMMLLEQLVTAGTTTSWCLRKFVRQYQQLRVKSKSSDIEAPP
VRLGQLEILYYNAVPRRRGMEDEPVVSHFKMTMVDHEYLVLGSGNMDRAS
WWTSQELGFLFYVPGVDWHKLWDGVLEKRMEVYYRSRDD
>Mb|QLI67691.1
MKLNISYPANGSQKLIDIDDERKLAVFMEKRMGAEVAGDSVGDEFKGYIF
RITGGNDKQGFPMKQGVMHPGRVRLLLSDGHSCYRPRRTGERKRKSVRGC
IVGMDLSVLALSIVKQGDGDIPGLTDVVHPKRLGPKRATKIRKFFGLTKD
DDVRKYVIRREVQPKGEGKKPYTKAPKIQRLVTPQRLQHKRHRAALKRRQ
AEKVKDEANEYAQILAKRVAEAKAHKADARKRRASSMRK
>Mb|QLI67693.1
MLLKTVLGFVATAAAQLTAIDHTKALVTRAGTERISNRDFSAIRFYSQHA
AAAYCNFNAAPGTRITCQNNACPLVMRNQPVVVASAIGDALGVGAYVAVD
YVRREIVLSFRGSNNIRNFIADLAFAWSDCNLTQGCKLHTGFAQAWYDIS
DAITKAVRSARSSNPNFRVVATGHSLGAAVATLSAAYLRRDGLAVDLYTY
GSPRVGNKNFATWFLTQRGVQWRVTNGDDPIPRLPPLVFGYNHISPELWR
PGGDVQTVWQPSTTAICKGVDNTDCNGSGLSPDPWAHRNYFGSVDACAGS
SLTLRDARGLPEDLINRLTDWSRQDRQLSGSLGHHGDVEFLLKPLDESPA
SAPGKHEYTIV
>Mb|QLI67695.1
MSVRVVARIRPLLETELDKDIIVRPDGAEAGGPHTIVKIPNPKNQAEDFS
FAFNAVYDQSTSQETLFTSEVQPHLKSLFQGYDVTIFAYGVTGTGKTHTM
RGGLKLADRGVIPRLLSGVFRRGKKIAKDSEGNTTVNVSLSYYEIYNDKV
FDLLEPPEKRTPSGLPLRAEASGRTVVAGLSERACEDLRDFEKLYIEANN
NRVTASTKLNAHSSRSHAILRVKVTQTTDEMIRESTVSAIDLAGSEDNRR
TDNGKERLVESAAINKSLFVLSQCIDAISRGDKRIPYRESKMTRILSLGQ
NNGITIMFLNLAPLRSYHLDTLSSLNVSSRAKRIEVREIENEVVFKQFPR
SAGSHTVQRQPLRPLAHALSAHNVHNGNVAAKGSADVTRPIKSFSVYTDK
ARQSPTKTISMATSLPRPAAVNVSLVPRLSAAYKRPSDHESGIARPRKLA
RPSIDEPSITVTASQIEAMVEKKVGEILAARTAAEKAVQSQNRGASSQHD
VSEAMQRRLEALERRIESEDARVDSKSDGLRFLLAARQHREKGDDASALR
AYEMALPFFPGQAKLIGKIQRLRNRLGISVEQTCVMRSPMRSPLRHDKKL
RRTRLTINPDGTSSGQDETDVEEEFLDDDTVRIKPRKSRSKSMAKLQVYG
DVNETTTVLGARDLAPSRGLVSY
>Mb|QLI67698.1
MPSTPRALAALGRASQLALPTRVGSRAYSTAYSAATGRHALRSAARPTVA
MRLAATGRVAFRRAYADEAPKPKPKPGRLRRTFRWAWRITYLSAVGLVGY
TCYIVYQDRHPEPQFEPDPTKKTLVILGTGWGSVALLKKLDTENYNVVVV
SPRNYFLFTPLLPSCTTGTIEHRSIMEPVRTILRHKKAAVKFYEAEASSI
DPDRKVIKIVDNSEIQGATSETEIPYDMLVIGVGAENATFGIPGVREHSC
FLKEIGDAQQIRKKIMDCVETAAFKGQTSDEIDRLMHMVVVGGGPTGVEF
AGELQDFFEEDIKKLVPEISPRFKVTLIEALPNVLPSFSKQLIDYTENTF
REEKIDIKTKTMVKRVTDTTVEAEVSRPDGGKERVVIPYGLLVWATGNAV
RPIVKDLITKIPAQKDSRRGLAVNEYLVVQGTRDIWAVGDCAVAGYAPTA
QVASQEGNFLGRLFNNMAKTENHESRIQELSSKMNLQAGDSAEAAHEIES
LEKQLRRIKDIKPFRYSHQGSLAYIGSEKAVADVSWWNGNLATGGSLTYL
FWRSAYLSMCFSTRNRVLVILDWLKSKAFGRDVSRE
>Mb|QLI67699.1
MSSDYALSASHKEMLEKPLLETDPEVATIMKDEVQRQRESIVLIASENIT
SRAVFDALGSPMSNKYSEGYPGARYYGGNQHIDRIELLCQKRALEAFHLD
PERWGVNVQCLSGSPANLQVYQAIMPPHGRLMGLDLPHGGHLSHGYQTPQ

RKISAVSTYFETMPYRVDLETGIIDYDMLAKNAILYRPKILVAGTSAYCR
LIDYKRMREIADSVGAYLVVDMAHISGLIAAEVIPTPFQYADVVTTTTHK
SLRGPRGAMIFFRKGVRSVDAKTGKETLYDLEGPINFSVFPGHQGGPHNH
TITALAVALKQAQTPEFKAYQEQVVSNAKTLENTFKSLGHKLVADGTDSH
MVLLDLRQFSLDGARVEAVLEQINIACNKNAIPGDKSALTPCGIRIGTPA
MSSRGFGEKDFERVAKYIDESIKICKEVQAALPKEANKLKDFKAKVASGE
IPRINELRKEIAAWTSAFPLPVEGWRLDAGI
>Mb|QLI67700.1
MEEELQQSVMLSEDGEYDTADTGDQAPSSLTHAGLQKNGDVDVSGSEDEE
LSDQDASGEELDQDASGEEDNDRMDQQIVVGSATPGHGEEYDEDAEGEDD
LDEEVGAVKVKPVDSEDEDMGSDGSDAQSAAEEEESDDEEDEEDEEGAWE
EGDAGDEDEESDAVQSNTCMFCKQDEENDPSEDFEAFLACTRCGENAHQQ
CARDAAAMTEKNNSKRWKCPECFNEEESESEDDDDDADDIDAISAEDGEA
PPGSERSHVSKTGVDSSPVALANGSSGDRSGTDELVLEDYPIDGPRPMRK
RKSASAEADDATLAIRKRRRNQSIDGTDRDDSAQDSRSGDAARAASSRIT
RLKVTRPPPVTIEKRSRTSLVIKIEVRPGNLKEILSRRKRDRRRVGGSSA
RPPQAPRSTPARPPPTPASTGNLPALISTMPTPFTSESYSQPFYSFFDKE
TEEMKGRPYGGILTEAEADTSKTLPSPEDRKKFEHAKQKAEDEWRARVLA
MQAEADLPIRKPKKASDNASQIECIEFGGWEIDTWYAAPYPAEYSRNRVL
YICEFCLKYMNSDYVAWRHKLKCGTKHPPGDEIYRHESVSIFEVDGRKHP
VYCQNLCLLAKLFLGSKTLYYDVEPFLFYVLCEFDDTGYHFVGYFSKEKR
ASSQNNVSCILTLPIHQRKGYGNLLIDFSYLLTKAEEKTGSPEKPLSDMG
LVSYRNYWRLELCRYFLGYMESDTRKREGLSIKKISTNTGMTPDDVVSAL
EGLRALVRDPQTHLYAFRVDLDYCRNYVSKWQSKGYVQLKPTALAWTPYV
MGRSNVVNFELGPPINTIAPREDDEAKIQEGATLTAAHARGMQSTLNGVD
KSDSQPEPTLASQQGSEQPALPVKSIEKVKTEDKENADPEDQGMVDATTK
VEPESWDAPYRDIPASRFEIFPPVPGGRRDRIRPSVSRPSAPRNGSSSSA
RPRPRSRPSGSARRPAASRPRNNSSRRKSGGTGRGPGRWPKGTKKSDYGN
ADSGPGLPPGWREKQARLKALAEGRDPGAAENPDEEPAQDEVRVLVSTAG
TNQSKAANGSSKVSRRDSDSRSEAGKAASDGEDVDAEGEDE
>Mb|QLI67701.1
MPPKTGKKVAPAPFPQGKAGSKKAPKNPLIEKRPRNYGIGQDIQPKRNLS
RMVKWPEYVRLQRQKKILQMRLKVPPALSQFQHVLDRNTAAQAFKLLNKY
RPETKVEKKERLLKEATAIKEGKKKEDVSKKPYTVKYGLNHVVGLIENKK
ASLVLIPNDVDPIELVVFLPSLCKKMGVPYAIVKGKARLGTVVHKKTAAV
LALTEVRSEDKTELSKLVSSVKDGFMEKHEQARRQWGGGIMGAKAQMRIT
KKQKAAEAATKI
>Mb|QLI67702.1
MAPPKRTAAPKENISLGPSVRDGELVFGVARIFASFNDTFVHVTDLRLVS
PETADGRETITRVTGGMKVKADRDESSPYAAMLAAQDVAARCKELGINAL
HIKIRATGGNGTKTPGPGAQSALRALARAGMKIGRIEDVTPTPSDSTRRK
GGRRGRRL
>Mb|QLI67707.1
MKRKLDQNGVPSPEPESQKRTKSESAGDQELTFADLGLDPRLVQAVAEQA
FLKPTLVQRKAIPLALNGKDVLCKSKTGSGKTAAYVLPVLTAILKKKTAE
TVAETSALILVPTRELADQVFKVIEQFSSFCAKDVRAVKLTDKLSDAVQR
SLLSTNPDIVISTPARAWYNVKSNQSSLSLDKLTHLVLDEADLLLSYGYD
EDLENLSWSIPKGIQTIMMSATLTAEIESLKKIFYRDNAPTLLDLEEPDA
EGEGITQLVTKCAEDEKFLLAYIIFKLQLVKGKCIIFVGDIDRCYRLKLF
FEQFGIRSCILNSELPVNSRIHVVEEFNRNVYDIIIASDEKEVLGNEEKA
DEEENNEGEQEEDSDAEKEERRPKKKRKASKRDKEYGVSRGIDFKNVAAV
INFDLPTSASSYTHRIGRTARAGKAGMALSFVIPKEQFRKHMPTSIQSAE
NDEKVLARITRQQAKKGKEIKPYNFNTKQVEAFRYRMNDALRAVTKVAVR
EARTRELRQELMKSEKLKRYFEENPSEINHLRHDGELRTARQQAHLKHIP
DYLLPKDGKKDLTENDIGFVPLRKPGGPRGHRKGKGTKRGGSRVGARKGD
PLKTFKARRKTK
>Mb|QLI67714.1
MPGSSATEPLLIHMPPFPQPDGSASKLPTFLGKRPVASINYRWRPFSEPS

PEVSTVNKVFSPLYWPTPVHDTSFAFAWLIENFPPPTHKRRDIYIYGSYL
GASLAASLALTEAHTHARFGVRGLIAYNGIYNWTMFLPDHRINRPPKRAK
ATTAPPGPQEGSQLHRLQEQMPALFDMPSNLFDPFASPSLFFHSPGLIVP
QSFFMTVEHSALIDNMTSDEEITPNPIKSPRKSHLVFPPRQSTLKIPDTL
LLFDSAALPVPKPTTKPKSARARKRAKPKGHSFEAQAAELAELMRRSVNV
VELKERSKWDEDVHSRVDEAIRRVRVVDVGLERDSTELSDVGCDAVTTWL
ENR
>Mb|QLI67715.1
MGEYIDSRNASDELWKHDAPGDTPMWQFIQQVNKKHALSINGYPGLYQWS
VDSVSSFWEEVWDFVGIVASKKADKALPENAPMYPRPDFFSAARLNFAEN
LLFCPTWPIDPDSVAVITATELPGDIKPTTWRQLRESVRQCSNALRGAGL
QSGDVVAGYVSNHEEAVVAMLAAASIGAIWTGISPDNGVSAVLDRLAQIG
PKVLFADNGTVYNGKEWPSTSKTNEIAAALKGVGLELVVVIDNISADLGI
QDIKSQGVTALDYKSFLRDAPPDPIKFEQLPPSHPLYVLYSSGTTGLPKA
IVHTALGTLLQHKKEHMLHCSMTAASRMLYYTTTSWMMWHWSVGALAVGT
TLVLYSGSPFRPSSHMSLPKLLSQLKVTHFGTSAAYLTALEANNVRPVED
ASIDLSRLEAIYSTASPLPPSTFRFVYEAFPKTVNLASITGGTDIISLFG
APCPLLPVRVGQIQCAGLGMDIRVVDSISGEDMPPGEQGDLVCVKPFPCQ
PLTFFGANGQTKYRSAYFERFENVCGVKGPVWHHGDFVKIPDPSTGSLVM
LGRSDGVLKPAGVRFGSAEIYNILTRFFAAEVEDAVCVGRRRETDRDETV
CLFVVMVPGKPFSQELKTQIMAKIRSELSPRHVPAVVEECGAGIPKTGNG
KKIEVAVKQILSGLNIKTNASVANPEALEWFKEWANKN
>Mb|QLI67716.1
MLRARLLPFGLWTLEMRMKPRTIHSQALRKILSYSTLQNIVVDSIMGSYT
ANLVETLDAVDASTLDGDDASRLQLAMAARKLFHKLETKEEKTIRLAIEE
PVVFSAIQALIDVGLWEDWTGVGGGEKTIEDLATITKKDIDPELLRHLLR
LLATNHVVLEAGEDRYAPTAFSLSMGDKSTLVAPALRIRTDHIAQCAHHF
PEFLADTNYRKPLDDNASCYIDTFPEKKNFWQRCKANPMHQESFSSFMLL
WGKHKRPWPQFYDTKALLEGADLSDGSAFIVDIGGHHGVDLLRVLDEHPD
VPAGSLVLEDLPEVITSVSLATDKIKVIEHSFFEPQPVKGSRAYYLHAVL
HDWSDKVSIEILKNVAAAMKPGYSKVLIHDIVLPATGTTCYQAALDCLVM
QASANERTEAVWNKVLAEAGLKLLKLWPDGRGYESLIEAELA
>Mb|QLI67717.1
MAQHPTLKATYASRAQTATHPLNAYLFKLMDLKASNLCLSADVTTARELI
YVADKLGPSIVVLKTHYDMVSGWDFHPQTGTGAKLASLARRHGFLIFEDR
KFGDIGNTVEMQYIGGSARIIEWAHIVNVNMVPGKASVTSLANAATRWFE
RYPYEVKTSVSVGTPTAEEFEDHDVGSSGRDDTNDSAPRRTDDGRKGSIV
SVTTVTQQYEPVNSPRLTKNMAGDDEVLFAGIEEAPMTRGLLILAQMSSA
GNFMNKEYTQACVEAAREHRDFVMGFVSQEALNTQVDDQFIHMTPGCQLP
PQDDDRNGSVQGDGKGQQYNTPQKIIGIAGADIVIVGRGILKASDPEGEA
ERYRSAAWKAYTERVR
>Mb|QLI67718.1
MTEAPPSALMPVCSSAQLGSPQLEKAAQHKSDTAVTGSRAQTSSRPRDET
AGNFSSRRGHASQLSISDPSHHVTEAIGTLYGDEDDSGSETGGRPLSFID
GSSYHEQLQRPAHLDSQHHAHARSRHQQQMEYDSRKPLTRSMSDELYATQ
SSYEEHGESLKKSQTMNIPSPNRRTSMQYEGGSKAPASPALSLRDVQAGE
AGSQFPLTNIDNPNDIAQELSNLQALRRMSMDVGNSIDPDMPPIGLSAMP
AIAPSGNDDENDPSRLLWVPARVHPELAPSEFKSFLENRVQSIRRRSGES
FLSADGSDLNRSNPGSLRRQKSMLSRQIDNSSGQGAVGYVDGADRLERKR
STAGSHNRELSLDELVKDPTRAVQKLAKEVHNTPSGEEGSTDDMPILPMA
PGMGLRRSTRTTYRKGGSLRSGDRVPFSKRLAQKQQDGELPDLPSVPPRD
ALRGHTPDSVQEPLSENYSRPSRSVRKQWDFSQDSPGNDGSSPNEEDVRV
ASPPVQEQLHVRAISMPTVASPPEGEPRNDEPGHASNQFSGTFPQRSSSQ
TTGSLKPESTPKTPEVVVEDSLGPHAARHSTPVTNNYPLQKQNQQAQYSS
KPLPPSQQPSYNDKFASASSSLLTGDASRTDNLTVIPTFSQIDKKSDKRS
KKDRDDDASSTSSKSGGAWKWFKGVTDDKDKKKEENKRAKAKALAEKTSD
NARLDVIQSSIDKTPAKGRESLVLDRDNMDNKLLEERKKESHRKSDSKKD
KDGNIFSSIFGGSKRKEEKEKSGKKSQHLQVPEEPVYKPLQPDVDYNWTR

FPLLEERAIYRMAHIKLAHPRRPLLSQVLLSNFMYSYLAIVQAMHPQMNV
PTSPQQKRLEEEARRKRQEQEYLAQQQMDDEDADNSLEQYNFDYHRAAVQ
YAESGSENQVDYVDEAQIYEDEHGNDNQEDYSYDNQDGYGHSVKDYYQYQ
GDDSDHRHHGGNSMW
>Mb|QLI67721.1
MDLAYDHIQENAYPQDRKESNETPKPEQQESSLNNDFQDAYKAISSSPWG
MKIGGFLGNVMKQGETVYAQAQKELAEVGEDATKGLSDLRESILNRTRSL
SVNTATAVSTDGGKEGDDTQSTPTKTRATASDDASEQSETVLSRIRSEAA
KRLKDLQQAEDAADEALLRFGGSVRDFLRDAISVAPPDESSQDSAVLFES
KDAQGKRVIHTSRFDAQMHVIHTSVDGFAEDPTSDEYEKWTTEFDVEKKT
DDISSDLAKYPELRTTMEKLVPDQVSYADFWKRYYFLRHGIETAESRRRD
LLQAASAEDEVGWDDDSDEEVGEAEKTSSGQKPSSGASSTTIQRPDSLAK
PPEPQKSNDEKSQADSEASYDVVGATSGKTSQAPNSPKDPRQGDDSDDDW
E
>Mb|QLI67723.1
MAGSRAIVPTLDTVRSIVERPHAGPGKKPTLIPIYRQISSDLITPSAAYL
KISAHSSSEHSFLFESAATEQVGRYSFVGAGPRRILTTGPGYGPETDPLP
ALEKELAEHVVAHVPGLQLPPLTGGAIGYVGYDCVRYFEPKTARPMKDVL
KIPESLFMLFDTIVAFDRFYGIIKVISYVAVPEDGKTGLEAAYETAKRTI
DDLVDVLNSPEIELPEQGPIELGQEATSNIGQKGYEAHVTKLKEHIVKGD
IFQCVPSQRFARPTSLHPFNIYRHLRTVNPSPYLFYVNCKDFQIVGASPE
LLVKREAGRIITHPIAGTVKRGKTLEEDQRLANELSSSLKDRAEHVMLVD
LARNDINRVGDPFTVRVDRLMVVEKFSHVQHLVSQVSGVLRPDKTRFDAF
RSVFPAGTVSGAPKVRAMELIAELEKEKRGVYAGAVGYFGYGSEDENGNT
VEGAMDTCIALRTMMVKDGVAYLQAGGGIVFDSDEYDEWMETINKLGANM
QCIKSAEELYYDQQQAAKSTK
>Mb|QLI67726.1
MKEGYEHEDDTQLAAMGHRPELQRNFSTLSMLGLAFAVLNSWTALSASLS
VSLTSGGSTSIIWGLVTAGVCNLCIAASLAEFLSAYPTAGGQYHWVAVSW
PKWVPILSWITGWINVAGWIALVATNALLSSQLIAYDPQRWHQFLIYIGL
TLLSFVINASMNSLLPVIYHGAFMWSIGGFVIVSITVLACASPDFNSAYF
VFCNFINQTGWPDGIAWLLGLLQGGLGVTAFDAVAHMIEEIPHAAAEGPK
IMVVCVGIGTFTGAIFLIVLLFVSGNIDDVISSSAGPLLQILIHATKSNA
GAICLLMLPLVCLVFATLSVMTTSSRMIFAFARDGGLPASKFFAKVHTRL
GLPLNALILTSVIVILFGLIFLGSTSAFNAIISASVVALDLSYAMPIAVN
FIQGRNALPDRKWKLPRWFGWTADCISLSYISLTTVLFLFPPGLPVTGSN
MNYCIVAFAIIITISVFQWIVDGRKNFTGPRVNLVPERAMQPPTQS
>Mb|QLI67729.1
MKAIQVSQTGGIDVLDYKDVPVPTPKEGQVLVKNEFAGVNFIDTYFRSGL
YKLDLPVILGSEAAGQVVSHHKDVTSFPVGTRVVYMDSQAYAQYTAVPAS
KLIAIPAGLTTEQAAAAFLQGLTAWTFIREAGNVQPGQWALVHAAAGGVG
QLLVQMLRSLGAKVIGTASTQEKCDLAKKLGAEWTVSSRDDVVARVNEIT
GGHGVDAIFDGVGKATFDADLQMIALKGHLVSFGNASGPVPDFSILRLSD
KNVTLLRPKVHGYVSERKDLEKYSAELFDKLISGQVKIAIHDIYPLKEVG
RAHSDLESRKTKGKLLLSVE
>Mb|QLI67731.1
MGLLPEFDDRHIEAEQQEYHPYEYQTDNNDSWAGALPVKQGLYDPAYERD
ACGVGFACHIKGKPSHKIVSDARNLLCNMTHRGAVGSDARDGDGAGVMTS
IPHRFFIKNFEKEQDIKLPPPGQYAVGNLFFKPDEETLEESQRQLEDVAE
SLGLRVLGWRRPPVDSTLLGPAAKSREPIIAQPFVVLASAYGSGVAPEMT
DPQKFDERLFERQLYVLRKRATHTIGLKNWFYLCSLSNKNIVYKGQLSPV
QVYSYYHDLVNADYEAHFALVHSRFSTNTFPSWDRAQPLRWAAHNGEINT
LRGNKNWMRAREGVMNSDIFKEELEQMYPIVEDGGSDSAAFDNVLELLTI
NGVLSLPEAVMLMVPEAWQGNDQMDPKKAAFYEWAACQMEPWDGPALFTF
ADGRYCGANLDRNGLRPCRFYVMDDDRIICASEVGTIPVEPESVIQKGRL
QPGRMLLVDTKAGRIIDDKELKAAVANRHDFRSWLSKELLTMPKLLEKLE
SAIDLAAKPNAIPLQEDPLLLSFGYTHEQVSLLLAPMAADEKEALGSMGN
DAPLACLTPAPRLLYDYFRQLFAQVTNPPIDPIRESIVMSLECYVGPQGN

LLEMDPSQCGRLLLPSPILSIPEFNAIKNMSTVQPEWTVKTIDLTFPKSQ
GVQGYIKHLDEICNEATAAIEARDRIIVLSDRKTSADRVAVSAALASAMV
HHHLVSNKWRSMAALVVETAEAREVHHMCVLLGYGADAINPYLAMECVLK
LNREGLIKKKLSDEALIRNYIHSCDGGILKVMSKMGISTLASYKGAQIFE
ALGLDETVVERCFKGTASRIQGLTFELIAEDAFRFHERGFPSRYTVDIKA
LPESGEYHWRDGGEPHINSPAAIANIQDAVRNKNDKSYEAYSKAEYEQIK
NCTLRGLLDFNFEDATPVPIDQVEPWTEIVRRFCTGAMSYGSISMESHST
LAVAMNRLGGKSNTGEGGEDPERSERMPNGDTMRSAIKQVASGRFGVTSA
YLADSDELQIKMAQGAKPGEGGELPGHKVSKSIARTRHSTPGVGLISPPP
HHDIYSIEDLKQLIYDLKCSSPRSRVSVKLVSEVGVGIVASGVAKAKADH
ILISGHDGGTGASRWTGIKYAGLPWELGLAETHQTLVLNDLRGRVVVQTD
GQLRTGRDVAIACLLGAEEWGFATAPLIAMGCIFMRKCHLNTCPVGIATQ
DPELRKKFQGTPEHVINFFYYIANELRAIMAQLGFRTINEMVGHVEHLKM
RDDLRTHKTANIDLSLLLTPAHKLRPGVATFNVRKQDHKLYVRLDNKLIS
EAELTLDKGLPSRIECDIVNTDRAMGTSLSYQISKRYGEAGLPMDTVHVN
IKGSAGQSFGAFLAPGVTLELEGDANDYVGKGLSGGRLIVYPPRNAVFKA
EENILVGNVCLYGATAGTCFFRGIAAERFAVRNSGATAVVEGVGDHGCEY
MTGGRVIILGRTGRNFAAGMSGGIAYVLDIHSDFRSKLNTEMVEAEPITD
PSEIAFVRGLIEDHHHYTGSELAARILVDFNRALPRFVKVLPVDYKRVLE
EEAAKAAEAKRAEYNLPVVSGVKHKKEEKAAKLQDLEEAIGDAKAEKKRA
LVLDKTKGFMKYARRSEKYRAASTRTKDWAELSSRLDEDELKYQSARCMD
CGVPFCQSETGCPISNIIPKWNELVFSNQWKDALNRLLMTNNFPEFTGRV
CPAPCEGACVLGINEDPVGIKSIECAIIDRGFEMGWMIPHPPEIRTGKNI
AIIGSGPAGLAAADQLNRAGHSVTVYERADRLGGLLMYGIPNMKLDKRIV
KRRTDFMAAEGVNFKTGVAVGEEGQPSLSDLRASNDAVIIATGATVARDL
PIKGRELGGIHYAMEFLHKNTKSLLDSELVDNAYISAKDKHVVVIGGGDT
GNDCIGTSVRHGAKSVTNFELLPQPPPERASDNPWPQWPRIYRVDYGHTE
VRQHTGKDPREYCIMSEEFVDDGSGKVKGINTIRVEWTKSPSGGWDMKKV
EGSQQFFPADLVLLAMGFLGPEARVLGDEIEKDARKNVKTPAGKYCTNVE
GVFAAGDARRGQSLIVWGINEGRMAAREVDLYLEKNTNLPVTGGIVKRTA
QEILGRVAEVN
>Mb|QLI67734.1
MRSELEASETERDTTVLKETLDTEDVWACENDSASCQDDSSQWSETLRAS
SSSPSVLSEESSNLAVEIPDFQLSSPRSCYEPFHPPAPPKSEEDALECFG
PSVQRQSSPSSGHFELQLDEFAVYLDTENYPCEMRCLHHLYARPGYSNFF
FDGILSNNGQGKTFMKRVPISAVPVGQYGLQYHNVGNQIWLQSTYCDQSD
IFYKLCLPAREYRRFFKAFLWVANLAKHFVDFLVFMEEKGTRVTIRHFQS
AFGDWLLVVHGDAPDLMDWLAQHASSDFRTSVNANISFLYKEYRGIITDR
EYGYHDIWAEIWDFTKYPDLSTGTAQEPTIVTDYVYHRFSDLPFGNRLHN
FPLSRRASELRERASLKHGLELACTSHGPATSSFVNETGGDISDITRAIK
PGDTISTRRDGEVSRSMRRRELSNDFAGVDRRFALVQKVCERSDGGRYFE
VLWYYRPSDTLCGLMKYPWRNELFLSDHCSCAEENKIQDCEALRVHSVQF
RGDSTTTSEFFCRQRYLSKEKKWIALDDSDLMCSHLNPRLQDSTSGKYRP
GDTFLIHVNVASSTSEACELISVNTRGAGTRLCFRKLLRRSNVDSMAVTS
ARNELVYSNESIECDPSGIVGKCHVRFFAHDENIPAPYNRAGVGGYFYIT
HRVSPAGLCVPLRSVPSSLRQGFDPAVVISELRGLDLFCGGGNFGRGLEE
GGAVRMSWANDCNARAIHTYMANCSHPNLMSPFLGSIDDFQREAFSGNFS
KSVPTIGSVDFISGGSPCPGFSTLTNDRTTDEQRKNQSLVAAFASCVDLY
RPKYGLLENVPGILPSKANREQDVLGQFMCAIVGIGYQAQVFYLDASSCG
SAQRRSRVFISFAAPNCKLPGKPQQTHSHPSETRYTSLGKLPTRRHVLAE
RDFLRATPFSYSVARQVSSGLPKIYNGQTDTCIPFPDHRLARGLTKGLKA
RISLIPNRPWGMNLRSASRGILTPAERGVFVTINRKTSRPNTEPHKLDRQ
SSAYGRLYPNQLIGAITTVQVPNDRKHGRQLHWDENRGLSVMEARRAQGF
LDGEVILGTPAEQYKIVGNSVAREVALALGLSIREAWAESCADDQDTEAA
KPLSSSLGTGALGGNADHGPRHTSAAEASIRTRVIPQKRRRRATSRMVVV
TGRAKEKCPPECIDAR
>Mb|QLI67735.1
MRAATFARSAVWAASRSSRTLQASRPLRLFSIKAEAASDSKQKALDPNRL

SITQTSSPKPLSQPETLVFGREFTDHMLAIEWTQEDGWLDPKITPYQNLS
LDPATCVFHYAFECFEGMKAYKDKDGKVRLFRPDMNMARLNKSASRIALP
TFEPTSFIELISKLVNLDSRFIPNQRGYSLYLRPTMIGTQKTLGVGPPGS
ALLYVIASPVGPYYPTGFKAVSLEATDYAVRAWPGGVGDKKLGANYAPCI
VPQLQAASRGYQQNLWLFGEEEYVTEVGTMNMFVVLKDKKTGQKELITAP
LDGTILEGVTRDSVLALARERLVPEGWKVTERKYTMNELYEASQDGRLVE
AFGAGTAAIVSPIRTIAWKGKHVECGLKETEESGETALRVKEWIEAIQYG
EEEHEWSVKIA
>Mb|QLI67736.1
MPLQKSYSESIGLSKNRGPWQRSHSHPANLQAQRPRPLPPVSDVTKKKLS
RFQYRPAEDESVASAPGEASGTPTKPQDAAVARAGVVGNAATPVTRLNWR
DLLEPNAAPAENEADISSPNDKLLWNNQQDTLYAAALSPMMNRKGRKRAR
SSSPISSPATDKGGLPAVNVDKLRTALRSPRVDPTVELWDRYSLNGSENI
TTPNGAMNPSLAQLTVSSSPKPSKDMSARRGDANLRRAISCGLNWPKRRK
VERSKSGSQSSSQQREMEAASKSSLVTALLDTVTGTINEPGPSRAQEQLM
RSPSPKKRKRSPERMETPRRAEKSAEPSSSSSDYGDDFDDDVFVELEASM
QATGTTNTPLPDKIDQGQPGRRNHDGKSQSVIDLVEEFNDMDDDDSVCDN
IVQSRQALGISTPKRHSKNLPAAKLEESPGDEFGNDLDGDIDFDAVELAA
TQSIQQLEPPSKSILIIQADNSKVTRTVHLRGAWFETPAHTNSYVHVIGS
FSARGQCIIDDAQNLLILHPDQLISSTVVADSFGCMRRAVLQDRVKATSE
ASPPLVYGTMLHEIFQEALMANQWDLPFLGCIIDKITENHVEDLYTIKVG
LPSAKEHLLSKMTELSYWAKAFVAPKPQDDAVVEDRNGKKANMAITKLLD
VEEHVWSPMYGLKGNIDATVEVAMSDGEQTRTLTVPFEVKTGKHANSSHM
AQTALYTLLLSDRYDIDIAYGILYYMETSKTLRIPAIQHELRHMILQRNQ
LACYIRERSVQLPPMLKSKHMCGKCYAKTSCFTYHRLADDGDGESSGMHE
KFDELVRHLTPTHQDFFVKWENLLTMEEKESQKTKRELWTMTSTEREKKS
RCFSDVIIEEGSASVDTLNPRINRFHYTFVKRNHQPSFSFLESELMTGEP
VVVSDEEGHFALAIGYVTSVRKQRISVAVDRRLHNARNRQLGFDEIDNQV
FSSIMDVTQGKSPDQEQSNIKHPPVRYRLDQDEFSNGMATVRNNLVQMMA
NDVPAATQIRRLIVDLAPPRFKTAPTQYTVTDGENLNVDQRRAIEKVMST
QDYSLVLGMPGTGKTTTIAHIIRALVSQGKTVLLTSHTHTAVDNILLKLK
TDKIPVLRLGAPAKVHLEVQDFAHLAGQPRKTFDEIKEAWHGTPIVATTC
LGINHPVFQERSFDYCIVDEASQITLPICAGPIRMARSFVLVGDHNQLPP
VVRNEDAREGGLDVSLFKLLSDAHPEAVVNLEHQYRMCEDIMTLSNTLIY
NGRLRCGTESLRRKRLHIPNINALGQVHFSASSLTHPGTPRSFCTGPGPS
RCWLYDLLESEARVRFVNTDTIRPLVREEAQGKRIVNSTEVRLVSQLVES
LLAVGVPASEVGVMTHYRAQLFLLKDRLKGYAGVEMHTTDRFQGRDKEVV
VLSLVRSNEACNIGDLLKDWRRINVAFTRAKTKLLVVGSMNTLKHSGKEN
MLSKFISLMEGRDWIYNLPADALESHYFEELSTPMTAGPTPKSKSPKKTV
KKGVFSLEGKENRRPSPKKARIGERALLKGKLVTRDILNEMTNGAYMGV
>Mb|QLI67737.1
MIDHVLGRPSSKSRRLQVLAVLSLWSFYLYRGHRHGPPGAQSLSKFFTKR
LTAWQISALTIIYLYAARNFSALVGLASPEPMANMYDTTYFRATWVLTAL
DAGFWTAMKIKVKWMRDLASILFSLFYLVAAERADEKVRKVRGMITVEHL
RVAWNKGTTPYLSVLQRLMRPRFTRWPPRQIRIPRPSNSDYKEPILGWLY
FDGPLADLLLHSRLVLDVPGGGFVAMDPRCNDDKLLAWASKSGLPILSLD
YKKAPEFPYPYAVNEIFDVYAAIINTKGRCIGLSGRETPRIIITGDSAGA
NLAVSATLMITETSDGEFRRFAGRGALPAPDGLVCFYPALDMNIGNWMTD
EQMSLIRDRKMRKTNRTIARRKSMQYNQLVGTPHHSDDEDEITPPESTSI
PPRSPHDCQPQFSHTGPRSFTSDTERNGPKKVDNMSHHSQPMQTRLATSS
MISYFNDRVLTPEMMRAMIILYIGPHNRPDFSQDYLLSPVLAPENLLARF
PKTYFMTGERDPLVDDTVIFAGRLRRAKEAVAREQNRPFDDTEAAEVALI
PGVSHGFMQFPGIYPPAWKHFDRCVLWFEQLFLEAEHLRTKNQHPTHLSP
RTERMAYHSEQRQHHQHHLAESSGDEDKPLEISMTKLNRGRGSNASGAEK
LEMVSNEKGNGDAQKHKKKILSKNKSLIKLGSTDDLLDRRMEGLAGGLTG
TGEDN
>Mb|QLI67738.1
MPFDDDDNLAAEPSAQTPNGGPTTEDALEDGVPDYKLFSAMFSKKPVSSK

VIRKGEKDFESHGTQAQDNTLEASRQALEEVLSYTRVHRPENWIRAWYFP
DWWTQQRPEGSQEDALLHERVVAVEQERGSWMKDIGRAVSGSKDQLGVGR
LWLLPEEALYLIERGNVDIWWPDKEFGELLPLGVGNGPGRNQFGPDDYDA
GVPLTVEAAYAMMVGYEGERGKTTLSKYQVYTHLKRAGFNVMRAPIHAPP
EPTAPSSSSQSLWQWLFSFVSRESKSRHGPYGPLVSPGLYRAYLPVYQQL
AVIPRHKPSAHPEEHHHVQEPFRVHFHVWKPAGIPFSKKNPPPPDFRIAV
ADTADSKLPTLEQIEALLESTPFDLPNPKWQGPGKLYQRLKHGHRNVLVA
VVDRGLVNFMRFGEAAFGAERLFERFDSRGSGTRGGRKGGRGRGRGRGRG
RGGGRATR
>Mb|QLI67742.1
MQIFVKTLTGKTITLEVESSDTIDNVKSKIQDKEGIPPDQQRLIFAGKQL
EDGRTLSDYNIQKESTLHLVLRLRGGGKKRKKKVYTTPKKIKHKRKKTKL
AVLKYYKVDKDGNIERLRRECPSDTCGAGVFMASMPDRQYCGRCHLTYVF
DKQ
>Mb|QLI67745.1
MQGFNMGRYVPPDLEGTTSANKLHKKHGLGQRASKLASHGILTVRFEMPF
PIWCTSCPKPTIIAQGVRFNAEKKKAGAYYSSPIWSFRFRHAACGGTIEM
RTDPKNTAYVVVEGATKRDTGEDKGPREGDEALAIMTDQEREALRKNAFA
SLEKTIEDRERLKAATQRIDGLLDAQAKFWDDPYAQNQKLRRAFRAGRKE
RGREAERAEDLKDRMSLGIDLVPATEHDATRAALVDFGPGQDGRAAQRAL
ARPLFQTAGGDGNRTGDRDAARNKSGFVADIMNNTRWAKDPFLQKKDGVK
AARIQGVKRKRTDDEDRNPSSDKDARSASALVEYDSD
>Mb|QLI67746.1
MSASGGTLPAGGNPSNGEDTEILRRIHQALDVVHSASSGNDARRQAQSFL
EEVKDIPEAPFQGYRLAADKTQPPVVRHYALSLLEHAIRYRWSTYNQEQA
TALRHWILELSQSVSKQDASYLRKKTAQLWVEIAKRCWGTEWMDMDSLLV
QLWQIQDSAVHKELVLFILETLSDEVFTGDDSVVTMREGVLSKACVEIFT
PTSVLVEAFPNRQPGPDVRHGPEGWLERISQFLNYCVSSEAKDTADAKSC
AVKALSVFVSLMPWAIPKAIAAAQCVNVMTAGLASSHIDVQKASLEALNA
LYYRSNFTDDEFCDLVAPMYSPSSVDLCKGLYRWATVDPEDIDEDRYQIL
KKLSEMLSYLGDYFERKFAKVPQEAASSEFLGLLIQVTQSPSLMVSIPIL
VTWTRILGHRNLGPSDLVSKTVGPLLDVCTSRLVRYENLPENSTDPTYLF
LLDDTDTIPERHAFLGNYRRYSSQIIEQIVQLKLVDAVSYILGRTEDILQ
KLYDGQPPFSKQTYTKHSTPALKVDSHFTVIEATLKGYVKWRRYHGRDHE
QQVAEMESNLEVWCNKLLAMSFEDPIIRKRALQLLVFFSTTALNKDAGFM
LKVLEHILLTWPALEPEYRAYNDAIKDLQSESMIELQRLAAEMPDHLLGV
YDQIESRVNDMMTSGALDEKRSLAYRSFLFLIIHRASGIDTQTKIQKLAE
FVEPVKAQWQSEPIRTSLKSYADFCQYLGLDKAQKYLASRHAHELKDWGS
CELDSEGLLLQNELEERLKTLPLRPTKSFLAFSVERLDKSSPAFQASYAL
WQQGFSNILADLLEYLKFAHATHNPDSWVELPTEMRSMVERVLSDRFWQA
GISEGSKDDFYARVMDKKNTIEGLASTIRGSFYGFNGLSGPLSEALFSDS
IWLSTHQQSNLLNLVRYLVDDCPVDYREQFLPQLLAACFRQMDAKVNGEW
ERMEKQQTCVVDGEAGLKEEMKAESILRQVTYTAVMMVADFLDPTKSNSH
TLKSRSRKEGLEDEPSRPYPSLRKFCLMRQEIVEPLLVFCTHGIRMRDTR
CCGMVLRLFISLVPEFQLVQGQEQYGSTGNIHDGKAGSSHTDTTPVPAEI
ASVIREYIAQDVLKACLTSFHEPYFVEVQKELASLIATIVVYYSPITATP
KDIILSLPNVNPAELDRLAPYMAKPASHPRQQRAIVLELLKDLKGVSVSE
MGKLAKTAGFGGSSRNKRGNRSKMAQGFMNAPTEADGRVTRGNGVVGARR
TTPDALEGVSNLFEG
>Mb|QLI67750.1
MGCAAASRTASLNTVNAKKVQINASGGLQDAVNYLFSRVPPQIVKISSQD
INNIYPTQSSSRSAHLSVDLDFSTAIESNSSSSPLTTATISAQDFPVFTT
DTQSTWLPNSPSALPAPSAHQLQSPVENQQDFVLFDNPQPRQVPNRSSVP
LSSQRRHSYHNRRDRLVSSAVQNQRVAQLLQAIGHQSLSSVNANRFTNQF
YASSAPSSSVSLSSQQNIQQHQQGSARPPVPLFNQSIGSVHQSAKMMNAA
DVDLDEFTAFEGGASTAFSSPAAPSVMDFGGSMSSSTSNLGTVSPQDLLV
QDPFMSAPNSSALTALTSPSIYNESPDFDGYDVSPNFGSADFETSGDPWY
PLFPQDSSHSGPELKLEDSPAQNSDDIDSVGPVSGSGNRKKATNSPTSSG

RHSSVAGVGSRKRDKPLPPIIVEDPRDTVAMKRARNTLAARKSRERKAQR
FDELEDRIAKLEAERDHWKKIALSQSGAQT
>Mb|QLI67752.1
MAAANLNPKKPNSSSSSDGSDLDPLWQNLDWAVGQMLIMGWDGTEVTPQI
KSLIEDHHLGSIILTAKNLKSAQETAKLVQELQTIAKNAGHPQPLLIAVD
QENGGVNSLFDEDYVCQFPSAMGIAATGRVELAYEIYKATASEISACGVN
LMLGPVLDVLNNARYQPLGVRSTGDDPQEASQYGLAALNGIRDAGIAAAG
KHFPSYGNLDFQGSNSAVPVITQTLEELSLSALVPFRNAIATGKLDAMFV
GGCGISNPSMNVGHACLSDQVVDDLLRNELGFKGVAISECLEMEALSHDL
GVQNGVVMAVEAGCDLVLLCRAYDVQLEAIKGLKLGYENGIITKDRIFTS
IRRVQHLKSTCTSWAKALNPPGISLLSQLHPSHVSLSRRAYDESITVIRD
KEKMLPLSSSMHPGEELLLLTPLVKPLPASAMTKSLLESKKTWSQAPTQH
DSWAHKDRERSAIMSGEGVFREFGKNLARYRNEKLLHTSYTANGVRPVHE
NLIARASCIIIVTADANRNMYQAGFTKHVDMMCSMHRSRGNKKQLIVVAV
SSPYDFAMDKSIGTYICTFDFTEDAMSALVRVLVGEITPIGSMPGTLRKS
KKVLKSRQHWLVEEYDRARDYAGLEDLLKAVHRASAPDLHFLKSTRAASM
ELHLPNIKESHFVVRNSSTGALYGFVATYFIAGVGIIGALLVDPSKRNVS
IGRSLHRRALKSLSQQRGLKKVQVGMSFPGVFLGIPLDPEANTVKEWFSN
SGWDTQFPRRLTNMIIHDLPNWVAPEGLLQSIQRANISFDLIHGLENAES
VLHHVRANANPEVLELYTYALSENKTCGIVRAKDPSGNLIGTVIICRQGS
PLVTYIPPLLSDREDVGGIIAPIVPSTPQATLVLQGLALMGVRQSKGHRA
SKAVLSWIVDDAFEPLVAMGFDILQAFEEITNSPEVCQT
>Mb|QLI67754.1
MPRESLADGGACRSECSNKLFRSISQDLRVNGAGAAVADNIVSTSLTGTG
PKVGPQRAFAKQNNLRHATSDGPRRRTRPDGTRFHGPSIVRNSTRQLLEH
MPSSQLLEKVLGKNPYADSWRKRIARFRTIRLPILLLQEILRQEELQSAG
ESGLLELLPTLQENKTILKHPSLAGHTQKAIGRYVYILRGQNDEERCKRY
LEEPTSVPLFIFHFLLRQSSGISDPVTLQRMIESSHAYYNNRRADNTISE
GNRQSSRDGSEKMLDIDRANFNLIMRLLVHHCRRLEPRFVVKLADSAAQY
IQNMPISAQGERKVFTTQCDIFNGCLQILRPLPHVHVAQRSTPNAYFWEA
QRILLSMSAGLSKPLLVDRGGFRAIREVLSGQAKNHAEVYSAARHASSWP
PYLQPGHGMDERTDPEDNWSRAVSAGMLMQEAGFAKDEVDDAVDILQGMT
QDGTPTIQQRAMISKRRRIGVWEASIRATRNAQEAWDRFRNPPKEGMKPG
LHQYSAMFEKLVLREVEPDGSISPGDKALNFPTQHEANLAEFERARLRPP
SISELYQHMRLNGVEPDGSCLCILVANAASLEIAHQYLRDSPEKSRIIRN
LIAEEPDAGLLRLVGMGLFAAYLQVCLRVEGRRAGNQLQHVIRLAESRLS
HEQSRWAPFMWGTILKELSRHHHALRISLHQQLDLMLRVMDAIEKVDGVQ
LSTFTQFHKGVRKAVRQQLNSVFTKDAEKEALAKSPLTQALFYQMCMASV
DNAHPRRSLLHQGLGQEDSFPMAGAEVLLLKEAKARIKYMFWTLEQRERH
AQAYLDGYQIAPLEAMNSRRDAARSDHAHDYMLTLGYLGEFEEMGRLLEW
LIQQWGQFNVASAVNEMDEAPPQADFFETLCVFRLVAEPMLGNGRVSTLL
QTLADSGLNWTWPDDEAVKTYAEVHHDESISTLRRVIELARDGNKESRQT
RN
>Mb|QLI67755.1
METLRLKPRQPVEDEIENWDDDDDLVLEGDELSFRTSLTTAAGPTNSRAL
SRPPSRRRDSTSSHVSFRSELESWHGEEKQLHLPADDESSTMDAIAAAEH
AGIPLPKNVPSSALMGGTIKRLGGRKIRRIIQDDWENDLELPEASQGFSI
KPKPDVEFPETLRQVSAGSVQTSPIRTMKRPSFPEPQRRLSAQSNTSALS
SALNLDKFKDTEEDDDFLGDGIATIKVAKGRIAPQPVSFITPPTPQKSET
TKETEDDFEKDLELPSDGKLKLSTRRDIPKTPSSNVDDLDWGEGSLGTRY
GGTRRDGRSNRSSSASALSPSISSSITAESEDETFEGLVLPAGPFNFRER
LQKRKKSFSPERIPEEQPSPLPPASKKPPTTQAEADREDFFEDLEIGDGN
VFGPGKLTLHRNIKVKETQPASPARPKTAVSLTFTNKPTATTTTTTTTTT
TQTRIPRLSHERAHSTSLEPVYESGSSAAPSRSSRRSQSRLGHSHQSSVV
SLPTPTTTSPGRQFPLSTPRGREVGSRSSFSSLRGDAPTTSSQLLKQKRS
LPAVRGSTTTAPKPVLQRSTTDRPPSRAETGRPQIGLRPKTPVERQRPGL
VAESPASLTRKPQPFLPAGASQSQSQHVASKTLRQFRRHDSENAIDLRPI
SRAFSRSGGRSPSPHRYKVAADTWERLSKPKNKKHFGDGHELDAFDDLPT

SRETETRFMKQPVASGTKTTIRNRLYQNILPDRTATPAPPSPFFPLKSAA
TPRFARDTAASRIARETSLAHRAPSHGPLSAITAQREGGGGGGSALTSRT
TNVGHQPHLPQSTIRSKKKTKRPQQLKPHLIANLNAGKESKVVNGMFYNA
ETFRWEGNENVLNAFDATVATPSTTTASTHVTREREATTPRPALIANFSA
TKGVQVVGGMVFDPENMCWLRMGQQDKTKSDAGDTLDGFDALDDDEDVFK
DIPDLDDNAAEEDRGEGGRVSDVKDDWLVGEEFDVGPEFVRRQREEEERW
RKKCEKWVGRGPRDQEMWRWTIRELVSQFDELPM
>Mb|QLI67756.1
MLHVAPKVDLPTQTSSSSVHLAPPSPSDIGIPSKTFHGYKRESRPTTPTK
GETYNDKYVVLYDFGGIDYDAAAKEFNCLLDNLEAAGLHTEVRPGYEQTI
LVFVKAPSQLLGNTVYKQRVKDWLYAITQSHPGGDKHTVVKAWFEAEDVL
ALYHLILWPTDLGGAGITPEVGEWENVKAIFPLHNEPVNQSILRHLSKTI
MLTAEDFDKIRDLHGSKIAFYFAFIQTYLTFLTFPAITGVFTWLFLPKYS
LAYAILTGVWCTVFLEYWKIQEIDLSMRWMVRGVNKVKINRPAFKYDKII
VDENGRTKHYFPKWKQIARQLLQIPFIILATIVLGLMICSVFVVEVLICE
TYEGPHQFYLEYVPTILLAVAIPRISSSLEGIATALTEYENHRTADEHEM
SLTQKLFILSIITNYLPILLTAFVYVPFGDVIIPRVKQLIVHLFPKFAAK
LVFRPFASDTDRLRNEVIALTVTGQLSDFFEENILPLIKYKFSDWYREYR
RAYTKDTMLLTLVSDDASEAEYLKRVRNQATRSQYNVQEDVAELVLQFGY
LALFSPVWPLISMGFLINNWIELRSDFAKICIEHRRPAPTRADGIGPWVT
ALETLTLLGSISTAAIVHLFGTDTIGAGGWTTLPITIFVSEHLLLMLRAL
TRWIFERYGSEQIRKERHERYARRLHYLEEIEANKRAGVNLSPAERERRK
SVLVASSGSFWTRQVEEGSSAAAGLRLIHLARQWKKSQSNETKVD
>Mb|QLI67759.1
MGVSGLLPLLKSIQRPTELKKCSGDTLAVDAYGWLHRAAYSCAVELGRGK
PTKRYVNAAMHRVRMLQHFGVTPYMVFDGDFLPSKAATEDSRAKRREEKK
KLANDLLKAGKTSQAAHEFQKCIDVTPEMASTLIQELKQMGIPYVVAPYE
ADAQLVYLERQGLVNGIISDDSDLLVFGAKRLLTKLDQYGNCIEINRRDF
CACREVSLTGWTDADFRRMAIMSGCDYLNGLPGVGLKTAYRMLRKSKSPE
AAVRLLQFDGKRISENYLTQFYQAELTFLHQWVFCPKKTELVHLTELDGT
RTADEMPFIGAYVEPELARAIAKGDVNPITKAPIVIATTPSKRRHSQTAT
QTQPPRKPITSYFKGHSRIPMGEMDPNCFSVDPQRVAQITEGGMVPRVFP
LPRPYVDDQSEITGRATSTPRRTSSAPTRTSPRLQRRRTEPISNMLARVA
SSPATATTSDPSLAPPLKAPLSEPQVRPVKKARLCDEGDEEDGSPKKSKF
FPLHKTKTSPVRAKSDAYLFSDDSIEETLRDLPDFEGWKPYNKRGKPIAI
FNDDSQVSCPDESQVSLDDSQNTSQDTEVTVPSETPDDLERPTNSMMPPP
KSTKSKAQALKTPSRAPNLSRFSFSSRPSSTPSSSMSHQSSVFSVASTPS
TAPSTTSSRLTPLQRLGAQATNLMASPKPARAKANMAKDSVKGLPVNPSF
VPLPKVDVTEVEALNKSCGSEDQMVPDSDGENEDEIELRPRNLNLSRFAF
S
>Mb|QLI67761.1
MVLHNPNNWHWVTKDVTAWTKKWLEDNLNEISATNGDVEVKIVEVESIEG
EAEVYQRKGKVKTVFDLELALRFAGSAPDADKVSGTIKFPEVQPDIEDYT
FEVKLEDPAPKENQPVKELVRSQLVKELEKKFNLITSVLIEEHGKDIQHA
AGSNPSSGFSTPKTHPQTSSPKPTEPSSAPSSTSGIVNTTTVTDKEEFRT
TADEMYQTFVDPQRLAAFTRSPPKVFEGANKGGKFELFGGNVSGEYLELE
PGKKIVQSWRLSQWPAGHFSKLHIEFEQNNVDHVTVMRVEWKGVPVGQEE
VTKRNWLEYYVRSIKQTFGFGTIL
>Mb|QLI67763.1
MSAADSPGSRPGPDKAIDEAAAERKQQLGRPARSRLPGWLNHFNANDLKV
AFRCWVAVWVATILIFIDPVLQNIGIATFFGAIVLYIVPPAGILMVYLLA
SLSLLLGMCLAWAWGLLSMKAALAVRPDEETQARLQSLQQQAGMLANQTG
QPPAAIVQKLVYDGFMLDARVVVIYFVMCCVFIYALARLRYANGKMILLQ
LFGTIVIDIFLLTGPILTRFNSKLAQVFVKPGAIGIGLGAVCCLFVFPQS
TSYVVLGQMEQLIRMLEAPVDSTRRYFAGDETLDDAQLRASKGSIISLFK
AMEPGLAFLPLDLSRGRWNADDVKCLQRRVRDAIAAGVFLLDFHITRVSA
AARAERLGMAQPTPTAQDPDDGSDKEKTSFDTGLVDALKSPEQSSIRHGL
LETLKDTTAEILQVNSRAIKAAADTVHIINTGRWYLGTARTKRLDGLKEE

LAGLRAELQSVRESCVINTNDAVVGAHADLFDEQGQLKNRKDTNPLSLNG
IVLAMVLEERIVATAATTEAIVDYIMELLELRTTTRVWAPSQLRYAFSWL
FSGRVAAPVAGASTVPAEDPDDILEQAEETHRRLRLVRKGNAAPRKQRGF
LTRAVIGTYKWLTNPGGMYALRMVIVTVALSVPAVLPNTAGFFYREKGIW
AVIMAQTCLLMYMADFTFSLVSRGLGTILGGALALAAWYAGSGNGIGNPY
GLGATTAVVSFILMWWRLYLPPAFAQASIMTASTFVLIIGFSWDQNHVTQ
YGLPGVGYVAFWRRLVTVLVGLAAALIVQIFPRPPSATRYVCKTLANTVR
SLTDHYALLVSQWSQSDREGQNLGAAAAAEKITLKVSETLLDLTDAISLL
KVEISSTPFDQKVLFTTREHCSRMNQCLGKLLVLSSTLPKRLQDRLTKTV
GVIDDGVVGNVMSVLTIIESSLRTGAPLPERLPAPLVRSCFVTWYTQHER
AELNIDLIKSEDYRRYCVAVSCYVTFLSTVDDLVDALKETLGEAHVIHQW
EEAV
>Mb|QLI67766.1
MTTTPQEVPLHSIDRTPEYEEFMTKLQEFHEKRGTILDPEPKVGLIYLDL
YKVFNHVVASGGYDKISEEKLAWRRVAAELGLHSNNEASTAFSLKEKFYK
NLAAYEIKTVHNKEPPPKEILEDITAKGASLLTRTLENFRPKRDSNVNAG
DSAASGDDGTPTRERSAPEAPSSSRASRGLREAPPQRVIFQPDTGPSRST
RQAVQQNAAQNASPAQANSQTTHHPNMPPMPPGGHQGRGPSIMHHPSNSE
NTSNVVLSYQPKHLKPLQLRVVATPSSAPSEFQRHRLPHRPALVDPTSRQ
PMQPGTGFDGPNIYTRCLNALRSGVPSEQAFALNHLVKISFERGDKYRFD
AFPGLAEGLVDKGLEVGSLFYHVCWTVVWDPYADSGGIGSLDGNNGTPDI
LDRIANLVEKDLPDALQTEAHTDQMVLITEAVLTLRNMVTLPENAVNMSD
FYPIKDLICIIVHLPAKDCLTELKHMALDIAEQITPFMALDSDDPLYKTL
LEQLSSDDRGAILTSLRALGRISMNLEATNQLSNVPAESLQRITNWLLLN
DDELMDACLDLLYQYTAVVSNVDNLVRSTTPENLVDHLVRLLAHGARKVH
RDFILAPEKKLPARDEIAPMPEDLLQQMIKLEEPDRVHKWVKCFFEEDND
SFVTQLAAWQAYQSAFVGPLKAIGQPMITPADFIRNSTSVYKDSNAQVLR
EPGESQQKFIIHGIRARPRPLNFEDAEYVRCLWATNPEKRNEKCGQFFLK
PEQMWNHIMTKHLDEQANEGGQFSNKERKFVCTWGECSKYAAPTSLHLQD
FARHVNTHIMATLPNDTNNSQKSWVVPAKTMAVTYEETMTVRDERNPNLP
PQAAGIPLSAVLVLRNIARNVVKTEAEEELLKMQEPNSSEKGGWNEKLFR
AVLPRLFEVLAENRALGPYISSLLDLIRPKTQPGDEEEY
>Mb|QLI67769.1
MSGHGDLQELLRLFTSRKVPMMAAMGHIKALQAKNLRSIEQIAEAPLSAV
EAAISDAKLAKSFHTACKTHGKKATKRAAEESTSPTSKKPRLEPHKRDLD
YSSMSAEELESSLSLPLVEDEETIRKTTLVTNRAPLVLAFAVELLRFTMP
EQPPSSRLSLGQAVVSANSRSKAISIGIEKAPPGGEEQVPEGQPKVRVLG
REIPVLKRSGYSWKSDQLEDKATGSTEGHSKPESSSPGEKKAWVASQKLT
SRASTFVAHAASLSSPSLRTSLMNGLMTDKPELETATHNAWALRTSYGNS
PLVQEASFDDGESGCGKFMLQTMREAHVTNTLVVLTRWYGGIMLGPDRWR
LMRECINDALSSRGRKSTLAGEALWGLDPENKTPSLTTVGMPIHRPEGAR
NYLLRSFGTAAVDGGGSETKKTLTAVSDEKQENLGRLLGALRLLYKSWAG
VLRKDELDRRAWSWYVSVRPDVEAGPSGWGAKGHLNLGKILDLRRAEGSA
GAKKLAES
>Mb|QLI67770.1
MASVRSLGLHHPSGLRHAVEEQLLPQSPPPSAYNWEITSDDKGDEAQEDE
LLATQDCVVWCRGGIFRKTFKFGLEKEPITQALLAYFATSEDALHNENHN
SGPPTKAAALAAPKLDKALVVFLKTQAHIFFLSGTSHVIHMPFEVESACA
GPIGVVIQRKHKAENLAPIALKFPRVPPNSFVSSQLTALNSSQQTAFSVE
GMGKPKPLHFGPSSTLETMWDMPSEQQQLSHWPRLVSLMDPLSDIGLVVG
EPEPYSRRPARQNKSKKPAFLDPAEEILHIEQINLPGSDCRAFDAPLILA
VTINRENGSYTVWRLKYLEHEDPFIGQQKKAKNRTSRRRSSMPPQGFTSG
TSTPVQQNYRESFGAPLPGKRQRKSEKAEKPLDLVSSLEQQDNEGAGVTR
RSSRRLSSMLARADLSASHERSVFADQPLTSSTGASKRIESHNGHHGRSS
SSFHHIHPSLGSLLEAPLAATLDEGLHNMGLDDHDFDGLQNEIRFTKIQT
IALDSSNVRYSTSTLPARCQTKVFIVTAPPFATDEQGRTQLLIGIQEPLE
KRLQMMTLHVKAQEKSDPATTASGKDQSRYSIVSISPGTQWKVQSVVDSS
KLVDGDLSVILVLSDSDDGQHELTAQAPWIELTKISLSLLFVDDTRNIQY

RGRVADRDVKQRKSEVMDLTSGGIVGIRHPRNCGVVDVVDAHGRLHQLRI
QLQPSCPQVKKVLDICKSVLPDALGGRLHAGWLHMTQWLQNHQDNLPNVE
WSATTVLLLSSFLNLGREDDSPFKTSQLPVRKRRPLSGSFSAIRESEDWR
ALELGETTNSLGCPAWMMNQGWQWALEEETADNASDQSDALLSSKFLTRH
IALSKEYLSSPLGEAAMGRQGYMPVSLARSSATRRKVAVDIFMALHLLLD
EEKLDITTPEYVSPGRTDLRVLLCQLARWLKWQTFVTIYELGIQEDVDTR
HDLELNLRPAIPQPPVRPDILEWIQSRLSGIRGKPCMTPADIYYASSRVS
EAEKLQDKRWDAIATRTLMFKRFFKIIRPKSTAVEMVEAMQDCGFTNYIL
ETLPEAVLAPLQDAISLCQPHPPSQWPHHLLDLVRRSDISLIVKPSRRPR
QSVANILAPTHMASWDFKMLCQSVEEANNTGYDEGEGTERQAVIRALFKD
DRRLNEAQHLLSTHKARVVRLNPDPSWPESEYLEKQKELVSRIATGTLAI
PAGRGLLYYSLRFPLITQKFHIGGFNLNCIVKPTNVTVGVDKSLFTEEKV
CWGFFHQGVAAGLAISPQAKGIDTSWILYNKPTQELSNRHAGFLLALGLN
EHLKGVAKWVAFKYLTPKHTMTSIGLLLGLAASYMGTMDSLITRLLSVHA
TRMLPRGAAELNLSPLTQTSGIMGIGLLYCNSQHRRMSEIMLSEIEHIDE
EDEEEPLRSECYRLAAGFALGLINLGKGGDLKGLQDMKLTEKLIAHATAT
KNVEIVHILDRASAGAVMAIALIFMKSEDQIVARKIDVPDSVLQFDYVRP
DILLLRAVTKNLILWSKIEPTLTWVRDSLPLPYRARYKLLGTTKLRSTDL
PFFSILTGICFSIALRFSGSASTRARDLLLHYLDQFIRISNIEATPRSHP
DAAPLYDEELARSNARMCQDILALSCSIVMAGTGDILVLRRLRSLHGRDN
PDIPYGSHLAAHLAIGSLFLGCGTVTFGSSNKAIAALLISFYPIFPSNVM
DNRSHLQAFRHFWVLATEQRCLVAKDVLTGQPVSVSVQIKKKQDSSSEHI
LYRTTPCLLPPLDQISSLSTSCGPRFWDIELDFSNDELRQAFAQTQSIYL
QRRPPSEGAFTSTLRTLGSDGKGNNPLEWIFGLGKLRSVSYAERAALMES
NEKEQDAGSAVDARMEMERGILQGGDRERLEGARLLFEWGAARERLLRSA
APSVHDSQATVRPGDSQETIGEQVTPHAPGGDVWWMRDSAIEALKGQVWL
AVRDGDI
>Mb|QLI67777.1
MLASRQVLGNLARGRVLNGASTSLRRMATVSDSSLDKKVRQNIWEDNNFI
NYKKMSENLAVVRSRLNRPLTYAEKILYSHLDDPHGQDIERGKSYLKLRP
DRVACQDATAQMAILQFMSAGMDQVANPTTVHCDHLIEAQVGGERDLARA
ISINKEVYDFLSSACAKYNIGFWRPGSGIIHQIILENYAFPGGLLIGTDS
HTPNGGGLGMAAIGVGGADAVDVMANLPWELKAPNVIGVKLTGQLSGWTA
PKDIILKVADILTVKGGTGAIVEYHGPGVDTISCTGMASCANMGAEIGAT
TSVFPFNDRMYDYLAATKRKDIGDFARGYASELRADEGAEYDQLIELNLS
ELEPHINGPFTPDLGTPISKFSQAVKENGWPEELKVGLIGSCTNSSYEDM
SRAASIARDALDHGLKAKAAFTVTPGSEQIRATIARDGQLKTFEEFGGMV
LANACGPCIGQWDRQDVKKGEANSILSSYNRNFTGRNDGNPATHSFVTSP
DLVVALTIAGSLHFNPLTDKLKDKDGNEFLLKPPTGDGLPVRGYDPGNNT
YQAPPKDRMGVTVQVAPTSDRLQVLQPFQPWNGKDVKDIPILIKAKGKTT
TDHISMAGPWLKYRGHLDNISNNMLIGAINEANDEANKIKNFTTGDWDAV
PAVARDYKKKGIKWVVIGDWNYGEGSSREHAALEPRHLGGLAIVTRSFAR
IHETNLKKQGMLPLTFADPADYDKIKPDDKVDILCTQLAVGEPITMVVHP
KDGKSFEVPLQHTFNEAQIEWFKNGSALNTMAKTA
>Mb|QLI67779.1
MAAIVTPSNNRLKLTPSNSPFLSRPSRSLMRGRSMHEPRLSLRRVVGTTC
RSPTGFDTVNSSFAYIAGGAVVVVDVEGQLYSQRFYRARPTAVPMYSVSS
SQNAPSTPTATTPKANDSRNRVAPNCRDSGYSPVDWATDPPSGSRTWTSR
ERIKAATCLALSPDGRYLAVGETGYAPRVLIFSLLDVSSDTPLVSISEHA
FGVTAVAWSEDSKYLASLGAANDGFLFVWKIDPRTGAAKLFQQNRCTSFI
RDMVWIGNSLITLGVRHVKAWKIEDGASVSPTKPKFSGDGTPSTPTSQKT
LPGRNMLLGSLLEATFSCAAVDGKSLILCTEAGDVCILDDDDRQMKLTKV
LNLEFTITTITIRNSVAYIGGKDGHFATLDVEGVMDGCAASVLTTSQTSA
GVVALGFLPDKLVTIDSKQSIDIWNPDYLLGQHVEAVAHIPIPGHGEPII
GVHSLYRPNKANAAFVTWSASGNVTFWDVDGQVKLTLDVPVDNSDSDNEL
TLPNQLTSARTTKSGKLLVTADRQGIVKVIDVDSKDYLLDIKAHSSDCLC
ISIYDEESKFLMACCGRDRTAQLFHRDSTGRIEHFQTLEFAAKVIQVLIP
TDNKVITCSLDRTLQVHDIVSREGEPDIIAAIPSKVISLKSSPTSMTMGP

DNRTIFVSSLDRSIYQYDLASGRQIFCFKCMDEGGVETAVLDSLFVGQWP
SKDLDFLLGSSNTDKSIRLYDANSGAFLDREWGHTEAINGVSLVEDDDGT
RKVVSVGSDGTIMVWTLDLNDPSPRSMSRDPSPVKEANNNSSRPTLRKVL
SKAELAEFQRPSPSPAGRRSPPRSLRRRTSRLNLGTAPSVNRTPAGNLAT
SPSDSAISEDTPSRRRPSEINRGDSPPASPKSRVSRRPSMPVLSAVAKRK
SSSNLRGVGSLNMATEQACRTLRAYRKKLSSAEPITAEGLTELDQELRLT
AAALGDRAIRSKAMNETVLSGLLDQYSERLVTLLDEKLRLTNQLPKDREI
ETTSSDDRPRSADGSSSSSSP
>Mb|QLI67780.1
MSSTDAKHEALQPIHPSMAGKLDPVFEKLYNDNVANTPLKPIDLAVLRSK
YSVLYSYGTGPAPDVGRIYDRQIPLDDGSGANLDIRVYEPDTKGPWPVHI
DYHGGGWGLGDLDTEAHICKHICKKANVVVIDVAYRLVPEHPFPVGITDS
FAALKYIHAHGARDFNIMPESISLGGVSAGGFIALALAHLARDNKPNPIP
LKLVAVGTPVIDDLSQYSSAAESPFPSMQENEMAPTLNWGRLAWFDKLKW
SSLPADPKEHKAAREKVGWFANLLDAPNFKDLAKTVIYTAGADPLRDEGE
HYARKLVENGVEVTLKRFPGVPHPFMHMDKDLWQASQFIDKTAREIRLAL
HD
>Mb|QLI67781.1
MSPHGEHNGESATNGAQNGAHTNGAHTNGAHTNGTQTNGTHTNGHDAREK
GVSEKVVQPTETQKSGSKKTSSTGALMQLRRASRRPLPTEMGDGTYRNIL
RRPTLMQDLKSFGLGDLKTLRDIVKAKLKRETQQDDKTMIMERTIQLVST
MPNKSKRQEILTNSFIDTLWNSLDHPPLLYMGDEYKYRQPDGSNNNPLMP
KLGAAGTPYSRTCKPGPANMGALPDPELIYEAVMARDGFKKNPNNVSSIL
WYWATIVIHDLFWTNSKDPNQNDSSSYLDLSPLYGKSKEDCDSIRTFKDG
KLKPDTFADRRLIGNPPGVCILLIMFNRFHNHVVANLASINEGNRFAKPA
AHLEGDALAAAWKKYDEDLFQTARLVTSGLYINITLVDYVRNIVNLNRVD
TEWTLDPRQEMGVAVGTAKGAESGVGNVVSAEFNLCYRWHSCISEMDDKW
IQDFYTQILGDNYGEMNLQTLIAAVKRYETTIPEEPSERTFGGFTRGSDG
RFNDDELVDAISTAIEQPGGAFGAQNVPRIMKPVEILGIIRGRKWNLCGL
NEFRKHFGLKAYDTFEDINSDPGVADALRNLYQHPDHVELYPGIVAEEGK
APMVPGVGIAPTYTISRVVLSDAVSLVRGDRHYTTDYHPGYLTQWGFNEA
NYNLEINHGCVFYKLFIRAFPNHFRYNSIYAHYPMVTPKENRMILTNLKR
ADRFDFGRPEFIAPPIHLTSYGAAQHVLTNQGKYKVTYREAFQFLVGSSG
TKCMLAGDDPAHAEQRKVMHSLLYEDGWKASIKSFYAMITERLFAEKSYK
IAGKAQVDVVRDIGNLAHTHFAARMFNLPLKSTENPKGVFSEQELYEALA
MLFICIFLDLDPVKSFPLRQKAKEVTEQLVKAVEMNVKLTKSLGIRGLYT
GPSPKSDDLSMYGVNLIKGLSKAGLGAHDIVCSHVLPVAGAMVPVQAQAF
AQAVDWYLSPEGQEYIPELQRLAVAESSPENDALLLGYAMEGVRLASAFG
LFREAAETETIKEDDGREVHVKAGDRVFVSLASAAREEAYFPEANKVNPR
RPIDSYMHYGAGPHSCLGRQVSQVALVEMFRALFRKRGLKRVPGPLGELK
KIPQPGGLYLYMTENWGGVSPFPTSMKISWDE
>Mb|QLI67782.1
MSTSSQTIDVDALLDFSACDGSYNSPSLSPSTASKPTFASPVTAAVSTPS
LPSTTQTLSGPSHNYDMYRQQTGFVPGALTNTMAVNQTNNTGYQDFGSLD
YLSSFSPENDVFDFNASPSQGTMEMEFESPADSQFFPTVNPSSIEHDTSA
LSQTSSVGRLWPGAHSQAALAKAQAQQRQQQQQQLIQQTQRQTSPKSRGK
APQPTDPIVEQKITQLLNSMRAKPASNEPESQSVLNNLPRTKKDEEEMDE
DERLLASEEGKKLSSKERRQLRNKVSARAFRSRRKEYISQLEAEIANKVN
ENGDLRSQNRALMDENKRLTDLTRMLLSSPSFSTFLDNLSANPTGLPQGS
PVKIEQNPQQEQNQVPKDVNAYNSQFSSQQQIGMAMIPEQTMDFSLLSLG
NTYNFQPQVFVVDTPEVPNAIDASVLSGKTSNFVEESFSSDDEKVEVPVI
ERPLKTKAIETPEAPVDEEFESDPEFALFHSEPATTTTTPKDIDTENLAG
IDLFGGIESEKMFARYELVNATEEEATAAFAMARVQRISANIDSVVSRLE
LLTMDI
>Mb|QLI67784.1
MGIKQLFQIIKEEAPEAIKEGEIKNHFGRKVAIDASMSIYSFLIAVRSDG
QQLMNDSGETTSHLMGMFYRTLRMVDNGIKPLYVFDGAPPKLKSGELAKR
FQRKQEATEGLEEAKETGTAEDVEKFSRRTVRVTREHNAECQRLLKLMGI

PFIIAPTEAEAQCAVLARAGKVYAAASEDMDTLCFNTPILLRHLTFSEQR
KEPIQEIHLDKVLEGLNMERSQFVDLCILLGCDYLDPVPKVGPTTALKLI
REHGTLENVVDAIEKDSKKKYTLPEDWPYKDARDLFFEPDVRKADDPLCD
FKWDKPDVEGLVTFLVTEKGFSEDRVRGAAARLEKNLKTSQQARLEGFFK
PIPKTDAEKAAHKRKLDEKNEEKKKKLKQDKKDKAAQKAKPRGTA
>Mb|QLI67786.1
MYLLLILGGALVAFALAWLSSAVITAVFSPLRYLPGPFWARFTRLWYVKR
VYEGHFEHDNIQLHRRYGRVVRIAPNMYSIDAPEAVGTVYGIASKMPKSD
WYEGWKHPSPDRWTLFPDRDIQRHAETRRRFQGLYSMSSLVSYEGYVNEC
TDILQRRLSEFARHGSIIDMTHWFQCYAFDVIGNITYSKRFGFLGRGDDV
DGIMRALHGSMIYSTLVGILPALHKYLYHAMNKLNVGGAVGRTYLMKFVS
ERIQQRRSERGQYADKSVSLDENAPQDFLEKLMVQNEDNPQKVTSYHLFM
MGLSNIIAGADTTAISLSAVLYHLIRNPDSLQKLRDEVQQRFSEGLFDGT
HLTFKQSQEMPYLQAVIKEALRLHAATGLPLWRVVTDGGLELDGHFFPPG
SVIGLNTWVAHYNEDVFGPDARQFRPERWIETDDNGPDIKAMNAYYLPFG
LGSRTCLGRHISFLEMSKLIPLLVRNFDFELVGRRAAWKTENYWFVKPTD
FFVRVVDRKS
>Mb|QLI67787.1
MTQVGDAGLDFTGEDDLIEIHSSPPTRSYNPLEDLLNPLQPIPAQPVRLR
PGRRSDRHPPPLGPMSRQPSQPQQMQSTEFIDLTEEPDSPVEIRHSQPPS
QIGRNPRRTNSQRTTPPSLSRSDSTFMGTGASVIDLTVDSPEDDRAPEPR
VTRSQRHHHHHHHHHHHHRTRNFRQDQLIELEFINANSGSIYSNLSNNVR
RIAGLLGGSFMSRGFTATPIEFPPNFAAREPSPKPPMEPVPPTRDGFTRD
TSLADERVVICPACGDELAYDPTGTGPQQGSASTSGKKRKRAPGEHHFWA
LKKCGHVYCADCFENRRPTKANPLGVGFRAPSGKTPGIAPNDIRCAVESC
DSKVALKTEWVGIYL
>Mb|QLI67788.1
MKEPEAELQASANLSPVSPSPVHTAAPLVVPALQDTVDTIDAMVAAATAG
VPEALVDQPVVDPALADATDNDDVVDDDSFNDPYGEDTEPAQPAQAHPPP
EPSEANDDYAKTFDSPIEPNEGGEQDIDQLVSSSLQESNLVPIPSEQLNV
SRSSEVSPATVHDPSATSTPPVSNSHQNAQHEPSANPPVPAPSDHRGDDQ
QDQDQDQGAQPKPTDGPNPAVTESQSSPSLDIQKLVADLTAKPTESNADS
DPSPASPAQAEPPTGSASLSSSTTLPSTSSLPPRPPQPHSASPLYASQHH
PAGANPNATAAVVAQPNTGQAPSYHIGSAPGTSTDPVASLPLPPASGLNV
SPYSTQTAPGYSADQGRDVEYQRQWDQFMADERQYMSEAKWDRFPEGSRI
FIGNLSSDKVSKRDVFELFHRYGRLAQISLKSAYGFVQYHTIDEGQRAME
NLQGIEIKGRRIHLEISRVQDKSKKDRNRSPDRGRGRENGRRGDKHGRDD
YRPGRNHSPRRNDYHSRDDSFGGRDRGYSDSSRGGRGRSRSPPYGRSDRD
PYRRRSPSPHGRPRNEPELDIPRRYGADVPDVQIILQPDVNRDFVNWVEG
AFKVKGLKTQVMYLHPRFPKDQVIQRQAAEGVHGVVDLDIRAQNLARVPV
QVFDRSAGSNNVRFDQYVDLDPGTAAEVILRAKSSAAPANYNQGYGSNGA
YQNPYGTQHQQPAYGGGQQPTAYPLQPQQPPPTSAADIASLMGHVDNATL
QRLLSTIQGQSSAPPAAGSYPHPAVGGAHTAHNPPVDIQAILGSLSGTNP
QQHAPSQTPYGAPYGGQQSNGSSNQGANGNNGDAASQVQNIMAQLARYRQ
>Mb|QLI67789.1
MSSRNYPRPPQRDSWFAPLSVDLIVKVLNVTFLHPFVCWMIPLCFRAQTV
RWEAPRMIASIAWATFITLCSMAGVVNQRIAYGLPREVDLSEEVIVITGG
ASGLGMLVAEVYGMRGASVAVLDVNEMENGEARGVTYYKCDVGDKAQVKK
VADEIERDLGTPTVLINNAAVVIGKSLLNLTLDEIDSSLSTNLLGPFYCL
KTFLPAILRGGRGGTIVNISSVIGHLGAAQLSDYAAAKAGLTAMHRSLTA
ELRETHPEIRTVLITPGQLSTPLFYGVQTPNSFFAPVVEPVDVAKEIIGA
IDNGRGATVGMPLYARWVDWYNVLPAGVQVLARWVAGVDRGMRTFIGRQG
LSDELTKR
>Mb|QLI67793.1
MDLFRRASRMVPTTQNLPLPAHDEKRGTAKKNTLASRLAFFRRPLRLRGN
STVSVPLGVVLIFPLLVVVLILVLFVRHPSSPGRILMPAGAPPAIRKISE
KHDKVFVTGCLEPDTSQPRANATFVVLARNKELDGVIQSLKSMERHFNRW
YHYPYVFLNDGEFDDHFKETVRNHTSGSVEFGRVGPDMWGFPDWVDHKVA

KEGIAKQGDAAVMYGGLESYHFMCRFYSGFFYNHPLLLKYEWYWRVEPEI
TYFCDITYDPFLKMIENNKTYGFTIAVKELRETVPNIFRYASAYKRLNNL
TSQGLWEMFVEPRDPAEEEKKKEQLPDEILKNDPANNAPPKIDPESMESE
KYNMCHFWSNFEIAKLSWFRSKEYNDFFDMMDRSGGFWMERWGDAPIHSL
AAGALLGVKDIHYFRDIGYRHTTIQHCPANAPSRQLPRTPYLEMTTLDEK
KRIEEDKYWEDWDEVKENGVGCRCRCDTDIVDVEGKEGSCLAEWVDVAGG
WAP
>Mb|QLI67796.1
MLSRRIATAARMAPLRSMHPLRSNYLAMLPPLMQTVRTYSDKVVKVPQMA
ESISEGTLKQFSKSIGDFVEQDEEIATIETDKIDVAVNAPEAGTIKEFLA
SEEDTVTVGQDLVRIELGGAPSGSKEEPAASKDEAKEATESEPVKTETKP
QQSQTSLNPEPKQTEKPATTRAASKEAQSAQSEPSQTSTASASTGSREER
RVKMNRMRLRIAERLKQSQNTAASLTTFNEVDMSNIMEFRKLYKDDVLKK
TGVKLGFMSAFSRAAVLAMRDIPAVNASIEGPNGGDTIVYRDYVDISVAV
ATEKGLVTPVVRDVESMDLVTIEKAIADMGKKARDGKLTIEDMAGGTFTI
SNGGVFGSLMGTPIINLPQSAVLGLHAIKDRAVAVNGKVEIRPMMYLALT
YDHRLLDGREAVQFLVKVKEYIEDPRRMLL
>Mb|QLI67800.1
MAEDANAAWPLADAALTQEILDLLQSATHLRQAKKGANEVTKALNRGTCE
VAILAADTEPLAILLHIPLLCEDKGTPYVYVPSKTLLGRACGVSRAVIAA
SINSNEASELAGQIKALRDKVERLAI
>Mb|QLI67803.1
MASPRGVANILAKSPADVVILSSVRTPVTRAYKGGLKDAYPEELLACVLK
HVRERSVPGTDLGALIEDVGVGVVLSELGGSKAARAAMNHVGYPSSTALF
TVNRACSSSLQAVAAVAGEIRGGMARVAVAAGMESMTRNYASRAIPVDAW
PALAASPVRDARDCLMPMGLTSENVAERYAVSRPDQDAFAAESHRRAAAA
RHAGLFDAEIVPVATRFQELDRQGAKVGAEQRLTVARDDGIRENVSVEAL
ARLKPAFKPGGASTAGNSSQVSDGAAATLLMSRGTATRLGLSDRIIGKFV
SAAVVGCAPDEMGIGPALAIPKLLASHGLAVGDVDRWEINEAFASQALYC
LRKLGLEDAWAAGRVNPTGGAIALGHPLGATGARMTSTLVHGMRRDGHDL
GVVSMCVGTGMGMAGLFAREA
>Mb|QLI67804.1
MPPPKLLLLTGPPPSAALTESSCSIHNFDKPFQDLLGLPAASPHNLPPQT
QYTAWRSLPLARQPLHTGFTQAHHLQHDAPSSPSFFTTADTTTHGFATA
PDHDGTQDVLAQFCEQSLAAHNSSAAPDLDSFDTTESASFMTTSLLSEDG
QPAPPVPSHLSDLEDVPSAKQVTSLQPQTITLNLIVGVLSVAQPRTVTTK
WGTALSLIEVLVGDETAAGFAVTFWVPVDKVAESDAVQLRRQDVVLMENV
ALHVFRGKVYGQSLRRGLTRLHLLWRGEGGGSYSTRSLTRAGGDNPQVGK
VRVVRDWVLRFVGLDRTGRKRRRGVVDWDKPPDHTQ
>Mb|QLI67805.1
MQFESDFRFDMDDSFSMCSQTLPCPSAGSSFSSASSAYEPFTPTSRRSTP
NELSLDFEGAYNPFATHHSELTSPSGHMSKYMFGGPVKGEPEHMSFNEAI
PTTPMKKLDGMVTPDYDHMLEMNLASRHSIGSITPSGSFPMYTISPTTMG
PTSFMMTPTHSLSGSEIAESTSSWSCSNESPISFFPQKGLGPLELEGLDM
DRHPQSPLGTYHLHAPPSPGRLRAHRKMMVHEIQRKTNELQRAQIRASRR
VSGSKSDGGVDVVRRAMCKCDYPGCHKAFRRNEHLKRHKQTFHGEGPNRF
SCEFCGKDQFNRQDNLNNHRKLHARPNSRNRGVEFIPAAVPIIEQEERSR
KRRAPPKSKSADKRVDDY
>Mb|QLI67806.1
MSIGEAEPRRSVRATKGQHTKSFDELEPAVAPKRRQTKKTKKAQEKDQSQ
EPEEVIRCVCGATEQDEDSGEAWISCETCYAWQHNVCVGVSSFEDEIPDN
YWCEQCRPEDHKELLAGIARGEKPWEARRKAHEEEEAERKKKKGGRRGKG
KRSSDSKDELEKDGKAKAKASPAPEPASKDRKEPASKQGKRKIREESQDT
DGKAAKQRRVSERDVVPAAAKYTKPDDLASAISELAASRSGPAKALKKSI
AHVLAIIAKDGELRVPDGKTEDSMSEEYALQIERAVYDTHPASKGQKEYN
HQIKSLTFNLKNNPEVMQGLLHGIHSPTTLAVMTSEQLASAELQKQTAEM
RAKAEKQSILYTQETGPRVRRTHKGEEVVEDETALNEAPLPMAGGPRKGV
SQHPPLVKREPTGGDHGEVKARSPTQPEAQHSPSQSNFDINKVFSTVKSP

TLSQNRRPSAPVLNTSGPGFDPDVDRMLQDETESPPYSPTEESQDPDVIW
KGSLAMSSIADFPAMAKHVGGANFASFGPWSKLIPRRMTVAGRITQQSAI
EYLCSLRYSNLTDIVVVSITPTSAESQPDFRALVDYFISKGRYGVVGNKV
AGNVRDTYLVPVPAGDDGHPEFMLNLVDNYIPKTRTEPMLLAVFVYRNEP
DQLKQTNKDELTPVATPQPPSVTASPTPAPGGQRSSSTSGPAFSPATPQG
SFGQQHHGGPPQSTTPVPIPQPPHMQRPPPQTAVPPPSHSPPAGDQLTEA
QKYQAQQAGQAVAAQVLGDLISVPTVQFLLPQAFQMSRLEWEVIRSIYEK
DPRARQDLQYLGALLEKEGSEKRTQGSAVGGSQ
>Mb|QLI67812.1
MRTTAALRMFRPTARMMRPVPKEDQAGHTVSQRLRKLKQIPAELIPLAAV
VGFAIFAAGYSSVRKFVVDKNLRLARQGPGGRAVDHQDEHH
>Mb|QLI67813.1
MSEAYERERQNDSRLNELSAKVSALRGVTVDIYDHARAQDVIDNTVWLLP
HAPPMKKDLNPSRSTSNEPPE
>Mb|QLI67816.1
MQTVPSLVGTGTAIAAAASGLVFGQPTDLDLNLNHQAATASSRGARVHQR
SISIDRVLPAKSYSPSRTLPPPSRRPATSSAAPRSIGSELHAAGTSIHQT
TRPPLSRRQRPQTSQVAPSSTSSRRQSLVVLSQGDIYTGIPVVEGSRESI
SSSGSWLKRLSSIRPLSQHGSPRSSILADSPSVSFSYGSAAPILSRSGSV
SKPMPPNKLVKRSPSKNYTSEELAQRKSKGHLPTLRRPATSHQRSATLQQ
FRAGLDVAGSSADAKYSFDEPICPEKLLGASPIEELGQSPRTARAAKTSG
WVSFFHSKTASLMTKPSSHTRLGGSGDGGDAASSAKNLQSKRINPARSML
PGTGAHLIKPRMVSGSSTQSDSFASSHIEEEPNYQLVSRNGDRVGSPTTG
EMVSGMPPSARPRRSFSSTFSQTANWASKTSGSLRRTKRGATQGSGENKR
HVSAPVRALPSAVGRVDTMTSTPIAPVQQGLTLDSAATFQRPAPKRNASS
PLPPLSNLPGLHAELSRRSPVGGVAGHSIRPNQPSGSSTSSTSVAMTQRR
GSHYERLSALDSSDGDARDLISGDEYDTDFKSDTLFDSLRTMGSGRVRAV
ETPLESVYDESPPSTGGNKRSKRLSIQEILDRTWDEDGKIMEEDESNSTP
VHAPHRYMGSHVRRELREDPRFGLDPLTSNEVARETRNFGRFSLDDDFDE
DWTRDDDDAPVNALSPPSKGSSLSSSGINPNVRLALASIGGNLNAPSKPR
SSSSERPLSNLFDWSEPTPHDKQNPACHSRPKTAYAKQEIDSRGGRPVVR
KGPMPAHVRSQSVPVVHDLADESKPAGSKYGTWGLGSKTVSEDWDEDFEF
GGLNDGPGGKEPEDLFAVPESIRASQPSVKAHSGQIRELSLLVNDLKRLC
RHGRELNMLNGEQKPFWKEAEGIIALASPDEGSANEDGQSEASIYMDAFE
AANPPGDEDDIDDNLSFDRLDAVIERNEPVMSKTAVVRERQSPRRRSVFS
PDDDIFGANWPLSDDRPRSSRPSRPRTPENPPAKLQDVDGVVRSVIESVQ
YRSTLATAQPAAQGSRKRDTGTNRMHFDTNSLKLLVRRAGELRDILSDLI
RRADQLTQSPARTPRHERRLDSSPAFTRVFDDPGSSPARRPVKSRGNSTT
IVERPSSENSPPSSIGRRVPLMTVN
>Mb|QLI67818.1
MSRFSHYDTDEERLPEGMRRVGYDADTQVYTFQDADGSYWESAPGCQYGQ
LTRVGHGLPDDAADTEPFLMPHEISPSKSWRAELMPLLNFGVIIGVCLLG
LFWYLHYAASGEETSRPICISGTSPYTVKKDDTCWAIANGRGISVDSILS
ANEGLDCDMLRVGSIICLPD
>Mb|QLI67819.1
MVPPQRTGSFSPNPAQTLQTGSNHSPHPSHAGVLSAGASPSTSSPTGPSS
VAKIVVAQVYLLLSTIKEEKEEVKWDSKGAQLQKLIDENGMEVFSKYFTR
LVAGNASQIFPSLNRPVTGSGNYHILVAEMRKIAHEPDQARKIAESVESG
TEDIFRDFDLSTFMDNFKLDALEKTLLALAFKTGPRSDLKTKADAILSAN
FSTFLDIISRPDIEAHADLSPAFVALVVDRFIQYHPPSFNASSRRDLQIY
VTKRYLSVDQAPPSEVLAALDIGRVLADRPANALARYVQKTGSEFTRDEE
ACLLYLQSRPNNIQLNSEQVSAALTYATISQSPDHNPTVLVSALQRVLPK
SFDWNETVLFFDQPSARISSSQFLRLYTALLPIANESKYNFEIQRLWGGS
WSEPEAQLSFVSAYASLAPNQLDATTIPGLKRSISIEDYVNSPANVRERA
AAAVKHPLVSVAALSAIFNVALNSVHASQSVEAKRLFQEVVVPNLDIFLV
SALEVPRDTWAPMAVDTVTSLFENFLYKRSPEYDFVLDSLWRKDKEWVIQ
RLIDAHAVKPVDLPLIFEHALRHNWLDVLVYLPNGFGVDMAALAHTEGYL
DLSNWASINAERSVEMSRTLLQFLLIKAELEIRYQRPSDSQPPAKSSTSL

QVRTVSALLQILEDFLPKAPVQDLIMVQRLCITAYPRLINYGEGYDDVID
ENGKDGNSLPATANSKMEDHYKKMYGGEEDVRTIVDILREYKHSRDPLDQ
DTFACMIHGLFDEYNHYQDYPLEALATTAVLFGGIISHKLISDLPLKIGL
GMILEAVRDHSLDKPMYKFGLQALIQLYVRFQEWPGFCRQLLQIPGLQGT
EAFKRAEDAVHDHEGDLVHSRNGAGTPLALAFKSDTFTNGSSEEQPGADT
QAPPFTAIGVDPIPPGFEFEEPGEEEQGKIQFVLNNITEGTIQSMRNELR
EMLECKHQQWFASHLVEERAKMQPNYHHVYLELVTLLQDKALWDEVLRET
YISVSRMLNSEATMQNSTERTHLKNLGGWLGLLTLARDRPIRHRNIAFKQ
LLIEAHDTKRLIIIIPFVCKVLTQGAKSTVFRPPNPWLMDIIHLLIELYH
HAELKLNLKFEIEVLCKGLNLDHKSIEPSGEILNRPALEESTEVLGPEQL
EAFENISLNGLGSSVGAGISTQAVAATIPDLGPLITIPPTNEMVISTTRL
HEIVRNAMTRALQDIIQPVVDRSVTIAAISTAQMVRKDFVAEPDENRMRA
SAISMVKATAGGLALVTSKEPLRANLTNHMRNLSNEHTSGLPEGTIIMCV
NSNLDLALSIIEKQAEERAVPEIEDMIESDLETRRRHRLQHPNEPFFAAT
VNRWAMTIPNPFKLSPTSNGLNPEQMAIYEDFARQSRTTPAAAPSHATSA
SDATRTLANEVLQEPYTNIPNIPTPAETPSIQHLGTQMQPYAPVQTAAAN
AIPSGRNAGFHQMDVRGLADKVNRLLQELLRVAGEAREDHFLDLPRPHIV
LDVVDALVQLIIKTSQNSEEFAIYAAEQISALIFQQFEDNLTLESLVHVL
ETLRKISGHALNSRVRALFSQQPGSNFLSLQLLAALIRTDLLDWRNIDMA
MSKAIETRKDGSLEFLEHMLDLALLNNRPIALYADFVRTLETAWAWISED
PNSAAGQRLKTKLMGSGLPQPSRGPTDAESQGTAFRQDQMEYVFEEWVHL
WNNQNALDKSTAVFIQQLQAKQVIRDKNDFFVFVRTAIDLSVDRFEHILH
AGAIGDAYVMVDALAKLISMFISRNEDASTSRASFLDSVLVLITLVLNHH
HVKRGEQLNQRVFFRLLSMLLHEVHNESENLSEQEQRNMMLKFAARFNDL
GPLRLPGFTFGWLSLIQHRVFLPVILQMLDNAGWGLYANLVVQLLDSLSE
QLKAFNILTVSKEVYRATLKLLVVLQHDFPDFVAGNHVRLCASIPPHCTQ
LLNAVLSANPQQGYTKLPDGKEEIKTYPGLIEEAKSMLQEGGLLDLVDQT
LQVGPSEDVVAQIAHAMTQSEPQETAYGHIGVAANPHVIGSVVIYIGNQA
AERLSQTSSSISVTGSEPEVSTLSLLIHELAPEARYYLIASMVNQLRFPN
SLTEFFSQVLLYIFGKDLNDPEESDIRQEITRVFLERLVGYWPQPWGLML
TVLELVKNEKYMFFDLPFIKSTPEVADRFAAALQRA
>Mb|QLI67820.1
MAQRFGASSLHQRDSRSALFEGYTGGDHTPRGHSPNPYNPGYAAAYPNGG
HSERHGLGYRAATPNRKGQYSDAVLDELESQNEDQVAGILGKVRTLKHMT
VAIGDEIRESSALADKMNDAFDDARMRVRGTMNRMKIMAERTGVGWRLWL
LFFAAVGMLFFYVWLF
>Mb|QLI67821.1
MGATTVPTKEQILVPETLLKKRKSQEKARAERTASLQKKKAANKEKRGVI
FKRAEKYVKEYRDAEREKVRLHRVAKENDSAYVPAEAKLIFVVRIKGINK
MPPKPRKTLQLLRLLQINNGVFIKATKATTEMLKIVEPWVAYGYPNLKTI
KELVYKRGYGKVDKQRIALTDNAIIEESLGKYGIVCMEDLIHEIYSVGPN
FKQASNFLWPFKLSNPTGGFRPRKFKHFIEGGDLGNREEAINALVRQMN
>Mb|QLI67822.1
MTSLLNKTDSGPPPPRQIRFVNNQGQPPSKRRRINAACLTCRRRKTRCAG
ERPVCSTCTKNGHQCQGYPEDSRKDVPVESLEAKLAADDDDIPLDDIPTE
DDSKKTVHEKSDAIPTPPTTNSVVIVNGHETKKHGSLSPYRKPLVARTEL
DTLKPPISPTQHASHPSANSDDTPGSPTLRRNYNHRVPYFRYFGPTAIVP
GFKQMVVSVRDRRRSTGGSQSAASPLSNPSGLRGSSIAVGSDVAVEDMPV
YDPSSHLPVHPVILSLVDTFFLHLGCNYPFLKKPKFVQMVKEKRVEPILV
DAICAIGARFCDISQFTSGNDKMPRTERGQVFAQRAKHATVETFPCPSVG
AVQALLLMAYEGFGANQDSALWMYLGLAIRMAVDLGLQKRVGIQYQGEKD
PWYTRHRSRGNGDSESPEDRKTSDSDALSLDEQKEVEQERIDTFWAVFFL
DRVISSGTGRPVTFRDDDFELPFPAARVDPETGWPVLFPVLLEIIHLYGR
VSDILNNIRDVRDLTEDKWRKLATMEHEVTKMYKNWDARLQFNVSNFKAY
LNLRQGTTFILLHFWFHALFIVLHQPTLLTPFGGLWTEHQLLPDSRELSM
SSAKTICDILSFADLIDPISFIGNPFTSQPIYIAACAFLMESSANASEGS
SRESSPPAVTKRPATQTKTSNSKHSRHSLLASAANQNYQRCYNSLQQMHA
YWGGVKYILTALDQKAKGIWDCETYTSEEYESTKMPPLQPGVAGDFGHLT

QTSPKVGGPPIAWSLAGTANSPNSNLTLMYQNSNGHPVSNAPHQHARSVQ
QRQASTPPGNMLYDPIRQSMPAEPGSMYPPAVPPSNLAGPTTSPRWTRAR
RPSTLSNAAGQSHVAGASDGFSDPGAEGSKVYMPSGFTPVSQHSASYEGY
TASPTSNLADGNSQGRGGGTANNSVYYAQGMPYQSSWTFGNGSMDGITFD
SQDIDIGALGLQQPDLMSGWLEYIPSDGDVLGLFENHDINNGQGNQTGQ
>Mb|QLI67823.1
MEGIQTHPSNAAQAKAFTAPGSLSFPGATNELTPPSSNGDAMQRPAANGQ
NAANGNGVTPATPAATPSATQGPSGITPTLQNIVATVNLDCRLDLKTIAL
HARNAEYNPKRFAAVIMRIREPKTTALIFASGKMVVTGAKSEDDSKLASR
KYARIIQKLGFNAKFTDFKIQNIVGSCDIKFPIRLEGLASRHHNFSSYEP
ELFPGLIYRMIKPKIVLLIFVSGKIVLTGAKVREEIYQAFEMIYPVLQDF
RKV
>Mb|QLI67825.1
MTTIASLRRMSAKSLSEKILAEKDAANTSFAIIDVRDDDHIGGHIRGSTN
IPIGQLDAMMPTLVRRLQDKKTVVFHCALSQQRGPSAALKYLREKDGLLR
SLGSREIAAEQEVYVLDRGFVGWQQVYGDDERLTEGYVKDIWENY
>Mb|QLI67827.1
MNPAQGQAPQQAPAQGQPSAAGQQPRPPMYQPTQIRNLPMLSEEEKNKYE
QGLKGLWNKANTSPANSPDQLAARQKIIDFSKMLINKIQVRRNQAQQQQQ
AQQGQPGGQPARPSSRQSNPPGQAAGTPTTTQQKPQQTPATSQGSQGQAT
AASAAGPTQNQNPQPGAAVAAGAATAATAPRLKIPEHVLQHVNKITFHPP
PQFANKPAAEVTKWIEELKDKYARAIFTMDGSKAKVASMEKFFKERTDAG
RPLQDEELRQYQSRRDAQLKLYHEALKWVENARRQQEAFANHQQAGAQAS
QNGTAAATPGNQQTNTQAAPNTAENNAQAATQNATASVNAAVEAAKTQQS
LAAASRPSPANGTPTPTPTPAQSQPRPVPQPAQQPQVQAQVKTEQPHPPP
VNTVQAQAAAAQGQATARVQTPQSATPAIAGGPTRALSHSAAMNLANQRA
VSTPSTTPAQSQAASSGAGVGGAVNQGAVVPGAVQQQQSQQQLQQQQQQQ
QGHPHAHPTQTQKMPIPKQLPEKATAVPQGVTVGGGVTAGRPTMSQGSGT
LGGVMNQPPVARIPAYSHDAEGDHVLSKKKLDELVRQVCGGSGDSQDGNL
LTPEVEESILSMADSFVDTVLHSACRNAKERGSKLLEIRDIQLVLERAYN
IRVPGYSSDELRTVRKVQPSPGWIAKMSAVQAAKVMPGKGDP
>Mb|QLI67828.1
MFNYTSFHDVFPTEVQLPYDRRLQHDIEAHRKNADDVLFIDRVMRALGIS
KAKVYPPKTENALRQLHQQICEAGMSMHHKFSLLYYILLDFDDASKEVFV
SESFASLSGMPANYQLFMKGLWHMDREEYSRAIEYVAHPSLNPDFADDII
IALIKQASDQDYTLALSYFYSVQPILKSPVALELLYDAMARTNVTQALLY
SRTHPQHTREQLFRRWISCVLNNGGGDDLPSRTSELAFMPFDSLEETWFE
EYLTTGEGRTLKRAKDTLLIRKIACDRFSYVAKVRPSGQWAGILEGIKGG
TEGQGD
>Mb|QLI67829.1
MLGLRSKCLHPASKRPYIKSSMGGLDRSIEGCQHGSTHAFVVEFESEQDR
DYYVNEDPAQASFVLEVLQKLDKATILDFSPGVF
>Mb|QLI67832.1
MASASTIFIAVIGAGGVGKCFLTQLRDLAVRRPSPKLSLCYIATSRKALY
NDDYSAINLDNAIDVLAASSKAPPTLTDLVAYLSKAPGKAIIVDNTSSQD
VAETYPAALKAGVSIVTPNKKAFSGSYQLWQDIFQASAASGAKVYHESSV
GAGLPVISTLKDLVETGDEVTKIEGVFSGTMSFLFNSFAPLTGSGGKWSE
EVKKAKALGYTEPDPRDDLNGLDVARKLTILARLAGLPVESPTSFPVESL
IPKELESCSSGDEFLEKLPSFDQQMEEKKASAGDKVIRFVGSIDVGAKDV
KVGLQTFDPSHPIAALKGSDNIISFYTKRYGSNPLIVQGAGAGGDVTAMG
VTADLIKVIGQLA
>Mb|QLI67833.1
MATFNSMPAMSAAAPNEAMGLNHPAPGQPMTMEHFDQDFNFDEAVLDGAP
LPTLPFTPAYDFDNFTTTFEDPFAYSSRAFEPTPNQDALNEESSPQELDN
KLLGFSDPIMNATVVDKTGKLAEVNMSAELYGMFFVAEDVFGGDNSGRPL
ELTCYRRNLWQCSGQITLPKNLSHLMDERGRQFSIDELSASITAVESIEG
KSTEIISIPWKSSSSQAPEETKVAAAPPQIPLDLSTAQEVDAVRVTLPVS
WKRLQFKHATANNGRRKGLQQHYVVQINLVAKSKSNDMMKIAEIQSGPVI

VRGRSPRNFDSRKDVPLTGEKRLEKKNTASSDNTALKIERENIQATMQRF
QPLGNVATPAEWSANPQHVSHPNSQSPHPAKRMAVSPTVARPPVPSWSAD
GKSMTTGQPHRGSLSRQNTAVPINLSLSEDEKSPNRSSAELQSPQMGKSH
PVSGQNAGNSPVDDPDPLYEYFPLTLDDWMPPIDAVYRPHVVHHTIVPPE
IKAQQSQSKAKRYFSAE
>Mb|QLI67835.1
MGQGQSGMGGGDGREDKDKKKDKPKYEPPPRPTTRVGRKKRKAGGTSAAA
KLPAIYPTSRCKLRLLRMQRIHDHLLLEEEYVENQERLRKAKAAKEGAAV
GPDADVDRLADERGRVDDMRGSPMGVGTLEELIDDDHAIVSSTTGPEYYV
SIMSFVDKDLLEPGASVLLHHKSVSIVGVLTDDTDPIVSVMKLDKAPTES
YADIGGLESQIQEVRESVELPLLHPELYEEMGIKPPKGVILYGAPGTGKT
LLAKAVANQTSATFLRIVGSELIQKYLGDGPRLVRQLFQVAGENAPSIVF
IDEIDAIGTKRYDSTSGGEREVQRTMLELLNQLDGFDDRGDVKVIMATNK
IDTLDPALIRPGRIDRKILFENPDQNTKRKIFTLHTSKMNLNDDVDLEEF
ISQKDDLSGADIKAICSEAGMMALRERRMRVQMADFRSARERVLRTKQEG
EPEGLYL
>Mb|QLI67840.1
MATTGSILSLPFRRSTQLSLASVIRQYINSKYDQHPDMFKQDLEVIDALR
QDAINVREPHPSGIKKLQAYAGQLAWISGKFPIDIGAEFTWYPALGYNTE
RPMVRNNIKYELLNVLYNLAALYSQLAVNTSRGTAEGIKTAANQFSLAAG
VLSHMKKEVVPLLRMYDPPEDMDEHTLESLMQLFLAQSQECFWQKAVVDG
YKDASIAKLAARVSDLYNLAGEAAMKSEAISSAWIHHMTAKHHHFAAAAQ
YRAACDCLEKRRYGEEVARLTDAVACVTEGLKETKGGYLNKTVVDDLNGL
KRKVEEDLKRAEKDNDMIFLNPVPPKSELKMLERANMAVARVPPQVANPF
DFLGDRAEFGPALFSRLVPFSVHVAISIYEERRDRMVNQNIIQDLEGLND
KIHSLFSAIGLPGSLQALEKPLGLPPSLVQHAEELRQADAIGRLRKSFTD
IDKLRAADIAIFEEGTSALAAEREEDQKARRKYGTDRWSRPESQSDAQGG
KLWGHADEIEGYFASSASSDGVVREKFAANEELLHVLCGSDRGLMDFVPS
SSQRDTNPELKASVGKLRSAYNDILRLESRRRKKVETLRENARKDDIKPD
ILKEAARLERTYPSTAIVPAHFEDFFEKRLDRLYEPELDALDKESLEQDR
LLKEVERANAEFESQKRRCGDRGSREREQALQKLDSAYFKYKEIINNLEV
GRKFYNDLNKIVGQGFRDVVKGWVAQRRMEARVLEEELSMPPLSTLNLSR
DHAPLQGASTTQYQAAQPAYFAPEPVAQHHQTSPQRQNDHAAGQSTPNPV
EASIQSWAGEAVRQPQPVAPQTTNPMTTMWNPAMGIKFGGSGPPAPRTDG
GQQPGGGSATTWNPASGVKFG
>Mb|QLI67841.1
MAQPNPELRRQVIAIYKELLYLGREYPLGYRYFQPRLHKAFMSRAAERSE
DKIRAGIAQAEYVKKEIEALYYLKRYRTLRKRYDVGA
>Mb|QLI67843.1
MAPAKRAAKAKPSKSSGANGLPPAPFKQPPDVLNPFIQGLSEKHVYITHI
DSKPAAFKRKIFLVPVAMNIAVSLLFVWRMYAILPWYWKIVQSGFGWPNE
TTFPIAGSSWQDFAWEVVKRGVTMFIDFVLFVFVWPWPVEFAAGQAHGNP
MLWRRKVGFQDKEIYVRRSRDWDRVLGDIFKDDDSKRILLAYVNAATSPI
LQEQKTGYLLMNGQWDLDWASMVHAHALVDHKIAAMEAFRTVVLLHHKDF
GWLCYDMKADIAADGDDKRRQVFAFRNALTSMGQEDLFYRWVEMVQFDAT
QPGGFGPEQQEATAKRIREMFEAAGVNFDQLWKDAVGEARI
>Mb|QLI67849.1
MPSMPQTNGADSVSLGQPSFLHDLRAFLKEWIRLNWMDVLLMLTIGALSM
CVYYAPIKVVRTFPITFNGSGDIVYPEWAYPYRGWILPAWFSGLVSILGP
ILVYLLAQVRIKSAWDASNAIMGTMWAVSLGTIFQVTIKQLIGGFRPYFL
DVCEPDISLARRHNKTGLNAVGFQQVMYTTEVCTQTDTWRLKEAVTSFPS
GHSTAAFAGFFFLFLWLNAKLKVWADHKPAFWKLALTMSPLLAAVMIACS
LTIDAAHNWYDIVSGSMIGIVMAVASYRSTYAAVLDWRFNHLPLRETEPF
GYECDIVTQTVTRSVGWGRGVHGFNEPGERDSRDFCGHDPTEAQRSDIGE
TGVGGRPTRRAIARSYHRAGELGT
>Mb|QLI67852.1
MADHGIGAADELSQLSLGGHGPEPPMPPPNLRTAGIIATDISDNFAAAVR
TLEPGELVKDGFFTLFDSVAALEIMDPKMDSGCVQPGEEFEVLYDVSRPL

LPEEVLGIMDQLLCHEMSWHLGYPLSQTIFTSVYVDALLMPAPGSIRQAK
FIRNGHQNVNQEPMLDILRAYCLGMLKACGHVNERIKSEHFYEEEDFVPN
TYNRTLLTEFPTQDVKDVLKAARETVESLKGSIPDGVSEALTSRLDLRYI
FLDATESPQYLKNLPKARLPWEEAIAILPRISSTHSLTKSVEDAFSTKLQ
RKLASTMPPRPIVQLKFDDAFNHLSRLLKDGLDVIGVLEYTNSQCLQNFV
YTFQSKKPQPMVYVRTLLQTFLFNAMEILGSMSIRQLLDDDLSIITLPAN
TFLDRDNDEIEAVHDPRFVMSQQMELFRQKAAQPFLDIFRTACQNRCRVR
RTLCHLIRDWENLQVDAEDIDQILQIKTKEPPLLQRSTLGLSPVETYSLP
LSSWTYLYKLQQMEAIVQLGFELEVYQADELAGMYWYLNYLAKSRLQHTE
RIKTFVVRQATTVRSLHTIDPSQEAQLQRSLAYMRLSLLQAAVTWELSDA
LSCIYTVLNRYAIVKPPPRPYSNDQLRYDLRMRPFASIGLPSLPSFEEFT
LGTKQPDTSTEELLEYAERAATGAKRGFETLSKYSAEDSFSVGSHEAWTT
SVKGALKACIAAGVAISSLQKALRRTVGENGTGDVLHVTATVPTPDKAYH
EWWIVPRIVPVVTMEMGN
>Mb|QLI67853.1
MASSDTASKGSFPPTLKLNDGNEIPVLAYGLGTANYKSTSGYDQSVVAIT
KTAIDLGYHHLDGAEVYGNEEELGAAIKASGVPRSQLFVTTKAHGEQQKP
TQEAFELSLQKLGLDHVDLYLLHGPWFAASHEELQQKWADLEAIKASGKA
KSIGVSNFLQEHVEVILQTAKVVPAINQIEYHPYLQHGDLVAFHKKHNIA
VSCYGPLLPLTRAKGGPIDAVWSELAGKYGVSESEIGLRWILDQGLVALT
TSSNKGRLQDYLAKLGTFELTQEEVARIAEVGNRYHFRGFWLNKFGPDDR
R
>Mb|QLI67857.1
MEKLPTTPASTPPPANRRDIQQNRLTPTRPRSPLPGLSNSEPFLRFPRPQ
GNKRLANWISTSNPDIMRAATPTEDSGMAESTFELISSTDVESHDENYTE
SISESVGSLDFHRPDDVQSLAGTEQTFDDESLADEEVETSPQSAEHEAGT
ELINDETVVAQRASDASESDEEARSRCSLSYTQQSLKTPSILTPEASKII
ERPPENTNNKFYTPPVRFRHWMDITREGMFRTWEYATDATSAVLPGLLFG
VVFSLLISTLYPSTVEFMKPAETVVTSTVTATTTPLVIYTKQSTSTATVA
QQTTSTKGMGLIAIKEEASDEWLFGLKRPDVQFSPLGHGAVVVHIASHVQ
KTWLKKKNCVSITATRNGEAVEVEFYPSKDGFLVKFPKKEAFGVVKLLVE
ATCRPTVSKAVKVHFGKGIMEEAYERTMNLAHDISELVPVAAQEAERCIV
GAKKSFGAVSDTVASRVVTVSDTLIGRLKASSARMERLLSELPSSSIEYA
REVVDGVPQALESMYKQASEAMGSHNNIKLDIQDGILDAELYFLRTRISA
KMWWLKVTRQTAEHDEYGKKAQDFIAKRRCEGRNKIRGLLRDDGKDHGFC
GRTKRSWCKRHSRKGRDGTFIFTR
>Mb|QLI67859.1
MPDAPFAFLPLGAIIQSIKVNDTNIVLGFPTQEQYVQYNTAYFGETVGRV
ANRIKGAQLDSVNGKVYPLAANNGPNTLHGGNVGWGKRIWKGPAPVGIKQ
IPGVQGLQGGESVTFTLVSEDGDEGFPGTVAAKVTYTTGTQVVDGKEVLL
LGIEYEAELVDGADETPINLTNHSYFNLTGSADIEGTVVTLGSKLLLPND
EFSVPTGGPVPHASIDTTQPFTLGATDPQVDNCFTKTAEPASVPIDTRSQ
PLVRDLAAHHPQTGIHLEVLSTEPSFQFYTGDFTNVPAVEGASAKGGRSA
FCCEPGRWVNAVNVPDWKSMTMLKRGETYGSRIVYKAWSD
>Mb|QLI67862.1
MADLDHFDLLPIHMDAQSKAITASKQSRALNAELEALNTLHRALLNVDSS
SNGVPPPPIPVNPKRTGQVNKLRDNGNAEYRKGKYADAVRLYSLGIQMAL
GRPLWEPAALVREEVSGLLANRAQAHMATQNWPEGAVDAEASVEARRVGN
AKAWWRRGKCLMEMGRLEEARDWVKQGLEVEGEEGDLVSLLKEIEALVER
RKAH
>Mb|QLI67863.1
MDFWSRLLSPLAAGSPRKDQAKDPAKRLHRFEKEYASLLVRKRWPQSAAM
GFSLLCLVRVHQKLTVGQSTWRTSSNLSRDSEAAETLEIRLQELTNILSD
ESRRPLPHPCIQYASIKQIYVPIGKIATSSYNEWIIKEAVLFFATLIESE
EEAFVENPTFSGSLTNLLVRITGVNSVRLGLDTESRVVELAFNITTKIRL
DPAILPAWFKTPQFSASQNSLQDLDERERFAGRMQRVDFPLFYILMDYIH
HEGKVGDFARTGLLYIIEAASSSAALEQWIVESDLSTLMATGLGALYSQL
SRKLVIDHLSHDLPPILALSDYEHPTSNYEIISSCSPEFQSHLETFLSHL

LFWQDVLNHCRSVEVKSTLLEHFQVIFLQQLLYPSLLESSDIDGGSSVAV
LTYLRRILESLDHPDMIHLILHYLLALPDAAPPNKKPPPSLVSDARRRKS
MDLTKMMSEKAEEPATPLLFNLVDLILSCLRSRNQQTIHVTLQLVSAILK
RHHRYAVFTLLRTDVLPSHTKHRTVGAHEQEVNYLVHLAISIGGRTDFDD
MYAGILKDTSTRLESHPCSLRLIAPKVSTNNHKLPDLPDSLPGAPRDIRE
HTLRPDDPLLNIMLDLLGTFFVNPVETNLSVTETIVDLGICGYMSIEGWL
ARNPSTYVYTESVEELERGYETSVASESAEPPSRPIDSALSDSFDEARRM
KDMERCRTAPVWREASLPRVLNVLKRLCDQVAKYKQTIPRFDELLEQRRN
AFQTAESMLDNPPPAVRKASPIRSGTPERSSLDETLRTGSPPRPSAIEGF
AQRLLSELGTPSRSASPKGRKEQNRSSATSTSGGQPSAPEPIMTPKAIRV
PPKEFPINYNDPSKSGTRSFSPGSVRPDIGALSEERDMIAESQASAFAAI
DQSILSRRVGLPDEKIKPIPLDLTKKSKATEVDEAESPKQQEHGNASGAE
AMPDAVEDAAASGQTPEEVTVSVSHVLTNVIIFQSFLLELASLWQTRAGL
FDEVCYA
>Mb|QLI67866.1
MAEIQAKLQTLSDEYQKLQQDLQNTVNSRQKLQSQQQENAGVFKEFEKLG
EDETIYKLMGPVLLKQDKVEAESTVKGRLDFIAGEVTRLETQIKETQDEL
EKKKAEIIQIQASAQAAAGGKGPQK
>Mb|QLI67867.1
MPGYHVTPLYGGAIVCDIPEKFADVSKLRQVPDNQEVWIDKDGYTSIIFD
ITERVGEPGHGPEIDGRAMTTHLEDMVGSDIDTVKIWNTAETEFSRLESK
PPAYTLIATQTPRTGQSRQGGSPPDFTAIIMTLLRLEKYKSDILITINVP
HIKGEYDEDEVDLELGKQGKLIGDAVEYSARIWESLKIKDWGLFGES
>Mb|QLI67868.1
MASRPELKLDDEGGFIRFYKSLPDVGEDTIRIFDRGDWYTAHGDNASFIA
KTVYKTTSVLRQLGRNDHTGLPSVTMTVTVFRQFLREALFKLGKRIEIWE
SPSGRMNWKCVKQASPGNLQDIEDDLGGQIESAPMIIAVKISAKASEARN
VGVCFADASVRELGVSEFLDNDLYSNFESLLIQLGVRECLIQLDKGDKDK
DPELAKLRQIIDNCGVAIAERPAGDFGTRDIEQDLARLLKDDKSVNLLPQ
TDLKLAMGSAASLIKYLGVLQDPSNFGQYHLYQHDLAQFMKLDAAALKAL
NLMPGPRDGSKTMSIYGVLNHCKTPVGSRLLAQWLKQPLMDKDEIEKRQQ
LVEAFFTDTELRQTMQEEHLRSVPDLYRLSKRFQRGKANLEDVVRAYQVV
IRLPGFIGTFEGVMDEAYRDPLDIAYTTKLRELSDNLGKLQDMVEQTVDL
DALDRHEYIIKSEYDQGLQTIRKKLNQLDRDIRAEFHEAARDLGQEADKK
IFLETSHKVHGVCMRLTRQEAGCIRNKSSYQECSTQKNGVYFTTKKMQSY
RREYDQLSQNYNRTQSSLVNEVVNVASSYCPVLERLAGVLAHLDVIVSLA
HCSVHAPEAYVRPKIHTRGEGQTRLIGARHPCMELQDDVQFITNDLEFTR
DKSSFLIITGPNMGGKSTYIRQAGVIALMAQIGCFVPCAEAELTIFDSIL
ARVGASDSQLKGVSTFMAEMLETANILKSATKESLIIIDELGRGTSTYDG
FGLAWAISEHIVKEIGCFAMFATHFHELTALADQYPQVRNLHVTAHISGT
GKDNSKREVTLLYKVEPGICDQSFGIHVAELVRFPDKVVRMAKRKADELE
DFTTKHEDNALAYSKEDVEEGSAMLKQLLIQWKEQVGKGEMSQEEKVSKL
KELVAADSKLLANPFFQSVKAL
>Mb|QLI67870.1
MADYTQYHALGQGIADPNDSNRTAGLGQPPYQQQPPGYGYGAPGPYAAQP
QQSPPPAMGGPPPAGAAYGTPHGPDDRALQGFQGGVAQPGADVGLASQMG
AMSLGDGQTTIRKKKKDRHAYHTVEPTGSSQAYNGMGPPGSNPNQFIPTA
APGTPGAPAFGASAQFAGPQGTPHMMNAGEFPTPGHFGHGNGSADAAATI
STSGPSKVSLEDMPSVPVSRDSVQPYFLKNVYPTFERHVPPPATVSFVAL
DQGNASPKYARLSLNNIPATSDGLHATGLPLGLLLQPLAPLQAGEAEIPV
LDFGDAGPPRCRRCRAYINPFMMFRSGGNKFVCNLCTHPNDTPPEYFCAT
SPQGVRVDRDQRPELHRGTVEFVVPKEYWTREPVGLRWLFLIDVTQESFN
KGFLETFCDGILAALYGGENQEKDENGEPKRRVPEGAKVGFVTYDKDIHF
YNMHPGLDQPQMLIMPDLEDPFLPIGEGLFVDPYESKSIITSLLTRLPEM
FSDVKNPEPALLATLNGALAALEKTGGKIVCSCSALPTWGPGRLFMRDDG
NHPGGELDKKLYTTEHPAWKKVAEKMAASGIGADFFLAAPSGGYLDIATI
GHVAATTGGETFYYPNFIAPRDGPKLSAEISHTVTRETGFQALMKVRCSN
GLQVSAYHGNFVQHTFGADLEIGVIDSDKALAVSFSHDGKLDSKLDAHFQ

SALLYTTASGQRRVRCSNIIASVSETSKEAGMREQGIRACMKFVDQDAVI
ALLAKEAGTKLATTSSNLKDIRNWLSERTIDVLSCYRKHSAQQHPAGQLV
MPERLKEFCMYMLGLTKCRAFKGGIENSDRRVHEMRMVRSMGALELSLYL
YPRMIPLHNLQPEDGFADPETGHLKMPPSIRASFSCVEAGGVYIVDNGQQ
CLLWFHSQTSPNLVADLFGEDKDSLKSLDAYTSSLPVLDTHLNAQVRNII
EFIKTMRGSKGLTIQLARQGIDGAEFEFARMLVEDRNNEAQNYVDWLVHV
HRGVQLELSGQRKKESETDLNTLSNFTGLRPTMW
>Mb|QLI67872.1
MSLSDWDSTSSRDGERYLKHQHSASSDHGARALVPMWDSSDPERAPPPLP
LNPQSPVLTSRAGTSSAIQSAHAALNERARENSAVVRRGDGSPERALVKG
ASHRRLQSLQPGSVRDLSLMIEGNWSKQDLRPSTPTTPRELPPEPRSDGD
SANSTPIPGPSLTPILRPTARRLPQQSILGENTPPQSSTMLALQNMGSKS
ASREAESALSNITNGSTPKQPPYSDDVSAQLLTLTNIATSLQKEMSALSR
RSRDNATDLLSLKEATKSRDEDVRKSLRELVGNINEQSSRITSFNGPYLD
NKPFGSPPTHSRAFQLPRIPSPKSFAESIDRGSISTPSLSGVDTSASLIL
LEKIWNGLGTKEGQESLVGLLTQLSQKLSGLATSAKVDELAEYIRSQSEN
AVVLGSATGSGGLGNQDMELERGGLVTQRVDRLLFNGENRRSSAPSGRGA
ELVNEELMNIIRSVKDSVTQGGGLTAEVKALVRELRGEVLGMGRELGKRL
EKVGSKGIEEAEAPSKDEVSRVIDEGLEQMKEQLNNVLREHRRQSAQTQS
SQKTVVDYKEIYNAMRAALRDNEASTDETPDLSREDVIEAVRDAWENYKP
EIEVQQLGLERDEVLACLKEGLQEYAPRDERPEAATREEVFKAVVEGLKH
FAPPQVETPASLSRDEIIDAVRDCLEEFEFPVAASAVGTELSHRDVVDAV
REGLSGLDLPRNDALMAVPSNNDEIASRLQDIMEYMKLEFKAASESHRDV
VQQAVREGLSELDLPRGDALMAVPANTDEIVSRLQDMTEYMKLEFKAVSE
EAKDNVAANGRDTEQVLDATKDGLENLRIAIESYVDRVSGVAGHEEFMDN
MSRTMEEFKEEVSRAVSMANDSSREELQAHLEGLREVVNSSMVPASSPTV
PSVNNQEVLEALHNGLTSLRQEILRPRPETGDILDAIQDGLNDLRAALER
VINKPVDLTANDEVLDALKSGLDGVRSDIETLRDSSNERAVAAVATATEE
DTSRAIIPADMVKQDDIKNLEVLITQLRIKVEAMEPETETVQRDDLARLE
DMLRSVQEGVNEISTREPPAAAEPAKEVHEATDTNNSDAASKDDVQAIET
ILRNTKASLDDLVEGEQAVRKEHLDAIEALVLETRETMTSMTEQLATISQ
KEDLNALETLVTQLSLGLEEIKERSDKAPSSDEEMATKTGLEAVEAVVLE
IKTALDGLTATDFASISNKEDVSNLETILKEAKEKIEAHTESNTKALDDR
QAEIVGVADRVTAVKTLLEEFQEAIKSKLDDGSSGIDAVTKLLEGMGEKI
DKNENVGADLKEMFETMKTQFEENKEVVAGARLESNEKLQEAADSIGTKI
DDKITELNVKYDEFKATLNEKSEATEAREIETEAAVVGTKAIAEDLKLLI
DTLGSTVTDSLEKMEEASKTVFSKVEDLATRTEEIDTEGKTEHQKTRDHL
QQAVAAVESLQGEVKEHQPEILTAVKDILLLVGEHFEHSKTASTELQDKI
AEAKPVEQQMLPPPEKYDDSAVQEKLNLIAEQKYDDSAVQEKLNLIAEQK
YDDSAVQEKLNLIAEHKYDDSEIKERLDRLVEQQYDDSPVHEKLDRLVEQ
RYDDGPVHEKLDKLVDHSTTSAQAFAQLETLDKVHQSVVNTAAEISQFLS
SQTQRIENDHEDREKTLQDTVIELERKLAEREHMEAAILSLKDEEDRLRN
SVLNLRTEQESLIRQKTRLTGDVSSLETALRLRKEELSDMEYRAERLERR
IVEGVMDHSRVLLMAKDSKGRDDMNRKRVKKPVGEDGETGGTKSNRPALS
MALATRRNLGVPTQNGSARRIASLSQMNSSSPSGLVKRSQSVRTGAGNAL
RKRSWGGGMAKGVGEEDKENLRVGETVEELDEPDTAASSPVASTVAYTAD
NDSVLDSLVDDDGEGSDTGTLRRSSVGTTVMSSQYTESEAGYSEYDGTES
DWTESQVGTDVGAENGLVIYEQ
>Mb|QLI67874.1
MDGKQQTPQPQIQPCRYKVGKTLGAGSYSVVKECVHIDTGRYYAAKVINK
RLMAGREHMVRNEIAVLKKVSMGHQNILTLVDYFETMNNLYLVTDLALGG
ELFDRICRKGSYYESDAADLIRATLSAVAYLHDHGIVHRDLKPENLLFRT
PEDNADLLIADFGLSRIMDEEQFHVLTTTCGTPGYMAPEIFKKTGHGKPV
DLWALGVITYFLLCGYTPFDRDSDFEEMQAILNADYSFTPVEYWRGVSSN
AKDFIRRCLTIDHTKRITAHEALQHAFVAAEEPTDGAGENLLPTIKKNFN
ARRTLHAAIDTVRAINKLREASHLMDGARSKEPGRGAAPRSDGNGTRKDD
SGISLGKEDSGYGTQHDADVVMGNTTAAPALSSVPASLQPGNQANKVIQT
SKGLWSAPTSNR

>Mb|QLI67875.1
MDVTPRLSRSCSSASRKSGLTLDLSNLPPLTQPTPPSNTLLFTGLNNTDI
FRPDNLERVRGLVAKIAPIHAFAPLKSFRRIVISFFDIDSSIAVRQVWDG
EAILGDRCRVYFGQQTNVEAKDEHLALPDAGKLFFISPPPSPPHGWEVRL
EDAPNKQVHAEDLAEALSKLRNTRPGLESPISPVDGPAASGSTRSRSSTL
IYRPEENGCSPDLPAVFVEDMTEEPELISPIDGCRPIMAHTARPPVELMH
DA
>Mb|QLI67878.1
MTVDHSFQTVFAVPLSCNGCVKTVSDSIYKLGGVSKVEGSLEDQLISVEG
FAAPSAIVEAIQATGRDAILRGSGASDSAAVSILESFTGKLQREDDNGDR
EVRGLARMVQVSSGRTLVDLTVRGLAPGTYRATIRQYGDLKHGAKSTGPV
WSGDQSERNGVLGDIDVGQDGRGAAFVDHPFRIWEVIGHAMVLTRQNEAA
GPLENDDNTVVGVIARSAGVWDNDKTVCSCTGKTLWEERRDEVRKGML
>Mb|QLI67879.1
MPSSEKTGERRPSTSKGNRISQFFSSSPKSKDHTAAQQALLAMQQQQQQQ
QPAASQQQQTQANGVPAGISPPSTSLPIISLSTAVAGDHSSSEPPTTTLF
RPKSAEEMERKRRAEAQFGPLCHPSHLYVSKSHGRPLEPPVEDEPPYYFL
LTTYVSYLLLILVGHICDFFGKRFGDKKHYDSLKVQNGFAPLNDDFDSFY
TRRLKMRLDDCFARPTIGVPGRFITLMDRKSSDNNRSYQYTGTYTETLNM
SSYNYLGFAQSEGPCADAVEECVRRYGVTSASPRGDAGTTDLALEVEREV
ASFVGKPDAMVFSMGYVTNASSFPALASKGCLIISDELNHASIRIGARLS
NAVIQSFKHNDMYDLERVLRENISQGQPRTHRPWKKILVVVEGLYSMEGT
MCDLPGILALKKKYKFYLFVDEAHSIGALGPRGRGVCDYFGIEPSEVDIL
MGTLTKSFGANGGYIAAEKHIIDKLRSTNASTQYGESPAPCVLMQILASL
KLITGELCPGQGEERLQRIAFNSRYLRLGLKRLGYIVYGHDDSPIIPVML
YNPGKMPAYSREMLKRKISVVVVGYPATPLISSRARFCVSAAHNKDDLDR
LLAACDEVADVIQLKFSTGVAGGLEPLPAGVDVAASEEKEWRRASGIPLT
PPRWSMEDIVRYGVRDAKRPLR
>Mb|QLI67881.1
MTGAGKSGGKASGSKNAQSRSSKAGLAFPVGRVHRLLRKGNYAQRVGAGA
PVYLAAVLEYLAAEILELAGNAARDNKKTRIIPRHLQLAIRNDEELNKLL
GHVTIAQGGVLPNIHQNLLPKKTGGKSGKNASQEL
>Mb|QLI67882.1
MPPALSDDESSDVEPQPTTTRSSRSSRSLADDARSTGSDDEPGPVAANGG
SEVGEEDEEEDEDLEEDVFIVEAIKSHMIDEDGSLRFQVKWEGYSSKKDL
TWEPEENLEESAQEILDEYFRSFGGRENIFNQTEKASRGKKRGRGASNAA
AATAKRSRKNGTHPSDSAPPASAKPWQPPAGSWEDEIATIDACEDEGSGK
LVVYLIWKNGQKTKHETSVIYKKCPQKMLQFYERHVKIIRDDERVAAQGD
>Mb|QLI67883.1
MTDLLHILPSFRTAPFGALLPIIEQHALSTTDLLALHPADISKQTNLPLL
DLKRLIATIQVSLSDDLVPRHPLQQIVSSAPEHDHSHHEQPSESNSQDAS
KSPAIQPQKGNPSSPSFISTLCPQIDAALNSGIPTSSITEITGESGAGKT
QFLLSLCLAVQLPPPHGLRKQALYISTESSLATKRLSQILASNPLLQDID
FADRPSLDNILSTVTPDLESQDHILEYQVPVLLSRHNIGLLVIDSVAANY
RAEFERAGSSYGSNMAARSAELVRLGALLRDLARKHGIAVVVANQVSDRF
SSSPAPLPRTMSYRTSTSAQESPLASRSMPPPPPQSTLASTPSSPLPFSF
SDDAPPTHPALRLDHQQRWFTGWGDDPRSSHSLKTPSLGLVWSTQIGCRI
ALFKRPVYGRPRQIAAPIEADDDNIDRGATLRNWRRWMKVVFAGHVAASG
QGVTGAKEFEVTTGGIACVPGKNG
>Mb|QLI67884.1
MADKENIDVAQSSDVAPSKETTPISPETPSKASAASTSPTARRAGATTSS
ASARRTTATGTAASSRTAAGSTAGARATGGLSKPPTRPPTTTTATSRRPA
TATSTTSTHRSRPSVSASEDERRSTLTSTSARRTSTLMSSPGRTAEKKAT
TGTGTAGTARKPATATASTTGARPSTLGSRSSTAGTTASARSSTTAATRT
APKRLSTSGATGRVSATASRPTTARSDASSGAKEIEELKAKLLEGDSEIE
SLKAQIQSSETIISELRDQLANAAPAAETEAMQSSIAEEALANLKAEHES
ALAGLESRLSEVVQKLETAESELDQHKTNLATAEGSKAAIESEIEALRKS
LETVQLENDEKVKASESALQQALDGHATKLSELKDSLSEQHKTAIEELEV

SHKNELEKGQTDASAHEMAIAELKTSHESIVAQLQSKIDELTASQAALES
ASNEKLRSDQQAHNSRVAALEGEIAELKALSDVSGKSAESATTQLTELQN
SLAEKEKELSSIAEDRSSLSTKLTETQSTLADKEGEIAKLKTMHDERMKN
LSQDYENEIESLRGDAFFKRKFEELEGQHNELKTSSEEATKAHSDALAVS
KAEHAAAVEALAAREQEHQKNLDALRASHAEELESAKTGASADKDAHISQ
LESLKAQHAEELSILKKESEATLAKQLEALASTHAGVLNALKQEHADEHQ
KSQAAYDEALSVAKSAGNDAHVSELASIQAELQAAKEQLEQARDDGELAI
ETAKNELEEKHIGEIEKIITMNTEAMDKMKADGIQARKDLEELTATHAKS
IEDAIAEYRSTSSSLEEKLAEQKAANGSMESALKTAQEALTKAQAEVEEM
GQQLAQEKMERMTALAELDAVKSAKPDTSEADALRKELATLKKTHEDAHA
AAEGALKGKEDELESHKAELAGAQDSLASMKNDLALAQKELEAHRSEADA
KHKTALADYNDLNDSMTSLVEDANNKAKELEARLGEATRKAEEKEKKIEE
LEAQLKVRDAEIVEARANGGKSKGLSSSRFAGAESGSDEAVANEDVAEGE
DDTNGIGGDDDDDDADDHSSAALASVREPLC
>Mb|QLI67885.1
MPHHDVLVTGSSGHLGTALMLTLPSLGFNPVGIDILASETTQHVGSVSDR
ALVSGILQANPIKHVLHTATLHKPHVESHTKEQFIETNITGTLVLLEEAA
RLGNQVESFVFFSTTSSFGLALSPEPGSPAAWIDEAVVPVPKNIYGVTKV
AAEDMCALVQRQTGMPVLVLRTSRFFPEEDDDGDRRRAMGDDNLKVLELA
YRRCDVEDIVSAAVCAMRRARDVKWAKYIISAPPPFSRDAETLARLDSRP
EDVFRSVAPGVDEVFGARGWKHLARVDRVYDSGKAVRELGWRPKWTFVET
MERLARGEAWRSELTDRVGKRGYHAVSTGVYTKR
>Mb|QLI67886.1
MDGYPPGSLDHNVPFLVASGLNSAEPELDLQGELSTQGQLIKSDLPPLEG
REAQLLETYFEEIDARGTSWAVVPRDEPYRFRIKTVGRSYLLPPRKTIIP
DSVGPPDPSATIHSPFSPLSHVSALYPDGLIDSRWIKKHQHLVPSIYACF
YSLANDNRLKLDVNEIKATLARSGFKTRVAIILFGNQDSGTAVLTEEVQD
RLEGIRKGTALDPKSIFYIPTQESPAELKRKIDSILAILYSNAVEYYRDL
GRHARKKRSRGIAPQPTIPPTSGTSHTLSLPDWNFRYDMKTAILAEFRQE
LDPAIRSFEQAYEILLGQDVFDIIPSWSSRWNEARQLADIVSIRCLRLHL
WMGHTSLAVKRWQTHRDRIGDLVDRRGQGTNTYGWQAWEARWAMVMANLM
EKIEVSGLAPASMTIFLPPERAVLGERLQPWELLHHTGYWYRMAARHLTA
RRTLAHMIPDEDREPPESASAKSAKSTYDTYMCPPPYQENPLSGDGGVSH
AQLIIDCLIASRTQFEARGQLRIAAELSLECAKEMATLGSWEEAVAILQP
VWDSSSYRAENWLDISEDLCWLLRRAAKEIGRADLLVAADWELMNKKFSK
RQHWRYDLSQSLDGVTVEEKPSIQLSDASSSHLISSSFVFRSKENKAGDQ
CLAQFTLTSNAMADSMPISFSSVKILFGGSLKPILLRHGSSGEESSFITK
VALEEQFSTDGPGDLPSSLSGTCDLTLKPGEKRVFEMAVPLRESGEAEAS
TVVATYEHKSFDIEYTMSFRDTDQVTGWYIKTSDRPRQLRPDARILHIKP
RPPKLLVSLLESSGQYYTNETVELGVELRNEEEEPANVKLDVQLYGKSVS
SFKLQVGEHERNGETGEDESKALGVPVGVISSGAVAELKLTIDPTTAPTT
HDLHIQATYHLESDTATPIIQVLSLSLDLVGPFEANYDLLPRLHPEPWPS
LFDYEGLHLVTEGETAASPAKGFAQQWCLVCHYASFAMEDLTVLGMELQV
VSCVGGARCTVTRQPEVAGDGITIPPKTIHEAQFDLVAQKLSLDDRHPVT
LDLAFVIKWKRKTAPVDGQANVTALPVGQYLVLGTEPRVLASALYSNPTA
STNLLHLDITIENPSNHFLTFGLSMEPSDMFGFSGAKQTTINLLPMSRRT
ARYRLLPFVQGDYIRPGLVVRDKYFQKVLRIIPTEGMKIDKDGLLVWVPG
DGSKDSERGAKET
>Mb|QLI67890.1
MSHRKFEAPRHGSLAFLPRKRAARHRGKVKSFPKDDPKKPVHLTAAMGYK
AGMTTIVRDLDRPGAKANKKEVVEAVTVVDTPPMIVVGLVGYIETPRGLR
SLTTVWAEHLSDEVKRRFYKNWYKSKKKAFTKYAKKHSESSGASITRELE
RIKKYCTVVRVLAHTQIRKTPLKQKKAHLMEIQINGGSVADKVSFGQDLF
EKPVSIDTIFEQDEMIDVIAVTKGHGFNGVTARWGTKKLPRKTHKGLRKV
ACIGAWHPSHVQWTVARAGQMGYHHRTSVNHKVYRIGKGDAEDSASTEID
VTKKKITPLGGFVRYGEVNNDFVMVKGSIPGVKKRVMTLRKSMFTHTSRR
ALEKINLKWIDTSSEFGHGAFQTPAEKKQYQGTLKKDLASA
>Mb|QLI67891.1

MAPQNLNVLITTFGGLGLPSTLVVPVPPSTTITELREEIYERLPTTDTRL
IITTISNRQLPEVSQAPISAYLSSSQDEFLSLRLAVPLCGGKGGFGSQLR
AAGGRMSSRKKKNQEDHGSSRNLDGRRLRTVNEAKALAEYLAIKPDMDRQ
EKEKRKERWEQIVQISEEKQHQIRNGGQGRLEGKWVEDKEEYREKTRDAV
LAAMKSGSYRDNLLSTSHGSASSEQPEALSNSEDDAKSNSKESSSSDEAD
EPAKTPARTFFGFDEDDEFMSSSDEEEAQSKE
>Mb|QLI67892.1
MSLFGTSPTEEHNEPSTPSRQTPRSRRGGRGGGLFDETPSHKISSSSLFD
DGNAGGDDSSPWDMPTPRRQQTRADMVRNLLPASDVPDSYITVFDAIVRE
HGSSGRATAAGIADLFANARLGPEAQTRIMSLVAPGDGSDVSLGRNEFNV
LLALVGLAQEGDIISLDSVDERRRHLPHPKLPGLTAEPVLPPVAELAAKP
PQTPNESLGHVEVDAAQHQEGEASRQSRASVQVHTPPQPKALRPAMDDPE
DDPWNTPEVHKGHDHSKLNGNSSHAIANGQSSFRSSPSTNIGDQTLSQYV
SAEPISSSVHGSRSGNDAVQGQAAWGFYGSSNASSGGGGFADGSHNITPN
PSNTAHPFGGDGGAVDRSPAREPPTSVGRATSGRTGSSVEENIIVTLMPG
KEGVFLFQHHNYEVASQRRGSKVIRRYSDFVWLLDCLHKRYPFRILPLLP
PKRVAVNGNHLSNDGAFIEKRRRGLARFLNALVRHPVLGQEQLVIMFLTV
PTELAVWRKQATISVQDEFTDRALPPGLEDSLPPTLEELFSRTRSGVRRS
AELYINVCNIMDRLVKRTEGVAADHARVAMSLTSLTETSADTYATDTNEI
PLLNDGLVAMSKHLRTCQTLLEDESRGWDEGVLEDLKRQRDSLVSVRELF
DRREKLDRDNIPYLERRIQANETKLAGLRAKPEGIVKPGEIEKVAEAIIK
DKESIVQQHNRSIFVKECIRDELVTFQSTQYHVSRWNQDWAGERVKYAEM
LADNWRRLLDELEGMPLGE
>Mb|QLI67893.1
MAIREDLVASAAQFLQDPSVASSTVENKIQFLRSKNLTQEEINTALARTG
SPAPAGAVQYGSPAGPPQQYYPPYAQQAWQPPPPAPRRDWRDWFIMATVV
SGVGYGLYSLSKRYVYPLIAPPTPERLEQDKKSIEEQFDKAFALVEQLAR
DTEALKDAEKQRTEKLDNAISELETVMSDLKSANRRREDDAQRIREDVQS
LKDAIPKAMENQKTLADNRLSEINTELTSLKTLVSQRMAPSASPATGTGT
STTSSSFPRSSGSTLPAGRSSTSTATTPASATVESVPESSSTSTPAAAAA
TTTEAPKNPAQSFSRPPGLTSGSGSSTKASIPAWQMAAAQSASSATTEGS
GKESGTSS
>Mb|QLI67895.1
MSALFNFRSLLLVLLLLICTSAYVHHFTPGIMDRNKNGVMGIFWKCARIG
ERLSPYISICCVFMAASILMN
>Mb|QLI67896.1
MLRADCYGRTHDIFETEILSKICEVSRQSSHEGRAHVSHLLSKSKHKGPN
GEHVCLMFDVLGNHLGFQTLLLGLDFLHRECGIIHTDLKPTNILLELESP
NNAIAKYLSVVPPRMADPQRGAPLREVITTLPISEIKHPHVRIIDFGVAS
WRENHLSDLIQSPALRAPEVTIGAPWDTGVDIWSLGCLIVEFIQGIVLFS
GEGSILRIPNMKSTSLERLLNGTSKPFLKPEDMLDHEVDILIDFIKGMLT
IDPGSRKSAAELLQHEWIHSR
>Mb|QLI67897.1
MPQPIPRPPGVPLLGNIFNITPSNTWASLNKLSEEYGEIFQITVLGHTIV
FVGSVALAEELCDQKRFRKYVGGPIVEIRAAVHDALFTAFENESSWGIAH
RIIAPHLSPEALGQQNFTEMRDLANELFAKWKNLEANTVISPLSELQRLD
LETVTLTLFGQKLNCLEGPEHPMLKGMEDSTSEAMMRPTRLSLVNWLVYR
NKFNKSIQTMRRYAADVVEHRKDNPTTRQDVLTAIMNNEDPETKKKLTDS
QIIDEIVSMPIGSSTAPCLLTSAILLLLRNPDVISRARREIDAVVGAGDI
SIEQLDELKYVQAIVHESLRLSSPAPGFNIEPLPSDSKAPVQLAGGKYQV
AHNQPMIIVLAGVNRDPTVFEDPLAFKPERMMGEQFERLPDGAKKWFGNG
KRECIGKHHAWQWSVLVLAMMLQKVDFEMADPEYTLEKDGWFNVRPVGFK
TKVKPRE
>Mb|QLI67898.1
MACEPCIRGHRSTKCTHANERLMVPVRKPGRPLSSCPHPSSRPCSCAAVT
AAIPRKQKCRCGTSDPDAVVESKPEKDAPSSSSSATTPPSPSKFGSAGFR
VQKQGAKAGPSRKQSIDIAGLQRMDASQLNVLPPYSGVNQPQMSTPNGSS
TPMSEASLYGSMDMTPSEGSFGTEAGMFPMFPYAMGAPMMPHGPMRVTPI

GQNGSSVNGSVKPESETLKSCCGGSNGVKSHRDDASSVPAPSVNGNGGRE
PEAKSCCSSTPSSSGQEKKPVGIMPPPGVPFPPNGILIPPFQHPMAMANG
MYPFYAQPNIFNYPPHYGSYLQPLQPEQWRQLMAAMAFQHGGPQAFGMTG
PMPMQQPPPTPNGSSWTSHQCTCGDACQCIGCAAHPYNDATQNYVRSAWS
SMMEEAQKSHAHTNSNGSHLNGQTNGVNGANGETEESTKTSCNGTTTPIK
TNGDGTWSPAAPQTPSDAASGLSEEQALSASDFFFVSYPFGDTCAGETSS
CLCGDDCQCIGCTIHNNTDPAPSTDDTAA
>Mb|QLI67899.1
MAPITKETDYLVLGGGSGGLGSARMASSKFGAKAMVVEAARLGGTCVNVG
CVPKKVTYNAAAIAETLHEAKSYGFSVTETAPFDWTTFKNKRDAYVKRLN
GIYERNLGNDKVEYLHGWGRLLSKNQVEVTLDDGSKVVVNAKKILIAVGG
RPSSPPQIPGAELGINSDGFFDIDKRPKKVAIVGAGYIAVEFAGMFNALG
TETHLFIRGKTFLRHFDPMIQETVTNEYERLGVNLHKESQATKIEKDANG
KLTVTYKDAEGKESSVSDVDHLIWAVGRTPMTKDIGLEEAGVKLTENGHV
QVDEYQNSSVENIYALGDVSGEVELTPVAIAAGRKLAQRLFGPAEFSTQK
LDYSNIPSVVFAHPEVGSIGLSEPQAIEKYGKDNIKVYKTGFTAMYYAMM
EPEHKGPTNYKLIVTGPEERVVGLHIMGQGSGEMLQGFGVAVKMGATKAD
FDSCVAIHPTSAEELVTLK
>Mb|QLI67900.1
MAATMDAINTHVSSPLSTTAPPSASSSTGPGHGSHGYPSPPVTAIDEPSA
SEDESGRGRSKFPLLPDPAAALDHHSDTAPLPVGMNASRTPSVVKSRRRQ
TSSSTNKTSDSVQRLTAAEVQELTSSPDSLPIAPAPERHSIDQEIAAKTA
EQREMLRQRFQSRSPDPRRLDTLQTNGAAAESYAGAGYAGLWHVREGAQR
QCSPRTVSTPPTSRAQQYVLDTGSQPTSPRRYSYNPLPRPTPLNLNGEAA
NGSLNGHVLSPAIATSPAAINAQQASPAAPSQGYTGPSPPSPIPSMIPLP
PMSLPTHLQLELAAQRPSPLYIHQSYLNDIPYESYAAKLERLKNVLLLPP
YLERTLYFGALACLDAWLYTFTILPIRFFIASGVLIKWWGYVVSKEARWM
FGYVRDGLGRLWERGRTSQSRRPSDVSDRDRSRSRSRVRELLNDDGGLSA
PGLRDRSHHTKTDLHVDGNEGPKSPLPSKRHPVPQLGTFRHKRTKSIPSS
LSAFHKADLLQGAVIICSSFALMTLDASRMYHFIRAQSAIKLYVIYNILE
VGDRLLSALGQDILECLFSNETLSRNPSGRSKILLPLGMFILALVYNCLH
SVALYYQVITLNVAVNSYSNALLTLLLSNQFVEIKSTVFKRFEKDNLFQL
TCADIVERFHLWIMLLIIGMRNIVEVGAFSVPGAGFDSTHEDGSSAVPLH
PPSILPHSFTVLPSWLRSGEALSPFLIVVGSEMLVDTIKHAYVTKFNNIK
PNFYGRTLDILCKDYYTNAFVTPSLTRRLGLAVIPLSCLFIRASIQTYHM
FLATHVHMPIPPSTQTSLTEESAVPSSPAMIAALSRFDALIRDSLGRATY
GYPYGSPLNSRPWYSWTSDDLIAALTMVVVFFIVFLVLLILKLLLGMVLL
QYSRNRYAKMKQKESLVALGKAERESYDATGRRVGGRGDVEVTDDKARWI
HADKSEGLKGGKGPGGKKSDDKGGKKPDGDYMGVARYEMVAKRIW
>Mb|QLI67901.1
MPYNTRRKSLSLPSLGIHIPVTHAARAAAASTKAARRPPSPSSSSSSSSP
ATSAHLSDPSVDAMDSHPSKRLKRSHGAASAPAEGIVEQTPPPSPTPATS
VEMSDLDAIRKVDLEGINDDIVEASIVQLQSTANRPHLIKELATVLTQTL
SSVQQSANPCAIISSRLSSYMKRPCWTALVPCPLAKELETVHPRRTYFFL
TTCPRQPLPDSSITQPINLPAIVSPSVSLTDDSGSDDVDGRRRELSPSPE
VDLSSPEFGDMEDDVGMAITPMGSFKTHNNHLRHGRDLRRDSPPLETDER
EFTQTADVLQKRKFTHDNTPTSSETAERNPTIEYGYRDDMWFGDRAPPTA
AFLTSPAIKPSPLSTMRKDDDADSWLKLNLLFEWDRTAESIEIDELDCLL
DTC
>Mb|QLI67902.1
MKLPPLSFRSSLSPRNALSTRAPRLAKSGTINSLSIRAAFAENRQIIRSY
SAVALSAADLKFGQPVYETHPHILKAGELTPGIAAQEYADRRAALADSMS
EGGVAVLHAASLQYKSGAVFHPYRQESNFLWLTGWNEDDAVAVIEKTGPG
WGEYSFHMFVKRKDPKEEQWNGYRNGIEAAKDVFNADEAHSIDGVDSLLP
DILRSAKLVYADVQPVRDTSKNSLWRFFSSKDSTHPARTPLYPVMNKLRV
IKSAAEVANMRKAGQISGRAITEAMKHGWAKEKDLHAFLDYQFIVNGCDG
PAYIPVIAGGERANCIHYTVNNNTFKDGEFILVDAGGEYGTYITDISRTW
PVSGKFSAAQRDIYEAVLKVQRTSVSLCRESARLSLEDIHGITARGLVDQ

LRSIGFNVSMSNIDQLFPHHVGHYIGLDVHDCPGYSRRETLKRGHCVTIE
PGVYVPHDERWPKHFHGMGIRIEDSICVDDDSPFILTTEAVKEVDDIEAL
R
>Mb|QLI67904.1
MHILVTNDDGPPSSHSSPYIRCLVKHLEDAGHIVSVCLPHTQRSWIGKAH
MIGQTLKPTYYTPSSNIHGEDNEGTTRQLPSTAADADEWVLIDGTPASCV
QIGLYHFFQDKGPIDLVISGPNYGRNTTSVFALSSGTLGAALEAAVCRRK
AIALSFAFFSRNHDPLIIEAACRHGVRVIEALYKQWPTDESVDVYSVNVP
LVEGVEEHKTLWADMLQNYWKEGSCFQEIDGDTGDADEEEARIRQGEIDN
GPTLQEKKGHKHKHFKWAPRMADVYKSVEESGPGNDGRIIRDGNTSITPM
KANFAISHPVPAATEFEFGFEATSADAAFQAIIAYEDPYVQPLIVTALNA
IFPRGLVKLVTDVSMTDDFSIANFLDSPESKVVQITPYESIDFEFAATHP
TSCLINSYMIRKALIRKHYLSATVDHWVAKNPDSPLKTHVKRSESFEVDY
AEFLDDALVEAFDLRESMDRNAEMDESEPSKREWWILKPGMSDRGQGIRL
FSTMEELQDIFDRWEADRPDSDDDEDYTHDQDTHGEGGDYITTSHLRHFV
AQPYIHPPLLLDDRKFHIRTYVLCTGSLTIHVYKHMLALFAAKTYSCPWQ
SPDDIESFLTNTCLQESPNENSVRRFWDLPLPQTQLSGIFEQICSVTGEV
FEAAARGMPVHFQTLPNAFEVFGLDFMVDQGGKTWLLEINAFPDFKQTGW
DLSEIVEGFWRGVMRVGVGKFLGVDVAKCEDEGDMVLARDVDLGKR
>Mb|QLI67905.1
MDYVLLPKIELHAHLTGSVSRPTLHEIWKRKKASGQTDLEDPRVVMPEGK
HDYNLQTFFPLFSSYIYNLLTDEESIRYAINSVLQDFLADGVVYLELRTT
PRSTTTMSAEAYITVLLGAIEEFESLHPQLHTRLILSIDRRHSIRTAESI
LDLAIRLQDTNKCGVVGIDLCGDPTARPGGEVAVFTPVFEQASRAGLGIT
VHFAEAEASGSHDELRTLLSWNPGRLGHVIWEDEEARKVIVERELCLELC
LSCNVQAEMVHGGFEGHHFGHWKGVKGPKISLAILEWAFGKRMTPAERLR
KNQRMLDKAIRELDQTRVKLEKQEKTLIQQIKTSAKNGQMGACKIQAKDL
VRTRRYIEKFFSMRSQLQKISLRLQTYRTNEQMMQAMKGATMALGSMNKS
MNLPQLQRIAMEFERENDIMEQRQEMMDDAVDDAMDVGVEEEGDEVVEQV
LEEIGIDFNQSLGETPTAIGNPAIAEGKIAQAVGGGSGGDPVDDDLQARL
DSLKK
>Mb|QLI67906.1
MPSLNLTGARGVARLLLMTASLVSLADAIDVDICASLNTADMNRNLSVFQ
TNGLCHDLCTPQGFVYAITQSNSCWCSNYTPAKSAQVATSKCSLGCPGYP
DEKCGGPGVYGYVFLNIAQPSGTKGAGSSTTSESTEATSKPTSAISTLTA
DGTVKTVTIIPTETGHTAGDNQGASVQDSGLNTGGIVGIVVGVVGGILVL
AGLIIFFYLRRKKQQQENDYQDDPSIRGSSSGMMGSGRPEMSRAPGSPGS
TGNRSSTLQIDPRMDPFQQTLYARSGSRESVNTLRDDHDYSRRIQQPKVL
RTTNPDPA
>Mb|QLI67908.1
MSLLSAEVHAELTQLLQALQSADNATRSQAEEHLQNNWTSSRPEVLLMGL
AEQIQGAGDNAARSFAAVIFRRIASKTRKVESGNNVDVFYSLAKDQAAVI
RQKLLETLGAEPDRIVRNKISDAVAEVARQYTDNNDSWPELLGALFQLSQ
APEAEKRENAFRVFKTTPGIIERQHEETVLQAFQKGFKDDAVMVRLAAME
AFASFFSTISKQSQMKYYALIPDVLNVLPPIKDSQDSEDLSKALLALIDL
AETAPKMFKQLFQNLVQFSISVIQDKELDSICRQNALELMATFADYAPSM
CRKDPSYTNDMITQCLSLMTDLGEDDDDAAEWLASDDLDQDESDQNHVAG
EQAMDRLANKLGGQTILAPTFNWLPRMMNSMAWRDRHAALMAISAISEGC
RELMIGELSQVLDLVIPALQDPHPRVRWAGCNALGQMSTDFAPKMQTDFY
DRVLKAVVPVLNSPEARVKSHAAAALVNFCEEAEKSILEPYLDDLLSHLF
QLLQSDKRYVQEQALSTIATIADAAQAAFSKYYDTLMPLLVNVLQNQSEK
EYRLLRGKAMECATLIALAVGRERLGQDAMTLVNLLANIQTNITDADDPQ
AQYLMHCWGRMCRVLGPDFVPFLDNVMPPLLELAMAKADIQLLDDDEQAE
QMNGEDGWEFVPLKGKMIGIRTSTMDDKHMAIELLVVYAQVLEAAFAPFV
ANIMEKIALPGLAFFFHDPVRYISAKLVPQLLSSYKKAYGCPSNELAGLW
AATVGKLLEVLSAEPSIETLAEMYQCFYESVEVVGKDCLTPVHMNGFIDS
VHSTIEDYQTRVTQRAEEKAGATADDVEDEAEEIEREIEDDQTLLSDMNK
AFHSIFKNHGAAFLPAWERLMSTYESFLTSTDPTQRQWGLCILDDVLEYC

GPESNRYANYITQPLIDGCRDPSAAIRQAAAYGIGVAARHGGAPWTQFLG
GSIPYLFQVTQVPEARNDENVYATENACAAIAKILHYNAGSVGDVQAVVS
QWVETLPVTNDEEAAPYAYAYLAELIDQRNPVVLNQAGKIFVFVAQALEA
EALQGQTASRVASATKALLTATSVDPMPLLSQFSPEAQQTIMGYFN
>Mb|QLI67909.1
MDFVKSFLPEGKGVLPYYMLVLSVVSIGNCLQTYSTLHFTRRVYNGRFIR
NTRLPPETAVFNPEDSVDKLVSAQNDPKATDQVTPLAGRLFGTWTLITSI
VRCYAAYNLHIGPVYNMAYWTYIVAFGHFASEKFMFKSMTFGLPQVFPFA
LATCALIWMPLVRDHYVEIN
>Mb|QLI67910.1
MASVAKTIVATGLSSGIGLEVLKQLLEQATPYKVILGARDIKAASDAVAR
LQYDKTANAVAVLPLELANLKTVKAFAQQALDSVGGGKIDYLLLNAGMFK
PADKESPGKWCEAAVVNHFSQHYLTHLLREKLVASKARIVVVSSGAIRNV
EDPAVLEARLANGSGADGYSVYAGSKFTQLLGAHWWRRQLKGQCDVVAVS
PGLIPNTGLGRGSGFQIPPNLPDAKSVPEGAQSVIAALSRSDFPEDSGRI
FLTSWGEWWDKDVIGKTLDKELQDKWCPGKEVSERDAGLVP
>Mb|QLI67911.1
MNTDIVQSRFLSNPQDLGVVAVGFSKGQPKAGVDAGPEALIDSGLLTQIR
EELGYKLHGDTKVQKYEELISTTDADHRGMKNPLTTSAVNRKISQEVYEH
AKEGRLVLTLGGDHSVAIGTVTGSAKAIRERLNREIALIWVDAHADINTP
EVSDSGNIHGMPVAFASGLAKDDKEEYFGWIKEDMLLNIKKLVYIGLRDV
DKAEKQTLRENGIKAFSMFDVDRYGIGRVVEMALAYIGADTPIHLSFDVD
ALDPMWAPSTGTPVRGGLTLREGDYICEAVHQTGSLVAIDLVEVNPSLAA
TEAGAQETVRAGCSLVRCALGETLL
>Mb|QLI67912.1
MARRRKPPRAGALSELQPLKIATQIATLQALYYTTAGILMLFTALVAGMS
FSVDMILGWDAVRGDTTQGWLLSFIWLIDGGLCMAVAIVVLIARSKLVPD
FALTIHLLHLVFTTLYARSLPRHSMWWMTMMASAAVAVALGMWGCRYREL
QPVFFGGGRILGSNSAPAAASHGQVAAEEGQAGLDDDDDDGRGGAEDDGF
SRGRGRGRGKDGAGEYEMVGMKQAKD
>Mb|QLI67914.1
MARLNEVPMSSDSLETLRKKMLRQNRDLAKSNNVRALRIRELESECALML
SENLELRSRILELEKQVEDNEARRIADHALAIKAKLESQLTEWGTLLSGL
GLEPPMKRHSPRIQKSIKQRMSYIAGRPSPSQRRLRDVARDIEELGHISE
NRSSSRQSLNPEQIRALRSEADSAELEAPAKQPLIEQEPTKVDSPPRAAL
ATKDVSSPRRLIEPPISLPSPRSSKIADAPLPSPEKKRTEPSNRRQNPIL
QPSTPAPEHIAAPVKTGSKRKFAQDETENTAPRQLTNENIAPRGMADKVS
IREKAGGKTLKELAHMRKEAREKHVTTGNSRKPLASKSTNDDVSSPRKNP
KPIAIDEVGTGKAELAKSKPTKERTKSKAKMNAAAKLEIPPPVTKTVIAD
LATTVSEPAVTSPKSPEPALGGDDPRGDTPPPVDIFLNGETSRASRRNRT
TISYAEPNLRDKMRRPTKELFDAVAGEGKYARRASQCDLPTSEGPKIKRE
SDVGDSLRRIPPTSDEFEAPEPGSIPASPLAKKSSAQDASAAVITERRRP
ASRAGEFAADLNLDADENESGDVDVYEFTSSSPQIDNNAPTETTRATRRR
GANTRRRSAAVDSEEDSSVKERSVSRRRSMMV
>Mb|QLI67915.1
MDLIDPATGQPLPEDAIQRNLFIDGPVHVYQIPPLTSMKGYSAISWTDDP
SRNIFTGRLRVVETAFESPASDGGQGQSKLKVDLVLEDPSTGELFAAAPY
TNGSVVEPVIDSSRFFALTVRDPQGHKAVLGIGFEDRSKAFDLGIALQRA
RRALGLENAGADASAATQENKDYSLKEGQTITVNIGSKSRGRRQPEPPST
SSDPATLQSFALPPPPGAGSGLASAQQGAGFSLPPPPSAEDVKRKRQSLR
DWGFDDGENGEFA
>Mb|QLI67916.1
MSHCHDEHVGHGAHDHHHGDEHDHSDDITPALQSSLYQQINFDEITTLNE
STRDAGKAIVKKTWAERLSAEPELASDADEQLLMTVPFAAQVKLHSILIR
TSPSLSAPKTLHLYVNHDNLDFSTAEDMDPVQKIELSQTSDVQEIPVKRA
LFGRVQRLVLFFMDNFGDGDEDVSRISYVGFKGEWTKLGKAPTNILYEAA
AQPGDHKIKGTSVNQMGSGIGGRGPGV
>Mb|QLI67917.1

MGSKSSLLPADFEWGFATAAYQIEGAVAEGGRGKSIWDTFCHLEPTRTKG
ANGDVACDHYHRYEEDFDLLTKYGAKAYRFSISWSRIIPQGGRNDPLNEE
GISFYSRLIDSLLKRGITPWVTLYHWDLPQALHDRYGGWLDVQESQLDFE
RYARVCYERFGDRVKNWITLNEPWIQSIFGYSTGGNAPGRSSTNDLSDAG
NSATEPWIVGKAQILSHVRAVIAYNKDFKPSQGGQIGISLNGDYYEPWDS
ADSRDKEAAERRMEFHIGWFANPIFLKKDYPSCMREQLGDRLPLFTESEV
ALLEEAETDFYGMNYYTSQFARHRGEPASDTDFVGNLDELQQDKQGTPVG
EESGLHWLRSCPDLFRKHLTRVYNLYGKPIYITENGCPCPGEDKMTCDEA
VNDPYRIKYFSSHLDAICKSIVDDGAVIKGYFAWALLDNLGAFLSISQVF
VANRFTEWSDGYGPRFGVTFTDYKTLKRTPKQSALLLRKMVTDRQGLSVT
TS
>Mb|QLI67919.1
MQAIPRAAVRSAALRNVANRAYSSSSSPYSKTIENLRINGDTKVIFQGFT
GKQGTFHARQAIDYGTKVVGGTNPKKAGSTHLDLPVFKNVSEAVKETGAT
ATALFVPPPVAAAGIEEALQAEIPLVVCITEGIPQHDMVRITSMLKSQSK
TRLVGPNCPGIIAPGQCKIGIMPGFIHKRGRIGIVSRSGTLTYEAVNQTT
QAGLGQSLVVGIGGDPFSGTNFIDCLKVFTEDEDTDGIIMIGEIGGSAEE
EAAEFLKEANTVGGGKPVVSFIAGISAPPGRRMGHAGAIVAGGKGGADSK
IKALEAAGVIVERSPAGLGKALYDEFVRRDLL
>Mb|QLI67920.1
MPEKITSKNLSYHSSLPPFLARLHAQSAGAAGPDPLLAAQKRSAKKRSAS
EEAEDAPLVVDEHGNAVDLQIEKDGTVKETETNSDKDETRGAESDEKAVR
RDTDAAASSIGARKRKVGKVIGERASHGSGNDGKDKQDDKLGGAEAPGDK
KVKKKAKKIKLSFDEDEG
>Mb|QLI67922.1
MRRPQAIGVSLRRAYCLRQLQTSDYDAHLIRRFVPSPVRDTYAALRALNL
ELVRLPEVVSNPVIGTLRMKFWHESIDKTFAGMPPREPICILLHQALQGL
EARAGSPTKKSIKFWVSRLVKTREKYMDGRPYPSLAALEDYAENTYSTMM
YTTLASIPLRSMHVDHLASHIGKACGIIAVLRGIPVLAAPGRPVKTPTGA
DAPPTREPSLLLPLDIMAEEGVKEEEVFRQGPSAPGLQDAVFKVATRAND
HLITAREMLKRLKAGEDPGHEFEHEGEAEHIYLGESDTAKEIRQSFGVLL
EAVPASHYLENLEKVNFDPFAVKSGGWRLPWRIWQSLSSERL
>Mb|QLI67923.1
MKFTSVTALVASYLAASALAAPRLESRDEDKFAQGLPISKTGKGGPISGG
TNHELDLQNPDNLGRQSTDNGIVPNLKWSFSDSKTRILKGGWVREQVVQD
LPQSHDISGAQQHLKKGAIRELHWHRVAEWGFVYTGRVLVSAVDENGKYQ
AEELNYGDIWYFPKGVAHTVQGLEDENEFLLVFDEGDFDKVGTTFNIDDW
LAHTPKDILAKNFGVPESVFDNIPTPNPYILNATVSEKNVTGAVTPVASG
NSSFVYRTLEHPAEKIGGQGGVFRKIDSTNFPISKTIAATFVTLKPGGLR
ELHWHPNAEEWLYFHQGTARATAFIGNANARTFDFRAGDTAVFPDNSGHY
IENTSKTEDLIWIEIYKSDRVADIPLTQWLALTPPDVVSQALKVPIEFVE
KLKKEKQVFVE
>Mb|QLI67925.1
MPNMLYSFQQHTQHQHQTQPQHHQGLQHDHGMPSANGLAHHTSFGSSGLS
TPNAFNSSVLSNGHAANPRSGQAPQNEMWQEQLRMQKEAERAHSAMTDQQ
QPHYYARLKASENRGIGGPPPASGRTQADGENDPEDRRRPYVVDKESNRQ
DWHNMDMSGQGLRNLAPELFQYKFLNELFIASNKLVRLPKLIGELRQLRH
LDASYNQISELPAELGMCTYLKTLLLFNNEIRDLPFELGSLHQLEMLGIE
GNPLDPGWKQEIMEKGTKSVINALKEGAPVPDPPSPRKEIVIQEDVSANL
ERIKVFSWNVLCDKYATPQTYGYTPTNALNWEYRKSCILDELRLRDADFL
SLQEVSTDAFKEDLSPELAQMDYKGVHWPKSRAKTMSEKDAQTVDGCAVF
YKQSKFILLDKQLIEFATIAINRPDMKNQHDVFNRVMPKDNIAVICFFES
RLTGARIILVNVHLTWDSALADVKVIQTGILMEHVTKLAEKYARWPAVRD
KKMITVPLPDDAEAPEPQAEPGPSQEYRTNTDIPLLVCGDFNSTEGSSVY
DLMSMGRVPPDHLELTSYQYGSFTRDGIEHPFSLRDAYAHTKKTADEMPF
TNYTPGFADVIDYIWYSTNTLEVAELLGPPDAEYLKRMPAFPNWHFPADH
IQIMADFVIKARKDKKPNQDREQQADSGGPSGRG
>Mb|QLI67928.1

MASHVELVMNPWEAKALDYHYPAETLPPATGRPSSVSRGPWQAMSNPVFY
PGLYSATGFDIMGILTSLRDRPNPQFEFKIDCSAALVLCNLEEPDDPIVY
ASDAFCDLTGYSQAEIIGKNCRFLQRPHPSSWDTSKSKPKRDKHAASKMR
HALQAEQEIKLRVINYRKDGRRFINRVSIVPVTLNGSGYRYAVGLLEDDS
SPKDGSQKDR
>Mb|QLI67930.1
MASGGSLPPNFLLTPHQQSLLFAALNANKPQASASPTAKGLSVSPSSFQG
SPMQNVDNSAFQESPYLDNIDYDFGDSSFDFSFASNGGEHAKMVADAPGT
VSSESTENDGHEKRSHPDDEEDDSSPGNDSKRREGTEKIAKKPGRKPLTS
EPTSKRKAQNRAAQRAFRERKEKHLKDLETKVDELEKASEAANHENGKLR
AEVDRITTELNQYKQRVAVMSQHKPTPRDKVSFGSAALNNLSDVSFQFEF
PKFGALPGPPIKQPSSQPLSPQQNGQKNSPARSIASEGKSPQSSVQRPTQ
DDFAKFAGVFAPSMSNSTRTGSRASVDSVPFSVAAATSSPSASSNSNAGP
SSSCGTSPEPFTQSPMSFKPLETLTTIGEEQPATTTADQPFAQFAHVDIA
SPSFDWLVQQNGGGHFDPQLFGDYRESQENVLSNPTFDDFFFNDGVDTDF
FTPYNAAPNEHAPKKNLIAEIDAQQNSIDDDMGNKPNMSCNQLWEKLQEC
PKAQNGEFDLDGLCSELTKKAKCSGSGPVVAERDFDTILKKYMGKDVSAD
CMATKLGISVDPEKPNGVTTP
>Mb|QLI67931.1
MRRVPISEVGRYLLRGSISKRGCLVGRHTLGHGAVALRSPTPLSGHFRAA
STIEKPTASSNESVVPFRKQLKEEAKLAKKQGKKKSKGDYQTVDGWELTV
GIEIHAQLNTARKLFSPATTSFNDEPNSHVALFDVSMPGSQPLIQRETLL
PALRAALALNCEIQSVSRFDRKHYFWWDQPAGYQITQYYEPFAKNGHITL
YARDGIAAEDGESVTVGIKQVQLEQDTAKTLTQTGNVHWLDFNRVGVPLV
EIITEPDMHHPRTAAALVRKIQLLLNTVDTCVSGMETGGLRADVNVSVRK
ADDPSCKLGTRTEIKNLGTIKAVEDAIIAERDRQIQELEAGRSIAGETRG
WSLGSKETRRLRGKEGEVDYRYMPDPDIAPLVIGNDLVEHLRTSLAVLPD
AELDTLINDFGLTAKDALSLIILDNGARVQYFYKVLDGIDRKMQVELADK
TRPDLKSYATLAANWVTHELGRLTTYKAGPLASAELLFTADGECAQVPAA
DLSQLLYHLCLKQITGKVAKELLLSIYLEELQGSVEEAIEANGLWFREIS
ETEYRQLAKEAVEGEDKVLQEFIGYKKFPQGKLMYLVGKMMKMGPTERIN
AATAEAAMRAQIEASQGRG
>Mb|QLI67933.1
MASPLQFAYRTQKAIGVFDAAPAYEPLAGFTKPEGNLRCSAYSPCGRFFG
WASPETVTVVDASAGNVVLVLPIINVYELGFSPLGTFVITWERPAKDEAG
DATKNLKIWRTVEDGVAGADKQPIGRFVQKQQGGWNLQYTADEKYCARLV
TNEVQFYQSHDLVTVWNKLRVEGAANFALAPGSQNHAVAVFVPERKGQPA
VVKVYNVPLFQNPISQKTFFKGDKVQFKWNKRGSSLLVLAQTDVDRSGKS
YYGETTLYLLSTNGAFDARVTLDKEGPIHDVSWSPNSREFGVVYGYMPAK
ATIFNDRAVAKYSFPLGPRNTITFSPNARFVLVAGFGNLAGQIDVYDLEK
DYRKVCTIESGNPSVCEWSPDSRYIMTATTSPRLRVDNGVKLWHVGGSIM
YSEDMVELYNVLWRPSAPENVAGGDPLNPVPSPHASAAAYLGTVKTPSKP
AGAYRPPGARGLATPLHFKREDEGGAAHFVSNGTQNVGPNGFGRPRRGVP
GAEPAEPSQAAARVVPGAEPVGEDGAKKNKKKRGKKNNQEGRPEAEPNGG
ASLAPYDNRAPGSGRSPERRGPANGNGRDHRSRSRNNGGSRSRSNTQHGQ
RLNLGQQNNYYAQQQGGAAVAGAAADAGQSANAKKIRSLQKKIRAIEDLE
MRLAGGEKLEDTQVKKINTKSAVLRELEGLEKEA
>Mb|QLI67934.1
MLRQSLARSAWRTSRRTVNASRTFATTPRRQAEVELTVDGKKVSIEAGSA
LIQACEKAGVTIPRYCYHEKLMIAGNCRMCLVEVEKAPKPVASCAWPVQP
GMVVKTNSPITHKAREGVMEFLLANHPLDCPICDQGGECDLQDQSMRYGA
DRGRFHEIGGKRAVEDKNIGPLIKTSMNRCIHCTRCVRFANDIAGAPELG
STGRGNDLQIGTYLEQSLDSELSGNVIDLCPVGALTSKPYAFRARPWELK
HTESIDVLDGLGSNIRVDSRGLEVMRITPRLNDDVNEEWINDKTRFACDG
LKTQRLTMPLIRREGKFEPADWEEALAEVARAWAAKKPQGNEFKIISGAL
TEVESLVVAKDMANKLGSDNLALDTPTGSQPIAHGVDVRSNYLFNSRIWG
IEEADCILIVGSNPRHEAAVLNARIRKQWLRSDLEIGVVGETWDSTFEFE
NLGTDHAALKKALAGPFGKKLQAAKRPMIIVGSGVTDHADAKAFYETVGA

FVEKNAANFTTPEWDGYNVLQREASRAGAFEVGFTTPSAEVAQTKPKFVW
LLGADEFNEADIPQDAFVVYQGHHGDRGAQIADIILPGAAYTEKAGTYIN
TEGRVQTTRAATSLPGASRTDWKILRAASEFLDVSLPYDDLAAVRDRMIE
VSPALAAYDIVEPVALRQLSKVQLVDQNKGSKATGEPLKKVIDNFYFTDV
ISRSSPTMARCSASKATGDPRTNFMAPGMEEDKPMGQVAYGV
>Mb|QLI67937.1
MSNKNTKALAHEKRKGESALSDFAEYVEQQQNLRFPASRRTNVVATSSAN
DAEHHEELDELFDNLDLADTAPRIPLRDLVLGTDDYTLEKLHDVIAERIE
EGMGETVFELGYENNGDSMNLTADNWNTAYRRLAAAAKAAGADCELLLTK
NVGGELEAESTANKPDKGGGCNGKILIRRVPTNVEDVIETRIAVVGNVDA
GKSSMLGVLVKGDLDDGRGKARVNLFRHKHEIESGRTSSVGMEIMGFDSV
GKVITSDTPGRKLSWEDIGKRSAKVITFSDLAGHEKYLRTTVFGLLSSSP
NYCLLMVAANNGLVGMSKEHLGIALALNVPVMVVVTKIDICPPNILQETI
TQITKIMRSPGARKVPTFIKNREECINTATQFVSQRICPVFQVSNVTGEN
LGLVRTFLNILPHHGRYNTDAPFEFHVNDTFSVPFTGTVVSGIVKSGVVH
EGDNVLIGPDSLGQFTPTAIRSIERKRIRVPAAVAGQSASFALKKVKRKD
VRKGMVVLPKVEGEPNPKVHREFVAEVLILSHATTIKKKYQAMLHVGPVS
QTCAIIDIDRELIRTGDRATVAFQFVQRPEYLAPGDRLLFREGRTKGLGI
VKAVGYDPNHPLMEKSANGDDQKDQDKPHAGRQVRVSA
>Mb|QLI67939.1
MVATKTASAEPAPWESVPAHEQLFVLITGANSGIGLGTAQALIDDFLATR
SLSSHLIVIPTTRSGSKSLETIRQLREYATKAAKTSKVLSRGGADYKWED
AASRIHILSLTLDLCDLRGIWDFAHKLRYGTVRNPEGLEGEYLRNVRIPR
LDSIICNAAFGGWAGMNYFAAIRSFFTKGIIQTATWPDFKLSLPTCLLNE
RPVLNYPVKPLLGEVFCACVFGHYILAHKLLPLLSRSSENEAPGRIIWSS
SLEAVRKVFDVNDVQCFTRPEAYESCKRLTDLLCLSSSLPAARPFSSRFL
TIDDDAEAAKKQLVRPKIYLTHPGVVASTLFPLPWFLFWLYEISLVVCRW
VGSPWHNVTGYNGAKAASWVVLQTQEALDDSDAERVKWGSSTDVRLNVDV
KKTEVEGWGWEGKPETPDTIAADSAVGVLHKSVGRKTGTRDATAEDIAEF
EEVGARCWQQLEELRAQWEDILERDGK
>Mb|QLI67941.1
MNPRFSLAASSDTTYHSFHDIELQEPPSPPRSRGAIDSGRAADGVNQPIQ
PAPPPTAVKMQRQDSGYESHQSGSARNSLSHPRPVPPSRRTSTVSSTAGA
VTTARARARPSTRRSAKSYPQSGVPSPGYFQFPAPDLVELTETASRVEPP
PPNLPPPTTHYWTSDSTRRLEYAAIDAASRGVKGWIRRHLVPDCFTPRHV
AFDDDSGSVRRYRLELEEEQDEKACPQVRERRKGWNFFSLRKSSTI
>Mb|QLI67943.1
MDPTLLIGDIVERDSKEKRPVRFPAVPSSSTGFPSHKRRWNNSAFKQQRA
GNLDQKAPAFEENENVDIQGTGKQNDNPSQGFDRAERQRIDRENRQKLAA
MTPQEIAQAQEDIMTGLNPGLVQRLLQRATIDEPTGPSPFDLPDDVTKTT
GQAEIPLSNPPSIEISDSEKPQNVQPLEERHQPASGPHGREPAGLPSKKI
ADDYDEDKAPSQIPADLFPIKELPKTTHFPVPPSLPDLDPSDPNFLATLH
EKYFPELPADPSKLAWMAPLPSEDSTADKDSTYYPHPEISVSALRFDFRG
RFLSPRVSRSIASTKGLHHHGEAPEAAGYTIAELARLARSAVPAQRCLAY
QTLGRILYRLGNGEWGKTDNDPIAMGIWAAAKKGRVLDSLMEAAAGEGGH
RGSRAYATEALWLFERGGWKEKYQGR
>Mb|QLI67944.1
MLSVTSDIAHQLNLTGVWKRTGGRKKADAIRAVEPRRVNIVSEGLCDDVI
RYIAPSLERHRGCDLVDLNPGAGVWSRKLHEFLEPRKHVMMDLDAELYKP
FLSDLISKDNVELLPKSGLVWKDLLGMLHTSLSDQQEISQKDTPSRNDTL
LITANLSMFPKKAFHGFDSISSMVLYQFISSISTSALFQRYGLVRMLIWV
NDEDKRRILPRSTNRRKKSAFEAELSCEWVHEVAGLDAEVEDRNALRDEW
INMESASQTLERMKAAGLSMPKGRETLVYSKLQREPALLGQKLAGARPPS
LTRPFKQELELLEQGLSESAESSKRLKALRQRGKYDQKDALMYLGLLQER
DTVAKLAASSPTEFERANAAWNEKIDNLTKNSRNEFNGIKDSYHLFRQSP
PALLWDRRAYEPLSAKGEEFFPNAPTALLDIQPKAMHPVFRQHGPNSSRS
GDMSEVMLRFWFHHTLLPIQKAMEGLWGGFGDLFAECPSVRDPSRGGTPM
TGNGALTVRAMNADQWAEIMEAWMNWPFRPSYTQMLGRLVEEVDGDGDEE

DTKSGATGLAL
>Mb|QLI67945.1
MPITEFETKEKYRYQSGFNCHFESEAIKGALPIGHNSPQAPPFGLYTEKL
SGTAFTAPRHENKQTWLYRILPSCAHPPYQPSPEGTAPELVVRESSKMHY
IPNQLRWDPFSHDESKDHDFVSGLKLVAGAGDPTQKQGLGMYVYAAGKDM
DEKSAFYSADGDLLIVPQEGDLDIRTELGWLLVRQMEIAVIPRGVKYNVR
LASGPARGYALELYEGHFSLPELGPIGSNGLANARDFVAPVASFSEDFGA
TAHEGPNKYLVTVKFNNTLFQTTQAHTPFDVVAWQGNYYPYKYDLGRFNA
IGTISFDHPDPSIFTVLSALTPVAGTAVADFVIFPPRWLVGEDTFRPPWY
HRNTMSEFMGLIRGAYDAKKAGAGGFVPGGASLHNVMSGHGPDADSHEGA
RAAKLQPVKVGTGSCAFMFESCLMVGVTEWGLRTCKKVQEGYSEESWGGV
VTHFKLPEGAVANSHLAE
>Mb|QLI67949.1
MPLLVAVLAGLLSGASAAANLVQHERVPQLPQGWARMDASLDPDRPFRLS
IALRQPEIHRLASKFARGSGHLSLDEVRTLRAPDPKDAAAVVDWLRENGV
HDTVTKDDWIHVNTTVSTAESLLNTQLQRYSFDGKVSMVRAREYSVPSHL
SDAISFINPISNFMTPLREPMTPLPAQDARDRRATAACSGTVTPKCLRQL
YNMHYKAPGNSSEVRLGVSGYLEQHSNHADVRDFLRQQVPELPRYDFKVE
LVNGGTDPQKPPTAGSEAQLDLEYVMGLGFPSQVTYYATGGRGEKIGDDG
KPVTGKKLDNEPYLEFIQALLDKPDDEVPHVLSVSYGDDELSVPRPYAER
VCGMLGLLTKRGTSIIHSSGDGGSAGGRVGNCRTKDGTNKKVTMSTFPAS
CPWVTAVGATSNIAEPPSGASFSSGGFSQYFERPAWQAAAVDKYVEEING
HLDGYYNASMRAVPDISAVGTNFRVIVGARRKLIEGTSASAPVFAAMISL
INDARLRKGKPSLGWLNEILYSDKVTSLLQDITKGQSYSCTWDKVSPGGW
PAKQGWDAITGLGVPNDFAKFLEVLEDV
>Mb|QLI67950.1
MPPHNDVETFHEVLRSSRRILALCGAGLSASSGLPTFRGAGGLWRMHSPT
KLATMRAFTTDPGLVWLFYGWRRHLALKAKPNPGHEALAALAKKNPDFLC
LTQNVDNLSQRASHPPEQLCTLHGSLFDIKCTTEGCGWIQRDNFDDPFCP
SLQAGSEDPPPGESLPLLDPYHRIKHVPEEELPKCPKCQTGIQRPGVVWF
GEELDENMIENIDEWITRDKIDLMLVIGTSAQVYPAAGYISEAKLHGARI
CTINPEAEEETELYKIQPGDFAFGQDAADALPRLLEPVIGKLQENGEFSL
>Mb|QLI67951.1
MSSNPFRKKALGPIDTARASSPRSSLRSEDAEAGDRKNKPVKRVRVLSPP
PLSPDSPEWPFEAPQPPVPLSDDTYNHSNYYPSFDPFNGASTDESDGESL
ATPPTDKYQHQPQQQQQQHDAGSGGIPANPFSKTLRDLEGSKDLQRERK
EEGEVLKAANEGRKSLNVDSFKRLLMTGNADDFASSASTDGEEETAKGNA
LPTAQTNARGRNYTDSGSLADSSHEDEDKEESKILAVKSPASKEKKAPPP
PPSSRHGKSLRQREPKDVKDDGHATAEPVRRSMDDDEPRLDTQATNAAEG
EADARSQINSIPNLPGARKPAPAPPPPRGHARTDSKSHDNETPPRRTSSE
DMPSRSDSTRSAGHAPAPPPPRRPYLAPKQSSSSSPSTSSSNMASSTDHV
QDTARSSHDSPTPSKPLIAPPPPPTRQPSTRRPPSVTGVDAPSRRVSSEN
KSRETVPPPPPPPRHRGSSPNADGRKVSGEASAADPSKSADILADLDALQ
REVDALRGQMK
>Mb|QLI67953.1
METSDVQLQPLRASKISRLPGPAVHTGLAELNESQHNARAQTAIPAPPGF
KPHLKREIPQPAQQPDPKRKPLADRAAEYPASASMSISTSAASKLAIKGQ
TLPQLKRPTRHATNTAAPTSLTSFAKSFGPTRNQVDRSNPTTFRSSHARS
KSQAARPRTAHGHRPEEHFEDTTSTNAWDVDGRVDSMESQFKELKDMVNT
TLVERKGHEDALELAKTRVSELEEHRQRLEERNEALRTDLDVARNENRQA
KYELERLQWQQKRDIEDLNRRHRDTIDDLSRQHRTVVEDLSKELDQLKDE
ETKEHQARIDALNRHYQQELEDERQKKDREIQDLRARMGNEQQDLNLELQ
RKDREIRDARLECDSLRDDLEREKSLKGSLQTNITELSAANTTLEAKINS
LRSHVEFLESDSKAQSDSFASMEARLQEALAAADEARQKLIKEETERRVL
FNKYQELKGNIRVMCRVRPALTDDASVEAGILFPDEKTSAEIVLAGPEEK
SSLGVVSRKNYPFEFDRVFAPVVQNEEIFGEISQLVQSALDGYNVCIFCY
GQTGSGKTYTMSAEDGMIPRATHMIYDTMTKLKEKSWEYTMEGSFVEVYN
EELNDLLAPNDRSAEARSRKLEIRHDEARKQTTIVNCKTVQLNSASSVER

ILEEAQKNRSVAATKANERSSRSHSVFILKLVGENMATGERCEGTLNLVD
LAGSERLKHSQAEGDRMKETQNINKSLSCLGDVIEALGRGSGHVPYRNSK
LTHLLQYSLGGNSKTLMFVMVSPLETHLKETLTSLRFATKVHNTHIGTAK
ATKKIRGSE
>Mb|QLI67955.1
MPPAACLRCGNRAVIKRPKNHHKLCKECFLSVFEDEVHHTIASSKLFFPG
EKVAIGASGGKDSTVLASVLKTLNERHNYGLDLVLLSIDEGIKGYRDDSL
ETVKRNAVQYDMPLKIVGYDELFGWTMDQVVETIGKKGNCTYCGVFRRQA
LDRGANLLNIKHVVTGHNADDIAETVLMNLLRGDLPRLSRSTSIVTGSAS
TGIQRSKPLKYAYEKEIVLYAHHKRLDYFSTECIYSPEAFRGTARTLIKN
LEKVRPSAILDIVRSGEDMARLTPDKSQDACACDEGEGMGGCGSANGRTS
GGELAEMEAALKPKHKTTELETEISPNGTPTDDGSVKLPLRGRRQQKGQR
QRPTGPLQTLGACIKCGYMSSQQVCHACTLLETLNKNRPEVSI
>Mb|QLI67956.1
MATEKPNDHAGHIETTMSGYDSQGADRDQGLSSATSEKAAERGEVATNEY
GESMILFDPVAERKLRLKIDLYIIPTVAVLYLFCFIDRTNMGNAKIAGLE
AELGFTGYNYNEIISIFYISYILFEIPANLLCKYMGPGWFLPLATVLFGI
ASVATAYVHNFAQMAGVRFLLGVFEAGMLPGCAYYLSRWYRRSELAFRLA
LYIIMAPLAGAFGGLLASAILNLDSFANLHRWRMIFAIEGIITIGLGLVA
LFTLTDRPETARWLTPEEKEMATARVKSERVGQTAVLDKIATKKLVRGIL
SPVTLSTSFIFLLNNITVQSFAFFLPTIVRTIYGSKSTIQQQLLTAPPYL
VGAVFMIPFPLLSWRMDRRQIFITLSAPLVMVGYCMFLGSTASSVRYAAT
FIIAMSTFTNGAMCSAQVSANVVSDTARSSAIGANVMFGNIGGLLSSWAF
LPWDAPNYHIGNGLNLAAAGTIFIVALLTHLWMKSDNKKRATRDADEELR
GLSQEEIEDLDWKHPAFRWKP
>Mb|QLI67961.1
MSKLQGNGVAQCSSPGASGSESGLKALRRRNKAVKEDSKAAGPVAHRIIL
GQSSELPIRARFAPSPTGYLHLGSLRTALFNNLAAKASNGGAFVLRIEDT
DQKRLVDDAEERLIKDLKWAGLCWDEGPDCGGPFGPYKQSERLDIYKAHV
QTLLDHGHAYRCFCSPEQLELQKRELHKAGKSTAYPGTCRSIQHTESERR
ARDGDPHVVRFKGDGFGRPKFRDAIYGLFQKQDLEEDFVLFKSDGFPTYH
LANVVDDHLMNITHVIRGEEWLISTPKHLALYMAFGWEPPTFAHLGLLVN
PDGSKLSKRNDSVNLSTYHDKGVFPMALLSWLANLGSSFKPNVKPPRTIS
DIADALTFKFTRGGIKLNMEKLDHFDSKYRDAILWDPISDLSETETSLIS
DYITQRMLQEIDSVTEKAAASELLPREWRRALTPVPALASQENRATYVYN
IFATKKGGFLSAEALVRQHPYLLWRVPETVYKQSLASINPDERILLALSS
AIDDKKLWASQGAEVMDAIWTALEGQGIDNITVHNTLRLVGAGGHDVVSQ
SSSRMFMLLGRDEWRFRLNTVQRLLNHA
>Mb|QLI67964.1
MTAKVKRVFSPPNSLAERLAEPLLDPAIPRKLPESPPSLAAQLPLNWSAA
YTRMPMDSNGESHRDGGITFAAQDKLPKLPIPNLSSTCDKYLEALRPLQT
PRERAETEHAIRDFLSTDGPELQEKLQRYAQGKTSYIEQFWYDSYLNFDN
PVVLNLNPFFLLEDDPTPARNNQVTRAASLVVSALEFIRAVRKEELPPDT
IKGKPLCMYQFSRLFGTARVPTENGCQIVQDPEAKHIIVICHGQIYWFDV
LDDNSDVIMTEKDIAINLQTITEDAAQTPIQEAAKGALGVLSTENRKTWS
GLRDVLTKDHGSNNADCLNIVDSALFALCLDYTEPADVAALCQNMLCGTS
EVKNGVQIGTCTNRWYDKLQIIVCKNGSAGINFEHTGVDGHTVLRFASDV
YTDTILRFARTINGQAPTLWTSTSPDPSKRDPESFGDVNTTPRKLEWDMI
PELSIAVRFAETRLADLIEQNEFQCLDFSSYGKNFITSMGFSPDAFVQMA
FQAAYYGLYGRVESTYEPAMTKFYLHGRTEAVRTVSQDSVNFVQSFWADN
PIEQKIEALKKACQKHVSRTRECLKAEGCDRHLYALFCVWQKFVNDEIES
GMSSIGQSSPTDGYSPVGSPGGDSSGSMDQVVEVKPNRERGNSTNSRSRD
AHPLPFIFADGGWDKLNTTVLSTSNCGNPSLRQFGFGPTSGDGFGIGYII
KDDSISICVSSKHRQTKRFVDTLESYLLEIRRILRITNRKSSTAKSSRAR
EAEASRPKQMSRLKSRGRLIATDSAGRGPSRPESTSQTEESPIVSDDDEL
GGYGFFDAGMLLQALKARDRAADPVESAKASERAAVQARRRDVGKKLRLT
TE
>Mb|QLI67965.1

MHLLLIRHGESVDNAAGLYGGSRDAGLTAHGALQAQRLASSLAASKLHVR
HIFSSNLGRAAKTANAVCDSQNKTHQSALAVVQLAELREKHFGNWEGVKF
ASASAQHRPEQTGAETTESLQARCSAFLETYLAPLFTARMVGPEENCVVV
VGHGISLGVLLLALAKKLTSTGATRDASKDAALNQLASTRISWSNTGYAD
LVISKSNAAAEVAARGNPWSGLELRVAQINCTTHLSGLRKTRGGIGSAAH
DEKQKTIEGYFASSRKRKADKVTD
>Mb|QLI67967.1
MDDDYGADEALLAAMAAADPAQSSRRTVQQPVPQRIQQPKPQRMDKAPPS
KTSGSKVVQPTPQALPQKQTGGSTILVSPRQRGNPVLTSIRSLPWEYSDI
PADYVMGLTTCALFLSLKYHRLHPEYIYTRIRNLQGKYNLRILLTMVDIP
NHEDCLRELSKTSLVNNVTIILCWSAAEAGRYLELYKSYENTSSAAIRGQ
QASSYADKLVDFVTVPRSLNKSDAVALVANFGSLKNAINAEQEQLGMIGG
WGGVKVKRWSAAIEEPFRAKKAARRGLQPSKTGAQQASTSADGQHADRTS
RLEEAVPLSRVPLRDMPAVGTGSGAPSAAKGTPDGGLSSGSAPKQFQFLD
DDVENEDDDAEEAFMAAAIEDSRRTSQPTGGNSNSGRTAGAEKMDDGLSG
GVAAALAKLRENG
>Mb|QLI67969.1
MLSRAAARTTTSLVSRRGFHATRARMSSPYHYPEGPYTNIPFNPRSKWFG
AGYWAFMATGFLAPFGIAVYQTYKEQ
>Mb|QLI67971.1
MSGKVTAVGIDLGTTYSCVGVFDQEKCEIIANDQGNRTTPSYVAFNDTER
LIGDAAKNQVTKNPENTVFDAKRLIGRKFSDADVQADMKHFPFKVIDKSG
KPTVEVQYMGETKTFTPEEISSMILMKMRETAEAFIGETVNNAVITVPAY
FNDSQRQATKDAGLIAGLNVLRIINEPTAAAIAYGFDKIRPEDKDRDDIP
ERNILIFDLGGGTFDVSLLAVAGGVFEVKSTAGDTHLGGEDFDNRLVNHF
VNEFKRKHKKDLTTNSRALRRLRSACERAKRALSSASQTSIEIDSLMENI
DFYTSITRARFEELCQDLFRSTMQPLDRVISDAKIDKNQVDEIVLVGGST
RIPRVQKLITEYFNGKQPSKSINPDEAVAYGAAVQAAILSGYNSQTTSSI
LLLDVAPLSLGIETAGGMMTKLIPRNTTIPTKKSEVFSTFSDNQPGVLIQ
VFEGERQRTKDNNLLGKFELTGIPPAPRGVPQIEVTFDLDANGIMNVSAV
EKGTGKSNKIVITNDKGRLSKEEIERMLADAEKYKEADEAEGRRVGARNA
LDHYASNLRTTLQEPGVDEKLDAGDKEKLAAAADISSWLDQNEQASADEY
EAKQKEIEAIANPIMMKFYGAGGQAGGMPGGMPGGPGGPGGFPGAGGAGG
AGGDDGPTVEEVD
>Mb|QLI67974.1
MPPRLNIPPVTRTVLIVLLSQSFLSAAIRYRQWTENSHIVIPYLTLVPQL
SLVFPWTFLSSALVESNVFTLAISALTLYHGGRYLERAWSSADLAKFLAL
VTLVPNVLTFFIMVFFFTLTRNESWTLTTISGGISIQIAFLVAFSQLIPA
HTVTLFRGIVSLKVPRFPLLYIGVVTVLSLTPLLSRAALWQATFGFLASW
TYLRFYKKVFPDLESSQPASLRGDASETFAFAEFFPGPVKPFVAAVSDQV
FDILVAMRVCTPLSHADMSARGDRALQRGAPGTARAEAERRRAIALKALD
QRLNAAATRAPAPTQPSGPPIQTQPQPNAHTAMASQPGAVLGETKFEPEH
DDATASKN
>Mb|QLI67975.1
MSTTTPSRSTYHNPTGSASRTAAAAGAECTPEMRDRQARGKDPYKDSESD
CDDGPERRVRLGRGVKTEPFEDKERRGFALSVLDSPESLMMYALSAGDSV
PGQRLRFTAMLCGFEERRKGKEVAGRERERERDRDKERERERRR
>Mb|QLI67979.1
MASNPPARCCSLGSLFEGKPTGKLTKIDNKIDAYVATPPEGKARNGYGIL
YLPDVIGIWQNSQLMADLFAEQGYVTVVLDLFNGDPVKLNDKPAGFDIMT
WLKEGTDGNNPHTVPYVDPIVEAGIKYIKGLGVTKLGAVGYCFGAKYLVR
HYKNGIDVGFVAHPSFVEEDELAAIGGPLSIAAAETDSIFPSDKRHKSEV
ILKATKKPYQINLFSGVEHGFAVRADLKVKVQRFAREQAFLQAVAWFENY
LLEDQ
>Mb|QLI67980.1
MDRGLSLTAPREATDGRTGRLTAKKNTGHATSQRGFRHVQSPHGRGDKSQ
VAEAETLRSLSISPAPGGRLSPIPHGPYHPPGGIHVTTGMKFSPLKRFWT
ANKPAILVALSQLFGATMNLSARFLELEGEGMHPVQMLLLRQAVTSICCL

AYMWWVSIPDYPFGKKEIRWLLLVRGCTGFFGIFGVWWSMMYLPLADATV
ITFLAPGVAGFVCYFLLKEPFTRLEQLATLVALLGVVLIAQPVALFATTA
DAASSSSGRARRASSIPGSDHETTPRERLLAVGVALIGVLGAAGAFTTLR
AIGKRAHPLISVNAFAVICSIICVTALGLGPVLDIGQPSLRWIAPTSLKQ
WLLLLSLGGLGFVMQYLLTAGLAADKSNRANAMIYTHMLFAASFDRWIFG
RRMGLMSFAGCTLILGSAVGVIFMKRAPAPPKAEDVERQSNLSGEAEGSP
MLVGVAGHTESVNLERIR
>Mb|QLI67982.1
MALPNSTPPLLLTKRLNAFLHANQTPQLPTLLLTTCHGKLLAHASPHPVS
VLRTHATVAASLLAIHTSSSVDVPSALPGSRTPDPITSSPILRPDTSDKE
DEDDDDDVAPHDVAASKRAAARRRAARSVKPVTITVQLSGGTVIIRRLKC
GLLFVCVGPSAHDLQSDVHLQHENGDVTGSPSEVESLVSAGAQTTSSLDS
VGAASVVAMRRHAADLARWLDDKLGTLRVPEEGVGSSD
>Mb|QLI67985.1
MATNFEDIAKQFIEFYYNTFDTDRKGLASLYRENSMLTFESASSLGANSI
VEKLAGLPFQKVKHQVSTLDAQPSNSDGGIIILVTGQLLVDEEQRPMNYT
QSFQLSRDASGQYYVYNDIFKLVYG
>Mb|QLI67986.1
MKRGAAKAGGLSRRKPKVDAFENLPHDASVDQLKAAAVHVSLEELEHRVA
DVRDSWETESLFEELTQGSPEEQSPDICTPEESARLRRELREYGPIIFCK
RTLDCGRYTAKKLLTAFSIYSSDSLEERTEEYYSHLVSLAITRELSKRIK
LTDHNTVDDAVDLIQKCKNIIVLTGAGISTSLGIPDFRSEGTGLYSKLAH
LGLNDPQEVFNIDTFKEDPTIFYSVAKDIVPATDRYTPTHKFIAMLHQRG
KLLTNYSQNIDNLEVKAGLPKDKLIQCHGSFGTASCIECGYQTQGEAIFP
DMKAGKIPRCPKCIRSLRVNGGATKRKRSAGVEKRRSRRWSDIDDDDDDD
SGYDIPSAGVMKPDITFFGEKLPDEFSKRLTEHDRDKVDLVVIIGTSLKV
TPVSEVSNWLSPNVPQIYISRQAVSHINFDIDLLGDCDVVVTELCRRLGW
PLRHEMVPPNQVINVTPETGYKNRHVFEAVVPKANGVKKSP
>Mb|QLI67990.1
MPSKIASPISPPATVIVTGANGFIAQHCIAALLNQGYNVVGTVRSASKID
PVKEAHKSHPGLQLVVVDDITSSDCYLKAFDNLKPSAVFHLAAPFHYNAS
NFETELMIPAVLGSTAILEAATQIPSIKRVVHTNSFACIYDAAAGPSPEK
TYTSKDWSPLTYEDGVNASNAPTAYRASKTAAEKAAWSFISERKPAFDLV
SLCPTMVFGPFLPGSTPKSIQEVNTSNLMVWSVVSAGKNQPVPPTKGPCW
VDVRDVAEAHVKALLVPEAGGGRYMLCQGVYCNQELADVSRKVTSKYAEK
IPVGEPGKREKHTHFGVDASDAERVFGLKWRGLEDCLDDLVPQLFEIERS
SVSA
>Mb|QLI67991.1
MSFVAKPVIISGAGVVGLTLAHGLKKANIPFEVYERDDHIDARGHGWAIT
IHWALPFLVELLDDATLEAVENVQVDPEVGRNDNGNFLFINLETLDSKFR
IPPNKRRRVNREKLRKALLQGVTDHVRWGKRLDSIELANGEKGGVRAHFT
DGSSVEGSMIVGAEGTNSETRKFLVPDNYRNHQLPVRFIGAAVDMTPEQA
KPLRDIDPLLFQGCHPVTGHFLWVSMMESPEVNGTKGTYDERYRVQIITS
WMVKSEKDEVPPSDAGLVAEMKRRAAGFHPLLRQAVEQIPESAPALEITI
QDWPCFDWDNRDGRVTLVGDAAHAMTMYRGEAANHGMLDAYNLVQCLKKM
YSGKVSRKAAVDEYEEEMRDRTGGEEGAVMLSRQACLDAHDFHGLNEKSA
VLKKRAIKAKW
>Mb|QLI67993.1
MALIVPGIRRVALQAPSRFFVCNQCLRQTAASRPRFASRILNVVRSRSFA
DAKPTTPLGTMADHVASRSASMHAQELSKTALPKTNPKSVAYWLIGSAVS
VFGIVVWGGLTRLTESGLSITEWRPVTGSLPPMSQEDWNSEFEKYRASPE
FKLLNPHMTIDEFKKIYFMEWAHRVWGRFIGLSFVLPTVYLIARRRVTTK
MAFNLVGISALIGFQGFIGWWMVKSGLKDDLFAPGSHPRVSQYRLTAHLA
TAFVCYSWMLLSGLAILRTHRLLADPKAAMSALRALQNPALNAFRRSVFA
LATLTFITIMSGALVAGLDAGLIYNEFPKMGLGLTPPKAELWDKFYCRKE
DGSDLWWRNILENPSTVQMDHRILAVSTFCAILALFAYSRTGRVAAALPK
DAKKGATGLVHLASMQVALGISTLIYMVPVPLAAAHQAGSLAVLTGALVL
AHRLRVPRPTLRMIEQRLKQLKQ

>Mb|QLI67995.1
MEVNGVRVAVEGCGHGTLNAIYAAVAESCKVRGWHGVDLLIIGGDFQSVR
NAADLTIMSCPVKYRHLGDFPDYYAGTRTAPYLTIFVGGNHEASSHLWEL
FYGGWVAPNIYYMGAANVLRFGPLRIAAMSGIWKGFDYRKTHHERLPFSG
DDVKSFYHVRELDVRKLLLLQTQVDIGISHDWPRYIENHGDAAWLFRKKP
DFRRESQNGQLGSIAAEYVMDRLRPPYWFSAHMHIKFPAVKKYTDAAPVA
PSTNVNGDEATVPEGGANPDEIDLDMEDDENKSTEKPATGAEASESKATN
QISAELRAQLPASFAKPPPRDNRTPGQPVPPGIKNREVRFVALDKCLPGR
HFLQLCDIAPFNAADLAAYPPKSSGPRYQLQYDPEWLAITRVFHSTLSIG
DKTARPPPDLGEEHYLPLIEAERAWVEETVVAKGRLDVPENFELTAPPHA
PGSPEIVDEQPEEYTNPQTTAFCDLLGLKHLWNASAEERKARRDQGPPPS
EYQGRRGGAGGRGGRGGGRGGGRGRGGHRGGHGHRGRW
>Mb|QLI67998.1
MAAVEVEEMQGGGLQTAKDLFSGAVGGVAQVLIGQPFDIVKVRLQTTNQY
SGAVHAAASIYRNEGALAFYKGTLTPLLGIGACVSIQFGAFHAARRWFEA
RNAADRGLKANGLSYGQYFAAGAFAGVSNAALSTPIEHIRIRLQSQPHGD
ARLYAGPWDCVRKLSAHQGLLRGIYRGTAVTVYREAAAYGAWFTAFEYLM
NRDAARNGVDRKEIPAWKIALYGGLAGEALWLASYPFDVIKSKMQTDGFG
PDQKYPTMRSCFAATWRADGFKGFWRGIWPTLFRAMPVSAGTFAVVEMTM
RAIN
>Mb|QLI67999.1
MEVMEVVDLTADAVEDDYEDVGFETSTPSGNAMETSSFEGQADDSAMNIE
TLLGDLEQEDSDSVTSGGAPEEAPEEAPERQPESSEADSDSSHSDEEFNQ
EVDEFICFDDGDPNNDSDSDEELDWQGDCVQSCQPKYEKIHEKSQIRKFR
IGESRQLVAGFRAKITRMKAKIAGLKTQNRRLRADLAAARSSASGPPRRT
KAMWPEYLKHFLRGQRTRLPPGIRCYSDIYKLSCRELNIPPHPNRVLPGL
VLREKDDDEDGAIESIAVTGVFNFNQLPPIAQFRVLQMLLCFYGKKVHVL
TRLDPYHVPSLSSGVLGNDPERPQPLRRFHIGDSPVSLTYATLPNDLLAP
LVVCKKWLFWGAHLFYGENTFAFSSLGE
>Mb|QLI68003.1
MLSLRSFARSAPRAMTRIASSSLRSGVARPSMLAKSSAISALRPARAATF
STTVGRRAADGETDDELSAKLESEIQIEEDMKAQEQQPASIKDFLDNSPF
ELIDTPGQEVVKLVRSFGNEKITVSFSIADITSYDPYAEDAALEDDEFDE
SAQTSGKQSNNAAEETDDMDELDEEAAAPINLSIVVEKPGKTAGALNIDA
TAQDGNIVVENMFFYEDAKVAKVESPESAQKRADVYPGPPFGSLDEDLQV
LMERFLEERGITQAMAVFVPDYVDVKEQREYLRWLSNVKGFVDA
>Mb|QLI68004.1
MPLEQTITIVNNSGKIISTGKQLFSIFKEAKASYDEKKAEIRGLKRAETF
DQSQIPYRHDGYSRPRGHGSHSGRSVYEPEYYTEYDYGKNPGSYDGRRQS
FDVYSEAGSRRSHHTSRSHSKHYHHSERNRPALTENNLKTLSEISSTTPS
RPPMTYRSPYAETLPKDMAVSRMNLAQYDMKSIVEDDWRQSALLRRASES
QISRRDEPKDKEIDMNLAYGNIPPDLEYRVDLDPSSQGEEHKANQLVRKV
EGLLDEAHCLQHSATSIIKHLQQKPDSAAAVALTLAELSSVVGKMSPAFL
GFLKGGSPAVFALLASPQFLIGTGIAVGLTVVMFGGWKIVQRVKESQVPR
EAIAYESAPMDRPAPLRTQSDYNASFDEALIVDEELSTIETWRRGIMPFG
VDNESADIELITPEAERAQREKHKDDFDLRSFRSAHTAARSTKTAKTTKT
TKTSKTAKSSKTTKSQKTSSKDKEREVPERRSSRNPTVESINGDSELGRH
RSSRKERERDRPREREVRMIEDGRSNNMDIVFRPKAQRQGDNMLKALFKN
KDRHESNAEYVLA
>Mb|QLI68005.1
MADARSLLRQQKASRRIDHPHAAYSAAGKLLCTLCREQVKAESLWDGHLL
SQGHKHRVQQKKVTQTTSTSPMPEPAPNDAVPIHKRKLEPVDEQEQDREM
EDAVPVKRSKPDIAPLTGIAFNGTPAAAAAAAQEHAAKSGSTSTPDQVYS
AGSNNSKESTKTPPNLIRRLSTTPSQGVELQIPSRPATPAHREGGSASSA
SASGGYFALQSQTGPVTPLHRNASAANVTGPAPTAASAAASAADKTGSAA
QQVDESEWAAFEADIAAAEAPYDQGAVISAPAMTTEEVAAKEAADADRKA
QADVDIEDEKEEATRALEDEFAEMKELEARVKSLKERRNQILKKRRESQS
LGTAPPVEGASLAAAAVDQNGAEERVSGDEEDGDDDDDDDDEEEDDWDGF

RFRT
>Mb|QLI68008.1
MAFKRGSRIHANQRPSTDEGHQSQMASAYLSRRSSWNSVHDDLIDATQDR
EENQRNDIINWESQPTTSQSHSLVSDDKDTSPGASSGHSENSAASTSSSV
SVSDSSDYLAPASNGRPANFGVVFPGVYRSSYPKPEGYDFLGSLGLKTVV
TLVKKDEPDHDLESFLTTNGIRQVIFNMKGTKKEAIPMSTMRSILELVLD
QKNYPLLLHCNHGKHRTGCVVAAIRKLSGWQLDAVVDEYRVYAEPKVREC
DVDYISAFPCGPLQSMYDLYGEPARFSPVQVRTFFRGLLFTSFVMVVWLV
SGSHISLGPHDSVV
>Mb|QLI68009.1
MVKKRKNNGRNKKGRGHVKPIRCSNCSRCTPKDKAIKRFTIRNMVESAAI
RDISDASVFAEYTVPKMYLKLQYCVSCAIHGKIVRVRSRVGRRNRAPPPR
VRYNKDGKKITPTAPKA
>Mb|QLI68010.1
MAPTFLKELRRRSRASFRTDNSTDASSDGATSQGTSPSSGSVTPPSIAHQ
SDPALDLQIKKTNTSQQNQVQMSQSTRPMISPGSNTSRHSVSGMSGLGAP
PSNSRGSVPLSQYAPRIHSVSENSWVYQKVLLLHGTIGEPGQYSMDGNVV
ISRYDDSFPAISWPVCESHFKALVYLQPGPNKFRLDFSSPKLANSSSSNP
IHASYLTLHMIPPMNAPPLQLAIIVAKDSPQTYDATPARAEKEGNGLETA
VRKFRMAAYLWQAFTAEQMWRHKLGRRVFRYEEEWTLGTANYRDRENGTM
RSEARIHIIRSDKTVAEILDLNRAQQFEKATDKNALFGMAADAVKEYFKP
LPGQKNYVSVLFLDAHWDVQEKVIRGHAALGGNAGDLQLAIFGSHCLQSY
PSSFEEVVPAFTDCTPTDTKYVANDCNEAGSSWEAVNIGVGAHLHETGHL
FGCPHQESGIMLRDYVVLNRSFVPREAFSTRTKSKGGLVHQADECGWHRL
DCLRFRAHPAFRLPNDAPLNSDDSVQAFAIEGGTVLAMAATGVSFVEILG
EGDDVCHAWLEYTTETGPVQRQLALSEQDLRARLPEAKRKGRISISIKSH
GGGSLTITDFKEFTSKSSLVKLGNGRTASRSQKLGESKMEGSEAQEFVFT
SATQQDRIMSRVTVYHGSALNGLEFVYDDNTSQLFGNRGGKEGGDTFEFD
IRRGEYLSGLLVRAGFWIDGVQILTSLGRKSPIFGNAHGGSAHNIIPPRG
YSICGISGSCGSWVDGLSVLIKR
>Mb|QLI68011.1
MDTNIQRALNDKLYDKRKVGALELEKVIRDLVAAKDYQRVHDILEQLCND
YAYAVHQPHARNGGLIGLAAAAIALGPDLPRYLSRIVPPVLACFTDQDAR
VRYYACEAMYNIAKVAKGEILVYFNSIFDQLCRLGADSELSVKNGAELLD
RLVKDIVSESAASYVSVLEAPPRFDGDDKAVVEDQQVTLPTAFSLSRFIP
LLKERIWVINPFTRQFLVGWITLLDSIPDLELVTYLPDFLGGLLKFLGDQ
NADVQTATQSCLDKFINEIKRISRVKKGIVESKKSREGGKRKRQESMDTE
SLRPDLEEGDEIESDAFDDDEEDSAEEDWIPGQDVEINYKNILEILTATL
DSPLEEDCLLESLRWIVEFLDICPEEVLPFTPKILAHMLPAMASSKETIH
QAATRVNTCLMDYVVSISDDSGLSYQGISAGQSQQASLTLAVDRPDDSHS
NRISLSSIREQGTQSPGHGLGRSASVSGGLSANSPQTDLDYAAAVNSLTL
LFLNDHEATRVAALTWLIMLHRKAPRKVLAFNDGTFPALLKTLSDPSDAV
VTKDLQLLSQISRNSEDDYFANFMVNLLQLFSTDRKLLETRGNLIIRQLC
ISLSPERIYRTLADCIEKEEDVEFASIMVQNLNNNLITAPQLAEVRKRLR
NLETKDGQTLFVALFRSWCYNAVATFSLCLLAQAYEQAYNLLQVFGELDM
TVNLLIQVDKLVQLIESPVFTYLRLQLLEPEKYPYLYKCMYGILMLLPQS
SAFAALKNRLNSVSSIGYLTVAPRSAATPSGSSNYDRPNRLKGRDDGGIR
WVELLEKFRSVQDRARRVQRHNLEGEDIAMTSLGELRMADGPLDLKNSNR
APAPVISPAASKELAPTPPVKKSGLGRQFGRLGGAVSGKSKRS
>Mb|QLI68012.1
MSDQKGAMVEFRETLEEAQQRAMASCREWPRMGFQDFMELVQVPENIHSQ
GPGVKRYMSRQPAWMAGNELPWDELFRNLKGPRPRHRGLGVFGGCVYAQA
PLAAARAIEEEERRQTAATGLVKPVPGIHSIQGIFTVPGLGDRPFVFDVT
NIISGRSFFGRQVNVRQPKQPSSNPAGPFPDSDAELPLGDICFSCITTFK
RPAEGVDDVQSPISAQKRYANILSQRAPDEWEPAPQSDIDIITSLFKDFK
GHGGFPLLDMHKVDMSEYNSDKEVPDRIQLMLYRPMKPIPKDDVNGHIVC
HAFEADRNGLIMLGNHMGYGFNMDKAASLSYSFYVHTNPDEAVMDGDGWW
IQEINWPRVSANRCMMESKIWSPEGKHVASAYQDGMLVPAKEPMEVKESK

LVYSLDEAGSVYKAPNSLRGVPVEKVTENSFYWEKSWDSLDDFIAYEEEE
RFLKEKYQAMKHAEPNNTDIRKRAKLHQDNMSKHVKIREIFGLHTPYHPN
QLVAKQHLPPEGLCRKDLMYRVACKISDLKVLNARGILAMDAWDFIRWRL
GRKLEEQLALPGQSGRHFVRTLIYKLCDESECGSTGYEDSIMRQAVLVSA
QYQNRIASFKTNTTKAGYSAARSLGSGLPRRSTNEANRKRNRRTGGSQSG
QTASAQRAEERRQRRAQLAAQSGSYQGVNAFRGMQRDQQVLQQGDSL
>Mb|QLI68016.1
MKFSSASFLVVSAAAVVLGVPVPAPPGIPTASTARTLLAGLKVATPLSGD
GYSRTLFPTWETIEGTCNAREFVLKRDGTDVQTNTACVAQSGNWVSPYDG
VSFTAASDLDIDHMIPLKNAWISGASQWTTDKRKGLANDITRPQLWAVSA
HANRAKGDSSPDEWKPSLKTFWCTYSKSWIQVKSHYSLTITDAEKGALSG
MLDSC
>Mb|QLI68018.1
MGESAPSADAESSDGPSKPSKRHIPPDVVNITPIGDIILSVTFETSTETL
RKTRKAALSASRKASLPNGPSTPPQVLKPKVTIAYRVQLDMLKTHSQYFG
NLLTNPQFREAKLITAVYADLAGRKIKTSEADPAELPHVKITDDDEATQS
AGRESVFEDMLRIIHQVPIKTTRTVMSYATTLALLADRFDCRAPVSRALM
EIKFKWPLTNTRPYLDESGRVTETEKVLRQKILVAWLLGQPMRLHQASRE
LVIRGSRLWGVYGEEDEAQGAWWHLPEGIEEELRYRRECILDTIASIQRH
FLTLYASRNRQCKLGYDSSTACDSFQFGQMLKFLLSKDLLHLVDFSPSSV
DDVPDTSMVDVDELLGTLKQCPNYQIDKHHTNCGLRIRIDPILDYVRAMV
GAGVVAIPLADWKRRRADVSWLNEKEGLRNVRDAEEGSNAFAFTRALATD
QRLRYEGALYADGMARRLFTADTWDWTPEA
>Mb|QLI68019.1
MIPSAAPANMMATRASAVPDEQDDSRSSSSTASPVPADNEESDFFLGAND
SESSLGVPNFQDMQVEDSCWPPINRLPNEILISIFAKLGATSDLYHCMLV
SKRWARNAVDLLWHRPACTNWRNHSSICQTLGLERPFFSYRDFIKRLNLA
ALADKVNDGSVLPLAACTRVERLTLTNCRGLTDSGLIALVENSPSLLALD
ISNDKNITEQSINTIAQYCKRLQGLNISGCDGISNESMINLAQSCKYIKR
LKLNECVQLRDNAILAFAELCPNILEIDLHQCMHIGNAPVTSLLFRGTCL
RELRLASCELIDDSAFLNLPDKRVRTYEHLRILDLTSCTRLTDAAVEKII
DVAPRLRNLVLAKCRNITDAAVHAISKLGKNLHYVHLGHCGQITDEGVKK
LVQSCNRIRYIDLGCCTNLTDDSVKRLALLPKLKRIGLVKCSSITDESVF
ALAEAAYRPRVRRDASGVFIGGEYYTPSLERVHLSYCINLTLKSIMRLLN
SCPRLTHLSLTGVAAFQRDDFQAYCRVAPVEFTQHQRDVFCVFSGSMVSQ
FREFLNTSSQFAELRESLPYRAGGSRIRGAAARRSQAMANYLNAAEGEGF
DDEMPDEENDFEGLEGTDMAVDPAAQNPPHIPAALASSAPSIPVPPPPPP
APGIMQYPFASASHAHPAIPSLEQQYAALVGPLAFSSFITNEPATGNPPL
SANGRAQASIANLTSQSIPQRARTSSASGLANGTGASTAPLHQHPPDDEQ
Q
>Mb|QLI68020.1
MDGPASSFNGHSQRRKLVKKPPSHTYTRSSSGLDDSHSLGSRRSSQSLRR
APSAPAVRSSIANTSSSSSPRHPTASQWPSNASPSIAQGDFLATSNPPSG
PGLITSSLSSPSNPAASPYPALAHRPNRLSDSLARRHSKEPSDDLLGAPF
DGAAILSRIESVKYTAPRESVQRPPASPALAKPIAGTRNVNPALRASQSF
SNMEPAITEKIQGGKAPDGAAAAPKRYSDEGKDSKPPMIRKKSGFSGFVT
SLVGSQKKPTISAPENPVHVTHVGYDSATGQFTGLPKEWQRLINESGIPE
KERRENPQTMVDILQFYKETTERPPEDQILEKFHHAGPADNRSYNTPTSP
NMYPTNYMGSFENPRAPPPVPPSRSHGRDMMPSRPAPKPPVSISNRSGPS
SAYPTKDSGIGLSQQPDDYSPQSKDSSPMLPEEHRSRSNSRANGQSPYSP
LTPSSAQVAAYQQQQFVQQQHEQALAQAQAAMSGHVGRAPSKRHPQPQAS
VPAPHQTAAYARAVDANGHSNASRQQAGPGAVPGARPRQRARQSNGIDVV
TSLKRICSEGDPREVFRGFQKIGQGASGGVFTGFERGTNRLVAIKQMNLE
QQPKKDLIINEILVMKDSSHPNIVNFIDSYLCAGELWVVMEFMEGGSLTD
VVTFNIMTEGQIASVCRETLKGLQHLHSKGVIHRDIKSDNILLSNEGSIK
LTDFGFCATINEAQNKRTTMVGTPYWMAPEVVTRKEYGRKVDIWSLGIMA
IEMIEGEPPYLTESPLRALWLIATNGTPQIKNEAELSHVFRDFLCFALKV
DPEKRASAHDLLRHEFMKQCVDLAQLAPLVRAAREQRAQEKARKQ

>Mb|QLI68022.1
MPPPSSAQQKVLTAQFLSLTGATERQATKFLKSTGYKINEAVDAFYSSGN
EVKGPSPTESKLDAQFDTLRDAKNDEKDKMELESTMEYLSSKLNISLENA
ELFVALELLQAPSVGEITRRGYVDGWKTAGAGTTHKEHASHIQRLSSALA
TNPVLFKRVYKYTFVAGREGDQKALSLENALIYWGMLFSPPGMLWKSENH
DWLDLWKTFLDEKWTRSVNKDMWNMALEFALKSVADESLSFWNEDGAWPS
VIDDFVEWCRGKGIGKQETMEVDAEQ
>Mb|QLI68024.1
MSILSGHLEQISYSCQGIDSLPFPPPKLFTNALLSNHDITSLIRDTEAHE
RALFSVPPPPSPATQSRRSAEPESKSKGRRQTVFNVASGEVTTGPPTRAS
SNPRRHTAVAAVLGGEMHSQLRRGETDRKGDVDVEILLRGAEKLCGVYEL
PGARDRISALRNKHRNGKNTTAYYEARVAEQAEQLASMNKSWMGEDGSEE
DDAGDESELWTEEDLRREEAEAKEMEAKKRELQARLRQMEKDLGGLMDL
>Mb|QLI68028.1
MKFGLILLGGFIPAAVAVPFDQYKVHEQRGPLHQQWIKGQQASGSTVVPV
RIALKQSNLEKAEDYLLQVSDPASPKYGQHFTTQQIVDLFAPSEQAINQV
KTWLVSSGIPAGSIALSNSKGWINFNTTASELESLLKTKFYLYTNNATDG
VYFGTEAYSLPHDVSSLVDFVMPGISFTQMQKKTARIAHPSSGKLQGASI
DPNGTDHCDTYVTPRCISALYQIPPAKLANPNNTLGIFETEGDVYSQEDL
NQFYAAAAPGIPKGTGPIIHLINGATAPNPPSSAGAESDLDFEIAIPIIY
PQKTSLYQIQYPGNAGYKNFNYIFNDFLDAFSGPYCHDNGDEGSGKECND
LTPPNVLSVSWGDSEDPSLVSFHKRQCTEWMKYGLQGTSVFVASGDYGVA
ENTCLGPKQNIFVPDGLCGCPYITAVGSTYLPKGAKVGDPEVATERFSSG
GGFSNIFATPEWQHSAVSDYLTKHKPSYKSYNTTDGKLPSSGGIYNRGGR
GYPDISAIGDNGVVVVGGSQGTIGGTSMSAPLVAAIFNRINEERLNIGKS
PVGFINPALYKAYSKKAFNDITKGDQPGGGGCGTTGFSAASGWDPVTGLG
TPKYTQLLEYFLSL
>Mb|QLI68033.1
MRHPSRLARPLARASACPSRVPTTSRALLSQRASASARATQDFDLNKLNS
KRRDYERNRTAFLTAGAVAGIISFIYTAWKLKKALDVQGEKDKIAVKCDA
PIPTEAFKTEAGEKRKVVLHDEEGREIVPTGYSVVPAFPRTMELDLPGAS
TAAGPVAASVANSAGVEYTLVGLGMRTVTFIGIQVYLVGFYVATQDIERL
QHYLVKKINPLATTLIPSEKDALRKALRDAAEGEQTWDSILREAGCRTAF
RIVPVRDTDFHHLRDGFVRAIQARSGRGNAYSDEAFGAAMKEFKIFFNRG
QVPKRKELLLSRDANGTLAVLYGEGGHKQQVGRSLMGTLSDERLSRLLWL
NYLAGDKVASEGARDNIIEGIMEFVERPVGTVAAQIL
>Mb|QLI68035.1
MAKDVFSVPIFLVVFRETLETVIIVSVLLAFLKQTLDGPERDVRTYKALR
RQVWLGVAAGFFLCMVVAAALIGVFYTVGSNSWDKNENYYEGAFCLFAAV
IITVMGAALLRIGKMQAKWRVKLAKAIESPIKAGSRGCFSHWLEKYSMFV
LPFITVLREGIEAVVFVAGVTFSAPAYAVPLPVVVGLLVGGIIGYLLYKG
GSTAKLQIFLVASTCLLYLVAAGLFSRGVWHLEAQKWNNAIGGDASETGN
GPGSYDIDKSVWHVNCCSPTSTSDGGWGVFNAILGWQNSATYGSVISYNV
YWIFVIAGFIVMRFKETKGHWPFMKAKAPAAREDSVSGSSSPQESEVVKS
TNVTEKTTPA
>Mb|QLI68036.1
MLCGISGEAPQEPVVSKKSGVVYEKRLIEQYINDHGTEPSTGEALTADDL
LSIQSSRVVRPRPPTLTSIPALLATFQNEWDNLALETYNLKEQLARTREE
LATALYQHDAAVRVIARLSKERDEARDSLSKVTVTDGAINGEEMVVDSVE
GLPEALAEKVDEVHASLSKGRKKRPVPEGWATSDDISAFDTTASSALPVS
ETTSIDLEGGYAALGGLKGEAAIYSIEADKLERQLPINEAVTNTLWNGPK
LFFATSQGNVKVYEGGNEVASVSEHSGPVTGLSVHPGADILASVGTDKSI
VFYDLATMKRASRAFADASLTTCTFHPDGHLFAAGTVSGDIKLYMTKNLD
QAAIFKLGAPVQALVFSENGFWLAATAKGQTTVTIFDLRKEGDAATAKVL
ETGGSVQSLAWDYTGQYLATGGASGVTVQQYTKSSKKWSEPFRNSTPAVG
VRWGESARKLVAVNGEGVVSVFGVKE
>Mb|QLI68037.1
MTTQNFAHPLCLLRKQKTASGTQKCFLVSDRKTPVLKDLQLQPTDRAPPR

ELKMDSIEQKQKQLKSILKNSISEASAAARSENEAREIAIAHAKLLQHRK
ELEAEILDSVVLLSEYPLVRAAPYSASNPAQSDVSGFKTHVRLFQPSDYD
DLIEERNVNELCGYTLCARSRRKVGTGGEWKLTGSGDIVKRKDLEKWCSP
TCAKRALFVKVQLNETAAWERAGIPDIQIDLLDEDKSTETQADKTARQLG
ELRLEEQRQAVRDAKTLALERGERGIDDGADKINVELKEKETKAPKQEDI
STQAVDDHLRVEGHRFDLPSRPKA
>Mb|QLI68038.1
MSNYYGGQPNNPQYGAPGAGGGAQNLQFYPSSYSPAVSGAATPQQASYGY
GAGASGSFESGAQAGFSSAGFGGPGVSGRMGEHGGLRTGWLAAFSTEGYD
GEPPLLEELGVNFGHIQMKTLAVLNPFRHIDQHIMDDSDLAGPILFFLLF
GTILLLSGKVHFGYIYGLALLGSTSLHMILSLMSPTEPPSSESYHPQYND
PSSPPQHQQGGHFSATLTFPRSASVLGYCLLPLVVTSLFGIVMPMDTPLG
IVLSTAAIMWCTYSASGMFCAVGRMKGMRGLVAYPLALFYVGFGIMGIFS
SRGSGSLANAAAKINA
>Mb|QLI68039.1
MPAPSTSVDVCMYDGFGLNSGLTITGGNGALLVNGEAFAWRPWEATGSME
LVNRKGQFDLPKEAFGIFDLLWPRPDLLVIGVGKHNLPLSPETKKHIAEQ
GMRVEVLDTRNAAAQFNLLATERGVSEVAAALIPIGWKEGVGAEA
>Mb|QLI68041.1
MPGQPSSQHNAPSQYNPTSSQAGTGRTSIDRDGSGLQPLNGVPSRNLAAS
AVSFAPRGSSLNPTIGPGSFSSELRSQTISSRAGSRMDVGSMYTGSIMDK
VEEDGTEAAMQQALSTLRDKLNREMKIKEGSENMLEALNIKKAKQTKEQR
QRVEVELSASNLRIKELRQKISEAQRTRAPPPTTPTRNRAQETIFQSNGL
RSPPSISRSAAGSDLDDAAESPTFALAELLQALEVEGMTPEYYVSRANQL
VDLFKRHPALKYDLVWAIFGLRMQMMLLSESREVVAVGYRVARYAISDSA
SIKRIRSFNTDYLVIRSLSNYRKADVEREQALKFVRAFLDVKDGIKEVSR
AIVRTIVAVAEQGEDRRPGIHAADQSIDRLRPICIETLAEILVRDPALLI
ASGGLGTLSEALAEGTYKSPESLTSAFLFLLDSPQKRKYIIPGYGLDVVF
TAFTDLLAGADGILKQTSGAISVALRSWSGLMTLSMYDFRPIRSLIASIL
FPSQNVRETVLDLLFSLLRIKPPAWATSFLAGRRLTTYGRVANMKSAARR
RSGTLDMEEDSGEQSFVDHYTALLLAVFIKSGLLQSLLRMVQTEEDPMLK
RKSTLLIGEVLKLSSRLLPPSWSADLQLLPELFSAAAEFDNEGHFNASGI
IYQISSVSRTLYRSAPSESLAGGLPSSDSMRNLARFDEQPKSSNPAVALD
DATFRQLLIDSGVLNSSNYMKWNWEVILKIIDGPLQTGKRLDEAVKASKF
MKRLMSFYRPFKYKFAEVKNTRGTQKYVRAGCALVHSLLQSPEGIKFLAD
SKLLRQIAECLAQCDPTSGLTAQFPMFSKDRLTDTLCGGYFAMLGVLSSE
PKGLLLLERWRMFNMMYHIVDFKQRPDLIKLLLSNFDYGLQGHPRVLLSK
ALTAGTKDIRIHATNALRKCTIGPVANISGDNEVSDSKWAIRLLVTQLYD
PEIEVCSTAIKILESACNKKVYLEYIVQCRPALDHLGEIGAPLLLRFLST
SIGYHYLDGLDYISNEMDDWFLGRNDSYVGVIEAGLARAFLADPDDNSHR
LSLFDESDPEAGFHDSHVPPHFYRELTRTREGCKLLRDKGHFEEFVTTIR
EHGMQTEDAELITKVKGCLWAVGNVGSMELGAPFLESSDVVERIVKIAEG
HEVMSLRGTAFFVLGLISRSTHGLEILSEHGWDANTTSMGTSLGLCIPND
LSKFFSVVPWQHVNAASITIPDTQRTIQAQPPIQKARPPLELSELPPLLS
DEDVNGRILELVVDLGNMVLYKKALMELQKLKQRKPNAFRSTDFFKKVMG
LMEWNHYRLGIRRLVIDLFEKNVMRQIVFGDDDTEDSESTSGQGGNDNGD
GSSGDDRTERQRSISEPAEMPSDLMPAPLRVRK
>Mb|QLI68043.1
MALKQRDVAIAAVAAFVAWGYAVSWFSVLRWAGYAFVAGILVSIIGLVAT
LLLTAQRPQRKPYQLPRASFLGAGRWKKEVSALRKRQAYEKQRLELGCSP
HVLDAVDGLLDLILRDFVCSWYSHISRNQVFPNEVDRAVRLAITNLLESL
RHKDLAEIVTSRLIPILTAHFRDFYDAEKSVRGRKLNRSVTESEELDLAI
ASKYKDGNLHPAASLSFPDTKMVQQDYLRSLVERIVPKMLPEKMLASRSV
SIIIREIVSCAVLSPVLQLLSDPDTWNQVMESYGRTMLQDRSTVRKLRAA
LDQHASPTPRTVKMAPAPRLAPGDSERKFEKFIRAIRKVNNLSDARRFRS
EVASQLKRDSLQVNQDPAYLRRLEMGKRLLDQRVQHLAAGGDRLPISFNP
APAPAAAVTSKLENVSLVELLRDASGLSYFMEYMDRQRLMSLVQFWLVVD
GFRNPLEDDGQEDEAVSSNLSMWSESDRMDLQQINRAYLSKPELHVPDSS

RREVRQFLEAGKSATPLQYHRARKSILKAQSAVLEEMQSQHYHGFKKSDL
YYKCLASQESSTASLAPPPLPPTHPPTTRASQSYQAKPKPVSRLATSASS
YTTRRSDSASDLRGMDGNSNGPDPLTQSRRSLDEPGSAPLFDDEDVDNDG
LMDSVHSAGEPEPPLQVTPDTHIVQAVEEALNNIMDDDRPQTAEDLRASL
FGDAEDAEGSLLTSNDRESNRDSIDSGRPIIAAKDQEKPSLSSLGLVSAA
SRIGVFVDDDLFGDEDKFLADEVEDAEEGNTDKDEDEEVHEAAPGDLGLA
EAISALTNDIDRLVAQEAIVDSLTRKAELTNNTSELRILRKSKASLQREI
RRKELQRQQYVVQESDNSLYGRSSVKIKSIQVGREDDGREFAMYVVEVQR
NAGEQMPAASWIVTRRYSEFHDLHQKLRSRYPSVRNLDFPRRRVVMKFQS
EFLRKRREALEKYLRELLLLPEVCRSRELRAFLSQSVITPGQDILDREDK
KDIVSRLYDSVADGMDDILGNIPVLDQISVAGQNLIAAATNQLNAMPLNV
NEEAFPAAEAEAELNAFENKELEPFIKPICDIFLEVFELNRGNNWLRGRA
VVVVLQQLLGGTIERKVRDIVKMLLQDESILRYIALVEDSMWPNGVLQRD
RKPRSEAQKKQTRREASLMLATLVPDLAGNVVGRANAQTASRRIFATLNN
SRLNAHLVFTMLEEIISIMFEDS
>Mb|QLI68044.1
MGAQQSLPGAQSENSTSNPSKICYYELIGVDTDATDAEIKKAYRKKALEL
HPDRNLNNVQEATRNFAEIQAAYEVLSDPQERAWYDSHRDSILAGNDFAG
DDTEPATFRNVRLTTTEEIFSLIRRFNATIPFNDEPTGFFGVSRETFEHL
ALEEETAAEIGQIDHSYYPTFGSSDDDYEAVVKKFYAAWSGFSTKKSFSW
RDKYRLSDAPDRRIRRLMEKENKKCRDDAIREFNDAVRFLVTFVRKRDPR
YLPNSQTDAERQKQLRDAAAAQAARSRAANRLKFESYVAPEWSRPQDEHG
LDDYFSDIEEEDSEVEILECVICNKSFKSAQQFEAHERSKKHVKAVHHLG
KQMKKEGIALELDVAPVQETGQRPTMESVDLFTTEASPQETDPTMYRPAD
GRAYQEEQIEASDSSTDQQTDEYAPRDEVEERLRLSGPEGCDVDRVEDMA
EAHLVASVQSVSLDEVSVSTQGKIGKAKAKRQRKMARQLEEATAQVR
>Mb|QLI68045.1
MSFGSGGFSGFGQNNNNAQSGFGGFGSNNNAPSAFGQATSGGFAQNNTTG
GGMFGGNTGNTGGGFGASTGGFGTNTGGGFGSKPAFGTSTSGGLFGGNTS
NTNTTSAFGGFGGNTAVNSTSSTFGGGGGTTGGMFGANKPGFGTTTNTGN
SLFGGGNTTSGGFGTTSTGFATGSVGDPPGTATTPFNAYTEKEPNTSTTN
SFQNILFQEPYKKWSSEELRFVDYAQGRRHGNATGGGAFGVSSGFGGFGS
NANANQTTNAFGQTNTNTNTGGLFGNNAGNTTTTGTGFGSGGFGANANAT
NTATGGLFGGGNKPAGGLFGNTNTQTQQTGGGGLFGGGGTGAFGSSNTTG
AFGQANTTNNAGGGLFGNTANKTTGTGFSFGNTNAANTNTGSTFGANTAG
TAFGSNTTNTGGGLFGNNAGNTQATGGGLFGGQNNQQQNAGTGFGGGFGQ
QNQNQQGGGLFGNQQKPATGGLFGNSTATGTTGGGLFGGGNTQQPQQNTG
GGLFGAANNATSGGGLFGGGNKPATGGGLFGASNTAQTTTGGPSLFGGGN
QQQQSTGGGLFGASTNQQKPGGLFGGSAQTGGLFGAQNNQAQGSSLFGGS
TNQQQIQNALGNSLLGNTQQNNNAPQSLTANLSDVSAYGSPSLFAGLGGN
DVANPGPLATPLNGNSKPRKSSILPMYKLAPATAASRFATPQKRGFGFSY
SSYGTPGGSPASSISSTPGAMGRSLLGASSSGSLSKSISASNLRRNFNAE
DSILAPGAFSSSSSARWYGSTGSKKLVINRDIRSDLFSTPQKDKTEVNGS
ARKLSKRVSFDTSNVESEDGTPVRNALPAPEDTPKSQPDEATPRQNRTTN
GVNGSQTPEMEQVKGNELAIVHEEDNSATPEARAPLGFENAPGDYWMQPT
RDELQNMNRMQRQRVDNFTVGRDNVGSIKFKVPVDISSIELKDLCGGIIQ
LEPRSATVYPVQAKKPPVGKGLNVPAQISLEQSWPRGGRDKRITSDPKRF
NKHIERLKRIPDTTFESYDKETGVWTFGVEHFTTYGLDDSDEESDEEMVP
PPGLATPPQRVSNDAAAIDVSNREDGRPRFRQNAALPGAFDEQNDLYERE
NIDKQSFLGISSADSAPNDVRLSLEEDYASERGDEYDLSEDEDMTRSFIG
QHLAAELDDTSSENEQDAKRSTPGGILRARMRAMKDQVGPVTLEVADGDD
WMEMLRKTVSPVKRDRQLLREMNDSPLKFALGMDGGDDNVEQDLRKSSIW
GKSMSKTQRTSGLASTQMAADKGRGFATSIDLMNSLFEKPKPMRQNLRAS
VPAKGFPQWPYERQDKNFPVNSEEKAYHNATRPTWGPDETLVVTRPLDTM
QSRRSFRGNSDLLQFQRSGIQTDRQDIRLAKLSAESSKKFLRAQDKVTEI
RLVGDIPMATLRAGSLQDVFHHQEMNDPGHVNEKRVWELASILFDGAANS
GEPGPDHVSRKQKLSQFWTELVEQASTTNVGLAGSSEEKAVASLAGHRIT
EACKFLLDGKNFRLATLVPLIGTSVVAKKDIREQLKAWHDSKMLSEFSEA

IRTVYELLSGNVCVCEGVKNVPVEDRMESFVISKKFGLDWRQAFGLRLWY
AISQQDSPALAVLKFKDDINQDKEELPRPWYHEQGLKPVWNDTEEGTRQD
LLWGLLQLYADKNVDLEAILRPENSQLSPLNMRLSWQLGQALVATGQVSF
GKDGEEKADASTIAYASQLTAAGEWLEAVFVLLHLNNSSVRTKAIQEHLC
RHAGMIGPDTGATFTLLTEKFRIPASWLWEALALYMRSVKKDASAEVHCL
LRAGEFVEAHRVLVQQVAPQAVIERDYATLSSLLSQFQGQAESIPEWTQG
GEIYGYFLSLVQHHGKGESAPHPLLEKLVAGLNVMNEHVGETEVLRYAAV
SDMADDTAREILKLAKKKQDVELRSRILNLPLTQDRLLAYSVDLGMDRYR
EVMSH
>Mb|QLI68046.1
MASAVTDLEAGLQAMLNLKPPGVSGSRITSLTSLCVANIQSESVLIQKIY
THFKKAPGTHKLGVLYVVDSVTRKWLEQAKAQGQPVNSSAPDGTYAAGVH
RVTELMPVLMNDILQTAPEEQKEKIKKLLDIWEKGQTFPTQMIESFKEKL
TAPSSKTSTTPPGSPPASALAALQSHIPASAAPAPNGSSILEALANMARQ
SSAPGSANNNASNPPAPVASASYSIPPSALPQPVSSSTIPQPQHQAQPSY
PPTSQPVTMPSLPFSLPQMAGQSPALPNNASNPPNPYAAANPAAPGVAGG
ALDPAVQHQIMLIKALADQGVPFDKIPSLIQSMTTNSNNAAGNSAAAPHT
GFQPPVPAAQGTFSTSGQQPWGGPATVSGDGRDRGYEDGIRSPRYRGRSR
SRSPDRGWGSRDSPRGGRGHGRNSPPSGRHDRDRNGRGGNDYRQRSPPGR
RGRSPTPPDSLPHVERWVDYDSSVPSGHIRVYSRTLFVGGVTCSEAELRR
VFSQFGTVQTCIVNKDKRHAFVKMLTRKDAAAAKESMEDSRSSELPLRTR
WGVGFGPRDCSDYATGISVIPIHKLTEADRKWILTAPYGGSGGRPIESGL
SVEEPDIEIGAGVSSKAISRRMQTDKGGSNGPKSTRNREDDTGRGGRRGR
DQGNGGQGMASGFPFGIGTLPNGMPNFPSGYEFSDPSGR
>Mb|QLI68047.1
MSPHSTTPRSLPRMPVKQKMAVSLKSLMSSVLGGGKASTPAYDELFEKTD
PQKDGEECLHDCDGCTVRYPRGFKIDEEDVLYGQVKGWSTHVLVGTGKTD
WVRDVGDEKGSVMEAISKADGPTNGRLMLSASNMPTPHDTSDYSEPTTVL
LLPAFTLVENVHPTNVTTLITELINKAPTTMSPLTTPSLPKSLPGLDADV
PVLETKACPHSAVILMCSHRTRDARCGQSAPLLRKEFERHLRPLGLYRDL
HDERPGGVGIYFINHVGGHKYSANVMIYRRPNAFGQDEVVEAAGHSDSTA
TNGSSNGTNGTSNGASNGTSNGTSNGTSNGDGAKPDVGAAQGIWLARVKP
EDCENLIRYTVLKGKLVKPESQLRGGFDRVKGLTSW
>Mb|QLI68048.1
MASSPPASPLHHRERPLSAIVRPGPRSASRLSMSSKAGVGSRASDDESRT
AVKVAVRVRPPLKPNDPGYDLIPQRFQRSMVQTTSNTSLAIDSPQGRKIF
VFDRVFSPEVLQDGIWEYLADSINAFTQGYNVSLLAYGQSGAGKSYTMGT
SNPGEHEDESMTGVIPRAATALFQKLDLPNAGGTGSSSNRQSMSSLRTPR
SYNHQNLLGDRNWSLKATYVEIYNEQLRDLLVPESTPANERTNIAIREDT
KGNIILTGLRQVDIGSVDDLMNALNFGSSIRQTDATAINAKSSRSHAVFS
LNLVQRKSKLQTATGAEKRLSMPLEAMTGQDLSITTDSKLHFVDLAGSER
LKNTGAQGERAKEGISINAGLASLGKVISQLSSRQSGSHVSYRDSKLTRL
LQDSLGGNAITYMIACVTPAEFHLSETLNTVQYAQRARAIQSKPRIQQVD
DGDSKGLIERLRAEVSFLRDQIRSSQGSDHNPRRGNAAPNERSDRSSERE
SELQNQLLDAQENYSALSQRHAKLIAELARAREHESEQAGQNDSTSADDR
LNRSNSFAQAVEQVVLEYEKTIQSLEQSLSSTRSTLSNTETSLLEKETKC
AYVDTINSQLQARLQKLMDREASTENYLHDLEAKLDGHTSGEEKNATIIL
ELRKEIARVRENEAACEDYISTLEERLAEADQDIELMQREIDRLESVVER
QRSLGKLDALLYELDHVDSGKADEPEPTMMNGATRGRRFTDHSRSQSHVS
RKSQLDGPIPEENDEHSQDLNTENRVHDAAADEETAQDPDQENDVQHGVE
VEAKELTSGNTARSAKQSKYVADKLENVTQELLDLRVEHETTIQDYDQLQ
AKYEEALRSLSELQDAVDEARHPERSIVRDSTVFESPLPKVRPDSFTSDG
KSVDLKDEPRSSMSRSLSSELSSAMESPATMNSSAVDLSSEADTATTKPA
VSSEDLGHREENEQLTAEIERLRKLAEEKDVAEKTLAEKYAELESAHNDT
LDMVEELKTEVSKARAVEATSPRTSTPVIRRKSSQNLLVIDRAHRSFASL
RNIASENFENQPEVMQSFELNLNSAMHELHSRSERIQELEADITSVKKEM
ETKMTIISGLTRERSSLKASPMDMSMVANLRDQLERNEKQMSQMREAHES
REKELTTQLDELRESWRNAAAHPTIGEGVNSTIVDDSAKEREQRIVELQE

ELSGWEETHRLVIDAAEKTEADLRANIAKLEAQIASSNNELNNSIAQDKS
ANQELVKDSEQKQKKLVDFLRTEIDEYKAIININSTKIVELANEHADAKS
ELEKLNNSHESAIKELEQHRELVASLETQVASHSETAKSHQEALDLLKDG
HAKEMLELKSHEQKSYEEQAEVLMQEHTESMHKLEDELSRARDELSEIAT
QVATGMGVDVNIELLSERIDKLLVSQKTLAEEQQKAEEMKNQVAELTSIN
ENVLGDIKAAKSSLTEMLSDSADTVPTSVVEQLGAVKTKLVDLEGRNKKN
SRLVEELEEQLQNNFDEAQMTNNRLSTLQTERNVQLEEATAARAKLQGDL
DSIREEYAVLQAKYQELSSTGDIKRSNSNSTIRKSASHTSLPSPPPAIPL
PPLPNVAARSGSPTNGVPNSPTSGRPVSKDNVNISQLTEDQEARIRTIEK
HLYAERQLTQTLEEALTDLEKQSKKVKGDCDAWKKRSQELEAELKELKER
QMEPQADNRWSMQAVEEERKKRQAAELARKQLEERMNAINKSKKKKGSLN
CF
>Mb|QLI68051.1
MASRYGLWEVPNPGHSYEHHNIVKVLIPISVVGWLLFGAICILCINGRGR
AGRWVPEWYLDSEGKRWDKAAVGAWWLAVLFGWPLILPGVVVGCLSRKLR
MWRAEKKLKTQKSEKELEPDDKV
>Mb|QLI68052.1
MSDRGGRGGAYRGGGGGRGRGDFQGSFRGSRGGSDRGGRGGFRGGRGGAP
PNESRSKVFSIDGRIPSPDQTVTELENRIISQHESAVKGLTTKMGALTVQ
QKSASPDTFPERPAFGSSGKSVLLWANYFPVHFKIPVLYRYVLRVTEEVS
SAAKGKEKTEVKDVKGRKLHLAIEHLLRHFTSTDKSRALASQFKDQIVSV
KPLDLHKNPISISIPSENDDREPDVVDILIDGPTEIRISDIMTYTQTMDD
HGDRNTYPKFPEMIDALNMILGHNARSKLGEITAIGGSRFFPFNKDSTTA
SLAQDYHTLIAARGFFQSARLATGRLLLNTNVTHGVFRVAGKMDQLMKSL
AIQQVARDDHRLKRLVGAFAKFLPKARVWVTFTIADGTKVRRSKALQGLV
TKITASTAEGPNRPTINMDYEYPGPKNVRFWLEEESRFISVHDYYKRKYG
MTLQDFPVLNLGTPKRPSFFPAEVIEIQPGQSVRAKLTGEETTAMLAFAC
RTPYENALSISSDARNVLEYDGNETLQKFGVTVDKNLATVNGRVLNVPAV
AYIDATKKKISVNPFNGSWNMRAVKVVKKGDMISRWTYMNLLSRDTDRQV
GLETMEDFAKFMANDLGMNIAKSPVRLPKHFMTKDGALYGPGLVNFFKWA
EDNKIQYIMFILGQKDSGGLYTKIKRLGDCVHGIHTSCLVAKHLFKEKNF
SYYSNVGLKINLKSGGVNHKLTNDFGLLKEGKTMLVGYDVTHPTNMNVVK
GNEPPSLVGLVASVDQDLGQWPAMAWEQKSKQEMLGSTLTDAFKSRLSLW
RAHNNKTLPENIVIFRDGVSEGQFAQVIDQELPMIRDACRQTYQSGKQPK
ISIIVSVKRHQTRFYPTNTEDMSRSGNVKNGTVVDRGITLAPYWDFFLTA
HEALQGTARPAHYTVLLDEVFRYKYGERAANELERLTHELCYLYGRATKA
VSICPPAYYADIVCERARAHRPEYDVSDVESVSTSTSSESSGAGSIRQVH
KSLRDTMYYI
>Mb|QLI68053.1
MSLPSYQEATAGTDWLLLVAPFCDFDDYRSLCLVSRRFRQIFAPRLWNDL
LCSVRRIGLDPGDDLTWWFDFVFNKLDRVGRATRDLVQALDARHFAKDAF
DFSSDQNERRLTSSLQRALTLLPHVNTILLDGHADIDHAVLLGTQSLAAL
GLNNLRILSIKDCSCQLPNVFFNTPHLQKLIYLDMSNLPGSLISLLQSDL
LPALRILKVRGRELDDSALCFLMRRFGPQLWSLDVGSNKISDDAVQIFKD
WGLPASQLRTSDHFQVEGKIVTQSWGTADYGHFLCIQESDLSGSFCHPER
YFVDAPAYTSHANMAPQEYQVHRLDGNQAVRRDSADAVKEAMSEEHQSRL
GTDDYLFSTGITHLRLSYNRLSAHGIQKLLRISNGCIEDLTCDAMPLLPN
VGTYRKAWPPKSNLDGILGAAHLFRPVFSSNLRVLRIHHSLVTNIPSLQM
VGLSSLARLFLSETAILRRVERAYPQTFTPDMNPRLLSLTLTCLPRRSSG
PLISRLLQFLKLLAIQERGIQEALLSASTRRSPGVLQGLRHLRLEFEPDS
MEEGFSTSEDLDPEELMNSGEKVFSFFEDERMESWSRSRARQMLHSKRVA
VHSPAAVDNQASGSDRDNPEFVTYDADWSGDRFSIPVWAGPNDPDAPEVL
KDYRRLVLERHVRDGVGPASPAQILAGAPSRSFIFHVAWSAAVMPSDLRP
PTRSELAGMSDVLDELKTYRLNGRANYMQHKELAQMAGRHVSLGETHFFW
TGRLEVSTEATLPLAQSSQYWR
>Mb|QLI68054.1
MTVKSLGIRVSIDRGGTFTDVHAAIPQQKDIILKLLSVDPANYQDAPTEG
IRRILELVTDQKLPRGEPLDLFHFESIRMGTTVATNALLERKGEKVALLT

TKGFRDLLAIGNQSRPNIFDLSVSRPEVLFDQVVEVDERVTMEDYTEDPQ
SAKTIANGDDAHLVTAITGETIRLLQVPDLAVVRSNLQQLWDDGFRSVAV
VFIHSYAFPDHELLVGRLALEMGFSVTLSSEVQPMINVVPRGTSAVADAY
LTPIIKQYINSISANFRGGFESASTRVEFMQSDGGLADYRKFSGLKAILS
GPAGGVVGYAQTSWDDEEKCPIIGFDMGGTSTDVSRYAGVYDHVFETTTA
GIAIQSPQLDIHTVAAGGGSILTWKNGLFNVGPESASAHPGPACYRKGGP
LTITDANLFLGRLLPDYFPKVFGPKENEPLNREIVESKFADLTSDINKDR
ELTGLSPFSPQEVALGFLKVANEGMAGPIRALTEARGYDAADHHLACFGG
AGGQHACSVAEVLGISRIIIHKYSSILSAYGISLADVVHEVQRPAAITYG
KDTQAGIQAQLELLAEQARLELLKQGFSPDNIKFDAYLSMRYQGSSSSLM
ILKRDDWDFQREFEESHRRGFGFHFPEKPIIVDDFRIRAIGTTGSKQANT
PFSQLKSVNAAESSAPASSGTNRVFFEGFGSLDTRVYELQSLPVGARITG
PALIIDNTQTILVTPDSIATILDSYVVIDRKLQKQERSSDGVEEEFSPIQ
LTVFGHRFMSIAEQMGRTLKKTAVSTNIKERLDFSCAIFSPDGGLVANAP
HVPVHLGSMQFAVQYQHKLWEGKLQDGDVLVSNHPSCGGTHLPDITVITP
VFDGDNLAFYVASRGHHADIGGILPGSMPPTSSALWQEGASIESTKLVSA
GRFNEDEIKRLLLVEPAKYEGCSGTRRLRDNLSDLKAQIAANAKGITLIR
ALISEFGLASVHRYMYAIQHTAEDAVRGLLRDTLAKFGPEPLTATDYMDD
GTPIALKITISPSGSATFDFTGTGPHVLGNTNAPKAITHSAIIYCLRSLV
KSSIPLNQGCLAPIQIIIPEDSILNPGAGLAVVGGNVLTSQRVTDVVLRA
FRACAASQGCCNNLTFGTGGKDPITGEHKEGFGYYETIAGGSGAGADWVG
QSGVHTHMTNTRITDPEVFEKRYPCILRRFQLRTGSGGRGRNKGGDGTVR
EIEFRVPVQCSILSERRSRRPYGMDGGQDGQAGLNSVHVIDQMTGKMRIV
NLGAKATTKLGPGESVIIESPGGGGWGSE
>Mb|QLI68055.1
MVVKEFLDSDRVNFLIWRYLLEGNYRETAAKFQKEWHVNEPHRDFPFAPH
VKSQALVSVVNRGLIYYALEREFALSKRSRDAASVGADAEAEAEPVQVGV
FGPLNSQQPAKVEEGEGAAVGGDVESSRKRIQQQLQLPNGSPAKKPRLSN
GYTDASASASAAVATAATTSTTTTNTTSTSATTPMDLDPSHHHHPHPQPD
SHDGQHDASNNHAYPSPLEGEQLPPPIIHTDGPEQGTQVDKVEELSPDTT
FIHLMDDERSLLKASEATATPSPSPVGAENAPVLLQCEWNPCDPSMLAAA
GTDALARVWTICRSTDATTESAKDGRDHVAPLANTLIDPSTPRTTTVTAL
SWTSDGTAIAVATDLETSAAVSVWSADGVLMQTMDVAEPPIIKLSWNPSS
SALLAVSPENGGALITVYSFSGKVLNYHLPGHDIETTALDAAWIDDDEFL
LSGGDLLLCLRFTNSAISQSRKFETKEDDSFTQVLFDRHSKLAATSSDKG
ILDLWDESGQRRSISAHQGAITTMAWQPLPPNQAVPDDERLIATGGDDCA
ILIWNARKPESKAKCFLTMDLPIVRLAFTPDGAFIAGASSRQVLIWKVDN
HTMPRASWNRPPHPGWLSPKGSSDAEEEDEHCLCWDVSGQKLAYGSNSRL
AVINFRR
>Mb|QLI68056.1
MSEEKKIPKVAEAHLVDTFHPPEKMLANIEEYQKLYKESITNPNKFWGER
ARELLSWYRDFETVSSGTLADGDVKWFPEGQLNASYNLVDRHAFKDPDRV
AIIYEADEPQDGRNVTYSELLRQVSKCAWSLKQMGIRKGDTVAIYLPMIP
EALVAILACVRIGAVHSVVFAGFSADSLRDRVLDARSRVVITTDEGKRGG
KLIGTKKIVDDALKQCPDVTNVLVFKRTGADVPMQAGRDVWWHEELEKWP
SYIAPEVMNSEDPLFLLYTSGSTGKPKGVLHTTAGYLLGAAMTGKYVFDI
HDGDRYFCGGDVGWITGHTYVVYAPLLLGVSTVVFEGTPAYPNFSRYWDI
IEKHKVTQFYVAPTALRLLKRAGDEHVKGDISHLRVLGSVGEPIAAEVWK
WYFEVVGREEAHVVDTYWQTETGSHVITPLTGITPTKPGSASFPFFGIEP
VIIDPVSGEEIHGNDVEGVLAFKQPWPSMARTVYGAHKRYMDTYLNVYKG
YYFTGDGAGRDHEGFYWIRGRVDDVVNVSGHRLSTAEIEAALIEHHAVAE
AAVVGVADELTGQAVNAFVAVKDVDVVNDALRKEFIMQVRKSIGPFAAPK
AIYVVPDLPKTRSGKIMRRILRKILAGEEDQLGDITTLSDPSIVERIIRI
VHEAKAK
>Mb|QLI68057.1
MRSLGERLHLLRKGTPPPSAPVRPRPLPASNKIQQLINLRCGPGAAILPP
EVTRIHMDFATSMSNGHMGARKFWRQYLPRLKYHNPAVPMILNRHDDNAA
PPIMTIYMRASAAAAAAETSQPASSRTGLSKAQPPGADEKVVTIDMRNRH

SSDILEFFLAETRARALQPTSAEIAEIQSLEAIKTQGGVDRERVRALRAE
KKKEEDMLKRARAAGGMAEQEEA
>Mb|QLI68058.1
MSQDGQSQQSRVSEPFTIEENIHHLNAIDKSIAQLMSQTATALNALTTQA
SNTSAPNGETSSLDPPAQKEAFKSATDSFLTTLHSIDVRMKRQIMALEEA
GIVRLSNTPRQDPNGATKGSLKPNGVGTVGNLDVGWLNSRSTRVERDMEA
ELWQDAKEFLQKQQSGTGS
>Mb|QLI68061.1
MSHPRVEEVSDSDPDVASDPEEGDIDDFIDADIMRRIPQQQPPSRSSAPP
ASAPSNLPSQMLGPDADRSAYSSFQCIYPVYFDASRTLSQGRRVPASLAV
RSPLAREIANACSRLRLPTLLEPEKTHPKDWANPGRVKVGLKQTPQNPSG
IKSKHHLYILIAKHLQQNPTTENSPGLRVRMGGQVQPPEGKPYPKPAVPR
GWKMSEYLPYLSPAMTGGGVSENLFKDMMKEMQSGGDPMSALMAGAAGHG
GDEGSAGSGGKKKKDKKGKGRS
>Mb|QLI68062.1
MPPARLRSIYRSILRELPPRPVLASPRSHLHNRLRASFTDSSASSHAEAE
QLVAYLRSQRLYVTLVERYNPGMDMDEEERVRLTARRVGMDMPELYARGK
KF
>Mb|QLI68065.1
MVPQSKKRKVSPTTGPEPKRNKNLQKAGTRPKAHAKTSGKFKNKTKPTPV
GRAVLDPSALAWESVGEDFGGLQVLSGVDVVKNGEAVQFVVKSDKTNSQP
VVSDSEDEEESFEGFGDDIVAREPVDSGEAGDSQDSLEGEKSQETISAVK
QGKASKTQKGQQQSDNGTQKKSKQQKIADKKLQKSASHGAGNEFLALAAM
ADEEAETDDLDMGDWVALNLSPNLVSAIAKLGFSKPTAIQEKSIPEIIAG
GDVIGKAQTGSGKTLAFGIPIVEKWLDTHEEQEDQAGDDDDQPNKKNREG
PMAVILSPTRELAKQIGDHIKAVCDGLPASPYICVVTGGLSIQKQQRQLE
KADIVIGTPGRLWEVLDGDAKLQEQFTRIKFLVVDEADRLFKVGQFKEAE
DIIGALDRENPEENGYSDTSDAEDETRERQTLVFSATFDKNLQTKLAGKG
KSAKTGSDEEKMAYLMKCLKFRGEPKFIDVNPVSQMADNLREGLIECGAM
EKDLYLYTVLLLNPGRRTLVFTNSISAVRRITPLLQHLNQNALPLHSQMI
QKARLRSLEKFTAARNTILVATDVAARGLDIKEVDQVIHYHVPRAADTYV
HRSGRTARADRSGVSIILCSPDEVLPTRRLASKVHAERASSGKKQHIIES
LPIDRKIASRLKPRLDLAKKITDAILAKEKAHSNDAWLRNAAEELGVEYD
SEEFEATESSGWGGGSRGGGRRRKEKEAKALTKAEMGVLKAQLRQELSQR
VNLGVSERYITGGRVDVAQLLKERDNSVRGIFLGGDNLGLGLGL
>Mb|QLI68066.1
MPCFKGIAVSIHANGAPLPEYGMQKQSRVSRISTYIPVPQPNLTADSNKP
EPAKFAISITLLTAGLAIPYSAPKPTDSNPYPKPQYVGSLQPASGRDRPK
FGGMVNPYIPMTNSENETIAAYIYFDGRAKEEVATLLRPGEETWVNSRWV
QVPDAEGGGLAEREFLFREVGLERWLNGLDLQGHDAAEKLEKRRQKFEKR
RRRQTLVQEAGTGTDTSGGQKDTLRYGADDASPIEAVFDSDSWSDDDDEP
PEATGQIKVAMFRVLASGEIKKGEYSPQFDAHDGDEETGNGENVGADVEH
TTSFAKPKTLDPKTISTQTVTGIDGPDRPYAVFTFFYRGDRQLQKIGVVQ
SSKNPQATPGSAKRRSGQLDFSNLGPLKAGGTVGFSAFRDQTTETTRRRK
ARKKSNGNIAEDSDDDDDDDDDSTDNLNKMDDDDKDGEEKEASEDSKFGG
ELADGVNRIRLKRAHSADPDSHSTPETSQRGQAEDSPVPQVPPSLQPPSF
VQSGSDVPVDALIGSPLKKARPSVDVTNTGEKLSTTQSLSAALDAAVSVT
SAPATTSTTTTTTPPSKVDEEEEL
>Mb|QLI68070.1
MSEAEEMRVDPTRAQALISQLSSVKERIGGLAHGRDVRLVAVSKLKPAND
ILALHQAPASHRHFGENYTQELTQKAQLLPKTIQWHFIGGLQSGNNCYSP
ASHRQVHSGPLTHLPGHCKTLAKIPNLFCVSSVDTLKKAALLNTTRGALL
ASDPSLPKLSVHVQVNTSGEEAKSGCAPGEETVALCREIISNCPNLHLLG
LMTIGAIARSKATTADNENDDFIALKQQRDLVARQLALDDARLELSMGMS
EDFEGAVVLGSGEVRVGSTIFGTRPAKADAKIKE
>Mb|QLI68072.1
MPPRTRKNANDRQRKVKVVTEQHVIDKPSPVAEFPMREWSLKLHLLDEEG
NERPADVFTKVVYNLHPTFENPTQTFIKPPFLCSNEGWGEFEISIDCYTT

EKTKLASIIHDLNFLQEKYEATHTVTFKNPSQALQERLRETGPLPNDDDR
QKKKTVASKKGGQKYDYEKIAEALEKLDEEDLLRVIQLINENKGPDTYIR
SDVEVDNLAEAGEFSIDLYTMPDMLTAKLWEHLSKKGLVN
>Mb|QLI68073.1
MSGYPGGHNDHYDDGYGHHGGNAQGNADSYYQDDQYYDNGYDARGGHGGQ
NGDGYYDESGYYNADPNNPYHQDGGYYDGHDQYQDEYYNGQGGYYEQDYN
QGYGGHGGRHGSEEDSETFSDFTMRSDMARAAEMDYYGRGDERHNSYGDG
QRGYRPPSSQISYGANRSSGASTPNYGMDYGNVLPAGQRSREPYPAWTSD
AQIPLSKEEIEDIFLDLTGKFGFQRDSMRNMYDHLMTLLDSRASRMTPNQ
ALLSLHADYIGGDNANYRKWYFAAHLDLDDAVGFANSSTKGLRRKAKNKK
KKKNAEENVNEADALQDLEGDDSLEAAEFRWKTRMNRMSQHDRVRQIALY
LLCWGEANQVRFMPECLCFIFKCADDYLNSPACQALVEPVDEFTYLNNII
TPLYQYVRDQGYEILNGVYVRRERDHKHIIGYDDCNQLFWYPEGIERVVL
QDKSKLVDVPPAERYMKLKEINWKKCFFKTYKESRSWFHLLVNFNRIWII
HLTMFWFYTAHNAPSLLVGKDYQQQLNQQPAGSRHLAAVSIGGGIASLIQ
VLATLAEWAYVPRRWAGAQHLTKRLLFLLLIFVINVAPAIKVLFMPTKLK
QSGDVDVDYIIGIVNFIIAVITFLFFSVMPLGGLFGSYLTKNSRQYVASQ
TFTASWPRLKGNDMAMSFGLWLTVFGAKFGESYVYLTLSFRDVIRYLSIF
KPVCIGDSILQTYLCSNHTYILLGLMAFTDLIFFFLDTYLFYVLLNTIFS
IARSFYIGSSIWTPWRNIFSRLPKRIYSKVLATTDMEIKYKPKVLISQVW
NAIVISMYREHLLAIDHVQKLLYHQVPSEQEGKRTLRAPTFFVSQEDHSF
KTEFFPSQSEAERRISFFAQSLSTPIPEPLPVDNMPTFTVMIPHYSEKIL
LSLREIIREDEPYSRVTLLEYLKQLHPHEWDCFVKDTKILADETAQMNGD
PEKDEKDTAKSKIDDLPFYCIGFKSSAPEYTLRTRIWASLRSQTLYRTIS
GFMNYSRAIKLLYRVENPEVVQMFGGNSDKLERELERMARRKFKIVVSMQ
RYSKFKKEEMENAEFLLRAYPDLQIAYLDEEPPLAEGDEPRLYSALIDGH
SEIMENGMRRPKFRIQLSGNPVLGDGKSDNQNHSLIFYRGEYIQLIDANQ
DNYLEECLKIRSVLAEFEEMKTDNTSPYTPGVKNEIRTPVAILGAREYIF
SENIGILGDVAAGKEQTFGTLFARTLAQIGGKLHYGHPDFLNGIFMTTRG
GVSKAQKGLHLNEDIFAGMNAILRGGRIKHCEYYQCGKGRDLGFGSILNF
TTKIGTGMGEQMLSREYYYLGTQLPLDRFLSFYYAHPGFHVNNMFIMLSV
QLFMLCLVNFGALRHETIPCDYNPDKPPTDPLYPTGCANTDAVMQWVQRS
IFSIFFVFFLSFVPLIVQELTERGVWRALVRFLKQFFSLSPFFEVFVCQI
YANSVQQDLSFGGARYIGTGRGFATARIPFGVLYSRFAGPSIYFGARLLM
MLLFATVTAWQAALTYFWITLLGLTISPFLYNPHQFAWNDFFIDYRDFLR
WLSRGNSRSHGQSWIAFCRLSRTRITGYKRKALGEASAKLSADVPRAAIA
NIFFTEILTPLLQVVWTLIPYLFINAQTGVEQFNNEDINNVDDYLKPTAS
LIRVLAIALAPIGINAGVLAAMFGMACCMGPLLSMCCKKFGSVLAAIAHA
MAAIVMLIMFEGMFFLEGLNFARTLAGMIAVISIQRFIFKLIVAIALTRE
MKTDQSNIAFWTGKWYSMGWHSLSQPGREFLCKITELSMFAADFILGHWL
LFMMLPVILVPQVDKLHSMMLFWLKPSQQIRPPIYSMKQSKLRRRRVTRY
AILYFVLLVVFVGLIVGPVVLKKANVLTPDNFKKWYISLQPGKMRLMQPN
FLNNKNTHQSSETGTANPTYSYPSNYGPSSESGSAQTTS
>Mb|QLI68075.1
MGSRDTPGWRRLDVGVIGGGIGGMSVAIALRRAGHAVTIYEKSDFAGEVG
ASVSCAANGTRWLHEWKVDVAKGDPVVLKKLINRDWKTGEPVSVYDLEDY
EERWGYVYNMFHRQYMHSMLKDSALGEEGEGTPAKLLVNHKCKNVDVEAG
IIAFDNGVTAHHDVVIGADGIGSAVRSAIGIRPEKRPSEQSCLHVNVTTE
EAVRLGLVDYSQHSALEYWGGQEGKWDKIVLSPCNDGRLLSYYCFFPREQ
GDYASQTWGGADRPVEELLSPYPELDRQVFRHLEIGREIRPWRLWIHDPY
PYIHRGSVCLLGDAGHPMMPHQSQGACMAIEDAAALGILFDRDHFGGSIA
EALAVYDAVRLPRVTRVQAAAAKAAYNINERIGFSVNKSTTTYKVEDPKK
ILTIEEMNTYDMYKDIEQHLAQRRGEAYTERFVDGLPIGLELPSGVVVGA
>Mb|QLI68076.1
MSDVEKIYVTYNDVHKLCQKSADRLLGEFQPQLMIAIGGGGYIPARILRS
FLKRPGSPNIPIQAIGLSLYESLGEDTEVEQIGKKVTRTQWLDLTALGQM
ENLVGKRILIVDEVDDTRTTLEYAVKELEKDVEAARQRMGGDQPKTEFCI
FVLHNKDKPKKGVLPHDMIATRYEAAVTGDAWINYPWEAIDIDEHDRNA

ETKSGI
>Mb|QLI68077.1
MEPASLTSLPDDLLLDIVEYLDTARDTSHLGRVAKHTHRLVQQAGWKTFV
KTRFPSLRVPHGNGCSWSDAADTISYLDRCWDKRAIQLSLFRTQPQPRTK
DAPRQPRQAIDFHGIVDAHYMPSLDEEVVAWGAGEDLHVRWGRDATQTWK
SVLGASTSTKYAPGTGDATCLSIIERRAGLPEVVVGRANGDVQVLSAANN
KSFGQPTKTVIKLDGRQDAHSQTPFSISPGRLAVSCTEWQPDTDMLATCR
SSHLHLYNIPPERDADPKPLAYHDLSKGRPGNEESLIRDIKFLGRTNIAV
ALGASSQPIQYGTIRPTGVDFMPASRNPDACDIRDGRLELTAPGEVTSVW
SIQAVGHRANSNLLLSSWHDGSFRLMDVRTPSPYDAIYRDNFQPYHAGGP
LLVYGTERFVSGNNTAPTLRLFDFRFPKPYFHSTALPCSPREPRPRVACS
SGKPLDDTTAVWRCEPGSSRRCNWHAAMESPCFRQDTTLWLGHRGMDRVF
SLAKASDTSDKFYLGLRGAIAEAQLVLEQDVPTRRQSVPRCPAGWRVSAA
PNAAMADTGISLSTSSVHGGTHGDLHNGMPEMLYHYDGSQRKQGVRELLE
PPEGTRFDTAWRQQVAARHRGHSSRGL
>Mb|QLI68078.1
MGSQADRKNSGKTDISNLGFTPMVKVSGEWEVEKVVDAIPGDAPKSGGQS
PLAFFHMIERLKTTKREGWRRFGIERGESIADHMYRMSIISMFAPPSLAK
RIDLHKCMKMCLIHDMAELLVGDITPVDGVPKPEKSRRESLTMHYLTKNL
LGNKDDAAVGEDIRAIWDEYEDSKTLESQYVHDIDKMELLLQMMEYEKRA
EGALDLGEFAYVSSKMVLDETKAWAKELIEEREKFWGTKQHVDGVKGVEG
GVTEEKTKMQDEYYSRD
>Mb|QLI68079.1
MTSSRDRRVAKELQDIQADRDNSGVYAAPIDDVNLKHLKGTIPGPPDTPY
YGGTFIIDIRIPDNYPFKSPSMSFSTKIWHPNVSSQTGAICLDTLGSGWS
PVQTIKTALLSIRMLLEVPNPKDPQDAEVACMLMQQPEQFARVAQEWAIK
HAGAPRQELNLSQYKNSDAPKSDDASRYKGFSKDLVDRFVNMGFDVDAVV
DAFLFVGIDRNNGQDYELEEAYMGDITARLLGEQ
>Mb|QLI68080.1
MKFLKVGRVAIITRGRYAGKKVVIIQPVDNGNKPHPFGHAVVAGIERYPS
KITRRMSKTRQEKRSKIKPFIKVINYNHLMPTRYTLELEGLKGAISGETF
KEVSQREDAKKTVKTVLEDRYKSGKNRWFFTPLRF
>Mb|QLI68083.1
MPSRNDAAPPWVPPTWSPEPSMGVLPVITNPEDYPDRAPRSNAATAASAA
GVRALSAQLVAFYFRAPAKAFFRTRVDYLAYARTVHHQQLKSMLDAVAKD
GSASRMRVFLQHTWIWMRSTTPGVLTSAIKQQGWSVIPHQILPPLIANVG
VGAVLYTSYLHILGHLHEESAKATKRVYPPPSPTHTFTAGFLAGGLQSVV
AAPLDALQARYDHHDLMPSDGSSRPRSMWTFGAEKLREIGLRGIFAGWGL
SFAKDSLGSAVFFSMFEYVKAQGYYRFVTWYYGGLGQDIVDFLAMKRPSR
QDGDSQVTVIRPHYAIEPMFLFIAGFSASFSQQLLLHPLTHFQVKHWDHL
EDLDEKAARYRAARAANPDRPRRPWRMLRAYYHAYQETWAGCTAEAAAEG
QGLARWLYRGFWWNTIRQVPSTSAGLIIFELIRRKYGYGADEVRITKDGY
DILLN
>Mb|QLI68084.1
MARGSSSSGSRARPRFQLGWQRFIPFLGYHHVLMILIAVAIILLSLLLAG
CSSSSPLIPDIFLLSLYYQKYQAVPDTAQVDYNVVTAIANIAGSASLQAR
VGYFGICVNPDGGSWLCSNNATALAKEVSVDQDPLNLIWLAGQFKDMIVF
PYLIIIAIIFAFLCLLLLATFPGWHDEEDSEGSEREVKPFPSRAVSQVAL
AIIFIASIFVLVSVLWQHTASVAASVIAQDFGNGSVKSGVGTSAMVMGWF
SFTLLIIVTIGLLVMILSMRVLKQMMRD
>Mb|QLI68085.1
MGWVANATPEVDAQSQWATILAVCIVLSVLSIATVSCRLWIRGTARGLAG
DDYMSGLSMIFAFIYSILCIVQTKYGLGLPLKLRPKANIGPYSQVNFAGR
PIYQIGISFFKIALLISYLRLLQGTDRKVYRMVVWTTIVIVFLAHLGCTL
SLVFACTPVEKSWKPWVAGSCLQPGPSFTGYAVVTIVSDVVVAVLPIPVL
LKLNVRPEKKLGLIGIFMLGLFTTLCSIMRYVQIDRIQNGDGNSTMLVLW
GTIEFNVGNMVSSLPFLAPVCMKKARDYRSKYSGGSNSGPSRSRGLRNGE
RYKLSDRSHDKSAFVSANKSGSEENILDNGAILKSVTYSVQIDEGSKSRP

SGRRDDEV
>Mb|QLI68088.1
MEAASARAAARDRWSEFGHAPSQLQRFIQGACSPENYEPNLALSLEIADL
INSKKGSAPREAATMIVSYINHRNPNVALLALSLLDICVKNCGYPFHLQI
STKEFLNELVRRFPERPPMRPTRVQAKILEAIEEWRVTICETSRYKEDLG
FIRDMHRLLSYKGYMFPEVRHEDAAVLNPSDNLKSAEEMEEEEKEAQSAK
LQELIRRGTPEDLREANRLMKVMAGYDTRSKTDYRAKAAEEVAKIQAKAR
LLEERLEAFRPGDKMSDGDVFSELASALQSAQPKIQKMCEEESDDHDAVA
RLLEINDSIHRTVERYKLIKKGDVDGAAKIAAGSLIPSGPAKTPSSSGDA
ANELSLIDFDADAANDGNGIGNNQNNGGPAGSTSQASGLENDLLGLDIGS
SSSNFGQGGNIVLGFGANQNIPGPALLSSMTQDNSASGNVSNQTPSFSQF
ASFTSPAASQPGTPQPAVHQQPKPPSYQPASDPFAAVGAASALTSAPTPP
PQPAAVSNDDDEWNFSSSLPPAAPNKPKEHKGTINQGLLRTDFLAGRATS
ASNSIHITFAFSNNSAQPITELHFQLAVTKGYELQLKPQTGRDLAPKQSR
GVTQEVDIWHAGNRGHRVDSVKLRWRATYNVGAEKKNEMGEIPEFSLA
>Mb|QLI68089.1
MSHIDYALYESPVGYALFQVVHQSDAVGLKLKETQAAVNDLAKFGKMVKL
TNFSPFRGHVEALENINLVSEGIVSEYLKSVLELNLPKAGKKTKVVLGVS
EKNLAGAIKSEFPGLECETADTSDIVGDVIRGLRLHADKLLKDLKPGDIE
KAGLGMGHAYSRAKVKFSVTKNDNHIIQASATIDFQDKGVNQFFMRVREW
YGWHFPELVKIIPDNLTYAKLVLAIGDKKTLNDEKLHDLAAILGEDGDKA
QAIIDAAKVSMGLDIAAADLEIIAGFAEAVVKQAENRKTTSAYLEKKMGH
VAPNLQALIGTPVAARLISHAGSLTNLSKYPASTLQILGAEKALFRALKT
KSNTPKYGLIYHSSFIGKAGVRNKGRISRYLANKCSMASRIDNFSEEPST
RFGEALRQQVEDRLEFYASGKKPAKNADVMKSVMSVLGDGAGKDSDDEMA
DAPASAKKEKKDKKDKKDKKEKKEKKRKRDEEEAPPADDDAEKKKKKKKR
KSKAADEE
>Mb|QLI68090.1
MSSHGQQPAVPISITICGDGGCGKSSITLRLVRSQWTSEYDPTIEDSYSV
TRRIDGTTYHLSLTDTAGQEEYRGMWASSNLGADAFLMVYDITSRDSLDA
LQYFDDLINMEAETRLDNAARARRAGVNPGHVNTGTASGSKTVPPVKIVA
GNKCDLQESRQVPAAMGLDWARKRGCGFMETSARLEVNIEETFALIVRRV
VERRRLAEMGVMDNTEMNARGMTKPLTPLPVESDSEKRAPGLRGPTLSGQ
TSVGRGQGGFWKKLRCW
>Mb|QLI68091.1
MSSPSLPRGTWLLVLAVCLVLLPGRAAAFGAGNIPSIAQVEGHNWRHGDI
EDMLASVAFLHGKKWTSMLIKRTYFGNWLRDYSQAVDVGSLKGVNAATIR
ILVWVLSFMAFGYATEEFEVTEERLGCYRPEEHIDNPLGYADGVDARTFD
PRLRGPVQPVETEIDMKTGMKNYIANETGQWATSTGYIRFSFARSIHFGR
LYSCGERGKGKDENLCEALRCLGQALHCLEDFGAHSNYCELALRELGYHN
VFPHCGTQTEINIQGKRIYPIVTGTFGAVDFLHSVIGEANDHFTQSEVDE
VDVALKNAEGARSGPVATGDRGLFDGSGPSDFISLLSKLPDVGTGFASEA
KDLKEASEAQERENRQNQQRTRANNANVVPGMSPDFDPVKTAKRIYPILV
FRDKIVKAISRGIAKVPGLEKLLEHISETLTAFVLGLLAPFIRPIINSVT
KVLKDGSSGVITASSKSQLEPWENNRCSDPTHSMLSKDHFTNVLNSCAGR
VAATILQYAVPRVIFAWENPNVPVDEVVNDVIRAFHHPALRSENIEIQRD
MFQTVRMWADQHPRRHELERILSSESVKTGKNHILNGTKSGSSGGGHSHG
AFDALSQLGHGKVAGSLWSQVRTRDLDSMSGNDGEQASNYVSNSPAPETS
KMPTPPSYTSTSGGGEAASYMNEPNTFQAGYNAPPPPGQYYQGYGQQPPP
GGPQGYPGTQQYGAPPPGQWQQPPQGYGGPPPQGYPPYGQGQPPSWNQYP
GQGRY
>Mb|QLI68093.1
MTPSPLPCGQASEPRRPRSDPKVSVNIAEGSQSHGRRFQLEMSECVETKT
VTTTTRLTRKFPQVFVRDPTPLESLDTKEYPLAMKPTPPELLDFSYYVPE
DDQSEEQDIHCDDEKDTALTGSSQLVVTKPSRQLVTGAITPTAVKAEPSL
HQTPLPNRTSRQASRSPSEYYPRRTRQTRSSVPDSPSSSASQGGTGSAVT
PRTSQRPARSSTSHAESDKLRRSLTQLYSLYDASCSSGNTAAAGMPPSSR
RDVGERATRGGSGFLATPDTSEVANSAMDRSFQRLRSLNSERPQSLQDSS

SAPAVACDAASPAGSDSHTVFSSNVATPPVTDADPEPFGDADDSLQHSLR
TAIHHRPTIDVVAAQDASLPSPRLSPTLAASQLHSAEPDDGAQSSFQTSA
SDTTMPWVEGTQATDDSKALQLTSNSQLTKDRALRRLQLNDDFGPVVLDT
QTLLEAFDSMKTEMKTFMMYQFLRRCPRPTLRLIADTVNPTLKCDFLRQL
PLELSYHVLAHLDHRDLCRAAQVSRHWRNIVDRNETGWKELLDSDGYCMA
PGELHKAIVQGWGWQDPVGPLDYEHDLSMQGRLTSSEFELTRAFRSETAP
KSRSSKRKRGLNTYSASERSKRRAGTSQESSTTAVRDPRVETELKQHKSE
GPLSAANAAAAAVPDPQLGLPSLRQLHLFKSIYRRHHMIRRSWTSGEVAP
GHVAFPAHPRHVITCLQFDDDKIITGSDDTLIHIYDTKTGKLRKKLEGHE
GGVWALQYEGNILVSGSTDRSVRVWDIERGLCQQVFYGHTSTVRCLQILM
PTETSRDLSGQAVMQPEKPLIITGSRDSQLRVWRLPEVGSRRYIQTGPPA
QESDCPYFIRVLTGHTHSVRAISAHGDTLVSGSYDSTVRVWRISTGESLH
VLRGHSQKVYSVVLDHKRNRCISGSMDSLVKIWDLATGACLNTLEGHTLL
VGLLDLRDERLVSAAADSTLRVWDPENGRCRHTLMAHTGAITCFQHDGQK
VISGSEKTVKMWDVNTGECVKDLLTDLTGVWQVKFDERRCVAAVQRDQLT
YVEILDFGAIRDGQCREELGRRILLNEVEVGDILDGGV
>Mb|QLI68095.1
MPASTIPASQFDSIPDTIEAFRRGEFILVLDDPGRENEADLIIAAEDVTT
EQMAWMVRYSSGLICAPIMPSRADALQLPVMVSASQDARGTAYTISVDAA
HDSVTTGISAHDRALCCRFLADPKSTATSFIRPGHILPLRAAPGGVRQRV
GHTEAAVEFCRLAGKNPAAAIAELVEDGEPTPGQPIITRAGMMRGEANIA
FARKWGLKVCTIADLVEHVEKTEGKLKPKLNGA
>Mb|QLI68096.1
MFGPFPVSPSLTPRVDSGDISSPTPINLPRYQPFAQDDVIIGGDSQSLPA
LLEQTGRDSLVLGPAGLLAIGLDLKLDADESDLIPDTCCLPDFRQWDRFT
AEEARNQDGKDRFPLRNGNLSPGCQVYLERRRELSNTNDDAFRTVRRISP
PKGKQQARLGNTYEFFRCLELFTSFWDDPSRPPELPPSPELTTASDTSGC
IECDTQTHAEPPHAPTMGRTSSGHSMPPEYRQNLLNAFIKLVSYDFGCNV
SRARLEPRLHLNSPAGRKQRKSYTPSNCHFVFQSPMTRETARAGVVYGPV
AAVSARPTVNFTGPDAETAQSSDLAREVLAALITAQHRHREGREEVRFGH
DQWWTTQRRWGGGLGGPIGREVEQDVPPVDDRQQGKDLNGRRNGPAMKRA
RRTMPVYDNYRMVRPPASSWDRKAKYEAIGRVEGSVYDDIFVISSLFHHV
SILRVRVPTRLLEVLDGSPEPDLSKRSWGKVQAWKSAWYDFFDVDQRIAA
MRLVWAAVAYQMRKPPSRDQRV
>Mb|QLI68098.1
MATEDERYRQSSQFRLWSFSPATLLELREKTNSLAKQQITPRIDPVPEFL
TSDEEAQLVKFFTIELIRAAQFCELPTEIRSTAAMFLRRFYITNSVMTYP
PTELLKTSLFFGCKAEGFYIRLAKLAEKFPNTTSDQILAGEYLLCQGIRF
AFDVRHPFRALEGAILQLRIRLPEEETRINNAHARARETLKFSALVTDVY
FHFTPSQIMLASLLMVDPGLVDILCPRPAEGGDSDGDKAGGMRTDVYDKI
MTTIESCRAMLEDEPPERMTEYWGTPEIVKSMKPLRKKLQRCRDPDRADL
VALQRARREQAVAKPKKPNTTNDGAVFGDAESRDPKRRKVDNADDMFGPA
LGQ
>Mb|QLI68101.1
MADEDTANHFGFEDPNFAFINESAAATEASANPSSLAEADPSAQQQQRQQ
QQQQPPQPQQQQLYPPSTSTSNWDFQATPAYDNVDIGASTAPTTQPGVSA
PAWSFPVFDQSPDGPFNFTGFTPSDVNSNVVFGGPNAEALNTARQQAAVP
MSPALQQKLRNIAMPPHLQYSSPKSASSPDSANNDSKGGTASSPEQGDGL
KRDSRKRKVSSDPEDEDDVDDDDKPVKKTAHNMIEKRYRTNINDKIAALR
DSVPSLRIMCKSARGEDTTEDREELHGLTPAHKLNKATVLSKATEYIRHL
EKRNNRLLEENAAMQARIAAFEKLFMAGAMNGSISPMQHPPTPMQYAQDN
PQQFSSSPMGTPQPSNPASSGMIQVPDDMKRIISAQMAVGQPYPVPQQSF
RGGNPSMMRQQQIQQQHQQAQQAQQNGFFQGNPYFGKLMVGSLAGLMIIE
AVRESEASTETTEGRGLFALPLHLLGSVASSLDVHIMGYHALSSLKTLLL
LGTFLWIFVPSLFASSDSKSRKPQAAVLRKAPSLASSIQIRRQAWLTSIQ
TVWVPRRNFFLEAAALILKAMKLSLRNVFGTHVFHMLTGMTREQEIARVK
AWSIALDSQLAGGDVDICKSRLVLTLLASGTLPDTPIRLMLKALHIRVLL
WRVTSSGIVQAGVNIIAAELARSKWNEARQLNQLLAQLRRGDSSSVPHDD

ELPEHLVALVEQDCDDVLSPQVIQRAHNLAFNMDTTQNVTEPIDNMDSVV
DDVAISSPLDAVAAWWSTQVLHRVLGDALDRDAPSKPDTAEGAIDIATRV
SPIGSYAQMRAILARAVLVKQSRGAHIAAALQALKSDRLTGPFSKSNLIV
GHRFDDFSADFSIALRCAMAIAHLTRVASTPNAKLANVDSLNSIVRPENM
ACMSLLGFTSVMELMDYLLLHKDHDQSIEFILEKLAATLRLWVGGQFASH
CGIHAELRQRVVERCLAVTKSLVGMEIDTGYGSMSDDETVQE
>Mb|QLI68102.1
MSGAENTEPKVEETKPIETTEQATGSASDPAPAAADEKPVTSSAVFSMFG
GGAKKEKKDEDEDRGDTSGSAKAQREAAKAEAGEEDAPESEDVHFEPVIK
LTEKVETKTNEESEEQLFKMRAKLFKFSKESSEWKERGTGDVRLLKHKEN
GKTRLVMRRDKTLKVCANHYIVPEMKLSPNVGSDRSWVWNAAADVSEGEP
EACTLAIRFANSENANLFKDAFLKAQKENEVIFGKEEGSTAKSE
>Mb|QLI68103.1
MSSKRHSILPAIDRSGPKPPVNFSSSLTISENAILQGTHSITMQSETVVH
PRSRFESNLGSILIGRRCIIHERAYIGARPADLDTAKPGGVALGDYVVVE
VGTVIEAGDTEIGEGTTLQVGCKIGSGAKIGRHCTISQKSVIPPGEHLPD
YTVVYSDGLRRLDSRGVTDMRKLGLVKQIAVLKKMIPSNPDKFK
>Mb|QLI68104.1
MSLRSALVAVWAASATAQFIPSILGSRDVSGCPNTLAVSYPAPVAAKGWT
YRLVAKGFKKPRSIVFDDNGGLLVVDAGVGLYRLTVDQDKGETCVVMSSP
KTLVNSTELNHGLALSNDGKVLYASSSSRVYAWTYDSKAATVSDTNYTVV
ANMSDTDKTTRTLLMSQRRPGMLVVSRGTVNDQDNKARNPNSGHAQIRAF
DVSSQRKGSEPHDFMDGVLLGSGLRNSVGVAEDPATGGIWSVENSVDELA
RNGRDIHAGNPGEELNYHGVLDDPSTDKDRGGNYGFPMCYALWNTTGFPD
LGGLKPGDQFPGPDAETFTDATCAKDYVAPRLALQAHAAPLDIKFTKDGA
KAFISFHGSWATTNPVGYRISSVAFDTSKGEPSAQKSSTDAAVDVLSTPD
LAKCPDACFRPVGLAWDSQGRLWFSSDSTGEIFVLHQNGTSSGGGGSTGS
PSLVADKAAAWAVVLAAIVAGLFLA
>Mb|QLI68105.1
MRLLTLLAALQWVLVAVAFDKWNVWLDHSDVLGDQWLGDTQLGLLALRIA
EQEGRKLCILRPKSLKRDNAPMIMKTLNEVCREKSLVYMPGEVYNINSPM
VTTNIVDVKIILTGRLQWSGNIDHWLRVSMPIGFQNQSTVWHFGGHNVLF
DGYGIGTLDGNGQLWYNWAVSEGNLPHRPININFKGLSNSVIRGLRFVQS
QMWTMAISHSRNLELSDIYVNSTSNSRWSTLNTDGCDTISSDNITFRRWW
VANGDDAIALKGNSSFIYIYDSIFHGGQGIAIGSMGQFDGKYEYINNLYA
RNLTFVNTAHVAYLKTWAGVSRGVPPNGGGGGIGLATNITMEDIKIRRLR
QQPFFCWQCENYSGWAGRDCNSSKFKMSRVTWKNVTGTVNKDVKDVGSFQ
CSSAAGGCDDFEVSDIDVWVDGGDKLDNWHCENVHGNKGFTCNDPPPQKS
G
>Mb|QLI68112.1
MELLELVEYEPTTRPFQCDWESCNKSFNRKSDLQRHYRIHTNERPYTCTT
PGCGKSFIQRSALTVHIRTHTGEKPHQCQHIGCGKRFSDSSSLARHRRIH
TGKRPYKCAHDGCLKSFCRKTTMVKHQRRSHQRGLHPNDMLDDCTSESDM
GESPPTPGQTMSWPMQGAITHPAMAHSHPMHRAASFADFGHHVNQYGMSQ
QMGSRHSMPAEVHEYHAQDAGMQIVHRPTGMPQQAYYVIDQNNPGIATMN
TNVPHAYHIPRQHVERPAIDMTYSAGSMTSISSSPASFSPASGHSPAIHD
GMYTHQPPAAPTYSLQDAASVESQNNMVPYAQQMPQTQGSQPENEWGYPY
QSPVEVATIGQIPAYGSGVYDMYSGPKIEFDDPTMQLPSSRVETM
>Mb|QLI68113.1
MSVRIGAMRGLLRCNRQVRSSRYCSIRPSCAIRQYSSSGTLLSSKPANAS
NSSVSIRGGTYKTDPAWFNVPSNVLDATSRKLHLQKDHPVYITRQIIESN
FPSPTYNYYNEYNPVVSTHQNFDSLGFSPNHPGRALSDTYYINDKTLLRT
HTSAHQADTFRADESEGYLISADVYRRDAIDRSHYPVFHQMEGARSWDRN
KVPNGNIAAAVRADLKTLPKHNVEVEDPNPPYHDERNPLQDCHSAAEAQA
IGEHLKRSLENMVVDVFSRAKAAALREDPTFVDEPLRMRWVEAYFPFTSP
SWELEVYYAGDWLEVLGCGVVRQSLYNNAGVPSQLGWAFGIGIDRIAMLL
FKIPDIRLFWSQDQRFLSQFTGVSDNLDKLKRFAPFSKYPPCPRDVSFWL
ASTSPAGGNTKGNFHENDVMEIVRNVAGDVVEDVRLIDEFVHPKTGRKSM

AYRIVYRSLERTLTSEEANSFHESVREALVRELGVELR
>Mb|QLI68114.1
MHLCYAEIRRHTTEYKNIFHSSTITDINLHQDLASKMTTLLVYDFEAAIS
LGQFEDLQTIIGNAKLYKDLQTFKCLGDILLQYPIPAQVLTTTLKTISNE
IHRLEQFDAAKLCRYLRIILQTTVSVNDTAALQIIGQIMKVAHESRDAGT
LLPRADLEWIAAITFNHAIDYYALSEETSCRVWAAKSMELAEYLNDRGRL
AKTLRDRFGQLRFESEICSWQVDKAAA
>Mb|QLI68115.1
MGARAVSPGGALLRTSRMFSVPKPLPEPPSTSLHIGDHKSATMTRQYPQH
QSITTPLSSREKGDWGYKRPFPLKSTMTTSTPLIRVKKVDSVENVTDFAS
AADHSLSLEKFQELHVAMSVPRGKIPGEARSASLWPKSVFEEELDSTESQ
SGRSDDKRWKFRGPWLARMGEGEFVRYLRKTVHPKRAQFRALLKRKLAED
ITTSQNKLALEKGTPAPPKVEPRDITEAHFSEYLRTLRNDRTTLYSLVSE
FLDLAPLAVPIGLFQSFQHMNPPLESGSLYGNSGPPPSHPSAGISYLRTN
SYMENHPVYGPQGRRTPVLARVVYPRAGHKPAKLGVGGFVADVPIGDNEF
NIRYGRGRTQANKMLNGIAHLDTTTYGGAKAYIEPKTATVDPSGKVVLQL
RETHPEAQVIAKESKGQSHIYHDAETKAKQATQFQGDKSTGRRIERITDE
ILNDDSSKVRDS
>Mb|QLI68116.1
MGHTSEPDSGMKRAAEVSEAMLSVPGYADDSMFFTVRYGHKAKETLRKCD
YDELMETLNKMTALWVKSGGGGAKPEPGAEERHAQIMELRRHCLEIIKDF
PDLVRDFDRFHASSKAALSAVMRSNT
>Mb|QLI68117.1
MAPTSQQIPEGMENFVVGLIGMGDMGKMYADRLSAAGWRILACDREDRFD
SLKEQYIGKSNIEVCRNGHYVSRASDYIIYSVEAAIIDRVIAQFGPSTKM
GAIVGGQTSCKSPEIKAFEEYLPPDVHIISCHSLHGPGVDTHNQPLVLIQ
HRAPDEALRKVEAVFSCLRSKYVYLTAQQHDRITADTQAVTHAAFLSMGK
AWHANSQFPWELNRYVGGIENVKINTMLRIYGQKWHVYAGLAILNPEARK
QVAQYAESVTALYKLMLKGDLEGLRVRVYNARDKVFGSASNWGARPLIEP
SILSSFSLGKPTDAPPRPNNHLSLLAMVDCWAALGIVPYDHMICSTPLFR
LRLGVTEHLFLNTALLDETLKTAVEDKMYRSDDLEFTFAARGWAECVSLG
HFETWEKRFVSTQEFFQPRFAEAKVVGDQMMKKVLESYVENGK
>Mb|QLI68118.1
MGINNPLPASLKSECRKCGKILTSFVNPRQAFGPDKVIPPSILSNAKGFA
IITILKAGFLGSGRVGSGLVVARLADGSWSAPSAIAAAGGGFGGQIGFEL
TDFVFVLNDASAVKTFAQMGSITLGGNVSLAAGPVGRNAEAAGAASLKSV
GGIFSYSKTKGLFAGVSLEGMAIIERRDANEKLYGTRFTAQQLLSGAVPP
PPAADSLMSVLNSRVFSGMRAGSVTDDAMYNDMPVYEDRHDDVVWEGRRG
DAYGQGVQRDRTGSGLGQGEFGRPSRSSTWKDDVYDRQDFNPPSRSNTFA
SGQGYNDSNGGANEKRVGPGRPTAPKPKFGADKEASLKQNEAIAVFNFDA
DQPGDLGFKKGEIITVLKKTDSDNDWWTGMIGTRHGIFPSNYVKMK
>Mb|QLI68119.1
METRSSRARLRRTFRYPSDSDTPSALDEQEQETLIADLAAQNRQTNTSFA
TVLLALPALSTIPYVPLLLRGPPNPIIAVLSLTSLLSTAYILYKLPPPET
GIAPLDAWVQRGRVPAASETIRRLKRGAGPDKSPLEMYLPYLNCVLSGVL
VVLGLVLGRGDGSFAWIGMGNLPAIVYGVVLVAKVVMGSVDPERELGGLK
YGFKGA
>Mb|QLI68125.1
MTAPKDTELERVELGSLAGKVSLDIAPGASEAERDLFRTFASRDPEWVAA
QDKRLVRKIDTRMMPCLIVMYLLNFLDRSNLAQARQGTLEKDLHMRGTDF
NLATSIFFVGYLLMQLPSNIIITRVRPSIYLASAIVLWGVVSTCNAAAGS
FVHLIVIRFFLGFAEAPFFPGAIFLMSSWYTRAELTRRVAWLYTGNSLAN
MFGGLISFGVLRDLNGVHGIAGWRWLFIVEGVATIGVGIMSAFILPDYPQ
TTRWLSDEERAFASWRLLADINEMDDGKSTTVWQGLKLALKDYRLYLFIL
LAHMSLLSQTFQYFFPSIVQTLGYDPLITLLLTVPAWFATFLVSITVNWT
AAKTEDKSIHIFCLMVVAAAGNAIATGTTAPGARFFAMFLMPMGSIAAYT
IIVGWVANSFPRPLVKRSAAIAIVNMIGNTASIYGSYMYNKDQGPQYIPG
GSSNAAISFLVGVLALILRYLHKWENRKLERAENEAAANGTLTGRGPEGF

RYIY
>Mb|QLI68126.1
MSPNSDNAMARFLFAILKQKNLKDIDWNQVAQDPVLLQPITNGHAARMRY
SRFRATVTGHEPQKRGRHGEKDRVSKPSKRDHASKKDAIIKSESGVSLAS
LAQQYSPASLPSPYLGDNDDLNARFLTPCSDDMMSIRPAVIEKRRHRDSN
AFSPMMDNNSDFFTDSGSLTNSPAFSAFDAAYDLGSYSTVAADFQSHDPS
DLGGPQSVLDCGHEWNERFHDPQLF
>Mb|QLI68127.1
MAEKRYSGAPGSPVVVGKMNSQEADSDDRHAKPQHIEDGTMAKESDHLKR
ALSARQVQMIAIGGTIGTGLFLGTGKSLATGGPASLLICYAITGAIVFIT
MLSLGEMAAFIPIAGSFCSFAGRFVDDAFGFALTWNYWFNDAVSTAADLV
ALQLILEYWTTNFPGWALSLIFWVVLIAVNIITVRAYGELEYWLSLLKVI
AIIVFIVMGIVVNCGGNRDGHYIGGENWHIPGAPFVGGIGGFASVFVTAS
FAYGGTESIAITAGETKNPTKNLPRVVKNVFCILSVLLIGLNVPYNYPNL
SSKKTSTSPFTIVFEMTGAHAAGSVINAVILTSVLSAGNHALFAGVRLLY
TLAMEGHAPRVFGKLNRNQVPWVSVLATSLVAGLCFGSSFIGAGQLWSWL
QNLVGVSNQLSWIAIGVASIRFRAGLERQGKTHLLPFKNWTYPYGPWLSV
FLNSFLVLVQGWSCFSPEFDAVSFVSFYIELPVMLIMYLGWKLIKKTKWV
KLDDMDLVTDVHTADEAESETKKGWQSKTRRVLNWIF
>Mb|QLI68128.1
MADSVDRVFVHALNTVKKIPKTGASRPPPSDRLRLYGLYKQAMEGDVDGV
MEQPTAGPFLTAEELHRERDKWDAWNSQKGLSRTEAKRRYVEALIETMYR
YANTSDAIELVTELEFVWNQIKHNSPSSTDSSPKAPASSKAPRTFQEPLS
GTDGPMKILSPMSEQDEAELRSQRMMDLDDAVEESREPESQDQKSSRWQK
KVERAVTKLSAEVAALREQISTGREWRSKKERSFPAWFGWLLWIFLKHLL
LDCVILSIVLLWMRKRKDRRLEDLVRSALKIIREYVRNILPSR
>Mb|QLI68129.1
MPLCTSCRCLLRRALRPWQHFLSTSCKPSPAEMLSKPTWSVRSLRHPPPT
TTAAAPAATEKITSSQLHHLLRLAALPLPQTPSEENTMISTLQDQLHFVR
AVQRVDTTGVEPLRAIQDETEEARRENTITLADMQGLLDAEEHVGHYQRP
RRVRTRIEDESSEKWDVIGTAGRKAGKYFVVESQKT
>Mb|QLI68130.1
MDVEESPWADSGDSSQQISQTGPPSSQPAGSQATIAAPRPSRGPRRLVAQ
PTRLEAVEDPLGPLSALADDNDGTSDAPPVPPQKEQIVIRTTMPQQQQPR
RPIDPHNVNDDDFKSPGSPRAPPPVDAARPSSVQSSTQPSVSIEQASKPS
FHITVGDPVKIGDLTSSHIVYSVRTKTTSRAYKQPEFEVKRRYRDFLWLY
NTLHTNNPGSIVPPPPEKQAVGRFDSNFVEARRAALEKMLNKTAAHPTLQ
HDADLKLFLESEAFNVDIKHREHKEPIPAESKGVFGSLGISVGRGDKFVE
QDDWFHDRKVYLDALENQLKGLLKAMEIMVGQRKMMAEAAGDFSASLHAL
STVELSPTLSGPLDALSELQLTIRDVYDRQAQQDVLTFGIIIEEYIRLIG
SVKQAFGQRQKGFYAWHSAESELQKKKSTQDKLLRQGKSQQDRLNQVNAE
VGEAEKKVHQARLLFEEMGRSLKTEIDRFEKEKVEDFKSGVETFLESAVE
AQKELIEKWETFLMQLDAEDDESVFYRPPVYQQQSKPPGDTAIDRARARM
DEDSD
>Mb|QLI68133.1
MDLFSSSQPIRSLTALFIAWKGFLLAVSLGAAVAPDYDTSTSLFFDRMYG
HNITIPTIAARLTRWDALYFMHSTIRGYTYEQEWAFGLGLPTTVGAISRS
LSSLTPANYALEPLVAIALAHASHFVAMLALHRLTMILSGNAKLAFVSSA
LHILSPAGLFLSAPYNESPFAGLSFMGNLLFAVSLKSKANGVKRNVTMLA
SGILFGAATAFRSNGLASGLLFAVQAVDALLLFARAPSLCTLLSMIAPVL
GGLCVAAGSVVPQTVAWMRYCGSDGVGRPWCEKMIPSIYTFVQDHYWNVG
FLRYWTLNQLPLFLLASPMLAILIKSGLDLISKPQRITTASRNGAAESDL
NTFVRALAGSQALIAILAITTYHVQIITRISSGYPAWYWWVGSCLMDKRR
QGYGVAIVMFMVMYGSIQGGLFASFLPPA
>Mb|QLI68136.1
MALVLLGGAFAGLTIALMGQDSIYLQVVSGDPMEPQYKNAKRVLSLLNKG
KHWVLVTLLLANVIVNESLPVVLDRTLGGGVAAVVGSTVLIVIFGEIVPQ
SICVRYGLPIGGYMSTPVLILMYLMSPVAWPTAKLLDWILGEDHGTVYKK

SGLKTLVTLHKSLGELSERLNQDEVTIITAVLDLKDKPVSEVMTPMTDVF
TLAEDHVLDEKTMDNILSSGYSRIPIYRSGNATDFVGMLLVKTLITYDPE
DRIPVREIPLGAIVETRPETSCLDIINFFQEGKSHMVLVSDHPGSDHGAL
GVVTLEDVIEELIGEEIVDESDVYVDVHKAIRRLTPAPRARRIHAEAGAA
AVGRKPGDGAALVHLAENLDLPEPLVGSPGAMSEGVPERVAKTATFMKRR
GSAGPDGTPNNKPVPFKASLDEMKQQLRHLAPSNRAANPRNTRTNVFKIK
QGLSVTTFVAGDGNGRPVDGPVLKQDDVTEGDESTPLLNTISLNGGGQEN
GYGSDKSKKDKAKSKTPERQ
>Mb|QLI68138.1
MSTHHTHPDREYRRRDDRHGNPSNDDKPSRARPPSPTGRSERRSASPDRE
PHRTHRSHRHHHHRREHNRPRHTLEAVSSPVALPFSARMLSKSDYRTFEP
LFAYYLDLQKQKYLEDMDEREARGRWKSFVGKWNRNELAEGWYDPDMFAK
CTANYARPEGDVSGGERRDERSQKIPTGREEATPRAGNSDDDQDYGPVLP
SSDPRRQVVGARVPTFQDLSLRNELAEEQRAADREELRRARKADRTVQKE
RLEELVPRAEPGTRERKLEKKKEVNDKMRQFRDKSPGLEAGDERELMGGG
DELEEYKRTKEREHKRKSERQVQREEFERAKREEMEERRRAWQEREEGTV
SMLRELAKQRFG
>Mb|QLI68139.1
MPSAVSPALEAASAEAKGTKSPRSLNIDDFPHLVDASALDTASRILGVID
RYRLQKSKDDPVGAGEGGLKFLAVIYSHIKAGKAVPMCLPAFPFKSPNRS
AKVLGKNPDRAEELALSHLNGLCQAITDIYPPGAKLTIISDGLVYNDLLG
VPDKDVWSYGENLRLLAKAKGFTQIDFCRLRDLVPISLPNELDEMTYVAN
ASNFRRSLLNTFGNPNWEWKEVCQIEDVCLTYRGYIKFLETDLAEVYPLQ
EDRSKSRYKRGIEYIAKQMMYRGDAFASAVRQKYKDHVRLSIHPSTGANK
LSVSLLPTTTMFTTPWHCTVGYKLDGTVVSGMRSDFEKDETYELVLENGH
PSYYREKSDLFSWGTEKGGVTFEPIYPSGWLLKPAAGPGSLTINDVDAKK
VRSLSEISSPVIMRGFFKNPKKEAFIEKSKEFGEPLPWKFGLVLEVKDQG
TDTRGLNNVLSAEWMPFHYDGLFKTATQVNDKGEEVCVSVPPHFQFFVGA
TSSPRDTGFTLFSSSTMFFKYLPKWLTVEDLAKKTWAVSTSSFDATKLKG
LPLVISHPTTGKPCLRYHEPWPQTKTQFEATLVTLDDHDEASNQAICEAI
DATLHDRRVAYYHIWEKGDLVVSDNILMMHTRSDFTAGSDRELWRIHFD
>Mb|QLI68141.1
MASVNSSAFLNAFRGLRICASIPLRQIRTPILRQSRTLEAAALLPNVRAF
STTSPVLGTWLEPSLNRKKKMAKGRPRVATGGSTKGTTVIWGDYGLRMID
HHRRISAKQLKMAEDTIKVRLRGEKYRLYKRKCCNVGVYVSGNEMRMGKG
KGSFDHWATRMAVSQVLFEIKGRIHEQIVRDAFRLAGNKLPGQWEFVKKG
DAPVVGITKLDGITLEDLKRPRRQIAPTELLQASASTPVTESGSTSDASR
SP
>Mb|QLI68143.1
MDYIPMPYIEETSAAGRKTWDIFSKLLQERIVCLNGEINDYMSASIVAQL
LWLESDTPEKPITMYINSPGGSVTSGMAIYDTMTYIKSPVSTVCVGGAAS
MAAILLAGGEAGKRFSLPHSSIMIHQPLGGTRGQASDILIYANQIQRIRE
QSNKIMQYHLNKAKGTDKYSLEEVNDMMERDKYLSVDEALELGVIDEILT
KRTDKKEGQEKKTDG
>Mb|QLI68145.1
MSACMRPRPLGNVLHRLPPLSGPSPQATSRRTYAVHSTGAPRFQVFNRRT
KWLQKERAASRPQESRQADYLKDEVAVRVSERLLDINRHFPKVLDLGANS
CNLARALVRENPDPDPSTPTSPPLSARISELIAADSSETLLYRDSDHDFN
RKLHITRQVLEDEESIPFGPDSFDLVMSSLSLHWINDLPGVLSQINSILK
PDSPFIGAMLGGDTLFELRTSLQLADLERRGGLSPHVSPLADVRDVGGLL
QKAGFKMLTVDVDDIIVDYPDTFALMRDLQAMGENNAILGREMGPIRRDV
LLANEAIYRELHGNPDGSIPATFRIIYMIGWKEGQDQPQPLARGSGEVNL
KDVLEKKQ
>Mb|QLI68146.1
MAAERHPLALPPNLSPDDLDALSELSIVLAKVRAGIQSSNGLTTATGVTP
GGVHTHGQQLSFKDVPGATDGLKHKVQHARAQVRALPDMDRSVEEQNREI
KDLQARIEKQRALLETLREGGVTFGKRDRAASDDKMEI
>Mb|QLI68147.1

MSSSSSSSPYNAASLFGVKELVAVVTGGGSGLGAITTHALVANGAKAVYI
LGRREEALLTTKKNASNPDVVHTIVCDVTSKDSLAAAANLVREETGYINV
LYANSGVIKAATAPHDASDDIKSLQQKLWKPDMEDFTTTLHVNVTGAFYT
AIAFLDLLDEGNKRAIVAAKSQIILISSIASFSRTPLAGFSYSASKAAIT
QLTKQLSSTLAQYKIRVNAIAPGFFPSEMTQDMPYMKTEKDPREEGALHS
SVVPLERVGSEEDFAGVALFLASKAGGYVDGAIILADGGRISLLPSSY
>Mb|QLI68148.1
MVPEQDTGGPVERPRRNLSTSAPGPNTETEQIQDADVQMQQAPQDEAQNA
GKNQSTSQESPRRCDPALRSSTPAAAVLSRNPSSNSPSSSPTPEPVVTRH
SRRHSLHQSLPVEPPVTKATLSELDVSKIIHNPKLRHDINFDPELHFRPN
LDGEKGRKKQERANQFWRTLKEELTLFIIDRPAFHAKHGESDDWTLPLLL
KAVKEIIQTLVPQRDRQFLDEGFNIELLMQQFYKGIADLEKLALWLSQVL
KSHCAPMRDDWVDTMYTQLRDGNRDSDLDQLVTGMRSLLSVLEAMKLDVA
NHQIRCLRPVLIEDTTHFEQKFFVKKIQSRKVDITGAREWYREFERNYSA
TLLNATQTFGDMGVFFEALSRLVLPSSGEKRVPSTFLFDEERIMKIRSDM
VDAINLDICMRMYEDLERVARYSSRMFGRGCRPLDEDTFNSSTMPNSEFN
FNTPLSNSRPSSLALSSSSSSASSSPRSSLSMPSYVAPENCESRIKAKNV
YNSLVALLQSTPPSSRYCNRWQTIAPAMALQIFRYTNAPSEFLPTFEGKL
MDNVCRVDSQIYKEVEQAFHSRLMTALAGRVREFKALSGVSLFAVATGGR
VANNNHLSLCRDSESSLRDGQDEGGVEDMATRLAHLGVLHWRVWAQLAYL
SEPEDMSVDVPSHI
>Mb|QLI68149.1
MAFNLWAHQFCLACDKQVQVDGAAYCSESCRLADQEKTSTPSSQASSPAF
APAGYPWSTPSPSLCHRSSSSLSASRPKFYLSPAYDFGNPQAYGASSAAR
SLSLSSNHTASTFEQSTSTYAANLTPSSSHSSLCSMQSTSTTGEPNQLSD
KAKQELKAYAISFEHVRLQRRRSY
>Mb|QLI68152.1
MSRAVALNSSHRHEAQSFDIFDDRSLTPDKHAHVDATGNSDVLKELSHGQ
SQNRVTPAVLAKSPRRDPTSPENKAAKAQSNRRASRARRPQIDSETFEPL
GAVPEETSSLIDPEENLVAISPATKDDKVNGYHTKLNEDHLPAILDPKSA
LEGQVEKHLAGQFEWIMTYPAEASSQTANGPQDEYNAGSESRSQLYGCNL
LPVRRRRTSQTGSGIATDIYTLLATPLNKDSYPTPQTMDGTRSADSLVSL
AMSQAPPPPYPGTPLKAPEVVTMVEQAISPSLTIMSENSPSVVAGSSRTG
SFSIPRIEDSLEELDRLEEELEAIHDLTLPRGSTTANTQASEPNPVTPSG
VTPSAPAKSKEASVAGCSATVRARPSQKARPSLRRSASLTLRGKRRAQQE
SSESQKSESSVSRSQSSINRLSAPKHPIKSAKPPTVPKFELPGEAVARRL
KEQREARQAQQQAEAQRTQAAIPKPRVTNKPLPKPTFELPGEAISRRKRE
EREAKLKAEEEEQRKRREFKARPVRHSIGPSTLPRETLTSRARQSRASPE
ANGENDSQGAQRKRMSVGVVRSVPVPSQVGNSSQTRGRNSTIASATEESR
AASTSTGSAGGKRISVSLEELHVQRQRGKDIFTRDNCYTQDREKSKRERE
LAAKTAREQAAERSRIASREWAEKKRLRELAAKRAKATQEAGKEVKA
>Mb|QLI68153.1
MSPPGILLVTMTPKPGLSLSQFHEWYNNEHGPTRLRLPKIFPNGLRYKAT
SDDQPAFMAVYDVTAMNHLETETYTTLRENRSSREANTIGQVDVKRYFYD
LLYTKQSPLFTSIETLTDEEAEGIVTVAVSITVSEADGAGHQYQKWFVQE
HAELISKVPGWLRSRLFKTSSLEDPGKVVYFCLHDYTKQNGLGGAKHKAS
MDTPWRTEVFSKYVVSKGRQTWSLFYVFGSGPRDLASLSELPASAAFTST
DGKTTTTPGSDPVISSYVTTKDSLNIPYRLEGNPSPTAPTVAFSNSLLTS
LHMWDSFVATLKKNRPDLRILRYDTRGRHAIPQPPVPATLDMITDDFKLI
LDSLRIPKLHALIGVSLGGATTLNFSIKNPTRVGKFIACDFNATSSPANT
QAWKDRVAIAQGDAGQGIKKLAVQTVERWFHPTTMEKKNPAQEMTDMLAG
NDVDGFANSCTALWDYDMKPDMKSCSVPGLFVVGETDAKGAVVKAMEGFR
GLLGQGDAELRIVPSTGHLPMFEDPDAFYDAVKDYL
>Mb|QLI68154.1
MSGSEVPAATGGSNNASEFVRKLFRMLEDPSHQDVARWGKDGDSFVVVEG
EKFTRSILPKHFKHSNMSSFIRQLNKYDFHKVKPSSDSESSNPGGNVLEF
KHPYFRADSKDGLDNIRRKAPAPRKPQVTEDFTTSQHVSVVTEQLTATQQ
QVQQLQELYAEVSQANRLLVSEVMTLQKMVNAQKQAQYEMLNYLSPYDER

SRGIMGQSMNSNGSNDVEDGVPELRRARELLSSVAPDTVSDRELERLHDI
YAAPTESATLITPVTMPLMHDPMTDLSRYPVYPVGQTVGIDPFQPDHIHK
IPFAIPNEGTPSTTLSSETTISAPAPISSVPATTGLGTDRATPMWGPKKP
QVLLVEDDQTCAKIGIKFLNSMGCEVEHALNGVDAVTRVSNVGRDHFDLM
FMDIIMPRLDGVSATMYIRQHCPTTPIIAMTSNIRPDEVNGYFEHGMNGV
LAKPFTKEGMLKSVRTHLAHLLKDPPEQSEAGFMMSNVSYVGAAPPSLKF
ESSTPPGGNSSGWSPSHMGPSNADQWNGMMNGGNQYSVGRSSFTATDHDS
PPEKRQRLNVSQANYG
>Mb|QLI68156.1
MENLSFLPPRLTSSASSCSSLNDMSLRNRKLRLLVAATGPRDTSWAQALV
VRLSKNPHIEMRAIVDDVVPRLTQTIIVLENRSLAAGQCGRADDVEFFRQ
QAFDLVEWADLLVCVPLDADGIAKMLAGIADTFLGEVFRGWNIQKSIVLV
PGMSTHMWSNPATKRQLSKIHRKWNWIRVMTPILWHYEGQMNPKRVPNWN
GFNEVLGIIKNQADLLGLGRDVEIATSTAVVPDTNDVQIRSKLPPEIWTM
VLDHAGDWELAKALGMYTNLPMPTTWSLEPKDPTDPLKVYEHQLEWTVLT
GNPAAICKKLSQSPPNFQYVPALVVKLVIRFALIDVLAYMEANRPELFRA
FDGTTLPTNASAYYPRTDVLDFWKQSKWFRDEHLYDAEAVDGASKNGHVR
ILDWWWRRSGLPLRYTETALEQASGRGHLLVLEWWRDAAAQDEEIVLRPG
RSLLWASQYGQADVLQWWDASGIPVAHGDGVAKVASRWGQVQVLETWRRL
KGDNKLVFDAEVLLSSTIHQHVDVLEWWRRFAHGELEGMEGRKQPVEFRT
CNIEEALEDSIGDPDQNPARKWWAQYGLDLRMRNEEWLQTRYL
>Mb|QLI68158.1
MRSTLLPRFASHRHTIQISRSRQRCYSARVIGESQPLIKTVADLRAWHPE
TNVPDVQLCGWVRSVRKSSGARFVDITDGSSMRPVQVVVNKALAADIRPG
SAVCLQGRWHNAADMKQPSTEGSSVSDISSQHTQSVSGADGMEQGAGRPP
TIQELQVDEVKVLGVSDPQTYPIQNKYQTPESLRYISHLRSRTPLNSTLL
RLRSDATFILTQFFFNQKFQQTHPPIITSSDCEGAGEAFTVKTGSKAEFF
RDPKYLTVSTQLHLEALAQSLGDVWTLSPTFRAEQSDTSRHLSEFYMLEA
EMSFVNDMNEVMDLVQSMLKNLAQGLKGLRAANELVQNRLDSRDPAERLA
FADLVEQEELDRRWTGLVSPSQWPRITYTEAIERLQPVANQFEHKPIWGS
GLQSEHEKYLAKEIGFDKTNNAYLPVFITQYPRGIKAFYMLRSASSPPQG
ETVECFDLLVPSLGELAGGSMREHRLSELEDNMKLHGLEVPGKKQDRSSG
LGWYLDLRRWGCPPHGGFGLGFDRLLSYLTGVANVRDVVPFPRHYQRCDC
>Mb|QLI68159.1
MGSSSALRLAILECDTPQPQTRAQFGGYAGVFTALLSSAAKTLSPPQDLS
KMVTITAHDVVNDLYCYPALEDVDALLLTGSRHTAFDNDPWILKLVDFTK
RALETGRIRVVGICFGHQIVGRAVGAGVGKSDKGWEVAVTEVDLTDKGKE
MFGLDKMRIHQMHRDIVQNFPPDAIPLGGNAFCPVQAMYLPGKYITVQGH
PEFTNEIISEILFNRHTVGIFTDEVYEDAIKRAPVPHDGVAIGRAFLKFL
REG
>Mb|QLI68160.1
MDKLPRLRRKPKPPAIETSVDRTSSDTAESTFTTEITSQPSPQKASMRKS
PLRNLKLRVNAKRARTHSPVARLSSSPVVAVFSQDGVAPRSVTDEGSSVN
GRPRPIMPAFLNTSMHGIEAKFQELTWAERNRLAQGARPDAPTNFRWSSF
KQTDGLQRGVMDRYINIKPWNHNRVKLQVPEEEFDYVNASTIVLNPLSDN
SLPPLRYIAMQGPTLPSFNYVWRMVAEQTSSPAVIVQLTSMTEGGAIKCN
QYFPDSKEDATWDLNKDNIWNDNWRAQLSFESLEELANGAIEKRKLLLDV
EGEAEPRIVWHLLYTRWPDYGVPTVDDMDSFFELMTISRDHTEPSNPRII
HCSAGVGRTGTFISLEHLMRELDGGKLEATETNCKTLPDLIYDTVDNLRQ
QRRCMVQSEIQYRFLYEVMKKLWQDRYGAVLDGHDADRTGNSEPAAKRLE
VADSFPVSDDDDST
>Mb|QLI68161.1
MSTNVDEKSKDAHVRQTGSSSSSDNDPPAGFLPATAGVTNVDAAWKFLNE
HRDAEGIDAVNLTALRHKIDWHIVPLMFLCYTMQFLDKVILNYAAVMGIN
KDLKLQGNDFSNVATFLFVGLLCFEVPNIYFLQIFPAAKWLGANVTLWGI
ATACGAAAHNYQSLLVSRIFLGIFEATIAPSLMLISSQWYTKSEQAPRFS
FWYLGLGLGQIVGGLVSFGFQHMTPGAALAGWRTMFVVLGCLTVVFGICT
LFFIPDTPMQAKWLSDTEKVALLKHISVNQTGIQSRKFRVKEIFEALLDP

QIYLLLILSVVLLSVSSGVVTTYSATLIRNILSDEPPAWAPKKAALLNMPS
GIVSIFFTLMVGYGIRKKSHRWAWIIACIIPAIIGGALMSFLPKSNKAGL
LAGIYLVNAVVAPLTIFYNWTVANCAGATKRAFAAAIISGSFSLGNIIGP
QTFQARDAPDYHPAKLAVMGTQAGCAVTTFVLFLYYVWANKRRNDRSKET
EEQFLSPEVWASLTDKENKHFRYTY
>Mb|QLI68162.1
MPERSETPESDAGSRSNGDGDQPPARKRQRVRLSCLECRRRKLSCDRGFP
CERCIKSGTPDRCSYESRNGEVVNASSGIPPPFAQLDSRRFGIGGGDGPS
GFTSREDHERIRRLELEIAQLKNHLVRPGGTGSFDGSTVAATDSPATRKD
ENLAETEGVPRPDVQECIEAGSMSGDKGELRFFRGKGFRTRYFGPHNASM
AFVELTGLCPFMRETADEWLRPVILHDRKDRKRRMEDREALFAKQDPELE
ALLPTKEETDALVSVYLDQFEQIHRIVHIPTFRKEYAEFWKPDSKQRYAA
FTALILAMMAVTSCVHTHDSLKFIGMMSNARHWAEKWIWACDAWLSRQSQ
KHRKLIHFQIACLLYLGKRVNTIKKKRFWTSSGALIQDGISVGLHREPSH
MGGKITVYNQEMRRRIWTTVQEFDMQASFDHGLPTLLSQLHYDTNAPRNL
DDEDFDEETTTLPPSKLAKEYTFSSFQNLSRQSLPLRLELSRLLTGPLSD
IDYDQVIRYTNDLTQEIDALPSWDMNMEGSKEKKNPLIAYTLLHVQLRQY
IIPLHQPYLKLRKQNSKYQYSEIIYYNAARDIVLLHDKLYEQGIRSLNFL
REDALTTAINLCSVTMLQPRGSTNMIMINSHHTLKLIEKCIAMKEDRLLR
CGNNEPWGYSIMCAALGLLEAHLGTKTTEVAKSTSAERFVNLHYRLLANQ
EPPVPSENMPETPISGATTLSAPGLGPSPGLEVPDRPKSVTPFIFPPSIP
TVPVDPTTAPATAWMIGVGDQAQPFNMDPSLELLGLNLNELWGESWELG
>Mb|QLI68165.1
MPQQSVLMLGAGFVTKPTLDLLTKAGIPVTVACRTLKTAKELAGNNKLAT
PTTVDVLNDNALDAEVAKHDLVISLIPYIYHVNVIKSAIRNKKNVVTTSY
VSPAMMELDQQCKDAGITVMNEIGLDPGIDHLYAVKTIDEVHAAGGKIIK
FLSYCGGLPAPESSDNALGYKFSWSSRGVLLALRNAASIVQDGKIFNIAS
KDLMGTAKPYFIMPGFAFVAYPNRDSTPYAQRYNIPEAQTIIRGTLRYAG
FPQFIRCLVQVGFLEETPIKALESPITWREATQAVLGTASSDPKELEAAI
VAKAEFDSPEDRDRILSGLRWIGIFSNEKITPRGNPLDTLCAVLEQKMQF
EEGERDMVMLQHKFEIEHKDGSKETRTSTLVEYGDSTYSAMARLVGIPCA
VAVQQVLNGTLSEKGVLAPMTAKINNPIMKELKEKYGIEMKEKTVL
>Mb|QLI68166.1
MTAAWKAAGLTYNRYLAIAARAVRRSLKEDQRIAAERRGEMDLRFAKWSN
GKQGDAKDLGAANAAAMAESASS
>Mb|QLI68167.1
MAPAGETLLLKSTDFQETGSVHTAVSHTEDIHALQGQRQSVNVALSQNVE
SLQRFCHENEFQLPTVFKAAWGLVLGKYLGLDHVVFSSLVQDGTIDAAPA
SCQLLLDETVSILSLLRNLELQTKTLEVEKTPKTGDNSPLSDSQLVFQVH
QPGKVVAAVNRSSQSTGNIVLLVQASDSMLNITLSYSTSTLIEEEAVNIG
ATFAKAASLIVLNPNEKLHDLNLISDRDLASIWCWNEPQPRVIDSCVHHI
IQSRVEQQPDALAICGWDASITYSQLDDYSNRLAHHLLGFGVGPEVIVPL
CFEKSAWAIVALLSVVKAGGAFVFIDPSHPKGRRDDIISQIASTLVLTSV
AQADQWTDSSLEVVPLGPQLVDALALQVQPPVTTVSPNNILYVIFTSGST
GRPKACVVEHSSFCSGAVAQAEVANMDSSSRVLQLASFSFDVSLLEIMTA
LMHGACICMPSAASMNEGVAKLINKFQITWAFLTPSLVKVIDPRDVPNLK
TLILGGEALSKSDIDTWAEHVQLGNGYGPSECSVAAAGNPNLTPSTSPSN
IGHAVGGLCWIVNAEDHNKLVPVGCVGELLIEGPIVARGYLNAPEKTAEV
FITGPDWGPLTRQGAPRRLYKTGDLARYNPDGTIHFIGRKDTQVKLRGLR
IELGEIEHHISVHGLTQHAIVILPKSGPCKERLVVALSLKDFLPDTSKTS
NVRLIPEEARERASKQVADIKEQLSRHVPHYMVPETWIVVEAIPLMLSGK
MARSQVSKWIAEMDDETYDRAVGVQSQGQGATEMTEHEMQLQLVYSDVLN
LALSRIPLDRSFLSLGGDSISAMQVVSRCRSRGMAVTVKDIIALKGISEL
ALRVVHVANSAHDQEESFDTPFALSPVQRMYVATTHLASNGSSTRYNQSF
FLNVTRQIGDEELRKGFQMVVAKHSMLRARFMESQDGTWQQVVLKQVEGT
YNFVVHNVIAVEDIDAIIEDAQSSLDLIRGPVFAAHLFSVKSQGQFLFLV
AHHAIIDLVSWRVVMKDLEDAVTSGEIANHVSLPYQKWLQLQADNARDHG
SPGEVLPFNVTPPDMQYWGMEHQKNYISDTSEDAVKLSLDLTKSLMGSQC

HTALRTEPIDLILSILMYSFAHVFKDRATPPFFRESHGRESWTDEIDISE
TVGWFTTMFPLCTSATFEDDIVEFTRQIKDNRHAIPANGRPYFASRFLNQ
EGMEAFADHSQVEICFDYLGLYQQLEKPDALLRQEPRTMTANTSDIGPNV
RRFALIEITAEMISNQMQFSFVYNHNMKHMPKLKKWIASLPDSFSECIDQ
LSNATIQHTLSDFPMIKMSYEGLERLSNDILPQTGISFEQVQDIYPCSPM
QQGLLISQSKSTGVYKYCHTAEIISRSANSVDLDRLTSAWEKVVQRHSVL
RTIFIPSVGTEGVYDQVVLKHYRGRVSIFHDDGADSGNKFKNQAPIEFND
IEPPNRLSLCQTNEGRVFCQFEFNHAIIDGGSMPIILQDFILAYDGRTPD
RPAPLFHEYISYLRSLPKGPGLTYWMNYLRDIEPTYLPSIQGSSKDPQFL
EMEVNINMETSVLLDFCQQNNLTLSNIFHVAWGLVLRAHTGRNDVCFGYL
LSGREAPIRDIERAVGPFITMLVSRLGLENDVPILDILQKVQGDFLLGME
HQHCPLADIQHELGLAGQPLFNTVMSFQKAQEGAEEDESTISVVGLQAYD
PPEYEVTLDVMVSEADIVTTLGYWTSSISTEQAKNLGLIFSRAIDCIVKD
INSTVDDIDLVGGEHMKQILQWNADLPPAHYHCIHHLFHEQVLKQPDAPA
ICSFDGNFTYAELDDLSTKLAHHLVSMGVGPQVYVPFCFHKSAWTTVVIM
AILKAGGACVPMDPAQPVSRLEAISTMCSAKVAVTAPQNAHLLEGLVDVI
ISVDQGFINGLPTVAGIPCASVQPDNVAYVIFSSGTTGTPKGIQLYHYSL
ATFALGNNLIVKGNGPGQRVLQFAAYTFDVSISDILGSLMFGACICNISD
HDRMNNLSQAIRDVNATAIDLTATVAALIRPADVPSVRILQLGGEALTKE
VVDIWAGNLDTLVNVYGPAECSVTCSYSCNILDDTDLANIGRVVGGLGWV
VEPNNHDKLAPLGCVGELLIEGAILSAGYFGDAEKTKKSFICNPIWSRTQ
PGVDRRFYKTGDLVRYNSDGSLHILGRGDTQIKLYGQRLDLTDIEAQVID
HQPRLRQITVEAIVPKGTTRKTLAAFFVMDDTDDGSTVNSLDSTPISLTP
QLQKDLQGLQSSLALSLPRYMIPATYIRMSQMPMNSSGKTNRAALRKMAA
QFSETQWSDYSLENKVKEPPSTDLEKRLQQLWSAILCIDIDMIGAKDSFF
GLGGDSVAAMRLVAAARASNILISVMNIFKYPNLCDMALTVEIHETITRD
LVELDSFALLEPSSREETIEDCADRCKVDKDFIEDVYPCTPLQEGLMAIS
TRLPRAYVARMVFKIPPTIDLNRFRNAWQQLISLEPILRTRVILTNTNSV
QVVLRQENPWKEGLTLESYLEEDNGLPIEYGGLLHRLCIIDGLGQDSYFI
WSVHHALYDGWSQNLLFERVKQLYMGDAISKSPSYNRFIHFLAQEDTESA
NKFWKSQLLREQAPTSFPVLPSPSYKPRADHVQTLRLPLSRQKQSAITSA
TLIKAAWAMVVSQYSRSDDIILALTLSGRTAPVPGIMKMTGPTITTVPLR
IELPSRNTTVYKYLELVQNQAMEMMPYEHVGIQSLRRLCLEAEPDYELDL
KHLLVIQSLGEADGFLDLELLPTEEDDFNTYALIVQCSVEEESVCIEARY
DKDVIETGQMERMLWQFHHVICQLNDESHSSIMEGVNLVSPQDLEQLNVW
NSQKHESVDMCIHQVIEKQMRSRPVDAPAICAWDGQLSYQELDRLSNALA
THLVDLGVGPEVMVPICFDKSAWTIVTMIAVLKAGGAYVSLSSAHPLSRL
RGIIHDIDAGLILVAPQYAELFVDVVPRIIVVEPSWLTMLPTLSISAPSF
GRPDSAAFVVFTSGSTGKPKGVVIEHRSMVTMALAEGPSMQFDSDTRTLN
FAASTFDVSNSEVLTTLMFGGCVCVPSETERLNDLTGVINKWKVNWLFLT
PAMADLLDPAKVPTLQTLALGGEAIRQDLVERWATKVHLINSYGPSETTI
WTSNSHLSPGKQPANIGRGYGAHTWVTDVTDHNRLAPIGCVGELLVEGPI
LARGYVKNPEITSAAFIFDPLWAADSQKKPRRMYKTGDLVKYNVDGTLDY
IGRKDTQVKLRGQRIEPSEIEDHIRQSLPDVQHVAVEIVSREGHKEDRVL
AAFLQHGQKDQHLVHDTNSNLVLITEDLRKSMLHLQQLLMGSLPPYMVPQ
LFIMLYQAPTTSSGKLDRKAMREMAAKLSKAQLAPYMLVDVEKVAPSTPM
EKKLQSLWAGLLRIDVSAIGANDNFFQSGGDSITAMRLVAEARASHLSIS
VADIFRFPKLSEMAKAIQDVVFTPATDIEPFSLLRDEHSLDDIIQMAASV
CEVKVEAIEDIYPCTALQKGLFSITIRDPGAYIAQKVFQLPEALDFDRFK
RAWQTLADMHPILRTRILDTDLQVVVREEPAWQSASTLQAYLEHDLAENM
NFGRPLCRYAIVEGHFIWTAHHAVYDAWSNQLLFDQLRGIYDRDEIPKAV
PYSKLIEHLEKIDQSAAMAFWKGQLSGDKPTSFPEARSLTYKPRPNQAFT
YSMAVTHNYKSGTLMSSTLQAAWALAVARHSNSSDVAFAMALSGRNLHMQ
GIESVIGPTITTVPIVMNLDMEQTVADYLGNTQKQMTEMIPFAHVGLQNL
HSLDNYVDLKHLFVIQSAANEDAEGVLGMIPVPYDIAGFDSYGLVVECVQ
GTDKVDVEVRFDTAIISHDRIQKMLNLFEHLVGQLNQDESRSLPLKHVSF
FSPQDEEQVAAWNREPPQEKNETMHNLVQAQVSARPLAPAILASDSNFTY
AELDSRSTQLAHHLVQVGVKPEVMVLVCYNKSAWTIVAMLAILKAGGACV

AINPEHPPERLQAIAQDVKASIVVTQQQHRHIFQGWNVNVVLPTESLTNK
YPATGTFTSAGPENPAFVVFTSGSTGAPKGICLEHRALCASAQAHGKAMR
LGTDSRVLQFAAYTFDVSIGEIFTTLIYGGCVCVPTEEERLNDLAAFINR
MQINWAYLTPTVASFLQPADVPTLETLSLGGEAVTKENVAVWANRVHLIN
IYGPAETSIWSTALCGLEPDTPPTNIGYGVGALMWITEIANHDHLCPIGC
VGELLIEGPILARGYVALDETTRARFITNPAWAKGQGDRRFYKTGDLVKY
NFDGTIDYVGRKDTQFKLHGQRIESSEIEHHLSTDRHIRHAMILLSKSKS
GQQRLTAIISLESVPPSAARDMPQLIQAESAQLEVSLIRKKLAESVPGYM
VPSIWLVVDHMSMTVSGKLNRLEMTKWVEQMDEDVFSTSADAEDLEGSHI
PATVMEDKIQKMLCQVLNLQTVRLDRSFLSLGGDSITAMQLRAKCRTEKI
ELSTQHILRAKSISQMALLAKSTSETALAHPEILDSSFGLTPIQQMYFDI
ASNAEMSHHFHQSFLLRVTRRIGTLQMARAIEKLVGQHSLLRARFSQEEN
NQWTQRITGDVADSHRFSMHQLSRREEMMPLIQASQRAINIREGPVFCAA
LFTIGSNDDVGGSHQLLFLCAHHLVIDLVSWRHILRDLEQLLDSGRLSSP
KPPSFQTWATMQAEYAKELLPKDVMPLQIPPADFGFWGMLNQPNVFEDQL
ETAFSLDEETTDKLFGVSNEALQTEPVDIILSALIDSFHRVFADRPTPTI
FSEGHGRESLAPESDAAEIVGWFTTLNPLHVPVMKEQDELKILAHTKDLR
RQVPSSGYPYFCSRFLSPQGQQTFGHHTQMEVILNYQGRYQQLERDDALL
RPEPLAENEVHKDQGSDMHRFSLFEISVEVVQGKTRVSFAYNRRMHHQDL
IRQWVSESKECLGTLTNRLSVTELQLTRSSFPLLSLEDWELSQFQSHLSS
LAIHPSQVEAAYPTSPMQESMISAQQKVPGIYGVRNMYDVIPGPQTGPVD
HCRLMTAWQRVVDRHVSLRTIFVPSVSRKDSFDQVVLHTHTARISVIECD
DDNVSERMRSQEMIDFLEPVPHHRLSIFTTASKVMCCLDISHALTDGMSM
RLLMHDLYMAYESNLQLAPGPMYSDYIAFLQQQPASQDAIFWRQYLDAVE
PCHLPRPDAPLCSEREQRSVHVHLKEDFAQIQGFCKTYGLTVANLIQSTW
GLVLRRYTGCDDAVFGYLTSGRDMPIDGVMDLVGALIHMLLYRQVVDSAT
TVLALLQQTRNDFLQSLPHQYGLVPAIKEHTRGAAQGLFNTIMSMQYSNG
AGEESESADSKEPLLKFENKGGQDPNQYDVSVAVHVDGVSVEISIAYWTD
MMHDGQAAGLAADFSKMLTAIMAQPTMKIMDLDV
>Mb|QLI68169.1
MATTLRRRAPRETQPVDDGSPTREDSPAIEPTAHIHVVEKAPRATRKRRN
TFIFLLGGICGLLAAGFFAKTNDLIDFPEIGELSMDSFLDVLPAGLIKDM
RDLVQGEREVTDAYDSFSVGLKARSEGLHAHHPVIMVPGVISTGLESWGT
ANTSRQYFRKRLWGSWTMMRALVLDKELWKKHIMLDKKTGLDPPGVKLRA
AQGFDATDFFITGYWIWNKIFENLATIGYDPTNSFTAAYDWRLSYQNLEV
RDRYFSRLKSHIESAVEYDGEKVVLASHSMGSQVVYYFFHWVQSEQGGRG
GEDWVDRHIGSWINISGCMLGAVKDLTAVLSGEMRDTAQLNAFAIYGLEK
FLSKEERAEIFRAMPGLSSMLPLGGNAVWGDLNWAPDDRPNQNFSYGSVL
NFKVGANWTAPDKNMTVDGSMKYLFETTEDWYQKHVKNSYSHGVAHTAAE
VEANEKDPKKWINPLETRLPNAPNLKIYCFYGVGKPTERAYYYRAPDQPA
STRLKITIDTALTEGEVDHGVVMGEGDGTVNLLSTGYMCNRGWHMKRYNP
GNSRITVVEMPHEPERFNPRGGPKTADHVDILGRQNLNELILRIAAGKGD
TITDYVVSNISEYADKVKIYEEGEQKAAAKS
>Mb|QLI68170.1
MASTSTSPAQPQPPSQPPDASFRFPREYHFPAFFTPQPNLTTHHAQLTKW
SSLILSYAKHHRLFKLSLSSASDSALFSNIRINRRLVPADIRQVIDFMRK
DGRAEYAGGDKARDGDVVYVYWKKPDEWAALVEAYVEDTAQKGSVLTLYE
LTDGEGTRGTELHGMDSEVLLKALNVLVKRGKAQIFGSEDSLGVKFF
>Mb|QLI68172.1
MADASCSGPSPFKRLVDHQSRDVSHHQDRLVDRSAGQPHGSFRSVRHESP
GQDSFGAFMDATPSTIPGMSHDPANRLAVHAAALQQPVAPLHHPELSQQA
RASPVSDVSNWAADFNRFSSQQMRSPPTTAPSLPMQMNQHPMQMNFQSAF
GQAFGGPMYGPGAAGFMGPQAPVEADFDKEMSQWMSNHGGGNMSEVDAVM
EQMARELELNEATLPAQAESTAPQEILQVESSSRYTDLETPEIGNLSLQE
REVDHLPAAEVEQVIHDEAAAKGKSAVSEAAERLLESVQHENGEKWQNSV
FLSLMRDFRDGRKDIVDNEIRAMQDEEQPSDHQQPTT
>Mb|QLI68173.1
MALDEYYHNKIEAMKLEILKGQAALRRLEAQRNDYNSRVRLLREELGLLQ

QPGSYVGEVVKVMGTKKVLVKVHPEGKYVVDVSDSVDVAKLTPGKRVTLL
SDSYKLEKLLPSSVDPLVSLMMVEKVPDSTYDMIGGLDQQIKEIKEVIEL
GLKHPELFESLGIAQPKGVLLYGPPGTGKTLLARAVAHHTACKFIRVSGS
ELVQKYIGEGSRMVRELFIMAREHAPSIIFMDEIDSIGSSRVEGSSGGDS
EVQRTMLELLNQLDGFEPTKNIKIIMATNRLDILDPALLRPGRIDRKIEF
PPPSVEARADILRIHSRKMNLTRGINLTKIAEKMNGCSGAELKGVCTEAG
MYALRERRVHVTQEDFELATAKILNKHDDKEVSLGKLWK
>Mb|QLI68174.1
MAAYPSAFVVFDELVKDLFGSSPTLDLLHEHAEYPFAHEAGVFIQEDNSL
FITSNQFTDPKTKNRRIQITRVQLPKNGDDSVQCQEIHPDQVPMANGGVN
YKDGILFCAQGNQGGPGGLAFMESKPPYKSHLMLSSFYGRDFNSLNDVVV
HSDGSIWFTDPIYGFEQGIRPKPRLPCQVYRFDPDRNAVRAVADGFGRPN
GICFSPDEKIVYITDTDWIHGDGTTDEFRASHIYAFDLVNYSGQPFLTNR
RLFAMADTGIPDGIKCDVYGNVYSGCGDGVNIWSPGGVLLGKILLEGGAA
NFCFGRDGEMFILNEHRLWRAQLGSSTKGALLGI
>Mb|QLI68176.1
MGDPLARGTLLSFFLLITTLGKMADGHDHGDSHIPEGQTVSLEPLDTTLW
IHIFINMFAYGVIFPIGMVLGITKSRWHVPTQVVGSALALLGFFLGHAHS
GREFVSHNIHSVFANILQLLLIGQVVLGLYLKGHWEKGVNGKIRKLIRPC
HSIIGKAMPLLSWAQMIFGGITALGFCQGEHVGQCAAHFIMGGAFIAYGV
ILTIILLVGQIWIQRTGRSQEFFDSAVIAAWGCVNTFTEHRWGTAWVKND
WQHTTMGIIWWCAGLAGMWLSRDRDGNPKRNFIPGFVILVTGWAMSAHPQ
ELMVSAMTHSTFGKTLMAAGLSRIIEIAFVLKDKQSLSESGRSWNSFQYI
PVFLLYAAGFLFMGATEEQMNLVAQSSMDGVSYILIMYSLAFLVFLFTNM
LVHLYDRLANPDINTKDFPNGHSRASGHAAEEGQLRDAEQFELDGLMSDD
EGGEGQAMLRESLDRPSIDTPSTLGKNNHGRV
>Mb|QLI68177.1
MSRRQLLQELLASTRQSGLGGQPRAPASLPRRRTTSETPTCSSRHNFSTS
LPLRSAQVHSVNELPDRTFPRYLQSTKPGSSLLSLHWPRPPRNLLLVQKL
YAPDVTESVIKFAKHIRTDYPDVNLVVEPRIAAVIRNHLPFPIYVSDSRS
NTAGKIDVIATFGGDGTVLRAASLFKLHGSVPPILSFSMGTLGFLGEWNF
SEYRKAWREMYMSGSDVASVQDAAVPIDNGRDERMAANTPADVAGWERLR
GKSLGVGRASKILLRHRLKADIFDRSGDNINHQVSDTLAHQHKSSIAGTQ
EPSPSLRAINEISVHRGSHPHLAIVDIYQNGHFLTETIADGILISTPTGS
TAYSLSAGGPIVHPLVKSLLITPISPCSLSFRSLVLPLDTKVTLRMSPKN
RGRELDLNIDGKRCAGVLPGSEIRVEGEFVGRAGPGEEWHGGVPCIIRTE
DDDPWVGGLNGLLKFNHPFGREPPSDEFSD
>Mb|QLI68179.1
MSYTLYDGSIATAQDNLTSLKAILKKAETAPNAASLPDARIHEDMLPLSF
QVHVVSDIAQKMVARSTGAEPQKLENDLKTFDDFYKRIDQVQELLTKTDK
EVVNKRVGDLVPIGLGPGKDAELPSHAYVTGYAIPNLFFHLVTAYNILRK
EGVPLGKMDYLTPFIGKHVSE
>Mb|QLI68183.1
MDSSHRPQQQSSQPSPEPTALQPLHTHLPQSTSLPLRTASSTPVSSPGLF
SPSASRHNLANLPYTSVSECNTPAPIGSSPFLHPLQTHRVRETHKALIDS
DNITGRKSINQYEVIEEIGRGMHGKVKLARNLETSDNVAIKIIPRFSKKR
RLGKVMALSPQDKTKKEIAILKKIRHPNVVALLEVIDDPELKKIYMVLEH
VELGEIVWRKKGLPHICQYERRRVEREMGGPAPSAEEERYNQHLERRQAI
KELKRAKMAQTFAGPSDYWSVEHGAADEGSSVGHWSRISSHEDFALTDGP
SSPSGSRRSSAAPSRSMSIASTGLMPSELAEDISWVDDMETPGPLHSAPG
STTALDGTMHGAYTEDAPRRQFRERSPSMADSIISHMSSVDYNPYAHDPF
GEDYSFVPCFTFDQARSTFRDTVLGLEYLHYQGVVHRDIKPANLLWTKDH
RVKISDFGVSYFGRPIRDGEPDDTVSESEAKDFDDDLELAKTVGTPAFFA
PELCYTDVDSKHQPKVSEQIDVWSLGVTLYCLIFARIPFLAEDEFQMFRK
IATEDVHIPRRRLKPVDPSTSPAEHSLYKRQNAHPYRDDNDLEYEDVDNL
LYDLLRQMLTKNPEKRIRLRDIKRHPWVVHGINNVVGWLDDTDPARPSQG
RKIQVDEKEMSYAVVPLTFLERARSVVKKAVGRVMHPLVERSDSKSRRRA
TSSVASSAGDSIFKNGGANLHPGFERRPSLRGEDYFGSVAKDVLAGSSQP

AITAPSTATVQTDGAYDPLATVLQPVEGAKDHRHHQLWTAPGNNDASNGS
SDFASATACSRRFQRHAGHRKMAPQFLQLAPALPETQSTPVTPHVEVHPQ
LPRDGSRRSRALESQSEDNSRSASIERGVFMSADKRTNGSCSVSTATASG
TMHRHPGPIRSLDLGRGSRGGYNTPSPLSSSPQASSATYQHGQPRSDPNL
QNRFGFGVNAQQTAAAQGHGAKASVSLVDQSGFISMSPETIQAAVEPLCQ
SQILSPRKVEPANVPCPPSPGSQDWTQHPAAPPRDDTMTTTKSSSLESMD
GMGTPLTSPSETTSPLHTDPPKSASGRLLVFQSDPSLPALLSGASSVSAD
MEAEHLARSGSGGQTCRYEGSSEAITPPALVKEPASGFPMTSMFKHSQSL
GGDSSLHLQLEHDRTHTTEDMDSRTPRVDEDDDSDEGIFLMTKSKKKASP
TSASHSSRPFEAKRRDTGASTASDETAKKVSVYDEDSMRLRDS
>Mb|QLI68184.1
MDGMDYDPMVMDGEPEQPQVTISAADSTRVDFALSRTNLSFANSIRRIIQ
AEVPTIAIDLVEVEVNTSVLADEFIAHRLGLIPLDAKGVNELNYSRDCDC
EQYCEQCSVNLTLHARCTSDEIMKVYARDLIVDGRHASQVGTPVINDPEG
MGCLIAKLRKDQELKLTCIAKKGIAKEHAKWMPTSAVGFEYDPHNKLHHL
DLWYENNTDPQKEWPKSKYAEWEDPPQDGEPFNYDAVPDRFYFEVETSGS
MEPDQIVQGGIRALQQKIGALLKGLDPKKYGGEEPAEFDGPRSPDMNMDG
GTTPWQDGGYTTPYGGNITAYGGGNTTYGGGNNTAYGGGAGAYGTTPYGQ
SSWQ
>Mb|QLI68185.1
MREVISINVGQAGCQIANSCWELYCLEHGIQPDGYLTEERKAQDPDQGFS
TFFSETGQGKYVPRAIYCDLEPNVVDEVRTGAYRNLFHPEMMITGKEDAS
NNYARGHYTVGKELIEGVLDKIRRVADNCVGLQGFLVFHSFGGGTGSGFG
ALLMERLSVDYGKKSKLEFCVYPAPQTATSVVEPYNSILTTHTTLEHSDC
SFMVDNEAIYDICRRNLGLERPNYENLNRLIAQVVSSITASLRFDGSLNV
DLNEFQTNLVPYPRIHFPLVAYAPVISAAKAAHEANSVQEITMSCFEPNN
QMVKCDPRHGKYMATCLLYRGDVVPNDTHAAVASLKTKRTIQFVDWCPTG
FKLGICYQAPENVPNGDLAKVNRAVCMLSNTTAIAEAWSSLSMKFDLMHS
KRAFVHWYVGEGMEEGEFSEAREDLAALERDYEEVAADSMGEEELEAEY
>Mb|QLI68187.1
MSHSIHGSLSPRQGPASPASNRANDDNSGLTQSLSQLSLTNEPQTLKPLP
SPRNSWGTPAKPPPRRSSSASPHPRTPSRSPSNGPGSRRASHADTPPLLR
KASMNSLHSANGVAPGRPLARRSSSSHIISTSPANRTPPEEKPPLTARSI
ASDYFAAELAEYHGATPTLPTDTVVILNDAVYGHRFSRPRTSRSALSTIV
ERPERIKAAVLGISTAYVRLGGRHCQGHLPIHPKRNLPDINSIPFRIHKT
TRQLSLLSSAVTNVHGTKWMEELKLMCGSAESILAMGGKELQRPDISRGP
EHPAPQKLHEGDLYLCEESLNAMQGALGAVCEAVDTVFGSGPRKAFVGVR
PPGHHCSASHPSGFCWVNNVHVGIMHAALQHGLTHAAIIDFDLHHGDGSQ
AITWQHNARANAANKSHASWKRTSIGYFSLHDINSYPCEMGDEDKVKNAS
LCIDSAHGQSIWNIHLQSWKTEQEFWTLYESTYAILLEKTRKYLKNETAK
LRASNQVPKAAVFFSAGFDASEWESAGMQRHHVNVPTEFYARLSQDVVKL
AAEEGLAVDGRVISVLEGGYSDRALCSGIMSHLSGLVSGSHDKEQSTIKT
DQESSGLAYEMGQRIGAINLNAVPIMKPPPSMYDPAWWATNELDLLEEPE
EPLAVPRKPRNSTPPTYTSPTQASTARVADPIKMKNSLSGLSTSYSKVPQ
RPTSPPPPEVPWHIAAKELCKTLIPSDRQTDSCTAEDLNAEASRARRDRQ
SLLLGNQPPTPSGSERSLSRMSLRERKSRPIGPIDEEMSEVLPKTRRKPA
STPISSPAKAGRSTPRPAGAQSARKSSRRLSVASTNLSPPREEPPPVPPI
PATAPVPSRTASRPNSRAGRTQSSNKVPVKKTQAVPSRIDPAPKGGRNAA
RASTPAKGAKQSTIAADTPTPATGLPLNAVGNDAKSDIDSLTDGVKKIRI
NLITKAQRDGKQKAKPEQSVEPHRQLGQDSSIVKSEKPPTPEGMSSIPNA
PEQLPTQLAPASGLPEAPPTPSLSGGISTPVEEMHTSNTSSPTPTPSIPT
GSSPDVPHVTIQPASDASDVFIPYQPEGPEPVTMVQHEHLKWLPPNIQTP
SSVTPVHTPLPSATKTKNNLFHYTSGIRFAPRPDAELQGRPGSVDKPRTG
HEQ
>Mb|QLI68189.1
MAILSFTLSEEGVSAFRDALICLNKFSDDVSLEARKDSFVLTTLNSSKSA
YASFKFATNRFFSKYQCQPMGQFRDRFYCTLYIRVGDSLALEIISTTTLS
ALIPLSLISLFRSRSSSESQRDAEKQTLIDRCDITVEDGEGIKSRFIARI

VFRNGLTSTHRLPFEVAVPVHAKFNRQDAPHHWTISSRTLRQLMDHFGPG
IEYLDINTDGDHVNFTCFSEKTVNEDAVLKKPLQTSIAVETDEFDDIDVE
DKLHVVISVKDFRAIVQHAGITGNAISARYSLPAKPILLSYVGDALACEF
LIMTVGERGSNPAQKTKKGRKATAAAASGPRLEAASRRTSVAPNETTTNI
QPYQLSQSAPSIAKPTVPNPTPHPHISAARASASRIGAFDLRPSQKPPPP
TIRSESLFVDDDGWEPVRDEEEDVEQDARLEWDHSADPNPSALQLSRIEE
RHAASVGRQEAADAQTAPESTYLEPTQKLSDVKSLALFPD
>Mb|QLI68192.1
MKKRRTSAWKQNATLLAVSLAALRPAAAAAHAFPYVPTQILLPTACVSEA
TCSGADLAYVFSQGDDGRVRFSALNYSADMTAGAQPAPLTTDLPFLKDQP
ATTAFGAARTSNGSVVVYSGACDGAAGSLWSFSREANGDGGGAWSKQTTT
PGRSQQGSPRGPYFLGGTLAFSSKLSPAMDQPTVYTYGGMCRAPEASAET
WQSDANYTKAMVSLTPSTQAADTTYSLGVASTAGPRTPIAGFTLTQLPAS
VTNISGAVTQQAGFVLLGGHSQKAFINMSTAAVWNLPEESWSYINVGGPD
ASAGGENVESRSGHTAVLTEDGTSIVILGGWVGDVSNAAEPQLVVLQLGQ
TYSSWKWKVPREQPGGKGVYGHGAAMLPGNVMMVYGGWETSASGGSSKRQ
ASSGGALRFFNVTSMSWSDSYSNPRPGKIPTGTGSHDAPGAGSDAVQSRK
LGLGLGLGLGLALLLALLVLAFCMWRRRLNKRRDSRRDEAAQVMAQDARY
FSHDADEMTEREDAFSWHVAGHSGYQGASEKSMGYESLRGARATLDDGHG
RAITRKPVMSRSMRGGYVPAETRFNALASPPGTIHPILEDEEDEYHQAPQ
HGTEPLTPTSEEHSDPFVTPTATATAASVLSPPSNRSSTTASPEERRRYD
ADVQDWVCDVDAADSLLERYNSSRQSRRSPTRKNSTRSAALRDDESRSGS
NLSESNRSAADSLRRSQSNRKSGSFLGGMLLGGDHPKPSSSSSSSYNTAK
SGFGALQAEGPALLGRTTPYTAANAHDDEDLTAPSSPSKSKPRRSWLGSL
GRVFSQSGGSSPRASSENSAMRPEMEQYGGDYEPRAGLPGELLRRKQGRS
DWEDGPEAGPVAGGGEQRPENDWDIERAVEQRLVQVMFTVPKERLRVVNG
DAESQTDMEADDTRQKGARFKETDELQEPQVAELVDPDKNSSLSTVERDE
RGRDDRPYSEVEDPDLLHVDFAEPRLSHSTDGDSERRSSGAVFTAEAIRF
ERPRTRVLQMVDTIESRSQSSSPTRGQGHGEE
>Mb|QLI68194.1
MSGNLEKSGEERKASVVLHTAPDVEHIEAPVTWKAYLLCAFASFGGIFFG
YDSGYINGVLGSKIFIHAVEGPTVDKISESHTSLIVSILSCGTFFGALIA
GDLADWIGRKWTVILGCIIYGLGVVVQMITSPDHALGPIVAGRVIAGLGV
GFESAIVILYMSEICPRKVRGALVAGYQFCITIGIMLASIVVYATQNYDN
TGAYRVPISIQFPWALILGIGLLFLPDSPRYYVKKGQIQKAINSLSRVRG
QPEDSVYVQTEVAEIIANEEYERALIPSTTWFGSWANCFKGSLWSAKSNL
RRTVLGTSLQMMQQWTGVNFIFYYSTPFLQSTGAIDNTFLISLIFTLVNV
CSTPLSFWTVERFGRRTILIVGAFGMLICQFLVAIIGVTVGFNHTHPDPS
DPTKSLADNIAAVNAQIAFIAIFIFWFASTWGPGAWIVIGEIFPLPIRSR
GVGLSTASNWLWNTIIAVITPYMVGEDRGNLKSSVFFIWGGLCTCAFVYA
YFLIPETKGLSLEQVDKMMEETTPRNSAKWKPTSTFASTLGAGDYIEKPA
AEHV
>Mb|QLI68195.1
MPLTFPTPTIVYHHQYQRQHHHRRCICCLSVFVEHPRRFPQSHIQHAAWS
AGSFCFTPGPSSSLLLIFASLHLLYQGLHERAVSPARLYNITASRNQSLL
SISVSMTLDFNTNGGGEALINLNFDQHHRDQTAYAYSTTPATYPSPPTAS
GIMTNTRPWTFPVDMASSNPQATSTASPEDHQGSSPLSHASSPQSQHSPL
QQSPFQPAANPLTDWALQQQQQRHQQAAAAHQQPQQPLPAQDLAQFIQDS
ALLGFNAFPQNFQPSPLDYLPPTTQAALQANLLESPFTSMPPMEETAHAM
HWGSSGMDNWHDFQSTLQMDGLPRLDSVGSNSPTGTYLEVLSLPSSSGEG
WAMVDWSHHGLDHFQQAQNAAIFNPGQTLHLRTNSDSSEGANSMEFGSFE
EIPRFPYSPFSPESDGYADQNCNHNHRNCVAETRNHTLDTVSPTASVAPV
ATKVESSSSVRHSPGSGAGSVSPASTTTSRRNSGPRKSPITKTTTKTIIR
RSSNGKKDGTVEKKVGRRKGPLLPEQRKQASEIRKLRACLRCKFLKKTCD
KGEPCAGCQPSHARLWQVPCTRIDIKDIGYFMKDWKADYERHVDLGMSVY
NVKGFSSKETLMWITHGYGFALPVMVREVYVADEGCFSVEWVESTVAHQD
PIDFETRTEKLDVGAEGIRLDALQEYLDKHVDGPFEDFIDDHFEGTPFIT
EILKTAHRYYVKDRMPVIKKALKLVLAYNLTMHITMVENQGSEVTLDGQI

DDEDSKFYGRTVAPVMINFQIKCALADMWRELQKDILEELSALYSGVYSG
EKMKNWPTIFMLASILLAVWEEMQFDCHYRVPDPAAVNKFCHDMETTPVG
VIVGLFHAISQKLPAFTDWDTRQHGHLLHNNVSVCEAMTEVRQHVIKHEA
YLRTRKEQTKFDRYDFDSLSNKFLSKLVIRAN
>Mb|QLI68197.1
MVVTTIQLVSLLAGLISLAVYVTTRHIQLSQARRSRTWPKPPPFQAETRL
PQNEYYLLVLGSGGHTKEMLMMMDDGTRDFGNSHRRYLISSGDKMSLNHV
EDYEAELKATSENPGTYDTRVVTRARRVHQPLWSTPFSALRSVLDIFPVL
LSPPKNEVGRQLKYPGIVFSNGPATGFFVALAVHLLKLFWVVPDDRLRFV
YVESWARIATLSLTGRLLYYTGLADAFYVQHGEVAERYGLVNAGEMVFNS
RRARQTRPGLDRQ
>Mb|QLI68198.1
MNVKNWNDRADKDLFFTILSVKNIGVISGAEWTTIGNHMRTLGYGFTNEG
CRQHFQGLRRSQNKAEANGTAPELSRRVDPTLNPITRRPGPGRGRPRKPE
NEAVSPVDGGIGPASVSMPASTASAASASASAAAAAFHAVHAVHAVHPAH
APSITLKQEDTADQQRAQQSQQQQQQPLPQQPQQQPQQQPQQQQPPLQPQ
QQPPLQSLQPPPPPPSPPQPLKSESIVETTPEFTTPPVEALATAPATETT
DAADGTPVVEQDGDDDADAPEEPPTKRQRLDTQELEQQQPLDDEAVLALA
AHNGATESDYPEEDDNYGDEEV
>Mb|QLI68200.1
MSSSKRRKLDGQGPPQSPVSAVSAIAARRRQAASVAQSQATEPANLAEPS
TPSINSFSALQTLRPDRNVTRPPKKSSKQSVQLRNDGRRLLAADDPKAAE
RLPNNQISEDVQSSFSATLSQTISYSSFSLTKSNCRTKAGGALELTLDEG
ERFLVLGSFGIRVVGGEVTLAGATLHAAENIHWVSAPHCVALPVIRTIQD
TQLELHNDPNTSGLRKLGRLSPLFRRIWNEPSEAVDGIMAGGQTFQIIVS
SSDAPRRCAIQELASPPEWNKKLASLLSISETGQRTTFVCGPKSAGKSTF
SRLLTNRLLTSSNGENPVKRVAVLDLDPGQPEFSPPGTVSLVCVSNPNFG
VPFTHTAFDDPSNTILRCHSLASVTPASAPDLLVSCATDLYGTYQRSLRT
CPLIINTPGWILGTGLDLLVELITRIRPAEVIYMSEDGPADVVDVLKNAT
KNSFSMLPSQPSEFTSRTAAHFRSMQMMSYYHSHIDIENKRLRPPRVTWS
GHAISSARPFVVSYHGSNSGVFGIISYDYQCTPELLAASINGSILAMVEI
EGPEAFRQFTGTPEIRGDRDIAMTPEVVVTRTAEDLPFIPNPNDATLDPQ
YCRTIGLVLIRGLDLKRKCLQVITPTPYASLEKIKGEGRNIVFVHGRFDT
PYWAYTEDLFAKSSQEELVDKEVHITDEETSEDESREEKAQPGGTDHAMI
STIPWIEVLDGTQKRPVGSRVWRVRRDLGRHNTD
>Mb|QLI68201.1
MPSPHGLVVRPTLPDRDVNPATIEEAYVRFIFYCNPALQLDCDTESLRDA
FRTPPRSGGKSFDTFIIYDLVRKFYNKEIKTWTELTTTLGVEPPDPSKDE
SAQKVAQYGVRLKKWMNSMHVKAFFEYLMDIPNDYWMRIPTDPNPIASGV
RDGVALEDDMALRALLPHIRPKRGRKRPEADDALASPAQRQRLSPASAAE
DYCQGQSGPWSAHPDAHGGTPLDAQRAHNPASWGQNESLQTPVGRWPQSA
VTPTTRGSFWDDALEPRSAAAPTKGKVPNLRRGAKNVSSAWKTAGKDPGT
RPRGRPPINRTPVEGPGQHHAHGWTPTNETQPEHSYYPRPVPAMHPEQPQ
PQPQQQQQYHHQFPENQSRVTPAGQHDQHAPPYEGGSRPSRPSISLQVPE
RAGGSVRLATPPAPAPAPALTQAPAQAPTLAPAPAAPPPQPRQVEVNGSE
PNGQVPGDDSQYHDGWRKFAREAADAYEQPDQSHGPAGSGDLGDKDSDMP
DYYFDRMDDRTNVDVLTAYFTRSMTEADWRDADGNSTKTVSLEESAAMVH
AMLQTMYKTATSPQAFLINLAALAGSTTLVTTRPKCTRLSENDGQFRYKC
EWEYRFGHVKGGFTFDQVVPASMWAKTKRPPTPDEDRDRNQEQQQQEEEE
EGKFTKEYWQKKYEALTAELESRDRNLADLRDKVTKALGRNFM
>Mb|QLI68202.1
MEDQFGGRTDDDLFYDDFEPVDSEPIVVQEEAPYIQEPSPVAPVAPAAPV
APEPAQARQSKPRKSSPPAQAPNSNAPSPSPSPSSSQPPQSQSQSQTQPQ
SKGGNLSSSRFARKGNFNMRQQSTPPPPPPPPATKSPAPPKESKEKEHGQE
REKDTKGDKEKDGTPPPNAPTAPAKDKKPQAPSQNTAANAEARLQSGANP
RQKLTEEELAAKMEKMKLLSAEKTRQFERAEKDEQQHAQAYAKGMEEARK
RRAEEAERRRRGEEEKRKLDDERAKNRERKLKAMGMKEGGWDEGKEAILE
EEARRGFKGANGGIRGTKRGGLGGSRYSREVDDQPDVDRFLDDQQRSRGR

GRGGGGRGGGRGRGSGRGGFDGSLPKPNDSSAPAPSLNVEDFPALPSDGT
RKPTGPIAPVAYPRKAAPAPIPALPSPSPAGGKWDDEMEALDELKQQGES
>Mb|QLI68204.1
MGALKYVEELQKKKQSDVMRFLLRVRCWEYRQLNVIHRASRPSRPDKARR
LGYKAKQGYVIYRVRVRRGGRKRPVPKGATYGKPTNQGVNQLKYQRSLKA
TAEERVGRRCANLRVLNSYWINQDSTYKYYEIILVDPQHKAIRIDPRINW
IVNPVHKHREARGLTATGKKSRGLNKGHRYNKTQAGRRKTWLRHNTTSLW
RYR
>Mb|QLI68208.1
MSRDQILSPEAVDSMIADGDTIVIFEEYVLRLNSWLDRHPGGRLAILHMV
GRDATDEITAYHTDRTLRTMKAYRIGRKPMGPWTNRTPPIRGGVFQTGTQ
SESVPDLSDIQLLDQDKDFLDEGYGSGVSRVASRDQSHRSSPSPGPEYAT
GSQATLERAVPRAKASHVQDGVVKGKKPVNGNKVAEAQVALNPEEWTEWA
IQQGKVNDSRDFPSLDSDVQGAIAAKYRLLHRRVQDEGLYSCPYIEYGKE
MARYTTLFGLFLYTLCHGWYLTSAVFLGLFWHQIMFTAHDAGHRAITAVF
AVDTLIGLFIADFCCGLSMGWWKSSHNVHHLITNMPEHDPDIQNLPLFAT
CPSFFKSLRSSYYDFTFVWDAAAEFLVPYQKYTYYPVMGIARFNLYLLSW
LHVLSSKSSSLGSSKAWWIRPTEIAFMMCYWFLFGYLLLWRTLPTWTLRV
AFVLISHIVTMPLHVQITLSHWGMSTSDLGEDESFPQRQLRTTLDVDCPA
WLDFIHGGLQFQAIHHLFPRVPRHNLRKVQAMVKEFCADTEIPYSILGFL
DGNRKVIGRLEEVSEQVKMLVNCQKYMAETGESGLH
>Mb|QLI68209.1
MAATTNTNTYKFNHSMIRVKDAKESVKFYEFLGMSLVKKYEFPEAKFDLY
FMGYNGPQAVSHGNSHVNREGVVELTHNYGTENDPNYTVNTGNKEPHRGF
GHTCISVDNIQAACQRLEDAGYKFQKKLTDGRMKHIAFVLDPDGYWVEII
GQKSLEETENIKETDPNTYRMNHTMIRVKDAEKSLKFYQEVMGMTLIKTA
ENEAAGFNLYFLGYPGAQGTAQANREGLLELTWNYGTEKDANFKYHNGND
EPQGFGHICVSVDDLDAACQRFEDLKCNWKKRLTDGRMRNVAFLLDPDGY
WVEVVQNDKYSDKYSV
>Mb|QLI68210.1
MSALRQSDAGPGGNSDGHTSDNNCIPDILDLEAGLWQEAPFPRLPDDAPP
ELRDYLQDVENPRRVWAIYRASRRHDFQLLVERYIAQLRVGCGSPQCNTA
TCFTCRKRLVGKAPIRRYSPTSARTLAVYLASQDNPDRGICPYLRRSNSP
PPAVNNLIFSTQRPRSQTFSDLPSPRASGSPGPRKASVGAEAKPKQSSSG
RRCQVSIIESPGSDPVKPDAQPPGGDEGPSKHIGRDQGRHTVGSSKINIA
EKPVSRDYRSFAATTFGTVAFKMLEWLTPQGLRAISGQISDVCDPESTFT
QQQSNETTQEGTDLTSAPPPSLIRQSSSKSSSQPLITPELEQSHQALPEH
AGRTDESMLPRDSSRRKRNSKGSIKTATTSKPQKKTSLEPLPPPASAEGP
KPTGKSSSFHGDKRLRGPKLTPGVVGRTVPEIPLKPAFFENVPCLSPPPV
DEVDPVSSEHGDSEEDSSANTTIAVVRAQRRKQVPEDHHNASVETEECQK
NYPLPQALSQLNVELIDFICDVFQEDHTNEKHFFGPLTLMESHPRPQTRS
NRLARRRSNNQVKSTGVLSGKWRVFNEQTIFNVLSDPHLLVKSFTKDGKL
YDSQTLWYCMLRMTRAAPSLVLHSLWLAAKSLFAPPEGLNPARLSSKRLF
GECPNPALTNFEAGCIMAICLHALVAVAPCAEDSKTLYEMSRIRSYGYVL
GGQGTAARQPSSVCLEYDDVFSNELALRLARRLFCAITARNCFADIAEWD
GQVDEASFDVLGLLLNQLDLLGSGPIRILEFTQADRLLHETRVPTLLLDW
ARAVLLQEWNGRPEYSTDGPFHGALSLITTLYENRNMLLLGDIQFRVDYF
SERLDAMDIPVVWVGFASTRHRHHLLDHPYLFSPETLTSFFRSINFSRMS
KNFEESSSLKTRMTAIVDPGSLVTNAHHKHVLQDLLKTASSRYLILAISR
DHVLRDAFDQLWRRQERELLRPLKIHLGEGAGGEQGFDSGGVQQEFFRMA
MAECLDPNYGAFTVDDRTRMAWFVPGSLVEIWKFEMMGLLVSLAVYNGLT
LPITFPKALYRKLLGQPVEELYHIADGWPDLANGLTALQEWDEKDGLVED
VFARTYEFSVAAFGADVTLPMSKDKSSWPQGIPTPPARNSPAALEDEAPL
VTSENRDEYVSDYVRYLTDVSIRPQFQAFERGFRACLDAKSLTLLTPSLL
QSIVEGVQEIDVSELRRYARYVGWDSSHHTVKDFWSIVKRYDDRMKRKLL
EFVTASDRLPVGGMRNVQFTVQKNGEEEGSGGHLPTAYTCYGTLLLPEYN
DKEVLRERLGMALENAQGFGFA
>Mb|QLI68213.1

MFRAQAAGPFDDVVAKATDENLTSEDWGAIIEVCDKVASDSNGSKEAVQS
MIKRLAHRNANVQLYTLELAHALCQNCGKPMHREVSSRAFTDALLKLAND
RNTHSQVKAKILEKMKEWADMFSRDPELGIMNDAYHRLKQTNPTLQAPSA
PQKQGLTDADRQKEEDELQMALKLSLQDEERKKATAQAGPSTQSSGTNQQ
ESAPVPAVSAGTTAATVSRVRALYDFVPSEAGELEFKKGDVIAVLESVYK
DWWRGSLKGNTGIFPLNYVEKLTDPTPDELQREAQMEAEVFAEIKNVEKL
LTLLSASNTAPREEDNEEISKLYHQTLAIRPKLIKLIEKYSQKKDDFTQL
NEKFIKARRDYEALLESSMSHPPQHSYQQYAMRPAAPGQGYPGGGYPQGQ
PPQDPYYNQAPPSDHPHYQASSPAPNNFQSQGNPAPFYVAGAEVPSQAPG
AQPQQQQPQQYPPRDDTPSLGPPSGKQPAPVNPSSPPPQSFTPYSPQPPQ
QNAAPYSQMPSQQRPQSTYGAQELATSVYDSPIAPHNPNPAPFGQQPYHS
YNQPEQAPPPAPTGQAPPPPNSMSPAPLQPGGAAAYDARHGLPSQAAPET
HPSQPQYKPYVPPGDGTSPNDYYRQGNPY
>Mb|QLI68218.1
MLSQNRNAQGTEPPPPTGPLGHPLPRRASPGNSVTLKHHQLARDASLRAS
PGSSPAANPSNVTSSPRRNSSGESHETGQSDPKKWFDLSNQNPTATFDNS
AMDGMDVDPPFFQKESDSSNEDKPYQFQHPAPPRLATGQSSSADDYRSVI
DDLTVEIQKLKEELKRYKQKGPETLRKDKLFEIKYHGLPRRRRRELETTL
RDFAASIEDSPDASSSQRKKSLRHATRDHMYSASGSASKHASSSSGSNAR
PVDSAYASMSSAANSTGASLGRPSVGSRVRSDQKVENYLRDIPEGLYPRH
MILTDRDRKKLVVRRLEQLFTGKIAGRHARRARHRQTDGVEADGVSADVD
SQAQPHLAKQQPTPATNNAAGGGAAAAGNSEPSREAKILPPEQQSGHSCK
KSRSRDNGSACHSNGDQTESRGNGNSSGSGTNTSPTQPPVPDQRPTRVKD
LDPDRTQVPSENMEYIRHLGLVPPELITESKKDNLDVQPDAEGWVYLNLL
CNLAQLHIINVTPSFVRSAVNEISTKFQLSPDGRKIRWRGGRDGTRFSSD
SSNESSQRSADTEDSESGQRKRQKTGNSTADDVQSGGSSKNRSKFGPQVS
ASSDSFHYKPLFVHNSSPNGETSLDETLSSFGPIDDSNGDDSRWNLSGSG
TSNRRKRRHDGAIIYYSGAPFCTDLSGGPGDTSPATYMLSSGQDRQDSQG
QSSRPMLHRSTSGSSLGYRPLSERNHCIDTSVKMDVDSSDGVPDLTGDSD
SESSIPDSEFPWSSAQQYIEVHPLEPCGLGGVMPDDHFMVVVTTKRPMTD
ATASEYQSNLPRKSEEATEFIISRLASMSTSSPAPLATSQQNVYPVEIDY
MSGRIKRLAPVPLPPPVIFLPPFSTDTSSSGDDEFGSEDDDDALESSEEL
MSRAVNPHQSDGYPDGVDLSSGDEDGEEPDEDDAANGMYERMDTDDVPNF
KRPIVSAKRPSIGSAEAAPGSIRCRSKSASADAVFRTGGSSAATAGGVES
GHSTSDEES
>Mb|QLI68219.1
MDRGNGAGPGATAASRAERFEDEKRRIIESCFSKKDTDGSLLETYITHIR
TIEYSSHPSTPPPPQARTPEVEKARVIIVAVRKSGRVRMHKSKENANGTF
SIGKTWNLDDLTHIGSFTGPQVKPNNREWGGDTGFLVTLGKPYYWEAQTD
KEKKFFIASLIKIYGKYTGGKTPELAGFDQKELDQVLGAGRRPAASASRP
PPLEQTSSQQSVVSGSPAPSIPPVPLASPLVPPQISSAPDPARFQKSPVA
VRQPLNGGNSPAGSFESAISRDRPGDRPGPRWTNQANRSQDSVANSFTTA
RSEDTSSQPPRSRNGMNGPGAFGRFGDSREPPEAPQEPLPTPPPPQGPPQ
SEGKPPPERRRPPMDPTRPQDRDLVPPPLNSPTVRKEPVAPPPRSSDRIS
PRTDTSQTTPAPADRVMPPLTIPPLDNSRSESINLETTVEPTNSSTTSLS
VPASNDTGPVLEPPPEPEEDTRPGLGPMIRQKKSKGDIAGALWKAASAAS
AFRPRPGGAGERLRQAQLKPEGPDGITGVVPAPPRPATPDISKPVSGHVE
TPTESPKPAAGGSPASTRTSTAVPEVTVTIPNASQATTAEPDKGKKEEAP
KEEPRKSIVTGNDAKYLQSLSVDPSLLDDRSEEFGKWLDYFGWVPGRQMR
SHSAEEIKTDVEREINRAQAGGWLARFQEEDDRVEAIKRGIDLAMGECEE
LDNLLTLYSVELSTLSDDIAYIEAQGQGLQVQTANQKLLKKELESLLETC
AITAGDLEALRLAPLDNLRGLEDVEAALVILFKAMIKIDPSLGGIEPTPS
TKSSTNQPPVFNSDYDNMRIVQEKKQMYLQQSAFFMQRLVEFMSRQFDEA
CAGSKRALEGALSKKVDSSHYDAGRELLWQYSPLMLYARDVDLNNWNHLI
QIYQDKSHPLYKGQFQNVVSIWRKNARKTTGEEAELLFSSQVEKQQEGVA
TAARKMTVKRSQTLARALRSPLADSGNRANPEKSPTADSRSFPYEVFSGV
LDDLLPLVEMEQNFIIDFFHATTLEQADFTDAVAVNAPRDRRGGDLKRHR
LMEPDRDLARRVTRSMEVIFAFLESELQRLMEWVVGQDPLQGVGVLAALE

RKLSEISQSNQDYLNTSLQKLHSLLEGRFSKFVEEQIKAIEETKVKINKR
KGVISFFRVFPAFMSAVENMMSGLDTNSALRRMVDREYDRILKSMFDSLM
VIARERPAVVGVSTGPADPEDKEALNFHILLIENMNHFLEETDTRGLEVL
EEWKDQANTEYHEHMDMYLSAVMRRPLGKLLDYLENIEAQIQSGKSPAAI
AQQPSNDKAIFNKILGNFDSKEVRKGVEALRKRVEKHFGDADDPALSRAL
VAKVTSECEKFYLSVETRIGQVTTDVYGGDVPFEWPRADVKLAFR
>Mb|QLI68220.1
MFVRKRDGRQERVQFDKITARVSRLCYGLDTEHVDPVAITQKVISGVYGG
VTTVQLDDLAAETAAYMTVTHPDYAILAARIAVSNLHKQTKKQWSSVVSD
LYHYVNPKNERASPMISKDTYECVMRHKDELDSAIVYDRDFNYQYFGFKT
LERSYLLKLNGKIVERPQHMIMRVAVGIWGDNIERVIETYNLMSSKFFTH
ASPTLFNAGTPQAQLSSCFLVDMKDDSIDGIYDTLKTCAMISKMAGGIGL
NVHRIRATGSYIAGTNGTSNGIIPMLRVFNNTARYVDQGGNKRPGAFAIY
LEPWHADVFEFLDLRKNHGKEEVRARDLFLALWIPDLFMKRVEKNGDWTL
MCPNECPGLADCYGEEFEALYEKYEREGKGRRTIRAQKLWYSILEAQTET
GNPFMLYKDACNKKSNQKNLGTIRSSNLCTEIVEYSAPDEVAVCNLASLA
LPAFVDYDEGRYDFKKLHEVTQVVVRNLNKIIDVNYYPVQEARNSNMRHR
PIGVGVQGLADAFLALRMPFESPEARELNKQIFETIYHAALTASMELAKE
QGPYSTFKGSPASEGILQFDMWNVTPSDLWEWDTLKQQIKEHGIRNSLLL
APMPTASTSQILGNNECFEPYTSNIYQRRVLAGEFQVVNPWLLKDLVDMG
LWSDAMKNRIIAENGSIQNIPNIPADIKALYKTVWEISQRQVVQMAADRG
AFIDQSQSLNIHMKDPSMGKITSMHFAGWKLGLKTGMYYLRTQAAAAPIQ
FTVDQEALKVADASVGKDRLPKKRTAPAGTSYMTSVSAVPRAAHLRKEDD
INAVNANGIPTPSNTPPPAGASRPIASPSKPAAIKADVDEGDSPKVLPTE
PSEVIKDEELAEPGTKRSGQSEDGDDSGERERDIYSEAVLACSIENPEAC
VMCSG
>Mb|QLI68223.1
MERPNDGEVHPPRMTRAEYTRNVRAAIEERRHKAMVNRQHYQPQIHEQIQ
RLFSDYEAPFYARMRGFGRTQTLALAESTVLGLAAGTDRNLSDTETQALT
EHFLSSVHNMLAWKWVMTGLAGYMAYRGRQTMRFPFFKPVITGGRFNPAT
GGPQVRIMWHTARFAAYYGTFWLIGEPIFQGVNFLRQSYSMDKDPRLASL
LRDGKKQTGEVLGAVQDSNSQQYGDAWRPESQQEYSSQINDGYQRPSQPT
QAQEQQTWASYRRPEPAQQSPRGWDAADDIDDASPVAPSPRSQAEGAQSY
GGSAWDRVRQQAQYSPRQTRRDQKSWEAPQGANSAWASDTDASAQYRGPR
DDYSYSSTDEEKSTAKVQAQREFDALVERERRGADQERGSWGK
>Mb|QLI68224.1
MASRTEQDHKTLMRRALALAEKSPPKPSNFRVGALLVNLDDGRVVAEGYT
LECDGNTHAEECCFIKLAEQHATKEEGLVDIIKTPHALYTTMEPCFKRLS
GKLPCVERVLRQKSWIREVYLGVQEPEKFVGQNPGRGMLKSAGIKVAAVP
GLEDEILEVATAGHASDA
>Mb|QLI68225.1
MEGDEAYGTGFLMDEPEDYYPPSPPPTKQIFGMQNGKQVTLHLVGHSPTE
AHHLWNGAKFISDYFEQDPSRVEGKSVLELGAGAGLPSLVAGILGARKVV
MTDFPDPDLVANMQKNIDECNATVEPEGHVARTIDAAGFVWGADPEPLLA
RLAPACQEGDGGDGMQGGFDVLVLADLLFRHSEHGALVKTIKETMRASPD
SAAYVFFTSYRPWKQDLDMGFFDVARNAGLEVEQVSERKLEKPLFEGDPG
DLDVQKTVKGFIVRWRVEDCNS
>Mb|QLI68226.1
MASAYSVYHGASNGSPYTITAMGRWSCLNKTLPPADKIKALHVYDFDNTL
FKTPLPNPALWAGQTIGLLATQEAFTNGGWWHDNRILAATGEGLEKEEPR
AWDGWWNEKIVQLVELTIKQPDALCVLLTGRSEKGFSELVKRMVAAKGLD
FDIVCLKPQVSPTNQRFQNTMHFKQLFLNALMETYKGATEIRVYEDRPKH
TNGFREFFSEYNRRQNASPTRGPIDAEVIQVADTSTTLDPVMEVAEVQHM
IKLHNNSIMRQPMHLRPNRLQIKKTVFFTSYMINSEDSKKLMKLVQLPSG
LAGNELKIHANNILICPRPCPSSILDKVGGMGSKMLWEVTGTACYDNSIW
AACVRPVPSTAAYHTDNPVPLVVLALRKGARPVDAGRIEHWQPLAAGQSF
VFETTVGEKVILRVEDEDPRKDEYESLFANRHSKRKHVGDDGRVANSTHG
QYGGRNESRGFHSGSRGGGRGRANPSRGFRGGARGAGRGGGRGRGAGYHY

RSLDDVEPKNQQPGYGQPVGYDDAHPHLNQPQSQAPPTGPKASLGRGGSY
QGRGGGRGGGRGGGRPMGGGQAANNTDLQNYY
>Mb|QLI68228.1
MTFSFSAREFALGFAAGCIVTIALGAFLAFALFRPKDIYGLGHWKLNVRT
PFRSLWMNLGFWTTEDGRHIDHFDVATRALLEKIVRAAGLGVENQPARAA
SKTKHSVAVLDVGFGCGDQTIALAELIQASSRPQFRYVGLTLNAEQLQAA
QERLSGALALEGGRENAIGLSKGAVKLFQADAAKPESWSSAVHASVDSLA
DEAFSERWLMALDCLYHFSPSRKSIFKLAAKTLDANVMAFDLILNENAPM
WQIIAVRLLGFLLSCPLYTFLTEEQYRNQLIECGYDEAQIEIRDISDHVF
GGLSGYLRKQEVALSRYGISLAGYNLTGKVFAWFDRTRVVKAAIVIGRTK
RNA
>Mb|QLI68229.1
MPDLNSVPPSPHSLALSRQQSSHQMAPPLSHPMVSSPSQSSSINILPSNQ
NAVSHSHSRSASLSQPHGASSSASPQLIAAQPLQSQLPPLSGDSSVGPGP
GPLRHPRPLTASELHMQLEKEQEAVVNRLTRELSLLRAAQNASVVSNASS
TSASASAHDPITESSLLSGSGFTIPTARRHHRNSSSASQNHGLQQLASSY
EARHPVPRPAQSIPLSRQSSHSRISRTNSPGPQQSALDPSSYFHSQRVPH
APSVVMSSVAATPGSGSLTDQMSPGLMPATMRYEETAFYRNELESAKKEN
ETLKRRIRDLERMVQTRRSTDVARPRSDSVSTTASVSIAPSGGVSIAGPR
DSLIPQRPERERGLTSQSVTSAAGSVAVGVPDDEVKVGESAASSGLVGQR
QQ
>Mb|QLI68231.1
MYEDSWYSFVPEVPKKPAAPSQSQSHGRKESLLQQPNNSLHLSQGDVKDE
RTERLDHVFEELEDTNTPPEPTIIRRAASYTDFYYVVRAQLTKDGQRRRK
KKPDKKSRAWEALLLKGEDDADRNYSASEPLDQAFERQLLEESQQEYLLY
CDQLSLTERHLDGLIDDADATLALLTSLSDSFQSVDAQTTTFQAQCEGLL
KEKQRLKTLADEVETDLYYYLYLDTATRRLNAPGASRLVDDEEFGTLIGN
IDSCIDFMNRHETYRERDTYLARYSALLTKALHLLDHGFSTRLEKSSTEI
ARQVAAAASDSARHALAYGRFAEMIADTYSLLPNVQQVVRNAYDQYGRAL
ESSTKADIYANSATNMFHTYLTTRDRDLKTITQRDLEEYQKAAKSLSVET
ASRNYVKQLFERIYNEDALFLKIFGIEPMWNTATTSAFQALKGINTMMVH
PGHIAPLGNTMQSVLQTAKLESVCSVVGWLANEYSVAEQDEEESYSGRKH
REYAARLLVDHLWPFTDNAFDAEITKSISKAALQDHDLKIGPVVDGVASS
NAYPLVKKAIELLSKFDHAMPKERSSKNSSVVFNIVRETIRVLQRAEARI
QSLKSGTDPDLFMVKNLLIVKNELVSLEIGDIRNHPQSMQHFGQIWDTLS
PTNWVSFFGSILGGSIWSRGAPSVTAKTLTAEDMSEQLDELLRQSIYAFT
KRWATLINDSTNRKTGVKPIAKVEAELESILDTAFSNQPEVIAKLKEAIK
LNAQAQNNVKDEKQGVKRY
>Mb|QLI68232.1
MPPRLSLRVAFPRPSAVIASHAPRRFLLPLTAQRGIQYGWSTAPPRSKHR
RFNQPSAGLPAPTTGPAAALKRRENTTPLRAGVLATKKGMTSMFVGKTRI
PCTVLQLDQVQVVANKTREKHGYWAVQIGAGSRQPRNVTSPMLGYYEAKG
IAPKSQLAEFTVRGTEGLLPVGSQLLPDWFQTGQIVDVRAKSRGMGFAGG
MKRHGFAGQGASHGNSKNHRTIGTTGPSQGSGSRVLPGKKMPGRMGNEWV
TVQNLKVMKVDNELGIVLVSGAVPGPKGRAVKLQDAKKRKAPALPYRERV
LREMLERHPGGEAQLEAARQRHLEMKEQRQSKAAEL
>Mb|QLI68235.1
MSKVFSTSDVASHNKGDDMYIIVDGDVYDITKFQDDHPGGKKILQRVAGK
DASKIFWKYHNEGILKKYKGKLQVGSLDSKPKPEPKAEPKPAAASAPKKA
ASPSSSNEQSEPLEPFGALIPFADPSWYQGNFSPYFNETHAALRAEVREW
MENEIEPNVTEWDEAKRVPEEIYKEMGRRGYLGGLLGVNYPTEYVPNGVK
SVPTENWDLFHEMLLTDELSRAGSGGFVWNLIGGFGIGCPPIMKFGSQAL
KDRIMPGILAGDKRVCLAITEPDAGSDVANLTCEARLSEDGKHFIVNGEK
KWITNGIWADYFTTAVRTGGEGMNGVSLLLIERGPGVSTRRMDCQGVWSS
GTTYITFEDVKVPVENLLGKKDKGFGVIMTNFNHERIGIIIQCLRFSRVC
FEESVKYANKRRTFGKKLIEHPVIRMKLAHMARQIEASYNWLENMIYQCQ
KMGEHEAMLRLGGPIAGLKAQSTVTFEFCAREASQIFGGLSYSRGGQGGK
VERLYRDVRAYAIPGGSEEIMLDLSMRQSLKVAQIMGMKL

>Mb|QLI68237.1
MGLRSTLQIPRLPHRFFSADRSIPSSSAQTQTQDSTNSNNHEEPQERTVE
AESAADNPWFLEVEPPRHPPSLHAPFVPNAPNESPELIAPMIKYIFEDMG
LDDISLMDLRDLDPPAALGPNLIMLFATARSERHLHISSGRFVRWLRRNY
KVDARADGLIGPGELKTKLRRLRKKAKLMGTNTMILPGGDNGISTGWVCV
NFSTSDKVSDETVSYDESGRFSGFGASPTGTTIVVQCMTEPRRNELNLET
LWQGMLKRNLEQTMKIKGQPIQDQTELETLVSSKIQLPDNSSRVQWQKLQ
QASQQQRFFSTNARRLQSQDYAAASSENKEPENPSLAAEPEDSELKTLQR
RITDLHISGTSLSQQVLERLISAAFHASHSEGDASTRLDLVDQLLQTAEE
RGISVQSKDMLVTLIESIVTSAAYGPELERAQSNLEFLLLNLQTPLEHGQ
TLRLMRAYAERQAWDRFWDTFRAPARFQIARPGELYELAYRVMVSTQDAR
MCIDALRWVYPEMLKEEPPVYPTGPVYNALKACILVADPAAEEMLHNPPS
MDSLGLVGRRRLQHREFVRVLREVEDAHRHLLGTQAREERAQALHRFTDG
NTPS
>Mb|QLI68238.1
MDRLVSSSLPQQTSDANLNRSASLPHSQSQSHPRQRQQREQEQRQEYNHR
RSTSQAPILEHQHAPGSKTASPAPLAGVGRFTEEWDASQRGSSIIDESNR
PGFAAMQRSNSVHSFAAGDDQQLPVRNNTLRKKSSMRRTSSLRRSSSRRS
SRPGSVRSLALHSASDRDDAHSAFYCPIPTSGTPTDVLASRFQTWRKVLK
DLIAYFREIQSHYEQRSKALVKLANVANNISTPPQFLRSAGIDDALQFLR
NYNTAAIQEANKSKEIEEDVILALTGLRSDLQQKIKEIKHLSGDFKNSVD
REMETTSKAVRALAEVLDKTEIDASSTTGKQDPYLMRLAVDRQVERQIDE
ENYLHQAYLNLEGSGRELESIVVGEIQKAYNAYAGILKREADNAYAVVDE
LREGPINMPKDQEWIHFVTHDDHIVDPTVPMRSSDQIHYPGQDHVSAQEI
RAGLLERKSKYLKSYTAGWYVLSTTHLHEFKSADKAQAPVMSLYLPEQKL
GSHSTEGGSSNKFILKGRQTGTMHRGHTWVFRAESHDTMMAWYEDIKALT
EKTPEERSQFVRTHSRSLSRSSRRSVSSDGIVDEDDEEPFSANQVDVNPE
PREDMVSRRPQPGGRFPSDLQINAQRGLQAPQSPSSVSSGHQDHSPDAQA
YAAAGALPAAALVAGREGQHLHHGQENHMGYGGTEQTPMEDMPSQAAIAS
QQAHHDGVNPYTCEPVPHPSNQDGGYFAGAFVGSQGHQDHDLDGKATNVV
PEASELSNAPRQNVDTEPVNSGEREVASNTTENDSQSHANANATADKISP
LVLAASNIPRSETQRELALQVVPEASATEHTTPAETSLGGTSRPKRETDD
RTESAATISNLQIPGKYPKGSGIVSP
>Mb|QLI68242.1
MTNLRKAFIAAIVKSLLRGKSLIQALLSFWVNPGIAEGGKGNSTKTLQDF
LKDAEAALLGPVSGNGLQELSAGLKKQFVERLQIDEQCMLPSYSHQLPLG
SECGQYLALDVGGSTLRVAVVELRGRRVDADKQSEIISMHNYRIGKDVKD
LEGMDFFNWMGQKIAETLAESGQHENSEAKPLPISCAWSFPIEQTSMGSG
KLLPMGKAFLADRGLLGEDLAGIIKQACKQSGLHVELRALLNDSSACLLS
RAYSYTSTRFGLILGTGVNLAAYMPVQSVGMKKFGVRPKGWYEEANHVIV
NTELGMFGHNILPLTRWDHALTKQHPRPDFQPLEHLVSGMYLGEIVRLAL
LEAIETTGLLGGVVPHSLRTGYSLGTDTISMIESDTSPNLEEAIKTFSDR
HPSSYTPTSSDLIAIKALSSFVSIRSSALVATCVYTLWDLRLDFEQQYVD
TLLESSPERKRVEDDMKLESTTVAFNGSVIENYPGYLANCQRYLDELIES
KCSPEPRSIRLIAAKESSLLGAAVALACVERGD
>Mb|QLI68244.1
MAGYFEQFVAFGTDIVGLERIMRFIQSVFSILTAYPALIPMLLSHQPSAV
HKTSALAMGQLSNHLNMTRRAVRLFWCLGSFQTSWNAYVAPEKSIETWLA
ILADSLFGLFGLMESVTLPDLLQVKGLSVFGLAEAVRIDDQSKSVWLAAL
ACSALCTGVKILKSYAHRAVPETGSGFGAPEHEKKKTGKGGENKAQAAAE
KRRQEREAASKEAGRKIGGLTRKLLADLLDIAIPAWATGLADIDAGVVWV
AMLFSTIFTGYAVWERCGQAIDKGRA
>Mb|QLI68245.1
MLLSTVPARRSKTTFTYLARDLSTSLAPVSSSAAVSLHTPPLTGKNKPRV
LSTVLEAPTPVANSPEPGQLPPGGATSVPPAPAPAPAPVPVPGSVPEPVP
VTVPPRVSSLGGAQAASSSTLTPELEQQTAAPTNPGTTTSTPTSTADKDH
RNHRHRRKARQHPAPKGALASSLSTPSIPSTGIASSETPVAAFAAASVRP
LVSASAPVSAPSPTPTPTPTPASTLRQALASASSRSRCLASNDSFLDKPT

ATVDANMADNATARTPPRPRDSHDLSMSPRDITRDSLVVNMLLSLDQMSF
GQSSADGPFGGNRGANTSTAVVYDEEYSPRPWSDDFNTSSRERNDSVNNN
RHWRRSSGSSDVDSIVVDDSAKRPSNPPASRGRHGNGTSNNVMGQFPRLN
SMRETAYKPQPGTPPRSSHTRGRRQSNKSSSSASLDMGYPHGTANQRLAP
RTGRSASFDYGPQSQSQIQQPSVSSPFLVDFAGAMARDEYGAAPTPTVPS
GPRREASGRPIPPPPPEPVEPRTLERKRSTSRSLRSSSGRNRRGGTARQA
SAQAQPSMRAEDLESAPAPSVGYGKSKESVTTANANHQPKERPGFFRRVF
GSSKSTAPSNSSISAPQTSSSRLPISSPDRTLLHQQQPTTPTTSTSVAPP
KDVASSHSHQHPTLQKKSSFFRRRKKSVADEAQPLPNPELIPPVPLLDSS
NFRTAKDGPSGQDMGSPTSSLRQVMNPYLRDGVTGLGLTGVAVSRPLSDI
TNTPSSKSDTIGETRNEEYKRDFSPDYEPSPNARIRKVNPESDSERMDGT
PCRPKSKQPYETLDANNSLLDFEAGSDDADHRHKHQKKSEKPSSTATTRR
KDTEDRDATIRARRKKPVLDFDNGSKLPTQTSLNLPADGVGSVSLASSST
ETGYRTAPSAPPSVLVEEIDESGSTALATLESMKSHKRLDEPGLAVGEPT
EDDRQKARRIFDGGEDFIQKDRAAAWMGEQGPVRQRTLQAYMEMYDFAGL
NILVSLRQICGRLVLRAETQQVDRILVAFSKRWCICNPMHGFKATDVIHM
ICYSIMLLNTDLHLADIESKMTRSQFVKNTMTTIIQAVAESAPGAFVRPS
ILPEKNPILSPEPTRQAMDPSRTNRHSFRPLPRNETNHNDIDECGPLVKS
PYNGPRKGWEEQVEIVLKSIYASIRDQRLPLFGAEQEKNLNVAPSQSNLS
VIGMLKRSPSVLSKAPSETLSTRGRVADGSRNTTSRWTSKSRSRPGLGRN
GFSSSRTSFDDGNSMWSPAMSSATWSKHSLGRTQGSMSQDSFASSMPRGD
YQKSIGFANALSQAIIRDVDTNGQETVPSIVSADISSAQFLEDESLELAG
PPWVKEGRVMHKHHLDGYGKRAKGRNWTEVFAVVQKGQISLFSFNASKSL
RQKSRSRQGGKLNGPVGGGNWQDNAVNLGTFNLRLTLASALPPPGYSRSR
PHVWALSLPTGAVHLFHVGTPEITREFVNTANYWSARLSSHPLVGGISNI
EYGWSSAIVNNSLVNAINESATAGESGGRNSRPGSSAAHARKSSAASGSI
RGPSFDQAAGPFTSSGRGKLPGDRIHIAEWAPPTQSMRSSNATETEQLET
LTTYVKSIEQELQSHNQLRSPMLLAFTPRGHNAGKAMANWERKSAYLLRE
IVKFRTYVDCLQQAEARKQEIYAERDAAQKASQDDLSDGDMELSGDEEGD
ETLRP
>Mb|QLI68247.1
MPTASATLLGAGSAFRTTSAEGATHPRLKPAGTAARRSLSSTSGARGGSA
SRQARAERRSTSPLVTSRHNYVPLPRYPDQQLRQYFHTYFVTHLPSSSLH
PDSHRTIGPGHKLPRDASTPHTPSPGLSPAAVSIPNMQSRDLTVVRIPLR
RAKHHFGSATARGSRPYNEDTDQAGTVDIPAFAKRGPMSVRQKPGEATPA
DSVLGDPQIFYFGVFDGHGGTQCSHFLRDELHGYIEEAAIEFGLQSSLRK
SKPGRDQQKPAIGEKPTSKQRALDSIDMKSAKDVETKMDVPDAENRRFVG
DPPNHGQALPSAKSPPAESQNMDKAIRLERDLVKAYGTTIGGYFRRFNPE
HFDTSPEAGKQASITVESSLAYAFLRADLDFVSAQARKADPDDSDQPLND
DEILGAPHATPSGHGIGGTTRFKGGSTASVALISTPTTAPFWHPAAHSTL
VAAHVGDSRILLCETATGLPHPLTSDHHPTTPTESRRLRRYAPAGSMVTG
DSFGEERIAGLANSRAFGDIKSKRIGVSAEPEITRVEMGPAEFSFLVLMS
DGVSGTLSDQEIVDVVKEAKTPEQGARNVVEYATEVSRDGDNATCQVVRL
GGWERRSEGGLGSMGTKEIRDIRKAEAQDPRRGKR
>Mb|QLI68252.1
MATLNETPAGDLATQILHALDKNSPLLSSAAFPTIPSTEVKGALDRLASR
DMVKYETIDREEAILEPEGEQIAANGSHEARVFEALRTAVEGLTIQELET
AIGDKNVVKIGQGRAFREKWISKTPDGKFKAAKESIKDTTKEQMQTIQST
KSYPDAKMLADLKKRKLVRTQKIISFNFQKGEKFALKIPEVATDLTADMI
ASGSWKSATFKGYNFKALGADQHPGALHPLNKVRSELRQIFFEMGFQEMP
TDKFVESGFWNFDALFVPQQHPARDMQDTFYVSDPKEADMPRGEDSSDTA
DYKTYWDNVKDVHQDGKYGSIGYRYPWSGEESKKLVLRTHTTAISANMLY
KLAQQKGPDGRPPPARYFSIDRVFRNETVDATHLAEFHQVEGVIADYDLT
LGGLMEFMDIFFGKMGITDLKYKCAYNPYTEPSMEIFSYHKGLGKLVEIG
NSGMFRAEMLESMGLPKDMRVFGWGLSLERPTMIKYGISNIRELLGHKVD
LNFMRNSPAVRLDKE
>Mb|QLI68255.1
MHNPFSPRDFVLGIVVGFLIAIALISIGTFAFLRTSDIYSLGHWKLNLRT

PLRSMWMNLGFWKTDDGKPIEHFDEATHTLLEQILEAAGLCAKDGSPIKS
DSVAVLDLGFGCGDQTIALAKLIRAEKRSHFRYVGLTLDEAQLQTARRTV
DRALALGHDGEAVGLSQGSFKLFRANAAKPETWNRTIRSSVDALADETFS
ERWLMALDCLYHFSPSRKPIFKLAAKALNAQVMAFDLILDEEASSWNTML
VRLVGLIMGCPLYTFLTEAQYREQLIECGYDREHIEIRDVSDHVFAGVSG
YLKRQEAALGQYGISLGGYKLAGRLFEWFDRTRVVKAAIVVGRTKGTS
>Mb|QLI68259.1
MFVLPPPPRYPIGGPYGATGITGIVPMIETNNIISNPTGPEWQFLVGEGT
YVLKEDLHLATPPPHPSEAPIVNPNPLATNPQPASVGTKMTLVRIQSRPP
PFFYRGPSATTLSLGGASSQDQSGDARYSTEGGMSSEDGRGTSTSDAPNS
ATLGFTPAFGEGNSLLVPSQTKDVNKRKKPKNNVTKSNSSFISRVIVNES
LSRRMTDRPGDGIFAFANINRAFQWLDLSSPNKQDYFTKILFTKAHCLSH
DVNMVTKGASHIDVIMGFSTGEIIWWEPISQRYTRMNKNGVINNTPVTHI
KWIPGSENLFLAAHMDGSLVVYDKEKDDSQFTPEDDETSVHGSESGHGQN
GVKPGTKMHIAKSVHSQNQKTNPIAAWKLSNQRINSFAFSPDNRHLAVVS
EDGTLRLIDYLKEELLSLFFSYYGGLTCVCWTPDGKYVLTGGQDDLISIW
SAADSALVARCQGHQSWVSSLAFDLWKCDERNYRFGSVGDDGRLCLWDFN
VGMLHRPKTSARQRGSVSGPAGISPRSEAASPLGTLLRSNSLYEADEYGD
GVSHPVEPRSRIPILPPVLTTVIDTHPACWLDFTEEAIITSCKNGHVRTW
TRPGASV
>Mb|QLI68260.1
MLSLYLTRAALRSPLLSQQSRLFTSSTRTLLPASFAGPLPSYFQKPRLPA
NTVVRFVPQQTAWIVERMGKFNRILEPGLAVLIPFIDRIAYVKSLKEAAI
EIPSQSAITADNVTLDLDGVLYTRVFDAYKASYGVEDAEYAISQLAQTTM
RSEIGQLTLDHVLKERAALNTNITAAINDAAEAWGVTCLRYEIRDIHAPG
PVVEAMHRQVTAERSKRAEILDSEGQRQSAINIAEGKKQSVILASEALRA
ERINEADGDAEAILLKARATAEGIDAVSKAILEGREGAKGAISLTVAEKY
VEAFGNLAKEGTAVVVPGNVGDIGGMIATGLSVYGKVGEAQAKSMAKTAI
DTSASTERGDKKEIEQPRSEEEAATIRKDAMKESMLDGFDQTTARR
>Mb|QLI68261.1
MSRRAPNPSADRAAQNQATIKNLLRLEPNKVCADCKKNKHPRWASWNLGV
FVCIRCSGIHRGMGTHISRVKSVDLDSWTDEQMQSILSWGNARANKYWEA
KLAAGHAPSEAKIENFIRTKYELKRWVMDGPMPDPATLDVDGDDDVPLSL
VKEKQVIEKKESIRKASIGKSHAPQQIRAPEADLIGGDDGPPSRSSTAGP
PVAKIAPKAEPAAPKTTNTRDSLLGLDFLGGSTAPPRPASTPGATSQGAK
SRPDLKQSILSLYASAPKPQPQQQPQQQAFGGMGHQQQGSMGGFHDAFGG
LNLSTTASAKPAAADPFASLGSTSSSNRAKPQHTSNASQFGGLSGGGFFD
PKPVQPSTTSHQQKPSNSGFEGFSPFSSPPTQTATAPAPKPLQSSSSAMD
DLFDFSAPTTQRSPPLPQTSSPSATTNSAFMISSPQTKSAAPAPATTSSA
FGSAAISAADVWGTNDWTTPAPAAPTASVAQAPPSPQQLKAPAPAPAPAA
DFGWGNTSSSFASTPIVPGASGGFNPAPKVTADEEFGGWASSTAPSNTGK
PAVPKSGFGGDDDLFSNVWQ
>Mb|QLI68262.1
MSQAVKRACDACHRRKVKCDGINPCRNCASAQLSCTYNAIPQKKGPKGSR
AKVISELRETQRQTSLSAKVQSRMNGIACPPTTSGLAPTPGLLTSELVKE
CAQFFFDNLYPQAPILDRRQIEQQVLYMEQNRDAYCLMTSLCAFIMLQPG
MSMPAGDPYSLDMVPGANIVSSQLLLEECLRVRKGYEYLDSITLNSLATN
FFLFGCFSGQEMHDKAWYYLREATTMIHMAGMDKEEHYMQFDAAEASRRR
RLFWLLFSVERAYAIQRRRPITLQATINLPTLGDDPADPQAHMLNGFVLL
VNMFRPFDDAFTATWNKTRAQLTSQHVNGLQKQLNEVAQSCACQDSNFQD
LHTNQQWLKNTAWQLTSGVVNGNSGDSMSFQYPMNMSRELLVNMASQFPV
QVVDLIGTGLIEKLLEMSSSMAEYLAMQPASRDPFAIGPREHLSQILSLA
AVARNGDYRFLPLLLNKLGEVLPRITNPMLQNAPENSNLANIDIFDGFGT
AGMAQPPSQMHMAMDNDYDRKFSVEEYEKKYAMEMNGNTPESATHSNHSH
SSPSIAQQGSDMGGSFIGSPNIMSPGMDYSHNMNGFPCNPMNEMVMSPMG
SSGQSNGMSQGQHMGHDHMPQRMNGMAGQNMRQQGINVMNPLSGMGHIQQ
RQSSFHMTGQPHMGDFHNLPPRGNPDTSNPMVGMNTMGAY
>Mb|QLI68263.1

MDVYRQTNTLTKKTLKIILTRHSTSTIYSALVLPIIASLYLGLGQKFNNP
NDQFGIGTPQNILSLGEALSKAQGGRDTVAFVNTGHAGGEIDRVIGSLSK
TVGDAGKNATKISNQDDLGYVCKSTIRGTSNCYGAVVFYSSPDEGSGGIW
NYTLRADAGLGLYFRNDHDDNDPEVYTLPLQHAVDAAIAGSSFPSQVQQY
PYTQETEDERQARVLREYQKTFINYLSVAFVIALIGVCYHMPGLIATERE
TGISQLIDAMMPATRGWHRQFVRLVSHQNAFIITYLPGWVIASVVCRVLI
WKNTSFGIMIIFFVLSGLAITSMSLLGACFWRKAQMSGVVTAVVWIALGI
VAQAVPNPSTATVAILGLLFTPCNFVYFITYVARYEQDGQGASLTKTHKD
SPWSLPGIVLWVFLLLQFLIYPFIAVFLERALHGVTTGVRSSSSPEKAAA
QAGHVIRVENMTKIYRPSLLRRFFSFVSPPRPEVVAVDNLTLTANRGQIL
ALLGANGSGKSTTLDIIAGISNFTNGNVSIDARGGLGIAPQKNVLWDDLT
VEEHITIFNQLKSPQRYATKEETMALIKAIGLTQKTKAMSKTLSGGQKRK
LQLGMMLTGGSSVCCVDEVSSGIDPLSRRKIWDILLSERGTRTIVMTTHF
LDEADLLADNIAILSKGKLRAEGSSAQLKDRFGAGYRVHVLNAKYMKHAP
EVPEVQRNELSNTITYIAPSSNLAAKVIRTLEHAHIPYQFSGPTIEDVFL
NLADEVRDDEGRNQKNNQARQLATKERLSDSEDAAKQGAAIDQELLSGRP
VNWASQMLVFLRKRITILKTNWIPYAIAFIIPIIAAAITQMLVNNKSAVG
CSPRESSTRADFGDFTKFYNSAHLVGGPASKFNESVARDLTKTIVPGSAD
VGDFSLKLVASRSEMQSYIETNRRTVSPGGWWLGDSGSVPAVAWRSDNFS
MYTSVLTQNLMNNMLANVTIATRYTHFDSPIAPSTLDALQLVIYFCIACS
IFPGLFGLYPNFEKRQQIRGLQYSSGARTIPVWAAHLLFDTCIILVSMAL
VAIIFATTSDIWYYPGYLFPVFMLYGMVTILWSYLFSLVCKSALATYAAT
AVLQGLGFAVYVISFLFILTYSEAASLDKNILISHWVIAVIFPTGSLIRA
FFVGLNTFSTTCDGDKLQSYPGAMTAYGGPVLYLAVQAILVFGVVIWLET
GSGKYATGKAKPTAEVDDAEIADELVRVDNAGQGQRANGLQVVHLTKAFK
KKTVVDNVTFGVDRSQVFALLGPNGAGKSTTISLIRGDLHPSRNGGDVFV
QGASVTKHRALARQNLGVCPQFDAIDSMTVEEHLQHFARIRGILDVKKQV
DAVVRAVGLGDYTKVMAHTLSGGNKRKLSLGIALTGNPSVILLDEPSSGL
DAAAKRVMWRTLETITPGRSILLTTHSMEEADALANRAGIMAKRMLALGN
TDALRHRFGDTLHVHLVCKTAPHSTLEEMERIRAWTAQTFPDAKIEHETF
HGQMRYSIPASSLTPHGKSDGQNDASASGSAIGQMLVMLEEHKDALGIAH
YSVTPTTLNEVFLTIVGQHDVLEEGYKEEEKPWYKKHIWEWF
>Mb|QLI68268.1
MSASGLVQATSPAQGVRLITFTRPEKRNALSRQLLDEFLVELEAASTDPA
VKAIVITGTGTFFSAGADLKDIADLDSAGARSCRYLENLCVGMTAVRKPL
IAAVNGPALGGGFEVALMCDLIFAAKSAYFALPETKRGLIPGAGGTQRLT
AAVGKFRAMRTILTGKPITSEEALSWGLLCELMEDGVLLQQTIVAATALT
THGAEALELAKEAICRADGLCRDDLFERQLYYTTFGTEEKRKGVDEFLAK
RGR
>Mb|QLI68269.1
MANLPAIKRATTAFGQGVSDNNVYHMQPRINLRRDLIPVETPIARSLWTV
FRRDKSCHDGSDYNTCEKGVNTNVTNLAIILGIVIPVVVATIVLIFLHRR
NVRRLRQEDAQDPTKGLDFGMGEGPAKTSKRKSLFGIGGEKTSHKGNQLS
MDMNLSSPYLLPPNIQQSHDSVHSLARTFPSDQDPYRTVGQYSSSDAGSI
KSFNPNGSRHGSMAYSTSVGAAGRMPPSRSDSMPKTPASPAARSDPFATP
TAPQSIHQFPLLEDAKEVVRPMEPVVPEIGTVSYPDEKTGEPQMPDIQPP
PLAVSRDSHYELPNSSLEQDRPILAELPARSERGPIGMAHSGVDGFDFQS
RESYPSGLGLDMGLQNVYSSDAPHAVDNIPGQALSHDDYGHPEIRTSQYY
DGDSLGAPAPADAPEQSNDALGVPQQQMKRLSVGFRPLPPDEVMESEDPE
YRANRIRSFYKEYFDDREPAPPLPGREAAGYYEDYDAGYLGDAAYYDAET
NAFVMPYAQPVTRRAMTPPPAGRFRGGPGGPGPRGDPRGPVRGPHGSIGG
MSLQGGPRRPRAGSAFGPRPGSSASAQMRGRPPKKNLPPPSVLHTLPTPS
KLKDDSFAIFNATDFAPPDSFREHVAGRSQSPMGERRAYSPKVPVASPLV
TSFDELSVLPSPHMLRKSSTFTGLDFAPPKKFKDADTMSDAGSIRSNTSG
ISAAQLHAIRGGAGRVSRLPGDTVFTSAALEDTLKPQWSMRP
>Mb|QLI68270.1
MAGPTSPSNEGPSFAPPQLPPGWIAQWDGASKKYYYVQLATGVSQWEIPT
QAAKTGTTPGQAVDHPYGTPPPELITHPDGSQTVKHPDGTMEPIMADGTR

GVDGPSGDRGLGTMAMNALLGGKKQSSGGGSGIGGLASQFLGGSSGGHGG
GSSGIAGKLAGQLASNLFSSSDKPEPPQNYHGGQNSSKPSHQSGIAGAVF
GGVAHMFGGKESSGNQNYGYSNTGAGSYAGGDAPTYHPPGSTHSSSAPKP
PSNTSSTPNQQHHSQGSQGHQSQGHQSHGSQSYPPPPPPPPQGHSPNQQSN
YQSQAPPHDTYQGASFPGQQQQQHNQPPYGQNYNSNSSSSGVQYGGQHGG
QGGYSGGQHGYGGQQQQQQQQQGYGQPQHQGGGYNQQYSSGNQSYQGHQQ
YQSGHY
>Mb|QLI68275.1
MTSYFYHLKFELYPTPNPTGASGDQQAEQAHLWLPSPDTNVFDDLPSHPH
PVRPATGAVPSSETSHSFSADGPCSSLASSSLDSLPDGVIDCGVSRAPVR
RSDKRLEIITERQWLERVNSYPPPPSSAALRPQTAVKDWRFGRVNIETID
PAAAGAAVGMAGESSRAGPSAAPALGPSYGGEGTATKASFIPCETKNTEV
GWGVVHFYREGDETTVFGASLQEQGQEEEQEETSSLQDCTTLCIPAVPVY
MSPSDLLGFVGERWLEDISHCRMVMTSSMNRYLVLLKFRDSRFAKKWKRE
FDGKVFNSVEPQPCHVVFVKSITFETPKLSQRADYSKAQSSSSAVSSSLK
PFPPPTPNLIELPTCPLEGIRLSRLSVHEYVRVGRHSQRSGQPVLTAIWI
VGIKPMYSGHAKDHWKDTAHSFALELETQHVWDYAGDLWVHRLIRDKGDG
KVVELPNRSNQSGRSLDEDVVPRAKLDNIGLEYTQLLMSQLESQRSYYEE
MLSKAVDKAAKAAAAAESMALQSSAATEKLNELEEKFKSLTADTIPQLER
DLERERNKASKSDKLARNMGKSLQEEKRVNEGLMKRIEHLGSENEKAMEQ
LGEMKSEVEELKEMNRDLSMFISGQEKLKQLENEGQLEEGELQEGSASVP
EKKGRRRKR
>Mb|QLI68276.1
MGFQLPSTVGRVVAVLGGGVLGRRIACSWAASGYDVVIRDPSPEQRAAAV
QYCNTSMSDYSDSAKRGTVQALDDLEKAVEKAWLVVEAVPEKLDLKISTF
ADLEKLTTQDALLASNSSSYKSREMVGHLQRETRRRVLNMHYYMPPGNRI
VELMTDGETDETIFPFLVDKLKETGMHPYVAHKESTGLIFNRMWAAIKRE
ALSILAEGVATPQELDGLWLEMWSGSQTGPVAMMDQIGLDTVAFIEQHYV
AERKLSDQPLKFLQKFLDEGRLGAKSAKGGLLPPQQATGPAHHFGDSGNI
LDPTLYVLDIGLSASNPLDAPNAGRIMVGSPDGRPFRTLVSNQHMPDGID
ISTSAKKLFWTSMGNPSQPDGAVFSSNLDGSHVKEIVPRGSVHTPKQLTI
DHKNSKIYFGDREGLKIWRCNFDGFGLETLIQNGDWRNDEEKADQMRWCV
GVAVSPSTGKFYWTQKGPSKGNKGRIFRANIDFLPGEGAGNRTDIEVMFQ
NLPEPIDLDIDEAANVLYWSDRGELPTGNSINSARLDAARPVQGNATSVP
GRDYTLLSRDLHEAIAVKLDLKKGHVYATDLGGSMYRLDADGGHKTRIYD
GVGALSGLALAYF
>Mb|QLI68279.1
MSPRTRLLGACRALAVCTRPVAQHGRESFRPATSVFRSFSTDANGHPTTS
RDAARNENISSTLKVNASATNFGKQPTPPTPQTSQGPDNLHIDTLSDAAL
EQILYGGRPTTSETEGGLTAAQEEALYREGTIPTPAEAEAEALIARSQAS
EPAQLSIGQGGDIQNPGHKFGLPRKPYPEGFNLKQRYHPVLEQLTRLLMR
DGKLSVAQRNVATVMNFLRTAPAPIYSPKFPLLPGTPPASHLPLNPVLYI
TIAIDSVAPLLKVRNIAGAGGGGRALELPVPLAVRQRRRMAVKWILDVIE
KKPSKGSGKNQFPHRIAEEIIAVVEGRSSVWEKRKQVHKLGTAARANVSS
KKLKVKKKM
>Mb|QLI68282.1
MAFLLPIILASFFTFAKNLFVPPSNYGIGQVRPIMTLPEAFRVAKGSGRS
KIVLVNNGYAGGDIDRVLDKIANDTTTTGAPDVPMDIVRVSHEDDLVTTC
RSTLRGVTTCFCAVVMRSSPTEGPGGFWNYTIRTDAAFWREPVKFDVRVS
TNVEQVYVLPMQRAVNAVIAAFNGSSSDLLAKTEGLPFASISQKERDTKV
LQAYHKSIINFMAVAFIANILWITYHLTGFVATERESGMSQLLDAMMPTD
KPWKAQAVRIVAHHLSFSLIYAPAWIIGSIIIRFGVFVNTSVVIVLLFNL
FSGLSFASMSILFASFFKKAQLSSITATITILLLGILAQALTRPAIGPVA
TLSVLFAPCNYVYFFTFMAKFEKWNTAADLAKSPPMSSWEMPGIVFFIIV
VAQIFVYPLLGAIVEKKLHGTTTEGRKIQLQDDGESSLVENAVELDKFTK
IYYPSFVSRLFCSRLKPREPVVAVNELSLQAGRGQIVALLGANGSGKSTT
LDAIAGLHKLTSGSISIDGRGGLGIAPQKNVLWDDLTVQEHLVTFNRLKA
PGAPASKDDILELIRAIDLFPKRNALAKTLSGGQKRKLQLGMMLTGGSAV

CCVDEVSSGLDPLSRRKIWDILLAERGRRTLILTTHFLDEADLLADHIAI
LSKGTLRAEGSSVELKDRFGSGYRVHVRGDTETAHLPSIPGVRKQQEFDL
VTYMAPTSNLAAQVIKSLEETGVTQYRFSGPTIEDVFLQLAEEINDEEAF
KNVNAGVPREVDSANEKDLSSSNGSSTLQGFKLLDGQRVSYVKQAMILYR
KRLTVLKRTWILYLIAFLLPIFAAGLTALYVRGKKQVGCTPADQDSSFGT
EDAFTQVRNNMTIVFLAGPTSKLPITTVNSLLQPILNGSRGGASVGSAAL
QNLKLVDTFDSWKQHINDEFRNITTAMWLGDENSNPAVGWVANVFISSSI
TAQQLLDVFLTNTTIATTWSSFDVPFNPGIGDALSLVIYMGLALSCYPAF
FALYPSNERRRFVRALQYSNGVRPFPLWGAYLLFDFTVAIISTALLTALW
AGMSNVWYHLEYVFVVFFLYGLASILYSYFISLFTKSQLATFAWSAASQA
VFFLGYLISYICVVTYVQVDRIDSSLLICHFVISVFSPIANATRAMFLAT
NLFATACDGDKISKTPTGLVQYGGPILYLIIQCLVLFGLLLWFDSGNVGS
SVRGLFDRGKPVTVAEELDAETADELTRVTSSGQMGDGLRVIHLTKSFGK
NTAVDNVSFGIKRGEVFALLGPNGAGKSTTISLIRGDIKPSRNGGDIFVE
DISVSKDLAAARRHLGVCPQVDALDQMTVREHFEFYARIRGIPDIEHNVS
AVLQAVGLEAFASRMGYALSGGNKRKLSLGIALMGNPTVVLLDEPSSGLD
AASKRIMWKTLAATVHGRSILLTTHSMEEADALAGRAGILSRRMLALGTP
DNLRHRFGDILHVHLVSRTAPRTTDHEMQRVISWIQTRLPAANVDTKTYH
GQLRFSVSASEVLALQKQSANSEPEDIKSHDSDTAHAAREGSAIGQLIVL
LEENKEVLGLSHYSVSPTTLDQVFLTIVGQHNVKEENYEEKKESRWVKMI
KPKHPKK
>Mb|QLI68285.1
MFDKSSGQVYREWTVPRQSLTVHGGDRDWSRRRPYPLFTPRGTKGPRSLM
DMATHVIANNIGDVTERHLLDSMPVRLLWRIWLFLEARGVCLHAWKLFSK
ILVADDEDKTLGLYRFRQHICHPQEELKIYTQPLMSPSTDFIAHLVISGG
CQFSTHELLCLAGMKSLGVLELIQPADEVRASFPEVSDRLIRGWAEMENP
FPLLRVLRIWRDQDATQESLRWVTKFPSLALYDVMASRGDWPDPSGSALE
HGWEMAGSASGMEDSLLRYLMLFAPLEQIRSSRLRDLARRVDADLSSLCG
DSRCAVKFVTDRQAPPLLDYLTDTANAYLPTWDIEASSRDVGSCHGIAFE
AWAFWLYAFIGQLGGDEDLRSRGVQSDTQAVVGPFILPSKPFASLFLGHN
GRGGITNTPSYVSRGLFATKRMTFTRPPNLQGRDEPPLPQKMSRKTTNLA
WDDQVEHSLRSKKRQRLDDILQSLSK
>Mb|QLI68288.1
MAAHLSSPAISGDGEATSSRVGEVVPDSAPGTSGLSDPSGTNHHGLSPPT
PLNRPSNSRLPRTNTSQNARDKEQKPLSKTTSSTNSASFGLDPLSTQIFL
RANSNAEPTIAQRLLYSARADSPTRDNQHRQSIDSQGKTLSAAVESNRDR
KKGPSFLSRLAIRGAWKKDDDMNDSDSELGELRTDGFNARALTSVVGAGG
GYIPLHKEPPRYIRVKPHNKKTRDFNHLFLAQELLGTKRASEDEPRSREP
ATAVGSKILKAGDAIWATEFSLDGQYLAVAGKDHTVRVFSVISTPEERRA
YEEEEEDRGRGDGERLSAPVFLSKPAREFEGHTGEVLDLSWSKNNFLLSS
SMDKTVRLWHMSRPECLCAFKHNDLVTSIAFHPTDDRFFLAGSLDAQLRL
WSIPDKAVAFSANAGELITAAAFSPDGKMAICGLLSGLCLFYETEGLKLD
SQIHVRSSRGKNAKGSKITGIRTAFLPSDNGEDVKVLISSNDSRVRIYNM
RTKMLEVKFRGLENQSSQIHASFSDDGAYIISGSEDRRAYIWSASSADND
LKDKQPYECFDAHPEVVTTALMAPIKTRQLLSASGDPIFDLCNPPPVMLL
SLEERNASQTRISEGEQTDGAVSARKPEESPAYLERSRHLDGNIIITTDR
TGTIKVFRQDCAYTKRQQSQWETGSKFSGRIAGGVGRSGSITTRTSTGSR
VHSRRGSLNLGSASQQQTQPASDRIMSWRQDVEGRHSIGLATSRSDRSLS
PMKASRTPLNSSAANLASEARKQPYASSLASKTNVPTSPTSSIHTGGDSV
RIRKDKNGGIPLTPSFSLISASESEQTEDKGEGGFWNLSKWKPNISSLRY
SLNASPLGSGSGNTTGSNSKMSGDSVKVGDRASGRRSLAVHDKRRLKGEE
EVSRRRSSGPTATSRPTFSESNSLGGSIEEGAEVSHILAPNKKRTDSGVA
EVSADSTDDSDTATCRECGGRSFQVKRLAKGKTVLSCQKCGTLAES
>Mb|QLI68290.1
MAQPEPSLPGADRIRSLKDEESIFQAFDAYPWTKDTMFLSGLSAILGPPD
TPGSPSDMAVHARIFYYAQRIGVQIDFAQYKDWLSRHPGHTPPQVLPEEY
RSPSAPSPSPSEPPLPWQQAAPKADLYVDRKAAAENATGDQPSYPMGFAEML
KLLQEGKPIPGIKQIPNTVVRDPSVKPVGSRAAPKKPWERDNPQMAPEVN

LPKALDTEFPQLDTETAEATSAADPVAS
>Mb|QLI68292.1
MASQAGETPNGTYKPRYVDIGINLSDPIFRGRYHGHQKHPDDLTGVIDRA
RQVGCTKLIVTGSDIKSSQDSLNLAEEYPGTCYATVGIHPCSSAIFGAAD
LKKKQSEHTTPCEGDPYAPMSEEHHPDAARSAHAISLLTTVVAQARSSSQ
PHMVAMGEFGLDYDRLNYCNRVIQKHSFEAQLRVAASLQPQLPLFLHSRA
AHGDFVAILKGVFGERLENLDKGGVVHSFTGTMEEMEELMDLGLYIGING
CSFKTEENCRVVKAVRLDRLMMETDGPWCEVRPSHEGYKYLIERKAAQAP
VENGTAAPQAAAPEPQAKQPKKQKNQKKQPEVPDRFKIVKKEKWEEGAMI
KGRNEPCTIERVAQIIAAIKDVSVEEVCEAAWKNTVHVFRLGETT
>Mb|QLI68293.1
MAQPQSQVPPRAFSPPQHSPSPAGSQPGFALPPNKRVRTDGGPASQPGSP
YAISPYAASPGAAGSPPLNAASPTFSQSTPMSSGYNTPYTNGHTAPALNL
PDARAGASTPPLNTPHPPPPPISIPQYTNAAMAPMPQAMASPAPLPNVMG
PPQRPAERPTKDYEYDVTDSLAGTGIDLRAEEQYMSELYSNAMDANSEAR
TGFAQHPAAGKSSFYGAGPANQAPETASEQNQKQLAAKAAEQAWADSSMR
LAVQRTQEINDPFLLVAILHRRAEKIAKENHISLNLDMKNNAQTMGKMRL
PEQFPTPKVTVKVEPGPDSAMVHTTGSYIPHDSYLVDQLALMSIATKQRL
REMVEDAGVVATNRQKTSHGEIPEEWTAAAAPMNAETLGAAEPTSTTEEG
EDGVAVPSDTAPLKRSADGAGLGNGAKPPKKLSKISSFMTLTMRELARQE
REWEEARLRRRQKRKDGVGDAATPSRSGSMAPGTPGSVAPESDKPMTKKE
MKKSQALKAAEANSHANQNLTSSMFAGLGGKSSLFGKKKGAKTYDWMNVG
RGGSGASTPTKAAAGGGKGINGTPGAISAANVAMTTEGRNRLGTWREDKE
KGKNIQLRDWVVVLERDGRESKALQQAYIHLDASNPK
>Mb|QLI68294.1
MPPATLARLEQYTACDISDALLKLKVPGAGFIADLNPYSTKSGKDADTLT
IAPVSTILFAAKGEVLSEPQPNVPKDTHWSDATEPGTFVVMKQPSGQTNA
ICGGIMALRMKMRDAKGIIVAGRVRDVQELKSTNLPILAHGLSTVGSGGG
SVPWAMQVPLDINGVTVSPGDIAFQDPVNGVVIIPKDKVGQVLEMLPKLI
AADDKVKQDVLKGMSVYDAFKLHRGT
>Mb|QLI68295.1
MTDYKFEGWMGLDAESAKGNMVWQEYEPKPWEETDVDIKVTNSGICGSDL
HTLRSGWGQTPYPCVVGHEIVGVAVRVGSKAEGGIKVGDVVGVGAQSDSC
LGRDGPCNECSHDAENYCTKVINTYASVHRNGGKGYGGYGLYHRCPSHFV
IKIPDGLAPEYAAPMLCGGVTVYSPLRHFGAGPGKKVAVAGVGGLGHFAV
LFAKALGADEVVGISRKASKKQEALDLGCDDYIATEDDKDWGKHNARRFD
LIISTISSEKMPLSSYIDTLRLDGTLVQVGAPEGPVPLNIFSIIPGRRRI
AGSGIGSPKEIREMFQLAVDKKIKPWVEKRPMKDANQAIVDLEDGKARFR
YVLQN
>Mb|QLI68296.1
MDENIGLHHEGSLAALPASSAAHLLSNFHPYEPISRATSPGIPYPKADED
DKKRYRPRTFAYFQQLPFDVEEEQQRDAALQGILKQLYIAIKAEDFSPGA
LHWTRELQAWLALKFEMTRELRAKLARLYYSLALAPGLDSNTADRFLRMV
LTLIRKNHYLKPGEDLTFDWRPIWHEVKGIVLPSEGPSLQSGRRRPAKQL
LKLCTHAHTYFDPRDRPAMLEEFLPFFSINDMPNAFIVFGLINTFLPSHP
APEKDVAAQPQEFLPTIFHLWSIMSRSKVADICLIDLFSRLARDHVHCGH
VPFGAHGIFTKAQSDLIFTSILRLTQIPVGQANSPYTALDYLAGAGAYLE
KDSKKHPVPYMMARLIVSSLSPRCMDQDDSIMASLEGLMESIDTFFHPSN
QGSWTNMLGQLTLYLTDAFVSRWNREQSGELELPKERRISPALKKRFVGS
LKEVTFMGLFSKSNRVSNCYYNALQGLAYLEPDLVLPGALQRFYPSLQGL
VEVHRTTSSLNSLQMIANVMSKLKGYRCHITALLALALPGIDANDLNKTQ
YTLNFIQSVAYSIPFVELTKEETHIHDTTLAMQWVQAEMDRMERDGQNVQ
IDYEKELSDEDEANILRSSTAGFGEFILTLLGKVFTLLENLPDANQVRGG
TPEDNVVNALPAALSPLFASLSPELFDMALEKIATFVSSHVVHQARDAMA
WILNALCKVNPEKTLKVFVPMFVINIRNEIDQNHAASDRTTGTDYLPRDR
ALVWHISMLAMTVVHVGREVLNYKDELLGIAVYMQEKCRGLPTILVSNYI
HHLLLNLTNTYPIDHALHEPDVIKRGLDIDDWGKTTSPADLTIRWHQPSP
PEVEFAVELFASQTKSATEQLELLFSDNPPVSRTGKNKEWSDEVSRLMQQ

MRLVISGMSTMFDPKRASGNGNEKLSNGNDGDGDVEMGEDDESLAEGAED
EELRPQFRYKAGYLLKSDDPVYGRIHELREQLGHLLSKAHSFLSENQEDD
VICSTALCNAYRTWITDVGIERSAHPLERHLRLYKADIAAFKIKGLRKVY
PRPLLIKRAEAYQLLRMKHNASARHKSELDRRLLLDLAKSCQSLYADVRR
AAQTAQDSSLKVLIGGKPLVVPVVLEGLRKAIEANDHDRIKGGMYTLFFT
SLLRTIVRDWRFAPDAMSLYIETAGIDKPSIQNLGSSALYSLIEFGKANE
GLILLDDAILNAIRPSGDTDDAVDNRHRVILERRKKIETAKAALGLQLTN
RAKGAHWKIATRCAIFALNLCLRFDDLAPAEFVELVANGTIDPHPGLRGY
YLNAFTSLLGAIDMRAVYGHDYANYLMEKEIGDRNRIDVPVEKGDAEFTR
NFLEAFNKPEEAEYMVDSDHPGWLVWGSKFTAFRARPLPFDSYDELERNV
RRQIGNILTKEWMSQCFEYLKQEPRDQSTDRFRMSNVYLLMHVFDLTYYG
STTVTLDEIKELAEGVFGDGSDKHQHRATAEIIGALLAGSSDDPVEMRNK
VWEFAAPMLLKILNDGLTPENLQYWLPCIHLILDSRDPRRSREILDSLKT
FRLDITSNAAFKDSSKVQLLEFVLADGGWHFRQEKPILDDFLAHIDHPYK
AVREAMGKVLCVIFRTRYHESFESVPKLIEANKKASSIGIRPYQPSEELT
SAIKDVFDRLEKWRHEREPGQQTQSSYTSGSKTVLTWLDCTLSSNECTML
VPFFATPFMEQLLHMMDVKEDPELMRIAYHVYRHLPNIPFREGEDAKFIN
ALIKIGRSATSWHQRLRALVNMQVVYFRRIFLTAASERDALFTAVSDMLS
DPQLEVRGCASTTLAGMIRCSPRHIRDPMIARLERRFKDELQQNPMPKRK
THLPGTETPVDIHRQINRRHAAVLGLGALIEAFPYATPPPEWMPEVLATL
ARKAAGDPGVVGKATKGILSEFKKTRQDSWSVDQKYFTPEQLEDLEGVLW
KSYFA
>Mb|QLI68297.1
MVIMLLTLLSMALRAISGPTSFHGRSGSNNMENLVTFGDSYTDENRFNYF
IQHHAAPPIGEMLPPSSDTWSGGYAWGRLVANATGAKYYNYAVAGAMCTN
NVDSRDLDAINGPFPSVLEYEIPAFEQDTRYPGLYPDRRPDNTVYALWIG
TNDLGIVGILADKQKAGTTITTYVECIWTVLDRIYSAGGRHFVLLNQAPL
ERAPMYATPESGGLGDTPGWSNKTAYNTTEYANKVLEYTTSVNTMYDYGG
PMYLLAKRRWPGASLSLFDTHSLIMDVVNSPASYLDAPADVVAPFRTCLL
NGCVDSKHPRSSFLWFDELHTSERMQEILSKNFIDVVQGKSKYGTYYK
>Mb|QLI68298.1
MHGKVALEEAFALPRLHEKTRWWASLFAVNPDKHAAEMTDITDIRIKYMD
KHGVGYTILSYTAPGVQDVSDPKEAQALAVEINDYVAAAIKTHPDRFGAF
ATLSMHNPQEAATELKRCVTQYGFKGALVNDTQRAGEDDMIFYDGPEWDV
FWSTVEELDVPFYLHPRNPTGSLHEKLWAKRKWLIGPPLSFAQGVSLHVL
GMVTNGVFDRHPKLQVVLGHLGEHIPFDMWRINHWFEDVKKPLGLDCKKT
IREYFQQNIWITTSGHFSTTTLQFCMAEVGADRILFSIDYPFECFADACD
WYDGVPINLVDKAKIGRDNARRLFKLPAFKDSEKHVGE
>Mb|QLI68300.1
MPHTAPSGQKPKKTRAKHACRECNSRRVRCDVTEVQPCTNCAAAGAKCEV
LPSRRGRYPRRSRRLEPAPPPDTSVSLSPTRTVDTNVRPSSSLAGDSHLP
DLEPRSSPVVASEDAGAGLSNATASAASAAGTLFFGESNFLTLVPGNEAA
ALEGAGSASNQKPRLTFPIPGSPGSQSQVEGAGISAGTMRYLRDEGALTL
PDLQTCLPALQAYFTWFHPSFPILDRAEVTRRLAAMDISRFLLQAMLFIG
ATYCDDDTIVAMGFKDRSEAKRILYTRARLLFHADWEKDEIILIQSLFLM
SFWRGGPADVRDVRYWLGVVITLSESYGLHRSPRSISKSTHMNRMRRRIW
WSIYVRERQVAAALGLPSRIRDEDCDIEPLSATDLESEALCANNPLFGSC
QPEHITYTIKMVEIARLLGRVIDLHFAPGKRASTAGDVKGLDNALEAWKD
SLPENMKYATEEGSESVWTCLLHLAYNHLRILIHRNSFLRCGEGDKNNQV
VTAAACRISRIAEDMSTHGTLRYGQMHLITSLFAALCIHVISIRRGMGVS
RRIAEHRAQMCLLCLKEIQKYWRINNNVLDLFLQYLDRSIAERLHAAQTD
GAGNDAATGSKRPDPFIGSNGDASAAPSRGDPVLSPSAEQLRSQDDLFQD
QYFNLVNGHWEGDDALGDLGLFLQADDFSPVKGLNFLGRSL
>Mb|QLI68303.1
MAAATVHVTNIASATSDEEVRNFFSFCGKIADFKVATEGDSKSADVTFEK
ETAMKTALLLNNTQLGPNHISVTGSGSTTDDEGHHDAKVDRDSDDITQEE
KPRARILAEYLAHGYVVGDAAIEHAIELDTKHNVSHRFLSTIQSIDQKYH
ATDRAKAADTSYGISQRANSLLTGIGSYFEKASNHPTGKKIAKFYTDSSR

QVQDIHAEARRLADLKKQEHGGSAYKAAGLERVFGKEAPKESAPTAAPTA
TATATSGGAPATTTPAAVAPTEAIPATKPVDEKTG
>Mb|QLI68304.1
MGDPRESSSYSVVPRIRYNTVGGVNGPLVILENVKFPRYNEIVSLTLPDG
SVRSGQVLEARGDRAVVQVFEGTSGVDVKKTRVEFTGQSLKLGVSEDMLG
RIFDGSGRAIDKGPKVLAEEYLDINGSPINPYSREYPEEMISTGISAIDT
MNSIARGQKIPIFSASGLPHNEIAAQICRQAGLVKQHGVTNKGVHDAHEE
NFSIVFGAMGVNLETARFFTRDFEENGSLERTTLFLNLANDPTIERIITP
RLALTTAEYYAYQLEKHVLVILTDLSAYCDALREVSAAREEVPGRRGFPG
YMYTDLSTIYERAGRVEGRNGSITQIPILTMPNDDITHPIPDLTGYITEG
QIFVDRPLYNRGIYPPINVLPSLSRLMKSAIGEGMTRRDHGDVSNQLYAK
YAIGRDAAAMKAVVGEEALSAEDKLSLEFLEKFERQFISQGAYESRTIFE
SLDLAWSLLRIYPKDLLNRIPAKVLNEFYQRAQKDAKSKGKAREDVAAKD
QQQREENLIDA
>Mb|QLI68307.1
MPALQRTSSYTDIGFTLRRQFNKNEFRPYQREIIEAALSGHDVYVQAATS
FGKSLCFQLPAVIDQGITLVISPLLSLMINQVEALRASGVDASSLNSNTP
YPEKDRIQRDLETGHPKTRLLYVTPELCSGFRFRERLQLVHKQKELARIA
IDEAHCISEWGHDFRKDFKRLSWFRETFPDVPIMCLTATANPQVREDVLR
ILKLDETPDRIKMFLMSPQRHNLHIEIRYTKDQEDNRLADFLKWMRAVYE
RRRIESRKKELQDAGERPEGVSGIIYTISRDECETLAASLREEGVGARPF
HAKLTKEVKEETLNRWINNEPGYDIIVATTAFGMGIDKNNVRFVAHWRIP
KSFEGYYQEAGRAGRDGNASYCFLYYSREDLERVMRMIRSDSKEGSNLDA
RLRSLQALATYCENTNSCRHAQICKYFGDSSSPACDFACDWHKDVHDLKT
RFLKGLASPDWVSSQVEQGTYDGHDYYDDD
>Mb|QLI68310.1
MGSESRPVCHRCAQIKQACDGSLPCARCVRLSLPCRPRNAAGITTDDTHG
DVPKARIRRVQTGCLMCKRRKKKCDETKPRCGDCRRLCLDCAWPPERHGK
PKPGSGFADEAVKEDSNCDDGIVEPIPSSSIISQLPEAVIRDIPVRKSPS
ASSSSSERHGSFDNQSQYSHASHNSQLGYSNYANFNQPVEQTAIMTVATS
SMAMPSSSTMPVTSGIIGELTPDMPPQKLSWDMSASPPAWLESITPISNG
SPTASIDSAPPSQSLSIYVPQLMPQLSAPQDRALLNHYSTIVSSILSRRA
STENPYNSYLLPMAQSNDLVLHCILALSANHWRKLQPAMGDRGLLHKSKA
TQALAGLLPHVDTTSADIALVSSLLLCMTELFDGTSEGWKLHLKGAKRLL
IALRKQQGDILTGHYKFLLRLARFLDSAATTSTCRPPLMGTEAAEAHALD
TWSSTPDEDDSAVYGIPKELFHLVDRINTLAELRSTRVDQASETAFRRHA
SEIEHRINNWSYEYGGMSRAVSTLTPATEDVLHATLAFEYAIRLRLHQIV
EGYELTDPKVGKYVDGILECVQKIRYGSPLESCILFPLVMAGGSCWKLEH
RVIIQDRLLVMERTCGFGYVYNARDLVERVWSRRDQSEGTGAIVNWARIR
YYEMHGLVVF
>Mb|QLI68311.1
MVRHKKDFAARGKKSGRGGGHGGGGSRQPRHQDGDQGQQPARPAFKAACW
DLGHCDPKRCSGKKLMKLGMMRDLHMGQRHHGVIITPNGRQVVSPADREL
MDQYGAAVVECSWARTQEVQWSKVGGKCERLLPYLVAANTVNYGKPWRLN
CVEALAAAFYICGHADWAEQILAPFSYGESFLHINASILKRYAACQDAAE
VKKTEAEWMERLEREYAESREQGHGDMWTTGNTNRMAMPPSDDDDDADDD
DDDDDDDEGEGEGEEGDSDARSDGRGSVDGIYLGKKPLDPKPGEIPANIP
DEQARRDKDPFDITDGSEDDDAAMEEIRRKVLASKTFSNPNPELEQEKKK
PASIPRPQQRHRYQVDLDMQPDSDNERSADNQDDSDAAGDDDEFDNIIEA
TPVTDRIGLAKLEKERRQATTTTKTFSSTSVSAPNRW
>Mb|QLI68312.1
MADLNIPIAVRRTRRSNAGIVKVEETESAALPPKTPRRARRAVRFSDPGL
SMSSGLTPMINRTCINTPRQRRRVSTPAISTRSGTPGPPNTIFLSHNHHQ
SSNSLHHTIDGRVERRIRRSNLRDLLNKLEQEKKRKTQQSQAEISHLKSE
IKSRDRDIYELQNATIVVDNERIWDLEQQIENLKDELAKRPSTPHHQTTS
YDWTLAARDPFSDEYTDMGMDEEEDQFGDATMAHFVASTPSRARSSFPTP
PATSPMLPSTPCSSRPCLPTPRSHTGTQVCLPDPEKRQAQEELASLQLEV
CKLTATLDSYKNLSAKLKEKLASVEPISPQSDDAPAAHVLESKIDALLQD

MSDRTEAVRLLGASIADLGFPGADASDMMAALSSGFRAARLELEYLTPGE
IALPLTSHGAEVLDLLLVRLRELAKKAKEDESSIDEYHELEQSLRKQLDA
RVSAMDQLRSEMAKAERLLHEKGLKVQDLEVANERLRGAVDGYIRDMSEL
EKLVQRMEEEHRDAAATSSAQREFDRHELQKRDAGIAELQERLADAVRQS
ARLQREMGDVQDSSTRHVVSLNRRHGAALALRDARVLELRGEIDRVNDSL
RAAHETIVVLRVDKGALEARVDDERARAKAAVESVKAELQRVLQMSHDFL
RSPGREGRQAGLRDEAEDELASSPSRVDGKAAAAAAASGSLVPMASGRKL
RRRYDSGLGLLEEDDVEMF
>Mb|QLI68314.1
MASIANQTYTPTEETEIQQWLTTSDRLKSGEDKATILGTLNSHLASRTTL
LGSKPSKADVAIYETLSPIVAKWSPDERTGEKGHPHIVRHLDFVQNSALF
GLELKEDQKIQVDQDDVRYVKPPVDAKAEKERLKKEKAAAAAAVDGNKEA
GLVDRTKEKVKEKVDEAKGAAAAAVGGAGGDKPKKEKKEKAPKQPKAAPA
AAPLSPCLIDLRVGHILKAIQHPDADSLYVSTIAMGDPAGNEDYTEYEGQ
ICRTVCSGLNGLIPLEQMQGRKVVVVCNLKPVKMRGIKSCAMVLAASPKV
KEGEVDEDKGPVELVAPPEGSKAGDRVFFEGWAGEPEGVLNPKKKIWETF
QPGFTTTGDLEVVFDAAVVEKLGKTGIGKLVTESGGVCTVQTLKGAAVR
>Mb|QLI68316.1
MSIKPKKAFAFYQPLPFEPFSDLESLPQCPITTFNDRKLLSYPACMSIGF
QCSTLCGIDLIPYPSMPPQRRVMMCLCRRRGWTDPAYECYRDTSGYTCLV
LVNGREYQTDLAYESDVLAQENAAMRAFMVCRNFSVNGGMLARNGIVQGL
PATSDSGRRRKSRHTSSRDGSERHGRRSGNHSSSSSTASFE
>Mb|QLI68317.1
MFATQQYLEAVDAGRKRFRDDEEIANAAAGFGEHRNKRIQSLPLRNPPRA
GQHSPSPSPSVVPLNASPACAGSRPHAEPFQSASHQGGPLQQDAEMDMMDVT
ISSPPDHDEAAQPPALLLNRSTGRMPTPIQPSFAAQVKGQYSGWAATVQT
QTTPNGVANLGHHVMGIAQDQTVPRAMTGGPDWQTLQNHRRLPSPISEMG
DPIMHDGSGIPTCTMMDSEEQFGQFGQPSPVHGSMSGQPSPLHAMEHPNA
MMDADCHHLLQHGESDTDPSSPSPNRKGHQRSKHTVNSWTWQPGMKKSFS
IGYRSDCEKCRLKVPGHFNHIVIS
>Mb|QLI68321.1
MAPVKVGINGFGRIGRIVFRNAVEHPEIEVVAVNDPFIDTEYAAYMLKYD
SSHGIFKGDIKKEADGLVVNGKKVKFFTERDPSAIPWKSAGAEYIVESTG
VFTTTDKAKAHLAGGAKKVVISAPSADAPMYVMGVNEKTYDGKADVISNA
SCTTNCLAPLAKVIHDKFTIVEGLMTTVHSYTATQKTVDGPSGKDWRGGR
GAAQNIIPSSTGAAKAVGKVIPDLNGKLTGMSMRVPTANVSVVDLTVRIE
KGASYDEIKEAIKEAANGPLKGILAYTEDDIVSSDMNGNTNSSIFDAKAG
ISLNKNFAKLVSWYDNEWGYSRRVLDLLAYIAKVDAGK
>Mb|QLI68323.1
MAAISVNAQSPVSAPSNIPQLGRPVSQGCFNTKGNMTALDPQIPGERMSS
GSCTLACKDAGWWLAGLHGPQCLCGMAIPPESASVADSKCNAPCSSYPQE
ACGGKDTYTIFNLGLNIDPPTYSESSSTTAASSTSSTPDATPTGSSPAHT
SATVTETASPSQTSKPEKSGPNVAGIAAGVVVGVIAAGAAIGGFFFYMRR
KRNAEIEEEHRRNAAVSAFISGSTPPSSHGSISMTDSRMDPVMAHRRMSD
GSIADNEDYSRRILRVTNA
>Mb|QLI68324.1
MADREILPDNLKPVHYNVSLRDLEFKNWTYQGTVTIDSEIVKPTKEIVLN
TVEIKITSAKVLVDHTKSSQAIQSTNFTYDERAQRATITFDQELPVSKKA
SVVIDFEGIMNNEMAGFYRSKYKPAVTPAKSVPRDDEWHYMFSTQFESCD
ARRAFPCFDEPNLKATFDFEIEIPTDQVALSNMPVKETKPTKDGWNMVSF
ETSPVMSTYLLAWAVGDFEYIEQLTDRRYNGKQIPVRVYTTRGLKEQGRW
ALQHAPKIIDFFSKIFDIDYPLPKSDLLAVHEFTHGAMENWGLVTYRTTQ
VLFDEKTSDARFKNAVAYVVAHELAHQWFGNLVTMDWWDELWLNEGFATW
VGWHAVDHLHPDWQVWAQFVNEGMEAAFRLDGIRASHPIHVPVRDALDVN
QIFDSISYLKGCSAIRMLANHLGVETFLKGVSNYLKAHAYGNAKTKALWD
ALSEASGKDVNALMGPWISKIGHPVVTVAEEPGQISIRQSRFLSTGDVKP
EDDTTTTWWVPLGLEGKKGETGVNTMSLLQKEETIRDIDDEFYKLNSGATG
FYRVNYPPARLAKLSTQLDKLSTEDKIAIIGSTADLAFAGNSTSAALLTF

LEGFSKETHPLVWTQVLDSIGSVKSVFNEDKEIKTGLENFALKLISDKVK
EIGWDAAENEEYLTTMLRKRIIGVAVASGHAEAEKEALRRFNAWHENAEA
NPLPPSLRLPVWRAAVKKDTARAVEILKNEWFNTKSIDGKLICLNALSAP
EDEQILKEQIVSFNFNESPPSNAVNAADMHVLGMGLAGNPVGRQVQWAFM
KANWDACVAKLGNPIVVDRFVRVSLGGFTDVSAVDDIEQFFKDKDTKSFD
RTLETVKDKIRGRAAYKKRDAAALKEWLGANKYL
>Mb|QLI68330.1
MASKAAHKRLTREYKTISENPPPYITAHPSESNILEWHYIITGPEDTPYH
GGQYWGTLIFPPNYPFAPPAIRMHTPSGRFQPSTRLCLSISDFHPKSFNP
AWEVSTILIGLLSFMTSDEMTTGSVSTTQTEKKYLSSRTRWWNSTGGGSH
AKNPALKGNVKAGDGGAKFRAEWPEVDLDNWEWMRKNKVDSTTGNRLDGE
GGSSCGPQLGIAGTSGHQAQAVVDAVVQQRDAGRGWIYRNKLLVAGAAIF
IYVLIARLVNGEGIA
>Mb|QLI68331.1
MKIAATLAVLAIGPVALADDVLYSSRFSKRGLLPNGNYNLSFFHVNDVHA
HLDQYTKAGADCTDATKGCFGGYARIKTKVHELQKQNPDHLWLNAGDEFQ
GTLFYTFYGGEKIAETINDLKFDAMTLGNHEWDGGDENLGKFLKNLTFPI
VSCNVKSTVKDLNETIKNYHIFEKHDLAVIGATTETTPNIANVGKGTTFL
DPIPEIQKAIYEIRNTTKVKRIVALTHLGYEVDQKLAQQTEGLSLIIGGH
SHTLLGNMDKAEGKYPTIVKDKGGNEVFVVTSYRWGEYLGSIEMTFDDNG
RALSYHGAPIHMDNTTSLEKNLQKNITAWRGPFEKYAAEVIGSTKNVLDQ
TACQKGDCLLGQVMADAMLEYRQNQTNGTADKPDFAIINAGGIRATIDEG
NITRGQVITSFPFGNAVTQLKYSGADLRKILEGCVSRVSQFNQKKTSSWF
QVSGNIVVEYNESRDAGSRLVRATVGGKALDDKTDYNVVTVDFVAGGGDN
LLKPATEFVTLESLDQVLVAYIGAHTPLQNALQKRVVSSNGTAGGGAGDG
GKASNGESKGASLRAPRLLVGLSFLEALVL
>Mb|QLI68333.1
MPDYDGDDRTRNGESSHHRNKKRARDEVQDDLPQPYNANSLQAAKRRAVS
PSEQPRKQKRPGARARISEAEREAIRQRQIEREREQAAQQAAAAEQNHMR
GVHDVVRQHYNSVPERGRQWRTTDSKIKGLRVFNNWIKSCIIQRYSPDED
HTPGSREMGRSSGKDLLVLDIGCGKGGDLNKWQQAPQPIQLYVGLDPADV
SIEQARDRYRILCSRGRGGRGGSHRRPPPRLFDARFHVKDCYRESIEDIE
IVQQVGFDPSPMNKRGFDVVTMMFSMHYAFECEENARTMLRNVAGALKKG
GRFIGCIPNSDVLGEKVRAFNEKAAAKKAEKEERDKGGDGATTTTTTPPP
AEPEDGELEEGEEEPTAEWGNSIYRVRFPGKTPQDGIFRPAFGWKYNFFL
DEAVEEVPEYVVPWEAFRALADDFNLELQFHRTFSEIWEAEKDDPELGPL
SERMGVRERGGGPLLVSDEEMEAASFYIGFCFYKV
>Mb|QLI68335.1
MAHAWLDSLSEDWVSQPGSDASDGQLPPLPSPDEIETPQIKQFPSRIPRR
AGNRSSLPSVDSNSPHVLSERSANDINVSIKRLPSKLSQEVSADVSRSIS
NETDGSVVHNRVPSQGSPDKNSETPEWKRRLVYGDVAYGEQRDLFCSAAV
GLQDMFKPPQEGEEESGLGGDTSQHVDMTMPSSPPTYPQPVTDEDLDGFP
DFDEDEEYPEDVTPSPSPRRLQKEIKYKLNESSPIPDSRKSGQPRVDDTL
EHQTEHDISRDESCLTAPLDKEAASRKPSGQSDTRNEDFSPILIGKHNGT
NGNVNFAPIEVPVDQLWQKLERLRVNQLLLDSQANHQAGIDASPRPPANM
ETTEDYIQNGGFINVRRGGRSGDGSFYNRGLSSELGADTSQMFPEESLQA
STPKEFPSVRTLGTNPYPTFQITQSPSLPRAPFPSPEKKQLVPEQTRPPA
TSPLKLFGPYDTFTNQTLLRRISQFEETSGSPSQRSTTSDVIDHHPEDES
ALSASTLQRRNISKFGGGDLDGFEFQGDISDEIYEDSDVVGKENVAPNEP
SLPSYPLRGIQAQDSSPDGYSGLVIERKRSKNETPSRVRDGETPISSNGA
SVTSGSPRRDLGSESKRPRTSPSKHPTPKRRRTLHRSDIAFGRESKEEAA
DTTHLQMHSLSSSKKRKDALPGNFEVADPNILASRPMLQPRSPTSSRGSS
ASYGQAQKSHLRNPANELPTPPSDAPTETDRKPSIRTQDFVDQAAQIMAM
IRNQVQPELASLEESEEYIEAEDRRPSSGAESYQDSTSEPFSRPPSREGH
PISWVPPRQDDPELANRLKQYEEFSDMGDVISSSVRSLGLAKNAMPASQD
PTRRIDGGNQLRSNIMPIPVRGEVISDLPNVRITTNPLANAEQGSSSQEY
PSHSSNRSTSQTYPSASSRGSESRRTIMPESVSHLIPDRVGSMCLDKDKK
VWIKRKESVSVPECDILPSEDSEEDPFASIPDLSVDMTREMHNLRLATAG

KEPAMDNPEERSSPRSPPRRVVSRGYVTLSPSQHMSPQDGAFTNEEVEKV
EGLSDYRKGDSTTKTQAGHHRGSNAPSLTKRRNLTISFSSPIASVIRDIS
LEDLDSIEESDDRDEANNAEASTHRSPHRSSSQVEAKPRKGSQHVRANPS
QAPLRQASFRGPAFIPRPVSRIDEQDEESTVELANEDRQLSVIGEQSMLN
HKTPDGRRASLSFVFSQTPGNRALALSAEDSEFIGRNVGKLSLSPLSEFT
LNNADHSFGFEVSYVMGPRHMATGDGSNKVLSMTIRELVDRLSEAEPHES
FWEDLSELDLHDKRLASLHMLDEFCGKLVTLDVSGNKLNHLEGVPSTIRE
LKVSQNFLTELTSWDHLMNLQYVDISGNEVTSLSALKNLVHLRSIKADNN
QLTSLDGLDAHDGLLHVRARNNMIEEVDFAASRFVRLEDLDLGGNRISSV
QNLDMLPALARLKLSKNKLTALPMAECMKSLRHLDVSDNELLVLDIASFP
SLHSIHADRNHISQVCGFDRTRRLDSLSLREQRGKTPLDLGFLSSAYEVR
KLFLSGNYMREFEPRVDFLNMQLLELSNCGLQRLPDKMGQLMPNLRTLNI
NFNALSDLSPLQFIPRLKKLLAAGNRLADSTRVTQLLTEFPHLTRLDVRD
NPVTLGFYAPLQVLVPTDGSGVADPFVLPDADVARDEMFASRLDEATRLR
RRLHQVVLVASCGRLRMLDGLAVRRREVLARDQMLQTLINDGLVPDPERP
QVQEKESEPIDANLNGPDAIDEETMADEQARAQLEEEAETAWHNTIHENQ
>Mb|QLI68339.1
MSYYSGVSDRPSERSRWTPLTRMLLSGEMTQEKQQDLSTREKFDRWMINE
GYRRVFVFVFMLTHALIFAFASVHYSMKDSLQGARDIFGFTFVIARASAV
VLHVDVGIILFPVCRTLISLLRQTPLNGIIQFDKNITFHITTAWSIVFFS
WVHTIAHWNNFAQVAAKNKLGLYGWLLANFASGPGWTGYVMLIALMGMVI
TSVEKPRRANYERFWYTHHMFILFFFFWSLHGAFCMIQPDVAPFCTNIGA
SAIGVFWQYWMYSGFVYLAERIAREVRGRHKTYISKVIQHPSQVCEIQIK
KQNTKTRAGQYIFFCCPAVSLWQYHPFTLTSAPEEDYISIHMRCQGDFTM
AVSKALGCEWGRKADTSKVVGVDRDQHGVNPVLQRVLPRVYIDGPFGSAS
EDVFKYEVSILVGAGIGVTPFASILKSIWYRMNYPQKKTRLSKVYFFWIC
RDFGSFEWFRSLLLAVEAQDLEHRIEIHTYLTAKIKADDATNIMINDANA
DKDTITGLRSPTNFGRPNWDMIFRGIRKLHSPGEAGVFFCGPKGLGSSLH
VYCNKYTEPGFSFVWGKENF
>Mb|QLI68340.1
MGTATEFYRSQIGGTSVINYSYTDFPFKLLLKDVHYFFVYCWALPWILLP
IRPCGSGELDELYPSGKNIFCILIHFILVLLQLAFILVLPLGIIFPVWIT
ALGVGGFMTLNWALCKLLNGKDITFHSDEKYAKARPEHAHEQWVFLNGVA
VGDHWMMANLNRLAITFGRPILGIHNRTSGILFDVIECLVQRNFTYATSD
VRICYKILRDVLYDPSKSKVVFILHSQGGIEGGLVLDWLLQEMPQDLLSK
LEVYTFGNAANHFNNPHRHVASQNLSATNPLVAIRTFVSESSFSTPVTSP
VGATNGHAKGGDPSALTTSGTSESFSSSRVSSAAKDRAIGHVEHYAHSTD
FVAMWGVLHFATNRMGSHQLPRFLGRLFNRATGSGGHQLNQHYLNGMFPL
ERDKKTGEFIGADEENAFMEEVIKFGKEGDAMENAREAFDISYLGTEGFG
TGHISTPVEVHGTRGRKRTKTDVMVKELSRLWSYRNGRSPGGEPPNLVTE
AGFCRNATM
>Mb|QLI68341.1
MSDSSRGSGAQTGGPETSLGQSPSPPSSVPSPSPNTTPSSPSPPSASSPS
PSPSPSSESPQPQPQPPATTSSSSSSPPPAPVPTTPSSSPSSTPQPETPS
PSPTPTSTSSTSSTPPPAPTPSTPEPTISSSTVVTVTPASSTTTKIVVNT
TTPTAGPSSTSASVSGSSTATPINPAGDGNSGGLSQPAKVAIGVVVPIAA
IALLALVGLFWWRKRRARQESEEERRKEVEDYSYNPNADPTIPAVGLGTD
NYEMRDEGGSGYRGWGSTTAAGSMGRKTSTTMSGGMTAGAFSDGGGHTRG
NPSETKTDPTVDGSSSPDGEIMGAMGPSAANNRGGDVHRGPSNASSSYSA
TGRSEGSDVGIYANGGGYYDQYGQNPYGDQRAQELGGQAVIRDNPARRNT
RIENPSHYPQQSAGIAQNF
>Mb|QLI68342.1
MSAPVPVSRCVHRSARSIARWTPRRAVPAIGFMSRTVRMTTTRPVPPQRL
AGVRLFTAGRRLYDAEEDDSFDPASIERESDEVDVCIVGGGPAGLSAAIR
LKQLANEAGNEDFRVLVLEKAGDMGAHILSGAVIQPSSINELIPDWLDEE
NPNRFEHATPAAKDQMKILTKNSSFPVLAPPQMHNHGNYIVSLNQFVKWL
AERAEEIGVEVYPGFAAAEVLYDADGSVKGVATNDLGIGRDGKPKDTFER
GMEFHARVTMFGEGCHGSLSKQVINKFDLRKDSQHQTYGLGIKEVWEVDP

EKFDKGLIVHTMGYPLPRDVYGGSFMYHFGDNMVSLGLVVSLDYQNPWLS
PYQEFQKLKQHPTFRSVLEGGKCISYGARALVEGGFQSIPKVAFPGGALI
GDSAGFINVPKVKGTHNAMKSGMLAAEAAWNALSQTDQGTVFLYDYEDSL
RKSPIWKELKEVRNMRPSFHSPLGAIGGVLYSALEAFVLKGRVPWTFKHK
TPDYAATKPADQFPKIEYPKPDGKISFDILTSVSRTGTNHEEDQPVHLQV
KDWDAHTEKAYPPFKGLENRFCPAGVYEYVEDESKPHGVRFQINSQNCIH
CKTCDIKAPQQDINWQVPQGGEGPKYYMT
>Mb|QLI68343.1
MPVPKKEGASSGGGAARADGRGQNGNRSNFRTDTAISNSRLGNERTLQPW
IPDAADGVDGSLEATAASGHWDQFAANEKLFGLRTDYDESLYTTAINKTH
PQFRERVAAADKKAKEIERSVATTAHVAEERVMDYAGGTGAGAQNEEDKY
SGVLRQDFPPLSGGGRENRYTPPAKRAPTSQSTVKGAPVDPAIISSQLKA
PVKRQAASTEQTRPQASEGIKNGVPVKAEPPKASTPKTTEETPTESKPSP
VLNLPAESQTPESKTTEKVTTTGPSSTSSSRNASSQPKEQTPSATATVEH
DVLNSFRSFASRERDNLEKVRSHKARADKEVKLNELKNFSISFKLSTPVP
KDLVSIIAKDPAKQKEIQAKAMKNAEDMAKTKADSGSKDKAQPVKDSVTA
KTPDTNTSANTSDGRSSRSAVVPQASGPAGNGSRHTAPRQYSQQPFHAQP
FRSNRGPQQQTGNLAQRLRNVEQQRYSQPPVTTSHNLGHEMRAPPTGPSG
NVDSGFNRRLSGAPGQMGAKLNPNSHEFRPSPFAATFNPNSHASAGSSPR
TSVNHVAESPVPTAPQTPVIRRKTKKVDVSKCLILSHIRTVAPPAGRNWD
DNGGLRPSFDTLPTWRQLKDDEKPESTMHLTYTEHFDRQPFNGPSAATPN
PTHVVPQMPHQHQLPFHLQHGAHGLGTRQSPHMAPMQMHAPQHGPVPIPP
YGTEDHRMMPSNSAQSFASPRMGQLPVAYPGQVPYQQPVFMGPGNPQMGQ
YRSFSNNPHYVPHQQSQMAGPMIMQPQFIHPQGMVAAPQMMYHGGHPQYM
PPTGPPQPGPGGSGYPSPGRPTAPVMMHQGSQQGQPVYGMSPSVQYNQPA
YGPGQPSGPSQ
>Mb|QLI68346.1
MVRSLIVAALAAGFANVALAHNIEVPPCGQECKTLEYVGCVKDGSPSALI
MRSNQDQGQMTVEKCGAVCKGNGFRYAGLKYYGICFCGNTLGGSPADEGS
CSYPCSGNNAQKCGGDSTLSVWEDKTYPKKPEEVSGDDYKAIGCYTDDTN
KGRTLSWPVNVDSASITPSSCIAACKANGFAYAGTEFGGECWCGSFLNPA
TQAVDASQCNIPCHGDSSVSCGGRGRLTVYEAKVLISTEPCIPVPPPIST
SVAPPASTAGPGTSAASSAPGSSGAPGSSVPASSNSGVPTLPGTGVPSEC
SAPPTVTVTVTPSSTGPVPTGPASSGPESSGPGSTGPGSTGPGSTGPESS
GPASTGPQSSGPETTGPASTGPVPTTTPATCTTVVTGSDCGPAATTAPNQ
STGPYTSGPVLPSGVVTCTKTLTGSDCPPAPTGTGPVPTGPETTGPQPSG
PETTGPQPSGPETTGPQPSGPETTGPQPSGPETTGPQPSGPETTGPQPSG
PETTGPQPSGPETTGPQPSGPETTGPQPSGPETTGPVPTGPQTTGPQPSG
PETTGPQPSGPETTGPQPSGPETTGPQPSGPETTGPQPSGPETTGPQPSG
PETTGPVPTGPQTTGPQTTGPQPSGPETTGPQPSGPETTGPQPSGPETTG
PQPSGPETTGPQPSGPETTGPQPSGPETTGPVPTGPQTTGPVPTTTPATC
TTVLTGSDCGPAPTTAPGQSTGMGSSPSGPAPTGPALTSTVITCTKTLTG
SQCPPAPTTTPNQSTGPSTTGPAPTTTPETTGPETTGPPETTGPETTGPP
ETTGPETTGPPETTGPETTGPPETTGPVPTTTPETTGPETTGPPETTGPE
TTGPPETTGPETTGPPETTGPETTGPPETTGPETTGPPETTGPETTGPPE
TTGPETTGPPETTGPETTGPPETTGPETTGPPETTGPETTGPPETTGPQT
TGPVPTTTPTICTTVVTGSDCGPVPTAPGQSTGPNPTGPAPSIITCTKTL
TGSQCPPTPTTTPGQSTTPETTGPAPTTTPNQSTGPVPTTTPVTTCTTVL
TGSQCTPAPTMPGESTGPVPTGPATIITCTSTLTGSQCPPKPTTTPGQST
TPETTGPAPTTTPNQSTGPVPTTTPGQSTTPETTGPVPTTSPVTTCTTVI
TGSQCTPSATMPGESTGPVPTGPATCTSTLTGTQCTPTPTGPVSTVPESS
TPATTVPASSTPATTVPETTGPVPTTCTSVFTGSQCVPSATLPGESTGPV
PTGPATCTSTFTGTQCTPTSTGPVSTVPESSTPATSVPESSTPVTTVPAS
STPATTVPETTGPVPTTCTSVFTGSQCTPSATMPGQSSGPVPTGPATCTS
TFTGTQCTPAPTTTPATSVPGSSTPVTTVPESSTPATSVPESSTPVTTVP
ESSTPATTVPQTTGPVPTTCTSVFTGSQCTPVPTTPGQPTGPSPTGPIPT
GPVTCTSTFTGSQCTPTPTTPVTTVPESSTPVTTVPESSTPVTTVPESST
PVTTVPESSTPVTTVPESSTPVTSVPESSTPVTTVPESSTPVTTVPETTT

PVTTIPTIPATTVPETTTPVSTVPVTTPTTPVPITSTPTTPTTGFSTTTL
CTTTATTTPDCEYQIGDWCAPPVPDWHDKLGCIKSAAVCKLQWASCFKKA
GLTGAIGCFKYVEFCKSIELSCLKCVLGKCDKSDCKPPTVSVTTIVTPCE
PTATNPPTPPPTVCPPKPTNICKDTGSICGVPLPIVSCNDDKSQFAAAPF
KLYNDADTSKCPIFPHPEVPNACAEACKEQYDQCTKTSLWSCHGLKRSES
AFEDDFVAPTANEGPHRRTLGLLTQLKCKKQYSACVAANKYIDTSNHCSS
WEGCY
>Mb|QLI68347.1
MSQATVNGQTREELEINTLSLFDVLHNSLILRTIAPYLPVYSLLQLSAAN
KEFRSLIRNTPGVFRHLDLTQVKKAQFDINPIDNGGEVWRNVQLDENLSE
DDFYSGPLRGVFNAIQRQDIWKHVQTLMLDGLSVTSDLCHELINDTSYSV
RILSIRDVKNLNQAKLRGALQYACRPTRPENSPRLKALYVFGSKEAHPET
EPAPQDRESQASISSGWNHKSHQALASSLERQGDSWWCKRGRVITRPISQ
EWVNCMAACEGIIAFDAVLCKGPRHRNSPAFGRPLMMADNAPAVATHAVG
GCGGCGKAPEGLVSEERCPPQCLPLLAPVPIMTSSIRAATCPSQPGQSFV
PRCLDCLRERYCTCCDKWWCESCFPLPGQGQDAGVNSFIVVDEDDSVASF
AQILDLQDSTPKIKNLVSKSCWECGNNCEECISQTQRSCKKCSAGYCIVH
NEGSSSEYCDWCISRGRGLGRQDSKSGARMQLASNGIPLSLARRRIRDAL
TRPLL
>Mb|QLI68348.1
MRELFSPRNIAVDTLDTLDTPLVNIIVAPCGEAKTPGRGSAKEVPLSTKS
LPQHPGNDPARAFQLPPPPPMSPPMAGGPSMSTMMNIPPPPPRYPGAPPG
AVNGIMIPPPPGPPPNSNLGPQQSWHGNYGKLYDGRTPVGIPPPPPTGHQ
HLPYNPKFHAQMAAGQTIAMPPPPRPSDGMAMGATYIPSRGTYGEGVGIP
GFDPNELASTSGSYMQASMNTMDTSHTGPMDDPNSRDRFYQSRNPSNAST
ATAGSTIPPELAAQWPLDTVLIWLAQNQFSKDWQETFRSLNLYGAQFLDL
GSLHGGRGNFGMMHQRVYPRLLQECSSSGTGWDQSRERDEGKRMRRLIRS
IVTGKPVDPSKMVSPHHRANSVASHGHNGSAGLEPVDSPNTPSGSTGSVN
FAGQRFSQTRSTTMPTLGSGTMNSDSSHRSALKYIDSEGGRKLSPITNEN
AEGGTFRGTTDSPGGSPVPQQVLFPSATTPVLSPSPASAKLGHRSRNSTD
SVTSNAAIYGSGVPADAAAILSRGLNLAEAMSASRSQDRSQYSPLNSGDR
SAGGDTSASARDSKSFLSFLSRKKRQREDGSFPSPDEFDSPESPMCVPKS
LADVYTSLNGSDSQLDRLGSAFVGTHEKDQVAMLERLNRSGGVRTFVLAT
TDYWNYRMCDITEAESATAIRQTICGSLGLTGFEQYSIFATELGKSDHAE
PLDDQQLLTDKRVKGDSTGALKFFISPAKISSATTPNSVPASLFPGYIPP
GMDEESYNRSRQRSSSSPPSSRANTIGDRIDEKTLAQEASDYRAEIERKQ
RAYLAKRKEANKKESPTAPDYVPSSSIKGRNVDFDQPRPSPFEDKKPDQL
FPQRRPPAPPSDPSATLIKANSLSRKPGHNLRNSSGSMDGFPSPRHPPLA
ANGDGESTDGQRRSKYAAQPAPASGGIGAALVGMGRNLGAIGQSTANGPK
ATSPGRIISPPYSSAGSGNAQQQSGKTALSSVNYASTSNRGRGSPKPANG
SPGSVTWSPGNLSFVVPDYSPGGTPALVAARVPSGSSAKNTEAVPRPVSP
GDVSPSSSHSKSRGSADVFPSKRKSHGPDLDFKESDIQFSNSPSSQPPTR
DIKPPPGDDSDDDSSDDGLFAIPLAGRKKDKPSSDTGTSASSSAQMGTDT
RPVLHVVTSRKEKGRSVTFMKSPDSFDSSNDGGARTSSTRRTPGTPQTDT
WDSDKESQINRRKSFIEKDVWANRPPTDDLLNNLDDFFPNLDLDQPVLED
HAGGDIPPLPSTDSGDGRNRSSSASLASMTTPGHSTSGAPPVAVPPSRQL
SLYTNDNDTLGSDESTLKATERPTSSHAVAHRSIHRSGGLGRMKSIREVA
SQANKRYTSISQPVGSKSQTNLMRRKSTKMFNANIVQIRPDQRGSMVVPD
IPQNTLPKRQTTFRWFKGQLIGKGTYGRVYLGMNATTGEFLAVKEVEVNP
KAAGGDKGKMREMVAALDQEIDTMQHLDHVNIVQYLGCERKETSISIFLE
YISGGSIGSCLRKHGKFEESVVSSLTRQTLSGLAYLHREGILHRDLKADN
ILLDLDGTCKISDFGISKKTDNIYGNDKTNSMQGSVFWMAPEVIRSQGEG
YSAKVDIWSLGCVVLEMFAGRRPWSKEEAVGAIYKIANGETPPIPEDIQE
TIPPLAVAFMMDCFQVNPFDRPTADVILSQHPFCELDPNYNFYDTELYTK
IKDAYKIS
>Mb|QLI68349.1
MLKTSPILTGSANASPTSFRRGHATPPSPQASPRLAPSTSTARLHRSATT
SSPKATSNSPRRRHAKPIEPAVDGRVHHDGVTSRQKINQLPPSPQPQPQH

IVTPAASGSLPSGTPAHPQLSTGSDNDAQKVLSPNKRRVSPGHLSDDTPA
TSQTGESIAGRAPSSAITTAPKRARTSGEPKILPQRYELCAVEDIVELIA
HMLAELISTNDAIRISSGGLTRFHSRTAPGISVRDYLHRLARHATLTPPL
LLSMVYYIDRLCALYPEFTINTLTVHRFLITAATVAAKGLSDSFWNNTTY
ARVGGVRVAELKMLELEFLYRVDWKIVPNPEVLVAYYRGLVERTPQYALE
SDGSSDDSSIGDDEDVDEEEQRRQQKEEAQTWQE
>Mb|QLI68350.1
MSRKGVGIGAFDRSRLTSAHYASHGSSLRANNAQALETQLAVFRSLLQQF
AQTHAKDIRSDPSFRAQFARMCTAIGVDPLSSSSSSGGGPGGSIWAQLLG
KTVNDFYFELAVRIVEMCSATRGENGGLIGLREVRERLSLGRVDSAGNSS
DISEDDVRRAVETLKPLGGSYGIVRVGRKEYIRSVPRELSNDQVAAVEAA
QVLGYVSVSMLCDNLSWERARCRTVIDDLVAEGMLWVDKQTGGEWEYWSP
TFMVDIDEGHAPVAD
>Mb|QLI68351.1
MAASPPAGCSTSHDGKFQVSIYSRGEAKRSVEKRACEGEGTLVMTLKDGV
LKDSHDRTGYIASNYQFQFDGPPQANALKSSGFSLCQNGSLALDGTTEFY
RCLSGNFYNLYDRHWAEQCSPIDIVATSCDGGNNGGSGGNNGGKPVGTSM
VQTTVVNVISDGQPQVHTTVVPIPMCQIGDGQVQVRTTPCASVPQAPTKT
AAPPAPPVSQISDGQIQGPPKTAAPPPPVSQISDGQPQAPTHTEVPPPPV
SQISDGQPQAPTHTQAPPPPVTQISDGQPQAPTNATTKAPTAVPTAAANK
AFPGVAAAVFALFGAVLYL
>Mb|QLI68353.1
MDGDWDEVARIPFPPPGVHAMPTPVTTMTFDTFQELLWTGNDYGRVTSFL
GSELQRYTSFKAHLSTDGAVRQILVNDKGVIVLGSKDVHMAQRRGPPIWH
IKHDDTKELRCMSFTSKGTAEIIVAGLQDTMLVIDLNKGEVVKQIATEHQ
YSIMKRGRYICAATKNGTVNLLDPVSFAVVKSWNAHSALINDMDAQHDFI
VTCGYSLRQGQTYMLDPFLNVFDIKKMSSMPPIPFPAGAAYVRMHPRMLT
TSIVVSQSGQMHVVDLMNPNTSNVRQANVLTYLSMFEIAPSGESMALTDA
ECYIHLWGSPSKLHFVDLPTPIEFASPEEHAPQIEWTPETPLNSVGLPYY
RDVLASAWPDLVSDVGAPPIRFDPQFLVSLKPTEFGLYGRNTRGFRRNQV
EDTRNSHKSANSGLKAPKFLSEKARESAISDSRTDDKTDDVPSPVAENEI
ESKKSDVPVMYRNVEIKYSKFGVDDFDFGFYNKTRYSGLEIHISNSYANS
LLQIMHFTPLIRNLALQHAATACVSEICLLCELGFLFDMLQKADGSICQA
TNLLRTLSSHPQAGPLGLLEEDPHGSSLNVMLQGLTRFLLDKIVSDYKSM
SPTSSAMEQILATSATSSIRCMNCRSEYTRPGSTYVNDLLYPPFKPPGRN
AKMPRVTFSQVLKSSVERETTSKVLMLNTAITSHDHRMLWSTPGWLPEEI
GIIVEQGQFFCYEGEDLKLHLQRGIHNITVYSLIGMATNIEGGQTQKPHL
VSMVNVAHAEQEAPGQSQWHLFNDFLVRSVSTDEALTFNTSWKVPSVVSF
QIKEANNKIDSTWKHQLDTSILYHDFPNPNSETKTYHILDPKTERPGPDT
IVALDTEFVAIRQPEIEMNSDGERETIRPIVYALARTSVVRGQGENEGRP
FIDDYILIQEPIVDYLTSYSGITREDLDPRISKHSLLPLKMAYKKLWILL
NLGCKFLGHGLKQDFRVINIHVPKSQVIDTIDLFFLKSRLRKLSLAFLAW
YLLKEDIQMETHDSIEDSRTALKLYRKYLEFEDAGILETMLQDIYRAGRD
VNFKPPRKDDVGVQRTDTPPLPVDSSTPGPSTPVRKIIGMAPGSGFGGGT
SWTPGKGSPLR
>Mb|QLI68355.1
MASDFPYVKKMTPEGYVFPSHRLRRTCDAGRVPLVLVACGSFSPITFLHL
RMFPMARDHARNEGYEVVGGYLSPVSDAYKKKGLAPANHRIRMCEIAAEN
TSKWLMVDPWEALSPTYIPTARVLDHFDYEINHVMGGIECSDGTRKPAKI
VLLAGADLIQTISTPDIWDAQDVDHILGNFGVFVLERTGTELDSALAALK
PWEKNIHVIRQVVTNDISSTKVRLLLKRDMSIDYLIPDDVINYIYDNNLY
RELDLAGGSKGKEKADVTAGPSSS
>Mb|QLI68356.1
MESGVKRRKIDRGGSSLRHDGLIDVEARNAALVSSASTFVLQTDELLRKA
KLDYSKALKDVDGTLFRLKTIVDSIEPHAPLPIAEATAKFEKQHRITVPY
PDPKPRKDTPYKLSYAKPSQCNVVGSYVSRTMVKTQSALGIDMVVEMPVS
LFQDKDYLSMRYFYRRAYFIAYIAAHVRNEMRDTMALSFQYLHENPLLPV
LVLKPSADGDAMDGTEKTISKSNKKDSNRHGYCIRLIPCAPDNLFPWSKL

TPSANCTRLGETQENEEKKRKGNNTSSPFYNSTLNAERTFIQYLRVLTFA
KNECPAFPDACILGRIWLQQRGFGGAISQGGFGHFEWAILIALLLQMGGR
NGQPALSGSLSSTELFKAGIQFLSTTDFNKKPFAFNTAKVDAKLIREPGP
VMFDPVRELNILSKMTSSSASLLQLYAKSTSDLLADEGAEKFEPTFIMKT
DITLQVFDAQFEINNSHISKYADSSDRSSIAWKLGSEVHQVLKKAFGNRA
QLVHIQLPSIKPWSLSDQPAKLATKLLVGVIFDPAHMARQMEYGPPAEEE
KEAAAFRQFWGEKAELRRFKDGSILECVEWNGKLPSQICEELTSYSLKRH
FNIANEDIVAYGDRFSSLIGLSHLDKEAFDSARRAFTTFESDIRNLDDLP
LQIRQLSAVSPSARYSSLELPMLGFHMNTIQLLDVNLYFEASSKWPENLT
AIQEAKIEFLLDFDRRLTAANDKIVTYLGRENGDVGIENLAYLDIVYNNG
AAFRLRIYCDLEETLLQRQVQNMVLDPHVREEASQALDTLKWYSATLPLH
TQIIATFCTRLQPLSQSIRLVKHWFASHKLCGHFSDELIELFVLHVFLQP
YPWVMPSSPMTGFLRTLFFLARWDWRDEPLIVDSAETVSAAERSAMHREL
DSWRKRDPHMNNSAMFVATSSDHSGLGYTRRGPSKLIAFRMTRLAKAACK
LVRDQGASLKPESLFETSLQDYDVLVHLSTKAIKNILHDAATESGARKHS
MYKNLDASTGKMPLPIRTHPAAVLAQEWQRVYDDTLVFFPGGPNESVLAA
IWNPKLQKKEKFRVGLPYNFRRVAVDDGDDAAADMVEVNRQAILLELARA
GGEMIKRIEVGDDEE
>Mb|QLI68359.1
MLSLLLYVGLANAAVVRNSECDFHMNAAGSHGGPLGELNGGQIRFGNIPQ
TTFHIDGDKIWDRNGRGCWWTPPTRVLQCDTNQPPDSGFTIGCDGRVSYH
GQTTFYQCETGDDNQYNIYLGPNEGINCGEVTLTADGCHIACPPPPQPAK
SCPANLNGAYEFPHLIVSVDKSNPDKAAGTSFFGEVTSSISSIFNFDIPA
ADAGKTCTLVFLFPRQDQLTTSSFNFSGDGKIDFSLLNSPATQGTTWNNQ
PGKKTDYGITTVAPGNSYTIASFPCPAGQAIGFEMSDCGDTNFRYFQDYN
PSPIGLYITKC
>Mb|QLI68360.1
MSKRVANENGDGPLKGGDRPEAMDVDDREMGDFEDEFEDEFESEDEIFEA
GVDGRPDAEREAEEKAAAMDVDQGTFIVGRNKLEPGQTLAPDLSTYEMLH
NLSTPWPCLSFDIVRDALGDNRKAYPATMYMVAGTQAETGKASDNQLMVN
KFSGLSRMEKQDEEDSDDDDDDEDSDPILESKAIPLNSTTNRIRAHQIPS
NEPGRPPTTLTATMTESGNVFIHDVTPHLASFDNPGTTITAQQNKPISTI
RAHKSEGYAVDWSPMVPSGKLLTGDNDGLIYVTTRTDGGGWVTDNRPFQG
HTSSVEEIQWSPSEQSVFASASSDGSIRIWDVRSKSRKPALTVQVSKYDV
NVMSWSRQTSHLLASGADDGTWGVWDLRQWKTSTDKPQPLASFDFHKEQI
TSLEWHPTDDSIMAVAAGDSTVTLWDLAVELDDEESKDTAGVKDVPPQLL
FVHYLKDAKEVHWHPQITGSLVATGEEFSVFRTISV
>Mb|QLI68361.1
MSVPRARLLDLMKAQCQVFATTYNPEGVRMGNKVLRQRLKGPALAAYYPR
KVATIKDVKREFGPVLATWDEAEEDRFEYIEELKQRGKSAPKKKNGPPAE
KPGRKR
>Mb|QLI68362.1
MHQQARPPPRPSVSPASSPQINQISQTRPNTARDAAQSSRQRAGSNVSGR
DTMASPAVENNPAMGPPADSIKKLDQIVQNFFNKAAVLILESRMKVKPTR
NANGQRKTNKWFQIETDEIDDFRDELKTWKTCGSLDHRPPPLIIEIFLDT
SSLKESQSLVILDDDGKRWDVMEQLNSSESSSGSYHTAPTSRNAEVVLER
WRIDLKTTGTLLTDDFGPILPTIYKKAIVFFRSLFITTKLLPAWKFASQS
PAKTSHPALTPRCRIRTSEPGKLATGLLKHPIDGRREPVTEYVFGDLEVP
VGRLSTAVTYRNYCGFRIDDSESLLSSRFMGADENLFKPSVTQRTDRAHG
RVAEVGSLRDHRRGTALSDLHQTYGSLSTFHGDGPLGTSPLSALRSIKVP
GSDTSSPPASLPRGAGTEVAPSSLPISGRISTPRAMASRPGESSSRRTSI
SFQPFKAGSLSGSPVPRQLEPESPSSAHSQSRPVNFPISSQPRNRSSLTA
GMPASLRGGPPSTSVETPTVGSPRPASTGRYSSSFTHRRGRMSIGRAGDD
EQNSSGRQSLASSVAQPGSGLLAETGGTSSGSLHAEEDAISDFLKALDSR
KTLKSFEPTKRGESATNRTVAQLSKFHLMRDSNNALTESMTSSMQMQRSS
SSSSRQLTSVPGMVAPASMSASSSPGKPVSPHTPHTPAIPSRLSENSIID
YTATGRITSRGGRRRQEPAVPEPRRESTITQEGTTAIDIPLSPRLGSYQR
RSSSAVQQTRAVIEDDDTELAFAGGNRSISLGADDREPPTLSILLGRQLQ

MEDEATGGEGITGSLRPTADVQPSDASAPDAMQRGSNEDNLPDGLMPSSV
QSGSLFPRRRYAGMGAASKATPPQSSRGSFTGSLSRLARADDESVGEEPL
VFDLSEMDAQGRRSLEEGRGGGNPSNERGAFEPRGTTRRGW
>Mb|QLI68364.1
MAPKQDTPFRSADMSMVQLYVSNEIGREVVTALGELGLCQFRDLNEDVSA
FQRTFTQEIRRLDNVERQLRYFHAQMEKAGISLRKLDLDTERLASPSTSE
IDELAERSQKLEQRIFALNDSYETLKKREGDLTEWRWVLREAGSFFDRAH
GNVEEIRASMDNDDAPLLQDIEQHHGAPEVERSFSGMNIGFVAGVITRER
VAAFERILWRTLRGNLYMNQSEIPEPLIDPTNNESINKNVFVIFAHGKEI
LAKIRKISESMGAEVYSVDENSDLRRDQIHEVNNRLQDVQNVLRNTQATL
EAELNQISQSLSAWMILIAKEKAVYSTLNLFSYDRARRTLIAEAWCPTND
MPLIRSTLQDVTNRAGLSVPSIINEIRTNKTPPTYLKTNKFTEGFQTIVN
AYGTASYQEVNPAMPVFVTFPFLFAVMFGDFGHAIIMLSAALAMIYWEKS
LKKVTFELFAMVFYGRYIALVMGVFSIFTGLIYNDVFSKSMTLFPSAWKY
EKPDGWQPGQTIEARLNDDGYRYPFGLDWAWHNTENTLLFSNSYKMKMSI
ILGWAHMTYSLCFSFINARHFKKPIDIWGNFIPGMIFFQSIFGYLVVCII
YKWTVDWTNDDPNIGGQPPGLLNMLIYMFLQPGKLDIPLYKGQATVQIIL
LLLAFVQVPVLLFLKPFYLRWEHNRARAKGYRGIGETSRVSALDGDDEND
GLVNGHGNSFDDDGEGVAMISQNINEEHEEFEFGEVMIHQVIHTIEFCLN
CVSHTASYLRLWALSLAHQQLSIVLWDMTLGPTLKMSGALGVIMIVAGFY
LWFFLTIAILVCMEGTSAMLHSLRLAWVESFSKFAEFAGWPFAPFSFNTL
LEETEELKEFLG
>Mb|QLI68367.1
MPKPRTKRGADREERKRKRRGEDADKSKYESKRQRRIADELQPGDQQDFP
IEEYQQQPNGPTEFFGMLADEEQEYFRRADEMLELNQFPTAEDRDMFLES
VFKEAQGKELKLASSQSCSRLMERLIQLSNTTQKKNLFNAFGGHFLSLVQ
HRFASHCCEALFLRSAGIVTQELAGFTVDIKGASADEHQPEASMEELFLA
TLDELEGNLSYLITDRFASHTLRVLLLVLSGRPLDDVSVKTLMKSKKKEN
ISVAGSAAADEQNKGLRAVPGSFTLAVQKIIADSIAGVDSTGLRVLARHP
IGNPTLQLLLELDMALNKTEKKSSEPSESETAPPTLLQQLLPGAPKSLSD
PASEASEFINGMIYDQIGSRLIETLVAHCPGKVFKPLNHNFFLPRIEVYV
RNDISCYPAIKVLNRIGKDDLVTAADKITPTVPQLVAKTRYNVLRALFER
CSARGINDQIKKLTKALKEGCGSQPADLVTTLCHLSEPAKSKEEVTRNEY
AVQSHGAALLTTLLSIPGSLKAVHESILALPTPHLLRLATTSMPTVTLLT
TALSTPSQNSLFQKSLVATVSPHTAELSTSQFGHNLINSIADVPSKGKDF
SVPFHVKESMMQRLANQEAELRESWMGRSVWRTWKGDTWKTRRGDWKTWM
REIDAQAAQPVTHAKGRDEGDKHNPAEKQDKKHMNTRKDEE
>Mb|QLI68369.1
MASNGLTNGSSAAPTSRFDPNFTSHVIKTMGPNMTPRNREVLSSLIRHLH
DFAREVELTNDEWMTGVHFVNFIGQTSTKTRNEAHRISDVLGLESLVDEI
ANKIIAEGDIDPTSSSILGPFWSPNAPFRDNGSTIIQNPNPKGRVCKMHG
TITDLLTGKPIPGAVFDIWQASANGKYDFQDPENQTPNNLRGKFTADDNG
KYWFYCYHPTAYSLPTDGPAYQLLTLMDRHPMRPAHLHIMVTHPEFRGCT
TQLYPKDDPWLETDTVFAVKDDLVVDFKPLKGDDKAVLELEYNVVLSPKG
YKGKTF
>Mb|QLI68371.1
MPNEVEDHSADLSDHLSDNEEENESPNKDDDNMTDTKKKYDPKDPQRPRR
KKARRACFACQRAHLTCGDERPCKRCIKRGLADACQDGVRKKAKYLHDAP
PEALRPVLGPNYDPEAERVRTNGQRQNSTHSDTVSPVTGGNGFLAPGNGP
SFPVFSAANQPQVGMPDSLPFNAQATSAFQTSPAATAPQMDNMMQGNSMD
FNALFDPSNPALYNFDLEGLNFGSQYAGWEFGILNKMGLAAETPPRENSI
SATPTTEANYASLFGNGPNQPFDTMMTGSDYSGVDSTTSIYTQGNLQHGL
PHAYAIAAGPNSLASPSTDATASPQAALDGSPHTAFNHLPGVASAGRMKP
KQDKMSQILIGKRRDSASVYANVKEPYPYTAGFHNMISVIKHRLPVNKLV
MIARSLGEIRPSFIACTKDLTREDLLFMEKCFQRTLFEFDDFLQHCCAPT
IVCRRSGEIAAVNKEFTALTGWTKNVLLGKEANRNVFVGPTPYGSDDGTD
GSTRPDATTPHLKNATLEPSGERPQPVFLAELLDDDSVVQFYRDFAQLAF
EDSRGKVQRSCSLNKYRTQDMLDGKETPTHRDRTGILSSRVTKIDSEHGI

SRIEKDGKVNCTYCWTIKRDVFDIPMMIIMNFLPRYYPNQEPHQLAV
>Mb|QLI68372.1
MTNLRPSPPKLAIPASHGSPSAVSATIQTGRPSMWSKSAQRKMTRLYVYT
TLPLAKIVALVHSHTPGTAPGRESGHKKLNILLDKEPRWLHPKTDEDMDR
RITELANSPTRLKSAEYAAFGPSRSISDNLSISPSPQRLETEDSVSPAPF
DVSPLHRPAAAFASHQPADGALNFWVSPGARSSMAMSPAEDAADPPHFAK
FLRQTTCLSNATSNTTGSFQRVLSDFSEPYVQTVKRLVKRFTAPMGSRPG
LSPYSEGSSAIETWADEEDCPPEFDGRPFPIPSDFLNLDEYQSTGVCYST
HDVHDRRRCLCFMPLDTCVLPWVTRQGLTPVGQRLLSNGPREQDFGPSDS
FGNTVLHFLAARGSYELVERSLRSDLCKSRIAAKNSAGQTFLHVLNASIL
RNPDHVEHLLDIVTARGYDIHMRDHYGRSFFHLLQAGGHPKELIQRVSRR
CDGNFHMARDAFGFTPARNSSTMMELDEPNFVGLQDSFGPAAFTDAGLTK
ESKLLENVRKAMLNPYLQDEEGRNGLHCLAMATLSEASVVNKYHLQSSQR
DKVTKKGAKPERELDSSSSKLGLRLGLLSGLLDAGVDPNQHDNNGNTPLM
TFVAELPEEDDYKIPPRILQLLIARGANIHARNRRGETALHIAVRCGRKL
AMRALFEKGANVHARDSAGRSLLDVADVKMLHCDSDEHMREYAHYEACRA
WLSGKGLALQGPSVLDEWGGGVRA
>Mb|QLI68373.1
MHRQPFIPARNSRHRIAALSLYRALLRSTRKIDVPKDAQRPARDAMTRAV
RTAFVRNRAYTSYRLVYASMVAGYKFLSLLFTAQKPGCPEHTQLIEHLRR
RGDLNPSHSKPKDTARRPREPFLKKVAGHDDGHPVYKPTYIPHSFRYRIP
SVSATADGQPFLRLKKPQPRALSKMIGVKGRIFINKIRKLVEIDGEPRWN
AAQEDQWDELMAKQMKAERVRGESERSGGVNGGGQKISESSFSWSVQLTR
LWWEWQVERTWRDWNARGEALHQVVEEQNIQLRRKDAVDSETLQKRSLKA
GSMPSSRNLGTPPNVDVFPARPYPLTASIPAVLAKQTDRQQMGGTGKSVD
PFTEPAWNALVRAQGNRLLKWAGKGGASKE
>Mb|QLI68374.1
MVRLREVPRTATFAWSPGSGKPLLVTGTRAGAVDADFSDESKLELWDLSL
DDQLQGLELQPLASITTESRFYDIAWGSADSDHPKGIIAGALENGSLELW
DAAKLEAGAPDALISQTNKHTGAIKALQFNPLKPHILATGGAKGELYIFD
VNDIENPFRLGNVAARSDDIECLAWNRKVSHILATGGAGGFVTVWDLKTK
KASLTLNNNRKAVSAIAWDPNNSTKLLTATPDDNTPVLLLWDLRNSNAPE
RTLQGHEQGILSLSWCSQDSDLLLSSGKDNRTLVWNPQTGDKYGELPEMT
NWTFQTRFNPHNPNLSATASFDGKITIQTLQNTNPDTSQTPAEKNLDGEE
FFRAAQTQPQGASWSLNKAPNWFERPVGATFAFGGKIVIFKANPSQPGQK
RSSKIAIAKFSADADVSDASAKFQTALESGDVAAICEERASQAHTEEEKA
DWKVIETLAGDNPRGQIVEYLGLQENELVNGESKADADADEKKTDSKADE
EGEQKSKEVSDFFGGDEGDDFLSSVAATKGAKTDNPFHLFSDGDTSVEKN
ITKALMLGDFAKATDICLKEQRIADAFLIANCGGQELVDKVQTAYLSQKS
GMPSYLRLLGSVIAKNLWDVVYNADLENWKETMAIVCTFSDPTEFPDLCE
ALGDRIQDGGSRKDASFCYLVGSKLEKVVSIWVAELEEAEKAGMKEATGG
STFSVHAKSLQNFIEKVTVFRQVTKFQDSEQSQTSDWKLATLYDKYTEYA
DILASHGQLEVAQKYLDLLPSSYPAAEIARNRVKLATKKPAAQAATRQTT
TRQATSSTASRVQQPTLGYGTPQPATTGSPANPYGGLGQPHAPVTQTQQY
PPQNQYQPQGYQPSMGYQPTTQPAYGGGMVPPPPMAGGPPRSSSSTPSAP
PPNRAKSAENWNDVPLVTKAMPRRSTPVAPITSPFPGSTSPTAGSSAPPT
GPPPPGGYGHATSAVPPPPRGAAPPRVQSPLTGQTHGIPPPRPSSTANQY
APPAPQPGAVPSPVPQVVPRTASPYNAPPAGAPPSNRYAPSPAAQRPNQP
PTSAMPPPPSAGSRPPPPANPYGAPPQQTPSPGQYAPSPYAPQQGQAAGP
PPAGRPPSGPPPSNLPQATPPPPKAAAPPAKAKHPAGDRSHIPAHAQRLV
DVLSQDMQRVAAKAPATFAAQVNDTQKRLNLLFDHLNNEELVQPATIDQL
TQLADSIQAKDYAAAQKLQVEIQRDKTEECGNWMVGVKRLISMSKATP
>Mb|QLI68376.1
MPNTSLRKPQKGFRRGGKSAFHGQRTRTFAATSRAEGTSADEKWERTRLA
QTIDENFGFGRYESGKKKEGWLVNVQPTAIEDERIPGGRAALDCYFIEHD
GATFKATVEYDPYFLIAVKKGHESEVEEWIKRVPGDGCIKTIRRVEKDDL
NMPNHLLGYRRTFLELKFANVSDLMAARRDIMPVAEKNKKNMNAMDAYAE
VVTANGNFDLFDDDLRTDERTNNPFAEASDYIVDIREYDVPYHVRVMIDL

DIRVGKWYYIDVKHGVTKILPNEDRSLPADPVVLAFDIETTKLPLKFPDA
AIDQIMMISYMIDGQGFLITNREIISEDIGDFDYTPKPEYPGPFMIFNEP
NEKAVIERFFLHIKEARPTVIATYNGDFFDWPFVEARASVNGIDMYQEIG
WKKDSEDQFKCNYGVHMDCFHWVNRDSYLPQGSRGLKAVTVAKLGYDPDE
LDPELMTPYANERPQTLAEYSVSDAVATYFLYMKYVHPFIFSLCTILPLG
GDDTLRKGTGTLCEMLLMVQAYQKEIVLPNKHQSPKEAFWDGHLLDSETY
VGGHVESIEAGVFRADIPVDFAVDTGAIDELLRDIDAALKFVITVEEKKS
LADIENYDEVKEQIVAKLMKLKETPRRRERPLIYHLDVASMYPNIMTTNR
LQPDSMIDESDCAACDFNRPGKTCDRRLPWAWRGEYLPAKRDEYNMIRHA
LENEKFPGKTPQAPMRTFADLGADEQAALVRKRLQIYSQKVYHKIHDSTT
IVREAIICQRENPFYINTVRDFRDRRYDYKGKAKVWKGKTDSLKSSGASA
SEVDAAKKMIILYDSLQLAHKVILNSFYGYVMRKGSRWYSMEMAGVTCLT
GATIIQLARQLVERLGRPLELDTDGIWCMLPATFPENFAFKLKNGKKLSI
SYPCVMLNHLVHDKFTNHQYQTLVDPKTFKYETHSDNSIFFEVDGPYKAM
ILPTSKEEDKNLKKRYAVFNDDGSLAELKGFEVKRRGELKLIKIFQQQIF
KFFLEGEDLTQCYSAVAKVANQWLDVLHNKGSTLADEELMELISENRSMS
KTLEEYGSQKSTSITTAKRLADFLGEQMVKDKGLNCKFIICARPRNAPVT
ERAVPVAIFSAEESIKRTYLKKWLKEEPTNTDPRALLDWDYYLERLGSVI
QKLITIPAALQKVRNPVPRVPHPDWLQRRINIKDDKMKQKKLTDLFTKEP
LGDITNTNGSRMEDLEDFGNKLLKPKQVSAAVASSQPAPTVQKRKSPEPS
ENTDPMAALPKVMPNPSENYPAFLKYQKQKWKLQKQARIRRRQLFGDRRG
LTGNNIQQSFMRQAHVTFMNTWQLLHLQATDSPGVVTAHILIDAKIHAVK
VKVPRQVFLNLKGSELPDVDIAGCEVEQVNYTLPNGHPSSHLFKLTVPED
VYFSEAAKFSLLFNHPSVEGVYEKQVPLNIRAVLRLGNQCVIDEQQNAVL
GKGLEQGFDLFGLKRPVKIRTYLESSQLAYIYISHITAGERQIFGIFSTT
SDQGHLIIMQKNKDSGQDLPNITRMYREMLAKRAEEAAGTNWQDCFAYQE
NMAFKITQVSTRRKVHLEIGDVVKKMRKDESRPQMMVIQSSQRNLLVHDI
PVLAEFPVLPLKYDATDSSLPPLGWQAVVARRLVGHYLGLGSWILHLTAL
ARYGDVPLCNLEREDPRFLIDVAYARRLQANGVVLWWSAGPRPDHAGYEK
DDILGPLDTVKMPNVNNPGTFASVCIDLDVRNLAINTILTSSLINELEGA
DSVSFNPSGADGSETLASDNAFANSGVLVLREMVKAWWAEACKGSTMADV
LVQHLVRWVENPDSFMYDRALHYYVQMMSRKAFQQLMADFRRVGSQVVFA
NANRLILQTTKAEVGNAFAYSQYIIKSIKSKPLFHFIDLEIKEYWDYLVW
YDEFNYGGKGCQEVIEADEQNLETIMHWQMATFLPVRLQPIFQEWVIAFI
QLMHNLKRPANHDPDSTPRLTQLPNRNDETGESQIILGKAYEKPLKKAIA
SLISQQKRELLHPELAEDYSFPSLPGSHLTPRAPVLELVKSLMQILSLDK
NITLEARLLRKELLALFDVREFSKEGTFANPSESLKLMQISCDSCTMARD
LDLCRDEDLQGSDDRGWQCSFCGTEYDRVAIEERLLSMVEAWTVEWATQD
LKCSRCGALRVNDLMEHCTCSGEWKEIVSRQETMRKLTVMRRVAKYYSLR
MLSNVVDGLFSDL
>Mb|QLI68379.1
MVSLKWAVTFILAVSFAVFVTFFGRLPVFRRTPIGFLHRLIWIHVPNMLL
SIDNRLTSGRITRSLSRFGHFIMHERHPAVVIFFLILMVGGEYLYLPPIW
HRLAPFTKLTVTVTVFLPYLFLYLSCAADPGYITKENHAYHMSLYPYDHA
LFHPGVECKTCGFVKPPRSKHCSLCKRCIAKADHHCIFINSCVGYGNHQW
FILLLLSTAILCTYGGLLGGSLLSSSIKQHFVDWSPWSPLKQGWGMYVVI
WGWGIQTNIGLGVITLLSGLTAPLVWGLLLYTMYLVYCGTTTNESFKWSV
YKDDMEDGYAFRRPMPSNRERDLVKEPICRRWPVEPEMVLAATDDGKPPP
AQMALAGEGEWERVWNLKDVENLYDLGLWDNLGDIFVADCAFGARVEDPP
AERGLRTKRTKSK
>Mb|QLI68383.1
MPPAQDPATLQVELGAVPPAGSPYGVPVPGSERPGRSAVYRGWRFRDREL
LTTFDPNVRSIHDLFEASARKWPKKRCMGTRQWNSATQTWGDKFEWMTYG
DVAERRKNFGAGIVEIHKRIGYPKDKYGVGVWSQNRPEWQIADLGASSQS
LYTVSLYETLGPDATEYIINHAELPSVVCSLPHISKLIKLAPRIPSLKLI
ISMDPLEHGELPGLSKADVLNDLAAQHGIKIYSMAEVEKIGVESGRSMRP
ASWDDIATINYTSGTTGAPKGVVLSHGNAVSANAAARLGGHVKDNDVHMS
YLPLAHIYGRLIDHIALSEGAAVGFFRGDVLGLVDDLKILKPTGFISVPR

LFNRFNSAIRTATVEAEGFKGALSRHVVNSKKAAMKAAPGQASNTHFFWD
RIWTRKVRAAVGLDNAHTMISGSAQLDPDVQEFLRAAFANTFQQGWGMTE
TYATGTVQMKGDFSTGNVGPPMGDVELCLESVPEFDYTVNDKPNPRGELL
VRGPAIFKEYYKNEEETKKVLEADGWFHTGDICEIDKLGRFKIIDRKKNV
LKLSQGEYISPERIENVYAANTNLVNMGYVHGDAKESTLVAIFGVDPENF
PAWASKVLGRTVSNDPSDMRAAAKDPKARQAFLKVLDNIGRKHKFNSFEK
VRGVHLDVEPFTIDNELLTPTLKLKRPQAAKKYRAEIDAMYAEINNAQPA
GSAKL
>Mb|QLI68384.1
MAIVEGWYPPTRENWQTTVLIFQILYPALSLMQLFISWYGMGKTSVRSAF
NLPGRVAWVLMEMPGFTTVLYMMKTLPRMHGIDDLPWQNRVLAGLFVIHY
IYRAVVFPLIQPSMSPIHVLVALMAAGFQVLNGVGIGAWLAAYGPVTAEA
WAARSSVAQFCVGIAIFYVGLAANFFHDEELREIRRAEKRRRQRVLDKQG
GAGGAASVEKHYQIPQAGLFKHVLYAHYLCEWVEWFGFLVAAGWGCVPAW
TFLVNEVFAMLPRAVKGKKWYVEKFGEDKIRKRWAVLPGVV
>Mb|QLI68385.1
MTASRQQRRQRSGPWLATNNTGIFYRPEAHIDRRSPSTDCFPTPLISPRR
SRPHAIHVVTYPSGYVPRELRPDAKTNRERSQRSRGERPPRSSPDSEVPL
AFSGATYPGIGQSSSINSSRRSSKIFHHKRNGSSTSSSQIFRPALGKLIA
SISGRSPNNTEASQPRQPLPTQATPAPGRAQVQTHSAVPATTLSASAGVL
ASPAYDDARQDSAAQIPHQPHHHLSPPGLSPHRPSHPHPSDEMAVASSNP
PAAYNIASHRNSLMSVRSAKSAVTSLVTEVPKPVASGSGVSCSILLAERN
IFLTGFDHDGHVHRDGQTGTALLRGKLQLNVTKNVKIKAIQLKLLGRART
EWPEGIPPLKQDVCEEESLRTQVLTFFNAMHDGWEGEYGNQCTYKLKSSS
ANSSSTNLAVPQAPHRPVSLLLSSSSRNGLTAKELKRLSLQNTQSRSFGK
SENGVATTTQVKGFKVFYPGTYNYTFELPIDHHQLETTKLQYGSVKWELH
ASVDRAGAFKPNLHGTKEVSIVRLPDQMSLETTEPISISRQWEDQLHYDI
VISGKSFPIGSKIPIAFKLTPLAKVQLHKLKVYVTESIEYWTNDKRVTRK
DPGRKILLLEKTAGKPLDANWATSDLTTVRGGELQPEQRRQARDIANLRR
ARDAQRRGRAAEPLPEPTNNLLGDLDLGLEHMWGSTEIEANVQLPTCQMM
AKNKDLRLNPDCSWKNVNVYHWIKIVLRISRIDPEDPSGKKRRHFEISID
SPFTVLNCRATQANTNLPAYAGPNNNQTPHQSVCGCPDSASLPCEGSQNS
STGTLPGTELSNDGFPLPPQAAHLAHSAMSPMSSISSPLRSVDDLGTQGE
PRPIHLLRVPSFNPPAFDDDTAPPPLLVNEVQGAANAPVMTPPPQYDAVV
GTPSVDGLADYFTRLANAGYGNEDDNDSGSDDSDELPTRILDRTGRVNVA
HPRTPGSRMPSRSFEISRPPVNLHLGDLPTRPGINNEANAVN
>Mb|QLI68386.1
MRGTALVAGLAALRAGAAYAAATSYCPVPGQVCFQWGVPEAAASSGSGSV
YIQIRATTSYEWVGLGIGEHMSGADMFVVYADGAGNVTLSTRRGLNHVMP
VYEANAGVELLAGSGETDGRMVANVKCGSCSQLMLGSSNAWLSAWKHGGS
LGSRDLDAQIGYHDGHALFSVDFAKATISSDRNPYVGGGDDAGNSTSGSG
AGPAPGGGVVVVQADPIQTLVNAHGIIMSVVFVLGYPIGAMLMSLVGRWA
IHAGWQLVVFLAMWAGFGVGYTLARRTDLFFRQCHSQLGIILVCLVSLQP
LLGYLHHRHYLRHKQRGIISYLHIWYGRSLIILGMINGGLGLQLAGLTTD
NHAFIVAYCVVVAVVAAARVSRIRCDGDQPCHGCRIRNRECSYTPSRRGG
SRKKRNVAAKPTSNEQPDLAFPSPPLSEIDHLTSPGAGLRLLSFDELDLQ
NGAGSLGTSESGAEDFVQHAVRIYDSEQAILNAYYIYLHQYFPILPPPVR
PVTPDNPIRITSLEGCFPLSLTSPLALAISAMLALIPFPDQEPTPVMSTE
EPLRRSIAHKLAQLALECVESDSELIDDTPGGHPSPIPFKGQPIPRFPLH
PRTPVMLEGLLALLLLSNYEHTQRGNMLKMTTRASQALIMARNMSLHQLD
GFEDEYSEAKRRAWWMSYFRAQLCSVVRQSASVIMEDDSLYTTPYPTIQG
DPEVWPLYIASLQVSFATVRLAAAGVPPCAKPDPDLLRSKSEKIAALDAR
AVQLIERAKTGPGSKGYPTNDDPASEPLVSKTMRSIAAIRSSSARIRLHR
YHAFSDIPLFSMNHCDLSSPDFVPDTDNVIPQTNPPESSPFTFDEAAEVC
VESALEVSRQLKKLPYPDKTLTTVPARMTPTILCCAMHASYVMMMQFYRL
YLRHQQESGDMMTTSFERRVDELRHALQDVIATLDGFANVLEAIRGMKID
VEAVFTVAFSDMMT
>Mb|QLI68387.1

MSTDSGAPESWISSFCALLGHEYFAEVSEEFIEDDFNLTGLQTQVAMYKE
ALEMILDVEPEDDEDEEEEEEEEDDNESGLGDGQERMGGRPGERRHHSRM
ASDLSVIESSAEMLYGLIHQRFICSRAGIQQMSEKYELGHFGCCPRTNCD
QARTLPVGLSDIPGEDTVKLFCPACLDVYVPPNSRFQTVDGAFFGRTFGA
LFLLTFPEYDLTKRGVEVLSSATSRIAADEELVNGMYTRNIAPGLGPGRI
YEPKIYGFRVSERARSGPRMQWLRARPIDVNDLDESRIYAEQCAESEDDD
ESMNLNGRAMVRRRPPGNARLRQRQNQNGSPMALSTNGAESEL
>Mb|QLI68389.1
MHRQILGPTPLLQISRCVQHHRPRTVIPSPTPPRPSIDAAVKMLYELIAI
VRPGNLGEVKEIAQTVGSLVLRNGGVVRGLDNWGIFSLPKPISVHQMKHT
HGHYFVMRYDSSTKVHQNVRTTLRLEPRMIRSAHVKLGDGKLSTLSGFGE
PKWRTQGSEA
>Mb|QLI68393.1
MSAAPTSTSAPPPARKVKFVASDPSRGGLPVKRKQVQQACAACRRKKRRC
IHADDAHAQEESTSEEHRGQDSSPSQASPSTGASVVTPRMRTAAVMHENG
TVNNTSHTALALHTSQPPYSGHRASVAMPSQTDHLKQQSSRFVGDLNPEG
MFLEVTSISAGASSQKGDVGIWLSSGAFGGTGQSSQFITSRPPPLMDHFL
LPFVREHCLSCLPPPEDFARLKNVYIQKVHPIFPVIPIADIDNDLDHPCS
IVLRQLVCLAAAADPELTEHYRLQNRGPGLLSPQDFSQSLSSSVRAILET
SLITDRVMHIQALIMLSLYTQPTCAEESDLPAQLGGRAIHHIQTLGLHLL
RYDAPNCDDLENLFCCVWALDRINAAEYGRPCLIHERDIGADLEACIRKR
PPCFRLFLSVIQWLDQVVELYRPGPSAEASGLEKIAYIDLPVLEAMIVDA
DALKVPSSLIATIETFYHAVIILSCRLARPGTVHAASTLPPPSANARRSL
AAERIACAVPRDHLSPMPFIPYAVSLALSVEYRKMRHSRLAMFRARAMSS
FKRNCEMLRKYGDHFWSANVVAGLGERVLKEMERAATSLTTKDAASPVPL
DVGSRSTSAGVQSQSVRQTQMLPGNTAGITSAVGMDNAVDFSLIDAISGQ
DVFGHIDPSFNLEAVDDVLEANLDIGLPLNWGDWGQFAIN
>Mb|QLI68394.1
MDAKGASASTPRGIRKSNSTRSAGAHSTTSHESVRVVGVHPLGTLRVLGP
ESSPRISEQNDVEDDDDQGPPPLESPVENAYGLSGNQHNSPQSSSASPTK
RSRDPQAHEMGGGDVAVILDLPDLFVVAYDSVSFTAQNFVGVRDVQAGPH
FFWVAHPSGVAARDGVWLLGSESQNHVHVVQWDSYNEMLTEATRSESRNS
AESMPNIHAQLVPYADPSRLTEGGGQLSVSQVERNRRIWNQLTTHVNATV
LNRITGPQVGGWIVHTLDRAQGALKFSPEVELERAVPNRNLQRRELHFAF
QSTTRLFSIESLGSERTREATDPSIYLMSQIEDTKNNLVYDDLLGEFQFA
FVVGVHLGNDASLEQWWFMLLKLLVKSHLLVQGQPVLVASMWHTVAAQIA
YSSQWMESSVLENNDTRCRELRIALIIYKRRMEEFLSDDENIVTPDHLAV
STAFSRLEAVLTDIGWDLSGDYLRKGIVVMEDGEEVELELTDLEAEDERG
EWAPEFVELDENGRERGLISWSD
>Mb|QLI68395.1
MRLDFSNLAVTASLLASATNLVAQDIPVDLPVSTLLTSAQSHLARGETSE
ALAYYDAAIAKDPSNYITLFKRATTYLSLGRSNQASEDFGRVLELKPGFE
GAHIQLAKIKAKVADWEGARSEYMAAKRNDESTELVELAEAQGAADLAEA
AERDKNWEECISHAGVAILVASRAPSLRERRSRCRFQRGEIEEGMGDLHH
VLQLRPGNTDPHILISATTVYALADFDNAIAQAKKCLHSDPDSKICQTLH
RQERRLQKAFQKVESQLNRGQNTLAGRSLVGSADEPGLLPSIREQIDNLR
RDGWIPMQARTKLYDRVVEMLCQAYSESNHKDASKYCEEAIELDPESFWG
LIYKGKSLLKREEYDAAIQTLEKAAEIRPDKSDKVHPILQKAQIALKRSK
TKDYYKILGVANDADERQIKAAYRKASKQYHPDKAEKQGITKEEAQKKMG
SINEAYEVLINPELRARFDQGDDPNSQERGSPFQGSPFGGGHPFMFHQQD
GGGANFKFHFPGGGGGPFGF
>Mb|QLI68399.1
MQSIPELLVEEKNDLPGVNFARDLAPFRVSRGASPISELEIRDKLPRGIE
MFGSHPGLTIVHIFTPLTVERMTNPGKADTIRILVATDNHVGYEERDAIR
KDDSWRTFDEILNIARTEDVDMVLLAGDLFHENKPSRKSLYQVMRTLRQN
CLGMKPCPLEFLSDAATVFEGAFPHVNYEDPDINISIPVFSIHGNHDDPS
GDGNYCSLDLLQAAGLLNYFGRVAEADNIEAKPILLQKGITKLALFGLSN
VRDERMFRTFRDHKVKWFRPNVQMGDWFNMLAVHQNHHAHTATSYLPENV

LPDWLDLVVWGHEHECLIDPTQNPDTGFHVMQPGSSVATSLVPGEAVQKH
VAILSVTGKDFKVDKIPLKTVRPFVTRELVLAQDKRFKGLDKKKDNRQEV
TRRLMEVVDEMIDEANADWEAIQTDEEALEERPLPLIRLKVEYTASDGGL
FECENPQRFSNRFVGKVANTNDVVYFYRKKTSQRKATATLPENVLEALAD
GADAVKVEALVQDFLSAQSLKVLPQGPFGDAVNQFVSKDDKHAMELFVSE
HLTGQVKQLLGLESDDEDLDGAMDIYRTRVEQQMTSGPGKGMFSTDKKRV
LKPKPDTWDSDFDGNWEDEPDAWTYEGSTHDLSLTTTQGPGRTGRSRAEA
HGEEMSDDEPAPRPARRTGRGTKAATTKAAEKKASAQKAPARGRSRKNVE
QSEEEEEDVVMESEEEPAPPPKSTTRGKRAAATRTAASTTAKKTTTRGAA
KSKQTKLDFSQKSGGASQKAFEISDDEISDDDAFEPAPPVSSRTRRR
>Mb|QLI68401.1
MFQPGFGGGFDPANPEHLYNMARGRRPVMQRFDEYYRCYPVVMAPGAERP
ELNYGSKIILPPSALDKVSKLHVQWPLLMEMINGEKGRHSHAGVLEFIAE
EGRAYIPQWMMETLGMDVGDMIQIRTTSLELAKMVKLQPQSVNFLEISDP
KAVLERAFRNFATLTKGDVFNFEYNDEIYEVAVLDVKPETDKMGVSMIET
DVSVEFAPPVGYVEPERRSGTNTPGSTRSGVPTGGLVQSQGTMAQAIGYG
SIAPSVTSNPTNFVGEGQRLTKKGSKTSTPKPATPVPAEPTAPKSRTGAP
APLRLAPNKLFFGYEYKPLKTKEEKEQEQEDSKRPHFAGQGQSLRGTVKR
KGEADDKSKAPDKKGPSEGYRLDGRRPK
>Mb|QLI68402.1
MKDLPGSVPGKPVLPSLWTENLTIAFLNRLPNRYHVGRRRARSKSKTMHP
GAEDITVLRTSFNFWDGLRDLQRHRWRASDLQYIFLIGLTLFSLWIAPPA
PALKFFALVAVSWIFLMPATRQFFLPAVTIWVWLVYFFCSRFIPYDYRPH
IWVRVLPALENVLYGANLSNILSAHKHAILDILAWLPYGIIHFGAPAVCS
LLMFIFAAPGTTPVFARTFGWMSILGVTIQLMFPCTPPWYENEHGLVPAM
YGMEGSPAGLARVDKIFNIDLYTTNFTNAPLPFGAFPSLHGGYAVLEALF
MSHCFPQFRMFFIGYVGWIWWSTMYLSHHYAIDLVGGGLIAVGFFYSVRA
RYLPQRQADKITRWEYEFVEIGDRHRLADEEYGHEYFGLGLLEQRRENSS
DGWTLGGSSSCSSSSGTLSPTISEDTVPGMMVMDISSNGQIWDGNAPPRD
VELSEVVVVRQN
>Mb|QLI68404.1
MPPSASKEKRLAKKAAEGKTKAGKGAKAKAKEPELDANGNVIRDDAPATS
GEKLDEVKRLAEQMDKHGISDRVTTGVLSSTHASKDVKITSTSLVFHGRV
LITDSTLELSFGRRYGLLGENGCGKSTLLKAIAAREFPIPEHIDIYLLNE
GAPPSDLGALEWVVKEAEMELERLDKLAEKLLEDEGPESPVLIDLYDHMD
KMDPSTFATRASLILTGLGFNKKTIHKKTKDMSGGWRMRVALAKALFVKP
SLLLLDDPTAHLDLEACVWLEEYLKKWDRTLVLVSHSMDFLNGVCSNMID
MRDRLLQYYGGNYDSYHKTRAENETNQMKAYTKQQEEIAHIKKFIASAGT
YANLVRQAKSRQKILDKMEADGFIQPVTPDKVFTFRFADVDKLPPPVLSF
DNVTFSYSGDPADDLYRNLDLGFDMDSRTALVGPNGVGKSTLLRLMTGKL
SPTGGSVTRHTHLKLGLYSQHSAEQLDLTKSALDFVRDKYKEKSQDYQFW
RQQLGRYGLTGESQTALMGTLSEGQKSRIVFALLAIDGPNMLLLDEPTNG
LDIPTIDSLADAINAFSGGVVVVSHDFRLLDKIAKQILVCENQTIQVWDG
TIGDYKNYLRKKMISAGAV
>Mb|QLI68408.1
MLLLRRVTPRELRLCQHTYTRKAPFSASPCPCIRQRPNRPSPSRPIQTPR
EKDSNLDRDRNARPNDDPLPAPSQPRASASSPGRPLAIQQARESATPDTS
TSGPYKQVLLTSKSIRHNPLATSPIHITTRTSKSYTQQLSAADKFFSSSP
CRFLYSAEALRHHSMNNVTPEIVVLGSSNVGKSTFLNALVGQPKAARVSQ
KPGRTTLMNAYGVGPLPKIPRGLVPKGMPPPRHSLVVVDTPGYGFKSQAS
WGDAVIKYLQARRTLRGAVVLLSSEKKLLPEDKWMLRTLAEANTRTVVVL
TRADKARNGWVETCSLLADVVQQEVDRLDQEFGGKWKDGQDAASRIYITA
AGMESPGRLRNGGGIGGVRTAILEMAGFTLQETVAKKADTVAYTGPVVSF
DDIEWRT
>Mb|QLI68409.1
MRLTRDNINEHVVRAQYAVRGELAVKSEEYRARLKDGDTTLPFNQVISAN
IGNPQQLDQKPITFFRQVLSLLENPELLKKEDVLINGLGYKTDVIERAKW
LLTQVGSVGAYSASAGAPAIKESIAAFLERRDGFPADPSKIYLSTGASSG

VNTLLHVICADKKSGIMIPIPQYPLYSATLSLLDAQAVPYYLDESKAWGT
SFETIKAAYEKGKADGLDVRCIVIINPGNPTGASLSEEDVRSVIDFARQE
NLVIMADEVYQTNVFIGQFHSFKSVLRKLQKENPGKYDGVELASLHSISK
GMVGECGHRGGYFELVGFDPEVEAQIYKFVSIMLCAPVIGQCLVELMVNP
PKPGEPSYELYSQEYSGIHEGLRERASALHKAFSQMEGVECAEPQGAMYL
FPTIQLPAKAHEAAAAEKRTADEFYCLRLLEATGVCVVPGSGFGQKEGTL
HFRTTFLAPGTEWVAGIVKFHKEFMDKYR
>Mb|QLI68410.1
MAVESARAPSPNYFGLVIEPSNDPRGSSALARENWSPGSSVKSFAAALPK
QITLDPNPEFEAFRKAADLNRGKSFSLATSHVMLPTSHPTPVRPRPPRWH
TYTSDTGSDASFPKSIRSLKPKLTSKSEVDHDSLHDSAYVSSDSKRNSES
SLIPPTQITASMPFESPRQIETPHRRPHQPRTDPHDSLLSLTLPQPDPPS
PELANITRATTLPPKLEMGPSSMISGSQLQALMETVAEGKMLLLDIRSSQ
SFAHSRIQGALNLCIPTTLLKRSTFDLKKLQQTFQGNLDSEHFSKWRDTD
WIVVYDAHASDKRDAVTAQNMIKKFTNEGYQGNTAILRGGFTMFSAIFPE
LVDTSSTNPPSSTQENCGNTSKSTLAPVIGGVNLPMTGNDHNPFFSNIRQ
NMDLADGVGQFEIGRPQDLESPLLPTWLRDASSRSDHGKKVSNKFLNIEL
DEQSRMRTAYAAFQPNQKPQDCRFQLSGVEKGTKNRYKDILPFEHARVRL
RETRSGSCDYVNASHLSASRSNKRYIATQGPLPATYEDFWSVIWEQDVRV
IVMLTAESEGGQLKCHPYWKGRDFGPIRLKALSEKKASLDIDKHRSESSP
TPTSSSSHEFGRKRANTTTTFPSSSPARAQSQGQSETPYVTIRKFALSHA
AHPFAPIREITHLHFAAWPDFGTPAQPSHLLALVELANVMQRAARPVETT
SMGSKIPTIDSIPVTWYDEPEEDANSRPMLVHCSAGCGRTGAFCTVDSVI
DMLKRQRQANVSGPQTKDGDGDVKMSTHDEFAFPMTTKDGASRGFLDRVT
GGHNSQTWESFDMDTTWIRDDGVDLIQKTVQDFREQRLSMVQSLRQYVLC
YETILEWLNRMQDKTWSSMGGRVRSGSLQQVRS
>Mb|QLI68411.1
MFSKSVSIVTLALATLSAGQTFSKCKPTERDDCPADSAFGGKSDCDFTKG
ACSIFSEAEGTKLSYKDNGAVFTIQTETNAPTIETGKYIFFGRVDVVMQA
APGAGIITSAVLQSDDLDEIDWEFVGSDDVQVQTNYFSKGDTTTYDRGKY
HRIGNPTGSFHTYTVEWTSKQVNWMIDDQSVRVLDAATVGAKFPQTPMQI
KLGTWCAGGKNSPEGTRQWAGGFTDFSKAPFNAYYKSVSIVDYAGKDSPA
NGGIKEYIYGDKSGSWESIKVVKGDSSKDPSPSASASGSASASASASVSA
SASGSSKASATQTETDSSKPSTTTAPESKTSSANSTITTVSRSASASSNS
TASATLTSSRGSATSAPTTVPTTVVPGAAPRSAAAIGGSLLVGAGIAMAQ
LFL
>Mb|QLI68412.1
MSLKEEFQTRNFSIYGQWLGILSMIICLATGISTIFTFKVVLIIFAAIAI
ASSFLILFIEVPLLLRICPTSGKFDDFVRKISTNYMRAAAYGIMAFLQFL
SNLGGPSSLIAAGVFLTLTGICYLLAAIKGQAFVGSKTLGGAGVAQMIV
>Mb|QLI68413.1
MQTPLLLAIALSGLASAHGNHGQKPLVSEDATWMEKHMAEEHHIDSWDTG
AFFALHDYNSDGAWQGEEIMRTYGLMDPSNRDMSHDKKLEVLQHLMGLLD
KDHDGEVSGKEFQEFIDGGETLPDMGTGPGHHGDDEYEYEIHHWEKYHDE
NTKLEDLTHPEDIEHFKHHEELEKAQEEQEAMDKKSIIEENIPAKFRRH
>Mb|QLI68414.1
MTEMSSLLSPVAPAVPTSAVIKVTDSPVPIQVFTHPSADDYSVFFPFHDL
AALNSGQQDLFSFKQLIKNGTEGQDLLLLFTDGCEVDGKTDCSKACSNVS
TFFGSLETFYNCIALSSISYWTRETNRYYISEEAERNASGIMGSSSLASF
DGRPVLSSFVACAQEACGADGLHKPCDDSIKTLSHEANATAIFQAMDDFC
PDLQAEINPDIFGPGVLISYVLQVTFSGTLYLFLKAFTLYVHYAHKKQET
EKRLQRASSRGGLTRRLTRIESIIWRDSSALSRTSIALATTLVEFQEAQC
WFVFAIQIASILAIVVNSQEGTFWGEIIVNAAVAFHVSQNGILPMFLIQI
CLHHEGIRNWHTFLGFFVEYLLAIVATRQKVYFRDAFNLFKQENKIDACG
QNPSPRSYCASTQGVEGLRLSFFPHPLLYKMVFLVLDTIAIVILVFDQLS
WTLRTHRSTKHITIRGYRVGRAPNSGRLKLAWIHFKQWVWRILEVMYLVI
NILYLISLIKVISADSFQANKWSYGQIIAMTVWGPVIVKLFDLAVSGPPK
NGQRLNSGPPRLRIDNVINRRHGTSADEELDAEEEAYGTGRERRIPAPPE

IDLRQRSRPTRDEEADLTDGIGDSIGDDIKRVGR
>Mb|QLI68420.1
MESEQSPLLGNENQSHENGRRHHSRRDSKWSRLREIGIFVWALIATAAVI
IIAITTQHDQQTSRPDPDSRPTKRNLVFMVSDGMGPASLSLTRSYRQYVE
SLPEHDTLVLDKHFWGSSRTRSSSSLVTDSAAGATAFSCGKKSYNGAISM
LPDFQPCGSVLEAAKRAGYATGLVVTTDITDATPACFASHVLIRQMQDAI
ALQEVGHGPLGRSVDLMLGGGRCHFMPNSTAGSCRLDDIDVVALAQEKYG
WSYADSRPAFDALHAGQNVNLPLLGLFASSDIPFELDRRNMNDVYPSLSE
MAQTALRTLEKATDKSDKGFFLMIEGSRIDHAGHINDPAAQVREVLEYDK
TFKAVLDFIDDTTTETILVATSDHETGGLATALQEPGHLPVYNWYPHVLA
NVSASCESLAKSLQSHVSNMPASISKQQLKSWINEKLVIPGLGVTDATDD
ELSLLADDPEASVNTFAAIISLRAHVGWSTHGHTAVDVNVYSSGGPETER
IRGNVENTEIGHFLSHYLEVDVDEITQELQEKARISGDNRFHATQDPKKT
GDWASDKYHTFED
>Mb|QLI68422.1
MVHLSAINKDDSGSAASIANDAVAHLTQKARSTLKLSSEEDSLTTSVYGS
RFAEQELPRFTMPERAMPREIAYRMIKDDLSLDNNPKLNLASFVTTYMED
EAEKLMAHSLSKNFIDYEEYPQSADIQSRCVNMIGDLFHAPSGNGIGCSS
VGSSEAIMLAVLAMKRRWKIRRQAEGKSTERPNLIMSSAVQVCWEKATRY
FEIEEKFINCTPTRFVIDPEEMVAKCDENTIGCVLILGTTYTGDYEDVKA
VNDLLIEKNIDVPIHVDAASGGFVAPFVVPDLEWDFRCDKVVSINVSGHK
YGLVYPGVGWAIWRAAEYLPQDLVFNIDYLGAQQSSFTLNFSKGASQVIG
QYYQLIRLGKQGYRLIMSNLTRTADYLSDSLQKLGFIIMSKRAGEGLPLV
AFRFPEPNEGGREDRHYDEFTLARQLRSRGWVVPAYTMAPNTNKMKMLRV
VVREDFSRSRCDVLIHDIKICLGMLEEMDRETIKRQEEYIKTHLTSSGRA
QESIHKPRHFKDDDHSLQGKTGKTHAAC
>Mb|QLI68423.1
MLVTKHAAELCALLVNDLFGELPSVGSSSTHFGFCGVVLVAGLTFLRFSQ
QRLLVALFTKGRCTLSQLVQHVSLGPRHLRNGLGVLIQQNLLYHYTDPDT
GGTSYEANSDACYNLVRSGKILEVINSQYGTAERDLVQTLMQLGYARVAD
LTHAFTSRAPKTNGHTNGSHESSSGLIESKNHLNSALSRLIQAEILETVR
PDSFRNPAEVYREIEADVTKTAPGEKASKNKIEQHMQIVERFKTFRDQSK
ALKRQLDQTSGPSTKRRRLQNGSRHSDPFDDDIELNPNVVVRVNHEKCLV
ELRNQRLADFAADMLGETTGHVYRSLLDLLTAKVSRCRADPLVGEENKGP
QPSVTTLQIYENLSDEVKVLGGIGKAPKDKIDFDSAEKIRSGPYSYDSDS
DEEDEPEVKAAPVPRGRLARAAAAAAAAAAASAKESEDEGSEDDIDVQGG
HTHTNGDRSTKVKFEDGAIAKDSRLDQMRQHLLLLAESKHRFVRHCGTQG
RGQWTVDFELLMDRLRESELDAYIEQSFGRHGLRLTRILREKGKLDEKML
PSAALMKKGDVQGKMLAMQMAGLVDVQEVPKDNSRLANRTLFFWFFDGER
VQSQLLDDVYKGMLRCLQTLDVERHKERNILTFVERKDVKGKEEEVMTAE
HYNKYNRHLEGQQKLLGQVMRLDDMVAVFRDY
>Mb|QLI68425.1
MSSNPLKAGTTSKLGQGSFPPGATTTQSLLLEKVQARTSTPDSEALASSD
DEGEHRNEASQAAPVQSHKPVRRSSWLNDTSQPLNRPRKGSFASSSMSPT
GSHPSTPSAEAGSGPWGSHSTSSVGRSANSSAFSWGAGIWNADRKDASSR
LSEVLPSPTSIIPPGAGSNSFFGSDSGLNQVSPGSRDSMSNSQIPFAIPL
HPTPKTYRSQSYSVGQMDPETAPPGGMNSSAILGRARHPALQHRPSRPSM
LSEMANDGSMLGKVKEVEDDDDESTSESLQGSYHQMSESKTIEMLTRENA
MLRQQQYNNSRIRPRASTGASFLGNGYETVPEESDFAVDELDEATDSIDS
LGRRASGRRMSEYGANAFRTPLGLNNQKADNLNLKKAALWSSSPGFFGGD
ISQSRRHSFANMPTRQGSISSIADSVSALDASGVGEGQSSHGFPAGFGDG
TGMANINNRKSLLLKSHEQLLSSATEILAGLPSTMQPAFSNPYAPHFGLQ
NQFTNRPPSPHRNVFGMAQPRHNQLLHVVLFKCVRADVFYIQEGTGLTVK
PGDLVIVEADRGTDLGTVAKVNVDWQTAKELKEHYAEEHYKWLMMYSQGA
AAAQEGSGAGLMASSSVLQGSAVGGMGPPTQHHLQEPNAGELRPKLIKRL
AQHHELQALCDKEGQEAKAKRVCMQKVKEHGLNMEILDAEFQMDWKKLTF
YYFADSYINFNSLVTDLFKIYKTRIWMSAINPASFASPTLGIQAPSGIGP
GAVGVGRASGGERRQNPQVQEQQQPQAFGRGYRPTFSQPFGSDRNVPPTS

AYPPNNYAYGGGVFGNARGTSAPYAPSLSPGADGFPTGFGQQGDFQSLRQ
RFPGAQNLPSPTPHAAGVSPITAQNDWNTAFQGLSLNTH
>Mb|QLI68426.1
MPAVMDDPAGPTVYRVSGQPPFPDPNCPGLPPDIVPRQVTLRDRQTVATI
VPFVSRHQLPASLLVYLCDQLNREIEGGDTYPMMEAFTVDGFADYWFQTF
GAVMLMGNIDCAQDVLDGKDWAKECLGSFFIKPNYPGRSSHICNASFITT
DASRNRGVGRLMGEAYIDWAPRLGYTYSVFNLVYETNVASCRIWDALGFK
RIGRIKACGNLRSHPDRLIDAIIYGRDLAPGESEELVSEERFDKIRFYLK
YGKYPNGADRAEKSRLRSAATHYKLLEGDKLMLKDKEVISDPGRQYEIAR
NVHAIHHGGINKTTATIAEKFHWSRIKETVSDVIRACAECKELGKTPNPG
GSRKNGSASTPTSSATPSGGLGSGSGDAQRRQAQSPVPQHDGDGDGDIDI
HVDAGRVLALQQQSRAGLMDSLSRPGPAPSQGPYANPSDISVLAPAHQDS
HMLHDAHSHAVPHPPGHGHHGHNPMLQDRPASHEAYQPIDPQIINHHGHQ
HASSAHDDMPFDQYHPHADFQALLNATEDVGPEPDSDAAAAEAAAAAAVD
RDLEMLIEHEDVDCHEGPMDCLDEARRTGHRDRLYDVGFDGG
>Mb|QLI68427.1
MKNKPDLEGDITYIKTPKAAPSAFEAVDCGVPITHSPSIHNAPLPAEGAG
SEGFSNIVLGVLLVGVPYFLSRFVGGGLKTTVFFGILTLFPILIAYWTYM
SNFSPRINEHVKLPGRPVESYVTFKDAADQAKWSGANKVPIQTFSEMYID
GKADFKGDVLDVLEYRHDWSKFSFTWDLFKYILLTFGPDVLFHTKDQDME
QIRPNYDSGNDHYAWFLGPRMIYTSGIISDTTREETLEELQDNKMAIVCE
KLELKEGETLLDIGSGWGTLAKFASVNYGAKVTGITIAENGAAWGNDVLR
NAGVPEEQSRILCMDYRDAPKQKYNKISQLEMGEHVGVRKLTTFFRQCYD
MLEDDGAMYVQLSGLRQAWQYEDFIWGLFLNKYIFRGADASTPLWNYVRS
LESAGFEVKSVDTVGVHYSATLWRWYRNWVGNGDAITAKYGQRWYRIWEL
FLAWSVIASRQGSATCFQILVVKNLNSVHRINGVGSQFGLSGALATSRAA
GKASLPK
>Mb|QLI68428.1
MFRHSLRTAARSTTSASWRPMAVRSFASAPKTTFDWQDPLASKNLLTEEE
LAIAETAERYCQERMMPRVLQAYRDEHYDPKILQEMGELGLLGANIDGYG
CAGVSSVAGGMITRAVERVDSGYRSGMSVQSSLVMGGIYEFGTEEQKQRF
LPEMAKGKLLGAFGLTEPNHGSDPGSMESVAKPHPTKKGYFSLSGAKTWI
TNSPIADVLLVWAKLQETGKIRGFLVERKDCPPGTLSTPAIKDKNGLRAS
ITGMIQLDECPVPEANMFPDVEGLKGPFSCLNSARYGISMGVIGALEDCI
ARARTYALERKQFKGNPLAKYQLIQKKLADATTDAAYGTLAAIQVGRLKD
EGKATPEMISMVKRQNCDVALRNARILQEIFGGNAVSDEYHIGRHVANLF
VTQTYEGQSDIHSLILGRAITGIQAFV
>Mb|QLI68429.1
MSSRDDASSASTYESQNATGLPPTYASFSPVTLSAKTPSRSARRSNILVH
QKSPLLLATPPQVTRALAYSHPFLLPLNKFVGLLTWSTGDPWQSFLLLSA
FWLVVLYGDVVVRRAGPVVIGMVIIAGMYGRRYSPLSSSGWAEPARPTKD
ASSVSTASAAASHGEGASSKKAKHQRAASEITSTKHQKTLDEIVETLKEF
TGRCNVLMEPLLEMTDFLSTQRTPTSATTKPALTAMFVRLLIVTPFWLAL
TMPPFHVITTRRVVLVVGTILLTWHARPLRVTRTLLWRSSLVRRSAAAIT
GLRFDGPLKTGHKLAAEAAKSHAAAAGKGNRKAGRSKSSGVKFTFIIYEN
QRRWVGLGWTASLFAYERPAWTDEHNNPVPSRDEFELPDVEDGSRMRWQW
VEGSQWRVDGVPDEKGPVDYDGEEGKNGWIYYDNKWQYGRRGQDGWVKWT
RRRKWYRDAELVEVDESDQHVQLVPGQNGTSKSPKKDYKKGYSTSDEKMV
PVRAEPQPTAGDQPGTDDDGASILSTSSKSSFWPSSFRRRAVEKSSTLAE
KGQPRNIAEPVPDDDASGLDLETELELQKQGKDGGRWGIGDEAHMNLE
>Mb|QLI68433.1
MGGRQIRPARVFQSVSQELNHQMLAGHTVAQPPWYQVMNSVPPAETLVRT
VTPRHRMPNPKAKKPKKLYRPQSISYPEDALRTSFYKDHPWELARPRIVL
ELDGKDYQHCDWSKGLRQPGVPLTGECVVQRQLHLMHAEKMSKRKAYDTA
RKEFYRLRQEEEIEKRVAVEEAKHVGAYFGKSRLDVGVQLEDQEFENWKI
WAGKETANRAARSNSEIETFGVEDSEEELAEAASV
>Mb|QLI68434.1
MAIPSPDQVPRGDVTAALNFYAPPADKAARPFNYVDSPPAGQPQRNYSDH

VQDTLIRDVRGRESAFTLDRDAFQIISDVAPSSEPSFADDASIRARYYPE
VEALLLAAIPGSRAVHIFDHTLRRADPAAPRTPVTRVHIDQTAASVEKRV
RRYFPDEADALLAGRHRIVNVWRPLNARPVESFPLAFASAATLDPADVVP
VEHRYPDGYTGETAAVRYSPRQEWYYLSGMTGSERLLLECFDSESLRPGS
RVGGRVPHTAFVDPRTRLGAEGRESIEVRALVFGP
>Mb|QLI68435.1
MRPCLTPLLASLVPGLAAAAAADYHELLTLRPLPFSQLLASFNFKSNSSI
ADFEAHNFRLFPRSLGQILEYAGTRELHLRFTLGRWDAETWGARPWDGTK
EGGTGVELWAWMDAETDQQADENWLTLTNALSGLFCASLNFIDGTRTSMA
IDVKPICDQSGDCVLQMEQTIDMVMDVNRSKRPRDNPIPRPPPTHELVCD
TSKSYHDDDHCFPADHLNGQDWTLSQIFGRPMKGTCPLADAQVPPVCLQV
PDSRVVYASEGSAEIKDENGMSRCYTIPPQSEFALVLPKSEAKTPEEAAK
ELVEPVQPLLYAERSFTGHGQEHGGVQAILTNPNNVEVEFVYLESLPWFM
RIYLHTLSTRISSSAAPTTNSSELIKQIHYRPALDRSRGTQLELLMRIPP
RCTVFLTYDFEKAILRYTEYPPDANRGFDVAAAVIRTLEPQVMNLRTTSL
LLYLPTPDFSMPYNVIIFTSTAIALAFGGLYNILVRRLVGADEVASPMAK
WKLRNLLSKFQKRGIGRQ
>Mb|QLI68438.1
MSAGTEGDAGDAAELTYPSPGAEGMESGPFYHNARDGAPEHHDQAEQQVA
DEVHGQGHDQTQMSAQDDAASHHHVSRPANLEELQLAAQLGQGLAGTSIL
PATDPNMTVEDPNMRNIMPHPDPDHHHAASYVQEAPPPDPMVQHQMPVAV
GPALPPQYAMGDGIPPRKRSKVSRACDECRRKKIKCDAQSDSGETPCSSC
ARSSIRCLFSRVPQKRGPSKGYIKELADRIHSIENKLESEGNLSQDDIDK
LFATDRSRHSNGLEDSTRKRPFSSISTGDYTTPTTVRQAPWGSEARSAQP
ASGQGEGLVQEYNNNSLAPQPTPIKPDETPIKPHAAPADVPMVDGDEVPA
VDEQAFLEFLASVQPLYPVLPTDLGRLQSLLLQTNAAVRSAFSLALSCIG
QPSSDSPKIASSLLHEWESNDSPSSRATDIVHAQTLLILIIDADWRGTSS
LPFLLARAVALANSMKLWKCSPVDSTTEPDSDDELCVRIWWSLVLMDRWH
AAGTGQPAQIPDRSVVVPASLENTIGEVSFHLLRLSKLLNRISFVISTLQ
AGLYTADASMAAILTDYIENYREDLPAHIDAAAYPMIHLAYWHCRLLVTM
LTPGATPKEIIWPTKELAHLLSVTPELKGPLFSHFVSLATMSLRKLARTE
ATREEAAEIIKEIAEKPSGGHWDGVKDKLADGLRPASSSAAEAAASQGLQ
HLADLATASECLAAGEEVPFGAALTTGYLEVAS
>Mb|QLI68439.1
MAPRFQVIVFGDLSVPYHAELQRLLSDKDSCTLVSLFSDAHRALRSEIAR
LPPSQRSCFPHVSNLAELLIARQSSQVPNCALDSALVCLCHIASFIAYTN
EPGRCYPQSSSSCLLGSCIGLLAAVAVSCAENVLDLVEAAPHFVSLAFRL
GHMVQNRTTSVVSGTDVHSSCSSVVSGVDEETMPPRIEAYCVSRGLAALS
RVYISAVGSGSVTISGPPAHLKEFLLHNPAFKAGKIGVAGLFHSCCLYTR
ADVDALVSGVGVHTRSRAVRLHVVSNSEEAGEGSCEATCEELLQRAVAEV
LLRRLRWDLVIQRAVGTLESSKLSEADIIPFASSSVESLATALRASEAVE
SLDVLSTLPLSRSEGSSVSGPKSNRSKIAIIGFSGRYPEADSNEAFWELL
AAGLDVHKEIPKDRFDPWLYYDSTGKKKNTSGVTMGCFVRNPDLFDSRFF
GMSPREADQADPAQRLALMTAYEAMEMAGFVPDSTPSTQRSRIGVFYGTA
SDDYRETNAAQNIDTYYVPGGSRAFLPARINYHFRFSGPSFDVDTACSSG
LAATHIACNSLWAGDCDVAIAGGTNILTNPDNWAGLDRAHFLSRTGNCKT
FDDGADGYCRAESVATVLLKRLDDALLDGDPIYGTILGAYTNHSAEAVSI
TRPHSGAQRAIFSRILTSANVESSRVSYVEMHGTGTQHGDACEMDSVLNV
FAPEGSRRDETLYLGSVKANIGHGESASGISSLIKVLLMMQKSSIPRHVG
IKTKINRNFPLDLARRNVHIAMKDTPWPRPDPRDAPSGRIAFVNNFGAAG
GNSSVLVQDAPATSAAADDDPRPLHIVAVSAKTQTAFKNNLAALEKYLDA
HPDVPLGSLSFTTTARRVHYGYRAAVTGSSIRDIREALASAANADKCLST
AGSTTPVGFCFTGQGAQYLSMARELLAHPQFLSLVTGLEDVVRLQGFGSI
LDVLQGMADDPMEQLSPVKVQLAMSCLQMALGKFWMALGASPDVLVGHSL
GEYAAMNVAGVLSDADAVHLVGTRAALLSKYCSVGTHAMLAVKASADRVA
SLLGRPGLEIACVNGPEETVVAGPRDQIDSLAAVLNTQSVKTTLLKVQFA
FHSAQVEPMLESFRQSCGSVRFKDPAVPLLSPLLGKVVRSAADLASPADY
LTRHCRETVNFRGCLLSAAEAGVVSDKTVWVEVGPHPVCGNMIKSTLGAS

SRTFPSLRRGEADWKVFTSTLTSLYNSGLAINWGEYHSGLTHNKQVLCLP
FYKWELKSHWIPYVHDWCLTKGDAPPPPQPLASPAPVARQKPLTTSVQDI
IHEQYSGDEAQITARSDIQHPDFREVLLAHKVNGQPVCSSAVYADMAMTL
FTRLLEQSAVGFDKSDIGVEVSDMSADKALVLNDDPSHLLEMKAAVKWST
RRATFTMSSISSAGAETAHHARCTGFFVDKSQWRADFKRRDFLVRARIDD
LRRSVHDDSGSVHLIKTGMFYKLFSALVDYRPSFKGCRELVMRSAELESS
ARVKFNTPPGTADKWMTPPYWLDSLGQITGFTMNANDEVDSKNQVFINHG
WENMKLLEPLSQDVTYTTYLKMRPKDKGSYIGDVYIFNEGARDIVAVYEG
VTFSAVQRSILDKVLPKPKPATAKPVPSQPVFGSQAPAADDEPSTEPPAL
LPPHKYGVPADKLKAIIAEEVGAPVSDAQDGAELAGLGVDSLLALTISDR
MLEELGVKVDSTAFITCKTVGEVIQLIAGSSTPSSDSEPSTPPVTPPPPR
EPEFENAHLSRGVEAALSALSMDPDEYTPIASMNDKAKPTPAPAFGVVSP
LTVLPPPSHVPPASSVLLQGNPATCSRKVWLFPDGSGSATSYLPLPDVDA
DRVAVYGLSSPYVRRGDCKPCRFEELTAAYVAEVRRRQPAGPYIVGGWSA
GGICAYDAAQRLAAEGDAVDGLILLDSPRPVRLKKLPPRLYAELDRLDVF
GSGGRRPPAWLAAHFVLFADILDTYRPRGWAAARPLPTWALWARNGVDEG
GLIDIWPGDPDNLLWLLHRRAVVDANGWDELVGGENVVMDVVEGAHHFNM
LKEQGARQVTDFLRRAVTCMAGGVL
>Mb|QLI68441.1
MDRPRRTPKRKALENIDAPDVPHHQLLEKACSPLTSQEIEEWEGWIELES
EPVRVCKITQRPPSSSLPLQAFFNIILRDLGVKHVKTQELFTIDQASLDA
LPKPVFGLIFLFQYGPGYEEDEQPTNTESEIWFANQARFHPFPLSHSGFT
KSTTNSQVQTTNNACATVALLNIVMNADNLDLGERLQSFKESTKDLCTAL
RGHSISSNKFIRKIHNSFTRRMDQLNADLCLENDVSDSMKKTMSRKAGKQ
KRKSNKTSLEEYGYHFIAYVPSGGFVWELDGLRSNPLKLGPLESQDWTAI
ARPQIEARMLQNEDSQLSFNLLAVCRGPLLQHSRTIATMLAALDYIRSRM
TDSEAFSKLISGEEPVLDMDDQAQLAEFNLTHSDIQNAEIPPQLLAAAAR
SDWEAPDVYQLYQRFCAEARAAVREFRAELIVMDEDERRVTGRRRDYGSA
LHSWVQKLAEKGVLEDIIKMT
>Mb|QLI68442.1
MIPRRSLATVTRYPGIVRSTVSLRQPLLGAVAWRKSGHVFPRRLADRAWE
STAAPPTPPATPFPSSPASEPASSSSSSSANKEQETGHFDVKPNESLFFF
ENIFPRKLSSLLRYPTETDGDMTALLRRFYSASGSGGSLDPIAFVKRAIP
EDMPVKVTEILPRLKDGGAFVKVQYDASISPSEIESTLLEKLEQHPLKPW
FSPFRGIKARLVKGTPWLEDLYRFPSSLVKVEFVPPEPGAAAEELSEENL
YSMFRRYGKIADILPQPFDSKVVPKYAQIGFPRLSDAIMARNCMHGFVVT
ESMGGGKTGTLLRLSYVKKVKAHSIWNWLTSHPRIVIPILAAILAGASVM
IFDPIRTFFIKLHVSHSLRFTESRLYKWFKSQTDSLSFKSSRHMDGLNTV
WNHRRDVIDQLQNWLDGSTFIVITGPRGSGKSEMVMDQALAGRKNVLLVD
CKPIVDASGEAGTIKRLAGAVGFRPVFSWANSISSLIDLAVQSTTGVKAG
FSETLDTQVSKILHTTTGALKQIGLSSRSKHDKDAQLSEDAYLEAHPENR
PVIVIDNYLHKNEEITIVYDKIADWAASLVQNNIAHVIFLTSDSSYSKPL
SRAMPDRVFRTLSLGDLEHKVAKNFVTSRLAEDNPESAKASVEDGKKTAK
HMDLTGLDECIETLGGRLTDLEFLSRRIKAGQTPRQAVDEIVGETATDIV
KIFLMGKTTESDKKWSAEQAWHLIKSLADAPSLRYHQVLLSPAFASSTSS
NAADGEAALEALASAELIALKSHQGRPKLISAGKPLHQAAFSVLVGDRVL
KAKMDMAIMKEMAKTEAKTIDAVETELSLLGQLPRQTLETAGRVTYLLEK
LDASQRKIDDLEKQMVGLKKVLVEEY
>Mb|QLI68445.1
MDNDLRSKAWPDQEGRLTRHNSLEAFSNLPPLRTDIDRSYPGALNEKADP
RSMASTPTLEAQDEAKGGHDHDHHDHAGGVDIFDPNRPKLGFKQRLHHFT
WAWFTLPMSTGGLSLLIFAQPHQFPGLKTIGMVVYIVNIIIYVTLAALMV
ARFLIHSGDMSKSLKHPREGFFVPTFFLATATLITSSDRYAIPKNDVGLV
WAVQGAFWAYVVATLILAVGQYSYVFAAHSFGLQTMMPTWILPIFPIMLS
GTIASVIAETQPAAAAMSIVVAGLSCQGLGFAVAVLMYAHMVGRLMQAGL
PNREHRPGLFMCVGPPAFTALAVIGMAKGIPATASQNHIGMPVDLTTIRT
MALISAIFLWSLSLWWFFIAVIAVVQSLPKYFHLGWWAMVFPNTGFILAT
ISIGNELGNEGVLWFATGLSICLLGMYFFVLFFNIKAVITQDIMYPGRDE

DVEDH
>Mb|QLI68446.1
MPDFKPGQIVQLSDGRKATVRFAGQTNFQVGEWIGVELEDKTGKNDGSVQ
GVRYFDCPAGYGMFVKPMMATIIAQAPSTKPVGRKPARPSSFHPASVKAA
PSGDVALAKRRSLNAPSPSPVPKSRPTSLVRSPAKSPTKQLSAASSASAS
RTGTPSNARVPSTSAVGAARARTSASGQRTPMGPPTVPAPRTARVSSISS
TTARVGGVHARSVSGARPPQSMRTVSRPDSGRRPSTDSQAGRDSGSADDA
GPGSPIKSDGEILSPQPTSPVAGRAKALEKIAAATSPGTSSTPPLSKRTT
AAVTTQRSAPNSALNRENEDLKAKLKVLERKRLEDRDKLNQLDRVQSERD
KFEGIIQKLQQKYQPQQQENAELRKQMKEAEARFISIEKEQADLEAELEL
SVVNRQIAELDLEDSQSQVEALKLKTEELELKVDLLEAENSDYCKGMSPE
ERASAGWMQMESTNERLREALIRLRDITQDQEKELRDQIAGLEEDIKELG
PVKEHHDLTKEKLIRSEQVVEQLKEQLNDALGAQSMIEELSEQNMDMSSM
IEKLKLEITELETLKEIDDELEANHVQLEKQMQEELDIRDTIISEQVQRA
GRQQGELDDRAHTILRFRELVESLQADLDDMRASQAVTEGESEKLNDRSR
AMMDLNMKLQISVAKAQVKTIDLELRHLDAQEAEQHLEIVKLFLPDTYRE
DQDSVLALLRFRRLAFKANLVNGLIKERINGQPQPGHEDDLLAGCDAVDK
LVWVAAVCDRFINDISHCTTEQFSAYQNSLLELEPVERALNSWIDGLRRD
DLKEKKCAEELQRTIALMSHLAELHISNGLPNFADYVYMSSLVVQSHLDS
AAIAFNATRVMVQRAISSEAEEDEMSQLFAKKFDMVITQTRSTKVIAGKA
VRALEDLKTRSLSLLPDTKPVFEQCEHNSRELANIARAIGNHVHLLLTQD
EGRVEPFTYGEIQSAVHQAVLEATSASESEMFSAYFSKLRIVTGQINDVA
ALATDLDQTEEFDVHPAPWKLRSQELKVLKTVPADAEEELRRLREEHNEA
RRTIAQRDEHLSTAVLKIDTLESRMRDAQTNVERISSLQKQLEASGEQVT
ILKEDIEKQDRELKGLESERDKWKKIASDSRAYADGADAAGMKAGQERAV
ATAREMDALKKNIEGLQSAVRYLREDNHRARMTEQQNYDWLAEPLKKPTS
TREQRSALVVAEGKDVLGELVKMATASRMFDFGTLPKDKLAWKPAKSTPQ
YLAAKQMEDFAAWNAWQRSVLRKSSMLRTPDAGKSLLRNPAAQLRIRLPG
PDGKIKTGSALGCDVQVVGSREWEALQSSSIAV
>Mb|QLI68448.1
MTTAARIHPLLLTIRFSASIPDLDLDIPSPQTTTVLALKHLLRTRLATRN
RLRLIYQGRLLPDSSALSSVLKPPPPPPPQPPSSSSTSAKAKGKAVDGAP
ARVYVNCSIGDELAAQELAAEEASAANPPQEAGPEPPTNPAGRTRPRPRG
FDRLLQAGFTSSEISTLRTQFASIQTERFTPDSVPSPDGLRRLEDAWIDT
NAGELPSAASPLEDELSNMSTVLDVLIRGMMIGFFFPLGSITWLLRQGLW
SEKWQIFVGSGVVLSVTVGIVMGISGER
>Mb|QLI68452.1
MAPREGAHRSSRRRHTEGAGESSKRHHQHRKKRSPTKRHEATSNSEESRE
ERRHRRPSSSQNLSSGALAQLNRENDRRKRKAEKDRRRHHRGEYQEVDTT
PRSTPQKPRKHHKSKKRRVVSGAIMEEGRAGRSGLRGGGSWGEDEFEKGE
LYRRHKKKKPRKKLWMILGIASVVVVIIIVVVAVVVSKKNGGDSGSSSQS
SSLDGKDPNEVPAQWKNTYLDPWSWKTTTDFNVTFTDQMVGGLPVMGLFS
EWDDSAKANDKVPALNEDWGSYGSRPARGVNLGGWLSLEPFITPSLFNYD
LKMGIIDEWTLCQHLGASAASTLENHYASFVTEDTFKAIAAAGLDHVRIP
FSYWAVEVYDGDPFVFRTSWRYLLRAIEWARKYGLRINLDLHGLPGSQNG
WNHSGRWGPIGWLNGTNGQLNGKRSLDVHDKLSQFFAQDRYKNILTHYGL
ANEPRMTFLQTAEVIQWTEDAYKTVRKNGVKALVIFGDGFMGLDNWKGLM
AGYDDMVLDVHQYVIFNEEQIVYSHQKKIQYACDGWTQQAQQSMNTQTGY
GPTIFAEWSQADTDCAKHLTNVGWGNRWEGTLNTGDPKTSVLTPRCPTKD
SSCSCAQANADPSKYSGQYKKFLQMFAEAQMHSFEKGWGWWYWTWKTESA
PLWSYEAALKAGIMPTKAYSRDFNCDTDVPDFSKSGLAENY
>Mb|QLI68453.1
MSNYGGGRGGGYGGGHGGYGRDRSDRGDRSGYGGGYGGGRSNGYSNGSHG
NSNGFGGGYGGGHGGYGGGGGDRMSNLGAGLQKQNWDLSTLPKFEKSFYK
EIPEVANRSSAEVDAFRRKHQMTIAGRDVPKPVETFDEAGFPRYVLDEVK
AQGFPAPTAIQSQGWPMALSGRDVVGIAETGSGKTLTYCLPAIVHINAQP
LLAPGDGPIVLVLAPTRELAVQIQQEITKFGRSSRIRNTCVYGGVPKGPQ
IRDLSRGVEVCIATPGRLIDMLEAGKTNLRRVTYLVLDEADRMLDMGFEP

QIRKIIEQIRPDRQTLMYSATWPKEVRAMAADFQTDFIQVNIGSMDLSAN
HRITQIVEVVSDMEKRDRMIKHLEQVMENKENKILLFVGTKRVADDITRF
LRQDGWPALSIHGDKQQNERDWVLDQFKTGKSPIMVATDVASRGIDVRNI
THVLNYDYPNNSEDYIHRIGRTGRAGAKGTAITFFTTDNQKQARELVNVL
QEAKQKIDPRLAEMARFGGGGGGHRYGGWGRGRGGGRANANSQPLGNRRW
>Mb|QLI68454.1
MATAAPIVPESLPFDNRSPTGAGCNHIPMNNSNNVPKINAAYSSNTSPLS
TMTTNPAQSVTSPPPVTGKRARNSDESDVKAGHASTAEEDGRKKRSRGRP
RLDTKDETAADRRRTQIRLAQRAYRHRKDTTITTLEQRVKELEKANDDMS
KGFNDFYDIILSERALEAVPHAAQQLSAIANRISTVADKARNNPGEGSCS
EDSDEHPVSNHGNHPNLQSAQATTSAVTAMPTNNGFHLDTSQQANILGTS
TGSPGVPGTAVATTSGPVIHVPASLSYEVVTAATPDNATFPFYSSMEPSI
EDGFNTNLPAGHSPYSSMAPPLSYASNELTFGRRLQRQTTENGLRLISMS
NPPPERFAAVFGFCLFFESKAAIIQRLQLSLSRNQHEDLSHWRFPFTSLG
GAGTFFPPQQSIEGTGGDPMAMRIGNQGTRSYGKPQEMTGQSMGPFSPEV
QATRDERIDHKMRMMWPGFEGEFFDPDEVETYLRQLGIFIPQRAEYVEAD
VDLGDLDRDGSSARASSDSAMMPNGQQAGFATADSGYGGSSHSGTWTNSS
NSPVSGSSDVSGLQVAVDVAQQPVMGLRGHDGGNEAAGDYDHGMANFILH
SGPSQMWSQPMAWAVKSKIGLNVSLLIQEISNKSVCLGRSPGVRRRDVNK
AVKVAAGLAAVPA
>Mb|QLI68455.1
MPPKDTDNIKALESLVQTASALLTQLQAVLSEIHKAPDSASASSSSPSPE
PDPKINTLSLAHDSASLIRAHGTKISLLIINEPFTPSAVSTVVRELVAGP
IPGLVSAAEACAPKQYTAVVRRELAWRAQRVLSELQQLLHKIPNDGKILS
ESKREGFAKGEKGSLPATGLLWSACDNVIDLAKMGVGGFFIQKVDQWRDI
LNDVMEEMKEWGDEEPDSDSDSDSDPDVNNLADQLGSQSLSAQAMLDSLM
NSHQTIPSSDPDRIRPRLETSLKRLRLVTLLYQALTKRRLKKLPALPPQT
PDSNVPSRLDEMAKVLAKLPDSFGDLACAFYELQPSEIDKTMDQCFLDAF
AASELFAKDWQDGRDAFSEWTDKFQAEIKKI
>Mb|QLI68456.1
MVLTEYTYVFVIGTGFALLEAFNNGANDVANAWATSVSSRSVTYRQAMVL
CVVFEMLGALTVGARTASTIKNGIIPLSAFQNNPGVQLLAFTCAAAGASL
WVQWCTRHSAHVSSTYSLISSIAGVGVATVGASGVQWGWNNGKGLGAIFA
GLGMAPAISACFGAIIFMLIKLVVHVRKNPIPWAVWTSPFFFLIAGVICT
LSVVYKGSPRLGLDKKPAWYIAAVTLGVGWSLFVLAALFFVPFVHAKVLK
KDYTVSWKHVVYGPLLFSRQPPEDAVEAKVPDYAVVHHGEVSDDNSQSDA
GVFDTKGASGTVTDGEKPGTPVHPANNVAEKKLILAECNQQGYQRLMAEA
RERHHAKLRKTRGPLGWAMRTLHANPMGAGSLYETHNMIALCKRIPAMVV
VALLYGMHYDIHTAQVGIEGTPEGRRMQRVYDKATKYPNEVEYLYSFVQV
ITACTASFAHGANDVGNAVGVWAAMYSAWSTGQALTSSSPVELWQIAVIA
LTICIGFITYGYNIMKVMGNKITYHSPSRGSSMEMGAAITVLVFSQYSLP
VSTSMCITGATVGVGLCNGTFKAVNWQRVGLLVFSWFMTIPIAGLIGGCL
MGILLNTPHFNN
>Mb|QLI68457.1
MSAPGPESIPTSADPRSRRATKKRVLSPTSSQAASLTALFAKPDQNIPLP
SSAAVKRAPHIPEIYTNVQGSSAGAGSGEFHVYKASRRREYQRLREMDET
LKREKDQDHFEKSRAERLQRDEEKTRKNRERREKMKARKANKGKKTGGTG
VGRGEGAEDAAQGPSRHGGDPDVQEDRGEQDTTAGGAASSAPVGLVIHDD
D
>Mb|QLI68464.1
MKLSNPGTVPVYTISGASTARPLPDWLARRRKRSLKNDPEYQNRVELLQD
FEFEEASNCIRVSEDGEWAMSTGTYKPQIHVHNLPQLSLSFTRHTNSLNH
SFILLSSDYSKSLHLQTDRRLEFHTPMGCHYEVRLPRYGRGVLYDCHATE
ALVPAVGLDSEGKGEVFRMNLEQGRFMKSYQIEVGGDDELTGGGLQGGIG
VGSVNVAANAENTHNLMAFGTSIGTVEFWDPRTKSRVALLAGHDGEITAL
DFSSSGLSLATGSSTGMVQLFDLRRPVALLKKDHGYGFPVKKLFHMTNSS
QEKKIMSADKRIIKLWDEATGEPWTSVEPVVDINDVAWCKNTGMLLTANE
GKQQHAFFIPQLGPAPKWCVFLDSMVEEMAEEVHTETYDNYKFLTLPELK

QLSLAHLIGKTNLLRPYMHGYFVASKLYEQAKLIVNPYAWEEERMKRVKD
KVEKERASRIRGTKKVKVNQKLVDKILKKQENREKVDTNAGILGDDRFAA
VFEDEEFVVDETSKTFQLLNPSAQIEQPVDPKAPTRYQAKDSDDESGSED
EIRNSSKSGNDITMRVSSTQNEGQPLRDTALGSRIQKSGRVTKARQGDIG
GERQVTFIPESKKKKLQEQDQPSPRQQQRGERRSASSNTFRRM
>Mb|QLI68466.1
MRLPNVIWLLAGPLTLIPPVSAASSVKVGMNAAFDAAPYLLELVETAAGE
NSTAYFPLLDRIASGYFLTVSSESELYSRFLNIIHEDGHITRPDALSTFN
LALSLRSAAPRIEAHYQHYSTAMEPLKELRDCDIWVLLDGRQYCTPDFDA
EAKNEPVSAVVKPLAFDRTMGIGRDAILYADPTSDRFADYHQSLLKAART
MKLRYRLRYRRSKSHISRPLPVSGYGVELALKKTDYIVMDDRHSSQSTEE
SPKEPVEVLDGTDDVADLNPLSTSQLSSIGMKAASFIKDSDHPFDTLVKL
TQDFPKFASSIASRNVSITFAAEIKQNQAKKMRGGINFLWMNGAQLTDRE
IQPFALVNMLRSERGVVDGIRDLGFDGEQAVALLSHKAVSAAKEDDKPFR
YDWTDRLEAGRVILWLNDLEKDDRYASYPKSLSSLLQGTFPGQIPPIGRN
IFNLVIPADLSNTEDLAFISEVESIRERGIPIRFGLVPLQLSDEAKTRAK
IAYFLTENYGIESTISYLSQLAKAHQKTADSKTLLSTITENHTLLPGGED
MSLSMILQASDFTERLVKAEKWVKRLKADTVVRPLFVNGVLVPRDQSWMQ
SLSMTVSQDLQTVQRGIYHGVFDDDTWTVGVFLEGAASRRNLYISETNEK
TLRVLNIAKVYRENADLFNAVPVFDFYAESTQENWAVVTVLADMSSRAGL
DLILSALEFRRNNPAIRLDFVDTQDNTKISSQVNKALKANEAKLKDIETI
QDLEQLLSEASSYDASDDFAVFVARFLADTKMPTSSQVVIMNGRVIGPIE
PEASFDAEDFQQVLSYEQTRRILPVYAAVADLGLNEKVSDCMAAAKLSSI
IALSTLSDLPEGIFESSSATRSSIYTSWNSSHTVIESGDPKKSNIHIVGL
LDPVSEKSQKWAHVLRLLAELDGVYVKLFLNPKVQVEELPVKRFYRYAVE
PTPKFDENGSVKALAASFSGLPLNALMTVGMDVPPAWLVAPKLSIHDLDN
IQLSAANSDVEATYELQHILIEGHSRDDEGSAPRGAQLVLATESQPLLTD
TIVMANLGFFQFKANPGVYNIQLKEGRSAEIFTIESIGAQGWEAAPDEEG
SELALMDFQGTTLYPRLKRRPGMENQDVLEDTSNPSQGNIVSKGLKFAEG
LLSGGKGKSTSDVQHAEINIFSVASGHLYERMLNIMMVSVMRNTKHSVKF
WFIEQFLSPSFKEFIPHLAKEYGFKYEMITYKWPHWLRQQKEKQREIWGY
KILFLDVLFPLSLDKVIFVDADQVVRTDMMDLVDLDLNGAPYGFTPMCDS
RTEMEGFRFWKQGYWSNYLRGRPYHISALYVVDLRRFRELAAGDRLRQQY
HALSADPASLSNLDQDLPNHMQFQIPIHSLPQEWLWCETWCSDESLAKAR
TIDLCNNPQTKEPKLDRARRQVPEWTTYDQEIAALDLKRKQRLQSDAAET
DRTTSEDGKVEDGRSKDEL
>Mb|QLI68467.1
MEVGHLLTDPGTQILPSTTALMMVPPEDEELAVAISDMANAGAFIAETRL
KATDDQEAISIIINNTTTCKINKETKARRNLHRQPDSPFPHRLLSDNPLL
VPPISPVKSNYRYESLTDDIVQAWNERGRQEIVQHGVQAREDVDTTELSM
LFQEFIQAVVENRLDATDAGACLKEILGEDNADTIKDADAFAPHTLFLDS
LDIIMDSDSELYRPTLRDFLMATGVSPALMRRVLNAPILQQIGLIRQNFT
TLGVRQATNFLYRQANYNLLREETEGYSKLITELFATNSVTPTPPELAQQ
TFERIKGLIGTFDLDVGRVLDVTLDVAAAVLIKQYKFFVKLLRVSSWWPR
SHFKYDATYNGGLPPWANPGYSQWTATIEDEELIAKQKLLRDVAFWDRAR
EIHLGAFFELGGRRVNDVDLQQAKITNGRETKEEADFEQQWMEETKTLPP
PGNRVAAQLLGFKLLFYNSELRDKADVLPANLLYLAALLIKVGFLSLIDL
YPHLSPSDDDMEKVRKEEAGKLEREEREARGGGQMNALLMAGVLPQGDDD
NPNASNIPKKEAAKKVDTEVKQSVKEENDTKNRLPEPLEQKVSLLTQLLT
IGAIPESLFILGRFPWIPEVFPDVLERIHRILHVSLEKVYNDTRPMAMAS
IDCPNKQLPDLDQSGMPKGSVRLQRPAPKKQWRWPYPEKFDVNENQNYRF
YWDEWADNIPVCQTVDDIFTLCNTFLNLSGVNIGKDEALLAKLAVIGSKS
IADDKSESNLDRWYDLLRRLMLPALSHTKANASVVDAIWSLLKHYPLTTR
YSAYAEWFEGQISRLPAMKTAFARATSETRGTMKRVSLTNLGEMAKQLAK
TSYSSPGIVFRVAFEQLESYPNLIEAFVECAKYFTDLSYDVLIWSLLNSL
GKSRSRTQAEHALTTSKWLQALSRFSGKVFRRYPALNPTPVLQYVNDQLF
RGNATDLIILKEFVSSMGGIVDSADFNDYQVLSMAGGAWLRRYTLIRAQD
RRFDNVKSSKRLIQALVDSKLAANLLLNLAQYRRAAIFRVPEDEAHIKYL

SSVVDDSHQILIQYLDLLWSNLDPSTFDAIVPSISQLITTYGLDTSLAFL
IGRTSLGHRIFPWKEGKGKKKEPVAKPSTDKEGDVDMAEAKPQSEESKEP
GTQGAISNEEQKSEDVSLTRTTVNALQPIIESVRSTVRPEIWQKITPELY
ATFWALQLGDICFPEETYVRERQRIMNDWQMLSSDRSDMSRKAVDRRNEK
RKELMDLQGYLLDELSEHGLRKAKWKYYLTRQFQSSFPDATAKADIISDI
LLEQCFLPRVLVSPADAEYTFRFIKALHEWNAPVFKLMSLYDRLFNANRL
RSLIFTCTVREAEYLGRFLKLILEDLSRWHKNDPIPGDKDNKGTKDIPKL
GAYDKEGKGPSEQPHFGFSLTVNDEGKPETFVEHAQFRDLLFRWHKNLNM
ALKNCLGGSEWMHIRNGITVLKTVLDFFPAIDFMAAQFATQLQNITKREA
AAKTSPDCEEGERVDLSVAAQGAMSELQRRKSKWVMVQAFRQNAVTGSRL
EGNKSSVPALRPTAADFKPQGDKTSSARNAAAEEEDGEVQDKEATRETSG
STLQEPLPPKPTGSGRDSTKLGDTPGQSRSSTPKPAAAKSEPRGHKLPDR
PSHNLPSRPDVPIPGHFTPERFGQSRGHDRRDVRESRSHRDGRDVRDGRD
RDTRDSRDIKDHRESRQSDSERPGRGRDFGDRRATDYKEENGRAELPPRP
TQSERERPHREQRVSTKTHDRPLESSGAELAMPVEPHEPVMNPERAALFA
QDTPERLPRGTDPDRSLRGRRQGGSAVMDGINPERAALITDRDDMAHNAP
GRLNRDDGRERPARGQSPRRGSRHGNEQVSAPGAWEDRQGRNVSQDQRPI
GREPRDRSPLPGTFRGERPMDRDDDRLHLDTMRDGPGFQRPGSRPQELDN
RKPYQDQNYGRLNPVPQPPNSEIPSGPRGRGRNASRGGHGQPTLPGRPDN
RFGPPETPRPPSPERAGPPTGPSSGRNRRGYEPGGGPQTPGGTPAGPLGR
GRNFIQGGDKPSPANVNATTTSGVHPDRLAQLGGPPSGPQGNQRHQGQGV
SIPDRPHPSPRGGSGPDTDTPTGPSAVDRPGRGGGRRQLAGINNTLQQAQ
AKIIPEPPRTTGPRSNQPRQMLGNSDIQVLAGGSSTSTPNQERHDTMWAS
SNGVDNQARRDHDRSRNEREGRSDRSNRTSRRSSRERDRDGKDPNESRER
RSMGGPDAGGNREERESRRQLRDSMGPPLPAGGRDSRHRADGQPAGGNWG
GEDRGGHRGSSSGSRGTGPRDGANQASNRRDHREDRGRKRRSEEGVGNLT
SDREKRPRR
>Mb|QLI68470.1
MCGPEVTNRPRGGALAALSAPKNGHDVPSSCFTQGDLAFHTPGQPGSTEA
THARLTPAEACRLADVDKLRVVIHSSGDMTRSAHDCGLFRQFVFPAARLS
NNYVEFPLSSPLSLEVGNGGIIGRRVSVSSRSEAGPEVVIAEGIVGFNFL
EQPSL
>Mb|QLI68472.1
MPPPIDISSLNKDSSSEYEPRIISILSNAINELAPTELSAETTAGEIDKL
YPSDVSAAEGFLWSFWTLLVAVAKKIPADDARQQLLVTTIGKLKNKRDEE
VEMWGQKTRVWTELPMLGPVMRDEWNLSPDFDGSEKDNATIQEWISLNSF
AARMYGASLQPWVNLGIWELRSGLEEPPEDRPNAKDTRIATAYEWIVHAG
KALYANGRQAQQLDAMEQRALKPGSLLKIEASGLSSDRWNFWSERIGVLG
ATAGSGAAKEKAQKALETMKKIEGN
>Mb|QLI68473.1
MASNIFHRDETPSRRDRPIQSEAGASSHSEEVANLRESHRLVEENLDHNF
NYIDAETSATARRHAALDTSNNIAGGGSTQFSKFASTSRWPYHDEDVEND
VPASLLVESNEQEPKRTLQSMHTHHDTTPLHAGKFDRQPKAQWQATTVHQ
HMHPNSQQSDISIQQPRSLMNGLAPGGRREKALWRWVNISNLDSFMRDVY
DYYEGGGLLCILCSNALWLLETLFVAVLLTFLTQCVDYGKVPHSKSLSEV
VVDQCTRNMSTSWSLGIWMYSFFFIWKSVQYFFEIRRLIYVRDFFVYLLE
IPEQDMQTVSWQDIVARIMSLRDDNPKTATNMPRNLRRYIGSQSKERLDA
HDIANRLMRKENFLIAMINKDVLNLSLPVPLLGDRQLFSKTMEWYLHYCI
LDMAFNEFGQVRQEFLRADRRSLLSQKLRQRLYFAGFLNLIFAPVVLAYV
VIVYFFTYYNEYQKDPKLAAARKYTALAEWKFREFNELPHIFYERLYMSF
PFATRYIEQFPKRMTEEIARSISFMSGAITAVLAVGTLLDSELFLGFEIT
QDRPVLFYLGVFGAIWAMTRGMISEETTVFNPEYALRNVIEYTHYMPDHW
QGRLHSFEVKQEFSELYKMKVVIFLEEVLGIITTPMLLFFSLPKCAEQVV
DFFREFTIHVDGLGYVCSFAVFDFKKGIRQTQTQAAGPDLRDDYYSTKHG
KMAASYYGFLDNYVINPKTGIPGHMPPGPRGQFYPPPAFPSLNSPSLAAD
MHKSHISRTDLARNRARGTSTMPHKRAMVPTVPQLSPMASVLLDPHNQPT
APSLGARSWHRTRQARGAYHGENQIVEEGAGYGADRQSVDDEDDVYDEGG
ILGESTWETSPDKGLSRENSSTQHGELGEGGVLGLIYQLRQAQHRRRGGV

V
>Mb|QLI68474.1
MPPTIHLVRHAQGFHNLCVENESLRDPDLTALGEQQCADLRSAFPSHARL
TGLVASPLRRTIQTCDLAFGGADKLYPIVLLDTLQEVSGAPCDTGSSKAA
LRAEFGDKIDLQRVRDSWTEKGEGSAFEPTMEALMARAKTARRALREMAG
DGDDEIAVVSHGGFLHFLTDDWDGVPLDQATAWTNCMCRSYQFAESPDDK
DDVRLVETKESWARRQPASRQPLTATEQREIRAIVQKRLLPYLKIKA
>Mb|QLI68483.1
MGLAKLSLAFLAVAGNALAAADIEPIEVKGSKFFYKNGTQFYMKGIAYQQ
EVGAGGSSSSSSDTKKNYVDPLSDETVCKRDVPLLKELGTNVIRTYAIDP
KADHKACMELLQDAGIYIVSDLSEPDKSINRDTPQWNTALFERYTAVIDE
LAQYPNVIGFFAGNEVSNAKNNTAASAFVKAAVRDTKAYIKNNKKITRWL
GVGYAANDDKDIRAEIADYFNCNTVEESIDFWGYNIYSWCGKSSMQKSGY
DQQVKFFENYSVPVFFAEYGCNNDGAANRIFDETTALYSDDMTGTFSGGI
VYMYYQEANDYGLVKVSGGKATKLKDFDALKKKVNGATPKTLEMSEYKPG
GKMNECPKLTDNWQANKALPPTPDKSLCSCMAKSRACVPKSGLAVKKFGD
IFGFICKASPESCAGINGNATTGVYGAYSMCSDEEKLAYVLDAYYSSQKK
AKDACDFDGSAETQSANSDSSCSDALAKASDTNKKAATATSPIGGGGNAK
ATSSSDNFAVHGAPVARIFAIGDFAVGLYMLVALCVGAGMVAL
>Mb|QLI68485.1
MCGVSAILLGDPDATSAAVDLHESLYSLQHRGQDAAGIAVCQGSRVFQCK
GLGLASEVFSDGRRLQQLPGYMGIGHVRYPTMGTASASEAQPLYVNAPFG
ISLTVNGNVINTEELRRYLDEEAHRHINSDSDSELLDADGIRPLCIGSRP
SATIAGANDYFMASESVVLQQLGFRDIVDILPGQAVFCPKGGTPIFRQVV
PRRGYTPDTFEYIYVARLESWIDGISVYRSRQKMGEKLAEKIKLVLGEKG
VEEIDAIIPVPETSNVAAAALAQKLGKPYVTALVKNRYVHRTFILPDQAS
RLRSVRRKFSFVESEFQGKNLVIVDDSIVRGTTSRQIVQLAREAGALRVV
FVSCSPPCTHPHIYGIDLADRSALIAYNRTQREIADYLGADEVVFLDVDG
ENGLTAACIEAAQGETPIKNMEIGVFTGGYVTGLPDGYLENLSDLRSGRR
QQKAGFESQKAGEGEDGSVAARPAPAGLPCSGEAATPDHREDIKHVKLEA
LTLRVAADPNLNEIIRRYMEKERLSTEKALQFCTVLSEQIKQIQADFQKN
QDSDLTSGMLFGKGLQGCMHHMCFMLAHLEKYQKIVAERLNTSLNAESAG
EQASFDKLQNKAKMLQRCLAFCSDVDTYMESQISNIENHLEGDDIIRFLV
SIDGKRIGGKNRGTGKRQKQAGGHFREASLQQMSQLHQRGHTKHDTQSSA
SISGDEPPLATLQSLLGKCHGPGVILD
>Mb|QLI68486.1
MSYPTRSLSSHMRAPSSSTSSASPSPALLARIEEKKAELQSLRELRDLSA
AVSNQMEALEQKLTTLSDGTEAVAAVMGNWHNVLRAINMASTKLAKPSND
TTMEDASASNESLPQTLVRIPTEHAPTLQAQADAIEAAAVEGDSTANT
>Mb|QLI68487.1
MVKKNSKAVSSTREEPASAPPAALLNLVGSFLNQHSFAKTHSAFQKEVAK
NGWLCETSIDGDNSLVSIFKIWESGQNSKVESEEDSDDDSSDASSEESSD
ESSSSSSSEDEKTVAPATKKNVVETKNLKRKAPVESSSSESSDSSSSDSD
SDSDSDSSSDSDDEPQAKKRKTAKKAASKSDSDSDSSGDGKMDVDQISSD
DSSSDDDSSSEAASDSKIDVEIQVAAAAKVALPESDSSSDNSSSDSDSDS
DSASDSDSAKAKSKKTKGVASKDSSDTSVTLDNQSPAPKPKVEDPPLPPN
PVTNGRKKQNDPFSRIPKNIKVDPRFASNEYVSMDYSQRAHEDLIVTKGK
GFTKEKNKKKKGSYRGGMIDITQKKGVYFDD
>Mb|QLI68488.1
MASAGGLKSTYKPSAAAAAASAPLPPGWTEHTAPTGHKYYYNASTKESTY
QRPGLPPPSAVSAQDAYSPYANLPSLADPRVANAYLAQLNPQNQPRARGA
HSGRGGSRGGFEGRPKPQPVDKPRKAEKIPGCEPWLLVYTKYSRRFAYNP
VKNASYWRIPEKLMEGILELDKARIRGKATGTAEDAKSEKNAENKTGDLA
EKQHDYDSDEYEEIEVQVTDDEGGDSLDTEHPSKRRRTNKGESEEEVVEE
EGEEEEEEEEEEDEADGPVEFTEADIAAQLQAMGEEYGLEPGDYDDGNME
SWPEGTEGVEFSEEDAKLLFKDLLNDFNINPYSPWDKLIEEGKIMDDPRY
TALTTTRARKECWDEWMREKITELKEQRAKQEKKDPKIGYMAFLQEKATP
KLYWPEFKRKYKREEAMKDMKLSDKDREKAYREHISRLKMPQAKLKSDLT

ALLRAQPIHLLNNKSSPTSLPTPVLIDLRYISLDPKIRDPLVEAYLQTLP
PPPEDVAASEEDGEKKKKAREARGKREKALEERNRVVEEQKKRRERDLMA
SKARLRDEERELEIAMRVGKRGLQSQLQDEA
>Mb|QLI68489.1
MAASDNTVPSALGGKPERRSPPPASQSKRDRKRQALMERLSSMTDKFQRE
QDMTYRDQLQKIQYEIGLVQRFDPYDTNALEVAAELQREHRQTQGPPVHA
ESARSLMDMAGIKFPNFIDEIEDLIEIRDFQLTQSKNEYERKVQEYKNTH
AYKVETARREHMALTSTLRDRLINTLTQKKNRLNREKEVLEINDSNALLL
NPNHFSLTNPGSPGGTHGKRATRLRKDADDLQMFSDKKRKRNPGEDDGSP
GPMRRGLDPNSTTPLWQSEKARAVAKQNGPIYSIDKLFTDKELSLHYNSA
ALAAHQYVLRNRVNGNASSPDDSDSGNGDDNDADSVLSAPMMERQVSHAT
RSTRGGANTNFLDDKILGVEGIANFEVPANLDLMHAQEPPKMPPHVPQQY
LKPYPRSADQNFPVPLSQDDIMSDLSVMGFLKQYDHSHKPGAHLDAPTGL
RKILEAVSIPYQQGRFVAITSAAREDPENFRDSLGIPTSGLRDQLSPGHG
GSNQSVAGYSASAIPMSRQSSLGGVAMSRQGTTGSGRGRARRG
>Mb|QLI68496.1
MISDQESAAAMVPAAENNSARPSAVVETPEGNNPGVDGYDVGLMDDLLSA
LSSIKAPGSFASFGVLPQPPPAGLFVDDVGDITMPLSEVQARQLIAKARQ
APYGKGSATIVDTAVRNTWELDSGQFRFQSPHWPGFMRHLCAQVALNLGI
NAPITAQIYKMLIYEKGAMFKAHTDTEKIPGMFGTLVVCLPSAHQGGEVV
LKHCGQKKVFKTSEAPQSFASWYSDVSHEVLPVTSGFRWVLTYNLALHLT
GPGPSAGLPQFETGALRRTLKRWLSEPKESRETNCVYHVLDHDYTEANIS
LKALKTRDLAQVHSLKEISGELAVDVFLALLEKEEMGSCEHDPYEDRYDG
FRRYNRSYYDDDDGYDDYNDDESGYHHLEEVFETKYAVKTLVDLQGRVVA
TRLRLDEQDILQEDCFEDLEAEEEYEGPNGDALAAVIVPRDSLLSFFDTY
DMNRYCSPYHNFKTQVRYLARACLQPQPPKSFINALVILSQQAWNIRRTT
IGGFGAQEPWIDGDGIRDVLTAAIQHGKYNFFQEAAGHHEGLLPPEFFVW
MRQWLLTGDSNTNERFNVIQSGISAAISSYPYFADQFTAITRLVSMSSDD
LTPDTAATPDYILDWARGKLRSCMDVCASESLGREDGPAMVDLALFFNDP
PAFMSQIISPTIGQRHDAAAFFLAFLARLRQQSKNGILAMGNAIQFYQTK
ARSFITLADFTQMRGDAGGQAIPTRITPHNCEQAQKLDRRTAISYEVLAD
FFSAIIQSSTEADDLVTLFVSKLVVKAQQLPALEFHALWLPFLRRLIPIL
VSNTIPLDNPYCQQLFSALLHAYVNKYVGHKPAENTSLIRPGVRCQCSDC
QWLNRFLAGTERVGRFSINKKRRMHLHQQLDSGGVDCTHETWRSGSPQTL
VVTKTFRHNEKRLLDWKSRLMLAKEQIRKFEPLHLSLLLGPNYLTIVNME
HLSAAPERAQVQSPAVVSGSSRATDQPRGPLQAISSARLPPSPVAGVKRK
LPPTEIDIIDLTGE
>Mb|QLI68497.1
MSYPLYAARSARQATHNAICRRFASDISVTRTGKPILRVEGGRSSLGGHT
ATVFGATGQLGRYIVNRLARQGCTVVVPFREEMTKRHLKVTGDLGRVVFV
EYDLRNTPSIEASVRHSDVVYNLIGRDYPTKNFSLNDVHVEGTERIVEAV
AKYDVDRYIHVSSHSADLQSASEFYATKARGEQVARSIFPEATLVRPAPM
FGFEDNLLLKLASVLNLFTANNMQEKFWPVHSIDVGAALEKMLYDDSTTG
QTFELYGPKEYSMRQIATMVDKEIYKQRRHINVPKRILKPIAGVLNKALW
WHTMSADEVEREFMDQVIDPTAKTFKDLGIEPGDIANFTYHYLQGFRSQN
YYDLPPATEKEKREDKKYIHVLDEL
>Mb|QLI68498.1
MADPLLTSLCSICHVSTPKYKCPRCNIQTCSLPCAKKHKAWSECSGRRDP
TTYVPRSKLRTAAGVDHDYNFLHAIEVSAERSEKMLVEEKGLVQQAELRP
LTIQQVRWKMGRDGRKRKVLVTRVLREAKGRTFEKGLAQRLKRLNVQIVC
APLGMARQRENSTKLNRRTGRINWQVEWLVVGGEKETARSLSKVMDDVPL
FQAYPAMLEEKARAETGSRKQERHMRAWEVQSWADSRWNLGLDCMQDPVD
GKWSLFSGGRVEDWPAEKEKAQRQQFQFFLAGPLARSDMPAQVTALEAGD
CLRDILANTRVLEFPTIYVLKRGDRLPPGFVLGPKDSVPPQTQRTKRKDG
PSNTQFQASSKRRKQGRMDREEGEVGTDEDREDGLDEETESKGVSLEAAE
VIAEQSLGEEEDDDDTSSSGSDSESD
>Mb|QLI68500.1
MPAYNSIFNNDPNPPRLIGNFPILPLRTKTRGPAYTLPIPSPPLPANESP

DPDSDSYDILDEVISLFRANTFFRNFEIQGPADRLLVYGILFLSDCLQKI
KPGATARDAQKDVMNLALDLNFAIPGDPGFPLNQMYEPPRDRLDAEQLRQ
YMSQVRQELAARLLARVYADDETKPSKWWLSFTKRKFMGKAL
>Mb|QLI68501.1
MSETFEFQAEISQLLSLIINTVYSNKEIFLRELVSNASDALDKIRYKALS
DPTQLDSGKDLRIDIIPNKEAKTLTIRDTGIGMTKADLVNNLGTIARSGT
KQFMEALTAGADVSMIGQFGVGFYSAYLVADKVTVVSKNNDDEQYIWESS
AGGTFSITADTESQQLGRGTSIILHLKDEQAEYLNESKIKEVIKKHSEFI
SYPIYLHVQKETEKEVPDEDAEAEEVKEEEGDDKKPRIEEVDDEDEEKEK
KKKTKTVKETTIEEEELNKQKPIWTRNPQDITQEEYASFYKSLSNDWEDH
LAVKHFSVEGQLEFRAILFVPKRAPFDLFETKKTKNNIKLYVRRVFITDD
ATELIPEWLSFVKGVVDSEDLPLNLSRETLQQNKIMKVIKKNIVKKSLEL
FQEIAEDKEQFDKFYSAFSKNLKLGIHEDSQNRQQLAKLLRFNSTKSGDE
LTSLSDYVTRMPEHQKNMYYITGESIKAVSKSPFLDTLKEKGFEVLFLVD
PIDEYAMTQLKEFEEKKLVDITKDFELEETDEEKKAREEEEKEYESLAKS
LKNVLGEKVEKVVVSHKLGLSPCAIRTGQFGWSANMERIMKAQALRDTSM
SSYMSSKKTFEISPKSPIIQELKKKVETDGENDRTVKSIVQLLFETSLLV
SGFTIEEPAGFAERIHKLVQLGLNIEEDDSAPAEAPADAAPAAETGDSAM
EEVD
>Mb|QLI68504.1
MTSPPTEEVEPLDDLDASLRDFEPPISPISMRHSSALPSEPATEDLEDSD
TGSAGGFSPPAWRRLGNGDRSRGFWKQHPRLPKRQRRLSRETSPGSDVSD
SDDDDVEDDVLQQAIRTRLPAGSQSPEKGRSPSPERGEDVTLKVGNISPT
TPKANSRESPGPDNYFRFAVRAEVQQRTKPIEAAVLFAQRRHRALTNGWM
SIVSGILVAILSISTLRVLVKPAAPRPVGDLVKVAGIARSFEPLIYFSQP
AVAHVKDLQSTSIAVWDLAESVRVSGMADAEIIVDNLDSISEAMKKLVLE
LTKFHTHVDGDIDSILSVMEWAKMHLDRLNSRPPPSSLSHAYDNIHNFLT
TTAILEDAHGHPTRVGRLTTTIFGMSNPQREQRMVQLLFNSFLSTLEEAI
QAELENSVSLFALFDDIDAHFLKLARTVAHETSTQEEFQTELLSGLWARI
LGPRAAELRKFEKNRALLHDVRSKTIRNKGNLVGHHGKLLTLKSSLESLR
GKLASQLVRGLNSSTLTLEDQIRGIVQVRDYLSDVRSQQKTKVMETLYSI
DRSRKYVYHEIDEGRHVEAGPM
>Mb|QLI68506.1
MSEEKSRVSGEVPRAESSSPVLPTVNPAVEKPQPPKSVVHPAFYVIVWIG
FSSSVILFNKWVLDTLNFRYPVILTTYHMAFATIATQLMARFTPLLDGRK
TVKMTGRVYLRAVVPIGLFFSLSLICGNLTYLYLSVAFIQMLKATTPVAV
LIAGWSLGVSQPNLKQFLNVSTIVVGVIIASFGEINFVLVGVLYQIGGII
FEALRLTMVQRLLSSADFKMDPLVSLYYFAPVCAVMNGVVALLWEVPKVS
MTDVYNVGLFTFFLNGLCALMLNVSVVFLIGKTSAVVLTLCGVLKDIMLV
VASMMIWGTPVTPLQFFGYSIALGGMVYYKLGFEQLKGYMGEASRQWAEF
GARKPVLRKIAIIVLAVFLFFSLFTALAPSYAYDPTRLTNTVAGLGQTGA
Q
>Mb|QLI68507.1
MADNSNGEKVNTNIVTLTRFLNEEQTKHKEASGDFTLLCHALQFSFKSIA
YYIRRATLVNLTGLAGSSNTTGDEQKKLDVISNDLFIEAMRSSNKCALLV
SEEEEDIIYFENATGSRYAVACDPIDGSSNLDAGVSVGTIFAIHKLPEGS
TARKEDILKPGTELIAAGFTMYGASSQLVLTMKNSSVNGFTLDNGIGEFI
LTHPNMRIPKSRAIYSVNEGNSLYWEENTISYFNSLKQAQSNGKPYSSRY
IGSMVADAYRTLLYGGIFAYPADKKSPKGKLRILYECAPMAMVFENAGGQ
ALDSNMKRMMEVDPEHIHDRSGIFMGSYDEIEKVKSFHK
>Mb|QLI68508.1
MRGTPNRNQGRGNIPFTNSPANSNIPRPVLDSQPSEASSSVSASRQKQSK
RDEAIRRKMENDLSKKKHLTNRARHSRKAPPGTVLALKPSQALQIKPQTT
VSEAAQLMAAKREDCVLVTDDDDRIAGIFTAKDLAFRVVGAGHKAANITI
ADIMTKNPLCARTDTSATDALDLMVRKGFRHLPVMDENQDISGVLDITKC
FYDAMEKLERAYSSSRKLYDALEGVHSELGASQPQQIIQYVEALRSKMSG
PTLESVLNGIPPTTVSVRTSVKEAAALMKENHTTAVLVQDAGAITGIFTS
KDVVLRVIAPGLDPANCSVVRVMTPHPDFAPMDMTIQAALRKMHDGHYLN

LPVMNDGGEIVGMVDVLKLTYATLEQINTMSSGDGEGPAWNKFWLSIDNE
TESMMSGEGSHSHHHTNLGSRMMSPDVTRDRHGDSVAPGDSASHVGVESP
PPSIAPEIPEQNPADVPFPFKFKAPSGRVHRLQVIAAQGMGAFVSAVAAK
LGAEVDAIGGVPAVEDGKMSGAGFALSYMDNEGDTVSITTDNDLLEAIIL
ARQSRHDKVDLYAHDPEKPPVAPPAPVETPVIPTPPTSTAAGLRERRRLY
DDDDEDEDEDEDESDGAPVRRSRRTRSAPVQEQLIAGVPNDLLLPGAIVT
LAVVIVGVFTIARATSR
>Mb|QLI68510.1
MARIQALALILASLLALVSASSAPTFCKCTCFKNSTIIPLGPKGESSGSP
SNSFLSFSLFSESNPSGAAASDDATDDQGLSRRSLSTSCSECTKSFCLSQ
GIDFCKEAKEENVVTMCFQRDSNKDKIIVWGFILGTVGLLGWAAFKRVVE
VRGKRAMSRQDISYAPVSGERR
>Mb|QLI68512.1
MGISRDSRHKRSASGAKRAFYRKKRAFEAGRQGANTRIGAKRIHTVRTRG
GNHKYRALRLDSGNFAWASEGCTRKTRVIVVAYHPSNNELVRTNTLTRSA
VVQVDAAPFRQWYEAHYGQSLGRRRQKAQAAKEGKVEEETKKSKAVEKKQ
AERFAATGKVDAVLEKQFEAGRLYAIISSRPGQSGRCDGYILEGEELAFY
QRKLHK
>Mb|QLI68513.1
MDTTAQLLDSITSQCLPPPSESLLTSLTTRSPQPPLPSLVATAKARLLAA
DLTSSSYLDGAQLCPLPSGTDSANVKEAKLRHHTHVQVVDIENLSLSRWE
QVEEMETIERGERTRGREIIRVADEESADAAESLAARGTGTGTGPRPPVS
AAKKATHRLVLQDCKGERVYAVELTRIDGIGVGKTNMGCKMLLRAGTPVA
RGTVLLTPENCVLLGGKIDAWHDAWMNGRMARLKEAAGAGNAHTTTTTTT
T
>Mb|QLI68514.1
MGPPRTRAQHFLAQRGRFQEGSMNDRVSAVPPVHFLGPQDREVLERPVYA
EQSAYAAFDPAYFGRREERQPAQQMPQAQAQAQPQPRPTARKPTTKMNIF
GQVWEGVRGRLRLRRDADDKEKVREKKDPASSDAQDDRPSRDEVFASYQQ
LVASGFFSSHAIQSTRHGPPPNYRPSTSHGVPSRNAGFAPQWPLAPRPLT
PQSLPGAHEPTPQTVCSPVSVASSRGTKRAAESPEPSVDGDGDGEQGGRE
EERDDGEDESTLAHRFLPKRLRISASRDISLPKLRSVASRKSMRAAVALA
RRNVSVGAQEEDGKERELHKLSKKMSWAQQQQQQQQHGEVPAARASMDML
PRGRNSSEIHGSVTRRIRRPTSARNLRSAGMRDGNGGLKVVPDANRGIPR
VPDIPAKFTYGEDRENGAPWRGLRR
>Mb|QLI68515.1
MAYSTAGPSQGLGLSRSSIYSLFFLVALCVLSTLAACGSAAATGDKYLIG
VGKADITGPVVEIGFAGYANLDQKGTGLRQRLHSRAFIVADKNNPDDRFV
YLVLDSQSGDTATRYGVLDGLKNLGAEYSIYGHNNIALTGTHSHAGPGAW
FNYLLPQITTLGFDKQSYQALVDGAVLSIKRAHENLQDGYLDAGTTRVKD
GAINRSLYAYLANPESERKQYEDDTDTLMTLLRFKRASDNKDIGVLTWFP
VHGTSLLGNNTHAAADNKGVAAWMFENAMKDNSNAADGFVAGFSQANVGD
TTPNVLGAYCDDGSGQQCSLENSTCADGKSQSCHGRGPEFRALDLGVKSC
YEIGRRQFAGAQTIYNSLGTSGTPIEGSSVKAFHFFHDMRFWNFTLPSGK
QAQTCPAALGYSFAAGTSDWPGAFDFTQGDSGEPNANPIWRVVSGLIRTP
TEEQKACQGSKPILLDVGEMDEPYAWTPNIVDIQVLRVGQFLIIVSPSEA
TTMSGRRWKAAVATEASSFLPQKPIVVLGGPANSYSHYCVTPEEYEIQRY
EGASTLFGPHELDAYINLTVSNMHYLHPDSKDTPDQGPLAPDNRGKALSF
ITGVVVDGAPIGSSFGKVVHQPSASYQIGSVVNVTFQGANPRNNLRQEQT
FAAVEQQGSDGSWTRVRDDTDWFLVYSWRRTNFILGYSEVDISWETYGNA
QPGTYRIKYYGDSKPPIGSVNPFDGTSNSFKLTGGLTRQLRP
>Mb|QLI68518.1
MEQNILSTPELSPLEQEVLEEYERLAGNMKQLASVLDDLASNPSTEILDG
LRDLERKTSLAFTLLKASVYSIVLQQEIDWGGGREE
>Mb|QLI68519.1
MSKTMLWIGFSLEDIEPIPAVLTTFTIAFITLVVYLSYTPKVVGAPEFIT
DTVPFIGSWSFFTKKMSFWRGAVAKSKTGTFSFWLGQNHVIGLSGEAARK
MYLEDRRLHLIKGIAVIGHGPDFINGRSTVIHEIWKGTGPSGRTYAQRRL

LELQRSDLLAKRLPKVTRDARTAFEAMKMNESSVINPSKTCFRIVIIQGS
RIVATDEIADNPELLHSLFLYTQILQFTNTLHLLAFPGLSYLSPGYWKRR
YGRRGLQKIVEPIVNRRMAKGAAGGDDALKYLIDAGDSQDYISTFLDWQG
KIYNEIKSSAAIYATDKTRKLPLVDQLDQLPIEAWEKMSESLELCYKEAI
RMCVAFPMGRFNDTPDAISIPGSNQVIPPGSFTCYNTIDAHYNEKLYPNA
MKWDAERWSEGRMESDQEAYGFMGWGAGRHPCVGMRWAKLQQNIIIAYAV
ALYKWTGCDENGIPTAVFKQPTHGLDHLAPKLPQGTHCRFAPRGE
>Mb|QLI68520.1
MARFGRAKFLTCFYCGKRPGIKFDGTSRDFLCLHCDATNYLDENGEITDP
PVATEHEAAAIQYAAPQSPSPSQTDDIFCQTCLKNQHLFTKSLAQYFPDD
PSDPDYPELERNYYRYRRNLEKRYPQVCSDCAERVEERIRQAGYTAKTDH
LRRMMSLSRGRRTRRRTPLDWVNTLGKTLWRTSFLLQLLWHFVMITQALQ
QSNDGMYDPDDQSVYATAISWLKLAIGFMPSAATLIQWSIWTAILAVWWN
PHFVQVNRGFTRHLLGFTQWYSFQGLTIFFRFIFRGVVDANAQTAGSGTA
KLSAHVAMALVMTVIHVLAGRSIRVDTSPLFATEDRPLVPREAKPPKQRK
QDDSKTFSELVNDALDSANATPQKSTMTDQAPSPQSPFSPSRPQTSRRPV
FGGVRPAETPTKRTEQLQYDEEMDWSPIRSPHRALNDNATPLTSKFGRTN
DERLSSPEAKNPFWCKVPAAPVNPAQRLRNPQWSPAPKKHEAAETGKVMF
TRRGHPPTSSQTTLDSDTNGGVEFKQPSFFAPQKDHDASSLADLLSQSFT
LGQDQQEEDAETAAEQSGSTAPAKSMPQPSVKPSPESTVLTVLLSAWLLT
MFANVPYVWEVRLTTLFVAGVIALRGTGDTSYTPGNRTPGPAAYILSALG
AAELAAICWVGSKVWSGQSDQTGPYGVGVLVSMLGHQALRMVL
>Mb|QLI68521.1
MAQPGFGGALDTPRTNVGDATYLSRQPDFADISQEASFHSPGKDGDFFQQ
LRNGRSNGVSLRTPRQRGPLADRRNLPQNLGEVEFTPMLKFSARRNARKN
GKENGTTALNAPALDRIDEDDMTPIPRMDSSVYLGSRSQSFLDNTLPQLE
GSSVASTPLAMQPRRGGDKGPLQDGNQLSLREQENVIDRIEKENFGLKLK
IHFLEEALRKAGPGFSEAALKENTELKVDKVTMQRELHRYKKHVTAAERD
LESYRQQMQELQEKGRRKQNDDGQRLELEQLRKALEERKSDIDALQQQVD
QSHNDEDQLEKLRDNIEDLEADLREKDRLITERDDELDDLRDRLDAAEDK
QREAQRRMSEMEGAENQNEELEEARETIQDLELTIRRLEEQVEDFKEKAD
QAMSQKDRAESDLEELQEEMANKSVMTKGLSRQVEEKVSRLQEELDKSGK
EYADLEKELAGANEENEQLRAEVDELRNEQQEIRRGQHDDSAKVRGLEAE
LLAVADEKDLLQVRHETLKVESDALQRNVQQLEKEVEELQKNVSDEREYA
VEIEKDLRQQYQDELERLNDAVSDLQAEVRERDNLYDNDSEKWENEKQAL
VSERERAEEKAAGLQRTIERLREAEGNLSNKESTLQKAMESEMERHKSEE
AVMARQIDDLQDALETRQTLLTNLRNELSTVRDALRQTQIDYQGQVNKIV
ALENEVEVLRSKSNNQLTPGRRDAFVRDSQYLQDQVTRLKAELGSVQSSL
AETRAQRDELQRQLHQNGAQENDTLQVDQERLDLRAAKMKLVSEVRLLKD
ENKALSEGRKEAEKALEHEIVKVATLEDRLKQEVQQLQTKTKSSTATPFN
DQETVSARRTIRELEQRVKDYEDQLVKLQAADDGDDVGNISILRRDLSAS
RQKELDFLQKEAVHRDTIRGLNVQIAELERQLHESKVSQMAASPSSSAAN
SQRTLDELKSQAAESERMASQAIGELQKQMSDLEDQKLVLEEVLEEANQQ
AEEAAAQNEKAMRRIRHKLDKAERERDAAIASHSENNKQGKHLKRSQAEI
ENLEHDVRQQQELIDGLAAAEVSLRRKLERARSERAAFRMSAEKLQRDIQ
LLQVTNGPISGQEDGRAVAKRHDGTLDGDNHAFETLVRAAEGAEERHRKE
LKGMVMQMEWMQARWEREASLRSDAAYAKKFIQLQLDVANACNKAQLREL
EHIRTNLLHSKKPLALPAAPNGLAFGKVKPTSIRPFLTMARFIARMRIAS
RNWSQQEQVRLKLVAARDDQRRVKRSRLFKVVRMDA
>Mb|QLI68522.1
MDSTSRKRKDGPVPANGSTQPAHKKSKGGSAGRWKTPHQKAKIAEKVELG
TTLAIGDQGIWVTFARGMKYKAIREFQELCVEYGKTMYGIEPPANDDEIA
QGDEHQDIEASIEAELSSMKSGGKADTNQTFKPVSSGVECLFFMKTMDPV
QPGSLAKKMCEDARDCQDQRQRKCKYINRLTPVVNMDKATDKGIDKVARA
VLSPWFELKDEEKGDADKTEAPPSAVPSPPYTYAIRHNIRNHTTFKSGEV
IQKIAGLIDAKHCVNLSKPDKVILVEIFQLFCGISVVDGKEWEELKRYNM
NELYSLTPVIGKKD
>Mb|QLI68523.1

MTFTNAPVTRLLVLGLVAASIGASMLDVKHYFYISIDTHLWKYRQFWRLL
AYQLCYTNSTEVLFAAMSLYNLRVVERMWGSRKYASFVVVCFLFTAVIPP
LIMAVLRPLSAGFFNYMPAGPTPIVFAILAQYHSMVPHVYKYRVATSQNT
TTGGSSGVTLSDKSYQYAIALHLSLLQWPGSVLGAFVGWTVGNAWRGGLM
PASLVTWRLPGWMVGLRSHRRSAEFEGLRRRLEGENGSATGASSGVQGSS
QQAERRRTMGQQIVDQFREAL
>Mb|QLI68525.1
MSRSRTIVSALRPSAAVSGQGVRAFSVSAARLVNMPKEAENLRKAPRDQI
GVLKAPLVNPADKYQSKSDNMHRYGSWLMGCLPKYIQQFSVWKDELTIYI
SPSGVIPVFTFLKYNTSAEFTQMSTITACDFPTRDKRFEVVYNLLSVRHN
SRIRVKTYADEASPVPSVTSLYDGANWYEREVYDLFGVFFAGHPDLRRIM
TDYGFEGHPLRKDFPLTGYTEIRYDEEKKRIVTEPLELTQAFRNFEGGSS
AWEMVGPGEDRRPDTFKLPTPKPEKKEEPKK
>Mb|QLI68528.1
MSQVETVSAFVEGAPPGELADVIADIKALTVSSPDIVSELTPAFEKYNEE
QLVTVKLPGSSQPVVISSHNALGDGRYYDVESSSSFAVDHATQKASAVKT
HAIEGPQVDLVTSTLKGLSAYVKEHFPNASYGAYPVESDSKVAIIIVANK
YSPNNFWNGRWRSQYIFDPASGSLEGSIKVDVHYYEDGNVRLLTNKPVTA
SITSRTGAGIAKEISATERKYQEELNKGFVNLSENAFKSLRRQLPVTRQK
IAWDKVMSYRLGQDIGGGNSKGSSVGSMG
>Mb|QLI68531.1
MASLRSCAAPLRWATKSSTLSFVRYNSSHSSSTSAIAYKALRNRSAPLPV
TNAPPAWSAQAAVSNILYETPTPSMAPPKRHILNCLVQNEPGVLSRLSGI
LAARGFNIDSLVVCNTEVEDLSRMTIVLTGQDGVVEQARRQLEDLVPVWA
VLDYSNAALVQRELLLAKINILGPEYFEELLSHHREITAGEYDREHTPEG
AEQAQSLEETSADFHPSKLAASEALRHKHEHLKTITYFTHQFGGKVLDIS
TISCIVELSAKPTRIDSFLKLIAPFGILESARTGLMALPRSPLRGPAEEP
LVKEAEEVVDASQLPPG
>Mb|QLI68533.1
MDPATAGASKHQGDDAPELLIDGEAAQHAKGAAHKHSRGSIEDPTSAPID
SDSDDLEPITRRGTQAMMDDQDRSELVRIATVLSRRRSSIATHPIPTISL
GDIDENAPVFNPEHRDFDLEKWLRRFMEQLGEEGISEKCVGVSYRNLDVF
GSGEALQLQDTVGSMVAAPLKLGEFFSFNKKEHKQILHSFDGFLRPGELL
IVLGRPGSGCSTLLKTICGELEGLNIGEQTKIHYSGIPQKQMIHEFKGET
VYNQEVDKHFPHLTVGQTLEFAASVRTPQQRVQGMSRVEYCQYIAKVVMA
VFGLSHTYNTKVGNDFVRGVSGGERKRVSIAEMVVAGSPFTAWDNSTRGL
DSATALKFVQALRLASDLGHQANAVAIYQASQSIYDLFDKATVLYEGRQI
YFGPANQAKRYFEKQGWFCPARQTTGDFLTSVTNPQERVAREGFENKVPR
TPEDFERLWRQSPEYQALLQDIDAHNKEFLGERQGESIAQFREQKNLRQS
NHVRPKSPYIISVWMQIKLCTKRAYQRIWNDISATATQAISNVIMALIVG
SIFFGQPDATISFYGRGSVLFMAILMNALTSISEITGLYDQRPIVEKHAS
YAFYHPAAEAAAGIVADIPVKFVTAVAFNLVLYFLADLRRQPGPFFLYFL
ITYISTFVMSAVFRTMAAATKTVSQAMTLSGVLVLALVIYTGFAIPVPLM
HPWFSWIRWINPVFYAFEILVANEFHNRQFTCSSIVPPYSPNIGNSWVCN
VAGAVPGQYTVSGDAFIATNYEYYYSHVWRNFGILIGFLVFFLITYFITV
ELNSATTSTAEALVFRRGHVPAYLQKGSKHAVQNDEAPTTADEKTANGDG
KTEVKALAPHTDIFTWRDVVYDIEIKGEPRRLLDHVSGWVKPGTLTALMG
VSGAGKTTLLDALAQRTTMGVITGDMLVNGKPLDPSFQRNTGYVQQQDLH
LETATVRESLRFSAMLRQPKSVSKKEKYEFVEEVIKMLKMEDFANAVVGV
PGQGLNVEQRKLLTIGVELAAKPKLLLFLDEPTSGLDSQSSWAICAFLRK
LADSGQAILCTIHQPSAVLFQAFDRLLFLAKGGKTVYFGNIGDNSRTLLD
YFEVNGGRKCGDDENPAEYMLEIVNKGQNNKGEDWHEVWHASPQREAVMQ
EMETLHRDKQQEPRAEGETVKHTEFAMPLATQIQVVTRRIFQQYWRMPSY
IFAKFALGIFAGLFIGFTFFDAPPTMGGTQNVIFNTFMLTTIFSSIVQQI
QPLFVTQRSLYEVRERPSKAYSWVAFIFANIIVEIPYQIFTAILIWAASY
YPVIGIQSSARQGLVLAFVIQLFIYASAFAHMTIVAMPDAHTAGSIVNVL
AIMSIIFSGVLQTATALPGFWIFMYRISPFTYWIGGIVGTELHGRQITCS
TSEANIFNPPQGMTCGQYLQPLLEQAPGTLQNPDATSECRYCGVSNADQL

LAGSDIFWSERFRNFGIMWAYIAFNISMAVLLYYLFRVKRWHK
>Mb|QLI68535.1
MGRNGIGEPSSRKLHPFFANGASGLISTTSTSNDTPNTRLGSSDISTPVI
DAGDDGHPTRKYKSDLASQYERDTGPQQLSHAADAPGAALTRPLSGSAQR
LPPSKITGQDTCMSTKNQYELQCLSTPSNMPNKPLNDITQCALPTTSMDQ
SERNAPPQAKKVLRLNPRTGTLGSPPKTKQKSKRSLIVCIKYGRDEKSRS
EIGEKVTRVLDGSLQLPETPTEQRPHKSVTKTSDIIAKSSTSTKGTHPFF
SGKPNPRSSTPIQSTDSGTRSPAQKNSVFMSTPVSPRRPRDSFSSTTATA
ESQFGIRPRGTKVPGAKHPMWPAQGMSHVRGNSFRPLCTTECRVETGGYK
KFKGQVTTIGPNESVLAAVVDRLDVAGVRRSLPQDNDAFNPAPAELRIPQ
KRCESGSKLQRRIRPQLTITSPLALAGIEDPCLDELAGPPPTLAHPAISR
HYVSLGTHLSAYDRSTCEGSSWNQKYAPVSAAQVLQKSKDALHIKQWLEG
MKVQSVETGSGDVTGDKGRSKAESLPRKKRRKNKVDDFIVDTDEEGSDLE
EIFGSDGEEDGSSGRCLKSVVRAGGARFKEPGQLRNTVVISGANGCGKTA
AVYAIAKELDFEIFEINSGTRRSGKDVLERVGDMTRNHLVQQHRAAQPSV
DGGLDYEGNGSTKSGKQGRMTAFFKSKAAPETKARSKQAIGGQTQKPSSK
AQKQSLILIEEADILYEEDKQFWAVLTGMMNQSRRPFVITCNDESLVPLQ
SLNLHGIFRFMPAPDPIAVDLCLLIAANEGHSLQRSAVESLYRARDRDLR
ATISDLNFWCQIGVGDRRGGFDWFYARWPKGCDLDNDGDVVRVISEDTYQ
NGMGWIGRDVMISECDRYDREVEALQQSWNFWQVDMGDWCQSNELGSVVR
AAWDSGDTEGKRAAALEALDKFYQTMSDADICSAGMCATWLQEPVDPSLP
DLTSKAREDFIIGRTLLEADDYIRPVMPGKTISMTLRSQARARLRSRLQD
VQPSRDNAGLCAVNESSAISRLESSFHDTSKSVNRMDMAHAFDPIAIAPK
AQPSSHLDPSVFDRTMQLIVLDVAPWIRGIVAFENQLMQERLKLSHVLSD
GVTRKRMRNTRSAYSALEGSERRSTRREQYFGGCLSTGLVMRTGGDFWQD
AVAKHTKGDVLGTETRYSQQGIASRPEDEMDL
>Mb|QLI68536.1
MKLINKRVLKDVEEDQVSLLPEDPEDMWHAYNLIIPGDLVHAHALRKVVT
VNSGTGAAAAERVHTDLTIKVKSTFFDPVISSLRVSGVVASENDHVPMGS
HHTLDIEVNRSFTVIKPDGWDSVSKATLSQALSDDKNGAMAAVVMQEGLA
NICLITQFRTVVKTRVESVVPKKRDTAADQDAGLKRFFEKTLSSLLRAVD
FSDSRPLLLASPGFVAQDFKEFIAKRGRDKSDKVLTAVSKQATVIHANSG
HVYSLNEVLKSPEVIAKMKDMSYIKEAQYMDNFFDLLKMDDGRAWYGTKA
VEKAVADGAVGPGGGVLLVNNSLFRSQELATRKKYVALVDKVKNDGGEAR
ILSSDHESGQRLKMMGDIAATLNYPMMDLDDDGEDEEEPQAVVPERNHED
DYGMLDSVI
>Mb|QLI68537.1
MSYNKAEKDIGEGPKAHKIRITLTSRKVTVLEKVCTEILDRAKSKDLRVK
GPVRLPTQTLKITTRKTPCGEGSKTWDSYEMRIHKRLIDLHAPTEVVKQI
IVNIDAGVEVEVTIAA
>Mb|QLI68539.1
MGDSLGFDDGRLAKRVRLLSSPELHHNRETKASSQQHAQHPPPPLQLQQR
EHQPCSSSYEKQQGQPSSLHADPQPQHIPPPPTEYVAASAPSTGIAMVGL
HAADDWSRNNPWSKEPIFDPAWEWPASATVLAPPSSHLQLGACRNSLETG
HMPPTPSPSTTYGTSPAAATALYAPSVIPTTSQLDSPPAVSSESLYSSRP
TQFEAAFTPPIPRPSLPSDDFPVPSSGIVGDDHPPPQCSHASGQAAFHSP
FRDCIGTFGLYKSQFFSRPIDGKVNGYIKFSWPKEKHGKQRSIGRKSSIP
DATRVIEIDGSTEANDELTSQSSTSFIGPSPVSSKGGDDLLELTVDLSSF
ANQPSIQNFTFHTPVWLTSSPVSNSSHEVACMGAPKMLPSQYGYEAKMDE
TDRRFWMFYIRNWCPGRSVLEDTNLWLRDFAQMHKSVGVRSAIQSLAGIY
IYDYLPLESIRNRVNQRFFDAEERFSQLLNDPMTARDESQANELITIAVI
LSMQDIVLTERRLKKPFSPRWLQGFRQGEQFLQATDHGSRFWKSSNVQSS
SLRNSQSIIVGRAVILAQPMSSLPPPRKFDAQKEASRFGWLLYGTEKDMY
QIHGGCGFSKKLLHVLNQVTYCAARLQQEPESPIVPMTADYLQRELLQMR
QWSPESKSLEAAQSGPSVIDWVRSLSENYQIDTNAFMTDVTAEAWRFAAI
IYLQCRVMRLSRNHPDVVSNLDDLAKCIAIMPTSGSQFTAQAPLFPVFLL
GMLATTPEHKAVSGNWFDEVLQTPVRSSVPPLHTALKHIWVWIDKEIPVA
LLPHLETNQPIHLRHPWWERLVTLVQQQEQEVLCLT

>Mb|QLI68540.1
MEQIQTQASDLRYLAQAPAALSDTASISYDTPRPGPHASPSGSASYGDNS
AGIGASAAASNANKRKSMDDGAAQKQTRSKRNRYISIACNECKRRKIKCN
GETPCQRCGNLNLACLYAPNCCSNNFKDSDEFRSVTSQLSRLQEEVNWLN
QTMKTLLQAEPTRIVAPSTDRILNGRLGSTLAPSSSAQSSASMARPDLSH
SKVGEFRGPTSMAYSLDIANTTISSMGYRGIDETSDQEQHGSDVAMQIGT
SNTGHDPLLEFDKDEMIRLCRLHEEEVGIMYPVLNIESVTAHAKNIAPLL
DSMRGQQQPTGLVNDEKTLQLKIVLCCALVTEFHGNSDKAVRLYESMEGV
VNKKLMSDPTDVASLPLLCLLAGFKYLSYEEVLAWRIMGQVVRLCLEAGI
HQMAGLMRIQDDAERRDALNSFWSAYVLDRRWAFATGLPYAVMDEEIDPR
LPLPEEYPFLVAMITYSRLGAKVWRQVSRLGPALARESHQEEIDNLDREI
LQWYDSVPEEVKVQNWDKEKQITSIPSYNLQRLKIFTYLRLNQIRIWLYT
PILHSATSIMQHPGQAQRVVDFAKDTIQYLNNLNNTTNLYRRSQSFYHQF
LASAISVVFLASVHAPIRFSAVCREEFYLALDLVRDLSAKSFVSKRLWRT
IKSLKDVAPRFGLNPDDDAHSNAALGMIGLARGHMESTPMTQSPFPNIHM
PQSHQTPEQPVEQNGKRIQSELSRIFEGYVGLNGFQFGDEQVPPHNDLGT
ADSVGGMFTPDGTVFPHLKEMF
>Mb|QLI68541.1
MGKIIKTMQSKHKETLSPWLKNYVETASAIDLPSLPNHLSTFPSRWPFGR
GDLYHWIPLLNRFDSILEHFNKTYALNEGPQSRDFAADVLLNRAKDSAFS
SEKQWTYEELQELGFSADGDRVLIEAVLLFTRMLLEHCGNRSIYASSPHL
NDLLNTTALSVLISTLEVGSELAQRYQASVKRIGNASRQISAALLANHYN
IDIERVQQIALPFVKTPIVSLADPASSITPVSTKGKEKAHGSSSRNSTPT
HANDLVAIATCDDAMWQGWGDVKVLYYPQDSNVGVDVSNDAGRTSLPSTP
TPLRRSHTVGSQQNTPRARNIGTDDSSPIIPRTPAVSEEHAIPGQKSFDL
PKSVVSTTSIYDLVKRCPDDMPSSAKYELFHRVRVAKALTESLESRQKIL
AIRLLAITNLAYIHTESNFVDKVLRHDIDETRRYQLVYQLAELIHPSADG
HSKIPLWLQTFALALLEAVSGFHARCQDVLSALNANVNHGILLYVIRKAV
AGMQVDEATDIGDQASEADDWRNNLFSLTLHLSMATRVGSEMVSAGLMDI
LVEILHIRSQVAQRHHSMVLAFLDGLIWTYQNAFTAFFNASGLGAVAQLL
VDAVTEAQNSLKSSKGVKPDQQSVVVDYEIPYYQQQTLKWLLKFVHHIMS
NSYSYGGNTERLLRNLAENSDLLHSLREIMANKQSFGSVVWTNSVTILSD
FINNDPTSFAAISESGMIKTYLEAITGQPVPEQQQGGESKQAEQHQEDQG
DAESPDSSAAAASIADEDDRPHPPSDDDIRRTRRNKPAEGILASSEAINI
VPQVLNSICLNNMGMKLVVASRAFDSYLEIFESSQHVSCMEADSDLAGNV
GANFDELARHHPPLRTSISNAVIDMVARVRWLGIERGMESDWGARLYVTT
AEGTVVTVHPNEHFMKAPTVESAPERQGATTEGADVDMSDAASTNETITE
KNKKPQKLAPAQSLTPYIHGLANFLTGYLSNTLLKVNFVRKGGIELLLDL
CESPSLDASFGDSSASRVLNQVIAQLVEHSPVRGLPSLLRRAQSNIDVLK
PLAAKTEALPPYFGAFLATDLHVWSDDPEQSKVVLQATQKIKALLNAQNL
IKIISDCFTTSRSNALQFYPVNIYDYYLKLVKSIGPLLRGILAEEAGELS
VVPQHWSLRRQGFLGVTNTSEGRDAAGSDETSIPDVLNDAARSWPSADGT
DTSAPVEPTEQERSSARFMNYEILHTLLHPMIPTSFPLFQSLGKALLPRR
EPNHLDPFPRPRQLEIAMALADTVLSHLGPSVAQAEPTSKDFHYWIIMLH
TIHEMLIDHPSPRQSDRSNVQIIMPVLLAFKEQGGLDVLNSILRILASSV
KAGPDSNIDETSKVKVAAFGLKKVLDLYYILVNGKYLSESSNHFNLHRQA
DRSQNIPNVAQQIVVEFRAAILPAIMELWTSNFVEKVPDATVKRLLDILK
IVCAAEHEPQAIPKDAKAPLMLFKYTDARFNWASVQSVVKEVIDTGFEED
LVHEAVYRANGNTAPVIEYCRAHTDGLAGPRNPIPPEDADISALQQESSA
VQEDQSNTVTAPDSDQMILDALPDISADDMVEYVDRHIGNIQADNDNSSE
ATGVTSSNHRDDTTDKIPTMNFVVNFNVVKDELDNMRSKIRAQLIDQCLD
VIRTHPETAIEVSDLVSTTILRRQNQEAQEEVASTLTFALSSLSLDEDEK
RRNGKCIAAYAHLLALLLQDENFFEHNKENLADKVDEYVGFLKVPLSSST
DELPPWIPYILLVLEALLCHDERPVPVQWKPPKSLEDPVSEPVIRLGLPL
VGDENCNKILESLLNLLPRIGKEEILATAVLRVLLILTRKRRLAKVVGEK
KNLQRLFLMAKQLSGSGSERLKQTRLTAHIITILRHIIEDEDIIKQIMRA
EIQADFPNLIRNQRGHPDVSSFLRAMAPVALRAPDLFVEVTNEVLRFQRW
MAPSGDSNRSQALVWKDQQSDSKTEEEKPTADGSSSDTVKPSTELADKEM

ADVPKAHHDIKRPVVENPDGVIHFLLCELVNYREVDDKEPSAPSDLNTEV
TGTANLRDPSPKDISAADGKDKKQSKPLFKAEEHPIFVYRCFLLNCLAEL
LQSYTRTKVEFINFKRSAPPLSTTTPIKPRTSILNYLIYDLLCQGNLNGT
TDTIVSKKKAATSSLTQKVLVALVSKTSEKAADRRADKFVYDDEPDLLFV
RKFVLDTILKAYEKAPLTDEPLETRYSRMQCLAELMNHMIGERDRDQNPN
SRGSDSLQSRSQAQLRRLMYEKGYVDKLTSSIAEISLSYPGVKRAIKYIL
RVLRVLTSTAKELSQSSILPADPAGDHSDEEFASSSSLSDVDDDDREETP
DLYRNSALGMLEPRGEDDESDEGDEDDDEDMYGDEYDDDEMDYGENDITD
GEDDISDDDEELGGMGEIEGLPGEPGVVEVIMDEDEDDEEDSDDEEDDDM
DSADMEDVEDRVEIVDEDGNPLEEDGDSGWESESDVEEDDDPDQDALDYD
AEVQEEGEAHMHGMAPGDLLDNMARAIMGDDGGYDPDLGLDDHYLDDEHE
DGEDEDEDEDIDDEEYIYDEDYPDDQLPPMPSLGWDAVNPDMDDRHRNML
LVDGHRRPVLARMDNRSSFPGRFLGGSHRDQLGEFRSYFTSRNHRSTGAQ
SNAEDGLNPLLRRSDQANEGLPRPGQTHALDLRIPPGLLGAGRHSMDGPM
GLLGELMEFLPMMSQSGNGQQAFHFHITGPGAPREFRDLGPARWVRTDNR
RDATAQEPHQAVSFTAESTLDRYQEEARMVFGVNHTTEAFKLINIITAQL
TPAAMELEKKIKAEEAEHQRRADEERKKREEEERLTQEAKAAEEKAAREK
EAKEREEQERLARERLATAETTENEQPDRSSEEAMEGIETSGPSSEPQSA
VEPGSTSAAAEQPRVMTTIRGEEVDVTELGIDPEYLAALPEEFREEVIAQ
TVSERRSQAREEAAAGESTDVFQEFLDALPDELRQEIAQQERQEERRRNR
EEARRQATVPSGQPPPAEMDAASILLTFPPALRQQVLMDQGEDIMDQLTP
EMAAQARALSQHHPPMPQHQGVRSIVTPGQGPPQAAARDNSEGKAQRRAV
VQILDKSGVATLLRLMFIAQQGSIRNYLFSVFADVCENRQNRLEVVSTIL
QILQEGSTDMNAVERSFGQLSIKARKHKERETDQKTPQSLKKTLTNLGTT
ATQVQQTNSETSPLLIVQQCLDLLVDLCTRSQHIPWLFLTEHEVVGSTLK
RALSRKGKIKDSKAHKYAINSLLTLLDREIVIESSVVMTHLADLLNRVTL
PLLSLDRKRKDTTEEVRKEAPVATEGEQRSASAEQQPPETGTSTEPPAGQ
IPTSEQNADAAEGEPHQQKKEPGSKKPRQLQPPVIPPQNLTLVVKIFVAR
ECSSKTFQNTISTIKNLSAIPGAKAVFGQELVKQARLLSENIVSDLDDLL
PHIEQATSGTEIQGVALAKFSPGASEQNKLLRVLTALDHLFDGRKKGGDG
ETEESKEHDKQHLVTSLYHNSTFSAMWEKLSACLKAIRERENMLNVATIL
LPLIESLMVVCKNTTASDDLSQSQASKGMVLSSPPPEARTASLFFAFTED
HRRILNELVRNNPKLMSGTFALLVKNPKVLEFDNKRNYFNRSVHSRSSQN
SRPSYPPLQLSVRRDHVFHDSFRSLYFKSGDEMKFGKLNIRFHGEEGVDA
GGVTREWFQVLARQMFDPNYALFTPVSSDRTTFHPNKLSGINPEHLMFFK
FIGRIIGKALYEGRLLDCFFSRAVYKRILGKSVSVKDMESFDPDYYKSLC
WMLDNDITDIITETFSVEDDEFGVTNVFDLVPNGRDVAVTEDNKHEYVRL
VVEHKLLSSVKEQMEKFLQGFHDIIPAELISIFNEQELELLISGLPDIDI
DDWKSNSEYQNYTPSSQQIQWFWRAVRSFDKEERAKLLQFVTGTSKVPLN
GFKELEGMNGISRFNIHRDYGDKDRLPSSHTCFNQLDLPEYESYDMLRAQ
LHKAITQGSEYFGFA
>Mb|QLI68543.1
MAHNSVQRTAPIAIAPKPPRRDPAPQRQDSLCKFEIGPSSVQTGASIESS
SLAGSTFAPCQTCRFSGTKCVLSDSEDGCAACQANGSECSFLSSSLASPQ
SRKRKMNGVLAREASFGKRSSPNISTRKLGNSSLSSTAASSSFLEDMANV
GGPTILKRTLGMQNDRFSQYIGLTTDFEPSLINLSAFDPQDESLLSRGTL
RKVSDNDTFLMLPDNSTPGYDHVMEDLDAIEAVVSPNGRHLIDLYFRIVH
PAFPIIQKHVFLEKYERSHREFSPPILAAVYILAINWWDHSEKLSNLPRP
DVRELERLICSTLADAMNRPKLSTIQAGLLLSQRPEGDQWAPTAQLVAIG
QELGLHLDCSIWKIPPWEKGLRRRLAWALYMQDKWGALVHGRPSHIFSSN
WGVRALTANDFPDVEWDEDDSDEKQDIEKGRLLFTRFVQLSEILAEILET
FYTLNSTQNIADAGPQGTQLVLSLAKPVQLKLKEWYSGLPANIRLESYSM
TSYSPVSPPSTNRLSSIGYLHLGYFATEITLHRRIIRSLASNPTTVDPYV
QHICRSAAKARLISAMDFVNRLTPNHLRSFWFFASKTNFALIGTFGSLLW
ATSPGREEADWYRRRLGEYRWTLSVSSKPGESKALTQFAISMLDISTGLL
KKLPEKPSMSRSGSLADMSAPSGPPSFPGSFSGGGSLMGQFSNIHSADVS
SAQSPRSDDSSSDDEMEDSYATPI
>Mb|QLI68544.1

MPTTASSPPRHASPPTSLSIPPSTAAPTQPHAITAHAPPAPLQQTLPSQE
TQRHVAEARAAVVASMGNLIDSQLQSRAGILHDNAAALDKQERDVVRATE
GLRRETEKLAREADAAARRVKELGNVQNWAEVLERGFLVLEETVRLANRQ
DGGSESGSGSCSCSCSECGGDESGGGDEEEEEEDDDGEYGEEGMMDVDVD
LANSMHRDGNKVSLEARLAVGADADMVTTEAEAEEGGRSTWSDASRSLME
PDSSTGTGRAKGSETASLSTES
>Mb|QLI68546.1
MAERNVSQILGQLKHSPSMSYPEANALLSKAKLALLSLNALTPSQSTSPQ
LLALARETFEQGALFAIRARNPEAFTRYVQQLQPFYELPSATLPPNLPER
NKVTGLSLLLLLTQGRYAEFHSELEGLANRDGGGSSGDVEGDRYLGYPIR
LERWLMEGSYDRVWKAMKSSEVPCDEYGVFSEILKNQIRSEIASSSERAY
PSLPISSTKSLLFLDSEGDVIQFAKYRGWVVRDGHIYFPDAADSSEDSGQ
SKDISQVIIENTLGYARELETIV
>Mb|QLI68547.1
MAASVDEMLPPPMAPSGLQRTYGGNRAFHNFYNDYSHITDPNLRRRLALS
EIDKVPFGLYHVRAILVAGVGFLLDSYDIFSINLITILLGMVFWSGEEVV
NGFGGNNGVLPDTTNQALKAATSAGIIIGMVVFGWLADALGRRRMYGVEL
AIIIFGTFSCALASSSPSIGSAGLLLFWRVLMGIGIGGDYPLSSVITSEF
APTRWRGAMISAVFSMQGIGQLMSAIVALVTTVAFKSSFIRISDEAHCDA
GCQIAADRAWRIIVGVGSIPACLALYYRITIPETPRYTFDIQHDVEKADA
DIKAYVSSKSNSDYERRHSRSKISEDSLDLPRASWADVFAYFGEWKNFKV
LVGTTMSWFFLDLAFYGLGLNQNVVLHAIGYGSGSNMYYKLYNQAVGTII
LTAAGSLPGYWTAIFTIDTVGRKPLQICGFLLLTIVFGVQGFRLHDLSEG
SMLALYIIGQFLFNAGPNTTTFVVPGECFPTRYRSTGHGISAAMGKIGAI
VAQCISIPLLKNGEVAGCVGNACSPYLDRLLKLFALFMLLGTFVSLLIPE
TKGLTLEELSGESRTSYNAGCNGSISLGSPRLRAWNPFHGGQPAGFAYPR
AHSGYFGKHHRSPRDDIIASPELVAENMRQRRSRFWKRNRKARIASDGSN
EIALRNRTSGTTAGSSDAESSTHASVPQQQLPTWGAGWGRIDRGPLPTTN
SVQLNDVGMLLKPA
>Mb|QLI68548.1
MPGGPSGRGCQGCRKRKKKCDLAQPSCSRCLRYGIRCVGSGERVYRFKNA
TVTGSVPVARTAVTARSRGGILADGQIKLSPPRPLGNELTSTTGAFISTL
QISDPKFSVLGFGPFLEDVPCRLGRSQILRTAAMAFSSAVTAVHSRQATV
QALKDYGTALACMRSSFINDPSQVGKAETLCGVYLLLLSQYFLGGHNDEC
LVHLQGLLYVLNNQAARDYQDPFSSKMVDLASIIAITECVIDPRVQIKRW
HADLRKTSYYGPLNNYSVVDIRSTNLTVANLIDLPMFLQEPEYHLVQLRS
SYDLMRVEVKKIIPITKQLHEACATSLDMNYYKGYTICESSICVLHTLMA
IIRKTLLVFHPYDSTLKEHEESATNVVLASAARAWNFRPLGTTYMPKTLC
LLWATTDDPNVKAKVEDMIDNYREDFRGDSWTKIALFFEQRFERLRKRVQ
TTMPYHLRQDTTSPGSTNDETESFVEV
>Mb|QLI68549.1
MAGGSAAGSRGRAGKFNKPTRGGGKHYSRSLQPVDADGNQVSMWSAGAAD
ERSEEDSEEESSEEESDDAGPSKATADAENRDDRKALKKARKEAAIAKQK
SREVQVGDMPSSDDESEDGMPANPNHSRSARKQANSSDVDDITEGVQNMN
VPGNRKEREAAEAAAAKERYMKLQAEGKTDQAKADLARLKLIREQRAADA
SRRQAEKEEREEQEKTRKAEIEAKESKKREAAAGPKKASKRK
>Mb|QLI68552.1
MAANANPRHSMPVGGYHARNGASSGPPRSSAAPFPHIDDLVSMPKDINPN
QSIRKLLEQAESSFRQSELHRDFNRPAIALKDYVRAFVIAVEVIRRHPDF
PTMASKQGDASLLHKALLSKINRQSEIYNQIKQDIMADNRRTGVRPTVET
SPVVTTVNGLSNAPLPVTSNNGVRPVQQEERQQQKKLPNGHVPHLPNGGP
PAKSKPAVNPKPAALHGNAIPRHNRTNSANNMTLDLAARFANLRGPQPSP
GQDPRIKTYPIIPQRPAGPRQMPPSPPKVNTNMSYDTALPKPPDAIYSPV
RGSVSGEAARLPTSTPRGLFSRKGSSTSISGTPSANQPRQSDEYFPLVPP
SAPKPSDVAEKALKIPEGNTITPRELITVREAKPSILYIDVRPREDFDEG
HIMAVSIICIDPNILERGDVTCSDISEALVLSPPTELSLFEKRQTYDLVV
FYDQDSESFPKSDKDPRDIALWSLNRALVELSYDKELRNPPKLLKGGINA
WTDLYGAGSLRSTPTTDAKTQPRASKRHPFIQRRGSKYVFNPLPEKDVET

FRKGLEKDAAPPSFPRTRDEFLRFPPVSLEQQSMSSGISAEQRYKHDFAT
GFGSPVQMLTPPTRPQAAVQRPSHISLSEDGHDDHEITEQVPATPVKVHT
GLNNPGNWCYANSIIQSLLASPSFGREIADSEWARRYKNQVPRKPGEKMD
QPQLMIQMLSNIFYWMSSGKFQTMKAQMLMDYSRHLCATGDPKTKFGGKD
QQDAQEFMSFLMDQLHDETNLRRGQQGTVDQPNVQGIQLVQAAERYWENH
KRLNDSIVDQYWRGLQLSTVQCHHCATRTYTFSQFESLGVNVSGDRNMTL
SEALRQANTGDEIDDFRCNCCATSRPAKISESLARMPPLLCVSFRRFQVN
GRGVVKSTAEVTWDFNDFDFTPYFLNSAEDWQGASTHDRAFCGPFRYQCY
AVIVHSGRSIDSGHYYAYVRDSSTHDPYAWYCCNDSVVTKVRIGSREPTD
IQNTVFKSGSDAVPYLAFFRRKSE
>Mb|QLI68553.1
MASRIGPRSVKDATRFTSTIPHATSKTAGAAGGRAAAATPKPSPRVPGET
PEQRVRRLRQAHIAAQKAQISRTDRVIDASRRFLDVAHRWTVGGIVVFTA
VAGVVSVYSVWDMLQYNRARRAEWVEAQKKLEADSLAAARIAYLKGTATE
EQILLVEEANREAKESGVKLPPLLAPAEHRTHFEEHVKPKFSGDAVDEEK
AGKGVLGVFSGFFGGSTAAGETPSNATAPLAAEGGVVQSIETKAKDAWEA
EKQNQLKGGSLDQLGLDTGSSGQKTGKRGWWPW
>Mb|QLI68554.1
MYGMNANYDATANRDGVPLSTPRPTHEDEGVAAVNNDPTKTEATSITEGH
NDEKGASSATSLGKEDDDIKAEMVRRSSAVQALARSYSRTSAAAAPGQNP
FFAKEDSPLNPNSPKFSSREWAKAIVELVSQDGKSFRSSGVCFQNLNVHG
FGAATDYQKDVANVWLSAAGAARQLAGSGKQRIDILRNFDGLVRKGEMLV
VLGPPGSGCSTFLKTIAGEMNGIFVGDGSYFNYQGISAKEMHTRHRGEAI
YTAEVDVHFPQLSVGDTLTFAARARQPRHLPEGLHKNHFANHLRDVVMAM
FGISHTMNTRVGNEFIRGVSGGERKRVTISEAALSGAPLQCWDNSTRGLD
SANAIEFCRTLRLQTELFNSTACVSIYQAPQTAYDFFDKAAVLYEGRQIF
FGRADEAKQYFINLGFECPARQTTPDFLTSMTSSLERIVRPGFEGKAPRT
PDEFATAWKNSAEYRALQVEIEEYKEAHPVGGPDAEAFRAAKRAQQAKGQ
RKKSPFTLSYTQQIKLCLWRGWKRLVGDPSLTIGALIGNFIMSLIIGSVF
YNLDDTSSSFFQRGALLFFACLMNAFASALEILTLYAQRPIVEKHARYAL
YHPSAEAIASMLCDMPYKIANSIIFNITLYFMTNLRREPGPFFFFLLISF
STVMVMSMIFRTIASASRTLFQALVPAAILILDLVIFTGFVLPKQYMLGW
CRWLSYIDPLGYAFEALMVNEFHDREFKCTEYVPTSKAPFAGAAQFLTKY
ANVGPENQICSQVGAVAGNPNVNGDAYTSSNFDYAWNHRWRNFGIVIAFT
VFFLICYMVAAELVSEKKSKGEVLVYRRGHKPAAAAEAEKRHNDPEAAMA
NIGPIVTAERSRGQSKEGGILQQQTSVFQWHDVCYEVKIKSETRKILDHV
DGWVKPGTLTALMGVSGAGKTTLLDCLADRTSMGVITGEMLVDGRPRDMS
FQRKTGYVQQQDLHLQTTTVREALNFSALLRQPAHVPKKEKLAYVEEVIK
LLDMTEYADAVVGVPGEGLNVEQRKRLTIGVELAAKPPLLLFVDEPTSGL
DSQTSWAILDLLEKLTKAGQAVLCTIHQPSAMLFQRFDRLLFLAKGGKTV
YFGDIGENSKTMTSYFERNGGHPCPEEANPAEWMLEVIGAAPGSTTDIDW
FQTWRDSPEYKSVQNELETIKAEKQGSSPVNAIEEDPGSYREFAAPFMVQ
LKENIYRVFQQYWRTPVYIYAKTALCTLVALFIGFIFFKAPNSKQGLQNQ
MFAIFQLLTVFGQIVQQSMPQFIIQRSLYEARERPSKVYSWKVFMLSQII
VELPWNSLMAVIMYFCWYYPIGLYRNAIPTDAVAERGALMFLFLLMFMLF
TGTFSTFIVAGFETAEAGGNLANLLFTLCLIFCGVLATPETLPRFWIFMY
RVSPFTYLVSGMLSTGVANTKVICAANELLSFEPPSNQTCWQYMEDYIKV
FGGELTNPNTTSTCEFCTISDTNVFLAGVSSNYSDRWRNFGLLWVFVIFN
IGAALFVYWLARVPKNKLGGKKAKKE
>Mb|QLI68556.1
MELRGAASSKGSPDTRSILTSTMAKISTGTSSPRKSSFLQLQSIPPPLRP
LVRAYLLGYASAVLPRLLTLVLQHLSNRKRKTPNYALPERDENTFLESAK
HVLKTGLDPCRFPTFCAALVGGATLLQAPLNKIITRAATNLGSVAKLRLS
RFLATFVSAWFSLKLLQSKKPTLRRRAGSSTTRYVDPATGKKLAGRTVDL
TLFAATQAIDVIVGELWARHKSRRLAAQKWTKVEAFISQMTDPVSFVASC
ALIMWAWFYAPDNLPRAYNKWITSAAHVDIRLIEALRRCRTRELSYEKET
GQAHLLGTMCTDHSLPYEWGDPCKTIPFPCDIVHMGRGPSCEYHAWRRFW
LSWKWSMYTYLPLALALQLRKPNRDSLRTALLSAARSSAFLGTYITLFYY

GVCLGRTRIGPHLLGKDVACRQKIDGGYCVGVGCFLCGWSVLIETANRRK
DMALFVAPRAMATLLPRQYKMEVQWRETLAFALSTAVVFTCARENPRRVR
GMLGGILGLAMKE
>Mb|QLI68557.1
MEIPHPETILNSLNEAQRRAVTSNSPTVAILAGPGSGKTHTLTSRVVWLV
QHVGYQPADVIVATFTVKAAREMKGRIGKRLGEECEKKIVLGTFHSIARR
YLAVYGKRIGLDPKFGIADDGDSRAIIQRIIKRLDLQIEPAHAKSWISKK
KSKGTTPVPKQSGRRPNETPSLNECYTAYQDHLARSNLLDYDDLLVRCVE
LLQEHPSCVSNVQAVLIDEYQDTNGIQYDLMKLFAQARQRITIVGDPDQS
IYGWRSAEIKNLYRLLRDYPETDEISLEENYRSSQLILDMSLQVIQQDSK
RYQKVLLPVHTKGTKPVLRSLKSSSREGEWIVSELRRAITMFGGAIKNED
VAILLRSAYLSRHIESALGKAGISYRMIGGHKFYERKEIKILLDYLRVVY
QPNMNDALARIINVPRRGIGETTIKSLLEEAEMSKLSLWALLWRHCRGGR
RATTKLRPNMEQKLNTELLRIISSLRQNMEDINASGSMSLVDLIEQLINQ
LSFKKYLEAEYQEDHEQRWANVQELLSLANDFVRDYDNEGEEEALPEIEN
LDQVKDNDILGKFLANVALASDAQSGDKEQDKASQVTISTIHASKGLEWP
VVFIPSVYGGSIPHSRAEDMDEERRLLYVAMTRAKALLYLSCPLYASQGL
GNKVELSPFVSAFAGSFAQKGPSFDRPVVEGVGRILGRAIPSEKSIFDKI
PIMFCPEDDQYPIDPFDHENPDYLDPHDGNSYGRPPKKQKTQHYVNNGGT
AQEKPWKPDYSTTMEQSSGFTMSSLPGFASASTHRTALAASGVEAEMSRN
AAGKAYSNRSVKGTTNRRPDQRSLLGFVTTESRQLATAAGMKPAPRDFSL
TRYQAAASRAAHRTSQPAVNVNTEATIDPTFAKHKVTGAKPISRPNVAQS
TMKEPSAKHYACFSSSPTRPLSPEPQHDEGKAATTVSQRPATVFHSTTFT
SMKPAGGIRRPVGLGPPPSMDRLRKPFKPLTINRPKGPPGNS
>Mb|QLI68558.1
MAPPNGSAKGRKAVTPKNMPKPVVPALPLKRKPAPVTVATKDDAATAPGT
KANGANSEASQTKQVANGVAPETVAGNATAPVALISQPKTESLPGSDGSA
NGGPSTNGHVTVVAEAVESETPAGNKSTMTTTDEIDSDKKPNGATVSGQT
AAGVSQAESTSPEPTSEPATDSNVASKNEPRQKPVQHHAVKAAKGQPPPG
ISPSRYQMPPPFQPASRPYAVVNNNDISRGPPVPLPNGPVHMHHSHPSNS
SIHFGAFPGSQSPSPAPPHTGGMVPPPGMTTADARPAYMAPAGSGFPPMM
PPYGAEMMQPGHFDNYGRPGMAYGPLETFSPYGNNFGPSTPHSFHGSQSS
GHPDDSGMYHQYPPNGNRNGATGPGDSDGHVHSHQGRMFGHPEYLGMMPR
GSFPHQGAILDDGSDGFLFFIQQQFGNAELADCTLELRYVDDRAAPVRIP
GHRLIFARSGPLAEQVKNQMHQVSDRGMQILLLETRSGWIRSDSFYMAVQ
RLYGHPLLPIPPRNRAEPADVTDAGTSHEQLDFALSYAAAGHLLGWMPVV
RRGCEVATQLLSWQTLEKVIEFALEGSSDNGSHMSYKYGEGSKVLLNAVV
TFIVHNLPPTFSLDTGAGHPEQYARLPECPPPSPASKKRGPIVAKGSSVH
LGMGRRSQQLTGIQFGDLSLAEEAGGSDSVTPKAAQQAQPVPLVVLSRAL
INLPFFQLKMILESAGSGNVNGWANAESRYRIIRSAVEEREARRHRTLDA
VLRGRLQSPLSLRSALCSPTPQDMGPWSPLGWQEEILPYGNADGPSLGRK
WVPFVERQGNPEAAYP
>Mb|QLI68559.1
MPRASGPGKRQQGASSGHRDSKHDNGLVSPAKRNAGRKSHGHQLDGSARP
PDHSAGHAGPSPSPPLLPLHAAAHPHASSTKSNSDAAIDTRSADFTRRGS
LGTFSETSSDSCPSQAGANGNTPIEPGHRQIDLNAMKNVDVHKDSGPLEM
AVTVVKSLPMQDTLAILIIILMHVPHLSLSLIYAFFALVTFVPPVTTRTGM
NINLAEILDSHSHTLNFVTLLCIDFFFFLIWVFLWQPIQDGILEFAKPVI
AITLGGGTNAKDGTSRGVTTCFTWILLHQTIRATRSHWAKLARHLPENWP
LPTLLTQSLEPRSVAYDKRSTHEWIQSILAMHILTQGIVRYVREWYLKRE
KYNAASGLSDPEAGKSSSLGNSSGSLGGGHQNSSQSLESSNDAGLAGADA
DASVPHPPTSTNLTTTKKRRKQNAQVRLQQPLWAAVASTKIVVMKEMELS
KSVRPDGIVNIHTNEGTTFEKKDRQIWISYIGSDEVCFNTSYFSDSKTDI
PQEPMKSNGHVKTTRPSGVDSSKPFYVRINNAFWQPTRIFPIDEVDEDDE
DKDGKRGKRIRWTGDIYGLRPASKYVCEFVDVETGAVIFSTSIRTVKELL
REDDSLSPVFPPSQQPLRPDSPATILRTSIAASEAKLAEEKSRLRTLRKD
LKIKINAVRKENELVDNQLSSAGNSDEKHKQKIRQQETQKAQAERDTESL
TGELKNFDTAPELFERKKKMERTYAAEKKTFDAEQKEFKKHKSSLDAEVK

TKELEMSNLNTRRNKIATRISRVETELNHITDANNRGLGEAERRNQQRAL
WLSHAASIEHNISERLSVVHGNNRAKMRQYQLLQGQTQAYSVYLNSANGM
PGDDGPASGAQPATWNPNPVVTPNYPPGMWPVSSSDLIPPVAALSTHASA
PGWQPPPSAPPFEPRGARSRGRSSSMLSDLSGFTEPSDEGSVKSPPIILR
RPGPGLLGRNGTSGGSSASSGSASFGDPASPR
>Mb|QLI68561.1
MGHTGVATKEDVLPGDGSARRGSDQACVTVTRAATGSACDDAQHLTARIE
ALSESLRNSLPVGLVPYKLAEALRHDSNHRLQLAREQGIKLEHLHIGADC
LPTWSTLYWDPENESLHSTIQMHLVHLDLAQKLGEFLQRQIRDKSNQRLR
QARALGIKLENIFIGESLLPEWTANKGGSDREEVSPSAVDDTLSGSTRVG
TGHDSLGTVVGPITDLSGTDAWQHSDSEGSVSFPGVAPCNVHDGDEDDQH
PDEIYMNNHVDTRPIYDEYGNSNIVHDVEIVGGHGEHDTVDLGVYMAVDD
EEVDDLPEVELAMDRKGFEQHFGDEDAVDVYIPDMEIFCFDKIEEYDDDD
DDDDDMHEDDDNPQDTLRLEPSRYMELDDDDGHGEDDAISQGNAMDICES
DDSSSDSEMLSEEE
>Mb|QLI68565.1
MAPADFYDDDDLYDGNDYEEDQEEELSPEDKQAMEEGTADVKKALGANAS
KVTVKQIQEALWHYYFDVEKSASYLTKTFIAPPPPKPTPKKAPESKLHEF
SFSSSLGLFVRSAGAASALPLSNSPQTPETLPLPPCRTFSYRTNSIILQS
EFDDMPWLNTPESRQATLIAPFEPRGGLLGGAGDAPKMSKLQALAAARKK
KNDEKKEQAKSSETESGLKRLSITDDSQKGNTKGASSPAKRLRGPDMQPS
SRPILGRGDSRSESSQNTHSSAAPVEVIAPQKESGKPNQYPPVQREEQEE
DAEPAPACAPSAFAKTLFGPAPEDRQANRPDFFAMPYAASSSFLATAFSN
PSPDDIVLAAQAKAGKKTAAPVKAASIVVKKDKNVDAVSKVVDVANGVSN
LRVSDAPPPKSKGLDVAKEYEKSNRKKSISFVVVGHVDAGKSTLMGRLLL
ELKYVQERTVDKYRRQAEKTGKQSFALAWVMDQRTEERERGVTIDIATNH
FETPNTKFTILDAPGHRDFVPNMIAGASQADFAVLVVDANTGAYEKGLKG
QTREHVLLLRSLGVQRLIVAVNKLDMVGWSKDRFDEISQQVMGFLTGLGF
QSKLVSFIPISGLNGDNIASKIKDATAAWYQGPTLLASLEDSEPSSARAI
TKPFRMSISEVFRSQQQGTTTLAGRIEAGNIQIGDAVIVQPSGEGTYIKS
IMVDTEAQEWAVAGQSVTIALTDIDPVHIRVGDILCGTVNPISVGDTFTL
KVMAFEHLMPMPVDLHRGRLHAAGQIQSIPATLDKATGEVIKKKPKVVQP
GSVARVTIKLGSKVPLEKGQRVVLRSGGETIAAGLLE
>Mb|QLI68567.1
MSRRYDSRTTIFSPEGRLYQVEYALEAISHAGTAIGILAKDGIVLAAERK
VTSKLLEQDTSAEKLYVLNDNMICAVAGMTADANILINYARQAAQRYLLT
YNEDIPCEQLVRRLCDLKQGYTQHGGLRPFGVSFIYAGWDPRRQFQLYLS
NPSGNYGGWKATSAGANNASAQSLLKQDYKEDCTLKEACAMAVKVLSKTM
DSTKLSSEKIEFATVGQTEDGKIYHRLWNADEITALLKEHDLAKSEDAEE
K
>Mb|QLI68569.1
MTPQEVAALLQEYGGGDKQSGLEGSSDPSDIRLKRAELLSNVLAACRDAW
STKSEQLDLIAEKLGDGSRDVAWRLPYGDSGILDFFLDLLDEGGLRQKLR
IHSLRLIGNSCADTDENRARVVQDNRLLAITRHVKDESIIPFNIPVIYNI
LVDYDPAQVLASKSRLSQQLVHLLSLPQISKYAPFVPYICKILALLVSQE
GECAVADPSTVEVLLKLANQPKAKEDVDEFIAIVGVAVAYLANKDFQSRV
VSEMQMGLFMDAFYHAHAGLNVADVDDEDTVTQLKQLRTSLLSTLADISG
NDAFATAYPLSDAVPQSFLAWIRGNNPFLQSAGCLALGNLSRSDKTSIAL
VDTYGAHEPLVRLLSNSAVTETQLLHAACSFLKNLAIPTANKPQLKDLLL
PHCVPRIYSLDALPQIQFAAVSLTRLLLLNCPSNVRQICASADTAPTELS
SASGIISLFRRSDAEPTKLEAARCVAGICRVLHSSPVSEILENLGSDDNT
DESKLSNFYKENHVENPLRFLITQGKWPSLRSEAWFVFALMSRSESGARV
VLSVLGDKAAESALIETITGQELENKEGAAAHGESKDSEPHPELSSAAGE
LQLEPQQVDPKQKENMAKVDRENALVLCTQLLKVAKSELAPEKLASLQRL
VRDGTHLVVTDRTKAHSS
>Mb|QLI68570.1
MKSAKPVAADTPSHRRSQPARQARTNPQRSSANADRSGDGRGPVNGRPLG
CQPIEIFPAITHFADTISALPKELVRHFTLLKEVDAKIFAPEEQLFQLVA

AISNSSLPETRANNDAAGAAQPASAPMSTQNSSSGMALNPARASSAPSLD
EASAAPTSVFDPSNIPRRQLFRQTAFKIQEMLVSLEEKNHVLSTANEALQ
RQLARAEDVWPYLENEFSDEAKWGSTTHWAYPENRTGSKVSHTERARRDG
AAAISAAAQALAEEAAARSDARKQAVQAKKGLKNQQQDSDADDLEARHKE
PAKKTTAKSRKTVTADPNSTALGIPAPSTNGNPPQKRRKVEKTVNGANTG
TGGAMSTVFGTNAPKPKTGSPRGTPAPDGPKKRKALPTGSGQAKKKNGLS
PSVTSSPVMGIFPDPKVPARASPPPTTSAPRPASSRARQNSIQSNAENGK
ARPAPSAANKPSGNGQGTPEMASVAAFPRAVQEAKPAKEESAPVKTEPAK
KESERPETSSNSTPATATKDAKPDEPDRKSESATPQTTPATATTKTGRAS
KPSTPALATFQEAARSRPSRSTETGSKKSHKKSGPVAQVVLIQQAAAAAD
TDASSSMQGDDEDGDIDGDEPTYCYCNSVSYGEMVACDADDCKREWFHLA
CVGLKVAPGSRTKWYCEDCKERLKMGAKKSGGR
>Mb|QLI68571.1
MAGHFDLSSQAQEASPYATLPGTSQAGFVPHNMTPMKSNLAPGREIAGCN
PSRWVYELLNNDFTVCDLFERARHAKLESTYTWDHAHKLVMNGNQSFSTA
SSRVLSSICLDCHFHFVFKMTWDEAHSDGLCHQNQSSWPLRDDQFPWHHL
VWVGSSSDPDVAQDNTKYCPLLAREYFVCSAPPCTFQLTLEISEPRMPQS
WVELLLDREAICQQLQAAKEQEPGRYEAATDEWAYQATLNLNTYLKNLLE
STPEDARSISKRNKRFAVLFGPRCFAMFRELEFTEKVELRDGVDEGSFTP
SPPPPAGGPSETTEIGTYRSYLEDVRAEVQCLIHKAGQAAEKPTIITPVL
HADLGCNEVPNAAANALVNVERYKLIGVLPNQTREAVVNAYKRQWDLLPS
RRRALVDSIMGIANDSGDELLSDYAITQSSVFDNQTHRPATNDDDGLVPQ
ALNFLGLQPPNNYSADSLIQAFRQKLARDPADATTARSMLLLIAQNSNDD
MYQAQLLMEADAKMSLETSRSILELKAVEVPWETTYEAAKNKIEASKSKE
ATQVFLDALEAIADHTYSPALKQAAMELRQENGIYGVKAKSEDEPVNYAL
PVGLHNIGNTCYLNSLLQYLFTVKPVREIVLNYDNVKLALDDESIKARLL
GGNKMQMDRGEAVVAQAFAKELSELFQNLKASKRSATKPSQRLANAVLLS
THTLLHNTKQSSEPPIGPRPPPLPARPSPAPPTQSVEDVEMTNGSVANAL
EPSEMASNSSSRTLIDQEDARSDHSYERVETVHVSGQADGDTEVLSKTDD
PMDQSSKSPPQPTKETITTEATNDAQGSFQDVDMADAEIETVDQKVLNAL
EHQKRSSGTDQQDVEEVMGSIINRLQAAIQPTSVDEKTGIQLEKIMETFF
VTTVNYTKKFDEREYQSEISFDRSITAFPAPDGPCSLYDALGRNFDQQIL
EESKLSRYTAIKTLPPVLHVLIQRSQSMGSKNGNAVVIPETLYLDRYMDA
PHTSPVFQRRVEDWALSDRISDLKVQISKVDSNPTYTSYLEKYAAGVLET
EVADGTANAESAVGEAPEIVAAPEENWDFDGPVDEDFLLVTTSTSTGSEQ
PIPHEPKPLNIRASDAAIRQMMEAELQKREETLKEHQESLKGTPYRLHAV
ICHRGQLMSGHYWVWIHDFEENTWRWYNDADVKENKNTAEVLETLSTSGE
PYFLCYVRDEDKAEYVNIPKRQPPTPPLELQESPASKMVTDKDGDIDAVA
EVVAPVIEGIEPPGDETDPGYKKGNDDPQRMQE
>Mb|QLI68572.1
MDQMLQHFPPGILTYPDPSEFDYSTFLQPTEDIPFPDPSHSTSAGTISES
SPGQATPPSSDGRSTSAVVDMSVTRRQSAQKQRLERRGHTKSRRGCYNCK
RRRIKCQETRPACGHCVKTGLKCEYPSMPQITHQPHHQVPLFSLQDMRFF
QHFLTQCYPHHPLKQEEIWTHEIPCIAHNHEFLMHAILGFAASELVPTDS
SFVQAAMNHRIKAIKAIKKRLSESARAQTSYEEANALVATCFALTFQSVC
LEDGLAEYMTFIRGILIVGMQMMFKGIKPIFEQLFEDQQNEMMEPYLRDL
PLIQRGWADAAVEAISNLRPLCAEPVEVEYCEQLLGIAEKLNVNSFDAYK
SNGRQYAWWMMLPHSTFQQLINLDRQSMILLHAHWIALSQIMAFITEREY
DLREKRPTASTDAGPAPGFVKWLKFLNARVDYEHQMYNQWPLWVEAQLDK
DLTFFGRRL
>Mb|QLI68573.1
MGNGQGKPVDLNGEVNLNHFRLLRVVGRGAFGKVRIVERKDTNLSFALKY
IRKDEVVKSESVRNIIERRMLEYVNHPFICNLRYSFQDIEYMYLVVDLM
SGGDLRFHISRKTFTEEAVRFWIAELGCALRYIHEQNIIHRDVKPDNVLL
DADGHVHLTDFNVASDIVPGRVLTSKSGTLAYLAPEVYSGKGYTVCADWW
SLGVLFYECIYNKRPFDGSSESTLSQQIQNSSPRYPVTQPPVSLTCLYSI
RAALDPNPRTRMGSTWESFIYNDFFKAIDFNLLEQKRIEPIFIPSSDKTN
FDATYDLEELLLEEAPLEARARRQKPRERLKEDATDQEIREDELYRMIEK

DFRPFDYTVAAYKKITEASRSLNGDPQSDVQPNNHPQALTTDKATPIPAG
GTVQQTSHPVPAGTIPNPDPRQGRPVRPAPPPPLFHQSEYVSRALPPTSK
RVASSTGGVSVTLDGGGSWSELARQDATLPTDANTPADSKAESSSGMFGF
LKSKKGRNNSPKPKERGVLGKEGARVIIS
>Mb|QLI68574.1
MAPTASTPSGQKDQSIPTTRYHSTSSALGKLYQPFKCPGAACPRTSSSDR
SSRKRRRVDYSGTDGDSSEDKSYTNNDRLALATRDVNKYPVYRAKEKHLV
FRKAFSVPLKDKSTASYNPNRPPPTLGLRQGAVVSPRPLHDPSGEFAIVL
YDPTVDGKPDNSQESEKLNTTANDIQKPQLDAPLVHKSLAEILGIRKKVT
KEQPQVPVVLDPKLAKILRPHQVEGVKFMYRCVTGMIEEKANGCIMADEM
GLGKTLQCITLMWTLLKQSPEAGKPTIQKAIVVCPASLVKNWANELTKWL
GANAITPFAIDGKASKEELTRQLRQWAIASGRSVTRPVIIVSYETLRLNV
EQLKHTKIGLLFCDEGHRLKNSDSNTFNALNSLDVSRRVILTGTPIQNDL
TEYFSLTSFANPDLLGSRLEFRKRFEIPILRGRDADASESDRQRGDVCTA
ELLGIVNKFLIRRTNDILSKYLPVKYEHVVFCSVSPFQESLYNYFITSPD
IQALLRGKGSQPLKAINILKKLCNHPDLLNLSDDLPGSECCFPPEYIPKE
ARGRDRDVRPEYSGKMMVLDRMLARIRQDTNDKIVLISNYTSTLDLFERL
CRSREYGCLRLDGTMNVNKRQKLVDRFNDPNGEEVVFLLSSKAGGCGINL
IGANRLVLFDPDWNPAADQQALARVWRDGQKKDCFVYRFIATGTIEEKIF
QRQSHKQSLSSCVVDSAEDVERHFSLDSLRELFQYRPNTTSDTHDTFKCK
RCKPNGKQHVKAPATLYGDTSTWNHFVNTCLQSIQDLLLRQEHGLGQVSA
VFQYISH
>Mb|QLI68575.1
MVAEYNGTTGTADAGYSTNVNDLNIFYDTGNLAFLTISSVLVLLMIPGVG
FFYSGLARRKSALQLVLLSMLSVAVIGFQWFFWGYSLTFSRTGNAFFGDL
TQFGLMKTLAQPNGSSFLPDILFCLYQGMFAAITPALAIGAVADRGRILP
AIIFIFIWSTVVYDPIAYWTWNANGWLYKLPSYDFAGGGPVHIASGTCAL
AYSVMLGKRSGYSKTQGLPYRPHSVTNVVLGTVFLWVGWFGFNGGSALAM
NLRAVMACYVSNLAASCGGLAWVLLDYRLERKWSTIGFCSGAISGLVAIT
PAAGFVTPWGAVIVGLCGGACCNFATKLKFLTGVDDALDIFAVHGIGGMV
GNILTGIFGASYIAALDGNTYSPIGWIEHNWVQLGYQLAGICAAFGWSFV
LTCIILFLMNLVPGLSLRVSATEEDVGIDDAQLGEFAYDYVELTRHVADS
SSPGSMSSSSSTHEKNTREVV
>Mb|QLI68580.1
MAWEQLDITKPHLVYIILGGFTSLFMLCSSFIKERMYIGEATVATLCGVI
FGPHVANLIDPNSWGNTDIVTVEFSRIVLVVQCFAVGVELPKFYMEKHWK
SVIFLLVPVMTVGWLLTSLFVWWMMPGLNWLDSLVVAACVTATDPVLASS
VVGKGKFAKRVPKHLRDLLSAESGCNDGMAFPFIYLSLFLIQYNRNAGEV
SFHFVVYVVLYECIFGAVYGFLIGYIARHGIKYAESHDLIDRESFLVFYF
VVALFCAGSGSILGVDDLLVGFAAGVGFSNDGWFGEKTEESHVSNVIDLL
LNLTYFVYFGTIIPWEQYNDGAFGMAAWRLVVIAIFVLLFRRIPIMMALK
PFIPDVKNWREALFAGHFGPIGVGAIFVAILARAELEHDEPVPLGELPPP
SAPHFALIYLVWPIVTFLVISSILIHGSSIAVFTLGKRINTLTLTMSYTA
APDDGPSWMNRLPRISSQSRSMAKTMSETSLDDLKDPEFPPGTLAPPTGF
LRRQRDEEGHSRSGSRASSLVSRKRKHKKWDDGIGPGGPISQSAIFPNRP
SQTEASEKEPSSPTRETTDSTMFSDEQRTEKQGETDEHGESPRDHSPQIN
VYDEGSHLVFENEDGDVIDMQPTPQGEQERNSKTKKEDQPASSKPEGEAS
SSWRHGGWRRKMSEYYTSELEKRKDKGKSDRRHEPARAYQFGNTIIVEDE
DGEVVKTYELPSSKPDGQESDLIGASLKYLGLGARARPSEKNTAAAAEEG
QAGVESSNKAQFKPGWAGFGQRGDGVRRKSVAQQQQDDDDDKKIRFTIGG
VGRRMTKEDFIREVQKLDAGTRNEVIDQSTASNALKSIARQDAPVEPKIT
QSQRPVPVIREHDSESGSGSGSGSSNGESSRAAEKRSVSMSPTRKQPQVT
EPPRGRRDHPSEEQGETSVERRRRLAVLTRQAEDDDKGNDDTGETPAERR
RRQAALGMGGPAAEEDSDSEDEGTERVPPARKGIRFAEPQRKAT
>Mb|QLI68582.1
MSPDRYDRGTSRRGVWSHWVPLAITLTVATAGVAAWIWSQRKEDEEDNEH
EPGLDYENADYGDNPPYGASQRDSRGPQQPPRLYQQGDDAASYGVATAPS
DVSAGGWGARMSGALRRTPSPQHFFDSTGKTVAAGMAAAGAAVGKALASI

REEDKAYGENPWQEEADAKKGRAPATGEKSRKTVAIVVSADTKISDISDD
GTHELASILSHIPRHNDFSKIKLYILIYAPDLKEPMLDGTSSNLPPPSLS
SSFSNIGHDQAQTPGEETKSPLIAPSNTNAAYDAVYSQALSLVDKETTIL
PFTTPNGHVHILRHIQPDILYLQESLSGDNGSIITTLQTWLRHDITLVVG
AESGSGGLADSESEAEKTGKQVEKWWQRPERVGRGRGVVVVDGMRVHDDW
ARRVQGRE
>Mb|QLI68583.1
MEDPGVKNNKTTSTERRSQSPASTPAQAHTASEGADTAAAPSANRATISG
ASSEEPPASESVPQTKQENGVSHETEGASSRSGGAEQPDKGEQTPPRRLN
GNKTRQEDNEGDSELSDPDSNVAFNELEDEIVVSGRSSRGAPKLPSGRQT
SEGNEDEAMEDAPEESAGSHYPKRKRTSIFNDLSESKLESAHTAAPERSP
TSPTSTIKFKPNRQSLGGPGVKGVLLGTWRDSHVPDEDKRHAVIGFIDVR
DRLRTRIQPNTKYGESLAEDYPLPPGPGGSWVTFERVVFSDHLVGLDHCQ
VKEYTRLRSEVAPEETEDGRIAAEHAAVKEAIRRVKENPALENPVQPPPI
AYGIDMPDHLQGPARPDLKRRRVSGGFAAINPAPPEMVPEPTAIPASQLA
IAAHQNRFSVDPLPGTRPTRIIIGYWKPSSEVDPKDRHAVYGILGQNDMF
RVKVVRETRDGRFVDGNFPSGAGALWIAYEEVEFEPHLKALNRQEVKEYC
RVRQYQLDHGESSADRIENETKAVYEAQTRAGTMPYKQPHNVTVPTFTAS
PQGDGDERMNGRHGYGGHELRQSRRTEPRPIRASLSESELRASRLQSVEA
VERTNALARREIARAEAAQGRADRHAIQRERAAAAAAADAANAAAAAAAA
ASNMPLEHHHNTNGRMVFHESDDMQRLNKVWARQESLRMKSSSDEAKIYD
GIKYERKATGPFMGKLVSQGTIINIDGEDYVEYRVLTKPSFF
>Mb|QLI68584.1
MKFNNAILLALATTALAAPASDVTTPKRQDINTVTDQLLFSSTLPQFEAR
RNAKNPPSLDWSSDGCTSSPDNPFGFPFLPACHRHDFGYQNYRIQKRFTK
AAKAKIDSNFKSDLYYQCQRELAKGACDRLADVYYEAVKEFGGGDATKRD
RSDYDRAVAAYNAAVKEAQEQGLLPVLD
>Mb|QLI68586.1
MDGAVLVPDDVLDSRPPPPPRLFERLRQIAGYTWDETKEPIHSSYDFWLV
FGTRFVSSKPSPPSSGHNESSLGSITKLQFGRPSPSEHGNHVPPSETQST
ETPKTPLPHRPSTPISSPPSSADGPPIVEESVVARVSSHNLREERAFQIA
KNVAGTADPLGEHIAKPIDLLRLNPGPGDRNHITVAIYQYPGANYLGKVL
DFGPAFYQARKEGDAYVSYRPATSSHLRPPINLEYFLDFAIGAVQCLEIL
HHGQGIIHGEIRGDAFHFNAEENKVRIVAFGTGLRSFEHGLTSTGWLTLS
KEVGVKNKLLYISPEQTGRMPAEPDTRTDIYSLGILMWTLLTQQPVYAGD
TPMDIVQGVLGRRVPNVATVRMDIPDVIGRIIQKCTAKNVADRYHSASGL
RHDLVKVQQLLSDGDSTALKDLEIGTKDISSFFILPTNMIGRTTEQSELV
KVIDRVSRNHSLGSNRNRFPDGSGLANELVVLDDVSSEGGASSVRSVEGT
NRRSGSFNMGGGSSEYKFPRNSFHPSILSDTQTLSNETVSSNQSGPQLRP
TRPWERHHSISVETASAVESTGGPESGRMGGMTESSGSSLSRQLGAAKFR
RRGQCEVVAIEGAGGLGKSFLVQSVLGEARRRGYCATAKFDTARRTAFGP
LLKLLSSLFKQVWGETNTETPFHQVLKQYVRPVWPMLHRVLGLPEFLLGP
PESASPMVGRPSTNNSQQAGQRSGVKSSSGKRRGSSPGSSPSPLSQHQAA
NAAAAATAGSQSSQDYLCAGTSTKTTRLMNTCLDILRVFTTHKFICFCLD
DLHFADDESLELITQIIGAKMKMLIIMTYRPEELPRERMDKIIYPAQLEE
NHRNGRPRVTRISLEPLSEAEILDYVSATLSRPKEEILSLALVIQAKTAG
NPFYMREMLSACHRKKCIWYDYRDSIWHFDLDRLFAQFQGEKDYDVLDTG
FITRRLDELPSAARAVLSWAALLGNSFSFEIISRLMKGEFDYGDDDIPNC
CPNLSKRSYTEAEAIAGLQAAIQAYIIVPSETDDRFRFAHDRYVNASAAL
KECNARRMHFIISQTLLKYYAVEARQRESTACHICEAASIIKMRVPVRQE
FRKLLIDCAQAATENGARPTAAKYYGAAIQLLQGDPWSDDADDASYEETM
QLYLRAAECHLFMGQLSLANDLLSTLFANAKSAMDKAPAYVLQSRIFAQN
GNSLAAFISLKECLAALGVPLEDEPTYEKCDKKFHKLAKQVQEMDREALM
NPKKNTAPVTASIGAVLSETASAAWWSDCLQYYHLTLVMLDMHLTRGAFP
QSGMAFLQMGVVGLARFNMTQFAVDMGTICQDLLYSARDAFSMARGQMLH
SCFIGHVQYSMSLSTSQMDDAIELASVGGDRLSTILSYGLCAVTKFFASE
NLSDLEAFCQYGCEDIVNWSLDTRGGALLVAVRQLSRALQGKTCTHEPLR
VMTDDQHDSVGYKSWLTSQTQESNRSSLFYESLELCALFLYGHYERAIDI

GQKCVEKLPMLWSARNSRLILLFYGLARAGQLLRLMQDPRSQTDDFTAQT
EEIVNELTGFVKMMEDWSVVSDVNYRSWSRLLNAQVAELSQDHGQAIQHY
EEALDHAAEHDFIFEEALGNYVMAGFFIRRRARRSARAALQDAVGLYRQI
AAAGVASAIEEEHSLLLHGPTRNHRTVEVGIQTDFVADPPPAQYRPVDGV
DGEELTQVPSNAMSEFRGERIGAWRGSMRMPTEDGVGLPALDMIDLHAIL
VSSQVISSVLQVNELLKTMCDVILQTCGGSATRAAIIIQDNDTESWCVAA
SGDPERGASAHEPGLPLSGSTLIAENVVLYCTRFRETVFIPDLFADERFG
NVNGGWLQRYPGGKAIISIPILHGTKPLLGVLYLEGEPGSFTDRNVTVLQ
LLVNQIGISYSNALSMKNVEKISAENRGMVSVQKRALAKALEAETKAKHA
EAEAKRNVKLAEEAAKAKAIFLANVSHELRTPLNGVIGNSELLRDSDLNR
EQLEMADSIRVSADLLLTVINDILDFSKMEADKMKLYIIAFNPEEMVREV
VRAVSYSNREKTSKKNVKIIQDINLPPMLIYGDPIRLHQVLGNLIGNSLK
FTEDGSVTIGARLDAETKDSATLTFWVRDTGIGIPAQQLAKLFQPFSQAD
ASTARKYGGSGLGLSICKSLIEIMMKGKIQLESEESEGTTAWFTVTFEKA
KADVSAGDALGKASPPIDRYSLMTSPFDRVASPNPFMDLSRVSKEDVRIC
VAEDNPINQKIAIQYAHRLGYPNVVAYENGLKAIEGLRKKAAEGEPYHIV
LMDVQMPVLDGYEATKLIRKDSIEAVRKVLVIAMTASAIQGDREKCLAAG
MNDYLAKPVRSEVLKKKLDAYVDVSKAGVIDTVQSPISPISPTTPNDLEA
NGTSLASIASSNKVFRDTRAPNSSIIDPMQEDAVYNMPLDSTSLQAADSP
VLSANNNDQRTLSMHLPELTRNSTNHSDSQPSTEASTPEAVSTGDPVALQ
PKRQPRKLTKTRDNSDAHWDAEAKPIDKDREKHKGVLTKKQPLADDPDGD
AS
>Mb|QLI68587.1
MVVNTLVYHPSVKHYLSFVATTVGRDKLLRTIQYFARFYAWYLLRTNATK
AQIAPWDVTKKQFGLTRKILRVGKNVEHLKAAVETADNPKPLDAFLRYAG
IGRQIGYAGYLTFDSAALPDAAGIKKWDKAKTVQAYGYRFWAMGLVFSIS
AQLYTLYQLKQREAKIDRKDGEGVINSKHIARERAASQLQLISDLCDLTV
PSSALGWAGFDDGFVGLAGTLSSLIGVYNQWKKVTAKPASK
>Mb|QLI68589.1
MGLPEERSGNDSSSDGAPLGRRATMEDLASASRGVDERKLVRKLDLYLIP
LIMGIYLFSFVDRVNLGNARLYGLEEDLGLSTSQFQVAISIFFVTYMVFE
VPSNLVLKLFTPRRWIAFITVSWGLVATLMGLVTSYGSLLACRLLLGAVE
AGLFPGLNIYLTFFYTRNELALRVGYLFVSAAAAGALGGLLAYGIGHMDG
VQGLGGWRWILILEGIPSVVLGVVTYFVLPNDAETAWFLDDAEKALMEIR
RRREYGSTKGGGGIEKRDVLRAVTDWKAWAFYVAQFGVDTMLYGFSTFLP
TIIQALGTWTTAQVQLLTIPCYFLGAAAYMGTAFLSDRMQMRGLFCVIFG
SISVVGYGVLLADVATSVHYFACFLVAGGLYVVVGLPLAWLPNNSPRYGK
RATSTGLQLTFGNAAGVMSAFIYPKPDAPRYIRGHAVCLSMVGMGTAIYG
FLWFWYWRQNKRREAGVIEEKFRDMEEEELMELGDDSPRFRYMI
>Mb|QLI68590.1
MSSSITIPTTRSSASPDAFHASSKTLGGSSMSSYSSSPSRSSPSTSPNTK
MLHTRRPSLLSSAISKQESTVINIGDPDGPPRLISYLSSSQGFVWNPEIF
LPSYVDCDYVPLENQREPVHEIILSDEESRNMLPH
>Mb|QLI68591.1
MDDLLNLELLSLVSKVTSELQNHIGISDKTLAEFLISQRLESSSTDDFRK
RLDKIGADFPPSLVDSLDRLVLAMHPKFKGNGDQINGEEHHSRTLEEKEK
VFSGLALADKAVAYDSGADAIDDTLALLEGLEGKAKKEKASRKRSRSPEY
DGKESRRRRRDRSRSRNRRTRERFRSRSKSQDRGDEDWRDGFKESRNDRR
GRRRHDEDESDDRLRKAPYPEIDDAPKLHKVYQGHVTGVRDFGAFVNLHH
VRGKVDGLVHISRIIAGQRVNHPSDLLSKGQDVWVKVTSVEGNRIGLSMR
DVDQETGMDLEPQARLTTGANMEALGGGVKNGFVNDTSAMPRDVLGPPRR
HKKRMTSPERWEIRQLIASGVAKASDYPDLEEDYNATLQGDGEMALEEDV
DIEVREEEPPFLVGQTKQSLELSPIRVVKAPDGSMNRAAMSGTSIAKERK
ELKQQEADAAAKEKKSNQNLSSQWQDPMADPDKRTFASDLRNARMQAESE
DVPEWKKAVIPKGQSLGKRTNLSIKEQRESLPVYAFRSQLIKAVQENQIL
IVVGETGSGKTTQLTQYLAEAGFADKGIIGCTQPRRVAAMSVAKRVAEEV
GCNMGEEVGYLVRFDDCTSPSTRIKYMTDGMLQREILMDSDLKRYSCIML
DEAHERTIATDVLFALLKKAVLRRPDLKIIVTSATLDADKFSSYFHECPI

FTIPGRTFPVEILYSREPESDYLDAALVTVMQIHLTEPKGDILLFLTGKE
EIDTACEILYERMKALGPSVPELLILPVYASLPAEMQSKIFDPAPPGTRK
VVIATNIAETSITIDEIYYVVDPGFVKQSAYDPKLGMDSLVVTPVSQAQA
NQRAGRAGRTGPGKCFRLYTEAAYQSEMLPTTIPEIQRQNLSHTILMLKA
MGINDLLHFDFMDPPPVNTMLTALEELYALSALDDEGLLTRLGRKMADFP
MDPASAKVLLSAVDHQCSDEALSIIAMLSLQGAVFYRPKEKQTQADQKKA
KFHDPHGDHLTFLNVYNSWKQNAYSNPWCFENFIQARSMRRAKDVRDQLV
KIMERYKHPIVSCGRNTDKVRRAMCSGFFRNAARKDPQEGYKTLTEGTPV
YLHPSSALFGKQAEWVIYHDLVLTTKEYMHCTSSIEPKWLVEAAPTFFKV
APSDRLSKRKQAERIQPLYNKFAGEDDWRLSAQRRGGRGGGGGTWG
>Mb|QLI68593.1
MNETGAWGAWQVQPSKFTRAVVDAMRTLYPEELADRSWDNVGLLVGNSEA
EGRQPKVLVTNDLTWQVAADAIGQNVSVIVSYHPFIFSGLKSITNDDPQQ
ATLLRLAQAGVAVYCPHTAVDAAPKGLNTWLADIVAGPHASRRSVAVPCP
AAPESHSPAGYGAVGHFEAPVSLAEILRRLASELGGLKYVMVASPVGADM
KTATVGSFGVCAGSGYDVLKKADVDLLVTGETSHHSALRAIQQGRTLVQV
FHSNSERGYLQKFLGPMLEEQLKKAVPEAQVVLSQFDKDPFTIYNVDHLG
>Mb|QLI68594.1
MSSGEFHEFSDDEFAASNLFDFSNSPDTLQSLDAIGSTDQKSFLNPQELT
TAGPFPDSPNDSYHDSSSESASSSKRTGSSDSTKTPITTGDSTMDDGADI
KMEWGAANYSSYDDDENAFTFGREADSSAMEGLYGFGEQDDSFMDRSFDF
ESASSSPEAQNAGQTAMASPAMPTIKTNNSSAKVARKPNSKPKTVTHKKQ
SSQYSTLSTGVKTSGSREVSPMSAMVTSHNSSPAANFLNSPSPLGNHING
NTIWPNRVDVTPATAPDALPTPVQFSNQMPQPMLMPQHIPLYNSTPVYNF
TGGYELRILPTPLKSRVETQIPIKMTLSPLPPGVTKLHLPTHTISKPKLL
ARPCAERSPDTLELHVNLVCTSAMEQGDFKKKALERAAVVPQGYLPELDD
EKNSPQNGGDVRICAGCISRERKRAARKKIKKPEEEKIWGQDEERRVIVF
NTQEVKEWQPLSGVLDARGRPDPVVTRAMQIDAPMRIACYCRHHSEKMGF
NVIFTIKDFRDRLIAQAMSNPIMITDDHKTHPMAQASNPQVSETVTSTTI
PIHMPQPMESNHIAPAPGVQNGSFQVSSPNADGNSQQGGQSPYSGSVVSG
KTTPTMSTPAVTGRTLSRPSSPNQGGPAKKRKSSSSRVPNGLTMTRLDTS
PPPGSQITSAQISTATSPFSPNPSPFQQPEQIFGPMNGPAPFANEPPTPG
SNDQLPFFSNRSASMDNLAIAQMYSAPASSHPSRAPSPNGLRNSVAGGMQ
NQFTQSLAANLYPLPLGVNQARAPPTIHKIIPNEGPKIGGIEVTVLGAAF
FQGLEVFFGDQKATTTTYWGDSSLVCLLPPSPVAGAVAVTFKHQGIPGAL
NFAMGKQPPTFKYIDDNEDKLIRTALSVLGHKMSGQMVDVSDLARRILND
GNSTWASGGGSSSSAGGPMFNHAATSREHLESQLLKCLDLIDLDDSTHKT
RLDLKRSTGHTMLHLACSLGYHRFVAALLARGANPDARDKGGFTPLHMAA
IHNHPEIVRRLMLVGADPTIRSLSSLTPADVAQSRAVLRAIRHSERHVRS
RSGGSLHSRTSSATSLRSLWEPMTRAHTHDEPISIDSSEESPEYTSGDFE
DEDPDEDVYLTMRRASSMRQDEESSEHIEEPARPDSGMASPTTALAAALK
DQVQQQLQQFQQSMTLHLQNLAHFPQMPALPGMAMLPDYQAYFQRVQQLM
PGMSGARPGSAGSTGENQGKMDGRWWDISSYMNNASAVPPPAYEDIFPQQ
DVDRKQESAIRAAAEAEADMKCATLYDQTTSTTTTSTTRAKAEAPEILKI
GRKNAITKEQQEQFLRAREIKLKSLRSDRNLFFVWIPLLLVVVCAMLYSY
FPSLFPFLWTSVRALVQAGHNRASRLVSTAPERVVEI
>Mb|QLI68595.1
MAGEKAAVASGADVGEGLRKRPVNNGTPAPVAPQPTDNKKQAAQKAEKNL
IDTFVEWEHIIAPLIFTALAFFTRLWKIGLSDIVTWDEAHFGKFGSYYIK
HEYYFDVHPPLGKMLVGLSGVLGGYNGSFEFKSGDKYPEDVNYTFMRAFN
ALFGIVCIPMAYYTARELNLRRPAVWLVTLMVLCENSYTTISRFILLDSM
LLCGTVATTLCWAKFHNQRHNSFEPEWFFWLFMTGFSIGCVCSVKLVGLF
VTALVGLYTIEDLWNKFGNTKMPITTLTAHVGTRVVGLIVVPFLIYLLSF
ALHFAILDRTGPGDAQMSSLFQANLKGTEVGKNSPLEIAYGSRATIKNMG
YGGGLLHSHVQTYPEGSQQQQVTCYHHKDANNDWFFYPNRREPDYDAESP
DLRFIGDGTIIRLIHAQTGRNLHSHDIAAPMSKSDKEVSSYGNLTVGDEK
DHWKVEVVRDVASRDRSRIRTLTTAFRLKHEVLGCYLKGTNKNLPQWGFK
QIEVSCTKENNPRDAYTHWNVEAHWNDKLPAGDPGVYKSPFFHDFIHLNV

AMMTSNNALVPDPDKQDDLASKWWQWPILHVGLRMCGWDDNIVKYFLLGN
PLVYWGSTIGIGIVGLVVVWYILRWQRGFADLNNKEIDQIHYSGIYPVAG
WFLHYLPFVIMARVTYVHHYYPALYFAILTFGFLVDWTVRNRNTIIQGTT
YGVLYAVIIGLYVLFMPVCWGMVGPNQQYSYMRWFDNWRISD
>Mb|QLI68599.1
MERGLQTSMWAAGMPSSRRTYDLARHDTRSRSLPPPMSGFAAPSATSTST
PGSHTTTTTTTITPFLPATQAPAAANAGCPSRVKSFRRFEQACRRVRWKC
IDLGNSYSRALDPADCHFTAADAEMNFKVDFHEFYAWIEQAIVLLLRIFG
VSVKGGSSWWDGTRPAGNSSHAYHHNVLKALEEEENPLYGILGSGDVHKA
LWEAKKLRNRWKDASEDKETPPLKMYNLHWIVAEILEGLEKAYLLAKEEV
GDDLQNSNDDGMAVENGRDWEWMVESMDWEA
>Mb|QLI68600.1
MATERSYGPVQPTPDYLQLLHHYVQPHGQLKKAGYIFSRLSSTDLQRKER
CEKCCKVLKNRRQRNQPAEPKLPSASDAAVSRPNHLELHSSNRASGAVVT
ESQINPHTKQALDKAAKGTERTQDGSSGNTSFICSYHPGHVANRKWTCCN
GPLFSKPCAGQHEHSPRHYNVGELEKNWQFYETKTAPLPTHVAAVVLDCE
MGTASSGESELIRLSVVDFFTGAVLIDSLVYPSVKMAHYNTRFSGVTRHT
MDDSRRRRKCILGRDSARQALYKFVGPDTVVIGHAGHQDLTSLRWIHHRI
VDTLILETGKRRLEEDLSRRREWEESENTEMGESANPSSAVEDKDNTAAT
NLQEGGLSLKALTLKRLNRTIQVKGRGHDSLEDALATRDLLHWHIDAILR
SDTEGLL
>Mb|QLI68601.1
MDEQSSSVPPDFNSGRGRIPENYVEYDLFLVQQKKGCRTLLQELEMLRKS
AINICNTVTKDHIWQRDDFHLEMKSSEGMTYLHGMTDYGDAIEDEWLIVY
LLRQLTQSFPDLWVHVADADGEFLLIEAANVLPKWITPEMDQNRVWIHDS
KLLIIPLSSAADAQQEGNTANMTLRNAFDYLRTKPNALLHSPLIEAEAFY
RLEKYPDYIRHSTHYSLVTIPRKLAYILHLIPKSVSIAVEMFYLRDAVTM
KSISSASAPLVFPPEDFVTTSIHFTRVLFAQLKSQRLDPPPRWRALIKDR
SEASPSDFQKLARQDMGMKLTCGYEMLSVDADKSRHRVVRELALLLRDLA
EDGDAGLPTDRDMESWLNHQRDDAEDWMDINYLDFERELDGKHQGTSKEG
GSGFGDPQAQDHLRKIVSRFENFLSDNKAGIDGVNLDDSDGNDNEDADDS
DGDEDSEFEDKAVSFDEEAFQQMMREMMGMPENGTTNNCGVDAGASDLTQ
QPICEETENNPDDLQQLSSEMEAELRRHGALDLKSETPRHVKAPAAEQCH
DEQQPSSAISTVEESMEELNIDYNLAQNILESFKGQAGMAGPTGNLLGMM
GFTLPRDEDDRRRED
>Mb|QLI68604.1
MSPRITRSSARQAASQAAHQASSLASPSAAGTNSTSTAPVPTAQGDRKRK
VSSSTSQDKPSTPVAAPHPNSGRRSKRQKISEPPSKGTSQDTLNLTLQGT
RRKGKATAAMDGNDGQASPAEPPEVSGRQVASSRKSSRSKKSDSTTPTSA
RRRSRRNDSNNADQDTAMTGTDDNDEPGPPPSPPIENPVDDSSEDNDEDG
HENDQPRSDNHDDDHEDEDEDEDEDEDDEDDDDDPFGGFGGSGGAVAGLS
STLRALTGMMSGLTGRLRELLNNLRDDDFSVQVIALQELSEILLVSNEDN
LSGHFSPDSFVKELVLLMNKEESPEVMLLACRCLANLMEALPASVANVVY
GGAVPVLCQKLLEISFIDLAEQALSTLEKISLEYPANIVREGGLTACLSY
LDFFATSTQRTAVTTAANCCRNIPDDSFPVVKDVMPILLNVLNSSDQRVV
EQASLCVSGIVESFKYQPAKLEELVSVDLLRAVLRLLVPGTTNLIGSSIH
TQFLRVLAFTARASPRLSAELFKLNVVETLYQILTGVSPPSGTENVASKL
DSVVVMQALIHRPREQIVETLNVICELLPNLPRNADPAFGDFVELHASTE
PITPSSVGGRSRRSPNDKRLDLLEQCKPEVRRFALIIFPTLTDAFSSTVN
LSVRQKVLTAQLKMLSNLDEEILVEALVPVPYASFLASILSQQDHPSLVM
LGLQATELLVSRLESIYRYQLYREGVIHEINKLASQEDPLDLKKEESDDD
EEQEDEDEDENDEGDGEEKPQDTTKDESGNHLQSSSNVVGVEERENNGDE
AGADDDNNSSGDDDEDERDDEDADDDEENDGDHVPGDMSPASSRGSTMSV
DMPPHRYISDMKSMTSRIRNVAKKFLETHEGGEHAQIMKNKATLILHDLS
DLATELETYYLKRPSATLGGAQKDKELFSQLASYFDTDVLESVTSAELLA
SGLVQTLLDIFSNPDEELARSAQSTFLEIFMGYSLKSKPKTATADSPATP
FSVLVHKMQDLLSRSEHFEVLTVHHNTFDGNRSSPASMLGKQIRLRLVAD
DESNIPRPYRNIMVSIHAIATFKSLDDYLRPRISLAERPPIGRRDGLSRA

LAAVAGMGGLQFSTTAGAAGARLTERSSFGSVPPPPPPPPAATTTPSSSR
YSRRSKNRSMPQPNTPATSQDPSVGPSHDKGILRRSSRRQVVSTEETPLS
RPPPTDGDDTQNTLECADEKQMSDEDDLEDGSALDAIVGELDEDMDDADH
TGSRPDPSAVNLEVATGGKITARKDDGTRVPTPSSSALFTSRSGSSGATV
PGGPPPPPPSSGQGTPTPATSRPPLSYSLALQAVPQDWHIEFSLDSKVIP
NETTIYRAVHTSTSTSEELVSRSIWSATHPIKFRRVPGPPTTEPATFGSN
PDSEGETVNGIPASLAKNPTTASILRLLNILHELNANIEDVLAENRNSSI
SLNVEPLSQFVNTKLTAKLNRQLEEPLIVASSCLPGWSEDLARLYPFLFP
FETRHLFLQSTSFGYARSMARWQNAQSEDSRRDRNNDRPFLGRLQRQKVR
ISRLKILESALKVMDLYGASQSILEVEYFEEVGTGLGPTLEFYSTVSKEF
SKKKLKLWREMDSVGSDEFVTGQSGLFPRPLNQEELSAPNGERILHLFRM
LGKFVARSMIDSRIIDIHFNPIFFRIGDAPSTGIKPSLGAVKIVDPGLAR
SLKTIKKFALAKKEIDEDPNRTAAQKVADTESIVIDNVRLDDLCLDFTLP
GYPNIDLEDHGSHKRVTIENVDIYLEKVIDMTLGSGVRQQVDAFRSGFSQ
VFPYSALSAFTPDELVSLFGKVEEDWSLETLTDSIKADHGFNMDSRSVKN
LLQSMSEFDVQQRRDFLQFTTGSPKLPIGGFRSLTPMFTVVCKPSEDPYT
SDDYLPSVMTCVNYLKLPDYTTIQTMKKQLYKAMKEGQGAFHLS
>Mb|QLI68609.1
MSSYLEKQSSAFRGTLASAAAKLSNPSSTSAGKATSLAPPSPSPSAASDS
TTPTAKRKRDLAPEVPFSQPQLTGYGAEVKTQMAFAVEYLKKKGEPKSIT
EIIDHLSLRGYSEEHKRELTEGLRGHPRVDWKPDASLSEQTWKTGMYSYR
PIILNVKDGTSLLAYLQRKTDASGVSVKDLKDGWPDCEETLASLEKQHKV
LVVRTKKDNFPRYVWADDPSLHNSVQPEFQVMWHRVPIPSVDDMHRKLVN
VGQKPTSEDPLKIQQAAGNKPKVQKKRASKRTGKATNVHMAHLLQDFSHI
RR
>Mb|QLI68612.1
MAAYADERSPLLATTNEQENETEADARNESSPLLGNRRDSATDGDHEHHH
DISPDSPHKGEAPHRKRRWPSLIAMVILASLVIVVMVLGFVVPPAVQQYV
ESAVVLEPTDLSIESLTANGVRARVKGTFQLDGSRVKDENARRVGRLATG
IMRQLGTASTKLRVHLPHYGNALVGTADLPPITLNLVDGQVTNLDFVTEI
APGDTDTMRKVVNEWLKGNLETLQVTGATALSLKSGIFPLGTHDVSESMV
FEGQSLYRAFASLYFANELPSMPEYTMQSLLFHDVPTGDNGQMGVGANVS
MTAYNEFPIDLTIPSLGFEVLVPNCNLSQPNIKIATAATSAIDVHAKSNV
TVEAEGIIRELPESLTKACTPSEFSPLDNFMKRYLHGEDAEVFVRGKAPE
GGDLPGWIGEFIESITVPVQFPGRSLDNFLRNFSLDDVDFKLPSPFADPS
DADSKPRVSGTVVILAAIPADLDINIEVTSLRANGDLLYKGTKFGELHVD
EWQKATSNMIRNPGDGEDLLKVVSRIVDTPIDITDSDTFSNIIQELLFGD
EDIILDVNSTVDVKVSTVLGNLVIRRVPAAGKVPVKHVPGDTLAALNPQV
GEVHILNTSSTGVHIQASVTVTNPTPYTASIPYVSIHILEDNLLIGEAIT
KNLHFKSGVNTNIIVEATWDPPSFGGKKAHKAARRLLSEYLSGENTTITL
RTHNGTIPTVPIIGEALSKINLTLSTPRLELPGDEDTEGQGFIRDATFHI
LSSTATFMLASPLRYDTVSIEYINATAFYNHTEPIGQIIRDEPFDVSPGV
SQTPRLPVEWSADHMGLDKLKKALGGKLKLDAVADVTVRVGYWFETVHYV
GKAIGARVSL
>Mb|QLI68613.1
MGNDGGSIPKRRELVKNAARAPTVSELKATALESLTHAWTHCALSGAILE
METAVSDWKGRLFNYEAILSGLMPSDEPVETTPATFGIKSLRDVVRLKFS
RTDGKWACPISMKEMGPSTKAVYLVPCGHAFAEVAITEIQESSCPECGET
FDKDNTVTILPTTEKDVRRLEKRLDDLREKGLTHSLKKDKSEKKNKKKRK
GDEVNGNDDKAMKADALQPKKDADSRISGINNPMAASLTAKVLAEQDERN
KRRKLVAEVMSRNGEVVKG
>Mb|QLI68615.1
MASATGLPERPPARESIVAAETEPLLGKPGDAVLPTGASIMRAFVIGTGT
LAQLGVAIICADIWAHMFFSPIIFFSGHPIAMSAAVFTLTQSVLVQQPIS
SDTPHQKRVGQYVHAALNLFAFAAIVTGFAIIEINKFRSNGPHFHSAHAY
LGVFTLVVLAGQYVVGLTMWATPRLYGGEERAKSLYKYHRYVGYLILLTL
LATVVAATETDYVRSVLGVNFWTVLAGSVLVVMGVFPRIQKQKLGLGPRQ
EERPVHDFESATS

>Mb|QLI68617.1
MENGTVLDIGGSSLTTDIGARLRDVLKSKDEGGKKPVLPDEYLYNDLGLE
LWRRFISQHEFYQTRDEVAMFQANGKDIAKQFARDSKKLFLLDIGAGDTG
KVHHLLVELEKHAQVPITYCALDISKASLETNVEKLARQHSGPGSMVNIL
GLWGTFQHGQKFAREKNFDGRMIYLSLGSVLCNDEWDNAVDHLKAWKDVM
RPDDLMLIGMDGHTAKDEDQKKKLWDSYHKREDLLKPFFDNGYDGMNRSI
GEQIFNDKNFEYHAELEDEPTTRHRNWITAKKDIWCEATNSMIEAGQEFD
WFDAHRYGPEKVKGMCSQVGLEVMKVWQAKDSHFYQYLIQSISIPVLMDS
DSGVSGVA
>Mb|QLI68618.1
MDPQPAAIPTTICSAADAAPSASNVGTGPCLSVTPSPSSKPPVLVLNLLV
FHPHFAADVDTGRKPRCTTNVASLDLSPDDYDRSPSPIVDASTHPSICPL
PIARQSGCSDLRRQPLEPAFARYVFWRRRGGNHPPNLRMTMADCLAAGLY
GGSWLTLLAMFFAMSAVSAQGDITVVTSAQKDVPDWANPSALNMAITLQP
GGKPSSSMYTVIPLTASVGLNGTGVIRGTINIMGHLKAANVSSYNDLSGG
DTIAFLSCDKPASDGFIDPDKMLSRLMKASLKAIILYTIDQNICLISNEG
TLPYTSILSMADSGEAGGVLSYLNGTQPVASADAQISGKAGQPSPNSGSG
DGGGSNSAVAMSILYSITGIITLLFLVIIATGAIRAHRYPERYGPRGGLG
GRPRQSRAKGLARAVLDTIPIVKFGNQEPAKPDPELELDGAMTDGHETTS
HRAVTNTGESEPPEAKTAAAAAAALADKKDKSGQSSSEQTSKAPDAASAH
AENEEGDEHIGCSICTEDFTVGEDVRVLPCNHQFHPHCVDPWLVNVSGTC
PLCRLDLRPGQQGPNDQSSFNPEENLAPPLVLEGDDGDSSHATHSNRLSR
LLDVNRLRQAGVEERIAALRQMRAHSANHGTREPETVEDRPQASRLTDKL
KDKFLVFAIYADSYIFVFASAMLQHSIGLNASFKTCDSAILICLVCYVTT
KFIYLFLVEKAHIIRGTPKKRIGSRLYMFNSLGMIAVYLAVVILNFVFRI
AKLQDGECIIGMKSFAMIPLISFDAVVNVYLTIIFLIPLRNLYSYKNMPR
THTNMRLRTIAFKTFCGAVCTLLSSIVNLCALMSLDGEPGWVCLVSCNCD
IIFSAAVIQWVTSKDNMGTSTASSTNGVINGREALGHESIRVLAPYAADF
STVSSPVDPSLVASARCSTQADMHAANATSAGGVIITTTIERETKLGGGS
KSQGSPVGIPMGRGYQECYAGEDGCATFQDDPQGAYGSRVNITAG
>Mb|QLI68619.1
MSSRRVPLTSNPNVANSPLRGPGSLHAYAKQKRSYANVQREEAYGQPPPV
KKQVLEDGTQRAVRSPSKAHRTQVVVQRSSTRLVAKDRATRTTQASARTV
QDVDTEKEVWKKHHRAKFPKMVFYFESIPDDIRAKLTKRVNYLGARQEPF
FSIDITHVVTTRSIPPEKSGTQHEESLEQEQQEPEEQPQTINPSLLDRNT
AAGRRKLLFDFRQTQIPSQQADDPTRRTKGTRNNDVLHKAREMGKKIWSL
DKFQNMLAVLLESETRGATYASGTTSVRSQYGVTKGSHEPNLLQLLHNER
LNGPSDRDPTAVNRELVYFKGPYIYVWDMDDKYKPIMVREYAKAVNKQDG
EWPQFRSVGNGRCPFVEEIEIPEKEQRRNREREKDARLVKREDPVPILKP
PEIPIPKPVTGKRTLTEMEDGQNRVRTGPAMDVFNPAKAVLSKQAELKSQ
NAFTSRAEGTRLFAGEPVASGMQPSNVTSAIRSQMISSTSGINGGKAGTS
KEVHGLQRKVLQKANPASHDVSSRRFAEVSMDVASSRSTTMSRQTSKAVQ
PHDDESQRTECREKKSNSQSLKSKRDLKPGYCENCQDKFPDFDEHILSRK
HRKFAENDDNWTELDLLLEQLKRMPKYAGAESDNDDNGW
>Mb|QLI68620.1
MKGINSGSEGSGDKIGGRFWRGTKLGSQDYSLGERNIGENGTIDVGGEGE
GSVANGTSTEYRTYKRRWIGLATLTLMNIVVSWDWLTFAPVADFSSQYYG
VSKSAINWVSTAFFLAFVAIFPITIAILHRGPKLAFMIAAVLVIIGNWIR
YGGSTSSSGGHFGAIMAGEILIGFAQPFILAAPTRYSDLWFTNRGRVAAT
AVTSLANPLGGALGQLINPLWVKQASDVSQMVLYVSIISTVCCLPAFIVP
AAPPTPVGPSAETPKLSLRASVKVLTHSLELWLVLIPFAVYVGFFNSISS
LLNQMMTPYGFSDDEAGIGGAILIFVGLVFAAITSPILDRTKKFLFALKF
FMPIIGICYLVFVWMPETREVTGPYVVLAILGAASFALVPVALEFLTELS
HPLSPEVTSTTAWAGGQLLGAIFVIISDALVAGDDANPPKNMKNALIFQA
VVALVVCPLPLFLGLFGRADKVVLRRVRSDEQGARTNVQTVV
>Mb|QLI68621.1
MRATAADVEAVESHVHEQDECLKNNVFSDCTAVVDHAAERSLCFKFDFRI
LPVLVVMYLFNALDKGNLGNAQTNGLSQNLNFKDGQYNLVVSIFFVPFVI

FAPPFAMLAKKYGAARALPVMMFTFGSMTLLTAACQNFGGIFAVRWFLGM
AEAAFFPVVIYYLTTFYRRGELARRLAIFYAASNIGNAFSGLLAFGVFHI
KSSMYPWRYLFLIEGSGTVLFAIFAFWYLPKSASEARFLNEEEKALAFHR
MQVDSSSVVLEKFSFKEALQIFKYPSSYAFLAIEVCLGVPLQSVALFLPQ
IIQRLGYSTVKTNLYTVAPSVTGAAVLVLLAFASDFTRVRFPFIVLGFAL
TFSGFVIYASITDVQAQIQVAYFATFMMCWGTSAPSVLLSTWYNNNIAHE
GRRVTLTSVGVPLANVMGLVASNIFQQHDAPKYMPALITAAGFGASGALI
ACLLGLYMIFDNRRRDRGAGIKLKAQDVSTLKLRDGPNSPEFRWYL
>Mb|QLI68622.1
MTSYLIIGAGNFGAATALTLAKREGTTHVTLVDTTNFPNPRAASHDINKI
VRDDYPDKLYMQMLKKAMPMWRNHGLYKPWYHEVGMLRADPSNFGEQSIA
SYKEMGIESKAHFLTVKEVRQRWNGSFATANFDGLDKILFNPAVGFAEAD
KALGAVVQEAVDQGVEYVVGVVTKINIGATGECTGVTLQSGETLHADKIL
LATGARTAALLAQSAPENQQLQVGDRLLATGAVSFYAKVQGAQQEKLSSI
PVLKNCLPQVKGEGMSILADGTIKFNCDMCFTNYVECPLTGQRLSMAPED
SAFNVWTGPKFIKFFQERARKTLDGLYGKEVEDVAIDAYRMCWDASTPTH
DFLITPHPHSHGLYVATGGSFHGWKFLPVIGDYIADMMQGSLETEFADRW
AWDKKGGDGHSANPTYQIVGDLQDWLA
>Mb|QLI68624.1
MLVKSATALLAIGLAPLLASAQFSITGAPVAANGNVPLRRNVNDLYSEGG
PQWDLYIQALASLQSMNASDPLGYFQVSGIHGLPFIEWNRGGARNNNGWG
GYCPHGEALFLPWHRPFVLLFEQLLVEHAAGIASQYPARYRDQYVAAANN
LRSPYWDWSSDSNVPPCTVPERVFVNVPNGQNLGRIEIKNPLSTYNYPRE
ALDGQFGYFTQSRQMVRCPAPERYPDTASQELQDYRLKQATYDVFSTANN
FYQFAVRGRVHHLEELHNDVHYSAACRGDFFDASLSGFEPLFMLHHTQVD
RLWAYWQFINPSQASFSGRYRGQSRFSTPEGTIIDQNSPLQPFFDSKRKY
YTPKSVSSIKGMGYTYEGLEHWRKSPAQLRQDSVQIINSLYAPASAFAKR
GGPQTKTRHFAHVELDRAQVERPCSIKVFVGGKQASTIPVMLFPANGNLR
SSLAIDKFLTKGSSSNGTLESIEHLIQVEISKSDGTVIPLDKVDSLNITL
EQASITPAASRNEFPKITDAKEHAAKIQAYSKS
>Mb|QLI68626.1
MAHKLPQIGVLQSLSEDAQTKTLDLLFEPSPAIHSTLLPVVRNAEYSSYS
ELIDECRPALFDLSSKSTPSNPNPVLLSVVGSHPRLGSKKVDSAQSASEQ
ANLQGQGEQLTELNREYEEKFPGLRFVVFVNGRGRPEIMKDMRARIDRGD
FAMEVDAALEAMCDIAKDRASKLLA
>Mb|QLI68627.1
MEMKIALGHLGMYLGLLAGAGLLYAFGLVVYRVFFHPLAKYPGPFLAKIT
DAYQLYHAYKGDRHLEFWRMHQKYGKVVRFGPNSVSFNSNQSLKEIYGFK
SNVRKAEFYDAFVHPAANTHNTRDKDVHARKRRVLSHAFSESAMKEMQRY
ILGNVRTFCDQIGLNDGGADAKGWTKPRNMSDWCNYLAMDILGDLSFGKA
FHMLERPDNRFALELVEAATTRHLICGTMPIVDKLKIDKFLFPKLAAGRA
RYMAYSKGQLTERTKLGEETDRRDFFYYLLKARDPETGHGFTTPELWGES
NLLIIAGSDTTSTAMAATIFYLVRNPHALKRVTQEIRTKFSDVEEIVHGP
LLGSCTYLRACVDEAMRLSPSVGGILPREVLPGGITIDGQTLPQGTVVGT
PHYTIHHNETYYPVPYGYAPERWLRDEVNPLTGKTTTEDDVALAQSAFCP
FSIGPRGCIGKGLAYVEMTTTLARTIYLYDLRRAIGVVDPGEGRADLEWG
RHRVTEFQLVDTFTSAKKGPMVEFRKVEKA
>Mb|QLI68629.1
MEKHLTLVTDGETLWASDRGMSSDDGAWEGVVLPSPSPTEAAWKGAPLSR
PVQTGQLSPVSSGVDTSLDSSSVPTTAHSASSSCTDSWSIGYVDQDEGDN
EVTWETWEHAQDEVMTAPKLEPNDDDIKLGDVTVAPLTPIQQIALQHNSE
IKQKRPRGRPRKHPQPQAVNVNKVTKGRSKTGCITCRKRKKKCDEAKPRC
MNCEKNAVVCEGYHEKQIWKSGKEKAEEERMRHECQPIITMQPIFHGVET
AEDMIFWKHYINHLSNVLTVEGEAKNAFKDIILPLANQHQGLMHSILAVS
SKHIDLDSPYGAKILQDNSAVTPESLQQRGDYHHDEALRRLYKDMENPVP
KEDPAHDTVLAARYGQILCLLLQTRAEGNPRGEHRVHLQAYQTLIQHSPP
ENKTFHTFITEFFQYHIYADDLLWHSGANSSRLSTDSSGPPSQIHPPRLI
GVGDGLFRCLSQITTLRNVIRANVAASVDPVVDYTSLYRAAEIDAAIREW

VPRWPLGDSRHRVGPLYKQMMWVYLFRTIYPPSTTPSRRSTFMTLPVTSS
QHNTGHLQRRASMVASVGSSTSSSAILSNLTSLTGSHTAQSCPSSRNPSR
TSSMHEQDTIPLVGEECQDGHKQSSPPPARRPAQDDRRITLAVEESLAIL
ESFKPCDPAQTLLLIPCTVIGTACFDSTQRSRIRTAIKVIRGYTGLRSSD
RVMELLEEVWSLMDQGDWVSVWDWQFIAKRLGLDFLCA
>Mb|QLI68632.1
MAAKAPVPQNDNVAHALAGAGGGILSMVLTYPLITLSTRAQVESKKAESK
FIDAVQKIVAREGIPGLYAGINSALFGISVTNFVYYYWYEWTRAFFEKAA
ATAGRASKKLTTVESMIAGAIAGSATVIITNPIWVVNTRVTTRQQNSEAD
LESGKPARKPTTLGTLMALLKNEGPQALFSGVLPALVLVINPILQYTLFE
QMKNYVEKRRKVTPTVAFFLGALGKLFATSVTYPYITVKSQMHVAGSHSN
KKEGMTEALRRVIREEGYAGLYKGIGPKVTQSVLTAALLFAFKDALYEQT
VRLRQSRKA
>Mb|QLI68633.1
MAPIAVSPEREPLPETLEALTAKFDDTLRFYLNGTKVVLDDIDPEITVLE
YLRGIGLTGTKLGCGEGGCGACTIVVSQYNPTTKQIYHASVNACLAPLVS
LDGKHVITIEGIGNTKRPHPTQERVAKGNGSQCGFCTPGIVMSLYALLRN
NETPTEHDVEEAFDGNLCRCTGYRPILDVAQTFSVERSFPNGPPKLNGDA
GCCRQNGNNNVQASDANGCCESDTNGALSENCATNNSTTNGTDKSSQING
GGCCMQNGGRPPSGRCCMQKKGLDDQPMKRFTPPGFIEYNPDTELIFPPA
LKKQELRPLAFGNKRKKWYRPTTLDQLLQIKRVHPQAKIIGGSSETQIEI
KFKALQYPESVFVGDIAELRQYEFKDNHVEVGGNVTLTDLEGICEKAIKY
YGHERGQVFEGILKQLKFFAGRQIRNVGTPAGNLVTASPISDLNPALWAA
DAVLVTKSATKETEIPVSQFFTGYRRTALASDAVIASIRIPVTAPKNEFY
RSYKQAKRKDDDIAIVTGALRIKLDDHGIVSECNLIYGGMAATTVDAKTA
TAYMVGKKFAELETLEGVMNALGIDFDMQFSVPGGMASYRKALAFGFFYR
FYHDALTILDGQSEHVDKEAIDEIERSLSKGTIDETSTAAYEREVTGKAN
PHLAALRQTTGEAQYTDDIPPMANELHGCWVLSTKAHARIRSIDYSKALD
MPGVVDYIDRNDMPSAEANKFGPPNFDEVFFAEGEVHTAGQAIAMILATS
ANRAQEAARAVKIEYEELPAILTMEEAIEKESFHPVYREIKKGDTEGAFK
DCDHVFTGTARMGGQEHFYLETNACLAVPKREDGEMELFASTQNANETQT
FAARVCDVPANRINVRVKRLGGGFGGKESRSVVLSSAVALAAHKTGRPVR
CMLTREEDMVTMGQRHPFLAYYKVGVNKDGKIQALDLDVYNNAGWTFDLS
TAVVERSMSHSDGCYYIPNVYIRGRICRTNTVSNTAFRGFGGPQGMFIAE
TYMEEIADRLGMPVEKLREINFYEPHGITHFNQVIEDWHVPLMYKQVKEE
SDYDLRKVVVSKFNDEYKWRKRGLSIIPTKFGISFTALFLNQAGALVHIY
HDGSVLVAHGGTEMGQGLHTKITMIVAQALQVPLETVFISETATNTVANA
SATAASASSDLNGYAAFNACTQLNERLAPYRAKLGDKATMKDIAHAAYMD
RVNLSAQGFYKTPEIGYVWGENKGKMFFYFTQGVAAAEVEIDTLTGTWTC
LRADIKMDVGQSINPAIDYGQIQGAFVQGMGLFTMEESLWLRDGPMAGNL
FTRGPGAYKIPGFRDIPQEFNVTLLKDVEWKELRTIQRSRGVGEPPLFMG
SAVFFAIRDALKSARKMAGVEATVGADHSEGLLRLQSPATPERIRLACED
EIMRKARVHAKEGEQSFFVVI
>Mb|QLI68639.1
MTGTAEAASSSNDAAAAAQRASEQARLRRERREAKIKAGGAARLSKINGM
AGRVVHDDDQATPAAADKPTTPSKPQTHGDPDEVDISQHFYTPQTTARPA
PASSSPGPEAAISEAQLRQMMLGFDRPSNPNTPGSSTPSSMPNMDDDPLM
KMMTQMMGQAGASPFPGMPPMPGQQAPQAAAAASAPSAYHTTWRVIHALI
ALSLGLYLTLFTTFAGTKTERDAAALAHVQHTPADDDNEHHKRTFFWIFA
TSEAVLLTTRVFLDRGRAPPPGILWTIVGYLPEPIRGYVSVCLKYGQIFT
TVRTDILTCMFVLGACSWLRA
>Mb|QLI68641.1
MSQEKPLPFIYQFAAGAIAGVSENFVKILVMYPLDVVKTRVQLQTGKGSG
AESYNGMLDCFRKIIKNEGFSRLYRGISAPILMEAPKRATKFAANDEWGK
VYRKMFGMNTMNQSLSVLTGATAGATESFVVVPFELVKIRLQDKASAGKY
NGMVDCVVKTVKNEGILTMYQGLESTLWRHILWNAGYFGCIFQVRQLLPK
ASTKQGQMTNDLISGSIGGTVGTILNTPMDVVKSRIQNTQKVPGQVPKYN
WAWPSVITVFREEGAGALYKGFLPKVLRLGPGGGILLVVFTTVMDTFRKW

QS
>Mb|QLI68642.1
MATSSKNDRKRQRSEVEEPLPSTADTQIVVGQSTKRPRVEERRSLFVRSL
PANVTNDTLADFFSQHFPVKHAVVVVDQKTKESRGYGFVTLADSDDALAA
KTTLDKAEWEGKRIRIDIAEPRKRNAANSEDTVPKRPGREESQRPPKLIV
RNLPWSIKTSEQLSNLFRSYGKVKFSDLPQSKGKLRGFGFVTIRGKKNAE
KALEGVNGKEIDGRTLAVDWAVDKETWEKQQDLEAEDEVPNREEENCTDD
KALPETALSSDDDKDDNDNDDDGTASKPDKNNELDADLENFMKNHMQNME
DEDEDDEEDDKVDNSKSSAEKKTTDNSSTVFIRNLPFTTTDEQLKGFFSH
FGAVRYARVVIDKITEKPAGTGFVCFVKQSDAKSCIKEAPRPNASAAGSK
PSLLLDENADPSGKYTLDGRLLQVAQAVNKAEAANLADNSLAKRREKDKR
RLYLLSEGAIGRGSPLFDLLSPSEIQMRQASAAQRKKLVQSNPSLHISLT
RLSLRNIPRNIGSKELKELARKAVVGFAVDVKEGLRQPLSKEENARDGKD
AKEKERQRKLKGKGIVRQAKVVFESNQGSKVDEMNGAGKSRGYGFIEYTS
HHWALMGLRYLNGHQLQGDNGKKQRLIVEFAIENANVVQRRRAAEEKSGE
SQREGRNTRTELADTSKGRAPKLERRGKGAKHESEETRSHGDDKQNHGAS
KAEDMQQRLIARTRLMRKKKAKARGKS
>Mb|QLI68644.1
MNLLLSDDYLLQDYPESITNTIRSGHATVVRFNRKGDYLASGRVDGAVAI
WDLETMGVARKLRGHRWKAILWDLQDGKRLREVRFRAPAYMAELHPWNHL
QFVASLFEEQPVLVDMTDLVDVKHILPSVPKRTNSEGSAAQREKQAKEDA
KEKTTSAIWTSTGDHIIAGTSKGKINFIDTKTFEIIYSEKVCSGVITTLR
ITGAGRDLLVNSQDRIIRTFRIPNLSVDDLDLDTIQLPLEHKFQDVVNKL
SWNHVTFSATGEYVAASTYNNHELYVWERNHGSLVCMLKDPKEEQGVIEW
HPSRALLAACGLETGRIYIWSVISPQKWSALAPDFAEVEENVEYIEREDE
FDIYAQEEIHRRRLDAEDEDVDVLTLDPTKFDDDIDSFRMPILFNLGESD
SEDEFIAVSTGTMRRRSPGEGQSDIDGPVDTVTTKKGGTAKGGGRGRKKV
LP
>Mb|QLI68650.1
MASRRLALNLSQGLRSRAGLSAAGSLRRGFATPSSVGKTQTTTLKNGLTV
ATEHSPWAQTSTVGVWIDAGSRAETDENNGTAHFLEHLAFKGTAKRSQQQ
LELEIENMGGHLNAYTSRENTVYFAKAFNSDVPQCVDILSDILQNSKLEE
SAIERERDVILRESEEVEKQVEEVVFDHLHATAFQHQPLGRTILGPRQNI
RDITRTELTSYIKNNYTADRMVLVGAGGIPHEQLVELAEKHFAGLPAKSP
ENQAYLLSKQKADFIGSDVRVRDDTMGTANVALAVEGVSWSSDDYFTALV
TQAIVGNYDKAMGNAPNQGSKLSGLVHRHELANNFMSFSTSYSDTGLWGI
YLTTDNITRLDDLVHFTMREWMRLCTNVGEAEVERAKAQLKASILLSLDG
TTAVAEDIGRQLITTGRRMMPGEIERRIDAITEKEVMDFANRKLWDKDIA
ISAVGNIEALFDYQRLRNTMKPKF
>Mb|QLI68653.1
MDSIPSDIYANDDKLPDTGSLRRFPDEIIQHLLRFVPPRDILQCFQLLCH
RFYRLANEGLLWRYHCQSTFRYWHPGHGFGEKLQATVASVDWKYLFLLRT
RSNHTISELLDGIVETKVGRLKKIEKICRLGYDAKDFLLVQCHIHESASD
VLARRYFSNAILDNIHRSIALEEWSRLGLDRDSLSAQDAAQRLERALGAF
DMFVLHDQTGDLDDISQMLDDTEQNYRNLRNCLIGQALRYEEHESIPLIS
SAIFCCLAARLGLNAECCAFPNHVYAIVFAQSGYTLDGDVDCYREQPEGM
YLDPYGLDNEVPASDLHNLLADYAWQTSTDAFFAPVPPITMVLRTAQNIK
ATFLRILELQGDAHPELSQLLRGNDAMNVDAALYSAMWSSLLLTPPNTFE
WNDRLASFLHRFAGSWPEDAWLVEKYLWPMYNSFTALRHGFARHVNRGLG
DPWDHWRLVRDQDDVIPPMSRRDLDRNQSVPYKIGQVFRHRRYGWIGVIT
GWPDQGTRHLPVAASRDPDDEADTAAPGEHSRLPVWPPTQFYFICFPSNG
SEPNVVAADNIELIHDSRNIDDTMFPMAGKFFRRFDPESCRFVSNIKEQY
PDD
>Mb|QLI68655.1
MIHFDVFDARDLLFRRDASFPPGDNSSDTVIGNIHFNATALDNNNYTFYP
GNNTLSNISDCYLVFAPYSPVLVRANGSFLNATTCYSSINPIGKRGYAGI
GLGVFYALSIVLTLVALAKHGALYLRTTKRFYPIGRRWQWYWACLVAACA
LVSLFSNVEIERYYLQDLPIILCVFFWFLMCMGTVALVWESVRHWGSWLE

RQFIDPNPFVYREDDRRAKLEFWLPLWFYFWFWMNFFMVVPRSWNFVKSQ
HSVEQTNAVAIPNATSVRFKVGAFCLVISWLTILFSLRHSIRHYKPRHRG
IFNRALGFIQAVPLRFMLTMPLALALIAYQAVISFIWDFSLVKADGNIPI
IMAWGYGPSLLIIYIQIVYGYASPNEDKELIRQRRERGDIVDRELGVVHK
PAWWRRVRGDHLRTMRDKIDQNVNEVGGERGIGRRVENDAERDARLEAER
SAINNDGIELSSLRDDPHNPRVDRAGIHSLNKTTSVTDDPLANPYLGKND
RRHAERLMQSAATVLFPSQELAMERARRATELMEDGPPPPYNDRQRGESE
TQRPLSAQRTNSTSTTLSINAEPQQVRSMLDI
>Mb|QLI68657.1
MASQIPRRLFSTTARRLQSNGDLKSESKRNPELMILGGVMVAALGGAGFY
FGRSPTKSTSESPVTIASRPWEGEGEGKYKYHPGGDISKEPKDAPSALNV
VVVPNVTLPAELHEKYNKWGKEGYP
>Mb|QLI68660.1
MSRPSRVQARCVGNPSFRPEDIAQNQDRLKQARDLLSKNDLYQASRMLLG
LPEPDVYTYHAIASVKLAEAQHVVDRGGDNGLHGWYKAEDGSTRDPPSQA
DIEAYISIFSPTTSTAAALKNFTTNAKKGSLRSETAAHISRNKFIHASLL
TQLTIPKCKSPPIQNPYFDFWAWSCRNLEWCGPNACSERTSMGHHILPIF
MHHFGCVTPSHEGLEALRILADGRPIADVGSGNGYWSFMLRGYGLTVHAV
DNMQSEWRVNWVDDTSITDGVKWLQRNNGGRDMVLLLVYPVVGGGVGGGT
EGSFTRGLVGAYEGDTIAVVGTQNRNGYTGFKSMTMDEYMARERKDWVKV
VQMPLPSFAGKDEALFVFQRGERAPKADQ
>Mb|QLI68661.1
MLAPRAAIRGTKNSPLSRLSARPLRNIHNTCSRSKKLNLPSGQVTHATAP
SPSKYWPKYEIPQGAPQLIYDPAASDPVTISWTDELLFIQDGPDAAPRAL
RPATLRDSCTCSACKDPSSGQKSFASIEIPPNVGISSVRPTDSGLAISFS
NDIPRFPGHEMILPWHQLGSSLKRTSSSPASKNSILRRTGVQYWDAATLS
KHVRKIDYAEFMQGGSRFWDAVIDVCRLGIVYLKNVPRDEASVVHVTTRI
ANIRETFYGRTFDVRAKPNAENVAYTSGYLGLHQDLCYLQPPPMIQVLHC
MENSCSGGESLFSDGERVGRLLWPFVAGSGSKMASLAQHRVPYQYDKHGH
FYHADRPVVDVGGDGGFAGVYWSPPFQGGYTDGHAEGNAGQDITGWIEAA
RLFEGLVNDAAAVHSYKMEPGECVLFDNLRTMHGRTAFDAGRGGSRWLRG
AYIAKEDFLSRAVYIPEGQAEMYRGTSEEWSGDRAQGELRRSAWWQDVVR
RVREVDGPC
>Mb|QLI68662.1
MATKQDIQVASVAAGFTLGFGFLTAWKAIQQTRRNKRPWKSIYVYMIWGE
IMANLAIGIIGWVFLDGLLSPTVPVLFFILFLWVFEIQLLMQIIVNRIAI
IAEHTRTVTRLKWGTAVVITCINIAVFCIWIPAHIEPPASELYVKLNEIW
DRISKVLILLVDAGLNYYFLRTVKRRLVLTHGLTKYAPLVSFNAKLMVVS
ILMDAMLIGLMSLPNQIVYIQFHPVAYMVKLNIEMTMADLITKLARGENS
DTHPPSTSNHRTFTHEDADDDNQTRTYPMHTVNKTQAEDSLSELTKVVSH
HGKLNGGIQVETVITSTSTRNNRGERERNMAPSFDVDDEYSLAHGGSDDG
TRHMQ
>Mb|QLI68669.1
MSQVKNLRAMFENKGDTSPPDRGRSPGITPSRHIGNASSNGSPRPLSKVR
TNFVAIEKDGRMGLRRDHSGESSLSRRRMSTETADTELNSNYLENPNTVP
SDDVSKASRRNIASETIPESPRTMSHEQAGVSVDAGSQLDKAHMEVKMKL
GSPPHLQSEERGTGSAGNAGLNSVSATNRSHGIKHNGHLSIQAEMMTTGP
TKGFSATSAARPESRANNRGLKISTLSADKTQKANTGATSSTKARTVPSG
VVAKSQPAKTKSQNDAGFIKPKPKSPTKQVQMPSSLTAPTASSVSKVHVT
RRPLSRQSGTQNNPAQPSIRPNMSNSTASHSFNKGQVSSSSRSRPSLGPP
PNKQSQVTASNKKQSNVDEGFLARMMRPTQSSSSKATDKAPVTPPKRTAQ
RPVGGENGNQALPRSASSRGLHGKRIPVPASLQSTSAPLAGPTDEPDPPE
SEEECNELHIDANNRSEIANADKEARSPPNPATKYTIEEKGVSAVPEGPK
RFEALITGSTCNTNSARNDLVHNSQLREEADDSKASVAAGPTPLVELMEA
SSSVGSDEIASKDKRPGAIEGVDDAAPEATSKQQNCKADLVSETKEIPQG
PAGNSLANEEPVTAEGHESSDPVEMDLAAQHTPETCTSPFDVNQPYEQSI
QQVIQSTRNHIVPEINSPSPLEIIQTDLGGDKPDGIPKMEAVSDEQKQPT
VQGHEGTAEVALETEHA

>Mb|QLI68672.1
MDYDLVMEDEFDALCLDVRRHRDSASKTKYPAKLHARKVVRELGVDDGLI
YLPGQPEISLEDSDQPRLFRQRRYFFYITGANFEDCTATYEVKHDKLTLW
IPYVEPRQVLWFGSKPSAAECKRRYDVDEVRYTTQLSGFLRSFAAQPSPP
VTYILHPKQAPDLGHGSQSQLCLDSSLLLPAMDRARVVKSDYEVAMVRRA
NNISSAAHRKVAERILRLTNEREIEAIIQAVCIASGSRSQAYPIIAGSGA
NGATLHYGSNNASLGGKQCVVIDAGCEWNCYASDITRTLPLSGAWTPKGA
AIHAIVQRMQDECIAKVGPGAAWREIHLHAASVGMEGLLGLGILKGRRED
VARAGTVAAFFPHGLGHHVGLDVHDVSGTLALSAAEGRGQQLDFGKRAMV
TPSMLADMTCASSSQDAVGGGRSKTQLLLPNMIVTVEPGIYFCREYLEGY
FRSDPAHADFIDWDLLEEYYDVGGVRIEDCILVTEDGYENLTVAPKGDEL
LDVINKGKK
>Mb|QLI68673.1
MSMYSHRGMGAVPPGNSARLNELLDQIRAEFETQLRQTEGFEHQISAQVS
EMQLVREKVYAMEQTHMTLKQKYEEEISMLRHQLENARKGGPQPGMPGPP
QHPGPSQQPPSIAPGNGLFSGIMAGGNQAGLAPPQQQPHAPPQEQQMGPQ
HQMGQGPPGLPVPPPHPNAQQPPYQQGYPQGPVSNGMGPQPPQSTASPGP
GRRGVGRPPNAVGPATPQINTPVPYPGNAQSPQVSHPTPDHARMGGPRAP
PVGNALGDLEVDAVAPHNKKTGSDWYAIFNPAVQRVLDVDLVHSLTHESV
VCCVRFSHDGKYVATGCNRSAQIFDVQTGEKVCVLEDHNAQDMTADLYIR
SVCFSPDGRYLATGAEDKLIRVWDIQSRTIRNHFSGHEQDIYSLDFARDG
RTIASGSGDRTVRLWDIEQGTNTLTLTIEDGVTTVAISPDTQYVAAGSLD
KSVRVWDIHSGFLVERLEGPDGHKDSVYSVAFSPNGKDLVSGSLDRTIKM
WELSSARGGQSAAPKGGKCVKTFEGHRDFVLSVALTPDANWVLSGSKDRG
VQFWDPRTGTTQLMLQGHKNSVISVAPSPQGAYFATGSGDMKARIWSYRP
F
>Mb|QLI68675.1
MPPAATGGLSLKPNIGVYTNPKHDLWISEAEPTARSVTAGSDLKPGEVTI
AIRSTGICGSDVHFWHAGCIGPMIVEGDHILGHESAGDVIAVHPSVTSLK
VGDRVAVEPNIICNACEPCLTGHYNGCENVAFLSTPPVPGLLRRYVNHPA
VWCHKIGDMSYENGSLLEPLSVALAGMQRAKMQLGDAVLVCGAGPIGLVT
LLCCAAGGASPLVITDISESRLAFAKELCPRVITHKIEPGSAEDTAKAIV
KAFNGVEPALTMECTGVESSISAAIWATKFGGKVFIIGVGKNEINIPFMR
ASVREVDIQLQYRYSNTWPRAIRLVESGVIDLSRLVTHRFPLEDALKAFE
TSADPKSGAIKVQIQSFD
>Mb|QLI68676.1
MSKQSYKQQKEDFVSNLSGGSVPEIAAVTAVVPVAILLWSALQARQSFFR
PYTPLSYAVDFLLCVGAFLLATTLYASTPVLLILFLLAPVASVYMLPAQT
ARKKKPIVPRDDKNAARPLDVLPAKPFLTSYRGAMMVMTCVSILAVDFRL
FPRRFAKVETWGTSLMDMGVGSFVFTAGVVAARPVLKERASGKSMSLVKR
LVYSARHSAPLLVLGVIRLLSVKGLDYAEHVTEYGVHWNFFFTLGFLPPF
VAAFQAVLRYVPSYAALALLVGGVYEVLLENTALKAYILTAPRTDLLSMN
REGIFSFFGYLAIFLAGQDLGMFIIPRAINPRSKATPGTQRNTLLMTIAV
WAAIWSALYYLCTGYSFGLGLVVSRRLANLPYVLWVASFNTIQIFACCLI
DTVFFPATYNATDGRTEREANEFATSRVLRAFNRNGLAVFLVANLLTGLV
NMTVRTLDVGPSVTMAVLLGYMGAVTGFAVLLDMYDVSVKL
>Mb|QLI68681.1
MKFARAFSTNGVPANKVKFVPTSGTYPKGFSAAGVFCGVKPGNTSKPDIA
LVTSDRDCAAAAVFTKNKFQAAPVTFSRTLLQRKSNAGLRSVIVNSGNAN
AVTGIGGLQDAARMAETTDQRVGTENSTIVMSTGVIGQRLPIQKIVDHIP
LAIHRAGATHKDWLNCATAICTTDTFPKLMSRPFELPSSPGIEYRIAGMT
KGAGMMAPNMATLLAILATDAPIAPGIMTNVLRRAVNRSFNSITVDGDTS
TNDTVALLANGAAGGKEVSSEQSPDYDAFQTVLTDFATDLAKYLVRDGEG
ATKFVTIRVVNSASEESARQVARAIALSPLVKTALYGRDANWGRILCAAG
YSLISKPGQPVNDVPEIVPEKTSVSFIPTDGSKELKLLVNGEPEVVDEKR
ASEILEMEDLEILVSLGTGDESAVHWTCDLSHDYVTINGDYRT
>Mb|QLI68683.1
MVKETKLYDTLVVKPEATQDEIKKAYRKVALKWHPDKNKGSPDAAEKFKE

CSQAYEILSDPEKRKVYDQYGLEFLLRGGGAPPPEGAGGFPGGMPGGGFD
GFDFGSGGMPGGGTRTFHFSTGSGPGDGFNFNNPNDIFAEFMRQQSGGMH
GDEDMPGIFSSFGSGGGSRSGRTRMRSSGFGEARQREHTPEISTVERPLP
LSLEELYNGVTKKMKIKRKTFDETGKRVQTDQILEVPIKPGLKKGSKIKF
NGVGDQVEGGRQDLHFIVEEKEHVLFKREDNDLIHTVVLDLKEALTGWKR
TVTTIEGKQINLDKSGPTQPGSEDRYPGLGMPISKKPGQRGDFVIRYKVN
FPSSLTAAQKQQLREIL
>Mb|QLI68684.1
MAAPKKKIAVMTSGGDSPGMNGVVRAVVRTAIHMDCDAYAVYEGYEGLVR
GGNFIRKMEWHDVRGWLSEGGTLIGTARCMAFYERPGRLTAAKNMVLNGI
DALIICGGDGSLTGADRFRSEWPSLLEELVTSGELSEDQVAPYQHLNIVG
IVGSIDNDLSGTDATVGCYSALSRICEMVDYIEATASSHSRAFVIEVMGR
HCGWLALLAGVATGADFVFIPERPQEHDWRKDMRLVVNRHRKLGKRKTIV
IVAEGARDKDGNKIVPEEIKDLLADKAEGGLGLDTRITTLGHVQRGGTAV
AYDRVLSTLQGVEAVHAVLEATPDTETCFIAMNENKIVRKPLMKAVQETK
ELAEAVDAKDFEKAMSLRDAEFADQYNSYITTTNVMIDDHKLPHKHRMKV
GFINVGAPAGGMNAAVRAAVAYCIGRGHEPIAIHNGFAGFARHHDDKPVG
SVRPFDWLEVDGWASKGGSEIGTNRELPEESGMELIANLIEQYEFDALFI
IGGFEAFHSVAQLRKHRDEYPSLCIPICLLPATISNNVPGTEYSLGSDTC
LNELVGYCDKIKQSASATRRRVFVIETQGGRCGYVATLSGLSVGASAVYI
PEEGISLSMLNADVNHLKEVFKNDRGQSRAGRLILVNEKASKVYDAKLIA
NIIREEAHDRFESRESIPGHVQQGGVPSPMDRCRAVRLAIKCIQHLEDFG
CNAHNRVKKDPHSTSVIGIQGSEVVFTPVKALEEQGTDWPNRRPKAAHWL
EVRDVVDMLGGRPDYPRPEKSLTGLIAKDTKRGIA
>Mb|QLI68686.1
MLRQLSARLSPTTRFLARGNAWRRGMATEPTELDQITTLPNGLRVASEAL
PGSFAGVGVYVEAGSRFETPSLRGVSHIMDRLAFKSTSSHTADAMLERVE
NLGGNIQCASSRESMMYQAATFNNAVPETVSLLAETIRDPNITEDEVAEQ
IETARYEIAEIWGKPELILPELVHTAAFKDNTLGNPLLCPEERLGEIKRD
TVLKYREAFYQPERMVLAFAGVDHGVAVRLAEQFFGDMKSTSLPGAREVT
GSETESDTDSASSSSSYTSASSSSSATPRTHIMSKIPFIKNLSTSAPRNA
AVLNNLPADIAAFPAHYTGGFLSLPAQPPSLNQTNFTHIHLAFEGLPVGS
DDIYALATLQTLLGGGGSFSAGGPGKGMYSRLYTNVLNQYGWVESCVSFN
HSYTDSGLFGISASCLPGHTSAMLDVMCQELRALTLDTGFSRLQEGEVSR
AKNQLRSSLLMNLESRMVELEDLGRSVQVHGHKIPVRDMCARIEALTVRD
LRRVASMVVHGTLGNPGRGSGAPTVVVQEAQAYGLTSHSMTWDQIQDRID
AWKLGRR
>Mb|QLI68688.1
MPSSCQELRDALAQCLQESECVMIQRNKASDCLREPLASTLPTKCQQLKK
GYGDCKRGMVDMRKRFRGNMPVTYKTMEGAETGKGYQLYAGKPAFAGGVK
ETSGNEPIPQDWRDVENEKYRAEQAQLLQQQQNK
>Mb|QLI68689.1
MADRGRGGGFGSRGGDRGRGRGRGRGRRGGKSEEKEWQPVTKLGRLVKAG
KINSMEEIYLHSLPIKEFQIVDNFLPKLKDEVMKIKPVQKQTRAGQRTRF
KAVVIIGDSEGHVGLGIKTSKEVATAIRAAIIIAKLSVIPVRRGYWGTNL
GAPHSLPSKQSGKCGSVTVRLIPAPRGTGLVASPAVKRFLQLAGVEDAYT
SSAGSTKTLENTLKATFVAVSNTYGFLTPNLWKETKLIRSPLEEFADTLR
DGKRY
>Mb|QLI68691.1
MVKNLPLGLGGLVMLATSILFLFFIILAGVTDSTPLNKTYFLRAHTDGIT
GARAYSQWTYFYICGDNNQDCTSAKAGLPLGWAWAANARNVPDDLGGSYG
GGTTSYHYFYMWRFSWVFILIALFFEVLAFFSGFLACCGRLGAAIAFIVT
GVALLFHAVATSLFTATFVGARNAFNDAGRSADLGAYGFGWIWGSFVAAL
IALVLFGLGIRGDKSGGGAGGGGLFRRKRSTRSYEGRRVKDEYS
>Mb|QLI68692.1
MGKPLDAASPNPSSSSTPSCLTEASASMTLHPPPPHRYFDEDTAELTNDD
DDDDLPPLYSDHEEFNHNTDSFTPFDPLLPRLPHGDIFETVHPFRRDETS
AYFLDRRLDRDPDFLAGHIYKLCQVPPRPFVKLVGTHTESGSGGGGGDDD

SNSRGSRETKTVTDFDIEIELTHLLYSDARTAQARRSIRTAGNLEKVRRG
TVFATRAPGFGGAGPVPEEGTPALREWCRRYCASASWLRCFTVQRRVEGC
DWDALGARLEGLARAANYRGRVEVRFPVRQARVHVYSDCRVNRWRLTPWV
VALFTFSLAFVLAWPALFLATRRWETVTAEWRMSDGHARVRVRVEEAWYA
VWAGTIQRAMLERRRGLLDQGDLQRTWARGGGGRQGDAVAAAVEAMGVVN
RSFGWGEDSC
>Mb|QLI68700.1
MATDAGLRLSAVLVAIANILVPLSIATFGIGFFPYKPVLPGLAEYEPLEF
GEPPRAPFDRLVFMVVDALRSDFVFSQGSGFEYTQSLIRKGVAMPFTANA
RSPTVTMPRLKAITTGSIPSFVDLILNFDEADTSSTLAAQDTWLAQIRAA
GKGKLLMFGDDTWLKLFPETFDRYDGTSSFFVSDFTEVDNNVTRNIPGEL
ENKDWGLMVLHYLGLDHIGHKSGPRSNNMPPKQREMDSIVKLIYEGLESK
DHLKSTLFVLCGDHGMNDAGNHGASSPGETSPALVFMSPKFKGVLPKLAA
PTQPKDEFDYYTMVEQSDITPTIAALLGFPISKNNLGAFIPDFLSFWPSP
KDQIQILTRNAKQILGIVTAVSGKELFDPKTNSKPCSLEDTDMNALACDW
HRLSKEADSLLGSSTLDPIWLSGMANWLRKAQDLMSSMASNYDIPKLALG
QTLALVAVVCNVLAMTRLGTAGVGNILPLVVVTVSYGGMMFASSYVEEEQ
HFWYWSSTIWIGHLGLGAIRRTRAASSGLRYLLALGVIRLIRSWNQTGQK
FAGEADLVKLFLVPNPPLLWALIIASYFVASLQLMSSIHDLPYVAVTSVT
SVLVSSAFTFKLAFTAEDSPELVTAFAKKMNDAFHGQSLLSRARVVFAVL
LLLSSFAVYQARKGGSKAISSAKLIHHIYAFLAMTQSRATNIPLLLLSSI
LLHLLDFSELTVAEITTTSILFQYASFFAFGGSNAISSVDLSSAYNGISG
FNVVAVGALTFISNWAGPIFWVIATNLLLLEKFRQGQRHIFRHHLVLLTL
FATASVAFVMAACTALRTHLFIWTVFSPKYLYCMAWNLAQHLAINVGLGS
LLFSLGAR
>Mb|QLI68704.1
METELPLPFLVSVAVPPGLANLDGLTREEVSLLGNPFLQATPKIQDKLLR
FFNRHSYEFHAHLDATSLESQDDVIALLDRGARKVFVAPDALSHYTEFGD
RVLPVVSKPDISSASEHGLLVRNFDLSTPESEKFLEAAQSAKVKNLYLLP
TDGAPLEKFIEAAQKANAIPILPSTGLTTDKSAAGKLLVSKVIAKYWKSD
RQDGLIPTVVTDEAGVALGLAYTSEESILEALKTQTGVYQSRKRGLWVKG
ATSGDTQDLVRVGLDCDNDTLKFVVKQTGRFCHLQQFGCFGNLNGIPALE
QTLISRKKSAPEGSYTARLFSDEKLLRAKIMEEAEELCDAKTPQDVAFEA
ADLIYFALTKAIGAGATLADIEANLDAKSLKVTRRPGNAKGKWAEKEGIK
TEAAVPKAALAEKSADGRITMQRINSATVSEAELLKALQRPSQKSPEAIL
KIVTPIIDDVRKNGDKALLSYTHRFEKATSLTSPVIKAPFPPELMKLDPE
TIKAIDTSYENIKKFHAAQKEDKPLQVETMPGVVCGRFSRPIERVGLYIP
GGTAVLPSTALMLGVPAKVAGCKKLVLASPPRADGSITPEIVYVAHKVGA
ESIVLAGGAQAVAAMAYGTESVSKVDKILGPGNQFVTAAKMHVSNDTNAG
VSIDMPAGPSEVLVIADKDANPAFVASDLLSQAEHGVDSQVILIAVDLNE
QQLKAIDDEVHNQATALPRVDIVRGSIAHSVTVQVNTIQEAMRISNEYAP
EHLILQINDAAKSVDQVMNAGSVFIGEWTPESVGDYSAGVNHSLPTYGFA
KQYSGVNLASFMKHITCSNLTAEGLKNVGSAVMQLAKVEELEAHRRAVEI
RIKHMNGQ
>Mb|QLI68705.1
MPALATSKFPFRDINLRVTNDAYTFTSPSSPDAPALVIDRPTGDIRLSDS
NPQTTKRATRVSSIAGILGIIQLRLDKYVIVINKAQPVGRLKGHMVYKVI
AAEILPMRERQIHDPDEDTFIGLLNRFLQRGPMYFSYSIDLTNSFQRQSQ
ADTSNPLWMRTDDRFFFNKHLQSDLIEFRTRGSRSQPGKQAAVDPYILPC
IFGMLEIKPTKFKNTPLTIIIISRRSRYRGGTRYFTRGLDEDGHAANYNE
TEQVVILNDSSSGLGGYAGSSDMQSGKLGSGPGQEMQIMSYVQTRGSVPT
YWSEINTLKYTPKIQVRSTDAALAASQKHFDEQIRIYGDNYLINLVNSKG
RECKVKESYEQMYRSLASAPKEHREADTLTDEKFHTIQPGSRNQEFDRLH
YVYFDYHTETKGMKMHKAYAITERLREELIAQGYFRGVDMPANVDGKIDA
RSLQTSVMRTNCMDCLDRTNVVQSIFGRYTLNRIFEDLGLMSRGAQFRDE
DPAFEFLFRNIWADNADIVSTAYSGTGAMKTDVTRTGKRTKLGALQDGRI
GLTRYFLNNFRDGPRQDSFDLFLGAYDPSATNVGTNLIFVDRRPTLIQSI
PYVLAFSIFIVLVGIFTKPAPDAKVLPMRLFILFWSAVAAWCFHFVWTHG

MLYVNWPRLNPRPFAVDGYTEHYGKARKDAVIGTFVARHERGLSTARYLN
AEEGKKRIE
>Mb|QLI68708.1
MPSFSQRPESPKKGRIIKSAYDSESFESDGSIHVEGLVGSATISPSGRDI
ALASPEGLAIIDLDSPYNPPRRLRSHGLPWLVCDVQWSPFAARDYWVVST
ANHRALVWNLNLREDSNTGAIEHSLHGHSRAITDVNFSAHHPDMLATCSV
DGYVHWWDLRRPRQPVLTFCDWFAGATQVKFNRQDPHILASAHDRFLHIW
DDRKASEPVKSISAHTSKIYGIDWNRTRSTGIVTCSLDKTIRFWDYGKDD
NELEHVIRTDFPVWRARHTPFGWGLLAMPQNEPGDLYLYDRLWGEDSPVE
VAPNPVAVFPGHGDHKAKEFLWRSRGGVSDDNIDHREFQLVSWGTDNELR
LHCVDANILAAVGHVKDGPARKGLNLTRQGAAYKTFRTIDDSASRDRKAG
TMSDHRASTGLAQYKQGGASSGGRSGLNRNLRPAWRGPSMKAKIDSSKHV
DRSQAQLGWMKGITMTKRKPSTDGYRRRASKDHGILSHHHYPDDEWGQPD
TIQEELLRISTQIPKVKWENIDMDNLTLNASLTGPWGVDGEPIFVKVKID
IPAGYPKLNAPTFYIEKTSFMPEDTHKKIMREIHLLAEQFLQRKQNCLEV
TFTYLLGEVDLESSTTFFKNVRDLDDDLDGLADESSSEEDEDIPAGGSAS
MSQELPHADADTTLAPLPNRNIIPPPPRTCGARFSPDGRLVCFFPSKEEK
ARALFSTSAEFYNKERSKGEPFFAGFGRLTYDLPSKQKYAMDEASATDDQ
SDESDSEMSSSSSSGSESTSMHKMNLWYSSNRQLRKTWSEDRSVRSSGGG
TGAGTGTGTGTSRRRFGRPRNLIAIHDLRADLPSKREFAEEYAIFGDGAD
VCEHNAQVAEKYGNAELVDVWRYLALLLTKGIPLEVLGRDHNEASILIIA
RDAISKHSKINKSQVDSSSKSDSGLVGRVKWGQHPLAKEFIMDLFDHFER
LADIQMLAMLSCVFNEPYTDDGVAYVESHLTKQETPLPLKAPSFSLDYFP
TDASGWRWNIHTRSHTTSAATTPGTLHTPVHYPGSHASDEFLWSGDPGTS
SYSCGETPPLKGKAFLGDSDTPQPPMSRSPTSRGILRGNTGLASAFTATL
PKSFGGGGSSSPPNQGRKRPSPAEYIMSSLVPTAANSGTSTQGPANASGD
SGLARTSLSDEDYRRDDLLSLVPISVNVIQADQSIFDDDGWLNTPLLDSS
RSSIYARYRYAYAEMLQMWDQPLARLEVMKFNILRDDSAMAITDGSFHDS
TTLRDGASGTIGHHKTGSSPIVMGKKDQLHNLVASGRGLDVTGICRTHET
QLEPARYTSSHNNIGGAVGTCDRCHRIQTQLQCVYCLEPVDALFPPCLSC
GCASHEACLAEWHASGETLCPAGDECNCVDEASNGQVESWAALRGAMMKG
RDKPAKLHLLPPAAMDGSGDEKLGRGKNDWEQVELNIPSREQGGLTVPQS
PAALSFGRLKKAGTWSRTASLRGPGRRS
>Mb|QLI68709.1
MASVMSEPVSRSSLVDEKVEPESGYASGQSDYSPEEEPSRPDVFLTKAHV
EYLNKQMEPMHPMDILRFCKILFPNLFQSTAFGLTGLATMDMLSKIQDEN
PESKPVQLIFLDTLYHFKETHELLARVKARYPNVPVNVFKPDGMDTVEQF
EETYGQELWNTADEMYDWIVKVEPLQRAYEELKVTAVLNGRRRSQGAARG
SIPIIELDEERNVIKINPMATWSFTQVNEYIKEHSVPYNALLDQGYKSVG
DWHSTSPVKEGEDERAGRWKGKSKTECGIHNKKSRYSKFVAEMEAKQVAP
VS
>Mb|QLI68710.1
MAKFKVPSWLQTSTAAEAEKEKKKQNRRSFGGFPQNIRTRQHVPAATRPQ
AVHEDPKPVAKTQTTETTRLVTLAKRIAAEAEKLEQYLKENDLPQPGFGS
DAPGDFPNLPPEIQKSRQDIVYATRELGTLVRGPRESVRWGVWSYLDTLS
LQIINNYGIAKLVPLNTPIPLSDLQEKTTLDPINLARILRHAMTNYIFCE
PEPGLIAHTAASRKLAEDGALQDWVGFNSEDIFPSAAKVLKSLKEHPEAT
SLTTTGFNYAFDTVDQEPMFVTFGKDPARAKRMGGAMASLTGGEGYEVRH
FVDNYDFSQEDERGGTFVDIGGSHGFVSVDLAKKWKNMKFIVQDLPKTVS
SAPKPISDDESVAERIQFEAHDFFKEQPVKDADVYFFRWIIHNYSTPYAV
KLLKNLVSALKPGARIVINDHCLRESGSENPWDEKLMRSMDMIMLTLLNA
QERAEQEFRDLFKAADERFVFKGVTRTPGCRMSVIEAVWEPTKPTANGTA
KHADSESTE
>Mb|QLI68711.1
MGSFVEIVAVICLIWAVAVVVVQGIGIAAIFRHFSRAASPPVSSKLAQDA
PSITIIRPVKGLEPRLYECIASTFQQDYPADKLSVRLCVEDETDPAYPVL
QKLVHDFPSFDAQVLVESRDPILHGTKGRVDNLGPNPKVRNISRAYRQAK
EGDIIWIIDCNVWVASGVLGRMVDKLMGYGPAGTATTPYKFVHQMPLVVD

VVDYSRPGTEDNQVLLSSPSEAGASSSTAKESQDLLTRICRHGGGRLDEM
FMATTHVKFYGAINSVGIAPCIVGKSNMFRKAHLNRVTMESSNRMLRDGD
ERPKGVDYFSYNICEDHLIGDILWRSAIPGYSNHGIVWGDLVIQPMAGMS
IVSYAARRVRWLRARKFTVLAATLVEPGIECFLCCAYFAFALTTLPWFHE
TLHIPQTMSAMALIWLSAVTAWMVVDWQTFKLLHSGWSVQCGRDSPQFVR
GTSQAGGMPRRGFLEWLLAWIGRESLALPVWTWAVLCGTTVTWRGKSFRV
NMDTSVVEVAGGVTRKSSRTPELERSRQSSKDRLD
>Mb|QLI68714.1
MAQNHEDMAAELHEEFNERVPNKGTDLTGGYIEKPSLDQKRDLAVGDTET
GPTPDGEEPNEHERHTLRRIGENLPASAFMIAIVELTERFTYYGAQGLFQ
NYVNNAKDGSDGQKGLGMGHQAAFGLNLFFQWFCYVTPILGAIISDQYLG
KYKTILIFCCIYWVGLLILWTTSLPVAIENGAGLGGFVTALVVIGLGTGG
IKSNIAPLIADQYQRRTMAIKTEANGERVIIDPAITYQRIYMVFYWCINV
GALSLMATPFMEKYEGFWTAFLLCFCMFNVGIAILIFRRKSYVIRPPQGQ
IITDAFKAIGMMITTRSLDGAKPSWRAEHGKSGKVAWDDHFVDELKRALR
ACKVFVFYPIFWVCYGQFSSNFVTQASQMEVHGMPNDFMQNFDPLSILIF
TPILEKIVYPVLRRRGIELRPITRITIGFWLAALCMAYAAIVQKLIYSAG
PCYDHPRNCPAGLSAENIKLPNHVHVAIQTPAYVFIGVSEIFISVTGLEY
AYTKAPPSMKSFVQSLYLFTNAFGSAISEGFVPVAKDPKFLWMYTGIAVA
AAVTGCLFYLLFHHYDAQEEAMYALDRDVPVLTLPGQEQTEKPGDKEVDP
K
>Mb|QLI68724.1
MVHRIAFWSCFGLAVRFWQVGIEMRPFFNKSSLWAYPAYALGGASFGYWL
QGIDDKQSAMLSERKAFLLEKRKRKAEREAGDARGDAAALQ
>Mb|QLI68725.1
MTNPNTININDIVFPSQPSLSTLLTSLKRSTLSIHNRLTSIVSDAEFVHQ
AAEALKQPPSNNNANQETQERAPRPLVANERCGSWYIPPGDKYASAYFKS
TDGHERAWKFSTRRLNLHLLDMIEKHDGIIIVDSTRRGKRMPDALSTTIP
IWCTVLNTVLLPSHPRSSQLFLPPHLLPTTHAQITALVPQFVASFKALHL
QDLPKITKPLRPFWVTQNSALGPETHALPPEPSAEAQDQTAPGTIYASYR
PVICLTASRRNTSSSEIDSNGYIQGAADDTENWAHGLTPAAFWANRDLLL
STPEPDLPGLIASLSLSPSPAAQATPASAPDNNPTTRLTPQISVCALPPP
PGAGCCISLTSATTPRDQWAKSAASMEVGLGRHKTASRNLRTALPYICDF
ASACLQRDPQARVTVACESGKDTSVGAALALSCYLFDDEGQFRVPDETAR
FTKTLVKMRLARIMTAYPEANPSRQTLQSVNSFLMDWTK
>Mb|QLI68726.1
MSNSNLFRPIAFGKGVLQHKIVLSPMTRFRADDNGVPLPYVKTYYSQRAC
LPGTLLITEAVAISPRAKGFPNVPGIWSPEQIASWKEVVDAVHSKGSYIW
LQLWATGRAADMDTLKANGLELVSSSATPVGPDEPTPRALTEDEITSYIA
DYVQGAKNAVFGAGFDGVEIHGANGFLIDQFLQSSCNQRTDGWGGSIENR
SRFGLEITKQVIDAVGKDRVGMKLSTWSTFQGMGTMEDLVPQFQHFLTHL
AEMDIAYVHLANSRWVEEEDPTIKTHPDIHNETFVRMWGDKKPILLAGGY
DADSAQRVVDETYAKQNNVMVVFGRHYISNPDLPFRLKMGLDLEKYNRDT
FYIPFSDEGYLDYPYSEEYLAQEQGVAA
>Mb|QLI68728.1
MSEPDTKRVKTANEAPYELIYWPGIPGRGEFVRLLFEEAGVSYTDTAKSG
GDAVPTVLKLMAADNKGDDMNPPSFACPALKHGDLRLSQTPNILLYLGPK
LGLAPTEGNGIYHINQIALTILDCFSNEVHDTHHPVASSDYYEAQKPEAK
RRAKSFIDERLPKFLAYIQRVLDGKASGDGPWLYGGKLTYADLVLFQGID
GTEFAFPKTMKKMRDSGKYGGVFKLYEAVKERPNIKEYLASDRRSQYSDG
IWRHYPELEED
>Mb|QLI68731.1
MSNSLDQLKATGTVVVSDSGDFASIAKYKPQDATTNPSLILAASKKAEYA
KLMDVAVEYGKKKGGDIDSQVDNALDRLLVEFGKEILKIVPGKVSTEVDA
RLSFDTKASVDKALHIIDLYKEQGISKDRVLIKVASTWEGIKAAEILQRD
HGINCNLTLMFSLPQAIGAAEAGAYLISPFVGRILDWFKAHNKKEYAKEE
DPGVASVKSIYQYYKKFGYKTIVMGASFRSIGEITELAGCDYLTISPNLL
EELINSSEAVPKKLDASQAASANIARKSYLKDEALFRFDFNEDQMAVEKL

REGISKFAADAVTLKDILKQKLA
>Mb|QLI68735.1
MAPSGPITTPLTTLLGIQHPIMLAGMAHTAGGELAAAVSNAGGLGVVGGF
QYTPEQLREIISEMKARFKQPNLPFGVDLALPQVGGSARKTNHDYTHGKL
NELVDLIIESGACLFVSAVGVPEQEVIDKFHKHGILVMNMVGHPKHAVKA
LERGVDMLCAQGTEGGGHTGDVANSVLIPAVVDVARRYKPAMLKGQTALV
LAAGGIHNGRGLASSLMQGAVGVWVGTRFVACTEAASAEEHKEAVVACDF
SETDRTLVLTGRPLRARLNPYLKSWQVRGNAAQELCDKGIVPIEHDFEND
REDIEFPHLMGQVAGAITKIQPAKEIVDEMVAEAVDQLNLANVYLSSKSK
L
>Mb|QLI68738.1
MIPRKRQRPNPAEASSISLPTTALSRETATSLSQSLSMHAPSPAAKAPQP
APQPDHPDHPRRPRQPAASEAEAASTRLNVLVGPQLLPSLPPSIPPMPPS
QSTHPHGAPPTHPLAQKQVRKAKSWYGPWPKTPRKATALTSVARENILGG
TVRSAAAPNLDRFDSSKKSAAETASIRGDSRSVFLSKVFEDQMPAVATDT
ATVDDNAQHEETSIPQPPHTPKDIHATKDRNDTKEDEVIKPQAETSNSPT
APPAASAAPPPPSGWLVWWTKTPTTEQQTVTVTSTTLDNTDNPTPENRPF
TPPEQTAPPDSVPREPPPSAPTVSSTWRFGFWSSTNNKMDKTDPEKEGKV
EGAQPKEQQDIAMEDAPAPAAAPENEPSPKAGSTWAFWSRASPSKSSSKI
SSSGSGEMAVIGEGSEAHPKQMSEVDVSSPQSKASKQETQAREAQPQAKS
TWRRTKRVRPSSIEAATDSDASTATRTATPVIEEPQGLPPPPKEPKPPAP
STKAESSSKSAAEAESVAKQPPNLLLPSFTSTYQMKENPSILQQITQLLL
RTSQPPANHVFRVKDPPKIRKAIAIGIHGLFPATYLRPMIGQPTGTSLRF
ANLCAEAIRRWTDAHGSPDCEIEKVALEGEGRISDRVDNLWKLMLNWIDH
IRRADFIILACHSQGVPVSIMLLEKLIDMGIITNAKIGVCAMAGVALGPF
PDYKSSILMGSAAELWEFGNPQSTNSQRFEASLKRVLDHGARVTFIGSID
DQLVPMESAVYSPASHPYIYRAVFIDGRIHAPDFIAHLVGFALKLRNLGV
TDHGLVRELSVALAGSLYSGEGHSRLYYDPAVYDLAICHALETTSTPSPV
TCEVGPRGGLASANPYVLPWIMRGMLEESFVKTELSSETEDLLQQFDDWK
PSNKALKDVKYRLEAVRSKL
>Mb|QLI68740.1
MADKQAETPAPREKLEAQIKSADMTEDMQQESIEVAQEAMSKFTIEKDIA
QHIKRTFDERKGPTWHCIVGRNFGSFVTHETKHFIYFYLGHCAILLFKTQ
>Mb|QLI68741.1
MSSTLLPFLYQTRTLQRAICVQPVTQFIRLAHAPARPKSRRRDNFIPFEW
DHDTPYEEIANVPGQQSTITPSEAEIFKGIFDDIAQGKMPVAKNRPTPSE
GMQIQSENSQESKPGMARSIVEQARVMEFREKFLGRYPPSLRNAAQIALG
LYELEPSDSSASEMVELEEADQAKWEERARYERARVEERERVDGLMKACT
TDGELWAVMEKEVFSLPAKLGIWQRKAVRAKNKKVKDTKKSATNTDGGET
MESTRTADSKQTAEAAKKTKRKAKGEAAGNTEPKAREESTRNTKAKGPEK
SAAAGKPATQAAADEEKRVMDVHGPLYPYFINTAMGLFDCAFARPSDYAF
QILPRVKDLGLPSYVLGVSTPFYTRLARMHWNRFGDASSALDVLQEMNSA
GLYADKDVYDLLITIRDHLHGCTWGAQGPFVMGMMEAPPYDGMLTQRLEE
MERYAVQSMSPTGEQVEFAA
>Mb|QLI68743.1
MSRSIARRIYADAFAKWPKQDLRPDYQLQDVLKAAVEERYKNYNPAMEAE
ETLKARALQFLVQDKFNNRYKLKGPMLEPKSQPTYFQDLVREIEEAPRRT
WLERLGKRLSGMIRLQ
>Mb|QLI68745.1
MATSFSPIQRQDVTTLVRLVQGNQLLNRQLSSICQVNGLKSTGVKAELQR
RIVDLIQDTVNSNDASRFQQVRQSITNAASQRPSPSSKACTQRSTMTTAA
GLPTTQFAPPMTSYNQAASPYQQRGLHGSTSAVNGRTSNNPFTSSTGNII
FHPSPFYSIETAVSNLRTCETMAQHRNSINIPIKASDHPALLQCLTDESY
RVMIFCAGDIYGVQNVAFPHQSELKVNNQEIKANLRGLKNKPGSTRPVDI
TAALRLKLPLYINNVEFTYALTNKKFYLVANVCKITTVKELVSIISTRRR
IPKESVVSELNKKAQDPDVVATSQVLSLKCPLSYMRLDVPCRSLSCTHIQ
CFDATSYLQLQEQGPQWLCPICNKSAPFEQLAVDEYVRDILANTPKDLEA
VTIEPNGHWLTKASRDENPKSSNDPAFDDDDELEISEINIVGRRLETPKT

ITPITGTPASGGRDSSASAPRSVPPNSAKRPATAVIDLTLSSDDEEPLEK
PRKRQNTAANGFQNSSSMGFLSESPGPSAPIQQHVYSSFCCRYTPDREII
ALPASTTSIPSCHRFVQKQQEHLIAAIAMSLDPVMPPFNSSDPSANFLSS
SCLDFLLIELVPLAYRVTNERDSTQDPSEPAGAQTAEAASTSHRGASLGS
GGAHHASSSGTRKLDDEEELDAVHFRLESLGYRVGQGLVERFSRDRPRFN
DTLDVIKFLCKDLWSLVFGKNIDNLKTNHRGVYVLTDNVFRPFSRMSTVA
GGQAVVRAQPFLWFPCGIVRGALAALGLRATVQAEINELPGAVFQIKTLN
SKP
>Mb|QLI68750.1
MASALFFLDLKGKTLLARNYRGDIPMSAVEKFPILLSEAEEESSAVPPCF
SHEGINYLYIRHNNLYLLALTKRNTNAAEILLFLHKIVEVFTEYFKALEE
ESIRDNFVIIYELLDEMMDFGYPQTTESKILQEYITQESHKLEVQARPPI
AVTNAVSWRSEGIRYRKNEVFLDVVESLNLLVSSDGNVLRSEILGAIKMK
CYLSGMPELRLGLNDKVMFETTGRTTRGKAIEMEDVKFHQCVRLARFEND
RTISFIPPDGEFELMSYRLNTQVKPLIWIECVVESHSGSRIEYMLKARAQ
FKRRSTANNVEIIVPVPDDADTPRFRTNIGSVHYAPEQSAIVWKIKQFGG
QKEFLMRAELGLPSVRGDDEHGGGMTGGFGGSMGGVGGMGKGAKRPIQVK
FEIPYFTTSGIQVRYLKITEPKLQYPSLPWVRYITQSGDIAVRLPDAV
>Mb|QLI68751.1
MSDSPPPSPSQHATAHAYASNGLEILQAASDAAQAIQNPQALQHAAAAVA
AEAVSQVSQVSQQPQGVGMPGLAQGPDMAQQHLVAGTGGIMRDPTVNPKL
TRLRRACDMCSMRKVKCDDSNIPCRPCRELGVECTYERETKRRGPPNKHA
EAAKAAKRQRLEMAAVVFPPASPSPQTAAKALMNISSDGILDAESIAPMP
VLELLVDDFFTYIHPLAPFPHEPTFRQSFANREDRTRPEFLGLLASMVGA
LVASFPRSAREHLKAQHSTHLFPKAIVLVEKCRDIALLTRGSKWILKQPK
TLDDACTSYFLGLAGAYTLQWNPARHFLAETLILMRELGFMRPKHAGDLP
TFGTDPYSTEPLPFNHVKDQIGKRLFWCLLLGIRSMTQLGITGTDLVIAP
STPSLPYPSYPENVDDICVLANEIIHQAEGTVTLLTGFRFGIDIYTTMNG
VVSLELAYGMNTLPWADQRILLRDGLMAAKGIVDNLPPELQLSAQGEDAN
SLATLDESGLQYVPPVWPNAQPSHDVRNVIKTQPAQRRRLQYEIQKANIF
MSQLATRSYFVELYFNLRDVHLADEQNNPVGDRSEEERALQDADDKEIFA
LMAGERELIVQNLLEVLGSISQRNMEPNGLSLINKIRQVASTLLNDSADR
KGPVAIKSEEALSQLIDILIKLEKTGPAAASRAGDPNHMTAEDEEEELRH
WADLREYQLRFAASGGYAGVGI
>Mb|QLI68753.1
MSKSWKATLKLPKSSFPARPNPKLQRRHLRDCSDDLYQWQTNNRPQNDQF
VLHDGPPYANGPLHIGHALNKILKDMIVRSQVQNGKRVVYRPSWDCHGLP
IEMKALGSNTAGGLSPVKVRESARKLASKTITEQMKGFQSFGVMADWENK
WTTMDREYEIRQLRVFQKMVRHGLIYRKHKPVYWSVSSRTALAEAELEYR
EDHISHAAYVRFPIVADLSSIPALAGFDGPIYAAIWTTTPWTLPANKAIA
VHDKLLYSLLRVGDYGLLVGSSRTDAMRELLPPFEIVADSIYGSEIAGLR
YRNALLGKSAAEQPIVHADFVSADSGTGLVHMAPGHGQDDYEACARLGIE
AFAPITDEGFFTQEAYPDQPDLLTGAPSILEGGGKAVLELIKEDVLATHD
IQHKYPYDWRTKQPVVVRATAQWFTDVGRIKEGSLKALSNVRFVPESGRS
RLESFVKGRSEWCISRQRSWGVPIPALYDEAGNAVMDEETIEHIISVMQQ
RTSQAWFSDPIDDPAWVPANLQGGYRRGTDTMDVWFDSGTSWMEIGQPVD
VYLEGSDQHRGWFQSSLLTYVASQKADGVSDADIQPPFRTLITHGFTLDA
QGKKMSKSLGNTIVPEEVIDGRLLPPLKDKSKGGSKGAPRFDALGADALR
LWAASCDFTSDILIGPSILQPIHNALIKYRTILKMVLGSLHESSRSAPLT
KLDQMAIAQLSDTMAQVWEAVNNYEFHKATGLINRWVATDLSAFYLEALK
DRLYCGDGGGALEPIFIGFLRMLAPITPVLVEEAWSHRPSWMVDDVNLEN
PARQLYKSPLIDPSRLTVDPDKLRRDAVSLGIVHAAVKAGLEQAREAKAL
GSSLQCSVLINTENNELATLLDEYADELDAMFVVSHVDVNRTVDDERAWC
YTREFELPAAPGGIVHVLPPRQAKCSRCWRYLAAQEDGLCDRCEDAVADV
AAAA
>Mb|QLI68761.1
MGANASKQEANGQYEWKASGPPSVSVEVLDSLKSSPETDASRAKLIEQHV
QARVAEELKKLQKKESEALSVAHEKIASSENSDENKLSSFSLGKEVDEMR

RKLDERKKVRELPESVENARGAVIRCLREHDRRPLDCWQEVEDFKAEVKK
LEKTWVDKITS
>Mb|QLI68762.1
MGDFQMSLPYPNPSLSQMPSIQNPNISQADQTAPSRARNRHNVSGIRQKV
LMASLLVGLLFSSLDTSIVATSLVTISHELNDFANAPWIILAYLLTYMGF
AVCISKLSDIYGRRNMLILSWIIFIAFSMGCATAKGMIELICCRAFQGIG
ASGLYSLTQIGLVEVGPVHRPSLIGAMIGATLAAAFVLGPLVGGVISQLS
DWRWLFNLNIPFGLITILILTNFWPQEDVADLLSWTAFRSIDFLGSATLL
CSSGFLVFALQQAGSQALAWLSPAIVISFIVSTLSCIVFVTWEIHLARKR
HRHIEPIFPIGLMGKRVYAAGLLVTLFTGFPYICFSIILPERFQMVNNQN
PLMAGVRILPMLSACAFGSFLGGAISSKRNNTSYTLVAASCLQLLGVGLM
TTVSGDSETAAGQYGYQAIFGLGVGLSFSAATIMTNILATEPNERASAQG
AVAQARVLGGCIGLSLCTILFNAHANRLLSGRLTDQELYMLHRSPLSGLR
LPDNLRGLVRQVYIGAFRREIEVIGVACAVMVVISLFTLEKKPTPIERIT
APVKDESSSYRRGSDSGTEINDVTHARHMA
>Mb|QLI68763.1
MVLQDLGRRINAAVTNLTRDQNLDEKAFDNMLKEICAALLEADVNVKLVM
QLRKSIKSTVNFKDLPPAVNKKRLIQKAVFDELVQLVDPHAEPFKPKKGK
PNVIMFVGLQGAGKTTTCTKLARHYQTRGFRACLVCADTFRAGAFDQLKQ
NATKAKIPYYGSLTETDPAAVARAGVDQFKKEKFDVIIVDTSGRHRQESA
LFQEMIDIQTAIRPDETIMVLDASIGQQAESQAKAFKEAADFGAIIITKT
DGHAHGGGAISAVAATHTPIVFIGTGEHMLDLERFAPQQFVQKLLGMGDM
AGLVEHVQSLNLNQKDTIKHIQEGIFTVRDLRDQLSNIMKMGPLSKMAGM
IPGMSNMMQGMDDEEGSAKLKRMIYICDSMTDKELDSDGKILIEQPTRMT
RIARGSGTSVREVEDLLTQQRMMAGMAKKMGGNMRNMQRAQQAMGGGNKA
QQMAAMQKRLQSMGGAGGAGGMPDMGSLMKMLGGGGGMPGGMDMQAMMRQ
MGMGGGMPGMPGMPGGSGRGRR
>Mb|QLI68765.1
MSSSTVMSSPLRSSSMHVSCPRFDDFLPARRLRKALTSKGCSFGLDIPSN
HFAAVLAPFACLTTANSRYHSFALLHVMVLDSQSLEGPPSLQHMTQNGYI
VAPAAASAVPRALPEICYTLRRKVMAFLEQQPADNDVALKNTQKQARASI
KIVEEALRRYQPDELSLSYNGGKDCLVLLVLILACLPALYDPSITTNGDA
PVSKNPPPTIQAVYIAPPDPFPEVEEFVSSTTKEYHLDLTRYALPMRQAL
EAYQADKPAVKAIFMGTRRTDPFCEFLTGFSPTDKGWPQFMRVNPVLDWH
YTDIWTFILRLDIPFCSLYQQGYSSLGGVKNTRPNPALAVGSEGTKFRPA
YELVRDDEERLGRDK
>Mb|QLI68770.1
MGSVRVLLIGNGGREHALAWKLSQSARVDTIFAVPGNGGTASCPKVTNVT
SVSAEDYPGLVTFAQSNGVNLVVPGPEAPLVDGVEGFFKKAGIPCFGPSK
EAARLEGSKTYSKDFMKKYNIPTATYENFSDYQKAIEYIDSVSHEVVIKA
TGLAAGKGVILPQSKQEAKDALKQIMVDKAFGSAGDEVVIEELLAGDELS
VLTFCDGYTIRSLPLAQDHKRIFDGDQGPNTGGMGCYAPTNIATKELTER
IDKEILEPTILGMRREKQPFRGVLFTGLMITSAGPKVLEYNVRFGDPETQ
TVLPLLSADTDLADIMLACAEGYLDNCHLKVEQKFSATVVVAAGGYPGSY
AKGIPMTVLQPPRGSTIFHAGTRLDGDQLKSSGGRVIAINSVGESLRAAV
DASYSALASGIISFEAMFFRKDIAHRAFRDQSKEAMTYAQAGVDIQAGND
FVEKIKKAVASTKRPGAEAEIGGFGGELDLSQCGYQSVPILVGAIDGVGT
KLMIAQAMKKHDTVGIDLVAMNVNDLIVQGATPLMFLDYYGCSKLDLPSA
AAFVEGVADGCRQAGCALVGGETAEMPGMYQGEDYDAAGCAVGAVTSEER
LPRQNTMTEGDVLLGLGSVGVHSNGFSLVRRIVQSAGLAYESQAPWDISK
TVGESLLTPTRIYVKSLLPVLSEIKGLAHVTGGGLIENVPRMLPDGLAAE
IIYGSWDILPVFQWLKQAGNVDPAEMCRTFNSGVGMVIAVDASKADAVAQ
ALSASESVYKIGRLVRRNQGQPGCEVKNLELWA
>Mb|QLI68771.1
MKTSLWALTTTLLLLLGLCRSHPPEPGRSIETTLDRRQLGDVDAESPSHV
YRGEIGRTPEDVEKDGGFYSRGLQKSQSYGGSSLSDVEMQEGSSLFRHAA
GDTAEYTRYVSTSTDPGVSLTFAINDEKPEEKGFVYKIHADKKLVNVNKS
LGKYSPYPAQKEHAAVGFIPFEQIEGWWQVTYLDDFSDPKIGKVTQERLR

NEKFKAFQKNTKFSKNTFQNQRGAGVAPQLAGFPRLSTAWEEDPWKQFKA
QDVQKNLDDLITSACAGKDSCMTQLGHSSKQKSAKVPWTANAADGPDARP
AKPKPKPAAVKSGFRVHGRAGTLVGFSILAPYLQDVLRRLRDWDHPIGQT
VKLFDDGVNAAQEAIGGPPRNDISGNDNQAALINFFKRVFWALQSVTPGQ
ERPADLALLSYGEKSRRRLDSVNDVLRTCERVDAVPPDEQEQQQQQQQQQ
LGAKVHNTCADLRKRAMEIEAPAAAKLVTGQAACLVCGLAWDVTGSRCLD
TTGAVRWPPKSPESYSPETPDASSKCRDATGIIPCGGGQTAAGLKIGRAV
CAICGFAWDAEGAKVANLDFEARVPVPFSIFPSTYRDSESQTAVQTHEEV
DVKLPQRQEPAGRQDQYSSYSAHVDLPPRGEHREHREHRFSEEEVRFSEE
RHRRPGFQQEQFVKEDFRPGRTEYTDTRIEIDTHQQPYSSPIDVAEREYR
ERFRPVYHTTVDAPTRPQYHQTEDVRVNQYTVEERPVQTSVHQQVKFSDE
TVEPARFSQAQQKSNMGYYDEDGHYHSFRQGIHKLADKIAHPHHHHHDHV
DVDVDIKEDIRVTGTRPRPVQSGGSYVPNTVTIPCHHIRLGDFLMLQGRP
CQVIRISTSNATGQYRYLGVDLFTKQLHEESSFVSNPAPSVIVQTMLGPV
FKQYRVLDMQGGHIVAMTETGDVKQSLPVIDQSNLWARLSTAFESGRGSV
RVLVLNDSGRELAVDMKVIHGSRL
>Mb|QLI68772.1
MTASTESNGAAVQPAKTNGGKAEVKILMLHGYTQSGALFRAKTRALEKLL
AKALSPLSLTPVLVYPTGPNRLLPQDMPFYSPPSDPAEEDDYQPDTWAWW
RKDEGSGEYLYLEKGMEAVAQAIREAGGVDGVCGFSQGGAATGIVAAALE
VERAVPEGPAGEWVRKLREANGGRAAKFAVSYSGFWATPESLQFLYRPKI
STPSLHYLGSLDTVVDESRSQALIERCESATQVVHPGGHHVPVSKEWVMP
LAGFIKQHVYDAAPKAGL
>Mb|QLI68774.1
MGSTASLTDKEGRNDSLLSLSDAERDELWTEKNPFFRPADRGSHAWLFLT
ASFMIEGLALGFPGAYGVFQAYYSTHEPFKNAAGLPAVATCATAYQGIMY
LGMPFSMGIQRQYPRLSTWSPVVGVLIMCLSLIAASFSTKVPHLVLTQGV
LFAIGSSLSYCPCVAYLNEWFDKRKGMAYGIMWAGTGLSGSILPFLLEFL
LGRFGFRTTLRFCAAGLVALTLPLVYFIKPRIPPQLQKRHINPFNFRFAL
SQTFVLHMLANSVQALGFFLPSIYLPTYARSVLGSGEFPAAATVLLLNLA
STVGCPSMGWLSDHLHVANCLFIATVGASLATFLIWGFTTSIPLLLTYCG
FYGLFAGSYTSAWTGLMRQITTDNTISAKYRCEDIDPVMVMAVLAAGRGI
GGVLSGPLSQALVEHMLWKGEAYAGYGSGYGPLILFTGVTAAVAGIVLPW
RYMGWIR
>Mb|QLI68778.1
MAWQASSAWKKSLSKASSDDSSFPGLKPTTPDATTKIEYEELQQNELLAL
EAIYGDDFVKHSDVHSAWKKTEPSFDIRIKASIDSDFAVTVGFVMTATYP
KTPPLLTVKGLDDLRESTQFKIQKFVDTEPREFAKDQQEMVDRIVEGIRD
ILEDAAQVKASGKQLPSLEEERERHEAKLAEKARRQKEDEERKKMEETKE
EERVMSEMLQQQIDRQRQKAKESRLSRRPNGLEGLPSDNSAASIERIDFD
QFCHVKNKEGNILSFKSVTGKCDPREGKVTVVYTVRPVLGNGQGDQTLAL
KEAILRPNGRDPKEFKKQLQSVESRLQELKNTKSIHHNRLVEILDFKVES
GKSDDAAVANFWTVRILMPMAVKGSLEELLDLAGHLELGKVRSWTRDLLD
ALSFLHSKNIAHQDIHANNVLLFRESTGEFVPKLSDCWYQREIHNASTPK
PGLPGLTSAKSAYWLPPEIAGQSKPQYSYKTDVWEFGIVFTQMIFGLDVL
QKYSSPRNLMQELTLSQPLEELVSRFFKEDKQKRPRPFELGSSEFLATDA
PVFLESDSSRALSATPSLTSLHVVPARLRRDSMSRGAVVSRYTEDFVEEG
RLGKGGFGEVVKARKKLDGQIYAIKKVTQRSHASLTEILKEVRLLSQLSH
PAVVRYYNTWVEEVPDSTDTEGDTSTEGCTEETEETVSDGIDIQFATSTG
GLDFMSSNAAIEFGYDDDSDDNAIEDDSDDDSSIVADQAGNGVLSPGKER
AGQILRRARQHRAFRTILYISMEYCEKRTLRDLISRNLYKDTSEIWRLFR
QILEGISHIHALGIVHRDLKPENVFISSSLEGVDNVKIGDFGLATSGQFS
VDKVAANSLENDSMTRSIGTAYYSAPEVRSAGNGLYSAKVDMYSLGIIFF
EMCYHPMIGMEKADVIGKLRRPKPELPSDFKPAERNQTDIVLSLVNHNPK
ERPSSADLLKSGKLPVQMESETIRRTLAGLGDPSSPYYSKMLSTLFARPC
DAAKDYAWDMLGTQPSASELLNQGVVRSTLISIFRRHGALEMPRTSIYPR
SSQYGENVVQVLDPNGTVLQLPFDLTMGNARMIAKQTGGPILQRSFYFNN
VYRDKLDTGQPSVFGEVDFDIVTTDTLDLALKEAEVLKVLDEIVQAFPSL

SNAQMCFQVGHSDLLQLIFDHCGVESSCRRQAAEALSKLHVHGHTWQKVR
AELRSTTVGVSATSVDELQRFDFRDTPNKAISKLKVLFEGSDLYQRAMPT
MAHLKEVAEYCKRFGVISKFYVSPLNSLRENFFMGGIMFSCVYDRKTRDV
FAAGGRYDHLIKEQRPKIGGNFEERHAVGFSLAWERLARVPNKSGSKTFL
KKQESDSNTMFSGRRCDVLVASLDAAILRSTGIEVLQLLWDHDISAEMAK
DARSPEDLLSKHRNENYTWIIVIKPEAMLKIKTMGRKDVQDVDIPLGQLM
AWLRPELKERDSRALAKLRGNVSHGSESSGTHNSDRNQDQDVKVLVAGTK
SKKFNRRTVMDQAQVSASNLMRSYLDGPILAIETTDQVMDLLRETRLSEH
ESWKSVEHAVTMTEKKYIRELHSQLDTWRSTYEMKNISKHAFIYNFRTGT
CVYYDLSA
>Mb|QLI68787.1
MVPSQSLDCLYRVPTPRQQARFQRRPKTSVNPLYFWPAFRQYRNRQAHKD
TQKDKGGVWRRPELEDAFVDSVLLMPHMGRRKFSMGGKLHGRNMLISEYI
FVICIALLGSKEIFRIDNSNESIEQMGRKQVSSHMQVVKKFFEDLRCFHF
LFPSEEKKEPGSTNSDDGYDEEEQESFKSNPVLTALAEGRVPDVKPNYEY
FSQLLALQSSICTRPKTCEIFVSSSDIKIRDDVAYDAHDTPLDQASFPHL
NKYTNCDDSPNVLGKDVLLHEYTRSLDRTTSACAKSVTRRWQNDAPAMFE
TLELPSRDEECLLLEMCATLELHEHAKFPSGSELTGFVEVAITQPALQNH
RWKCITRLTRPAELHSDEGKHGVYTNDSGIHRRGCSDSKAECECHTRPRQ
DIHVPFPAVEWASILSMAVQYPDVEHQRLKEKRRKYADGDGKKELERAGS
KRKRSEDEGDAASWTRRELTGSDLICKVAMYQELWSCAPDSTQWTRQAII
FWRFNTTNQWHKYNPVFKPAGTTWRWLTINDPMSRYHQQKALVYPTANVS
RDALMSPTPTIHQHLTAAMNETFNSWDQGSSAPHVPHVPPLQTPNNSIGL
FDSFSNGLATPPPTATLPPAYPTGNFDSRSGSFDARSASFDTRSGSFDGN
LGPNNGVAFLPTTGPPPQHERQPTALGNNNQPFFDGQQSFSEVKPINTAV
NSYMTASTSLELPNQLVYDNSGVDASNMQGWDMSALDGWSAAASAPGSAT
AEWGPNTKVESHAGDHHGAMWAPPHWPLSAGAAPSSHERGASPRPLKRRR
ETMDVHVPVTAGW
>Mb|QLI68789.1
MVKAVAVLRGDAKVGGTVTFEQESESAPTSITWDITGNDPNAKRGFHIHT
FGDNTNGCTSAGPHFNPHGKTHGAPADEARHVGDLGNVNTDAQGNAKGSV
TDSQVKLIGPHSVIGRTVVVHAGTDDLGKGGNEESLKTGNAGPRPACGVI
GISS
>Mb|QLI68791.1
MALQPCRAHFPRARCALRALMPSLRTYATESTSEPFKASSQESSVPRWSQ
TPPGMKAPMQLDFAKSFKNKVWKVNNDPAKLDNMYNRLLGPGGSKMLPEE
LKWLAVTHKSFDQGRRGFNDKLALLGRLTLVMEATKEIVSKAPLPGSRQT
DEFDRVPFEHEQLASVDNLSVEEPKDVVSKEKLFSLASDAGILDVLRWKP
RLPKSLESSGVEVVVNSAVLAILGAITLQHGSVVASKVVRERILARLSKN
H
>Mb|QLI68793.1
MRSIRANRLPLRPLPRYTSTASSCAPKTTALSSVLIANRGEIAIRINRTA
ERMGIRATTVYTDVDAGSWHASTGYQSLGLGPASGYLDGDKIIALAKKHG
IQALHPGYGFLSENSQFAEKCEREGIVFVGPPATAMADMGNKARSKEIMT
AANVPCVPGYHGPEQGEQELLQYAKDITFPVLLKSVRGGGGKGMRIVMTE
DEFMTQLKSARAEARASFGEGGEVMLVEKYIVRPRHVEVQVFADKHGNTA
ALGERDCSVQRRHQKVLEESPAPDLDEATRQDLWDKARKAASAVGYVGAG
TVEFILDKDSGKFYFMEMNTRLQVEHPVTEMVTGLDLVEWQFRIAAGEEL
PLKQAQVEEQMKKLGAAIEARIYAENPEKGFMPDSGKLVHAVLPESVQAD
PDVRLDWGFGSGSTVSEAYDGMIAKLIVRGENREQAIAKLAAALRAFEIV
GVSTNVEFLKRLCYSPAFIEGDVETGFIDKWRDELFKPRHIGDELFAQAA
LGVLSLQLNGAEPHGQTLGFGDASASSERKLAFKVLDGYSEKEGEIVEAA
VTQTGHNLYNVTVSRKGQEAPAVFNNVASEHTPEGAVMKLQTYFPAERIL
SSVVPQTQDNDTKITVFQHGVKTDLSLLPPSWYEKALGLKEIAASVVAPM
PCKILKNEVVEGQEVKKGTPLVVIESMKMETVIRSPQDGVVKKLAHKEGD
ICKAGTILVLFEEDPAKGEA
>Mb|QLI68794.1
MATDHAPPPPGTPPSEVPTPGFDRKFESTEETFYFYHVIDTEKATLQCQV

LGPNVSNTFSHTGAITHYKASDIEFALARKTAGFLALLQPGTSPSRTILA
WAESGHGHNPRGDFLEPKSSVLSNRRWTARAMRLAREIGINMGHPYDRLT
LGGKVGMFRASHVEVKLAVHAVYILLKMAAMPTDRVTKADLKRLRGRAWC
GGARPRFEIYFSKKNCRACAQYVRRLEELTGAEITLCWKERLVKIEYPKS
KMGSVEDAQREVIPVEGSESEEQAQAQEQEDVNEMPMVDLTDDTDDVGSQ
EVSEVVEVTPEPLGVYVNGLAYCVGQMGDRGRVMRAVVGLAKIWKRQTAT
RASMRRRPQNSVSGGEPTTGRRRAWLATPPASNVPVQTQGTARAGLESRG
AENEDAVVGETWPRQYSFVCRDKMSLGR
>Mb|QLI68797.1
MAKAIEAKSLSDINDTANNPPQYFESAAGAKKQDPLVLYISRVVGLNVVI
LSPVIPNERVVSAEDVKSALYLVHVDNPVPAPVRGEAECRSSQDTRRLPQ
SIIPRRPVGGSDRPLTPESSPSASQSHQPSAIEAPGRTLYGLHTAMPPKK
PVGSQSSPRTSTETSASAQIVSKDSSDPQEPSGNTQTHCINSISVPRPDT
SLPPTNSANQSSLSNSQPISQAHSRSPSPRKHSTASTDMPLTLTLTRQNP
AAQKWTVGYIRLPELESSSGDGDTASTSTTSPMQHHPIDIEIIQSGYAKF
REHCVRKYSPTESGDLKAAAGSTARGSMDHQSSMYGNKEAFYRRVAMNYS
NSLLTNAKEFARNLTRNRSDSAASVSSYKSDTAVKPDTICTFGSPPEGMK
ARGYTFVSPWEGQCHFSTGKHGNSLQCFYIPPATATVYSNSLVKTSHSES
TLISTLSYNLALSPVQAKGPTGVLGKVIDCCSKSRKSNIPSDSRYESSFT
SLGAERAGGGLNGREAKLATLTIYDDGLKMLDLLVAANIGVFWRTWEKFA
PASTEQKHVSEAQIPFPSIEDHS
>Mb|QLI68798.1
MANESAKALKALHVPSKPIIFANIWDVASLNAINSLNTPSSKLVQGVATT
SWAIAAVNGQLDEDLTAEQTLESIKRIAPFAAKAGLPLSADLRDGYGSRI
SEVVTDAVNAGIAGANIEDSIPSPLFDKGLYPIDEQVQRLKTALQAAEAA
GTPDFVLNARCDVFCLEDESLDDETRLREAIERGKAYLELGATTVFYWGG
PRGGMTKHHLETLVKELDGRVAASFSAYPGIKTTKELADMGVARVSIGPG
LFKVAMGAVRAAAENMLTGGSPAP
>Mb|QLI68800.1
MSQERVNPFSDPISEDDIPQPQNLETRDTAKSLEHPSWKPSQDSGSRISH
YPKPILQDDDNTNSEWETVAPDEDVILRPSVHLRQRLEIDRRAYPTHNAL
GCASEAAEHAQYRTALMPTSSSRNLARTLPMHTSNRPKHASQWLRASSVY
EDDSGFPKITDQSQQQGCQPETSRALTKNRPATLGFDRNAQGELVASYSK
SSSRASNDPFKFDGAGYSMFLQSSAEKEVSRALYGAGTWSEVHSTPVYTP
QRSERRVFQGQFHTNEHPLSELAQLVNNTAAEKDTGSDWQTITTEQTTHR
ALELTLRLDKDVGSSLADVSDGSEYESFDHFLGATAGSQDSRESHQGGDV
YSNANPRRAFFYRFSQPTCRNSRVSSARPVATVRHLSHLSRLSSDTSNKP
KFSSKLSQMRKPIMSYSSLDSEPEQDNFDTTTPVSPDTVVEVVTPRHSNR
SSPRCRQDSREILGLGTQDGSMIYKSRDFAPRNTADYNLPSLPFPLISLQ
EAALRQSHGIARDEADYTTAGSMCDSKARPDTISTISSYGPKTPITPVGG
IPGTSGIPRPPRALRRHSPARALPYDPTQGQLNSSAIIDTHSGSFFRNSL
PTNTSLLSARFFRLSGFSARTTRERRDEPKHVFLTSSETDLIHSAREDIL
CRRRRCPEEDAQQRRVFLVIFVMAVFFPPIGALALYGKFDATIAWYGKGE
RGCLTQEQRSTLKQQLLVEGVLYLGLLTSLVVYYSVHG
>Mb|QLI68802.1
MEILDEKQRLAMQNGQPPEIQPYVPTQAVVDCDDGAAALVTDGFLVPSLD
GPNDAEPPAYGEQHNHVRFSQPGFDAGAEITDDGRININLHTKNRRLANL
LAPTIDSQISVEPQAEPALPPAYIPPGLSAEPGQPPPPSLNVVIQIVGSR
GDVQPFVALGKVLKESYGHRVRIATHPTFKSFVEENDLEFFNIGGDPAEL
MAFMVKHPGLMPGIDALKSGEVSKRRRGIQEMLLGCWRSCIEAGDGLGPP
PKPHRINEPLDLAAGMPGDGHDQPFVANAIIANPPSFAHIHVAEKLGIPV
HMMFTMPWSPTRAFPHPLANIQSSNADDVMTNYMTYTLVEMLTWQGLGDV
INRFRQKALDLPSLSFIWAPGLLNRLKVPYTYCWSPALIPKPNDWGRHID
ISGFYFLNLASSYIPDPELAAFLEAGPPPVYIGFGSIVVDDPNAMTRMIF
DAIHLAGVRALVSKGWGGLGADDVGLPEGVFMLGNVPHDWLFEHVSAVVH
HGGAGTTAAGIKAGKPTIVVPFFGDQPFWGAMIARAKAGPEPIRHKDLTA
EKLAEAIKFCLETDTQERAKELGHKIREEAGTDVGAKSFHKHLDIDSLRC
SVAPSRTAAWRVRRTKVRLSPLAASVLIDAGLIAYKDLKLYRPVEYNTED

QPPDPVSAGAASLIGDLSGIGMAIADVPRELFKSRRPKDGQSSETSTLLG
SSSKLTLATTDTISQNESTTDLFSPTSREREGQEESRVPTADSNRSLTEL
SSASQSLVEPRSVARSGGDAESGSSRTPSRTGLRARASSDQLRQTFSRRD
PSPLGISLDATVGAGRSVGRAVSTGVKSPMNFCLGLAKGFRNIPRLYNDD
TVRPIEKVTDLNSGIKIAGKELGYGFFDGIAGLVTQPIRGAEKEGPGGFV
KGIGKGIGGLIAKPAAGIWGVPAYMMQGVHAEVNKMFTKSVQNYIITSRV
VQGTQDLTRASEEEKSDIIIRWNNSKWDLKNYYMLKRKEKTAEKAPAGSE
QSTQSDLPFSRPKTSWLHTRGMSAEERKRLQTQKRGFVDAPVPPSSSSSS
ARPSSSDDAEFEQAIQTSVRETSRGNIEEDAMIEAAIRESIRAMRARGAL
LETLPEDSEEIPEKDLSIFEDEEYQITDEEYQSLVEQAIQQSLASHIEDL
FHPQETGISELDAFTTEIHSAQGSGTAQADEHDADLQRAIEESKNVPTPP
SFDDEEELQRAIEASKQDMAREHNQQTEEDIVLEYVKKQSLAEAEFHSQL
NKGKGKAAAAETDSEDEELKRALEESLKLHRGDCAGPSGSGSRSRSRSAS
RACDEATEGK
>Mb|QLI68803.1
MSSTITTVQPIAVSEPTSHDDIRLARLEDHDEHDTTPMRLSDNDTNWDTK
EGWVVVAAGSAIFFVFLGLIYSYGIVQLHLTESRLASISTLSFIGSVGAA
MSPLTGMIVARIIKLAGYRVTALIGSLFLGLGEFTAGWSTKSVPAMFVTQ
GFLFGIGSALLFLPAATVPSLWFKRKRGLATGVVFGGAGIGSAVISVSME
KLIGVVGLESALKILGALAWAICITASYFLRAPAGRGRAVSSVQWRLFRS
VKFNMMLVMGAIATFPLFVPPFLLPLYVANMGFSGQAAAAILACWNIASA
LGRIGMGFGADAYLGPVNSLLISLTVIGVSAMALWPFASSLGLLVFFAVL
NGIGSGGFFSLMPVVVGAVFGDGQLANVMSMLTTSWTFGYFMGSPIAGYL
LDAYGGAEAGLAAFRPTFFYAGSLTLASAGLILAVRLMMNRKIFARV
>Mb|QLI68805.1
MADQEEHREIAPGSATDGSTAVTTTGEKKIIKKIIRKKKKPARVQIDPSE
FTSEAPPQTGTTYNIWFNKWAGGDREAYQQTKAKGRCIIATDAGYTTADN
VSGSYFCLRFARGICPLGQDCTNLHRLPGTFDLFNPNVDCFGREKFSDYR
DDMGGVGSFMRQNRTIYVGRIHVTDDIEEIVARHFAEWGQVERVRVLNSR
GVAFITYTNEANAQFAREAMAHQSLDHDEILNVRWATADPNPMAQAREAR
RVEEQAAEAVRRALPAEYVAEIEGKGPDARKRRRIESSYGLEGDEAPDDV
HFARGKTAVNPVGHEGLQVEYQQPLMLENGQSTEEPETTTAPPPAPETAG
IFSSSTLAALNKAKVAVTSKPKAAAPTGPLVSYDSDSDDE
>Mb|QLI68806.1
MPIVLMRRKIVLAVGVALVLAALLYTSGHLDTSRFMNTIRPLNKSNGQEK
TSRLHFLVPASVSNLRLCYNLVSAAVNRYPVPTLLGWNGQGELDAAQTHL
AKLRTMKRYLDSLDPEEDDDLVIIVDGYDIIQQLPVEVMVERYFDVAQKA
DIHLAKRMGISVTKLHERDMKQTVFWGPDKICWPQDPAAGRCWAVPPSFL
AENAFGTDKGPHDIFAKDPRWLNSGTVMGPIGDMRKVIDATMDEIAATHN
AYFRHSESDQYYLANVWARQEYWRSKQIANGSEVKGGPPNRILPEKISES
QETELHIAIEYDSALFQPQAGNDPFLGYLEYNNKSYTSRVHVDMFERGRK
FVPYTVQMPNNVRMALMKLYNSIPKTLAGAPAENWVRQVRLATNFVTKQI
YGTWHCTGPKDSIDSEFRKMWFYPFVPVLLRESVRSSQKEDIISSNVIDG
RRWVAKSVYPRDEKLVGEYGGAWSDEEVGGRFIPWKDMCGDHEDFLFWGQ
QDTGIQEDEYMMHT
>Mb|QLI68811.1
MNFQDLNFEYVEAPHWFPPREIVPSVHTTGDGSESARRLRLSQDSHKRLD
YRQSQSFSQRSLSPQPSILPKQPSSASPTHSSPELYGPDRLFGLSGAFQS
GRQPQPDTRALMSNQAVRFDEAQLAMFAADNAAPMDGLKDELALAAGKVT
PGVDDTQYIQYALEALTRERESNTASQIPIPSPPSETYNEGYQPRSPKFM
VKRFAAPPTASARGERLSSHEMVNSPLLHQCQLDTPELPQDRGFSYIPQP
TLPTAAPLSQKPSSTDHWIPVDKNMLQTIDPRGRTYPPLTFKPRILRPFS
MLMLMTLCALMMAALIFSAVYSRKNDGLTPYPGSIYSGQYFVFRILPQLL
AGVVLIYAQNIMAASLRIVPFATLAEEDPEKRYLALFRSLYPTTFLLPQL
SGPWQLKTFGIATWLANFTLPLLSATFTCIYVDGQGQWIWSATQGLIWVS
VALYAILLVAACILMTFWFGHWTGLLWDARSIADLIPLLHRTNTMNSYRR
KGLYERNCDYRAELRERWFDRLGYWQNGDMTGGVWHTIGTSAVPADGGAD
TGAEPRSERRSNNLSVGSHLTASSALLDFEGGTYLPWCLRDVPLVSFILV

TSGLLLALLIVSFLPQTRLESGFAPLLTARPDDAAFSAANFLYSFLPATL
GMVLFLLFQSLDQALRILQPWGDMASLEGAVASRSILADYAACLPLHAPV
RALRNGHWRVAVISLMSTLFVFIPILAGGLFMALTAPTQQVRMFPSMPVY
GVLVAFLVLYVGCASLLVPRRRQFHLPHGVTTVASLISFCTARDLYEDPA
FRSVRSRKDLAARLGVGRKDAREESVWFFGVLAGRDEKRISVRRMKRYTE
KMAMTRSVTSMV
>Mb|QLI68816.1
MTGRGGGGGRRVLLPPINMIFKLLQSNATVSVWLYEQLSIRIEGKIRGFD
EFMNLVIDDAVEVSQVTKTNDKETRRQLGQILLKGDNVSLIQSLSN
>Mb|QLI68822.1
MPSSQQPAWLKTLIADQGPPPNLFAWTPENLDLGAKHNTPAISPTGGRND
LDRRVFILGVGNIGRLYASCLARQPRPPPITLVVHRKELLTQWVQGDGVE
LTRDSVAIKNKDFDIEWWTRTAPDHGPVREVADGKKLRNLFISTKASAAM
PEADRLRGYLDRHSTVVFAQNGMSKLWPPHGQEYIAHRYEAGNAPSFLAC
IVNHGVLSVGPFKSVHTALADASIGPVLVNPHPPPSVEFFMTQVATAPLL
NSKSVSAGELWLLQLEKLVMNAIINPLTALLRCKNGELFAGGELNNPLGQ
VLDKLLAETSAVIHALVNHESSADILASYMDQNQPSKSDIPTENIHELQK
GLAERFSLPYLKKKLYRFGVQVGENRSSMLQDVEAGKPTEIRDFNGWLVD
MAAFLDQRLDVSTHRALIELVEGNVILNQAELANRLL
>Mb|QLI68823.1
MEDPSMMDTESRTSDRDTSQLSDPPSDLDSSDRDTPDTPAQQTEWLATTR
KRRSTAGNRMKSMLANEEPDSDLELLFAEDENDQGFSDVGEDGSDVHMDS
SSDDEDDNNANDDGLEGEKELERQAKEKRAAQRKRRAQEAIPAKFRKKVR
IDPTTRASVPAPAPRPKKKSERASWLPSPADLPTRASSRQTTRISKEQLH
QQMAEREARRLKQLAQMEKKAARLEALKKPPMTQEERLAEAAIVEKRNSK
SLNRWEEAEKQREEERKAKLAALNQRTLKGPVITFWSGRGQWDDVELRSL
RPYVTEVEEKPKKKRERVDKTAKGKGKDKDGGKTDEKKVSKSTDTGNLQG
AIRAEDQNSETPKAVGNSTIDKPAMSDDRNMESGSAIAKDNKPCVPPKAD
AGTIEAVNLPTPTEASPAPAADDASASREVSGKEPNNEPMDAKPEITDDA
ALNAQEKVIIVGGSTKSSRESPTASTPILKEETVASTDSTVPQPPASAPA
PTPSYSPASIQISTAPSSNRPSGLAAPPPPPLASSLAATAGPTRPPEARP
SSVLAAPMLVPPPGIDVNVPSPVHAASKSNVLALPNTIQSTFTPPFVQAV
ESTTVAGPSASSATATATAKEVDAKPTMPADSDLQPARNQAAHKGKSGSP
IESEAPDVPEVPRDMNATRNGIIYQNFDGNAIRDKNIQTQILFGRKMTKL
AKPTPAPICVITNNPARYRDPKTNLPFYNMFAYKEIQRLSNGDYQWSRIL
GAWVGNPKHAARGVPERFLNPGSSGKLKTPERHEDSKIKQRERPEKNKEE
GQEIHVAAAGNNAAMKTEQSGGPGQTAQDIHVVT
>Mb|QLI68825.1
MGISRRPKDKAVRNAQSGAPGTQGAAGGRPKKATFEATKKKEIGVSDLTL
LSKVSNEAINDNLKKRFEGAEIYTYIGHVLVSVNPFRDLGIYTDHVLESY
KGKNRLEMPPHVFAIAESAYYNMKAYNDNQCVIISGESGAGKTEAAKRIM
QYIANVSGEQSGDIKKIKEMVLATNPLLESFGNAKTLRNNNSSRFGKYLQ
IYFNSTGEPVGADITNYLLEKSRVVGQITNERNFHIFYQFTKGASRQYQE
LFGIQKPETYAYTNRSKCFDVDGIDDIADFQDTINAMKIIGLSQTEQDEI
FRMLATILWIGNIQFREDDTGYAAVTDQSVVDFVAYLMEVTPDQLIHAIT
IRILTPRNGEVIESPSNPAQATATRDALAKAIYNNLFDWIVERINKSLKS
RQDTANTIGILDIYGFEIFEKNSFEQLCINYVNEKLQQIFIQLTLKTEQE
EYAREKIQWTPIKYFDNKVVCELIEQIRPPGIFSAMKDATKTAHADPAAC
DRTFMQSINGMSHAHLTPRQGSFIVKHYAGDVSYTVDGITDKNKDQLLKG
LLNLFQHSGNQFVHTIFPHQVDQDNRKQPPSAGDRIRTSANALVDTLMKC
QPSYIRTIKPNENKSPSEYNSPNVLHQVKYLGLQENVRIRRAGFAYRQSF
DKFVERFFLLSPATSYAGEYTWQGSTEEAVKQILKDTSIPKEEWQLGVTK
AFIKSPETLFALEHMRDRYWHNMATRIQRMWRAYLAYRAESATRIQRMWR
KKRTGAEYLQLRDHGHKLLQGRKERRRMSLLGSRRFLGDYLGVNAAAGPG
AQIRNAANIGSNERVMFSCRGEILEAKFGRSSKPSPRLIVVTNNKFYIIA
QLLINGQFQISVERAIPLGAIKFIGTSTRRDDWFSLGVGSAQESDPLMNC
VLKTEMFTHMQRVMPGGFNLKIGDVIEYAKKPNKFQQVKIAKDSQQPVDF
YKSGTIHTQQGEPPNSASRPMPKAKPVPPRPITRGKLIKPGGPGGRPSRL

TGNRTIQPRPGGGPRAVPQPPAAKTTASIGVANTSAPAAVNKPLPSHTRN
QNSTSGGASRAPPPPPPAAPPAKPKIMAKVLYDFNGQRDNELSIKAGDIL
EIVQKENNGWWLAKNDYGQAWVPAAYVEEQAAPAPRPPPAPPAAKSKPTP
PAPPAKRPVAGRKPGELAQRDSGLGLNGISGSEGSRSTTPTPSLGGSLAD
ALLARKNAMQRDKEDDDW
>Mb|QLI68826.1
MSDRYIGYVPTSSGRSPTFNPARASMPTSVGYSSMYAGDMHVVPSASQRH
YITPRGYSTSTTASGVPTTTRTYAITQDPRGNSRTRDGSRTRRSTLDSTS
RPPVIITTTQLDRSHGSSSHSGNARSGSPVRDEYRASDGQVYSQPASSLR
SRNTASRSYHAPSPSEDFGRYRDRADSHLSSRDIEAYRNSRPSVVYPSDP
RHSTAAIDYGDDGYQYTNAGELVKYDLDQSKPSRSRRHDRHDSFDAGYYR
PNVNYDLDRRNLNINTSHDLNRPPNGPSSRQHDGRPGPPPSTRGFDKINR
AYDSRDAPPAAPVPPSPTSAASLLEIPGSSGSVRRPRPLSLHQDAGLRSS
HHDELYRSREDERVMRAMRDRDFDRRSEPPRFYDESVTSRGFGIRTDVLN
HPEEIKERRRESRLDDQRKRSDEEVSYGSDKDREGRRWSRQDPTDDRRDR
RQTKRGEENQDQERSRTRETLTGGLGAAATAASLVASTKSGEKKDPGAPG
EKRRRSPPEDREIHKEVIVEQDARQSLERASKPMGGKEGTRERESLREKE
PLQERNPTTKERPPASERDVFVPKELVREPAPIRERDPVRESVRDSLRDK
ESMREEPRDGGSFERRRPDADTRTIREPVRASGSDSDDTNRPPRRGRASQ
AFNPNDASDLKQLKEQLASMEMSNKRQEKEVERPALAEKPEKPRARSPSP
SKEQAAAGSSREPSQEKSSEESRGRELVVPEAEGKQVRVVSPPKDKSEGK
PLKGILKQPKASFPEEANPVREGVAPHKEDKKLKDAPPGARWTKINRKIV
NPEALTIGKERFEVRDDFVIVLRVLSKEEIQAYAAATQVLRERRRTKIEG
KKEGDNDYGRERDTMRDEAGEEDEERHHRHRRRESEEESDEKERHKNRD
RDRDRERERDRQRERDRESEREGERQRHHRRDQADDYDNRARDDHYHHHH
HNNHRSFRERERERELEAKS
>Mb|QLI68827.1
MLSRILHRRAPAVAPRSVLRALSTTAARQSKTPSMGDINPSRAQIDSFNR
KQHEFRERLVEAQKEQGARALSANNFSASETSDQGSSKTDAGVTSEAKKH
EAERKSGPLSNLIYGTKEGREMEAQIQASFSQVLARGKYVHSIVFHEVKP
EKVDEYMELVGHFYPKMANLPENKVHLVGSWRVEIGDCDTFVHIWEYQKY
EGYHQSRYSITHHPDFADFDSKLKLLINSKKISLMQEFSFWPTTPPRQLG
GIFELRSYTLHPGNLLEWETHWRRGLKARREVMEGVGAWFVQIGELNTVH
HLWQFANLEERRERREKSWSVEGWSDTVHKTVPLIQNMKSRILIPMPWSP
VA
>Mb|QLI68830.1
MPLFPASLISPEVASSLPEGFTIRPLEKGDYAKGFLACLHDLTWTGDQTE
HEFNERYDEMDTHGKGPYYYVVIEHAGRIVGTGAVVVEKKFIWNRASVGH
VEEICIAKAYQAKGLGLKMINALDSVARNVGCTKSLLNCDAAKSGFYRKC
GYASAGMEMQHPFKSDEPTQ
>Mb|QLI68831.1
MDLLNTIRKTGSRGGADFSWDEVANSSHRENYLGHSLKAPVGRWQKGRDL
NWYAKADDTPANSAETDEERLARVRKEELRKIKEAEEDAIARTLGLPIPQ
RNTSGANAVDVGQQRQIGPKVGPPPGEDGDGKVKLERSRRDEGRRERRRH
RSRSRDRDDDRDDERHRRRRSRDRESRRHRSRDRRGHDRDERPLRPERNR
SRSRERRYRREDRDYHSGDRRRHEGRRRDHHRERSQSPRPMRQDD
>Mb|QLI68832.1
MQLGLVVLALAGLATAHMQMSSPPPFRSKYNPYTTSVDYDMNNPLPDSGS
AYPCKGYHKLLNTNQGRSVANWTAGEAYSISLEGSATHQGGSCQVSLSYD
AGASFTVIRSFVGGCPLTKDWGFTLPADTPAGDALFAWSWFNKIGNREMY
MNCAHVTVQGGGQGNYSSSEAAWASRPSIFVANVNNGCGTVEGADVMFPQ
PGPDVSNISKKTAKPVGSCK
>Mb|QLI68834.1
MPPTQLPASGNPLVFFDMTLGGEPLGRITFELFKDVVPKTAENFRQFCTG
ESKDAQGKPQGYKGSKFHRIIPNFMCQGGDFLKGDGTGSTCIWGLKAFED
ENFKLRHDQPGLLSMANAGPNSNGSQFFITTVPTPFLDGKHVVFGKVVDG
IEVVRKMEHTKTGYKGKDVPNMDVVIAQCGEM
>Mb|QLI68837.1

MSGTTIDDVVKRLSTADIDARLKLEAATTLRDSLDHYTSGPIYTPFLKRL
MPIFINILRGPCIFQSNSPEQKLRHCILEVLHRLPTSSAPVEPFEPYAEE
IVDLLMQLVRTDNEENATLCVKITSDIMRHQHKVLQGKVQQFLSLIQELF
EQMEKVVREQLDNASSTATSQPGAPSTPGGSQTNFQSPRPGSPVASVSDL
GADPQQQNRPLLKGMQSFKVLSECPIIVVSIFQIYRNTVQQNVKAFVPLI
KRVLLIEASAQKQAHAEAAAKGQVHTGVSQGIKAEQRAAYGDFITAQVKT
MSFLAYLLRQYSQQLNDFLPTLPDVVELLIAIRHIINYNFRKIFLPKIDE
LLDERTLTGDGLTVYETMRPLAYSMLADLIHHVRDSLSPEQIRKTVEVYT
RNLQDNFPGTSFQTMSAKLLLNMAECIAKLPNKVDARHYLMMILNAIGDK
FAAMNRQYPNAVKLSKQYLAEAGTQEPYLADKETRPDWDETDIFSAVPIK
ISNPRDRGADPVVDNKFLFRNLMNGLKNTFYQLRTCNIGTPIDPQNAPSH
WTDVSYGFTAEEVKVIIKLFREGAYVFRYYEIEKPATESQYASPVEYMAN
FYMVSSTKEEKDLLETFATVFHCIDPATFHEVFQQEIPRLYDMIFEHTGL
LHIPQFFLASEATSPSFCGMLLQFLMDRIDEVGSADVKKSSILLRLFKLA
FMAVTLFAQQNEQVLLPHVVNIVTKSIELSTKAEEPMNYFLLLRSLFRSI
GGGKFEQLYKQILPLLEMLLDVLNNLLMAARKPSERDLYVELCLTVPARL
SHLLPHLSFLMRPLVVALRAGTDLVGQGLRTLELCVDNLTADYLDPIMAP
VIDELMTALFNHLRPHPYSHFHAHTTMRILGKLGGRNRKFMTGAVPLTYR
GYADDPSSFDMRLIGSKKDRAFPADLGIDSAIQKLMEAPKMGKNSQNLRV
YDEYYKKQALHLIMSQVKLRIGYDQLPDDLPRIVRLQAQDFLARKMEIHF
APFEVTNRDKSIPKKDVQDGITKQLLKAVMFAHSLPRFKDEADAFLMDLC
RHFALIEVGRSLVDLKRNFSPFDPKAGEGPLCIDTRILSDAIVESLASDH
PDVREAAKKAIRELYKSTGIIFGSESNVGRLPLFIHLCGTFCHSCYEEEW
FTKTGGSLGINFLLTELDFGDAWVASKQMEFIRALLYVIKDMPQDLPEKT
RCLAETSLEVLLKRITKNIKKEDALPVQAQPGQPPVKQSRLAQICMLFNN
ELSHMNKFVRESAKNSLELIAKAAECQVWELLEPYKERFLQPIYSKPLRA
LPFSIQIGYINAMTYHMSLKNDWVKFDDNLTRLLMESLALADANDDSLAN
KPAEYRTHDHIVNLRVSCIRLLSTAMTFEEFANNPIKGKILSVFFKCLYS
ESKPTIAAANDALRSVLAVDRRLPKDLLQGGLRPVLASLSDPKRLTVHGL
DNLSRLLKLLTSYFKVEIGARLLDQIDSIVEPNAWQQISFTFFEQHQQMK
VITAILNIFHLLPAPAEAFKERLIDCFLGLEEKLRRTIQSPFRQPIYLYL
NRYPKEVWTFLLGKLDELKYGRLLAQVLRHPESGPLRDAAVDGLGALMTK
CNGLAGQGVEKKFIGIVNAVHILDSLSYFENTNSWMDKKENTTWMKSMGK
ELEGHIRNYTLPATLRLPAYQAAEQLMNVLVKSLERTPKDLDVLFSLVES
VTSNELRPTHCLFAFIYEKILMSESIEFWKSAFLRCLDTYSGRSASNKTK
QFLLHYVANPIVAMDVMRHWNQPDQAKGQRFMDRSVIDAVSSKIWKVHPD
MSPDDQSQPGIDHTRFEVLQLSAMLVKYYHTVLQEARKDIIKFGWTFIRL
EDVINKHAAYVVIGYFIAHYETPPKIVSQVYLSLLKTNQNEGRALVTQAL
ELIAPVLPKRCGGGPNERNAAWAVAPRRILADEGQNVQQMTSIFHFLVRH
PDLFYDARDKYAILLITSLRKVAAPPNPSYESKKLALNMMWLIWRWEQWR
VEGKTGSPSVGRDPSESPTSKKRKLDSDLPMSSPPTVRQNAPGGERNGYQ
IPQIFRQKMIKYLVEFIAQLNERYQLPSAKPREHSTPSLPLPPQSTELCK
KAMTLLYNLVQPQYWGDLELDLFPNVTDVILGSEKTQGILNADPTDKEKY
DEKFLTNIINTLQVVRIVLNFKSDDWVQKNLAAVQKVLEKCLKCENPEIQ
DCLHLADREYDDDRDLKSIIKRVLDAVPEDTPMEDADADGETEVQTSEII
AYLSQAATEAMNGGSYVSGVNLLWSIGNRKPAVIDQHIPSLMKALQSKHA
REHVQHYNNAAGQAINQGNRGQDGNSSSGGEMSAYDLEIQTKLMIKEIQT
VALRMETLGDSRRPFLSVLATLVEKSMHIDLCTEILNMVEGWVFRSEGTW
PTLKEKTAVLHKMLSFEHRSDSTMLSKFLDLVIRIYEDPKVTRTELTVRM
EHAFLIGTRATDVEMRNRFMSIFDKSLSKTASVRLSYVLTSQNWDTLADS
YWLAQASQLLLGAVELNTNIQLHQDDFRIILASQLAAVYPKDKDAREPNM
MPDDKFEAFMTSHRRFVSELGDVKVRDVIEPLMQLQHVDNQLAHQLWVAI
FPMYWSATMRDERADLERGMVALLTKDYHSRQIDKRPNVVQSLVEGAAKT
WPNCKLPPHVLKFEAKTYDAWYTALVQLENAAIRPEVESTTVRESNLDAL
VDLYASLQEEDLFYGTWRRRCQFVETNAGLSYEQNGMWDKAQKLYENAQI
KARTGVIPFSQAEYMLWEDHWVLCAQKLQQWEILQDFAKHENFQDLLLEC
AWRNTEMWQDAQHREALDNVIKGVMDAPTPRRAFFQAFMSLLKFHNQQEG
GNDFARVCDEAIQLSIRKWHQLPERLTNAHIPLLQNFQQLVELHDASVIC

QSLANTNSSNLDVKSGELKLLLGAWRDRLPNVWDDITAWQDLVTWRQHIF
GLINQTYLQLLPQQGQQNAGGASSFAYRGYHETAWIINRFAHVARKHSLP
EVCISQLSRIYTLPNIEIQEAFLKLREQAKCHYENPEELSSGLDVINNTN
LNYFNPQQKAEFYTLKGMFLEKLNQKDEADHAYGTALYFDIGAPKAWAEW
GYFNDRKFKEDPTDLNAARQALTSYLQAAGSYKNAKSRKLLARILWLLSL
DDSKGTIAMGFDDFKGETPVWYWITFIPQLLTGLGHKEAPRVYQILLSIA
KSYPQALYFQLRTNREDMNLIKKNQEAKERARQQRAQSIASNGKPSLSPS
QAKSEPPKTSDGGTPRAGTATEGDAASQVKTEGGDAATAPASLPPTPANA
PTPAPAASGQKQEQQPPAPNPNQKKMPWELTEEIMSVLKTAFPLLALSME
TMVDQIQKHFKCPPDEDAYRLIVALLNDALTQASRTPASFAKSVKLPSAT
ETNITRFAETILPSHIKKSFEADFVDVKPTMYEYIHKLRRWRTKFEEKLD
RRVCHAHLEAYSPHLSEFRYQKFDEVEVPGQYLQHKDKNQDFIRIERFLP
NVDLVRSYSTSYRRLKMRGHDGSVHSWAVQHPAARHCRREERILQLFRHL
NQTLGRKKESRRRDLQFTLPLMVPLAPHIRIVQEDTSYVTLQAVYEDHCR
RMGFSKDEPVLFTLEKLRGVLESKSQQGKPELTPPARLEVFNAVQEKWVP
SSVALDYFQQIFPQFAEFWLFRRQFSYQLAALTFMTYILYMHNRYPAKLN
IARGSGKIWGSELMSFMSASKPFFHNPEPVPFRLTPNLQVLMGPLATEGI
YACSLMAIARCLTEPEHELEHALTLFVRDEMIFWLTSSHRNGISESQLRE
SVQVNSDSIVKRAASLAHNPSGNLPANQTVIDAIAKAVNPMNLAQCDALW
MPYL
>Mb|QLI68838.1
MLLFCPQCANILTVSLTNQRTNRLECRTCPFEHTITEPVFSRRVFERKEK
EDVFGGPGAWDNAQKGRAQCPAEGCDGDEAAFFQVQIRSADEPMTSFFKC
MTCGNRWREN
>Mb|QLI68841.1
MPREVADIKKFIEICRRKDASSARIKKNKKSNNIKFKVRCQTHLYTLVLK
DTDKAEKLKQSLPPNLSIAEVGKKKN
>Mb|QLI68843.1
MVNITEKIKEIEDEMRRTQKNKATEYHLGLLKGKLARLRAQLLEPGPGAG
GGGGSGFDVSKSGDARISLVGFPSVGKSTFLSKVTKTRSEVASYAFTTLT
AIPGVLEYGGAEIQLLDLPGIIEGAAEGKGRGRQVISAAKTSDLILMVLD
ATKKAEQRALLEAELESVGIRLNREPPNIYLKPKKAGGMKITFQTPPKNL
DEKMLYNILRDYKMLNCEVLVRDENATVDDFIDVIMKDHRKYIKCLYVYN
KIDSVSLDFLDSLAREPQTVVMSCELDLGIQDVIDRCWKELKLIRIYTKR
KGVEPDFSEALIVRSNSTIEDVCDRIHRTLKDTFKYALVWGASARHIPQR
VGLGHLVADEDVVYIVSGWRA
>Mb|QLI68846.1
MASSPPIKKVCLIGANGTVGSVITRELVQAGCFDVSILRRSNSSSASPAG
VTEIPVSPALELQELTRALSGQDAVVAAFPLKDVSEHLRLVEAAFNAGVR
RYIPADYGSCDAASPQAQHHLRLYRDKTAVRTRCEELSQMAAEDQTGASP
FTWTSIICGHFFDYGLRSGLLHFNLDAERAQILDAGDIKASASTLRRVGE
SVVRVLQRHEDTRNRAVYVQSFCPTQLEILASLERATGNKWHTQHLDSNA
FLERESKRLEAGDGEATEEIVFVLGTVDADWTRKDGFAMELLGLQDENLD
QVVREVVAAHKTRASS
>Mb|QLI68847.1
MAAADATATSKLASYFARLSPVPAFPEYTGPFKVGTVDVEIPVTDLHSPS
PAPANAVDIPTVQFRVFYPAVPESSEKRITWLPNPQRHHVSAYTKFVGIG
STLADILSFLPRHLHYTTIPVHKNAEAADSETKRWPTMIFSHGLGGGRNS
YSYIAGSLASHGVVVFCPEHRDGSAAVSFIRLADSSSRFFSSAARRIVPY
NRIPHHVTPEVYEAREDQLRVRLWELGLLHDAMLAIDEGRGFTNMNLTTP
TLKQFIGRLDVHEPGRIIFGGHSFGAATVHQFLKCNYYADVAEVANMKKP
LFTPAKDSSIRKQITEKNVTMLLDMWCLPVLGPNSKPLYDLPLPVYADVP
SAPGGTALLAVESEAFHKWTEHLHVKAKLLSPDPTSKTVTPPLFERPSGI
KLPEPNFFYVVNSAHLSQSDFGILFPWLTKKIFDAEQPERALRLNLRAQL
QLLRANGIPVARTYAGDLVDGAHVDKLNARKDGQIPLTEEGIDDDRAIFD
RSGNDSVDHWKWIDLIGLGDFVGENFGKSVEEEVEEGEEQMQGEMEPSEN
EAVQPLVRTLSAAA
>Mb|QLI68852.1

MHASVILGAGLAIRAAAAVAVPTPSEPPVLRRDYDNNDNLPAAPWVTVDD
NGNPSKTFTPVFTTISGTPSGIDPAPHDLTASVYTLTSFGHLSTSTGLPP
NPTATNAGTNQGSFSRCYNKNGPYAPFCRPSYNSSIYTGNTYYVTWDPDY
YNKSAATLNSTYFVSVRLDYLNTTSNEYVLLETIDKSVPAKWGYFPLAVD
GKYLKGQKHTNNITITLMGHDTENSIAYNNKSIPLPVVVSNPPLDPTAPS
NAPKGKTLTIALPVTLGVIALILVGGCLWNRKTRRIELGNIMSRSRQGYT
GRRTRKLFNRSRKDNGIQLDTAPLSPPPFDYRDTVPERARRDSDALGSLT
GSPVRGTFEEPGSTGGRNTFRDEVRRQERERRGDF
>Mb|QLI68853.1
MEESSAPTSKKGAKKAEAKAKKEALKAQRAAELAAAAAAVNLEDDPAKDN
YGNKQTTTSAFSQHAQEVEIRSIDETHNEKTVIVRAWLQKSRVQSAKMGF
VELRKGGNWDIQGVIMASDEEPLVSRRMVKWVASINPESFVAVEAKVKKP
LEPVKSCRVSGFELHITKCFVLAPAPAVLGMTIGTASQPIVNFSDETDQA
GDVDAGKAAEAASEHTAPAASMLTHLDNIAMHKRAPVQQAIADIRIEVKR
LFRSYLDSRGFKEFEPPCLIGAASEGGGNVFRLAYFGEEAFLAQSPQFYK
QFEIAGGRERVFSIGPVFRAENSNTPRHMTEFTGLDLEMEIKESYTEVIS
ILEGVLLSIFRGIQERCANEIETVRAVYHSEPLLLPEPGKEVRLTFAEAQ
KLLREEGPPEFANVRDDEDMSTPQEKALGQVVRAKYKTDFFVIDKFPETA
RPFYAKLDDAGTTVGDGVRVTNAYDMFLRGQEVLSGGQRIHDPVELEARI
RSKGVDPKSGGIKEYLAMFQQVGVPPHGGGGIGLDRVVAWILGLPSVHLA
AYYPRTPKRLLP
>Mb|QLI68854.1
MKATPLIINWHDQNAPLYSAHFEPGGKGRLATAGGDNHVRIWKVESDGLE
RKVDYLSTLSKHNQAVNVVRWAPKGETLASAGDDGNVILWVPSELPPSNF
GGDTQDDKESWRAKHMCRSSGAEIYDLAWSPDAMHFIIGSMDNIARIYNA
NSGTLVRQIAEHSHYVQGVTWDPLNEYIATQSSDRSVHIYSLKTKDGQYT
LSHDDKPPRLASHIKADLPPRRISSSSPAPPDFGHRAQLSMAESATSVGS
PVPSAPGTPTSIPLPMNPPSVVSHSRRSSFSSRRSVSPAPTLPLPAVMPM
EASPKPQLGTKNASLYANETLTSFFRRLTFTPDGSLLLTPSGQYQTQHQS
EKDSKPTYEVINTVYIYTRGGINKPPIAHLPGHKKPSVVVKCSPVYYTLR
PSPPATTHITIDTSSAEEQLPSLPEPISKSSPGAAVMEPPPPPASVSSEI
SASQSKPLGVDTDASTPGPKPAFSLPYRMVYAVATQDSVLLYDTQQKTPI
CVVSNLHCATFTDLAWSSDGLTLVISSSDGFCSTLSFASGELGEIYKGEV
GPPKPTAASTAIPSNQNTPTPTPTTSFAPPSPFPNGSHHQHRNSASSFTA
PSPPATAPAGSQRPASPTRSNSTSSIATQSSSVPTGVVSNPPLIAGSVPA
LTATNSSKAQGVPITTPPETPRSAAGSVAGTKRDASESEREEIKEIKKRR
IAPTVVEKL
>Mb|QLI68855.1
MKGFMTVRFDHVNPASEKSNQNDVRSSLAGLDYSPLPRLTMRSLAMGCLV
SMGGLIFGYDTGQISGFLEMPDFLERFGMTNPDNGQKYFSAVRSGLIVSL
LSIGTLIGALAAAPVADRIGRKPSVSIWCLVTSVGFVVQIAAVTAWYQVM
IGRFIAGLGVGALSLLVPMYQAETAPPWIRGAMVCAYQLFITMGIFLAAT
FNYGTVTHHGGDSASWRVVIGLGWVWTLVLGLGILGFPETPRFDFRHGRA
DRAADTICKVYGAGTNHWAVYTQLEEIEAKLRAEDTVKLGPVREFAAMFA
APKMARRIALGVALQMFQQLTGANYFFYYGTTVFRSVQINSFVTQIILNT
INFLVTFIGLYIVEHFGRRKSLMAGSAWMFACFIVFATVGHFRFDREDPQ
RTPAEGIVLIVFACLFILGFATTWGPMVWAIQAELFPSRFRAKAMALGTA
SNWIWNFCIGFFSTFIFNAIDFQYGYVFAACNVVGGLIVFFFVVEGQGRT
LEEIDTMYLEGVTPWKSAAWVPPPAEHMARIRREAGADLETAAAAAAPGP
RDGGAGGARTEGKETGDEAPHHEETV
>Mb|QLI68857.1
MATSRAFTQCRLRPLHAYSSRPSTRSTAPRPRTSPIRAQRPMSVMSRSYQ
DALQKLAELQSNRTVTQLFGSTPEQSNAKARDLNAVAIPEVIRWLGRAGY
STDDLCRMRHIHVAGTKGKGSVCAYATSMLRRYGAVGTYTSPHLVSPRER
IAIDGEPVSQELFAAAFFELWDRFTEAARREGRDAAAAEGPGSKPFFFRF
LTIMAWHIFLGRGVRNVVMECGIGGEYDATNVLPARAVSAAVISQLGVDH
VAMLGDTVEKIAWHKAGVLKDGVKGFTVRAEPSVMGVLRQRAAEKKATLV
EVDPDEVAAWRGVEGNLKGGFQKMNQALAVLAVREHLNMGGGGWEDLPDE

MASGLREATLRGRCEVVHDGEQTWLLDGAHTRESLAEVARWLRENVQEGQ
EVILVFNQQERNAGELLAGFLREVNEVTGWEVFKSAVFTRNELKGGGDGD
VAVQEGAAKTMRDIVPACDTAVFDNVEDAVLHARNLVRNKAKGKVLVTGS
MHLVGGVLRVLEPDGLL
>Mb|QLI68858.1
MDAGLVREQPEPIAIVGIGCRFPGGASTPSKLWDVLKEAPNTAKVIPQER
FNLDRFYHPVGTHHGTSNVKETYFLDEDVRRFDAGFFNIPPPEAGAMDPQ
HRLLMETVYEAMETSGTTIEKLQGSNTSVFVGVMCSDYYILSAQDLDSVS
TYNSTGIANSNASARLSYFFDWHGPCMTIDTACSSSLVAVHQAVQALRAG
TSRVAVAAGTNLLLCPLGYISESLLGMLSPNGRCQMWDEKADGYARGEGV
ACVLLKKLSDAIADGDHIDCVIRETGVNQDGKTKGITMPSAVSQASLIQR
TYHQAGLDPRNKKHRCQYFEAHGTGTPAGDPQEAEALDTAFFGDSERDAN
DILYVGSVKTVVGHTEGTAGLAGIIKACLSIKHGIISPNLHLDSLSPSVK
PFCKNLEVVTSIMPWPELPAGQVRRASVNSFGFGGTNAHAILESPWPSDD
LQAQVEDRTPVSADKEALHLPFLFSATSEKSLIALVDAYSEYLESNSDVD
LHRLAYTLGCRRSAFAFKTCFSAASQEELCSKLSESTSDQGNLSFVRSTT
TNPSLLGVFTGQGAQWPRMGASLLQSSNRAMDIINELDASLAALPASDRP
SWTLRDELLADASASRVGEATIAQPLVCAVQILLVTLLRDAGVQFKSVVG
HSSGEIAAAFAAGFINHHDAIRIAYYRGLHSKLARSASGDIGGMLATGLS
YDEGIELCDHEDFTGRITVAASNSPTSVTISGDLDALEQLQLVLEDEETF
VRALRVDKAYHSHHMKPASGPFLESVRNCAINIMTPPNNAPTWFSSVYGG
QVMEASDIIKGSYWVENLLNPVLFSHALETALLSGDAFDAAVEIGPHPAL
KGPATDVMKAVTGKIIPYSGTLTRGKHDVESFSDALGHLWANAGPSSINL
DSYGQAMYQDWKTIKILRDLPAYPWNRDRILWTESRGTKLWRQQEGKFHD
ILGSRCADGTEEEWRWKNLLHPKELPWVLDHALQGQTVFPGTGYLCLALE
AAMQIAGNNSVQLLELTDLSIRKAIAIDESAGTETLVSMTKIIRTEEDIT
MAFACFSTVGKEGSGLALNAMGNVRVQLGQPMPDILAPRPPPAHGMRPVS
TDHFYSEVAKLGYNYGPTFRGICNLERRLGGSRGDIVGPPNDETGTTLLF
HPGMLDAALQGMLVGFSSPGDGRLWSLHAPSSIRRVTLVPSLCGANMTPK
VSFDCAVTDIEFNELTGDVEVFQDETGYKSISVEGVAFIPFTAATASDDR
GLFAHNVYGIESPNGQAALGSYRATKSEIQKGVDCERVAFYYLRRLHEDF
SQAERTTLHLPRHHQALFDYAEYIYNIVQRGEHAYVKKEWMEDTYEQICS
IMKRQVYIYGEGFVELGPGRRENLPAVVRGETTILEHLTKDNKLNNYYQH
ALGFHALNDLIAATVKQIAHRYPRMNICEVGAGTGGASRRIFAELGTAYS
SYTYTDISSGFFSKAQEDFAAYKDRMIYKTLDITVDPAEQGFEHGAYDLV
VAANVLHATPDLVATLSNARKLLRPGGFLVMMEFTDKSVLRLGVIIGGLP
GWWIGADTGRKHSPCVSLPEWNDILLGSGFAGIETSTPVLDPVVMPASII
VGQAFDSDLSLLREPTAVSPGLGMAKNAELLIVGGAGAASAKVVENVAAA
VRPHYQNIVHVRSWDDPSADHGLKNPTVLSLQDLDGAFWRDMTVRRFERL
KAVFLTAANVLWVSWGTDCDNGDGAMTVGFFRSLAYELPQAILQVMDLED
VSKADGSVIAQRLLRLDMSSYMNKTGSLGRTMFSNEPELRFRDGKVLIPR
CLPHVDQNHRYDSLKRVIDKEICLDESIATLNWSRSEYTLREDECAVLPC
LADHRRVRVDSSLASSIVTPAGRLFFSLGTDMDTGRRVVSASGKNSSAIS
VPHKWSVHVDVGRSIDSQYLSFLSGYLLSRHLSSRMPAGGTVVVHEPDPG
LASMMSRQLATMGCRVLFTTAATGVVRRNWLAMHPRIPDRQLIRALPTTC
DLYLDLSATTPCPEGSSFLSQRISKMLPPLCERHDAGVVIARQSTVFAGA
HDDAIHELLNRVNLFAAAQLNGVPDGMPLPMIPLSQILSSGASATSNALR
GIIQWHGEDSVPARVEPVVNRNDLFAADKTYWLVGLAGDLGRSICDFMVA
QGARYIVLSSRNPEINHEWVKDHAARGATVVYIAADITSESSLRASYDQI
RANLPPVSGVANGTLVLKDRGLVNMDLETFHANTRCKVEGTEHLDALFPD
NTLDWFIAFSSISATVGNMGQMAYTAANMFMKAIISQRRRRGVAGSVIDI
SQVFGVGYVERELNRQARSDREQAMRLMGKSGTLVMSEPDLHQLFAEAVI
SGRAGSGHDPEIISGLRIMTPAESKDALWGGNVRFGHMIQETGTAKPPSA
TKTAAIPIRAQLEAAKDAEQISAMLGAAFKAKLKAALQVGEETLSVTTPL
VDMGVDSLVAVEIRTWLANEVGADVAVLKILGGPSIQDLVEDIAVAIKGQ
ESSSDSAESETSSNSEDKTSTPATEGSSDGSGQEKTE
>Mb|QLI68859.1
MSLSLGLGVGGAAPPALNAYWGQTGGRFLRDICDSGVNYATVSFINNSPE

NGDGYPGSNFGANCAGEVYTNTDGKKTKLLSACSFIQRDIPYCQKKGVKV
LLAIGGAHIPGTSEYAVSSEEKGVEFAEFLYNAFGPYKSSWTGPRPFDST
TAHVSVDGFDLDLEDRTPKFKNGPYIAMVNWWRQQSHKMFITAAPECVMF
GNQNDELIANAEFDALFIQFYNNPVCDSIPNNTPGDKFSYDEWAAKVATG
KSKNAKLFIGLPASTDSAGSGYIDHEAMKDLVCRYHDHKNFGGVSLWDAT
RAMNNVGAEGKSFLQSAAEAVQYVCGEPPKTTSSAPTSTKITSTTSSTTV
KTTSASSEKPTTSNTASEKSATTSTSSASSSAKLTTSSIQSGNVTTSSIS
SGKPTSSSATSKKLDTTSVIATASATKSATTSSVGMTSSIHWSNSTATRT
IQTTSKPVAITTSKPVSMTTSTVYTTSVHTVTECPPTVTDCPVGHVTTEI
IALYTTVCPVTETAQPPKPTTTKAAVMTTSTVYTTKTYTITECPPTVTDC
PVGHVTTKVIPLYTTVCPVSETAQPPKPTTTKVPAMTTSTVYTTKTYTIT
KCPPTVTDCPVGHVTTEVIPAYTTVCPVKETETGNVPKPTGPAPSSAATK
TTVVSKTVKVQQPSSLATSTRSAAVVPQPSGKGCSGPSCPGATNAPGSGC
TGPECPGVAIPTTAPGSGCTGPECPGVAIPTNSWTNKHSSGCTGPQCSGV
AIPTNAWTSSSVGPSAVPSSPVTAGASTLALGLTGLVAIVAAQVLAL
>Mb|QLI68867.1
MQLLILLMSLFAMATAQFGFFDQMFGGHDAHEQQHQHQHQQRNNPSDAGH
YRSQFEQSHCDNYLCPDTLACVHFPHHCPCAWDAYEEKFELGEGKKVCVS
KGGFKPGEAARKIELARKGLL
>Mb|QLI68869.1
MSDKIPAVVSHLVSDEAKRMIDVVAKFVEEECIPADPVLEAQVGQGDDRW
ENHPAIIEELKEKAKKLGLWNMFLPKGHYKESPGWTNLEYGIMAEWLGRS
RSASEACNCAAPDTGNMEVLAKYGNDAQKQQWLKPLMDGKIRSAFLMTEP
QVASSDATNIELDIKREGNEYVLNGQKWWSSGAGDPRCKIYIVMGKTDKA
NKDPYRQQSVVLVPADTPGIKINRMLKVYGYDDAPHGHGHLTFTNVRVPV
GNMVLGEGRGFEIIQGRLGPGRIHHAMRSIGASEVALDWMLMRVNDDRKK
PFGKLLREHGVILEWIAKSRIEIDSARLIVLNAAHKMDLLGPKKALKEIA
QAKVLVPQTALTVIDRAVQSFGGAGVCQDTPLANSWAGIRTLRLADGPDE
VHLQQMGRNENKRGKEATQKIQWQREKTAGLMKKYGTQTAQPGANIRHAK
I
>Mb|QLI68870.1
MALGRGFSSYLVTPKELHEALKKNPPSPISTDPRVIPLCASWFLPNDERS
GIQVFREQRIPKARFFDLDKAIDKRSPYPHMLPDAKAFAAALSELGIRKE
DTVVVYDSKELGIFSAPRVGWTFKIFGHPNVHILNNFKLWVEEGLPTESG
ELYSVECCQYPIPKLDESKVASFEQVKEVALDFNKEGAEGVQVLDARPSG
RFSGKDPEPREGLSSGHLPGSMSLPFSALLDPETKAFPSPETLRSILQGK
GVDPNKPIISSCGTGVTACIIETALDVAGYGSPESRKVYDGSWTEWAQRV
RPSDNLILKTES
>Mb|QLI68871.1
MQRGAYGQSPPLHHPVPQHVSTVPQLRSPPPPPGSAQSMGQTYGGASTPY
QQQGGNSGNVFGAYGQFMNDPTAQVAAQFGQTAFKHGQEYVEQNIGRYVN
VSALKHYFNVSNSYVVNKLFLVLFPWRHKPWARKQATGASGQEGWYLPPR
DDVNSPDMYIPVMALVTYILLSTLIAGLKGQFKPELLGYTATTGMVVVIV
EIVALKLGCYLLSISSQSQLLDLIAYSGYKFVGIIATIVVAEILNGGKGT
GGWIGWGVFLYTFLANSLFLMRSLRYVLLPETSGNTGGPMQTDSRAKRNQ
RTQFLFFYSYIVQLFFMWLLTMGI
>Mb|QLI68875.1
MVNGTKGSPVLFAMANTTLFDSALPPLPSYTLEPRGDFFSWISDFWLSLI
LPVFVYWVMSLFFHVIDVLDLFPQYRLHTPEEILRRNHASRYEVARDVVI
QQIIQVATGTVLALSEPPEVIGKADYDVAVWATRLRLAQRALPGFLSLLG
VNATAISKSMSATHPLLAGALAGGYYPFLSSAESTAPAFAPWEMAVAKVL
YHFIIPAIQFFVAVFLLDSWQYFLHRLMHVNRWMYTTFHSRHHRLYVPYA
YGALYNHPLEGFLLDTLGAGIAFKLTGMTVRQGTCFFAFSTIKTVDDHCG
YAFPWDPLQLITSNNAAYHDIHHQTWGIKTNFSQPFFTFWDGLLGTKYKG
PRADRLADKKRTEKTK
>Mb|QLI68877.1
MAEPQPRNVHEGATTGDVEDELQSTAKSAEDRKAASALASLDAAAANDDA
AGRDVDQEAVNKAMKSLGGAKSAPVPAAAPAKSVKVDAADVSLLVDELEL

SKPKATELLKAHDGDAVKAMRAWVKA
>Mb|QLI68878.1
MSQFKDIMKKGWHPEKSGTGIRGSVSGLMGRNKDNKDDSSLNHVARPLTD
LKDPASFAPPPRRTGSGLARAPSPVSTKRNVVAAPSKYADPRARPAELPP
RRTGSGVESDEASPTRDAADRRSPPSYDEATSGGVVSKAPPRSLPPRLPP
RGAAGGGEKSGHLNEGAVSRLGAAGVSVPALGIGRGGAAHSTSPSPPPAS
SRASPSAEASGAGKWGAQVNQLQGRFGKMATPSAASSAQEQQPSQGTSWA
QKQAAFKTASAFQKDPSSVSFSDAKAAASTANNFRQRHGEQVAAGAKAAN
KFSQKYGIADKLGAYAGSQSTQPTQNKSEPPPLPGPSQPYTGATNDAAQA
AVSGLAGKKKPPPPPPKKKPGLAAAAAPDPASAAQGDMPPPIPMSTRPQF
>Mb|QLI68879.1
MAKAHPSEPSPVNKNPPIPQGCKIQKRPLNRPQVPSSSKSPVIYISSHTP
FLSAVSRIRKLLTKSLRNTAAAPKNASTHARVEALQRSTNTAYGNASPLL
VTVLGTGKAIEKTLSLASWFEQQGDCDVRLRTKTVGTVDDVIVEEGEDDG
LSRIRRVSCLEVVITLK
>Mb|QLI68880.1
MEIKTVLTTVISGSRYLVHPQVLGTIQETIKPDVIIPVTVLEPEQLYYHL
EGTLRLFDFYDDVFTPEFGNILIEKPGGNSGVAVSSVGNGKSVMRLRNDS
VAAAKRSVSDLPAGPYILHGPNVHQAWKIYNDTLDAFAFGVYPERVDEVD
LFRVLQLPADSETNYTSIPVPSKLYSTVPPEQAPLKGYRFTIPDSMSLKG
IPTTLSSSAWRDLHDNPADSTAAFAKRLIDLGATIVGKTKSSQFGSGREW
TDVPSPKNPREDGHQDPGGGATGAASSLAGYEWLRATTGIDSVGQVFGPA
AAQGLFALRTSSGLLPLDGIQPSFTTYDSMGIFGTDLSELFHDAVAVVHK
SLASPAISFPKTITYLTDFSSADEERNDKYEQFLDAVEAFLGVKSTRLSL
TQTWASKPPAEAKGQGLQEYMRDAPFLSFCYEFYHQYDEFRKDYKAKFGK
DPATEPTVKQRWDWGSNVTKSLYDEYQARLDVFRNWFDSTIMSTNHGGDA
ILVAAYPSHAPSYGVPKPSKINGVTPDLLASVLRVPQILAPFAQFEYQSK
VSGRPEYHPIYGAVLGPKGSDTMLVKLVQAAFQQAQWRTRVDKGSIRRRQ
LNGESIPVFFTAYQVPNTNPPLIGVSASNKVLDIPYTLGQTSVDVAANDF
DPNTDLNNTMYMIQCSPNEGILFDTLSSGSFFQEAVGLLQENGLAGMSNI
TLIASHEHQDHTGGLSAVDSVDKLRGSQLVAQENNNAVEQAQVRVGEEQT
LTCGNATINLFHTQGHTTAGVMACTGDYCLTGDECEDSVPFIVQAKTLDD
QARGLAQSIQTMRQLNVTRAPQAHGNMQAVANGNLGLRVCESNLLYKQKM
VDEFATLCPRGSDISLNELAEQINRDEAEITEEYYGVHKGNCDSVRKARR
DTRA
>Mb|QLI68881.1
MKTSLLALIATLALLLGLCQSQLESTRRTEIALDRRQAGDFDDEFPEYVY
RGETGRSPADVEKDGGLYSRGVQKQRAGAALSAVELQEGSSLFHHAAGET
AEFTRYVSTSADPGVGLTFAVNDDVPEQKGYIYRIHTDKRMVDVNRSLGK
YSPYAAQKEHAAIGFIPYDQIEGWWEVTYKDDFSDPKIGKESQEKLRQGK
FKGFKKNPKFNKGSFQKLRGTGAAPQLAGFPRLSPAWQDDTWKAFKTKAV
EKNLDDLISSICAGKSTKRDAGCMTRLGHQETTGLTRPKPPKKPSTASNP
DGKPPKSKVSSKRPIYKSSKVRVTKAVGKAAAFTLILPFARDLLEAIKQW
DNPIGAAVRWFDDAIASIQEAIGGPSRDDIDGNDLKASIICALKGGRESE
TIQGRKSHFCTPTKDEFTESLQRDFKQGKIDELIRGCKGVDVYNGGDPHV
WRWSQRRCEALQRTDEYAERIWEMGLTGLLDSCSELESNPPGNEDLRIKL
EDHCTAFQDEVERAEKPVKKPVVGIPKITAGKCKCDAYHLQPFSEHCGRL
CRASYVLGGWGAL
>Mb|QLI68882.1
MARFVPRQRKHKVLARQKAEEAAQHEVRDTNQELLPATQAERQEKKARMK
EELRQDGVKVSGKKAKRLEKYIENKLRKDENRELLAKLAARRIDTSLFSS
SRSLGQGKETKKQALSRALRESRAGLDGDDEILLEKRQEPAIELEDDGSD
ADESNVARSAKVAIESTIEKSIPNSEIPRPLVRIPDTEVIGSGLKRPLEQ
DDEGKPVIKKKQKRGGVKTKAPLETIEIIQPNKLEYEEWDGFDSDDEISE
KNVTQPGEDTEPEVDDDTGSEEDTSDDSEGDEESEGSEKDNDAEQPAQRS
SAFKAWALQQRNEALGYQPIDVTTGPSEVPKPENFVPRAPEQEPLPMELQ
PTANDSRKAFSVTVTRSPEIQEARMKLPVVSEEQRLMEAVHNNDIVVICG
ATGSGKTTQVPQFLYEAGYGSSDSPTPGMVAVTQPRRVAAVSMSRRVAEE

LGDHSGSVAYQIRFEGTVSDKTAIKFLTDGVLLREIAQDIALRKYSAIII
DEAHERSVNTDILIGMLSRVIKLRAELSEEDPTIKPLKLIIMSATLRIED
LTMNSTLFPTPPPVLEVEGRQHPVTIHFARKTQHDYVEEAFRKITRGHRK
LPPGGFLVFLTGRNEILQLSKRLKAAFGGMTAAEGPKVKISANEAPMEVE
DIEFGDVDDYGDEMGGVPSDDEEDEEEFQIEEDQEAAPLKMQVLPLYSLL
PTREQLRVFEPAPDGTRQVILATNVAETSLTIPGTRFVFDCGRSKERQYD
RQSGVQSYEIGWISKASANQRSGRAGRTGPGHCYRIYSSAVYERDFQQFA
DPELLRMPIEGIVLQLKSMNLQNVVNFPFPTPPERQSLAKAEKLLTYLCA
LSPSGDITQIGKTMSVFPLAPRFARILLVGHLHDCLPYTIALVAGLSAAE
VFLPEAQAIPALTAKGDVEIRTTADVIAEDRQANVRRVFNEVHKNFCYLD
DKSDAIKLLQVVGEFAHEPTEEWCEKHFVRFKVLKEIQQLRKQITELLRN
NIPSFANLKFQDKLDPPTPKQVSALKQMAAAGFVDQVAIRADLAPAPPEQ
YRKPKRAIDVPYIPLVPLHAVQGPEHDSLVYIHPTSPLAHCSTQECPEYI
VYSYLQRATPSGVDVPKKPKTRMHAITDVTGGQLSGLAKGTPLLSYGKPI
KEVKPPAASGEGSMTRECWVIPYLRAENTGGQGWPLPAKKVKQKKVPGKG
WIVE
>Mb|QLI68887.1
MSSPRASSPANSPAAASGANIGRPSSPTPPGGPRTAIRRRAAADQKEKIA
NARPTSTRSAGAGGSSSTMLRLYTDESPGLKVDPVIVLVLSLVFIFSVVA
LHVIAKITRRFSS
>Mb|QLI68888.1
MSFRGDDQRRYGSVPPVQYTVAGQQQDQHGTHQATSASGVGRRPSFNTGD
DAGYYTQNHNRTASFTRGEDELFLTGGPVGRGGNVPVNRYASTSSAMAGY
QHQYQDPTPPTPSQSGYNPQSFSPSSNSAYHRSQASTLPYHSAQRFSTSS
AGSYHTPSPPATNYTPQAYNPAAYAATNSVPQRQASHHGYGYAAHDQGYA
AGVGTAGSPSYGHSPSASHTSTFSQPVRSPSFSSSFQQSFPTSPSSFTGS
NPISQTPTYDPSNYHSSTQYSSYGSNGVAPYPTSTSQTQAPYPNSSHIPV
GPNYTASNDQHSYFTRHPSDSQSSPRTSPYMQAQSSPGLQRHPTNAPLPS
RPMEDVPEETTPWDEHGRPLANDDVDEERIAQETIMQDIEAELGGSEYHS
QRRPSPIGSQAFDDQTQRLRGYSSASAHSPGSRTSPGRGSSRPRFDDDDD
DDDDPEGTAGLLAMQQAELDDQRFGGGTFMYSGTDLAPAAPLPTTMAAPT
ATIQSNFPAPPPEELNHSSDSDLGGMDIGMLSGGYAGTLAYGADIGSPPG
SSSMLDGPRPLPTPGYYNSLRESYDHAAAFNNAGMDYVGTGGLQAPDAHR
LSFDEGGEERVSIHSQHSGTESPTRDDYQDLFYHPGLSNRPLPALPPGPG
SDSSSMLSVHNSVRSQHQHDHSINADARYYQADGPETFYQHGAQQTLHPE
RSVSLGGRSHTPQIQAPARSRTDAAEERRKMNRNHQITQQGPAFDYDRAP
VAASGAFDSITLPSGRKRRFVPSKLTSSDFNDCKAPWALSHIETWIRNMG
EGEPDLKEKAIEEAVTNLFVYKIPTMNVADAEALSSQVVSGMLESQVLLP
DEEWVKFGDGHISGVLWQLTGSGCYAPKLHDVEMSGRCYSHYCTRTLKKV
DLEVLSEEVQGADAWHTFYKLTKEVVDSKPKKEVERQNILHEIVTGEENY
IKQLDIFRRLYRDDLRTRAPPIIHPDKRDKFLTAVFGMLDTVVHINKDHL
LAQLKYRQQEQGPWISGFSDLFREWIRKAKSVYTEYAIGYPRAAYMVRKE
ADRNLLFKRFLEDKQKDKLSAKQDWTHFLITPVQRLQRYILLLQSVEHKM
IGDSEEKTNLAKAIQEIQTVTLECDAKVAETNKRVQMMELDRMLVLRPGF
QSVLNLDHLGRVLIMQGELQRMGSKGMRWVDSHALLFDHYLILAKVVVPK
DGKGERKYDVSREPIPMPLLFLESMNDEPVMKQKGLTAPLGRATAAPVGA
QLNKVASNGAGRPGLEHTPAAPGNTLVPTTSNDTEGKILYPFKVKHLGHE
VYTLYASSARDRLDWCNSIIEAKTRHAKALYAQNAEPFRLRVLADAAFHY
DTGSVYARASGVPVKGTPLDRAIQDLDKVLGSAQGTAAVCRAQVNCATGF
SAFGKSVIAIGTDYGVYISDSSNPRGWTRSVQISRVTQIAVLEEFSVCLI
IADKSLISYPLDVIAPVSEFAAPVNDNTRRAPQRLAKDVTYFATARMKER
LLVFYKRKEGMHTSFKVLEPILHKATEKKSRLFGGRKSGGGSTDTFRDFD
EFYLPTDCYSLSIFQTYIAVATSKGVEMLTLDKKQPMSIPDLKTHGIANI
AGRIREQKPLGMFRLDTNEFIVTYEDCAVYVDKHGEVSRTLIMEYTGKQK
KAKGATTYKQYLLLFNEDYVEVRNAGNGRLRQIIAGRDVRVIDFGLGGPT
GGNAAQSQQTYGPNGQSLLAGDTSKGSVKIAMCHPELPGRQIVLELLLND
GHCEG
>Mb|QLI68889.1

MSRYFPHTSYAEDQPVPHVVLATHVLTRALAAGSLAGLGVAAARQAVPAW
RRPRPGGGPALVSHRILRASSAGALWGLGVGAAALAGRMYGRELVEWQDR
SWRLLENRGQLEMDDWTYLGMGAGVAAAWAAGSLKGLGGRGVMGAVGAGG
VVGGLAWAGWRFGVNGGRFKEKPKEGDL
>Mb|QLI68893.1
MPSIGPQLPTSPPKRKRSSSPPKHTSLPPAKQARASNNADEIDLSDSDSD
LSFGPPAPPSTTTAPTKPCPPAKPDSTSLPADSDSSDSDSYGPSLPTATS
KRPTIGPSLPATDPAPQRDSWMLAPPSSSGYTERDPTRMRNRKFTSKPAT
TSSGPSSIWTETPEEKLKRQRDALLGRTAEASADGGSASQRTKHQEERDR
KIAATIDAHRGKSLYDEHSRKRRDDGAKNGRAAHDNDDDDDPSKRAFDRE
KDMAVGGKIGEKARRELINKSANFGGRFQKGSFL
>Mb|QLI68898.1
MDKSQAQEYLSSLLNQNLRVHTTDGRLFWGAFKCTDPDKNIVLANTYEYR
QPSRPRLVEGPDGTTTEDRTSRYLGLVVVPGHHIVRMELEEFASQVSGRQ
VI
>Mb|QLI68901.1
MSISAEADGLRSSYRTVSSPPPVTDSAMLRPNSVNRVSSNSMNILSPSDI
VGPSPVTSNGTETTEIEDDALEDVEQTHYGDMSQSSELLMLSTNLPENFR
QASGDEAVSVIHAPESFASWTSTESTAQQSQPSDRSFSGREEENSAALSS
PMQKPISQPPRPPISTDVKPVRYSIESATPRAQDLQDMLGDGSRLRSSST
SSLEKIDEQTEAEEDDDGNDDYSATPFLRPADDEITALRAALKECWTLCN
TLASLSAIHRARVFYSSGTPDAHEKAWKTCWKLCQRLYDNQDEDLASHNV
RMNLELCRDFCQALFDVRQKKDETSDSVLRVSFELNNHLYSAQDSRKLPE
PFRERTLDFYITLCHRLMKQRSELAEETDHLLSACWTLAEMLFSLRQNKR
DGNPVDEELLGSAVQACWDLCDIFRDGWTQIRPDRNTPRPSQTSFFPQQP
IEQSGRESRQSNRSSLHSKRESVKSSHQEERPREPPPVPETPVTEFEDTP
ISPESRSPQMPNIMVLGTPSDTGLGGRWSSNTSNISSYSRGSNRTSSTAT
TTTATEDPNISRSKLLVLRAAMNIGFNRDAIQDPMSESTVLHKFVQGLPA
GSFGSLSSHATLLQQYKSSVLSDKFLPRNHALPVRGKRVIAQDMAKSVLA
MSNSSPRYAYLRDLFKFVFQFGVDEAESRRNVSIAI
>Mb|QLI68903.1
MTTLIDPAGGDTSLINRLRPVLPKDIPFVVHHVSTPPWKTDAIYSAPPNR
RPDRTYCENHFLTVSIDIPHSGEPSQNGTEPNKQVIVFGIEIFIYTTAHS
TTLFVSKADSTGYLPLLNLPKGTPSPIRGVCATFIEHLVGARRRQGIPFI
VSLFARSQGQYLFPGSIDHGAKHVLDDRALVRWWCRVLDPLITSPPDKGT
TWKNAKGYLIIPGLDTHETRAFVPRTAAAASSWALTHPLERISHYTREYD
WVPPRCLIPRFPDDPKSRFRDELDEEAGQSGAMKAAGSWKTVKTLDVFWD
MMAFRQECSSGRMTGFIWVVFDNEAPAGAVQGEPSSAMPRAPFPNLGRPF
PPRANKFEKYARSKSQRKKSKLKGPIQPRQPRAKTGQRNYLLKTPIRSPY
YYWPVQGRGSRIINESSYKRAVEMMLRLDFSTADKAVASSRRWTSEVGFG
SDWGQTVVGAQDPPTLAAGDGEGEAGDLSGLVKRKRADTTDGGAVNVLGA
GLVKKKAKAEDDAPQVNLLTSGLVRKKPKAGD
>Mb|QLI68905.1
MEQTKALNTLEPFLALSKSATSPRAAADLVTRATSSPNTYIFAELLQQPQ
IQALSQNPEFASHLTLLQIFSYGTYQSYHDTPNLPPLSEPQTLKLRQLSL
LTLARDRSNLSYEALQKALGLTSARQLEELVVTVIYAGLLHATLDPARQA
VQVNSVAPLRDLSPGSIPDMIAALSNWSEKCASTLGDLEAQIRNIRAAAA
AREKEKRAAEHRIQALVSEAWDSDKKKDLPGRDALPRRGYNKRSMVDATN
VTSEETMDVDEPPAVEEQKKRSSKRKM
>Mb|QLI68906.1
MNGQEQAPAPAPAPTPAPETTHHELDTPEQPAAKRLKVNDAAHSRAGSNS
TEGPDGQESRDVPTNGNDETVAEPRAPIDRRVGTTPIKKEYLLGVSTSRN
SESEMADDDAAEGRGGDDRDRRDNHGGAKGKRGKKEKRKKGQNTERSFGR
FGDSIQLCNSRAMYPEFSPHPCKFGDECRMSHDLRKYLEEGRREDVETFG
GKCPVFEQYGVCFSGWKCRFVKSHMRELEHEDGRKELVLIDNSNKTESNG
TVPMATSTKVSGGDELDERRAGIYNNVAMSTKIDLNRKRIDFAKADEYIK
WMNDEATLNNSFHHRRKDQSNEGIEDLRAQFVDPPFKPSEKRRLYFGPET
PTLAPLTTQGNLPFRRLCVELGCELTYSEMAMGMPLLQGTKADWTLIKAH

ESEIQPPAFKPSSNNYVFENYDNSRDLRFGAQISGNQPWIVTKAADVLNR
YCPHLRLIDLNCGCPIDMVFKSGGGSALLESQGKLERMIRGMNAMSGEIP
ITAKIRTGVKSSRPTAPSVISKLAFGSREHRERLGAPGCAALTLHGRSRE
QRYTKRADWSYIGECAALIKTYNEQRDALADTAAEPDASTLPNSKDGRMY
FLGNGDCYSHTEYFEHVSKSRVDTVMIGRGALIKPWLFEEIQQGQYLDKS
ASERLTYIEKFVRYGLDAWGSDELGIGFTRRFLLEWLSFAHRYVPVGLLE
VLPPSLNDRPPAYVGRNDLETLMASGNYKDWIKITEMFLGPVHPGFEFQP
KHRSNAYEAEG
>Mb|QLI68909.1
MSADDQQFLDVVLAHIPPQLKQYSNDVAEYIDQNVDQAARIVRETLSQAK
WLPEYAKPSPPLKPAVAVVSMTRFEKVQGWVSRHKILAAVIVFTCGTMIF
KGYQTSRSMRKTRRAKRARNGGRTEVVVIAGSPTLPLTKSLSLDMERRGF
IVYIVCNAAEDESMVHALSRPDIRPLGLDTTDPPQAGAAIERFAAFLQSP
QAPGPHIKPNSLTLKSVILIPSLHYQTSPIATIPPSSFADLFNTHLLQPI
LTIQAFLPLLASRLQPIGEKWIPPKVLVFTPSIISSINPPFHAPEATVCS
ALSAFTEVLTAELRPLNIPVTHMQLGTFDFSSFVPSRAAISRQGLVSAGS
PQEPLVWPDGARHVYGRNFVAQTSSAISGGRIRGLKGSSLRRLHNAVFDV
MDGSITGDTVRIGLGSSVYGFVGRWAPRSLVSWMMGIRRVDELSAWKTSS
CDGSEREESEDGEAATASSNDFIAVPSERNVWSADATESSLWVPPVSNSP
RS
>Mb|QLI68912.1
MAPEERSTLLRPPPADPNKAPIENVLEVTEVGVLGPDIFTNTRRAWVPPG
ARGVYGGTVIAQCLASAQKTVPDDFYPHSCHCYFILAGSGSIPILFHVER
VRDGRSFATRTVQARQRGRCIFTTTISFVREDAGGERTVRHAVPMPDEAK
APPPLDWQEEPEWARGGPFQNYRMPAVRGADPTARPDEQRCQQWMRCRGK
ISEAGGRQAHLNALAYVSDSTFIGTVGRVHRLWRLPFKPEDYDGMAEEYQ
PHVKELAEFEGLGSTVEEWKTRPKLGMMVSLDHSIYFHEPGRVKVDEWMF
ADMESPWSGDGRGFVTQRIFAKDGTLLATCVQEGVVRLAPDEPDKSKL
>Mb|QLI68919.1
MARLRPTLPDGRLGNQQSVVDPSRLQIRVVPYSPPRISSDGSTPSRPVSY
VEVSPKRPPLASCQDRENQPKNDTGGSLCLLEVESQLQDSTGPDPLMDLT
NETAESADHVNKVENWSIVSPTTIPAPPSPSYRPPRRTIAVNSDKTFSLL
PQSCSSASAVEGLRSPRRSSTAPSSSADRTISGTFGDDQPSSPLTPPQEL
SRSPCSPCSSPFPASFIAASSPSNYTLVGGVRKVPDNDGGGKAKALSIPS
LPVERDLQAAFVGSRPSASGYTQPGMLSSKQSFPSESASTLSERSNYKTF
VSESHYSGPDRHFEAADDSNAKLSLLSSSHSNFEIIGETCSEQSAENRSS
SDTNDSNRNYVLHGDFPSSPPTAVASGCVLKTEYSRESLVVAPLRPSKRF
FSDQATVSHQKSRDSIRTGSLTSISSVFVEEAARSLFAGGSVITSHGGGI
RHTSPRRNTTIGPGNRHQNLSYHHHWSATLSPILSESDRGSAVPSLAFSH
TTAGDRRLQSQSFDVGTPDLPRNDGQPNVEAWMELPPPASYRVWNRDFNN
SSLRLIRDQDEHGDGLAELEELHRRPSRTRLHSYLSSLPSDRNLRSSGSS
RSNSFSRCHIPAWARLYYGSGERRFLTIQHSSESLFSEFSASVHNSPLIS
RSPSVKRFTPGTQLPPQHRPPPAPCEAPSAPQSASDGPVPCPPLNMVCRI
KKQTSSIWSPHLRRDRRSRRYNLWRPPSTVWIKEEHISRRSNIQAILFVS
GFVFPFAWMIASFLPLPPTVEADMKEKNHSTSHVDLEQGRSGSNLEQDSD
NCYNKVKWWRSLNRYMSIIGLLLVGAAIALIVTSTQQRKP
>Mb|QLI68921.1
MDLPLGPVDDEKADPTWDADGSHWSTSSDDEEESVTAIDDLPRVSGTGSL
ATATHVRVELSNRPHLALFNKAAGAPKMEARGNIKHEPLEPARAQSSDSD
DSSLASIPAIARLKETPVPLPPTVAAAGQLPASVSSQPRLAGVPSTTPRA
PPAPRASGPASTPTPVPLPPTVMQNASAPQVSGQTPSSSAPATSLPDSRK
PAATSSTPSSGLGGSLHLPFTNEKIEPTLPHGAHSTATPRKRYPVPMEIA
ADGRPYTVYVQPNGKSVPTKGVLIPAGYELHSDPQLPFSCPVRGCQRRFK
NLAALSGHFGSSHSNMTFNDNGDGSISKFGNYRNPSGRSPGIVVAQVPFP
RATHPALKRGLGEDGAGPSKRQLEESDAGQDRKRRAMSEDREHNQTTQFE
TDVSKYMATVHSGFRTANRRDISRMLALPRKRDLPEEFLDKHTYTDLAAQ
TYACAVAYVVGDEVSGRSACHAATGPLACLSKQCIVLPKSIQLDPKIVRI
FTPHHRCVGCAYLALMTSAPDRCELRTEPSNAGSGDKVVPTSSGRPLPDV

PTLRSSQPAPQQEQQSSPMPSGQGTEQTPSRRPVRQSVLNRLQMDKDQAA
RPKSKSPTEQPSTQVNRPQTDRDQTARSQSKSPTEQPGTQLNHTDKDQVA
RPQSKSPTEQHSSTRLERGTLPIKHHGQFGTPEYEMEDWEIAPGRVTNED
ASRNIAYSGAFLTSSTPITISHDIDFNILTIRPGQIFNVPVQKGKIQVFS
VASGKLKVTTGGKVVQLGPNGVFPVRPGEKCVIENRIYFEAVVHCTTVKD
YELA
>Mb|QLI68922.1
MSRIKTRILIISDTHASRPHPPPAPNEFETADLVRVTTGYRDPLPPADLV
LHCGDLTKRSSIPEYETTFSMLRSLNAPLKLVIAGNHDISLDPTYDSDPE
AEQESRVVQDIVQDAQRDGVVYLTEGTYEFALENGALIRVYASQYTPIYG
GWAFQYPGHHDFALPPGIDVAMTHGPPYGVLDEAGISKRLGQPHHAGCEH
LFKAVADARPRIHCFGHIHEGWGAYRGTWLDTDEPGFSVPRPTKECVMDE
ATSGPIQTLDGLRPMFAADTQGTCPEARQKLAHLSNQRGYHIDLTEQQTR
VDSGKQTLFLNASIMDIRYRPSQLPWIIDIDLPNAEKRHTSIRRT
>Mb|QLI68926.1
MASQPWTPSTWRSKPIKQAPSYPEPEKLTKVVKELSRMPPLVHPNEILAL
KAHLRDVAHGKAFLLQGGDCAELFDYCEQGAIESKIKLLLQMSVVLIWGT
NKRVVRIGRMAGQYAKPRSSPTEMYQGREIPSFRGDILNGYHVDERDIDP
NRLTKAYHYSAATLNYIRTSIASGIADLHRPLDWGLGHVRDPVLKEKYSS
IASSIQQTLRFLQVINSRPDELHTVELFTSHEGLLLEYEEPLTRLLESPP
ARPAPPASSRAPSPPLPADDDEPKTQQAYYDTSAHFIWIGDRTRQLDHAH
VEFFRGIANPIGVKVGPTTPTSDLLDLLRTLNPDCEPGKITLITRYGANK
VGELLPKHIRAVEDSEYRRCVVWQCDPMHGNTLSTPSGIKTRRFNDIYKE
LQESLRIHKEQGSYLGGVHLELTGDAVTECLGGSEGLEEDDLSTNYTSFC
DPRLNEKQALELAFLVADHFSQEQKSDS
>Mb|QLI68929.1
MFRNNYDNDSVTLCAPPRPGLCDVSQASITYSPQGRIFQIEYAAEAVKQG
SVVVGIVSKTHARNAEELSSYQKKLFSIDEHAGIAIAGLTSDARVLSNFM
KQQCLGHRLTYGRAIPLRSLVDMIGEKAQINTQVYGKRPYGVGLLVAGVD
ERGPHLFEFQPSGMTEEMAAFAIGARSQMARTYLERNVESFADCSREELV
RHGLKALRESLVQDKELTVENTSVGLVGVQGEGLKDIETFKLYEEFEVKQ
WIDSVGESQGSGEGEEGMDVDS
>Mb|QLI68931.1
MPTRTSKTRKLRGHVSHGHGRVGKHRKHPGGRGLAGGQHHHRTNMDKYHP
GYFGKVGMRYFHKQQNHFWKPVINLDKLWTLVPIETRDAYVSGAKKDTVP
VLDLLPLGYSKVLGKGRLPEIPLVVRARWVSKLAEKKINEAGGVVELVA
>Mb|QLI68932.1
MASEGKKAAPKGSKRANAAAKAALVGTHAHKQRKVRTSTSFHQPKTLVLS
RAPKYPRKSIPHQPRLDEHKIVIHPLNTESAMKKMEENNTLVFIVDVKAN
KAQIKLALKKLYDIDTVKINTLIRPDGSKKAYVRLTPDVDALDIAANKLS
LV
>Mb|QLI68933.1
MDPVHSLADATAAAAAASSSSLSMLTTVRDEDGCNPIESAAPTPGVVADT
VKSTSPSPPAAKVDESLVRHAMYSPTSPRLMHSGSPPPRKDTLSSISTQA
TTATTASAETNNTSYSADTSPSLHQSIFSVKDGSDVSNTRRASRRRTGPL
SQQSRERAALIRKLGACHDCRRRVACHPSHHNMTWEDLLHRFHRSQSPN
SQDVTPSFGAGRPLSPASSLSIIQPTFAHETQAMDIDSSPTAYHQPGRHP
LSEARIRTPLPSGPRLEKSLSLPGIESLKNELQHNVPKILSTTHRGRYNS
VQVLLLFWHDDEDATTIQDALQELAHVFDRSYHYTFHIQAIPSASDSSKS
SWRWLSRRLNDFAEDCDQRDVLKIVYYAGHTCLDGNREMILTSSMDKDQA
STIRWSGMQQILEEACADVLILMDAAYYPSSKIVRRKGVLELLAASASED
HATSLGRCAFTRALSELLSARAARSAPLSVVELHALLLSHYPKIVRDTTP
ERETLTTFPVPLHTMMSGNSRLPSVFLSPIHHPSPTRNSFSYENNPQLHL
SIRLTDDNVDIESWNEWLRLMPEGVKDVKIDGPFRPSFRPG
>Mb|QLI68940.1
MDPKQETVDSQDILQEEIADLERRLAAAKTRLSTPPKGSDTPQPSLQSPL
NSPTHYLLLLSDSQLPIGSFAFSSGLESYLAHNRRATFSSFLPLSLSSYA
ATTLPFVLAAHRDPCSLIELDDQLDAAVICTVGRRASVSQGRALLSIWER

SFRATLPKSADLPVLREFAALLKDGSADGEAPPVSAHLAPLFGAICAIVG
LTLHQTAYMYMVSHLKALVSAAVRASMFGPYQAQKVLAGEQAQRWIGRVI
DREWETRVDEAGQSVPVMDLWIGRHEVLYSRIFNS
>Mb|QLI68942.1
MASHSSTPTTTDTWTGSGKTGDVERDGDIHGSKTHRFIEKHSQAPHTPSE
PPSPTSDTGPSGNEALAEPEAGRTNVETFLVVLALCLALFLAALDMTIIT
TAIPTISSTFNSSLGYIWIGSAYLLGNATFVPTWGKISDIFGRKPVLIGA
VVIFWIGSLLCAVSNSMGMLIAARAIQGIGGGGTIVLPNICISDLFSMRK
RGMYFGILGGVWAIASAVGPVLGGVFTSKVSWRWCFYINLPLSGVGILIL
IFVLKLHNPRTPVKQGLLAIDWAGSLLIIGGTLMFLFGLEFGGVQHPWDS
PTVICLIVFGVITIGLFVVYESMFAKYPIMPISLFRHRTSVASFSLSFMH
AFTFMGGSYWLPLYFQAVMGATSLLSGIYLLPFVLSLSITSASFGIIIKR
TGNYKIPIMFGLAVMTLGFGLLVSLGDDRNWAKIVIFQIIAGIGVGPNFQ
SPLIALQTNVEPRDIGSATASFGFLRQLGTSISVVAGGVIFNNQMKAQAG
TLQRELGPQLASMFSGSRAAASVHLVRTLQGHDAAVVKNAYWNSLQKMYI
LFTCTAFVGFLMSFAVKQKSLSKDHTEHKTGLQSLKQRNDGSAAEPAGKE
AS
>Mb|QLI68944.1
MSSPGPALSQGFGYGIILGVGFAFAIFMVALTHILKRFNNEVQTSEMFNT
AGRTMKSGLVAAAVVSSWTWAATLLQSSGVCYRYGVSGPFWYASGATVQI
LLFATLAITLKKRAPNAHTFLEAIKARYGPPAHFTFITFGLITNVLVSLM
LIAGGAATINALTGVHAVAAIFIMPVPVVAYTFVGGLKATFITDYIHGFA
LFIIIITFALTAYATSDVLGSPAAVYDLLVEAAARHPVAGNQDGSYLTMR
SQGGAVFFVINIVGNFGTVFLDNGYYNKAIAAHPVHALPGYIMGGLAWFA
IPWLTATTLGLSALALESSPRFPTFPNRMTEAEVSAGLALPYAAVALLGK
GGAAATLVLVFLAVTSSFNSELVATSSIFTYDIYRTYFRPDAKGKHLIYM
SHVCMVVYAAVICCISVGLWYNGISMGYLYLLMGVLISAAVLPATLTLLW
RGQNRWAATASPILGTACAIVAWLVTAKKTCGALDVVCTGSNDPMLAGNV
TALLSPVVFIPVLCLVFGLDKYDWQSMMDIRRADDHDMLENEGLDPEAQR
VAEMAEQANFDQEQRKLDRAFKIACIITISMALSFLILWPMPMYGSGYIF
SKKFFTGWVTVGIIWIFCSFIAVGLYPAWESRDTLVKVVRLIVTGKRPQP
VLVAEQETSADSSGTATPTKSGSVSKTG
>Mb|QLI68948.1
MSDPEVTSPLGDSNSSGEQETAPGHDVDSDTPHLEEQEMGNGTELHHVDH
RRDLLESEANASEVSSIDASLVDSLPRRAGSPVDSVTSGRRGSIQGSFMS
SPGSSVLPSVASRAGLSSPSPSFRPFDRRFQSRISSSSSNTPRPLSPAFL
SPHSRNASLSSNFFLDQNETDTPSPPWEVVRWTRLRKMNAQAFSEAGRRN
FGSPTSLAVSASIVLGTSKGPGTKAVESGAITAIAISADHTTVAGGHANG
NIFTWDTGRASRPFLSIPHLDETQAENRTTDGHVPNAAVVHLGFLGTRHT
ALVSADDRGMAFSHLATRGTGALGRTVKTTRILGRYPNAPAPAGKSIKPS
TVLGFAPLPLGNVEQATDSMGLTAILTPYLLVVVSTTPVAQTQHKSARPK
DVPAHSAMAGCLAWFPAVKLKTPDTHTRSDISRVKLVYCWSNVLTILDVE
EMYGEDADQPPSLKFKARSRWKCEEAIAAVQWLGRSVLAVLTISQRLIIL
EDRSMRMTEAFDLLQKHIYHKDLFSKQLHTLVEQLDEEDTTMHGVVADAF
YMSFKAYKGRMFVLGFNEVSIGALSNWADRLIAMMENGDYIGAIKLATSY
YTGDADKLTVGLPEDAALRHVMVQDKIMEIMSASLKYVFAQRQTQGKQDD
DADHLRELAETCFAASQAIEASDFLFEDMYEWYSDGDVGGIFLETLEPYI
LEGTITVVPPTVVKDMVSHYVTKGWEGRLEEMICHMQTATLDLDQITVLC
KQHGLYDALTYVWNQALNDYITPMIDLLGLLVPLVTNGTNNGIIEDDYFS
VNALKIFPYLSYTLTGRVYPNGELMDEETADKAKAEIYWFFFSGTTIEWP
RGSKKEFITRPGDESEPAFPYLRMILKFDAPSFLSALNEAFEDSFLNDSP
ENQVNGGSRMDMPEEQIFGTTINRQYVVSILLDVMNPDEFAPEDTIYLDM
FIARNLPKFQQYLLFSGSTLSKVLTGLCAYPGDDLAEDAQLSAEYLLSVY
QPPDMPSFMLQFKKAGFYRILKRVFRTEKQYGKLIATYFEDHDDRESVFE
CIAQCLRPQKGLDVRQAEEVQQVIEQHARDLLEISPEQTAQTLARQPTAV
HRHIIDSVADDAALQHAYLRALIEPRKSGDDEGDIKDQELTERYVQLMCK
FNPSHVSDFVEAMRPTDLRLEKLLPTMEENGVVDAAVVLMARDGQVNQAM
DRLIKHLFTLESALQGLLTEANDSADDVDVDTSTVEMLGSLQKYVHVGIW

LCQGQTKTSKKVNAVKKGKTLAKDSLSEDEALWLGLIDACVQITKRLSPI
ILEQAQSSVLKDDKALALLRSLVQHTFTSLLTATSSPIRVQTGSNLLSNA
GNNLSFLKILRAFLANAAASSPNLADLRSVLSSIFAAYAYEESILRLSNR
LLERNLFVNVNQSVQLRQRGWRPRGSTCEACGRRVWGPGVAGGSVFEAWE
DNQAVEDDLRKQRKIRIAEKAKGKVGESDGKDKGKSLAVRPSSMLIESDT
AVAGKEAPLGRLVVLACRHIYHQSCLEALQEKQENGVGGREREYKCPIDG
>Mb|QLI68949.1
MAALRVSAKPTTLSKSISRSMATEAPARDLTRSANTQSLVQSWNPVSTVP
VTVHSFPSLEPTSLEQWSVQHLYLPLRRDILHHAITFEGDNTRQGTASSK
TRYEVHGSHRKVRPQKGSGRARLGTRQSPVLRGGGKTFGPKPRDFGTKLN
RKVYDKAWRTALSYRYRRGELIICEDGMELALPPDFEMLADKYMRDGLRE
TYLQKYMTGVLRTLGLGRADGRTLFVTGDPRDRLYEAMEQVPWEGRALDV
EDVDVKDLLETGKVVMERSVLKEMIERHQSDLITRIAVGGLAKSAPSTGE
VVL
>Mb|QLI68950.1
MSNNLSYGQGGHYSSQDVTPSNTNQNFDGSAQDGKIQTIQKDLAAATEIS
RHNLNSMKDRGENLDGLQDKTSNLADSSQQFRRGANRVRKQMWWKDMKMR
MCLIVGIIILLLVIIIPSVVATRH
>Mb|QLI68952.1
MSTMPATGGHSKPCCNVPPIVTKGYKPKGAYEEIGGYKTYVTGPSDATRA
IVVIYDIFGYFEQTVQGADILAHSDSEHKYKVLIPDWFKGNPAAIEWYPP
DTSEKKEKLGAFFGKFPPPATAAKLPTYVQAVKQQFASLTKFGIVGYCWG
GKVATLATKADNNVFGAIASVHPAMVDPADAKGINVPMALLASGDEPAED
VKKFEDTLKVPKHVEIFKDQIHGWMAARSDLSNDRVKEEYERGYKTLLSF
FGQHL
>Mb|QLI68955.1
MSPTGWSDMHILRDINGQLPLESDRPEFVGAAALSSRLEAFGNRRPSHRQ
SNFGRAAISPPPLRRLPDAAPGHSHRTQPLPTSFGRDSASSALNGVCDNP
LGDDFLETALSMARHTTPPLPPPPSSHLSSEAPAINGTTPKAQTDSIADW
TTPKIISQLNTCRQDVKEGHAKLTGYILESTKVTERRIRHGRDLFANVRT
PVLTEKKSTTMRIKSKQHLKGKRDQREGHYTPTCTKTNKDRVPPYRFHHV
EIKKNVLTPNTMLTFVPHLRDLESSEETKYNLWLKELEDIDLKSGFKPMN
REDKQQLTIQGERAATVSLYLDTWLENMAIPGCDKSALISYMAAREPDYA
ITPQQKSDILNSHRKIENVVPGANKAAQMFTDAFHQVFKNTQPASKQIEL
RRVLMMDEAVDNIMDSKPIAKDATSPQLHDEDEDELAESNLATYCILGCL
ICFSHSCDHGEYDNKNMKRTFSISSCSHLSDALKRRRNGMERDRPSPIKP
CRRQCYQRAGDHVRMHLHPRPWLEDERIVLRSIFTTASHSTYMGDPICLA
AEFLNRHCDEVYTEFKSMGISLPQPEPPEAPRIKNLSWYDRRRKALLGDW
QDHTTSHAHQRRENLEPCSHEGPCVPGVCTCVDANVLCEKFCGCTVENCA
YKFTGCACHSQGKTCLDSKKDKPCICVQLNRECDPQLCGSCGALERADPL
NADDAELHATGCQNCDLQRGVGKSLLLGRSQLDGVGYGLFTAEDIAQDEF
IIEYVGELITHDEGVRREARRGDVFDEESNVSYVFTLLENEGIWVDAAIY
GNLSRYINHASEHDNRGCNITPRILYVNGEYRIKFTAMRDIEAGEELFFN
YGENFPNLTKKLLDHKAAGKPETKSKAAKSRRPENPETIARKATKADDTK
RRPGRPRVKRDLPPGFALEAVDTDTAPGPQSRKRKRRLQDDSDEDDYDGV
VSVKTANGDGGRGGRESEEPGSASRLRRRARTSTETPTKRTEPPKKTRGK
RGGARPGSGRPRKHPRPVPKPAAASQSRGDDGSTVAGDAAPSPGAEDDAT
PASASVSVSAPVSLRHANTPEPKRITRHSEKMDMEIADSEDNMSTAALRS
SGAKTTDVSAAREDDDDQDVVIRQRSDRASRTRRPPAKFRDDDIWN
>Mb|QLI68956.1
MFRTALLRTARGAARPLAAAPLSARIALPRAAAVPKMLGVRMYSAAGGLK
KEEVEGRIMSLLQGFDKVNDTANIKPVAHFANDLGLDSLDTVEVVMAIEE
EFSIEIPDKDADSIHSVDKAVEYILSQPDAH
>Mb|QLI68959.1
MTGTKGPRPKDDKTKKRKRGITESETTSKRHRQQNRESKANGNNTPTPQK
SKQGESNHEVLAGELSSFRPQHVIRQSDDGEAGWRVSKPMGGRMLDIDPI
LTKDEQYLILTYNTSIQIYNANDSLLIRRIPISTLDTSASKGSIPAHIVG
TRLSKNNPQLVWVACSDGHIYCVNWSQGDAPSPAFQTSSKTAKALAVVPV

AFAEDKEILLVAESEKSNRMEVIAYQLVNGGKRKSNSVLSLQKPGYGLQI
LETSEDGQVIVGAFQDSLFLGTASATAESLDQLQYEIFSFDSPDLITSID
LRLHARFSGSRKSQAGADTAVDVIIGGARGGIYVYHDALTHVRAVGKAQL
VKDGIQVQKYHWHRKAVHAVKWSTDGNYFISGGSENVLVVWQVDTSRKDF
LPHLSGSIENIVVSQTGSSYVLHLDDNSAMILSTAEMKPTAYIAGIQSAA
IDVSEPKDLLVRRVWNVSEHVRQPIPASIRPSDPSKLYVCVSNGRQETLS
GNLSMPLLQSFDLESFTSVSKQALARTQPTDVNLSSKGHPIDEPVVTNIN
FSGDGKWLASVDEWKPAPRDVENISQDLRHQFMRERHEVYLKFWEVREGA
ESLELVSRINAPHSTTCPESVLDLASDPTSSCFASIGSDGVVRLWRPRHR
QQNGVSVKDANGRDAVSWGCTQAIGVGDGTGIENGTDLINPTRHIEVQGR
VVFSEDGSTLFAAFGAVDPGVVYVIDVASGQIVKTLEGLWDGKLQSIQAL
GSFIIVLSSDLRVYNVVADELEYGIVVPKLYGVTELHQLAVDHTSGHFAV
TLPIGGVSSIGIFDPEDPEPLLVRSTPHRIVSLVSAPGASGFIALDDAAQ
VWVIAEGSDPSSIATLQPLHDLQLDGLSGAVDAGPTTGEVLATDEMDVAS
EDDMADAKEVDEDVDMGDDDDDDDDAYGSVIPQQFLTEIFDATPAFAAPS
IEDMFYKVTGLLATKPHAEQ
>Mb|QLI68960.1
MGNEGNQLSPDEGATSKSGRGLLKVPSRSSSQQRNQSSTASTGLSGATVT
DQRSSIDGRSKESRGSVPGRQRNGSASTNRTGGDSERGHNPGSFQPSSPS
TSEQKRRRKKKKNGGLLSLLGCCGVPDTANALEEDESVNVQKVEKLSPRP
ATAKPKPHAAQEQQPGKSLNEKERSQSVPSLDETEIQPSSSTQEPQPNPE
QAKNESKQPVPPAVTVDYPPGSPDEGAESPDMAAATNTGDVEMQDEGKEE
PEETPVVEDSQDSQPRTIPPPPAASGPSAVSTVPVAETGASVPEPQSWLL
PPIAAEHKGRKCLVLDLDETLVHSSFKILHQADFTIPVEIEGNYHNVYVI
KRPGVDEFMKRVGELYEVVVFTASVSKYGDPLLDQLDIHKVVHHRLFRES
CYNHQGNYVKDLSQIGRDLKDTIIIDNSPTSYIFHPQHAVPISSWFSDAH
DNELLDLIPVLEDLAGPNVADVSLVLDVTL
>Mb|QLI68962.1
MKVVSKEEEKAHYNVVVKGGLIGGTASLALGLGGVVFASRRYPAFRSLTV
PFRAFLVTSTATFGAIVNAERYSNNFQRQNDPSYGYKDASQRAIQIAREN
ESTYERFMEWGKENRYSIVFVSWLASMAVALALVGRAPMSTSQKLVQARV
YAQGLTLAVLVVSAAFEMHDAKKGSGRWETVMVVDPNDPEHKHLIEKKIH
KEEYEGQDLWMDMVEAEERRLAAKKAAENRA
>Mb|QLI68965.1
MLEASHGQNLHFCAHGLIDRAPFRNFGSKLPNLSRRNLSTTNQILPNRTA
TMGRELQKKKRRSNRQPIKQSNKTKKILNPRGNNIIAKNWDKKETLAQNY
RRLGLVARLKAPTGGVEKKLGATTTGISKNDPFAITSVGKAIISEAKVER
DADGKIIRVLGTTRPNPLNDPLNNLDSDSEEDGAGEEWGGIAEREAEEED
TTDVVRSLIEESHNPAPKVARYQSEGEREWLEKLVEKHGDDYGAMARDTK
LNPMQQTARDIGRRIKKMRSE
>Mb|QLI68966.1
MHIGVMRDDYHGRSPDRGRHRNDPDNMGASRGRGRDREGQDRLDDHYRSR
HDYSRSPPRHNFYDDQREKAPQRHHPDLGGERDYDYRYDGPDDEYRRRDR
SPRHRSRHLDDRGHSYEDMYDDGNRDEGRQRGRYDDDEPRDGGEYAHHDG
RRGRSRSPYGRHYRSRSRSRPRDAGKPTDTVILEGLPYSISSAEVRDSLL
ANSIAAEFPSIDVRLSSSRGNRRAFVQFEDVDQATAFVEEHFPKIRIELQ
HPTDDAPDGKFEAYVHYARNRDDGDTRQIRAADWICPQCDFLNFSTRTRC
KICGTQPAATNWEQSLTGAADVADRAEAVSQILVVYPLASFVTEDMLAKD
LKLLEKSEEPKSSTNGPTKLKSTAPGADVSRYGAKPGSLHRVFLIREVTS
GESFKYGFAEFWTLEDAAAAVTKFRMSRSFTVAGCAVTVAPTHMGVFLPE
EREIAPNIEHMSFNPLFNPSLRVRYRDMHVFPSQLVVTSEAPADVNLNAA
KSTEDEQGDKKQKKRKAEGSLATSAAKKSVPMAGQMAVWQRKHDELRGVE
EGGDASNGQPDSTTKSAPIKISLSNSTKLGAPSAPPTSARHPSPRAKSPT
AQATPPPSSPQHAPESTTPKEETYMDRERLMCLICMRKYKSIDEVNIHEK
SRNHRSAMEDSEKVKAALPRLATRDKRQKQDAQVTQYRDRAKERRDIYNQ
PNRPSQAPKPKTESKKPAEEAKPAVKAASKGAGMLAKMGWTTGVGLGANN
DGRTEAIATNAYQEGVGLGAEGSNLGDAAELAERKTKGSYSDYLTTVQDK
ARERYNQMN

>Mb|QLI68970.1
MAPSHIAIPPRDAGAVGAWGVSKSPHVATTTLRVEGMTCGACTSAVEAGF
KGVAGIGSVSVSLVMERAVVMHDPQTVSADQVREIIEDRGFDAEVLSTDL
QSPVASRFTEQKGSVNDPGFVTTTVAVEGMTCGACTSAVEGGFKDVPGVK
NFSISLLSERAVIEHDPALLTPEQIAEIIEDRGFGAEVLDTTKSMREAGS
DEAGASQSDIATTTVAIEGMTCGACTAAVEGGFKGVEGVLKFNISLLAER
AVITHNVSKISPEQIAERIEDRGFDAAVLSTQFESSDLGPLASTAQFRIY
GNLDAAAAQALETKLKSTPGIRSATVSLSTERLTVTHQPGIIGLRGIVEA
VEQEGLNALVADSQDNNAQLESLAKTREITEWRTAFRTSLTFAIPVFIIG
MILPMALPSLDFGKLLLMPGLYLGDVICLVLTIPVQFGIGKRFYVSAYKS
IKHGSPTMDVLVILGTSCAFFFSVFAMLVSILIPPHTRPSTIFDTSTMLI
TFITLGRFLENQAKGQTSKALSRLMSLAPSMATIYADPIAVEKEAEAWAK
SAEEVKTPKTPQGPPELGGSSAYEEKLIPTELLQVGDIVIIRPGDKIPAD
GSLVRGETYVDESMVTGEAMPVQKRLGANVIGGTVNGNGRFDFRVTRAGR
DTQLSQIVKLVQDAQTTRAPIQQLADTLAGYFVPTILILALLTFMSWMVL
SHVLTNPPKIFLQDASGGKIMVCVKLCISVIVFACPCALGLATPTAVMVG
TGVGAENGILIKGGGALQRTTKVTQVVLDKTGTITHGKMSVAKSTLVPLW
RDNEWRRRLWWTIIGLSEMGSEHPVGKAILGAAKEELGIDPEGAIQGSVG
EFKIKVGRGVKALVEPASSAERVRYRVLAGNVKYLEENGVEVPSEAIDAS
EAINAASSKRTLSKSSSAGTTNIFVAVDGKYTGHLCLADVIKEGAAGAIS
VLHQMGIKTAIVTGDQRSTALAVAAAVGISADNVYAGVSPDQKQSIIKQI
QSQGEIVAMVGDGINDSPALATADVGIAMSSGTDVAMEAADVVLMRPTDL
MSIPAALHLTRTIFRRIKLNLAWACIYNVVGLPVAMGMFLPVGLHMHPMM
AGFAMACSSVSVVVSSLLLKFWKRPQWMKDAEAEQTGGLRWASGRGVLGW
VREMLGRRRRKGEVGYVPLQNLEAEHA
>Mb|QLI68972.1
MPSPTDDETPFPALSHAATFSVFGRSHLSPQDAHLSPPRRARDFDGDGTS
EMRRLGGYTERSRSRRRKRAWKKLMWVKQSFPDNYTDQATFLENLQRNPR
LKPYDFWPLVADSTVILQHVCSVTIFIVCFVGIYHERVSPVSVVSWSSFA
TFVGWILWERWVSEEEDKEDEANAAAAASAAAEGSAAPAPVVRAGSVRRR
NRAGSVRRPPHPLRVESTTAAAPKPSGSVPSGALSPASSTANLHGGRLVP
SAPSAAAPTSASLSRLTPKTSSEALRGSGGGEPLVPLDENRTHQRLGTIK
SALLIWSTLLGLSPILRSLTESTSSDSIWAISFWLLAINIFFFDYSGGVG
AKFPASLSTNAALMASTVLASRLPSTGQVFSLTLFSIEVFGLFPVFRRHI
RHRSWRYHVLQTVVLVLGAGLGVGMVVGAGQGRRWPWKSGIVGMILSVVI
AALATGGCSWWLIGLQKYKNEIRGPWDPARPIIMSRTRWNDR
>Mb|QLI68980.1
MSESYPSLTQCAVVAAAFKVLLFPAYKSTDFEVHRNWLAITNSLSLSEWY
YEKTSEWTLDYPPFFAYFEWVLAHLARLVDPAMVKVYNLNHESWQTVYFQ
RATVIATELLLVYALQLFIDSTLLPSRRAAQVAALSVMLSPGLLLIDHIH
FQYNGFMYGILLWSLVLARSKSTLLQSGLVFAALLCFKHIYLYLAPAYFV
FLLRTYCLSTKSYFQIKFLNCIKLGLGILSIFAAAFGPFALMGQIPQLLS
RLFPFSRGLCHAYWAPNVWALYSLADRVLIHLAPRLGLDVKSEALQSVTR
GLVGDTAFAVLPEISPRVCFVLTLLFQGLPLLKLFLTSNPSWESFVGAVT
LCGYASFLFGWHVHEKAILLVIIPFSLIALRDRRHLGAFRPLAVAGHVSL
FPLLFTPAEFPVKTIYTVFWLVLFLMSFDRLAPASSKPRVFLLDRFSTLY
IAVSIPLIAYTSLLHHVIFGKSYEFLPLMFTSSYAAIGVVGSWVGYMVVY
FTS
>Mb|QLI68981.1
MARLIQSLAAVAAILATGTSATGDLSVLTGEIGRQNNESLFWGPYKPNLY
FGVRPRLPQSLWTGLMWGRIEEYNDVKDGLRYTCEQGEDIHGYGWDEYDA
RSGGIQTIHDKGNKIDITTSFSKIPGGSNGGSWAVRIKGQLHEDAPADHK
TVVYYYIAQEGKGELAAQGEGDELGYKGDTTFIGNSEALGDYKIVVTEGN
GKYPAGDHEISASRPGDVTLVHSAEVSEDAIWQAVPLLFQQLQPAAMAVQ
EEYGMENPPPAWQVYRMAHQPGKGNSHILQRTYEGSFEFDVIFSSASAGK
ELTKEDVTNTIAATSSAFADRFSTIFELKTPFDGEQYQQFGKSMFSNLLG
GVGYFHGKQVIDRSYAPEYEEESEGFWEETRQARERQAQALEGPYELFTS
VPSRPFFPRGFLWDEGFHLAPIADWDMDLTLEVIKSWYNTMDEDGWIPRE

QILGPEARTKVPEEFQVQYPHYANPPTLFLVIEDFMERLRKTNGTQPSGK
EKLAQERKNPLHTAHLNNMELGEDYLRKMYPLLKRQYDWFRKTQRGDLKG
YDREAYSSKEGYRWRGRSESHILTSGLDDYPRPQPPHPGELHVDLMSWVG
LMTKSLKNIAEALDMKEDVAELAKTLDAIEHNLNDLHWSEKDGCFCDATI
DDFEEHQLVCHKGYVSLFPFMVGLLKPNDPKLGKILDLVADEEQLWSPHG
IRSLSKQDEFYGTGENYWRSPIWMPMNYMVVKQLQSVAKQEGPYRSKARD
MYNRLRKNLVDTVYKSWKETGFAWEQYNPETGAGQRTQHFTGWTSLVVKI
MAMDDLSEHGRDEL
>Mb|QLI68982.1
MYASRTVCSRCASSVRAAVPPTYRSAPGAGSGSVAWFSSTAPAASQQASS
SSSTPHSFRQTGEGTISKNGNPQPRQPPFSQKAGASSKRQPRRNKDASAA
VALFNDVVQKSEVAAPREFPARPTSRQSAASTVLGEWEIAAKMKELEEKD
VEPLERLRLFQNDIWPHVKELRGQIPKHLYLATTMFLARTFDTVAQQGLT
GASVALSKMCATIGKWDLDMRNQLVLNLCHTLISKKHTSAERNALVEELL
DMWKHISQLRRMSQLHNGRLCFVLPSEDEIIEDISSSSTSLTKQTEPGEV
KSSNMAPTTKALASIFIQFRFEQARELVSGLLATLAILSDPRLARDGSQI
KAAPLLNLVSIVLDHQPADTAYVNDIFAGKIRFPSSKLADLQSYVLAQWP
QARDMVMRHDSEWRKSTTSSHKPSRSSSESSCLSGFHKQLRAAYRSRNTG
AIVSIWQDLNATLAQDQELRRQMSEDQDFLDFWIFVWCAVRRPKKLQETL
DLMREVGIQPTVRSYTSMMHGWKMCKDADRIEALWNKLVESGLRLDAVIW
TERISGLIEAGKPQSGIQALAEMQALWKKAVASRGSVESAANVAVQPSTE
VVNAAFKGLIGLDRRAANEVLYWAGREGIEPNIRTYNILLRESLRNSAPE
DVQSLLKAMKKRGVEPDAATFTIILEGLLGSMENASAAEQVHVVSQILGD
IEAAGLRANHETYGKMLYAVASLANGGADEAIAAVQDHMKAAGLSATPHM
VTILIERALSRDPLPADAGATVRALLKEHGLSNVAQGDQTLWERVMSAHA
VTGDRASAMAVFDDLARAGRPVTSLPCLTDLLKALLASEDAQATQDARQV
VGVVLNHKKAAAEGSFGRDTRYWRHHFWYLARENGLIDWQEVPALLESKL
RG
>Mb|QLI68983.1
MSCPDCFRGQIHEGETKGKVIKHYGFDTYVSEPPSGTTPKGIIVVIPDAF
GWAFPNNRILADTYAEKGGFKVYLPDFMNGHAAPVSMLFSMQTALAPGNL
FSRFIAAVQTILAAVPFFIRCAPGRTYPGVKGFFEQLRKEEGQALPIGAA
GFCWGGKHTVTLAHGAEINGQPLIDAGFTGHPSLLSLPSDVEKIKRPVSF
ACAEDDNQISLKQAEQIKAIVTAFPEPYKGELRVYQKTGHGFAVRADLKV
PDVAAQAAAAEDQAIAWFTSHFSTAT
>Mb|QLI68984.1
MATISFDINDALKHYMSDPTTIPTPEADSALFDCENDPESLTNPVINPVL
NPIVDAVAHDPDAILRASNMDSLQFLLKYTSFLPTHALSKIFDLVMSGLS
AECDYMYNDMDTSDEQDSVPHHKQLLEIYGFLLQWTIAAVETKAAEKSST
APAARGRGKPKKGASKEDTWDSATQLQASLEIMCKVLKQKLSKIFLTTSE
RDTFIGLLTRPVYMILESEQRAKTTSIRMHCFKVLCMAVKHHGHGYAAQI
NVIQNLTYFEHLSEPMAEFLHILAETYDYPQLADEVLREISNKEFNSNDT
RGPKSVSAFIAKLSELAPRLVIKQMTMLAKQLDSESYTLRCALIEVCGNM
IGYLSRQDERSENYKSQLNAFFDVLEERFLDINPYCRCRALQVYIKVCEL
DQKFPKRRQKAAELACRSLEDKSSNVRRNAIKLLGVLIKTHPFTVMHGAQ
LSRKEWQERHDKVEEELNALKPPPGMPGFGEENANTTVDNELLDEATQVD
SPQKPKAMTEEEKVAAIKKAQEEAATSEAIEKLTLTRRYYNEALRFIDVL
HDATGIVCQLLGARNKSEVIEAIDYFEIGDAYNIEQNKVGIRRMLRLIWT
KGNSDEGKGVQTHLIECYRRLFFEAPDSFSPNDAANYVARNMISLTFGAT
PAELTSLEQLLSTMMKGGIIPEIVVTKLWQVYGVQRREISRTQRRGAIIV
LGMLATASPEIVVDEIETMLRTGLGAHGRADLQLAKYTCIALRRINPTGR
NAKESTAKFNRLPNDHAVLGRLAAIAEVQSESKEWYGVAEQAINAIYAIS
KHPDVLCSEIIRRKTKHVFAQSRPGSRPTSKDETKGVPDITMNQTMHQTV
QVDKPKSRDNAVNLSQLLFIVGHVAIKQIVHLELCELDFKRRKQEKEKQT
PAKTDKDKEDADELDLIGGTTEDDFTEAMAHIRERELLYGPESLLAIFGP
VVSEICANNTSYADKGLQAAATLCLAKLMCVSSEYCETNLPLLITIMERS
PDATVRSNAVIALGDMAVCFNHLIDENTDFLYRRLADNDASVKRTCLMTL
TFLILAGQVKVKGQLGEMAKCLEDEDRRIADLARMFFTELSTKDNAVYNH

FVDMFSLLSAGGRMEEESFKRIIRFLLGFVEKDKHAKQLAEKLAARLPRC
ETERQWNDVAFALGLLPHKNEEIAKTVSEGFKVVQAAA
>Mb|QLI68993.1
MGRNKNKNKNKNKNKRVDPFPDINDVPEDYDMEKSQQRLLKEGLHPSVTS
KTPEEVEKAWIARAQQLIKRPTREEFMGKMGDFYNSSFTTPRYLTTAMLL
LRYESKYVRAAKKPADTGDNLLATMLNTIDVGGEIANILFQQGWKSVLDT
AITSSTTWKAVANSAKIWDFNAASLRTDGSSGVLLVVTPECTIGELNMSN
DLDPYGIDTSNSLSSWCREAMKYERQIYFSTYLIHGHIKGTIYRSKADKQ
KREGRKIIPDFMADDIVSAVERIRETRDVWGSSQFVSNPQNQLFQPPVIF
SASRLRKMLMHISEQKTALQVLYFEKIPLLDRRMIAVILRECPNVRMVGI
YDCPLIHFGDVLCLIDLIYEINYKRREAGLPEIAALDFYPRYNHGTPFQS
ANSATYGLTWGPHKLDVVQRGFFNIILKAFMKAKRMKLGLLFDDDKAFCE
YLRRVPGPPLAVPTFLDALHRYMEVKDERSKKRVIYDLLKPVRLGLCRRM
ESDRQEGYMSIMAKNLVFCSSCGYETLEEFYSTTARQMLPHSRVCAGCNL
QRWLDEEEDHLRGHKKQALNTLYPNWNGLNFNHDAPMPQTAKGIIKLQST
ISVRPDTNHTTVDREGVMYTRQVMLDLIRDNKIHWDSLANLPTLADLLNN
RQAWRRFSTNCNNLDVYCRAVRCVAEQDEEGKGKNSFLRSRFDGGMPDTA
EELQPSGDTVAGIPSYDIKSAIMFHVGLTMKGWGAVEKPAGKQCNLTERD
DAFW
>Mb|QLI68995.1
MAVPPSCTVLVIGGGTAGSYAAAALAREGIETVVLEADKFPRYHIGESTL
PSLRHFFKFIDFYDTVDAYGFYHKNGAVFRLAQAQPDACKSPSPAYTDFL
EAGGPDGYAWNLIRSEFDDLLFKHAGTCGAQIFSETRVDTIQFEPVANGL
KPDNRNLEDHLNPGRPVSATWVREDGTSGSITYKYLVDASGRQGILSTKY
LKNRKFNNNFKNAAIWAYWKSDNVYGPGTHMEGSPYFEALDDASGWAWFM
PLHDGTRSVGIVQDQKMVTEKKHELGRPSTLDFYKQCLDMAPRIRELLSE
AELIPHVRAACDWSYTASTYHLPNARICGDAGSFIDPLFSSGVHLAITGG
LSAAATISASIRGDCSEAAAGSWHSKKTVESYTRFFLAVSSATKQIRTQY
EPIIKDMDEKGFQRAFDLFRPIIQGTADADEAGKVSQLEISKILQFCFKA
FTYVPPERKDALFDKLRKLDSKALQDDAKQVQTLNGIEKYLTADELQILE
ILRSRRMIREDPFEMDSFSLDTIDGMAPKLVRGSLGLVNYEQVKIDKAHF
YSQDFLDGNCPGIREGSVHLPTSRVQLG
>Mb|QLI68996.1
MSTLPRTLSNLRKVGIKDYFRQMLYIGDTKYGRLVGTDRAGNKFFENMEE
LPLRTRWVEYAKHDYDAAQIEPGWHAWISYSVDKPPTEDALIATGLRKFE
KPYAIPNYTQTRAAFKTYSTTKPKVSAWEPVAAQRT
>Mb|QLI68997.1
MKNNWLPLALWAVFISLCSGTGNSILHKVEHQGPHLYALQSDEPLIVWRG
DTRSPDNVRNIHRGFSPPNEIEHLTKQQAEDACSLYQHPYGGTRSFTKYV
SSSTDPKVARLFASDFDDPANRGYIYEIQADEKFIDVKASLGKYGRYPEQ
VEQAASKPIGWDQVEGWYDLAEFLPHEMAILEDAASGPLKDMSQFVHRFH
ENPDWDLHKYYGRKGAGARPELAGFRKDSPAWDEEPWSQYKAKPVSKAWK
DYTERVCASPVVTTSQGVGICPDDVLPQVEQEFTLADPLGARDLTGQLQS
ADPAQYKLDVSFTKASEAGEMIEADEAAQAAEAAEMAEAVADAAAIDAIE
LAELLEGETLVDIILAFLLL
>Mb|QLI68999.1
METPCGAVHFPKRGGGHNIESGHMSFVDPYFGARVVKQDISGTAVIGRAL
VKTSPLISSTTPAPGASAQDGTGESRSRHPSPSSPALSTASTADGEEEDI
ESSPALPPLPAPKLPSRVAQLLRTPPPESGEENSSFGTASWGSPYPRTDH
NLRRQSFSSEPSDDSPIHHLNIETPFLRPQPGENPDDQQPQTTISAAAAV
LANRARRQHRGLTEDWIRTHTTGVVNAEPRHWFSDGSDSEHSSLSGSQLA
WFDDSDPRTPRVIRKPKSASRKSSASHTRRRSSVETLKPKELPQIAAGGV
DSSMSDVELQQTLHEVDLTRDVASPIEPRTDVAMVDVFEKSKTSNAQNMN
GELETPITPTKSKEKPLPKEPMTTPRIKKKVPWKGKNIMVLVPRDDQRGH
AGGAPMPLRPEEVEKMFSSWRELGYSVDGFDLLVEGYQPPGTDDSQSRQD
WPTDNDMARERSERIYKVLLPDLNAWKNYVDELQEAKLRALGVSFAEEEP
PEPSISPPTTNPSRQASVQYPSLPFSPPIPTSSASSNHGLPSYPFPAQFL
PASASPGLPSGASPVSFAPGKFNPRQSISLPTGNSPFQLAQSHGWSNPAG

ILQGLNRTDSPLASLSGILSPQSPYGLDGLQQSASPGFNLHQRHQSLQYF
PQQMGSIASPRLQDVREDEEEDFSKSPSKTPEPTMHNGDNLQAEIEDAEY
HLEEQLRNQLEHEDYNPQSMVTKPFAAMHSRDQSSQGLTVAERFANEPGK
AMELHHPRPHSRGHSLTQTFFQEQVDEHGLGKLGSLKELPETHKDEDEAQ
EIETNPSNLGTPVQDFDFAAAFGQHQKTFSTVSNPWQDHESGASSTRRSS
HGSKPSLSKLNVQAPEFKFNPASTFTPGAMYNFSGGFQPATFQAGVSTAI
PQSLEPTKPVPQFGASKMHASAPSFSPGQGVFSFSTSGPKFRPDAPSFTP
FQPLATSPTGGRGHAARQDSIFGNITIPAEDIVRPAKKSKAIPIIRPSSK
SSAQSPSAEEDDQLRDGPDGRLADESRVKRAKSAAPDGDMLPAFAEQPEE
QEEDIQPGHADEVRDDRSIAEEGSIDGEQTQPGDTSLSSIVTSDQVDTKA
TTAAPSVTSPAEETTRSLSHSGVDPKVVTPELPSSGLFENKTQDSEDQKF
LPATATPFIPVAPPAAQFPSPGSSDKKEDSAQEEPAQEEATAESTPQASR
ARLTPKPRGLAASRFAKPQSPDVVEQPDVLQSIENEPVTLSMPEPATVPN
AFQTRPLVDISEDREPTFEEIDAVMQQMETDPSMGVNKLLESSNWQSQPP
NAQICGPVEALQPSIDEFSRDGTSLTPRQFNVIPDATPMLSTELEDPFVD
PPISSRSPDSANDPDDAEIASEPASDWEGAFTGDEHDKLENRAQFFDGRV
NEVVGTLLASRLEPLEKTLSSIQEALTLQHRRNQSTRREIRSMSAEMQES
DADDEDEEPAPQRSISPRRDRRMEHIRLAVTEAFAAQQRNQPIVSAVERE
APVNDDSAILKALEEMKEHFTASLKTASVPDEEKESEARGRELLTPGPDE
QAQNKINELQAMMMDLAERLSNEQRKTEKEIAERRAAEDAGAELNRKLQA
AETRVEVEIINRSVFDQRVADLEERLRHQEDKTEEEVKTRRAAEDRLAEV
QRLLRISSEEEGRLRDLLDEKDARIKSLEQTGGKNAMRMTVLEAAQTNST
KSQTEMTNKFNALDADLKAVRQDNHHWRLEAERADESARRTAGELAHTLN
ENKHLQKSLHTLTSQLEENERLRESWRSKFLSLQEDMGKAAREVAEENAR
HIKRDQAMCARQEVLEARLQAEAKTRERLEIEMERLQENERSGMRAVNEC
KRLEGLLGELRTENHKLQQAASRYQREFEEARESGASEVKRTRMSLQTEI
DAANNQVNVIREELEEQNSKLRTELDNVKLEADTAKAQNEMLLEEAQSTK
AAELEAAERKYQNEIEDMQARYERQVNNTTEDASRTEQQLLERLSLSSSK
IEHLQDRIMHLEDKLEIAKEAAAAAAQAAKTAGTESGAAAVVSRPQQAPK
SLELPEKISPQALRESIMVLQEQLQAREQRIEELEQSLSKSDPDAAAKIS
KRDDEISWLRELLAVRHSDLQDIISALSGDHYDRDTIKDAAIRLRANLQM
EEQERERALNGGSAISLPNIAQSIQAATPRVAQTIGPIAAAWGNWRKSSQ
PSFRTISGVLSSPVSGSNSTPSKSRGGPASQSSLLSGLLTPPASGLRQAP
TSDNRPQPTAFSSTGRRYTLQGSSSVDRDRRESNSSRRSEKTPAAPNTPP
RHQERVEQPRTPPMMRQSGYDSDAQPGDFDDHDFFEED
>Mb|QLI69003.1
MSPSAETISRPEFDALLAEYPALIDAMSTAKSTQKPGQKTLKDLDAYRYD
KAPLLFGKQGQEMTLPDVQSLVEWKLRHGKFRPTLMSLVSSNPAPLAQQT
ISSAVAQYRTSPSTHAQAALDALTKLRGVGPATASLLLNVHDPGNVVFFS
DEAYYWLCRGGNKDAPIRYTPSEYAQLRQRAGELARRLGVGMVDVEKVAY
VVVKGKSGTNIQGLAAGKAGKRAAAAAGKDKKKAVGRDAAETKQSAPKR
RTDGGGDETGLRRSKRHRA
>Mb|QLI69008.1
MSLSDHPKAYGSAAVALAFGAGILVTLGFKDLYPDLEYRYQHTRRRSQAR
PGDRRRSSLFWGQPLKLEDHESQPPSPSRGDPGAGTGIASCIGNTPLIEI
ESLSRATGRVILAKAEFLNGAGNSPKDRVALNMIQEAEKAGLLVPHRGDT
IYEGTVGSTGISLATLARARGYKAHICMPDDQALEKSDLLHHLGAAVERV
AVAPITSPDHFVNLARRRADAHTASSADGSRGFFANQFESEANWHAHLNT
TGPEIWSQTGGDLDAFVAGAGTGGTITGVARYLRLGAKASAVKIVLADPQ
GSGLFNKVKHGVMYSNTEKEGTRRRQQVDSMVEGIGINRVTENFEAGRDL
IDDAVKVTDEQACRMARWLVENDGIFVGSSSSVNCVAAVVTAMGLPEGSK
VVTVLCDSGTRHLSKFWKRIKEMGLEDEEASDLFGELGIARQEE
>Mb|QLI69012.1
MASATSFYDFKPLDKRGQEVPLADYKGKVVLIVNTASKCGFTPQYEGLEK
IYKSIKDKYPDDFTILGFPCNQFGGQEPGSNDEIQNFCLVNYGVSFPIMQ
KIDVNGEKASDLYEWLKAEKPGLMGLKRIKWNFEKFLVGRDGKVKGRWAS
TTKPESLEKPILDELEKSAGPAN
>Mb|QLI69013.1

MAGQLGYRELIRKQFTPQKMMFHILFWTFHWGIFAYGWYKQAADDRLAGL
NTLKFSVWISRGAGLVLSVDGMLILLPVCRTLMRWLRPKLRFLPLDENLW
FHRQLAYALLVFTVFHVAAHYVNFFNVEQTQIRPVSALQIHYAQAGGITG
HVMLLCMLLMYTTAHSRIRQQSFETFWYTHHLFIPFLLALYTHTVGCFVR
DSVDAFSPFAGEQYWTHCIGYLGWRWELWTGGFYLIERLYREIRARRETK
ITRVVRHPYDVVELQFSKPSFKYKAGQWLFIQVPSISKYQWHPFTITSCP
FDPYVSIHIRQVGDFTRALGDATGAGGAQAKLYEGVDPMGMYEVALQNGQ
QMPGLRIDGPYGAPAEDVFENEIAVLIGTGIGVTPWAAILKNIWHLRNSP
NPPTRLRRVEFIWVCKDTGSFEWFQTLLSSLEEQSNEAARIPGSSGVEFL
KIHTYLTQKLDMDTTQNIVLNSVGADMDPLTELKSRTHFGRPNFKRLFST
MRDGILDRTYLSGLEGSMRTTVGVYFCGPSAAGKLDLKLHPNTKIEPNV
>Mb|QLI69023.1
MASTTSPIVSKILHLSDLELALLLCLISREHGIITTPSASLSLLTTELCL
VATRTFGLTCSIVDCTPSTTLDDFASALLLPPKSAQQPQQPDYFAHGPRR
SLHASPLLPSSQIANCILAKNLNLAPKSVQIQALELLRTRRIFTRTSVQT
APKQFIFIPVLEAEAPGKGERVTPHLNDFFSLAHWHDPDQEGFVNIDEED
EVERGSAESVNRRNDSNSFLIPESEISHLAQLSQQVQVDIEVTRYQMNIV
SFLRMHRAVADGITPNATKHLDKLMRCLAPLHGVDFVAPALVGLAVKKVY
LHRIRITSPEDERSMQWGSKLEAVAAILADMGPEEVVEDVLGMVTAPI
>Mb|QLI69026.1
MPHLVANFLRTSSGNISHIKKSAITRSRTTSRNNSVSASPYASDSEDHAQ
HSVKMPELGDALKKHRLSLHFGRSSRHDGAAGSSHSASSGGPHAPASMDW
SIESPPIVFYGSPEESTGALVSGLMYIDVHADAIEVDSFTASLTMHVTQK
KPFQSHCADCQTKVTELKNWQFLAHPTSLRRGRHQFPFSTLLDGSLPAST
DTPIVSISYHFKAEAVAVRGPASTSCLPIRFERPLHVKRSLPEPLYPHHS
VRVFPPTNIKASAHYTAVIHPSGSNAVTLKLDGLMTHNERVKTVDLWKLK
KVTWKLEETIKTIAPACDRHAPASSAAAAADADDNEAKRGAPRTETRILG
EKQLHEGWKSDYSGNDGTVDFEFDYAVNQPRTHGKEPRYACGLKTQDGTE
VSHALHIELIVSKEFAPEGKTHLAAQTGTGRILRMHFAVNLTEYPGLGVS
WDNEAPPVYQDVPPSPPAYPAEPPIEYDDLEELDAQRTGSTPGSRRHSES
GRST
>Mb|QLI69030.1
MATYQNGVAPARALDDDSDVEEEALVAEYKEHVQYEDGEELSQTTSLTMA
QQADDIQARLVQAAQPLDFSAPLDVKFQSYDSYCSLFHFILNSEGPVDLE
PPSYYWAWDVIDEFIYQFNSFSSYRSRIARQASNEEEIQILRENPNTWGS
YSVLNVLYSLIQRSQITEQLAAMRRNEDPAAVAGDYGSKNLYKMLGYFSI
IGLLRVHCLLGDFGLALKTLDDIELNKKAMFARVMAAHFTTYYYVGFSYM
MMHRYADAIRMFSHILIYVSRTKNFQKNAQYDSITKKNEQMYALIAICVA
FQPTRLDDTIHSALREKYGDQLLKLQRGGPESLPIFEELFRAACPKFISP
VPPDFDNPESNIDPVEHHLAVFMDEVKTNMWSPTIKSYLRLYTTMDLNKL
AGFLEVKPDELRSWLLVTKQRTKQLRWNDQGLFDGELVNVSDLDYALQED
LIHISEAKVGRKLVDWYLRNLSRTYN
>Mb|QLI69031.1
MVLYKRKPVQFLPPADIQDENAEVWHIPQTGELFQNYEDYLSRMDFYKQR
RFNDQITGHSGLTFFEAFNSELTGGREVEASFPEALKGPILRKVQFQTIS
RLDNLVDLIYEEFKHDYYPGEEVTVTLDGGDRIHGLVRDKTTYGPRILPD
GSRTLPVTRYLVNIKDSDEETNVTNEHICRDRGVFTKAMLRSFIKKTVIR
EAWTGAPWLVKHDYANMYHIDTRVPPQLRHDTKLLERKQLQAQKRGITNE
LNGVSNAGPMRLPELKPATKHQKGKQSQFGIKGLKWPLNEPVNGVDGLIQ
HEQPRAPIREPTPPPPPPPTKYPIEDLSLPPKEGSVRPRLKFMCHDPPVK
IEPEIDESRPFFDKIDMATVGAFLETWDTLNVYCEIFKLDSFTFDDFVET
MSVASEQLKVQLFDEIHCSVLKVLVDSEREGGKVRINLPELEDEYSDEEE
GEEEDDEADEGQEAAAEPEEKPKGRATRSSLAKLEAERLAAEAAAAEKEE
IRAEQESRNRAEELMREFDWIDHLRKRNFSNGGWERIMVGLLHQLSKDDR
YKESCEELLEQLVPADSNPTQEAIRQSYAELDVNCRVKALQIICLLTMQT
KAVRGYMEDCSETMTKYRKDRIEWQRQKKQAIEDVRQLHEQRRELVPDPD
PYEQVDANVVKEEEDAKMADADESQIDKENAEEGNDEPSQPKGKRRGRAP
ADKKKKKDAELEKEKKEKEKAKEKEKEKGAEKEKEKTRKEMIKLTSQQQK

QYNKILKEIQKKEDIIKECEDEVAVIENDLREADCPRTRVLGKDRFWNRY
YWFERNGMPYGGLPTSSTAAAEYANGCIWVQGPDDMEREGYIDGSPDLPD
ECKAKFRMTVSERKTREENGTSVFTARQWGYISEPEDLHALIKWLDPRGF
NELKLRKELVNYKEKIAKHMENRKKYLAGEEDKDKKDEAVTKRSSSRIRD
KTPEPPTYRCLQWENTMALEELGHIHSDPPPPPRARKQTKKREAQTEAAG
RGAAKSRRRG
>Mb|QLI69032.1
MATIAKGAATGPAKLQAVADLVNRLCDDLENISLLPKDRNDALEELKIYG
RDPKYADPIFTKDGFTMLLRYSFQNPPDDTSRAALRVVANAMLLKPETRQ
MFVDQGYPAQACDRLRGGNWDDEFLLSRALFLSTYGTNIDLPELIDSHEL
AQHLINNLRRHVKILSDKQKEKLDPMEDMALGETLKLMFNVTHFSKTHVS
SFAPAVPLIVALILKHEIPEPKPLDPPFGPLINSLLNLDLRIELSRLALY
PENEPHKLTSRLVDLLDQGIRAYSGNDLETVVTPVVSLLARLYENAPQPV
QQKLRECLLPTAEDRQGVLGRSDSLPSHLLKNSTNPTAPALRDAISHLLF
DISDKDASKFVENVGYGFASGFLFQNNVPVPASSAATFRKGDGFGAQKLV
NPITGQFLDREKIADAPEMTDEEKEREAERLFVLFERLKKTGVVNIQNPV
EKAVQEGRFEELSDDCVEELD
>Mb|QLI69033.1
MAQDMPPRGGYEPVQYKRNLPAKGFRPGILLLGVGAVMGFGWYKLIGGIR
EANELAREKMWARINLIPLLQAEEDRDQVRRYWADQKREKELLGENTKVY
NNESRFVRPTFAVSPAPSK
>Mb|QLI69035.1
MVPTLRNPTSWDEYEGRASPSGSLWSSVIDEEASLLDSDGDCTQPPVAHH
DRRRSSVTTRLAAMADIGGVNSFRSFARSWQRAASFAEVIPRRPSFILPA
EAETVPVSSGDEIQYSRSHVDSQPPPQAGLLRQHLEASMPQDSASVQTIP
DSGIGVGPAGGIPQEDFRERERKALDTEMASGALHDGSPSIRSNIFAVPP
HLATPSLIGSYGSQYGTMGARRYRSRSSASYESAWGVRGDRGGDYGDDVT
LGEEQPILVKEIKQGDRVVLTVDGQSTLPQSIFNSINAIIGVGLLSLPLA
FKMSGWILGLFILTLTAAVTSHTGKLIGKCMEYDPSIITYSDLAYVAFGA
RARVIVSALFTLELLAACVALVILFADSLDLLMPTVANTTVWKCVCAALI
LVLNMLPLRWLSYTSVVGIFSTFCIVCIVIVDGLVKQHTPGSLWEPARTY
LLPSNWLSLPLAYGLMASPWGAHSVFPSIYRDMRHPHKWSKGVNVTFSFS
YVLDTCLAIVGVLMFGDGIKEAITSNILKTLGYPEFLTILMCIFITIIPL
TKIPLNARPLITTADVVCGLHRDHHNPHHHRSGSDAQLSTLANILRAGVR
VLVVLILLAISVLFPAFDSVCAFLGAALCTLISVILPICFYLKLYSKDIA
KWERIVSWILLIIFSIFGLVGTIWTFLPKQLVGT
>Mb|QLI69040.1
MEPASFKKPRTGASPPRRFEIPPAASRPAVPMTNRDGPFSPLFFSHTPSR
RLGRPTGSSPDVSASMLSRTREEPSSAVTTVKLPHANVKPVTSAMPGSTP
PSLGSSVDMSAPALSPELRIASPNLQLIQSIGAMELVEQDSRPTFLIDTL
TPANTPPNQPSLHILYGNASLRSSRDIAELVGMEPAETVDVANFERFASW
ATSSPSSRASQEPTSIFHVYGGINWTYVTVRQRFRFVSGNVTRAGHSTTR
PSSAAPISNDSIPIPTNTSIVSQPMPVTPVVGSVGSVGPPDYFGDAVPGA
QGDEQLMDAIQTKLDDAAAPLNHPDEFTNQVLQQVQPTKPSFDWTVIPLH
DNLPDHIKFAKTRDWTSTALGSMTYWSPDLRTMSSLVMASPHPAAMYWGS
DMVIIYNEAYISLAGKKHPDLMGMNFADGWPEIWGEMEPIFDNAWKSGQS
TMKHDNRLFIKRNRFLEETFFSWALIPLIGNDGEVVGLYSPCFENTRRTI
NERRMLTLREMGERVATATTVTGFWPQVQLGLEYNEFDIPFSIIYSVREE
YEGEVSSLHSGSLLPPSQLALEGSPGVPDGHVAAPPSLDLKSSDEGFAPY
MRQCMAMGGAPVVLSEEAGNLPKRLLDGLQCRGFPDPCSTVVVLPVVPTT
TKAIAGFIVMGTNPRRPYDEDYKLFVDLLSRQLATSMASVVLFEEEIKRG
QRAARLAALDRQELSKQLIQRTQEANESEYRFTRMAEFAPVGMFIADAVG
SINYCNDMWWQISRHTRLQDAESGWMDSVRDEDRPALEAAWSQLVKEKVT
ISLEFRFKCSQQSGCNTIDTWVLMSAFPEKTLDGALKSIFGCITDISSQK
WAEKVQNERREEAVELKRQQENFIDITSHEMRNPLSAILQCADQIANNIS
AFTVHKDKNELENLLEGCLDAANTINLCASHQKRIVDDVLTLSKLDSKLL
AVAPADVQPIKLVQSTLKMFEPELVAHDIEFEFNVDDNFERHNVAWAKLD
PSRLNQVLINLMTNAIKFTQGRERRCIIVKVSASRDISEVTSTGVSYFSR

INNQRAPSMDITNEAEWGTGDVIHIHCSVEDTGPGLAEEEMKLLFQRFQQ
ATPRTHVQYGGSGLGLFISRILTEMQGGQIGVSSKAGVGSKFNFYVQCRK
CVHPPAKHDRISPFKIGRKVIVPPPASPLPVPETAPKLNDRSLLRSSSAS
GNRSPFDVLIVEDNIVNQKVLQRQLRHCGNNTFVANHGFEALQTLQKSRF
WAGQEQDGVDISVILMDLEMPVMDGMTCARKIRELEREGTIVAHIPIIAV
TAYARPEQIESAKAAGIDDVISKPFRIPELLPKIEELVSKYKNLSFSSDA
>Mb|QLI69043.1
MAAAADMDNTLDQTTLATINLLESRLLRLEHLLHGPATSLPHVHKDSAAQ
KLKDMERRFSMIISHFRVYNELLKIYKSNPDLFHSPDASVPPSQLSSDAI
QSIVLSQAPAYSSALSSLTAVQDSPIPDPSESASLIALTDKMKAIEATQI
AQLAEISELRRRSEMVIRAWYENWVLNNSQSLADVETRVERIEKQIRQRE
RAFEDAKQI
>Mb|QLI69045.1
MKAVPFISIVTLALASPRITDTSIAPNSSTTPAPTPPDGTYCECGYTYCA
SVLMAMKKPWTTKQLAEAYCATQNAVCNSGKPSTSINSALYICLCEDPGQ
KYGKQLDLLCGCDECLVVGPDFRGRCETPCHAGQCKA
>Mb|QLI69046.1
MASRSRSPSRGRQRSLTRSASPRSGDSRSPRRRRYDSRSPSRSVTPPPRR
NGRYRSDSQGWGRGRGRDGRDASESPLARSTKIVVERLSKNINEDHLHEI
FGQFGPIKDLDLPMNRTFGTNRGTAYILFDHEADAEAAISHMHEAQVDGA
TINVSIVLPRRKLSPPPPTARRGANIDPRIPMSGPRGGAGPPGRGGGFNA
GGGRRRGSPSGRYGPRSDVYRPSSRSPSRSPGAPPSRGGGGGGGGRYRSR
SNASYSSRSRSRSPGPRRRGGGGGGGSGAGAGGGGGGGGGGGRFEDEDDR
RRSRSRSYDSYAGRSPRGYR
>Mb|QLI69047.1
MHSSITATALLACVYLVYRFYVRRQRKAQLPPGPKPWPLLGNITDLPPQG
VPEYQHWLKYKDTYGPISSVTVLGQTMVIIHDLEAIHELMEKKSLMTSER
PRFEFAHSLSGFGKYLSLRPYDDSFRRQRKVVHQQLGTKALVDKYHAVQE
EEVGRLLLRLLEDPQNLWQSLKTKAGAIILKITYGYTVQHKKVDPLVKLI
EEMMRRGSEALVPMSYAVDLLPALRHVPEWFPGASFQRVAKKSTDINKIV
TDVPYNFVRQQMAQQDHTPCYVSRLIQELQDEDLRLDPVAAETIKWSAGI
LYAGGSDTTVSSLFSVILALVKFPEVQRRAQHEIDGLTGSDRLPQFEDRS
KLPYVDGVVKEALRWFPVTPMGVAHAASKDIMLKGYLVPKGAIILPAVWW
LLHDPQVYANPESFDPARYLPPRDEPDPAKVAFGFGRRVCPGRYLADAAI
FLTVAQILAVFNVSKALDDDGKEIEPVVASGFGLIDHPALFPYKITPRSA
KHASLIRDNTMGFSGKEANADALHAAGFESLKF
>Mb|QLI69048.1
MKPSTALGAIALPFCHALSLGGRPYQIIHEVDRRSGALQDFVTWDEHSLF
IHGERAMMFSGEVHPFRLPVPSLYLDVFEKIKALGFNMVSFYVDWALLEG
KPGEFRADGIFALEPFFEAASEAGVYLLARPGPYINAEVSGGGFPGYLQQ
LEGILRSQAPDFLNSTDNYMAHVCDIIAKHQITNGGPVVLFQPENEYSSG
HNIPFPNGQYMQYVINQARSAGISVPMINNDVGPAGNYAPGKGVGSMDIY
GHDSYPLGFDCGNPTVWPPNGLPTSFHQLHERQSPKSPYSIIEFQGGAFD
PWGGWGFEKCAALVNHEFARVFYKNNLAAGVSIFNIYMIYGGTNWGNLGH
PGGYTSYDYGACIRENRVIDREKYSEVKLEAQFIKVSPGYITASVGLAST
TAYSNNPGITITPLTSNKTGNFFVARQTDYTATGSVSYTVTLPTSNGILT
IPQRGGSLSLHGRDSKIHLTDYPVGDYVLLYTTAEVFTWKKYADKTVLVL
YGGPDELHEFAVKSPSATRILHVEGSSISSHEQASSTIVVQWTTSPERQY
IQVGNLAIYLVGTPTPITNLYMTPKLQFHPLTTETDRNSAYNYWVPVLPG
SNSSPYGTSLMNPDAAIVNGGYLIRSASINGNTLSLRADFNGSTTLEVVG
IPDGVTKLSVNGRPLKHTTSPTGTWLAQPDITIPTVQLPSLADLTWHTID
SLPEIHPQYDDSLWRTASQSPGNNRPQAHHKTSNATLFAAPPRASLYGSD
YGFNTGTLLFRGHFTASGKESLFRTWTSGGTAYASSAWLNDRFLGSFKGN
SAAENQNSTYALPSLTAGEQYVVTVVVDTMGFNENFNPGYEDMKAPRGIL
DYALSSPDGAPTAVAWKITGNLGGHDYADSFRGPLNEGGLFFERQGYHYP
RPPVDDAPFAPGSPFDRADRAGVAYYAARMPLDLPADAYDIPLSFVFANA
SRGGGDYRALLYVNGFQFGKYLSNIGPQTEFPVPEGVLDYGGDNWVGLAV
WSLDSTGARVPGLALRAGTPVQSSRNKVRLVTGPAYSIRPGAY

>Mb|QLI69050.1
MPNDNDYILTLSCPDKPGIVHAVTAVFAGHGHNVLDLQQFSDPASRRFFM
RVHFGPKAGAVTAEHLARPFDELAAAYEMAYDIRPVAQKMKVLIMVSKIG
HCLNDLLFRMKTGQLKMEVPVIVSNHADYEPLAASYGIEFHHLPVTKDTK
AQQEARVLDLVRRHGIELVVLARYMQVLSPTLCEAMSGRIINIHHSFLPS
FKGAKPYHQAYERGVKIIGATAHFVTADLDEGPIIEQRVARVDHSMGPQE
LVEEGSNVESQVLAAAVRWYADRRVFLNGSKTVVFG
>Mb|QLI69053.1
MGNGAKAQQKRERNAKDGKVAKSQLKVNQQAMNIQCQICKATFLSTTKGP
AYVPTLSLAFDAVLIPIPFK
>Mb|QLI69054.1
MAASSSSMLSLVKALFVPAVISLIIFLFLTFVIAPIWRRYRNRYSQYLPV
ESISNHTSGLRHRIVNRFSRFTLPSTWSRNRGSVDNPSADDFLEDGEELG
DVDEQTLNAIQRLHSHLGSGLDSNRRLSRDLEEGFMDDSDEEQARG
>Mb|QLI69056.1
MGTGKKEANRRIRQGKTGDGLANVRVKGENFYRNAKKVKTLNMYKEGKAQ
RNSEGKITKAASFQSRDVPDARIEPNRKWFTNTRVVSQDTLKAFRDAMAE
KANDPYQVLLKSNKLPMSLIRDGQDAKGIKQHDAKVAVETSPFAEVFGPK
AQRKRVKLNVSSLTDLAGDTEKSMDTYEVRLEQARLLSGISGQDEEERQV
AMAIEPVFDKGQSKRIWNELYKVIDSSDVVIHVLDARDPVGTRCHSIEKY
LKEEAPHKHLIFVLNKCDLVPTSVAASWVRNLSREYPTLAFHASINNSFG
KGSLIQLLRQFSSLHADRKQISVGLIGGPNTGKSSIINTLLKKKVCAVAP
IPGETKVWQYVSLMKRIYLIDCPGVVPPSSTDTPTDLVLRGVVRVEKVEH
PEQYITAVLDKVKRHHMEKTYDLKGWTNAVELLELLARKSGRLLRGGEPD
LDGVAKMVLNDFMRGKIPWFTPAPSRNTDDNGVTDRDGRLGEMPKKRDLE
DAGDTSNSVSAKTAANQPGSGRGYDREEVFEGFSSDYDAPSPTDLKDEAK
ADGNDPDFAPADFSQRLHEMGMVQPNVTFSPSSTAKNDVRKVASDPSMIQ
YPPNKQNATLSVLEARRHLQQQANDDIAKTGLHGRQYKRFVDMRILINTI
KLRDQGMTYAEIEERHCLEPGTVKKLGRHGVFSHVSATR
>Mb|QLI69067.1
MDILKTSSRGTKKTSKGSGLNAGNAQLPSAGTKTNPQLYHDEIRGMKKRK
RGVVQAENPVPAELPVVDFFAPKPEATENQKAETKKSRREPSPPPVAAKT
LTEDECRQLLRSHRLKITLLSKHEEQNKVKKSKKKKKKKTVIEGKKDDKK
QLFPQPLESFGHLRTTYGISRQIAENLVQQSYRLPTEVQMGSLPLLLRPE
VALGNEENLDHGVDFLAVAPTGSGKTVSFLIPTINSIIRRRAQADIQGVH
ELEAVIVAPTRELVHQIVNEGRKLVQGTRVKIVAMRKTTQLSAEEQVLAD
DSSDDELDGEDDEESESDDDKKHRTGRSKPHPLTKTDILVTTPALLLNFL
TAGPSGAQKVLPTVRELVLDEADVLLDPLFREKTLSLWTACTNSGLRLSF
WSATMGSNIEALVTEKLKDRTEALGISPKPFIRLVVGLKDTAVPNVVHKL
TYAASEQGKLLALRQLLHPAGSDGSDQPLRPPFLVFTQTIDRAMALHEEL
KYDIPLEAGGPSRIAALHSALADSARSSIMRKFRAGEIWVLITTDVLARG
VDFAGVNGVVNYDVPGSAAAYVHRAGRTGRAGRAGGIAVTLYTKEDIPFV
KTVANVIAVSDKQAGRTGDQAGVQKWLLDALPNVGKDDRKKLKQRGVESR
RSGSGSKAKITSKSGYERRKEHNRRGAMDANKRRRVDGGARDDAEWDGLG
D
>Mb|QLI69068.1
MLRFIHHPFEVVSKKLGASPDELKLIFSFILSYPLAGLLKRVPDAKPYRK
NLFIIGTSIFYLIGLFDLWDGLSTLALSAGGVYLIAKYMKNSYYMPWVGF
MFVMGHMSISHIRRQAANSPSSVDITGAQMVLVMKLSAFCWNVADGQLPD
EVLSDFQKDRALCELPSILDYAGYTLFFPSLFAGPAFDYVDYRRWVDTSM
FDMPPNVDPSKKPPSRKKRKIPRSGTPATFKAFLGLFWISLFVVLSASYG
HDQLLVDTYMHHALWRRIWIMYMVNLVTRLKYYGVWTLTEGSCILAGLGY
NGVDPITGKVFWNRLQNVDPWVVETAQNPRGYLAGWNMNTNSWLRNYVYL
RVTPRGKKPGFRASMMTFVTSAFWHGFYPGYYLTFVLASLIQTAAKNFRR
LVRPFFLDAITGGPSPRKKYYDAASLIVTQLTFSFATTPFLVLTFADSIR
AWSRVYFYGAAWTVIGLLFFASPGKAVLKAELEKRQGKASAKLVRSASTD
SLTGKDPILGISKDPERDVTEAMAEIRAEVEARQKKTS
>Mb|QLI69070.1

MSDELLDEVEAINSIYGDGTLAPTEDESSSTYILKLPGDASSLRIRFPDA
YPAQPPLVLGTHHSSGGVRGAGARDVRLFKDILPSVFQEGSVCLFDAVEE
FGRRVEEQAPGPEETQDGPEDEEPTPREEEEALPAPDWTLSAVVVENKST
FVARVARASSPEEARSHVAHLLASDKKVRLATHNITAWRIRGHGATQFQD
CDDDGETAAGGRLLHLMQVMDVWGVVVVVSRWYGGVKLGPRRFAVINGVA
RDGMVRAGVAGK
>Mb|QLI69071.1
MSQRGARATDFEERDYYPAPRRSGTRFEEADYRTSRVMTRSPPPRERESD
RGISFLRDDARRSEPGQMVLRKRDVETWDVHRSPSPPRVREQRIVHRPRS
ESPRYHDHEHERSRTRVVERERERVRSPSAVRMRSPSPGVRFVERRRRSP
SPPVREHIRTRIVEREKAKEPSPSPSPSPPPVIRGPTIEREVITHYTDVD
HGVIRARPPSPRPHTHRERETDIDISLSKNKTEVDVDIHRSTSRHRSGSH
ERRSHYHDDELIVRDGMDRLRIDDGRRSHRRARSAAPMASPVDEEAEYMT
GRIDARGRMGEAWHGHTKDWTIVDVPPGTERVRMDGVGGASTETNWSKYS
GVRRTTFVPERDGQLVAVPRAPSPRPAPVPVVGRDHTSVTVYDRDRDREI
DVDVDIDRRPGKPKPAPAPLPTRDMWTEITKDLVCREAIEQMGYSYEETK
WFFYIMEYLQYDEVFQLTQLTERIRRHRRHHREVEWERDYRDDWHHRYRR
HHRPRHEWDDERVREREVVYESRGPARGYFR
>Mb|QLI69078.1
MSGLDQVIRQQPEGQPQPQPERHGFNFNKLLLGGAAYLGLNLALSYFLAK
DQRGVQVTDPNTGKPITVASNLGDIPAFELRPSQLDEGAQYRPIPRKIAP
IWPQDSHVDIVVTLSPSFNPTPISETPAEYVVLQERNFQMSNSSEKRTVD
TKFTVPRAVQFNGTLWGHFYVGLTGSNLDPRRPGYDPAKAYHFAYPLTQY
LPKKKVAKTRNLLDSHSEDEEPEEEEPTGPIITNHYHPNASFAFVPAMGV
KDFNSIHPAVKHFLRLEATGARDGSGQNGWYYPVLFINNFWQLANHMTVL
NETVKELPLHIDLTTMAFWKFSTLASIELSSKENARQAAFGHSLPTGGDG
SEIEMVKEIFIDTNPILLGITAVVSVAHVILEMLAFGSDIAHYRKKKDNV
GISVRSILANVFMQTIIFLYLLDNSQNTSWVILGSQVVGIVIEFWKVTTV
VDVRFRPSAPGSLFPYTLVFEDKHKLTETEEKTKEYDAVAFKYMYMVAVP
LLIAYGVYSLVYDSHKSWYSFIITTLVGSVYAYGFLMMVPSLYINYRLKS
VAHMPAKAMMYKFLNTFIDDLFAFTIKMPFLHRLATFRDDIIFFIYLYQR
WAYKTDFTRVNEFGQGGDEGEPSSQNGKESETIEAKRATDAKPEGKATGA
DTGKATKRK
>Mb|QLI69079.1
MATLTETKNDRFQHHVGFDNVPSGEATKNNTLSLTLNVRHKGYQARRRSR
SFMVGVDEHSYSDYAIQWLLDELVDDGDEIVCVRVIEKEIRYNDKQYQED
AATVMQNILAKNASNHAISFVLEYAVGKLHATFQKLIQMYQPAMLIVGTK
GRSLGGIQGLVNARNSFSKYCLQYSPVPVVVVRPTEKRIKKKVKRANDTT
RQTYLGMLAASHGLHEADSETSSTYELEPQASPDEEAHQVAKALGLPAAF
DPTIKPLRDSLLPRSRPSSPHPPVNGTPERPPLDRSPGSAGGDTEEEEEE
EEEEFDVVDGMDVLNQQQKLELHKMEVGEAAALRKGVDDEEEEESDEAQE
LKEAS
>Mb|QLI69080.1
MVKKFFGLSGQALNWAIGAIAGCDFLLFGYDQGVMGGILTLPIFLEQFPD
INDTENGLTDAEKSNRSTYQGIAVASYNLGCFIGAVITIFIGNPLGRRRM
IFLGTAIMVVGAALQASAFTLEHFIIGRIITGLGNGGNTSTVPMWQSETC
SAHKRGKLVMIEGALITGGIMISYWVDLGLSFAPGSVAWRFPLAFQIVFC
IFILAFVLGLPESPRWLILKGRDAEAREVIAAIAEVENDDKYVENEFRAI
KDTVAEMSKGSFADCFARDNNRTLHRTIIAYVNQMFQQISGINLITYYAA
TIYRRLGMSPFLSRLLAALNGTEYFIASWPAVFLVERIGRRKLMLFGAAG
QAATMAILAGVGSQENNHACQIAGIVFLFVFNTFFAVGWLGMTWLYPAEI
TPLRTRAPANALSTSSNWIFNFMVVMITPVAFTNIKYNTYTIFAIINAIM
VPSVYFFFPETAYRSLEEMDIIFQKVKGWRGAFTVVHQAEIEPRRYGKNG
ELLISINEPGEKNNTEHRNGSSSETSADINNNMFTNIDEETQRRRS
>Mb|QLI69081.1
MSQTIASTQQICSPPYDAIELTRFSSRTSCGRGIDAQLKDTDTIRATAEA
KLGTTSGTTSDDSEPPADAQGQVERWNYPRSNVFKLGFACLSFVIAGMND
GAVGALIPYLEDYYNLNYTVVSLIFLTPFVGYSLAAFTNARIHVALGRRG

VSIMAPLCHIVTYAVLTSHPPYPALVVANSISGFGNGLTDACFCAWLGAM
DKANAIQGVLHSCYSLGALFAPLIATSMVVKAGLPWYNYYYVMIGTAALE
LGGLAAAFWDQPGSVYRAEHASDNEGSGAGTREAAKSKVTWLCATFFFLY
MGVEVGLGGWVVTFMLKVRHATPYASGISGSGFWAGMTLGRAALGFATER
YGERTCLTVYLAICIALELLFWLVPQFVVSAVAIAFLGFFLGPMFPGAVM
VTAKLLPKRIHVSAIGFAMALGGTGGTVFPFMIGAVASTAGVQVLQPIVL
ALIVVVTVVWLSFPKIRKKD
>Mb|QLI69083.1
MKSITFLSVLAASAVGLGAADGLKIDVTHSVQCDRKTQKGDKVAMHYKGT
LGDSGKKFDASYDRGQPLQFTLGAGQVIAGWDKGLLDMCIGEKRTLTIPS
ELAYGDRGIGPIPPGATLIFETELVGIDGVAPPEKETGKDEAKENKEADK
AGEKVITVVLKATEAPKTFMADNR
>Mb|QLI69084.1
MAPVQPPSRNSGTLAHHDGNGDGDHDHGASHATPVVAHERGNDSYSTNLT
RQAAPRRANSDPSNPSGVVRAVSFSGPASNNRPLVDSTSRNDQHVLLTIP
SRDAVTEMGSSFGDTRDRTDKTAHVSITSPANDKATQTLRDSIRRFRPGK
NRKDKANKALRNASWKDAFKVSLASTYQKLIMEGVLRRKPLRPSNDGRHI
PLNPSDALIDERSQRPYVSNFIRSSRYTAYDFVPKQLLFQFSKLGNFYFL
VMGVLQMVPGLSTVGRWTTIVPLSIFVAFSMLKEGYDDYRRYQLDKSENR
SETWVLNRGQGGSTTEKDATQTEVRAATGDADLESGVRDMSPRDWLCMQW
QHVKVGDVVRLRRNDGVPADMILLHATGPNGVAYIETMALDGETNLKSKQ
ACPLLAERCSTLDGIRSTTASIVSEDPNIDLYSYDGRATVGDETLPLSLN
NVVYRGSILRNTAEVIGIVVNSGEECKIRMNANKNVTAKKPAMQTMINKM
VLFQIVIVLMLAAGLTIGYSLWKKNIQDLSFYLVTTGQWTASVPFKEIFF
GYLIMFNTLIPLSLYISMEIIKIGQLILLQDVDMYDPVTDTPMVANTTTI
LENLGQVSYVFSDKTGTLTENVMRFRKMSVAGVAFLHDMDVQRDEDVKQK
KIEAAASWSCRSKGNQTQQTPPVSPHKTKFFHRDEDDILEEEEDEEDDGS
GPVPPRRTGSFASASHWKSTVRPNEEPEVKTEDMLDYVRRHPNTVFSRKV
KHFLLCIALCHTCLPEKRDDGEFSFQAASPDELALVEAARDMGWLLVDRP
AKTIKLQTNDDRGVVQVESYQVLDVIEFSSKRKRMSIIIRMPNGRICVFC
KGADNVITSRLKLSHLAEQKAKDISRRASVRKTFEQDKARTRMSMASGRG
TPRTSFALKRGESIDRLEGLRRSIGRLSTDANRLSYSEGVGSRMARRQAE
EPGTPRGSLDVLRARRLSLNPVSSVEDVDHRIDESVASNEAAVFEHCFQH
IEDFATEGLRTLLYAYRYIEEDVYASWKQLYREAETSLVNRQELVEQAGE
MIEQKFELAGATAIEDKLQDGVPETIDKLRRANIKVWMLTGDKRETAVNI
GHSARVCKPFSEIYILDGSRGNLQDVMTSTLNDVCRGMVPHSVVVVDGQT
LATIDASDDLAVLFYDLVLRVDSVICCRASPSQKAQLVTSIRRFVPSSMT
LAIGDGANDIGMIQSSHVGIGISGREGLQASRISDYSIAQFRFLQQLLFV
HGRWNYLRTGKYVLATFWKEILFFLVQAHFQRFNGYSGTSLFESWSLTVF
NSLFTSLPVILLGIFEKDLSAETLLAVPELYTFGQQNRGFNIRQYFGWMF
MGVAGSFVIYYLTLGEFWSALFTADTSLYAMGAVCFTVGVIFINIKLLIL
ELHSKTVITFGGFIISIAGWFLWCLFLSGMYPQRVGKDMVRRGFIDNFGR
QVSWWTTLLVALATLVIMDLVVQGVRRVYWPTDQDLMQRIEKDASARQAL
REHAALQEAGVEDDDVELQDLIMRRGRGSVAAEDGREDTR
>Mb|QLI69085.1
MEDPWSSPWATDTPAPPTIDLPSEPQQAHYGNSPRKSSRSPSPWGRGIDI
GMDDWGGWNEPPGGKASPCWGNSPNLKPLGSGTGVMLDPWALAVTDDKEG
IKESDSAIGLGDDGRADGHDNLTRHSEGNGVWGKEHIESNQEDAAVPSHS
VYMEPTRLLGEKQASKVQELVEMYDGMAKTLSVEATRTETVTTKDTQVTE
AKVSDHVDDGLVPDAPIPPATTSTRPMGNDEMAPVADKCEAGPVDLNPKT
EESGPDLQTASELGNGGVTSEVVLVPEKDAKPDQMTQHHGSTPPTDEATE
NNTLSHNPGTSIPYPIDFSSLNILFPNTQAAANTPEELPESIIKESFTSV
SQRKAWYRISRFGSIRKHNGGDDDNYVRVAWKNSTIRKDTLDTVRRWMEE
DRIGGRVVLGRRLGHGGGSMFNWDSEAPPVEIGELLARKKQKPKGHAQQF
SATLAGTVTSPIIASFGWSSSVPSSPATERISSEWHAKSSRLPETTVRDR
AEDKQVSADMPEPHQSPKPSAAQVVSSALAPIPVSQDEDDDWGDMVSCPT
LESTAALDTASHIEAAYPPTLEPDAEASIPKANEPVHASNALTEEPDPWG
GLEFFGGNSTGQTSQPMPLAQEDTAALLPAPENASMPAPLGQTREDESIH

TPLQFSSSTSSRPASIESATLNPQDDEAVTTILAGLPDLSYMMR
>Mb|QLI69088.1
MSSNRYAGGVPTRNGNGYGNFGRAPDAYDSYSDGRPNDRSDRYGAPLGAR
APPPALRNMPSRARVQETNAERQIAQVLEHIRSDWPSMCDNDSIPVQLAL
QLLDSSSVGRAHEYRKFQETHTYLQDSLKNIVHEHHQGFNSSIGTFHKIQ
GSIQASQKRVRELRESLASSKASLCSTDPELKKLSHTSTEYDELLQTLNE
LDDLRAVPDQLEARISEKRFLGAVEVLQNALRKLRRPELDGIGALNDLRS
YLANQETALMDILVEELHEHLYLKSPYCQDRWQSLAKAQGAHGDGYGDTN
HVPPFHSVLDSIDLEKAVTEDPTKNPEADTFNYVGLLVESLNRLGRLQNA
VETLKQRLPVELFGVVNETINEVDQRHPSSLRGTAGKTDGVFVYNSRETQ
MRADVIYDLLWTLYGKFEAIGEGQRAFHEFIKALIRREGAGNNSALLGSF
KELWNLYQNEIRSLLHNYVTTDADVYQFDSPNPGANFSGKKDAMREHLFK
FAEADAKSVDMVTEYDALEGIIHATVPGLTSGTRKQGLDSKKNRVALDAS
LARRDTGQGLVGSSKQSTGSYKSLVEPSVFNMSLLLPPTLTFLQRLKTIV
PPGSDLATSTLTTFLDNFLVNVFQPQLDETLSKLSDTVFGEADSFMQDQT
WKQVAPRPVFKGATQFFQIVTAFCKMLGTIPPDQALSSLIVTQMRRYHDR
CFTWYRSLVTKTQDHSEDANNLRASARFSIQPGPLQDIMKQVWAAEESSA
AELLSKEIQALIEQTNAPPLEMNDIIQDRDIISSLCLLYTSMKWLSARIS
SLRYITTHNTDSSRQAQPRQAKRRWSLMNDQGKAASDQNPVHLPLTQETV
QTFDNIVSSYQDLATTALLTLHAEVRTRIVYSLHTALSPSIAPYLLDQEV
REPDPQILSLNLELVLFDDTIVRYLRDKEVAFIRTGLGLLINNYLVSNAR
LASPMNAMGCGRMQLNILVLQQNLKNVEEGVDLARAANYFALFDGGPDAI
VEKAKEDKDKEAGSGAAAESFTYDELKALMELCFSEGMSNRERGIASAAK
RQLDDKLLGLSEHMWQS
>Mb|QLI69089.1
MALLQRIAAPTSKLRFSPSISLILARRASTMPPQLRDPSLLKQNVAYING
EWRAATSGETFKVNDPSTGTLIGTCAECDAQDTKLAIKAAADAFPAFRSL
TGRERAKMLRKWYDLMVENADDIAKLITWENGKPVAEAKGEAAYAANFFE
WFSEEAPRTYGDTIPATVPGNRVWTVKEPVGVCGLITPWNFPAAMITRKV
APALAAGCTVVCKAPGETPYTALAVAELGHRAGIPKGVVNVVTTLSNTPE
IGEVLTTDPAVKKVSFTGSTGVGKLLMKQSSGTLKKLSMELGGNAPFIVF
DDADLDTAVAGAIASKFRGSGQTCVCANRIYVQEGVYDEFVKKFTEKVNA
FKVGNGFEAGTTHGPLIHDRAVSKVEAHIKDAQSKGGKVVAGGQKMPDLG
ANFFQPTVIRDMTPDMAMATEETFGPVAGLFPFKTEAEVVEMANKVEVGL
AGYFFSRDLHRVYRIAEALEVGMVGVNTGIISDPASPFGGVKESGFGREG
SKYGIAEYQVTKMITFGGMGSPLQS
>Mb|QLI69095.1
MRASRPALRQGAFSAQASQAFRAQRFRFRESSKRWQSTADGAQQQHQSWF
KRAWESEVGVKTVHFWAPVMKWALVLAGISDFARPAEKLSFTQNFALTCT
GLIWTRWCLIIKPKNYLLAAVNFFLGLVGIVQVSRILMYESAKKKGLAEV
VEDVKKDVKDQVKA
>Mb|QLI69098.1
MASIAYSLRAVARPAVMRFAPRAAVRTFTTSRMALAKKYTKDHEWVDLSA
DRKSATIGISQYASDQLGDVVYVELPETTGDFVEAGDAIGAVESVKSASD
INAPIKCKVVQGNVLLEEKPSTINQVPEDDSNGGGWIVKVEVDEHGAKEF
DELMDAEAYKTFTSE
>Mb|QLI69101.1
MATISRQPFAPLDGARLQGLTSLKNRQNAIASPSNTKRKAELFDADDFEN
VDPLASAKRTKGASGTPTKDILKKPMSYVLTRAATTPTAHVRALASPVKP
STAPRRALQPKSPIAKLNTNVTKSSPISAPAGRSPTRGKRSGILSNRRRT
AGPYTRVDPPSFSLDSAAPFSLDAALKGTIPSYGSRPRSNALSQGSAPSL
GEPDMKASWFFDIHEDTPEQEMTNLLQHGTCVLDISSDEESEQKARRENA
EGRDKENIPPVDDVSQTSARRARALAADDMIVEKERIALGEMNTADFYAD
GCDKSSVIIVPADDEEQEQSADEAHTGQDFAFAPELVKTSSPSKDIEELM
AKSESPSKAAVLRPIEGTGESFDLWESGSAQDETELS
>Mb|QLI69104.1
MYSKQILQQLLVAVAIVSVAQSTWLDLNKIDVQALAARQLSDLVGGTSAS
ESSTQSTPTTESTPSSTSTPSSSPTSTPSSTPATPTTPSSSSSSTPSSTP

ASATPSATPEATSSSASTPSSTFVPVQTTSSSSTPTSSTPTPVVQTSVEV
WTTTNTNGSKTVLSSTTRTTSTPGLTSGDGETTGMSTKTRNTVIGVVVGV
GGAIVLGALGLVAWRIWGRKKHSEENDGLMAYDMSATGGVEKSERGSSAG
GAHTQRSPFQSTLENYHQPSQVTASSNF
>Mb|QLI69105.1
MAFNLNNSAQRRSNPFTRTGSPSPSSPTPLNRSRPKSALFSSPPPPPSVS
TPSPHGRSQSNSSYGTSLVATGNAVRHHRNESRNGTPTSNTFAPSFIKLE
EMRREHDTVKGIEGENDFSGKRYVWVRDSQHAFIKGWVVEELGNSRILVQ
TDDGTQRELDAESVDKVNPAKFDKANDMAELTHLNEASVVHNLKMRYQSD
LIYTYSGLFLVTVNPYCPLPIYTNEYINMYKGRSREDTKPHIFAMADEAF
RNLVEEGRNQSILVTGESGAGKTENTKKVIQYLAAVAPSETSARSKSQHT
NLSQQILRANPILEAFGNAQTVRNHNSSRFGKFIRIEFNRNGAIAGAFID
WYLLEKSRVVHINPHERNYHIFYQLLKGSSNRVKSDLLLSGLDTQNFAYT
RNGHDTIVGVSDKAEWDLLMEAFDVMGFSDDEQLSILRTVAAVLHLGNVE
VVKESRAADQARLAPESKQVVTTVCKLLGVPVDPFIQGLLHPKVKAGREW
VEKVQTPEQVRLSLDALSKGIYERGFGDLVSRINRQLDRTGMGLDDSHFI
GVLDIAGFEIFEENSFEQLCINYTNEKLQQFFNHHMFVLEQEEYAREQIE
WQFIDFGRDLQPTIDLIELPNPIGIFSCLDEDCVMPKATDKTFTEKLHSL
WDKKSTKYRPSRLGHGFILTHYAAEVEYSTEGWLEKNKDPLNDNITRLLA
SSSDKHVAGLFSDCAAADEDIGGSRSRVKKGLFRTVAQRHKEQLHSLMTQ
LHSTHPHFVRCILPNHKKRPKQFNNLLVLDQLRCNGVLEGIRIARTGFPN
RLPFTEFRQRYEVLCSNMPTGYLEGQAAATIMLEKLGLDKTLYRVGLTKV
FFRAGVLAELEEQRDALITEIMARFQSVARGFIQRRVAYKRLFRTEATRI
IQRNFNIYLRLVENPWWQLIVKMKPLLGATRTATEVKKRDAMIKQLNEKM
RLDEKVRQKLEDDRRNTHTEMVRIQQTLESERALALDKEEIFKRLQTREA
ELEEKLSGAIDDQERLEDQLDELMEAKSRAEEDVEKFRHQLEQAAGLIAK
LEEEKSKLSAKSVELENAVKEISRKQSERSEQEAALADEIKVLQSQLSVK
DRKVQDLEGKLLQLDQDTEVKLRAAQKESEAAKLRETRLSQENKDVKHQL
SQLSKTSTDYEDLVRSKESELALLRSDKRKFETERRTLEDQKKALTEEKN
RISSRSHDVQAELDAMKSKHSQLEREAEEAKTLLAARLSEDAQADKNRSL
LEAQIKDLKEQLYTTQMDLSRERQSRDDVLLLGEHKYQSLKDEYDSLNEA
KIVIEKELYVQQDTLRRAMEARTAVEKERDEARDEIRRLRVAKTQAEEAR
VQAEISGERQASKMALEREVSLRKDLDAAHDRLRWFEDECAKLNHEIEDL
NKLIASSGEFGLKNDQAKERMERELNTVKSRLAASENDNRALLNKLQQKG
LEIARSTSRANEASRGHIISLQKEKSRLEEQNANLNKQLGDAQVTVATLE
KKTEKLQLNLEDLNHEVAREVQSSRNAEKASSNFTVQLAEANRTIDSERQ
LRTQSQATVRTLQATIDARDKELQELRGQMLNALRSVDPEIRIPPPSDDS
NQKALLKNFDLARKVEELQQNLRVQSAARTNAESQLADLRATRHESPGRP
KLEEIHLNEAPFNGSPTQRRAAKINARRFSNTSTPRKANQDITEQHDTAR
SDKTVDTLAVNNRMDLKAEVEELQNQLQITQMQNRHLQSQIDRGTPGPDS
YNDQSPSLRRMQKLEKVNSRLHDMLDDSTKKVSALEKNIRTGELSLRDVQ
ARSHEEILDVFNSQEESRRALLHSHKDAVAELTDVKSHFDKMRHERAKLE
VELRDTKSDLQEMAMAREQEAQSRSQLLQEYTELQIRLDAETSKLADVSG
SLEVYKSRADEYFGKLEQAEIAVLKASRAEQFAKSQAKESEDTYSEVMAE
RQKMDATIEDLQRQNQRLEERIEDISTDLESVTQAKKRLQHELEDYRSQR
AMDIEDKESSMEQTRKKYQAEFTTLTKELDLAREEKLYKQAEIARLREEL
DELRSKWDDEVLNSSTWSKEKSRLEATLADVASSRDEAVNAHNEAQGKVV
SLLSQVRTLRSSVDEVTSERDLLLREKRSVEARLAEAKAGLEELVKGESP
SLRDAAILDKEVLDLKSSLAHQEDVAAAAVEKMRRAEALVSDVQKEIAAE
REASTELQKQKAALEKSLSEAQLKLIDLETKGFSSASQDIKFLHKRIQEL
ESQLEDHETERSKSQRSNRNIDRTVKDLQSQIDRKEKQNTQLSEDVNRMR
DKVDKLLKTIDELQASESTTQLSARRAERELREEKEKALRLEKELEGWKG
LRSEKASAVGSLRGRLDGPDVPNRSSSINRAPSLTKGFI
>Mb|QLI69107.1
MADFGEYPPNMAPEDAFVAQQQQTAKDHAVVPYSRESLALSPAGPANSLR
NDPTALKRKNLLTGHANETFLSEHTFRSKHRAIERNGGLEREYQNSATLK
AEAVRLRATRENKGDATVVEGAGAYIGPWAKYNRAKCDGIDDDEELRSDE
EYEVVDGDGDDNSDVVESGTILQAPEVALARRKEVAELGDETTTFHGSEQ

YDYQGRSYMHVPRDLDVDLHKENGSATNYIPKKQIFSWKDHAKAVTALRF
FPGSGHLLLSASADATVRIRDVYHDRMLLRTYSGHSKAISDVCFNSSGTQ
FLSASYDRMMKLWDTETGVCISKFTTGKTPHVIKFNPDPEHSGEFLAGMS
DKKIVQFDVRTPNEIVQEYDHHLAAINTITFVDQNRRFMTTSDDKSLRAW
DYNIPVPIKYVAEPDMYPMTNAAPHPSGKYVAYQSSDNQILVYGANDKFR
QNRKKSYRGHNNAGLAIDLDCSPDGQFLASGDSGGFVCFWDWKTCKMYHK
IKSGNQAVTCVKWHPQETSKVVSAGMDGEIRYWD
>Mb|QLI69111.1
MRPMPQCVYNSRRALYRVFVSPSEAPALSIVSRLQLSPLPAPHRNALAAS
QVRRYAAKPPARGPRGQTSVAAAEQLAHDNRGFDKRYTTQQDIARSGRDR
PPQDHEITDPQIMVIDNGATEGPLATPFVLTRLEPHESLRMVQPYVPADP
KNGRPGAQWAVCKIVNKRDEYERQKQLKEKKKAGVGKPKTKELELTWAIG
EHDLATKMRQMGQFLDKGMKVEVLVARKKGGKQVDGKEAEGVVRRIRDEV
DKVGGREGKSASGAVGGTYRLYLEGKQNQA
>Mb|QLI69115.1
MDKVKKMLNVGFEADAAKPAEPSSEAYAQLKAEYTKAGQDHVFTFYDSLS
TEDKAALYNQLSGFNPAYINEITKRALGETKSDTPDTLEPLPESAQASIL
DSSADDINKWYSSGLGLIGKNKVAVVLMAGGQGTRLGSSAPKGCYDIGLP
SHKSLFQIQAERIRKIQELAAKNAGTGSVVVPWYVMTSGPTHKPTEAFFE
ENKYFGLDAANVKIFDQGVLPCISNEGKILLESKGKVAVAPDGNGGIYQA
LIVSGVLDDMRKRGIEHIHAYCVDNCLVKVADPVFIGFSSSLNVDIATKV
VRKRDATESVGLILSKNGKPDVVEYSEIDKATAAAEDPKHPGVLKFRAAN
IVNHYYSFRFLESIPQWAHKLPHHIARKKIPHADTKSGETVKPETPNGIK
LEQFVFDVFPMLPLNKFACMEVRREDEFSPLKNARGTGQDDPDTSKADIM
GQGQRWVAAAGATVVADGGVEISPLISYGGEGLEKLSGTTITAPAVLERE
>Mb|QLI69117.1
MAAMPVIVPAAAKHTHTVVFLHGRGDVAENFVASLEYSKSSRDQTLQEIF
PSFRWVFPKAGVGASFSFGGNKVSQWFDIWNVADFSEREEMQTLGLKESV
AMIRKVLHIEAGILGGHWDRIILAGISQGAATATHALLNLKLPEAADDGT
KLSGGLAAFVGFSCRMPFPGRTLRETRAVLSLEDAPEDDEVIRRTPVLLE
HCVDDPLVLVENGRVLRDTLRGFGAEVHSREYPDGGHWLNSPAGMDDVAE
YLTHVLGLTPASAATNGTEMDLS
>Mb|QLI69120.1
MPVKKEPGEMSAFERKRLENIAANRAILTDISVTAKKIIPDKPKPAKSAP
PKRKSRGDQIKREPSRPTRMSSRLAGLEADNETLKRKLEVEAENQAEVAK
AKKLRVVGDLNLGDIAVEGKKWSAGLDSLKGIVRGAQPGVRTFTEDDIQE
TTDKSLKDLRQRMGGLKLYEHWLPNDIKITPQRVYALGFHPTEDKPIVFA
GDKEGNMGVFDGSQTPPEVDDDENPTSDPEISAFKTHSRTITSFVFPPTD
GNTVYSSSYDSSIRKMDLEKGTSIQVFAPSDIDTDMPISALDMAHSEPNI
LYFSTLDGGVGRYDVRAPGSEEIWTLSEQKIGGFSLHPLQPHLIATASLD
RTLKIWDTRKISGKGDLRHPALLGEHESRLSVSHASWSAAGHIATSSYDD
TIKIYDFTEASSWKAGHDISANTMEPKHKIRHNNQTGRWVTILKPQWQRR
PHDGIQKLVMANMNRFVDVFASDGSQLAQLDGEGITAVPAVAHFHPTLDW
VAGGNGSGKLCLWQ
>Mb|QLI69123.1
MSGIPPPPPPPGWGAAPPPPPPPPPPSSLPPPPDTPAPPAPPPPGYHPPSYP
QIAKFAQKKKEWLRDRRNRFGEKRKAGFVQTQKADMPPEHLRKIVKDIGD
VSQKKYTNDKRSYLGALKFMPHAVLKLLENMPMPWESAREVKVLYHVNGC
LTLVNETPRVIEPVFFAQWAMMWTFMRKEKADRRLFKRMRFPPFDDEEPP
LSWSENIEDVEPLEPIQMELNDEEDEAVYEWFYDHRPLLDTSHVNGASYK
TWNLTLPQMAALFRLSRPLISEVVDKNYFYLFDLKSFLTAKALNVALPGG
PRFEPLYKDIDPNEEDFGEFNAIDRIIFRTPIRTEFRVAYPFLYNSLPRS
VHLTWHSHPQIVFNRADDPDLPTFLFDRRINPISSRTVAPKNVEITLEDE
LFGPGNNEEPEEDAFELPAAVEPFLADEELENEHTSSAIDLWWAPFPFDR
RSGKMVRAQDVPLIKQWYLEHPPSDRPPVKVRVSYQKLLKNFVLNELHKK
KPNAHNNQNLLRSLKQTKFFQQTSIDWVEAGLQVCRQGFNMLNLLIHRKN
LTYLHLDYNFNLKPVKTLTTKERKKSRFGNAFHLMREILRLTKLIVDAQV
QYRLGNIDAFQLADGIHYAFNHVGQLTGMYRYKYKLMHQIRTCKDLKHLI

YYRFNSGPVGKGPGCGFWAPSWRVWLFFMRGIIPLLERWLGNLLSRQFEG
RHSKGVAKTVTKQRVESHFDLELRQSVMSDLMDMMPEGIKQSKVSIVLQH
LSEAWRCWKSNIPWKVPGLPAPIENIILRYVKSKADWWISVAHYNRERIR
RGATVDKTVAKKNVGRLTRLWLKAEQERQHNHMKDGPYVSSEEAVAIYTT
TVHWLESRKFSPIPFPSVSYKHDTKILILALERLREAYSVKGRLNQSQRE
ELALIEQAYDSPGTTLERIKRFLLTQRAFKEVNIDMNDNYSTINPVYDIE
PIEKISDAYLDQYLWYQADQRHLFPAWIKPSDSEVPPLLVYKWAQGINNL
SHVWETEDGECNVMVETELSKVYEKMELTLLNTLLRLIMDHNLADYITAK
NNVQLTYKDMNHVNSYGMIRGLQFSAFVFQFYGLVLDLLLLGPQRASEIA
GPPQSPNDFLQFRDRETETRHPIRLYSRYIDKIWIFLRFTAEESRDLIQR
FLTEQPDPNFENVIGYKSKKCWPRDSRMRLMRHDVNLGRAVFWDLKNRLP
RSVTTIDWDDSFVSVYSRDNPNLLFSMCGFEVRILPKIRNQNDEFPVKDS
VWSLIDNTTKERTAHAFLQVTQEDIQKFNNRIRQILMSSGSTTFTKIANK
WNTALIALFTYYREAAVSTVDLLDTIVKCETKIQTRVKIGLNSKMPSRFP
PAVFYTPKELGGLGMISGSHILIPASDKRWSKQTDTGVTHYRSGMTHDEE
TLIPNIFRYIIPWEAEFIDSQRVWTEYSQKRLEANQQNRRLTLEDLEDSW
DRGLPRINTLFQKDRSTLSFDKGFRARSEFKIYQLMKSNPFWWTSQRHDG
KLWNLNAYRTDVIQALGGVETILEHTLFKATGFPSWEGLFWEKACLANGT
MLLRYDHSLVAVEDVREGDLLLGPDGGPRRAFNVVSGKDRLYRITIGSGK
EDLVVTSNHILVFHEVKSDSDQYYDDIEMTTAQYAALESTERSKYRLFSC
PGLNSPQPTSPNSAAGRFIPQSHSFVVKDVVLETEATEWAGFRVDGDQLY
LRHDHLVLHNSGFEESMKFKKLTNAQRSGLNQIPNRRFTLWWSPTINRAN
VYVGFQVQLDLTGIFLHGKIPTLKISLIQIFRAHLWQKIHESVVMDLCQV
FDQELESLGIETVQKETIHPRKSYKMNSSCADILLFASHKWNVTRPSVLF
DTKDVIEPTTTNKFWIDVQLRYGDYDSHDIERYTRAKYLDYTTDSSSIYP
SATGLMIGIDLAYNLYSAYGMYFPGLKVLIQQAMAKIMKANPALYVLRER
IRKGLQLYASESNQEFLNSQNYSELFSKQTQLFIDDTNVYRVTIHKTFEG
NLTTKPINGAIFIFNPRTGQLFLKIIHTSVWAGQKRLGQLAKWKTAEEVA
ALIRSLPVEEQPKQLIVTRKGLLDPLEVQLVDFPNISIRASELQLPFQAA
MKVEKLGDMILRATEPQMVLFNLYDEWLKTISSYTAFSRLILILRALHVN
PDKSKLILRPDKTVITHEHHIWPSMSDEDWIKIETQLRDLILNDYGKKNN
VNVSSLTSSEVRDIILGMEISAPSLQRQQAAEIEKQQQEQQQLTAVTTKT
QNVHGEDIIVTTTSQFEQQTFASKTEWRTRAIATSNLRTRAKNIYVSSVD
NDLDDITYVMPNNILKKFITIADLRVQVAGYLYGSSAPDNEQVKEIKCIV
MMPQIGGLRNVQLPQQLPRSDFLDGMEPLGVIHTMSGSELPYMSAADVSE
HAKLLDAHSEWDKTNTVTVSVSFTPGSVSLSAWGLTPQGYKWGAENKDTQ
SDQPQGFTTTMGEKRKLLLSPRFRGFFLVPDDRRWNYSFMGSAFAGMEKK
PVHVKLDTPLPFYSDQHRPIHFHSFAELEDIWVDRTDNFE
>Mb|QLI69124.1
MAPMSTATMTTPGANSANVSSRNSPATGSSATQASSRPKRKVNTNGYHPA
NSQIPLSSMASIGLDLTSVERRGQPTAVRESLQKKSRPHGISEAPTYCPT
EEEWRDPLEYMKKITPEASQYGICKIIPPDSWNPDFAIDTEKFHFRTRKQ
ELNSVEGSTRASLTYLDGLSKFHKQQGSNLHRLPYVDKKPLDLYRLKKAV
ESRGGFDKVCKLKKWAEIGRDLGYSGKIMSSLSTSLKNSYQRWLCPYEEY
LRLAKPGVHQQLEQEYGGPLTPSPAHTPAKKSHVNTPSSVKGDSPARQAS
DVLQGSLNGLKRETDRDTPMLDAPPPAPTSAATGGFKAINTNGFTPVNAA
SKSANSESRSFTPDLKRLGSPATSASNTPDARSGLNPAASVKRQVGDSND
TTKKDVDTDMEDGESSSRRSKRLKREPVPTVAGSHMTPFRPSIPRIPKDE
TMAAGERCENCGRGESNGALLVCESCDNAYHGPCLDPPVKRKPDAEWNCP
RCLVGDGQFGFEEGGLYSLKQFQQKANDFKQGYFEKKMPFDHTLNCLRPV
TEEDVETEFWRLVADLEETVEVEYGADIHCTTHGSGFPTLEKNPNNPYAS
DPWNLNILPLHPESLFRHIKSDISGMTVPWVYVGMIFSTFCWHNEDHYAY
SANYQHFGATKTWYGIPGEDAEKFEAAMREAVPELFETQPDLLFQLVTLL
TPEQLKKAGVRVYALDQRAGQFVITFPQAYHAGFNHGFNFNEAVNFAPSD
WEPFGLAGVERLQLFRKQPCFSHDELLWTAADGNAATGLSIQTAKWLGPA
LDCIRKREQEQRAEFLAKHIEASPHKCPISGPGEEGCGCPLTFKIKERDV
LEEEEQCCTYCKAFAFLSRFKCGRTGKTVCIMHAGHHPCCDLAEEQRFAG
EDHALIYRVTDETMAETYEKVVDKARTPELWEEKYAKMLDEETSPSLKSL

RALLHEGERVPFDLPSLLILKEFVDRCNDWVEEATNYIVRKQQNRRKNEK
AWQSSMRKSIGSAYQDQKERESRNVSNIYRLLREAEHIGFECPEIGQLQE
RATAIKEFQLNAARTLENTATVSMETIEELLEEGRSFNVDTPEVDRLSRV
LEQMRWNQKARSSRGLFLSLKDVTELIEDGKRLEVPSYNDHLKFYSDQKL
AGETWEAKAKELIHAAIVHYPQLEALSNQVQANALPVSQETLAEVDRILH
KQREAHRQIMDITERCRNTNFRKRPKYAEVVDIIRNVDELNSKPTGTIDL
ENERKRHEDWMRRGKKLFGKSNAPLHILKSHLEYVLERNMDCFDVDNDTP
RQPAEPVSREVTPEQTGNRFDDRTRQVFCICRKVEAGMMIECELCHEWYH
YKCLKIARGKVKEDDKYTCPICDWRMKIPRDAARPKLEDLINLTDEIPQL
PFQPEEEDVLNRIIDNAQYFRDHIARLCHPLLSTEAEVETQRFFLRKLEG
AEVLLSYETNFFRQELHKWCPVAPEAPPILEVSLSTRKPRPTKLQKMLAE
YGVDNPDDLPEHAKGKANSLRRKAANAEAAAAAAQSPSGPLLPPSSSSAY
GGPTFCIGSGGNADSGTSPREHPSHAGVSSSEKQTRTKPMDVDSGLQPGL
LGPRGPVFTNNLALSLEDGLLRGLPEEEVDLESDIGKSRALEILGRTDRG
RQDAEHIWGPDVWAPKRGSISKTRRSITPIDVDETSRRDEGSVDQMFKEM
TNQEDDEDGKKKDDNNNTTETVTAESLESERNDLDAMIDGE
>Mb|QLI69125.1
MAPKKKEQQQKMSLGDFLTDSGFGGGSWADEVEETYVTGTQPLPPSDRPR
FGGGSSGMSNWQDRGYSSIRENLPQKLPDKPPFTAHLGNLAYDATNETVT
EFFEGCEIVSVRIIEDREMQRPKGFGYVEFADIEGLKKALTLDGESFNGR
MIKIKIADPPRGGDSRGGESSRDLSDWTRKGPLPDLPGRGGRAGSDFGGD
RERRPPRDPAAEPRAPREMNWERRGPLSPLPPQEGGPGPREGSRSRPAPE
GMGERGESHRGSRQGSASWGEGRQDGSRPPRREQPERAPTAAEKDFQWRS
SMRPDAAKAPEETETAVSPSAPAPAAQGGRPRLNLQKRTVTETAEAAPTA
SGGDSKANPFGAARPINTAAREQEIEEKRQIAIREKREADEKAKEERRLA
KQAAAEKEEADAAAAAAAAAAAAAQAEEAKEAELKSAEDEAGEKENGAST
EEKGAPRSREPREPREPKESIKSRATESGNWRSASNDQRVPRGPPGGGRG
RGGAARGHRNEGRGGPRANGARDQQASAAQGADEGETTPVDEDGWTTVPN
KKGRQGRPLA
>Mb|QLI69129.1
MHHPTLILAAASALSGITPPWFAHAAAVYPGSGPPQAFSLVERATPQAPD
GYAPAAVDCPSTRPEIRVGSSLSPQEKEWLPRRRAETISHIRDFLQRNAI
PGFDSEKYLSGATANSTALPNIGIAVSGGGYRAMLNGAGAVAAFDSRSPG
STSKGNIGGLLQTATYISGLSGGGWLVGSIYTNNFTTVQNAVNSGTIWQF
GQSILEGPKGFKDYYKTVFDEADKKHDAGFNRSITDYWGRMLSFQLVNAE
GGGPSYTFSSIADDSDFSAAKAPLPFLVADSRAPGEKNTTIDDALFEFNP
WELGSTDPSLNGFVPLKYVGSKFNNGKLPDSEKCIAGFDNVGFVMGTSSS
LFNQIILRLKDSNGSKYIPADVPKFVVKIILEILTALGDADDDIADWTPN
PFKGWNKAVNKAATSDRLTLVDGGEDGQNVPYHPHLLLERKVDVVFSVDS
SADTNAWPDGAAPMRTYERSLSPVSNGTSFPVVPGRNSFINLGLNTKPTF
FGCNATNTTNAAPLIVYLPNYPYQFHSNISTFSLSVNNSERDAMISNGWA
VATQLNGTRDADWPTCVSCAMLQRSFERTKTPVPDKCNQCFSRYCWNGTI
DERPPAKMYEPQLYGTAVDVKGTSKSAGLRLLGSTSAALATVAACLVIT
>Mb|QLI69130.1
MNELFEDFAKAYALRNGYLLAQTISPVPPPNEPHRLKRIWQSTNSHSAKG
DIKHFIKSQTSSRKTISHDEVNGWVEVYVAYWNAIGEILAGESGKSSWTK
VYEAWKELTSMLIRGYNNSGFEAWTIPTLYVVGKYLRLFAIKSDDERSRS
STDNSGTASLMQDDFDPELEKQGQLRDCEQHLKRIFTLCLNDRAPIEESR
KWGIYFVINLLFKTYFKLNSASLSRTILKTLAVYNDKGDMPPLEAFPRAQ
RVTFKYYEGVLFFLEENYVQAEKHLIEAWQLCHKDAKSNAERILTYLIPC
RLLTSHVLPTKALLQPYPRLQELLLPLAECIKRGDLHNFDLALQKGEDEF
VKKRIYLTLERGRDIALRNLLRKVFIAGGFDEPKEGETTPVRRTRVPVSE
FRAAICMGSGGELVDTDEVECLLANMIYKDLMKGYIARERGIVVLSKKGA
FPGTGV
>Mb|QLI69133.1
MGCRVCRARKVKCDGRPNGCRNCERLQLECVADDGSTPNGNRISLSPISL
RKIRTYRSCQSCRLSKTKCNGDRPKCARCAAKLIQCVYDGGAAPRWTRNL
NRSQAASGDDDDGSPTERDGMTGITPSETDERGSVRASLSKVSDDATEKA

SVVSEAAATCVTGAASQNGSNGQLSSHPLAWLLSSALPATRNLRRVVEQY
FSNVHPLRCFAFVHKPSFMRQLDKGLSSDDESALLHIICAHGAKFLVLSA
DQGEANHPPSFLKNAGNQWAKRAEFLMLANFGKISVQRLMTAILLHDFHF
RLGEYGQALMISGLAVRMAHALKINTEYNSDILCTEEDSNAPSVASRESR
RRLMWACYVLDAWAGSGVDQLTLLRENDIKIQLPSNERNFGLRIPSVTET
LGVGHVLQFLPPAIVPRRPAANMGIMAYYIRVVTLWKRIVRYVNHLHAGP
PPWQPESLFAALDADLLLWRRELPDFVEYTTETIYARLDSNQLGALVLIH
CTYHHNYLELFKLTMPDLFKLPKPFTFPPEHQEFLQTAQANAYYHAQQIA
DILAEAAEHGSRLLSDSLLPFFVYDSSRVMLYYVARLLDPNRPDAEVKLR
DAVKAVESNNRILGMMSALFPIAQSLSATIERWLSKVQKTVARDDLLSSL
EADDHLESHHSALPNNASGKQDKPGEYDLPPLSLHSLARSGTTESRGMDG
DAAARTASWSTQNPHAGGPASWDGTSAAAHQLVGLSQEQSVRSSLQHERP
VPLHPLRKFGATQPMCNALDLDDLQSFLSWDMYGIMEMGGSVSNEDMDDS
VSQSWTEAI
>Mb|QLI69135.1
MAADYLDAVNLMAYDFFGMWTPKSGHHSQLYAMSRDEPSGSSGVAHLMSH
GFPSGGILLGIPTYGRSFQHATGPGQKFKGGGGNDGTFEYNQLPRKGCKE
SVDKRHISAQCVGGDGGFVTYDNPDTVKAKAAFAKQKGLGGLFYWNGTAD
SKETSRSLVAAGFRALHTS
>Mb|QLI69136.1
MNPVALRSVCARARPEVQRTGYALKNYFRDFSTSQSLAADEASPPTPPSQ
PSVRQRTRDATSEISSLVKRGRGGSRGSSRGGSLSGQRQSAQPPAGSAPK
VIDVKSLPKGLSRGRGGFRGRGGGGRGGQVGAVPSQGQPHRAQPPAGNRF
SRPSGGRGSALRGGRGGRERGAARGAARGRDDDKDKQKRLGRRQDPFETM
DPYEEQFDRDMRFGAATAYAPRLTMGSLAPFAPATPSSAAGRRATVLGNL
SALGTADPVGVPQDLQARSYAEDLESEGLRFFADAKAREAAERYLQEKRA
EGEGEGEGEIIRDAGESVRSVVLDKAVRGEHEKMVFREGPVGVARNWHLR
AETYTGKDVESFENKLNSLVGKGAGKKGEKKAATTA
>Mb|QLI69137.1
MTLRGKAGVYAGAAVLRLLLTVAFPGLPDLLTGRVEISTPVTSFKRLQEG
LFLYNHNVWPYDGGVYHQAPLLLPLFSLLPSVKTWPIFTSVLYILVDLLS
ADALCTIANSGEAGQSRLFTSPRRAKRACGLAVAAAFLFNPFTIASCIGR
STSIFTTCAILHAISKAIQGSAFNAMIALSFASYLSMYPILLLPPLVLLS
FDRQPEARRTASAVAFGAKCVAITAVCLGLLLGMSFVLTGNSWEFLSRTY
GIQLTLSDLTPNVGLWWYFFIEMFDSFRAFFLAVFWLHLAAYVGGLTIRL
RTQPLAVLTLLLGIFSIFKPYPSIADASLFLAMLPLFRHVFPLMRYTYVA
SATLLYATFLGPAFYHLWIYAGSGNANFFYAITLVWSLGQSLLVTDLTFA
VLRDEWEVERPDMIGKEVKQI
>Mb|QLI69144.1
MASREEKPQSYPMTNTNLTVPEKTAKGPSTRVSPLERTSSGTTARSDNSN
PFETDVEAMATNSSLDKPRSSVILTRKKDCQVWPNKRDWKQRAKAGKQNR
TCAFMQRFNRRTRIMMKVLIIVLVVGIAVAVGFGVSKPLGAPIWGDQTTR
S
>Mb|QLI69149.1
MVIHTPNTISDSDGELCKMNFIYFSNEFPKGDLQDLLRLLHSHSKDKHHP
VLAQFIDEATRAVKDEVRALPSELKDLIPSFTTILSWAEDGGLREGLICG
AVDGVLLVVVQLAAYIGYTESHPEESVDVSNSGLLGLGIGLLASTAISLS
STLSELPLAGADAVRLAFRLGIHVLSVSENLEARDVSQKEDSWAYVVHNV
DPVAAQNELDIFHSRLNTPSTAKIFVSAISQNSVTVSGPPGRLKGLFNKS
EFFRVSRCIPLQVYGGLCHAPHIYNQQDIHAVVYGSSLNTIVSKARPALL
VYSTRTGLPYTANNAAELFECVISELMTKAICWDEVIRVVVDQVQCAGPR
GVVLNCFGNSIPLNDLNLALTSTLPDCKLLIKDFIPWLSLAAPRSNTPRG
AAQSKLAIVGMSCRLPGGATSTEKFWDILEKGLDVSRQIPADRFDINTHY
DPTGKELNKTMTQYGCFIDEPGLFDAPFFNMSPRESLVVDPQMRLSLVTA
YEALEQAGFVANRTASTALPRIGTYYGQAADDYREVNQGQEVGTYYIPGG
CRAFGPGRINYFFKFAGPSYSIDTACSSGLAAIEVACQALWAGDVDMAVA
GGVNVLTNPDGFAGLCNGHFLTKGHNACKTWDATADGYCRADGVGSLVIK
RLEDAELDNDNILGVILGAGTNHSAEAVSITHPHAGHQAYLSRQVLRQAC

VNPLDVSYVELHGTGTQAGDYEEMQGIMDVYAPLAKRRSKDKPLHIGAVK
ANLGHGESVAGTTALIKVLLMLQKNAIPPHIGIKTEINPKFGRDIDKRHL
KIPLEMTPWPHVPGKKRIAAVNNFGAAGGNTTMVLEEAPVRPADETDPRQ
THVISVSAKTRSSLAGNIESLLGFLDKNPNTNLGNLAYTTTARRYQHTHR
VAIAASNITQLRKQLTSRLDQIDSIKPVAKSGPPPIAFTFTGQGASYKSM
SLELYRDVPTFQHHIRHLDSLSQSQGFPSFIPALDGSHPKEHEHSPVVTQ
LALVSTEIALAKYWASLGVKPDVVMGHSLGEYAAMHVAGVVTASDTLYMV
GKRAQMLQEKCESGSHCMMAVRASLEEIKARSEGKPYTIACINGPSDTVL
SGTKQQIDEVSILLEQAGFRCIKLDVAFAFHSEQTDPILDDFEAICKSGV
TFQEPKLPVISPLLGKVIFDGKTLGANYVRRATREPVDFLSALENAQRVS
TISDETVWVEAGPHPVCTGFVKSIIPATQLAVPSIRRGEDNWKTMAESLA
ALHLAGIPVRWNEFHRPFEVRLRLLHLPAYSWNNKTYWMQYNGDWCLTKG
NNFYETGNDATKAKAALKSPPPSELQTSTVQQIIEESFNGSAGSVVMQSD
LMQPDFLAAAYGHRMNDCGVVTSSIHADIAYTLGSYLHRKLLPKAKQANM
NISNLVVTKGLVANSDTKSPQLIRVTASTVDAHSGVVDLTWQNVDNNGYV
HEPFATANIIYGDANEWISSWNPIAHLIHSRIETLERLAVEGRASRFSGN
MAYTLFASNLVDYAEKYRGMQSVVMHQLEGFANIELTTKEGGVWTVPPYF
IDSVAHLAGFIMNCSDAIDTKNNYCVTPGWNSMRFAKPLVPGAQYRSYVK
MIPTVEDPTIYFGDVYIMQDDMIVGMVGGIQFRRYPRILLGRFFSPPDKA
AAMEGKQGIVAPQPKTTRPALKSAGPERDHGKNNLPPVKPAAEVADEPSK
DKLPPTSAGVPAVESATGSNSVASKALQVIAREAALEVSDLEDDASFDNL
GIDSLMSLVIVEKFRIELDIKVGGSLFLDYPTIGDFRKWLEEYYG
>Mb|QLI69150.1
MAAPFRIILRSSCKWTPKYSSIQPQAPILRIPKRFLCTTHTRKEARAFPS
IFIAIGLIGTAAYYVLAPTSHKTLNSDSFIPYTITSRQPISPTSAIFTIS
PHHHDPSLPYLLPSSQEWKYPLWSVEFKQPEVQIARHYTPLPPADGENTK
DGTLSFYIRAIGGGEMSTYLNRLGVGRDVYLRGPHKGFDVLKRLGEQKNV
VFLAGGTGVVPGMQVAKAVLERDATTSVELLWAVRKREELQQCRQGGSSK
PWWNFWSTAAPAELTAEVEEPSAMGKQLARMKARYGERLTIRVAVDEERT
RFTERDIGEALASGGRALPSPGCQLHDQCLHVHASEFEDPDTKCQCSPAD
RPGKNLLMVSGPDGFIAYYAGDKKWIGGTLTQGTVGGVAGVLQRKNPVLA
NDWLVLKL
>Mb|QLI69154.1
MAPKIAIVFYSMYGHIRQLAEAEKKGIEKAGGTADLFQIPETLSDEVLGK
MHAPPKPTDIPTLEDPAILTKYDAFLLGIPTRYGNFPGQWKAFWDKTGGI
WASGGYFGKYAGLFVSTGTPGGGQESTAIAAMSTLTHHGIIYVPLGYAKT
FPQLTNLSEVRGGSPWGAGTFAGADGSRQPSALELELATIQGENFYQTVS
KAFG
>Mb|QLI69156.1
MYVLEEQRYIHEDLERLEQGIADRVREEPKPIRDRLNRDHEIAQLLDQIQ
RQSSSLLNLYKDENGTRAGELQELNTGDPFDAFYKQLGDIREHHARYPNE
QAENSEQRYKQKRGDPSAPVATVVDTIFSGEEAFGRFFDLHTCYDSFSNL
PNVKRLTYLQYLEYFDNFAAGYGGVKRGDKMTDQYFNYVGELAQYLEDFM
RKTRPLENPDNILSGFDEEFGKLWEKDEIEGWQIESATNGSKATLTDAEA
IWCEDCEKEFKNENVYKNHLTGRKHIKAAEQRKQRQQDAAAGSADSKSVV
VSSARLKERAIAEREYRVKKMASLMNTERSDTRVNVERKQGMTKREREQE
LENLLNASDTPREAAEDGEGEGDDDEDKIYNPLKVPLGWDGKPIPFWLYR
LHGLGVEFPCEICGNYVYKGRRAFEKHFNESRHIYNLKCLGITNPTLFRD
ITKMDEAVKLWEKMQKEKKKSKLDEGSVVQMEDGEGNVMPEKVYYDLQKQ
GLL
>Mb|QLI69159.1
MSELKWPSALVRKAFFDYMQQRGHTIGMQNCPSFMMNLAQTAIVDEVPSG
SVVPYDDPTLLFTNAGMNQFKPIFLGTINQSDPMANLKRAADTQKVIRAG
GKHNDLDDVGKDSYHHTFFEMLGNWSFGDYFKKEAIEMAWEILTKVYGLD
PKRLYATYFEGDKELGLPPDDEAKQLWLDIGMPEDQIVPGDMKDNFWEMG
DQGPCGPSSEIHYDKIGGRNAAHLVNQDDPMVVEIWNLVFMQFDRQKDGS
LKPLPAKHIDTGMGFERVVSALQETDSNYATDCFTPLFKQIQEVTGVRPY
SDKYGKDDADGIDTAYRVIADHIRTLSFAITDGAVPNSDGRGYVIRRILR

RGVRYARKYLNAEIGSFFSKILPALVSHMGDQFPELVKKQLDIKEILDEE
EEAFARTLDRGEAQFEKYAAKAAKAGVKKLDGDVVWRLYDTFGFPVDLTQ
IMAEERGLEIDESEVSVAKEKAREASKAVKASVDTFAKLDVHQIAQLDQQ
KVARTDDDAKFVKGDSKGKVQLIFDGKTFHNSTKELPEKTAIGILLDKTN
FYAESGGQVADTGRIVIDDVVEFKVMDVQNYGGFILHSGYIEYGALSSGD
EVICEYDELRRSPIRNNHTGTHVLNHSLREVLGDDINQKGSLVDNEKLRF
DFSHKSQVKIEELRKIETLSNEYIRRNSKIFSKDVDLDLARQIEGVRAVF
GETYPNPVRVVSIGMDVDTMLKDPKKQEWRSYSVEFCGGTHVEQTGLIKD
LILVEESGIAKGIRRIIAYTGEAAHQVQREAAEFSKKLDILEAMPFGPAK
DAQTKVVSQELSQLVISTLTKDELNTRFQKIATSVIAEQKKRQKAEAGAA
VGCVVKHFEEKKDVDYFVGRLPISANAKALTEVFKHFQAKDKTKSVYIFG
GSQEEGAVVHGVYVGSGLASKGVTAEQWASVVSDVVGGRSGGKEPTRQGQ
GTKPENIDDAVEVARKWLEEKL
>Mb|QLI69161.1
MTDASSSPVSQPQGSSPAANANQPALPPLTTSTPTPSHRSNLPRPMSHAS
KSRLSQYSSGSVPSRSRPGSHMFPLFPSSLSYTLVRDFAYPAAHPMHYGP
PPEPSRPPSGLTTPASETRRLSDPPASWEPRMPWDSWTSDGFSRGHDIPP
IQFGDGPPYSEDDDLQSPVVATRHRKHKSSSATLGQSRGRAGREGDIMGT
TNYDSERGYYVGTSGDGSERYYVTHGGEANGPGGEYVTYPPDQARHGHGY
HFPQQGHQRDCGRPGSENCSSPSSSGYHDLDESRYSRDYQFTITSPDEEF
HGKAVALFDFERENENELPLVEGQIIWVSYRHGQGWLVAEDPKTQESGLV
PEEYVRLLRDIEGGMNSLTGHLVDACISLNEAETPTQAEHGGKERQPSIT
STNGYHQPVVSVFSTSSKDLNPYPTGQLGIQAGQTPPQVIHYHGQRGGSQ
TSTPTIPQHNEPGMARRGSQDSTNDGEAGTTPVQEHPTATSSQAAAR
>Mb|QLI69162.1
MTAPLSQQTVSVKDAAAVRKRRRRAPAGGAADDCFTCSKRNVKCDRRRPY
CSQCLEIGNECSGYKTQLTWGVGVASRGKLRGLSLPIAKAPPVSREPKKS
PVTRGRANSTAAVMAAHWAEQEEARRHRGPIDIPSVTHSVSTPANSYPGP
GYDYLSMSHPENGTATVPQHGWGNIHYSSGLVHSPDATPKYSKFPLPLIT
DGLSSSVDSVSDVDYLSPLSQTYSRDEMPFGSGSSVIYDGYGRNQHSPVS
QSPPSGLVLDHTRVPTSCPGLVYAASEHSSSINSHLDPFDTHLSQKLMRE
CDSLSVPQIDVYNSSCSPVANSWTSSSHQREEELQSPQSEHTSNRWPTTL
REPDPLRVSPDLAARMPFFMDYYKNTIAPSMVYIDGPHNPFRDHILQLAV
HSQSLQHAICALSACNLRMKRKLSLGQDTQELLEKLTTEKNAIDSLGDRQ
PDDPSLAEEFQHRNLAVNLLNEHLNDPLKSTHDSVLATILLLCHYRMVES
GVAKFHTQFAGVKKILSMRTEQFGHSRGSAWIEALFTYFDAISASINDRE
AQLAPAVDDGLPLLPLGAENLVGCDRELFKTISKLGRLNLLSQHRPVQNM
ASSRRPSADTAPTHRMVRSPVGHPYGAGITSVGSVHMQQNQHAQQQPQHQ
QSQQLSDMYPIPAHRFDGNGFGTTLNDDEILASAMCTSAAFDDSRSLFWR
EWKDARLALQNWQFDAAAVAASLPGSPCPSQVRDLGSLSEAFRYAALLYT
ERLAGPSTPSSHTNFQNLVSQVVYYATSLDAGSTAEKFLLWPLFVAGSEC
VNELQQNIVRTKCRDIMARSGYMNNLSALDILEKLWAGEFNAATPDLRHR
SGPAKMAGCRGPFNWAKCFGGPGAEAEWIMF
>Mb|QLI69163.1
MGASDSKIVFKQGIFKLSEERHIPSGDPYWTSFWELPESSEDVFSLFSPN
DIRRTRDKALENIETLILALTSRLFLLRYHPSFPDPELAPEKDALNCIRV
LTRLLPYLYEKESLSSWQERFFWGQRRRKTRRAIIANEVLFDGAENDKEE
SKPNAEEYEEVKPLAEELIDTLIDLLFYSDLTITRQPHGHDKVTYAIWQS
GVGCNNAVATTKELESNRAEILRLLLALAGQSMYSTPGVLAQAGTRTLTY
LCTCQDKQIVLSVLCSLLNTTLKFNPASWRVPYNTLGFKDAKQILVTYSL
EFLLTLLVYPIPEQTTQSSISSKNFYRHFLGRLHRPQDFQFIVDGMSRIL
SQPLQEKTSYLPGAQATTNFAPEILMFFWEITQCNKRFRSFIIDTNRVHD
FVVLTIFYALEYKNEPAKQGVVRMCAFLLQTLSVDSKFGPSLNKQFEGQD
SLPVGIRINGFKGTYCDFLIHSIYNLITSSQGSFTAIYPALLAVINNISP
YIQGLGAAGSSQLMHLFTSMSSPSFLLANETNYQLLHSLMESISAIIDHN
YRKNPELVLAIIKNRKRIEALRSFTLESGQEEIERRKRLKKDRVDSVDSG
SIRSSFDSTRSPSVAPVKSPILDEITEDGTFAIGDSDDDSDEDPQPTPTQ
STTSENVSQASSAGNFDDAVPTQLRGMSEKARGKMPAGSRSFSRQNSTTS

LGGQSSSARMQSGSFEPTSQWIDGWLPELPLHSILTLIQQVSSVLPRQAG
REVLTPELVRRIQEIDLIGIEPSPARVHSFEWSPLALGWYESLLWGVVFS
NEMQIAKGTMGIWSGTAIKLFRVQETAPAGPTLTSPRGAVDAVGSNIVSR
IGQINLRGGQPAPNANPSTSPRMGP
>Mb|QLI69167.1
MAQQPLSTSAVPTDIYGGDEVSALVLDPGYCNTRAGFAGEDVPKSVLPSF
YGHVTGEPQRDLFGDECIIPRADFEIRNYMNRDSVVEDWDVAPKIWENML
IKRLQPEHQTLPSKNGLNDDVKEQDGEGDIAMDEAENQEKPLQENPILMT
EAPWNTPKSREKAIEIIMENWGCPAFWLSRTPVLSAFAAGKATALVIDVG
GANTSVTAIHDGMVLKRSIQRSPAGGLWLSSQIRNMWETSEPKVQLTPTY
MVENKSPVDALAPAQARLREFPFQITDSFRAYEEERVLTEFKESVVEVWR
GPGRYSAPNNEDYIKTQPGRVFEMPDGYNQMWREQRFKVTEGLWDENAGY
PVPEAERLTKAQTIPELIRASLGAVDVDLRGNLLANVVVTGSTSLINGFN
DRLSNELTAMYPGLKVKIHAAGLSSERRFGAWIGGSILASLGTFHQMWIS
RKEYEENGAGVVEKRCK
>Mb|QLI69169.1
MSQLDPLPKELPFRIVSKTVGRGAYASIKKAIPLDAANPVFAVKLIHKGY
AVKHGRISTKQLAMEVSLHSHIGQHPNIIEWFASGEDAVWRWIAMEFAEG
GDLFDKIEADIGVREDIAQLYFTQLVSGVSFMHSKGVAHRDLKPENILLS
SDGSLKLADFGMATMFEYKGQRKTSSTLCGSPPYIAPEILACGRVDKKNA
GSGKYSSDLVDIWSCGVILFVLLVGNTPWDEPSSNSWEFNEYVKTSGHSS
DALWGRIPQEALSLLRGMMSIDPQKRFSFRQVRQHPWYTRHNSLLTPDGK
ITDPINLATQMLENLRIDFTQLPSASQQQYLAGGDDMDVDTGLNSGKFSS
TQPEVPIIDRDWDWERPVLRSMAAPASSLPSHASDSRRMVLETLADEPSM
SQFSHTPGPSMTLTQQARRFRDICPPESLTRFFSHVPPTHLVQMLSDALH
QLNVPLAPVTPNLYGSPMANLKVKAIDGRQQNLHGEIQIDKQPLPDGSEV
LDVRFVKVKGDPLEWRRFFKKIVVLCKDGVYVPEV
>Mb|QLI69170.1
MSSLPNVHVSTHPSLRTKLSQLRSQTAGSRQVKALVNDISLILACEALAK
NLVTGPGPKGATPLGFEYTATAAHPQTICIVPILRSGLGMVDAVQTILPD
PVPVHHLGMYREPSTLDPVEYYNNLPHHSPQSSAENSTASSLAVLVDPVI
ATGGTCAAAIQTLREWGAKKVLVMSIIGAEEGVKRAAEEWPEGTELWIAG
VDAELTPDGMLKPGLGDIGDRLFLTTAKQA
>Mb|QLI69172.1
MSYMNSRLKGFGFGKRKSTASIQTTDGVPPAGTPPPGHPPAQIPQHQAPL
PGQTPPIGAASSTTSLPMNHPGAGNRPPSYTANYPPAGPGDRTSPLQGSN
RTPPSQMVGGPPPINTGAPVGYPPPNMAAMGQGPPPMGGPPPGGPGGPPP
GFGAPQGYPPGPPPTQGGPIAQQFQRPNPNPAAEVEGASKSKAQLIVGID
FGTTFSGVAFAFATNNEAKEDIITEWPGAGSYTKQKIPTVLYYDQYQKVV
GWGPDIADALAPTGYPKPGVQKVEWFKLQLMLSGNTYIDPINLPPLPPGK
SEIDVAADYLFKLRQAMRSALQKTLGEVFNREERNIRYYLTVPAIWNDAG
KAATRAAAIQAGFLRDENDNRLTLVSEPEAAALFCSKTGLLNLKVHDAVL
IVDCGGGTVDLIAYEVEEENPFTVAECTAGSGDSCGSTALNRNFSNILRT
KIRKMKLPDGSKTAGRVYAKCIMDFENRIKADFRNNGQKWAVDVGIEAEF
PEAGIEEGYMTFTNEEILQCFEPVVNRILELVRNQIIAIQAQNRTLQNIL
VVGGFGASEYLFQQIKLHVPPQFQSKVVRPMDSVAAIVKGAVTAGITERI
ITHRVARRHYLMATLQPFKEGYHPEAYRVPSLDGKDRCKFTRQIFVQKGQ
KVKNGEPVKVSFFRQVAPGATLMYEDVLYACDDDVCPEYTKDPRIKEVVT
LTSDLSRKNLEKDFERMDTPQGTFYRVYFDIYLTLDGSEFSAELVCQGEV
MGRCRARFR
>Mb|QLI69175.1
MPVVTPEKLASLQRQSRDIRNICILAHVDHGKTSLTDALLATNGIISPKL
AGKIRYLDSRPDEQLRGITMESSAISLYFSMLRRAAPEAAPEAKEYLINL
IDSPGHIDFSSEVSTASRLCDGAVVLVDAVEGVCSQTVTVLRQTWTEKLK
PLLVINKMDRLVTELKMTPAEAYVHISKLLEQVNAVLGSFFQGERMEEDL
NWRERMEERVNAAVAAKASADEDFSSDAGELQFEERDDEDIYFAPEKNNV
IFSSATDGWAFTCRQFASLYEKKLGIKRGLMEKVLWGNFYLDPKTKKILG
QKHLKGRNLKPMFVQLVLEPVWTVYQATCGGDNGKVDPSLLEKVTKSLSL

TISPHVIRARDPRLLLTTVFASWLPLSTALLVSVVESLPSPPAAQAERVP
DLIEQSPGSGAVDRSIKEAMVSFKREKSDPVVAYVSKMVSVPESELPENK
RKPGQMSGEEARDMARKKRAEAARAAQEANGDALDGLTQALDGAHINDDE
PGEHRADPEHLIGFARIYSGTLSVGDKLYVIPPKWSPAEPKAEPQPQEIT
VTALYMLMGRNLEALESVPAGVVFGIGGLEGKILKSGTLCSKLEGAVNLA
GVSMAGKPIVRVALEPVNPADLDKMIHGLGLLVQSDPCAEYEQFGSGEHV
LLTAGELHLERCLTDLKERFARCDIQAGEPIVPYRETIVRVDEMRPPVNK
DLGRGAVVGTTTSKQVTISLRVRPLPPDVTDFLLKNADAIKRLYTETKPG
ESGEEQEQEQPDGQEDKIVADTAVTAVDSLSIDELRKQLQKQLETGKGRD
VWKNRVDSIAAFGPRRTGPNLLIDATKNSILPKAFGTEARETTRAPRAGE
ALDPSHLSDKITYAFQLATAHGPLCNEPMQGVAVFIEDVSLDGGGGEDDG
SARDKLSRLTSEVIKTFQASLRAGFLDWSPRLMLAMYSVEIQASTEVLGR
VYDVLTRRRGRVVAETMKEGTPFFTVRAVLPVAESFGFADEMRKRTSGAA
QPQLVFTGFEVLDQDPFWVPFTEDDLEDLGELADRENVAKRYMDRVRRKK
GLLVEGRNVATDAEKQRTMKR
>Mb|QLI69176.1
MMKSSPLEWVMVKTTLPSSPLPPLDTRPEIRTERLVLRSTLESDLEGWHA
LRLQPEVMKWTGQGKPDPDIDWSREKLKQRLSPEGDAKYEYIVCLADTGE
MIGIAGSHMLVGELGWPVIGYMLRKEFWGKGYATELVHAFLKAWWALPRA
DVDVRVEKSTAVEGDDGKVRECIVAVTLDNNTPSQRVLAKANMYFVKAWD
EVDKQDQSLTETLYGYVAKRPMS
>Mb|QLI69177.1
MLSRQLPRLVARRARFHQPACQLRCQSTTTASSSSSGSNFATGILGGVVG
AAVVFGAYSFTPAGRTASKLNKAAAEASKKYDAAAKTLQSNPPSADQAVD
SIKQFAYSYAAWIPGGRGYVDAAFKDWETVRDSHKDEVDAIVNDTHKKLG
DVSKAGLSLETASRAVDVLAEMSRRIAELSKDALSDILDNHPRLKDKFGA
SIDELKSMAESYGPEAKRQVDQTWAQVRDILASGVSEESLAKARKLLEDK
AQQVKGLGDEAWTKGMEAARPYLDKNPKVKTLLEENAESLKKGSLSQLFD
RLRSAEKSGSVDELQDYVRGAVDKARAKAARAVEGSGLEKYFDAVPQGGE
VMKKLRQIGDVAEKHWSEGEQLLKDTVEDLKKVLEDKEKRAGEIVSEAEK
KVAREGKKKTD
>Mb|QLI69181.1
MEENQPTNWDLDTDEIASVTSEDLYENRPNRWTGPKSTWREFTREERLLW
RSMQQLVDKDLAVHLYDAFALKRQGRDQATAQSLTVKTKDVQDIVWAPPK
VWTAWPLKEKHISAEGLFQKQNDDEEKFTLRKVENGMPSTELQDELAATV
LRLAKERYRRRSWHRPLHPSIESPSSPLPSKSIDQSEDELSLPSSPPLSN
MPAGSEEMSLDHGEDESSNDDEDEDLENVHVGNEETYEPAISSNDDLSNL
LLRPCVRHILTQLDCTLTILHNARVAGLNHLSESESSTEGESEAGTPRKR
GKRGRPRRTPQPGEELSSSEQRNVQGPRRRGRPRKIHTPREGETEEEMWN
RVAREGHRRLPITAADRDAAFEAWVEEGYRREREQSRTSAQSEEHVAQAI
TGETKFERKLGKWGLRDWSDIVGAAALAGFSDAVISRTTKRCADLFGEGM
VIRRLDEVPSTRAPAFTSREYRPSNIQLPSSPAESESEPSATLLQRRTTS
RRASAARSSTASSPATPSGRSLSRSGSRSCSRSSAGFQFCPVPTCDRASR
GFSRRANLRRHMTLVHPGHTSEENDSDDEVVGGVHVDGFLKTFYPQRGWR
GEDVLARKRKRYYRGRSLSVNEGDDEGASDEP
>Mb|QLI69185.1
MVVKIRLARFGRRNSPFYNIVVAHARTARNSRPLEVLGTYDPVPKPDPYD
NSGKLHKDIKLDTQRAKYWIGVGAQPTDTAWRLLSMVGILPKKVFGPKPS
EKNETVDAEKVKIR
>Mb|QLI69186.1
MAGSFDVDPQLCQHYAQFASNLDKQNFRIIYWTMFITNLIVLFFGSWVYS
KAQKAVETLGTHSNQRAKVLRRYIFICLGCLAVSTVVVVMEAYALLALQF
CDGEDLMSLYWSTWTMLQVGSLIAIAGVILALCHNLRNRQHPPWALALGT
PVLVIAGILHLFHDCTRKRIKKLRRPAGSASDDGQEPRISQANTIENCSH
EDKCSDIQAEFIGFTVEGGPIVRFTQPVAPMPQGQLLGRDNGGGSTFAFP
RGMVRFETEDEIP
>Mb|QLI69195.1
MTDSVPTRQLGRNGPRVTGIGLGLMSFAGWYGQKDTSVESSLALLDRAHE

IGERFWDTADVYTGSEQRVGEWFRRSGKRDDIFLATKFAVRAGADGSRTV
DNDPAWIREACAASLARLGTDVIDLYYVHRIDDKTPIERTIQTMAELKRE
GKIRHLGLSECSAATIRRAHAVHPIAAYQVEYSPLFLDIESDRTGILQTC
RELGIAVVAYSPVGRGLLTGAVTSLDDLPPDDWRRGVPKLGGDNFPRIMA
LVDRIREVARRHGATPAQVCLAWVAAQGDDVIPIPGTTTLKYLEDNTGAL
KIKLTADEVAELRRYAEETELPGDRYPSSMASLVFRDSVPLE
>Mb|QLI69196.1
MEEYIVFDKALTKADHVVLQSLARDIKTASSVRSRKSKGRDSDCDSGIGS
EESSSSGDEAKAFPPQDDATTDQDIAALKASRNPKSAAFEPTIVLSVDDC
DAHLHPLLNNYLLQPYIQLARRIVRHETDVAMLTHLIIYFTTSVPSALVL
FRHFTYTHAVLHFVMQMYYVGTYTLMQHQHIHQRGILAKTYGLFDRAFPY
ILDPLMGHTWNSYYYHHVKHHHVEGNGPDDLSSTIRYQRDSVPDFLHYVG
RFFFLVWLDLPLYFLGKNRPGVAIRAAGSELATYAYYYFMYRLVGTKATL
FVYILPFVMLRMGLMVGNWGQHAFVDSDEPDSDFRSSITVVDVASNRFCF
NDGYHTSHHLNPLRHWRDHPISFLEQKHTYAKEGALVFHDIDFLMITFRL
LRKDYEHLAKCLVPMGNQIHLTMDGRVKLLKRLTRKFTEEEIAEKFRKVQ
>Mb|QLI69197.1
MTNVETATTEMANVEKEIGRLGRPEAFRWSQWVALGLEHAAFWRVGNAVG
RPLLERLSPPESLFHDPHETRDHEPTSYPKQRQQHPTLLPTQQTFHHQDP
DPVSRLSTPASSVQRSLRRTPRFESAPSPSPASTEPRQVHIAAASIGFSP
SPVPFARTRPDSSVTGLSSSLFEDRARLHRNNSQDLKEILDKDISSDRPH
SHHHSGSHSPPRRDRREPHPVVRVSRNTHSAILFALEEALRYPNPFTPDV
VEESADMADLMAATSGLPATSGGNGAASTRRPTAGPAPTSSPSGIRGPRM
IMQERAAREARQRAEAEQLALERSRAEQEARLLDETRRRNAERPSAGVAA
GQLSGTEPPQLQQQQHSPPRGRGQAAQEATHAHRVPAAVPTAKPSAEQQI
PAQFAPNTTQQPTRSPTYVTSSGRPRPQQQTIAPAEPTTQPTEPAKPRNS
FPHAFERWETLSAHWEGLTSYWIRKLEQNKDDINRDPLSQQLARQVTDLS
AAGANLFHAVVELQRLRASSERKFQRWFFETRAELERAQEVNGMLEKALE
RERRDRANAIRDAIVHERDTSKAEKQLAEMRKELQISKEEARRAWEELGR
REQEERDRTLSLQSGHPTVVGGVQVVPMTHGGVSRQGTTRREPEAYQAEY
PQSYGPEHPQAPSAASPTTSGPSGTYYRPHEQGQSHARGRSGGSEGAFSE
GEYIIDAAGNFVLDSRGQKIPFVAAPPSGASVSDPEADDYDTPATTNPPS
GAHEPASAPEGEGRWTGDYSDPQDYSGQGYGAPGWETVPRHHHPTRLSDV
IEEEDERSRTSASQSRGH
>Mb|QLI69198.1
MAAEPAGSWHPAMRPNSAADIVVPSDKSEAASSTDGNAAARQRTQSEEHD
ATESWFDNGHENENEANAWLISEDTEPAKLIDPAQPTDPLQPTEAAEVTG
LAETHTQEEQQKSEDAAIAWLTNGDGDGGAKLLSDKGGHSPSAKEPERTS
QDDHADGALACEVLNKEQARPEVDEAKETKHVAKGPSSTIAQHSSSMSFA
RTVSHEITFGDDDDGEWMLGRSDTDTFGFMPPSDRTNSFPVVPPISSSCE
PNDALPLPPNQALDVMEEDEKEAEVEEDEYMMQSPEGDSSASWHRHVATR
RNHAPSRSIGGEIADVATTAAASRFEEGIPLIPRREEEGEAPEATPDLFA
DEEANEDDFFSQVQNNDWDSNAKPLERKSTMQVMAAMNDAGTFSRQSTVE
EEIIEEHADESAEGVALGVSETIEPASATDDLASKWEQAFGGDDNDDFLL
DDPTVENKDIDAAAFLGSDDEGLLDDVLDEPVPAIQTSQPQKPAANPYAS
INSAVHQSTPYNATAATAVSAQYPSVIPYGGTAVHYGQTPALPQLEATKA
QSFADKSKAGYASPYDLPTELVAKTVKPRKRPSLQHIPPERAPPPPRSAS
MYSPGQVTPGASALPASSAPPALAGQPPLGLTPETTEKAPAPALRPKGGF
FEELPISSKPRPASRQSQRAPSPGQYAPGPPQHGPPPASRTAPALASTVN
QPPTAQHSAGETQTTPGMAGLVAPPRVNPYSTLQSQSPSVTPPSSSSSRY
SPAPAAQQGGGVSLSSRYSPAPSAGSRPNSSYGTGSSHNLPHLPRTSSPL
AHFESSGTTDGQHHDRRTSSSFEPRLNRVSSLPPTREVDEEEDEEAVSPG
NRSVSANHAAAADIAESRYSPASPVCTARYTPSLPSATNRPAAALSPTKR
TNSYAPQPVTSSAQASFAPPPRAQTQSPGAMQNVGRKGSKSSESGPRPSS
SHASTAPAMVKPAQSAYAPITRARGQSITNLLPPTDGREHDPLERWKGAP
VMSWGVGGTFVTIFPKSIPRYAIGQSTPVTVRTVGEVKVRNIKDLDPLQD
HLATFPGPLKGKSKKKEAISWLSAGIESMEKELPDVTFQSHLSLEDKRSI
ERLLLWKLLRVFIEHDGVLEGSPTVDKSVREVLTPETVGMQSEGAPLFTA

ADTTTAVTSMQADGVDSGAVEKIRLDLLKGDRETAVWAAVDKRLWGHAMI
ISQTVSSDLYKRVAQEFVRKEVNYPGHNNESLAALYKVLSGNYDDCVDEL
VPSHARAGLQLMSTENSSGPTKDATEGLDKWRETLTLVLSNRSNDDVRGL
NALGKLLSSYGRAEAAHICFIFSRQLSVFGGLDDPKADFVLLGSDHHKQS
DQFAKETEALQLSEVYEYGLTLAGGVTATAGAPHLAAYKLQHAVTLAEYG
YRDRALQYCDAIASAIVSQTRRSAYHHPILEGSVEDFLARLKQAPKEASS
SWISKPTMGKVSDSMWNRFNKFVSGDEDGNGQTGSDGDNGPFARIASSPN
NSRPPSTSNFDVYASSSSFPSASAPPAAGAAATRYAPVPTAQFPTAHANP
YVPAAQGQHVPLPTSTGSAQRYAPNPYEANYSGAPLAQPQTDGYQPVSYP
EAAATSGHQPVESAAAHTQQQPAAAVRAATSAGYRPYSLQESPNIQPQPG
ADDADQGYQPPSYGYEPPQIVPVPVANDAQGTGDNSGYEPPSFQPHGYEP
PPYQAHVEGDDAPKPKKKGFMEDDDEDIPAQRQPQESSKAEKDRENEEMF
RKAAEEDAKRAASAASAKKGWGFGGWFGGGKKPEVNIGEAQAGKAIKAKL
GEQSSFVYDPDLKRWINKKPGAENVETKKAAPPPPRGAPRTLSGTPLPAA
PGTPPPAAGRSSVPPMPFRSTAGRLLTTAQSMENLAGASAMGAPPTGLSR
SVSSNNTTSAPPSTGPPSRPATSMSNASSIDDLLGAPGPRKAGQKKARKS
GRYVDVMAK
>Mb|QLI69202.1
MAQHSRGHTAPLGQNGGAAAAGEGPPPSTLAAQLVENISTSTKSSRSDES
NELKELFATIQLVKDHPELLKSQADRVEHNHMLIYVYCRVALEGIKLDDP
FVNRAHAHAEALKAINFLRFTIKETPSVLAHTHASKTLLYRGTEPLWVWL
LPHLLRLLGHALFQELEGSLEGFLQYLLLLVSRTNELWSHGPELVLYLRA
CVSSTIDQLQDPSLDPSELNSFIPIHLPSQFALRQILGEQSMGHGPTYQI
SSSSQAIRQITSLAKILAYPLIQPDSTFSSLASLSQTVPWLLDTWLDLRN
AQKRWDVAGSSSPIPIIETVLNMSHGLASGSEMTATFKDKSHVLLVLLCS
EMVSCPEALLQEDETGDTSRQLYCQALLAISGATLESYSIGRMAASKLVQ
ELTLVSSQHALIGEGTDIWRCTRLLRQIIYAAPQHTFDEDTSPSHFRDKD
IQKAVEGLNLEYGRSSPSETVAKKRKVVAANSNPLLSLFRAIYDTLNMAW
TENEAGFMPEELFLESFTKVPNSSQCLALDLLSRVSCVCDGLGEVTTHSL
TENQCPVCELGQQSKPLIDLQVSKTASRLIFTRLIQEPSFLESRQPRVAA
MIALRRLVIHCEDAAFLNIETSVPGQWCLQSLNSSVRELRVAAGRTLGAF
MPVRPVSNVDEDLIARNRKNTIALLKSASDQDPAHLVETRILAWGQVGRV
VSEDELNLVLIKLVDYLGNSNNIVSAFTFNELLNLAEARNTTPRRLLEPF
WKSLAYMTTKDMTQRPQRSRAIAELLQISVNDLLLLIQTHALPWLVLDKR
QDVIQKIAEARQEKEVWRQLMDGSNLAATLALLLVQDTGDIEEFTKSRLN
EISPHFHSLSLLVLFQSEPVLIALELLKTAATADETKRSLVHQALQLMAT
TILSTNKDPKSKKGNVIGRFLQSHVLGLMARLTDVINDSITLQPLIMEQR
SAIATLEEMLKVCKQHVRVARPQISACLLAVISQESLREACFSCWAAMLA
YLDEEDVEALLETTFFIIRRYWGVLDDATATIGKNMLLSLLEKHGAMVEM
YISKLPSLSYCKGLESVEKTLKAMRPTLAMEEALGVFAQRVSHDNAGVVH
QALTELAPYLRDNQSALYTSAVSQRPDNAITTILRSLLDCACKYSGAQMD
IARLCVQCIGLIGCLDSNQIETVREQRSIVVLNNFVAPEEMTDFGLFMLE
EVLVPSFLSATDTKLQGFLSYAMQELLDKCEIKAACAMNETGMLGGNDIY
RKWIAIPDHAREVMTPFLSSRYMVAPMPPVTVEYPIFHPGKPYGNWLRSF
VIDMLREGQHPHADLLFEPLMRVIRVKDLSTAEFLLPYLVIHILLGARSS
ENEKNRIVNELLGVLRHQPADDASYVEREEMKRYCHAVFRCLDYAMRWIQ
EKRAAGRLSAEGKESVARIQRVLDQIPAELISQRATDCNEYSRALFHLEQ
HAQNMEQQKQDPGDRSRLLEKLQNIYANIDEPDGLEGISSQLHALDINQQ
ILSHKKAGRWASAQTWYEMQLAENPQNTEVQLDLLNCLKQAGQHDVLLNH
IEGMQADPSAENKILPFAVEAAWVTGRWESLAKFTGRFQEDVAQDFNMSV
ACLFDSLYRRSGPDAFGKAIQSMQDKIASSMTASATASLNAAHEILLKCH
VLTDLDVITNTKSGNEAERRRTLGLLDGRLEIIGGRFSDKQYLLGIRRAA
MELSRPTFADLDISGLWLSSARLARKTNSLHQSFNAVLHASRLGDDSATI
ENAKLLWRDNHHRKAIQMLQGAIERNKFMTQTGPSTSAGTAGSSKLNPQQ
KLLTARAQLLLAKWLDAAGQSHAVALREKYQQPPKTYSTWEKGHYYLGRH
YKKILEAEQTLKVEDQSDNYVTGEIARLVIENYVRSLNSGTKYIYQTLPR
ILTLWLDVGAQVDKAPEGKVSLSRELHKRRVEQLNMLHAFLDKYIYRLPA
YIFYTALPQIVARIAHPNTAVFERLSHIITKVVEAHPRQALWSLIGIMTT

KQASERKVRGAQILQALRGIQRKVDGTSHDVKYLIRTGEKLAEQLLVACQ
NGDFNSNKTVKASLSRDLRFQHKCTPCPLVVPVESSLTATLPAVSEHVKK
HNAFSRDVVTIDCFLDDVLVLSSLAKPRRLTARGSDGKSYMLLIKPKDDL
RTDQRLMEFNGIINRSLKRDAESSRRQLYIRTYAVVPLNEECGIIEWVPG
IKTMRDILLNLYASQKIHPDYAALRQLMEDASASESKIRIFTDDVLGRFP
PLLPLWFIQQFPNPSAWFAARLRYTRSCAVMSMVGTILGLGDRHGENVNL
EEGNGGIFHVDFNCLFDKGLTFAKPERVPFRLTHNMVAAMGIYGYEGPFR
KSSELTLSILRQQEETLMTILEAFIYDPTLDLQKEKKSSRRGDVSVRLQP
QSVVDSIKRKVRGLLPNENIPLSVEGQVEELIKQAVDPRNLTAMYIGWCP
FL
>Mb|QLI69209.1
MAPLCADPRQIVIALNPARRAALLHLIEEIIAFMALQLVNPPDELGSLTG
SDEDAQSSSPARKATPQKPTGKASQQIEHIQKAAMKYMQDWRASFLPKLE
EIIRVEDTPKIKEERKNRQDALDKKKLDTPEEGENLISFGDVQVDRSEDV
AALQKLYHPIPTRLTTVPAQDRREALGCVLLLLLSTGKYSAHSRTMILHL
ASALEIPQTFVNKEEAEIAHSLMESSTADKDKKETMSAEAEAEKRRQQNK
FSRFWKVGLASVAGAAVIGVTGGLAAPLVAGAIGGIMGGVGLGGVASFLG
IFWMNGALVGALFGAYGAKMTGELMDKYAKEVEDFKFIPLSTTGYGPDFQ
RPPDENSSAKETRRLRVTIGINGWLNDESDIVTPWRVLPSSETEVFALRY
EMKTLLSLGTALQDLVQSFAWKTLKVEIIKRTVLATLWAALWPIQVLAAA
SNVDNPFNRASNRSKKAGRLLADALINKIQGERPVTLIGYSLGATAIHAC
LQSLAERKAFGLIDNVVIIGTPAPSDAAHWRTLRCVVSGTIFNVYSENDM
MLGFVYRMHSLALGVAGLQAIEGVHGVQNLDLSEKVSGHLRYPSLTGEIL
KECGFTGVHAGGEIEKDDLIRMKDDHAEGKLVDLDAVAAGEPQRKDSRVA
HDLQGLTISPPALSPGESERPPLPARPAGTAEPRRREHEPDSDDSPVRAE
HGSLHEGFDDPPLVERGGRWKREQTPESNDGDDGEFMAIKMEDNDDDDDD
NGHDKPTKH
>Mb|QLI69210.1
MAARAGEENVATLFGDVHYFYEPENARPRHHRFDKGSYVYLFENAHDRRC
RIEIANQPGTDEQDAFDGFLDQARVRYSYKQHCAVSLIVADSVDHNEWHL
PTYDPRNDKKYHYKLHSLDIYFWTQSDALRFVNGVRRVLPSVQVEVLDEP
CHQQTIGMSSVVQKLENVAVSDPQYGNTTSSSSEIVSQEPTVDPAPAASL
DVLVNPLPPPPPPPASFAPMAYNPAAPAAPETIRHREKTPPPDEDPLNPL
AVAVAHDYQKQPFTPGFPPQPQLSLGMASPGLPPNISNTSSLPLQFAGPP
NQPGTQRAATMPANSGSAFSGPPGSPGFIPPPPPPQPSTQIPGHGTVAPP
GGFSNYTYSHQSTLRQDGQDYSIHQQLYRPTEIEFGMNKPHQYQPKTETR
GRLEENAGRLERGVSGMLKKFEKKFG
>Mb|QLI69213.1
MDVSQAISSYISKIVMPSGDASSTKMKILLLDRETISIVSTAVTQSSLLN
HEVYLIDRLDNTGREKMRHLRCLCLLRPSSESIQLLIDELRDPKYGEYYL
YFTNVAKKSSLERLAEADDHEVVKAVQEHFADYTVINSDLFSLSVSLPQW
RIWGPNPDAWNADSLQRCSEGLLAVLLSLKKKPLIRYEKSSPLAKKLASE
LRYLMSQEEQLFEFRKVDTPPILLILDRREDPVTPLLTQWTYQAMVHHLL
GIHNGRVDLSNVPDIRPELREIVLSQDQDPFFKKNMFLNFGDLGSTIKDY
VEQYQSKTKNNANIESIADMKRFIEEYPEFRKLSGNVSKHVTLVSELSRR
VSAQKLLEVSEVEQSLACNDNHNTDLKNIQNLIQTPDITANAKVGLVALY
ALRYHKQQSNAIPMLVDLLVAAGGVSPAQSSLVNKILAYHLSLHTSPSQG
GISDIFDSAGIFSGASSRFKLKGVENVYTQHTSLLESTLQNLIKGRLREQ
QYPFVEGGGSTRDKPQDVIVFVVGGATYEEAKMITGINATTPGVRVVLGG
TSILNAATFFNEVDDAVSSWPEVTGRR
>Mb|QLI69214.1
MRPGSLLPLLALGPVVQAAAAASSQESRDLFARDFEMLNSRDLEQLSPRD
VELLTRALVERGLLDDIWQKLKDATTCAGGELLLGALKVLAFFGDGAFVK
VIQGICKLAKVQPGDVCDGAVALEGPIIAADIRKISVGSRTSKAFCTTFL
GVCGYPDVQTWDIAYPSAQPAGGRPKPGGKDPIKIVHYSDIHIDPLYVPG
SSTQCDGRPICCRPYTKDDQPGNTKFPAGPNGDHMCDVPFTLERSMYDAI
NSIVPDAAFTIFTGDIVDHAIWNTSQPYNTNLIQHAYDTMNSSLKLVYGT
AGNHEAHPVNAFVPNAIGHDSQWVYNLLSSDWEHWIGESSTAMVEKIGAY

STKYPKGNLRIISLNTNLYYRHNFWMYQSYEDKDPNDQIAWLIRELDAAE
KAGERVYIMGHMPLGEADALRDGSNYLDQVFKRYQNTIAASFFGHTHVDH
FEVSYSDYAHRSASNAFMTSYIAPSLTPTSGMPSFRVYTVDPDTFAVLDH
TTYMADMTNPSFQTTPVWTKFYSAKESYGPLVSPPLTDPKAELTPAFWHN
VTVAFENDDAQFNAYMARKSRGWQPASCTGDCKTDEICQLRAARSQDNCW
KPKPGINLTKRSGVQDEKGEHDECGVPITLKTLSAVGADDAALQELKKIM
DEVIAEAKAKGIKGAEDA
>Mb|QLI69218.1
MSSVTVEATELTVQSERAFQKQPHIFLNSKTKAKSSRPGKGGRRWYKDVG
LGFRTPKTAIEGSYIDKKCPFTGLVSIRGRILTGTVVSTKMHRTLIIRRE
YLHFIPKYSRYEKRHKNLAAHVSPAFRVEEGDQVTVGQCRPLSKTVRFNV
LRVLPRTGKAVKKFSKF
>Mb|QLI69219.1
MAELSSPRITAPYLDSFVGRLVTIVGKVTQLRGDQATIDSDGIVTVVLNR
DAHLTNGNAVQLIGKVNPDLSIKVLSSLDAGTDIDFGLYNAVVQATHRHK
AIFISEN
>Mb|QLI69220.1
MESLSDTQWDVVISGTGLQQSLLALALSRSGKNVLHVDPNEYYGGAEAAL
SLQEADDWAARHSGPDATPFAGAQITKESEGLAASRSYNIPLAPQLILAQ
SELVNQLVSSKAFRQIEFLAVGSFFIYQAAGASSEPTLSRIPSTREDVFS
NTTIPARAKRSLMKFLKFVLDYDTESQAELWKPSESEPLAEFLEREFKLD
GDLQSYVVTLTLSADGKISVGDGLAAIHRHLTSMGMFGAGFAAVYPKWGG
LSEVAQVGCRAGAVGGAVYMLGTGISEVQRMDSDGETNVQVSLANGVVVK
SKTLVQSTKETSTDSVLLSRLIAVVNSPVPSVLQVVVEGAPTPCAAVVAF
PVGSISTDDGTASGFPIYALVHSSDTGECPAGQCIVYLSTVSSSTSKSLL
SNALSSFLNAIPTSEPPECLWRMHYEQVTGTGSFASEDNIGTFDWITSDL
ALNDSILQYVKKAWQFVGGADEENYMQFEDREGVNDDDDAFVN
>Mb|QLI69221.1
MSFHERNNKKRKTAHNGQQDRTSFKPSASFAPTEGGRDWTVSVAIPTSIL
TNLATADQRMSIPGRIARALAVFAVDEVVVFDDSPASSRPRQTDTAGYTG
DTDPAHFITHILSFLEAPPFMRKALFPLHPNLRLTALLPSLDMPHHPNPK
DWISYREGVTVPGKTSTGSGTLVEVGLDEPVEIEEDIPPKTRLTLLFPDD
QSQYPQCVDPQAPRTEGGYYWGYTVRKSPSLSSVFTESPYEDGYDFSIGT
SERGTPISKAFSAKPLKFKHLLIVFGGPRGLEFASMNDEELVKMDIQGVR
TRELFDQWVNVLPGQGSRTIRTEEALFIALTALRGLWDGK
>Mb|QLI69222.1
MSLPSAKAPPAQLSIRSFFQSKQTKYAPPPSSRETTTTTTTTTMPTNSPP
SAHPSPHPPVENLRLPPLPTTLPPEAVIRPITESDIAPLRRINSLLLQVS
YPDTFYQKAIDPSSSGPFSRVITWTHEGEEPKIIGGIVSHVEPDIDTSAA
QVPQNLYIRSLCLLSPYRSLGLMNAALEHVIATAISAPNMDVRTVTAHVW
TENEEGLHWYDARGFTRLEAPVKGYYLKLRPDSAWLMKRDVGASVRSSLP
GTNGNGVAASSRPVGASTTAAVVNLPPPPASGGPPSSKSTTPPSSAPTPP
PPPPPGARPKAVSGQSYQNHRPDMEWNDLPADMAPTLGALRKTGSESASA
TSSRSSSQAPRRKKDRSYPAAAFGN
>Mb|QLI69224.1
MSPVTVDTVTQARAIVNARLDELDDGLHQVNRTLHANPELAYKEFIAHDT
ITAYLEKLGFQVERSAWGLATSFDATVGSGDRQVIFCAEYDALPEIGHAC
GHNLIAISSIGAFLGAAAAMSALKIDGRIRLLGTPAEESGGGKQDLVEAG
AFNPPESVVAAIMAHPMPIEYVSQGGFSGLAGMKLIACQGTLVEFKGRTA
HAAGEPWKGVNALDGAVAAYNNVSLLRQQMHTEDRVHGVFEVGGTAPNVI
PDYTRMNWIVRSPTAAGCDKLMERVTKCWEAGASASACEMKTTRFVRSPF
LLLPSCSYLLEGAKKKLLTLDIPNRQHHYLNLRANDSLCQIYVDEMAAFG
QNIHLKIDRALNASTDMGNVSHFVPSFHGGIGIPAEPGVAMHNPRFAAAA
GTDEAHGAAIQAAKGMAMLGLRVLLDSKVAAQARLDFERDDEEAHL
>Mb|QLI69225.1
MTANVNKPTDIKQKEADINRKLQIYGIFSAFKQGKVPSNDQIDVALNSFL
ASKALGTPPENLSEDGKILVADTREVVRLAKNLLLSKNEGNLIQDFIWQT
TQFDPKSVQGPNAPVNKDQAKRDGDEALEGLRTLGTLLITNGQFRKLLKD

TTILFRDLAGDAATSAAGRIRPSEEQLGQMDRAAEDNTWHDKPDFSKENL
RKQAKGFYGGSPKKDAQDIANSTTNAALPQGVNSDRVDTVAAQSAVQQKV
DEKIDPETKEKIKKSKEEYRRRTKEYFNKKMPEERKDQIIFRLKKMVLEC
QQHPEYSKAIQTLLRLAETYGSHGRGLGKESTGSAKQARSGFAAAEADLR
TLIERFANGTSTSNLWESIGQIYKDADKDAELKDWFKSLDNFIRRCLLEQ
GYILDESSNKQWNRLYEKGRFLLREKYRSHTDRVIDETKFIADQFDQDSQ
NKAFGEAVQKLFNHLGNDSAGKSVFKPHLVKDLADTILPAVLMNINYIPI
PRIEYSDSQVDAIIENLVLESDNFMPNIAEVSSENFFRWGRKKVANKHHN
VIDVKISGIQMDLRDVNFHVNRKKGFPSLSDTGVLDILLPGNGLSFRMKV
ATAHKSDSQNIFKVEKVDVDFKNLKIKVKKSNHKLLFAIVKPLALKVLRA
PIQKALEKAIKDECNKFDSLLWQIKKDADAAGAGDVEDKANFFKRYYDAA
QKRYLEGKEKTKEVASDKKVNIAMTLEDSIFPEVKMAGGVSSKATEYKEL
SRKGDKWESPVFSIGSAKRSTDIPPAPKIENKARPATSGRTNGHTNGSTN
VHPNANANIVNGKAPLSSTPLAAPLEVNAGNVNQTIGGR
>Mb|QLI69227.1
MRSFLFSSYPAQPAPSSPPPSHPFKAYRSHNQTTSAKPISYDALSVEPSA
LRRSRLTPKYAPSTAEDVQDEQDIAVSVTIQSVGSTSSSCSSTSSASSDF
DAAMSIRTAVNWEASITLPPSSSPGTTTGLRRPTTPTSPVQIPKRSHRDA
RHHVKLETSHHRRTPKNFHTPDSISPSVAALLAVTDIPRPQRRRRKADSP
LTVDEIVNDQHVSEKELSLSLSRRPMDLLLSPPEDLMDDDFSATESNMGS
MLARTTSADSVPSLGGESFATDVCSSVDTPASMGVSARRKHSPVRRSLEP
IQSPSEDGAEEHPLAVVDNLGLEDLKMPAAELSDEGSTSFLQPFKPLRSA
FKSNLTASLRALRSAAKSFSSINFPSIPPDDFLTRSILTIDPNVPYMDER
RPPMTEEMPSAELRRYLNPTTSAHIDPQPKTRAGAFSASIQMQTYKVQRS
QSTPPSQRTAYPAGNPSKSASAPNQHPGRPGQQAFAVPGMRQREMRENPD
FIRIAVMEMAMRRRGKLDNQKPGRARWALPPRKASLKPYEVGSNGVPARW
VPLTHPC
>Mb|QLI69231.1
MSFRGGRGGGRGGGGFRGGRGGSFAPAGPPAEVQEMGTVEHAVEGEMFCR
SSNVKIPYFNAPIYLENKTPIGKVDEVLGPINEVYFTIKPQEGIVATSFK
SGDKVFIGGDKLLPMDKFLPKPKVAGEKKPKRAGGARGGRGGPRGGGRGG
RGGSGFGGGRGGGRGGGGFSRGGGRGGGRGGGGFSGGFSGGRGGGRGGGG
GFRGRGRGF
>Mb|QLI69232.1
MSLHSGHHDAVPTQEPKRDPKLPKMPRPFTSSSQLHTPFDSSPMEQLSSA
SVADFLGPALESPPSPQKLRLLNKQMKRASHLQRHHGHRTASSESSSLSS
MNTAERLPWELSLDNLSLVRRSSIRSNDSNAPSRDRPESIHNFGRSFFHR
RGKSKRESSTQNSSASSIYSGDMPSENIGHGGREGLIPSIFSRRKASRDE
TAQRRPYISGPFNFQHVAHTQRDNVVNDDHHVNGPETLNDAFTGGFETSS
YSRNMSWESIGEVYQEQTSSAVSRPPLVPRHTAPASGPRRLLQHIRSQER
LRKTPSQPPPRPPRSPIQANGFCTLGSLPPPRGSSRQSSYQDITDVINMN
AAARAQTSNEFCQPFPFGYATSPECIIDDLNSYEHSYSQTQEQSPPDETR
YSRVMLGARDSTWPLAISTPASYETLPDVPEEEEQHGMSRRSRLSLASNN
SSLRGSQSVPMLRSLAESRRPTSVVSETLGGLGIMGLDHIVDDDDHQSLV
QLRTPTRESWEDIIDYCYEHEAEANCDYQWDRPSLDTARDSLTPPAKLVA
LDDNGIEPASNGGLSAESHGSLSLVWQEAPSLSPASHTSTPVEHEVVTPN
PAVTNNFSLPRGDKKSIRPLNMKEPRHSSGPATFRESQTFTLSPSFLIPV
DYQQPMLLSDDTEKQAYSDCDLVGGHFEQSALHEDTNSSVGVNGSSPLLD
QRSSTSTNSTSRSNSTGRQYRSTNSSWTTLARRTASTTSLNKMAGSLTDD
SEPLPSAQPPDHDQDGPEWAGTYPATQDHVPDMISFPPSNGLKKYYHKSH
ASESQVRDDAPPLEESVKPRRPRAKTTSLPVQSPPPVGQYALFPRTYMKA
TGEHI
>Mb|QLI69236.1
MSTGQITLLDLPSKEPCSSWSLNPWKTRMLLNFKGLDYKTEWTEYEDIKP
KVQPHLPPNESGTPYTIPTVILPDNTWIMDSRKIANHMEESHPQPSVHLD
SPVLPKVEQLTSDVFHHLLAVCVARIPVNLLNSHSADYWYTTRGEAVGMP
VGEFEKKYGGEAAYSRAEPFLQKITALLRDNDAGPFFMGSTVSYADFVWV
SALVFFKRVDEDIYRQVLQRTGDQGVHVKLVEACEPWLQRDNY

>Mb|QLI69237.1
MVNLSVPKNYTFSPSSATPHLTINHNVAADLDSSNAFEGPEKLLEVWFAS
GPTALPSSAAPNGLKSVPADTWVGMLDMVNCKILSVLESDDVDAYLLSES
SMFVFPHKLILKTCGTTTLLLGLQRLLHIAAVNAGFPFHNADSVNDVRAA
ATPYRVFYSRKNFLFPDEQKGPHRSWKQEVKYLDDTLEGGGSAYMVGKMN
GDHWYLYITSPNQTLTPPLTPDSEINCQPTPAMTISSNGGSSAGGSYSQN
DETLEILMTDLDPENAKQFFLSHASAVASDKLAAEAKNARKKAQDSVDCL
TAATPEEIDVFGHCDEGDFETAKAQVTEELTTEGHALGSVVSESCGLNDV
YPTSVYPDARVDSYLFSPCGFSANGVVPAPPSAEDDTAGATHYFTVHVTP
EPGFSFASFETNVPGGQKGRTTAEIIEHVVNIFRPGRFSVTLFEAKGRGA
NPYGMGDGTFKSLSMQRLVDPVRGYRRVDRIVHDFEDYDLVFRFYEREDW
LGDKAARVGEIE
>Mb|QLI69238.1
MAGTRGGGSSRPDEEAAHAGSSERTPLLKPNDDVNDRADTPVTEAPQQND
SGVPGHQDEEQTVLAKEESFGRLALIMGTTWVGVFLGAIDSTIIATLSAP
IASEFRSLSLMSWLATAYLISNAACQPISGRLTDIFGRGPGLVFSNVFFA
LGNLICGLARDEKAMILGRVVAGIGGGGLMSISTFLGSDLIPLRKRGVVQ
GIGNICYGSGAMLGGVFGGLINDHTKLGWRLAFLIQVPPVLLSAVAVHFL
VTVPPKQSDKSYLARIDFLGVFLTSSFLILLLLGLNSGGNQVPWTHPLPL
TTIPLSLVAFVSFLWWESRARQPIIPVRLLMTRTVLAACFTNLLGTMVVL
TLIFYLPMYLQVLGESATDAGLRILPSPIGVSISSVLSGYIMKRTGRFVA
VGIASMVLIGTGIVLLTLMGRDPSVWLSSSAFFLVGGGYGAMLTTTLLAC
IAAVDHSQQAVVTSATYLARSLGGTLGITIGSAVYQNILKNRLWDRFGHQ
PNAADEIRRIRDDLGELKHLPDGWYDGVIDSFMEAFRGVWLTMFVLAIAG
LICVSLMRHHTLHSTLDRR
>Mb|QLI69239.1
MPPTPDDKGDPLALAMDARKSSKLGLLSGVYIPVCLNIMSILMFLRFGLI
LGQVGFLGILGLLATAYCVDLLTTLSLSAIASNGEVKGGGAYYLISRSLG
PEFGGSIGILFFLAQALNTAMNIVGLIDCIRLNVGPGFPGGYWAGYGLQT
AALVTCTCMCLLGSATFSKASNLLLAILTVAVLSVPLSAIFKAPFRDDDA
GIVYTGLSLSTFVNNLLPSSSKHVYRGLPTFRDLFGILFPATSGIFAGAS
MSGDLKNPSKAIPKGTLWAMLTTFVVYFVVILSMACTISRDSFLANDNII
SLTNLSAPIVLAGECAVTFFSALMGIIGSAKLFQALARDKLLPGLSIFGR
GTKKADEPILAIFLTYVIAQIALLADLNQIATFISMGYQMTFFVMNLACF
LLKIGSAPNFRPSFKFFNWQTACIGSLLSAAAMFFIDETYAAIAICVLIF
VFLLIHYLCPPKRWGDVSQNLIYHQVRKYLLRLKPEHIKFWRPHIILLIN
NPRRQTRLIQFCNSMKKGSLYILGHVIVTDDFNSGVHEARLQQQAWTRYI
SEFSRIKAFVQLTMSPSITWGVRNLVLAAGLGGMRPNVAVLGFYNMDDLR
KSNPAVPIPDVPASPSDKMRRPSKSQGKVAKRRRGDTSARLLEGTLPTDV
IRTEDMVSPTDYMIILEDLALKYRLNVAIAHGFDKLETPRHDGSNSKKYI
DLWPIQMSAELSSDGKNVLTTNFDTYTLILQLGHILRSVQAWRRAFALRV
LVFVEYEHEVEEESARVKALLEKLRIDAQVLVFYLASGHLNTYELIVNGN
TNDIDAEIIVCEALKDEEWWEDLQILRRQAEELSSSEELSQLAQFLDSTN
GRPGAYNPHDDAGNTRRHSMAEFVEMHRKPGIATLSKLGVSMGIHTTHIN
EEVLHEPESDTEFDTDVEGSTDDEEFEVGSRDFFWGSRDELGNPTLRLLL
QSSQDSGAATEQSPTKSGRGRRLGAIESSAQIPSYGTMNTSQTLADNPES
SATWHVPEVAEIVASPPEAETSVKIDSFPILDPLQVPKFPQGGSRRSRSM
SPSRDVIRDGTVTPTRPNFSRQSSAARFSSRPVPETKIIAEGEGSKISFA
PAASNPPTPRAERPAFSRQSSLGKFSSRPLPDTKVSGEEGARTISFAHQP
ASQPPSRAHSGSHSRHHSRHGSQYSVHGQDQANLVIPERSEPSTNHAPKK
SIVEDGGSATSGHDVALSFNDLPSRAQHLILNELMRQHSKDTGVLMTTLP
IPSEGTSLDEMSTIQYLSDVELLCNDLPPTLMVLSNNMTVTVSL
>Mb|QLI69240.1
MGSASRNEDMYEPIAIIGLACRYGGDVKNSSELLRHVMKARSVYGAVPEG
RLDARHLYHPATGHIGSIYSNGGYFLKDDINNFDSAFFQLPENDVVAMDP
QQKMLLEGVYHALENAGMSMKEIASTTTSVYVGCSNNDSLAIANSDLLST
LQCTSTGTSPSILSNRISWFYDLRGTSQTIDTACSSSLVAFHQACQSIQH
GHSMAIACGVNIMEHPGATQNLCSLGVLSPDGQCFTFDERANGYGRGEGV

GTVILKPLRAALRDGNHVRAVVRSTGSNQDGRTAGITLPNAVAQEQLIRS
VYAAANLNMADTGYVEAHGTGTFVGDPLELTAISSAFAPNRESDLFVSSV
KSVIGHLEGGAGIAGLISATLAVESGSIPPVTNLQKLNPNIPNQKRIRFT
KEAIPWPRSDLRRASVNSFGFGGTNAHVVLEDIGGFLSQNEGFAAESLNT
MKPFTNGHAHTNGLNHQDSDIESSVNGLTHKPNGAVISSPAKKRILTLSG
FDEDGIQRNNARLVEYLQSVPVKGQFLENFLHTVNEKRSSFDWRCYYILD
SHDSLLSSLRQVPHPIRVSKTPKIVRFVFTGQGANWPGMAQDLLVYRIFE
TRICEAAKYFMDLGADWDLREILARSDLDMTEPTLAQSTCIAVQVGLVDL
LMSWNIVPTTVVGHSSGEIAAAYCAGKISRQAAWKVAFCRGVVCSKSMGN
GTMMAAAVGEKQAEELLSRLGGIEQPLQVQIGCFNSPKNLTFTGKRESLD
TLKQELEQSGIFCKLLGVKVAYHSAELKAVAGEYLTSLGDLNFGDRVNQN
TGIRMHSSLTGECIGPHEDIGPEYWVKNLVSPVRFTSALLHSIGADSDLK
RLDKVTDTIIEIGPHSALRSAIQDTFADSQILQSIGYASILKRGETSGET
VLQTMGLLYTLGYPVDITAVNEREMANGFSTKHVLNDLPPYAFRHTSKRA
TTRQIEALKFPAFGRHELLGVPLLDSNPFEQRWRNFIGPQQVPWLSSNRL
NDKILFPGVAYILMAVEAALLRFGALDAGAVELRNISILESLFVPDDAVG
VEIIFSLSQLHEATELSGGWSRFRFVSHNLDGDSWTEHCVGSIRVEKGTA
SPPRPLFSEAMRAATASTEAENRYFTEMIEPDKLYQSFSSAGTNFGDYLK
GISEFRRSQDRMAYMSHVLSPDIPRTAHDRYTIHPCALESILQGLLCLGS
PEDTTVNGSMVANRLGHVWITNPLSDETATSRQFKTFAETERKTSTIYTS
DVAIYAQGSLEPNFLIKGLDLVLLPPQEDGQAADESFYVETWKPDAKMLT
SFEKVCSSQELSGSLIRAFTREDHQGLQLTCSVFVLDSLQKISGLNEDDL
PLHLRSFVDWIKAQAEIITQGRAPFVDLTALEQVRVKDELKQALFDSVAR
RSARGELLIRVGVNLVPILTQQVDSLELMFGSDDIMSRTYNEGLPGDVAI
LEVGAGTGSATKIVLDAFRNSGGQDAESGILPIDTYDFTDMSGAFFERAK
SRFADWSDVLRYQTFDVEKDPALQGFELASYDLVIATHVLHATANLQVSL
KNIKSLLKSGGDLIVIENVKPQLMCTPLAFGILPGWWRGIESYRQSNPLI
DQKQWDAELEQAGFKLRMQIQDTDEAANQETSATIASTQQLLDETCQHGY
KCAIIYHADYVSQLQLARECASTLTKVLECDCSLVSLREADAGELDADFC
VSLLNLQGFDLSDITDPDFQQIKLVLETCINMIWVSGNPDDDSKAAMCRG
LVRSARWERDQDAVNFITLGICNPLPPNSVISEQVARVARGAFISQGVIA
ANGEYATKDGVLLTNRLLPAKKINTAVNRRSDPKTEPIQLGAISQGIKLT
SIDPHKPQAFHFIRDELAESPLGHDEVQLKVHAAGVRREDIDEASRLIPG
QGIGRDCVGLVTEIGSSVTDVVVGDRVMAVRTIPSSGALSSLFRTHCSAV
QKIPDGVGYLEAATIPWNFCTAHYCIVKVARVQPGETIMIQCADNAIGQA
AIQLAKSIGANLLCTVSSNEDNLVLASRYNLDQAAVLSNDKVPLGVEAIL
SSMCKGGINVYLNCDPMGISDAYQSQVTPFGRVVDLHGAATANHLSLKDL
SKASISYISVAMDPVAQSRPELIQEALLDTSRRLSCSQFKPLSHVEVLGY
SELPKAILRCRDRQSKNPSMFVVQPKDDEVVPMRLEPIGDYLLRGDASYL
LVGGFGGIGRRLALWMHSRGARNFIFLSRSGPTDPRARELCETLKSSGCR
VESIVSDAADTESVNKSMQRCRDIMPPIKGCVQCSMVLVDSMICNMSHSQ
FMAAIRPKVQGTINITNNLPEKLDFFILLSSSSAIIGNRGQANYAAANVF
LDTFARHLTAQGRPTTSISLGSVLSFGWVAENQDRLPIALAYGTITEDRL
LSILEYHMDPRLGAAENSDNCHTIAGLRSASDFQRHSVPLPAFMSHPLFT
HLRASGRQGRAKEAEATISIAQGLAGAASTEEAVDFVSDAIIAKISRIMA
ISPKDMETYRTLSSYGVDSLVTVDMKSWFKRELGATVGTKDILGELTIYG
LAGKVVTGSSLRKESVI
>Mb|QLI69241.1
MAALDAFGTGKVLDHTTALDVLKEYKTADGLDIHELMDTTKHGGLTYNDF
LLMPGYIGFPASEVTLDSAVTKRITLKTPFVSSPMDTVTEHEMAIHMALQ
GGLGVIHHNCSPDAQADMVRKVKRYENGFILDPVVIGRNTTVGEAKALKE
KWGFGGFPVTEDGKLGSKLLGIVTNRDLQFEDETDATVANVMVTDLVTAP
NGVTLVEANKILAKSKKGKLPIVDKDFNFVSMISRSDLTKNQHFPLASKL
PDSKQLLCAAAIGTRPEDKIRLQKLVEAGLDVVILDSSQGNSMYQIEMIK
WCKKEFPGLDVIGGNVVTREQAASLIAAGADGLRIGMGSGSACITQEVMA
VGRPQAAAVYSVSSFAARFGVPCIADGGVQNVGHIVKGLALGASTVMMGG
LLAGTTESPGTSFVSREGKLVKAYRGMGSIDAMQDKKAGGGGKDSQKSNA
GTARYFSEGDSVLVAQGVSGSVAHRGSINKFVPYLAAGLKHSMQDCGMKS

LAELHQGAANGTVRFELRTSSAQLEGNVNMEAYEKKLYA
>Mb|QLI69247.1
MWLQAALSLAAWVTAAVADDSKDLGSVLAANKNLTKFYELIKKYPDVLLE
LPSDNGVTIVAPSDKAMANIAYTALQPLWDDDDKDKTTAILQYHVLDGAV
SAAALRDGPTYLETSRLTSPALTNVTAGQGVLVTRQRDDVVFTSGGGSRS
SLVEADIPFAGGLVQVVDNLLVPPAQLAGTADAFGARSFLGSLYAARLMP
ALAVRRDVTIFAPLDDAYGLVGGTLRGLNASRLARVMGYHVVPGRVLSSA
QLANGTGYDTLATDAAGKPARVLVRQEGNSKFVNAARIVQPDILLANGIM
HIIANVLNPDAERAAPDPSAATQAPAFAVSTVSGVFTSDLPCTADCPATT
TAATATGGADSSGATPTTTSSLFSSTSRGGGARCTAHVVGAAVGMVGLGA
GVAWM
>Mb|QLI69250.1
MSATTNPDPEQGAPAFIPLEANPELMTKLIHKLGVSQALQLHDVYSLTDP
DMLAFIPRPALALLLVFPITAVYESHRMAEDNTVDEYKGAGEQEPVIWWK
QTIRNACGMMGLLHAVSNGPARTLIEQGTTLDELLQKSTPLPPRERASLL
EKTPAVAAAHKEAASAGDTAAPDAQDNVDLHYVCFVKGSDGALWELDGRR
KGPIRRGELQENEDMLSERALGMGILRFLEREGGDLRFSAVALAGSLD
>Mb|QLI69251.1
MNIRTRLPNLVIETVRKQFPGLNKGLICLNNGSGALVHKGVMESITRTMS
SPHMNLRGLDSKSRIDVRERAAQYVKLASFMNADPDEIAFGPSTTGLLRT
LTNSLRPNLNADSEIIVSVLCHEAGITAWVALAKSLGISIKWWVPAGGMG
NNRDPKLTLESLRPLLSSKTRLVCCGHVSNITGTIEPIQEIAELVHTVPG
ALICVDGVAWAPHRPIDVRKLNVDFYVFSWYKVFGPHIAQIYARRNVQQR
YLTSLNHYFLDSSSLAVKLGLGNSCIELEHALVPIINYLVDEVGWDSIIA
YEEVITRYLLDYLTANPDLYTVYGSRTSDPKTRVALISFSVNGLTSDKVA
NEIHEKSNCRLLTGDCWSPRTVHDILGLADDGIIRTSLTHYNTLDEMKIF
TKKLDQVVHALKQ
>Mb|QLI69257.1
MGFFSRKDKAPKGNSSILASQSTTSVASGNSRGIGNRTSAGSFTSAGTPL
SPMSPIKLPKVDLPRPPDPQLDPVGYLRSLGAVRERSKIIMDKATRNELN
HFDVDLAKLPNVVTFVSGLIKRDYEAPFNTIPGHGRYQHFCVGDRDRVKE
LLDSWPESVDSTERCRRLIDLFLVSVLLDAGAGTQWSYKSTENGKIYRRS
EGLAVASLEMFKEGLFSGNASNKFQVDKDGLKALTVEKLEAGLQSRPGAE
LAGLKGRTELLIRLAGALSDKTEYFGADGRPGNMIDHLLSHPSTQASSML
IVPLPVLWNVLMNGLAPIWPPSRTALNGVSLGDAWPCQAMPNPGAASWES
ILPFHKLTQWLTYSLMQPMQSLLNMHFAGTELLTGLPEYRNGGLFVDVGV
LNLKKDDMERGLQNYADYCRRTGHDGVEVAPMFEPSDDVVVEWRGATVGL
LDLLCAEVNKHLKNELAGNEMTLPQLLEAGSWKGGREIAEINRPNTKEPP
ILIDSDGTVF
>Mb|QLI69261.1
MSTLKRKGESLTNGGDGKKPKANGSIASFFSAPKPARGAASAPASPAVKF
DKQKWLAGLTAEQKQLLKLEIDTLHDSWLALLKDDITSKEFLELKKFLDR
ETNAGRKWFPPKEDVYSWSRHTPFHNVKVVIVGQDPYHNDRQAHGLAFSV
RPPTPAPPSLKNMYIALKKDYPTFVPPPNRGGLLTPWADRGVLMLNTCLT
VRAHEANSHSNRGWERFTQKIIDLVAQKRTRGVVFMAWGTPAGKRVQKID
RQRHLILQSVHPSPLSASRGFFDCGHFKMANEWLIKRYGDEGEIDWALTP
GVTTKAVAATVAGASVNGTKSVKVEVKEEIKSEVKEMNGSTAVKVTKKTE
EDVDSEDEAALEEALRLAEEEVKKAE
>Mb|QLI69262.1
MSDDDSHAPPRPKLPLQQLTILAVARFAEPLATTSIYPYLPQMVRDFGVE
EAEVAKWAGLTSAVFSVFQSFAAVPWGKIADSWGRKPSLITGLVCTMACF
VVWGVSTSLPMAITVRAIQGASNGNVGIIRTMVAELVPEKELQPRAFSIM
PLVWSLGSVVGPAFGGFFADPAKQYPSVFGGVWFFEKFPYALPNLIATVF
FLISVTSATLFLKETLAAKRDERDWGLLFGKRLGRALKWNKKQDRRRRSF
VDGEATAPLIPNQIRHKTVHDSSTPGTLEIFTQQSVLIMLAYFFLAFHSV
AYDQNVTVFLNYPVKEHTPENTRLPFYFDGGFGLESGRIGTIFMLYGVTC
GLVQFILFSPMVTRWGVLNCYKACCVIMPMVYILTPYTSLFPTPETRLMG
LAFVLMLKAFSIIVAFPSVTILLTNSAKSLRILGTLNGFVTMFSGFGRAL

GPASTGLAFTWGAKHGYVVTAYFFLAVIAVLGAIPVFWVVEGNGPTASVE
NSDTEENETPVSSRILVDESAIDDSDAESHESEPLLGNSKKGAPYGAAKI
GCQD
>Mb|QLI69265.1
MSLSAPGSHIRLQLRRALSISHANFSTSAPAQTIRVPPESPSYIRLPTPP
QSDETKPPRVRGHLPVPREIFPRVEGSRKIQPEYIKKTAPRSTKSSRESP
SEAQEWKTALAETRRTNLKEGLDALWLRRREMDRSRNERVSHKFYKHNKA
GAAPERDDDKLTRPTVLTAIMDTKVYPDPDRFARADRSRTKVLAKEKQKM
EARRDALMELYTSASNFIVHENELKAKIDEVFAEDYFRKRSEDGHRHGTT
GNMWGSYGKPPSIANMMESAIGTSTRIMEADQTESDRSAKRQKRIAEDLT
GGKMI
>Mb|QLI69267.1
MKQRFSSLDVKVIAHELNQSLVTLRLANVYDLSSKILLLKFAKPDNKKQL
VVDTGFRCHLTEFSRTTAAAPSAFVARLRKLLKTRRLTSVSQVGTDRILE
FQFSDGQYRLFLEFFASGNIILTDADLKILSLARNVSEGEGQEPQRVGLQ
YSLENRQNFHGIPPLTRERVQVALKSAVDKAAATPASKKLKGKPGGDLRK
CLAVSITELPPVLVDHILQSNNFKTAVKPVDILENEVLLDELVKLLSEAQ
SSVQDITSSEICNGYIFAKRRDGNPIQEAQGSEAATNRDELLYEDFHPFI
PHKLQGDPSIKVLEFKGYNQTVDEFFSSLEGQKLETRLNEREAAAKRKLD
ATKADQTKRIEGLQDAQTLNMRKAAAIEANVEWVQEAMDAVNGLLAQGMD
WVDIGKLVEREKKRKNAVADIIVLPLNLGENLITLSLAEEEEEEEEEADP
FETDDSDSEDENEASTVSKKGEKPVKGLNVEINLKLSPWSNAREYYEQRR
TAVVKEEKTQQQASRALKNAEQKIAEDLKKGLKQEKALLQPIRKQLWFEK
FLWFISSDGYLVLGGKDAQQNEILYKRYLRKGDVYCHADLRGAPSVIIKN
NPSTPDAPIPPATLAQAGNLSVCASEAWDQKAGMGAWWVKADQVSKSGPA
GDFLPTGSFMVRGQKNFLAPAQLLLGLGIMFKISEESKARHVKHRIHDVD
SALGSDTATDRNDMQSIASVADTQEKEPEDDVTQSDNESDDGQEKEDARA
NPLQAPPDAAEDDRVDEATGAVSSLNLTEQPTGEADGEDEAAETGTNRDE
SELATEASEAPTKTSDTTTQTAGTPPSHSKKGPPKRGQKGKAKKVALKYK
DQDEEDRAAAEALIGATVGQKRQEAEAKARADRQAELDAARERRRAQHQR
QQKEVAEHEEIRRVMMDEGIEVLDADEAEKTTPLDALVGTPLPGDDILEA
IPVCAPWNALGKFKYKAKLQPGAVKKGKATKEVVERWKADSGKKGAVDES
SLDSERMWPREVDLIKGMKVEEVVNCVPAGKVRVMMSGGSAGGSGGGKGG
QKGGGKGGGKGGKGKAKK
>Mb|QLI69269.1
MEHLNMLFSHPSTVSSISQVSQTLKAHQNALSSEINSLELEQAYKPDSSL
ERMQSAQTELAELFRKIETVRSRAIETEQNITSMTAEIKRLDGTKRNLTL
SMTALKRLQMLTTAYEQLRGLAKTRQYRECAGLLQAVIQLMKHFNSYRSI
EQIATLSREVSELQRELLEQVCEDFEMTFVKGEVATKRSMLVEACLVIDA
LGDSAKSRIITWYVNTELREYRQVFRGNDEAGNLDNIGRRYAWFKRMVKT
YEDEHAAIFPSHWHVGELLTTAFCDGTRDDFKGILERSMRRGDGGKVDVN
LLLRCLQETLDFEQSLEKKFSDGPRASIDTLSSTEEKTHNFNGLVSVAFE
PYLSLWVDSQDKQLASLMPKYRNQPLIPADEEFSPQGIIPSAIELFHFYK
LTLSQCAKLSTSDRLLDLSKVLSKYLDEYAQQVLLHILQAGGSHGPSLHN
AILVLNTADFWYINTGQLEENIKKRIDNDLVPKVDLSSQADAFLGVASAA
VLALVHLVELECEGTWREMKNTNWSTMDTAGDQSTYVGELVKHVNTKTEE
ILGLVGKQQYARAFCDNLVEHMASAYINNIVVCRPISEVGAQQMLVDKYA
LTKSFGSLLSHHNPSSPTQQAPPSSFVRRVEHSMNRMDPLLKTLQVRPSP
PEGLVQAYLIHIGDRSDTNFKKILDLKGIRKQDQSHLIELFGIHRDGSSN
EKLVQSSPLLTPLMTTAGMGSGAGIAGMNPGAALTAASGARFDAGFLGER
LLSAARDIGNTTQSTEKATINENLRNFGKFFKRDIGGLGARFGKGSADDG
GR
>Mb|QLI69270.1
MGPQPLEPAGGHPTLLGFALHHEPRTITHLAAVETGTQAKPPDHVDIEVL
RRAFLSHNPRSLILVHRRRRNSCDTSRTGTMARVYADVNQNMPRSYWDYD
SVNISWGVLENYEVVRKIGRGKYSEVFEGINVVNYQKCVVKVLKPVKKKK
IKREIKILQNLAGGPNVVALLDVVRDSQSKTPSLIFEYVNNIDFRSLYPK
FNDLDVRFYIHELLKALDFCHSKGIMHRDVKPHNVMIDHENRKLRLIDWG

LAEFYHPGTEYNVRVASRYFKGPELLVDFQEYDYSLDMWSLGAMFASMIF
RKEPFFHGNSNADQLVKIAKVLGTDELFDYLDKYEIELDSQYDDILGRFQ
KKPWHSFVTSENQRFVSNEAIDFLDKLLRYDHQERLTAKEAQAHPYFDPV
RDPEVFKQNLANPGSNGTYKS
>Mb|QLI69273.1
MGDASAKDKFDIIRENLAEVLNPEIIEKILDEGRHPKIYWGTATTGRPHC
GYFVPAIKIAQFLAAGCDVTILLADIHGFLDNLKAPIELVEQRVHFYRYV
ITAMLEAVGVSTEKLRFVQGSSYQKSPEYIMDIYKLSSLISEHDAKRAGA
EVVKQTANAPMSGLLYPVLQVLDEQYLDVDAQFGGLDQRKLFTAAKEWLP
KIGYKQRAHLLNPMVPGLQGGKMSSSDQDSKIDLLDAPEVVSKKIKKAVA
APQVVEENGVLAFVEFVLLPASGLKNGKRSFTVDRERDGLEPLVYDNIAQ
MQEDYKNDVLNPQLLKPAVAKALNELLAPMQAAYQASKDWQEVALKAYPP
PPKKEKKVKNKGTRHPGHVVEVDGVAEKVKELNV
>Mb|QLI69274.1
MASNTTTALAGPLDTLNTTANVTTASTSPLAFLQDLDFVVLELKLVVSAL
GIIYLGSHAAIRRPPSATPPKKKHPGQKSDDDDRFSQGLEPSDAIMFPVM
AGIVLVGLYYLIQWLKDPAILNKILRYYMTAASMASILTLYAHGMDLVTS
FVFPSYWRGRNGSLRRVDQRRKTVHVCDDFGNAGAVTEKNPLPGIFSVFS
VTERARKAVWELRGLLTRHWTIKLFIHGVGEEATKIRFSHMMALFLSLAT
ALVYFSTNSTFLSNMLGYGMCYGSFLLLSPTDFLTGSLVLWGLFFYDVVM
VFYTSPYMITVATTLEVPIKLTFEVASRKSILGLGDIVIPGMVIAWALRL
DLWLHYVRKIKYEPTDLTIVTKDDSSGEIVRRSETKHKEVKARYVDVKNK
WGEGLWTRENFFLSSPSELPVELAGARFPKTYFYASMVGYTLGMAVTLTM
LLVFKHGQPALLYLVPGVLGSMVITALARGEWKDMCKYTEDGSLDTVDVV
VDLDGEGNAITTIGLLEDGIVDTTKDKKGDECEKKVAEASSKEGAAKTGH
KVFLLSIEAPPEDEEE
>Mb|QLI69275.1
MAHLNVKPDPAYLKHQAMQKTRHHFFRWTPRTARITFMYVAVVPAIFGYV
AYKTDGLYNFRAKRRGDTIYER
>Mb|QLI69279.1
MRNWRAPIGALDRGNRAARQAAIPDGQAAAVIPPTLALARRQDKTPTGLP
KSTMIAQRVGIAALRRGARPSLVPFIKAATGGMQARPVATAKISQDDGHQ
ILVKQRLQRPVSPNLGIYKIEQTWLGHSAWTRITGCTLSAAAYVYFGAYL
VAPLAGWHLETASLASAFATLPFAVKGGVKFFLGFPFAYHFINGLRHLWF
DLGKGFAKPSIKRGEMALWTSSILSGLYLAFGL
>Mb|QLI69285.1
MDVLSLISSPTYAPTTLRHLPQPVPNHLPTGVDGSIFTFEDAFEDLLAVS
QGQSLPDITTRYQQRRLLRDMFPSGEPTWFWLRRLESQGLVQVPSRAQFF
KAHQPDWTSFHEELDRHAADAWRSVSKGYGHDAPGSGNFFEDIGRAFKQL
ERGFGEDSAKNSYTGSEQKMELDKGPDTFEELFSEISSKFKESASSWDTF
VKSISEEAKLQSREGKHGAYIDSHDKNKVVTNENEHVDRFGYLHKTVTRK
MLDADGNEVGSETYVTIRPADKHLDNENNIEAGNNEPLEIGADSKKSSWF
WK
>Mb|QLI69286.1
MSLFTAQLYPGRALGFLILGASLHDVLTRLKAEPQRFPRLEVLHSPDRPV
TEPVCVVLPQNGLRLRFDGPEQRLRLIEVVDFTKIHITFKDRDLLKHTTN
GTSAPGSPVPGESPLGPTFRHIYHRLLGPTYAGEFIPPSSGQEDGTYVLS
YPGVAFNFSLAKSAYSPDKDVVTLLSASSSQVATSMAVFSGTSWAEARQT
LWTEILPSVKQTSVLPKTKDVYPDEVSLIRIHGGGKLDLFRKWTNNAFRI
QLGETTPQELVMELGPPNAIYRKNDQKMVIHKMRAASSSRGRPSSSDIGR
PDDLTDTDQSSMNTGSDDSDNDEAIDEQTASNPLEECFYNYFYLGFDILL
SLPTQPSAVPPADDTSEDPAENPIKHHHPDRLVATKVVLHGNVPGSYEFN
RHRRCRWEIAYLDDVTSETQFKYVEEGMNSKWEGAYSLGKVPQGQRGMVL
NRGWGDSPGSSCEFLGGWEESGAKRAEANGDSTTTLYGFPGLVFEVLRND
YVNTVTIF
>Mb|QLI69288.1
MATPPAEPSAPQPTALKPIHSLVLDTGPLIKNDPSVSALLSQAEKLYTIP
SVIPEIRDPATRARVETTLLPFVTVRAPGPASIKFVTDFARRTGDLAVLS

RPDIEVIALGYELECERNGGDWRLRNTPVQKRLNGRPKEAETQADAEDSP
KDDLGNLAAPPAAEGDETESPALEDKLDGLSLQSTENATPAEEEAAAGEP
SSTTDAPAANPDIPETQEDSDDEGWITPSNLKRHQAAESAGSTPSQPAQK
TLQAAILTSDYAMQNVALRININLVTPSFSRITHLKNWVLRCHGCFAITK
DMEKQFCPKCGQPTLTRASCSTDQHGNFQVHLKKNFQWNKRGNVYSVPKA
VHGSANGRLPKGAGGKNGWGRDLVLAEDQKEYVRASEEHKRQRNKDVMDE
DYLPGILSGKRSGGAAKVRVGAGRNVNSKRKH
>Mb|QLI69290.1
MATLPLSNMSSDLVWEIARNNNCFLSKSNRNGGVQFSHDPLNLTNKNSRK
HAGFVNDKAVGIIPNEKGGVTVISKKTTDATKPAKAFIKTTHGANKSSRK
TYKAVANLTAKSGYRSDLRSAAVERVSAIRRAQRPVKPEPEPKLRGNKAK
KAEESS
>Mb|QLI69297.1
MAPSFQTISIRDAAAQVGRSLSHLTKRIKIPLTPTSPPGLLQANAVDPWS
QSGKYGLGWTYFALSLMGCVLIVRIWHYWQDKIRQAIYKQEVEDHYRNLY
SIEADYAMAVQTGQSQQFFPDGPGLGNRQFRPKAHFSSVGIVNDTLALFR
WVFYRPIPDIVWGKNRFTFSSLAVLACGFIALIFVTLYCFLQQPLYWDSI
RFGSPPVAIRSGMLAVAMTPWIIITSMKANILTLIIGIGPERLNVFHRWL
GYLCLFLSVVHMVPFYIQPVWKDDGMTVFNQLFPDGSGMIYGTGIACIVP
LIWLCVASLPWIRRVAYEMFVILHVPAGVVYVGLLFWHTKNALLTWDYLY
ATIVIWVFAYFMRVFKLNWLKPWRNAFLVGDEAAVTLMSENAIKITIPTQ
MRWRPGQYVYLRMPGISVLDNHPFTISSLCSEDFPSEYGENYRDCVLVFK
PFKGFTRKVLDTAIAKGPFHTYRAFLDGPYGGMRRDLAAFDTCILIAGGS
GITSLMSQLLNLIKRMRDGKAITRKVVVVWAFKRFEAMDWFREELRICRE
AAPPESVTCKFFITGAVRHRDAGQMTAPINGQNPRALNHMLHDKLDGFVA
GIASKRNSALIQAEAEGDPERERELREENQDRITALPQQKYLQPHQYPPP
PPGPPSSRLGPNGYPEDKKLEDDDVKQPEEFHFPSLHKENPPHFNYAPPN
PSKPALSIPEPQEPYPVRAPELAHLRQPANPENAKQRPTSTIGPPAGFDF
GFPETPTEFQKNLMRSAFPIPHEIDGGWSMEYGRPDLGYMLKEWATGGVD
GRGILGRRTAVFVCGPPSMRVGVANTVAMLQAEIWGDDELEEIFLHAENY
AL
>Mb|QLI69298.1
MAATRSAALKIDWVKVTSSLGLRGQTVASLQAFKKRNEDARRKVQQLSEQ
PTTVDFAAYRSTLKNQAVIDEIEKRFNAFKPTTYDVSRQLKAIDAFEVEA
VKNAEATKQAVDLELKDLAATLKNIEEARPFEDLTVDEVAAAEKSIDEKT
TQLISKGRWMVPGYKEKFGDLALV
>Mb|QLI69300.1
MSLPKDTPNLDPGLSSPFPGQPRSFSSSLPREELTARLAEPVLPAPPPVR
GASPVPGGDALQATEAPFTGGSSTQGPGVSALAAALSNSLGQSPPRHGTP
AARISSPPPPSQSPALPTRADTPTHYGSLDSRSRLGPGSATGPYEDHEIV
KRHLVQPSDAENPTSEESSPRGTIKGKQPTDVGAGKVNEEEFSSLQLQGG
DVTRGIYKWTEQAEAKSKLNRSKSYDHMRPEPEEEVTDISAIKVPGGFRR
DHLRRRAFSPSGQHAELGSSGAGSPGTLDQPRLFTSSFLEFLSIYGHFAG
EELEEDDENLGPNEIWDSGEDRGGGDEEPTEDSALLGKKKRKRKVRGGSG
KNSPMNAALLLLKSFVGTGVLFLPRAYLNGGMLFSNLVLLFVSILSYYCF
VLLVTTRLNVEGSFGDMGGILYGKWMRGLILSSIVISQIGFVAAYTVFTA
QNLQAFIHAVSDCKASITIPLLILMQTAIFLPFSLLRDIGKLGFTALIAD
AFIMVGLAYLFYYDIITLNANGLADIIMFNQKDWTLFIGTAIFTFEGIGL
IIPIQESMKHPTKFPRVLFLVMVIITVLFTVMGAVSYAAYGSKTETVVLL
NLPQDDKFVNGVQLLYSCAILLSTPLQIFPAIRIIETELFTRSGKYNPWI
KWKKNVFRFFMVMLCSAIAWGGANHLDKFVALVGNFACIPLVYIYPVSQI
FP
>Mb|QLI69301.1
MGLTNYFKTGNKSEPAQAQLDAPTSRPIRSLQPSSLSDRPLSSFNIGGND
MELQPPTPRFHSRPQSSSGRSGRSTPSTAGSSMFLDDIKHEVMVNYLYQQ
QCSQLWVSDGSGEIEGVLLRKVRGHYMACPPQLGSSPFALACSAMNVQAA
MTVNSRVIKTFLQWSPDAVDVPLMNGLRVQILPTIEDLPRARKHQFAAFV
ASEGLLIVWDDDPLHLVQRAKAIESELMELVWKAGNTDDEEDEKRGGNIA

EAEIDEESGEVKPEKRPIHLQNTVLVSLTLILVTVSLGAAWRELAIEVSV
DGNYVRLALVALFPIQIFFTLFFAQVIVGCLAQIFGPIRQLTMNSKFYSA
RPPPRLQTPVLPHVTIQCPVYKEGLQGVIIPTVKSIKQAMSTYELQGGSA
NMFVNDDGLQLIPEEERHARIEFYADNSIGWVARPKHGENGFQRKGKFKK
ASNMNFALMISCKVEERLQAINRPLEWSQHDEAQAYEQALKDVLEEDGRA
WADGNIRVGDYILLIDSDTRVPADCFLDAVSEMEQSPDVGIMQFSSGVMQ
VVHTYFENGITFFTNLIYSAIRYTVSNGDVAPFVGHNAILRWSAIQQVSY
QDEDGYDKFWSESHVSEDFDMSLRLQCNGYIIRLAAWAGEGFKEGVSLTV
YDELARWEKYAYGCNELLFHPIRTWLWRGPFTPLFRRFLFSNIRFTSKIT
VVSYIGTYYAIGAAWIMTTVNYFLMGWFNGYLDKYYVDSWKVWFSIIIVF
NGLGNIALAVMRYRVGERNILYALFENFKWTIMLAIFLGGLSLHVSQALL
AHMFEINMTWGATSKEAEFSNFFIEVPKVLKKFKFSMLFSLVAIAGMIIL
AVAPFVPHDWRITDFVAILPMATVAASHFLLPLALNPALMTFSW
>Mb|QLI69304.1
MSHITQANQGLAAAEAQNWDEAITKLSKALQSSTNPAWLLARSKALVNVK
RYEEALDDANLAFHKSYERNKRDLMIDAHYRRAVAYFRLGQYANADCCAI
YAMRLVKGHAALEKEDVRAANSDHDGFWKPTAADAMAEAREDPFNQTKPE
GAMSAQPAHVGDWRRASTLRIQALAAMKKLPADDEARQATAPLVPERKEL
AALKADDKDMEGTTKNDVAAQKPAIPSDAPLRLQDFQTNTAMSVSIFSKG
VDKEKLQVKFLPESVHLNPLVYPNGDEKEFLLETFAEIEPSSSGYTVTPS
KVELRLVKKLPGKWSRVTKESPGTKEATEKDEESQALKDARRRAMDEADA
KTKDEKPTAATTQPEASKVKATTDSGPAYPSSSRTGPKNWDKIGADEDEE
EETGPNDFFKKLFKGATPDQQRAMTKSFVESNGTSLSTDWSDVGSRTVET
IAPEGVEAKKW
>Mb|QLI69305.1
MDTSYLAQQVNGSIAQLHGLFDEIGVPNHEREARESELFTALSEALNSQV
RLVTTEKKDMIDEAKKIITTIRQMEASLDDSKPRRGEYQEDEEFKITYPL
NRCVQGLREKHIQISRLHKERFEQVKTLVEALESYSSHLEPTFVQIALPP
TGPNQSLPTTFDISNTYVEKLDAEFTRVYDEYMRRIASAQALSNQIIQLW
AELGVPQAQQDGAIVKYYRDAPEQLGLHQEDLSRLHTKRDRLSDEKKNRE
RRLKDLRSAVEALWLKLGIVEGETKTFLNKNRGCGVRQINEFEDELARLN
ELKRQNLHLFVEDSRVKLQELWDSLYFSEDEMLEFTPAFSDVYSDALLEA
HEREVARLEALREQRAPILAVVDKHKSLIKDRDDLAASSQDASRLMMRGQ
KGEKRDPGKLLREEKMRKRIAKELPKVSAEVRKMLERWEDEYGRPFLVFG
ERYLDELEPEETKRAGPASRSKTPAKAPPSTVKKSAGLTRVPSTKTVPPR
SMTKTPTAAGRTATKKMQSPGKTNAISGRQSPSRIPARVPLSNLKTGGNS
PERPSRPESRAETLRNGAPVRAPPPKMRDLKSVPELQTPKNPYRGTGIGS
SIVRQVEPEDVYDDGAQPSRGVRSNSNLSNHTQPVYYEDAYDDRYDDRYD
DRYDDRYDDRYDDRYATIRGPRSAYPQGAPSRQLSNNSHTSTAISGSENW
ETYDDNSEPEEDVSEDYYNKLRAARGKRYEPEQGHGPTSSQAKRLRGIPG
TPYAAAPVMIDNDGNRIISGSEWTDEDVF
>Mb|QLI69306.1
MASEDPSSHLAESGITMHSDSEQYSPGEELSTSPESSGSPVILYKPPTVL
SLIRGAAINLLLPFINGMMLGFGELFAHEAAFRLGWGGTKVFPMSRRRAH
PIGPGIEVRERRRGPGPSLDDLASLE
>Mb|QLI69308.1
MALTTLESLKQSLRHKATTTSSSAKRPLSDSQYSAGFEILLGSSGWLTYQ
DFIIPQLSALLDPLVNSGVRISVLEVGPGPKSVLGYLPSYMRRSIGRYSA
FEPNELFVLRLKEWLYSTSGTGIPLPCLESPPDIHRIPFVVDSSASFDGM
GDGADKFNVILFCHSMYGMKPKSSFIKRALELLVERPGSGFVVVFHREET
LHLDGLTCHQTTSFPTGVTRVADDDMVLDRFAPFIAGFAMQDEHADKSTR
AEWRKVCRTLGHREQGRQDQLLFSSPEIMAAFTRHATALTELTSQVPLGS
GDGAKNRQARLRRPASIVRPTDIRHVQQCVCWALDHGVGLTVISGGHSGH
CLWPNVVSIDMGAFDQVHIVQARGEGGSGPNIDSGFLVVAQAGCKTGDIV
RKAMAAGLTVPMGARPSLGAGLWLQGGIGHLARLHGLACDAIVGAVMVSV
ANGRVLCIGRVPSQHRPIDAVCPDNEDDLLWAMKGAGTNFGVVISVTFKA
CLAPVYSIRNWVIPLSDRREAQLKLGDFDKLVAAKSPRNSSVDAYLYWER
DMLCLGVSMIESSTANKLAFGAQTHTPTPMSRLFGPEDNYNTVNGVGLFE

AEMYMSGMHGGHGGSKTSSFKRCLFLKHIGEASMIEILVTAIKTRPSPLC
YLHLLQGGGAVCDVAADTTAFGCRDWDFACVVTGVWSRDQDGTEAVQAAV
QWVYNVAAELLPLSSGVYGADLGPDPRDAALAAKAFGPNRPRLIRLKQIS
DPRKVLAYTCPLVKAPMAPTLIILVTGESCAGKDYCADTWVSVITSCAST
KLTARVIAIGDTTKREYAEATGANLQRLLRDRRYKEQHRAALTAFFQEQL
RHRPQLREEHFLNAVKDAMDMDVLLITGMRDKAPVAAFSHLVPSSRLLEV
RIQASNETRRARGSCRISGDNDDGTECYPPSLLFHNDLTGNEAAKKFAQT
HLLAFFHENLQRLASMVRPVPGFPRPGIEFRHVLDISQQPGGLGLCTSLL
QAHFTGDWAKVDLVACCEVGGFVYASASALRVGASLALIREAGKLPPPTI
SVRKSSSHISSCASHDAGEKGIEVGLNIFPRGTSVVVVDDVLATGKTLCA
VLQLLDEAGISADKVDVMVVAEFPIHRGRELLRRRGFGGARIQSLLVFDG
A
>Mb|QLI69310.1
MATSNLRHVGEDAFDSSADAADASHRRLPSGHRGEAVEMITSTDSLGSQS
PLPTKRPALLAHMGLGGVARRTLGICLLLVTVFLWTLSNFLASFIFSDHT
YDKPFFLVYINTSIFAISLVPMFTKYLLRNGVRGMRHDLIQMWTEYRQQS
SYTKAATEDDDLDNERLMAGSETAVEAIPRLDEKLSLRETAILSLEFCML
WFSANYFASACLEYTSVASVTILTSTSSVWTLVFCALLRVDPFSVRKLIG
VLASLVGVVLISTVDLTGKSDENRGSFPHKTTGQIAVGDSMALLSAVIYG
MYITVMKRRVGNEEKVDMRLFFGLVGVFNLVLLWPLFFILHWTNIEPFEM
PPSGNIWMIIIVNSLASFVSDIAWAFAMLLTTPLVVTVGLSLTIPLSLIG
EMIQYGQYSSFMYWIGAVVVFMSFVFINNESMENGDNEENGRAER
>Mb|QLI69313.1
MATVTISTPIKSHKGLFSTRTAGGRIPLTPSPRQSGNGVNSSPFTPDKHT
KSTYGGNLMAHLARSSSKNPHRESPKSNIARTVSTPRKALELGVSDFTLT
GTGLRTPGSAKSRKAPLRQKPAKATINYGGDRFIPNRGASSAIANAGSSK
LPRSDRQRPKTTPADSSSVLSSAADDAAAALEGLSINDEEADNYSRPSPN
TVAYQDSLASACGVKLNTRILEFKPAPPESSKPIDLRQQYNRPLKPNGAS
SAQLRRRIATAPERVLDAPGLIDDYYLNLLDWSSGNQVAIGLERSVYVWS
ADEGSVSCLLESPADTYVSSVKWSDDGAYVGVGLGSGEVQIWDVSEGQKI
RSMFGHDTRVGVMGWSKHLLSTGARSGLVFNHDVRIAEHKVAELVSHTSE
VCGLEWRSDGAQLATGGNDNLVSIWDARSLSVPKFTKTNHKAAVKALAWC
PWNMNLLATGGGSYDRHIHFWNSTSGARVNSIDTGSQVTSLRWSPHYREI
VSSSGFPDNSLSIWSYPTLVRNVEIPAHESRVLHSCLSPDGQMLATAAAD
ESLKFWKVFEKRAGTTSGIGGSGASGKADMAKHMTIR
>Mb|QLI69316.1
MEAKQERKQLDSRIKALISNGMQEKKRSFFVVVGDRSKDTIVYLHHIMTN
ANIKHNKSVLWAYKNKLLGFTSHRKKREQKIKKEIKRGIREVNDSDPFEL
FVSLHNIRYTYYKETDKILGQTFGMCILQDFEALTPNILAKTIETVEGGG
LVIMLLKGMNSLRQLYNLSMDVHSRYRTEAHDDVVARFNERFLLSLGRCE
SCLVIDDEMNVLPISGGKDVTALPPPDLDDTKTEQQIELEHIKDENEEKQ
PVGSLIKLARTVDQAKALLTFTEAIAEKTLRSTVTLTAARGRGKSAAMGV
AVAAAVAYGYSNIFITSPSPENLKTLFEFVFKGFDALGYADHADYSIIQS
TNPDFNKAIVRVNIHRQHRQTIQYIRPQDAHVLGQAELVVIDEAAAIPLP
LVKKLMGPYLVFMASTINGYEGTGRSLSLKLIKQLRDQSRLGAATNGETE
IADRSTGKASKTEDFQTTRKLREITLAEPIRYAKGDSVEKWLNKLLCLDA
TLPKMKSNIQGYPDPNLCELLVVNRDTLFSFHPVSEAFLQQMVALYVASH
YKNSPDDLQLMSDAPAHELFVLVPPNVESTGKLPEPLCVIQVALEGKISR
QSVLNSLSRGQRPHGDLIPWLVSQQFQDEDFASLSGARVVRIATNPEYVS
KGYGTKALELLVDYYEGRFASISEDDDQIMDEKIHRATDAELAKSSLQSE
VIAVRDQSQMPPLFAKLREKRPEALDYIGVSYGLTKALHKFWKRSSFSPV
YLRQTANDLTGEHTCVMLRPLENSNDRSWLGAFSRDFHKRFLSLLSYNFR
TFDTQIALSLDESANMGAKLDSIEAEALVKADLDRQFSPFDLKRLESYAN
SMLDYHVILDMMPIIATLYFTGRLKKSISLSSVQQCILLGIGLQHKNTET
LAQELNNMPSSQLLAMFIKILRKVTSHFGTLVSSSAEAELPRASNLGVTR
ENASSAHDDEIVDDRFVPLTTSLDDELEEGGDEALKALRQKQRELIDSLP
LDQYAIEGDAPAWADAEKQVRNASKDGKTNPVVSVKSKQKRKAGQTAAEV
YEEVLGEKASKKAKKGKKSH

>Mb|QLI69317.1
MSPPPPSPPGSPADESGESSFEDGDEQAGGDDTVTISDMGPRAQPPAQPQ
DSPPEARESHDYSTSSPSPPPADVAPFDWDYFESRYEKALQEADEEEKEI
LKEAEALAKYFRVWASAASAHDDDRAVKRLRTRQRFVNLAEEKMAQKQKH
YEEVVRAFESALALLKST
>Mb|QLI69319.1
MDFATEDNQNSAPGSVPATKLNTSRKGPDSQSVTKRLQTELMQLMTSPAP
GVSAFPSADGNLLSWTATIEGPDDTPYAGLTFKLSFAFPSNYPYAPPTVL
FKTPIYHPNVDFSGRICLDILKDKWTAAYNIQTVLLSLQSLLGEPNNSSP
LNGEAAELWDSNAEEFKKKVMGRHRDIEEE
>Mb|QLI69320.1
MDERDAAAAPPSVQPAADPTQTNVEAQIASREQVTGAMSGLAPGLGSGPS
AEEPPPSAQPPTAARAQAQASNPATRSITTAPVPGRAPATATAPRSAPAP
VAAPSHGHGHGHEQPALRVPRFVAPSSYLRFKTSHGSAMAAAASEPTSNP
STLSPPSPLDKEQRQGLKAIRDFLKVRTSYDVLPLSFRLIVLDTDLLIKK
TLNILIQNTIVSAPLWDSQRGRFAGILTATDYINVIQYYCQFPDEMSKLD
QFRLSSLRGETILHQATVEKAVGPAVCMLRQVGLDIEKAIGATPIETVSV
HPSRPLYEACRRMLKTRARRIPLVDIDDETGRETVISVITQYRILKFIAV
NNEHNTVMLKKTVREIGLGTYSNLATMHMDNTVLDAIHLMVDRNISCIPI
VDSENRVLNAFEAVDVIPCIRGGAYEELDGSIGEALCKRPDDSPGIYTCG
EGDRLDSLFDTIRKSRVHRLIVIDDDNKLKGVISLSDILKYVLIQGEEDS
PQS
>Mb|QLI69323.1
MSQPSRWRRPAQNFPLFPHLAIVPALLAIATAVALFLHSDVNAALLWSQC
HSHALLPALSRVPILGTPACYLVSFFYAALDSFRSKAVMSVILSYVGALL
TVSTVESARKVNEKNAVVSKPTLPWLVFNLLGGALVWQLVYAPAFILGAK
AWPFPSTGRLITDEGHGDPPVARDEEERDPLQLDKGRQMKLVETIAIPIA
VALGYYVPSTLMLSLNKPAAIGTWLFFPAYVSLIRQFVRYSLNKLRRFQD
GPMIHMEANTKAFIAVYAVPVLCSLLAHGFIIGNLTKADDRQEVTRSTVK
FIEIDSQFIGLTMLYWVFTEVGWRAPLAMVVASIVLGPGAGTVLGWLYRE
KLITSDMITQLLEQVNDEERGDVGEETPLIQ
>Mb|QLI69325.1
MSGSDSIPVTPGDMDPSKDVESSTSPACDGDAASEPGCANESVPEEPCAA
DKPKRMPSLRSVSDPRNMNSNSTSMGLLGRARNPSNTPPTLATGAGGVMN
NDVMARARALHQSRMGRSPIASSPNSPSPSFPGPGRNGFALAMPAAAMHR
PSPVPHPNSDPVVPKPSLSQRRFGMKLSDMGAGTSTQPPSGLRRAGAPKL
ADMEVGAPGGQQKPNGSGLGSKLDDFKKYIDAEKGWITFDGAATITKTGV
NFASGQTFTISLDEVQILDELGKGNYGTVYKVKHAKPSAPRFGHGLSGVR
LPHSLSDTVVDTQETKAAATAGEASSPDSGNGSDSTTGKLMAMKEIRLEL
DDAKFSTILKELVILHECVSPYIIDFYGAFFQEGAVYICIEYMDGGSIDK
LYAGGIPENVLRKITYSTVMGLKSLKDEHNIIHRDVKPTNILVNTRGQVK
ICDFGVSGNLVASIAKTNIGCQSYMAPERISGGAMAPTGSSDGTYSVQSD
IWSLGLTIIECAMGRYPYPPEVSSTIFSQLNAIVEGDPPDLPKEGYSETA
QNFVASCLHKMPKMRATYAMLLNHPWLQPFSKPKTITEEVEEGEEAEKAA
EAVGNLQLGGGDTEDADVAAWVKGVLEGGKDKSGDGSRPALHAVPLDSVS
PSSSPPLNRKP
>Mb|QLI69328.1
MANSKAPAEPLLVVLGSTGTGKSDLAVELATRFKGEIINSDAMQLYKGLP
IITNKITQAEQKGIPHHLLGHIPLHHLPWDVDDYKREANKVIGQIRARGN
LPILVGGTQYYVDPLLFTDVILDEVQQTGPSASFPILEESTEVLLEELRK
CDPVMAERWHPNDRRKILRSLEIYLHTGKPASQFYAEQEARKAAAASGDI
PATPWEKLLFWVYSDRQILTERLDTRCDKMLNSGLLDEVHELYAFKKKSR
ETGQEMDMTKGIWQSIGYKQFEPYLNALEAGADATEAEKLKLSGLEDMKT
ATRRYANYQTKWIRIKQMRRLQDEGPEAVSSLYLVDSTDVSRLQTNVVEP
AAKLTELFLKGEQRPAAVDISEMAREVLGGALELRPKETPIQRTCDMCRT
VLVTEQAWERHVKSVGHRRAVKKKTKLALVAVDAQKGGEAKESADPDGSR
ASSPDIGSIFSN
>Mb|QLI69334.1

MPTNAASNEFDEAAFNKEFVRCVLRIMPVKKSENVGEKCVRFVGFYLRHS
NDKDNEILGDVEGDTSLMPETPSTRLTSKILEEVLPLMTAKDKFVRYRST
QLISHIINSLDAIDDDLFQKLRHGLLKRIRDKEAMVRSQAVLGLGRLAGN
QAEGCTNSEDSDNDQGTDLLGKLLEVMQNDPSADVRRSLLVNLPILPNTL
PFLLERARDQDAATRRAVYSRLLPALGDFRHLSLSMREKLLRWGLRDRDD
NVRKAAGRLFRERWIADCAGISQQDDGTSEEAVPISFDGLLELLERIDVI
NSGVENGVALEAMKGFWEGRPDYREAVTFDDNFWETLSAESVFMARSFND
FCRSEGGGRYESLVEEKLPEVTKLAFYLGRYMDALIEALKRASQPAADEE
EEEDTVEQEFIIEQMLHIALTLDYSDEVGRREMFRLLRQSLSIPELPDEV
TKLTVEVLQYICAPDAAGEREFCSIVLESVADVHDTIVDDPLADERDDSF
HSAKSEVSRGSTPTNDKKRGNDSELSEEDAREKAVREIVVNMKCLHIVQC
MLTHVAGNLKDNADLVSMLNNLVVPAVRSHEAPVRERGLVCLGLCALLDR
SLAEENLGLFMHFFNKGHPALQITALHILTDILNVHGAQLLSSTPGLLKV
YVKAVKGGGKAPEVQAAATVAASKLLLGRVVSEQDACEELLKALVVAYFD
PSSATNHTVRQALNYFLPVFCYSRTANQDLMQAISLQALHSLLNLREGLE
DDDVDVGEDMLSMTTIGACLVDWTDPRKCYTPGAALDTEKKNVNGDVHLR
LGQDIMEKLRSNVNKEEKKLVAGLLGKLYISPGSSEAMIRDLYADVSEAV
EEGLLSDATSRNALYKIHVSLGKIVNTLDEQQPSNRRTSRSVSVAATDRQ
AAEDRTVLEEPKIKEEELNDSDEGTVVLKKDDKESATDDASSDEEDTKME
TA
>Mb|QLI69335.1
MANSKFEYVRNFEQPDPLLPNTWIVVRIDGRAFTKMCAKYEFQKPNDRRA
LDLMNVAAMAVVTDIPEIIIAYGVSDEYSFVLHKSCDLFERRASKLVSTI
VSTFTANYVFSWPTCFLDTPLSFPLPTFDGRAVCYPSVQNLRDYLSWRQV
DCHINNLYNTTFWSLVQLGGLDNKDAERTLAGTLAADKNEILFSRFHINY
NNEPEIYKKGSVIFRDYELVDPGSHSVAAEIDDLAEPVTQSKTQTEKDKK
RRAKARVVVQHLDIIKDDFWDRRPWILSNKPGKAPKET
>Mb|QLI69338.1
MSDPVVRDHAFSEHVKKEKDSSSSDHTPSDNVLSGPIEGEVTDFEERRTD
LEKQKTAEGNAHFNRLGWKRLTVVLIVEAIALGALSLPAAFASLGMVAGV
ICCVGLGLIAIYTSDIVGQVKIAYPEVAHYADAGRLLAGRLGYEVVGAMF
VLQLTFLVGSHCLTGTIAFLNITENGACSVVFGVVSAILLLLLAIPPSFA
EVAILGYVDFVSILVAIGITIIATGVRAGQAEGGLSAVGWSAWPKEGLTF
AEGFIAITNICFAYSFSMCQFSFMDEMHTPTDYPKSIWALGLIEIFIYTL
TGALVYSFVGVDVKSPSLLSAGHTVSKIAFGVALPVIFISGSINTTVIGR
YIHGRIYKDSITRFINTPKGWATWFAVISAITVAAFIIAEVIPFFNDLLS
ISSSLFISGFTFYLPAWMWFKLLKKGSWHSRENLLRSIANAACFVIGIVV
LVCGTYASVDDIILSFQTGRTRGVFTCAPIA
>Mb|QLI69341.1
MASSQPQTNGVTAPANTSSQTAPNTQSTTAAPAGAPQTPAQPPGASDPRP
RDARLIELLLTSQGVTAYEQRVPLLLLDFAYRHTSSILSDALHLSGDPYV
TQAGSKPSAASGASALAAGDAPITANGVKLAIGARLGYQFRGGSSGGGIS
KDYMQELARERNKVALPRIVPNEWGVRLPSERFVLSGTSWGLKDMWDEAG
DDDEEEEQGGDAMEGIEAPEPEDVGGDGVEGGTVDDVFGDDVDQEMAE
>Mb|QLI69345.1
MGEAVIEGSNWRLVEVGRVVAINGDHPFAGRLAAIVEIIDHKRVLVDGPS
ADPTLAVPRQAVPLSKCLLSQFVVDGLIRGSRHGAVKKLWEKNEIDAKWK
ESNWAKKREQIQRRKNLTDFDRFKVLRLKKQRRFEERKALAKVKASA
>Mb|QLI69346.1
MAGAFRPVNSSLLGESIKEEPMTTCSSSSVTPRPSAASHHPPQPQQSRTL
PDDSKTPTRSTFSSAVSDQKPLPNAPFPPTGQASESADGMPRRENSQHST
KSSRRDSMDVDMDDSDGDTGAGGDDGTISDGDSVGADGSKSNKKKKSQRF
YCTDYPPCNLSFTRSEHLARHIRKHTGERPFQCHCSRRFSRLDNLRQHAQ
TVHVNEDIPTDSLAATGSRFQRQMRTTERVRQAGNRARASTGGSAGGPVR
GHSKSLSTSSIASISSVGSGYGPQMDVRRRPPPLVMAADPRSRMSLESYR
SNADSTFSYRPPSPGDFSTPTSATFSTAQSSPRWMSGMASPTSSHSRSQS
MYSSGNRTPGRRLSVPSSANPFQSAPGRALFSAGQMNSSHPSAFSPAGSS
LVPSPTSTTSGWSTRRDSVSSAGDDAWRRRTWHPDSRNYGNQPPQTQSNQ

LAGVNQSVGPNPPPPIANPSSSQSSLRLPGIESFDPLPRRHPTPPPPQPQ
QQHGPSAMAIDSETAYRRSFQPGVGETIQPDERRNLNMYDASLQRGLNRL
DISHKTPPRDSAGSWASEANRAVQAQAERVQMQVNQPSVRFQDQVPPSRQ
FPASVHSGRSLHQHTMSAPSIATPRENKRHGWYHGPAPSQAHSEHVMQDP
RGAHVDRMVHPNFTGFNGFPGREQPAQTQQQDGKANPDSLRRLEALVAVA
TSEGTTATAY
>Mb|QLI69348.1
MAPLNVLMIGTGEYTTGFVGTGASGSDKKVGVVGLTLFDLRRRGKVDKLG
MVGVNGSKFPAIRQHLDKNITQVYNGLDTSFDSFPANDQKDPDAYKTAID
RLSPGDAITVFTPDPTHYPIALYAIERGIHVLLTKPAVKELSHHQDLLRR
AREKGVYVFIEHHKRFDPAYSDARFKAKTLGDFNYFYSYMSQPKYQLETF
KSWAGRESDISYYLNSHHIDICDSMVQQLGYLPVKVSASSSKGVATDLGC
VAKTEDTISLLVTWAKTGDPSKRAVAVYTSSWTAPERAGVHTNQYFHYLA
SNGEIRVDQAHRGYDVADDAAGQIQWFNPFYMRYAPDEDGNFNGQTGYGY
ISLEKFVDGCTAVNEGRLKPEDLDSKGLPTLGNTIATTAILEAGRRSIDE
GREIGIVVEGNTWKLM
>Mb|QLI69349.1
MLSQDPADGAVSPGESSGIPPPSGGSSNHRPSSVVSSRMTDIASDDGGEP
RAKNADPSRPETARTGTSSKATWAGPATRKGLAPGLQPKRGSVASTIASA
RAPSLTTRSHVPSLTSGAFFRPMSSQKLQAQRGAGSRPVPMNQQLHPPPA
NLDDNETDMGGSLIDGASGGENTQSIAQLQHRISAGQSVRPPPSRGTEMT
DQETFDRFIANASPTTGHYPTASMSDSVRPLQGPSDQNRELSVHVDKSYR
DLANLPSPVRSTRTFRSSFLLPGKMEQAQSSRHRDTTGAEKLSSTASSPR
FPRTESHSGPRPLTTDSQRKSRGRVHQYFDGNTVFCFGGRWQNTKHTPIN
IATGAFVVIPCALFYGFEAPWLWHNISPAIPITFAYLTYICLSSFIHASV
SDPGILPRNLHQFPPVADQDDPLRLGPPTNDWTLVKSADISAAAMEVPIK
HCRTCNIWRPPRAHHCRLCDNCVETHDHHCVWLNNCVGKRNYRYFFTFVT
SATILAAYLIGTSLTQILIHMNRESISFGDSIDHFRVAFALAIMGVLSIV
YPGGLMGYHLFLMARGETTREYINSHKFAKKERYRAYSQGNWLKNMIAVL
CRPRSPGYYRFKGKYRPGDQRLGQHYSLRTRKNSQGLELDEVPPGSRGFQ
GPAALRGESRAS
>Mb|QLI69354.1
MAEIQQPPKENGQVMAPPAQASTKSANSRKRGPPAHQIYALPAPIRTFPL
PAFYPNNPLSFFAVAYAWLRQVFAPPPAEPAVVHQGVWSKKTFSVHVTDE
KSMRALWEQGFYGKGSLSRSEPNWLRREQVRRGMQETHVSEILTVQRREE
RAQAKWERARLEQEVIRQTRLKEAQQAEDNSQSKTATMQLSPSPVIFAAP
TGPIELLSLPNSAAEAFEKPEVVSGTVIAVRAATEAVMMLPVDIAPPSI
LETGNGIIDTNVVSTASFVADSPRVRGQSSGSGVIETPHPPASETSSDSA
EDKTLKRQKSVRFSPKVESTTFDLADPPNPYHSLNGKGKRLLLNGGIDGS
DTAEEAKKLSADESEDIIVNREHLQLMPEEAFFLTFGFGALSVTDPASGK
SLTTKEMLTLFRQYSYFPPRTGCPDEPELGPDDGFLVHYAVYHHFRSLGW
VPRAGIKFGVDWLLYTRGPVFDHAEFGLIIVPSYSDVWWKDRGKQGPQNT
WPWLHSVIRVLSHAVKSLVLVYVDIPPPPKFDAALETGFAEALQLYRVRE
VMVKRWSSNRNR
>Mb|QLI69356.1
MTSHPDHPPADAGPSEVRNEVKKPKKKKVLLMGKSGSGKSSMRSIIFSNY
IARDTRRLGATIDIDLSHVKFLGNLTLNLWDCGGQEAFMENYLSQQRVHV
FSNVGVLIYVFDIESRDVDRDLATYVSILSALLQYSPAAKIYILIHKMDL
VVPTARETVYDERVRTVKQKTAEYINSVGGDTSTVELTPFATSIWDQSLY
KAWASIIHDLVPNLAVIERNLANLGVAIEAEELLLFERTSFLAVSSWTST
EGRRNPTEDRLERMSNIMKHFKQSISRFTGTPRNAEQFIRMEHKAGTRFN
LFILKFTTNTYLMVVLPPGEARFNAAMLNCQIAIEHFKFLDGPAPHAITA
TVVPPASS
>Mb|QLI69357.1
MTLEVWPHISPEELQIKAAESLDRELEWIVQETMTLCHELKHGIEDCYAL
LAPIEPGSTLVMSTHRNEKVKGTITRVGTRLVKGTLSLQLRTLPPQQLTI
SALEPIHIPPLDAIFTNLTQSIDLLGLLLDSKPAPTADNIASALAALAEC
LAESAGLLKGSTSSEPDPAWQTASCPAHHFSPAMPPNLSFFTTLQESSIV

LWLRALEPADAPVHFGVKLGLAIGTVRRLEHDEMDTVFRYDPDGDGSCEP
KRGLGGSRTSGKRDREENVFVREKVRIESADPSLISLYSKLGFLSHMLGQ
ARYNLAAVMGVELDS
>Mb|QLI69358.1
MASTLEAEAPHNSFDTILVLDFGSQTSHLILRRIRALGVFAELLPCTTKV
AELPWKPKGIIFSGGPSSVYDEGSPHVDPAVFELDVPILGICYGCQEIAW
RIDSSNVVRGEAREYGHADVTITKVNSHADRLFAGLGDGISVFMSHFDKL
INLPKDFVVIAATKNSEFAGIAHEEKPIFGVQFHPELEHTPRGTDLLFNF
SVNICGAQPNWKMGNFVEIEIARIRDLVGPKALVIGGVSGGVDSTVGATL
MREAIGDRFIAIGINNGCMRLNEFEEVKKNLRDHLGINLTVVDAGELFLS
RLAGVTDPEKKRKIIGSTFIDVFEKEAIRIEKEAENTPNSGKVEWFLQGT
LYPDVIESLSWRGPSATIKTHHNTGGLPERMMNGQGLRLIEPLRLLFKDE
VRAIGRQLGIHESLVNRHPFPGPGIAIRILGDVTKERVEIARKADNIFIN
MIKEAGLYDQMSQAFAGLDTSRSVGVFGDMRVWGYIVILRAVRTKDFMSA
EVFNFDNSFLQEVSRAICNQVDGVSRVVYDLTSKPPGTIELE
>Mb|QLI69363.1
MDIVLELTDTFIADYAYAYLYPFRPDLYDFPHANTANSSTHTFSSWTYKP
ATQFLQVQPSRAAYMSSLTRDNPYRQLATLFFITWFFGILVYFVFATLSY
YLIFDKRTLNHPKFIKNQIWLEIKQANKSMPFMAVCTAPLFLLEVRGFGN
LYDTTEEGPGMWYNILQFPLFLLFTDFCIYWIHRYLHHPLIYKHLHKPHH
KWIMPTPYASHAFHPLDGFAQSLPYHIFPFIFPLQKVAYVFLFVFVNFWS
ILIHDGEYLTNNPIVNGAACHSLHHSRFEVNYGQFFTAFDRLGGTYRMPE
AWMFEKERKMSEKQWKKESKTVDEFVKEIEGDDERTYGPAEEEAKKTK
>Mb|QLI69364.1
MALEECPPRSEPTLSFTQGLVVGQLSVVLILAAFIKFFIFGDPPSSDVTA
SLRATERRSRTLAHKQSLLSLRSPSQRQSQHLKRKKSSVLRNPPTLTIGS
ILSKTYYNVDSHQPESLDWFNVLVAQTIAQFRSDAQHDDAILNSLTKALN
GASRPDFIDDIRVTELSLGEDFPIFSNCRIIPVDEDGLGLGNGIKFDATA
AARDGTRLQARMDVDLSDMLTLAVETKLLLNYPKKLSAVLPVALAVSVVR
FSGTLSISFIPSNPSQSTPTMMTFSFLDDYRLDFSIRSLLGSRSRLQDVP
KIAQLVEARLHRWFDERAVEPRFQEIALPSLWPRKKNTRGPDDTIVDGGL
SISRSKGKDIGRDVRDEISWRKETSTEDRKWDMASLRHRNPVKDTDIPDI
PGSPHSP
>Mb|QLI69365.1
MAPEEVQRLSKVRNIGIAAHIDSGKTTVTERVLFYTGRIKAIHEVRGKDA
VGAKMDSMELEREKGITIQSAATFCDWKKKENGKEETYHFNLIDTPGHID
FTIEVERALRVLDGAVMILCAVSGVQSQTTTVDRQMKRYNVPRISFINKM
DRMGANPWKAVEQINSKLKMPAAAIQIPIGAEDEFEGVVDLIEMKAIYFE
GPRGTKVRSDIIPTALRELAAEKRQVLIEKLADVDDEIAEIFLEEQEPSN
EQIKAAIRRATIARNFSPVLMGSALADKGVQPMLDAVCDYLPNPSQIENT
ALDKSKDEQVVKLVPYNSLPFVGLAFKLEENNYGQLTYIRVYQGTLTKGT
YLFNSRTDKKVRVPRIVRMHSNEMEDVSEVGAGEICAVFGVDCASGDTFT
DGGLPYTMSSMFVPDAVMSLSIKPKRTADADNFSKAMNRFQREDPTFRVH
VDPESEETIISGMGELHLEVYVERLKREYKTECITGQPRVAYRETISRRA
EFDYLLRRQSGGPGDFARVAGWIEPNETPEDNHYESQVVGGHIPDKFLTA
CAKGFDLTCEKGPLLGHRVIGTKMVINDGATHVTDSSDYAFSLATQMAFR
KAFTDAGGQVLEPLMKTTITAPNEFQGNILMLMNKRNATIHDTEIGNEDF
TLICDCSLNAMFGFSSQLRAATQGKGEFSMEFSHYAPAPPHLQKELIAKY
EIELEAKRTK
>Mb|QLI69367.1
MAQASSTSNRSIKLKREVSQVPITSVGLYTTLSGKVYIIAGEDGDLTVYD
GPPTDTEEPIHRICVFEDQPIHGIRVHKDETSSAVAILLWGASYVGLLDG
TPLDDGNQPLLLATTSAPDWIYDAAISPWDSSIAALATAHNEVVPMTYHP
DARALTTGDVTSPSRPMLYASHLRWISPTGVLVAAGTVFGNVIVWKYHAE
TASHTMLFVLRGHEGSIYGVDISPELALPNGSTARLLASCSDDRTIRVWD
ISNHEGPHESTLDCVTETGFKAAERYDEVQAACVDGQVVPVAVAMGHASR
IWGVKFGVDEGHVVRDGRLPMYSFGEDSTAQRWELSLGPSLSGTISHRQT
HSLHDGKHLWARALDIRNGWIRIVTGGADGRISFIEDAAAVEEAGAQLTT

IDVPHLLQSPSDSASAREMISRYDFILDDTMLAITNAGRLFLGSLSGTQT
WEEVSVGQDVAPDLKSCYVLRKIGHGAAVIGTTSGRIFFFQTRQVRPVGS
VPGRIVEINHLASTADTIELLVHLHGSPASRYLTLSPAGDLLSEHQIAGL
DARFVAISATRLDDLLLVGSRHGWMSILVQRDDTWHPILDLATRSRDAIT
SIVPLPPSRNPSSDSRHILVTSRDGKYRIYQIRRTDAANAALALVHETSP
PFGPMIEGAWFSNTPTPELLLYGFRSRDFVIWNESTREEVASINCGGAHR
TFRLDYSAEQPELYRFAYTRTSKLSIYSQTRVPHRTVKSGTHGREIRALS
SNKRYIASGAEDTSIRIWEYVAADEKKERGAAMRYLACLKAHITGIQKLH
WFEDDYLLSSAGNEEFFVWRVRTLDSAYAGLAVVCEGSFADKSPTRDLRI
MGFDVSRAVDGQRMAITLAFSNSTLKTYEYGADGSFKLFATGLYTGACIT
QLRHLGQMDEQLWVLTASTDGHMALWKTRAASSALGSLTLQAAAQVHQSS
IKSLDMVRQNGIFHILTGGDDNGLGFTEVAEVRDDAGHTEYRFAGRGIVR
GAHAASVNGVALLAQGQDDGRDGLAVSVSNDQRIKIWRIAKSNPQMVTLA
SCISSGVADPGDVAVVDQGSSRQVILGGIGVEAWSVVQ
>Mb|QLI69373.1
MKLPSFVLFAVAATQTGGSAVLSRAPCTGNTATTRSQWCDFDINTDYYDI
VPDTGVIREYFFNLEQVTTAPDGFSRVAYAINGTIPAPTIIADWGDIVVI
HVTNNLGIAQNGTSIHFHGIRQNRTNEMDGVVSITQCPTPPGSTITYTWR
ATQYGSSWYHSHFGFQAWDGAAGGMIINGPATANYDEDKGIVLLSDWNHR
TASELYSETLTKGPPGISNAIINGTNIWKDESGTTVGTRFNTSVTAGKSY
RLRLVNGGIDTHFKFSIDNHTMTVIAADFVPIVPYETTLLNIGMGQRYDV
IVQANQHSVADNFWIRAEPQTACSSQNSNQGNILGILYYGNKPDTPTTKG
YAYKVAACEDESLDNLVPHVARSAGKEHWSDLELADLIQQENVFLWALNE
TSFYVDWANPTLLQVYNDEKEFTHRSHVIQLDEKNQWVYVIIQAQNAASH
PIHLHGHDFYILGQGTGTYNSTQQRSLSNPTRRDVALLPGSGYLVLAFVT
DNPGAWLMHCHIGWHTNTGLALQFVEQYGAARGLINHDFLNSTCEAWSRY
VSRTSIKQGVYEDGI
>Mb|QLI69374.1
MASMAPSEEQISQVIDFAGLNPHDDRTMVIQALKDTGGNVEAVVMQYFDN
PESFRQKFTSVWDESLFQADRDGTVNPAGISFHIESINKNDGIKSNTPPL
EGYGPMAPSRPPSRSNGRSPLGRMVDWAAGDTPAGSAAPSTREDEDMQRA
LRESAQEAGIALPEQQSGLMSTQSTSTPYFGPANRNDYDQGSWAMVPAGP
SESQVPSAPAPSLRKRMTGAPAFLVQGISTVGNHGLGGLLTVLHQIPLAR
NALLETGAPAASYGFNSEWWKGQEILPTHALARLQAGDLQWGNQDESKPD
VEEEIHRLMAFLDSTERSYGSVSVLTDLIPYPSLGPEKQFYEYLGPKNEE
KLKPLIHSAALSTVFGDDLGDEEAKFGLLEMEHLRSDYTYIKTVYESLDH
TMWSDVLSWNELHEGSKMAMFTEMGEVLAIKINGDGPQDSIDIPEKLYLE
KYQTNRKDEARRIQMSWCDTKNAIMQIAKEEQQLFEWRNDWDHQMMDKKQ
MIEKARNQWQVYSDYLLGLGKFRGMVASGFDTDSYPDYRKAPCELSESEN
SALGRVDEVLQTLLRMLTDIDKRLSSLNSQMEQIMSKQRFLGRLLTQPEK
QNRPRPMTCKEFLLRGVVTPSDVTYVCQRAEPDLIELGEQPKQLDQWWRL
AYAPNGEQPVQAEKVEIERVLRDVWQETKTPLMVYATETALNAVRAPLTA
PLQRFATAENKAFRQELHHEATQAADAEAGEGKKTPGSGPIDPISPSKRK
HRADSVDSMDSNRASIGSDDAQNGFDNPFEDGQTSTATMTASATPVTTTA
NETHPDLDTEATEVRMVAAASTVEQ
>Mb|QLI69377.1
MAHDTNTMLAGVYCTDNIHLVVGTNPLAAARCTASLAAGACPVLVAPAGA
DLHYNLQTKIEQGLVRWHQKAFEDEDLFTLGRDEVGRTVDAVFVTSAPRE
ALAARISSVCRRNRIPVNVVDAPLLSTFSLLSTHTDGPLQIGVTTNGRGC
KLASRVRREIASCLPPGLGAACNRLGELRRRIQHDDALAPGDLDDALDQS
SQFNRLVGDDEARTRRMRWLGQMCEYWPLKRLAAVTDHDVEKLLLAYPGH
NSSSSSWPVQLDAAAASPRGRIVLAGSGPGHPSLLTTATLRAVQTADVVL
ADKLVPSGVLDLIPRRTPVQIARKFPGNADQAQAELLETALAAVRSGKTV
LRLKQGDPFVYGRGGEEVAFFRARGLGDRVTVLPGVTSALSAPLLAGIPP
TQRGVADQLLVCTGTGSKGAPPAPPEYVASRTVVFLMALHRVAGLVAELT
ARLGGDDGDGDDARRALWPPDTPCAVVERASCPDQRVIRTTLRHVVEAVE
AEGSRPPGLLVVGAACEALFEREKGRAWVVEEGFGGFDSGPDDNWMAALQ
GAMGGDA

>Mb|QLI69380.1
MPVPFLGRLHPYEYIALVGSFVFVGLEAVIRVLTLALPSFAVSFFYLVSR
RLFNRFTTEEHKKAERKKKTISTSVRDAPDFVELCRIWGYEAEEHIVQTK
DGYLLGIHRLQWRRGEEGLKVNCGVGSTRKRVIYLHHGLLMNSEVWVCLT
DERRCLPFELVERGFDVWLGNNRGNKYSKKSIHASPTTVKFWDFSMDEFA
FHDIPDTISYILETTEQKSLSYIGFSQGTAQAFATLAIHPLLNHKVNVFI
ALAPAMAPAGLSNGIVDALVKASPSVLYLMFGRRSILSSATMWETLLYPP
IFCHLIDRGLGFLFNWKTKNISASQKLAAYPHLYSFTSTKSVVHWFQVIR
NKCFQMYDDDVHQPMNVATSRKYSKVAKYPTRNIRTPVVLVYGGSDSLVD
IKTMLRELPPQTVATEIPHYEHLDFLWARDVDSQVFQHVFDALDSFTDAE
HRAEEYDRYYTVRNESLMGSAFSFLHRGSESEASTTAAGSEGSNGQSPQH
VAREASELTENTSRNRIIGRADGVCDMTDKLKGVGVRRGGTKSAANPWQD
IGLLDKTK
>Mb|QLI69382.1
MRCAALPRRLRAASPSPACKSPLCRRGWPRNPSTKPALGLPAAPRWAHSD
RRLTILEHVHLKGDPLTGLAPYEQAEDAQEQHRARFLQWKASSEEQRLSA
PSCHVPRPRLLSFEATPTFTLGRRQEDLTAEQATRLQQPLDIGLGHRRNP
IAHRFTPQVRKTSRGGLTTYHGPGQIVLWPIIDMHSSLYPKYGVASYANH
LETTTQRLLSELFGIQTYTARDEPGVWVTTSSGQERKIAALGVHHRRYVT
ALGIAVNIDVPVTGGEAVNPWARFVPCGLDGRLVTSVAAEVGVDSALGWD
LEDLAGRWATLFDEGLVDEAKRSVGGGEARSRLQR
>Mb|QLI69383.1
MGFAQCTWPIWRVDDLTLCFRQDYLKVLLPLAVIVVSFLRLAWSNIGRLV
SSGKPKGYKPIANDHTALPPDIDDEDDDDDTEGDPYLEINGNRLALAKTI
SRGSVVEADTPHGQRLSQAVEELAIVGLIAVNAIALIKGAYGSAGRLVAI
TGLLTWIYVLVLASLRLLVGGTKWAIPHLWNHTASIYAINWVLNIFIFRS
ALINSTSQLVKILACTEFGLITLLFMLAATTRKGNKTVLMEWEDGIEPSR
EPLASLFSLATFAWVDAIVYKGWKKTMEIDDVWNLLPKDKAAAVIADYRQ
LKKTGALAWHMLKYFKGMLFFQCFLAFVSGFFTFAPTLLLRAILEYVEEP
DRAPINVLWLYVIGLPILDTIRSYADGMALWTGRKICIRVRAIIIGDIYA
KALRRKAAAGKDKVLGESSKKTKTDADPDSEGTIAKIKRALGLRKRNKNK
NASASDTESAGSINVDDSKPAGDDDQANLGTIINLMSVDSFKIAEITAYL
HFLCAAAPTQLIISVVLLWQVMGLSAIPGLVVMVFLLPVNYFLAKGFNIT
SKNIMKATDKRINVTNEVLQNIRIIKYFAWEERFGRSIDEKREAELNALR
SRFTLWACAVAIWNSVPVLITFFSFLVYTLIEKKPLYPSIAFTAISLFML
LRVPLDQLGDMFAHVQEAKVSIDRVEEFLNEEETEKYEQLGQDNVDEQGV
KHIGFKDATLIWGGKDTVAEDGSRAFRLMDLNVDFEMGKLNIIAGSTGSG
KSSMLMGLLGEMTLMEGKIFCPGGRSREDVKPDPETRLADTVAYVAQSAW
LVNANIRDNILFSAPYDPQRYRDVIVACALERDLEILDHGDETLVGEKGI
TLSGGQKQRISLARALYSNSAHVLMDDCLSAVDSHTAQWIFTNCIRGPLM
ANRTCILVTHNVTLCVPSADYVVYLDNGRVISQGSAQKVIASGVLGEEIQ
KSRPASSAGSQGPSRVPSRVPSSVGDGETMVNSNGESQDGSAASKKAKSK
KPQIDAMDEGKASGAVKWPVMKVYVQAMGPWWFWVLAVIVFNLQQLATVA
TNVWVRQWANQYVEEESMVAVGTLSQSYGSGAFSKGSWASISRLGSNALA
AESSSGKVYGQVSFSALVTPQVNVAYYLSGLAIIGILGSFAAFVRDVWIF
YGSLTASRKLHDRLMGAVTRAKFKFFDVTPLGQLMNRFSKDLEAVDQEIA
ATAIGVMSCALSLIVTVVLIAVITPGFLIAAVFIGGIFYFVATFYLRASR
DLKRLESVQRSPLFQQFGETLSGMTTIRAYGDERRFIRDNLAKVNTQSRP
FIYLWACNRWLSFRADLLGNLVSFSAGVFIILSLGKIDAGAAGISLSYAM
NFTENVLWLVRLYGMNEQNMNSMERVKEYLDLDQEAAAVVEKNRPPESWP
DKGNVEFVDYTTRYREDLDPVLKGITLKINAREKVGIVGRTGAGKSSLAL
AIFRALEAEKGRIVIDGINIGKIGLRDLRENITIVPQDPTLFMGTIRTNL
DPFDQYTDEQVFEALRRVQLIGADEPATNPTTPTIQVAKDSQVTRSPVPS
VMTNKNIFLDLSSPVTESGSNLSQGQRQLLCLARAMLKKPTVLVMDEATA
SIDYNTDSKIQETIRELTGTVVTIAHRLQTIVDYDKVLVLDHGEVKQFGH
PWELLSDEEGQFYSMCEMSGELDVLVKAAKRKWDEDRLVDVGDA
>Mb|QLI69385.1
MTGGISVRDVDAQKFINAYAAFLKRQGKLPIPGWVDTVKTGPAKELPPQN

IDWFYVRAASVARHVYLRKTVGVGRLRKVHGTAKNRGARPSKHIDASGSV
DRKILQALEKIGVLEQDEEKGGRRITQSGQRDLDRIAQTTAEADEEDEDE
E
>Mb|QLI69389.1
MSEHHETTDERNTSSSTMAARKNSQRPPCRHSPTSSSDIDAEERQVGSTS
TYGSGKSSQPAPSDCIPSVARELLRAGQVEENGIRPLVVGERTQKRFWSI
FTIWFSINSNILGITVGMLGPLVHNLSLRDSALVIVCFNLLAAIAAGYLA
TFGPKTGMRQMIHARYSFGRYFVSIPVLLNLATMTGFNAIICVVGGQCLS
AISSGTITPNVGIVVISVLSLIVSFAGFKVLHIFETFAFIPALISIMIAA
GVGGSRLKQQQEPAAPTTAADILSFGMIIAGYQIPWAAISSDLTTYFDPK
VPSWRVFHYTYWGLAIPTVLLMSLGAAIAGALPSNPTWEEGNEAYGVGGV
LAAMVSSAGGFGKFVVVLQALSLLGNTCGTFYAITLNFQALAPILFKVPR
YVFAVVITAIIIPVAIYAYRDFYAGLNNFMSMISYWSAAFVGIVMTDHVV
IRRCRFDSYDQAIWTKGSKLPLGVAAISSGVLALGLVVPCISEEWYVGPI
AEKAGDIGFEVAFSLSAMLYVPLRILEKRFTGR
>Mb|QLI69391.1
MANYHPPFSSAPLKTVEEIQFGLMSPEEIKNMSVCHILYPETMDESRTKP
RDGGLNDPLLGSIDRGFKCKTCTQSMSDCPGHFGHIELAKPVYHPGFIKK
VKKILEIVCHNCSKVLADTSDPEFVTAINTRDAKLRFNRVWAVCKKKRRC
ENEDRNEKNDDEFAPGMKPVAHNHGGCGNVQPQVRQAALQLKAAFDVAQE
DGPKRRETVPITPEMAHGILRRISEEDLRHMGLNSDYARPEWMILTVLPV
PPPPVRPSISMDGTGTGMRNEDDLTYKLGDIIRANGNVKQAIREGSPQHI
ARDFEELLQYHVATYMDNDIAGQPRALQKSGRPVKAIRARLKGKEGRLRG
NLMGKRVDFSARTVITGDANLSLHEVGVPRSIARTLTYPETVTPYNIGKL
HQLVENGPNEHPGAKYVIRADGTRIDLRHHRRAAQISLEYGWKVERHLMD
GDYIIFNRQPSLHKESMMGHRVRVMPYSTFRLNLSVTSPYNADFDGDEMN
LHVPQTEETRAEVKELCLVPNNIVSPQKNGPLMGVVQDSLAGVYKLCRRD
TFIDKEMVMNMMLWVPNWDGVIPQPAILKPRPRWTGKQLISMVIPQEISL
HAPEGDSDIPLKDTGLLIQSGELLYGLLKKKNVGAAAGGIIHLCYNELGP
EGAMAFLNGVQQVVTYWLLNTGHSIGIGDTVPDKQTIEKIQVHIDTQKAE
VAKLTAQATANELEALPGMNVRATFENKVSMALNSARDQAGTTTQKSLKD
SNNAVTMSESGSKGSSINISQMTALVGQQIVEGKRIPFGFKYRTLPHFTK
DDYSPEARGFVENSYLRGLTPSEFFFHAMAGREGLIDTAVKTAETGYIQR
RLVKALEDLSARYDGTVRNSLGDIVQFLYGEDGLDAMCIEKQKLGILKIS
DAAFEKKYRLDLANPPDWFKKDYEYGNELAGDKESMDLLDSEWETLLSDR
QTVRLINKSKMGEEMMQLPLNIGRMIETAKRVFNVRATDRSNLRPADVIP
RIQNLLSELRIVRGSDPISMEADLNATILFKALVRSRLAFKEIVKVHRLN
KLAFDHVLGELQNRWDRSFVSPGEMVGVLAAQSIGEPATQMTLNTFHFAG
VSSKNVTLGVPRLKEILNLAKDIKTPSMAVYLNTKLGTQEQAKKLRSMVE
YTNLRSITSVTEIYYDPDIQGTTIAEDMDMVESYFLIPDDGQDNTDQQSR
WLLRITLDRQKLLDKEIRIDDVAQRIKEEYPNDLAVIFSDNNADEQVIRI
RTVRQSDEKDEDGEKKIEDDVMLKRLEAHLLDTLTLRGVPGIERAFLTKG
TRLTVGEDGSELAIKDDPRCTQWYLDTSGSALRDVLAVDGVDASRTYTND
LWQIVEVFGIEAARSALVKELTNVLAFDGSYVNHRHIALLVDVMTYRGSI
SAVTRHGINRADTGALMRCSFEETVEILLEAAATGELDDCRGISENVMLG
QLAPMGTGNFDVLLDPKMLETVISDNSRMGLMPGMPVKDGEAEGAATPYD
TGSPMSNDSGYMSMNSPAAGNFSPIQGAGSETPAGFGTEYGGGGFGNVGS
MSPYSRGATSPFSTSPTSPFSSGMVGYSPSSPNAGYSPTSPLLDGGAGRY
ATSPSFSPSSSPSFSPTSPMLRPTSPASPNYSPTSPSYSPTSPTSPRHYSP
TSPAQFNSPTSPSYSPASPNYSPASPNLHGAAGATSPSYSPASPSWSPTS
PEAYSPTSPSFQRSPGNQQSPTSPSYSPTSPAFSPRTPGPGNSGNQ
>Mb|QLI69393.1
MALADKTKRVVSQFDFSDADANLHVQEFLKQMKEGLETDGTSISQIPTYV
TGVPNGTEKGLYLAVDLGGTNFRVCSIHLNGDTTFNLTYNKVAIPKELMV
AKTAKELFAFLAKQIELFLREHHAEHFESHVRRRATASTPMGYRDEQIFR
LGFTFSFPVKQLGINKGYLIRWTKGFDIPDAVGKDVCRLLQDEIDNLSLP
VKVAALVNDTVGTLMARSYTSTGKHRSLLGGIFGTGTNGAYIEKTANIKK
PIEGEYDASTGEMVINTEWGSFDNQLNVLPSTPWDKALDAESVNPGIQMF

EKRVSGMFLGEIVRLTVVDMIKDDGISLFRDANSSFNDRSTTTSIGPKSS
IFEAWGLDSAIMSVAAADNTPELSTIRLELEKSLDIYAPSLEDAQAFKAI
SDAVARRAARLSAVAIGAIALQSGRLDDPEEEVLDIGVDGSLVEHYPFFR
DMIYEALGAIDGIGAEKVAKIRIGIAKDGSGVGAALIALIAARMEKPGDF
LADLRSDIKRRLEKIPSNVSTSVEDPVQSWTPLVVGGLVAVAAIAGIWWS
RRR
>Mb|QLI69395.1
MAKSSRSSTRKENNRRKAASVFSPAEVARNERLSAKLLELARQPKPESSD
VNMDANASEDVAEEVGSAQAGDDTGMEIDSKPSKSRIGKKRIDKRKQKKS
SIVFAKYSDKAAKKQKGSR
>Mb|QLI69398.1
MPPKRKHSETHPTLQRAEAPRRRSTRRVKDDDGIDAGSNESRAKYTDGLS
SKENVERAMHNLWEMEHKLLNEAKRQRLAIEASNLEALAAKEDEAYGSQD
VTKSSIECMDAVATSTGTARQTKSDNDMNSVEETASLENEVGERGAKRPP
PVNSDQLPLPWTGRLGYACLNTYLRTANPSVFSSRTCRISSILEHRHPLQ
DPSQPEHPTKNRPDKSKPADPNRGLRYIQDIGLSNARDIVKMLRWNDKYG
IKFMRLSSEMFPFASHAEYGYSLSFASEVLATAGKVASELGHRVTTHPGQ
FTQIGSPKKDVVTASIRDLEYHDEMLSLLSLSEQLDRDAVMILHMGGVYG
DKQATLNRFRENYQKLSEGVKNRLVLENDDVSWSVHDLLPICEELNIPLI
LDFHHHNIIFDPSVREGTQDIIGLYDRIRATWTRKNITQKMHYSEPVASA
VTPRDRRKHSARVKTLPPCATDMDLMIEAKDKEQAVFELMRTFKLPGWKL
FNDIVPHERADEPRREVPKKAKRSVGGKAQVNGTTETISAAPEHILSADD
LNMGGPERRVYWPEGMEEWLKPRKREIKTGTKNGCDK
>Mb|QLI69399.1
MGVKEFTLLGTTQCQNVKLEVALSDEMSDLKSDLANLFAITDATAISLHG
SADILETIENVIDHQGKIGLMVDGSPIRAPHAPKELPFLGNHFEIYPDHM
GNHDRLFTQYGSVIKTVNMGTTTYLTNSPSVSEAALSESEYFTKTTSDPS
HPLYHMRDSTALFMCDTSSPAFHLAHKFIPPSMTPNAVRQYTPNMQQAIE
ECFSVFDELDRQDKAFHVYQYMFKLAGQIIYRIVLGLDVGHFKTIDTPAH
EIIYLLGEYMLLMKKTSLSPQWYKYLPFGDHRRLQEVKKRAWALVDEAID
NAEVGGGGKDAPIQEVALDSTCIADYLKRAVDDEGNKLPPEYRLTNIVVL
IGAGFVTSSSLLSWIIYALAKYPGNQERLVQELVDHGGSSTKTWSYDEIN
SLPFLDHFVKETHRLHNPSFQTARNTKKDVIVPGGWQMPAGAIVIPTFPS
IHKNKDHWENPERFDPDRWADDATSKKRHRRAFTPFAAGPRGCVGLNVAK
LEAKLALANLVYRYHFTDANKEPMQYDPEFLVIRPLNCYARARKRTSWPA
KLPTGA
>Mb|QLI69400.1
MQYVRNLSDSVSTAWNSINPATLSGAIDVIVVQHEDGSLTCSPFHVRFGK
FSLLRPSEKKVEFKVNGIKQEYSMKLGEGGEAFFVFETTENVPASLQTSP
LVSPVSSPGLGPEEAPAGLNDPDILELDQELSNSTRPPAAVLQTQPDENG
MMTPLSASPNMSKPRPMSGDWTSSLTRPHSDDVLRQSARAVNRDDEDESR
DPNESERSHSPPPMGMERARTLAKELSAVNIPSRVTETGDLMLDMTGFKS
SEEDMLQAEILARKILSEELEGNYDIGSLFGFDEEGNLWIYSSEEAKQAA
MNKTIESSLKAHRHNTAADAVSDPGYHSDGSDVTTSPHVPSHRRSESDAG
PSGLQTPPRTPPGYASNPNVNYAKTLRLTSDQLKALDLKPGENSMSFTVN
RATCAASMYLWNHETPVVISDIDGTITKSDALGHVLNMIGRDWTHTGIAK
LYSDIAQNGYNIMYLTSRSVGQSDTTRAYVNGIVQDGCRMPHGPTILSPD
RTMAALRREVYLRQPHVFKMATLRDISSLYGPDHNPFYAGFGNRLTDQIS
YRTVNVPRTRIFTINSNSEVSLDLLSLNKLKMSYVNINEVVDHYFPPVAT
LVKGGGEDYTDFKYWRDEPLELDEFSASDSEELDVPGDNESTYTVDDDEE
IGDGLVDSYISRESIDESVDDDNVENDEEYDEDDEEGEYEEDDAGSDDEA
QLVDHMARSMISSKGVEEDIGAELITGLSGNGVAIESQEVYVEEPVEIRP
TPTPTPERAY
>Mb|QLI69401.1
MADHDAPRPPGHFDSRTGLLTPESQGRPRFSYETGESSRSGGRRELISGT
SLSPSYTGDSQQHHGSGLAQADQNNRDGLHRGGSTRRRPKYKYGGGFLLH
DSISGGRRHASRGSARDNLQPNGKAPQSPRTPQPSRSNNRQLLQPSERSG
GSLRDKSSISPQSVESRSGGSAQKDVTSEESEQPFQPPRTSGEAVNDAES

TQIVQMALNLSESRRTASRRNVSRNTPPRLGPLPDGSSGSNLQQHFQQQR
KSSYNGSPRYQQAQSARRSSGKFDSSFEGGGHDSHYRYHFSSSTLARAQK
AKDHLELMAQYRRLLDVLPPLKPGLERVTSSPPVSPMASRPFQFGLGDRV
PLGRQYNPLQYIRNRKVRARERKVIDGESQGFGDLECVKPWVDKVVERAT
SFAAHPEDNESLLPGFPGADDPNSQISPDNSSRAAARVRRPRVDWFIEPC
DIVADAYWLEQENHKELIEDRTWRKIFPSTTNMTQPMSRETAVDTLEVIP
HFSMQDDHDLNPSISSTSKVDTAKQTLHNIRAFPHRHGGQLHNIHHDLGW
HVKDTISDISSSENDTGVSKRTSREGNRGGETISRTNRDFLQEQMLEMVA
RDARDRDLSDAISDQELVVSPSSIKTPEKNTLSQLPTRFHSSSKSSLVNT
SDSDRRPVLDRPYLGSPPRYELGRQSLDATDGPQKSPFYKGSSLPTSPDL
KPTRGSTDPTLGTNLSPGWSRSGSPTRNPISKIKQIIRDNKASEATPEPD
GSHHVSAKDASLPSENPNLLEKLQSSPPRPGASEPTPFGQTKMHLRSGST
RLRSEDQGAGLRGIFKGPRIDTVIRGGVSKLGDMFWRRDGTSESPPDNET
TDESESEKARGRSRSSTNVSLMPSKRDVAGHQSQPKHFLDSMPQFHPIQD
PNRAHSADNSKVDLGSPDSANRSRQQSRIDMLRPPHFDPRRASSSALLGR
RSTKPGDSDISETESRQGSAPEGVGDAGNHLSSRLSSSRPEDDGRSFKNR
PWSIADKATPTERTRLTRREIARMRALVLSTGVKAMEINRRAQEAHKPFC
PDSLAATGPSSGNSANIGWADIAKICPEIAQQGDRETAYYELYSLAGRAL
ATAIQNSGRRWQASADQLTYRTSPQLQNRIGDVRSSIADDLSERSRKAAD
LADETNRDLALGQPLKVKVVIDIMEKMLRRRRRRLRWIRRALWLTVEWLL
VGFMWYVWFMVMILRVFLGMGKGIWNGARWLLWL
>Mb|QLI69403.1
MAETARRKPSADRPQMDKMRRSSSYLSDHQQYRPPKKPETYHGIDEVVEG
SSPPKPSSPKSVLPFRGVPQDDMPATIVESGVSHSLSHPNCAPPPGHKSD
RLVATLFYKGASPSSAPASTRSAGLAESNETLPPNMAPTTNMDAFPLEPP
TQEAEQLDHIYGSYISPLCIASFLHLMSTFPQPRGADEPHSSHRCLDNPD
TPRIVELTLSPAPSPDCLSLSDLRKHEMIYRFEQEWNVDVVLQPDLVWRR
HPRLVVFDMDSTLITQEVIELMAETIKEPADLAARVADITHRAMLGELEF
DASFRERVALLKGVSATVFDDLRPVLDVTRGIPELIRALKRLGVKTAVLS
GGFQPLTGWLAEKLGIDYAFANEVVVENGKLTGEAVGKIVGKERKRDLLV
EIAAKENIHLSQAVAVGDGANDLLMLGKAGLGVAWNAKPRVQMEADARLN
SESLLDLLYLFGFTAEEIQMLSV
>Mb|QLI69404.1
MDQQQSSQHERDAPLPEEQVKRRRRNPEYTRSFLGLPSILEREHANNPTL
CTRAQLRQLLAPSFSDNIGISIANGLGLDENLDIPGPFDVRVNTVIMPSD
GVQQKQQETRNATSTAESQTIPTPPLNSTTQQKEAQVTPLPSSNSQAESD
LPIESSSQKPPPQDLATAESQTQQHDSAYCPGEEEDPKSSHTHTEEVGHG
ASPSQGHERLHSILSLPLIHPNGISMFPHLSTPEQEIMSQEALTQAEESI
IADNFTGSDVADGESDGGYDSDGFSSGSTSAESSVRDYMFENGRRYHRFR
EGTYNFPNDDVEQEREDMKHAMVKLLCNQKLHFAPIGNNPQEVLDLGTGT
GIWAIEMGDQFPSAHILGIDLSPIQPDWLPPNVRFMVDDMESAWLHPRNH
FDYVHSRHTVMAIKQWDRLYRRAFEHVKPGGWMEMQEIHHRPRGAGVDGT
VPPDHPVAKFWTLVTEGLAALGVDLDISSGGVLAGKMQEAGFTNVTERVF
HVPIGTWPKNKVLKTVGLYWRTILLDGLQAIALGPLTRGLHWTRDEVELF
LMDVRRAYHDNSALMYMPLHIIYAQKPTTAY
>Mb|QLI69416.1
MAMSLARPMIRSPALRMASRRFESTAASKASEAAKDTASKAQQGLSRVTS
AAGPAIAGAARGVGNALGRIGGRTGRLIAFVERQTPFVVYYSKVGAEVAK
LVFQGQKMSPPSVATFQNTYQTLWRSFQRGTLFQTPQNILQQARNLSNAQ
LATAGVVAAECVGFFTVGEMIGRFKIVGYHGETASHH
>Mb|QLI69417.1
METHFGNLPMRLCGVLVPSLPSFDFLNSTTFWVYLFWILWLHRYVRLLVH
CLSHWTYKSKPLPANPKYTNQDVTVVVPTIHDNPEELKPSLSSLLACNPA
CLYLVTPYDKFEALKKAAEQLGVDNVQVLSNTIANKRRQLCEAIPSIETP
ITVLADDDVTWPSKMLTYLLAPFEDDKMGGVGSCQRVKRVWYEPWSTRIW
NWLGAAYIERRNFEITATHNIDGGTSCMSGRTVAYRTEILKGHDFLSEFQ
NEKWGEGTLNTDDDNFITRWLVKNRWKTWIQYDPECELETTLENSMELFV
LQCIRWARSNWRSNWTSLFIEGHVWKQQMWCTYALHIATFTSLAFLVDPL

LLLSCWQATADWDTAQRERAFWAQFIFMFGFTKVVKLVGLFKRNPSDIVF
LPVSIIFGYFHGLIKIYALLTLRTTTWGSRPDGDTNNDSRLQPLPKPSEY
VASPKTKEHSDLTASSASGISDGFKIRQKAVYDAETPSPWNILAGSEPTH
RSRGSGLPKLGG
>Mb|QLI69418.1
MDCFGLCGSKDDDDERQPLLPQYNQDTTRQDRLHEKLHTYQMLRAMSNGY
MPSNKQVIIHLRTLLCATILNPSEQSTLSNSGRALIRTTRLWLQQFMDLL
EQKNAKDQIQDFIWYLSKARLDVDVANVRKNMSKSKARADVSATVESLRT
VVSLAMLNSDFRVFVADAATIARQVLRDTALALGDVSKEVGDKLDSPGED
MEALKKTDQDAQEPPSSEYVKDQAKDVGETAYKEAAHVGEEAYTSFSEHM
NAEAKDILVNRLRNAVTNLRKRTDYSESVSTLSGILQKYLKIYLSVGSDA
VDTLEGNLHNNTQVENAVHNFWLFLSSFGDGESWEKARASFSDFAEHNRT
DENLDEFVSQFVNLIQGMLSKPEFFDNMDERLDELRERLKELTSGSSMGE
DASNLLRSIQQALQSTAEDKDVKNMTSASLRLVHILFPPGEMGNTDLVRD
CVNTFIPLLVQAVQYIPIPRLEVSTPAMDLLMENLILEPGQTVNSSSFLP
YNLQFSSRNDIDITKSPFGTSSSVASLVTIKLAGMSIAADDLGYWLRLHS
GLLRFVDEGLTSFHLDHRGIDVTLDVEIGRERLEELVTLRSVHVKVHHLD
YSLRKSKFSCIAWLLKPFIRPLLRKVLEKQISSAIDEGLRTLNRELVFAR
ERLRATRVCNPGDLWTFIRAVAARLVPPPDPDIHTRVGVEPGKGVFRGRY
APGSLVKVWEEEARDAEQNIFEYRQDGWRNGIFDVQTTPAP
>Mb|QLI69421.1
MSGVNDSFLSVEKRRLQLEENIGQLKKTLQHWQTWDAEYEALKEEVEAVE
EPYTSDDLRHIHDEFDGELLVGREINEVFGIQELKPRSQIINVLQRRIDY
VGKNVASLEKQLEAAEDKYAAINVLNQPDAVDETGQSITEIIEELDDEDN
VVSYRLNQPEEALPKVKEALEKAGVDSLEEKTPAAEAKSIVSSKEKRSSP
ASLEPKPAASRQPAPSETHPAASVKKTVSFSEDVEVSNPPAEMSMRAKRV
DRIMRAAKEQESISHGTPIIPEDEDPDDAALRKQMLQYSMGEVGAVVAEL
ELEDVDSDDDSGFDYGDDDFDDDEDEDEDRFGRSTGRIVTDEYRQRMLEL
EQKLGIKSRFTQQAEQKAEDDGSKDDTEGIGRIVVKREPETAPSASKSAP
SKSNVKERKNGDGEGKKGVRFAQNLDIAPEDENPMSKPARETPVVEPLSD
VVERTTSANSTETKSTRKPSRFKKGRDNVPNNSLPKGPLDVPSQLVQQQP
RVVPTGPEGSTIVDKLVERDTSANVNIPDEFDNSITHQEVADEYQRMRRK
FIHRDGGFLKEDESVIQPLDEADGGEPMSRFKAARLSKQ
>Mb|QLI69423.1
MGSPLPVRSAALARDTNETKIQLAINLDGGEFPADTDARLLKATTGHASQ
SSKSQIISVNTGIGFLDHMLHALAKHAGWSFAINCEGDLHIDDHHTAEDC
CIALGYAFNQALGSLAGVARFGYAYAPLDEALSRAVVDLSNRPYSVIDLG
LKREWLGKLSTEMVPHCLQSFAQGARVTLHVDCLRGDNDHHRAESAFKAL
AVAIRMATTRVKGKEGEVPSTKGTLSA
>Mb|QLI69425.1
MSKINTLLFDCDNTLVLSEELAFEACAGLINQICAERGVKKEFTGESLIV
EFVGQNFRGMLTTLQKENGIEIAPDDMEKYVRMEEDAVIAKLKASLRPCP
GVDEQLEKLVASKKYLLAVVSSSAFRRVKASVDKVGQDKYFKNEKGEDLI
YSAATSLEKPTSKPDPAIYLHALKTLGKTAGEAVAIEDSKSGTLSATRAG
IKTIGYVGPYADDKKAEMEKVLTDAGAVIIMRDWKEFPAALEKIESGAL
>Mb|QLI69426.1
MASPDGPPLSLRPFPVADKAPQNLAEFIARVNTQSGGFRDVTENKIQDEI
KSNQVVNGADTDPEDVDMSDLGHDEEPVKDAALVRMDVLKNIEIAGNTAM
LTLDSLSLLLSKQNPTQAGLTLSQQLREMVGIGTLGADKLDEPILNVNKE
KDEEEVATGWTLMQINQARDAADEAGKFLQREVDAESKYWEDVMAVKKSG
WSICRVPHERHTLGVKFGFSEASPEFKNNGLAPMRRGDSGSVELDLGRLG
GVSEGLVVTYEKDGQVVGRSVPRRRAHDDASLESRVLEARNTIFSQELWH
ELTREARTLAAYGVRPEGSTLTCSVDSSSKIVLELVPLTSCPVADDSLPD
NSIAEAIFISLHVLLSYAHRYNELMRIRPIPPHISRSRGQQVYALLRPVI
TCMASTRAVLSCTTYIGSITKALQKSGLPASFTLKTTPFSAADPSSQGPN
QLAGAQSLIRNILQTIEFNITFTILPNASLTIRGRTFLFPVTTTFYQVAL
PPSSTLQGICAPYADGYSNPKALFSYIRTTTERAVTLHFLNALSASPSPA
QWIQSGTSIRDPEDDSRALQFTIAKQPVALVLTSSFSNNPKGETKSWTYS

AHLDSEPTRLEDVVAREASRPRP
>Mb|QLI69428.1
MALLNPTFLLGVLVLLYLSSFVFFAVIRILTGVSIQRIGYLSLRRLAYTP
RDGCRVEPTWISLVLNELVVTVDVRELEGHKPPNNDDGGGDDEKSGSEAS
PDESAPPPKTQRPRRNADTQSRSETWERLAKLKERIKKLHTNIRWLRMID
VVATNSTINVVDVGSIQVGSLTVAVDTRRKIVDHARLFKFHSKKKEQRQA
EWIISLRSVLFTADGSESLEVLDHCLLNVHGFLYESRQGLRDATISLKLG
RVHVPYDDLNACASRLRTCRRRPNAVDFDEPVDIVLSHVTEDSGDKGTQQ
DDLMKAVTDSREFVSSILRGIKEVQFAVSYVGMTQRIESVKPEGSPMLLN
ASMKEVGIDLHRLDPKSPAHRMYFSPKDIAHEALAAALSLSVGIDDGHGA
PQRLLYIPMTTTTVRTTLPSKTIQLAEDGAIGEKNANIMFANSVITSPSV
DLDPAHLPLLIAMMQPKPKAPKSSRVRNHSVLSRLLPKASIKFSMHEPVL
RMKLKPLQDTEDADDFDLIISSISSVSLDVEASHSTMEDMHYFLDGAMRL
QSHHLYYQTCTGDRFNLITTESFDLKAHLAARPDVCVELTGNIQSFGISM
VRPEITDGLRQIVRQLRIDVEPEKRFKSKTAKHQNFLRSLPDWLVRYQIE
ATDFNIEVAGVDEDISDDTRGVTIHLDSFSAEYRAHRLDGLQRRPGRRRT
HSRSVTQSDSDLTPSPASPRFKKKPQITGDGRRLALYAKGIEAQIVEGSD
HLEVEPFVNVPRLELAFSATSDNHGPMFHIQSTIKTVMLQYSLYRHYAVG
VAVLTLRKAFMRTGRDVPRPQQHGTYEKDHLRPPGSLRVPDADGSSTTPS
ELITVDVKAALVQIKAEMPHEPLLMLRVYGLETGRHRWSTPFLTSKVIRL
YVETPRMRNVWSRMIGIKHGRVDLRESKHKTPQGGYVEEKMFDVAMEALR
IAIPHEVIVARITDNFINVIKSVKQLHHRFKTGTNEYILEKKPEEPKHVP
KVSIRSRSFLFELEDGAFEWKLGIIYRTGRVEQTQRLARQEAFEAKSRKV
QDEELRKGGGKLRARSTFPRGRGEHSASSWFRSRSADAYKADDTGHEIKG
GTMPRYDPDREHVGIHGNSKLSEAEAHTALHRFNAQSWKKRIDRAYETSQ
KSMRELRETFWGPNHLPDDVEETENILEVPQRPALMSALISELHFMIDKP
SFAMKDLPNFIHTVGKGMPKDMQYGLLVPMHVAIQMGEARMTLRDYPLPL
LHIPGLKTGQSSRLQSVSLKANFVLAEEFRGHESTKKVRVDVVPPRSSDP
KSSKEGSFAIEVRRTIGPVKSFSEVFMDINTALPTRITWGPCYQPAIQDM
MMVIESFTKPQVDPSERVGFWDKLRLNTHSRVHVAWKGDGDMHLSLKGTR
DPYLVIGNGAGFLMCWRNDVRWHINAEDNPKRFMTVDSGEYVLAIPDYSH
QVREAARRHDHGTARVVGDTCADGAIFKKVIMKLSGKVQWMAGLLFERAI
ENGERSFSFKPHYEVVLKNPIYAKPDPDGLPYDALRGFRSRHIHLSIAVR
APVDRDWMSEKTEPSRSYNTVHLTPRFFTHFFQWWGLFGQPMSLPIRQGN
LWPGREKSSKKFGRHLATVKYNLLLAPLFLSHIYKHKDAEDYAENAVSAT
GLKVRFDSFMLDLHQRREEFNTLAKGKSTQSRTSGIKIHAAQLDLTNADV
RAVSASLRGTTTEAIRKGTISTLITEQDDKPDLSRFTIPDNDFSWIDMDD
FVELDWILPTEPHPDTKILPLAFAPRLTYFRQTDIGGIIAGDPARTSPFG
NEPTHFCIMPHDDDPRRVQSQLIQERLDQLAIQMQTHSRNIGEAELRVIQ
TNGEEPELVEHFDALKKHVSVLKDKELFLNTMLDQMQNKVRASSNGHRRP
SEPSSSSSEDLVDMPPTAEFESEFKNRFVIHNLQLKWNNLLRNIVLRYIH
QNSQRRGFVYYLSRPAVKFILDIVEEQSRAKANKTAKGGAPSTTNGGTSS
PSPSVPEKDSAADLEDRIKSILEDGKHFTDTSFDVNGDVNCGSIEDLEGG
IAKEFSPQSSYHVRLIAPQIQLQSEKNKKHVVLVTAKGMELKVLEVMDKE
RISDTVSGLVQRRFLVNMDSTQFFVTHQKWFLTSLVSMYAGSKYGAPNGS
AWPPWVPMEVMFNFQTDPFGFKRVVQKTSAMLRYDKYNNLRLKYNDEVNE
DGGEDVASSENAECRSDNLWVEFPQARALCNSSQYYAMYVIVLDLLMYSE
PLEKTRSERLEKIMLASDFSDLSGTPRMVQKLQERIRQLEDIKHHFQVYS
KYLDKKGWEDRLALERDLASCEDELFFMMKAITTSQRKYEIDSDTSALLK
WSISARDIVWHLIQDSAEPLVELQLKDVEYDRTDNSDGSHMNLIRVGKVL
GLNLLPDATYPEIIAPYSESDRNATTGDTEEDMIRVYWHMLEAIAGIPVM
DRFEVKLVPMKVQLEREAGKRLFEYIFPGMDKDKGTKDDSPLMVKHQAPS
ADEVDIESTNSSAIRLGIPAVDKDSSGFSTRAGSLELRLRPTLTSNNAQE
SSPKKALSVKSSEGGGHAFRLFRSGHTGKTVISKKASHESLRGTGAPRPG
MMVRSSTGFSSKDSKDSTETKKGSRWRGGKSQAEDKQQSDDLTKMINRAN
NYMTFAYIRMPSVVLCLSYKGKDQRNFEDVHDFVFRLPTIEWQNKTWSNL
DLALALKKAVVKALISHTGAIIGNKFSKHRPNAEQRSKLREMANSSVLIT
TSGSSQHYAASINDSDDSSSFYGTSPIDFSRSPPRSTRAGSTHSSIPAPR

STSRSSSVASGRSYRTGGSSHNGPSAPSFLSMTTPPTPIEGRNEAGIDLM
RPSSSHGFGNSNHRRALRNVSAGDSRPSTSAGSVESGDRRRTGAPSLRDR
LSVFTGRKRESSDVPDESNDVTGDDESTNDGKTPRGKKLSWAQARTKTG
>Mb|QLI69430.1
MIVPRHKCAVLLAVVIANVAICMLAFLYTTQHWELEGLAVSSSKHRRPKP
PNLTLTASVPKHYVPTVENGRRLLVIGDIHGMNTELGNLLDLAKYDAATD
HVITLGDMVNKGPDSRGVLSRLMSLNASAVRGNHDDRLLLALREDGAQAA
SPTGQEDAVLAARKRDKKILKVAKTLLPEQITWLSGLPVILKSPALRLYF
VHGGLVPGVKLDKQDPWAVMNMRTLRYSQDDELRRRDGHSSAQAEPEDED
EYDDDDDDDEGSEVTPAQKVVAIPIDDHSGQRWDKAWNKYQRKFVKKSHR
RTVMYGHDAKRGFTEGRYTVGLDSGCAAGGHLTGLIVRARGRGGISYDKI
QVPCKAA
>Mb|QLI69433.1
MALAATKSQTSRRCLQSLMRLFCCISWWWLAARTRETHDVGSSPTIHGQP
RPASNFNPPSPITITMDVAHPRRILAVSLDSQADQLGRVIKDLTGSSPCS
PSPSLAGTTHNLSLKTQYYTATVPVWLDLIASPSDWSASFLSDEAAEVLA
VLGGLVLIFSLPPSTSSESIDRVRDVIHHVGHVVNNGLGGWEWDGVRLAV
GVGDSDAQEWDELCAEAGLEFVQIGGKQLHLNEFGEKTGISRVKEALESN
DWAQDAPSDFGDFEDASDAGEGEGGHLDPESLDFGFDRADFEGLKKAIWE
ARPGDQEPSAPTAGSGATSKAEADNQGDTQDELGHEDVDKVEGMMRKLQA
VRDAGEGLPEAQRKRMAARAVGEVMKEL
>Mb|QLI69434.1
MREFMDYVHSAFYEATGWRRDNSYAALNATSDALLDFQTPRGLRLTLSSL
ASANFATSYQLGSVGVVDGSISYLFSSVPLRVHMTPRSEDVSLPDLLRSY
RPLSPLLPRTPSPPDAATAGPEASLLYGRLYLPQSQLEALAVRRLSPALQ
LQLSAVSAKHLRDGGTVLGLAQYDVGRYAFEGLASSDGGLLGLRGLYNFG
GGDAHPPPAGAEGEKERVYGRFSTGAEVYYGTLNKSGGVSFGTRFATLPA
HSGTPLSASLTVNPLMGNVAASYAVVAGRHCSLATRMEFNVFSYESAWAV
GMELWRRPLRRVAGGGGRPGGEYKERSFRAKMEWRLDEPELVPPPQPSPP
LSEPAEPDRVLAVHRGNGGEEYAGVLKARLDQNWRIGILWEGRVKSLLFS
LGSGIDLRKLDKPFRTLGLEIQFSS
>Mb|QLI69438.1
MSSNSFFLPLAPTTSASYPRAPASRTSPRVVAADMAPAPEPVRRRSSSSS
SGGFRVLKLSPVHWGEHLDDHKEDFHEATTV
>Mb|QLI69442.1
MVKAVVAGASGGIGQPLSLLLKNSPHIDELALYDVVNTPGVAADLSHISS
TAKVTGYLPANDGAKAAFKDADIIVIPAGIPRKPGMTRDDLFNINAGIVK
GLIETIAEVAPKAFVLVISNPVNSTVPISAEVLKAKKVFNAQRLFGVTTL
DIVRAETFVAEIVGQKEPQKLTIPVVGGHSGETIVPLFSKANPSVNIPAD
KYDALVNRVQFGGDEVVKAKDGAGSATLSMAYAGFRFAEKLLRAVKGEKG
LVEPSYVYLPGVPGGDAIAKETGCDFFSVPVELGPNGAEKATNPLEGLTE
KEKELLGKAVEGLKGNIKKGIDFAHNPPQK
>Mb|QLI69445.1
MRDKMETPSRPKGKKLPFKTTVLCKVASQNVETIHDEKLSAEDDGLDLFR
RSKEMEPIIAAELQRSLKKRQRLEDERRQSAKSKRPLDDNIEREVIANAE
SGTFSQAARSTPPVVSELVKDSREVVSKFVTPLASKRMRLDSTLLRNASL
DMDNADTTLNNLSSTRFLRSHPKQPTINNLPGDTLTSNGLPMVLLDSDDE
DSIQVAPTPTNRRDSVCFIARDEDDEFAHYIRKAEEQRVHDGSYEAAAKE
KIDILVTSIVPGTKPCCFKFLYDKQLRLVRNAWLTLQKHRGVLVDIEKDD
DIVLTWCRKKAYAFSTLLNMGIRPHGDGRAIVDGHSTSGLAHGRTKVHIE
AWTLDLFREMEREEALKRRRETVELLDEGGPVPAEREPVTPEVKIRVILK
ARDLDDVKMTVRPETTVGTLITSFRTQRSIGPDKYIGVWFDGDILEEHAT
MEEAEIDDMDTFELLHTFDALIGQSRNEIDEYLSRHPKLASNIRSLQSIS
AAEISNDIPTPPPENPQQENPPLDEVGATDRGACVKDNASLKTKIEALSG
RSEYRKLRPRPSETPHPSVPGPVSHDDSCKPLRKKGDGNAEVGQEKTKRS
CSQHSDGLKRDYILAKAAADVVANELTAPDTLIELREVILQERNRMSFNA
QEPLSAFTKGKTASEGEIAEQLECLHTHFEKLRQNEHIKTIADRYHSALI
VQLYDHYNKPKRTKYSLGVIFTKILLKDWVRKKLKNTTERTRRKKMQTKW

KAEVQMHRIWLDFNDEFGCGALSLIPKQFRNTHGLRLNRPDEQELLLRIK
AQHPYLKKDIEDLSFLMWHLLTYGVLPPQKLLLETLNELEIREMSQKNYR
ELLTFVDFPVPADVNGALEWPNRPHDNDFFSEDPAMLPQTAEQNAAEQQS
AVAGSGSMRQFDDLMEECIDPAVLGPNAATPYDGAKM
>Mb|QLI69446.1
MADVEMSDAPSASKRADAANGKGSEAKKKFEVKKWNAVALWAWDIVVDNC
AICRNHIMDLCIECQANQASATSEECTVAWGICNHAFHFHCISRWLKARS
VCPLDNRDWEFQKYGR
>Mb|QLI69447.1
MSFIYLTESQVGHPQPPVKELPTAAPALARRPSSTQESISTSQQSSRGDE
MDRINRLFEILSARSDIDHPVCVECTDLLVDGLQRKLEVASKERDAYVRH
LRQVKTERPSTDEMKARQERLQKAEKDRVAAMDELRRLEKEKDVLDEEIL
ALEEESRQLDKEEEIFWRGRNAFAAKMSDFQSERDSINAKYSHDSQLLEK
LQRSNVYNDTFCISHDGSFATINGLRLGRLSNKPVDWPEINAAWGHALLL
LVTVAEKLDYRFHGYDPQPMGSTSKIIRYDVPSPSSSRLGSRAMQAPPKK
HVLELYSSGDMPLGLTFMHRKFDNAMVAFLELVRQLGAHVQRQTQQSLNA
LSLPYKIEGDKIGEVSIKLGIAQDDGWTKACKLTLTCCKFLLAHASNIAS
TARSTAS
>Mb|QLI69453.1
MAKSKGPSKHSRAARRATSPSIDTDKSLKDIPLPKRSTSSAPRPSVLAVH
RAAGISKKSKPVRKSRLTSKMRKRHEKGLEMAEAVTERTGKKIEQSIGRA
KNVQRRSRTWEDINKDAVEGGAGDEQTGGSRFGALMEEADDDDDGDAVED
EDGFNPANPGASKPAAITDDDDDEIL
>Mb|QLI69457.1
MQRRPHLPGPSQAFAEKKTKILDRLAVPEAEYTDLSPKGTVDKGIRRLID
EINSTDGFVTTSSCAGRVSVFLEGRKAAESSDADAGAGADEQPPAQVAGV
GGKGAGGTWLYVSHDPVQGQDWLQTLALEDGEARRTGRLIHFKFEPMILH
VLTASLAHAQLLLRSALQAGFRESGAVNLTADSEAAAAPMVAVRSMGLGF
ESLVGHELEGKRYALVSGSYLRALMDIGNERFSENTRRIERFRGAFRESL
ARMGSDGRQWEDPAARRERMRAEGLRRKATAAEGVVDEDNGGRESADDLY
VALDFGLA
>Mb|QLI69458.1
MDDDIAVIGLGLRFPGDATDPESLWKILEAGESQWSEIPKERLNIDGYYH
PSGDRQGSISFKGAHLLKGDIRAFDATFFSIPSEDAKAIDPQQRMLLEVS
YEALENAGLKKEDVYGSDTSVYVGSFVKDYEQVCLRDPDWSPQYAATGNG
IAIMANRISYFFNFHGPSVTVDTGCSGSLVSVHLGAQSLRNRESSIAIAA
GAGMILTPSTIMPMTALNFLSPDGKCFTFDERANGYGRGEGIGVVIMKRL
SDALRDNDTIRAVIRGTSVNQDGRTTGITLPSKEAQVANIRSVYKSAGLD
FDQTAYVECHGTGTQAGDWRELKAVSETLAADRDIRKPIVVGSVKPNIGH
LEGAAGVAGIIKGVMVLEKGKIPPNINFKTGNPDINFENWKVPTSLMDWP
MPGLRRVSINCFGFGGTNAHAILDEAPKYLQSRGLIGNHSSADMTPSSPD
KLMLDEDATKSRENEYQLFCYSAHEKSGVSRIMGSHSEYIKARAKSGPRE
LLTDYSYTLSCRRSTMEWKGFIVAKSITELAAKLETAGSSETVRSLRKSQ
PRIGFVFSGQGTQWARMGQDLMQFQVFRSSLEEASEYLLHCLQSPFNLIR
EILRDESDSAISYPHIAQPATTAIQVALVDLLHAFGVSPTSVIGHSSGEI
AAAYAGGAVSRFHAWEIAYFRGLAAISMPFRAPKLKGAMLVLGMSFDETR
KYLEDGHFSAEIACVNSPASTTISGRVDAIEAISQDLAERKQKVFCRTLK
VQTAYHSSHMRLVEHDYRDSLVSIQPRELQTQVQMFSSVTGKSVRGEELG
PHYWSDNMVNPVLFVAAVTSMMQQPVEARPEILIEVGPRAALRSPTTETM
ASIFHEDLIPAYYSIMQPKINGILKVLGLVGDLWTEGCSLNMEQVISRGE
IQRMKCLSNLPPYPWNHDKSYWHESHLSIANRNREYPRQDLIGALTADST
PFEPRWRGFLRVSENPWIQDHQVQKTIVYPAAGMVSMVLEGAKQIKPAMD
GFLGYEISHMHLSKAMIVPNTEHGLEVALNIKITTDISGEVQPKASHQFS
IYSKYLGRDWEQHATGFIQFKHTTAYSSAPSTAHDKQLDKLSDSCKKGMN
SRQLYEDLDTIGMNYGPTFQNISNIRKGEGACVCKVGVPDTKSKMPAKFE
YPHIIHPATLDSMFHPLFVIETVPMVPTFIKSLFVSTGIDQDGPKVFSGF
ATADRVGLQGATAKIVMNGCGTSKTRVIVNGLYLTALQSTGLEEKFLPSF
RNLCTEIVWREDATFDSSTDLIENIRVLAHKYPGLAVLQAGGSTEDATEL

LKVLAPAKDDAPSLSKFTLLGHKDSDTFHEVINKVKETPAECFVECKTNM
ADVNSNYHLIISFDDLGLELKKLKDKLQVGGVLMKLKSSSLNHYTPINGN
IDEQHSRNPVVYRRPAALLGSPAPPIILLVPEDVNDEVSALVDLVKSYQT
KPAQEYEISVLNTQEILKCASLVEKSVIISLLDVFPSHDQHYSILQWDEF
HFNLLKDIQKAAKGMIWLTRGSHMNPHNLQGSSIIGLARTLMSEDPRKAI
VTFDLDVATNICDNTVVKNIMAVFSQTFCEEPRPGCYEMEFAEKDGKVFI
PRLRTIQALNQIIEGMAPCILNNRPFHNDAAGPEDLLDVKLSLVKAGLCD
DSWHYTEFPQTDILPDEVDIKFSQTLLIPQDVETALGRNFESSIGLDVRG
TIISVGRNVTEFLPGDEVAGLVSDGSIQSTLRVKLPFIQRNSTSLFPSQL
ISAYYSLVYMGRGKPGKSVLIHAGASAYGLAAIALARILGVKLFATIVGQ
DPDNQRKILESCGLTSDTIFSAEDEFTPCLLTMTDGKGVDILYSTSQDRL
NGVEKCVKDCKPISPRPSLFRIFGSDLLGGLIVRLVGGSPYSIGRNFESM
SRATAVNFDLRRLLREDADFVTELLASASKFLDEKQFDDSVVTSSAKDFS
IDSLDKALKHVQEHPYQGVCTVSANSGENHKVNVLCDNNFKSLSEAIDPE
GTYLLAGGLGGLGRSIAELLIANGAKHLAFVSRSGISNSQSRDFIEGLQN
EGVDARVYQADLCDKASLSAVMKNQLFVQMPPIRGIFQCAAVIQDSVFDN
MTYQMWNAAFLPKTQGSWNLVKNMAGLDSKPFFVFLASSAGIIGNRGQAN
YAAGNSFIDALAHHCLLHGVPAVSIDLGPVLGAGMLAEDDDMLDKLRAAG
FYGIRHDDFLTVIKHAITREIGSLGDTTPAQVILGVGTGGLLRQNKPADP
YWSRTALYSYLNLIDMPPPGLDGSNGLQGMDMKSLLARCGSTEGAVDLIR
LGLSNMLAKATNLLPEEVDVNKPPSAYGVDSLVAVGVRNWVLSNCNVEVS
VFEVLSNDTIMEMAHMIAKRGGYEKA
>Mb|QLI69462.1
MAPIPITIITGFLGSGKTTLILNLMPQLRAKNPAYKLALLKNEFGDLAID
SQLASSSAISGVQEMLNGCICCNLVGQLGPALEELARTVTPDRIVIETSG
SAFPATLALEVNRLARDAGRYELDGVISVIDVENWQGYEDTSYTAKIQAR
YTDLIVFNKWETAGEDRYEECVDRVGDLEVDVARAKSDKGWVDMNLVFGV
DGGLARDLTETDMELEVNGGQHDHDHKHGGQTNGHSHSHQNEVEVLSVEL
AAPSAATSTPKLLAFLRAAPKDEVYRIKAVATLSSAPENSDADAPRPGGL
PGGRYILNWAFGRWTFTPVGQGQQEHPSSRDVTLRMTVILARHEGNRWRK
KLEAGGYLEAEGVNKGELSVKRIL
>Mb|QLI69465.1
MASTQVHPGSFTHPGGKISKSRSRTVVKPILKKLHSHHSERESLDLDRGW
DDQPSPGLAVGPDFGTYDSDGSYQYSIPNSHGARSARDVSFSLSASDMTG
SGLGVAPRSKYSHVRSTSGNSHTSSIATTTSGRNGGTFVHPFQQQPHAST
PPLSYANSRTSFENGVPTGYSPTITEDDADVDPYSSFHSASTAPRPALYQ
SAFYQHPNHRRPSMASQRTSSLSDGTQTTRLPAPRSNSGTTRPLAPPSLN
QSRSELDSSALSSLAVDSPLSSTAPLATAGTMLSSTQTQASATSTSAAPL
SPLRSSLDMGVFRLRSRSEVDTFTHQEQVREARRKFEAKERAKDEKYAKE
QLRKRERAETKEAHKLEKSHTRLRKGSAGHGSVSSSTMSSGTDLRISSSR
KRGAMSEDSREKIEFSSFGYNSTHGGQTAPSRADEVHFTSPKRTKTAKHK
TMGVWTAFVLWLRTRLLKIGRR
>Mb|QLI69466.1
MSWLGVAPFKKFPAPFLKPMWPFFAAGLVIAYGANSAQNAMMASDEWKND
PRNPKAKTGGH
>Mb|QLI69469.1
MATEGGQTAPVGDAAAKKDQKTRGQPKTPQQPDQAASSDAPGEKTLSNAE
LKKRAKEEKAARRAQAKVAQVAAPAGPPGGQGATGDAKSGKSKANNPKQG
GLPVPSHHRSASRSVLPPVAKEVVKPKISECYSHLSMARRIAITQADKDV
HPVVLALGQQMSTFAISDSTTRLEATLLAFKKVIDAYTTPHSNTFSRHFT
SHVLNPQIEYLTACRPMCFSMGNAIRWLKLQISKIDIDLPDSDAKKVLCQ
AIDTFIRERIILADFVIVKTAADMIQDDEVILTYAHHKLVERSLLQAKSD
GKQFKVILVDDPFDRVGLEHAKNLAAAGIPVTYASDMGALRSNLQEATVV
FAAAEAMFSNGSMYARAGTCDVATVATDLGLRVVALSETINLTDRVSIDS
LTYNEIDPERSTGEECRLLFDTTRDKFLSIVVTELGSLSAMSVTAILRKL
EEL
>Mb|QLI69470.1
MSSAKKTSGKAQRSAIADVVAREYTIHLHKRLHGVTFKKRAPRAIKEIKA

FATQAMGTNDVRLDPQLNKKVWECGIKGVPFRLRVRISRRRNDEEDAKEK
LYSYVQAVNVKNPKGLPTVVVEE
>Mb|QLI69471.1
MLGWMLKRGENAPHADDGDTTQIDQPDTPAPVFAARAFKSALFGTPSKRN
EERRGFRSAASRKPTARSSETPSKPQGILLTPGTATSKRKRVSFGHDVPG
GPTTNLTRITEQDEPTESHQPTAPTHAPTKIAESQSRLAQPEPNVSDDEW
EEEEDCGPHDVTLDLNEPHSQSGRYWKEEFLKYHQEAKAEMEKLLKYKQL
AKSYAQQKDAEAIELAERLRDEQQRVIRMEKRIAENASQIASHRTEQSEE
VQAEMLAKLAKQSSQAAQYRHRVQELEAQMEELLTQRENGAGIETPRRRY
ASNSSIAATQKTLTETRRELRRARSQLKELDTLRDEVFTLKTQLKKAELK
LVVIEKEEARTNNGPRAQELRTLLKAAKEDSRRKDEEIRQLKSEFEAFRK
ENEVHETDTKAVLERAHNKIANLKKEVKALKSTAPAQTQARPHSWHPQSI
GEERMKESKSDEPIRAKVETAERRSLDEPLDMMKKRSQPRTLREKFVDDA
TLAPSLESTKAAERGSLGKSKWKPFVPRSPRHRAYTAEDAVNRHTRLNKS
KEIIAPELPPLANSANHEQSGWTSAVDPRVDLLSNRFARLGGPGPNAQIN
SSLVGNASKSTLPPERRAAALERIQKRMAEKKRLRNQSGTHNKENVRP
>Mb|QLI69475.1
MDEKSRLPPYAPYSPPQASAESHLPLGNNGCRARRRRGLRRSRAIKFFAL
ACLSLLVLAQWKQIRRSNRHSVKLSAEKLNENLATCKTLRHKPRDPIGSG
RDKSARFVDGGKPTLIQNATIWIGEPVEGTSNEDARAGKGWGWTKGDVYL
EYGLIKKVERHISLGSLPRDTQFYNAEGRLLTSGIIDMHSHAGVYSMPGL
RGNSDGNEFSSPVTPWTRAIDGLYVFDPQIEVIKSGGVTTSLILPGSSNN
IGGEAYLIKHAVGKKEGRNEFSAADMLADPERHWRYMKMACGENPKQSFS
SGGRMTSRLGESFEFRRAFEKARDLVQRQDDWCDKADAVGVDNMDSYLPE
ELAWESLGAAMRGQVHINTHCYTVPDLEAMVDHTNEFKFAILKRTWGGRP
PASALFADNMYYKMEAYVGSEFAGKILYQEGLTPVYVSDNPVLNAQHVLF
EAAKAYHYGLPYHAALASVTTAPADELGMGQRLGKVKPGYDADVVVWDSD
PLSVGATPVQVWIDGTAQFTAPVYLDKPIQGPIGPDATLADIVSEPTQVA
DALFKGVTKVLLSGDDAYNTGGEASNVAVSEGKITCIGTCTSEFEAATAA
GVKVVQLNNGYLTHSFTGVGGTIGLNAIDAEDSTDNGDTKEKFTRAVDGL
QLDNKKLRVGARYGVTRAISAPKFNGLKTHHGTSVGFTTSALTSLEKGAV
FAEDAAVHYTLDLNARTDGKSYSEAFGALRKKLLDAGKSDEEPEAYSEAA
YLKRVVSGNLVLALTINSADGIASALRIKSEVEGALKSKINMAVMGGAES
YLVAAELAAASVGVILQPLQPMPQTWDQRRALSGAPLTNGTAADWLVTAG
VTVAVGLPEDWYVRDLGFEAGTAYRNGNGRFTEKSALDLVSRNIYKILGV
RVDEDEDKGHFMISEGSPLEIGSRIRAVAAGNGRVSVFV
>Mb|QLI69479.1
MSTSRIFIKGLPPNITEAEFRKHFSTGNREITDVKLIPQRRIGYVGYKSV
HDAAGAVKYFNRSYIRMSKITVETARPISDPASRKGHSGSRNLGAPEGHT
APRSASDHDVNTKKRKREEQETSDPRLREFLQVMKSGREGAVADDSNAAV
GADHAFSHGDVTVPEGESDDEYEQIPSRGEKSRRIEPRQDKSDNVARQPP
PRVEKTPKDDIEEAGENKAEGIDSEMTDQGPSATGATDDDWLRSRTNRLL
DLVDPDDLASGAVLSPVDATTEQDMENDRLSSHSSDEVTPGTATEVATSK
ETATEDAVSAISRTSRLFVRNLPYSATEDDIRETFDKFGTLQEVHLPLTA
AGATKGFAMVLFTNSSDAVRAFQALDGVTFQGRILHIIPADAKREQGLDE
FGISKMPLKKQNMIRKKAEAASSSFNWNSLYMSQDAVNASIASRLGVSKS
ELLDPTSADAAIKQAIAETTVIQETKAYFAANGVDLDAFKSQRRGDSTIL
VKNFPFGTTMEELRKMFEEHGQVLRVLMPPTGTIAIVQFAQANHAKAAFG
KLAYRRIKDSVLFLEKAPKHLLRGDISEQTSRQQATGVQKFTVSDLLSSG
DKAEEVETTSLFIRNLNFATTTSRLAEAFESLDGFVSARVKTKMDPKKPG
QTLSMGFGFAEFRSKTQAQAALKAMDGHVLDGHTLGVKASHKGQDAAEER
RREDRAKKAAAQRTKIVIKNLPFQATKKDVRSLFGTYGQLRSVRVPKKAD
FTARGFAFADFVTPREAENALNALKDTHLLGRRLVLDFAEAEAVDAEEEI
EKMQRKVGGQVNKVALQRLTGGGRKRVTIGNDEEEFDT
>Mb|QLI69481.1
MSKVFVGNVKSVLSGDTLILTSPNNPSAERNFSLAYVTAPHLRREGDEPF
AFQSREYLRLNLVVGKPIQCTIQYTIPNSGREFGTAKLKDGGELPDELVKA
GWLKVREDAGRKEENEEVLERLEKLRGYESEAKAEGKGLWAGTGGVIEVQ

NDLGGPEFMKEWKGKTVDGVVERVLSGDRLLVRLLLSEKKHVQPMTLIAG
IRTPATERTVPSTGTTQPAEEFGNEARQFVESRLLQRQVKVEIVGASPQG
QLVASIIHPRGNIAEFLLQDGLARCNDFHSTMLGEKMAALRSAEKQAQSK
KLRLHKHHVAKAVGDNQEMTVSKIVGADTIFVKNKAGAEKRISFSSIRGP
RTNEASESPFREEAKEFLRQKLIGKHVKISIDGKKPASEGFEAKEVATVT
EKGKNIALMLVEAGWASVIRHRKDDTDRASNYDELLAAQEKAKEELKGMW
SGKPQKAKQYTDLSENAQKAKIMLATLQRQKKVPAIVDFCKAGSRFTVLI
PRENVKLTMVLGGIRAPRAPRADGEGGEPFGKEALDLANRRCNQRDCEVD
IHDMDKVGGFIGALYINRENFAKILVEEGLASVHAYSAEKSGNSTELFAA
EKKAKEARKGLWHDYDPSQEEADEEEETTEETPAESEVSLDKKPTDYRDV
MITNIDGNGKIKIQEIGKGTAALETMMNEFRKFHLDSKNNKPLGDAPKTG
DFVSAKFSADGQWYRARVRSNDRTAKVAEVQYVDYGNTEKIPWSDLRGLD
HGQFGIQKLKAQAIDASLSFVQLPTGADYFNEAIGVIAEMTEGKRLVGSF
DIVDSKENVSYITLYDPKSNNELPGLNDSINKEVVASGYGMVPKKLKAWE
RSKAFESYLKHLKEVESQAKQDRLGMWEYGDITED
>Mb|QLI69485.1
MDKIPLNYEASEGDTHKQTTNSLPPEVVQCLENARFLHLATCTDNVPHVS
LMNYTYLPSSPYSSVPVIIMTTNPASRKTTNIIANPSVSLLVHDWVSHRP
PTQGRRMSGGSPGPEHRSSLASLLLNLNSSAMSSISATIGGAARLAPSGS
DEEKYFTEQHLANNTFEEGESIFRQDSNTGADDRGSHFVAGEEVRVIIVD
IKDVRISDWKGTVRDWALVSDASLVNGA
>Mb|QLI69486.1
MRLPHPPLPRTSTSHIVTEPTRQLRRRDLNTKNLTFIIIFVVIALFAFIA
VLLVAFFRARRRRPDDQDAGSGGQWPVLHVFRRKRVGHRRYEQTSGENLD
GEAQIHQLEPTAVSANHVNTLQRDSRQSGSRAARANRTGASVDRNTSVRS
VMTLPAYRATAGNNEQVLGREGDRDGVDVIVDLPTAEEEESLREEEMEAI
YQIRIARREQIAQRDELRRQRRDARQRGDNNTLADDRARSRAASNNSNLD
ELRQEVSRIQDQRQRSVSSVSYADLGVARHDGTRIRASSNESERIGLLSD
AASIAISARSEVASSLGLHRREQSVSSLVSVDSEFIPEPSRARANSGSTT
PGLLSGEPAAGSSPEHVQDDLGVEPTPPPGYEVVSIEDDDREVAGPMTPL
NEPPPDYSGPNEHMPQTEQTSTEPAHPSPREGRTPSPLRTARGVGGAPQL
PSLRISRLPAIVIQPLNEESRDENVL
>Mb|QLI69487.1
METALKSWELDNNIELVDPKRDALYNYDAQGQKSINQEKPWARDPNHFKR
VRISAVALIKMTMHARSGGNLEVMGLMQGYVDGDTFVVTDAFRLPVEGTE
TRVNVQEEANEYLVEYLDLCRAQGRQENVVGWYHSHPGYGCWLSGIDVAT
EAMQQQFQDPFLAVVIDPDRTINAGKVDIGAFRTYPHSHKPEGAGAAVAD
GLQTVPLAKAAEFGAHASRYYSLEVSHFKSTLDAHLLELLWHKYWVQTLS
QSPLLTNRDFGSKQMLDLSSKIKEVTTSMARSRGQGMGGGGPAVKDKTIE
KLTQDTSLIATKERSGLMAAEIKAKVFNELGARNA
>Mb|QLI69488.1
MGDGIAKKRKRNGAEAVANATASSKKSKKVKNPPPQIEPEEDESESGDNE
SLSEEQNEQQDEENSEDAEEGPIAEDAEDEDADDTVGDLPTDGAPILAPT
AESDMFDQLKLSEKTMKAITEMGFTKMTAIQRTAIPPLLAGKDVLGAAKT
GSGKTLAFLIPAIEILSALRFKPRNGTGVIVVSPTRELALQIFGVARELM
QHHSQTYGIVIGGANRKAEAEKLSKGVNLLIATPGRLLDHLLNTPFVFKN
LKSLVIDEADRILEVGFEDEIRQIVKVLSNDDRQTMLFSATQTTKVEDLA
RISLRPGPLYINVDEETQFSTVDGLEQGYVLCDADKRFILLFSFLMRMKE
KKKKVIVFFSSCNSVKYYSELLNYIDCPVLDLHGKQKQQKRTNTFFEFSN
AEHGILICTDVAARGLDIPAVDFIVQFDPPDNTRDYIHRVGRTARGANTK
GRSLLFLQPNEVGFLSHLKTARVPVVEFEFPAKHIKNVQSNLERLIGKNY
YLQQSAKEAFKSYLHAYGSHSLRSVYDVQKLDLVRVAKSFGFATPPRVDI
NLGQSMSRNKVQGRRSYGSQPRQPVRQGGRR
>Mb|QLI69489.1
MSSSPANPFLSPTRTGRRKERRNPSVTPRRFGRFFTPRSNLPPESRAALR
SLDASATNCQLISPQSLASDVLSSDPICPSSPTEALGQVDGAGEKRKRLD
HCQVVRKRRRGLIDEDSTPPPLQFPGRSFHMSRDGDVQMVEARSDTREGL
DDRRRATLSHFFKASRSGSITGTEEKPFPAAPCIQSSEPRTNLTLEGYRP

KPIRKFRGRGFEAQLLDREHGFASHSGKQYLNFPAGDPRALTAPFYSKSY
DTHHCTSYTSQGNTIPFSLASCHNAPVTVIGDEQGFVRLFNTTTADGPVD
SKVDAHIKVHDNAIMDLDLSTDDFRLATACGDRTGKIVDVVTQSVAVELA
GGHWDSLRQVAFQPGTANGTALATSDRAGRVQIWDLRCSQVPIHCFSTAN
PNGTGNRDTTLDPITARTINTIDNAHERRVQGITSVASVTAIQWLPPGRE
HLLLTASEANASIKLWDTRYIKPRRQNEETPLAITPQPMSHAWRSYGITS
LALSTDASRLYAVCKDSTVYAYSTTHLMLGHAPELDNFAPKRRPTGTEGL
GPLYGLKHDQFRASSFYVKCALRHRTPMNNYQHELLAVGSTDSCAVLFPT
DEGYLYSSCAKHAHDLERSTSTSALTPLQSFSANTPASAGTGPSSSPIPI
FRVGTPLVRGHSREVTNLNWSHDGKLVTASDDYFVRQWQANEAEARHLRQ
VGEFGGERHMAGWAEVGPTWDDDEA
>Mb|QLI69492.1
MSNLKRKDAPGGQPPAKSAKNTKQGRPSKTDAPEIAKSKTAKTQPKKTSD
NDAHPKAPVVSLLKNEEPIFPRGGGSVLTPLEHKQIQLEAKADARREEEF
DTRGGKAQKIPKKRKVSLKGGKRSAENKDSEDFVKVESLNFKKLVKGSLV
LGQITRRNNLDLEVALPNNLTGHVSVVAVSEQLTNRVQEAAAEQDDEEDE
SSDETDVDLKSMFVVGQYLRVYVVSTMDESAIGNGKNKRRIELSLRPTET
[truncated: 521,458 more chars]
